# Supplementary material for: Community-like genome in single cells of the sulfur bacterium Achromatium oxaliferum
Source: Nat Commun. 2017 Sep 6;8:455. doi: 10.1038/s41467-017-00342-9 (PMC5587575; doi:10.1038/s41467-017-00342-9)

Description of Supplementary Files

File Name: Supplementary Information
Description: Supplementary Figures

File Name: Supplementary Data 1
Description: Bin duplication and completion data

File Name: Supplementary Data 2
Description: Copy numbers of “single-copy” marker genes in the single-cells genomes and metagenomic bins

File Name: Supplementary Data 3
Description: Binning information of 4 previously published single -cells of Achromatium from saline or brackish environments.

File Name: Supplementary Data 4
Description: Distance matrices between phylogenetic trees of individual marker genes occurring in more than one metagenomic bin.

File Name: Supplementary Data 5
Description: Phylogenetic trees of 102 “single copy” marker genes identified in the single-cells genomes and the metagenomic data.

File Name: Supplementary Data 6
Description: Phylogenetic trees of all proteins occurring in the metagenome in more than 5 copies

File Name: Supplementary Data 7
Description: Annotation table of the single-cells genomes and the metagenomic data.

File Name: Peer Review File

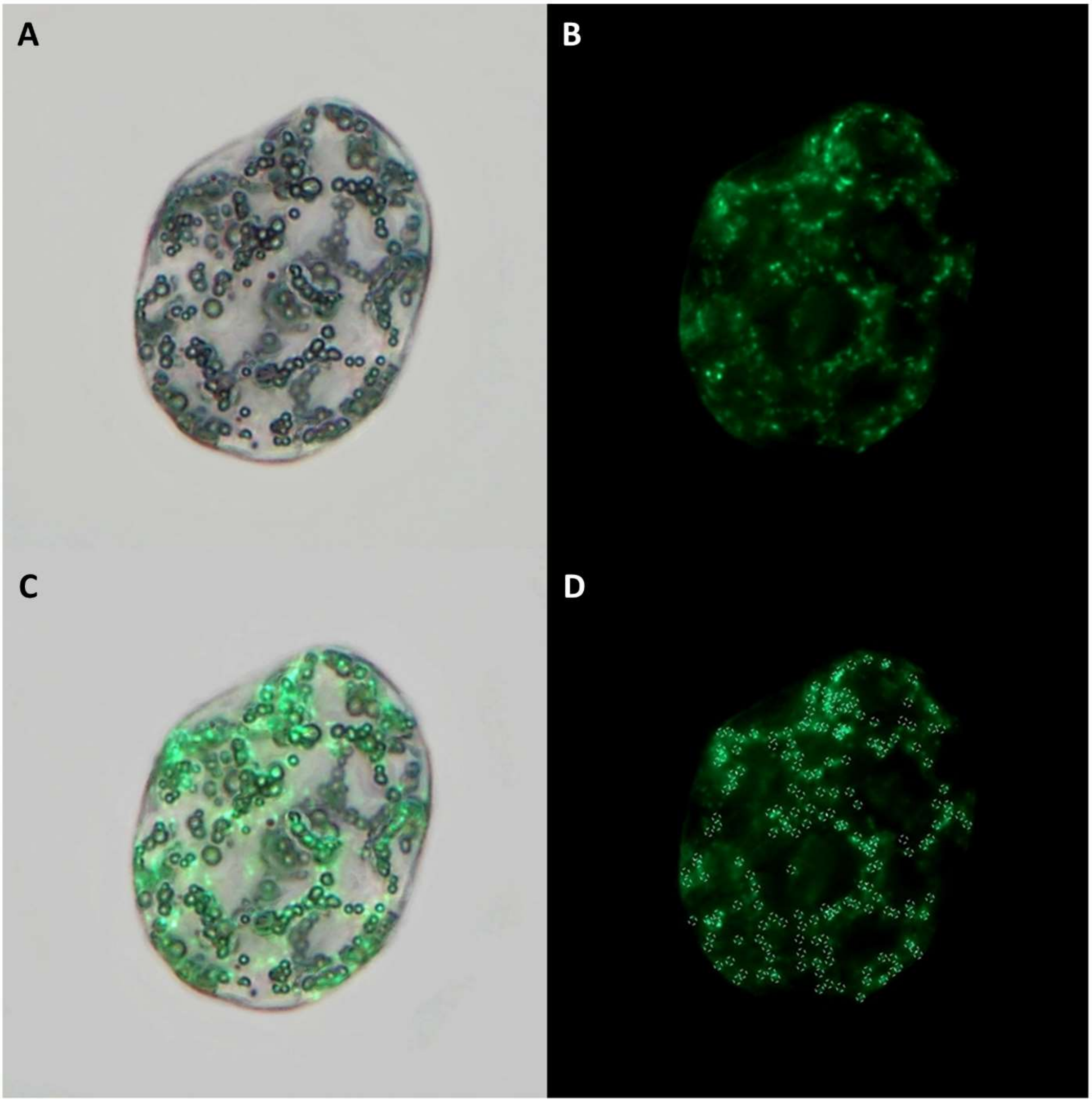

Supplementary Figure 1. A light micrograph of an *Achromatium* cell (A). The same cell labeled with the DNA specific stain PicoGreen (B). An overlay of the light micrograph and the DNA stain (C) and the counted DNA spots (D).

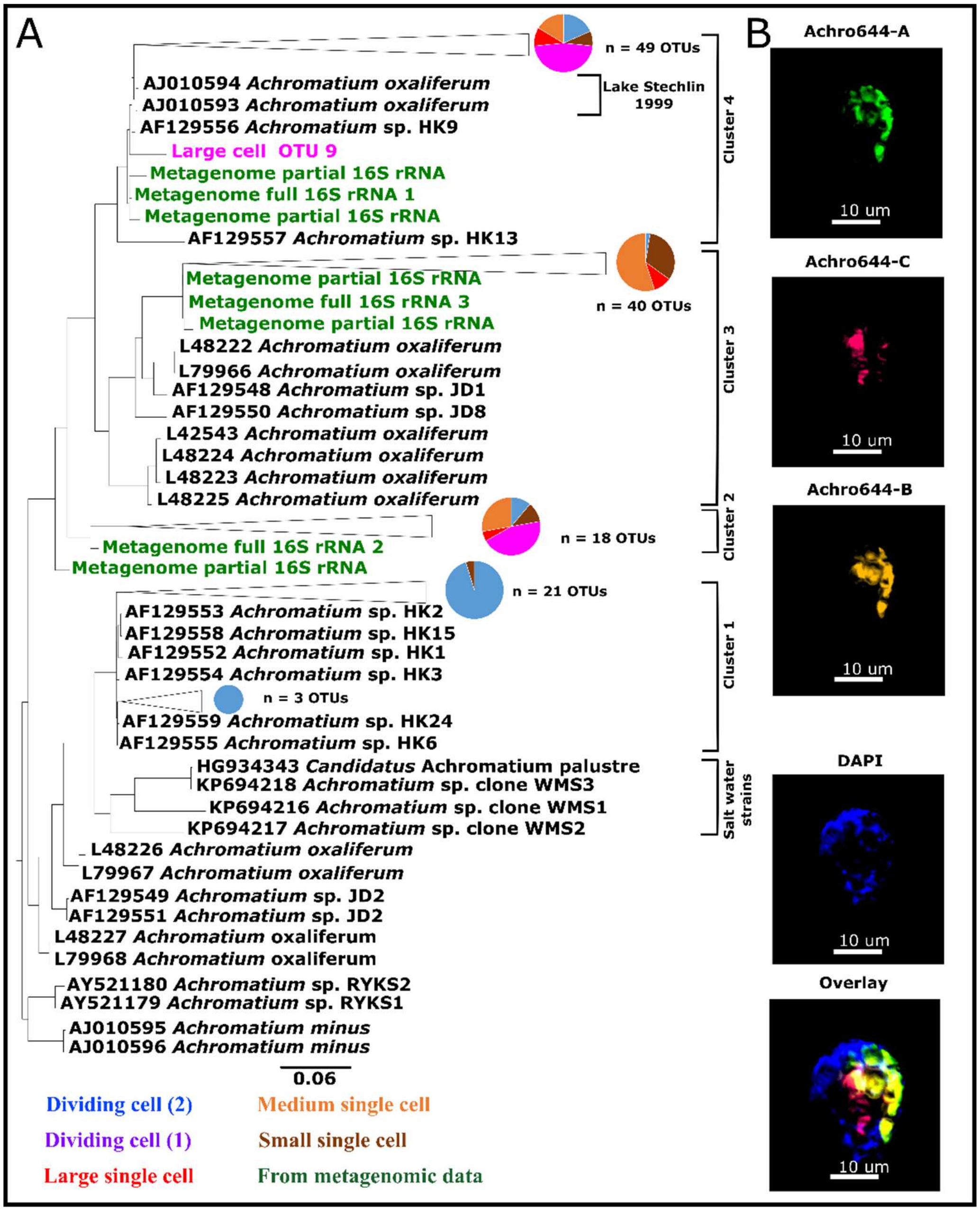

Supplementary Figure 2. A) Maximum likelihood tree of 16S rRNA sequences obtained from single *Achromatium* cells form Lake Stechlin, metagenomics data of the same cell population, and reference sequences. For reading clarity large clusters are represented by closed group. The distribution of sequences in each cluster is given in a pie chart with the color code explain in the legend. The full tree is presented in Supplementary data S1. The single cells are named according to morphological characteristics. B) Results of fluorescence *in-situ* hybridization experiments using specific probes against the three clusters obtained from the metagenomics data. The DNA was stained using DAPI.

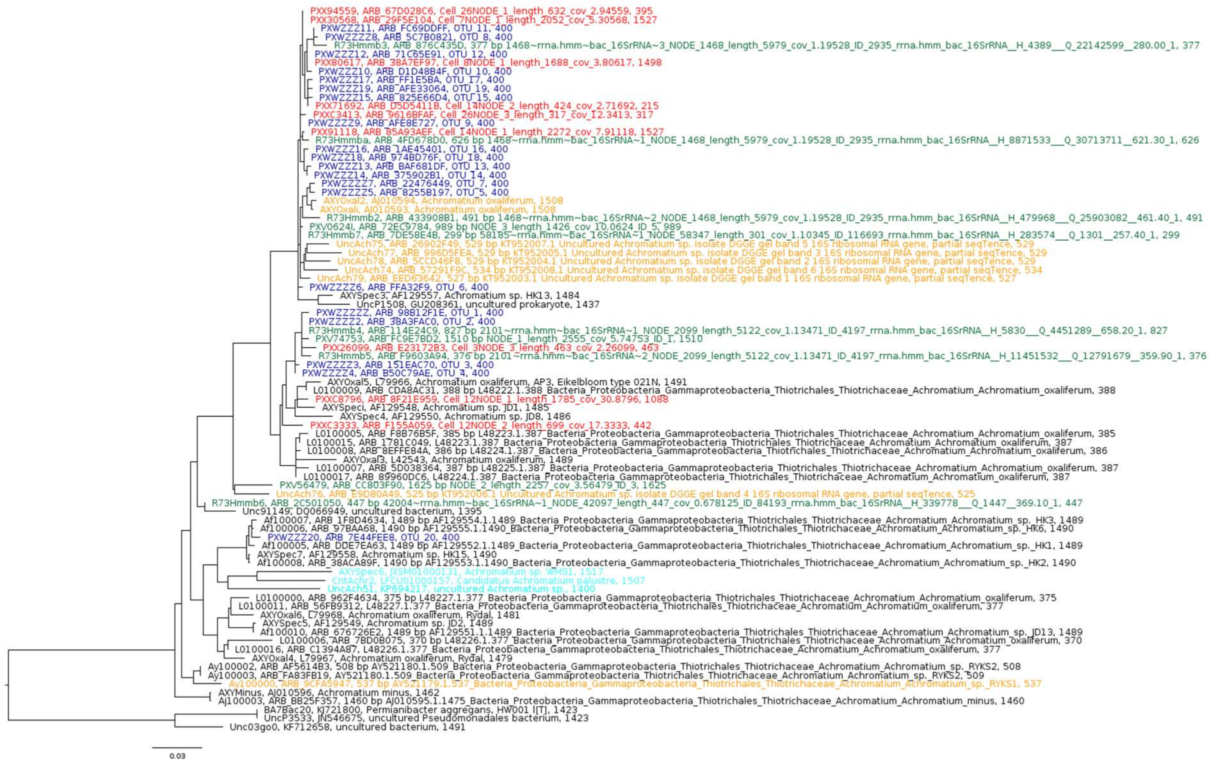

Supplementary Figure 3. A phylogenetic tree of Achromatium sequences. An initial tree ML tree was calculated using all available full length sequences of Achromatium. Amplicon sequences and short sequences from metagenomic data were added at a second stage using maximum parsimony. All steps were calculated using the ARB platform.

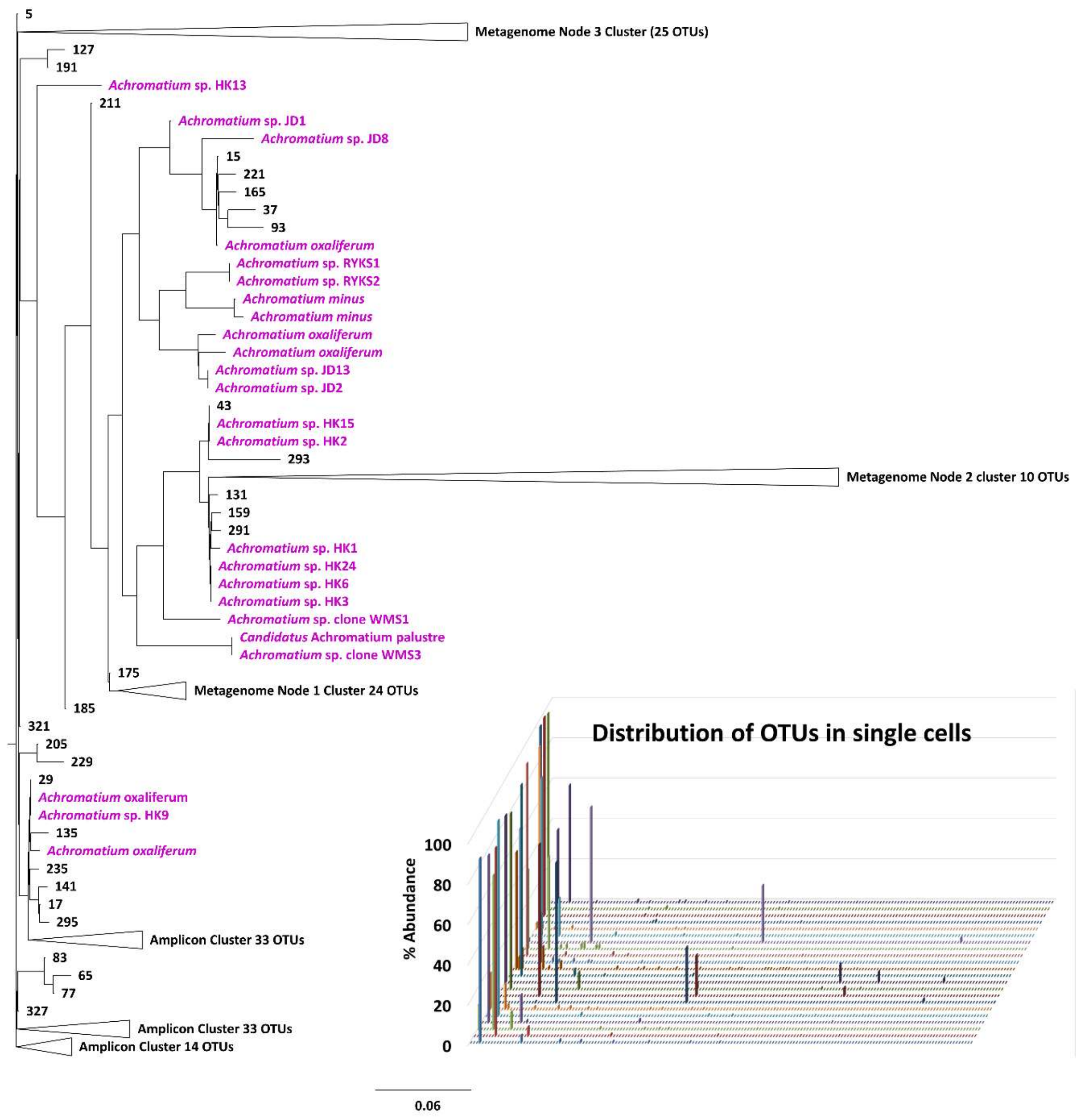

Supplementary Figure 4. A ML phylogenetic tree of Achromatium sequences including short ~200 bp PGM generated amplicons. The numbers represent OTU numbers as calculated by the DADA2 R package. Out of 177 calculated OTUs the majority are grouped within larger clusters. The distribution of the OTUs among 22 individual ells is shown in the insert.

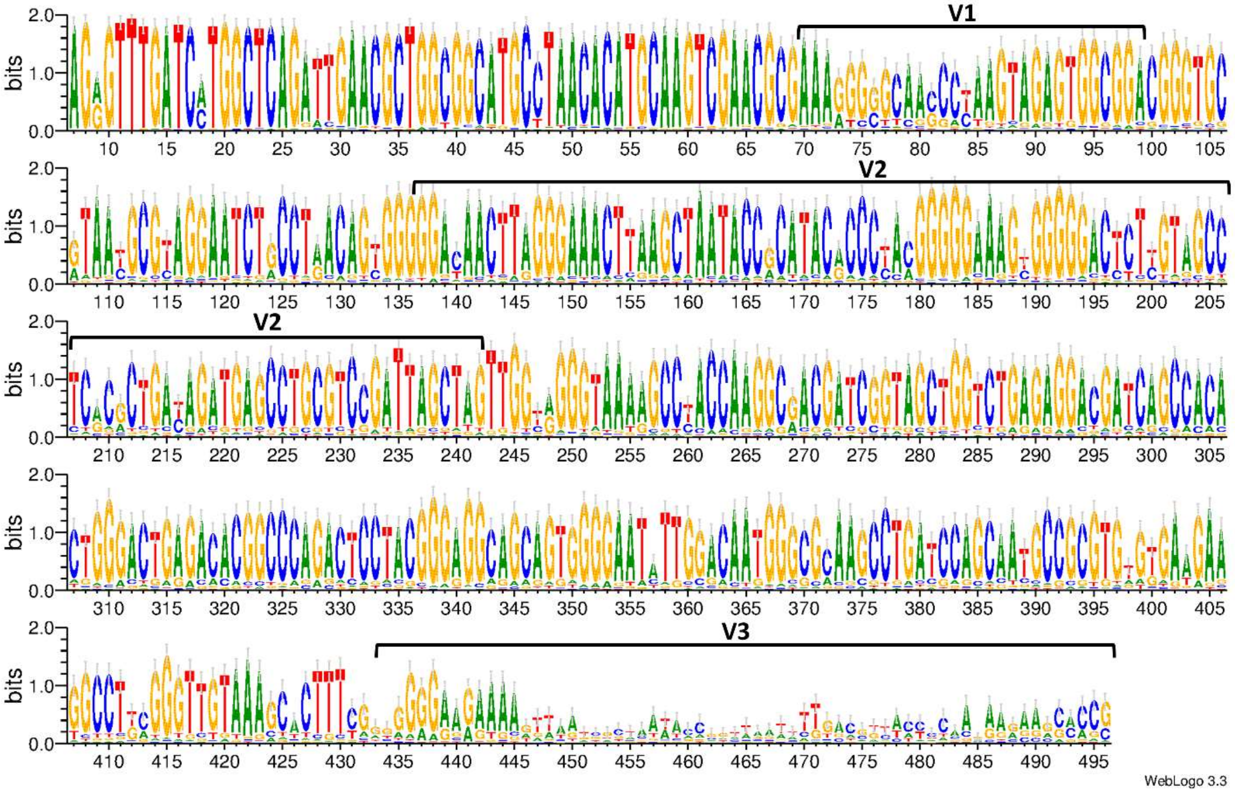

Supplementary Figure 5. Graphical representation of 16S rRNa gene sequence alignment from 5 different single
cells. Letter sizes represent frequency of occurrence at given location. Variable regions of the 16S rRNA gene within the sequenced area are marked (V1-V3).

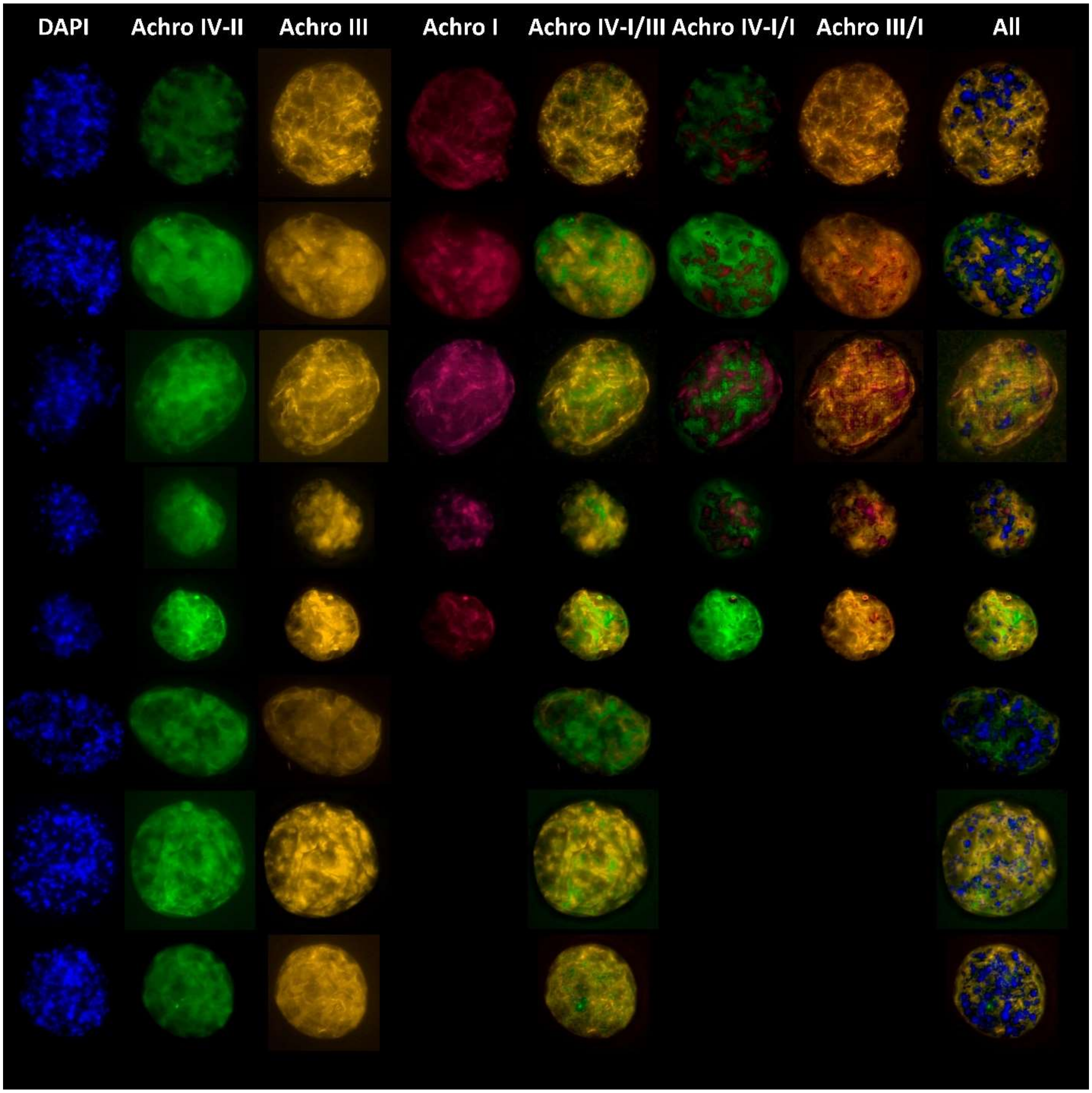

Supplementary Figure 6. Fluorescent in situ hybridization images and overlays of 8 individual *Achromatium* cells.

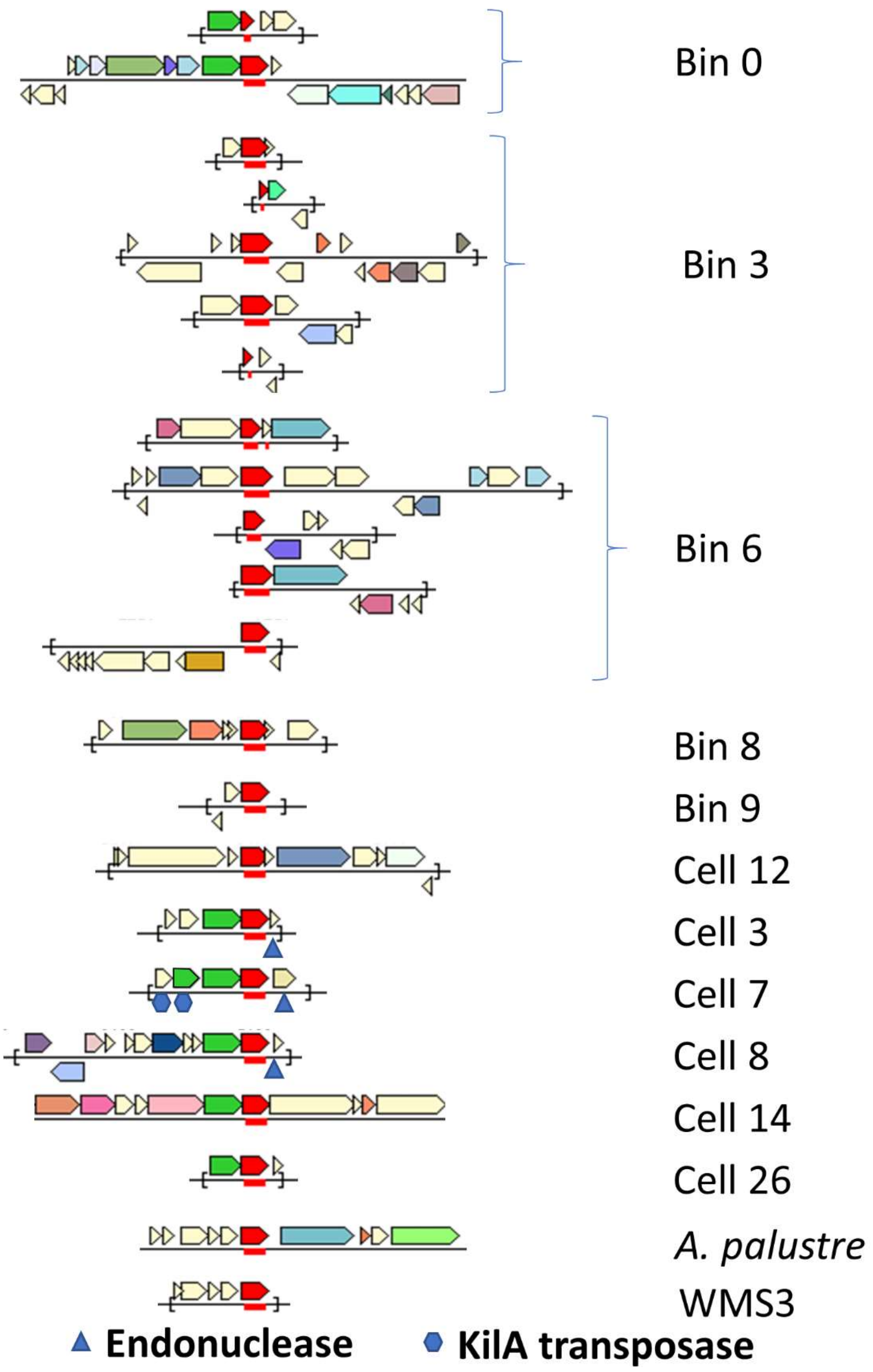

Supplementary Figure 7. Gene synteny of the *recA* gene in *Achromatium* genomes from Lake Stechlin and public data.

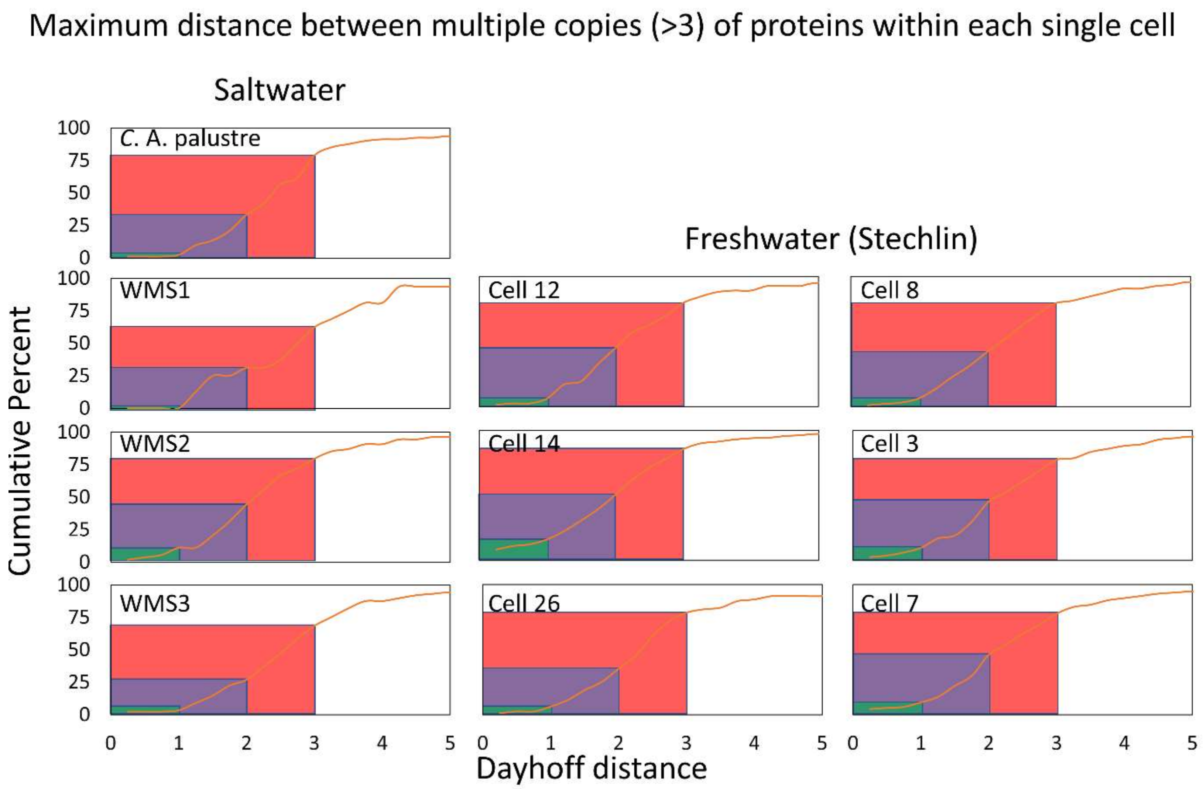

Supplementary Figure 8 Distribution of maximum distances between multiple copies of the same protein within the genomes of single *Achromaium* cells

Supplementary Figure 9 Average amino acid and nucleotide distances between single cells genomes of Achromatium from Lake Stechlin, metagenomic bins of Achromatium from Lake Stechlin and other public *Achromatium* genomes.

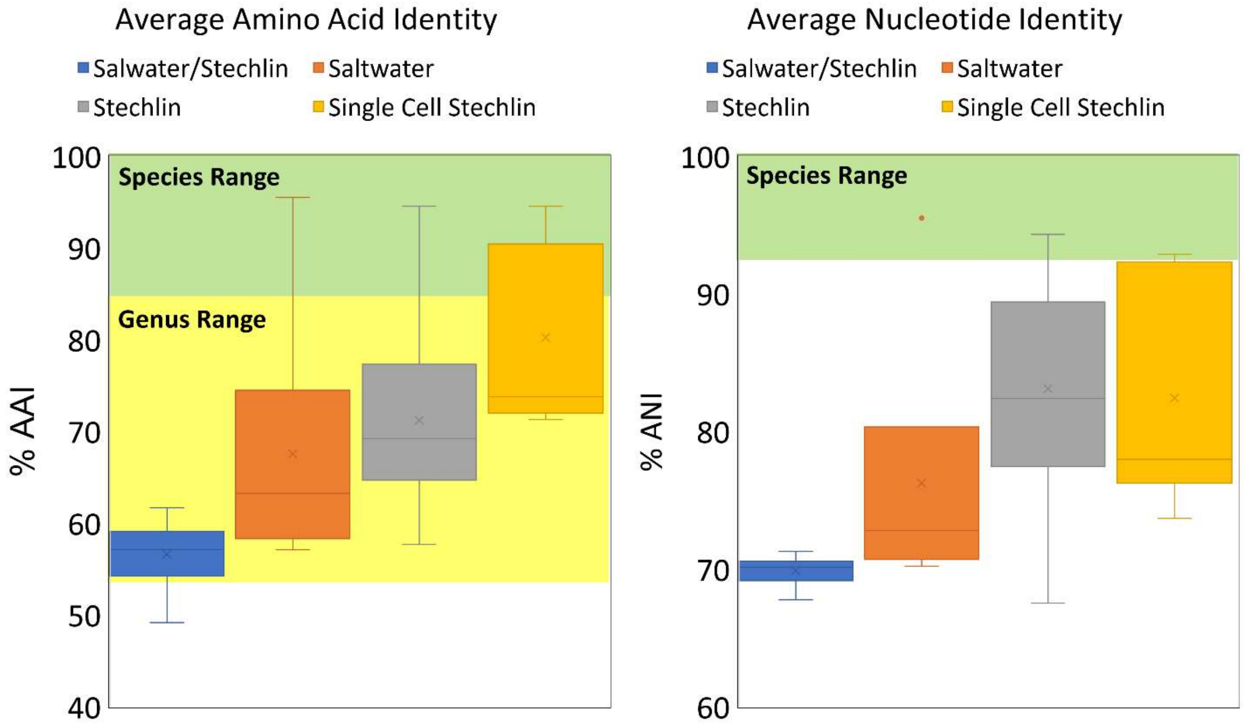

Supplement: Supplementary file 1 — Supplementary Information [file 41467_2017_342_MOESM1_ESM.pdf]
