# Supplementary material for: Community-like genome in single cells of the sulfur bacterium Achromatium oxaliferum
Source: Nat Commun. 2017 Sep 6;8:455. doi: 10.1038/s41467-017-00342-9 (PMC5587575; doi:10.1038/s41467-017-00342-9)
Supplement: Supplementary file 6 — Supplementary Data 5 [file 41467_2017_342_MOESM6_ESM.pdf]

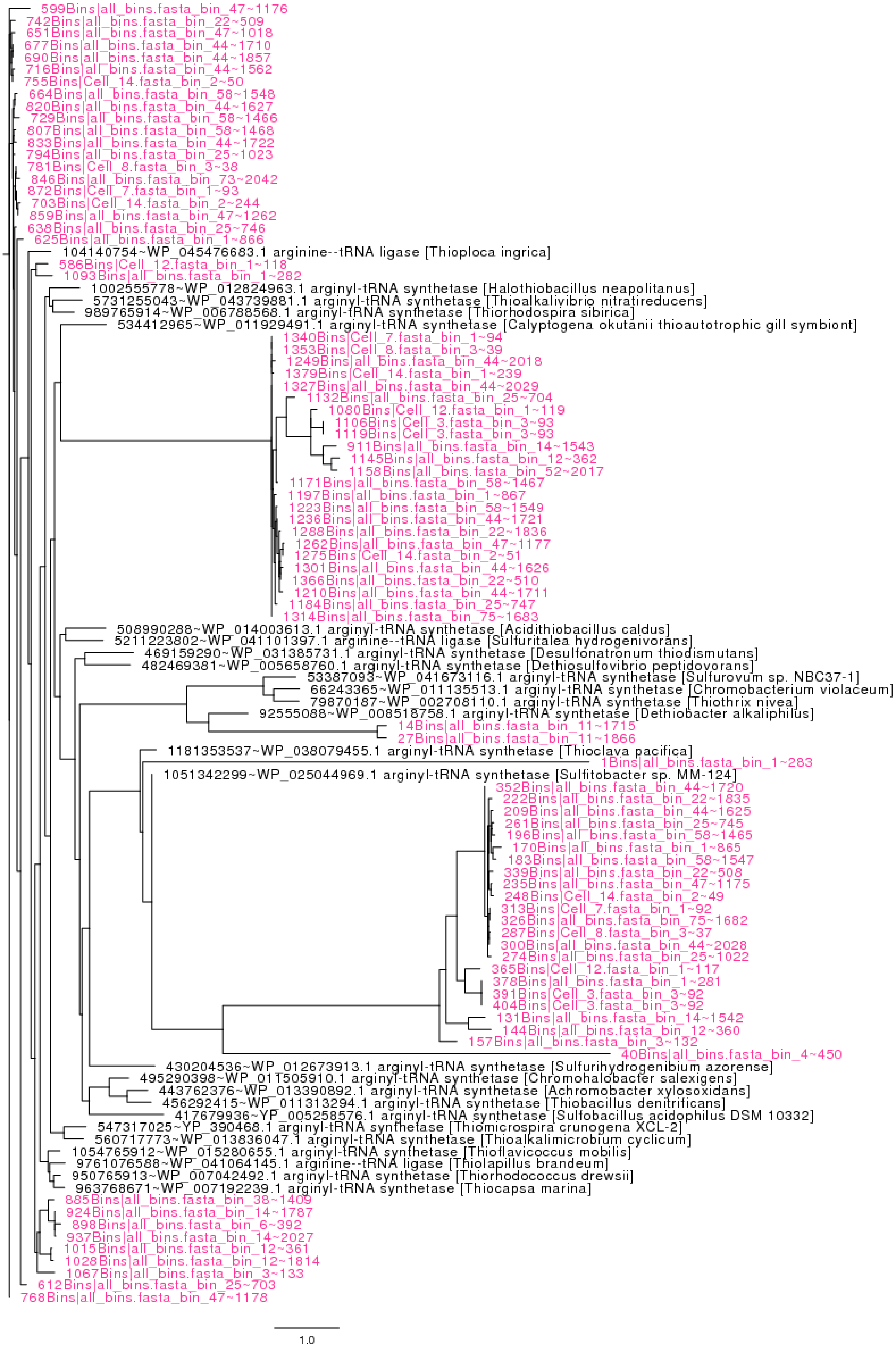
599Bins|all_bins.fasta_bin_47~1176
742Bins|all_bins.fasta_bin_22~509
651Bins|all_bins.fasta_bin_47~1018
677Bins|all_bins.fasta_bin_44~1710
690Bins|all_bins.fasta_bin_44~1857
716Bins|all_bins.fasta_bin_44~1562
755Bins|Cell_14.fasta_bin_2~50
664Bins|all_bins.fasta_bin_58~1548
820Bins|all_bins.fasta_bin_44~1627
729Bins|all_bins.fasta_bin_58~1466
807Bins|all_bins.fasta_bin_58~1468
833Bins|all_bins.fasta_bin_44~1722
794Bins|all_bins.fasta_bin_25~1023
781Bins|Cell_8.fasta_bin_3~38
846Bins|all_bins.fasta_bin_73~2042
872Bins|Cell_7.fasta_bin_1~93
703Bins|Cell_14.fasta_bin_2~244
859Bins|all_bins.fasta_bin_47~1262
638Bins|all_bins.fasta_bin_25~746
625Bins|all_bins.fasta_bin_1~866
104140754~WP_045476683.1 arginine--tRNA ligase [Thioploca ingrica]
586Bins|Cell_12.fasta_bin_1~118
1093Bins|all_bins.fasta_bin_1~282
1002555778~WP_012824963.1 arginyl-tRNA synthetase [Halothiobacillus neapolitanus]
5731255043~WP_043739881.1 arginyl-tRNA synthetase [Thioalkalivibrio nitratireducens]
989765914~WP_006788568.1 arginyl-tRNA synthetase [Thiorhodospira sibirica]
534412965~WP_011929491.1 arginyl-tRNA synthetase [Calyptogena okutanii thioautotrophic gill symbiont]
1340Bins|Cell_7.fasta_bin_1~94
1353Bins|Cell_8.fasta_bin_3~39
1249Bins|all_bins.fasta_bin_44~2018
1379Bins|Cell_14.fasta_bin_1~239
1327Bins|all_bins.fasta_bin_44~2029
1132Bins|all_bins.fasta_bin_25~704
1080Bins|Cell_12.fasta_bin_1~119
1106Bins|Cell_3.fasta_bin_3~93
1119Bins|Cell_3.fasta_bin_3~93
911Bins|all_bins.fasta_bin_14~1543
1145Bins|all_bins.fasta_bin_12~362
1158Bins|all_bins.fasta_bin_52~2017
1171Bins|all_bins.fasta_bin_58~1467
1197Bins|all_bins.fasta_bin_1~867
1223Bins|all_bins.fasta_bin_58~1549
1236Bins|all_bins.fasta_bin_44~1721
1288Bins|all_bins.fasta_bin_22~1836
1262Bins|all_bins.fasta_bin_47~1177
1275Bins|Cell_14.fasta_bin_2~51
1301Bins|all_bins.fasta_bin_44~1626
1366Bins|all_bins.fasta_bin_22~510
1210Bins|all_bins.fasta_bin_44~1711
1184Bins|all_bins.fasta_bin_25~747
1314Bins|all_bins.fasta_bin_75~1683
508990288~WP_014003613.1 arginyl-tRNA synthetase [Acidithiobacillus caldus]
5211223802~WP_041101397.1 arginine--tRNA ligase [Sulfuritalea hydrogenivorans]
469159290~WP_031385731.1 arginyl-tRNA synthetase [Desulfonatronum thiodismutans]
482469381~WP_005658760.1 arginyl-tRNA synthetase [Dethiosulfovibrio peptidovorans]
53387093~WP_041673116.1 arginyl-tRNA synthetase [Sulfurovum sp. NBC37-1]
66243365~WP_011135513.1 arginyl-tRNA synthetase [Chromobacterium violaceum]
79870187~WP_002708110.1 arginyl-tRNA synthetase [Thiothrix nivea]
92555088~WP_008518758.1 arginyl-tRNA synthetase [Dethiobacter alkaliphilus]
14Bins|all_bins.fasta_bin_11~1715
27Bins|all_bins.fasta_bin_11~1866
1181353537~WP_038079455.1 arginyl-tRNA synthetase [Thioclava pacifica]
1Bins|all_bins.fasta_bin_1~283
1051342299~WP_025044969.1 arginyl-tRNA synthetase [Sulfitobacter sp. MM-124]
352Bins|all_bins.fasta_bin_44~1720
222Bins|all_bins.fasta_bin_22~1835
209Bins|all_bins.fasta_bin_44~1625
261Bins|all_bins.fasta_bin_25~745
196Bins|all_bins.fasta_bin_58~1465
170Bins|all_bins.fasta_bin_1~865
183Bins|all_bins.fasta_bin_58~1547
339Bins|all_bins.fasta_bin_22~508
235Bins|all_bins.fasta_bin_47~1175
248Bins|Cell_14.fasta_bin_2~49
313Bins|Cell_7.fasta_bin_1~92
326Bins|all_bins.fasta_bin_75~1682
287Bins|Cell_8.fasta_bin_3~37
300Bins|all_bins.fasta_bin_44~2028
274Bins|all_bins.fasta_bin_25~1022
365Bins|Cell_12.fasta_bin_1~117
378Bins|all_bins.fasta_bin_1~281
391Bins|Cell_3.fasta_bin_3~92
404Bins|Cell_3.fasta_bin_3~92
131Bins|all_bins.fasta_bin_14~1542
144Bins|all_bins.fasta_bin_12~360
157Bins|all_bins.fasta_bin_3~132
40Bins|all_bins.fasta_bin_4~450
430204536~WP_012673913.1 arginyl-tRNA synthetase [Sulfurihydrogenibium azorense]
495290398~WP_011505910.1 arginyl-tRNA synthetase [Chromohalobacter salexigens]
443762376~WP_013390892.1 arginyl-tRNA synthetase [Achromobacter xylosoxidans]
456292415~WP_011313294.1 arginyl-tRNA synthetase [Thiobacillus denitrificans]
417679936~YP_005258576.1 arginyl-tRNA synthetase [Sulfobacillus acidophilus DSM 10332]
547317025~YP_390468.1 arginyl-tRNA synthetase [Thiomicrospira crunogena XCL-2]
560717773~WP_013836047.1 arginyl-tRNA synthetase [Thioalkalimicrobium cyclicum]
1054765912~WP_015280655.1 arginyl-tRNA synthetase [Thioflavicoccus mobilis]
9761076588~WP_041064145.1 arginine--tRNA ligase [Thiolapillus brandeum]
950765913~WP_007042492.1 arginyl-tRNA synthetase [Thiorhodococcus drewsii]
963768671~WP_007192239.1 arginyl-tRNA synthetase [Thiocapsa marina]
885Bins|all_bins.fasta_bin_38~1409
924Bins|all_bins.fasta_bin_14~1787
898Bins|all_bins.fasta_bin_6~392
937Bins|all_bins.fasta_bin_14~2027
1015Bins|all_bins.fasta_bin_12~361
1028Bins|all_bins.fasta_bin_12~1814
1067Bins|all_bins.fasta_bin_3~133
612Bins|all_bins.fasta_bin_25~703
768Bins|all_bins.fasta_bin_47~1178
1.0

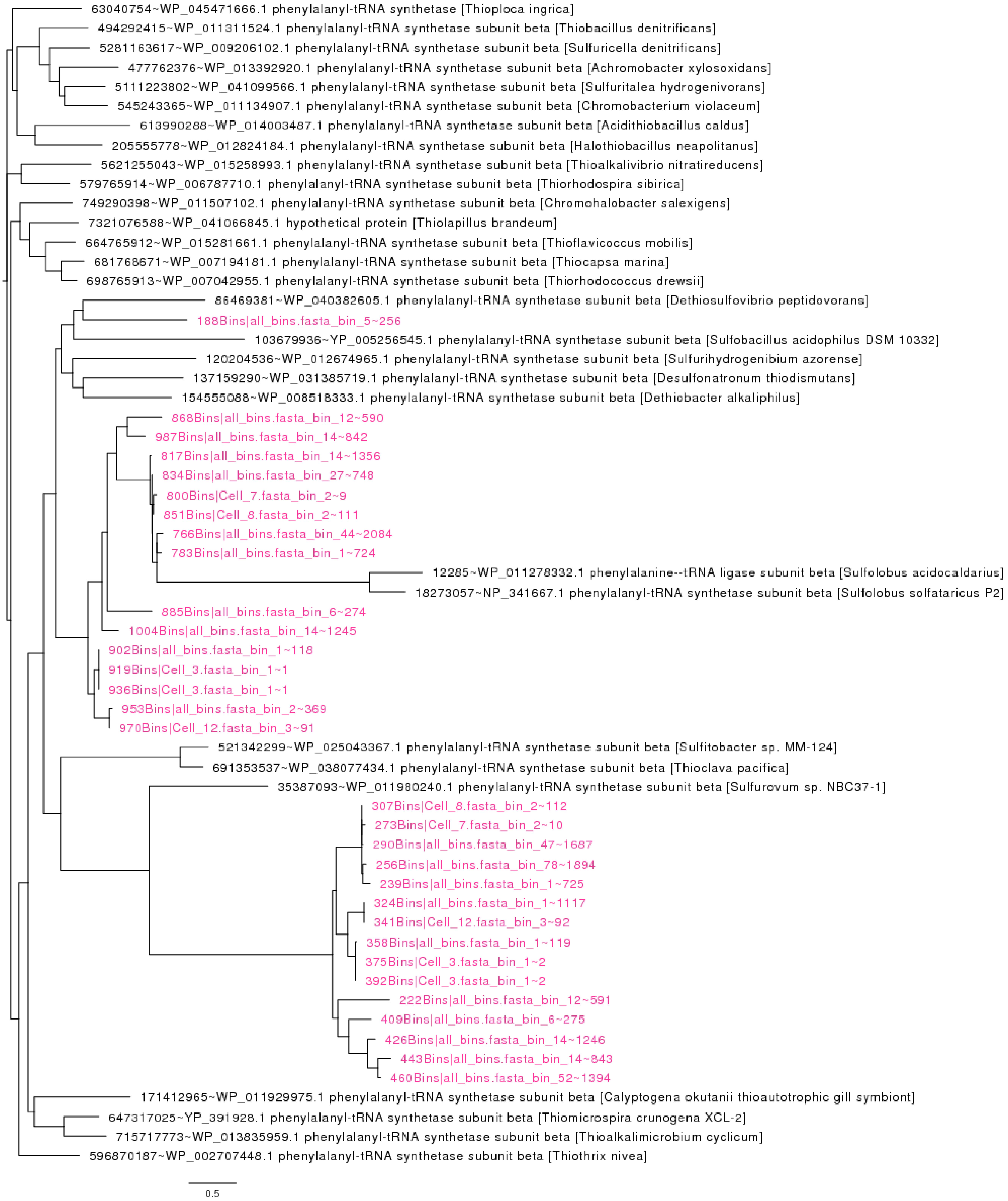
63040754~WP_045471666.1 phenylalanyl-tRNA synthetase [Thioploca ingrica]
494292415~WP_011311524.1 phenylalanyl-tRNA synthetase subunit beta [Thiobacillus denitrificans]
5281163617~WP_009206102.1 phenylalanyl-tRNA synthetase subunit beta [Sulfuricella denitrificans]
477762376~WP_013392920.1 phenylalanyl-tRNA synthetase subunit beta [Achromobacter xylosoxidans]
5111223802~WP_041099566.1 phenylalanyl-tRNA synthetase subunit beta [Sulfuritalea hydrogenivorans]
545243365~WP_011134907.1 phenylalanyl-tRNA synthetase subunit beta [Chromobacterium violaceum]
613990288~WP_014003487.1 phenylalanyl-tRNA synthetase subunit beta [Acidithiobacillus caldus]
205555778~WP_012824184.1 phenylalanyl-tRNA synthetase subunit beta [Halothiobacillus neapolitanus]
5621255043~WP_015258993.1 phenylalanyl-tRNA synthetase subunit beta [Thioalkalivibrio nitratireducens]
579765914~WP_006787710.1 phenylalanyl-tRNA synthetase subunit beta [Thiorhodospira sibirica]
749290398~WP_011507102.1 phenylalanyl-tRNA synthetase subunit beta [Chromohalobacter salexigens]
7321076588~WP_041066845.1 hypothetical protein [Thiolapillus brandeum]
664765912~WP_015281661.1 phenylalanyl-tRNA synthetase subunit beta [Thioflavicoccus mobilis]
681768671~WP_007194181.1 phenylalanyl-tRNA synthetase subunit beta [Thiocapsa marina]
698765913~WP_007042955.1 phenylalanyl-tRNA synthetase subunit beta [Thiorhodococcus drewsii]
86469381~WP_040382605.1 phenylalanyl-tRNA synthetase subunit beta [Dethiosulfovibrio peptidovorans]
188Bins|all_bins.fasta_bin_5~256
103679936~YP_005256545.1 phenylalanyl-tRNA synthetase subunit beta [Sulfobacillus acidophilus DSM 10332]
120204536~WP_012674965.1 phenylalanyl-tRNA synthetase subunit beta [Sulfurihydrogenibium azorense]
137159290~WP_031385719.1 phenylalanyl-tRNA synthetase subunit beta [Desulfonatronum thiodismutans]
154555088~WP_008518333.1 phenylalanyl-tRNA synthetase subunit beta [Dethiobacter alkaliphilus]
868Bins|all_bins.fasta_bin_12~590
987Bins|all_bins.fasta_bin_14~842
817Bins|all_bins.fasta_bin_14~1356
834Bins|all_bins.fasta_bin_27~748
800Bins|Cell_7.fasta_bin_2~9
851Bins|Cell_8.fasta_bin_2~111
766Bins|all_bins.fasta_bin_44~2084
783Bins|all_bins.fasta_bin_1~724
12285~WP_011278332.1 phenylalanine--tRNA ligase subunit beta [Sulfolobus acidocaldarius]
18273057~NP_341667.1 phenylalanyl-tRNA synthetase subunit beta [Sulfolobus solfataricus P2]
885Bins|all_bins.fasta_bin_6~274
1004Bins|all_bins.fasta_bin_14~1245
902Bins|all_bins.fasta_bin_1~118
919Bins|Cell_3.fasta_bin_1~1
936Bins|Cell_3.fasta_bin_1~1
953Bins|all_bins.fasta_bin_2~369
970Bins|Cell_12.fasta_bin_3~91
521342299~WP_025043367.1 phenylalanyl-tRNA synthetase subunit beta [Sulfitobacter sp. MM-124]
691353537~WP_038077434.1 phenylalanyl-tRNA synthetase subunit beta [Thioclava pacifica]
35387093~WP_011980240.1 phenylalanyl-tRNA synthetase subunit beta [Sulfurovum sp. NBC37-1]
307Bins|Cell_8.fasta_bin_2~112
273Bins|Cell_7.fasta_bin_2~10
290Bins|all_bins.fasta_bin_47~1687
256Bins|all_bins.fasta_bin_78~1894
239Bins|all_bins.fasta_bin_1~725
324Bins|all_bins.fasta_bin_1~1117
341Bins|Cell_12.fasta_bin_3~92
358Bins|all_bins.fasta_bin_1~119
375Bins|Cell_3.fasta_bin_1~2
392Bins|Cell_3.fasta_bin_1~2
222Bins|all_bins.fasta_bin_12~591
409Bins|all_bins.fasta_bin_6~275
426Bins|all_bins.fasta_bin_14~1246
443Bins|all_bins.fasta_bin_14~843
460Bins|all_bins.fasta_bin_52~1394
171412965~WP_011929975.1 phenylalanyl-tRNA synthetase subunit beta [Calyptogena okutanii thioautotrophic gill symbiont]
647317025~YP_391928.1 phenylalanyl-tRNA synthetase subunit beta [Thiomicrospira crunogena XCL-2]
715717773~WP_013835959.1 phenylalanyl-tRNA synthetase subunit beta [Thioalkalimicrobium cyclicum]
596870187~WP_002707448.1 phenylalanyl-tRNA synthetase subunit beta [Thiothrix nivea]
0.5

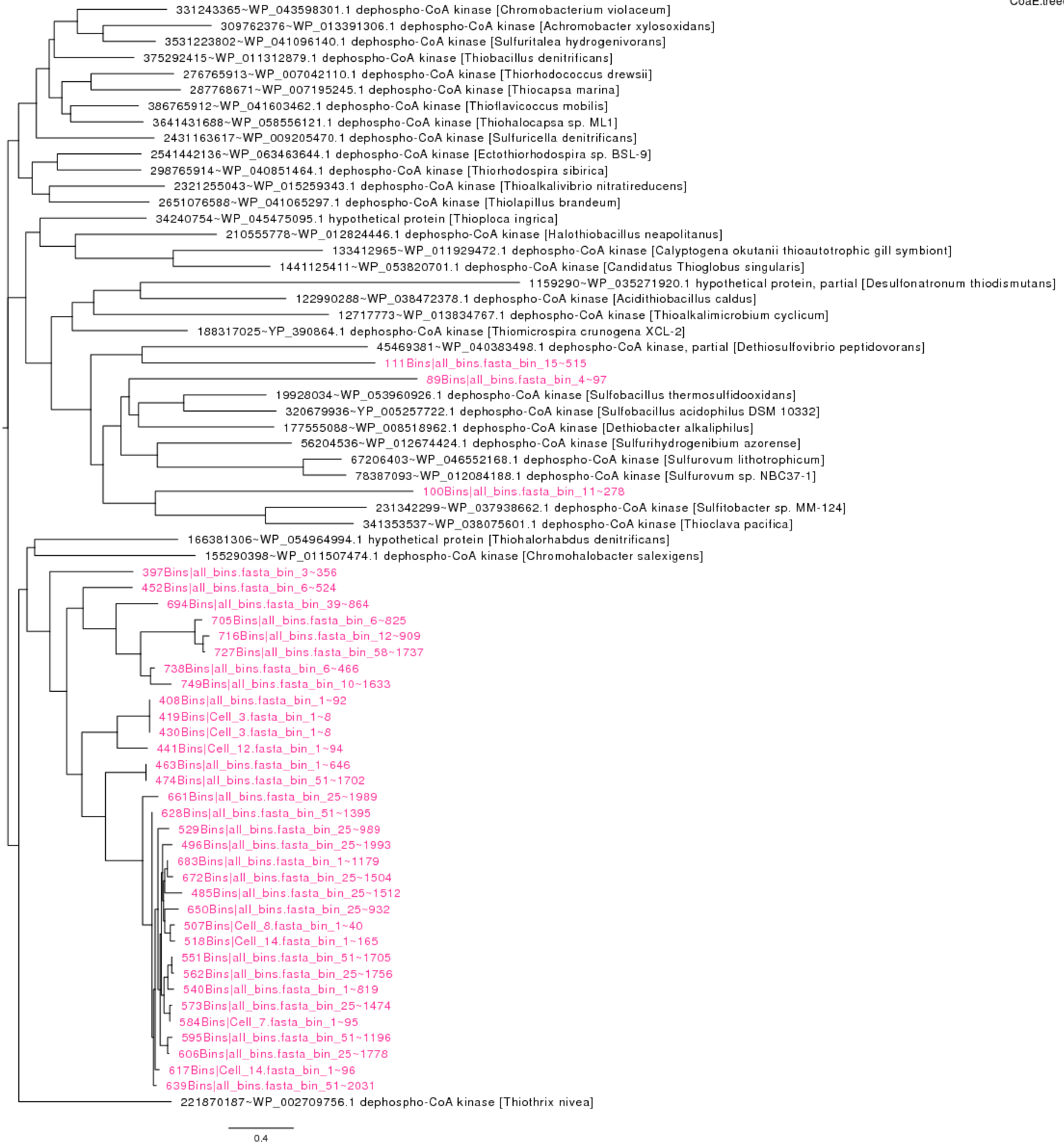
331243365~WP_043598301.1 dephospho-CoA kinase [Chromobacterium violaceum]
309762376~WP_013391306.1 dephospho-CoA kinase [Achromobacter xylosoxidans]
3531223802~WP_041096140.1 dephospho-CoA kinase [Sulfuritalea hydrogenivorans]
375292415~WP_011312879.1 dephospho-CoA kinase [Thiobacillus denitrificans]
276765913~WP_007042110.1 dephospho-CoA kinase [Thiorhodococcus drewsii]
287768671~WP_007195245.1 dephospho-CoA kinase [Thiocapsa marina]
386765912~WP_041603462.1 dephospho-CoA kinase [Thioflavicoccus mobilis]
3641431688~WP_058556121.1 dephospho-CoA kinase [Thiohalocapsa sp. ML1]
2431163617~WP_009205470.1 dephospho-CoA kinase [Sulfuricella denitrificans]
2541442136~WP_063463644.1 dephospho-CoA kinase [Ectothiorhodospira sp. BSL-9]
298765914~WP_040851464.1 dephospho-CoA kinase [Thiorhodospira sibirica]
2321255043~WP_015259343.1 dephospho-CoA kinase [Thioalkalivibrio nitratireducens]
2651076588~WP_041065297.1 dephospho-CoA kinase [Thiolapillus brandeum]
34240754~WP_045475095.1 hypothetical protein [Thioploca ingrica]
210555778~WP_012824446.1 dephospho-CoA kinase [Halothiobacillus neapolitanus]
133412965~WP_011929472.1 dephospho-CoA kinase [Calyptogena okutanii thioautotrophic gill symbiont]
1441125411~WP_053820701.1 dephospho-CoA kinase [Candidatus Thioglobus singularis]
1159290~WP_035271920.1 hypothetical protein, partial [Desulfonatronum thiodismutans]
122990288~WP_038472378.1 dephospho-CoA kinase [Acidithiobacillus caldus]
12717773~WP_013834767.1 dephospho-CoA kinase [Thioalkalimicrobium cyclicum]
188317025~YP_390864.1 dephospho-CoA kinase [Thiomicrospira crunogena XCL-2]
45469381~WP_040383498.1 dephospho-CoA kinase, partial [Dethiosulfovibrio peptidovorans]
111Bins|all_bins.fasta_bin_15~515
89Bins|all_bins.fasta_bin_4~97
19928034~WP_053960926.1 dephospho-CoA kinase [Sulfobacillus thermosulfidooxidans]
320679936~YP_005257722.1 dephospho-CoA kinase [Sulfobacillus acidophilus DSM 10332]
177555088~WP_008518962.1 dephospho-CoA kinase [Dethiobacter alkaliphilus]
56204536~WP_012674424.1 dephospho-CoA kinase [Sulfurihydrogenibium azorense]
67206403~WP_046552168.1 dephospho-CoA kinase [Sulfurovum lithotrophicum]
78387093~WP_012084188.1 dephospho-CoA kinase [Sulfurovum sp. NBC37-1]
100Bins|all_bins.fasta_bin_11~278
231342299~WP_037938662.1 dephospho-CoA kinase [Sulfitobacter sp. MM-124]
341353537~WP_038075601.1 dephospho-CoA kinase [Thioclava pacifica]
166381306~WP_054964994.1 hypothetical protein [Thiohalorhabdus denitrificans]
155290398~WP_011507474.1 dephospho-CoA kinase [Chromohalobacter salexigens]
397Bins|all_bins.fasta_bin_3~356
452Bins|all_bins.fasta_bin_6~524
694Bins|all_bins.fasta_bin_39~864
705Bins|all_bins.fasta_bin_6~825
716Bins|all_bins.fasta_bin_12~909
727Bins|all_bins.fasta_bin_58~1737
738Bins|all_bins.fasta_bin_6~466
749Bins|all_bins.fasta_bin_10~1633
408Bins|all_bins.fasta_bin_1~92
419Bins|Cell_3.fasta_bin_1~8
430Bins|Cell_3.fasta_bin_1~8
441Bins|Cell_12.fasta_bin_1~94
463Bins|all_bins.fasta_bin_1~646
474Bins|all_bins.fasta_bin_51~1702
661Bins|all_bins.fasta_bin_25~1989
628Bins|all_bins.fasta_bin_51~1395
529Bins|all_bins.fasta_bin_25~989
496Bins|all_bins.fasta_bin_25~1993
683Bins|all_bins.fasta_bin_1~1179
672Bins|all_bins.fasta_bin_25~1504
485Bins|all_bins.fasta_bin_25~1512
650Bins|all_bins.fasta_bin_25~932
507Bins|Cell_8.fasta_bin_1~40
518Bins|Cell_14.fasta_bin_1~165
551Bins|all_bins.fasta_bin_51~1705
562Bins|all_bins.fasta_bin_25~1756
540Bins|all_bins.fasta_bin_1~819
573Bins|all_bins.fasta_bin_25~1474
584Bins|Cell_7.fasta_bin_1~95
595Bins|all_bins.fasta_bin_51~1196
606Bins|all_bins.fasta_bin_25~1778
617Bins|Cell_14.fasta_bin_1~96
639Bins|all_bins.fasta_bin_51~2031
221870187~WP_002709756.1 dephospho-CoA kinase [Thiothrix nivea]
0.4

Competence.treee.tre.exp.tree.pdf

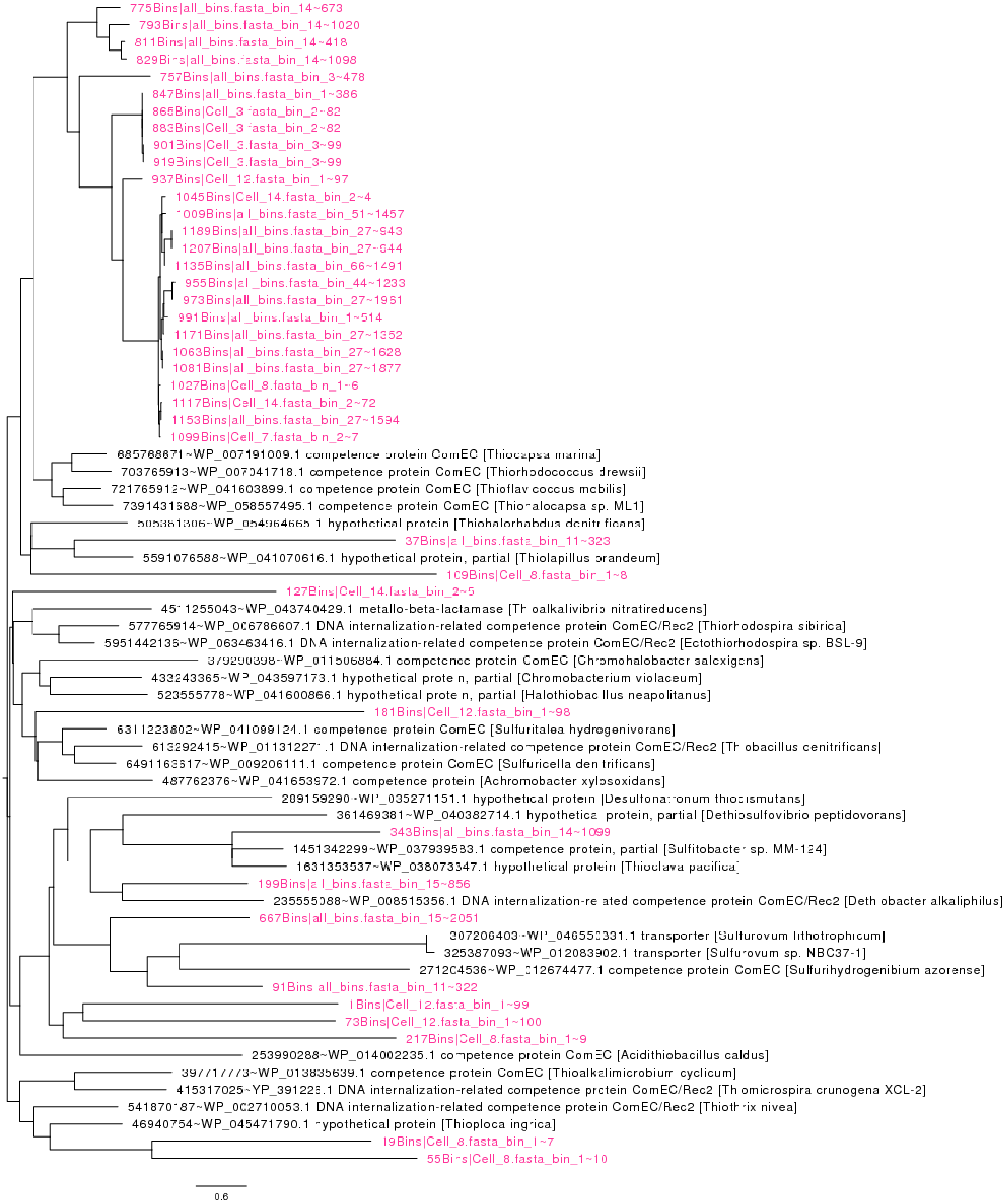

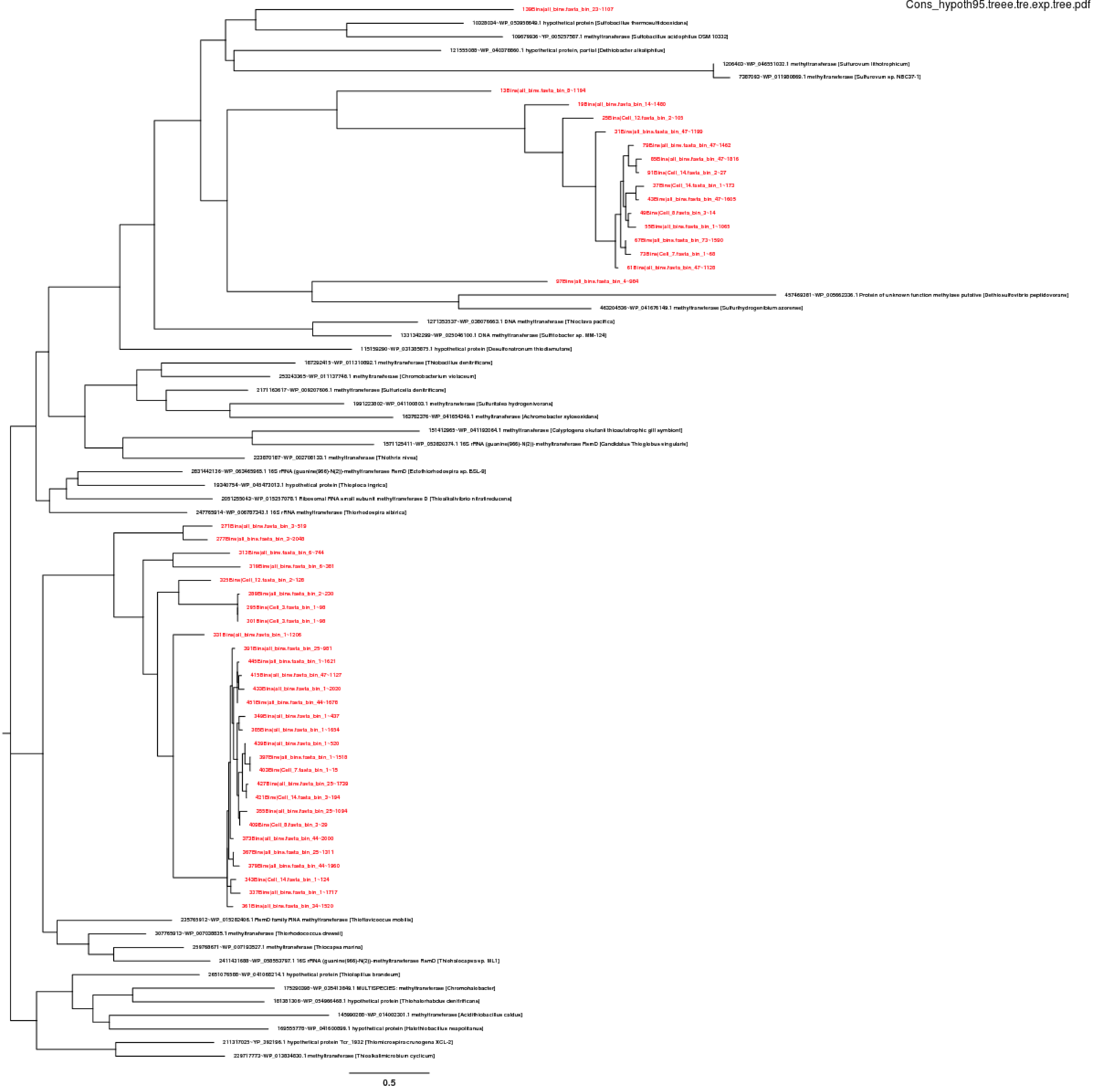
Cons_hypoth95.treee.tre.exp.tree.pdf
139Bins|all_bins.fasta_bin_23~1107
10028034~WP_053956649.1 hypothetical protein [Sulfobacillus thermosulfidooxidans]
109679936~YP_005257567.1 methyltransferase [Sulfobacillus acidophilus DSM 10332]
121555088~WP_040378660.1 hypothetical protein, partial [Dethiobacter alkaliphilus]
1206403~WP_046551032.1 methyltransferase [Sulfurovum lithotrophicum]
7387093~WP_011980669.1 methyltransferase [Sulfurovum sp. NBC37-1]
13Bins|all_bins.fasta_bin_8~1194
19Bins|all_bins.fasta_bin_14~1460
25Bins|Cell_12.fasta_bin_2~105
31Bins|all_bins.fasta_bin_47~1199
79Bins|all_bins.fasta_bin_47~1462
85Bins|all_bins.fasta_bin_47~1816
91Bins|Cell_14.fasta_bin_2~27
37Bins|Cell_14.fasta_bin_1~173
43Bins|all_bins.fasta_bin_47~1605
49Bins|Cell_8.fasta_bin_3~14
55Bins|all_bins.fasta_bin_1~1065
67Bins|all_bins.fasta_bin_73~1590
73Bins|Cell_7.fasta_bin_1~68
61Bins|all_bins.fasta_bin_47~1126
97Bins|all_bins.fasta_bin_4~964
457469361~WP_005662336.1 Protein of unknown function methylase putative [Dethiosulfovibrio peptidovorans]
463204536~WP_041676149.1 methyltransferase [Sulfurihydrogenibium azorense]
1271253537~WP_038076663.1 DNA methyltransferase [Thioclava pacifica]
1331342299~WP_025046100.1 DNA methyltransferase [Sulfitobacter sp. NM-124]
115159290~WP_031385675.1 hypothetical protein [Desulfonatronum thiodismutans]
167292415~WP_011310692.1 methyltransferase [Thiobacillus denitrificans]
253243365~WP_011137746.1 methyltransferase [Chromobacterium violaceum]
2171163617~WP_009207606.1 methyltransferase [Sulfuricella denitrificans]
1991223802~WP_041100803.1 methyltransferase [Sulfuritalea hydrogenivorans]
163762376~WP_041654349.1 methyltransferase [Achromobacter xylosoxidans]
151412965~WP_041192064.1 methyltransferase [Calyptogena okutanii thioautotrophic gill symbiont]
1571125411~WP_053820374.1 16S rRNA (guanine(966)-N(2))-methyltransferase RsmD [Candidatus Thioglobus singularis]
223670167~WP_002708133.1 methyltransferase [Thiothrix nivea]
2631442136~WP_063465965.1 16S rRNA (guanine(966)-N(2))-methyltransferase RsmD [Ectothiorhodospira sp. BSL-9]
19340754~WP_045473013.1 hypothetical protein [Thioploca ingrica]
2051255043~WP_015257076.1 Ribosomal RNA small subunit methyltransferase D [Thioalkalivibrio nitratireducens]
247765914~WP_006787343.1 16S rRNA methyltransferase [Thiorhodospira sibirica]
271Bins|all_bins.fasta_bin_3~519
277Bins|all_bins.fasta_bin_3~2048
313Bins|all_bins.fasta_bin_6~744
319Bins|all_bins.fasta_bin_6~381
325Bins|Cell_12.fasta_bin_2~128
289Bins|all_bins.fasta_bin_2~230
295Bins|Cell_3.fasta_bin_1~98
301Bins|Cell_3.fasta_bin_1~98
331Bins|all_bins.fasta_bin_1~1206
391Bins|all_bins.fasta_bin_25~981
445Bins|all_bins.fasta_bin_1~1621
415Bins|all_bins.fasta_bin_47~1127
433Bins|all_bins.fasta_bin_1~2020
451Bins|all_bins.fasta_bin_44~1678
349Bins|all_bins.fasta_bin_1~437
385Bins|all_bins.fasta_bin_1~1654
439Bins|all_bins.fasta_bin_1~520
397Bins|all_bins.fasta_bin_1~1518
403Bins|Cell_7.fasta_bin_1~15
427Bins|all_bins.fasta_bin_25~1739
421Bins|Cell_14.fasta_bin_3~194
355Bins|all_bins.fasta_bin_25~1094
409Bins|Cell_8.fasta_bin_3~29
373Bins|all_bins.fasta_bin_44~2000
367Bins|all_bins.fasta_bin_25~1311
379Bins|all_bins.fasta_bin_44~1960
343Bins|Cell_14.fasta_bin_1~124
337Bins|all_bins.fasta_bin_1~1717
361Bins|all_bins.fasta_bin_34~1520
235765912~WP_015282406.1 RsmD family RNA methyltransferase [Thioflavicoccus mobilis]
307765913~WP_007038835.1 methyltransferase [Thiorhodococcus drewsii]
259768671~WP_007192527.1 methyltransferase [Thiocapsa marina]
2411431688~WP_058553797.1 16S rRNA (guanine(966)-N(2))-methyltransferase RsmD [Thiohalocapsa sp. ML1]
2651076588~WP_041068214.1 hypothetical protein [Thiolapillus brandeum]
175290098~WP_035413649.1 MULTISPECIES: methyltransferase [Chromohalobacter]
181381306~WP_054966468.1 hypothetical protein [Thiohalorhabdus denitrificans]
145990288~WP_014002301.1 methyltransferase [Acidithiobacillus caldus]
169555778~WP_041600699.1 hypothetical protein [Halothiobacillus neapolitanus]
211317025~YP_392196.1 hypothetical protein Tcr_1932 [Thiomicrospira crunogena XCL-2]
229717773~WP_013834630.1 methyltransferase [Thioalkalimicrobium cyclicum]
0.5

CTP_synth_N.treee.tre.exp.tree.pdf

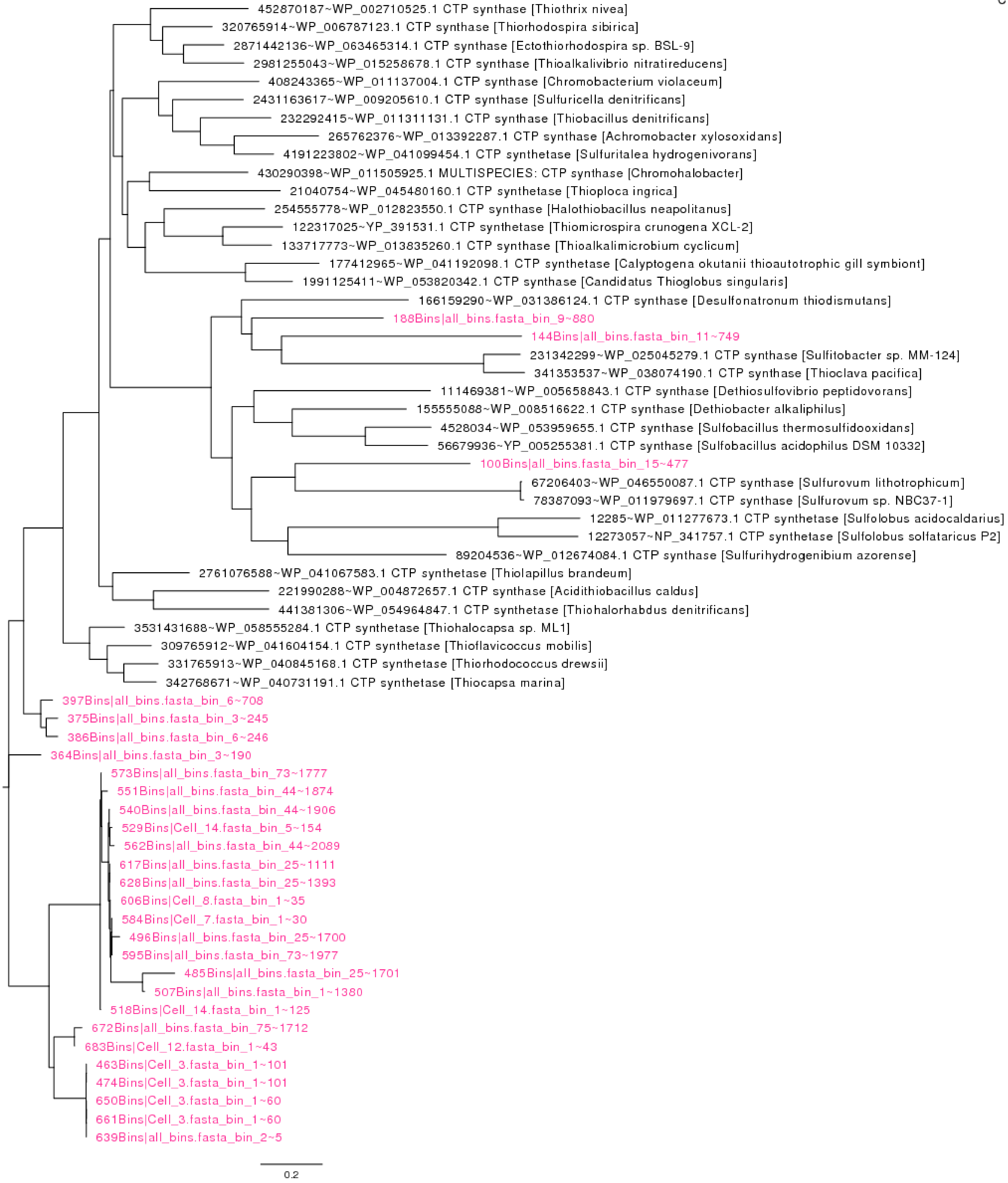

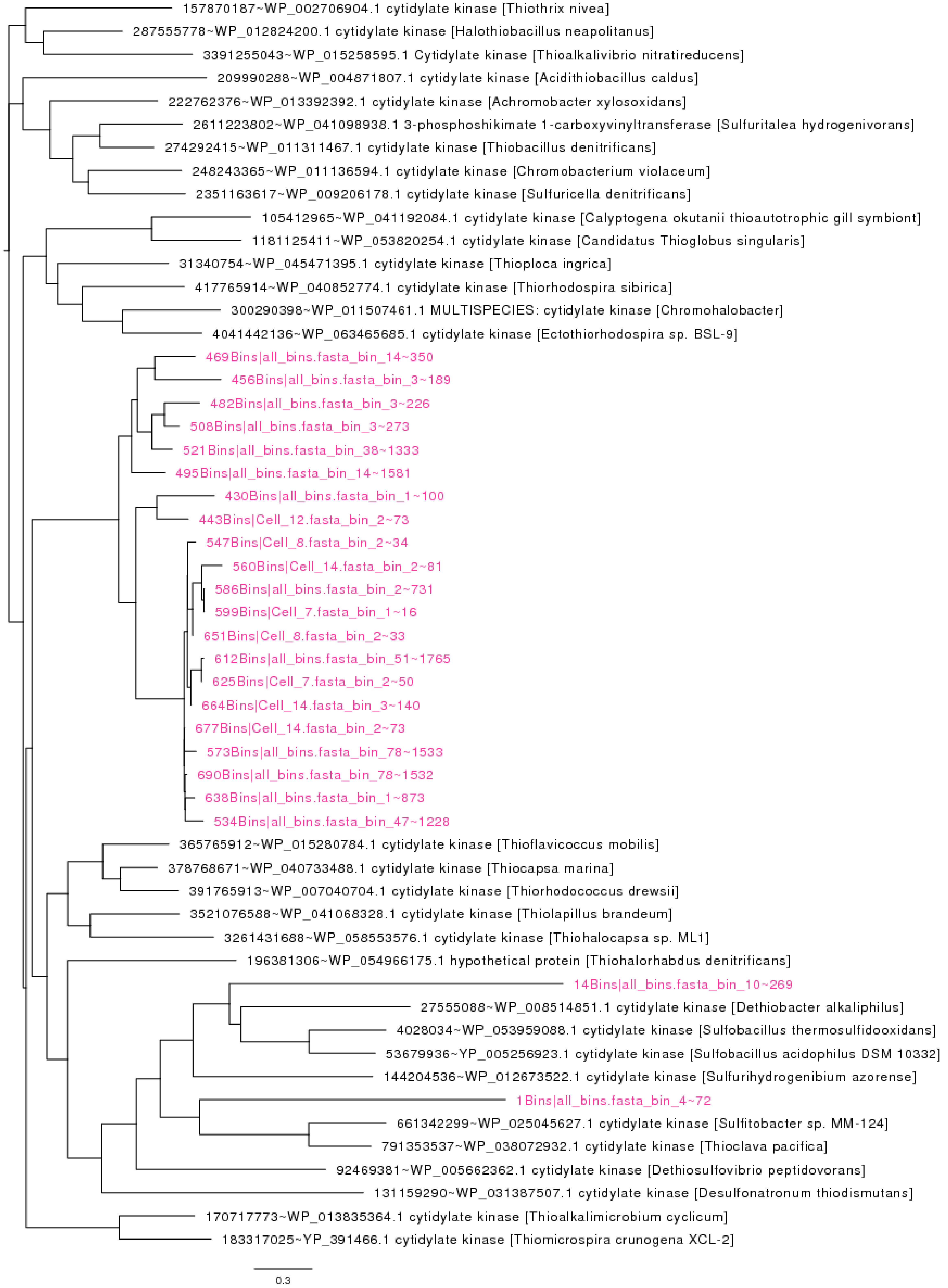
157870187~WP_002706904.1 cytidylate kinase [Thiothrix nivea]
287555778~WP_012824200.1 cytidylate kinase [Halothiobacillus neapolitanus]
3391255043~WP_015258595.1 Cytidylate kinase [Thioalkalivibrio nitratireducens]
209990288~WP_004871807.1 cytidylate kinase [Acidithiobacillus caldus]
222762376~WP_013392392.1 cytidylate kinase [Achromobacter xylosoxidans]
2611223802~WP_041098938.1 3-phosphoshikimate 1-carboxyvinyltransferase [Sulfuritalea hydrogenivorans]
274292415~WP_011311467.1 cytidylate kinase [Thiobacillus denitrificans]
248243365~WP_011136594.1 cytidylate kinase [Chromobacterium violaceum]
2351163617~WP_009206178.1 cytidylate kinase [Sulfuricella denitrificans]
105412965~WP_041192084.1 cytidylate kinase [Calyptogena okutanii thioautotrophic gill symbiont]
1181125411~WP_053820254.1 cytidylate kinase [Candidatus Thioglobus singularis]
31340754~WP_045471395.1 cytidylate kinase [Thioploca ingrica]
417765914~WP_040852774.1 cytidylate kinase [Thiorhodospira sibirica]
300290398~WP_011507461.1 MULTISPECIES: cytidylate kinase [Chromohalobacter]
4041442136~WP_063465685.1 cytidylate kinase [Ectothiorhodospira sp. BSL-9]
469Bins|all_bins.fasta_bin_14~350
456Bins|all_bins.fasta_bin_3~189
482Bins|all_bins.fasta_bin_3~226
508Bins|all_bins.fasta_bin_3~273
521Bins|all_bins.fasta_bin_38~1333
495Bins|all_bins.fasta_bin_14~1581
430Bins|all_bins.fasta_bin_1~100
443Bins|Cell_12.fasta_bin_2~73
547Bins|Cell_8.fasta_bin_2~34
560Bins|Cell_14.fasta_bin_2~81
586Bins|all_bins.fasta_bin_2~731
599Bins|Cell_7.fasta_bin_1~16
651Bins|Cell_8.fasta_bin_2~33
612Bins|all_bins.fasta_bin_51~1765
625Bins|Cell_7.fasta_bin_2~50
664Bins|Cell_14.fasta_bin_3~140
677Bins|Cell_14.fasta_bin_2~73
573Bins|all_bins.fasta_bin_78~1533
690Bins|all_bins.fasta_bin_78~1532
638Bins|all_bins.fasta_bin_1~873
534Bins|all_bins.fasta_bin_47~1228
365765912~WP_015280784.1 cytidylate kinase [Thioflavicoccus mobilis]
378768671~WP_040733488.1 cytidylate kinase [Thiocapsa marina]
391765913~WP_007040704.1 cytidylate kinase [Thiorhodococcus drewsii]
3521076588~WP_041068328.1 cytidylate kinase [Thiolapillus brandeum]
3261431688~WP_058553576.1 cytidylate kinase [Thiohalocapsa sp. ML1]
196381306~WP_054966175.1 hypothetical protein [Thiohalorhabdus denitrificans]
14Bins|all_bins.fasta_bin_10~269
27555088~WP_008514851.1 cytidylate kinase [Dethiobacter alkaliphilus]
4028034~WP_053959088.1 cytidylate kinase [Sulfobacillus thermosulfidooxidans]
53679936~YP_005256923.1 cytidylate kinase [Sulfobacillus acidophilus DSM 10332]
144204536~WP_012673522.1 cytidylate kinase [Sulfurihydrogenibium azorense]
1Bins|all_bins.fasta_bin_4~72
661342299~WP_025045627.1 cytidylate kinase [Sulfitobacter sp. MM-124]
791353537~WP_038072932.1 cytidylate kinase [Thioclava pacifica]
92469381~WP_005662362.1 cytidylate kinase [Dethiosulfovibrio peptidovorans]
131159290~WP_031387507.1 cytidylate kinase [Desulfonatronum thiodismutans]
170717773~WP_013835364.1 cytidylate kinase [Thioalkalimicrobium cyclicum]
183317025~YP_391466.1 cytidylate kinase [Thiomicrospira crunogena XCL-2]
0.3

DNA_pol3_beta.treee.tre.exp.tree.pdf

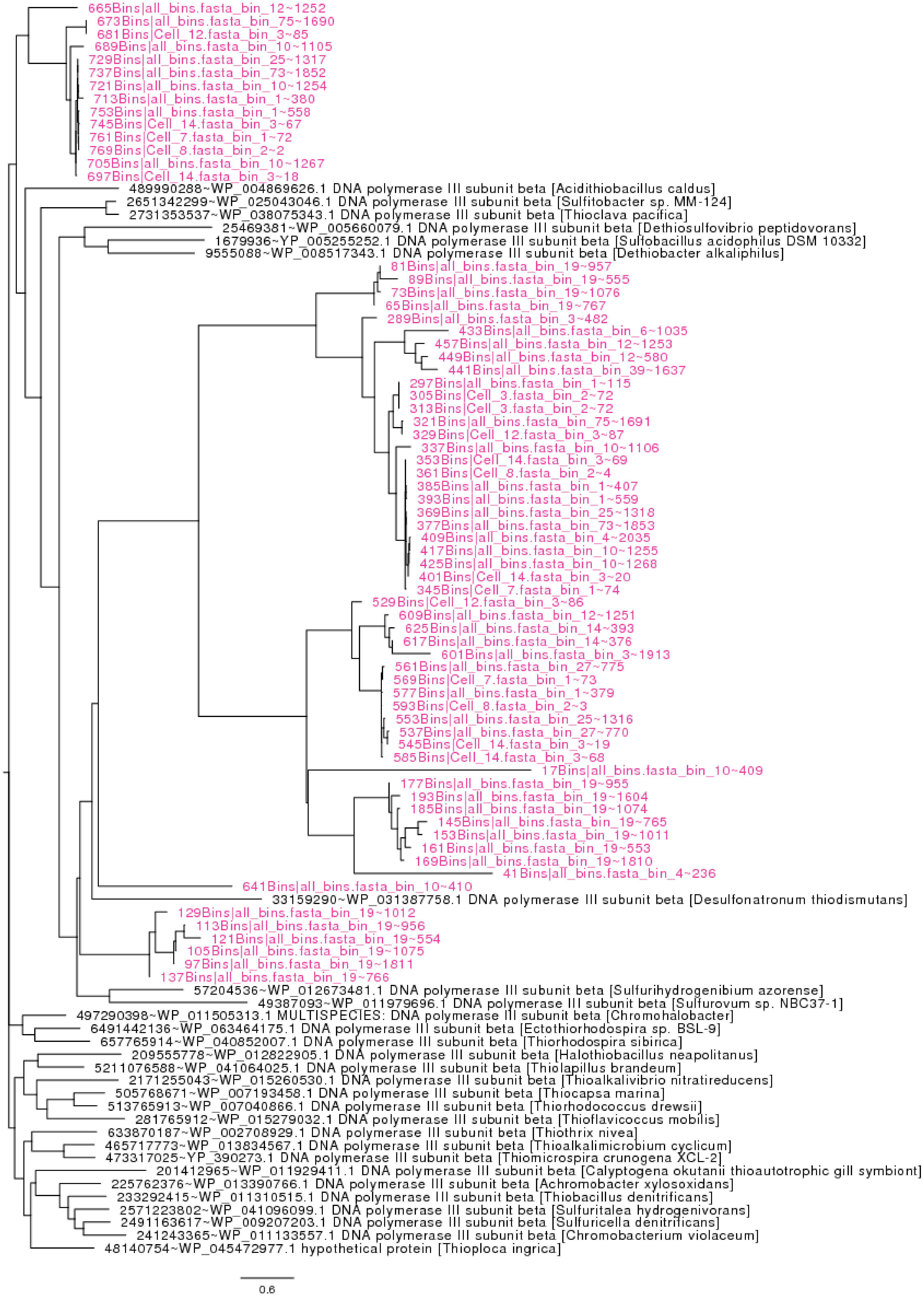

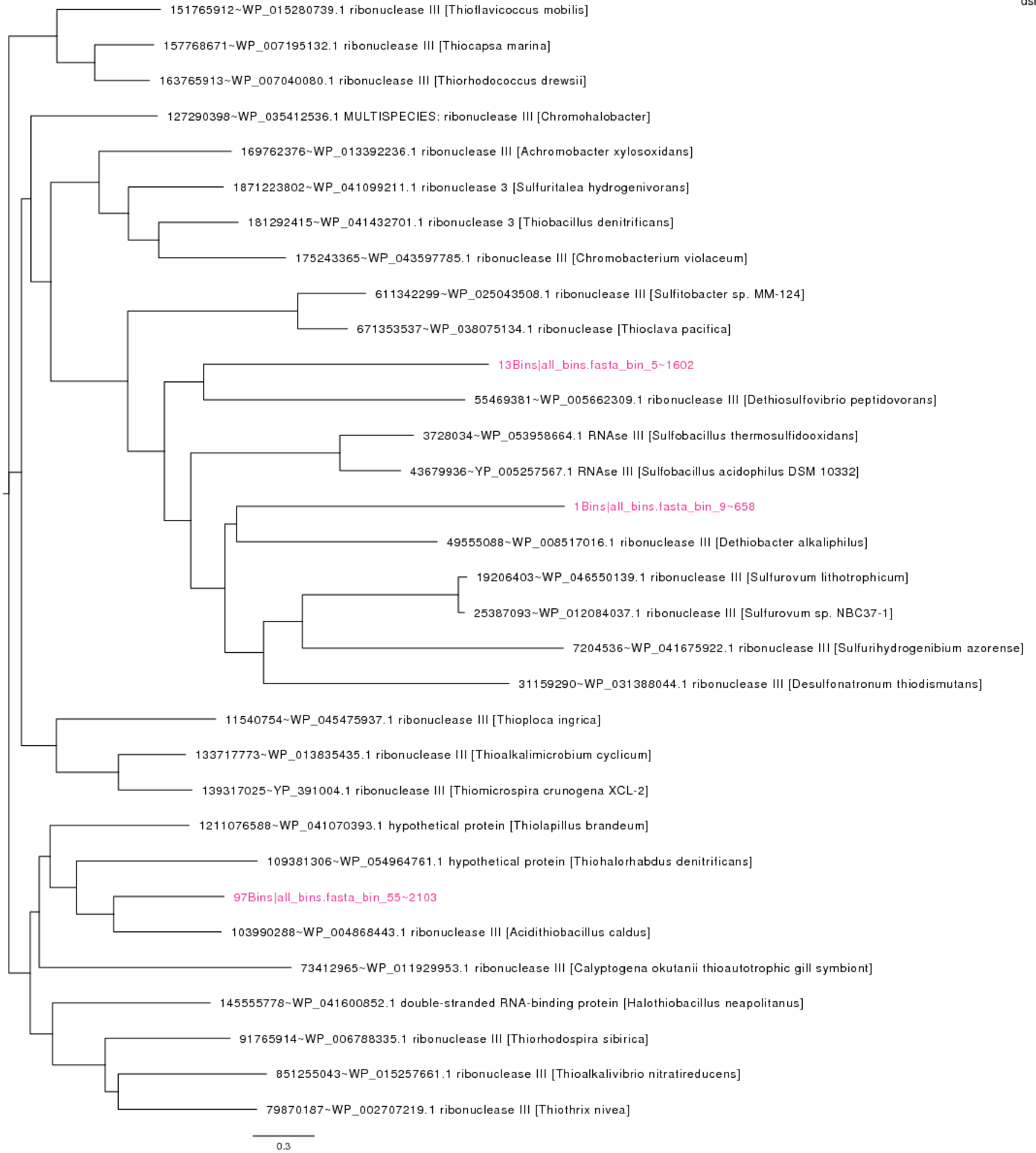
151765912~WP_015280739.1 ribonuclease III [Thioflavicoccus mobilis]
157768671~WP_007195132.1 ribonuclease III [Thiocapsa marina]
163765913~WP_007040080.1 ribonuclease III [Thiorhodococcus drewsii]
127290398~WP_035412536.1 MULTISPECIES: ribonuclease III [Chromohalobacter]
169762376~WP_013392236.1 ribonuclease III [Achromobacter xylosoxidans]
1871223802~WP_041099211.1 ribonuclease 3 [Sulfuritalea hydrogenivorans]
181292415~WP_041432701.1 ribonuclease 3 [Thiobacillus denitrificans]
175243365~WP_043597785.1 ribonuclease III [Chromobacterium violaceum]
611342299~WP_025043508.1 ribonuclease III [Sulfitobacter sp. MM-124]
671353537~WP_038075134.1 ribonuclease [Thioclava pacifica]
13Bins|all_bins.fasta_bin_5~1602
55469381~WP_005662309.1 ribonuclease III [Dethiosulfovibrio peptidovorans]
3728034~WP_053958664.1 RNAse III [Sulfobacillus thermosulfidooxidans]
43679936~YP_005257567.1 RNAse III [Sulfobacillus acidophilus DSM 10332]
1Bins|all_bins.fasta_bin_9~658
49555088~WP_008517016.1 ribonuclease III [Dethiobacter alkaliphilus]
19206403~WP_046550139.1 ribonuclease III [Sulfurovum lithotrophicum]
25387093~WP_012084037.1 ribonuclease III [Sulfurovum sp. NBC37-1]
7204536~WP_041675922.1 ribonuclease III [Sulfurihydrogenibium azorense]
31159290~WP_031388044.1 ribonuclease III [Desulfonatronum thiodismutans]
11540754~WP_045475937.1 ribonuclease III [Thioploca ingrica]
133717773~WP_013835435.1 ribonuclease III [Thioalkalimicrobium cyclicum]
139317025~YP_391004.1 ribonuclease III [Thiomicrospira crunogena XCL-2]
1211076588~WP_041070393.1 hypothetical protein [Thiolapillus brandeum]
109381306~WP_054964761.1 hypothetical protein [Thiohalorhabdus denitrificans]
97Bins|all_bins.fasta_bin_55~2103
103990288~WP_004868443.1 ribonuclease III [Acidithiobacillus caldus]
73412965~WP_011929953.1 ribonuclease III [Calyptogena okutanii thioautotrophic gill symbiont]
145555778~WP_041600852.1 double-stranded RNA-binding protein [Halothiobacillus neapolitanus]
91765914~WP_006788335.1 ribonuclease III [Thiorhodospira sibirica]
851255043~WP_015257661.1 ribonuclease III [Thioalkalivibrio nitratireducens]
79870187~WP_002707219.1 ribonuclease III [Thiothrix nivea]
0.3

EF_TS.treee.tre.exp.tree.pdf

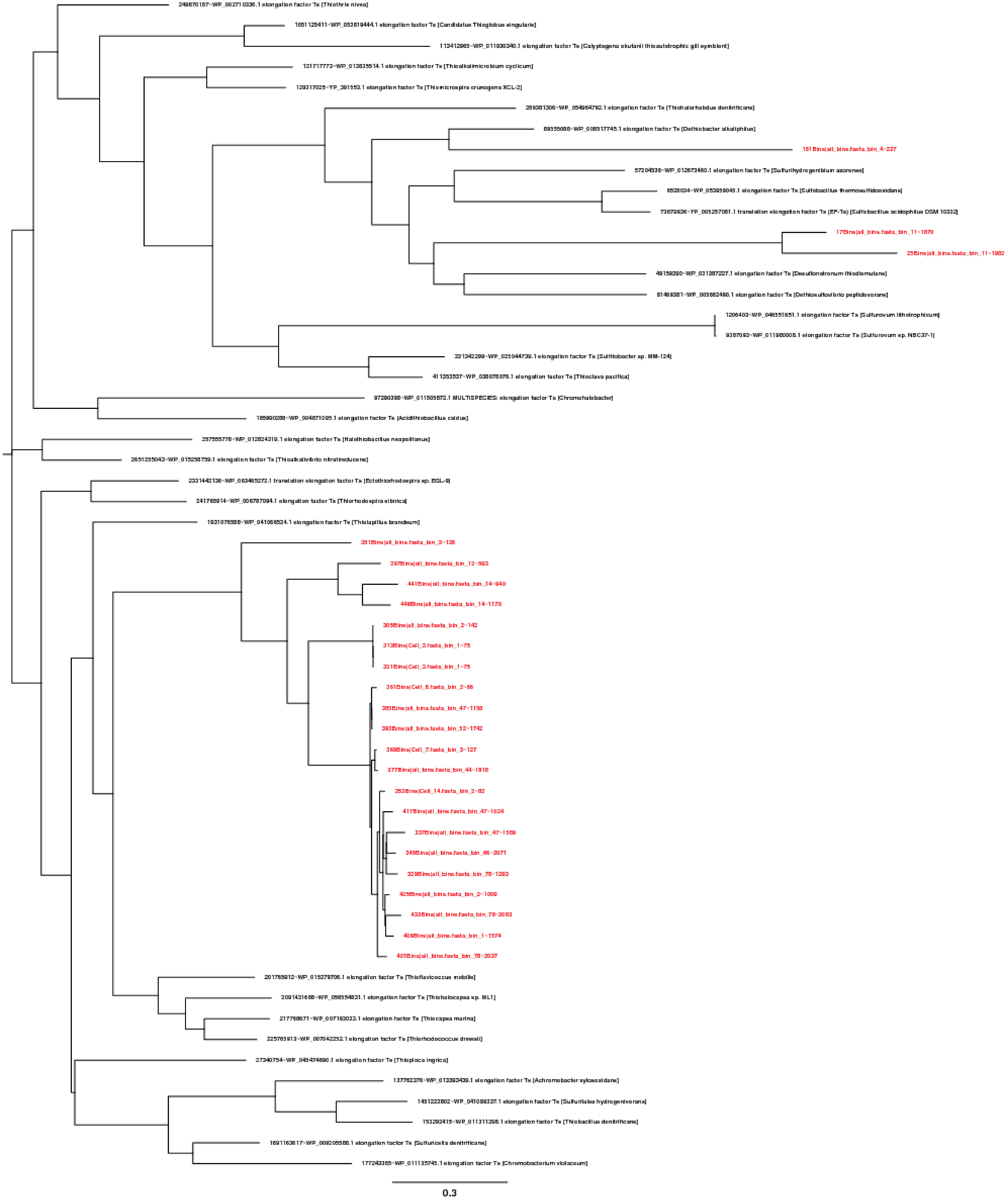

eIF-1a.treee.tre.exp.tree.pdf

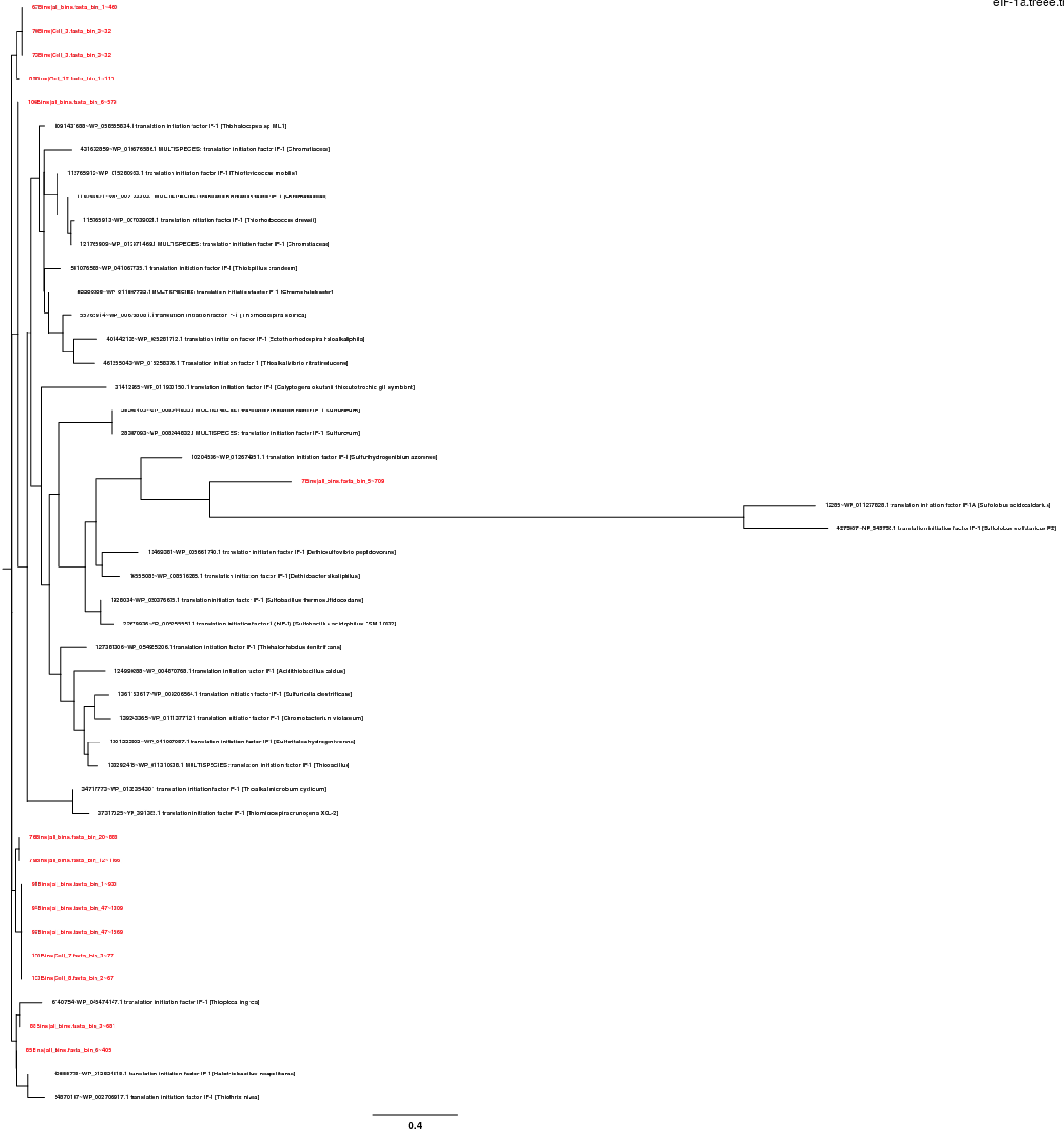

Enolase_C.treee.tre.exp.tree.pdf

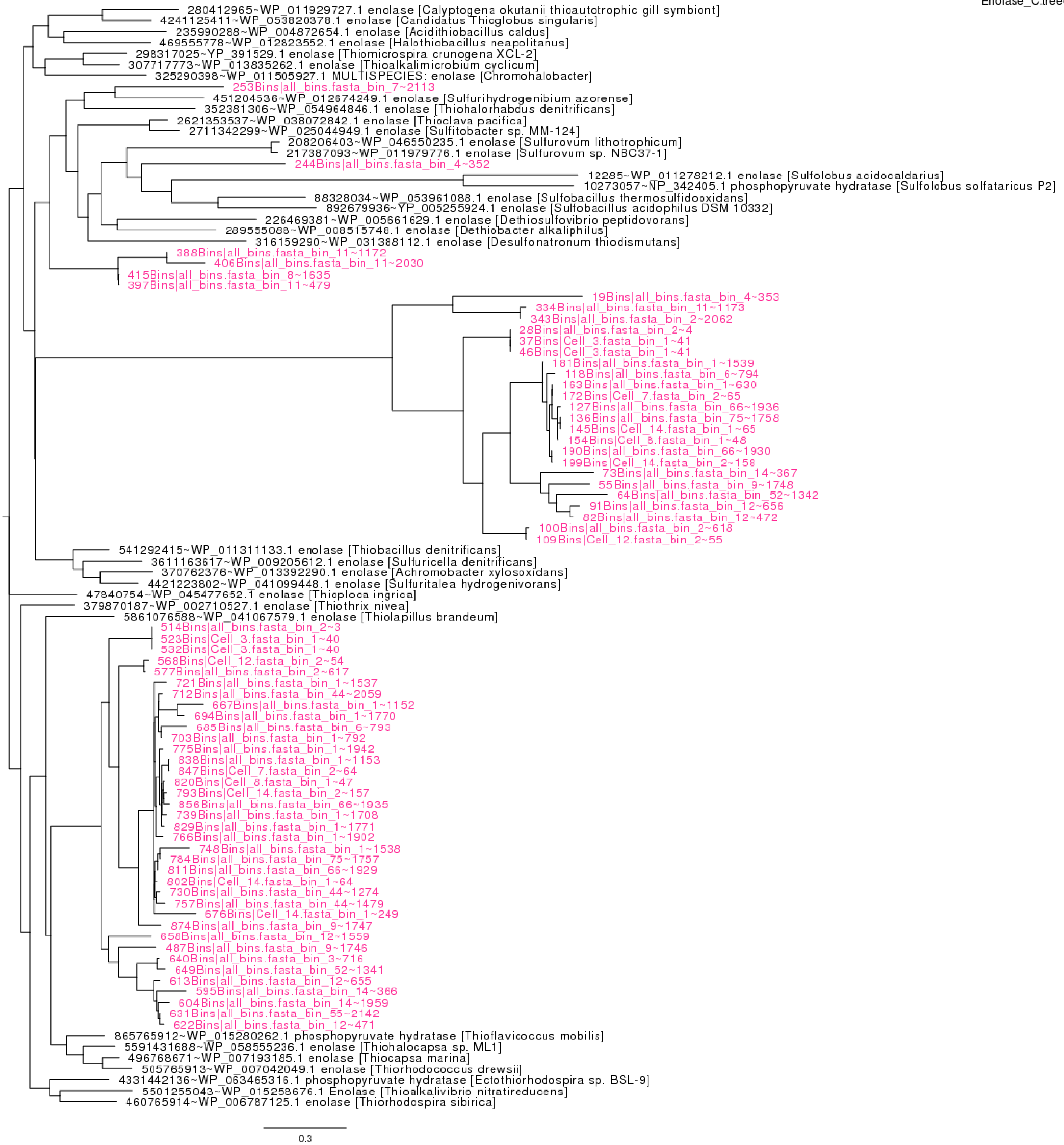

FAD_syn.treee.tre.exp.tree.pdf

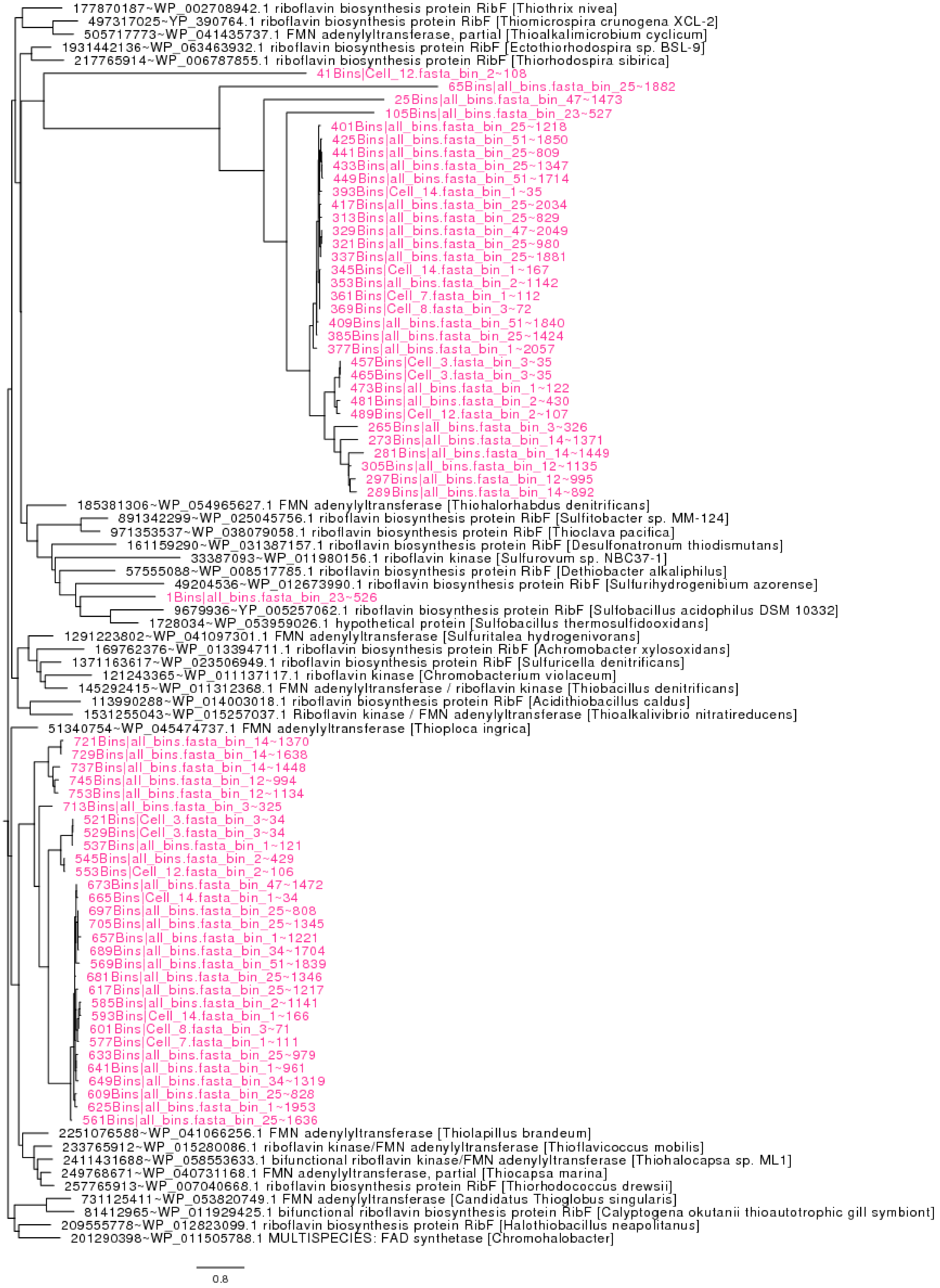

GAD.treee.tre.exp.tree.pdf

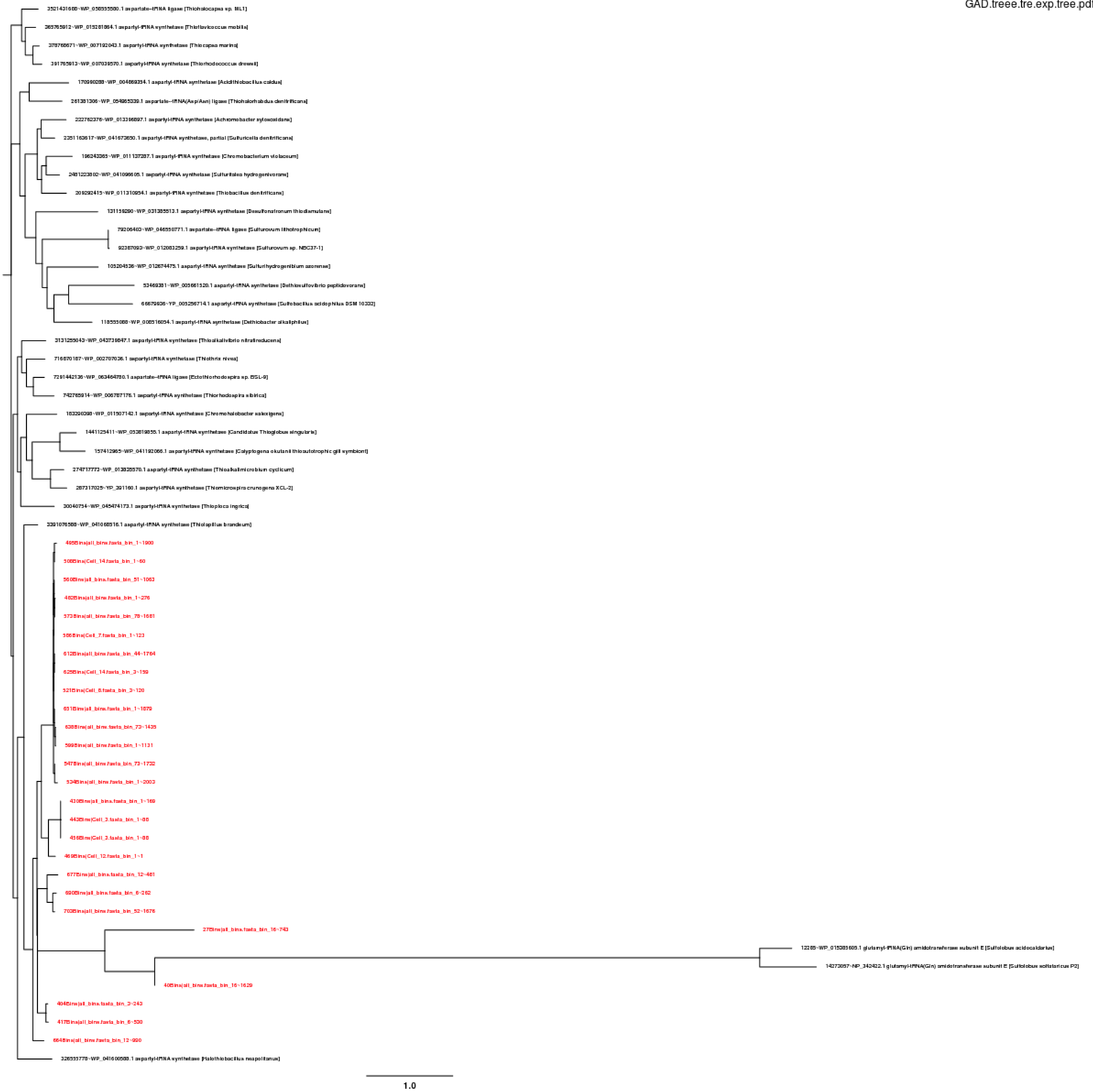

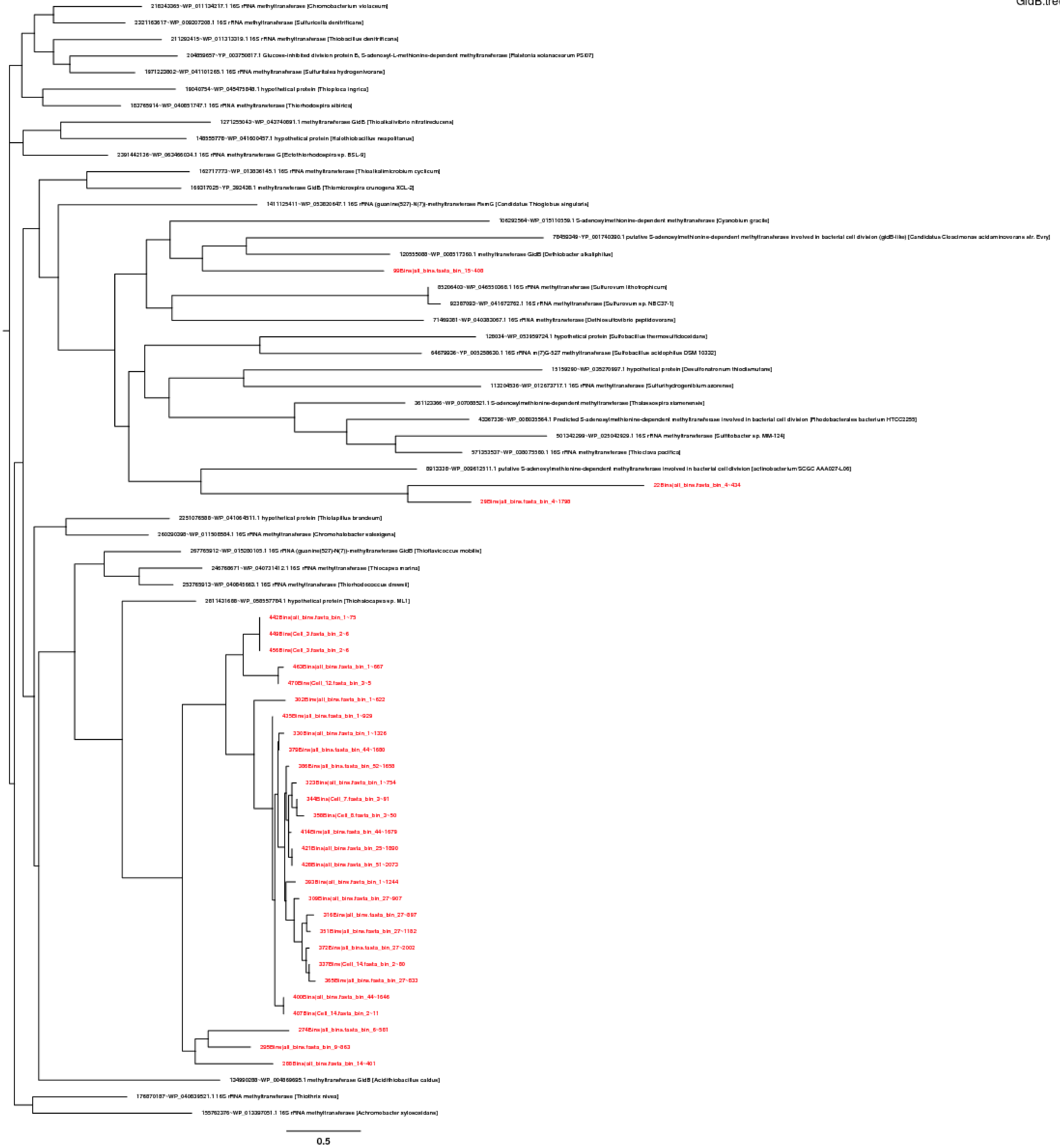
216243065~WP_011134217.1 16S rRNA methyltransferase [Chromobacterium violaceum]
2321163617~WP_009207208.1 16S rRNA methyltransferase [Sulfuricella denitrificans]
211292415~WP_011313019.1 16S rRNA methyltransferase [Thiobacillus denitrificans]
204859657~YP_003750617.1 Glucose-inhibited division protein B, S-adenosyl-L-methionine-dependent methyltransferase [Ralstonia solanacearum PSI07]
1971223602~WP_041101265.1 16S rRNA methyltransferase [Sulfuritalea hydrogenivorans]
19040754~WP_045475848.1 hypothetical protein [Thioploca ingrica]
163765914~WP_040651747.1 16S rRNA methyltransferase [Thiorhodospira sibirica]
1271255043~WP_043740691.1 methyltransferase GidB [Thioalkalivibrio nitratireducens]
148555776~WP_041600457.1 hypothetical protein [Halothiobacillus neapolitanus]
2391442136~WP_063466034.1 16S rRNA methyltransferase G [Ectothiorhodospira sp. BSL-9]
162717773~WP_013836145.1 16S rRNA methyltransferase [Thioalkalimicrobium cyclicum]
169317025~YP_392436.1 methyltransferase GidB [Thiomicrospira crunogena XCL-2]
1411125411~WP_053820647.1 16S rRNA (guanine(527)-N(7))-methyltransferase RsmG [Candidatus Thioglobus singularis]
106292564~WP_015110559.1 S-adenosylmethionine-dependent methyltransferase [Cyanobium gracile]
78459249~YP_001740390.1 putative S-adenosylmethionine-dependent methyltransferase involved in bacterial cell division (gidB-like) [Candidatus Cloacimonas acidaminovorans str. Evry]
120555088~WP_008517360.1 methyltransferase GidB [Dethiobacter alkaliphilus]
99Bins|all_bins.fasta_bin_15~408
85206403~WP_046550366.1 16S rRNA methyltransferase [Sulfurovum lithotrophicum]
92367093~WP_041672762.1 16S rRNA methyltransferase [Sulfurovum sp. NBC37-1]
71469381~WP_040383067.1 16S rRNA methyltransferase [Dethiosulfovibrio peptidovorans]
128034~WP_053959724.1 hypothetical protein [Sulfobacillus thermosulfidooxidans]
64679936~YP_005258630.1 16S rRNA m(7)G-527 methyltransferase [Sulfobacillus acidophilus DSM 10332]
15159290~WP_035270997.1 hypothetical protein [Desulfonatronum thiodismutans]
113204536~WP_012673717.1 16S rRNA methyltransferase [Sulfurihydrogenibium azorense]
361123366~WP_007088521.1 S-adenosylmethionine-dependent methyltransferase [Thalassospira xiamenensis]
43367336~WP_008035564.1 Predicted S-adenosylmethionine-dependent methyltransferase involved in bacterial cell division [Rhodobacterales bacterium HTCC2255]
501342299~WP_025042929.1 16S rRNA methyltransferase [Sulfitobacter sp. MM-124]
571353537~WP_038075580.1 16S rRNA methyltransferase [Thioclava pacifica]
6913338~WP_009612511.1 putative S-adenosylmethionine-dependent methyltransferase involved in bacterial cell division [actinobacterium SCGC AAA027-L06]
22Bins|all_bins.fasta_bin_4~434
29Bins|all_bins.fasta_bin_4~1798
2251076588~WP_041064511.1 hypothetical protein [Thiolapillus brandeum]
260290398~WP_011506584.1 16S rRNA methyltransferase [Chromohalobacter salexigens]
267765912~WP_015280105.1 16S rRNA (guanine(527)-N(7))-methyltransferase GidB [Thioflavicoccus mobilis]
246768671~WP_040731412.1 16S rRNA methyltransferase [Thiocapsa marina]
253765913~WP_040845663.1 16S rRNA methyltransferase [Thiorhodococcus drewsii]
2611431688~WP_058557784.1 hypothetical protein [Thiohalocapsa sp. ML1]
442Bins|all_bins.fasta_bin_1~75
449Bins|Cell_3.fasta_bin_2~6
456Bins|Cell_3.fasta_bin_2~6
463Bins|all_bins.fasta_bin_1~667
470Bins|Cell_12.fasta_bin_3~5
302Bins|all_bins.fasta_bin_1~622
435Bins|all_bins.fasta_bin_1~929
330Bins|all_bins.fasta_bin_1~1326
379Bins|all_bins.fasta_bin_44~1680
386Bins|all_bins.fasta_bin_52~1658
323Bins|all_bins.fasta_bin_1~754
344Bins|Cell_7.fasta_bin_3~91
358Bins|Cell_8.fasta_bin_3~50
414Bins|all_bins.fasta_bin_44~1679
421Bins|all_bins.fasta_bin_25~1890
428Bins|all_bins.fasta_bin_51~2073
393Bins|all_bins.fasta_bin_1~1244
309Bins|all_bins.fasta_bin_27~907
316Bins|all_bins.fasta_bin_27~897
351Bins|all_bins.fasta_bin_27~1182
372Bins|all_bins.fasta_bin_27~2002
337Bins|Cell_14.fasta_bin_2~80
365Bins|all_bins.fasta_bin_27~833
400Bins|all_bins.fasta_bin_44~1646
407Bins|Cell_14.fasta_bin_2~11
274Bins|all_bins.fasta_bin_6~561
295Bins|all_bins.fasta_bin_9~863
288Bins|all_bins.fasta_bin_14~401
134990288~WP_004869695.1 methyltransferase GidB [Acidithiobacillus caldus]
176870187~WP_040839521.1 16S rRNA methyltransferase [Thiothrix nivea]
155762376~WP_013397051.1 16S rRNA methyltransferase [Achromobacter xylosoxidans]
0.5

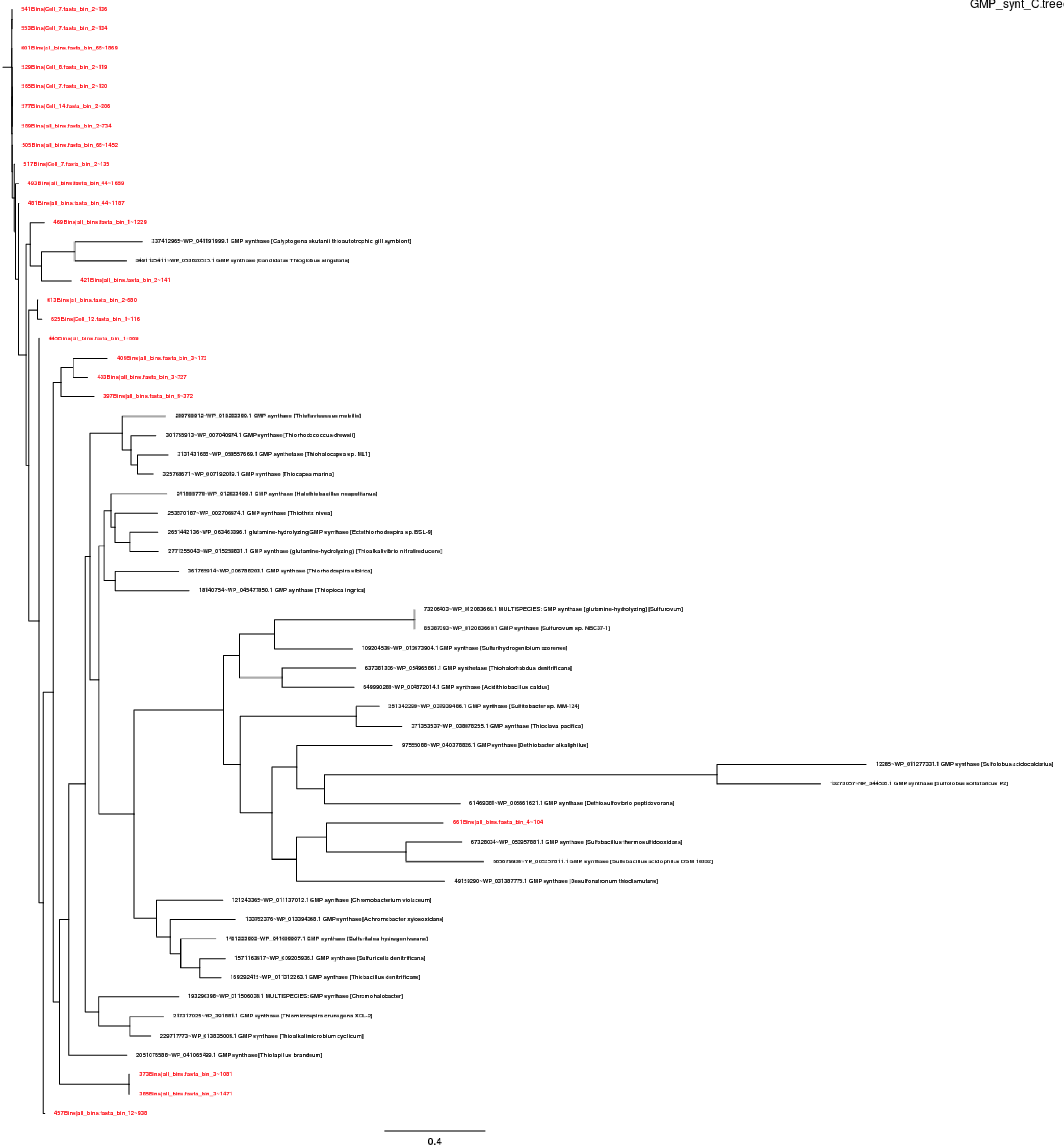
541Bins|Cell_7.fasta_bin_2~136
553Bins|Cell_7.fasta_bin_2~134
601Bins|all_bins.fasta_bin_66~1869
529Bins|Cell_6.fasta_bin_2~119
565Bins|Cell_7.fasta_bin_2~120
577Bins|Cell_14.fasta_bin_2~206
589Bins|all_bins.fasta_bin_2~734
505Bins|all_bins.fasta_bin_66~1452
517Bins|Cell_7.fasta_bin_2~135
493Bins|all_bins.fasta_bin_44~1659
481Bins|all_bins.fasta_bin_44~1167
469Bins|all_bins.fasta_bin_1~1229
337412965~WP_041191999.1 GMP synthase [Calyptogena okutanii thioautotrophic gill symbiont]
3491125411~WP_053820535.1 GMP synthase [Candidatus Thioglobus singularis]
421Bins|all_bins.fasta_bin_2~141
613Bins|all_bins.fasta_bin_2~680
625Bins|Cell_12.fasta_bin_1~116
445Bins|all_bins.fasta_bin_1~669
409Bins|all_bins.fasta_bin_3~172
433Bins|all_bins.fasta_bin_3~727
397Bins|all_bins.fasta_bin_9~372
269765912~WP_015282380.1 GMP synthase [Thioflavicoccus mobilis]
301765913~WP_007040974.1 GMP synthase [Thiorhodococcus drewsii]
3131431688~WP_058557669.1 GMP synthetase [Thiohalocapsa sp. ML1]
325768671~WP_007192019.1 GMP synthase [Thiocapsa marina]
241555778~WP_012823499.1 GMP synthase [Halothiobacillus neapolitanus]
253870187~WP_002706674.1 GMP synthase [Thiothrix nivea]
2651442136~WP_063463396.1 glutamine-hydrolyzing GMP synthase [Ectothiorhodospira sp. BSL-9]
2771255043~WP_015259831.1 GMP synthase (glutamine-hydrolyzing) [Thioalkalivibrio nitratireducens]
361765914~WP_006788203.1 GMP synthase [Thiorhodospira sibirica]
18140754~WP_045477850.1 GMP synthase [Thioploca ingrica]
73206403~WP_012083660.1 MULTISPECIES: GMP synthase [glutamine-hydrolyzing] [Sulfurovum]
85387093~WP_012083660.1 GMP synthase [Sulfurovum sp. NBC37-1]
109204536~WP_012673904.1 GMP synthase [Sulfurihydrogenibium azorense]
637381306~WP_054965861.1 GMP synthetase [Thiohalorhabdus denitrificans]
649990288~WP_004872014.1 GMP synthase [Acidithiobacillus caldus]
251342299~WP_037939466.1 GMP synthase [Sulfitobacter sp. MM-124]
371353537~WP_038078255.1 GMP synthase [Thioclava pacifica]
97555088~WP_040378826.1 GMP synthase [Dethiobacter alkaliphilus]
12265~WP_011277331.1 GMP synthase [Sulfolobus acidocaldarius]
13273057~NP_344536.1 GMP synthase [Sulfolobus solfataricus P2]
61469381~WP_005661621.1 GMP synthase [Dethiosulfovibrio peptidovorans]
661Bins|all_bins.fasta_bin_4~104
67326034~WP_053957881.1 GMP synthase [Sulfobacillus thermosulfidooxidans]
665679936~YP_005257811.1 GMP synthase [Sulfobacillus acidophilus DSM 10332]
49159290~WP_031387775.1 GMP synthase [Desulfonatronum thiodismutans]
121243365~WP_011137012.1 GMP synthase [Chromobacterium violaceum]
133762376~WP_013394368.1 GMP synthase [Achromobacter xylosoxidans]
1451223802~WP_041098907.1 GMP synthase [Sulfuritalea hydrogenivorans]
1571163617~WP_009205936.1 GMP synthase [Sulfuricella denitrificans]
169292415~WP_011312263.1 GMP synthase [Thiobacillus denitrificans]
193290398~WP_011506038.1 MULTISPECIES: GMP synthase [Chromohalobacter]
217317025~YP_391881.1 GMP synthase [Thiomicrospira crunogena XCL-2]
229717773~WP_013835008.1 GMP synthase [Thioalkalimicrobium cyclicum]
2051076588~WP_041063499.1 GMP synthase [Thiolapillus brandeum]
373Bins|all_bins.fasta_bin_3~1081
385Bins|all_bins.fasta_bin_3~1471
457Bins|all_bins.fasta_bin_12~938
0.4

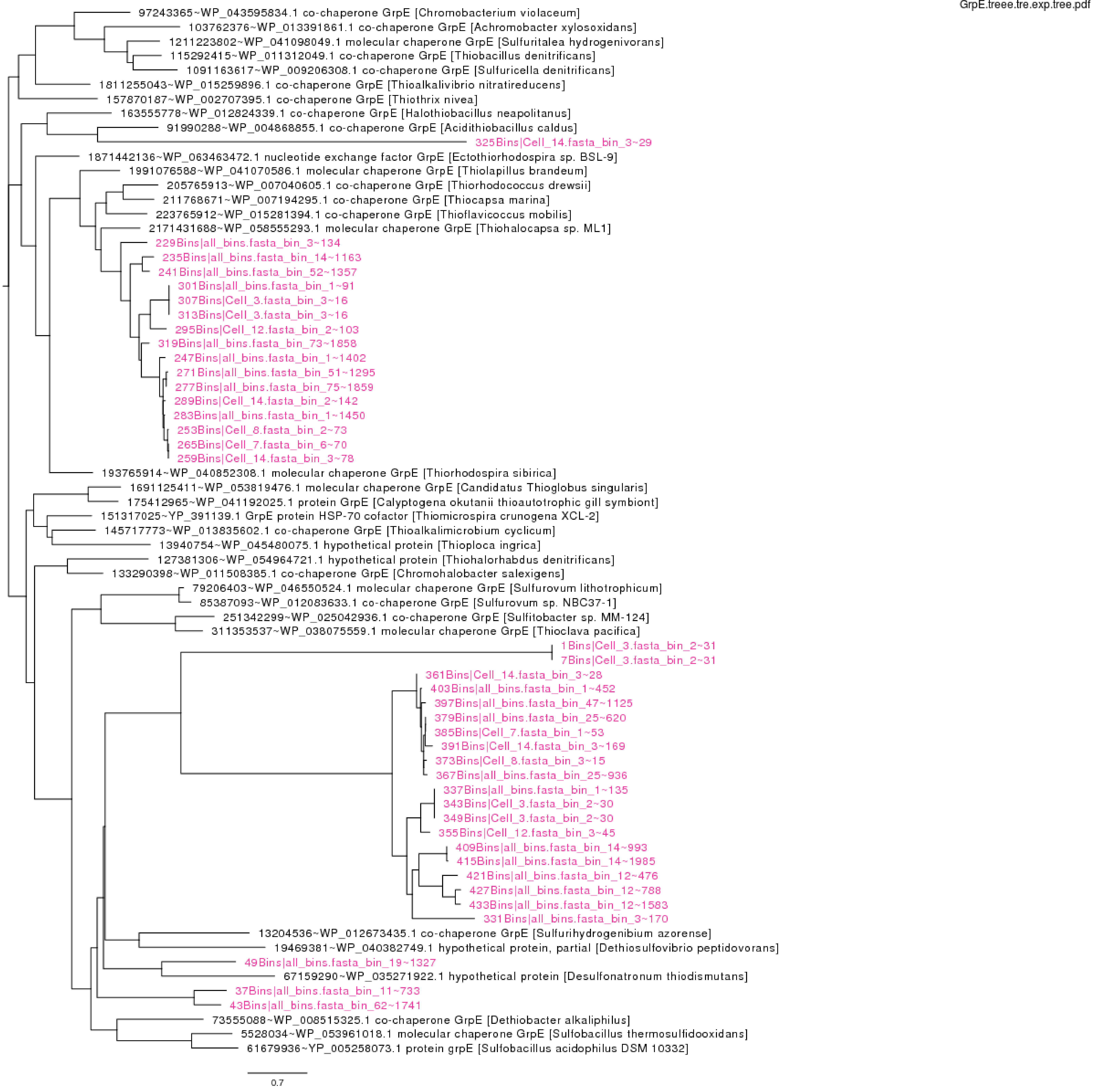
GrpE.treee.tre.exp.tree.pdf
97243365~WP_043595834.1 co-chaperone GrpE [Chromobacterium violaceum]
103762376~WP_013391861.1 co-chaperone GrpE [Achromobacter xylosoxidans]
1211223802~WP_041098049.1 molecular chaperone GrpE [Sulfuritalea hydrogenivorans]
115292415~WP_011312049.1 co-chaperone GrpE [Thiobacillus denitrificans]
1091163617~WP_009206308.1 co-chaperone GrpE [Sulfuricella denitrificans]
1811255043~WP_015259896.1 co-chaperone GrpE [Thioalkalivibrio nitratireducens]
157870187~WP_002707395.1 co-chaperone GrpE [Thiothrix nivea]
163555778~WP_012824339.1 co-chaperone GrpE [Halothiobacillus neapolitanus]
91990288~WP_004868855.1 co-chaperone GrpE [Acidithiobacillus caldus]
325Bins|Cell_14.fasta_bin_3~29
1871442136~WP_063463472.1 nucleotide exchange factor GrpE [Ectothiorhodospira sp. BSL-9]
1991076588~WP_041070586.1 molecular chaperone GrpE [Thiolapillus brandeum]
205765913~WP_007040605.1 co-chaperone GrpE [Thiorhodococcus drewsii]
211768671~WP_007194295.1 co-chaperone GrpE [Thiocapsa marina]
223765912~WP_015281394.1 co-chaperone GrpE [Thioflavicoccus mobilis]
2171431688~WP_058555293.1 molecular chaperone GrpE [Thiohalocapsa sp. ML1]
229Bins|all_bins.fasta_bin_3~134
235Bins|all_bins.fasta_bin_14~1163
241Bins|all_bins.fasta_bin_52~1357
301Bins|all_bins.fasta_bin_1~91
307Bins|Cell_3.fasta_bin_3~16
313Bins|Cell_3.fasta_bin_3~16
295Bins|Cell_12.fasta_bin_2~103
319Bins|all_bins.fasta_bin_73~1858
247Bins|all_bins.fasta_bin_1~1402
271Bins|all_bins.fasta_bin_51~1295
277Bins|all_bins.fasta_bin_75~1859
289Bins|Cell_14.fasta_bin_2~142
283Bins|all_bins.fasta_bin_1~1450
253Bins|Cell_8.fasta_bin_2~73
265Bins|Cell_7.fasta_bin_6~70
259Bins|Cell_14.fasta_bin_3~78
193765914~WP_040852308.1 molecular chaperone GrpE [Thiorhodospira sibirica]
1691125411~WP_053819476.1 molecular chaperone GrpE [Candidatus Thioglobus singularis]
175412965~WP_041192025.1 protein GrpE [Calyptogena okutanii thioautotrophic gill symbiont]
151317025~YP_391139.1 GrpE protein HSP-70 cofactor [Thiomicrospira crunogena XCL-2]
145717773~WP_013835602.1 co-chaperone GrpE [Thioalkalimicrobium cyclicum]
13940754~WP_045480075.1 hypothetical protein [Thioploca ingrica]
127381306~WP_054964721.1 hypothetical protein [Thiohalorhabdus denitrificans]
133290398~WP_011508385.1 co-chaperone GrpE [Chromohalobacter salexigens]
79206403~WP_046550524.1 molecular chaperone GrpE [Sulfurovum lithotrophicum]
85387093~WP_012083633.1 co-chaperone GrpE [Sulfurovum sp. NBC37-1]
251342299~WP_025042936.1 co-chaperone GrpE [Sulfitobacter sp. MM-124]
311353537~WP_038075559.1 molecular chaperone GrpE [Thioclava pacifica]
1Bins|Cell_3.fasta_bin_2~31
7Bins|Cell_3.fasta_bin_2~31
361Bins|Cell_14.fasta_bin_3~28
403Bins|all_bins.fasta_bin_1~452
397Bins|all_bins.fasta_bin_47~1125
379Bins|all_bins.fasta_bin_25~620
385Bins|Cell_7.fasta_bin_1~53
391Bins|Cell_14.fasta_bin_3~169
373Bins|Cell_8.fasta_bin_3~15
367Bins|all_bins.fasta_bin_25~936
337Bins|all_bins.fasta_bin_1~135
343Bins|Cell_3.fasta_bin_2~30
349Bins|Cell_3.fasta_bin_2~30
355Bins|Cell_12.fasta_bin_3~45
409Bins|all_bins.fasta_bin_14~993
415Bins|all_bins.fasta_bin_14~1985
421Bins|all_bins.fasta_bin_12~476
427Bins|all_bins.fasta_bin_12~788
433Bins|all_bins.fasta_bin_12~1583
331Bins|all_bins.fasta_bin_3~170
13204536~WP_012673435.1 co-chaperone GrpE [Sulfurihydrogenibium azorense]
19469381~WP_040382749.1 hypothetical protein, partial [Dethiosulfovibrio peptidovorans]
49Bins|all_bins.fasta_bin_19~1327
67159290~WP_035271922.1 hypothetical protein [Desulfonatronum thiodismutans]
37Bins|all_bins.fasta_bin_11~733
43Bins|all_bins.fasta_bin_62~1741
73555088~WP_008515325.1 co-chaperone GrpE [Dethiobacter alkaliphilus]
5528034~WP_053961018.1 molecular chaperone GrpE [Sulfobacillus thermosulfidooxidans]
61679936~YP_005258073.1 protein grpE [Sulfobacillus acidophilus DSM 10332]
0.7

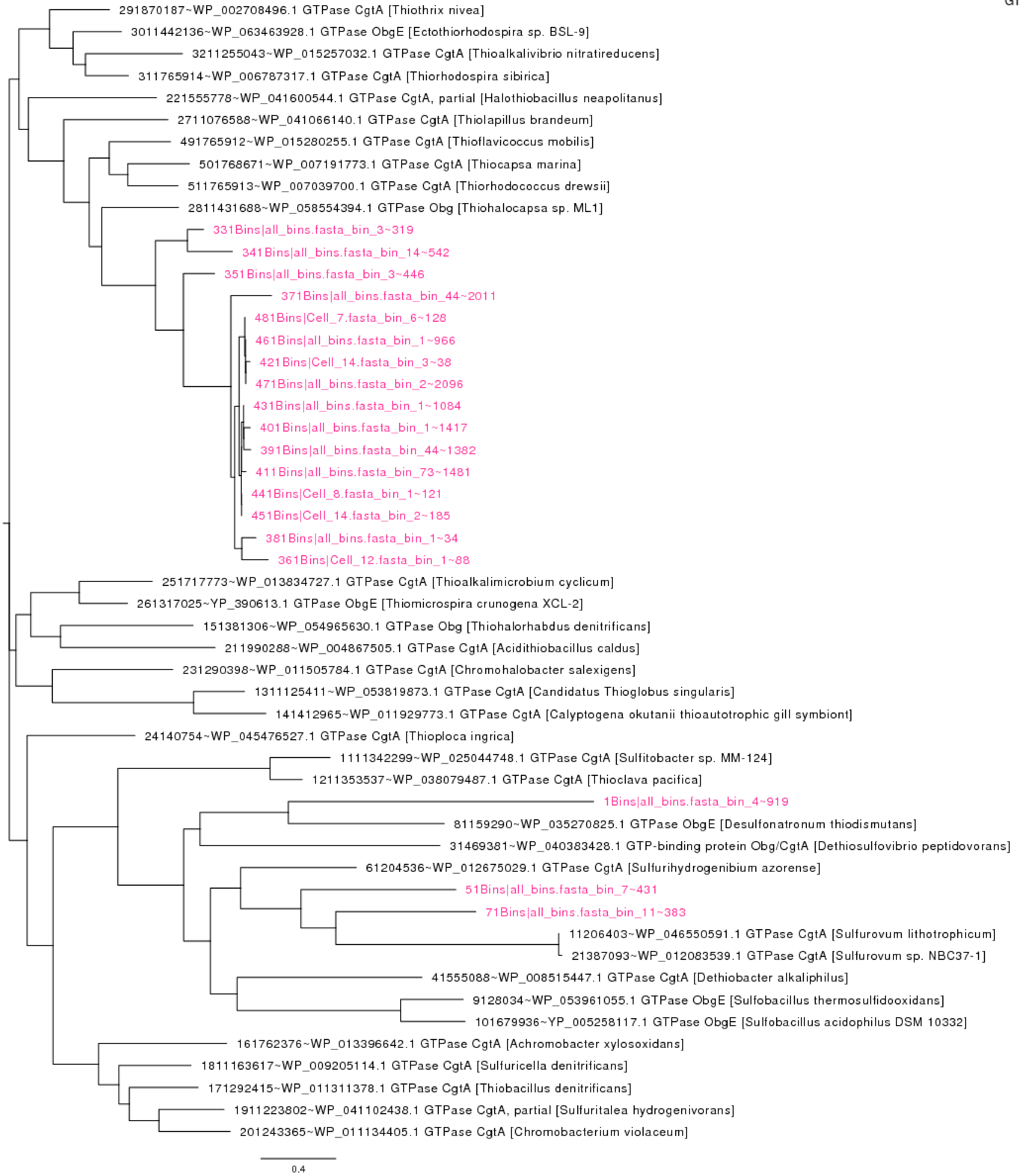
291870187~WP_002708496.1 GTPase CgtA [Thiothrix nivea]
3011442136~WP_063463928.1 GTPase ObgE [Ectothiorhodospira sp. BSL-9]
3211255043~WP_015257032.1 GTPase CgtA [Thioalkalivibrio nitratireducens]
311765914~WP_006787317.1 GTPase CgtA [Thiorhodospira sibirica]
221555778~WP_041600544.1 GTPase CgtA, partial [Halothiobacillus neapolitanus]
2711076588~WP_041066140.1 GTPase CgtA [Thiolapillus brandeum]
491765912~WP_015280255.1 GTPase CgtA [Thioflavicoccus mobilis]
501768671~WP_007191773.1 GTPase CgtA [Thiocapsa marina]
511765913~WP_007039700.1 GTPase CgtA [Thiorhodococcus drewsii]
2811431688~WP_058554394.1 GTPase Obg [Thiohalocapsa sp. ML1]
331Bins|all_bins.fasta_bin_3~319
341Bins|all_bins.fasta_bin_14~542
351Bins|all_bins.fasta_bin_3~446
371Bins|all_bins.fasta_bin_44~2011
481Bins|Cell_7.fasta_bin_6~128
461Bins|all_bins.fasta_bin_1~966
421Bins|Cell_14.fasta_bin_3~38
471Bins|all_bins.fasta_bin_2~2096
431Bins|all_bins.fasta_bin_1~1084
401Bins|all_bins.fasta_bin_1~1417
391Bins|all_bins.fasta_bin_44~1382
411Bins|all_bins.fasta_bin_73~1481
441Bins|Cell_8.fasta_bin_1~121
451Bins|Cell_14.fasta_bin_2~185
381Bins|all_bins.fasta_bin_1~34
361Bins|Cell_12.fasta_bin_1~88
251717773~WP_013834727.1 GTPase CgtA [Thioalkalimicrobium cyclicum]
261317025~YP_390613.1 GTPase ObgE [Thiomicrospira crunogena XCL-2]
151381306~WP_054965630.1 GTPase Obg [Thiohalorhabdus denitrificans]
211990288~WP_004867505.1 GTPase CgtA [Acidithiobacillus caldus]
231290398~WP_011505784.1 GTPase CgtA [Chromohalobacter salexigens]
1311125411~WP_053819873.1 GTPase CgtA [Candidatus Thioglobus singularis]
141412965~WP_011929773.1 GTPase CgtA [Calyptogena okutanii thioautotrophic gill symbiont]
24140754~WP_045476527.1 GTPase CgtA [Thioploca ingrica]
1111342299~WP_025044748.1 GTPase CgtA [Sulfitobacter sp. MM-124]
1211353537~WP_038079487.1 GTPase CgtA [Thioclava pacifica]
1Bins|all_bins.fasta_bin_4~919
81159290~WP_035270825.1 GTPase ObgE [Desulfonatronum thiodismutans]
31469381~WP_040383428.1 GTP-binding protein Obg/CgtA [Dethiosulfovibrio peptidovorans]
61204536~WP_012675029.1 GTPase CgtA [Sulfurihydrogenibium azorense]
51Bins|all_bins.fasta_bin_7~431
71Bins|all_bins.fasta_bin_11~383
11206403~WP_046550591.1 GTPase CgtA [Sulfurovum lithotrophicum]
21387093~WP_012083539.1 GTPase CgtA [Sulfurovum sp. NBC37-1]
41555088~WP_008515447.1 GTPase CgtA [Dethiobacter alkaliphilus]
9128034~WP_053961055.1 GTPase ObgE [Sulfobacillus thermosulfidooxidans]
101679936~YP_005258117.1 GTPase ObgE [Sulfobacillus acidophilus DSM 10332]
161762376~WP_013396642.1 GTPase CgtA [Achromobacter xylosoxidans]
1811163617~WP_009205114.1 GTPase CgtA [Sulfuricella denitrificans]
171292415~WP_011311378.1 GTPase CgtA [Thiobacillus denitrificans]
1911223802~WP_041102438.1 GTPase CgtA, partial [Sulfuritalea hydrogenivorans]
201243365~WP_011134405.1 GTPase CgtA [Chromobacterium violaceum]
0.4

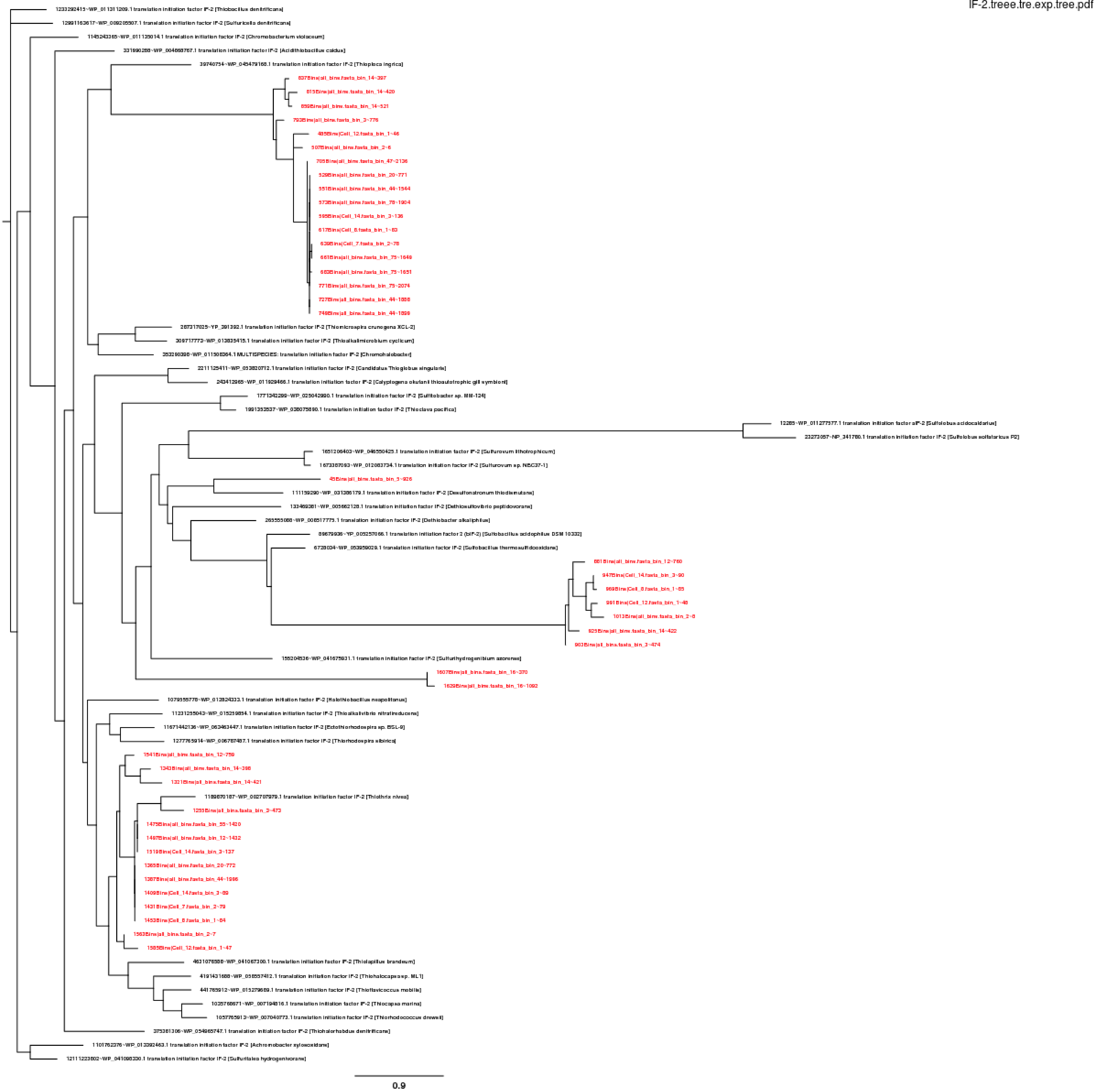
IF-2.treee.tre.exp.tree.pdf
1233292415~WP_011311209.1 translation initiation factor IF-2 [Thiobacillus denitrificans]
12991163617~WP_009205507.1 translation initiation factor IF-2 [Sulfuricella denitrificans]
1145243365~WP_011135014.1 translation initiation factor IF-2 [Chromobacterium violaceum]
331990288~WP_004868767.1 translation initiation factor IF-2 [Acidithiobacillus caldus]
39740754~WP_045479168.1 translation initiation factor IF-2 [Thioploca ingrica]
637Bins|all_bins.fasta_bin_14~397
615Bins|all_bins.fasta_bin_14~420
659Bins|all_bins.fasta_bin_14~521
793Bins|all_bins.fasta_bin_3~776
485Bins|Cell_12.fasta_bin_1~46
507Bins|all_bins.fasta_bin_2~6
705Bins|all_bins.fasta_bin_47~2136
529Bins|all_bins.fasta_bin_20~771
551Bins|all_bins.fasta_bin_44~1544
573Bins|all_bins.fasta_bin_78~1904
595Bins|Cell_14.fasta_bin_3~136
617Bins|Cell_8.fasta_bin_1~63
639Bins|Cell_7.fasta_bin_2~78
661Bins|all_bins.fasta_bin_75~1649
683Bins|all_bins.fasta_bin_75~1651
771Bins|all_bins.fasta_bin_75~2074
727Bins|all_bins.fasta_bin_44~1888
749Bins|all_bins.fasta_bin_44~1899
267317025~YP_391392.1 translation initiation factor IF-2 [Thiomicrospira crunogena XCL-2]
309717773~WP_013835415.1 translation initiation factor IF-2 [Thioalkalimicrobium cyclicum]
353290398~WP_011506364.1 MULTISPECIES: translation initiation factor IF-2 [Chromohalobacter]
2211125411~WP_053820712.1 translation initiation factor IF-2 [Candidatus Thioglobus singularis]
243412965~WP_011929466.1 translation initiation factor IF-2 [Calyptogena okutanii thioautotrophic gill symbiont]
1771342299~WP_025042990.1 translation initiation factor IF-2 [Sulfitobacter sp. NM-124]
1991353537~WP_038075890.1 translation initiation factor IF-2 [Thioclava pacifica]
12285~WP_011277377.1 translation initiation factor aIF-2 [Sulfolobus acidocaldarius]
23273057~NP_341780.1 translation initiation factor IF-2 [Sulfolobus solfataricus P2]
1651206403~WP_046550425.1 translation initiation factor IF-2 [Sulfurovum lithotrophicum]
1673387093~WP_012083734.1 translation initiation factor IF-2 [Sulfurovum sp. NBC37-1]
45Bins|all_bins.fasta_bin_5~926
111159290~WP_031386179.1 translation initiation factor IF-2 [Desulfonatronum thiodismutans]
133469361~WP_005662128.1 translation initiation factor IF-2 [Dethiosulfovibrio peptidovorans]
265555088~WP_008517775.1 translation initiation factor IF-2 [Dethiobacter alkaliphilus]
89679936~YP_005257066.1 translation initiation factor 2 (bIF-2) [Sulfobacillus acidophilus DSM 10332]
6728034~WP_053959029.1 translation initiation factor IF-2 [Sulfobacillus thermosulfidooxidans]
881Bins|all_bins.fasta_bin_12~760
947Bins|Cell_14.fasta_bin_3~90
969Bins|Cell_8.fasta_bin_1~65
991Bins|Cell_12.fasta_bin_1~48
1013Bins|all_bins.fasta_bin_2~8
925Bins|all_bins.fasta_bin_14~422
903Bins|all_bins.fasta_bin_3~474
155204536~WP_041675931.1 translation initiation factor IF-2 [Sulfurihydrogenibium azorense]
1607Bins|all_bins.fasta_bin_16~370
1629Bins|all_bins.fasta_bin_16~1092
1079555778~WP_012824333.1 translation initiation factor IF-2 [Halothiobacillus neapolitanus]
11231255043~WP_015259854.1 translation initiation factor IF-2 [Thioalkalivibrio nitratireducens]
11671442136~WP_063463447.1 translation initiation factor IF-2 [Ectothiorhodospira sp. BSL-9]
1277765914~WP_006787487.1 translation initiation factor IF-2 [Thiorhodospira sibirica]
1541Bins|all_bins.fasta_bin_12~759
1343Bins|all_bins.fasta_bin_14~398
1321Bins|all_bins.fasta_bin_14~421
1189870187~WP_002707979.1 translation initiation factor IF-2 [Thiothrix nivea]
1255Bins|all_bins.fasta_bin_3~473
1475Bins|all_bins.fasta_bin_55~1420
1497Bins|all_bins.fasta_bin_12~1432
1519Bins|Cell_14.fasta_bin_3~137
1365Bins|all_bins.fasta_bin_20~772
1387Bins|all_bins.fasta_bin_44~1996
1409Bins|Cell_14.fasta_bin_3~89
1431Bins|Cell_7.fasta_bin_2~79
1453Bins|Cell_8.fasta_bin_1~64
1563Bins|all_bins.fasta_bin_2~7
1585Bins|Cell_12.fasta_bin_1~47
4631076586~WP_041067300.1 translation initiation factor IF-2 [Thiolapillus brandeum]
4191431668~WP_036557412.1 translation initiation factor IF-2 [Thiohalocapsa sp. ML1]
441765912~WP_015279689.1 translation initiation factor IF-2 [Thioflavicoccus mobilis]
1035768671~WP_007194816.1 translation initiation factor IF-2 [Thiocapsa marina]
1057765913~WP_007040773.1 translation initiation factor IF-2 [Thiorhodococcus drewsii]
375361306~WP_054965747.1 translation initiation factor IF-2 [Thiohalorhabdus denitrificans]
1101762376~WP_013392463.1 translation initiation factor IF-2 [Achromobacter xylosoxidans]
12111223602~WP_041096330.1 translation initiation factor IF-2 [Sulfuritalea hydrogenivorans]
0.9

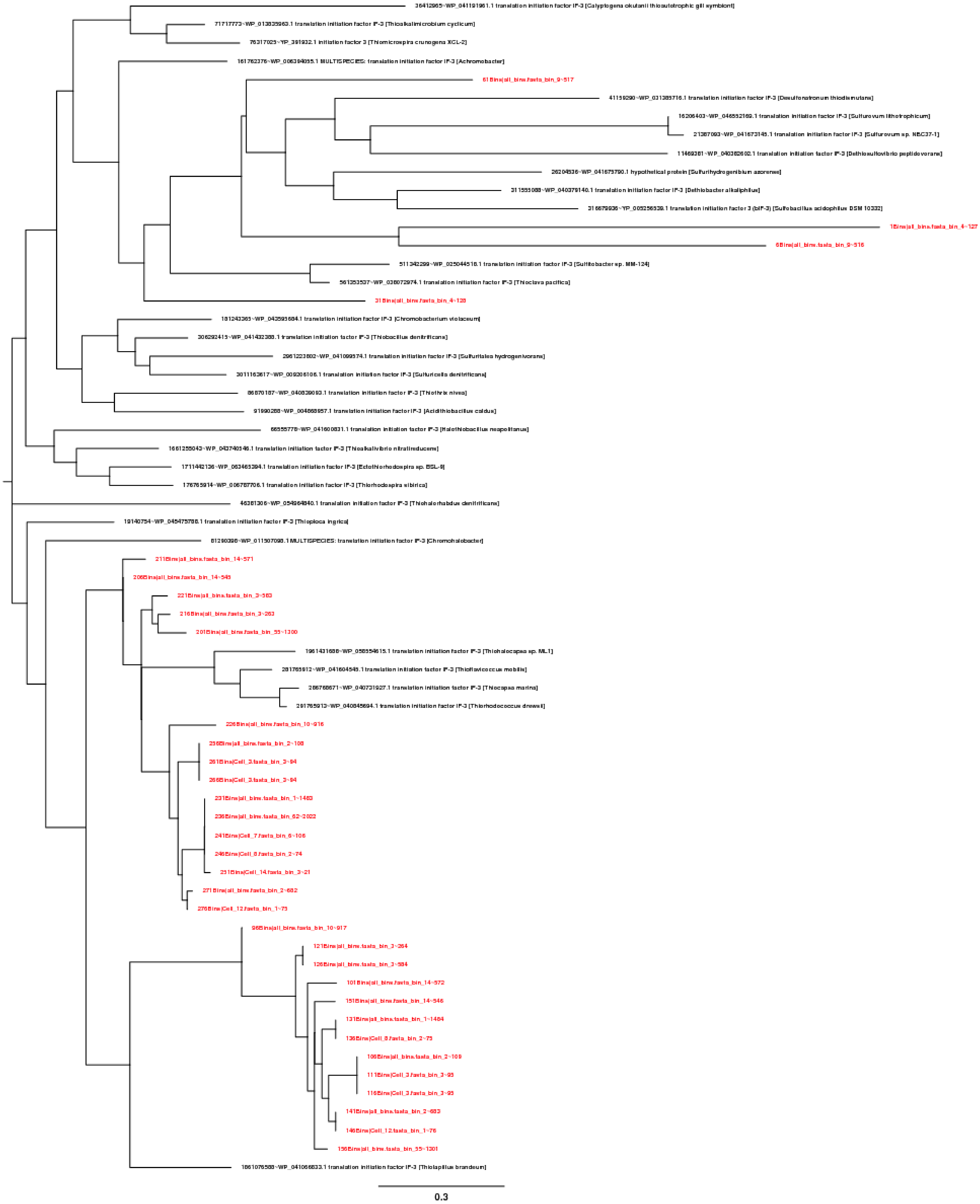
36412965~WP_041191961.1 translation initiation factor IF-3 [Calyptogena okutanii thioautotrophic gill symbiont]
71717773~WP_013835963.1 translation initiation factor IF-3 [Thioalkalimicrobium cyclicum]
76317025~YP_391932.1 initiation factor 3 [Thiomicrospira crunogena XCL-2]
161762376~WP_006394055.1 MULTISPECIES: translation initiation factor IF-3 [Achromobacter]
61Bins|all_bins.fasta_bin_9~517
41159290~WP_031385716.1 translation initiation factor IF-3 [Desulfonatronum thiodismutans]
16206403~WP_046552169.1 translation initiation factor IF-3 [Sulfurovum lithotrophicum]
21367093~WP_041673145.1 translation initiation factor IF-3 [Sulfurovum sp. NBC37-1]
11469361~WP_040382602.1 translation initiation factor IF-3 [Dethiosulfovibrio peptidovorans]
26204536~WP_041675790.1 hypothetical protein [Sulfurihydrogenibium azorense]
311555088~WP_040379140.1 translation initiation factor IF-3 [Dethiobacter alkaliphilus]
316679936~YP_005256539.1 translation initiation factor 3 (bIF-3) [Sulfobacillus acidophilus DSM 10332]
1Bins|all_bins.fasta_bin_4~127
6Bins|all_bins.fasta_bin_9~516
511342299~WP_025044516.1 translation initiation factor IF-3 [Sulfitobacter sp. MM-124]
561353537~WP_036072974.1 translation initiation factor IF-3 [Thioclava pacifica]
31Bins|all_bins.fasta_bin_4~128
181243365~WP_042595684.1 translation initiation factor IF-3 [Chromobacterium violaceum]
306292415~WP_041432388.1 translation initiation factor IF-3 [Thiobacillus denitrificans]
2961223602~WP_041099574.1 translation initiation factor IF-3 [Sulfuritalea hydrogenivorans]
3011163617~WP_009206106.1 translation initiation factor IF-3 [Sulfuricella denitrificans]
86870187~WP_040839093.1 translation initiation factor IF-3 [Thiothrix nivea]
91990288~WP_004868957.1 translation initiation factor IF-3 [Acidithiobacillus caldus]
66555778~WP_041600831.1 translation initiation factor IF-3 [Halothiobacillus neapolitanus]
1661255043~WP_043740546.1 translation initiation factor IF-3 [Thioalkalivibrio nitratireducens]
1711442136~WP_063465394.1 translation initiation factor IF-3 [Ectothiorhodospira sp. BSL-9]
176765914~WP_006787706.1 translation initiation factor IF-3 [Thiorhodospira sibirica]
46361306~WP_054964840.1 translation initiation factor IF-3 [Thiohalorhabdus denitrificans]
19140754~WP_045475788.1 translation initiation factor IF-3 [Thioploca ingrica]
61290396~WP_011507098.1 MULTISPECIES: translation initiation factor IF-3 [Chromohalobacter]
211Bins|all_bins.fasta_bin_14~571
206Bins|all_bins.fasta_bin_14~545
221Bins|all_bins.fasta_bin_3~563
216Bins|all_bins.fasta_bin_3~263
201Bins|all_bins.fasta_bin_55~1300
1961431686~WP_058554615.1 translation initiation factor IF-3 [Thiohalocapsa sp. ML1]
281765912~WP_041604545.1 translation initiation factor IF-3 [Thioflavicoccus mobilis]
286768671~WP_040731927.1 translation initiation factor IF-3 [Thiocapsa marina]
291765913~WP_040845694.1 translation initiation factor IF-3 [Thiorhodococcus drewsii]
226Bins|all_bins.fasta_bin_10~916
256Bins|all_bins.fasta_bin_2~108
261Bins|Cell_3.fasta_bin_3~94
266Bins|Cell_3.fasta_bin_3~94
231Bins|all_bins.fasta_bin_1~1463
236Bins|all_bins.fasta_bin_62~2022
241Bins|Cell_7.fasta_bin_6~106
246Bins|Cell_8.fasta_bin_2~74
251Bins|Cell_14.fasta_bin_3~21
271Bins|all_bins.fasta_bin_2~682
276Bins|Cell_12.fasta_bin_1~75
96Bins|all_bins.fasta_bin_10~917
121Bins|all_bins.fasta_bin_3~264
126Bins|all_bins.fasta_bin_3~564
101Bins|all_bins.fasta_bin_14~572
151Bins|all_bins.fasta_bin_14~546
131Bins|all_bins.fasta_bin_1~1464
136Bins|Cell_8.fasta_bin_2~75
106Bins|all_bins.fasta_bin_2~109
111Bins|Cell_3.fasta_bin_3~95
116Bins|Cell_3.fasta_bin_3~95
141Bins|all_bins.fasta_bin_2~683
146Bins|Cell_12.fasta_bin_1~76
156Bins|all_bins.fasta_bin_55~1301
1661076588~WP_041066833.1 translation initiation factor IF-3 [Thiolapillus brandeum]
0.3

IPPT.treee.tre.exp.tree.pdf

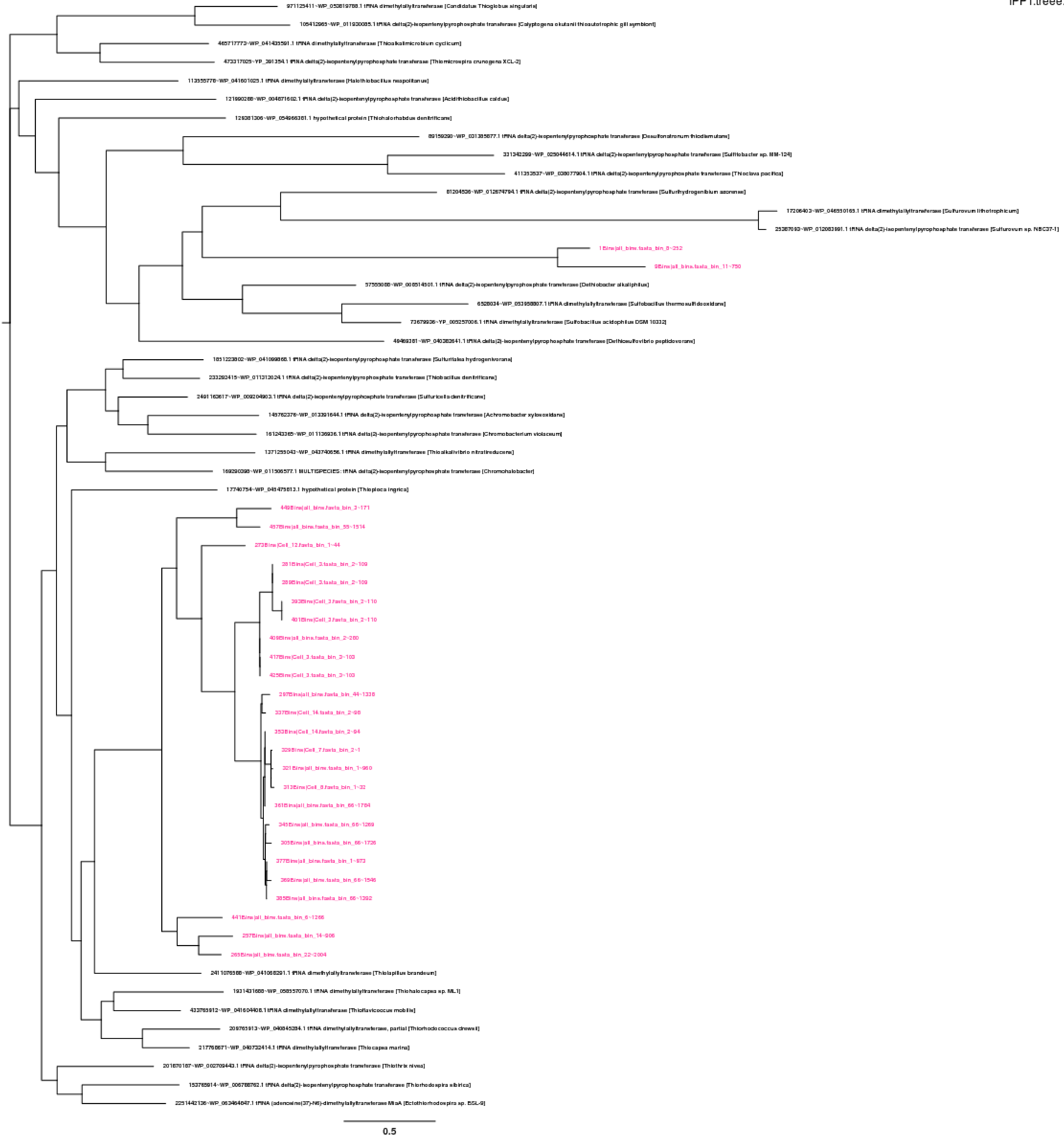

LepA_C.treee.tre.exp.tree.pdf

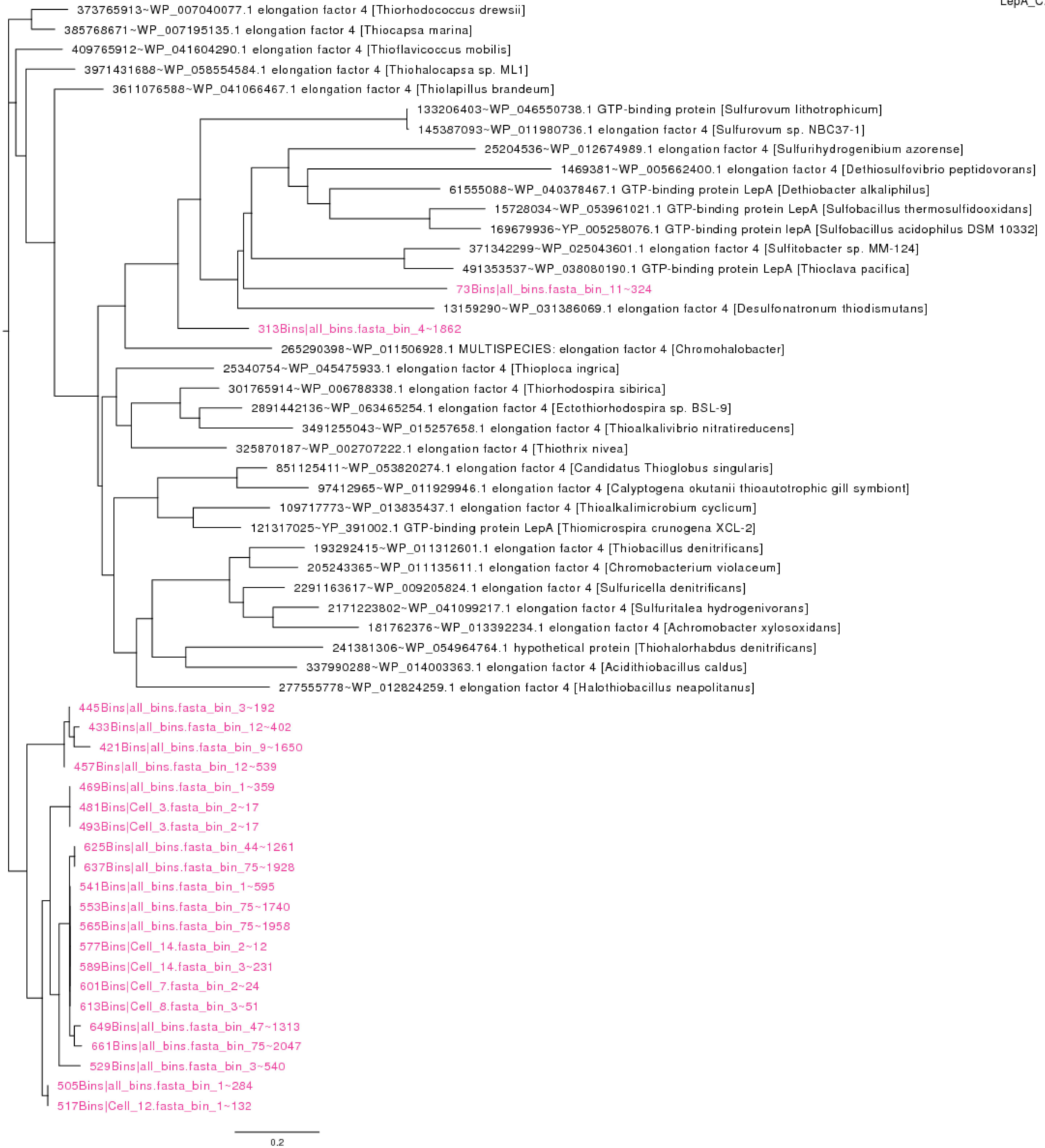

Methyltransf_5.treee.tre.exp.tree.pdf

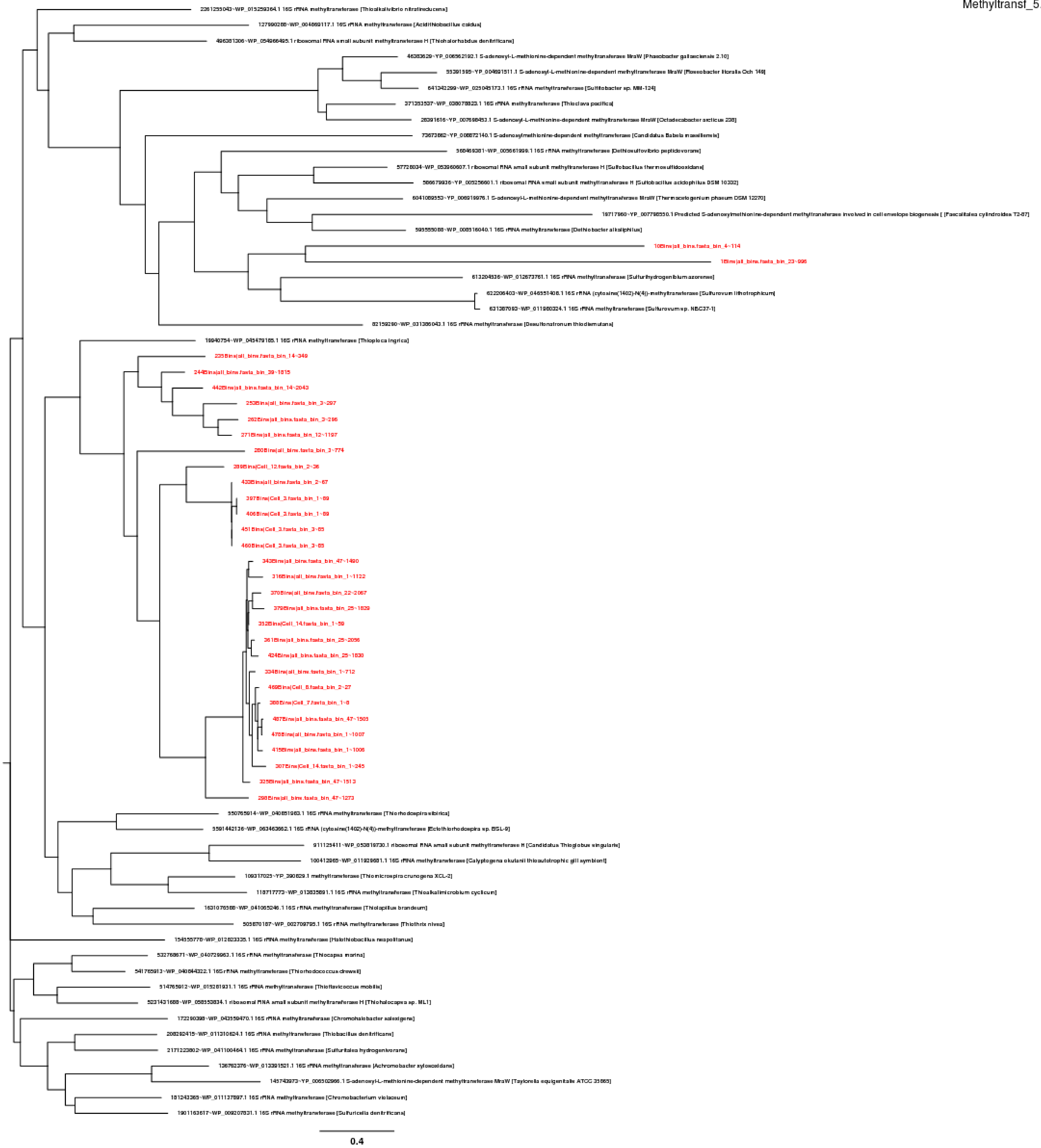

MurB_C.treee.tre.exp.tree.pdf

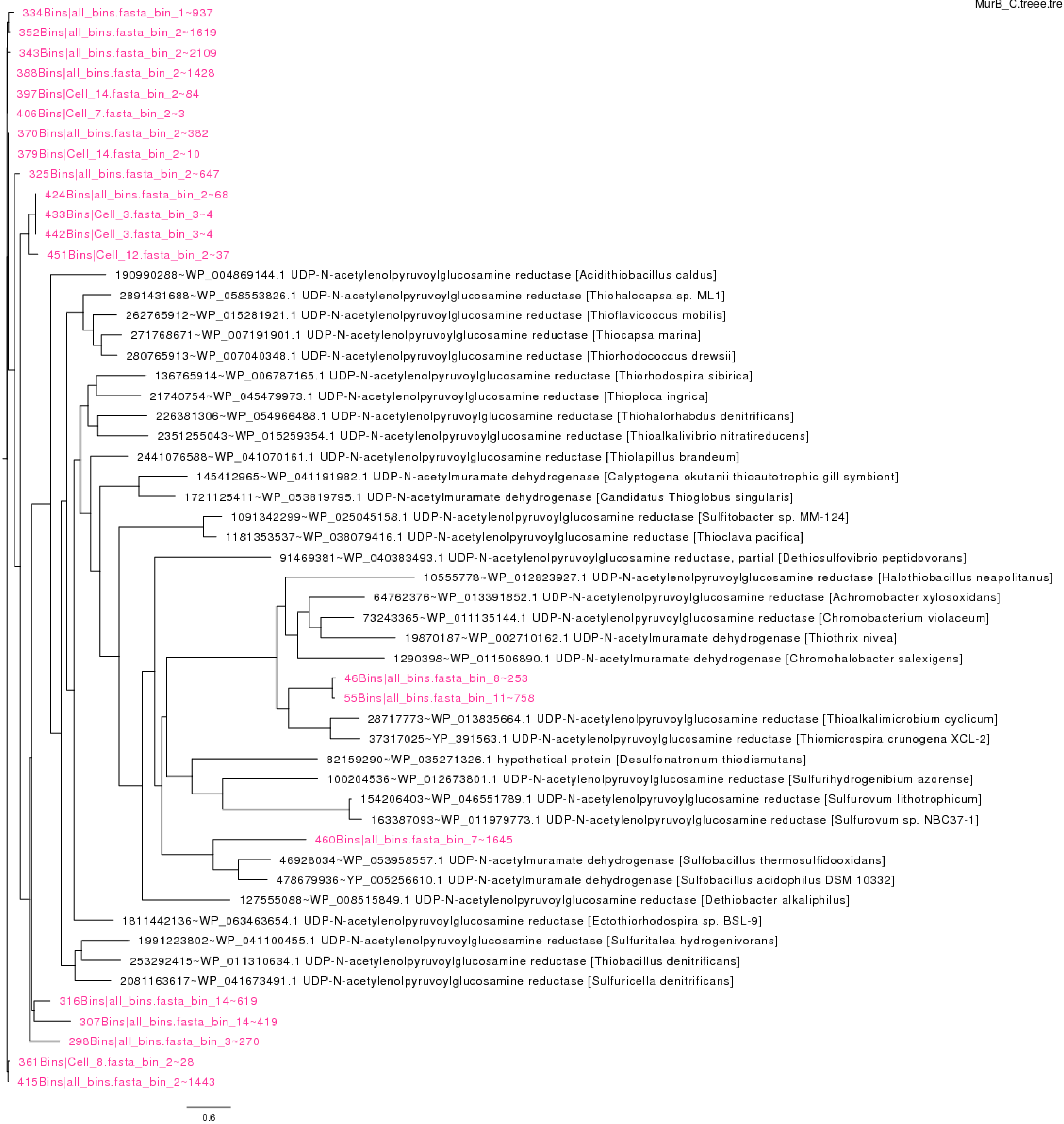

NusA_N.treee.tre.exp.tree.pdf

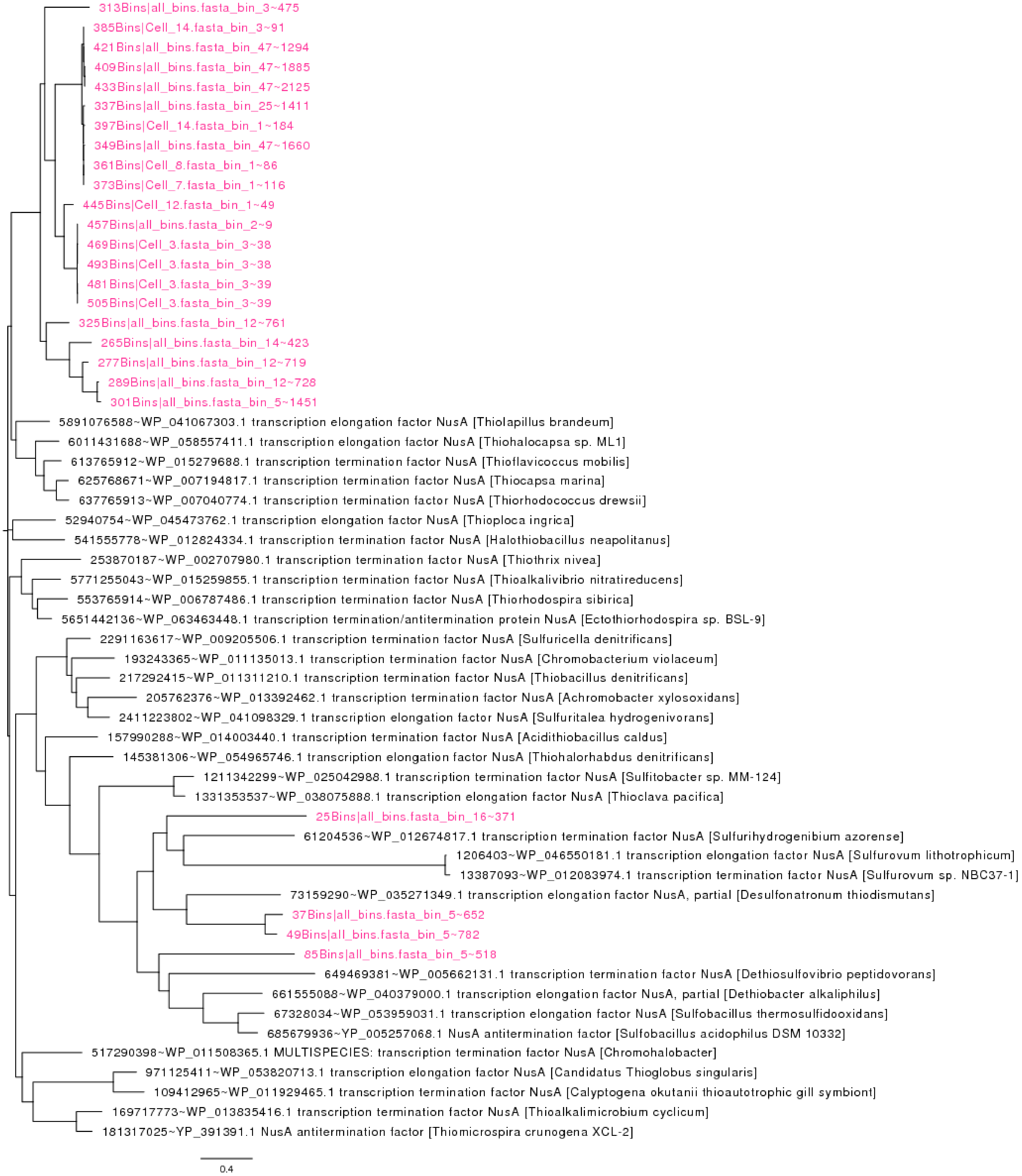

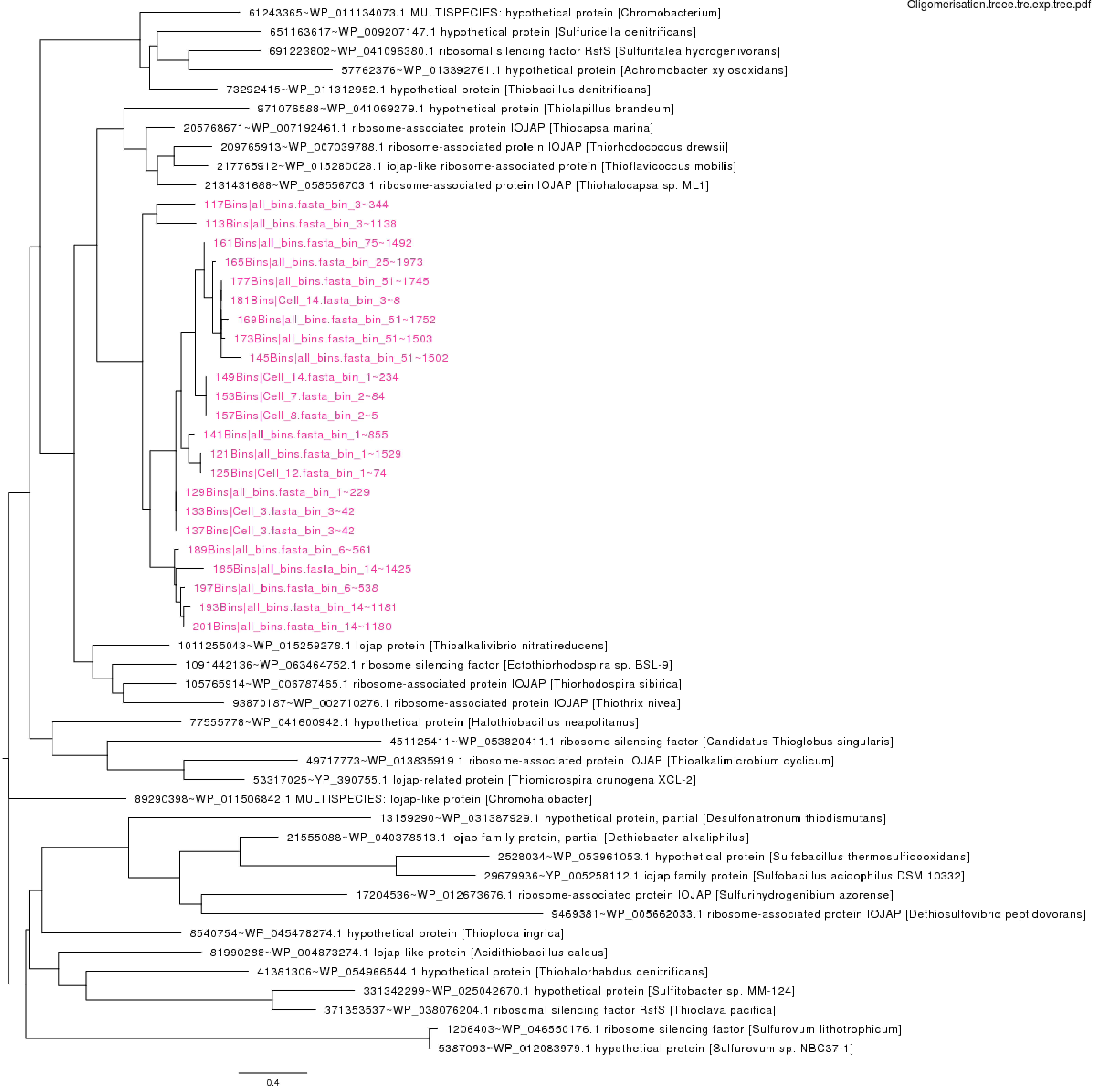
Oligomerisation.treee.tre.exp.tree.pdf
61243365~WP_011134073.1 MULTISPECIES: hypothetical protein [Chromobacterium]
651163617~WP_009207147.1 hypothetical protein [Sulfuricella denitrificans]
691223802~WP_041096380.1 ribosomal silencing factor RsfS [Sulfuritalea hydrogenivorans]
57762376~WP_013392761.1 hypothetical protein [Achromobacter xylosoxidans]
73292415~WP_011312952.1 hypothetical protein [Thiobacillus denitrificans]
971076588~WP_041069279.1 hypothetical protein [Thiolapillus brandeum]
205768671~WP_007192461.1 ribosome-associated protein IOJAP [Thiocapsa marina]
209765913~WP_007039788.1 ribosome-associated protein IOJAP [Thiorhodococcus drewsii]
217765912~WP_015280028.1 iojap-like ribosome-associated protein [Thioflavicoccus mobilis]
2131431688~WP_058556703.1 ribosome-associated protein IOJAP [Thiohalocapsa sp. ML1]
117Bins|all_bins.fasta_bin_3~344
113Bins|all_bins.fasta_bin_3~1138
161Bins|all_bins.fasta_bin_75~1492
165Bins|all_bins.fasta_bin_25~1973
177Bins|all_bins.fasta_bin_51~1745
181Bins|Cell_14.fasta_bin_3~8
169Bins|all_bins.fasta_bin_51~1752
173Bins|all_bins.fasta_bin_51~1503
145Bins|all_bins.fasta_bin_51~1502
149Bins|Cell_14.fasta_bin_1~234
153Bins|Cell_7.fasta_bin_2~84
157Bins|Cell_8.fasta_bin_2~5
141Bins|all_bins.fasta_bin_1~855
121Bins|all_bins.fasta_bin_1~1529
125Bins|Cell_12.fasta_bin_1~74
129Bins|all_bins.fasta_bin_1~229
133Bins|Cell_3.fasta_bin_3~42
137Bins|Cell_3.fasta_bin_3~42
189Bins|all_bins.fasta_bin_6~561
185Bins|all_bins.fasta_bin_14~1425
197Bins|all_bins.fasta_bin_6~538
193Bins|all_bins.fasta_bin_14~1181
201Bins|all_bins.fasta_bin_14~1180
1011255043~WP_015259278.1 Iojap protein [Thioalkalivibrio nitratireducens]
1091442136~WP_063464752.1 ribosome silencing factor [Ectothiorhodospira sp. BSL-9]
105765914~WP_006787465.1 ribosome-associated protein IOJAP [Thiorhodospira sibirica]
93870187~WP_002710276.1 ribosome-associated protein IOJAP [Thiothrix nivea]
77555778~WP_041600942.1 hypothetical protein [Halothiobacillus neapolitanus]
451125411~WP_053820411.1 ribosome silencing factor [Candidatus Thioglobus singularis]
49717773~WP_013835919.1 ribosome-associated protein IOJAP [Thioalkalimicrobium cyclicum]
53317025~YP_390755.1 Iojap-related protein [Thiomicrospira crunogena XCL-2]
89290398~WP_011506842.1 MULTISPECIES: Iojap-like protein [Chromohalobacter]
13159290~WP_031387929.1 hypothetical protein, partial [Desulfonatronum thiodismutans]
21555088~WP_040378513.1 iojap family protein, partial [Dethiobacter alkaliphilus]
2528034~WP_053961053.1 hypothetical protein [Sulfobacillus thermosulfidooxidans]
29679936~YP_005258112.1 iojap family protein [Sulfobacillus acidophilus DSM 10332]
17204536~WP_012673676.1 ribosome-associated protein IOJAP [Sulfurihydrogenibium azorense]
9469381~WP_005662033.1 ribosome-associated protein IOJAP [Dethiosulfovibrio peptidovorans]
8540754~WP_045478274.1 hypothetical protein [Thioploca ingrica]
81990288~WP_004873274.1 Iojap-like protein [Acidithiobacillus caldus]
41381306~WP_054966544.1 hypothetical protein [Thiohalorhabdus denitrificans]
331342299~WP_025042670.1 hypothetical protein [Sulfitobacter sp. MM-124]
371353537~WP_038076204.1 ribosomal silencing factor RsfS [Thioclava pacifica]
1206403~WP_046550176.1 ribosome silencing factor [Sulfurovum lithotrophicum]
5387093~WP_012083979.1 hypothetical protein [Sulfurovum sp. NBC37-1]
0.4

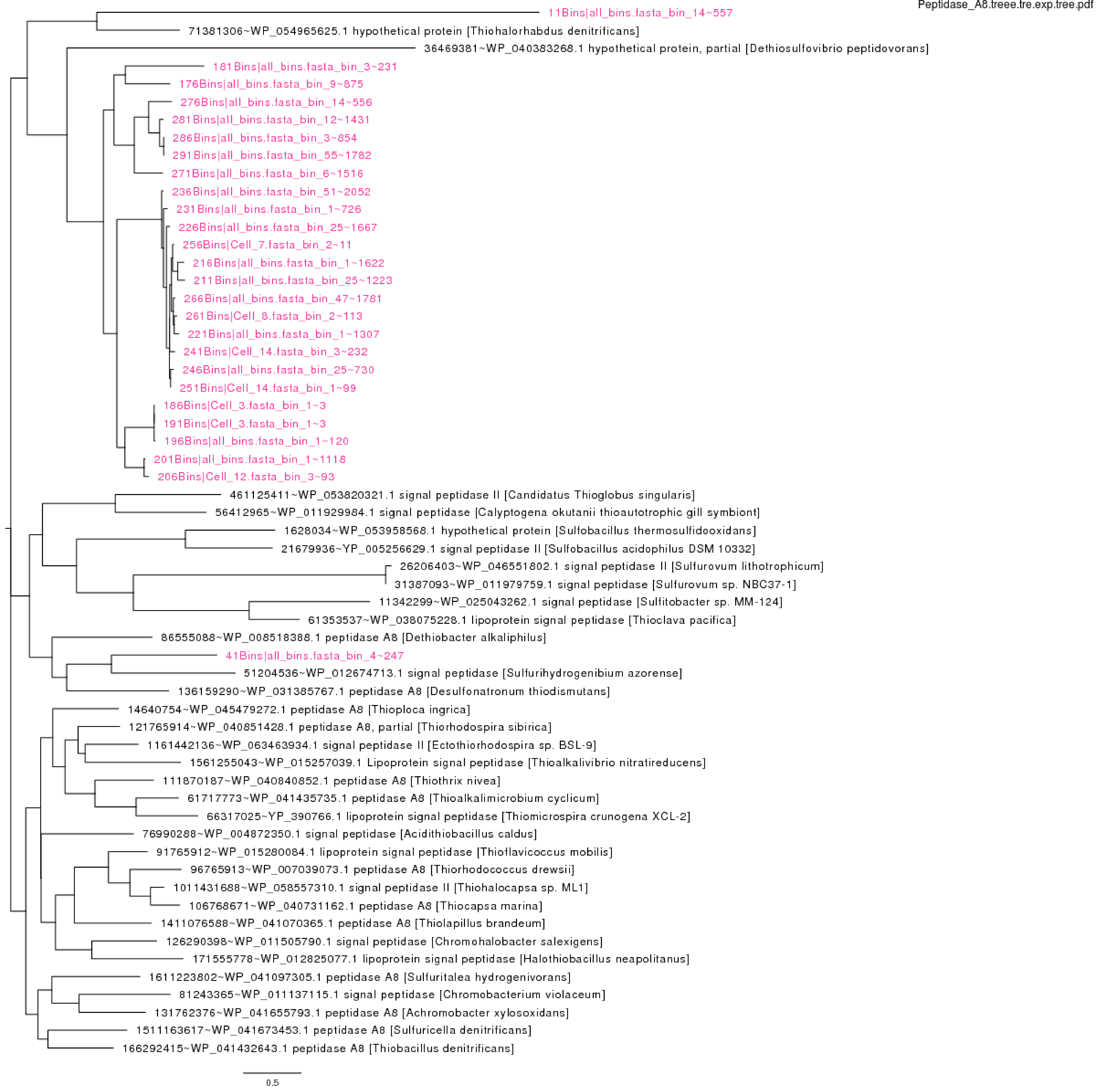
Peptidase_A8.treee.tre.exp.tree.pdf
11Bins|all_bins.fasta_bin_14~557
71381306~WP_054965625.1 hypothetical protein [Thiohalorhabdus denitrificans]
36469381~WP_040383268.1 hypothetical protein, partial [Dethiosulfovibrio peptidovorans]
181Bins|all_bins.fasta_bin_3~231
176Bins|all_bins.fasta_bin_9~875
276Bins|all_bins.fasta_bin_14~556
281Bins|all_bins.fasta_bin_12~1431
286Bins|all_bins.fasta_bin_3~854
291Bins|all_bins.fasta_bin_55~1782
271Bins|all_bins.fasta_bin_6~1516
236Bins|all_bins.fasta_bin_51~2052
231Bins|all_bins.fasta_bin_1~726
226Bins|all_bins.fasta_bin_25~1667
256Bins|Cell_7.fasta_bin_2~11
216Bins|all_bins.fasta_bin_1~1622
211Bins|all_bins.fasta_bin_25~1223
266Bins|all_bins.fasta_bin_47~1781
261Bins|Cell_8.fasta_bin_2~113
221Bins|all_bins.fasta_bin_1~1307
241Bins|Cell_14.fasta_bin_3~232
246Bins|all_bins.fasta_bin_25~730
251Bins|Cell_14.fasta_bin_1~99
186Bins|Cell_3.fasta_bin_1~3
191Bins|Cell_3.fasta_bin_1~3
196Bins|all_bins.fasta_bin_1~120
201Bins|all_bins.fasta_bin_1~1118
206Bins|Cell_12.fasta_bin_3~93
461125411~WP_053820321.1 signal peptidase II [Candidatus Thioglobus singularis]
56412965~WP_011929984.1 signal peptidase [Calyptogena okutanii thioautotrophic gill symbiont]
1628034~WP_053958568.1 hypothetical protein [Sulfobacillus thermosulfidooxidans]
21679936~YP_005256629.1 signal peptidase II [Sulfobacillus acidophilus DSM 10332]
26206403~WP_046551802.1 signal peptidase II [Sulfurovum lithotrophicum]
31387093~WP_011979759.1 signal peptidase [Sulfurovum sp. NBC37-1]
11342299~WP_025043262.1 signal peptidase [Sulfitobacter sp. MM-124]
61353537~WP_038075228.1 lipoprotein signal peptidase [Thioclava pacifica]
86555088~WP_008518388.1 peptidase A8 [Dethiobacter alkaliphilus]
41Bins|all_bins.fasta_bin_4~247
51204536~WP_012674713.1 signal peptidase [Sulfurihydrogenibium azorense]
136159290~WP_031385767.1 peptidase A8 [Desulfonatronum thiodismutans]
14640754~WP_045479272.1 peptidase A8 [Thioploca ingrica]
121765914~WP_040851428.1 peptidase A8, partial [Thiorhodospira sibirica]
1161442136~WP_063463934.1 signal peptidase II [Ectothiorhodospira sp. BSL-9]
1561255043~WP_015257039.1 Lipoprotein signal peptidase [Thioalkalivibrio nitratireducens]
111870187~WP_040840852.1 peptidase A8 [Thiothrix nivea]
61717773~WP_041435735.1 peptidase A8 [Thioalkalimicrobium cyclicum]
66317025~YP_390766.1 lipoprotein signal peptidase [Thiomicrospira crunogena XCL-2]
76990288~WP_004872350.1 signal peptidase [Acidithiobacillus caldus]
91765912~WP_015280084.1 lipoprotein signal peptidase [Thioflavicoccus mobilis]
96765913~WP_007039073.1 peptidase A8 [Thiorhodococcus drewsii]
1011431688~WP_058557310.1 signal peptidase II [Thiohalocapsa sp. ML1]
106768671~WP_040731162.1 peptidase A8 [Thiocapsa marina]
1411076588~WP_041070365.1 peptidase A8 [Thiolapillus brandeum]
126290398~WP_011505790.1 signal peptidase [Chromohalobacter salexigens]
171555778~WP_012825077.1 lipoprotein signal peptidase [Halothiobacillus neapolitanus]
1611223802~WP_041097305.1 peptidase A8 [Sulfuritalea hydrogenivorans]
81243365~WP_011137115.1 signal peptidase [Chromobacterium violaceum]
131762376~WP_041655793.1 peptidase A8 [Achromobacter xylosoxidans]
1511163617~WP_041673453.1 peptidase A8 [Sulfuricella denitrificans]
166292415~WP_041432643.1 peptidase A8 [Thiobacillus denitrificans]
0.5

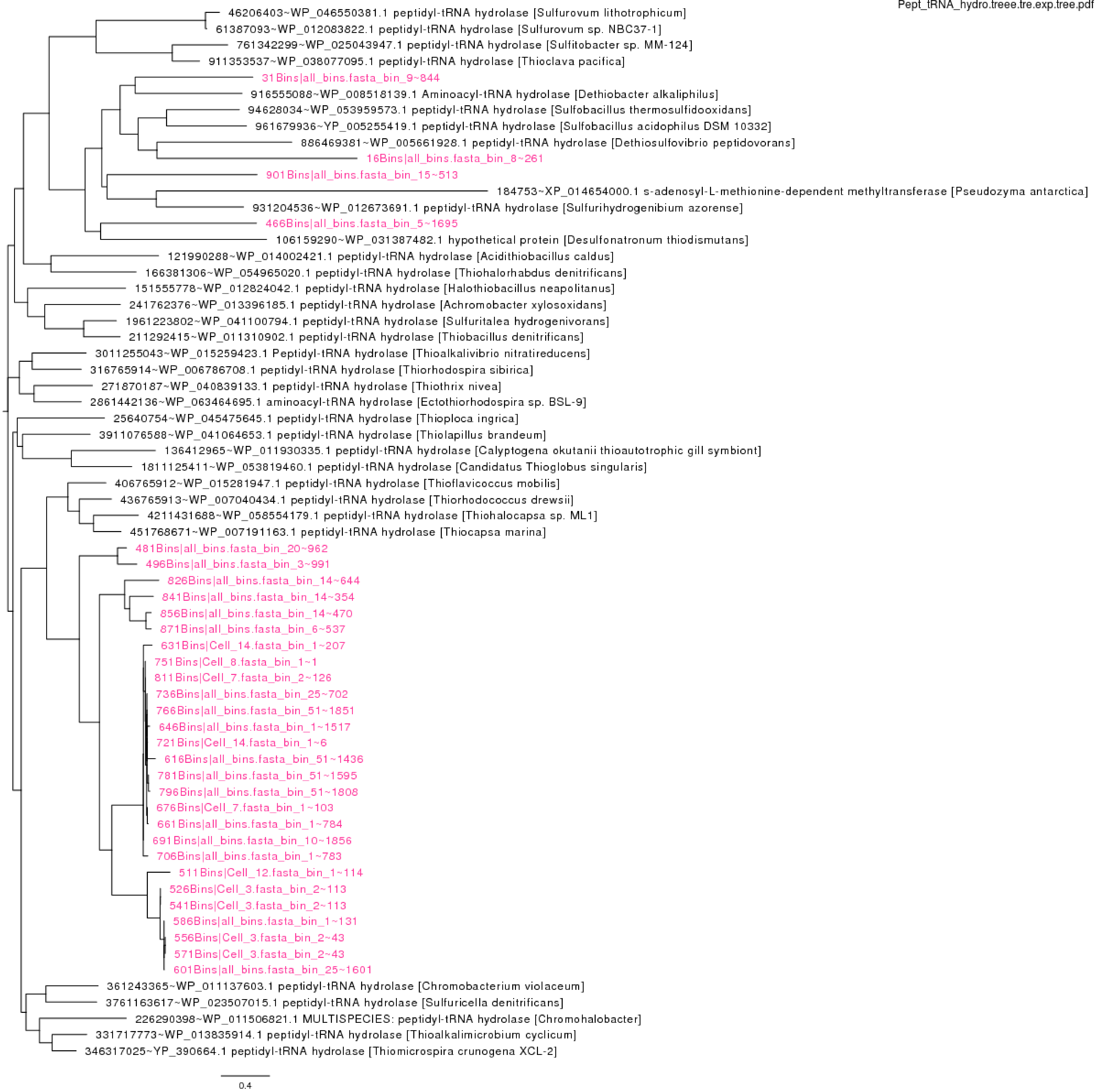
Pept_tRNA_hydro.treee.tre.exp.tree.pdf
46206403~WP_046550381.1 peptidyl-tRNA hydrolase [Sulfurovum lithotrophicum]
61387093~WP_012083822.1 peptidyl-tRNA hydrolase [Sulfurovum sp. NBC37-1]
761342299~WP_025043947.1 peptidyl-tRNA hydrolase [Sulfitobacter sp. MM-124]
911353537~WP_038077095.1 peptidyl-tRNA hydrolase [Thioclava pacifica]
31Bins|all_bins.fasta_bin_9~844
916555088~WP_008518139.1 Aminoacyl-tRNA hydrolase [Dethiobacter alkaliphilus]
94628034~WP_053959573.1 peptidyl-tRNA hydrolase [Sulfobacillus thermosulfidooxidans]
961679936~YP_005255419.1 peptidyl-tRNA hydrolase [Sulfobacillus acidophilus DSM 10332]
886469381~WP_005661928.1 peptidyl-tRNA hydrolase [Dethiosulfovibrio peptidovorans]
16Bins|all_bins.fasta_bin_8~261
901Bins|all_bins.fasta_bin_15~513
184753~XP_014654000.1 s-adenosyl-L-methionine-dependent methyltransferase [Pseudozyma antarctica]
931204536~WP_012673691.1 peptidyl-tRNA hydrolase [Sulfurihydrogenibium azorense]
466Bins|all_bins.fasta_bin_5~1695
106159290~WP_031387482.1 hypothetical protein [Desulfonatronum thiodismutans]
121990288~WP_014002421.1 peptidyl-tRNA hydrolase [Acidithiobacillus caldus]
166381306~WP_054965020.1 peptidyl-tRNA hydrolase [Thiohalorhabdus denitrificans]
151555778~WP_012824042.1 peptidyl-tRNA hydrolase [Halothiobacillus neapolitanus]
241762376~WP_013396185.1 peptidyl-tRNA hydrolase [Achromobacter xylosoxidans]
1961223802~WP_041100794.1 peptidyl-tRNA hydrolase [Sulfuritalea hydrogenivorans]
211292415~WP_011310902.1 peptidyl-tRNA hydrolase [Thiobacillus denitrificans]
3011255043~WP_015259423.1 Peptidyl-tRNA hydrolase [Thioalkalivibrio nitratireducens]
316765914~WP_006786708.1 peptidyl-tRNA hydrolase [Thiorhodospira sibirica]
271870187~WP_040839133.1 peptidyl-tRNA hydrolase [Thiothrix nivea]
2861442136~WP_063464695.1 aminoacyl-tRNA hydrolase [Ectothiorhodospira sp. BSL-9]
25640754~WP_045475645.1 peptidyl-tRNA hydrolase [Thioploca ingrica]
3911076588~WP_041064653.1 peptidyl-tRNA hydrolase [Thiolapillus brandeum]
136412965~WP_011930335.1 peptidyl-tRNA hydrolase [Calyptogena okutanii thioautotrophic gill symbiont]
1811125411~WP_053819460.1 peptidyl-tRNA hydrolase [Candidatus Thioglobus singularis]
406765912~WP_015281947.1 peptidyl-tRNA hydrolase [Thioflavicoccus mobilis]
436765913~WP_007040434.1 peptidyl-tRNA hydrolase [Thiorhodococcus drewsii]
4211431688~WP_058554179.1 peptidyl-tRNA hydrolase [Thiohalocapsa sp. ML1]
451768671~WP_007191163.1 peptidyl-tRNA hydrolase [Thiocapsa marina]
481Bins|all_bins.fasta_bin_20~962
496Bins|all_bins.fasta_bin_3~991
826Bins|all_bins.fasta_bin_14~644
841Bins|all_bins.fasta_bin_14~354
856Bins|all_bins.fasta_bin_14~470
871Bins|all_bins.fasta_bin_6~537
631Bins|Cell_14.fasta_bin_1~207
751Bins|Cell_8.fasta_bin_1~1
811Bins|Cell_7.fasta_bin_2~126
736Bins|all_bins.fasta_bin_25~702
766Bins|all_bins.fasta_bin_51~1851
646Bins|all_bins.fasta_bin_1~1517
721Bins|Cell_14.fasta_bin_1~6
616Bins|all_bins.fasta_bin_51~1436
781Bins|all_bins.fasta_bin_51~1595
796Bins|all_bins.fasta_bin_51~1808
676Bins|Cell_7.fasta_bin_1~103
661Bins|all_bins.fasta_bin_1~784
691Bins|all_bins.fasta_bin_10~1856
706Bins|all_bins.fasta_bin_1~783
511Bins|Cell_12.fasta_bin_1~114
526Bins|Cell_3.fasta_bin_2~113
541Bins|Cell_3.fasta_bin_2~113
586Bins|all_bins.fasta_bin_1~131
556Bins|Cell_3.fasta_bin_2~43
571Bins|Cell_3.fasta_bin_2~43
601Bins|all_bins.fasta_bin_25~1601
361243365~WP_011137603.1 peptidyl-tRNA hydrolase [Chromobacterium violaceum]
3761163617~WP_023507015.1 peptidyl-tRNA hydrolase [Sulfuricella denitrificans]
226290398~WP_011506821.1 MULTISPECIES: peptidyl-tRNA hydrolase [Chromohalobacter]
331717773~WP_013835914.1 peptidyl-tRNA hydrolase [Thioalkalimicrobium cyclicum]
346317025~YP_390664.1 peptidyl-tRNA hydrolase [Thiomicrospira crunogena XCL-2]
0.4

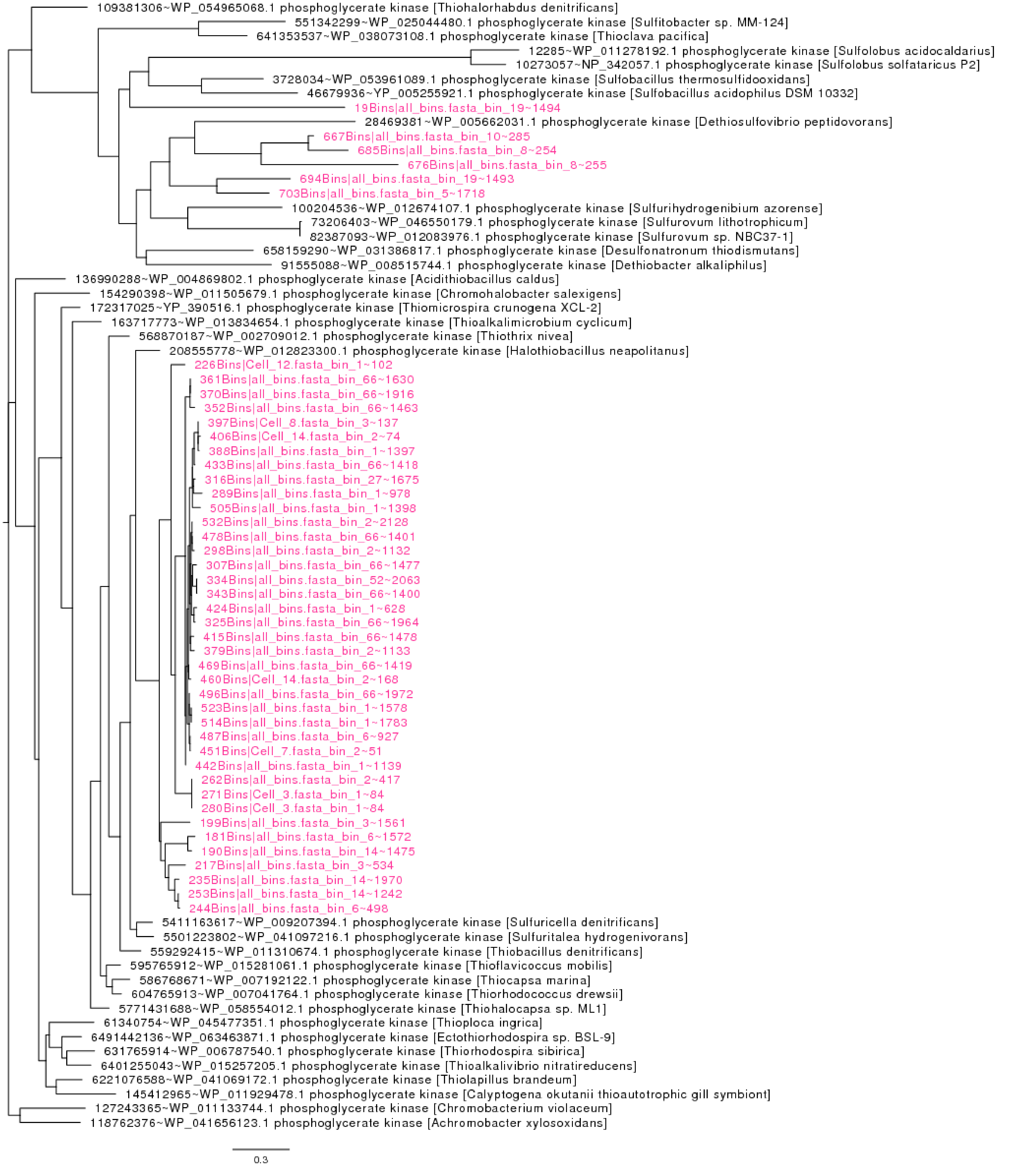
109381306~WP_054965068.1 phosphoglycerate kinase [Thiohalorhabdus denitrificans]
551342299~WP_025044480.1 phosphoglycerate kinase [Sulfitobacter sp. MM-124]
641353537~WP_038073108.1 phosphoglycerate kinase [Thioclava pacifica]
12285~WP_011278192.1 phosphoglycerate kinase [Sulfolobus acidocaldarius]
10273057~NP_342057.1 phosphoglycerate kinase [Sulfolobus solfataricus P2]
3728034~WP_053961089.1 phosphoglycerate kinase [Sulfobacillus thermosulfidooxidans]
46679936~YP_005255921.1 phosphoglycerate kinase [Sulfobacillus acidophilus DSM 10332]
19Bins|all_bins.fasta_bin_19~1494
28469381~WP_005662031.1 phosphoglycerate kinase [Dethiosulfovibrio peptidovorans]
667Bins|all_bins.fasta_bin_10~285
685Bins|all_bins.fasta_bin_8~254
676Bins|all_bins.fasta_bin_8~255
694Bins|all_bins.fasta_bin_19~1493
703Bins|all_bins.fasta_bin_5~1718
100204536~WP_012674107.1 phosphoglycerate kinase [Sulfurihydrogenibium azorense]
73206403~WP_046550179.1 phosphoglycerate kinase [Sulfurovum lithotrophicum]
82387093~WP_012083976.1 phosphoglycerate kinase [Sulfurovum sp. NBC37-1]
658159290~WP_031386817.1 phosphoglycerate kinase [Desulfonatronum thiodismutans]
91555088~WP_008515744.1 phosphoglycerate kinase [Dethiobacter alkaliphilus]
136990288~WP_004869802.1 phosphoglycerate kinase [Acidithiobacillus caldus]
154290398~WP_011505679.1 phosphoglycerate kinase [Chromohalobacter salexigens]
172317025~YP_390516.1 phosphoglycerate kinase [Thiomicrospira crunogena XCL-2]
163717773~WP_013834654.1 phosphoglycerate kinase [Thioalkalimicrobium cyclicum]
568870187~WP_002709012.1 phosphoglycerate kinase [Thiothrix nivea]
208555778~WP_012823300.1 phosphoglycerate kinase [Halothiobacillus neapolitanus]
226Bins|Cell_12.fasta_bin_1~102
361Bins|all_bins.fasta_bin_66~1630
370Bins|all_bins.fasta_bin_66~1916
352Bins|all_bins.fasta_bin_66~1463
397Bins|Cell_8.fasta_bin_3~137
406Bins|Cell_14.fasta_bin_2~74
388Bins|all_bins.fasta_bin_1~1397
433Bins|all_bins.fasta_bin_66~1418
316Bins|all_bins.fasta_bin_27~1675
289Bins|all_bins.fasta_bin_1~978
505Bins|all_bins.fasta_bin_1~1398
532Bins|all_bins.fasta_bin_2~2128
478Bins|all_bins.fasta_bin_66~1401
298Bins|all_bins.fasta_bin_2~1132
307Bins|all_bins.fasta_bin_66~1477
334Bins|all_bins.fasta_bin_52~2063
343Bins|all_bins.fasta_bin_66~1400
424Bins|all_bins.fasta_bin_1~628
325Bins|all_bins.fasta_bin_66~1964
415Bins|all_bins.fasta_bin_66~1478
379Bins|all_bins.fasta_bin_2~1133
469Bins|all_bins.fasta_bin_66~1419
460Bins|Cell_14.fasta_bin_2~168
496Bins|all_bins.fasta_bin_66~1972
523Bins|all_bins.fasta_bin_1~1578
514Bins|all_bins.fasta_bin_1~1783
487Bins|all_bins.fasta_bin_6~927
451Bins|Cell_7.fasta_bin_2~51
442Bins|all_bins.fasta_bin_1~1139
262Bins|all_bins.fasta_bin_2~417
271Bins|Cell_3.fasta_bin_1~84
280Bins|Cell_3.fasta_bin_1~84
199Bins|all_bins.fasta_bin_3~1561
181Bins|all_bins.fasta_bin_6~1572
190Bins|all_bins.fasta_bin_14~1475
217Bins|all_bins.fasta_bin_3~534
235Bins|all_bins.fasta_bin_14~1970
253Bins|all_bins.fasta_bin_14~1242
244Bins|all_bins.fasta_bin_6~498
5411163617~WP_009207394.1 phosphoglycerate kinase [Sulfuricella denitrificans]
5501223802~WP_041097216.1 phosphoglycerate kinase [Sulfuritalea hydrogenivorans]
559292415~WP_011310674.1 phosphoglycerate kinase [Thiobacillus denitrificans]
595765912~WP_015281061.1 phosphoglycerate kinase [Thioflavicoccus mobilis]
586768671~WP_007192122.1 phosphoglycerate kinase [Thiocapsa marina]
604765913~WP_007041764.1 phosphoglycerate kinase [Thiorhodococcus drewsii]
5771431688~WP_058554012.1 phosphoglycerate kinase [Thiohalocapsa sp. ML1]
61340754~WP_045477351.1 phosphoglycerate kinase [Thioploca ingrica]
6491442136~WP_063463871.1 phosphoglycerate kinase [Ectothiorhodospira sp. BSL-9]
631765914~WP_006787540.1 phosphoglycerate kinase [Thiorhodospira sibirica]
6401255043~WP_015257205.1 phosphoglycerate kinase [Thioalkalivibrio nitratireducens]
6221076588~WP_041069172.1 phosphoglycerate kinase [Thiolapillus brandeum]
145412965~WP_011929478.1 phosphoglycerate kinase [Calyptogena okutanii thioautotrophic gill symbiont]
127243365~WP_011133744.1 phosphoglycerate kinase [Chromobacterium violaceum]
118762376~WP_041656123.1 phosphoglycerate kinase [Achromobacter xylosoxidans]
0.3

Phe_tRNA-synt_N.treee.tre.exp.tree.pdf

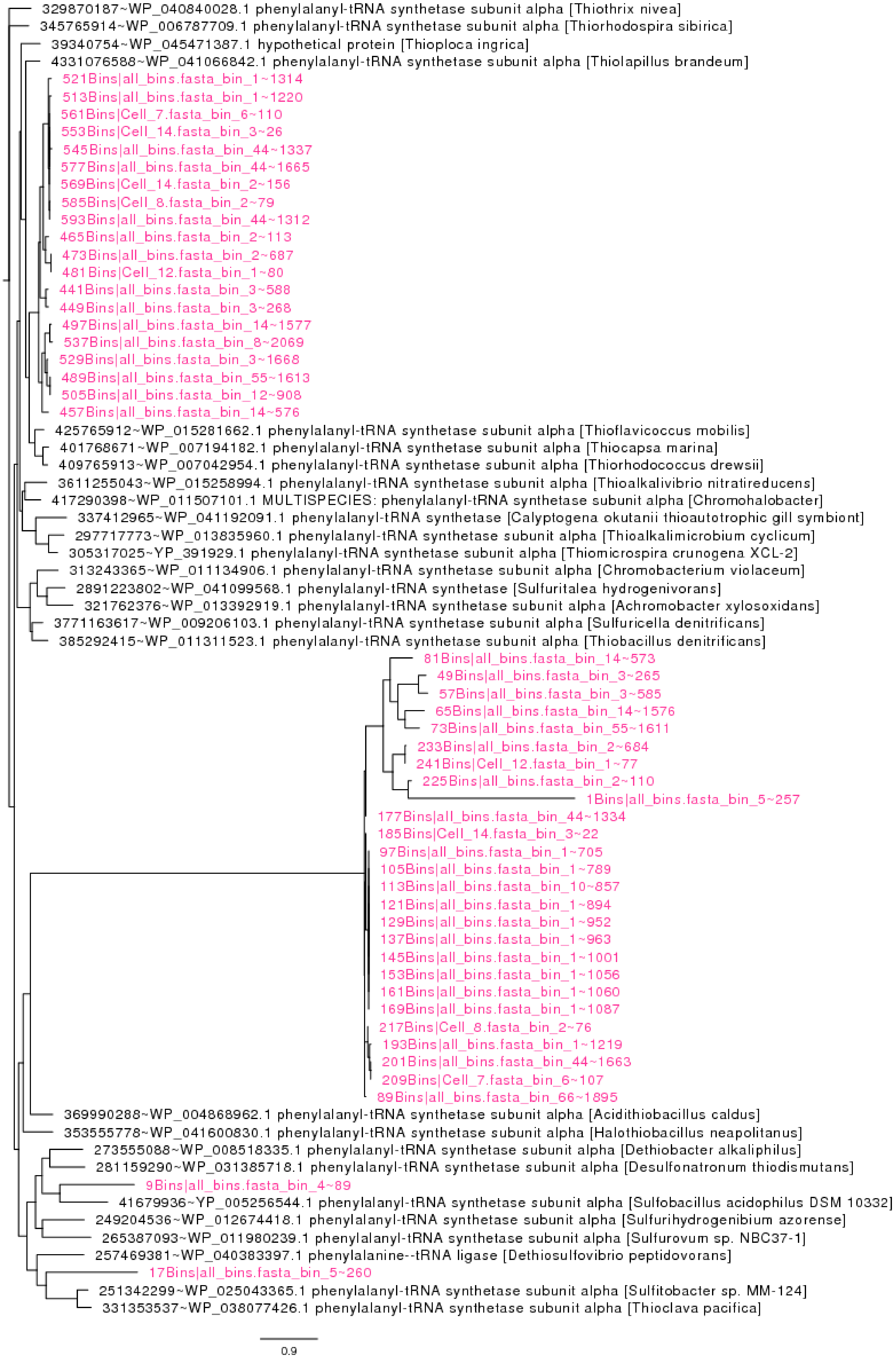

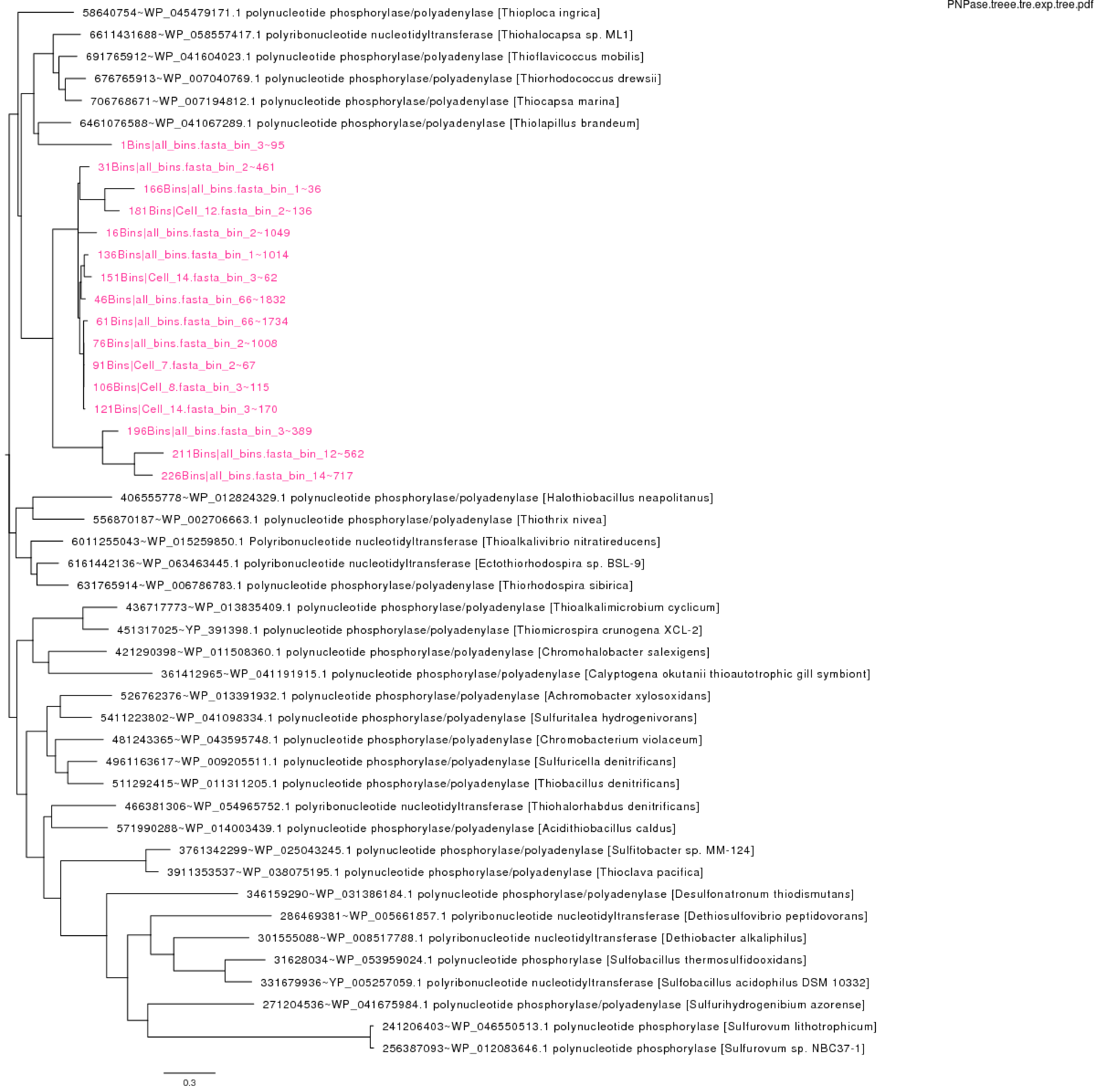
PNPase.treee.tre.exp.tree.pdf
58640754~WP_045479171.1 polynucleotide phosphorylase/polyadenylase [Thioploca ingrica]
6611431688~WP_058557417.1 polyribonucleotide nucleotidyltransferase [Thiohalocapsa sp. ML1]
691765912~WP_041604023.1 polynucleotide phosphorylase/polyadenylase [Thioflavicoccus mobilis]
676765913~WP_007040769.1 polynucleotide phosphorylase/polyadenylase [Thiorhodococcus drewsii]
706768671~WP_007194812.1 polynucleotide phosphorylase/polyadenylase [Thiocapsa marina]
6461076588~WP_041067289.1 polynucleotide phosphorylase/polyadenylase [Thiolapillus brandeum]
1Bins|all_bins.fasta_bin_3~95
31Bins|all_bins.fasta_bin_2~461
166Bins|all_bins.fasta_bin_1~36
181Bins|Cell_12.fasta_bin_2~136
16Bins|all_bins.fasta_bin_2~1049
136Bins|all_bins.fasta_bin_1~1014
151Bins|Cell_14.fasta_bin_3~62
46Bins|all_bins.fasta_bin_66~1832
61Bins|all_bins.fasta_bin_66~1734
76Bins|all_bins.fasta_bin_2~1008
91Bins|Cell_7.fasta_bin_2~67
106Bins|Cell_8.fasta_bin_3~115
121Bins|Cell_14.fasta_bin_3~170
196Bins|all_bins.fasta_bin_3~389
211Bins|all_bins.fasta_bin_12~562
226Bins|all_bins.fasta_bin_14~717
406555778~WP_012824329.1 polynucleotide phosphorylase/polyadenylase [Halothiobacillus neapolitanus]
556870187~WP_002706663.1 polynucleotide phosphorylase/polyadenylase [Thiothrix nivea]
6011255043~WP_015259850.1 Polyribonucleotide nucleotidyltransferase [Thioalkalivibrio nitratireducens]
6161442136~WP_063463445.1 polyribonucleotide nucleotidyltransferase [Ectothiorhodospira sp. BSL-9]
631765914~WP_006786783.1 polynucleotide phosphorylase/polyadenylase [Thiorhodospira sibirica]
436717773~WP_013835409.1 polynucleotide phosphorylase/polyadenylase [Thioalkalimicrobium cyclicum]
451317025~YP_391398.1 polynucleotide phosphorylase/polyadenylase [Thiomicrospira crunogena XCL-2]
421290398~WP_011508360.1 polynucleotide phosphorylase/polyadenylase [Chromohalobacter salexigens]
361412965~WP_041191915.1 polynucleotide phosphorylase/polyadenylase [Calyptogena okutanii thioautotrophic gill symbiont]
526762376~WP_013391932.1 polynucleotide phosphorylase/polyadenylase [Achromobacter xylosoxidans]
5411223802~WP_041098334.1 polynucleotide phosphorylase/polyadenylase [Sulfuritalea hydrogenivorans]
481243365~WP_043595748.1 polynucleotide phosphorylase/polyadenylase [Chromobacterium violaceum]
4961163617~WP_009205511.1 polynucleotide phosphorylase/polyadenylase [Sulfuricella denitrificans]
511292415~WP_011311205.1 polynucleotide phosphorylase/polyadenylase [Thiobacillus denitrificans]
466381306~WP_054965752.1 polyribonucleotide nucleotidyltransferase [Thiohalorhabdus denitrificans]
571990288~WP_014003439.1 polynucleotide phosphorylase/polyadenylase [Acidithiobacillus caldus]
3761342299~WP_025043245.1 polynucleotide phosphorylase/polyadenylase [Sulfitobacter sp. MM-124]
3911353537~WP_038075195.1 polynucleotide phosphorylase/polyadenylase [Thioclava pacifica]
346159290~WP_031386184.1 polynucleotide phosphorylase/polyadenylase [Desulfonatronum thiodismutans]
286469381~WP_005661857.1 polyribonucleotide nucleotidyltransferase [Dethiosulfovibrio peptidovorans]
301555088~WP_008517788.1 polyribonucleotide nucleotidyltransferase [Dethiobacter alkaliphilus]
31628034~WP_053959024.1 polynucleotide phosphorylase [Sulfobacillus thermosulfidooxidans]
331679936~YP_005257059.1 polyribonucleotide nucleotidyltransferase [Sulfobacillus acidophilus DSM 10332]
271204536~WP_041675984.1 polynucleotide phosphorylase/polyadenylase [Sulfurihydrogenibium azorense]
241206403~WP_046550513.1 polynucleotide phosphorylase [Sulfurovum lithotrophicum]
256387093~WP_012083646.1 polynucleotide phosphorylase [Sulfurovum sp. NBC37-1]
0.3

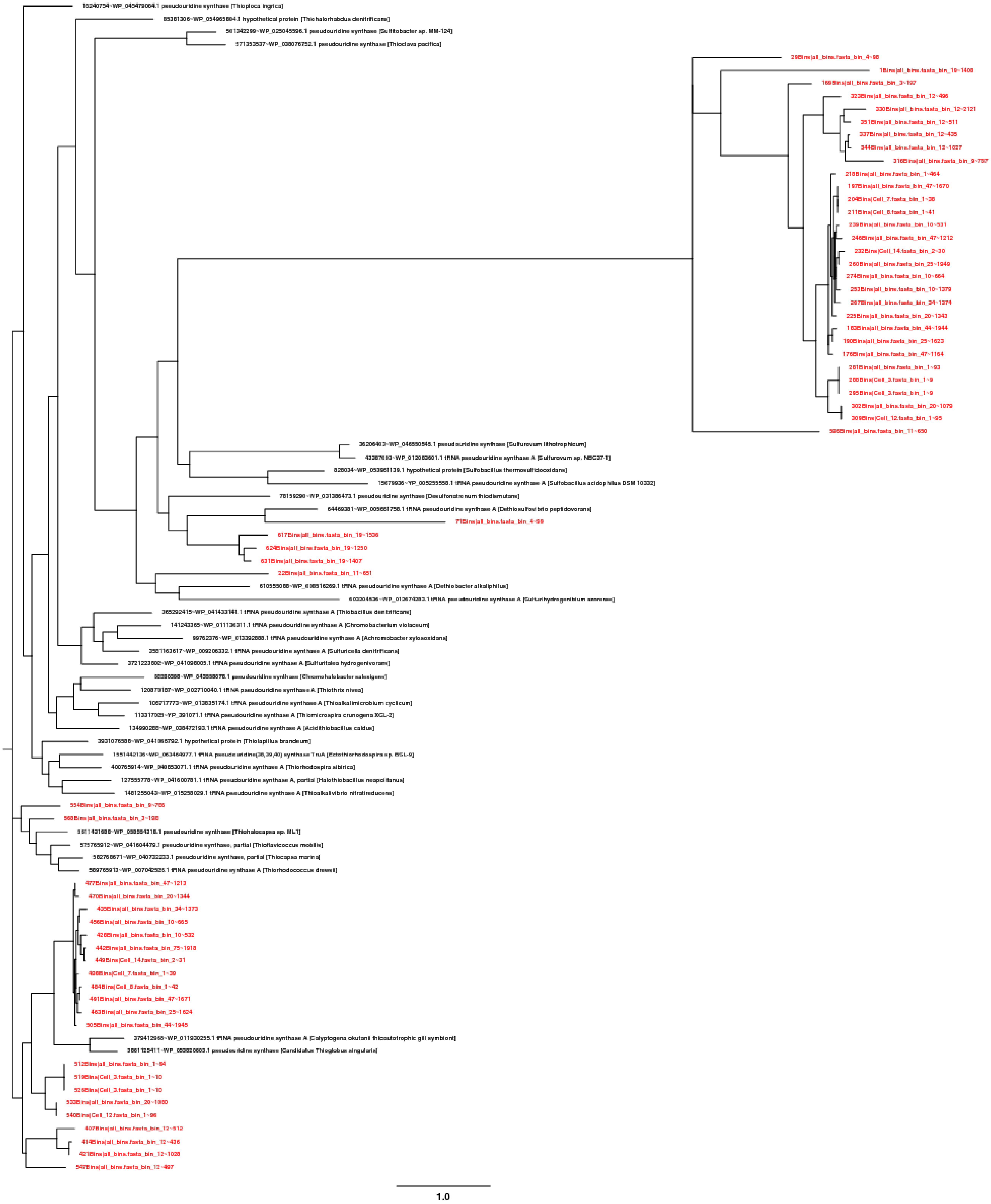
16240754~WP_045479064.1 pseudouridine synthase [Thioploca ingrica]
65361306~WP_054965604.1 hypothetical protein [Thiohalorhabdus denitrificans]
501342299~WP_025045596.1 pseudouridine synthase [Sulfitobacter sp. MM-124]
571353537~WP_038076752.1 pseudouridine synthase [Thioclava pacifica]
29Bins|all_bins.fasta_bin_4~98
1Bins|all_bins.fasta_bin_19~1406
169Bins|all_bins.fasta_bin_3~197
323Bins|all_bins.fasta_bin_12~496
330Bins|all_bins.fasta_bin_12~2121
351Bins|all_bins.fasta_bin_12~511
337Bins|all_bins.fasta_bin_12~435
344Bins|all_bins.fasta_bin_12~1027
316Bins|all_bins.fasta_bin_9~767
218Bins|all_bins.fasta_bin_1~464
197Bins|all_bins.fasta_bin_47~1670
204Bins|Cell_7.fasta_bin_1~38
211Bins|Cell_8.fasta_bin_1~41
239Bins|all_bins.fasta_bin_10~531
246Bins|all_bins.fasta_bin_47~1212
232Bins|Cell_14.fasta_bin_2~30
260Bins|all_bins.fasta_bin_25~1949
274Bins|all_bins.fasta_bin_10~664
253Bins|all_bins.fasta_bin_10~1379
267Bins|all_bins.fasta_bin_34~1374
225Bins|all_bins.fasta_bin_20~1343
183Bins|all_bins.fasta_bin_44~1944
190Bins|all_bins.fasta_bin_25~1623
176Bins|all_bins.fasta_bin_47~1164
281Bins|all_bins.fasta_bin_1~93
288Bins|Cell_3.fasta_bin_1~9
295Bins|Cell_3.fasta_bin_1~9
302Bins|all_bins.fasta_bin_20~1079
309Bins|Cell_12.fasta_bin_1~95
596Bins|all_bins.fasta_bin_11~650
36206403~WP_046550545.1 pseudouridine synthase [Sulfurovum lithotrophicum]
43387093~WP_012083601.1 tRNA pseudouridine synthase A [Sulfurovum sp. NBC37-1]
828034~WP_053961139.1 hypothetical protein [Sulfobacillus thermosulfidooxidans]
15679936~YP_005255558.1 tRNA pseudouridine synthase A [Sulfobacillus acidophilus DSM 10332]
78159290~WP_031386473.1 pseudouridine synthase [Desulfonatronum thiodismutans]
64469381~WP_005661758.1 tRNA pseudouridine synthase A [Dethiosulfovibrio peptidovorans]
71Bins|all_bins.fasta_bin_4~99
617Bins|all_bins.fasta_bin_19~1536
624Bins|all_bins.fasta_bin_19~1250
631Bins|all_bins.fasta_bin_19~1407
22Bins|all_bins.fasta_bin_11~651
610355068~WP_008516269.1 tRNA pseudouridine synthase A [Dethiobacter alkaliphilus]
603204536~WP_012674283.1 tRNA pseudouridine synthase A [Sulfurihydrogenibium azorense]
365292415~WP_041433141.1 tRNA pseudouridine synthase A [Thiobacillus denitrificans]
141243365~WP_011136311.1 tRNA pseudouridine synthase A [Chromobacterium violaceum]
99762376~WP_013392888.1 tRNA pseudouridine synthase A [Achromobacter xylosoxidans]
3581163617~WP_009206332.1 tRNA pseudouridine synthase A [Sulfuricella denitrificans]
3721223802~WP_041098005.1 tRNA pseudouridine synthase A [Sulfuritalea hydrogenivorans]
92290398~WP_043558076.1 pseudouridine synthase [Chromohalobacter salexigens]
120870167~WP_002710040.1 tRNA pseudouridine synthase A [Thiothrix nivea]
106717773~WP_013835174.1 tRNA pseudouridine synthase A [Thioalkalimicrobium cyclicum]
113317025~YP_391071.1 tRNA pseudouridine synthase A [Thiomicrospira crunogena XCL-2]
134990288~WP_038472193.1 tRNA pseudouridine synthase A [Acidithiobacillus caldus]
3931076586~WP_041066792.1 hypothetical protein [Thiolapillus brandeum]
1551442136~WP_063464977.1 tRNA pseudouridine(38,39,40) synthase TruA [Ectothiorhodospira sp. BSL-9]
400765914~WP_040853071.1 tRNA pseudouridine synthase A [Thiorhodospira sibirica]
127555778~WP_041600781.1 tRNA pseudouridine synthase A, partial [Halothiobacillus neapolitanus]
1461255043~WP_015258029.1 tRNA pseudouridine synthase A [Thioalkalivibrio nitratireducens]
554Bins|all_bins.fasta_bin_9~766
568Bins|all_bins.fasta_bin_3~198
5611431686~WP_058554316.1 pseudouridine synthase [Thiohalocapsa sp. ML1]
575765912~WP_041604479.1 pseudouridine synthase, partial [Thioflavicoccus mobilis]
582766671~WP_040732233.1 pseudouridine synthase, partial [Thiocapsa marina]
589765913~WP_007042526.1 tRNA pseudouridine synthase A [Thiorhodococcus drewsii]
477Bins|all_bins.fasta_bin_47~1213
470Bins|all_bins.fasta_bin_20~1344
435Bins|all_bins.fasta_bin_34~1373
456Bins|all_bins.fasta_bin_10~665
428Bins|all_bins.fasta_bin_10~532
442Bins|all_bins.fasta_bin_75~1918
449Bins|Cell_14.fasta_bin_2~31
498Bins|Cell_7.fasta_bin_1~39
484Bins|Cell_8.fasta_bin_1~42
491Bins|all_bins.fasta_bin_47~1671
463Bins|all_bins.fasta_bin_25~1624
505Bins|all_bins.fasta_bin_44~1945
379412965~WP_011930255.1 tRNA pseudouridine synthase A [Calyptogena okutanii thioautotrophic gill symbiont]
3861125411~WP_053820603.1 pseudouridine synthase [Candidatus Thioglobus singularis]
512Bins|all_bins.fasta_bin_1~94
519Bins|Cell_3.fasta_bin_1~10
526Bins|Cell_3.fasta_bin_1~10
533Bins|all_bins.fasta_bin_20~1080
540Bins|Cell_12.fasta_bin_1~96
407Bins|all_bins.fasta_bin_12~512
414Bins|all_bins.fasta_bin_12~436
421Bins|all_bins.fasta_bin_12~1028
547Bins|all_bins.fasta_bin_12~497
1.0

RBFA.treee.tre.exp.tree.pdf

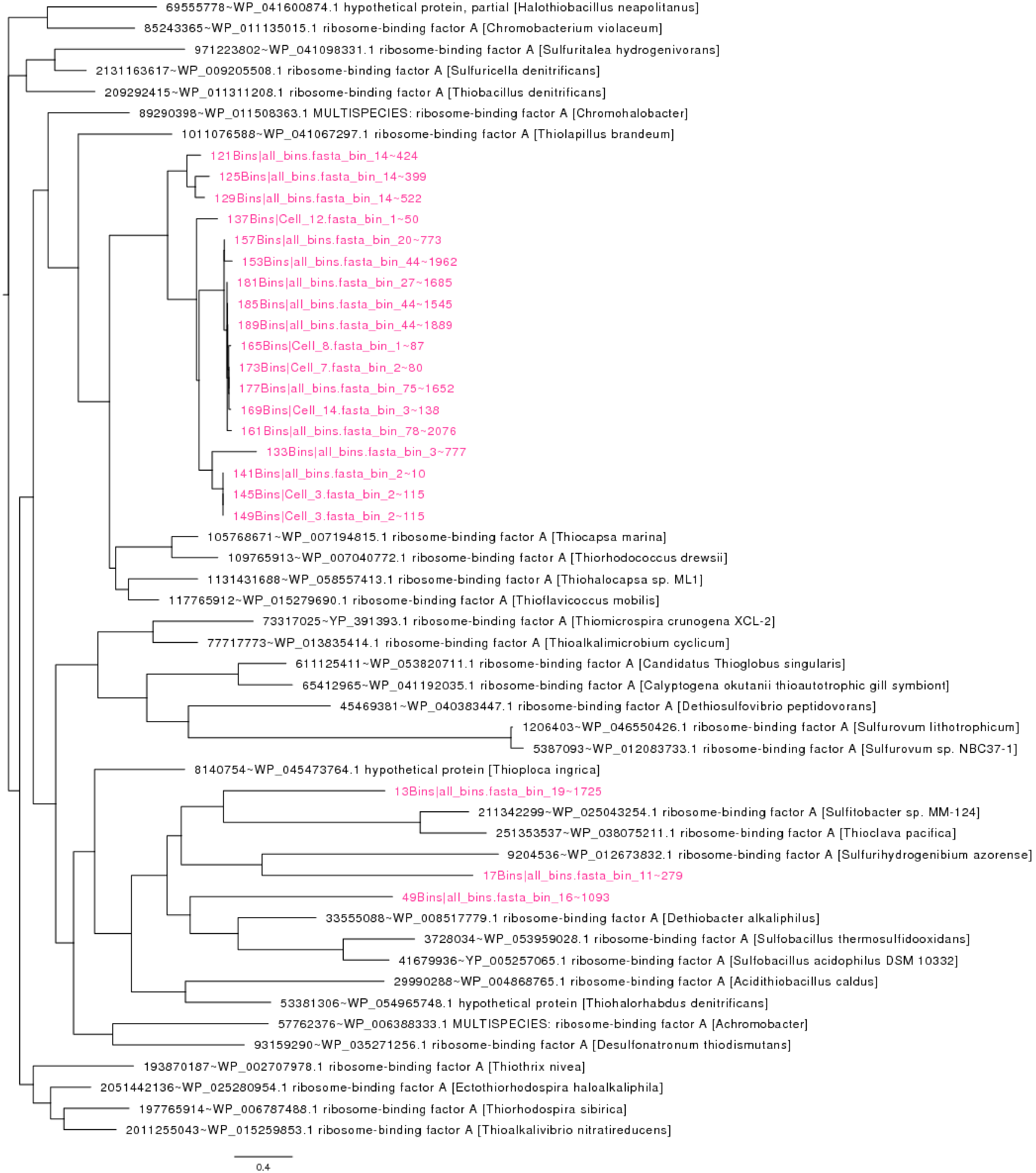

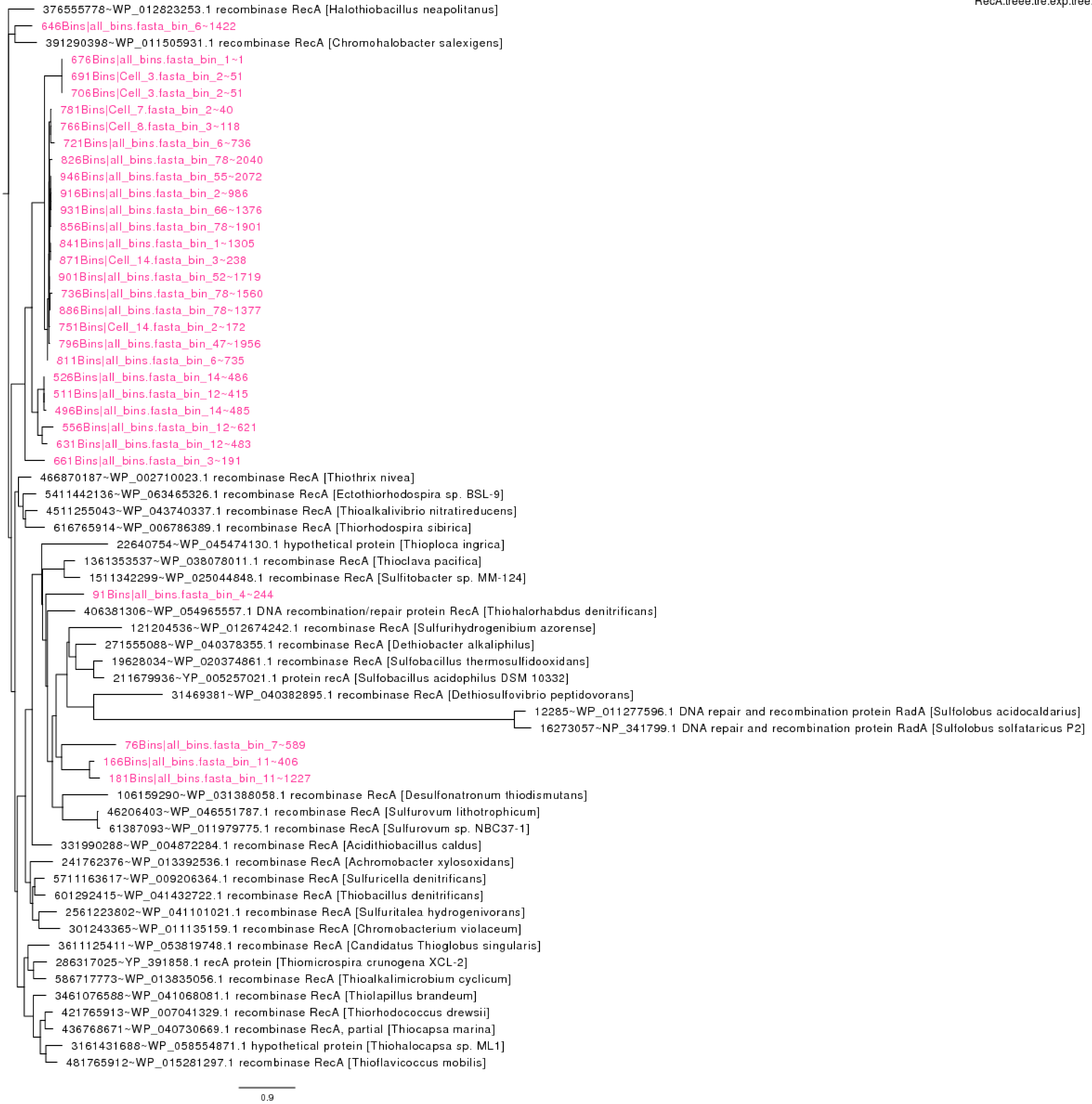
376555778~WP_012823253.1 recombinase RecA [Halothiobacillus neapolitanus]
646Bins|all_bins.fasta_bin_6~1422
391290398~WP_011505931.1 recombinase RecA [Chromohalobacter salexigens]
676Bins|all_bins.fasta_bin_1~1
691Bins|Cell_3.fasta_bin_2~51
706Bins|Cell_3.fasta_bin_2~51
781Bins|Cell_7.fasta_bin_2~40
766Bins|Cell_8.fasta_bin_3~118
721Bins|all_bins.fasta_bin_6~736
826Bins|all_bins.fasta_bin_78~2040
946Bins|all_bins.fasta_bin_55~2072
916Bins|all_bins.fasta_bin_2~986
931Bins|all_bins.fasta_bin_66~1376
856Bins|all_bins.fasta_bin_78~1901
841Bins|all_bins.fasta_bin_1~1305
871Bins|Cell_14.fasta_bin_3~238
901Bins|all_bins.fasta_bin_52~1719
736Bins|all_bins.fasta_bin_78~1560
886Bins|all_bins.fasta_bin_78~1377
751Bins|Cell_14.fasta_bin_2~172
796Bins|all_bins.fasta_bin_47~1956
811Bins|all_bins.fasta_bin_6~735
526Bins|all_bins.fasta_bin_14~486
511Bins|all_bins.fasta_bin_12~415
496Bins|all_bins.fasta_bin_14~485
556Bins|all_bins.fasta_bin_12~621
631Bins|all_bins.fasta_bin_12~483
661Bins|all_bins.fasta_bin_3~191
466870187~WP_002710023.1 recombinase RecA [Thiothrix nivea]
5411442136~WP_063465326.1 recombinase RecA [Ectothiorhodospira sp. BSL-9]
4511255043~WP_043740337.1 recombinase RecA [Thioalkalivibrio nitratireducens]
616765914~WP_006786389.1 recombinase RecA [Thiorhodospira sibirica]
22640754~WP_045474130.1 hypothetical protein [Thioploca ingrica]
1361353537~WP_038078011.1 recombinase RecA [Thioclava pacifica]
1511342299~WP_025044848.1 recombinase RecA [Sulfitobacter sp. MM-124]
91Bins|all_bins.fasta_bin_4~244
406381306~WP_054965557.1 DNA recombination/repair protein RecA [Thiohalorhabdus denitrificans]
121204536~WP_012674242.1 recombinase RecA [Sulfurihydrogenibium azorense]
271555088~WP_040378355.1 recombinase RecA [Dethiobacter alkaliphilus]
19628034~WP_020374861.1 recombinase RecA [Sulfobacillus thermosulfidooxidans]
211679936~YP_005257021.1 protein recA [Sulfobacillus acidophilus DSM 10332]
31469381~WP_040382895.1 recombinase RecA [Dethiosulfovibrio peptidovorans]
12285~WP_011277596.1 DNA repair and recombination protein RadA [Sulfolobus acidocaldarius]
16273057~NP_341799.1 DNA repair and recombination protein RadA [Sulfolobus solfataricus P2]
76Bins|all_bins.fasta_bin_7~589
166Bins|all_bins.fasta_bin_11~406
181Bins|all_bins.fasta_bin_11~1227
106159290~WP_031388058.1 recombinase RecA [Desulfonatronum thiodismutans]
46206403~WP_046551787.1 recombinase RecA [Sulfurovum lithotrophicum]
61387093~WP_011979775.1 recombinase RecA [Sulfurovum sp. NBC37-1]
331990288~WP_004872284.1 recombinase RecA [Acidithiobacillus caldus]
241762376~WP_013392536.1 recombinase RecA [Achromobacter xylosoxidans]
5711163617~WP_009206364.1 recombinase RecA [Sulfuricella denitrificans]
601292415~WP_041432722.1 recombinase RecA [Thiobacillus denitrificans]
2561223802~WP_041101021.1 recombinase RecA [Sulfuritalea hydrogenivorans]
301243365~WP_011135159.1 recombinase RecA [Chromobacterium violaceum]
3611125411~WP_053819748.1 recombinase RecA [Candidatus Thioglobus singularis]
286317025~YP_391858.1 recA protein [Thiomicrospira crunogena XCL-2]
586717773~WP_013835056.1 recombinase RecA [Thioalkalimicrobium cyclicum]
3461076588~WP_041068081.1 recombinase RecA [Thiolapillus brandeum]
421765913~WP_007041329.1 recombinase RecA [Thiorhodococcus drewsii]
436768671~WP_040730669.1 recombinase RecA, partial [Thiocapsa marina]
3161431688~WP_058554871.1 hypothetical protein [Thiohalocapsa sp. ML1]
481765912~WP_015281297.1 recombinase RecA [Thioflavicoccus mobilis]
0.9

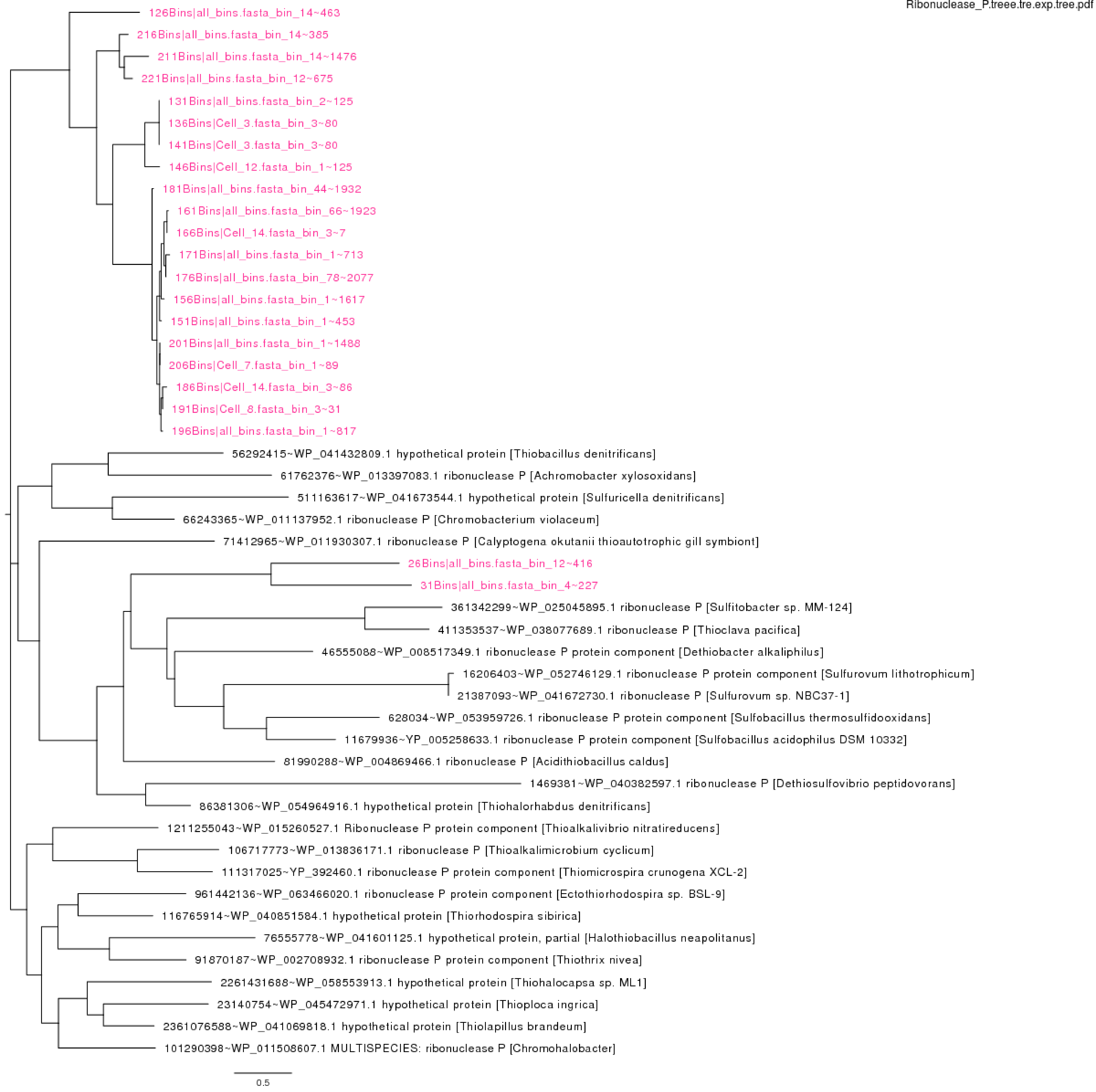
Ribonuclease_P.treee.tre.exp.tree.pdf
126Bins|all_bins.fasta_bin_14~463
216Bins|all_bins.fasta_bin_14~385
211Bins|all_bins.fasta_bin_14~1476
221Bins|all_bins.fasta_bin_12~675
131Bins|all_bins.fasta_bin_2~125
136Bins|Cell_3.fasta_bin_3~80
141Bins|Cell_3.fasta_bin_3~80
146Bins|Cell_12.fasta_bin_1~125
181Bins|all_bins.fasta_bin_44~1932
161Bins|all_bins.fasta_bin_66~1923
166Bins|Cell_14.fasta_bin_3~7
171Bins|all_bins.fasta_bin_1~713
176Bins|all_bins.fasta_bin_78~2077
156Bins|all_bins.fasta_bin_1~1617
151Bins|all_bins.fasta_bin_1~453
201Bins|all_bins.fasta_bin_1~1488
206Bins|Cell_7.fasta_bin_1~89
186Bins|Cell_14.fasta_bin_3~86
191Bins|Cell_8.fasta_bin_3~31
196Bins|all_bins.fasta_bin_1~817
56292415~WP_041432809.1 hypothetical protein [Thiobacillus denitrificans]
61762376~WP_013397083.1 ribonuclease P [Achromobacter xylosoxidans]
511163617~WP_041673544.1 hypothetical protein [Sulfuricella denitrificans]
66243365~WP_011137952.1 ribonuclease P [Chromobacterium violaceum]
71412965~WP_011930307.1 ribonuclease P [Calyptogena okutanii thioautotrophic gill symbiont]
26Bins|all_bins.fasta_bin_12~416
31Bins|all_bins.fasta_bin_4~227
361342299~WP_025045895.1 ribonuclease P [Sulfitobacter sp. MM-124]
411353537~WP_038077689.1 ribonuclease P [Thioclava pacifica]
46555088~WP_008517349.1 ribonuclease P protein component [Dethiobacter alkaliphilus]
16206403~WP_052746129.1 ribonuclease P protein component [Sulfurovum lithotrophicum]
21387093~WP_041672730.1 ribonuclease P [Sulfurovum sp. NBC37-1]
628034~WP_053959726.1 ribonuclease P protein component [Sulfobacillus thermosulfidooxidans]
11679936~YP_005258633.1 ribonuclease P protein component [Sulfobacillus acidophilus DSM 10332]
81990288~WP_004869466.1 ribonuclease P [Acidithiobacillus caldus]
1469381~WP_040382597.1 ribonuclease P [Dethiosulfovibrio peptidovorans]
86381306~WP_054964916.1 hypothetical protein [Thiohalorhabdus denitrificans]
1211255043~WP_015260527.1 Ribonuclease P protein component [Thioalkalivibrio nitratireducens]
106717773~WP_013836171.1 ribonuclease P [Thioalkalimicrobium cyclicum]
111317025~YP_392460.1 ribonuclease P protein component [Thiomicrospira crunogena XCL-2]
961442136~WP_063466020.1 ribonuclease P protein component [Ectothiorhodospira sp. BSL-9]
116765914~WP_040851584.1 hypothetical protein [Thiorhodospira sibirica]
76555778~WP_041601125.1 hypothetical protein, partial [Halothiobacillus neapolitanus]
91870187~WP_002708932.1 ribonuclease P protein component [Thiothrix nivea]
2261431688~WP_058553913.1 hypothetical protein [Thiohalocapsa sp. ML1]
23140754~WP_045472971.1 hypothetical protein [Thioploca ingrica]
2361076588~WP_041069818.1 hypothetical protein [Thiolapillus brandeum]
101290398~WP_011508607.1 MULTISPECIES: ribonuclease P [Chromohalobacter]
0.5

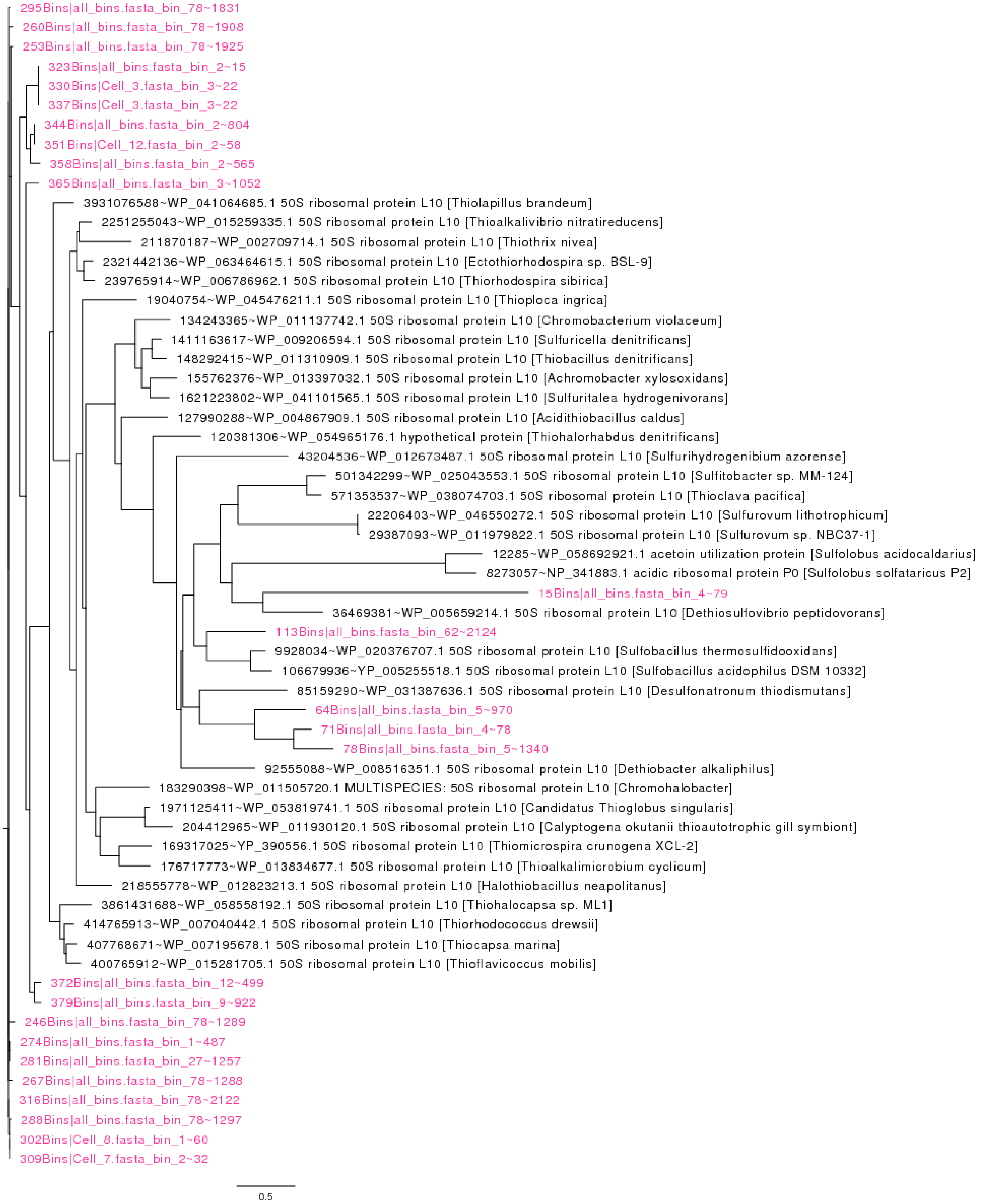
295Bins|all_bins.fasta_bin_78~1831
260Bins|all_bins.fasta_bin_78~1908
253Bins|all_bins.fasta_bin_78~1925
323Bins|all_bins.fasta_bin_2~15
330Bins|Cell_3.fasta_bin_3~22
337Bins|Cell_3.fasta_bin_3~22
344Bins|all_bins.fasta_bin_2~804
351Bins|Cell_12.fasta_bin_2~58
358Bins|all_bins.fasta_bin_2~565
365Bins|all_bins.fasta_bin_3~1052
3931076588~WP_041064685.1 50S ribosomal protein L10 [Thiolapillus brandeum]
2251255043~WP_015259335.1 50S ribosomal protein L10 [Thioalkalivibrio nitratireducens]
211870187~WP_002709714.1 50S ribosomal protein L10 [Thiothrix nivea]
2321442136~WP_063464615.1 50S ribosomal protein L10 [Ectothiorhodospira sp. BSL-9]
239765914~WP_006786962.1 50S ribosomal protein L10 [Thiorhodospira sibirica]
19040754~WP_045476211.1 50S ribosomal protein L10 [Thioploca ingrica]
134243365~WP_011137742.1 50S ribosomal protein L10 [Chromobacterium violaceum]
1411163617~WP_009206594.1 50S ribosomal protein L10 [Sulfuricella denitrificans]
148292415~WP_011310909.1 50S ribosomal protein L10 [Thiobacillus denitrificans]
155762376~WP_013397032.1 50S ribosomal protein L10 [Achromobacter xylosoxidans]
1621223802~WP_041101565.1 50S ribosomal protein L10 [Sulfuritalea hydrogenivorans]
127990288~WP_004867909.1 50S ribosomal protein L10 [Acidithiobacillus caldus]
120381306~WP_054965176.1 hypothetical protein [Thiohalorhabdus denitrificans]
43204536~WP_012673487.1 50S ribosomal protein L10 [Sulfurihydrogenibium azorense]
501342299~WP_025043553.1 50S ribosomal protein L10 [Sulfitobacter sp. MM-124]
571353537~WP_038074703.1 50S ribosomal protein L10 [Thioclava pacifica]
22206403~WP_046550272.1 50S ribosomal protein L10 [Sulfurovum lithotrophicum]
29387093~WP_011979822.1 50S ribosomal protein L10 [Sulfurovum sp. NBC37-1]
12285~WP_058692921.1 acetoin utilization protein [Sulfolobus acidocaldarius]
8273057~NP_341883.1 acidic ribosomal protein P0 [Sulfolobus solfataricus P2]
15Bins|all_bins.fasta_bin_4~79
36469381~WP_005659214.1 50S ribosomal protein L10 [Dethiosulfovibrio peptidovorans]
113Bins|all_bins.fasta_bin_62~2124
9928034~WP_020376707.1 50S ribosomal protein L10 [Sulfobacillus thermosulfidooxidans]
106679936~YP_005255518.1 50S ribosomal protein L10 [Sulfobacillus acidophilus DSM 10332]
85159290~WP_031387636.1 50S ribosomal protein L10 [Desulfonatronum thiodismutans]
64Bins|all_bins.fasta_bin_5~970
71Bins|all_bins.fasta_bin_4~78
78Bins|all_bins.fasta_bin_5~1340
92555088~WP_008516351.1 50S ribosomal protein L10 [Dethiobacter alkaliphilus]
183290398~WP_011505720.1 MULTISPECIES: 50S ribosomal protein L10 [Chromohalobacter]
1971125411~WP_053819741.1 50S ribosomal protein L10 [Candidatus Thioglobus singularis]
204412965~WP_011930120.1 50S ribosomal protein L10 [Calyptogena okutanii thioautotrophic gill symbiont]
169317025~YP_390556.1 50S ribosomal protein L10 [Thiomicrospira crunogena XCL-2]
176717773~WP_013834677.1 50S ribosomal protein L10 [Thioalkalimicrobium cyclicum]
218555778~WP_012823213.1 50S ribosomal protein L10 [Halothiobacillus neapolitanus]
3861431688~WP_058558192.1 50S ribosomal protein L10 [Thiohalocapsa sp. ML1]
414765913~WP_007040442.1 50S ribosomal protein L10 [Thiorhodococcus drewsii]
407768671~WP_007195678.1 50S ribosomal protein L10 [Thiocapsa marina]
400765912~WP_015281705.1 50S ribosomal protein L10 [Thioflavicoccus mobilis]
372Bins|all_bins.fasta_bin_12~499
379Bins|all_bins.fasta_bin_9~922
246Bins|all_bins.fasta_bin_78~1289
274Bins|all_bins.fasta_bin_1~487
281Bins|all_bins.fasta_bin_27~1257
267Bins|all_bins.fasta_bin_78~1288
316Bins|all_bins.fasta_bin_78~2122
288Bins|all_bins.fasta_bin_78~1297
302Bins|Cell_8.fasta_bin_1~60
309Bins|Cell_7.fasta_bin_2~32
0.5

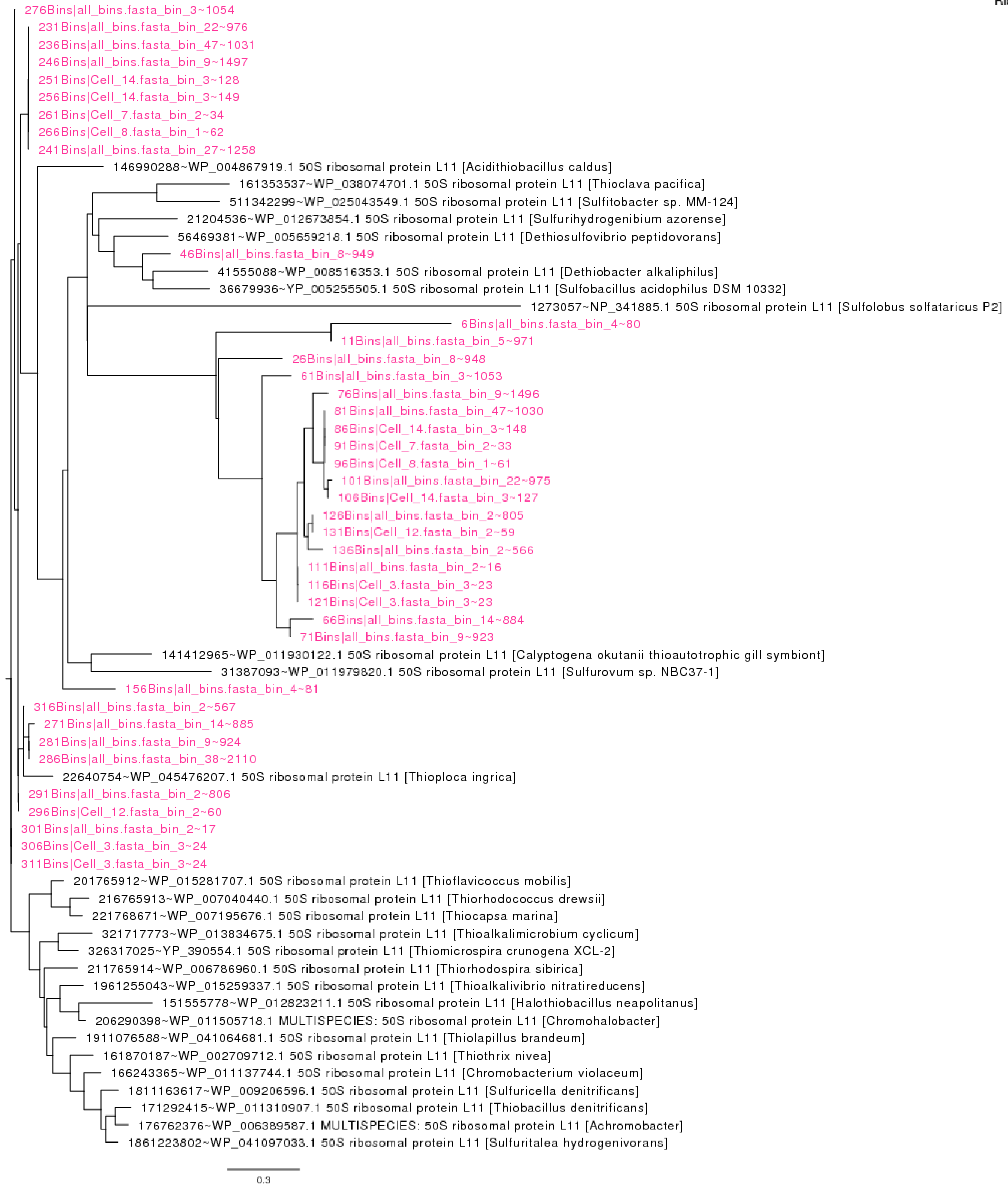
276Bins|all_bins.fasta_bin_3~1054
231Bins|all_bins.fasta_bin_22~976
236Bins|all_bins.fasta_bin_47~1031
246Bins|all_bins.fasta_bin_9~1497
251Bins|Cell_14.fasta_bin_3~128
256Bins|Cell_14.fasta_bin_3~149
261Bins|Cell_7.fasta_bin_2~34
266Bins|Cell_8.fasta_bin_1~62
241Bins|all_bins.fasta_bin_27~1258
146990288~WP_004867919.1 50S ribosomal protein L11 [Acidithiobacillus caldus]
161353537~WP_038074701.1 50S ribosomal protein L11 [Thioclava pacifica]
511342299~WP_025043549.1 50S ribosomal protein L11 [Sulfitobacter sp. MM-124]
21204536~WP_012673854.1 50S ribosomal protein L11 [Sulfurihydrogenibium azorense]
56469381~WP_005659218.1 50S ribosomal protein L11 [Dethiosulfovibrio peptidovorans]
46Bins|all_bins.fasta_bin_8~949
41555088~WP_008516353.1 50S ribosomal protein L11 [Dethiobacter alkaliphilus]
36679936~YP_005255505.1 50S ribosomal protein L11 [Sulfobacillus acidophilus DSM 10332]
1273057~NP_341885.1 50S ribosomal protein L11 [Sulfolobus solfataricus P2]
6Bins|all_bins.fasta_bin_4~80
11Bins|all_bins.fasta_bin_5~971
26Bins|all_bins.fasta_bin_8~948
61Bins|all_bins.fasta_bin_3~1053
76Bins|all_bins.fasta_bin_9~1496
81Bins|all_bins.fasta_bin_47~1030
86Bins|Cell_14.fasta_bin_3~148
91Bins|Cell_7.fasta_bin_2~33
96Bins|Cell_8.fasta_bin_1~61
101Bins|all_bins.fasta_bin_22~975
106Bins|Cell_14.fasta_bin_3~127
126Bins|all_bins.fasta_bin_2~805
131Bins|Cell_12.fasta_bin_2~59
136Bins|all_bins.fasta_bin_2~566
111Bins|all_bins.fasta_bin_2~16
116Bins|Cell_3.fasta_bin_3~23
121Bins|Cell_3.fasta_bin_3~23
66Bins|all_bins.fasta_bin_14~884
71Bins|all_bins.fasta_bin_9~923
141412965~WP_011930122.1 50S ribosomal protein L11 [Calyptogena okutanii thioautotrophic gill symbiont]
31387093~WP_011979820.1 50S ribosomal protein L11 [Sulfurovum sp. NBC37-1]
156Bins|all_bins.fasta_bin_4~81
316Bins|all_bins.fasta_bin_2~567
271Bins|all_bins.fasta_bin_14~885
281Bins|all_bins.fasta_bin_9~924
286Bins|all_bins.fasta_bin_38~2110
22640754~WP_045476207.1 50S ribosomal protein L11 [Thioploca ingrica]
291Bins|all_bins.fasta_bin_2~806
296Bins|Cell_12.fasta_bin_2~60
301Bins|all_bins.fasta_bin_2~17
306Bins|Cell_3.fasta_bin_3~24
311Bins|Cell_3.fasta_bin_3~24
201765912~WP_015281707.1 50S ribosomal protein L11 [Thioflavicoccus mobilis]
216765913~WP_007040440.1 50S ribosomal protein L11 [Thiorhodococcus drewsii]
221768671~WP_007195676.1 50S ribosomal protein L11 [Thiocapsa marina]
321717773~WP_013834675.1 50S ribosomal protein L11 [Thioalkalimicrobium cyclicum]
326317025~YP_390554.1 50S ribosomal protein L11 [Thiomicrospira crunogena XCL-2]
211765914~WP_006786960.1 50S ribosomal protein L11 [Thiorhodospira sibirica]
1961255043~WP_015259337.1 50S ribosomal protein L11 [Thioalkalivibrio nitratireducens]
151555778~WP_012823211.1 50S ribosomal protein L11 [Halothiobacillus neapolitanus]
206290398~WP_011505718.1 MULTISPECIES: 50S ribosomal protein L11 [Chromohalobacter]
1911076588~WP_041064681.1 50S ribosomal protein L11 [Thiolapillus brandeum]
161870187~WP_002709712.1 50S ribosomal protein L11 [Thiothrix nivea]
166243365~WP_011137744.1 50S ribosomal protein L11 [Chromobacterium violaceum]
1811163617~WP_009206596.1 50S ribosomal protein L11 [Sulfuricella denitrificans]
171292415~WP_011310907.1 50S ribosomal protein L11 [Thiobacillus denitrificans]
176762376~WP_006389587.1 MULTISPECIES: 50S ribosomal protein L11 [Achromobacter]
1861223802~WP_041097033.1 50S ribosomal protein L11 [Sulfuritalea hydrogenivorans]
0.3

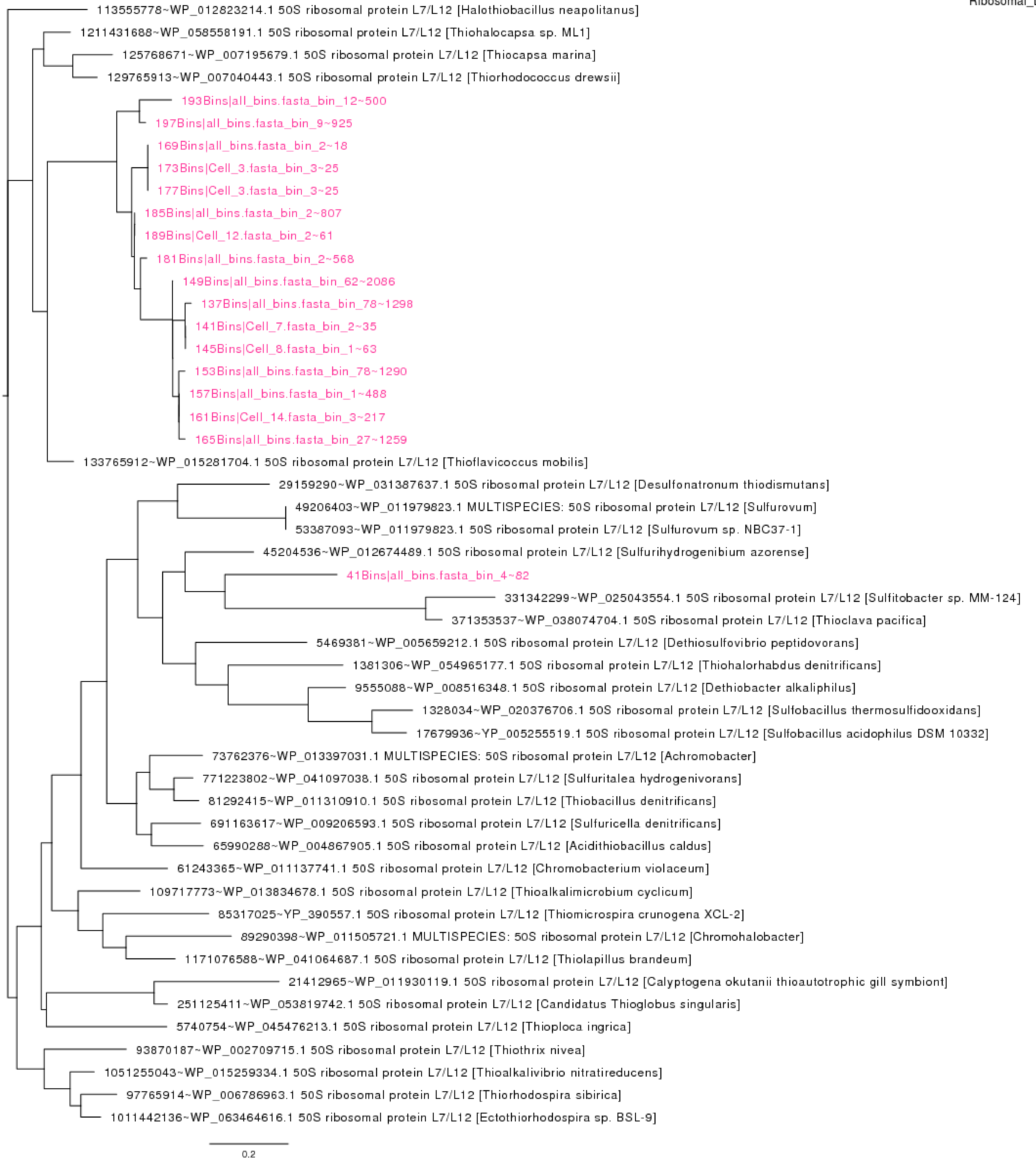
113555778~WP_012823214.1 50S ribosomal protein L7/L12 [Halothiobacillus neapolitanus]
1211431688~WP_058558191.1 50S ribosomal protein L7/L12 [Thiohalocapsa sp. ML1]
125768671~WP_007195679.1 50S ribosomal protein L7/L12 [Thiocapsa marina]
129765913~WP_007040443.1 50S ribosomal protein L7/L12 [Thiorhodococcus drewsii]
193Bins|all_bins.fasta_bin_12~500
197Bins|all_bins.fasta_bin_9~925
169Bins|all_bins.fasta_bin_2~18
173Bins|Cell_3.fasta_bin_3~25
177Bins|Cell_3.fasta_bin_3~25
185Bins|all_bins.fasta_bin_2~807
189Bins|Cell_12.fasta_bin_2~61
181Bins|all_bins.fasta_bin_2~568
149Bins|all_bins.fasta_bin_62~2086
137Bins|all_bins.fasta_bin_78~1298
141Bins|Cell_7.fasta_bin_2~35
145Bins|Cell_8.fasta_bin_1~63
153Bins|all_bins.fasta_bin_78~1290
157Bins|all_bins.fasta_bin_1~488
161Bins|Cell_14.fasta_bin_3~217
165Bins|all_bins.fasta_bin_27~1259
133765912~WP_015281704.1 50S ribosomal protein L7/L12 [Thioflavicoccus mobilis]
29159290~WP_031387637.1 50S ribosomal protein L7/L12 [Desulfonatronum thiodismutans]
49206403~WP_011979823.1 MULTISPECIES: 50S ribosomal protein L7/L12 [Sulfurovum]
53387093~WP_011979823.1 50S ribosomal protein L7/L12 [Sulfurovum sp. NBC37-1]
45204536~WP_012674489.1 50S ribosomal protein L7/L12 [Sulfurihydrogenibium azorense]
41Bins|all_bins.fasta_bin_4~82
331342299~WP_025043554.1 50S ribosomal protein L7/L12 [Sulfitobacter sp. MM-124]
371353537~WP_038074704.1 50S ribosomal protein L7/L12 [Thioclava pacifica]
5469381~WP_005659212.1 50S ribosomal protein L7/L12 [Dethiosulfovibrio peptidovorans]
1381306~WP_054965177.1 50S ribosomal protein L7/L12 [Thiohalorhabdus denitrificans]
9555088~WP_008516348.1 50S ribosomal protein L7/L12 [Dethiobacter alkaliphilus]
1328034~WP_020376706.1 50S ribosomal protein L7/L12 [Sulfobacillus thermosulfidooxidans]
17679936~YP_005255519.1 50S ribosomal protein L7/L12 [Sulfobacillus acidophilus DSM 10332]
73762376~WP_013397031.1 MULTISPECIES: 50S ribosomal protein L7/L12 [Achromobacter]
771223802~WP_041097038.1 50S ribosomal protein L7/L12 [Sulfuritalea hydrogenivorans]
81292415~WP_011310910.1 50S ribosomal protein L7/L12 [Thiobacillus denitrificans]
691163617~WP_009206593.1 50S ribosomal protein L7/L12 [Sulfuricella denitrificans]
65990288~WP_004867905.1 50S ribosomal protein L7/L12 [Acidithiobacillus caldus]
61243365~WP_011137741.1 50S ribosomal protein L7/L12 [Chromobacterium violaceum]
109717773~WP_013834678.1 50S ribosomal protein L7/L12 [Thioalkalimicrobium cyclicum]
85317025~YP_390557.1 50S ribosomal protein L7/L12 [Thiomicrospira crunogena XCL-2]
89290398~WP_011505721.1 MULTISPECIES: 50S ribosomal protein L7/L12 [Chromohalobacter]
1171076588~WP_041064687.1 50S ribosomal protein L7/L12 [Thiolapillus brandeum]
21412965~WP_011930119.1 50S ribosomal protein L7/L12 [Calyptogena okutanii thioautotrophic gill symbiont]
251125411~WP_053819742.1 50S ribosomal protein L7/L12 [Candidatus Thioglobus singularis]
5740754~WP_045476213.1 50S ribosomal protein L7/L12 [Thioploca ingrica]
93870187~WP_002709715.1 50S ribosomal protein L7/L12 [Thiothrix nivea]
1051255043~WP_015259334.1 50S ribosomal protein L7/L12 [Thioalkalivibrio nitratireducens]
97765914~WP_006786963.1 50S ribosomal protein L7/L12 [Thiorhodospira sibirica]
1011442136~WP_063464616.1 50S ribosomal protein L7/L12 [Ectothiorhodospira sp. BSL-9]
0.2

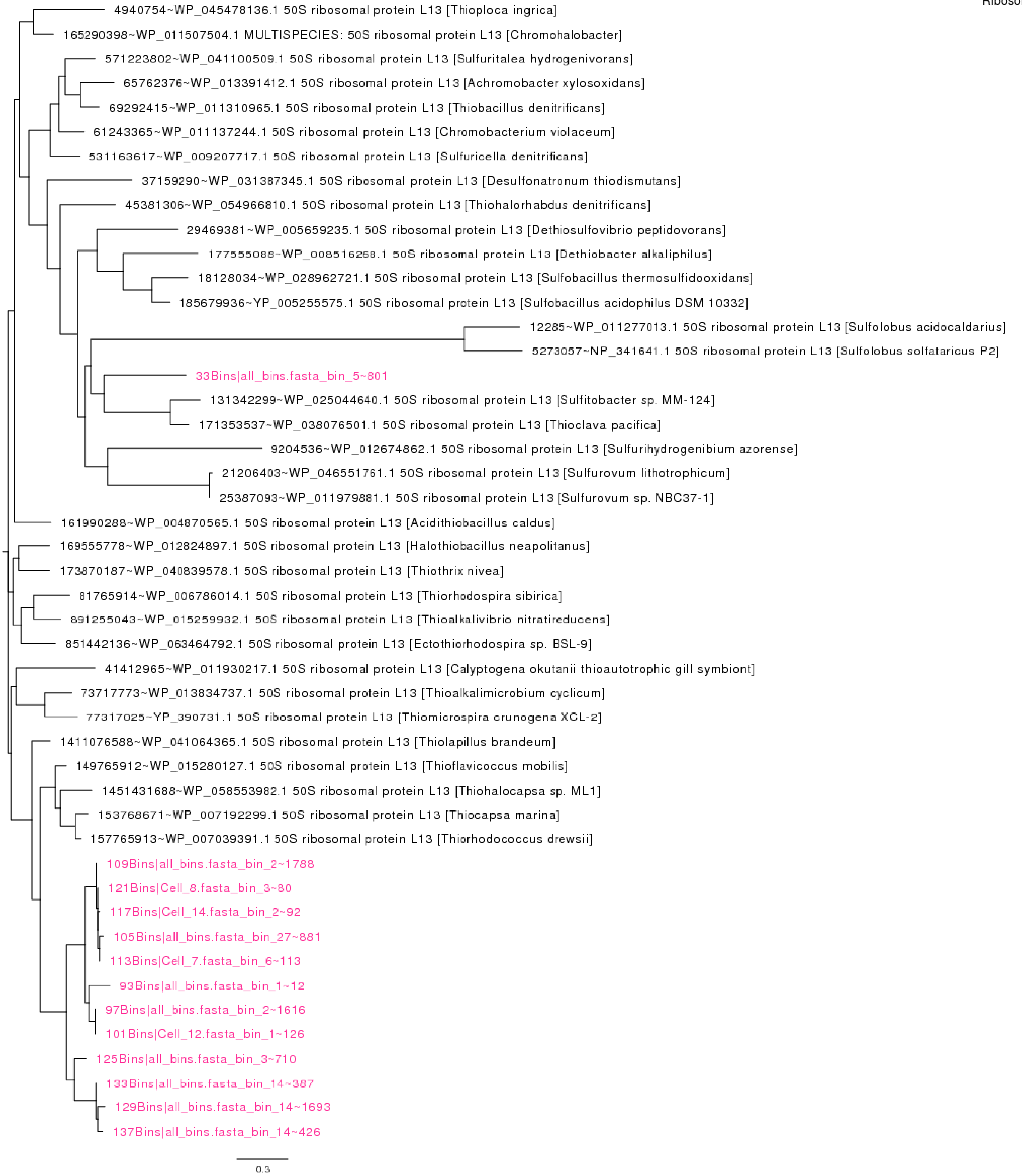
4940754~WP_045478136.1 50S ribosomal protein L13 [Thioploca ingrica]
165290398~WP_011507504.1 MULTISPECIES: 50S ribosomal protein L13 [Chromohalobacter]
571223802~WP_041100509.1 50S ribosomal protein L13 [Sulfuritalea hydrogenivorans]
65762376~WP_013391412.1 50S ribosomal protein L13 [Achromobacter xylosoxidans]
69292415~WP_011310965.1 50S ribosomal protein L13 [Thiobacillus denitrificans]
61243365~WP_011137244.1 50S ribosomal protein L13 [Chromobacterium violaceum]
531163617~WP_009207717.1 50S ribosomal protein L13 [Sulfuricella denitrificans]
37159290~WP_031387345.1 50S ribosomal protein L13 [Desulfonatronum thiodismutans]
45381306~WP_054966810.1 50S ribosomal protein L13 [Thiohalorhabdus denitrificans]
29469381~WP_005659235.1 50S ribosomal protein L13 [Dethiosulfovibrio peptidovorans]
177555088~WP_008516268.1 50S ribosomal protein L13 [Dethiobacter alkaliphilus]
18128034~WP_028962721.1 50S ribosomal protein L13 [Sulfobacillus thermosulfidooxidans]
185679936~YP_005255575.1 50S ribosomal protein L13 [Sulfobacillus acidophilus DSM 10332]
12285~WP_011277013.1 50S ribosomal protein L13 [Sulfolobus acidocaldarius]
5273057~NP_341641.1 50S ribosomal protein L13 [Sulfolobus solfataricus P2]
33Bins|all_bins.fasta_bin_5~801
131342299~WP_025044640.1 50S ribosomal protein L13 [Sulfitobacter sp. MM-124]
171353537~WP_038076501.1 50S ribosomal protein L13 [Thioclava pacifica]
9204536~WP_012674862.1 50S ribosomal protein L13 [Sulfurihydrogenibium azorense]
21206403~WP_046551761.1 50S ribosomal protein L13 [Sulfurovum lithotrophicum]
25387093~WP_011979881.1 50S ribosomal protein L13 [Sulfurovum sp. NBC37-1]
161990288~WP_004870565.1 50S ribosomal protein L13 [Acidithiobacillus caldus]
169555778~WP_012824897.1 50S ribosomal protein L13 [Halothiobacillus neapolitanus]
173870187~WP_040839578.1 50S ribosomal protein L13 [Thiothrix nivea]
81765914~WP_006786014.1 50S ribosomal protein L13 [Thiorhodospira sibirica]
891255043~WP_015259932.1 50S ribosomal protein L13 [Thioalkalivibrio nitratireducens]
851442136~WP_063464792.1 50S ribosomal protein L13 [Ectothiorhodospira sp. BSL-9]
41412965~WP_011930217.1 50S ribosomal protein L13 [Calyptogena okutanii thioautotrophic gill symbiont]
73717773~WP_013834737.1 50S ribosomal protein L13 [Thioalkalimicrobium cyclicum]
77317025~YP_390731.1 50S ribosomal protein L13 [Thiomicrospira crunogena XCL-2]
1411076588~WP_041064365.1 50S ribosomal protein L13 [Thiolapillus brandeum]
149765912~WP_015280127.1 50S ribosomal protein L13 [Thioflavicoccus mobilis]
1451431688~WP_058553982.1 50S ribosomal protein L13 [Thiohalocapsa sp. ML1]
153768671~WP_007192299.1 50S ribosomal protein L13 [Thiocapsa marina]
157765913~WP_007039391.1 50S ribosomal protein L13 [Thiorhodococcus drewsii]
109Bins|all_bins.fasta_bin_2~1788
121Bins|Cell_8.fasta_bin_3~80
117Bins|Cell_14.fasta_bin_2~92
105Bins|all_bins.fasta_bin_27~881
113Bins|Cell_7.fasta_bin_6~113
93Bins|all_bins.fasta_bin_1~12
97Bins|all_bins.fasta_bin_2~1616
101Bins|Cell_12.fasta_bin_1~126
125Bins|all_bins.fasta_bin_3~710
133Bins|all_bins.fasta_bin_14~387
129Bins|all_bins.fasta_bin_14~1693
137Bins|all_bins.fasta_bin_14~426
0.3

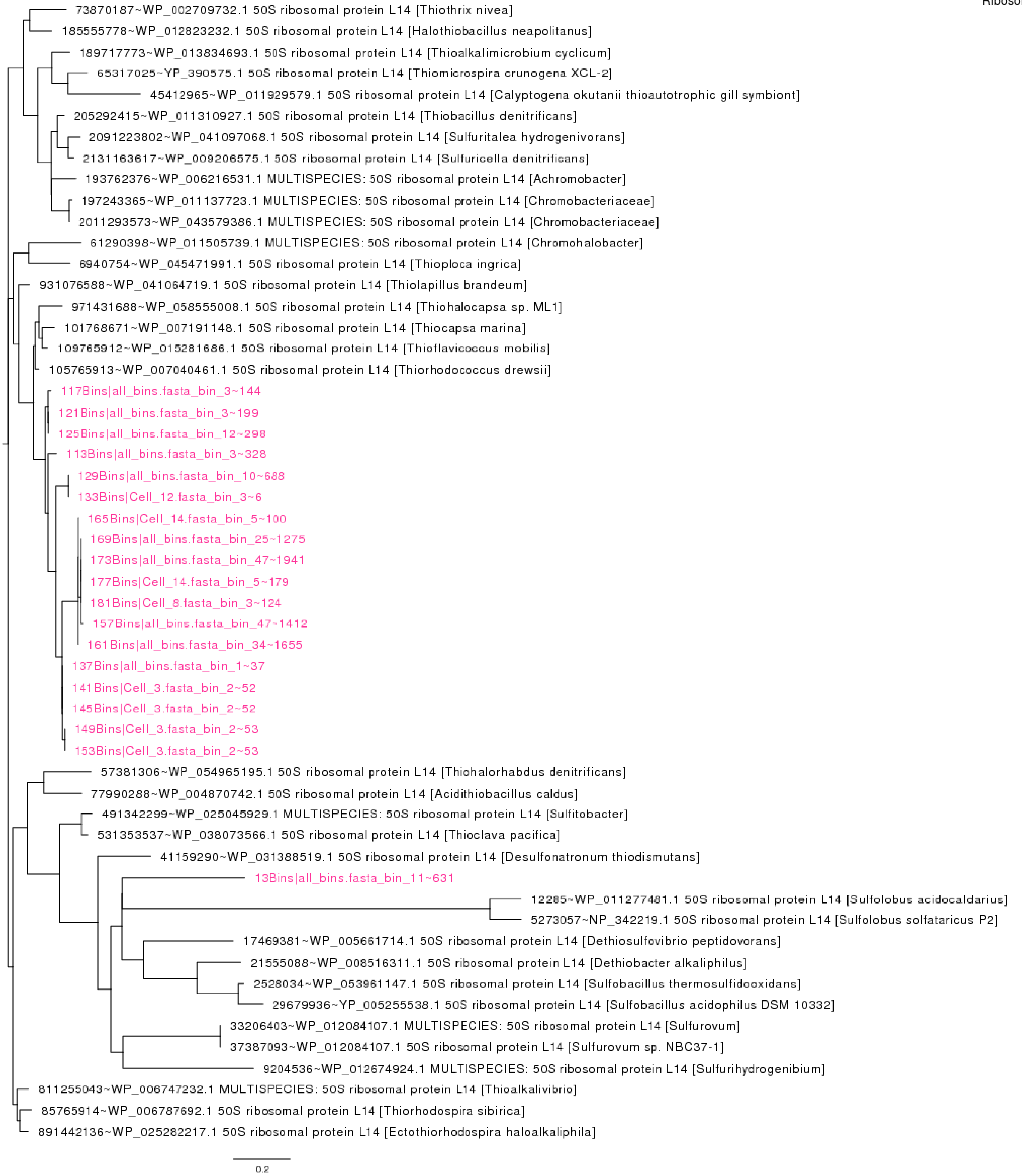

73870187~WP_002709732.1 50S ribosomal protein L14 [Thiothrix nivea]
185555778~WP_012823232.1 50S ribosomal protein L14 [Halothiobacillus neapolitanus]
189717773~WP_013834693.1 50S ribosomal protein L14 [Thioalkalimicrobium cyclicum]
65317025~YP_390575.1 50S ribosomal protein L14 [Thiomicrospira crunogena XCL-2]
45412965~WP_011929579.1 50S ribosomal protein L14 [Calyptogena okutanii thioautotrophic gill symbiont]
205292415~WP_011310927.1 50S ribosomal protein L14 [Thiobacillus denitrificans]
2091223802~WP_041097068.1 50S ribosomal protein L14 [Sulfuritalea hydrogenivorans]
2131163617~WP_009206575.1 50S ribosomal protein L14 [Sulfuricella denitrificans]
193762376~WP_006216531.1 MULTISPECIES: 50S ribosomal protein L14 [Achromobacter]
197243365~WP_011137723.1 MULTISPECIES: 50S ribosomal protein L14 [Chromobacteriaceae]
2011293573~WP_043579386.1 MULTISPECIES: 50S ribosomal protein L14 [Chromobacteriaceae]
61290398~WP_011505739.1 MULTISPECIES: 50S ribosomal protein L14 [Chromohalobacter]
6940754~WP_045471991.1 50S ribosomal protein L14 [Thioploca ingrica]
931076588~WP_041064719.1 50S ribosomal protein L14 [Thiolapillus brandeum]
971431688~WP_058555008.1 50S ribosomal protein L14 [Thiohalocapsa sp. ML1]
101768671~WP_007191148.1 50S ribosomal protein L14 [Thiocapsa marina]
109765912~WP_015281686.1 50S ribosomal protein L14 [Thioflavicoccus mobilis]
105765913~WP_007040461.1 50S ribosomal protein L14 [Thiorhodococcus drewsii]
117Bins|all_bins.fasta_bin_3~144
121Bins|all_bins.fasta_bin_3~199
125Bins|all_bins.fasta_bin_12~298
113Bins|all_bins.fasta_bin_3~328
129Bins|all_bins.fasta_bin_10~688
133Bins|Cell_12.fasta_bin_3~6
165Bins|Cell_14.fasta_bin_5~100
169Bins|all_bins.fasta_bin_25~1275
173Bins|all_bins.fasta_bin_47~1941
177Bins|Cell_14.fasta_bin_5~179
181Bins|Cell_8.fasta_bin_3~124
157Bins|all_bins.fasta_bin_47~1412
161Bins|all_bins.fasta_bin_34~1655
137Bins|all_bins.fasta_bin_1~37
141Bins|Cell_3.fasta_bin_2~52
145Bins|Cell_3.fasta_bin_2~52
149Bins|Cell_3.fasta_bin_2~53
153Bins|Cell_3.fasta_bin_2~53
57381306~WP_054965195.1 50S ribosomal protein L14 [Thiohalorhabdus denitrificans]
77990288~WP_004870742.1 50S ribosomal protein L14 [Acidithiobacillus caldus]
491342299~WP_025045929.1 MULTISPECIES: 50S ribosomal protein L14 [Sulfitobacter]
531353537~WP_038073566.1 50S ribosomal protein L14 [Thioclava pacifica]
41159290~WP_031388519.1 50S ribosomal protein L14 [Desulfonatronum thiodismutans]
13Bins|all_bins.fasta_bin_11~631
12285~WP_011277481.1 50S ribosomal protein L14 [Sulfolobus acidocaldarius]
5273057~NP_342219.1 50S ribosomal protein L14 [Sulfolobus solfataricus P2]
17469381~WP_005661714.1 50S ribosomal protein L14 [Dethiosulfovibrio peptidovorans]
21555088~WP_008516311.1 50S ribosomal protein L14 [Dethiobacter alkaliphilus]
2528034~WP_053961147.1 50S ribosomal protein L14 [Sulfobacillus thermosulfidooxidans]
29679936~YP_005255538.1 50S ribosomal protein L14 [Sulfobacillus acidophilus DSM 10332]
33206403~WP_012084107.1 MULTISPECIES: 50S ribosomal protein L14 [Sulfurovum]
37387093~WP_012084107.1 50S ribosomal protein L14 [Sulfurovum sp. NBC37-1]
9204536~WP_012674924.1 MULTISPECIES: 50S ribosomal protein L14 [Sulfurihydrogenibium]
811255043~WP_006747232.1 MULTISPECIES: 50S ribosomal protein L14 [Thioalkalivibrio]
85765914~WP_006787692.1 50S ribosomal protein L14 [Thiorhodospira sibirica]
891442136~WP_025282217.1 50S ribosomal protein L14 [Ectothiorhodospira haloalkaliphila]
0.2

Ribosomal_L16.treee.tre.exp.tree.pdf

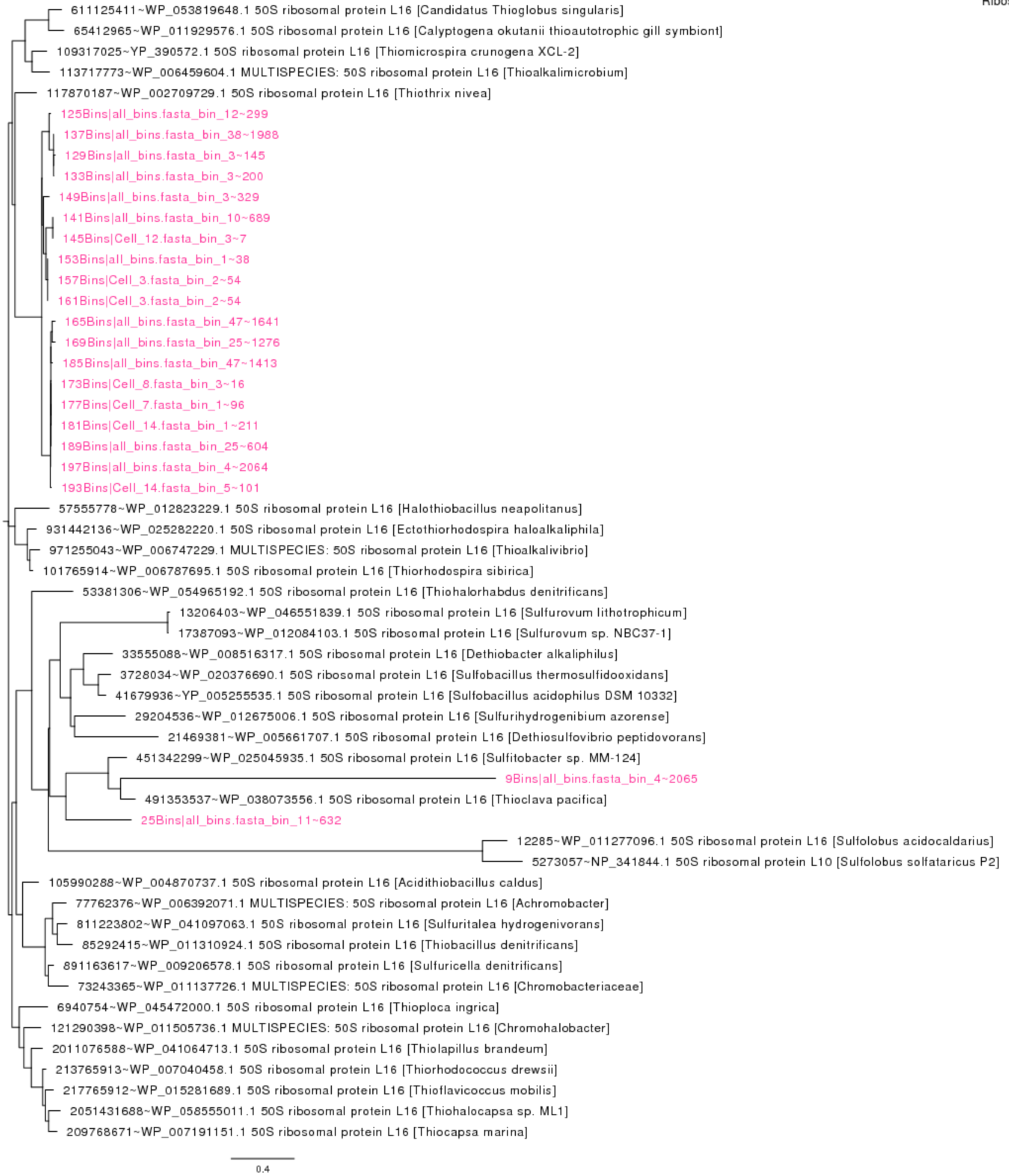

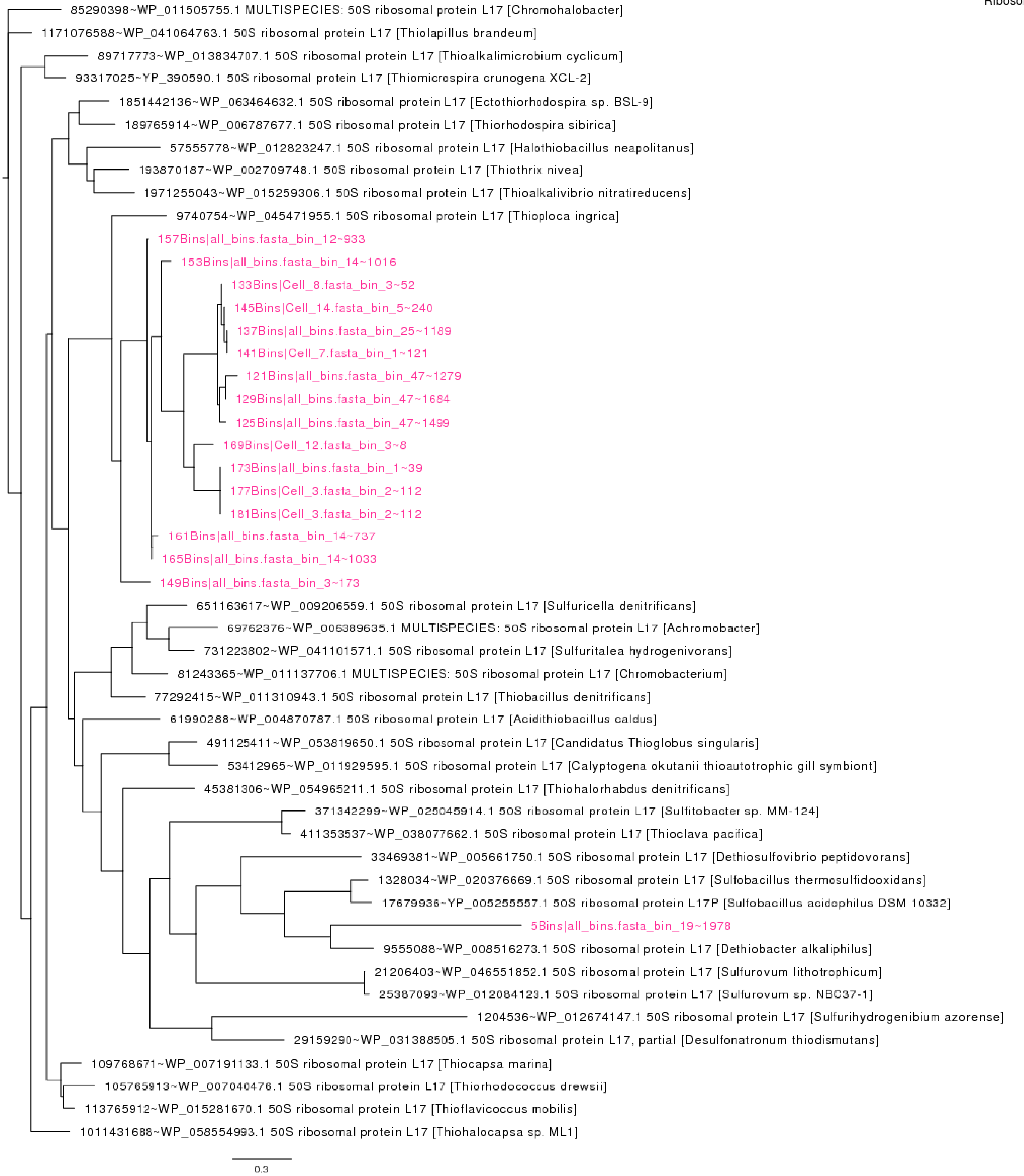
85290398~WP_011505755.1 MULTISPECIES: 50S ribosomal protein L17 [Chromohalobacter]
1171076588~WP_041064763.1 50S ribosomal protein L17 [Thiolapillus brandeum]
89717773~WP_013834707.1 50S ribosomal protein L17 [Thioalkalimicrobium cyclicum]
93317025~YP_390590.1 50S ribosomal protein L17 [Thiomicrospira crunogena XCL-2]
1851442136~WP_063464632.1 50S ribosomal protein L17 [Ectothiorhodospira sp. BSL-9]
189765914~WP_006787677.1 50S ribosomal protein L17 [Thiorhodospira sibirica]
57555778~WP_012823247.1 50S ribosomal protein L17 [Halothiobacillus neapolitanus]
193870187~WP_002709748.1 50S ribosomal protein L17 [Thiothrix nivea]
1971255043~WP_015259306.1 50S ribosomal protein L17 [Thioalkalivibrio nitratireducens]
9740754~WP_045471955.1 50S ribosomal protein L17 [Thioploca ingrica]
157Bins|all_bins.fasta_bin_12~933
153Bins|all_bins.fasta_bin_14~1016
133Bins|Cell_8.fasta_bin_3~52
145Bins|Cell_14.fasta_bin_5~240
137Bins|all_bins.fasta_bin_25~1189
141Bins|Cell_7.fasta_bin_1~121
121Bins|all_bins.fasta_bin_47~1279
129Bins|all_bins.fasta_bin_47~1684
125Bins|all_bins.fasta_bin_47~1499
169Bins|Cell_12.fasta_bin_3~8
173Bins|all_bins.fasta_bin_1~39
177Bins|Cell_3.fasta_bin_2~112
181Bins|Cell_3.fasta_bin_2~112
161Bins|all_bins.fasta_bin_14~737
165Bins|all_bins.fasta_bin_14~1033
149Bins|all_bins.fasta_bin_3~173
651163617~WP_009206559.1 50S ribosomal protein L17 [Sulfuricella denitrificans]
69762376~WP_006389635.1 MULTISPECIES: 50S ribosomal protein L17 [Achromobacter]
731223802~WP_041101571.1 50S ribosomal protein L17 [Sulfuritalea hydrogenivorans]
81243365~WP_011137706.1 MULTISPECIES: 50S ribosomal protein L17 [Chromobacterium]
77292415~WP_011310943.1 50S ribosomal protein L17 [Thiobacillus denitrificans]
61990288~WP_004870787.1 50S ribosomal protein L17 [Acidithiobacillus caldus]
491125411~WP_053819650.1 50S ribosomal protein L17 [Candidatus Thioglobus singularis]
53412965~WP_011929595.1 50S ribosomal protein L17 [Calyptogena okutanii thioautotrophic gill symbiont]
45381306~WP_054965211.1 50S ribosomal protein L17 [Thiohalorhabdus denitrificans]
371342299~WP_025045914.1 50S ribosomal protein L17 [Sulfitobacter sp. MM-124]
411353537~WP_038077662.1 50S ribosomal protein L17 [Thioclava pacifica]
33469381~WP_005661750.1 50S ribosomal protein L17 [Dethiosulfovibrio peptidovorans]
1328034~WP_020376669.1 50S ribosomal protein L17 [Sulfobacillus thermosulfidooxidans]
17679936~YP_005255557.1 50S ribosomal protein L17P [Sulfobacillus acidophilus DSM 10332]
5Bins|all_bins.fasta_bin_19~1978
9555088~WP_008516273.1 50S ribosomal protein L17 [Dethiobacter alkaliphilus]
21206403~WP_046551852.1 50S ribosomal protein L17 [Sulfurovum lithotrophicum]
25387093~WP_012084123.1 50S ribosomal protein L17 [Sulfurovum sp. NBC37-1]
1204536~WP_012674147.1 50S ribosomal protein L17 [Sulfurihydrogenibium azorense]
29159290~WP_031388505.1 50S ribosomal protein L17, partial [Desulfonatronum thiodismutans]
109768671~WP_007191133.1 50S ribosomal protein L17 [Thiocapsa marina]
105765913~WP_007040476.1 50S ribosomal protein L17 [Thiorhodococcus drewsii]
113765912~WP_015281670.1 50S ribosomal protein L17 [Thioflavicoccus mobilis]
1011431688~WP_058554993.1 50S ribosomal protein L17 [Thiohalocapsa sp. ML1]
0.3

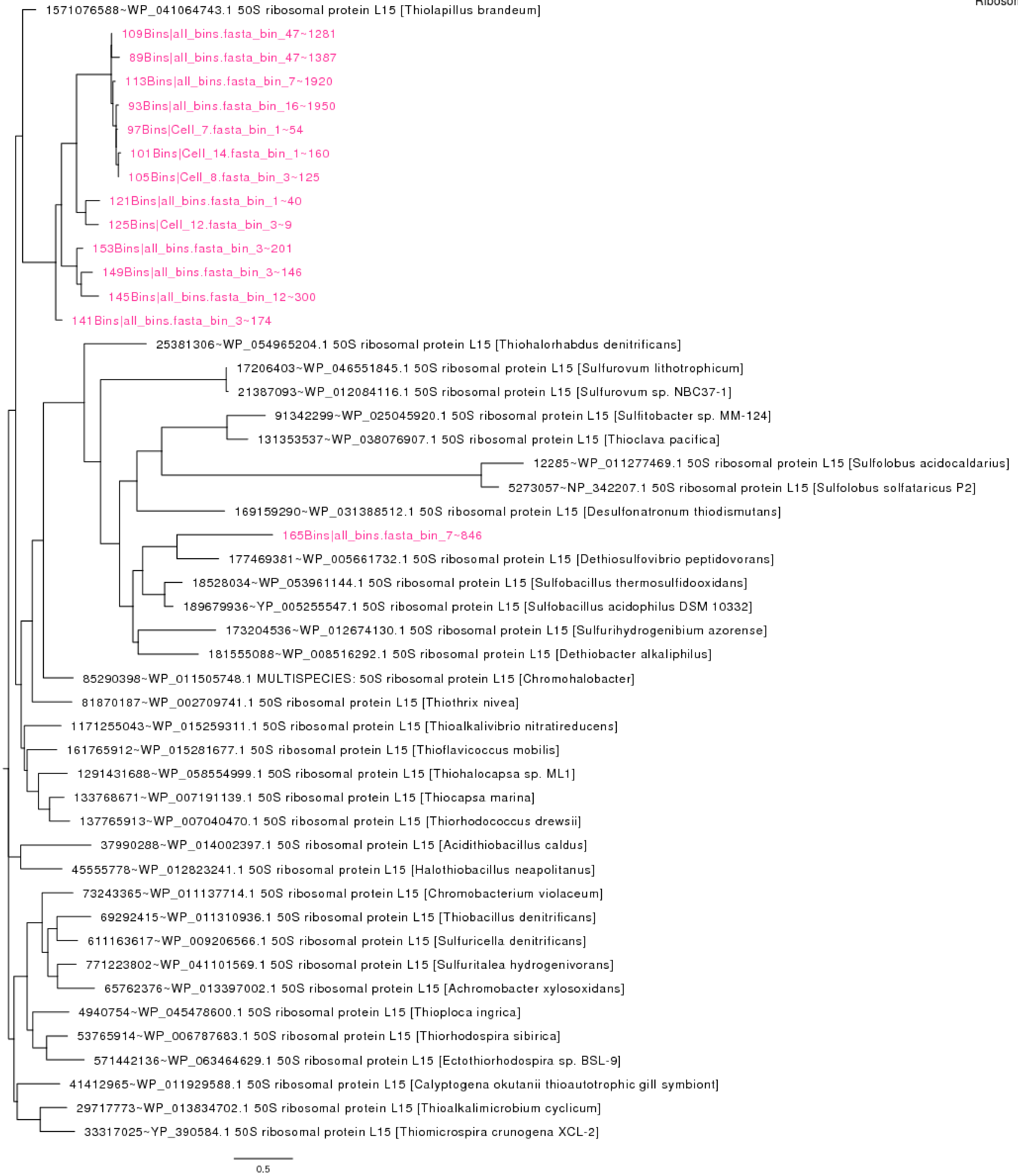
1571076588~WP_041064743.1 50S ribosomal protein L15 [Thiolapillus brandeum]
109Bins|all_bins.fasta_bin_47~1281
89Bins|all_bins.fasta_bin_47~1387
113Bins|all_bins.fasta_bin_7~1920
93Bins|all_bins.fasta_bin_16~1950
97Bins|Cell_7.fasta_bin_1~54
101Bins|Cell_14.fasta_bin_1~160
105Bins|Cell_8.fasta_bin_3~125
121Bins|all_bins.fasta_bin_1~40
125Bins|Cell_12.fasta_bin_3~9
153Bins|all_bins.fasta_bin_3~201
149Bins|all_bins.fasta_bin_3~146
145Bins|all_bins.fasta_bin_12~300
141Bins|all_bins.fasta_bin_3~174
25381306~WP_054965204.1 50S ribosomal protein L15 [Thiohalorhabdus denitrificans]
17206403~WP_046551845.1 50S ribosomal protein L15 [Sulfurovum lithotrophicum]
21387093~WP_012084116.1 50S ribosomal protein L15 [Sulfurovum sp. NBC37-1]
91342299~WP_025045920.1 50S ribosomal protein L15 [Sulfitobacter sp. MM-124]
131353537~WP_038076907.1 50S ribosomal protein L15 [Thioclava pacifica]
12285~WP_011277469.1 50S ribosomal protein L15 [Sulfolobus acidocaldarius]
5273057~NP_342207.1 50S ribosomal protein L15 [Sulfolobus solfataricus P2]
169159290~WP_031388512.1 50S ribosomal protein L15 [Desulfonatronum thiodismutans]
165Bins|all_bins.fasta_bin_7~846
177469381~WP_005661732.1 50S ribosomal protein L15 [Dethiosulfovibrio peptidovorans]
18528034~WP_053961144.1 50S ribosomal protein L15 [Sulfobacillus thermosulfidooxidans]
189679936~YP_005255547.1 50S ribosomal protein L15 [Sulfobacillus acidophilus DSM 10332]
173204536~WP_012674130.1 50S ribosomal protein L15 [Sulfurihydrogenibium azorense]
181555088~WP_008516292.1 50S ribosomal protein L15 [Dethiobacter alkaliphilus]
85290398~WP_011505748.1 MULTISPECIES: 50S ribosomal protein L15 [Chromohalobacter]
81870187~WP_002709741.1 50S ribosomal protein L15 [Thiothrix nivea]
1171255043~WP_015259311.1 50S ribosomal protein L15 [Thioalkalivibrio nitratireducens]
161765912~WP_015281677.1 50S ribosomal protein L15 [Thioflavicoccus mobilis]
1291431688~WP_058554999.1 50S ribosomal protein L15 [Thiohalocapsa sp. ML1]
133768671~WP_007191139.1 50S ribosomal protein L15 [Thiocapsa marina]
137765913~WP_007040470.1 50S ribosomal protein L15 [Thiorhodococcus drewsii]
37990288~WP_014002397.1 50S ribosomal protein L15 [Acidithiobacillus caldus]
45555778~WP_012823241.1 50S ribosomal protein L15 [Halothiobacillus neapolitanus]
73243365~WP_011137714.1 50S ribosomal protein L15 [Chromobacterium violaceum]
69292415~WP_011310936.1 50S ribosomal protein L15 [Thiobacillus denitrificans]
611163617~WP_009206566.1 50S ribosomal protein L15 [Sulfuricella denitrificans]
771223802~WP_041101569.1 50S ribosomal protein L15 [Sulfuritalea hydrogenivorans]
65762376~WP_013397002.1 50S ribosomal protein L15 [Achromobacter xylosoxidans]
4940754~WP_045478600.1 50S ribosomal protein L15 [Thioploca ingrica]
53765914~WP_006787683.1 50S ribosomal protein L15 [Thiorhodospira sibirica]
571442136~WP_063464629.1 50S ribosomal protein L15 [Ectothiorhodospira sp. BSL-9]
41412965~WP_011929588.1 50S ribosomal protein L15 [Calyptogena okutanii thioautotrophic gill symbiont]
29717773~WP_013834702.1 50S ribosomal protein L15 [Thioalkalimicrobium cyclicum]
33317025~YP_390584.1 50S ribosomal protein L15 [Thiomicrospira crunogena XCL-2]
0.5

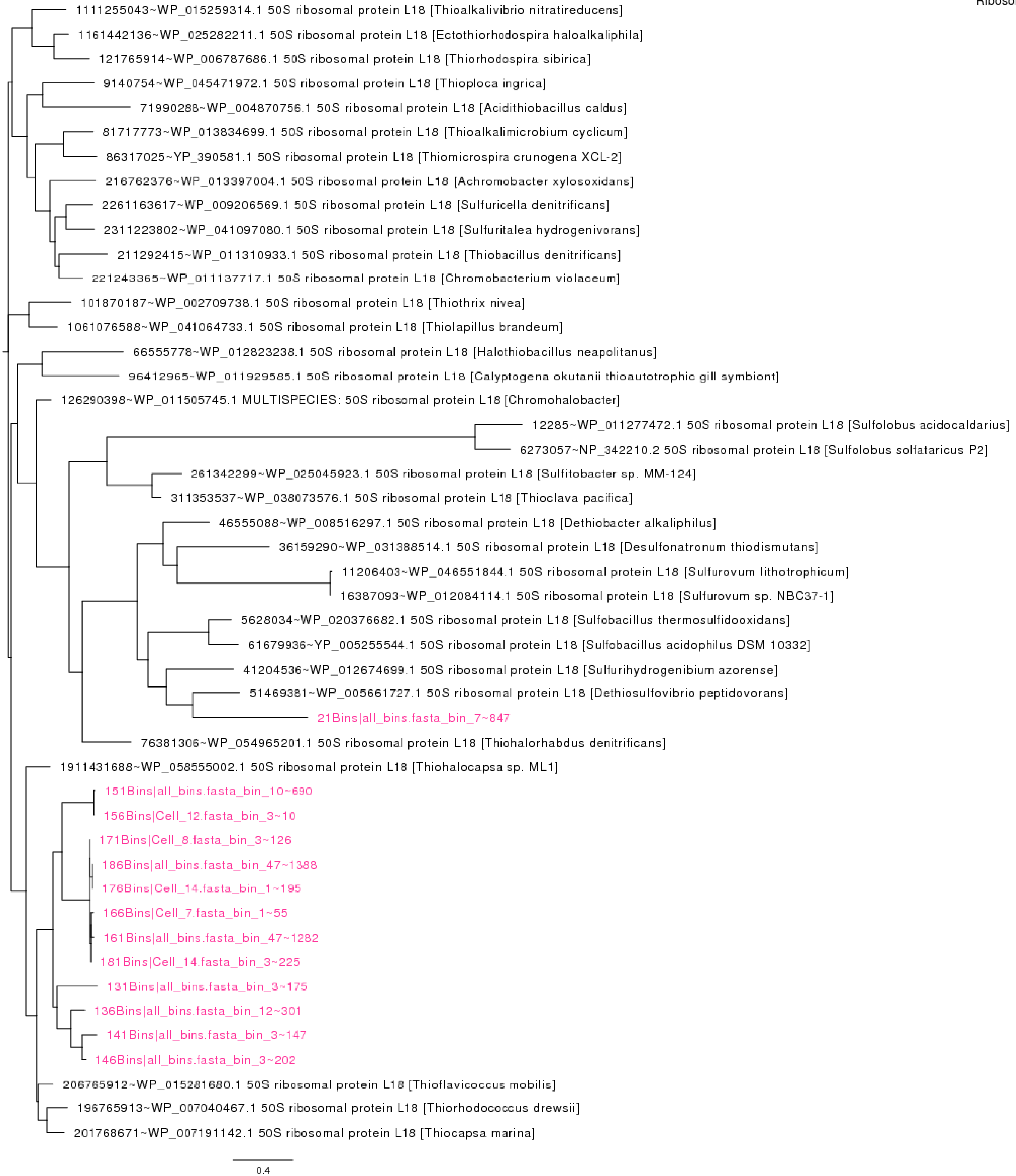
1111255043~WP_015259314.1 50S ribosomal protein L18 [Thioalkalivibrio nitratireducens]
1161442136~WP_025282211.1 50S ribosomal protein L18 [Ectothiorhodospira haloalkaliphila]
121765914~WP_006787686.1 50S ribosomal protein L18 [Thiorhodospira sibirica]
9140754~WP_045471972.1 50S ribosomal protein L18 [Thioploca ingrica]
71990288~WP_004870756.1 50S ribosomal protein L18 [Acidithiobacillus caldus]
81717773~WP_013834699.1 50S ribosomal protein L18 [Thioalkalimicrobium cyclicum]
86317025~YP_390581.1 50S ribosomal protein L18 [Thiomicrospira crunogena XCL-2]
216762376~WP_013397004.1 50S ribosomal protein L18 [Achromobacter xylosoxidans]
2261163617~WP_009206569.1 50S ribosomal protein L18 [Sulfuricella denitrificans]
2311223802~WP_041097080.1 50S ribosomal protein L18 [Sulfuritalea hydrogenivorans]
211292415~WP_011310933.1 50S ribosomal protein L18 [Thiobacillus denitrificans]
221243365~WP_011137717.1 50S ribosomal protein L18 [Chromobacterium violaceum]
101870187~WP_002709738.1 50S ribosomal protein L18 [Thiothrix nivea]
1061076588~WP_041064733.1 50S ribosomal protein L18 [Thiolapillus brandeum]
66555778~WP_012823238.1 50S ribosomal protein L18 [Halothiobacillus neapolitanus]
96412965~WP_011929585.1 50S ribosomal protein L18 [Calyptogena okutanii thioautotrophic gill symbiont]
126290398~WP_011505745.1 MULTISPECIES: 50S ribosomal protein L18 [Chromohalobacter]
12285~WP_011277472.1 50S ribosomal protein L18 [Sulfolobus acidocaldarius]
6273057~NP_342210.2 50S ribosomal protein L18 [Sulfolobus solfataricus P2]
261342299~WP_025045923.1 50S ribosomal protein L18 [Sulfitobacter sp. MM-124]
311353537~WP_038073576.1 50S ribosomal protein L18 [Thioclava pacifica]
46555088~WP_008516297.1 50S ribosomal protein L18 [Dethiobacter alkaliphilus]
36159290~WP_031388514.1 50S ribosomal protein L18 [Desulfonatronum thiodismutans]
11206403~WP_046551844.1 50S ribosomal protein L18 [Sulfurovum lithotrophicum]
16387093~WP_012084114.1 50S ribosomal protein L18 [Sulfurovum sp. NBC37-1]
5628034~WP_020376682.1 50S ribosomal protein L18 [Sulfobacillus thermosulfidooxidans]
61679936~YP_005255544.1 50S ribosomal protein L18 [Sulfobacillus acidophilus DSM 10332]
41204536~WP_012674699.1 50S ribosomal protein L18 [Sulfurihydrogenibium azorense]
51469381~WP_005661727.1 50S ribosomal protein L18 [Dethiosulfovibrio peptidovorans]
21Bins|all_bins.fasta_bin_7~847
76381306~WP_054965201.1 50S ribosomal protein L18 [Thiohalorhabdus denitrificans]
1911431688~WP_058555002.1 50S ribosomal protein L18 [Thiohalocapsa sp. ML1]
151Bins|all_bins.fasta_bin_10~690
156Bins|Cell_12.fasta_bin_3~10
171Bins|Cell_8.fasta_bin_3~126
186Bins|all_bins.fasta_bin_47~1388
176Bins|Cell_14.fasta_bin_1~195
166Bins|Cell_7.fasta_bin_1~55
161Bins|all_bins.fasta_bin_47~1282
181Bins|Cell_14.fasta_bin_3~225
131Bins|all_bins.fasta_bin_3~175
136Bins|all_bins.fasta_bin_12~301
141Bins|all_bins.fasta_bin_3~147
146Bins|all_bins.fasta_bin_3~202
206765912~WP_015281680.1 50S ribosomal protein L18 [Thioflavicoccus mobilis]
196765913~WP_007040467.1 50S ribosomal protein L18 [Thiorhodococcus drewsii]
201768671~WP_007191142.1 50S ribosomal protein L18 [Thiocapsa marina]
0.4

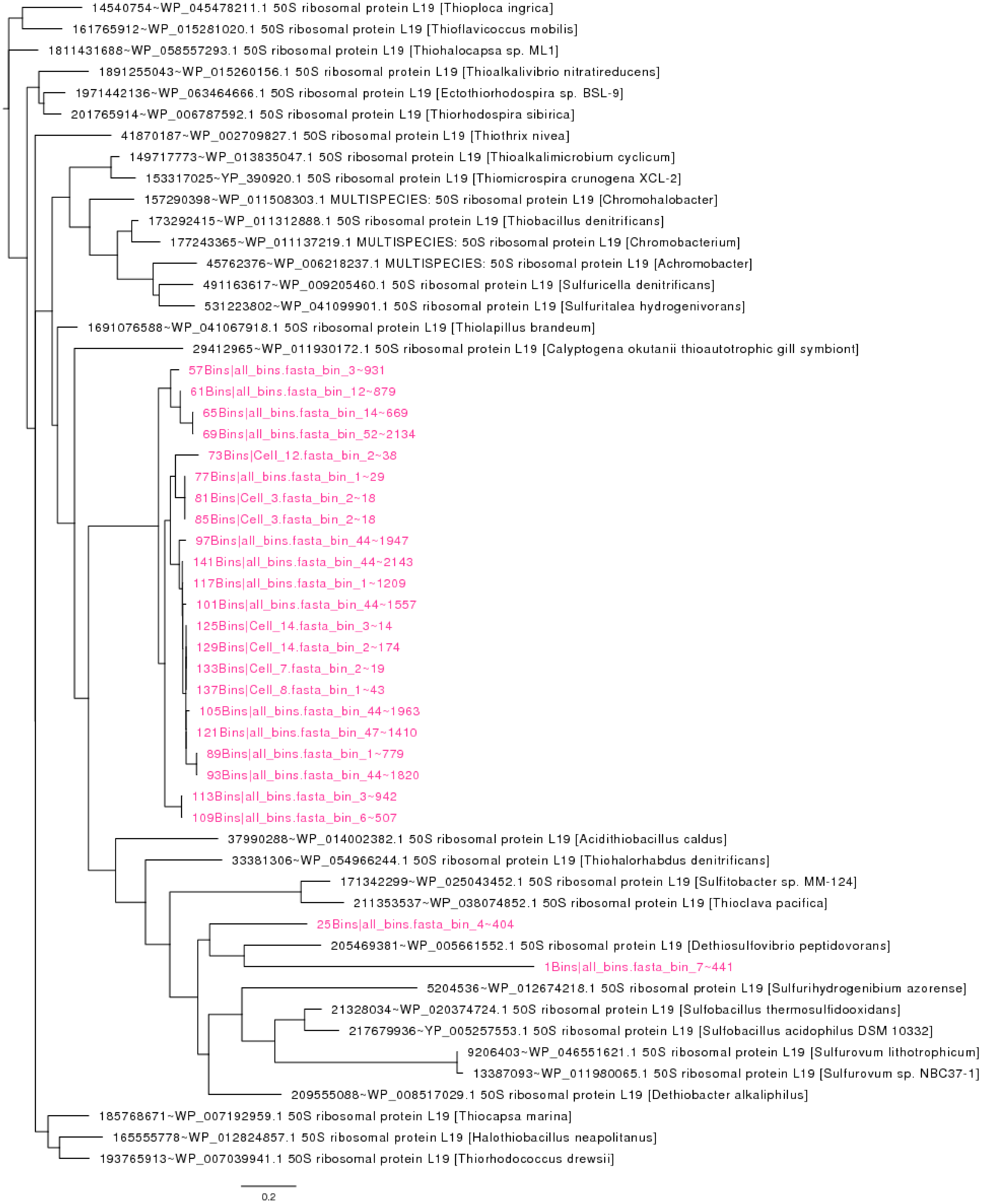
14540754~WP_045478211.1 50S ribosomal protein L19 [Thioploca ingrica]
161765912~WP_015281020.1 50S ribosomal protein L19 [Thioflavicoccus mobilis]
1811431688~WP_058557293.1 50S ribosomal protein L19 [Thiohalocapsa sp. ML1]
1891255043~WP_015260156.1 50S ribosomal protein L19 [Thioalkalivibrio nitratireducens]
1971442136~WP_063464666.1 50S ribosomal protein L19 [Ectothiorhodospira sp. BSL-9]
201765914~WP_006787592.1 50S ribosomal protein L19 [Thiorhodospira sibirica]
41870187~WP_002709827.1 50S ribosomal protein L19 [Thiothrix nivea]
149717773~WP_013835047.1 50S ribosomal protein L19 [Thioalkalimicrobium cyclicum]
153317025~YP_390920.1 50S ribosomal protein L19 [Thiomicrospira crunogena XCL-2]
157290398~WP_011508303.1 MULTISPECIES: 50S ribosomal protein L19 [Chromohalobacter]
173292415~WP_011312888.1 50S ribosomal protein L19 [Thiobacillus denitrificans]
177243365~WP_011137219.1 MULTISPECIES: 50S ribosomal protein L19 [Chromobacterium]
45762376~WP_006218237.1 MULTISPECIES: 50S ribosomal protein L19 [Achromobacter]
491163617~WP_009205460.1 50S ribosomal protein L19 [Sulfuricella denitrificans]
531223802~WP_041099901.1 50S ribosomal protein L19 [Sulfuritalea hydrogenivorans]
1691076588~WP_041067918.1 50S ribosomal protein L19 [Thiolapillus brandeum]
29412965~WP_011930172.1 50S ribosomal protein L19 [Calyptogena okutanii thioautotrophic gill symbiont]
57Bins|all_bins.fasta_bin_3~931
61Bins|all_bins.fasta_bin_12~879
65Bins|all_bins.fasta_bin_14~669
69Bins|all_bins.fasta_bin_52~2134
73Bins|Cell_12.fasta_bin_2~38
77Bins|all_bins.fasta_bin_1~29
81Bins|Cell_3.fasta_bin_2~18
85Bins|Cell_3.fasta_bin_2~18
97Bins|all_bins.fasta_bin_44~1947
141Bins|all_bins.fasta_bin_44~2143
117Bins|all_bins.fasta_bin_1~1209
101Bins|all_bins.fasta_bin_44~1557
125Bins|Cell_14.fasta_bin_3~14
129Bins|Cell_14.fasta_bin_2~174
133Bins|Cell_7.fasta_bin_2~19
137Bins|Cell_8.fasta_bin_1~43
105Bins|all_bins.fasta_bin_44~1963
121Bins|all_bins.fasta_bin_47~1410
89Bins|all_bins.fasta_bin_1~779
93Bins|all_bins.fasta_bin_44~1820
113Bins|all_bins.fasta_bin_3~942
109Bins|all_bins.fasta_bin_6~507
37990288~WP_014002382.1 50S ribosomal protein L19 [Acidithiobacillus caldus]
33381306~WP_054966244.1 50S ribosomal protein L19 [Thiohalorhabdus denitrificans]
171342299~WP_025043452.1 50S ribosomal protein L19 [Sulfitobacter sp. MM-124]
211353537~WP_038074852.1 50S ribosomal protein L19 [Thioclava pacifica]
25Bins|all_bins.fasta_bin_4~404
205469381~WP_005661552.1 50S ribosomal protein L19 [Dethiosulfovibrio peptidovorans]
1Bins|all_bins.fasta_bin_7~441
5204536~WP_012674218.1 50S ribosomal protein L19 [Sulfurihydrogenibium azorense]
21328034~WP_020374724.1 50S ribosomal protein L19 [Sulfobacillus thermosulfidooxidans]
217679936~YP_005257553.1 50S ribosomal protein L19 [Sulfobacillus acidophilus DSM 10332]
9206403~WP_046551621.1 50S ribosomal protein L19 [Sulfurovum lithotrophicum]
13387093~WP_011980065.1 50S ribosomal protein L19 [Sulfurovum sp. NBC37-1]
209555088~WP_008517029.1 50S ribosomal protein L19 [Dethiobacter alkaliphilus]
185768671~WP_007192959.1 50S ribosomal protein L19 [Thiocapsa marina]
165555778~WP_012824857.1 50S ribosomal protein L19 [Halothiobacillus neapolitanus]
193765913~WP_007039941.1 50S ribosomal protein L19 [Thiorhodococcus drewsii]
0.2

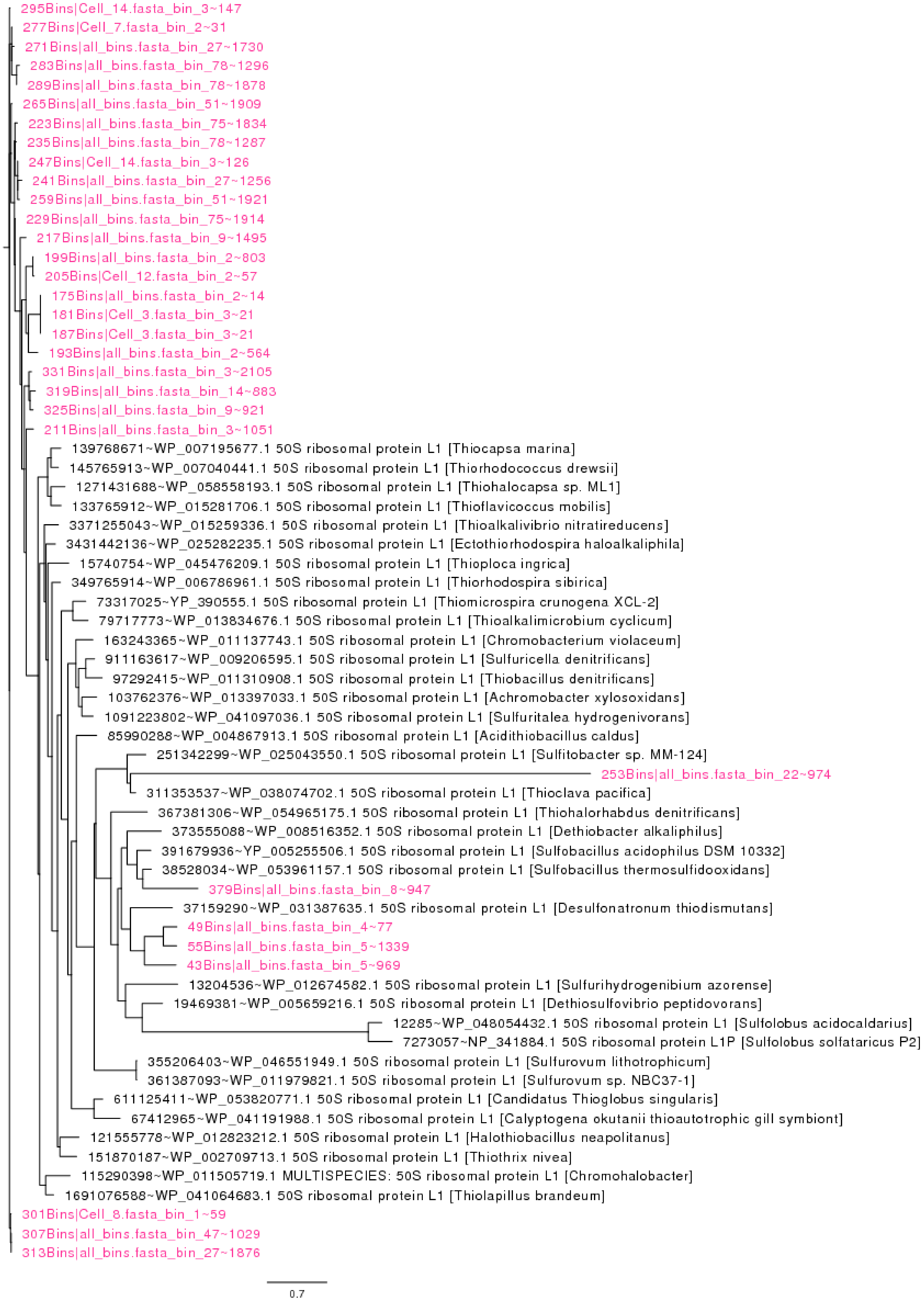
295Bins|Cell_14.fasta_bin_3~147
277Bins|Cell_7.fasta_bin_2~31
271Bins|all_bins.fasta_bin_27~1730
283Bins|all_bins.fasta_bin_78~1296
289Bins|all_bins.fasta_bin_78~1878
265Bins|all_bins.fasta_bin_51~1909
223Bins|all_bins.fasta_bin_75~1834
235Bins|all_bins.fasta_bin_78~1287
247Bins|Cell_14.fasta_bin_3~126
241Bins|all_bins.fasta_bin_27~1256
259Bins|all_bins.fasta_bin_51~1921
229Bins|all_bins.fasta_bin_75~1914
217Bins|all_bins.fasta_bin_9~1495
199Bins|all_bins.fasta_bin_2~803
205Bins|Cell_12.fasta_bin_2~57
175Bins|all_bins.fasta_bin_2~14
181Bins|Cell_3.fasta_bin_3~21
187Bins|Cell_3.fasta_bin_3~21
193Bins|all_bins.fasta_bin_2~564
331Bins|all_bins.fasta_bin_3~2105
319Bins|all_bins.fasta_bin_14~883
325Bins|all_bins.fasta_bin_9~921
211Bins|all_bins.fasta_bin_3~1051
139768671~WP_007195677.1 50S ribosomal protein L1 [Thiocapsa marina]
145765913~WP_007040441.1 50S ribosomal protein L1 [Thiorhodococcus drewsii]
1271431688~WP_058558193.1 50S ribosomal protein L1 [Thiohalocapsa sp. ML1]
133765912~WP_015281706.1 50S ribosomal protein L1 [Thioflavicoccus mobilis]
3371255043~WP_015259336.1 50S ribosomal protein L1 [Thioalkalivibrio nitratireducens]
3431442136~WP_025282235.1 50S ribosomal protein L1 [Ectothiorhodospira haloalkaliphila]
15740754~WP_045476209.1 50S ribosomal protein L1 [Thioploca ingrica]
349765914~WP_006786961.1 50S ribosomal protein L1 [Thiorhodospira sibirica]
73317025~YP_390555.1 50S ribosomal protein L1 [Thiomicrospira crunogena XCL-2]
79717773~WP_013834676.1 50S ribosomal protein L1 [Thioalkalimicrobium cyclicum]
163243365~WP_011137743.1 50S ribosomal protein L1 [Chromobacterium violaceum]
911163617~WP_009206595.1 50S ribosomal protein L1 [Sulfuricella denitrificans]
97292415~WP_011310908.1 50S ribosomal protein L1 [Thiobacillus denitrificans]
103762376~WP_013397033.1 50S ribosomal protein L1 [Achromobacter xylosoxidans]
1091223802~WP_041097036.1 50S ribosomal protein L1 [Sulfuritalea hydrogenivorans]
85990288~WP_004867913.1 50S ribosomal protein L1 [Acidithiobacillus caldus]
251342299~WP_025043550.1 50S ribosomal protein L1 [Sulfitobacter sp. MM-124]
253Bins|all_bins.fasta_bin_22~974
311353537~WP_038074702.1 50S ribosomal protein L1 [Thioclava pacifica]
367381306~WP_054965175.1 50S ribosomal protein L1 [Thiohalorhabdus denitrificans]
373555088~WP_008516352.1 50S ribosomal protein L1 [Dethiobacter alkaliphilus]
391679936~YP_005255506.1 50S ribosomal protein L1 [Sulfobacillus acidophilus DSM 10332]
38528034~WP_053961157.1 50S ribosomal protein L1 [Sulfobacillus thermosulfidooxidans]
379Bins|all_bins.fasta_bin_8~947
37159290~WP_031387635.1 50S ribosomal protein L1 [Desulfonatronum thiodismutans]
49Bins|all_bins.fasta_bin_4~77
55Bins|all_bins.fasta_bin_5~1339
43Bins|all_bins.fasta_bin_5~969
13204536~WP_012674582.1 50S ribosomal protein L1 [Sulfurihydrogenibium azorense]
19469381~WP_005659216.1 50S ribosomal protein L1 [Dethiosulfovibrio peptidovorans]
12285~WP_048054432.1 50S ribosomal protein L1 [Sulfolobus acidocaldarius]
7273057~NP_341884.1 50S ribosomal protein L1P [Sulfolobus solfataricus P2]
355206403~WP_046551949.1 50S ribosomal protein L1 [Sulfurovum lithotrophicum]
361387093~WP_011979821.1 50S ribosomal protein L1 [Sulfurovum sp. NBC37-1]
611125411~WP_053820771.1 50S ribosomal protein L1 [Candidatus Thioglobus singularis]
67412965~WP_041191988.1 50S ribosomal protein L1 [Calyptogena okutanii thioautotrophic gill symbiont]
121555778~WP_012823212.1 50S ribosomal protein L1 [Halothiobacillus neapolitanus]
151870187~WP_002709713.1 50S ribosomal protein L1 [Thiothrix nivea]
115290398~WP_011505719.1 MULTISPECIES: 50S ribosomal protein L1 [Chromohalobacter]
1691076588~WP_041064683.1 50S ribosomal protein L1 [Thiolapillus brandeum]
301Bins|Cell_8.fasta_bin_1~59
307Bins|all_bins.fasta_bin_47~1029
313Bins|all_bins.fasta_bin_27~1876
0.7

Ribosomal_L20.treee.tre.exp.tree.pdf

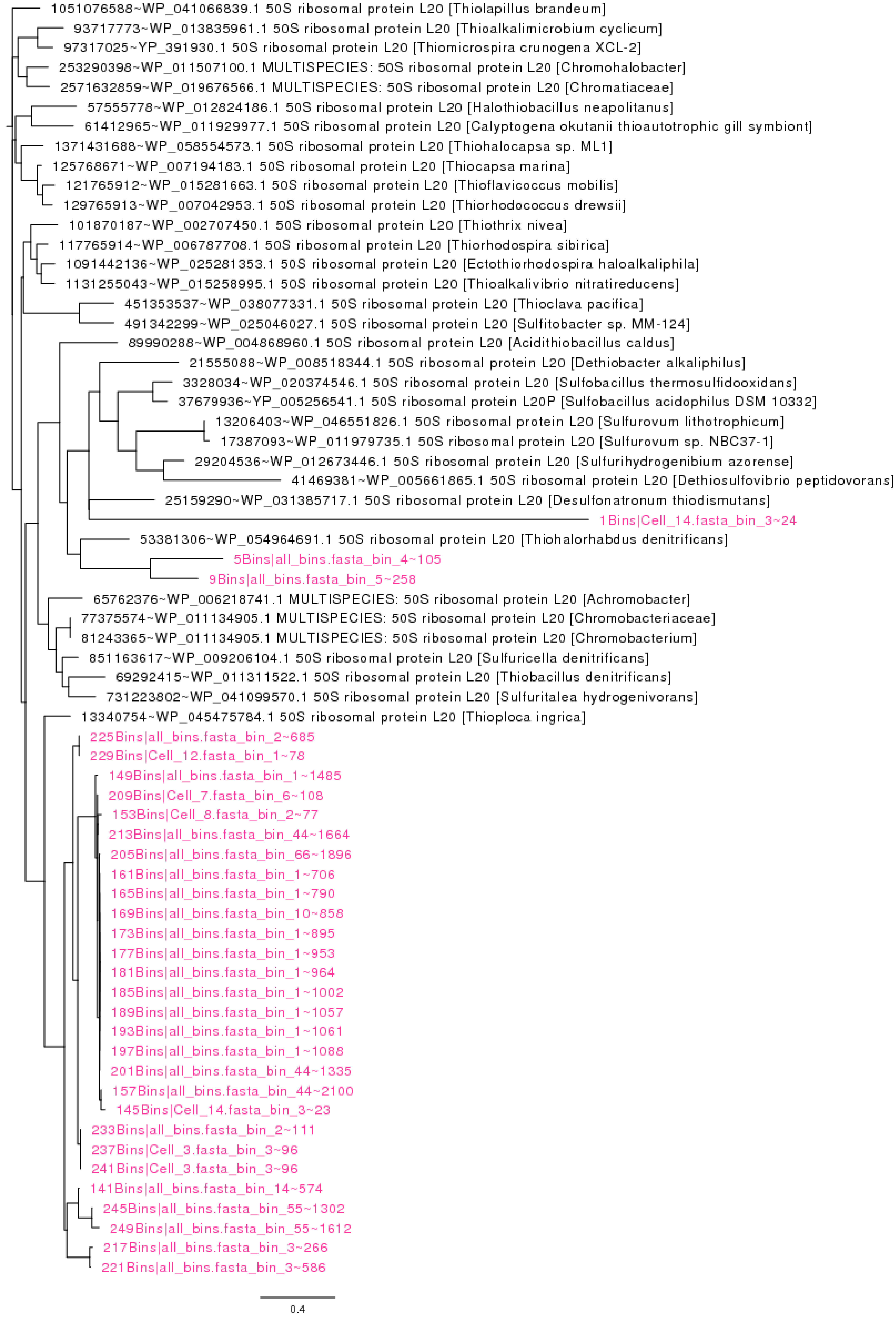

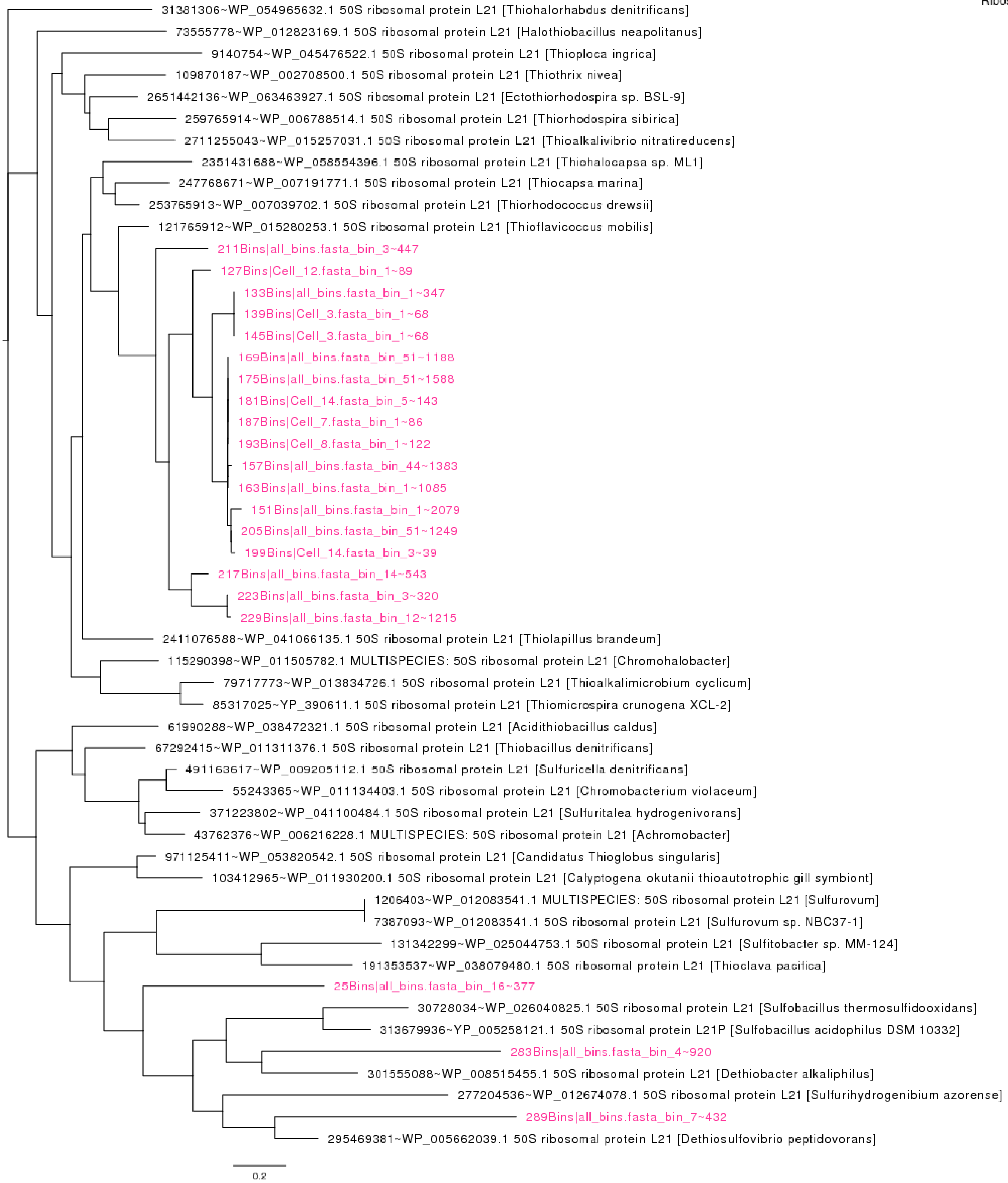
31381306~WP_054965632.1 50S ribosomal protein L21 [Thiohalorhabdus denitrificans]
73555778~WP_012823169.1 50S ribosomal protein L21 [Halothiobacillus neapolitanus]
9140754~WP_045476522.1 50S ribosomal protein L21 [Thioploca ingrica]
109870187~WP_002708500.1 50S ribosomal protein L21 [Thiothrix nivea]
2651442136~WP_063463927.1 50S ribosomal protein L21 [Ectothiorhodospira sp. BSL-9]
259765914~WP_006788514.1 50S ribosomal protein L21 [Thiorhodospira sibirica]
2711255043~WP_015257031.1 50S ribosomal protein L21 [Thioalkalivibrio nitratireducens]
2351431688~WP_058554396.1 50S ribosomal protein L21 [Thiohalocapsa sp. ML1]
247768671~WP_007191771.1 50S ribosomal protein L21 [Thiocapsa marina]
253765913~WP_007039702.1 50S ribosomal protein L21 [Thiorhodococcus drewsii]
121765912~WP_015280253.1 50S ribosomal protein L21 [Thioflavicoccus mobilis]
211Bins|all_bins.fasta_bin_3~447
127Bins|Cell_12.fasta_bin_1~89
133Bins|all_bins.fasta_bin_1~347
139Bins|Cell_3.fasta_bin_1~68
145Bins|Cell_3.fasta_bin_1~68
169Bins|all_bins.fasta_bin_51~1188
175Bins|all_bins.fasta_bin_51~1588
181Bins|Cell_14.fasta_bin_5~143
187Bins|Cell_7.fasta_bin_1~86
193Bins|Cell_8.fasta_bin_1~122
157Bins|all_bins.fasta_bin_44~1383
163Bins|all_bins.fasta_bin_1~1085
151Bins|all_bins.fasta_bin_1~2079
205Bins|all_bins.fasta_bin_51~1249
199Bins|Cell_14.fasta_bin_3~39
217Bins|all_bins.fasta_bin_14~543
223Bins|all_bins.fasta_bin_3~320
229Bins|all_bins.fasta_bin_12~1215
2411076588~WP_041066135.1 50S ribosomal protein L21 [Thiolapillus brandeum]
115290398~WP_011505782.1 MULTISPECIES: 50S ribosomal protein L21 [Chromohalobacter]
79717773~WP_013834726.1 50S ribosomal protein L21 [Thioalkalimicrobium cyclicum]
85317025~YP_390611.1 50S ribosomal protein L21 [Thiomicrospira crunogena XCL-2]
61990288~WP_038472321.1 50S ribosomal protein L21 [Acidithiobacillus caldus]
67292415~WP_011311376.1 50S ribosomal protein L21 [Thiobacillus denitrificans]
491163617~WP_009205112.1 50S ribosomal protein L21 [Sulfuricella denitrificans]
55243365~WP_011134403.1 50S ribosomal protein L21 [Chromobacterium violaceum]
371223802~WP_041100484.1 50S ribosomal protein L21 [Sulfuritalea hydrogenivorans]
43762376~WP_006216228.1 MULTISPECIES: 50S ribosomal protein L21 [Achromobacter]
971125411~WP_053820542.1 50S ribosomal protein L21 [Candidatus Thioglobus singularis]
103412965~WP_011930200.1 50S ribosomal protein L21 [Calyptogena okutanii thioautotrophic gill symbiont]
1206403~WP_012083541.1 MULTISPECIES: 50S ribosomal protein L21 [Sulfurovum]
7387093~WP_012083541.1 50S ribosomal protein L21 [Sulfurovum sp. NBC37-1]
131342299~WP_025044753.1 50S ribosomal protein L21 [Sulfitobacter sp. MM-124]
191353537~WP_038079480.1 50S ribosomal protein L21 [Thioclava pacifica]
25Bins|all_bins.fasta_bin_16~377
30728034~WP_026040825.1 50S ribosomal protein L21 [Sulfobacillus thermosulfidooxidans]
313679936~YP_005258121.1 50S ribosomal protein L21P [Sulfobacillus acidophilus DSM 10332]
283Bins|all_bins.fasta_bin_4~920
301555088~WP_008515455.1 50S ribosomal protein L21 [Dethiobacter alkaliphilus]
277204536~WP_012674078.1 50S ribosomal protein L21 [Sulfurihydrogenibium azorense]
289Bins|all_bins.fasta_bin_7~432
295469381~WP_005662039.1 50S ribosomal protein L21 [Dethiosulfovibrio peptidovorans]
0.2

Ribosomal_L22.treee.tre.exp.tree.pdf

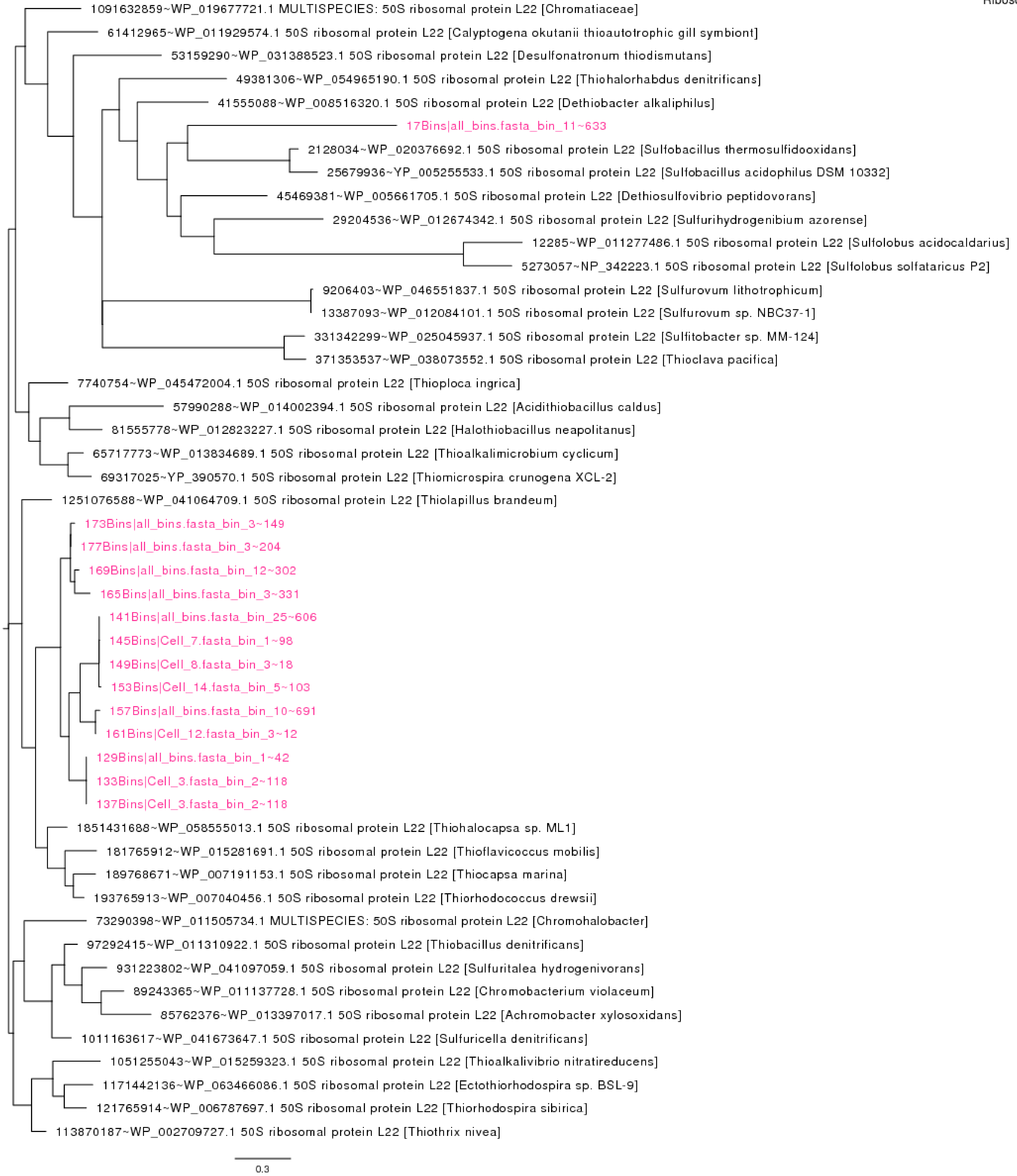

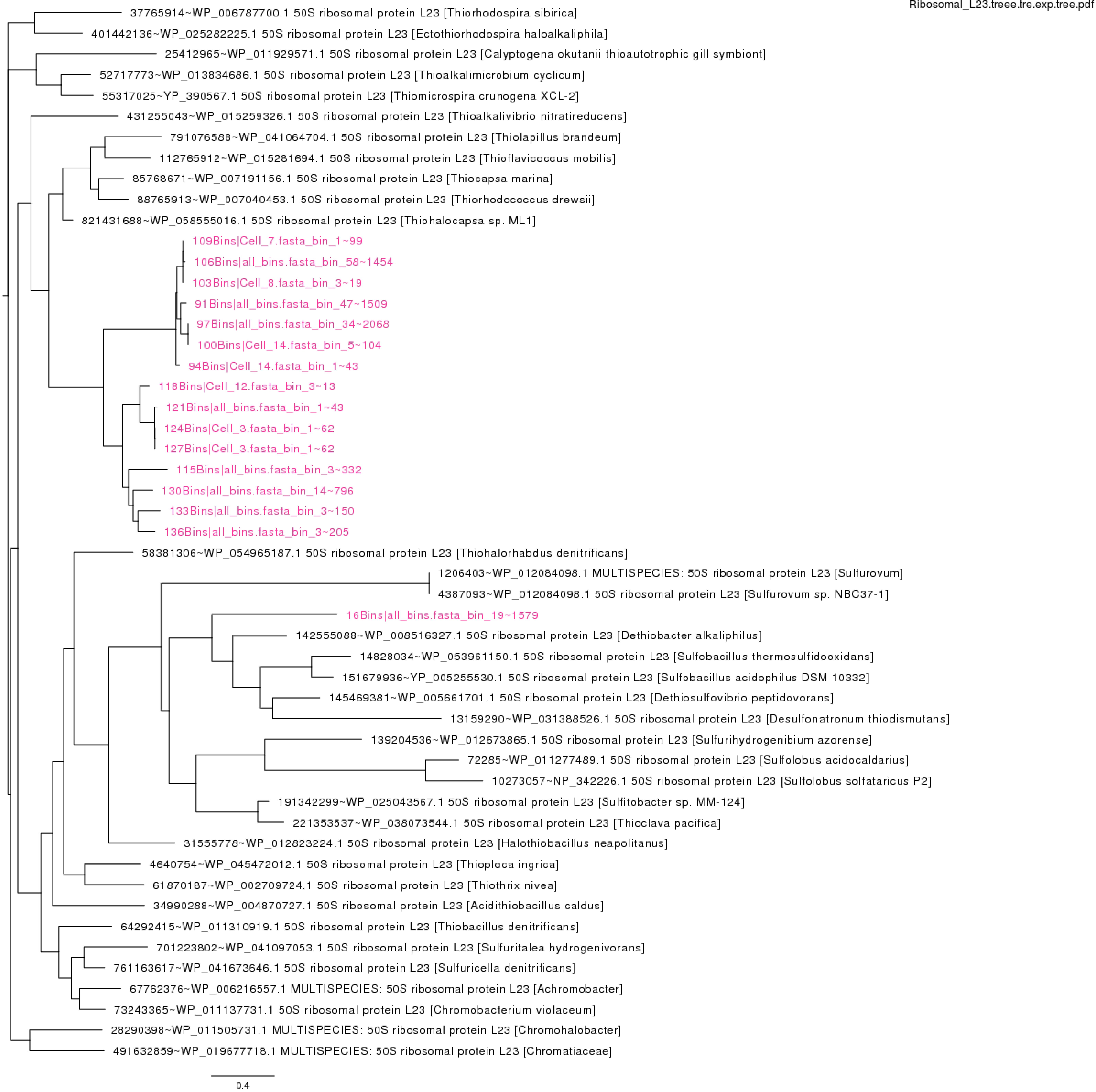
Ribosomal_L23.treee.tre.exp.tree.pdf
37765914~WP_006787700.1 50S ribosomal protein L23 [Thiorhodospira sibirica]
401442136~WP_025282225.1 50S ribosomal protein L23 [Ectothiorhodospira haloalkaliphila]
25412965~WP_011929571.1 50S ribosomal protein L23 [Calyptogena okutanii thioautotrophic gill symbiont]
52717773~WP_013834686.1 50S ribosomal protein L23 [Thioalkalimicrobium cyclicum]
55317025~YP_390567.1 50S ribosomal protein L23 [Thiomicrospira crunogena XCL-2]
431255043~WP_015259326.1 50S ribosomal protein L23 [Thioalkalivibrio nitratireducens]
791076588~WP_041064704.1 50S ribosomal protein L23 [Thiolapillus brandeum]
112765912~WP_015281694.1 50S ribosomal protein L23 [Thioflavicoccus mobilis]
85768671~WP_007191156.1 50S ribosomal protein L23 [Thiocapsa marina]
88765913~WP_007040453.1 50S ribosomal protein L23 [Thiorhodococcus drewsii]
821431688~WP_058555016.1 50S ribosomal protein L23 [Thiohalocapsa sp. ML1]
109Bins|Cell_7.fasta_bin_1~99
106Bins|all_bins.fasta_bin_58~1454
103Bins|Cell_8.fasta_bin_3~19
91Bins|all_bins.fasta_bin_47~1509
97Bins|all_bins.fasta_bin_34~2068
100Bins|Cell_14.fasta_bin_5~104
94Bins|Cell_14.fasta_bin_1~43
118Bins|Cell_12.fasta_bin_3~13
121Bins|all_bins.fasta_bin_1~43
124Bins|Cell_3.fasta_bin_1~62
127Bins|Cell_3.fasta_bin_1~62
115Bins|all_bins.fasta_bin_3~332
130Bins|all_bins.fasta_bin_14~796
133Bins|all_bins.fasta_bin_3~150
136Bins|all_bins.fasta_bin_3~205
58381306~WP_054965187.1 50S ribosomal protein L23 [Thiohalorhabdus denitrificans]
1206403~WP_012084098.1 MULTISPECIES: 50S ribosomal protein L23 [Sulfurovum]
4387093~WP_012084098.1 50S ribosomal protein L23 [Sulfurovum sp. NBC37-1]
16Bins|all_bins.fasta_bin_19~1579
142555088~WP_008516327.1 50S ribosomal protein L23 [Dethiobacter alkaliphilus]
14828034~WP_053961150.1 50S ribosomal protein L23 [Sulfobacillus thermosulfidooxidans]
151679936~YP_005255530.1 50S ribosomal protein L23 [Sulfobacillus acidophilus DSM 10332]
145469381~WP_005661701.1 50S ribosomal protein L23 [Dethiosulfovibrio peptidovorans]
13159290~WP_031388526.1 50S ribosomal protein L23 [Desulfonatronum thiodismutans]
139204536~WP_012673865.1 50S ribosomal protein L23 [Sulfurihydrogenibium azorense]
72285~WP_011277489.1 50S ribosomal protein L23 [Sulfolobus acidocaldarius]
10273057~NP_342226.1 50S ribosomal protein L23 [Sulfolobus solfataricus P2]
191342299~WP_025043567.1 50S ribosomal protein L23 [Sulfitobacter sp. MM-124]
221353537~WP_038073544.1 50S ribosomal protein L23 [Thioclava pacifica]
31555778~WP_012823224.1 50S ribosomal protein L23 [Halothiobacillus neapolitanus]
4640754~WP_045472012.1 50S ribosomal protein L23 [Thioploca ingrica]
61870187~WP_002709724.1 50S ribosomal protein L23 [Thiothrix nivea]
34990288~WP_004870727.1 50S ribosomal protein L23 [Acidithiobacillus caldus]
64292415~WP_011310919.1 50S ribosomal protein L23 [Thiobacillus denitrificans]
701223802~WP_041097053.1 50S ribosomal protein L23 [Sulfuritalea hydrogenivorans]
761163617~WP_041673646.1 50S ribosomal protein L23 [Sulfuricella denitrificans]
67762376~WP_006216557.1 MULTISPECIES: 50S ribosomal protein L23 [Achromobacter]
73243365~WP_011137731.1 50S ribosomal protein L23 [Chromobacterium violaceum]
28290398~WP_011505731.1 MULTISPECIES: 50S ribosomal protein L23 [Chromohalobacter]
491632859~WP_019677718.1 MULTISPECIES: 50S ribosomal protein L23 [Chromatiaceae]
0.4

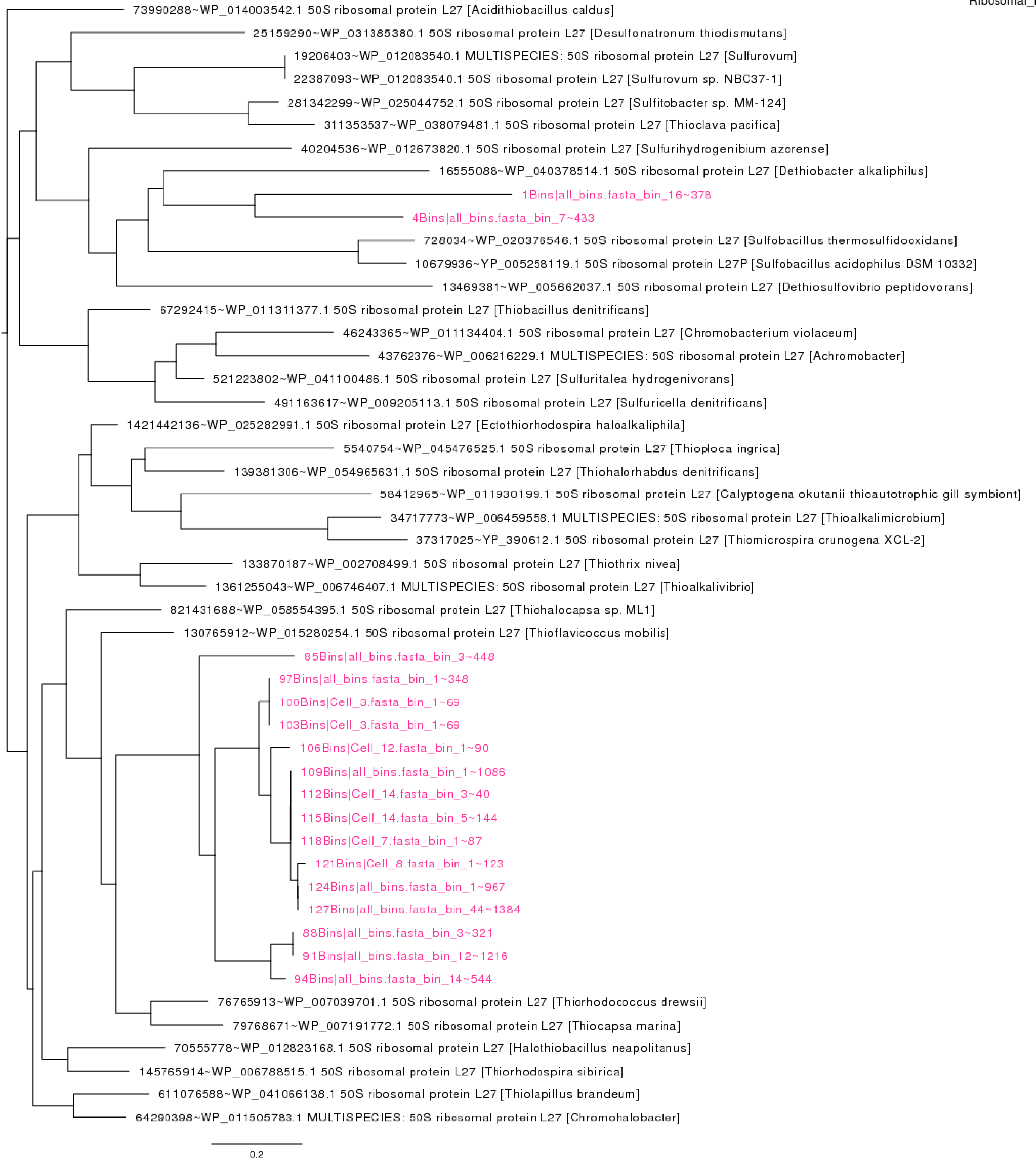
73990288~WP_014003542.1 50S ribosomal protein L27 [Acidithiobacillus caldus]
25159290~WP_031385380.1 50S ribosomal protein L27 [Desulfonatronum thiodismutans]
19206403~WP_012083540.1 MULTISPECIES: 50S ribosomal protein L27 [Sulfurovum]
22387093~WP_012083540.1 50S ribosomal protein L27 [Sulfurovum sp. NBC37-1]
281342299~WP_025044752.1 50S ribosomal protein L27 [Sulfitobacter sp. MM-124]
311353537~WP_038079481.1 50S ribosomal protein L27 [Thioclava pacifica]
40204536~WP_012673820.1 50S ribosomal protein L27 [Sulfurihydrogenibium azorense]
16555088~WP_040378514.1 50S ribosomal protein L27 [Dethiobacter alkaliphilus]
1Bins|all_bins.fasta_bin_16~378
4Bins|all_bins.fasta_bin_7~433
728034~WP_020376546.1 50S ribosomal protein L27 [Sulfobacillus thermosulfidooxidans]
10679936~YP_005258119.1 50S ribosomal protein L27P [Sulfobacillus acidophilus DSM 10332]
13469381~WP_005662037.1 50S ribosomal protein L27 [Dethiosulfovibrio peptidovorans]
67292415~WP_011311377.1 50S ribosomal protein L27 [Thiobacillus denitrificans]
46243365~WP_011134404.1 50S ribosomal protein L27 [Chromobacterium violaceum]
43762376~WP_006216229.1 MULTISPECIES: 50S ribosomal protein L27 [Achromobacter]
521223802~WP_041100486.1 50S ribosomal protein L27 [Sulfuritalea hydrogenivorans]
491163617~WP_009205113.1 50S ribosomal protein L27 [Sulfuricella denitrificans]
1421442136~WP_025282991.1 50S ribosomal protein L27 [Ectothiorhodospira haloalkaliphila]
5540754~WP_045476525.1 50S ribosomal protein L27 [Thioploca ingrica]
139381306~WP_054965631.1 50S ribosomal protein L27 [Thiohalorhabdus denitrificans]
58412965~WP_011930199.1 50S ribosomal protein L27 [Calyptogena okutanii thioautotrophic gill symbiont]
34717773~WP_006459558.1 MULTISPECIES: 50S ribosomal protein L27 [Thioalkalimicrobium]
37317025~YP_390612.1 50S ribosomal protein L27 [Thiomicrospira crunogena XCL-2]
133870187~WP_002708499.1 50S ribosomal protein L27 [Thiothrix nivea]
1361255043~WP_006746407.1 MULTISPECIES: 50S ribosomal protein L27 [Thioalkalivibrio]
821431688~WP_058554395.1 50S ribosomal protein L27 [Thiohalocapsa sp. ML1]
130765912~WP_015280254.1 50S ribosomal protein L27 [Thioflavicoccus mobilis]
85Bins|all_bins.fasta_bin_3~448
97Bins|all_bins.fasta_bin_1~348
100Bins|Cell_3.fasta_bin_1~69
103Bins|Cell_3.fasta_bin_1~69
106Bins|Cell_12.fasta_bin_1~90
109Bins|all_bins.fasta_bin_1~1086
112Bins|Cell_14.fasta_bin_3~40
115Bins|Cell_14.fasta_bin_5~144
118Bins|Cell_7.fasta_bin_1~87
121Bins|Cell_8.fasta_bin_1~123
124Bins|all_bins.fasta_bin_1~967
127Bins|all_bins.fasta_bin_44~1384
88Bins|all_bins.fasta_bin_3~321
91Bins|all_bins.fasta_bin_12~1216
94Bins|all_bins.fasta_bin_14~544
76765913~WP_007039701.1 50S ribosomal protein L27 [Thiorhodococcus drewsii]
79768671~WP_007191772.1 50S ribosomal protein L27 [Thiocapsa marina]
70555778~WP_012823168.1 50S ribosomal protein L27 [Halothiobacillus neapolitanus]
145765914~WP_006788515.1 50S ribosomal protein L27 [Thiorhodospira sibirica]
611076588~WP_041066138.1 50S ribosomal protein L27 [Thiolapillus brandeum]
64290398~WP_011505783.1 MULTISPECIES: 50S ribosomal protein L27 [Chromohalobacter]
0.2

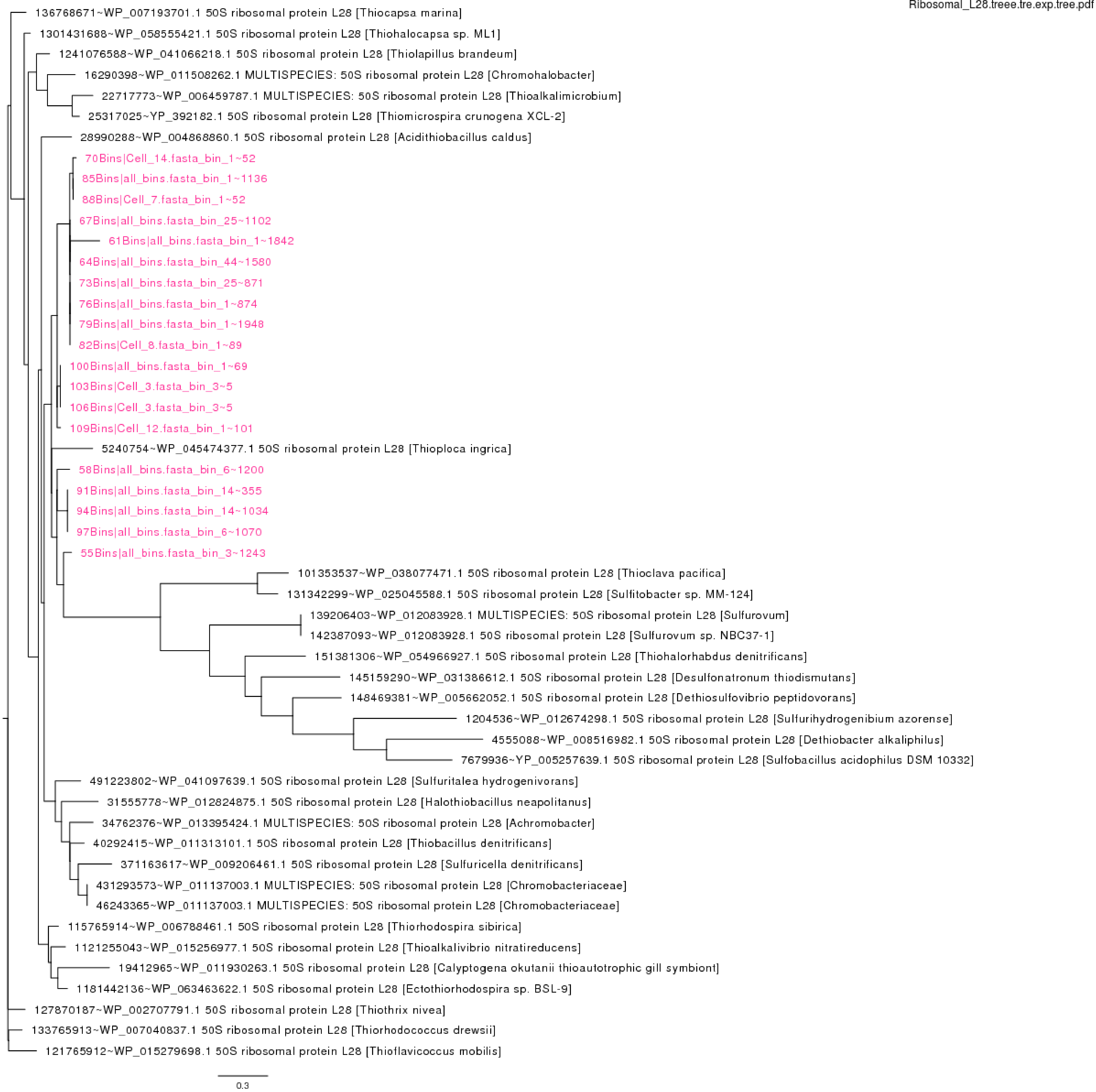
Ribosomal_L28.treee.tre.exp.tree.pdf
136768671~WP_007193701.1 50S ribosomal protein L28 [Thiocapsa marina]
1301431688~WP_058555421.1 50S ribosomal protein L28 [Thiohalocapsa sp. ML1]
1241076588~WP_041066218.1 50S ribosomal protein L28 [Thiolapillus brandeum]
16290398~WP_011508262.1 MULTISPECIES: 50S ribosomal protein L28 [Chromohalobacter]
22717773~WP_006459787.1 MULTISPECIES: 50S ribosomal protein L28 [Thioalkalimicrobium]
25317025~YP_392182.1 50S ribosomal protein L28 [Thiomicrospira crunogena XCL-2]
28990288~WP_004868860.1 50S ribosomal protein L28 [Acidithiobacillus caldus]
70Bins|Cell_14.fasta_bin_1~52
85Bins|all_bins.fasta_bin_1~1136
88Bins|Cell_7.fasta_bin_1~52
67Bins|all_bins.fasta_bin_25~1102
61Bins|all_bins.fasta_bin_1~1842
64Bins|all_bins.fasta_bin_44~1580
73Bins|all_bins.fasta_bin_25~871
76Bins|all_bins.fasta_bin_1~874
79Bins|all_bins.fasta_bin_1~1948
82Bins|Cell_8.fasta_bin_1~89
100Bins|all_bins.fasta_bin_1~69
103Bins|Cell_3.fasta_bin_3~5
106Bins|Cell_3.fasta_bin_3~5
109Bins|Cell_12.fasta_bin_1~101
5240754~WP_045474377.1 50S ribosomal protein L28 [Thioploca ingrica]
58Bins|all_bins.fasta_bin_6~1200
91Bins|all_bins.fasta_bin_14~355
94Bins|all_bins.fasta_bin_14~1034
97Bins|all_bins.fasta_bin_6~1070
55Bins|all_bins.fasta_bin_3~1243
101353537~WP_038077471.1 50S ribosomal protein L28 [Thioclava pacifica]
131342299~WP_025045588.1 50S ribosomal protein L28 [Sulfitobacter sp. MM-124]
139206403~WP_012083928.1 MULTISPECIES: 50S ribosomal protein L28 [Sulfurovum]
142387093~WP_012083928.1 50S ribosomal protein L28 [Sulfurovum sp. NBC37-1]
151381306~WP_054966927.1 50S ribosomal protein L28 [Thiohalorhabdus denitrificans]
145159290~WP_031386612.1 50S ribosomal protein L28 [Desulfonatronum thiodismutans]
148469381~WP_005662052.1 50S ribosomal protein L28 [Dethiosulfovibrio peptidovorans]
1204536~WP_012674298.1 50S ribosomal protein L28 [Sulfurihydrogenibium azorense]
4555088~WP_008516982.1 50S ribosomal protein L28 [Dethiobacter alkaliphilus]
7679936~YP_005257639.1 50S ribosomal protein L28 [Sulfobacillus acidophilus DSM 10332]
491223802~WP_041097639.1 50S ribosomal protein L28 [Sulfuritalea hydrogenivorans]
31555778~WP_012824875.1 50S ribosomal protein L28 [Halothiobacillus neapolitanus]
34762376~WP_013395424.1 MULTISPECIES: 50S ribosomal protein L28 [Achromobacter]
40292415~WP_011313101.1 50S ribosomal protein L28 [Thiobacillus denitrificans]
371163617~WP_009206461.1 50S ribosomal protein L28 [Sulfuricella denitrificans]
431293573~WP_011137003.1 MULTISPECIES: 50S ribosomal protein L28 [Chromobacteriaceae]
46243365~WP_011137003.1 MULTISPECIES: 50S ribosomal protein L28 [Chromobacteriaceae]
115765914~WP_006788461.1 50S ribosomal protein L28 [Thiorhodospira sibirica]
1121255043~WP_015256977.1 50S ribosomal protein L28 [Thioalkalivibrio nitratireducens]
19412965~WP_011930263.1 50S ribosomal protein L28 [Calyptogena okutanii thioautotrophic gill symbiont]
1181442136~WP_063463622.1 50S ribosomal protein L28 [Ectothiorhodospira sp. BSL-9]
127870187~WP_002707791.1 50S ribosomal protein L28 [Thiothrix nivea]
133765913~WP_007040837.1 50S ribosomal protein L28 [Thiorhodococcus drewsii]
121765912~WP_015279698.1 50S ribosomal protein L28 [Thioflavicoccus mobilis]
0.3

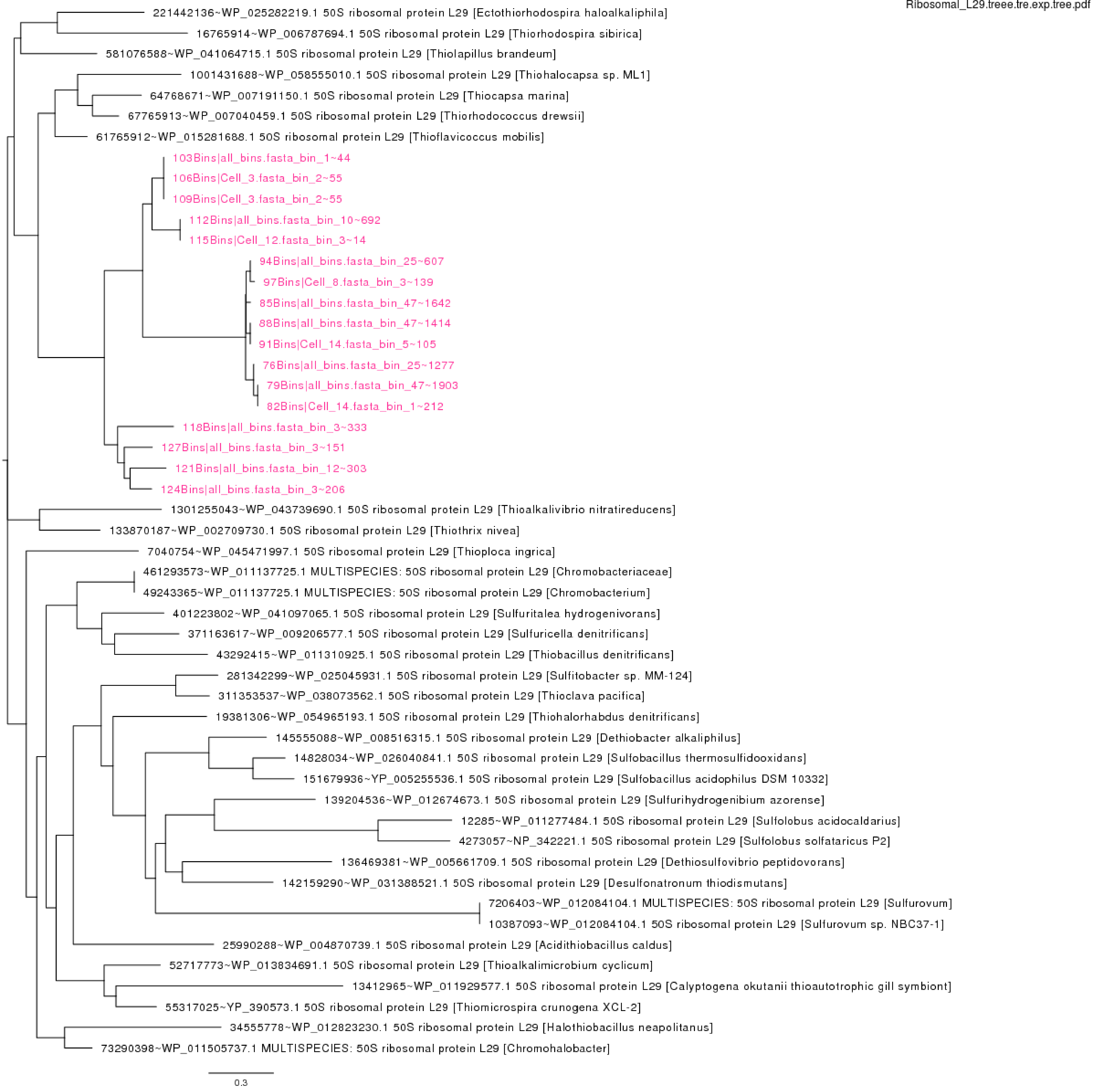
Ribosomal_L29.treee.tre.exp.tree.pdf
221442136~WP_025282219.1 50S ribosomal protein L29 [Ectothiorhodospira haloalkaliphila]
16765914~WP_006787694.1 50S ribosomal protein L29 [Thiorhodospira sibirica]
581076588~WP_041064715.1 50S ribosomal protein L29 [Thiolapillus brandeum]
1001431688~WP_058555010.1 50S ribosomal protein L29 [Thiohalocapsa sp. ML1]
64768671~WP_007191150.1 50S ribosomal protein L29 [Thiocapsa marina]
67765913~WP_007040459.1 50S ribosomal protein L29 [Thiorhodococcus drewsii]
61765912~WP_015281688.1 50S ribosomal protein L29 [Thioflavicoccus mobilis]
103Bins|all_bins.fasta_bin_1~44
106Bins|Cell_3.fasta_bin_2~55
109Bins|Cell_3.fasta_bin_2~55
112Bins|all_bins.fasta_bin_10~692
115Bins|Cell_12.fasta_bin_3~14
94Bins|all_bins.fasta_bin_25~607
97Bins|Cell_8.fasta_bin_3~139
85Bins|all_bins.fasta_bin_47~1642
88Bins|all_bins.fasta_bin_47~1414
91Bins|Cell_14.fasta_bin_5~105
76Bins|all_bins.fasta_bin_25~1277
79Bins|all_bins.fasta_bin_47~1903
82Bins|Cell_14.fasta_bin_1~212
118Bins|all_bins.fasta_bin_3~333
127Bins|all_bins.fasta_bin_3~151
121Bins|all_bins.fasta_bin_12~303
124Bins|all_bins.fasta_bin_3~206
1301255043~WP_043739690.1 50S ribosomal protein L29 [Thioalkalivibrio nitratireducens]
133870187~WP_002709730.1 50S ribosomal protein L29 [Thiothrix nivea]
7040754~WP_045471997.1 50S ribosomal protein L29 [Thioploca ingrica]
461293573~WP_011137725.1 MULTISPECIES: 50S ribosomal protein L29 [Chromobacteriaceae]
49243365~WP_011137725.1 MULTISPECIES: 50S ribosomal protein L29 [Chromobacterium]
401223802~WP_041097065.1 50S ribosomal protein L29 [Sulfuritalea hydrogenivorans]
371163617~WP_009206577.1 50S ribosomal protein L29 [Sulfuricella denitrificans]
43292415~WP_011310925.1 50S ribosomal protein L29 [Thiobacillus denitrificans]
281342299~WP_025045931.1 50S ribosomal protein L29 [Sulfitobacter sp. MM-124]
311353537~WP_038073562.1 50S ribosomal protein L29 [Thioclava pacifica]
19381306~WP_054965193.1 50S ribosomal protein L29 [Thiohalorhabdus denitrificans]
145555088~WP_008516315.1 50S ribosomal protein L29 [Dethiobacter alkaliphilus]
14828034~WP_026040841.1 50S ribosomal protein L29 [Sulfobacillus thermosulfidooxidans]
151679936~YP_005255536.1 50S ribosomal protein L29 [Sulfobacillus acidophilus DSM 10332]
139204536~WP_012674673.1 50S ribosomal protein L29 [Sulfurihydrogenibium azorense]
12285~WP_011277484.1 50S ribosomal protein L29 [Sulfolobus acidocaldarius]
4273057~NP_342221.1 50S ribosomal protein L29 [Sulfolobus solfataricus P2]
136469381~WP_005661709.1 50S ribosomal protein L29 [Dethiosulfovibrio peptidovorans]
142159290~WP_031388521.1 50S ribosomal protein L29 [Desulfonatronum thiodismutans]
7206403~WP_012084104.1 MULTISPECIES: 50S ribosomal protein L29 [Sulfurovum]
10387093~WP_012084104.1 50S ribosomal protein L29 [Sulfurovum sp. NBC37-1]
25990288~WP_004870739.1 50S ribosomal protein L29 [Acidithiobacillus caldus]
52717773~WP_013834691.1 50S ribosomal protein L29 [Thioalkalimicrobium cyclicum]
13412965~WP_011929577.1 50S ribosomal protein L29 [Calyptogena okutanii thioautotrophic gill symbiont]
55317025~YP_390573.1 50S ribosomal protein L29 [Thiomicrospira crunogena XCL-2]
34555778~WP_012823230.1 50S ribosomal protein L29 [Halothiobacillus neapolitanus]
73290398~WP_011505737.1 MULTISPECIES: 50S ribosomal protein L29 [Chromohalobacter]
0.3

Ribosomal_L2.treee.tre.exp.tree.pdf

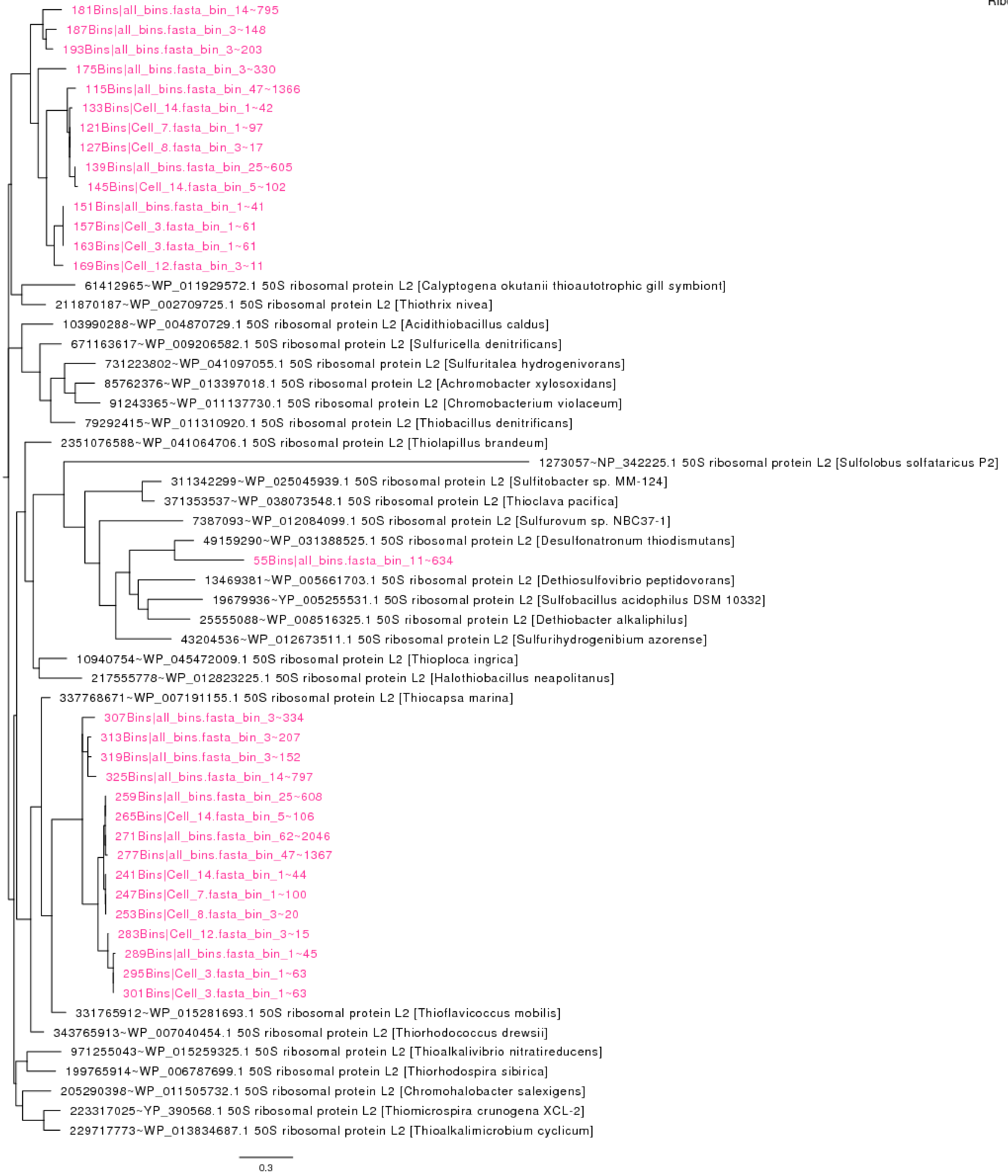

Ribosomal_L32p.treee.tre.exp.tree.pdf

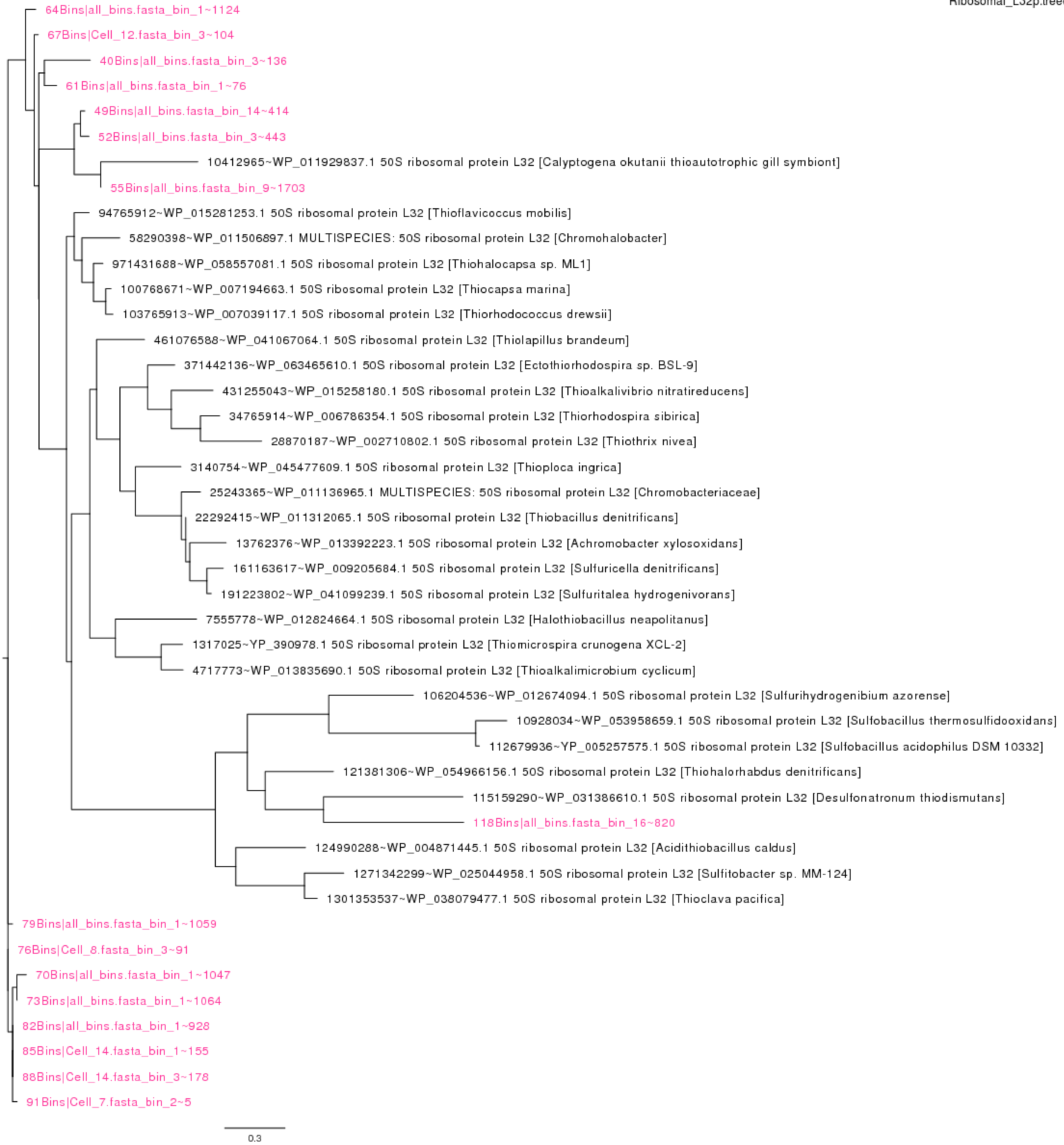

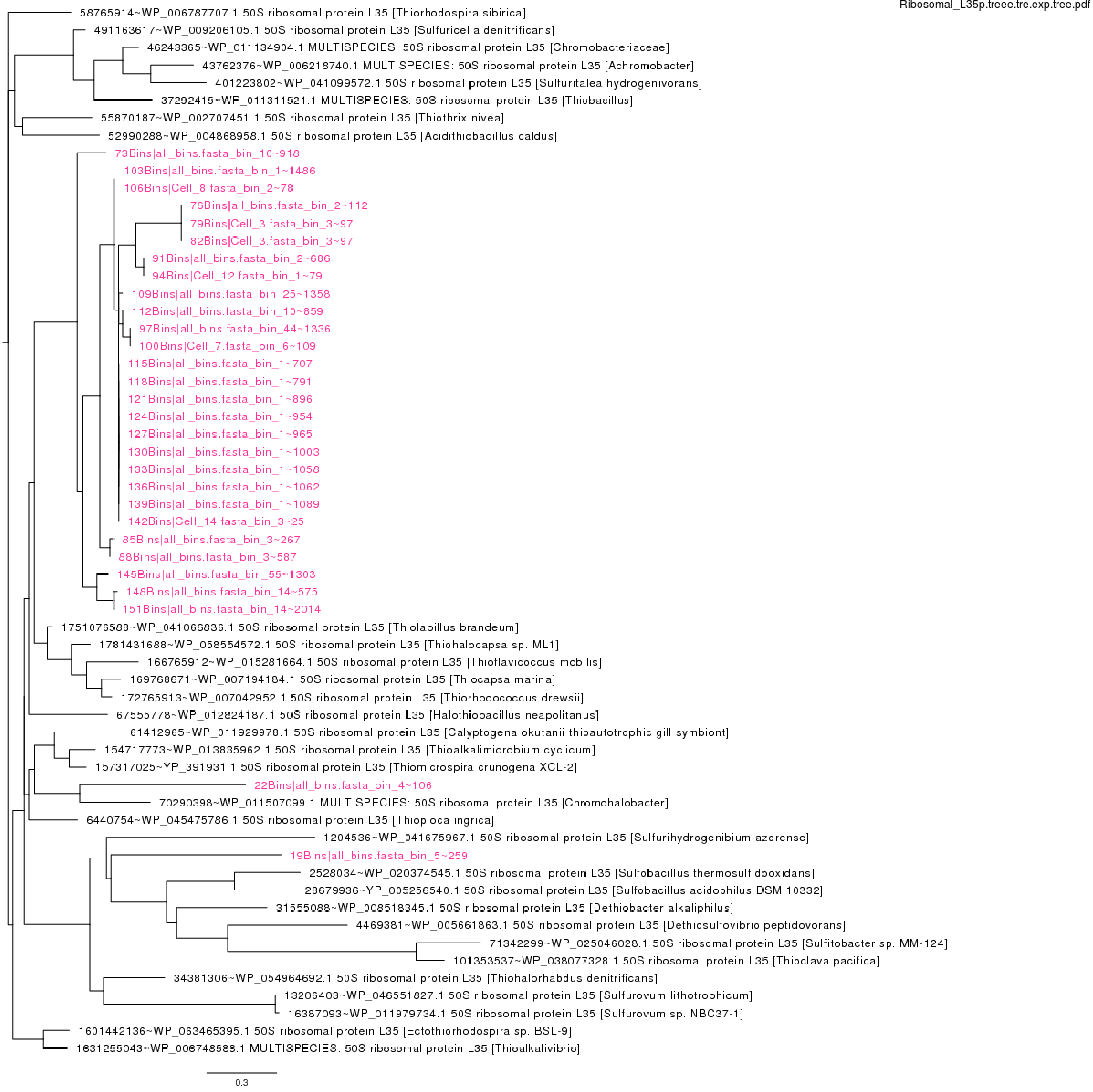
Ribosomal_L35p.treee.tre.exp.tree.pdf
58765914~WP_006787707.1 50S ribosomal protein L35 [Thiorhodospira sibirica]
491163617~WP_009206105.1 50S ribosomal protein L35 [Sulfuricella denitrificans]
46243365~WP_011134904.1 MULTISPECIES: 50S ribosomal protein L35 [Chromobacteriaceae]
43762376~WP_006218740.1 MULTISPECIES: 50S ribosomal protein L35 [Achromobacter]
401223802~WP_041099572.1 50S ribosomal protein L35 [Sulfuritalea hydrogenivorans]
37292415~WP_011311521.1 MULTISPECIES: 50S ribosomal protein L35 [Thiobacillus]
55870187~WP_002707451.1 50S ribosomal protein L35 [Thiothrix nivea]
52990288~WP_004868958.1 50S ribosomal protein L35 [Acidithiobacillus caldus]
73Bins|all_bins.fasta_bin_10~918
103Bins|all_bins.fasta_bin_1~1486
106Bins|Cell_8.fasta_bin_2~78
76Bins|all_bins.fasta_bin_2~112
79Bins|Cell_3.fasta_bin_3~97
82Bins|Cell_3.fasta_bin_3~97
91Bins|all_bins.fasta_bin_2~686
94Bins|Cell_12.fasta_bin_1~79
109Bins|all_bins.fasta_bin_25~1358
112Bins|all_bins.fasta_bin_10~859
97Bins|all_bins.fasta_bin_44~1336
100Bins|Cell_7.fasta_bin_6~109
115Bins|all_bins.fasta_bin_1~707
118Bins|all_bins.fasta_bin_1~791
121Bins|all_bins.fasta_bin_1~896
124Bins|all_bins.fasta_bin_1~954
127Bins|all_bins.fasta_bin_1~965
130Bins|all_bins.fasta_bin_1~1003
133Bins|all_bins.fasta_bin_1~1058
136Bins|all_bins.fasta_bin_1~1062
139Bins|all_bins.fasta_bin_1~1089
142Bins|Cell_14.fasta_bin_3~25
85Bins|all_bins.fasta_bin_3~267
88Bins|all_bins.fasta_bin_3~587
145Bins|all_bins.fasta_bin_55~1303
148Bins|all_bins.fasta_bin_14~575
151Bins|all_bins.fasta_bin_14~2014
1751076588~WP_041066836.1 50S ribosomal protein L35 [Thiolapillus brandeum]
1781431688~WP_058554572.1 50S ribosomal protein L35 [Thiohalocapsa sp. ML1]
166765912~WP_015281664.1 50S ribosomal protein L35 [Thioflavicoccus mobilis]
169768671~WP_007194184.1 50S ribosomal protein L35 [Thiocapsa marina]
172765913~WP_007042952.1 50S ribosomal protein L35 [Thiorhodococcus drewsii]
67555778~WP_012824187.1 50S ribosomal protein L35 [Halothiobacillus neapolitanus]
61412965~WP_011929978.1 50S ribosomal protein L35 [Calyptogena okutanii thioautotrophic gill symbiont]
154717773~WP_013835962.1 50S ribosomal protein L35 [Thioalkalimicrobium cyclicum]
157317025~YP_391931.1 50S ribosomal protein L35 [Thiomicrospira crunogena XCL-2]
22Bins|all_bins.fasta_bin_4~106
70290398~WP_011507099.1 MULTISPECIES: 50S ribosomal protein L35 [Chromohalobacter]
6440754~WP_045475786.1 50S ribosomal protein L35 [Thioploca ingrica]
1204536~WP_041675967.1 50S ribosomal protein L35 [Sulfurihydrogenibium azorense]
19Bins|all_bins.fasta_bin_5~259
2528034~WP_020374545.1 50S ribosomal protein L35 [Sulfobacillus thermosulfidooxidans]
28679936~YP_005256540.1 50S ribosomal protein L35 [Sulfobacillus acidophilus DSM 10332]
31555088~WP_008518345.1 50S ribosomal protein L35 [Dethiobacter alkaliphilus]
4469381~WP_005661863.1 50S ribosomal protein L35 [Dethiosulfovibrio peptidovorans]
71342299~WP_025046028.1 50S ribosomal protein L35 [Sulfitobacter sp. MM-124]
101353537~WP_038077328.1 50S ribosomal protein L35 [Thioclava pacifica]
34381306~WP_054964692.1 50S ribosomal protein L35 [Thiohalorhabdus denitrificans]
13206403~WP_046551827.1 50S ribosomal protein L35 [Sulfurovum lithotrophicum]
16387093~WP_011979734.1 50S ribosomal protein L35 [Sulfurovum sp. NBC37-1]
1601442136~WP_063465395.1 50S ribosomal protein L35 [Ectothiorhodospira sp. BSL-9]
1631255043~WP_006748586.1 MULTISPECIES: 50S ribosomal protein L35 [Thioalkalivibrio]
0.3

Ribosomal_L3.treee.tre.exp.tree.pdf

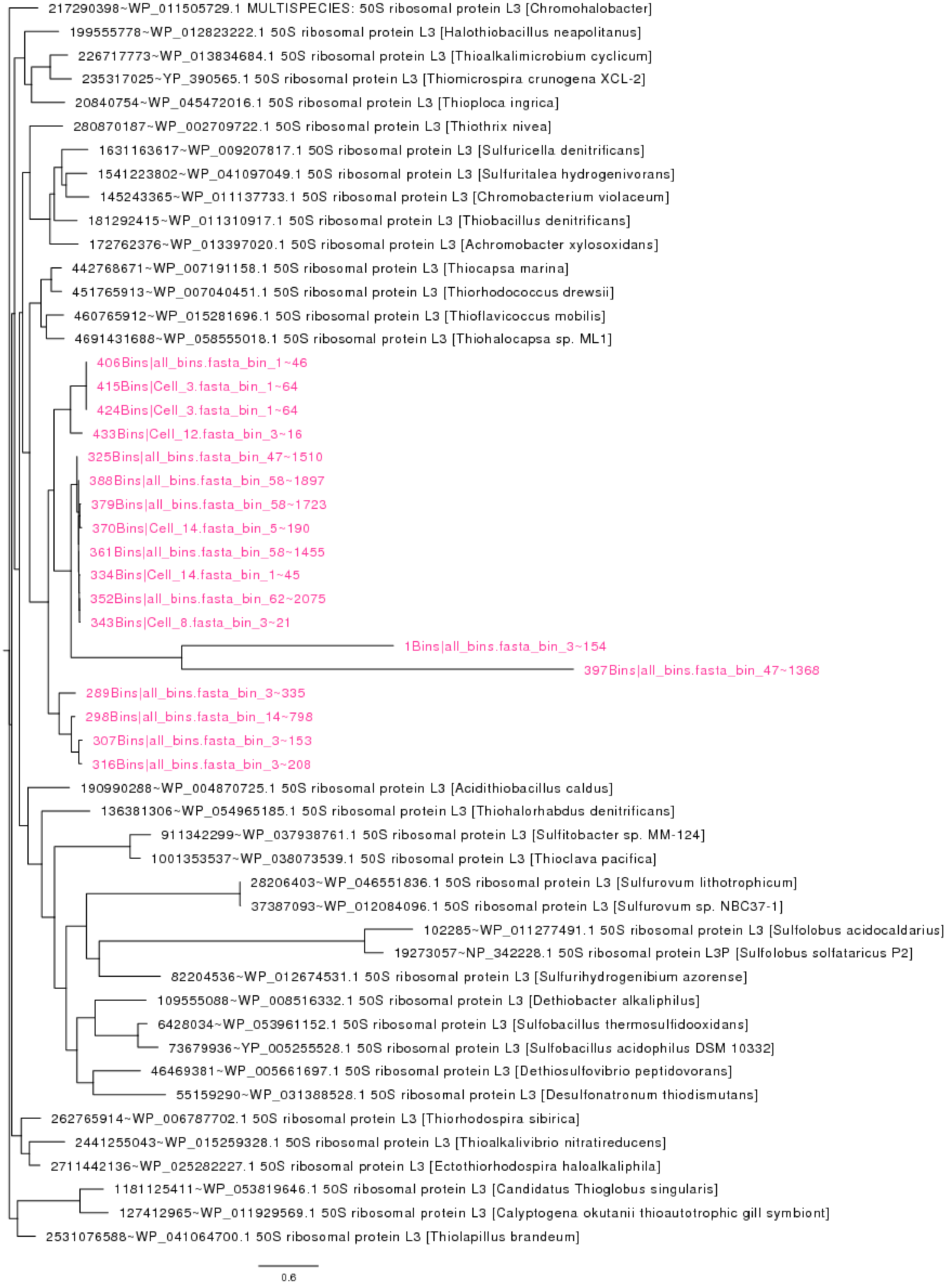

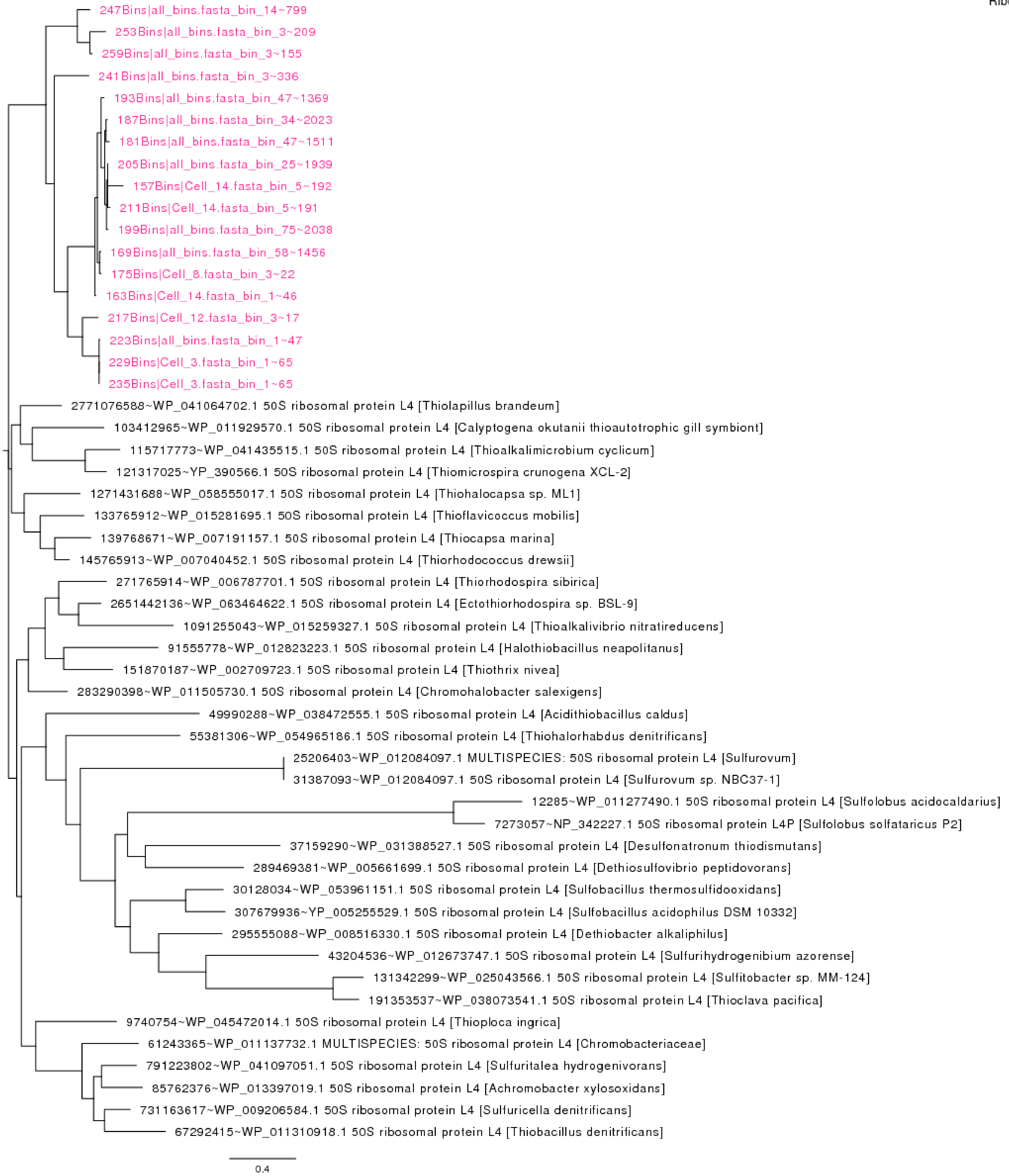
247Bins|all_bins.fasta_bin_14~799
253Bins|all_bins.fasta_bin_3~209
259Bins|all_bins.fasta_bin_3~155
241Bins|all_bins.fasta_bin_3~336
193Bins|all_bins.fasta_bin_47~1369
187Bins|all_bins.fasta_bin_34~2023
181Bins|all_bins.fasta_bin_47~1511
205Bins|all_bins.fasta_bin_25~1939
157Bins|Cell_14.fasta_bin_5~192
211Bins|Cell_14.fasta_bin_5~191
199Bins|all_bins.fasta_bin_75~2038
169Bins|all_bins.fasta_bin_58~1456
175Bins|Cell_8.fasta_bin_3~22
163Bins|Cell_14.fasta_bin_1~46
217Bins|Cell_12.fasta_bin_3~17
223Bins|all_bins.fasta_bin_1~47
229Bins|Cell_3.fasta_bin_1~65
235Bins|Cell_3.fasta_bin_1~65
2771076588~WP_041064702.1 50S ribosomal protein L4 [Thiolapillus brandeum]
103412965~WP_011929570.1 50S ribosomal protein L4 [Calyptogena okutanii thioautotrophic gill symbiont]
115717773~WP_041435515.1 50S ribosomal protein L4 [Thioalkalimicrobium cyclicum]
121317025~YP_390566.1 50S ribosomal protein L4 [Thiomicrospira crunogena XCL-2]
1271431688~WP_058555017.1 50S ribosomal protein L4 [Thiohalocapsa sp. ML1]
133765912~WP_015281695.1 50S ribosomal protein L4 [Thioflavicoccus mobilis]
139768671~WP_007191157.1 50S ribosomal protein L4 [Thiocapsa marina]
145765913~WP_007040452.1 50S ribosomal protein L4 [Thiorhodococcus drewsii]
271765914~WP_006787701.1 50S ribosomal protein L4 [Thiorhodospira sibirica]
2651442136~WP_063464622.1 50S ribosomal protein L4 [Ectothiorhodospira sp. BSL-9]
1091255043~WP_015259327.1 50S ribosomal protein L4 [Thioalkalivibrio nitratireducens]
91555778~WP_012823223.1 50S ribosomal protein L4 [Halothiobacillus neapolitanus]
151870187~WP_002709723.1 50S ribosomal protein L4 [Thiothrix nivea]
283290398~WP_011505730.1 50S ribosomal protein L4 [Chromohalobacter salexigens]
49990288~WP_038472555.1 50S ribosomal protein L4 [Acidithiobacillus caldus]
55381306~WP_054965186.1 50S ribosomal protein L4 [Thiohalorhabdus denitrificans]
25206403~WP_012084097.1 MULTISPECIES: 50S ribosomal protein L4 [Sulfurovum]
31387093~WP_012084097.1 50S ribosomal protein L4 [Sulfurovum sp. NBC37-1]
12285~WP_011277490.1 50S ribosomal protein L4 [Sulfolobus acidocaldarius]
7273057~NP_342227.1 50S ribosomal protein L4P [Sulfolobus solfataricus P2]
37159290~WP_031388527.1 50S ribosomal protein L4 [Desulfonatronum thiodismutans]
289469381~WP_005661699.1 50S ribosomal protein L4 [Dethiosulfovibrio peptidovorans]
30128034~WP_053961151.1 50S ribosomal protein L4 [Sulfobacillus thermosulfidooxidans]
307679936~YP_005255529.1 50S ribosomal protein L4 [Sulfobacillus acidophilus DSM 10332]
295555088~WP_008516330.1 50S ribosomal protein L4 [Dethiobacter alkaliphilus]
43204536~WP_012673747.1 50S ribosomal protein L4 [Sulfurihydrogenibium azorense]
131342299~WP_025043566.1 50S ribosomal protein L4 [Sulfitobacter sp. MM-124]
191353537~WP_038073541.1 50S ribosomal protein L4 [Thioclava pacifica]
9740754~WP_045472014.1 50S ribosomal protein L4 [Thioploca ingrica]
61243365~WP_011137732.1 MULTISPECIES: 50S ribosomal protein L4 [Chromobacteriaceae]
791223802~WP_041097051.1 50S ribosomal protein L4 [Sulfuritalea hydrogenivorans]
85762376~WP_013397019.1 50S ribosomal protein L4 [Achromobacter xylosoxidans]
731163617~WP_009206584.1 50S ribosomal protein L4 [Sulfuricella denitrificans]
67292415~WP_011310918.1 50S ribosomal protein L4 [Thiobacillus denitrificans]
0.4

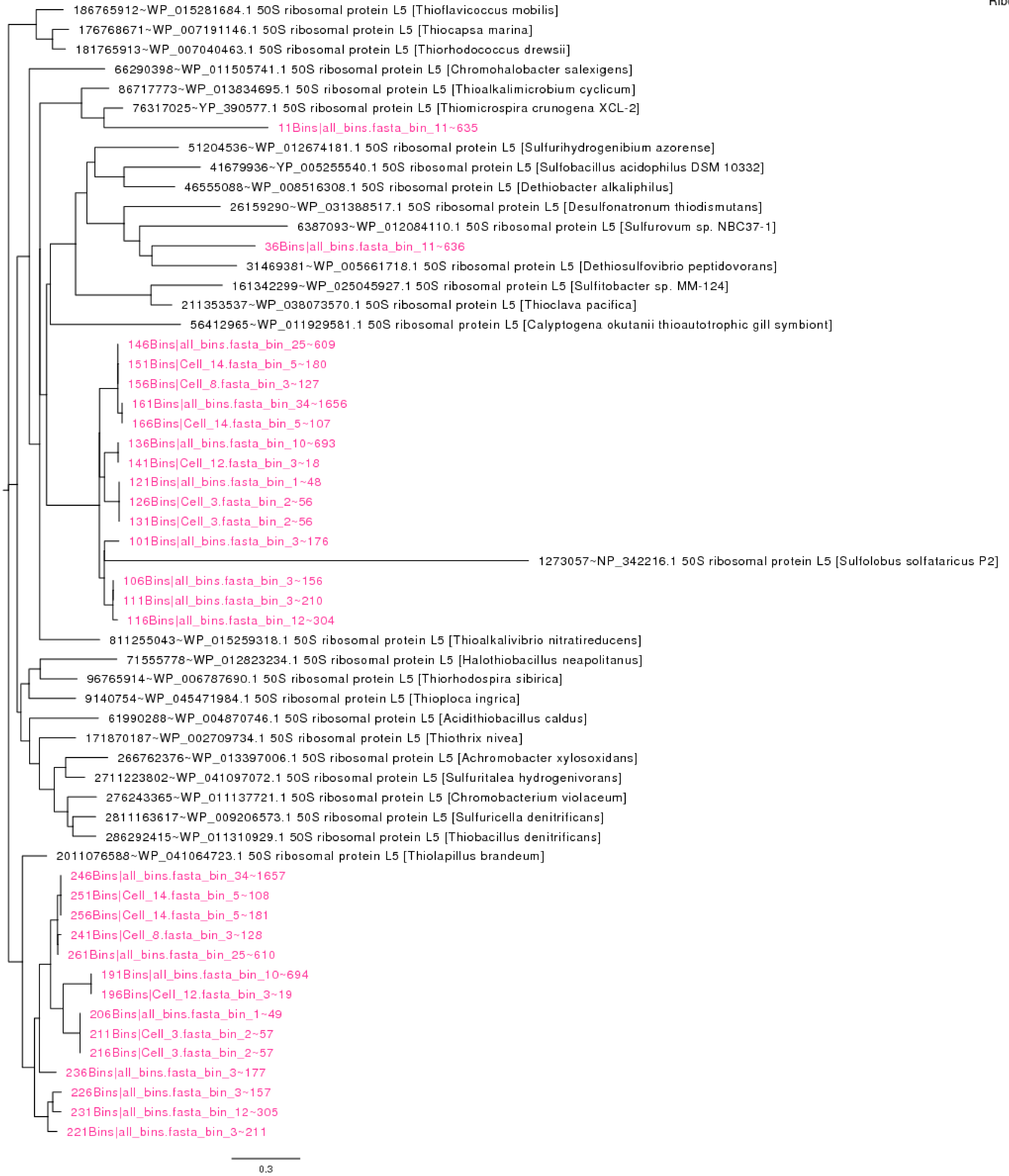
186765912~WP_015281684.1 50S ribosomal protein L5 [Thioflavicoccus mobilis]
176768671~WP_007191146.1 50S ribosomal protein L5 [Thiocapsa marina]
181765913~WP_007040463.1 50S ribosomal protein L5 [Thiorhodococcus drewsii]
66290398~WP_011505741.1 50S ribosomal protein L5 [Chromohalobacter salexigens]
86717773~WP_013834695.1 50S ribosomal protein L5 [Thioalkalimicrobium cyclicum]
76317025~YP_390577.1 50S ribosomal protein L5 [Thiomicrospira crunogena XCL-2]
11Bins|all_bins.fasta_bin_11~635
51204536~WP_012674181.1 50S ribosomal protein L5 [Sulfurihydrogenibium azorense]
41679936~YP_005255540.1 50S ribosomal protein L5 [Sulfobacillus acidophilus DSM 10332]
46555088~WP_008516308.1 50S ribosomal protein L5 [Dethiobacter alkaliphilus]
26159290~WP_031388517.1 50S ribosomal protein L5 [Desulfonatronum thiodismutans]
6387093~WP_012084110.1 50S ribosomal protein L5 [Sulfurovum sp. NBC37-1]
36Bins|all_bins.fasta_bin_11~636
31469381~WP_005661718.1 50S ribosomal protein L5 [Dethiosulfovibrio peptidovorans]
161342299~WP_025045927.1 50S ribosomal protein L5 [Sulfitobacter sp. MM-124]
211353537~WP_038073570.1 50S ribosomal protein L5 [Thioclava pacifica]
56412965~WP_011929581.1 50S ribosomal protein L5 [Calyptogena okutanii thioautotrophic gill symbiont]
146Bins|all_bins.fasta_bin_25~609
151Bins|Cell_14.fasta_bin_5~180
156Bins|Cell_8.fasta_bin_3~127
161Bins|all_bins.fasta_bin_34~1656
166Bins|Cell_14.fasta_bin_5~107
136Bins|all_bins.fasta_bin_10~693
141Bins|Cell_12.fasta_bin_3~18
121Bins|all_bins.fasta_bin_1~48
126Bins|Cell_3.fasta_bin_2~56
131Bins|Cell_3.fasta_bin_2~56
101Bins|all_bins.fasta_bin_3~176
1273057~NP_342216.1 50S ribosomal protein L5 [Sulfolobus solfataricus P2]
106Bins|all_bins.fasta_bin_3~156
111Bins|all_bins.fasta_bin_3~210
116Bins|all_bins.fasta_bin_12~304
811255043~WP_015259318.1 50S ribosomal protein L5 [Thioalkalivibrio nitratireducens]
71555778~WP_012823234.1 50S ribosomal protein L5 [Halothiobacillus neapolitanus]
96765914~WP_006787690.1 50S ribosomal protein L5 [Thiorhodospira sibirica]
9140754~WP_045471984.1 50S ribosomal protein L5 [Thioploca ingrica]
61990288~WP_004870746.1 50S ribosomal protein L5 [Acidithiobacillus caldus]
171870187~WP_002709734.1 50S ribosomal protein L5 [Thiothrix nivea]
266762376~WP_013397006.1 50S ribosomal protein L5 [Achromobacter xylosoxidans]
2711223802~WP_041097072.1 50S ribosomal protein L5 [Sulfuritalea hydrogenivorans]
276243365~WP_011137721.1 50S ribosomal protein L5 [Chromobacterium violaceum]
2811163617~WP_009206573.1 50S ribosomal protein L5 [Sulfuricella denitrificans]
286292415~WP_011310929.1 50S ribosomal protein L5 [Thiobacillus denitrificans]
2011076588~WP_041064723.1 50S ribosomal protein L5 [Thiolapillus brandeum]
246Bins|all_bins.fasta_bin_34~1657
251Bins|Cell_14.fasta_bin_5~108
256Bins|Cell_14.fasta_bin_5~181
241Bins|Cell_8.fasta_bin_3~128
261Bins|all_bins.fasta_bin_25~610
191Bins|all_bins.fasta_bin_10~694
196Bins|Cell_12.fasta_bin_3~19
206Bins|all_bins.fasta_bin_1~49
211Bins|Cell_3.fasta_bin_2~57
216Bins|Cell_3.fasta_bin_2~57
236Bins|all_bins.fasta_bin_3~177
226Bins|all_bins.fasta_bin_3~157
231Bins|all_bins.fasta_bin_12~305
221Bins|all_bins.fasta_bin_3~211
0.3

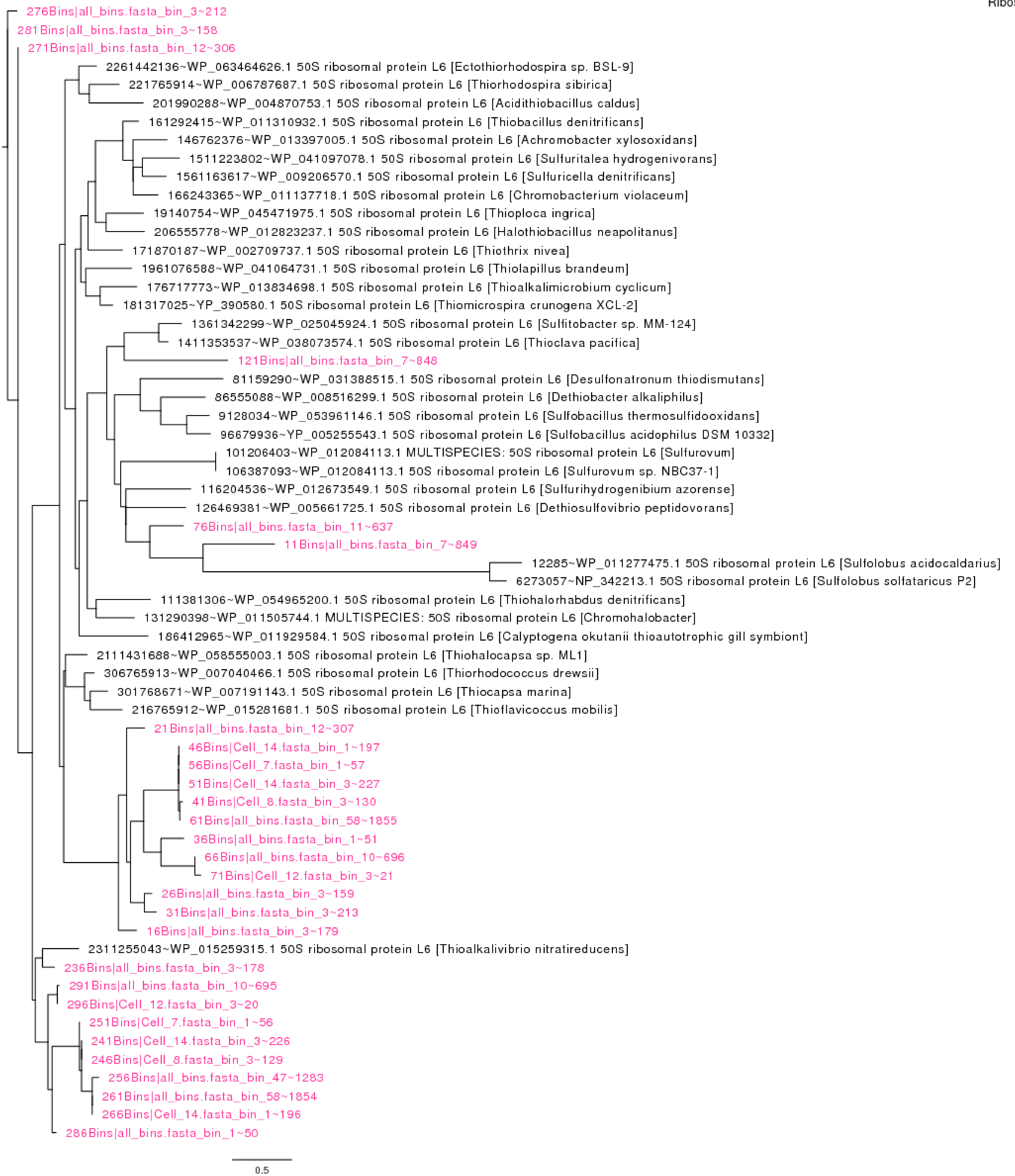
276Bins|all_bins.fasta_bin_3~212
281Bins|all_bins.fasta_bin_3~158
271Bins|all_bins.fasta_bin_12~306
2261442136~WP_063464626.1 50S ribosomal protein L6 [Ectothiorhodospira sp. BSL-9]
221765914~WP_006787687.1 50S ribosomal protein L6 [Thiorhodospira sibirica]
201990288~WP_004870753.1 50S ribosomal protein L6 [Acidithiobacillus caldus]
161292415~WP_011310932.1 50S ribosomal protein L6 [Thiobacillus denitrificans]
146762376~WP_013397005.1 50S ribosomal protein L6 [Achromobacter xylosoxidans]
1511223802~WP_041097078.1 50S ribosomal protein L6 [Sulfuritalea hydrogenivorans]
1561163617~WP_009206570.1 50S ribosomal protein L6 [Sulfuricella denitrificans]
166243365~WP_011137718.1 50S ribosomal protein L6 [Chromobacterium violaceum]
19140754~WP_045471975.1 50S ribosomal protein L6 [Thioploca ingrica]
206555778~WP_012823237.1 50S ribosomal protein L6 [Halothiobacillus neapolitanus]
171870187~WP_002709737.1 50S ribosomal protein L6 [Thiothrix nivea]
1961076588~WP_041064731.1 50S ribosomal protein L6 [Thiolapillus brandeum]
176717773~WP_013834698.1 50S ribosomal protein L6 [Thioalkalimicrobium cyclicum]
181317025~YP_390580.1 50S ribosomal protein L6 [Thiomicrospira crunogena XCL-2]
1361342299~WP_025045924.1 50S ribosomal protein L6 [Sulfitobacter sp. MM-124]
1411353537~WP_038073574.1 50S ribosomal protein L6 [Thioclava pacifica]
121Bins|all_bins.fasta_bin_7~848
81159290~WP_031388515.1 50S ribosomal protein L6 [Desulfonatronum thiodismutans]
86555088~WP_008516299.1 50S ribosomal protein L6 [Dethiobacter alkaliphilus]
9128034~WP_053961146.1 50S ribosomal protein L6 [Sulfobacillus thermosulfidooxidans]
96679936~YP_005255543.1 50S ribosomal protein L6 [Sulfobacillus acidophilus DSM 10332]
101206403~WP_012084113.1 MULTISPECIES: 50S ribosomal protein L6 [Sulfurovum]
106387093~WP_012084113.1 50S ribosomal protein L6 [Sulfurovum sp. NBC37-1]
116204536~WP_012673549.1 50S ribosomal protein L6 [Sulfurihydrogenibium azorense]
126469381~WP_005661725.1 50S ribosomal protein L6 [Dethiosulfovibrio peptidovorans]
76Bins|all_bins.fasta_bin_11~637
11Bins|all_bins.fasta_bin_7~849
12285~WP_011277475.1 50S ribosomal protein L6 [Sulfolobus acidocaldarius]
6273057~NP_342213.1 50S ribosomal protein L6 [Sulfolobus solfataricus P2]
111381306~WP_054965200.1 50S ribosomal protein L6 [Thiohalorhabdus denitrificans]
131290398~WP_011505744.1 MULTISPECIES: 50S ribosomal protein L6 [Chromohalobacter]
186412965~WP_011929584.1 50S ribosomal protein L6 [Calyptogena okutanii thioautotrophic gill symbiont]
2111431688~WP_058555003.1 50S ribosomal protein L6 [Thiohalocapsa sp. ML1]
306765913~WP_007040466.1 50S ribosomal protein L6 [Thiorhodococcus drewsii]
301768671~WP_007191143.1 50S ribosomal protein L6 [Thiocapsa marina]
216765912~WP_015281681.1 50S ribosomal protein L6 [Thioflavicoccus mobilis]
21Bins|all_bins.fasta_bin_12~307
46Bins|Cell_14.fasta_bin_1~197
56Bins|Cell_7.fasta_bin_1~57
51Bins|Cell_14.fasta_bin_3~227
41Bins|Cell_8.fasta_bin_3~130
61Bins|all_bins.fasta_bin_58~1855
36Bins|all_bins.fasta_bin_1~51
66Bins|all_bins.fasta_bin_10~696
71Bins|Cell_12.fasta_bin_3~21
26Bins|all_bins.fasta_bin_3~159
31Bins|all_bins.fasta_bin_3~213
16Bins|all_bins.fasta_bin_3~179
2311255043~WP_015259315.1 50S ribosomal protein L6 [Thioalkalivibrio nitratireducens]
236Bins|all_bins.fasta_bin_3~178
291Bins|all_bins.fasta_bin_10~695
296Bins|Cell_12.fasta_bin_3~20
251Bins|Cell_7.fasta_bin_1~56
241Bins|Cell_14.fasta_bin_3~226
246Bins|Cell_8.fasta_bin_3~129
256Bins|all_bins.fasta_bin_47~1283
261Bins|all_bins.fasta_bin_58~1854
266Bins|Cell_14.fasta_bin_1~196
286Bins|all_bins.fasta_bin_1~50
0.5

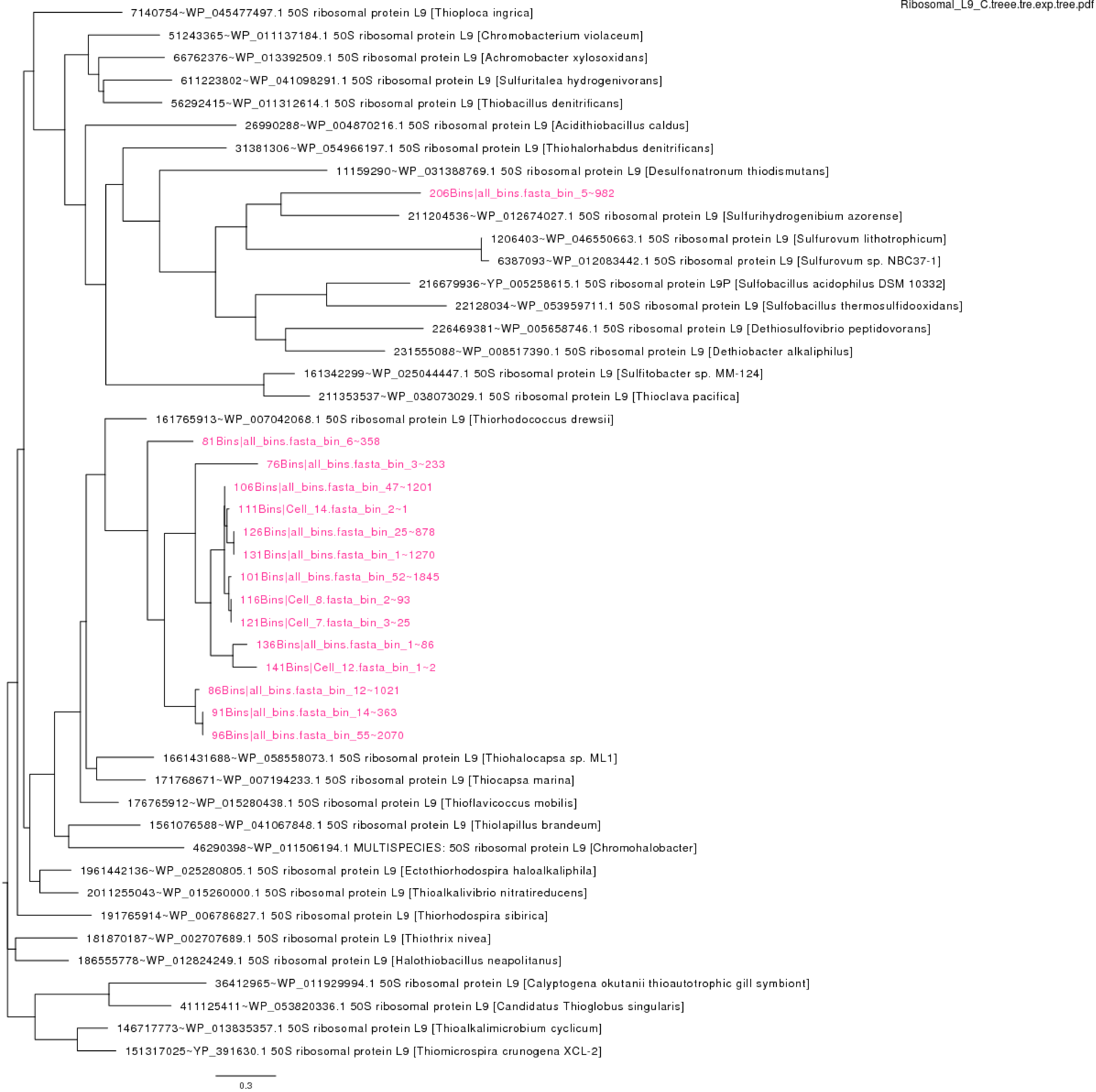
Ribosomal_L9_C.treee.tre.exp.tree.pdf
7140754~WP_045477497.1 50S ribosomal protein L9 [Thioploca ingrica]
51243365~WP_011137184.1 50S ribosomal protein L9 [Chromobacterium violaceum]
66762376~WP_013392509.1 50S ribosomal protein L9 [Achromobacter xylosoxidans]
611223802~WP_041098291.1 50S ribosomal protein L9 [Sulfuritalea hydrogenivorans]
56292415~WP_011312614.1 50S ribosomal protein L9 [Thiobacillus denitrificans]
26990288~WP_004870216.1 50S ribosomal protein L9 [Acidithiobacillus caldus]
31381306~WP_054966197.1 50S ribosomal protein L9 [Thiohalorhabdus denitrificans]
11159290~WP_031388769.1 50S ribosomal protein L9 [Desulfonatronum thiodismutans]
206Bins|all_bins.fasta_bin_5~982
211204536~WP_012674027.1 50S ribosomal protein L9 [Sulfurihydrogenibium azorense]
1206403~WP_046550663.1 50S ribosomal protein L9 [Sulfurovum lithotrophicum]
6387093~WP_012083442.1 50S ribosomal protein L9 [Sulfurovum sp. NBC37-1]
216679936~YP_005258615.1 50S ribosomal protein L9P [Sulfobacillus acidophilus DSM 10332]
22128034~WP_053959711.1 50S ribosomal protein L9 [Sulfobacillus thermosulfidooxidans]
226469381~WP_005658746.1 50S ribosomal protein L9 [Dethiosulfovibrio peptidovorans]
231555088~WP_008517390.1 50S ribosomal protein L9 [Dethiobacter alkaliphilus]
161342299~WP_025044447.1 50S ribosomal protein L9 [Sulfitobacter sp. MM-124]
211353537~WP_038073029.1 50S ribosomal protein L9 [Thioclava pacifica]
161765913~WP_007042068.1 50S ribosomal protein L9 [Thiorhodococcus drewsii]
81Bins|all_bins.fasta_bin_6~358
76Bins|all_bins.fasta_bin_3~233
106Bins|all_bins.fasta_bin_47~1201
111Bins|Cell_14.fasta_bin_2~1
126Bins|all_bins.fasta_bin_25~878
131Bins|all_bins.fasta_bin_1~1270
101Bins|all_bins.fasta_bin_52~1845
116Bins|Cell_8.fasta_bin_2~93
121Bins|Cell_7.fasta_bin_3~25
136Bins|all_bins.fasta_bin_1~86
141Bins|Cell_12.fasta_bin_1~2
86Bins|all_bins.fasta_bin_12~1021
91Bins|all_bins.fasta_bin_14~363
96Bins|all_bins.fasta_bin_55~2070
1661431688~WP_058558073.1 50S ribosomal protein L9 [Thiohalocapsa sp. ML1]
171768671~WP_007194233.1 50S ribosomal protein L9 [Thiocapsa marina]
176765912~WP_015280438.1 50S ribosomal protein L9 [Thioflavicoccus mobilis]
1561076588~WP_041067848.1 50S ribosomal protein L9 [Thiolapillus brandeum]
46290398~WP_011506194.1 MULTISPECIES: 50S ribosomal protein L9 [Chromohalobacter]
1961442136~WP_025280805.1 50S ribosomal protein L9 [Ectothiorhodospira haloalkaliphila]
2011255043~WP_015260000.1 50S ribosomal protein L9 [Thioalkalivibrio nitratireducens]
191765914~WP_006786827.1 50S ribosomal protein L9 [Thiorhodospira sibirica]
181870187~WP_002707689.1 50S ribosomal protein L9 [Thiothrix nivea]
186555778~WP_012824249.1 50S ribosomal protein L9 [Halothiobacillus neapolitanus]
36412965~WP_011929994.1 50S ribosomal protein L9 [Calyptogena okutanii thioautotrophic gill symbiont]
411125411~WP_053820336.1 50S ribosomal protein L9 [Candidatus Thioglobus singularis]
146717773~WP_013835357.1 50S ribosomal protein L9 [Thioalkalimicrobium cyclicum]
151317025~YP_391630.1 50S ribosomal protein L9 [Thiomicrospira crunogena XCL-2]
0.3

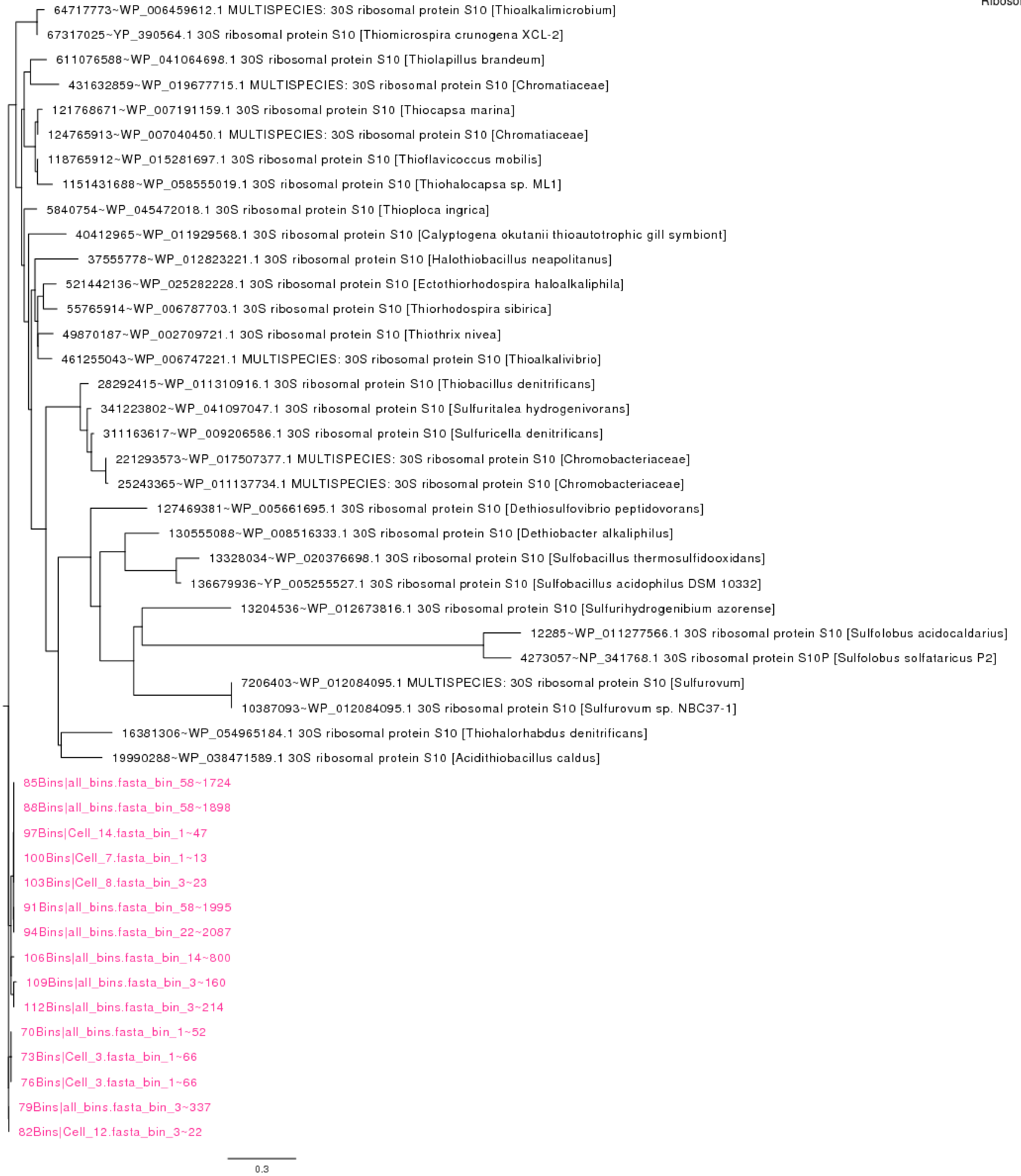

64717773~WP_006459612.1 MULTISPECIES: 30S ribosomal protein S10 [Thioalkalimicrobium]
67317025~YP_390564.1 30S ribosomal protein S10 [Thiomicrospira crunogena XCL-2]
611076588~WP_041064698.1 30S ribosomal protein S10 [Thiolapillus brandeum]
431632859~WP_019677715.1 MULTISPECIES: 30S ribosomal protein S10 [Chromatiaceae]
121768671~WP_007191159.1 30S ribosomal protein S10 [Thiocapsa marina]
124765913~WP_007040450.1 MULTISPECIES: 30S ribosomal protein S10 [Chromatiaceae]
118765912~WP_015281697.1 30S ribosomal protein S10 [Thioflavicoccus mobilis]
1151431688~WP_058555019.1 30S ribosomal protein S10 [Thiohalocapsa sp. ML1]
5840754~WP_045472018.1 30S ribosomal protein S10 [Thioploca ingrica]
40412965~WP_011929568.1 30S ribosomal protein S10 [Calyptogena okutanii thioautotrophic gill symbiont]
37555778~WP_012823221.1 30S ribosomal protein S10 [Halothiobacillus neapolitanus]
521442136~WP_025282228.1 30S ribosomal protein S10 [Ectothiorhodospira haloalkaliphila]
55765914~WP_006787703.1 30S ribosomal protein S10 [Thiorhodospira sibirica]
49870187~WP_002709721.1 30S ribosomal protein S10 [Thiothrix nivea]
461255043~WP_006747221.1 MULTISPECIES: 30S ribosomal protein S10 [Thioalkalivibrio]
28292415~WP_011310916.1 30S ribosomal protein S10 [Thiobacillus denitrificans]
341223802~WP_041097047.1 30S ribosomal protein S10 [Sulfuritalea hydrogenivorans]
311163617~WP_009206586.1 30S ribosomal protein S10 [Sulfuricella denitrificans]
221293573~WP_017507377.1 MULTISPECIES: 30S ribosomal protein S10 [Chromobacteriaceae]
25243365~WP_011137734.1 MULTISPECIES: 30S ribosomal protein S10 [Chromobacteriaceae]
127469381~WP_005661695.1 30S ribosomal protein S10 [Dethiosulfovibrio peptidovorans]
130555088~WP_008516333.1 30S ribosomal protein S10 [Dethiobacter alkaliphilus]
13328034~WP_020376698.1 30S ribosomal protein S10 [Sulfobacillus thermosulfidooxidans]
136679936~YP_005255527.1 30S ribosomal protein S10 [Sulfobacillus acidophilus DSM 10332]
13204536~WP_012673816.1 30S ribosomal protein S10 [Sulfurihydrogenibium azorense]
12285~WP_011277566.1 30S ribosomal protein S10 [Sulfolobus acidocaldarius]
4273057~NP_341768.1 30S ribosomal protein S10P [Sulfolobus solfataricus P2]
7206403~WP_012084095.1 MULTISPECIES: 30S ribosomal protein S10 [Sulfurovum]
10387093~WP_012084095.1 30S ribosomal protein S10 [Sulfurovum sp. NBC37-1]
16381306~WP_054965184.1 30S ribosomal protein S10 [Thiohalorhabdus denitrificans]
19990288~WP_038471589.1 30S ribosomal protein S10 [Acidithiobacillus caldus]
85Bins|all_bins.fasta_bin_58~1724
88Bins|all_bins.fasta_bin_58~1898
97Bins|Cell_14.fasta_bin_1~47
100Bins|Cell_7.fasta_bin_1~13
103Bins|Cell_8.fasta_bin_3~23
91Bins|all_bins.fasta_bin_58~1995
94Bins|all_bins.fasta_bin_22~2087
106Bins|all_bins.fasta_bin_14~800
109Bins|all_bins.fasta_bin_3~160
112Bins|all_bins.fasta_bin_3~214
70Bins|all_bins.fasta_bin_1~52
73Bins|Cell_3.fasta_bin_1~66
76Bins|Cell_3.fasta_bin_1~66
79Bins|all_bins.fasta_bin_3~337
82Bins|Cell_12.fasta_bin_3~22
0.3

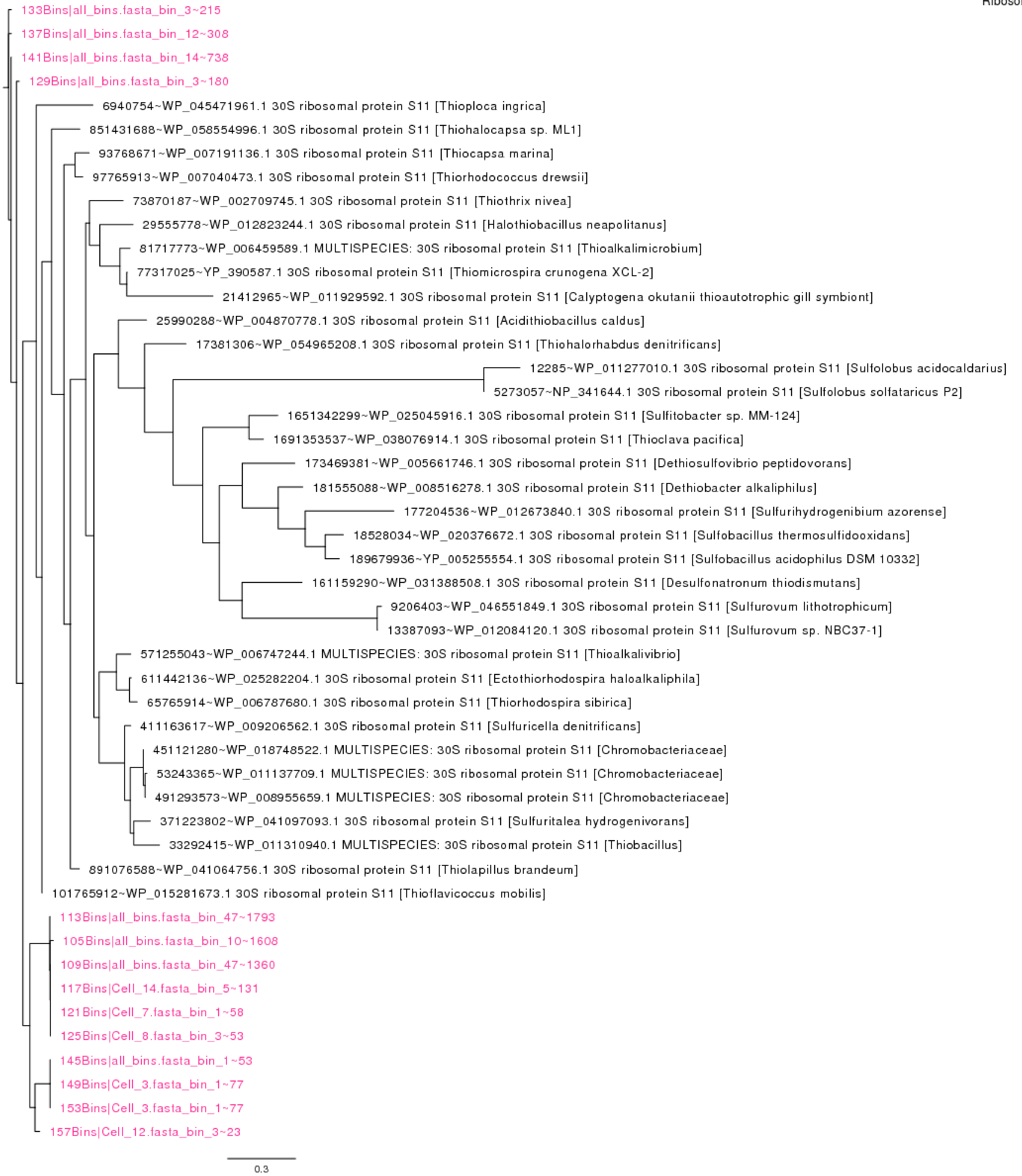
133Bins|all_bins.fasta_bin_3~215
137Bins|all_bins.fasta_bin_12~308
141Bins|all_bins.fasta_bin_14~738
129Bins|all_bins.fasta_bin_3~180
6940754~WP_045471961.1 30S ribosomal protein S11 [Thioploca ingrica]
851431688~WP_058554996.1 30S ribosomal protein S11 [Thiohalocapsa sp. ML1]
93768671~WP_007191136.1 30S ribosomal protein S11 [Thiocapsa marina]
97765913~WP_007040473.1 30S ribosomal protein S11 [Thiorhodococcus drewsii]
73870187~WP_002709745.1 30S ribosomal protein S11 [Thiothrix nivea]
29555778~WP_012823244.1 30S ribosomal protein S11 [Halothiobacillus neapolitanus]
81717773~WP_006459589.1 MULTISPECIES: 30S ribosomal protein S11 [Thioalkalimicrobium]
77317025~YP_390587.1 30S ribosomal protein S11 [Thiomicrospira crunogena XCL-2]
21412965~WP_011929592.1 30S ribosomal protein S11 [Calyptogena okutanii thioautotrophic gill symbiont]
25990288~WP_004870778.1 30S ribosomal protein S11 [Acidithiobacillus caldus]
17381306~WP_054965208.1 30S ribosomal protein S11 [Thiohalorhabdus denitrificans]
12285~WP_011277010.1 30S ribosomal protein S11 [Sulfolobus acidocaldarius]
5273057~NP_341644.1 30S ribosomal protein S11 [Sulfolobus solfataricus P2]
1651342299~WP_025045916.1 30S ribosomal protein S11 [Sulfitobacter sp. MM-124]
1691353537~WP_038076914.1 30S ribosomal protein S11 [Thioclava pacifica]
173469381~WP_005661746.1 30S ribosomal protein S11 [Dethiosulfovibrio peptidovorans]
181555088~WP_008516278.1 30S ribosomal protein S11 [Dethiobacter alkaliphilus]
177204536~WP_012673840.1 30S ribosomal protein S11 [Sulfurihydrogenibium azorense]
18528034~WP_020376672.1 30S ribosomal protein S11 [Sulfobacillus thermosulfidooxidans]
189679936~YP_005255554.1 30S ribosomal protein S11 [Sulfobacillus acidophilus DSM 10332]
161159290~WP_031388508.1 30S ribosomal protein S11 [Desulfonatronum thiodismutans]
9206403~WP_046551849.1 30S ribosomal protein S11 [Sulfurovum lithotrophicum]
13387093~WP_012084120.1 30S ribosomal protein S11 [Sulfurovum sp. NBC37-1]
571255043~WP_006747244.1 MULTISPECIES: 30S ribosomal protein S11 [Thioalkalivibrio]
611442136~WP_025282204.1 30S ribosomal protein S11 [Ectothiorhodospira haloalkaliphila]
65765914~WP_006787680.1 30S ribosomal protein S11 [Thiorhodospira sibirica]
411163617~WP_009206562.1 30S ribosomal protein S11 [Sulfuricella denitrificans]
451121280~WP_018748522.1 MULTISPECIES: 30S ribosomal protein S11 [Chromobacteriaceae]
53243365~WP_011137709.1 MULTISPECIES: 30S ribosomal protein S11 [Chromobacteriaceae]
491293573~WP_008955659.1 MULTISPECIES: 30S ribosomal protein S11 [Chromobacteriaceae]
371223802~WP_041097093.1 30S ribosomal protein S11 [Sulfuritalea hydrogenivorans]
33292415~WP_011310940.1 MULTISPECIES: 30S ribosomal protein S11 [Thiobacillus]
891076588~WP_041064756.1 30S ribosomal protein S11 [Thiolapillus brandeum]
101765912~WP_015281673.1 30S ribosomal protein S11 [Thioflavicoccus mobilis]
113Bins|all_bins.fasta_bin_47~1793
105Bins|all_bins.fasta_bin_10~1608
109Bins|all_bins.fasta_bin_47~1360
117Bins|Cell_14.fasta_bin_5~131
121Bins|Cell_7.fasta_bin_1~58
125Bins|Cell_8.fasta_bin_3~53
145Bins|all_bins.fasta_bin_1~53
149Bins|Cell_3.fasta_bin_1~77
153Bins|Cell_3.fasta_bin_1~77
157Bins|Cell_12.fasta_bin_3~23
0.3

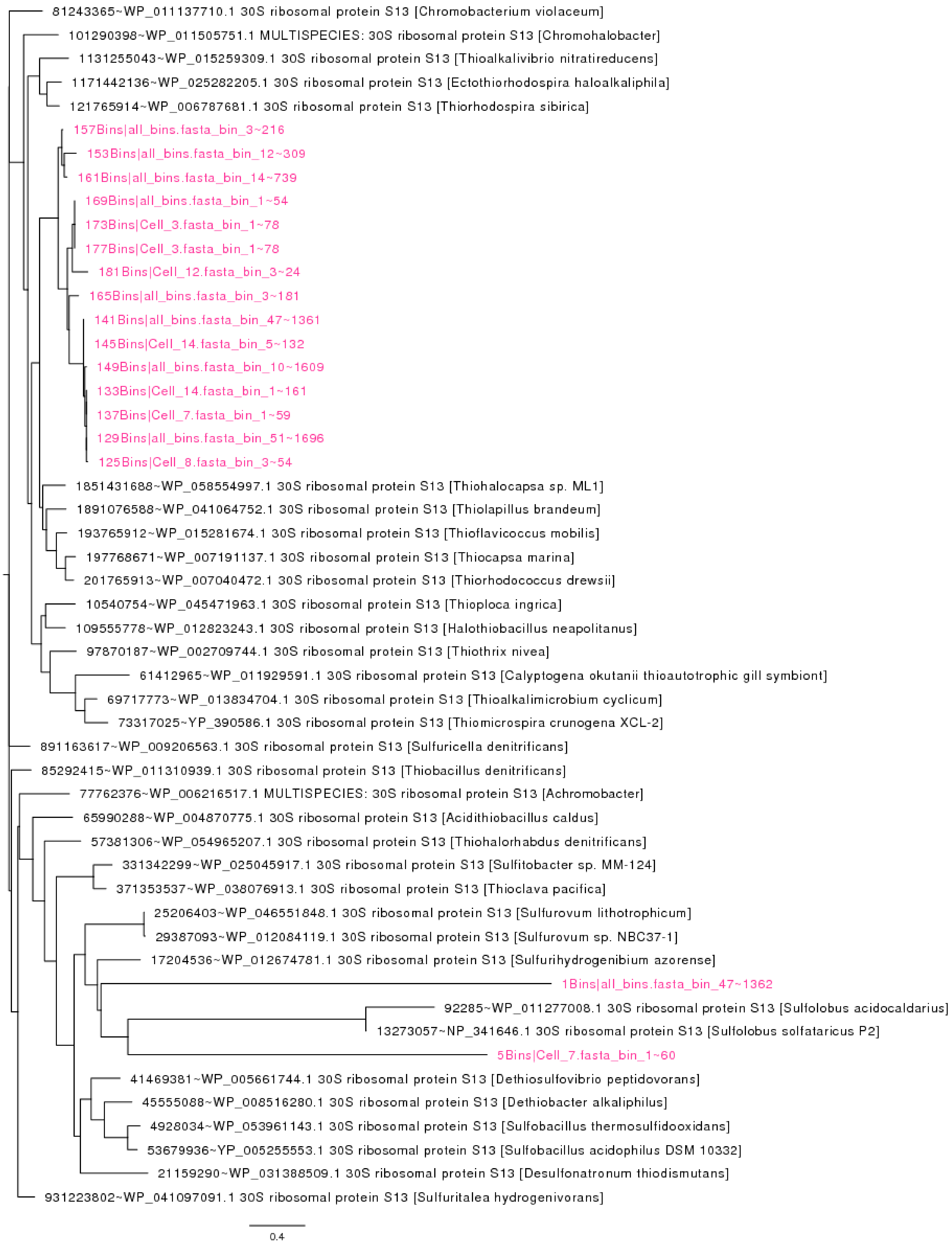
81243365~WP_011137710.1 30S ribosomal protein S13 [Chromobacterium violaceum]
101290398~WP_011505751.1 MULTISPECIES: 30S ribosomal protein S13 [Chromohalobacter]
1131255043~WP_015259309.1 30S ribosomal protein S13 [Thioalkalivibrio nitratireducens]
1171442136~WP_025282205.1 30S ribosomal protein S13 [Ectothiorhodospira haloalkaliphila]
121765914~WP_006787681.1 30S ribosomal protein S13 [Thiorhodospira sibirica]
157Bins|all_bins.fasta_bin_3~216
153Bins|all_bins.fasta_bin_12~309
161Bins|all_bins.fasta_bin_14~739
169Bins|all_bins.fasta_bin_1~54
173Bins|Cell_3.fasta_bin_1~78
177Bins|Cell_3.fasta_bin_1~78
181Bins|Cell_12.fasta_bin_3~24
165Bins|all_bins.fasta_bin_3~181
141Bins|all_bins.fasta_bin_47~1361
145Bins|Cell_14.fasta_bin_5~132
149Bins|all_bins.fasta_bin_10~1609
133Bins|Cell_14.fasta_bin_1~161
137Bins|Cell_7.fasta_bin_1~59
129Bins|all_bins.fasta_bin_51~1696
125Bins|Cell_8.fasta_bin_3~54
1851431688~WP_058554997.1 30S ribosomal protein S13 [Thiohalocapsa sp. ML1]
1891076588~WP_041064752.1 30S ribosomal protein S13 [Thiolapillus brandeum]
193765912~WP_015281674.1 30S ribosomal protein S13 [Thioflavicoccus mobilis]
197768671~WP_007191137.1 30S ribosomal protein S13 [Thiocapsa marina]
201765913~WP_007040472.1 30S ribosomal protein S13 [Thiorhodococcus drewsii]
10540754~WP_045471963.1 30S ribosomal protein S13 [Thioploca ingrica]
109555778~WP_012823243.1 30S ribosomal protein S13 [Halothiobacillus neapolitanus]
97870187~WP_002709744.1 30S ribosomal protein S13 [Thiothrix nivea]
61412965~WP_011929591.1 30S ribosomal protein S13 [Calyptogena okutanii thioautotrophic gill symbiont]
69717773~WP_013834704.1 30S ribosomal protein S13 [Thioalkalimicrobium cyclicum]
73317025~YP_390586.1 30S ribosomal protein S13 [Thiomicrospira crunogena XCL-2]
891163617~WP_009206563.1 30S ribosomal protein S13 [Sulfuricella denitrificans]
85292415~WP_011310939.1 30S ribosomal protein S13 [Thiobacillus denitrificans]
77762376~WP_006216517.1 MULTISPECIES: 30S ribosomal protein S13 [Achromobacter]
65990288~WP_004870775.1 30S ribosomal protein S13 [Acidithiobacillus caldus]
57381306~WP_054965207.1 30S ribosomal protein S13 [Thiohalorhabdus denitrificans]
331342299~WP_025045917.1 30S ribosomal protein S13 [Sulfitobacter sp. MM-124]
371353537~WP_038076913.1 30S ribosomal protein S13 [Thioclava pacifica]
25206403~WP_046551848.1 30S ribosomal protein S13 [Sulfurovum lithotrophicum]
29387093~WP_012084119.1 30S ribosomal protein S13 [Sulfurovum sp. NBC37-1]
17204536~WP_012674781.1 30S ribosomal protein S13 [Sulfurihydrogenibium azorense]
1Bins|all_bins.fasta_bin_47~1362
92285~WP_011277008.1 30S ribosomal protein S13 [Sulfolobus acidocaldarius]
13273057~NP_341646.1 30S ribosomal protein S13 [Sulfolobus solfataricus P2]
5Bins|Cell_7.fasta_bin_1~60
41469381~WP_005661744.1 30S ribosomal protein S13 [Dethiosulfovibrio peptidovorans]
45555088~WP_008516280.1 30S ribosomal protein S13 [Dethiobacter alkaliphilus]
4928034~WP_053961143.1 30S ribosomal protein S13 [Sulfobacillus thermosulfidooxidans]
53679936~YP_005255553.1 30S ribosomal protein S13 [Sulfobacillus acidophilus DSM 10332]
21159290~WP_031388509.1 30S ribosomal protein S13 [Desulfonatronum thiodismutans]
931223802~WP_041097091.1 30S ribosomal protein S13 [Sulfuritalea hydrogenivorans]
0.4

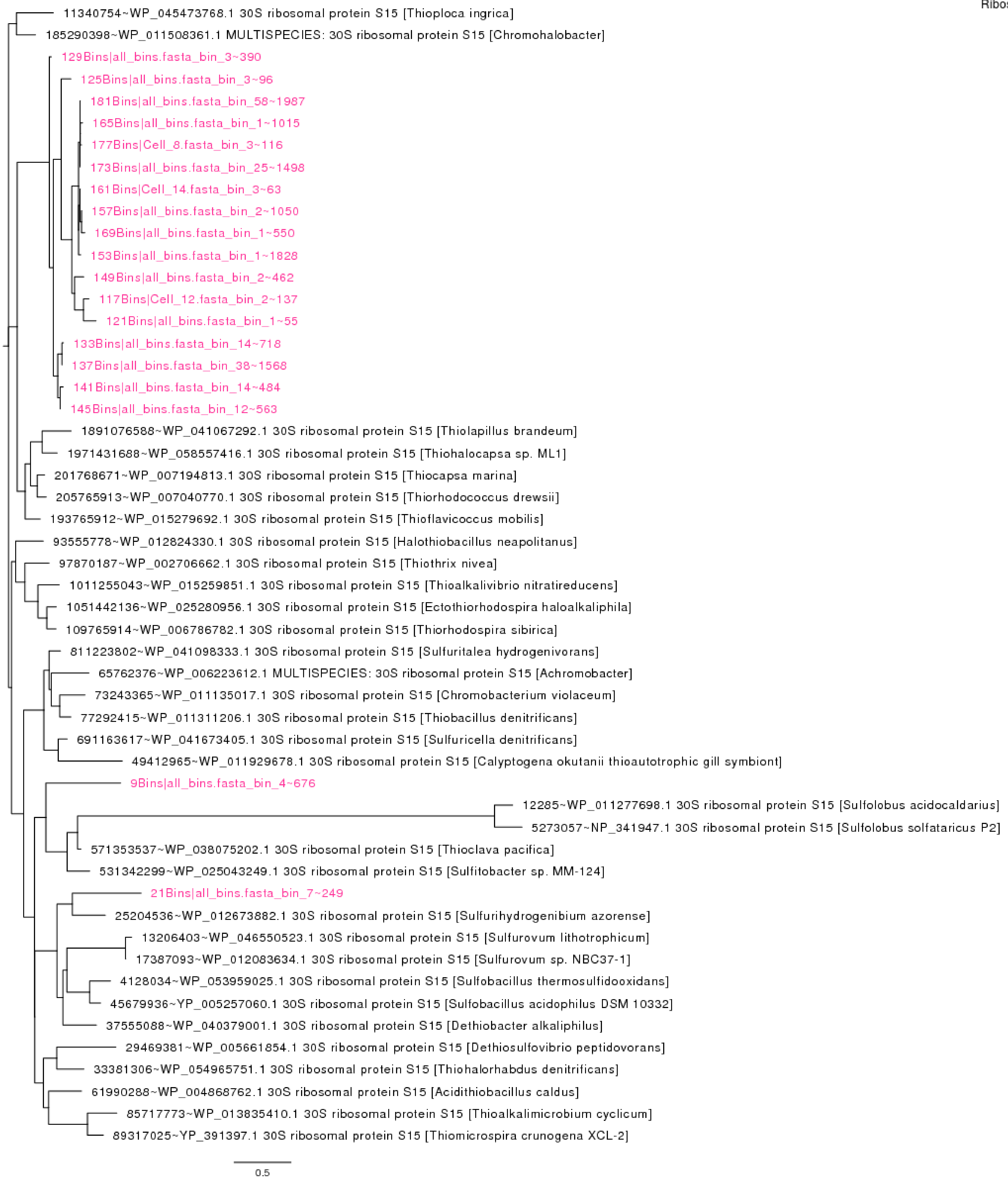
11340754~WP_045473768.1 30S ribosomal protein S15 [Thioploca ingrica]
185290398~WP_011508361.1 MULTISPECIES: 30S ribosomal protein S15 [Chromohalobacter]
129Bins|all_bins.fasta_bin_3~390
125Bins|all_bins.fasta_bin_3~96
181Bins|all_bins.fasta_bin_58~1987
165Bins|all_bins.fasta_bin_1~1015
177Bins|Cell_8.fasta_bin_3~116
173Bins|all_bins.fasta_bin_25~1498
161Bins|Cell_14.fasta_bin_3~63
157Bins|all_bins.fasta_bin_2~1050
169Bins|all_bins.fasta_bin_1~550
153Bins|all_bins.fasta_bin_1~1828
149Bins|all_bins.fasta_bin_2~462
117Bins|Cell_12.fasta_bin_2~137
121Bins|all_bins.fasta_bin_1~55
133Bins|all_bins.fasta_bin_14~718
137Bins|all_bins.fasta_bin_38~1568
141Bins|all_bins.fasta_bin_14~484
145Bins|all_bins.fasta_bin_12~563
1891076588~WP_041067292.1 30S ribosomal protein S15 [Thiolapillus brandeum]
1971431688~WP_058557416.1 30S ribosomal protein S15 [Thiohalocapsa sp. ML1]
201768671~WP_007194813.1 30S ribosomal protein S15 [Thiocapsa marina]
205765913~WP_007040770.1 30S ribosomal protein S15 [Thiorhodococcus drewsii]
193765912~WP_015279692.1 30S ribosomal protein S15 [Thioflavicoccus mobilis]
93555778~WP_012824330.1 30S ribosomal protein S15 [Halothiobacillus neapolitanus]
97870187~WP_002706662.1 30S ribosomal protein S15 [Thiothrix nivea]
1011255043~WP_015259851.1 30S ribosomal protein S15 [Thioalkalivibrio nitratireducens]
1051442136~WP_025280956.1 30S ribosomal protein S15 [Ectothiorhodospira haloalkaliphila]
109765914~WP_006786782.1 30S ribosomal protein S15 [Thiorhodospira sibirica]
811223802~WP_041098333.1 30S ribosomal protein S15 [Sulfuritalea hydrogenivorans]
65762376~WP_006223612.1 MULTISPECIES: 30S ribosomal protein S15 [Achromobacter]
73243365~WP_011135017.1 30S ribosomal protein S15 [Chromobacterium violaceum]
77292415~WP_011311206.1 30S ribosomal protein S15 [Thiobacillus denitrificans]
691163617~WP_041673405.1 30S ribosomal protein S15 [Sulfuricella denitrificans]
49412965~WP_011929678.1 30S ribosomal protein S15 [Calyptogena okutanii thioautotrophic gill symbiont]
9Bins|all_bins.fasta_bin_4~676
12285~WP_011277698.1 30S ribosomal protein S15 [Sulfolobus acidocaldarius]
5273057~NP_341947.1 30S ribosomal protein S15 [Sulfolobus solfataricus P2]
571353537~WP_038075202.1 30S ribosomal protein S15 [Thioclava pacifica]
531342299~WP_025043249.1 30S ribosomal protein S15 [Sulfitobacter sp. MM-124]
21Bins|all_bins.fasta_bin_7~249
25204536~WP_012673882.1 30S ribosomal protein S15 [Sulfurihydrogenibium azorense]
13206403~WP_046550523.1 30S ribosomal protein S15 [Sulfurovum lithotrophicum]
17387093~WP_012083634.1 30S ribosomal protein S15 [Sulfurovum sp. NBC37-1]
4128034~WP_053959025.1 30S ribosomal protein S15 [Sulfobacillus thermosulfidooxidans]
45679936~YP_005257060.1 30S ribosomal protein S15 [Sulfobacillus acidophilus DSM 10332]
37555088~WP_040379001.1 30S ribosomal protein S15 [Dethiobacter alkaliphilus]
29469381~WP_005661854.1 30S ribosomal protein S15 [Dethiosulfovibrio peptidovorans]
33381306~WP_054965751.1 30S ribosomal protein S15 [Thiohalorhabdus denitrificans]
61990288~WP_004868762.1 30S ribosomal protein S15 [Acidithiobacillus caldus]
85717773~WP_013835410.1 30S ribosomal protein S15 [Thioalkalimicrobium cyclicum]
89317025~YP_391397.1 30S ribosomal protein S15 [Thiomicrospira crunogena XCL-2]
0.5

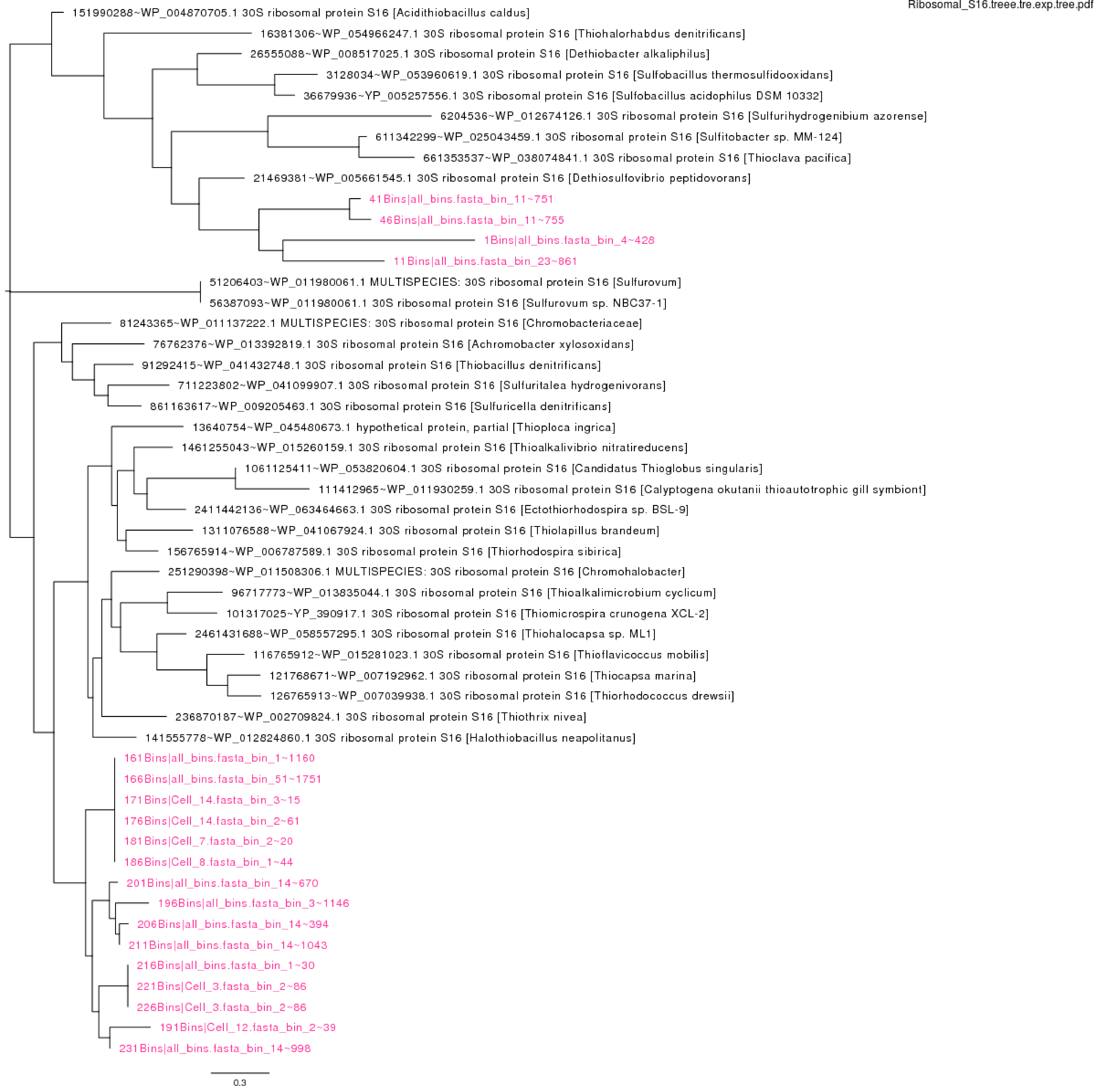
Ribosomal_S16.treee.tre.exp.tree.pdf
151990288~WP_004870705.1 30S ribosomal protein S16 [Acidithiobacillus caldus]
16381306~WP_054966247.1 30S ribosomal protein S16 [Thiohalorhabdus denitrificans]
26555088~WP_008517025.1 30S ribosomal protein S16 [Dethiobacter alkaliphilus]
3128034~WP_053960619.1 30S ribosomal protein S16 [Sulfobacillus thermosulfidooxidans]
36679936~YP_005257556.1 30S ribosomal protein S16 [Sulfobacillus acidophilus DSM 10332]
6204536~WP_012674126.1 30S ribosomal protein S16 [Sulfurihydrogenibium azorense]
611342299~WP_025043459.1 30S ribosomal protein S16 [Sulfitobacter sp. MM-124]
661353537~WP_038074841.1 30S ribosomal protein S16 [Thioclava pacifica]
21469381~WP_005661545.1 30S ribosomal protein S16 [Dethiosulfovibrio peptidovorans]
41Bins|all_bins.fasta_bin_11~751
46Bins|all_bins.fasta_bin_11~755
1Bins|all_bins.fasta_bin_4~428
11Bins|all_bins.fasta_bin_23~861
51206403~WP_011980061.1 MULTISPECIES: 30S ribosomal protein S16 [Sulfurovum]
56387093~WP_011980061.1 30S ribosomal protein S16 [Sulfurovum sp. NBC37-1]
81243365~WP_011137222.1 MULTISPECIES: 30S ribosomal protein S16 [Chromobacteriaceae]
76762376~WP_013392819.1 30S ribosomal protein S16 [Achromobacter xylosoxidans]
91292415~WP_041432748.1 30S ribosomal protein S16 [Thiobacillus denitrificans]
711223802~WP_041099907.1 30S ribosomal protein S16 [Sulfuritalea hydrogenivorans]
861163617~WP_009205463.1 30S ribosomal protein S16 [Sulfuricella denitrificans]
13640754~WP_045480673.1 hypothetical protein, partial [Thioploca ingrica]
1461255043~WP_015260159.1 30S ribosomal protein S16 [Thioalkalivibrio nitratireducens]
1061125411~WP_053820604.1 30S ribosomal protein S16 [Candidatus Thioglobus singularis]
111412965~WP_011930259.1 30S ribosomal protein S16 [Calyptogena okutanii thioautotrophic gill symbiont]
2411442136~WP_063464663.1 30S ribosomal protein S16 [Ectothiorhodospira sp. BSL-9]
1311076588~WP_041067924.1 30S ribosomal protein S16 [Thiolapillus brandeum]
156765914~WP_006787589.1 30S ribosomal protein S16 [Thiorhodospira sibirica]
251290398~WP_011508306.1 MULTISPECIES: 30S ribosomal protein S16 [Chromohalobacter]
96717773~WP_013835044.1 30S ribosomal protein S16 [Thioalkalimicrobium cyclicum]
101317025~YP_390917.1 30S ribosomal protein S16 [Thiomicrospira crunogena XCL-2]
2461431688~WP_058557295.1 30S ribosomal protein S16 [Thiohalocapsa sp. ML1]
116765912~WP_015281023.1 30S ribosomal protein S16 [Thioflavicoccus mobilis]
121768671~WP_007192962.1 30S ribosomal protein S16 [Thiocapsa marina]
126765913~WP_007039938.1 30S ribosomal protein S16 [Thiorhodococcus drewsii]
236870187~WP_002709824.1 30S ribosomal protein S16 [Thiothrix nivea]
141555778~WP_012824860.1 30S ribosomal protein S16 [Halothiobacillus neapolitanus]
161Bins|all_bins.fasta_bin_1~1160
166Bins|all_bins.fasta_bin_51~1751
171Bins|Cell_14.fasta_bin_3~15
176Bins|Cell_14.fasta_bin_2~61
181Bins|Cell_7.fasta_bin_2~20
186Bins|Cell_8.fasta_bin_1~44
201Bins|all_bins.fasta_bin_14~670
196Bins|all_bins.fasta_bin_3~1146
206Bins|all_bins.fasta_bin_14~394
211Bins|all_bins.fasta_bin_14~1043
216Bins|all_bins.fasta_bin_1~30
221Bins|Cell_3.fasta_bin_2~86
226Bins|Cell_3.fasta_bin_2~86
191Bins|Cell_12.fasta_bin_2~39
231Bins|all_bins.fasta_bin_14~998
0.3

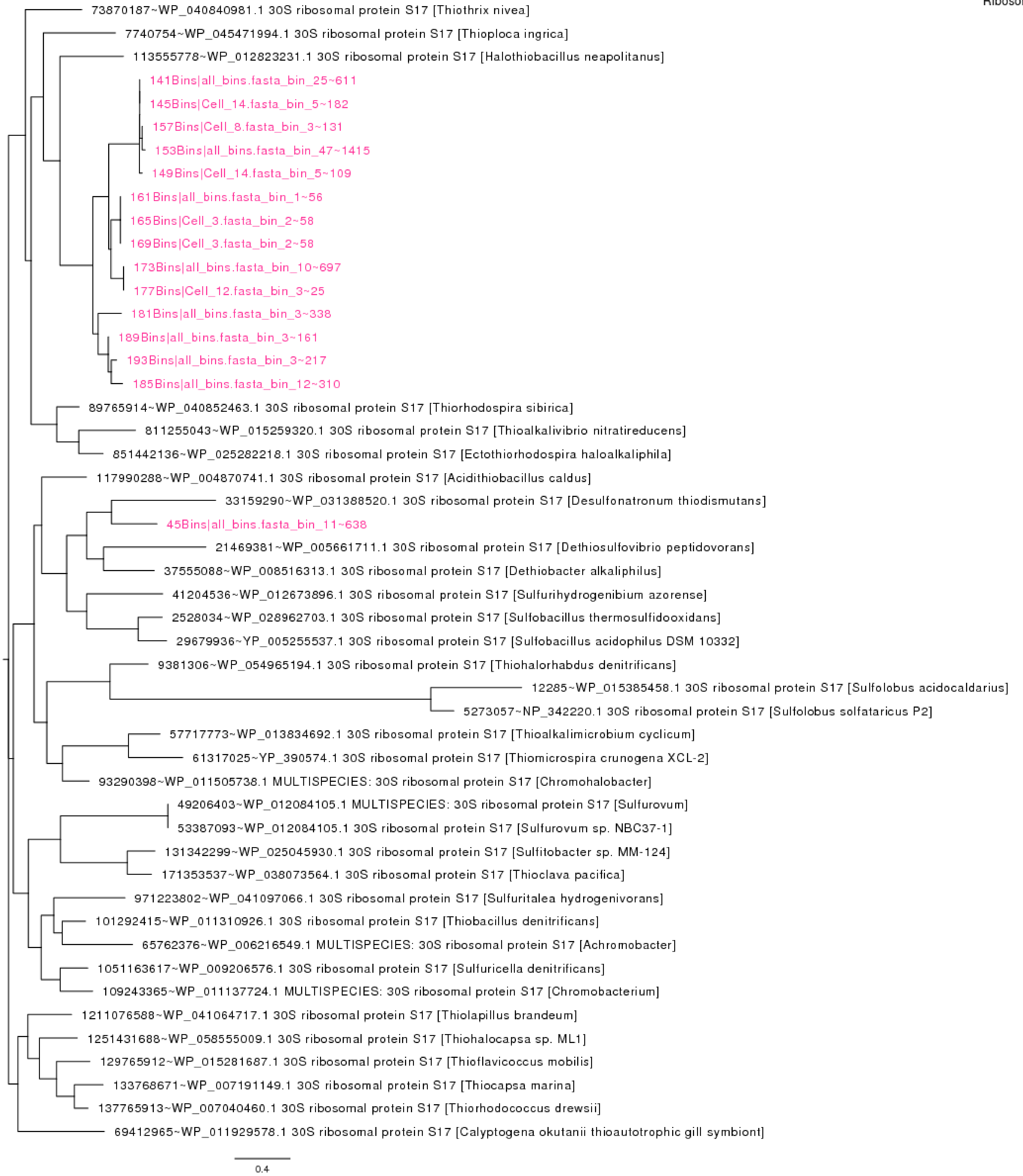
73870187~WP_040840981.1 30S ribosomal protein S17 [Thiothrix nivea]
7740754~WP_045471994.1 30S ribosomal protein S17 [Thioploca ingrica]
113555778~WP_012823231.1 30S ribosomal protein S17 [Halothiobacillus neapolitanus]
141Bins|all_bins.fasta_bin_25~611
145Bins|Cell_14.fasta_bin_5~182
157Bins|Cell_8.fasta_bin_3~131
153Bins|all_bins.fasta_bin_47~1415
149Bins|Cell_14.fasta_bin_5~109
161Bins|all_bins.fasta_bin_1~56
165Bins|Cell_3.fasta_bin_2~58
169Bins|Cell_3.fasta_bin_2~58
173Bins|all_bins.fasta_bin_10~697
177Bins|Cell_12.fasta_bin_3~25
181Bins|all_bins.fasta_bin_3~338
189Bins|all_bins.fasta_bin_3~161
193Bins|all_bins.fasta_bin_3~217
185Bins|all_bins.fasta_bin_12~310
89765914~WP_040852463.1 30S ribosomal protein S17 [Thiorhodospira sibirica]
811255043~WP_015259320.1 30S ribosomal protein S17 [Thioalkalivibrio nitratireducens]
851442136~WP_025282218.1 30S ribosomal protein S17 [Ectothiorhodospira haloalkaliphila]
117990288~WP_004870741.1 30S ribosomal protein S17 [Acidithiobacillus caldus]
33159290~WP_031388520.1 30S ribosomal protein S17 [Desulfonatronum thiodismutans]
45Bins|all_bins.fasta_bin_11~638
21469381~WP_005661711.1 30S ribosomal protein S17 [Dethiosulfovibrio peptidovorans]
37555088~WP_008516313.1 30S ribosomal protein S17 [Dethiobacter alkaliphilus]
41204536~WP_012673896.1 30S ribosomal protein S17 [Sulfurihydrogenibium azorense]
2528034~WP_028962703.1 30S ribosomal protein S17 [Sulfobacillus thermosulfidooxidans]
29679936~YP_005255537.1 30S ribosomal protein S17 [Sulfobacillus acidophilus DSM 10332]
9381306~WP_054965194.1 30S ribosomal protein S17 [Thiohalorhabdus denitrificans]
12285~WP_015385458.1 30S ribosomal protein S17 [Sulfolobus acidocaldarius]
5273057~NP_342220.1 30S ribosomal protein S17 [Sulfolobus solfataricus P2]
57717773~WP_013834692.1 30S ribosomal protein S17 [Thioalkalimicrobium cyclicum]
61317025~YP_390574.1 30S ribosomal protein S17 [Thiomicrospira crunogena XCL-2]
93290398~WP_011505738.1 MULTISPECIES: 30S ribosomal protein S17 [Chromohalobacter]
49206403~WP_012084105.1 MULTISPECIES: 30S ribosomal protein S17 [Sulfurovum]
53387093~WP_012084105.1 30S ribosomal protein S17 [Sulfurovum sp. NBC37-1]
131342299~WP_025045930.1 30S ribosomal protein S17 [Sulfitobacter sp. MM-124]
171353537~WP_038073564.1 30S ribosomal protein S17 [Thioclava pacifica]
971223802~WP_041097066.1 30S ribosomal protein S17 [Sulfuritalea hydrogenivorans]
101292415~WP_011310926.1 30S ribosomal protein S17 [Thiobacillus denitrificans]
65762376~WP_006216549.1 MULTISPECIES: 30S ribosomal protein S17 [Achromobacter]
1051163617~WP_009206576.1 30S ribosomal protein S17 [Sulfuricella denitrificans]
109243365~WP_011137724.1 MULTISPECIES: 30S ribosomal protein S17 [Chromobacterium]
1211076588~WP_041064717.1 30S ribosomal protein S17 [Thiolapillus brandeum]
1251431688~WP_058555009.1 30S ribosomal protein S17 [Thiohalocapsa sp. ML1]
129765912~WP_015281687.1 30S ribosomal protein S17 [Thioflavicoccus mobilis]
133768671~WP_007191149.1 30S ribosomal protein S17 [Thiocapsa marina]
137765913~WP_007040460.1 30S ribosomal protein S17 [Thiorhodococcus drewsii]
69412965~WP_011929578.1 30S ribosomal protein S17 [Calyptogena okutanii thioautotrophic gill symbiont]
0.4

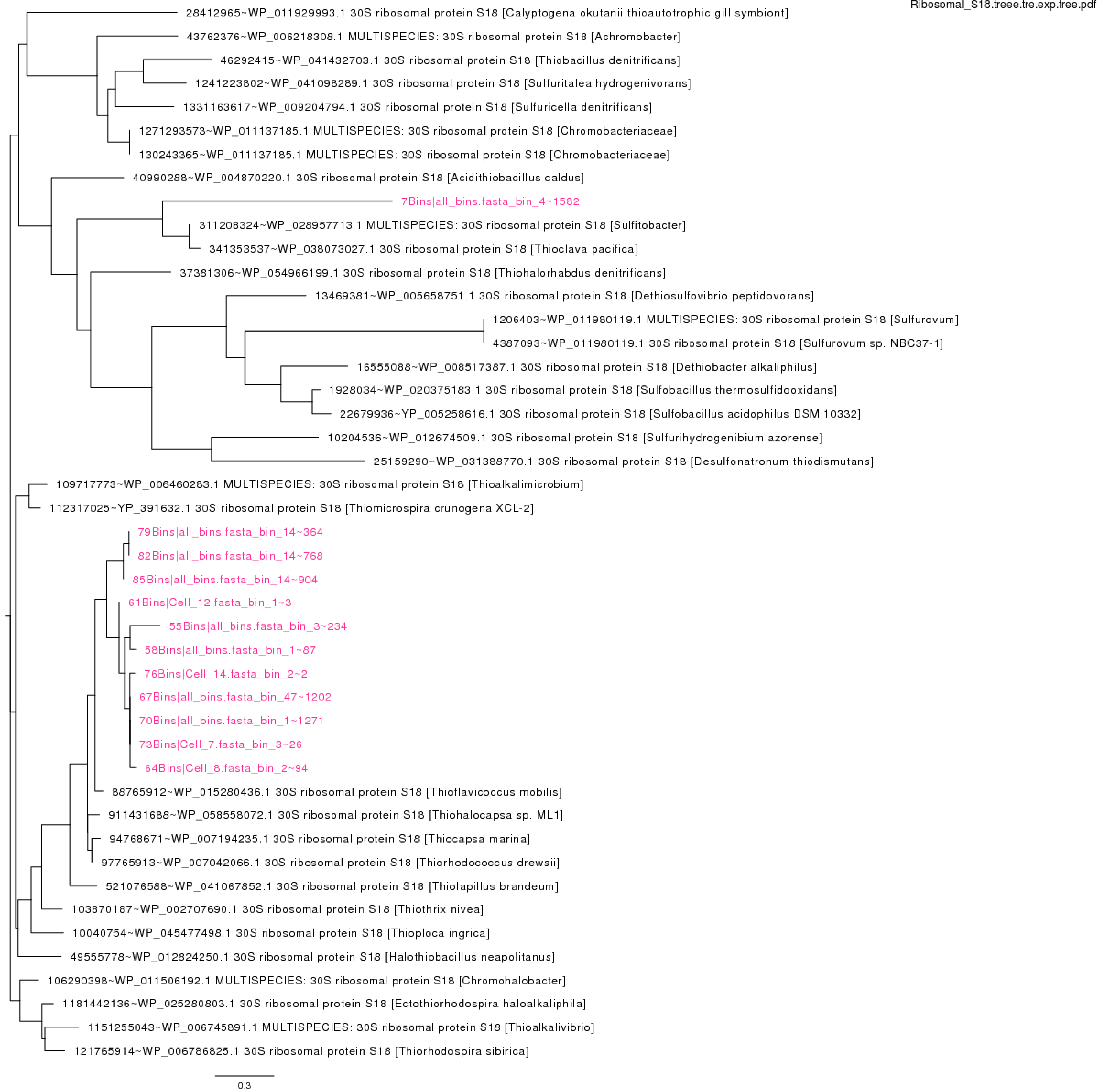
Ribosomal_S18.treee.tre.exp.tree.pdf
28412965~WP_011929993.1 30S ribosomal protein S18 [Calyptogena okutanii thioautotrophic gill symbiont]
43762376~WP_006218308.1 MULTISPECIES: 30S ribosomal protein S18 [Achromobacter]
46292415~WP_041432703.1 30S ribosomal protein S18 [Thiobacillus denitrificans]
1241223802~WP_041098289.1 30S ribosomal protein S18 [Sulfuritalea hydrogenivorans]
1331163617~WP_009204794.1 30S ribosomal protein S18 [Sulfuricella denitrificans]
1271293573~WP_011137185.1 MULTISPECIES: 30S ribosomal protein S18 [Chromobacteriaceae]
130243365~WP_011137185.1 MULTISPECIES: 30S ribosomal protein S18 [Chromobacteriaceae]
40990288~WP_004870220.1 30S ribosomal protein S18 [Acidithiobacillus caldus]
7Bins|all_bins.fasta_bin_4~1582
311208324~WP_028957713.1 MULTISPECIES: 30S ribosomal protein S18 [Sulfitobacter]
341353537~WP_038073027.1 30S ribosomal protein S18 [Thioclava pacifica]
37381306~WP_054966199.1 30S ribosomal protein S18 [Thiohalorhabdus denitrificans]
13469381~WP_005658751.1 30S ribosomal protein S18 [Dethiosulfovibrio peptidovorans]
1206403~WP_011980119.1 MULTISPECIES: 30S ribosomal protein S18 [Sulfurovum]
4387093~WP_011980119.1 30S ribosomal protein S18 [Sulfurovum sp. NBC37-1]
16555088~WP_008517387.1 30S ribosomal protein S18 [Dethiobacter alkaliphilus]
1928034~WP_020375183.1 30S ribosomal protein S18 [Sulfobacillus thermosulfidooxidans]
22679936~YP_005258616.1 30S ribosomal protein S18 [Sulfobacillus acidophilus DSM 10332]
10204536~WP_012674509.1 30S ribosomal protein S18 [Sulfurihydrogenibium azorense]
25159290~WP_031388770.1 30S ribosomal protein S18 [Desulfonatronum thiodismutans]
109717773~WP_006460283.1 MULTISPECIES: 30S ribosomal protein S18 [Thioalkalimicrobium]
112317025~YP_391632.1 30S ribosomal protein S18 [Thiomicrospira crunogena XCL-2]
79Bins|all_bins.fasta_bin_14~364
82Bins|all_bins.fasta_bin_14~768
85Bins|all_bins.fasta_bin_14~904
61Bins|Cell_12.fasta_bin_1~3
55Bins|all_bins.fasta_bin_3~234
58Bins|all_bins.fasta_bin_1~87
76Bins|Cell_14.fasta_bin_2~2
67Bins|all_bins.fasta_bin_47~1202
70Bins|all_bins.fasta_bin_1~1271
73Bins|Cell_7.fasta_bin_3~26
64Bins|Cell_8.fasta_bin_2~94
88765912~WP_015280436.1 30S ribosomal protein S18 [Thioflavicoccus mobilis]
911431688~WP_058558072.1 30S ribosomal protein S18 [Thiohalocapsa sp. ML1]
94768671~WP_007194235.1 30S ribosomal protein S18 [Thiocapsa marina]
97765913~WP_007042066.1 30S ribosomal protein S18 [Thiorhodococcus drewsii]
521076588~WP_041067852.1 30S ribosomal protein S18 [Thiolapillus brandeum]
103870187~WP_002707690.1 30S ribosomal protein S18 [Thiothrix nivea]
10040754~WP_045477498.1 30S ribosomal protein S18 [Thioploca ingrica]
49555778~WP_012824250.1 30S ribosomal protein S18 [Halothiobacillus neapolitanus]
106290398~WP_011506192.1 MULTISPECIES: 30S ribosomal protein S18 [Chromohalobacter]
1181442136~WP_025280803.1 30S ribosomal protein S18 [Ectothiorhodospira haloalkaliphila]
1151255043~WP_006745891.1 MULTISPECIES: 30S ribosomal protein S18 [Thioalkalivibrio]
121765914~WP_006786825.1 30S ribosomal protein S18 [Thiorhodospira sibirica]
0.3

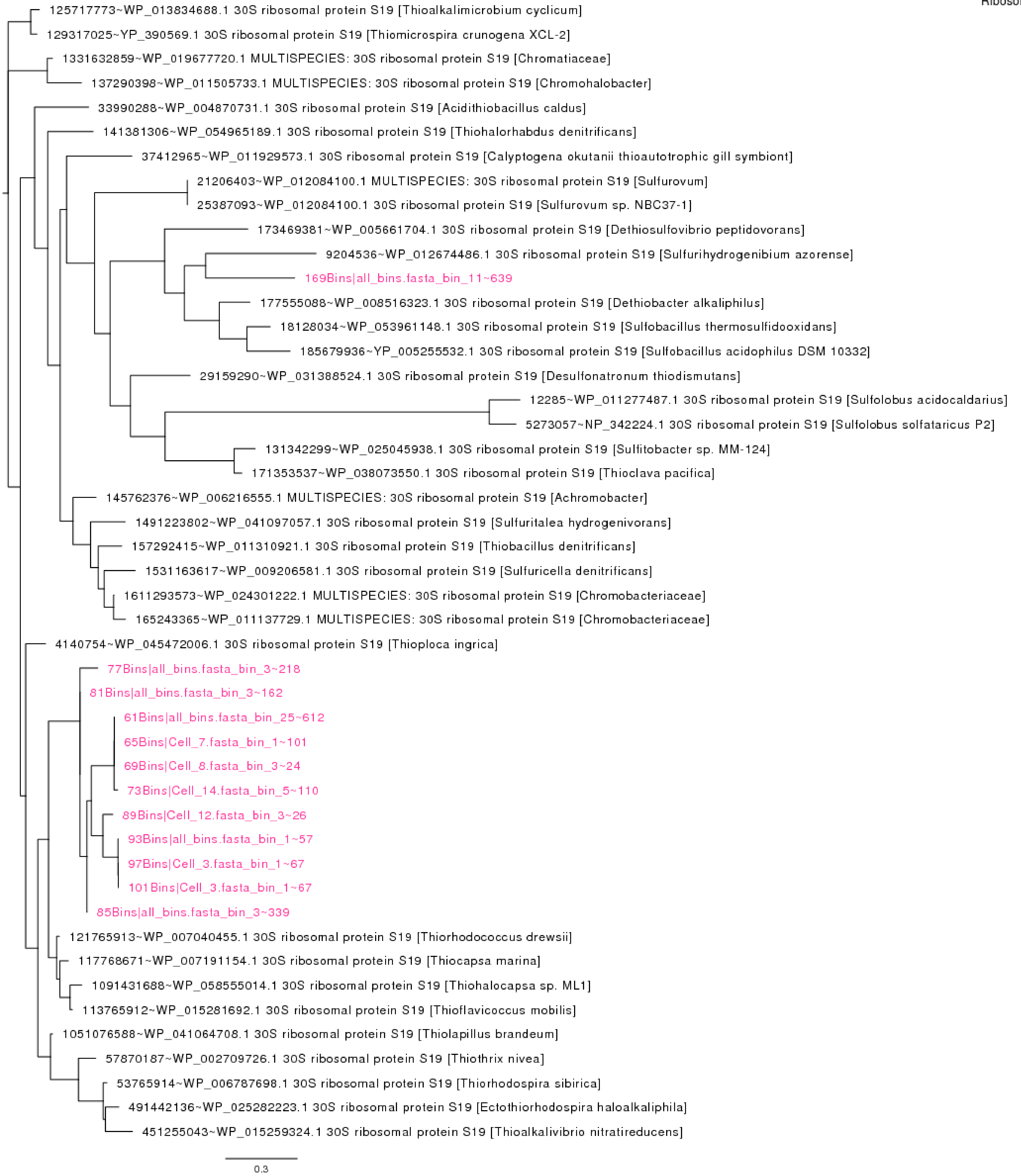
125717773~WP_013834688.1 30S ribosomal protein S19 [Thioalkalimicrobium cyclicum]
129317025~YP_390569.1 30S ribosomal protein S19 [Thiomicrospira crunogena XCL-2]
1331632859~WP_019677720.1 MULTISPECIES: 30S ribosomal protein S19 [Chromatiaceae]
137290398~WP_011505733.1 MULTISPECIES: 30S ribosomal protein S19 [Chromohalobacter]
33990288~WP_004870731.1 30S ribosomal protein S19 [Acidithiobacillus caldus]
141381306~WP_054965189.1 30S ribosomal protein S19 [Thiohalorhabdus denitrificans]
37412965~WP_011929573.1 30S ribosomal protein S19 [Calyptogena okutanii thioautotrophic gill symbiont]
21206403~WP_012084100.1 MULTISPECIES: 30S ribosomal protein S19 [Sulfurovum]
25387093~WP_012084100.1 30S ribosomal protein S19 [Sulfurovum sp. NBC37-1]
173469381~WP_005661704.1 30S ribosomal protein S19 [Dethiosulfovibrio peptidovorans]
9204536~WP_012674486.1 30S ribosomal protein S19 [Sulfurihydrogenibium azorense]
169Bins|all_bins.fasta_bin_11~639
177555088~WP_008516323.1 30S ribosomal protein S19 [Dethiobacter alkaliphilus]
18128034~WP_053961148.1 30S ribosomal protein S19 [Sulfobacillus thermosulfidooxidans]
185679936~YP_005255532.1 30S ribosomal protein S19 [Sulfobacillus acidophilus DSM 10332]
29159290~WP_031388524.1 30S ribosomal protein S19 [Desulfonatronum thiodismutans]
12285~WP_011277487.1 30S ribosomal protein S19 [Sulfolobus acidocaldarius]
5273057~NP_342224.1 30S ribosomal protein S19 [Sulfolobus solfataricus P2]
131342299~WP_025045938.1 30S ribosomal protein S19 [Sulfitobacter sp. MM-124]
171353537~WP_038073550.1 30S ribosomal protein S19 [Thioclava pacifica]
145762376~WP_006216555.1 MULTISPECIES: 30S ribosomal protein S19 [Achromobacter]
1491223802~WP_041097057.1 30S ribosomal protein S19 [Sulfuritalea hydrogenivorans]
157292415~WP_011310921.1 30S ribosomal protein S19 [Thiobacillus denitrificans]
1531163617~WP_009206581.1 30S ribosomal protein S19 [Sulfuricella denitrificans]
1611293573~WP_024301222.1 MULTISPECIES: 30S ribosomal protein S19 [Chromobacteriaceae]
165243365~WP_011137729.1 MULTISPECIES: 30S ribosomal protein S19 [Chromobacteriaceae]
4140754~WP_045472006.1 30S ribosomal protein S19 [Thioploca ingrica]
77Bins|all_bins.fasta_bin_3~218
81Bins|all_bins.fasta_bin_3~162
61Bins|all_bins.fasta_bin_25~612
65Bins|Cell_7.fasta_bin_1~101
69Bins|Cell_8.fasta_bin_3~24
73Bins|Cell_14.fasta_bin_5~110
89Bins|Cell_12.fasta_bin_3~26
93Bins|all_bins.fasta_bin_1~57
97Bins|Cell_3.fasta_bin_1~67
101Bins|Cell_3.fasta_bin_1~67
85Bins|all_bins.fasta_bin_3~339
121765913~WP_007040455.1 30S ribosomal protein S19 [Thiorhodococcus drewsii]
117768671~WP_007191154.1 30S ribosomal protein S19 [Thiocapsa marina]
1091431688~WP_058555014.1 30S ribosomal protein S19 [Thiohalocapsa sp. ML1]
113765912~WP_015281692.1 30S ribosomal protein S19 [Thioflavicoccus mobilis]
1051076588~WP_041064708.1 30S ribosomal protein S19 [Thiolapillus brandeum]
57870187~WP_002709726.1 30S ribosomal protein S19 [Thiothrix nivea]
53765914~WP_006787698.1 30S ribosomal protein S19 [Thiorhodospira sibirica]
491442136~WP_025282223.1 30S ribosomal protein S19 [Ectothiorhodospira haloalkaliphila]
451255043~WP_015259324.1 30S ribosomal protein S19 [Thioalkalivibrio nitratireducens]
0.3

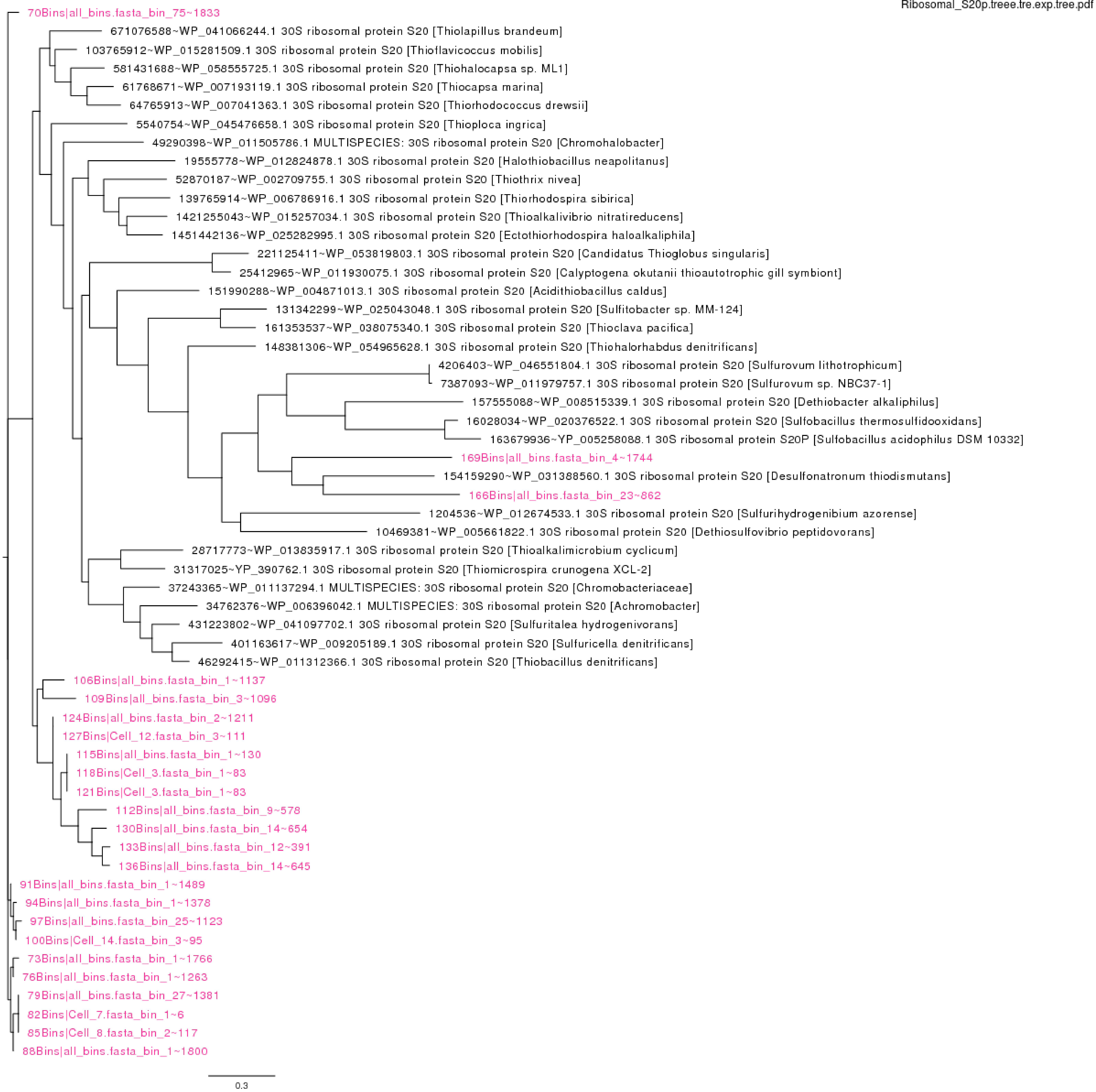
Ribosomal_S20p.treee.tre.exp.tree.pdf
70Bins|all_bins.fasta_bin_75~1833
671076588~WP_041066244.1 30S ribosomal protein S20 [Thiolapillus brandeum]
103765912~WP_015281509.1 30S ribosomal protein S20 [Thioflavicoccus mobilis]
581431688~WP_058555725.1 30S ribosomal protein S20 [Thiohalocapsa sp. ML1]
61768671~WP_007193119.1 30S ribosomal protein S20 [Thiocapsa marina]
64765913~WP_007041363.1 30S ribosomal protein S20 [Thiorhodococcus drewsii]
5540754~WP_045476658.1 30S ribosomal protein S20 [Thioploca ingrica]
49290398~WP_011505786.1 MULTISPECIES: 30S ribosomal protein S20 [Chromohalobacter]
19555778~WP_012824878.1 30S ribosomal protein S20 [Halothiobacillus neapolitanus]
52870187~WP_002709755.1 30S ribosomal protein S20 [Thiothrix nivea]
139765914~WP_006786916.1 30S ribosomal protein S20 [Thiorhodospira sibirica]
1421255043~WP_015257034.1 30S ribosomal protein S20 [Thioalkalivibrio nitratireducens]
1451442136~WP_025282995.1 30S ribosomal protein S20 [Ectothiorhodospira haloalkaliphila]
221125411~WP_053819803.1 30S ribosomal protein S20 [Candidatus Thioglobus singularis]
25412965~WP_011930075.1 30S ribosomal protein S20 [Calyptogena okutanii thioautotrophic gill symbiont]
151990288~WP_004871013.1 30S ribosomal protein S20 [Acidithiobacillus caldus]
131342299~WP_025043048.1 30S ribosomal protein S20 [Sulfitobacter sp. MM-124]
161353537~WP_038075340.1 30S ribosomal protein S20 [Thioclava pacifica]
148381306~WP_054965628.1 30S ribosomal protein S20 [Thiohalorhabdus denitrificans]
4206403~WP_046551804.1 30S ribosomal protein S20 [Sulfurovum lithotrophicum]
7387093~WP_011979757.1 30S ribosomal protein S20 [Sulfurovum sp. NBC37-1]
157555088~WP_008515339.1 30S ribosomal protein S20 [Dethiobacter alkaliphilus]
16028034~WP_020376522.1 30S ribosomal protein S20 [Sulfobacillus thermosulfidooxidans]
163679936~YP_005258088.1 30S ribosomal protein S20P [Sulfobacillus acidophilus DSM 10332]
169Bins|all_bins.fasta_bin_4~1744
154159290~WP_031388560.1 30S ribosomal protein S20 [Desulfonatronum thiodismutans]
166Bins|all_bins.fasta_bin_23~862
1204536~WP_012674533.1 30S ribosomal protein S20 [Sulfurihydrogenibium azorense]
10469381~WP_005661822.1 30S ribosomal protein S20 [Dethiosulfovibrio peptidovorans]
28717773~WP_013835917.1 30S ribosomal protein S20 [Thioalkalimicrobium cyclicum]
31317025~YP_390762.1 30S ribosomal protein S20 [Thiomicrospira crunogena XCL-2]
37243365~WP_011137294.1 MULTISPECIES: 30S ribosomal protein S20 [Chromobacteriaceae]
34762376~WP_006396042.1 MULTISPECIES: 30S ribosomal protein S20 [Achromobacter]
431223802~WP_041097702.1 30S ribosomal protein S20 [Sulfuritalea hydrogenivorans]
401163617~WP_009205189.1 30S ribosomal protein S20 [Sulfuricella denitrificans]
46292415~WP_011312366.1 30S ribosomal protein S20 [Thiobacillus denitrificans]
106Bins|all_bins.fasta_bin_1~1137
109Bins|all_bins.fasta_bin_3~1096
124Bins|all_bins.fasta_bin_2~1211
127Bins|Cell_12.fasta_bin_3~111
115Bins|all_bins.fasta_bin_1~130
118Bins|Cell_3.fasta_bin_1~83
121Bins|Cell_3.fasta_bin_1~83
112Bins|all_bins.fasta_bin_9~578
130Bins|all_bins.fasta_bin_14~654
133Bins|all_bins.fasta_bin_12~391
136Bins|all_bins.fasta_bin_14~645
91Bins|all_bins.fasta_bin_1~1489
94Bins|all_bins.fasta_bin_1~1378
97Bins|all_bins.fasta_bin_25~1123
100Bins|Cell_14.fasta_bin_3~95
73Bins|all_bins.fasta_bin_1~1766
76Bins|all_bins.fasta_bin_1~1263
79Bins|all_bins.fasta_bin_27~1381
82Bins|Cell_7.fasta_bin_1~6
85Bins|Cell_8.fasta_bin_2~117
88Bins|all_bins.fasta_bin_1~1800
0.3

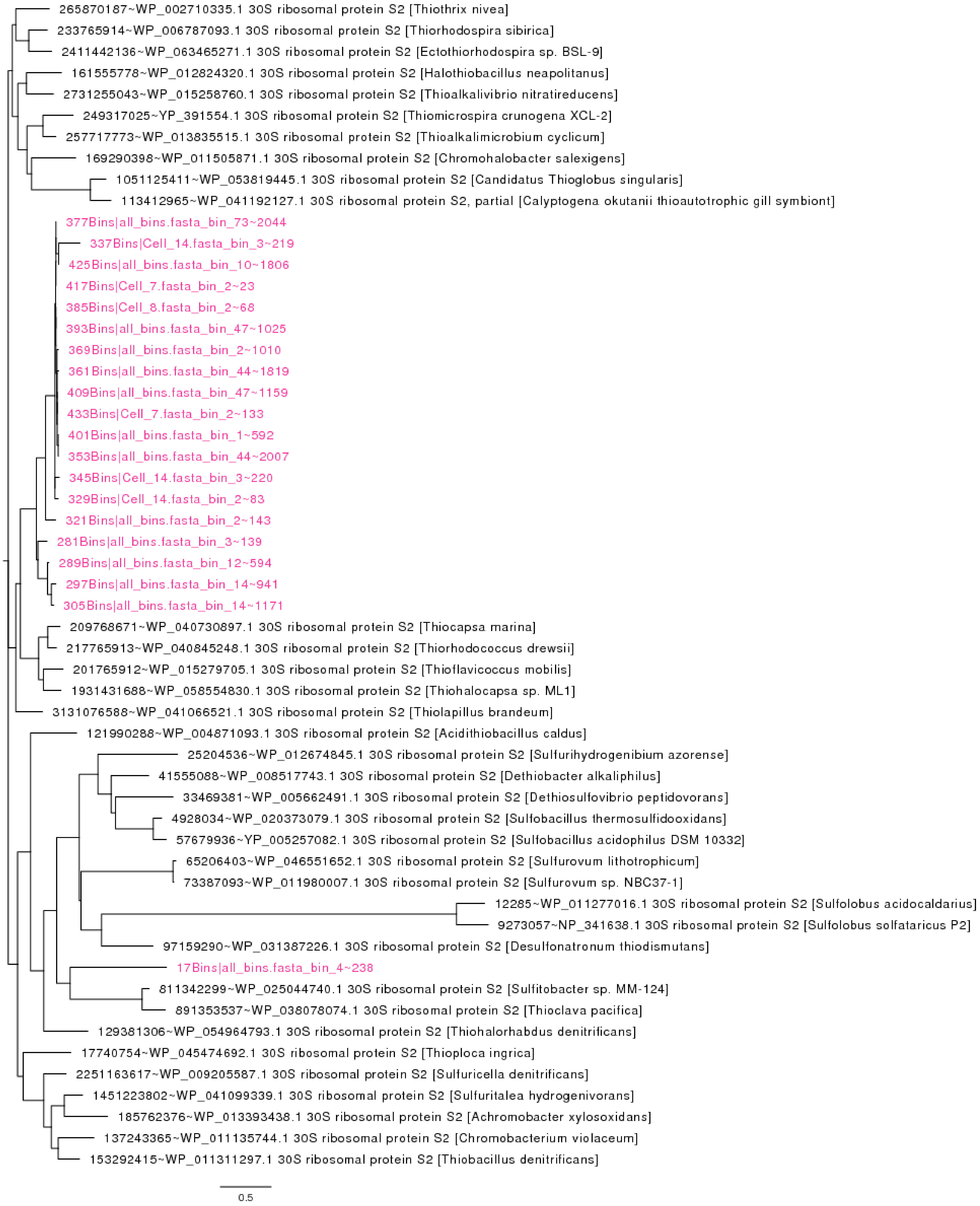
265870187~WP_002710335.1 30S ribosomal protein S2 [Thiothrix nivea]
233765914~WP_006787093.1 30S ribosomal protein S2 [Thiorhodospira sibirica]
2411442136~WP_063465271.1 30S ribosomal protein S2 [Ectothiorhodospira sp. BSL-9]
161555778~WP_012824320.1 30S ribosomal protein S2 [Halothiobacillus neapolitanus]
2731255043~WP_015258760.1 30S ribosomal protein S2 [Thioalkalivibrio nitratireducens]
249317025~YP_391554.1 30S ribosomal protein S2 [Thiomicrospira crunogena XCL-2]
257717773~WP_013835515.1 30S ribosomal protein S2 [Thioalkalimicrobium cyclicum]
169290398~WP_011505871.1 30S ribosomal protein S2 [Chromohalobacter salexigens]
1051125411~WP_053819445.1 30S ribosomal protein S2 [Candidatus Thioglobus singularis]
113412965~WP_041192127.1 30S ribosomal protein S2, partial [Calyptogena okutanii thioautotrophic gill symbiont]
377Bins|all_bins.fasta_bin_73~2044
337Bins|Cell_14.fasta_bin_3~219
425Bins|all_bins.fasta_bin_10~1806
417Bins|Cell_7.fasta_bin_2~23
385Bins|Cell_8.fasta_bin_2~68
393Bins|all_bins.fasta_bin_47~1025
369Bins|all_bins.fasta_bin_2~1010
361Bins|all_bins.fasta_bin_44~1819
409Bins|all_bins.fasta_bin_47~1159
433Bins|Cell_7.fasta_bin_2~133
401Bins|all_bins.fasta_bin_1~592
353Bins|all_bins.fasta_bin_44~2007
345Bins|Cell_14.fasta_bin_3~220
329Bins|Cell_14.fasta_bin_2~83
321Bins|all_bins.fasta_bin_2~143
281Bins|all_bins.fasta_bin_3~139
289Bins|all_bins.fasta_bin_12~594
297Bins|all_bins.fasta_bin_14~941
305Bins|all_bins.fasta_bin_14~1171
209768671~WP_040730897.1 30S ribosomal protein S2 [Thiocapsa marina]
217765913~WP_040845248.1 30S ribosomal protein S2 [Thiorhodococcus drewsii]
201765912~WP_015279705.1 30S ribosomal protein S2 [Thioflavicoccus mobilis]
1931431688~WP_058554830.1 30S ribosomal protein S2 [Thiohalocapsa sp. ML1]
3131076588~WP_041066521.1 30S ribosomal protein S2 [Thiolapillus brandeum]
121990288~WP_004871093.1 30S ribosomal protein S2 [Acidithiobacillus caldus]
25204536~WP_012674845.1 30S ribosomal protein S2 [Sulfurihydrogenibium azorense]
41555088~WP_008517743.1 30S ribosomal protein S2 [Dethiobacter alkaliphilus]
33469381~WP_005662491.1 30S ribosomal protein S2 [Dethiosulfovibrio peptidovorans]
4928034~WP_020373079.1 30S ribosomal protein S2 [Sulfobacillus thermosulfidooxidans]
57679936~YP_005257082.1 30S ribosomal protein S2 [Sulfobacillus acidophilus DSM 10332]
65206403~WP_046551652.1 30S ribosomal protein S2 [Sulfurovum lithotrophicum]
73387093~WP_011980007.1 30S ribosomal protein S2 [Sulfurovum sp. NBC37-1]
12285~WP_011277016.1 30S ribosomal protein S2 [Sulfolobus acidocaldarius]
9273057~NP_341638.1 30S ribosomal protein S2 [Sulfolobus solfataricus P2]
97159290~WP_031387226.1 30S ribosomal protein S2 [Desulfonatronum thiodismutans]
17Bins|all_bins.fasta_bin_4~238
811342299~WP_025044740.1 30S ribosomal protein S2 [Sulfitobacter sp. MM-124]
891353537~WP_038078074.1 30S ribosomal protein S2 [Thioclava pacifica]
129381306~WP_054964793.1 30S ribosomal protein S2 [Thiohalorhabdus denitrificans]
17740754~WP_045474692.1 30S ribosomal protein S2 [Thioploca ingrica]
2251163617~WP_009205587.1 30S ribosomal protein S2 [Sulfuricella denitrificans]
1451223802~WP_041099339.1 30S ribosomal protein S2 [Sulfuritalea hydrogenivorans]
185762376~WP_013393438.1 30S ribosomal protein S2 [Achromobacter xylosoxidans]
137243365~WP_011135744.1 30S ribosomal protein S2 [Chromobacterium violaceum]
153292415~WP_011311297.1 30S ribosomal protein S2 [Thiobacillus denitrificans]
0.5

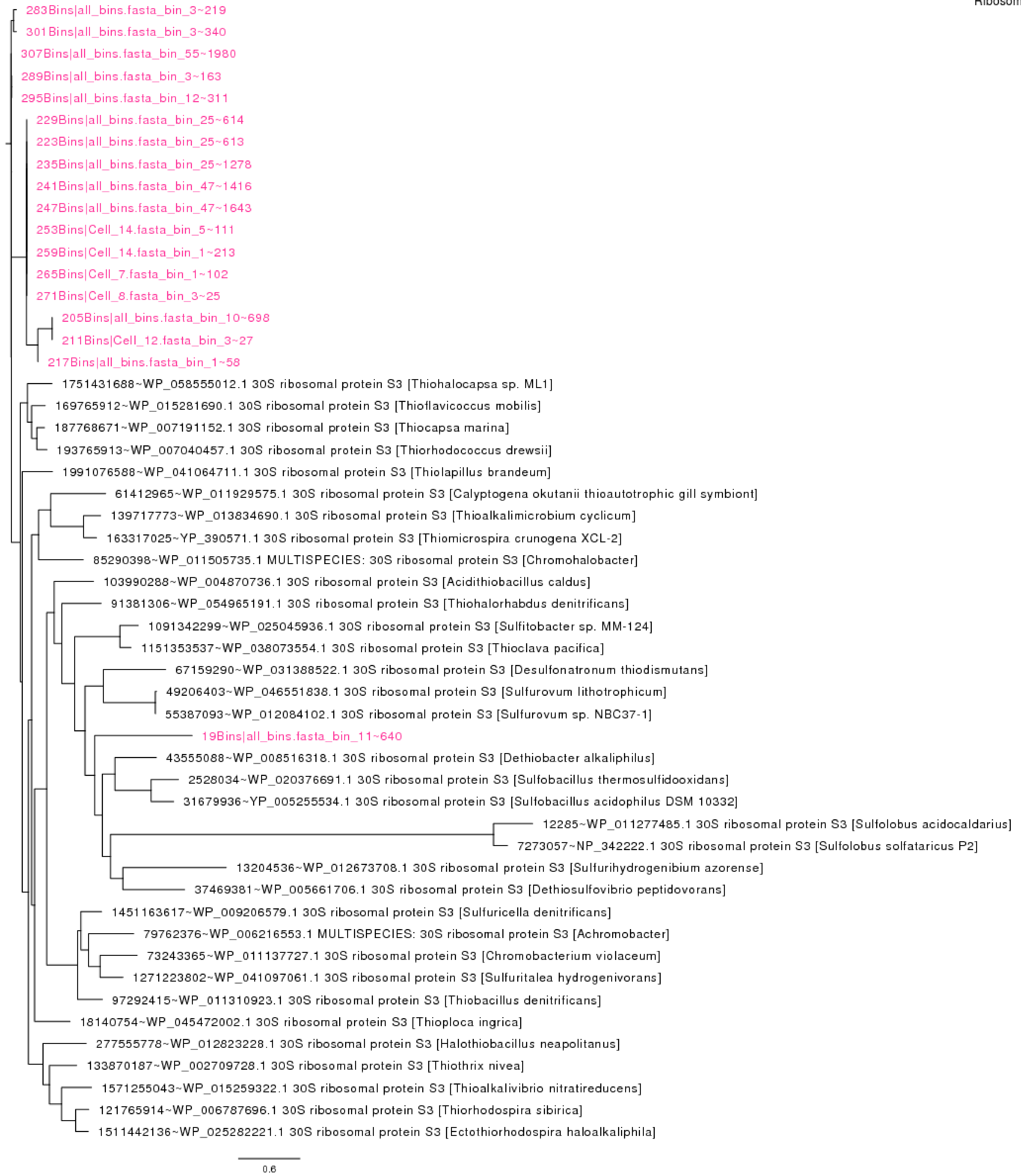
283Bins|all_bins.fasta_bin_3~219
301Bins|all_bins.fasta_bin_3~340
307Bins|all_bins.fasta_bin_55~1980
289Bins|all_bins.fasta_bin_3~163
295Bins|all_bins.fasta_bin_12~311
229Bins|all_bins.fasta_bin_25~614
223Bins|all_bins.fasta_bin_25~613
235Bins|all_bins.fasta_bin_25~1278
241Bins|all_bins.fasta_bin_47~1416
247Bins|all_bins.fasta_bin_47~1643
253Bins|Cell_14.fasta_bin_5~111
259Bins|Cell_14.fasta_bin_1~213
265Bins|Cell_7.fasta_bin_1~102
271Bins|Cell_8.fasta_bin_3~25
205Bins|all_bins.fasta_bin_10~698
211Bins|Cell_12.fasta_bin_3~27
217Bins|all_bins.fasta_bin_1~58
1751431688~WP_058555012.1 30S ribosomal protein S3 [Thiohalocapsa sp. ML1]
169765912~WP_015281690.1 30S ribosomal protein S3 [Thioflavicoccus mobilis]
187768671~WP_007191152.1 30S ribosomal protein S3 [Thiocapsa marina]
193765913~WP_007040457.1 30S ribosomal protein S3 [Thiorhodococcus drewsii]
1991076588~WP_041064711.1 30S ribosomal protein S3 [Thiolapillus brandeum]
61412965~WP_011929575.1 30S ribosomal protein S3 [Calyptogena okutanii thioautotrophic gill symbiont]
139717773~WP_013834690.1 30S ribosomal protein S3 [Thioalkalimicrobium cyclicum]
163317025~YP_390571.1 30S ribosomal protein S3 [Thiomicrospira crunogena XCL-2]
85290398~WP_011505735.1 MULTISPECIES: 30S ribosomal protein S3 [Chromohalobacter]
103990288~WP_004870736.1 30S ribosomal protein S3 [Acidithiobacillus caldus]
91381306~WP_054965191.1 30S ribosomal protein S3 [Thiohalorhabdus denitrificans]
1091342299~WP_025045936.1 30S ribosomal protein S3 [Sulfitobacter sp. MM-124]
1151353537~WP_038073554.1 30S ribosomal protein S3 [Thioclava pacifica]
67159290~WP_031388522.1 30S ribosomal protein S3 [Desulfonatronum thiodismutans]
49206403~WP_046551838.1 30S ribosomal protein S3 [Sulfurovum lithotrophicum]
55387093~WP_012084102.1 30S ribosomal protein S3 [Sulfurovum sp. NBC37-1]
19Bins|all_bins.fasta_bin_11~640
43555088~WP_008516318.1 30S ribosomal protein S3 [Dethiobacter alkaliphilus]
2528034~WP_020376691.1 30S ribosomal protein S3 [Sulfobacillus thermosulfidooxidans]
31679936~YP_005255534.1 30S ribosomal protein S3 [Sulfobacillus acidophilus DSM 10332]
12285~WP_011277485.1 30S ribosomal protein S3 [Sulfolobus acidocaldarius]
7273057~NP_342222.1 30S ribosomal protein S3 [Sulfolobus solfataricus P2]
13204536~WP_012673708.1 30S ribosomal protein S3 [Sulfurihydrogenibium azorense]
37469381~WP_005661706.1 30S ribosomal protein S3 [Dethiosulfovibrio peptidovorans]
1451163617~WP_009206579.1 30S ribosomal protein S3 [Sulfuricella denitrificans]
79762376~WP_006216553.1 MULTISPECIES: 30S ribosomal protein S3 [Achromobacter]
73243365~WP_011137727.1 30S ribosomal protein S3 [Chromobacterium violaceum]
1271223802~WP_041097061.1 30S ribosomal protein S3 [Sulfuritalea hydrogenivorans]
97292415~WP_011310923.1 30S ribosomal protein S3 [Thiobacillus denitrificans]
18140754~WP_045472002.1 30S ribosomal protein S3 [Thioploca ingrica]
277555778~WP_012823228.1 30S ribosomal protein S3 [Halothiobacillus neapolitanus]
133870187~WP_002709728.1 30S ribosomal protein S3 [Thiothrix nivea]
1571255043~WP_015259322.1 30S ribosomal protein S3 [Thioalkalivibrio nitratireducens]
121765914~WP_006787696.1 30S ribosomal protein S3 [Thiorhodospira sibirica]
1511442136~WP_025282221.1 30S ribosomal protein S3 [Ectothiorhodospira haloalkaliphila]
0.6

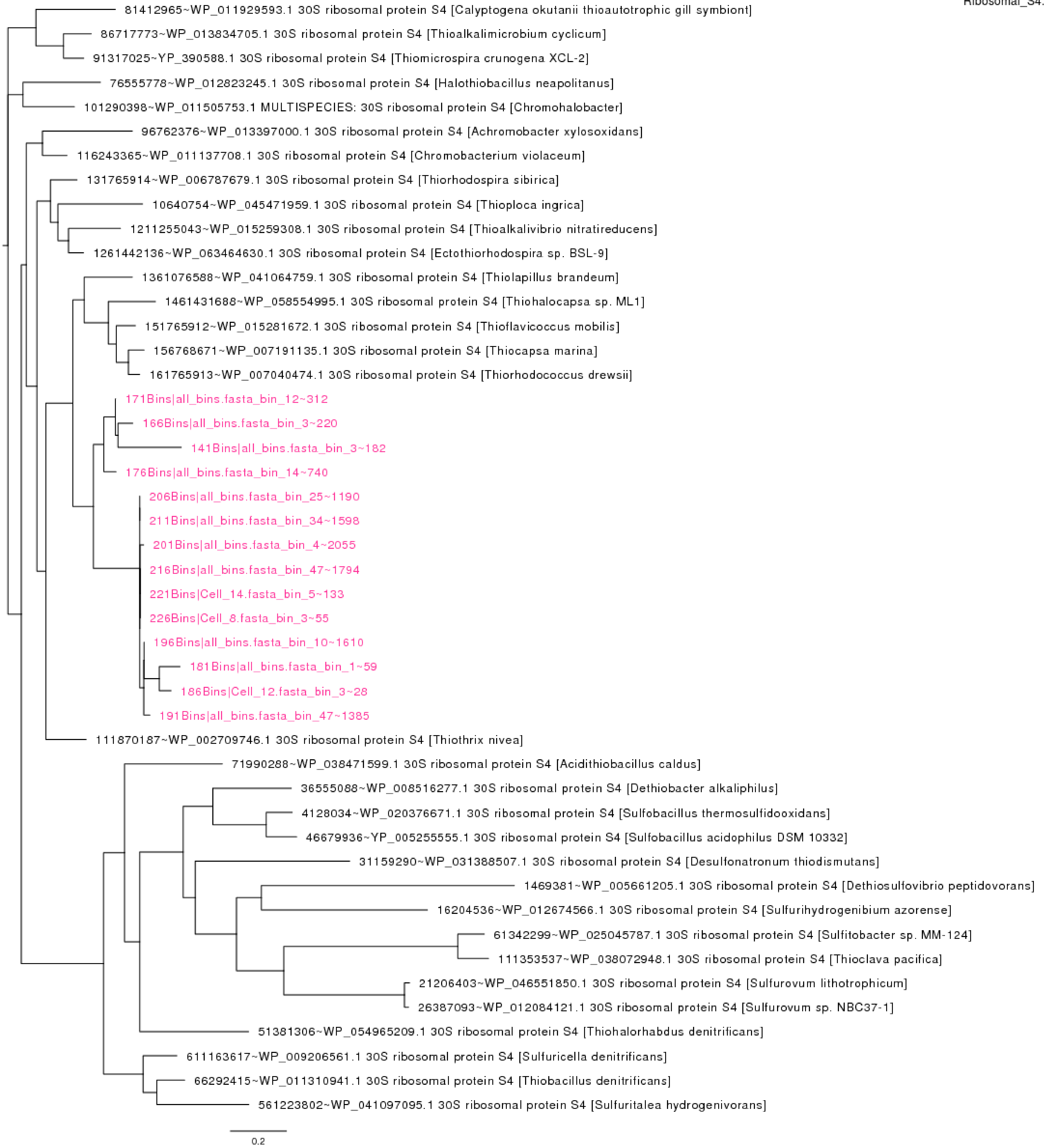
81412965~WP_011929593.1 30S ribosomal protein S4 [Calyptogena okutanii thioautotrophic gill symbiont]
86717773~WP_013834705.1 30S ribosomal protein S4 [Thioalkalimicrobium cyclicum]
91317025~YP_390588.1 30S ribosomal protein S4 [Thiomicrospira crunogena XCL-2]
76555778~WP_012823245.1 30S ribosomal protein S4 [Halothiobacillus neapolitanus]
101290398~WP_011505753.1 MULTISPECIES: 30S ribosomal protein S4 [Chromohalobacter]
96762376~WP_013397000.1 30S ribosomal protein S4 [Achromobacter xylosoxidans]
116243365~WP_011137708.1 30S ribosomal protein S4 [Chromobacterium violaceum]
131765914~WP_006787679.1 30S ribosomal protein S4 [Thiorhodospira sibirica]
10640754~WP_045471959.1 30S ribosomal protein S4 [Thioploca ingrica]
1211255043~WP_015259308.1 30S ribosomal protein S4 [Thioalkalivibrio nitratireducens]
1261442136~WP_063464630.1 30S ribosomal protein S4 [Ectothiorhodospira sp. BSL-9]
1361076588~WP_041064759.1 30S ribosomal protein S4 [Thiolapillus brandeum]
1461431688~WP_058554995.1 30S ribosomal protein S4 [Thiohalocapsa sp. ML1]
151765912~WP_015281672.1 30S ribosomal protein S4 [Thioflavicoccus mobilis]
156768671~WP_007191135.1 30S ribosomal protein S4 [Thiocapsa marina]
161765913~WP_007040474.1 30S ribosomal protein S4 [Thiorhodococcus drewsii]
171Bins|all_bins.fasta_bin_12~312
166Bins|all_bins.fasta_bin_3~220
141Bins|all_bins.fasta_bin_3~182
176Bins|all_bins.fasta_bin_14~740
206Bins|all_bins.fasta_bin_25~1190
211Bins|all_bins.fasta_bin_34~1598
201Bins|all_bins.fasta_bin_4~2055
216Bins|all_bins.fasta_bin_47~1794
221Bins|Cell_14.fasta_bin_5~133
226Bins|Cell_8.fasta_bin_3~55
196Bins|all_bins.fasta_bin_10~1610
181Bins|all_bins.fasta_bin_1~59
186Bins|Cell_12.fasta_bin_3~28
191Bins|all_bins.fasta_bin_47~1385
111870187~WP_002709746.1 30S ribosomal protein S4 [Thiothrix nivea]
71990288~WP_038471599.1 30S ribosomal protein S4 [Acidithiobacillus caldus]
36555088~WP_008516277.1 30S ribosomal protein S4 [Dethiobacter alkaliphilus]
4128034~WP_020376671.1 30S ribosomal protein S4 [Sulfobacillus thermosulfidooxidans]
46679936~YP_005255555.1 30S ribosomal protein S4 [Sulfobacillus acidophilus DSM 10332]
31159290~WP_031388507.1 30S ribosomal protein S4 [Desulfonatronum thiodismutans]
1469381~WP_005661205.1 30S ribosomal protein S4 [Dethiosulfovibrio peptidovorans]
16204536~WP_012674566.1 30S ribosomal protein S4 [Sulfurihydrogenibium azorense]
61342299~WP_025045787.1 30S ribosomal protein S4 [Sulfitobacter sp. MM-124]
111353537~WP_038072948.1 30S ribosomal protein S4 [Thioclava pacifica]
21206403~WP_046551850.1 30S ribosomal protein S4 [Sulfurovum lithotrophicum]
26387093~WP_012084121.1 30S ribosomal protein S4 [Sulfurovum sp. NBC37-1]
51381306~WP_054965209.1 30S ribosomal protein S4 [Thiohalorhabdus denitrificans]
611163617~WP_009206561.1 30S ribosomal protein S4 [Sulfuricella denitrificans]
66292415~WP_011310941.1 30S ribosomal protein S4 [Thiobacillus denitrificans]
561223802~WP_041097095.1 30S ribosomal protein S4 [Sulfuritalea hydrogenivorans]
0.2

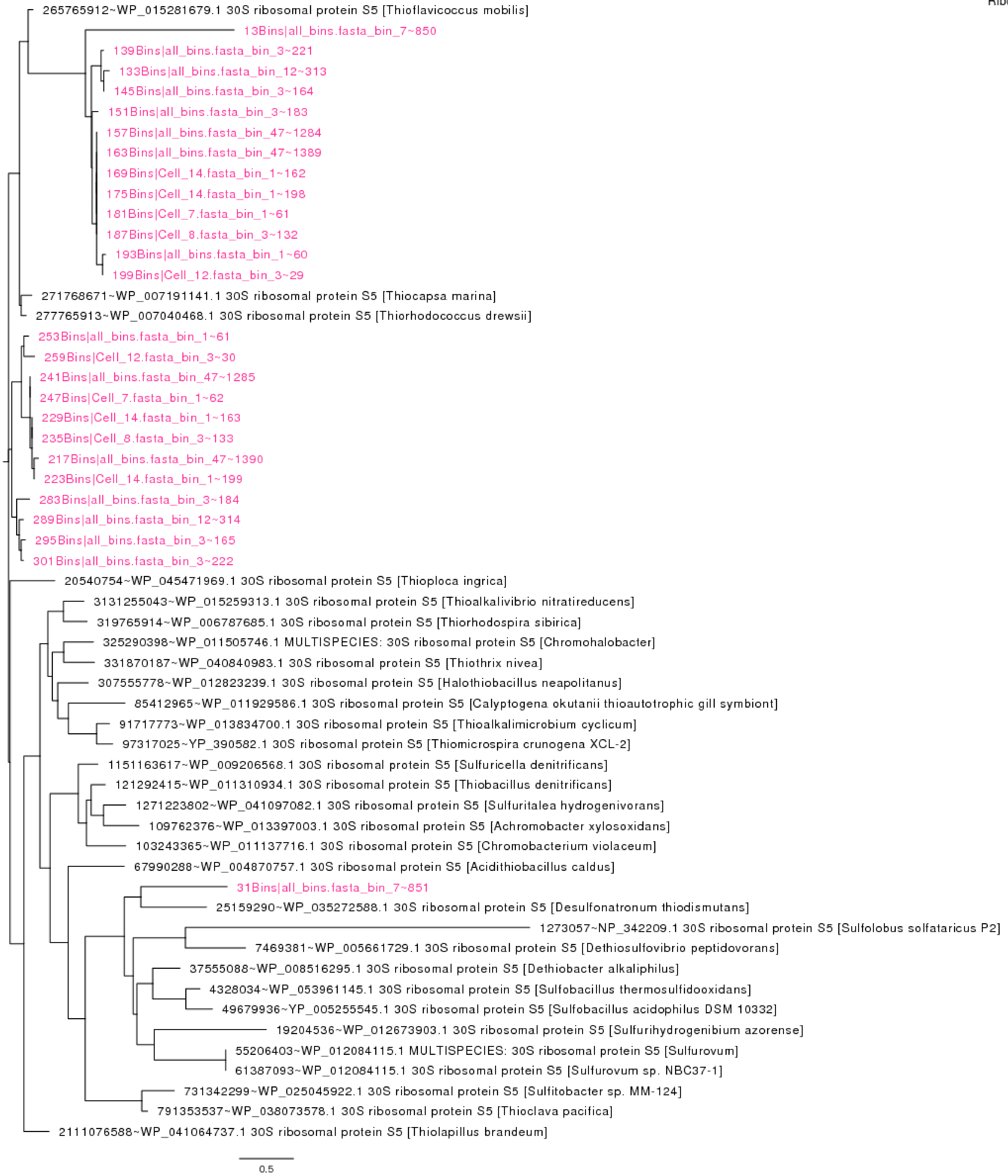
265765912~WP_015281679.1 30S ribosomal protein S5 [Thioflavicoccus mobilis]
13Bins|all_bins.fasta_bin_7~850
139Bins|all_bins.fasta_bin_3~221
133Bins|all_bins.fasta_bin_12~313
145Bins|all_bins.fasta_bin_3~164
151Bins|all_bins.fasta_bin_3~183
157Bins|all_bins.fasta_bin_47~1284
163Bins|all_bins.fasta_bin_47~1389
169Bins|Cell_14.fasta_bin_1~162
175Bins|Cell_14.fasta_bin_1~198
181Bins|Cell_7.fasta_bin_1~61
187Bins|Cell_8.fasta_bin_3~132
193Bins|all_bins.fasta_bin_1~60
199Bins|Cell_12.fasta_bin_3~29
271768671~WP_007191141.1 30S ribosomal protein S5 [Thiocapsa marina]
277765913~WP_007040468.1 30S ribosomal protein S5 [Thiorhodococcus drewsii]
253Bins|all_bins.fasta_bin_1~61
259Bins|Cell_12.fasta_bin_3~30
241Bins|all_bins.fasta_bin_47~1285
247Bins|Cell_7.fasta_bin_1~62
229Bins|Cell_14.fasta_bin_1~163
235Bins|Cell_8.fasta_bin_3~133
217Bins|all_bins.fasta_bin_47~1390
223Bins|Cell_14.fasta_bin_1~199
283Bins|all_bins.fasta_bin_3~184
289Bins|all_bins.fasta_bin_12~314
295Bins|all_bins.fasta_bin_3~165
301Bins|all_bins.fasta_bin_3~222
20540754~WP_045471969.1 30S ribosomal protein S5 [Thioploca ingrica]
3131255043~WP_015259313.1 30S ribosomal protein S5 [Thioalkalivibrio nitratireducens]
319765914~WP_006787685.1 30S ribosomal protein S5 [Thiorhodospira sibirica]
325290398~WP_011505746.1 MULTISPECIES: 30S ribosomal protein S5 [Chromohalobacter]
331870187~WP_040840983.1 30S ribosomal protein S5 [Thiothrix nivea]
307555778~WP_012823239.1 30S ribosomal protein S5 [Halothiobacillus neapolitanus]
85412965~WP_011929586.1 30S ribosomal protein S5 [Calyptogena okutanii thioautotrophic gill symbiont]
91717773~WP_013834700.1 30S ribosomal protein S5 [Thioalkalimicrobium cyclicum]
97317025~YP_390582.1 30S ribosomal protein S5 [Thiomicrospira crunogena XCL-2]
1151163617~WP_009206568.1 30S ribosomal protein S5 [Sulfuricella denitrificans]
121292415~WP_011310934.1 30S ribosomal protein S5 [Thiobacillus denitrificans]
1271223802~WP_041097082.1 30S ribosomal protein S5 [Sulfuritalea hydrogenivorans]
109762376~WP_013397003.1 30S ribosomal protein S5 [Achromobacter xylosoxidans]
103243365~WP_011137716.1 30S ribosomal protein S5 [Chromobacterium violaceum]
67990288~WP_004870757.1 30S ribosomal protein S5 [Acidithiobacillus caldus]
31Bins|all_bins.fasta_bin_7~851
25159290~WP_035272588.1 30S ribosomal protein S5 [Desulfonatronum thiodismutans]
1273057~NP_342209.1 30S ribosomal protein S5 [Sulfolobus solfataricus P2]
7469381~WP_005661729.1 30S ribosomal protein S5 [Dethiosulfovibrio peptidovorans]
37555088~WP_008516295.1 30S ribosomal protein S5 [Dethiobacter alkaliphilus]
4328034~WP_053961145.1 30S ribosomal protein S5 [Sulfobacillus thermosulfidooxidans]
49679936~YP_005255545.1 30S ribosomal protein S5 [Sulfobacillus acidophilus DSM 10332]
19204536~WP_012673903.1 30S ribosomal protein S5 [Sulfurihydrogenibium azorense]
55206403~WP_012084115.1 MULTISPECIES: 30S ribosomal protein S5 [Sulfurovum]
61387093~WP_012084115.1 30S ribosomal protein S5 [Sulfurovum sp. NBC37-1]
731342299~WP_025045922.1 30S ribosomal protein S5 [Sulfitobacter sp. MM-124]
791353537~WP_038073578.1 30S ribosomal protein S5 [Thioclava pacifica]
2111076588~WP_041064737.1 30S ribosomal protein S5 [Thiolapillus brandeum]
0.5

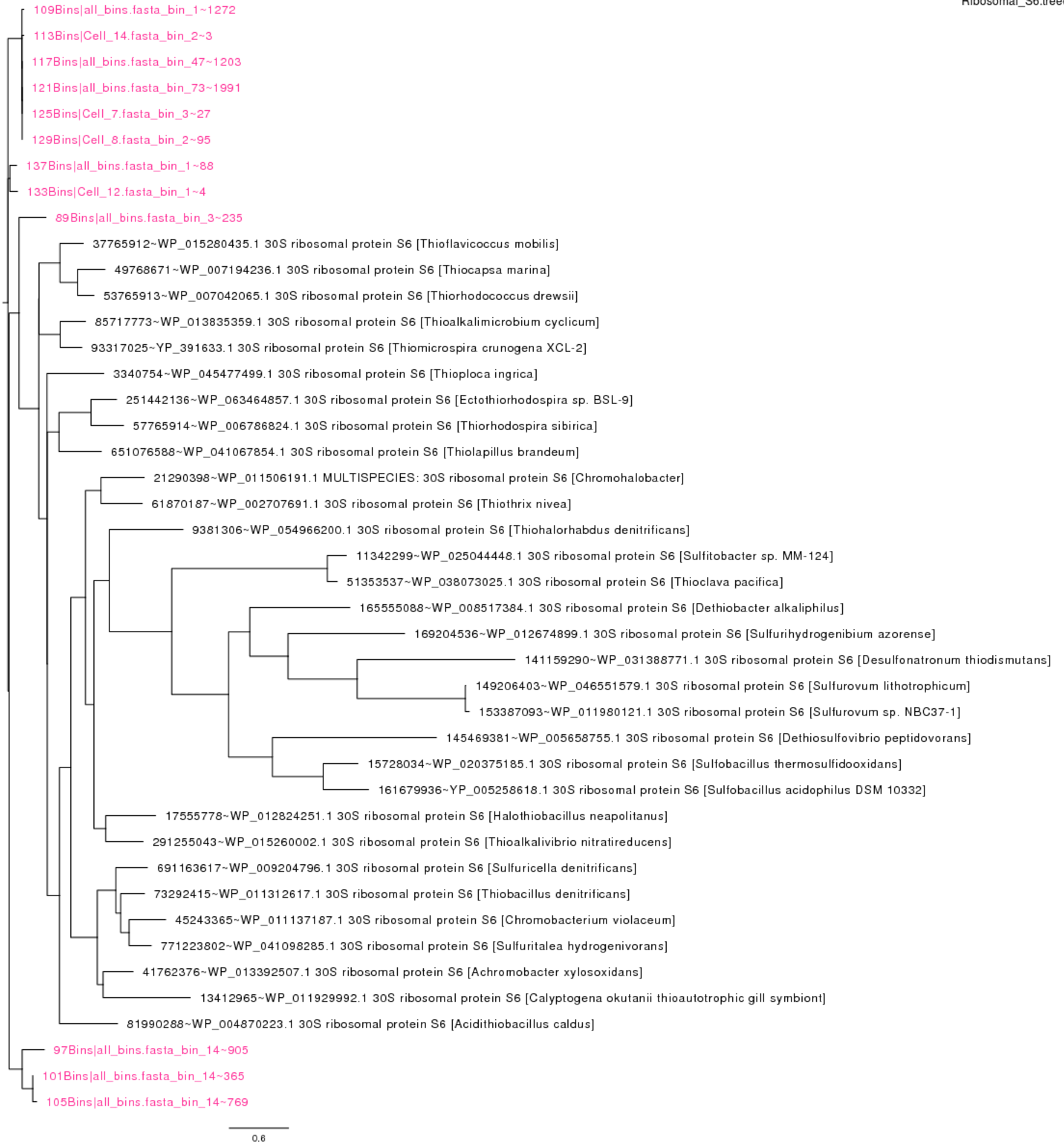
109Bins|all_bins.fasta_bin_1~1272
113Bins|Cell_14.fasta_bin_2~3
117Bins|all_bins.fasta_bin_47~1203
121Bins|all_bins.fasta_bin_73~1991
125Bins|Cell_7.fasta_bin_3~27
129Bins|Cell_8.fasta_bin_2~95
137Bins|all_bins.fasta_bin_1~88
133Bins|Cell_12.fasta_bin_1~4
89Bins|all_bins.fasta_bin_3~235
37765912~WP_015280435.1 30S ribosomal protein S6 [Thioflavicoccus mobilis]
49768671~WP_007194236.1 30S ribosomal protein S6 [Thiocapsa marina]
53765913~WP_007042065.1 30S ribosomal protein S6 [Thiorhodococcus drewsii]
85717773~WP_013835359.1 30S ribosomal protein S6 [Thioalkalimicrobium cyclicum]
93317025~YP_391633.1 30S ribosomal protein S6 [Thiomicrospira crunogena XCL-2]
3340754~WP_045477499.1 30S ribosomal protein S6 [Thioploca ingrica]
251442136~WP_063464857.1 30S ribosomal protein S6 [Ectothiorhodospira sp. BSL-9]
57765914~WP_006786824.1 30S ribosomal protein S6 [Thiorhodospira sibirica]
651076588~WP_041067854.1 30S ribosomal protein S6 [Thiolapillus brandeum]
21290398~WP_011506191.1 MULTISPECIES: 30S ribosomal protein S6 [Chromohalobacter]
61870187~WP_002707691.1 30S ribosomal protein S6 [Thiothrix nivea]
9381306~WP_054966200.1 30S ribosomal protein S6 [Thiohalorhabdus denitrificans]
11342299~WP_025044448.1 30S ribosomal protein S6 [Sulfitobacter sp. MM-124]
51353537~WP_038073025.1 30S ribosomal protein S6 [Thioclava pacifica]
165555088~WP_008517384.1 30S ribosomal protein S6 [Dethiobacter alkaliphilus]
169204536~WP_012674899.1 30S ribosomal protein S6 [Sulfurihydrogenibium azorense]
141159290~WP_031388771.1 30S ribosomal protein S6 [Desulfonatronum thiodismutans]
149206403~WP_046551579.1 30S ribosomal protein S6 [Sulfurovum lithotrophicum]
153387093~WP_011980121.1 30S ribosomal protein S6 [Sulfurovum sp. NBC37-1]
145469381~WP_005658755.1 30S ribosomal protein S6 [Dethiosulfovibrio peptidovorans]
15728034~WP_020375185.1 30S ribosomal protein S6 [Sulfobacillus thermosulfidooxidans]
161679936~YP_005258618.1 30S ribosomal protein S6 [Sulfobacillus acidophilus DSM 10332]
17555778~WP_012824251.1 30S ribosomal protein S6 [Halothiobacillus neapolitanus]
291255043~WP_015260002.1 30S ribosomal protein S6 [Thioalkalivibrio nitratireducens]
691163617~WP_009204796.1 30S ribosomal protein S6 [Sulfuricella denitrificans]
73292415~WP_011312617.1 30S ribosomal protein S6 [Thiobacillus denitrificans]
45243365~WP_011137187.1 30S ribosomal protein S6 [Chromobacterium violaceum]
771223802~WP_041098285.1 30S ribosomal protein S6 [Sulfuritalea hydrogenivorans]
41762376~WP_013392507.1 30S ribosomal protein S6 [Achromobacter xylosoxidans]
13412965~WP_011929992.1 30S ribosomal protein S6 [Calyptogena okutanii thioautotrophic gill symbiont]
81990288~WP_004870223.1 30S ribosomal protein S6 [Acidithiobacillus caldus]
97Bins|all_bins.fasta_bin_14~905
101Bins|all_bins.fasta_bin_14~365
105Bins|all_bins.fasta_bin_14~769
0.6

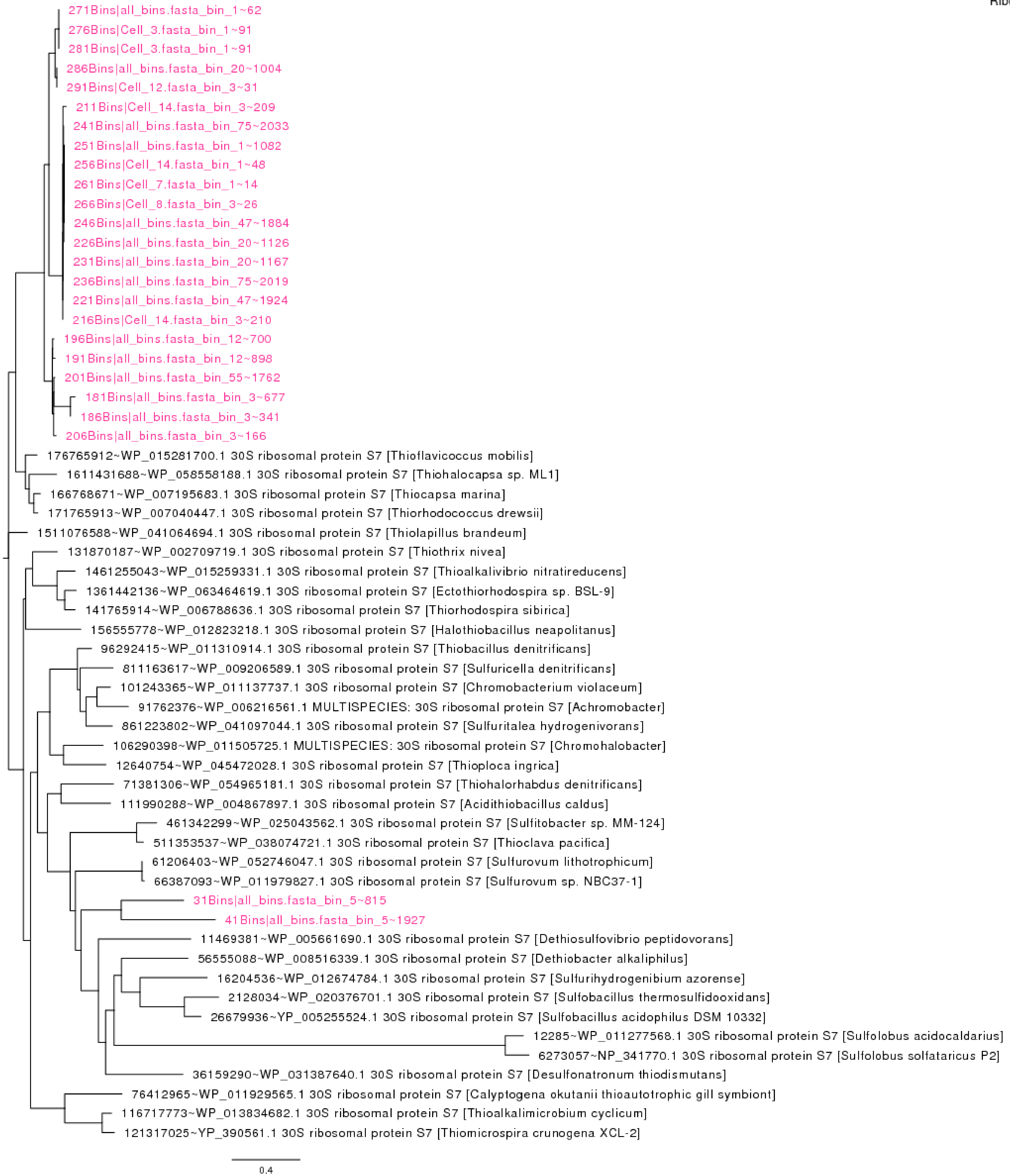
271Bins|all_bins.fasta_bin_1~62
276Bins|Cell_3.fasta_bin_1~91
281Bins|Cell_3.fasta_bin_1~91
286Bins|all_bins.fasta_bin_20~1004
291Bins|Cell_12.fasta_bin_3~31
211Bins|Cell_14.fasta_bin_3~209
241Bins|all_bins.fasta_bin_75~2033
251Bins|all_bins.fasta_bin_1~1082
256Bins|Cell_14.fasta_bin_1~48
261Bins|Cell_7.fasta_bin_1~14
266Bins|Cell_8.fasta_bin_3~26
246Bins|all_bins.fasta_bin_47~1884
226Bins|all_bins.fasta_bin_20~1126
231Bins|all_bins.fasta_bin_20~1167
236Bins|all_bins.fasta_bin_75~2019
221Bins|all_bins.fasta_bin_47~1924
216Bins|Cell_14.fasta_bin_3~210
196Bins|all_bins.fasta_bin_12~700
191Bins|all_bins.fasta_bin_12~898
201Bins|all_bins.fasta_bin_55~1762
181Bins|all_bins.fasta_bin_3~677
186Bins|all_bins.fasta_bin_3~341
206Bins|all_bins.fasta_bin_3~166
176765912~WP_015281700.1 30S ribosomal protein S7 [Thioflavicoccus mobilis]
1611431688~WP_058558188.1 30S ribosomal protein S7 [Thiohalocapsa sp. ML1]
166768671~WP_007195683.1 30S ribosomal protein S7 [Thiocapsa marina]
171765913~WP_007040447.1 30S ribosomal protein S7 [Thiorhodococcus drewsii]
1511076588~WP_041064694.1 30S ribosomal protein S7 [Thiolapillus brandeum]
131870187~WP_002709719.1 30S ribosomal protein S7 [Thiothrix nivea]
1461255043~WP_015259331.1 30S ribosomal protein S7 [Thioalkalivibrio nitratireducens]
1361442136~WP_063464619.1 30S ribosomal protein S7 [Ectothiorhodospira sp. BSL-9]
141765914~WP_006788636.1 30S ribosomal protein S7 [Thiorhodospira sibirica]
156555778~WP_012823218.1 30S ribosomal protein S7 [Halothiobacillus neapolitanus]
96292415~WP_011310914.1 30S ribosomal protein S7 [Thiobacillus denitrificans]
811163617~WP_009206589.1 30S ribosomal protein S7 [Sulfuricella denitrificans]
101243365~WP_011137737.1 30S ribosomal protein S7 [Chromobacterium violaceum]
91762376~WP_006216561.1 MULTISPECIES: 30S ribosomal protein S7 [Achromobacter]
861223802~WP_041097044.1 30S ribosomal protein S7 [Sulfuritalea hydrogenivorans]
106290398~WP_011505725.1 MULTISPECIES: 30S ribosomal protein S7 [Chromohalobacter]
12640754~WP_045472028.1 30S ribosomal protein S7 [Thioploca ingrica]
71381306~WP_054965181.1 30S ribosomal protein S7 [Thiohalorhabdus denitrificans]
111990288~WP_004867897.1 30S ribosomal protein S7 [Acidithiobacillus caldus]
461342299~WP_025043562.1 30S ribosomal protein S7 [Sulfitobacter sp. MM-124]
511353537~WP_038074721.1 30S ribosomal protein S7 [Thioclava pacifica]
61206403~WP_052746047.1 30S ribosomal protein S7 [Sulfurovum lithotrophicum]
66387093~WP_011979827.1 30S ribosomal protein S7 [Sulfurovum sp. NBC37-1]
31Bins|all_bins.fasta_bin_5~815
41Bins|all_bins.fasta_bin_5~1927
11469381~WP_005661690.1 30S ribosomal protein S7 [Dethiosulfovibrio peptidovorans]
56555088~WP_008516339.1 30S ribosomal protein S7 [Dethiobacter alkaliphilus]
16204536~WP_012674784.1 30S ribosomal protein S7 [Sulfurihydrogenibium azorense]
2128034~WP_020376701.1 30S ribosomal protein S7 [Sulfobacillus thermosulfidooxidans]
26679936~YP_005255524.1 30S ribosomal protein S7 [Sulfobacillus acidophilus DSM 10332]
12285~WP_011277568.1 30S ribosomal protein S7 [Sulfolobus acidocaldarius]
6273057~NP_341770.1 30S ribosomal protein S7 [Sulfolobus solfataricus P2]
36159290~WP_031387640.1 30S ribosomal protein S7 [Desulfonatronum thiodismutans]
76412965~WP_011929565.1 30S ribosomal protein S7 [Calyptogena okutanii thioautotrophic gill symbiont]
116717773~WP_013834682.1 30S ribosomal protein S7 [Thioalkalimicrobium cyclicum]
121317025~YP_390561.1 30S ribosomal protein S7 [Thiomicrospira crunogena XCL-2]
0.4

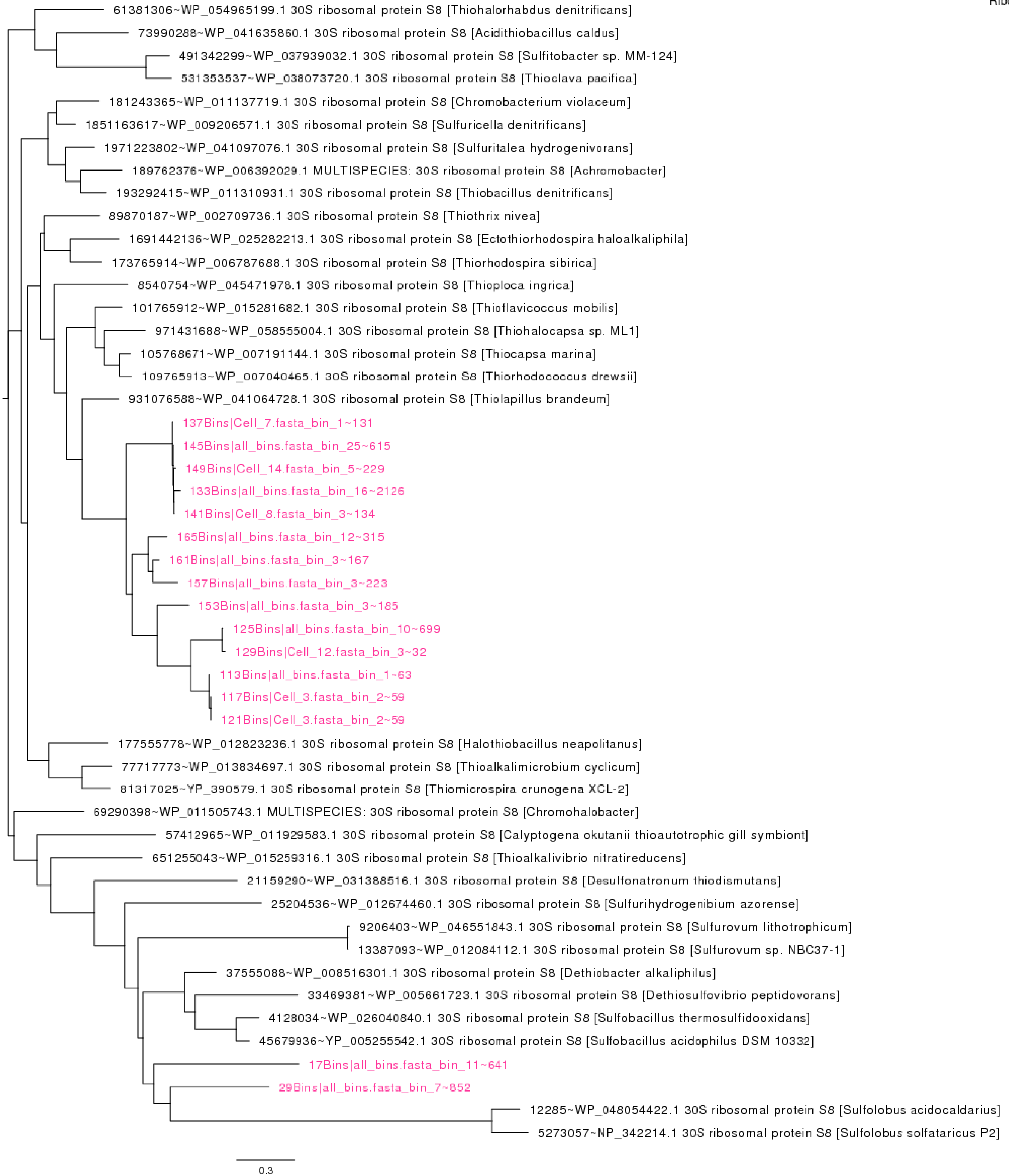
61381306~WP_054965199.1 30S ribosomal protein S8 [Thiohalorhabdus denitrificans]
73990288~WP_041635860.1 30S ribosomal protein S8 [Acidithiobacillus caldus]
491342299~WP_037939032.1 30S ribosomal protein S8 [Sulfitobacter sp. MM-124]
531353537~WP_038073720.1 30S ribosomal protein S8 [Thioclava pacifica]
181243365~WP_011137719.1 30S ribosomal protein S8 [Chromobacterium violaceum]
1851163617~WP_009206571.1 30S ribosomal protein S8 [Sulfuricella denitrificans]
1971223802~WP_041097076.1 30S ribosomal protein S8 [Sulfuritalea hydrogenivorans]
189762376~WP_006392029.1 MULTISPECIES: 30S ribosomal protein S8 [Achromobacter]
193292415~WP_011310931.1 30S ribosomal protein S8 [Thiobacillus denitrificans]
89870187~WP_002709736.1 30S ribosomal protein S8 [Thiothrix nivea]
1691442136~WP_025282213.1 30S ribosomal protein S8 [Ectothiorhodospira haloalkaliphila]
173765914~WP_006787688.1 30S ribosomal protein S8 [Thiorhodospira sibirica]
8540754~WP_045471978.1 30S ribosomal protein S8 [Thioploca ingrica]
101765912~WP_015281682.1 30S ribosomal protein S8 [Thioflavicoccus mobilis]
971431688~WP_058555004.1 30S ribosomal protein S8 [Thiohalocapsa sp. ML1]
105768671~WP_007191144.1 30S ribosomal protein S8 [Thiocapsa marina]
109765913~WP_007040465.1 30S ribosomal protein S8 [Thiorhodococcus drewsii]
931076588~WP_041064728.1 30S ribosomal protein S8 [Thiolapillus brandeum]
137Bins|Cell_7.fasta_bin_1~131
145Bins|all_bins.fasta_bin_25~615
149Bins|Cell_14.fasta_bin_5~229
133Bins|all_bins.fasta_bin_16~2126
141Bins|Cell_8.fasta_bin_3~134
165Bins|all_bins.fasta_bin_12~315
161Bins|all_bins.fasta_bin_3~167
157Bins|all_bins.fasta_bin_3~223
153Bins|all_bins.fasta_bin_3~185
125Bins|all_bins.fasta_bin_10~699
129Bins|Cell_12.fasta_bin_3~32
113Bins|all_bins.fasta_bin_1~63
117Bins|Cell_3.fasta_bin_2~59
121Bins|Cell_3.fasta_bin_2~59
177555778~WP_012823236.1 30S ribosomal protein S8 [Halothiobacillus neapolitanus]
77717773~WP_013834697.1 30S ribosomal protein S8 [Thioalkalimicrobium cyclicum]
81317025~YP_390579.1 30S ribosomal protein S8 [Thiomicrospira crunogena XCL-2]
69290398~WP_011505743.1 MULTISPECIES: 30S ribosomal protein S8 [Chromohalobacter]
57412965~WP_011929583.1 30S ribosomal protein S8 [Calyptogena okutanii thioautotrophic gill symbiont]
651255043~WP_015259316.1 30S ribosomal protein S8 [Thioalkalivibrio nitratireducens]
21159290~WP_031388516.1 30S ribosomal protein S8 [Desulfonatronum thiodismutans]
25204536~WP_012674460.1 30S ribosomal protein S8 [Sulfurihydrogenibium azorense]
9206403~WP_046551843.1 30S ribosomal protein S8 [Sulfurovum lithotrophicum]
13387093~WP_012084112.1 30S ribosomal protein S8 [Sulfurovum sp. NBC37-1]
37555088~WP_008516301.1 30S ribosomal protein S8 [Dethiobacter alkaliphilus]
33469381~WP_005661723.1 30S ribosomal protein S8 [Dethiosulfovibrio peptidovorans]
4128034~WP_026040840.1 30S ribosomal protein S8 [Sulfobacillus thermosulfidooxidans]
45679936~YP_005255542.1 30S ribosomal protein S8 [Sulfobacillus acidophilus DSM 10332]
17Bins|all_bins.fasta_bin_11~641
29Bins|all_bins.fasta_bin_7~852
12285~WP_048054422.1 30S ribosomal protein S8 [Sulfolobus acidocaldarius]
5273057~NP_342214.1 30S ribosomal protein S8 [Sulfolobus solfataricus P2]
0.3

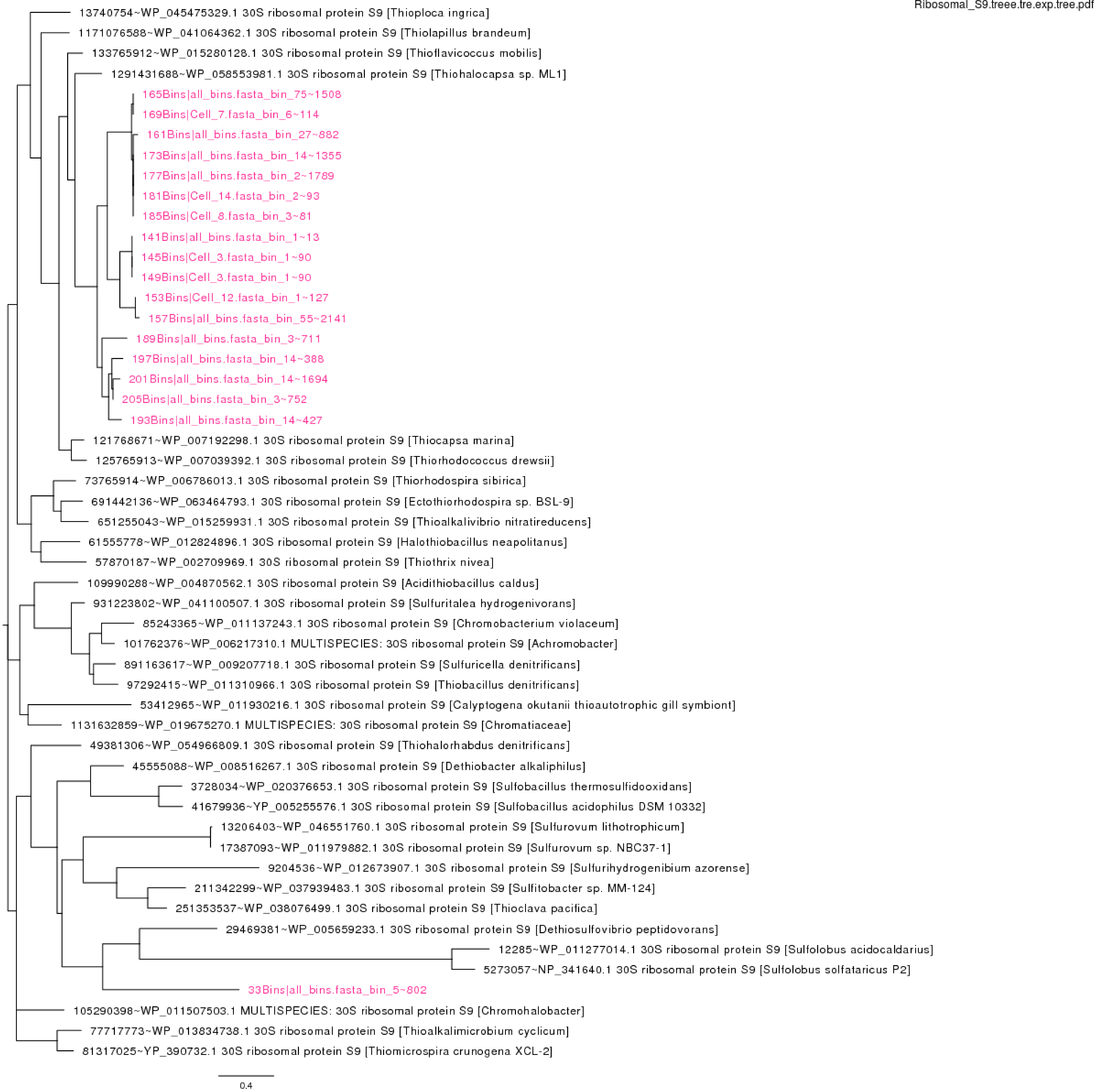
Ribosomal_S9.treee.tre.exp.tree.pdf
13740754~WP_045475329.1 30S ribosomal protein S9 [Thioploca ingrica]
1171076588~WP_041064362.1 30S ribosomal protein S9 [Thiolapillus brandeum]
133765912~WP_015280128.1 30S ribosomal protein S9 [Thioflavicoccus mobilis]
1291431688~WP_058553981.1 30S ribosomal protein S9 [Thiohalocapsa sp. ML1]
165Bins|all_bins.fasta_bin_75~1508
169Bins|Cell_7.fasta_bin_6~114
161Bins|all_bins.fasta_bin_27~882
173Bins|all_bins.fasta_bin_14~1355
177Bins|all_bins.fasta_bin_2~1789
181Bins|Cell_14.fasta_bin_2~93
185Bins|Cell_8.fasta_bin_3~81
141Bins|all_bins.fasta_bin_1~13
145Bins|Cell_3.fasta_bin_1~90
149Bins|Cell_3.fasta_bin_1~90
153Bins|Cell_12.fasta_bin_1~127
157Bins|all_bins.fasta_bin_55~2141
189Bins|all_bins.fasta_bin_3~711
197Bins|all_bins.fasta_bin_14~388
201Bins|all_bins.fasta_bin_14~1694
205Bins|all_bins.fasta_bin_3~752
193Bins|all_bins.fasta_bin_14~427
121768671~WP_007192298.1 30S ribosomal protein S9 [Thiocapsa marina]
125765913~WP_007039392.1 30S ribosomal protein S9 [Thiorhodococcus drewsii]
73765914~WP_006786013.1 30S ribosomal protein S9 [Thiorhodospira sibirica]
691442136~WP_063464793.1 30S ribosomal protein S9 [Ectothiorhodospira sp. BSL-9]
651255043~WP_015259931.1 30S ribosomal protein S9 [Thioalkalivibrio nitratireducens]
61555778~WP_012824896.1 30S ribosomal protein S9 [Halothiobacillus neapolitanus]
57870187~WP_002709969.1 30S ribosomal protein S9 [Thiothrix nivea]
109990288~WP_004870562.1 30S ribosomal protein S9 [Acidithiobacillus caldus]
931223802~WP_041100507.1 30S ribosomal protein S9 [Sulfuritalea hydrogenivorans]
85243365~WP_011137243.1 30S ribosomal protein S9 [Chromobacterium violaceum]
101762376~WP_006217310.1 MULTISPECIES: 30S ribosomal protein S9 [Achromobacter]
891163617~WP_009207718.1 30S ribosomal protein S9 [Sulfuricella denitrificans]
97292415~WP_011310966.1 30S ribosomal protein S9 [Thiobacillus denitrificans]
53412965~WP_011930216.1 30S ribosomal protein S9 [Calyptogena okutanii thioautotrophic gill symbiont]
1131632859~WP_019675270.1 MULTISPECIES: 30S ribosomal protein S9 [Chromatiaceae]
49381306~WP_054966809.1 30S ribosomal protein S9 [Thiohalorhabdus denitrificans]
45555088~WP_008516267.1 30S ribosomal protein S9 [Dethiobacter alkaliphilus]
3728034~WP_020376653.1 30S ribosomal protein S9 [Sulfobacillus thermosulfidooxidans]
41679936~YP_005255576.1 30S ribosomal protein S9 [Sulfobacillus acidophilus DSM 10332]
13206403~WP_046551760.1 30S ribosomal protein S9 [Sulfurovum lithotrophicum]
17387093~WP_011979882.1 30S ribosomal protein S9 [Sulfurovum sp. NBC37-1]
9204536~WP_012673907.1 30S ribosomal protein S9 [Sulfurihydrogenibium azorense]
211342299~WP_037939483.1 30S ribosomal protein S9 [Sulfitobacter sp. MM-124]
251353537~WP_038076499.1 30S ribosomal protein S9 [Thioclava pacifica]
29469381~WP_005659233.1 30S ribosomal protein S9 [Dethiosulfovibrio peptidovorans]
12285~WP_011277014.1 30S ribosomal protein S9 [Sulfolobus acidocaldarius]
5273057~NP_341640.1 30S ribosomal protein S9 [Sulfolobus solfataricus P2]
33Bins|all_bins.fasta_bin_5~802
105290398~WP_011507503.1 MULTISPECIES: 30S ribosomal protein S9 [Chromohalobacter]
77717773~WP_013834738.1 30S ribosomal protein S9 [Thioalkalimicrobium cyclicum]
81317025~YP_390732.1 30S ribosomal protein S9 [Thiomicrospira crunogena XCL-2]
0.4

RimM.treee.tre.exp.tree.pdf

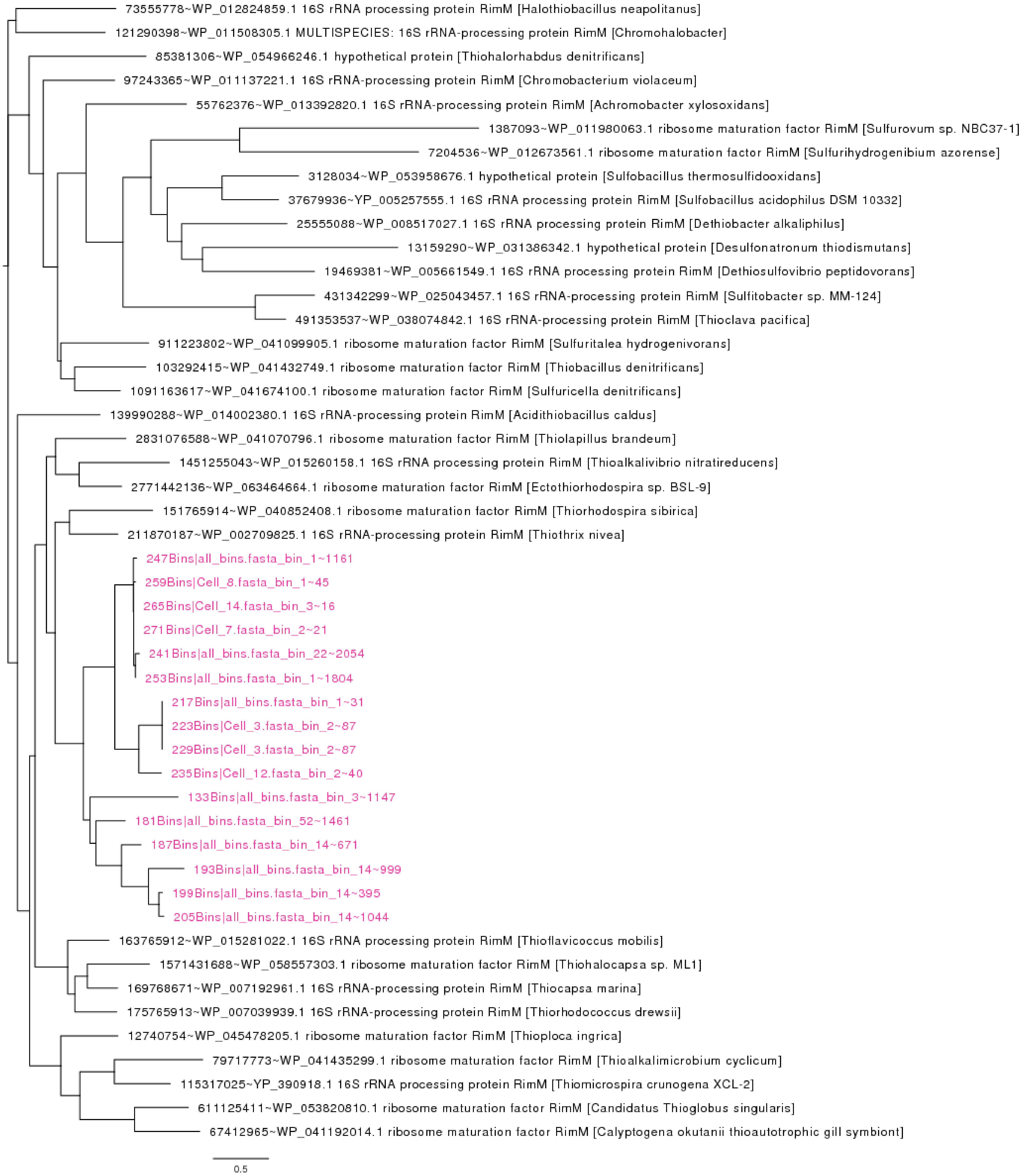

RNA_pol_Rpb1_1.treee.tre.exp.tree.pdf

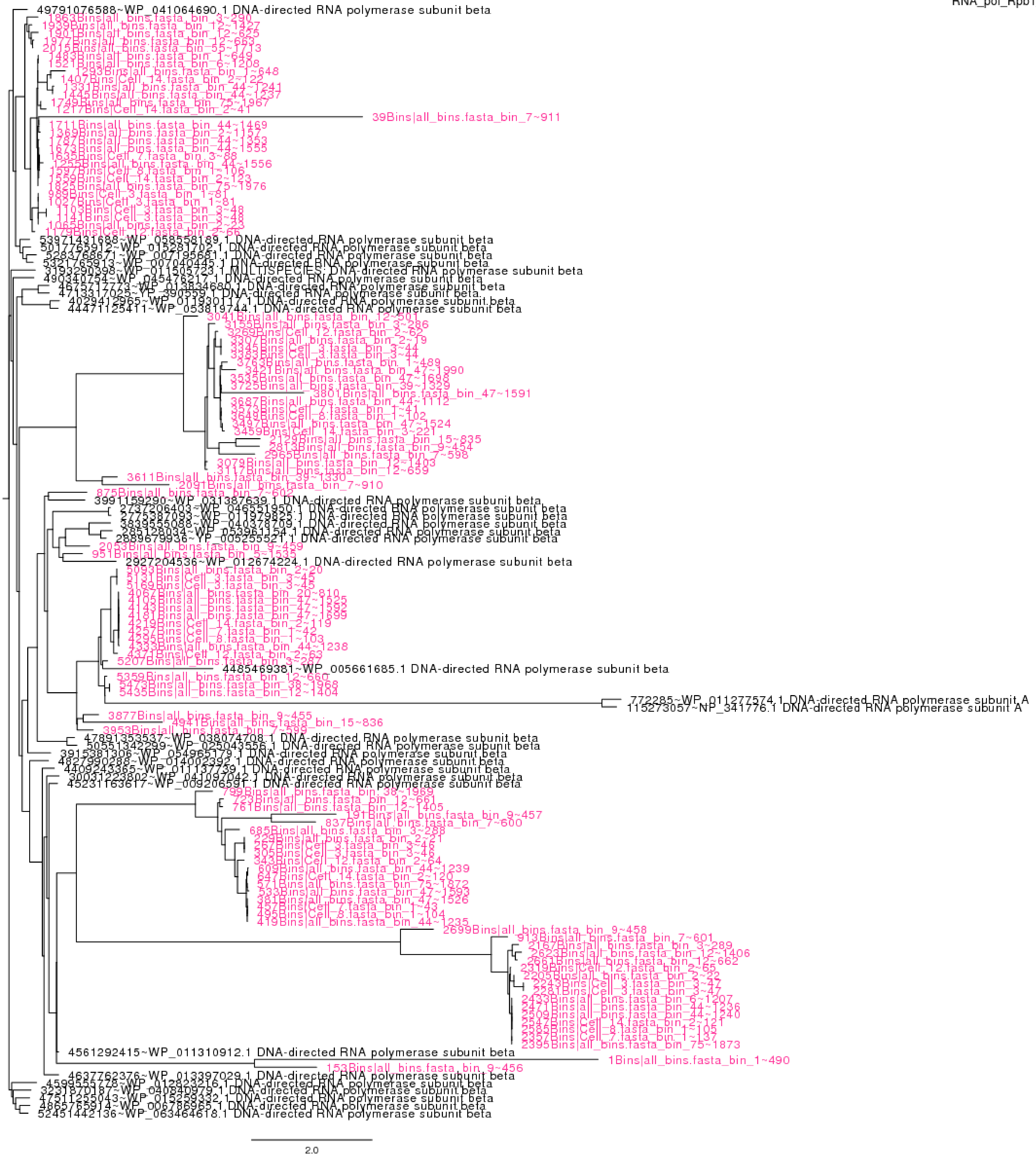

RNA_pol_Rpb2_1.treee.tre.exp.tree.pdf

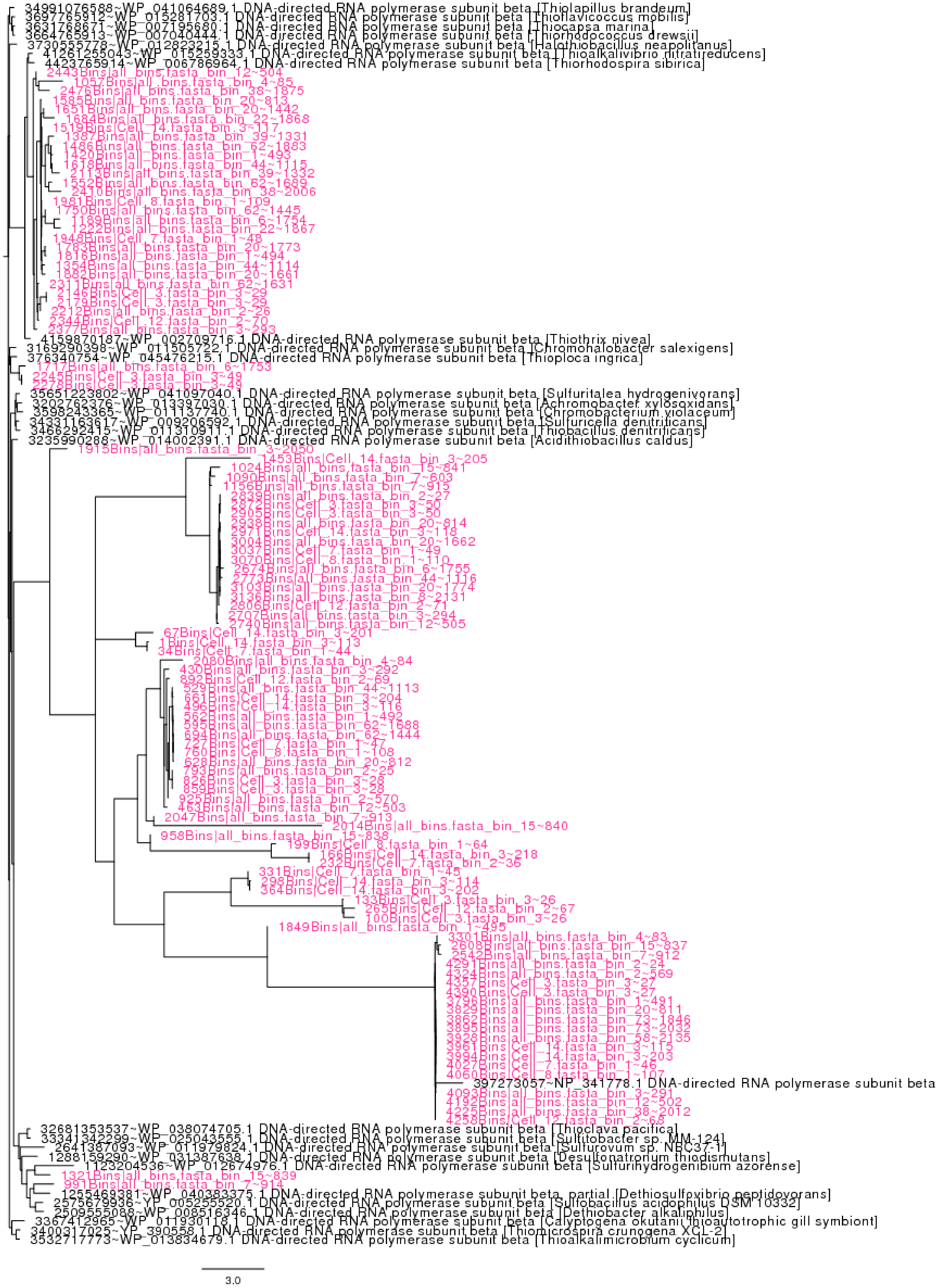

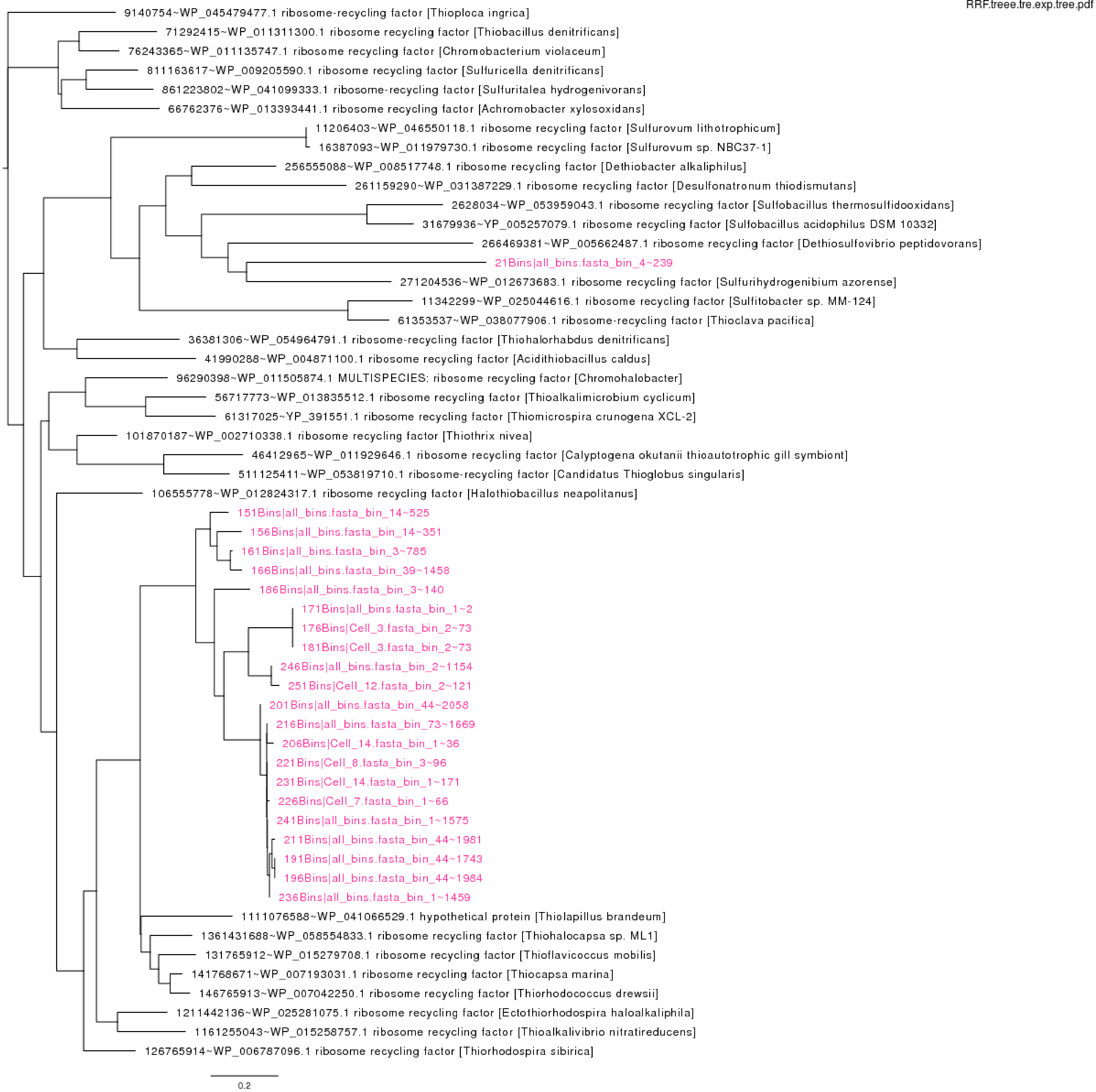

RRF.treee.tre.exp.tree.pdf
9140754~WP_045479477.1 ribosome-recycling factor [Thioploca ingrica]
71292415~WP_011311300.1 ribosome recycling factor [Thiobacillus denitrificans]
76243365~WP_011135747.1 ribosome recycling factor [Chromobacterium violaceum]
811163617~WP_009205590.1 ribosome recycling factor [Sulfuricella denitrificans]
861223802~WP_041099333.1 ribosome-recycling factor [Sulfuritalea hydrogenivorans]
66762376~WP_013393441.1 ribosome recycling factor [Achromobacter xylosoxidans]
11206403~WP_046550118.1 ribosome recycling factor [Sulfurovum lithotrophicum]
16387093~WP_011979730.1 ribosome recycling factor [Sulfurovum sp. NBC37-1]
256555088~WP_008517748.1 ribosome recycling factor [Dethiobacter alkaliphilus]
261159290~WP_031387229.1 ribosome recycling factor [Desulfonatronum thiodismutans]
2628034~WP_053959043.1 ribosome recycling factor [Sulfobacillus thermosulfidooxidans]
31679936~YP_005257079.1 ribosome recycling factor [Sulfobacillus acidophilus DSM 10332]
266469381~WP_005662487.1 ribosome recycling factor [Dethiosulfovibrio peptidovorans]
21Bins|all_bins.fasta_bin_4~239
271204536~WP_012673683.1 ribosome recycling factor [Sulfurihydrogenibium azorense]
11342299~WP_025044616.1 ribosome recycling factor [Sulfitobacter sp. MM-124]
61353537~WP_038077906.1 ribosome-recycling factor [Thioclava pacifica]
36381306~WP_054964791.1 ribosome-recycling factor [Thiohalorhabdus denitrificans]
41990288~WP_004871100.1 ribosome recycling factor [Acidithiobacillus caldus]
96290398~WP_011505874.1 MULTISPECIES: ribosome recycling factor [Chromohalobacter]
56717773~WP_013835512.1 ribosome recycling factor [Thioalkalimicrobium cyclicum]
61317025~YP_391551.1 ribosome recycling factor [Thiomicrospira crunogena XCL-2]
101870187~WP_002710338.1 ribosome recycling factor [Thiothrix nivea]
46412965~WP_011929646.1 ribosome recycling factor [Calyptogena okutanii thioautotrophic gill symbiont]
511125411~WP_053819710.1 ribosome-recycling factor [Candidatus Thioglobus singularis]
106555778~WP_012824317.1 ribosome recycling factor [Halothiobacillus neapolitanus]
151Bins|all_bins.fasta_bin_14~525
156Bins|all_bins.fasta_bin_14~351
161Bins|all_bins.fasta_bin_3~785
166Bins|all_bins.fasta_bin_39~1458
186Bins|all_bins.fasta_bin_3~140
171Bins|all_bins.fasta_bin_1~2
176Bins|Cell_3.fasta_bin_2~73
181Bins|Cell_3.fasta_bin_2~73
246Bins|all_bins.fasta_bin_2~1154
251Bins|Cell_12.fasta_bin_2~121
201Bins|all_bins.fasta_bin_44~2058
216Bins|all_bins.fasta_bin_73~1669
206Bins|Cell_14.fasta_bin_1~36
221Bins|Cell_8.fasta_bin_3~96
231Bins|Cell_14.fasta_bin_1~171
226Bins|Cell_7.fasta_bin_1~66
241Bins|all_bins.fasta_bin_1~1575
211Bins|all_bins.fasta_bin_44~1981
191Bins|all_bins.fasta_bin_44~1743
196Bins|all_bins.fasta_bin_44~1984
236Bins|all_bins.fasta_bin_1~1459
1111076588~WP_041066529.1 hypothetical protein [Thiolapillus brandeum]
1361431688~WP_058554833.1 ribosome recycling factor [Thiohalocapsa sp. ML1]
131765912~WP_015279708.1 ribosome recycling factor [Thioflavicoccus mobilis]
141768671~WP_007193031.1 ribosome recycling factor [Thiocapsa marina]
146765913~WP_007042250.1 ribosome recycling factor [Thiorhodococcus drewsii]
1211442136~WP_025281075.1 ribosome recycling factor [Ectothiorhodospira haloalkaliphila]
1161255043~WP_015258757.1 ribosome recycling factor [Thioalkalivibrio nitratireducens]
126765914~WP_006787096.1 ribosome recycling factor [Thiorhodospira sibirica]
0.2

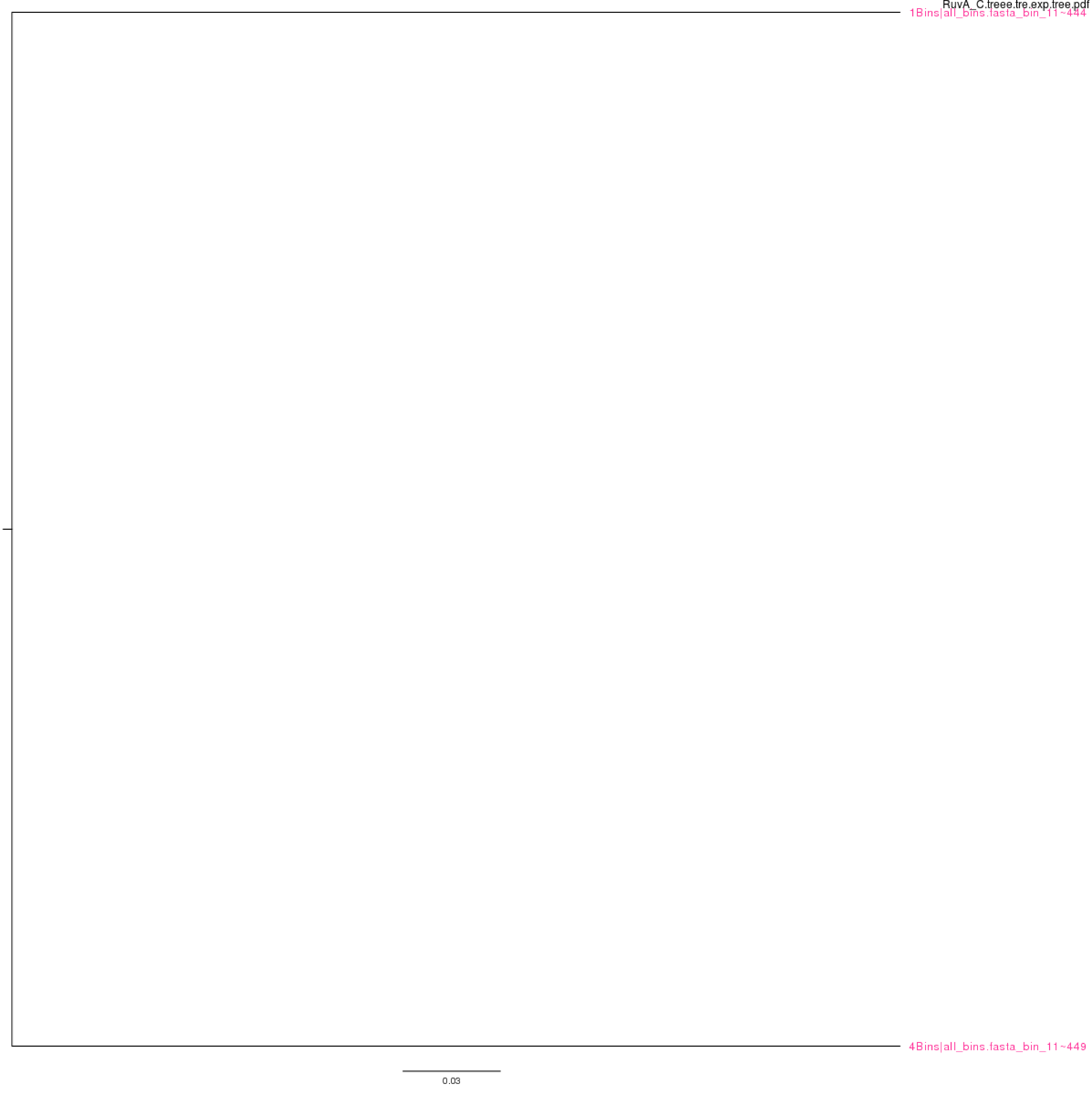
RuvA_C.treee.tre.exp.tree.pdf
1Bins|all_bins.fasta_bin_11~444
4Bins|all_bins.fasta_bin_11~449
0.03

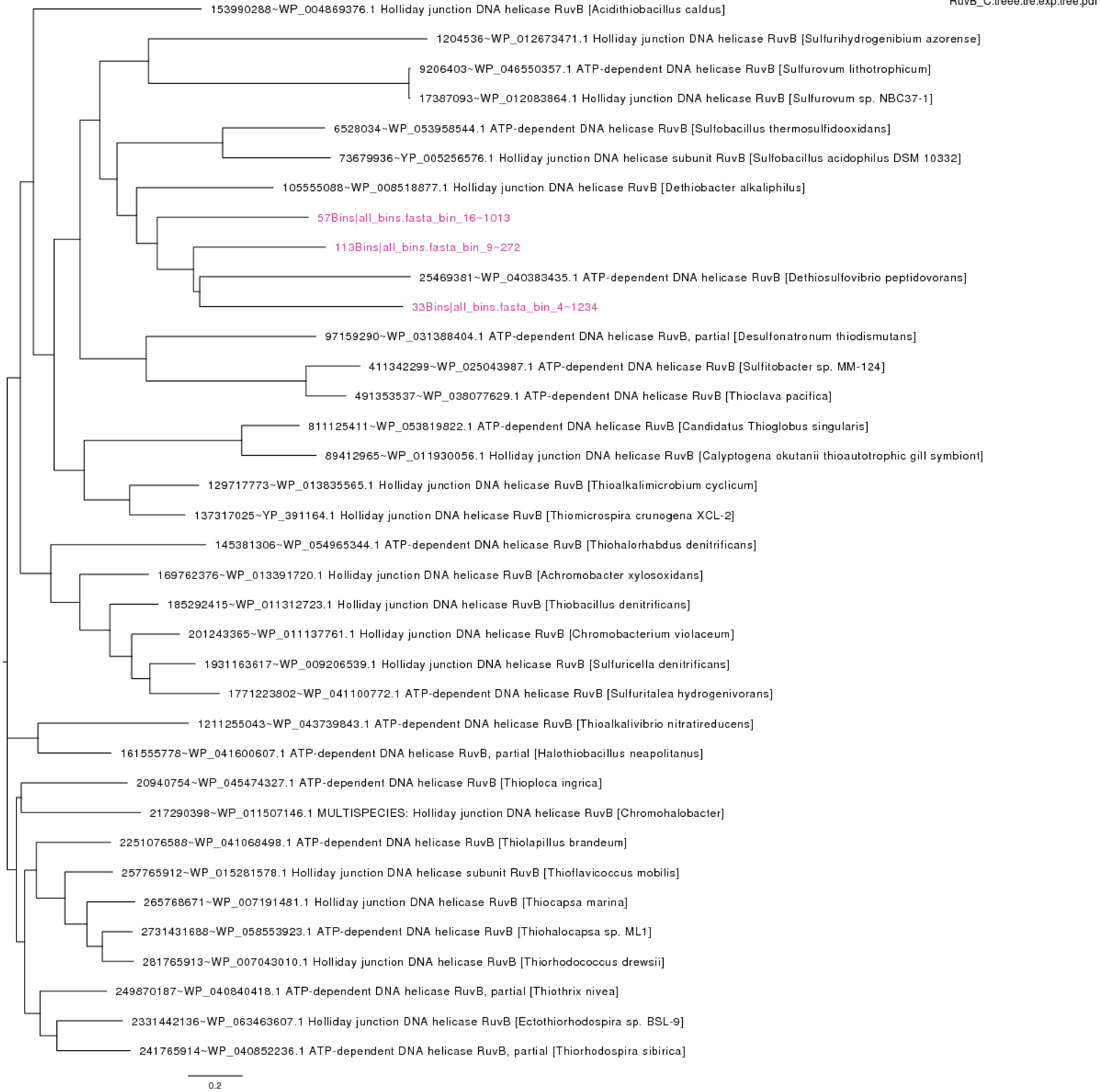
153990288~WP_004869376.1 Holliday junction DNA helicase RuvB [Acidithiobacillus caldus]
1204536~WP_012673471.1 Holliday junction DNA helicase RuvB [Sulfurihydrogenibium azorense]
9206403~WP_046550357.1 ATP-dependent DNA helicase RuvB [Sulfurovum lithotrophicum]
17387093~WP_012083864.1 Holliday junction DNA helicase RuvB [Sulfurovum sp. NBC37-1]
6528034~WP_053958544.1 ATP-dependent DNA helicase RuvB [Sulfobacillus thermosulfidooxidans]
73679936~YP_005256576.1 Holliday junction DNA helicase subunit RuvB [Sulfobacillus acidophilus DSM 10332]
105555088~WP_008518877.1 Holliday junction DNA helicase RuvB [Dethiobacter alkaliphilus]
57Bins|all_bins.fasta_bin_16~1013
113Bins|all_bins.fasta_bin_9~272
25469381~WP_040383435.1 ATP-dependent DNA helicase RuvB [Dethiosulfovibrio peptidovorans]
33Bins|all_bins.fasta_bin_4~1234
97159290~WP_031388404.1 ATP-dependent DNA helicase RuvB, partial [Desulfonatronum thiodismutans]
411342299~WP_025043987.1 ATP-dependent DNA helicase RuvB [Sulfitobacter sp. MM-124]
491353537~WP_038077629.1 ATP-dependent DNA helicase RuvB [Thioclava pacifica]
811125411~WP_053819822.1 ATP-dependent DNA helicase RuvB [Candidatus Thioglobus singularis]
89412965~WP_011930056.1 Holliday junction DNA helicase RuvB [Calyptogena okutanii thioautotrophic gill symbiont]
129717773~WP_013835565.1 Holliday junction DNA helicase RuvB [Thioalkalimicrobium cyclicum]
137317025~YP_391164.1 Holliday junction DNA helicase RuvB [Thiomicrospira crunogena XCL-2]
145381306~WP_054965344.1 ATP-dependent DNA helicase RuvB [Thiohalorhabdus denitrificans]
169762376~WP_013391720.1 Holliday junction DNA helicase RuvB [Achromobacter xylosoxidans]
185292415~WP_011312723.1 Holliday junction DNA helicase RuvB [Thiobacillus denitrificans]
201243365~WP_011137761.1 Holliday junction DNA helicase RuvB [Chromobacterium violaceum]
1931163617~WP_009206539.1 Holliday junction DNA helicase RuvB [Sulfuricella denitrificans]
1771223802~WP_041100772.1 ATP-dependent DNA helicase RuvB [Sulfuritalea hydrogenivorans]
1211255043~WP_043739843.1 ATP-dependent DNA helicase RuvB [Thioalkalivibrio nitratireducens]
161555778~WP_041600607.1 ATP-dependent DNA helicase RuvB, partial [Halothiobacillus neapolitanus]
20940754~WP_045474327.1 ATP-dependent DNA helicase RuvB [Thioploca ingrica]
217290398~WP_011507146.1 MULTISPECIES: Holliday junction DNA helicase RuvB [Chromohalobacter]
2251076588~WP_041068498.1 ATP-dependent DNA helicase RuvB [Thiolapillus brandeum]
257765912~WP_015281578.1 Holliday junction DNA helicase subunit RuvB [Thioflavicoccus mobilis]
265768671~WP_007191481.1 Holliday junction DNA helicase RuvB [Thiocapsa marina]
2731431688~WP_058553923.1 ATP-dependent DNA helicase RuvB [Thiohalocapsa sp. ML1]
281765913~WP_007043010.1 Holliday junction DNA helicase RuvB [Thiorhodococcus drewsii]
249870187~WP_040840418.1 ATP-dependent DNA helicase RuvB, partial [Thiothrix nivea]
2331442136~WP_063463607.1 Holliday junction DNA helicase RuvB [Ectothiorhodospira sp. BSL-9]
241765914~WP_040852236.1 ATP-dependent DNA helicase RuvB, partial [Thiorhodospira sibirica]
0.2

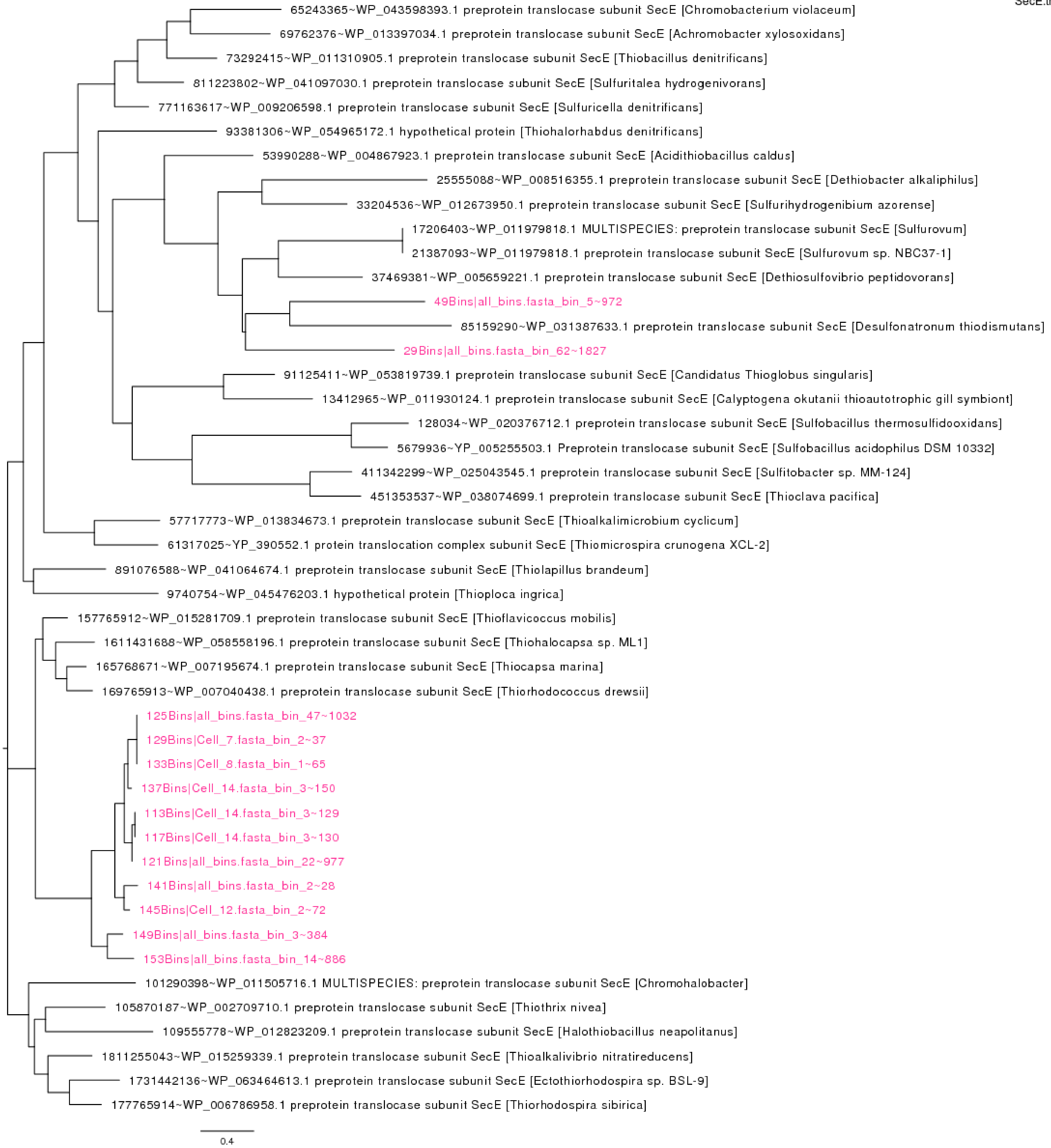
65243365~WP_043598393.1 preprotein translocase subunit SecE [Chromobacterium violaceum]
69762376~WP_013397034.1 preprotein translocase subunit SecE [Achromobacter xylosoxidans]
73292415~WP_011310905.1 preprotein translocase subunit SecE [Thiobacillus denitrificans]
811223802~WP_041097030.1 preprotein translocase subunit SecE [Sulfuritalea hydrogenivorans]
771163617~WP_009206598.1 preprotein translocase subunit SecE [Sulfuricella denitrificans]
93381306~WP_054965172.1 hypothetical protein [Thiohalorhabdus denitrificans]
53990288~WP_004867923.1 preprotein translocase subunit SecE [Acidithiobacillus caldus]
25555088~WP_008516355.1 preprotein translocase subunit SecE [Dethiobacter alkaliphilus]
33204536~WP_012673950.1 preprotein translocase subunit SecE [Sulfurihydrogenibium azorense]
17206403~WP_011979818.1 MULTISPECIES: preprotein translocase subunit SecE [Sulfurovum]
21387093~WP_011979818.1 preprotein translocase subunit SecE [Sulfurovum sp. NBC37-1]
37469381~WP_005659221.1 preprotein translocase subunit SecE [Dethiosulfovibrio peptidovorans]
49Bins|all_bins.fasta_bin_5~972
85159290~WP_031387633.1 preprotein translocase subunit SecE [Desulfonatronum thiodismutans]
29Bins|all_bins.fasta_bin_62~1827
91125411~WP_053819739.1 preprotein translocase subunit SecE [Candidatus Thioglobus singularis]
13412965~WP_011930124.1 preprotein translocase subunit SecE [Calyptogena okutanii thioautotrophic gill symbiont]
128034~WP_020376712.1 preprotein translocase subunit SecE [Sulfobacillus thermosulfidooxidans]
5679936~YP_005255503.1 Preprotein translocase subunit SecE [Sulfobacillus acidophilus DSM 10332]
411342299~WP_025043545.1 preprotein translocase subunit SecE [Sulfitobacter sp. MM-124]
451353537~WP_038074699.1 preprotein translocase subunit SecE [Thioclava pacifica]
57717773~WP_013834673.1 preprotein translocase subunit SecE [Thioalkalimicrobium cyclicum]
61317025~YP_390552.1 protein translocation complex subunit SecE [Thiomicrospira crunogena XCL-2]
891076588~WP_041064674.1 preprotein translocase subunit SecE [Thiolapillus brandeum]
9740754~WP_045476203.1 hypothetical protein [Thioploca ingrica]
157765912~WP_015281709.1 preprotein translocase subunit SecE [Thioflavicoccus mobilis]
1611431688~WP_058558196.1 preprotein translocase subunit SecE [Thiohalocapsa sp. ML1]
165768671~WP_007195674.1 preprotein translocase subunit SecE [Thiocapsa marina]
169765913~WP_007040438.1 preprotein translocase subunit SecE [Thiorhodococcus drewsii]
125Bins|all_bins.fasta_bin_47~1032
129Bins|Cell_7.fasta_bin_2~37
133Bins|Cell_8.fasta_bin_1~65
137Bins|Cell_14.fasta_bin_3~150
113Bins|Cell_14.fasta_bin_3~129
117Bins|Cell_14.fasta_bin_3~130
121Bins|all_bins.fasta_bin_22~977
141Bins|all_bins.fasta_bin_2~28
145Bins|Cell_12.fasta_bin_2~72
149Bins|all_bins.fasta_bin_3~384
153Bins|all_bins.fasta_bin_14~886
101290398~WP_011505716.1 MULTISPECIES: preprotein translocase subunit SecE [Chromohalobacter]
105870187~WP_002709710.1 preprotein translocase subunit SecE [Thiothrix nivea]
109555778~WP_012823209.1 preprotein translocase subunit SecE [Halothiobacillus neapolitanus]
1811255043~WP_015259339.1 preprotein translocase subunit SecE [Thioalkalivibrio nitratireducens]
1731442136~WP_063464613.1 preprotein translocase subunit SecE [Ectothiorhodospira sp. BSL-9]
177765914~WP_006786958.1 preprotein translocase subunit SecE [Thiorhodospira sibirica]
0.4

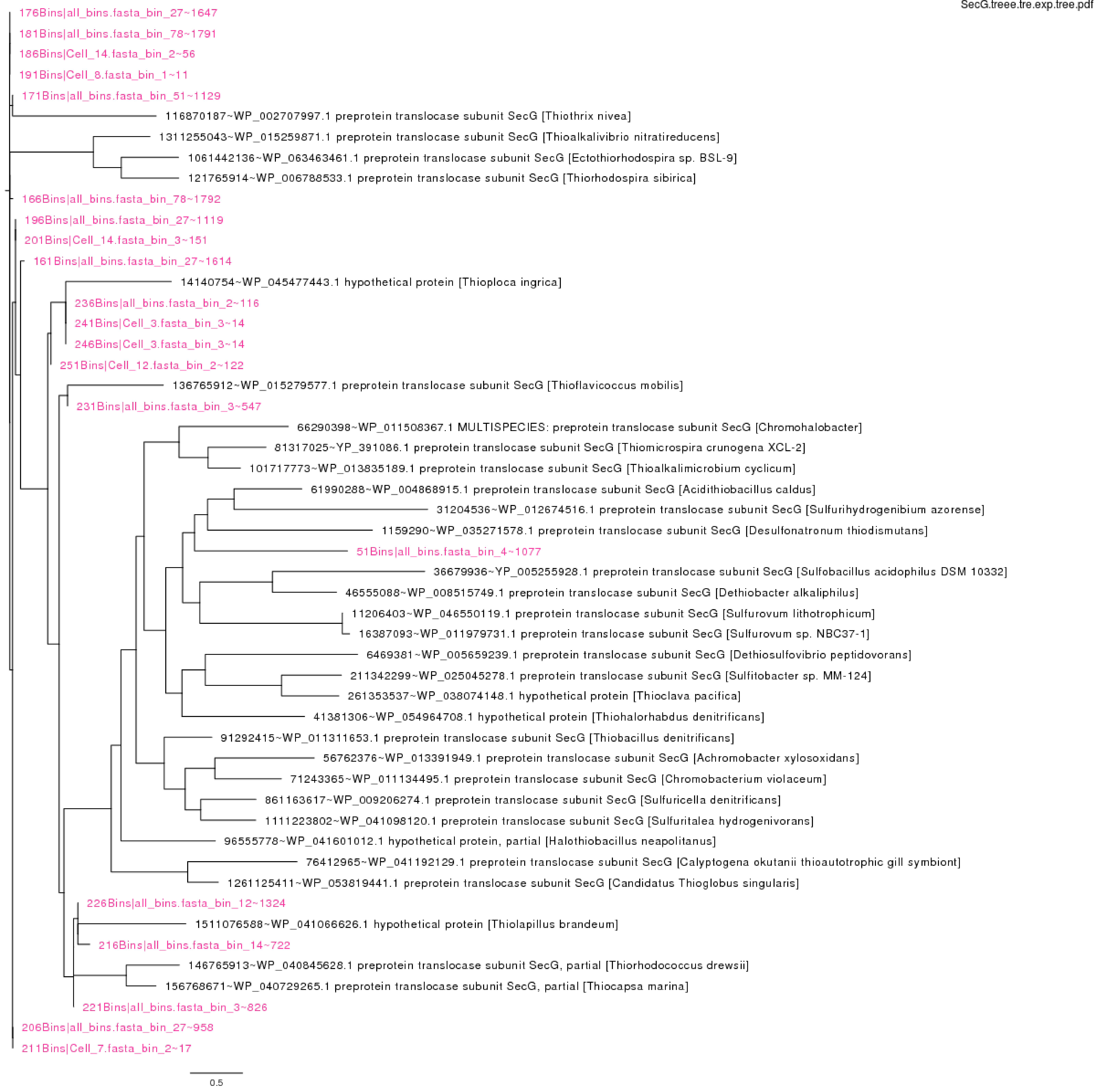
SecG.treee.tre.exp.tree.pdf
176Bins|all_bins.fasta_bin_27~1647
181Bins|all_bins.fasta_bin_78~1791
186Bins|Cell_14.fasta_bin_2~56
191Bins|Cell_8.fasta_bin_1~11
171Bins|all_bins.fasta_bin_51~1129
116870187~WP_002707997.1 preprotein translocase subunit SecG [Thiothrix nivea]
1311255043~WP_015259871.1 preprotein translocase subunit SecG [Thioalkalivibrio nitratireducens]
1061442136~WP_063463461.1 preprotein translocase subunit SecG [Ectothiorhodospira sp. BSL-9]
121765914~WP_006788533.1 preprotein translocase subunit SecG [Thiorhodospira sibirica]
166Bins|all_bins.fasta_bin_78~1792
196Bins|all_bins.fasta_bin_27~1119
201Bins|Cell_14.fasta_bin_3~151
161Bins|all_bins.fasta_bin_27~1614
14140754~WP_045477443.1 hypothetical protein [Thioploca ingrica]
236Bins|all_bins.fasta_bin_2~116
241Bins|Cell_3.fasta_bin_3~14
246Bins|Cell_3.fasta_bin_3~14
251Bins|Cell_12.fasta_bin_2~122
136765912~WP_015279577.1 preprotein translocase subunit SecG [Thioflavicoccus mobilis]
231Bins|all_bins.fasta_bin_3~547
66290398~WP_011508367.1 MULTISPECIES: preprotein translocase subunit SecG [Chromohalobacter]
81317025~YP_391086.1 preprotein translocase subunit SecG [Thiomicrospira crunogena XCL-2]
101717773~WP_013835189.1 preprotein translocase subunit SecG [Thioalkalimicrobium cyclicum]
61990288~WP_004868915.1 preprotein translocase subunit SecG [Acidithiobacillus caldus]
31204536~WP_012674516.1 preprotein translocase subunit SecG [Sulfurihydrogenibium azorense]
1159290~WP_035271578.1 preprotein translocase subunit SecG [Desulfonatronum thiodismutans]
51Bins|all_bins.fasta_bin_4~1077
36679936~YP_005255928.1 preprotein translocase subunit SecG [Sulfobacillus acidophilus DSM 10332]
46555088~WP_008515749.1 preprotein translocase subunit SecG [Dethiobacter alkaliphilus]
11206403~WP_046550119.1 preprotein translocase subunit SecG [Sulfurovum lithotrophicum]
16387093~WP_011979731.1 preprotein translocase subunit SecG [Sulfurovum sp. NBC37-1]
6469381~WP_005659239.1 preprotein translocase subunit SecG [Dethiosulfovibrio peptidovorans]
211342299~WP_025045278.1 preprotein translocase subunit SecG [Sulfitobacter sp. MM-124]
261353537~WP_038074148.1 hypothetical protein [Thioclava pacifica]
41381306~WP_054964708.1 hypothetical protein [Thiohalorhabdus denitrificans]
91292415~WP_011311653.1 preprotein translocase subunit SecG [Thiobacillus denitrificans]
56762376~WP_013391949.1 preprotein translocase subunit SecG [Achromobacter xylosoxidans]
71243365~WP_011134495.1 preprotein translocase subunit SecG [Chromobacterium violaceum]
861163617~WP_009206274.1 preprotein translocase subunit SecG [Sulfuricella denitrificans]
1111223802~WP_041098120.1 preprotein translocase subunit SecG [Sulfuritalea hydrogenivorans]
96555778~WP_041601012.1 hypothetical protein, partial [Halothiobacillus neapolitanus]
76412965~WP_041192129.1 preprotein translocase subunit SecG [Calyptogena okutanii thioautotrophic gill symbiont]
1261125411~WP_053819441.1 preprotein translocase subunit SecG [Candidatus Thioglobus singularis]
226Bins|all_bins.fasta_bin_12~1324
1511076588~WP_041066626.1 hypothetical protein [Thiolapillus brandeum]
216Bins|all_bins.fasta_bin_14~722
146765913~WP_040845628.1 preprotein translocase subunit SecG, partial [Thiorhodococcus drewsii]
156768671~WP_040729265.1 preprotein translocase subunit SecG, partial [Thiocapsa marina]
221Bins|all_bins.fasta_bin_3~826
206Bins|all_bins.fasta_bin_27~958
211Bins|Cell_7.fasta_bin_2~17
0.5

SecY.treee.tre.exp.tree.pdf

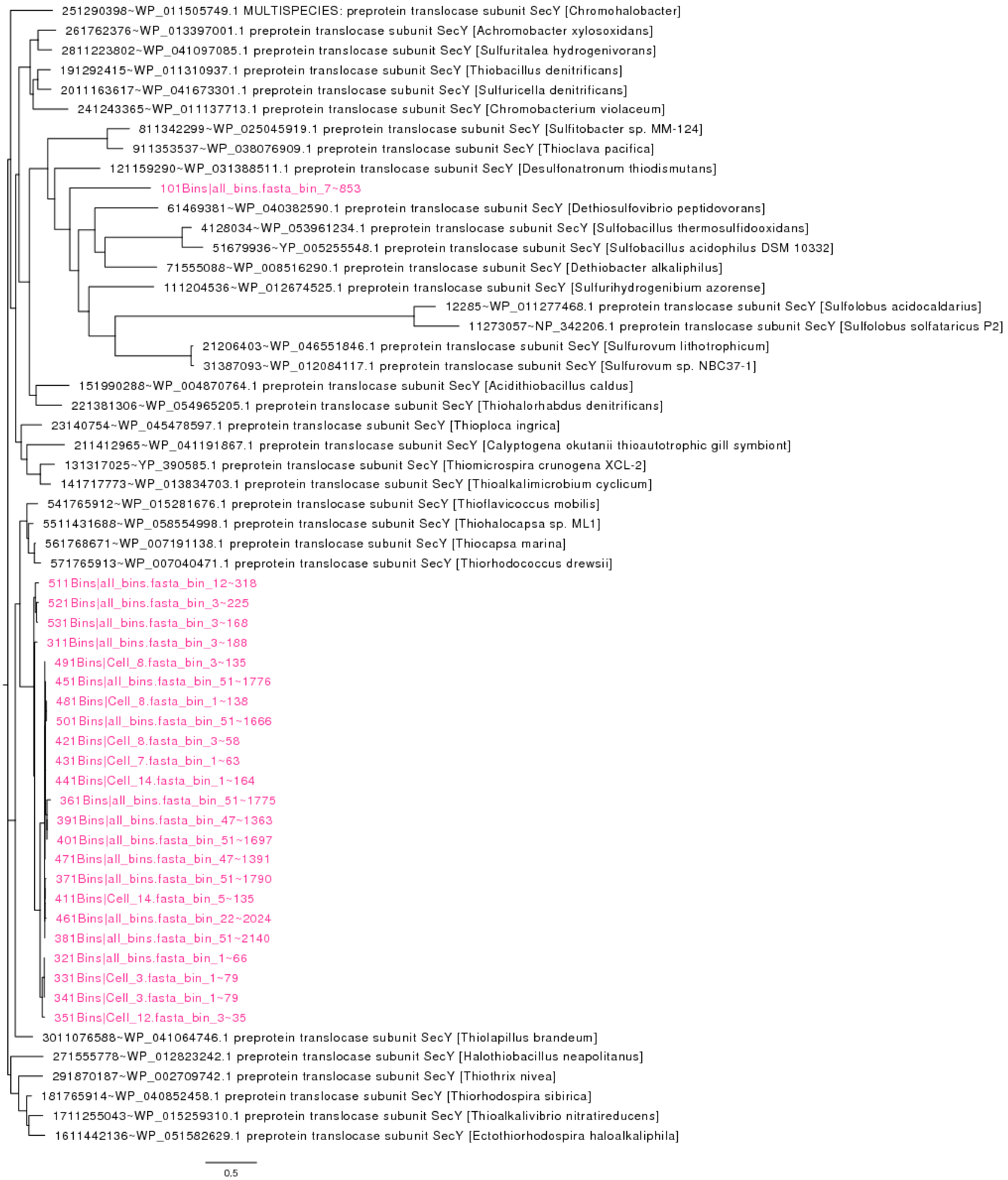

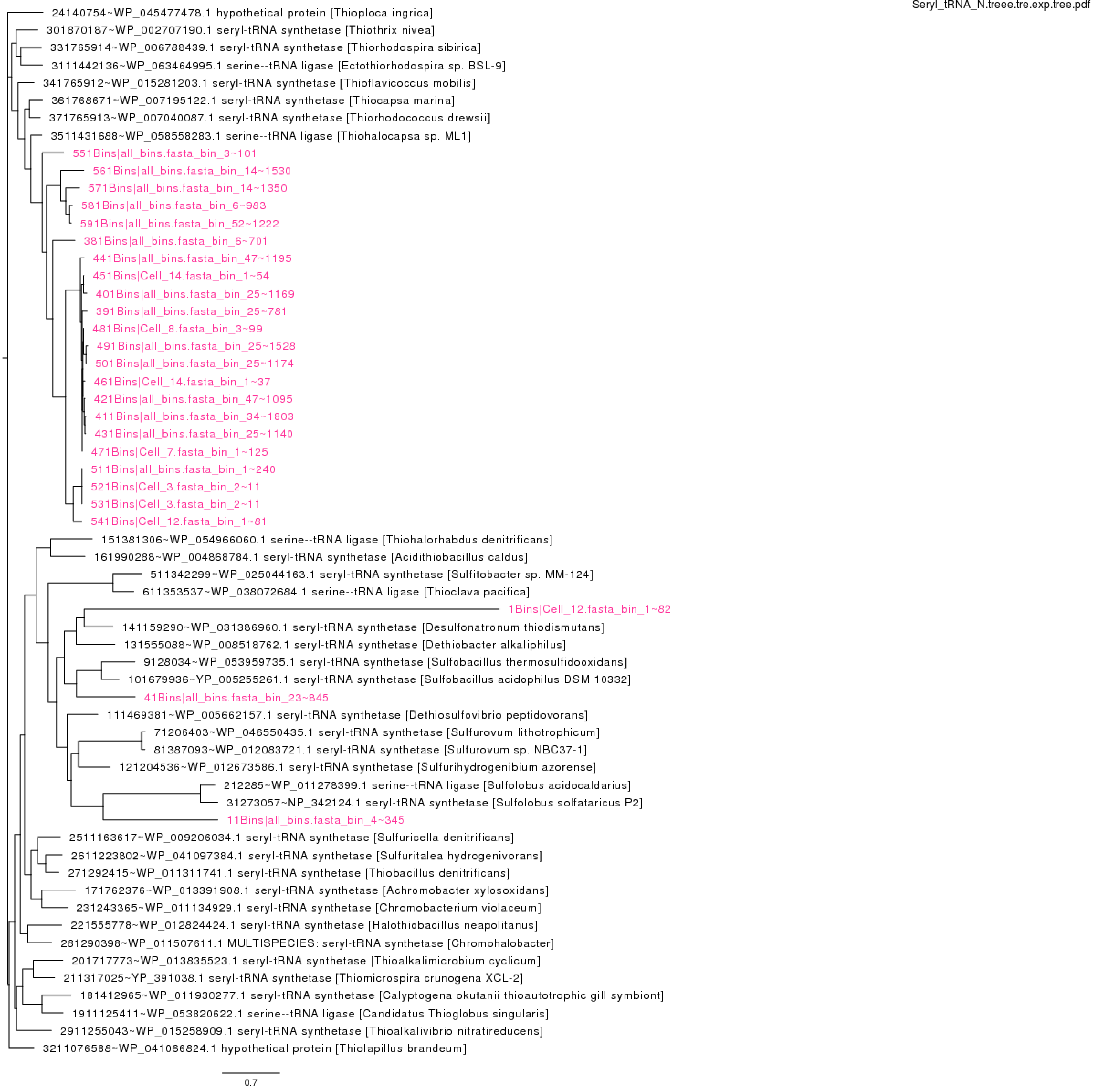
Seryl_tRNA_N.treee.tre.exp.tree.pdf
24140754~WP_045477478.1 hypothetical protein [Thioploca ingrica]
301870187~WP_002707190.1 seryl-tRNA synthetase [Thiothrix nivea]
331765914~WP_006788439.1 seryl-tRNA synthetase [Thiorhodospira sibirica]
3111442136~WP_063464995.1 serine--tRNA ligase [Ectothiorhodospira sp. BSL-9]
341765912~WP_015281203.1 seryl-tRNA synthetase [Thioflavicoccus mobilis]
361768671~WP_007195122.1 seryl-tRNA synthetase [Thiocapsa marina]
371765913~WP_007040087.1 seryl-tRNA synthetase [Thiorhodococcus drewsii]
3511431688~WP_058558283.1 serine--tRNA ligase [Thiohalocapsa sp. ML1]
551Bins|all_bins.fasta_bin_3~101
561Bins|all_bins.fasta_bin_14~1530
571Bins|all_bins.fasta_bin_14~1350
581Bins|all_bins.fasta_bin_6~983
591Bins|all_bins.fasta_bin_52~1222
381Bins|all_bins.fasta_bin_6~701
441Bins|all_bins.fasta_bin_47~1195
451Bins|Cell_14.fasta_bin_1~54
401Bins|all_bins.fasta_bin_25~1169
391Bins|all_bins.fasta_bin_25~781
481Bins|Cell_8.fasta_bin_3~99
491Bins|all_bins.fasta_bin_25~1528
501Bins|all_bins.fasta_bin_25~1174
461Bins|Cell_14.fasta_bin_1~37
421Bins|all_bins.fasta_bin_47~1095
411Bins|all_bins.fasta_bin_34~1803
431Bins|all_bins.fasta_bin_25~1140
471Bins|Cell_7.fasta_bin_1~125
511Bins|all_bins.fasta_bin_1~240
521Bins|Cell_3.fasta_bin_2~11
531Bins|Cell_3.fasta_bin_2~11
541Bins|Cell_12.fasta_bin_1~81
151381306~WP_054966060.1 serine--tRNA ligase [Thiohalorhabdus denitrificans]
161990288~WP_004868784.1 seryl-tRNA synthetase [Acidithiobacillus caldus]
511342299~WP_025044163.1 seryl-tRNA synthetase [Sulfitobacter sp. MM-124]
611353537~WP_038072684.1 serine--tRNA ligase [Thioclava pacifica]
1Bins|Cell_12.fasta_bin_1~82
141159290~WP_031386960.1 seryl-tRNA synthetase [Desulfonatronum thiodismutans]
131555088~WP_008518762.1 seryl-tRNA synthetase [Dethiobacter alkaliphilus]
9128034~WP_053959735.1 seryl-tRNA synthetase [Sulfobacillus thermosulfidooxidans]
101679936~YP_005255261.1 seryl-tRNA synthetase [Sulfobacillus acidophilus DSM 10332]
41Bins|all_bins.fasta_bin_23~845
111469381~WP_005662157.1 seryl-tRNA synthetase [Dethiosulfovibrio peptidovorans]
71206403~WP_046550435.1 seryl-tRNA synthetase [Sulfurovum lithotrophicum]
81387093~WP_012083721.1 seryl-tRNA synthetase [Sulfurovum sp. NBC37-1]
121204536~WP_012673586.1 seryl-tRNA synthetase [Sulfurihydrogenibium azorense]
212285~WP_011278399.1 serine--tRNA ligase [Sulfolobus acidocaldarius]
31273057~NP_342124.1 seryl-tRNA synthetase [Sulfolobus solfataricus P2]
11Bins|all_bins.fasta_bin_4~345
2511163617~WP_009206034.1 seryl-tRNA synthetase [Sulfuricella denitrificans]
2611223802~WP_041097384.1 seryl-tRNA synthetase [Sulfuritalea hydrogenivorans]
271292415~WP_011311741.1 seryl-tRNA synthetase [Thiobacillus denitrificans]
171762376~WP_013391908.1 seryl-tRNA synthetase [Achromobacter xylosoxidans]
231243365~WP_011134929.1 seryl-tRNA synthetase [Chromobacterium violaceum]
221555778~WP_012824424.1 seryl-tRNA synthetase [Halothiobacillus neapolitanus]
281290398~WP_011507611.1 MULTISPECIES: seryl-tRNA synthetase [Chromohalobacter]
201717773~WP_013835523.1 seryl-tRNA synthetase [Thioalkalimicrobium cyclicum]
211317025~YP_391038.1 seryl-tRNA synthetase [Thiomicrospira crunogena XCL-2]
181412965~WP_011930277.1 seryl-tRNA synthetase [Calyptogena okutanii thioautotrophic gill symbiont]
1911125411~WP_053820622.1 serine--tRNA ligase [Candidatus Thioglobus singularis]
2911255043~WP_015258909.1 seryl-tRNA synthetase [Thioalkalivibrio nitratireducens]
3211076588~WP_041066824.1 hypothetical protein [Thiolapillus brandeum]
0.7

SmpB.treee.tre.exp.tree.pdf

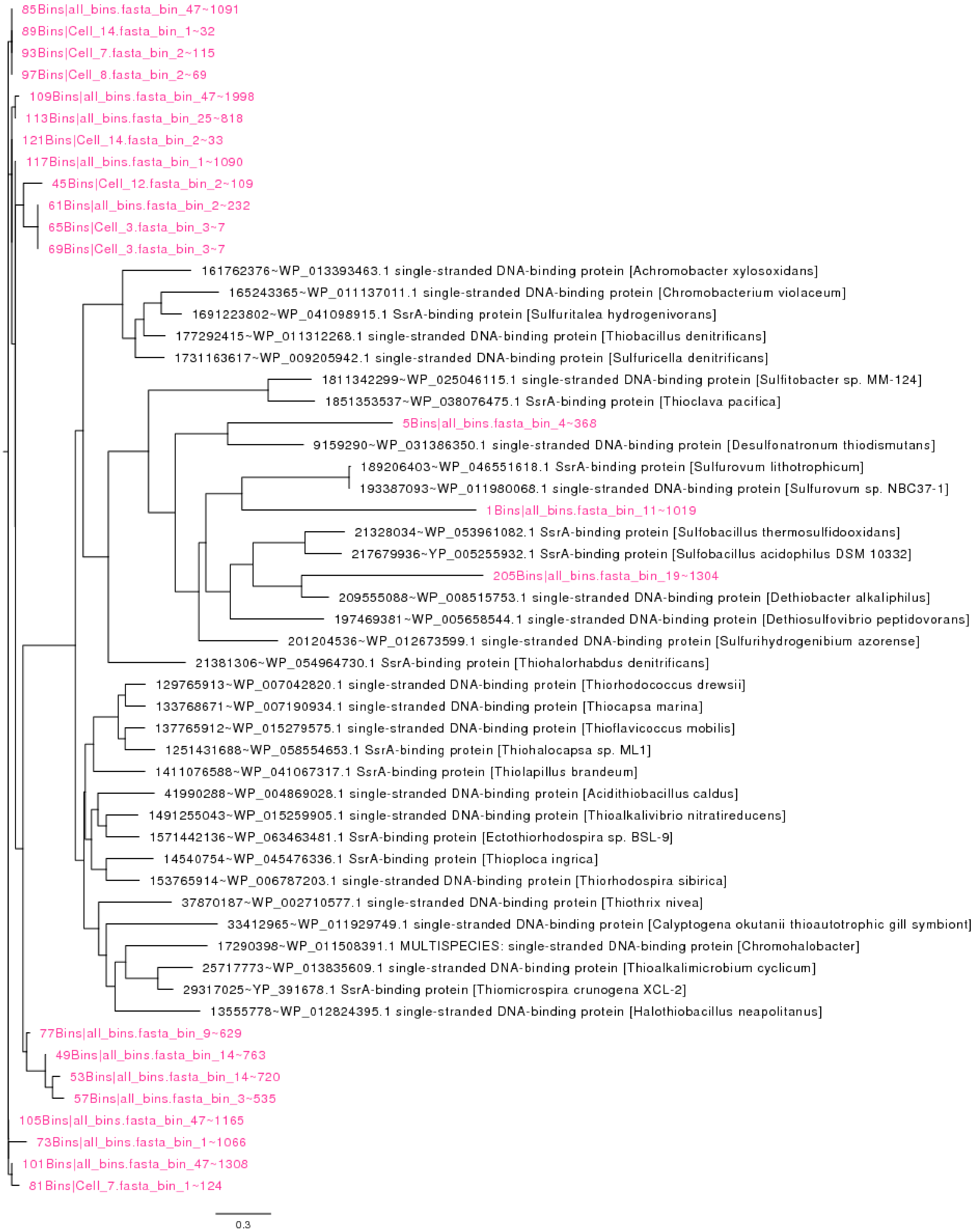

SRP_SPB.treee.tre.exp.tree.pdf

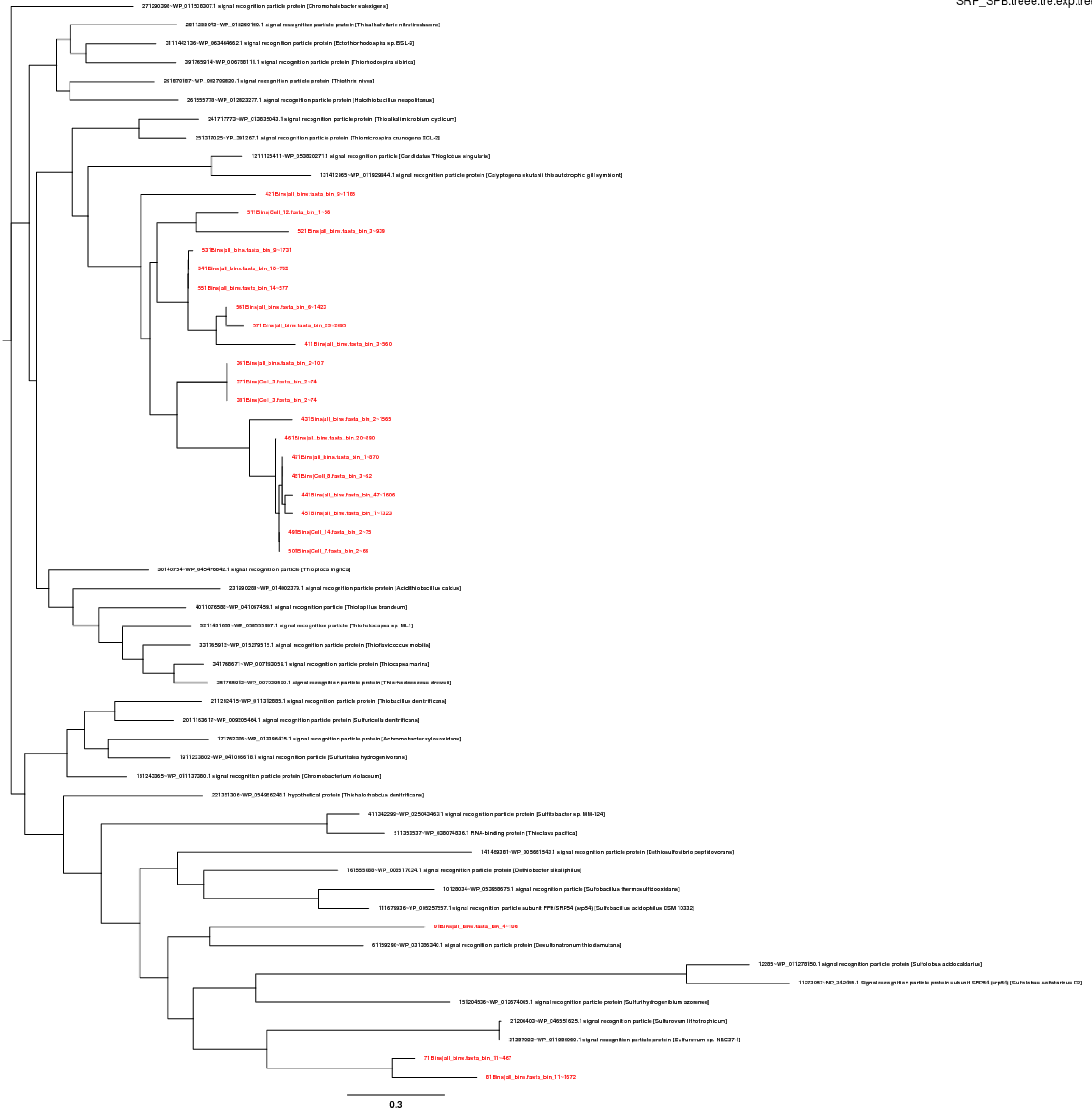

THF_DHG_CYH.treee.tre.exp.tree.pdf

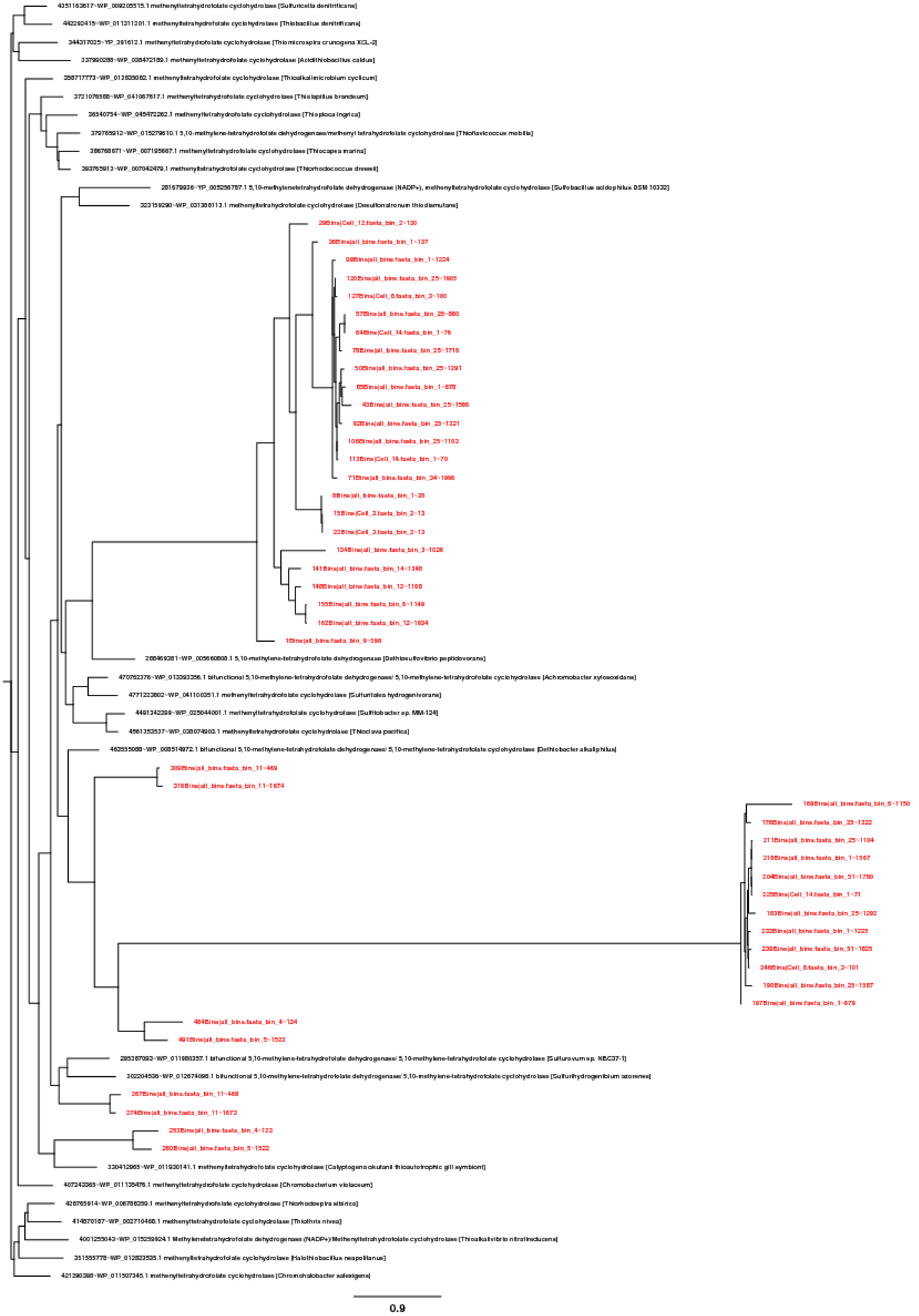

TIM.treee.tre.exp.tree.pdf

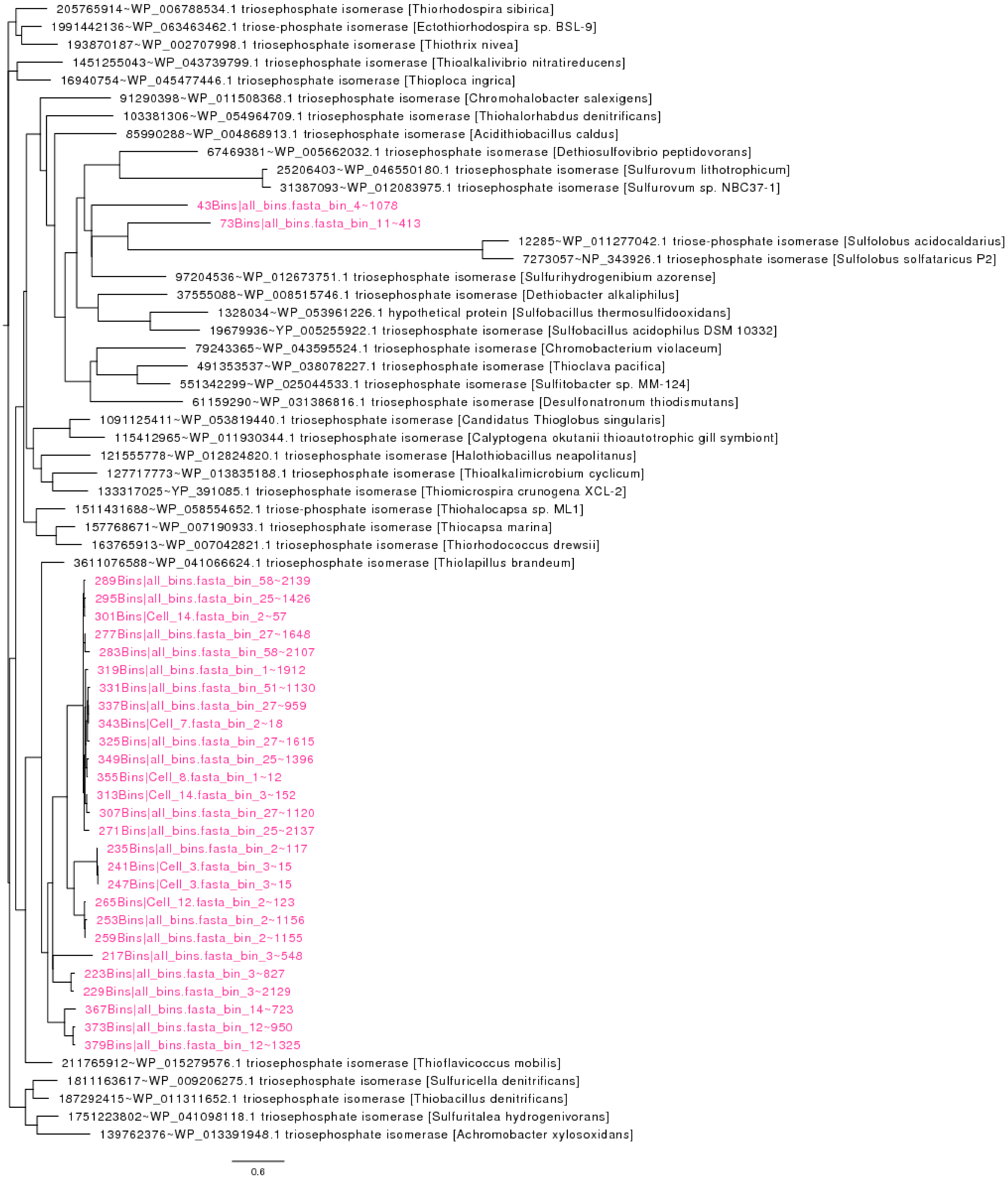

Toprim_N.treee.tre.exp.tree.pdf

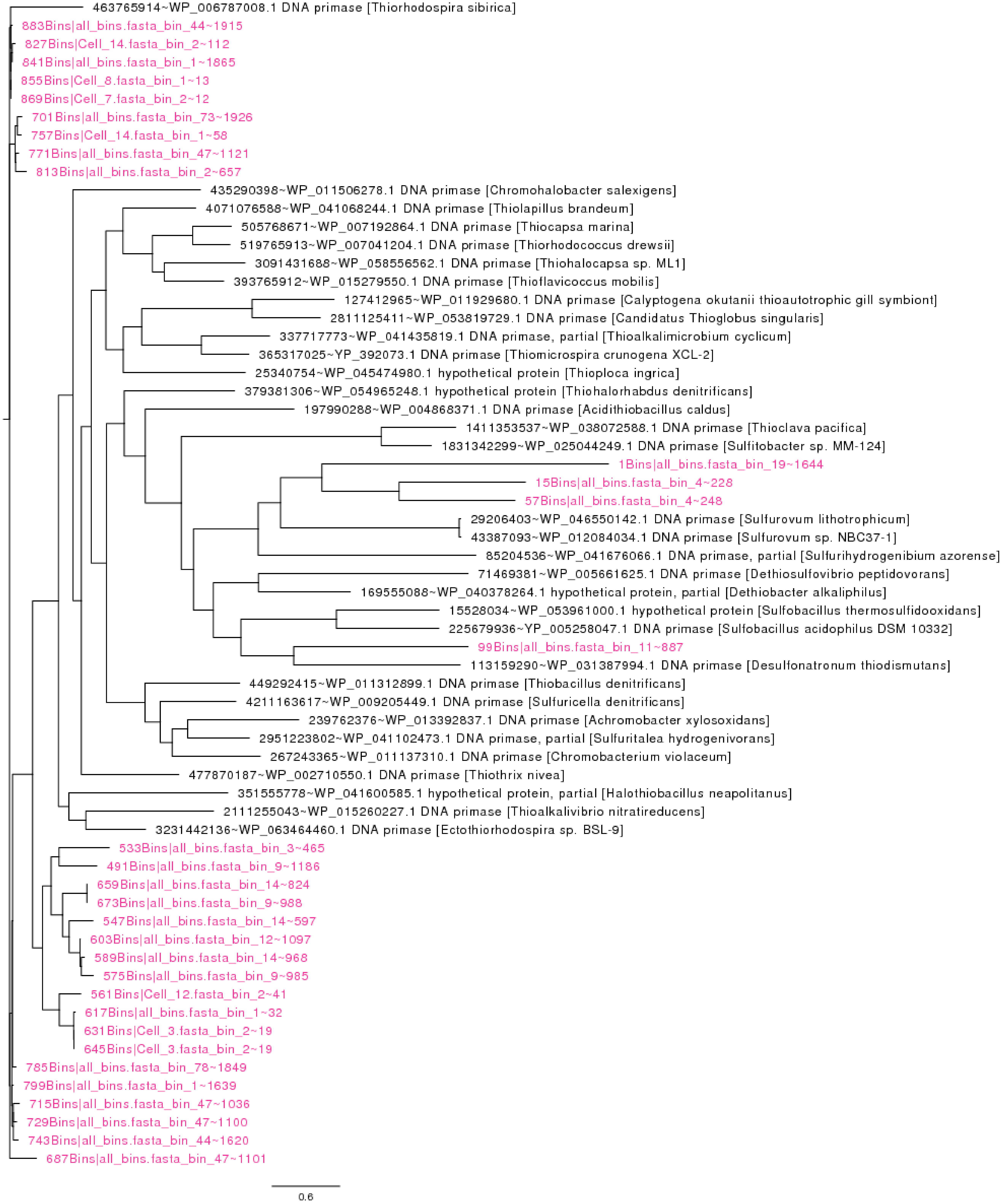

TRCF.treee.tre.exp.tree.pdf

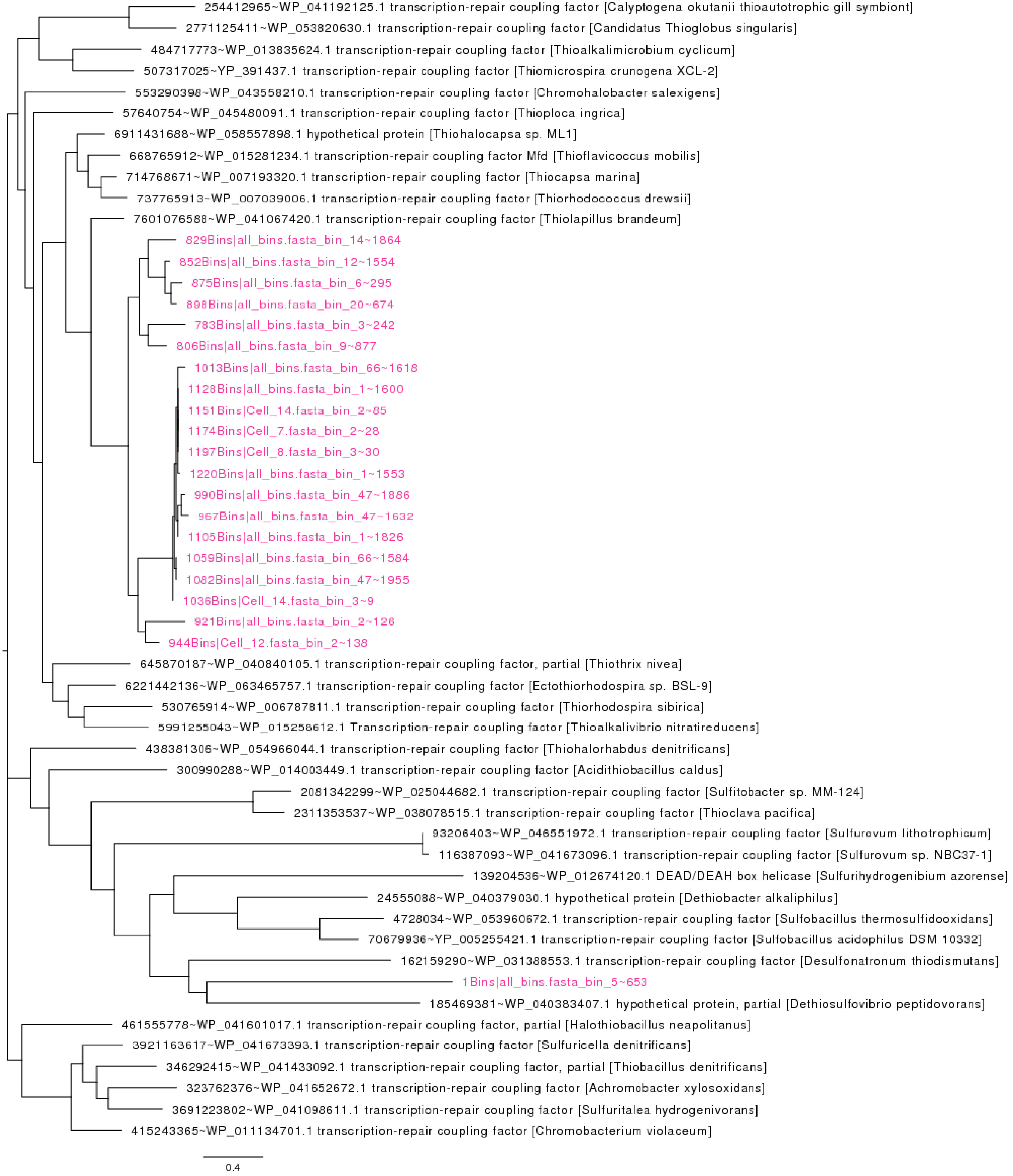

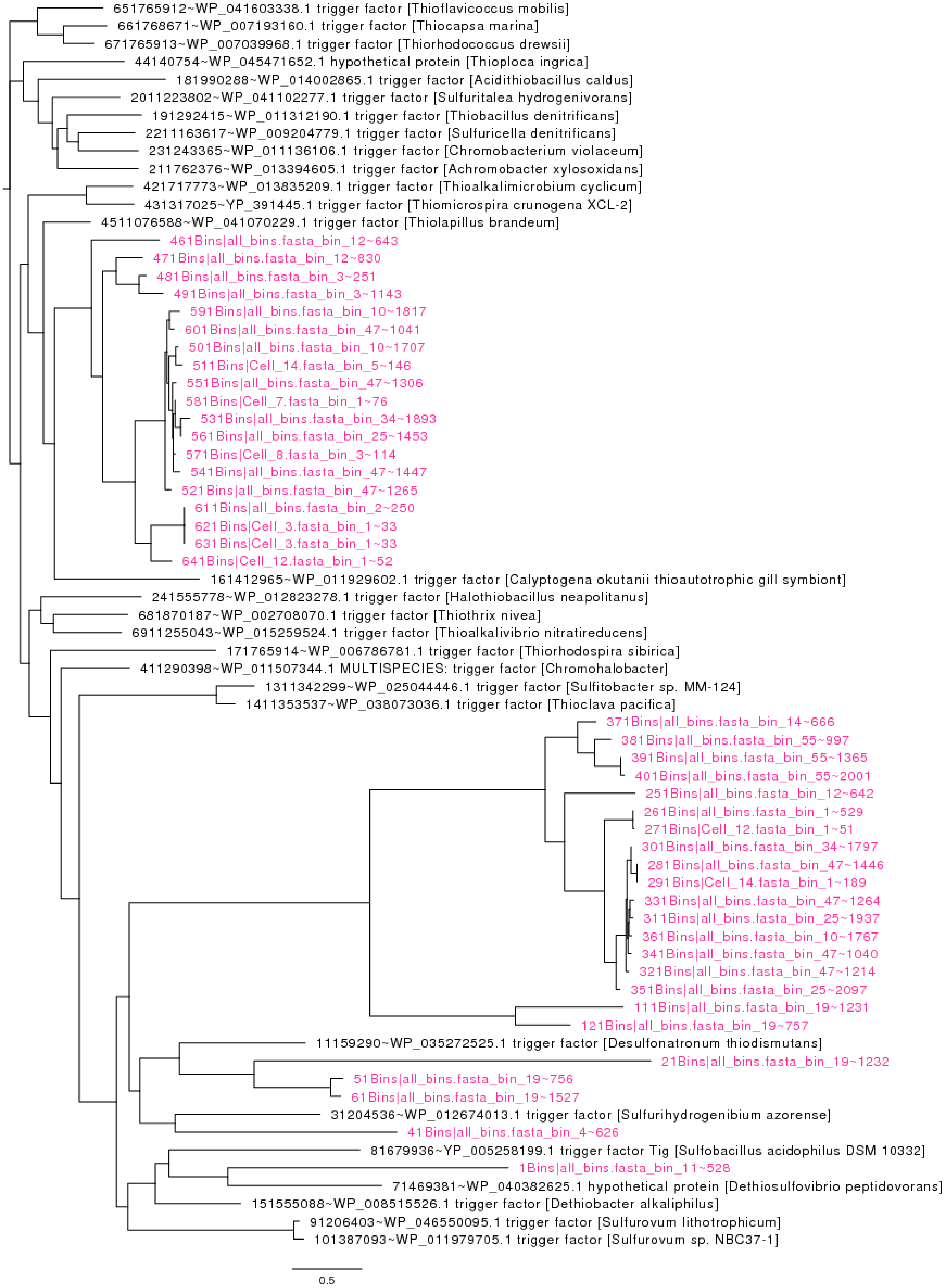
651765912~WP_041603338.1 trigger factor [Thioflavicoccus mobilis]
661768671~WP_007193160.1 trigger factor [Thiocapsa marina]
671765913~WP_007039968.1 trigger factor [Thiorhodococcus drewsii]
44140754~WP_045471652.1 hypothetical protein [Thioploca ingrica]
181990288~WP_014002865.1 trigger factor [Acidithiobacillus caldus]
2011223802~WP_041102277.1 trigger factor [Sulfuritalea hydrogenivorans]
191292415~WP_011312190.1 trigger factor [Thiobacillus denitrificans]
2211163617~WP_009204779.1 trigger factor [Sulfuricella denitrificans]
231243365~WP_011136106.1 trigger factor [Chromobacterium violaceum]
211762376~WP_013394605.1 trigger factor [Achromobacter xylosoxidans]
421717773~WP_013835209.1 trigger factor [Thioalkalimicrobium cyclicum]
431317025~YP_391445.1 trigger factor [Thiomicrospira crunogena XCL-2]
4511076588~WP_041070229.1 trigger factor [Thiolapillus brandeum]
461Bins|all_bins.fasta_bin_12~643
471Bins|all_bins.fasta_bin_12~830
481Bins|all_bins.fasta_bin_3~251
491Bins|all_bins.fasta_bin_3~1143
591Bins|all_bins.fasta_bin_10~1817
601Bins|all_bins.fasta_bin_47~1041
501Bins|all_bins.fasta_bin_10~1707
511Bins|Cell_14.fasta_bin_5~146
551Bins|all_bins.fasta_bin_47~1306
581Bins|Cell_7.fasta_bin_1~76
531Bins|all_bins.fasta_bin_34~1893
561Bins|all_bins.fasta_bin_25~1453
571Bins|Cell_8.fasta_bin_3~114
541Bins|all_bins.fasta_bin_47~1447
521Bins|all_bins.fasta_bin_47~1265
611Bins|all_bins.fasta_bin_2~250
621Bins|Cell_3.fasta_bin_1~33
631Bins|Cell_3.fasta_bin_1~33
641Bins|Cell_12.fasta_bin_1~52
161412965~WP_011929602.1 trigger factor [Calyptogena okutanii thioautotrophic gill symbiont]
241555778~WP_012823278.1 trigger factor [Halothiobacillus neapolitanus]
681870187~WP_002708070.1 trigger factor [Thiothrix nivea]
6911255043~WP_015259524.1 trigger factor [Thioalkalivibrio nitratireducens]
171765914~WP_006786781.1 trigger factor [Thiorhodospira sibirica]
411290398~WP_011507344.1 MULTISPECIES: trigger factor [Chromohalobacter]
1311342299~WP_025044446.1 trigger factor [Sulfitobacter sp. MM-124]
1411353537~WP_038073036.1 trigger factor [Thioclava pacifica]
371Bins|all_bins.fasta_bin_14~666
381Bins|all_bins.fasta_bin_55~997
391Bins|all_bins.fasta_bin_55~1365
401Bins|all_bins.fasta_bin_55~2001
251Bins|all_bins.fasta_bin_12~642
261Bins|all_bins.fasta_bin_1~529
271Bins|Cell_12.fasta_bin_1~51
301Bins|all_bins.fasta_bin_34~1797
281Bins|all_bins.fasta_bin_47~1446
291Bins|Cell_14.fasta_bin_1~189
331Bins|all_bins.fasta_bin_47~1264
311Bins|all_bins.fasta_bin_25~1937
361Bins|all_bins.fasta_bin_10~1767
341Bins|all_bins.fasta_bin_47~1040
321Bins|all_bins.fasta_bin_47~1214
351Bins|all_bins.fasta_bin_25~2097
111Bins|all_bins.fasta_bin_19~1231
121Bins|all_bins.fasta_bin_19~757
11159290~WP_035272525.1 trigger factor [Desulfonatronum thiodismutans]
21Bins|all_bins.fasta_bin_19~1232
51Bins|all_bins.fasta_bin_19~756
61Bins|all_bins.fasta_bin_19~1527
31204536~WP_012674013.1 trigger factor [Sulfurihydrogenibium azorense]
41Bins|all_bins.fasta_bin_4~626
81679936~YP_005258199.1 trigger factor Tig [Sulfobacillus acidophilus DSM 10332]
1Bins|all_bins.fasta_bin_11~528
71469381~WP_040382625.1 hypothetical protein [Dethiosulfovibrio peptidovorans]
151555088~WP_008515526.1 trigger factor [Dethiobacter alkaliphilus]
91206403~WP_046550095.1 trigger factor [Sulfurovum lithotrophicum]
101387093~WP_011979705.1 trigger factor [Sulfurovum sp. NBC37-1]
0.5

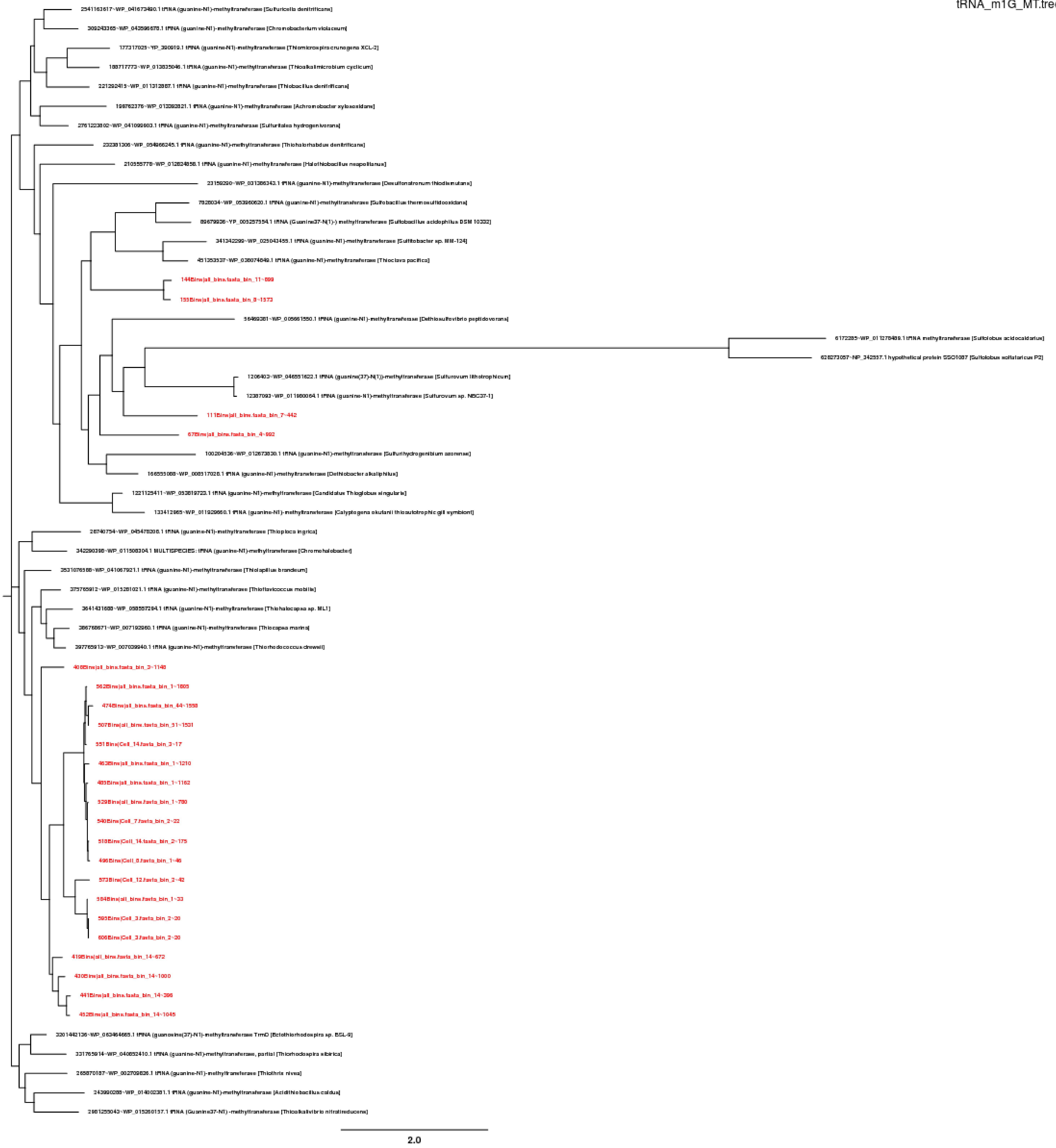
2541163617~WP_041673490.1 tRNA (guanine-N1)-methyltransferase [Sulfuricella denitrificans]
309243365~WP_043596678.1 tRNA (guanine-N1)-methyltransferase [Chromobacterium violaceum]
177317025~YP_390919.1 tRNA (guanine-N1)-methyltransferase [Thiomicrospira crunogena XCL-2]
188717773~WP_013835046.1 tRNA (guanine-N1)-methyltransferase [Thioalkalimicrobium cyclicum]
221292415~WP_011312867.1 tRNA (guanine-N1)-methyltransferase [Thiobacillus denitrificans]
199762376~WP_013392821.1 tRNA (guanine-N1)-methyltransferase [Achromobacter xylosoxidans]
2761223802~WP_041099903.1 tRNA (guanine-N1)-methyltransferase [Sulfuritalea hydrogenivorans]
232381306~WP_054966245.1 tRNA (guanine-N1)-methyltransferase [Thiohalorhabdus denitrificans]
210555778~WP_012824858.1 tRNA (guanine-N1)-methyltransferase [Halothiobacillus neapolitanus]
23159290~WP_031386343.1 tRNA (guanine-N1)-methyltransferase [Desulfonatronum thiodismutans]
7828034~WP_053960620.1 tRNA (guanine-N1)-methyltransferase [Sulfobacillus thermosulfidooxidans]
89679936~YP_005257354.1 tRNA (Guanine37-N(1)-) methyltransferase [Sulfobacillus acidophilus DSM 10332]
341342299~WP_025043455.1 tRNA (guanine-N1)-methyltransferase [Sulfitobacter sp. MM-124]
451353537~WP_038074849.1 tRNA (guanine-N1)-methyltransferase [Thioclava pacifica]
144Bins|all_bins.fasta_bin_11~899
155Bins|all_bins.fasta_bin_8~1573
56469381~WP_005661550.1 tRNA (guanine-N1)-methyltransferase [Dethiosulfovibrio peptidovorans]
6172285~WP_011278489.1 tRNA methyltransferase [Sulfolobus acidocaldarius]
626273057~NP_342557.1 hypothetical protein SSO1087 [Sulfolobus solfataricus P2]
1206403~WP_046551622.1 tRNA (guanine(37)-N(1))-methyltransferase [Sulfurovum lithotrophicum]
12387093~WP_011980064.1 tRNA (guanine-N1)-methyltransferase [Sulfurovum sp. NBC37-1]
111Bins|all_bins.fasta_bin_7~442
67Bins|all_bins.fasta_bin_4~992
100204536~WP_012673830.1 tRNA (guanine-N1)-methyltransferase [Sulfurihydrogenibium azorense]
166555068~WP_008517028.1 tRNA (guanine-N1)-methyltransferase [Dethiobacter alkaliphilus]
1221125411~WP_053819723.1 tRNA (guanine-N1)-methyltransferase [Candidatus Thioglobus singularis]
133412965~WP_011929660.1 tRNA (guanine-N1)-methyltransferase [Calyptogena okutanii thioautotrophic gill symbiont]
26740754~WP_045476208.1 tRNA (guanine-N1)-methyltransferase [Thioploca ingrica]
342290396~WP_011506304.1 MULTISPECIES: tRNA (guanine-N1)-methyltransferase [Chromohalobacter]
2531076588~WP_041067921.1 tRNA (guanine-N1)-methyltransferase [Thiolapillus brandeum]
375765912~WP_015281021.1 tRNA (guanine-N1)-methyltransferase [Thioflavicoccus mobilis]
3641431688~WP_058557294.1 tRNA (guanine-N1)-methyltransferase [Thiohalocapsa sp. ML1]
386768671~WP_007192960.1 tRNA (guanine-N1)-methyltransferase [Thiocapsa marina]
397765913~WP_007039940.1 tRNA (guanine-N1)-methyltransferase [Thiorhodococcus drewsii]
408Bins|all_bins.fasta_bin_3~1148
562Bins|all_bins.fasta_bin_1~1805
474Bins|all_bins.fasta_bin_44~1558
507Bins|all_bins.fasta_bin_51~1501
551Bins|Cell_14.fasta_bin_3~17
463Bins|all_bins.fasta_bin_1~1210
485Bins|all_bins.fasta_bin_1~1162
529Bins|all_bins.fasta_bin_1~780
540Bins|Cell_7.fasta_bin_2~22
518Bins|Cell_14.fasta_bin_2~175
496Bins|Cell_8.fasta_bin_1~46
573Bins|Cell_12.fasta_bin_2~42
584Bins|all_bins.fasta_bin_1~33
595Bins|Cell_3.fasta_bin_2~20
606Bins|Cell_3.fasta_bin_2~20
419Bins|all_bins.fasta_bin_14~672
430Bins|all_bins.fasta_bin_14~1000
441Bins|all_bins.fasta_bin_14~396
452Bins|all_bins.fasta_bin_14~1045
3201442136~WP_063464665.1 tRNA (guanosine(37)-N1)-methyltransferase TrmD [Ectothiorhodospira sp. BSL-9]
331765914~WP_040652410.1 tRNA (guanine-N1)-methyltransferase, partial [Thiorhodospira sibirica]
268870187~WP_002709826.1 tRNA (guanine-N1)-methyltransferase [Thiothrix nivea]
243990288~WP_014002381.1 tRNA (guanine-N1)-methyltransferase [Acidithiobacillus caldus]
2981255043~WP_015260157.1 tRNA (Guanine37-N1) -methyltransferase [Thioalkalivibrio nitratireducens]
2.0

UPF0054.treee.tre.exp.tree.pdf

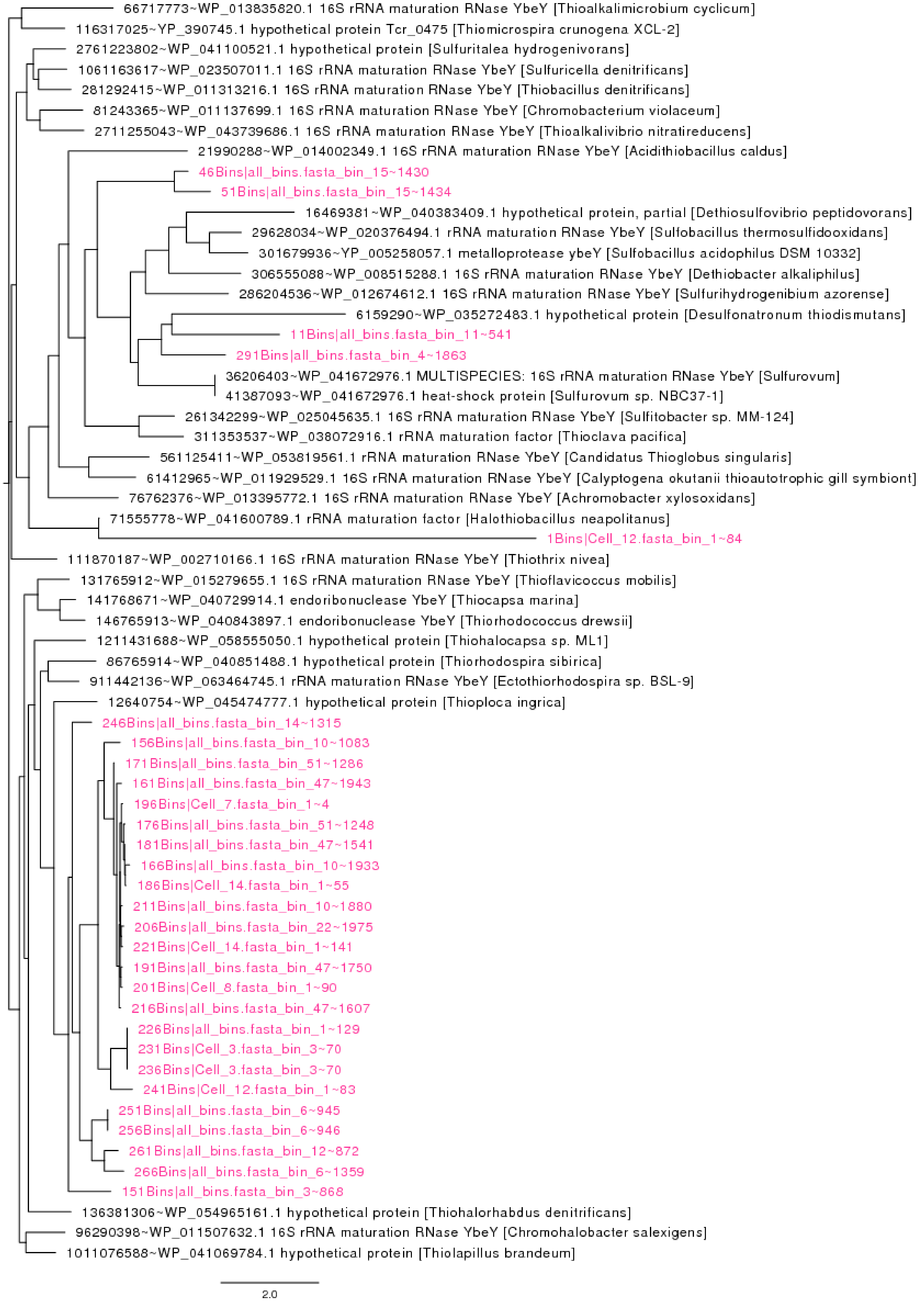

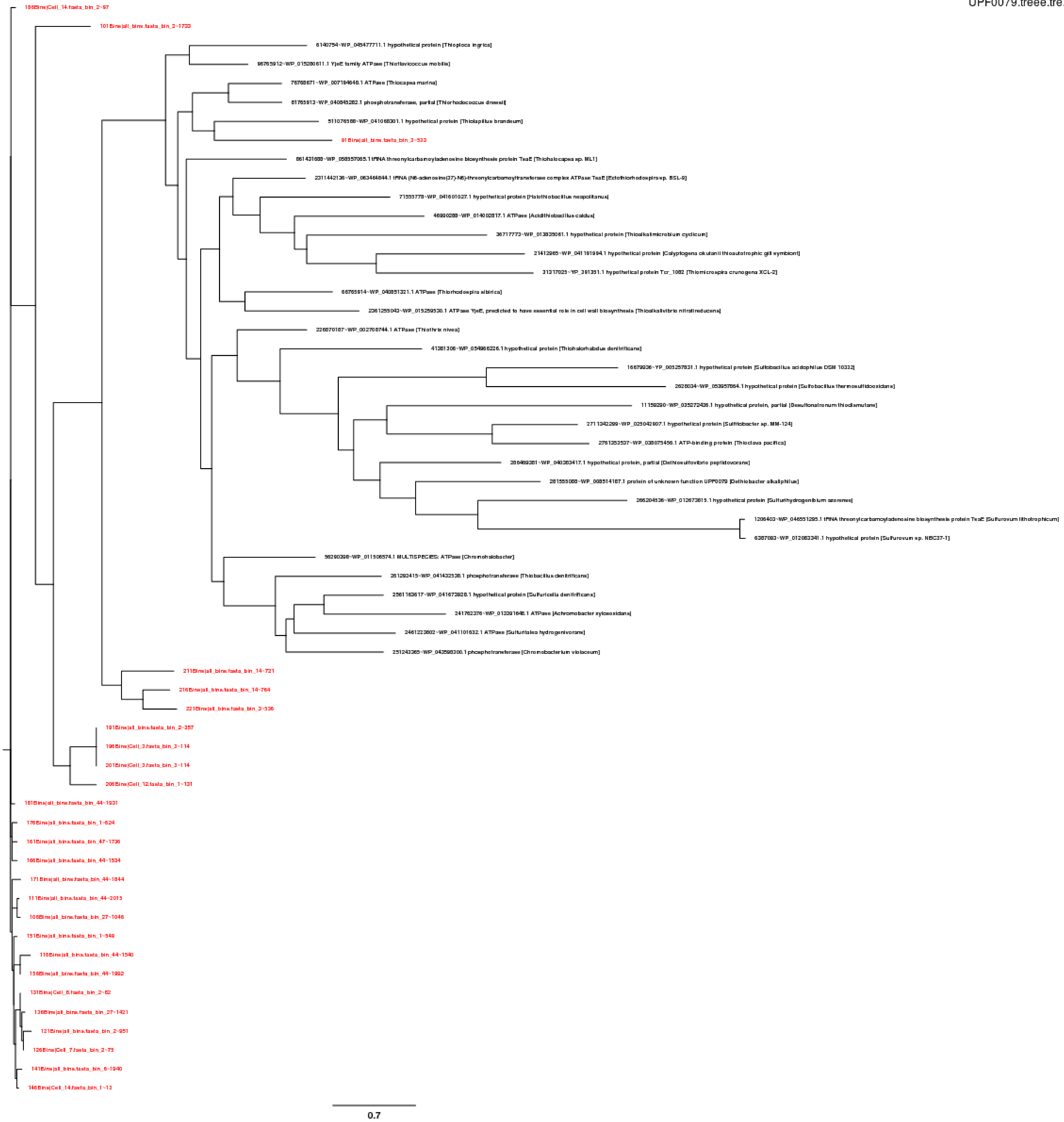
186Bins|Cell_14.fasta_bin_2~97
101Bins|all_bins.fasta_bin_2~1733
6140754~WP_045477711.1 hypothetical protein [Thioploca ingrica]
96765912~WP_015280611.1 YjeE family ATPase [Thioflavicoccus mobilis]
76768671~WP_007194648.1 ATPase [Thiocapsa marina]
61765913~WP_040845282.1 phosphotransferase, partial [Thiorhodococcus drewsii]
511076588~WP_041068301.1 hypothetical protein [Thiolapillus brandeum]
91Bins|all_bins.fasta_bin_3~533
661431688~WP_056557065.1 tRNA threonylcarbamoyladenosine biosynthesis protein TsaE [Thiohalocapsa sp. ML1]
2311442136~WP_063464844.1 tRNA (N6-adenosine(37)-N6)-threonylcarbamoyltransferase complex ATPase TsaE [Ectothiorhodospira sp. BSL-9]
71555778~WP_041601027.1 hypothetical protein [Halothiobacillus neapolitanus]
46990288~WP_014002817.1 ATPase [Acidithiobacillus caldus]
36717773~WP_013835061.1 hypothetical protein [Thioalkalimicrobium cyclicum]
21412965~WP_041191994.1 hypothetical protein [Calyptogena okutanii thioautotrophic gill symbiont]
31317025~YP_391351.1 hypothetical protein Tcr_1062 [Thiomicrospira crunogena XCL-2]
66765914~WP_040851321.1 ATPase [Thiorhodospira sibirica]
2361255043~WP_015259530.1 ATPase YjeE, predicted to have essential role in cell wall biosynthesis [Thioalkalivibrio nitratireducens]
226870187~WP_002708744.1 ATPase [Thiothrix nivea]
41381306~WP_054966226.1 hypothetical protein [Thiohalorhabdus denitrificans]
16679936~YP_005257831.1 hypothetical protein [Sulfobacillus acidophilus DSM 10332]
2626034~WP_053957664.1 hypothetical protein [Sulfobacillus thermosulfidooxidans]
11159290~WP_035272426.1 hypothetical protein, partial [Desulfonatronum thiodismutans]
2711342299~WP_025042907.1 hypothetical protein [Sulfitobacter sp. MM-124]
2761253537~WP_038075456.1 ATP-binding protein [Thioclava pacifica]
286469381~WP_040383417.1 hypothetical protein, partial [Dethiosulfovibrio peptidovorans]
281555068~WP_008514187.1 protein of unknown function UPF0079 [Dethiobacter alkaliphilus]
266204536~WP_012673815.1 hypothetical protein [Sulfurihydrogenibium azorense]
1206403~WP_046551295.1 tRNA threonylcarbamoyladenosine biosynthesis protein TsaE [Sulfurovum lithotrophicum]
6387093~WP_012083341.1 hypothetical protein [Sulfurovum sp. NBC37-1]
56290398~WP_011506574.1 MULTISPECIES: ATPase [Chromohalobacter]
261292415~WP_041432538.1 phosphotransferase [Thiobacillus denitrificans]
2561163617~WP_041673928.1 hypothetical protein [Sulfuricella denitrificans]
241762376~WP_013391648.1 ATPase [Achromobacter xylosoxidans]
2461223802~WP_041101632.1 ATPase [Sulfuritalea hydrogenivorans]
251243365~WP_043598300.1 phosphotransferase [Chromobacterium violaceum]
211Bins|all_bins.fasta_bin_14~721
216Bins|all_bins.fasta_bin_14~764
221Bins|all_bins.fasta_bin_3~536
191Bins|all_bins.fasta_bin_2~357
196Bins|Cell_3.fasta_bin_3~114
201Bins|Cell_3.fasta_bin_3~114
206Bins|Cell_12.fasta_bin_1~131
181Bins|all_bins.fasta_bin_44~1931
176Bins|all_bins.fasta_bin_1~624
161Bins|all_bins.fasta_bin_47~1736
166Bins|all_bins.fasta_bin_44~1534
171Bins|all_bins.fasta_bin_44~1844
111Bins|all_bins.fasta_bin_44~2015
106Bins|all_bins.fasta_bin_27~1046
151Bins|all_bins.fasta_bin_1~549
116Bins|all_bins.fasta_bin_44~1540
156Bins|all_bins.fasta_bin_44~1992
131Bins|Cell_8.fasta_bin_2~82
136Bins|all_bins.fasta_bin_27~1421
121Bins|all_bins.fasta_bin_2~951
126Bins|Cell_7.fasta_bin_2~75
141Bins|all_bins.fasta_bin_6~1940
146Bins|Cell_14.fasta_bin_1~13
0.7

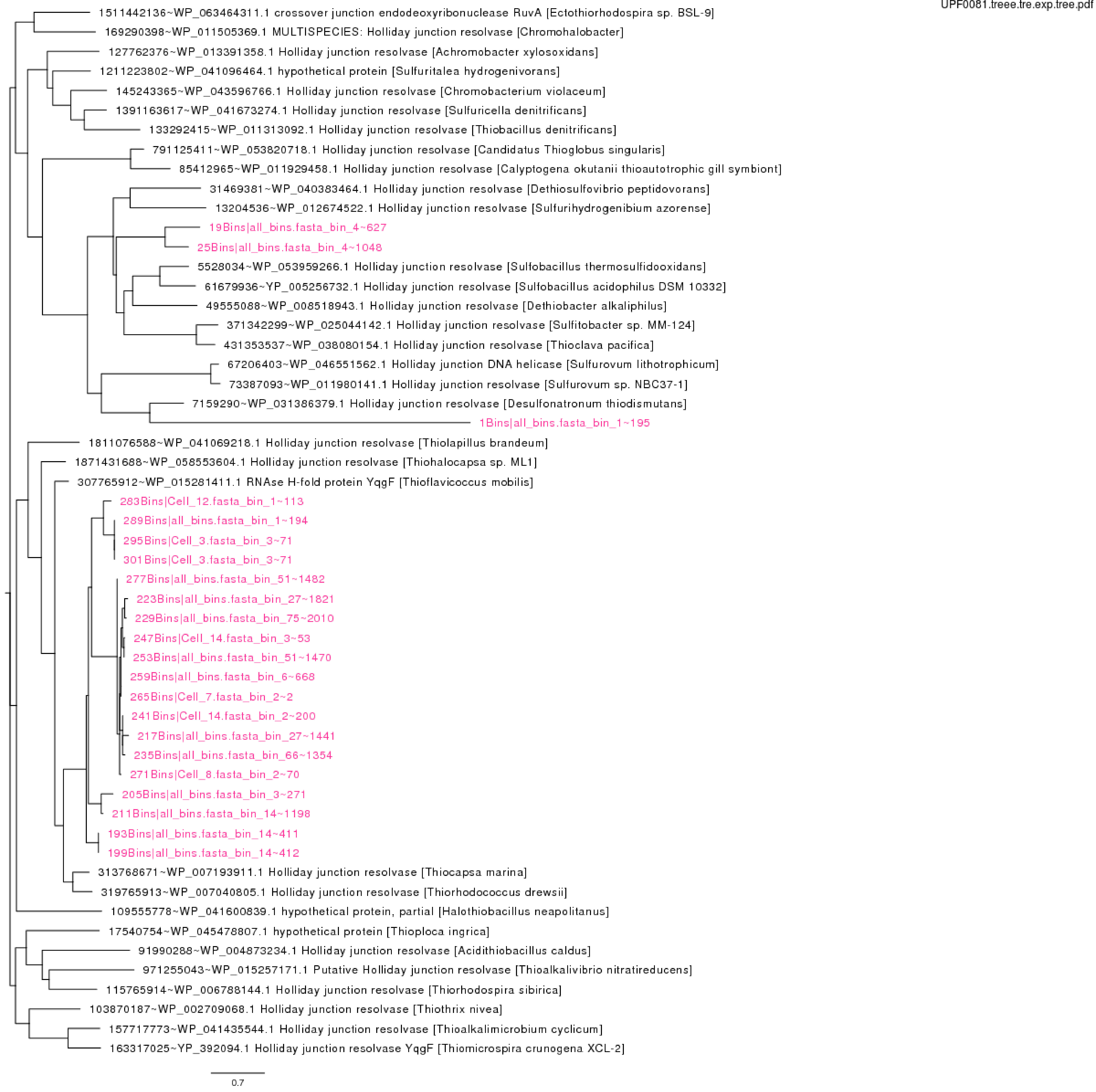
UPF0081.treee.tre.exp.tree.pdf
1511442136~WP_063464311.1 crossover junction endodeoxyribonuclease RuvA [Ectothiorhodospira sp. BSL-9]
169290398~WP_011505369.1 MULTISPECIES: Holliday junction resolvase [Chromohalobacter]
127762376~WP_013391358.1 Holliday junction resolvase [Achromobacter xylosoxidans]
1211223802~WP_041096464.1 hypothetical protein [Sulfuritalea hydrogenivorans]
145243365~WP_043596766.1 Holliday junction resolvase [Chromobacterium violaceum]
1391163617~WP_041673274.1 Holliday junction resolvase [Sulfuricella denitrificans]
133292415~WP_011313092.1 Holliday junction resolvase [Thiobacillus denitrificans]
791125411~WP_053820718.1 Holliday junction resolvase [Candidatus Thioglobus singularis]
85412965~WP_011929458.1 Holliday junction resolvase [Calyptogena okutanii thioautotrophic gill symbiont]
31469381~WP_040383464.1 Holliday junction resolvase [Dethiosulfovibrio peptidovorans]
13204536~WP_012674522.1 Holliday junction resolvase [Sulfurihydrogenibium azorense]
19Bins|all_bins.fasta_bin_4~627
25Bins|all_bins.fasta_bin_4~1048
5528034~WP_053959266.1 Holliday junction resolvase [Sulfobacillus thermosulfidooxidans]
61679936~YP_005256732.1 Holliday junction resolvase [Sulfobacillus acidophilus DSM 10332]
49555088~WP_008518943.1 Holliday junction resolvase [Dethiobacter alkaliphilus]
371342299~WP_025044142.1 Holliday junction resolvase [Sulfitobacter sp. MM-124]
431353537~WP_038080154.1 Holliday junction resolvase [Thioclava pacifica]
67206403~WP_046551562.1 Holliday junction DNA helicase [Sulfurovum lithotrophicum]
73387093~WP_011980141.1 Holliday junction resolvase [Sulfurovum sp. NBC37-1]
7159290~WP_031386379.1 Holliday junction resolvase [Desulfonatronum thiodismutans]
1Bins|all_bins.fasta_bin_1~195
1811076588~WP_041069218.1 Holliday junction resolvase [Thiolapillus brandeum]
1871431688~WP_058553604.1 Holliday junction resolvase [Thiohalocapsa sp. ML1]
307765912~WP_015281411.1 RNAse H-fold protein YqgF [Thioflavicoccus mobilis]
283Bins|Cell_12.fasta_bin_1~113
289Bins|all_bins.fasta_bin_1~194
295Bins|Cell_3.fasta_bin_3~71
301Bins|Cell_3.fasta_bin_3~71
277Bins|all_bins.fasta_bin_51~1482
223Bins|all_bins.fasta_bin_27~1821
229Bins|all_bins.fasta_bin_75~2010
247Bins|Cell_14.fasta_bin_3~53
253Bins|all_bins.fasta_bin_51~1470
259Bins|all_bins.fasta_bin_6~668
265Bins|Cell_7.fasta_bin_2~2
241Bins|Cell_14.fasta_bin_2~200
217Bins|all_bins.fasta_bin_27~1441
235Bins|all_bins.fasta_bin_66~1354
271Bins|Cell_8.fasta_bin_2~70
205Bins|all_bins.fasta_bin_3~271
211Bins|all_bins.fasta_bin_14~1198
193Bins|all_bins.fasta_bin_14~411
199Bins|all_bins.fasta_bin_14~412
313768671~WP_007193911.1 Holliday junction resolvase [Thiocapsa marina]
319765913~WP_007040805.1 Holliday junction resolvase [Thiorhodococcus drewsii]
109555778~WP_041600839.1 hypothetical protein, partial [Halothiobacillus neapolitanus]
17540754~WP_045478807.1 hypothetical protein [Thioploca ingrica]
91990288~WP_004873234.1 Holliday junction resolvase [Acidithiobacillus caldus]
971255043~WP_015257171.1 Putative Holliday junction resolvase [Thioalkalivibrio nitratireducens]
115765914~WP_006788144.1 Holliday junction resolvase [Thiorhodospira sibirica]
103870187~WP_002709068.1 Holliday junction resolvase [Thiothrix nivea]
157717773~WP_041435544.1 Holliday junction resolvase [Thioalkalimicrobium cyclicum]
163317025~YP_392094.1 Holliday junction resolvase YqgF [Thiomicrospira crunogena XCL-2]
0.7

UvrC_HhH_N.treee.tre.exp.tree.pdf

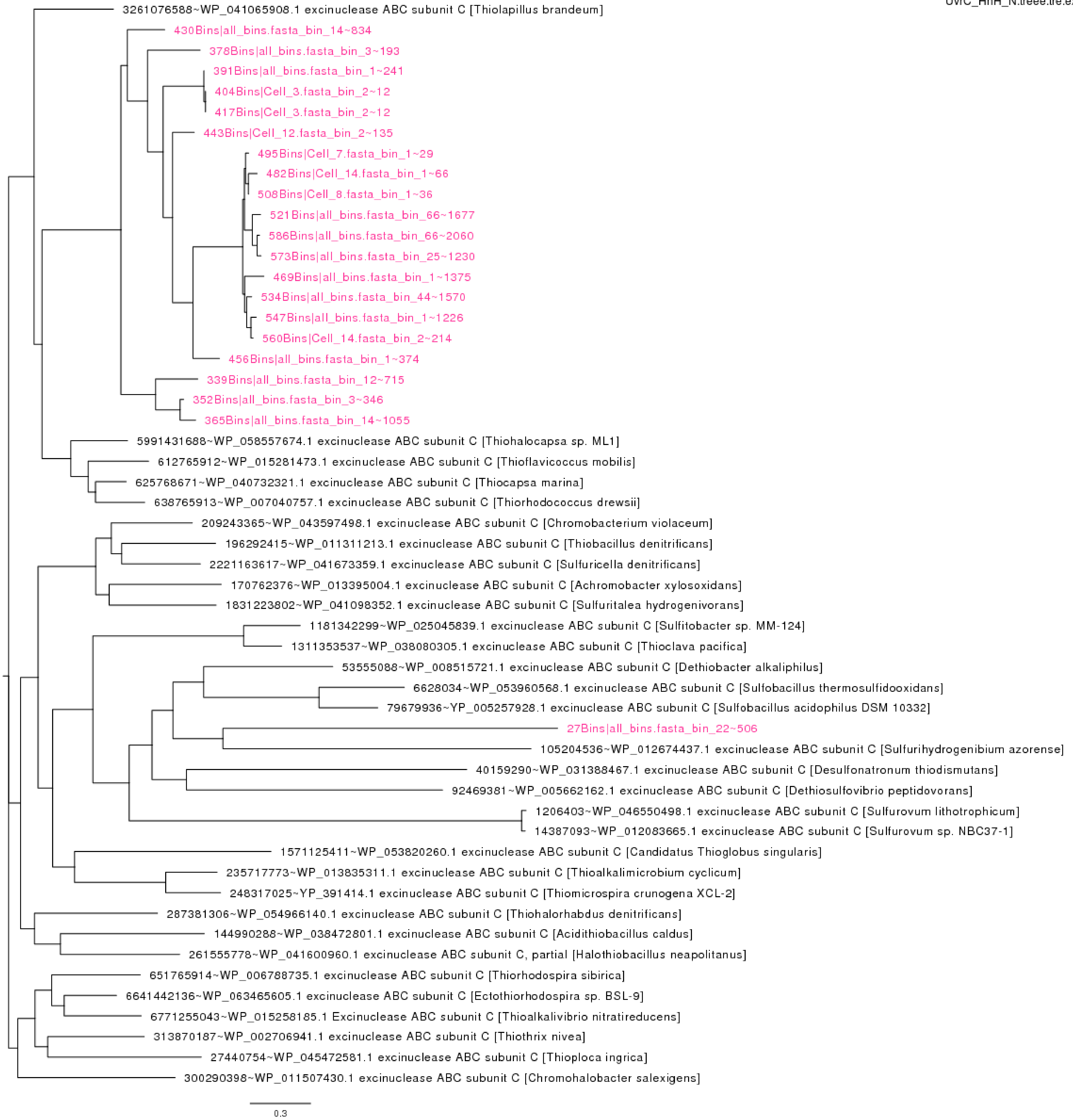

Val_tRNA-synt_C.treee.tre.exp.tree.pdf

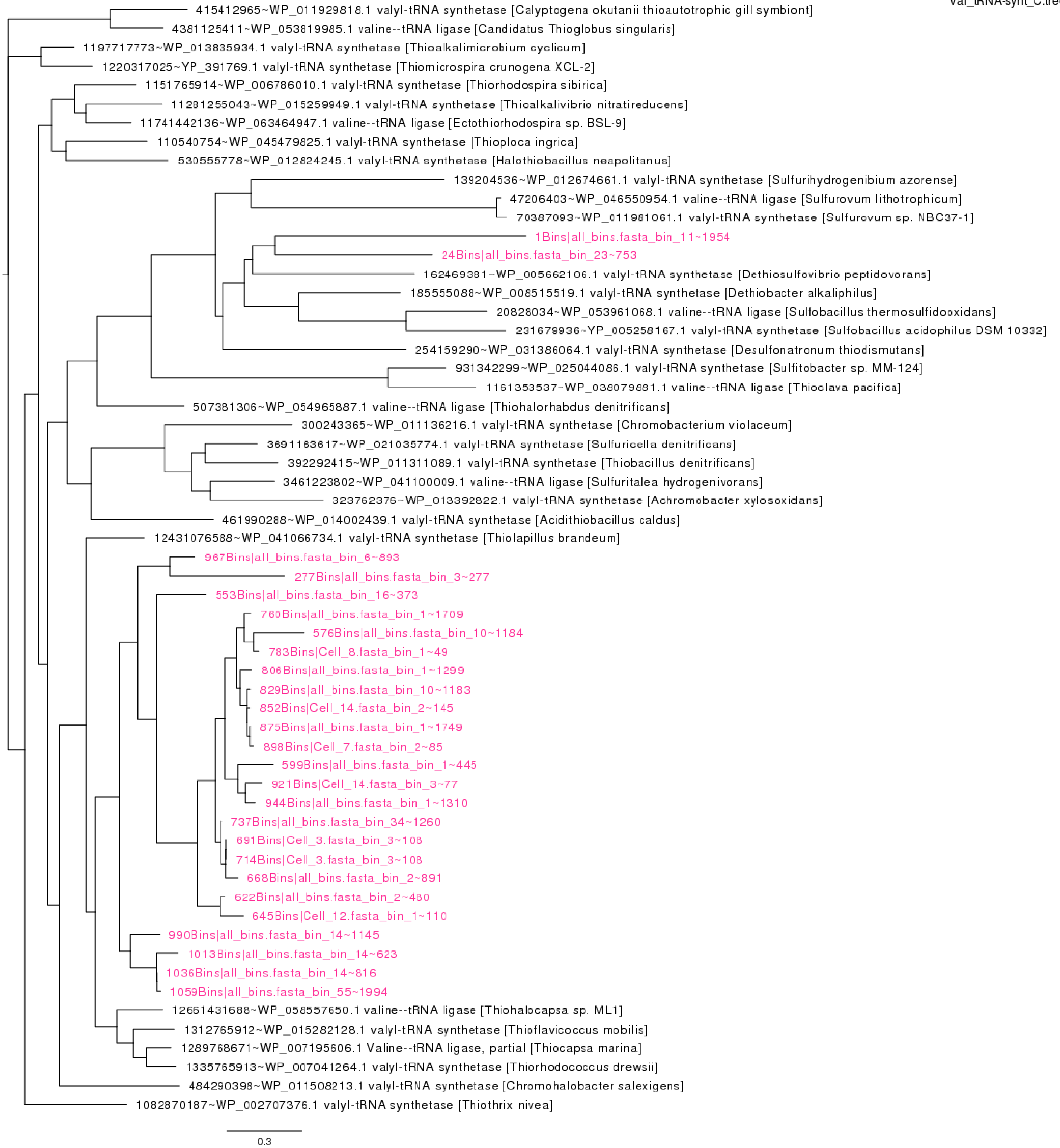

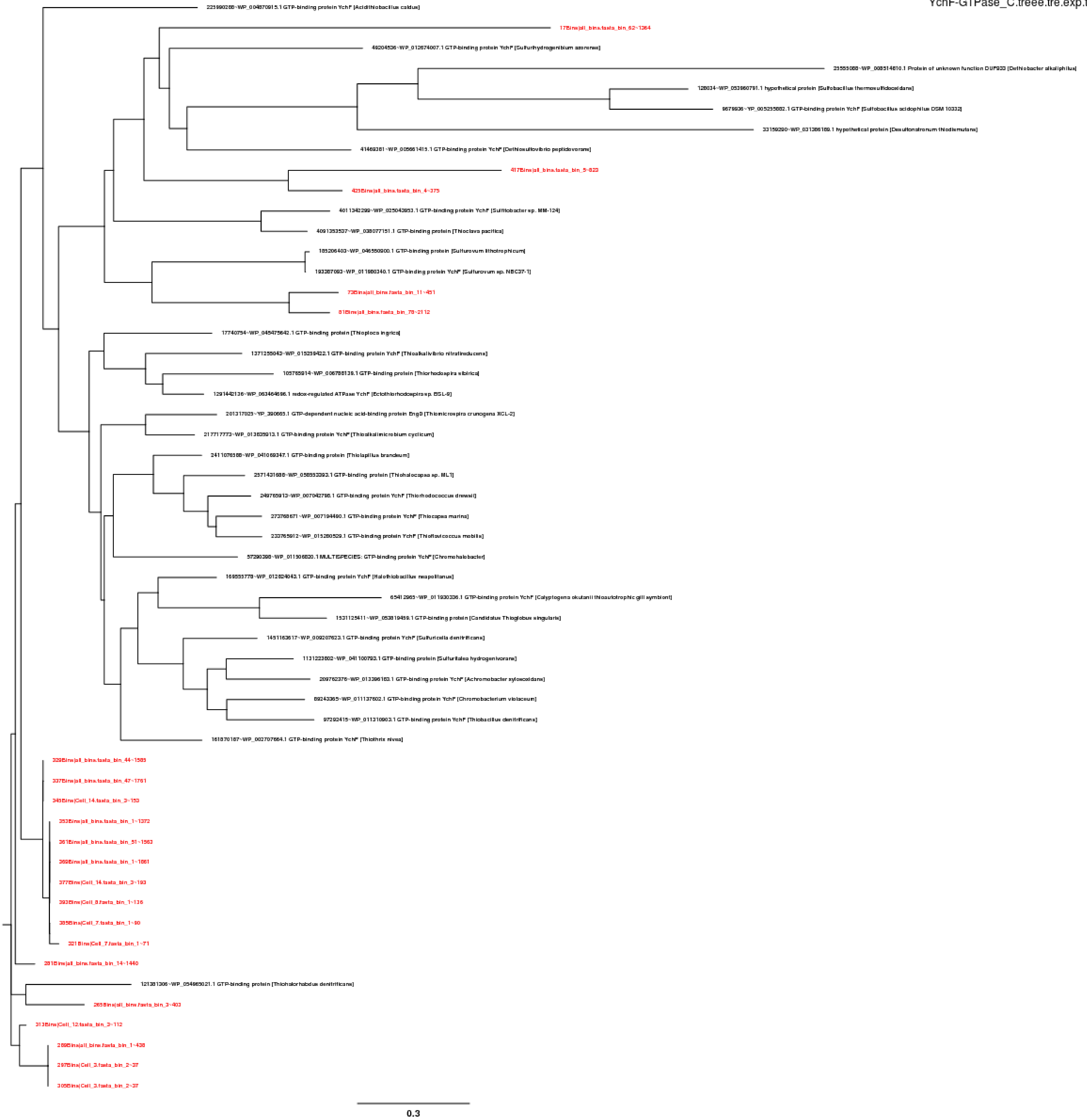
225990288~WP_004870915.1 GTP-binding protein YchF [Acidithiobacillus caldus]
17Bins|all_bins.fasta_bin_62~1364
49204536~WP_012674007.1 GTP-binding protein YchF [Sulfurihydrogenibium azorense]
23555088~WP_008514610.1 Protein of unknown function DUF933 [Dethiobacter alkaliphilus]
126034~WP_053960791.1 hypothetical protein [Sulfobacillus thermosulfidooxidans]
9679936~YP_005255882.1 GTP-binding protein YchF [Sulfobacillus acidophilus DSM 10332]
33159290~WP_031386189.1 hypothetical protein [Desulfonatronum thiodismutans]
41469381~WP_005661415.1 GTP-binding protein YchF [Dethiosulfovibrio peptidovorans]
417Bins|all_bins.fasta_bin_5~823
425Bins|all_bins.fasta_bin_4~375
4011342299~WP_025043953.1 GTP-binding protein YchF [Sulfitobacter sp. MM-124]
4091353537~WP_038077151.1 GTP-binding protein [Thioclava pacifica]
185206403~WP_046550900.1 GTP-binding protein [Sulfurovum lithotrophicum]
193387093~WP_011980340.1 GTP-binding protein YchF [Sulfurovum sp. NBC37-1]
73Bins|all_bins.fasta_bin_11~451
81Bins|all_bins.fasta_bin_78~2112
17740754~WP_045475642.1 GTP-binding protein [Thioploca ingrica]
1371255043~WP_015259422.1 GTP-binding protein YchF [Thioalkalivibrio nitratireducens]
105765914~WP_006788139.1 GTP-binding protein [Thiorhodospira sibirica]
1291442136~WP_063464696.1 redox-regulated ATPase YchF [Ectothiorhodospira sp. BSL-9]
201317025~YP_390665.1 GTP-dependent nucleic acid-binding protein EngD [Thiomicrospira crunogena XCL-2]
217717773~WP_013835913.1 GTP-binding protein YchF [Thioalkalimicrobium cyclicum]
2411076588~WP_041069347.1 GTP-binding protein [Thiolapillus brandeum]
2571431688~WP_058553393.1 GTP-binding protein [Thiohalocapsa sp. ML1]
249765913~WP_007042798.1 GTP-binding protein YchF [Thiorhodococcus drewsii]
273768671~WP_007194490.1 GTP-binding protein YchF [Thiocapsa marina]
233765912~WP_015280529.1 GTP-binding protein YchF [Thioflavicoccus mobilis]
57290398~WP_011506820.1 MULTISPECIES: GTP-binding protein YchF [Chromohalobacter]
169555778~WP_012824043.1 GTP-binding protein YchF [Halothiobacillus neapolitanus]
65412965~WP_011930336.1 GTP-binding protein YchF [Calyptogena okutanii thioautotrophic gill symbiont]
1531125411~WP_053819459.1 GTP-binding protein [Candidatus Thioglobus singularis]
1451163617~WP_009207623.1 GTP-binding protein YchF [Sulfuricella denitrificans]
1131223802~WP_041100793.1 GTP-binding protein [Sulfuritalea hydrogenivorans]
209762376~WP_013396183.1 GTP-binding protein YchF [Achromobacter xylosoxidans]
69243365~WP_011137602.1 GTP-binding protein YchF [Chromobacterium violaceum]
97292415~WP_011310903.1 GTP-binding protein YchF [Thiobacillus denitrificans]
161870187~WP_002707664.1 GTP-binding protein YchF [Thiothrix nivea]
329Bins|all_bins.fasta_bin_44~1585
337Bins|all_bins.fasta_bin_47~1761
345Bins|Cell_14.fasta_bin_3~153
353Bins|all_bins.fasta_bin_1~1372
361Bins|all_bins.fasta_bin_51~1563
369Bins|all_bins.fasta_bin_1~1661
377Bins|Cell_14.fasta_bin_3~193
393Bins|Cell_8.fasta_bin_1~136
385Bins|Cell_7.fasta_bin_1~90
321Bins|Cell_7.fasta_bin_1~71
281Bins|all_bins.fasta_bin_14~1440
121381306~WP_054665021.1 GTP-binding protein [Thiohalorhabdus denitrificans]
265Bins|all_bins.fasta_bin_3~403
313Bins|Cell_12.fasta_bin_3~112
289Bins|all_bins.fasta_bin_1~438
297Bins|Cell_3.fasta_bin_2~37
305Bins|Cell_3.fasta_bin_2~37
0.3
