# Supplementary material for: Community-like genome in single cells of the sulfur bacterium Achromatium oxaliferum
Source: Nat Commun. 2017 Sep 6;8:455. doi: 10.1038/s41467-017-00342-9 (PMC5587575; doi:10.1038/s41467-017-00342-9)

aas_2.fa.aln.tree.png

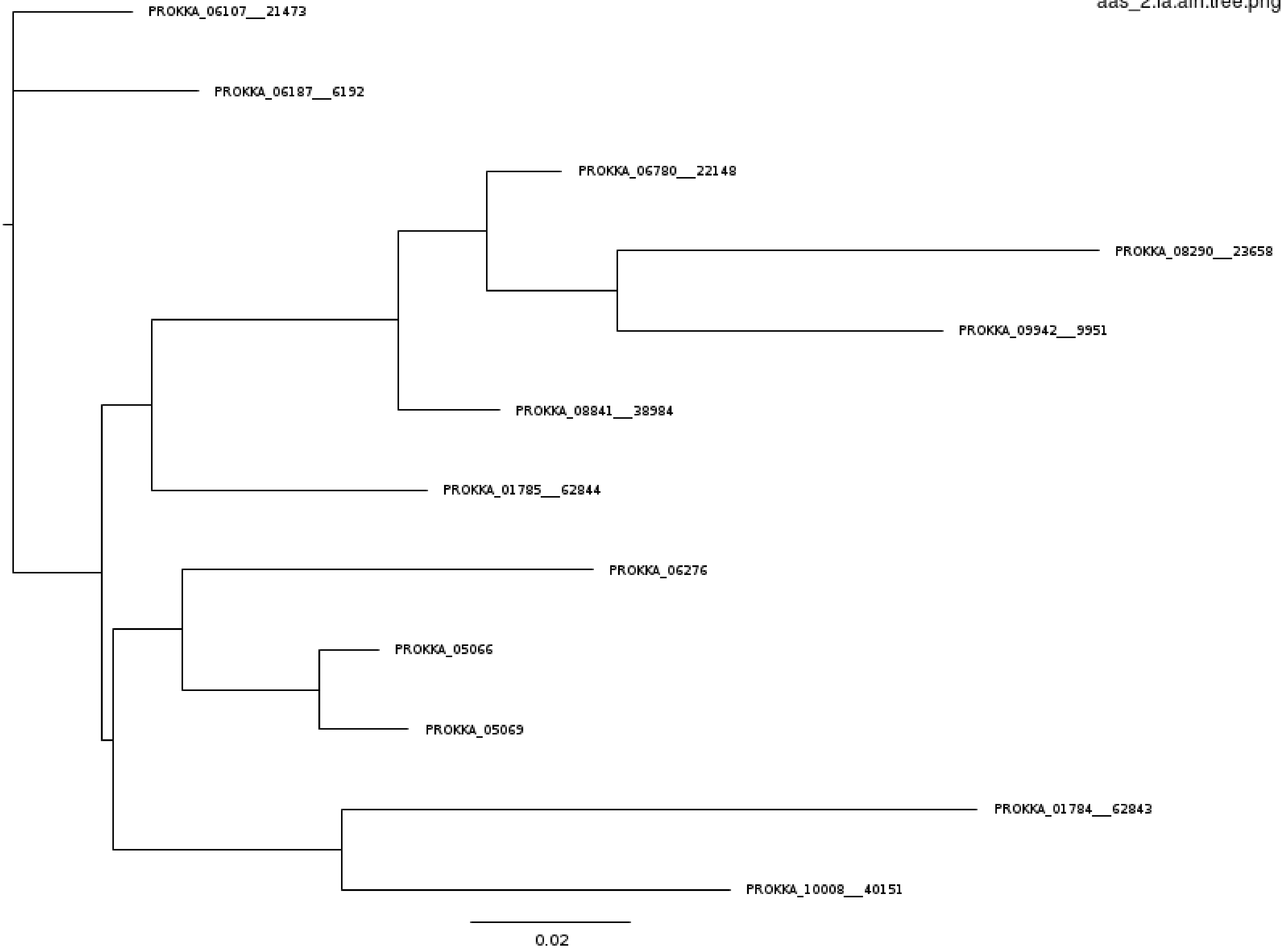

accC_2.fa.aln.tree.png

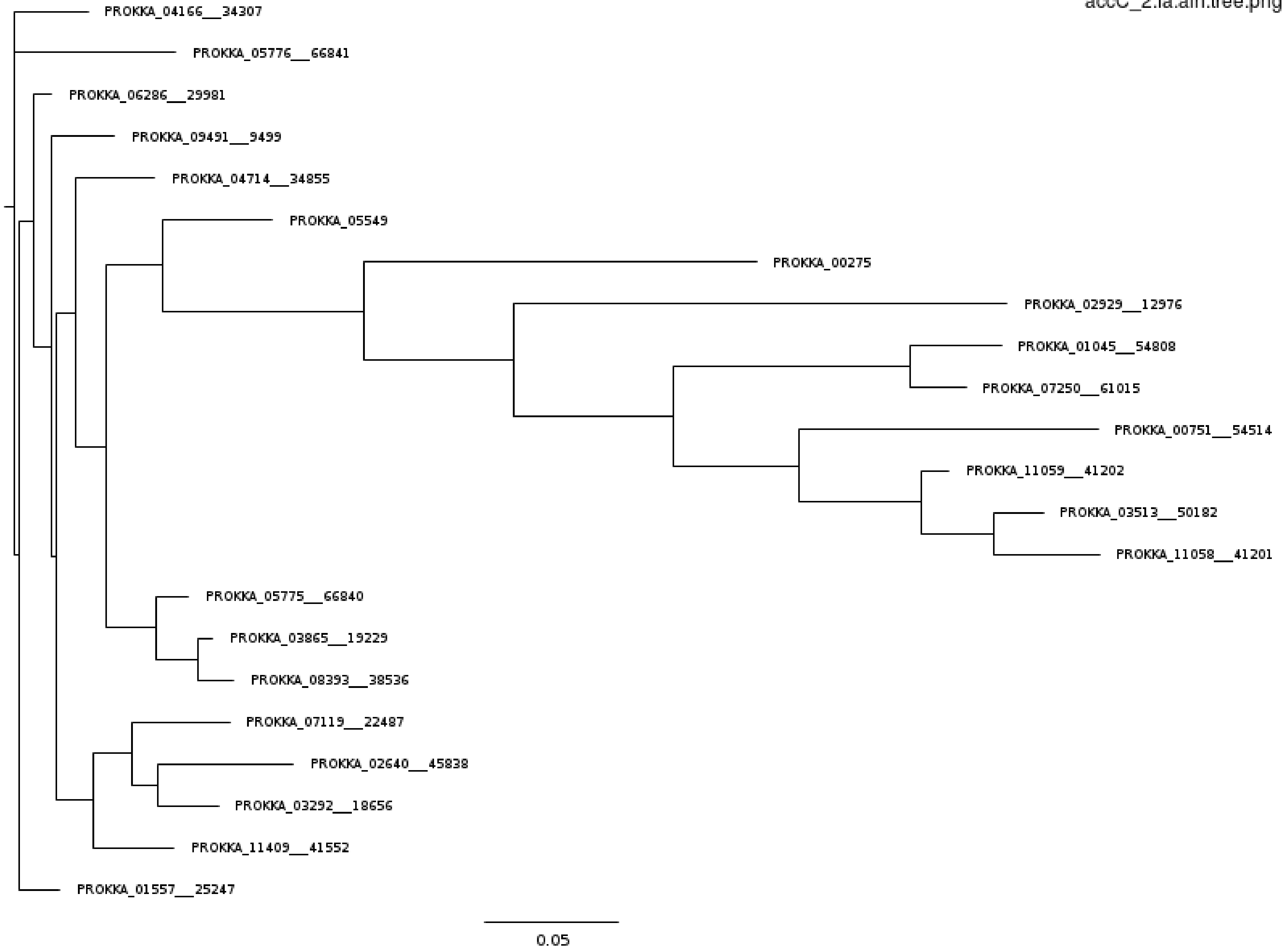

adk_2.fa.aln.tree.png

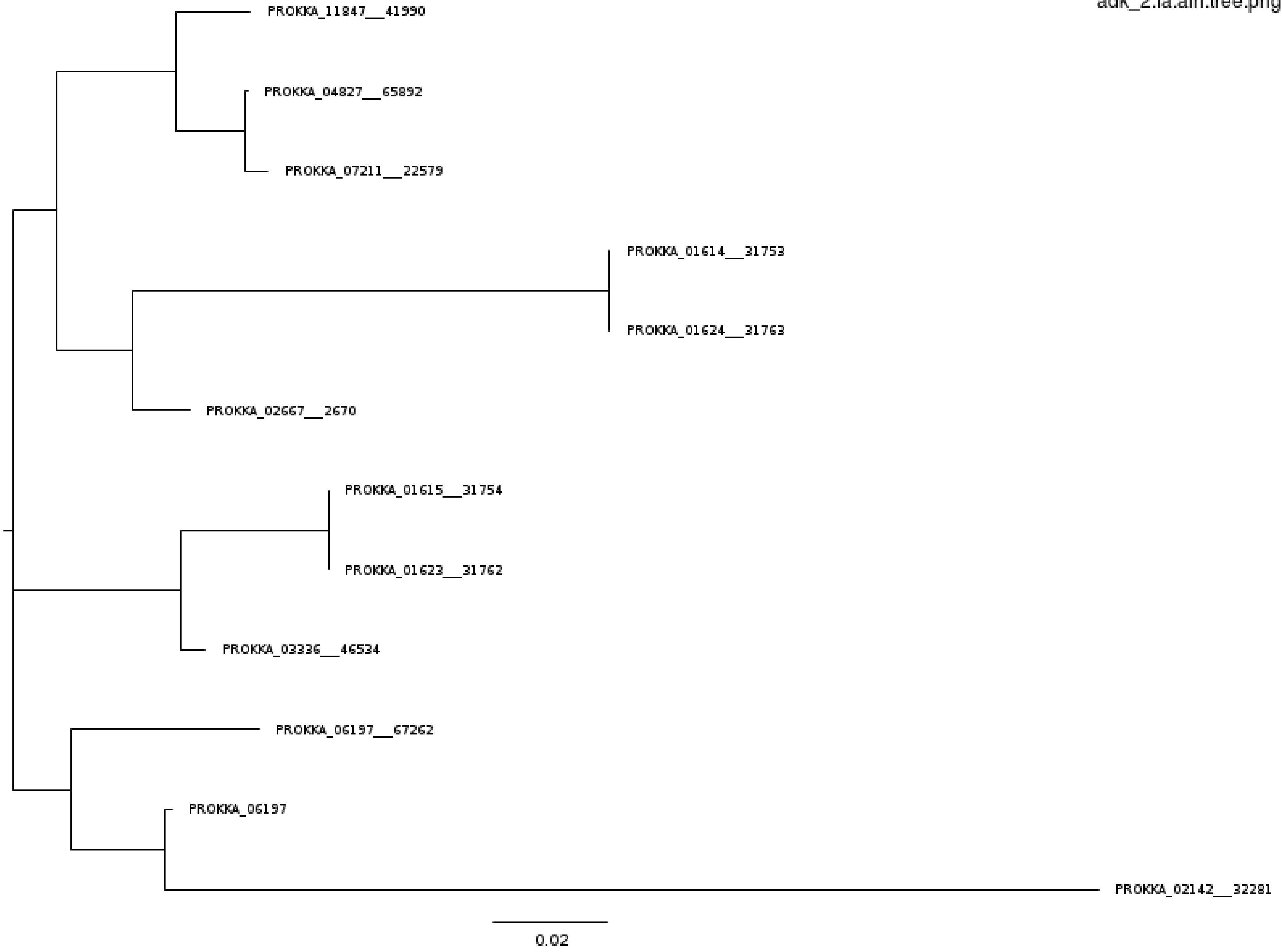

adoK_3.fa.aln.tree.png

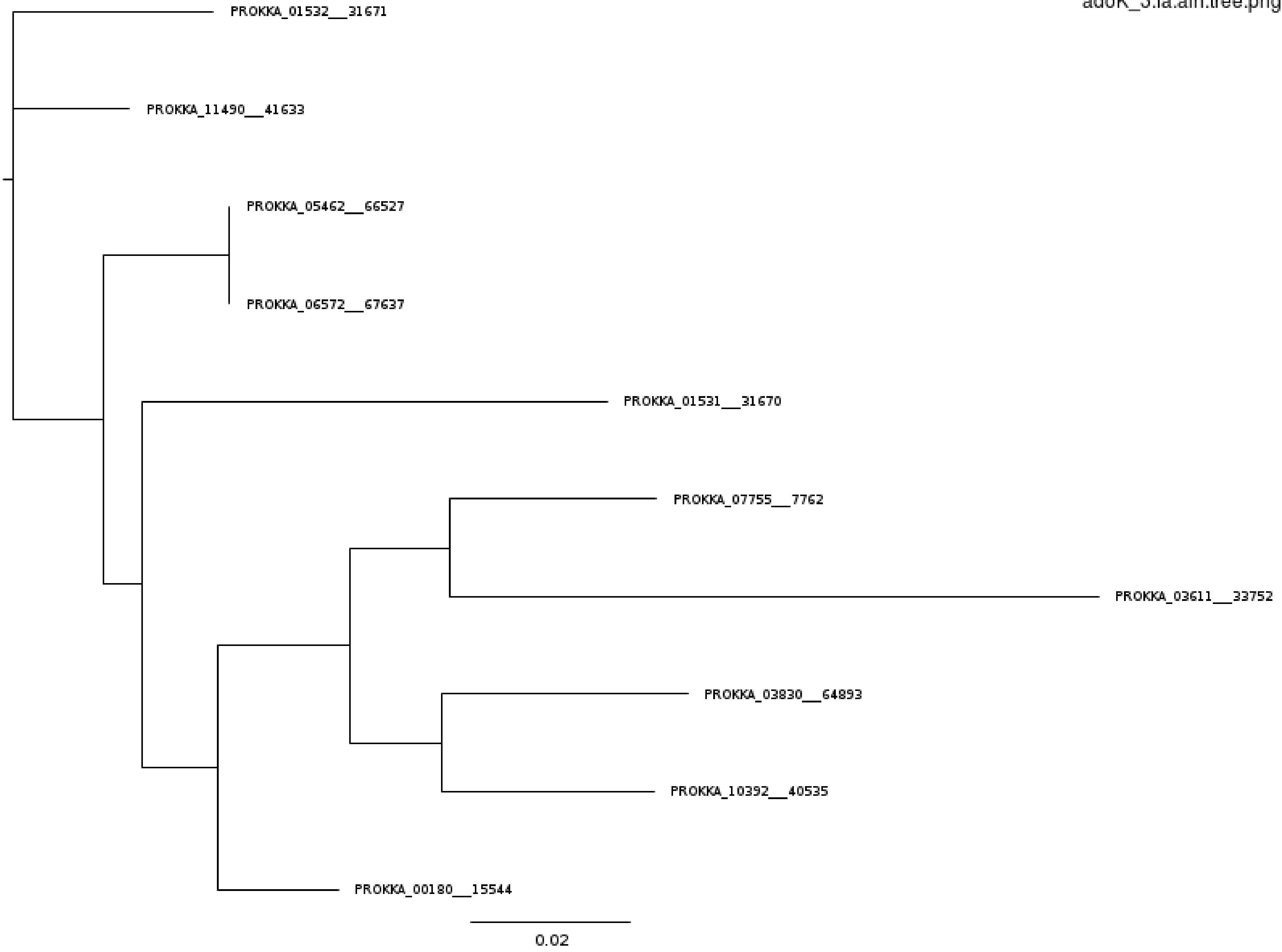

ahcY_2.fa.aln.tree.png

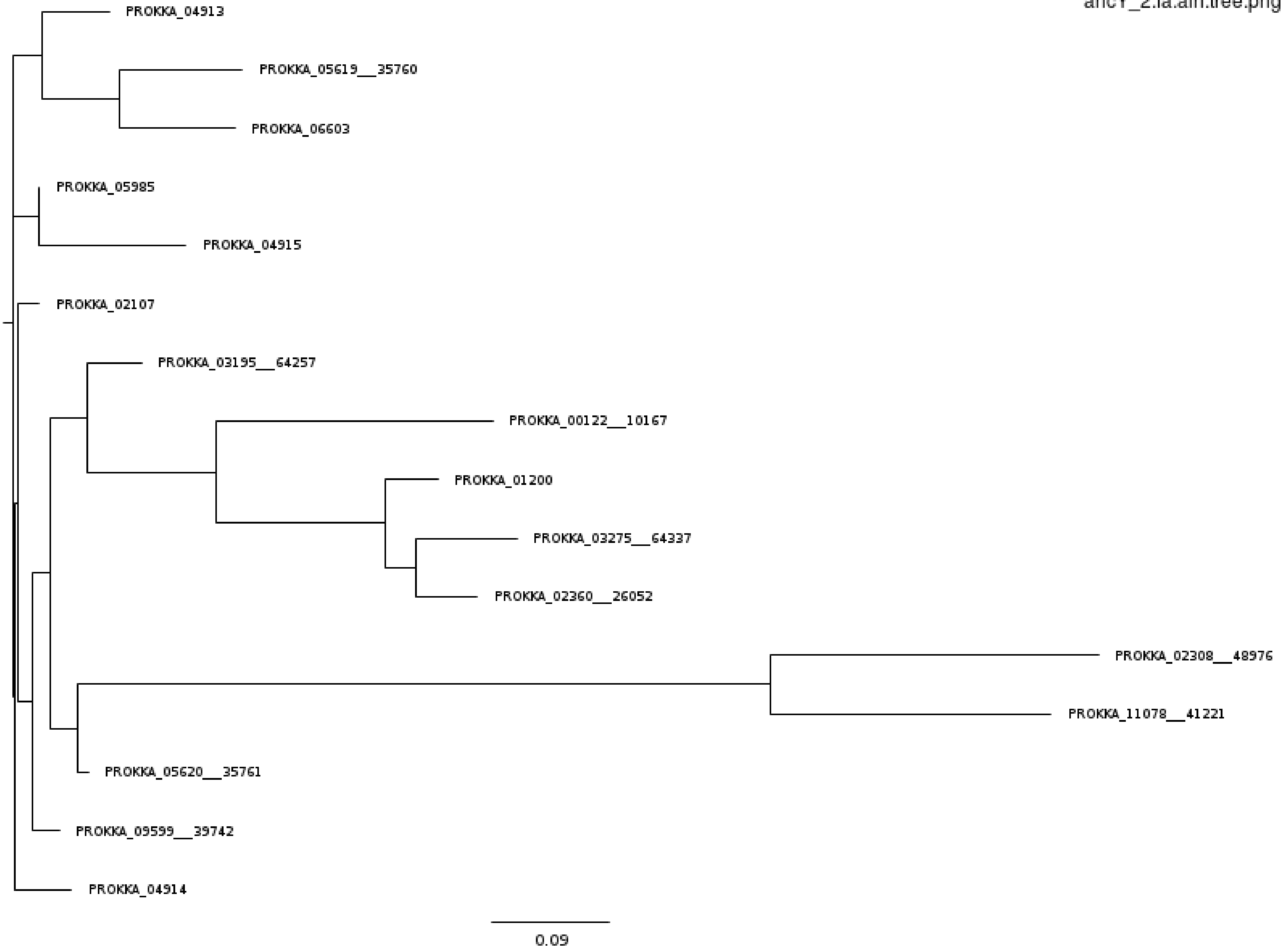

alaC.fa.aln.tree.png

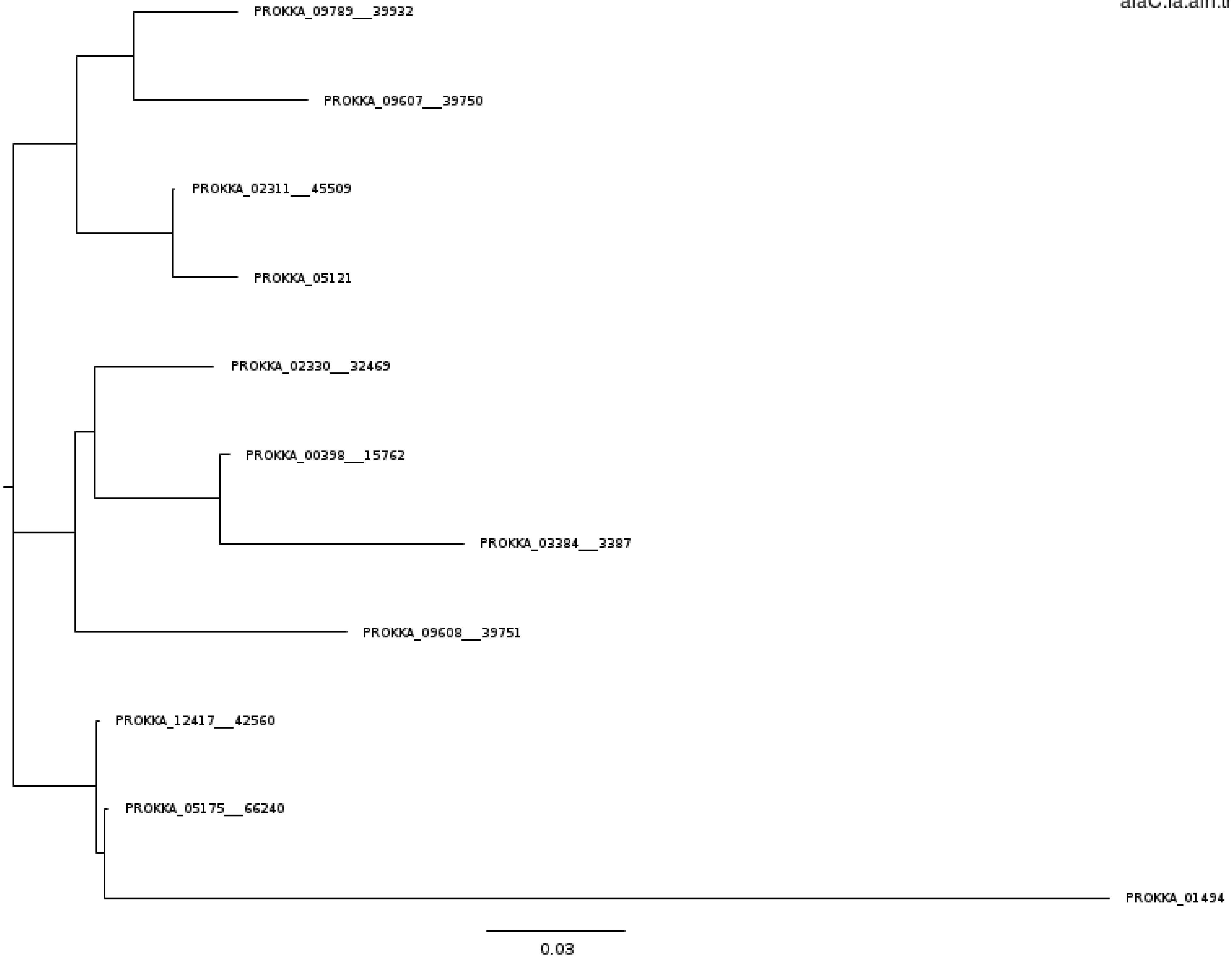

alaS_4.fa.aln.tree.png

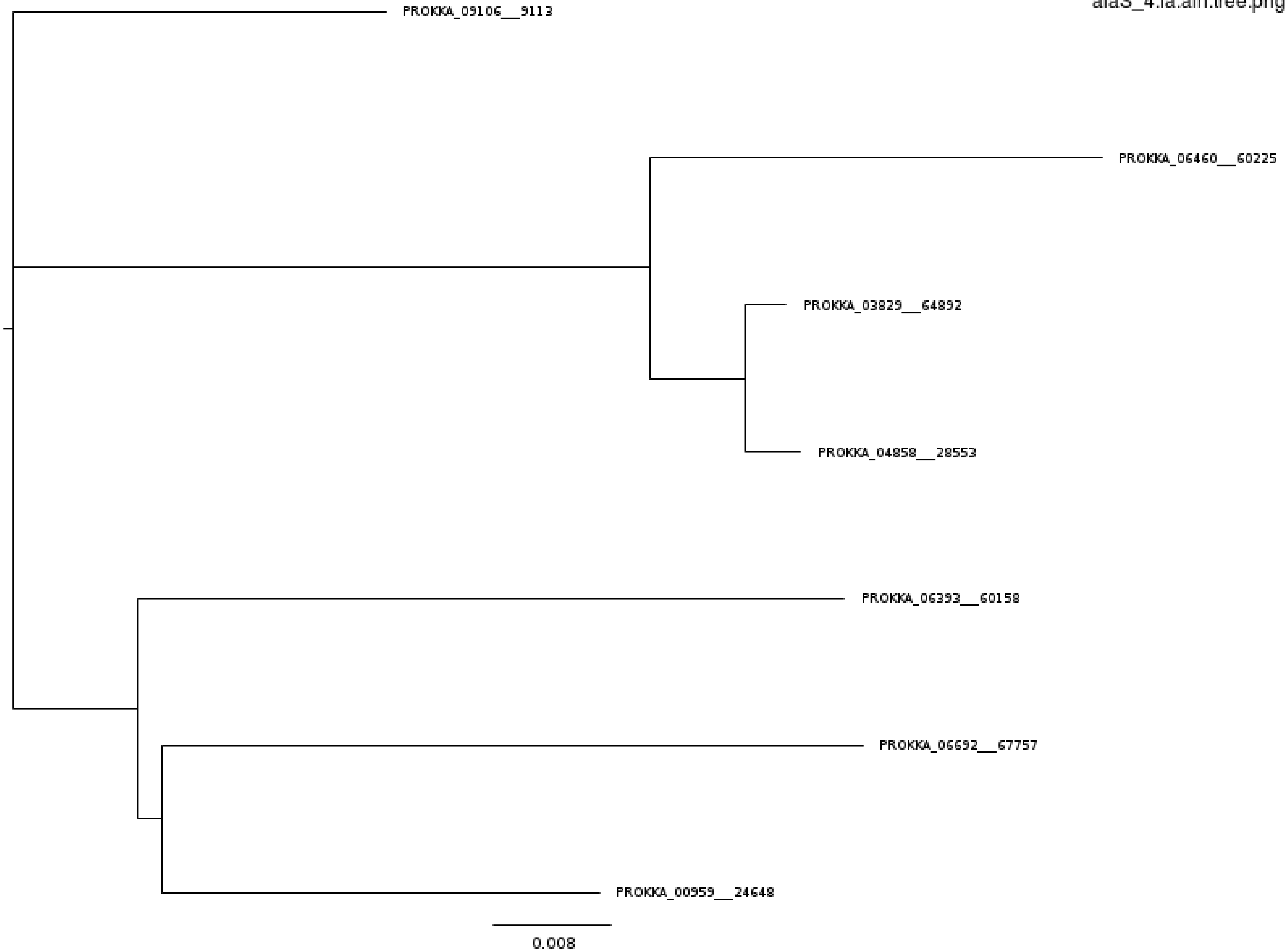

algC_4.fa.aln.tree.png

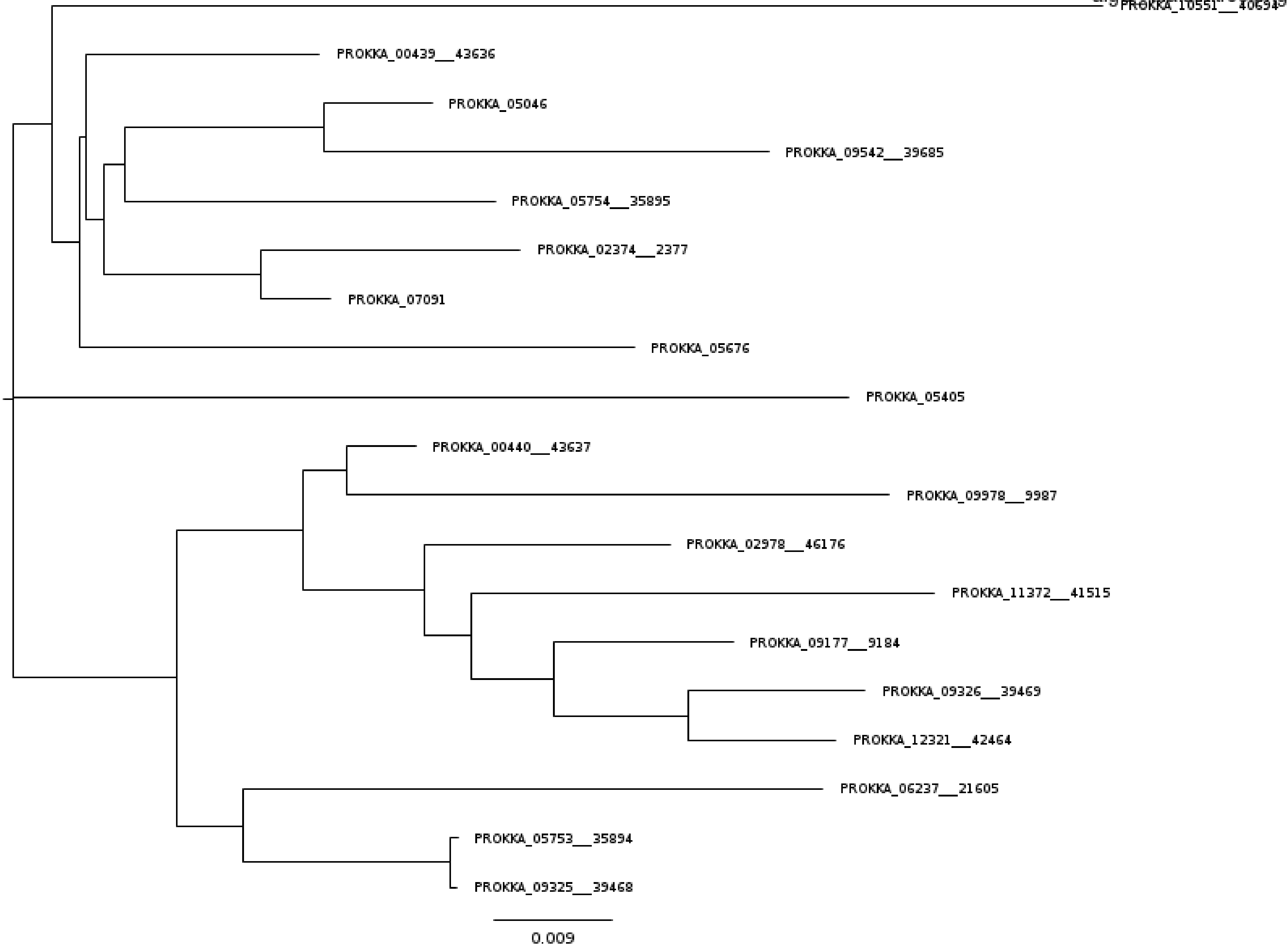

alr_3.fa.aln.tree.png

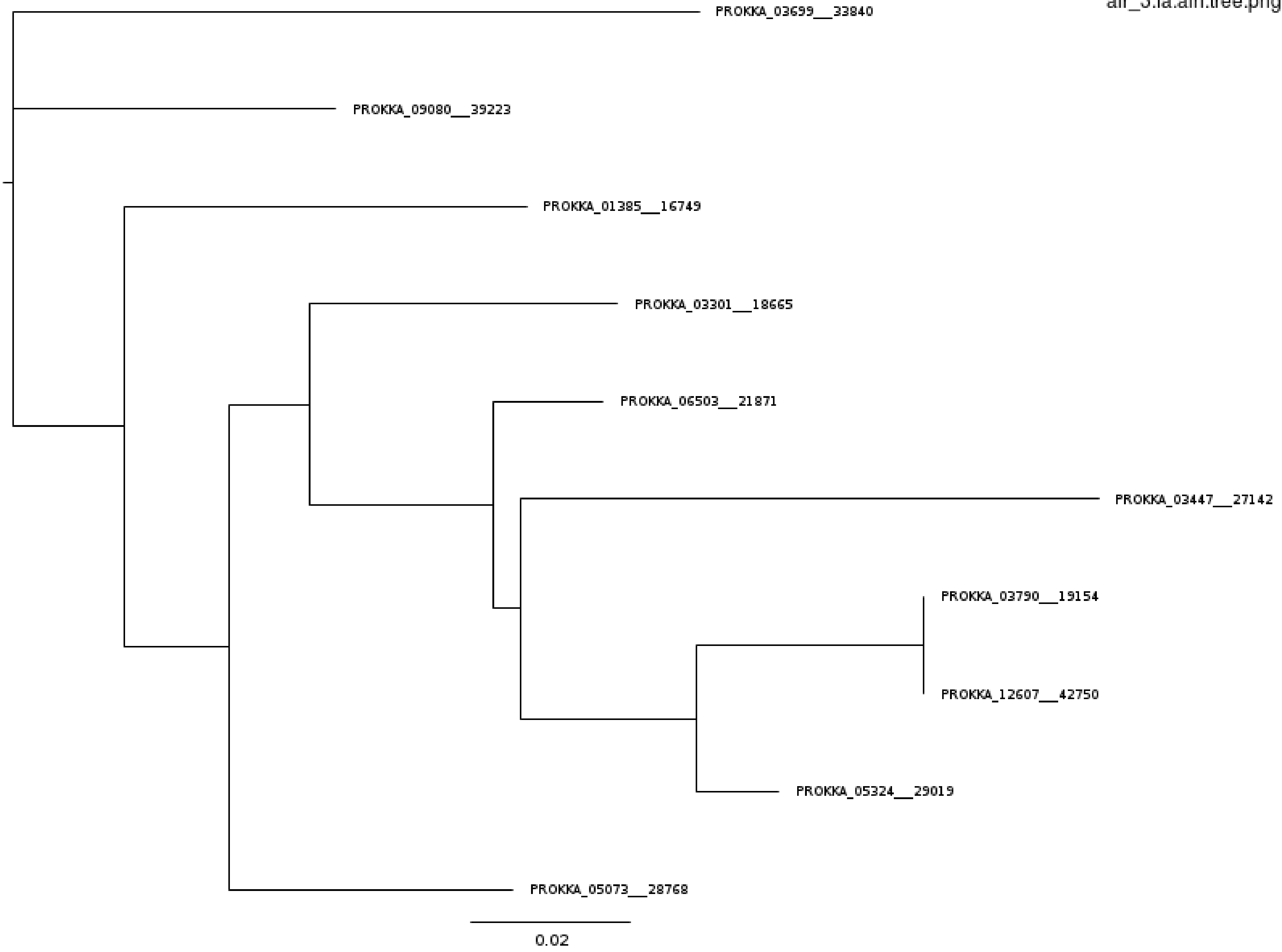

amiC_2.fa.aln.tree.png

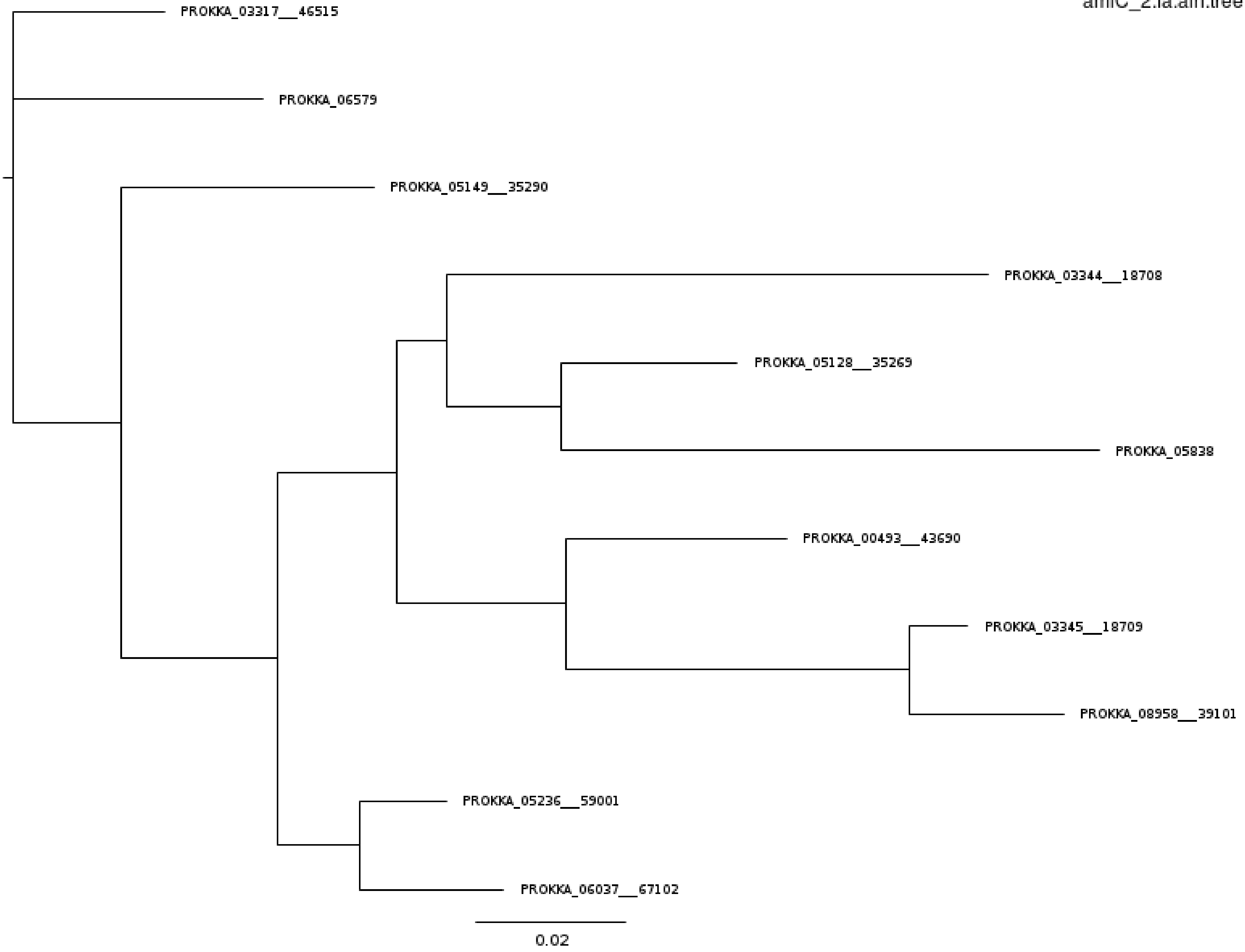

amiC.fa.aln.tree.png

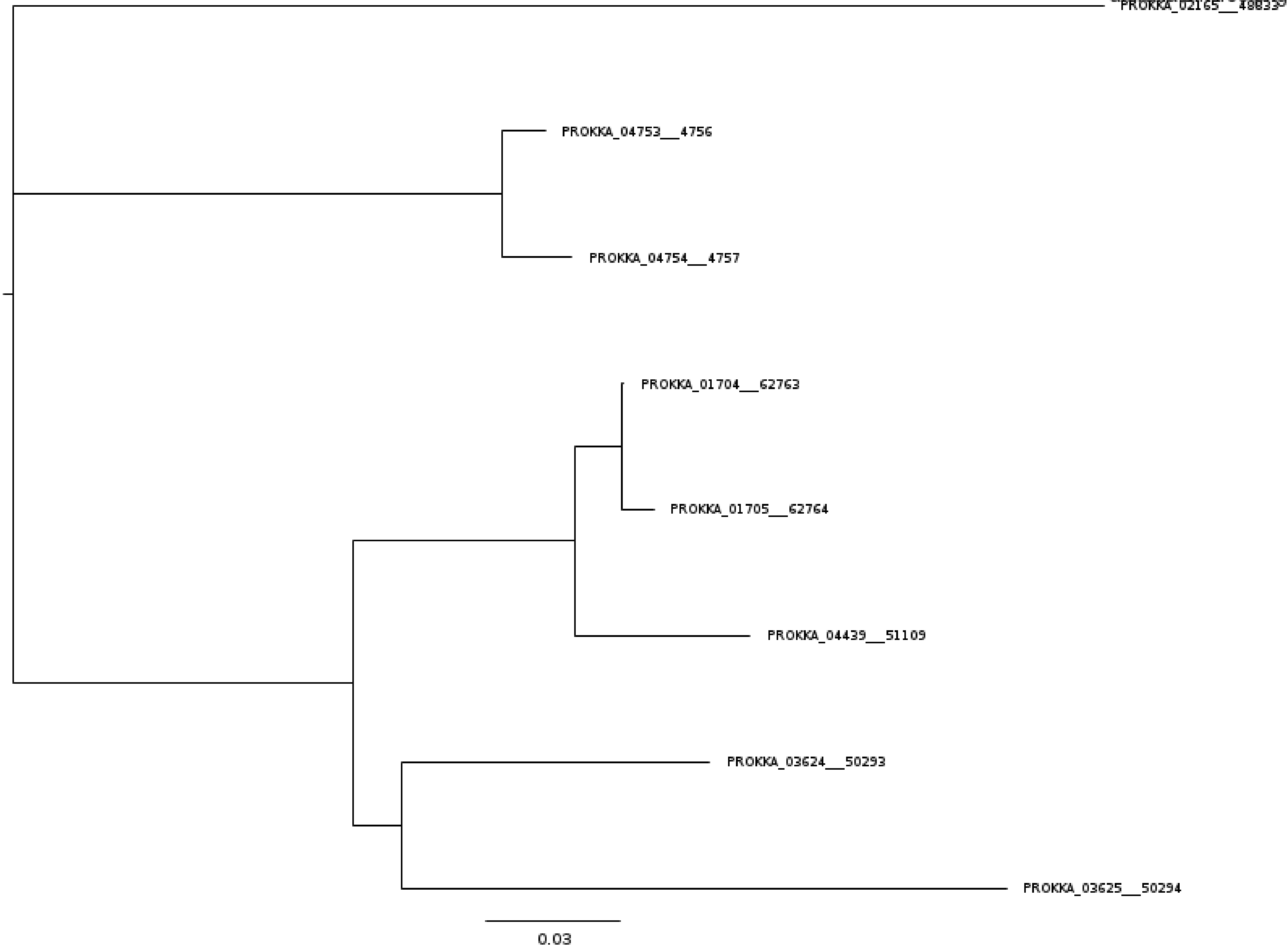

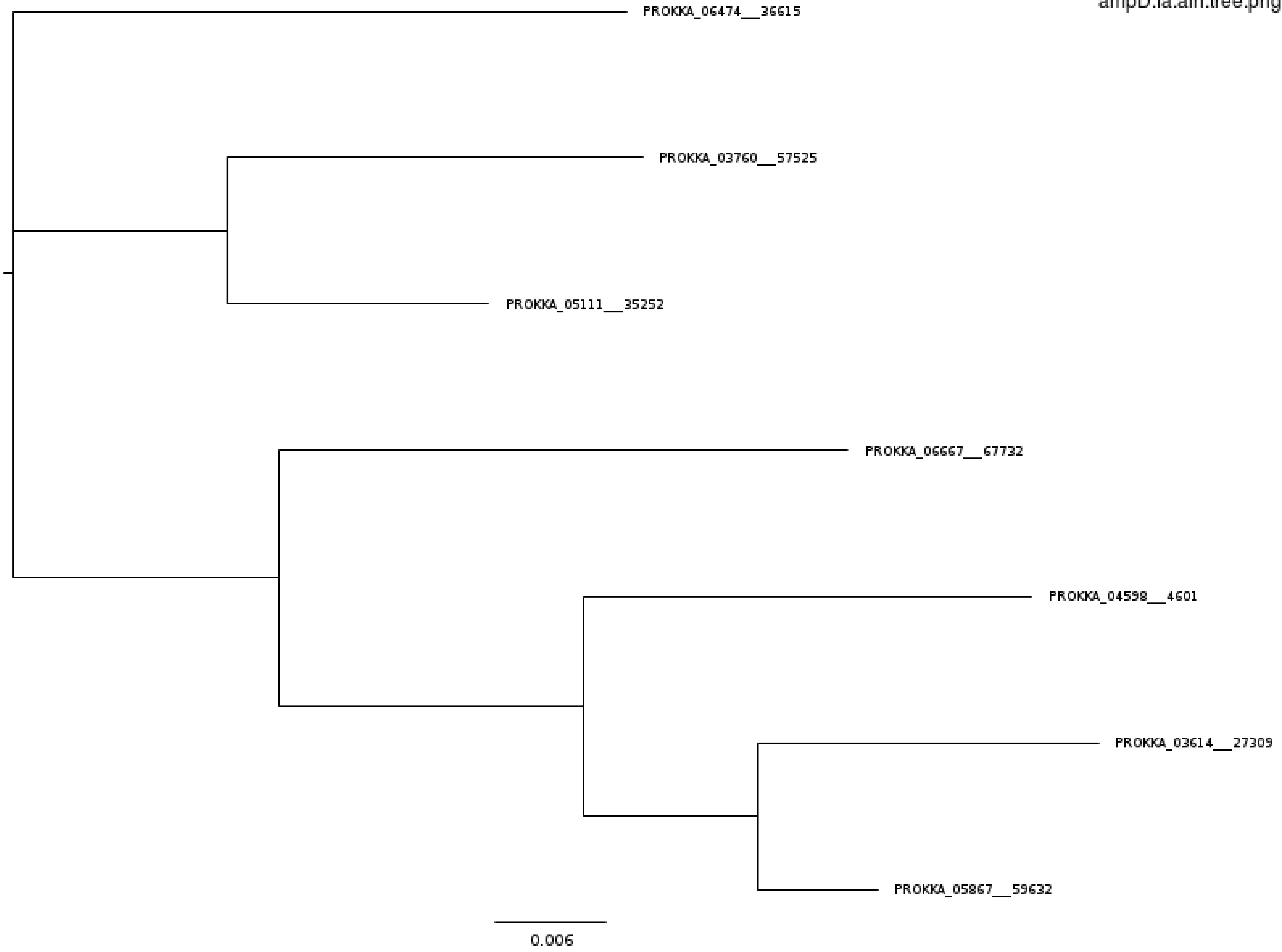
PROKKA_06474___36615
PROKKA_03760___57525
PROKKA_05111___35252
PROKKA_06667___67732
PROKKA_04598___4601
PROKKA_03614___27309
PROKKA_05867___59632
0.006

amtB_2.fa.aln.tree.png

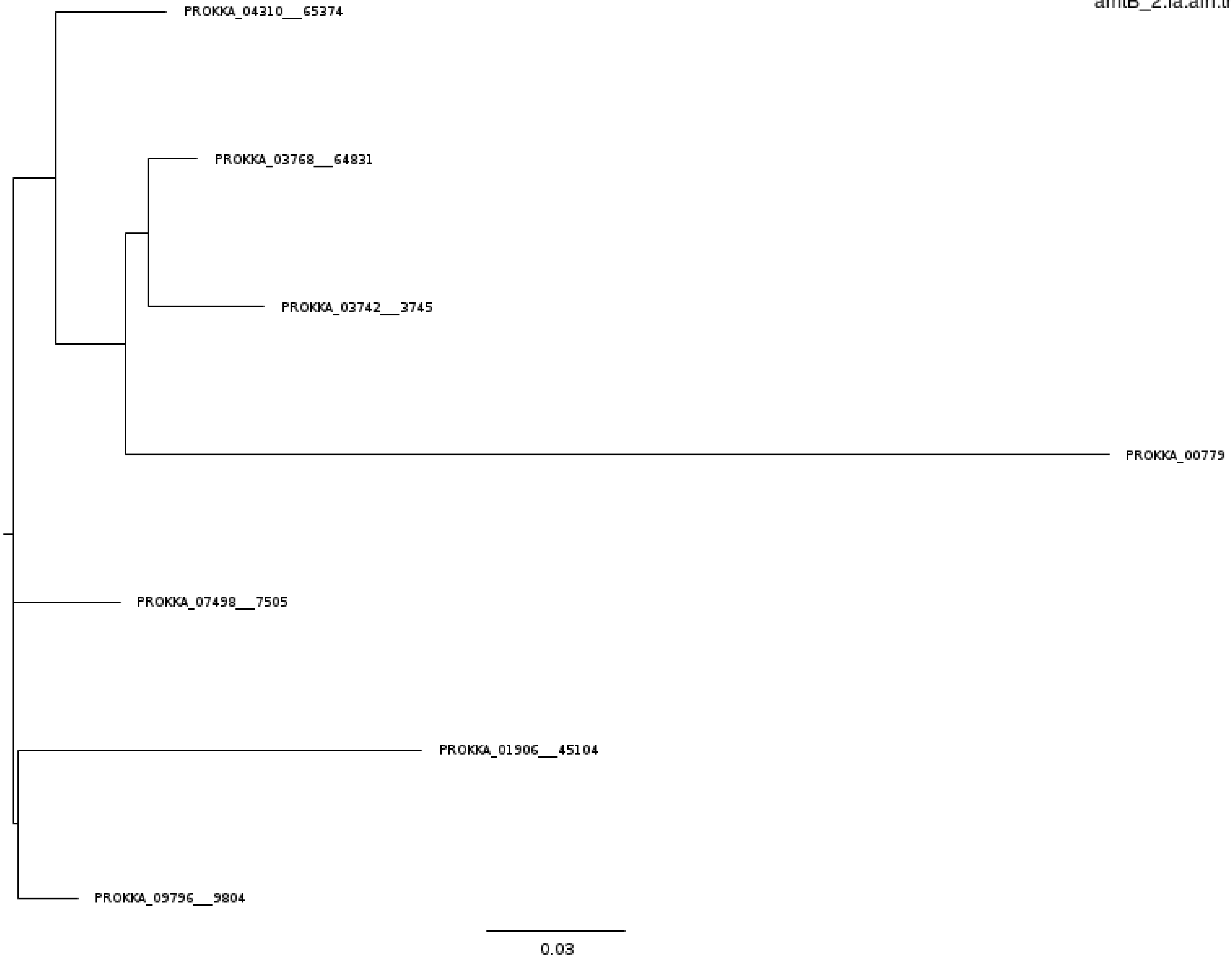

amtB_3.fa.aln.tree.png

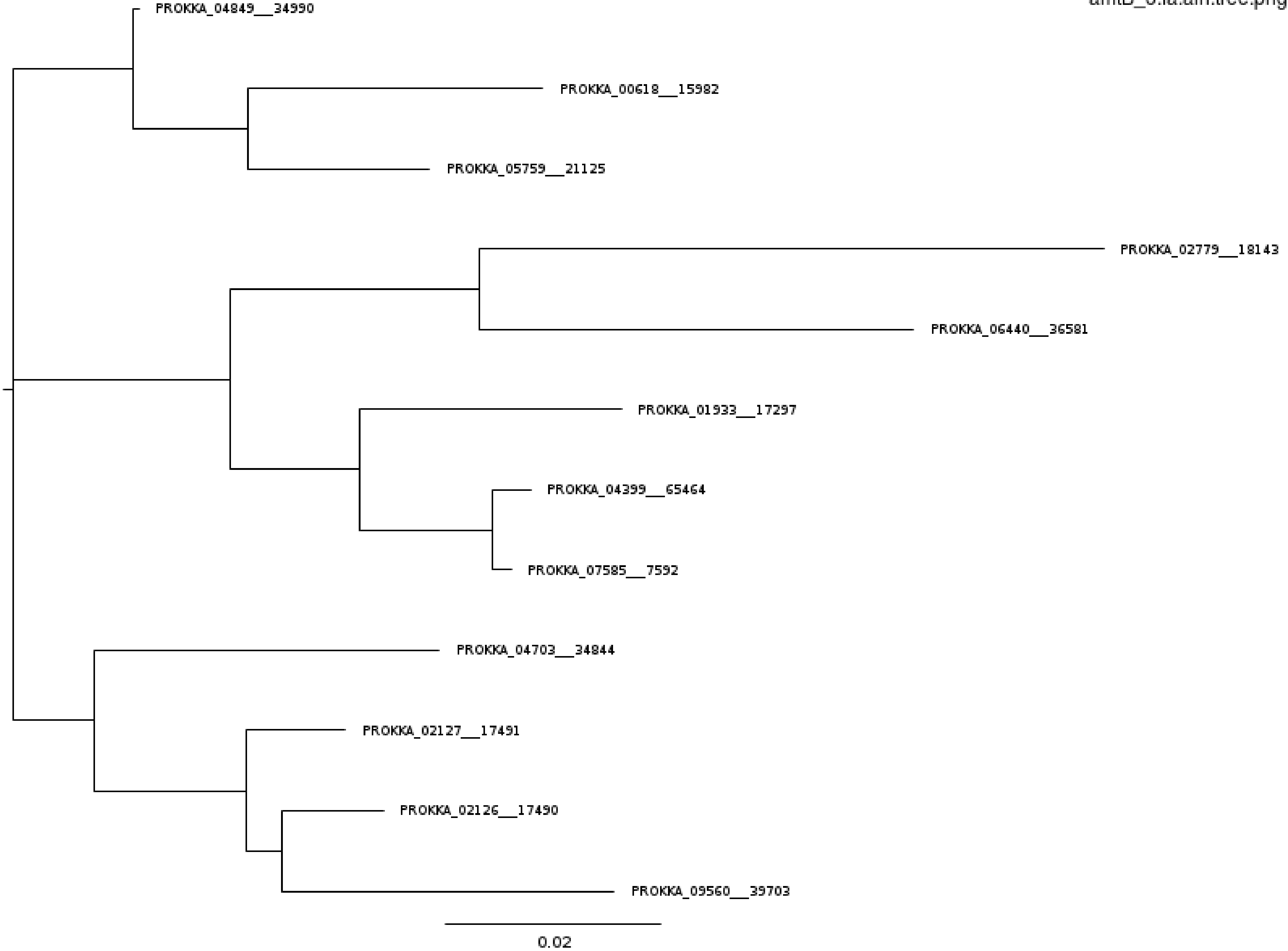

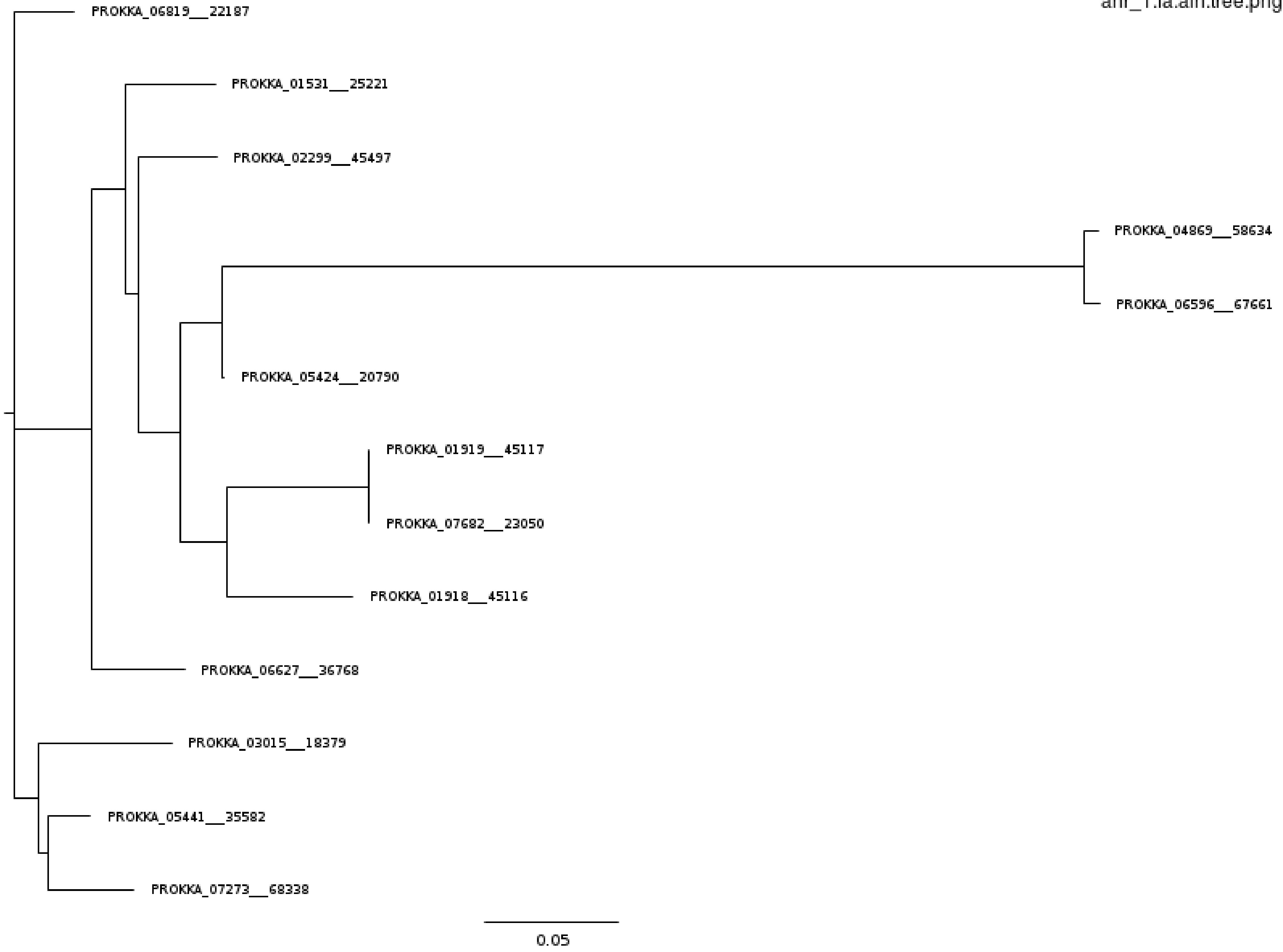
PROKKA_06819___22187
PROKKA_01531___25221
PROKKA_02299___45497
PROKKA_04869___58634
PROKKA_06596___67661
PROKKA_05424___20790
PROKKA_01919___45117
PROKKA_07682___23050
PROKKA_01918___45116
PROKKA_06627___36768
PROKKA_03015___18379
PROKKA_05441___35582
PROKKA_07273___68338
0.05

apaH_2.fa.aln.tree.png

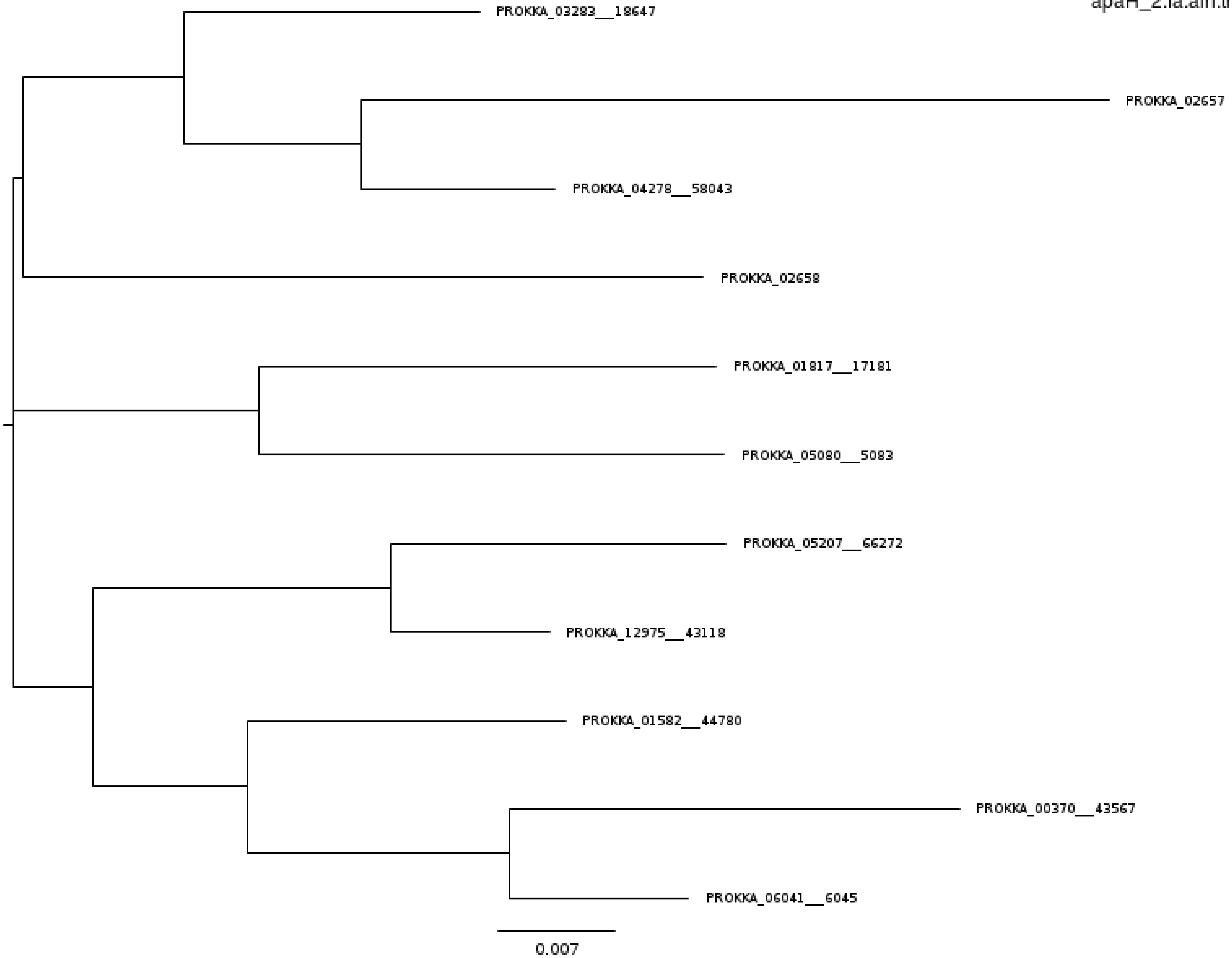

appA.fa.aln.tree.png

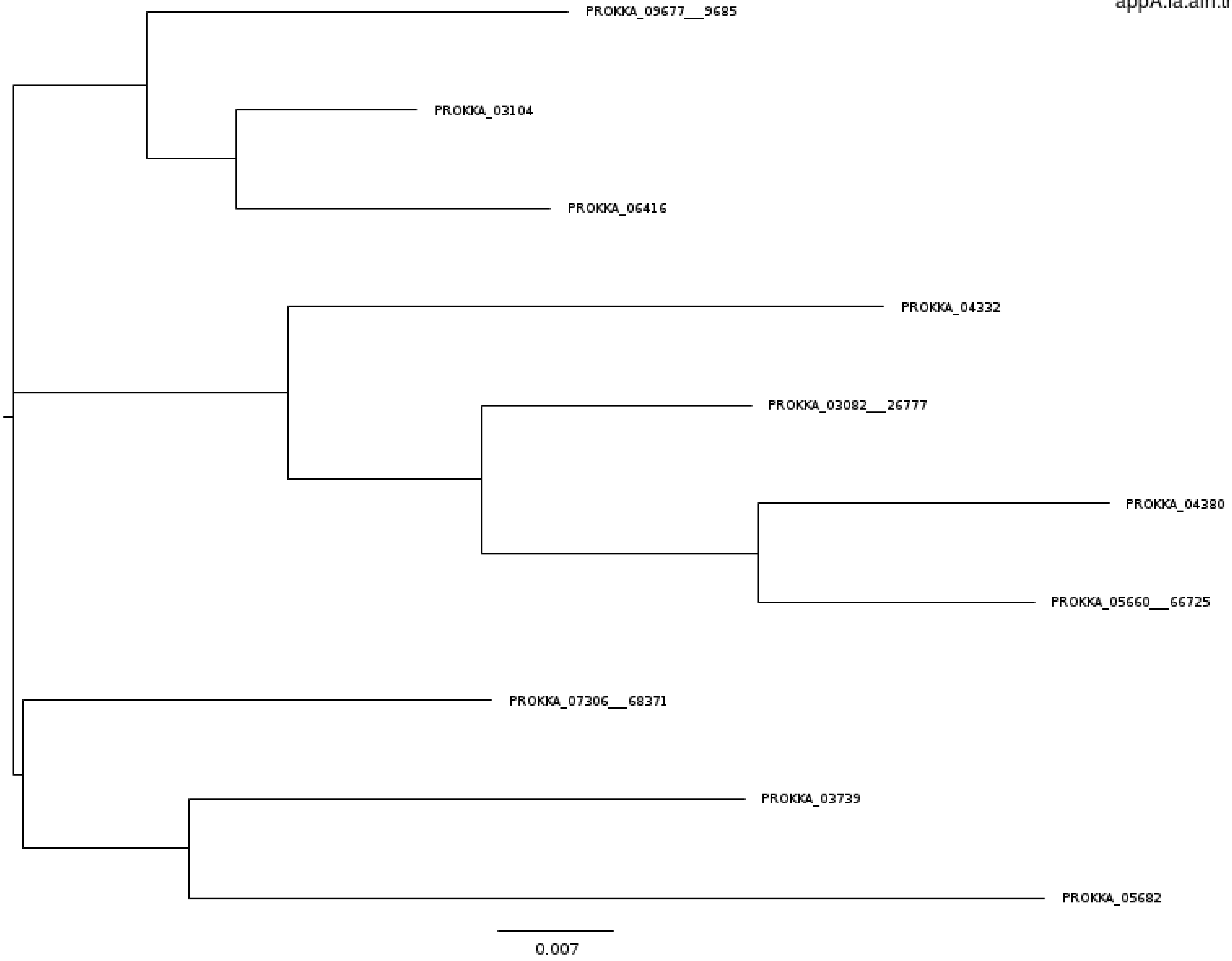

arcB_1.fa.aln.tree.png

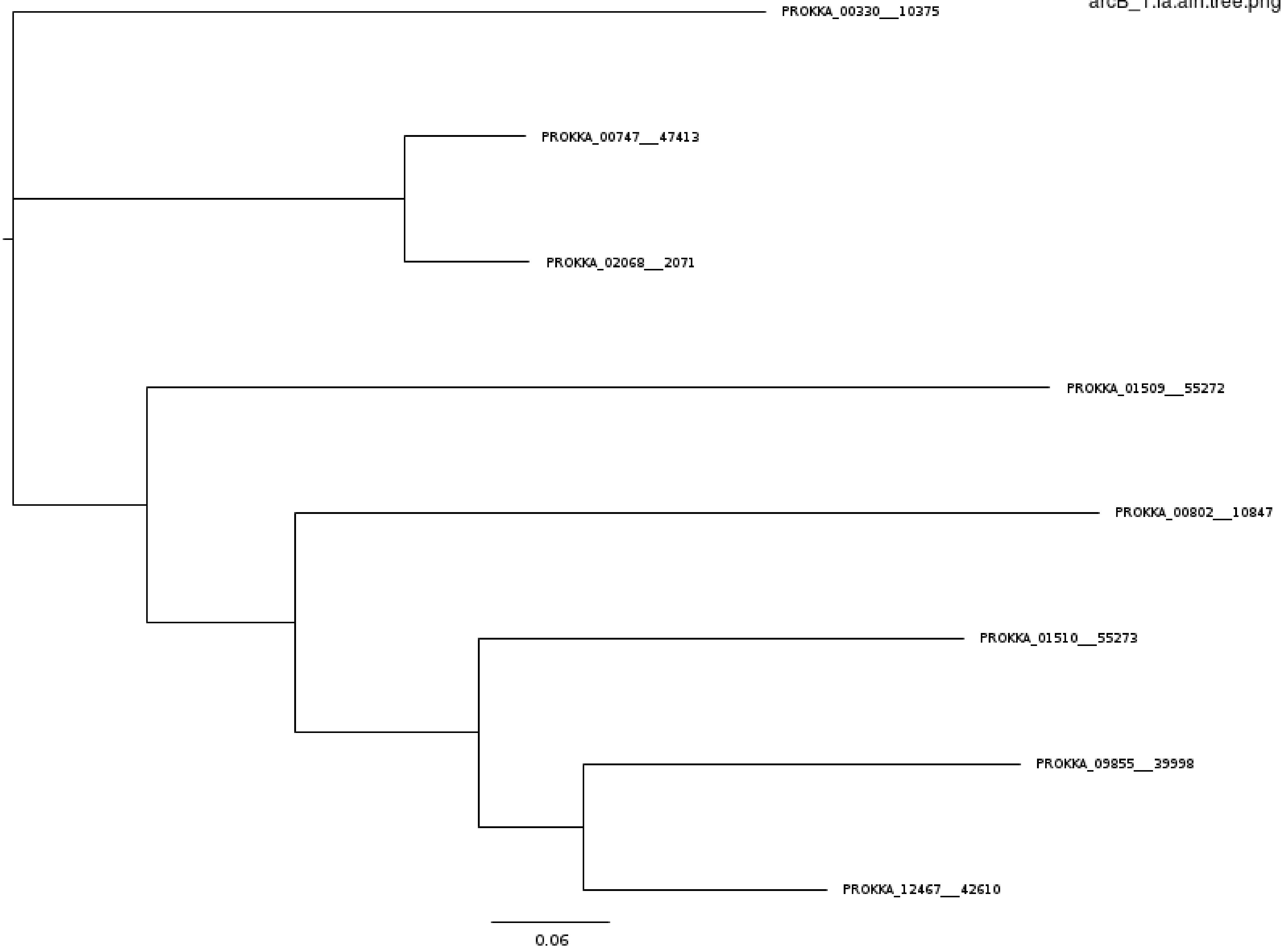

arcB_2.fa.aln.tree.png

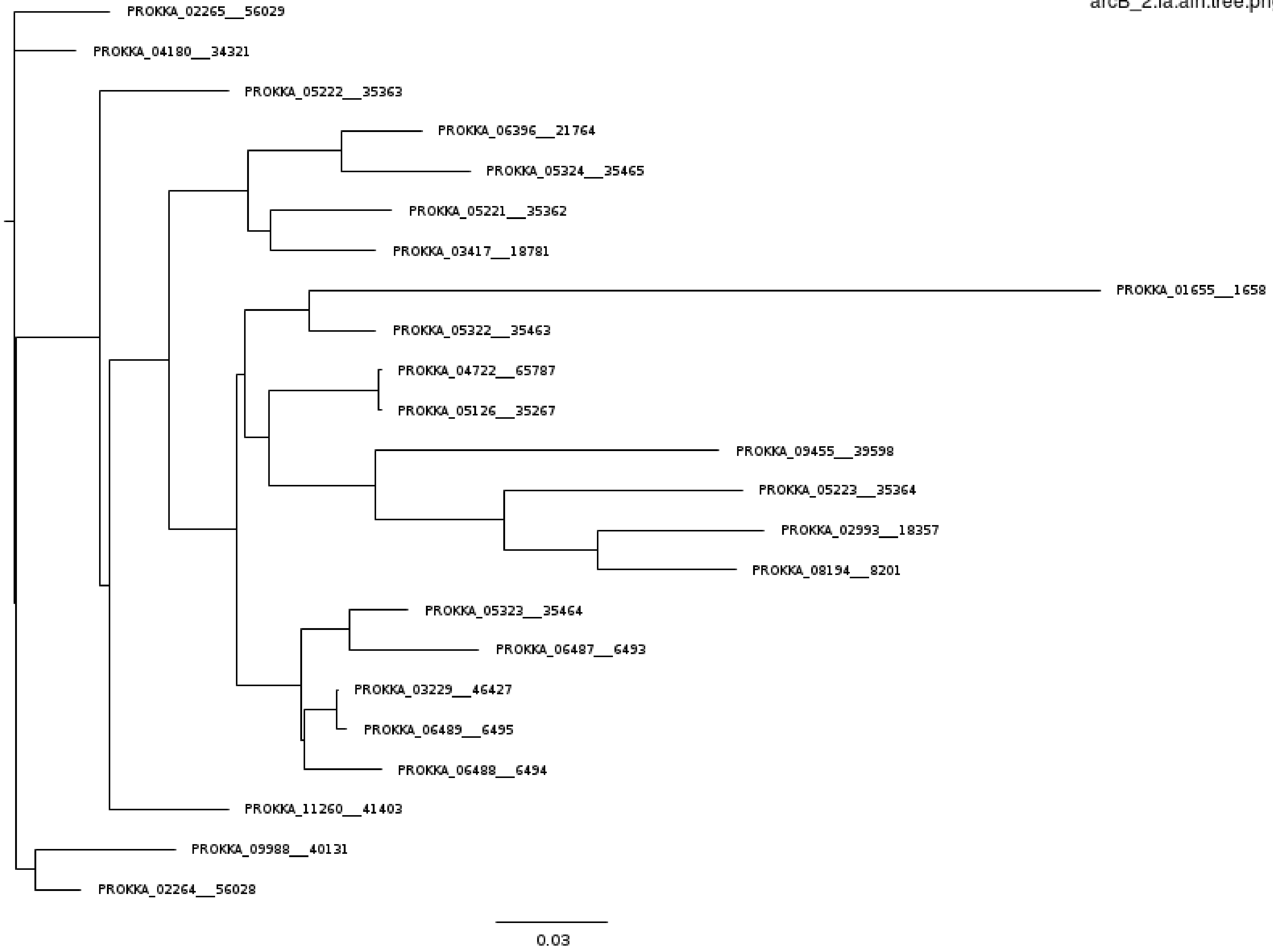

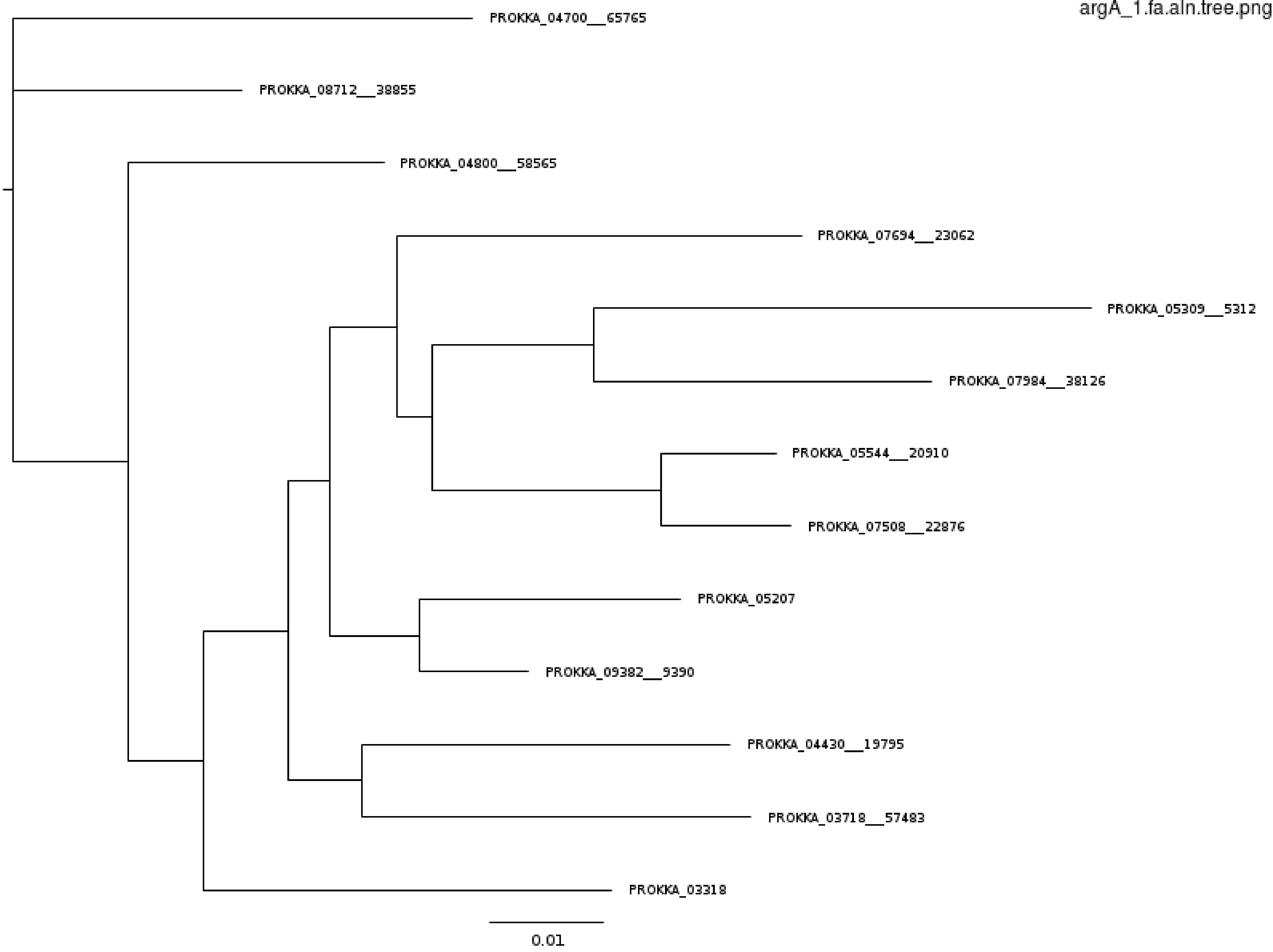
argA_1.fa.aln.tree.png
PROKKA_04700___65765
PROKKA_08712___38855
PROKKA_04800___58565
PROKKA_07694___23062
PROKKA_05309___5312
PROKKA_07984___38126
PROKKA_05544___20910
PROKKA_07508___22876
PROKKA_05207
PROKKA_09382___9390
PROKKA_04430___19795
PROKKA_03718___57483
PROKKA_03318
0.01

argB_2.fa.aln.tree.png

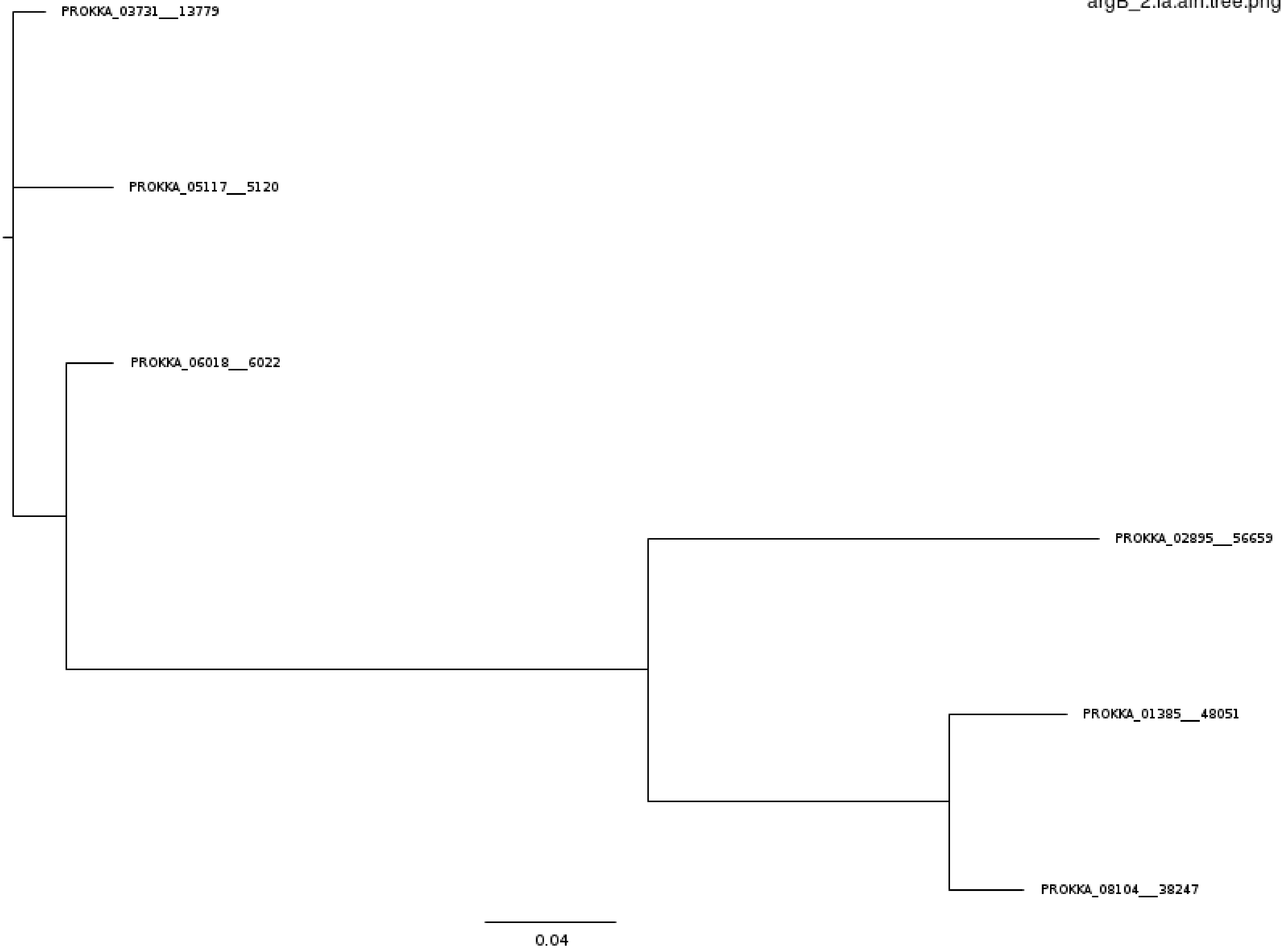

argF_1.fa.aln.tree.png

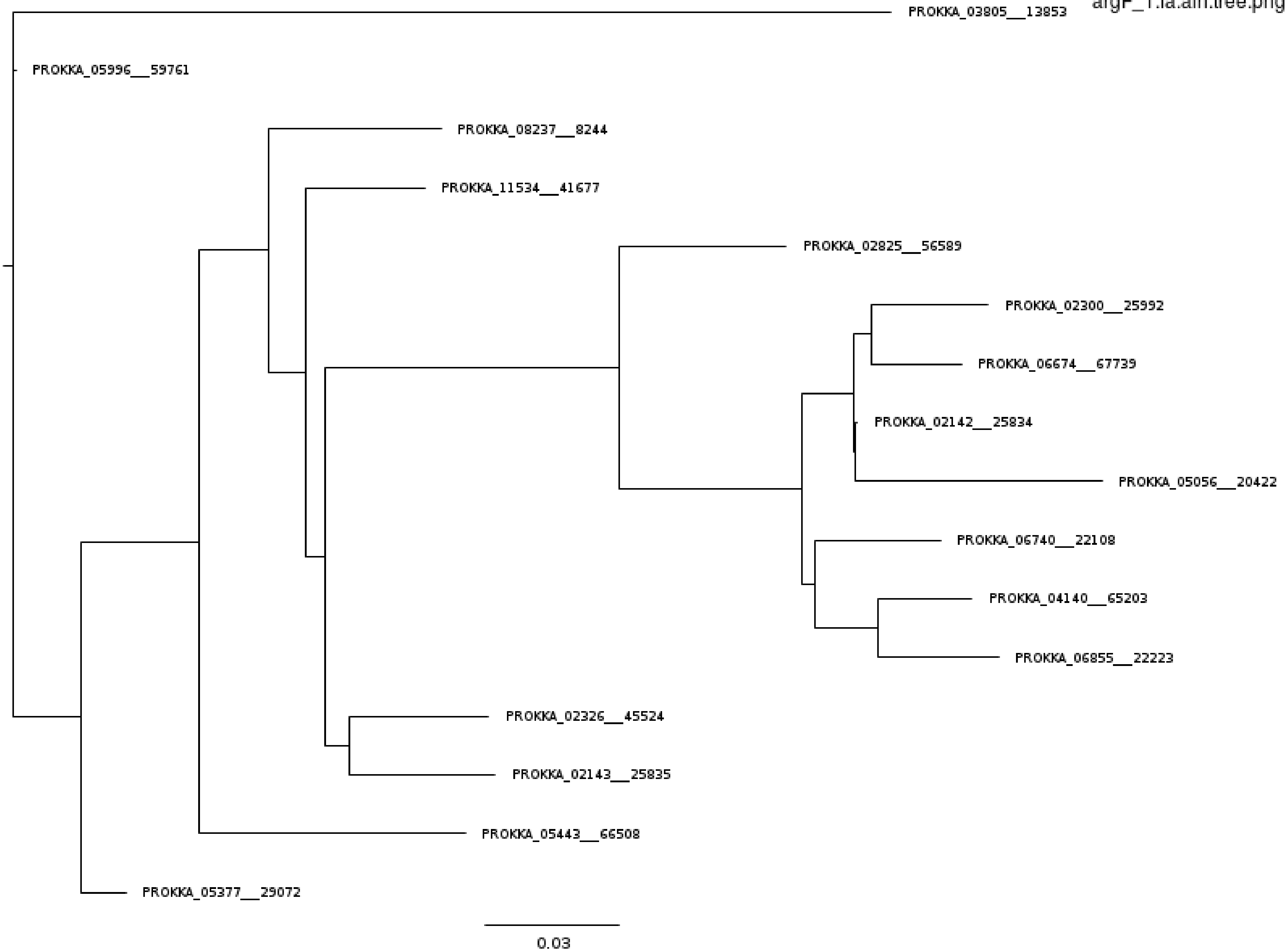

argF_3.fa.aln.tree.png

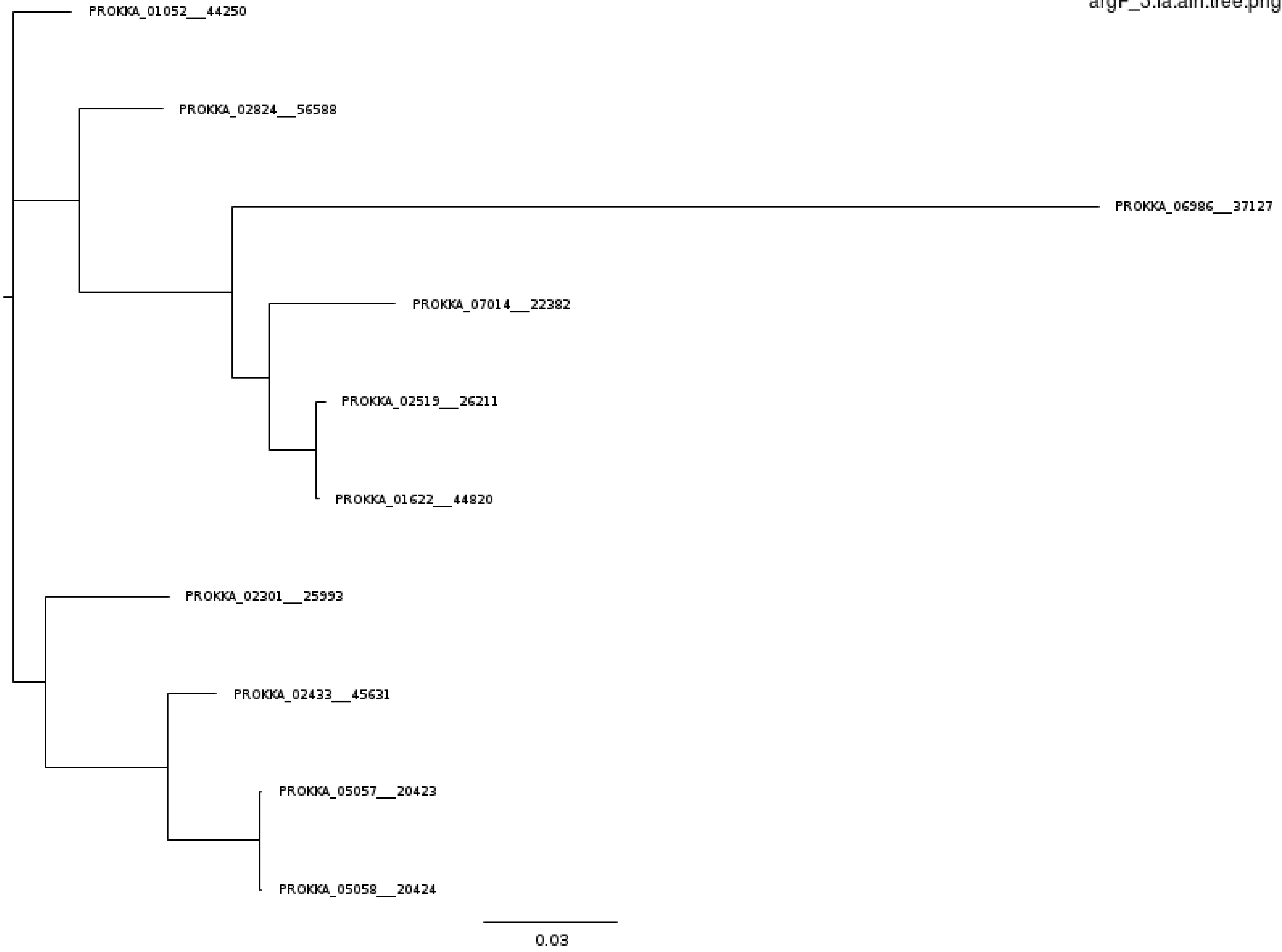

argG.fa.aln.tree.png

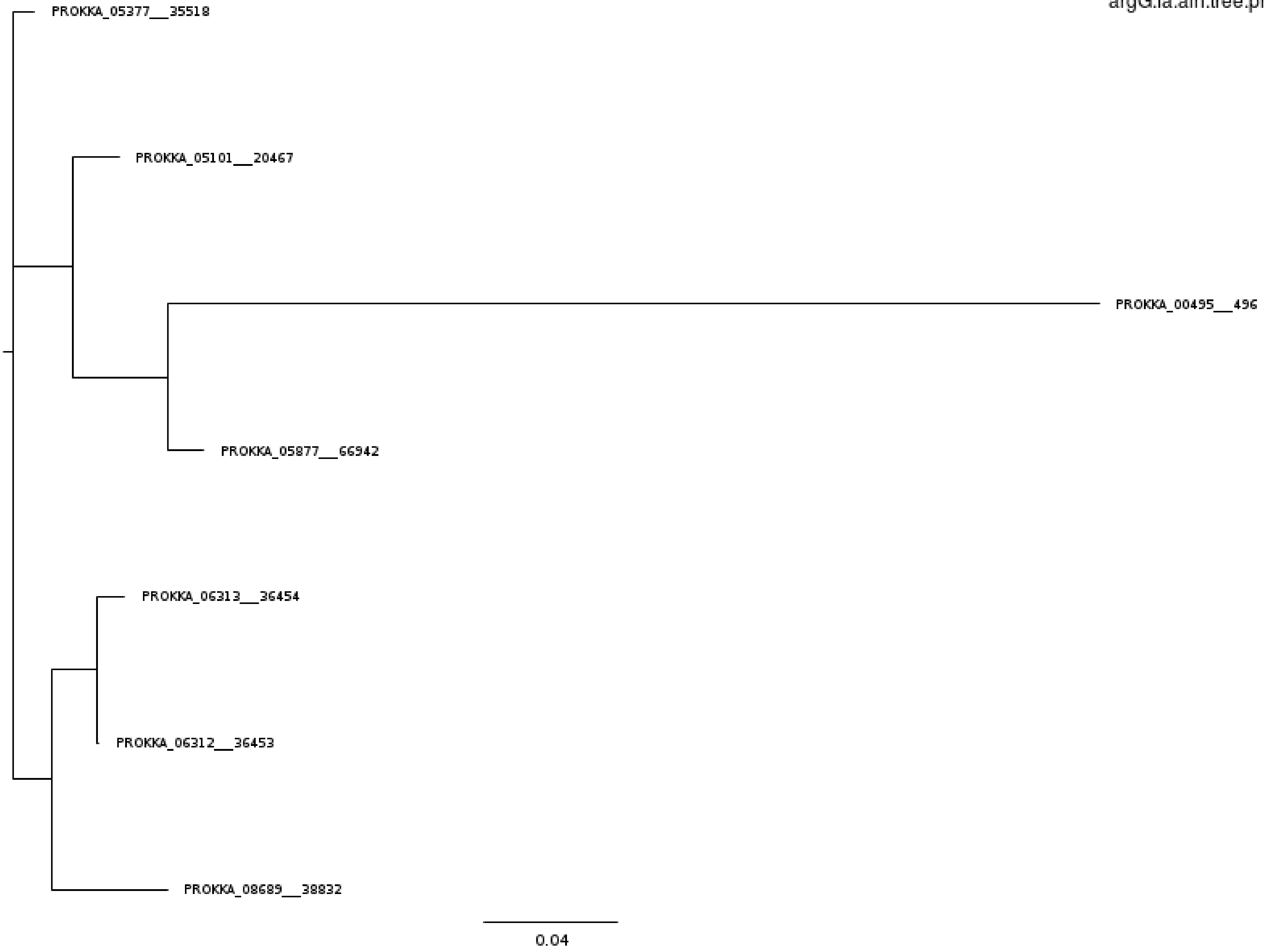

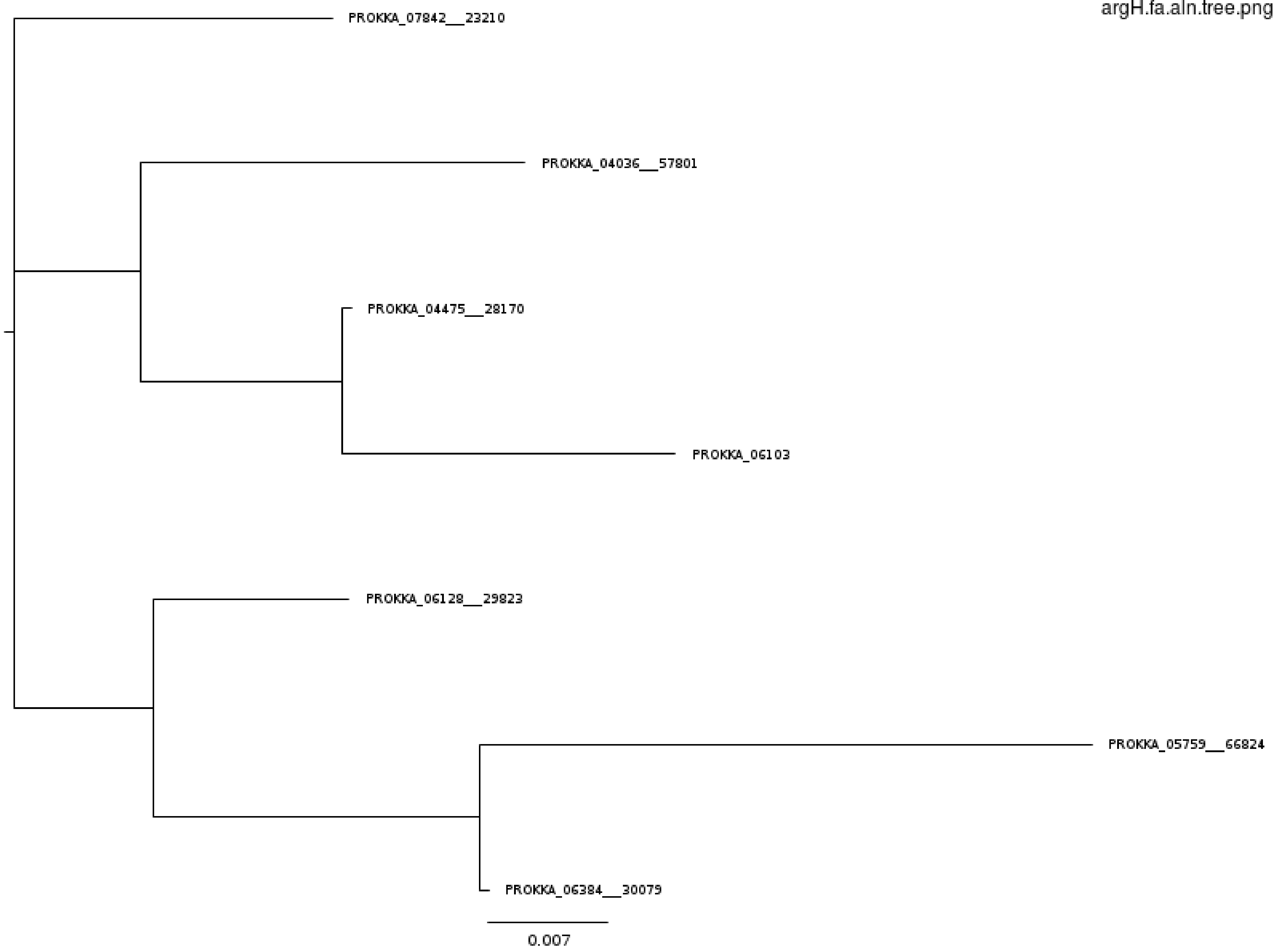
argH.fa.aln.tree.png
PROKKA_07842___23210
PROKKA_04036___57801
PROKKA_04475___28170
PROKKA_06103
PROKKA_06128___29823
PROKKA_05759___66824
PROKKA_06384___30079
0.007

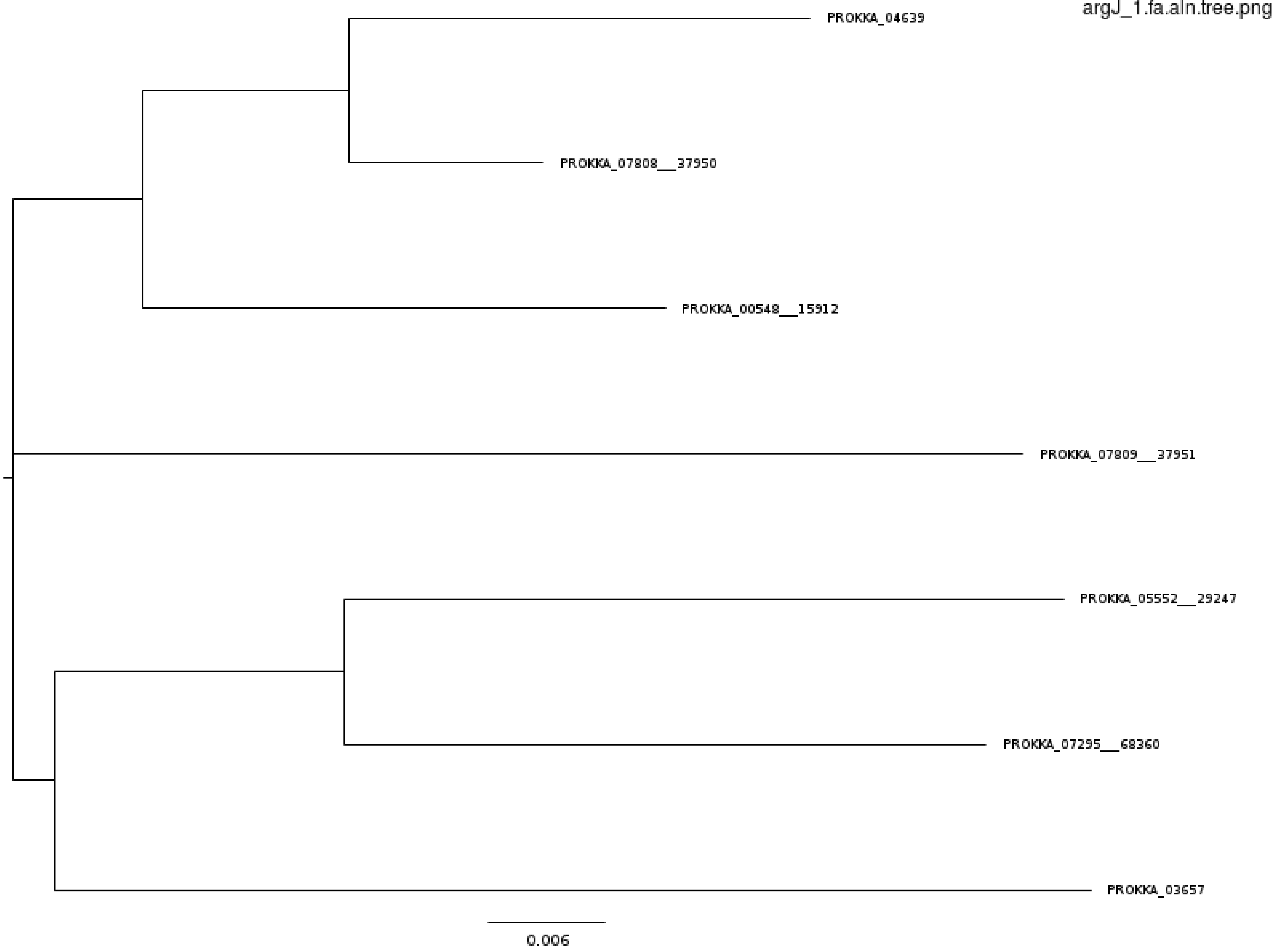
argJ_1.fa.aln.tree.png
PROKKA_04639
PROKKA_07808___37950
PROKKA_00548___15912
PROKKA_07809___37951
PROKKA_05552___29247
PROKKA_07295___68360
PROKKA_03657
0.006

argS_2.fa.aln.tree.png

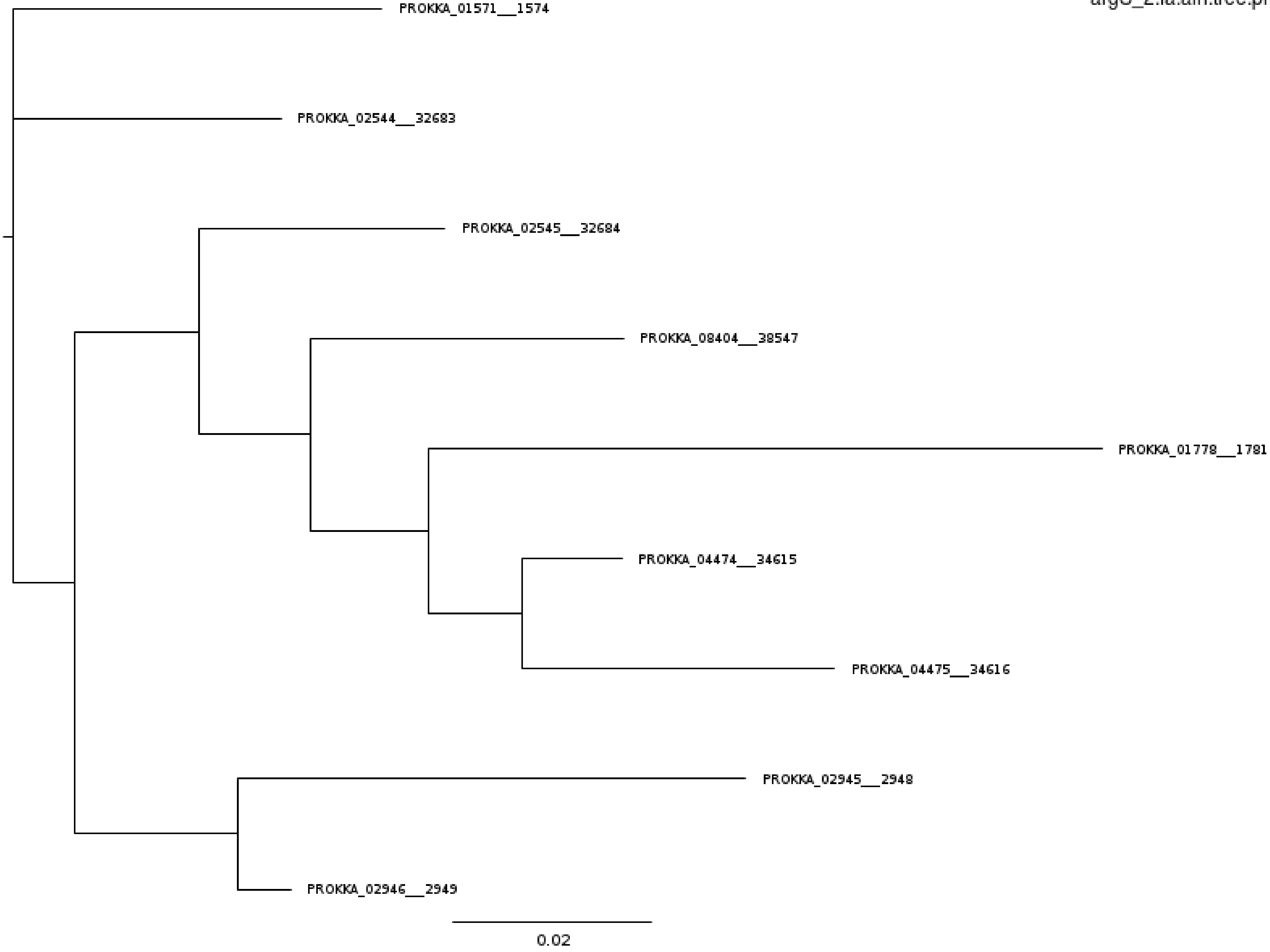

arnC_4.fa.aln.tree.png

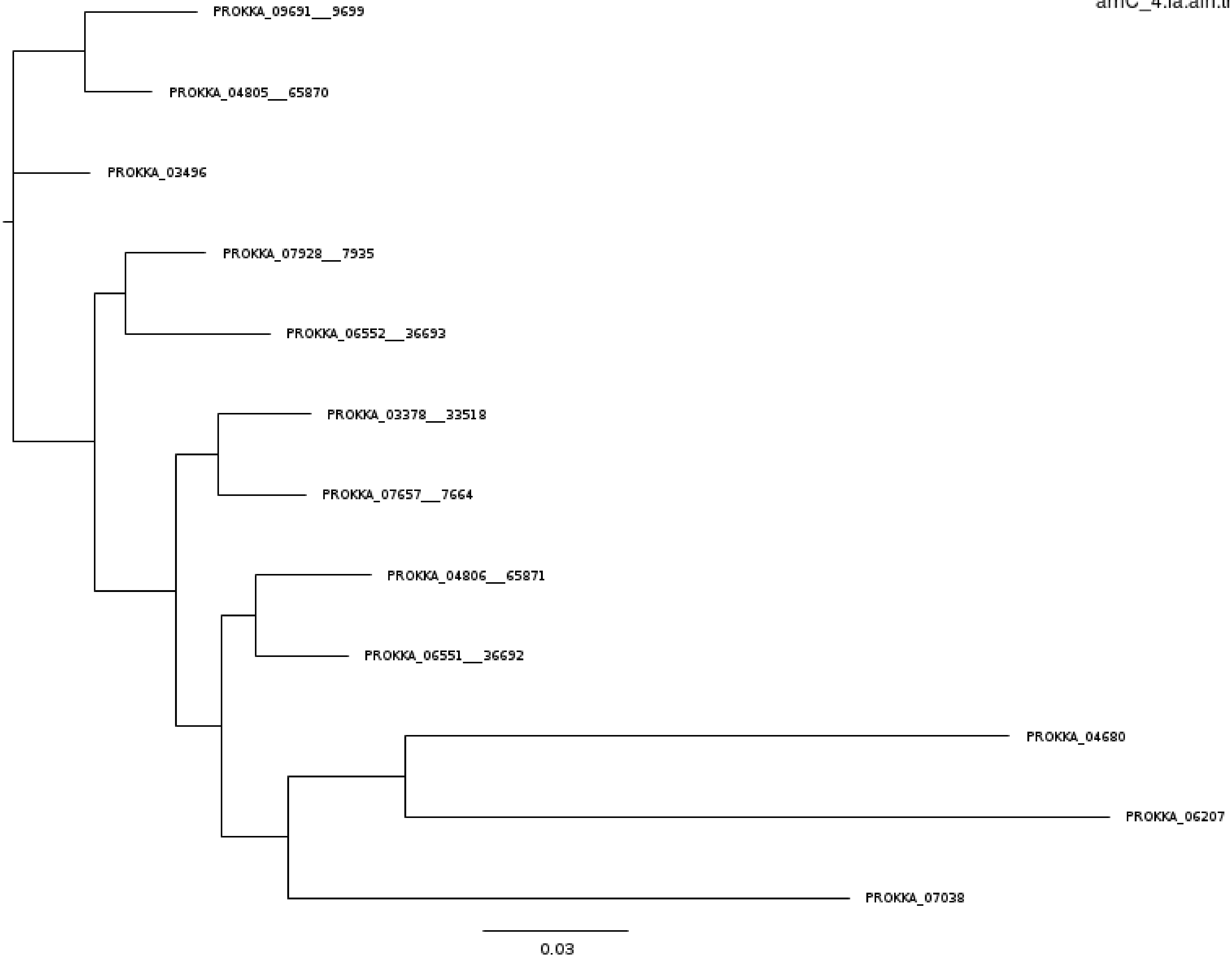

arnC_5.fa.aln.tree.png

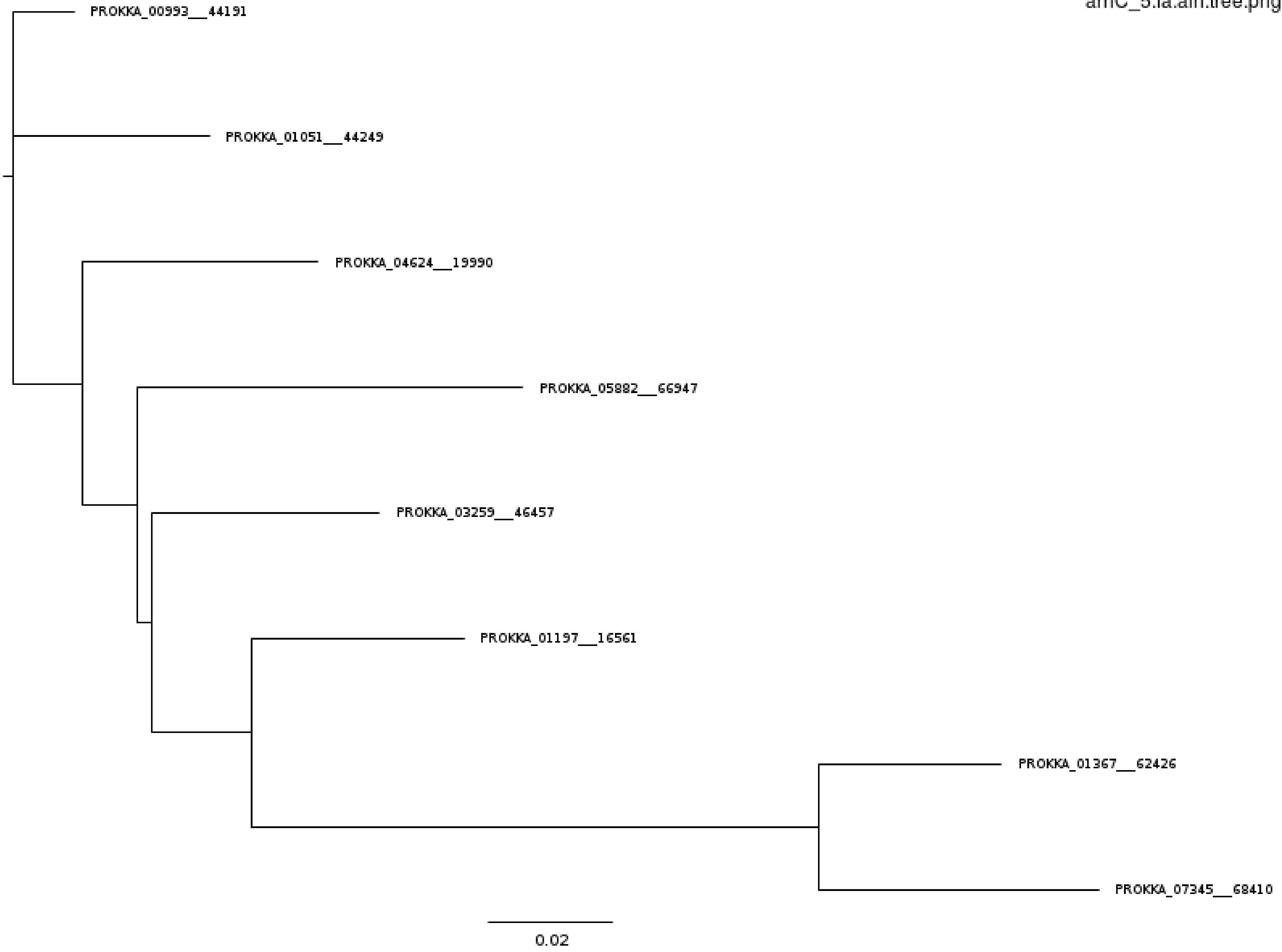

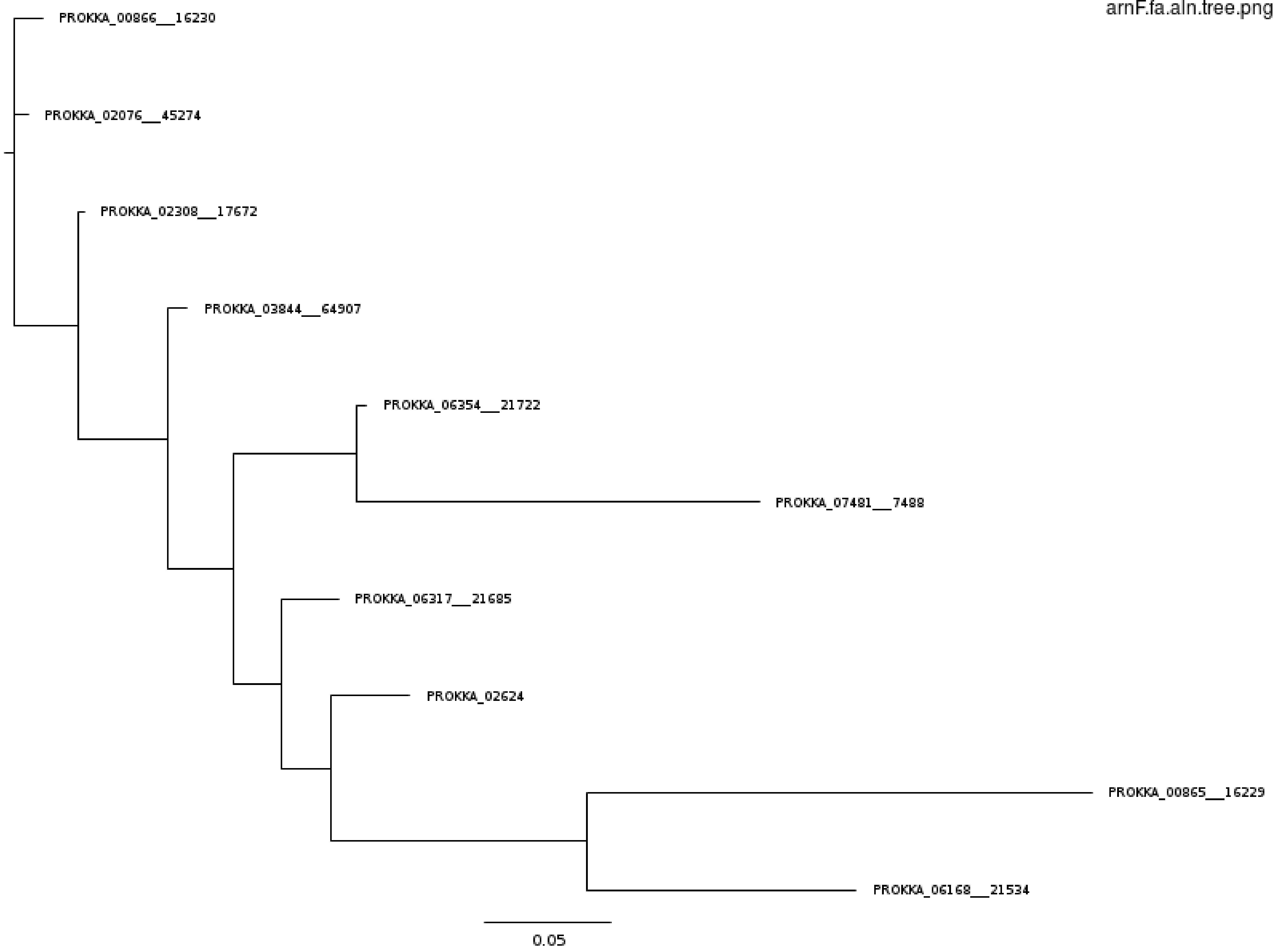
arnF.fa.aln.tree.png
PROKKA_00866___16230
PROKKA_02076___45274
PROKKA_02308___17672
PROKKA_03844___64907
PROKKA_06354___21722
PROKKA_07481___7488
PROKKA_06317___21685
PROKKA_02624
PROKKA_00865___16229
PROKKA_06168___21534
0.05

arnT_6.fa.aln.tree.png

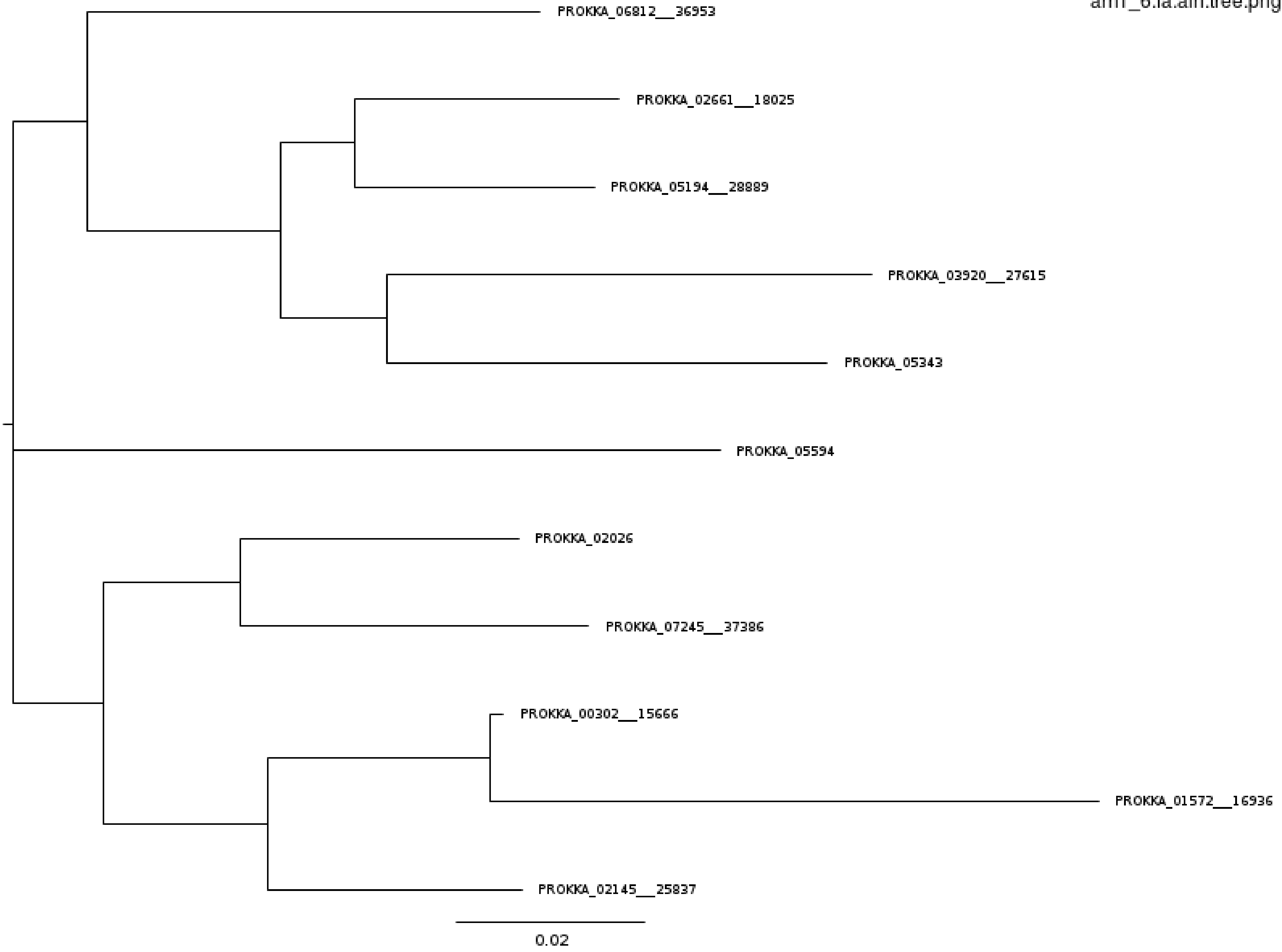

arnT.fa.aln.tree.png

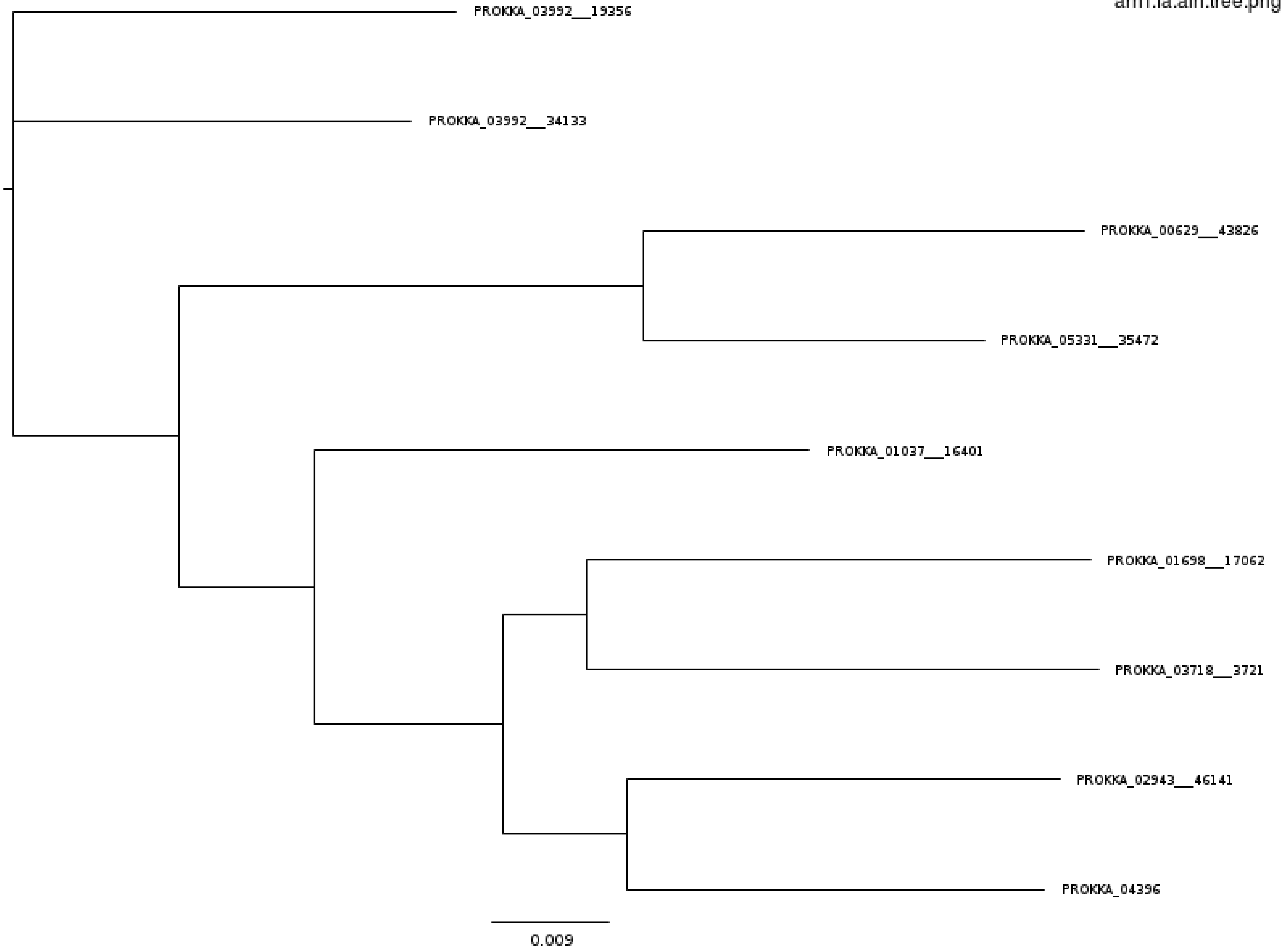

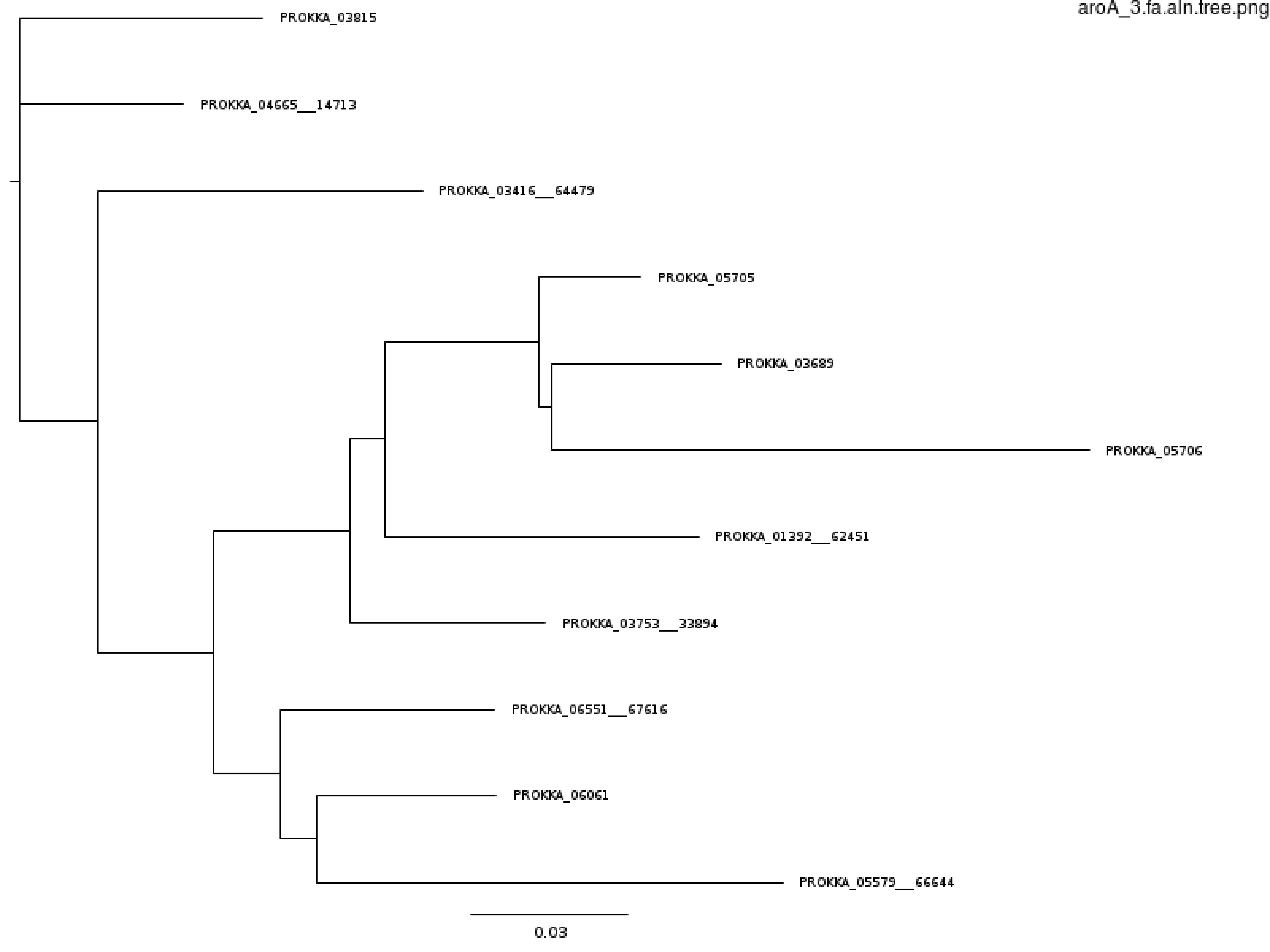

aroA_3.fa.aln.tree.png
PROKKA_03815
PROKKA_04665___14713
PROKKA_03416___64479
PROKKA_05705
PROKKA_03689
PROKKA_05706
PROKKA_01392___62451
PROKKA_03753___33894
PROKKA_06551___67616
PROKKA_06061
PROKKA_05579___66644
0.03

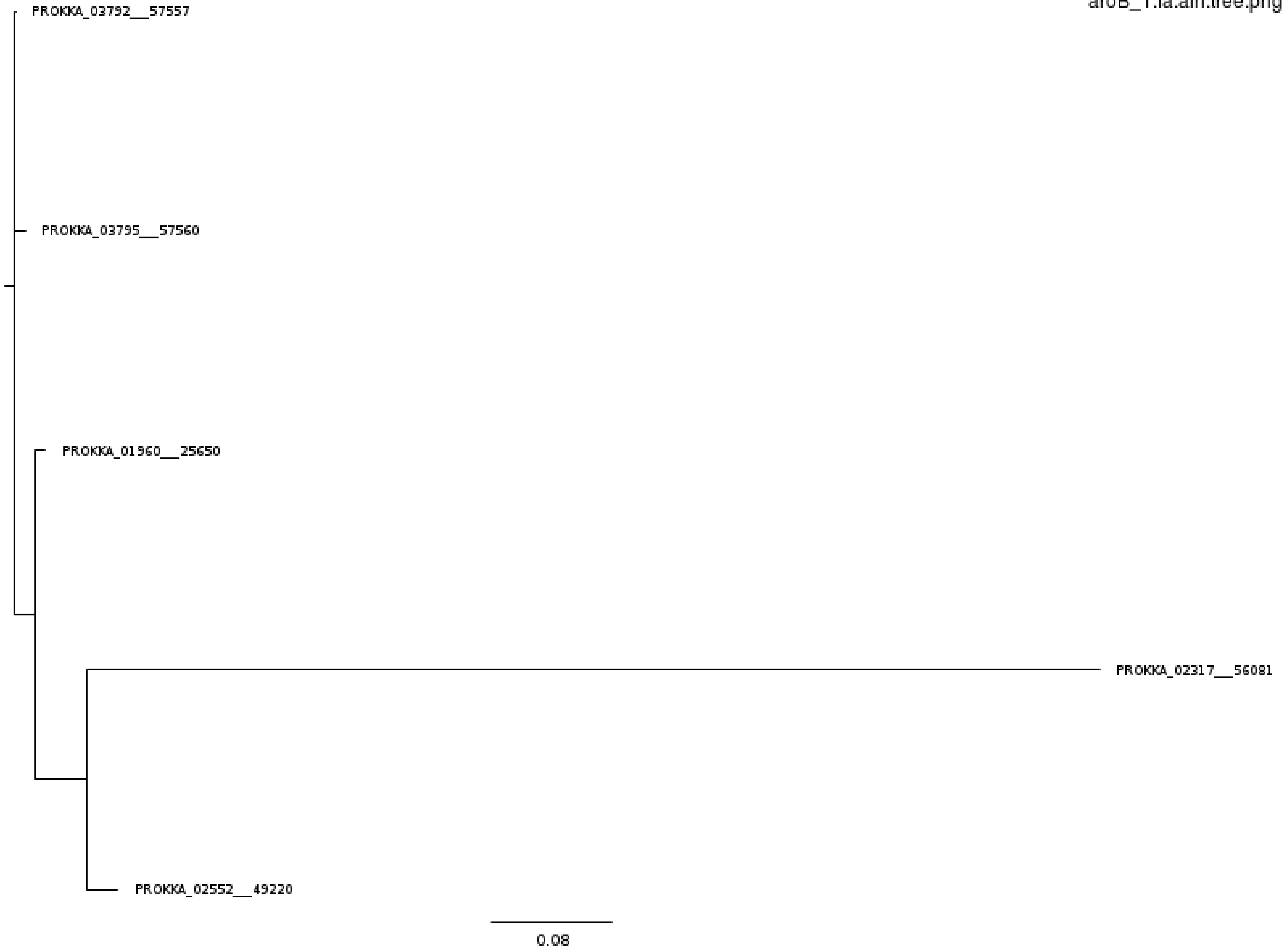
PROKKA_03792___57557
PROKKA_03795___57560
PROKKA_01960___25650
PROKKA_02317___56081
PROKKA_02552___49220
0.08

aroB_2.fa.aln.tree.png

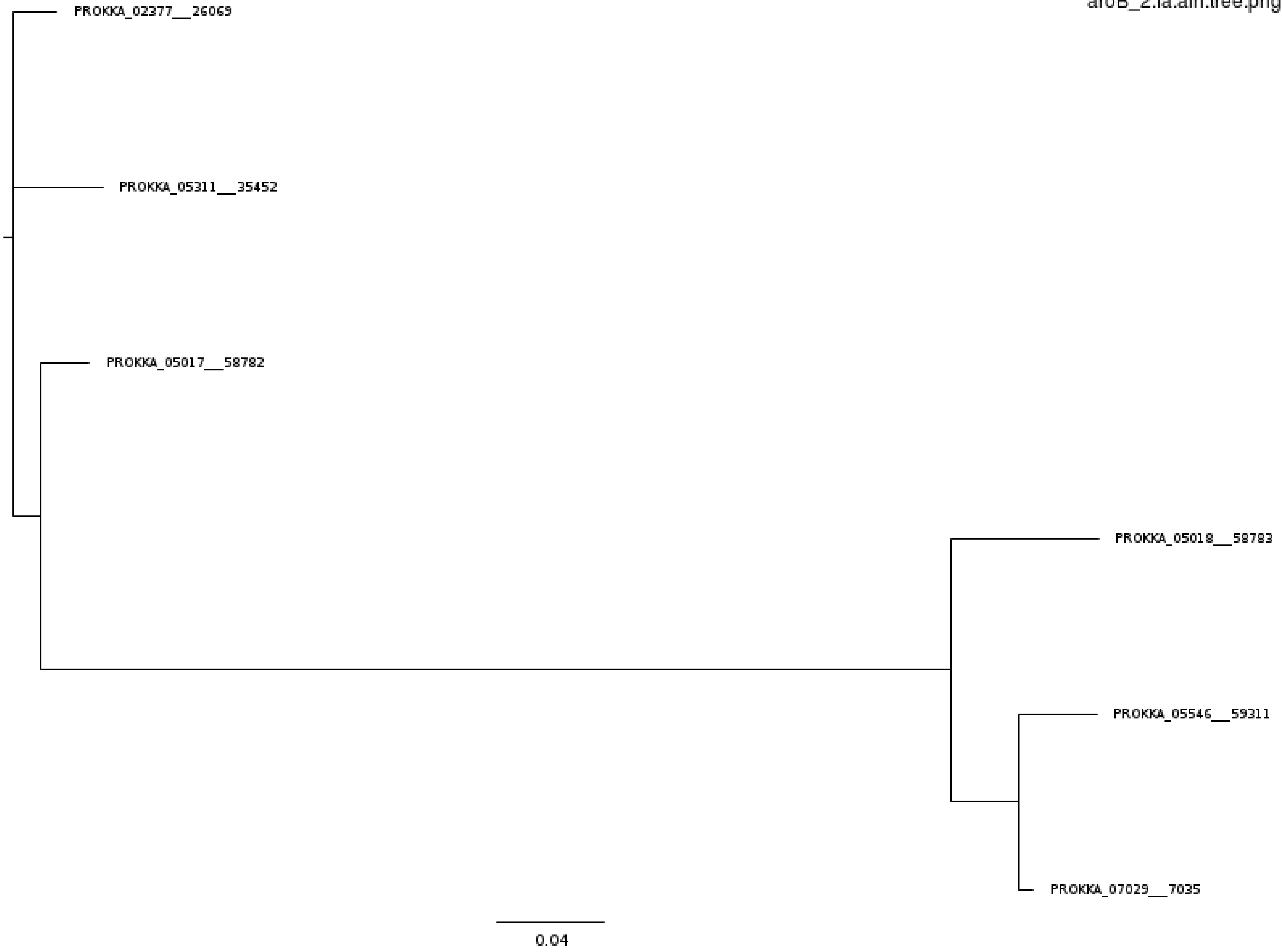

aroG_1.fa.aln.tree.png

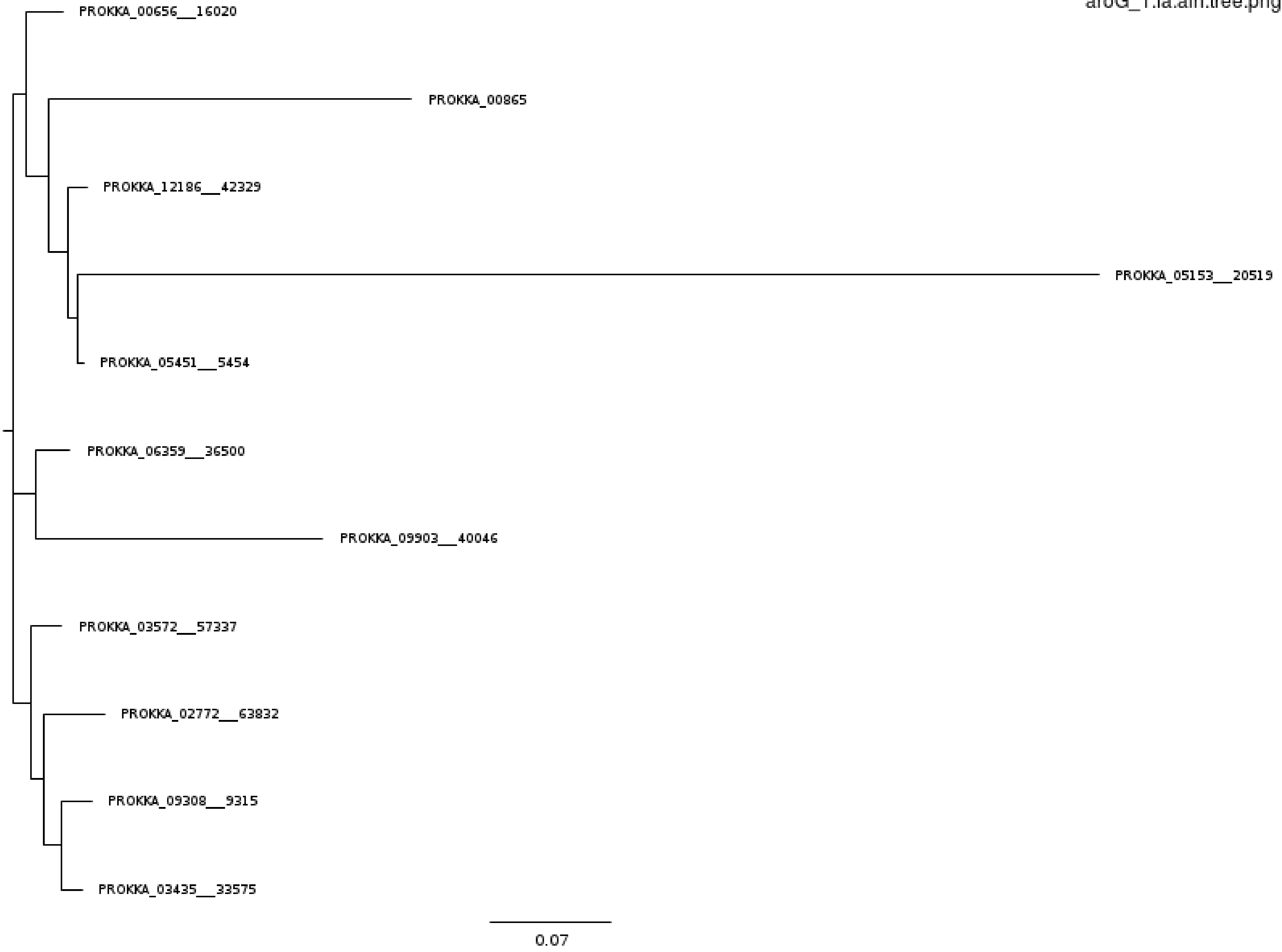

aroQ_2.fa.aln.tree.png

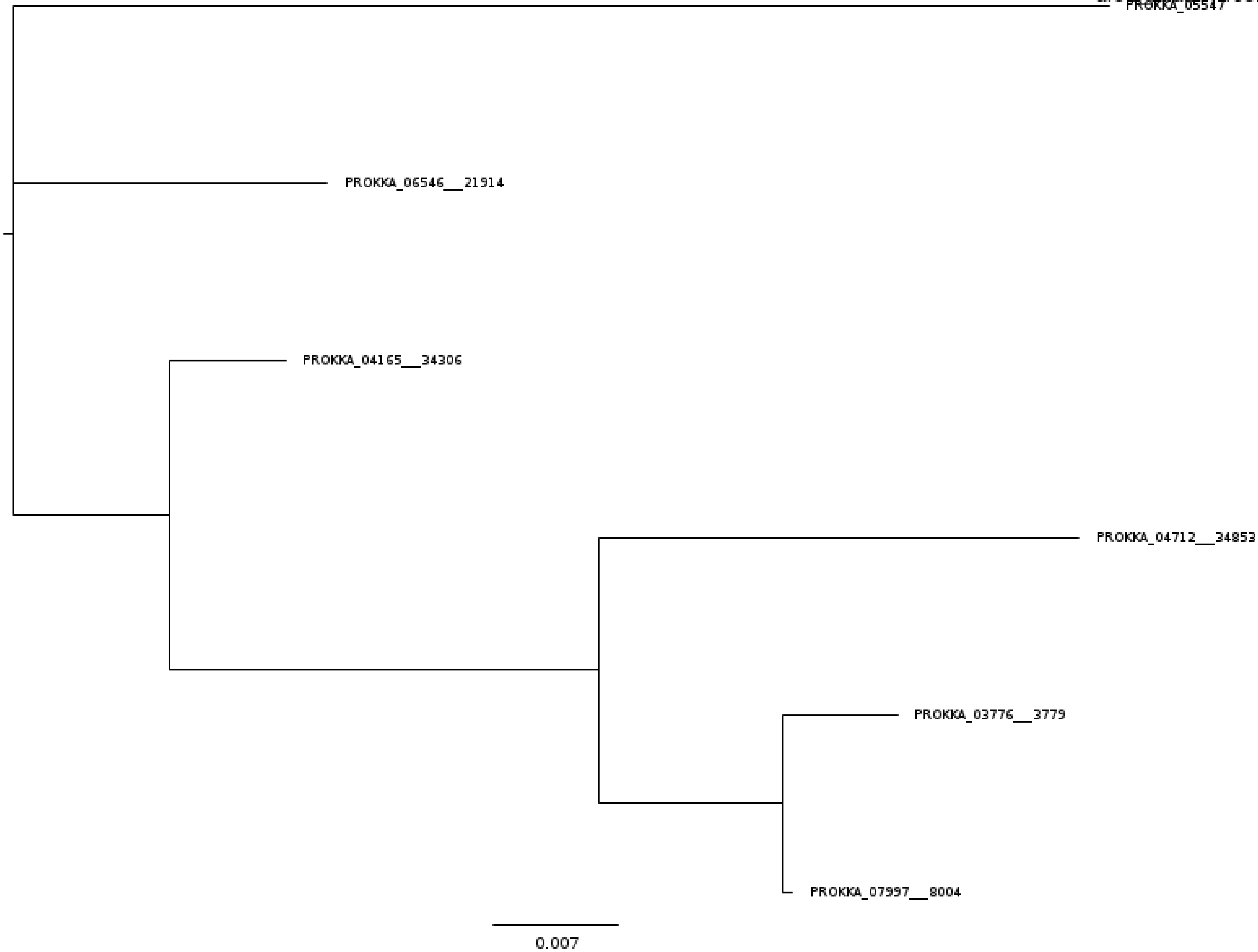

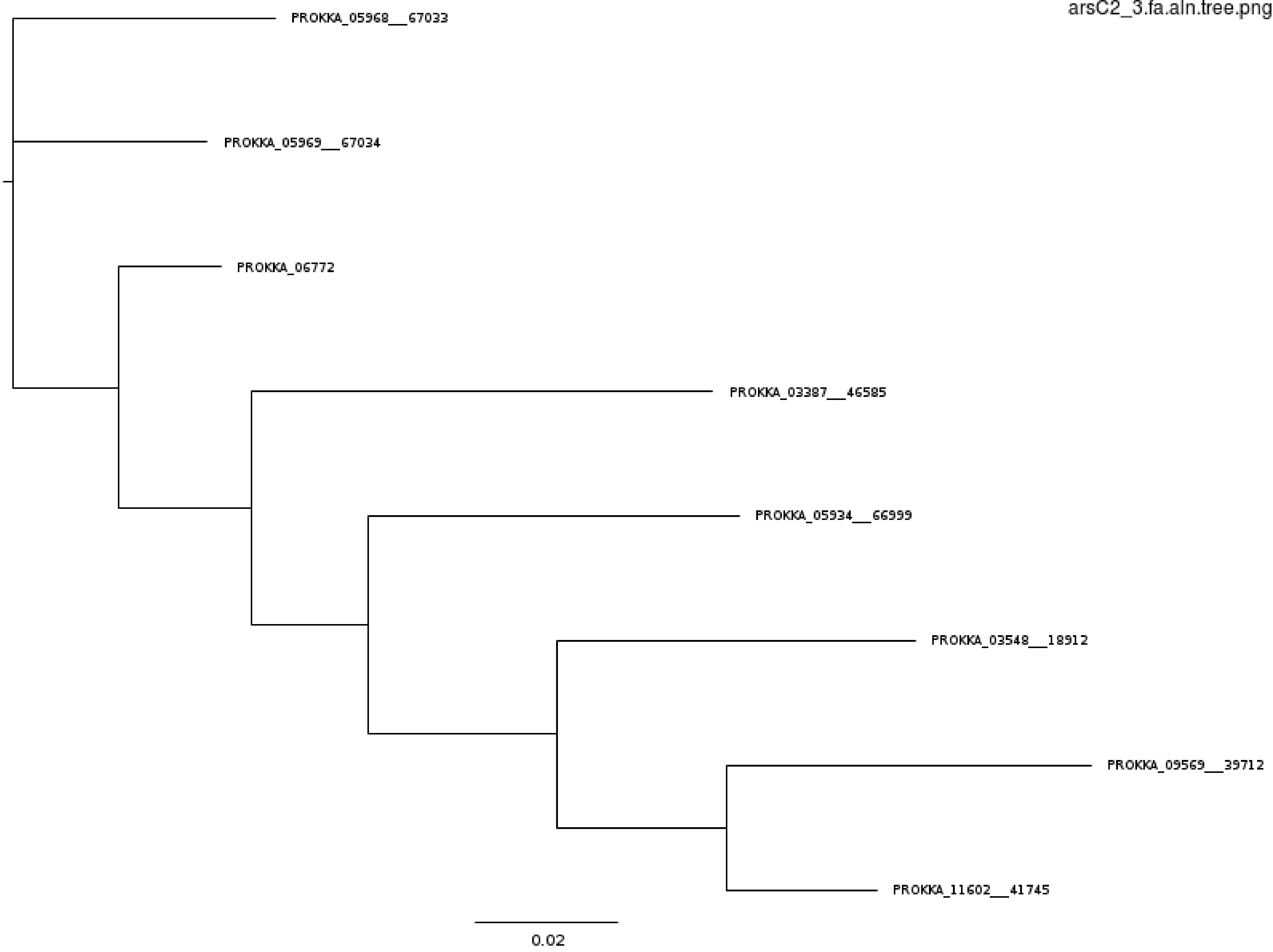
arsC2_3.fa.aln.tree.png
PROKKA_05968___67033
PROKKA_05969___67034
PROKKA_06772
PROKKA_03387___46585
PROKKA_05934___66999
PROKKA_03548___18912
PROKKA_09569___39712
PROKKA_11602___41745
0.02

asd_2.fa.aln.tree.png

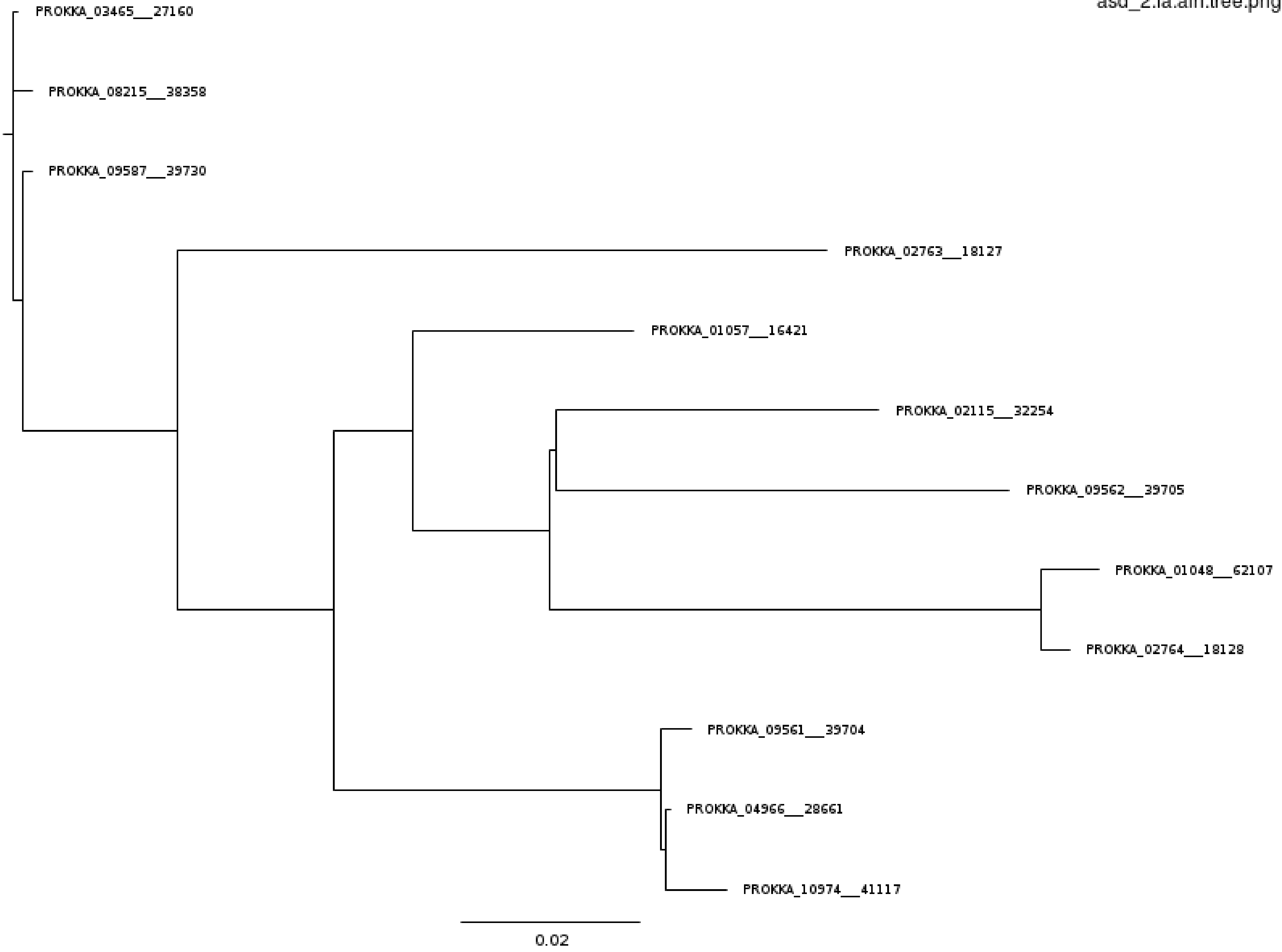

asd_3.fa.aln.tree.png

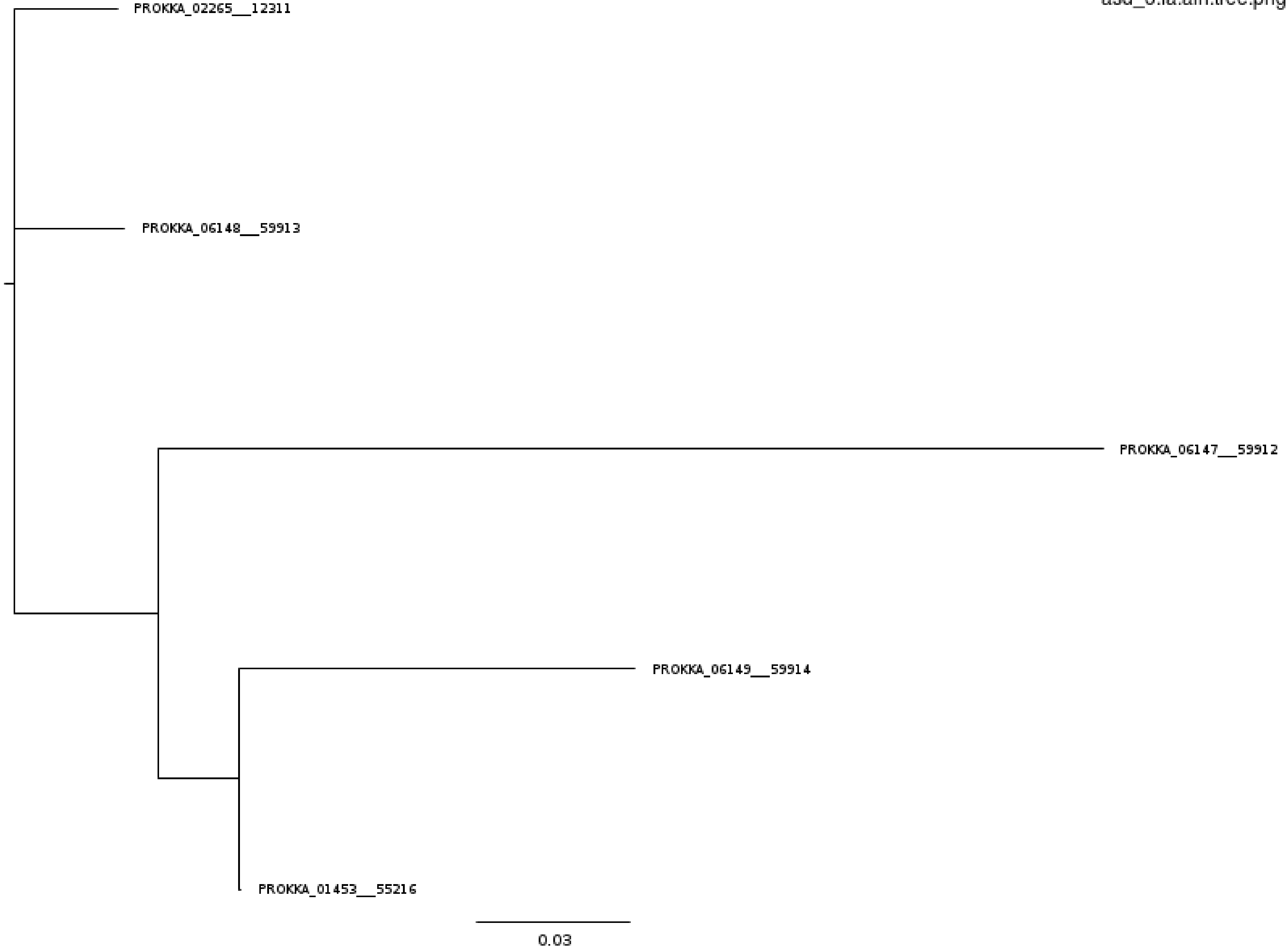

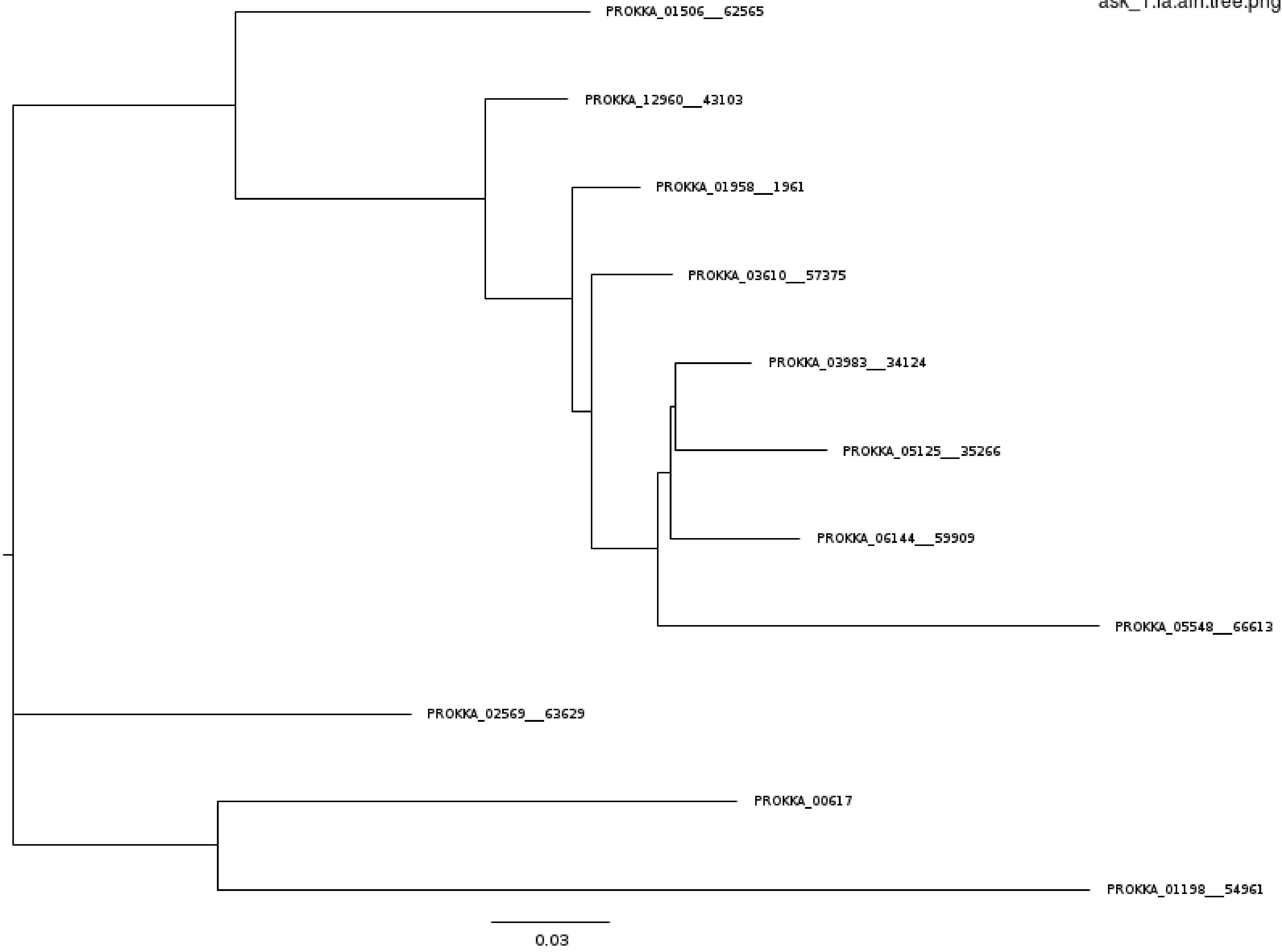
PROKKA_01506___62565
PROKKA_12960___43103
PROKKA_01958___1961
PROKKA_03610___57375
PROKKA_03983___34124
PROKKA_05125___35266
PROKKA_06144___59909
PROKKA_05548___66613
PROKKA_02569___63629
PROKKA_00617
PROKKA_01198___54961
0.03

asnB_2.fa.aln.tree.png

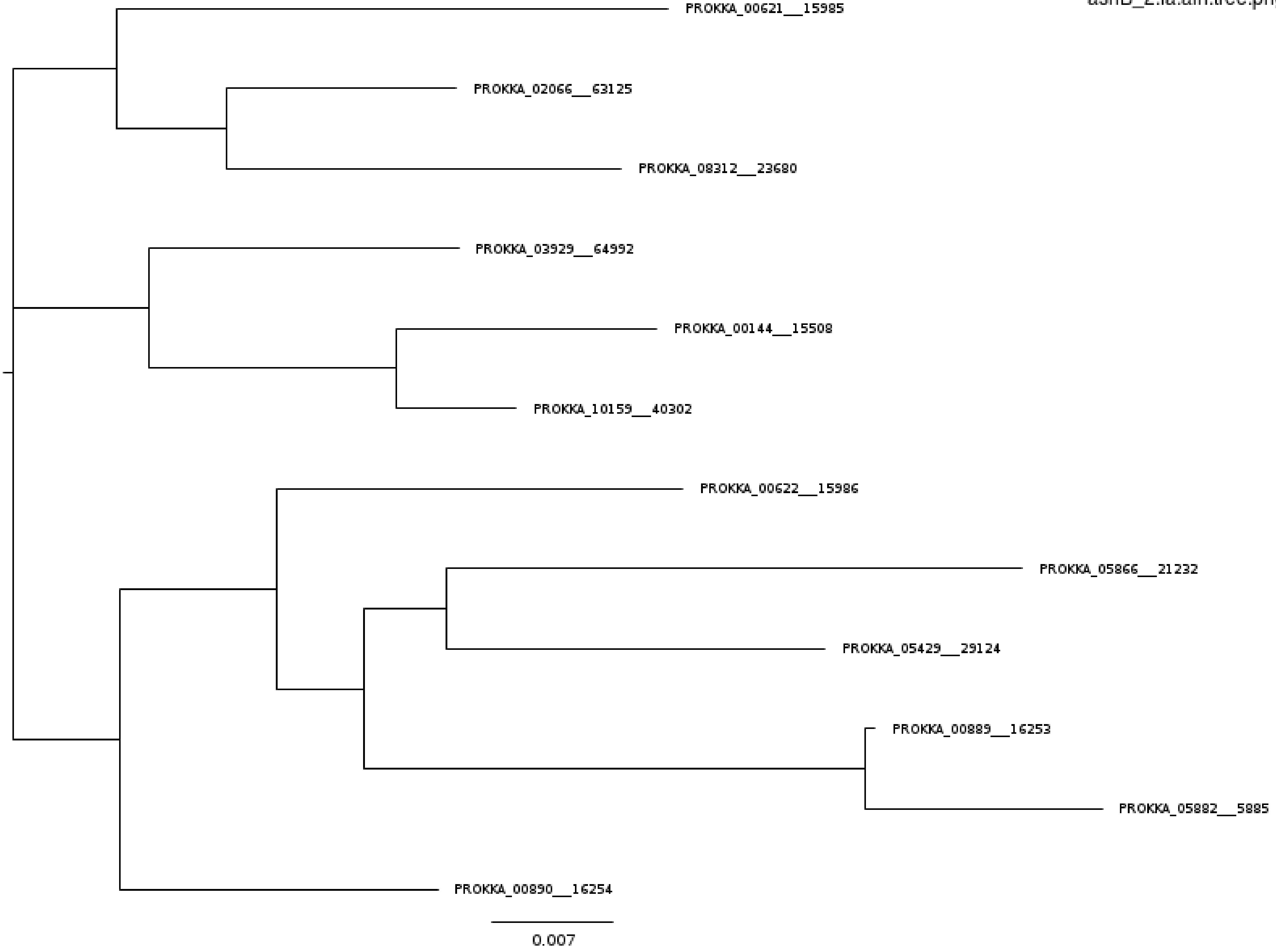

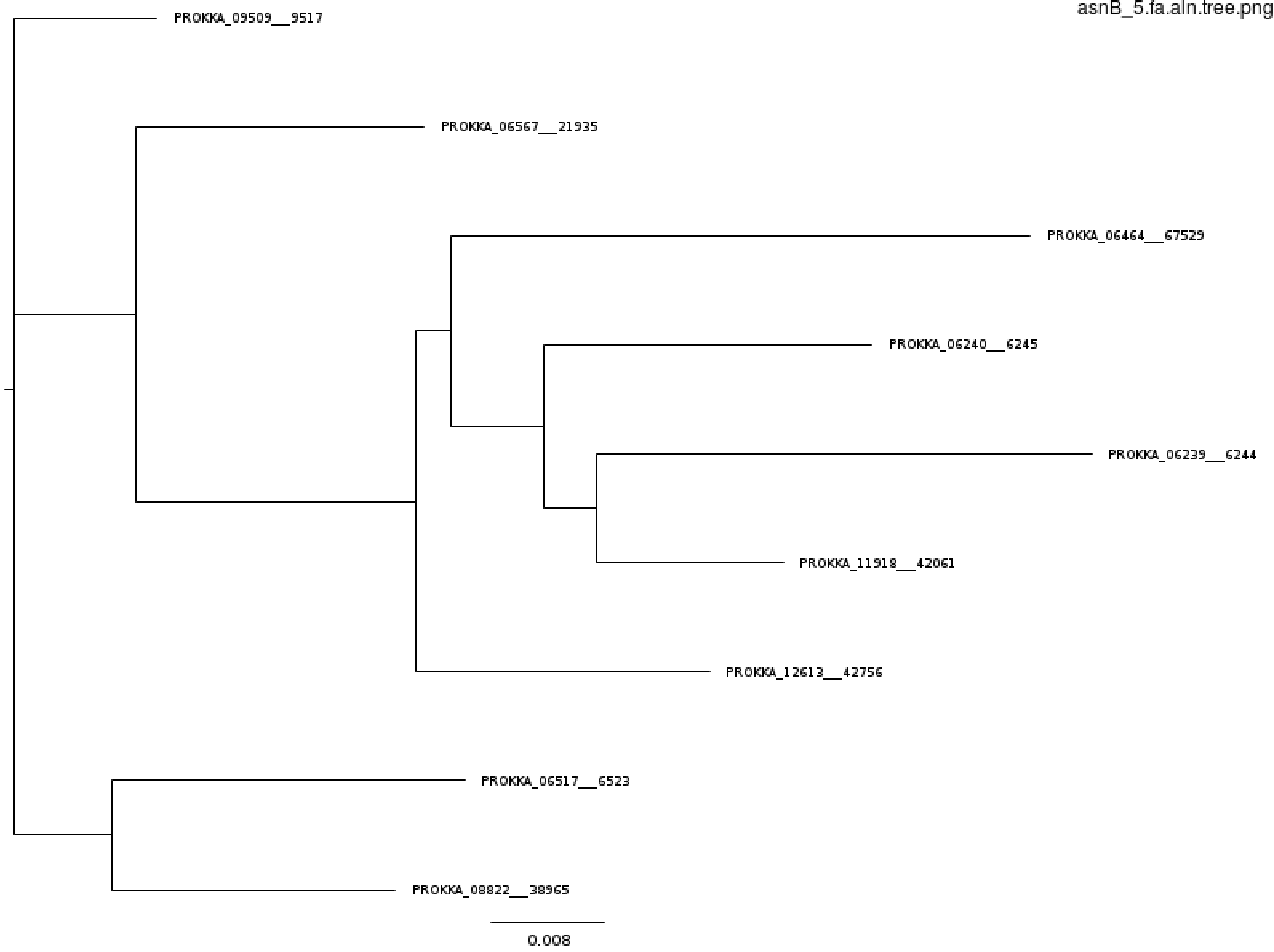
asnB_5.fa.aln.tree.png
PROKKA_09509___9517
PROKKA_06567___21935
PROKKA_06464___67529
PROKKA_06240___6245
PROKKA_06239___6244
PROKKA_11918___42061
PROKKA_12613___42756
PROKKA_06517___6523
PROKKA_08822___38965
0.008

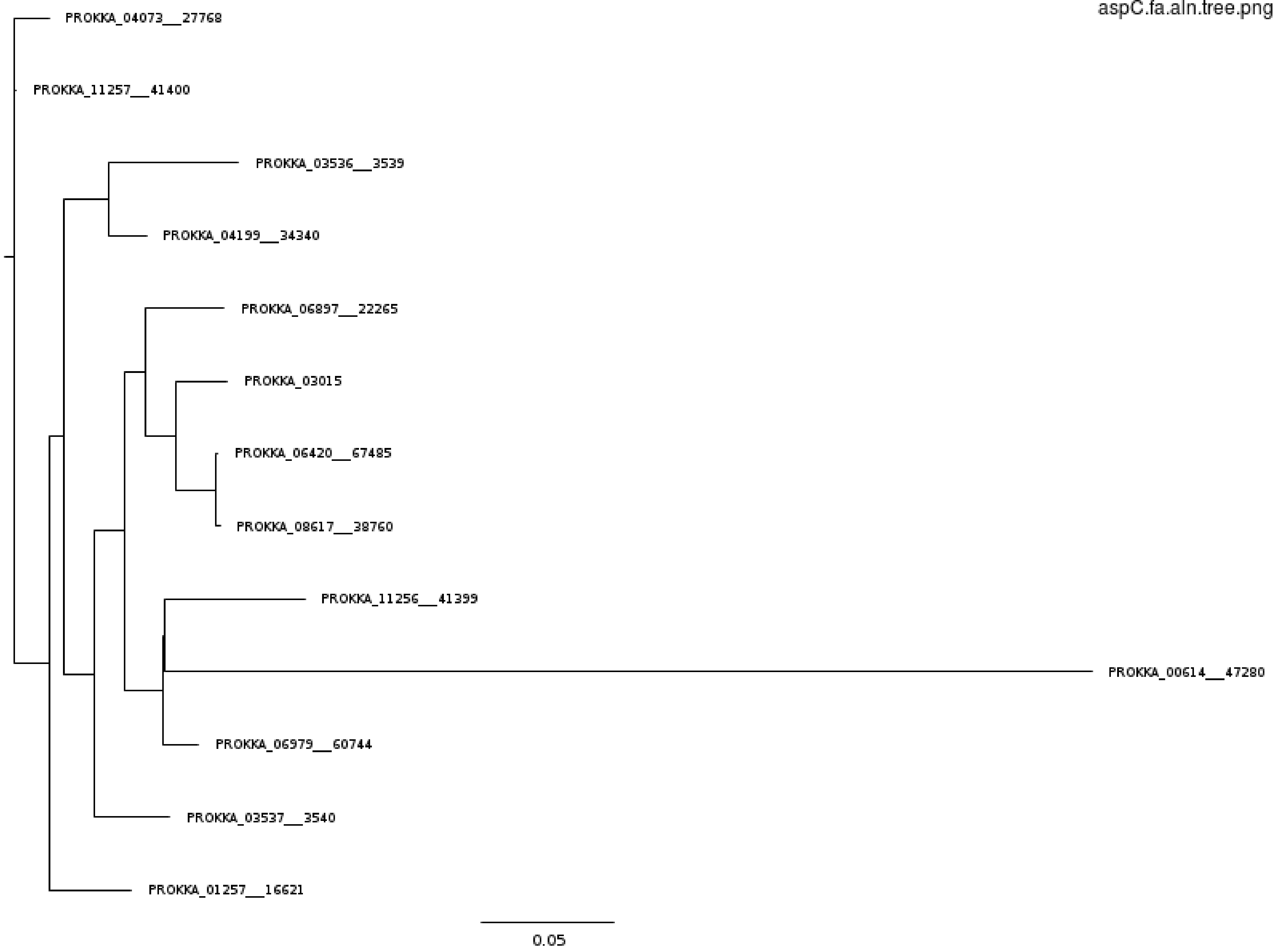
aspC.fa.aln.tree.png
PROKKA_04073___27768
PROKKA_11257___41400
PROKKA_03536___3539
PROKKA_04199___34340
PROKKA_06897___22265
PROKKA_03015
PROKKA_06420___67485
PROKKA_08617___38760
PROKKA_11256___41399
PROKKA_00614___47280
PROKKA_06979___60744
PROKKA_03537___3540
PROKKA_01257___16621
0.05

aspS_2.fa.aln.tree.png

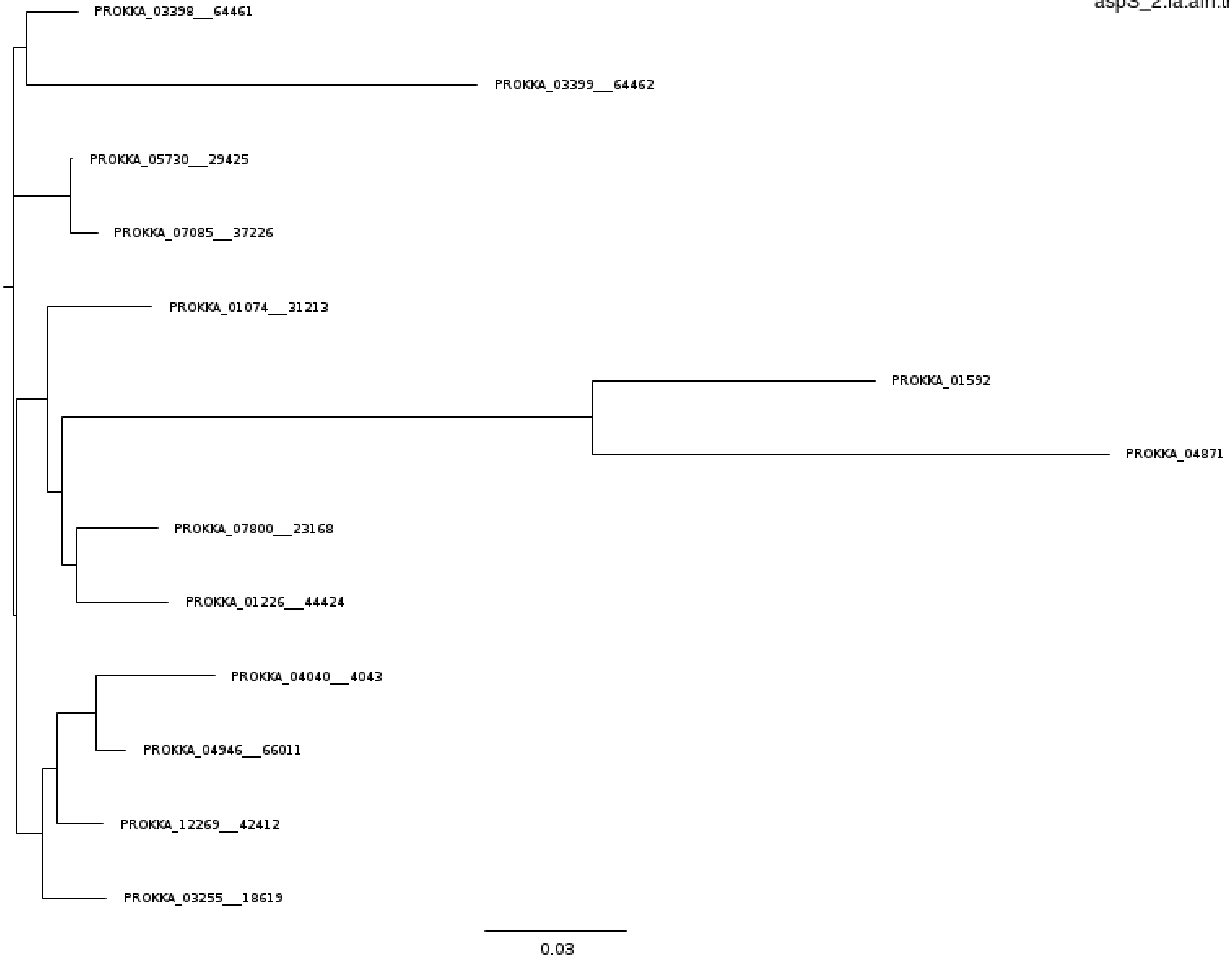

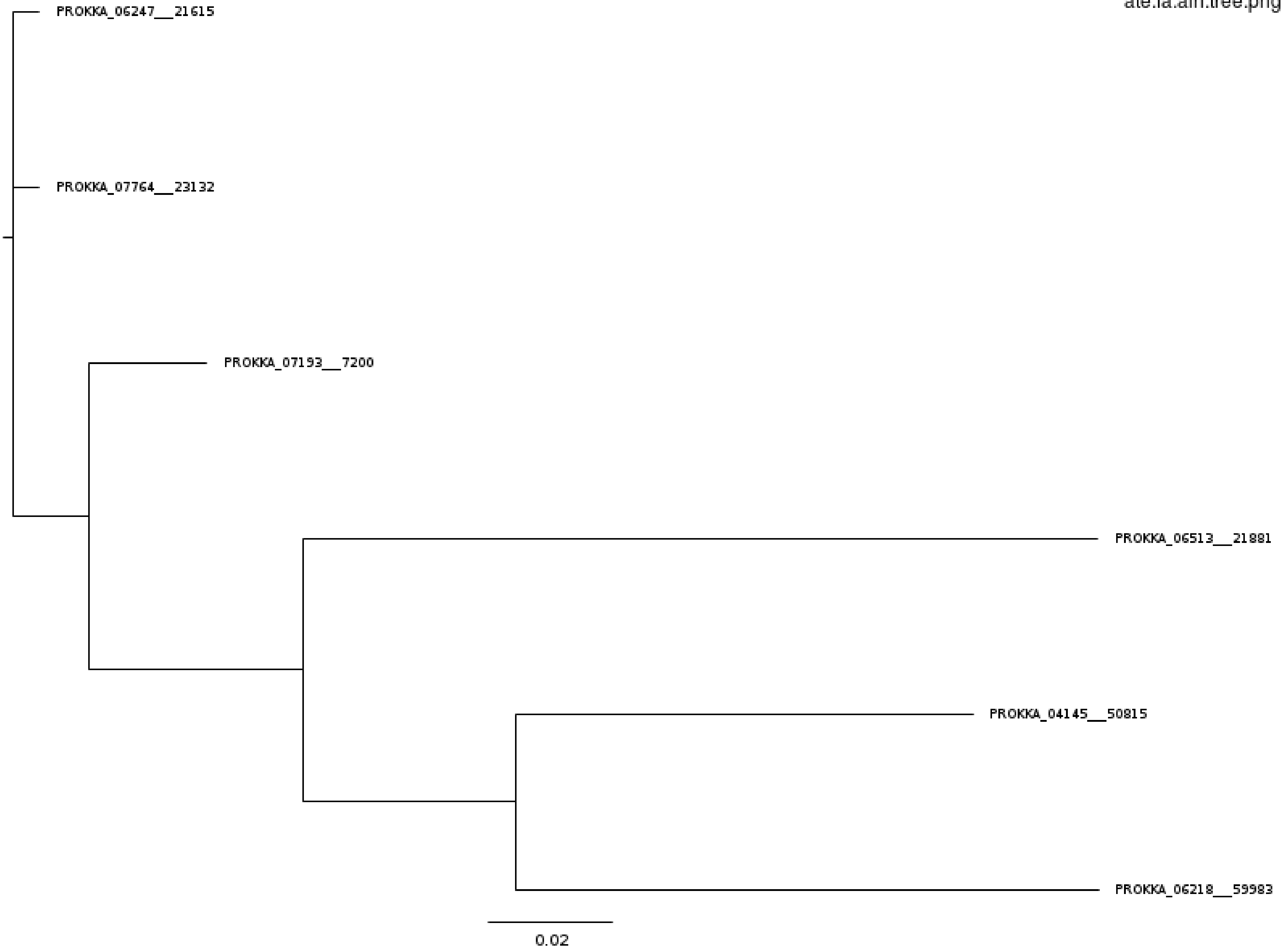
PROKKA_06247___21615
PROKKA_07764___23132
PROKKA_07193___7200
PROKKA_06513___21881
PROKKA_04145___50815
PROKKA_06218___59983
0.02

atpA_1.fa.aln.tree.png

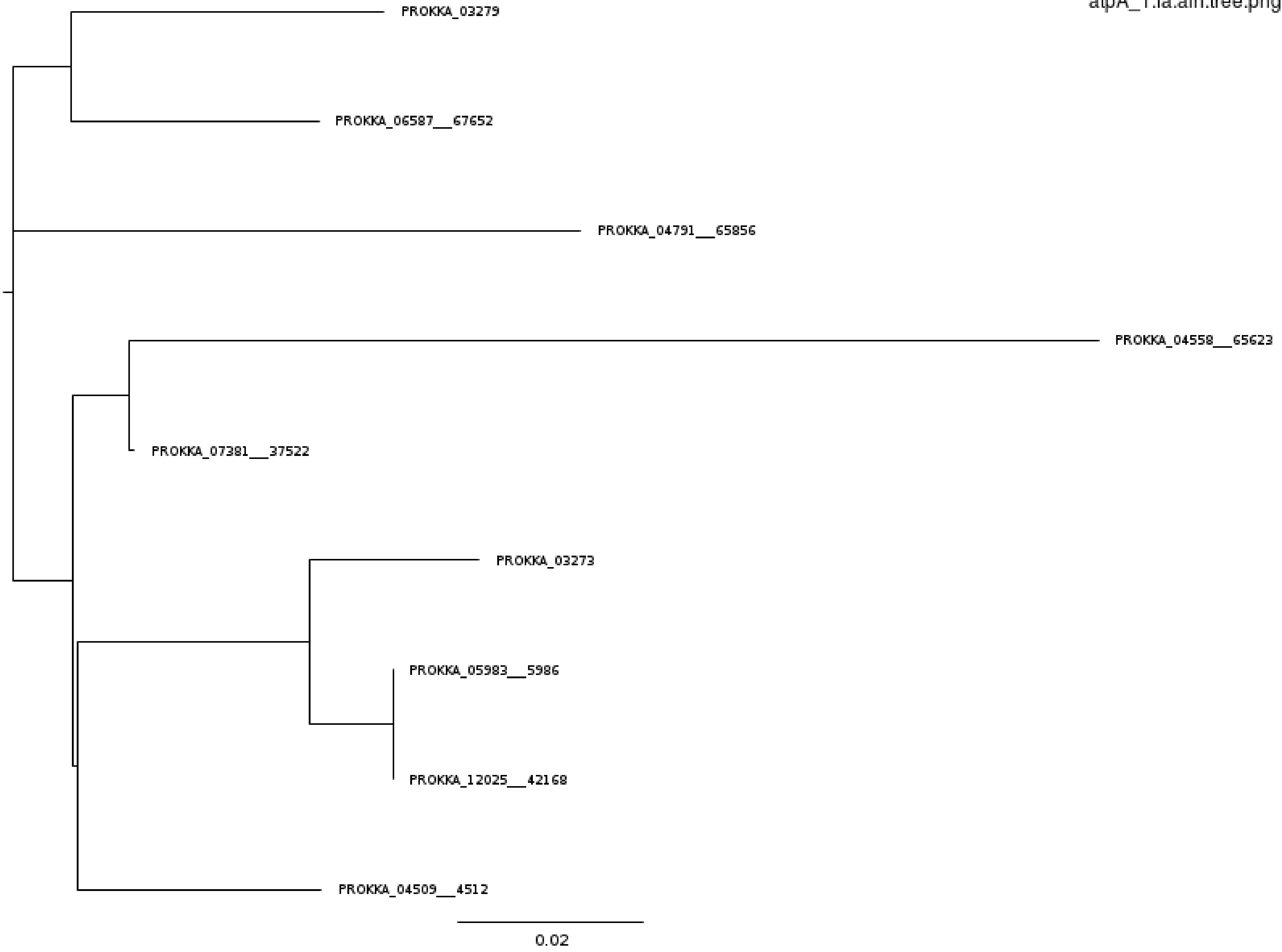

atpA_2.fa.aln.tree.png

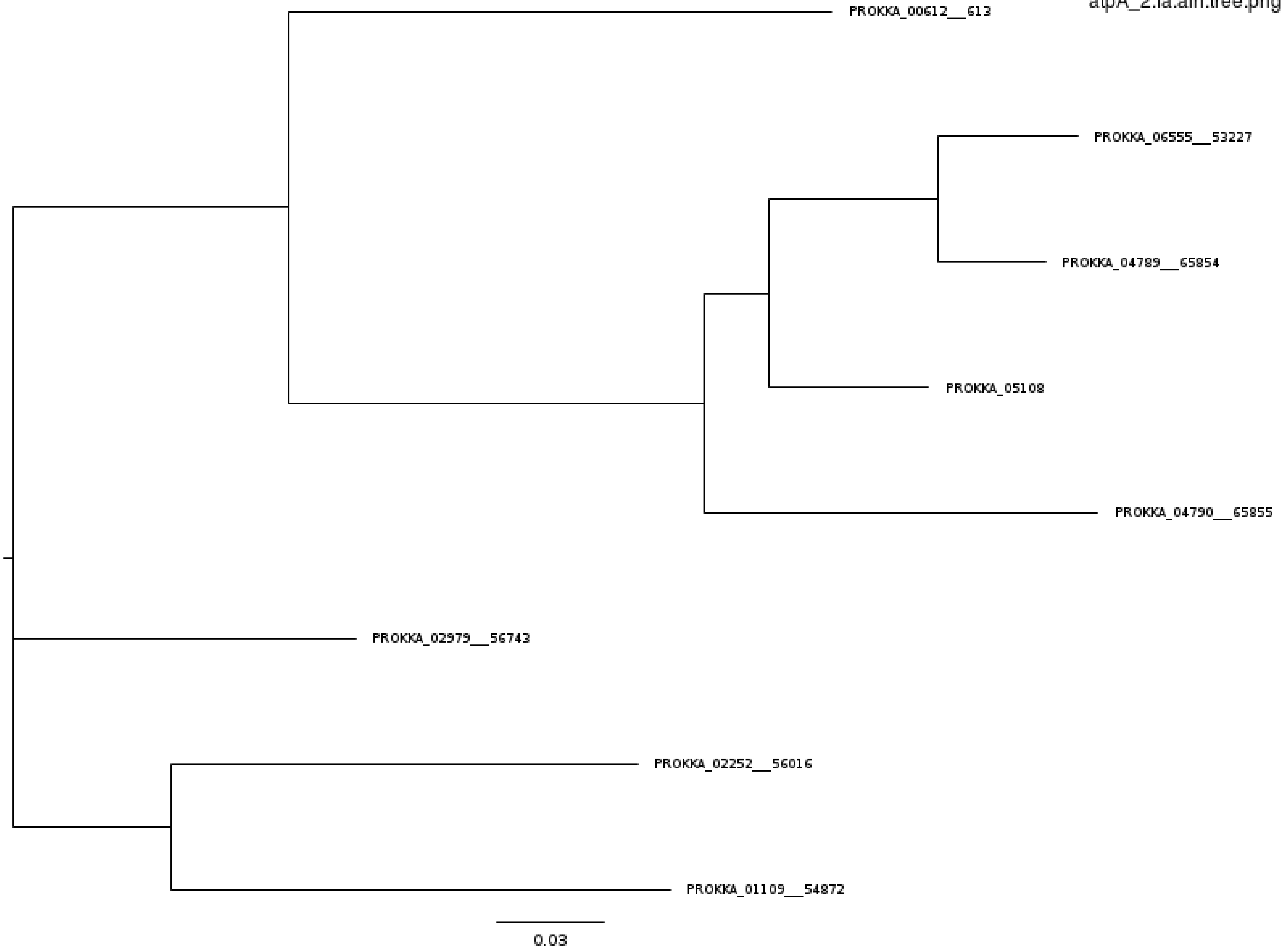

atpC.fa.aln.tree.png

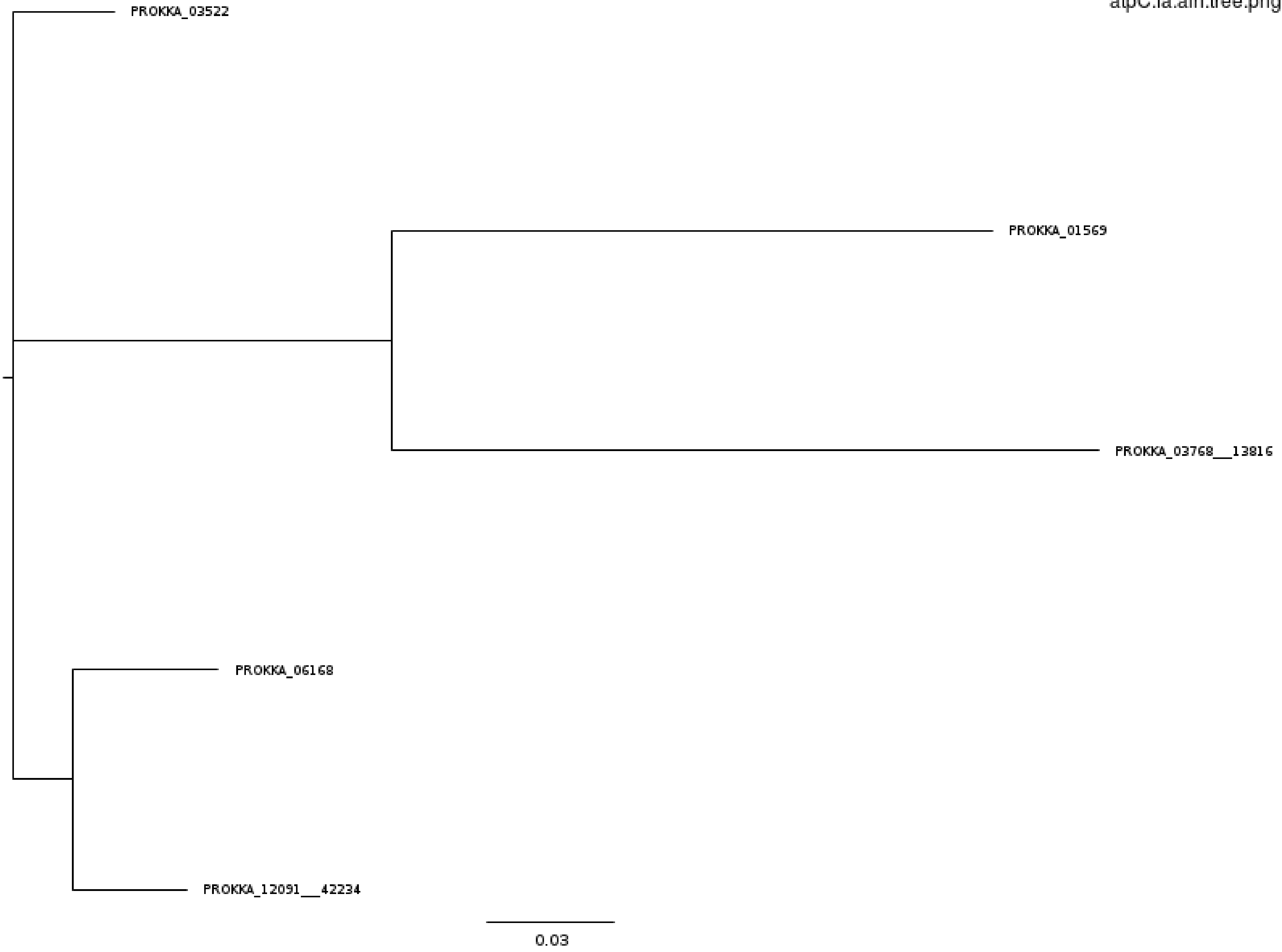

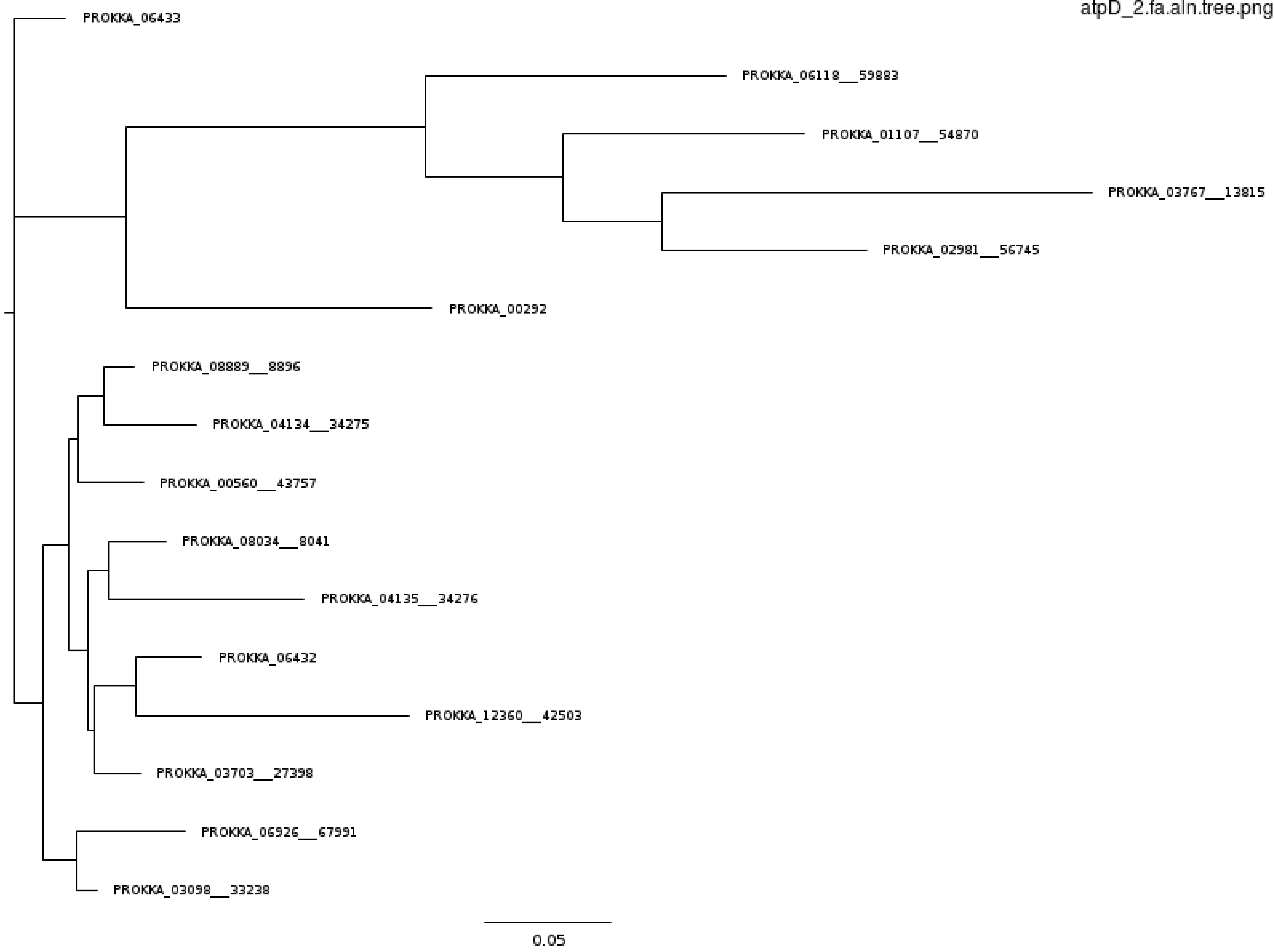
atpD_2.fa.aln.tree.png
PROKKA_06433
PROKKA_06118___59883
PROKKA_01107___54870
PROKKA_03767___13815
PROKKA_02981___56745
PROKKA_00292
PROKKA_08889___8896
PROKKA_04134___34275
PROKKA_00560___43757
PROKKA_08034___8041
PROKKA_04135___34276
PROKKA_06432
PROKKA_12360___42503
PROKKA_03703___27398
PROKKA_06926___67991
PROKKA_03098___33238
0.05

atpG_2.fa.aln.tree.png

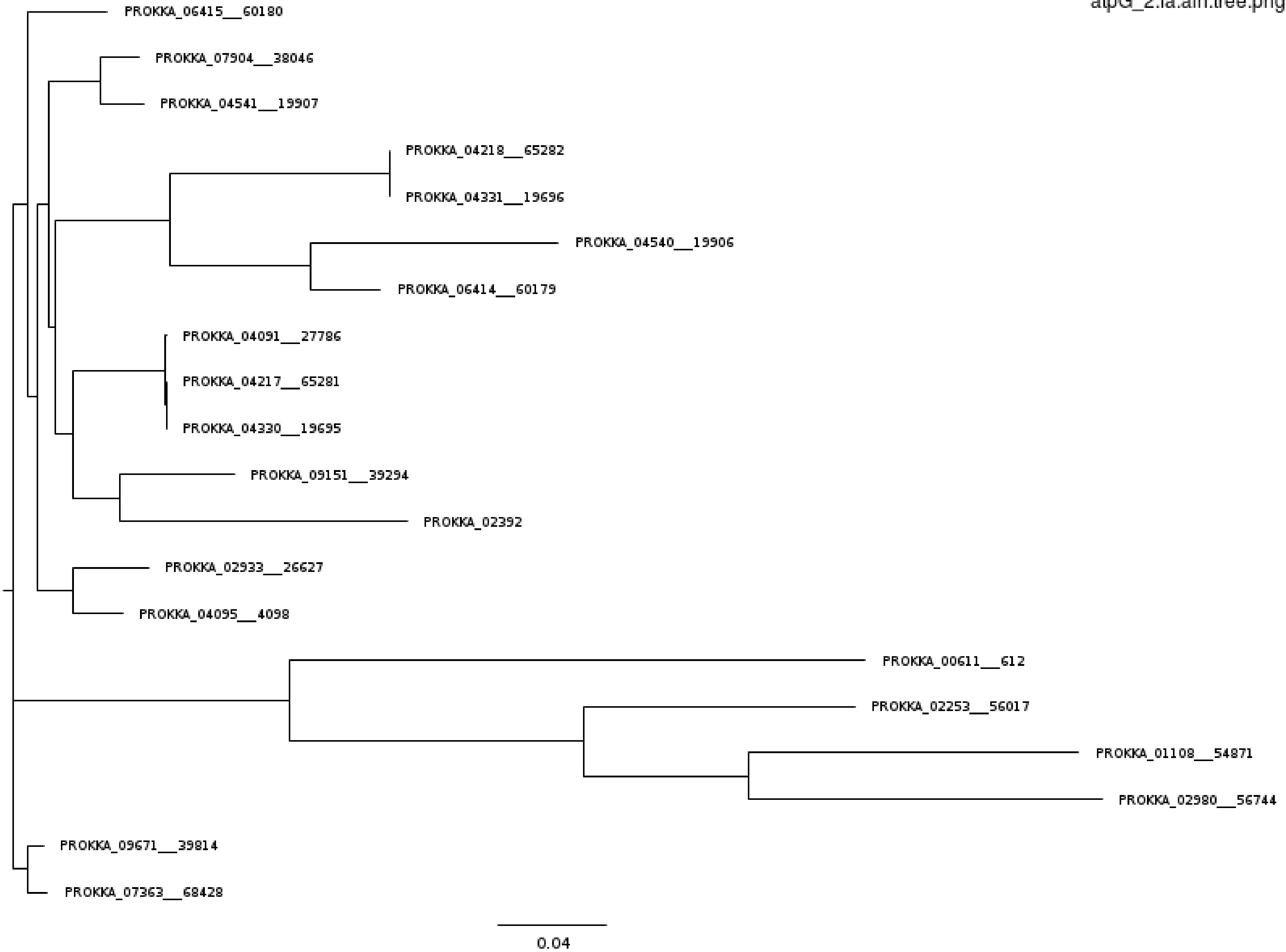

bamA_1.fa.aln.tree.png

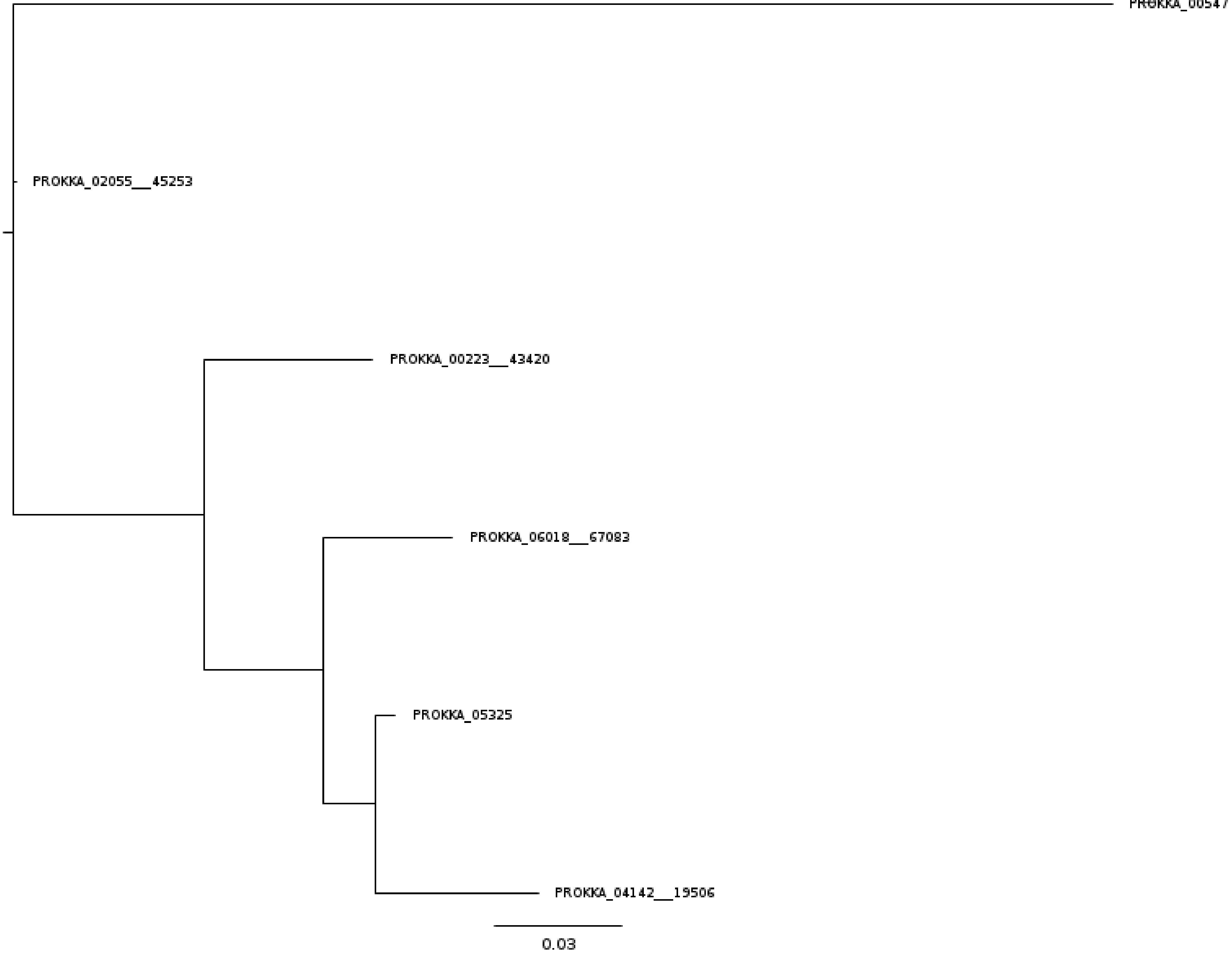

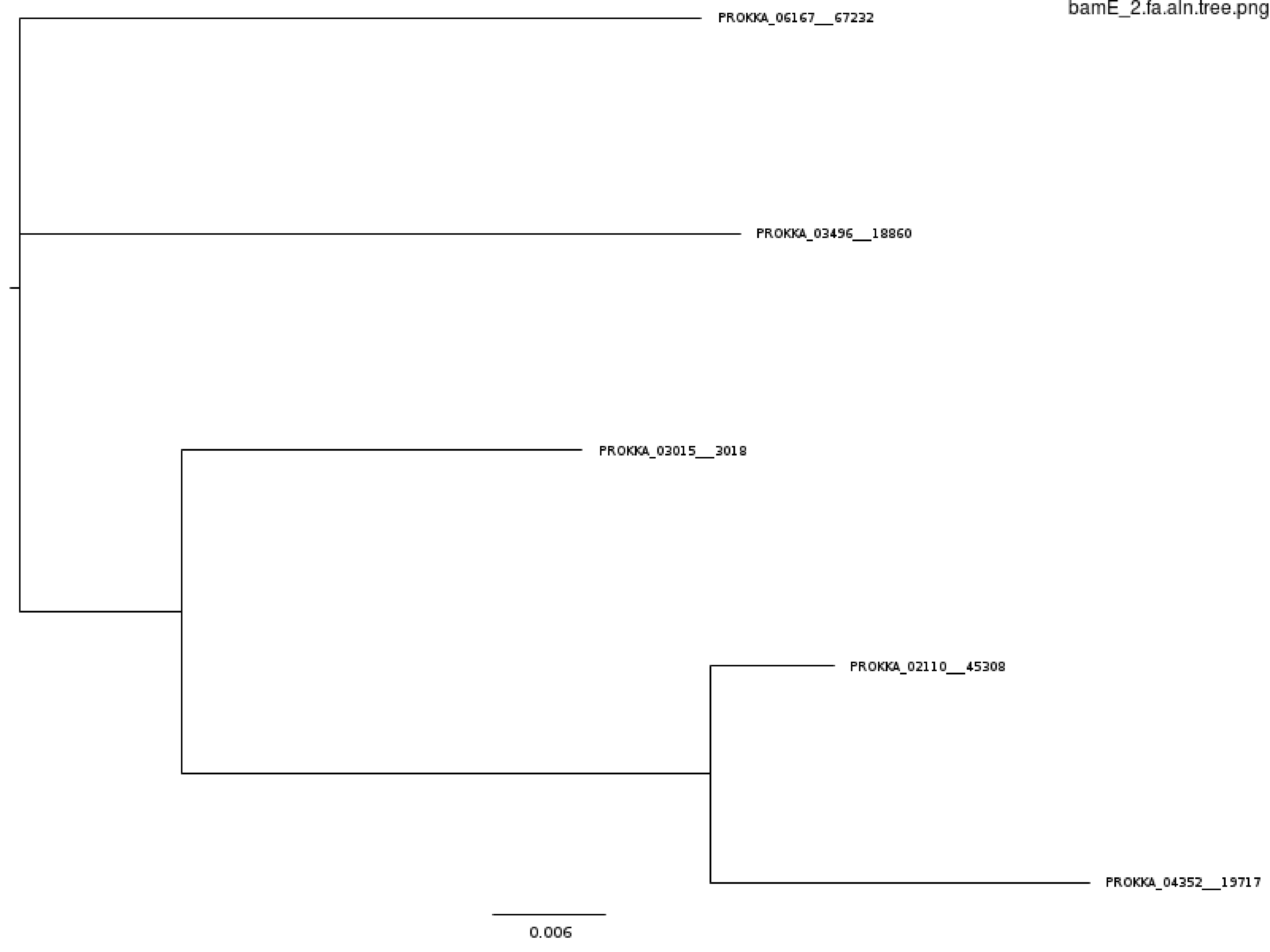

bamE_2.fa.aln.tree.png
PROKKA_06167___67232
PROKKA_03496___18860
PROKKA_03015___3018
PROKKA_02110___45308
PROKKA_04352___19717
0.006

bamHIM_2.fa.aln.tree.png

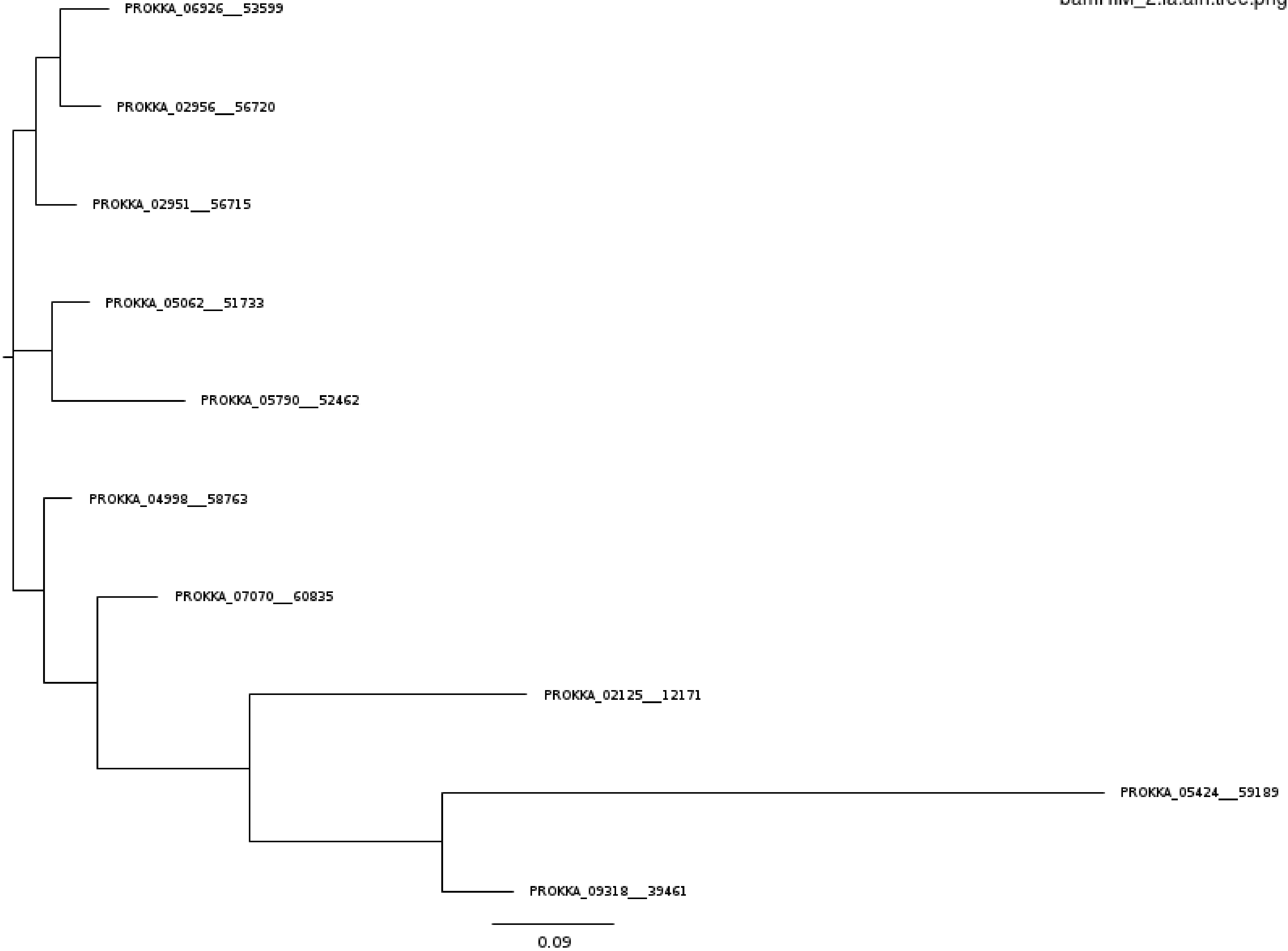

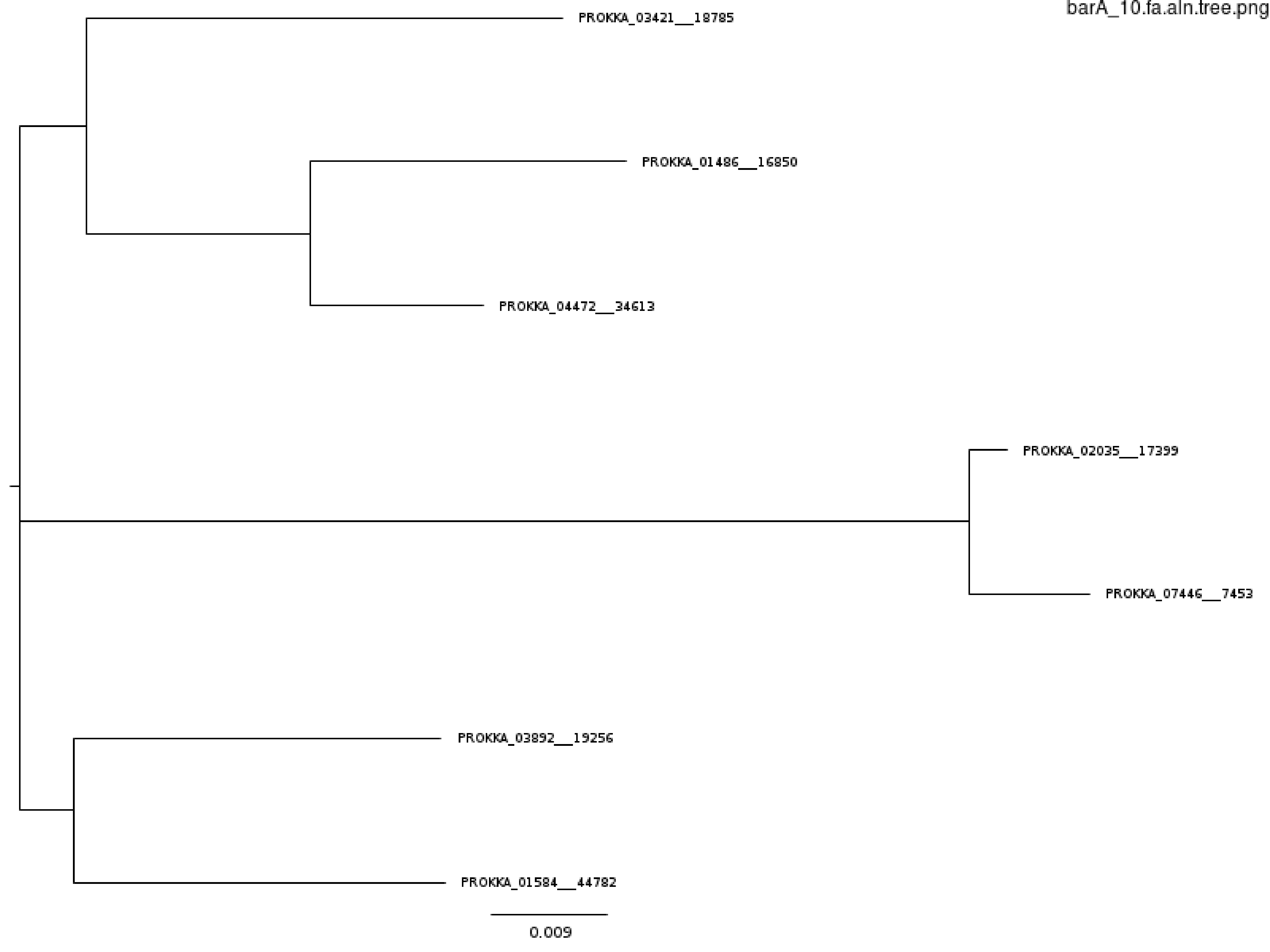
barA_10.fa.aln.tree.png
PROKKA_03421__18785
PROKKA_01486__16850
PROKKA_04472__34613
PROKKA_02035__17399
PROKKA_07446__7453
PROKKA_03892__19256
PROKKA_01584__44782
0.009

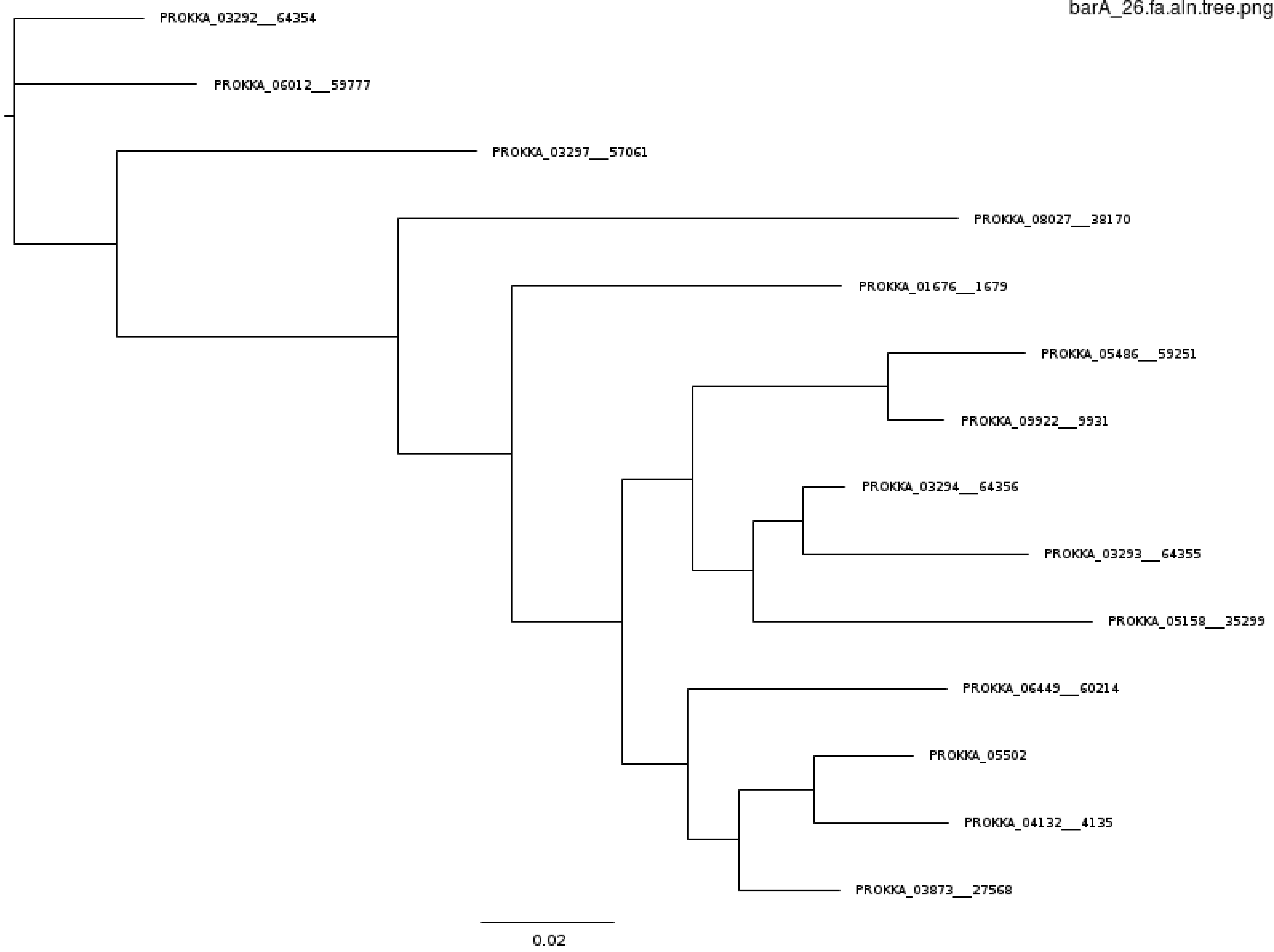
barA_26.fa.aln.tree.png
PROKKA_03292___64354
PROKKA_06012___59777
PROKKA_03297___57061
PROKKA_08027___38170
PROKKA_01676___1679
PROKKA_05486___59251
PROKKA_09922___9931
PROKKA_03294___64356
PROKKA_03293___64355
PROKKA_05158___35299
PROKKA_06449___60214
PROKKA_05502
PROKKA_04132___4135
PROKKA_03873___27568
0.02

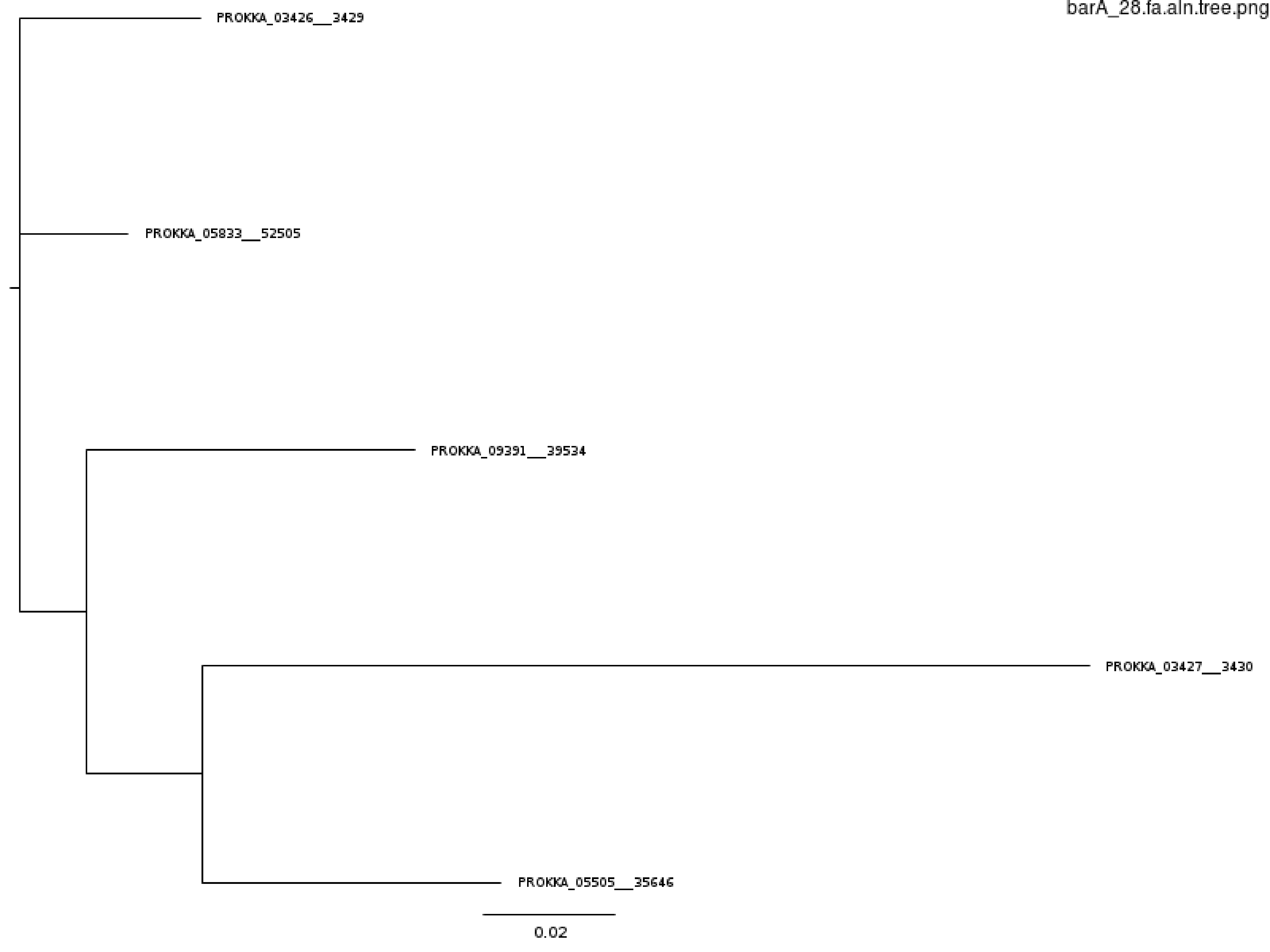
barA_28.fa.aln.tree.png
PROKKA_03426___3429
PROKKA_05833___52505
PROKKA_09391___39534
PROKKA_03427___3430
PROKKA_05505___35646
0.02

barA_2.fa.aln.tree.png

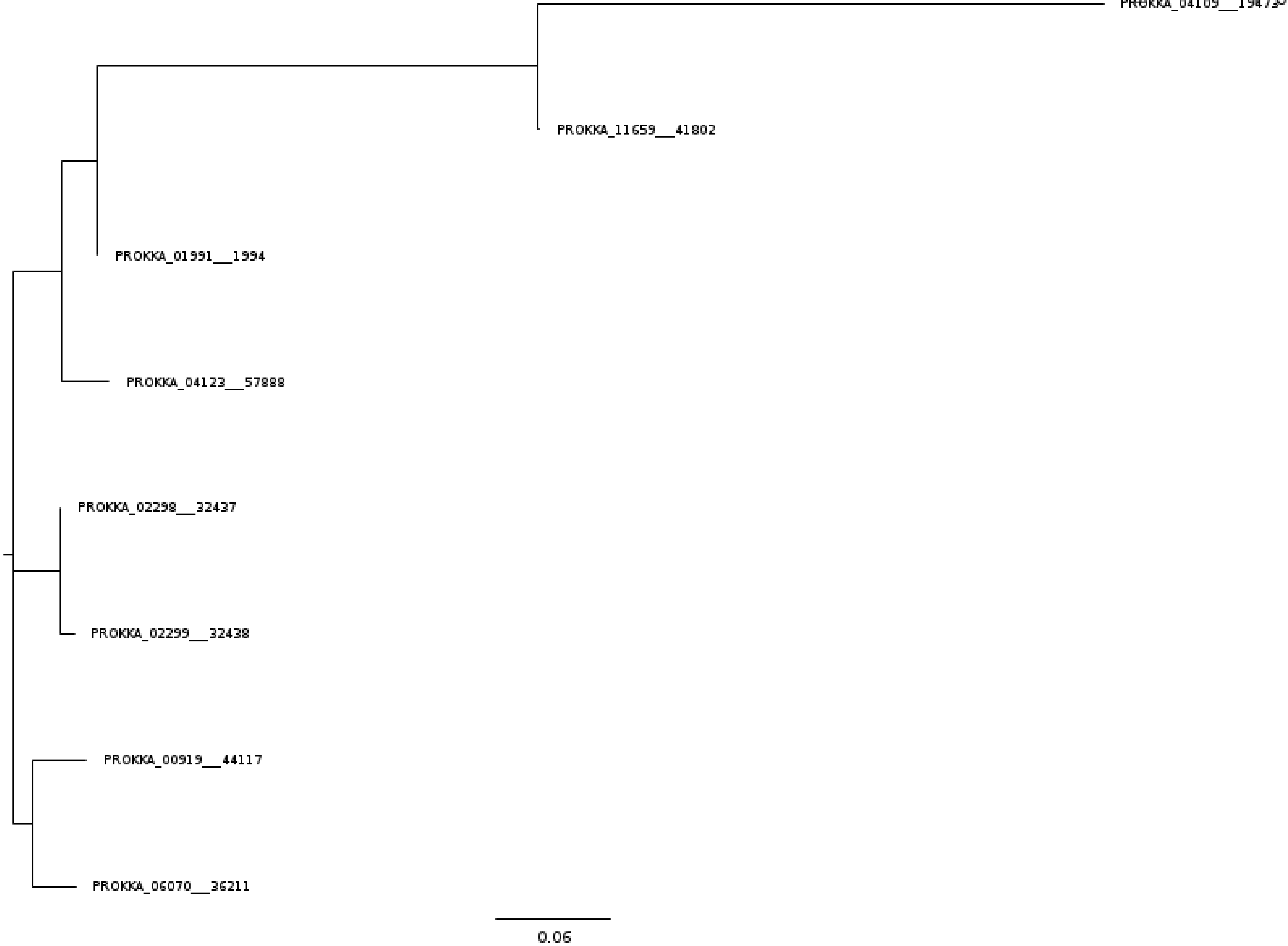

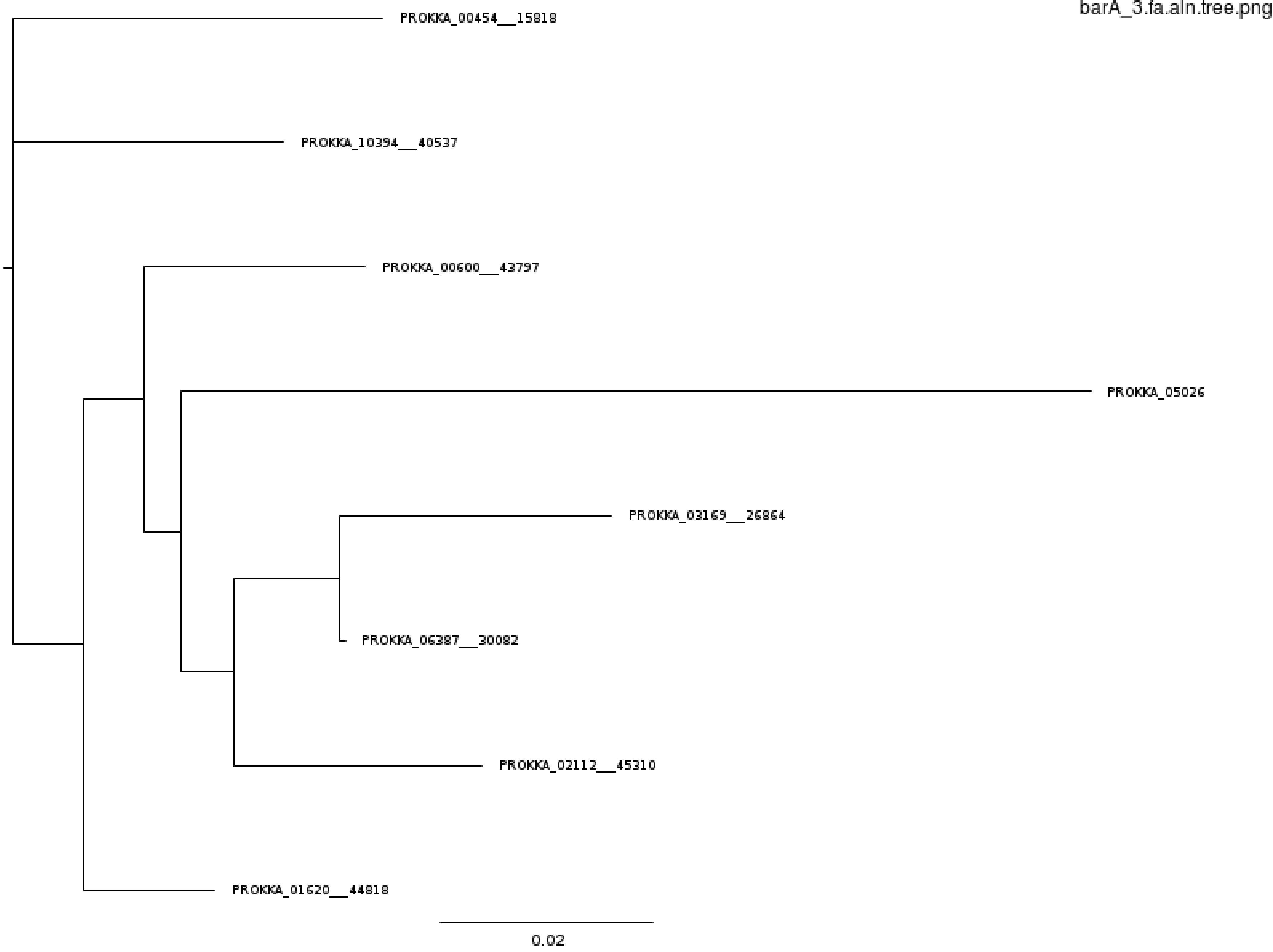
barA_3.fa.aln.tree.png
PROKKA_00454__15818
PROKKA_10394__40537
PROKKA_00600__43797
PROKKA_05026
PROKKA_03169__26864
PROKKA_06387__30082
PROKKA_02112__45310
PROKKA_01620__44818
0.02

barA_9.fa.aln.tree.png

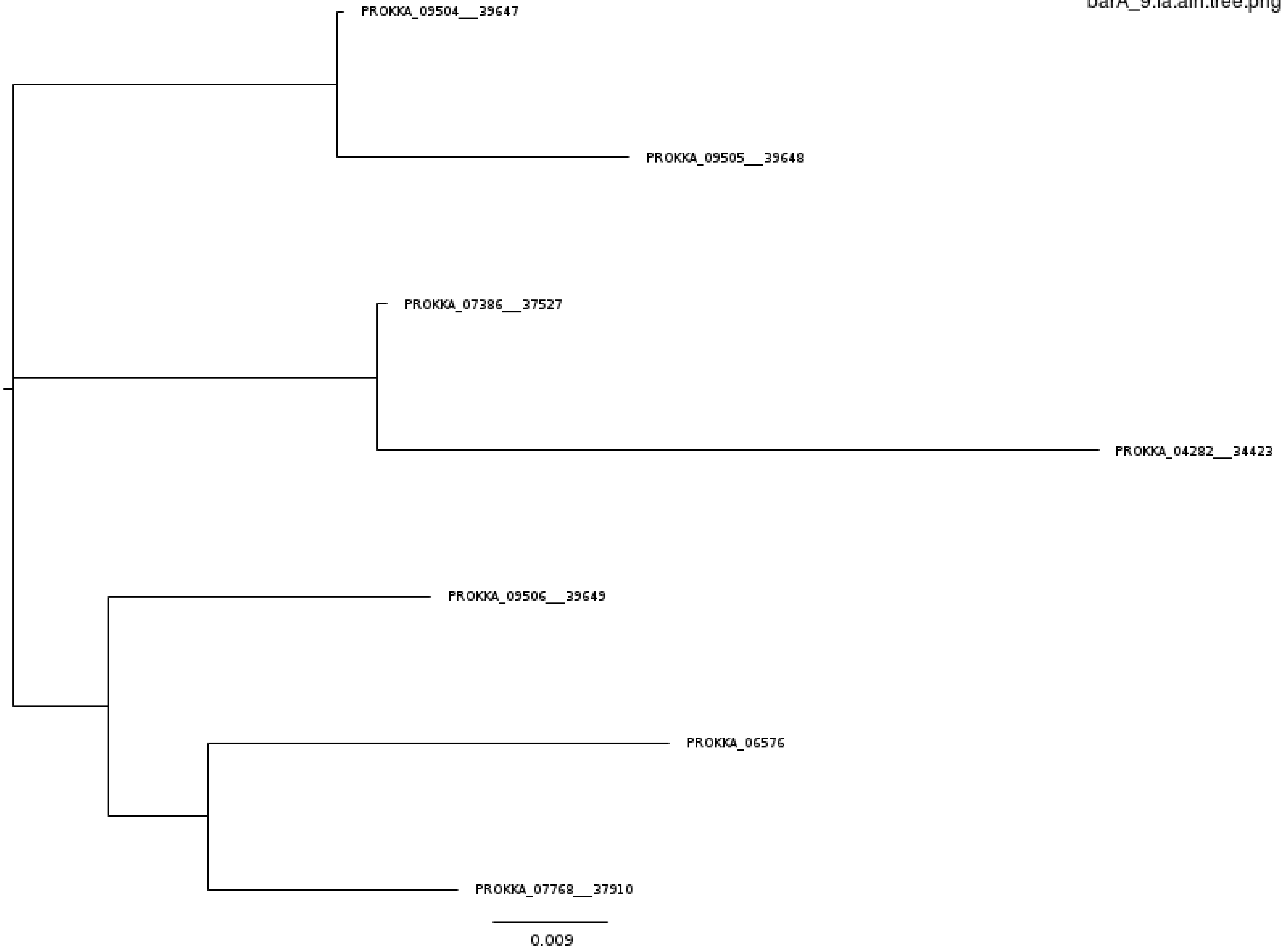

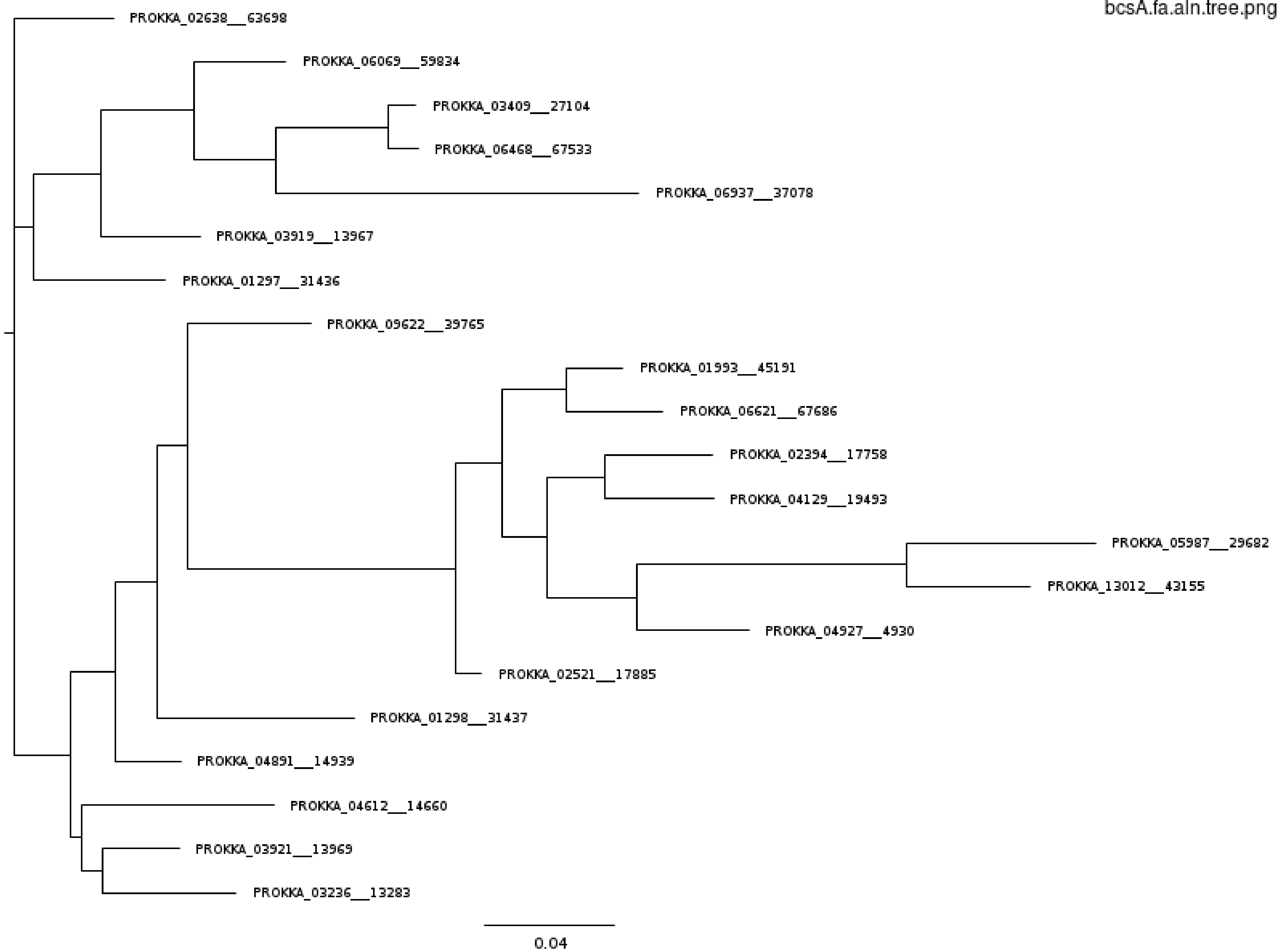
bcsA.fa.aln.tree.png
PROKKA_02638___63698
PROKKA_06069___59834
PROKKA_03409___27104
PROKKA_06468___67533
PROKKA_06937___37078
PROKKA_03919___13967
PROKKA_01297___31436
PROKKA_09622___39765
PROKKA_01993___45191
PROKKA_06621___67686
PROKKA_02394___17758
PROKKA_04129___19493
PROKKA_05987___29682
PROKKA_13012___43155
PROKKA_04927___4930
PROKKA_02521___17885
PROKKA_01298___31437
PROKKA_04891___14939
PROKKA_04612___14660
PROKKA_03921___13969
PROKKA_03236___13283
0.04

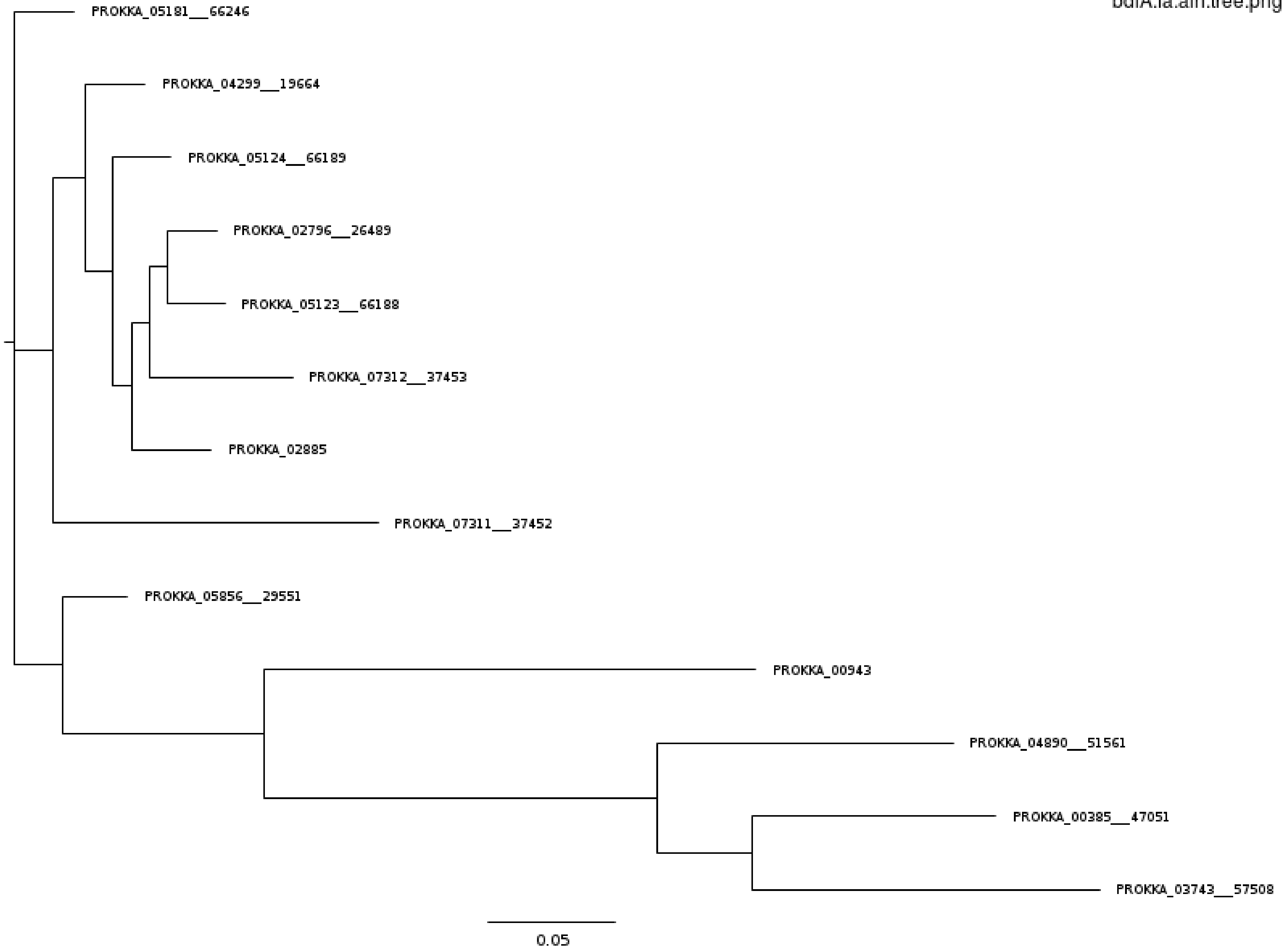
PROKKA_05181___66246
PROKKA_04299___19664
PROKKA_05124___66189
PROKKA_02796___26489
PROKKA_05123___66188
PROKKA_07312___37453
PROKKA_02885
PROKKA_07311___37452
PROKKA_05856___29551
PROKKA_00943
PROKKA_04890___51561
PROKKA_00385___47051
PROKKA_03743___57508
0.05

bfr.fa.aln.tree.png

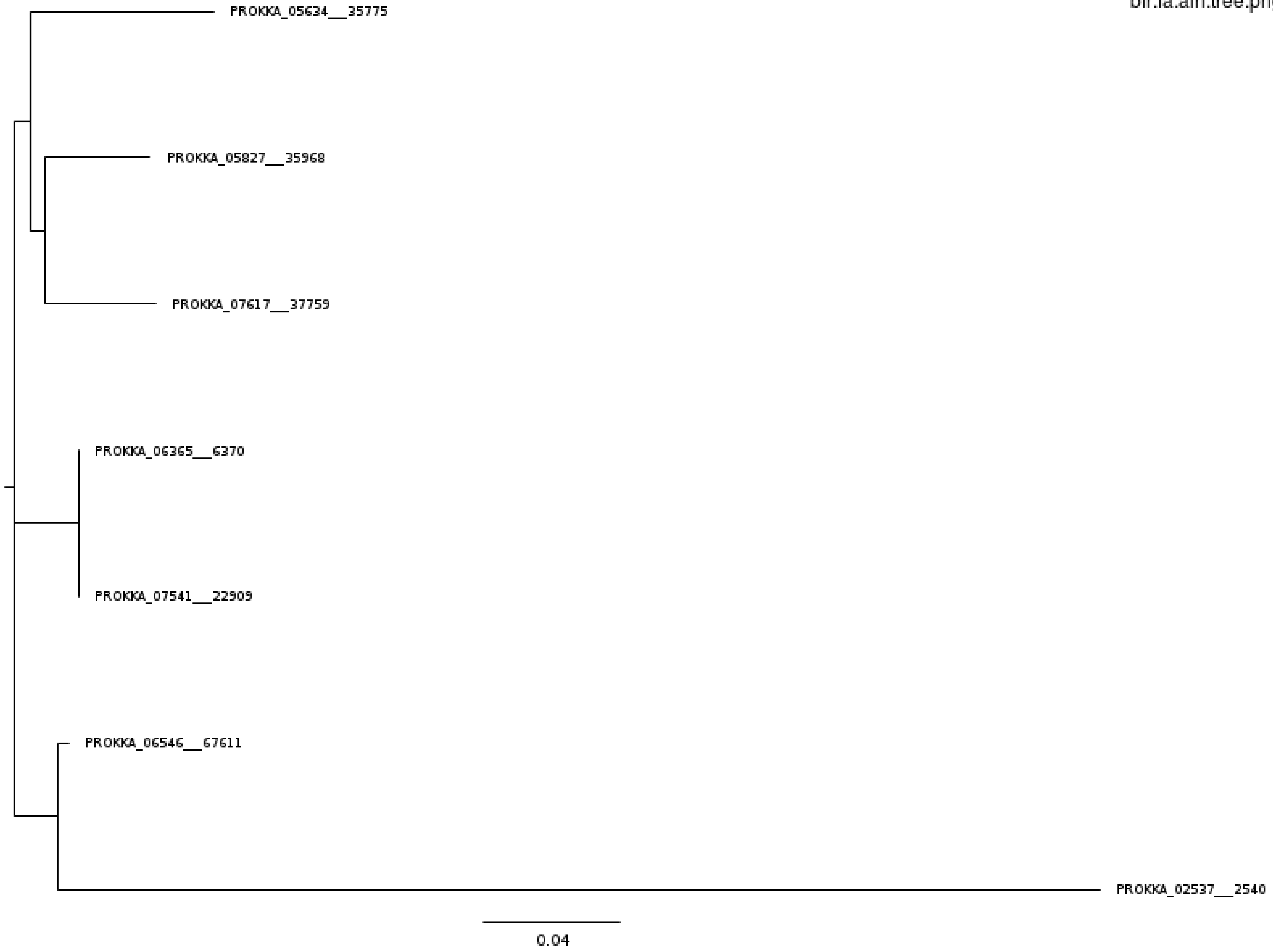

bglBB.fa.aln.tree.png

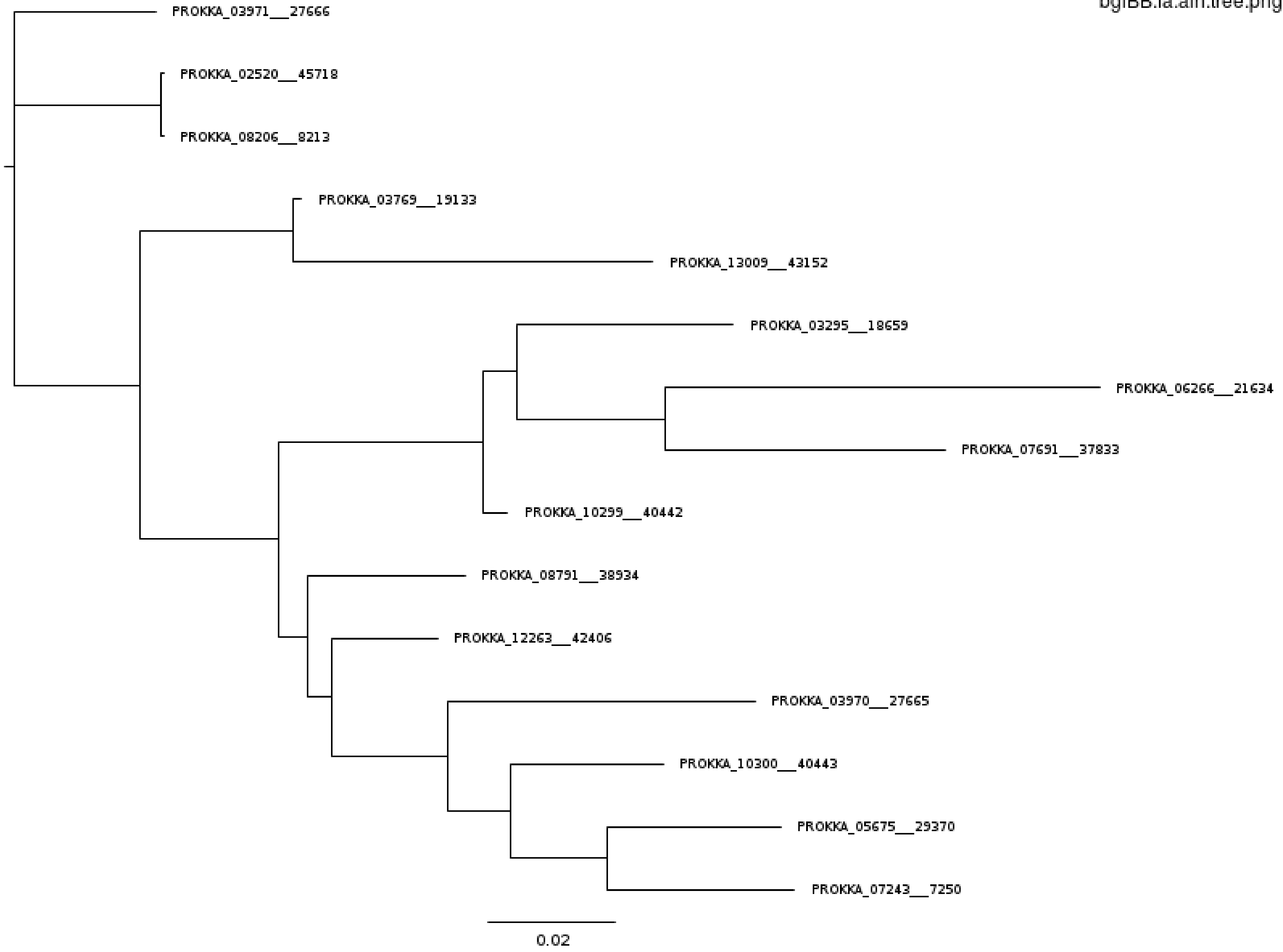

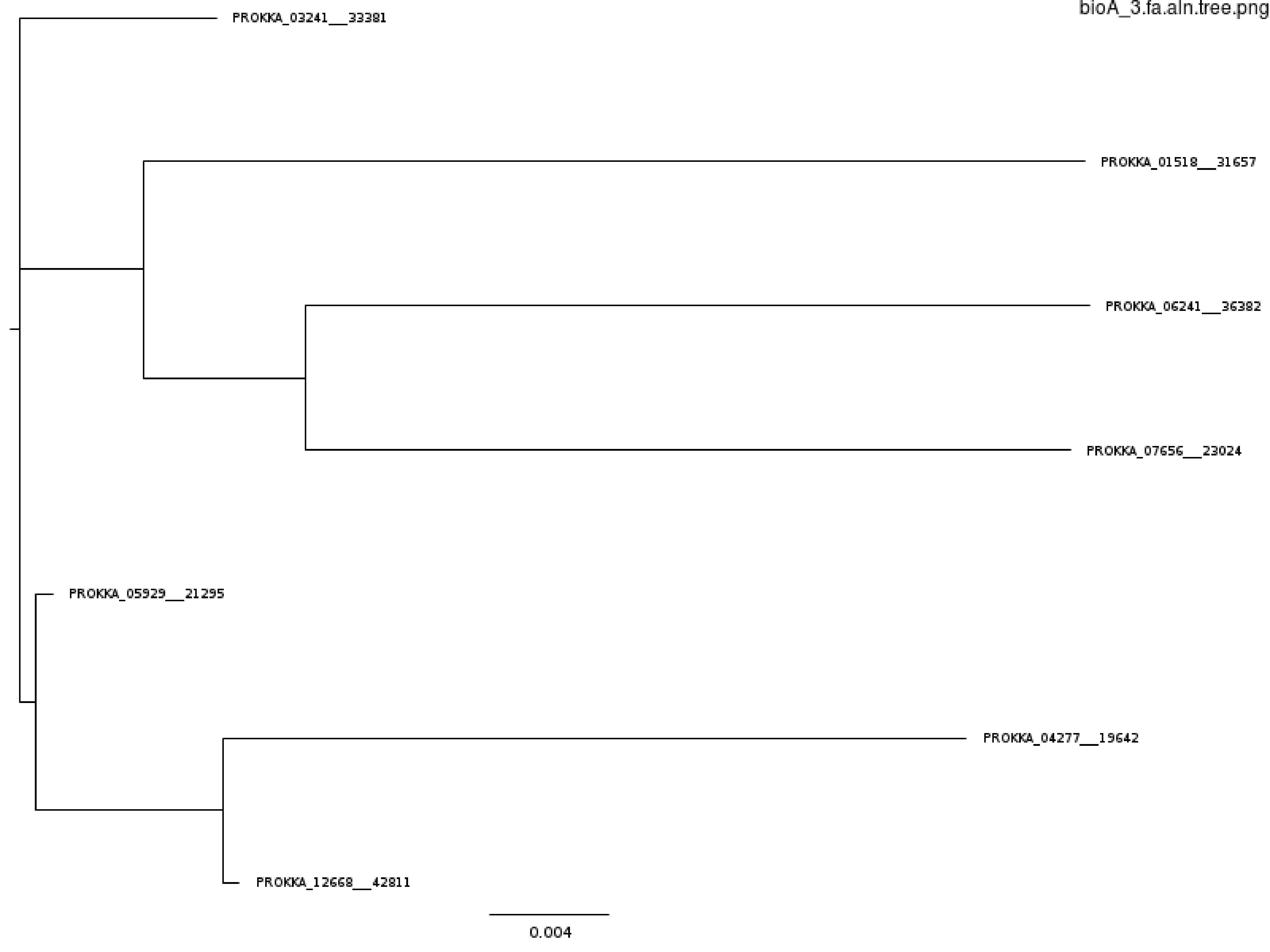
bioA_3.fa.aln.tree.png
PROKKA_03241___33381
PROKKA_01518___31657
PROKKA_06241___36382
PROKKA_07656___23024
PROKKA_05929___21295
PROKKA_04277___19642
PROKKA_12668___42811
0.004

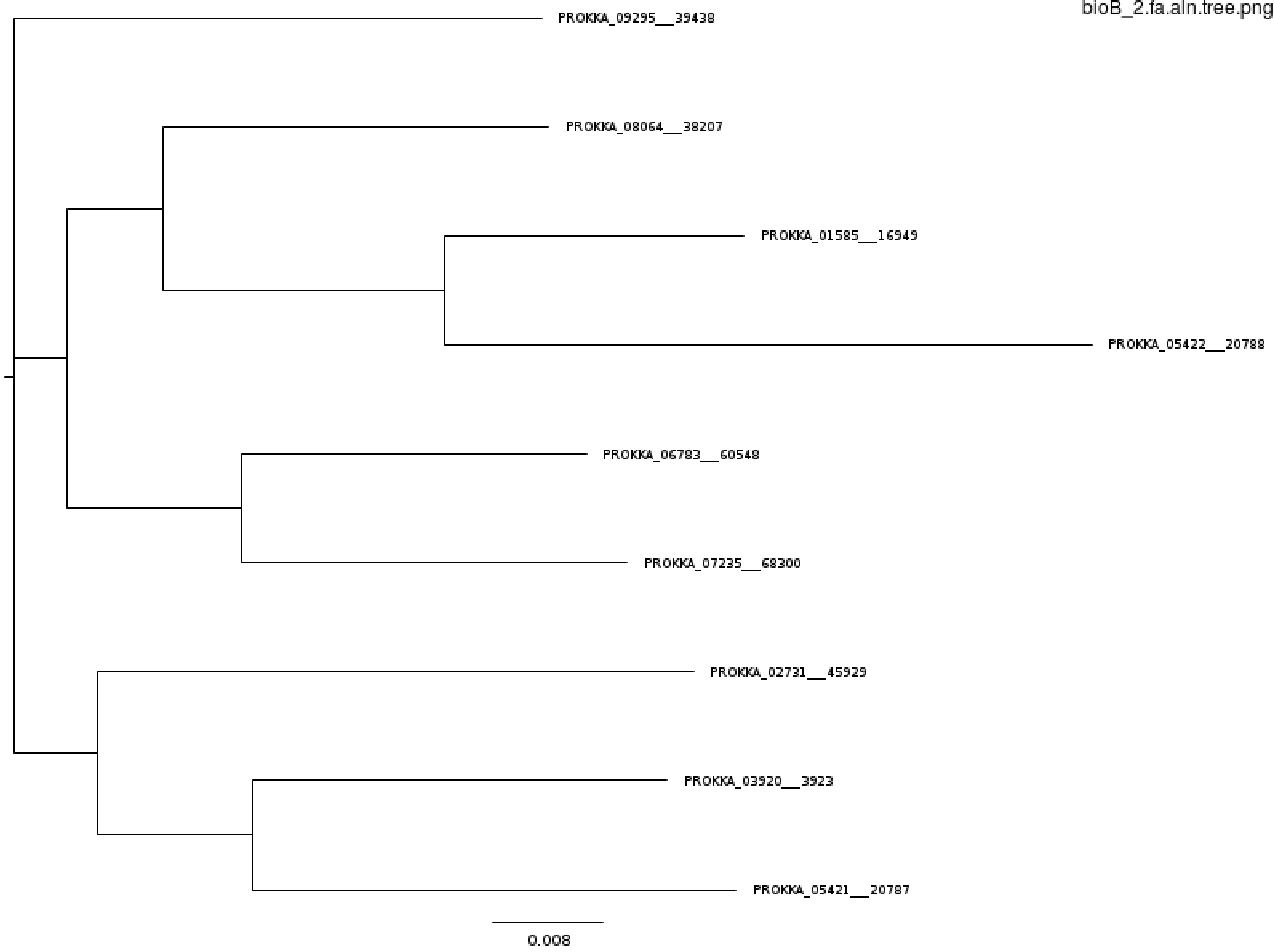
bioB_2.fa.aln.tree.png
PROKKA_09295___39438
PROKKA_08064___38207
PROKKA_01585___16949
PROKKA_05422___20788
PROKKA_06783___60548
PROKKA_07235___68300
PROKKA_02731___45929
PROKKA_03920___3923
PROKKA_05421___20787
0.008

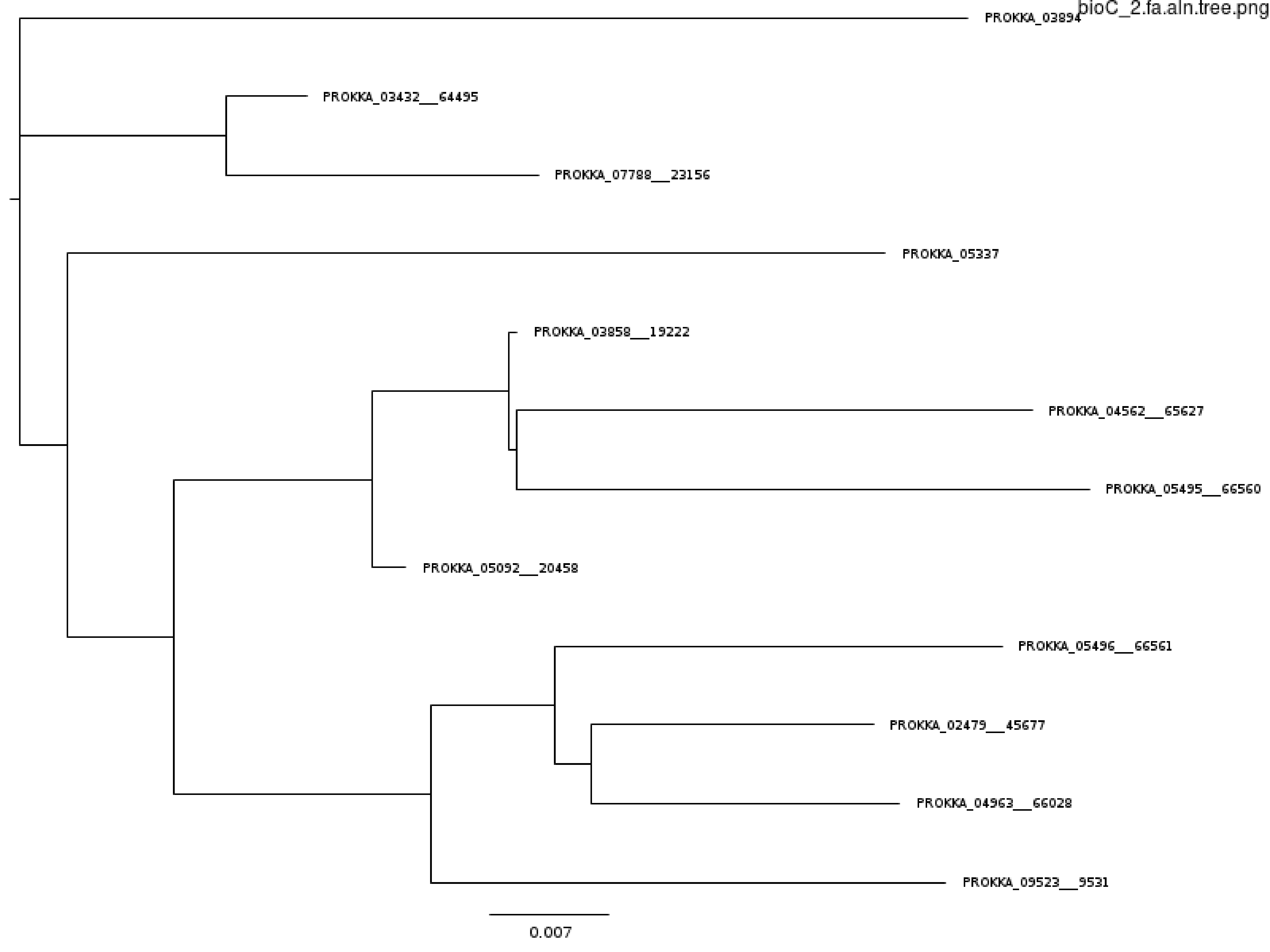
bioC_2.fa.aln.tree.png
PROKKA_03894
PROKKA_03432___64495
PROKKA_07788___23156
PROKKA_05337
PROKKA_03858___19222
PROKKA_04562___65627
PROKKA_05495___66560
PROKKA_05092___20458
PROKKA_05496___66561
PROKKA_02479___45677
PROKKA_04963___66028
PROKKA_09523___9531
0.007

bioD1_2.fa.aln.tree.png

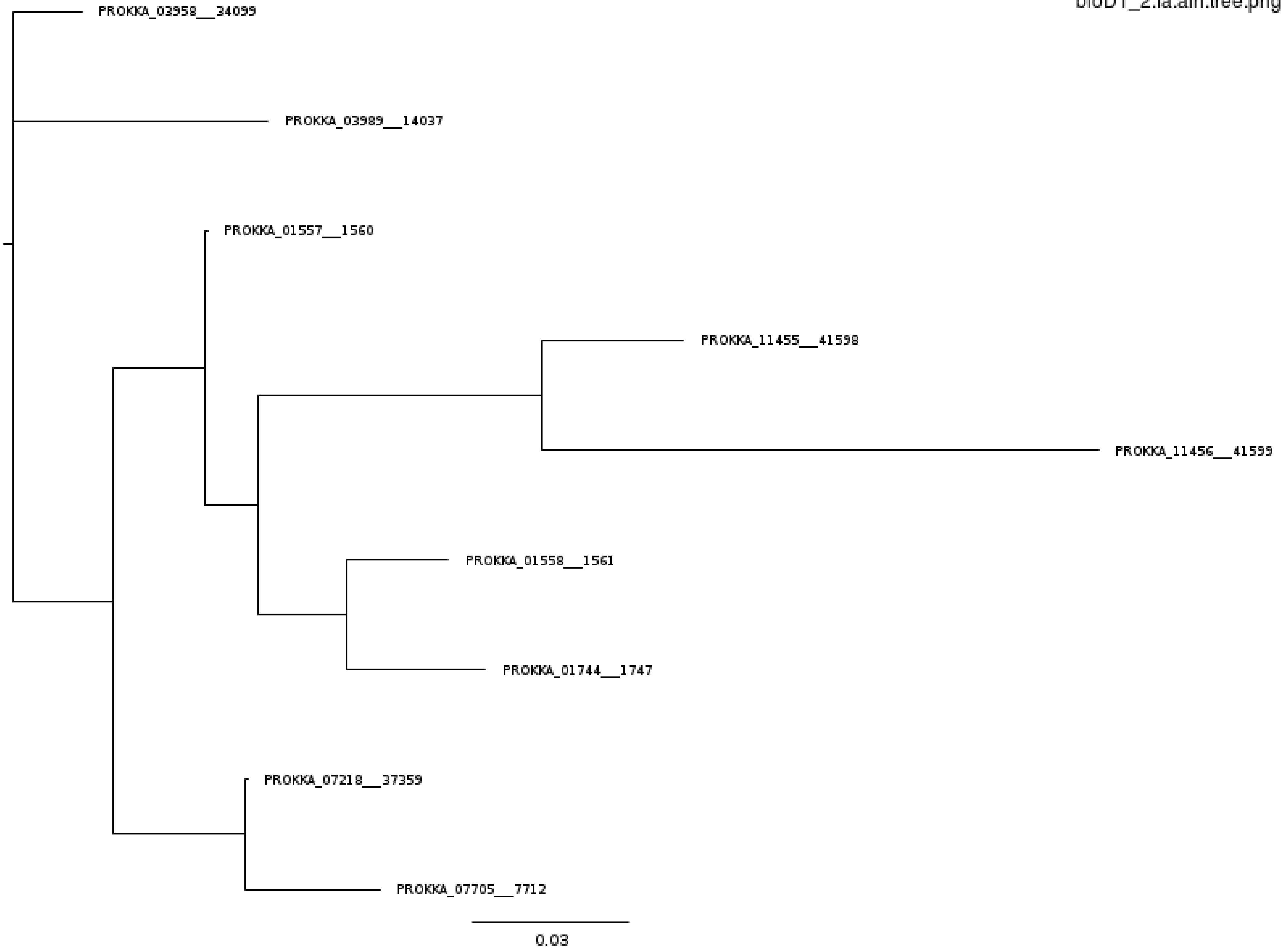

bioF_2.fa.aln.tree.png

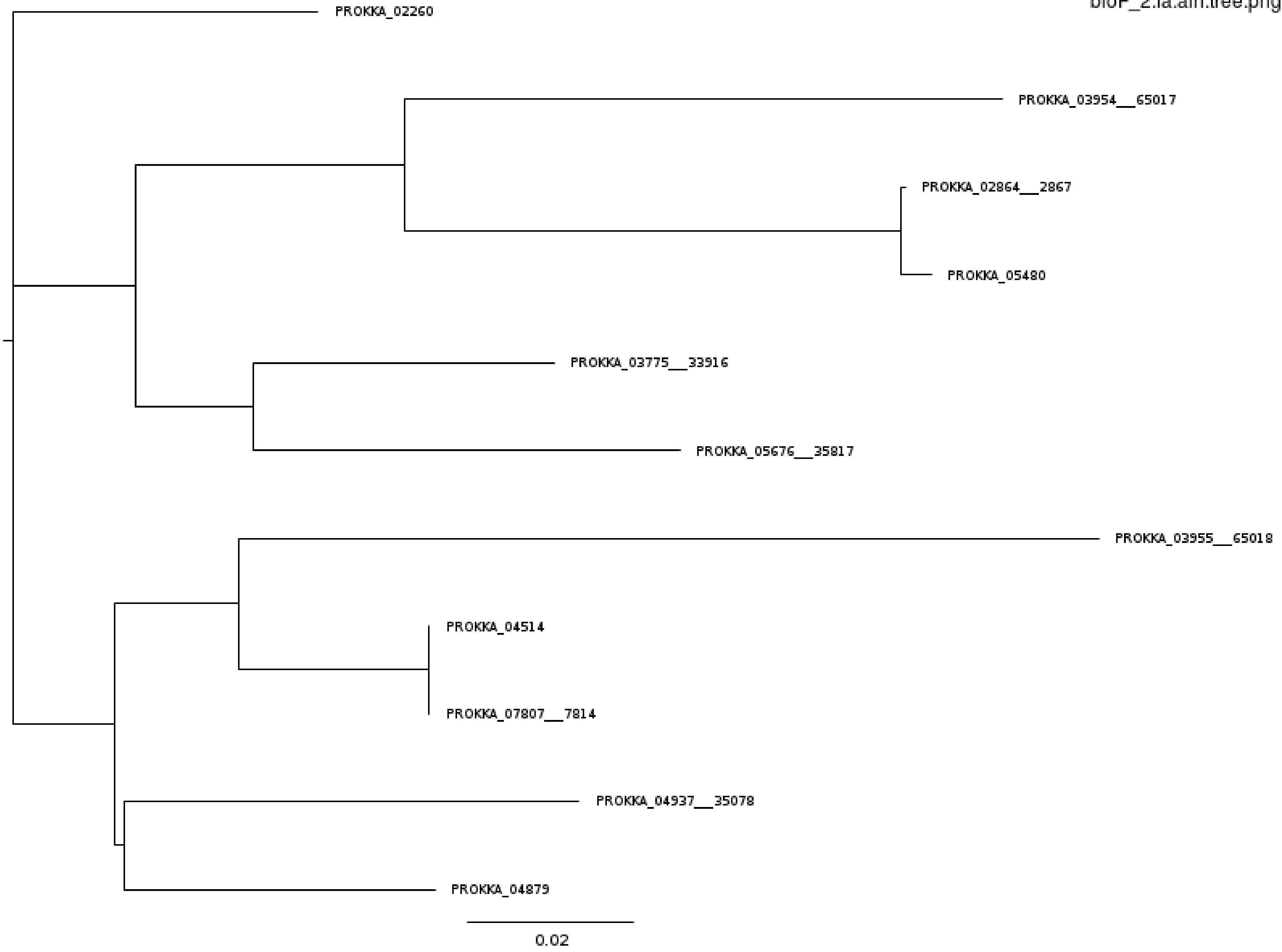

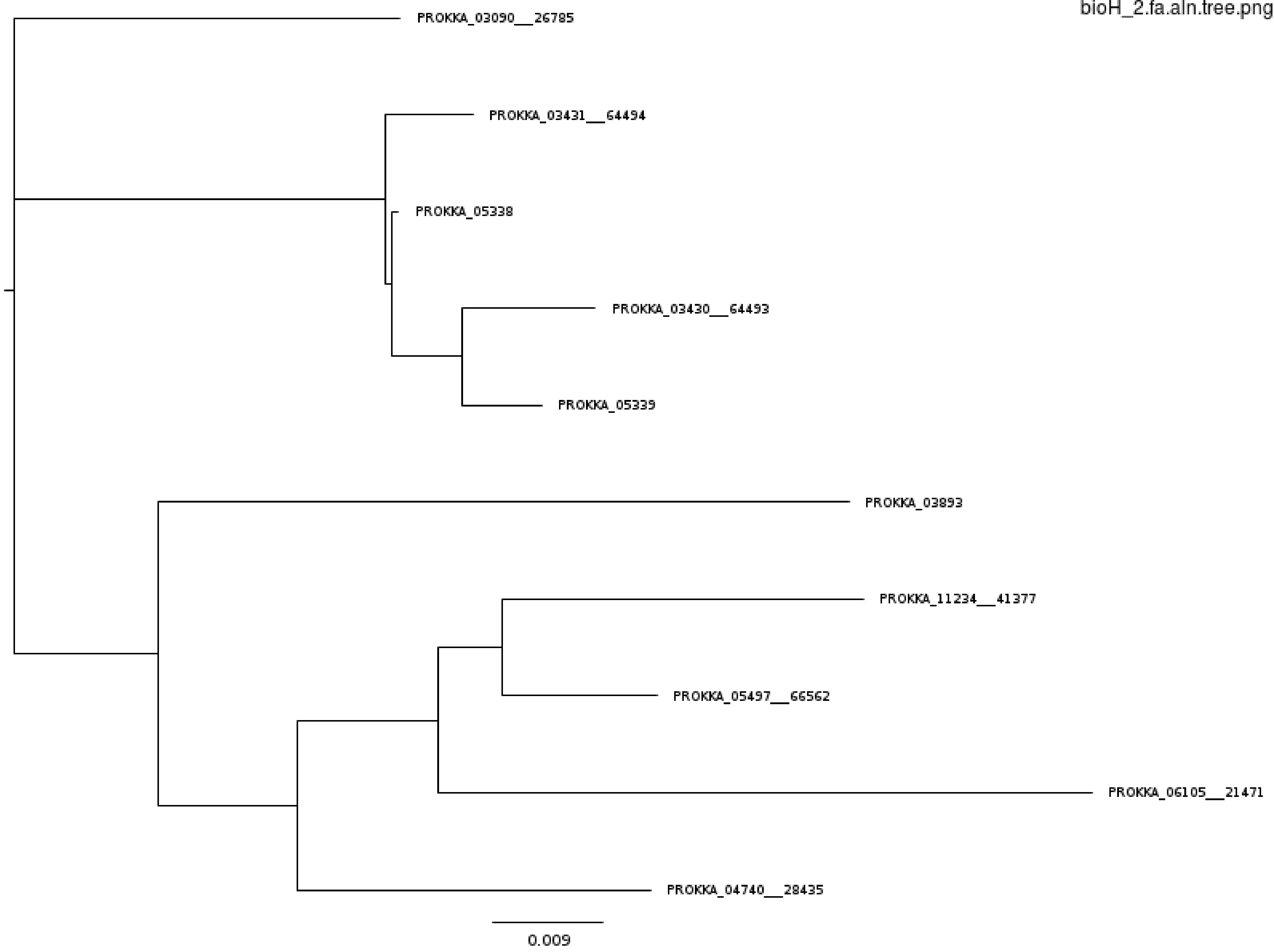
bioH_2.fa.aln.tree.png
PROKKA_03090__26785
PROKKA_03431__64494
PROKKA_05338
PROKKA_03430__64493
PROKKA_05339
PROKKA_03893
PROKKA_11234__41377
PROKKA_05497__66562
PROKKA_06105__21471
PROKKA_04740__28435
0.009

birA_1.fa.aln.tree.png

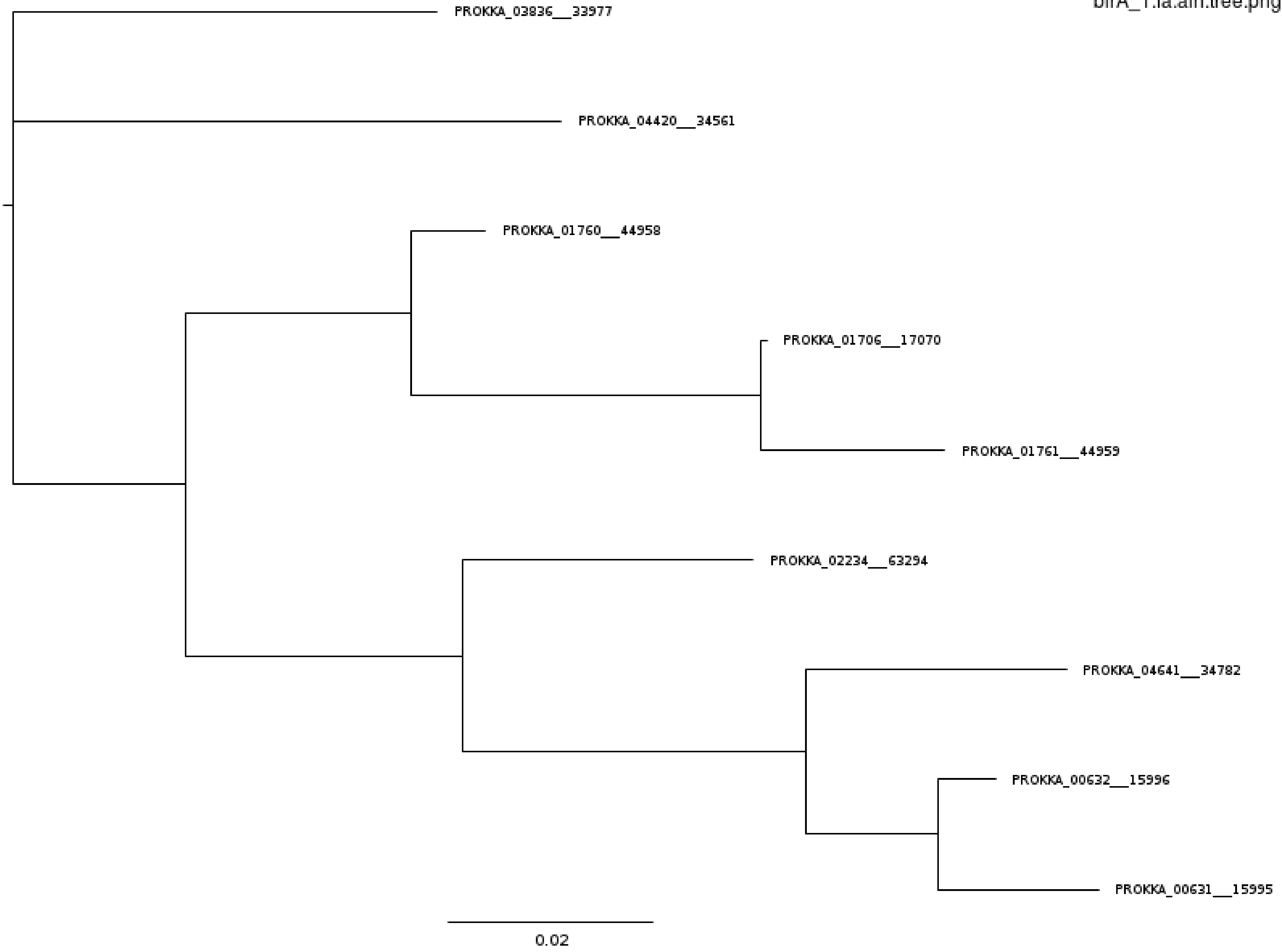

blc_2.fa.aln.tree.png

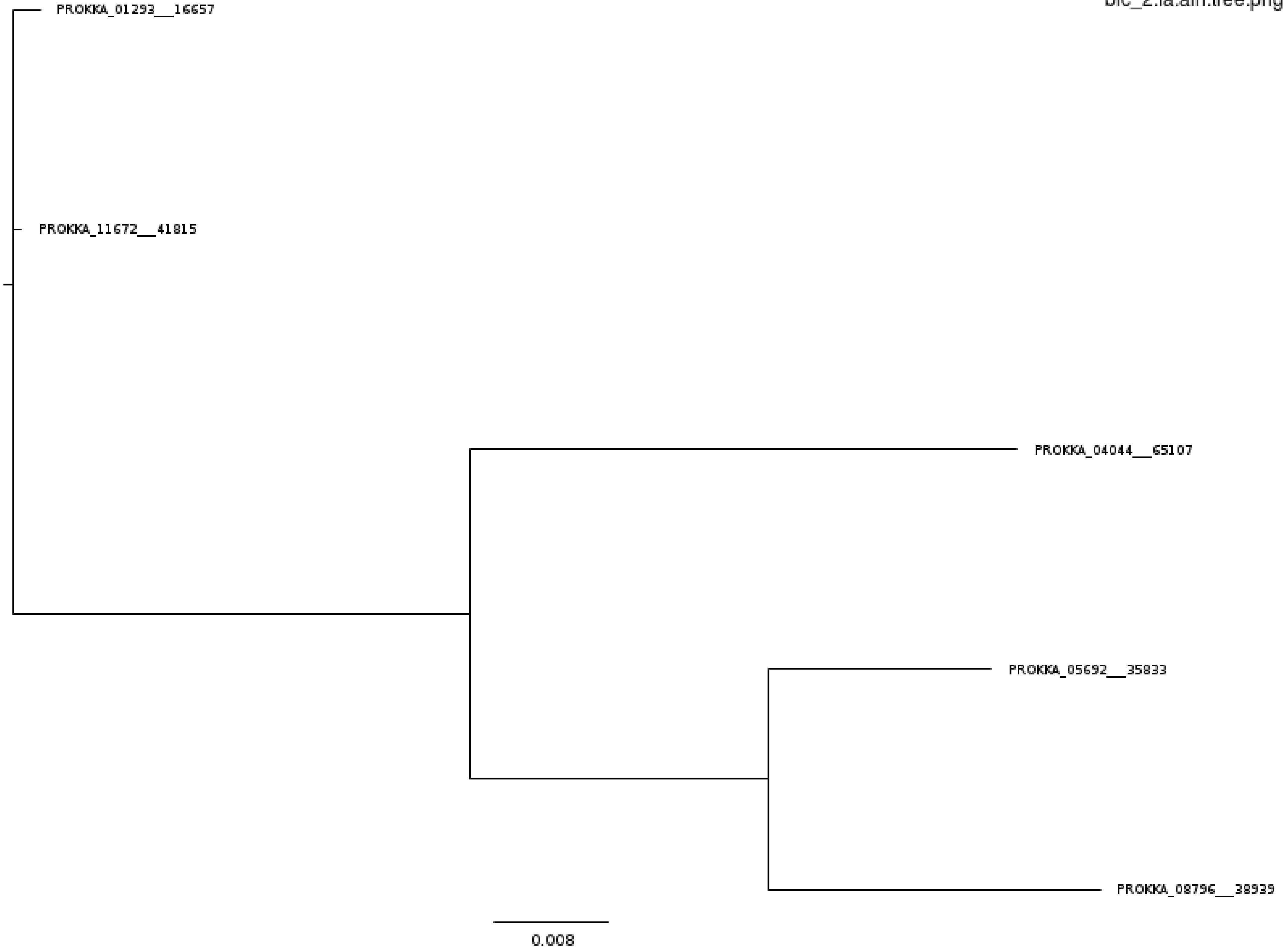

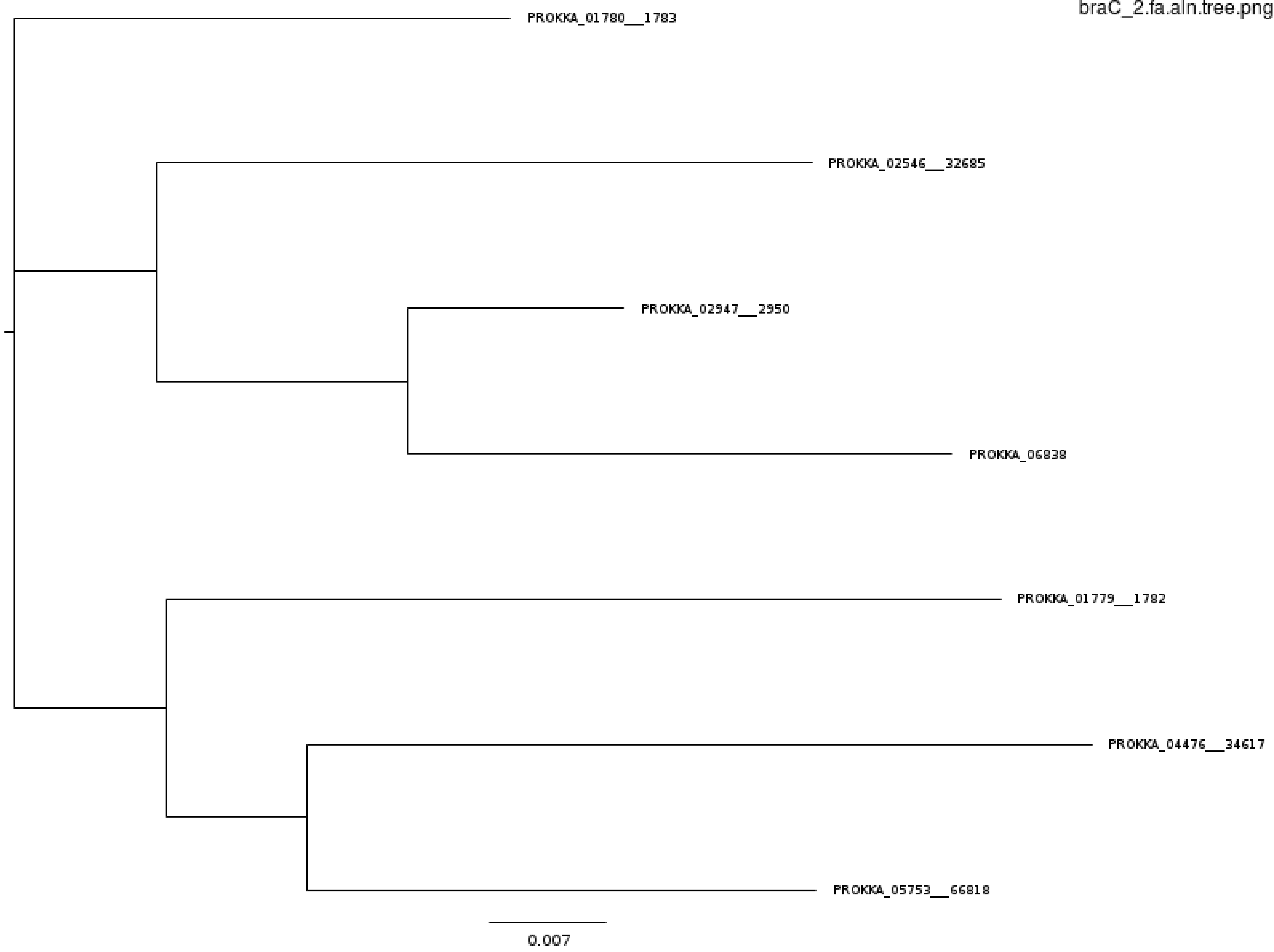
braC_2.fa.aln.tree.png
PROKKA_01780___1783
PROKKA_02546___32685
PROKKA_02947___2950
PROKKA_06838
PROKKA_01779___1782
PROKKA_04476___34617
PROKKA_05753___66818
0.007

braC_3.fa.aln.tree.png

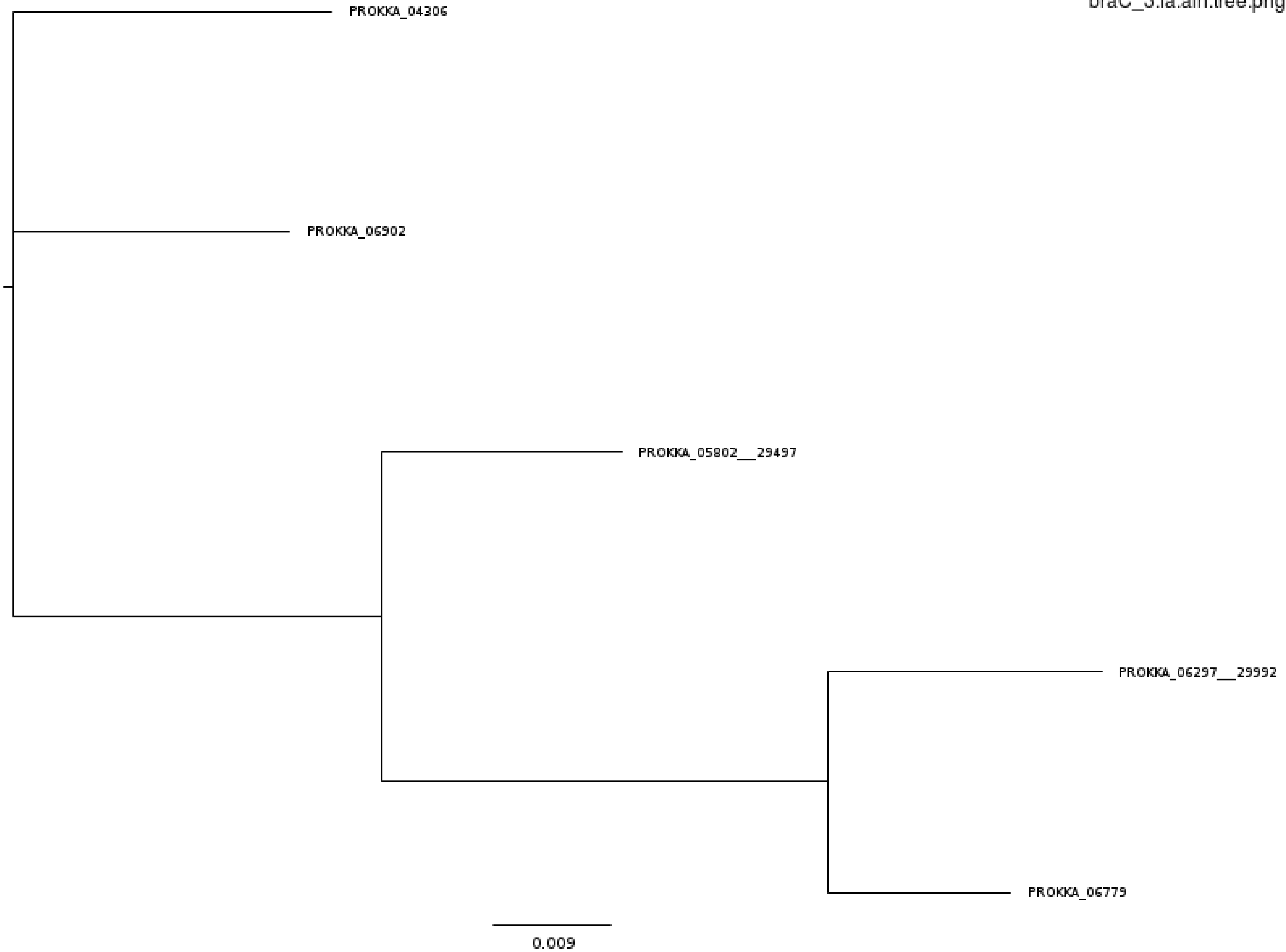

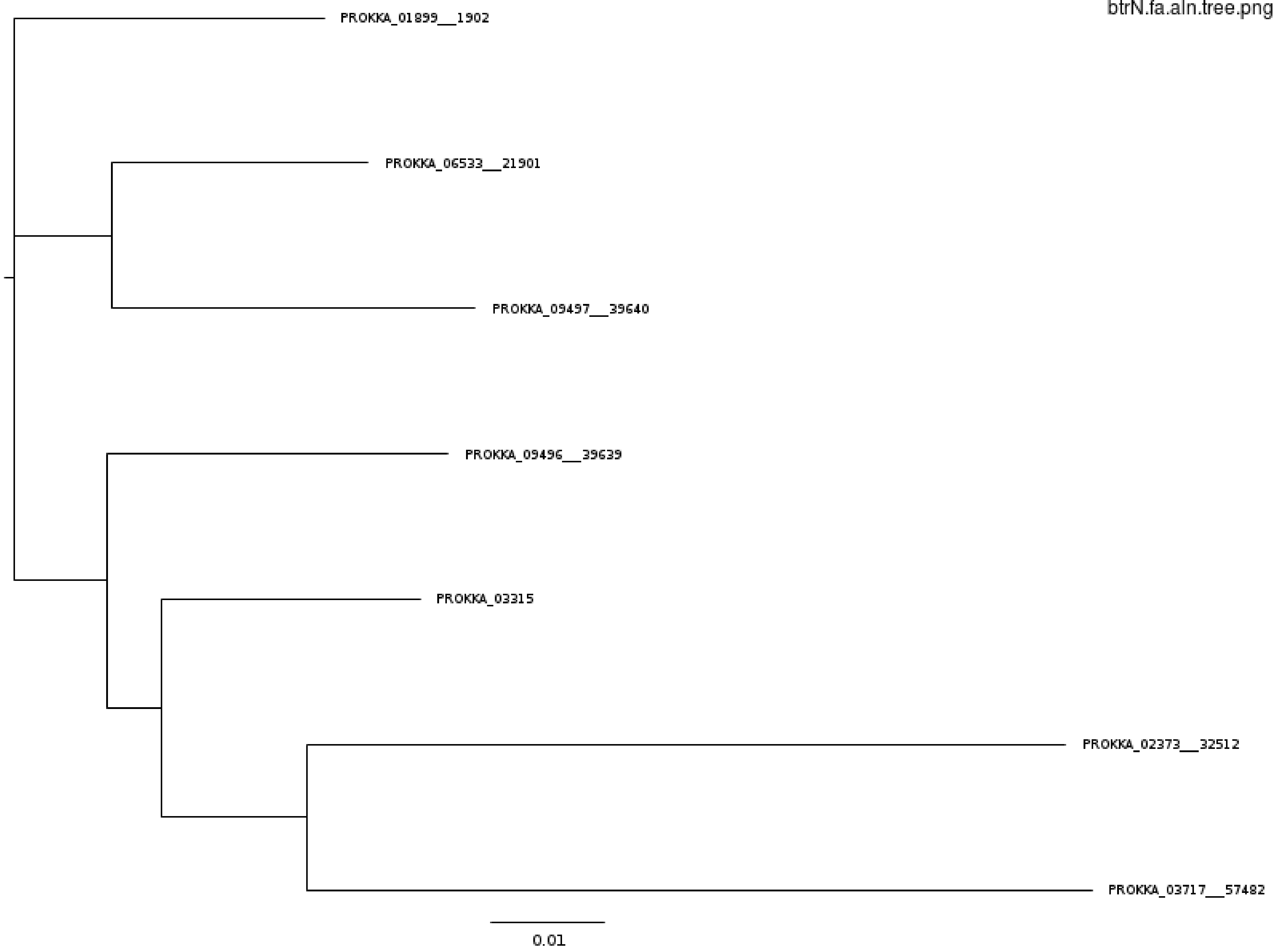
btrN.fa.aln.tree.png
PROKKA_01899___1902
PROKKA_06533___21901
PROKKA_09497___39640
PROKKA_09496___39639
PROKKA_03315
PROKKA_02373___32512
PROKKA_03717___57482
0.01

btuB_2.fa.aln.tree.png

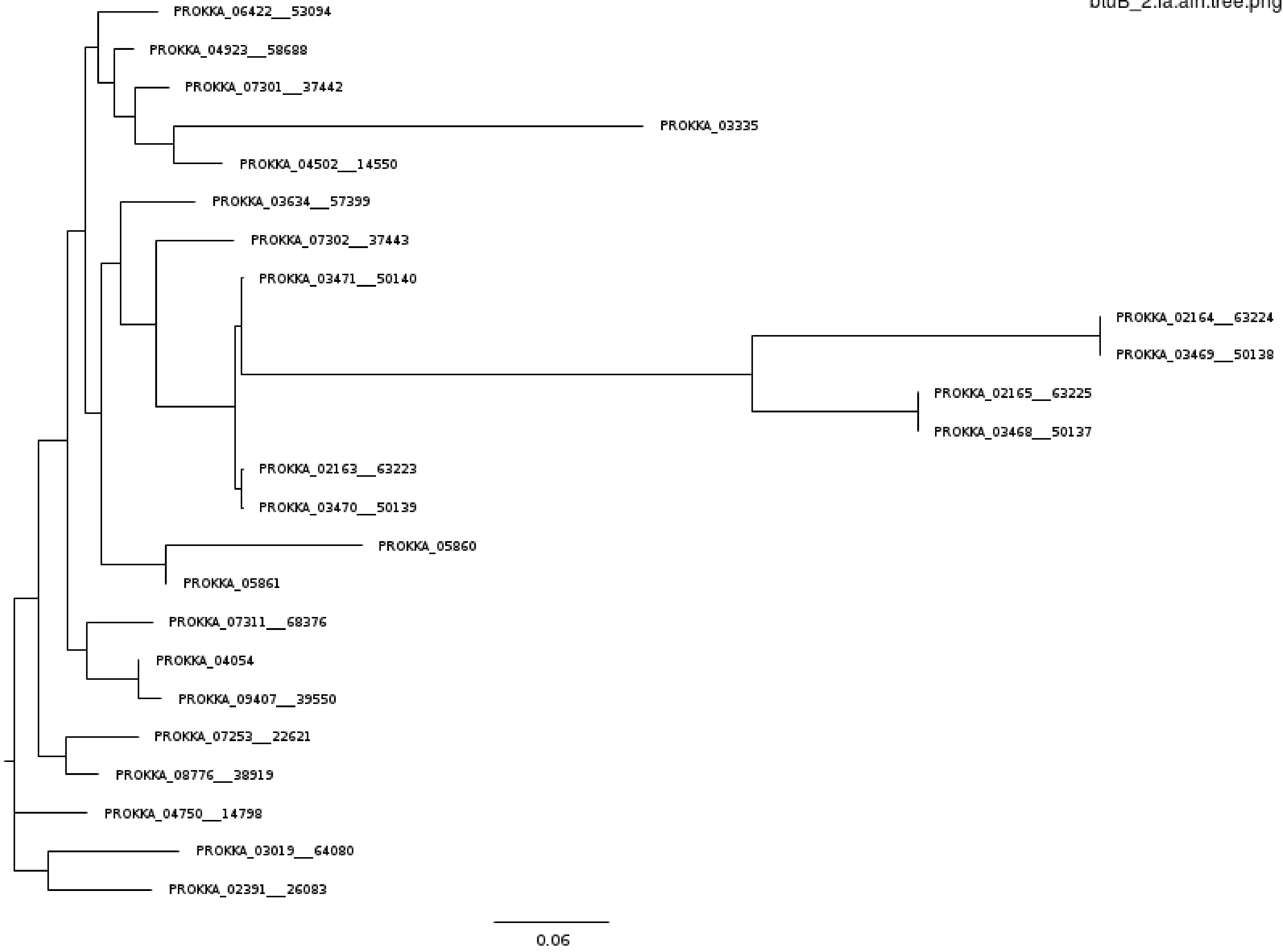

caiE.fa.aln.tree.png

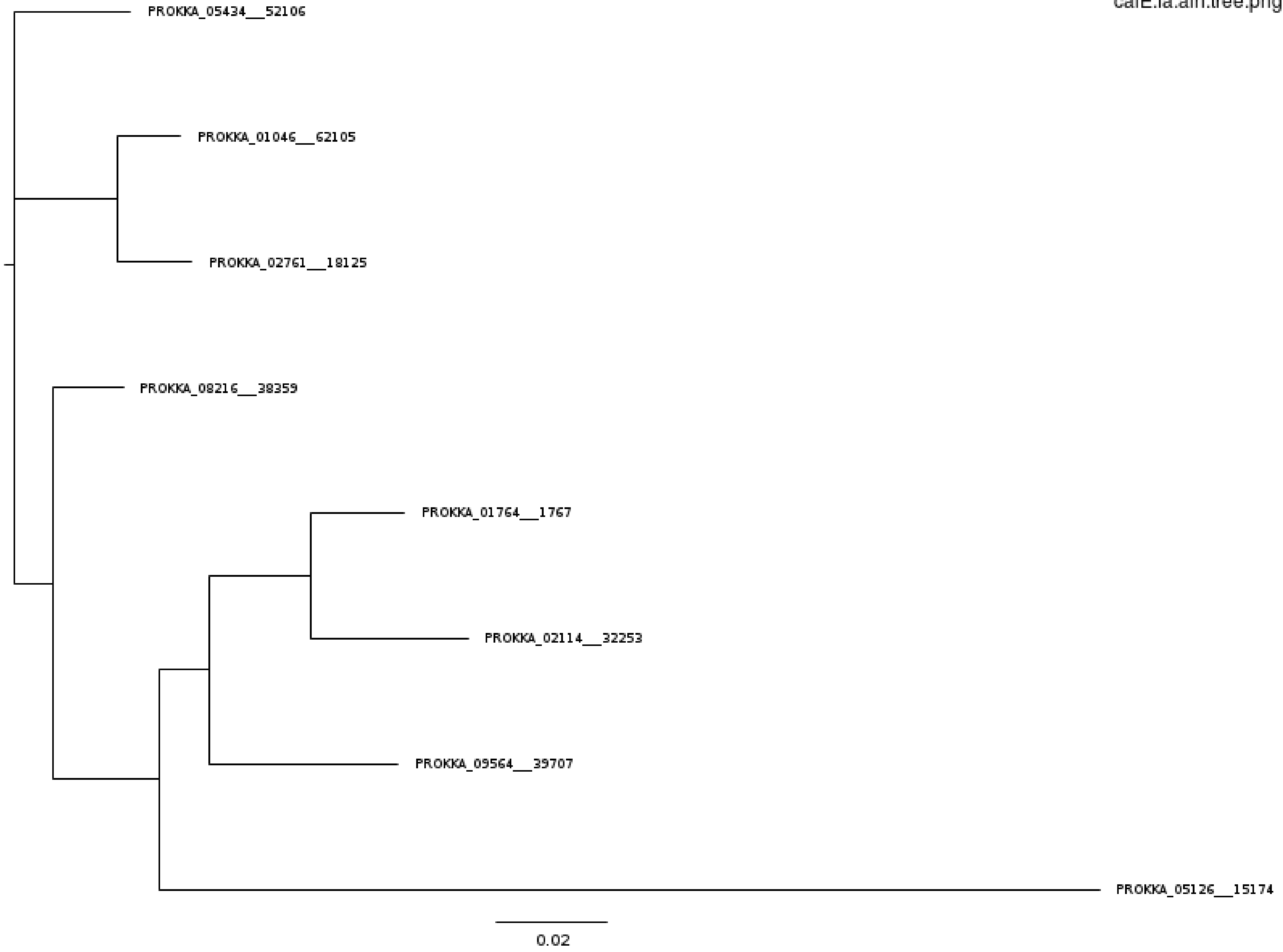

carA_2.fa.aln.tree.png

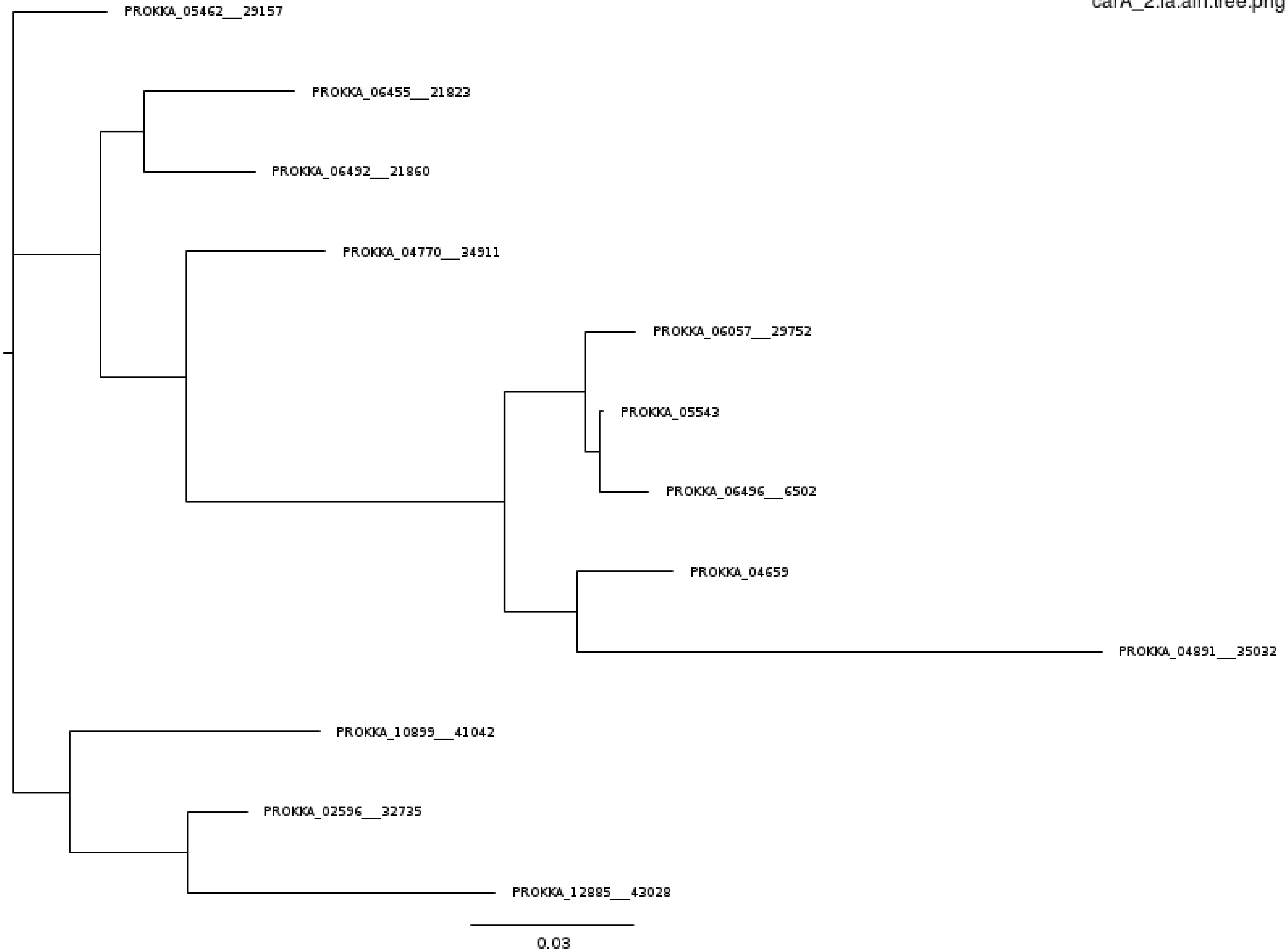

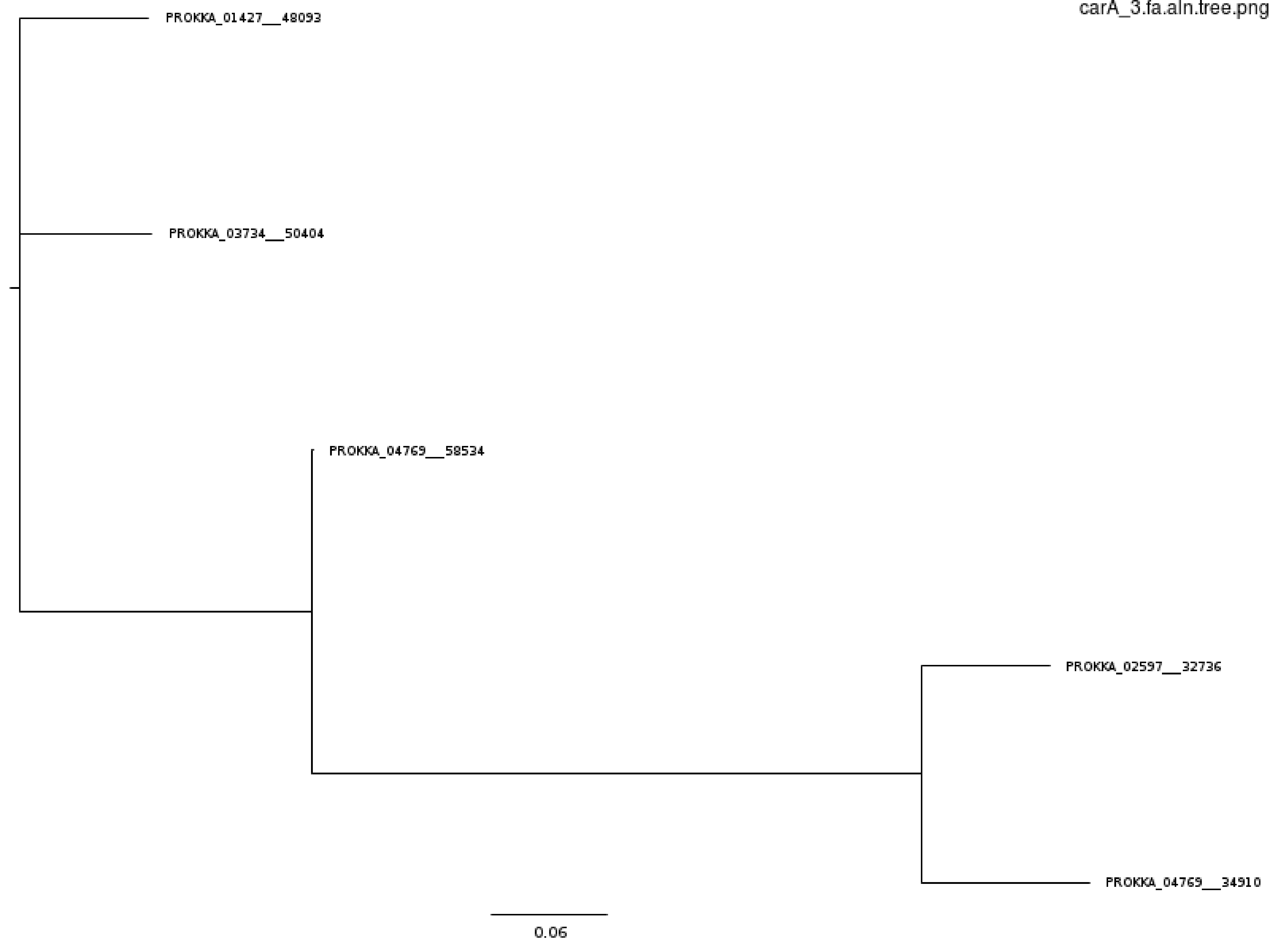
carA_3.fa.aln.tree.png
PROKKA_01427___48093
PROKKA_03734___50404
PROKKA_04769___58534
PROKKA_02597___32736
PROKKA_04769___34910
0.06

carB_3.fa.aln.tree.png

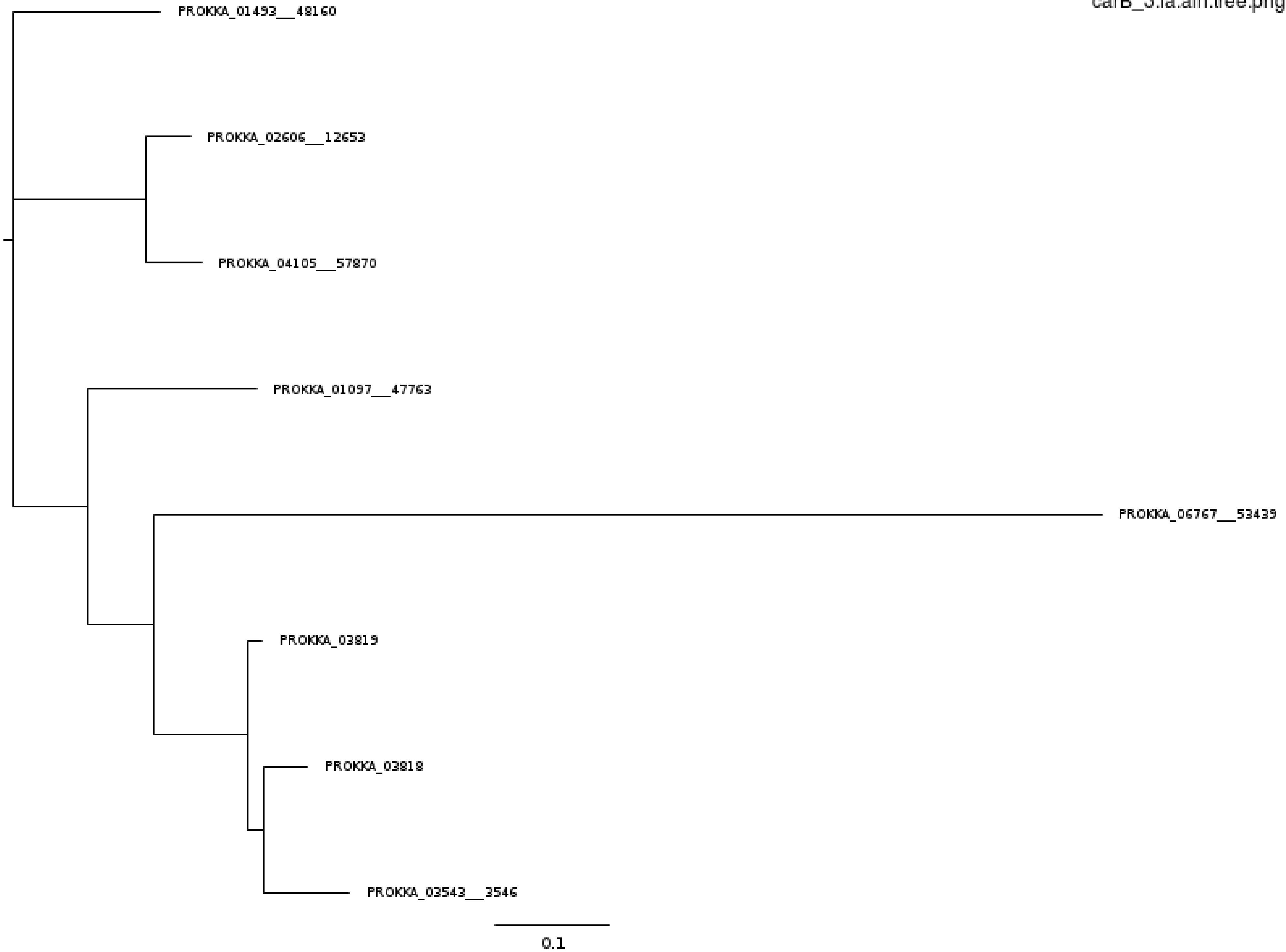

carB_4.fa.aln.tree.png

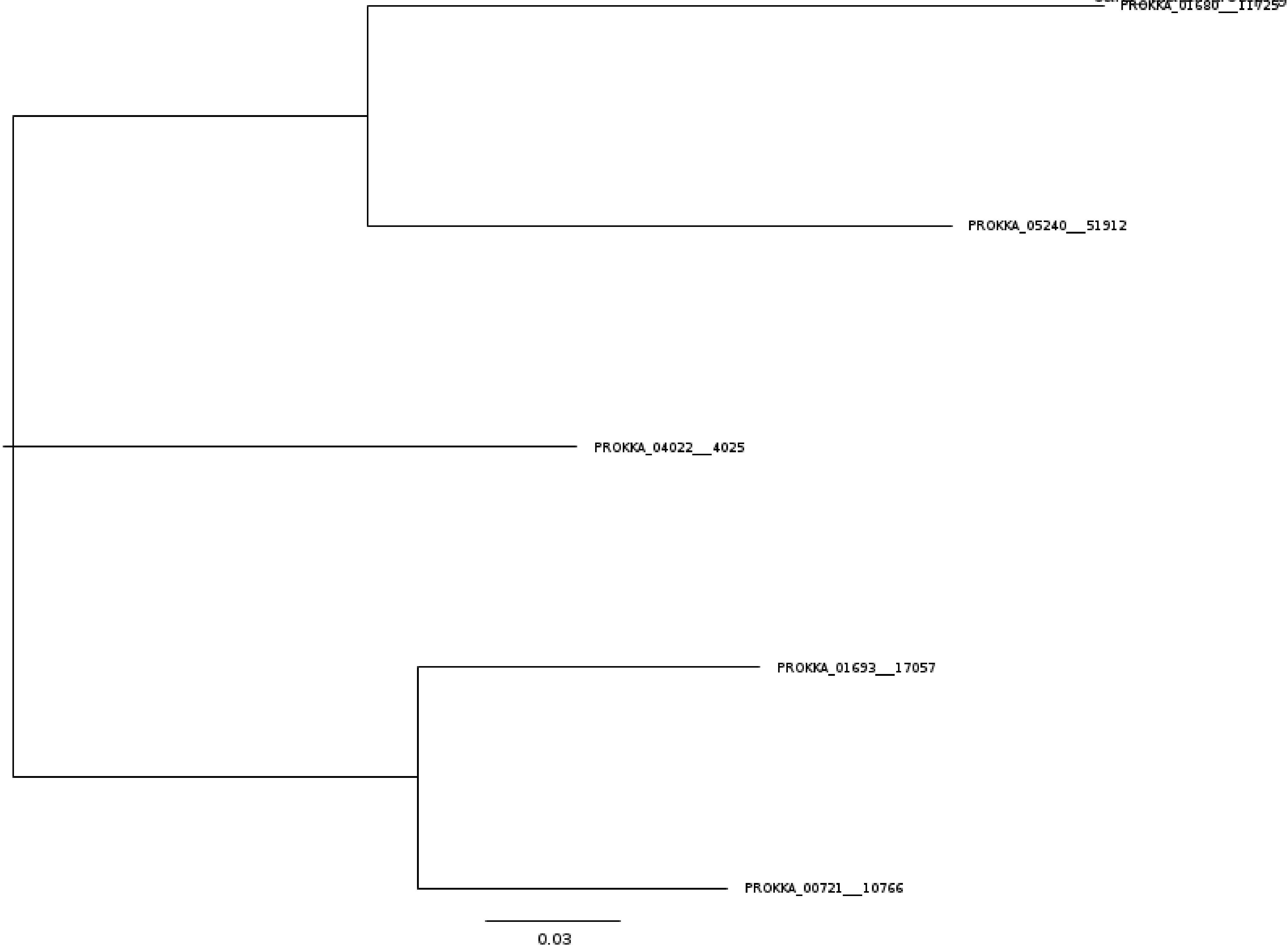

cas6_1.fa.aln.tree.png

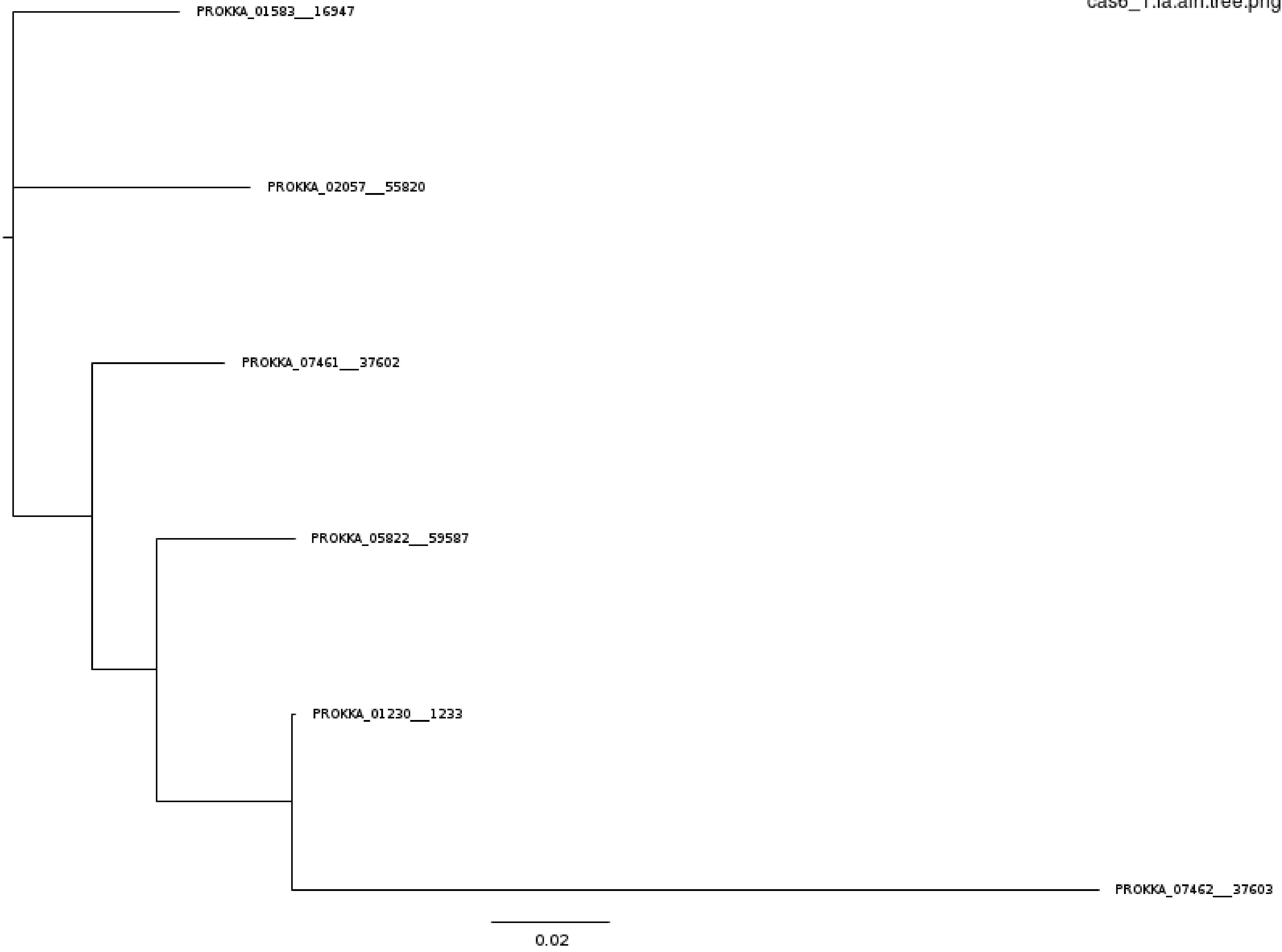

catE_2.fa.aln.tree.png

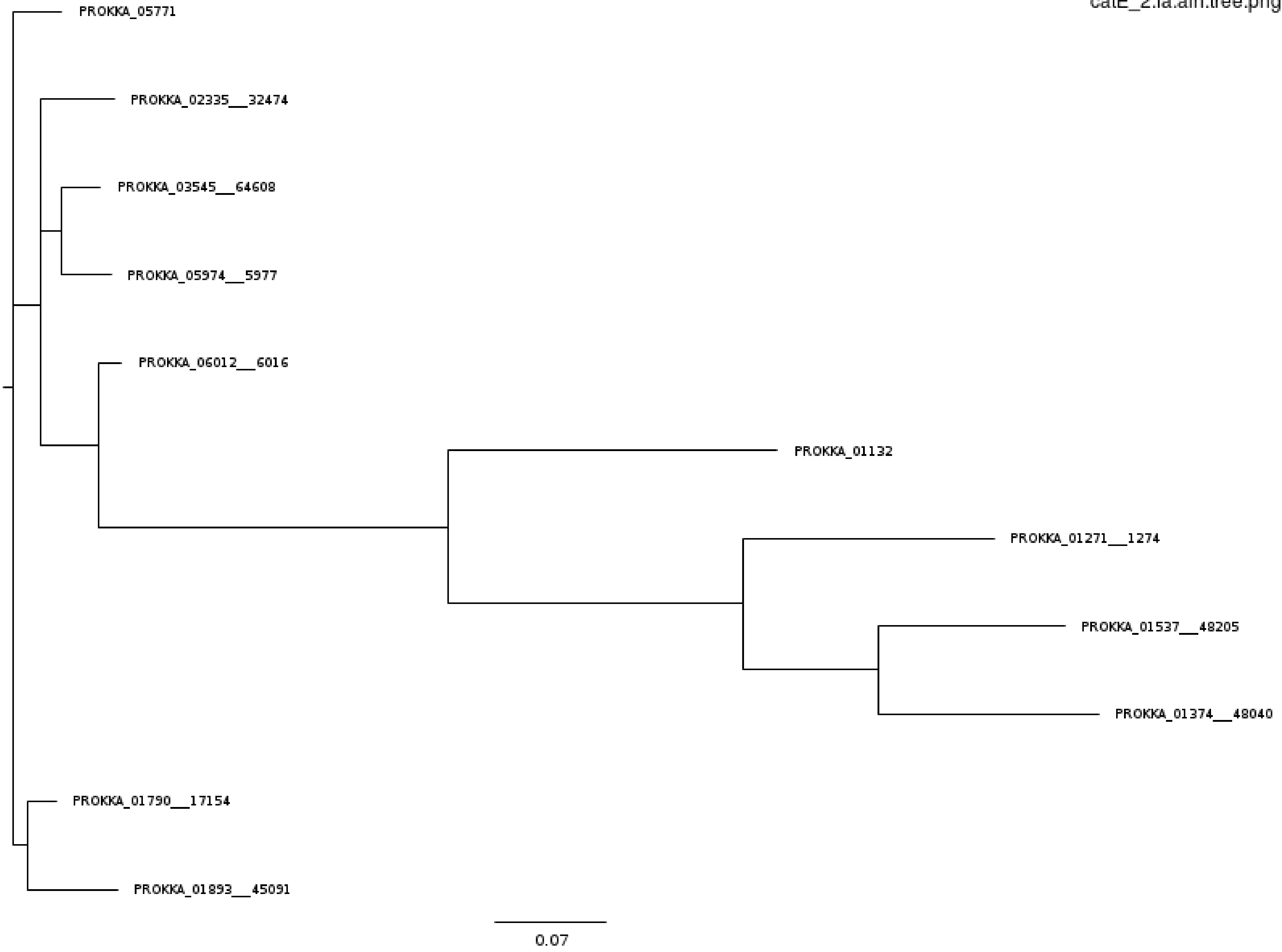

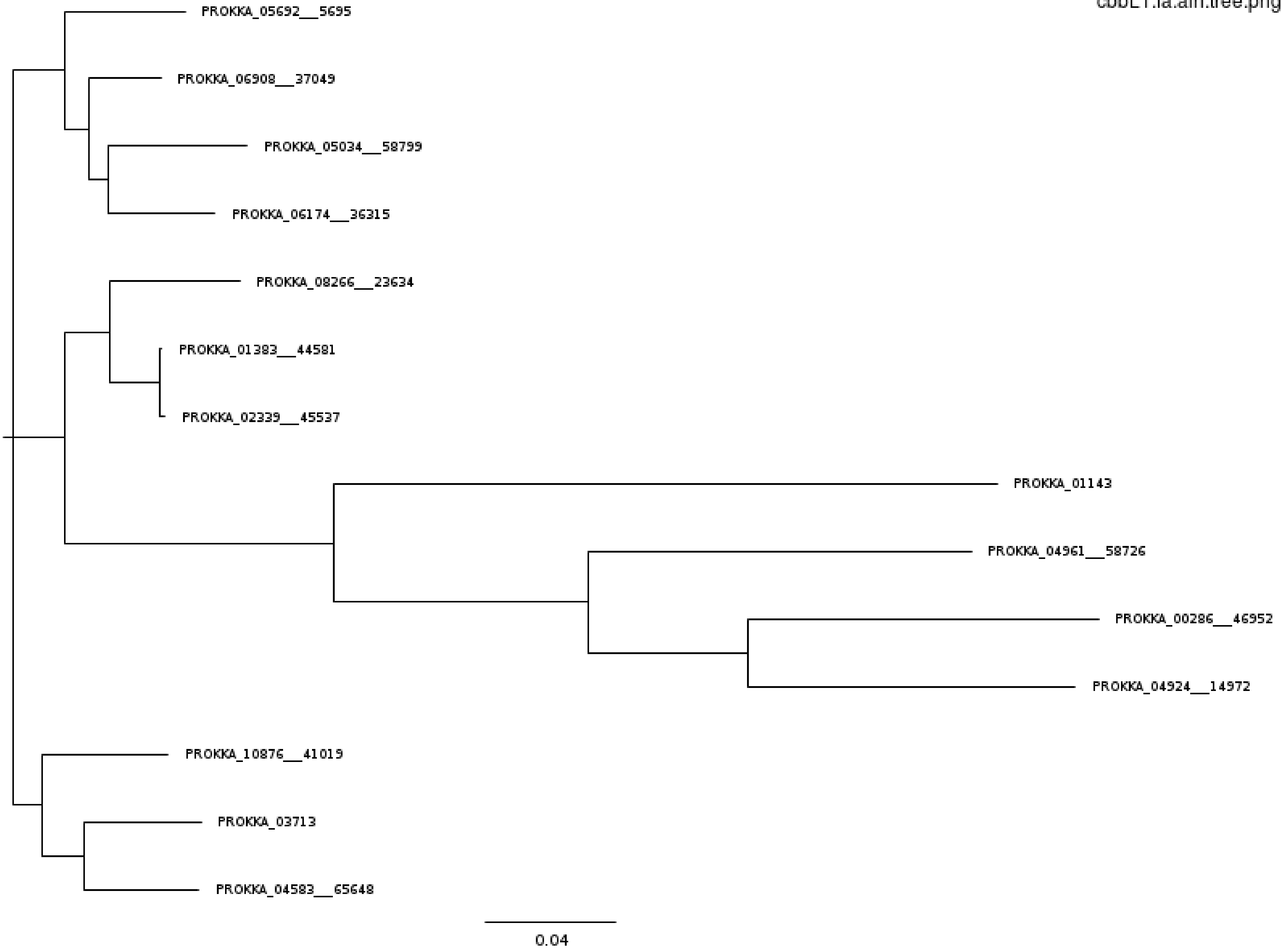
PROKKA_05692__5695
PROKKA_06908__37049
PROKKA_05034__58799
PROKKA_06174__36315
PROKKA_08266__23634
PROKKA_01383__44581
PROKKA_02339__45537
PROKKA_01143
PROKKA_04961__58726
PROKKA_00286__46952
PROKKA_04924__14972
PROKKA_10876__41019
PROKKA_03713
PROKKA_04583__65648
0.04

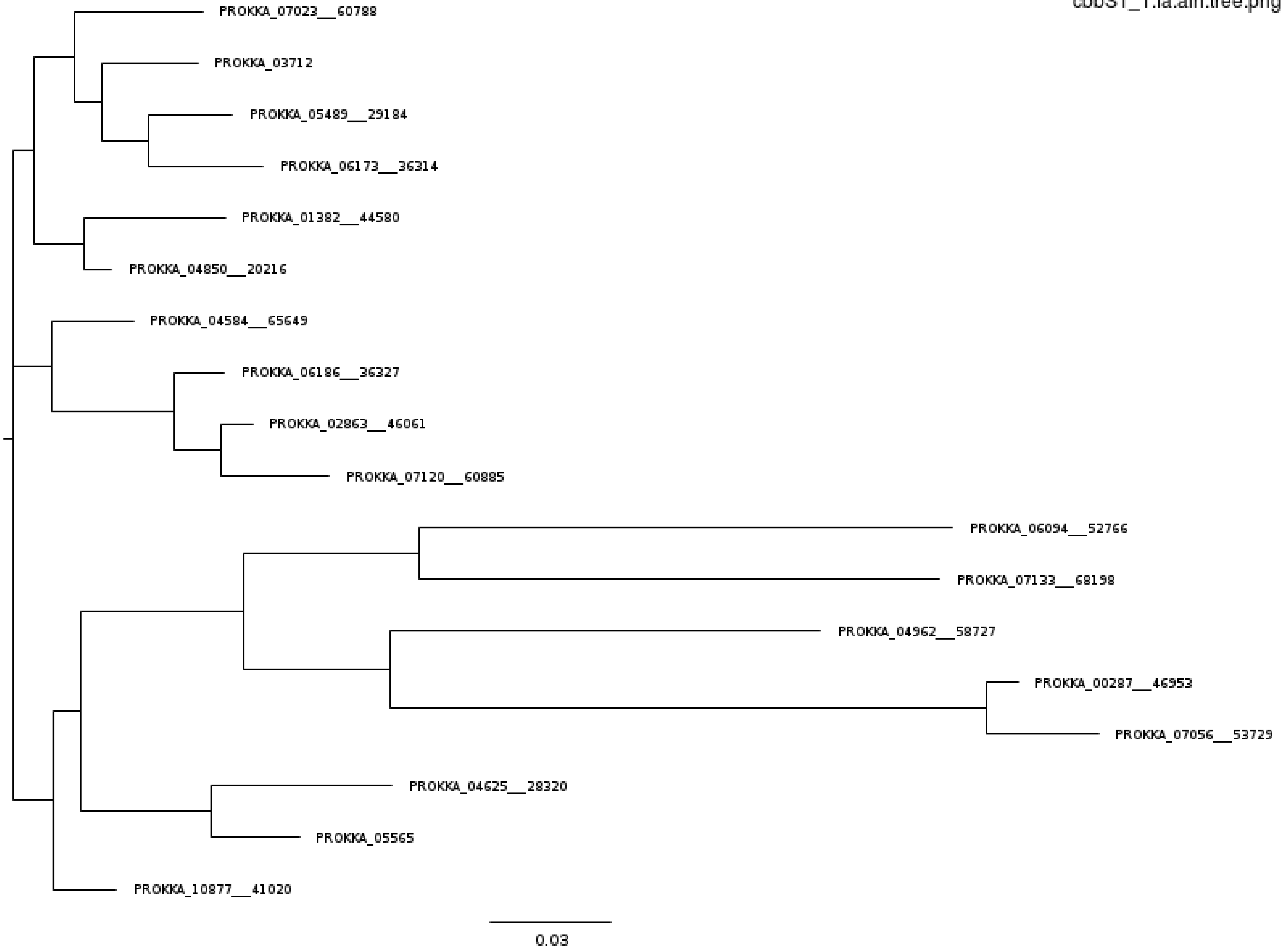
PROKKA_07023___60788
PROKKA_03712
PROKKA_05489___29184
PROKKA_06173___36314
PROKKA_01382___44580
PROKKA_04850___20216
PROKKA_04584___65649
PROKKA_06186___36327
PROKKA_02863___46061
PROKKA_07120___60885
PROKKA_06094___52766
PROKKA_07133___68198
PROKKA_04962___58727
PROKKA_00287___46953
PROKKA_07056___53729
PROKKA_04625___28320
PROKKA_05565
PROKKA_10877___41020
0.03

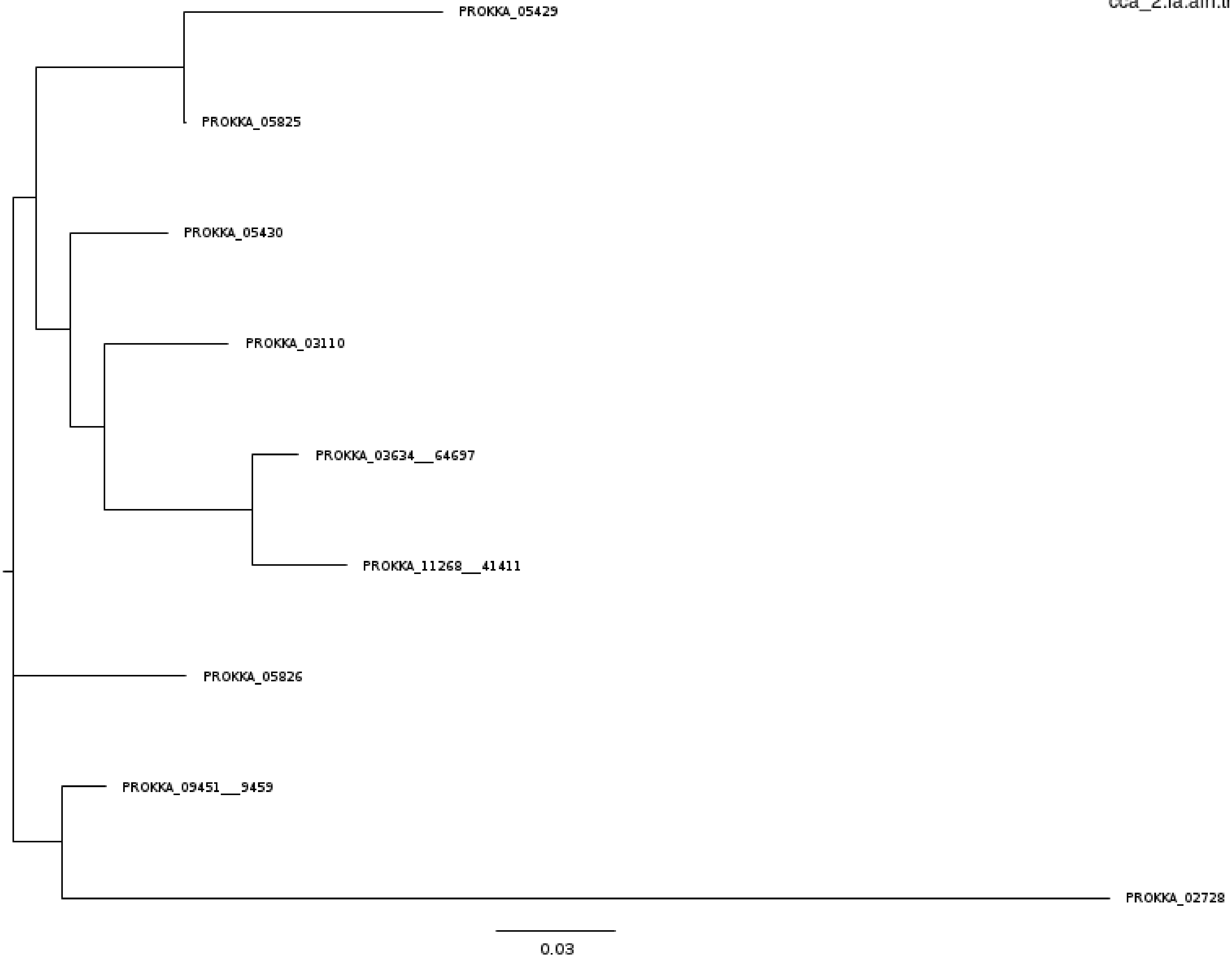
PROKKA_05429
PROKKA_05825
PROKKA_05430
PROKKA_03110
PROKKA_03634___64697
PROKKA_11268___41411
PROKKA_05826
PROKKA_09451___9459
PROKKA_02728
0.03

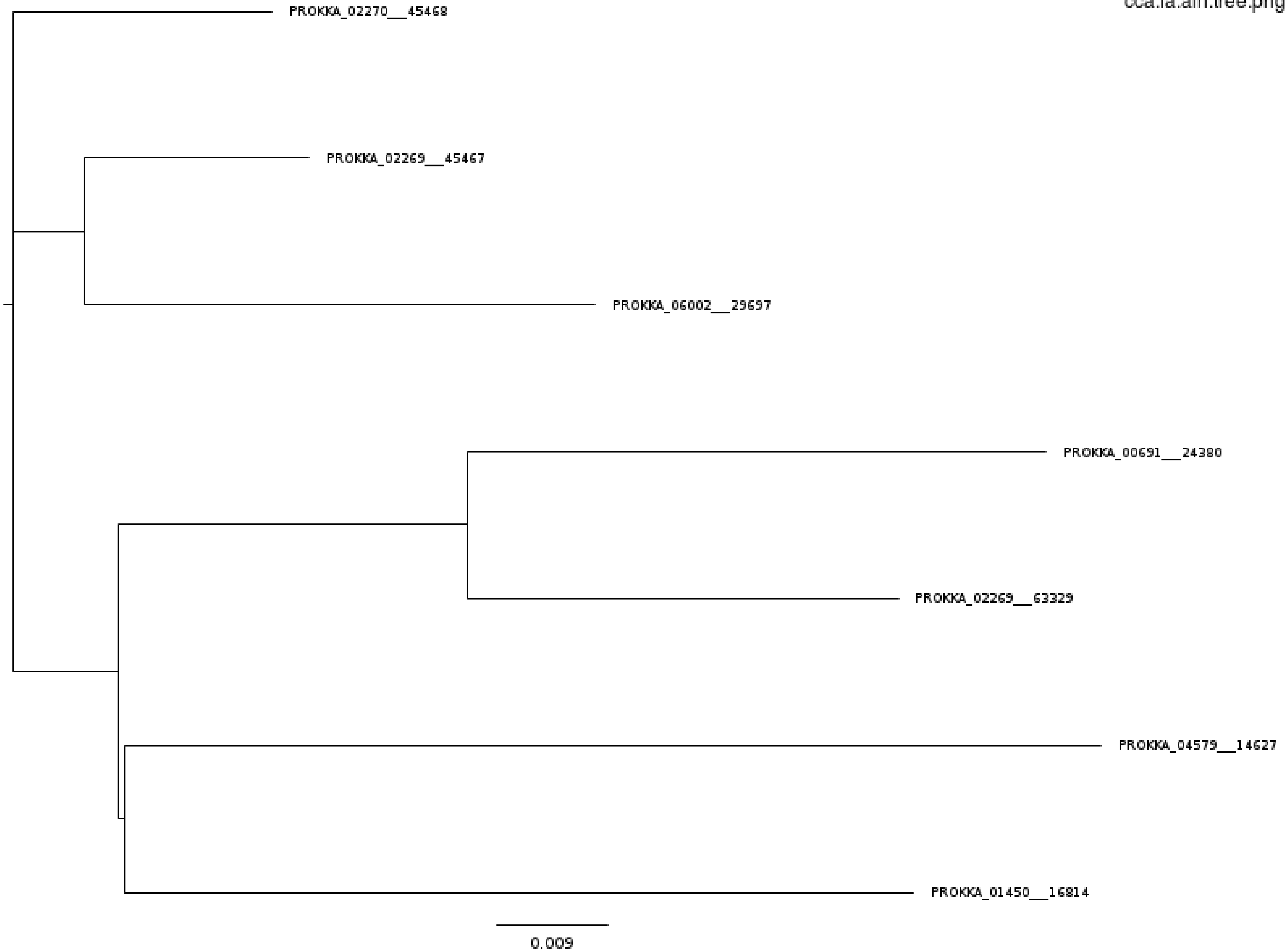
PROKKA_02270___45468
PROKKA_02269___45467
PROKKA_06002___29697
PROKKA_00691___24380
PROKKA_02269___63329
PROKKA_04579___14627
PROKKA_01450___16814
0.009

ccmA_2.fa.aln.tree.png

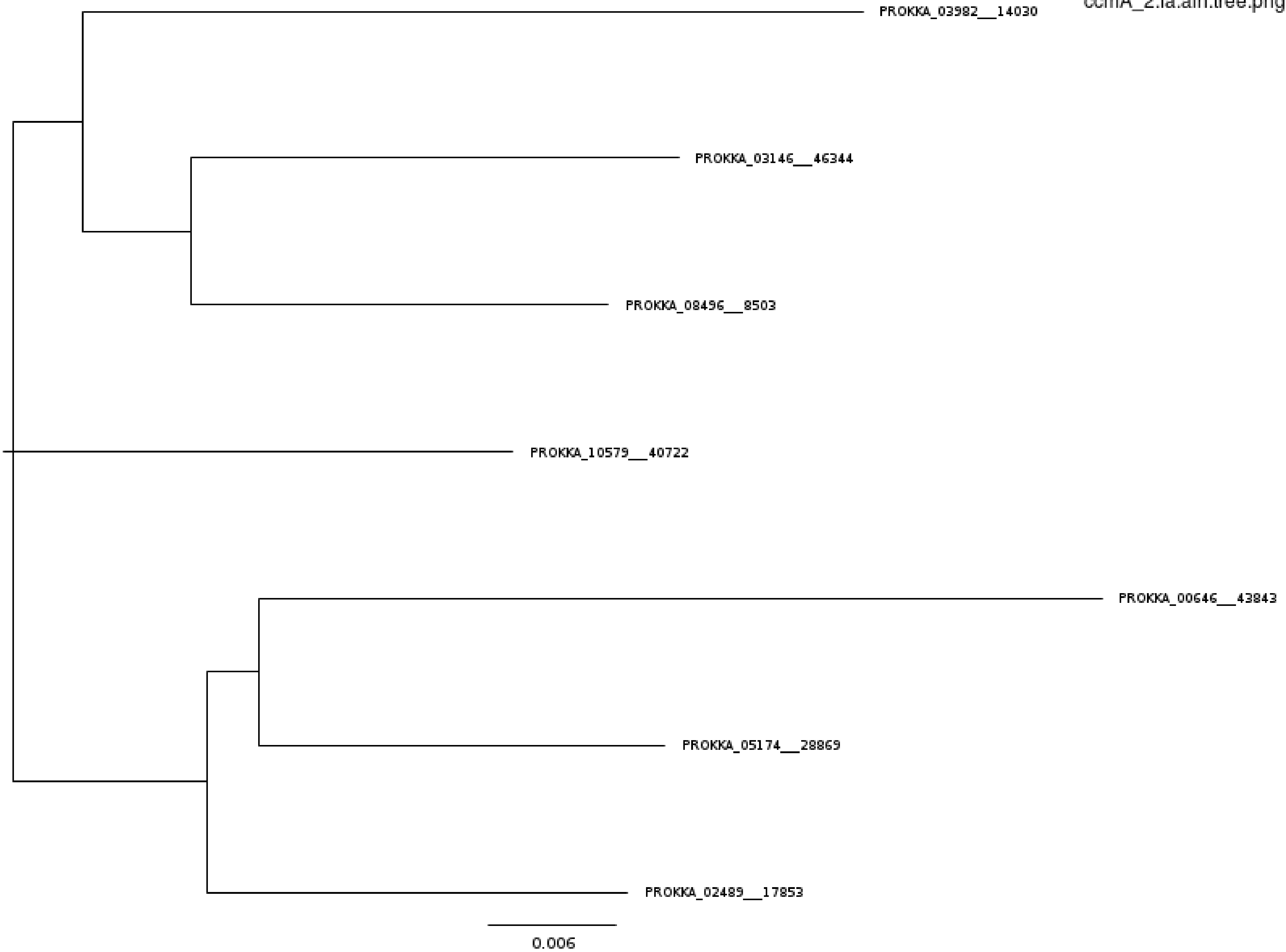

ccmC_2.fa.aln.tree.png

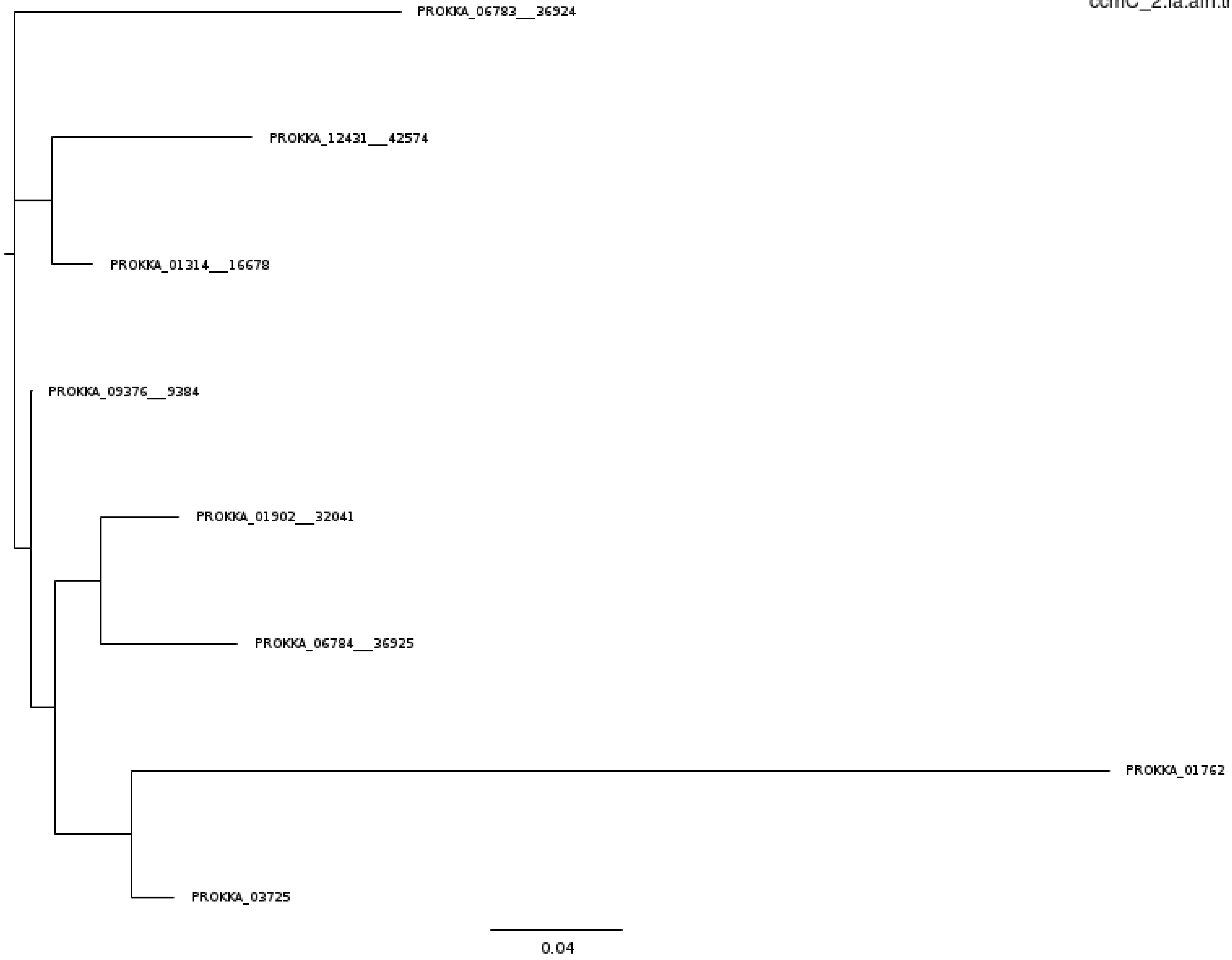

ccmF_2.fa.aln.tree.png

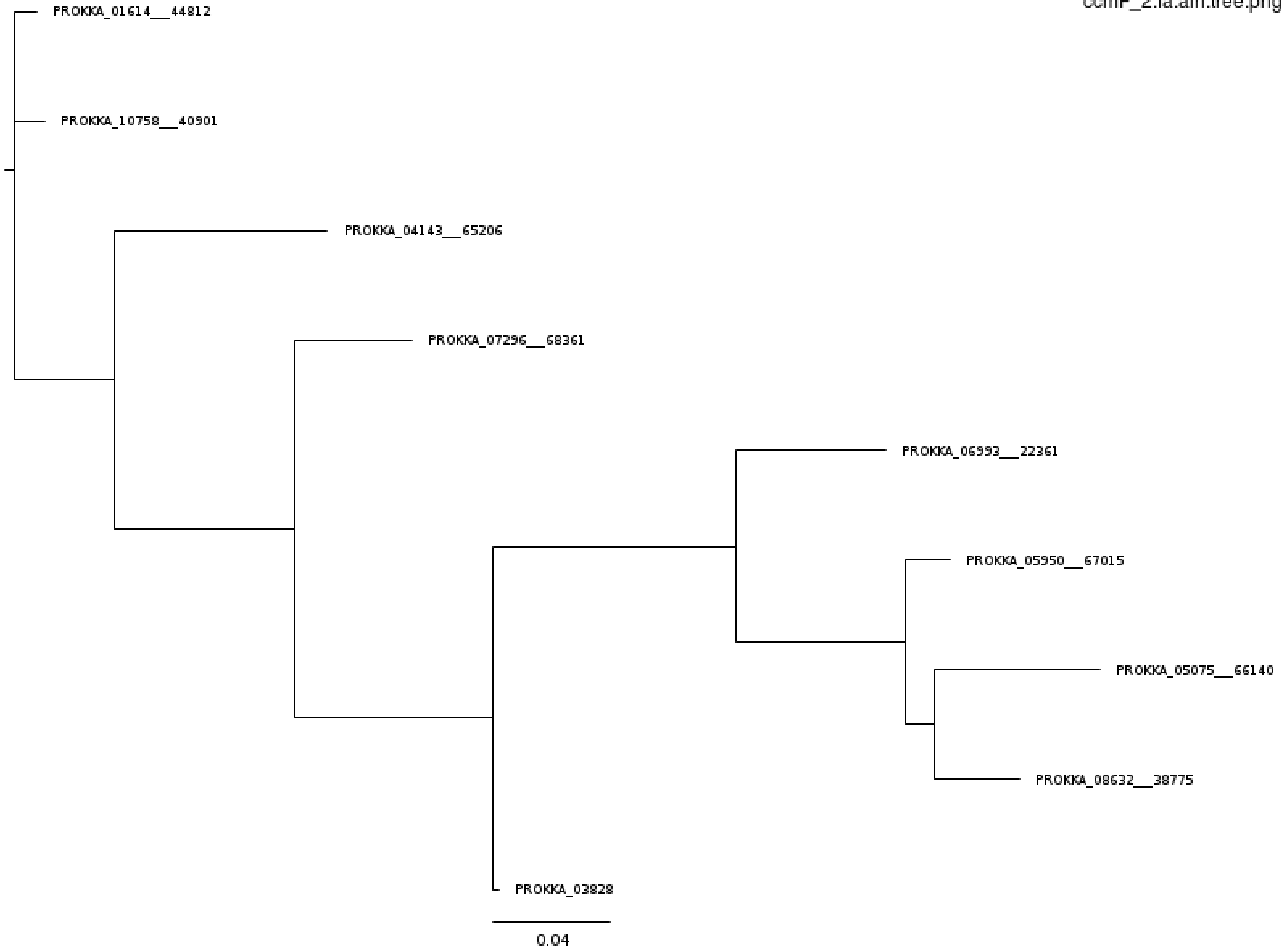

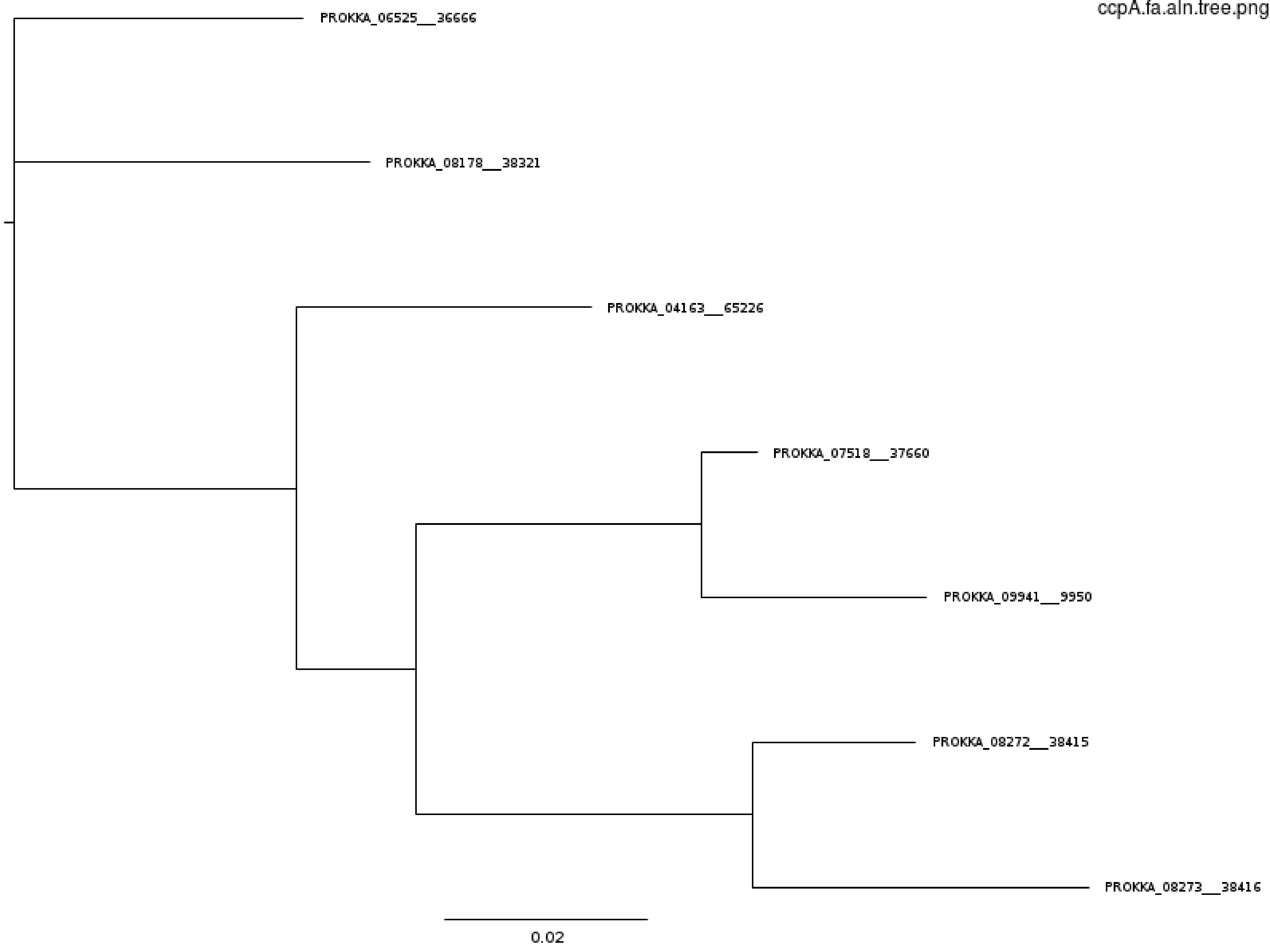
ccpA.fa.aln.tree.png
PROKKA_06525___36666
PROKKA_08178___38321
PROKKA_04163___65226
PROKKA_07518___37660
PROKKA_09941___9950
PROKKA_08272___38415
PROKKA_08273___38416
0.02

celH_3.fa.aln.tree.png

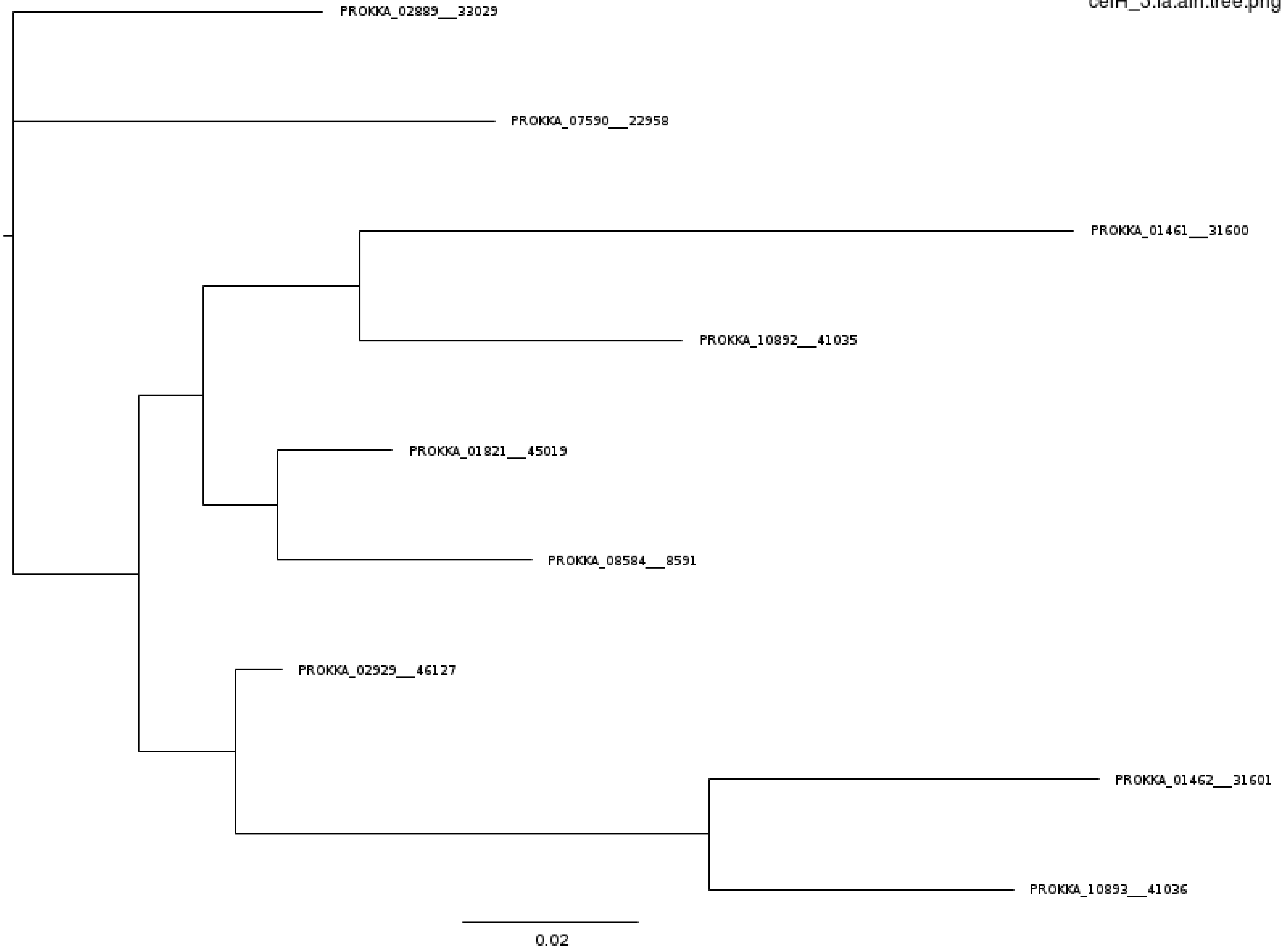

cfiB_2.fa.aln.tree.png

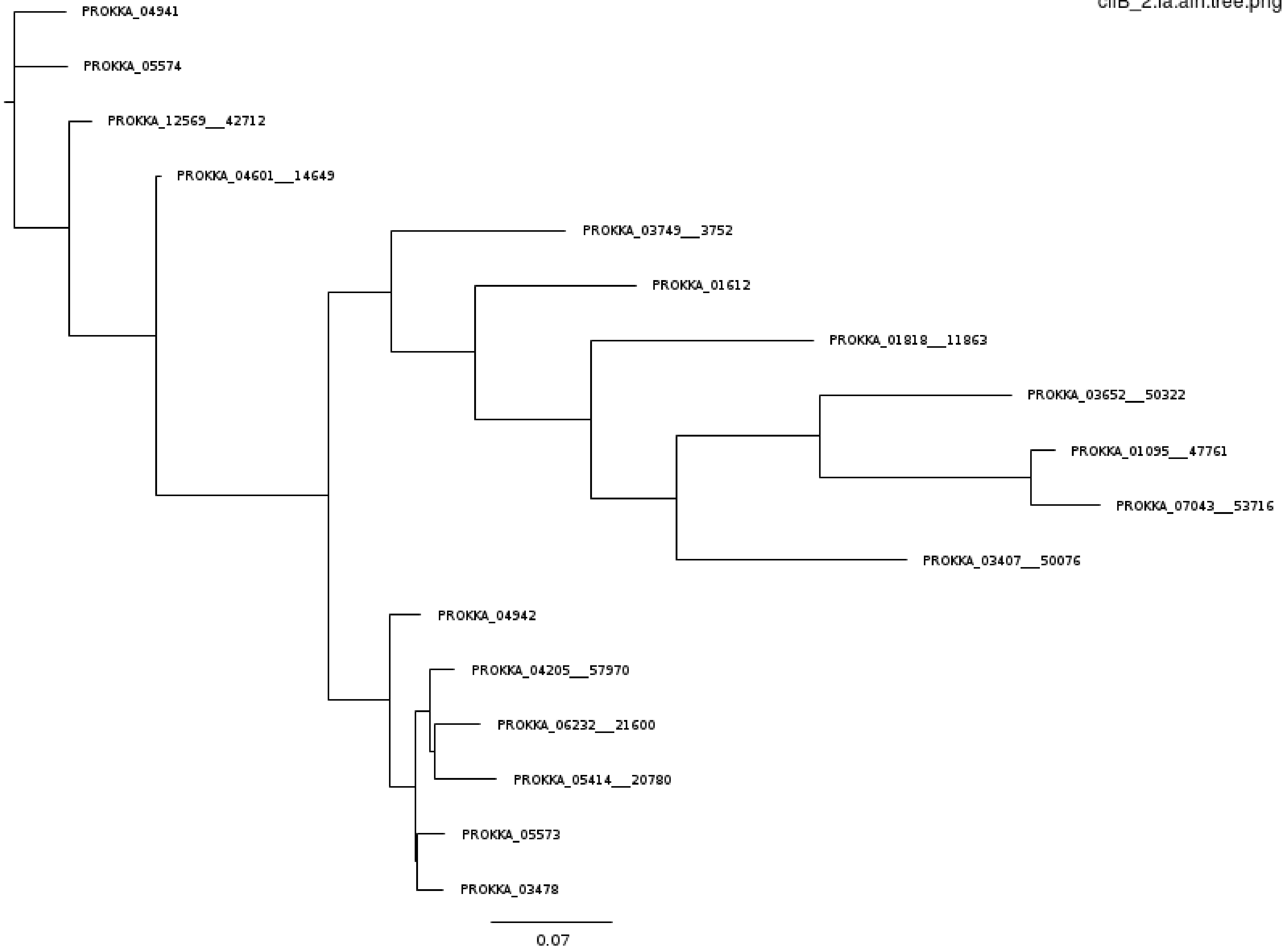

cfxP_2.fa.aln.tree.png

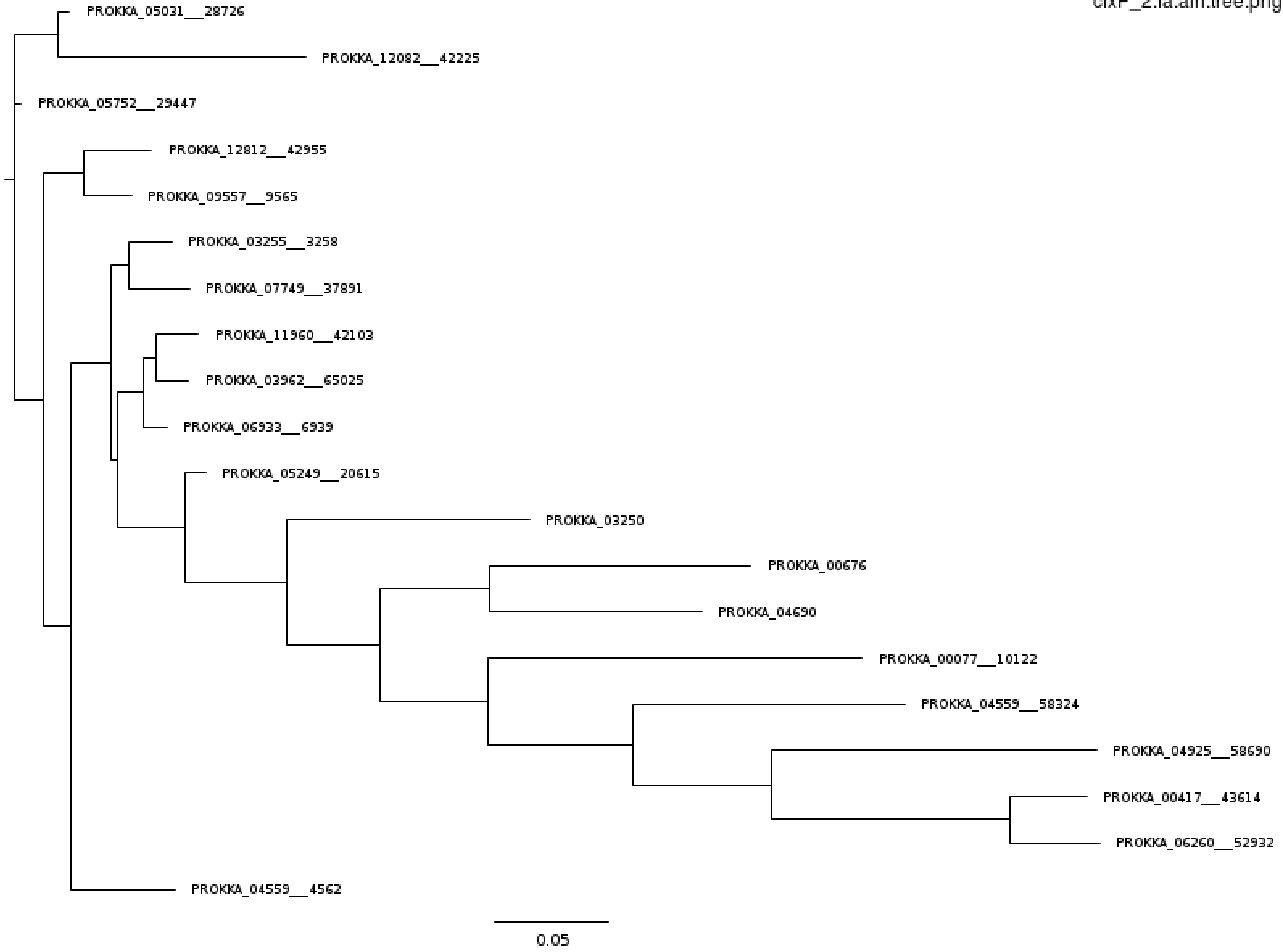

cheA_2.fa.aln.tree.png

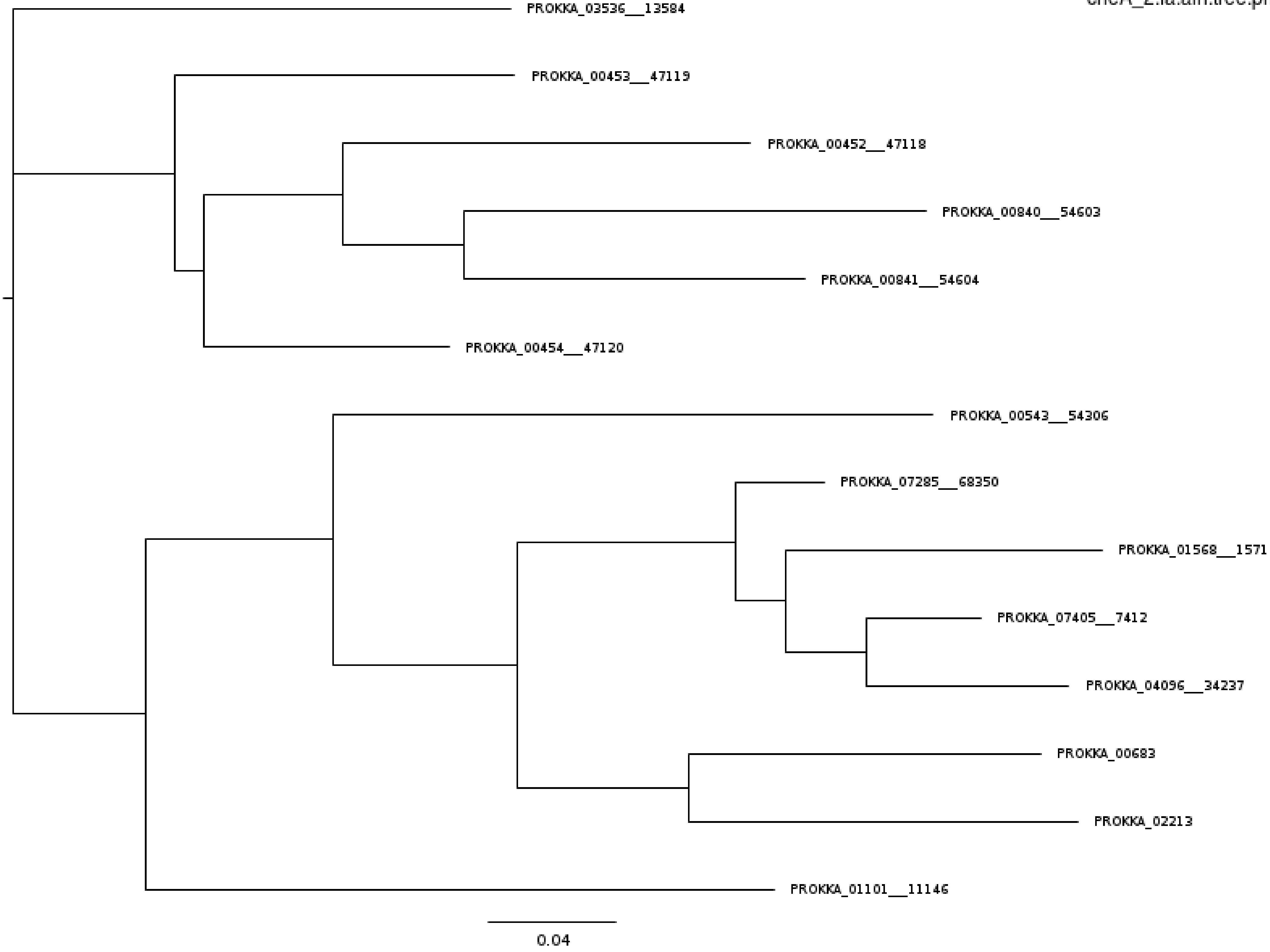

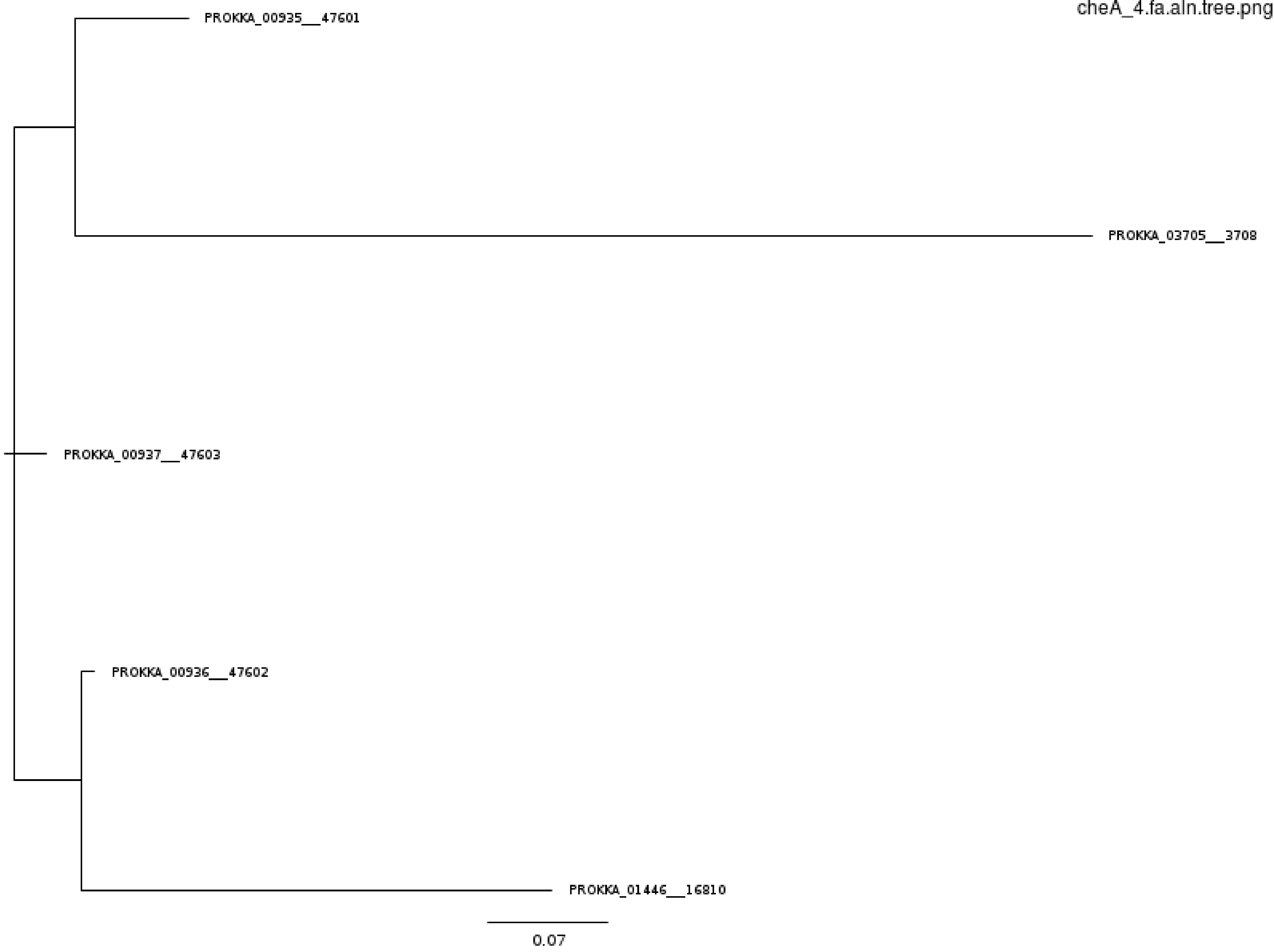
cheA_4.fa.aln.tree.png
PROKKA_00935___47601
PROKKA_03705___3708
PROKKA_00937___47603
PROKKA_00936___47602
PROKKA_01446___16810
0.07

cheB1_1.fa.aln.tree.png

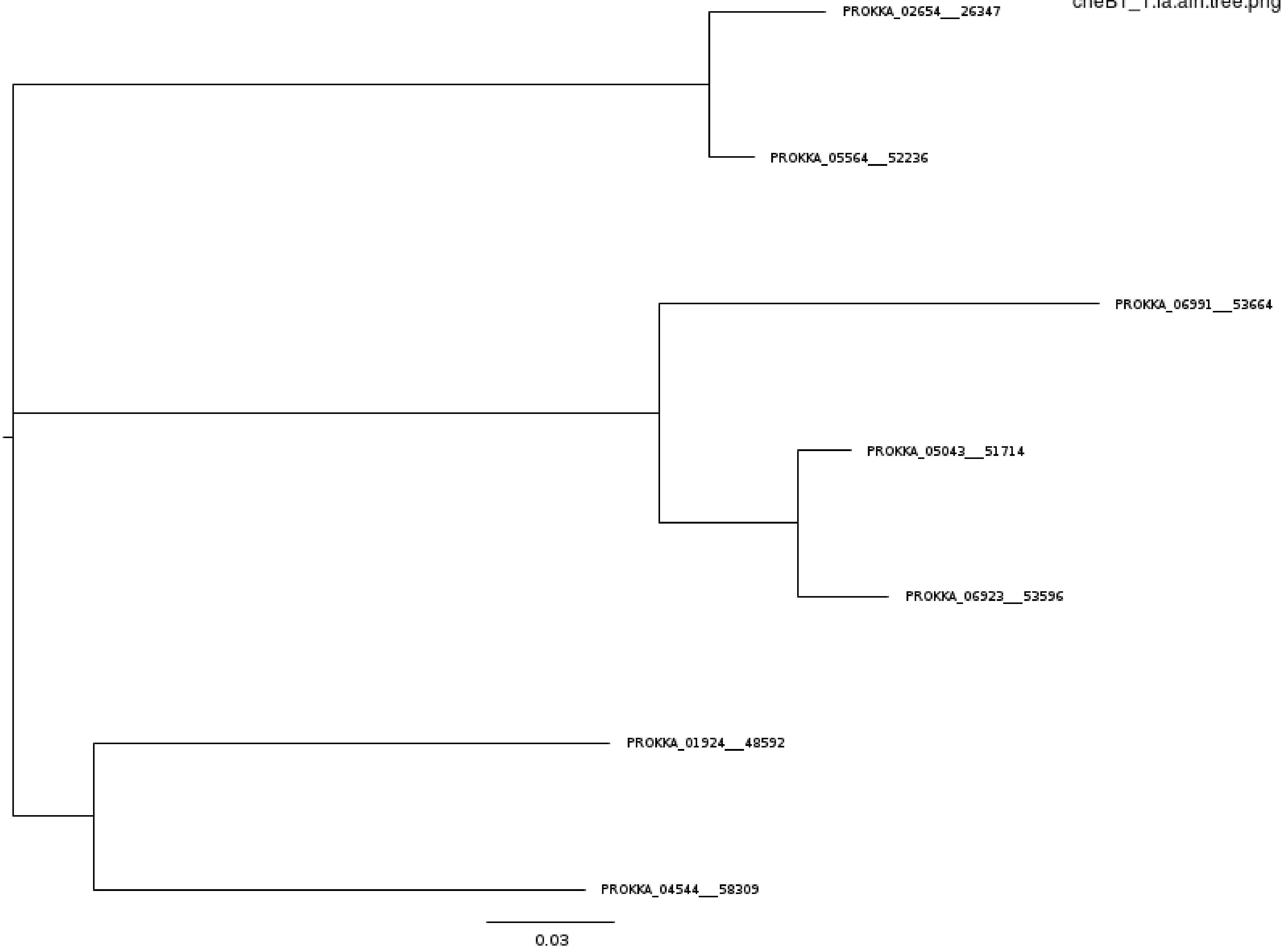

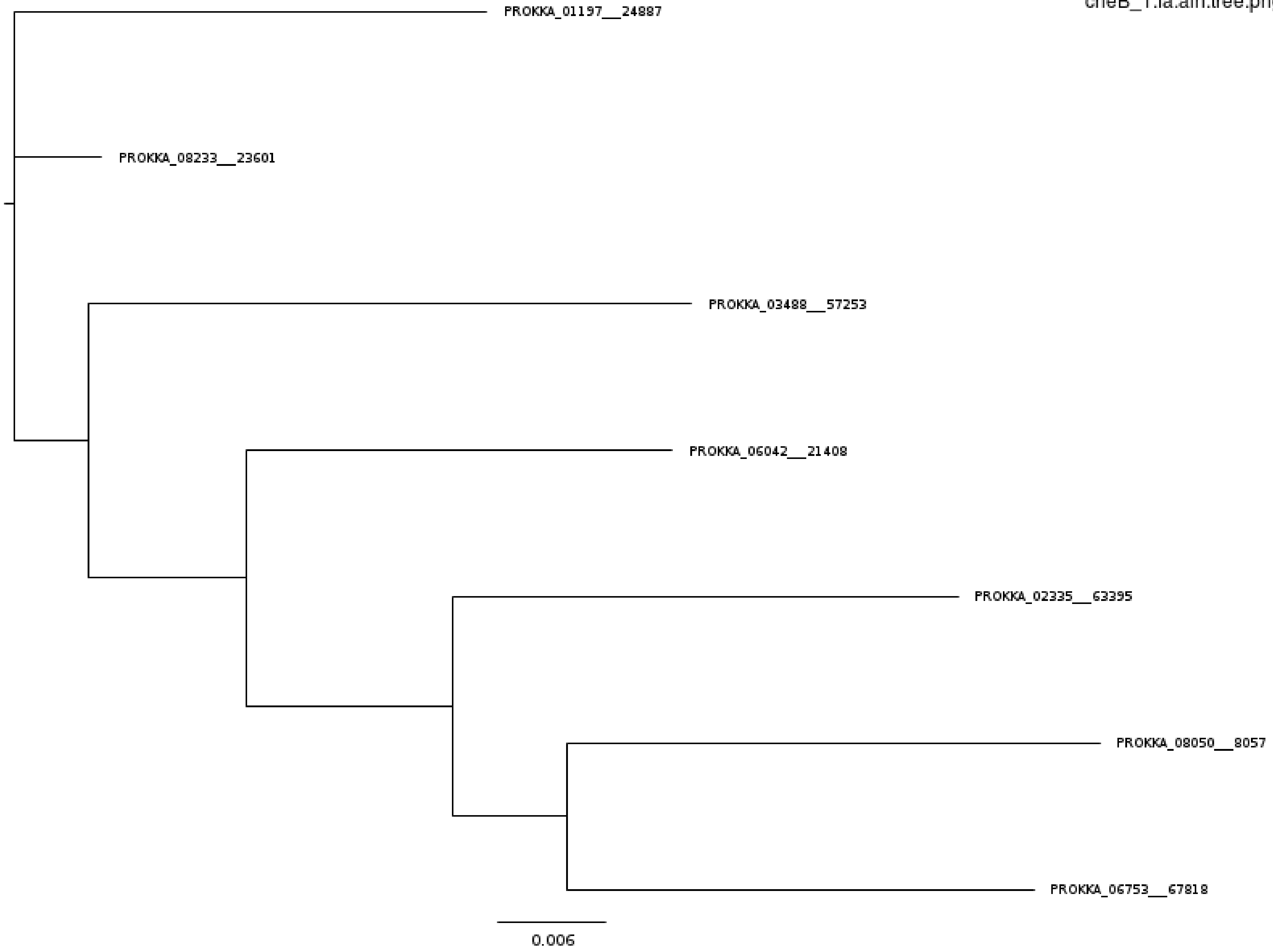
PROKKA_01197___24887
PROKKA_08233___23601
PROKKA_03488___57253
PROKKA_06042___21408
PROKKA_02335___63395
PROKKA_08050___8057
PROKKA_06753___67818
0.006

cheB3_2.fa.aln.tree.png

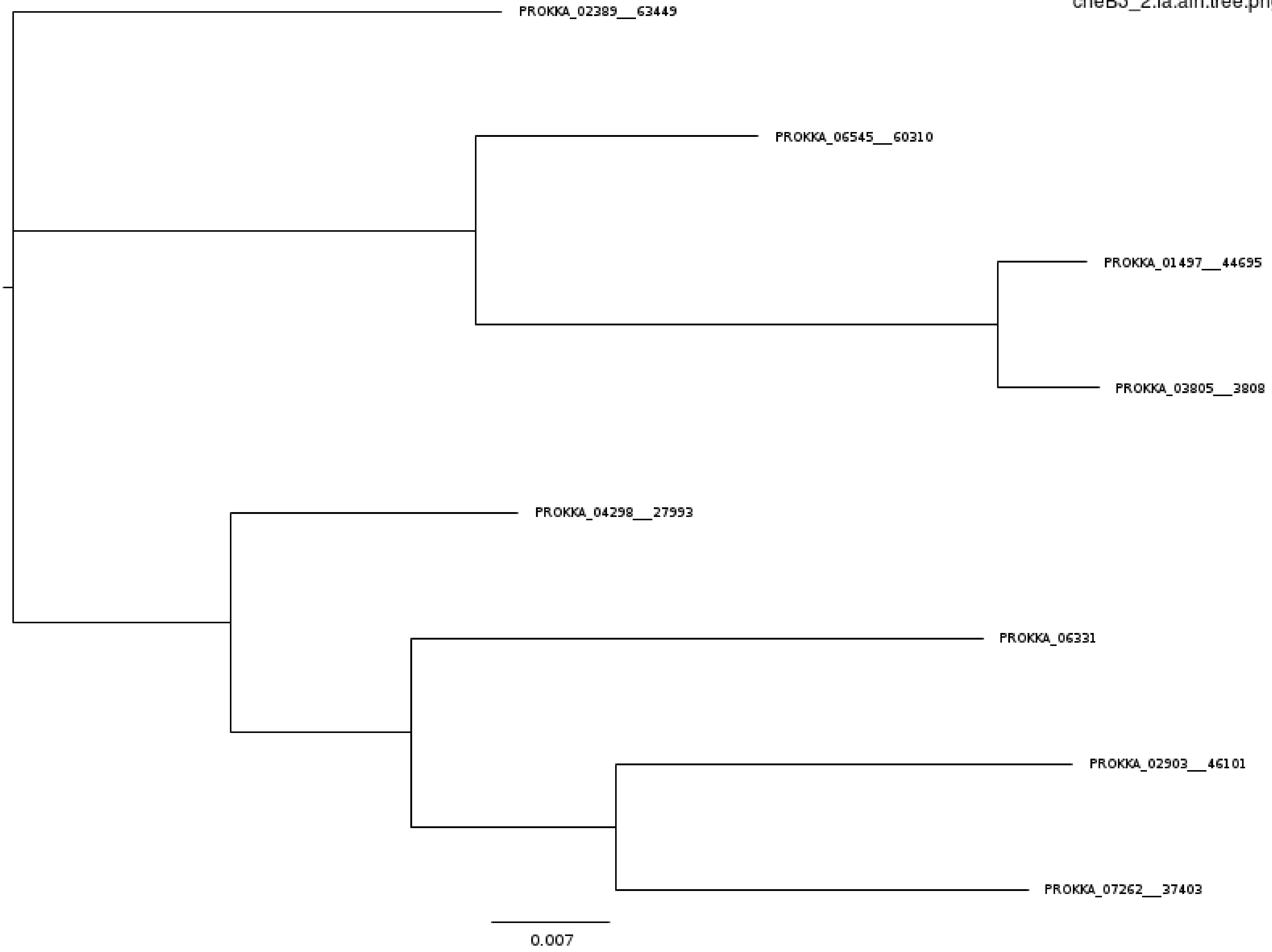

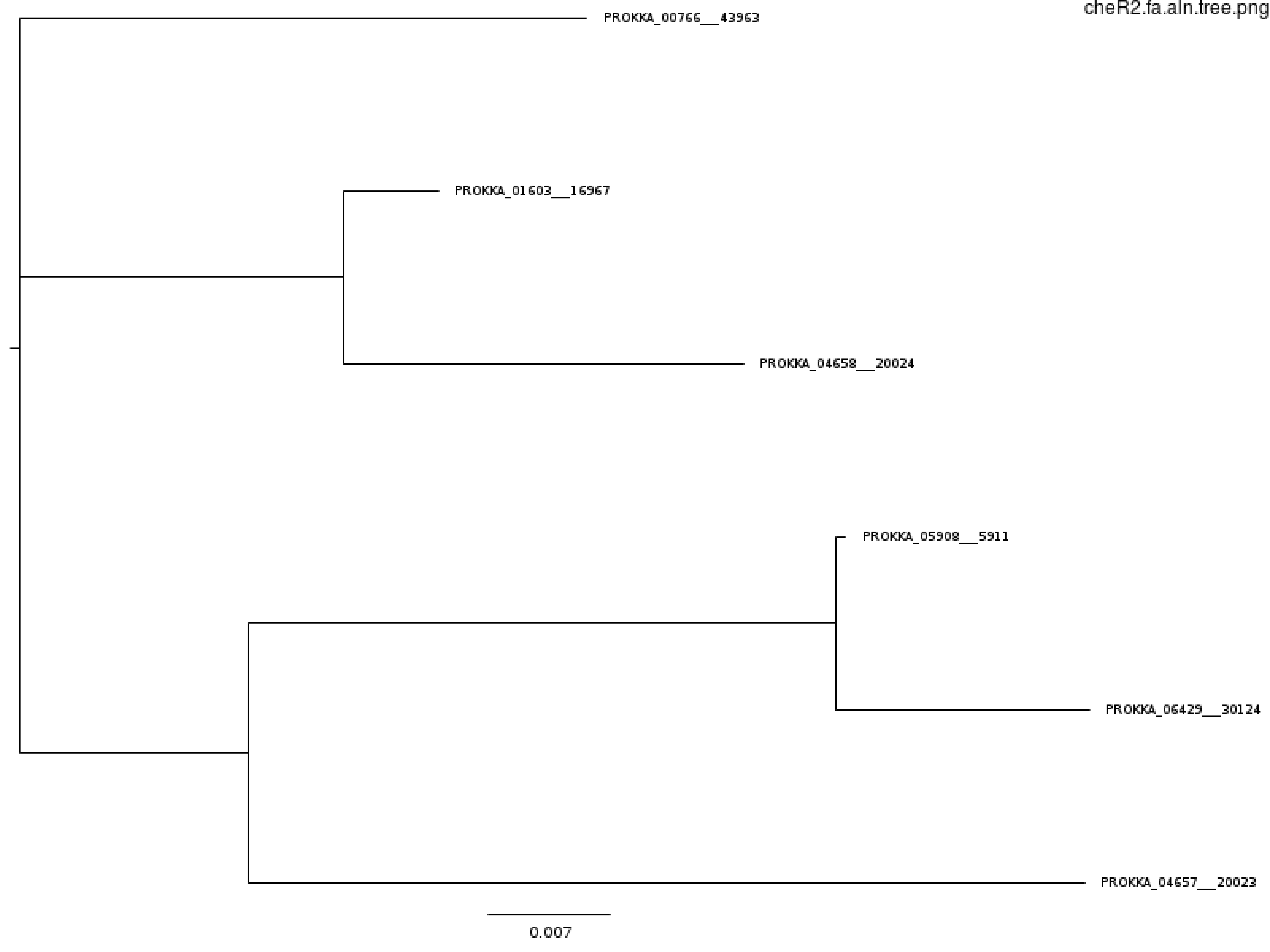
cheR2.fa.aln.tree.png
PROKKA_00766___43963
PROKKA_01603___16967
PROKKA_04658___20024
PROKKA_05908___5911
PROKKA_06429___30124
PROKKA_04657___20023
0.007

cheW_1.fa.aln.tree.png

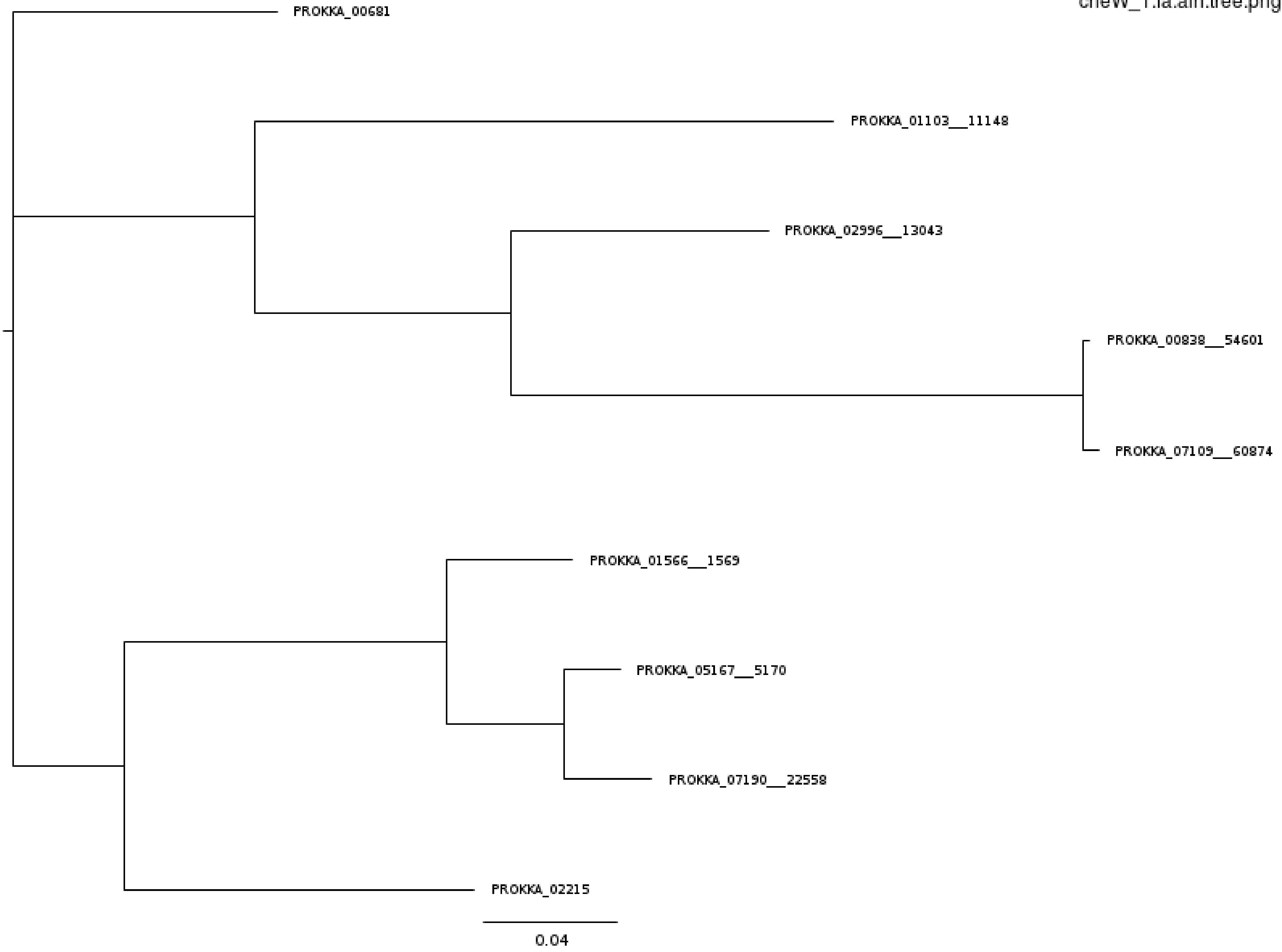

cheW_3.fa.aln.tree.png

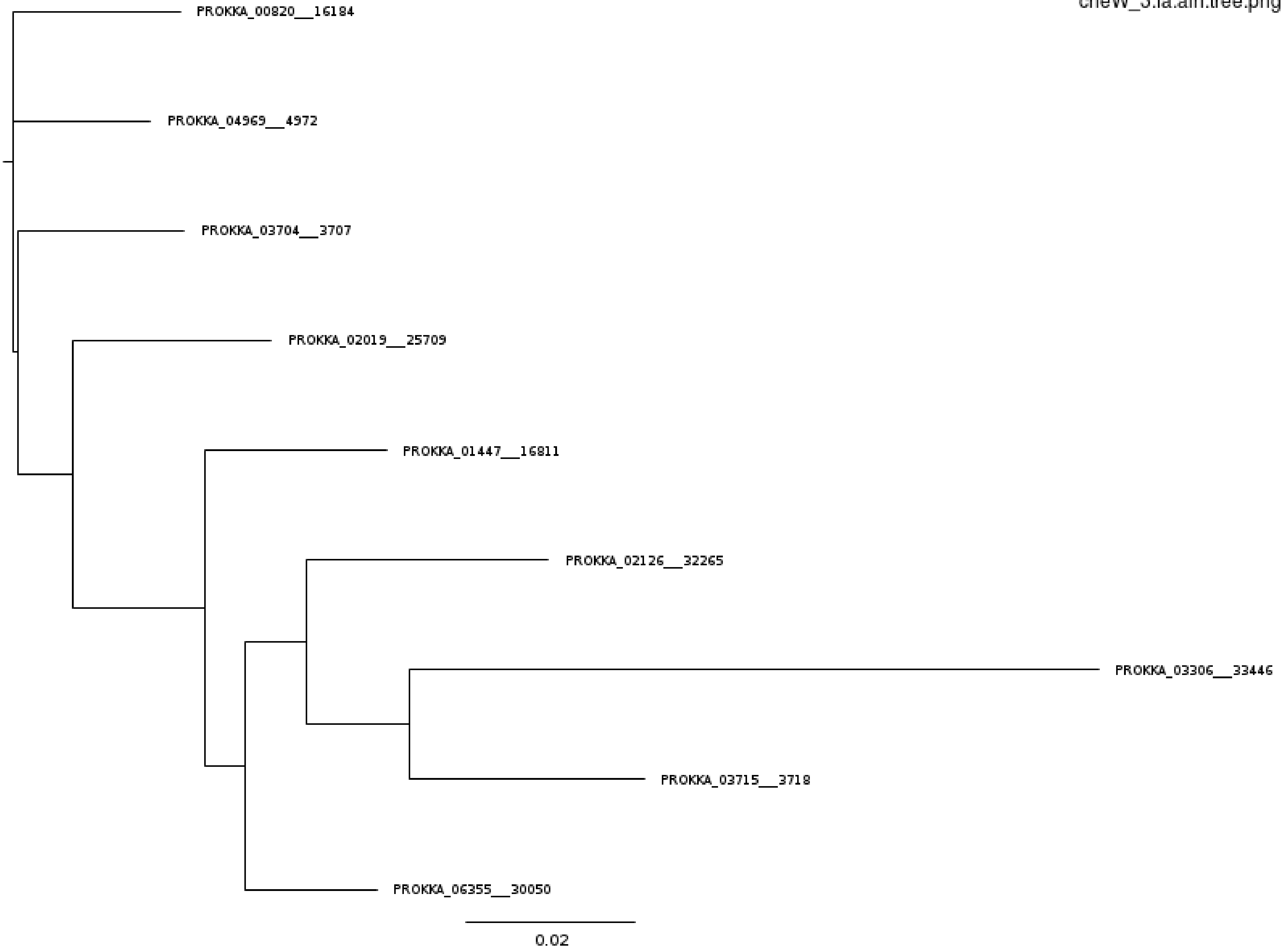

cheY_2.fa.aln.tree.png

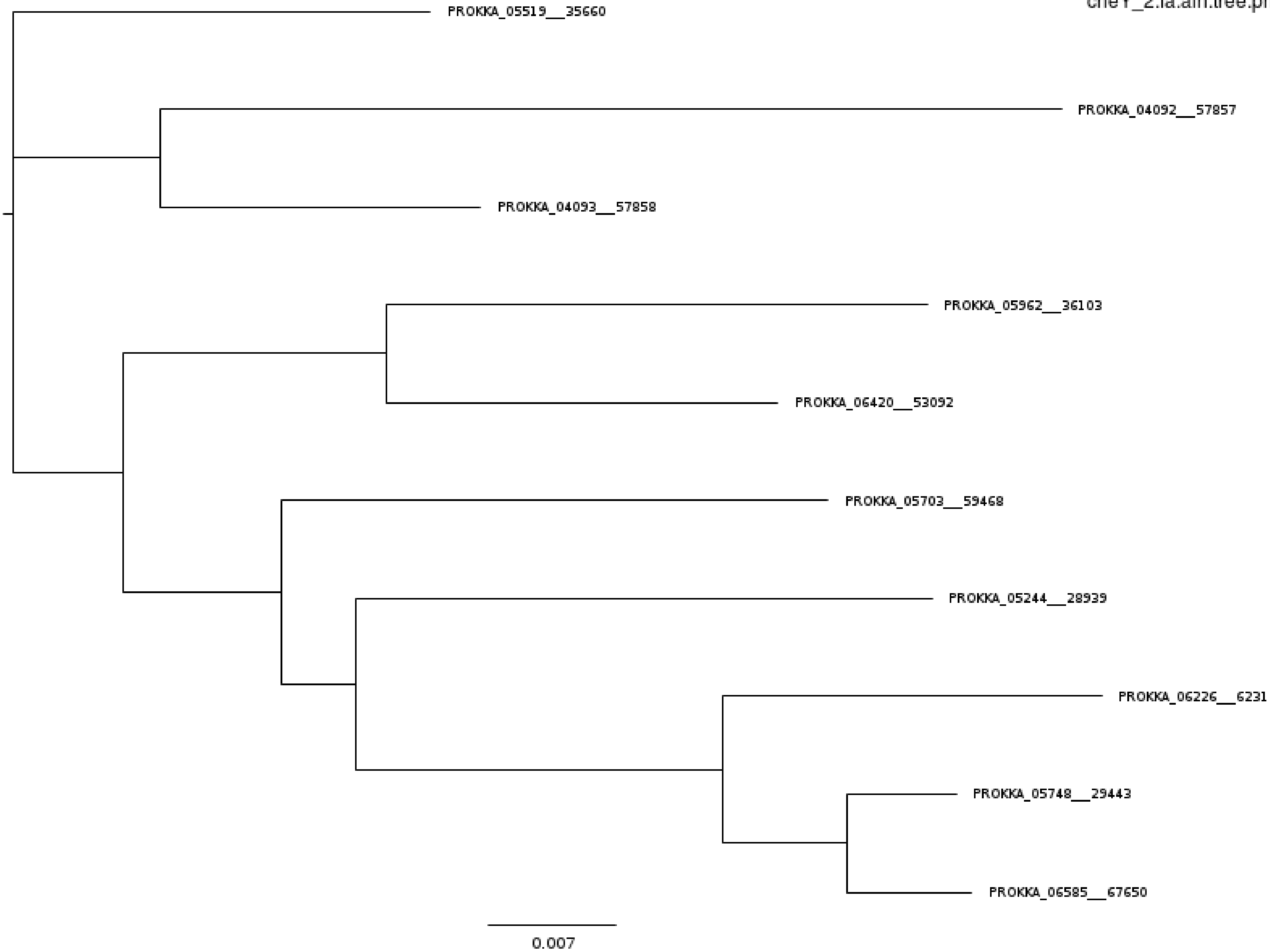

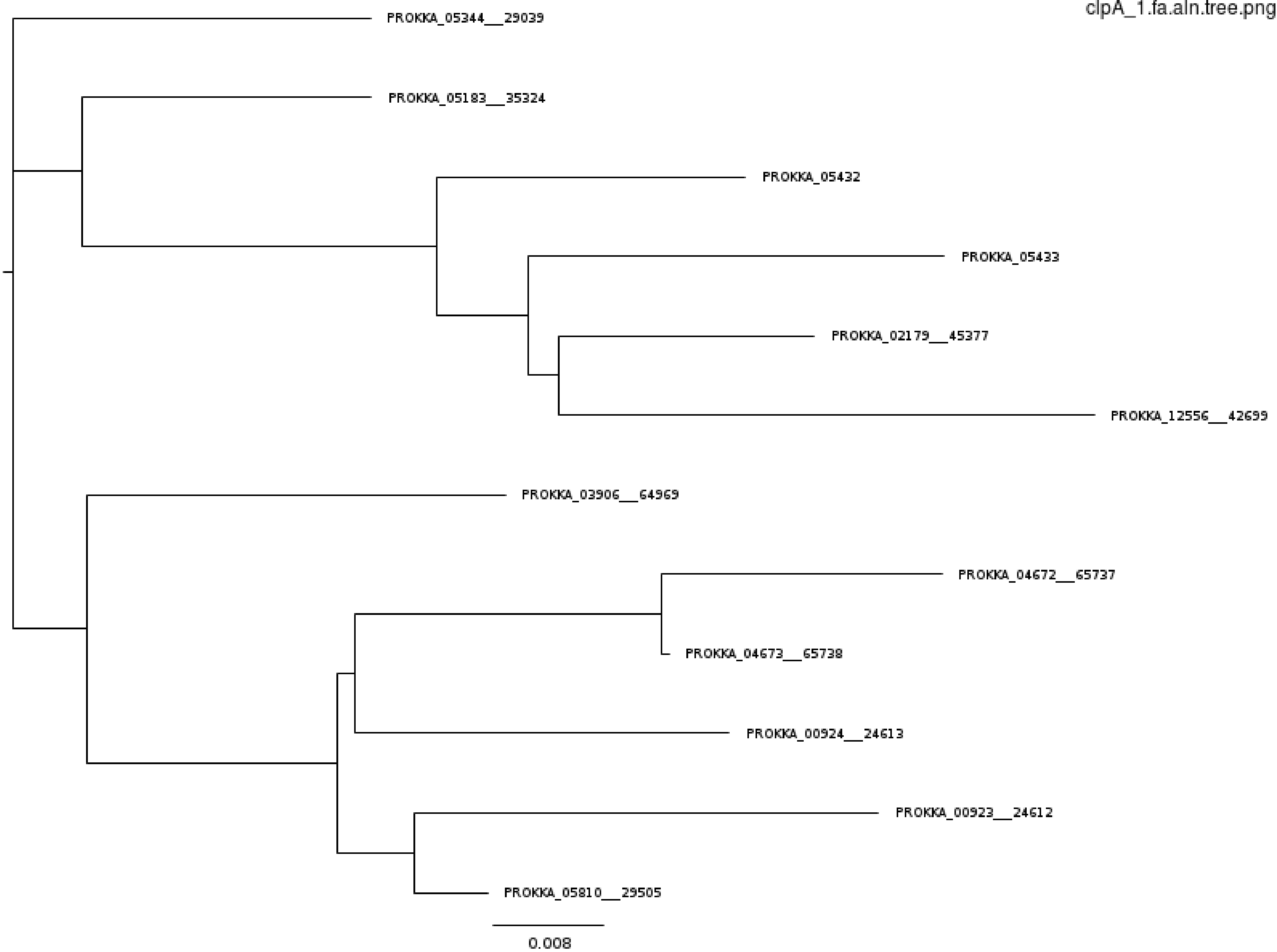
clpA_1.fa.aln.tree.png
PROKKA_05344__29039
PROKKA_05183__35324
PROKKA_05432
PROKKA_05433
PROKKA_02179__45377
PROKKA_12556__42699
PROKKA_03906__64969
PROKKA_04672__65737
PROKKA_04673__65738
PROKKA_00924__24613
PROKKA_00923__24612
PROKKA_05810__29505
0.008

clpB_1.fa.aln.tree.png

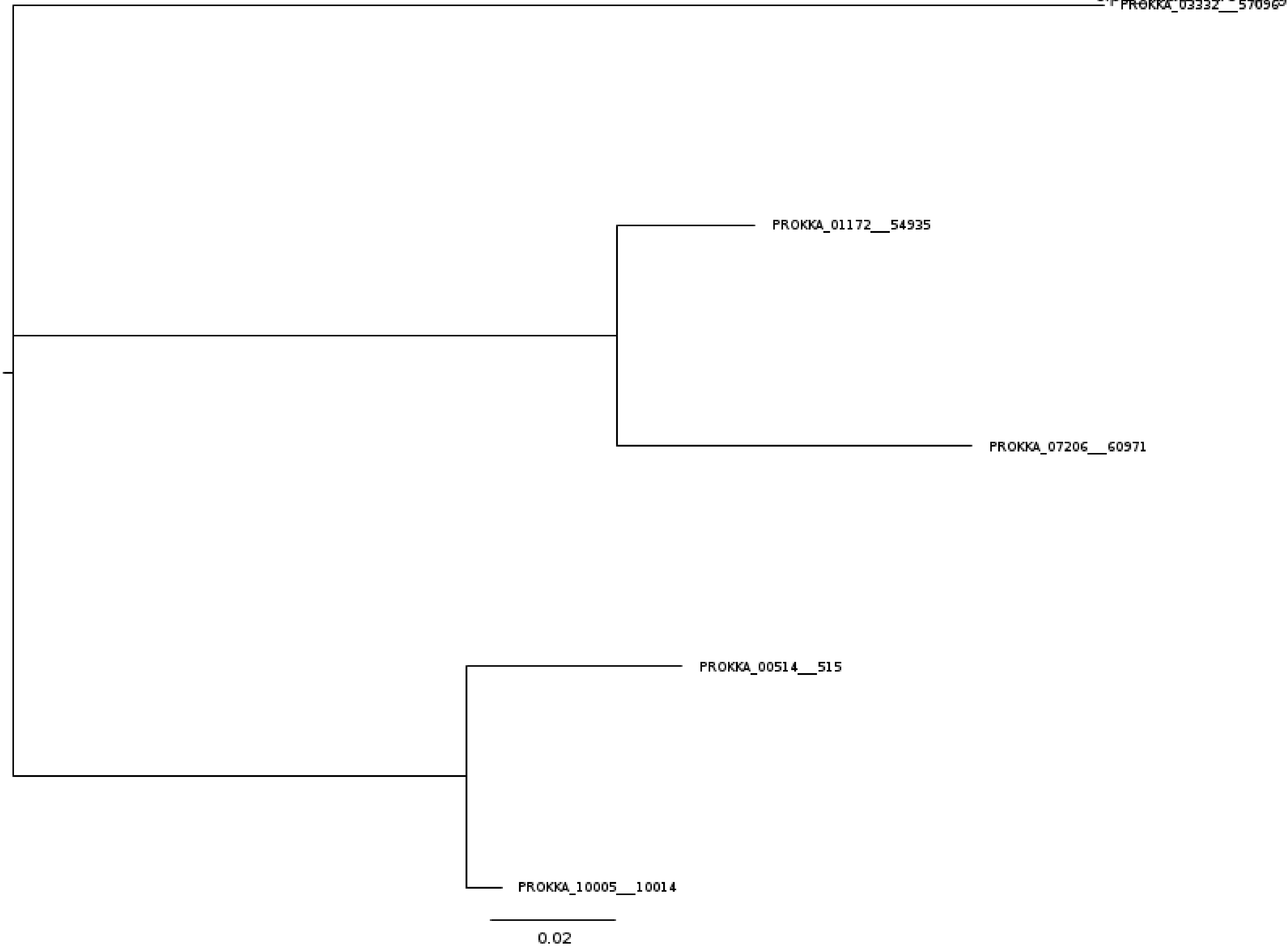

clpB_5.fa.aln.tree.png

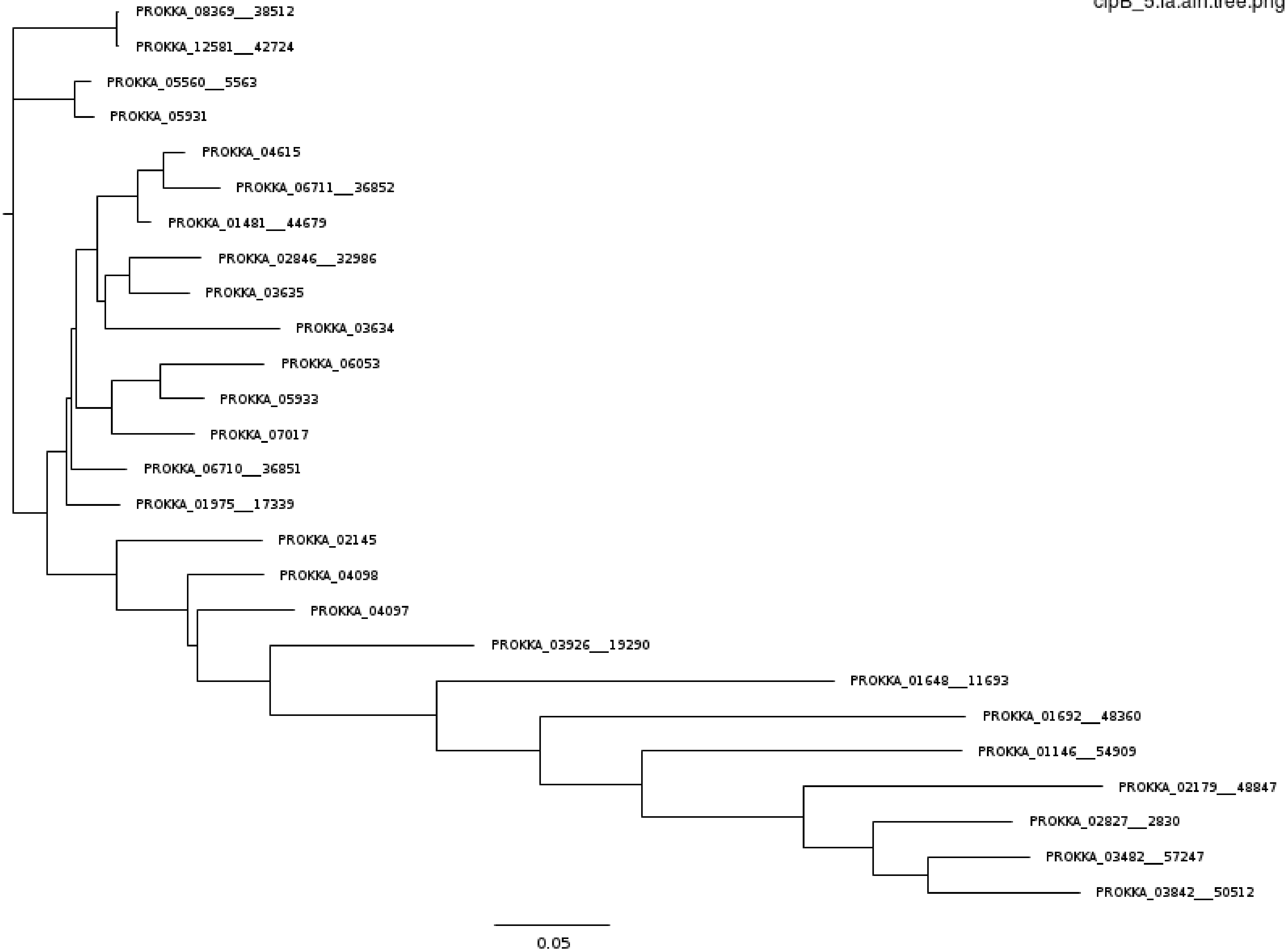

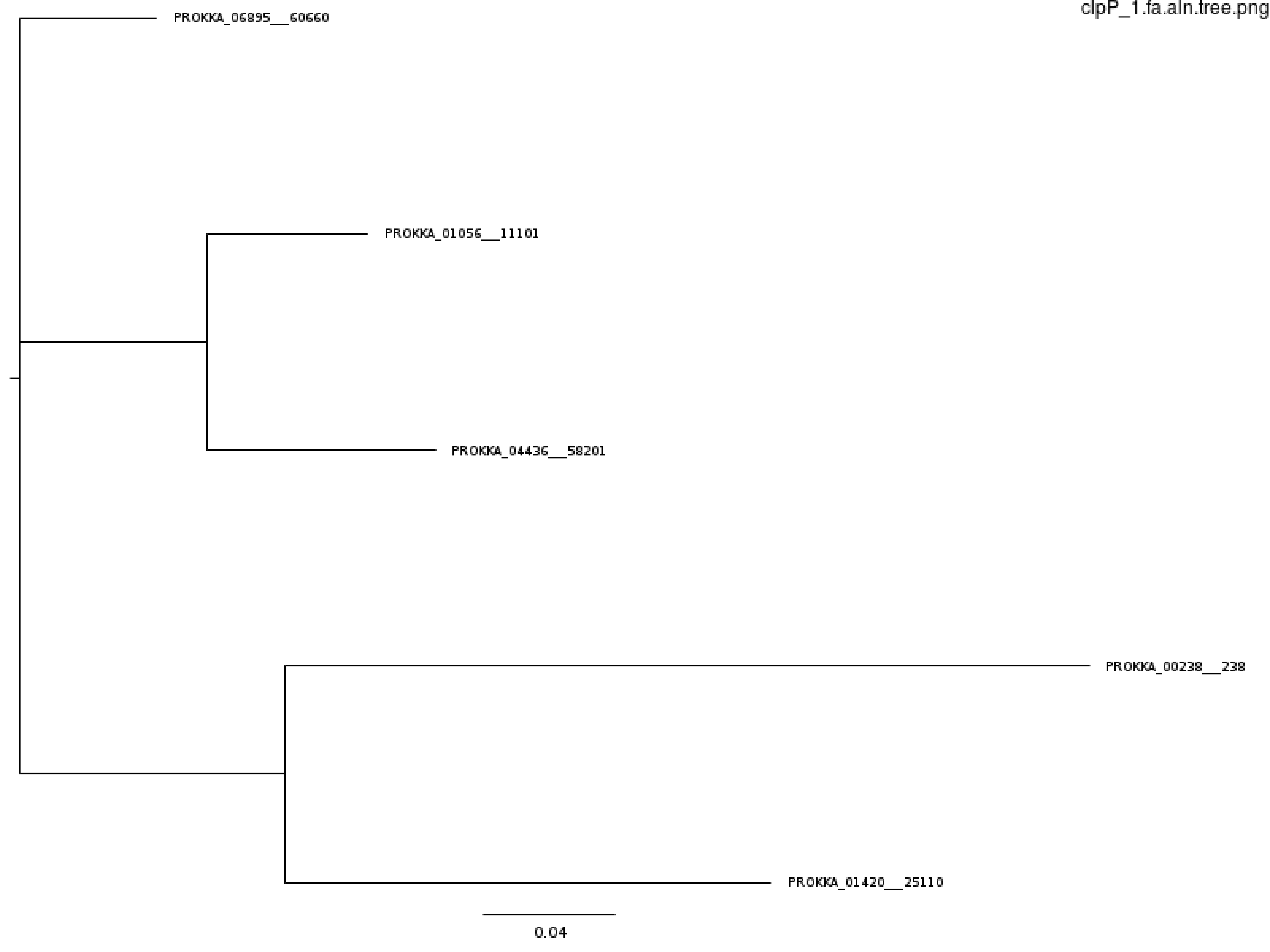
clpP_1.fa.aln.tree.png
PROKKA_06895___60660
PROKKA_01056___11101
PROKKA_04436___58201
PROKKA_00238___238
PROKKA_01420___25110
0.04

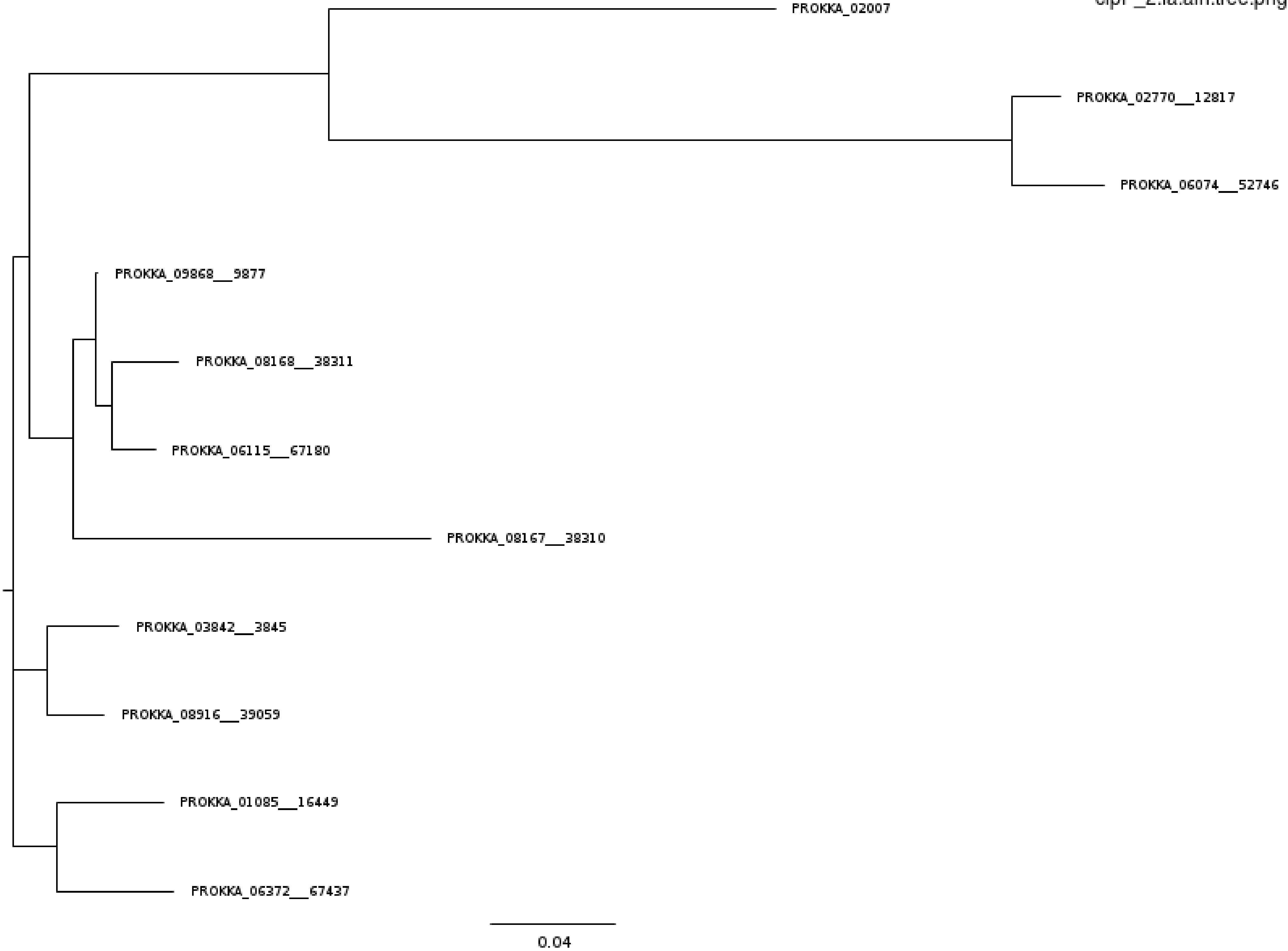
PROKKA_02007
PROKKA_02770__12817
PROKKA_06074__52746
PROKKA_09868__9877
PROKKA_08168__38311
PROKKA_06115__67180
PROKKA_08167__38310
PROKKA_03842__3845
PROKKA_08916__39059
PROKKA_01085__16449
PROKKA_06372__67437
0.04

clpS_1.fa.aln.tree.png

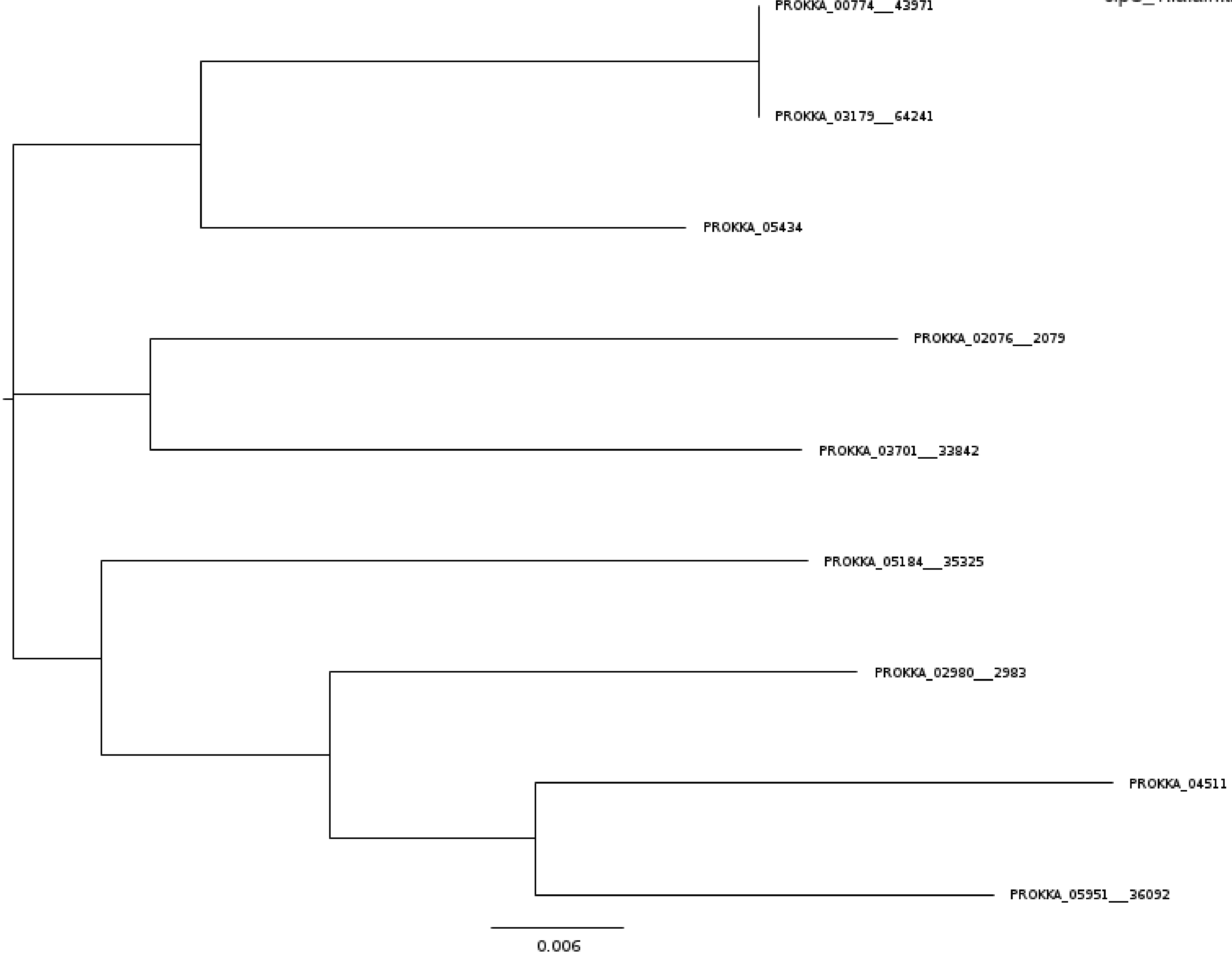

clpX_3.fa.aln.tree.png

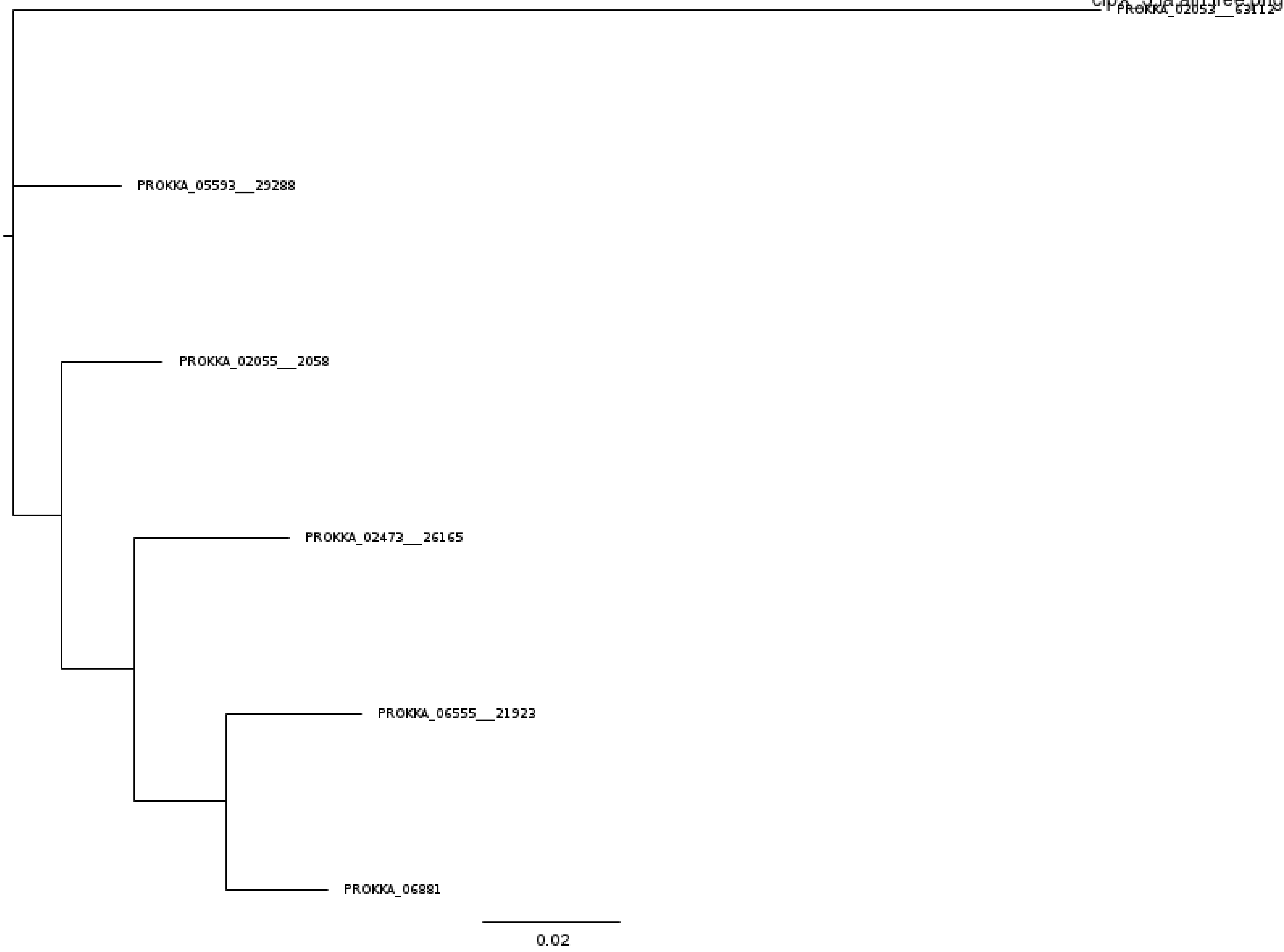

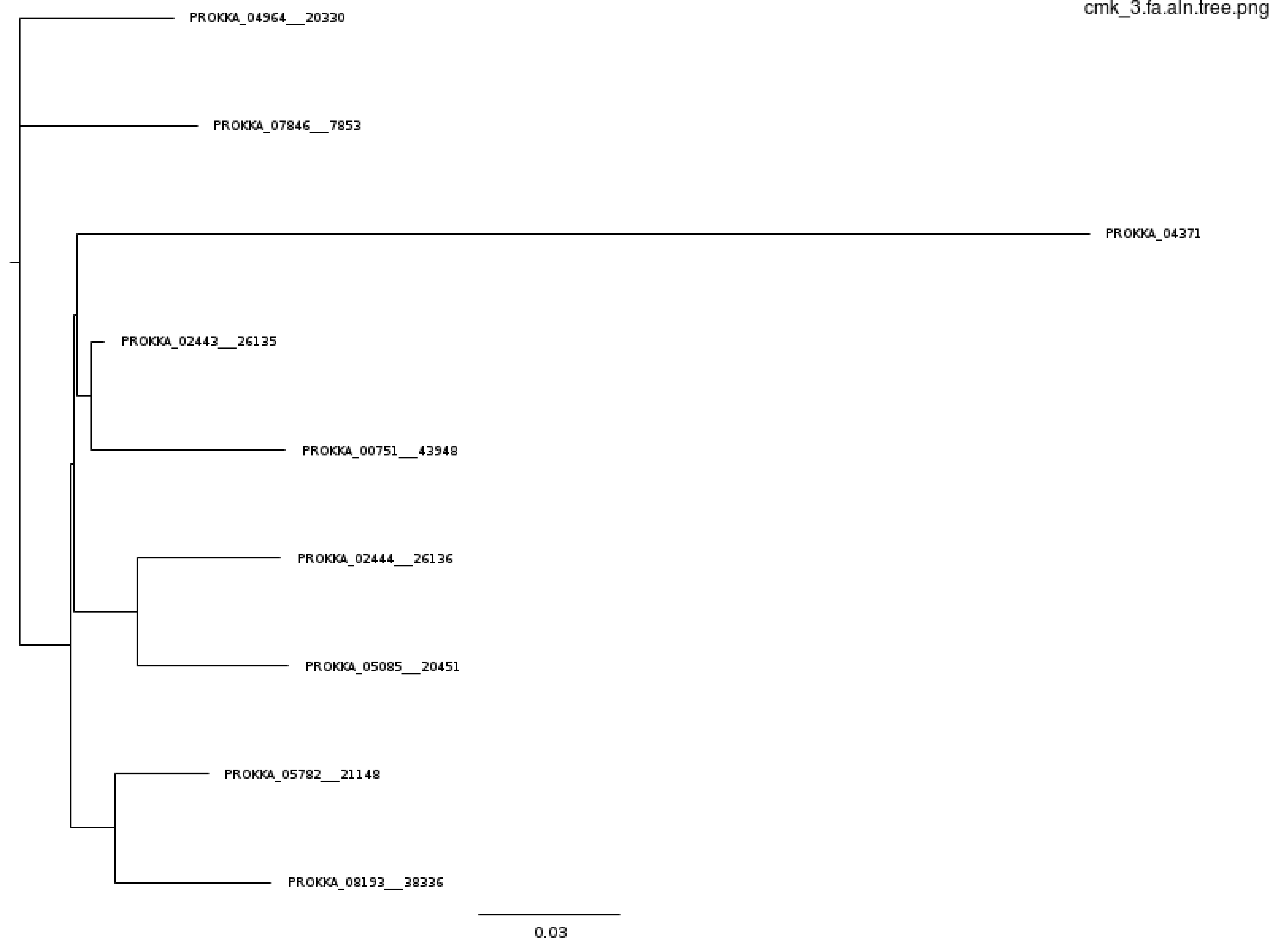
cmk_3.fa.aln.tree.png
PROKKA_04964___20330
PROKKA_07846___7853
PROKKA_04371
PROKKA_02443___26135
PROKKA_00751___43948
PROKKA_02444___26136
PROKKA_05085___20451
PROKKA_05782___21148
PROKKA_08193___38336
0.03

cmpR_2.fa.aln.tree.png

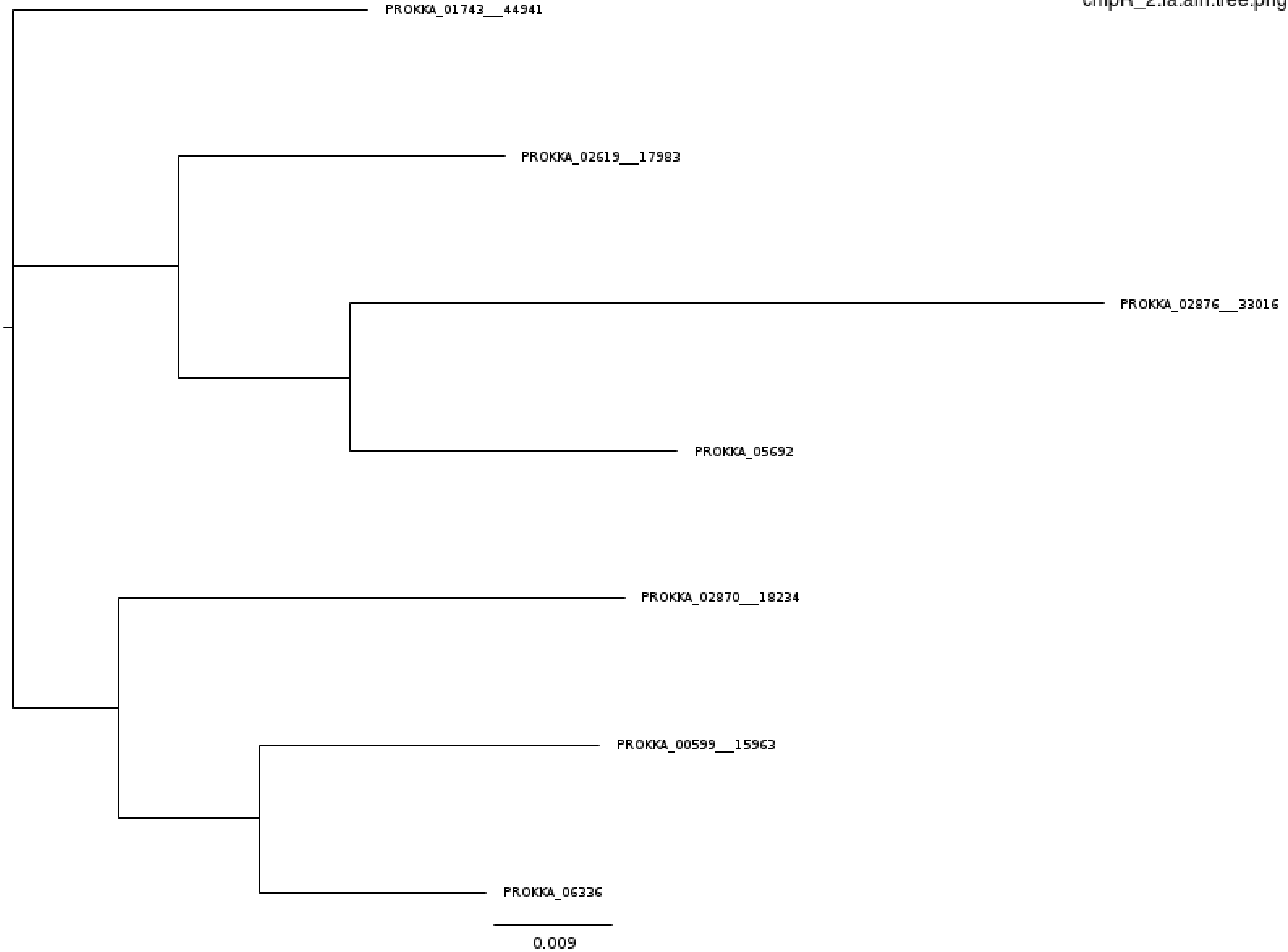

cmpR_3.fa.aln.tree.png

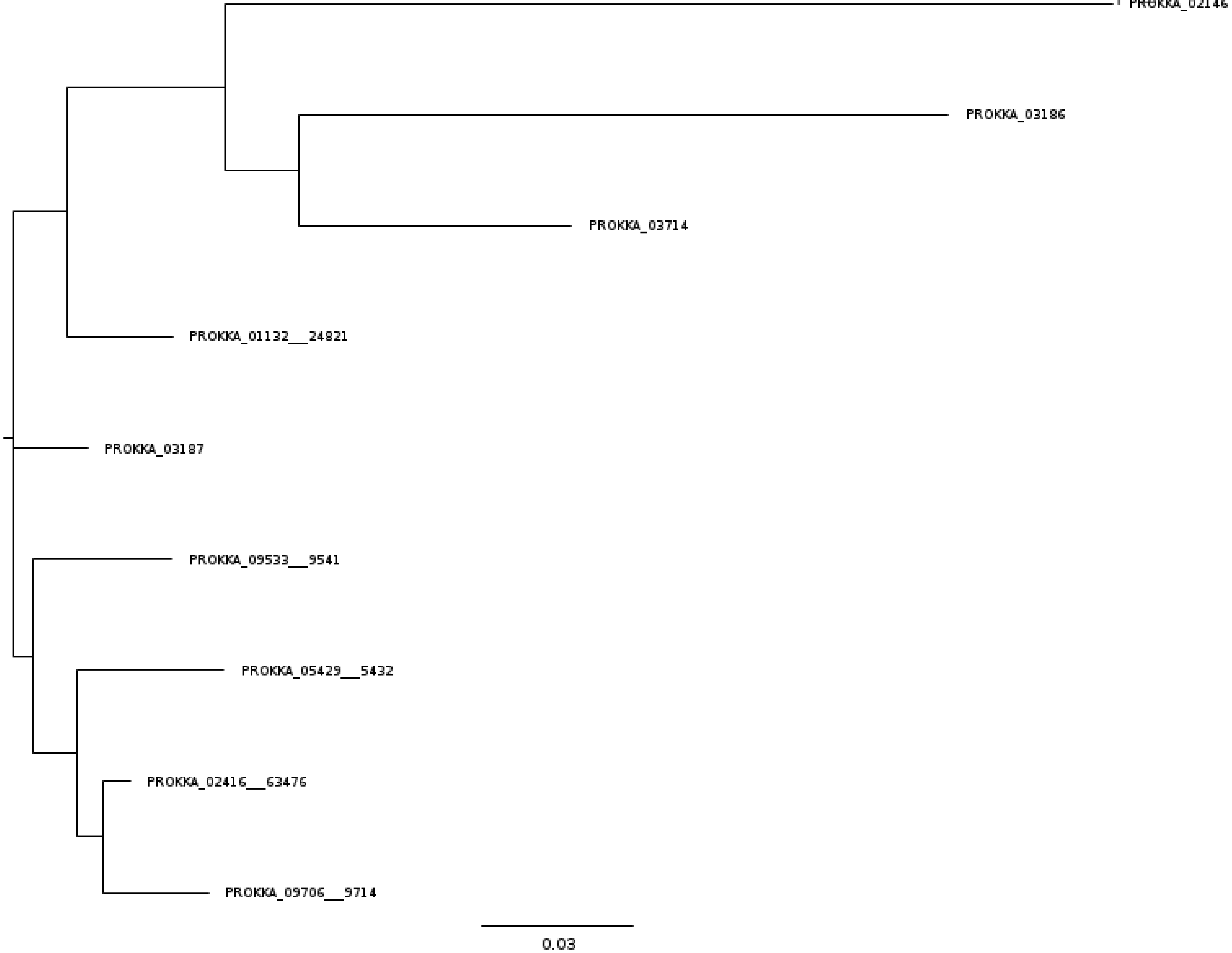

coaBC_2.fa.aln.tree.png

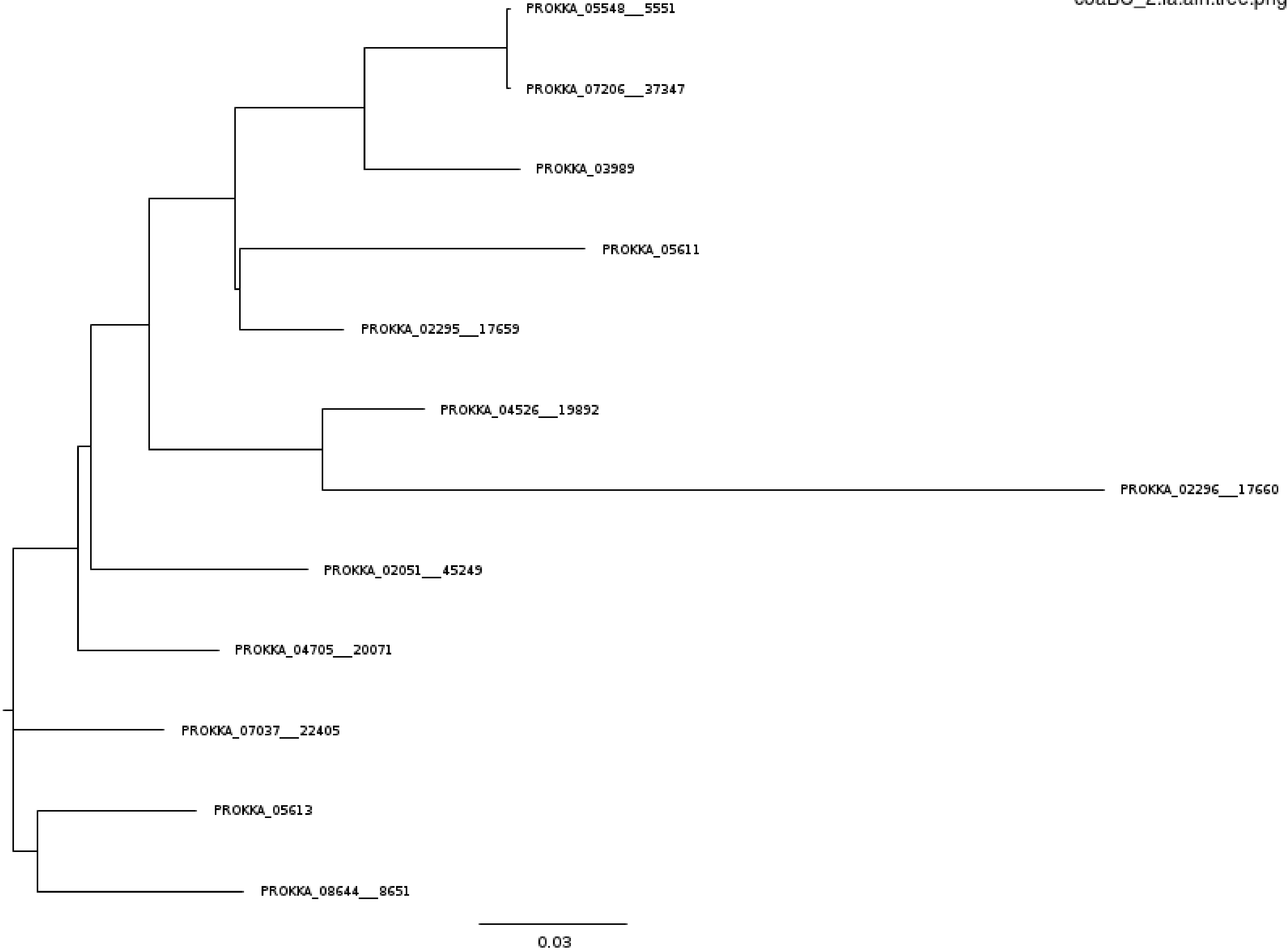

coaE_2.fa.aln.tree.png

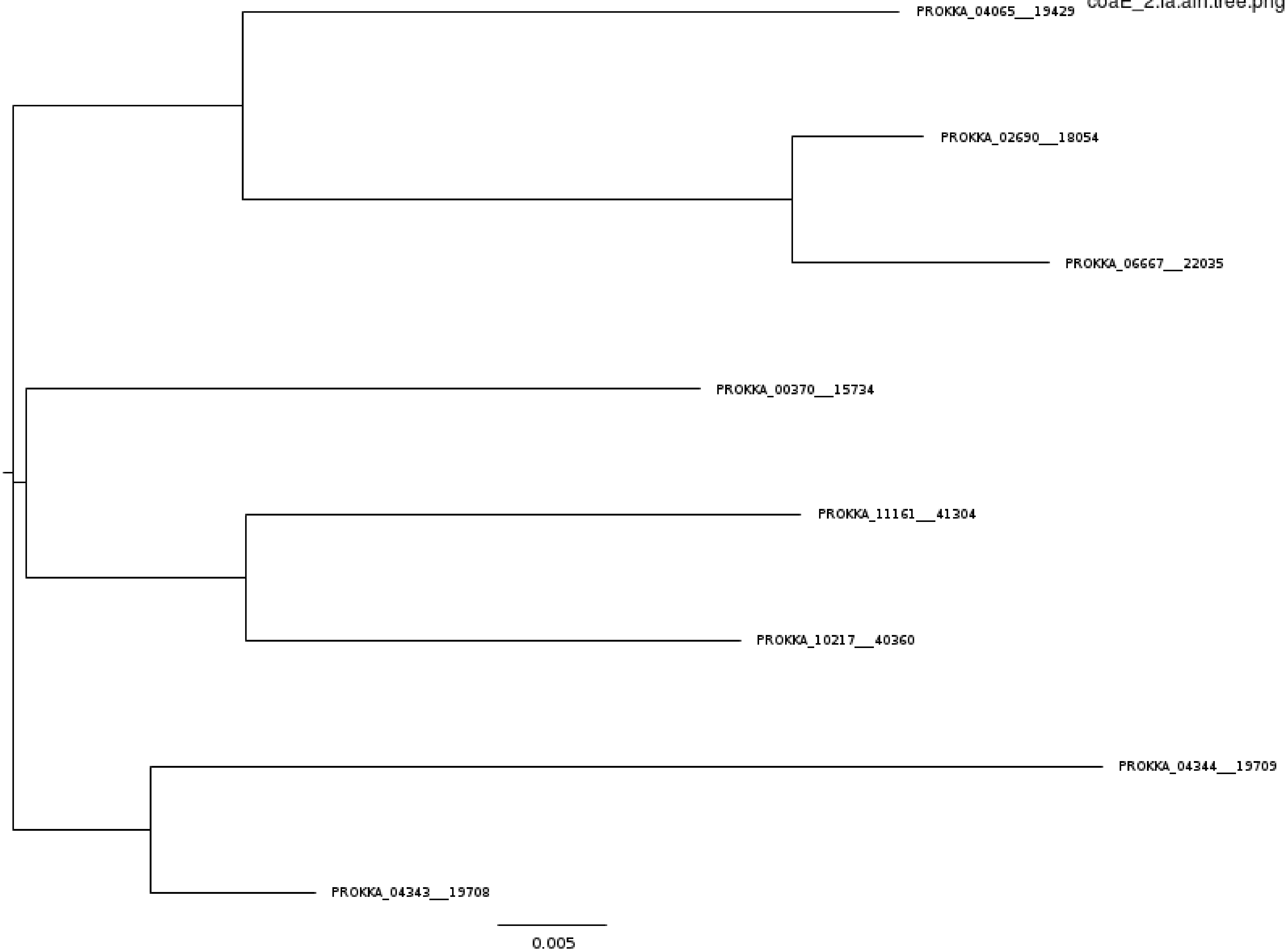

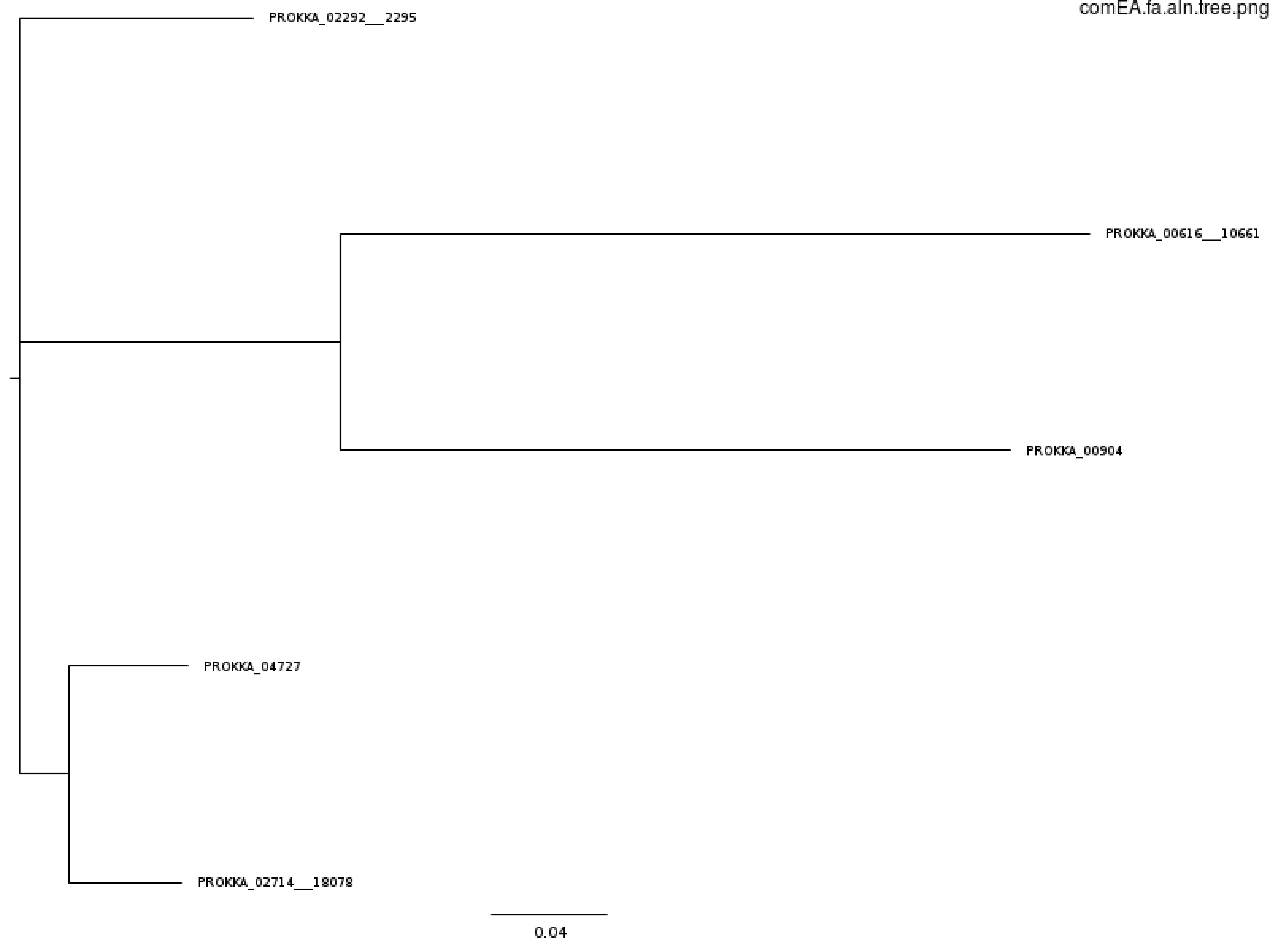
comEA.fa.aln.tree.png
PROKKA_02292___2295
PROKKA_00616___10661
PROKKA_00904
PROKKA_04727
PROKKA_02714___18078
0.04

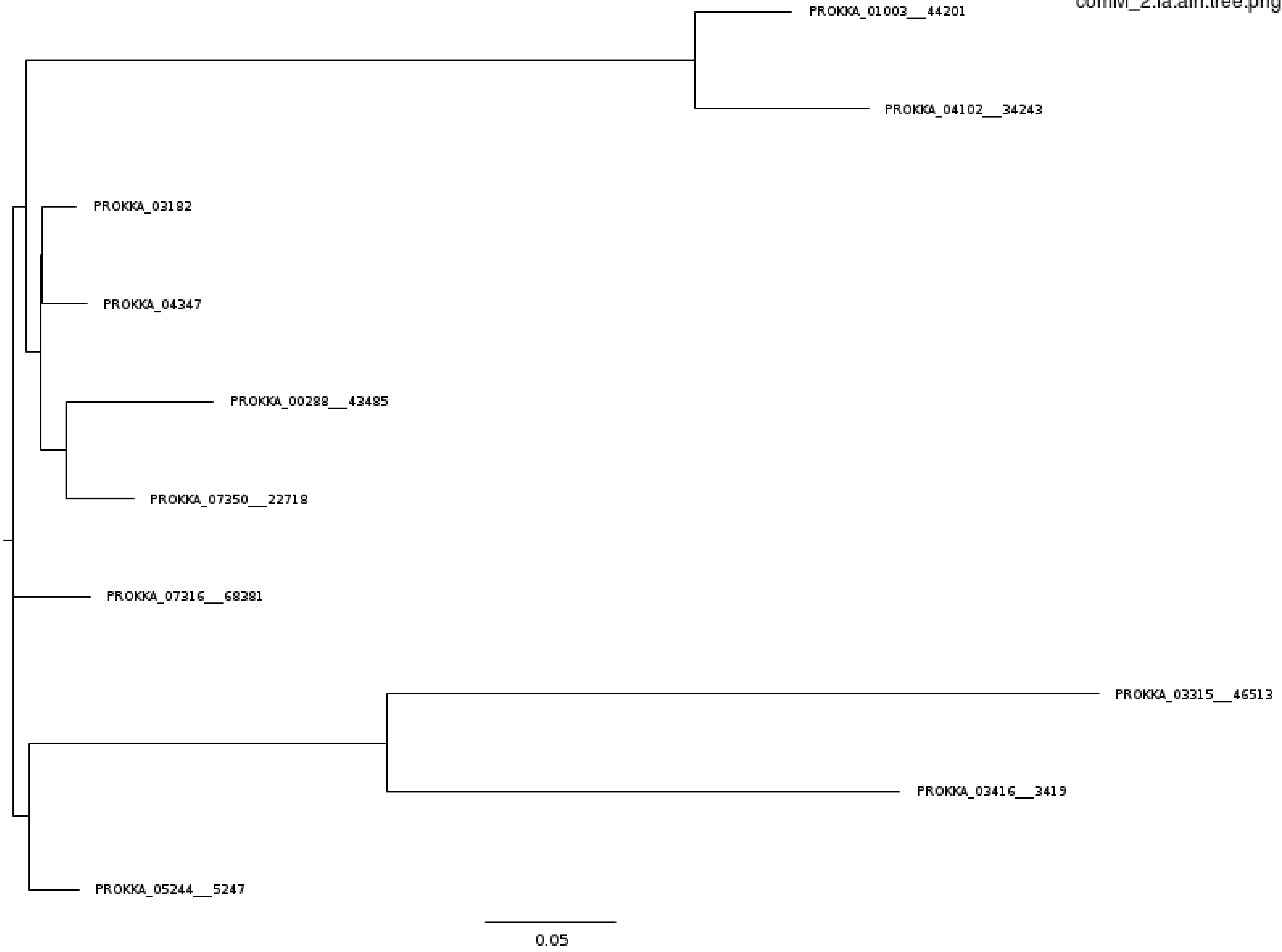
PROKKA_01003___44201
PROKKA_04102___34243
PROKKA_03182
PROKKA_04347
PROKKA_00288___43485
PROKKA_07350___22718
PROKKA_07316___68381
PROKKA_03315___46513
PROKKA_03416___3419
PROKKA_05244___5247
0.05

comM_3.fa.aln.tree.png

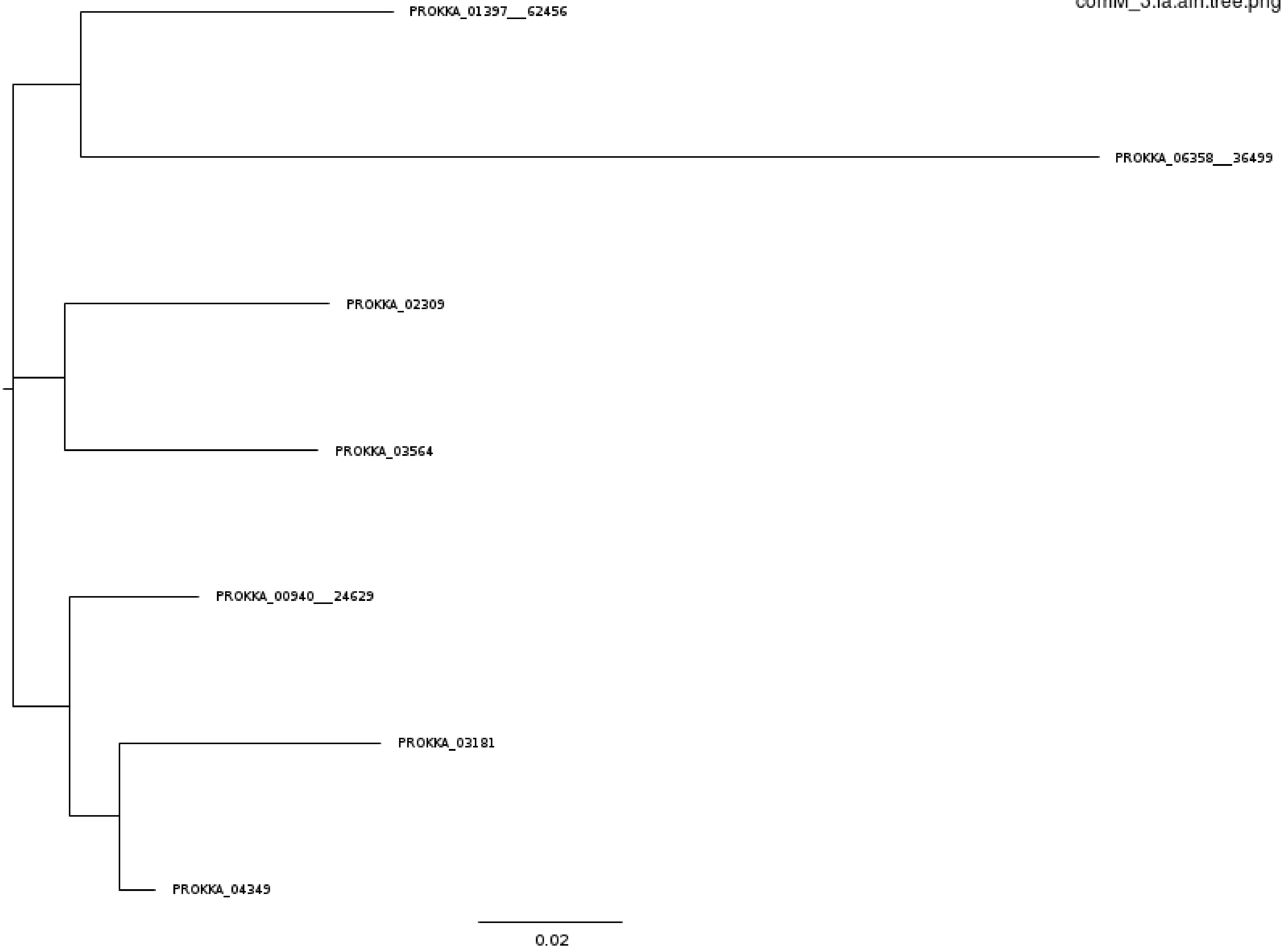

copZ.fa.aln.tree.png

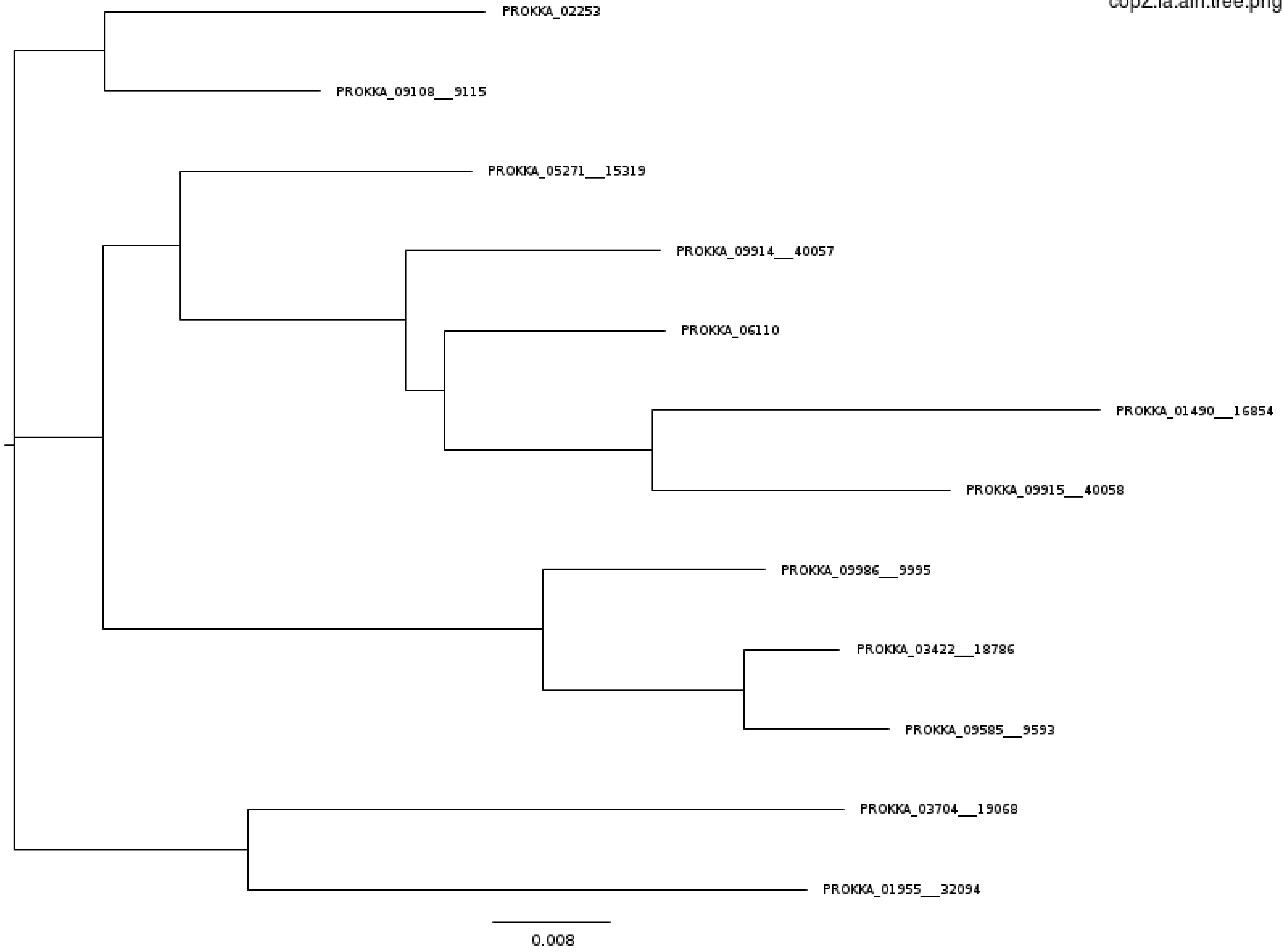

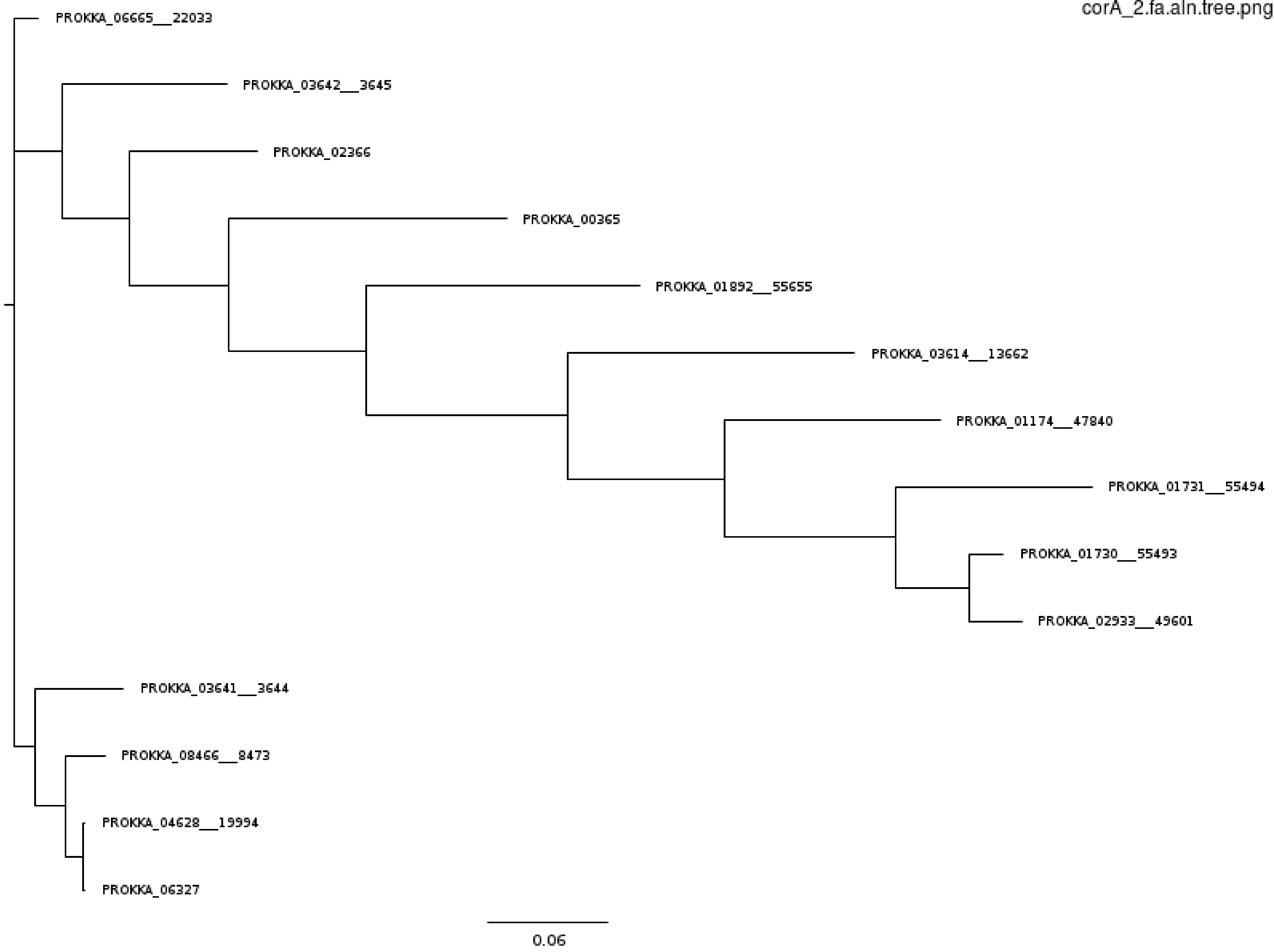
corA_2.fa.aln.tree.png
PROKKA_06665___22033
PROKKA_03642___3645
PROKKA_02366
PROKKA_00365
PROKKA_01892___55655
PROKKA_03614___13662
PROKKA_01174___47840
PROKKA_01731___55494
PROKKA_01730___55493
PROKKA_02933___49601
PROKKA_03641___3644
PROKKA_08466___8473
PROKKA_04628___19994
PROKKA_06327
0.06

corC.fa.aln.tree.png

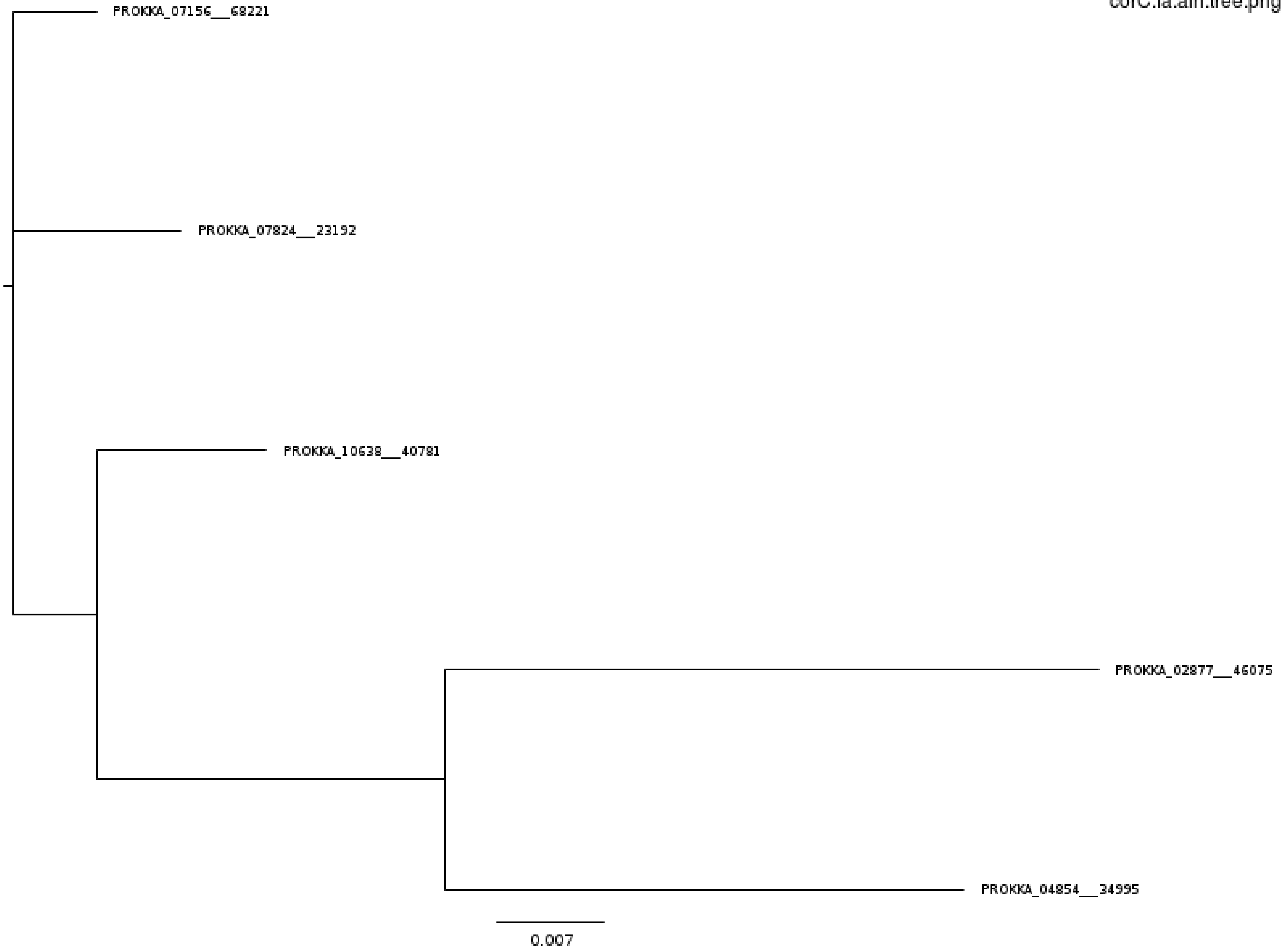

coxN.fa.aln.tree.png

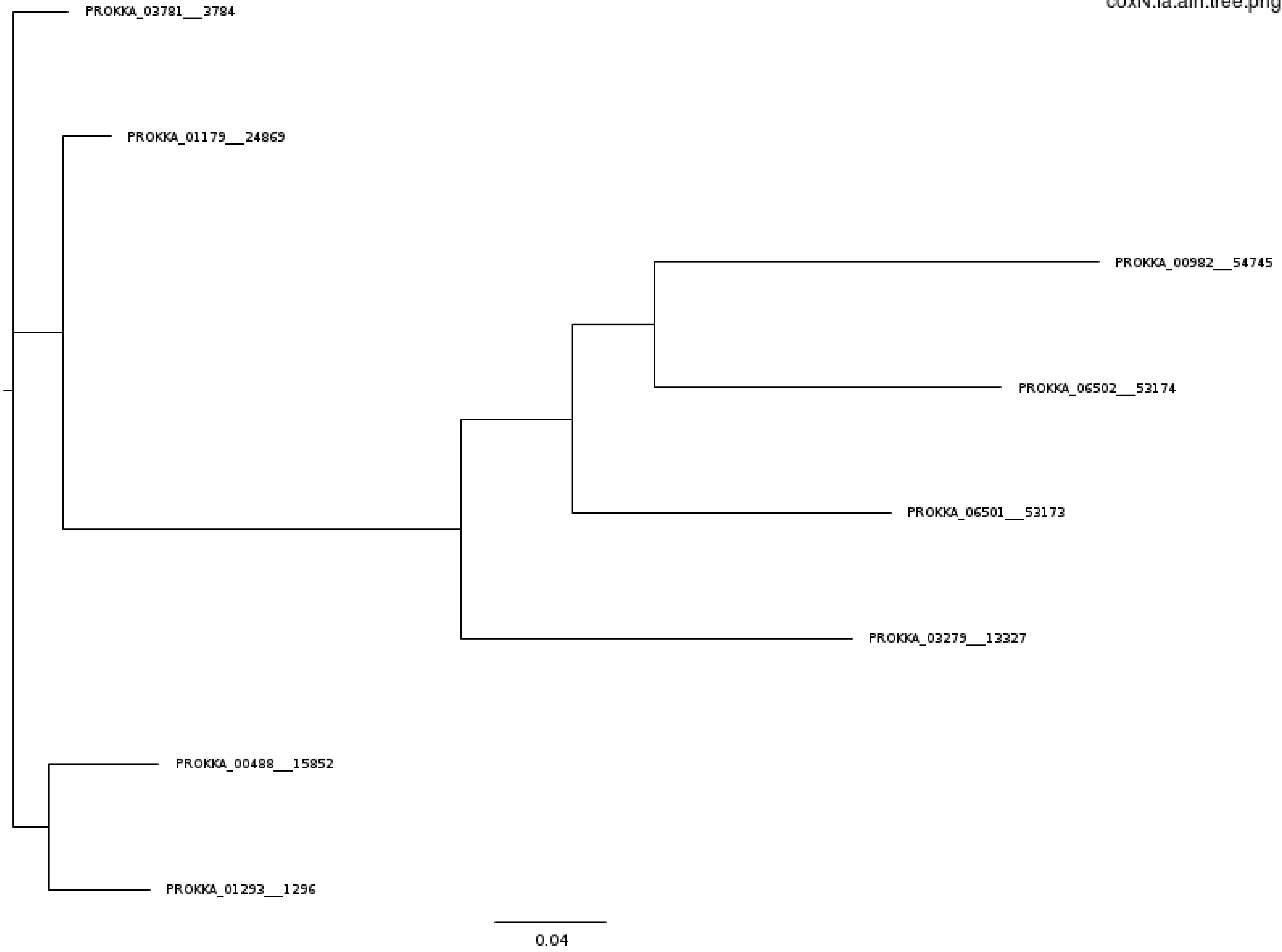

cpcE_2.fa.aln.tree.png

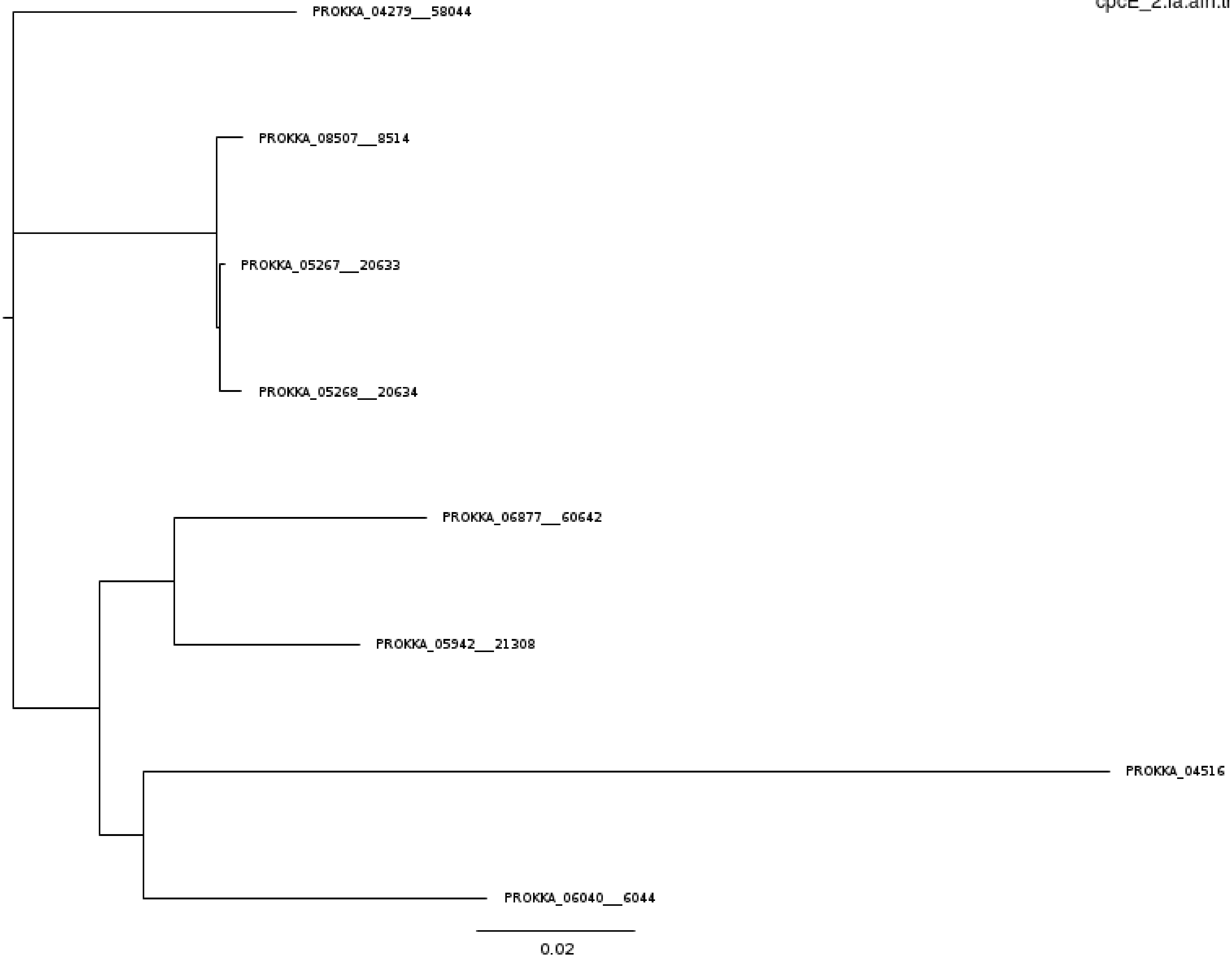

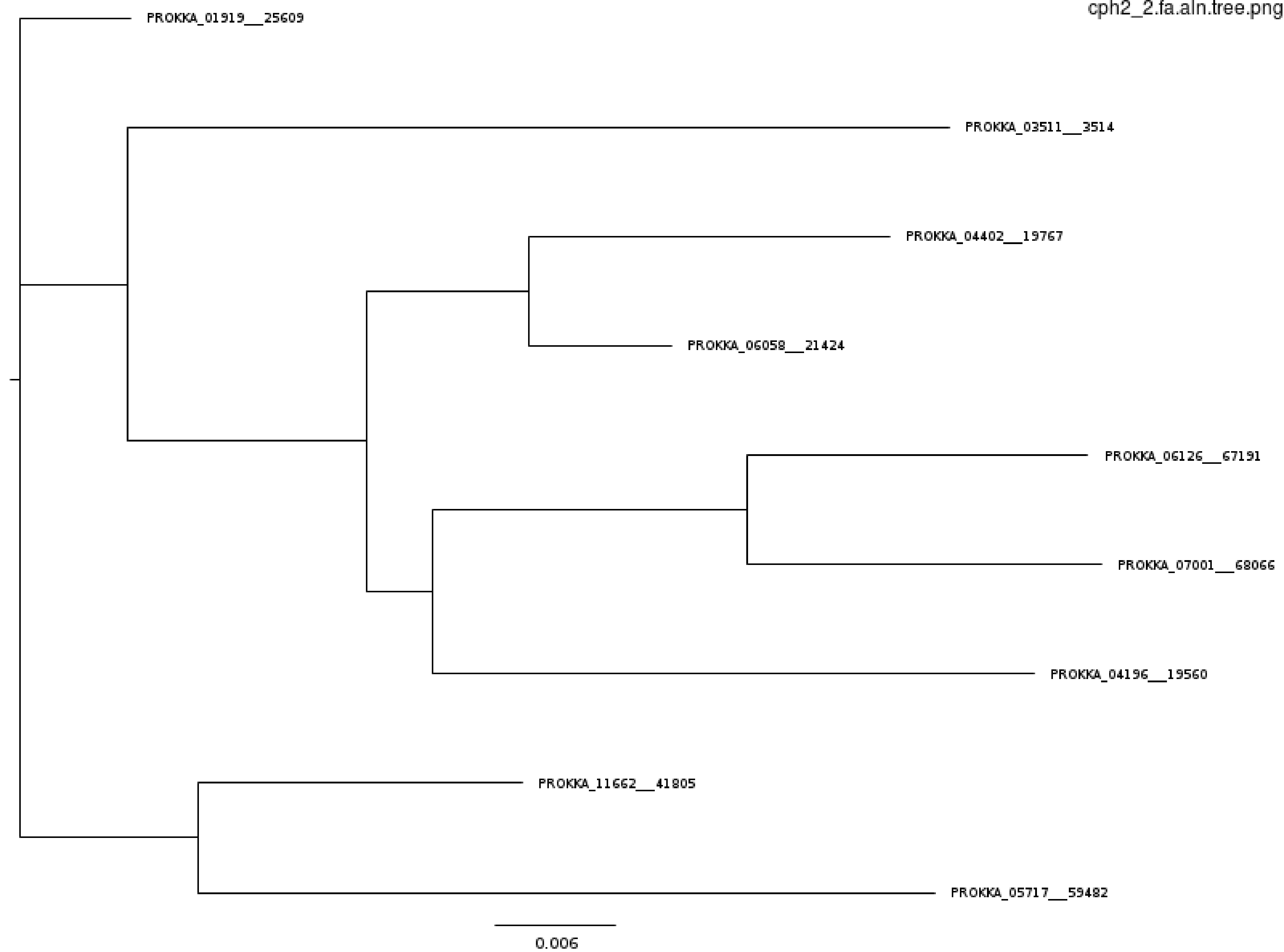
cph2_2.fa.aln.tree.png
PROKKA_01919___25609
PROKKA_03511___3514
PROKKA_04402___19767
PROKKA_06058___21424
PROKKA_06126___67191
PROKKA_07001___68066
PROKKA_04196___19560
PROKKA_11662___41805
PROKKA_05717___59482
0.006

cph2_4.fa.aln.tree.png

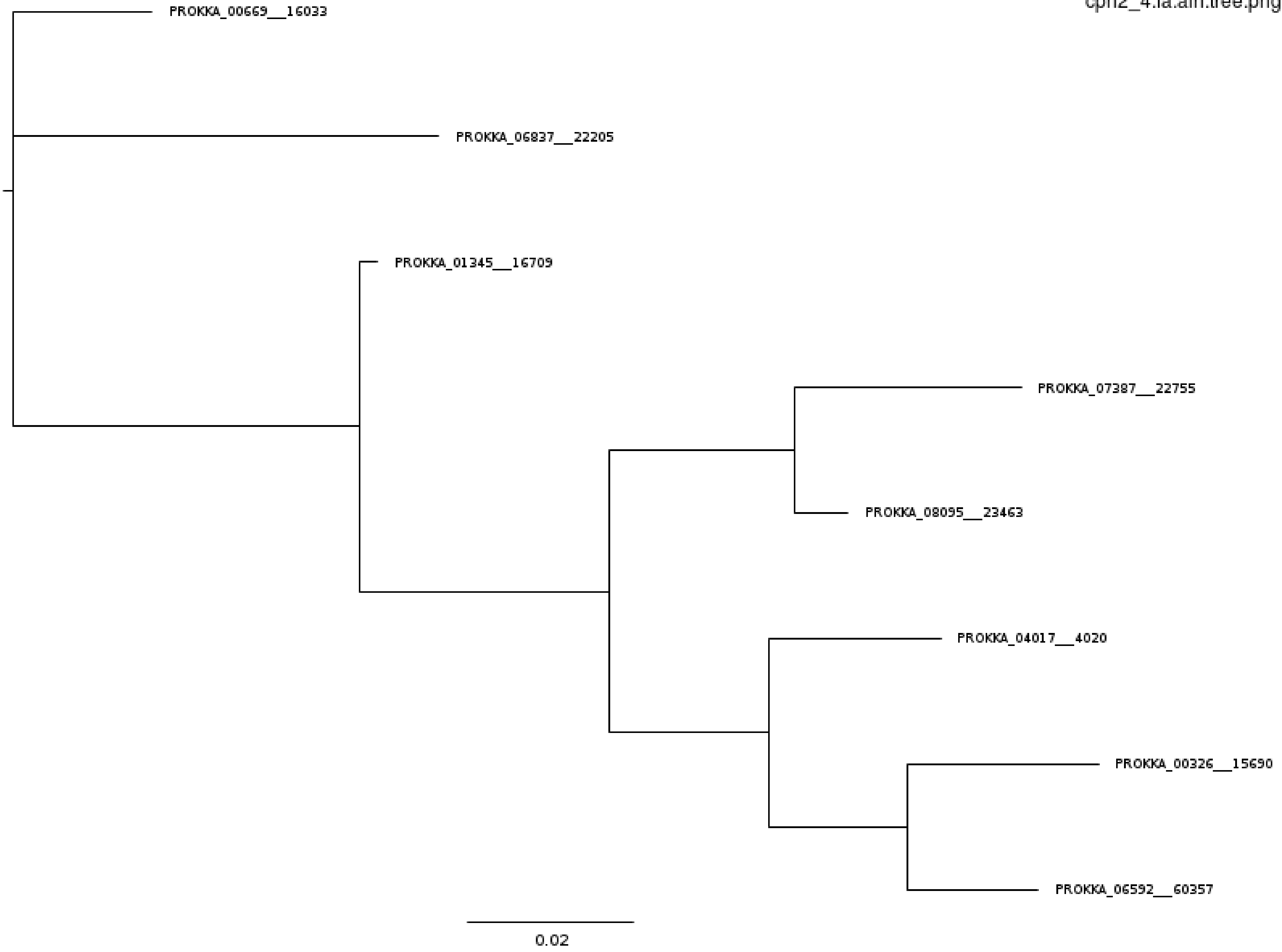

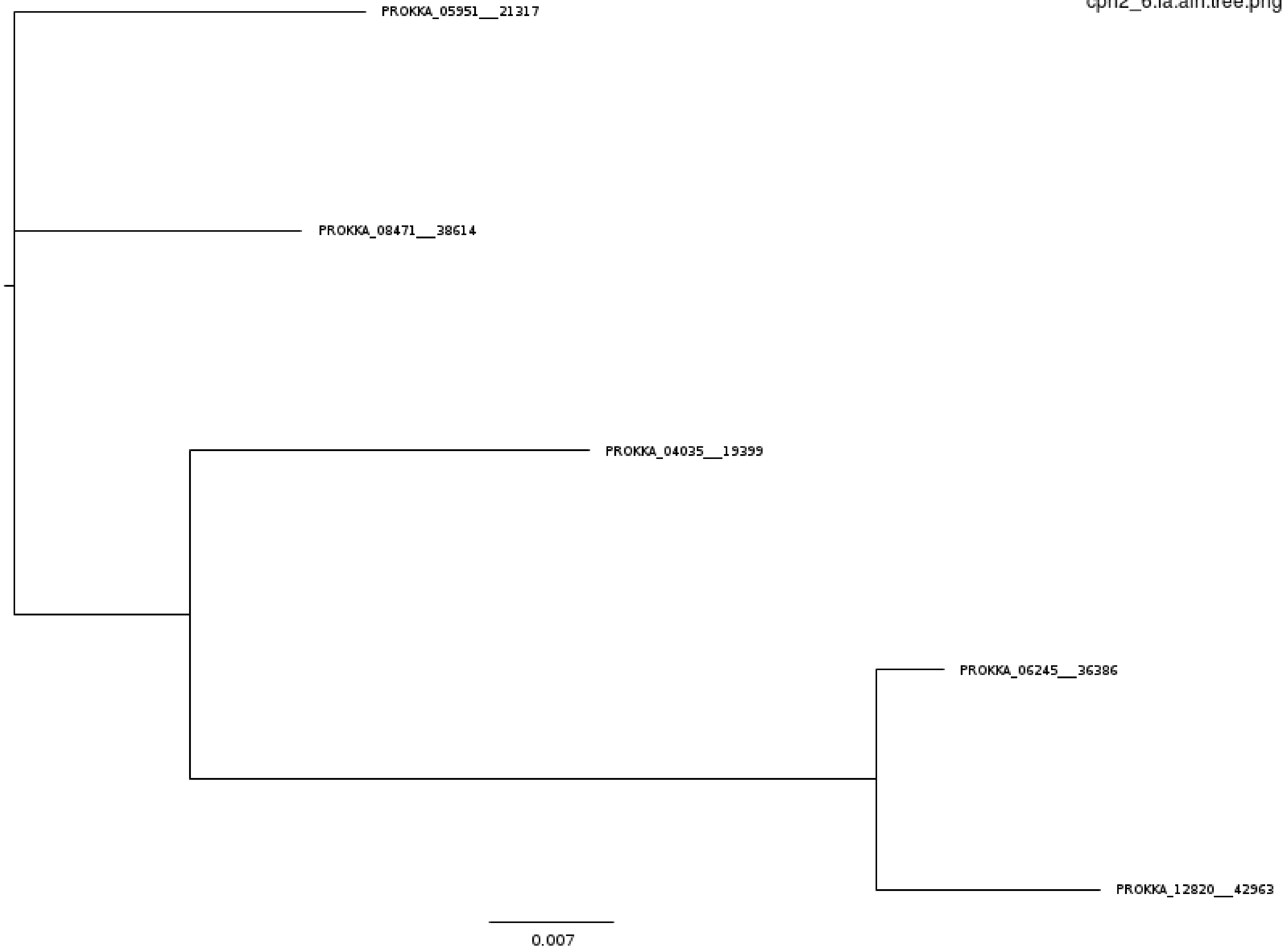
PROKKA_05951___21317
PROKKA_08471___38614
PROKKA_04035___19399
PROKKA_06245___36386
PROKKA_12820___42963
0.007

creD_1.fa.aln.tree.png

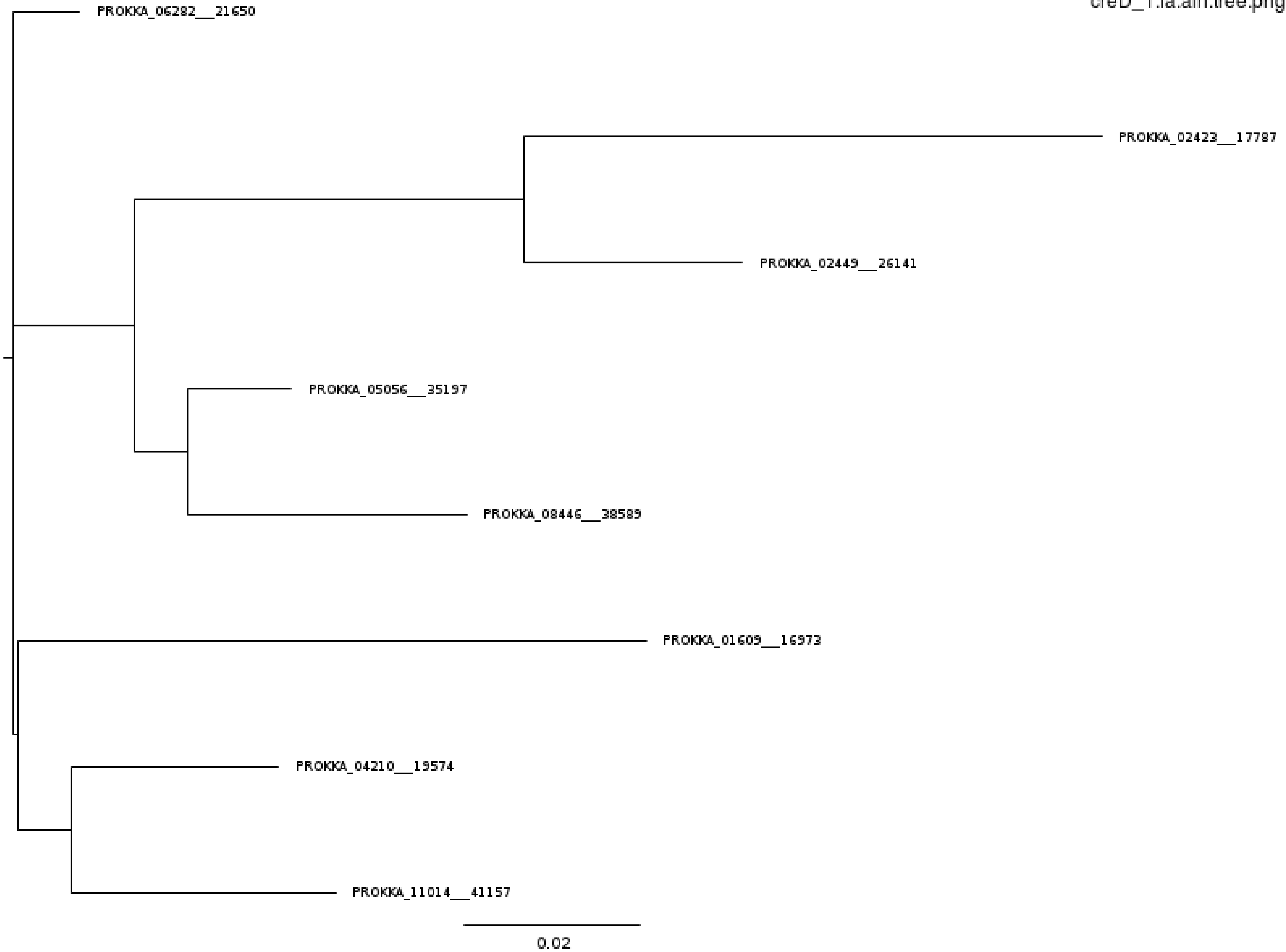

creD_2.fa.aln.tree.png

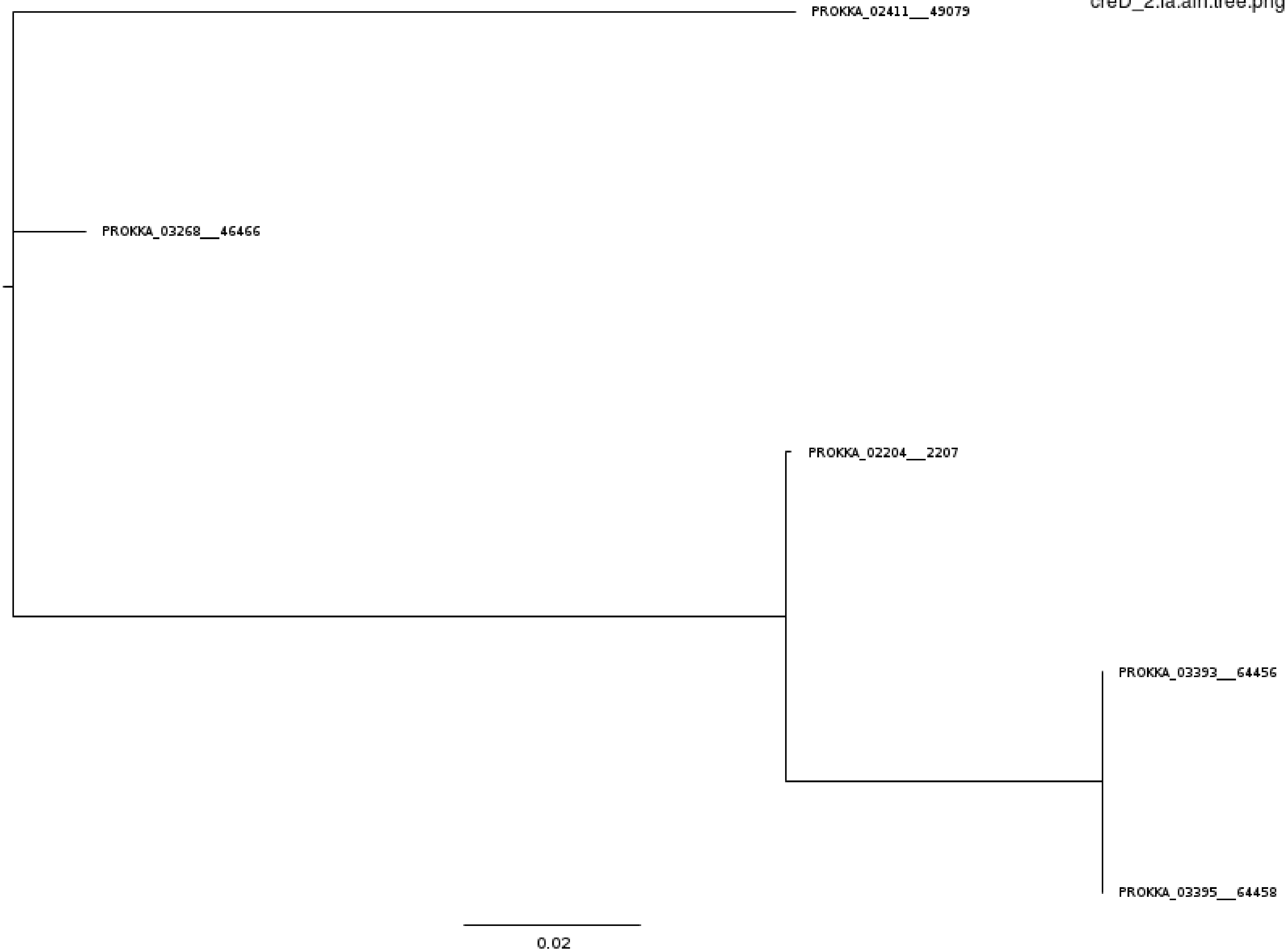

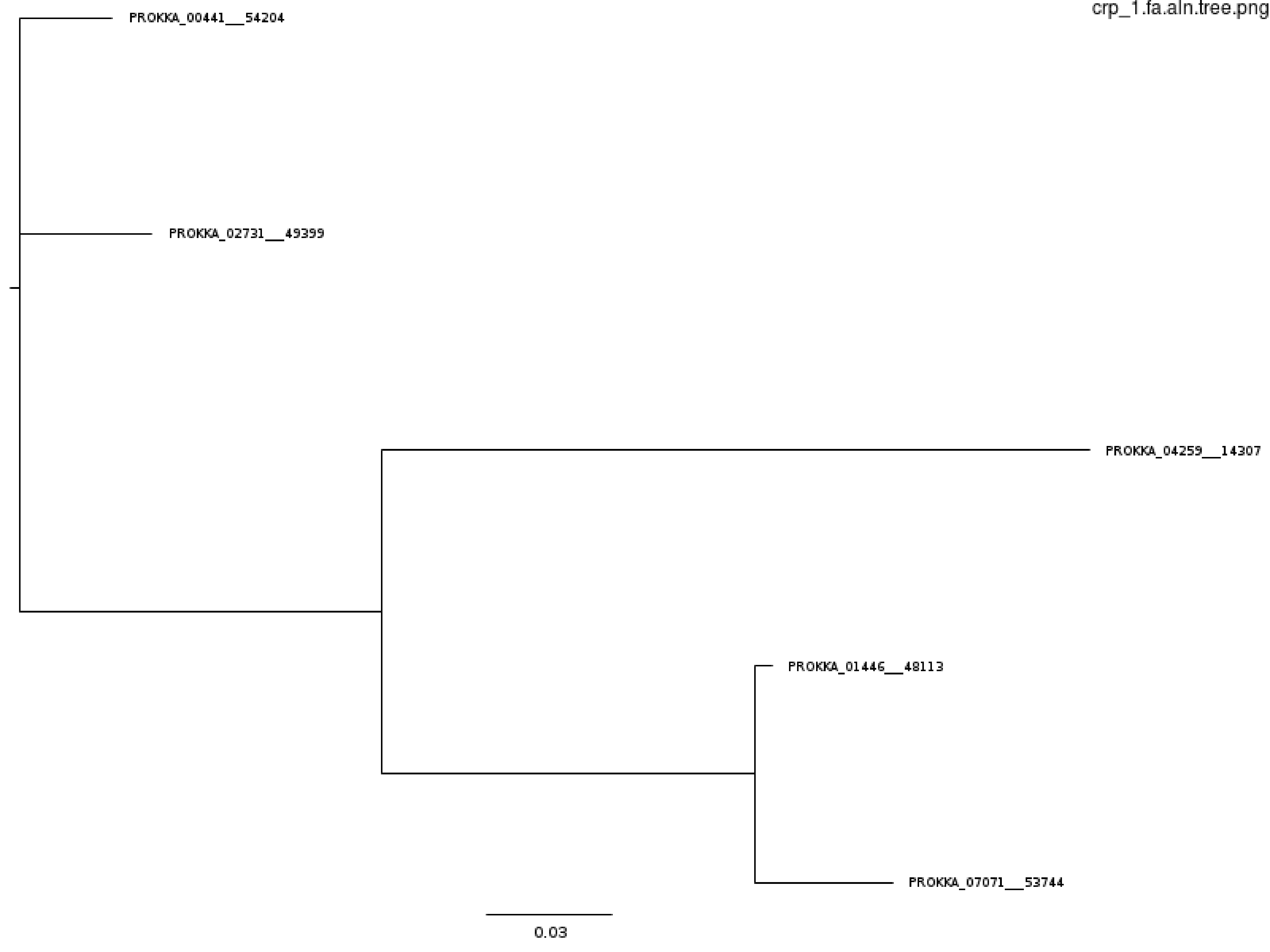
crp_1.fa.aln.tree.png
PROKKA_00441___54204
PROKKA_02731___49399
PROKKA_04259___14307
PROKKA_01446___48113
PROKKA_07071___53744
0.03

crp_4.fa.aln.tree.png

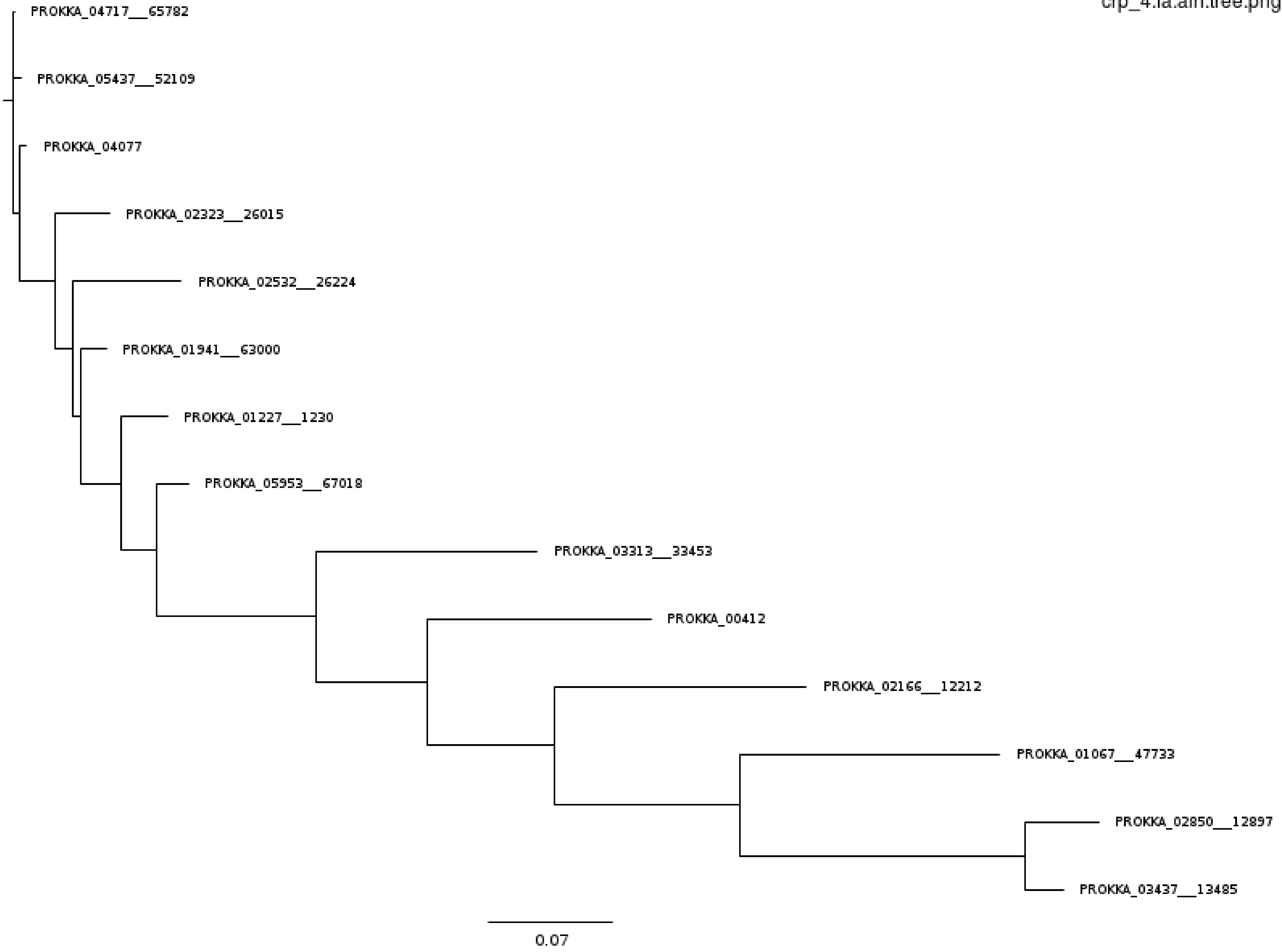

crp_7.fa.aln.tree.png

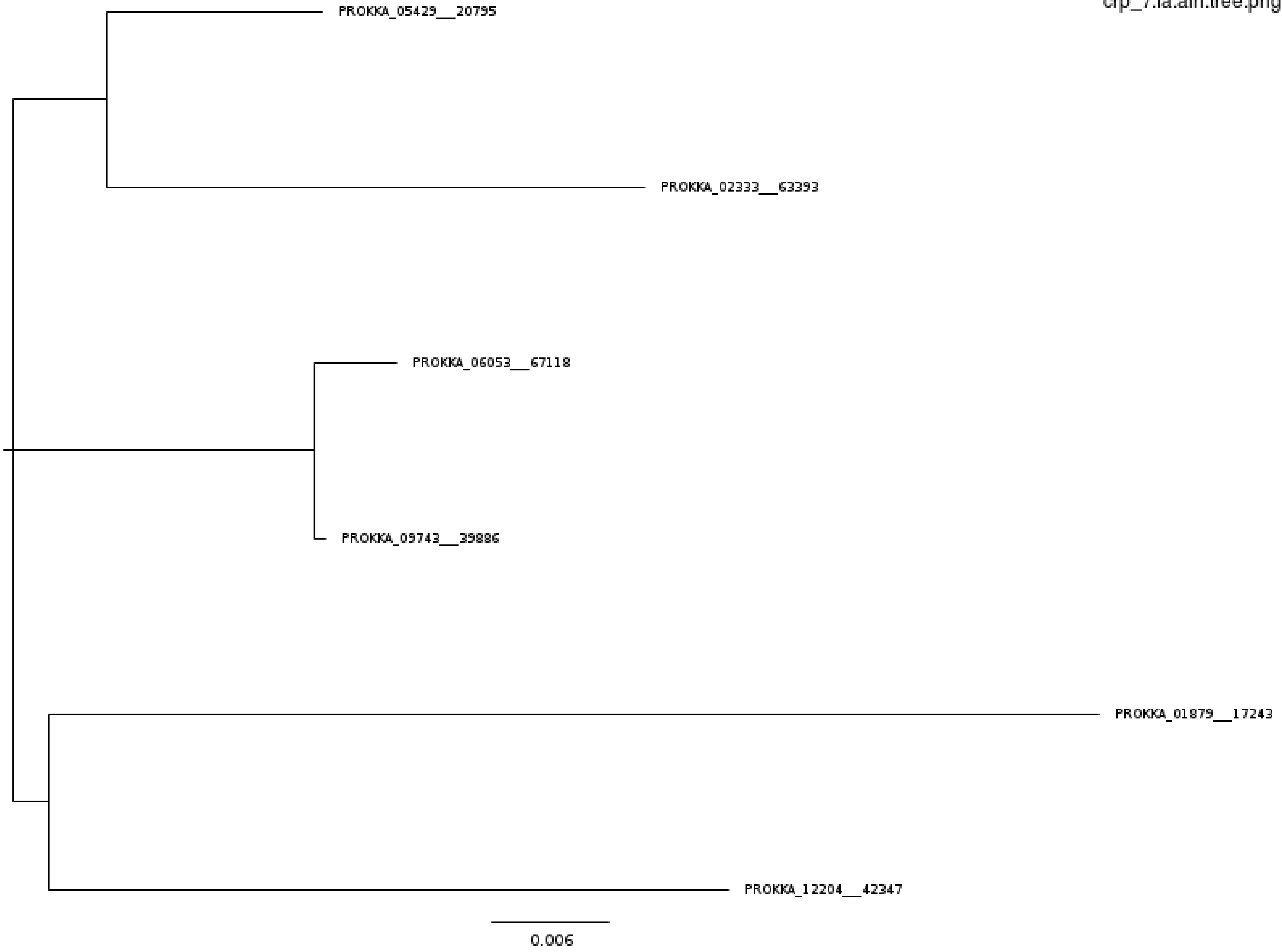

cspG.fa.aln.tree.png

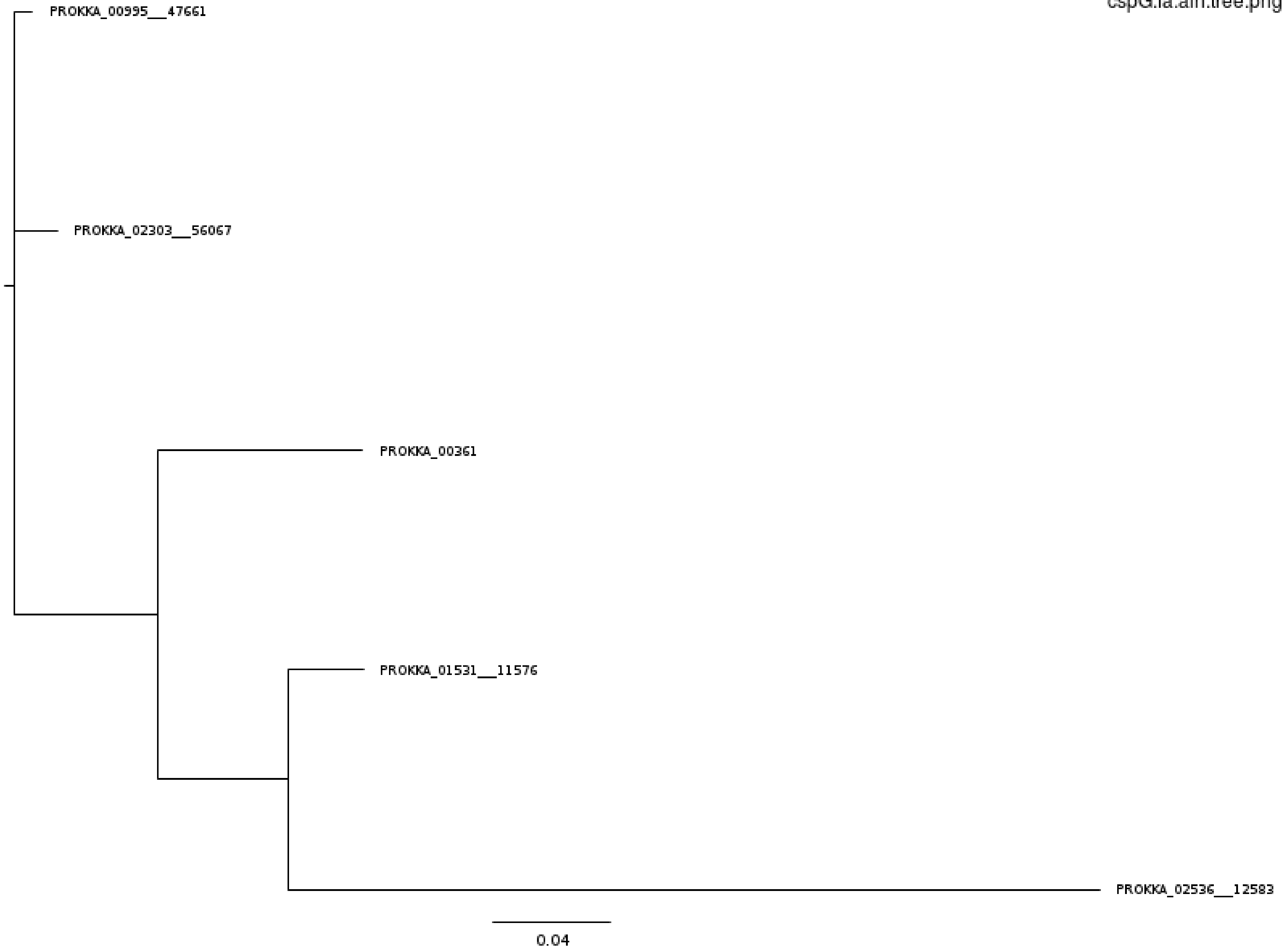

csrA_1.fa.aln.tree.png

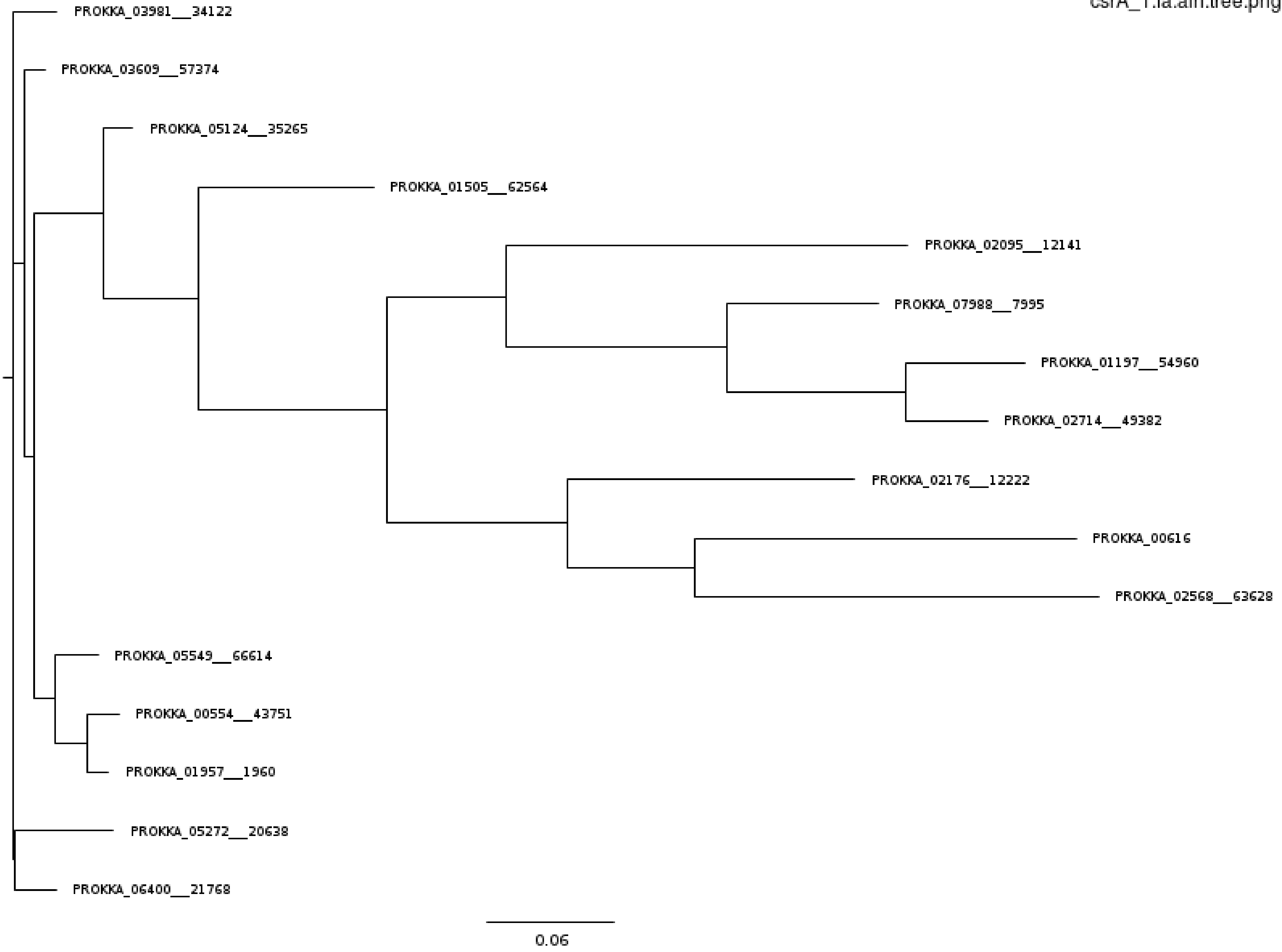

ctaA_2.fa.aln.tree.png

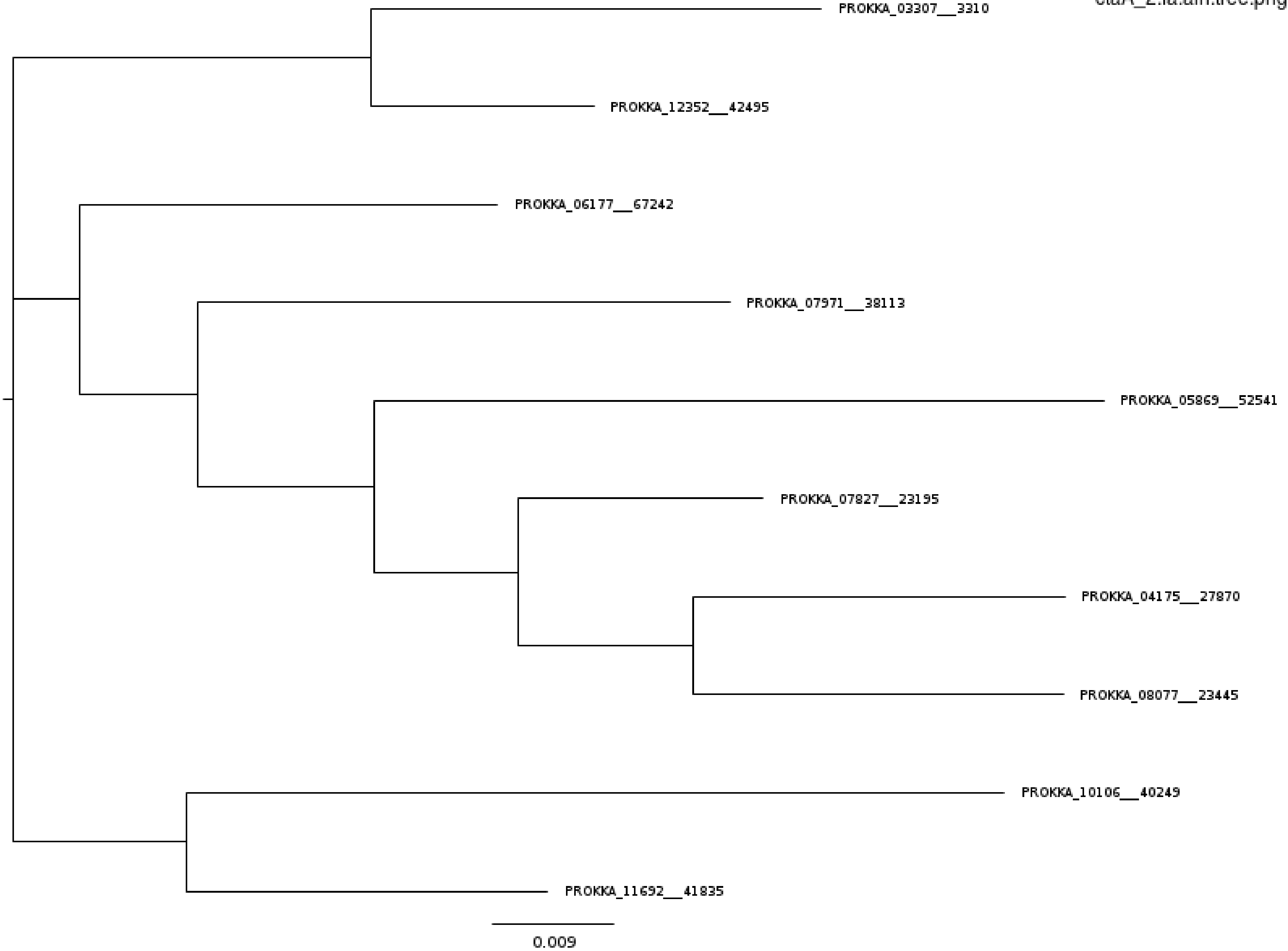

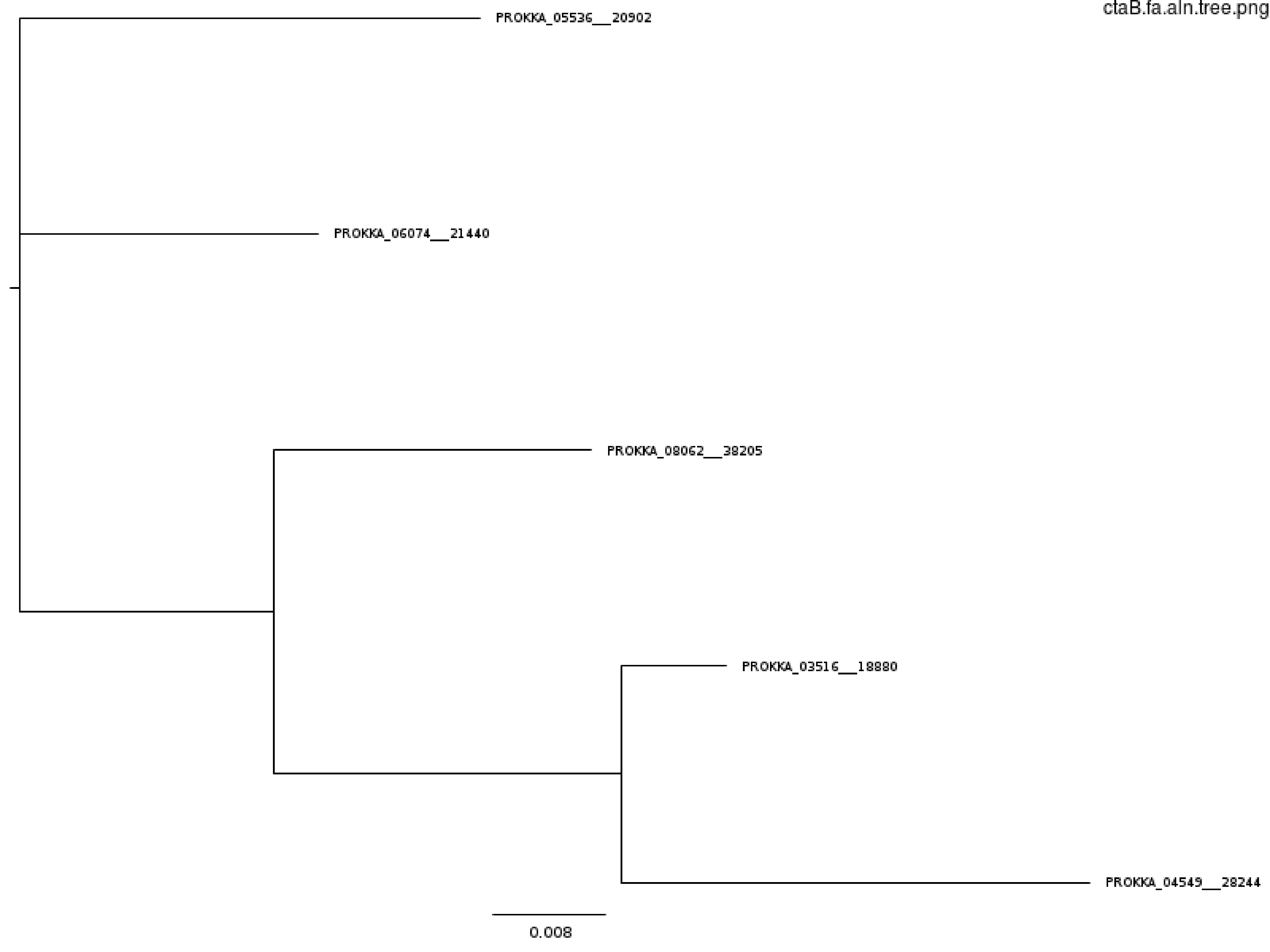
ctaB.fa.aln.tree.png
PROKKA_05536___20902
PROKKA_06074___21440
PROKKA_08062___38205
PROKKA_03516___18880
PROKKA_04549___28244
0.008

ctaE.fa.aln.tree.png

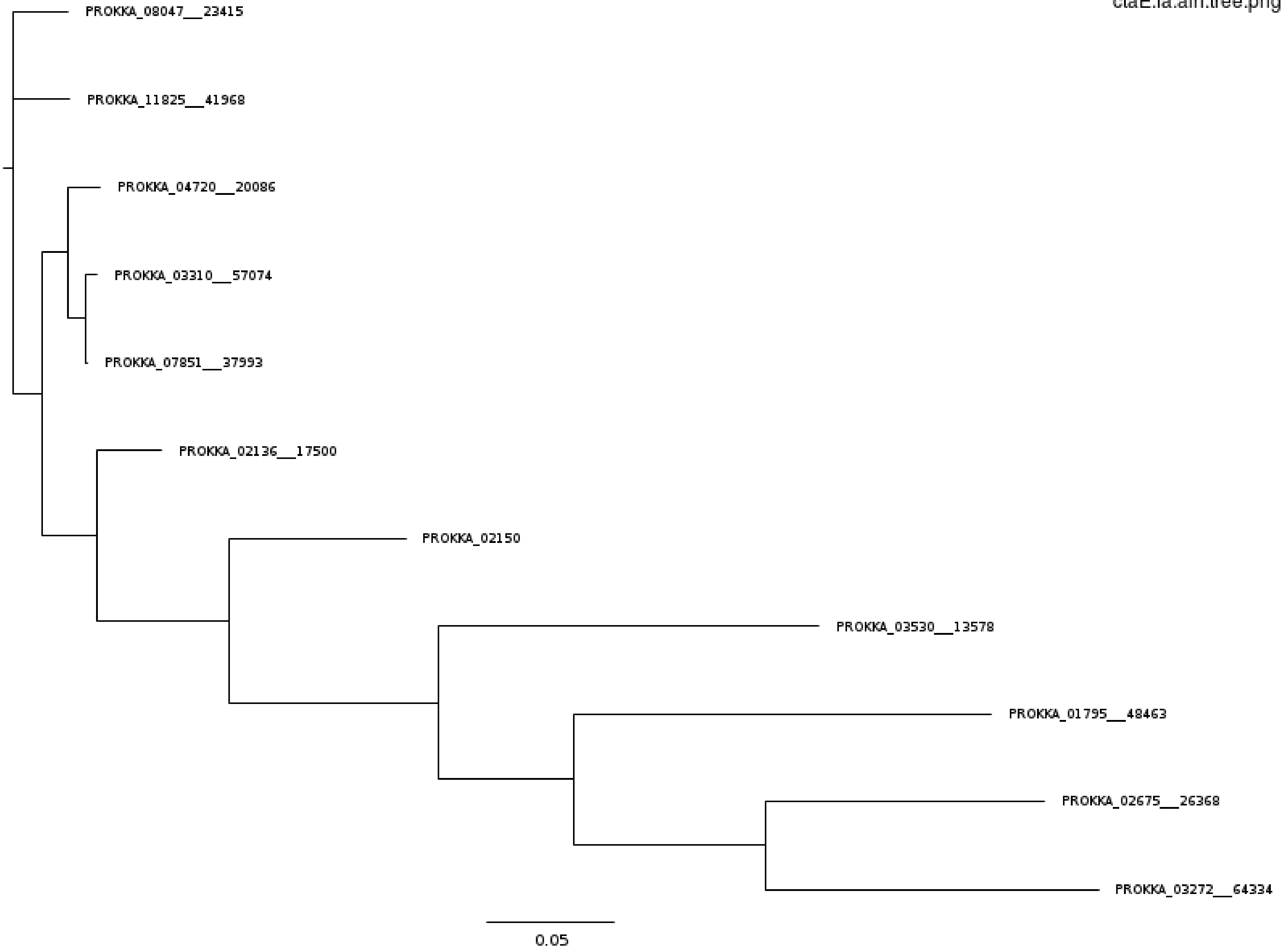

ctpF.fa.aln.tree.png

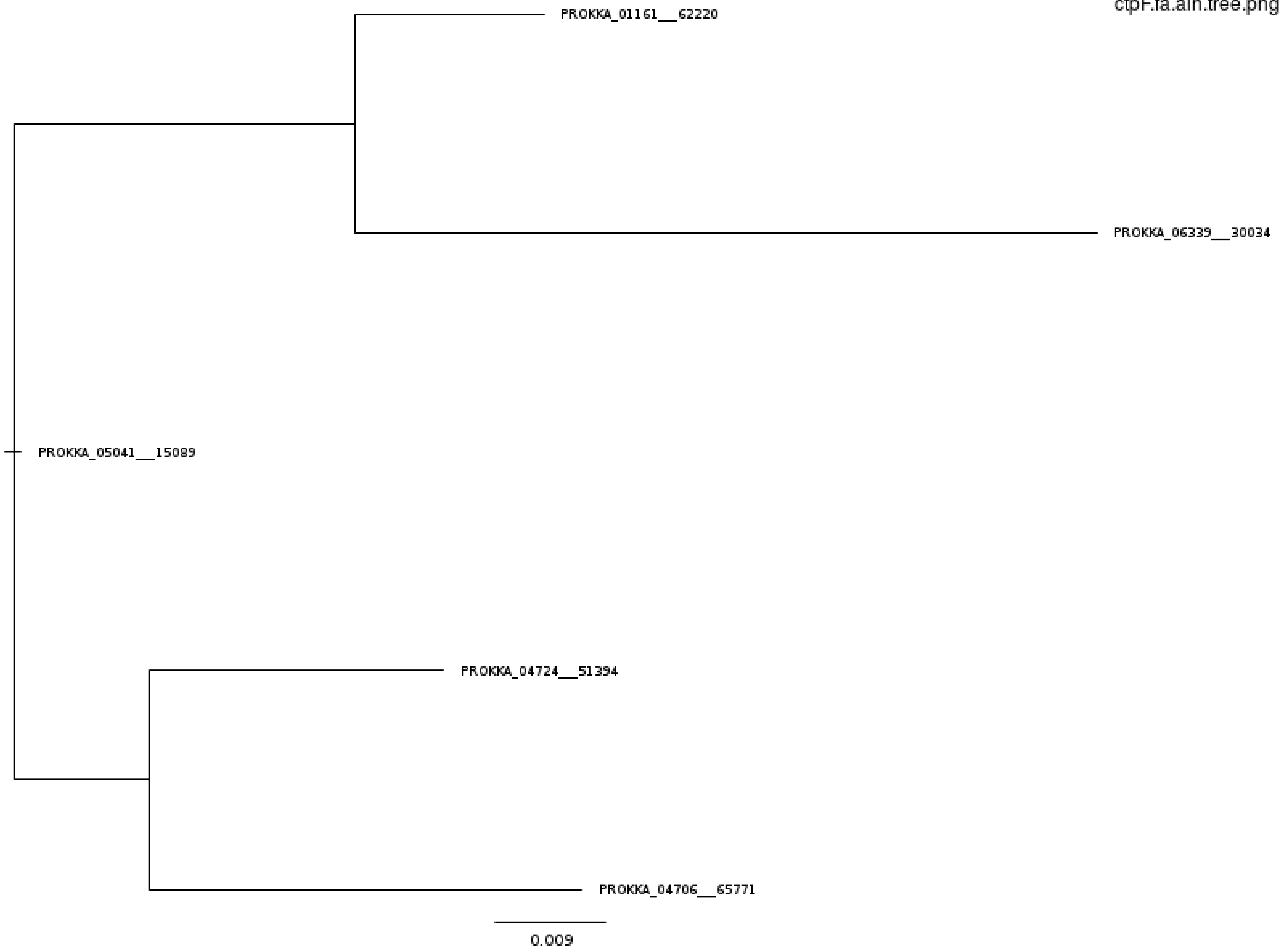

ctpL.fa.aln.tree.png

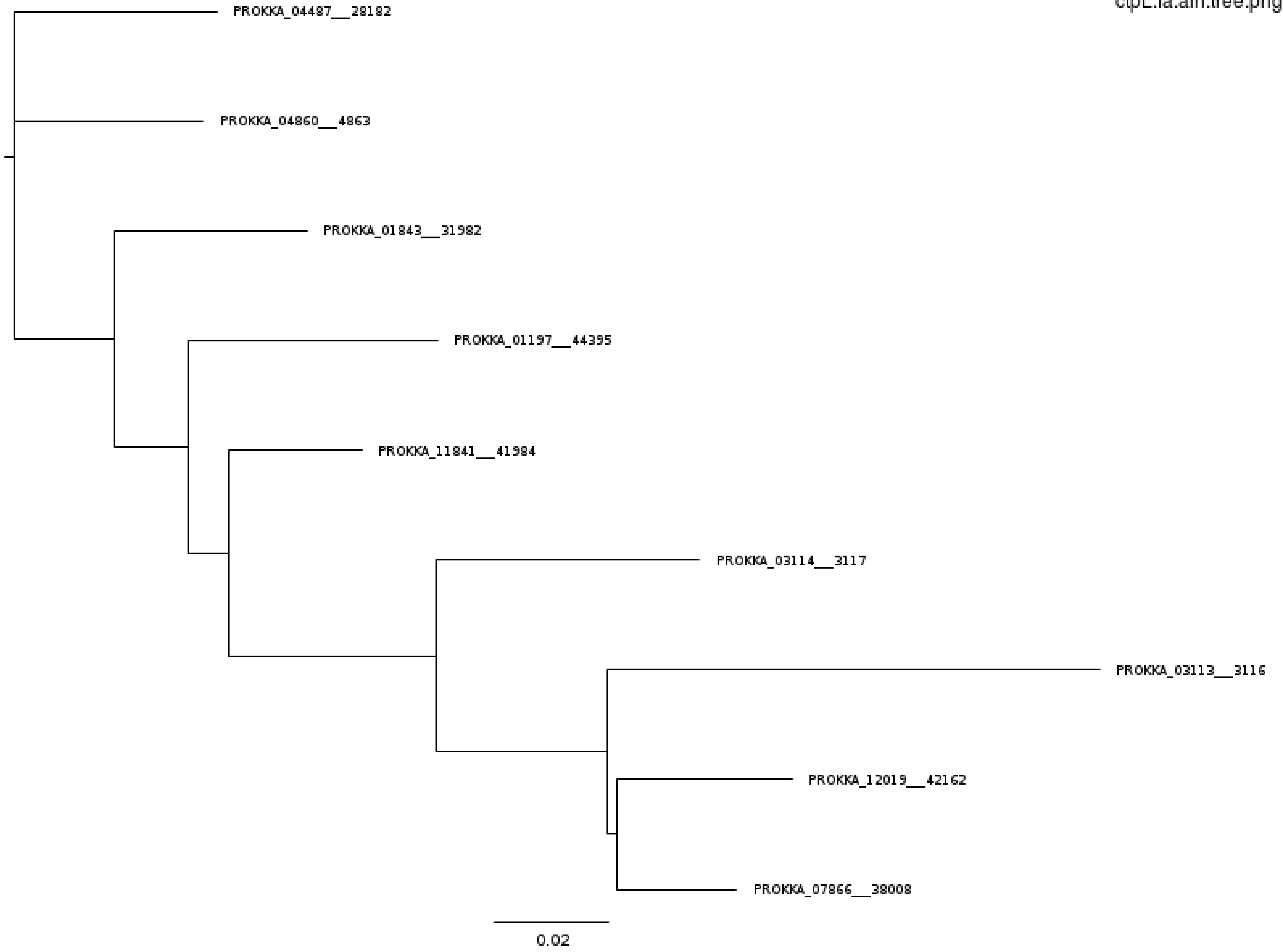

cutA_2.fa.aln.tree.png

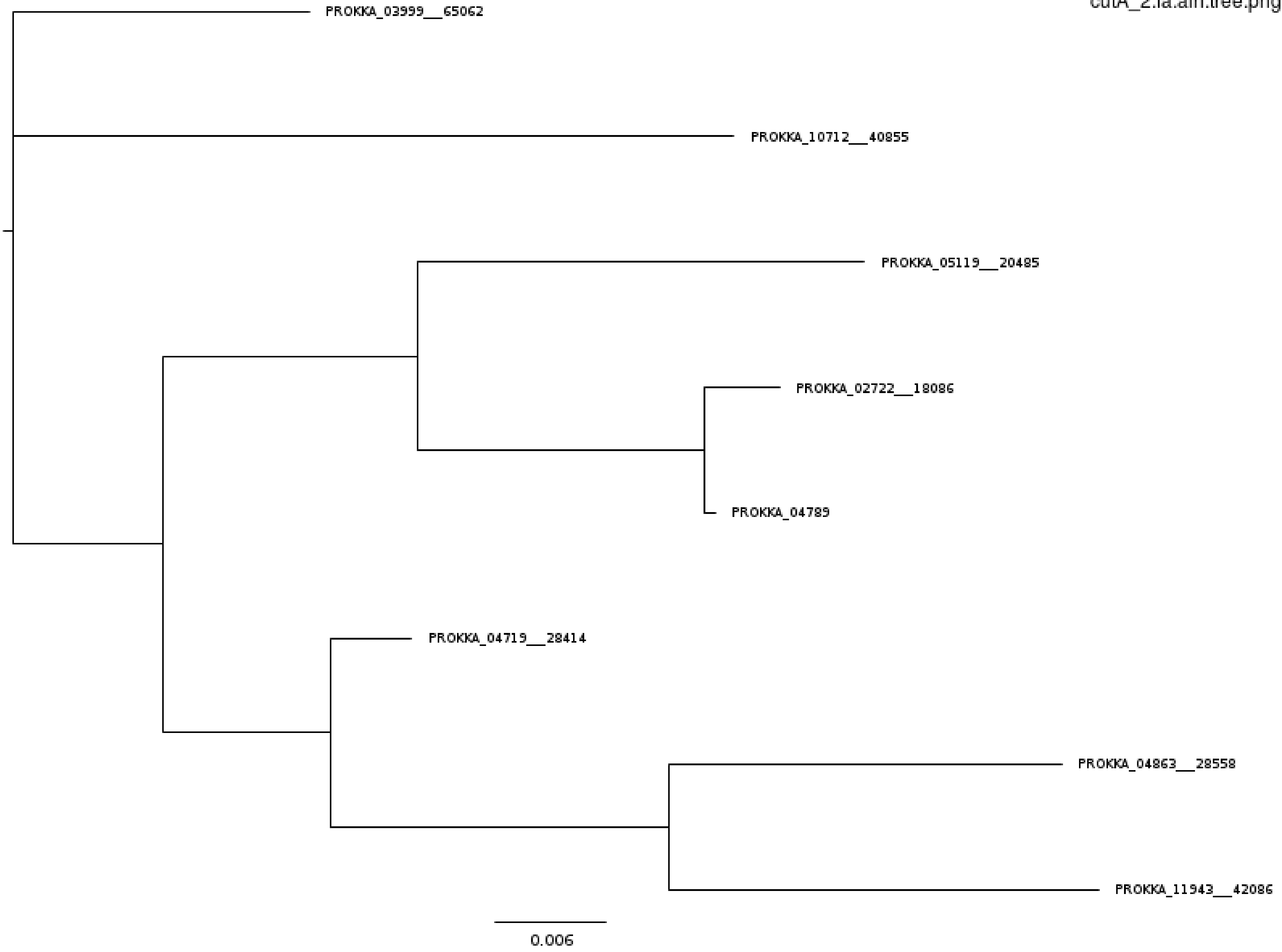

cyaA_2.fa.aln.tree.png

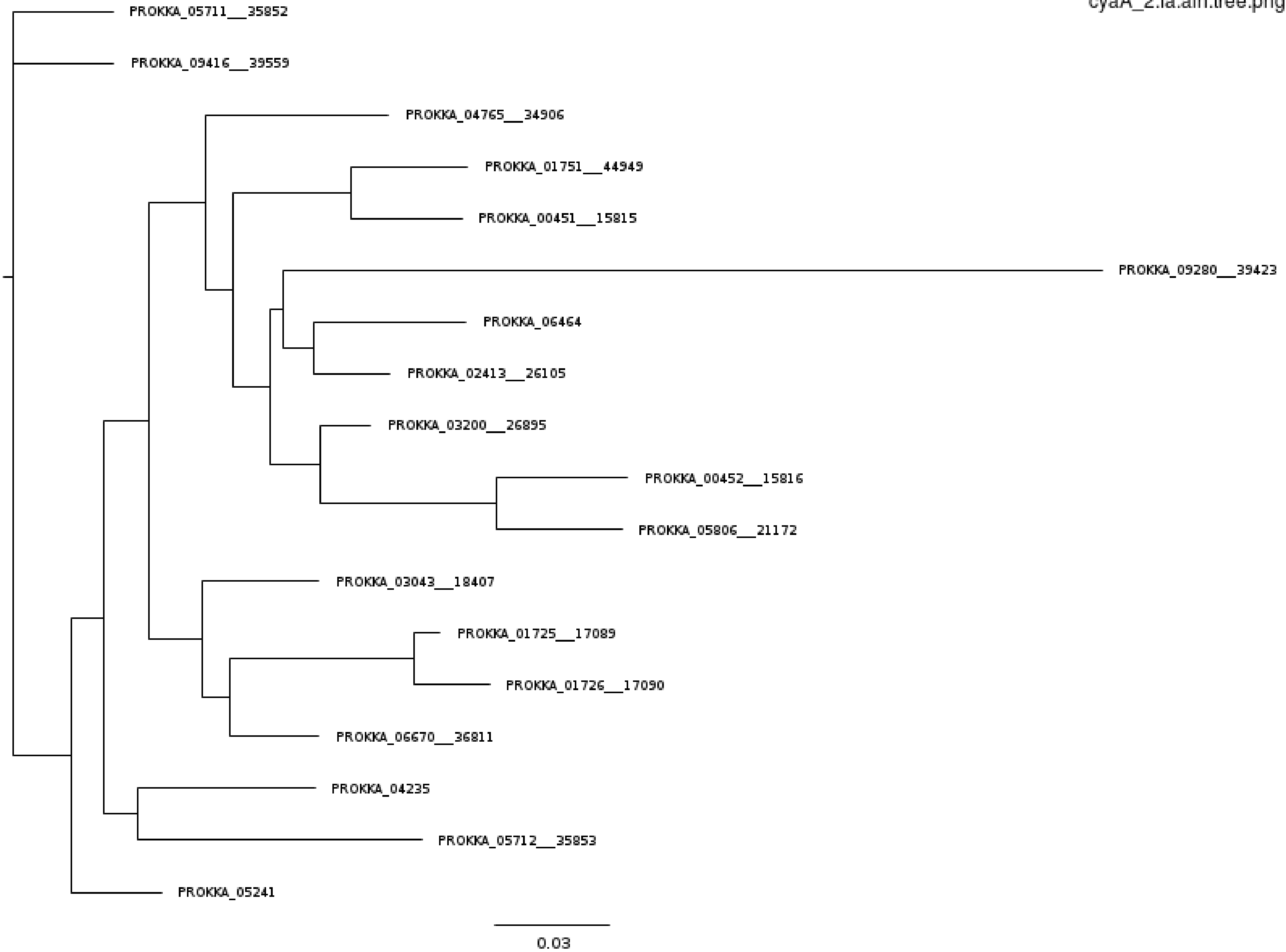

cyc1.fa.aln.tree.png

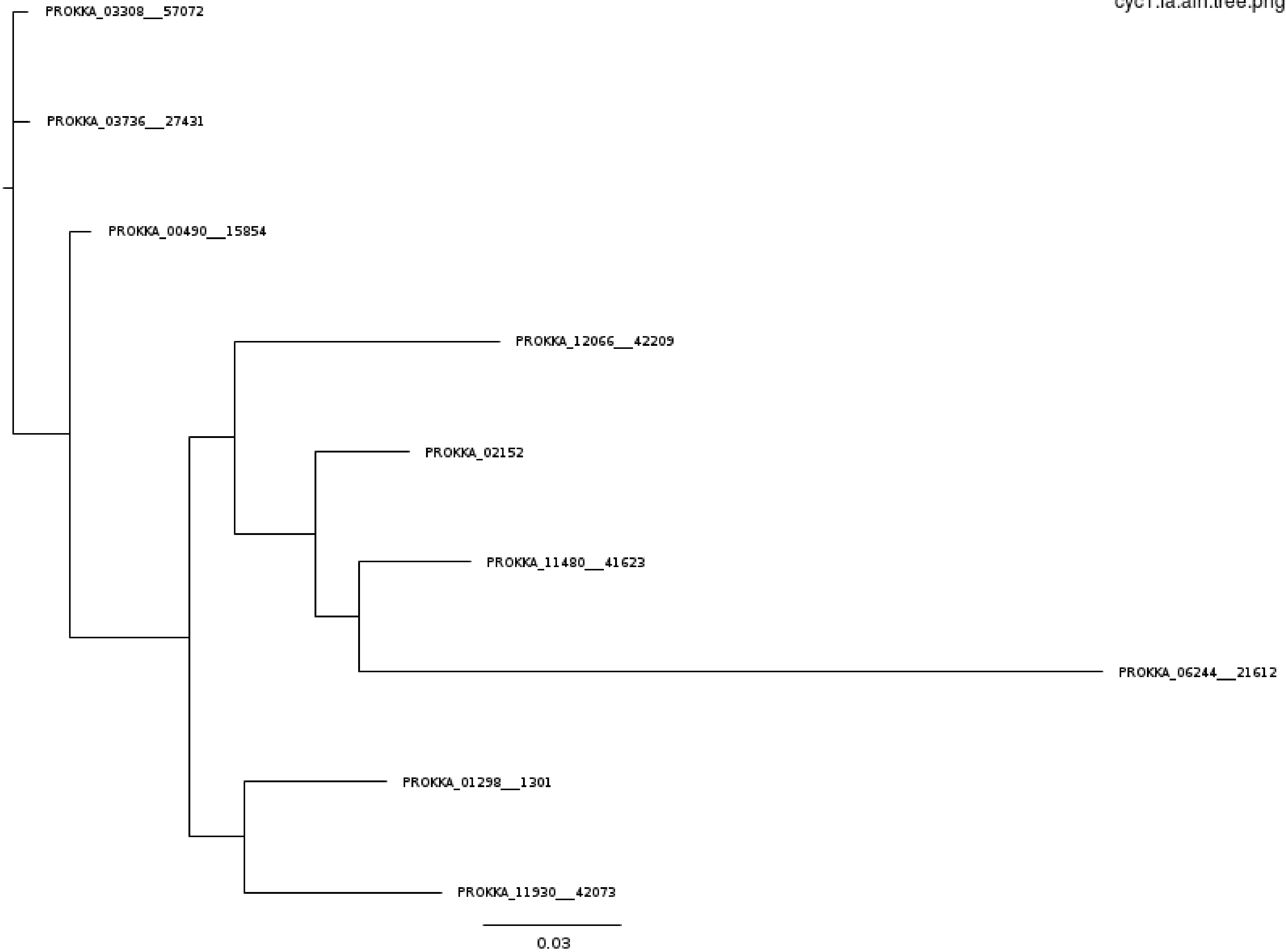

cysG_3.fa.aln.tree.png

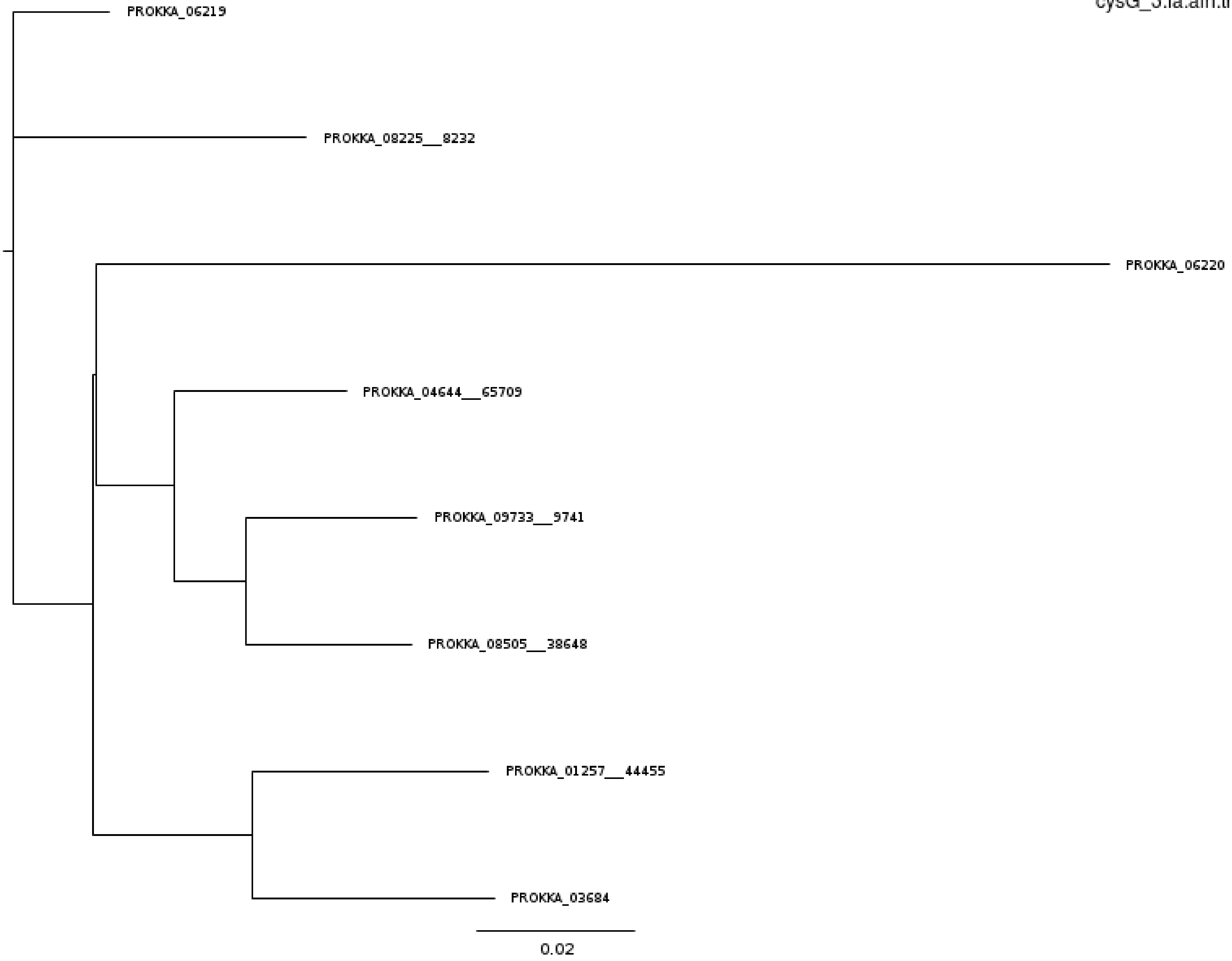

cysM_2.fa.aln.tree.png

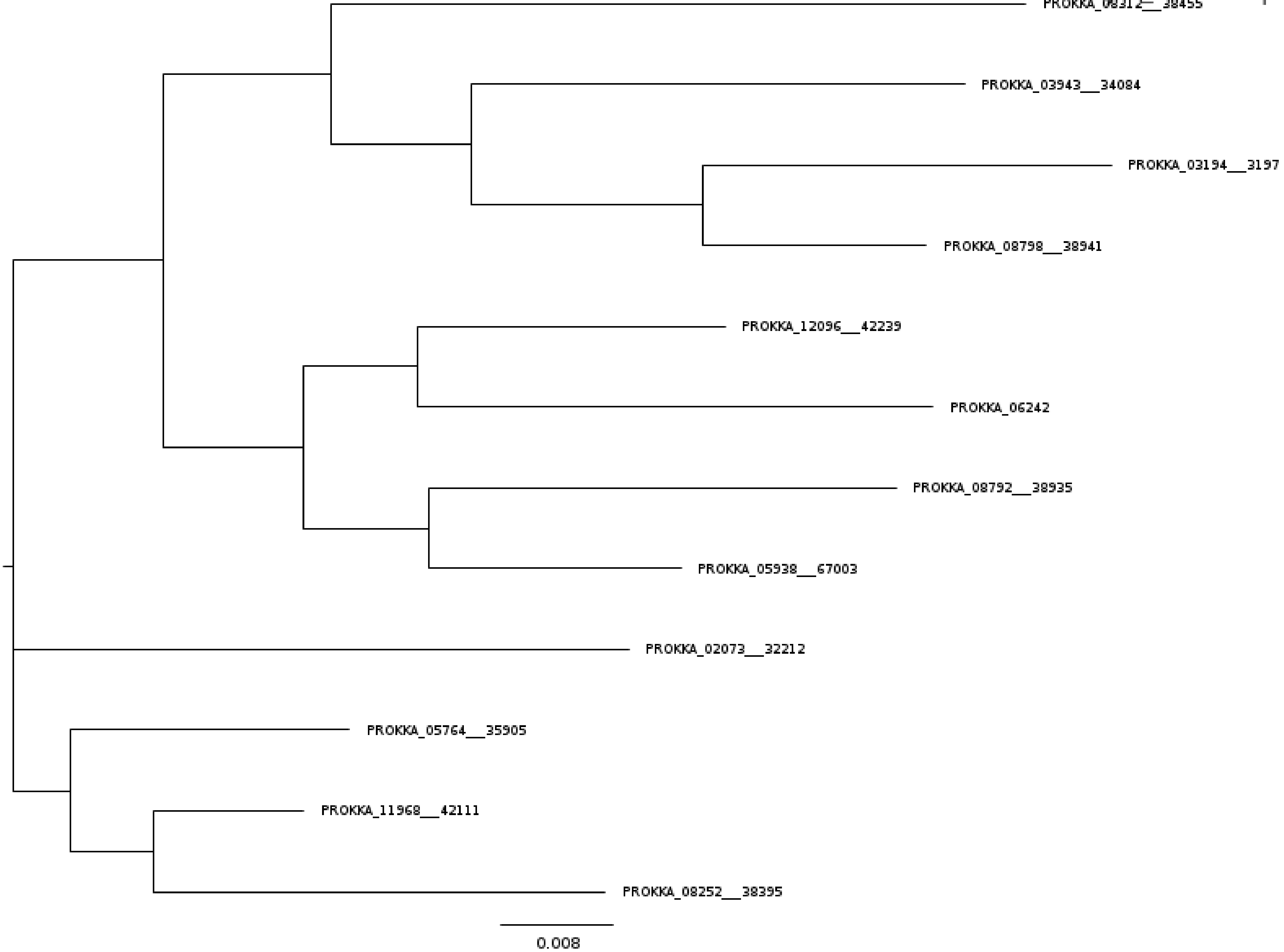

cysS_2.fa.aln.tree.png

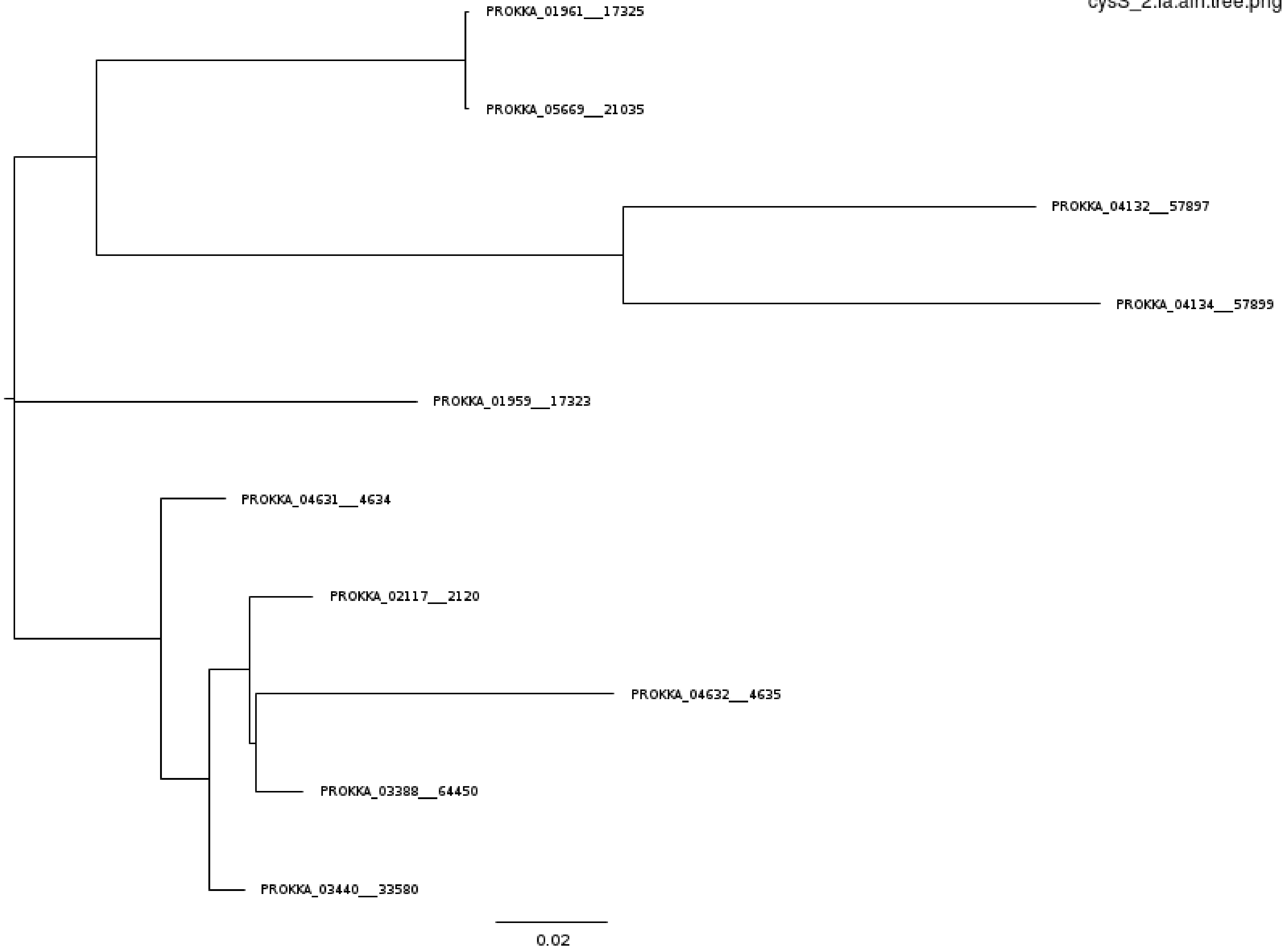

czcA.fa.aln.tree.png

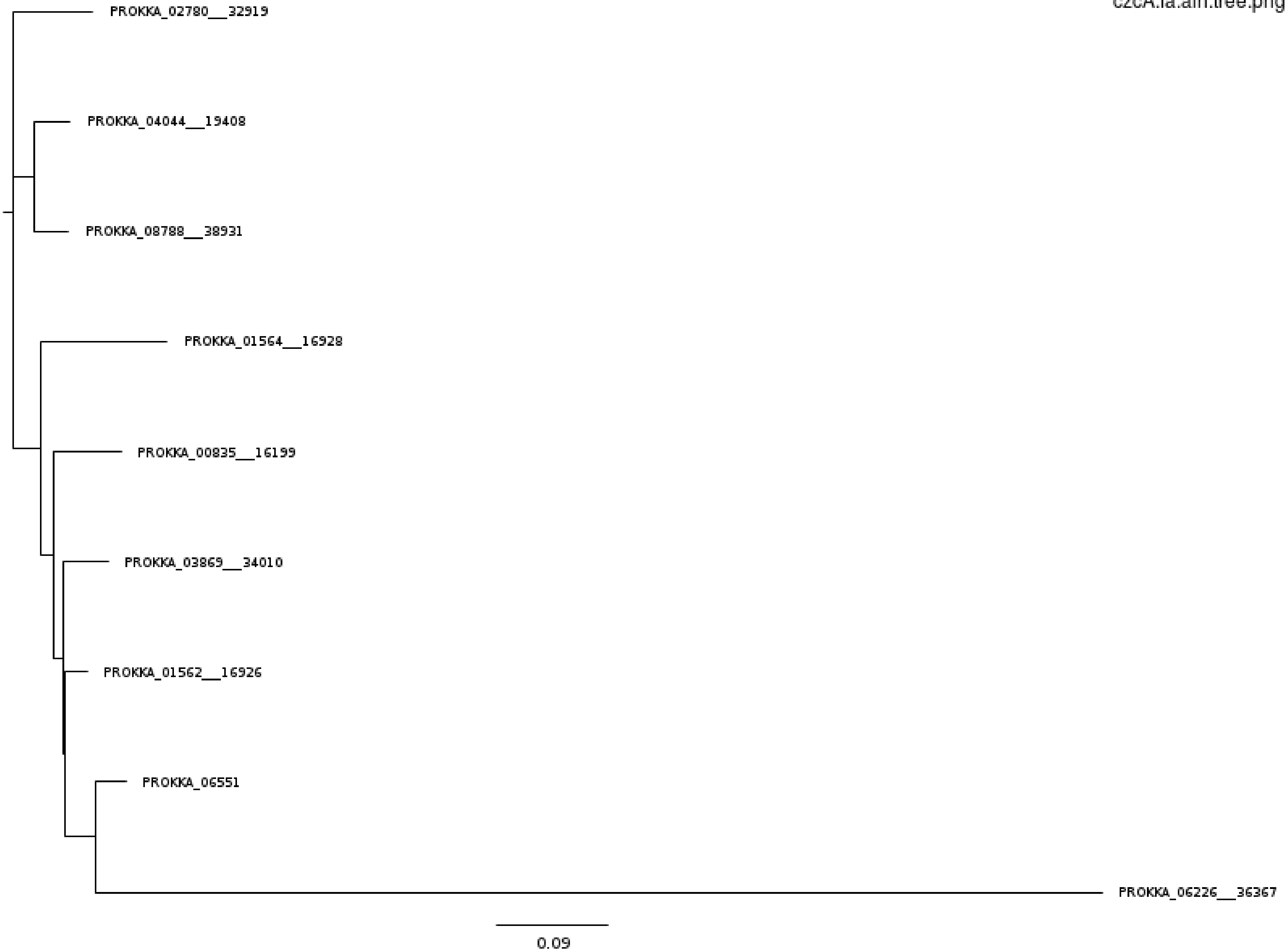

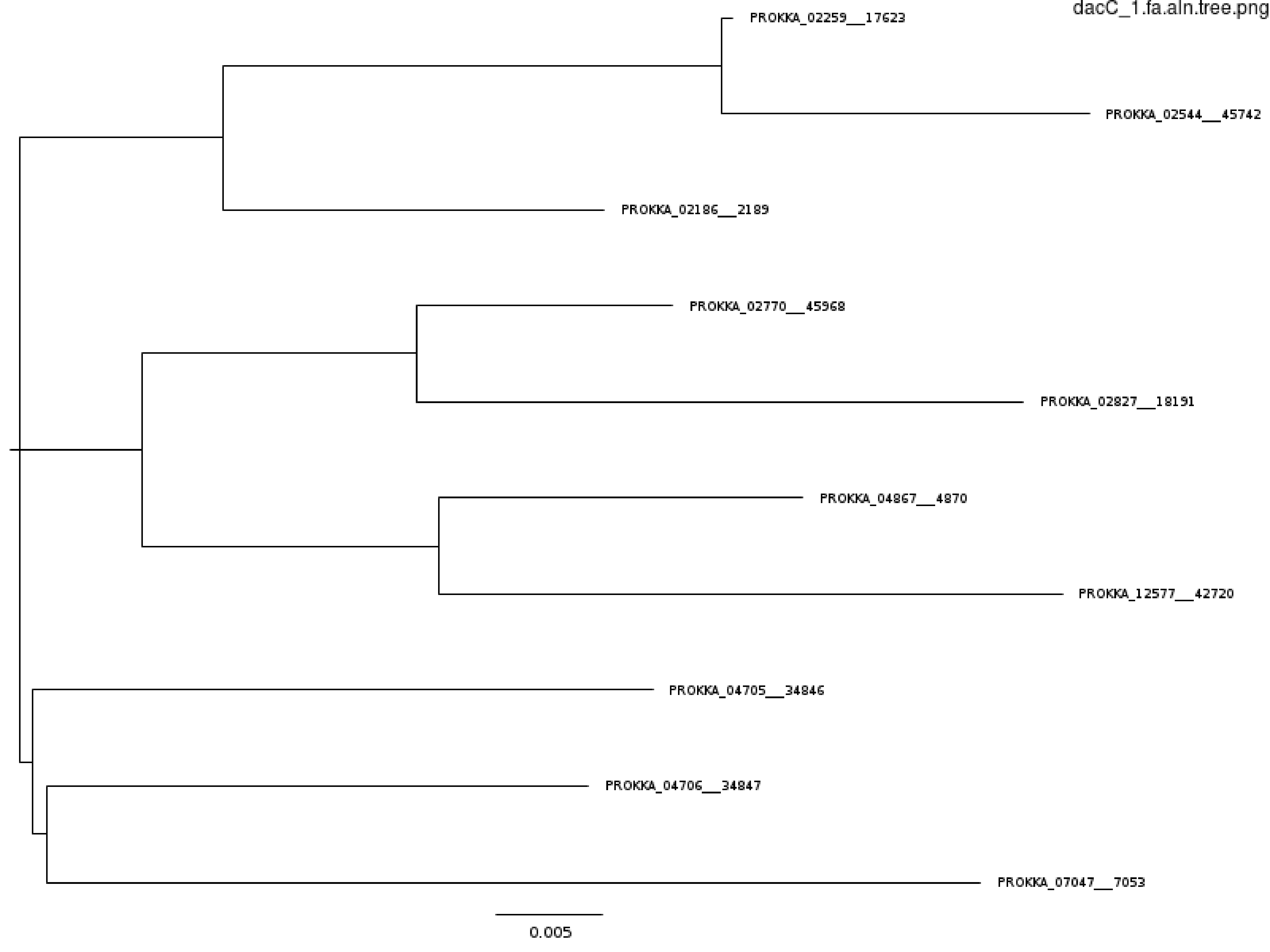
dacC_1.fa.aln.tree.png
PROKKA_02259___17623
PROKKA_02544___45742
PROKKA_02186___2189
PROKKA_02770___45968
PROKKA_02827___18191
PROKKA_04867___4870
PROKKA_12577___42720
PROKKA_04705___34846
PROKKA_04706___34847
PROKKA_07047___7053
0.005

dapA_2.fa.aln.tree.png

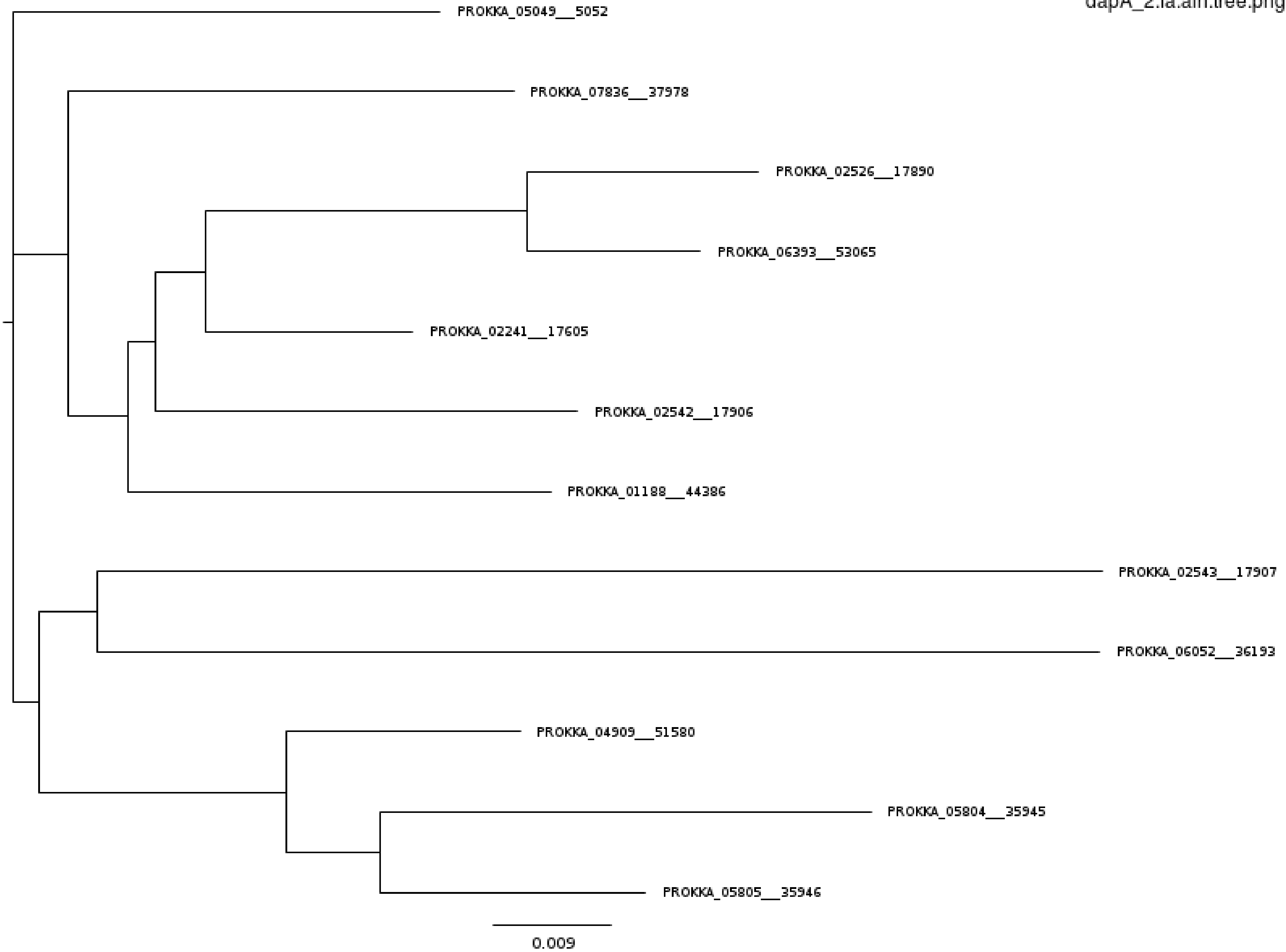

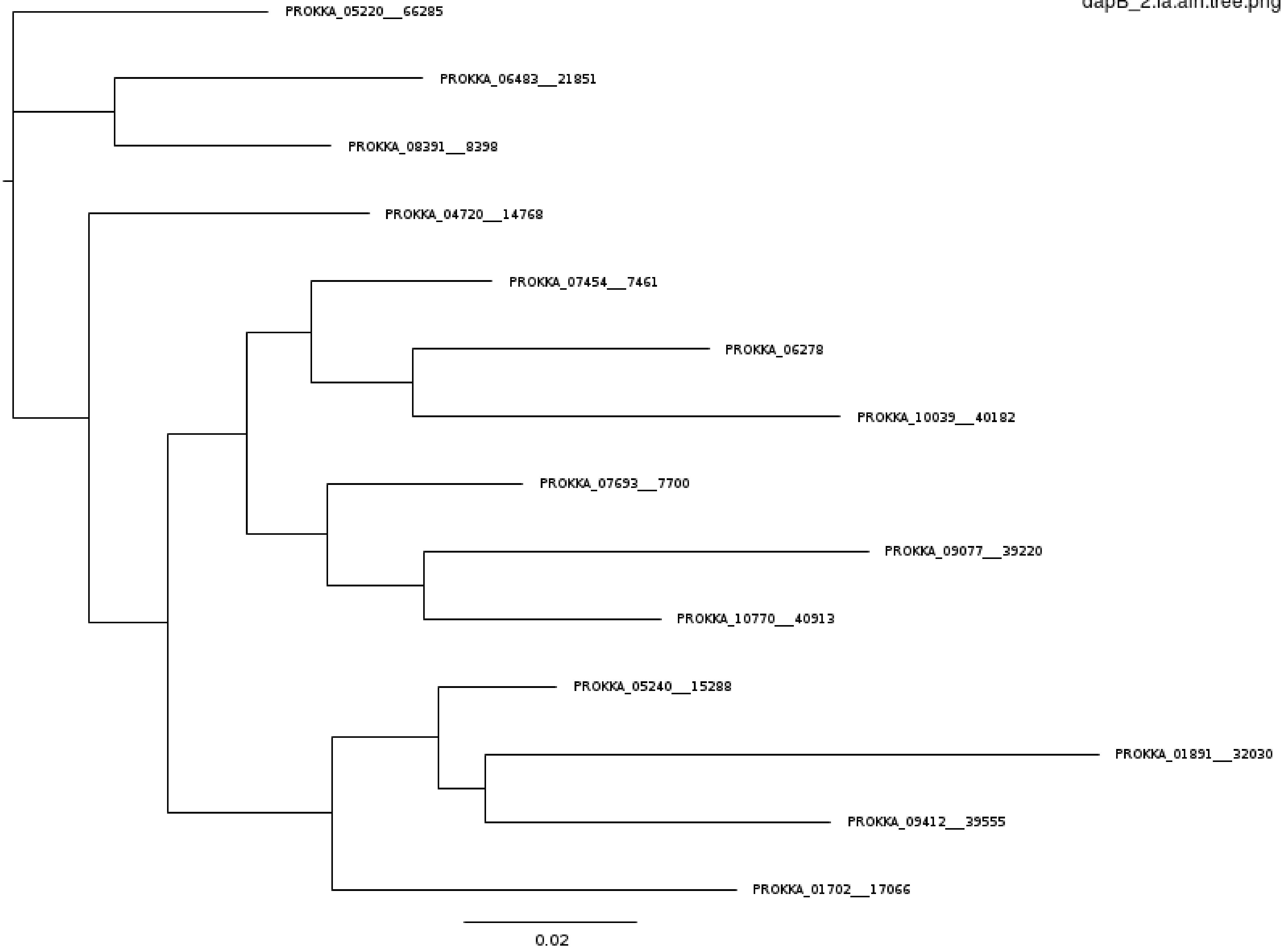
PROKKA_05220___66285
PROKKA_06483___21851
PROKKA_08391___8398
PROKKA_04720___14768
PROKKA_07454___7461
PROKKA_06278
PROKKA_10039___40182
PROKKA_07693___7700
PROKKA_09077___39220
PROKKA_10770___40913
PROKKA_05240___15288
PROKKA_01891___32030
PROKKA_09412___39555
PROKKA_01702___17066
0.02

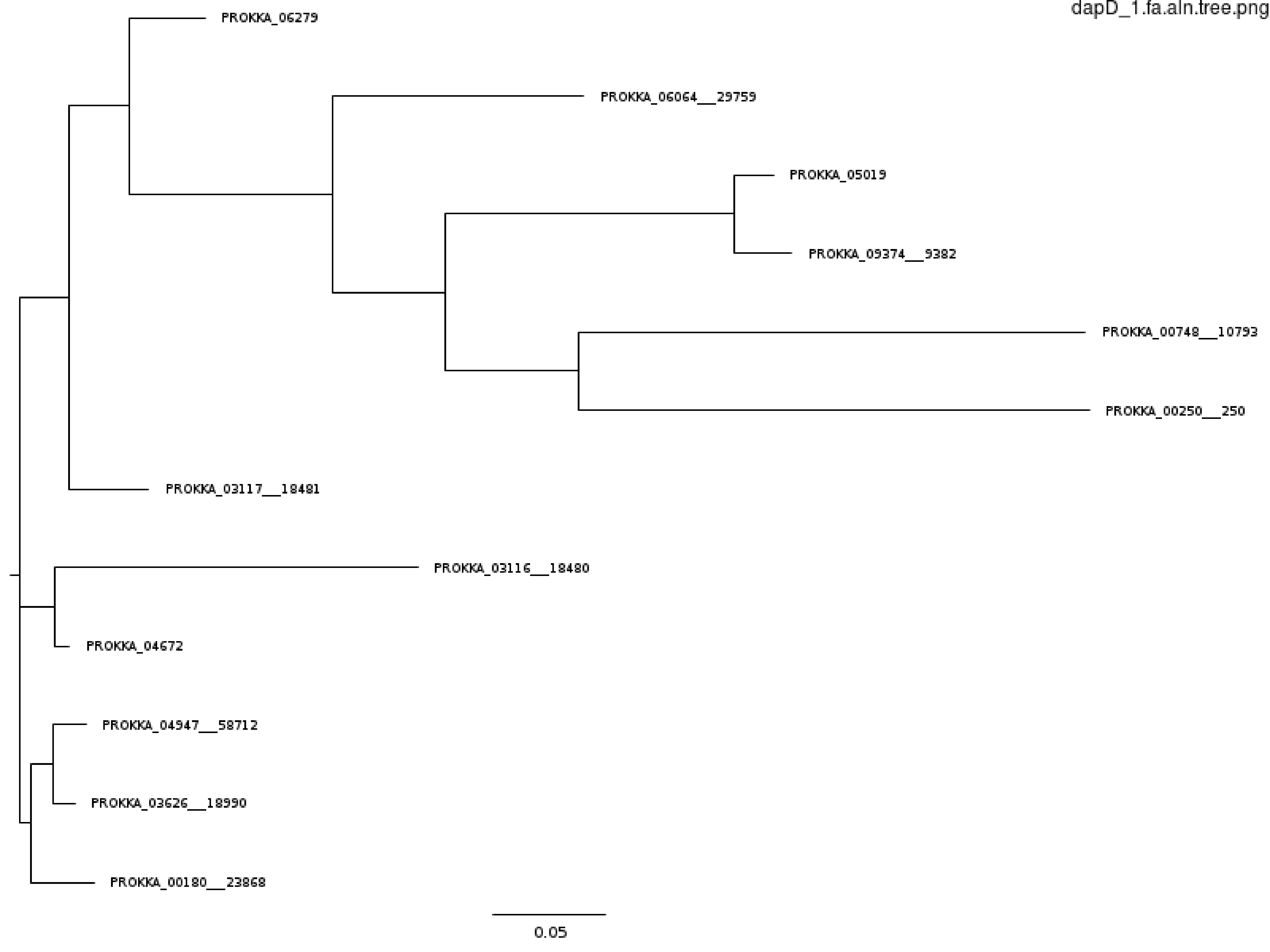
dapD_1.fa.aln.tree.png
PROKKA_06279
PROKKA_06064__29759
PROKKA_05019
PROKKA_09374__9382
PROKKA_00748__10793
PROKKA_00250__250
PROKKA_03117__18481
PROKKA_03116__18480
PROKKA_04672
PROKKA_04947__58712
PROKKA_03626__18990
PROKKA_00180__23868
0.05

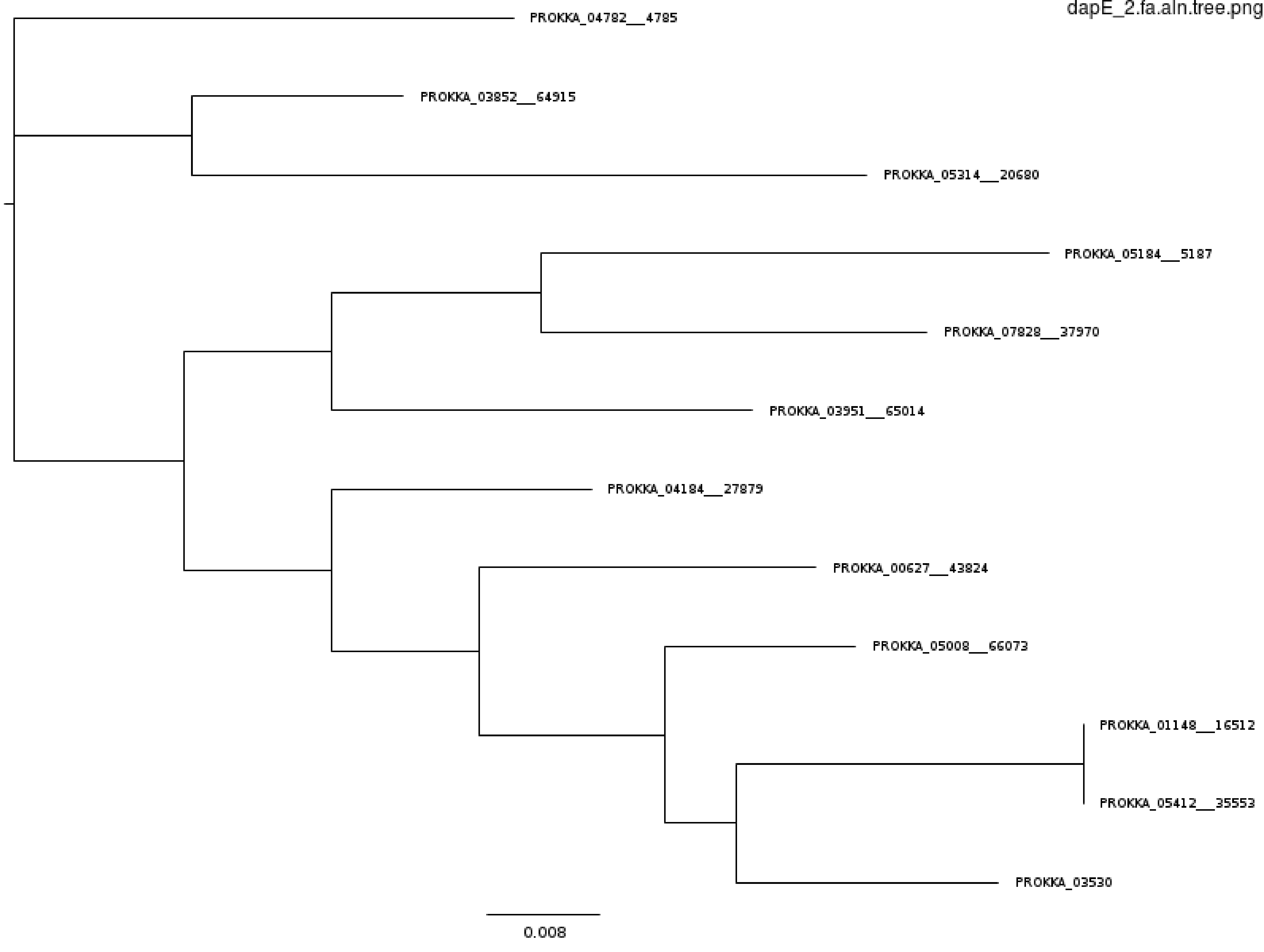
dapE_2.fa.aln.tree.png
PROKKA_04782___4785
PROKKA_03852___64915
PROKKA_05314___20680
PROKKA_05184___5187
PROKKA_07828___37970
PROKKA_03951___65014
PROKKA_04184___27879
PROKKA_00627___43824
PROKKA_05008___66073
PROKKA_01148___16512
PROKKA_05412___35553
PROKKA_03530
0.008

dapE_5.fa.aln.tree.png

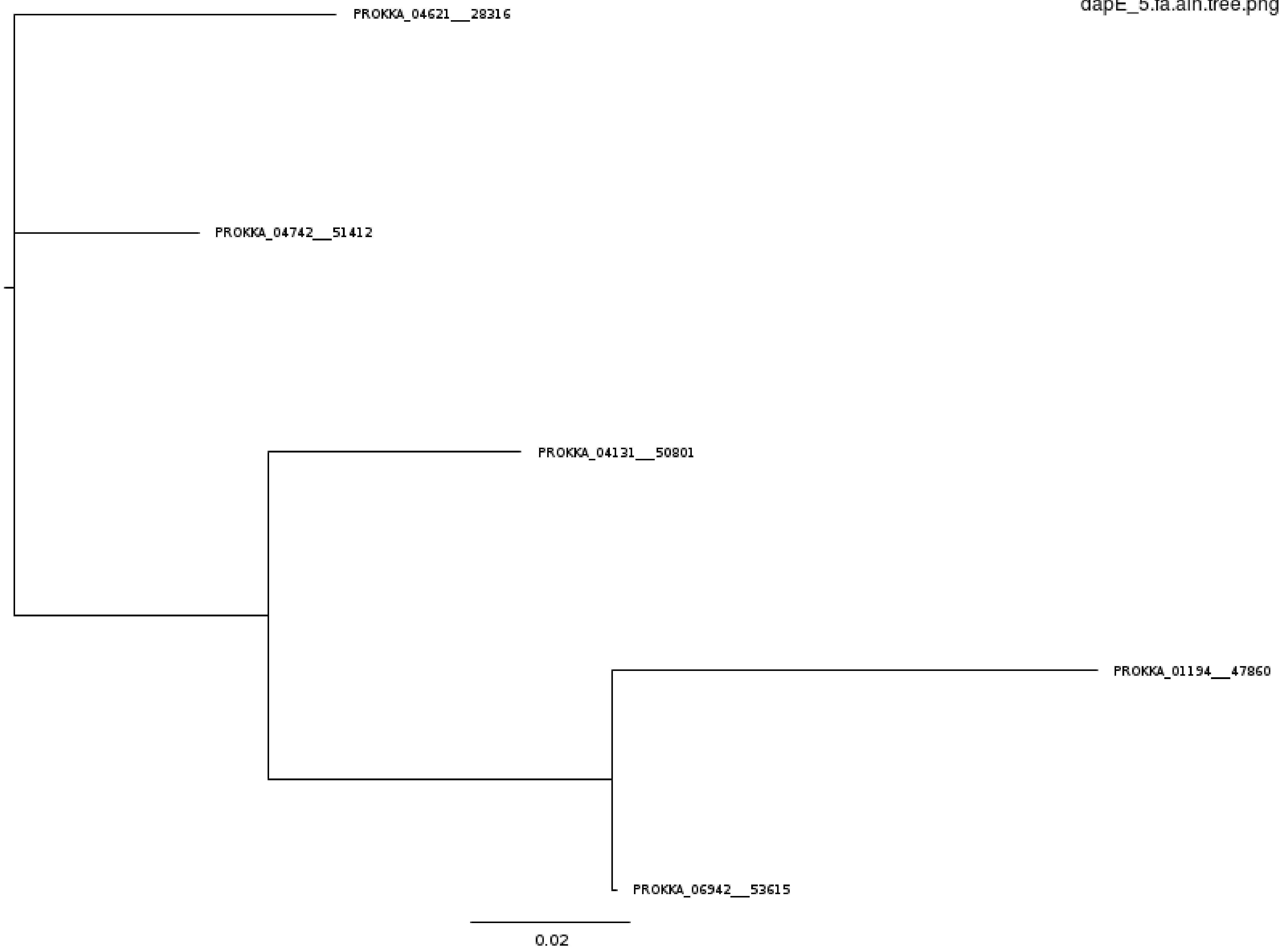

dapF_2.fa.aln.tree.png

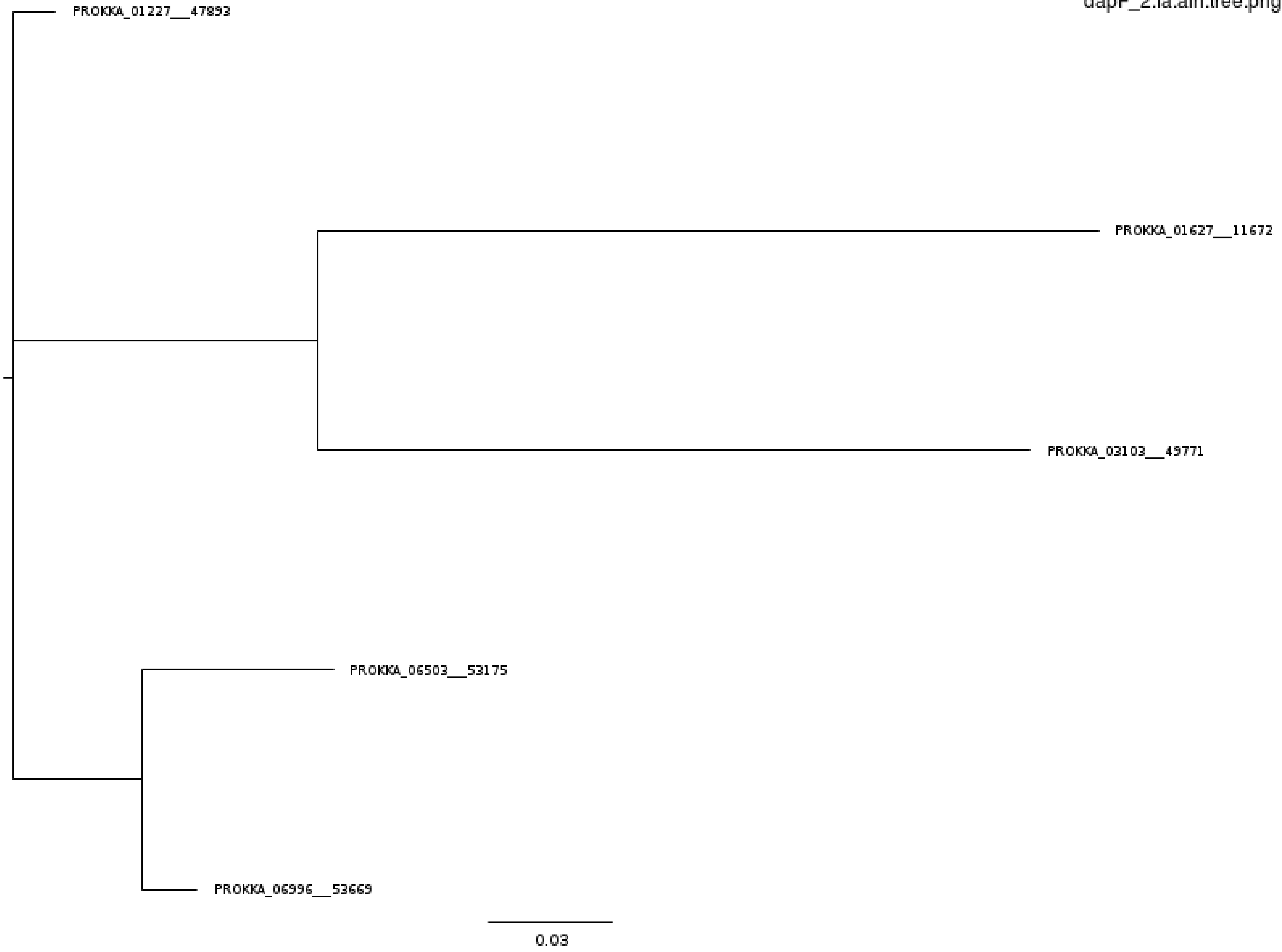

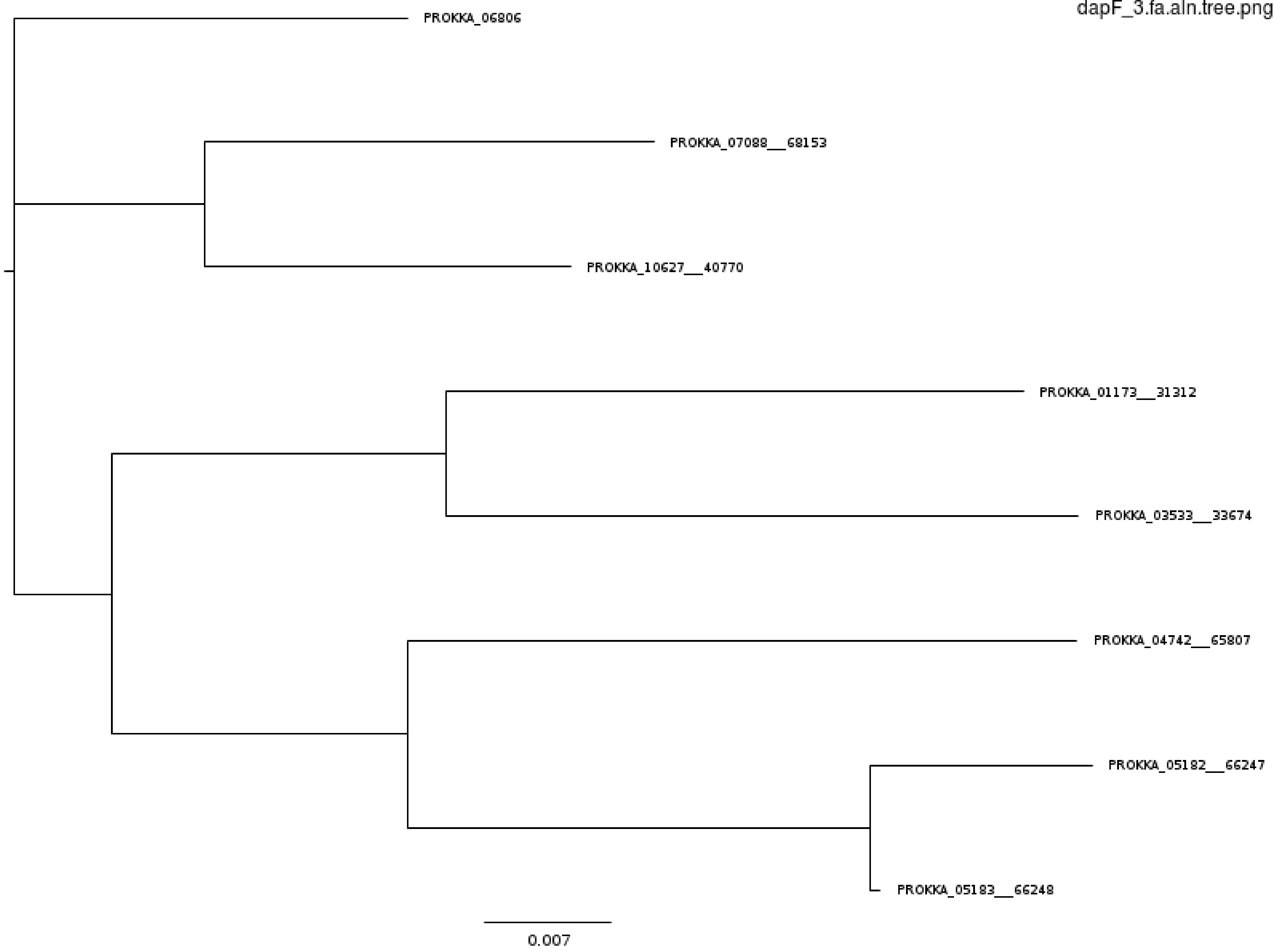
dapF_3.fa.aln.tree.png
PROKKA_06806
PROKKA_07088___68153
PROKKA_10627___40770
PROKKA_01173___31312
PROKKA_03533___33674
PROKKA_04742___65807
PROKKA_05182___66247
PROKKA_05183___66248
0.007

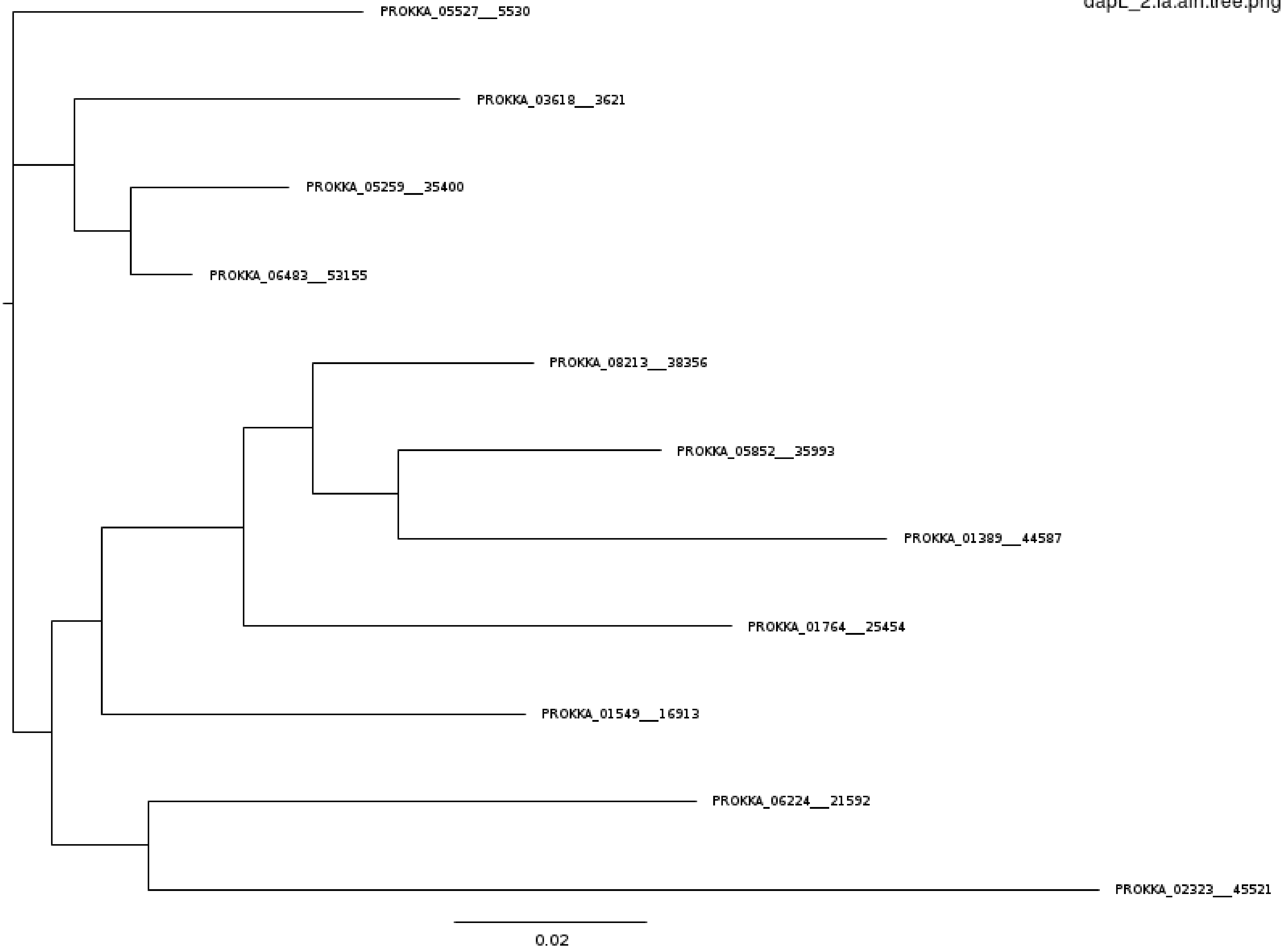
PROKKA_05527___5530
PROKKA_03618___3621
PROKKA_05259___35400
PROKKA_06483___53155
PROKKA_08213___38356
PROKKA_05852___35993
PROKKA_01389___44587
PROKKA_01764___25454
PROKKA_01549___16913
PROKKA_06224___21592
PROKKA_02323___45521
0.02

dat_1.fa.aln.tree.png

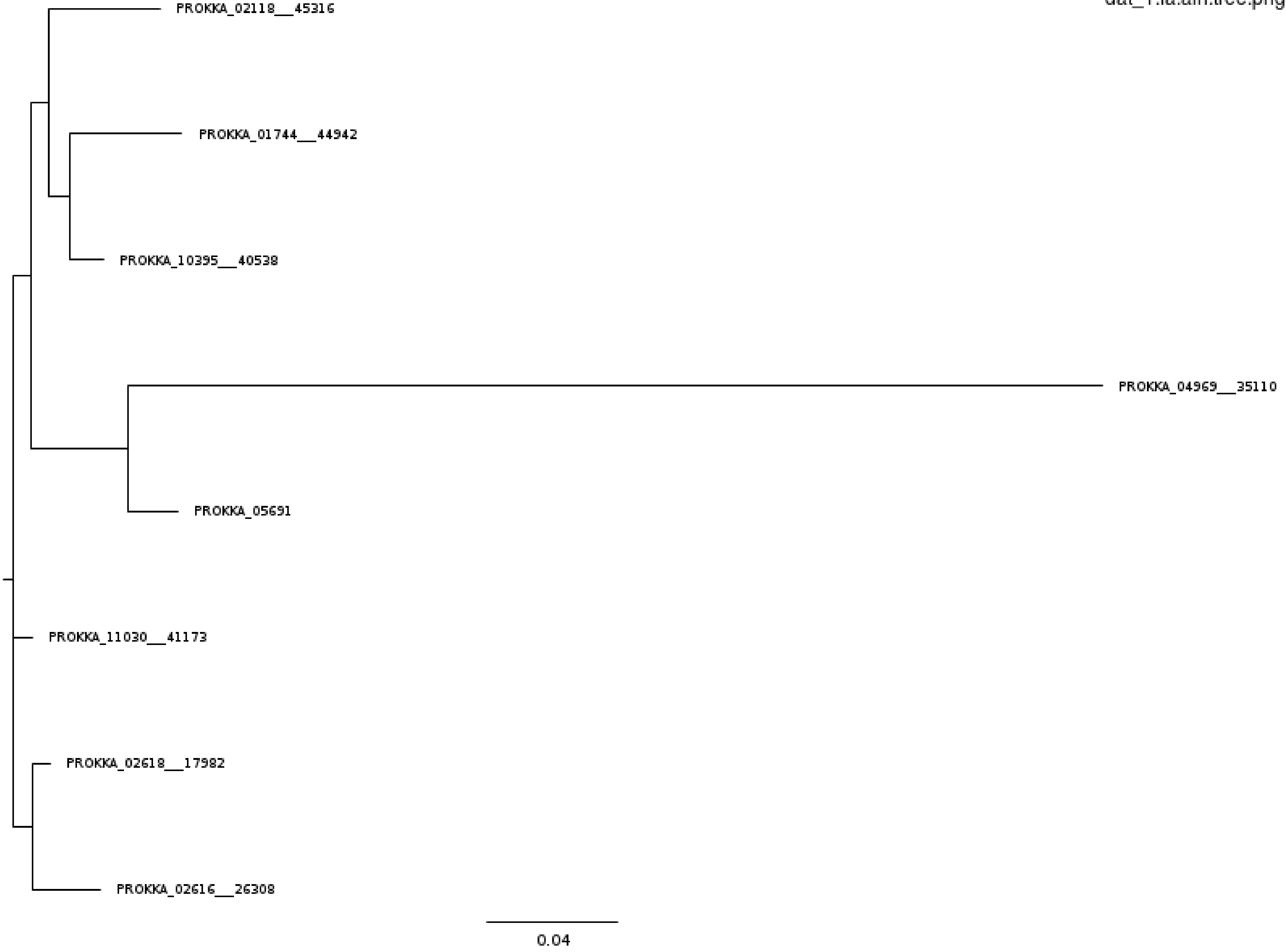

dat_3.fa.aln.tree.png

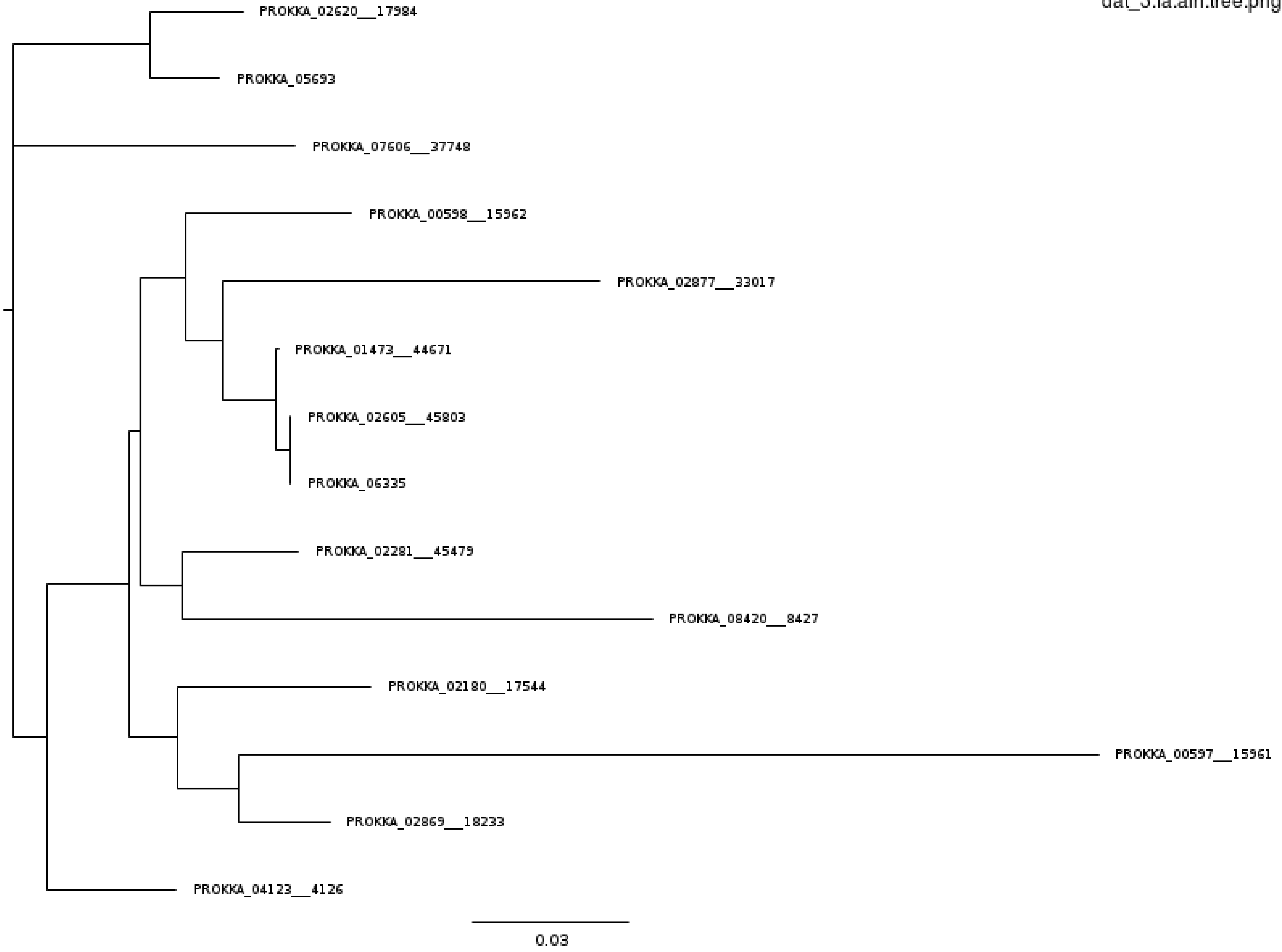

dauA_1.fa.aln.tree.png

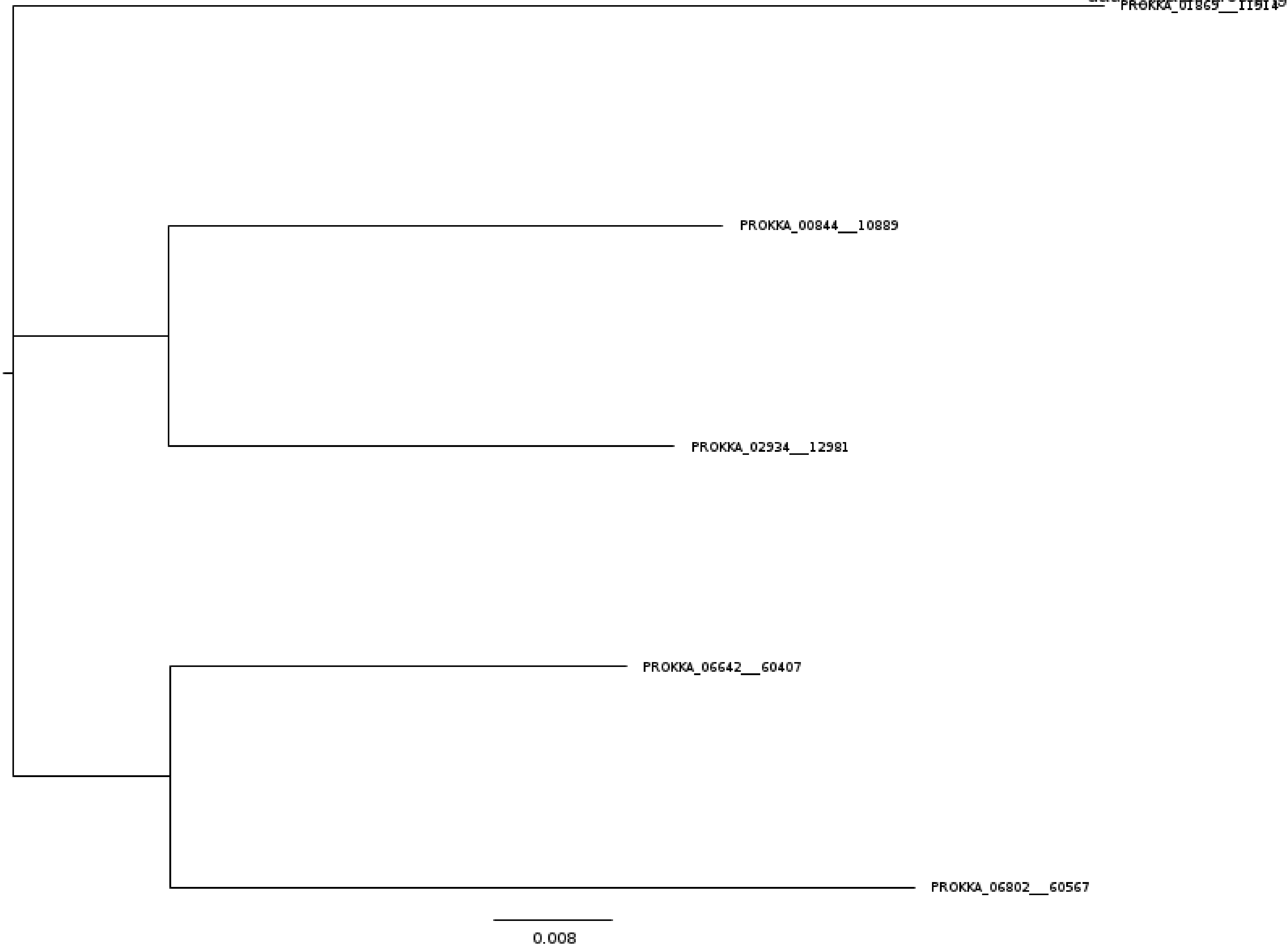

dauA_4.fa.aln.tree.png

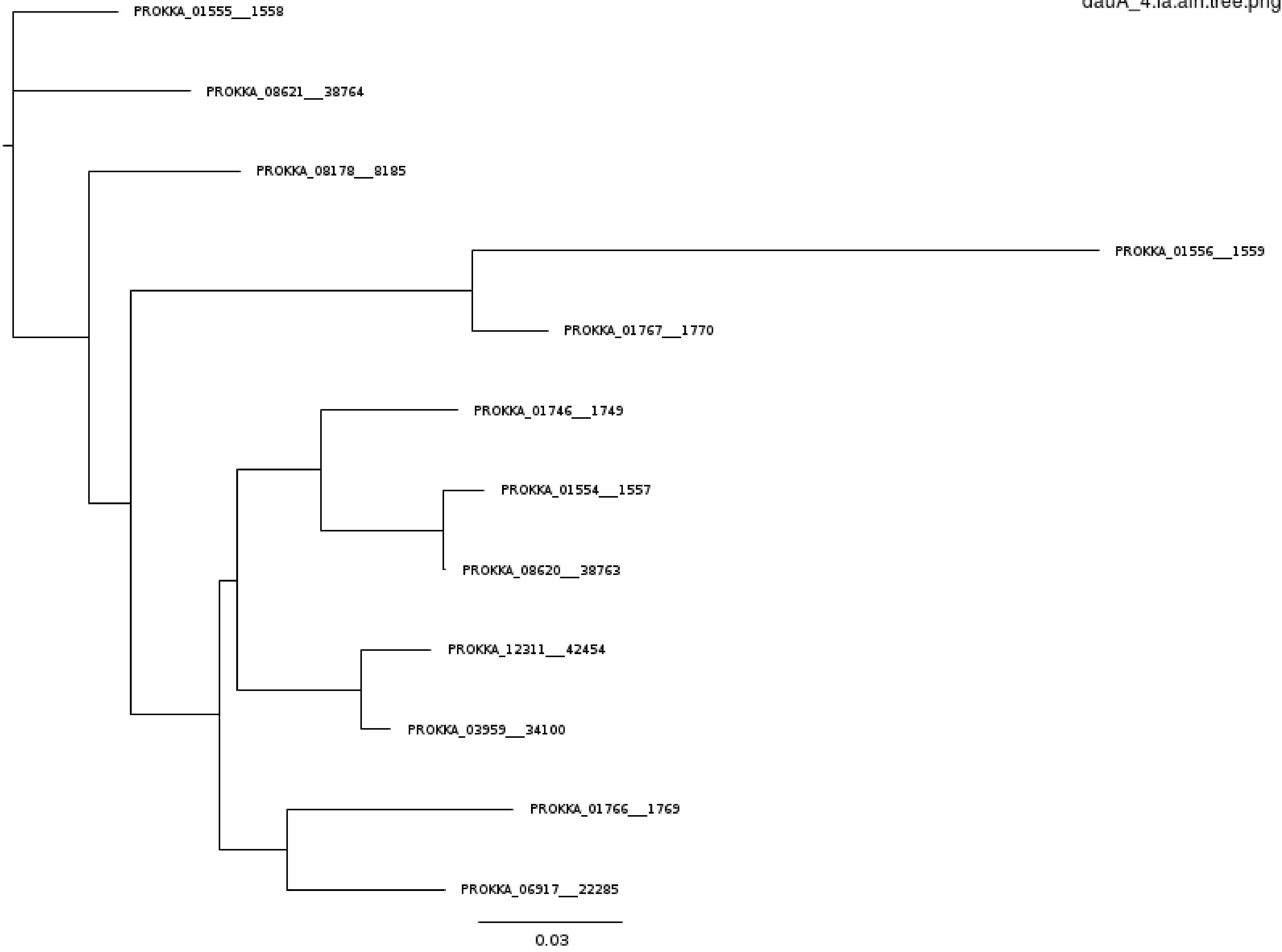

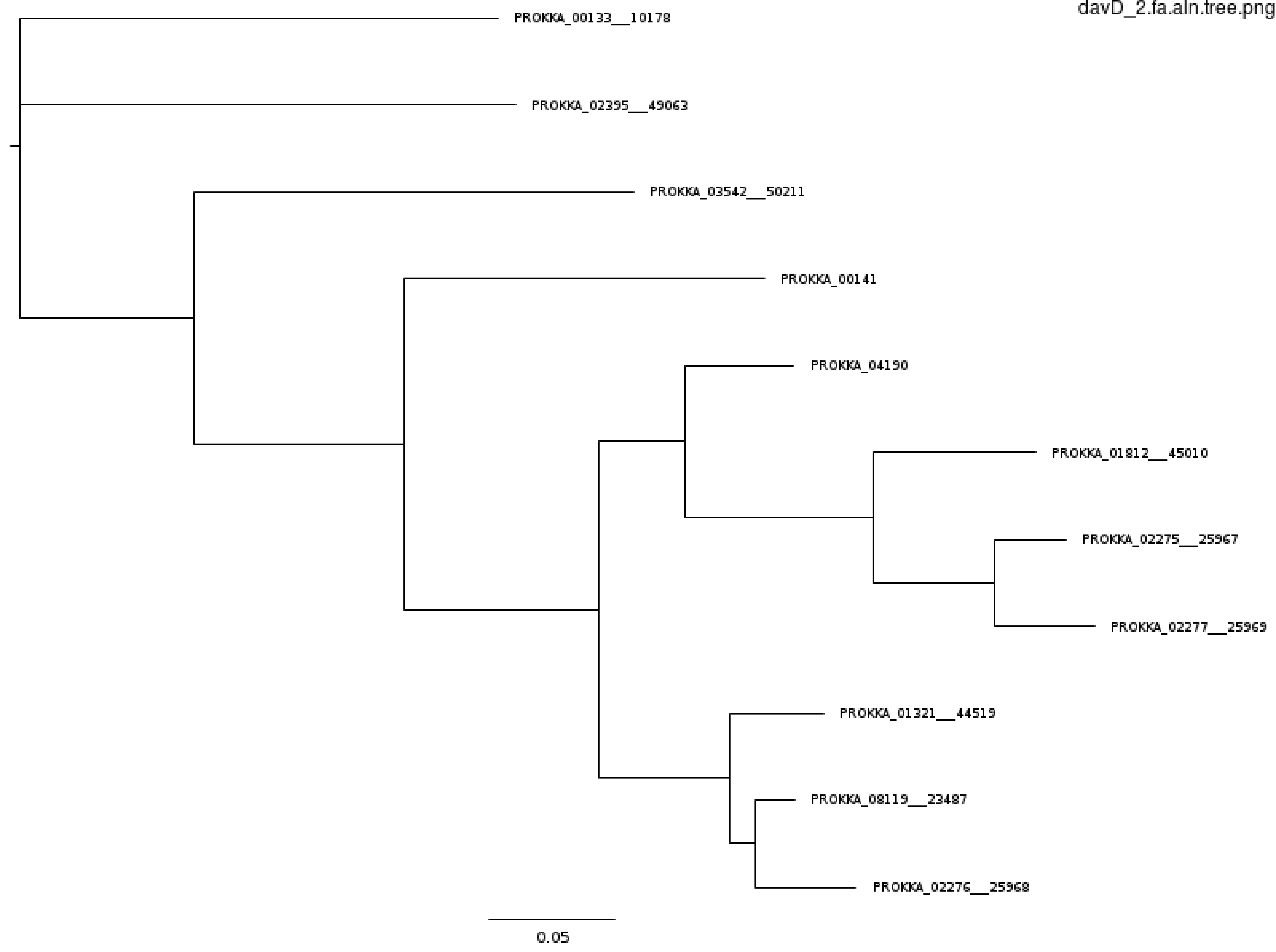
davD_2.fa.aln.tree.png
PROKKA_00133___10178
PROKKA_02395___49063
PROKKA_03542___50211
PROKKA_00141
PROKKA_04190
PROKKA_01812___45010
PROKKA_02275___25967
PROKKA_02277___25969
PROKKA_01321___44519
PROKKA_08119___23487
PROKKA_02276___25968
0.05

dcd_1.fa.aln.tree.png

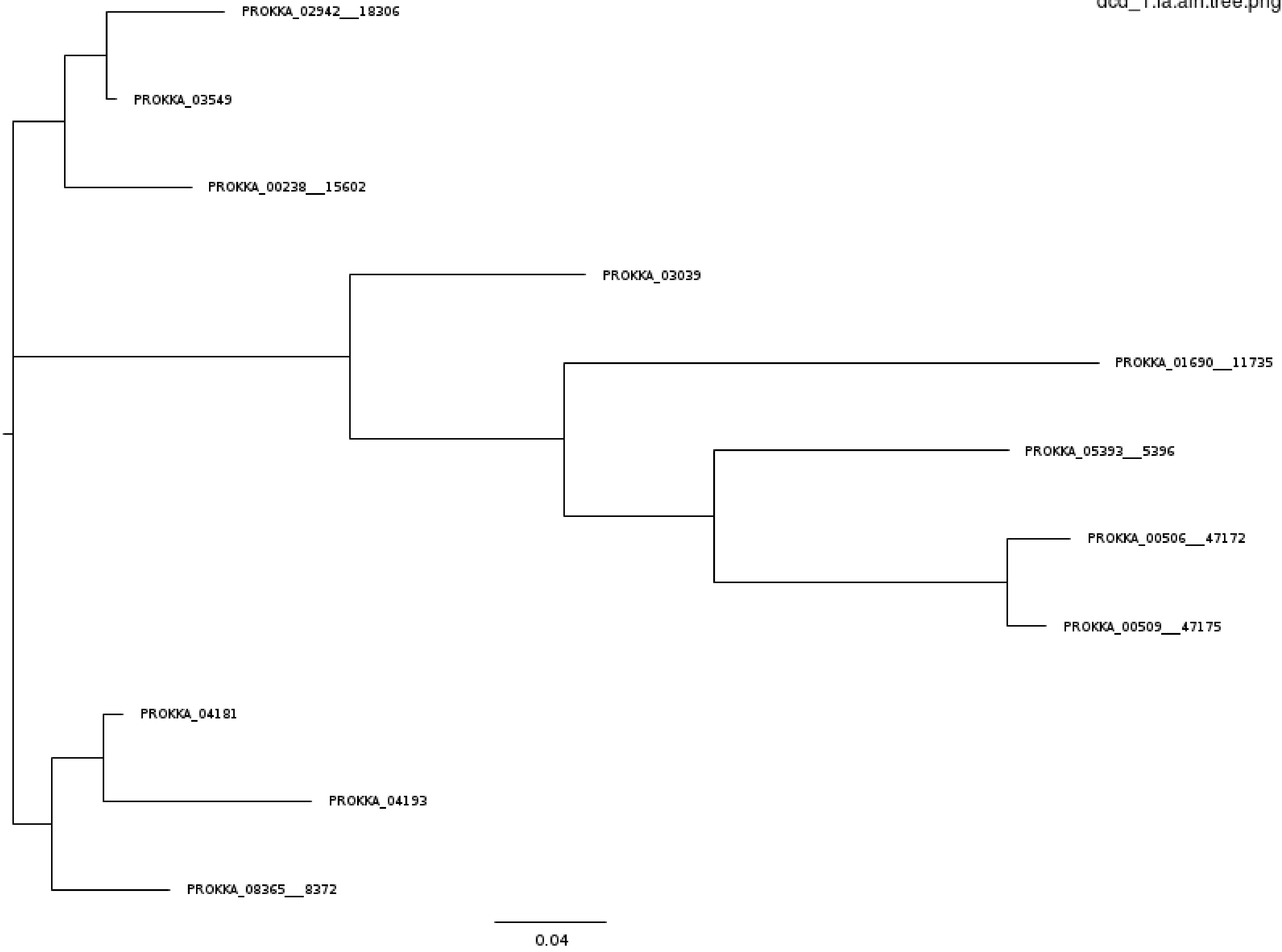

ddc_1.fa.aln.tree.png

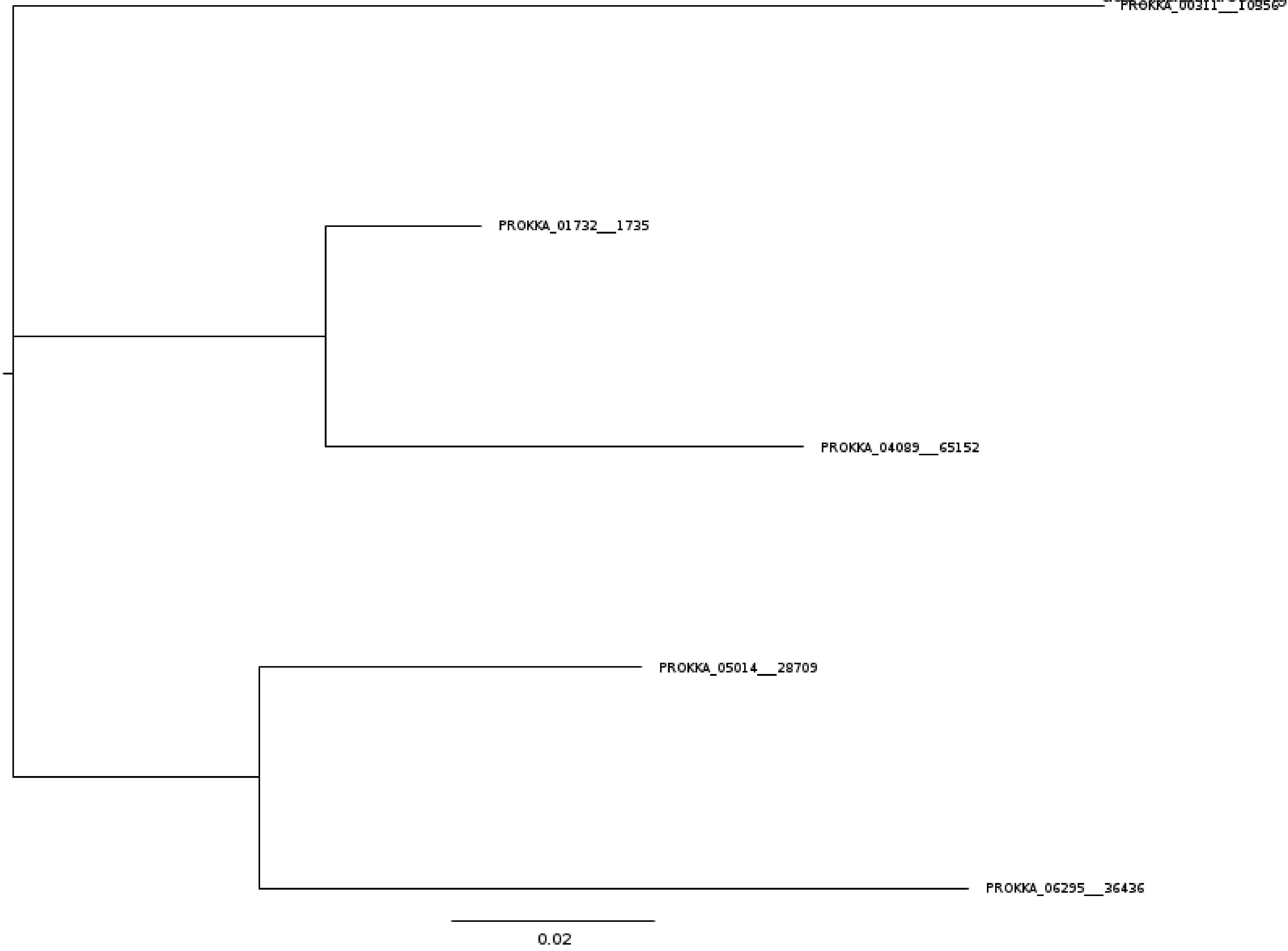

ddl.fa.aln.tree.png

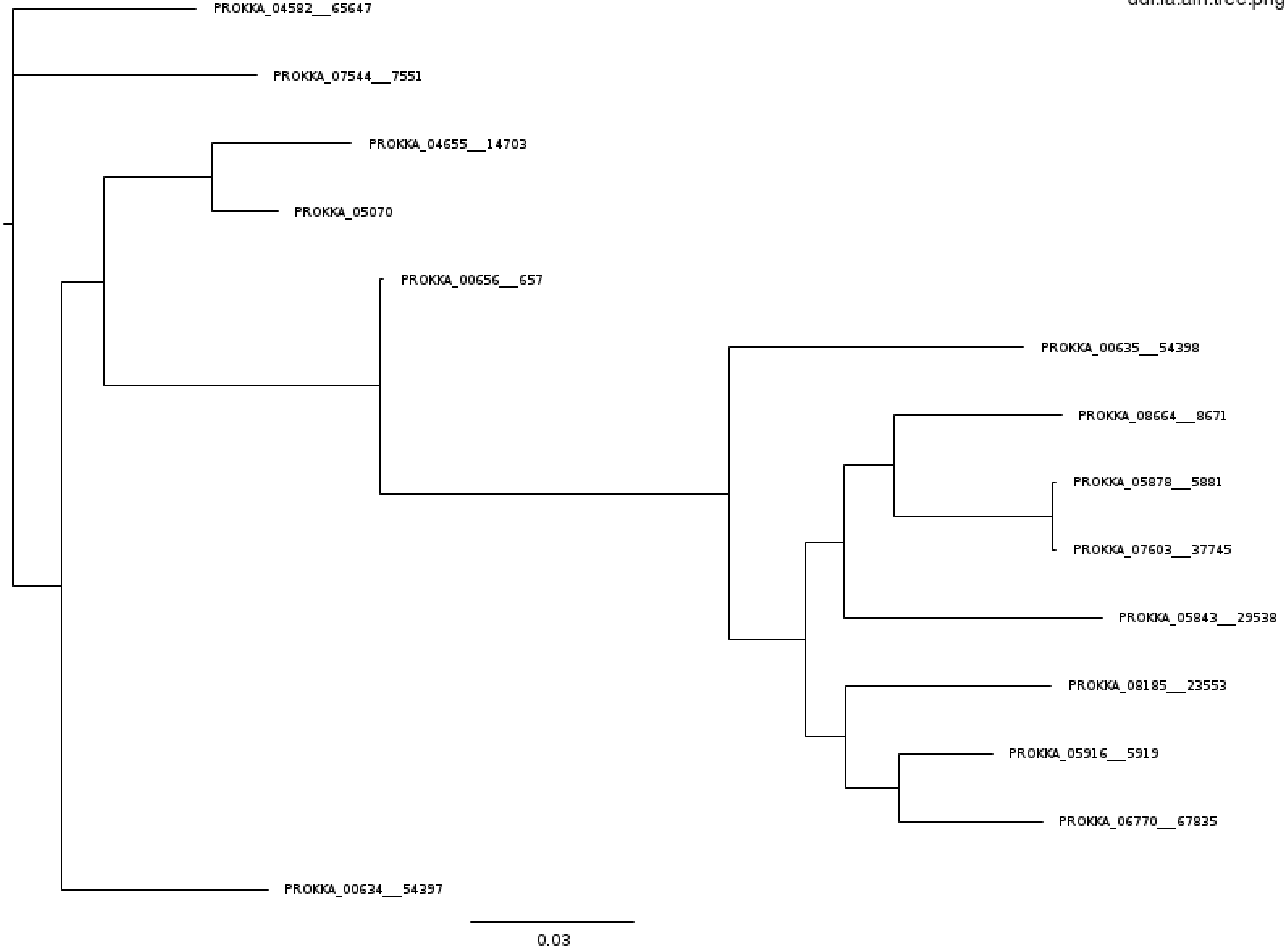

ddpF_1.fa.aln.tree.png

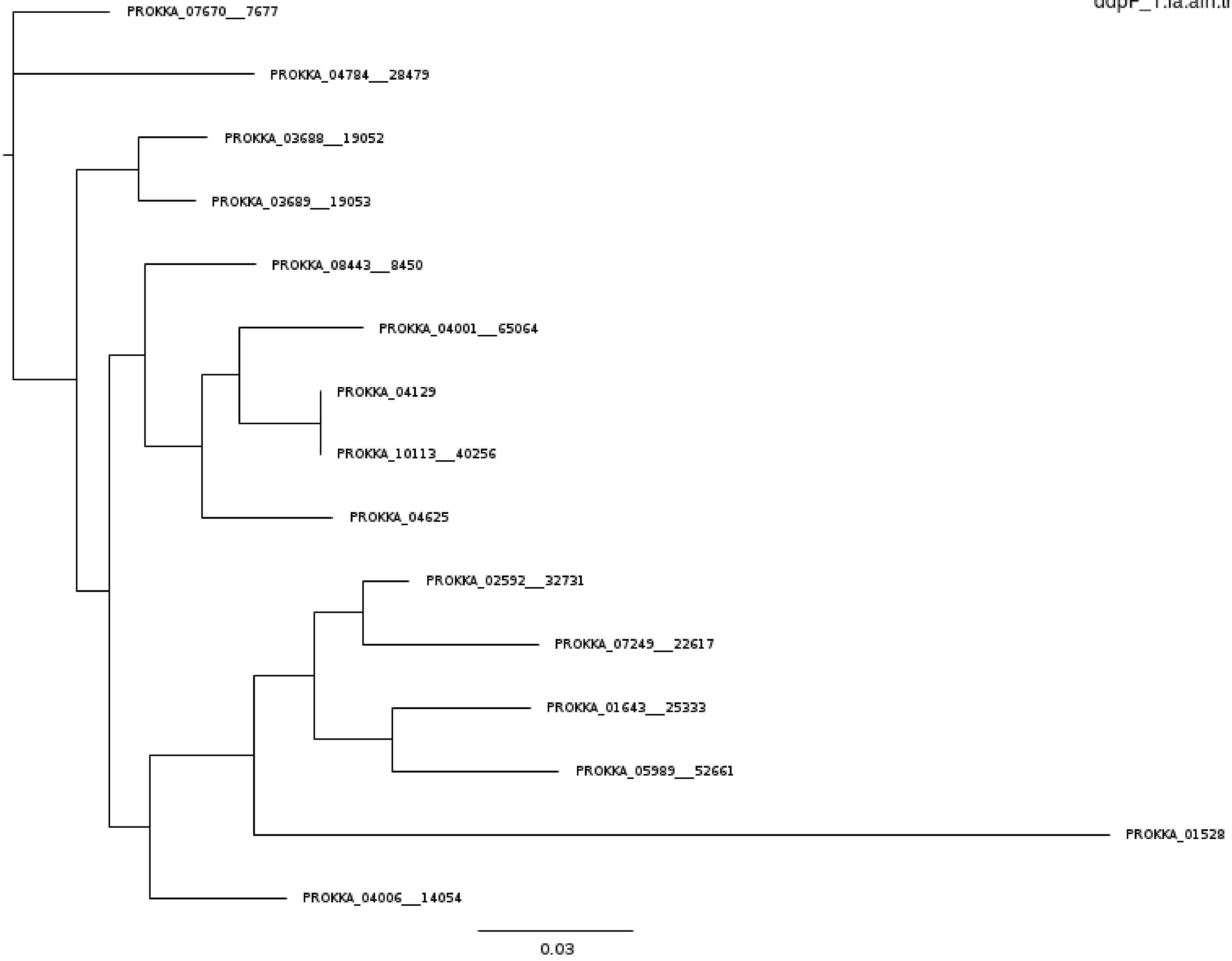

deaD_2.fa.aln.tree.png

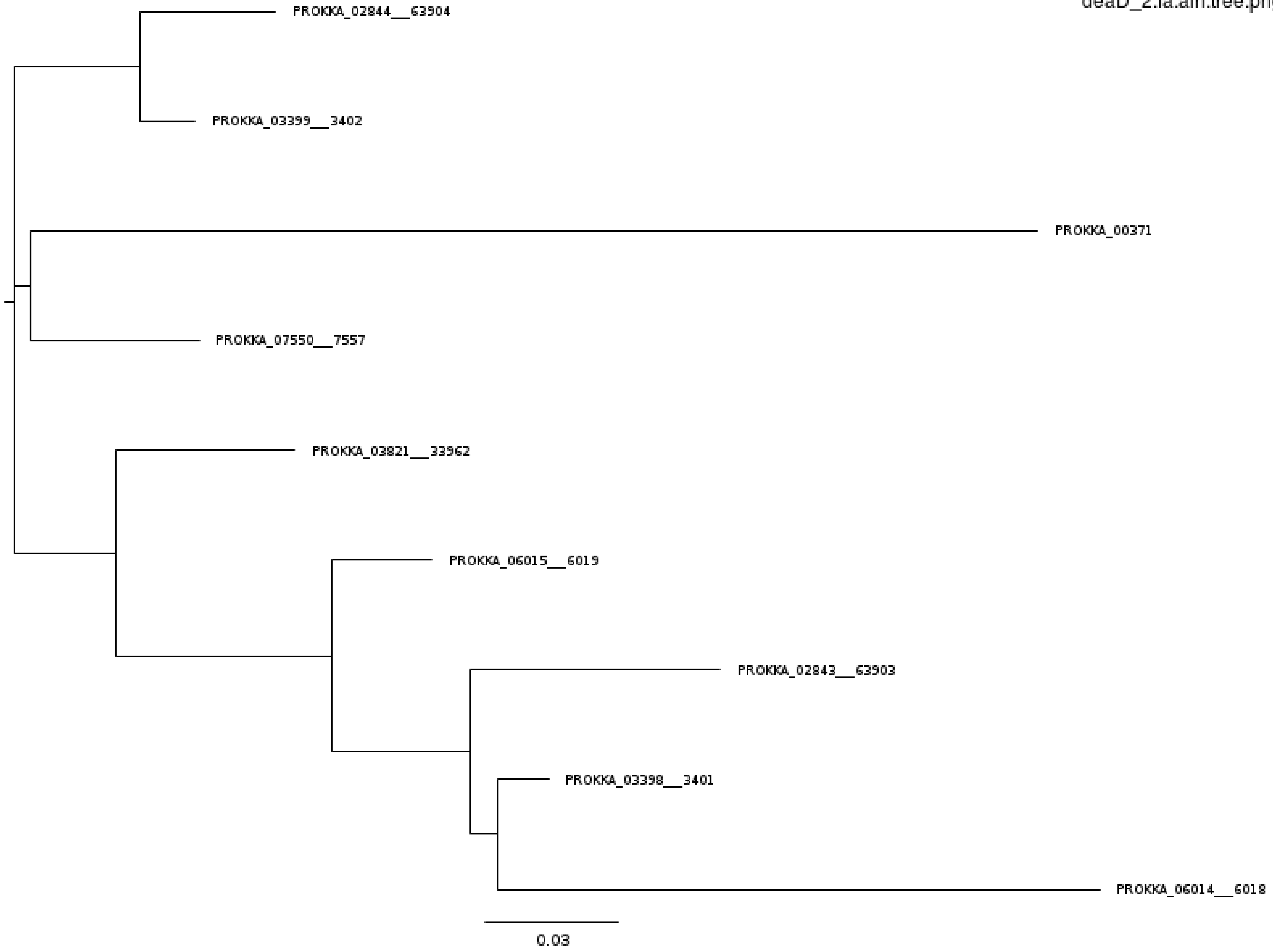

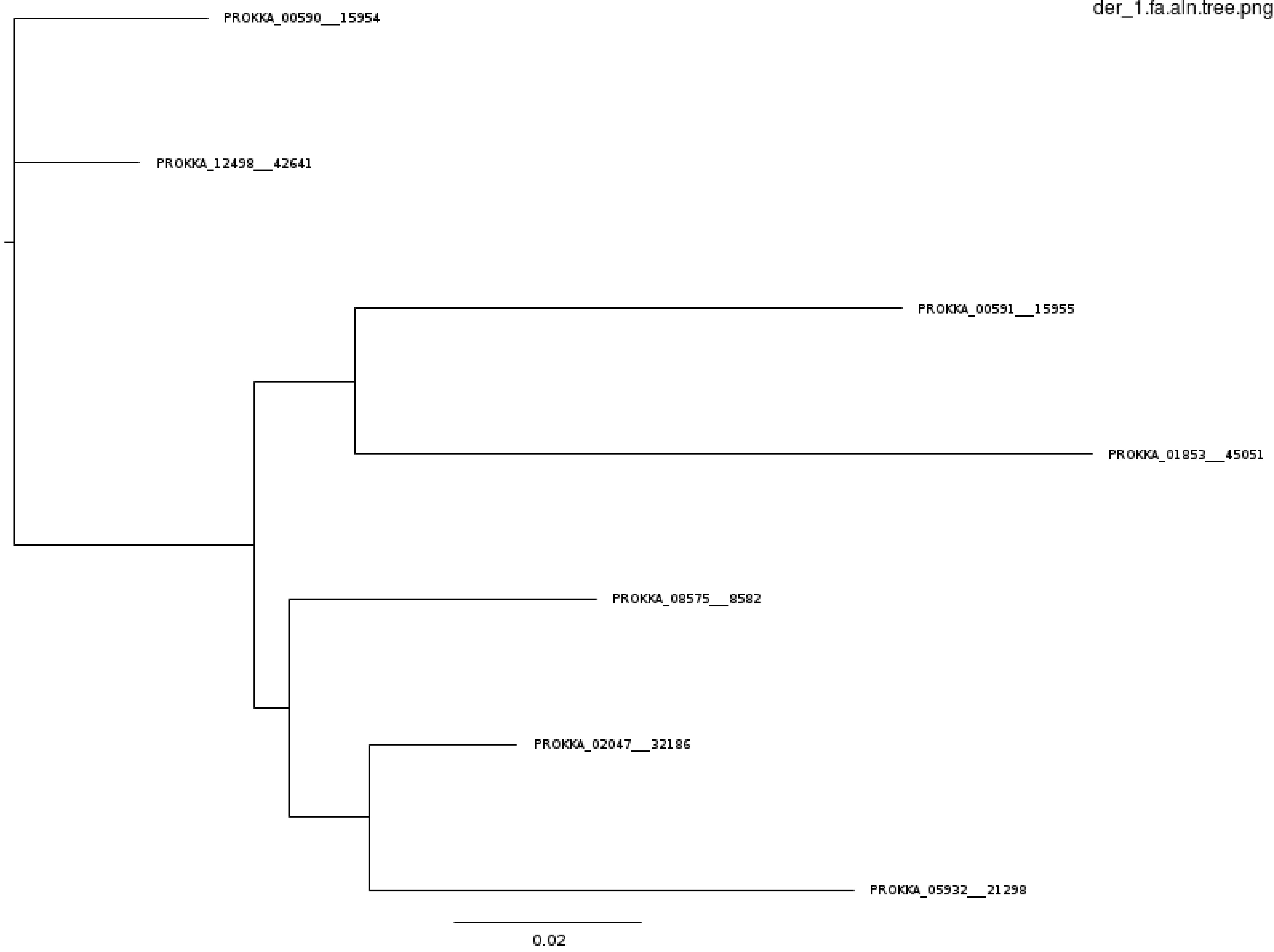
der_1.fa.aln.tree.png
PROKKA_00590__15954
PROKKA_12498__42641
PROKKA_00591__15955
PROKKA_01853__45051
PROKKA_08575__8582
PROKKA_02047__32186
PROKKA_05932__21298
0.02

dgt_3.fa.aln.tree.png

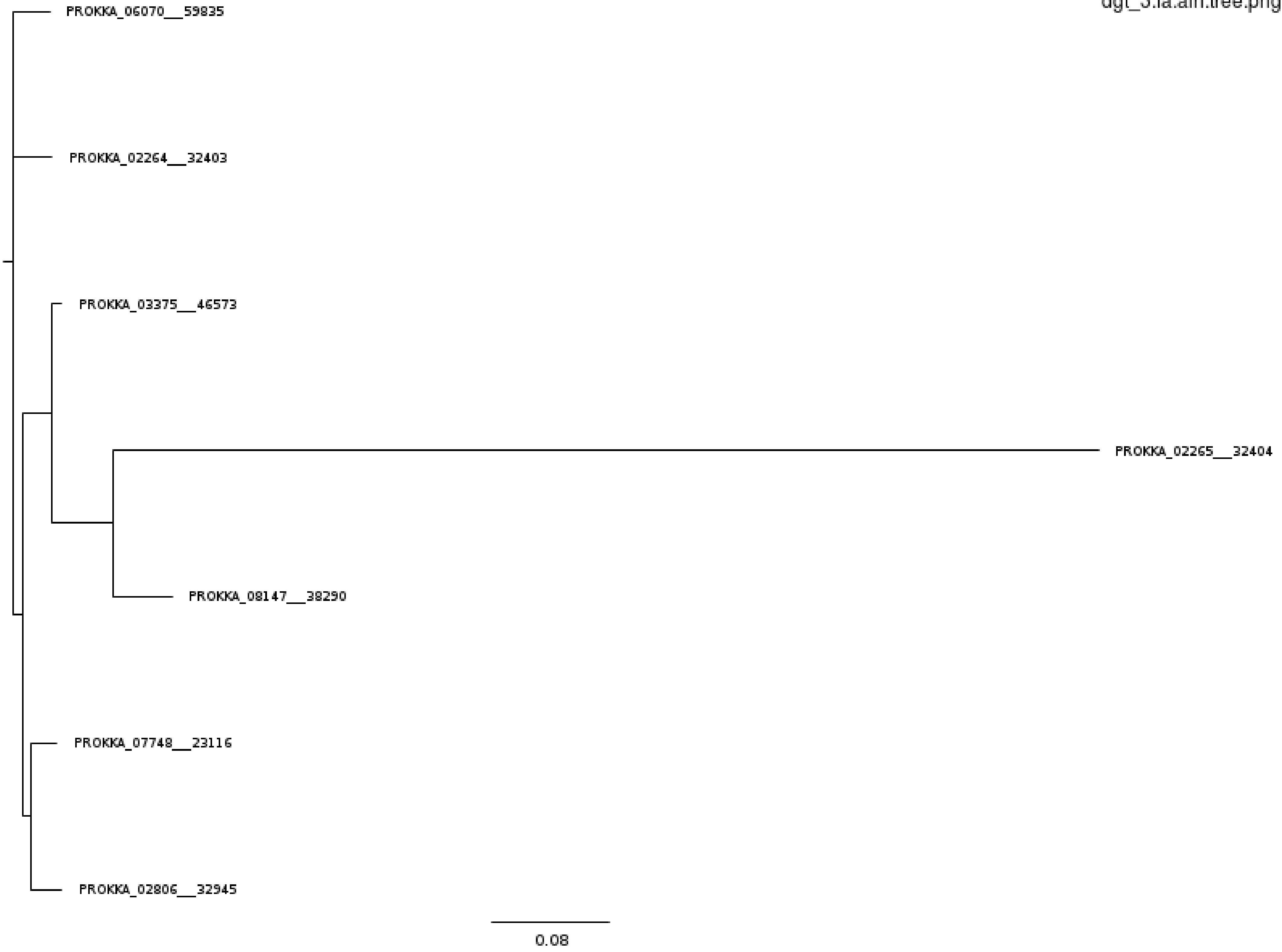

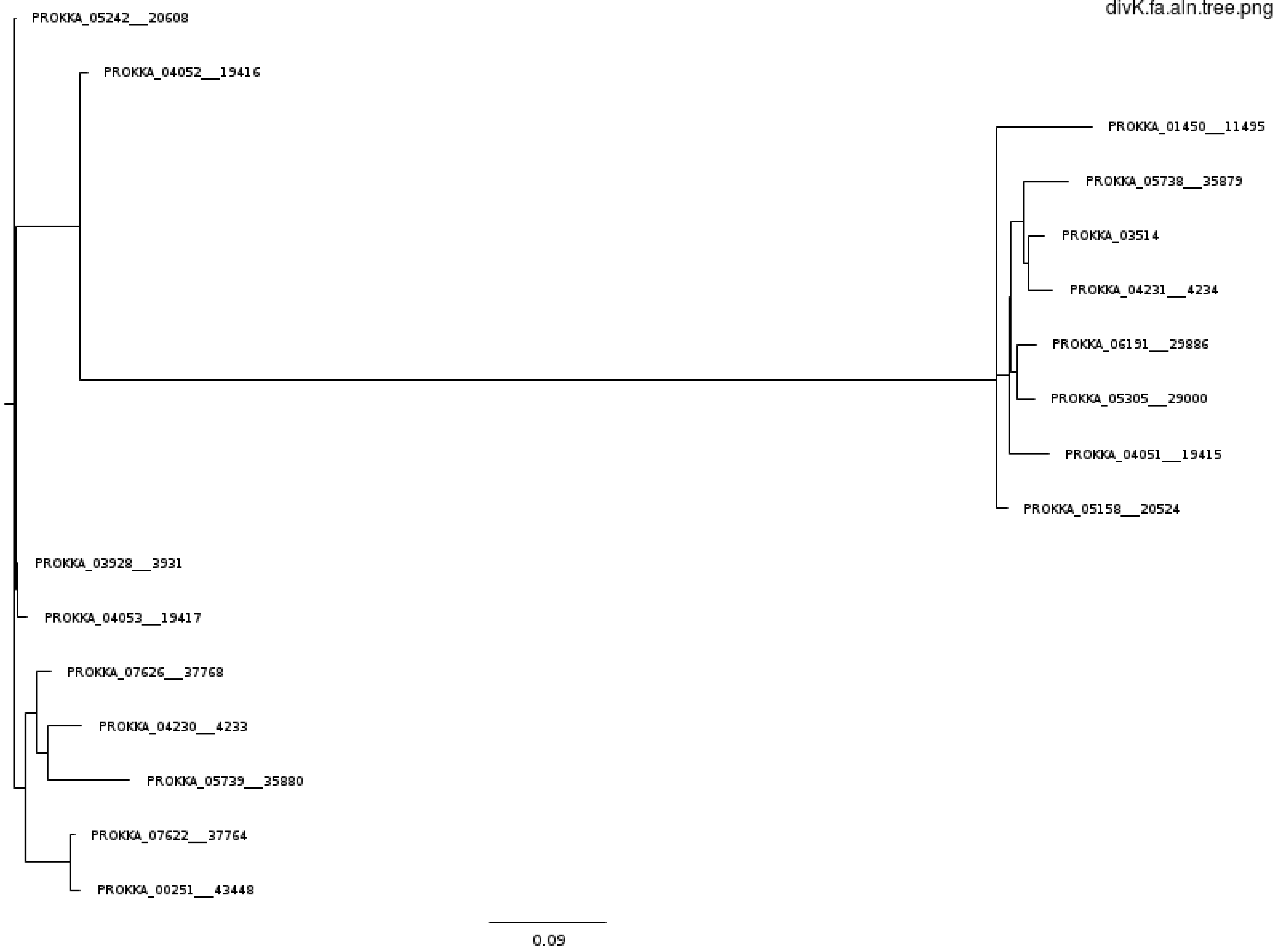
divK.fa.aln.tree.png
PROKKA_05242___20608
PROKKA_04052___19416
PROKKA_01450___11495
PROKKA_05738___35879
PROKKA_03514
PROKKA_04231___4234
PROKKA_06191___29886
PROKKA_05305___29000
PROKKA_04051___19415
PROKKA_05158___20524
PROKKA_03928___3931
PROKKA_04053___19417
PROKKA_07626___37768
PROKKA_04230___4233
PROKKA_05739___35880
PROKKA_07622___37764
PROKKA_00251___43448
0.09

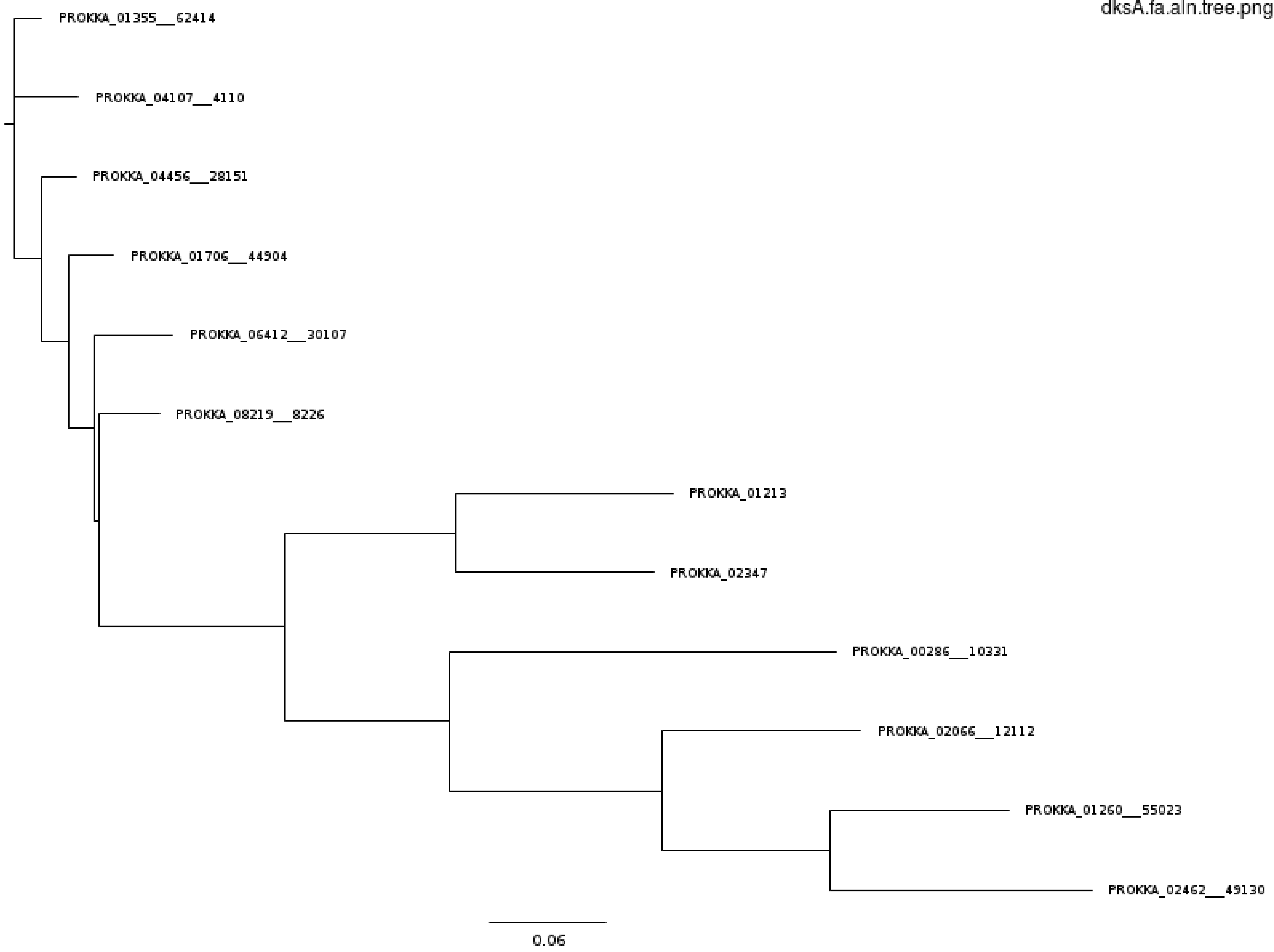

dksA.fa.aln.tree.png
PROKKA_01355___62414
PROKKA_04107___4110
PROKKA_04456___28151
PROKKA_01706___44904
PROKKA_06412___30107
PROKKA_08219___8226
PROKKA_01213
PROKKA_02347
PROKKA_00286___10331
PROKKA_02066___12112
PROKKA_01260___55023
PROKKA_02462___49130
0.06

dme.fa.aln.tree.png

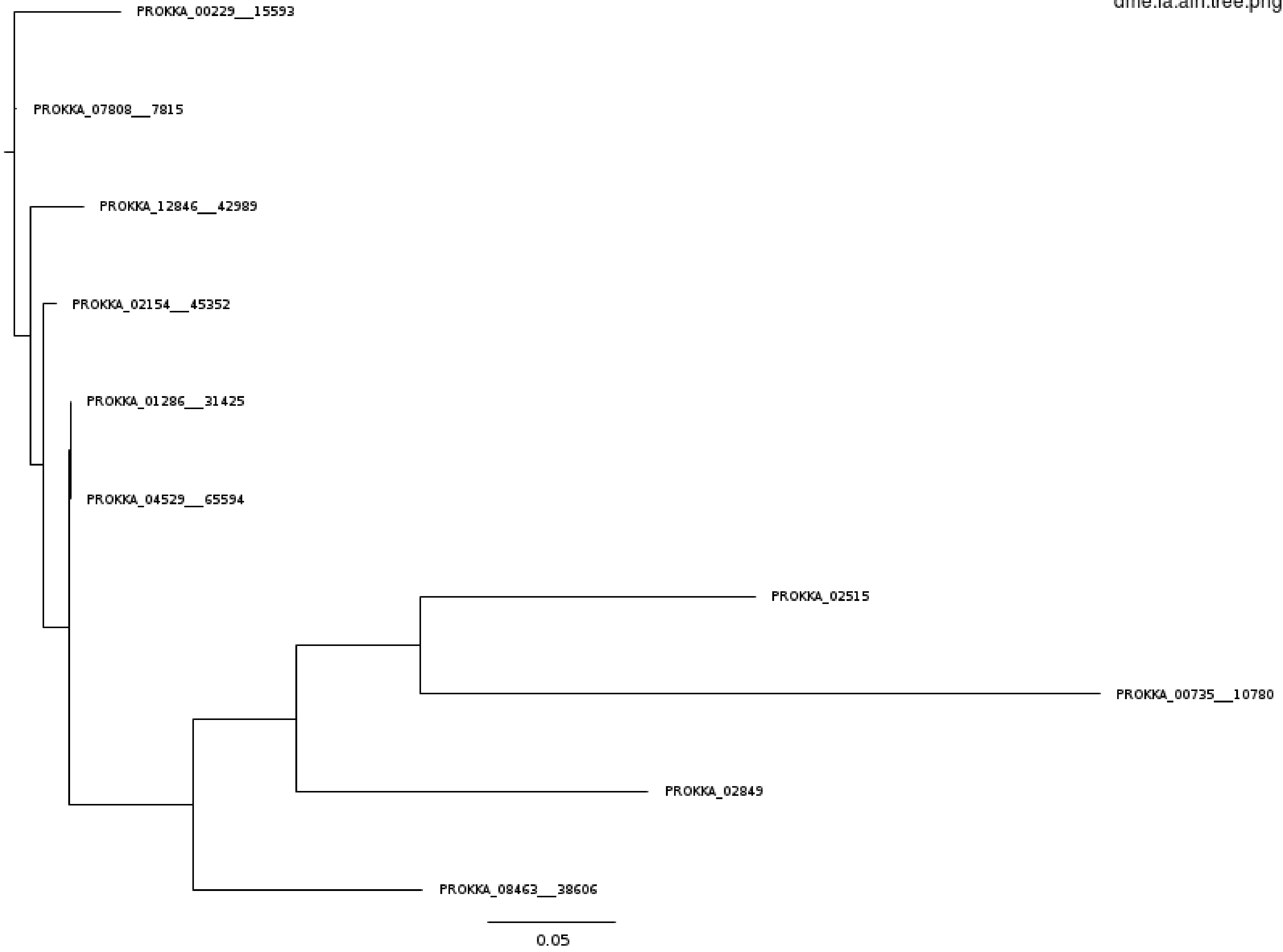

dnaE_2.fa.aln.tree.png

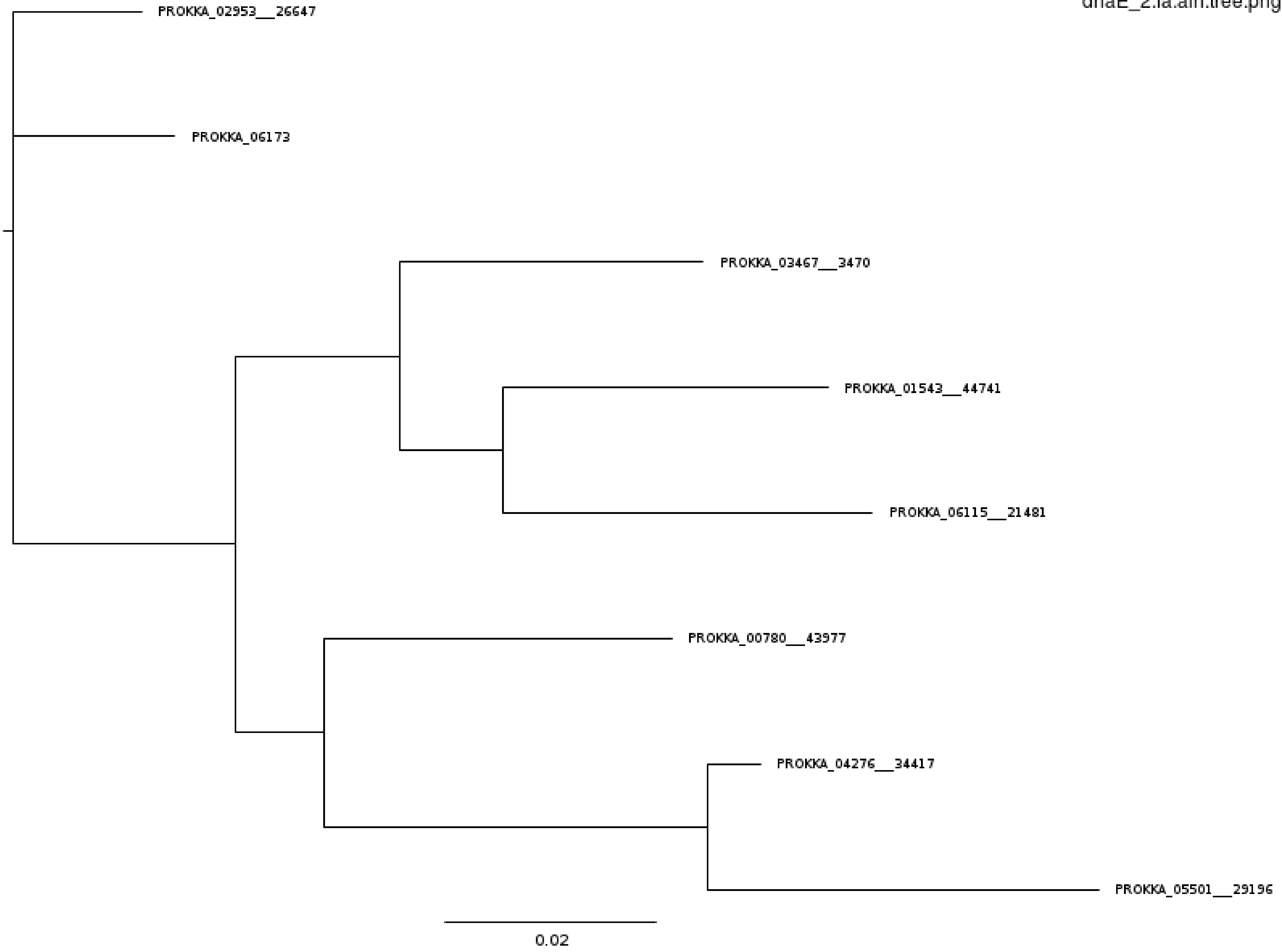

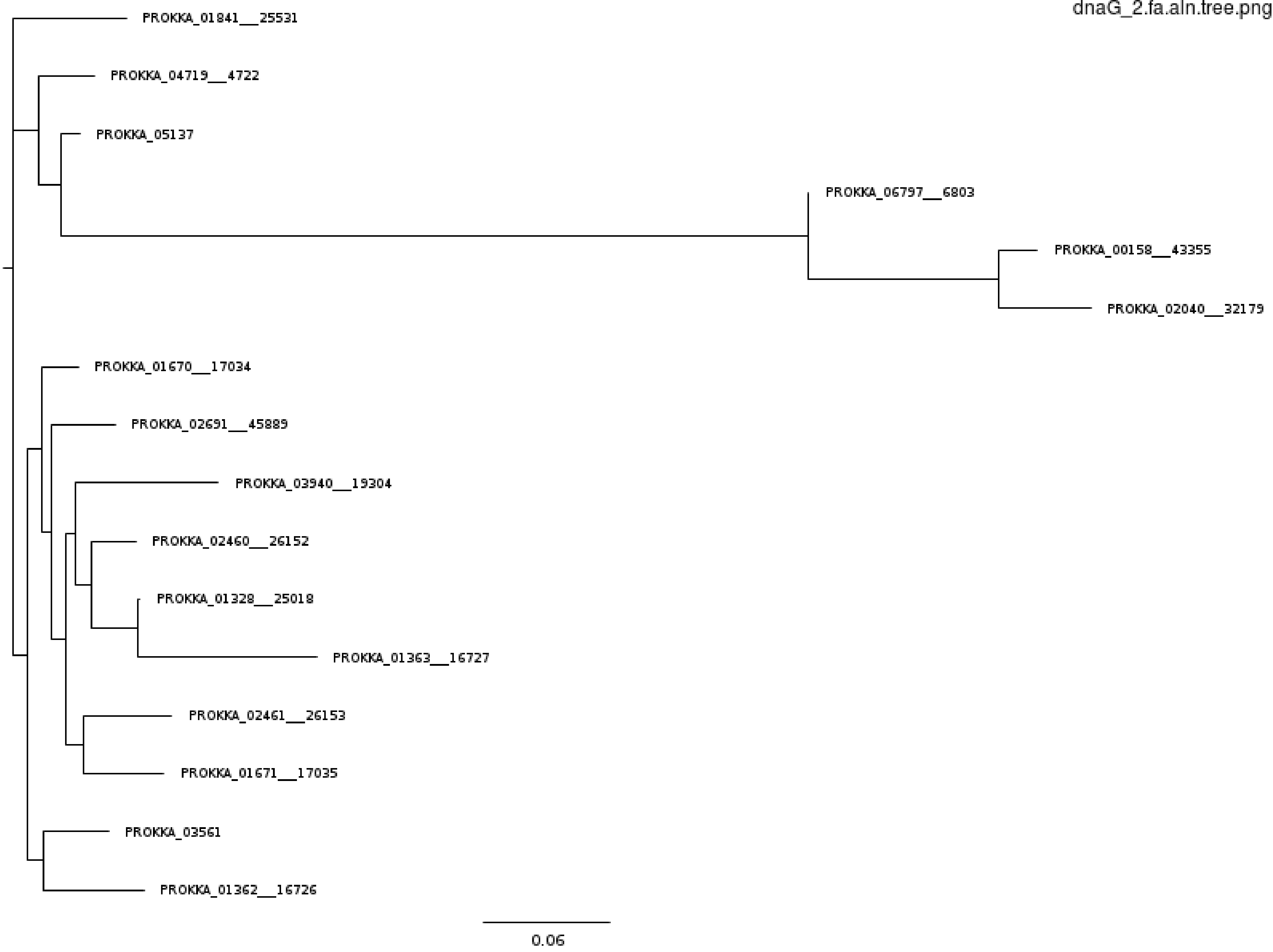
dnaG_2.fa.aln.tree.png
PROKKA_01841___25531
PROKKA_04719___4722
PROKKA_05137
PROKKA_06797___6803
PROKKA_00158___43355
PROKKA_02040___32179
PROKKA_01670___17034
PROKKA_02691___45889
PROKKA_03940___19304
PROKKA_02460___26152
PROKKA_01328___25018
PROKKA_01363___16727
PROKKA_02461___26153
PROKKA_01671___17035
PROKKA_03561
PROKKA_01362___16726
0.06

dnaJ_1.fa.aln.tree.png

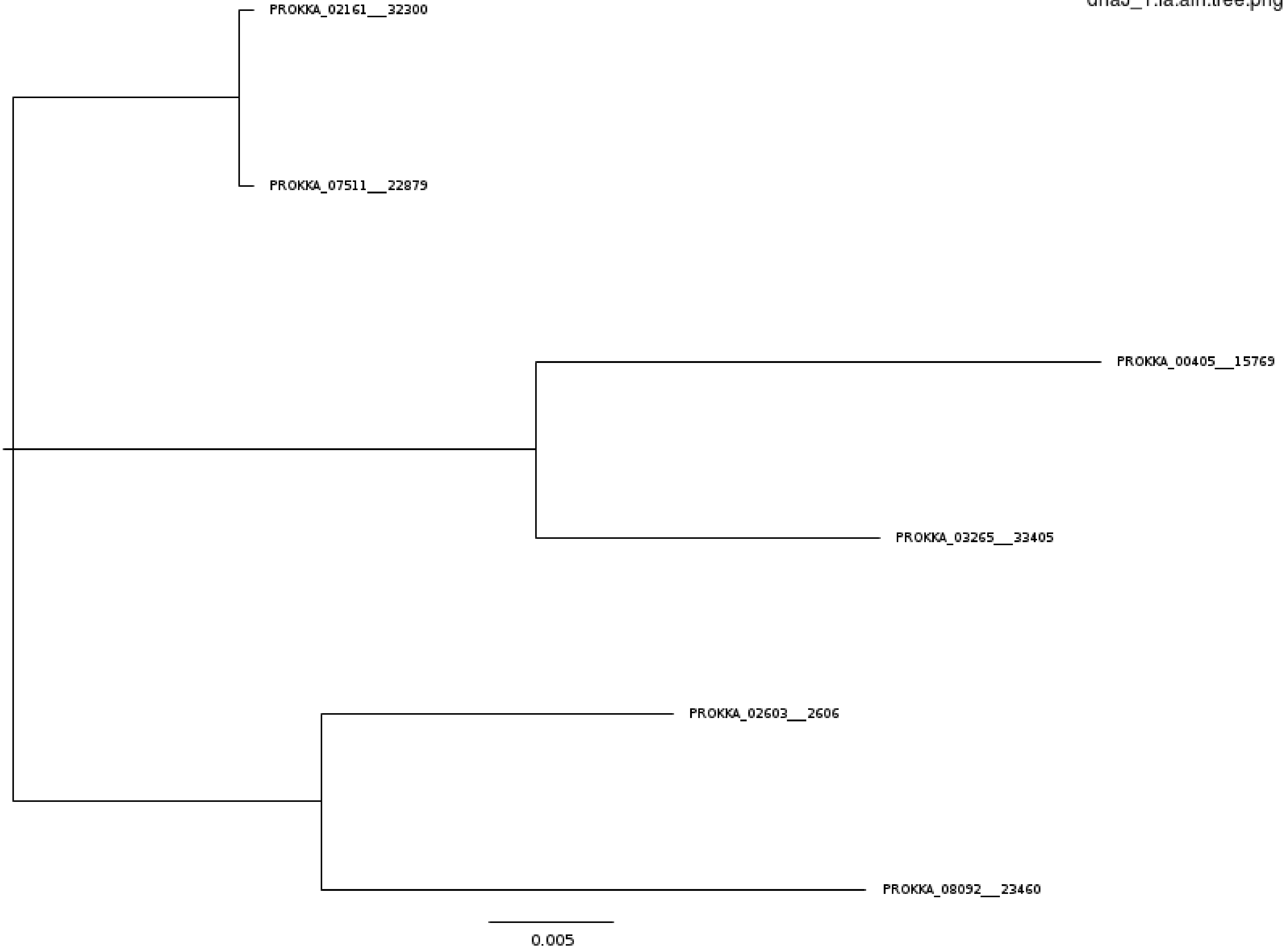

dnaK_4.fa.aln.tree.png

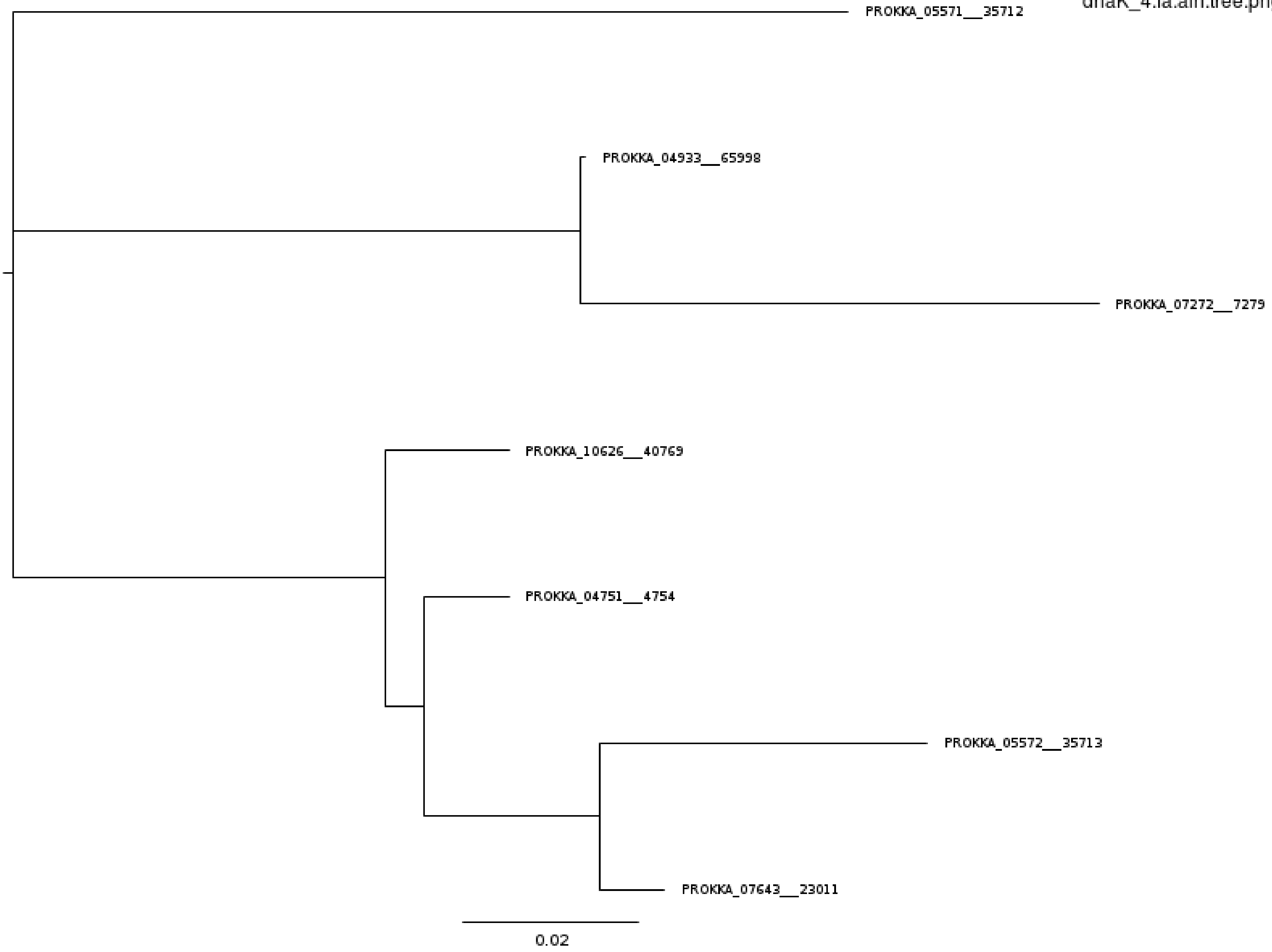

dnaK_7.fa.aln.tree.png

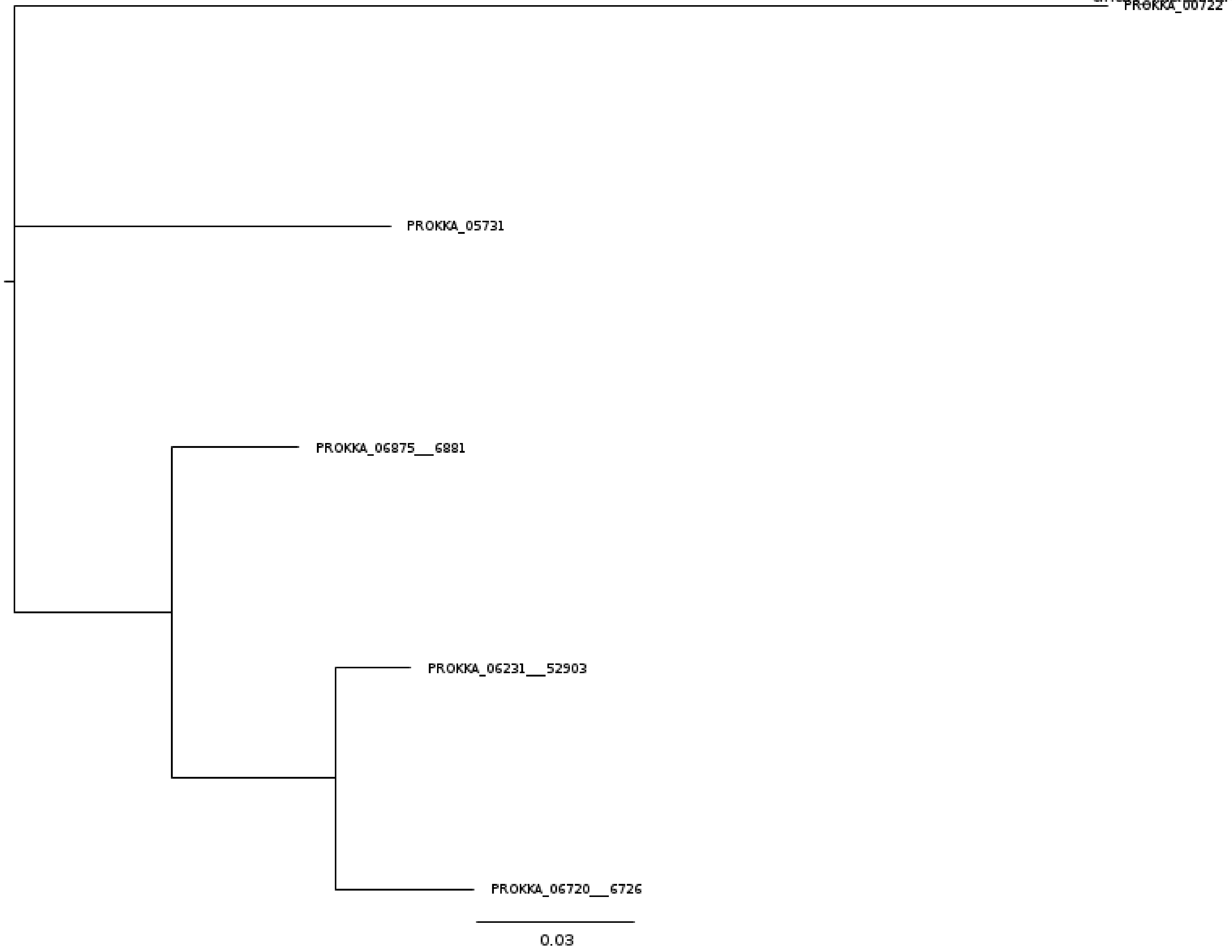

dnaN_2.fa.aln.tree.png

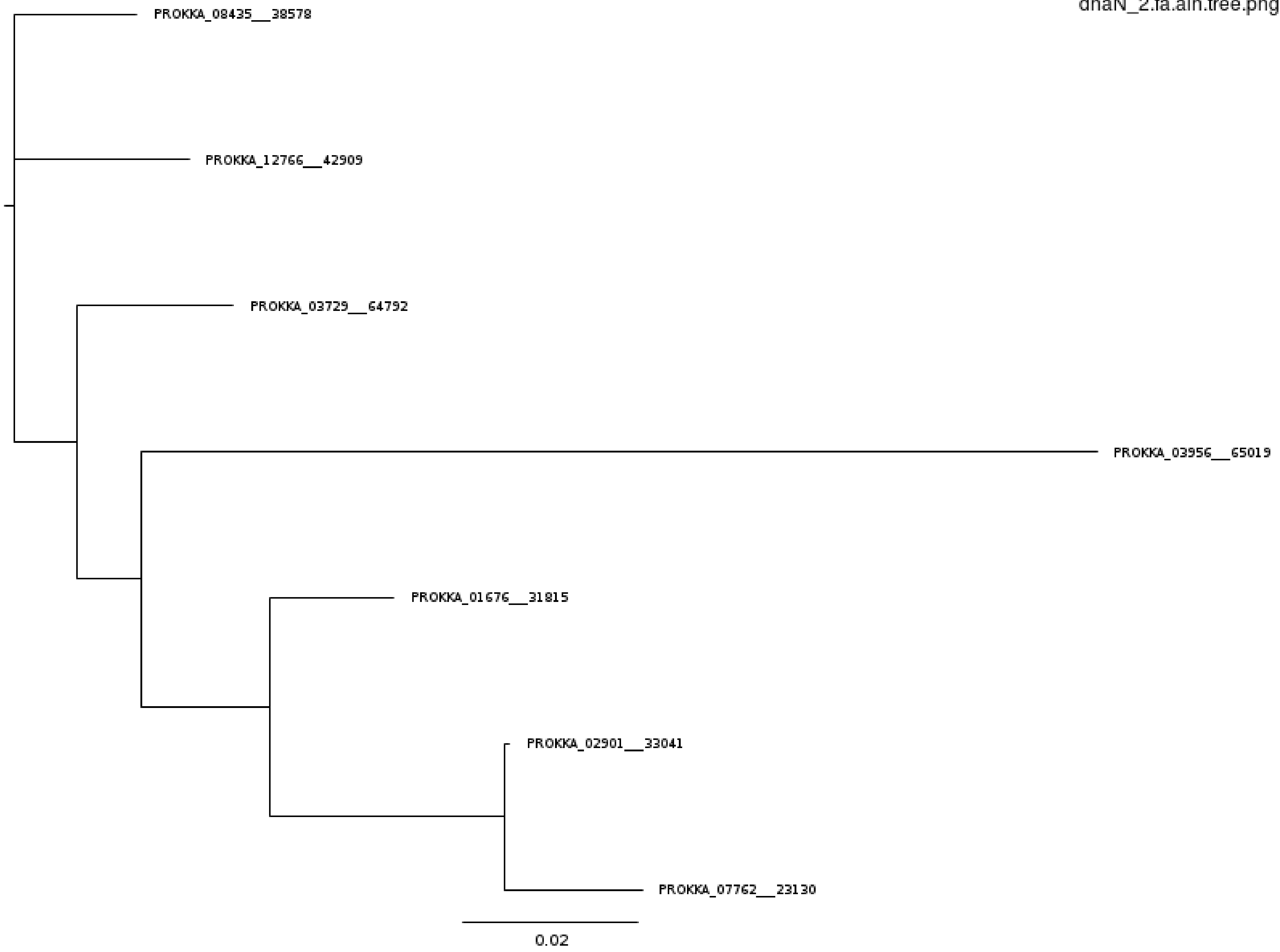

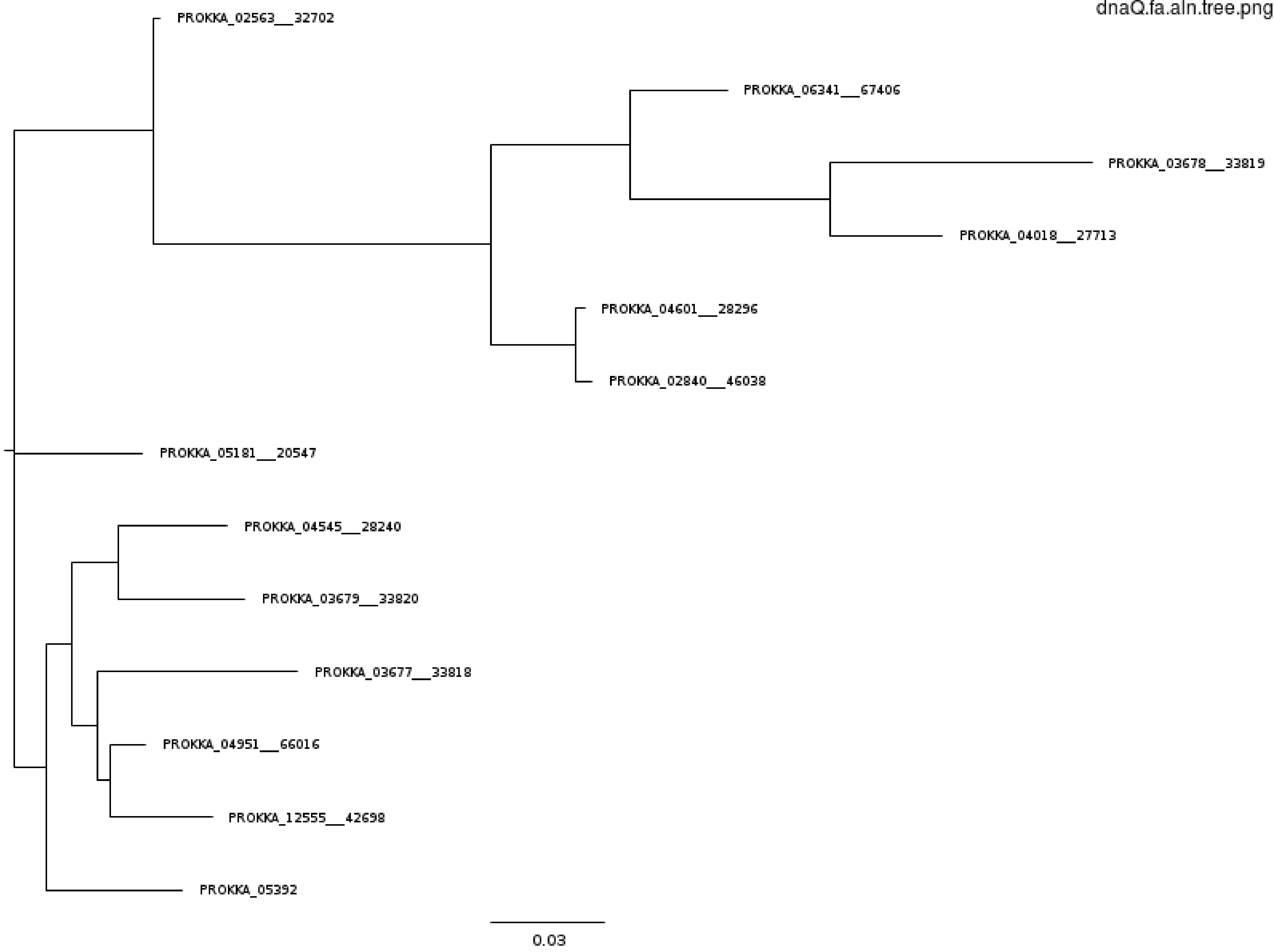
dnaQ.fa.aln.tree.png
PROKKA_02563___32702
PROKKA_06341___67406
PROKKA_03678___33819
PROKKA_04018___27713
PROKKA_04601___28296
PROKKA_02840___46038
PROKKA_05181___20547
PROKKA_04545___28240
PROKKA_03679___33820
PROKKA_03677___33818
PROKKA_04951___66016
PROKKA_12555___42698
PROKKA_05392
0.03

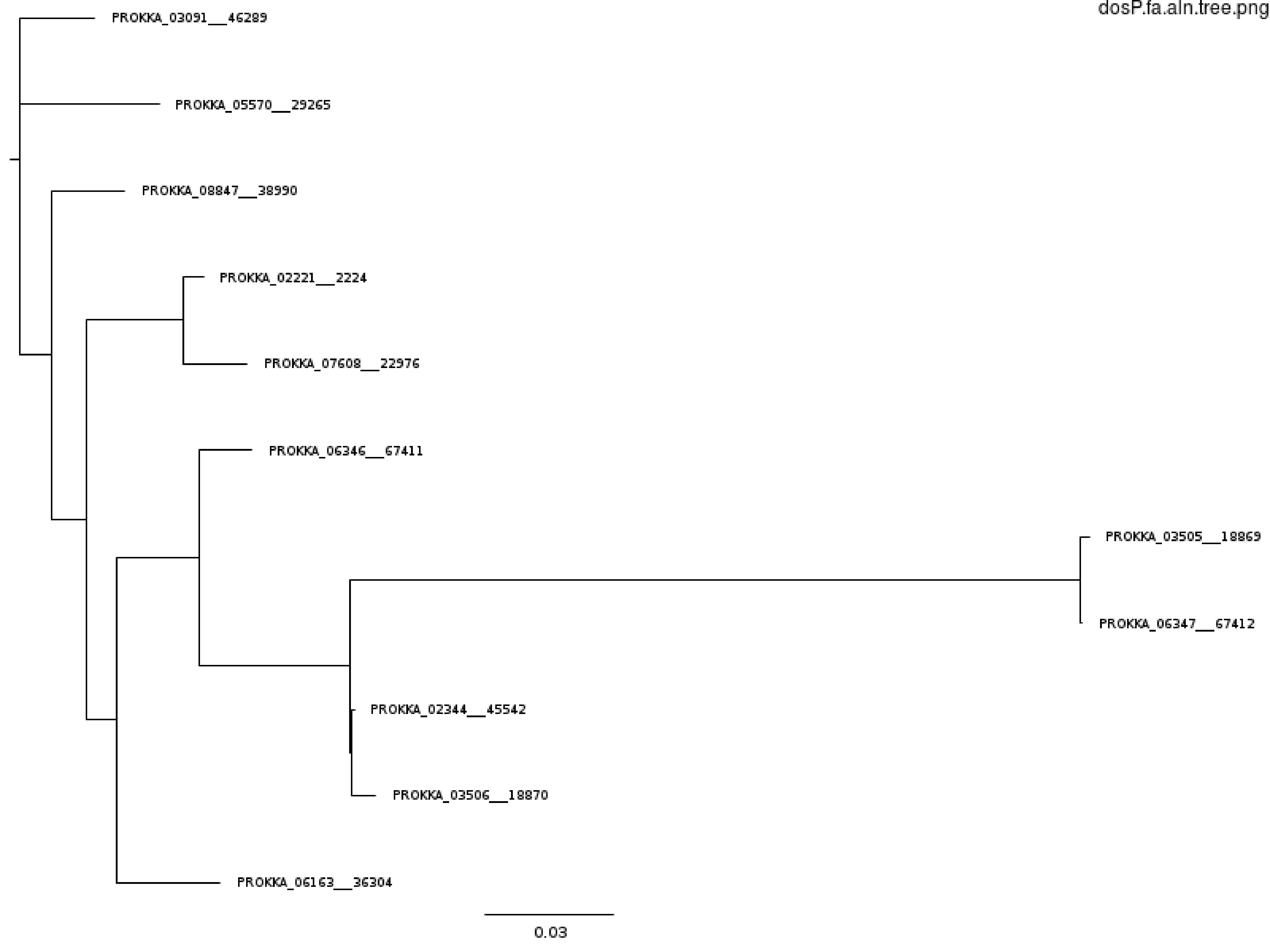
dosP.fa.aln.tree.png
PROKKA_03091___46289
PROKKA_05570___29265
PROKKA_08847___38990
PROKKA_02221___2224
PROKKA_07608___22976
PROKKA_06346___67411
PROKKA_03505___18869
PROKKA_06347___67412
PROKKA_02344___45542
PROKKA_03506___18870
PROKKA_06163___36304
0.03

dpnA_14.fa.aln.tree.png

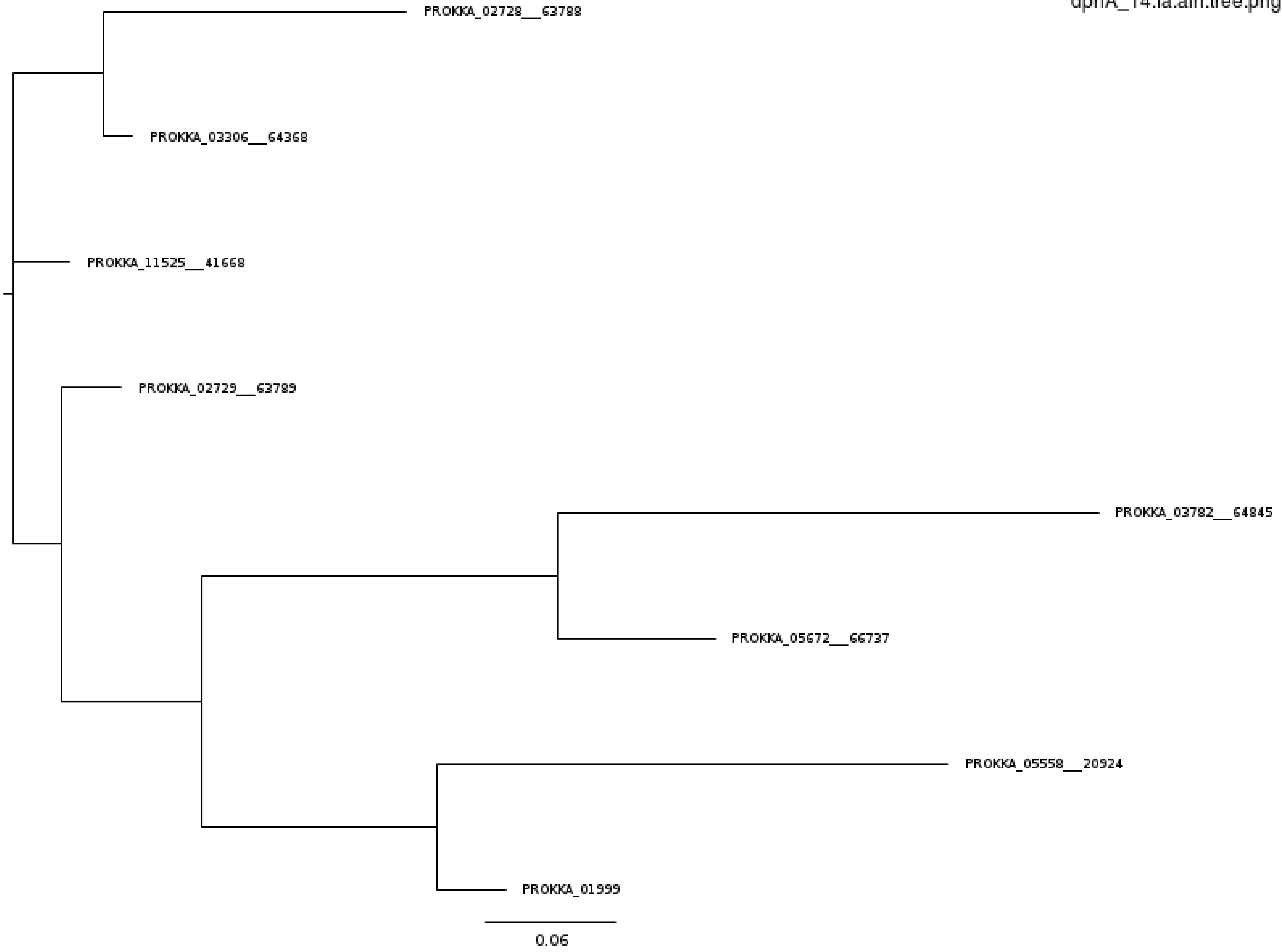

dpnA_1.fa.aln.tree.png

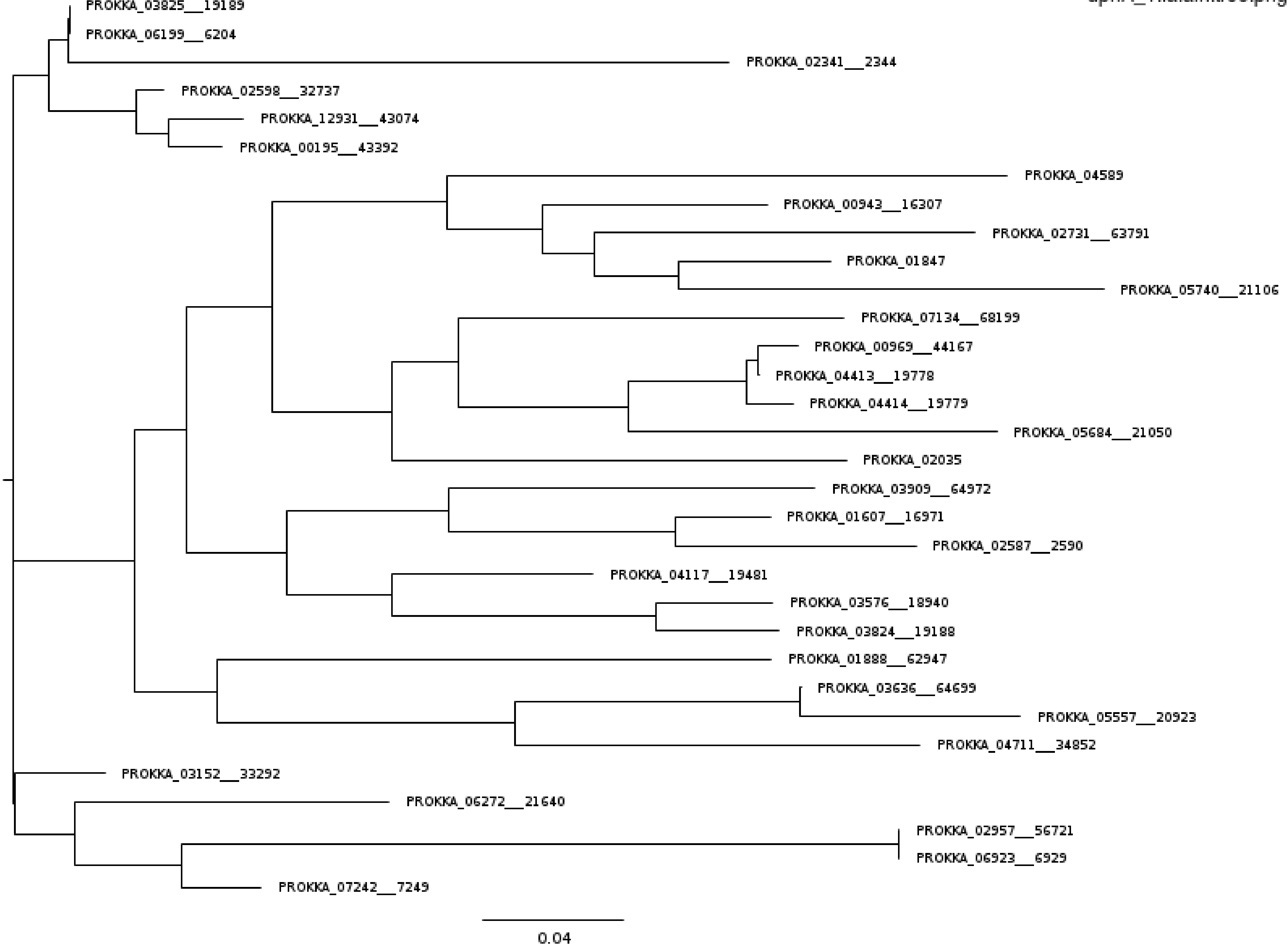

dpnA_3.fa.aln.tree.png

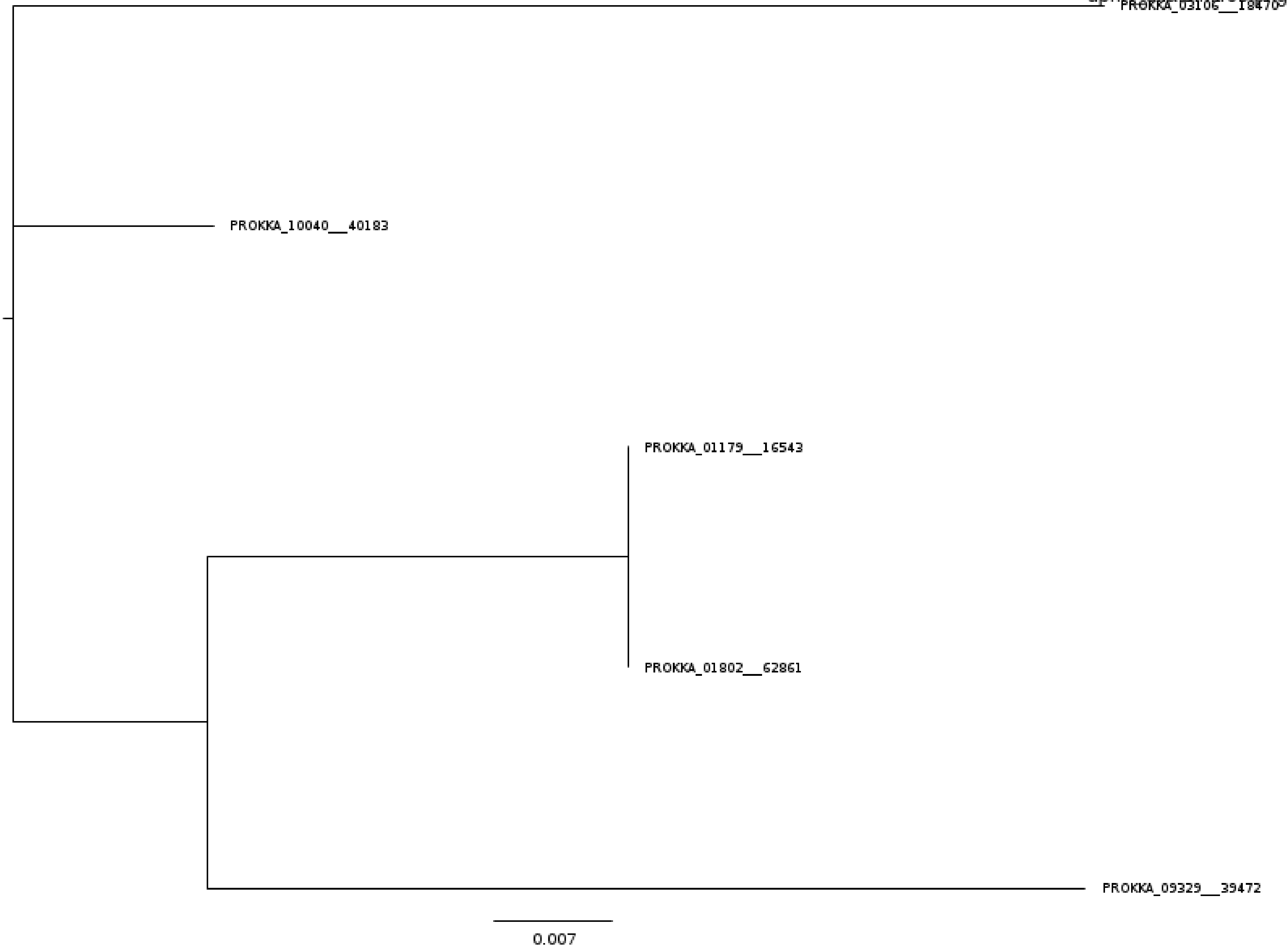

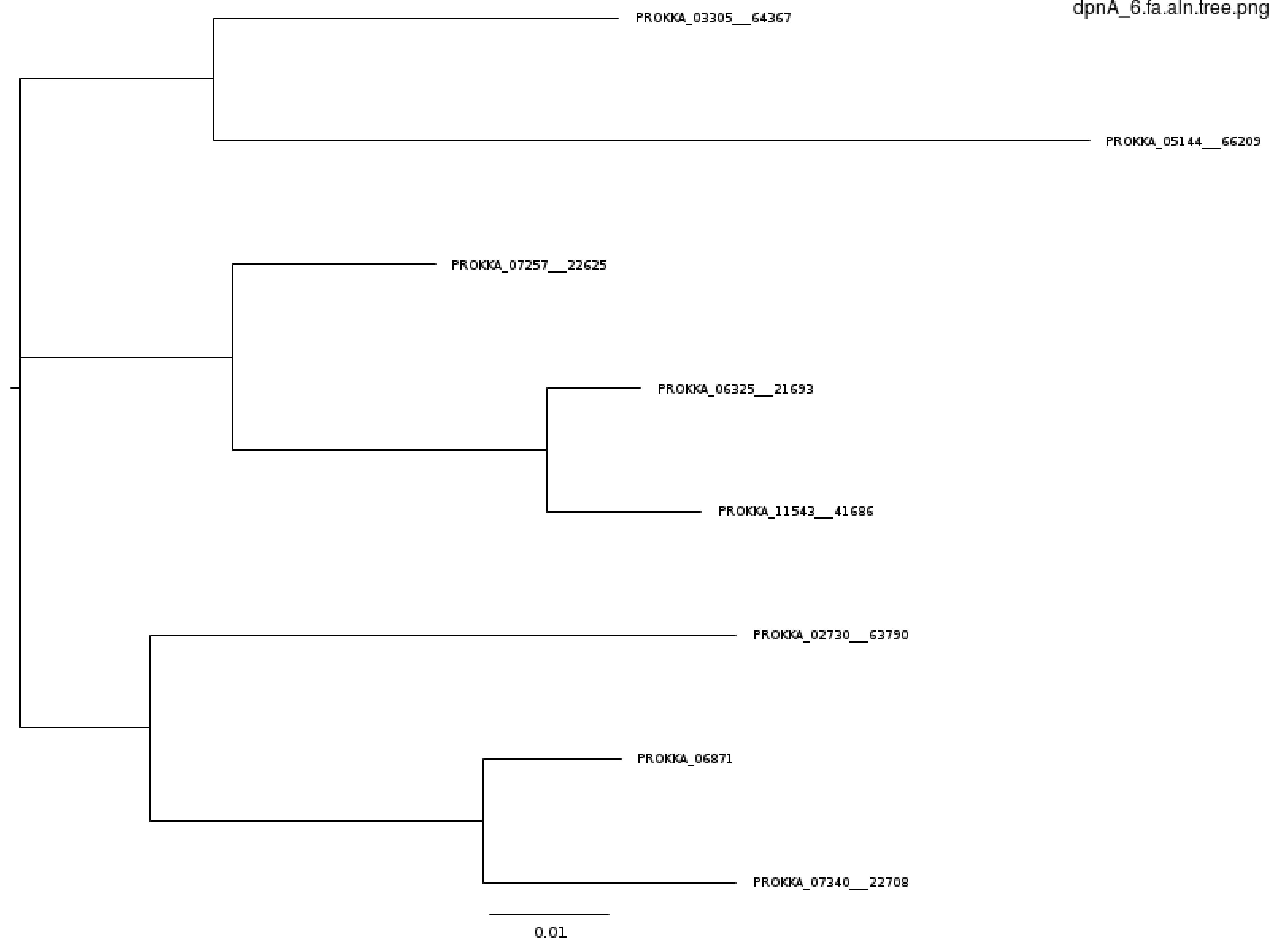
dpnA_6.fa.aln.tree.png
PROKKA_03305___64367
PROKKA_05144___66209
PROKKA_07257___22625
PROKKA_06325___21693
PROKKA_11543___41686
PROKKA_02730___63790
PROKKA_06871
PROKKA_07340___22708
0.01

dpnA_8.fa.aln.tree.png

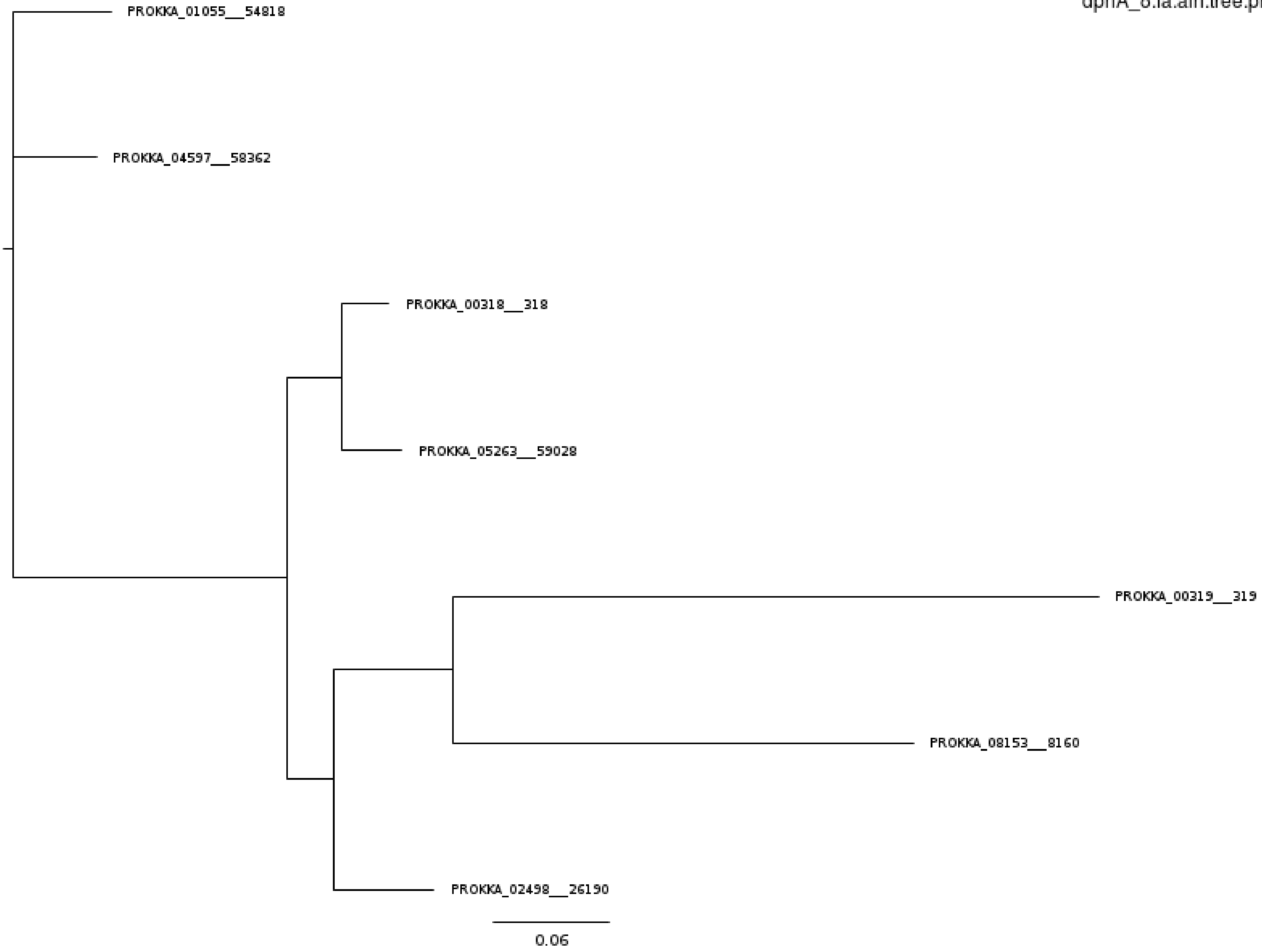

dppB_4.fa.aln.tree.png

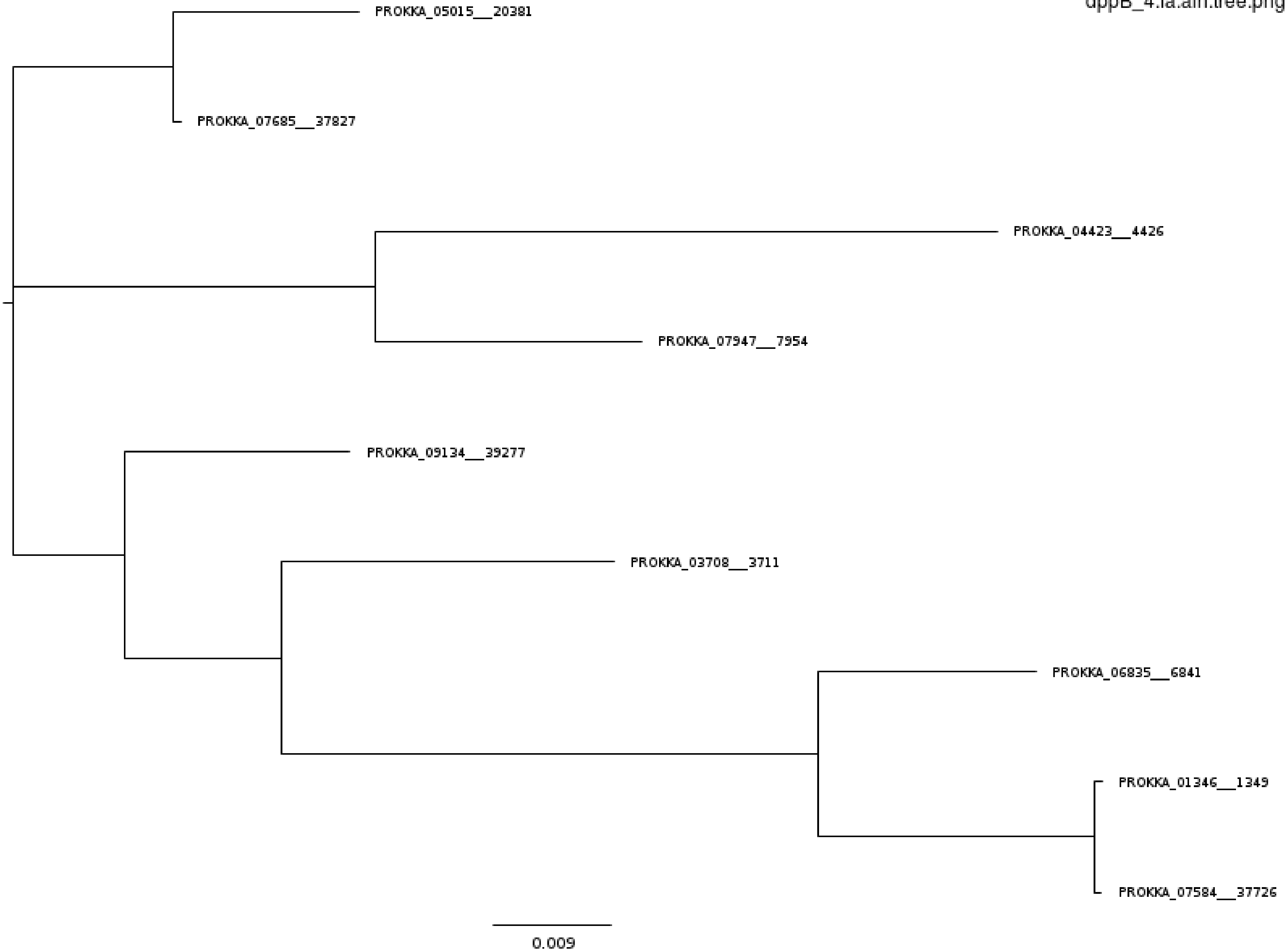

dppB.fa.aln.tree.png

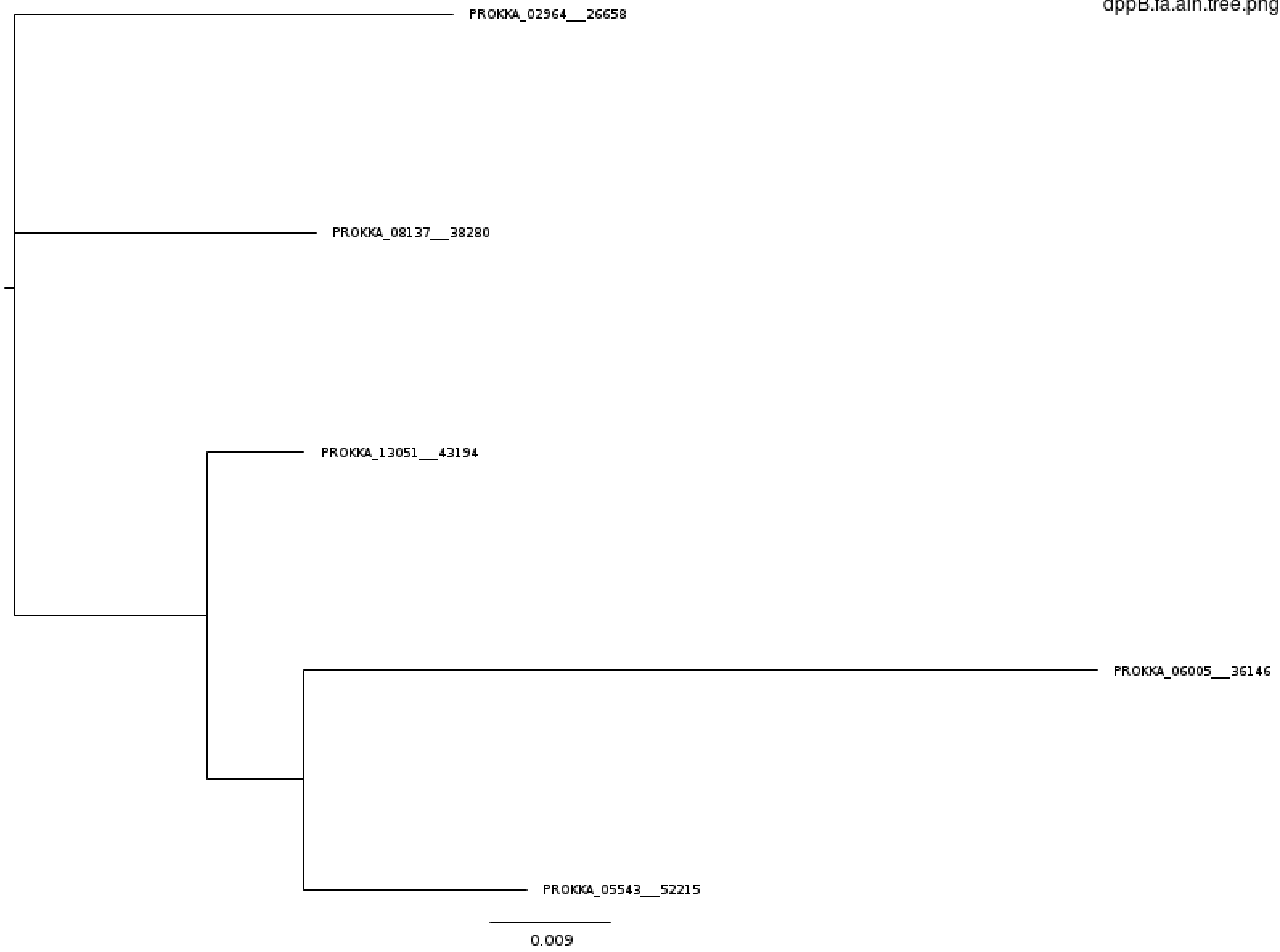

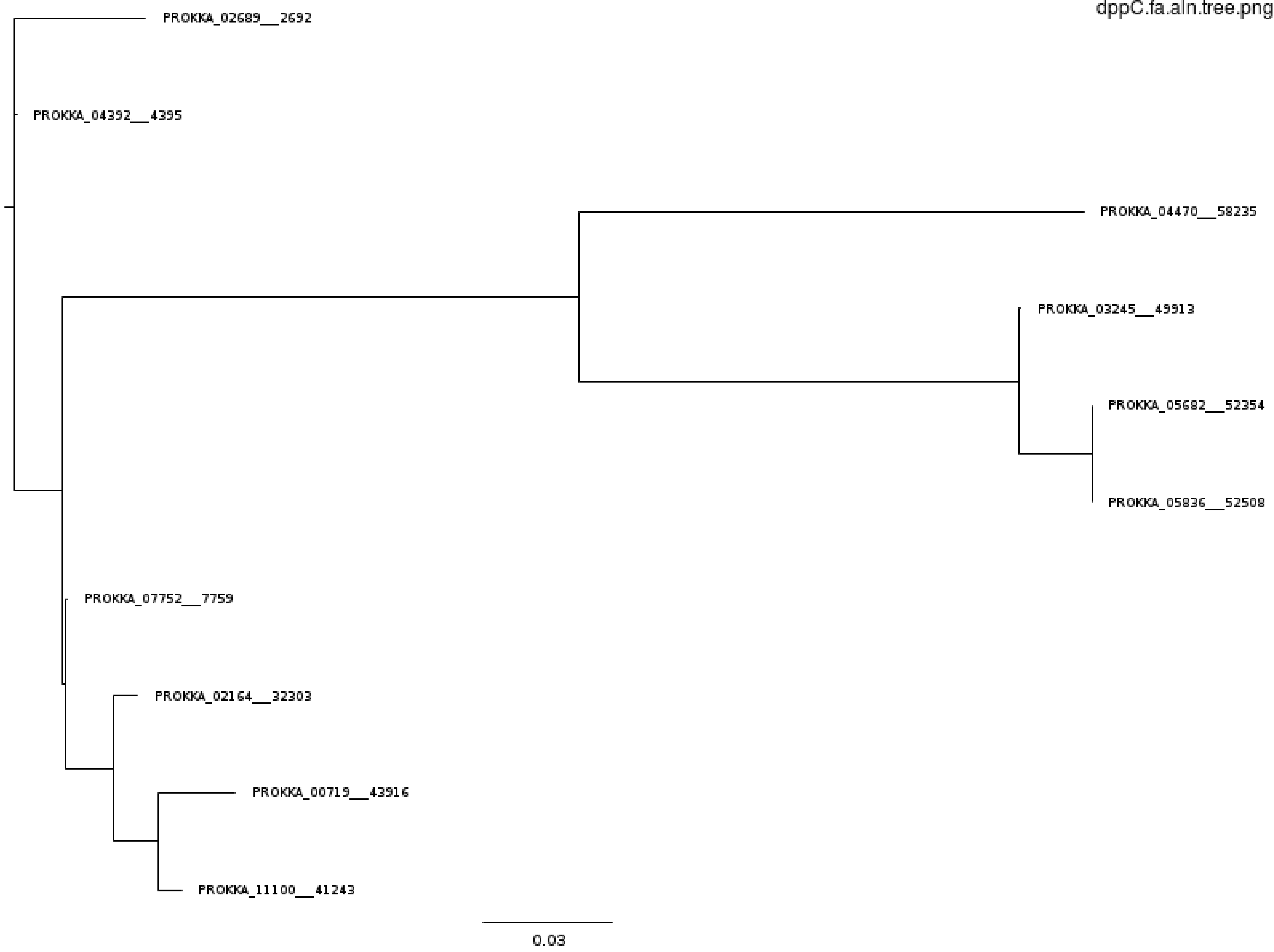
dppC.fa.aln.tree.png
PROKKA_02689___2692
PROKKA_04392___4395
PROKKA_04470___58235
PROKKA_03245___49913
PROKKA_05682___52354
PROKKA_05836___52508
PROKKA_07752___7759
PROKKA_02164___32303
PROKKA_00719___43916
PROKKA_11100___41243
0.03

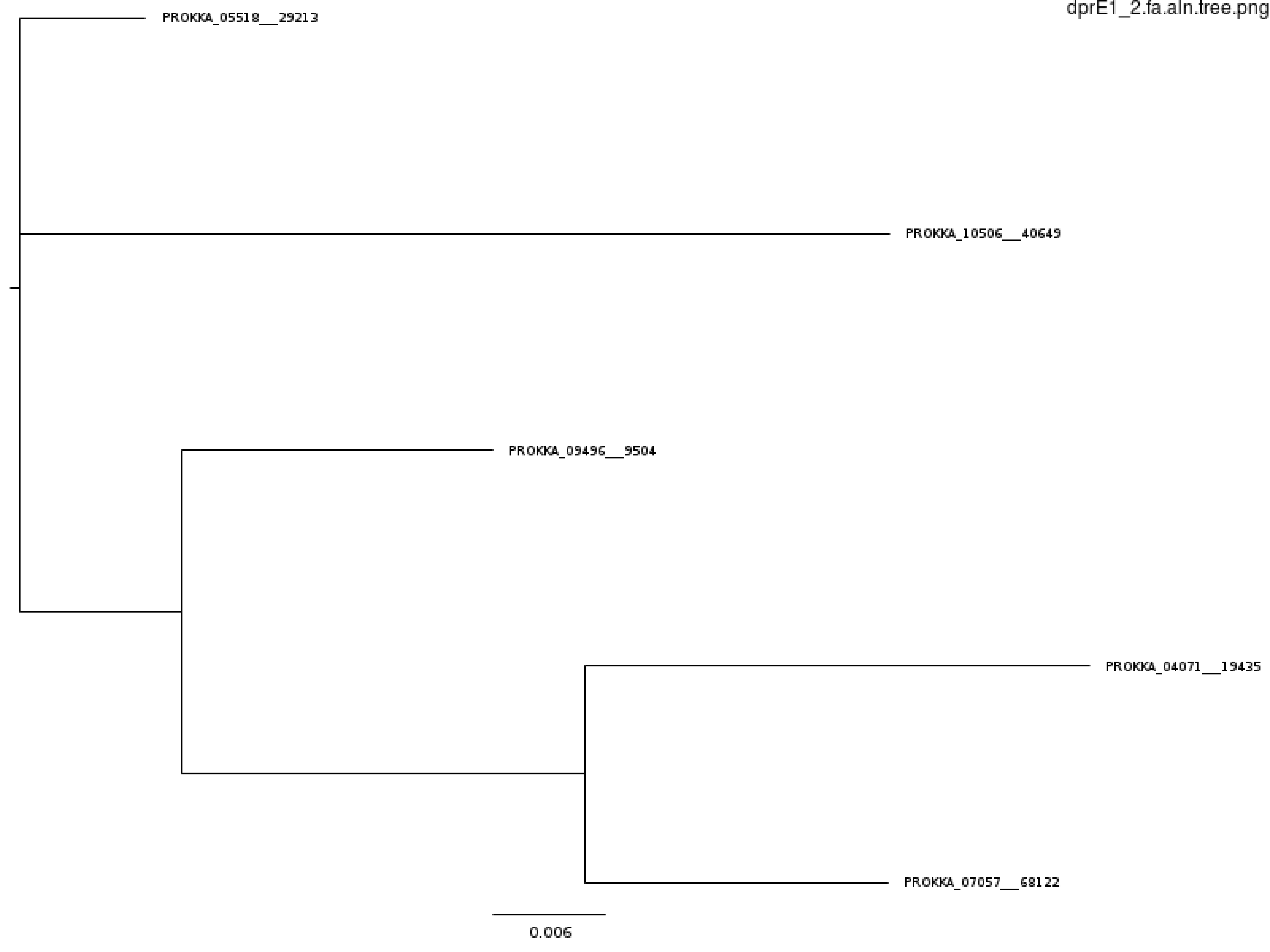

dprE1_2.fa.aln.tree.png
PROKKA_05518___29213
PROKKA_10506___40649
PROKKA_09496___9504
PROKKA_04071___19435
PROKKA_07057___68122
0.006

dsbD_2.fa.aln.tree.png

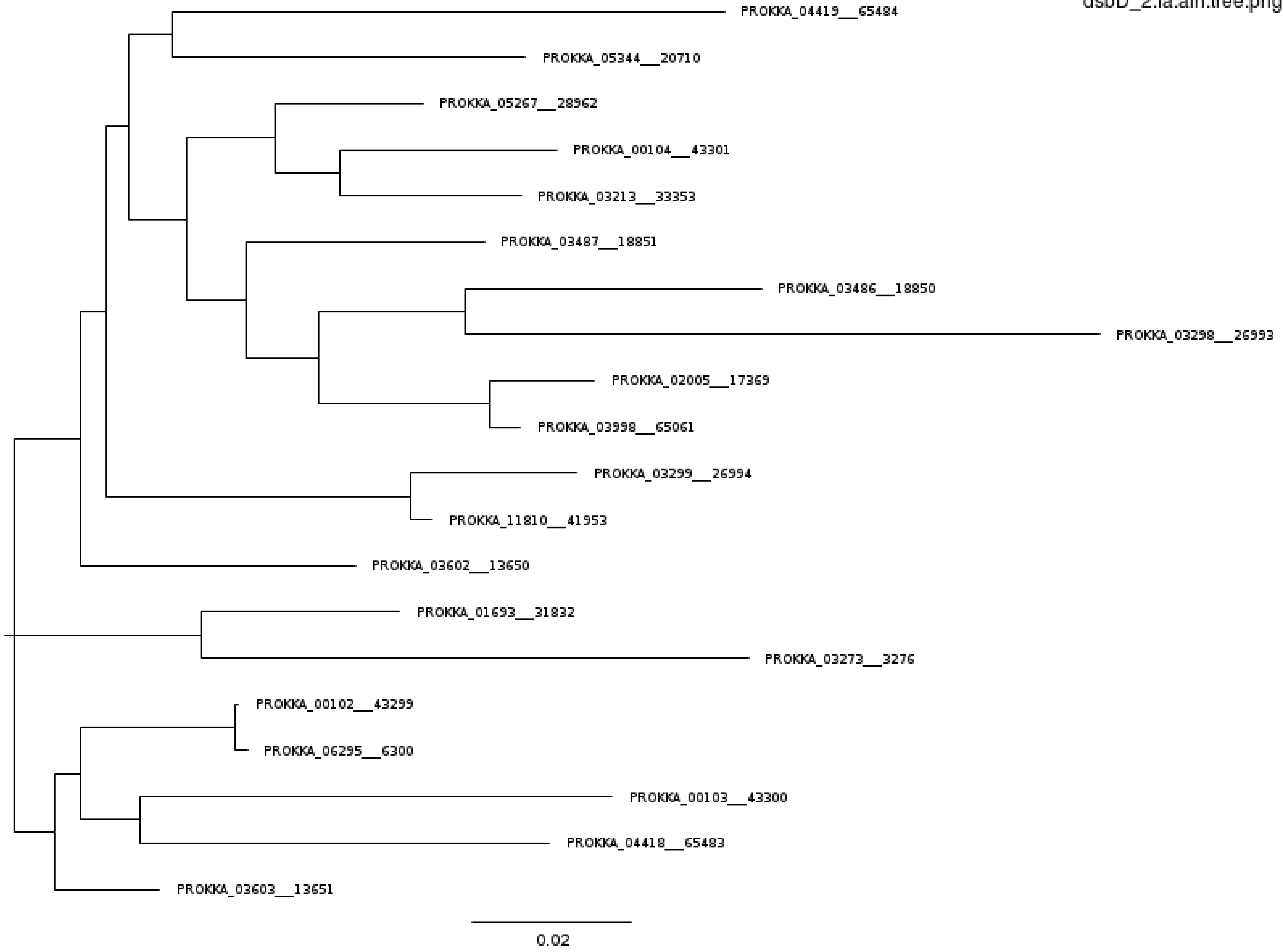

dsrE.fa.aln.tree.png

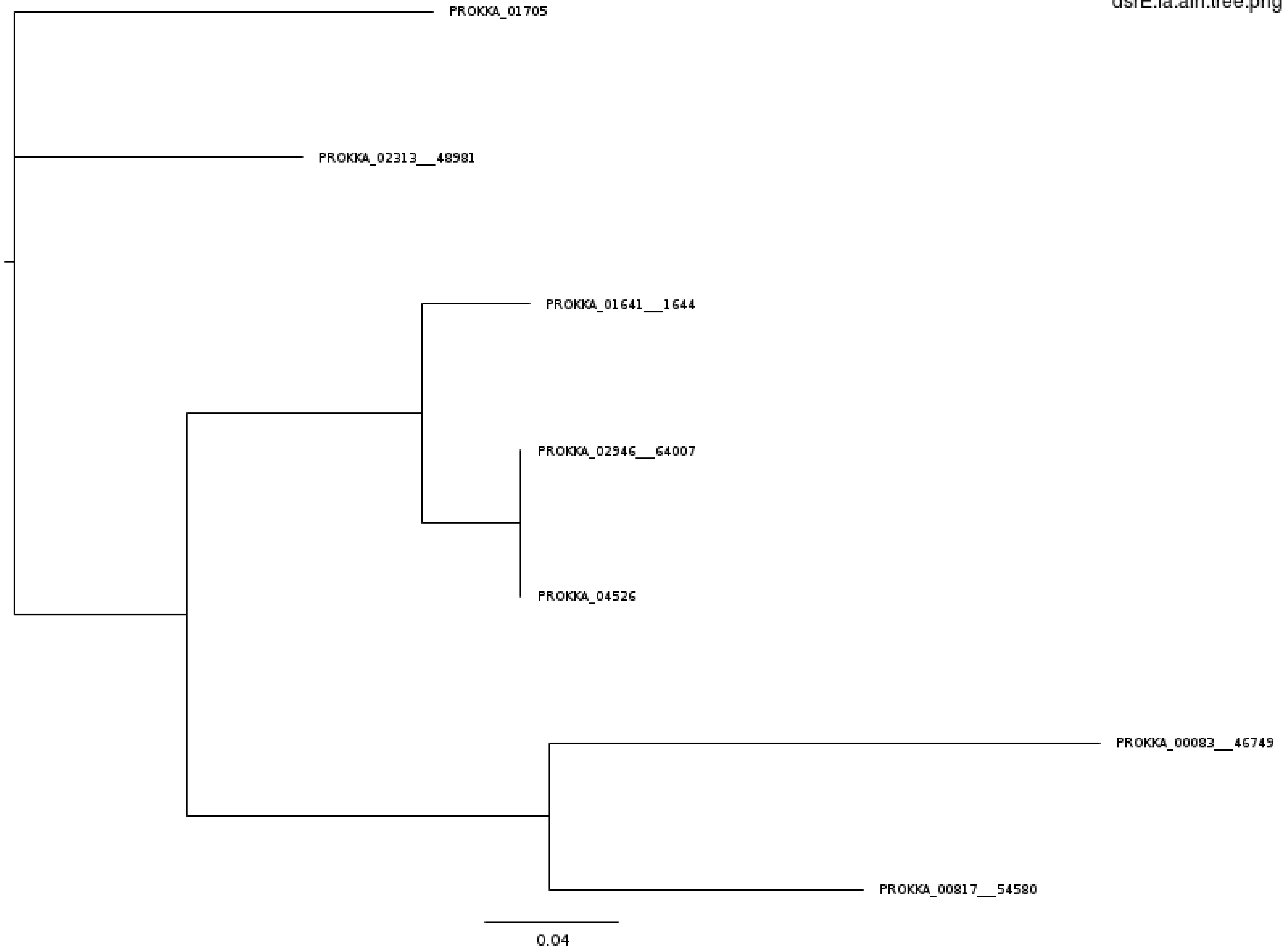

dsrE.fa.aln.tree.png

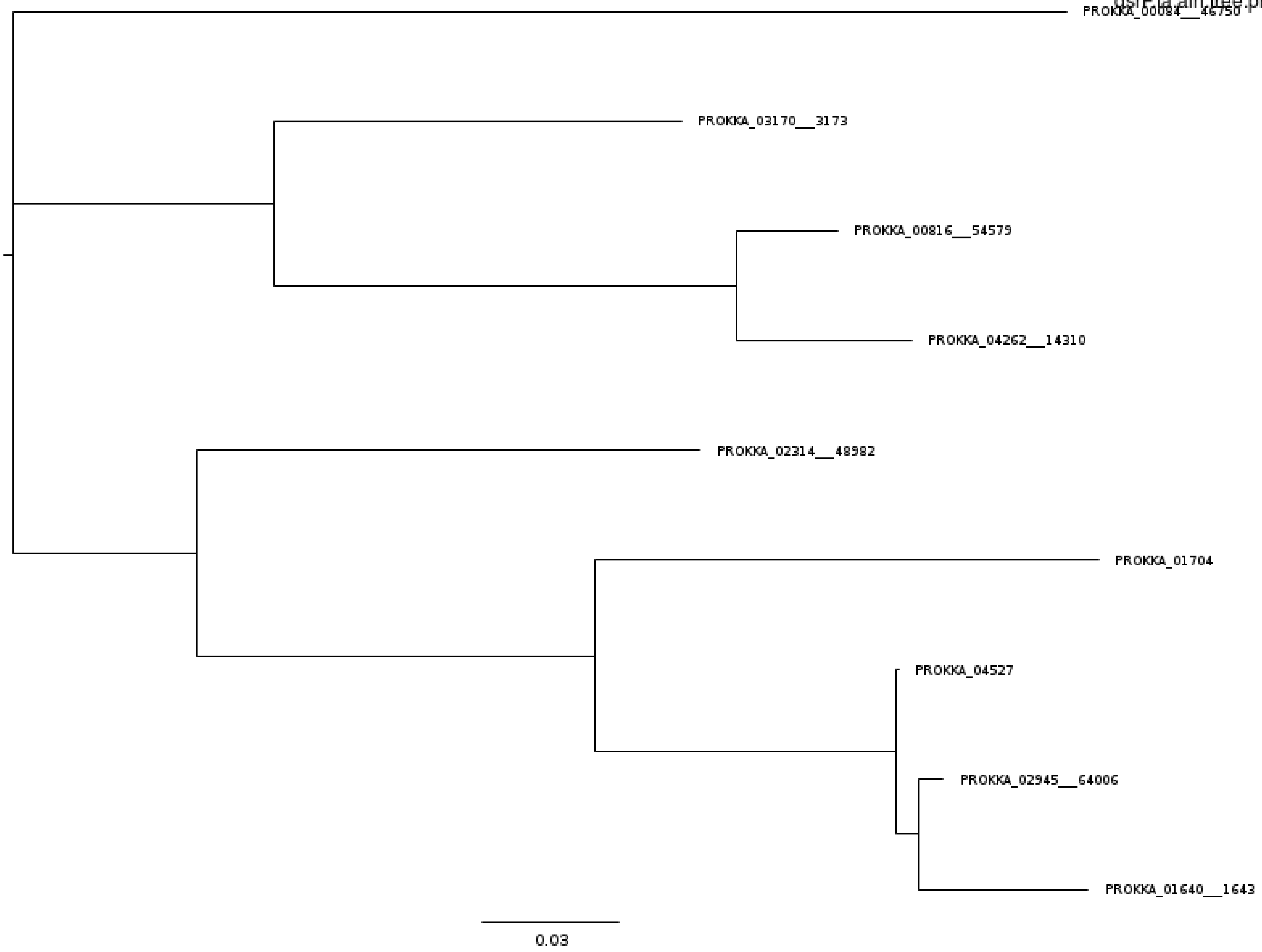

dsvB_2.fa.aln.tree.png

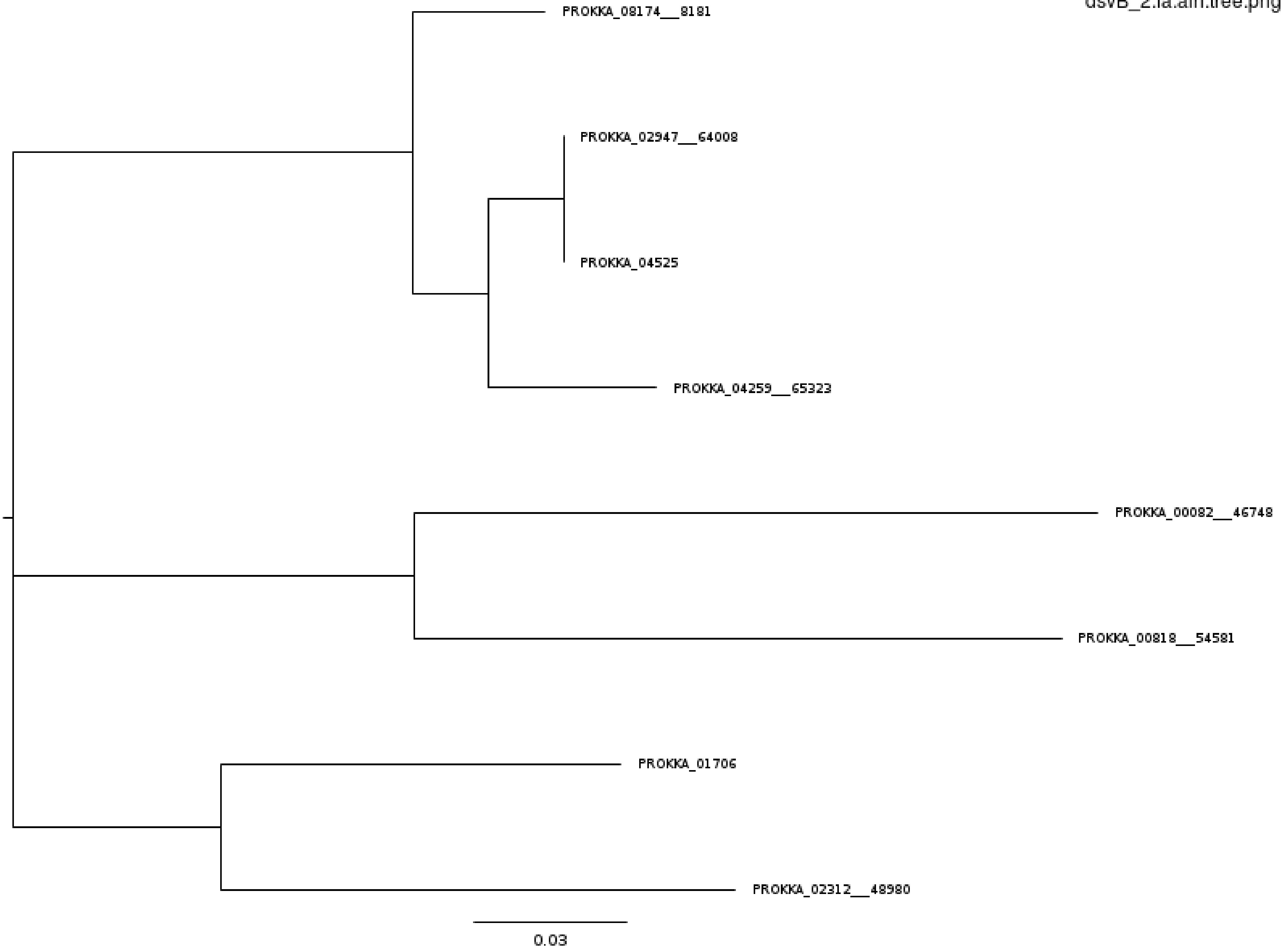

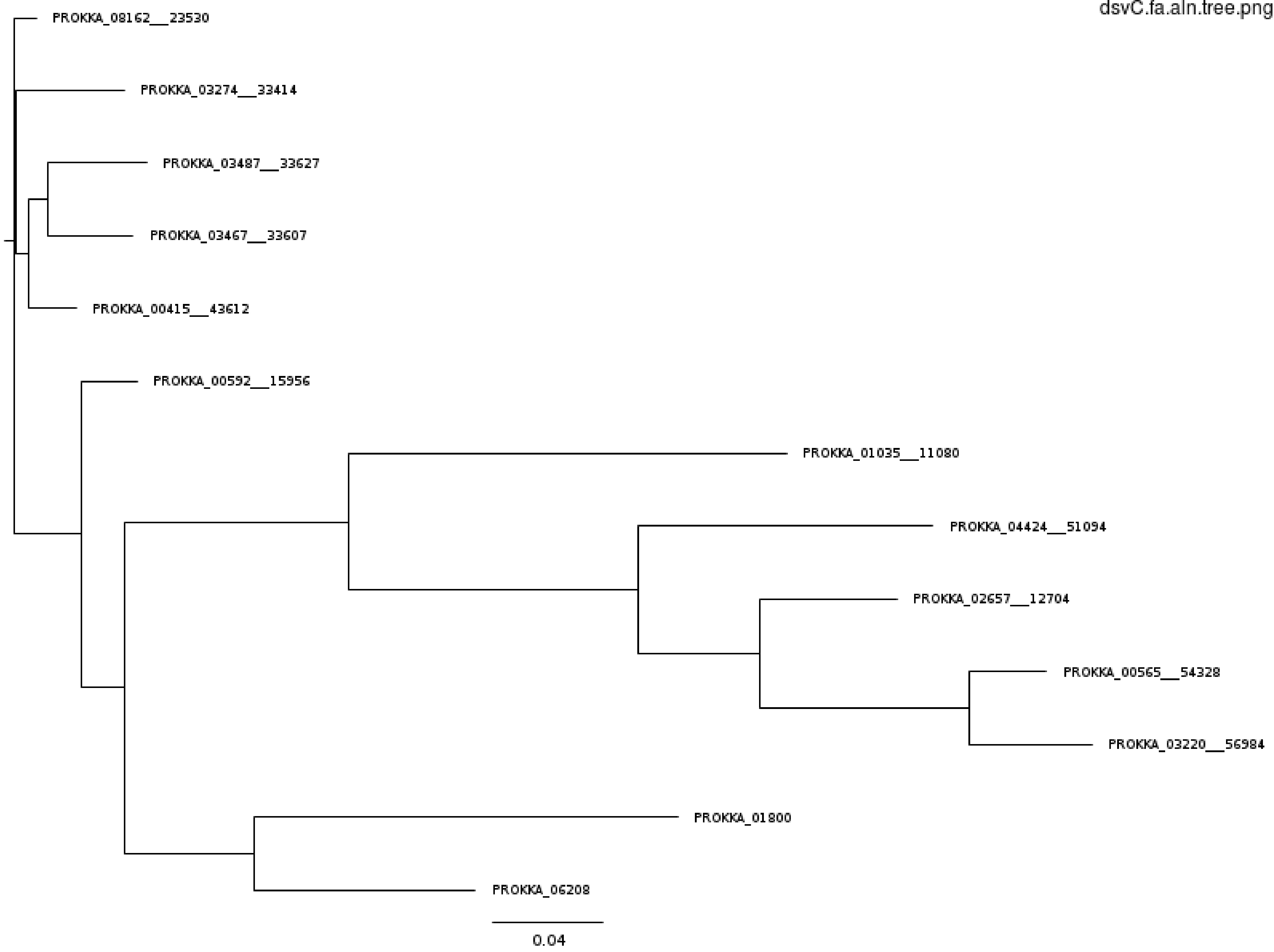
dsvC.fa.aln.tree.png
PROKKA_08162___23530
PROKKA_03274___33414
PROKKA_03487___33627
PROKKA_03467___33607
PROKKA_00415___43612
PROKKA_00592___15956
PROKKA_01035___11080
PROKKA_04424___51094
PROKKA_02657___12704
PROKKA_00565___54328
PROKKA_03220___56984
PROKKA_01800
PROKKA_06208
0.04

dxr_3.fa.aln.tree.png

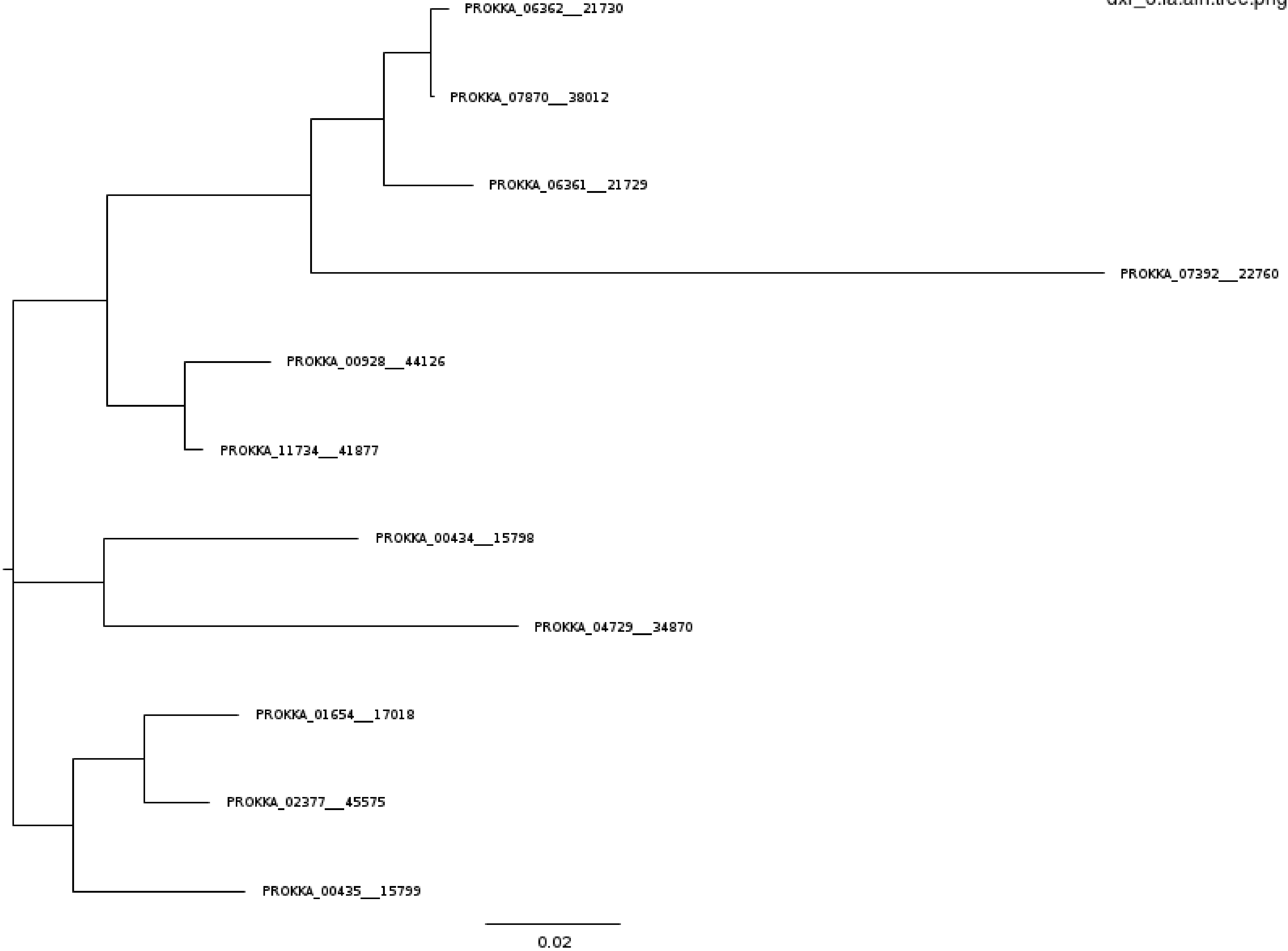

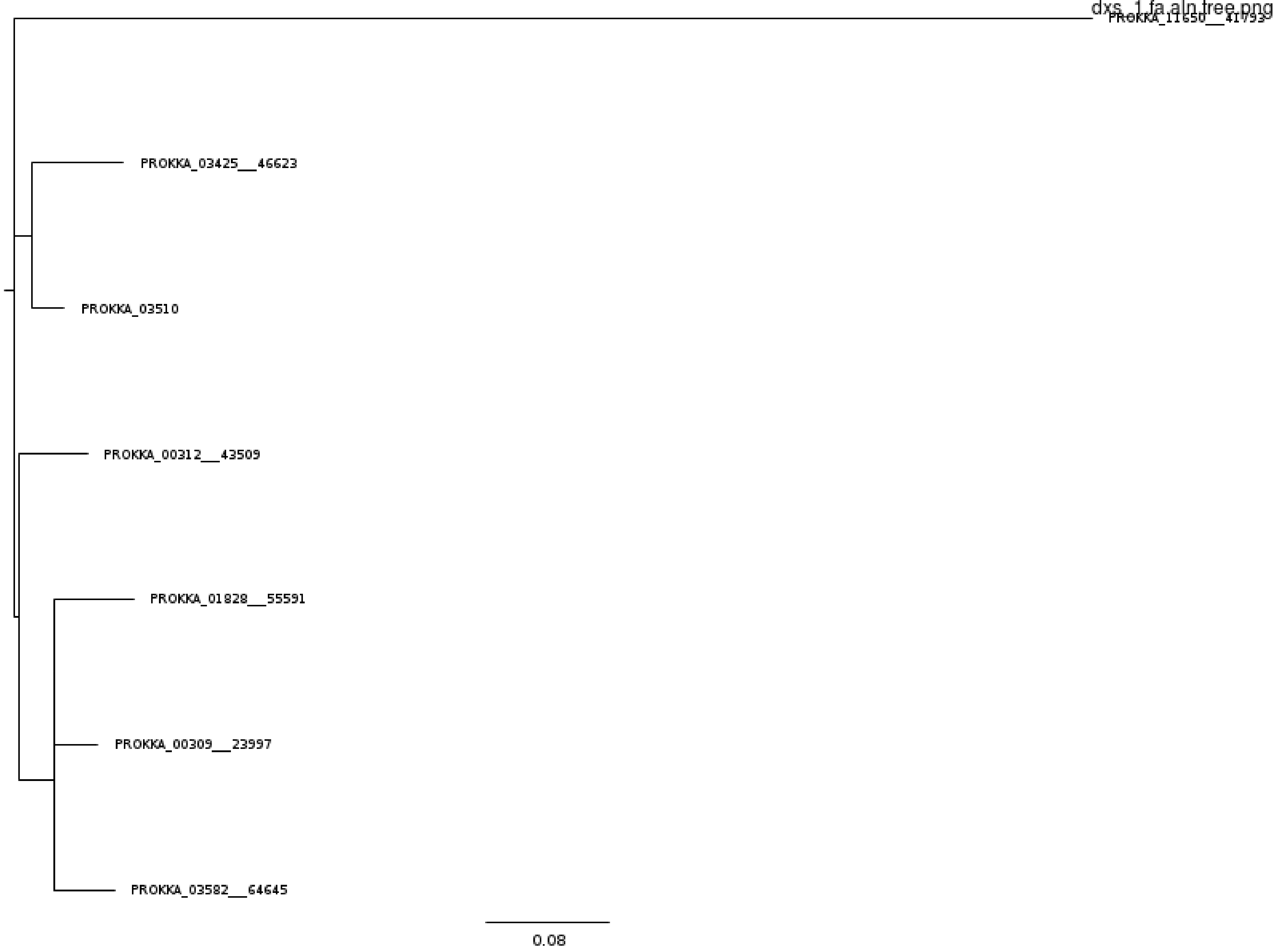
dxs_1.fa.aln.tree.png
PROKKA_11650___41793
PROKKA_03425___46623
PROKKA_03510
PROKKA_00312___43509
PROKKA_01828___55591
PROKKA_00309___23997
PROKKA_03582___64645
0.08

ebrA.fa.aln.tree.png

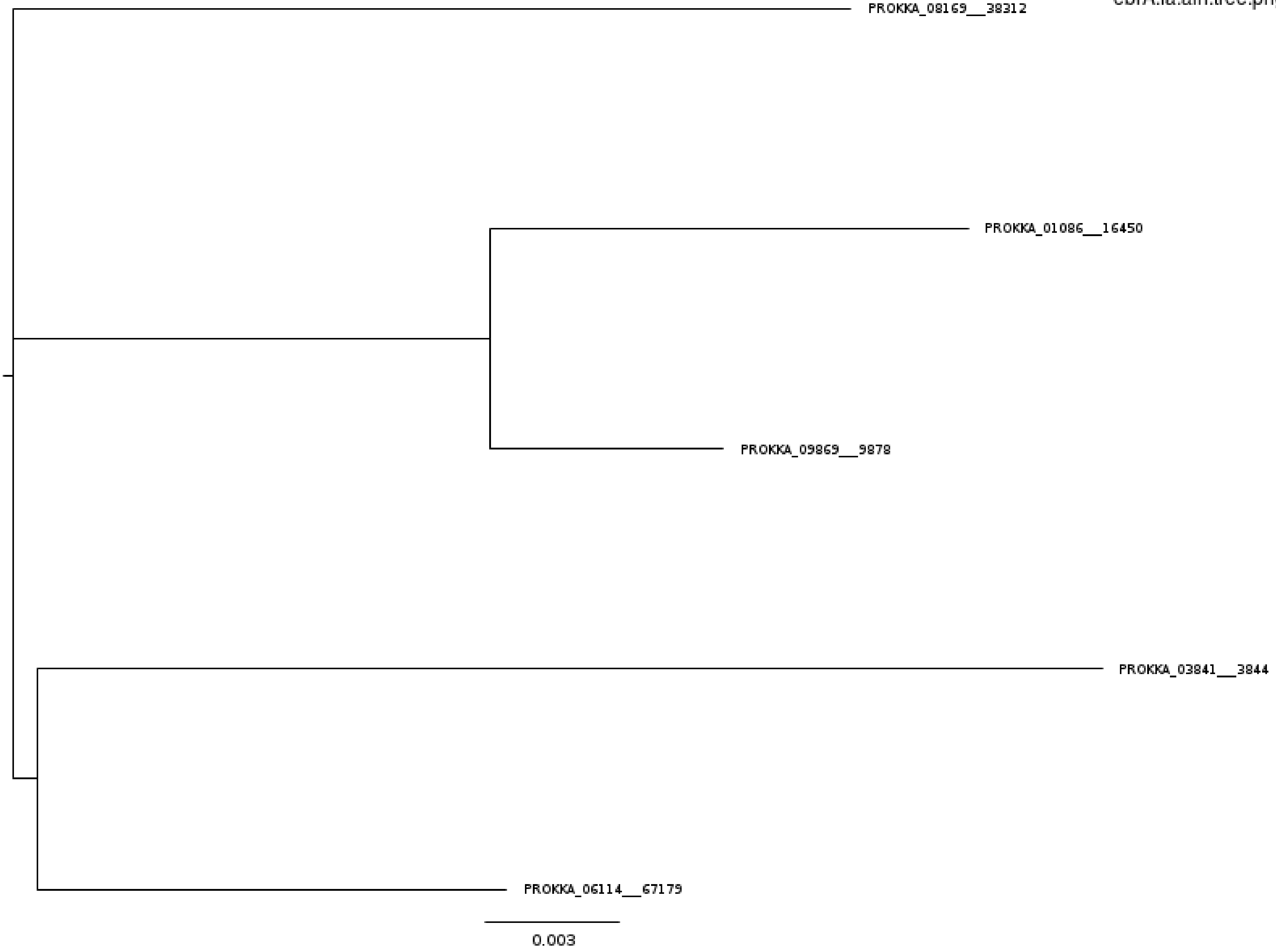

efp_2.fa.aln.tree.png

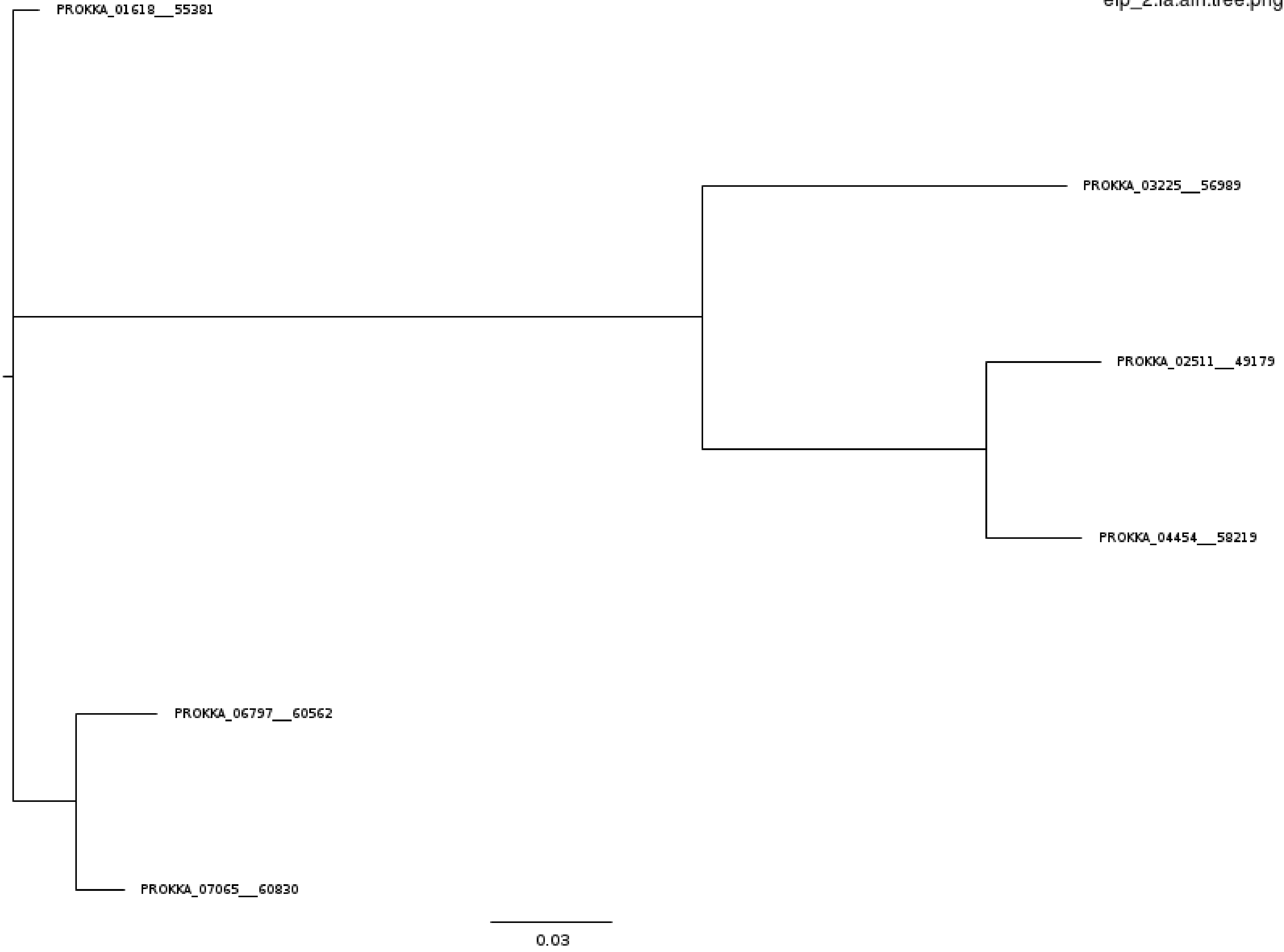

efp.fa.aln.tree.png

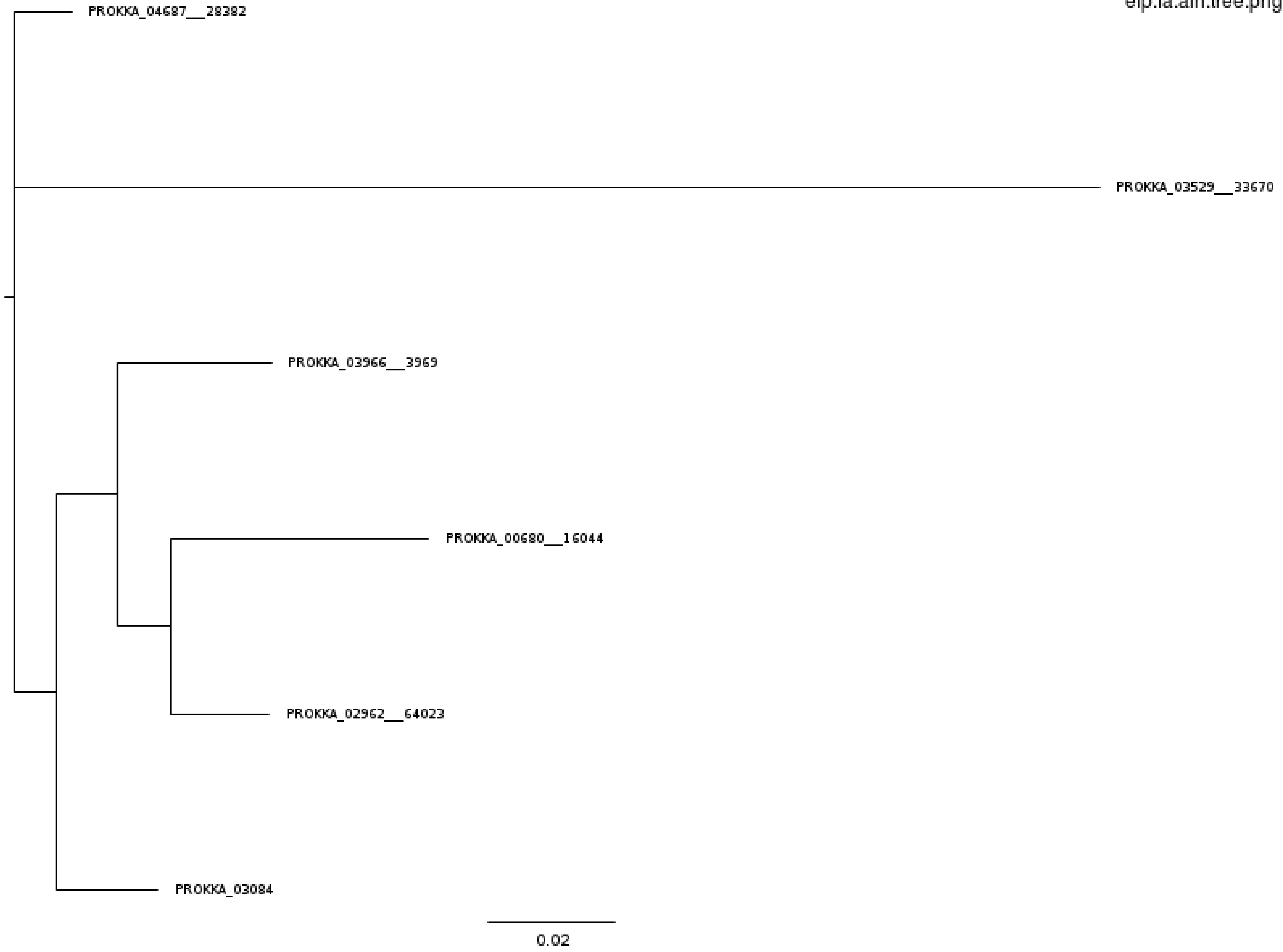

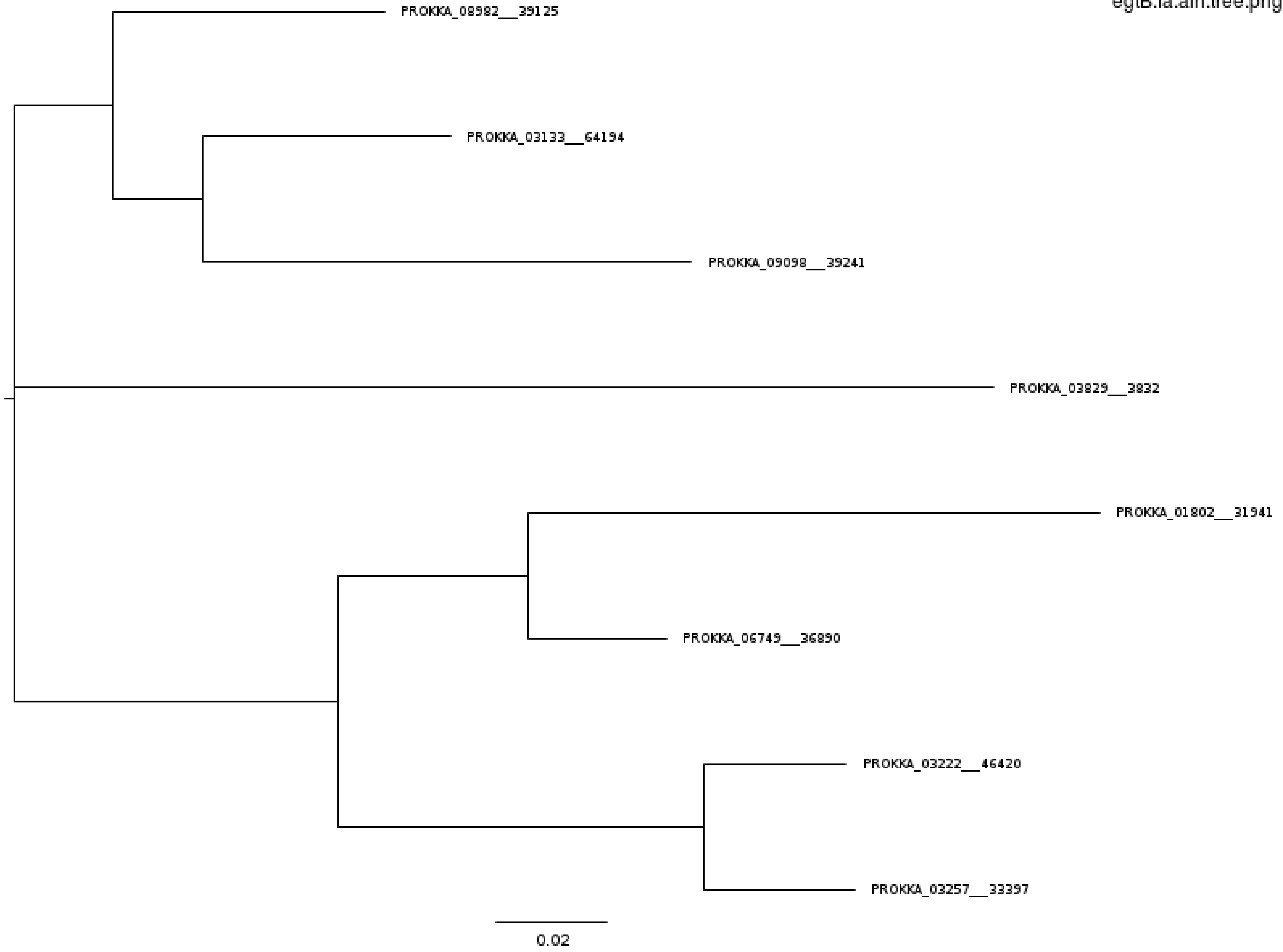

PROKKA_08982___39125
PROKKA_03133___64194
PROKKA_09098___39241
PROKKA_03829___3832
PROKKA_01802___31941
PROKKA_06749___36890
PROKKA_03222___46420
PROKKA_03257___33397
0.02

engB_3.fa.aln.tree.png

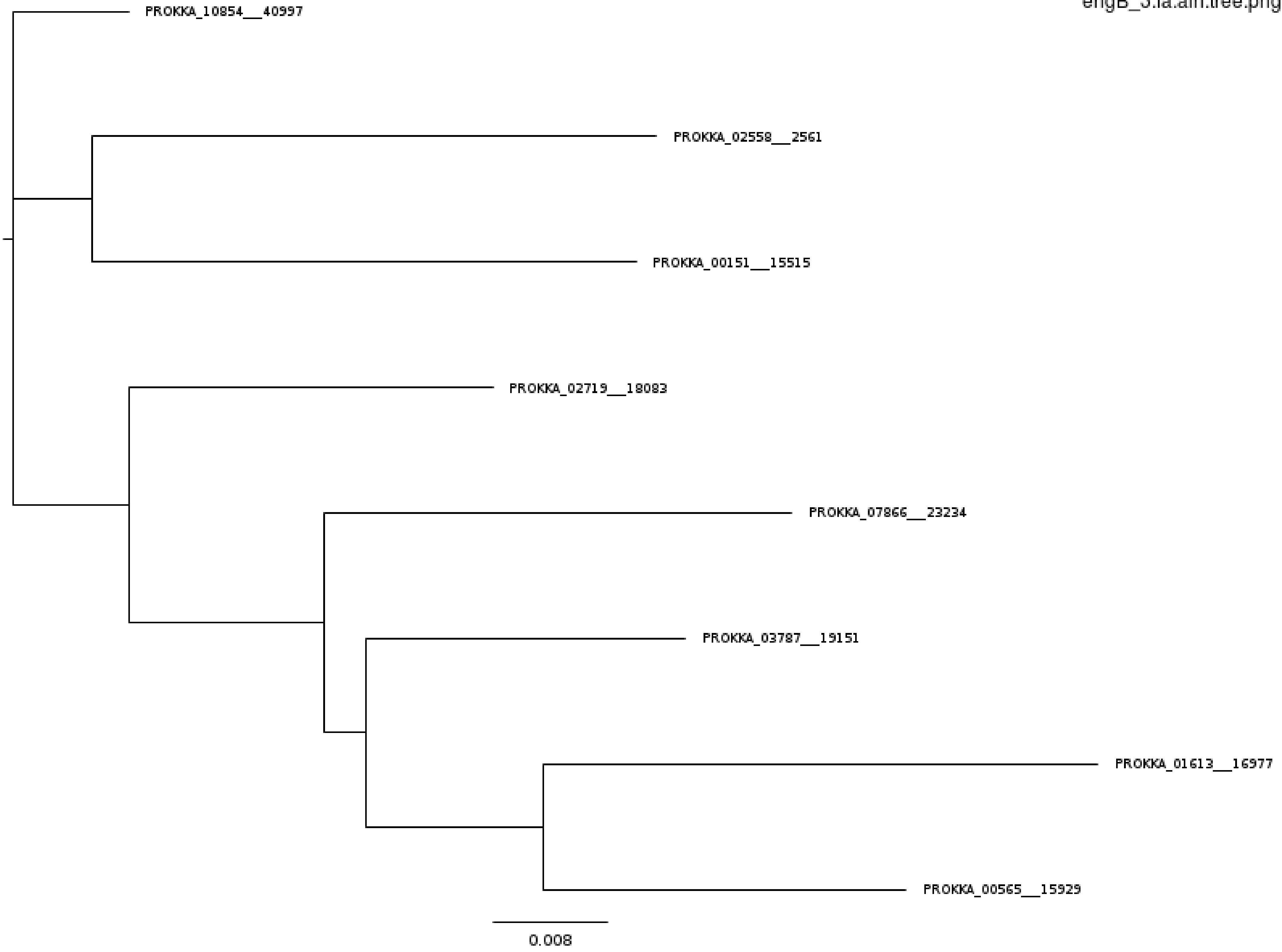

eno_3.fa.aln.tree.png

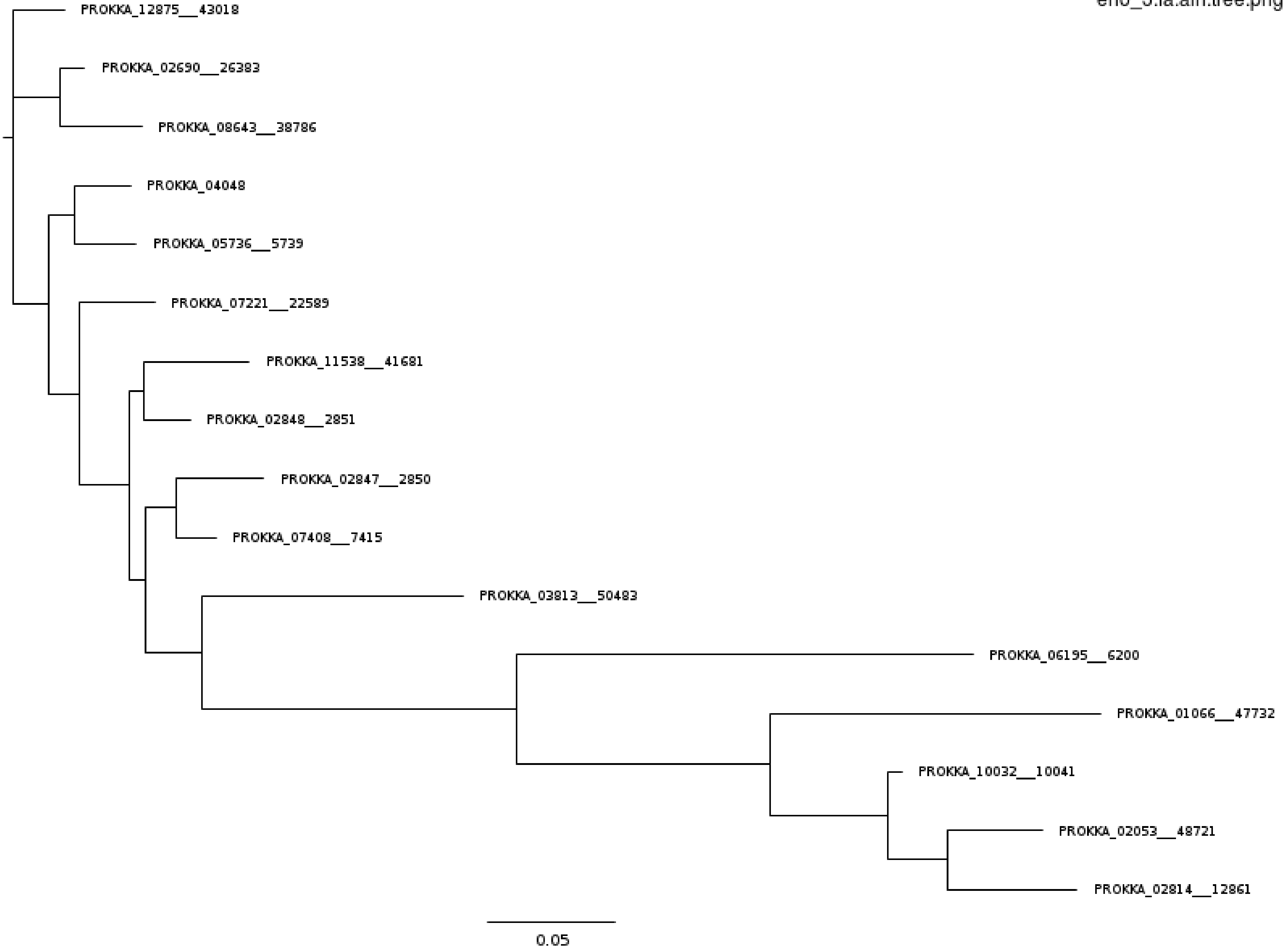

epmA_1.fa.aln.tree.png

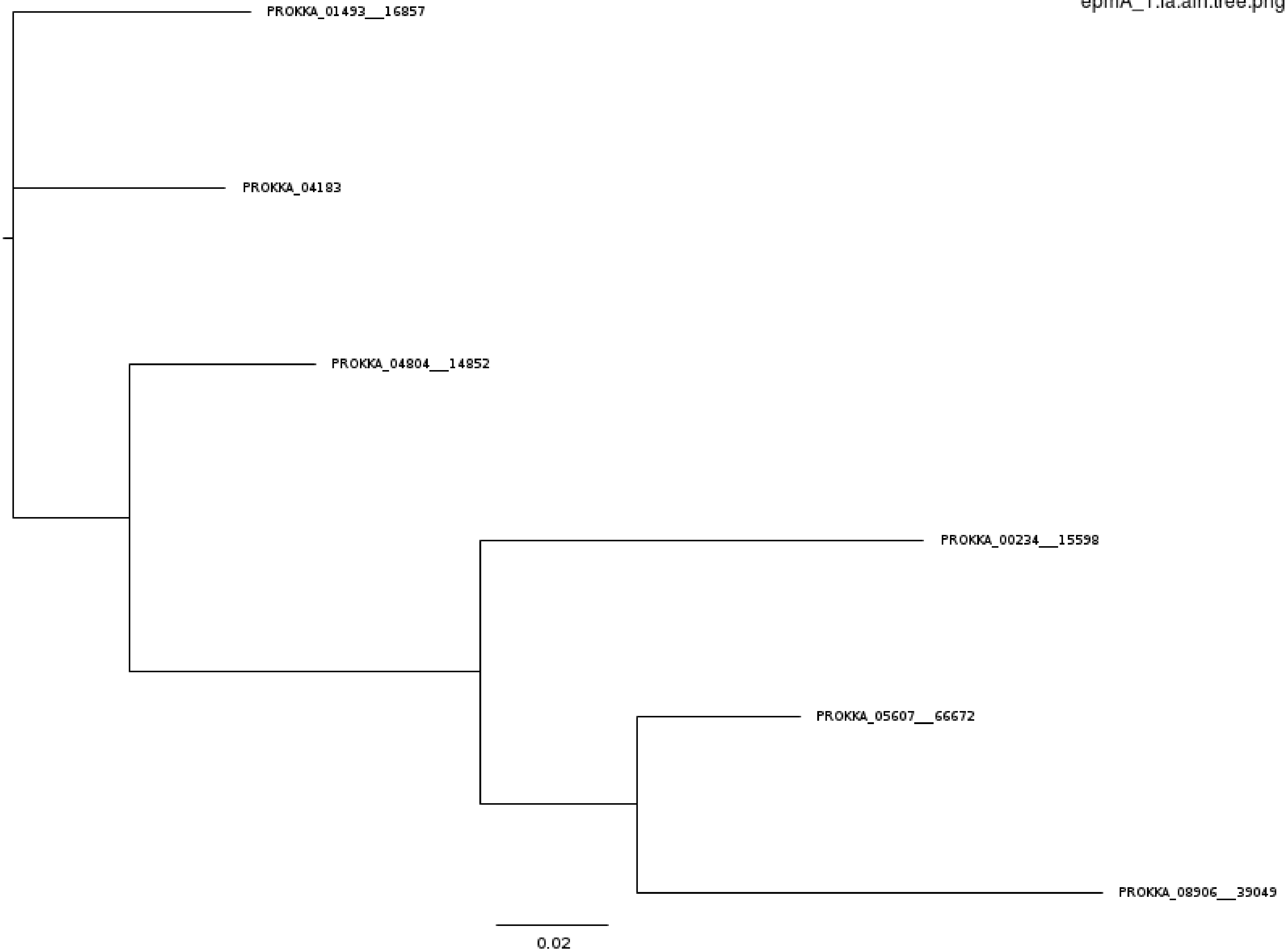

epsE_6.fa.aln.tree.png

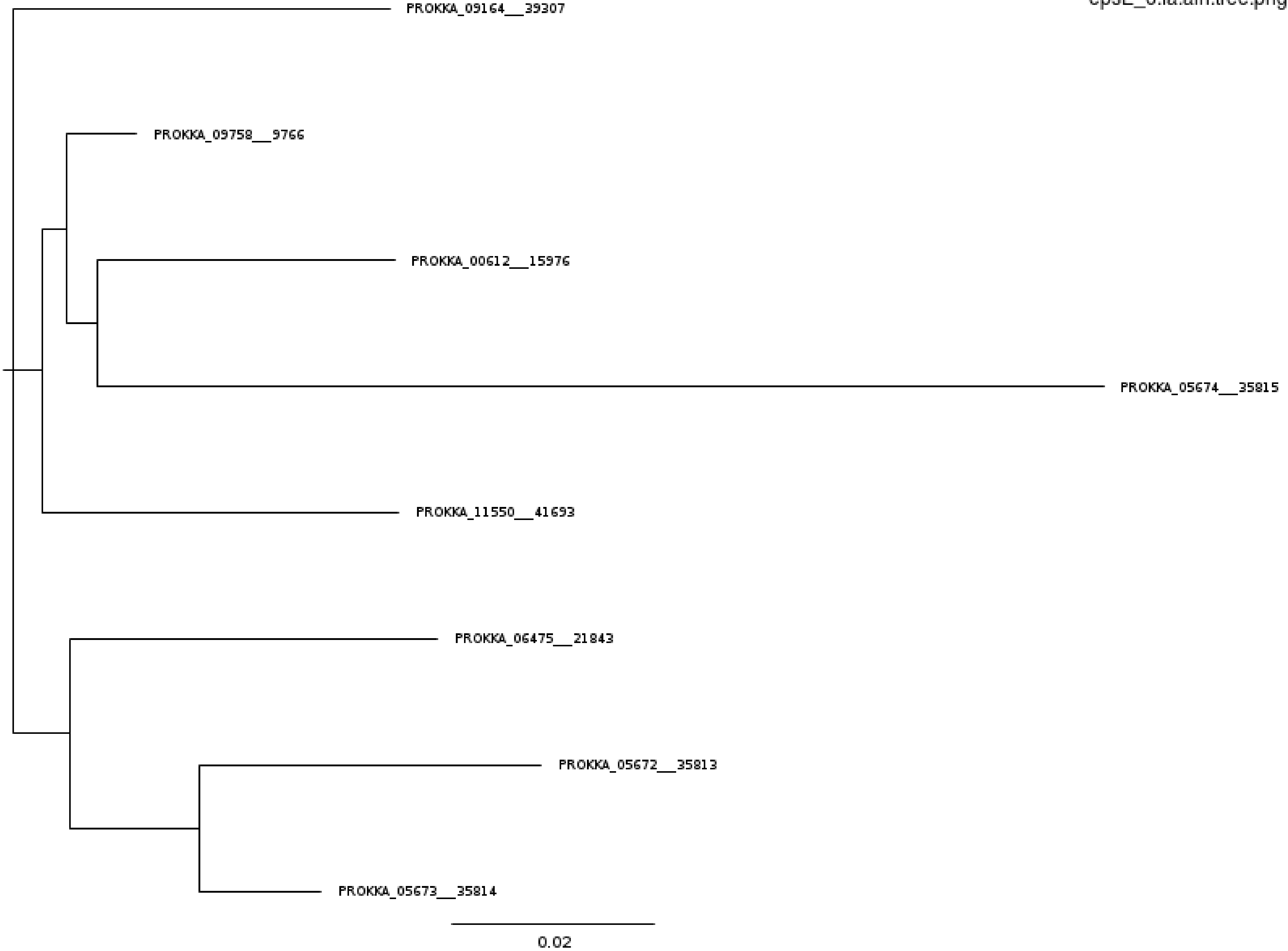

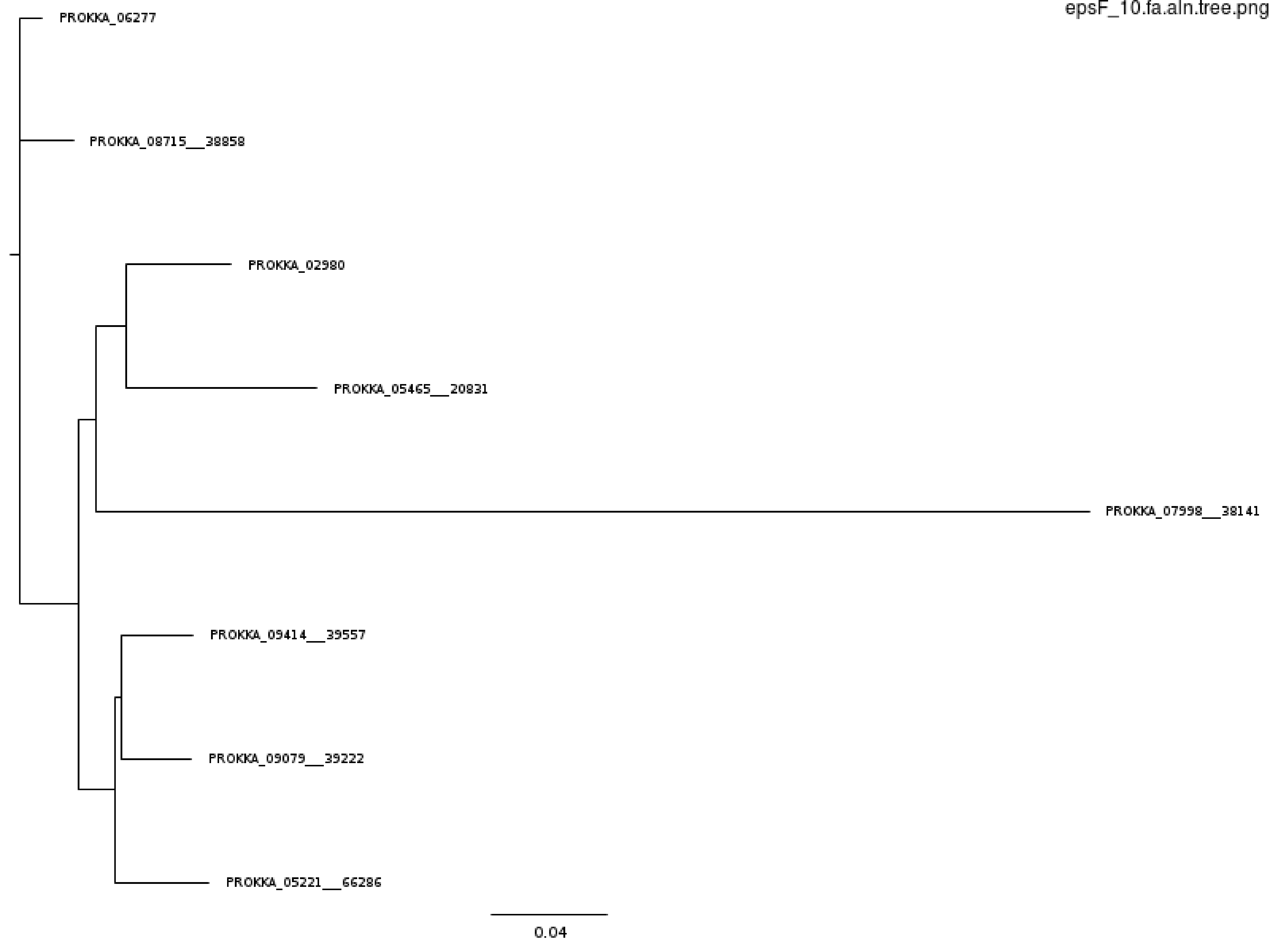
epsF_10.fa.aln.tree.png
PROKKA_06277
PROKKA_08715___38858
PROKKA_02980
PROKKA_05465___20831
PROKKA_07998___38141
PROKKA_09414___39557
PROKKA_09079___39222
PROKKA_05221___66286
0.04

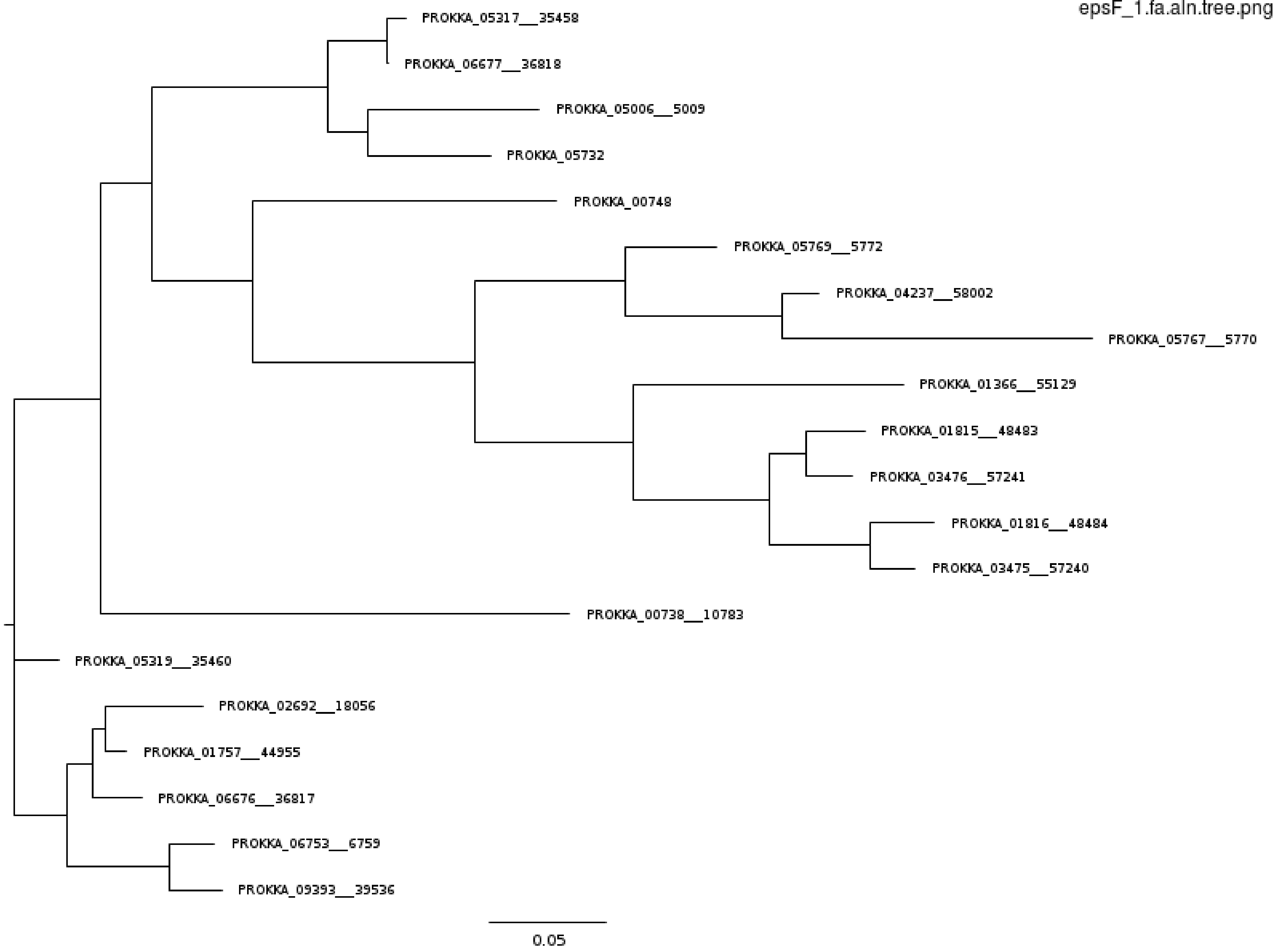
epsF_1.fa.aln.tree.png
PROKKA_05317___35458
PROKKA_06677___36818
PROKKA_05006___5009
PROKKA_05732
PROKKA_00748
PROKKA_05769___5772
PROKKA_04237___58002
PROKKA_05767___5770
PROKKA_01366___55129
PROKKA_01815___48483
PROKKA_03476___57241
PROKKA_01816___48484
PROKKA_03475___57240
PROKKA_00738___10783
PROKKA_05319___35460
PROKKA_02692___18056
PROKKA_01757___44955
PROKKA_06676___36817
PROKKA_06753___6759
PROKKA_09393___39536
0.05

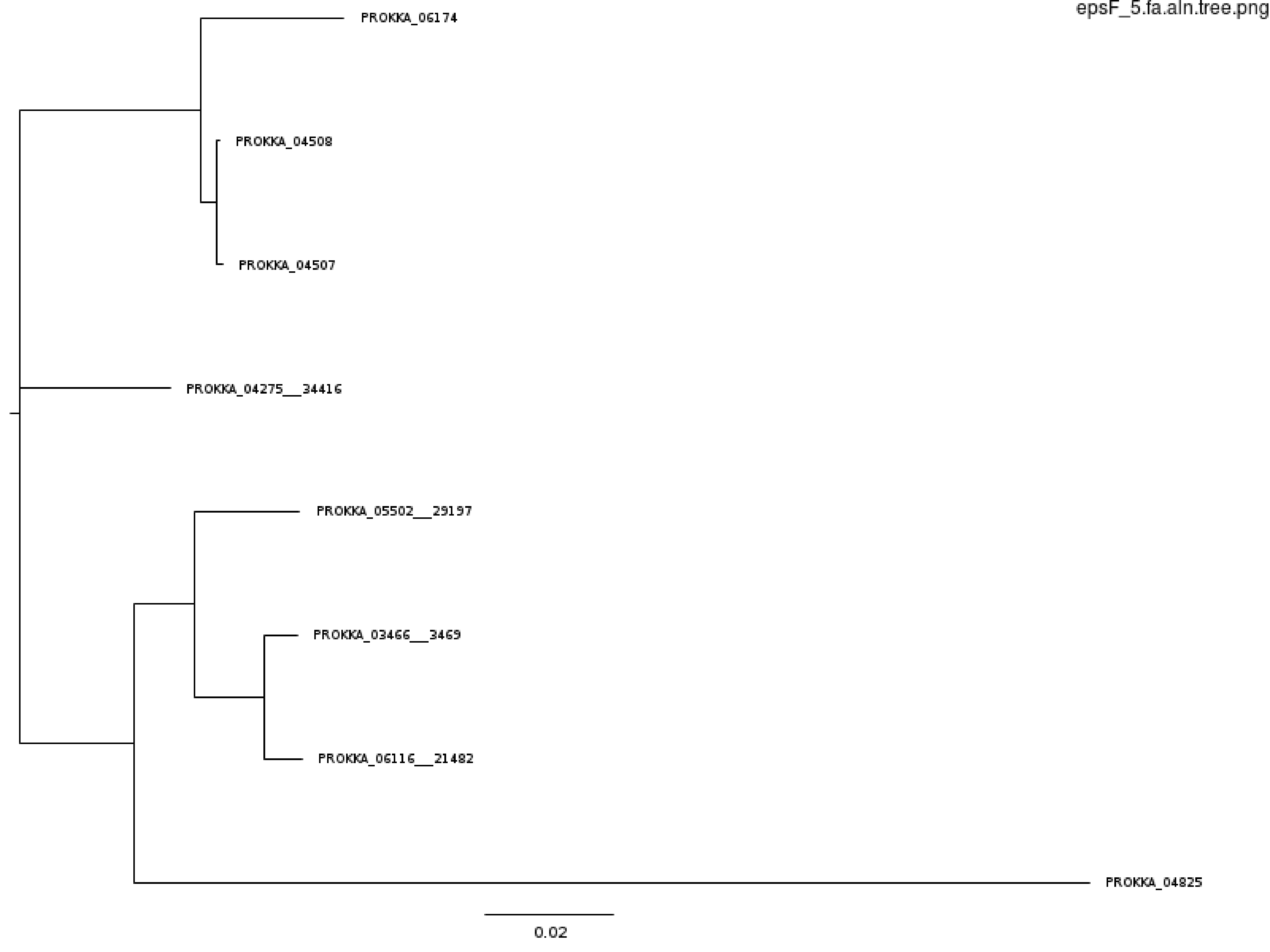
epsF_5.fa.aln.tree.png
PROKKA_06174
PROKKA_04508
PROKKA_04507
PROKKA_04275___34416
PROKKA_05502___29197
PROKKA_03466___3469
PROKKA_06116___21482
PROKKA_04825
0.02

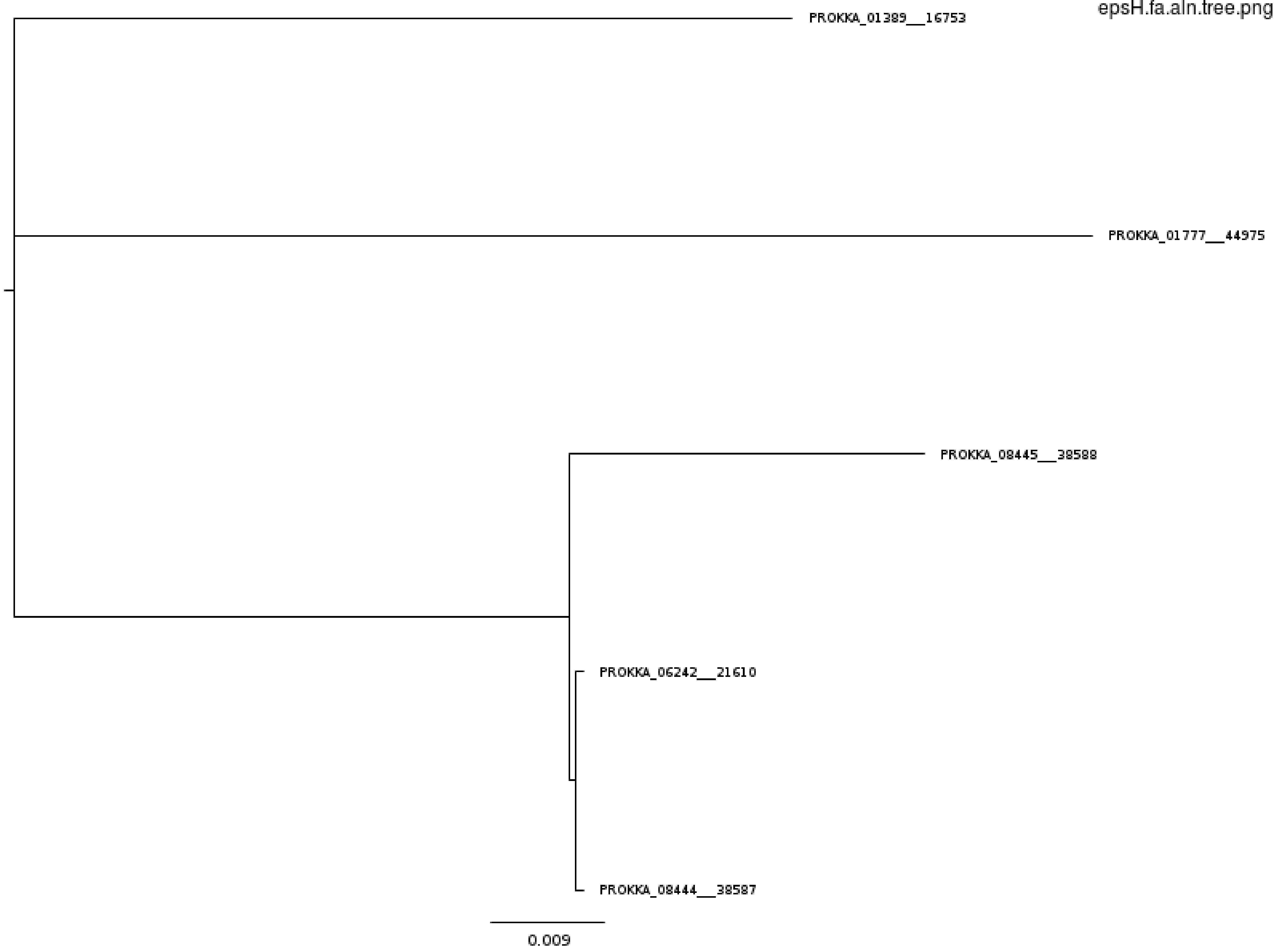
epsH.fa.aln.tree.png
PROKKA_01389___16753
PROKKA_01777___44975
PROKKA_08445___38588
PROKKA_06242___21610
PROKKA_08444___38587
0.009

era_3.fa.aln.tree.png

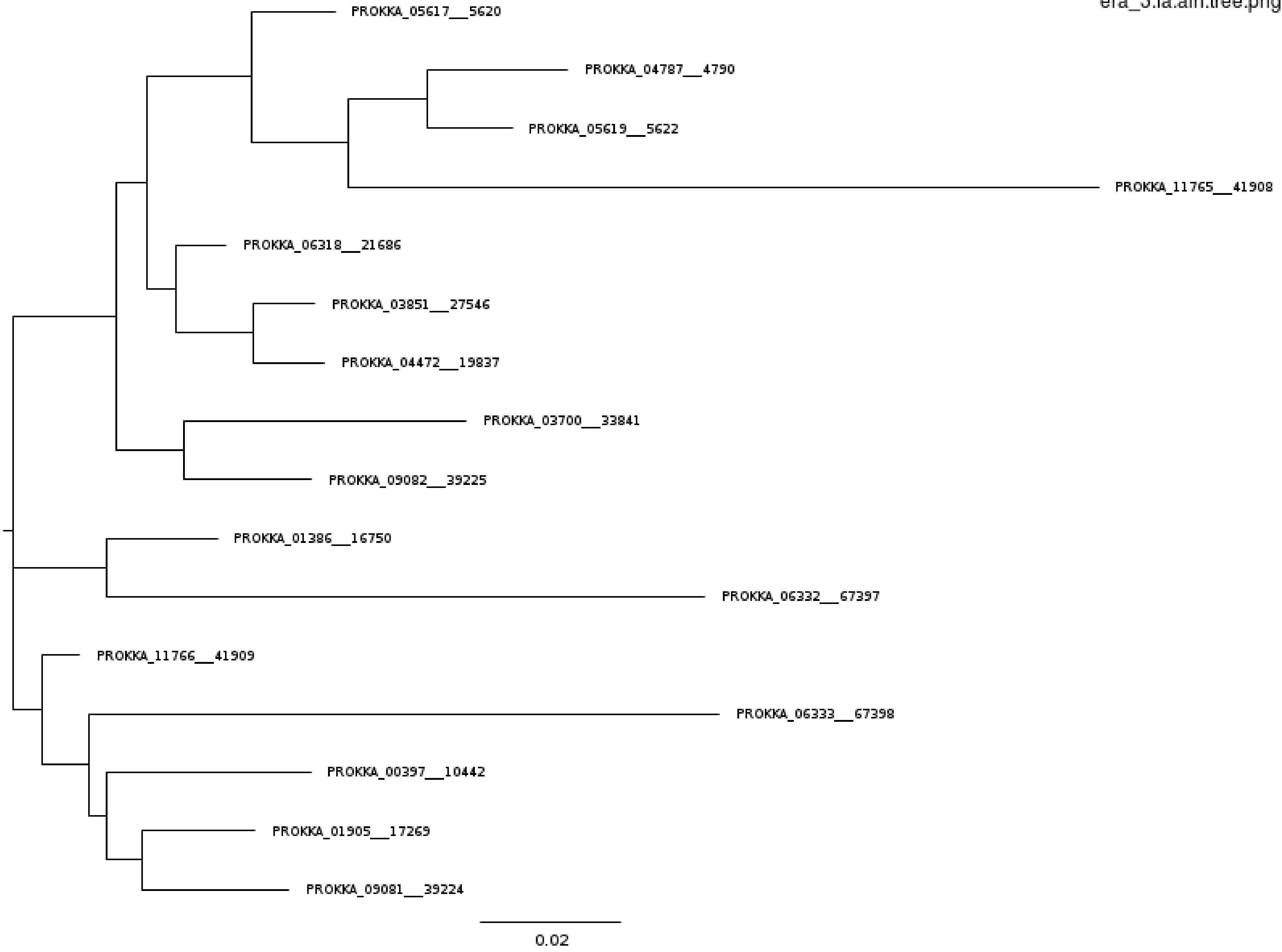

era_4.fa.aln.tree.png

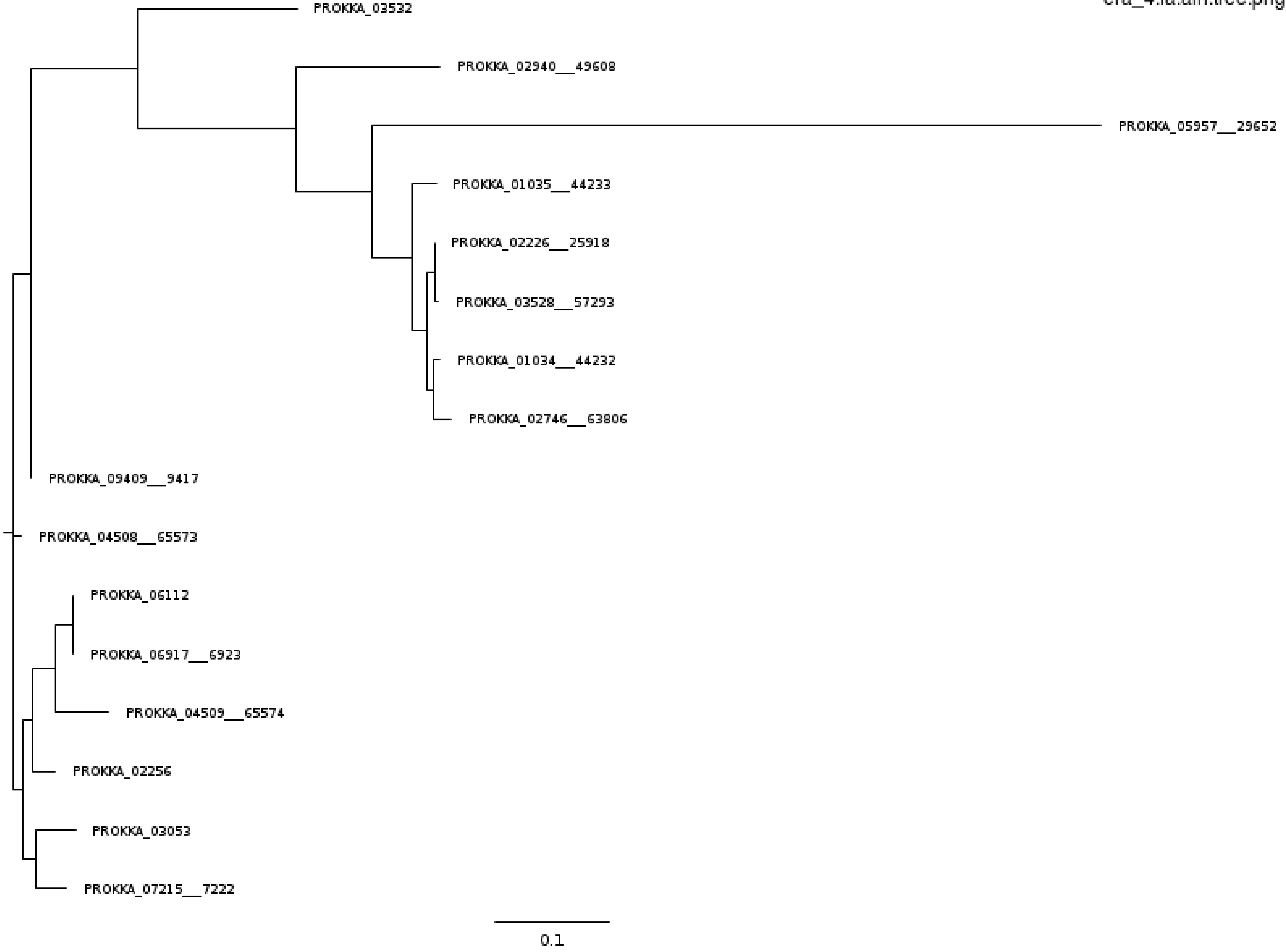

era_5.fa.aln.tree.png

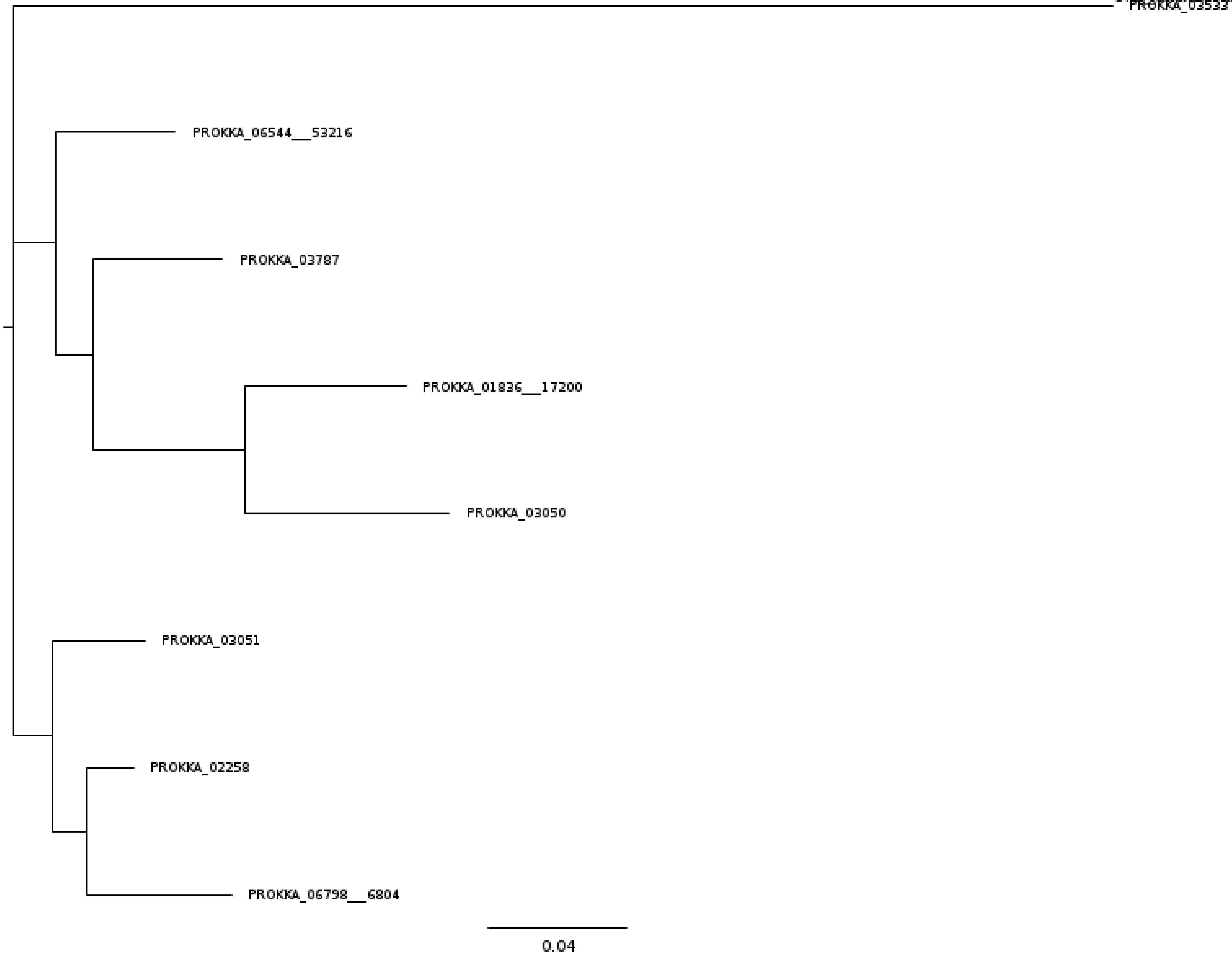

erpA_3.fa.aln.tree.png

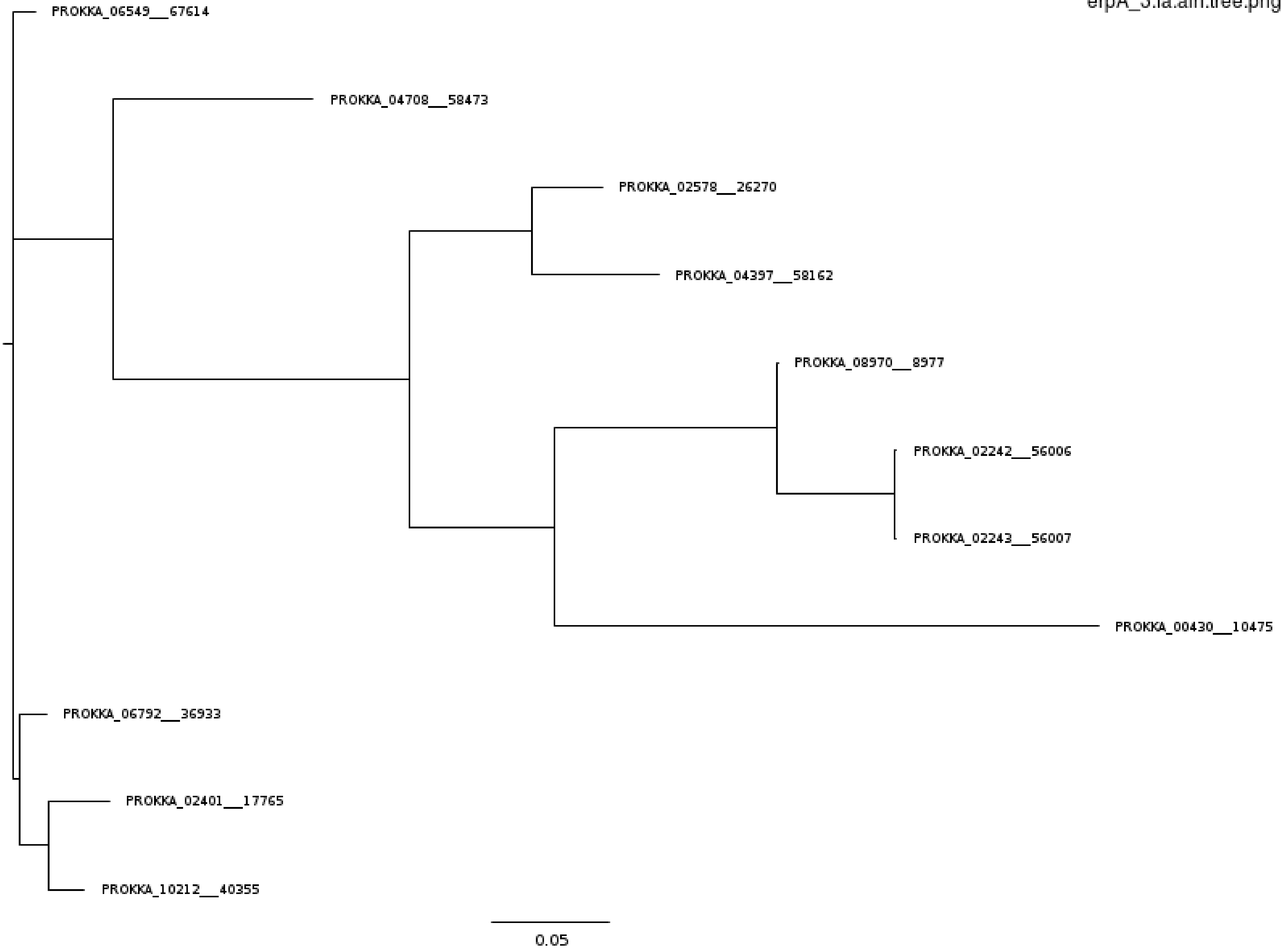

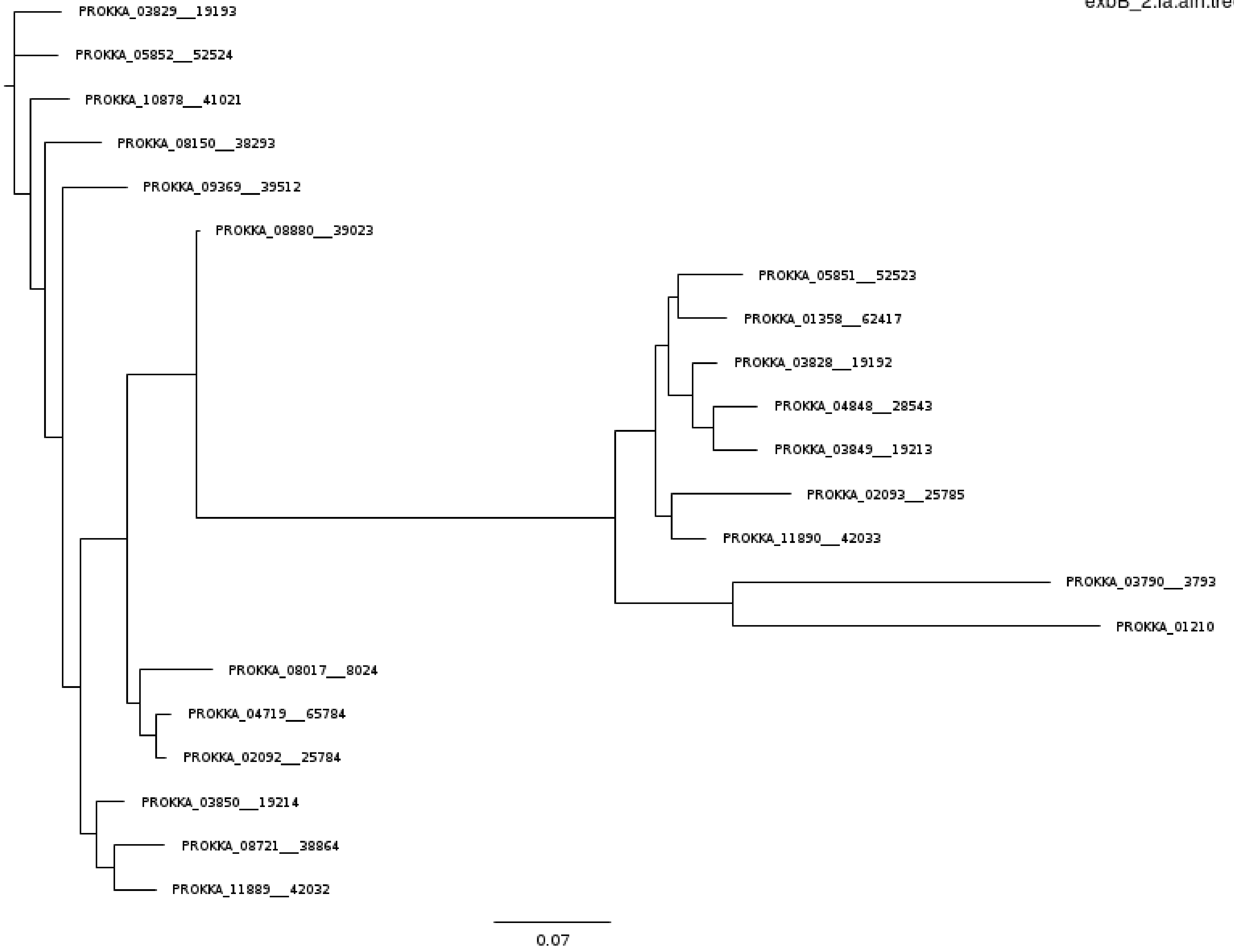
PROKKA_03829__19193
PROKKA_05852__52524
PROKKA_10878__41021
PROKKA_08150__38293
PROKKA_09369__39512
PROKKA_08880__39023
PROKKA_05851__52523
PROKKA_01358__62417
PROKKA_03828__19192
PROKKA_04848__28543
PROKKA_03849__19213
PROKKA_02093__25785
PROKKA_11890__42033
PROKKA_03790__3793
PROKKA_01210
PROKKA_08017__8024
PROKKA_04719__65784
PROKKA_02092__25784
PROKKA_03850__19214
PROKKA_08721__38864
PROKKA_11889__42032
0.07

exbB_3.fa.aln.tree.png

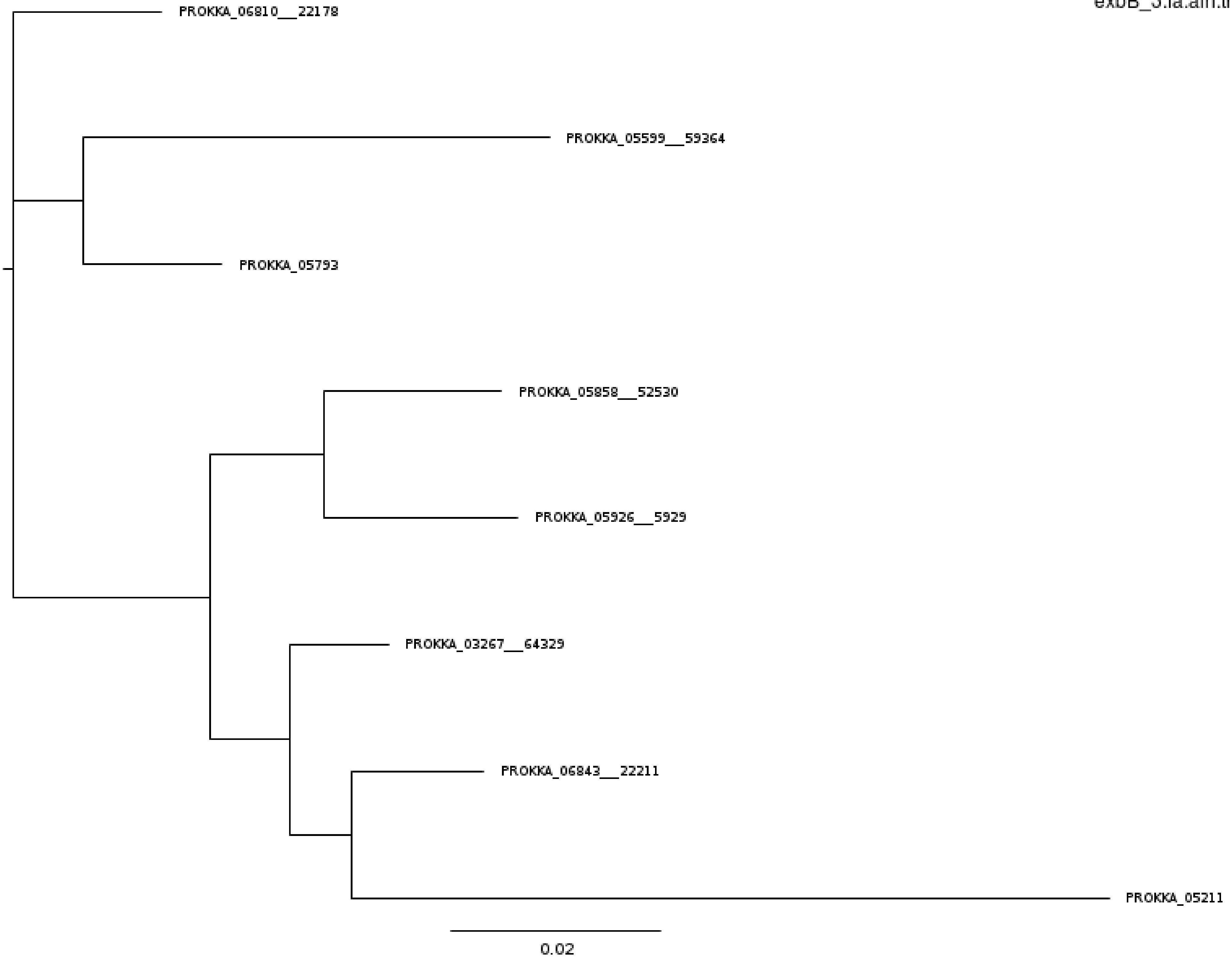

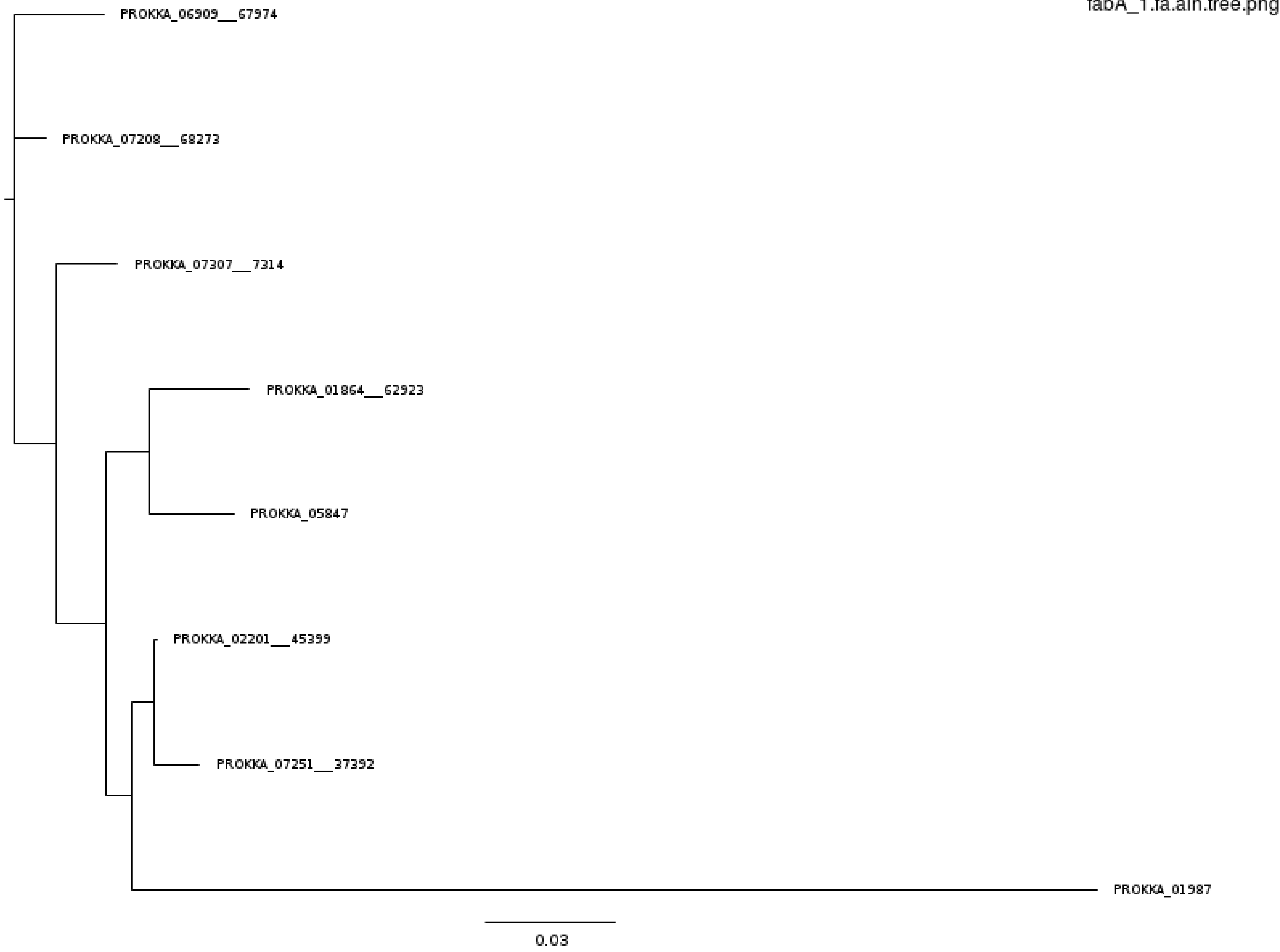
PROKKA_06909___67974
PROKKA_07208___68273
PROKKA_07307___7314
PROKKA_01864___62923
PROKKA_05847
PROKKA_02201___45399
PROKKA_07251___37392
PROKKA_01987
0.03

fabD_2.fa.aln.tree.png

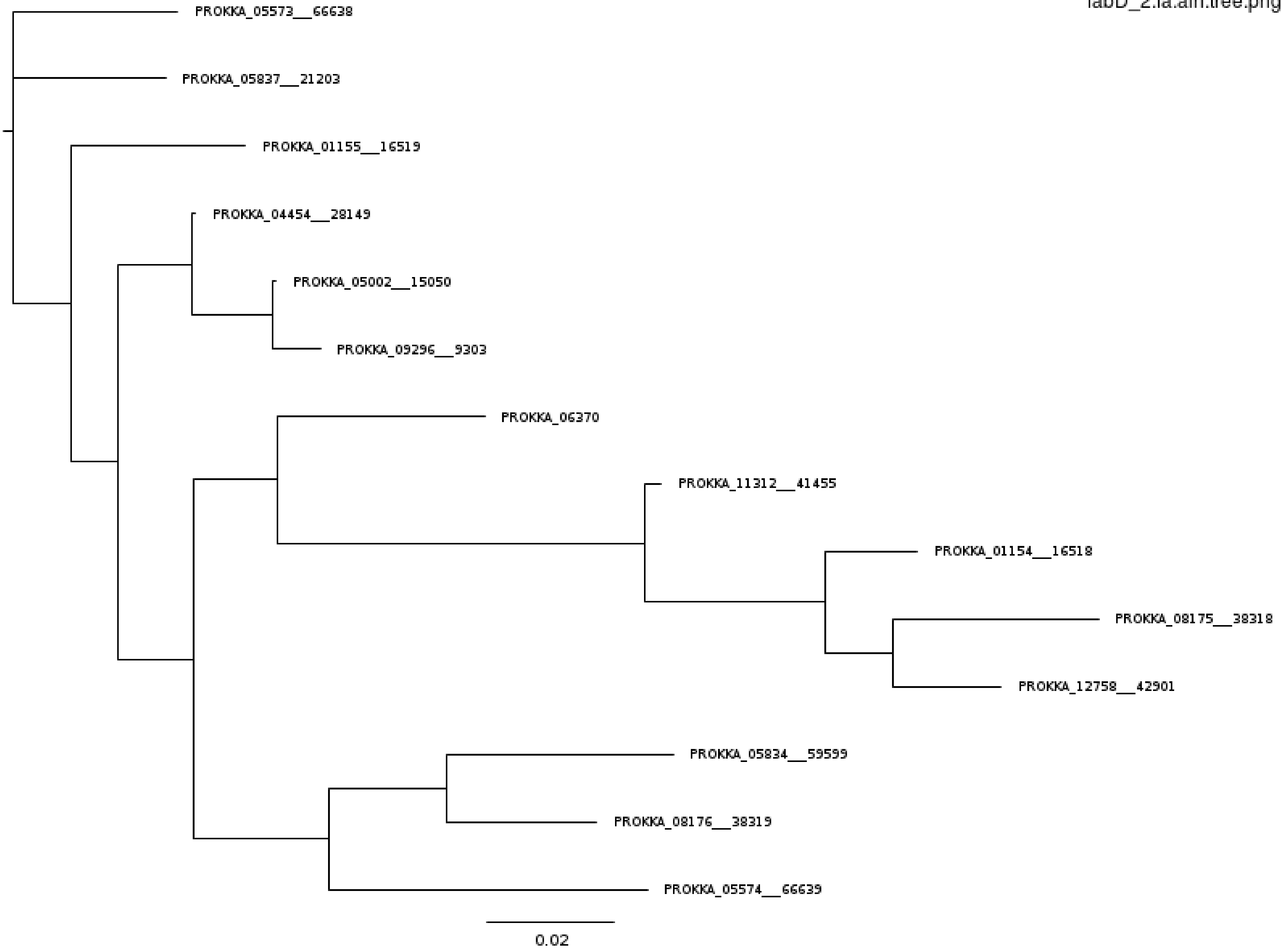

fabF_1.fa.aln.tree.png

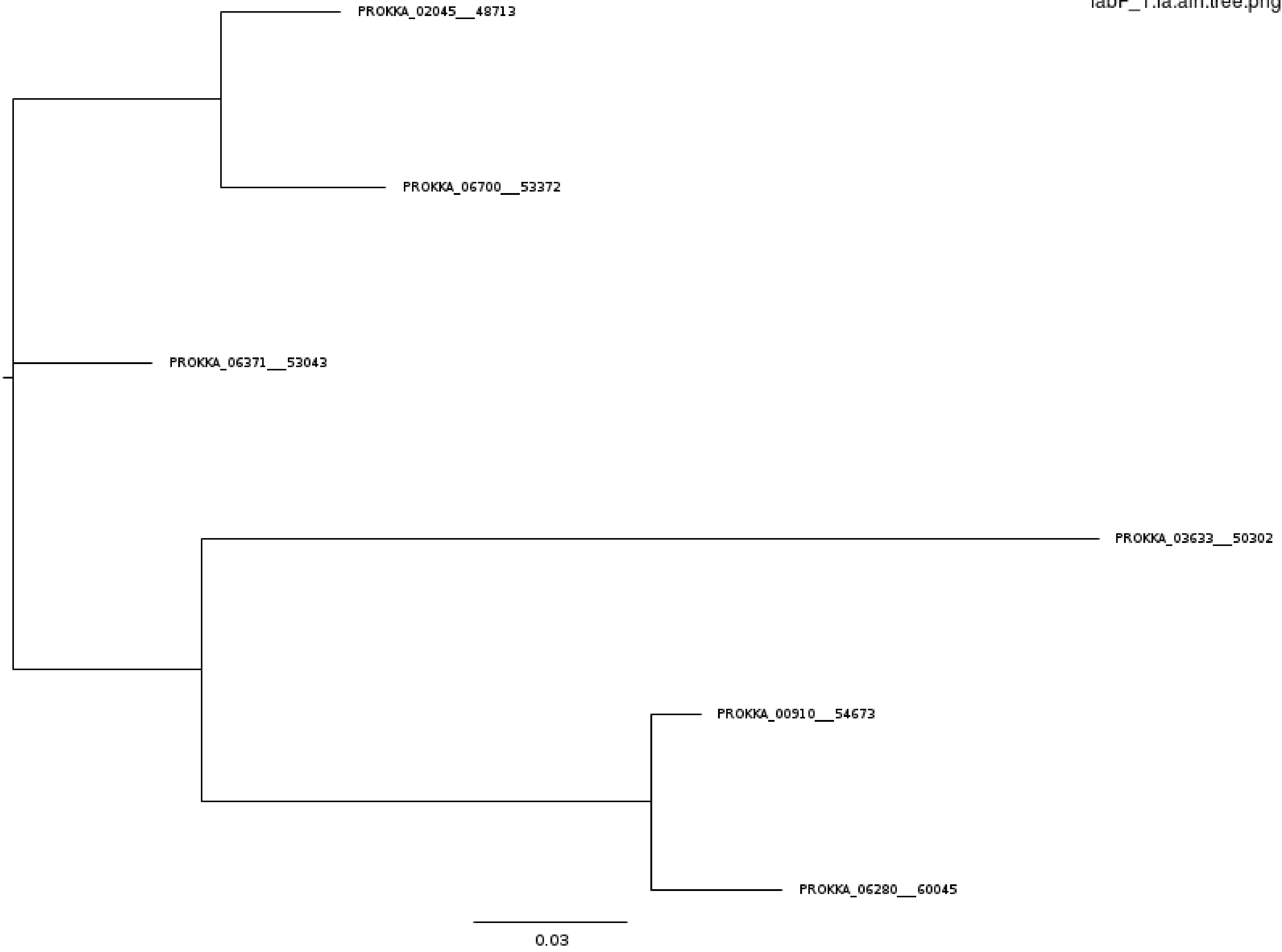

fabF_6.fa.aln.tree.png

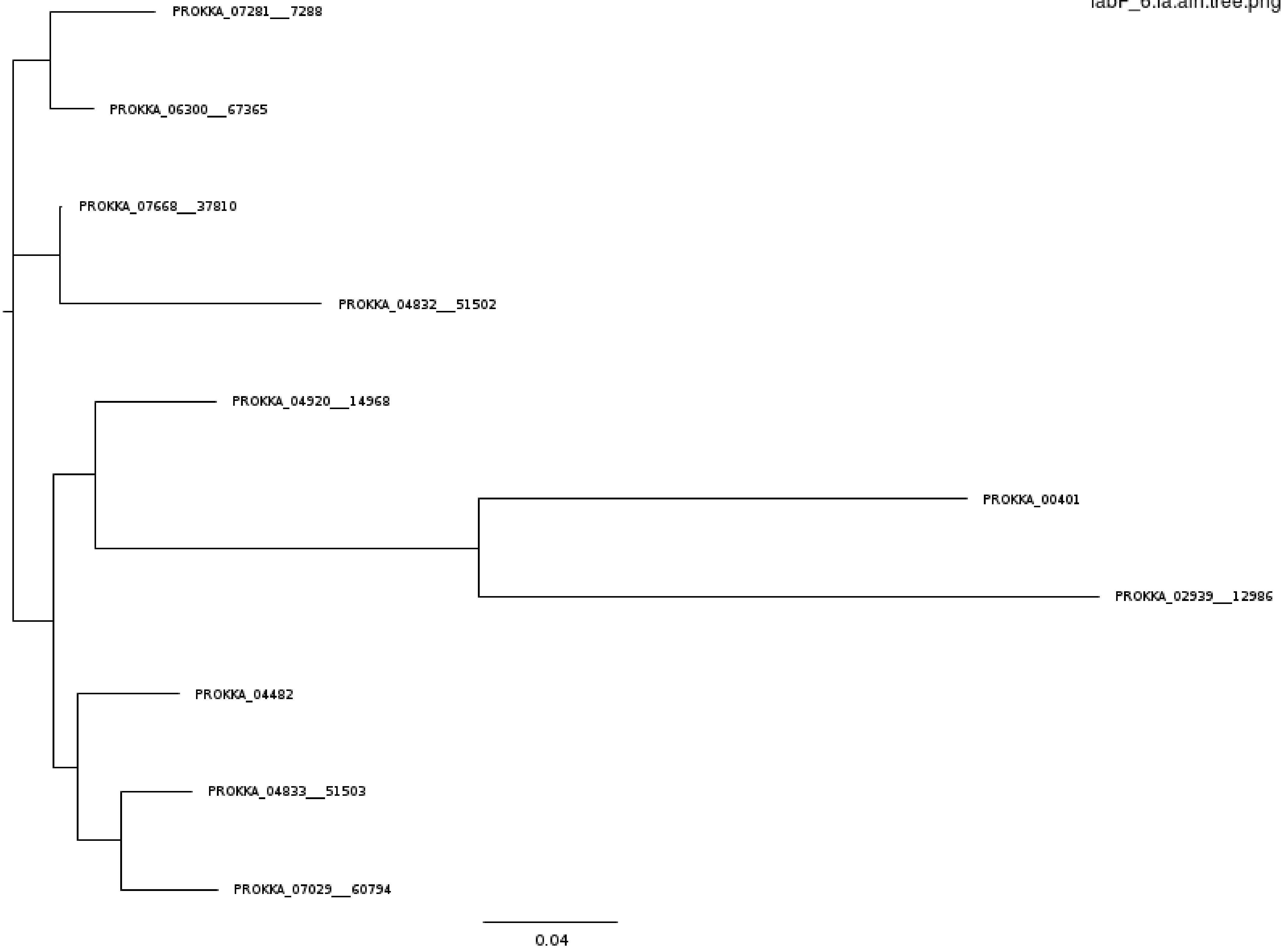

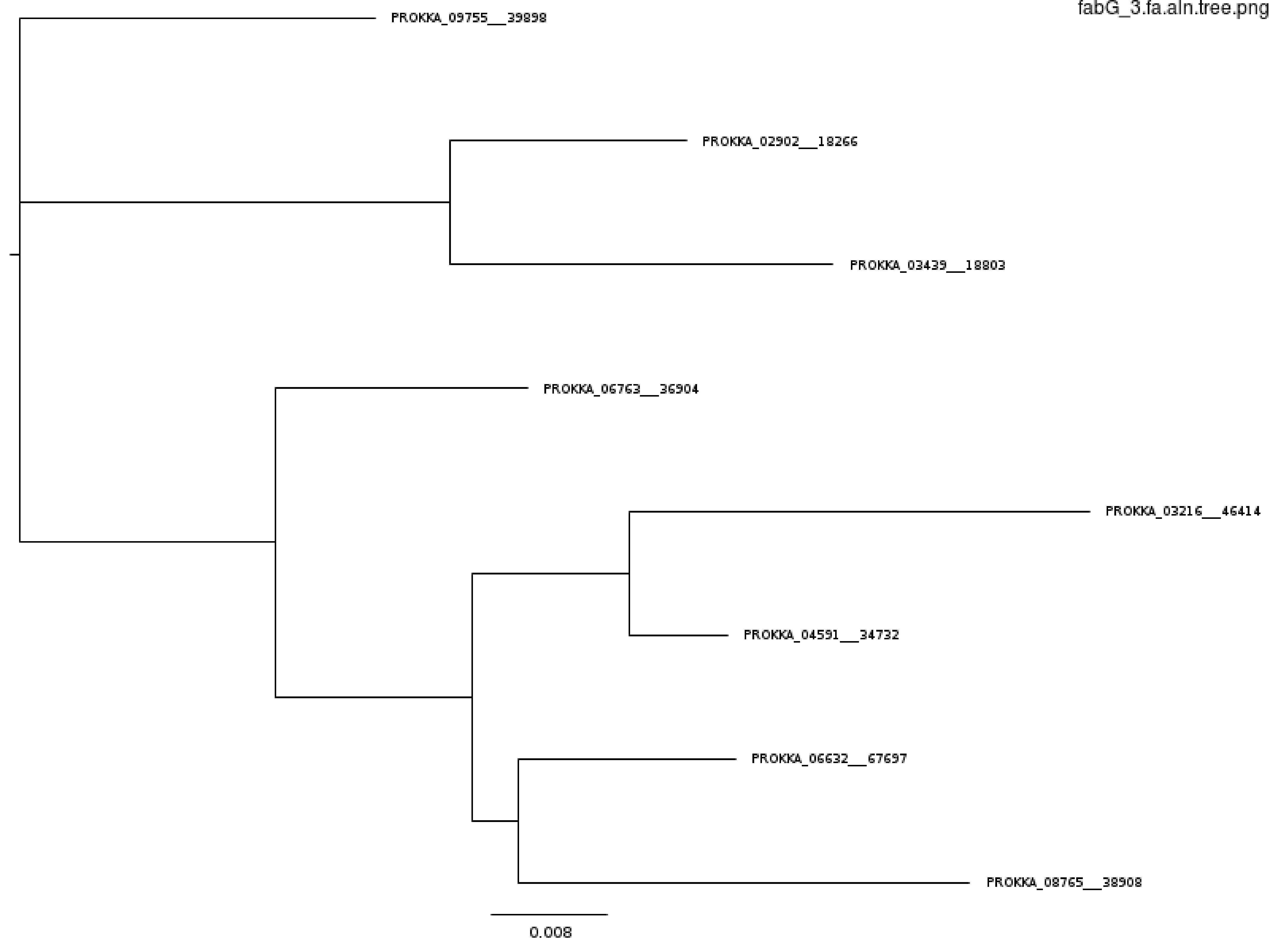
fabG_3.fa.aln.tree.png
PROKKA_09755__39898
PROKKA_02902__18266
PROKKA_03439__18803
PROKKA_06763__36904
PROKKA_03216__46414
PROKKA_04591__34732
PROKKA_06632__67697
PROKKA_08765__38908
0.008

fabG_8.fa.aln.tree.png

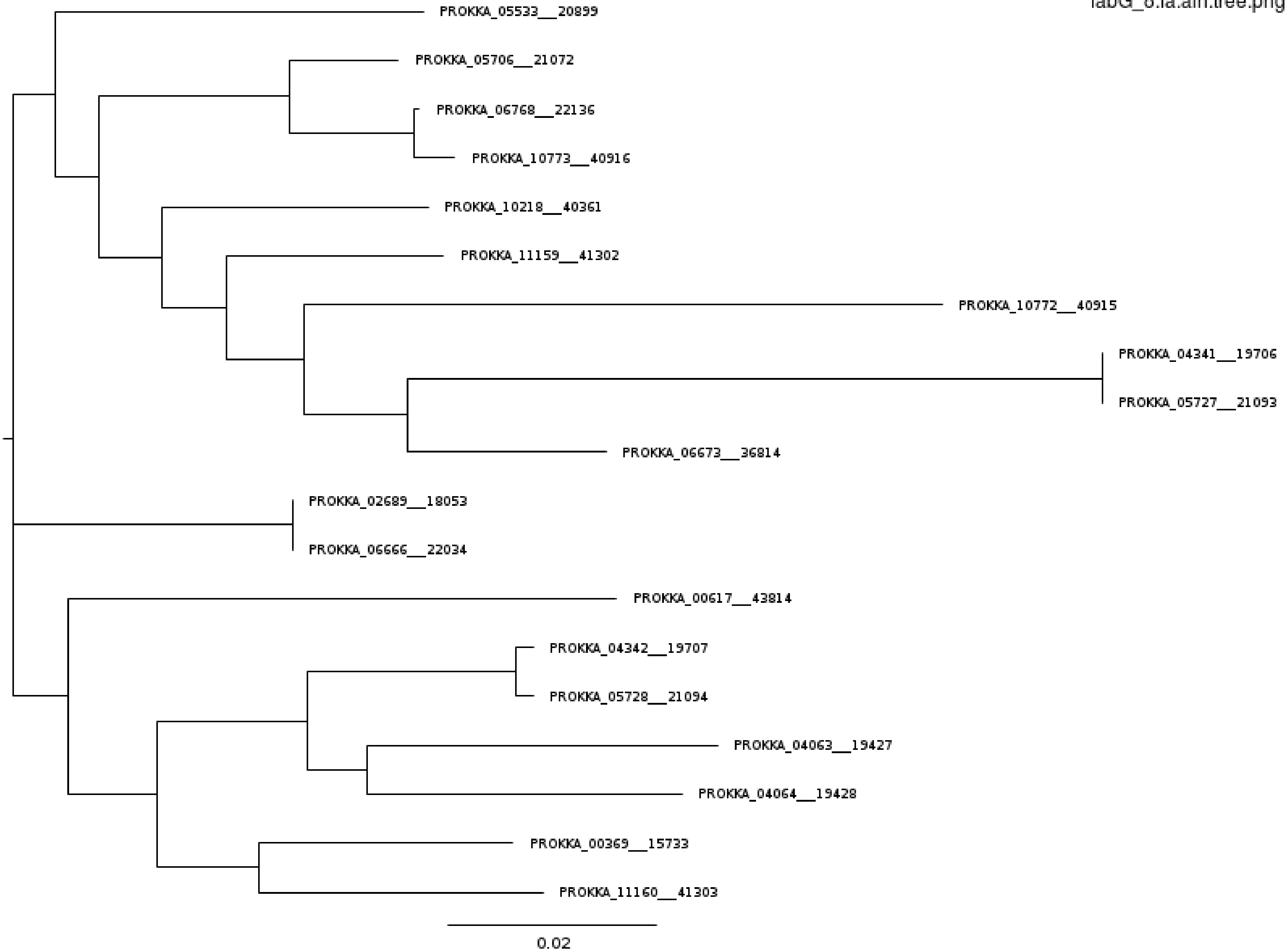

fabH_3.fa.aln.tree.png

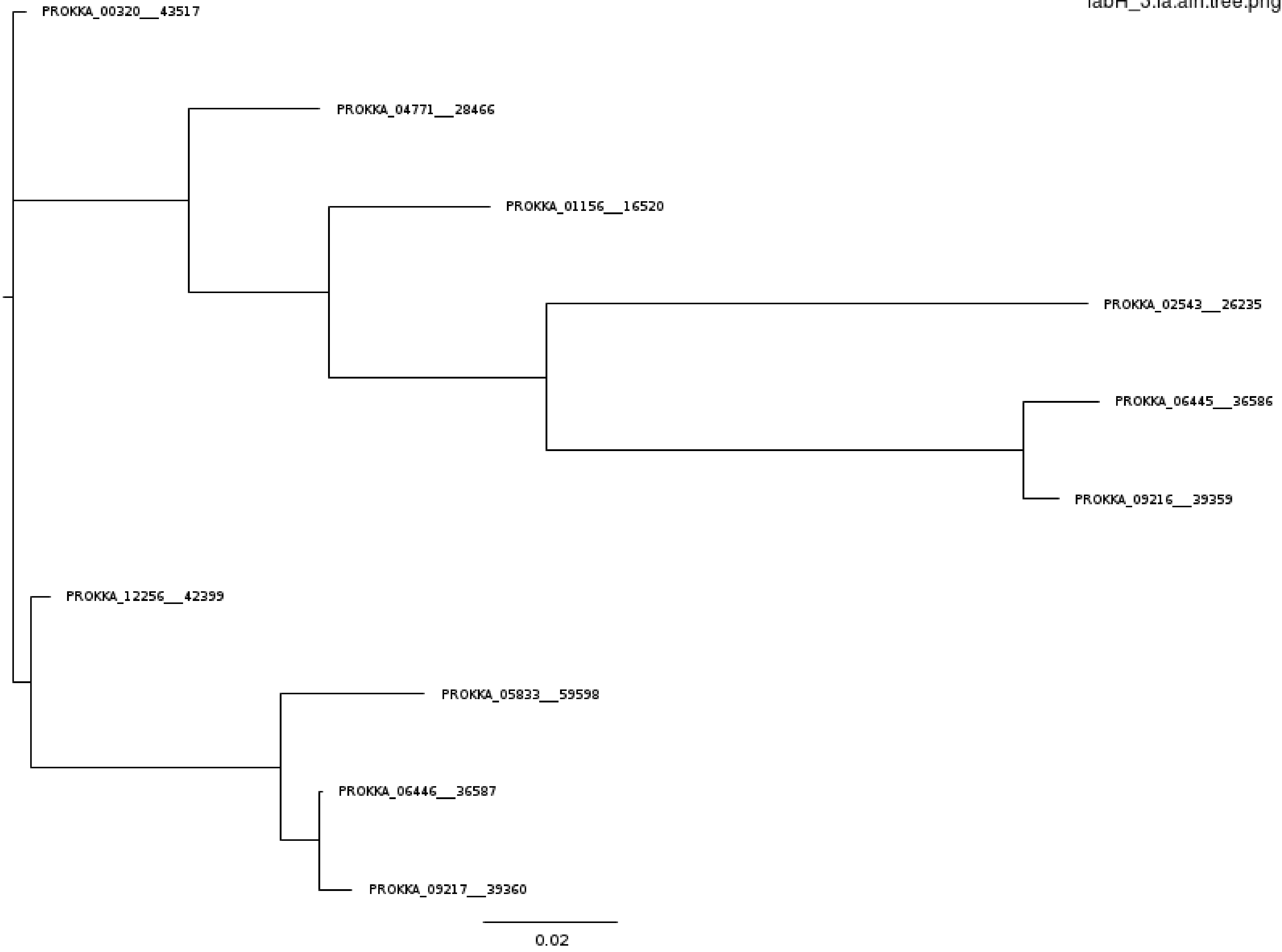

fabI_1.fa.aln.tree.png

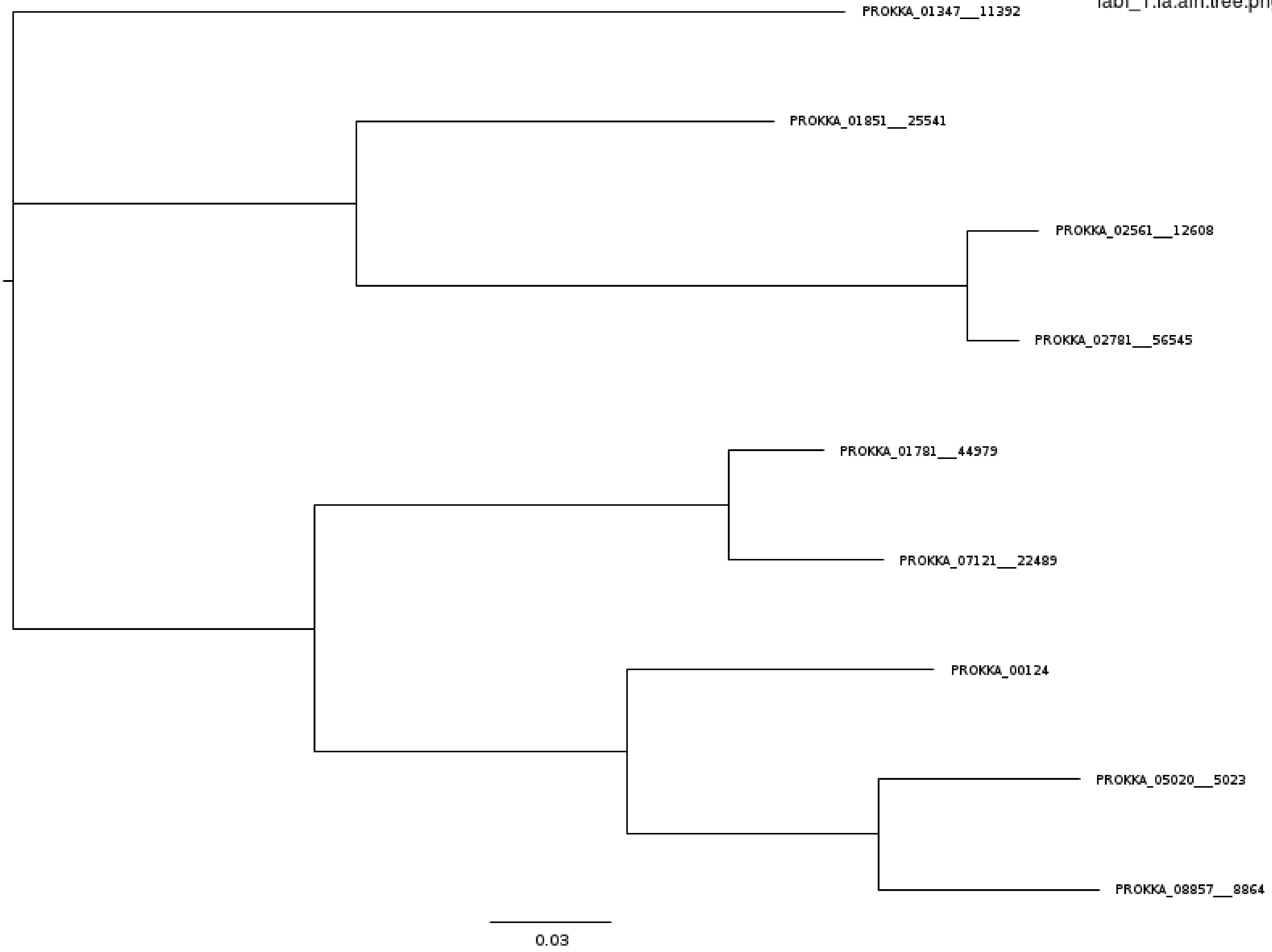

fabZ_2.fa.aln.tree.png

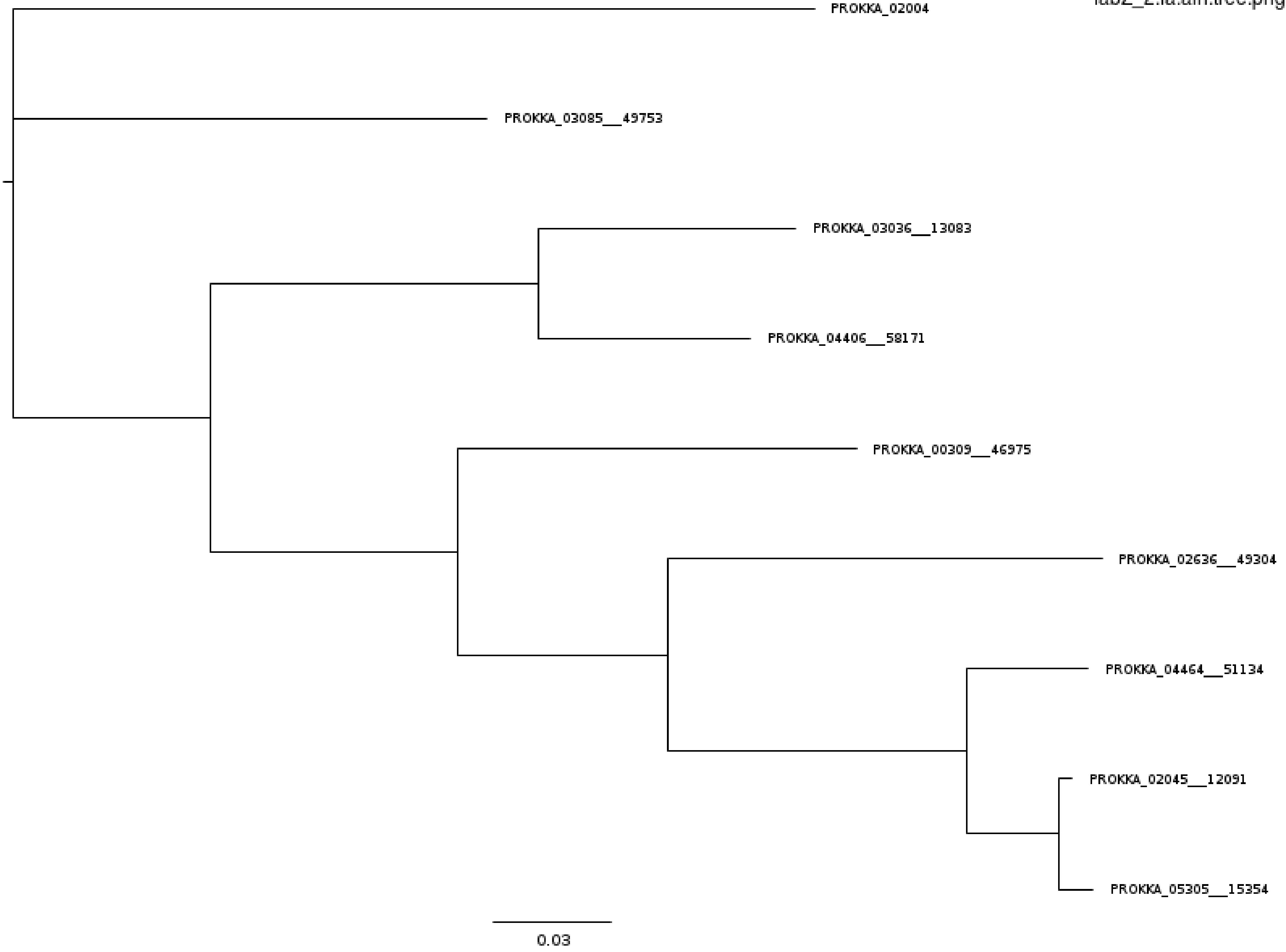

fbcH.fa.aln.tree.png

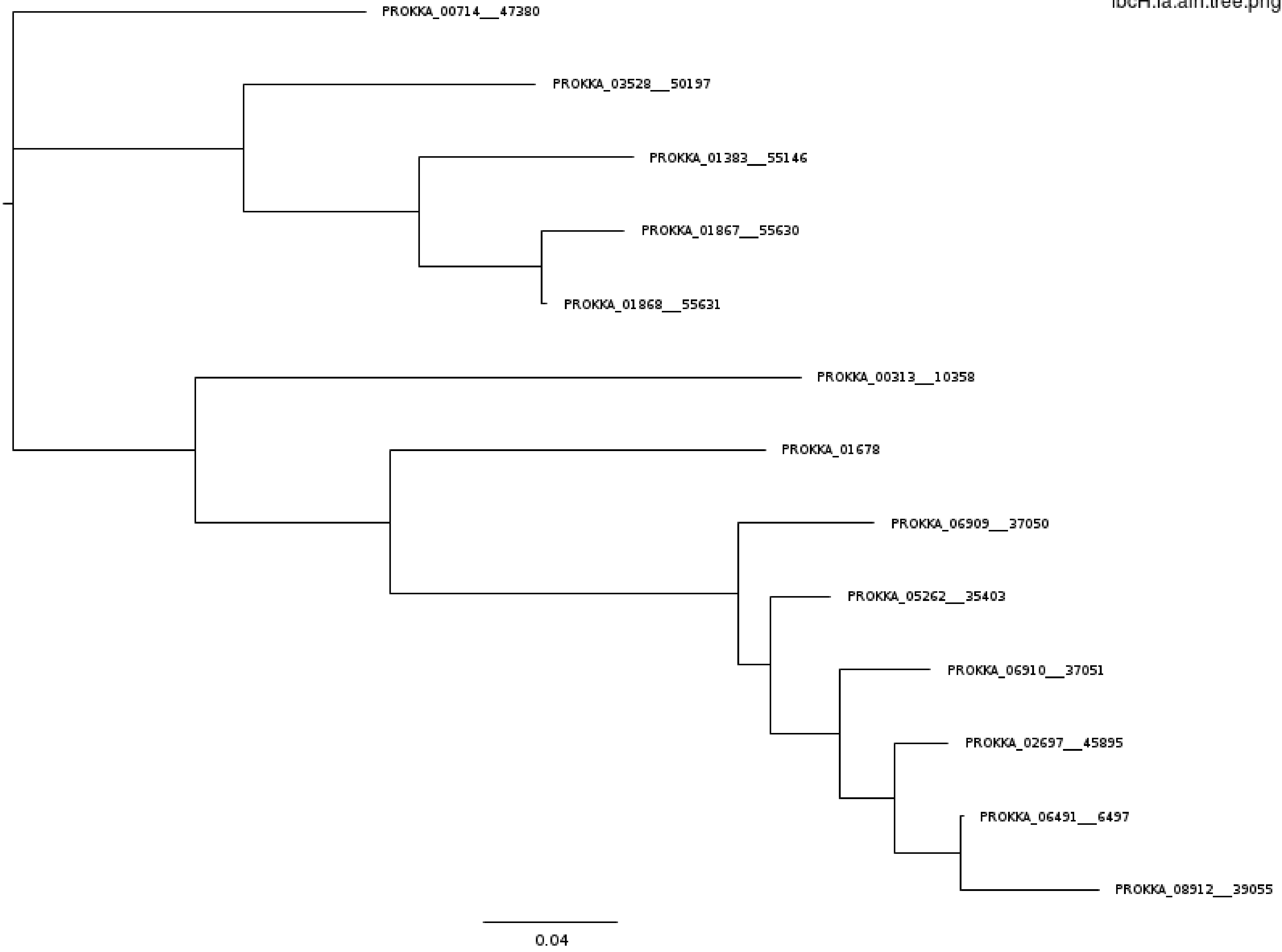

fcf1.fa.aln.tree.png

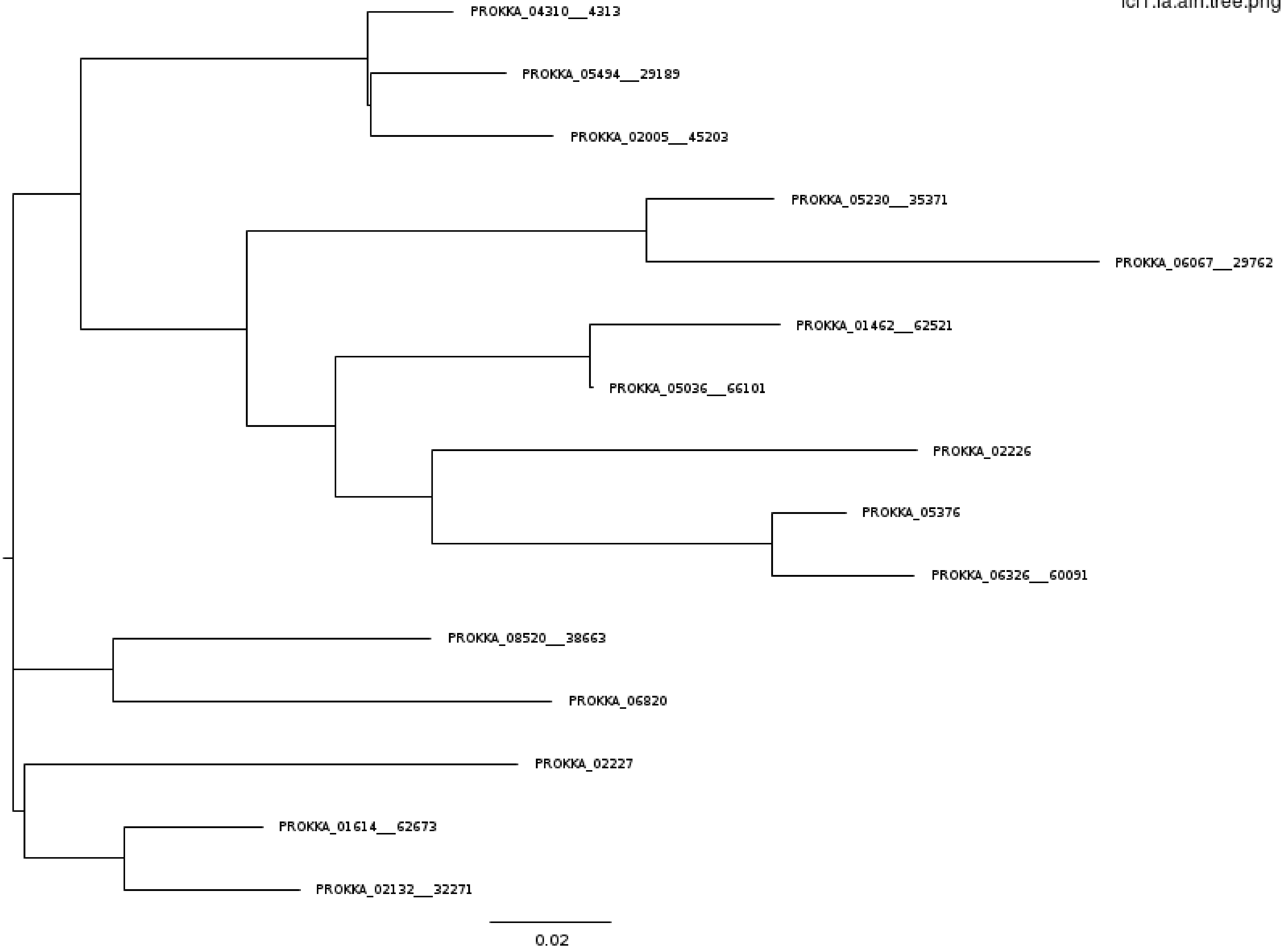

fchA_4.fa.aln.tree.png

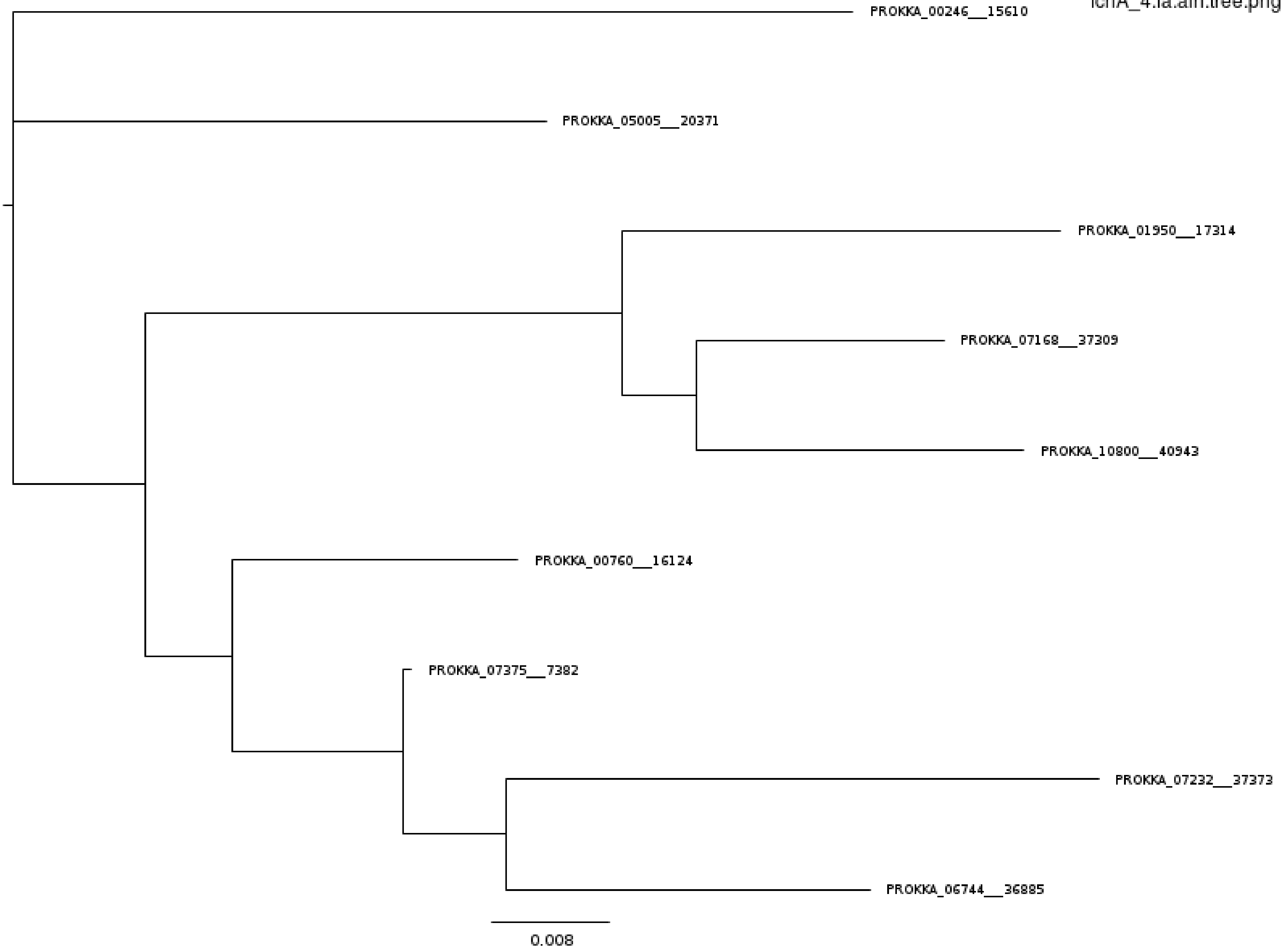

fcl.fa.aln.tree.png

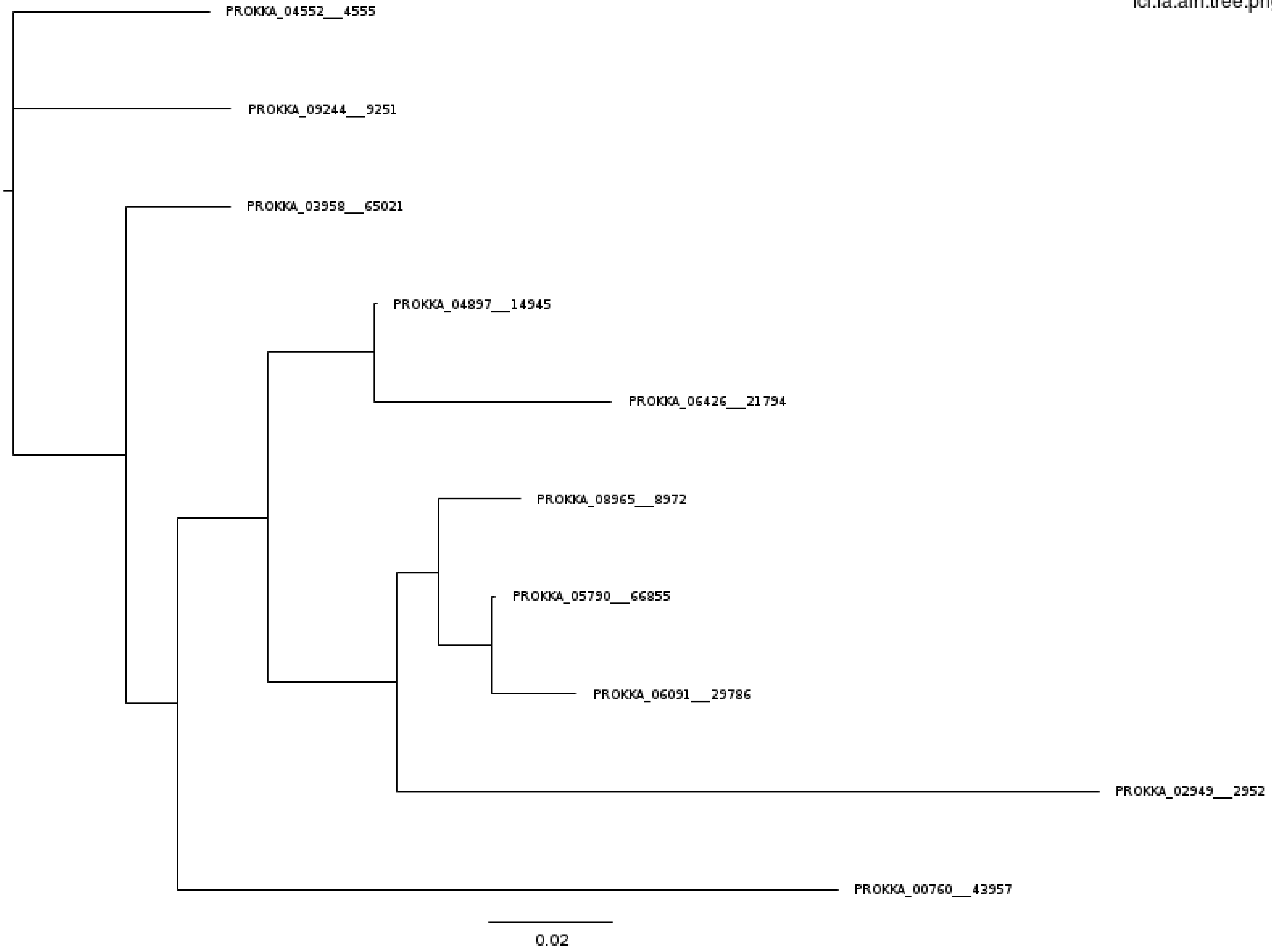

fda.fa.aln.tree.png

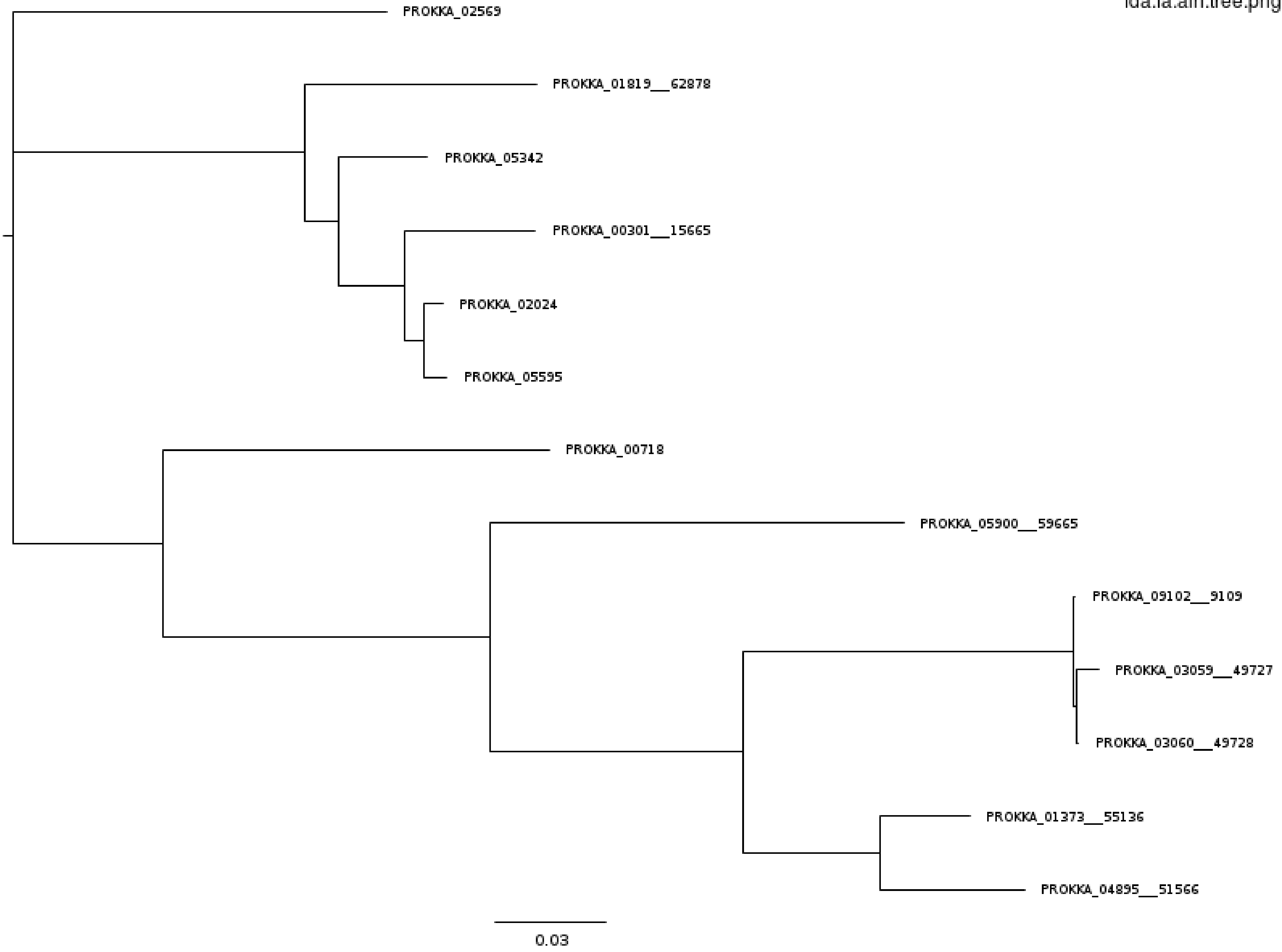

fdx_3.fa.aln.tree.png

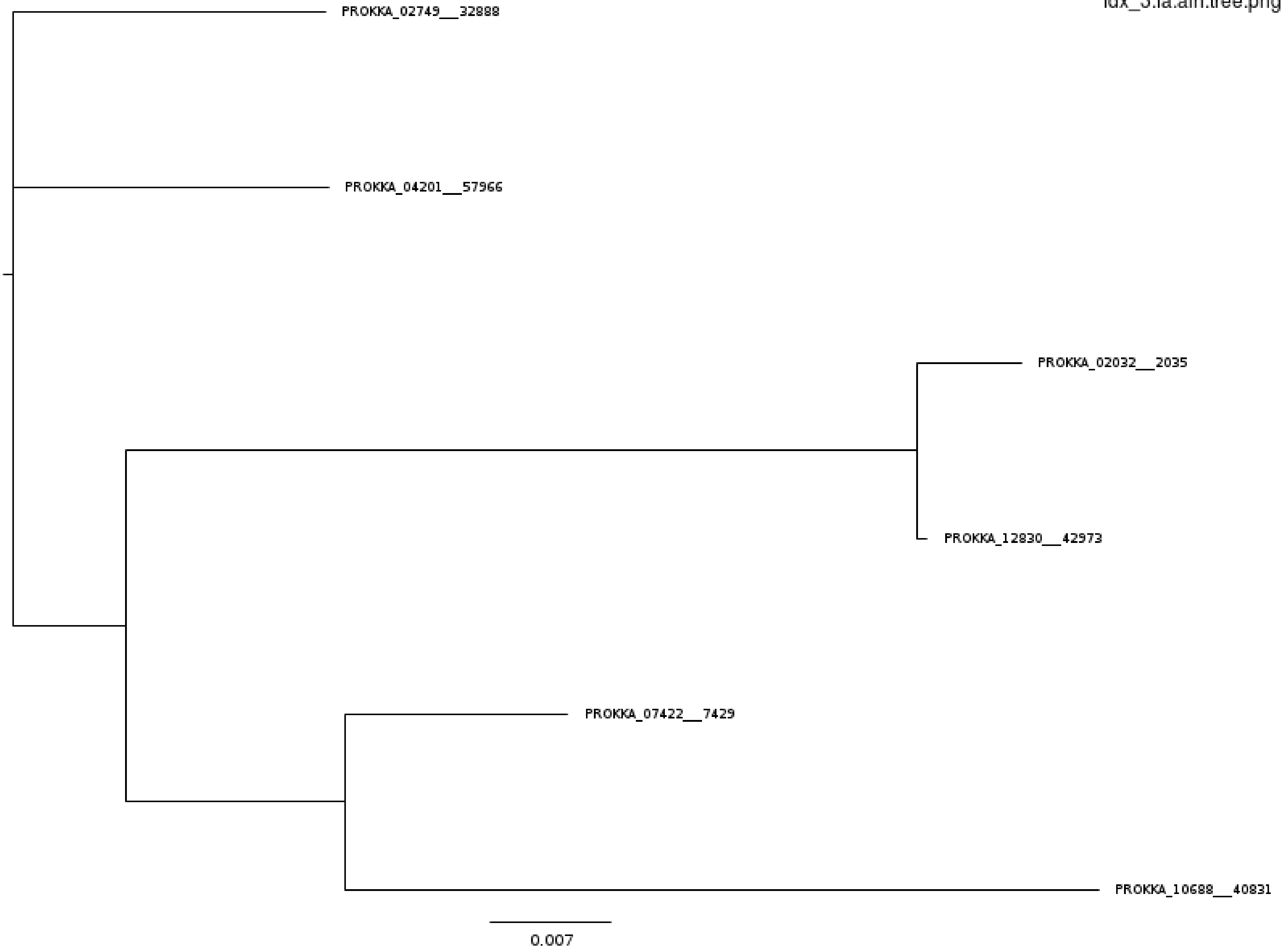

fdx_4.fa.aln.tree.png

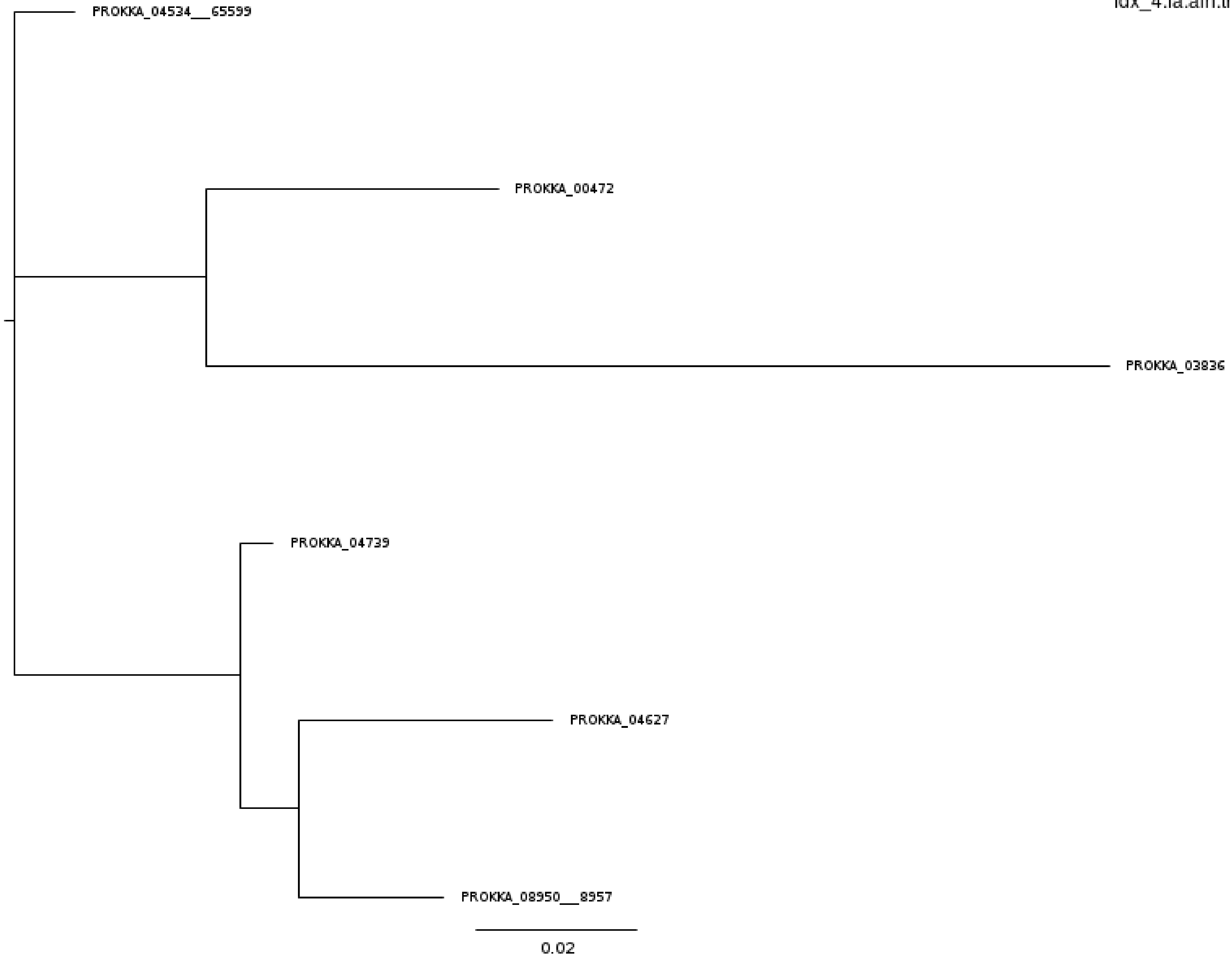

feoA.fa.aln.tree.png

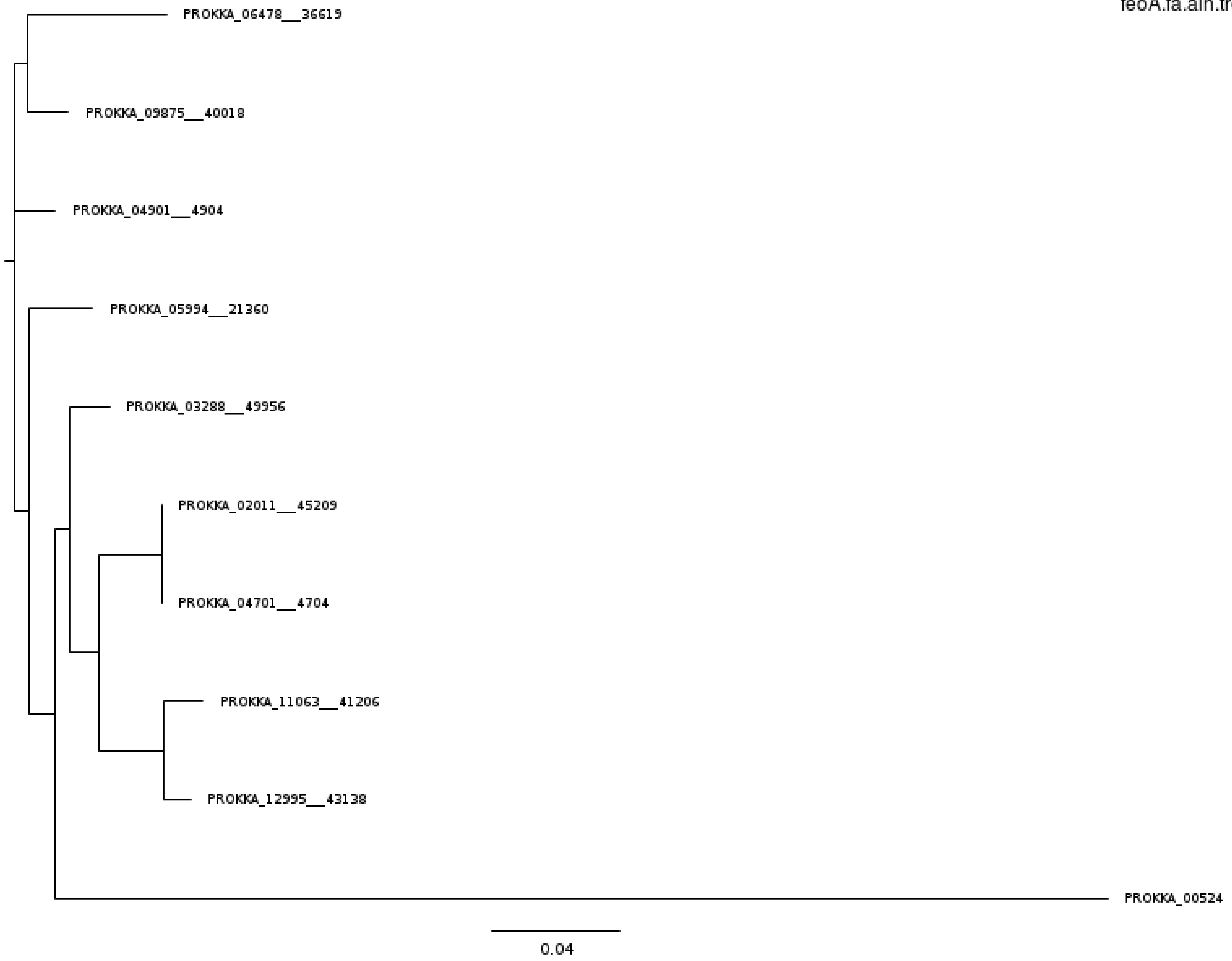

feoB_2.fa.aln.tree.png

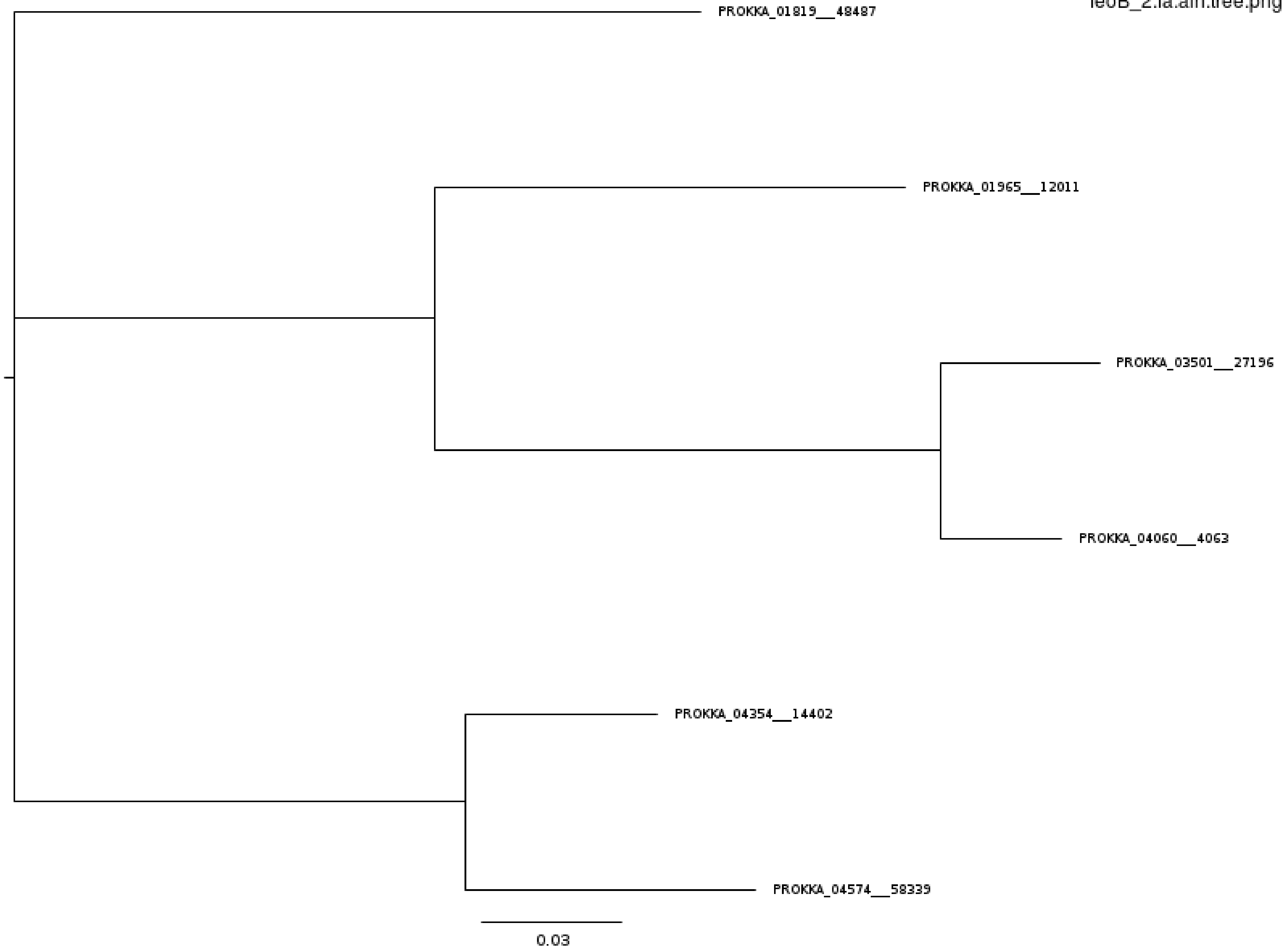

feoB_4.fa.aln.tree.png

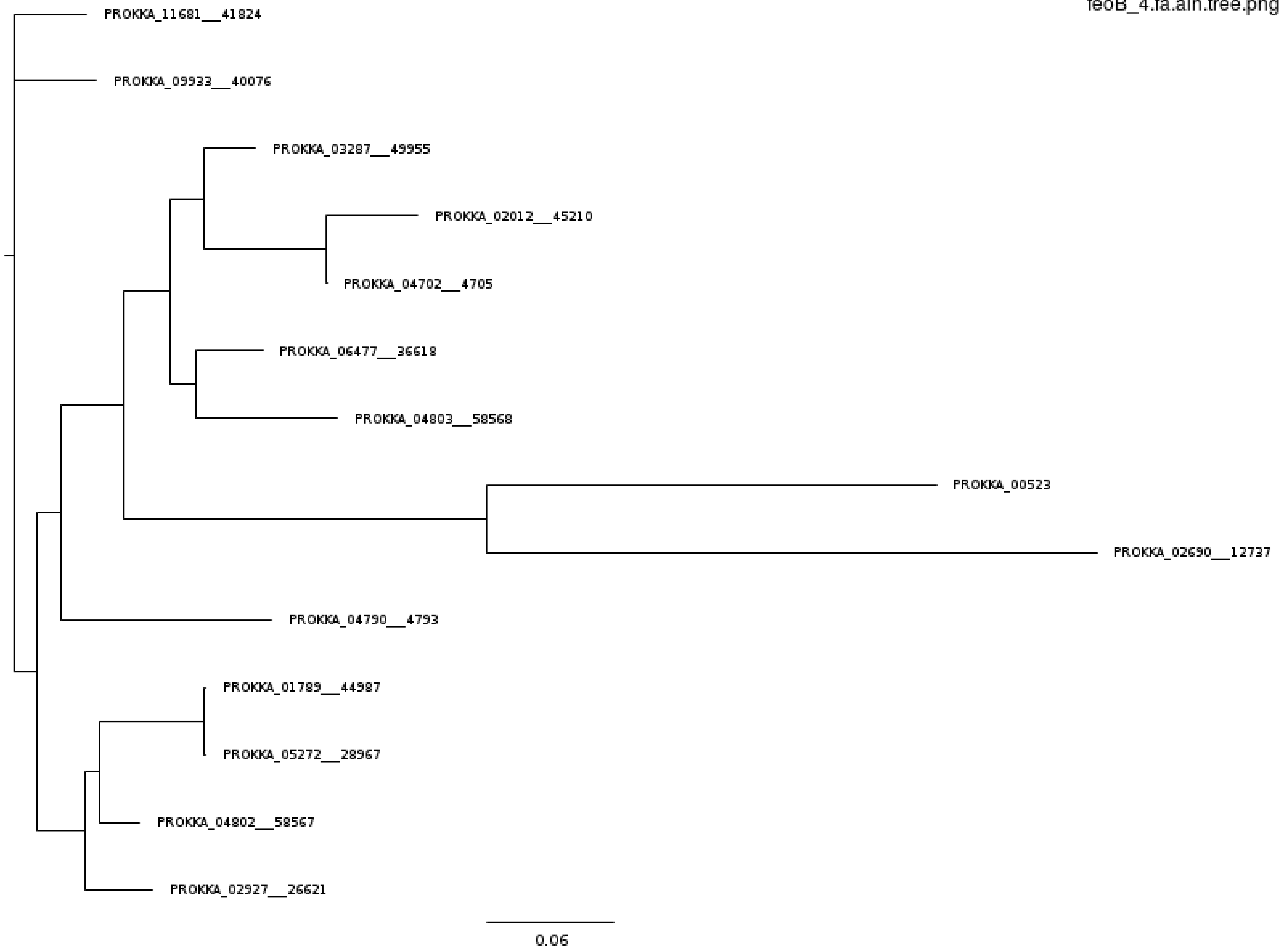

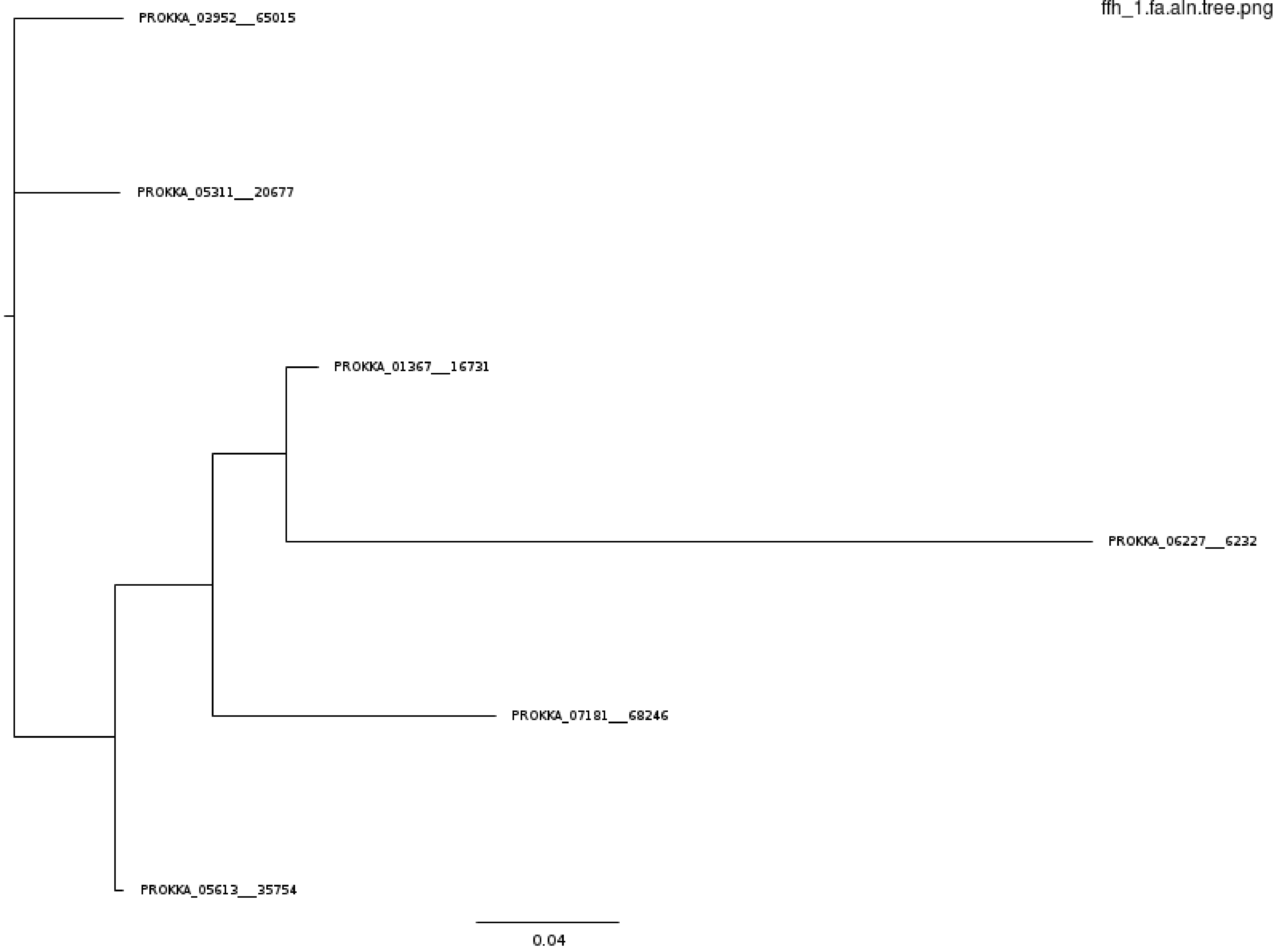
ffh_1.fa.aln.tree.png
PROKKA_03952___65015
PROKKA_05311___20677
PROKKA_01367___16731
PROKKA_06227___6232
PROKKA_07181___68246
PROKKA_05613___35754
0.04

fieF_2.fa.aln.tree.png

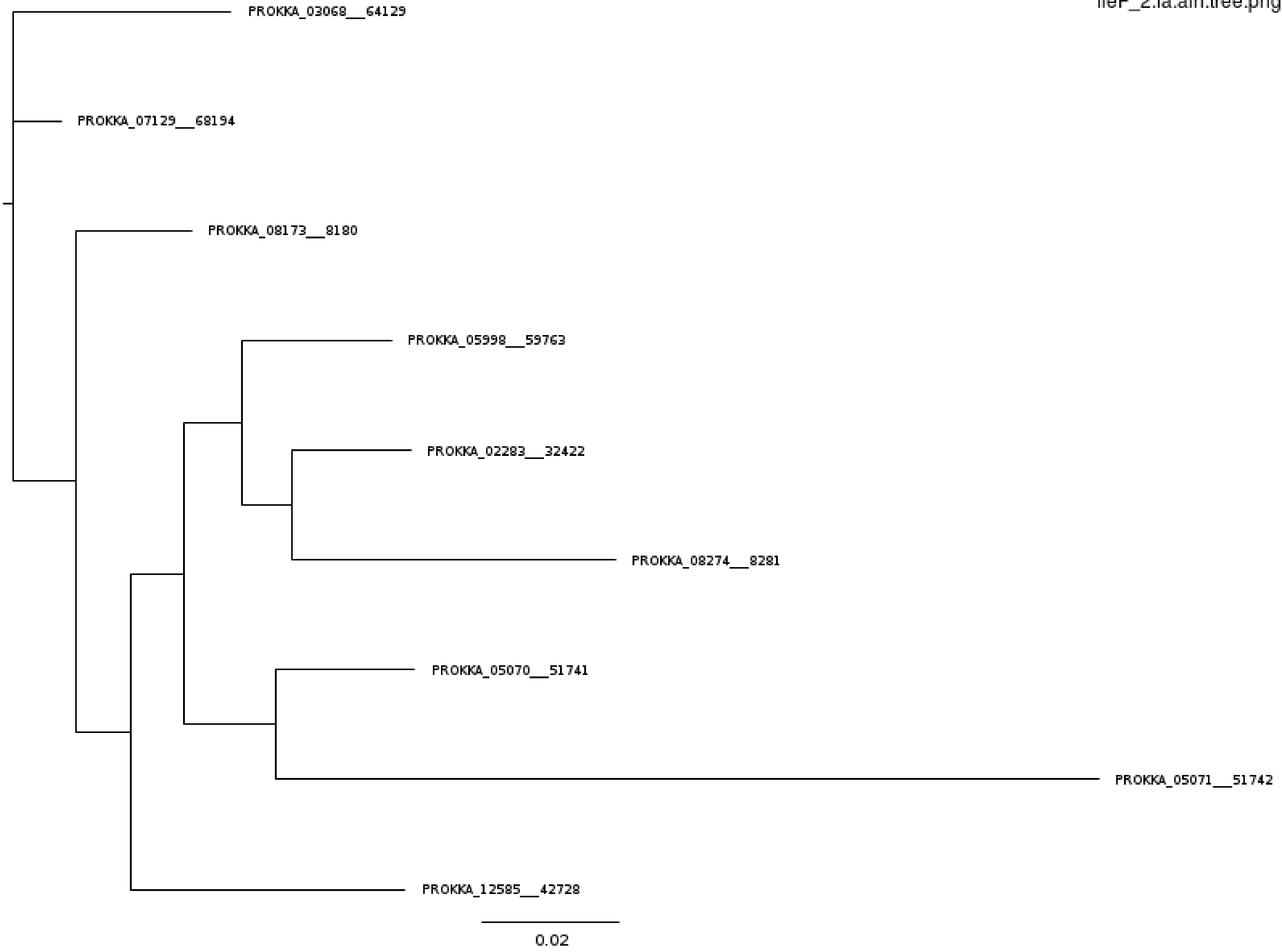

fitB_3.fa.aln.tree.png

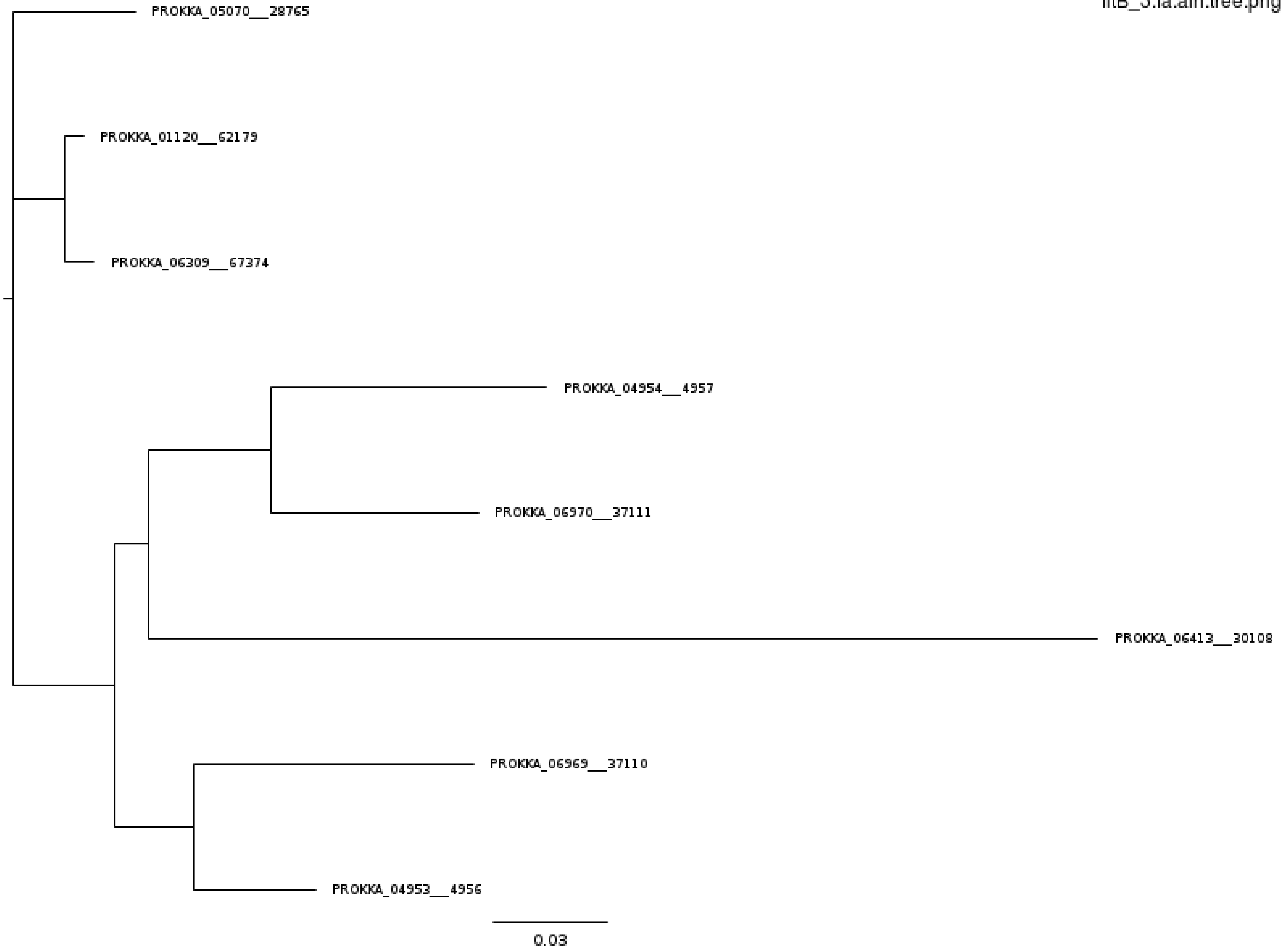

fitB_4.fa.aln.tree.png

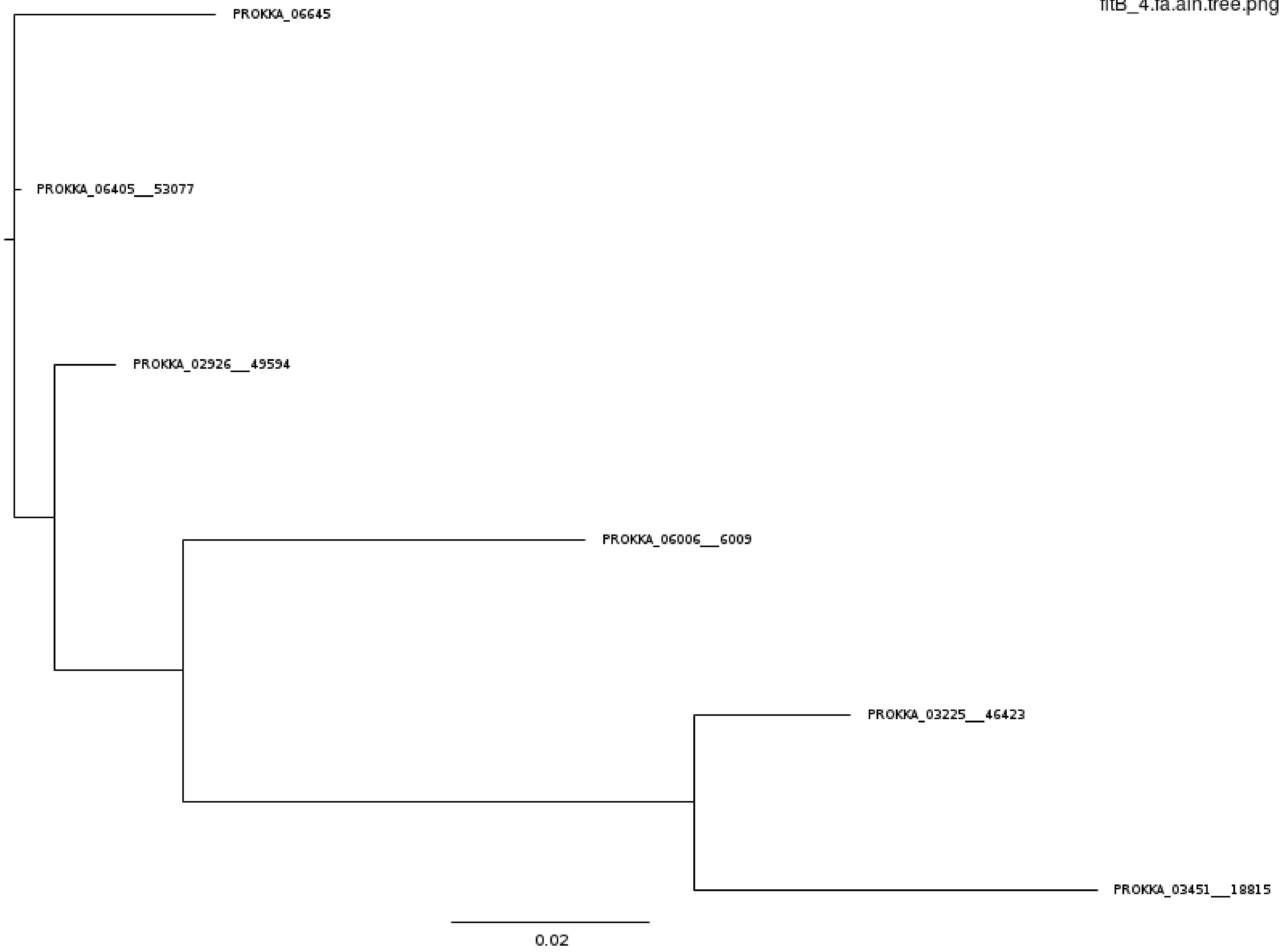

fitB_5.fa.aln.tree.png

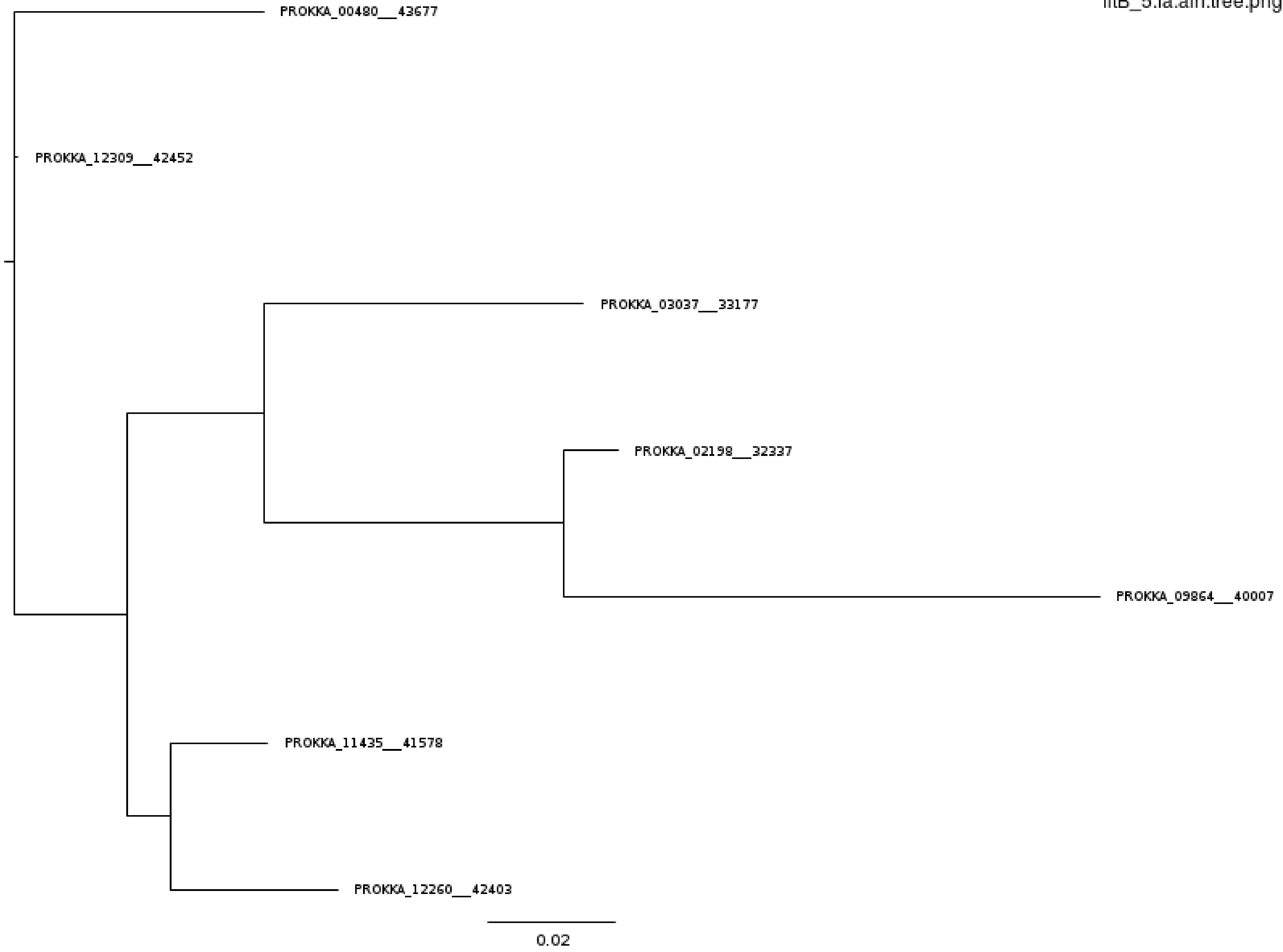

fixK_2.fa.aln.tree.png

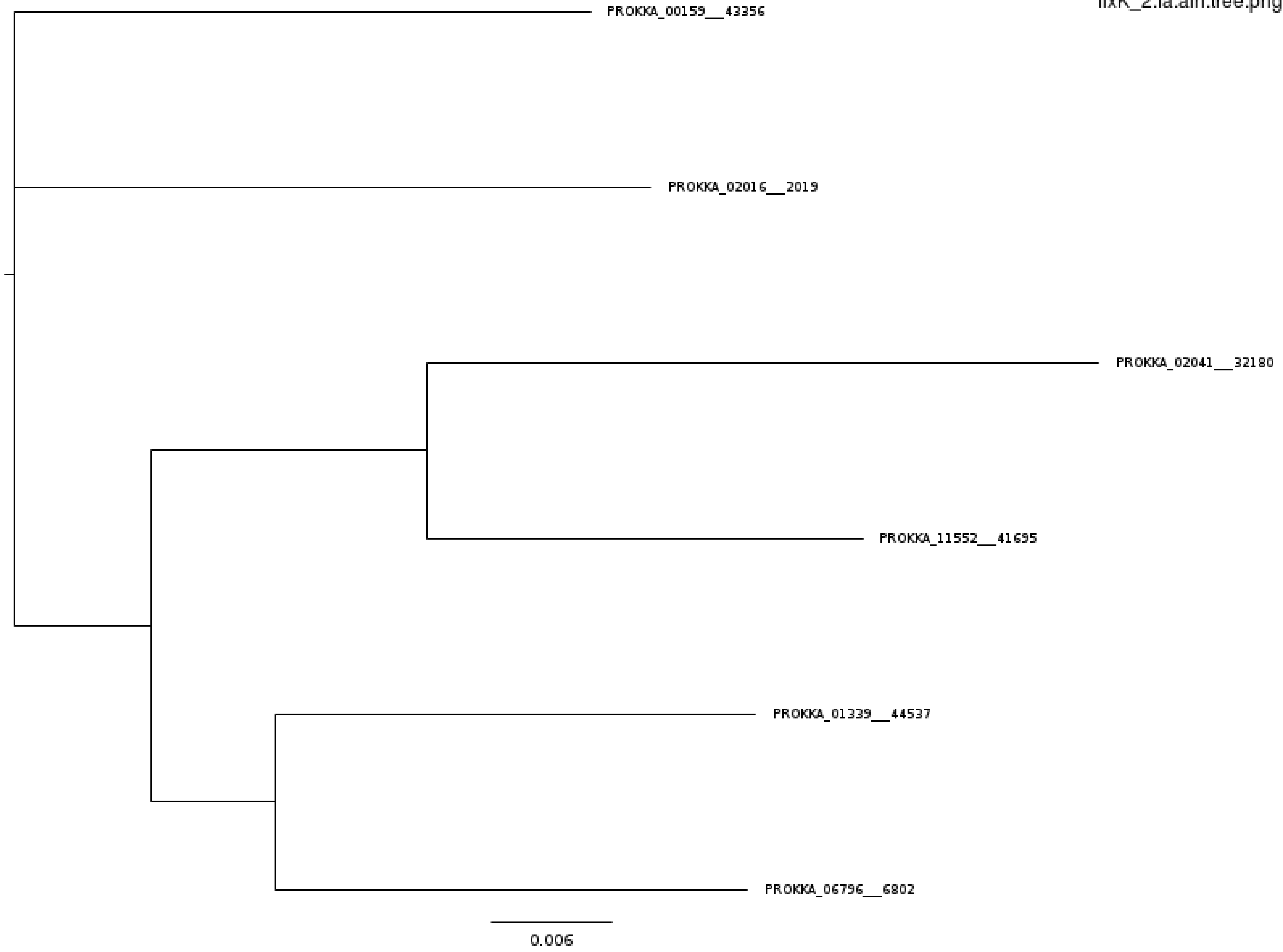

fmt_3.fa.aln.tree.png

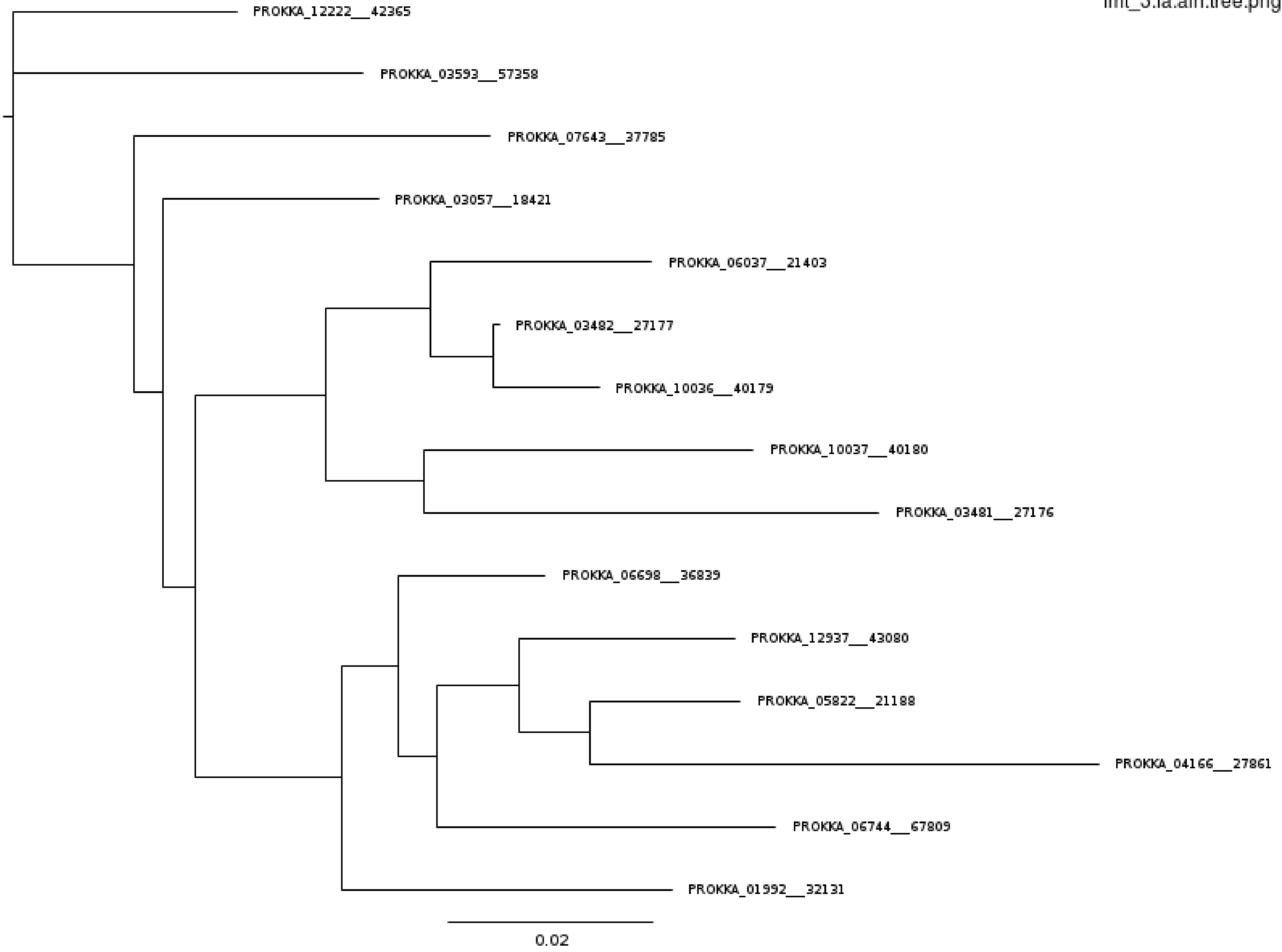

folB_1.fa.aln.tree.png

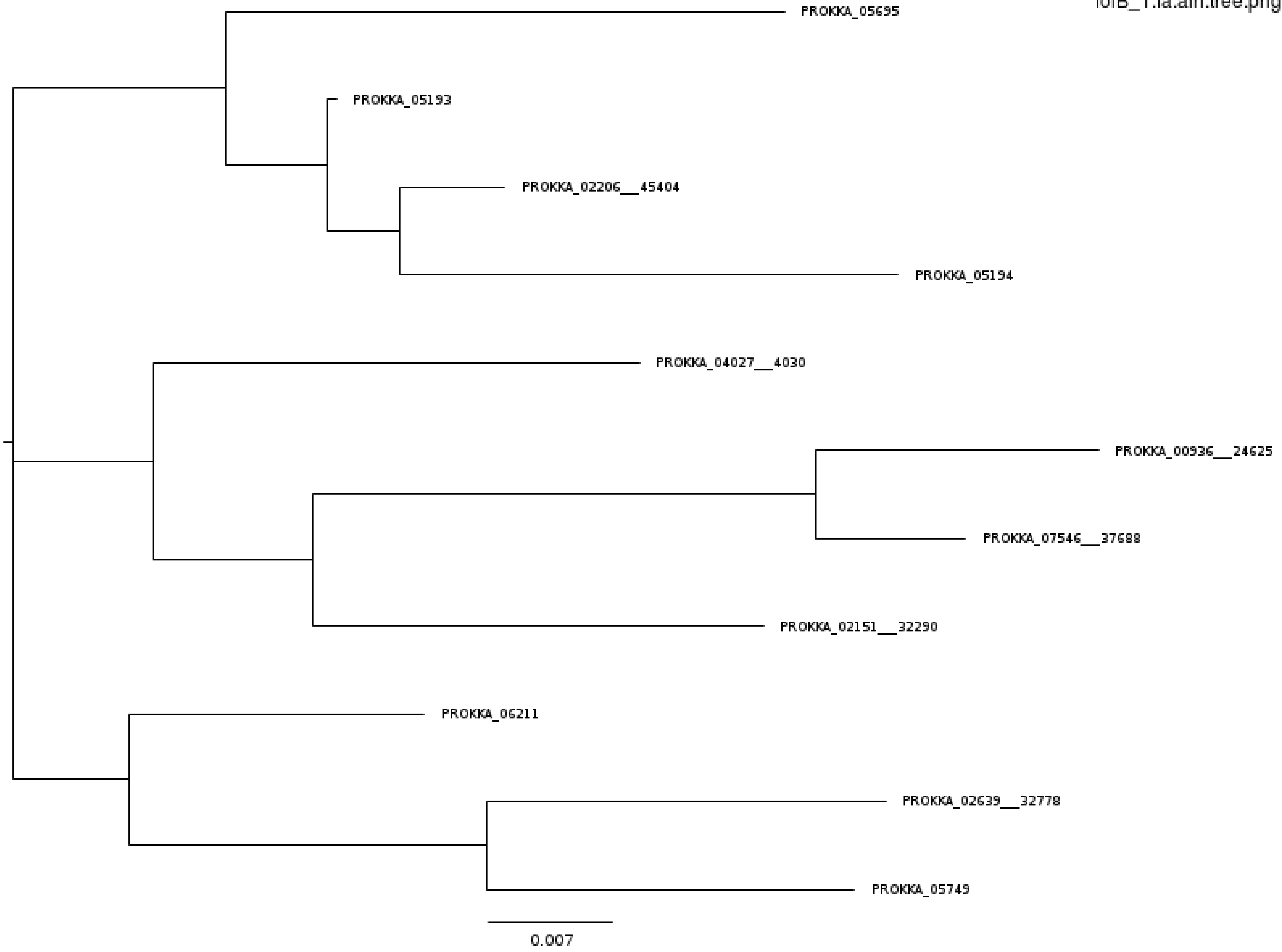

folC_2.fa.aln.tree.png

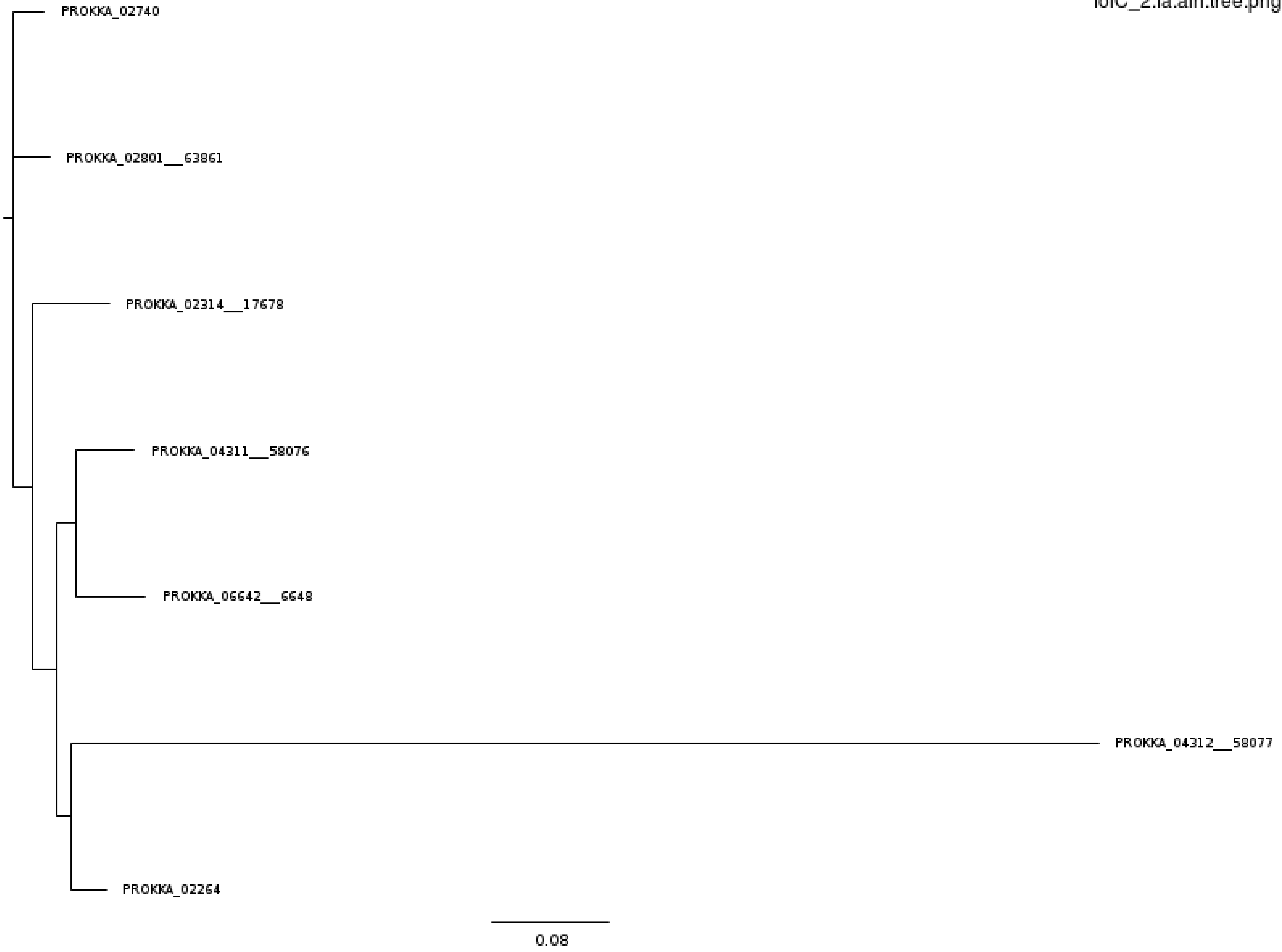

folD_2.fa.aln.tree.png

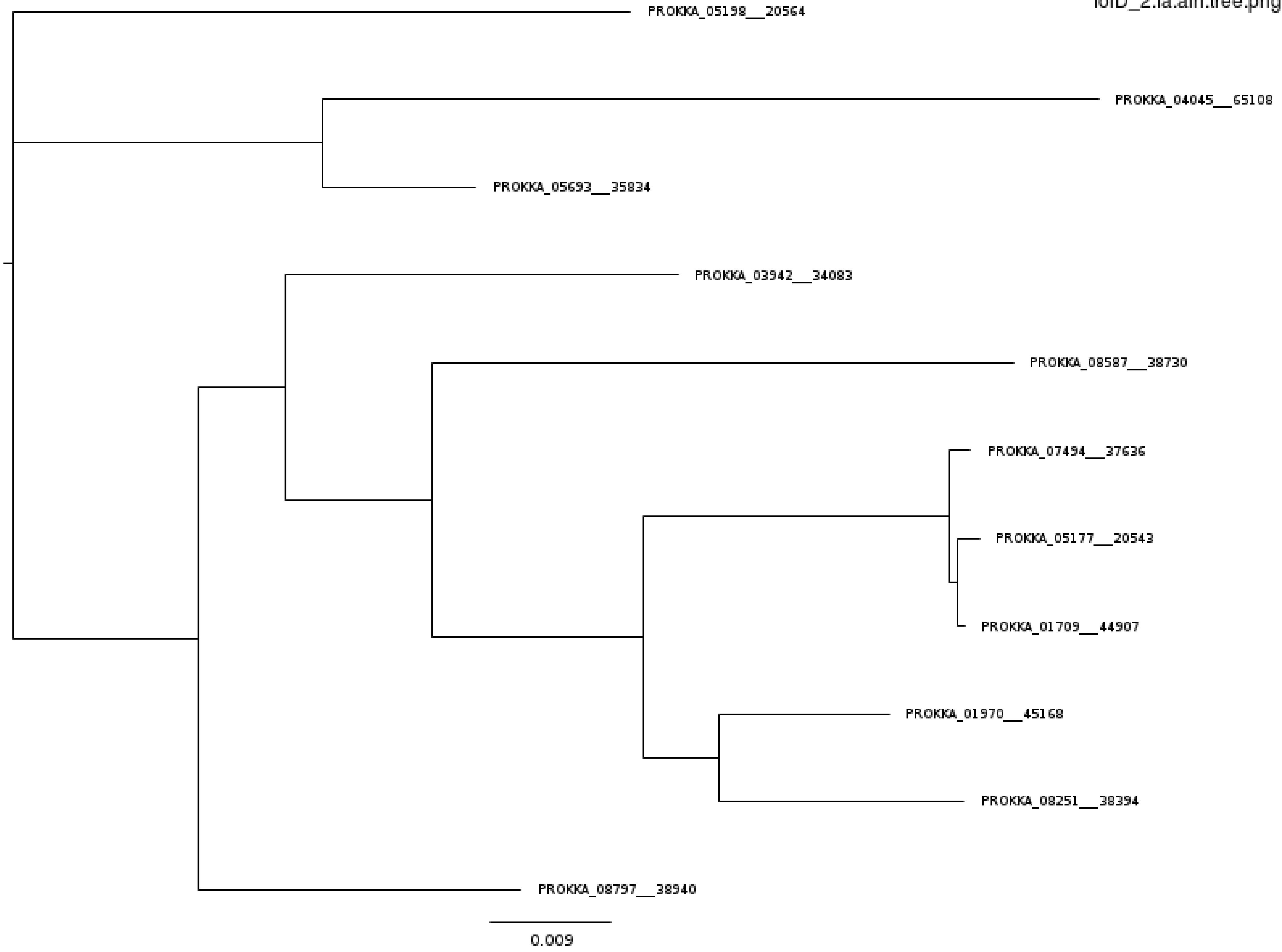

folE2_1.fa.aln.tree.png

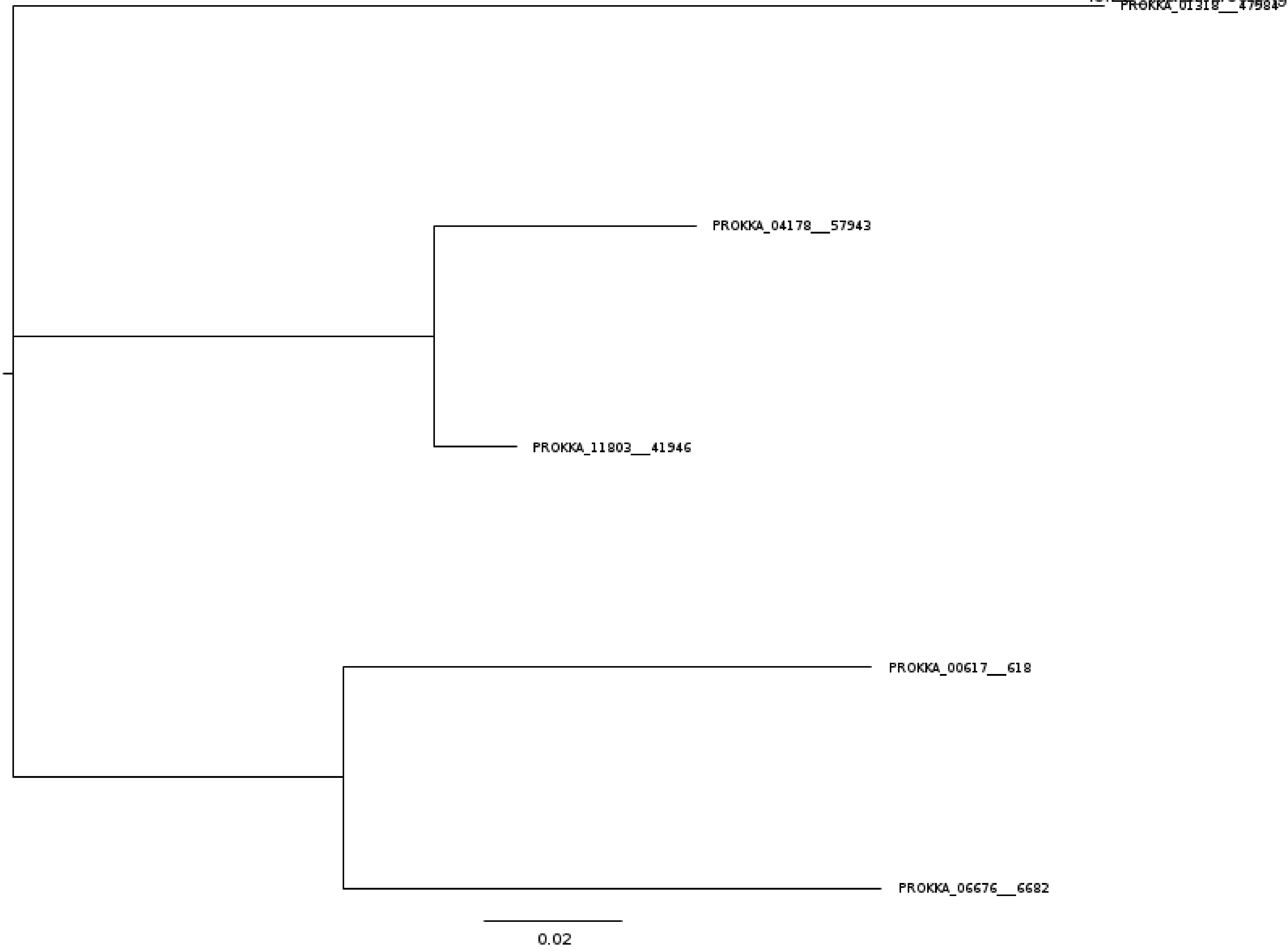

folE2.fa.aln.tree.png

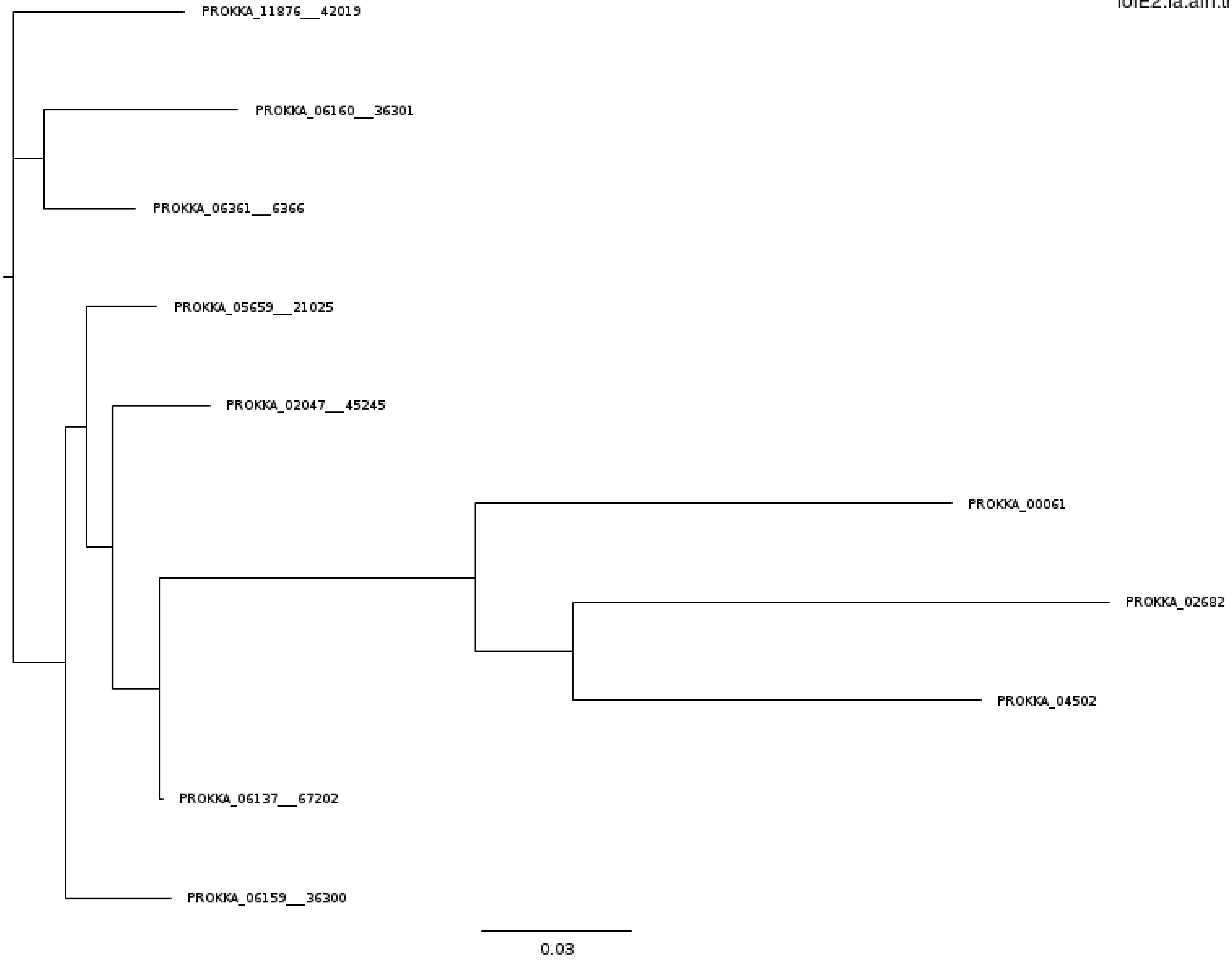

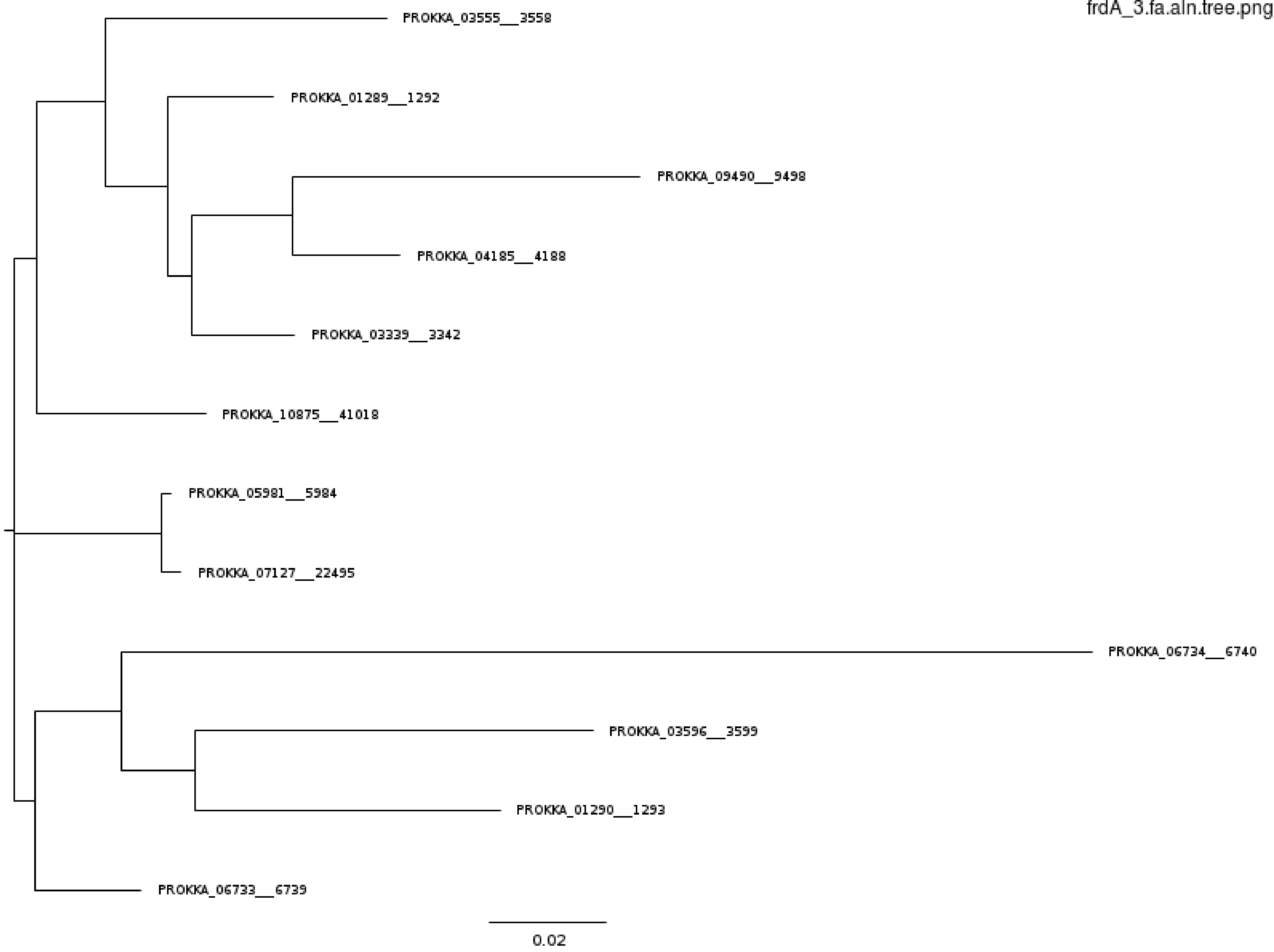
frdA_3.fa.aln.tree.png
PROKKA_03555___3558
PROKKA_01289___1292
PROKKA_09490___9498
PROKKA_04185___4188
PROKKA_03339___3342
PROKKA_10875___41018
PROKKA_05981___5984
PROKKA_07127___22495
PROKKA_06734___6740
PROKKA_03596___3599
PROKKA_01290___1293
PROKKA_06733___6739
0.02

frdB_1.fa.aln.tree.png

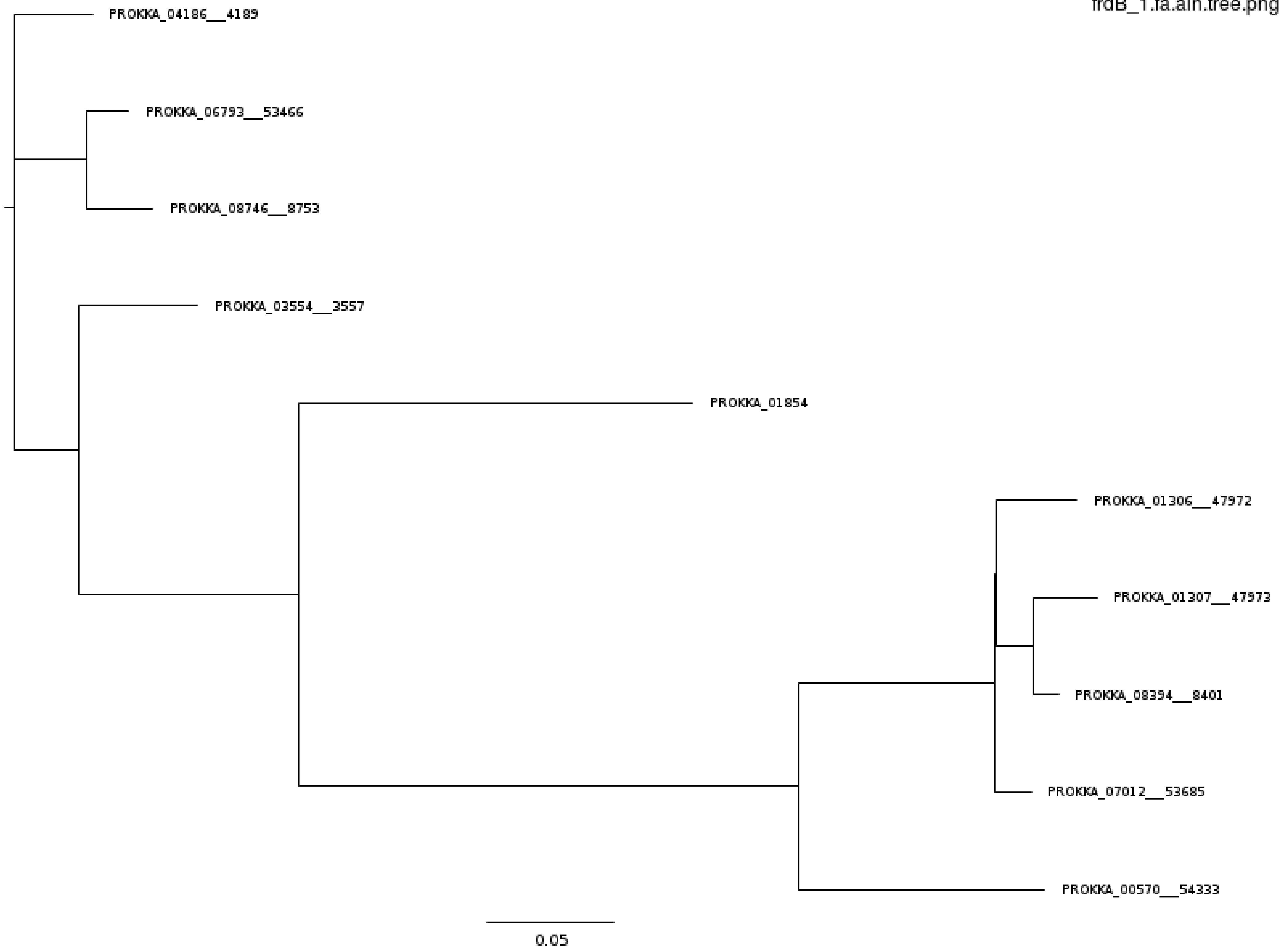

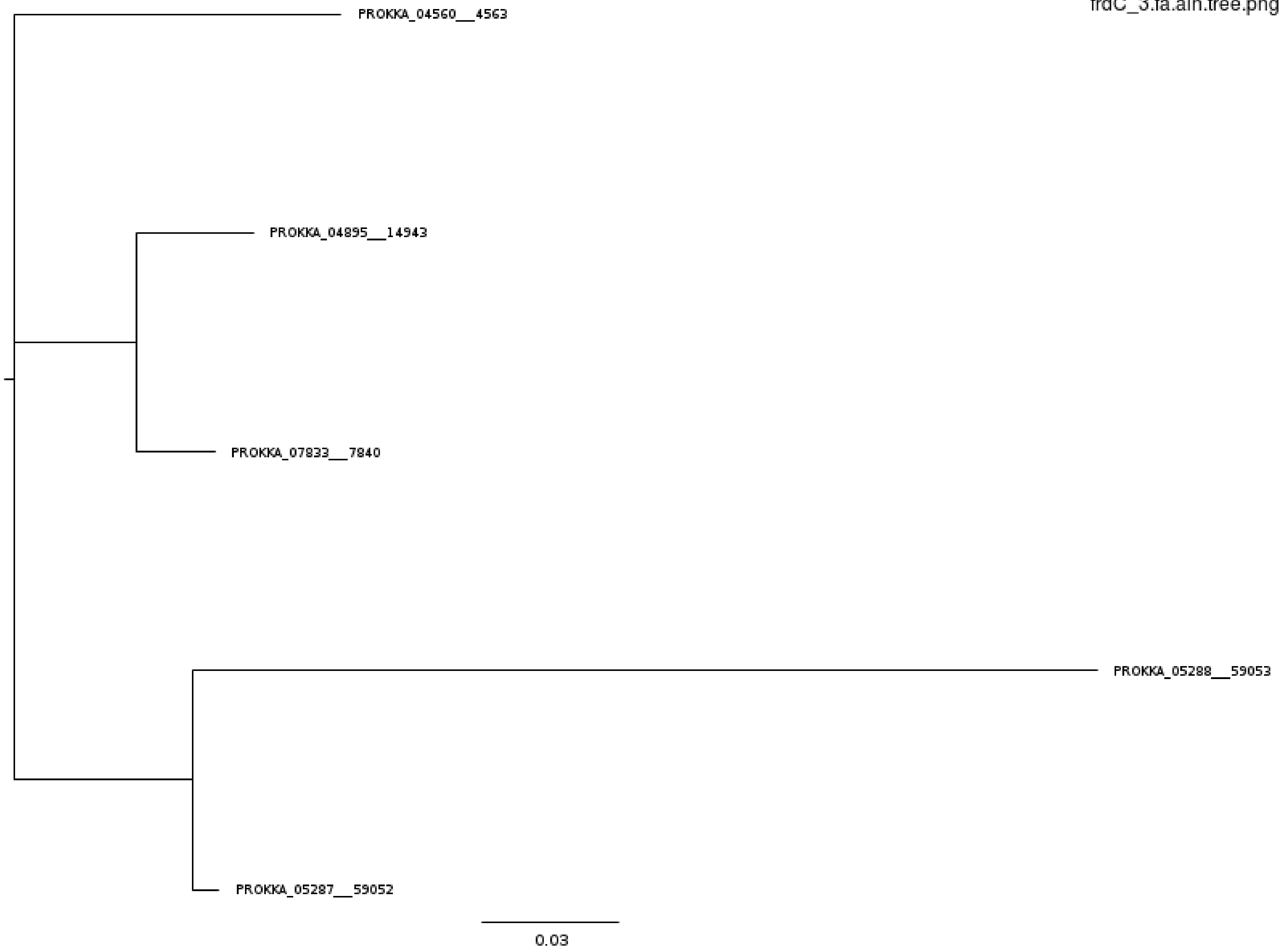
PROKKA_04560___4563
PROKKA_04895___14943
PROKKA_07833___7840
PROKKA_05288___59053
PROKKA_05287___59052
0.03

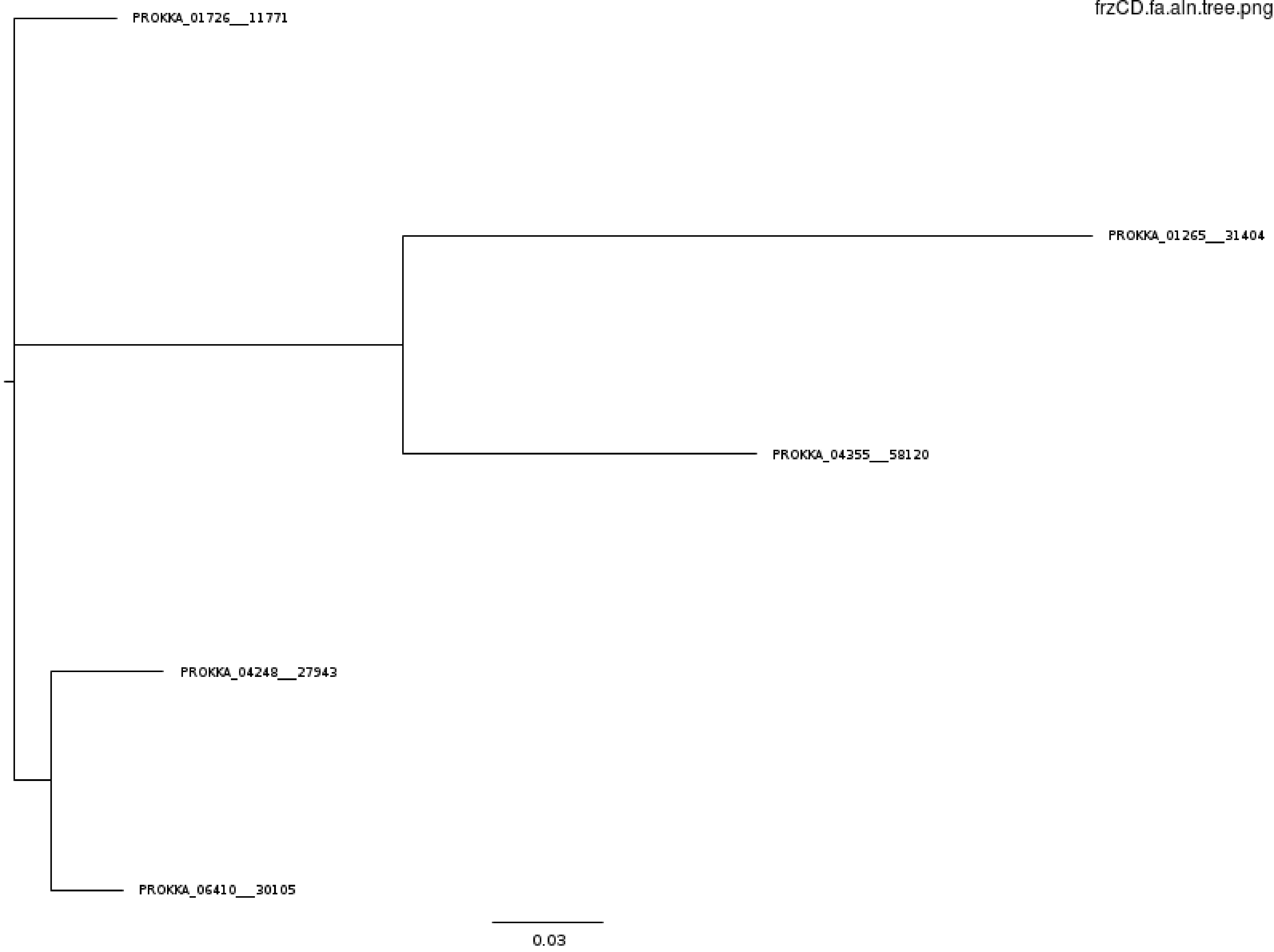
frzCD.fa.aln.tree.png
PROKKA_01726___11771
PROKKA_01265___31404
PROKKA_04355___58120
PROKKA_04248___27943
PROKKA_06410___30105
0.03

frzE_1.fa.aln.tree.png

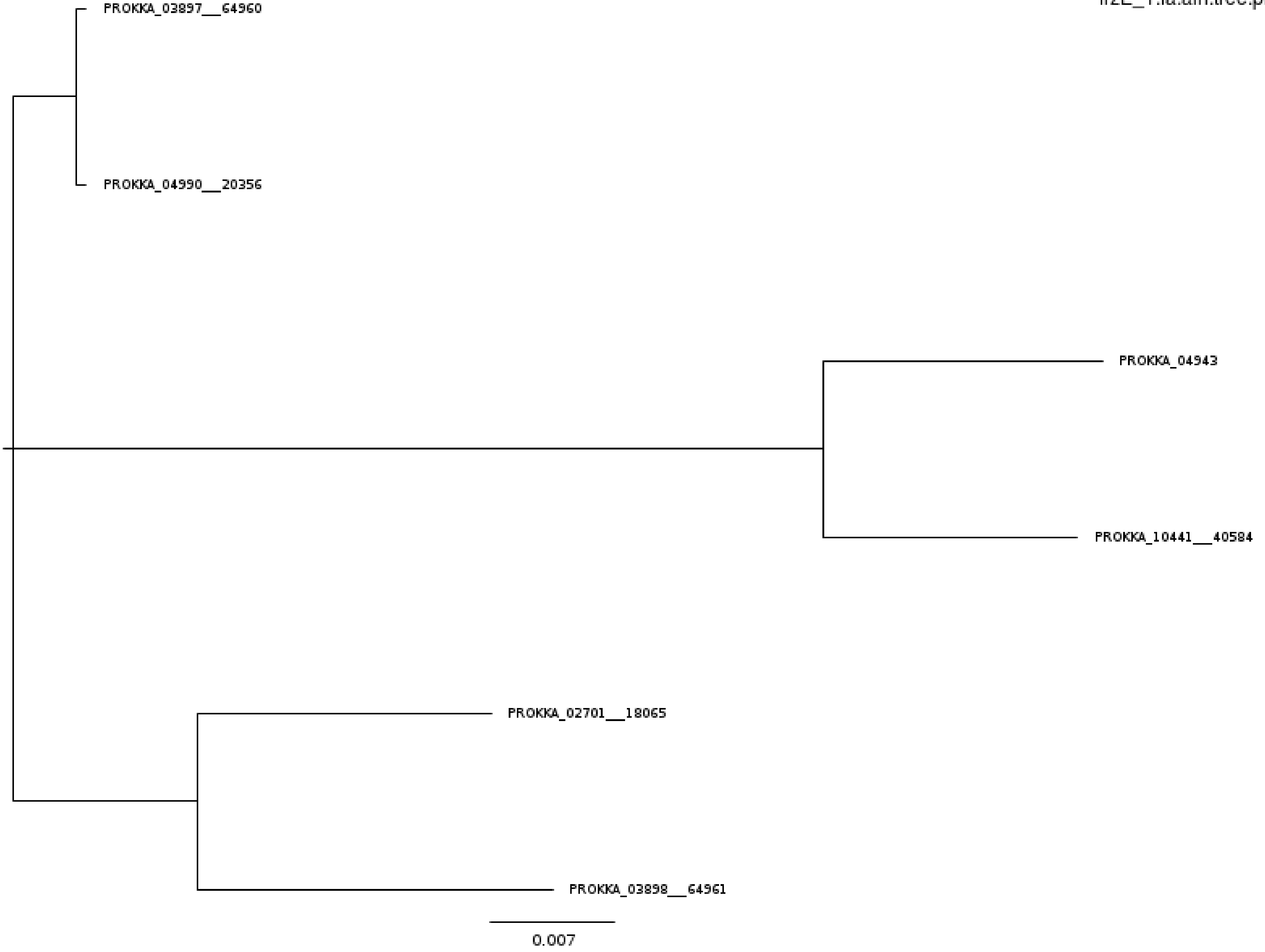

frzE_2.fa.aln.tree.png

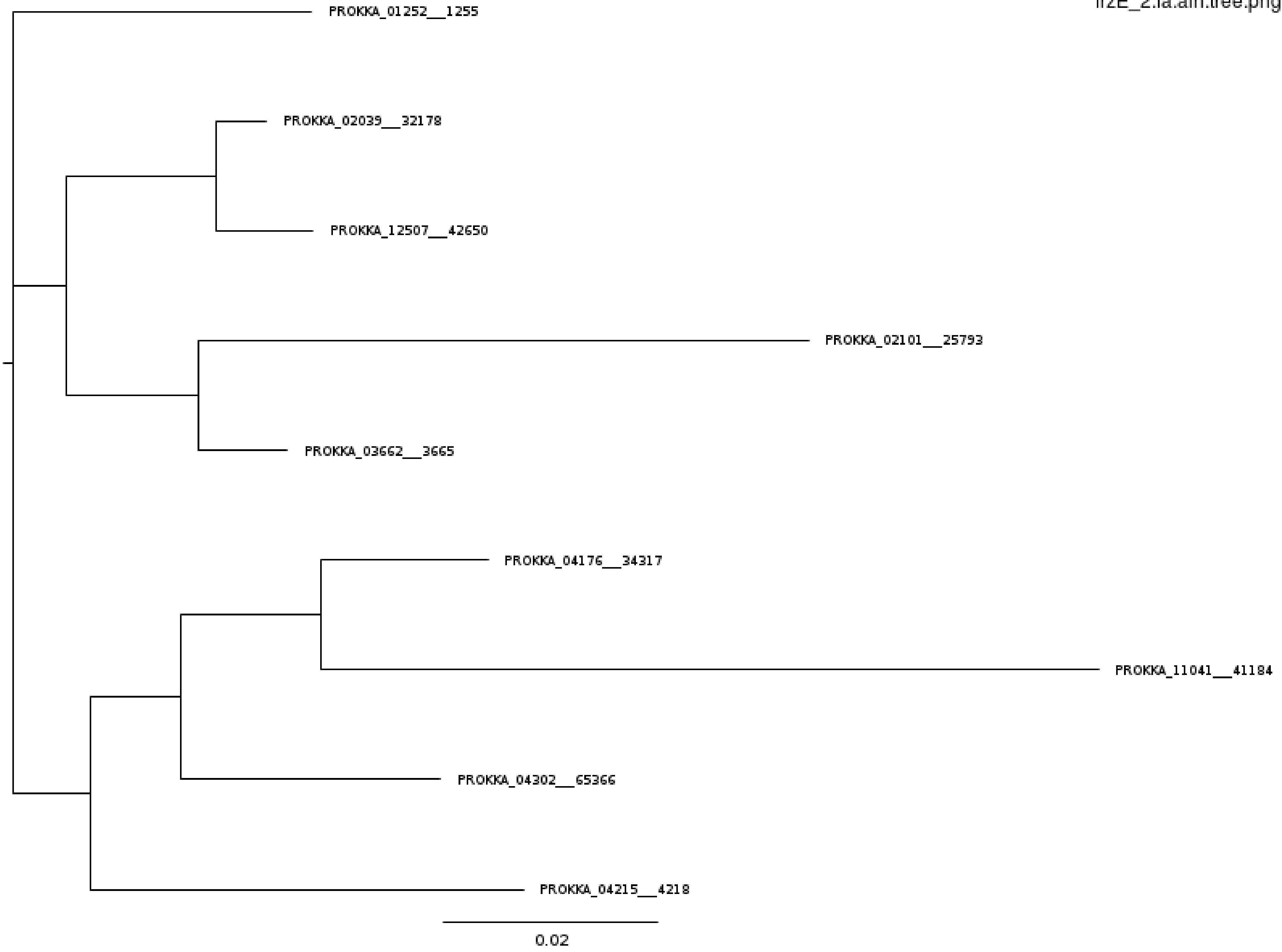

frzE_3.fa.aln.tree.png

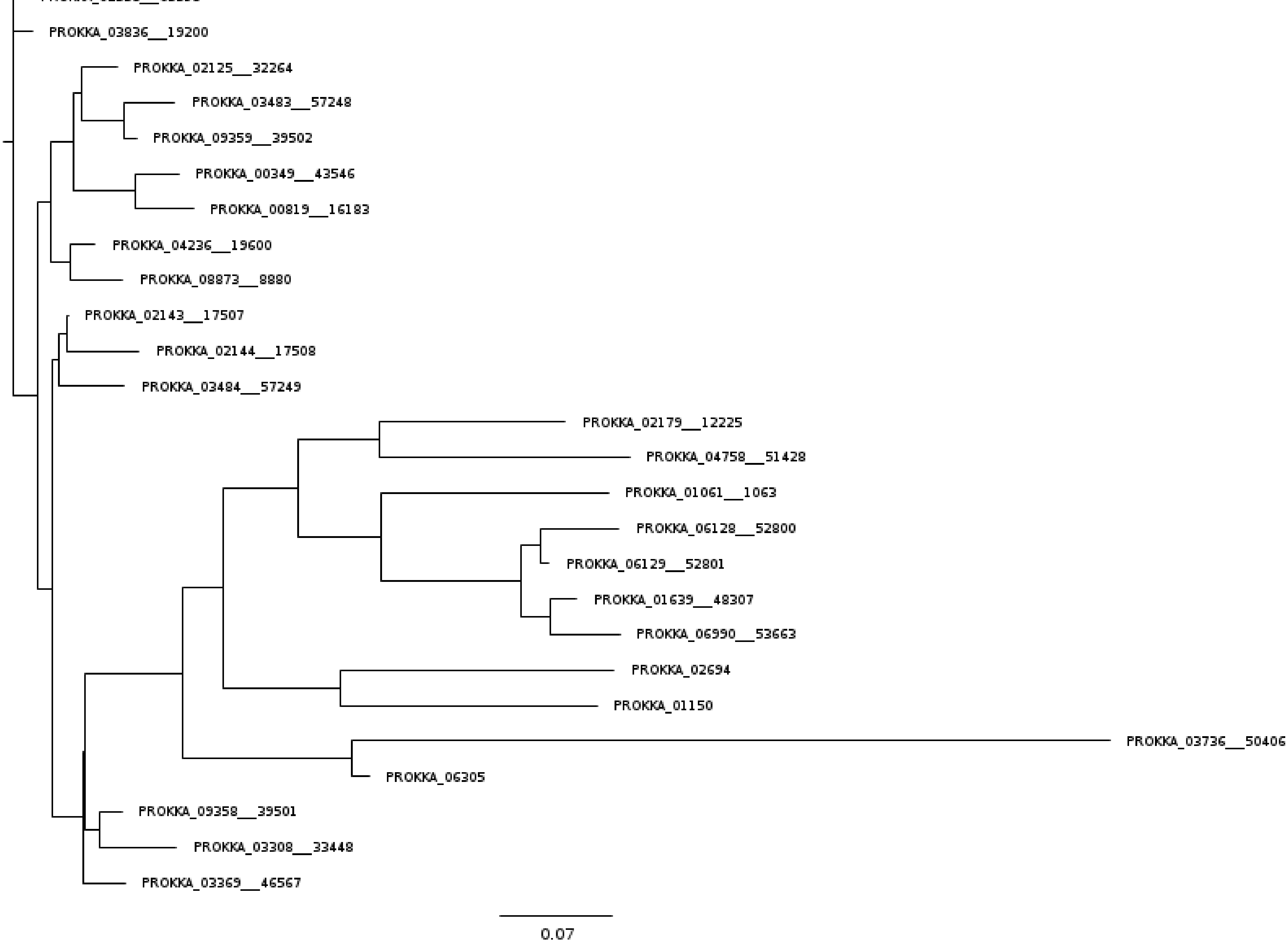

frzE_4.fa.aln.tree.png

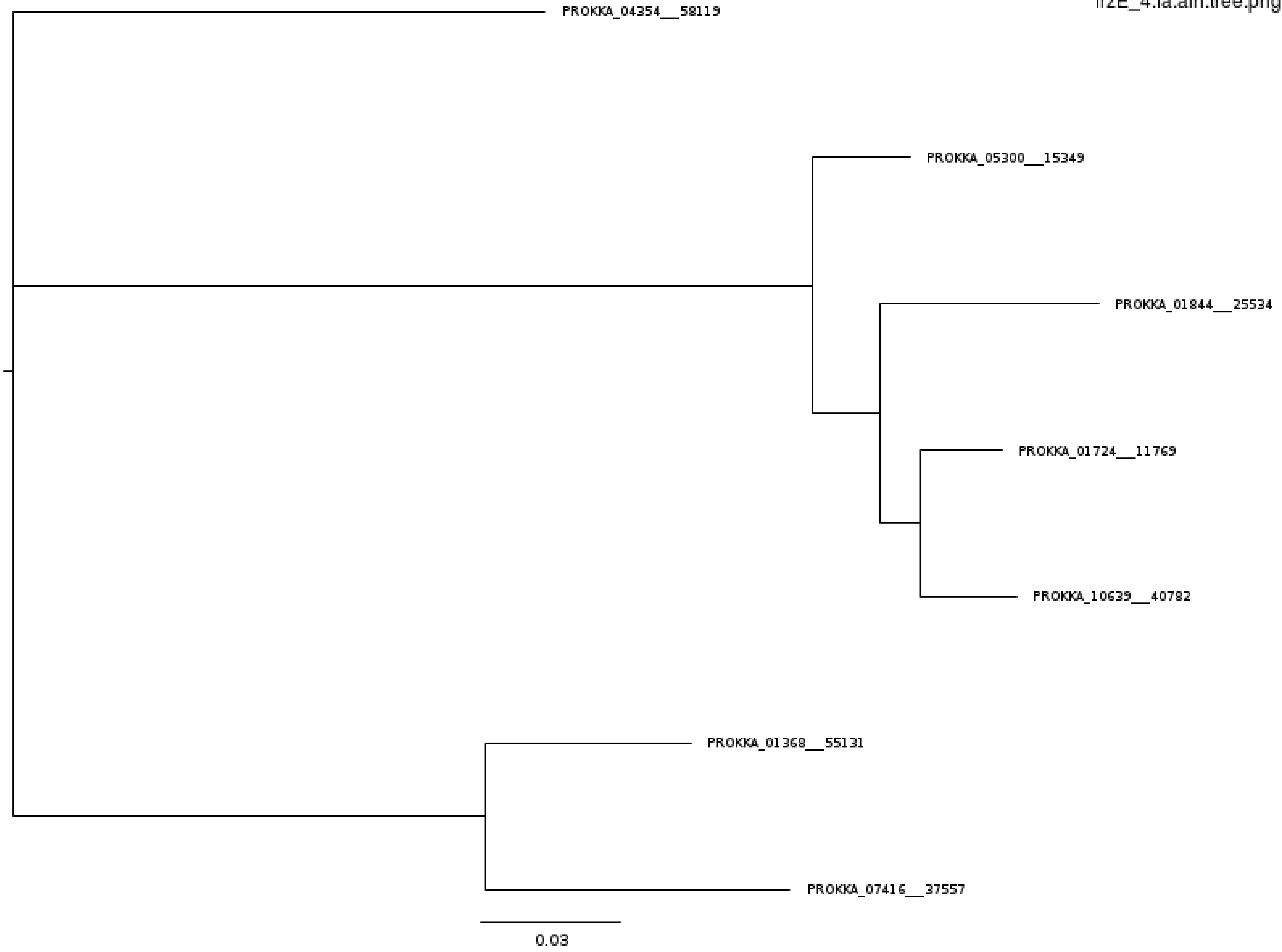

ftsA_2.fa.aln.tree.png

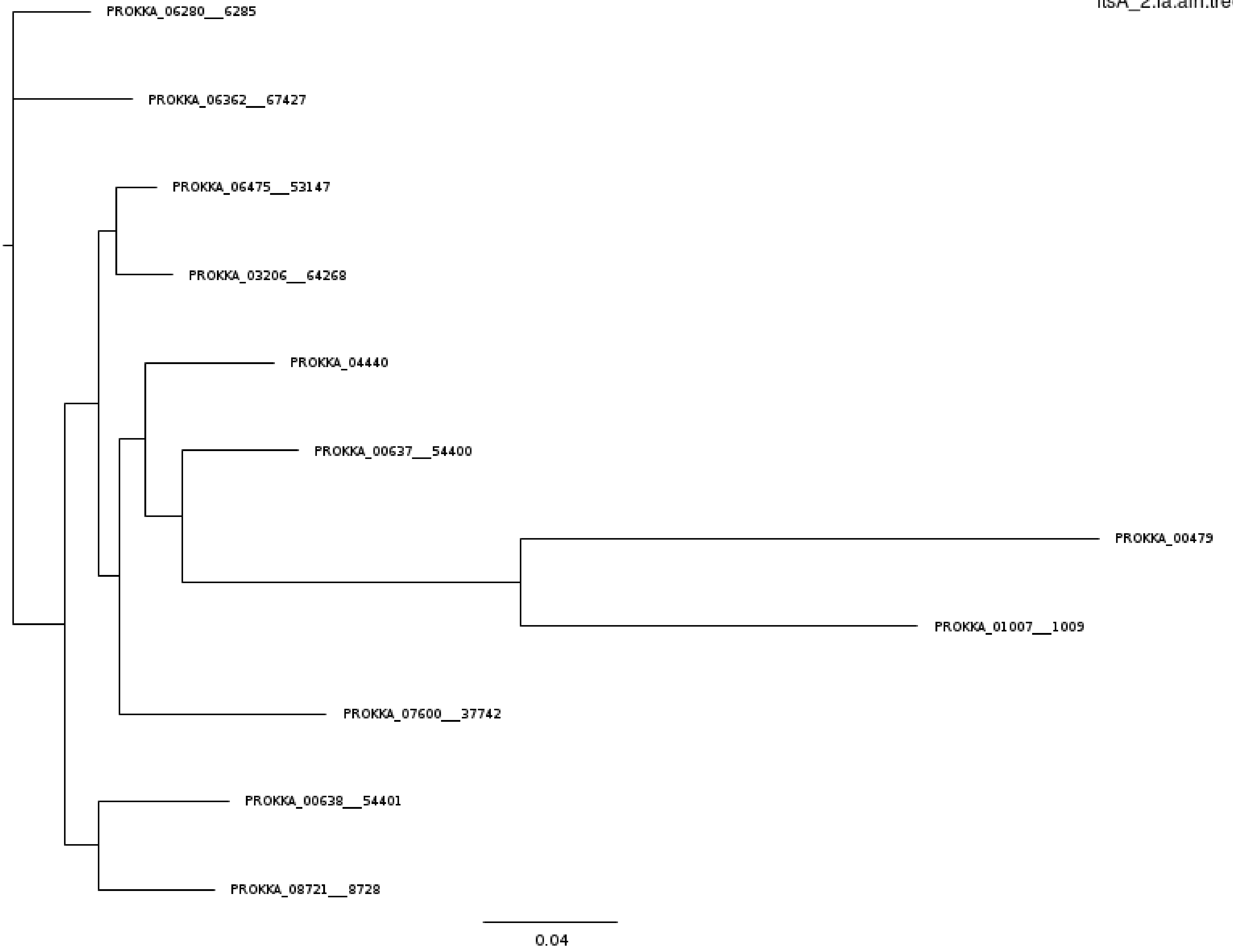

ftsB_3.fa.aln.tree.png

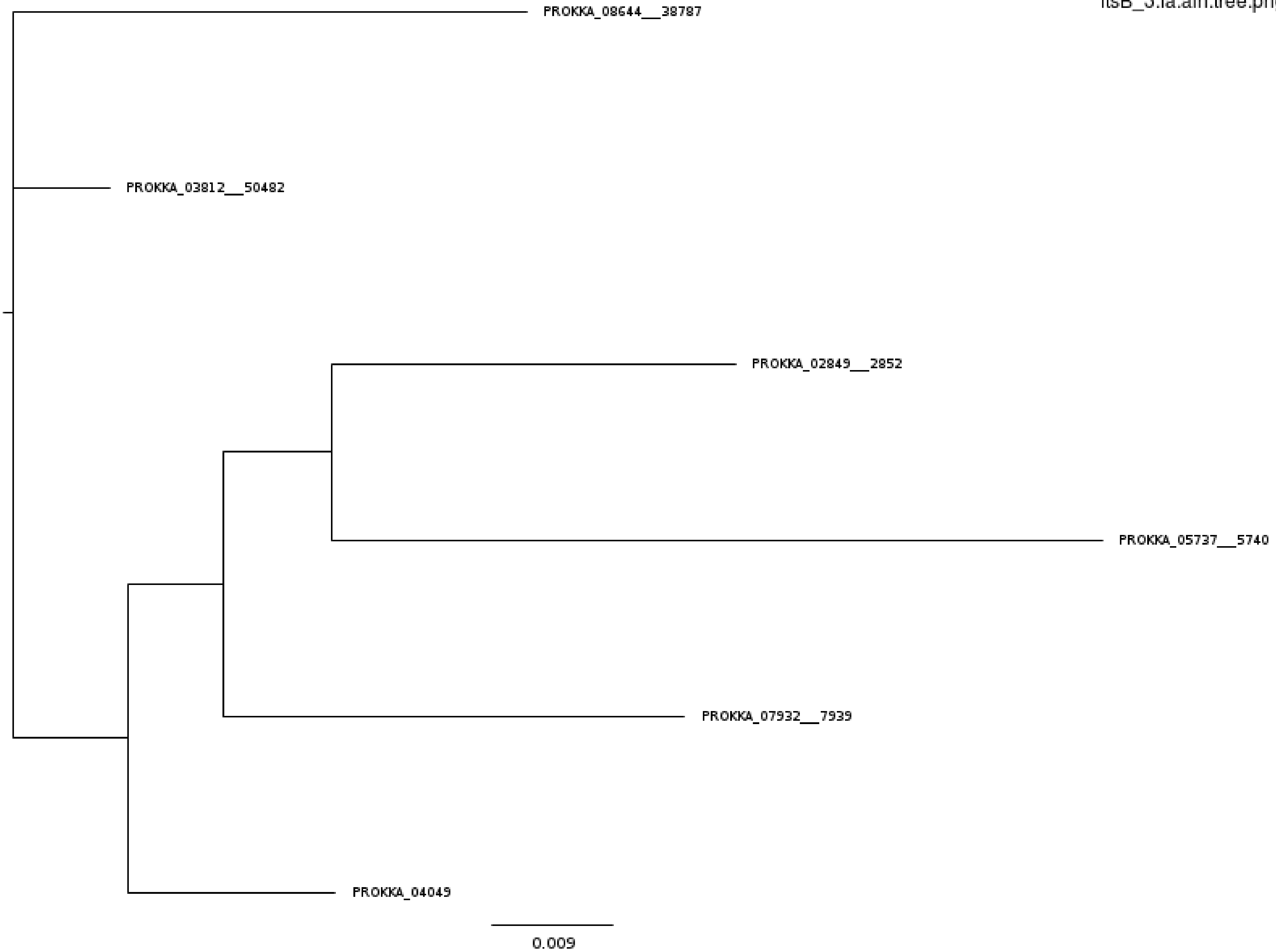

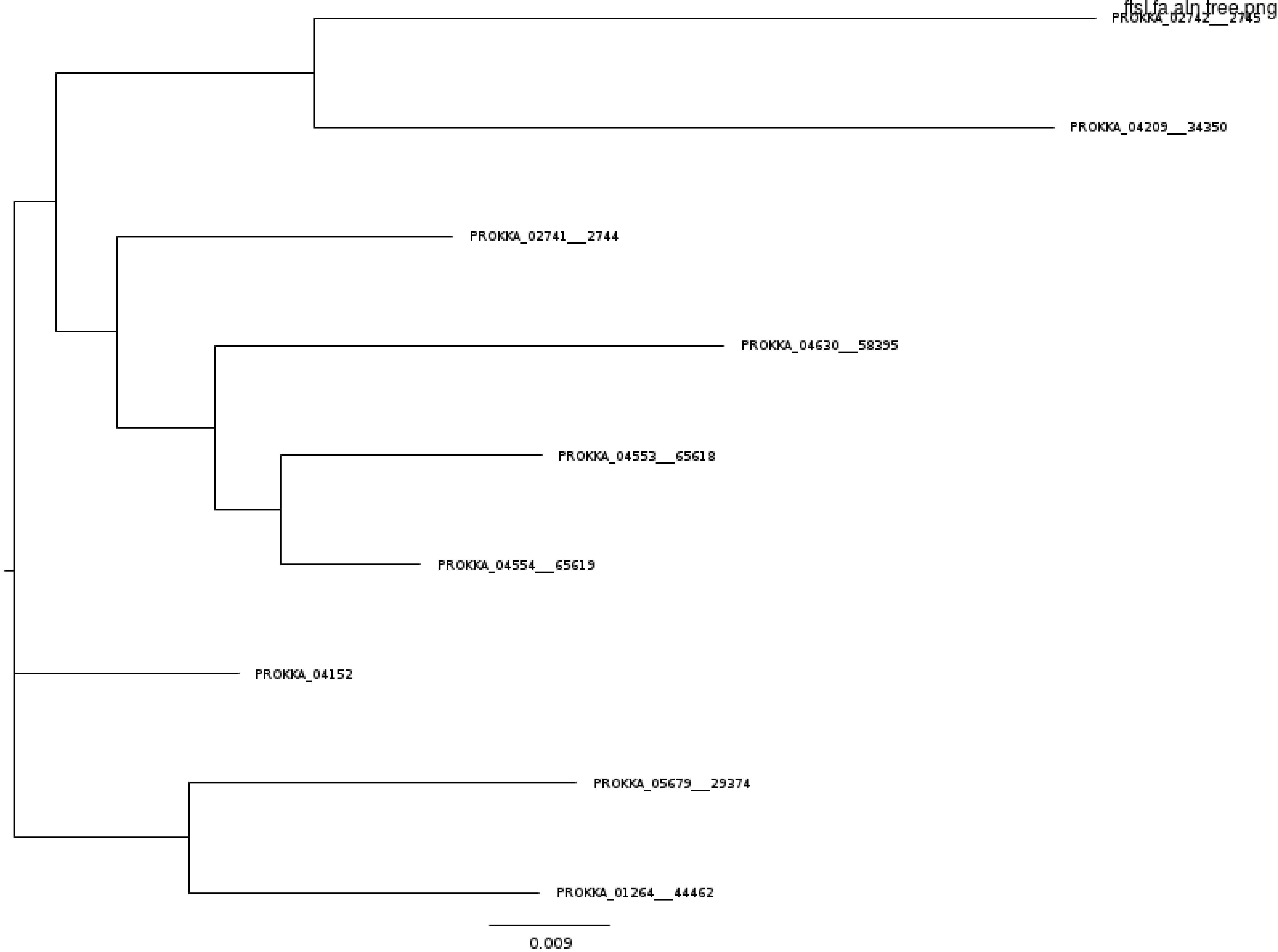
ftsI.fa.aln.tree.png
PROKKA_02742___2745
PROKKA_04209___34350
PROKKA_02741___2744
PROKKA_04630___58395
PROKKA_04553___65618
PROKKA_04554___65619
PROKKA_04152
PROKKA_05679___29374
PROKKA_01264___44462
0.009

ftsL_2.fa.aln.tree.png

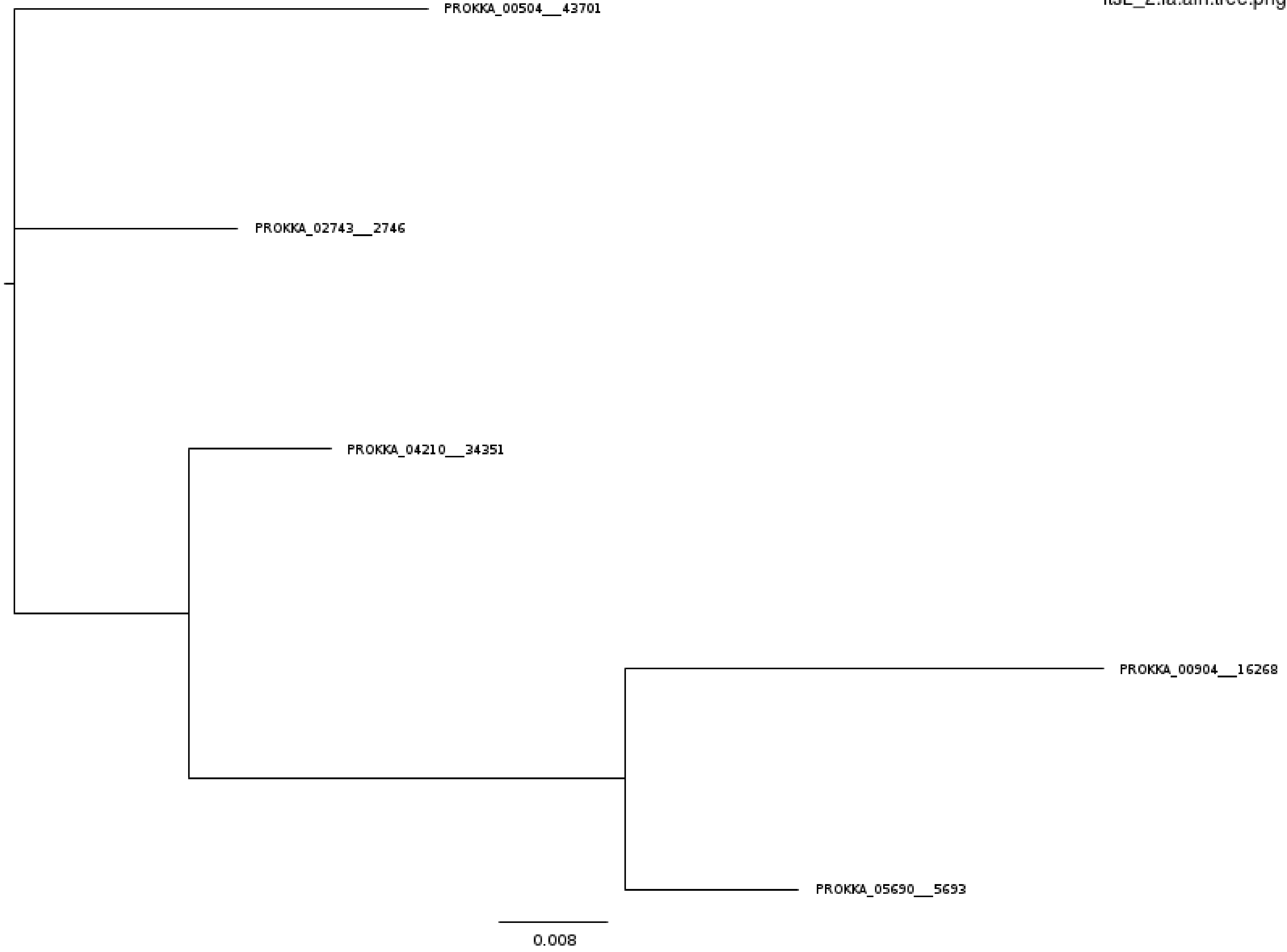

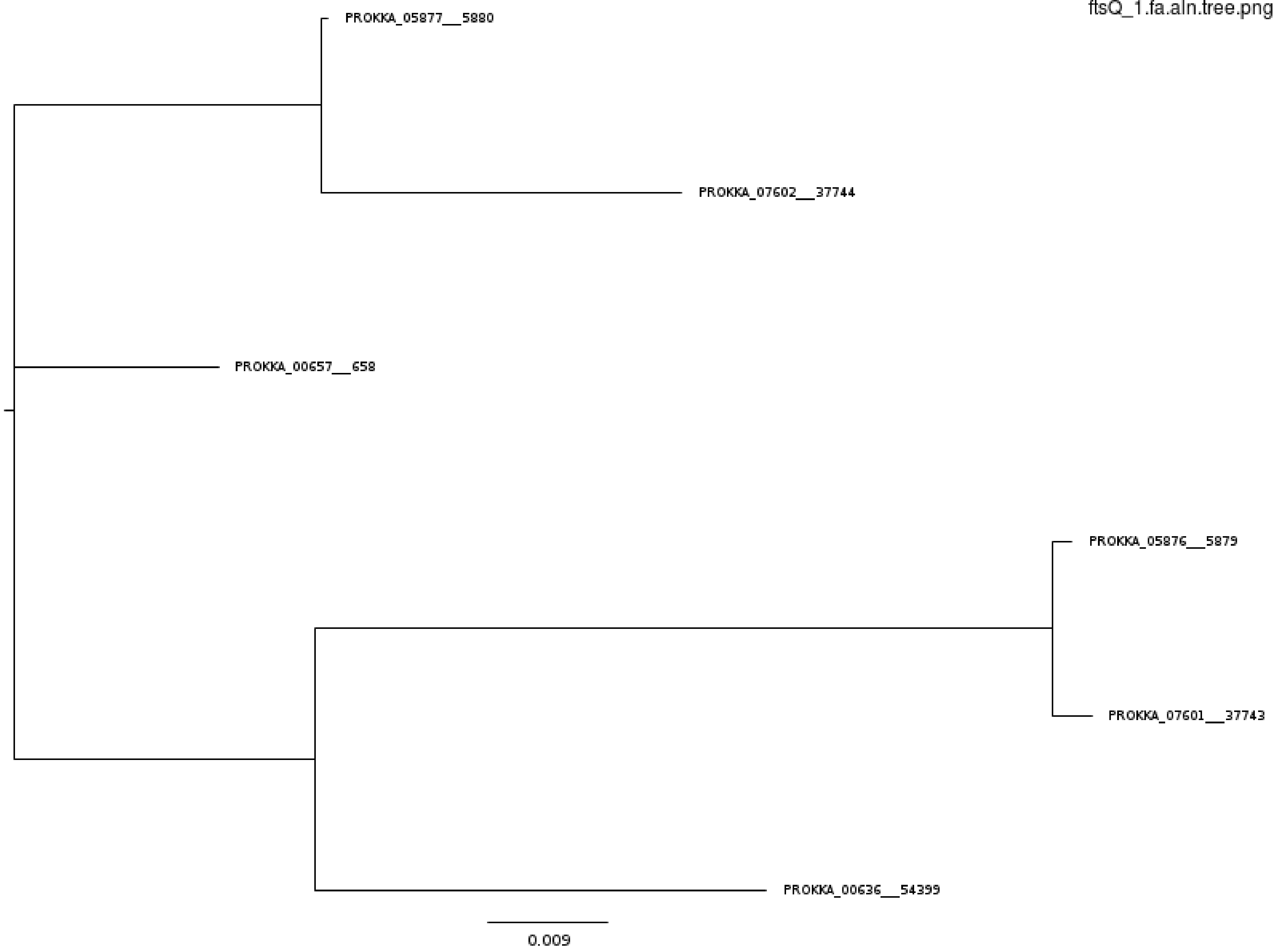
ftsQ_1.fa.aln.tree.png
PROKKA_05877___5880
PROKKA_07602___37744
PROKKA_00657___658
PROKKA_05876___5879
PROKKA_07601___37743
PROKKA_00636___54399
0.009

ftsW_3.fa.aln.tree.png

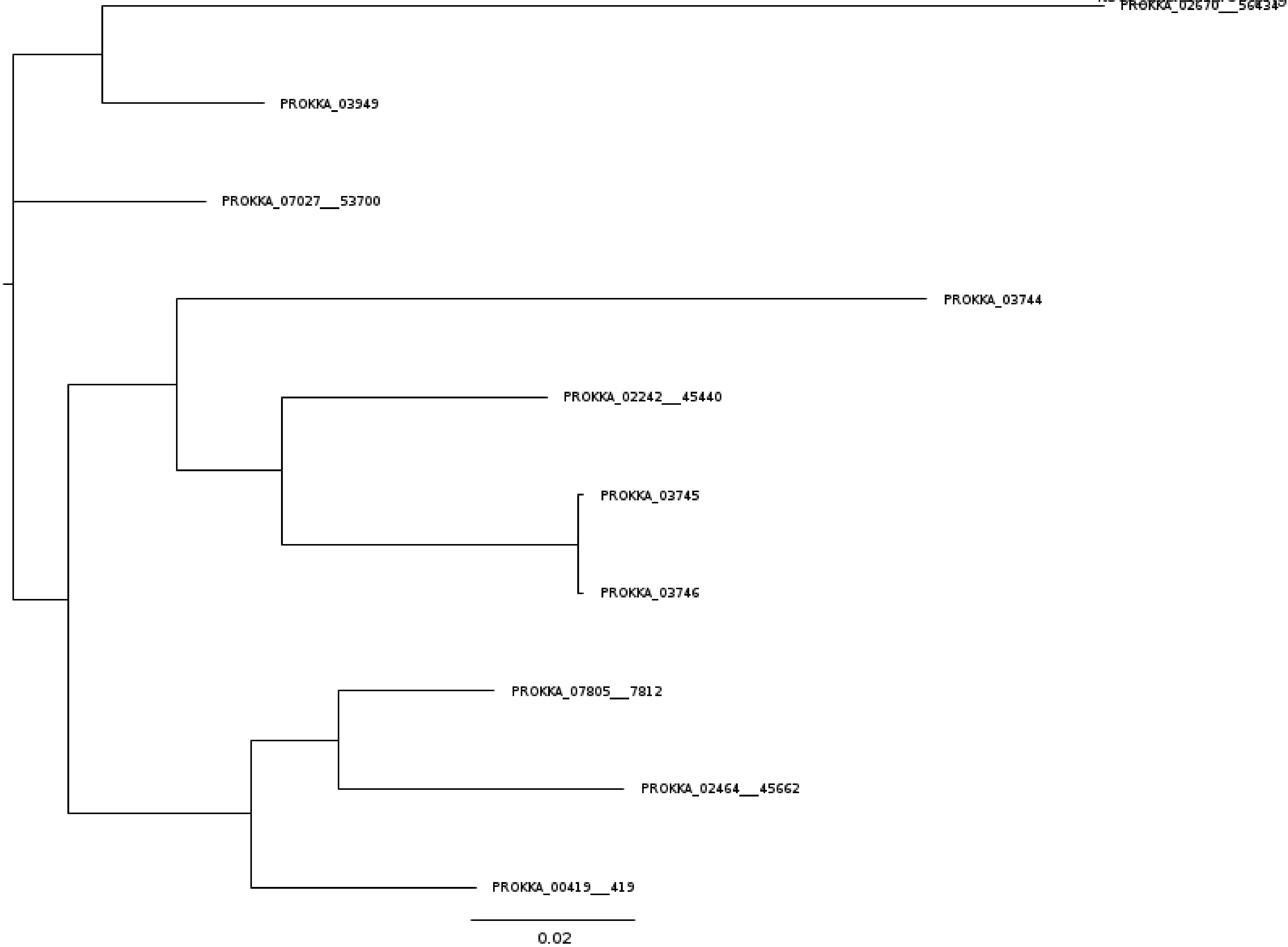

ftsY_1.fa.aln.tree.png

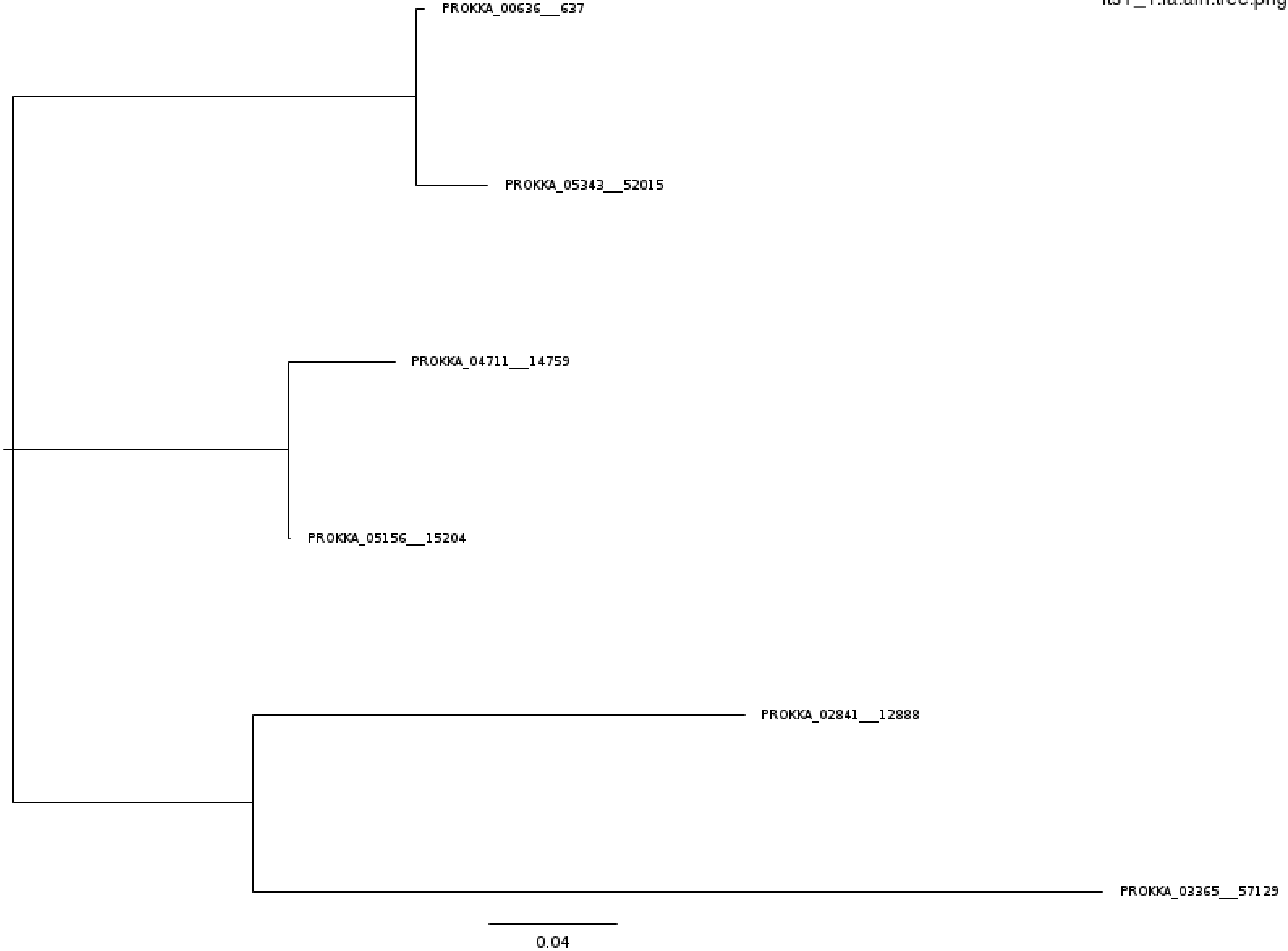

ftsY_2.fa.aln.tree.png

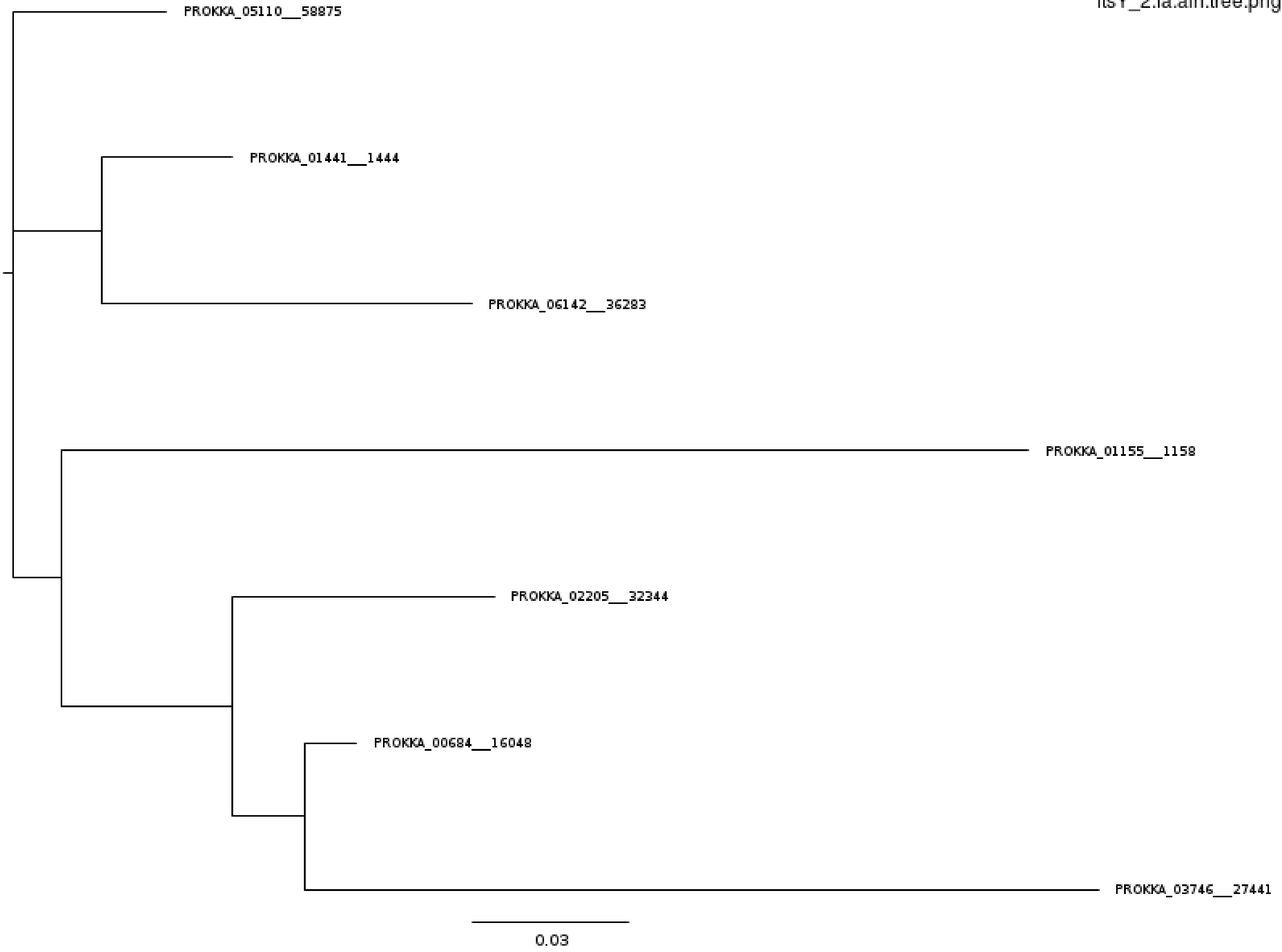

ftsZ_2.fa.aln.tree.png

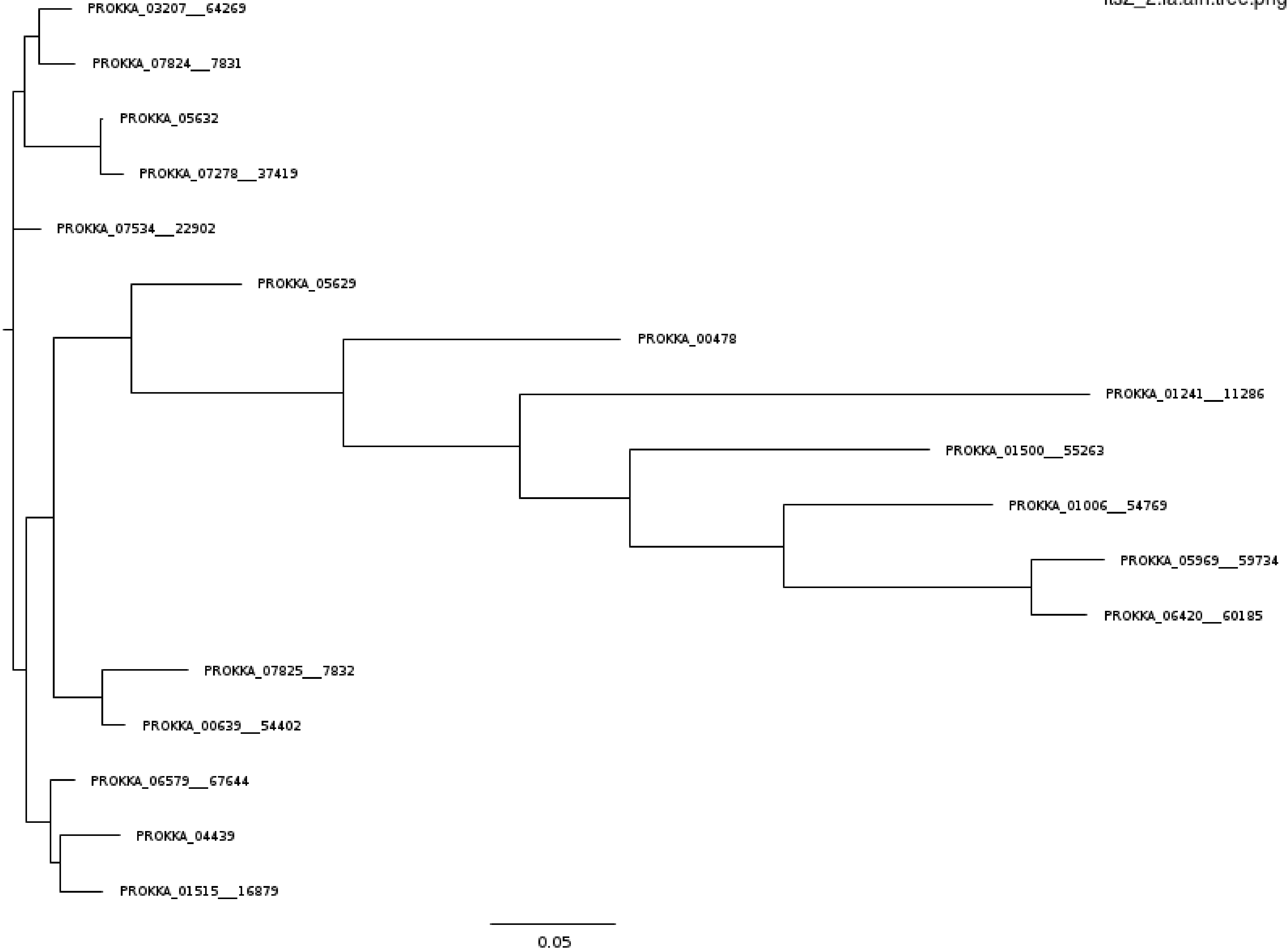

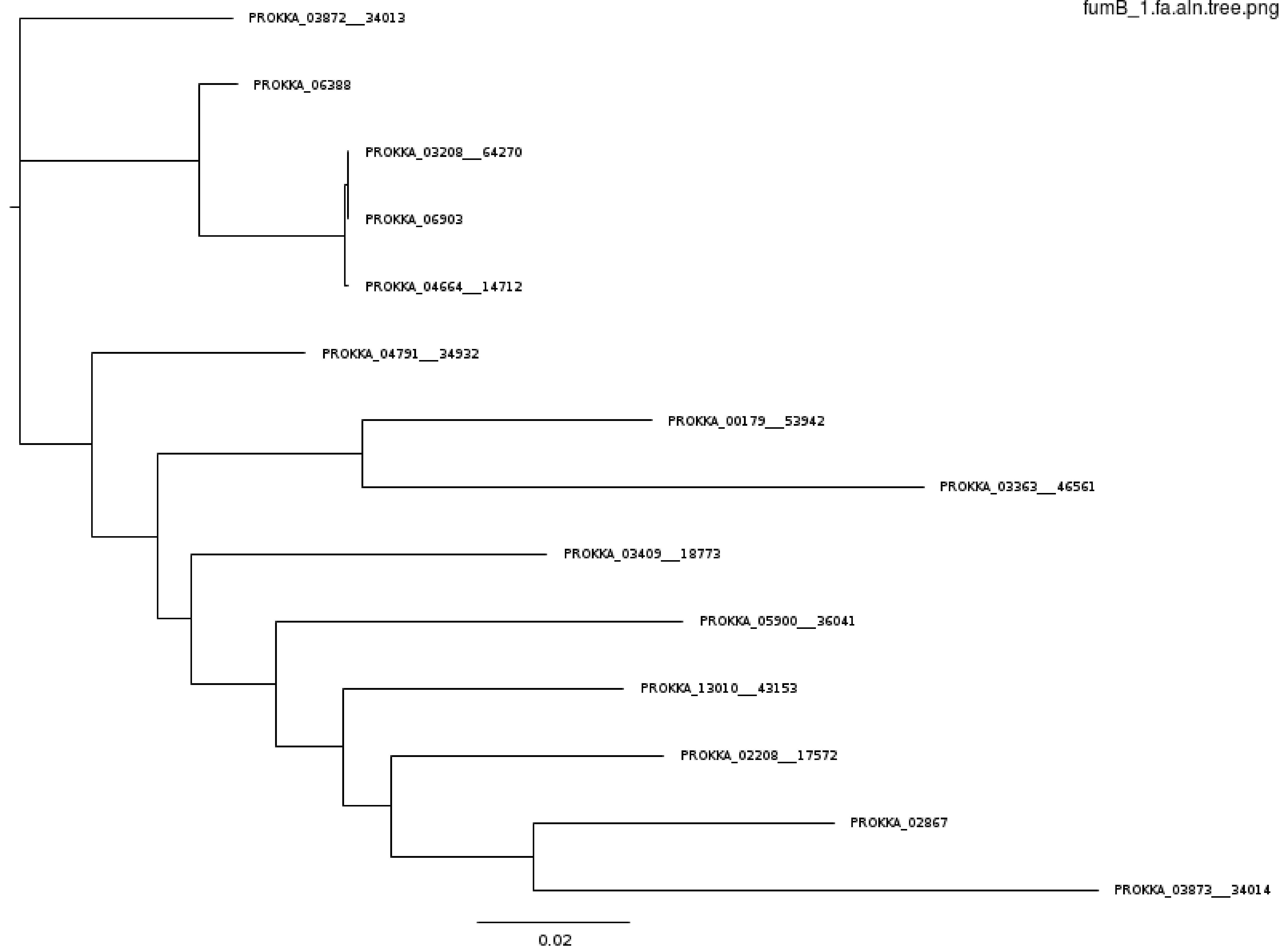
fumB_1.fa.aln.tree.png
PROKKA_03872___34013
PROKKA_06388
PROKKA_03208___64270
PROKKA_06903
PROKKA_04664___14712
PROKKA_04791___34932
PROKKA_00179___53942
PROKKA_03363___46561
PROKKA_03409___18773
PROKKA_05900___36041
PROKKA_13010___43153
PROKKA_02208___17572
PROKKA_02867
PROKKA_03873___34014
0.02

fur_1.fa.aln.tree.png

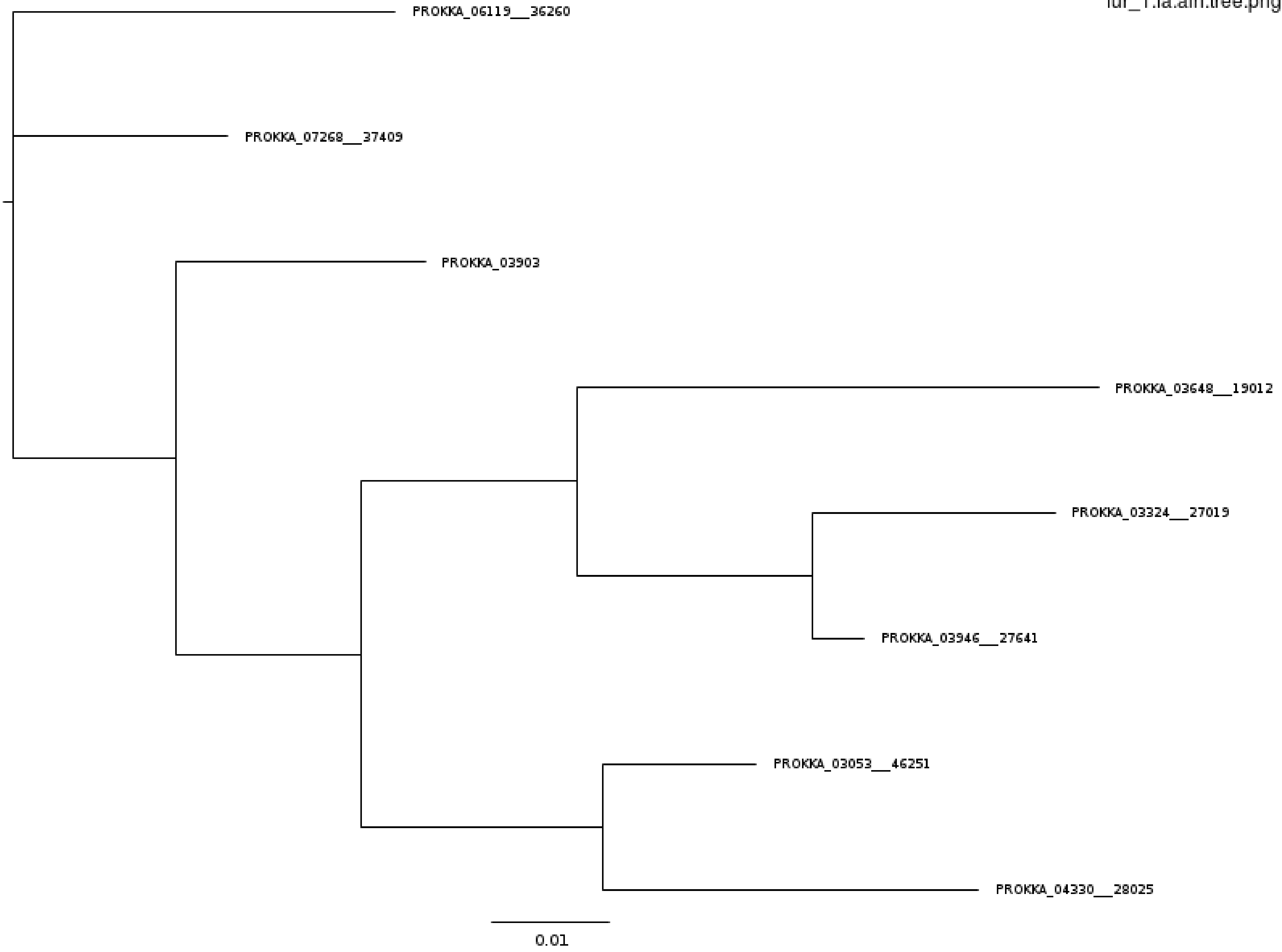

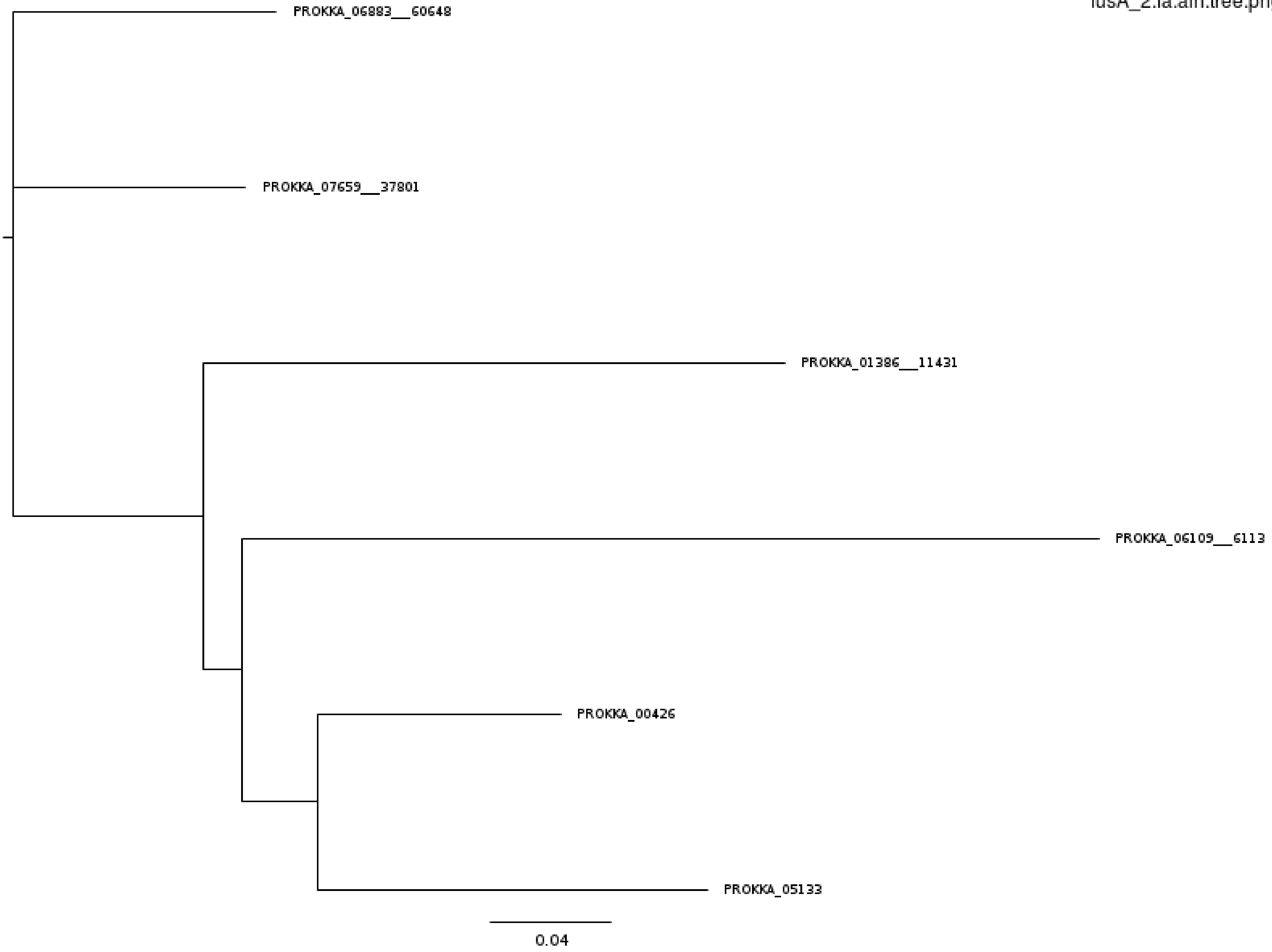
PROKKA_06883___60648
PROKKA_07659___37801
PROKKA_01386___11431
PROKKA_06109___6113
PROKKA_00426
PROKKA_05133
0.04

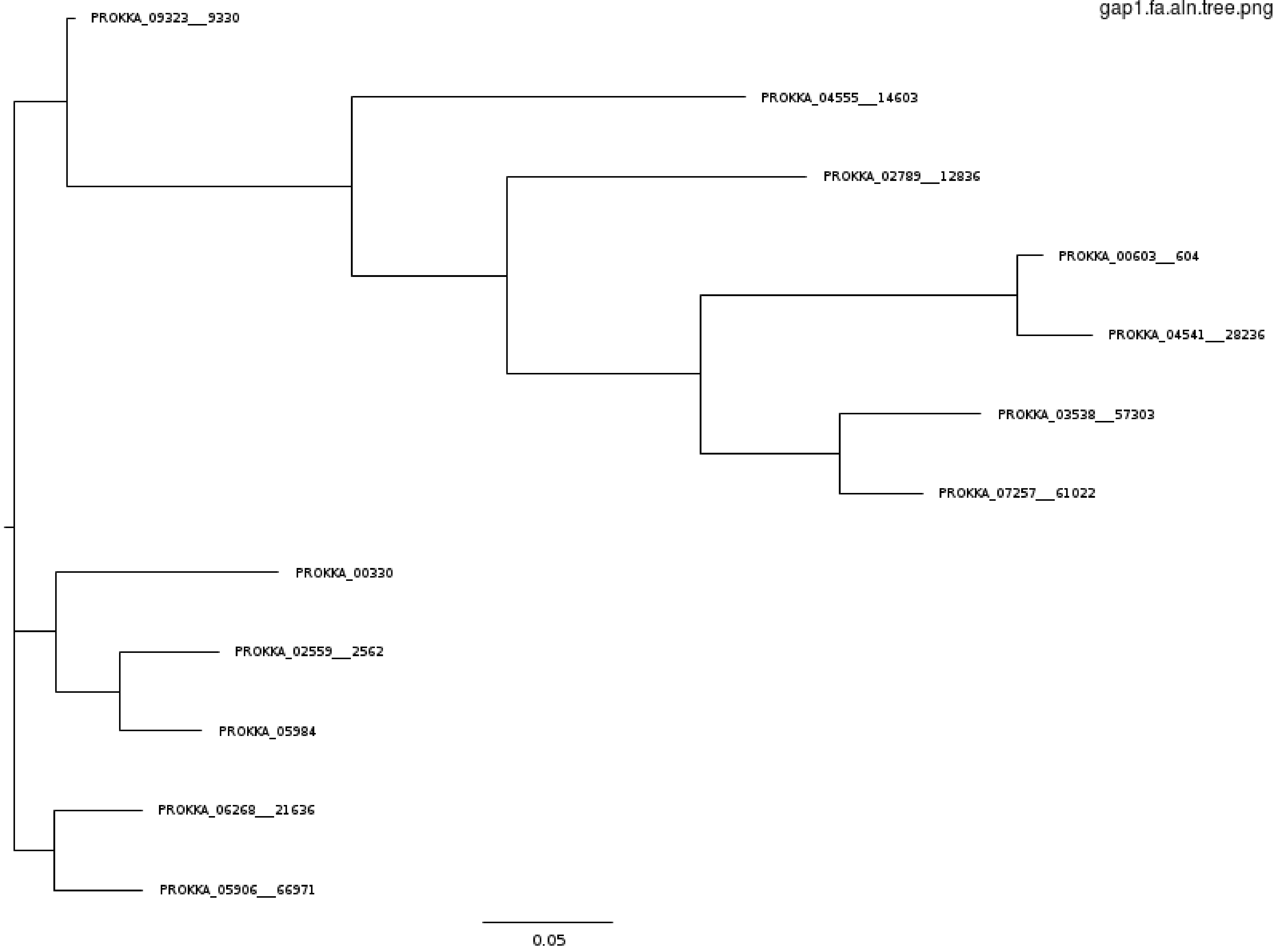
gap1.fa.aln.tree.png
PROKKA_09323___9330
PROKKA_04555___14603
PROKKA_02789___12836
PROKKA_00603___604
PROKKA_04541___28236
PROKKA_03538___57303
PROKKA_07257___61022
PROKKA_00330
PROKKA_02559___2562
PROKKA_05984
PROKKA_06268___21636
PROKKA_05906___66971
0.05

gatA.fa.aln.tree.png

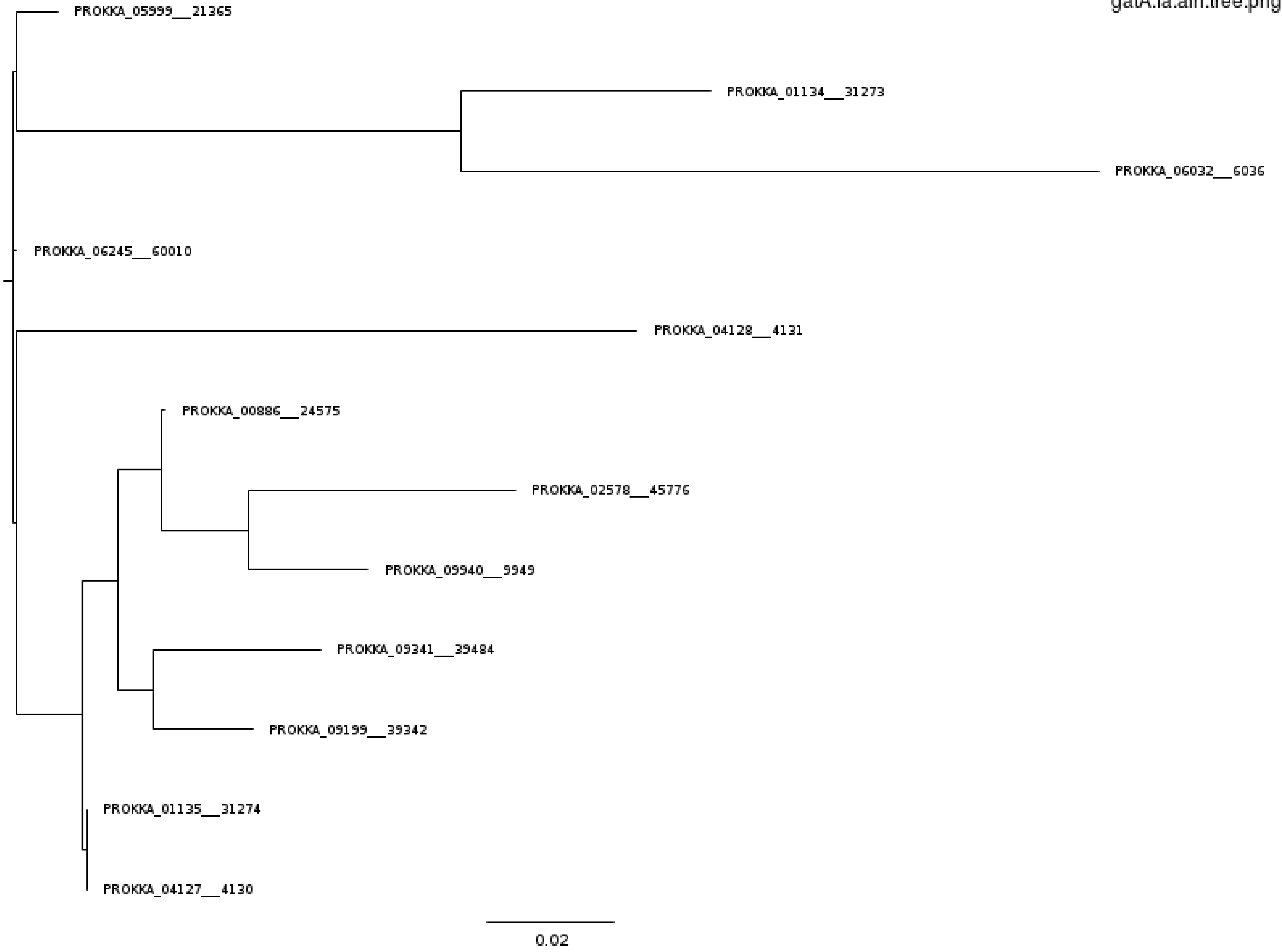

gatB_2.fa.aln.tree.png

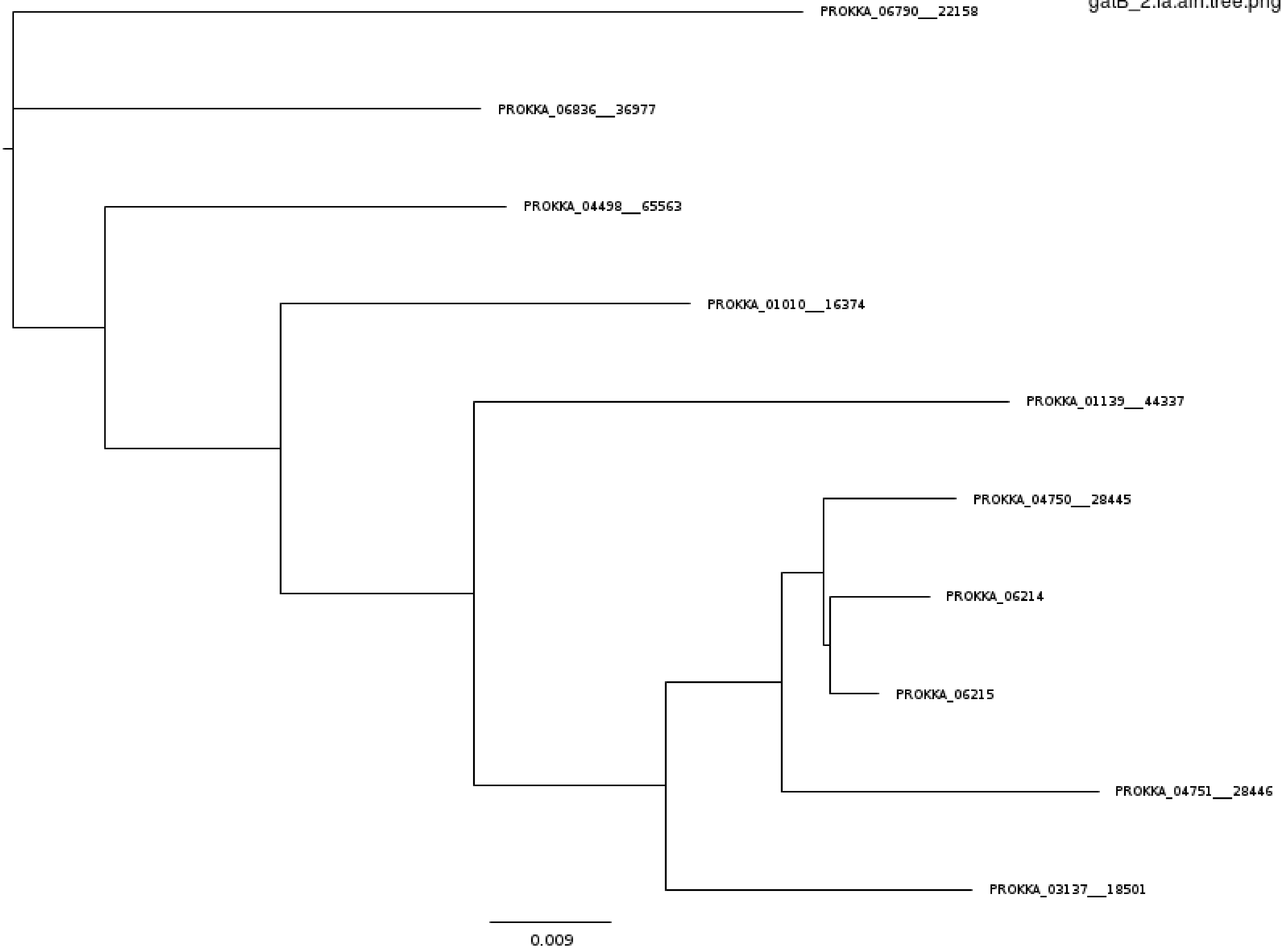

gatC.fa.aln.tree.png

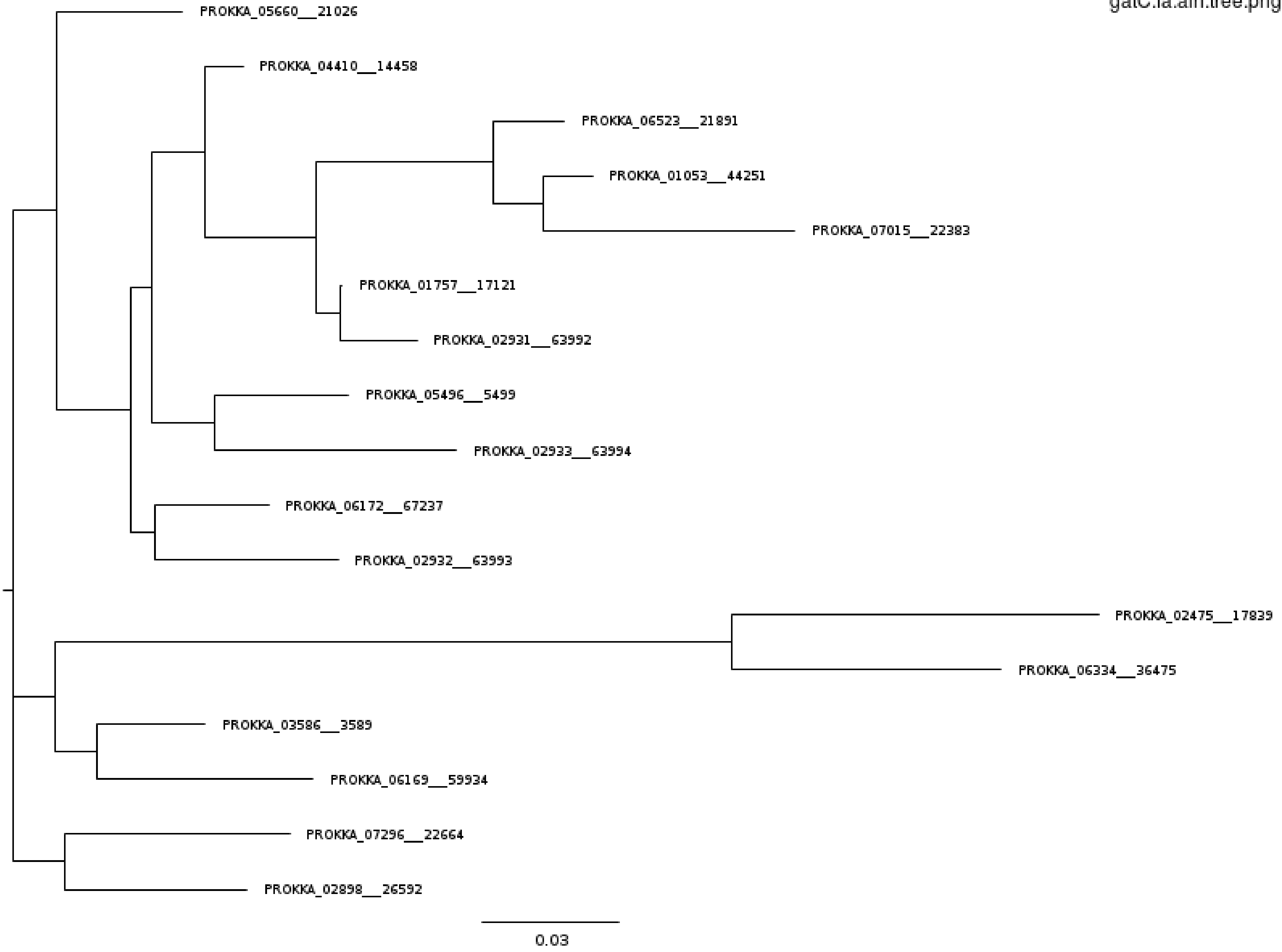

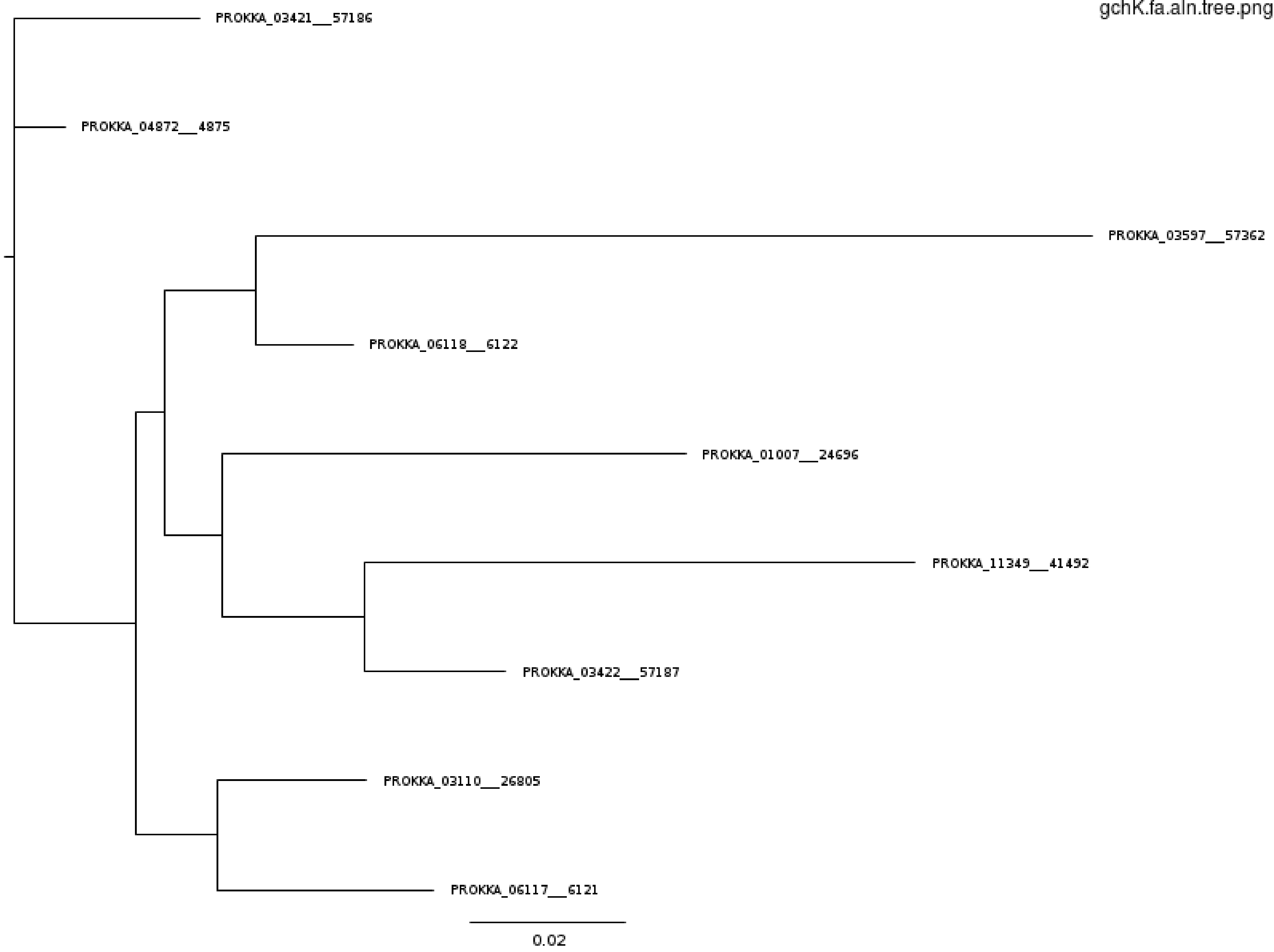
gchK.fa.aln.tree.png
PROKKA_03421___57186
PROKKA_04872___4875
PROKKA_03597___57362
PROKKA_06118___6122
PROKKA_01007___24696
PROKKA_11349___41492
PROKKA_03422___57187
PROKKA_03110___26805
PROKKA_06117___6121
0.02

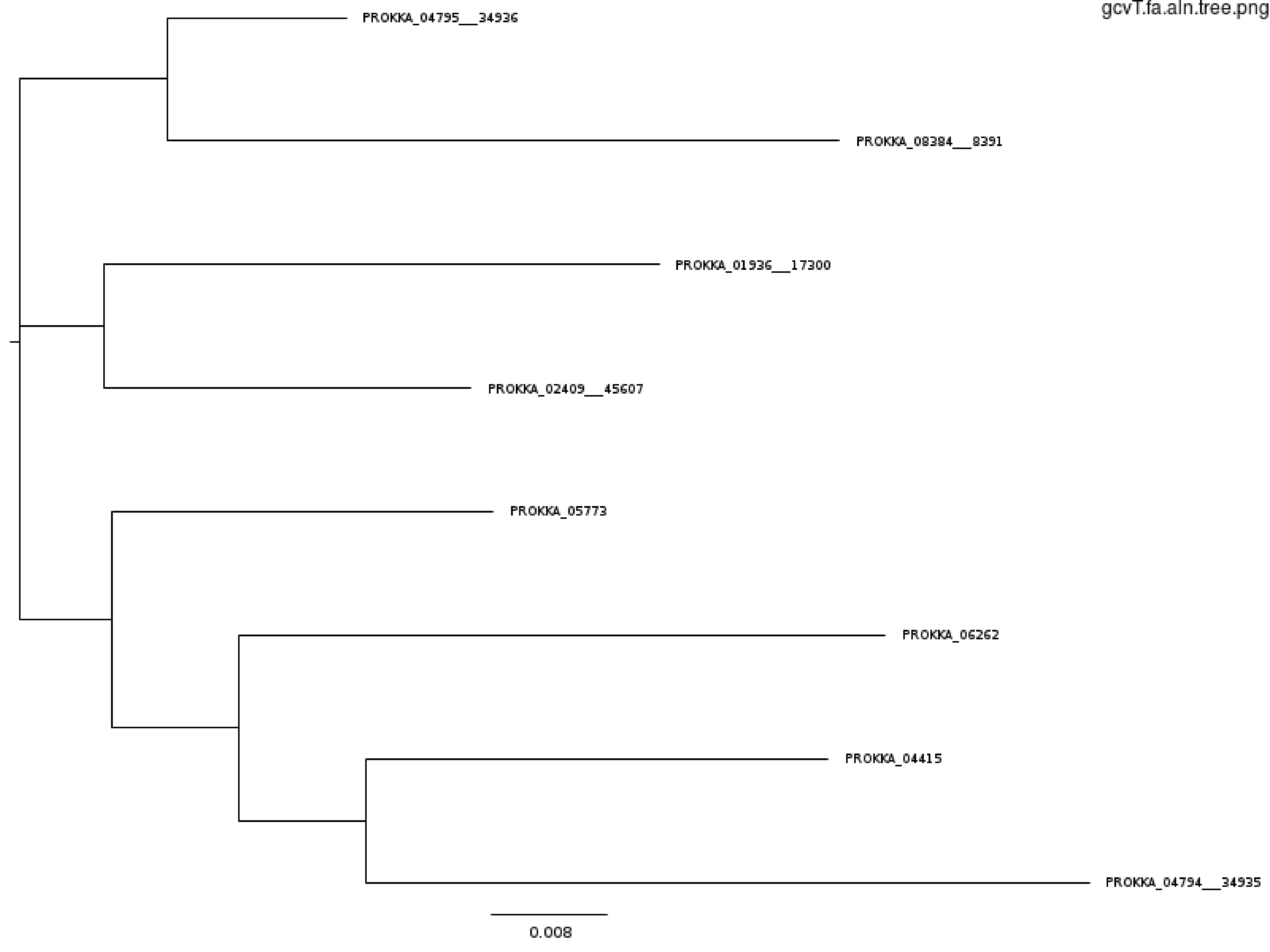
gcvT.fa.aln.tree.png
PROKKA_04795___34936
PROKKA_08384___8391
PROKKA_01936___17300
PROKKA_02409___45607
PROKKA_05773
PROKKA_06262
PROKKA_04415
PROKKA_04794___34935
0.008

gfo_2.fa.aln.tree.png

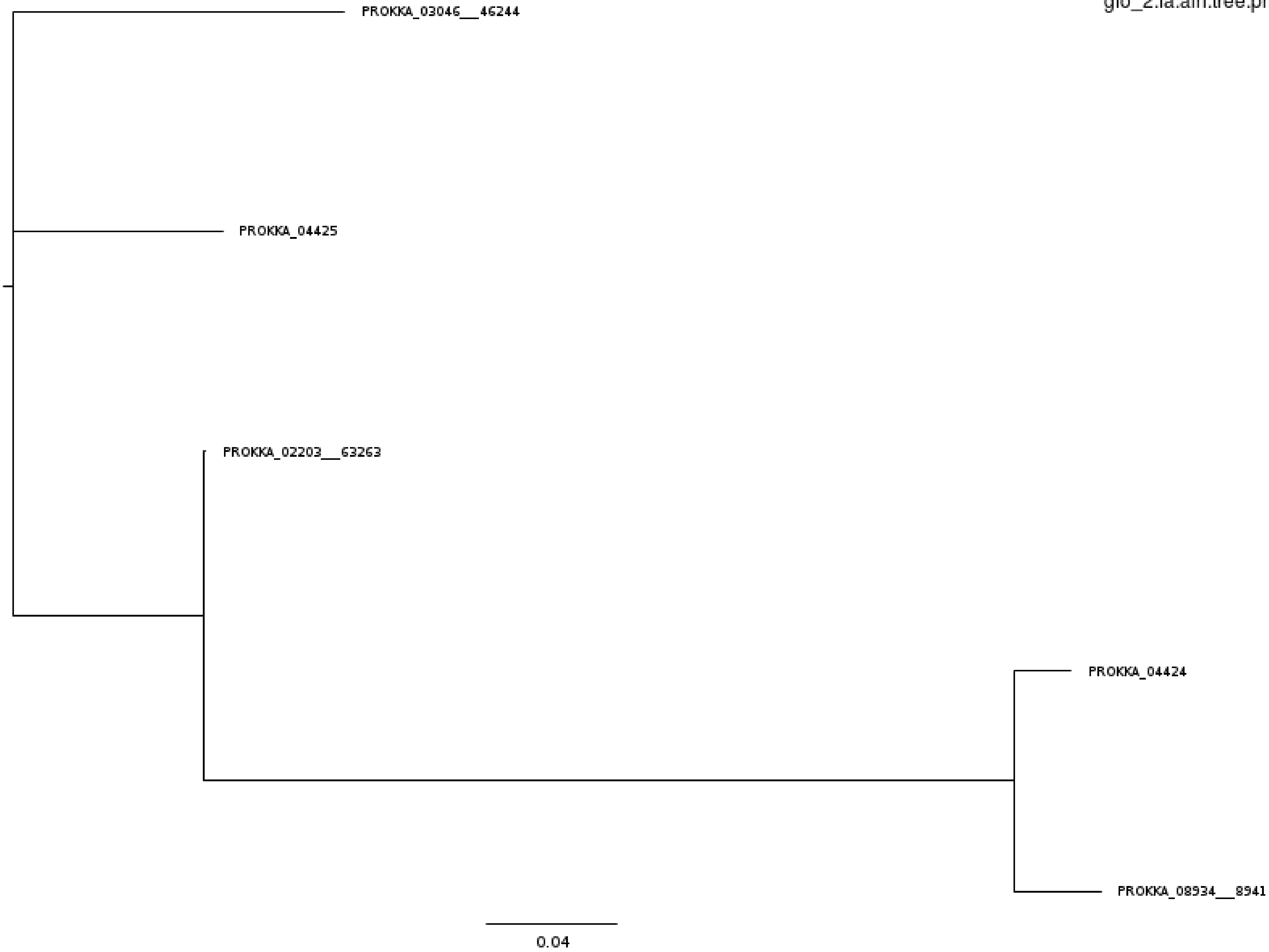

glgA_1.fa.aln.tree.png

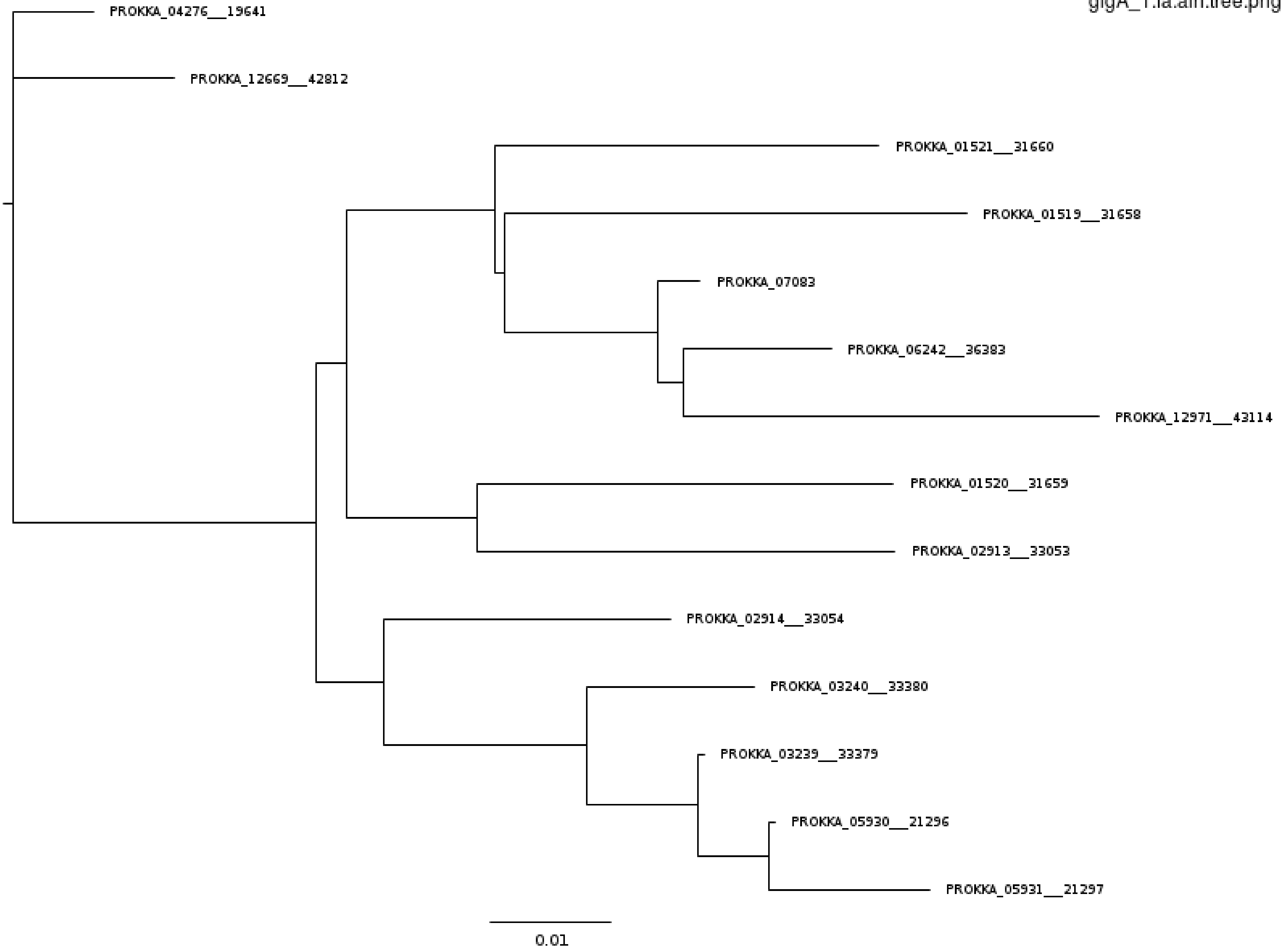

glgA_4.fa.aln.tree.png

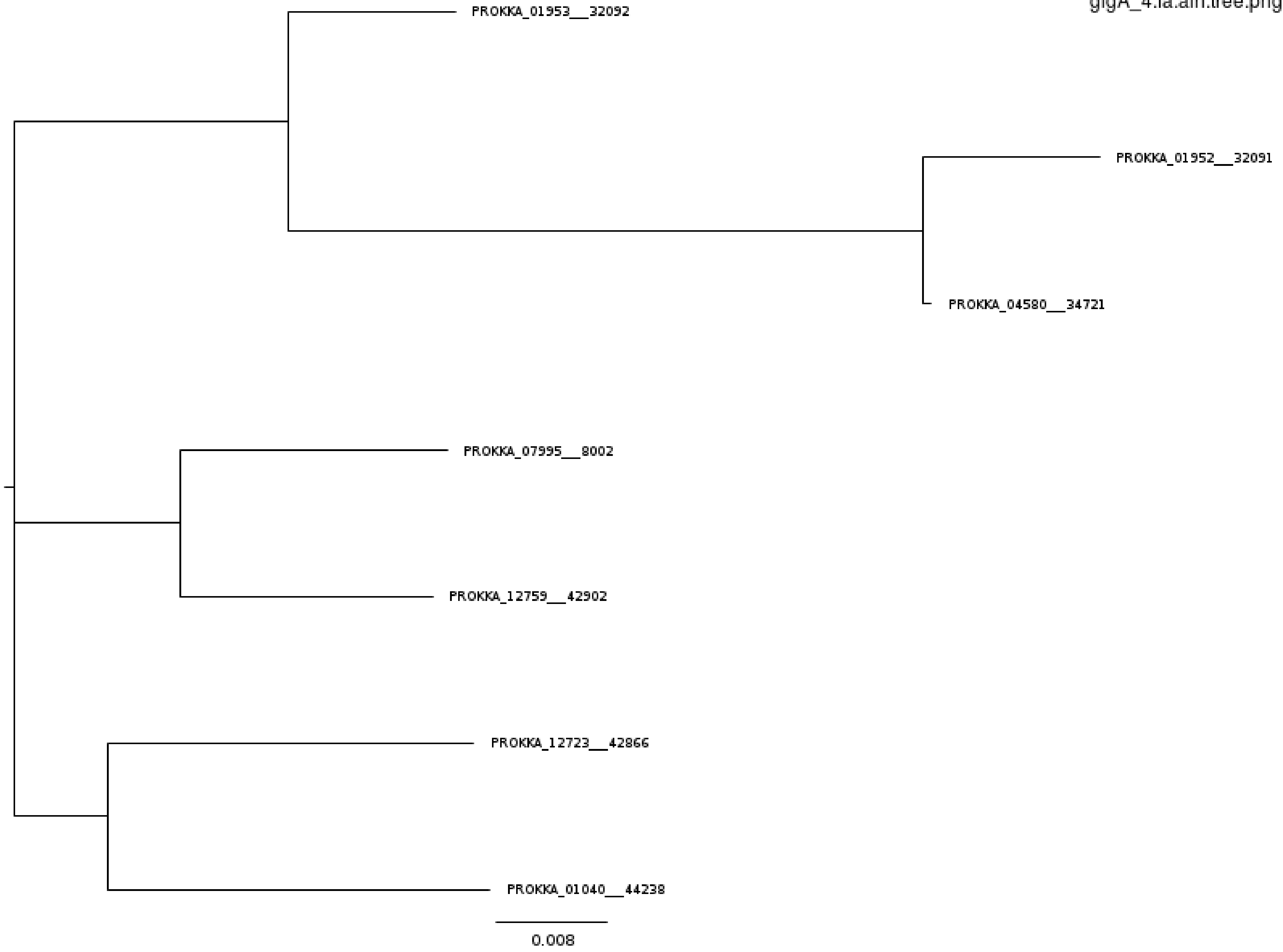

glgB_2.fa.aln.tree.png

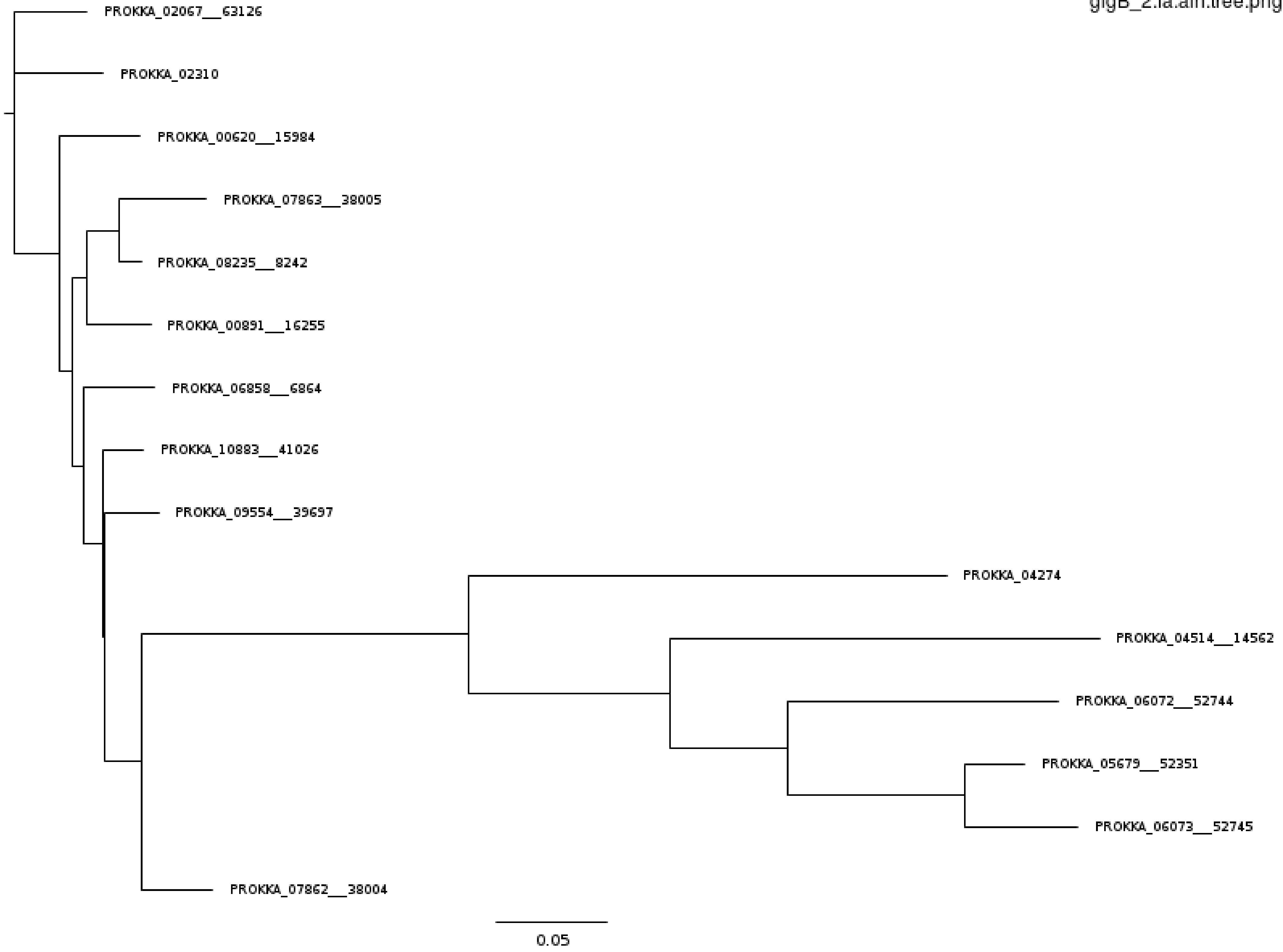

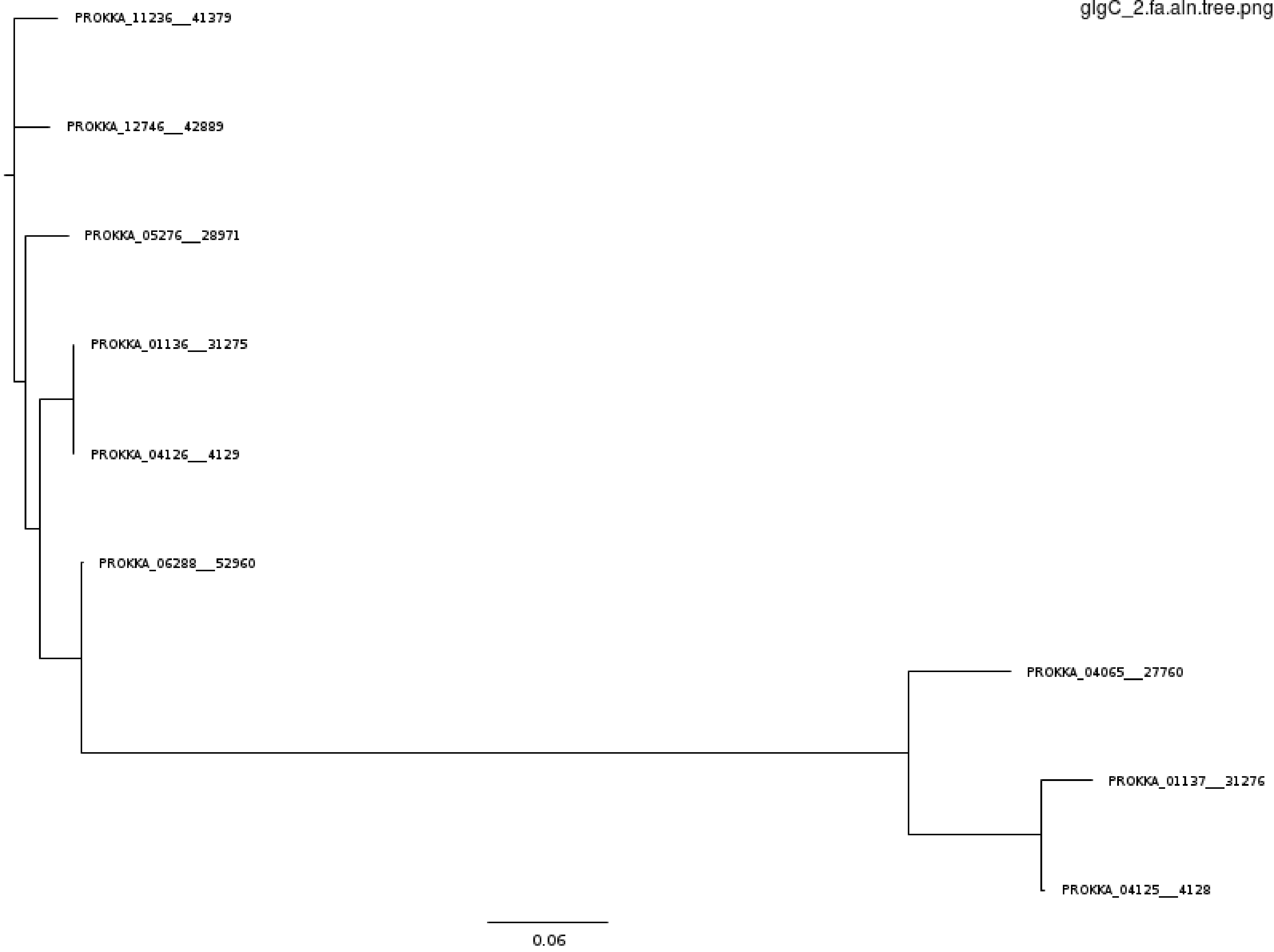

glgC_2.fa.aln.tree.png
PROKKA_11236__41379
PROKKA_12746__42889
PROKKA_05276__28971
PROKKA_01136__31275
PROKKA_04126__4129
PROKKA_06288__52960
PROKKA_04065__27760
PROKKA_01137__31276
PROKKA_04125__4128
0.06

glgX_2.fa.aln.tree.png

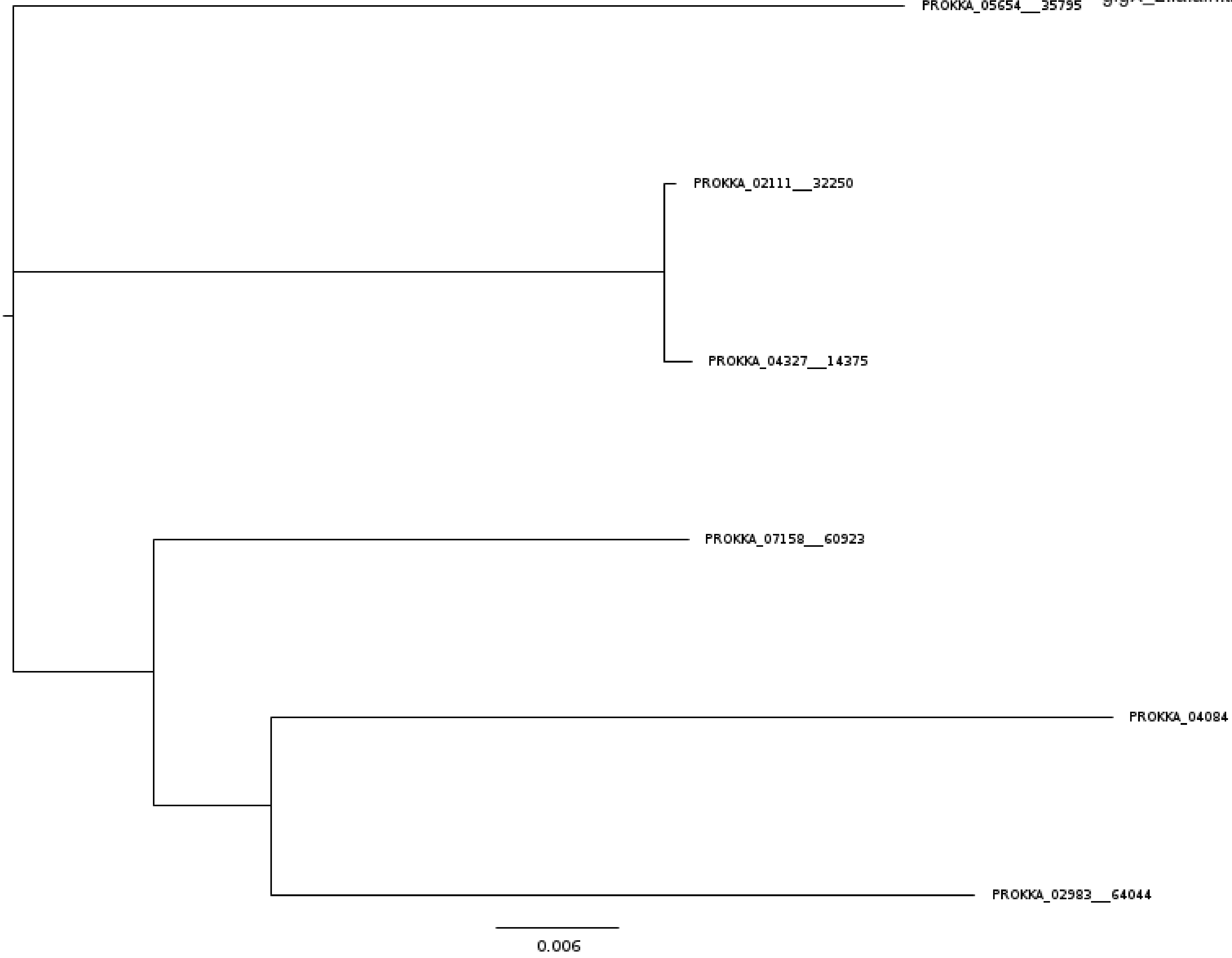

glmM_1.fa.aln.tree.png

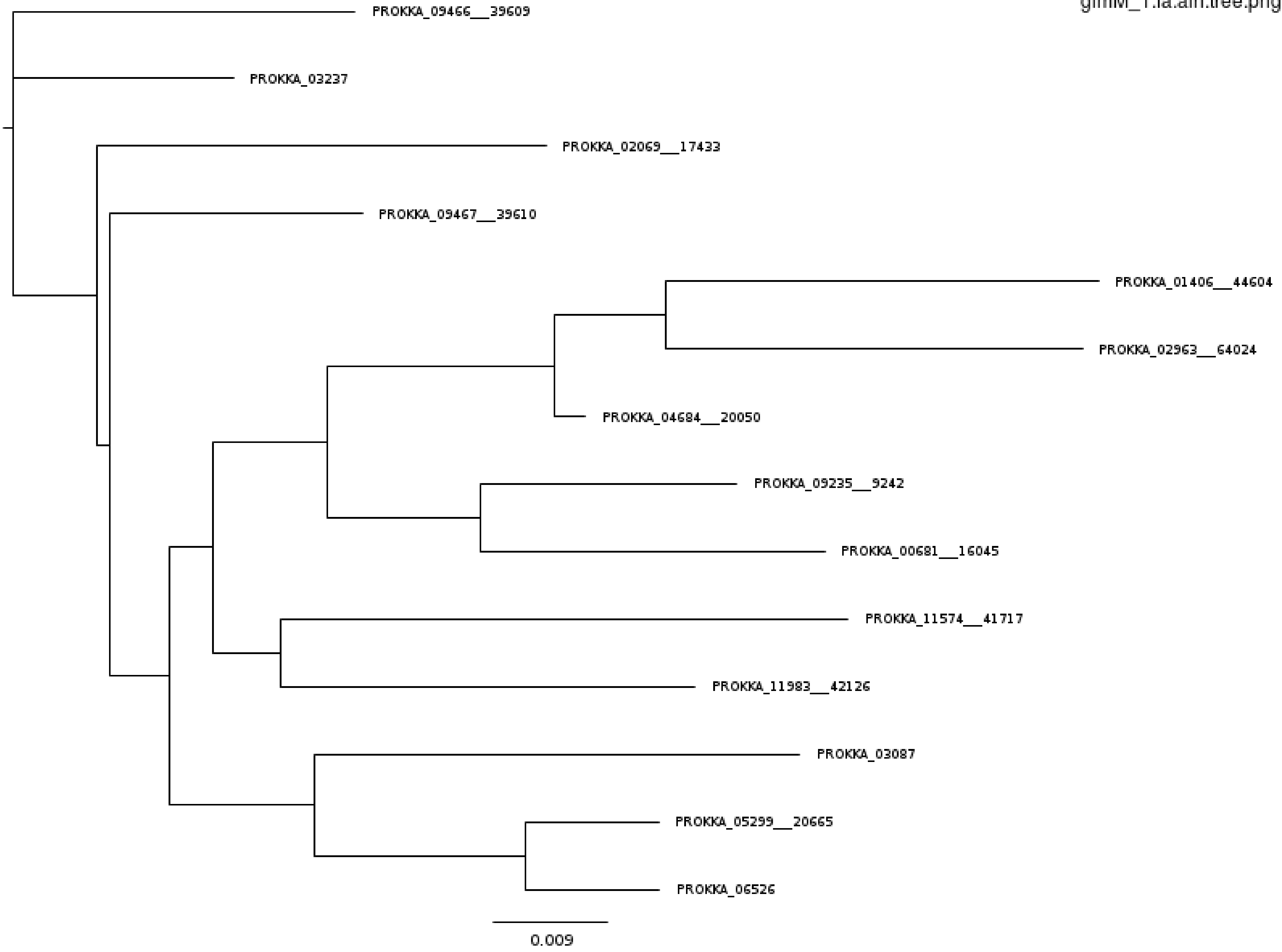

glmM_2.fa.aln.tree.png

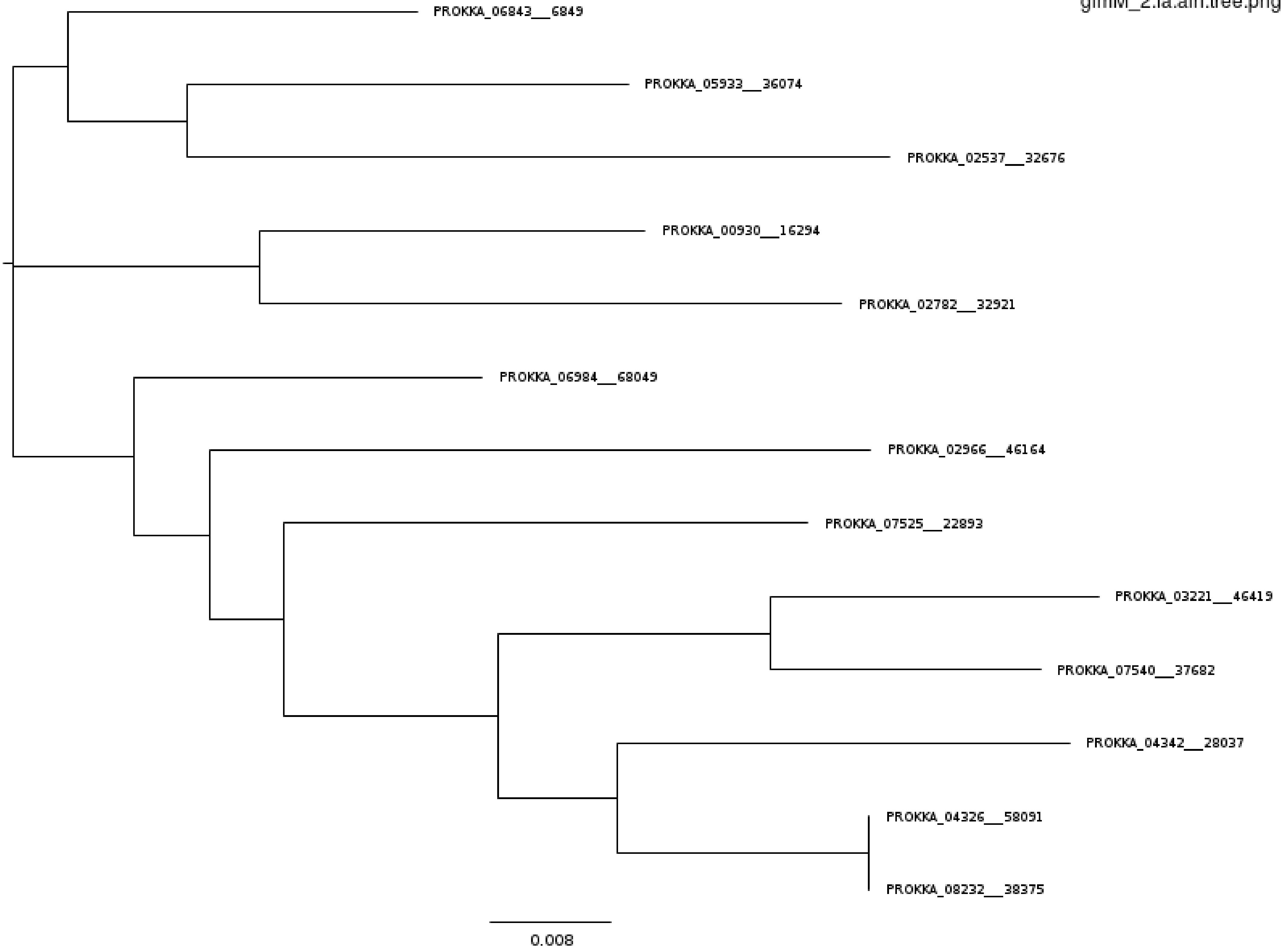

glmM_9.fa.aln.tree.png

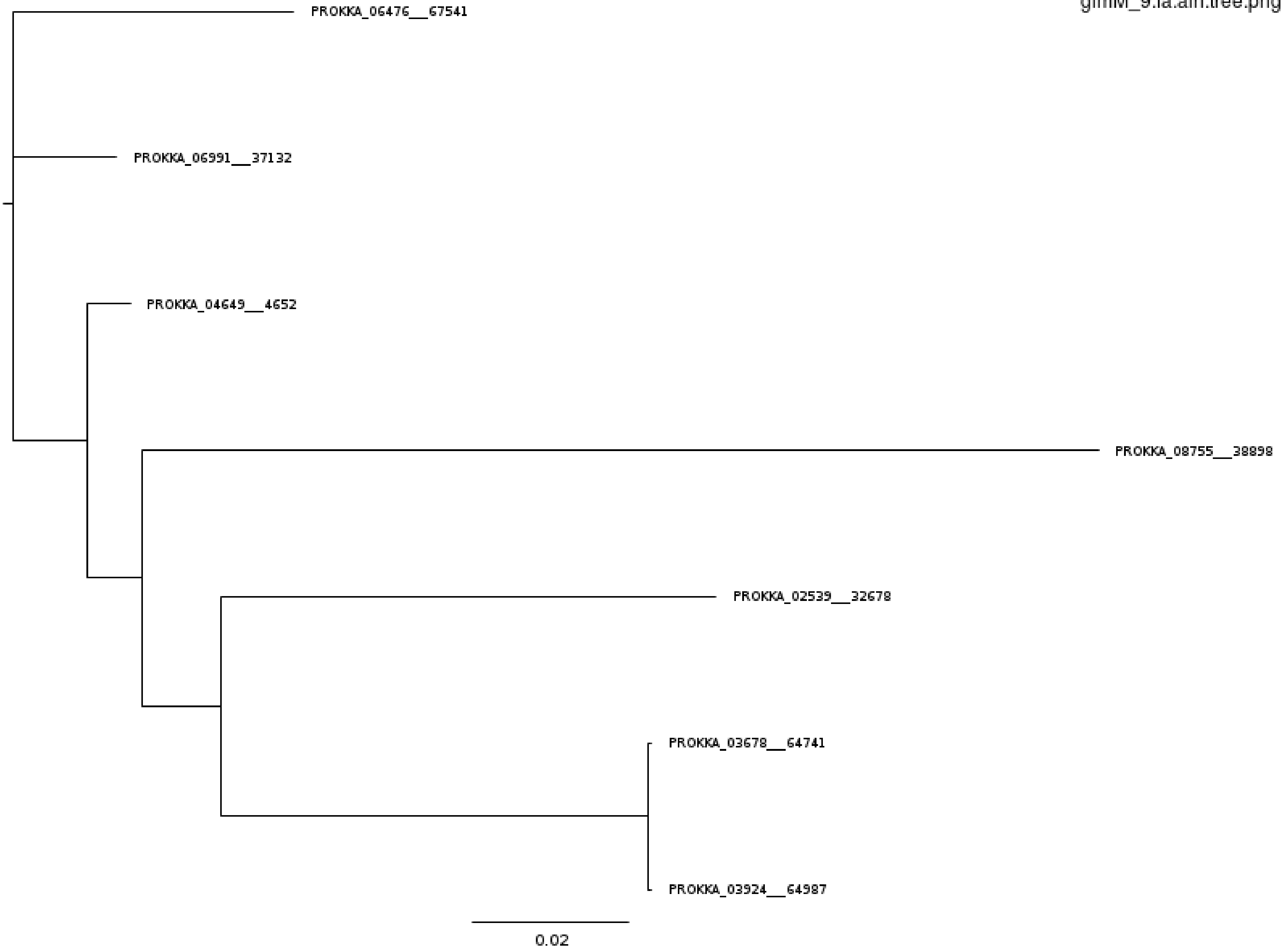

glmS.fa.aln.tree.png

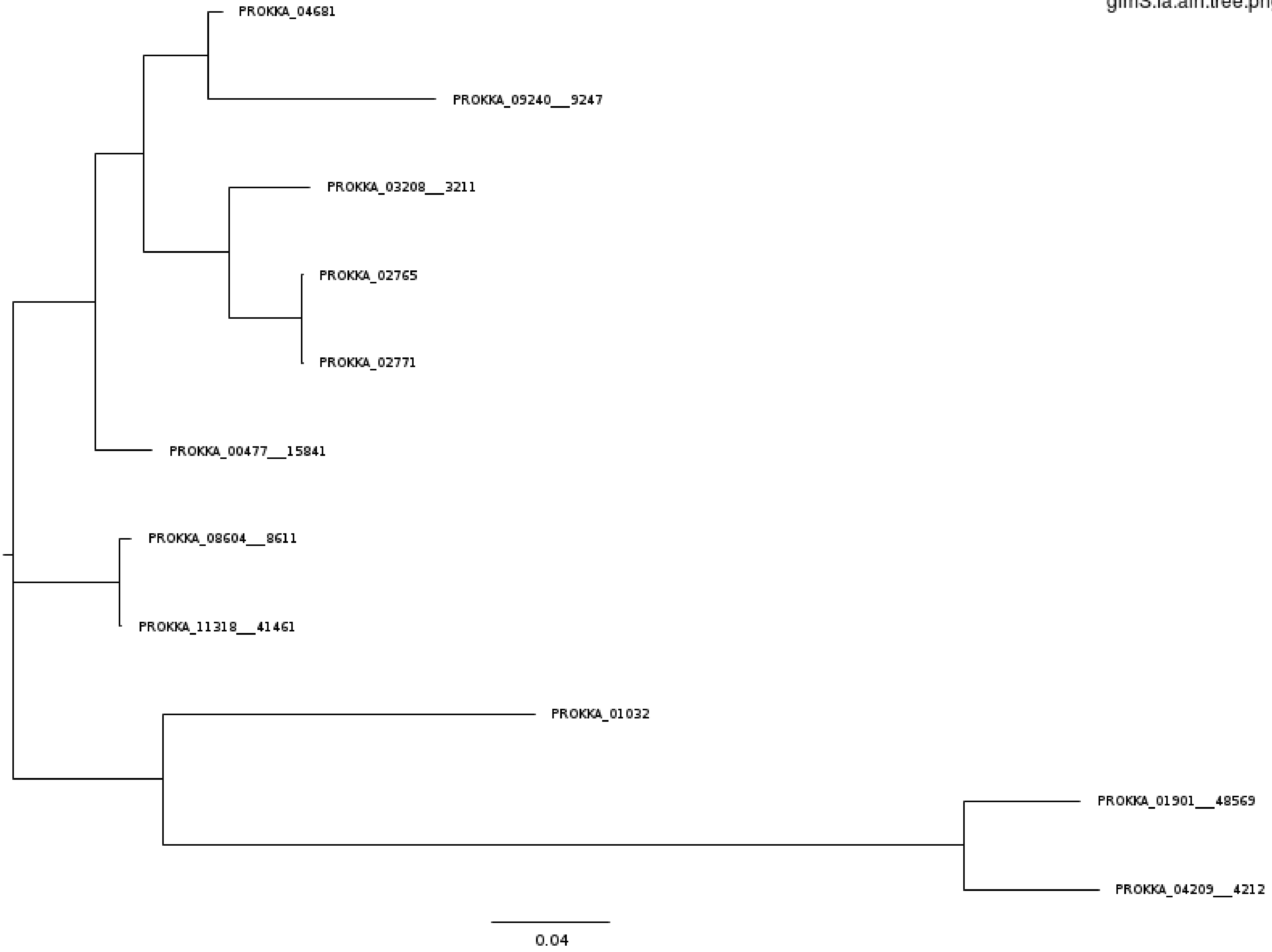

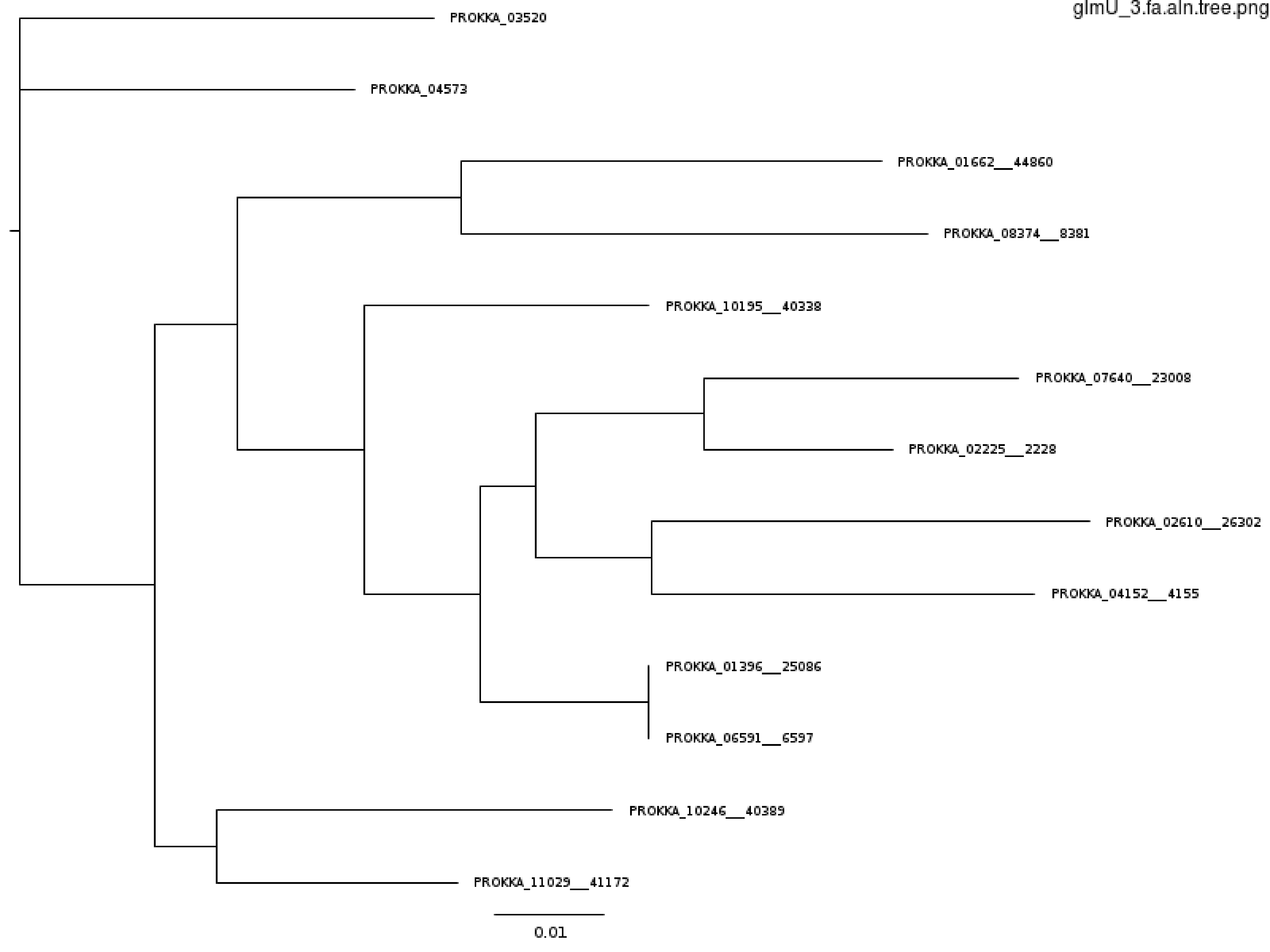
glmU_3.fa.aln.tree.png
PROKKA_03520
PROKKA_04573
PROKKA_01662___44860
PROKKA_08374___8381
PROKKA_10195___40338
PROKKA_07640___23008
PROKKA_02225___2228
PROKKA_02610___26302
PROKKA_04152___4155
PROKKA_01396___25086
PROKKA_06591___6597
PROKKA_10246___40389
PROKKA_11029___41172
0.01

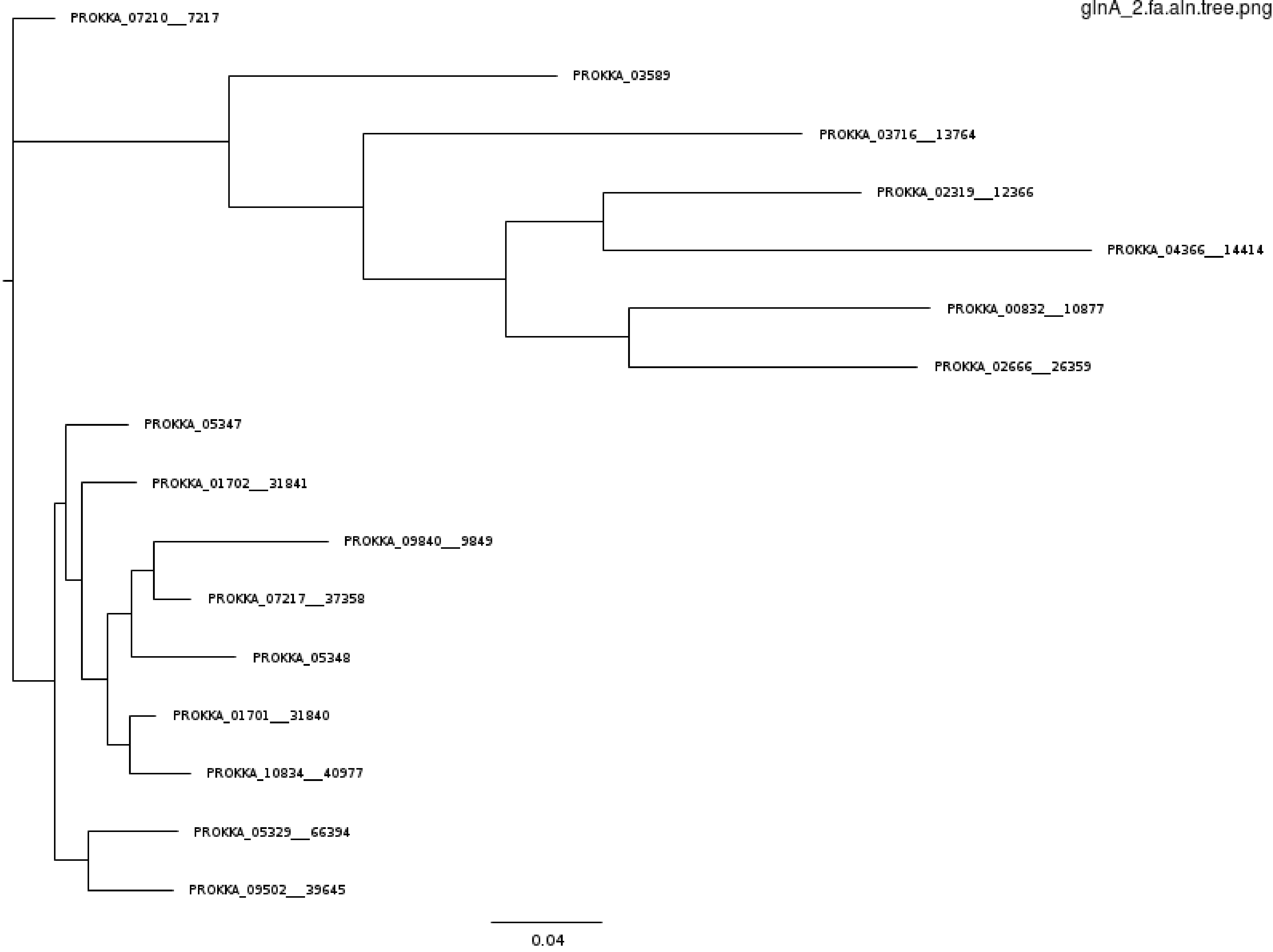
glnA_2.fa.aln.tree.png
PROKKA_07210___7217
PROKKA_03589
PROKKA_03716___13764
PROKKA_02319___12366
PROKKA_04366___14414
PROKKA_00832___10877
PROKKA_02666___26359
PROKKA_05347
PROKKA_01702___31841
PROKKA_09840___9849
PROKKA_07217___37358
PROKKA_05348
PROKKA_01701___31840
PROKKA_10834___40977
PROKKA_05329___66394
PROKKA_09502___39645
0.04

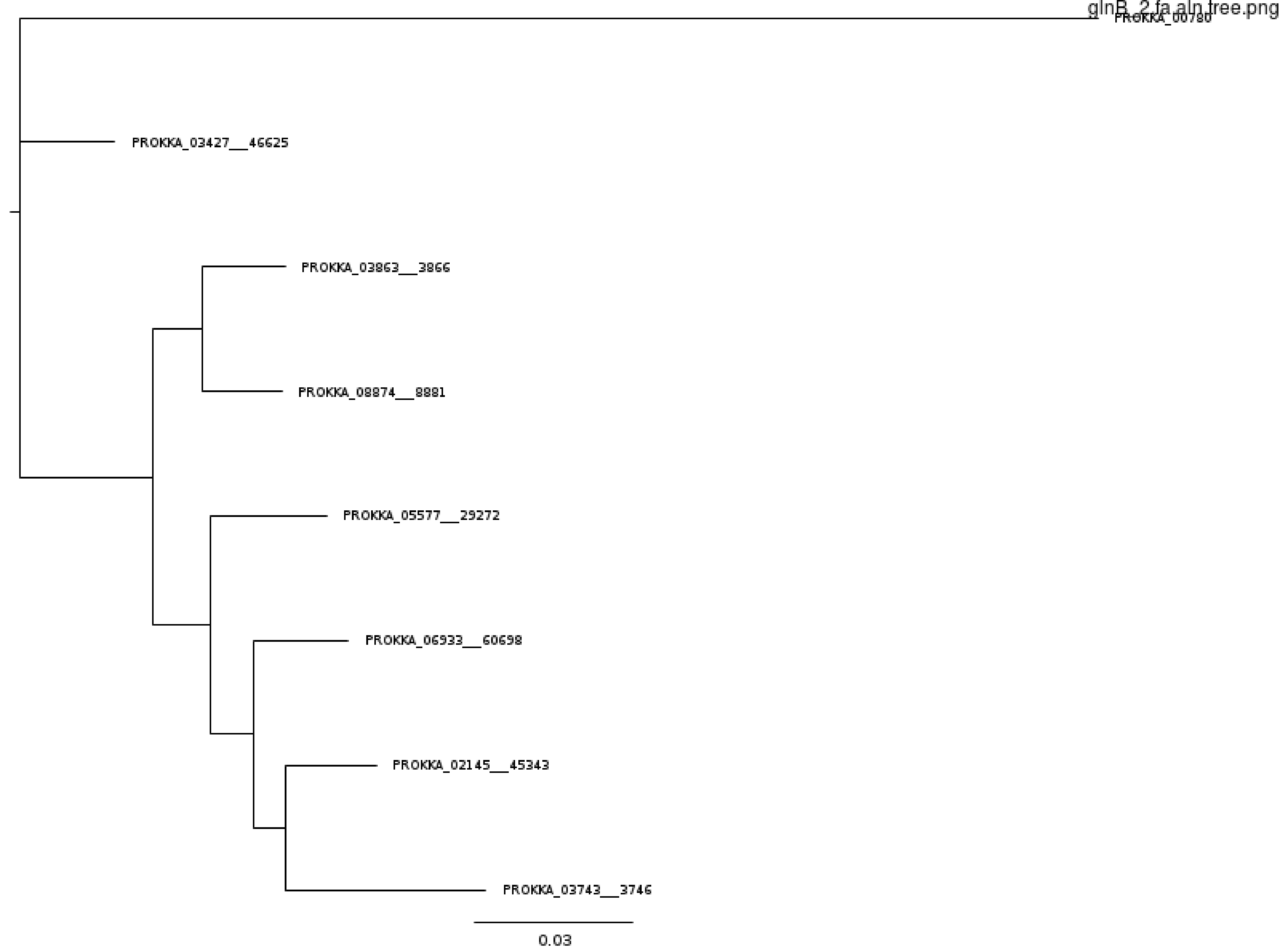
glnB_2.fa.aln.tree.png
PROKKA_00780
PROKKA_03427___46625
PROKKA_03863___3866
PROKKA_08874___8881
PROKKA_05577___29272
PROKKA_06933___60698
PROKKA_02145___45343
PROKKA_03743___3746
0.03

glnB_6.fa.aln.tree.png

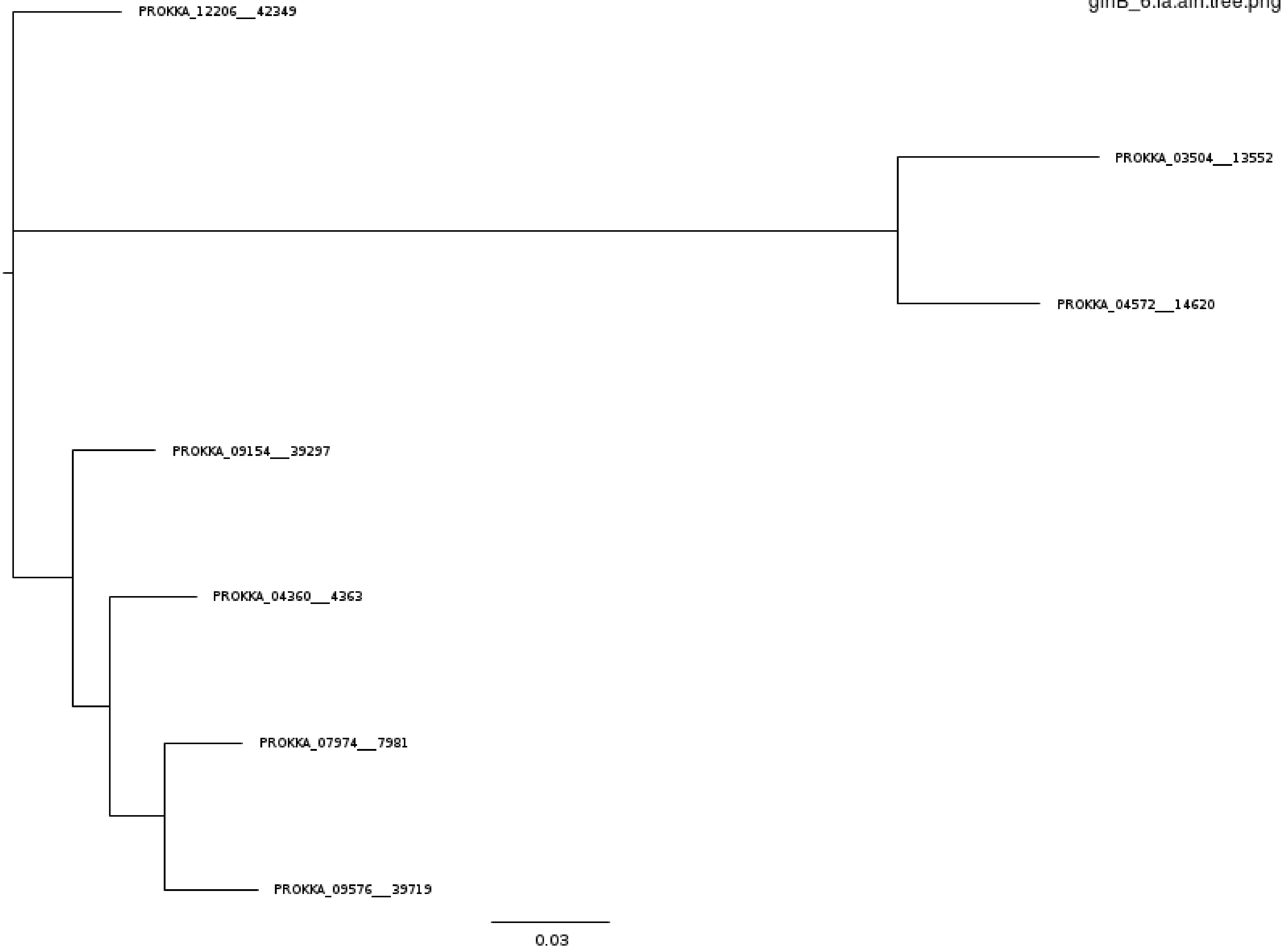

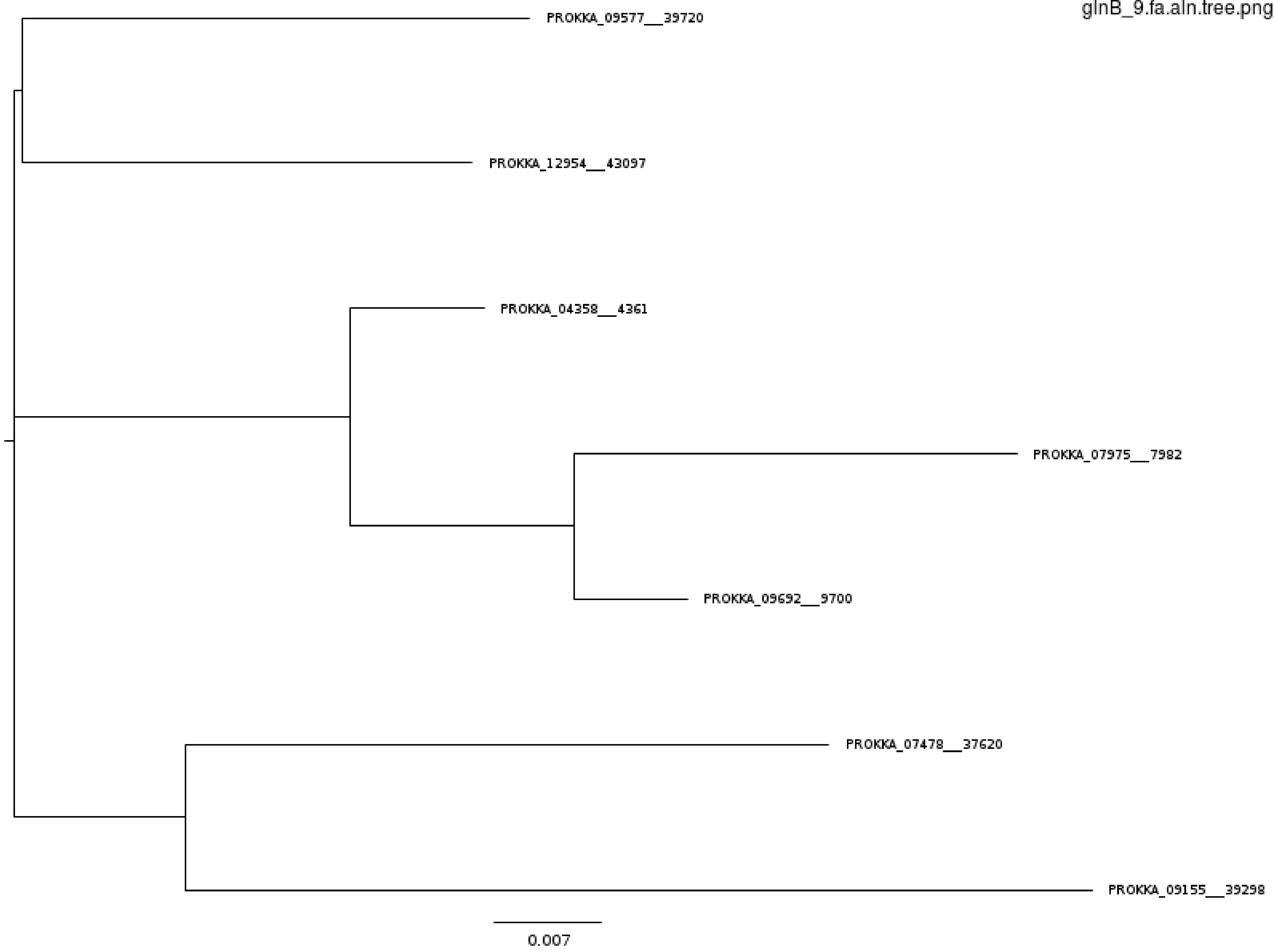
glnB_9.fa.aln.tree.png
PROKKA_09577___39720
PROKKA_12954___43097
PROKKA_04358___4361
PROKKA_07975___7982
PROKKA_09692___9700
PROKKA_07478___37620
PROKKA_09155___39298
0.007

glnD_1.fa.aln.tree.png

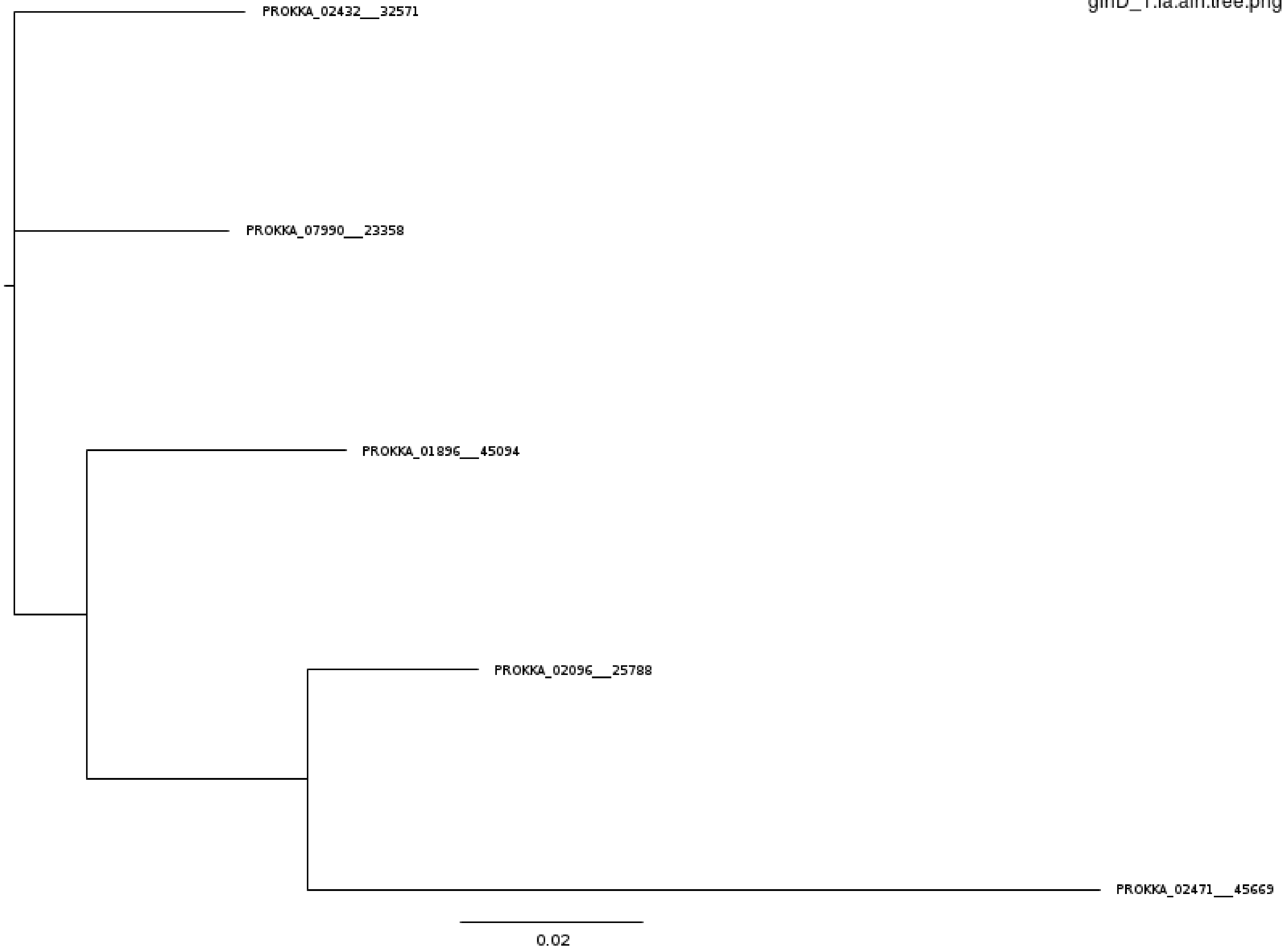

glnG_2.fa.aln.tree.png

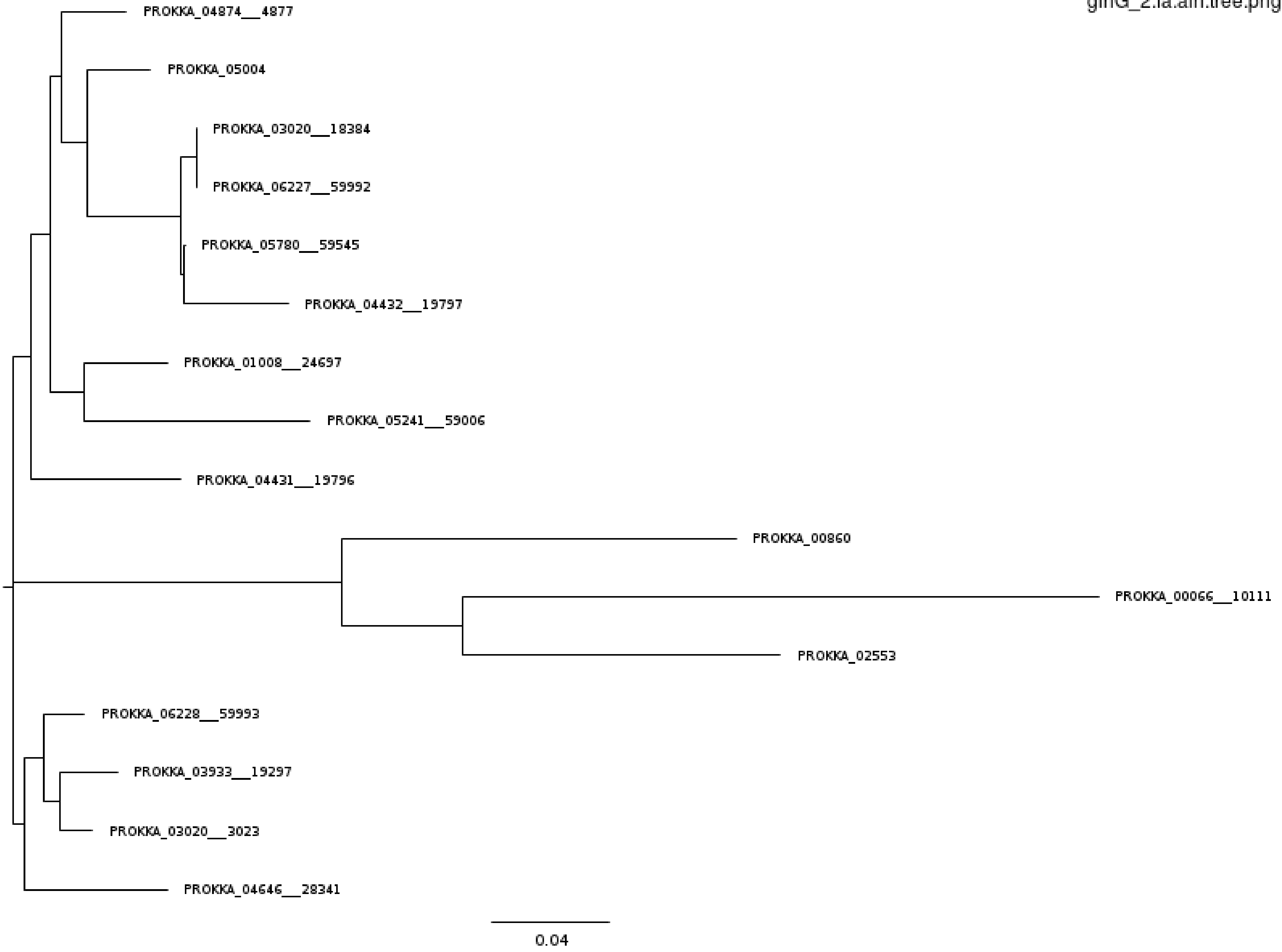

glnL_1.fa.aln.tree.png

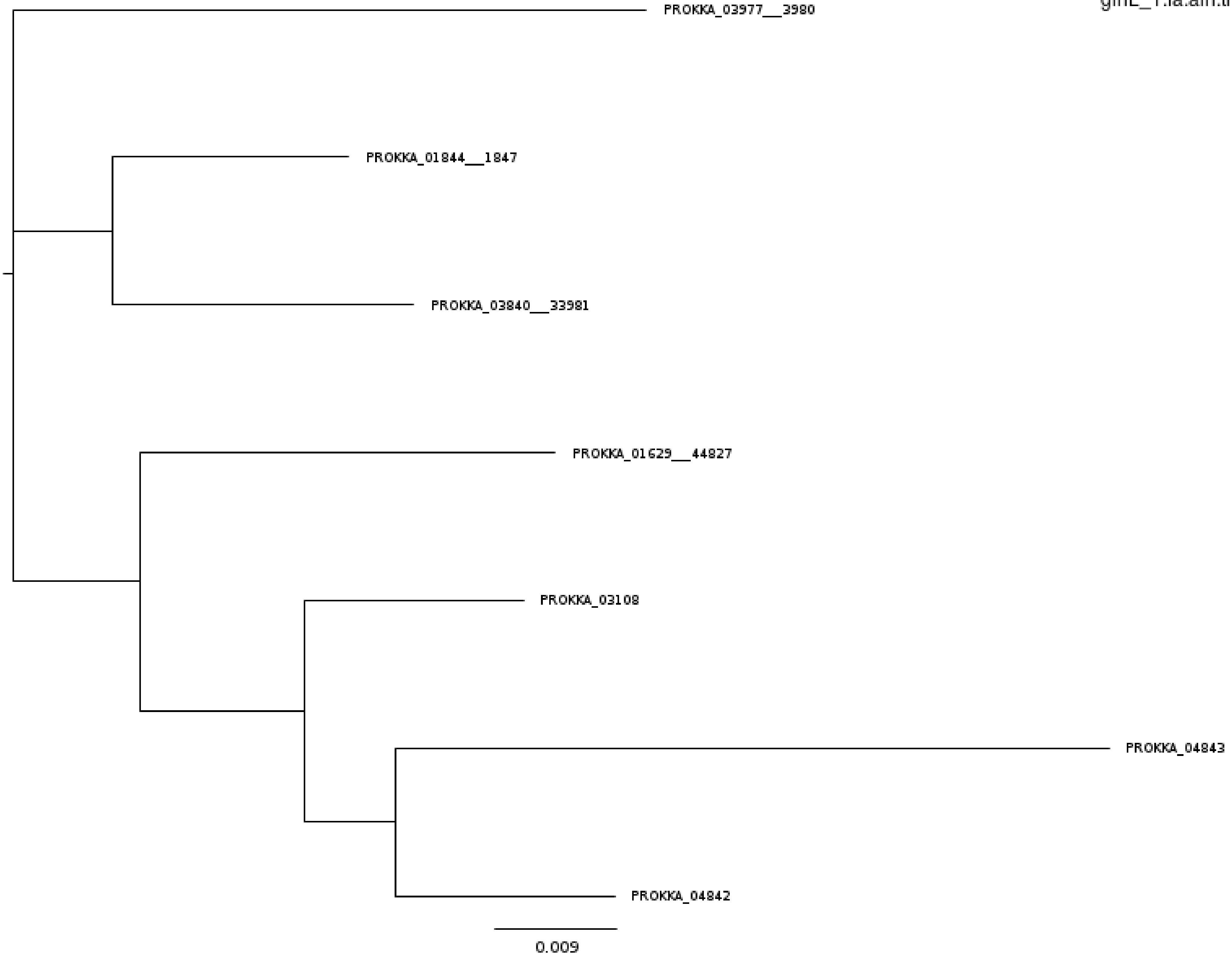

glnS.fa.aln.tree.png

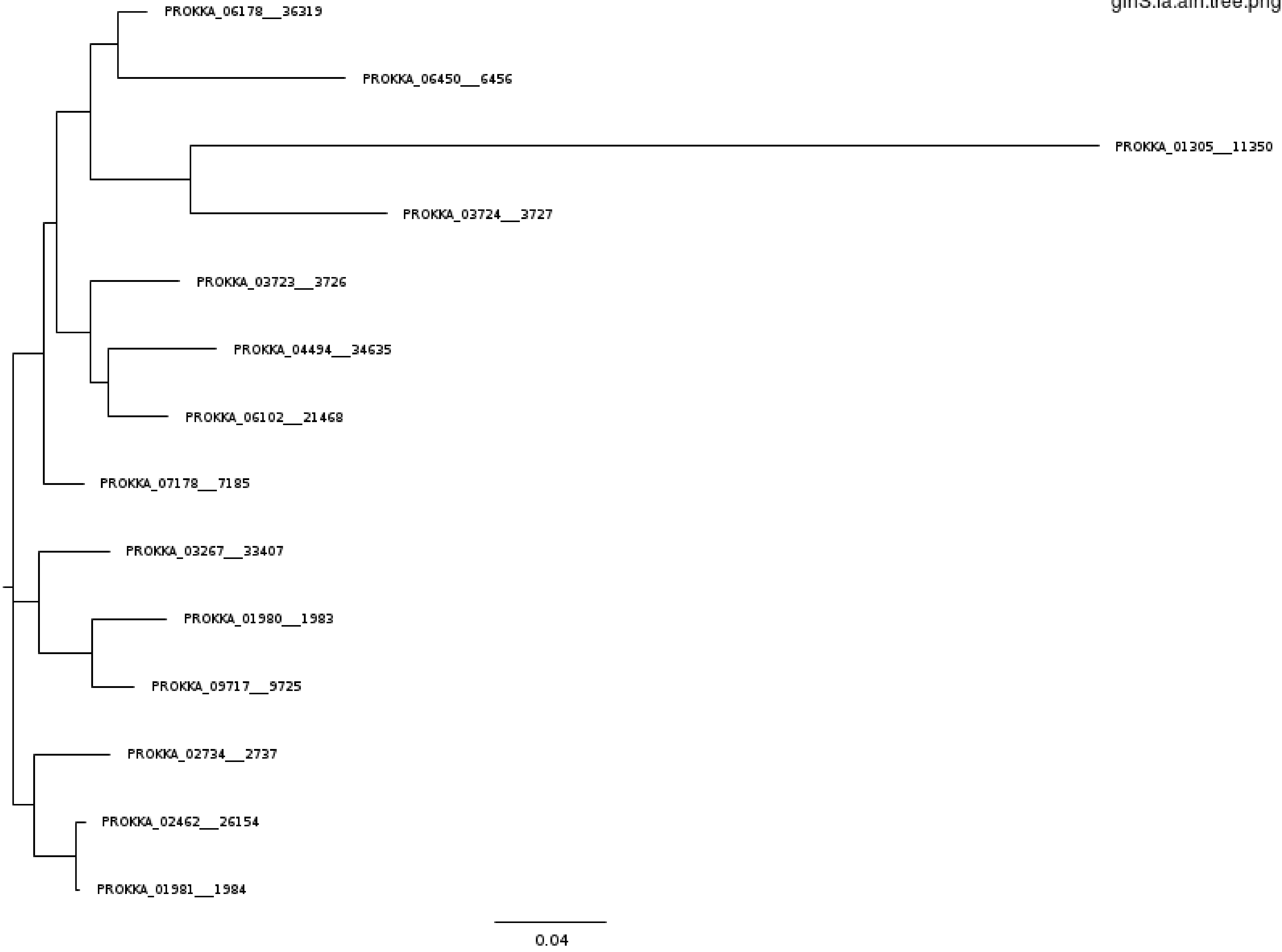

gloB_3.fa.aln.tree.png

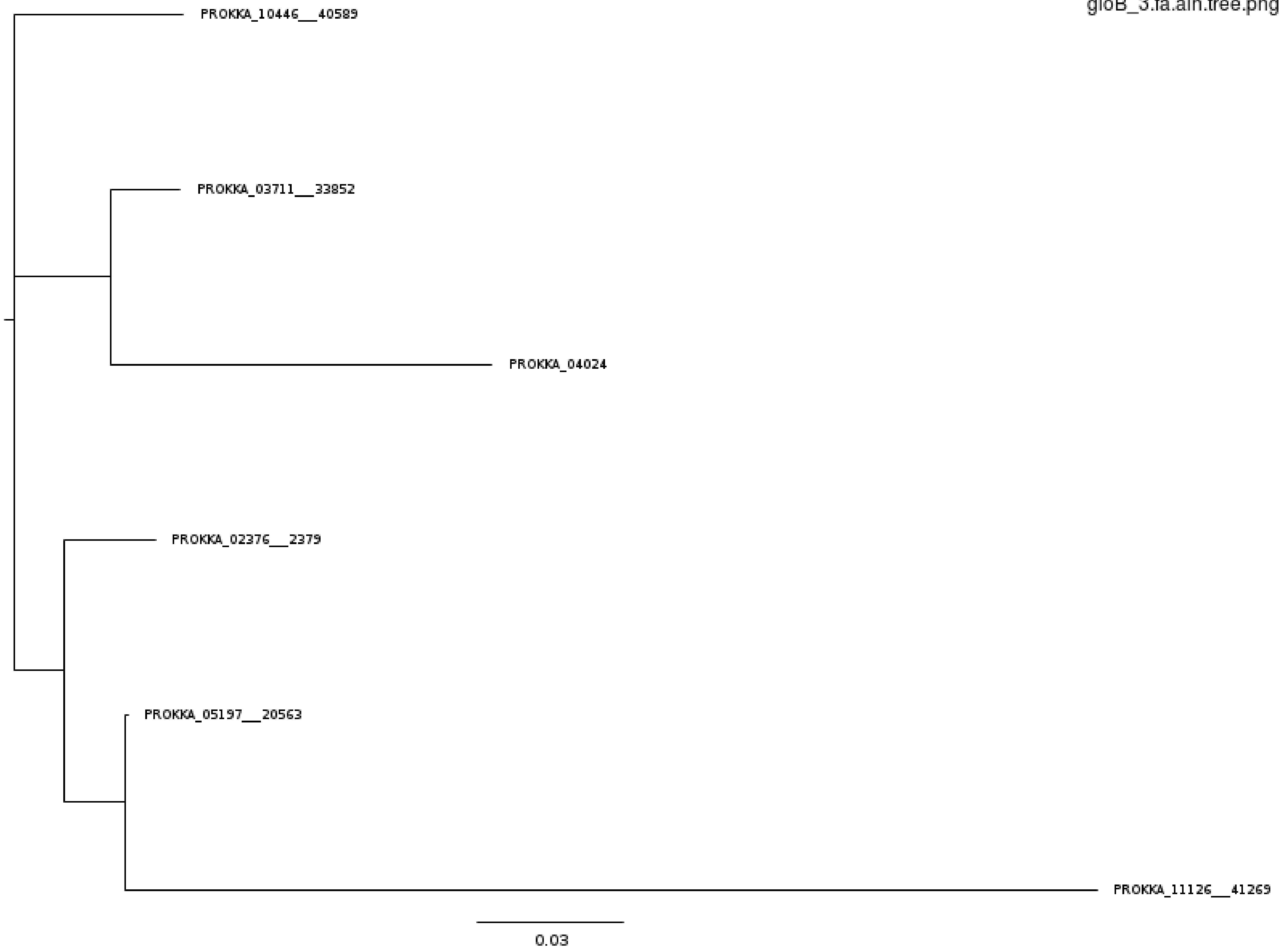

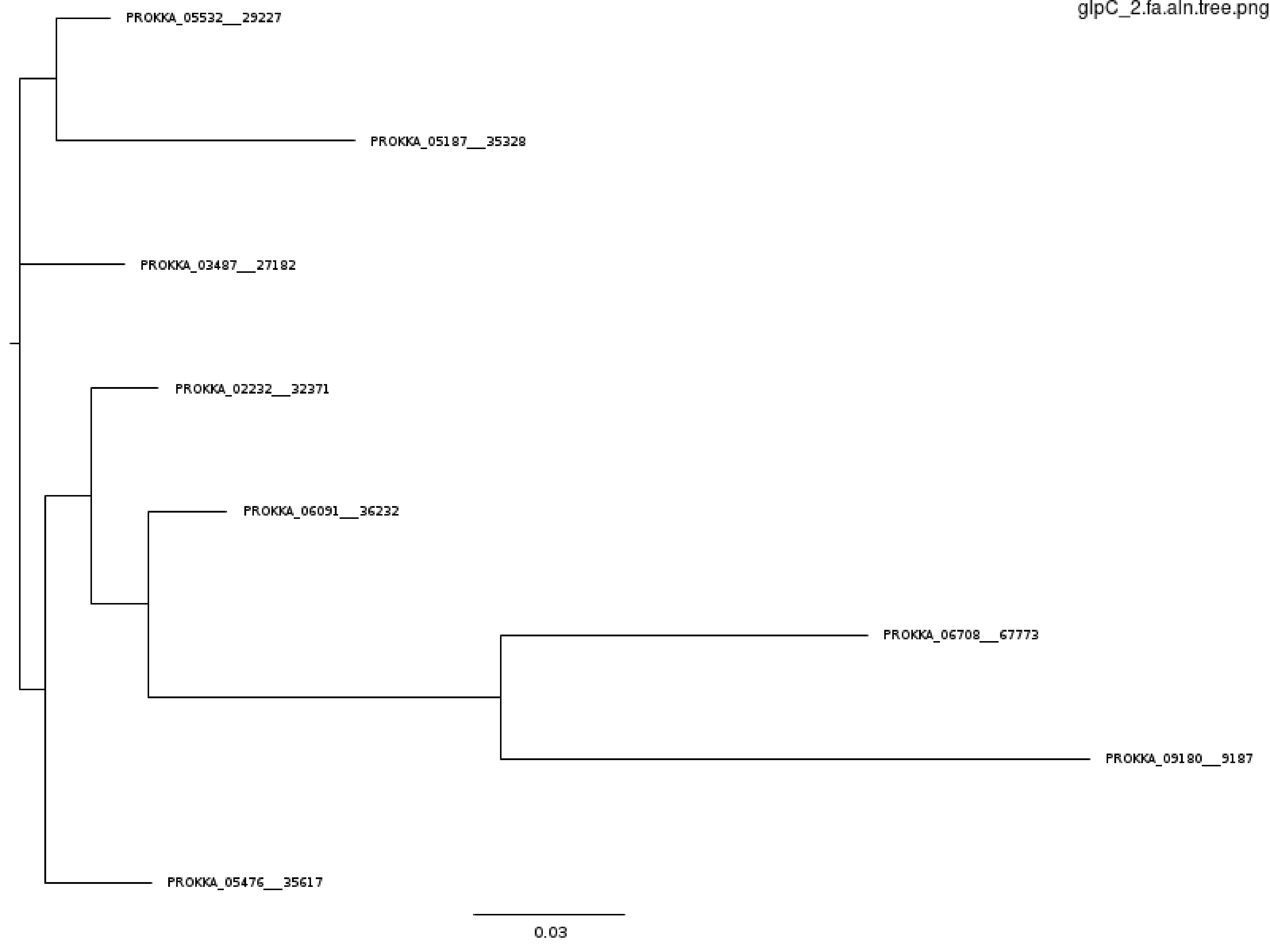
glpC_2.fa.aln.tree.png
PROKKA_05532___29227
PROKKA_05187___35328
PROKKA_03487___27182
PROKKA_02232___32371
PROKKA_06091___36232
PROKKA_06708___67773
PROKKA_09180___9187
PROKKA_05476___35617
0.03

glpE_3.fa.aln.tree.png

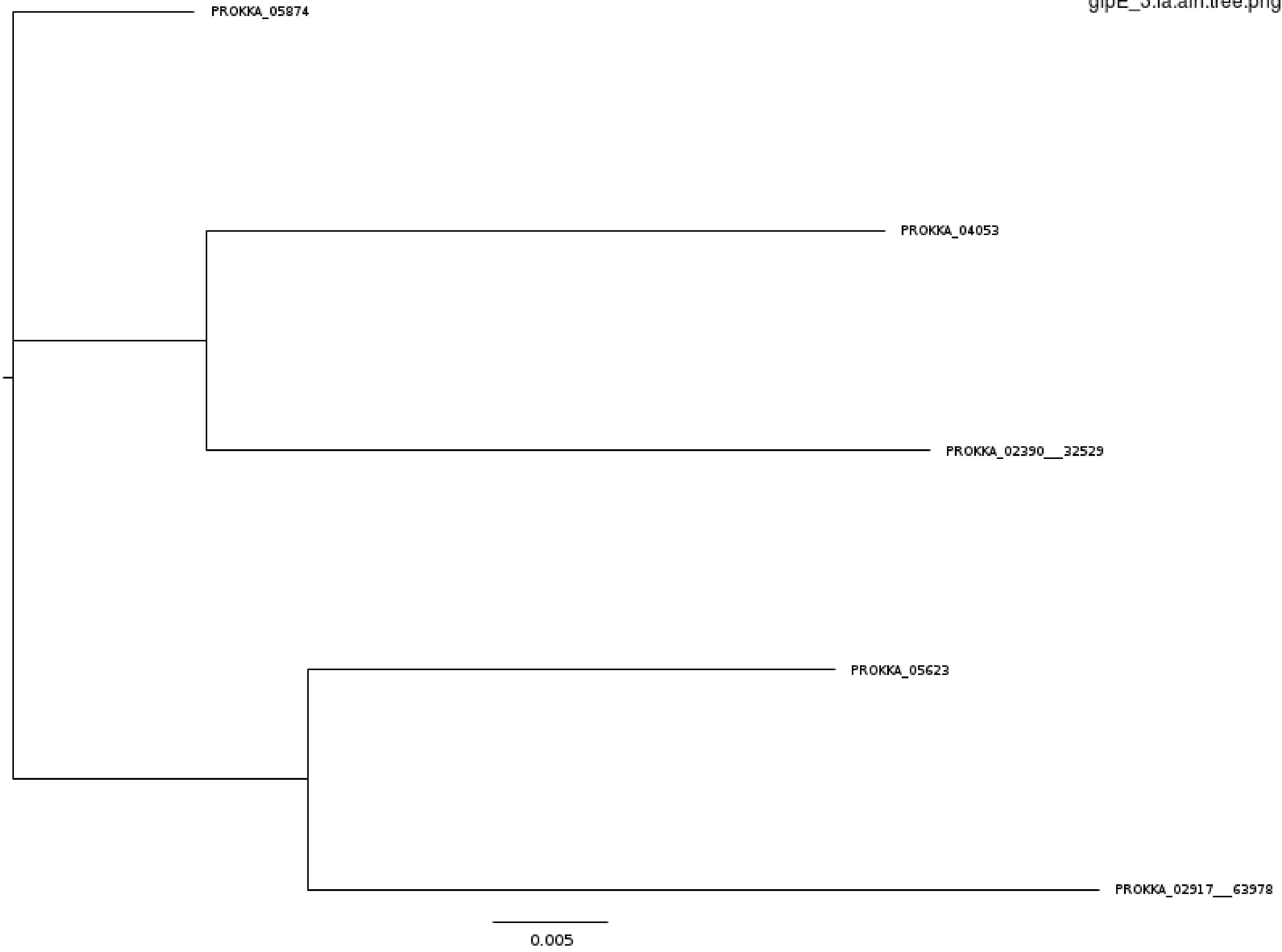

glnE.fa.aln.tree.png

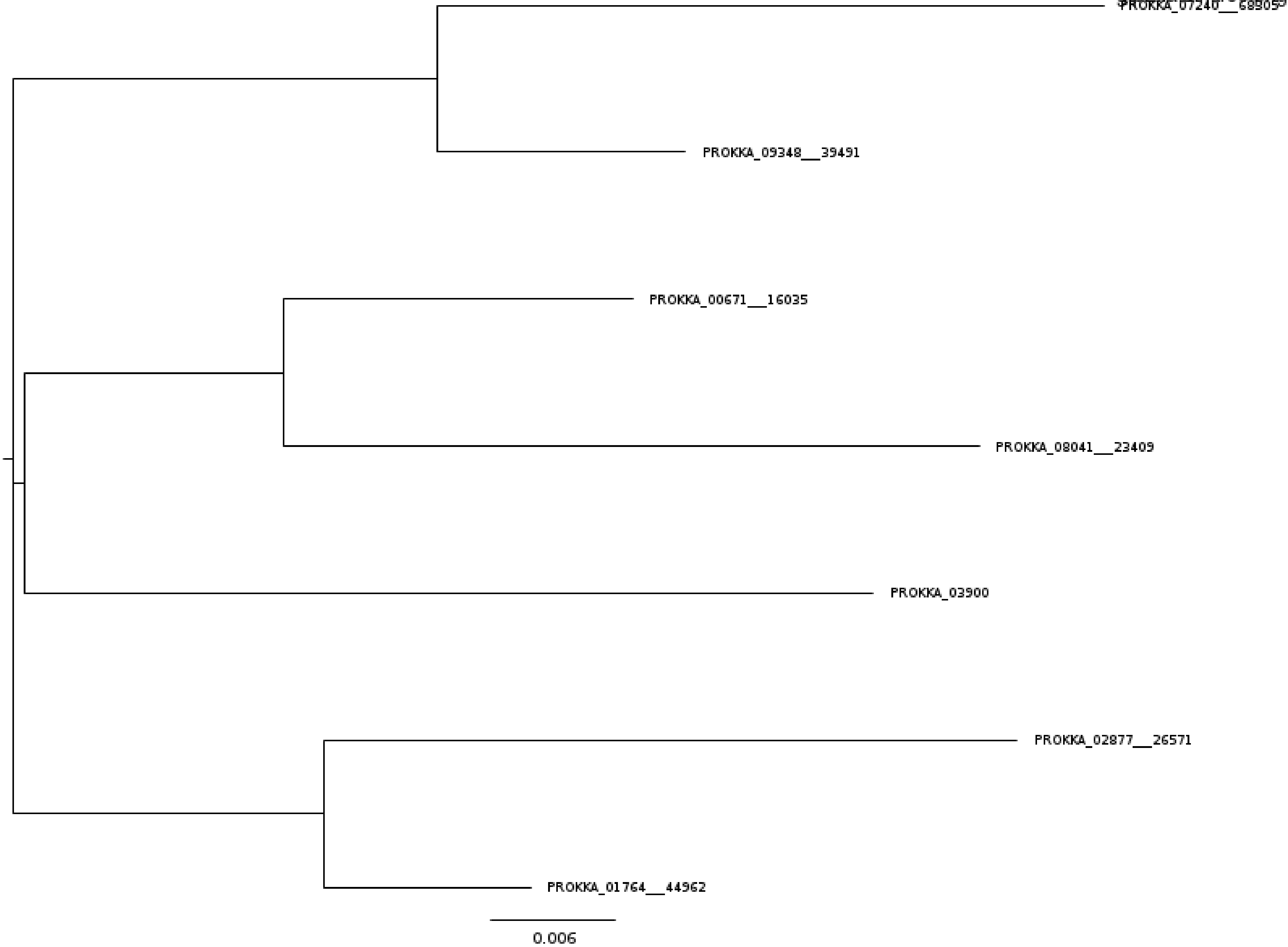

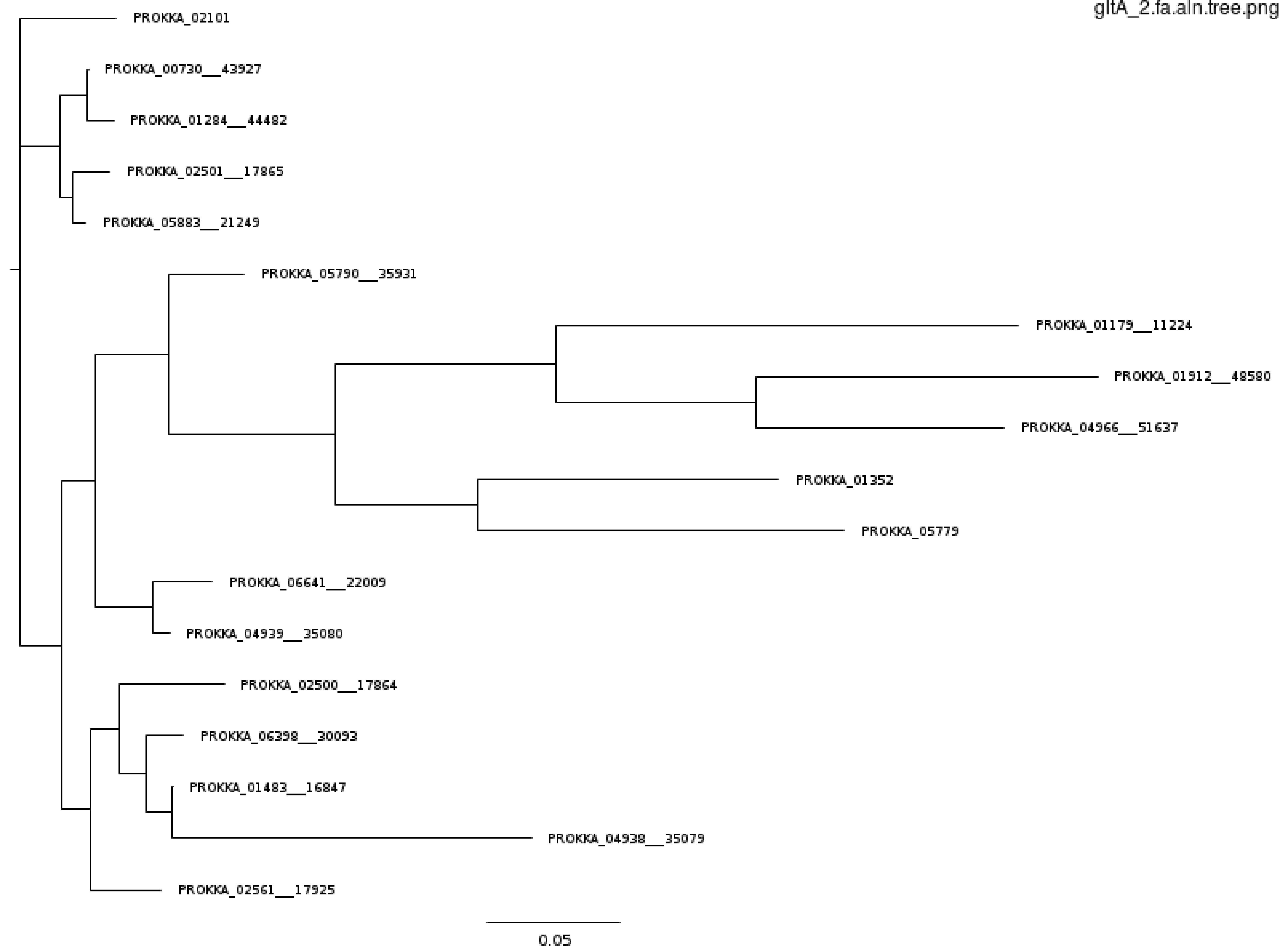

gltA_2.fa.aln.tree.png
PROKKA_02101
PROKKA_00730___43927
PROKKA_01284___44482
PROKKA_02501___17865
PROKKA_05883___21249
PROKKA_05790___35931
PROKKA_01179___11224
PROKKA_01912___48580
PROKKA_04966___51637
PROKKA_01352
PROKKA_05779
PROKKA_06641___22009
PROKKA_04939___35080
PROKKA_02500___17864
PROKKA_06398___30093
PROKKA_01483___16847
PROKKA_04938___35079
PROKKA_02561___17925
0.05

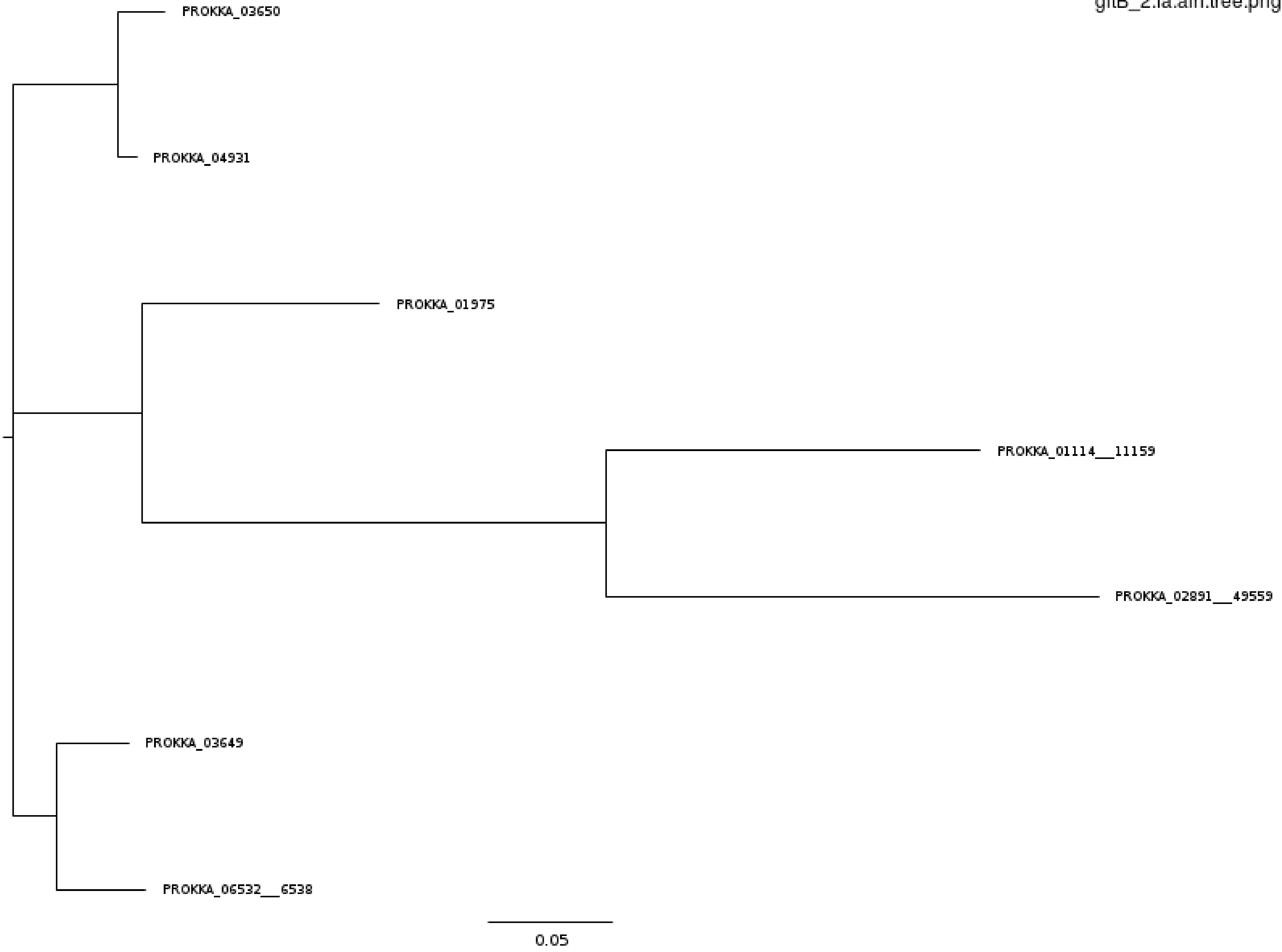
PROKKA_03650
PROKKA_04931
PROKKA_01975
PROKKA_01114___11159
PROKKA_02891___49559
PROKKA_03649
PROKKA_06532___6538
0.05

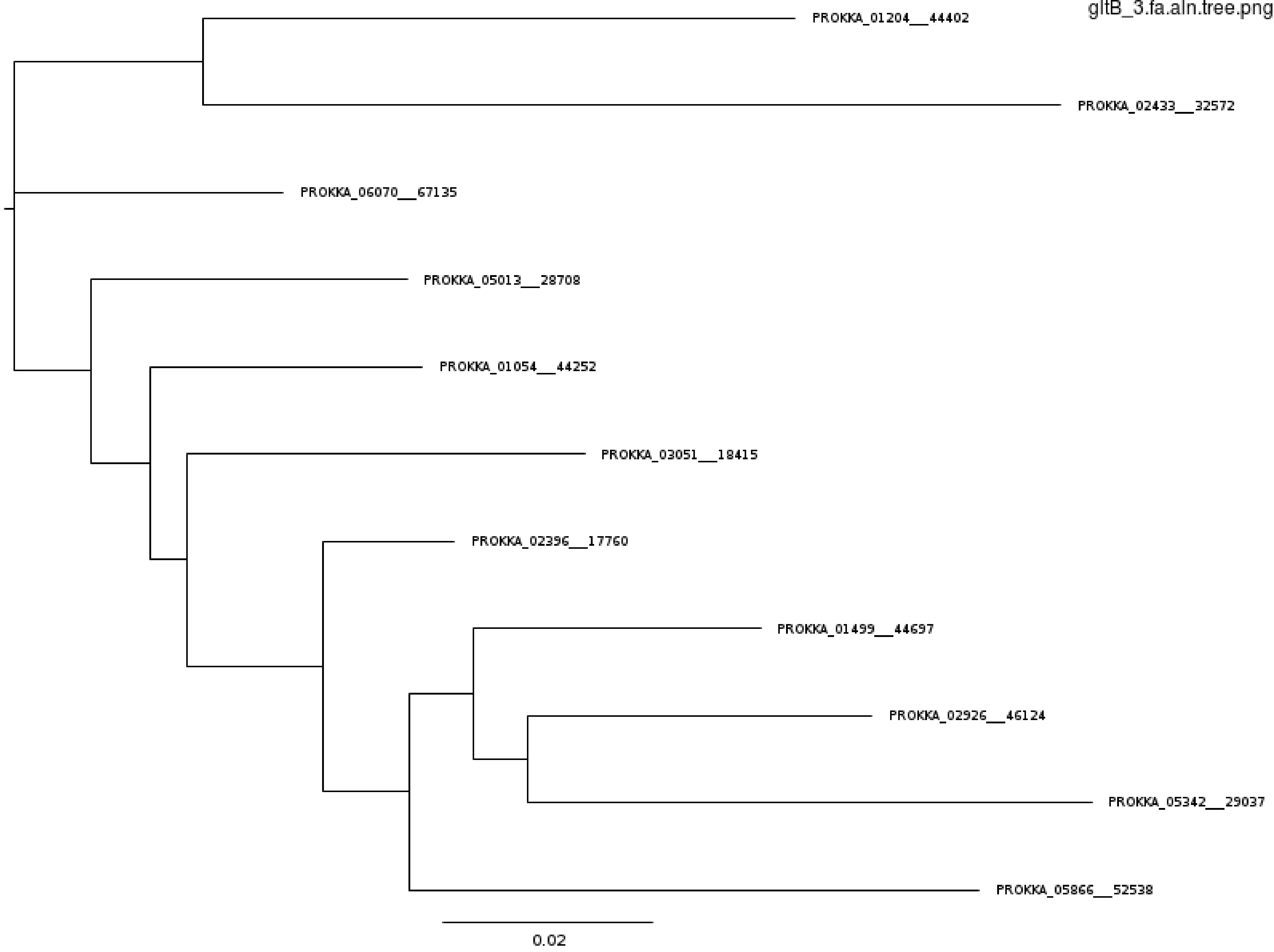
gltB_3.fa.aln.tree.png
PROKKA_01204___44402
PROKKA_02433___32572
PROKKA_06070___67135
PROKKA_05013___28708
PROKKA_01054___44252
PROKKA_03051___18415
PROKKA_02396___17760
PROKKA_01499___44697
PROKKA_02926___46124
PROKKA_05342___29037
PROKKA_05866___52538
0.02

gltB_6.fa.aln.tree.png

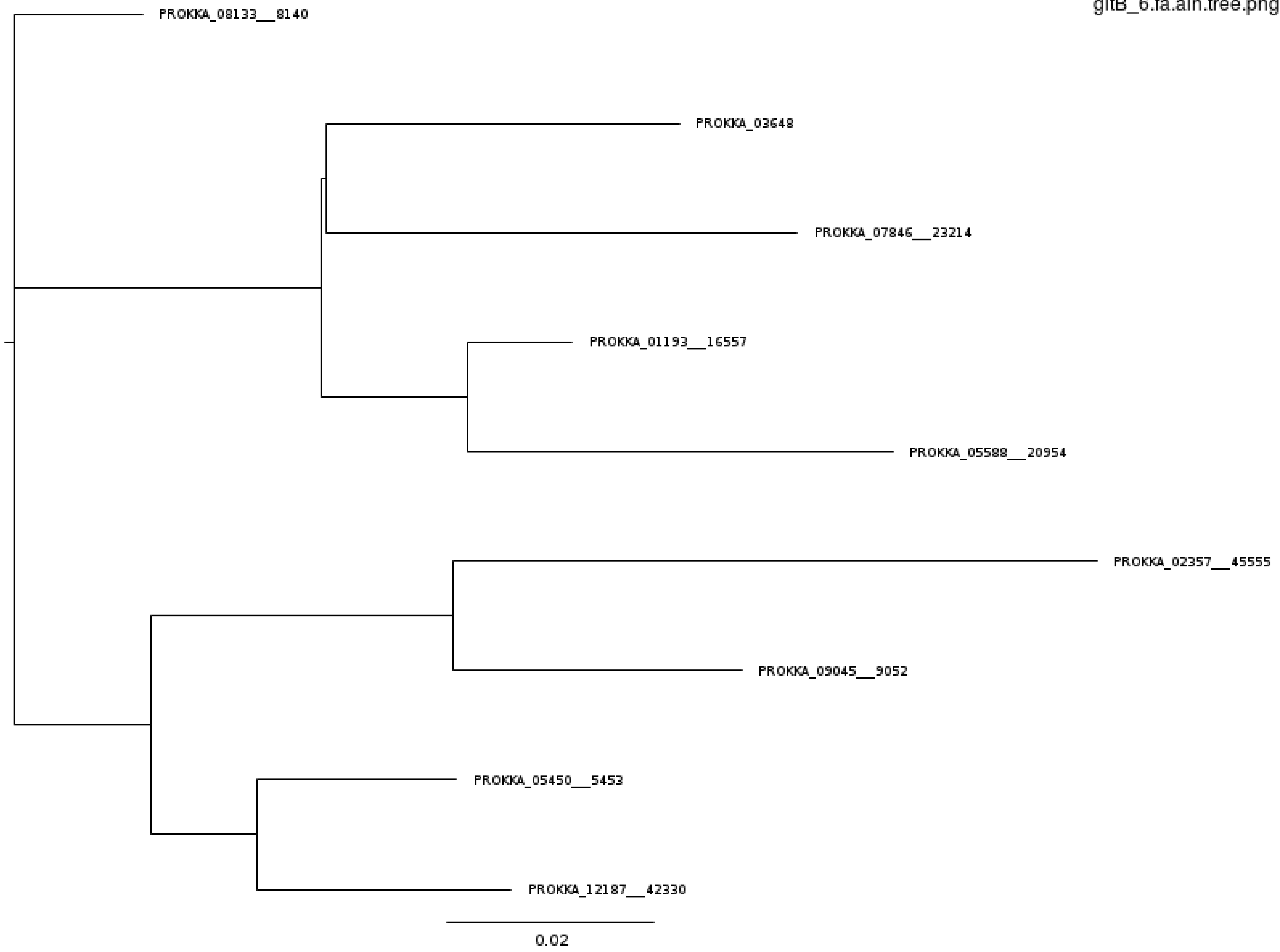

gltC.fa.aln.tree.png

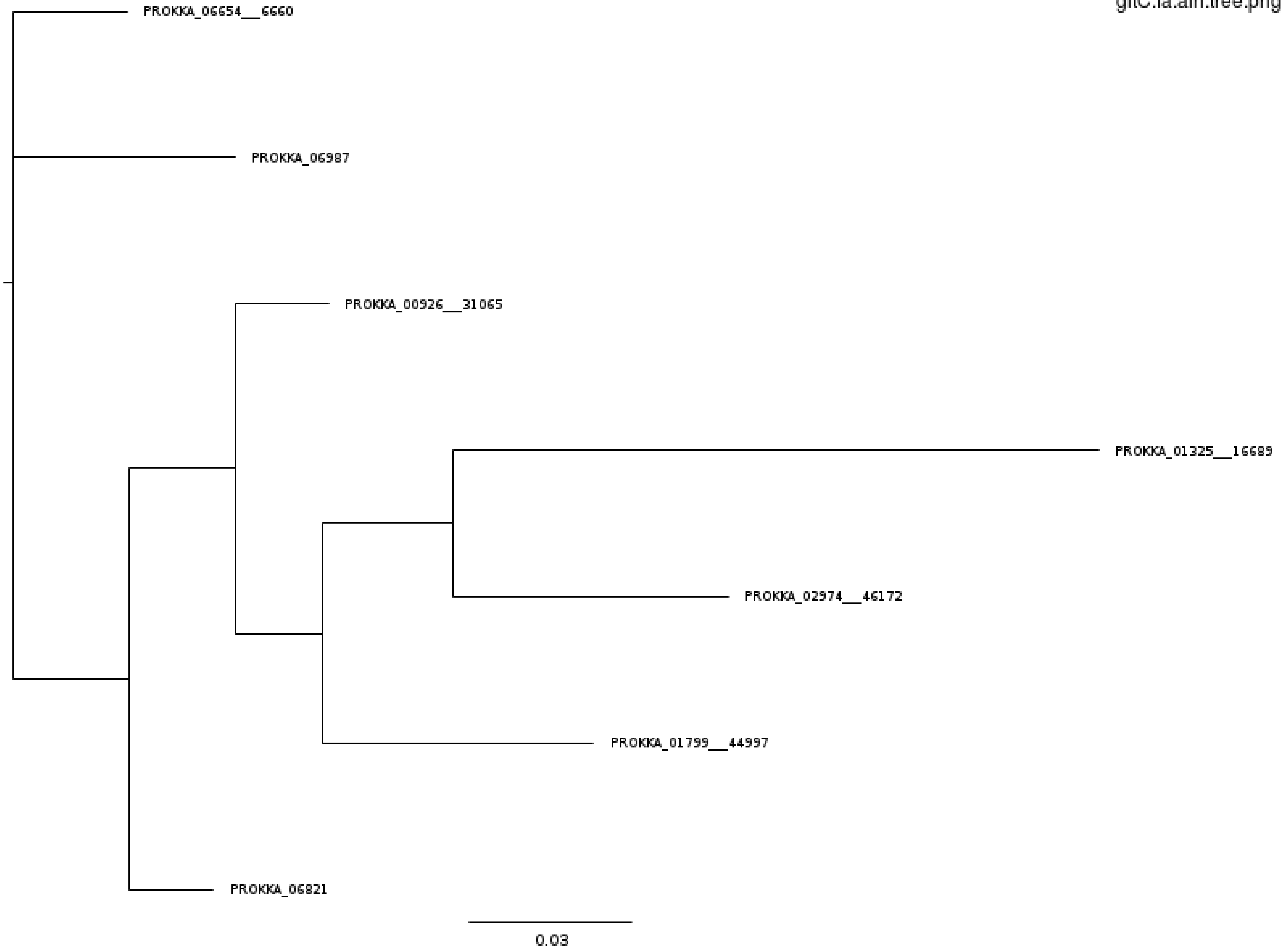

gltD_2.fa.aln.tree.png

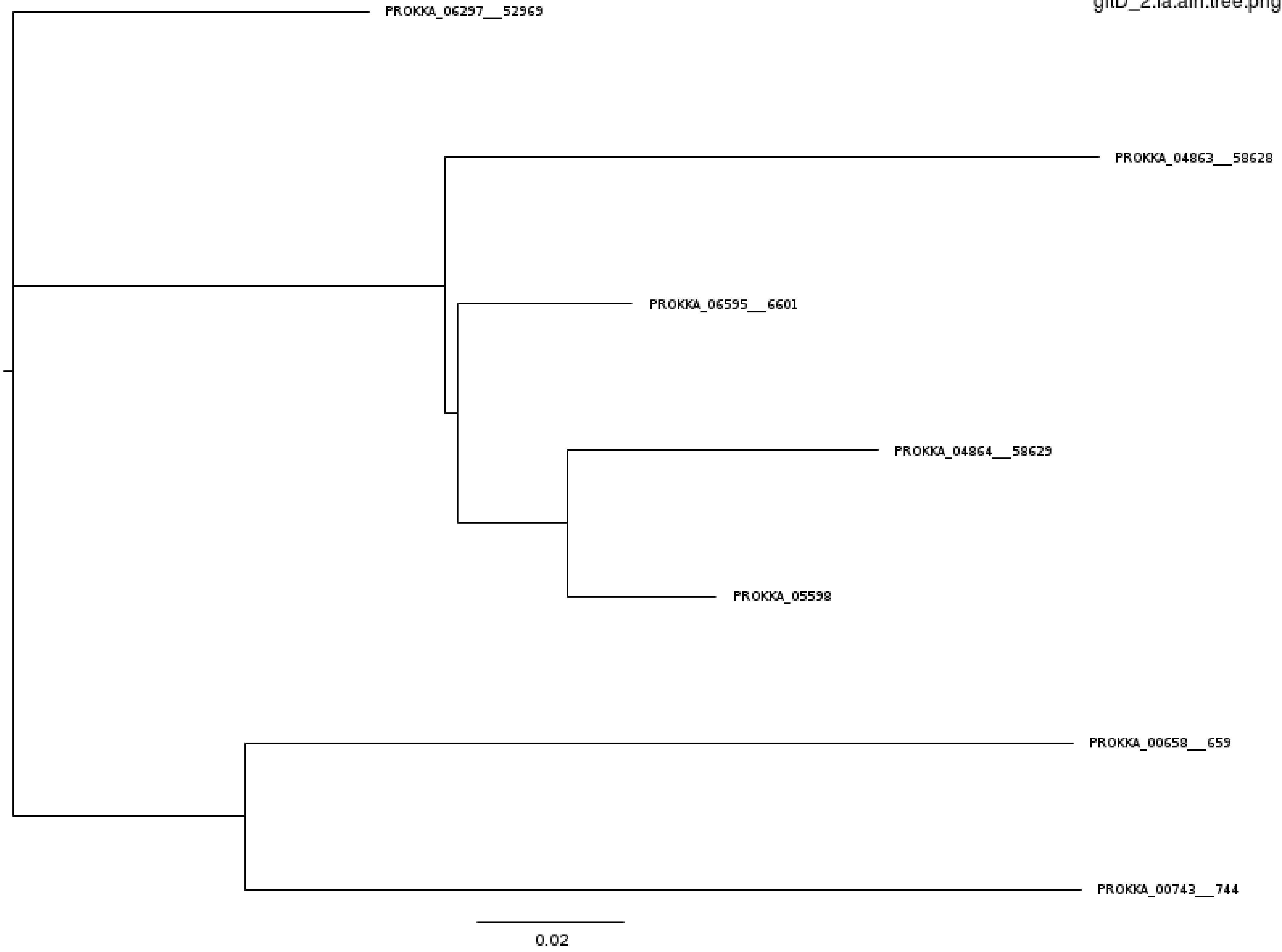

gltP.fa.aln.tree.png

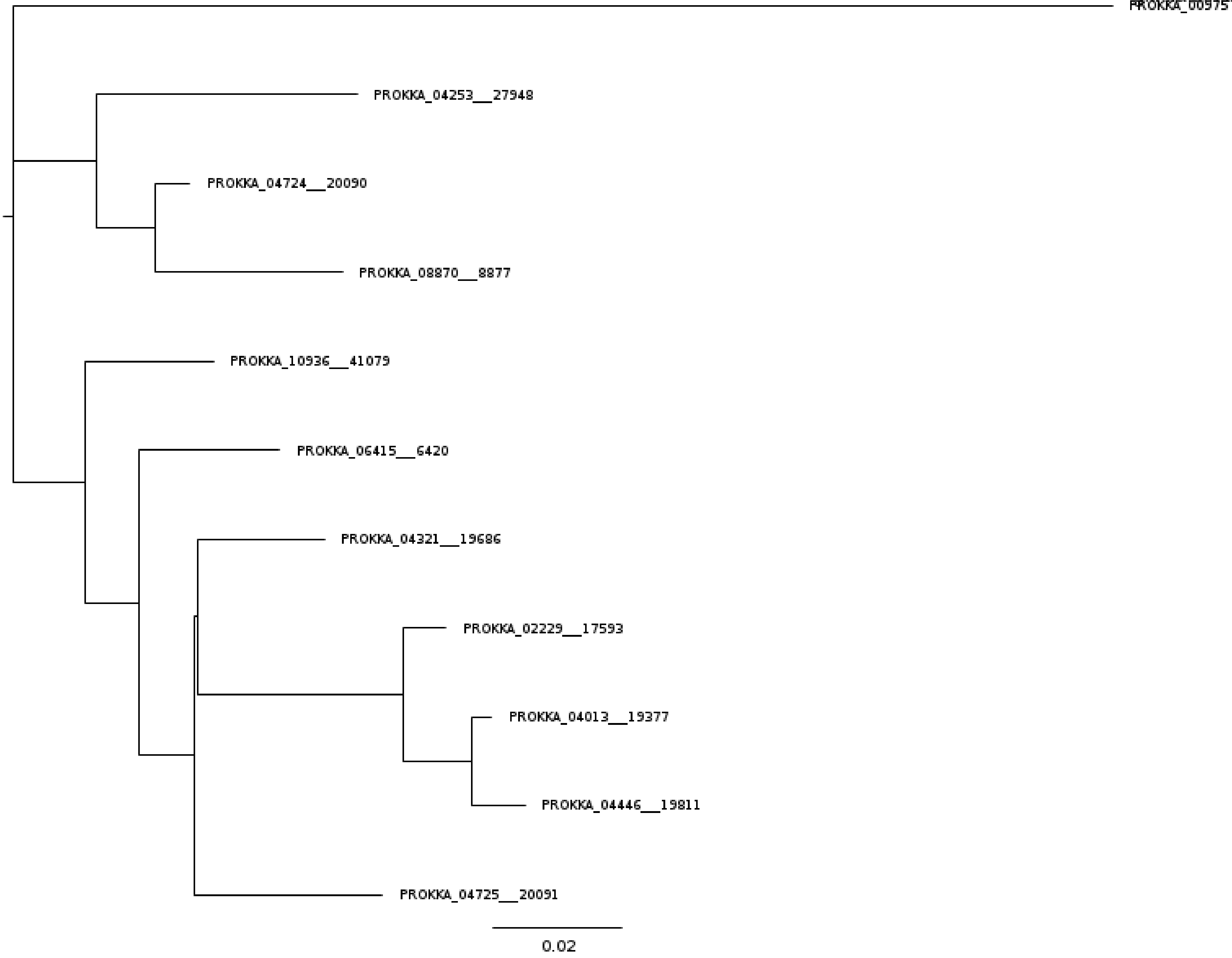

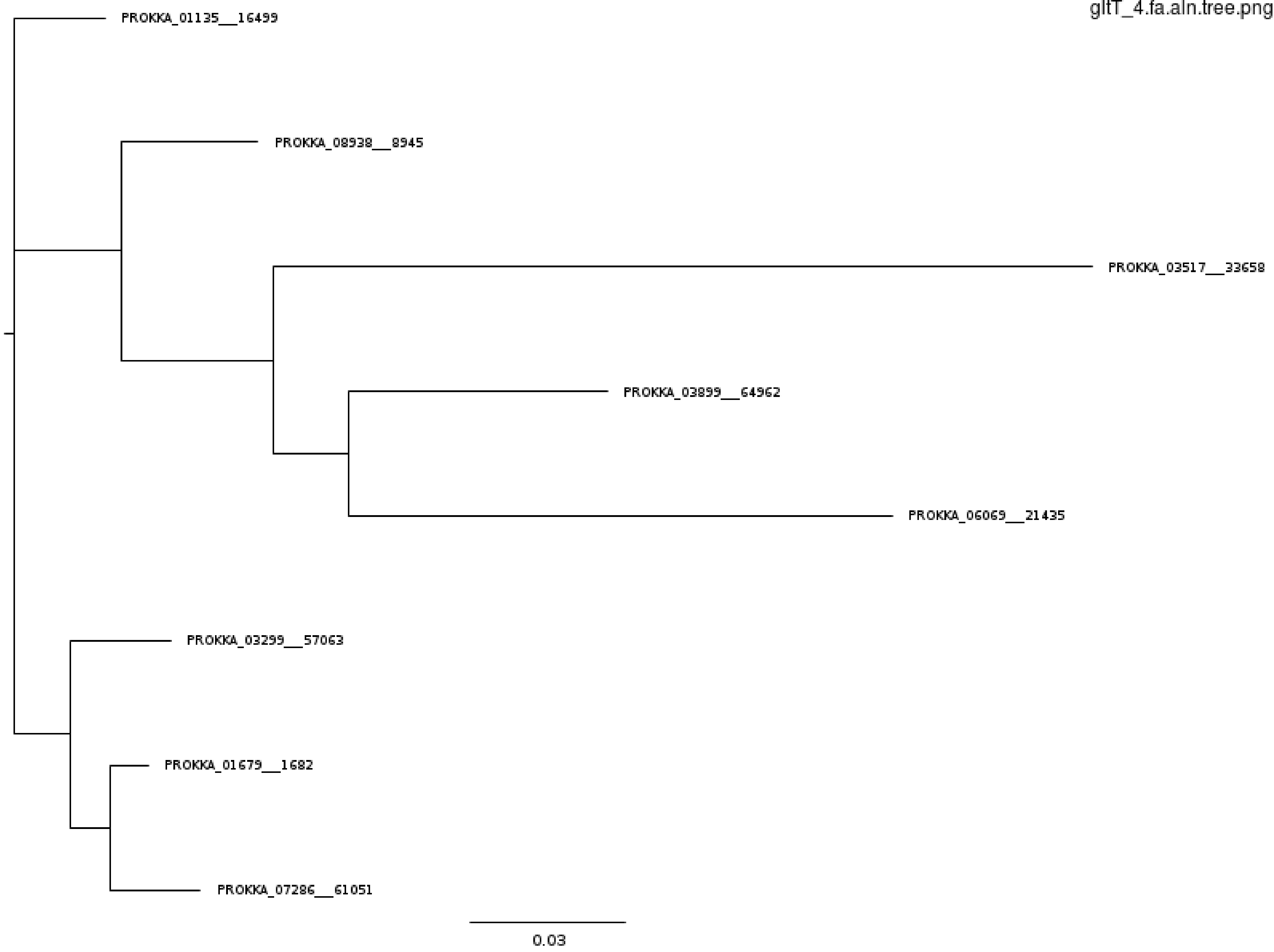
gltT_4.fa.aln.tree.png
PROKKA_01135___16499
PROKKA_08938___8945
PROKKA_03517___33658
PROKKA_03899___64962
PROKKA_06069___21435
PROKKA_03299___57063
PROKKA_01679___1682
PROKKA_07286___61051
0.03

glyA_2.fa.aln.tree.png

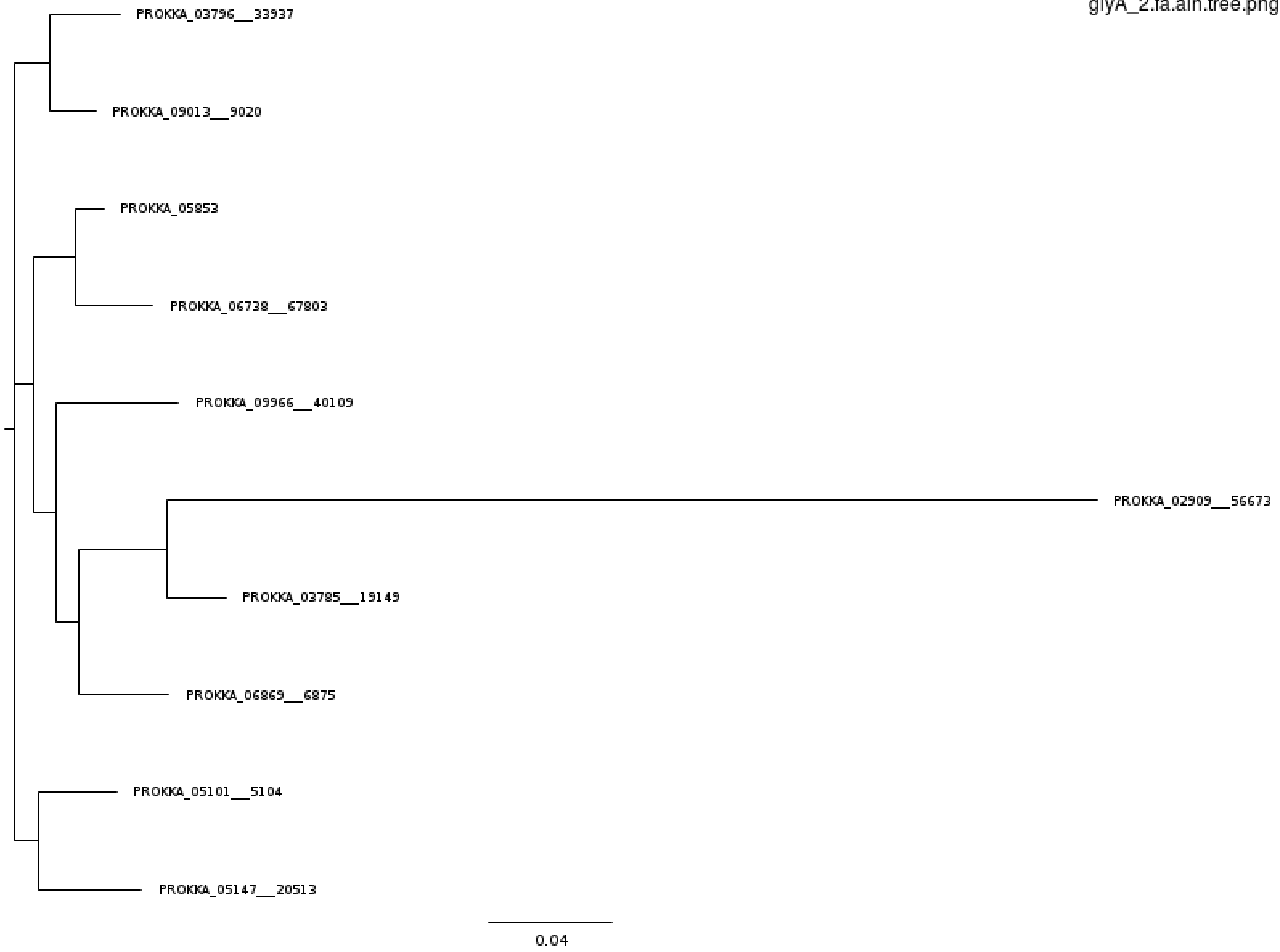

glyS_3.fa.aln.tree.png

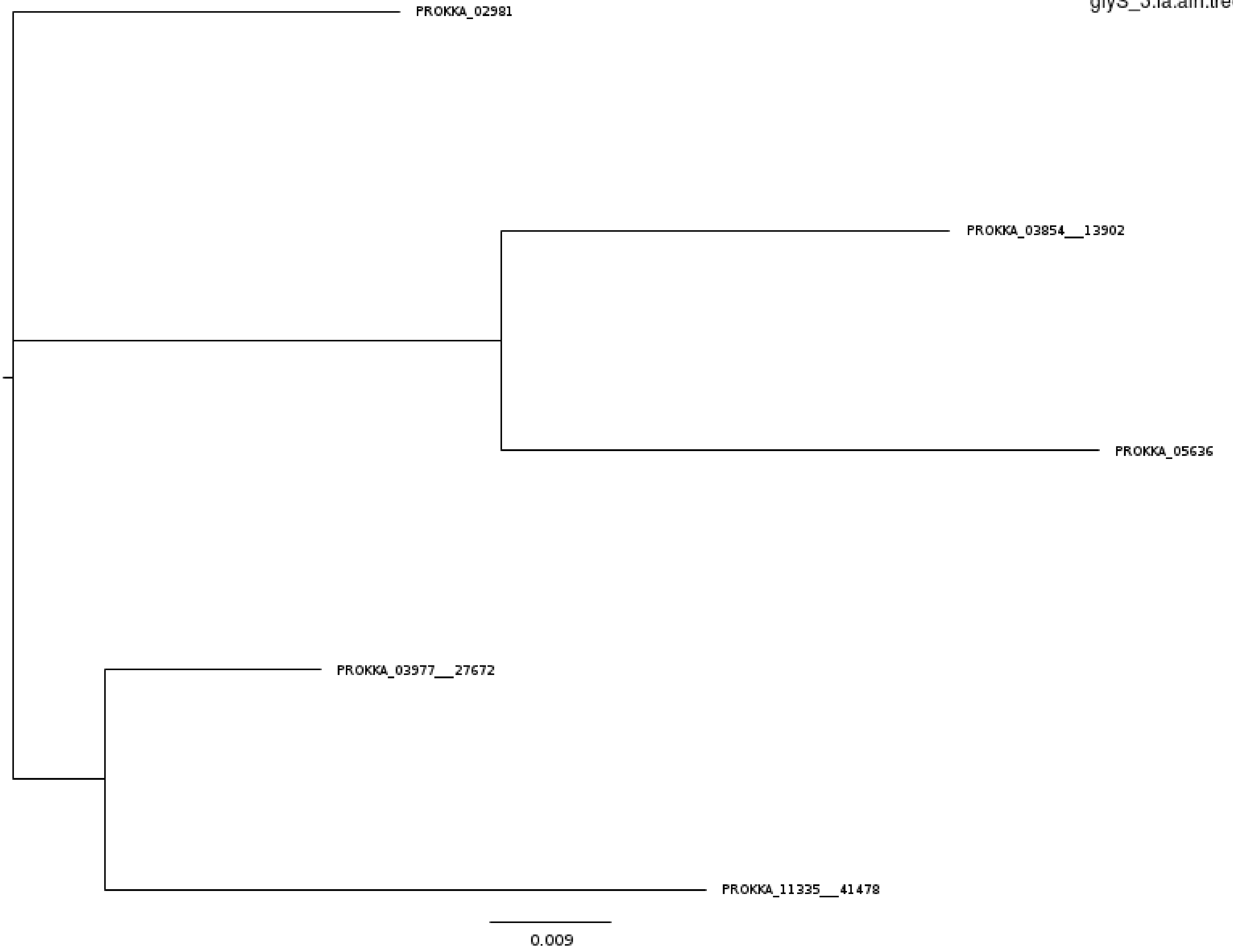

gmd_4.fa.aln.tree.png

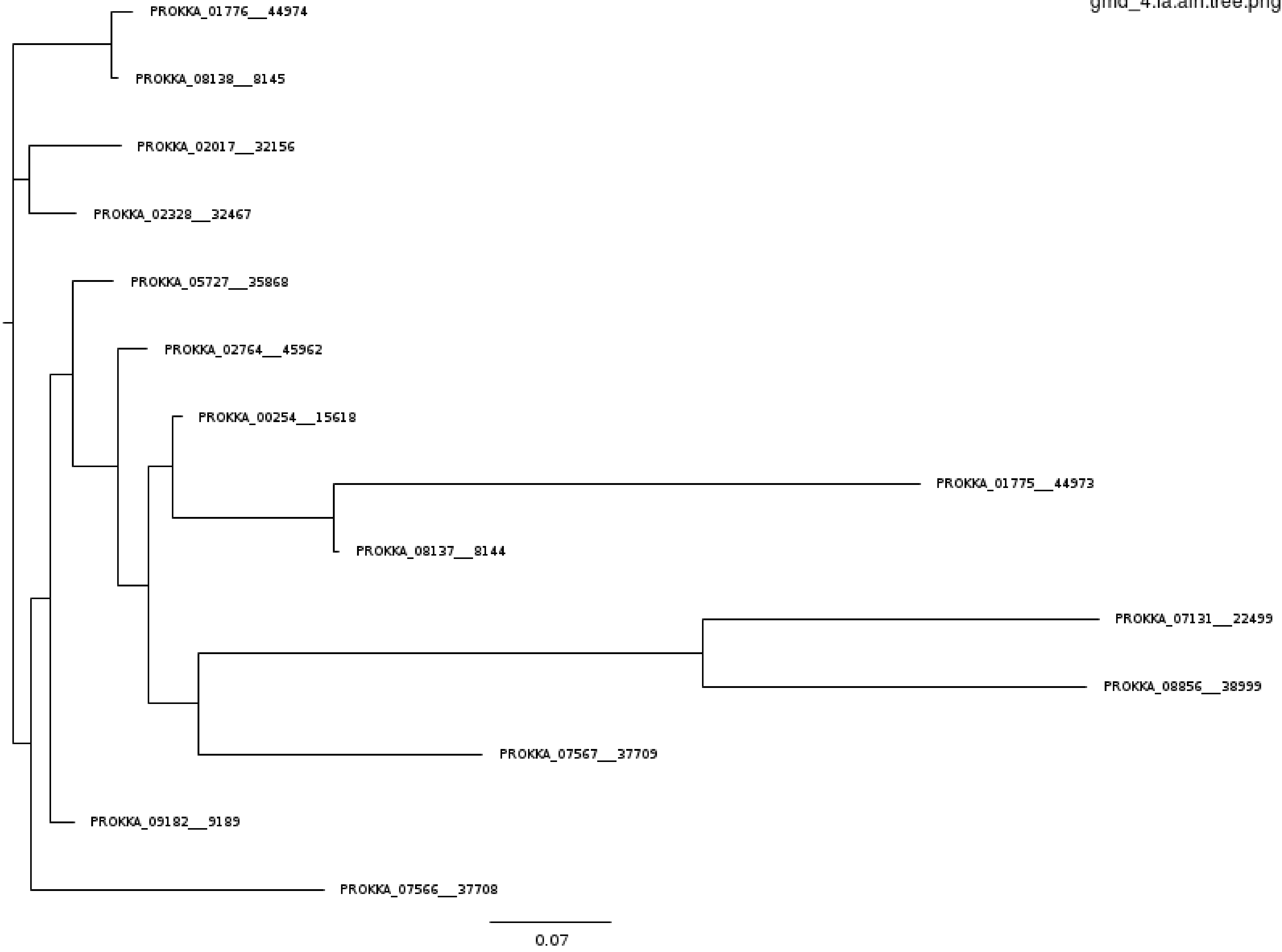

gmr_2.fa.aln.tree.png

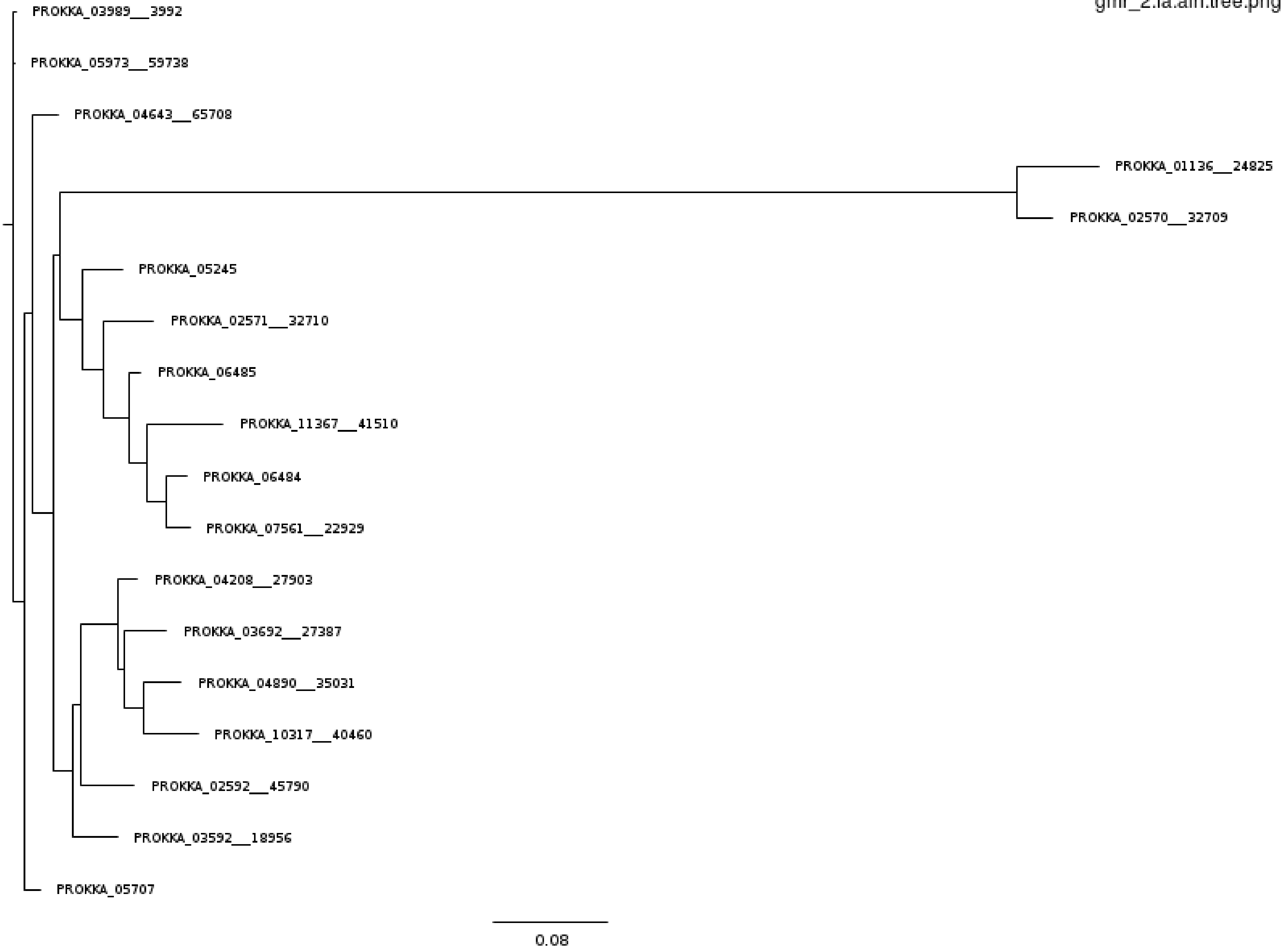

gph.fa.aln.tree.png

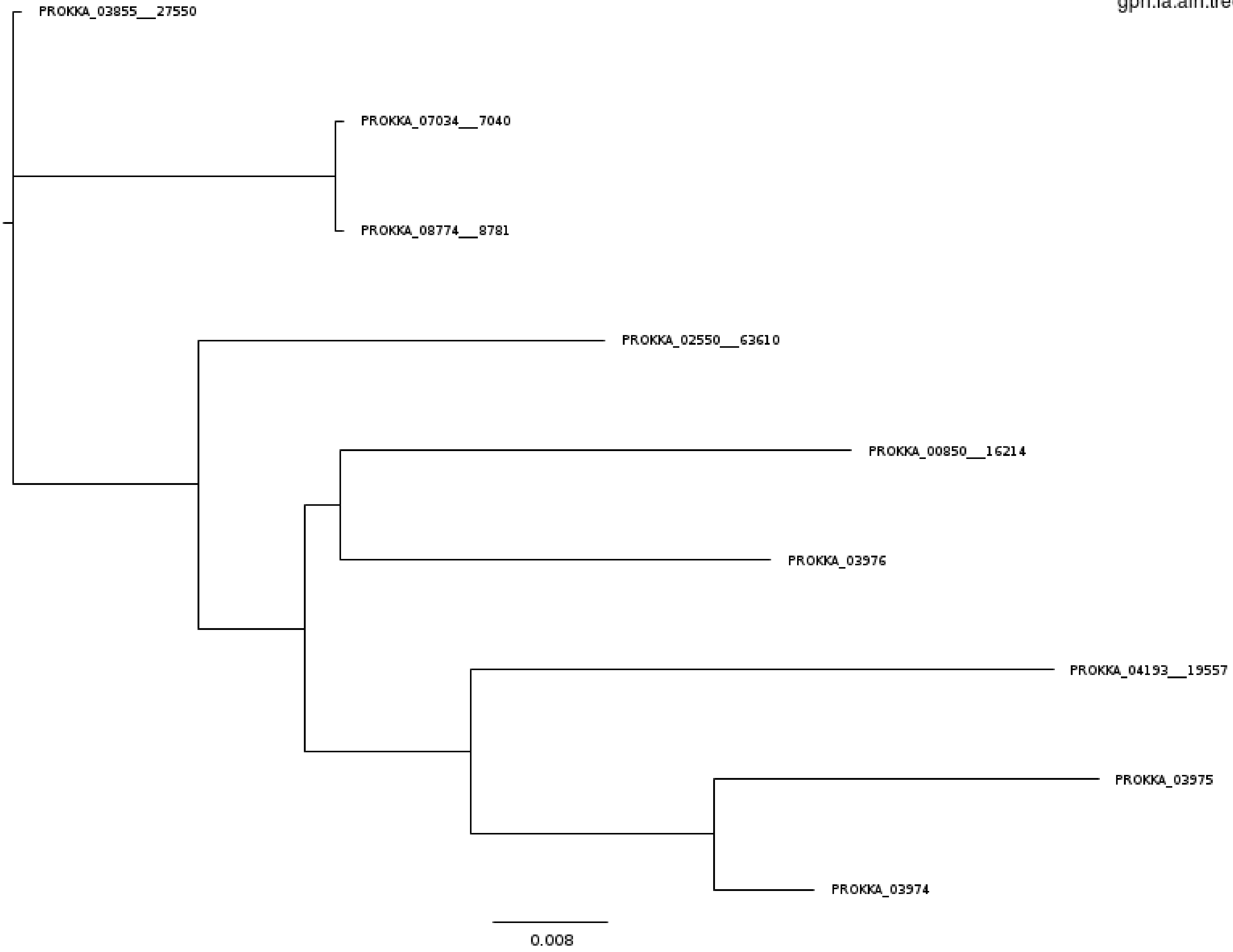

gpsA_3.fa.aln.tree.png

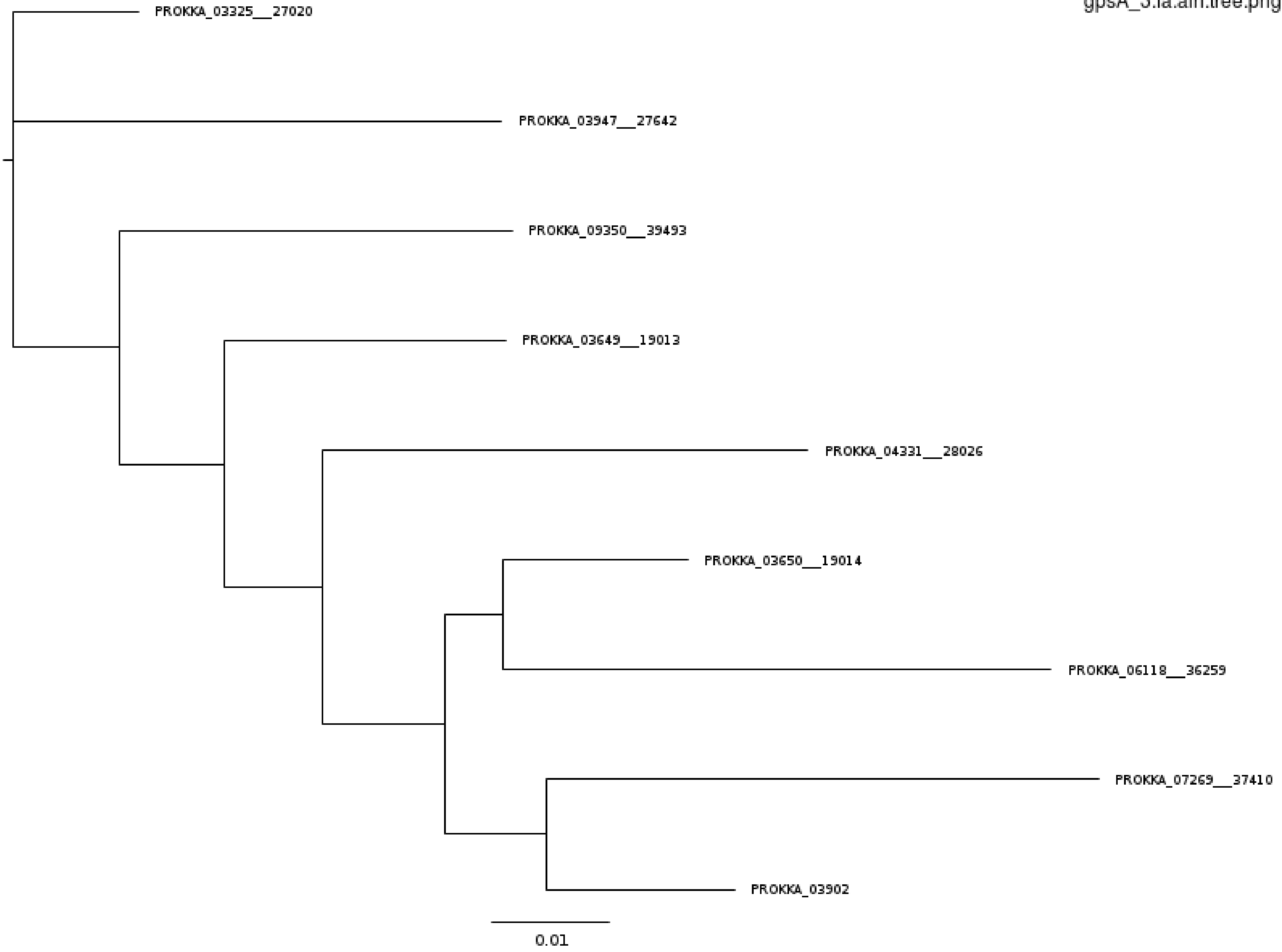

groL_1.fa.aln.tree.png

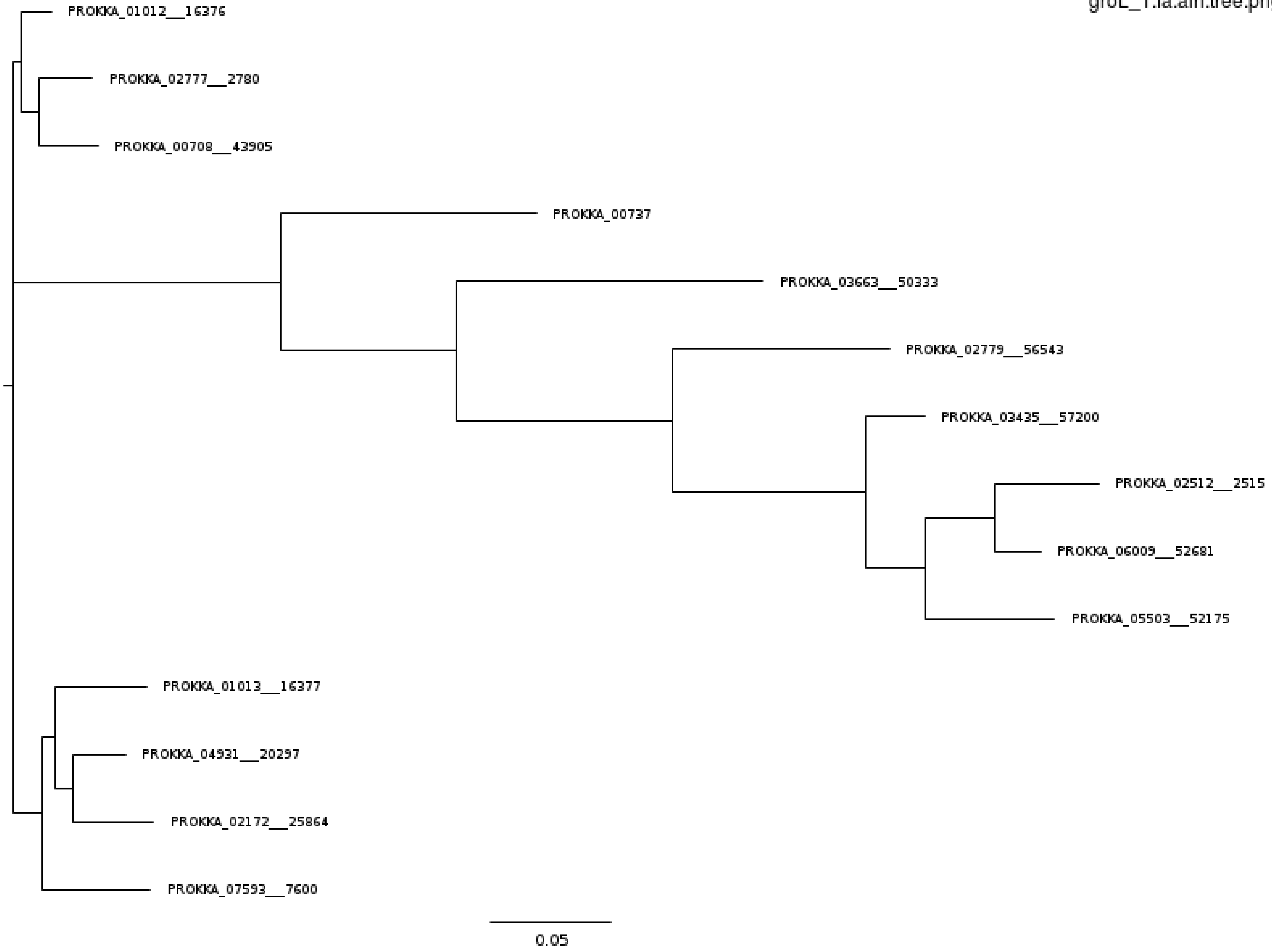

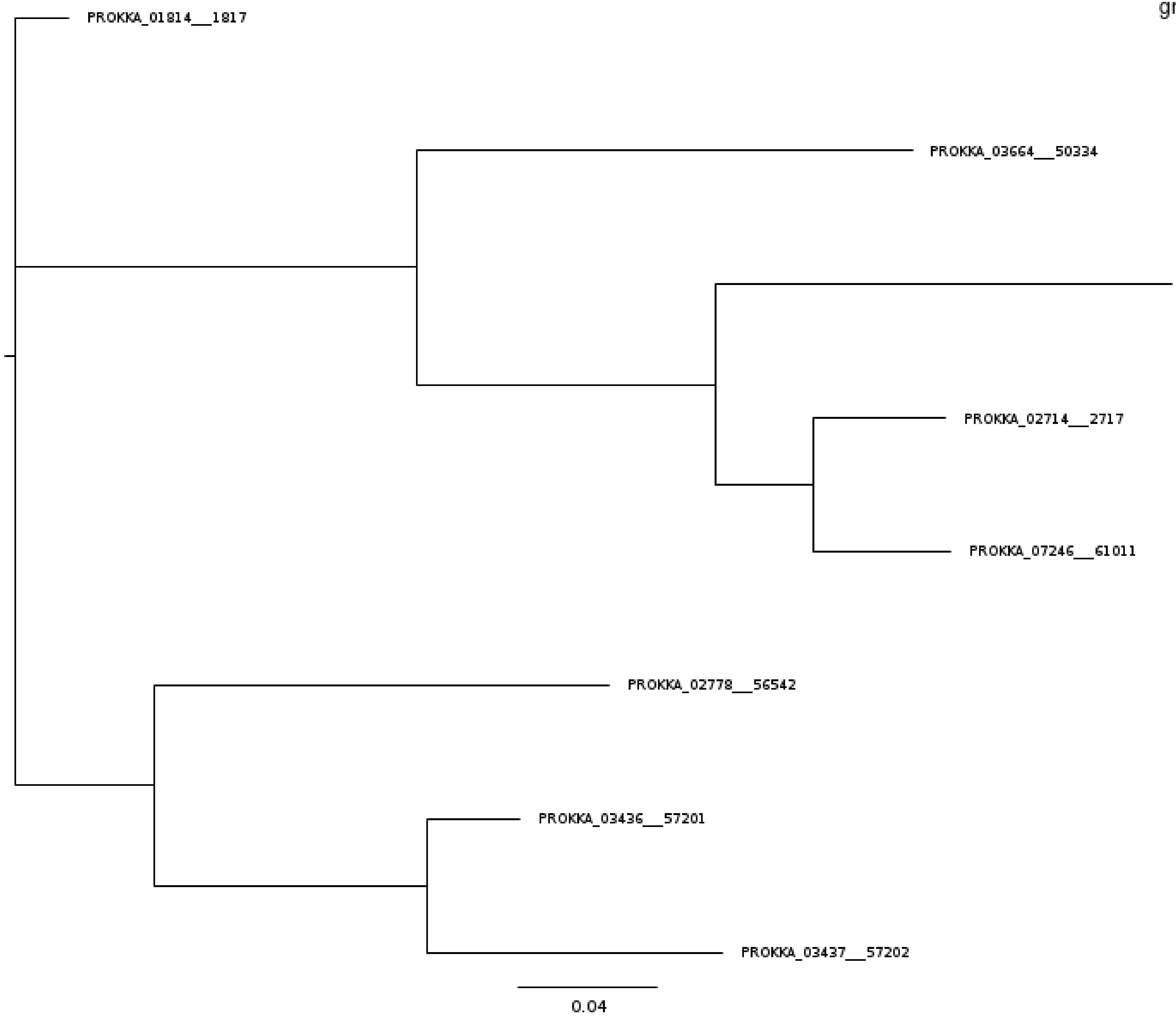
PROKKA_01814__1817
PROKKA_03664__50334
PROKKA_00736
PROKKA_02714__2717
PROKKA_07246__61011
PROKKA_02778__56542
PROKKA_03436__57201
PROKKA_03437__57202
0.04

group_1000.fa.aln.tree.png

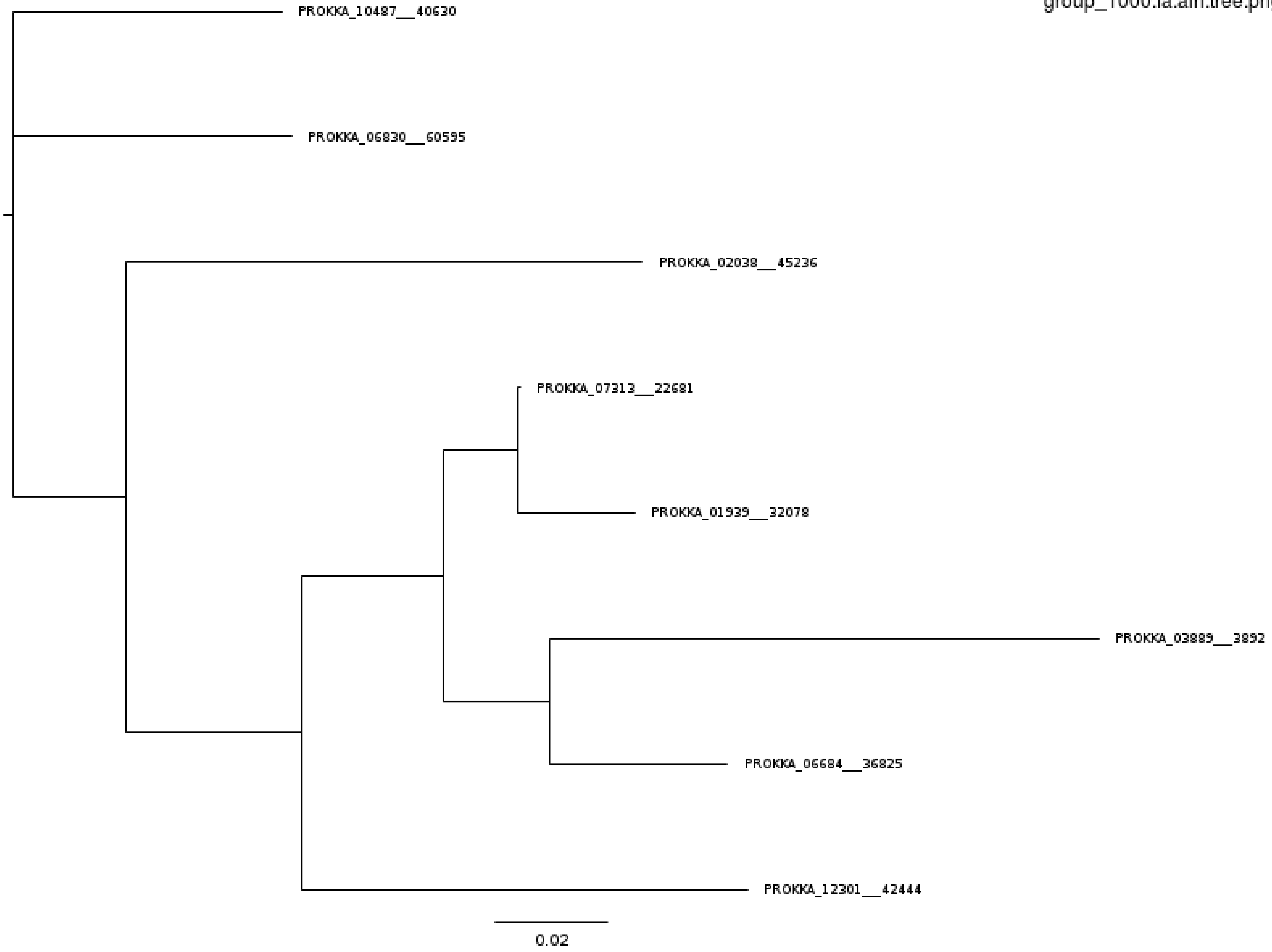

group_1006.fa.aln.tree.png

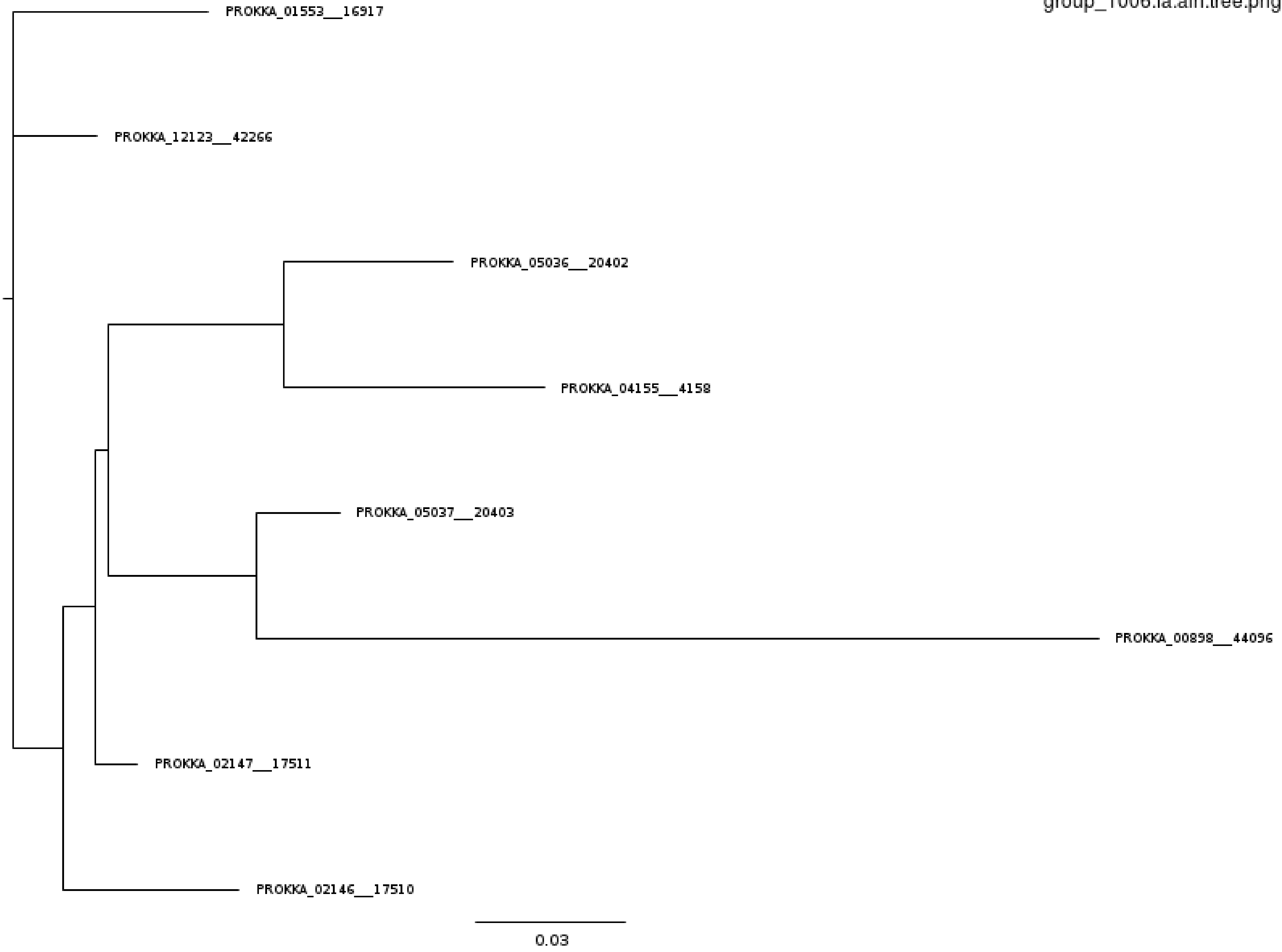

group_1007.fa.aln.tree.png

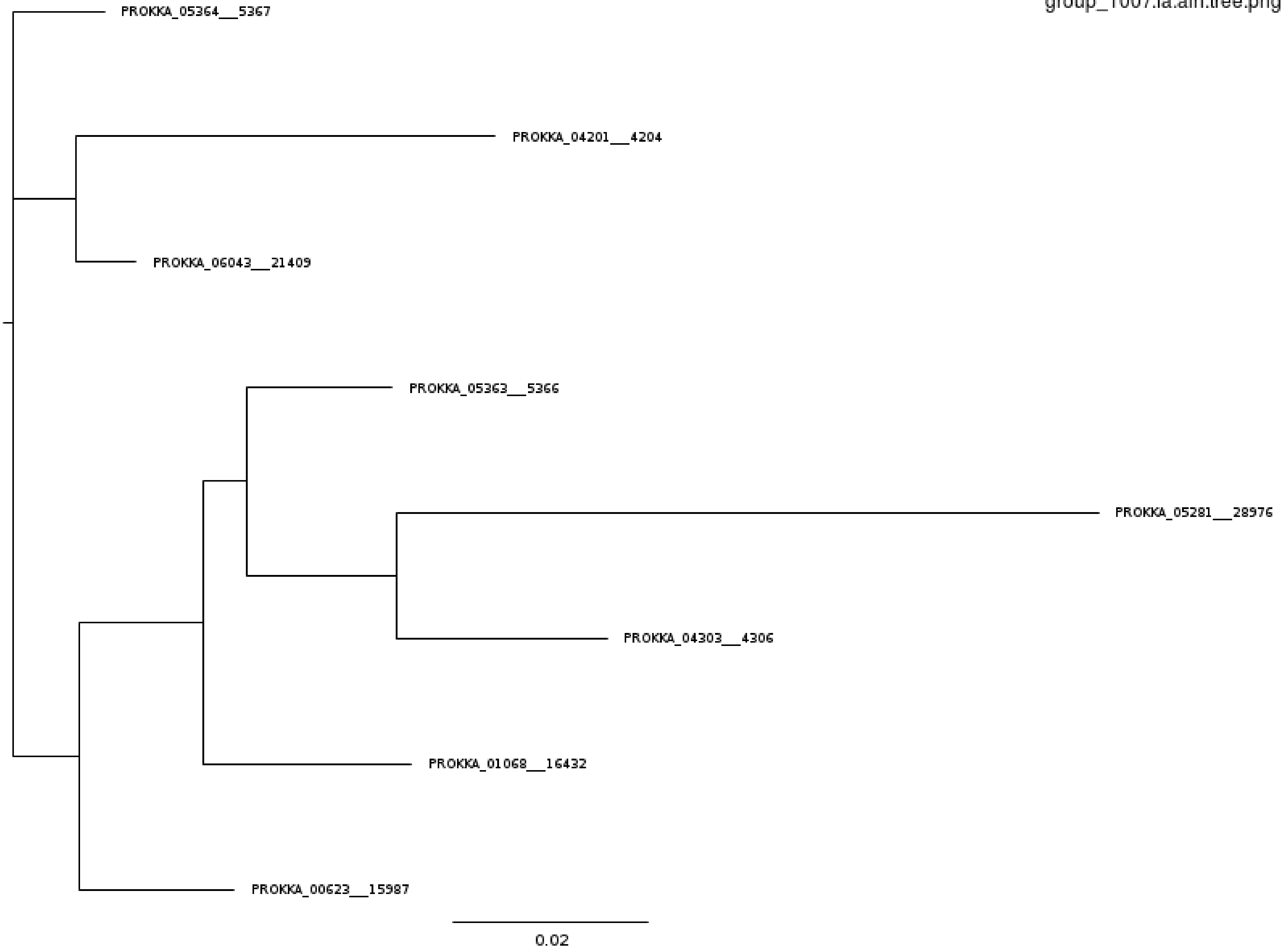

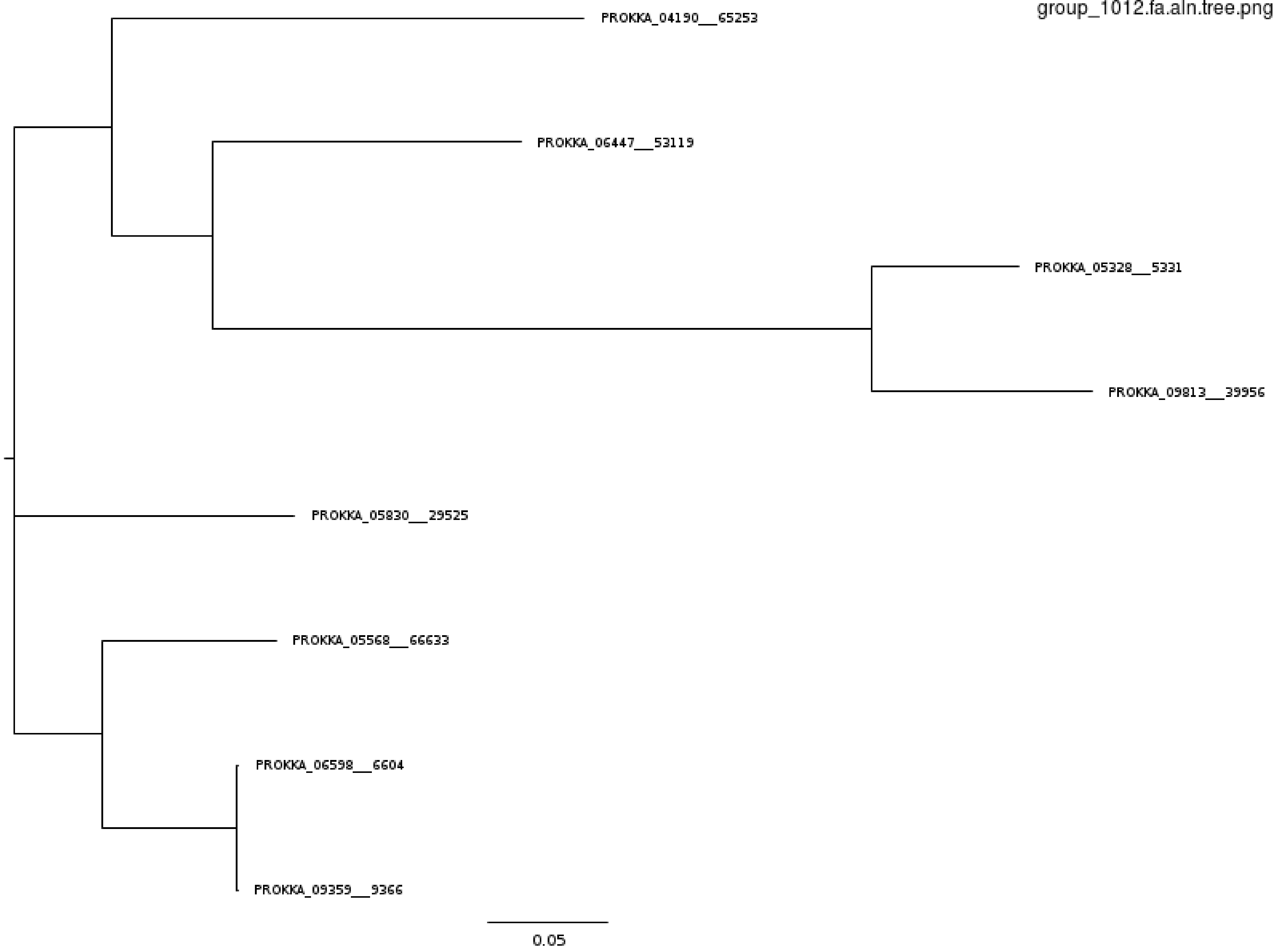
group_1012.fa.aln.tree.png
PROKKA_04190___65253
PROKKA_06447___53119
PROKKA_05328___5331
PROKKA_09813___39956
PROKKA_05830___29525
PROKKA_05568___66633
PROKKA_06598___6604
PROKKA_09359___9366
0.05

group_1014.fa.aln.tree.png

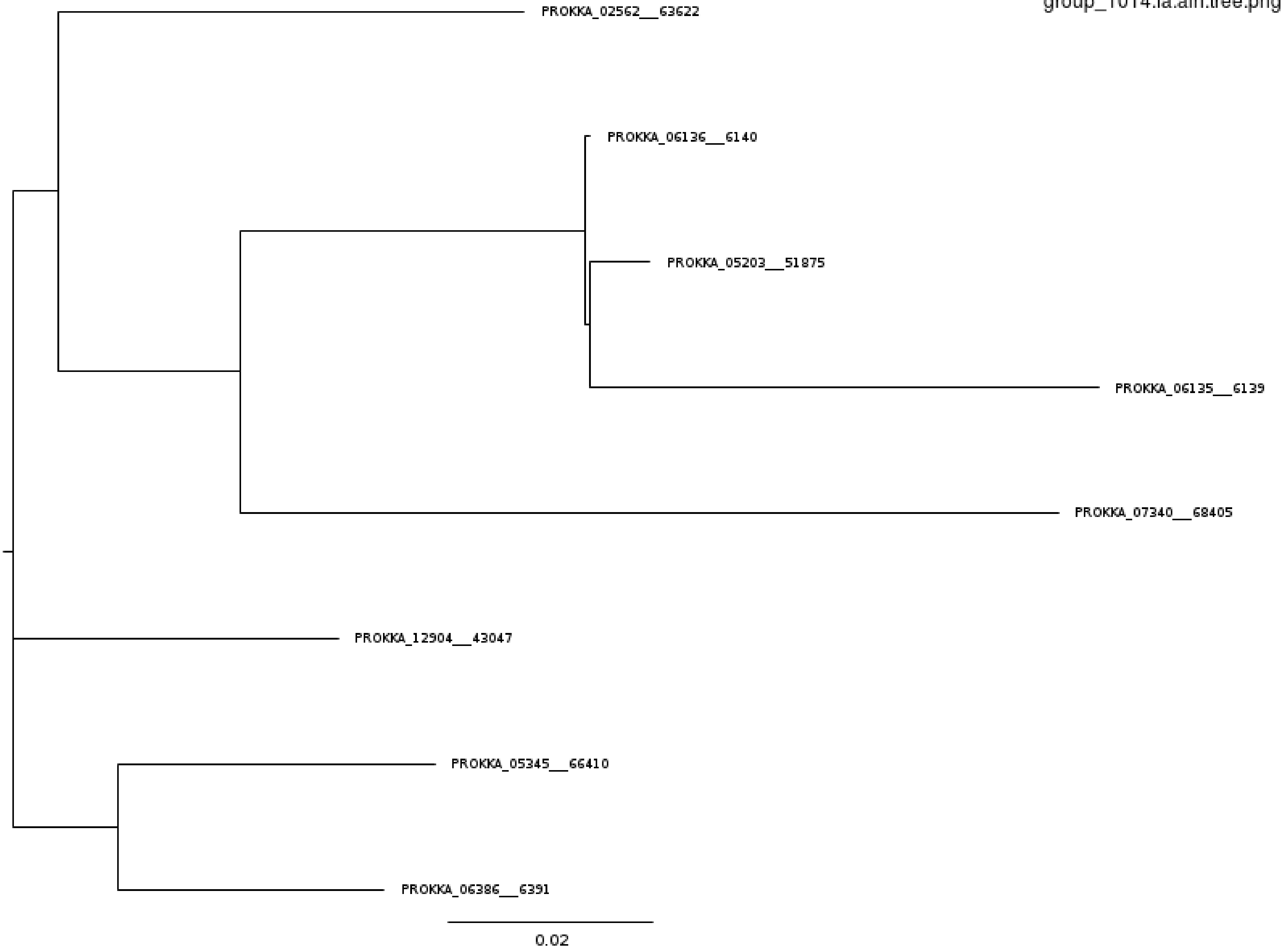

group_101.fa.aln.tree.png

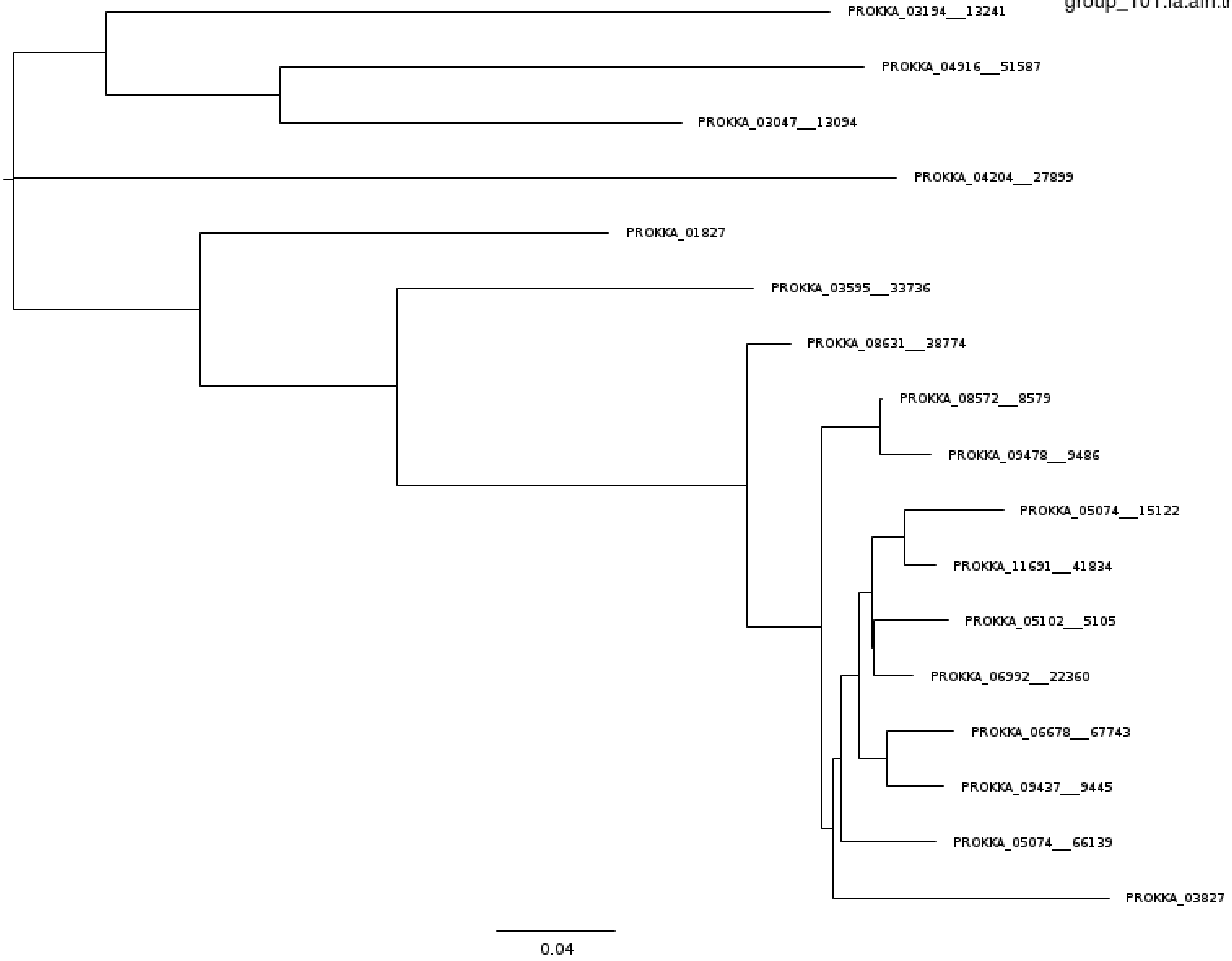

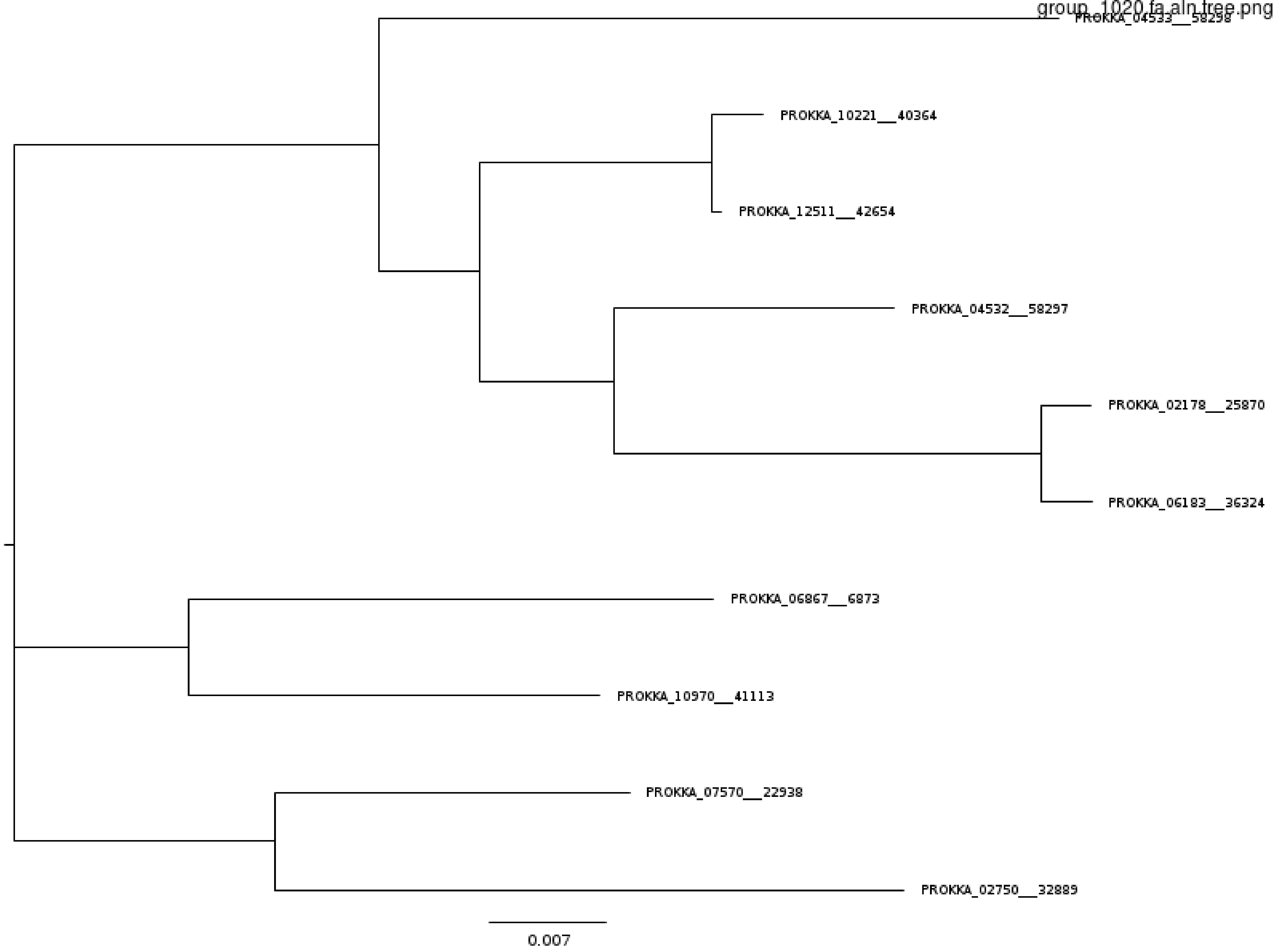

group_1020.fa.aln.tree.png
PROKKA_04533___58298
PROKKA_10221___40364
PROKKA_12511___42654
PROKKA_04532___58297
PROKKA_02178___25870
PROKKA_06183___36324
PROKKA_06867___6873
PROKKA_10970___41113
PROKKA_07570___22938
PROKKA_02750___32889
0.007

group_1021.fa.aln.tree.png

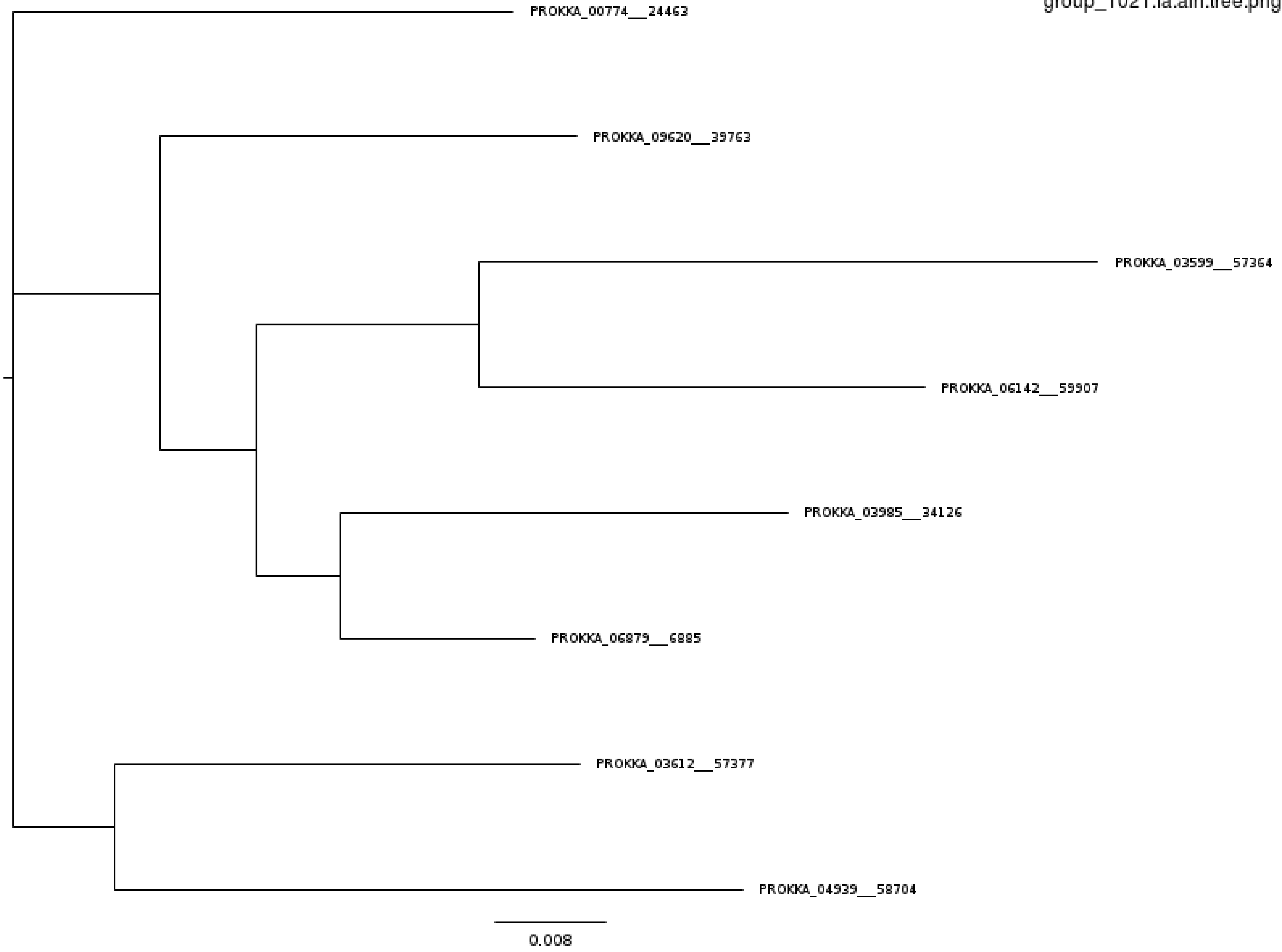

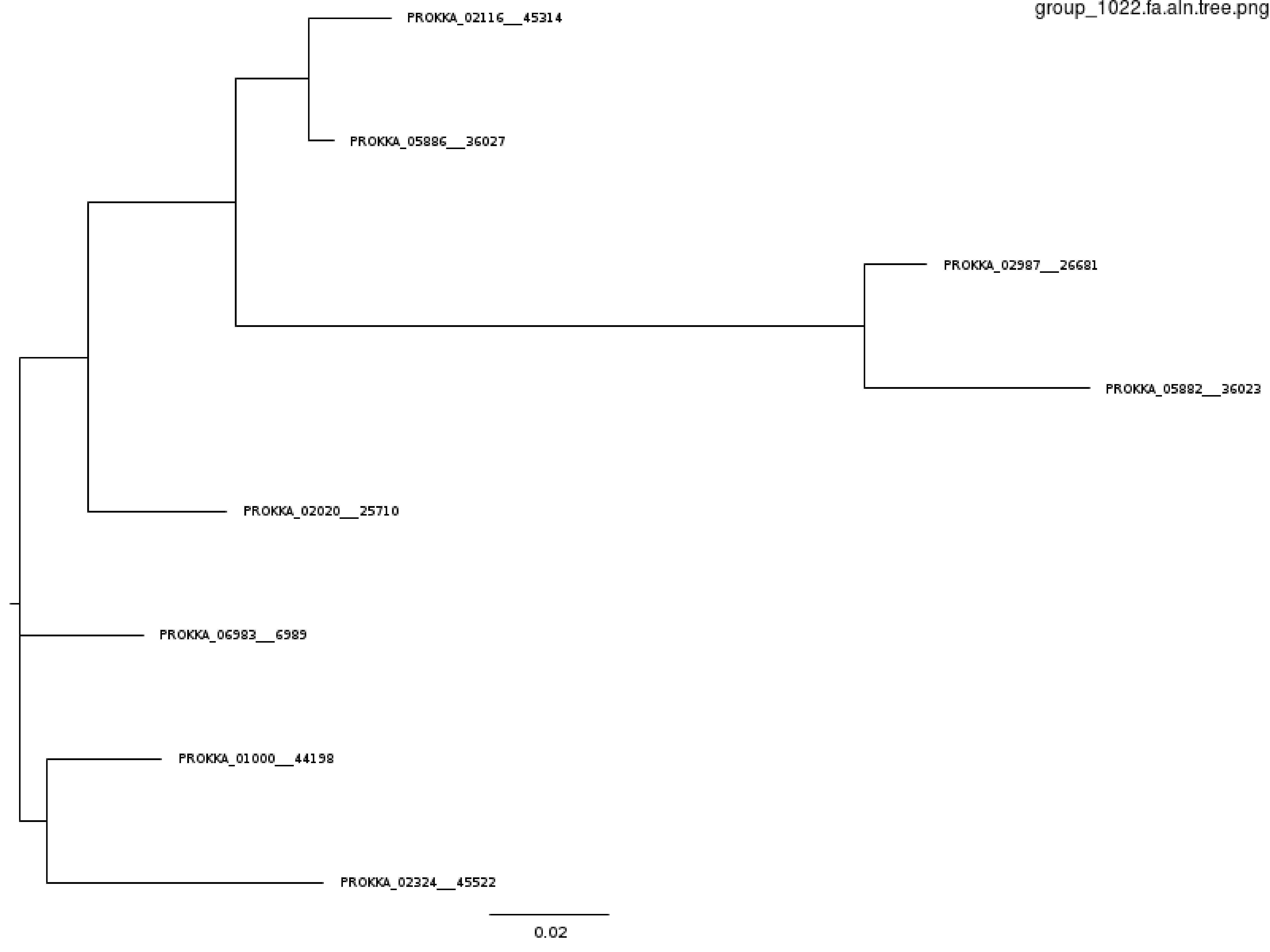
group_1022.fa.aln.tree.png
PROKKA_02116__45314
PROKKA_05886__36027
PROKKA_02987__26681
PROKKA_05882__36023
PROKKA_02020__25710
PROKKA_06983__6989
PROKKA_01000__44198
PROKKA_02324__45522
0.02

group_1023.fa.aln.tree.png

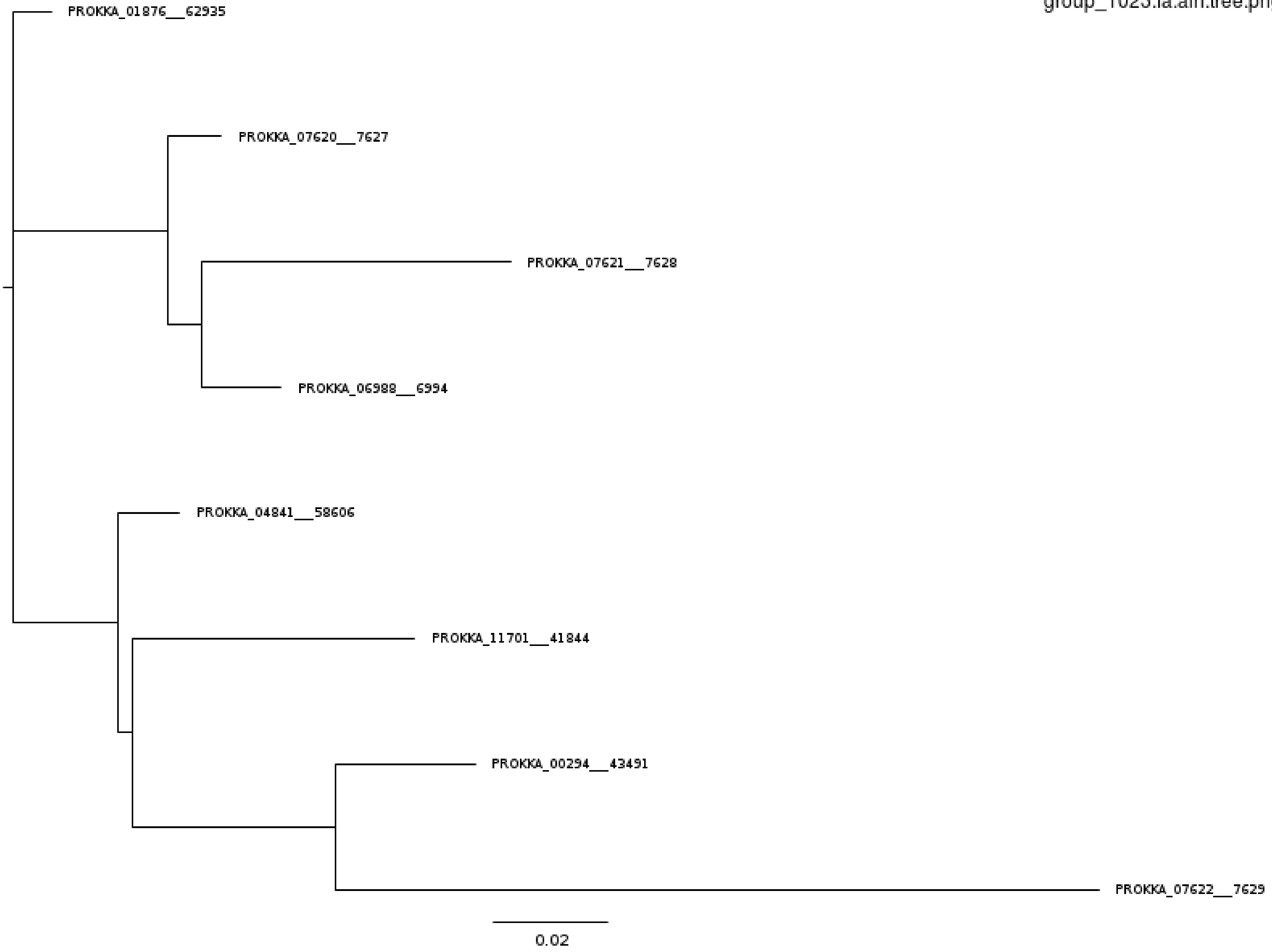

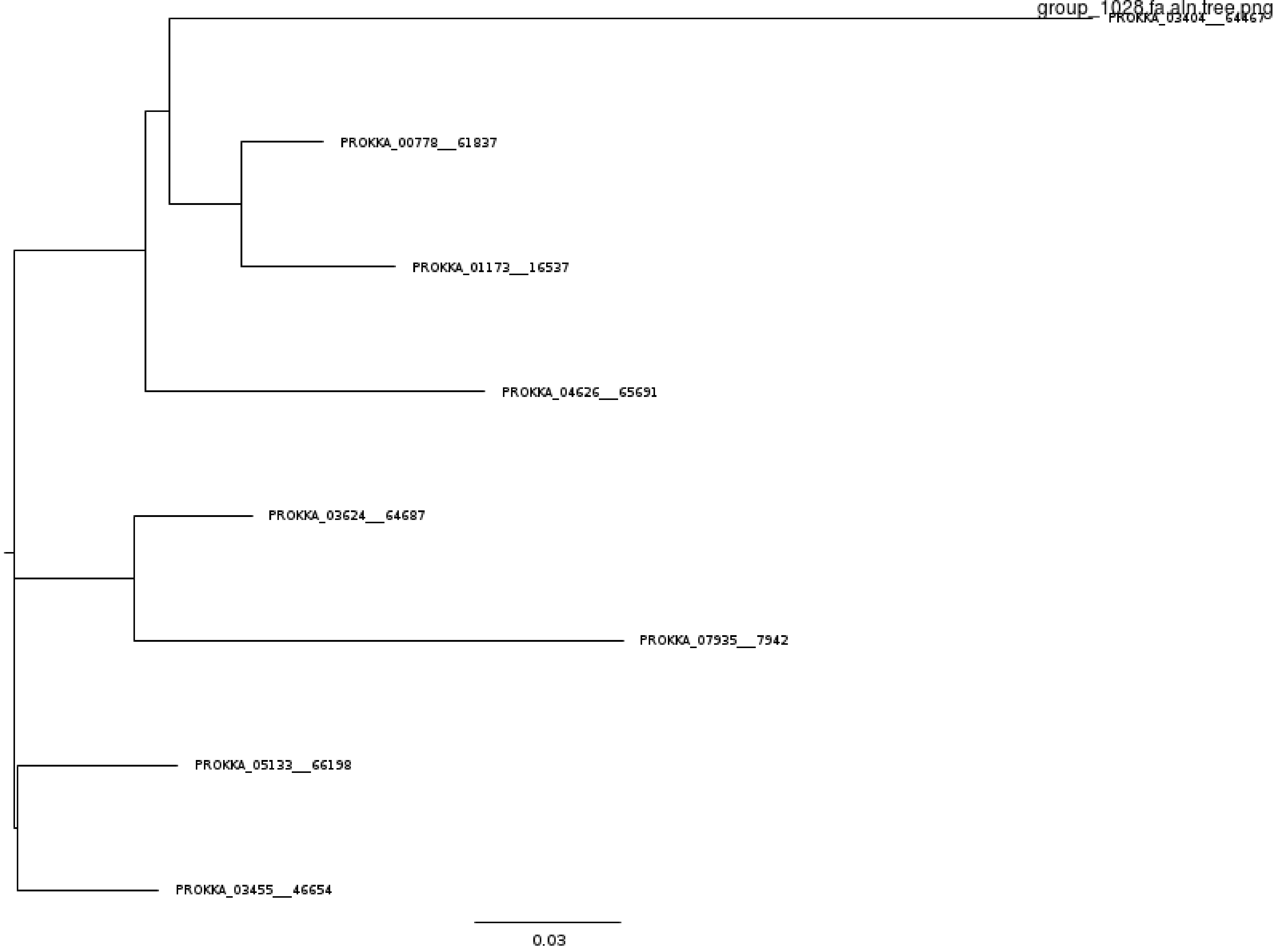

group_1028.fa.aln.tree.png
PROKKA_03404___64467
PROKKA_00778___61837
PROKKA_01173___16537
PROKKA_04626___65691
PROKKA_03624___64687
PROKKA_07935___7942
PROKKA_05133___66198
PROKKA_03455___46654
0.03

group_1031.fa.aln.tree.png

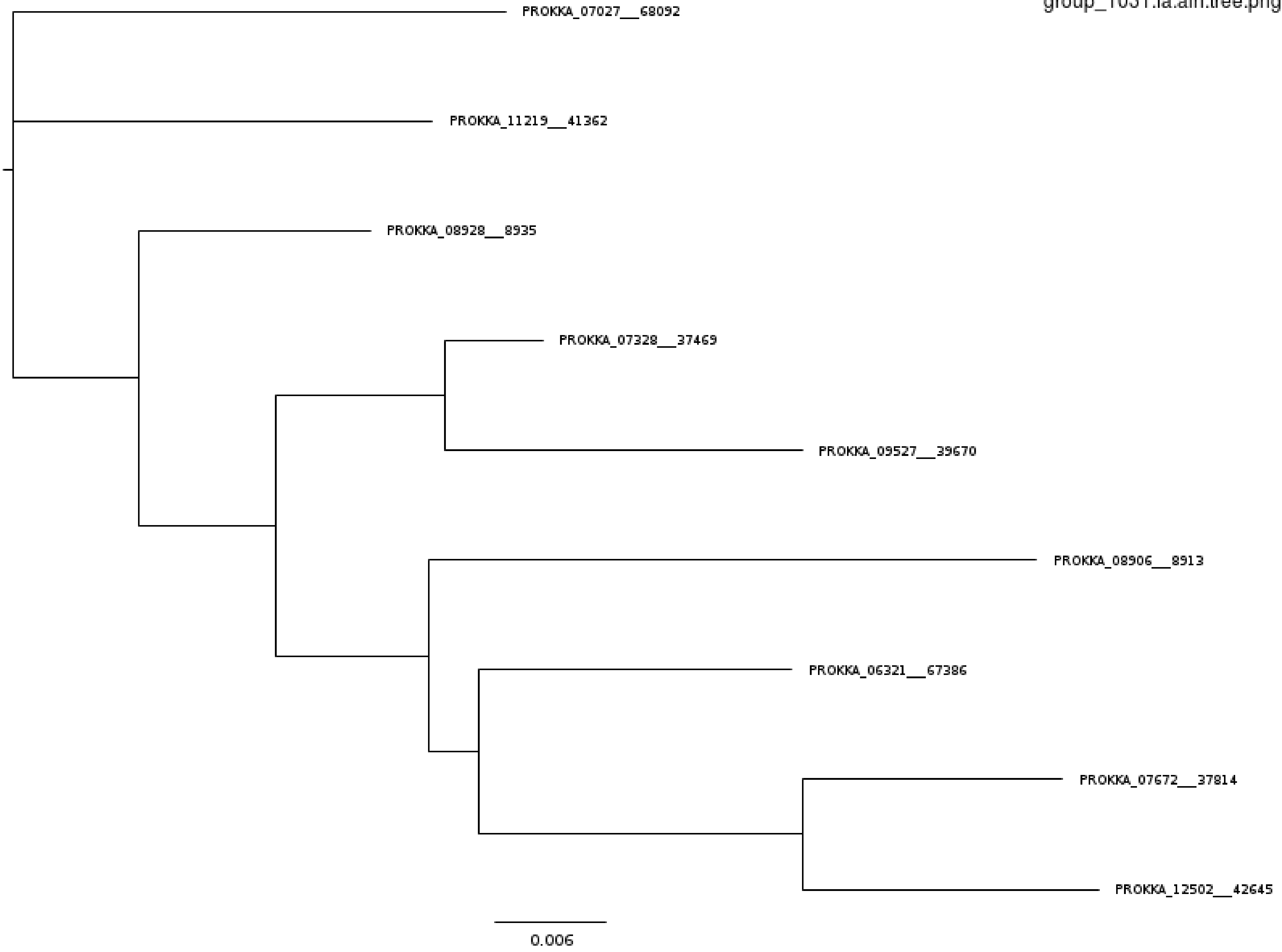

group_1032.fa.aln.tree.png

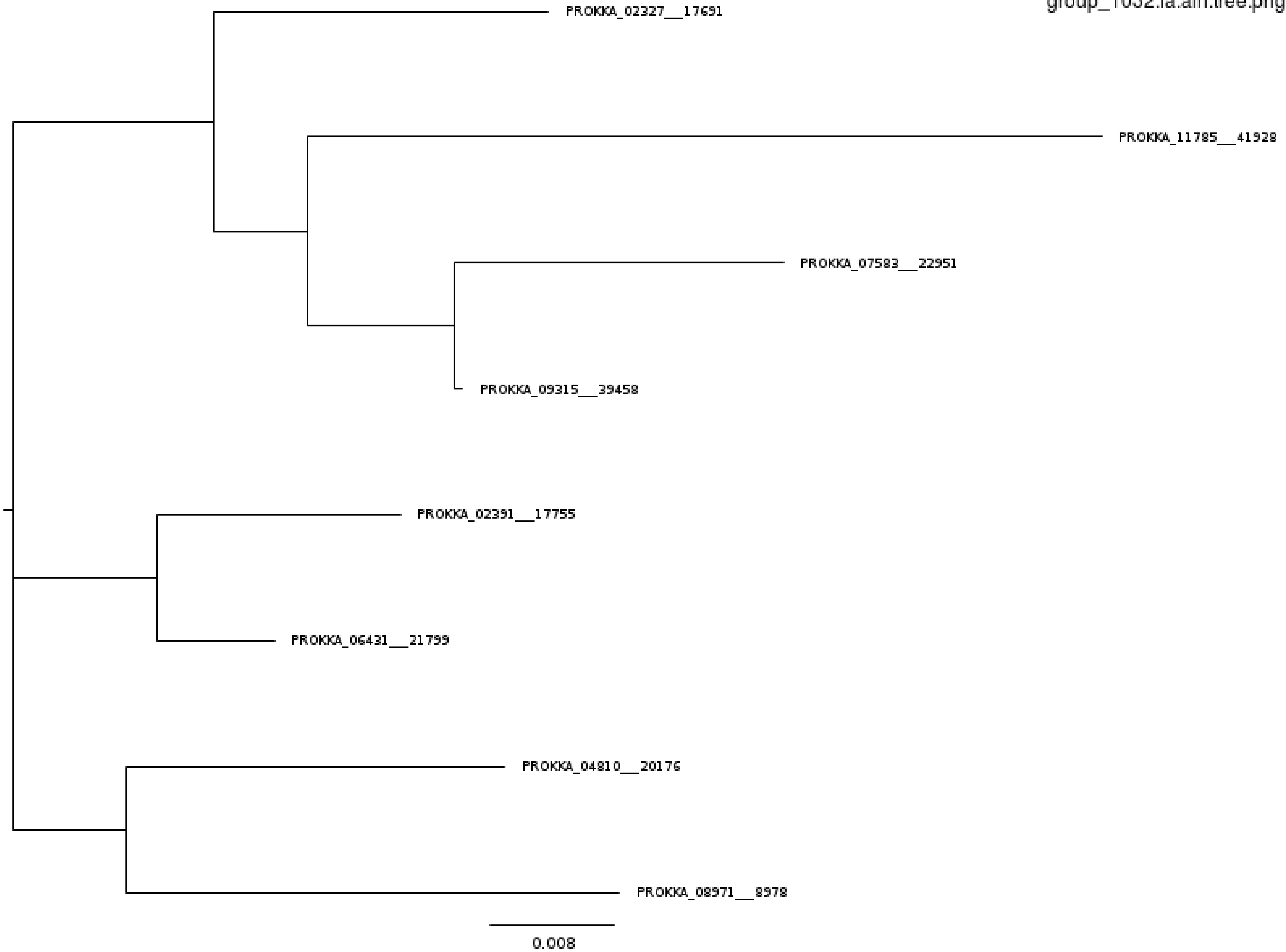

group_1033.fa.aln.tree.png

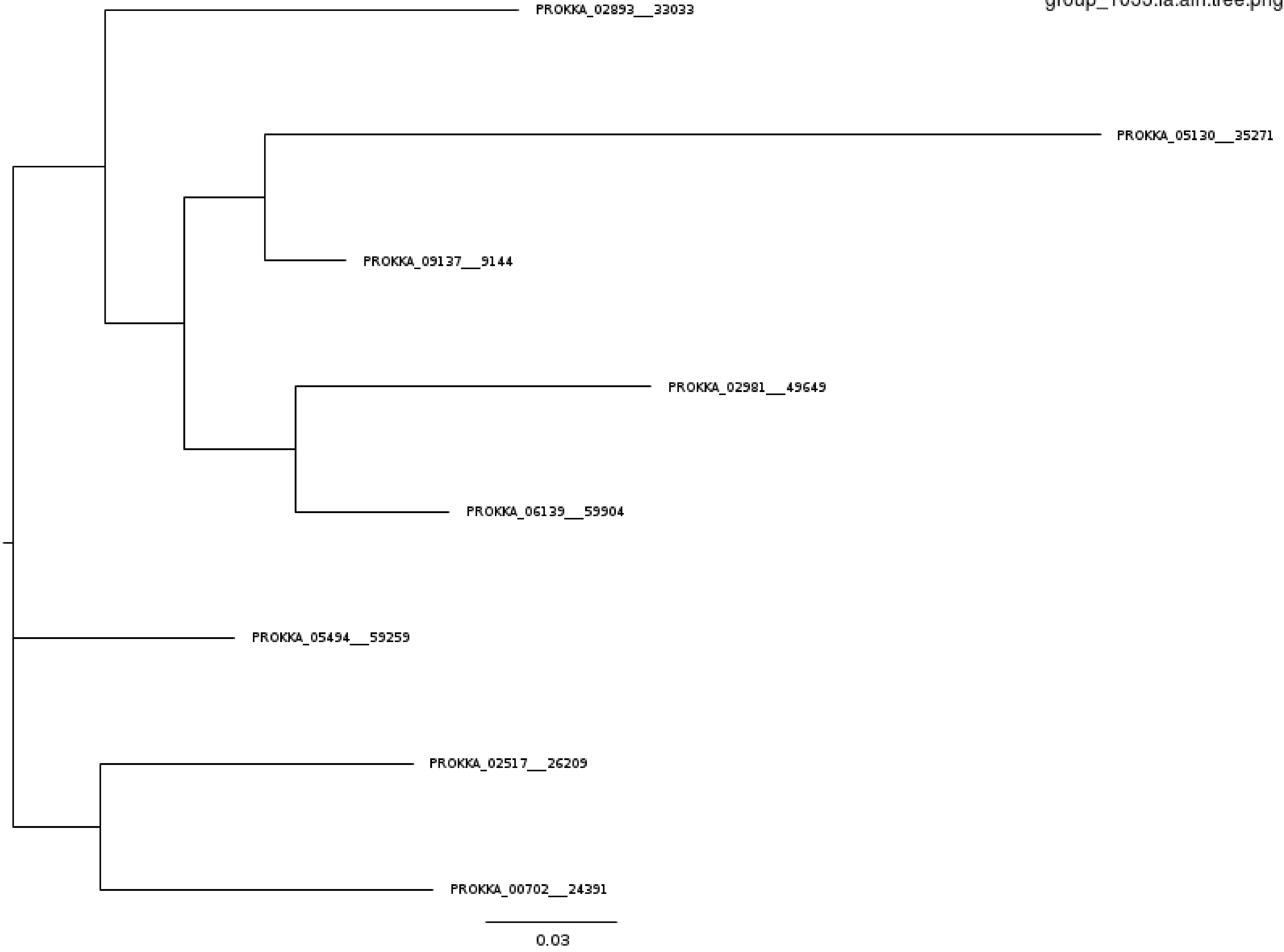

group_1038.fa.aln.tree.png

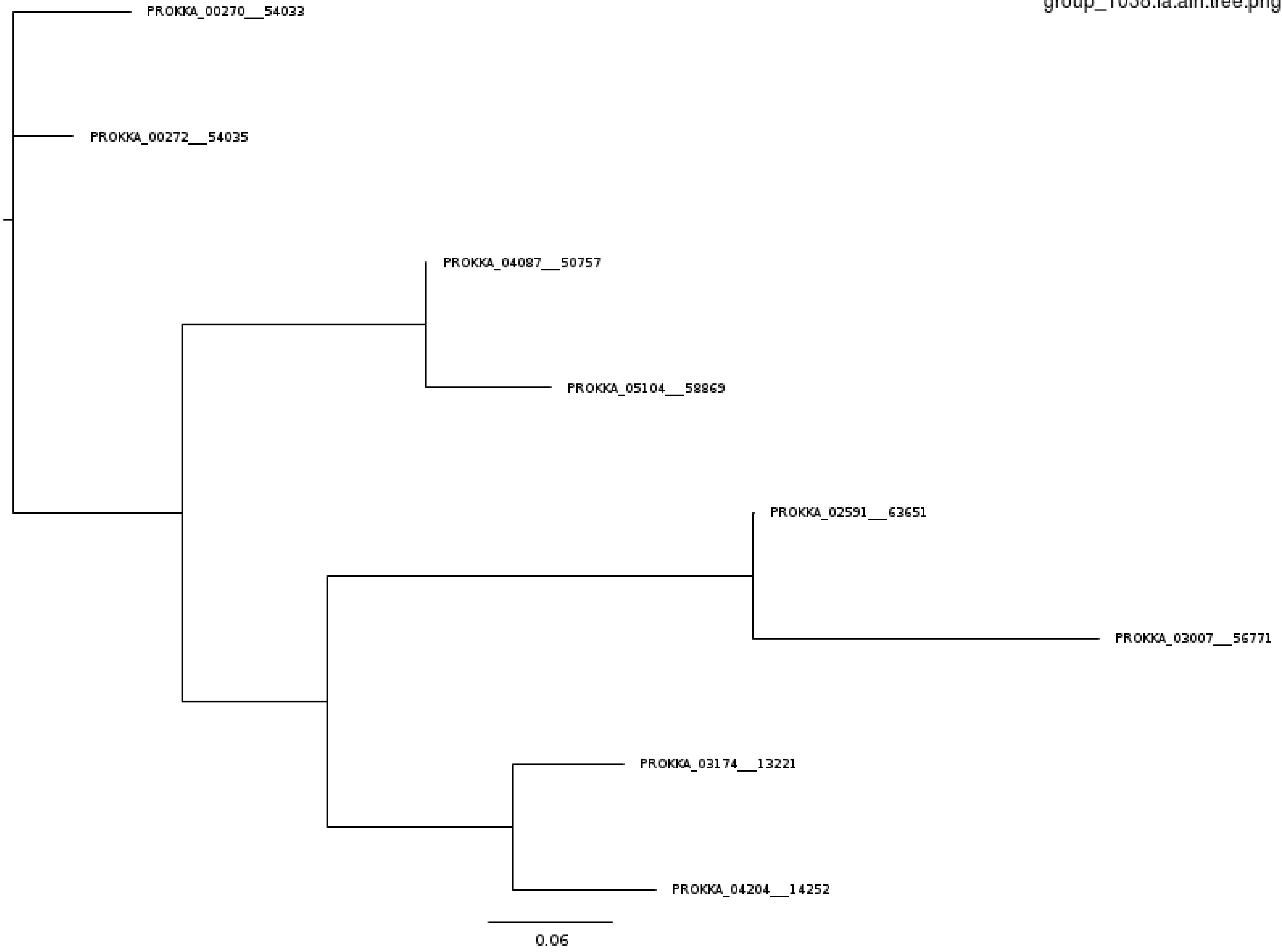

group_1039.fa.aln.tree.png

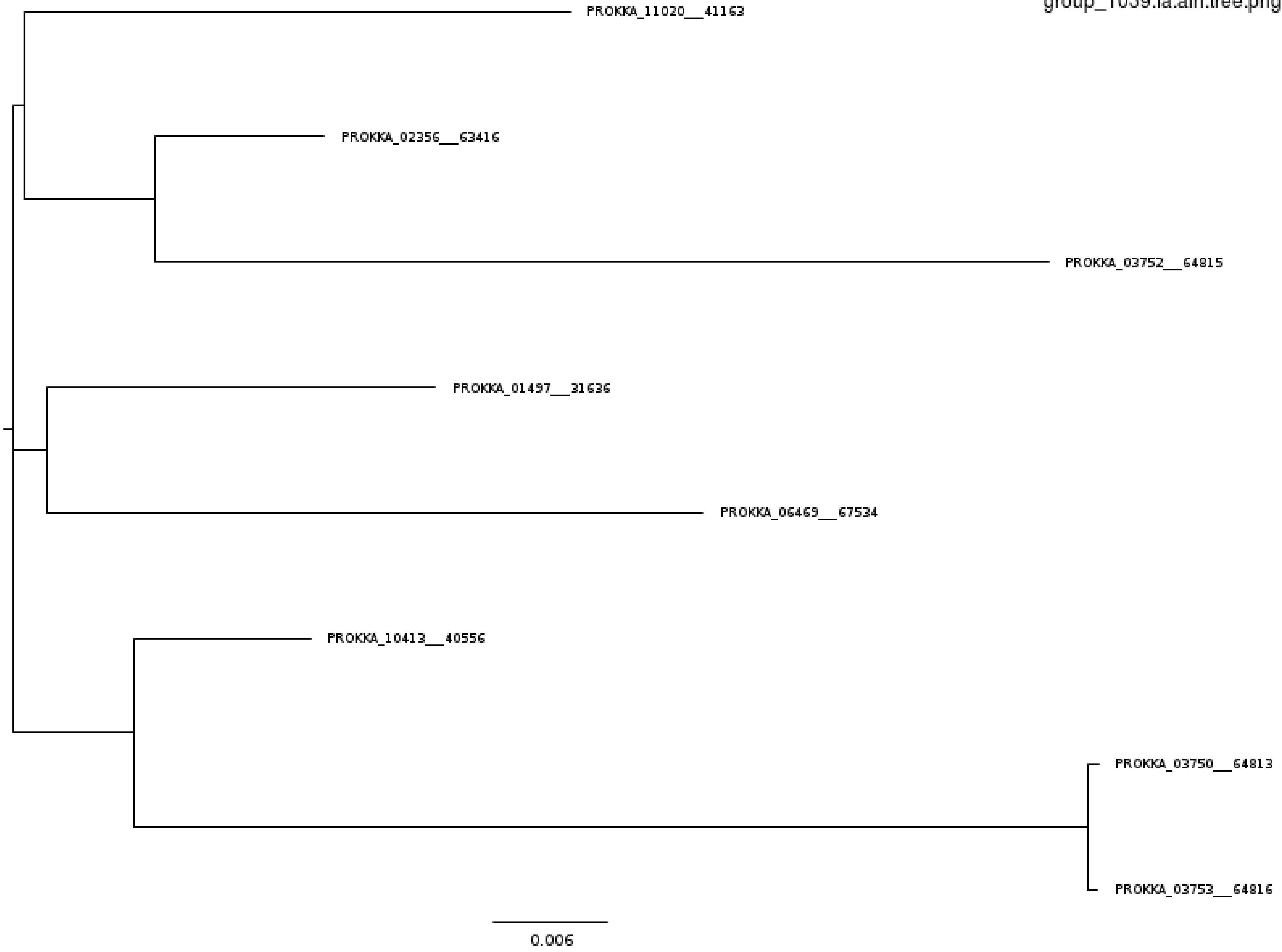

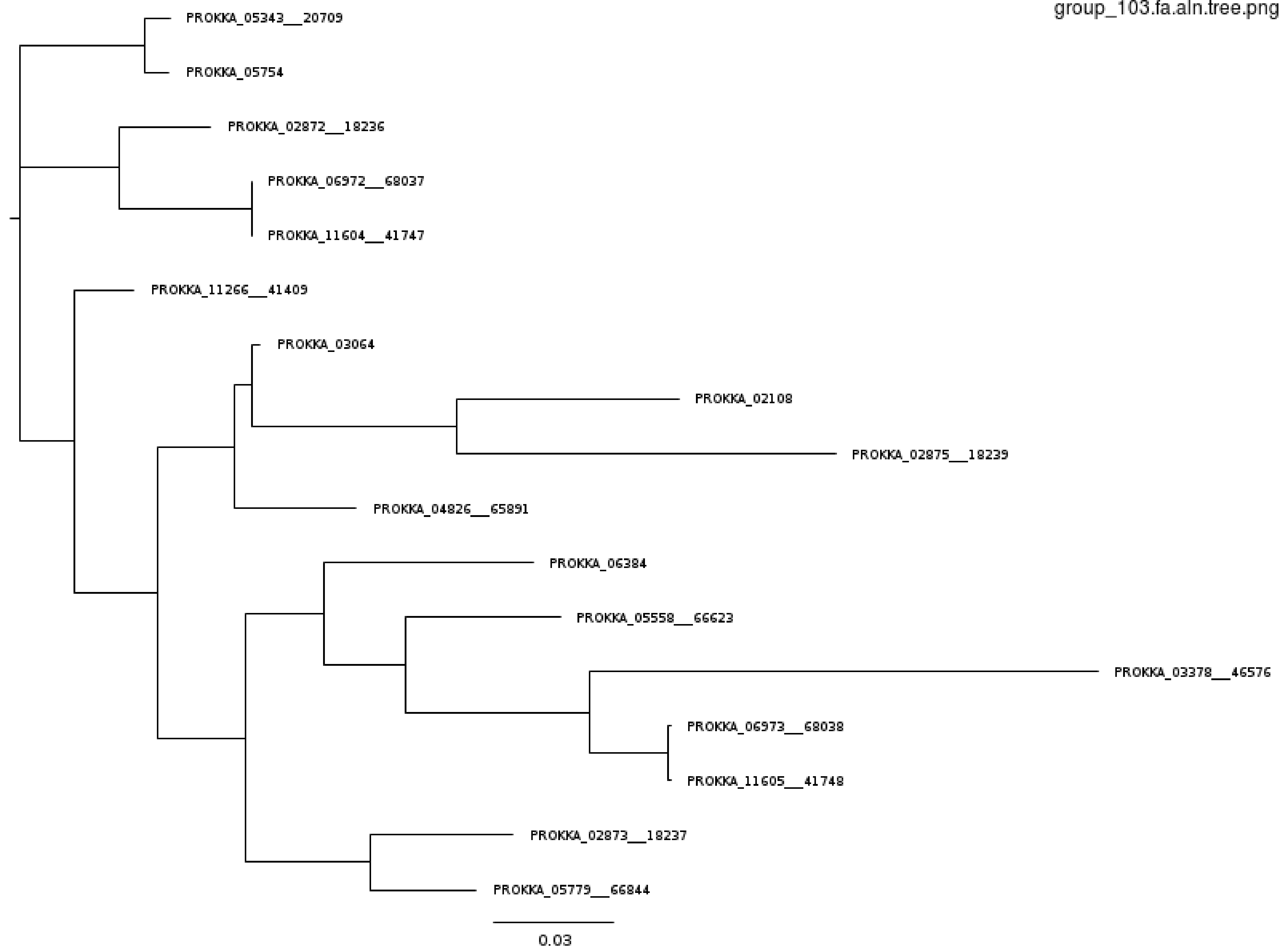
group_103.fa.aln.tree.png
PROKKA_05343___20709
PROKKA_05754
PROKKA_02872___18236
PROKKA_06972___68037
PROKKA_11604___41747
PROKKA_11266___41409
PROKKA_03064
PROKKA_02108
PROKKA_02875___18239
PROKKA_04826___65891
PROKKA_06384
PROKKA_05558___66623
PROKKA_03378___46576
PROKKA_06973___68038
PROKKA_11605___41748
PROKKA_02873___18237
PROKKA_05779___66844
0.03

group_1041.fa.aln.tree.png

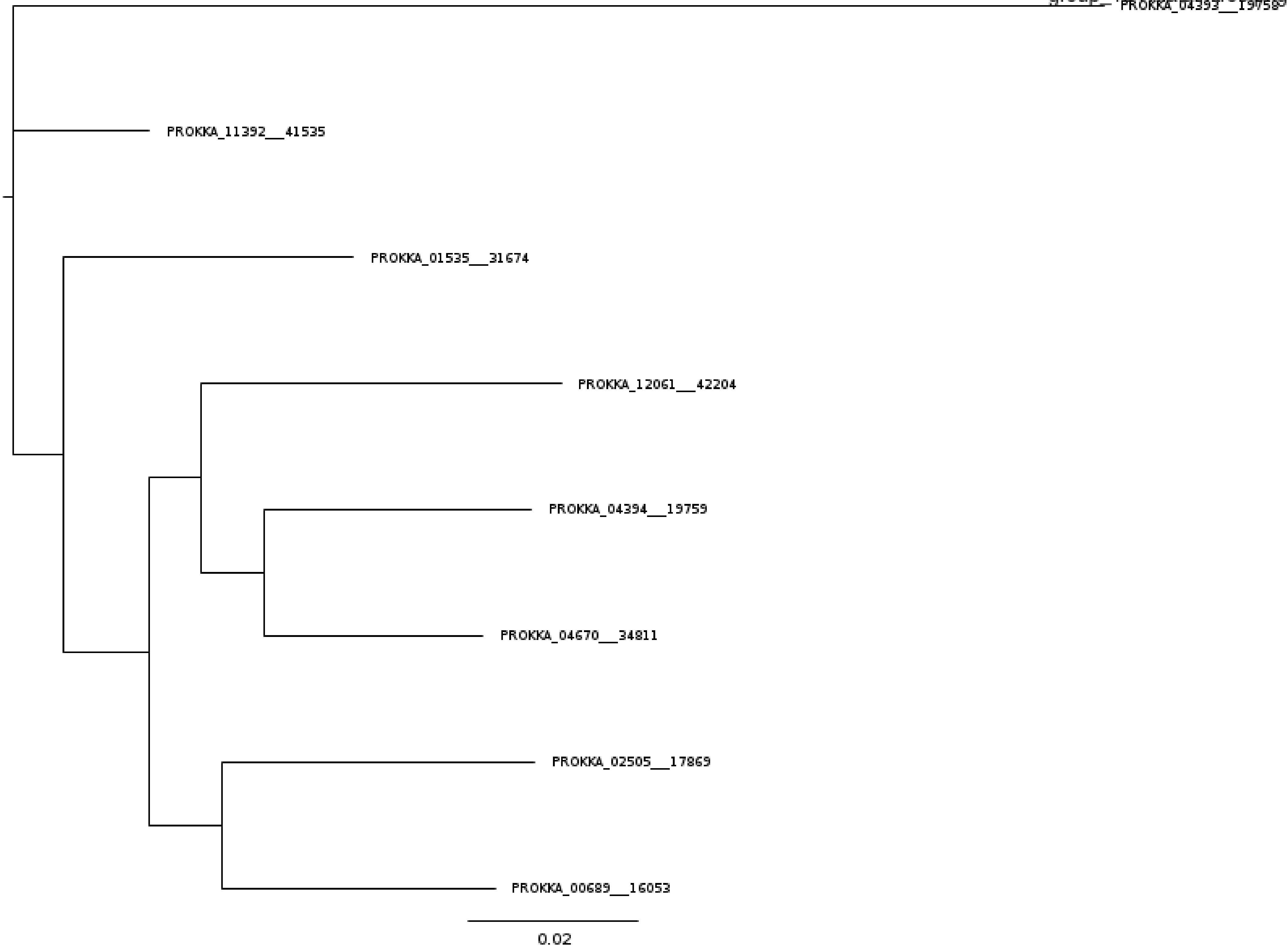

group_1043.fa.aln.tree.png

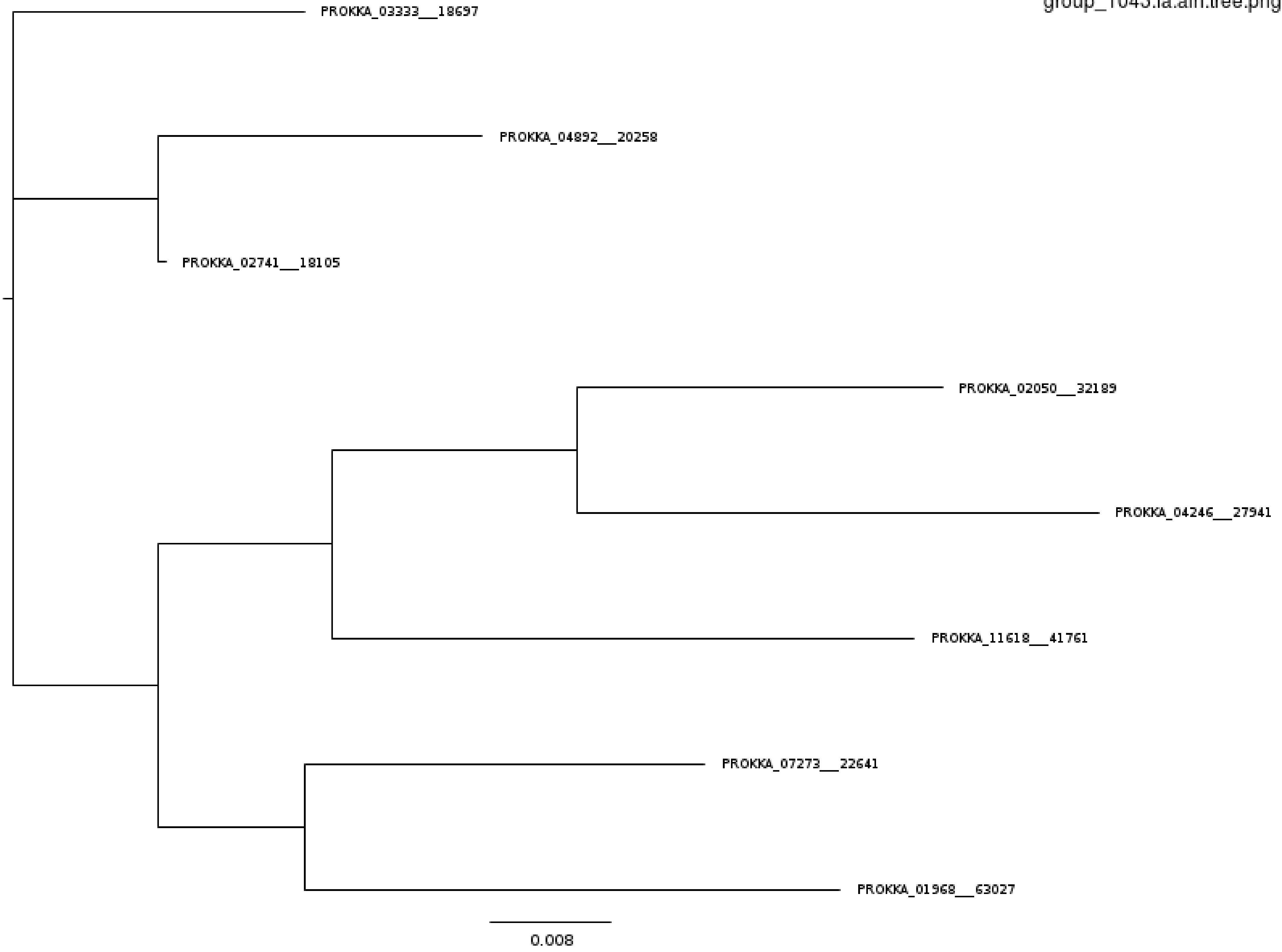

group_1044.fa.aln.tree.png

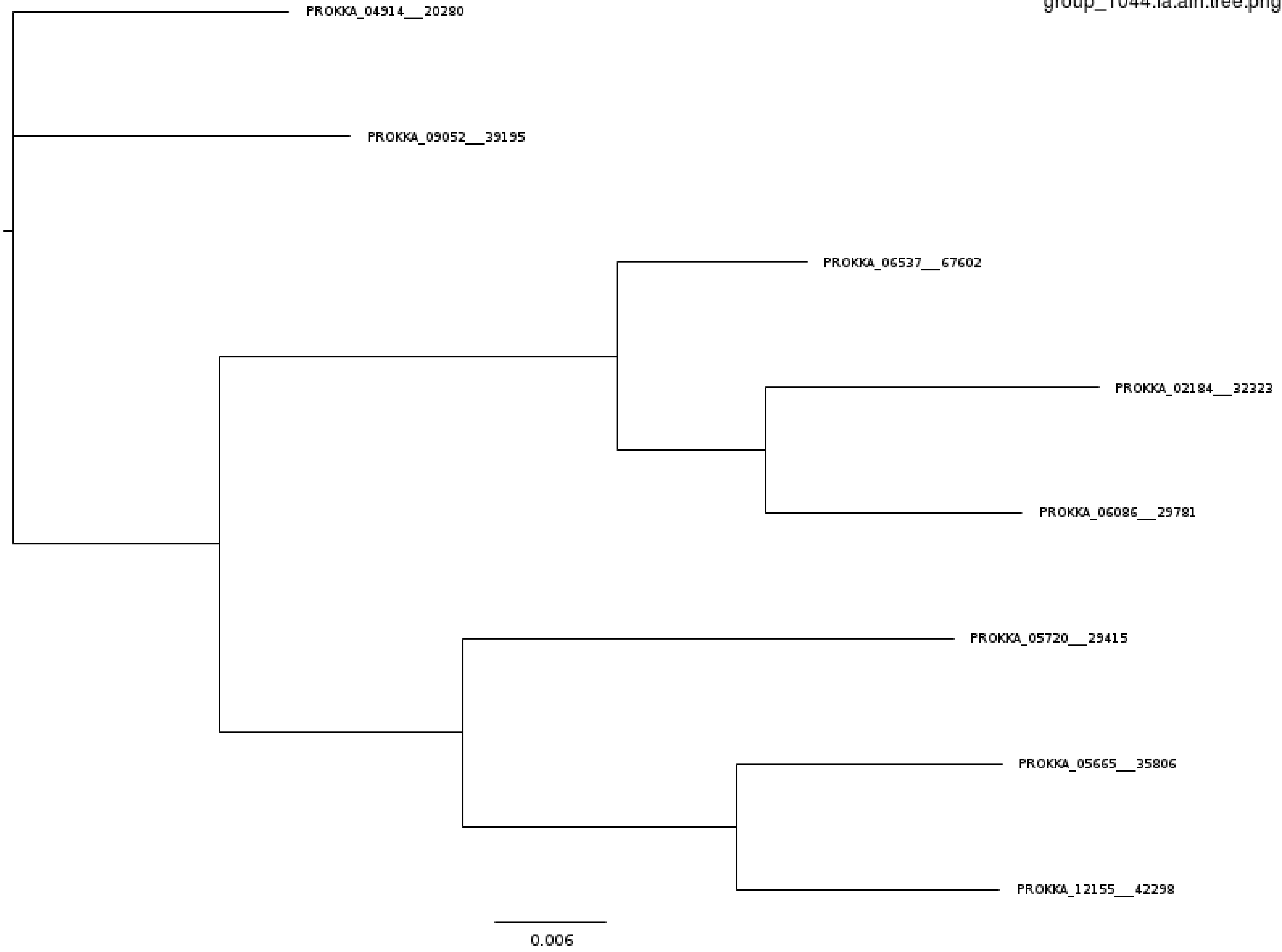

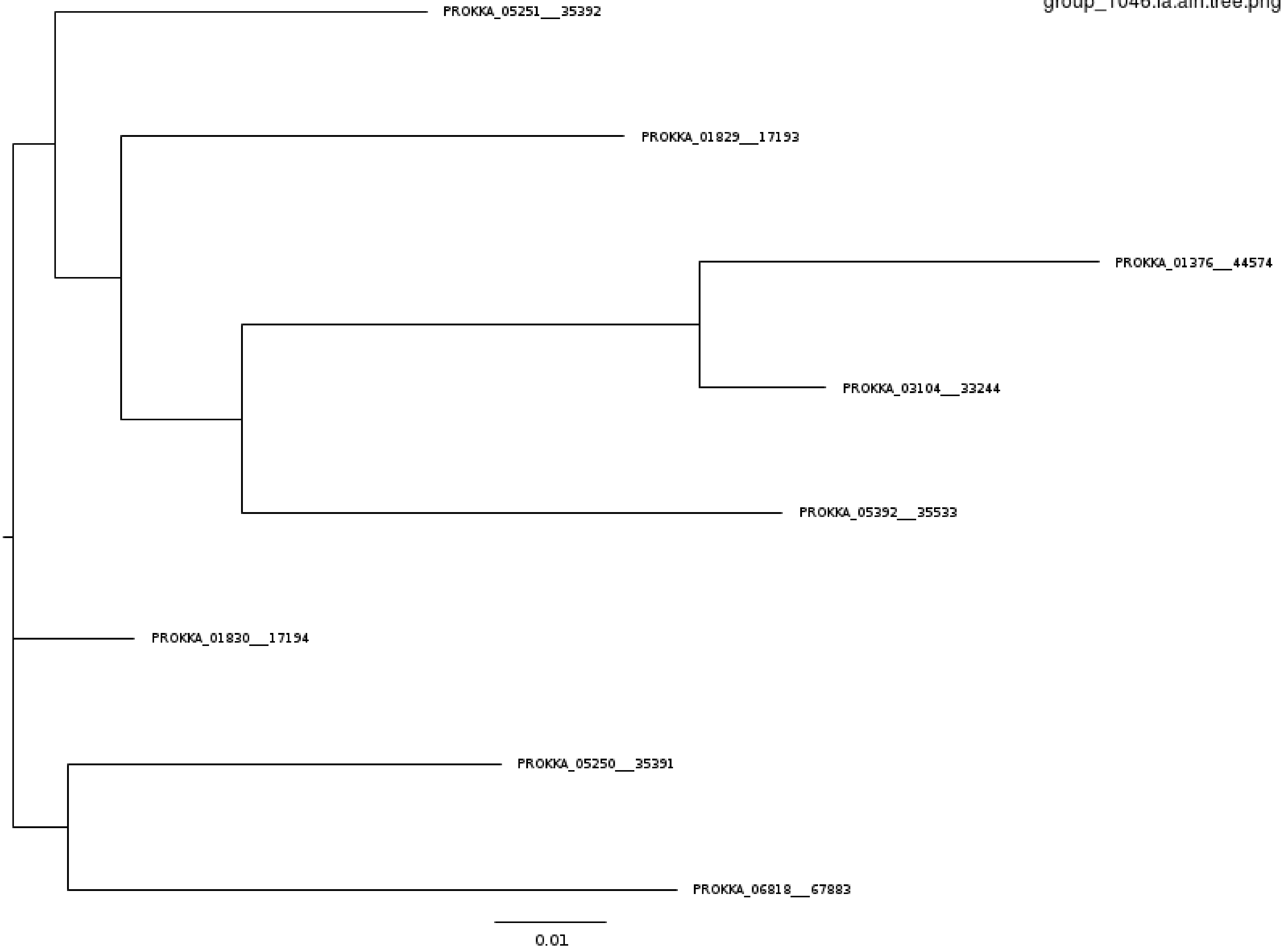
PROKKA_05251___35392
PROKKA_01829___17193
PROKKA_01376___44574
PROKKA_03104___33244
PROKKA_05392___35533
PROKKA_01830___17194
PROKKA_05250___35391
PROKKA_06818___67883
0.01

group_1048.fa.aln.tree.png

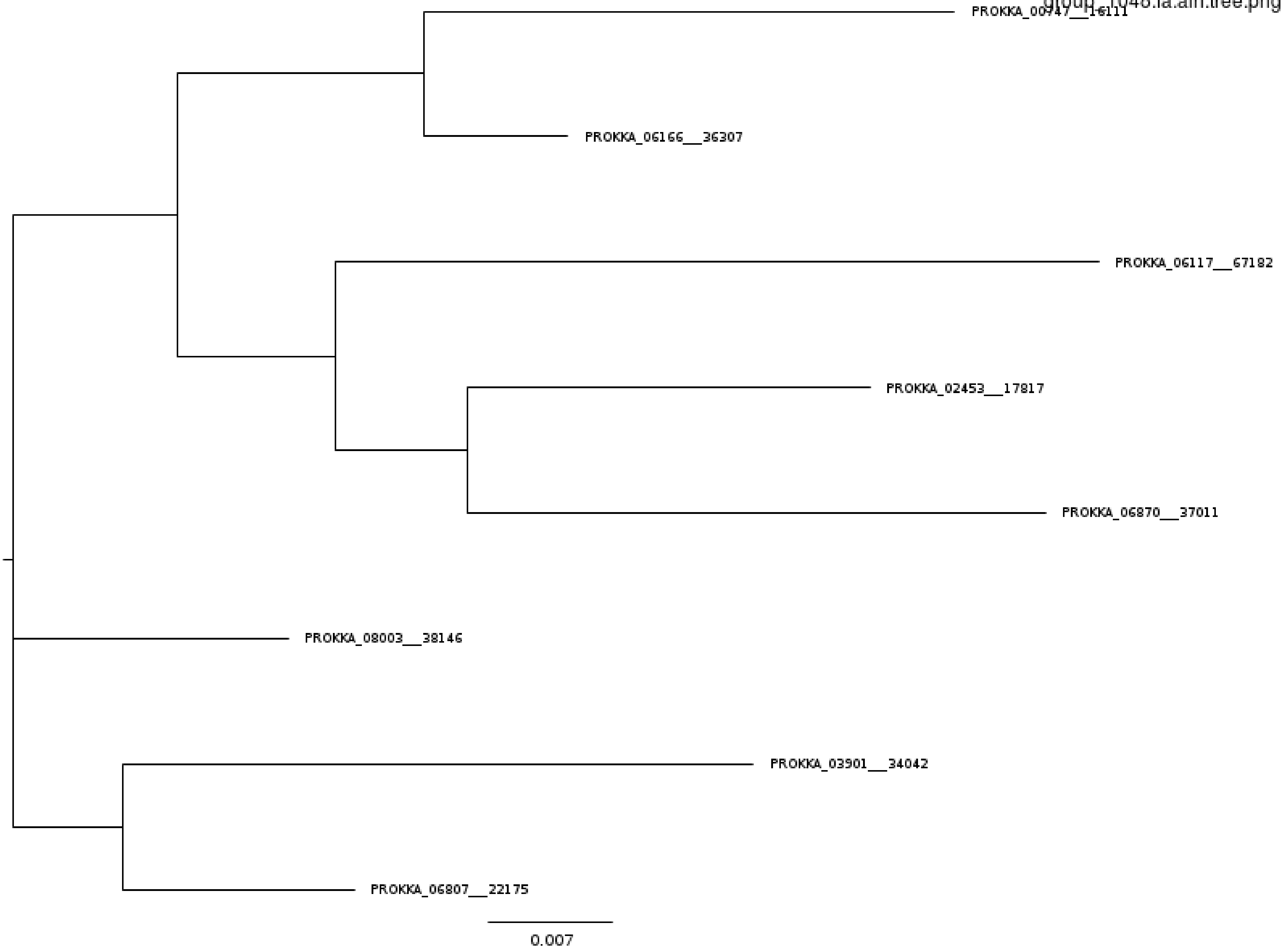

group_1049.fa.aln.tree.png

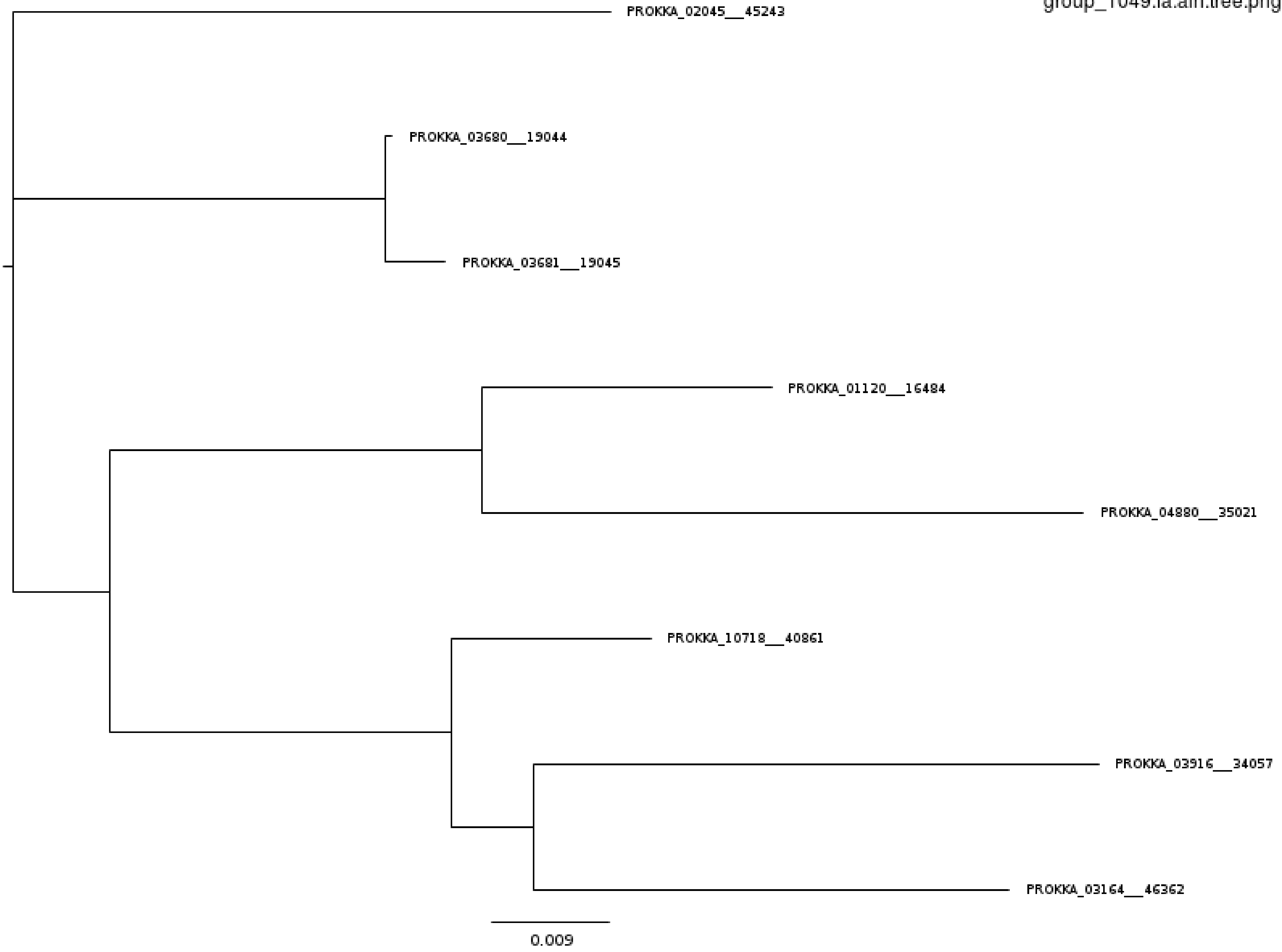

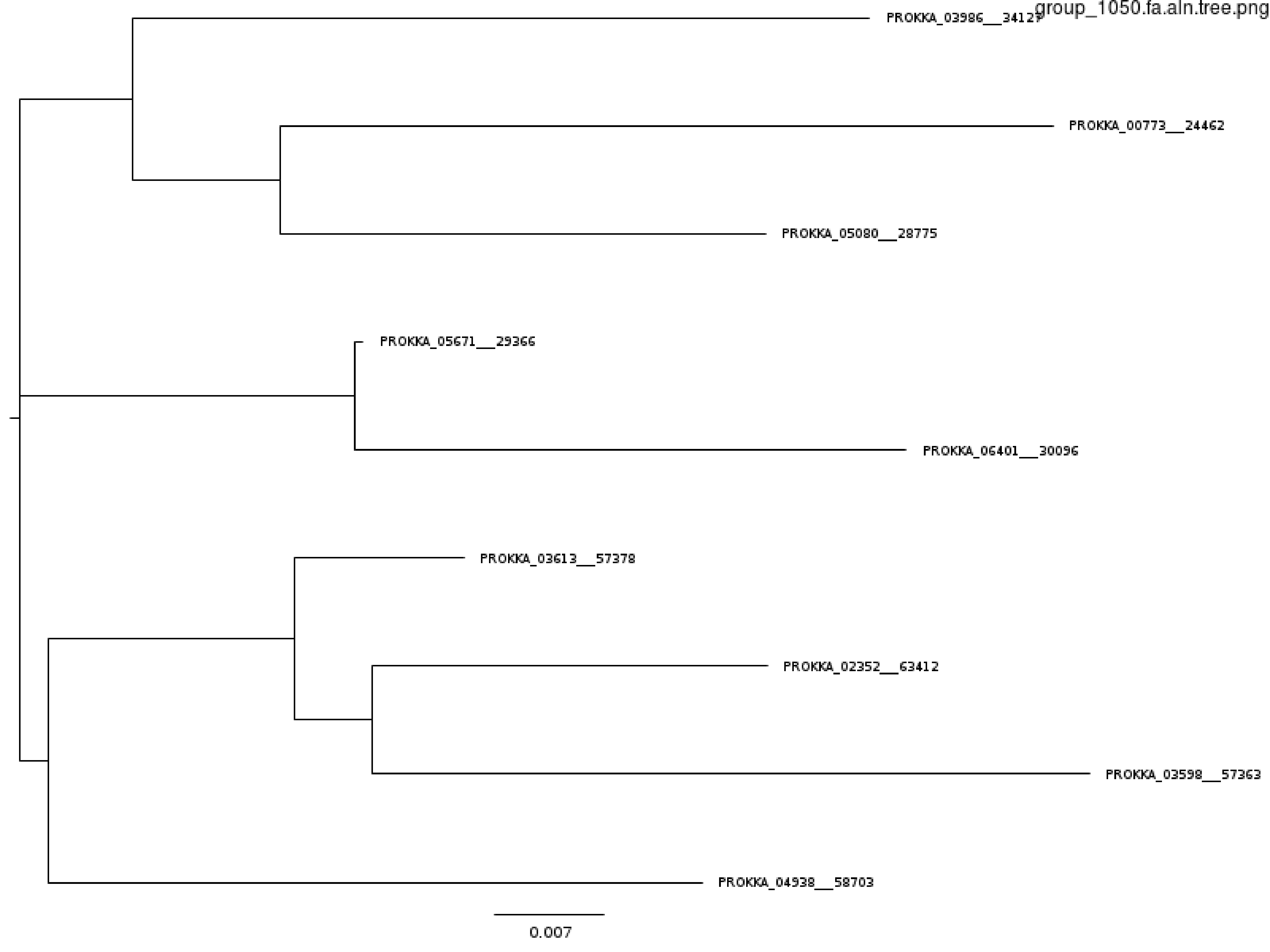
group_1050.fa.aln.tree.png
PROKKA_03986___34127
PROKKA_00773___24462
PROKKA_05080___28775
PROKKA_05671___29366
PROKKA_06401___30096
PROKKA_03613___57378
PROKKA_02352___63412
PROKKA_03598___57363
PROKKA_04938___58703
0.007

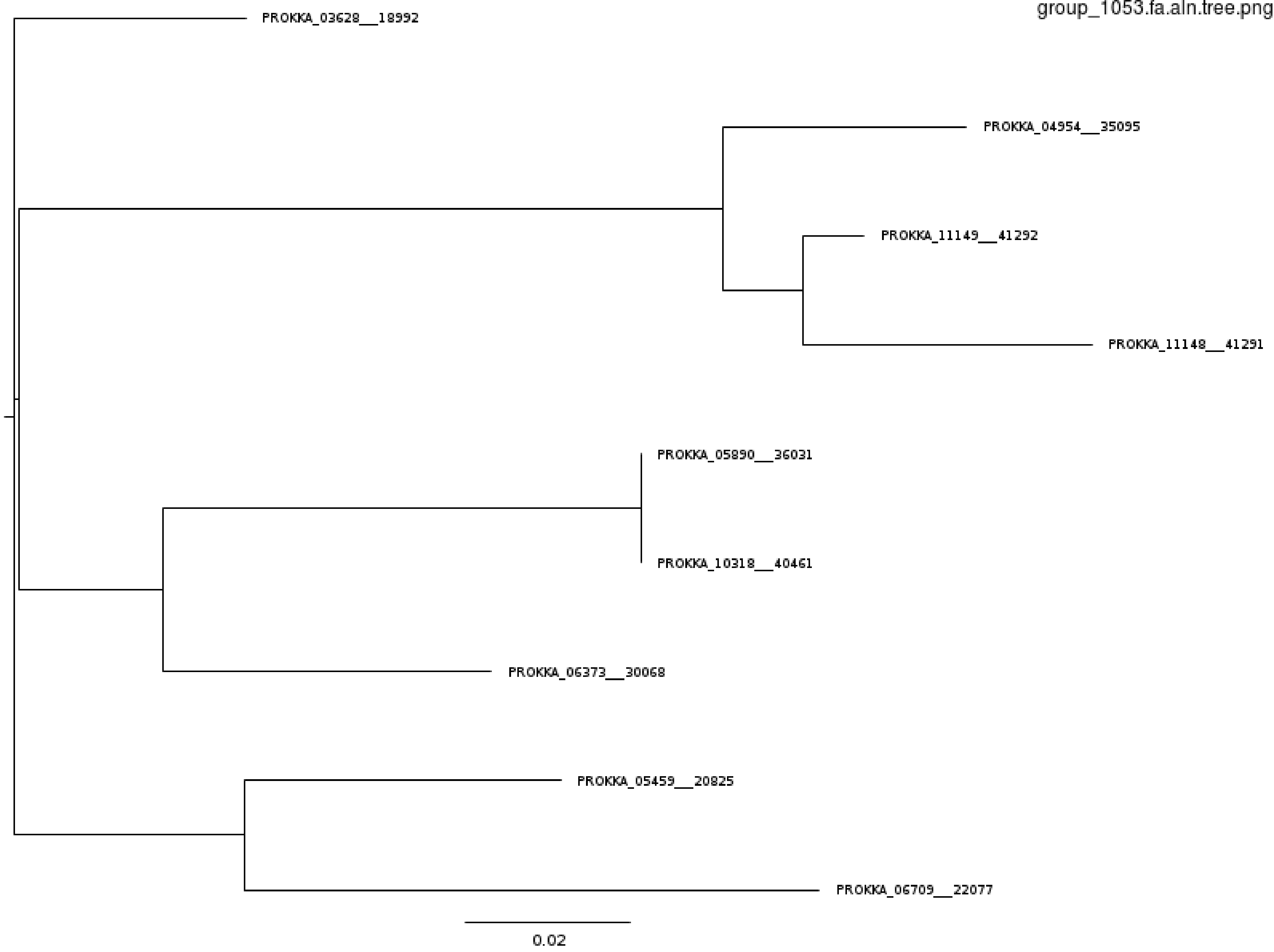
group_1053.fa.aln.tree.png
PROKKA_03628___18992
PROKKA_04954___35095
PROKKA_11149___41292
PROKKA_11148___41291
PROKKA_05890___36031
PROKKA_10318___40461
PROKKA_06373___30068
PROKKA_05459___20825
PROKKA_06709___22077
0.02

group_1060.fa.aln.tree.png

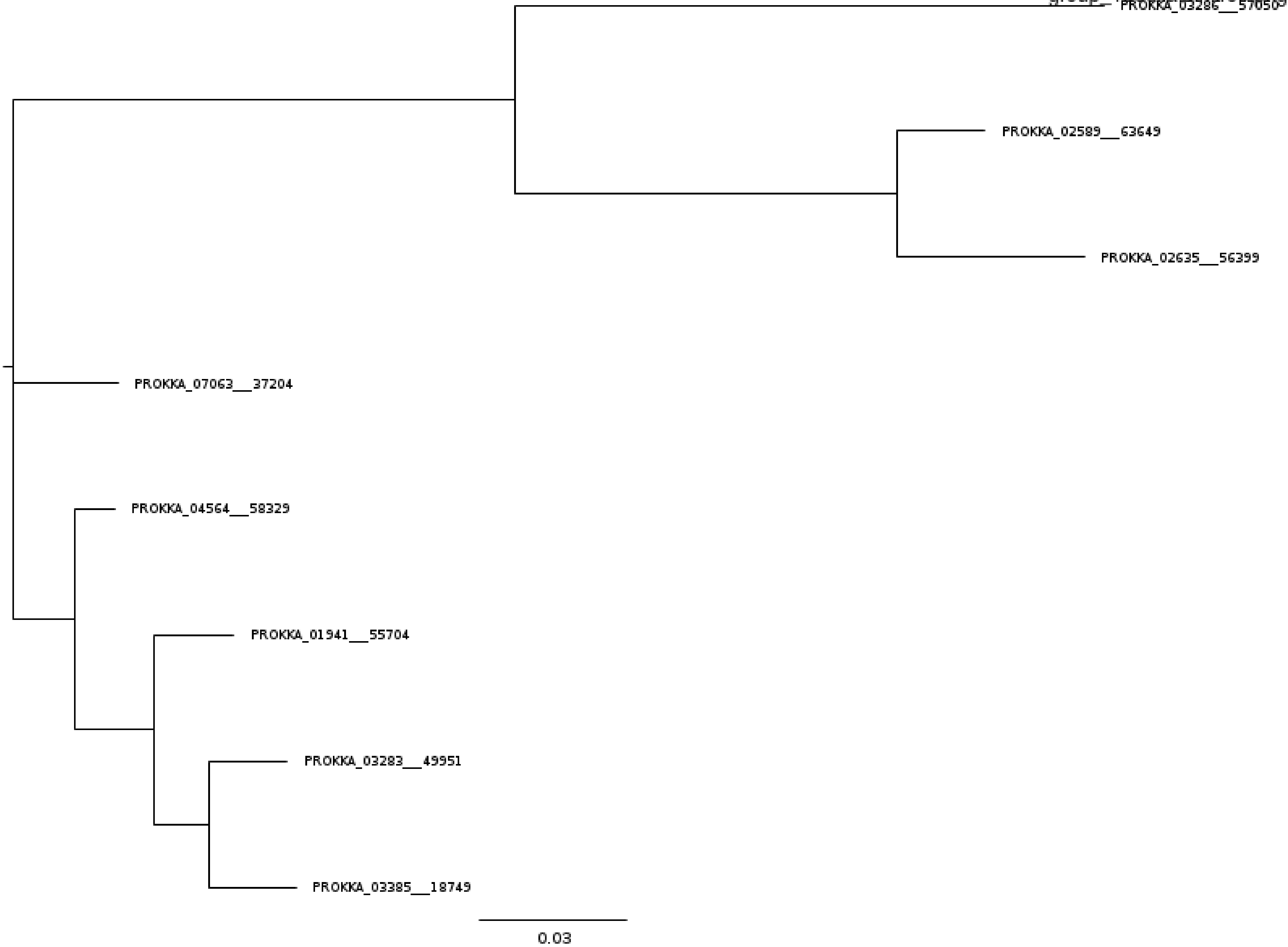

group_1062.fa.aln.tree.png

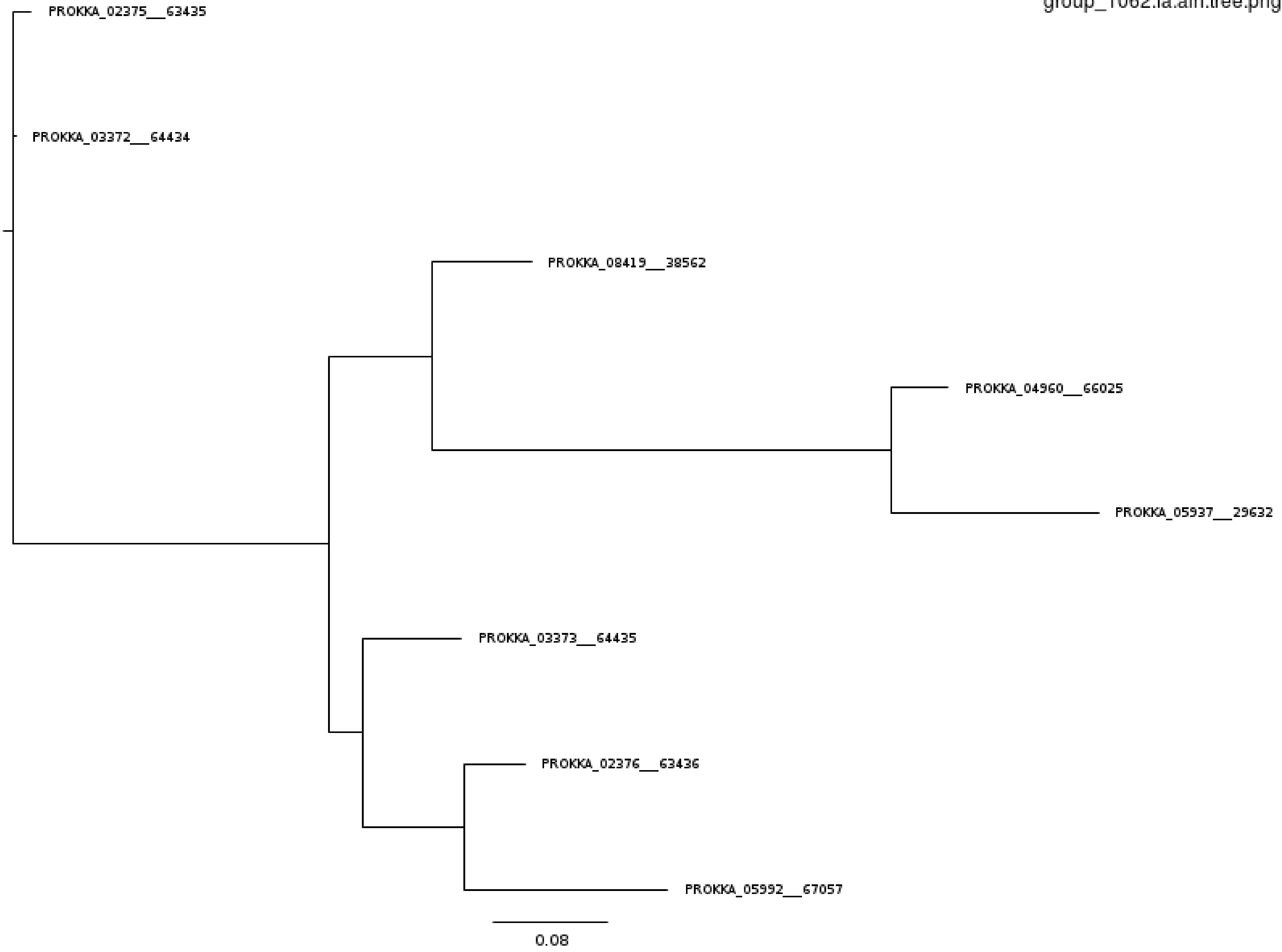

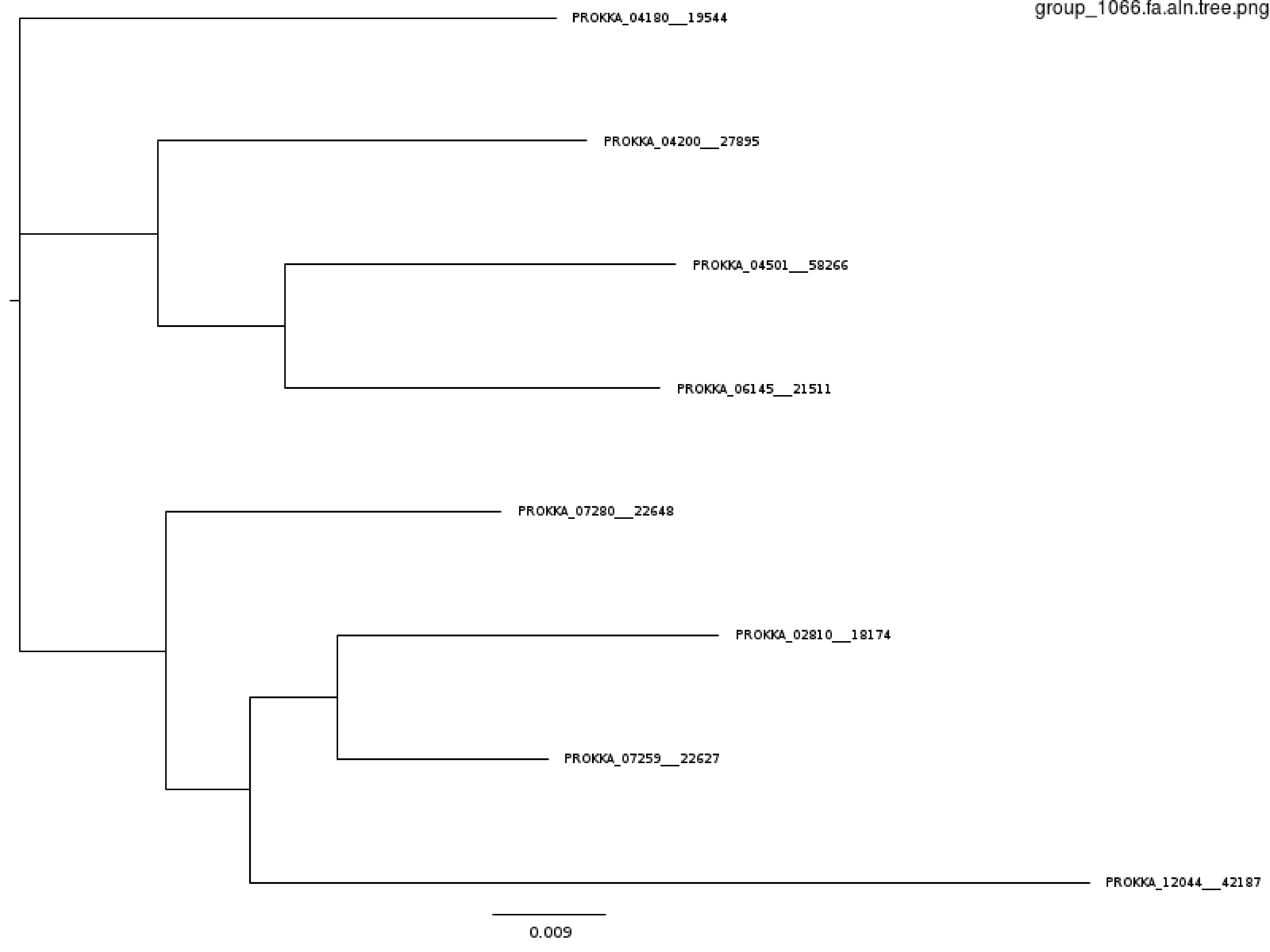
group_1066.fa.aln.tree.png
PROKKA_04180___19544
PROKKA_04200___27895
PROKKA_04501___58266
PROKKA_06145___21511
PROKKA_07280___22648
PROKKA_02810___18174
PROKKA_07259___22627
PROKKA_12044___42187
0.009

group_1067.fa.aln.tree.png

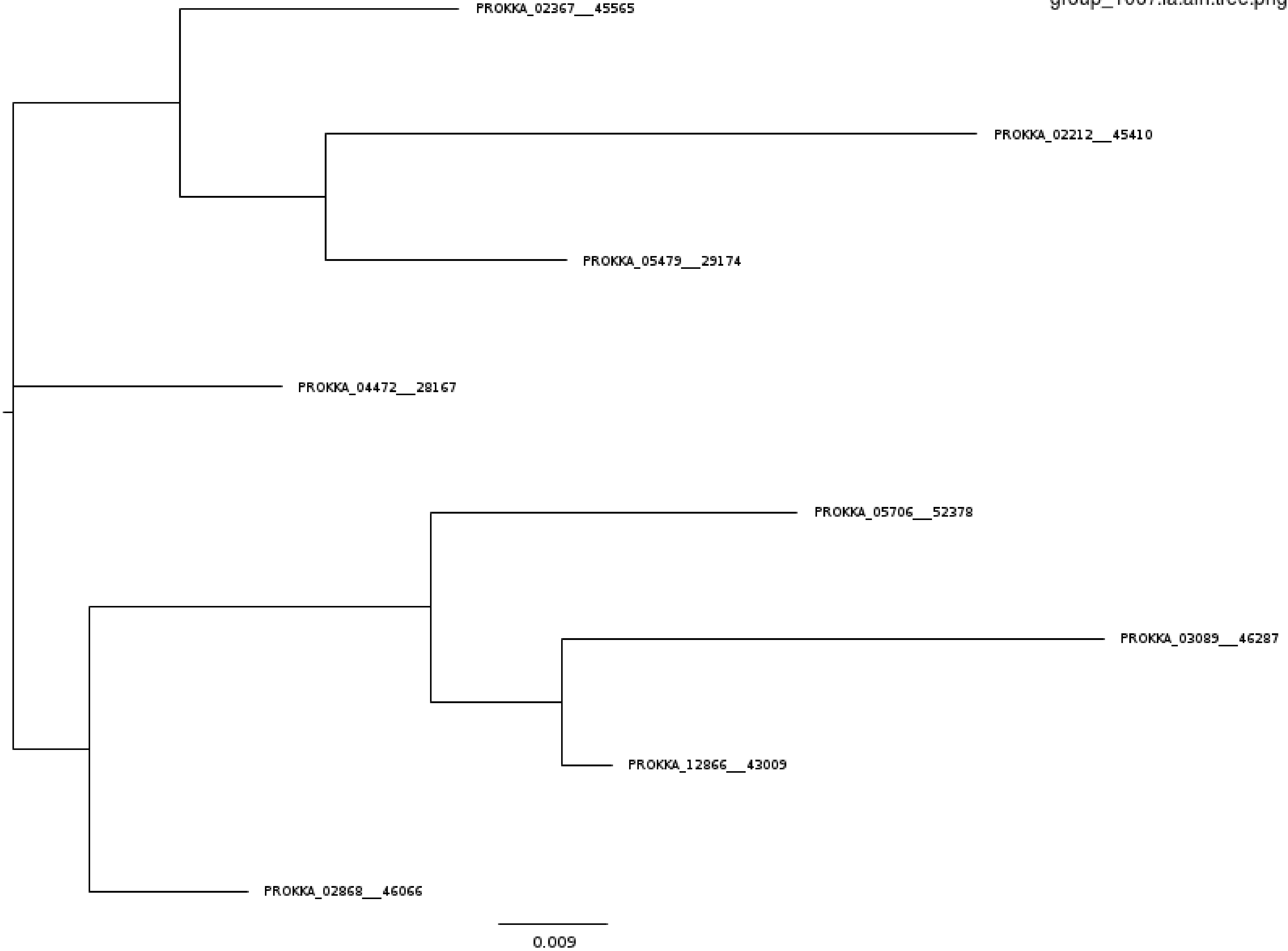

group_1070.fa.aln.tree.png

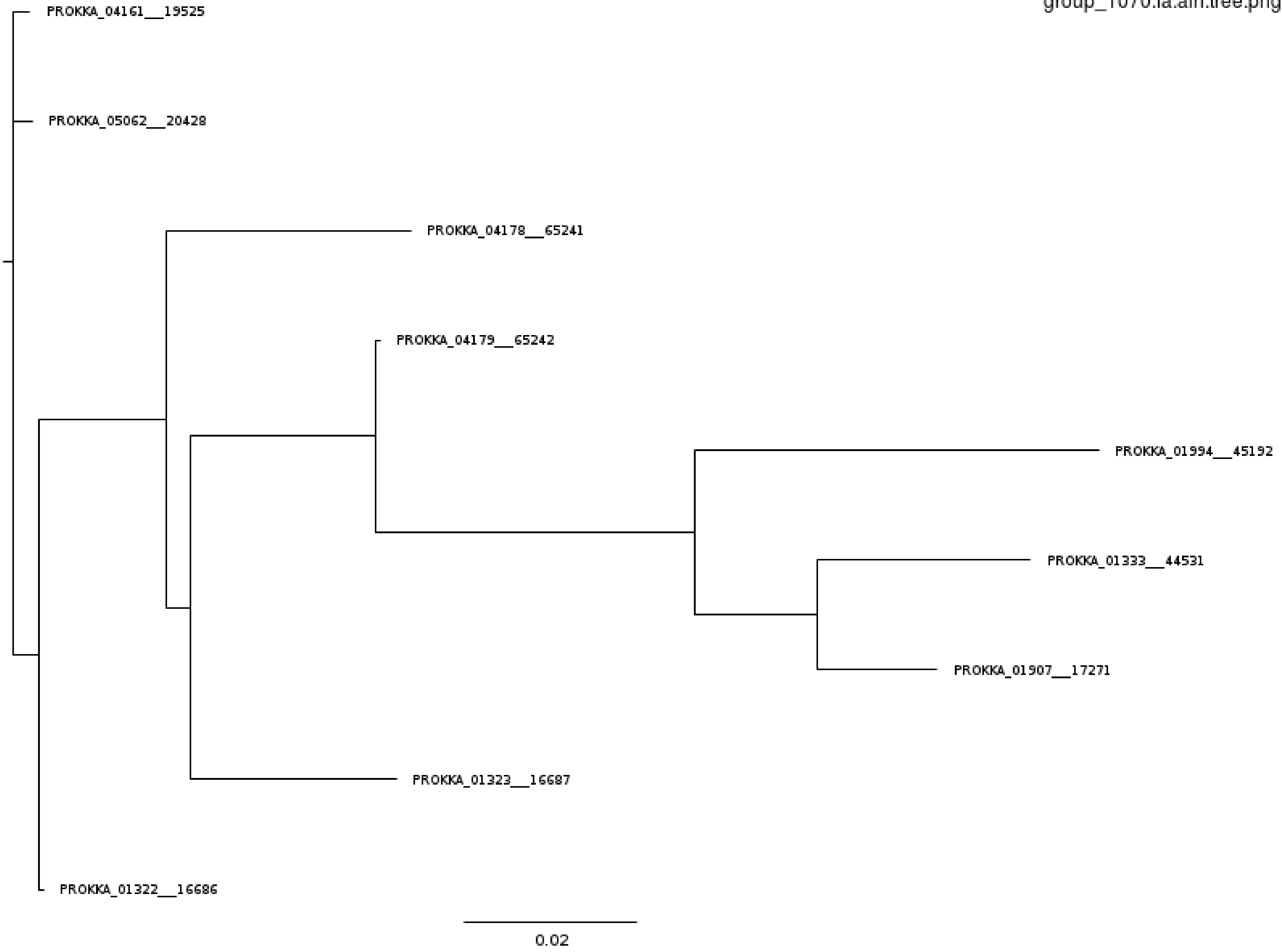

group_1078.fa.aln.tree.png

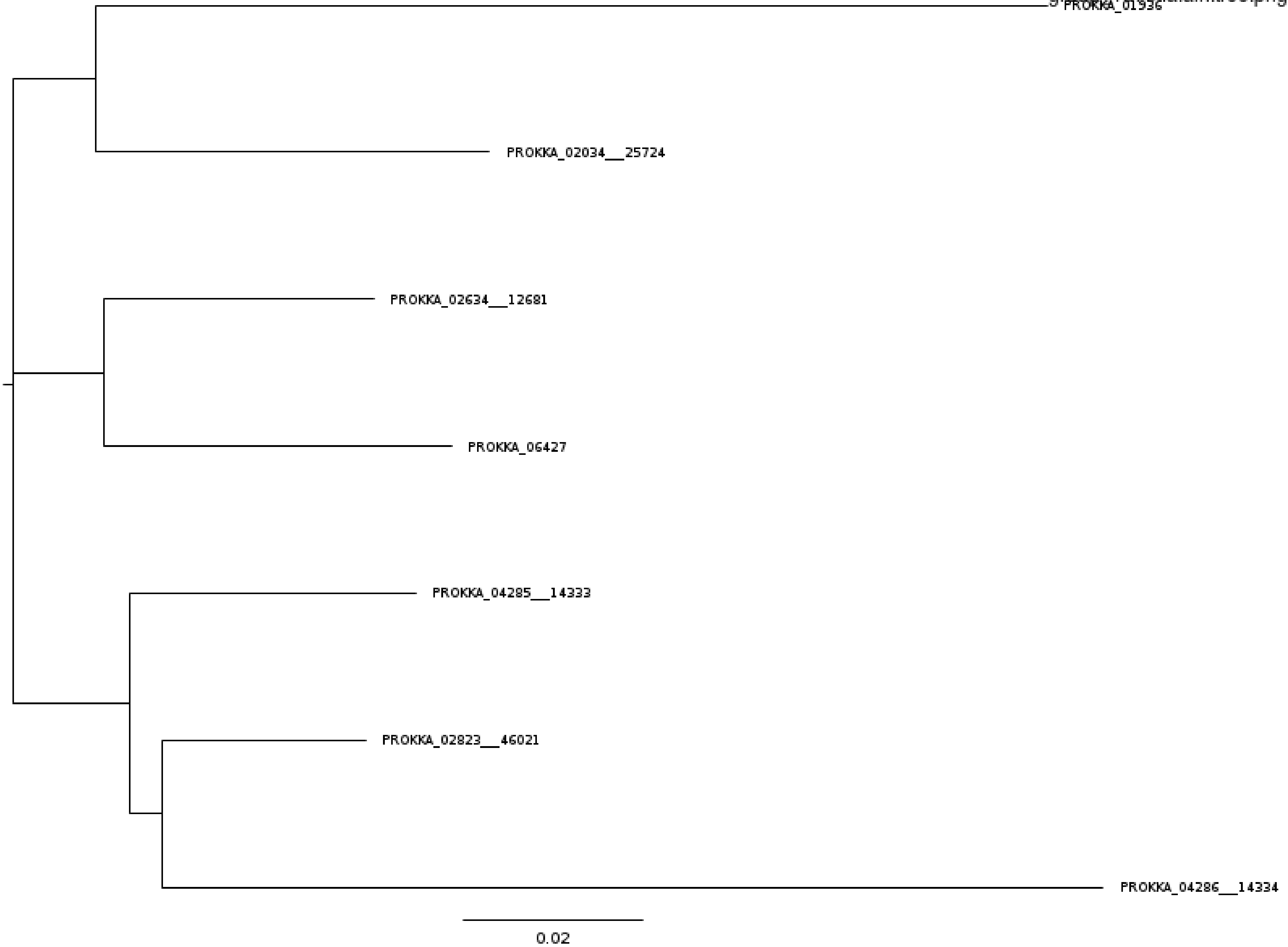

group_1079.fa.aln.tree.png

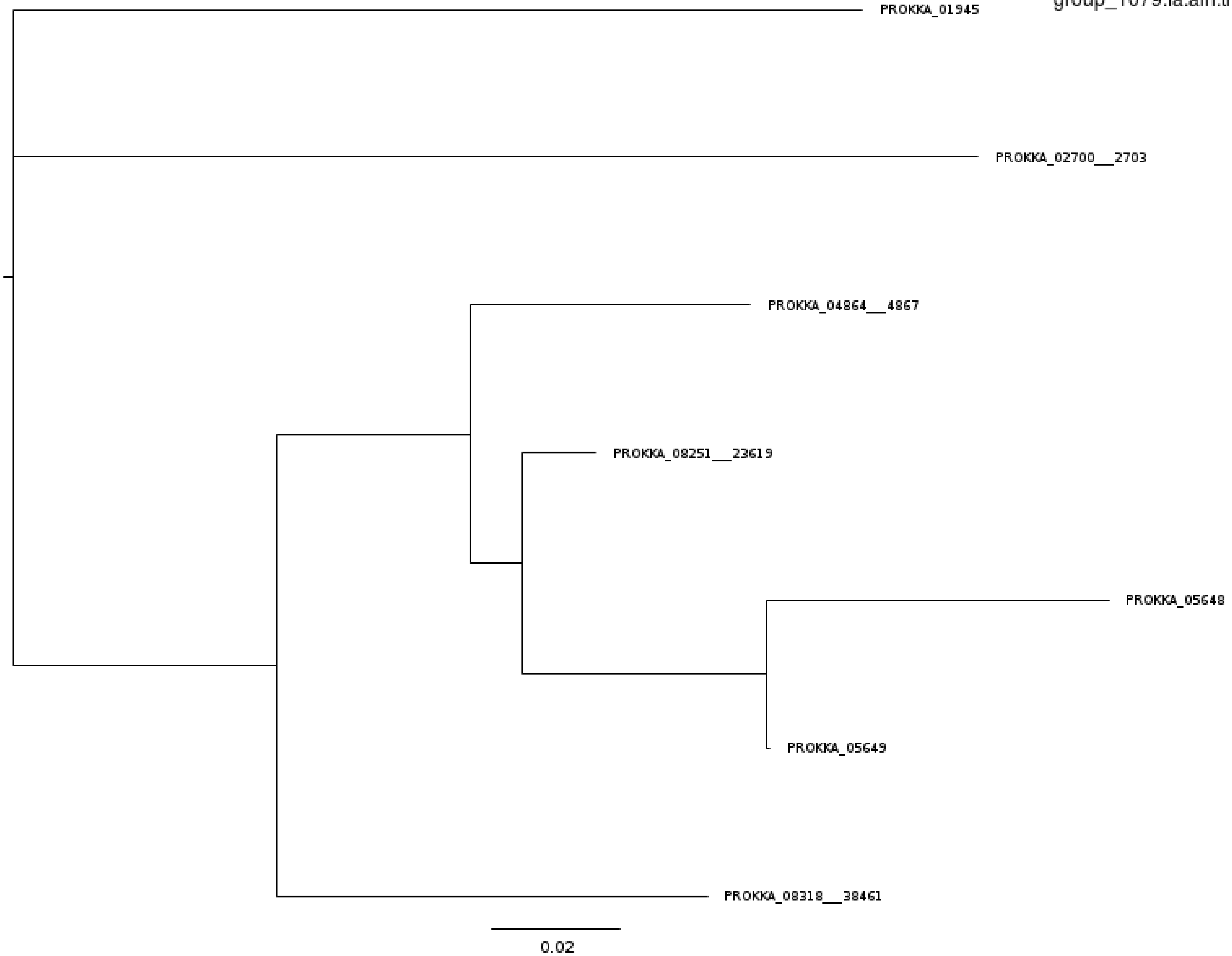

group_1081.fa.aln.tree.png

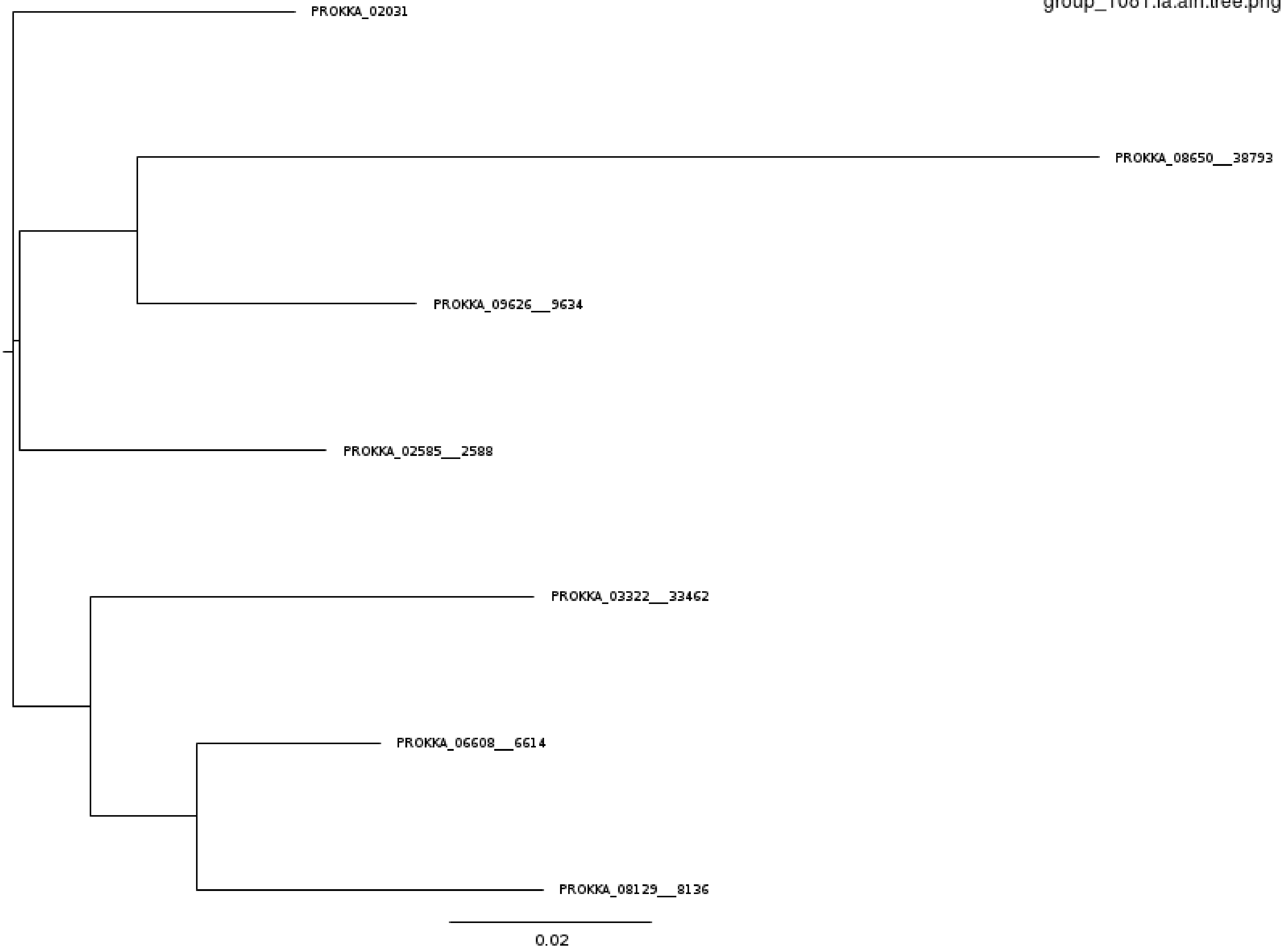

group_1087.fa.aln.tree.png

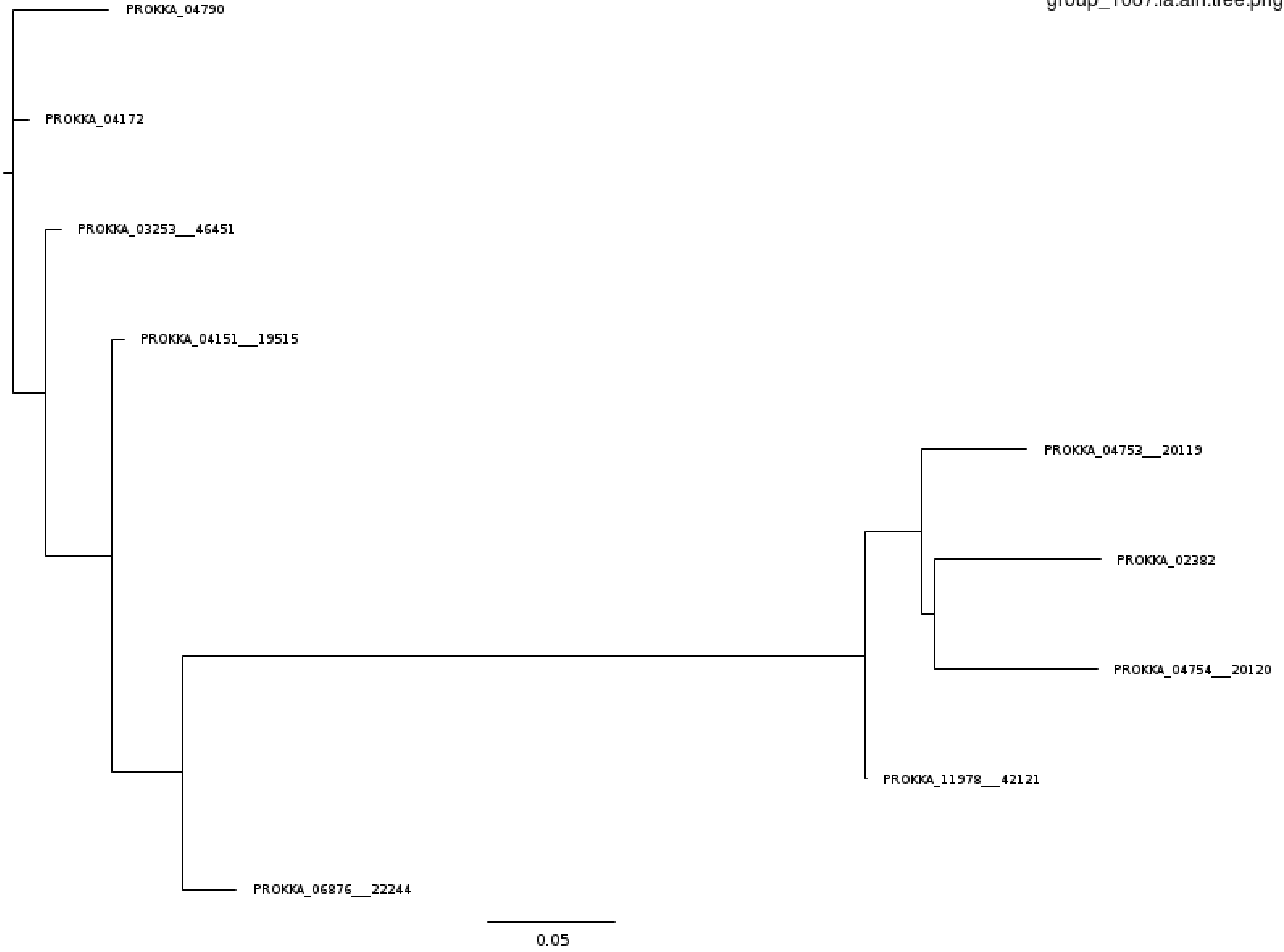

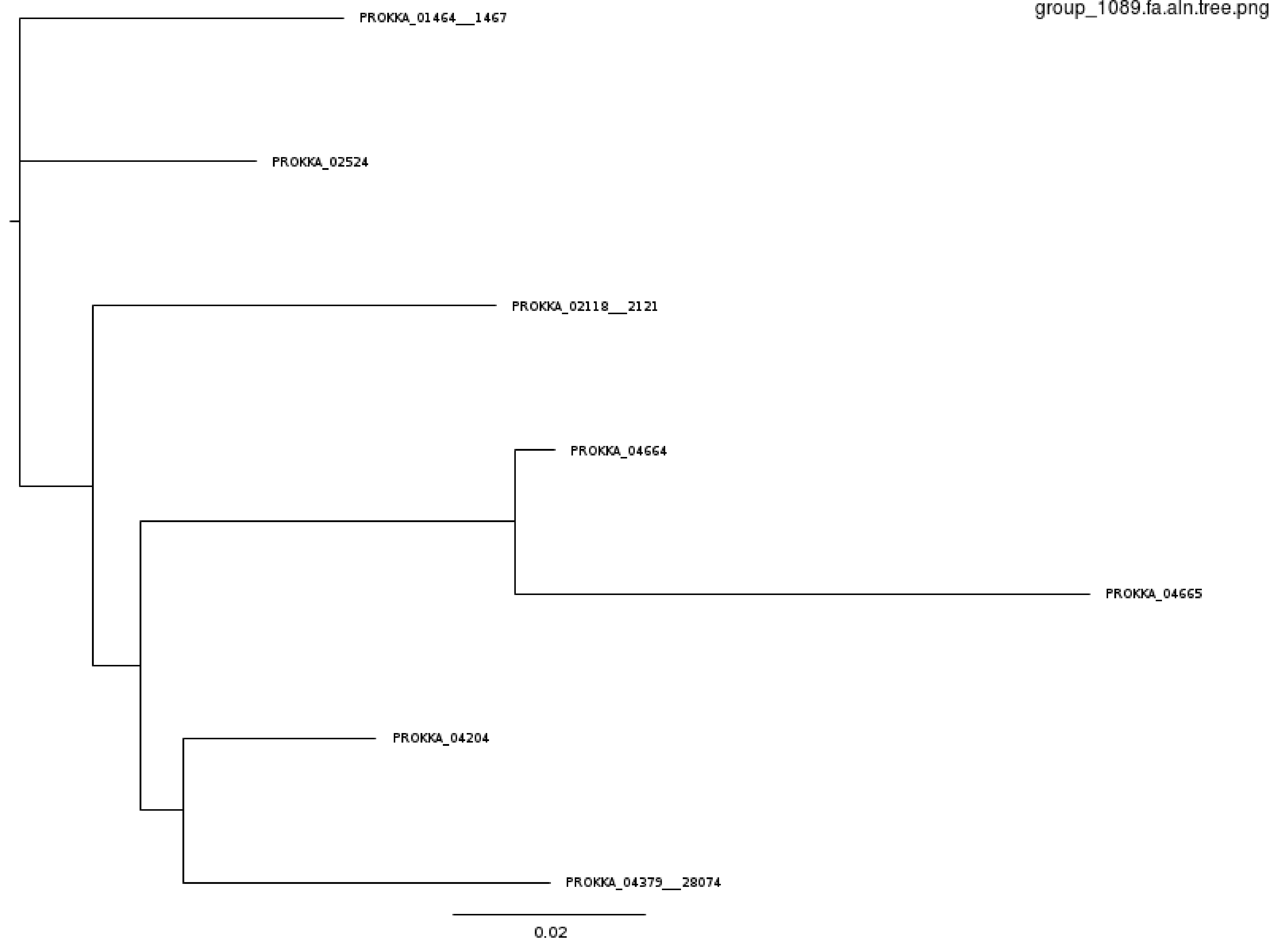

group_1089.fa.aln.tree.png
PROKKA_01464___1467
PROKKA_02524
PROKKA_02118___2121
PROKKA_04664
PROKKA_04665
PROKKA_04204
PROKKA_04379___28074
0.02

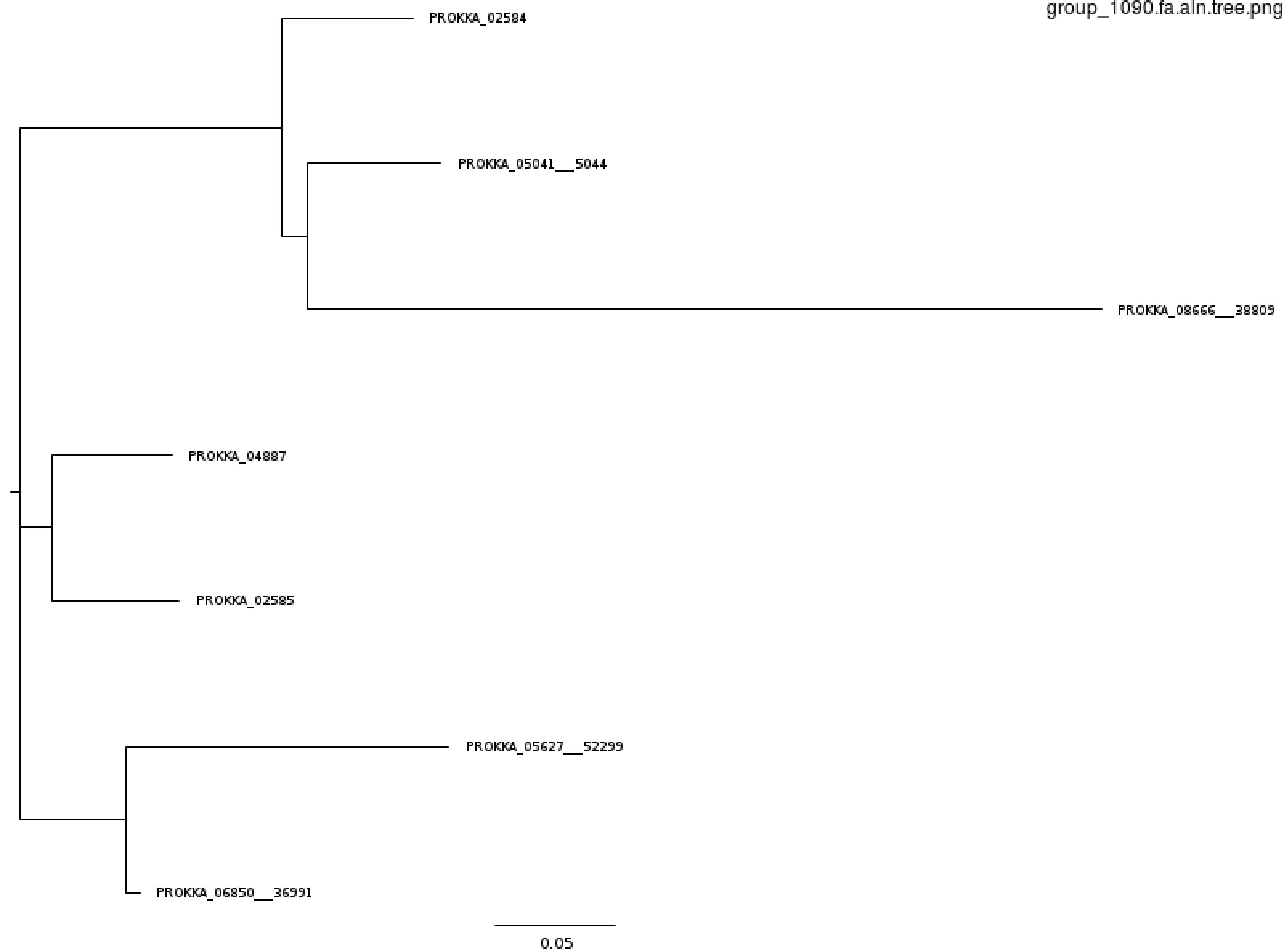
group_1090.fa.aln.tree.png
PROKKA_02584
PROKKA_05041___5044
PROKKA_08666___38809
PROKKA_04887
PROKKA_02585
PROKKA_05627___52299
PROKKA_06850___36991
0.05

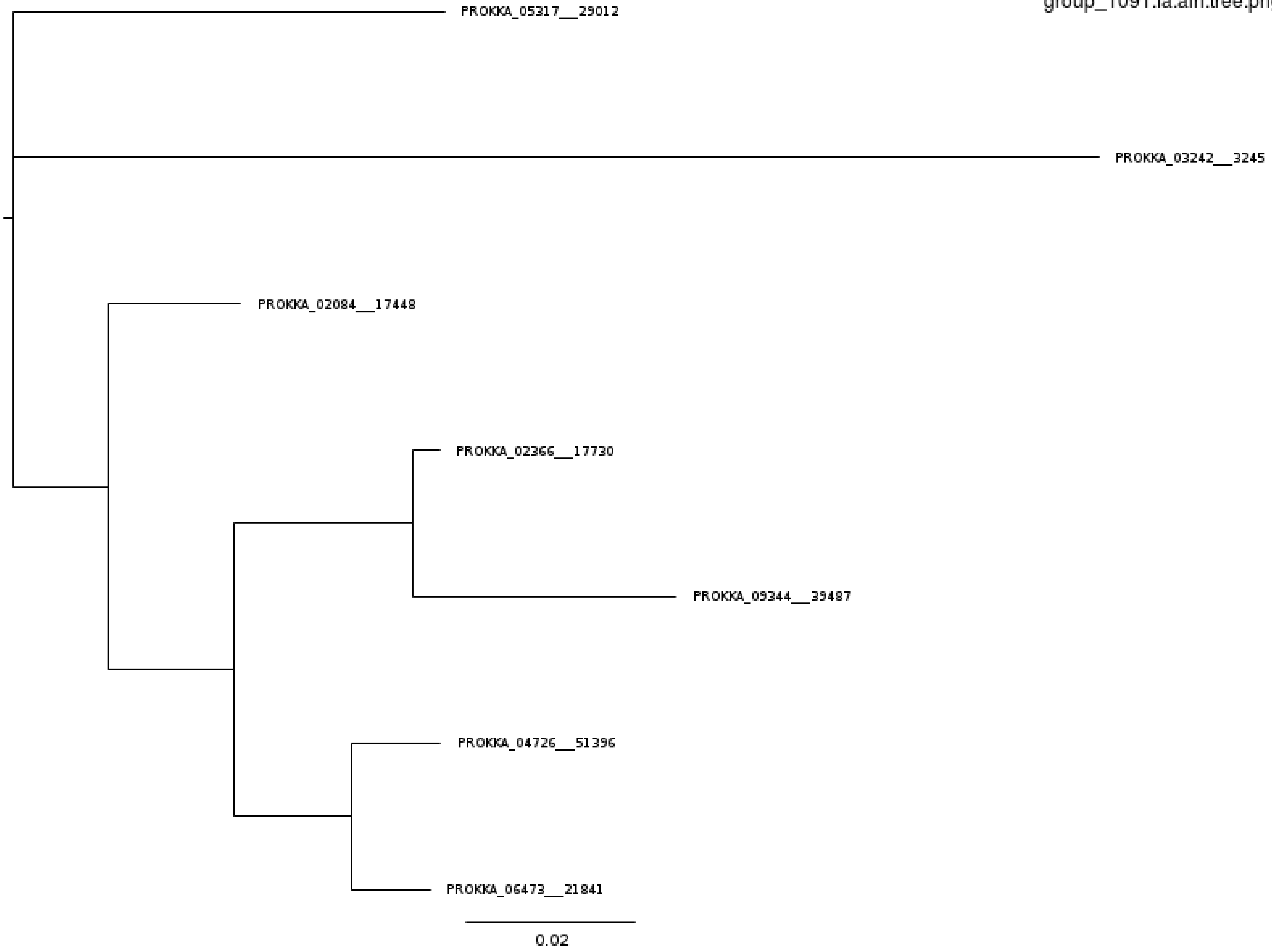
PROKKA_05317__29012
PROKKA_03242__3245
PROKKA_02084__17448
PROKKA_02366__17730
PROKKA_09344__39487
PROKKA_04726__51396
PROKKA_06473__21841
0.02

group_1092.fa.aln.tree.png

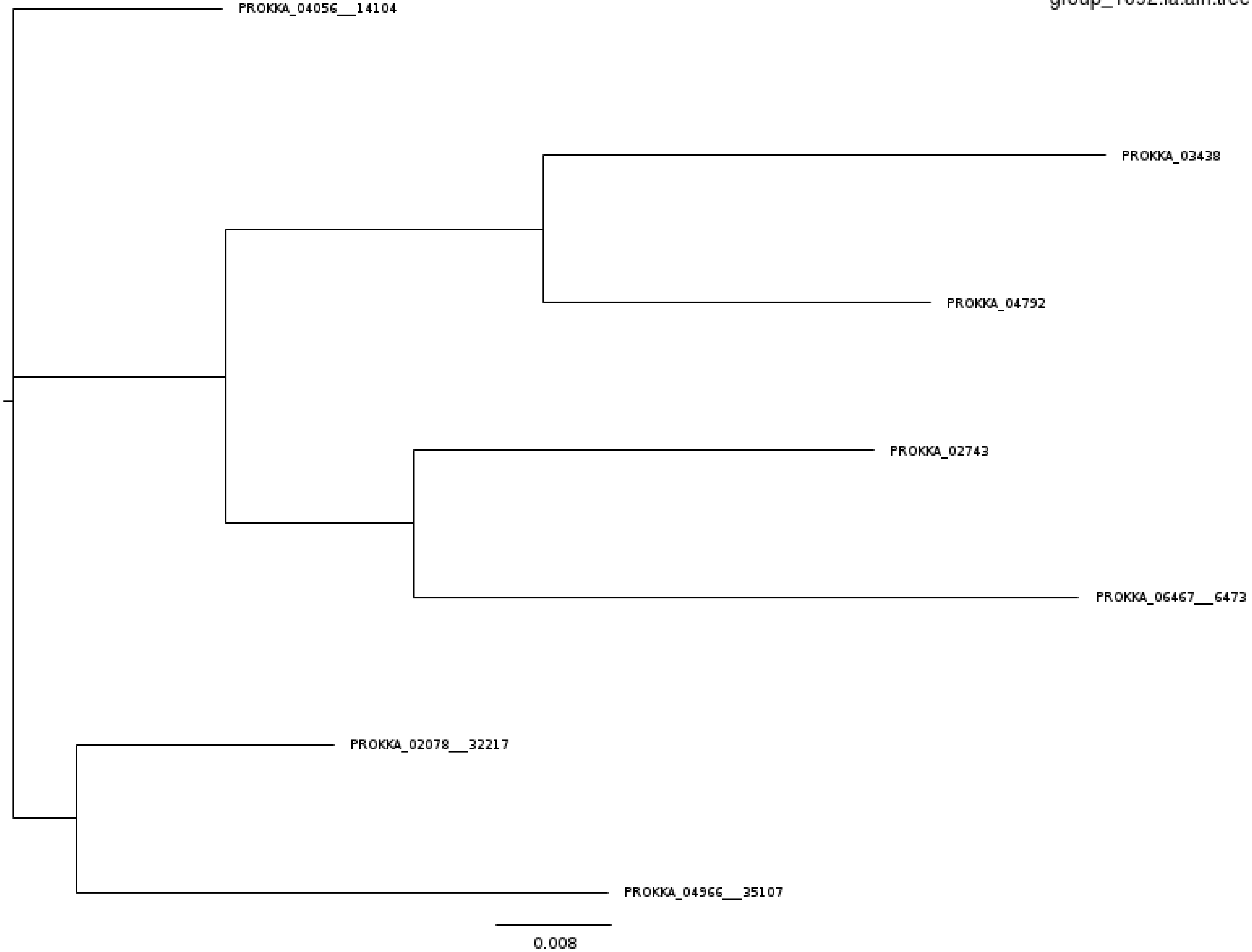

group_1093.fa.aln.tree.png

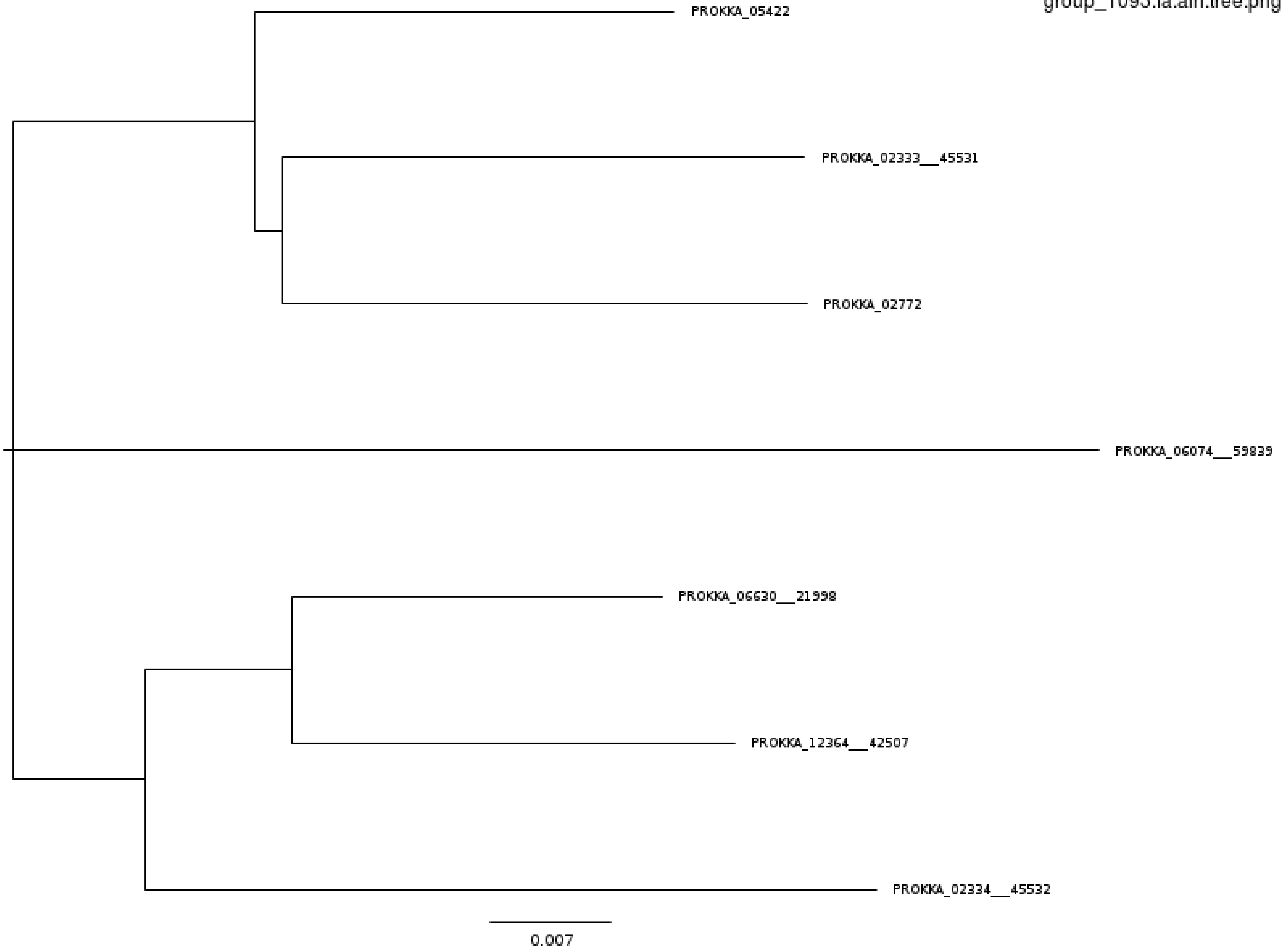

group_1094.fa.aln.tree.png

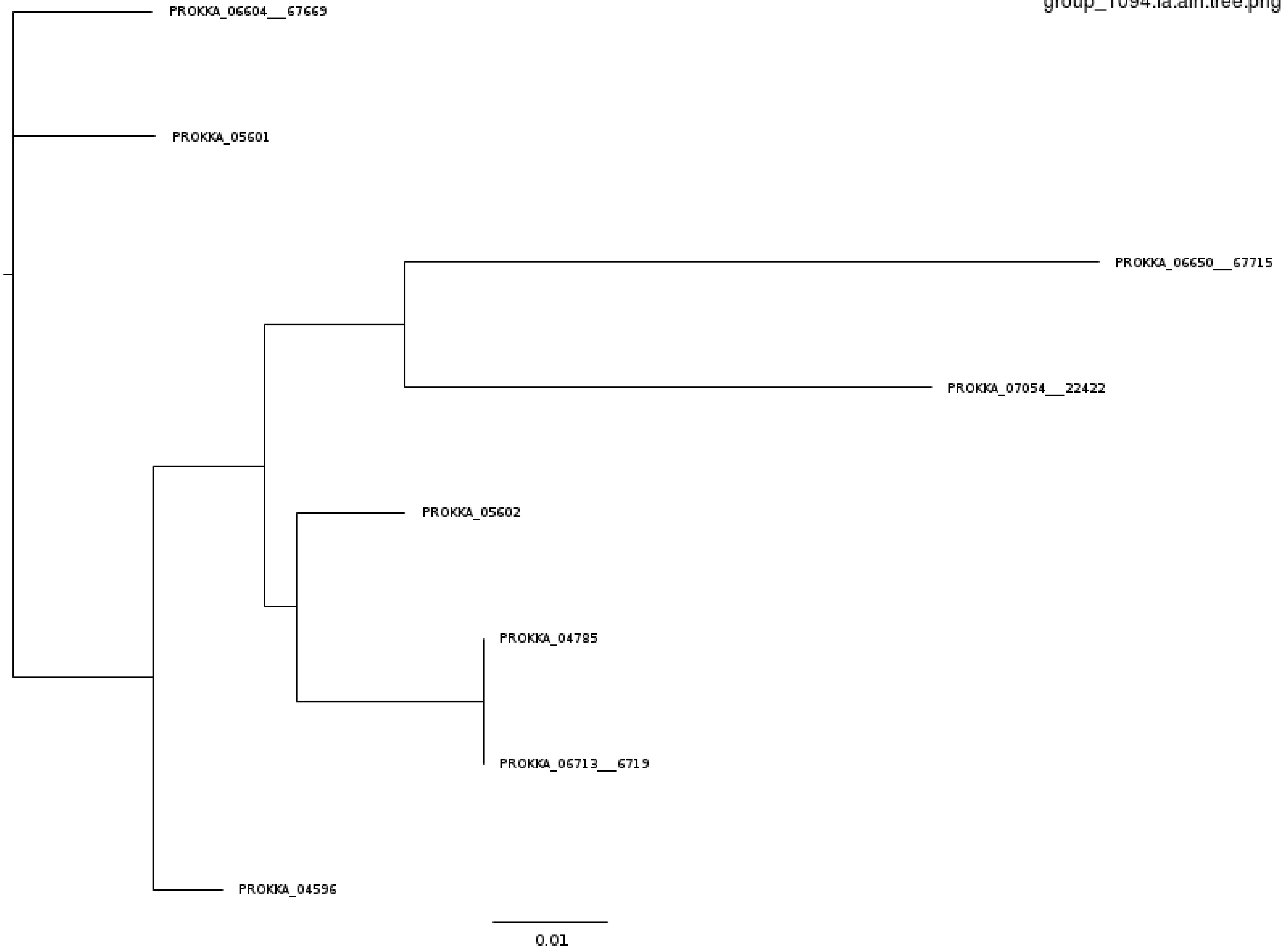

group_1099.fa.aln.tree.png

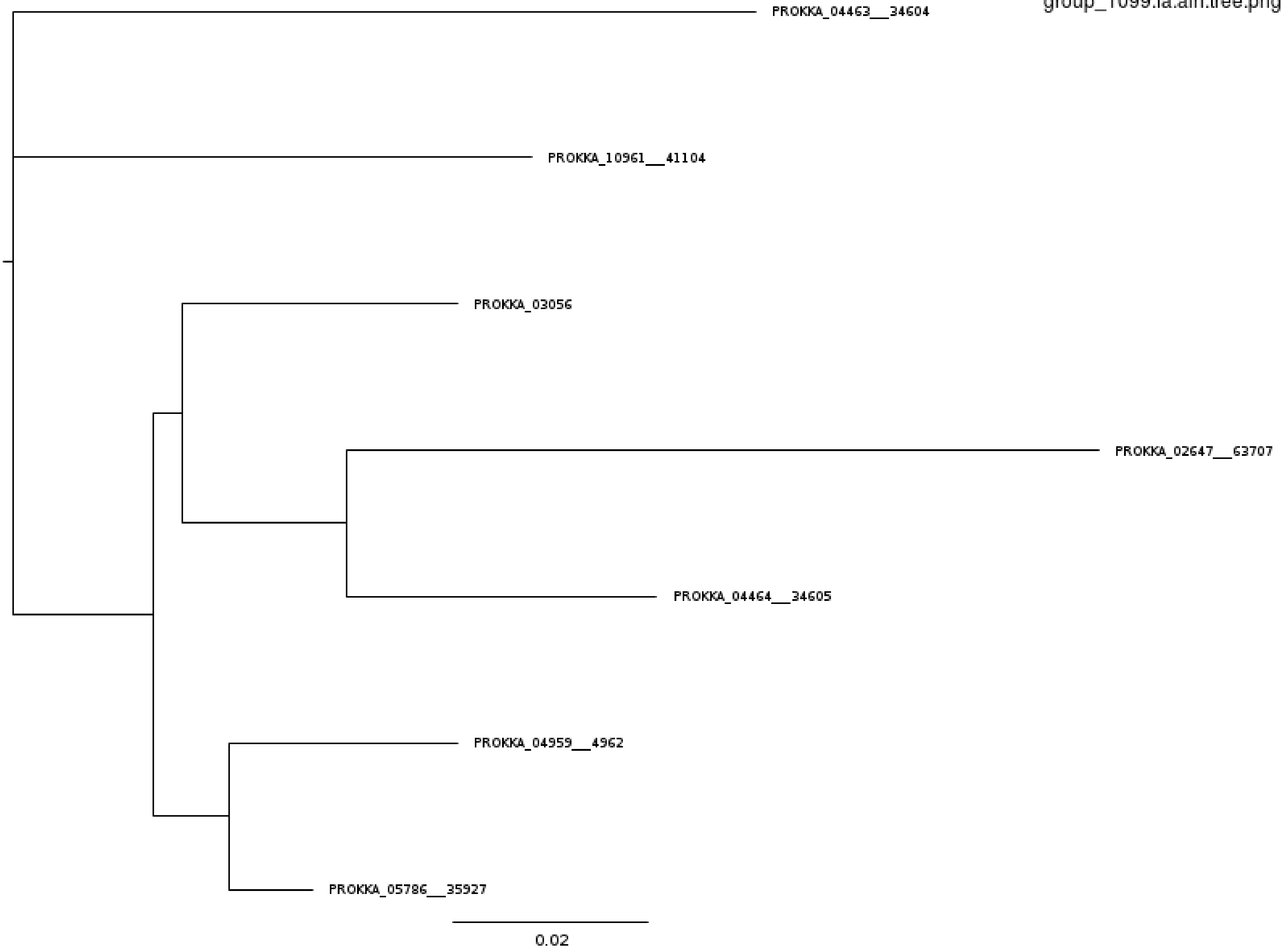

group_10.fa.aln.tree.png

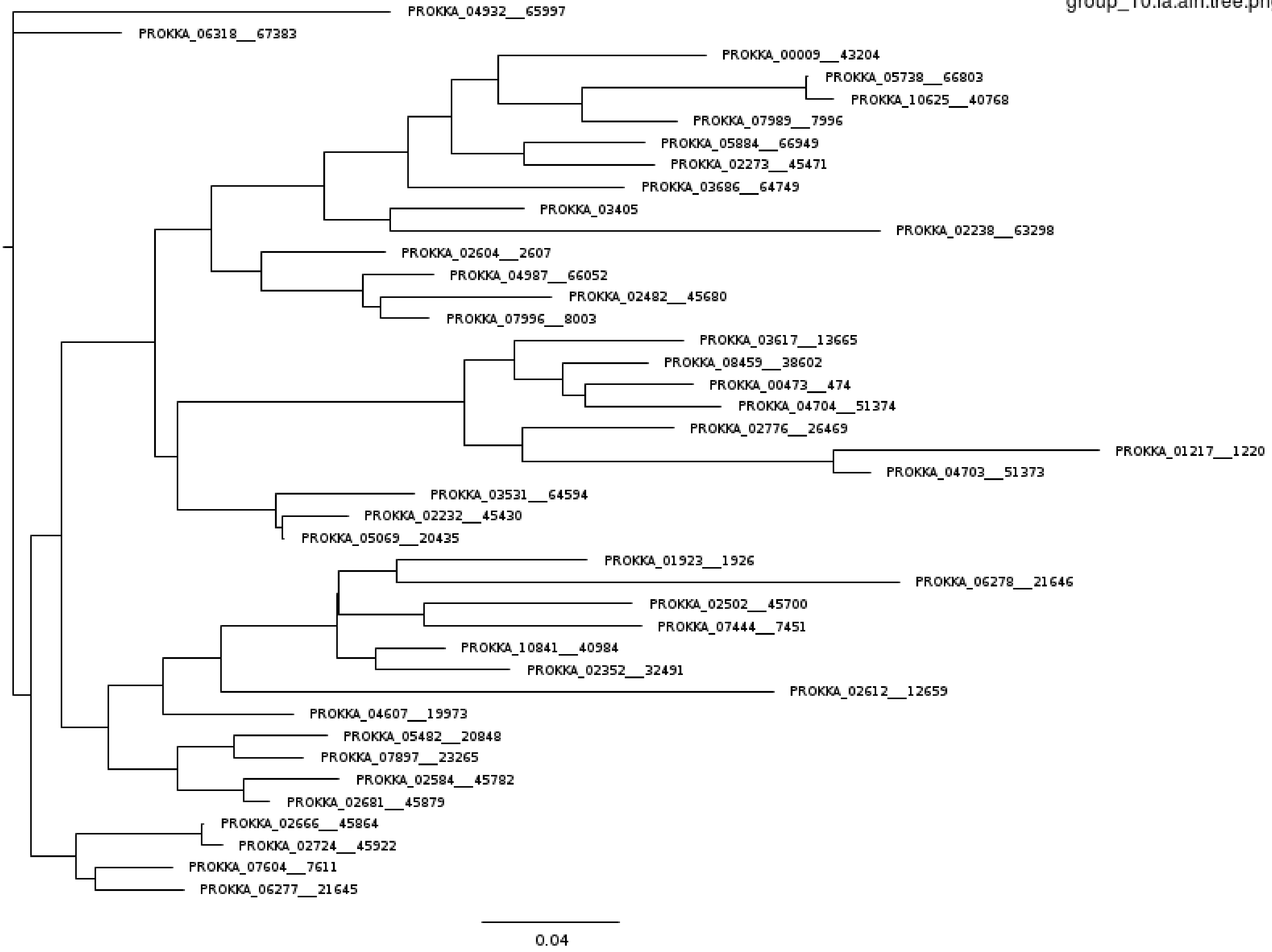

group_1100.fa.aln.tree.png

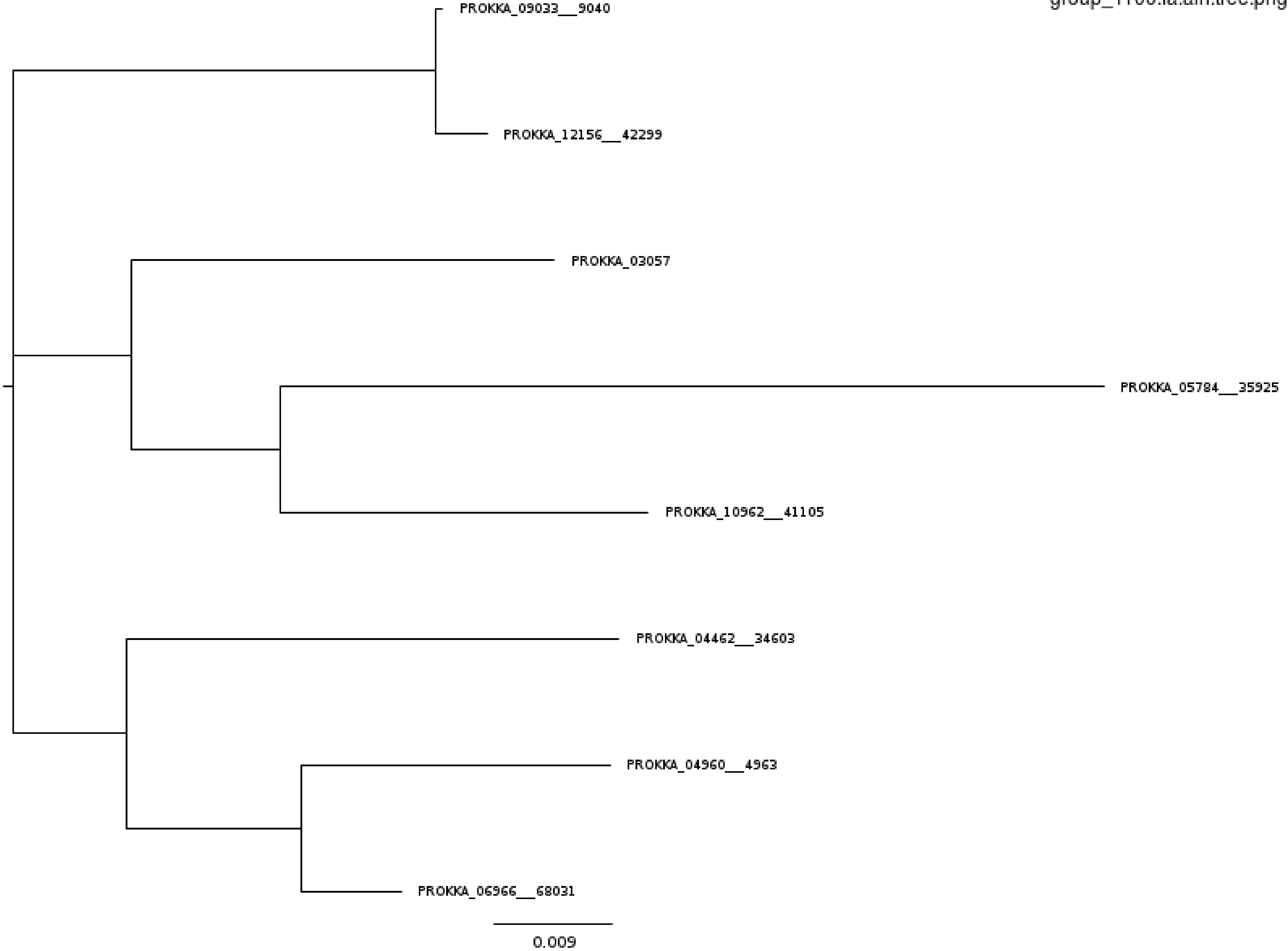

group_1102.fa.aln.tree.png

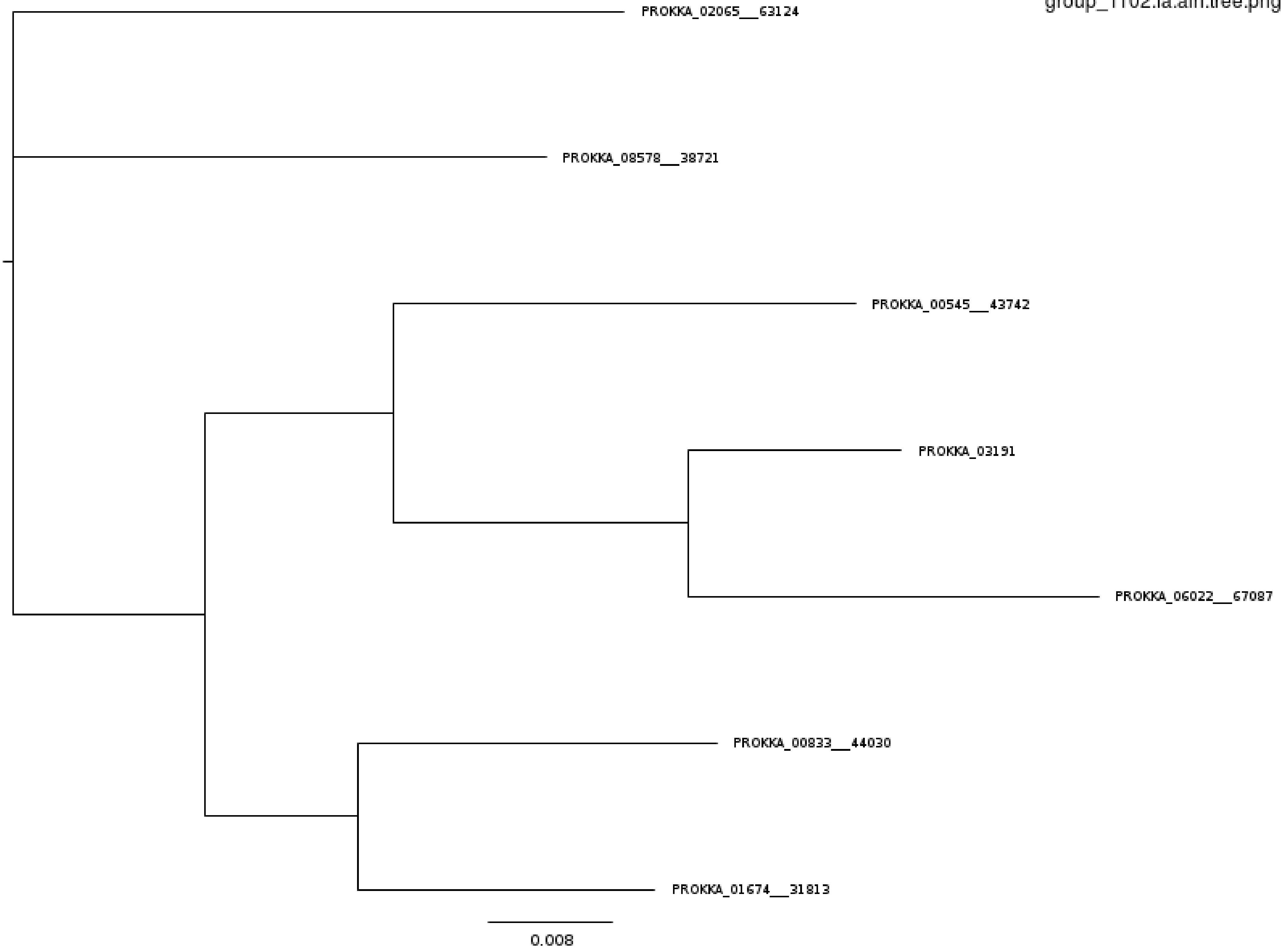

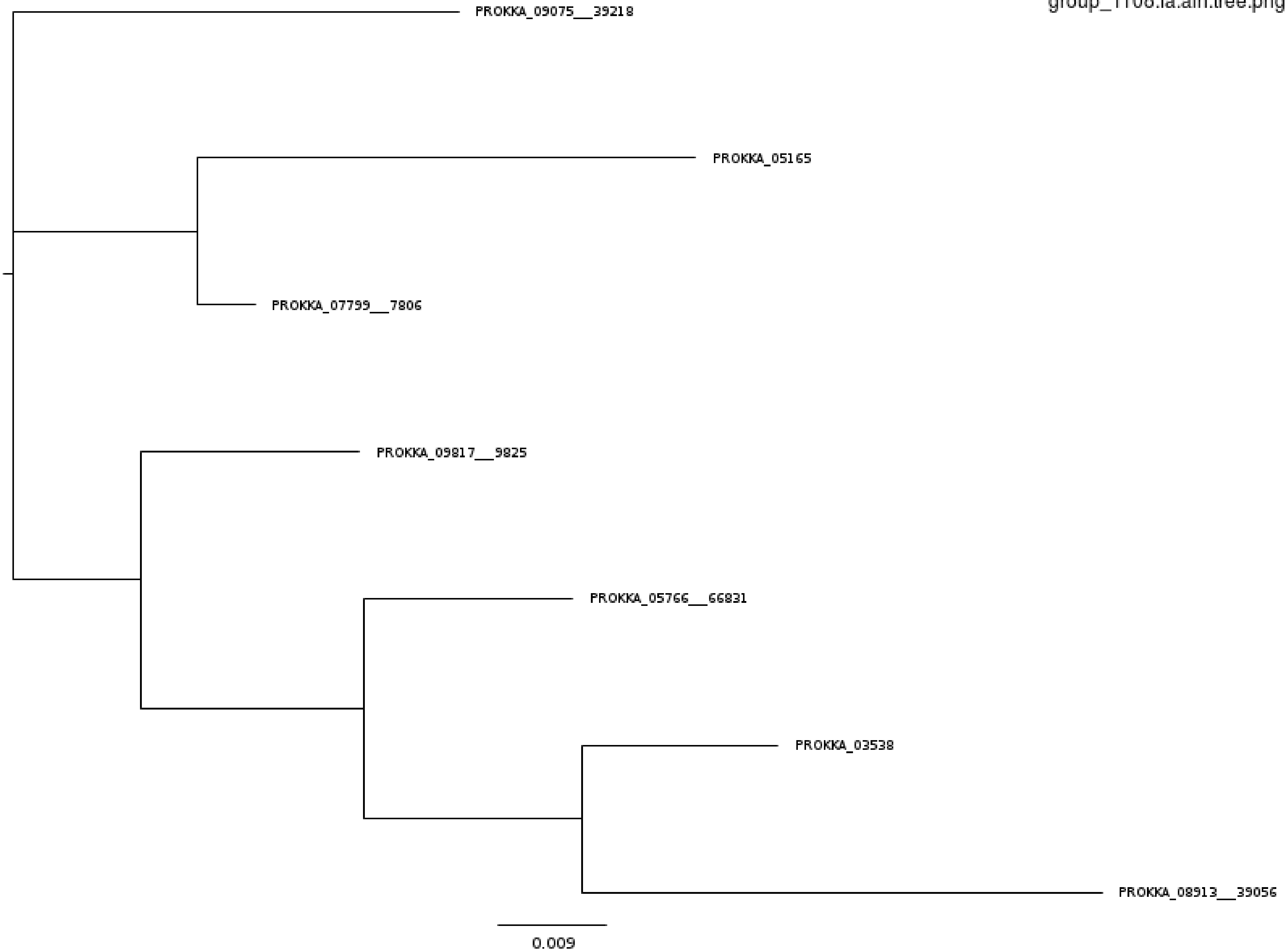
PROKKA_09075__39218
PROKKA_05165
PROKKA_07799__7806
PROKKA_09817__9825
PROKKA_05766__66831
PROKKA_03538
PROKKA_08913__39056
0.009

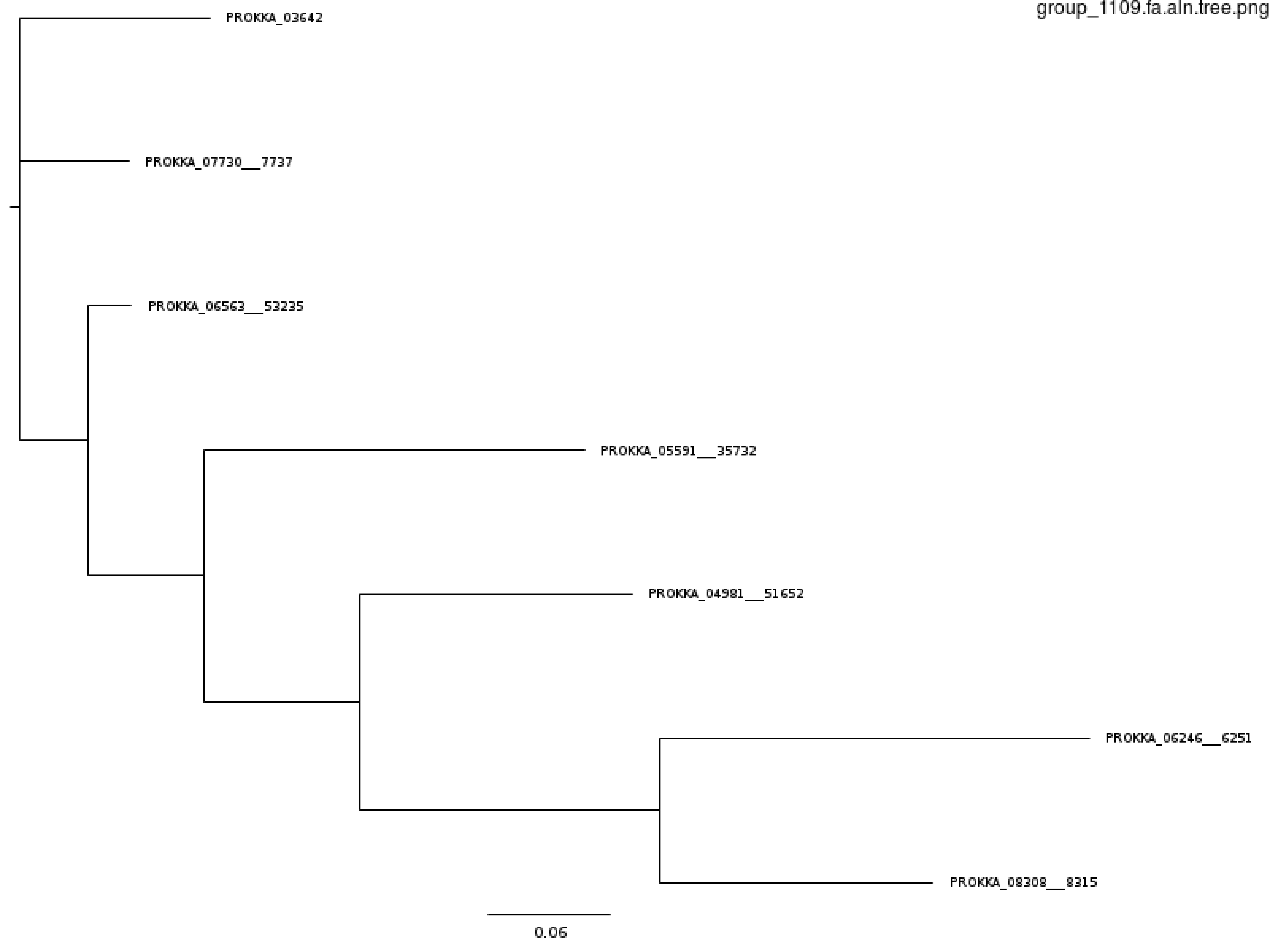
group_1109.fa.aln.tree.png
PROKKA_03642
PROKKA_07730___7737
PROKKA_06563___53235
PROKKA_05591___35732
PROKKA_04981___51652
PROKKA_06246___6251
PROKKA_08308___8315
0.06

group_1111.fa.aln.tree.png

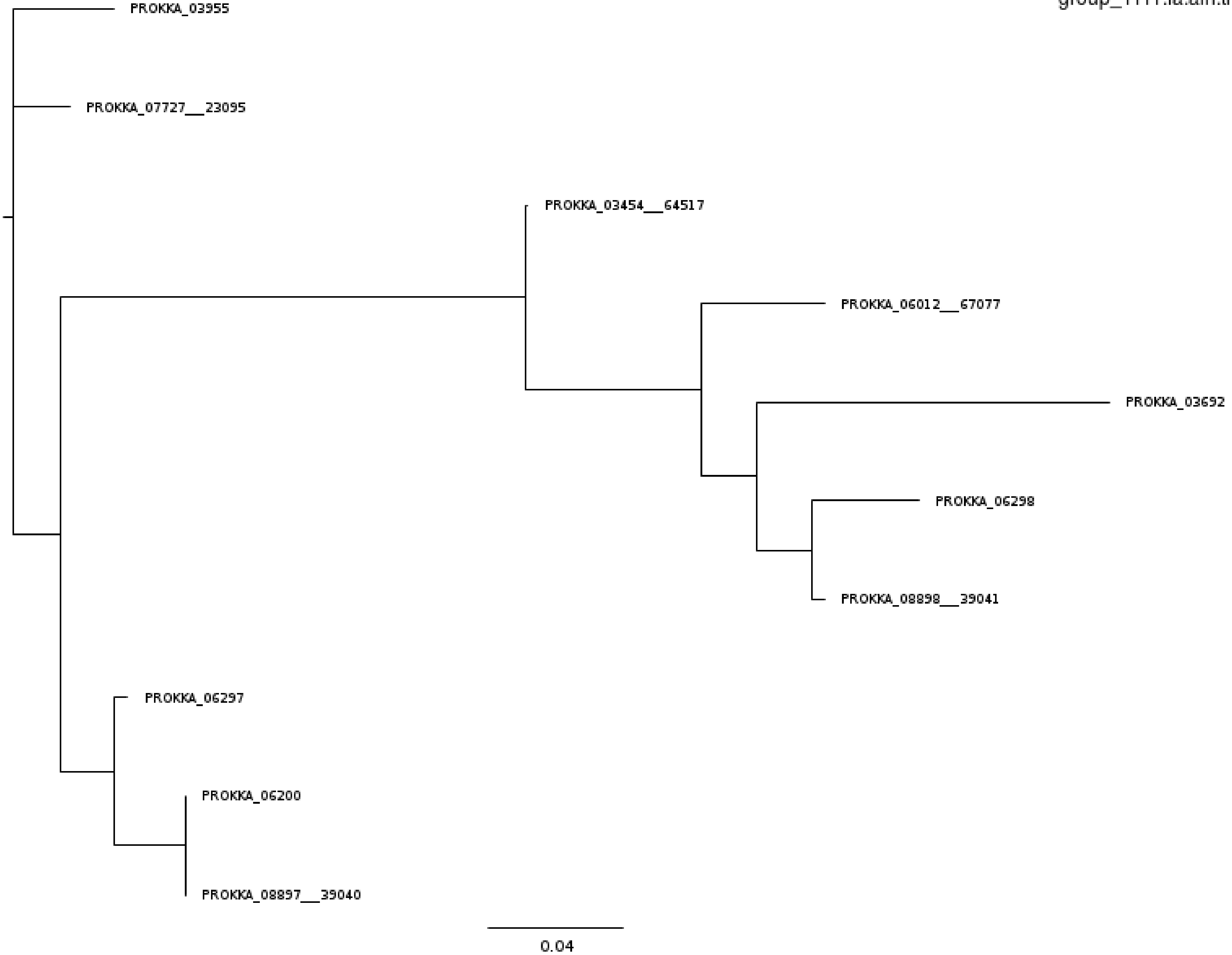

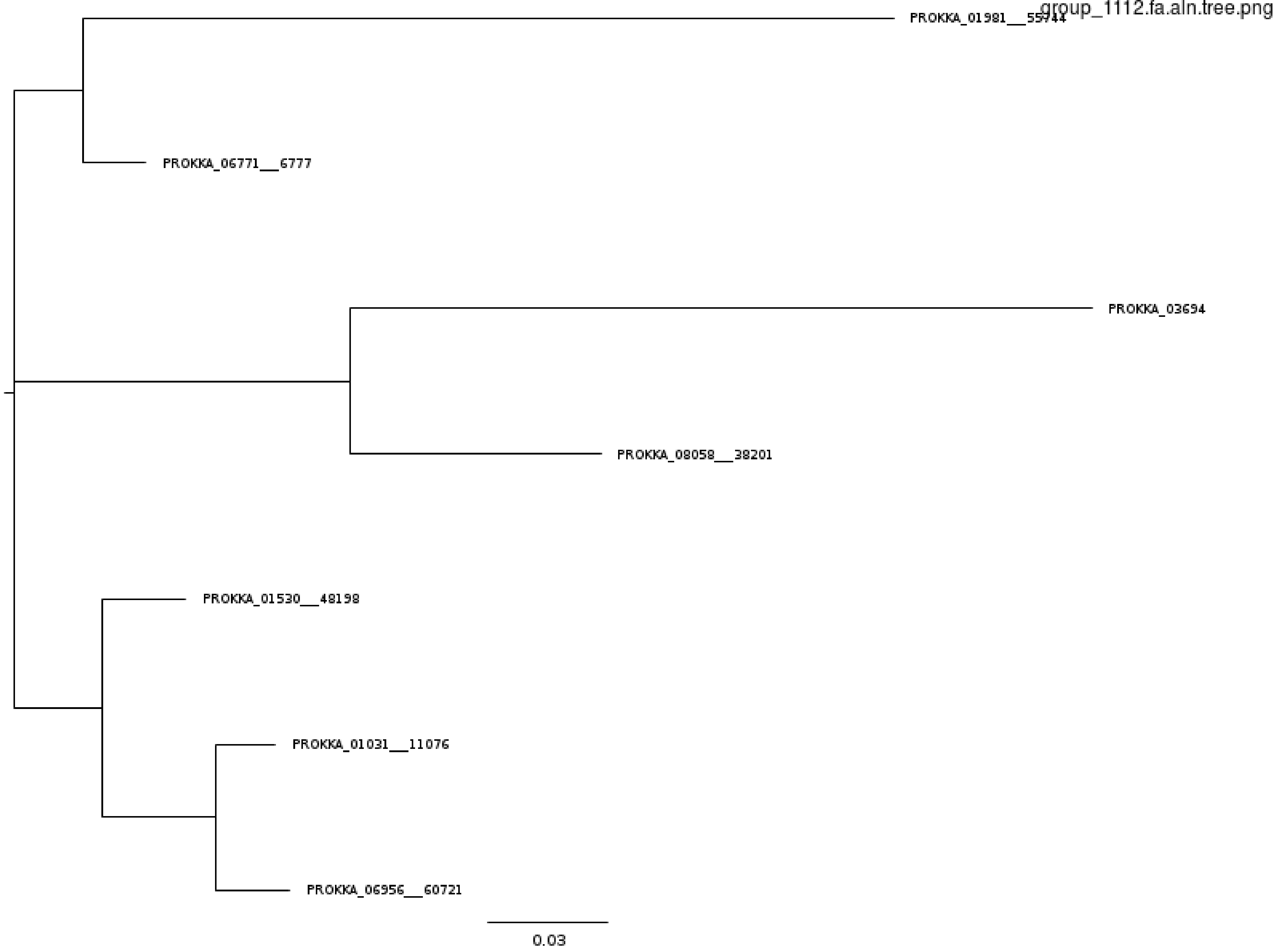
group_1112.fa.aln.tree.png
PROKKA_01981___55744
PROKKA_06771___6777
PROKKA_03694
PROKKA_08058___38201
PROKKA_01530___48198
PROKKA_01031___11076
PROKKA_06956___60721
0.03

group_1118.fa.aln.tree.png

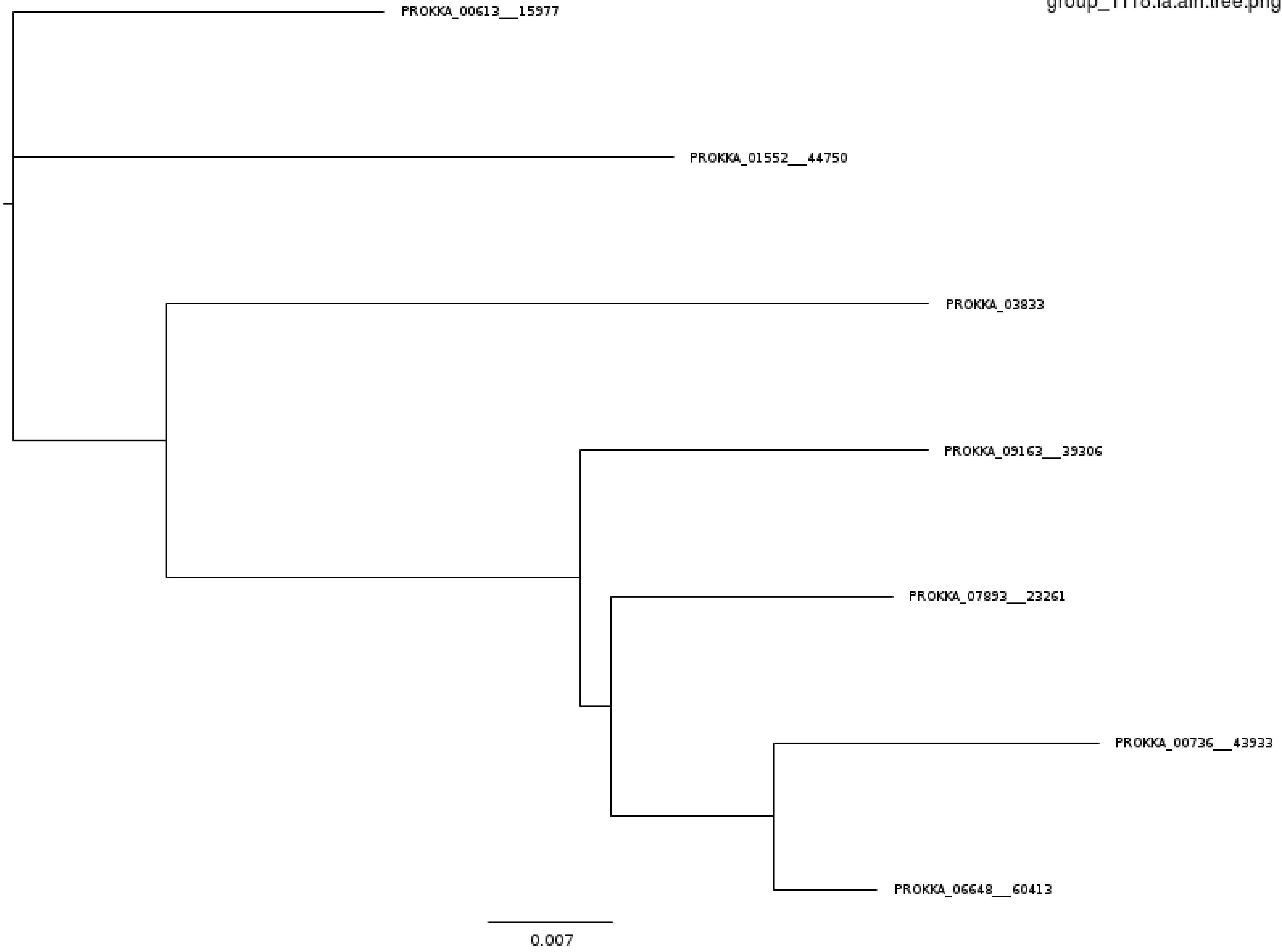

group_1119.fa.aln.tree.png

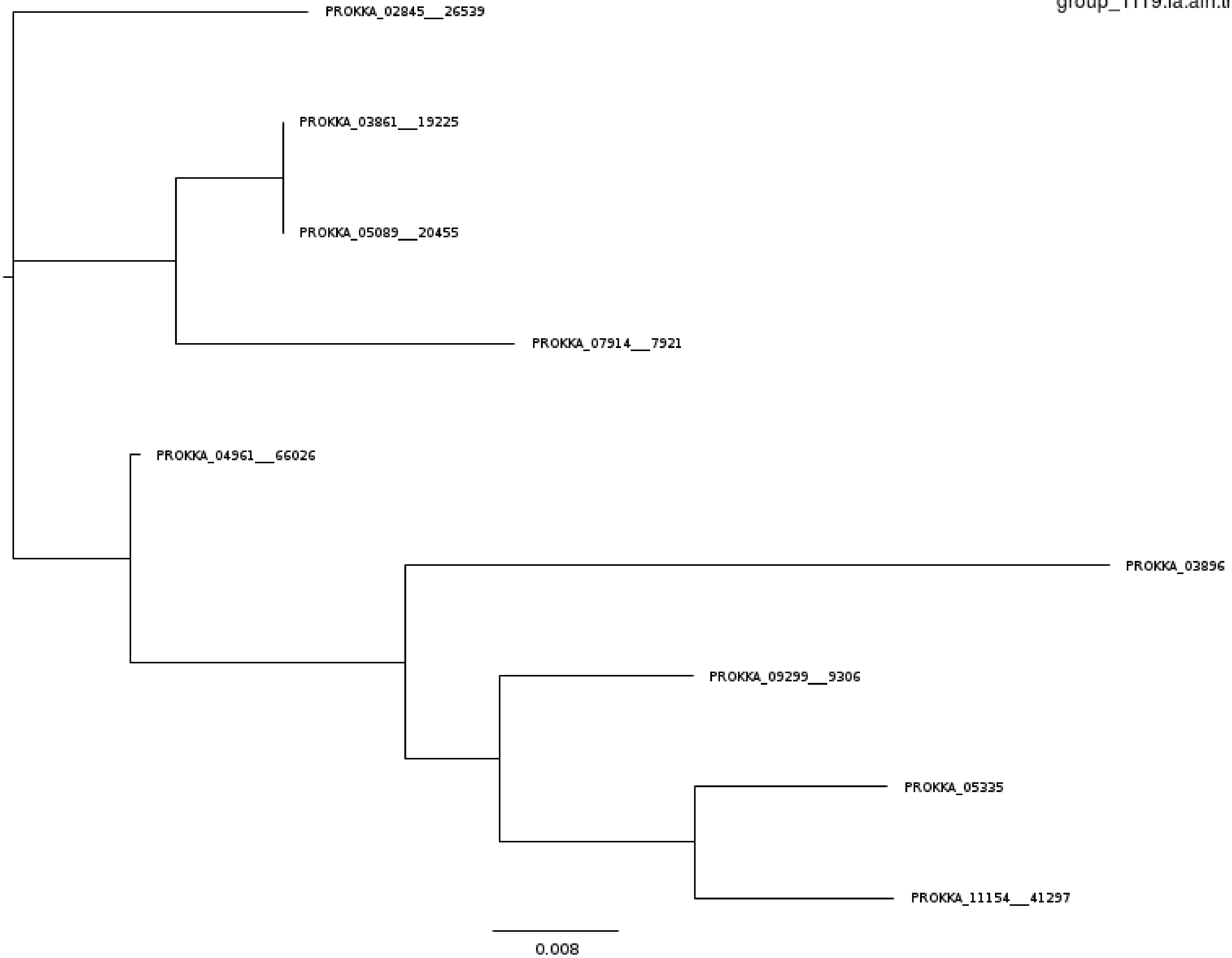

group_1122.fa.aln.tree.png

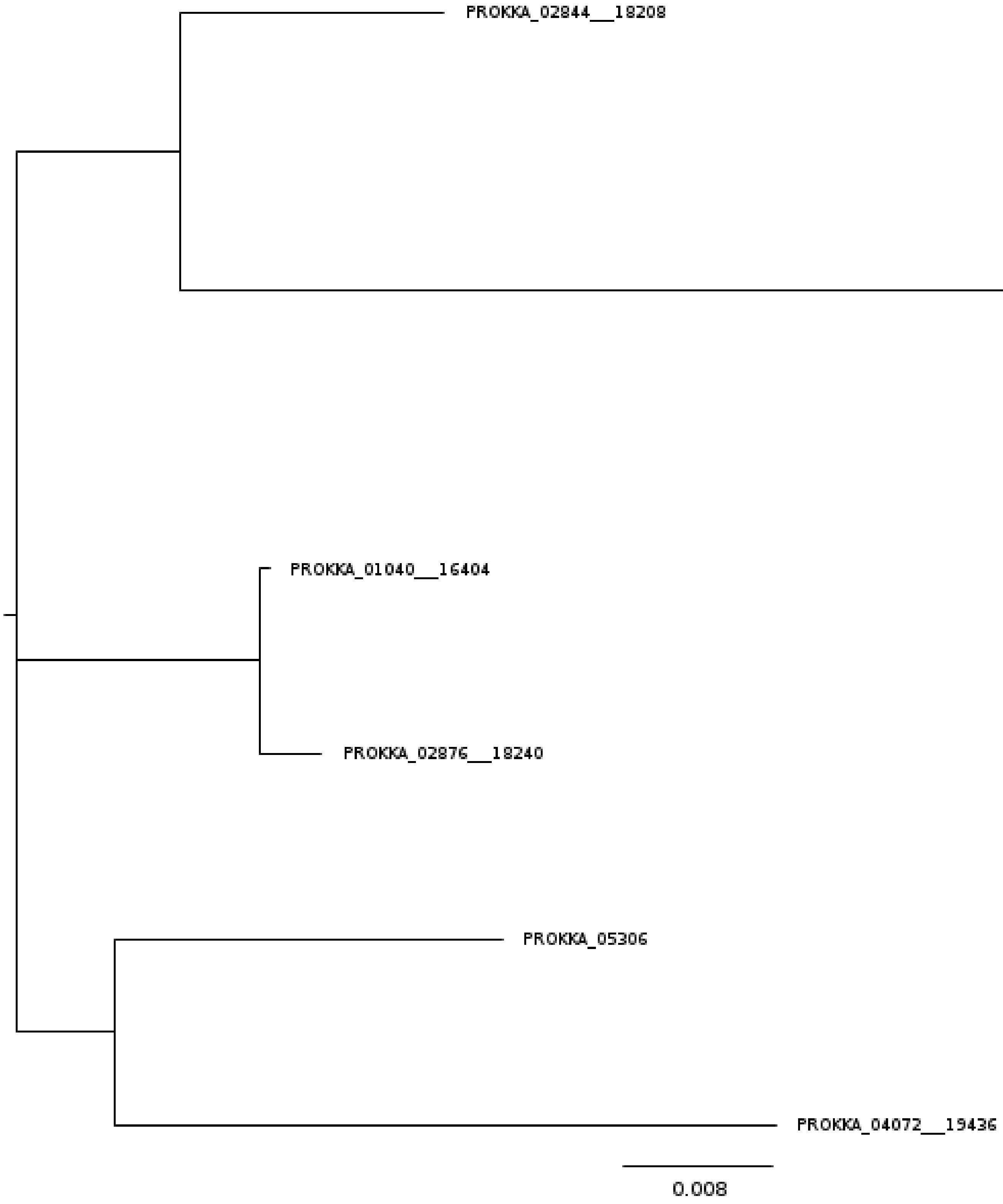

group_1123.fa.aln.tree.png

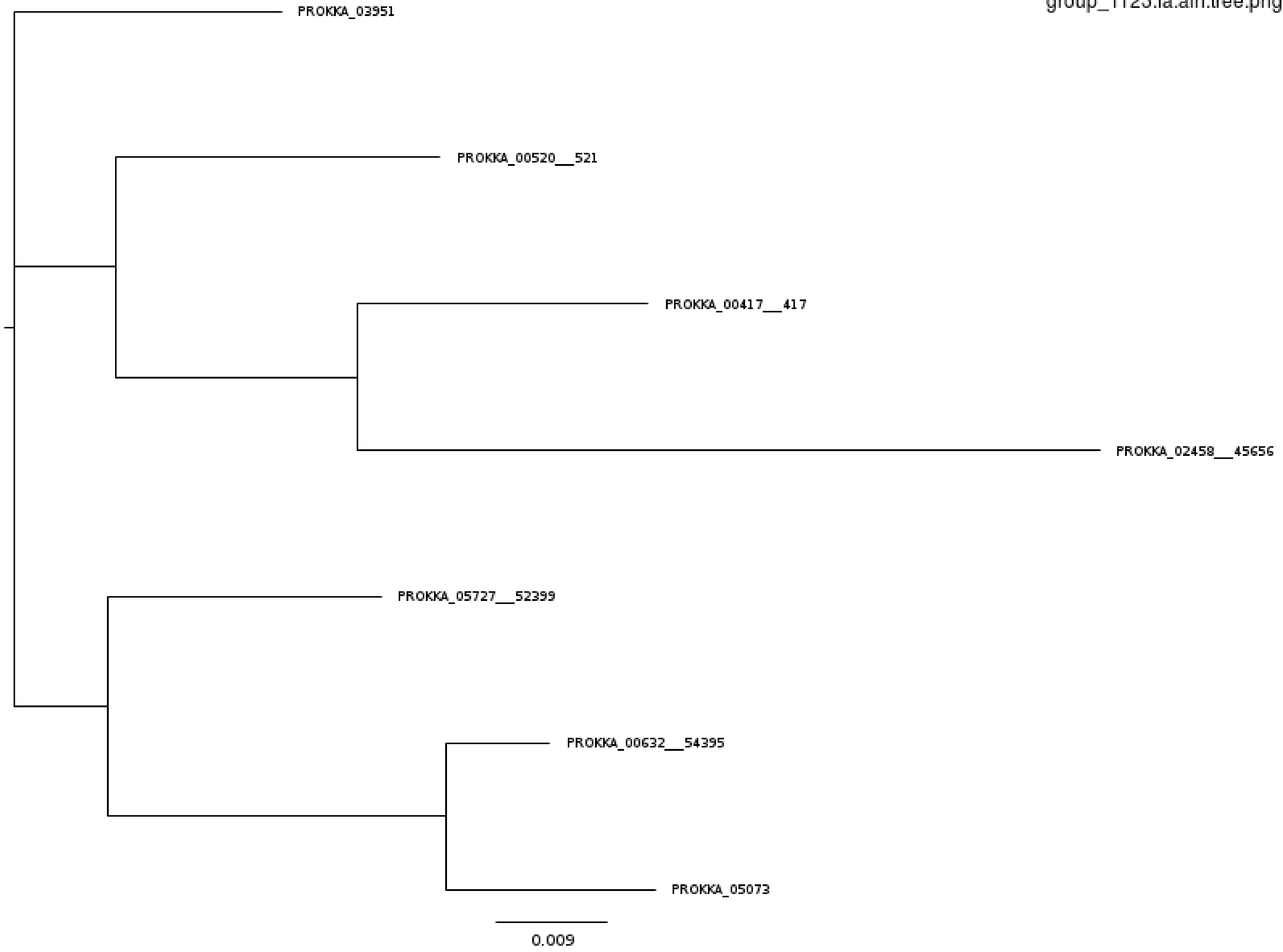

group_1124.fa.aln.tree.png

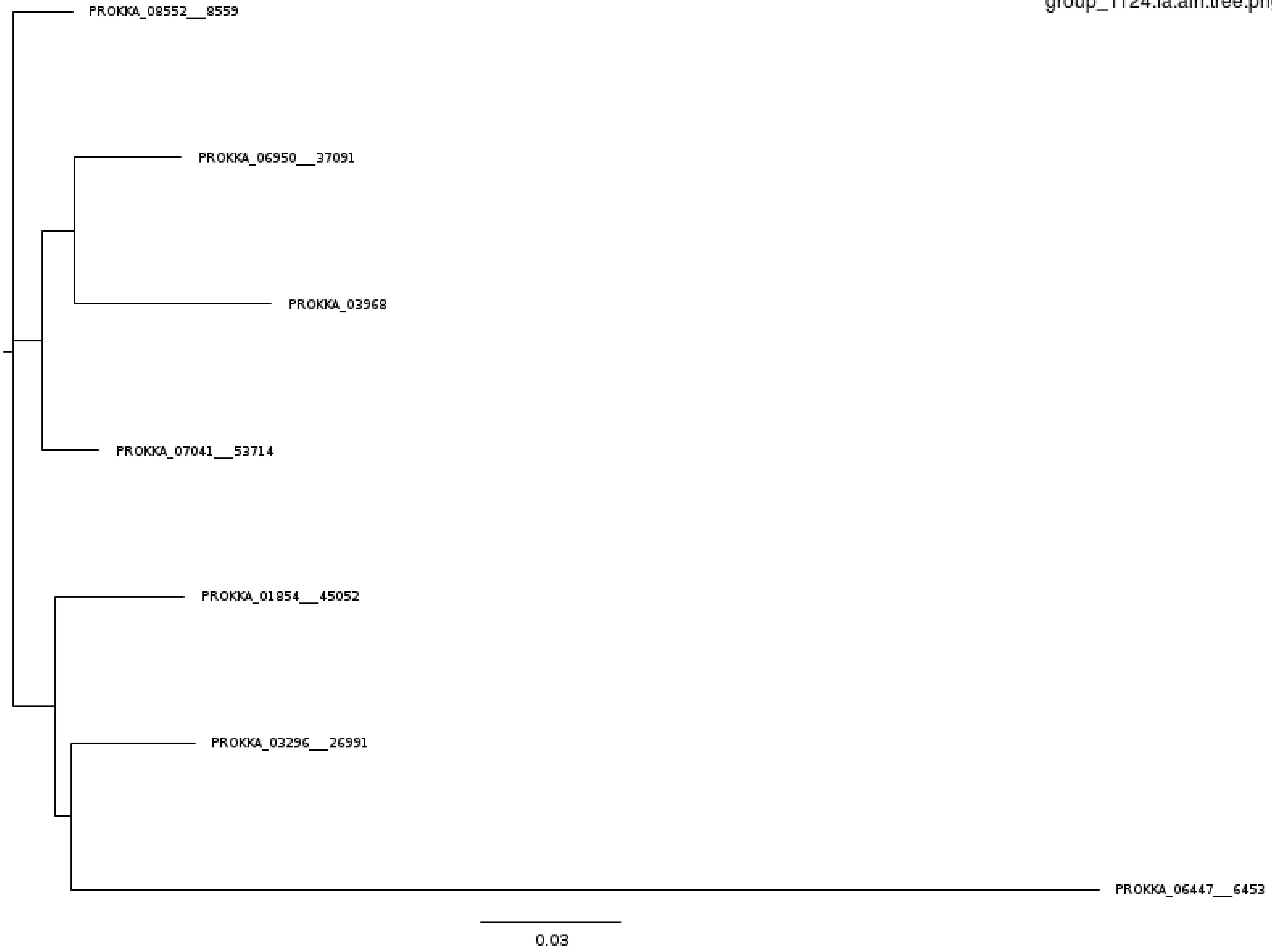

group_1126.fa.aln.tree.png

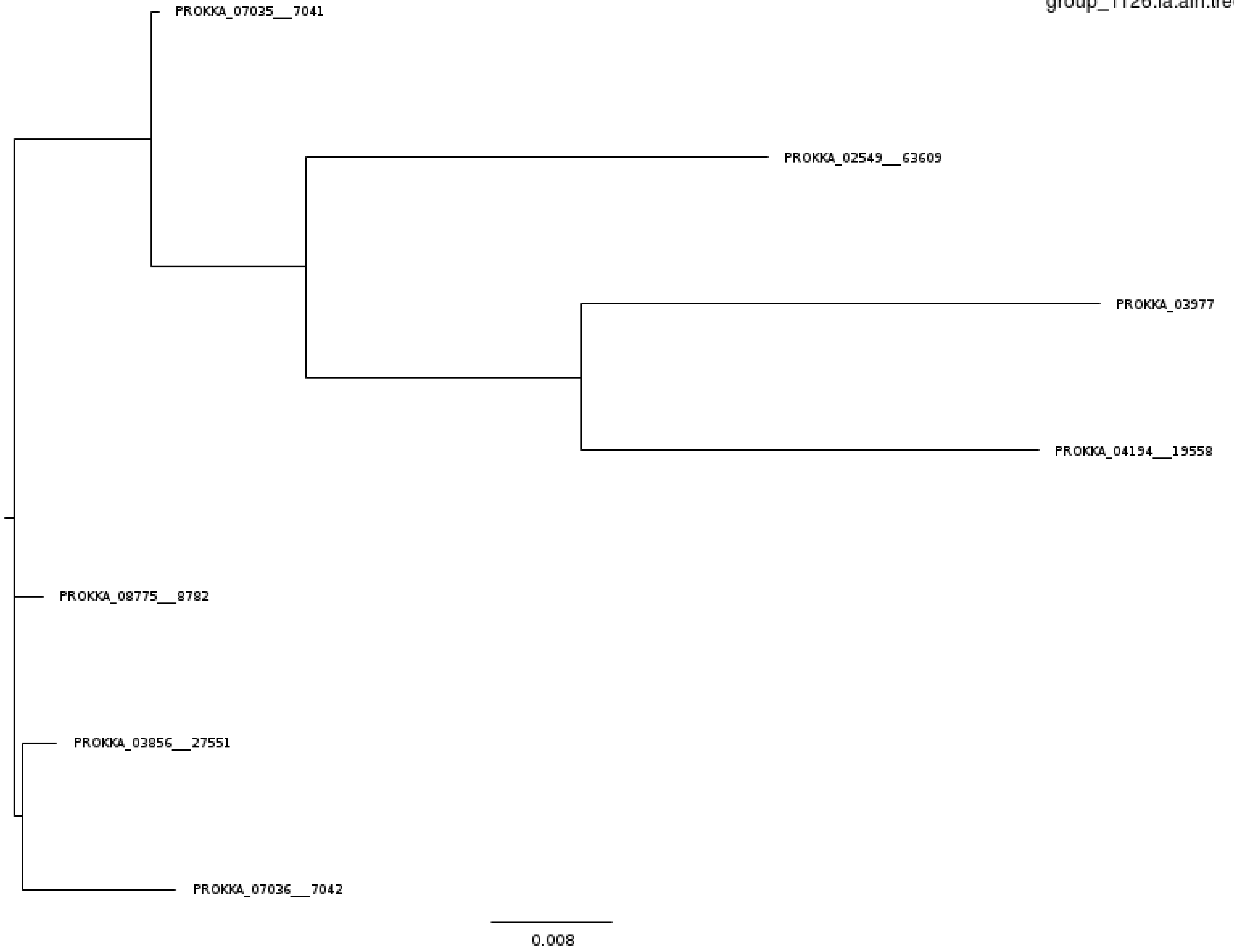

group_1127.fa.aln.tree.png

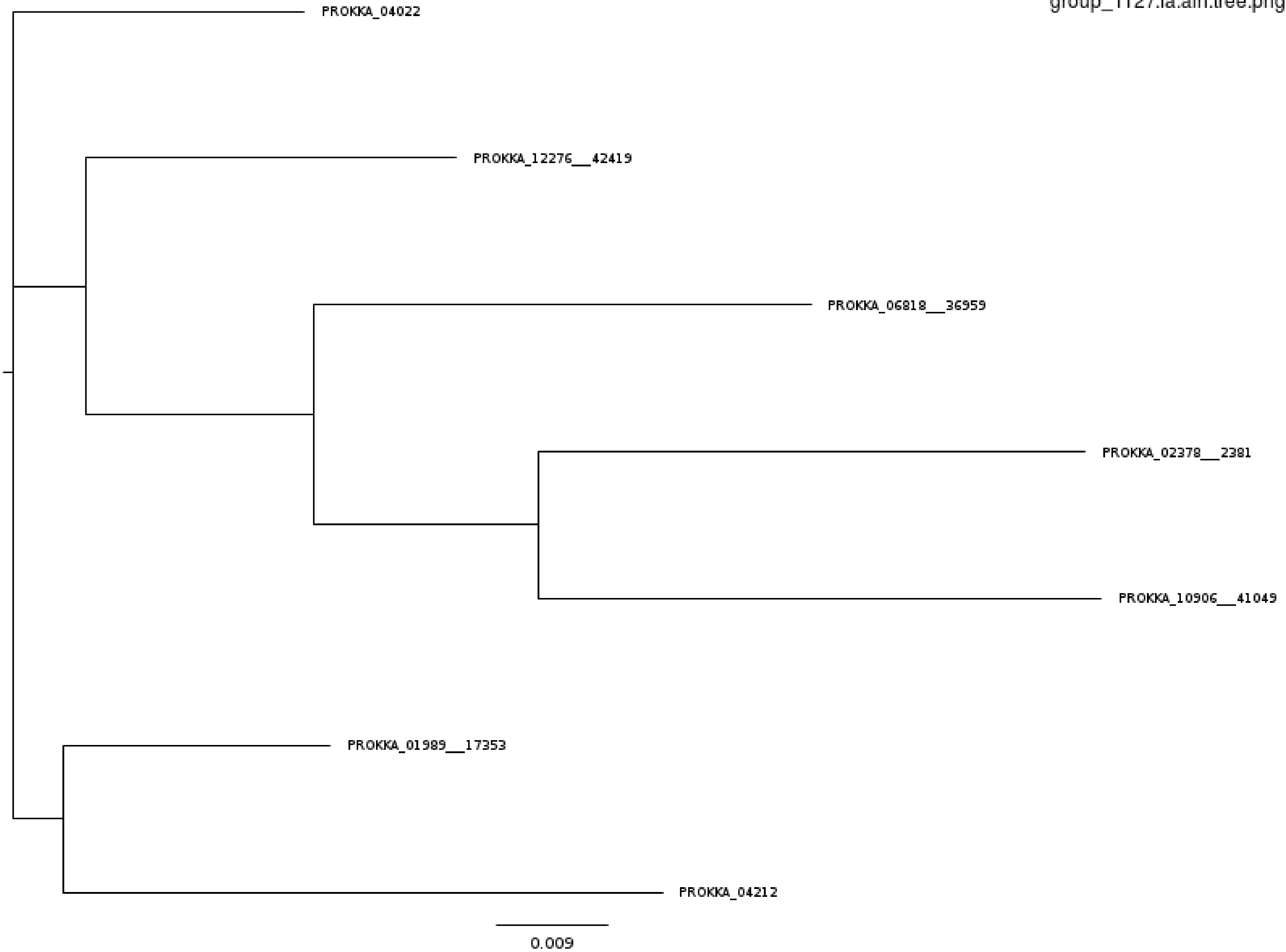

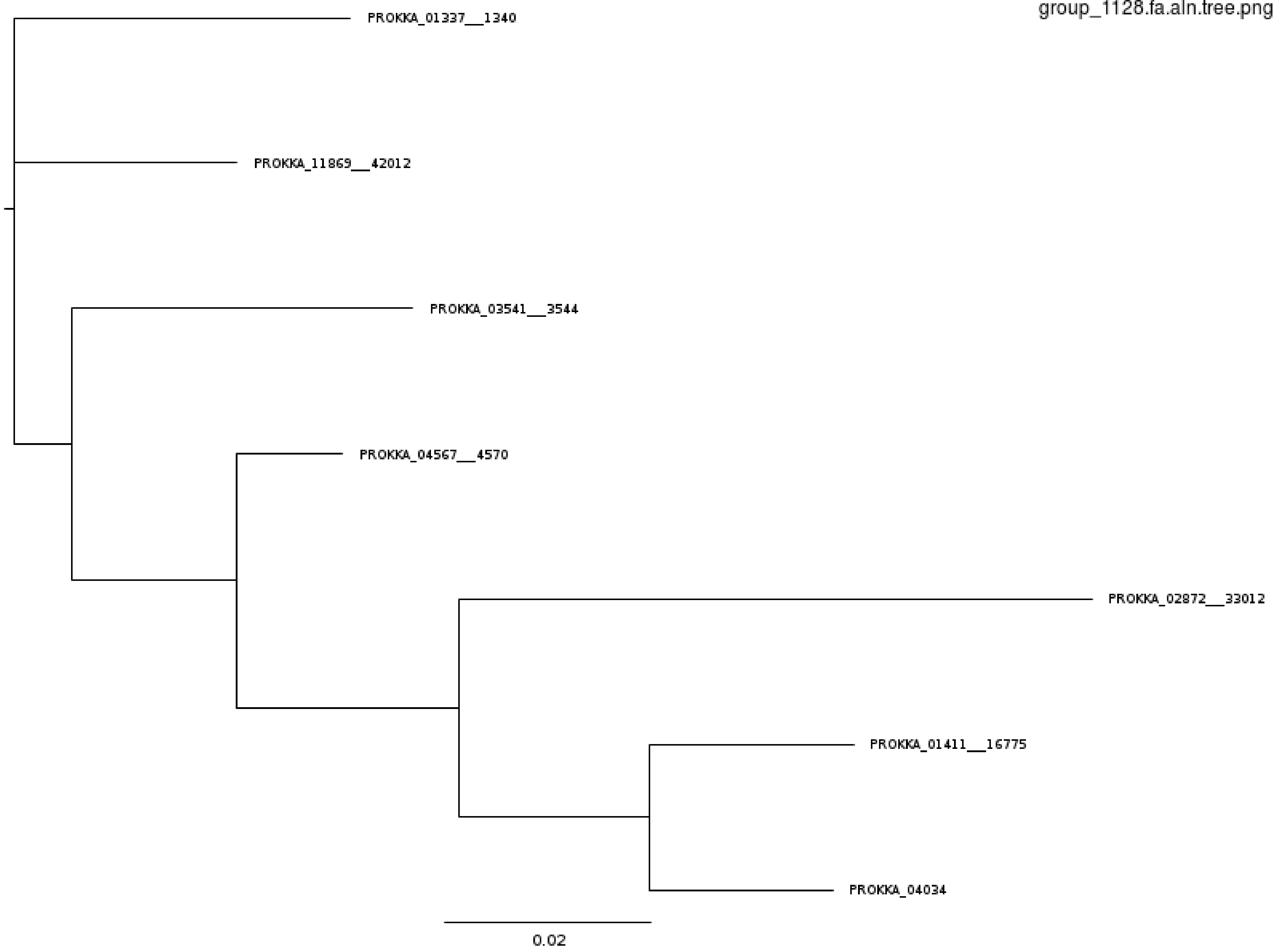

group_1128.fa.aln.tree.png
PROKKA_01337__1340
PROKKA_11869__42012
PROKKA_03541__3544
PROKKA_04567__4570
PROKKA_02872__33012
PROKKA_01411__16775
PROKKA_04034
0.02

group_1129.fa.aln.tree.png

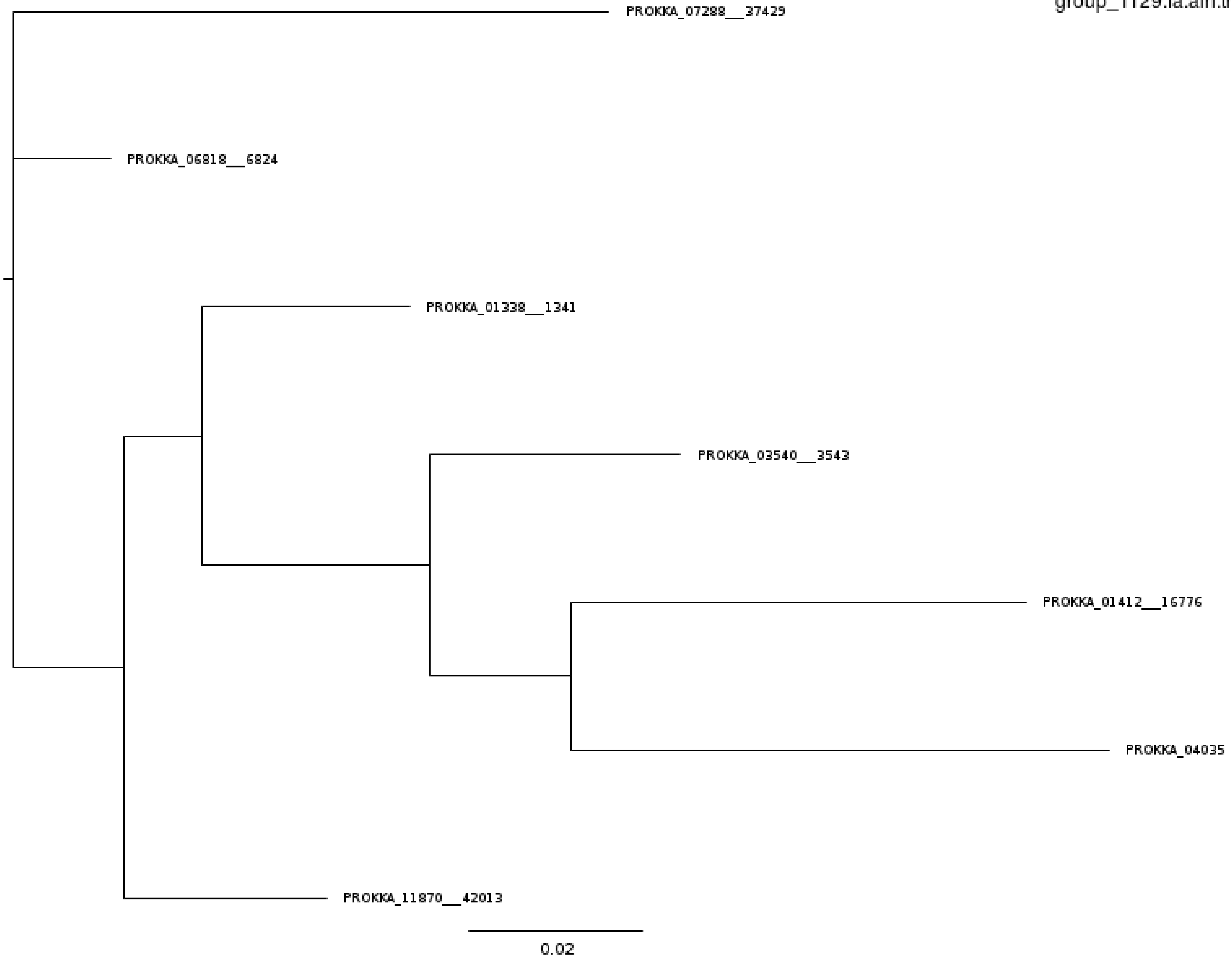

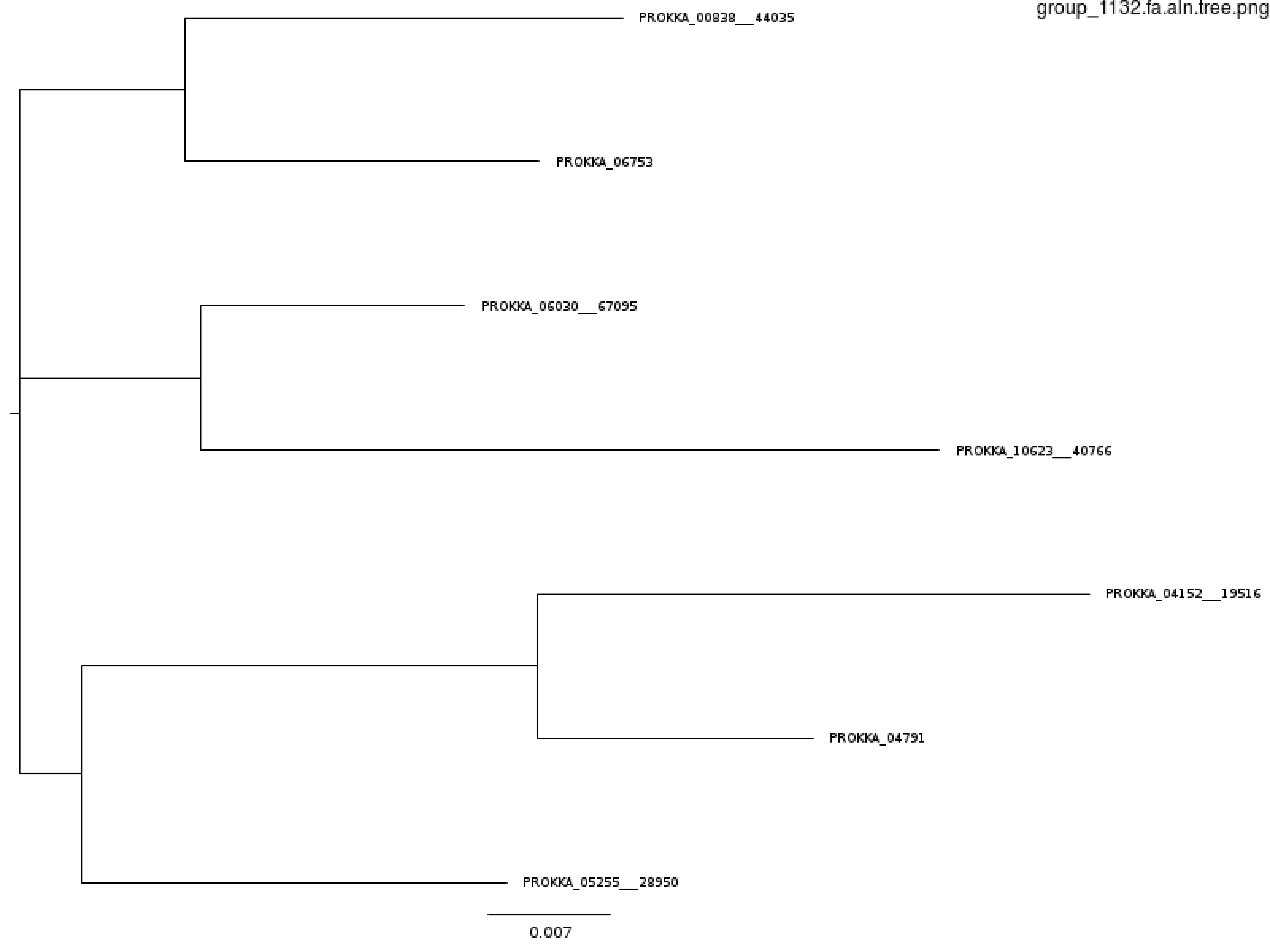
group_1132.fa.aln.tree.png
PROKKA_00838___44035
PROKKA_06753
PROKKA_06030___67095
PROKKA_10623___40766
PROKKA_04152___19516
PROKKA_04791
PROKKA_05255___28950
0.007

group_1134.fa.aln.tree.png

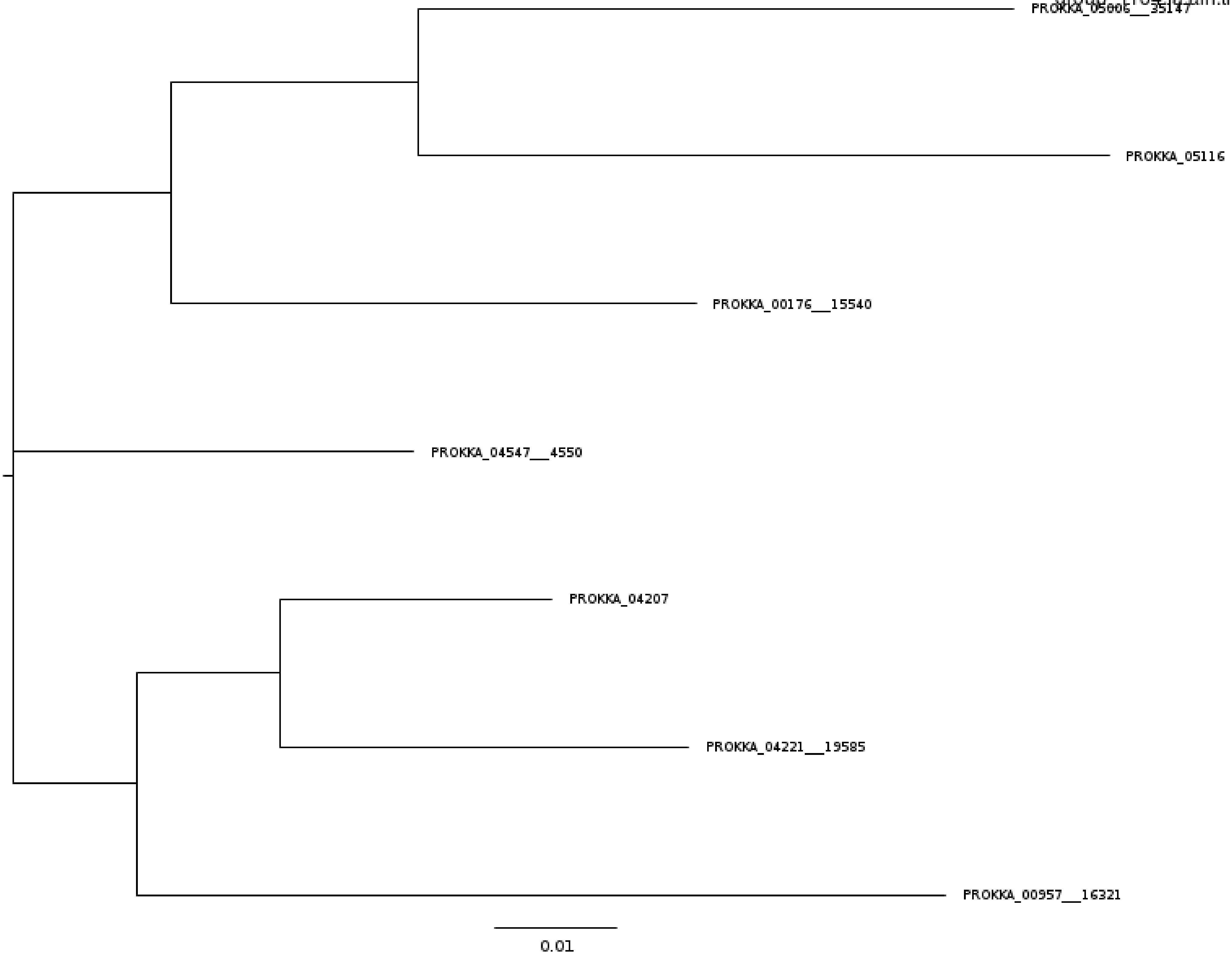

group_1135.fa.aln.tree.png

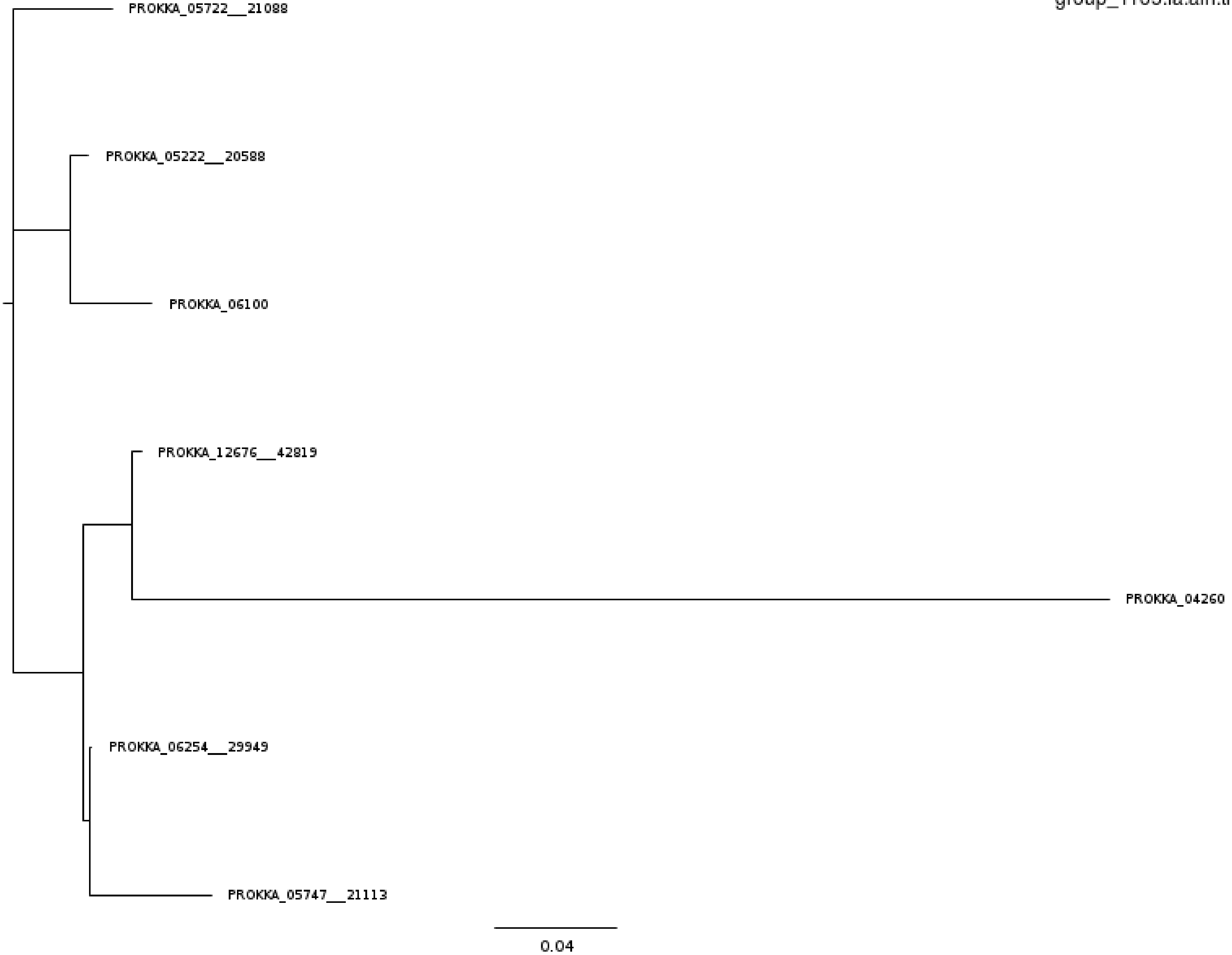

group_1138.fa.aln.tree.png

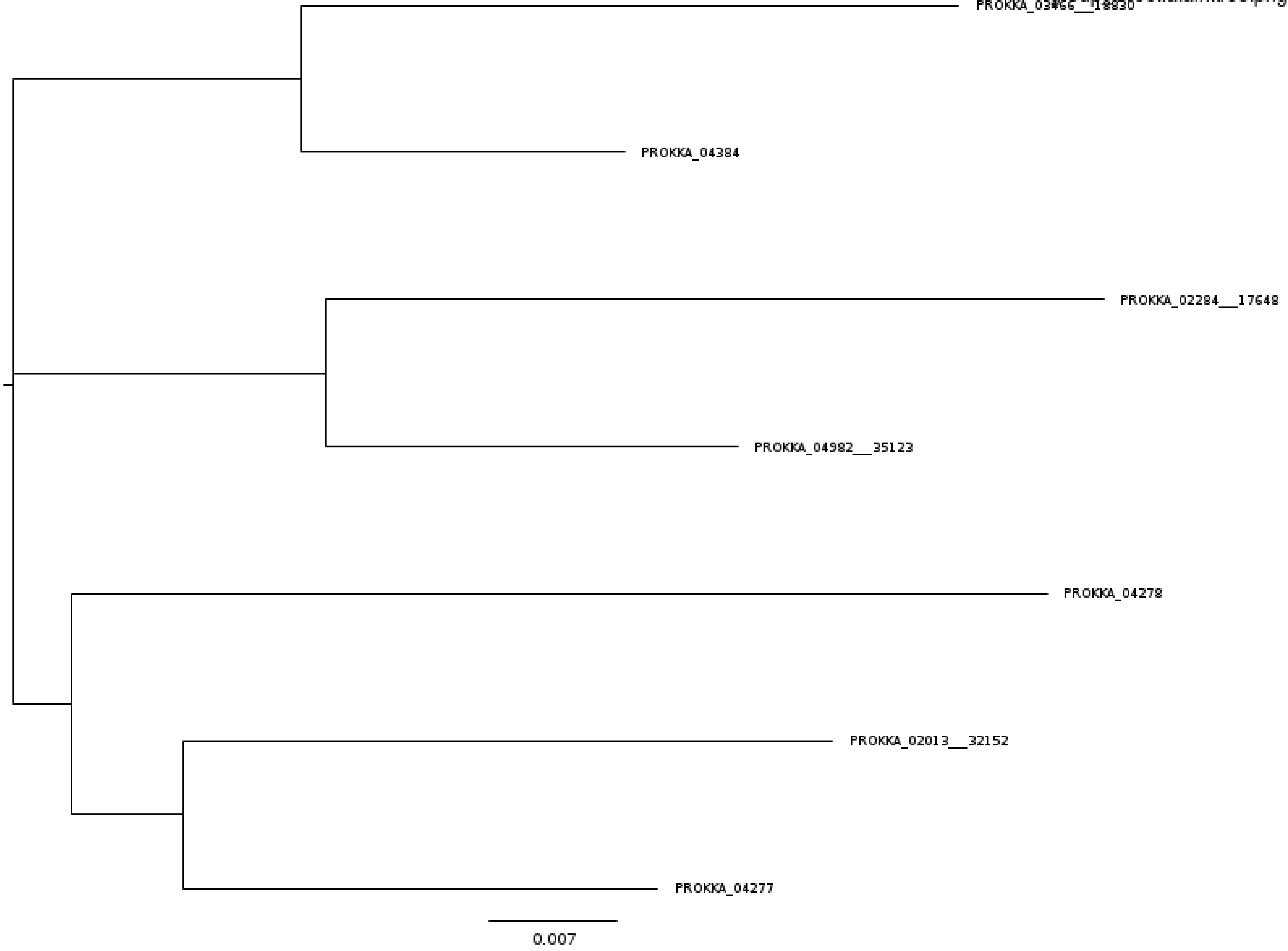

group_1139.fa.aln.tree.png

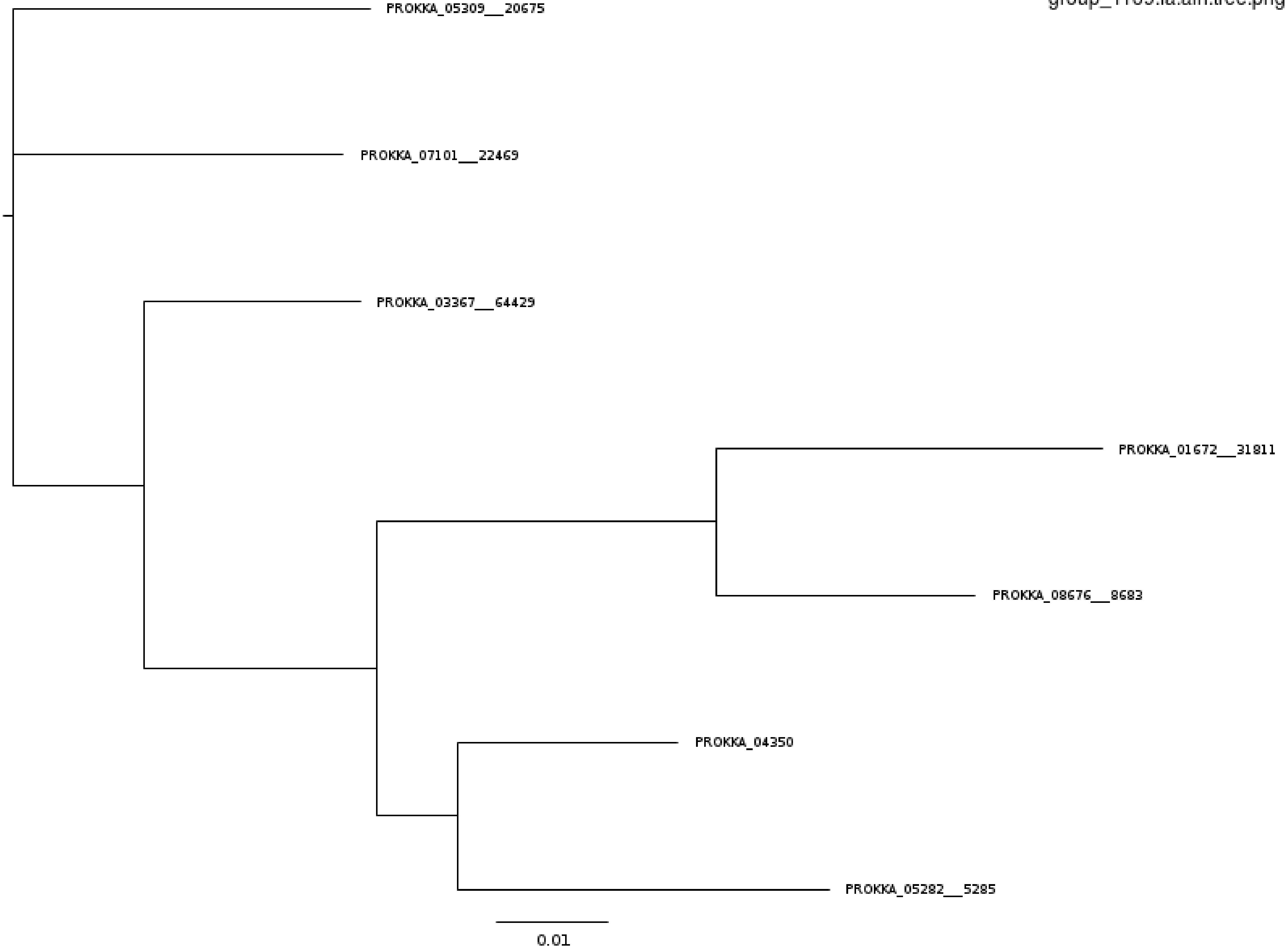

group_1140.fa.aln.tree.png

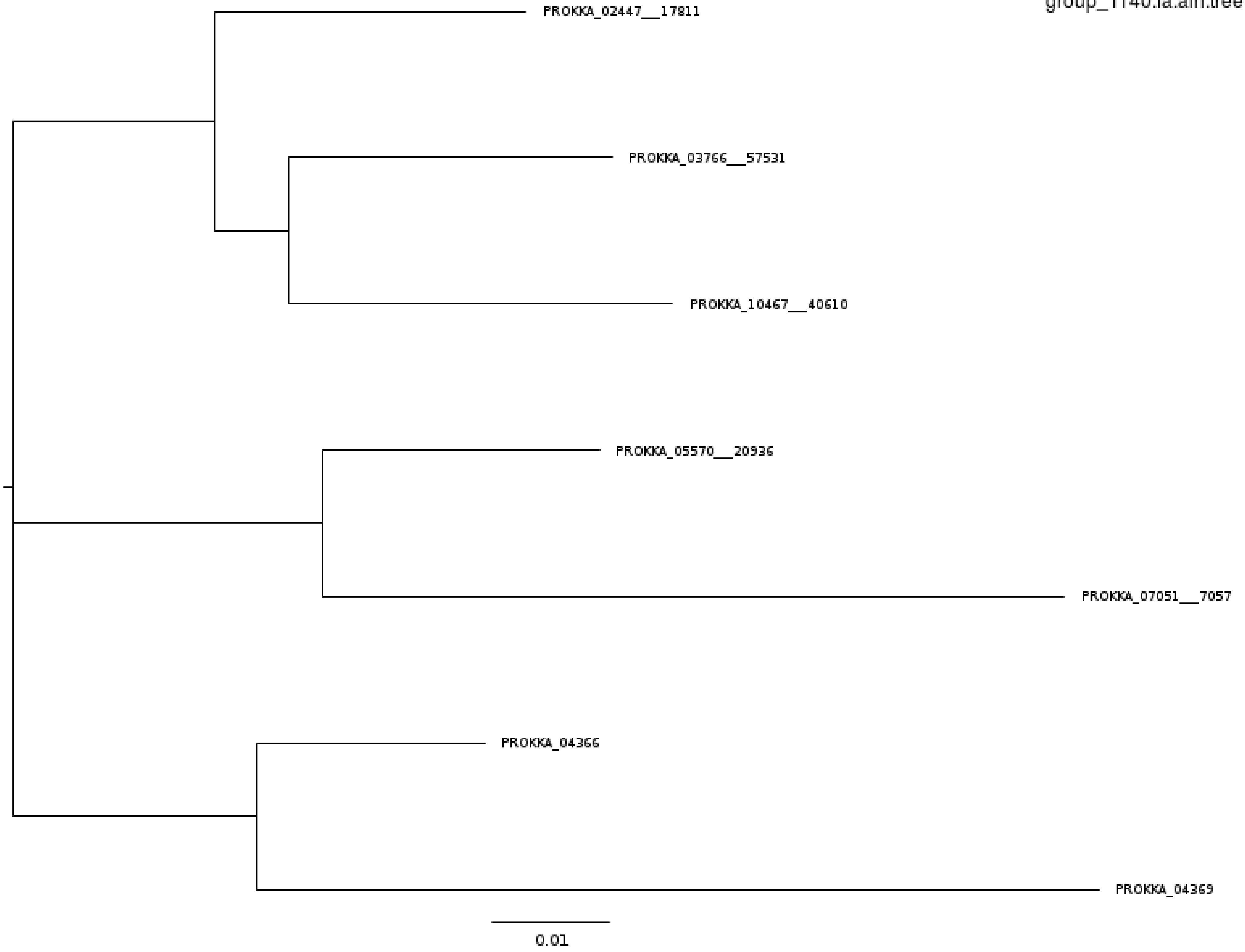

group_1142.fa.aln.tree.png

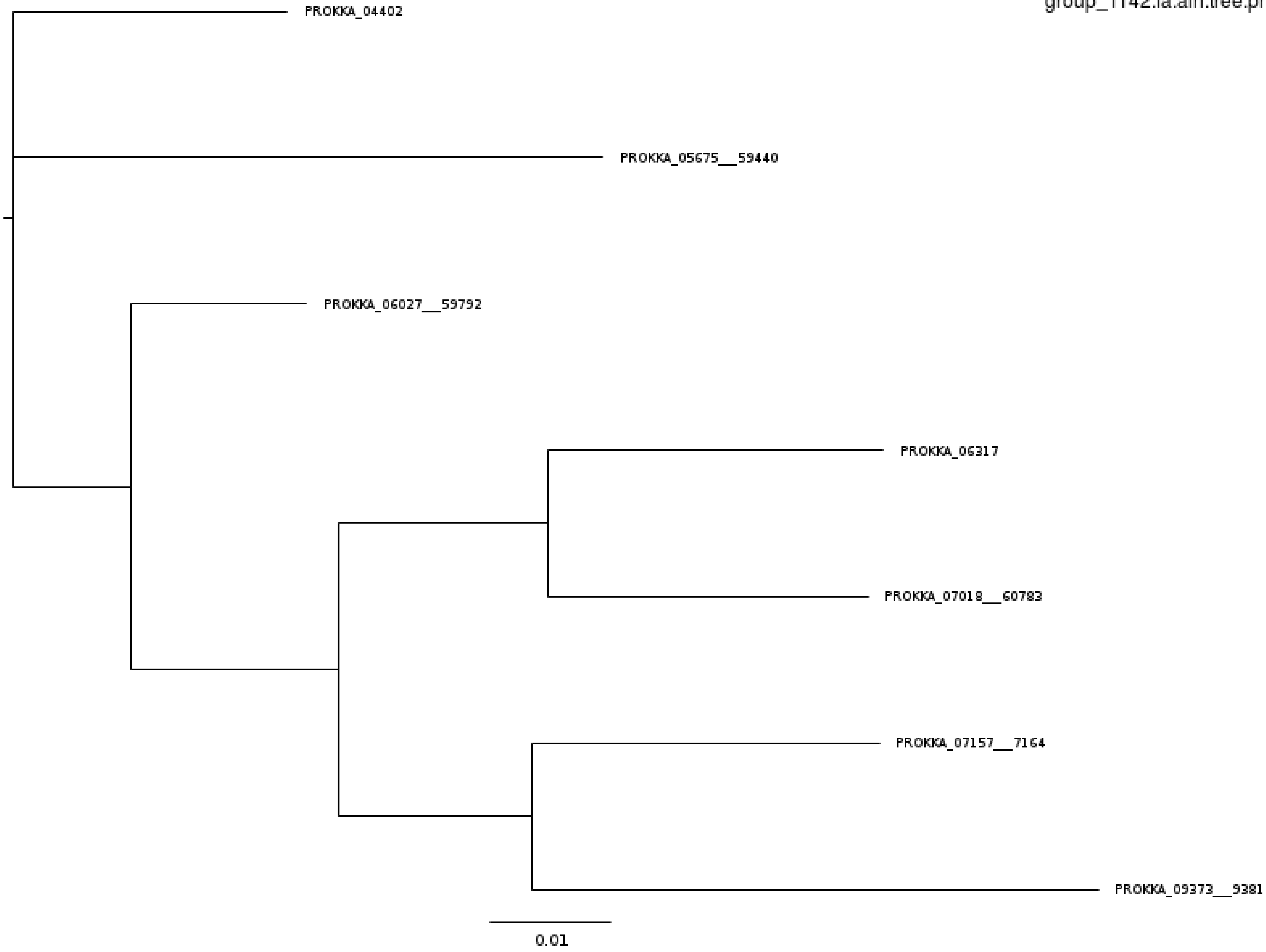

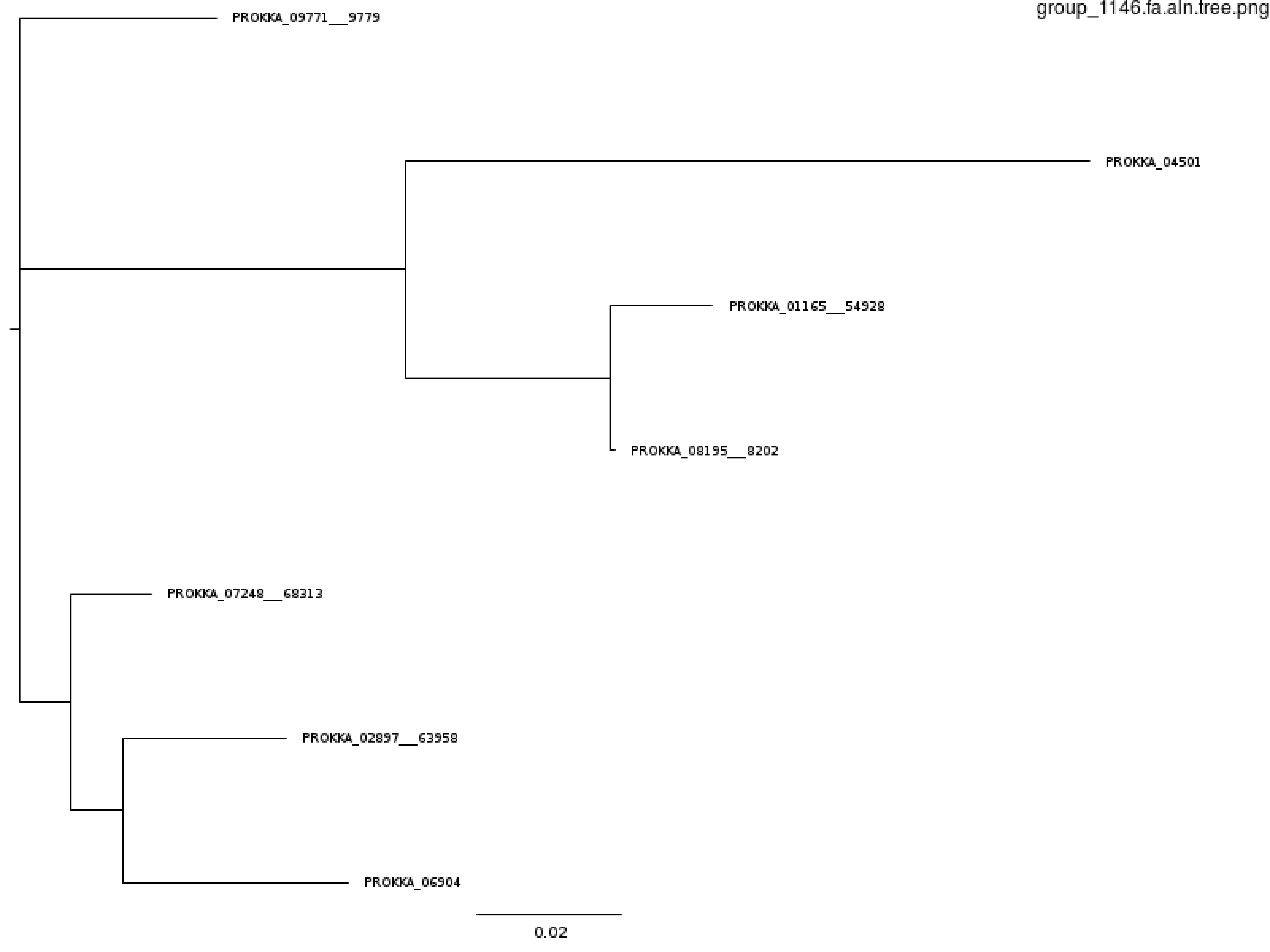
group_1146.fa.aln.tree.png
PROKKA_09771___9779
PROKKA_04501
PROKKA_01165___54928
PROKKA_08195___8202
PROKKA_07248___68313
PROKKA_02897___63958
PROKKA_06904
0.02

group_1152.fa.aln.tree.png

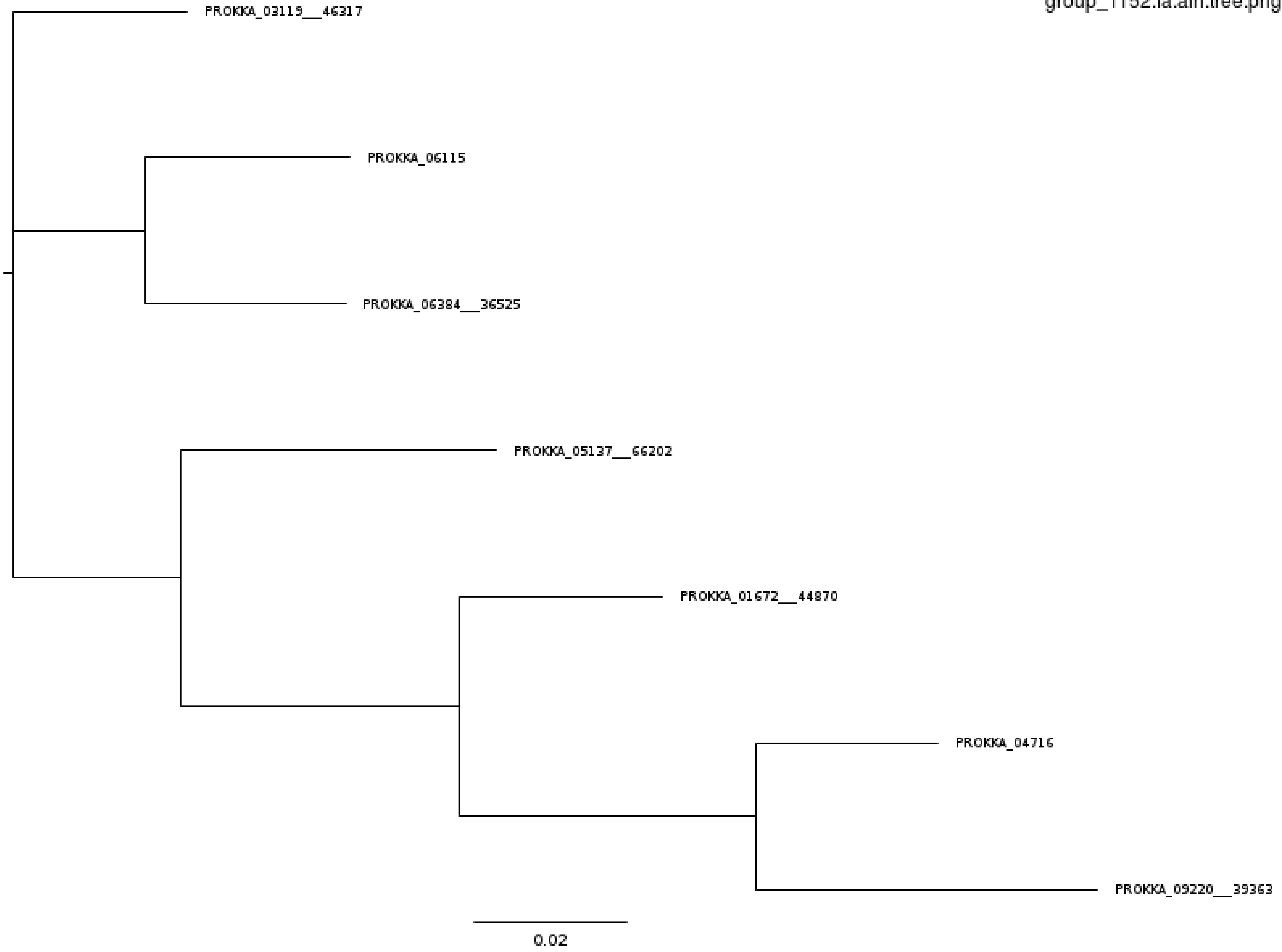

group_1155.fa.aln.tree.png

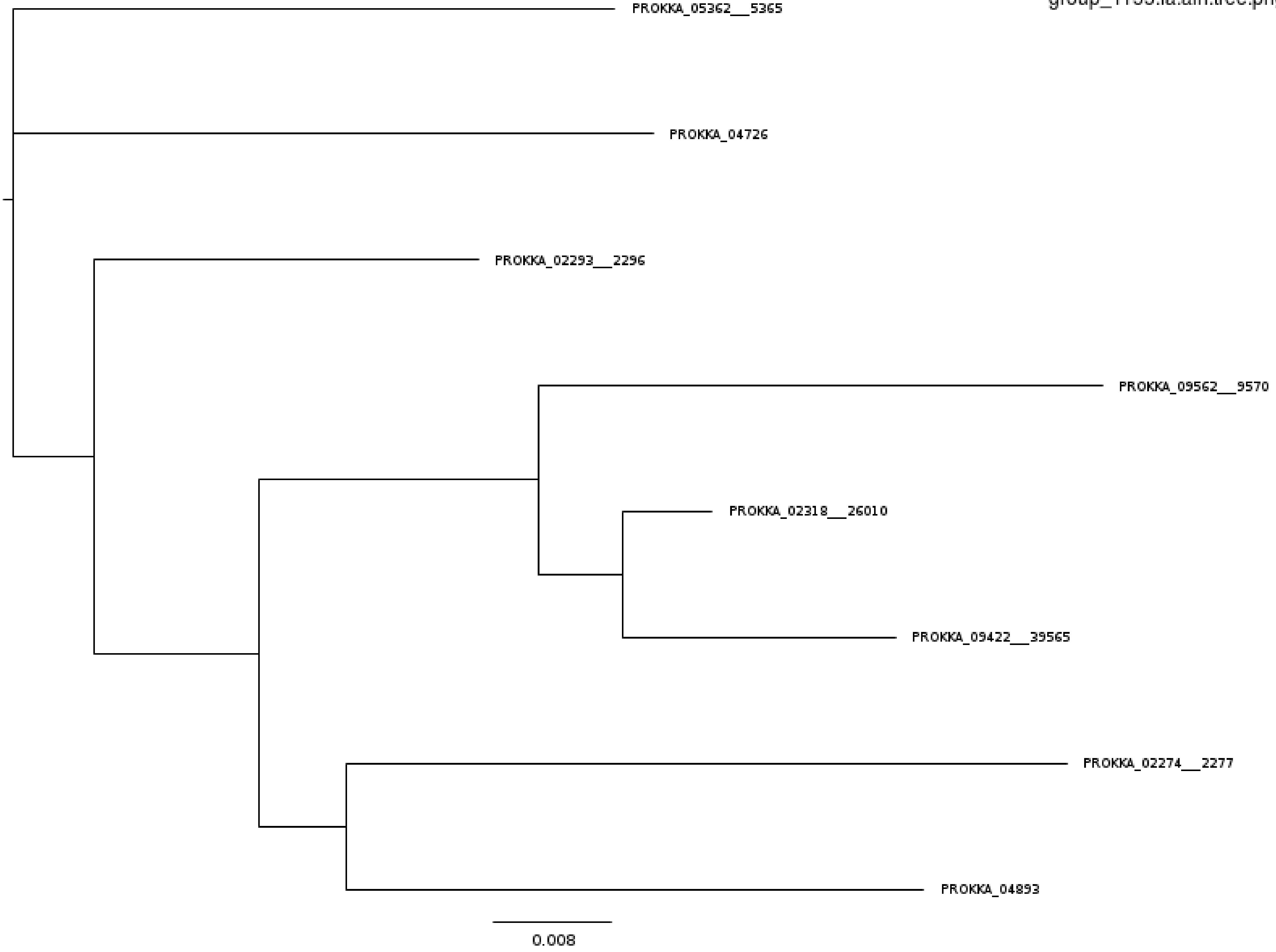

group_1159.fa.aln.tree.png

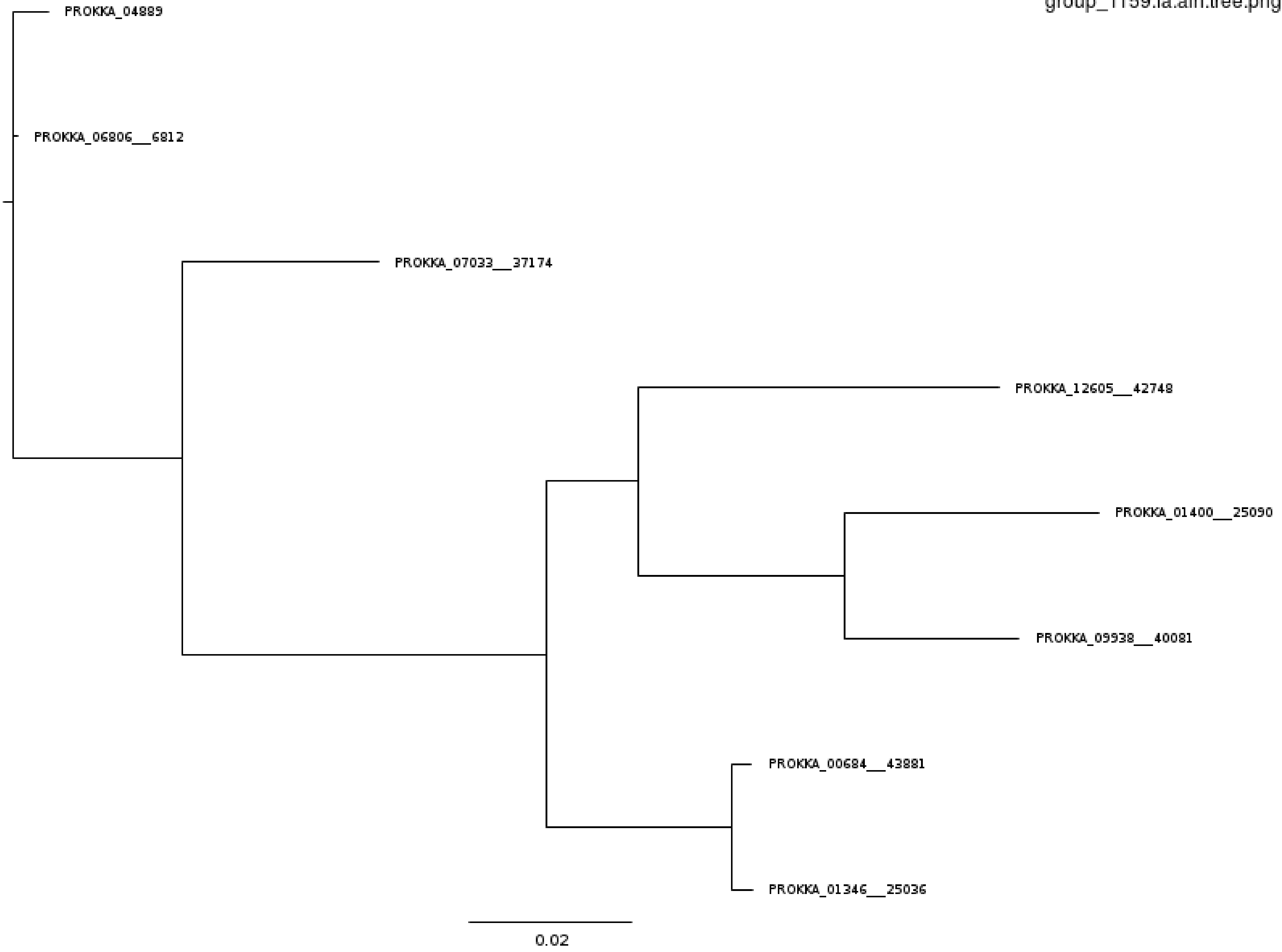

group_1166.fa.aln.tree.png

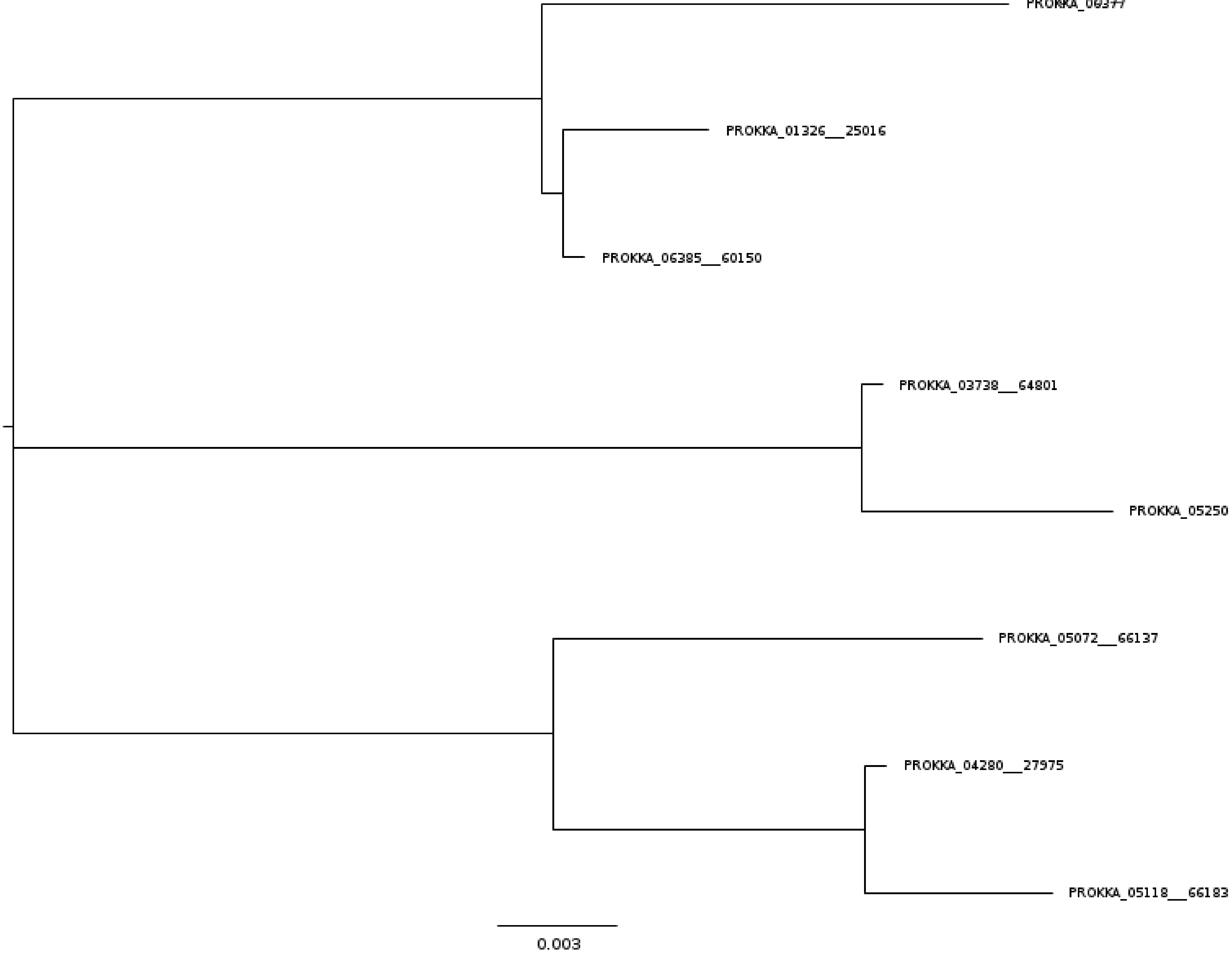

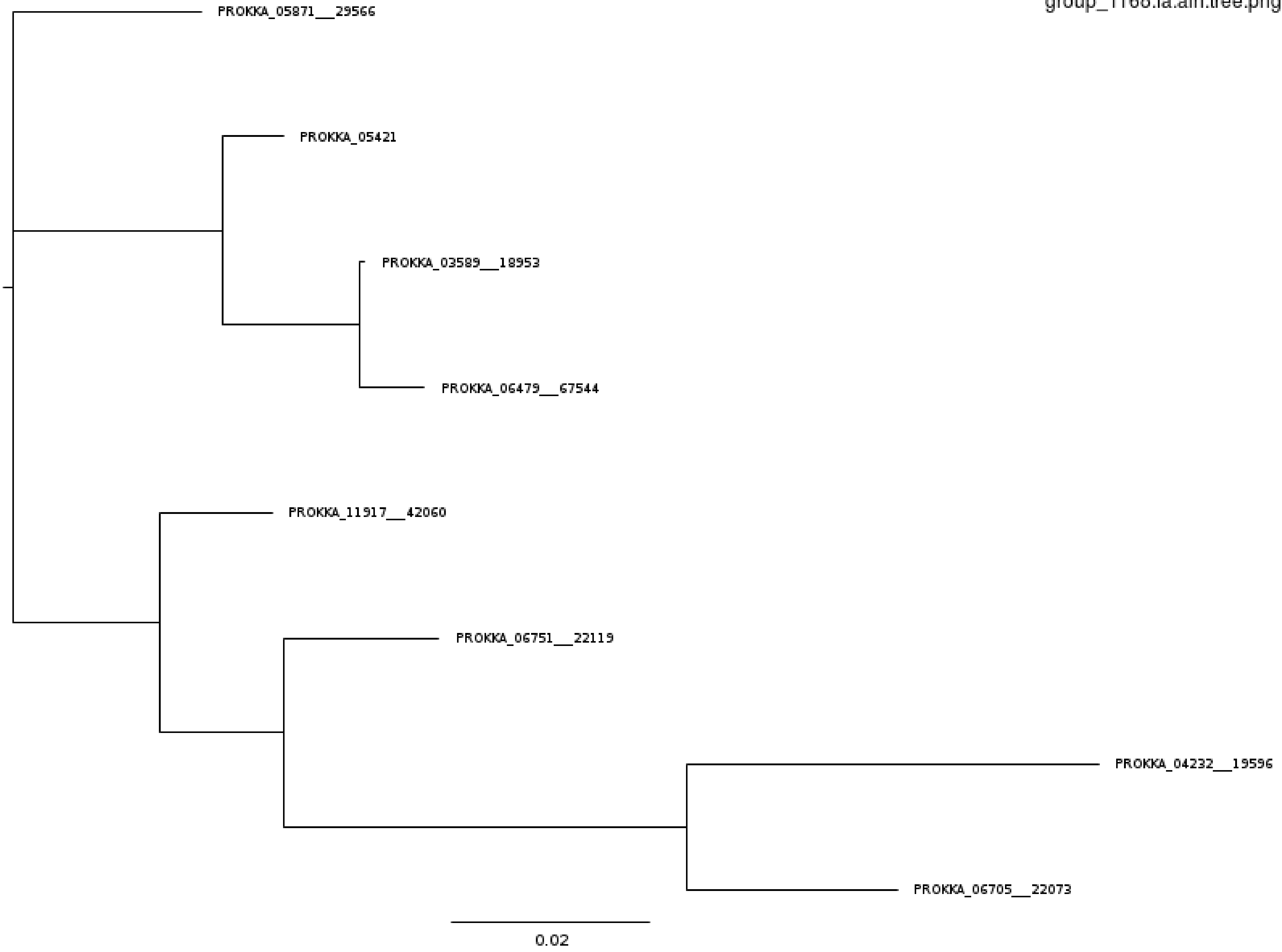
PROKKA_05871___29566
PROKKA_05421
PROKKA_03589___18953
PROKKA_06479___67544
PROKKA_11917___42060
PROKKA_06751___22119
PROKKA_04232___19596
PROKKA_06705___22073
0.02

group_116.fa.aln.tree.png

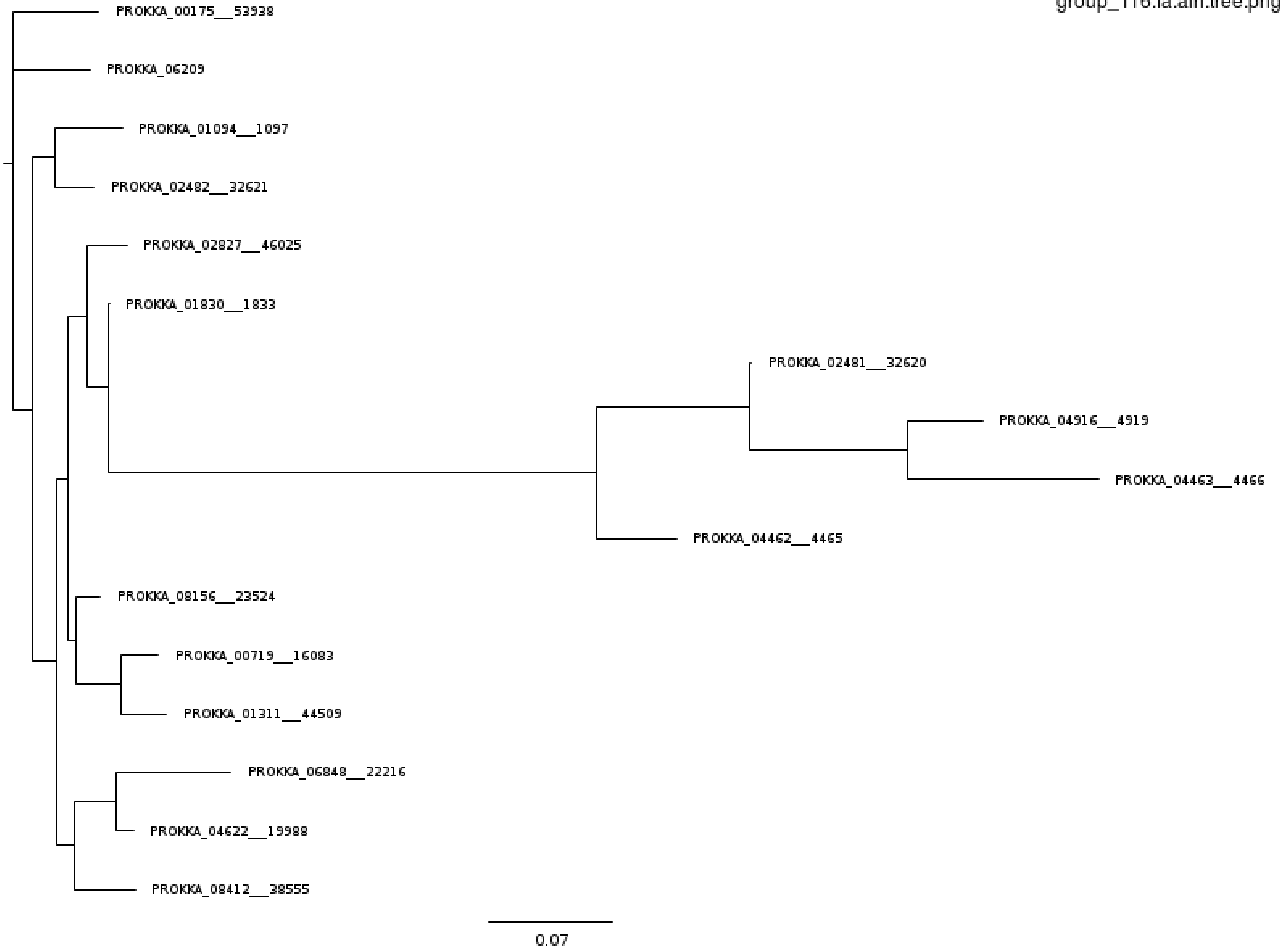

group_1171.fa.aln.tree.png

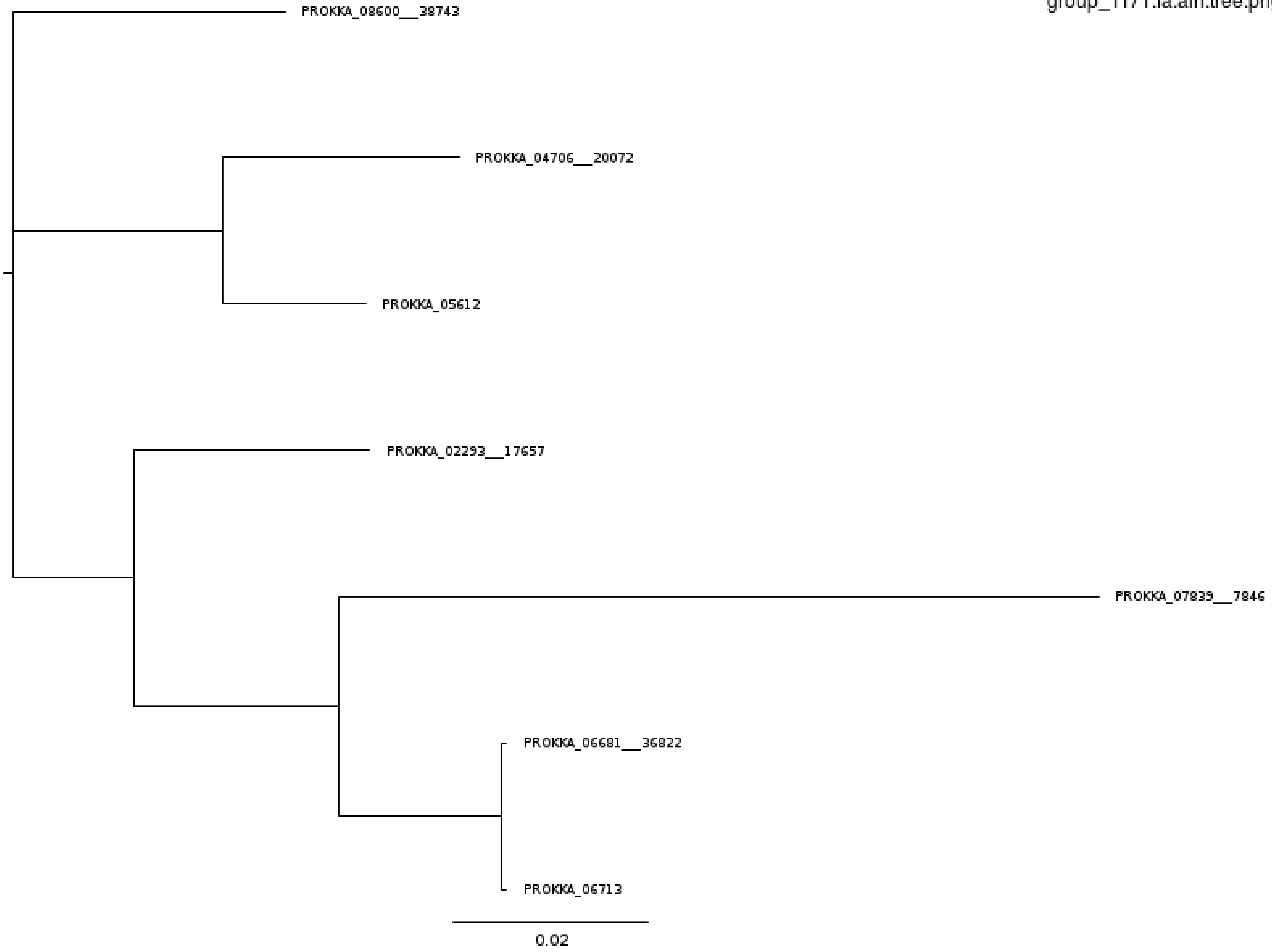

group_1172.fa.aln.tree.png

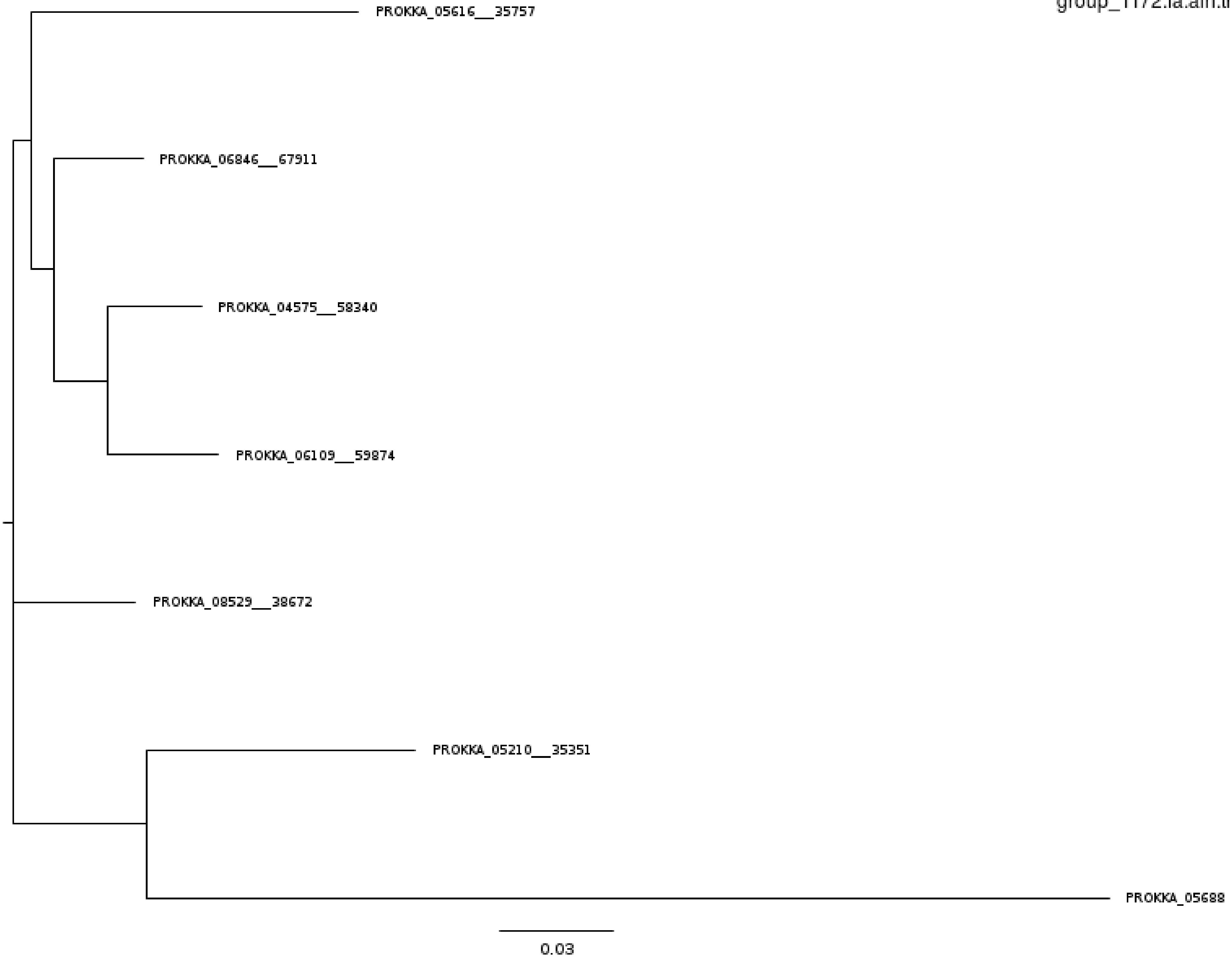

group_117.fa.aln.tree.png

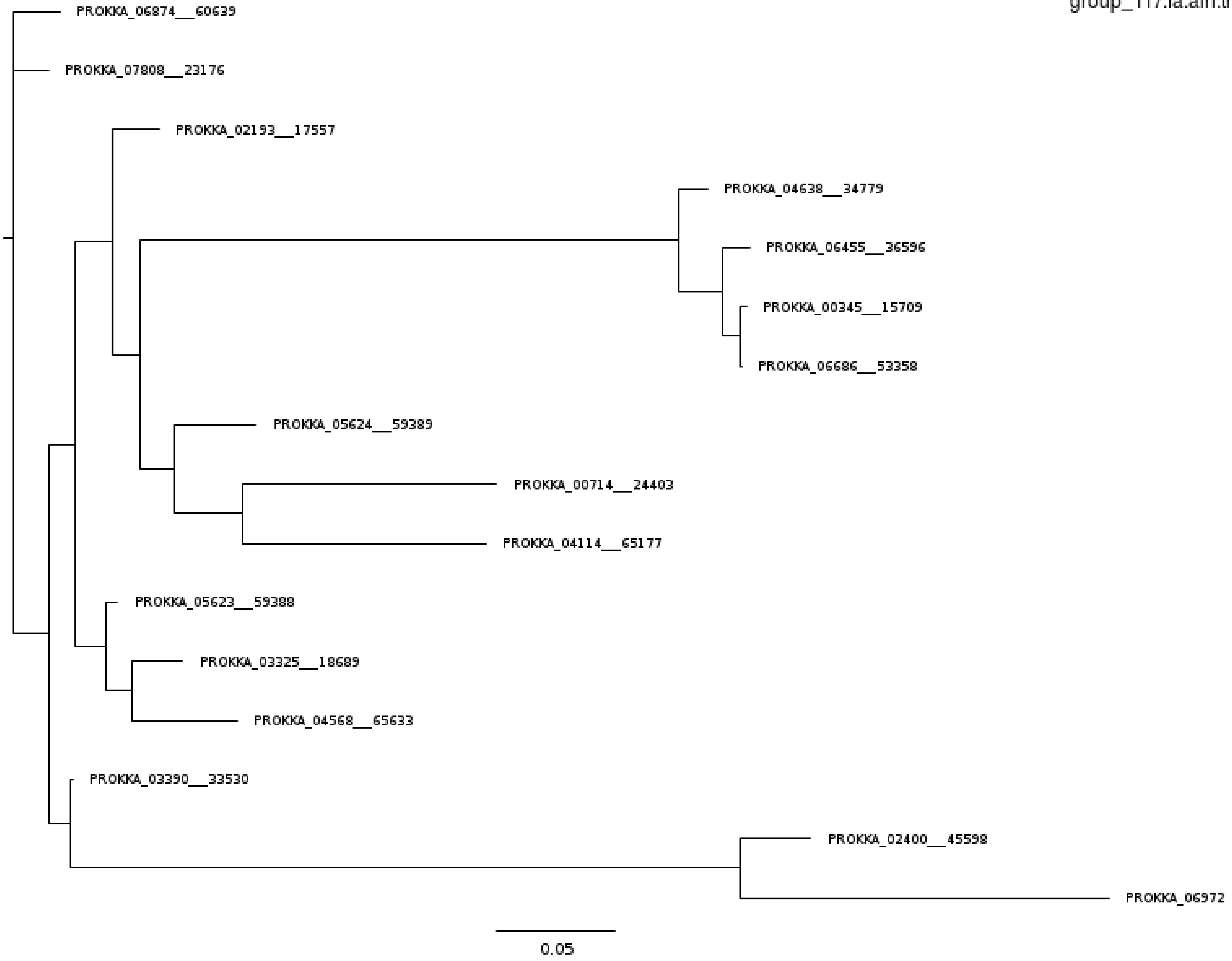

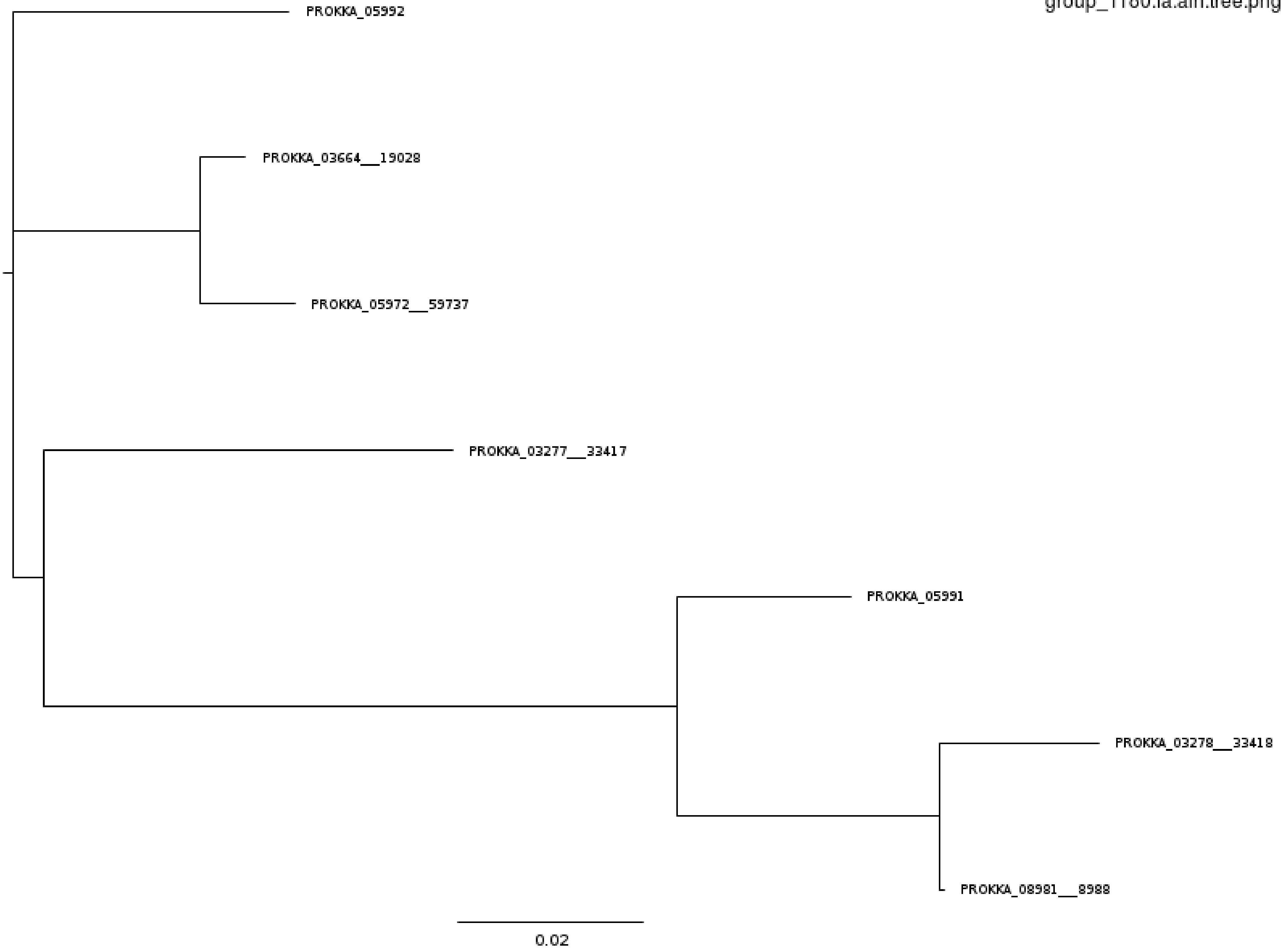
PROKKA_05992
PROKKA_03664___19028
PROKKA_05972___59737
PROKKA_03277___33417
PROKKA_05991
PROKKA_03278___33418
PROKKA_08981___8988
0.02

group_1182.fa.aln.tree.png

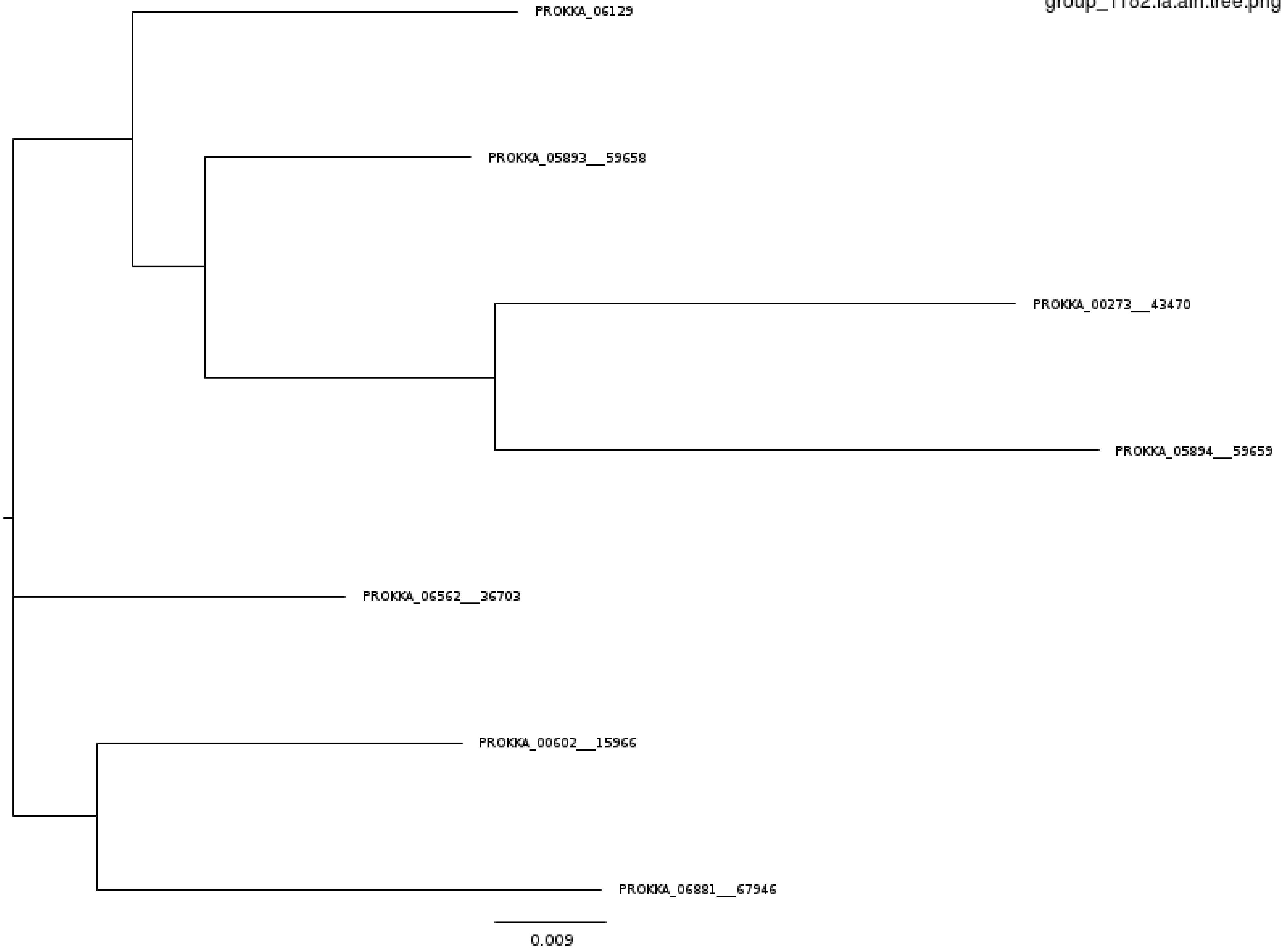

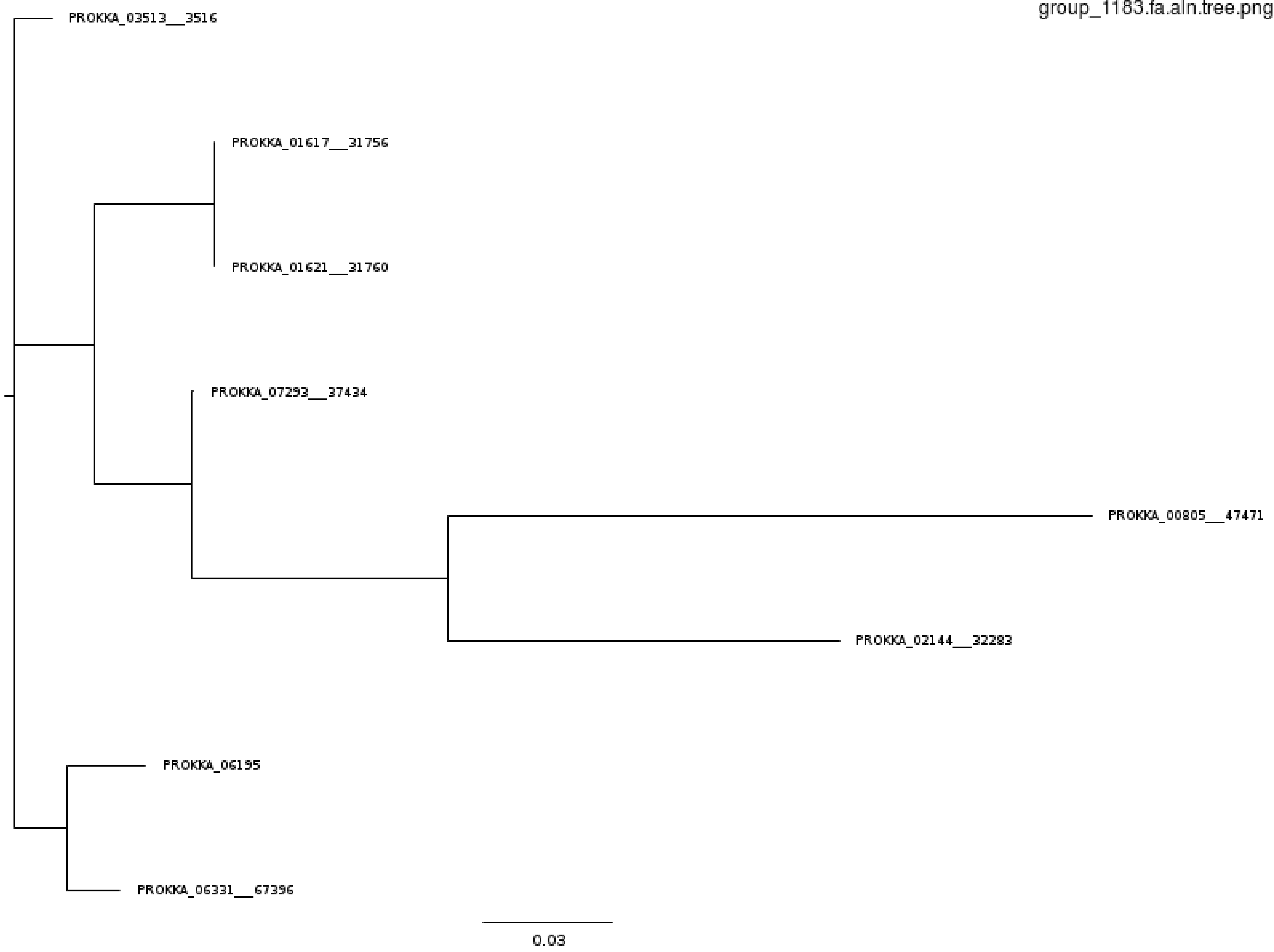
group_1183.fa.aln.tree.png
PROKKA_03513___3516
PROKKA_01617___31756
PROKKA_01621___31760
PROKKA_07293___37434
PROKKA_00805___47471
PROKKA_02144___32283
PROKKA_06195
PROKKA_06331___67396
0.03

group_1185.fa.aln.tree.png

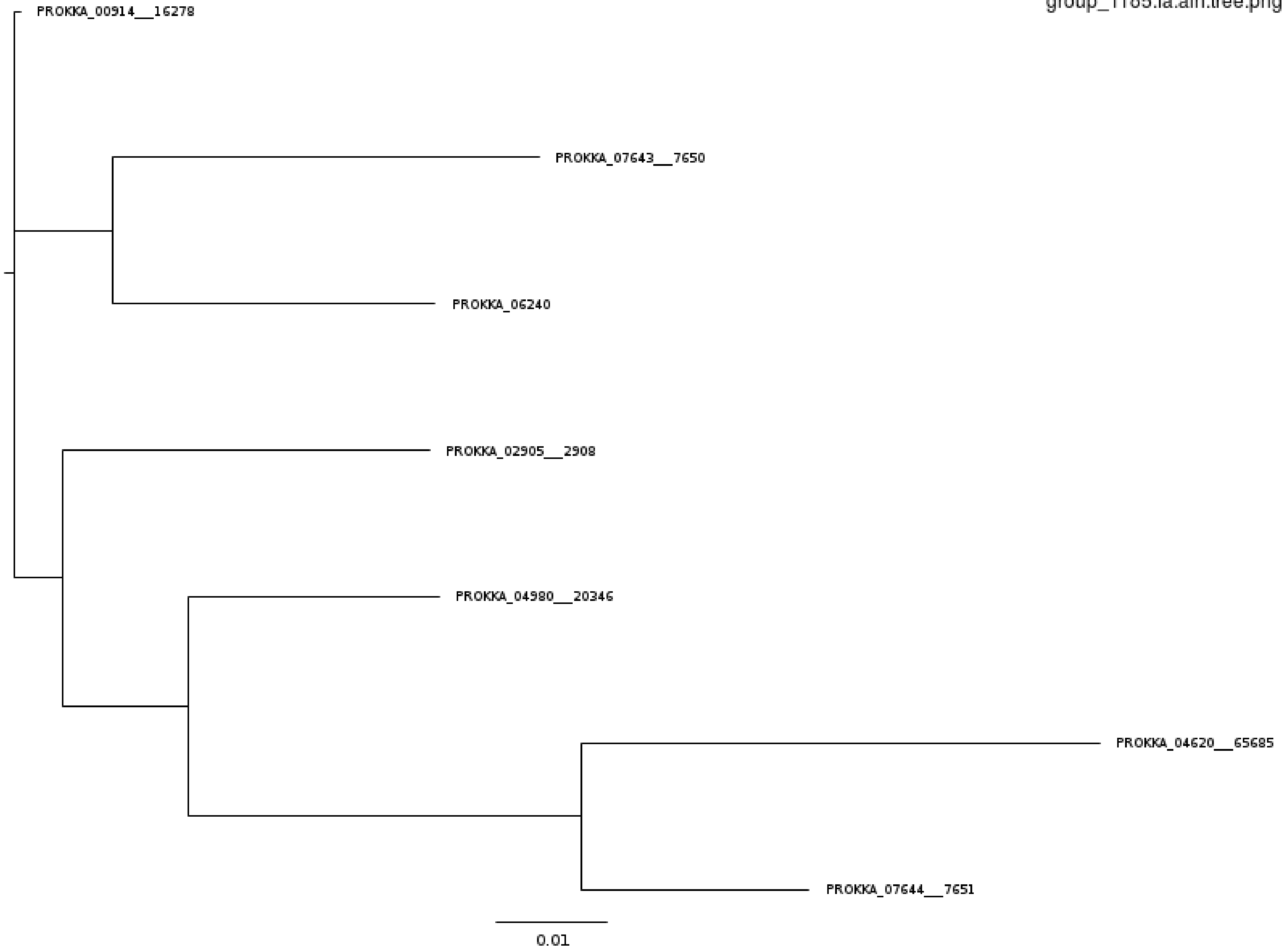

group_1189.fa.aln.tree.png

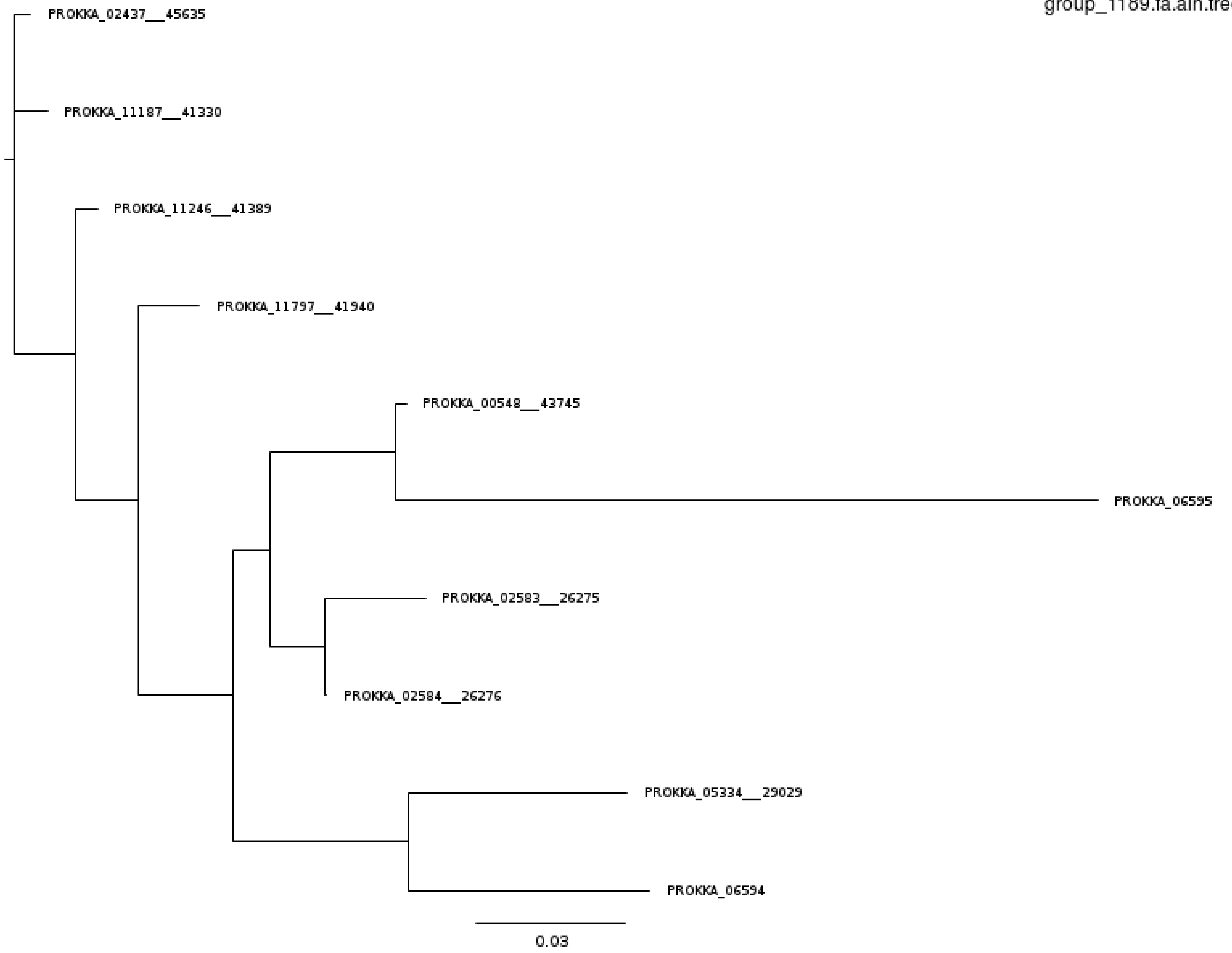

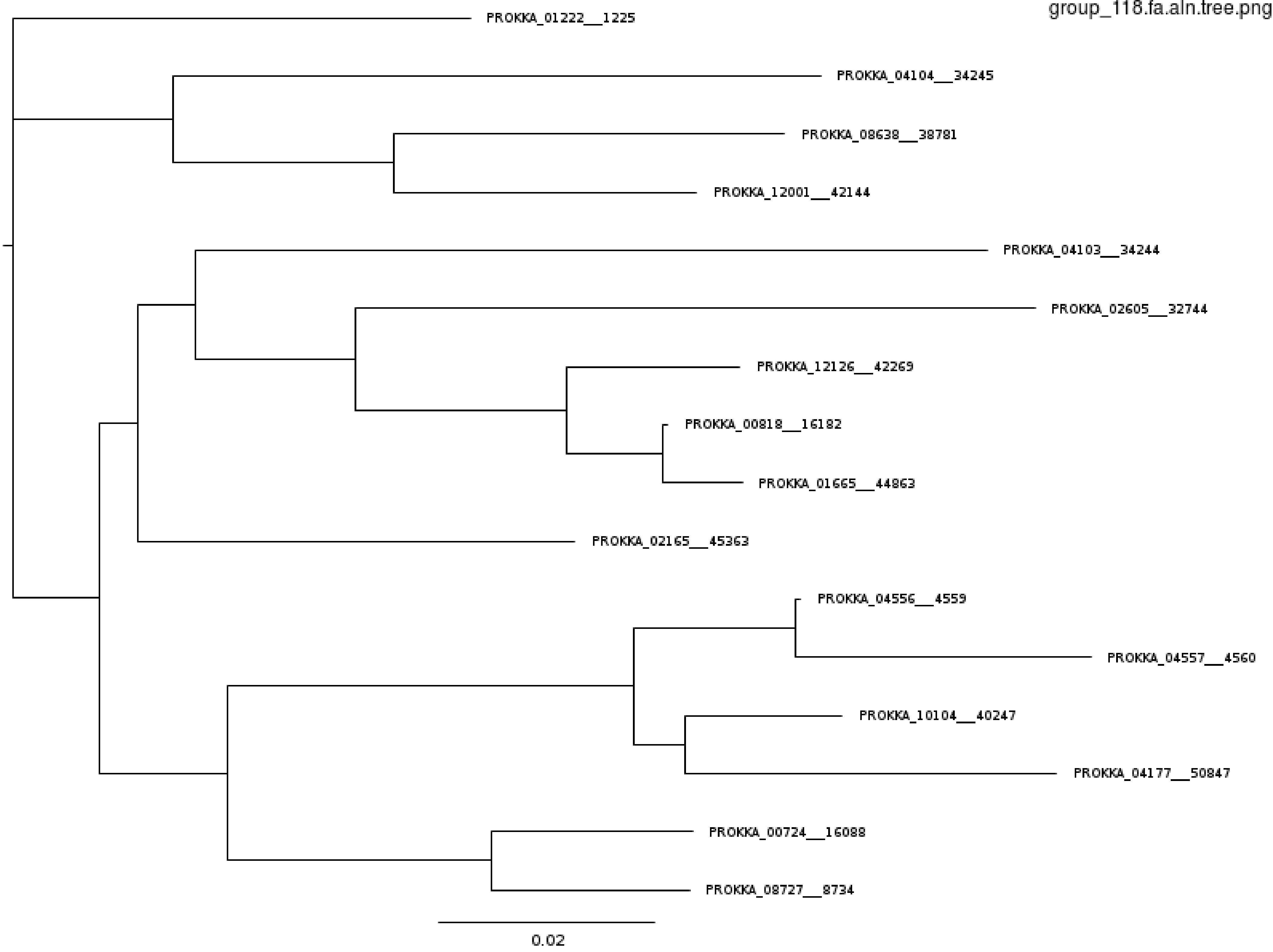
group_118.fa.aln.tree.png
PROKKA_01222__1225
PROKKA_04104__34245
PROKKA_08638__38781
PROKKA_12001__42144
PROKKA_04103__34244
PROKKA_02605__32744
PROKKA_12126__42269
PROKKA_00818__16182
PROKKA_01665__44863
PROKKA_02165__45363
PROKKA_04556__4559
PROKKA_04557__4560
PROKKA_10104__40247
PROKKA_04177__50847
PROKKA_00724__16088
PROKKA_08727__8734
0.02

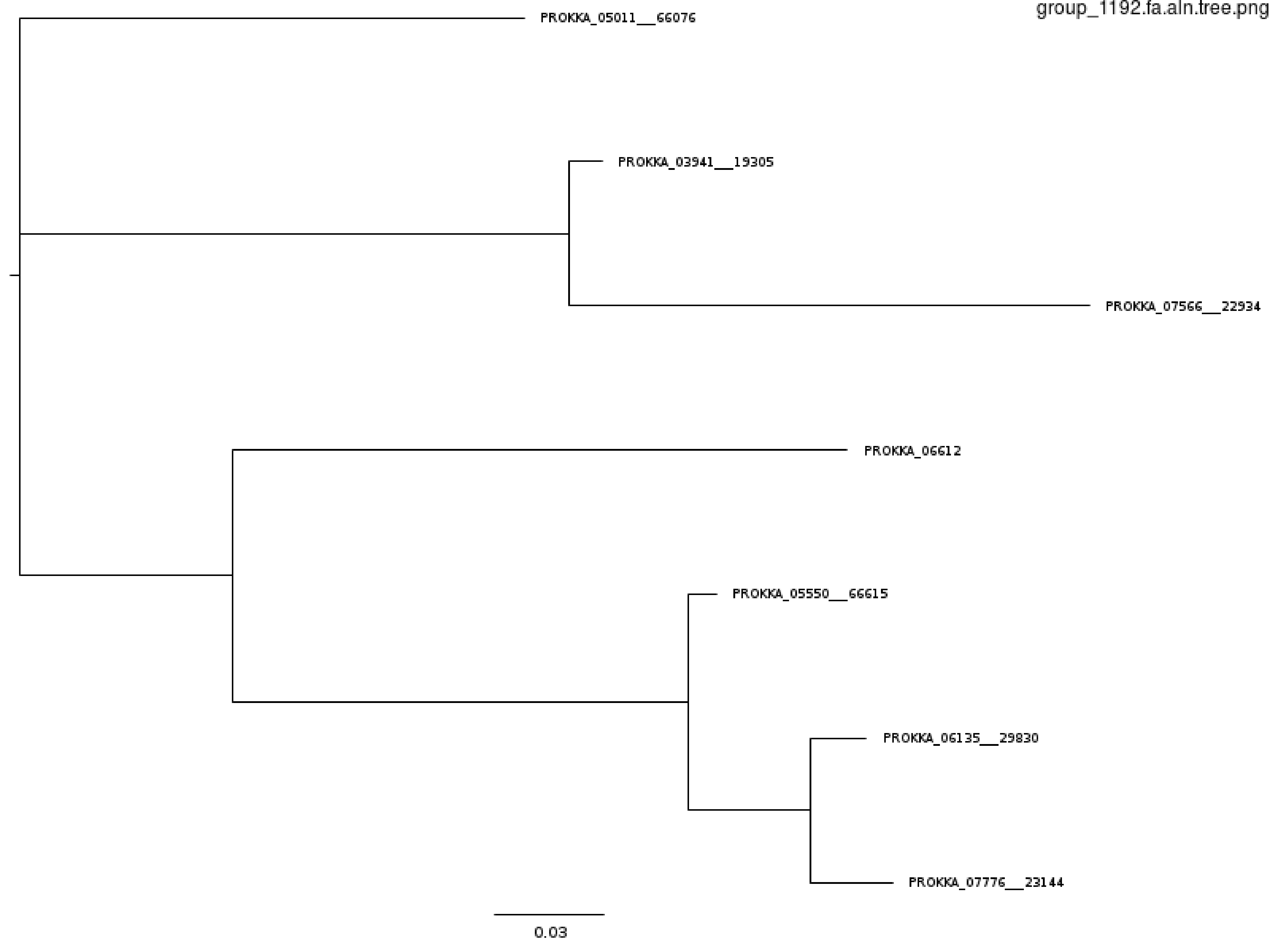
group_1192.fa.aln.tree.png
PROKKA_05011___66076
PROKKA_03941___19305
PROKKA_07566___22934
PROKKA_06612
PROKKA_05550___66615
PROKKA_06135___29830
PROKKA_07776___23144
0.03

group_1194.fa.aln.tree.png

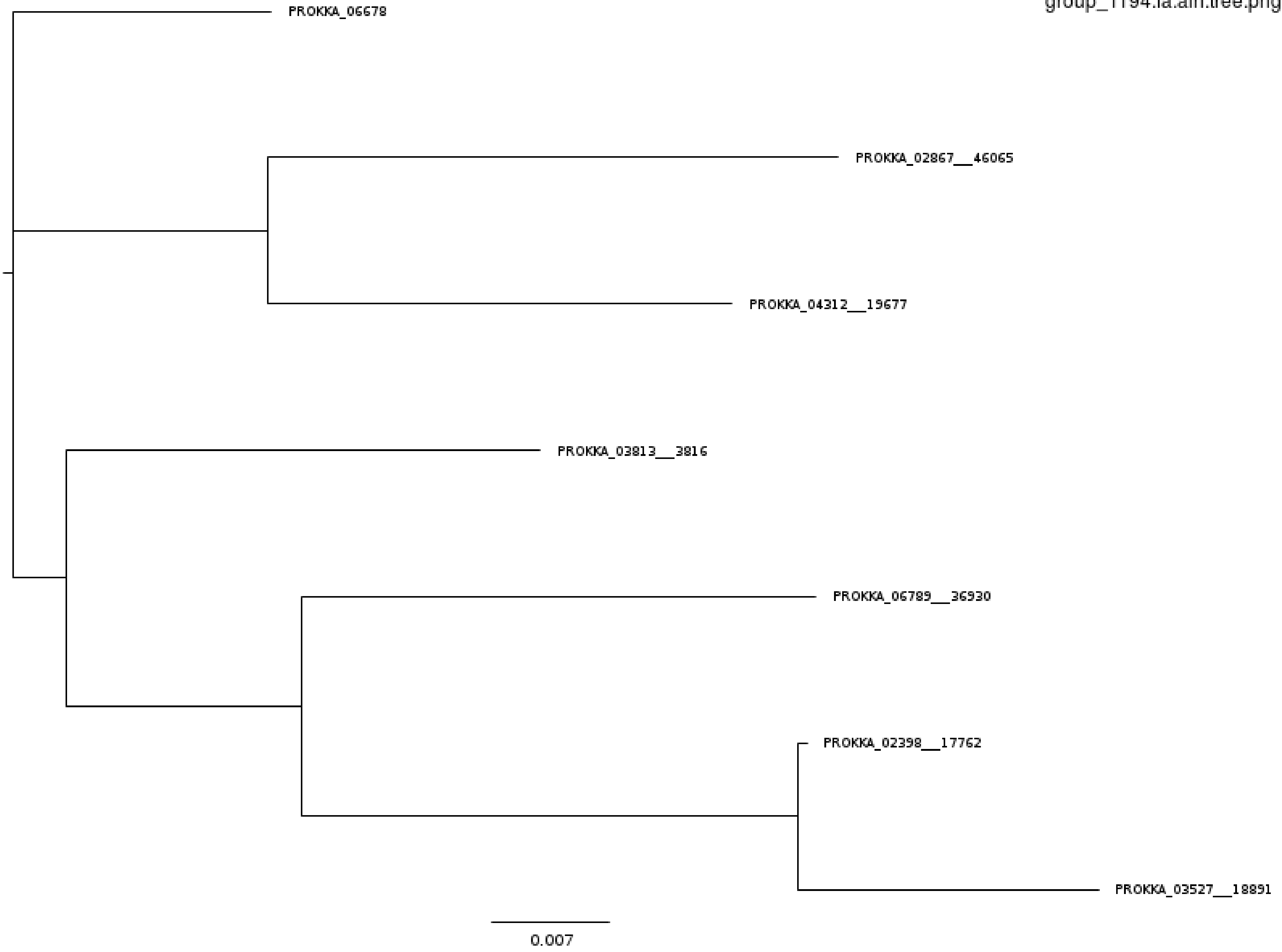

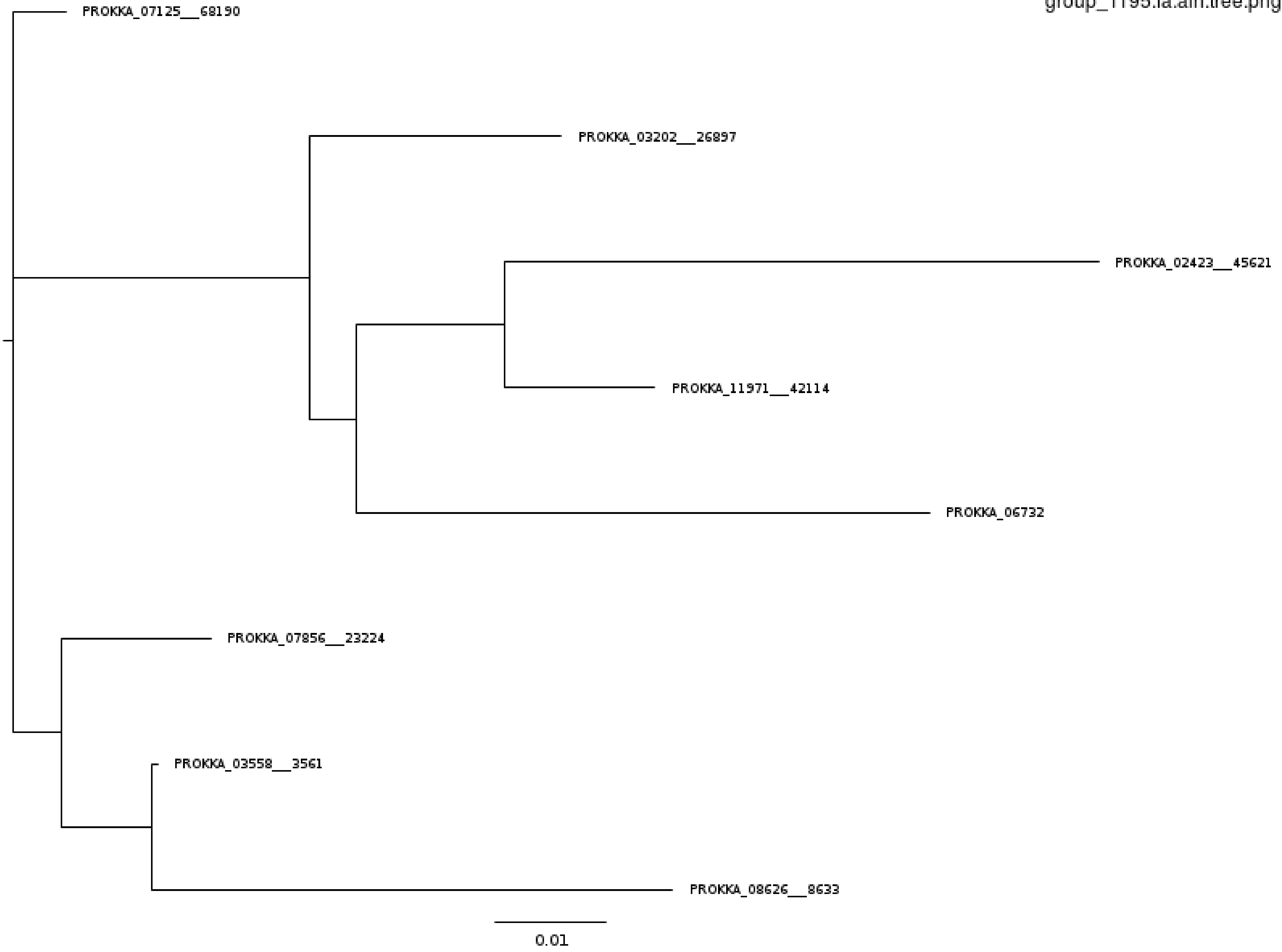
PROKKA_07125___68190
PROKKA_03202___26897
PROKKA_02423___45621
PROKKA_11971___42114
PROKKA_06732
PROKKA_07856___23224
PROKKA_03558___3561
PROKKA_08626___8633
0.01

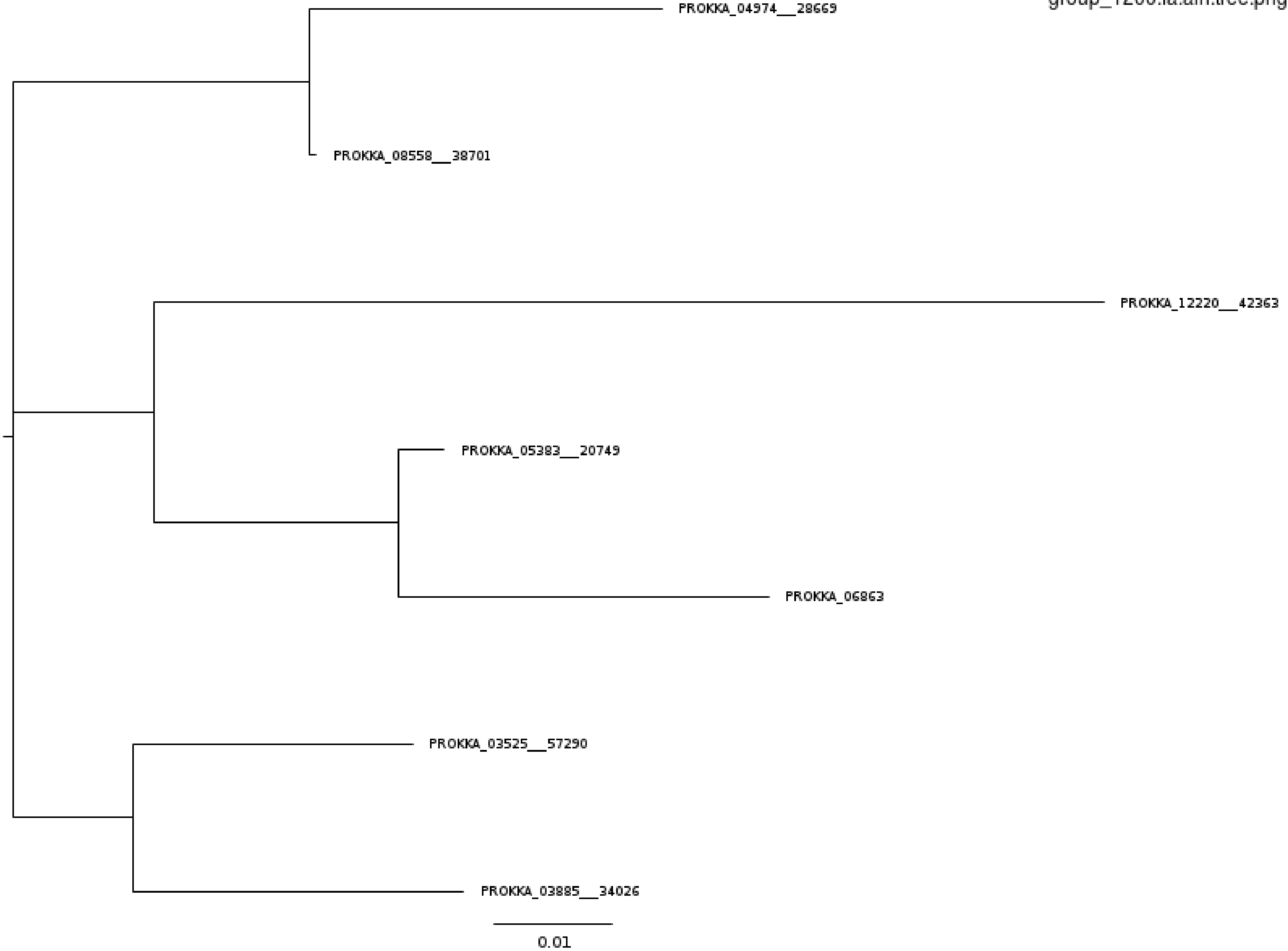
PROKKA_04974___28669
PROKKA_08558___38701
PROKKA_12220___42363
PROKKA_05383___20749
PROKKA_06863
PROKKA_03525___57290
PROKKA_03885___34026
0.01

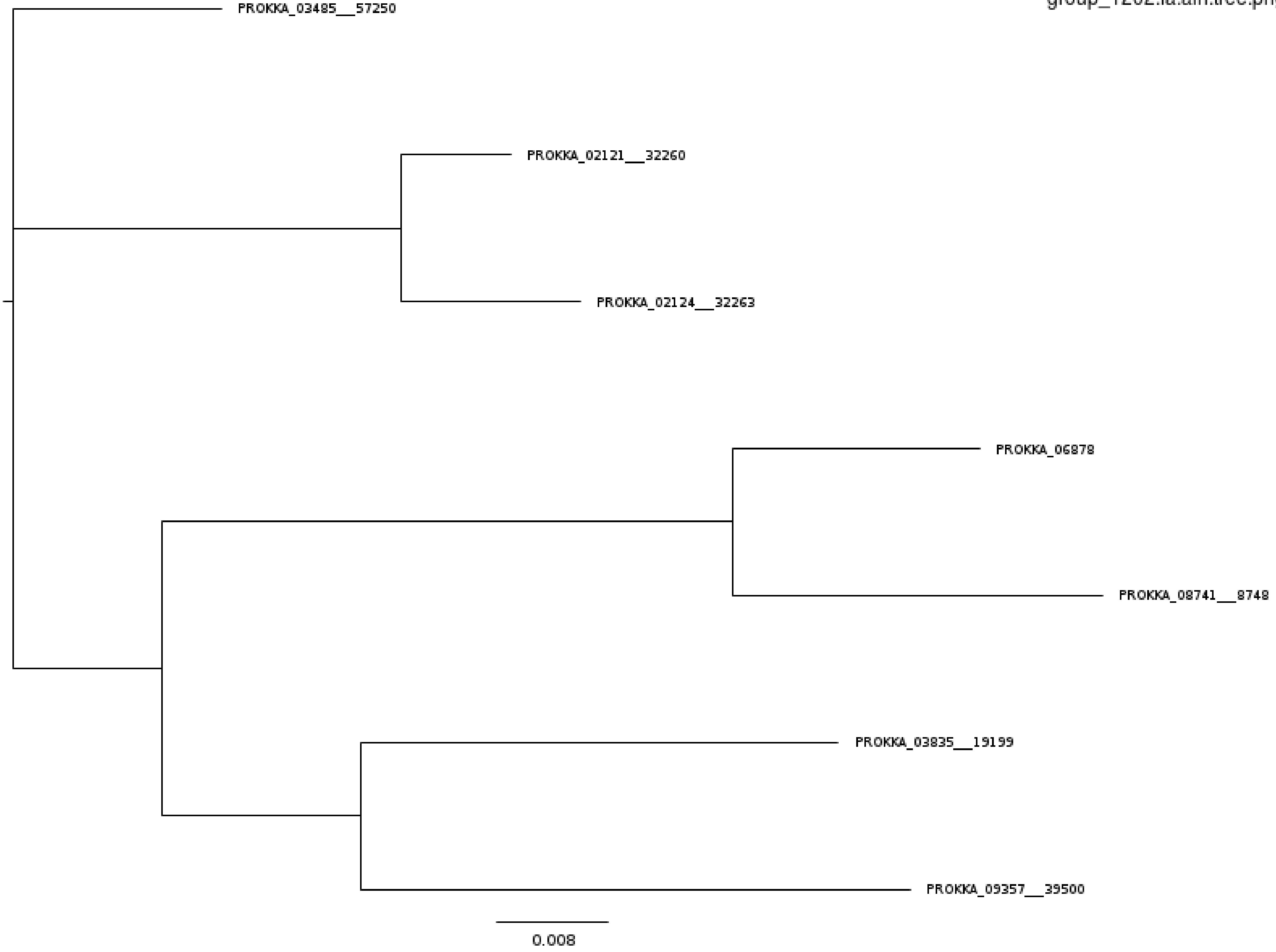
PROKKA_03485___57250
PROKKA_02121___32260
PROKKA_02124___32263
PROKKA_06878
PROKKA_08741___8748
PROKKA_03835___19199
PROKKA_09357___39500
0.008

group_1204.fa.aln.tree.png

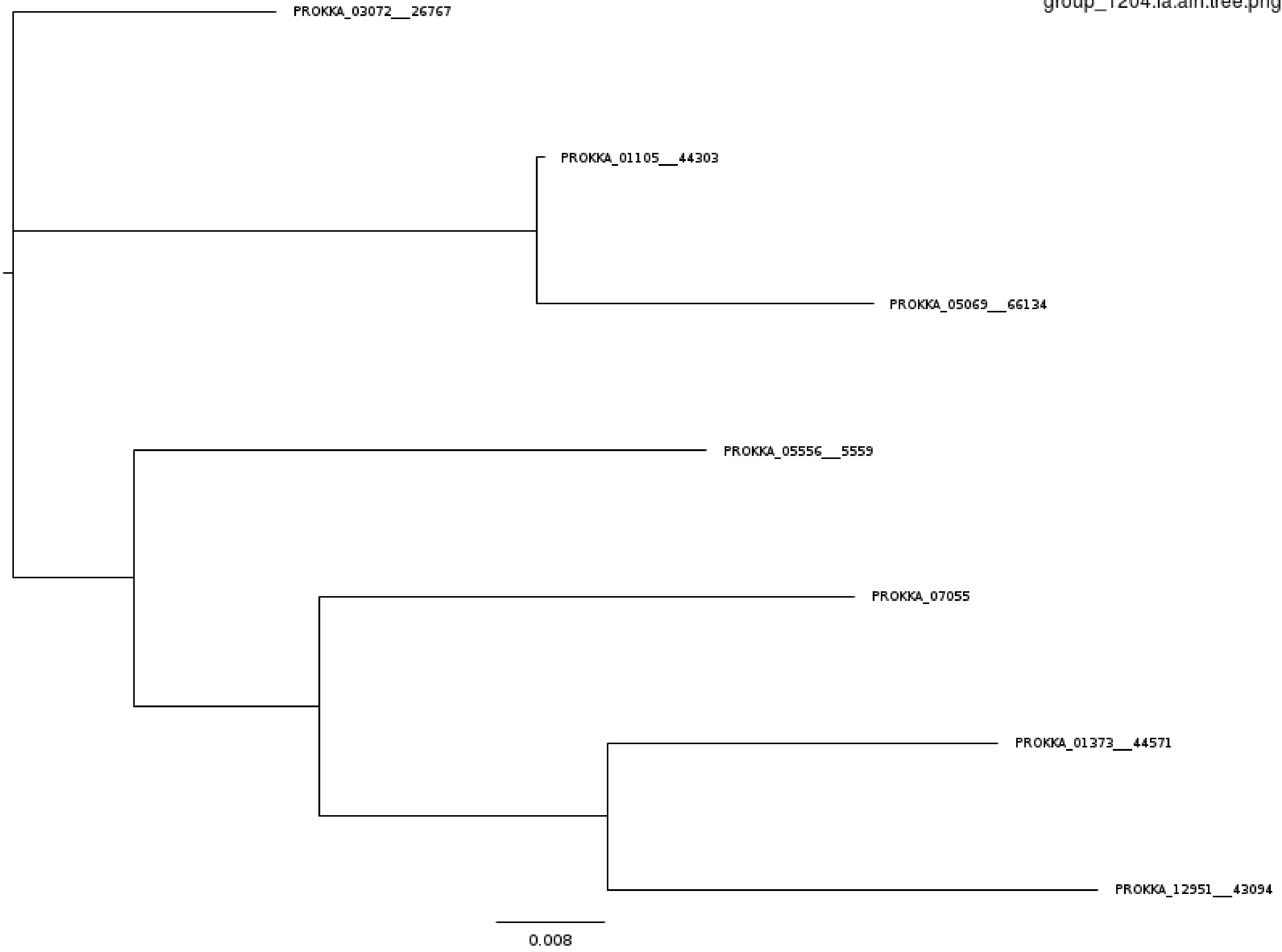

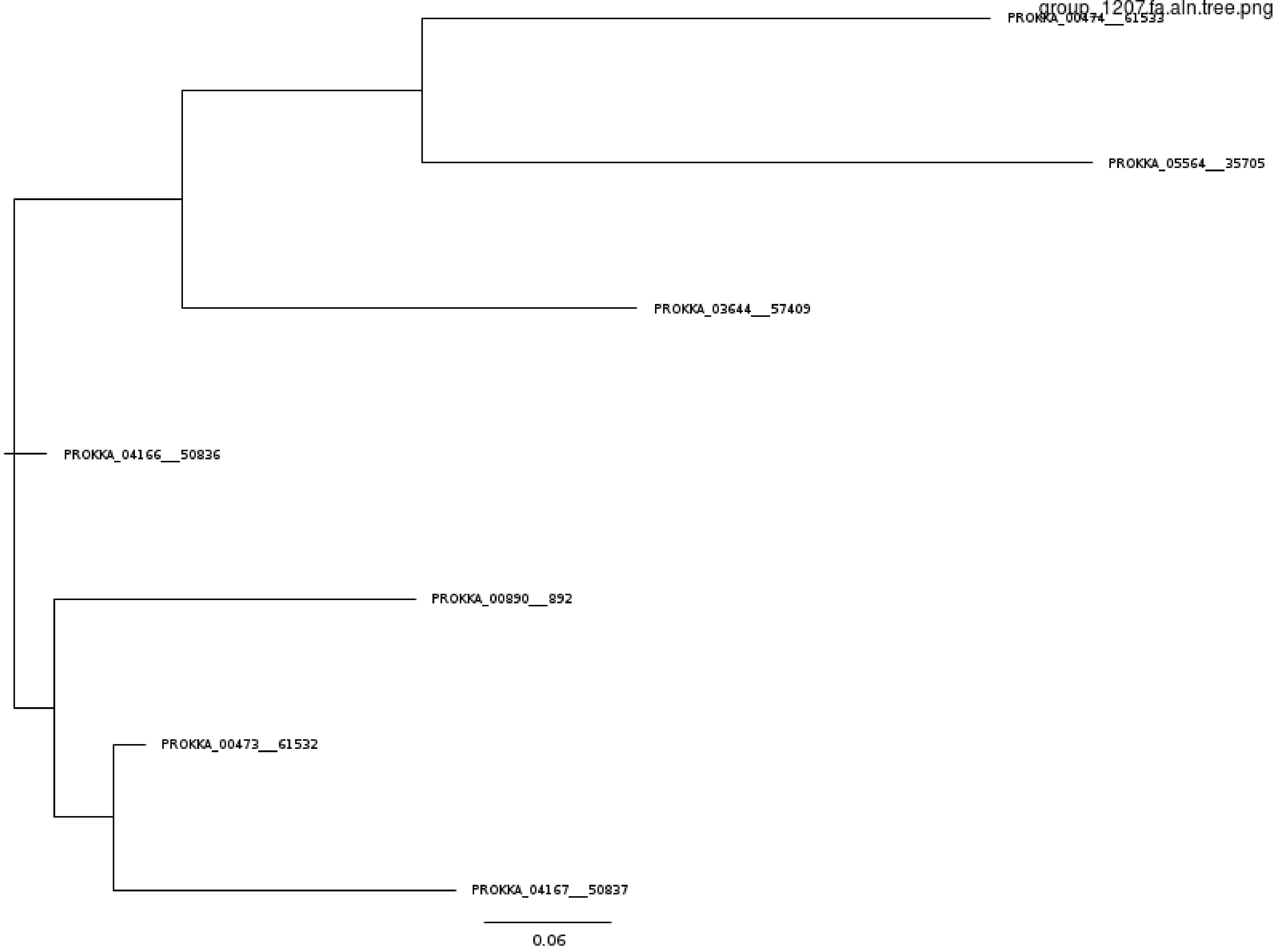
group_1207.fa.aln.tree.png
PROKKA_00474___61533
PROKKA_05564___35705
PROKKA_03644___57409
PROKKA_04166___50836
PROKKA_00890___892
PROKKA_00473___61532
PROKKA_04167___50837
0.06

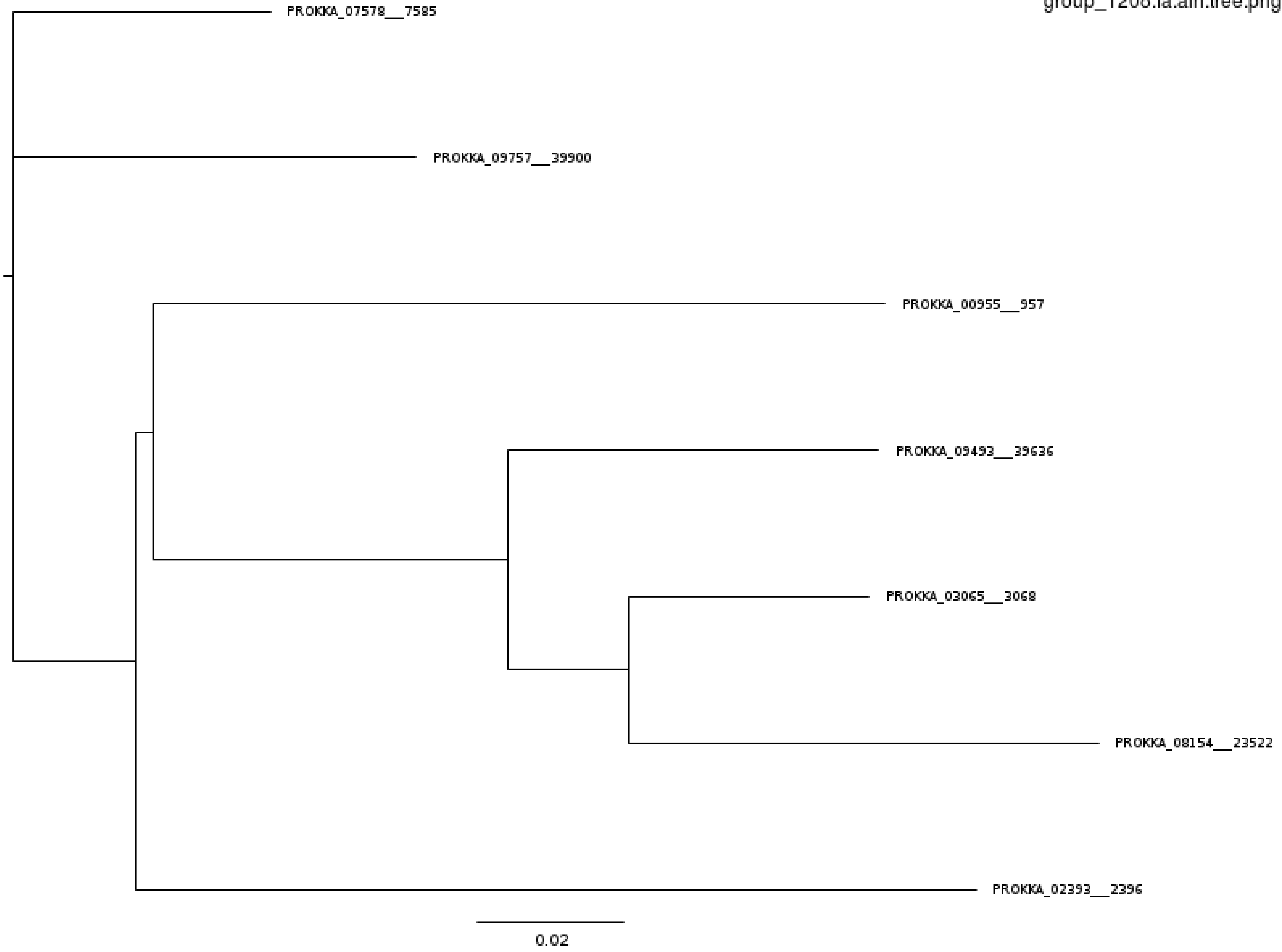
PROKKA_07578__7585
PROKKA_09757__39900
PROKKA_00955__957
PROKKA_09493__39636
PROKKA_03065__3068
PROKKA_08154__23522
PROKKA_02393__2396
0.02

group_1213.fa.aln.tree.png

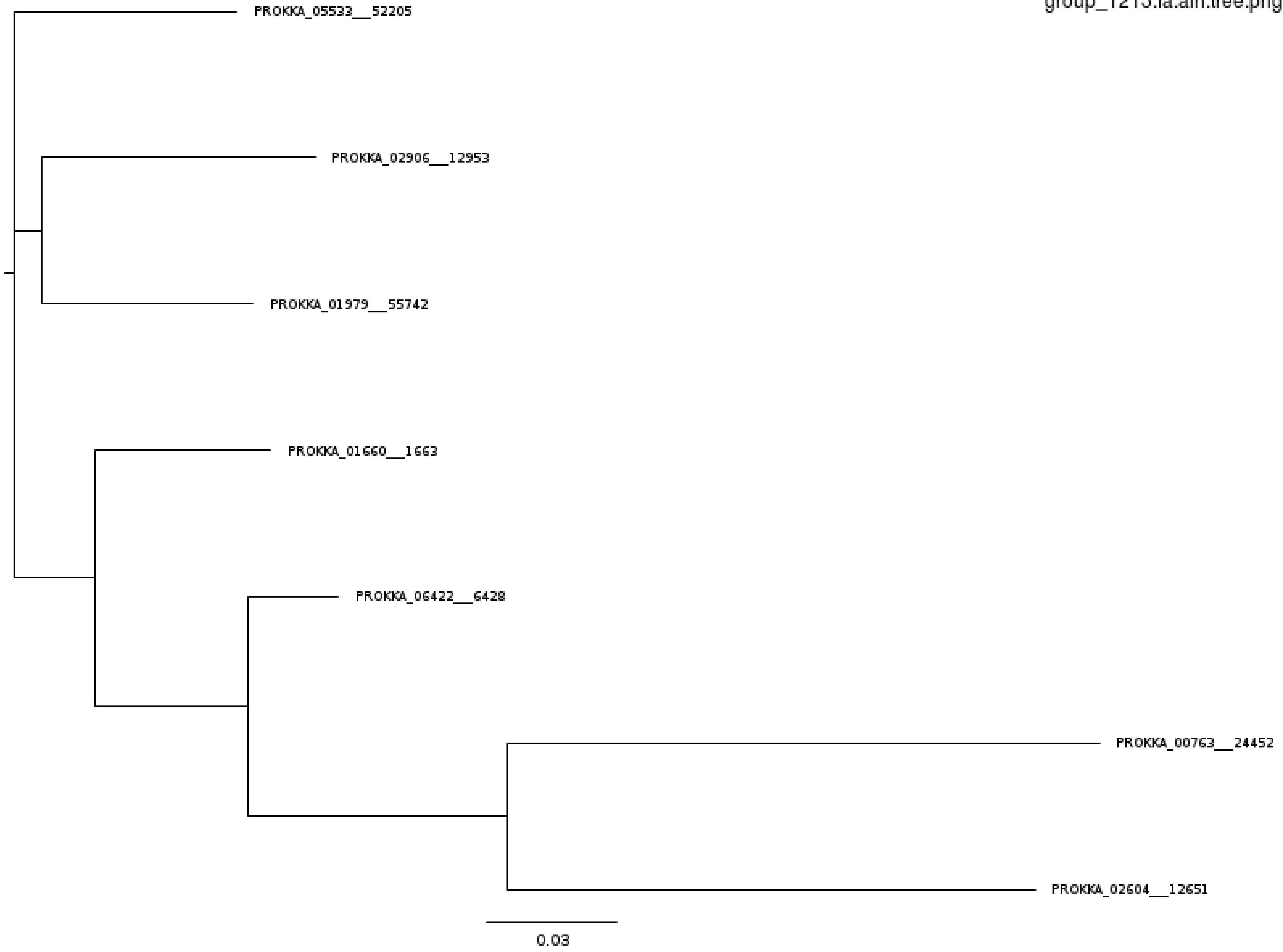

group_1218.fa.aln.tree.png

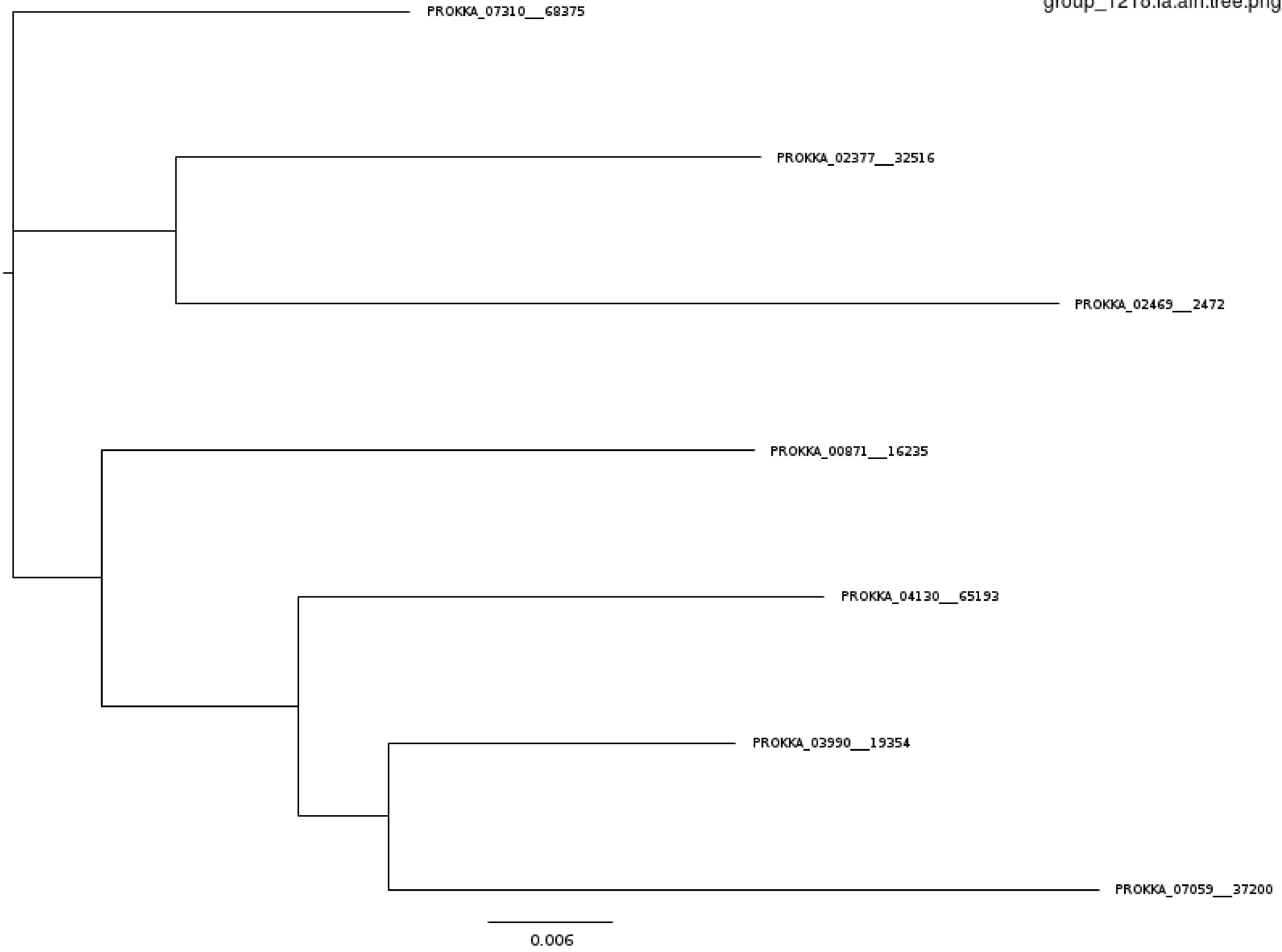

group_1222.fa.aln.tree.png

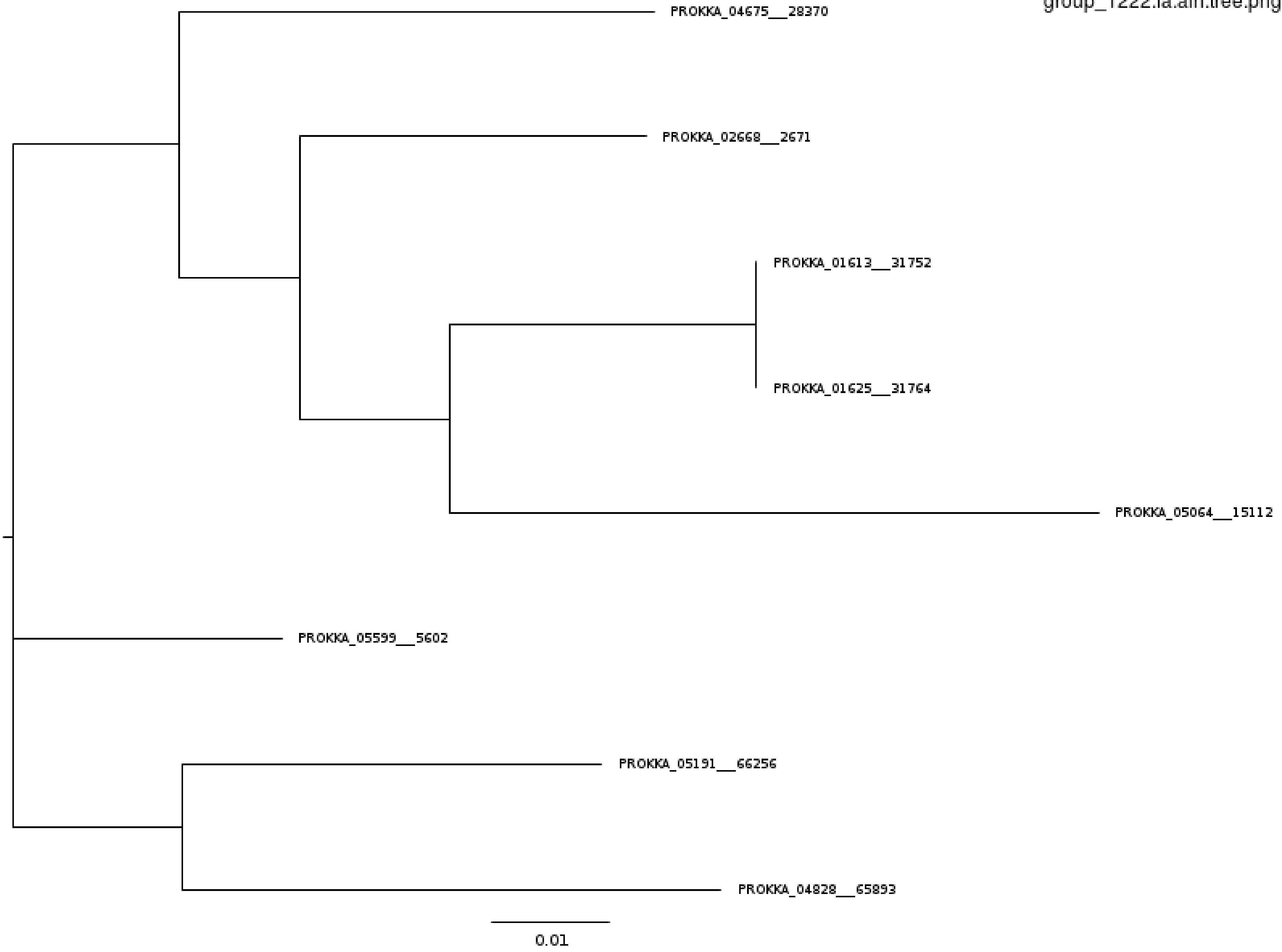

group_1224.fa.aln.tree.png

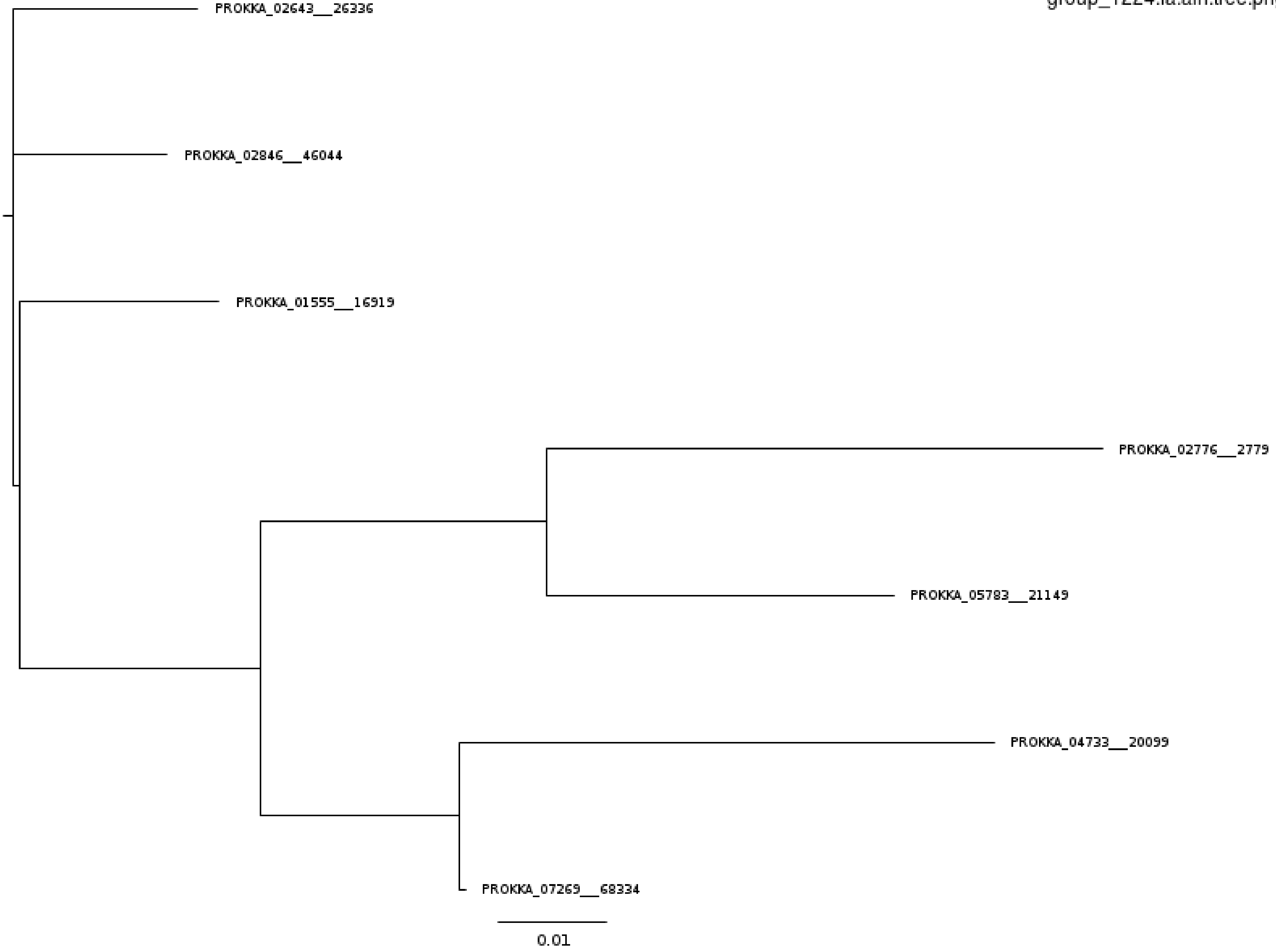

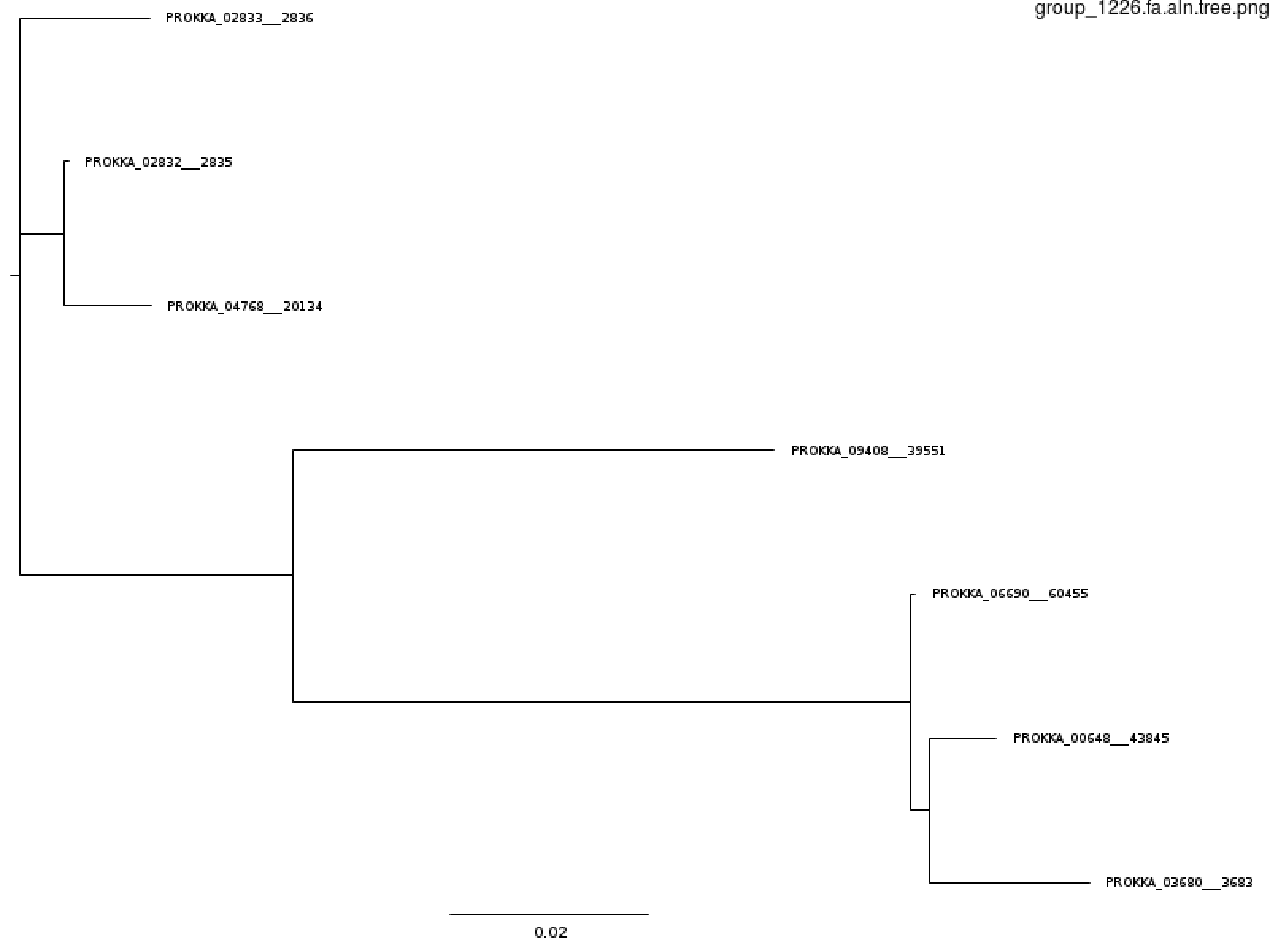
group_1226.fa.aln.tree.png
PROKKA_02833___2836
PROKKA_02832___2835
PROKKA_04768___20134
PROKKA_09408___39551
PROKKA_06690___60455
PROKKA_00648___43845
PROKKA_03680___3683
0.02

group_1228.fa.aln.tree.png

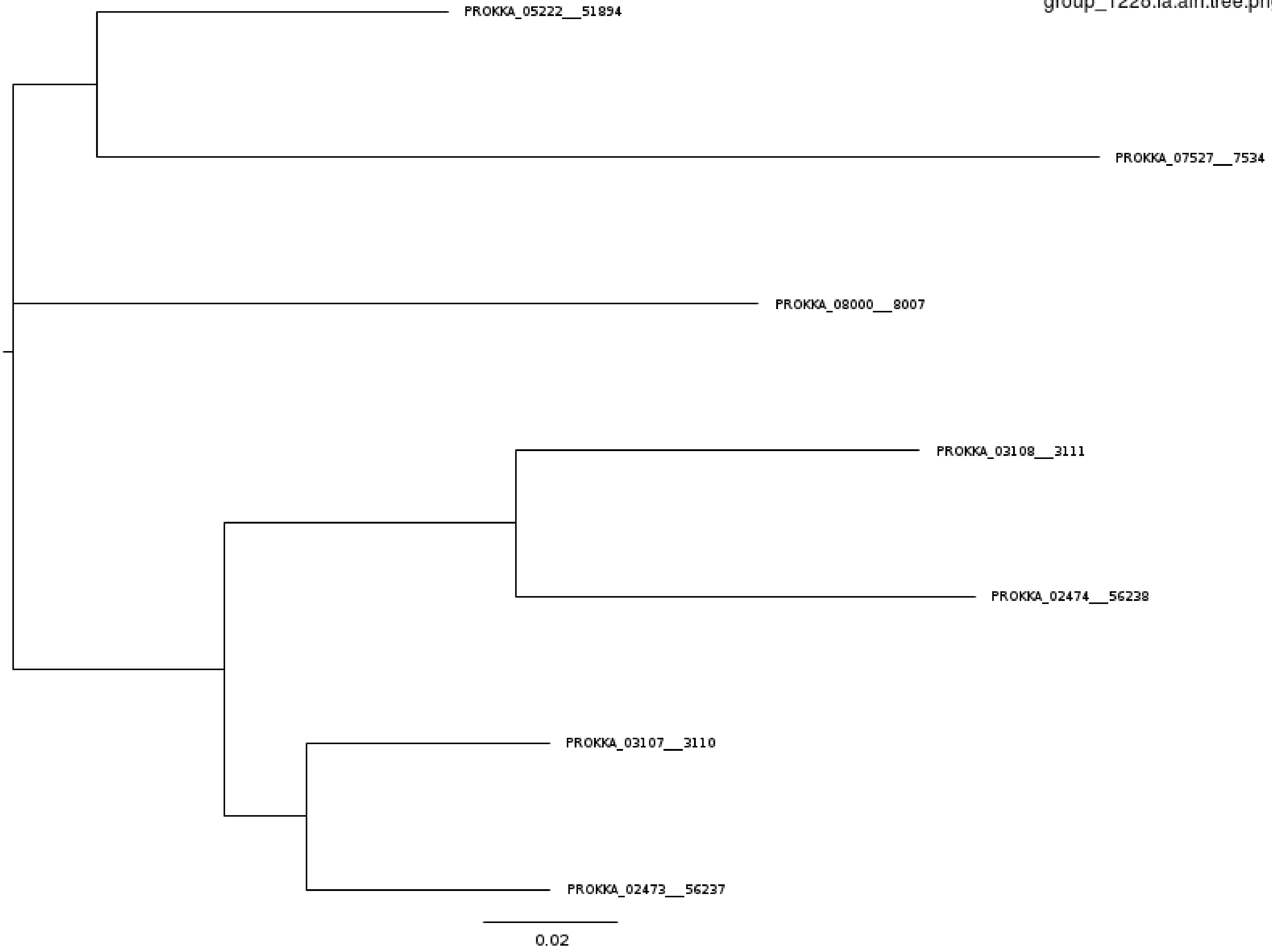

group_1229.fa.aln.tree.png

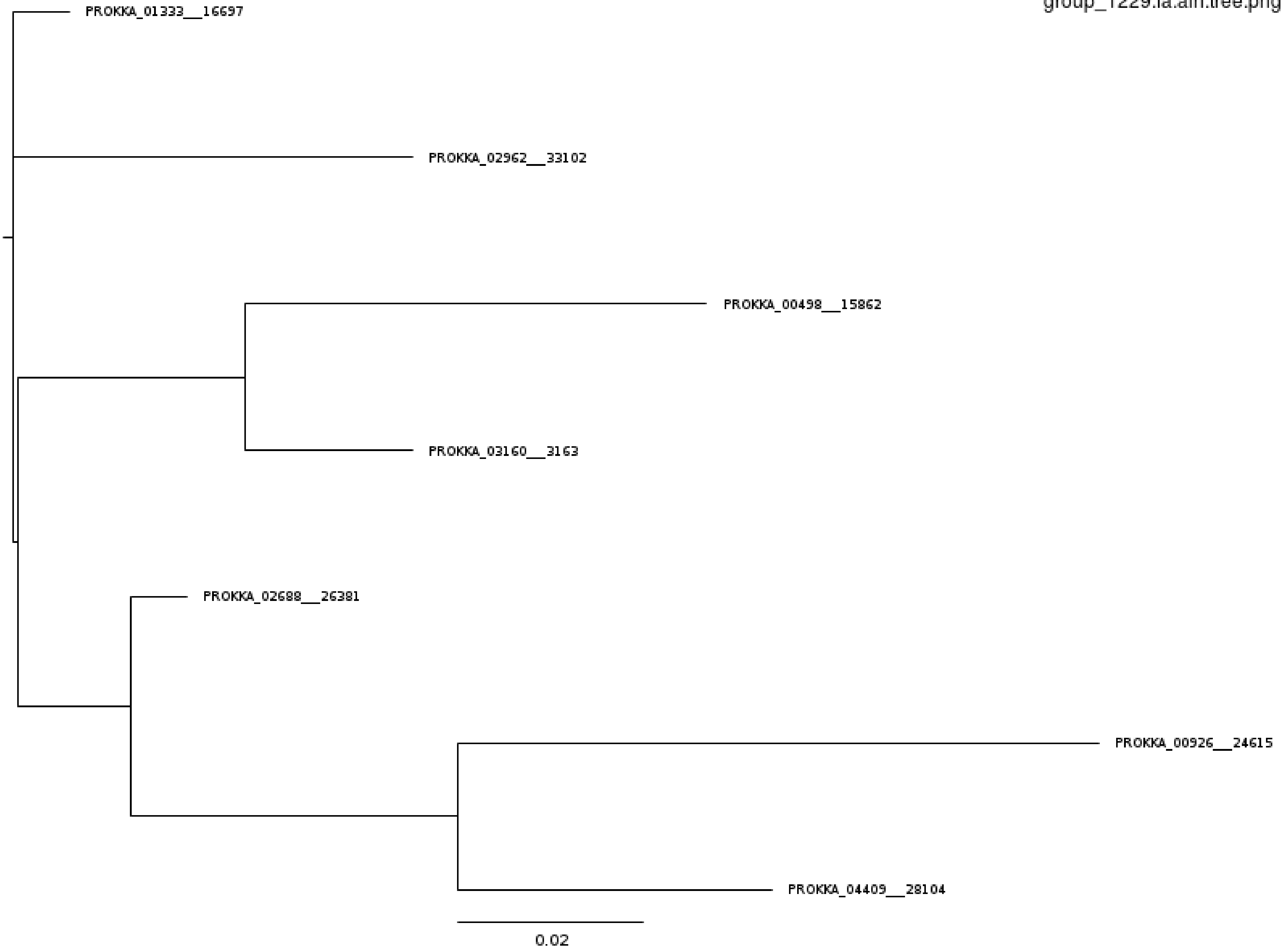

group_122.fa.aln.tree.png

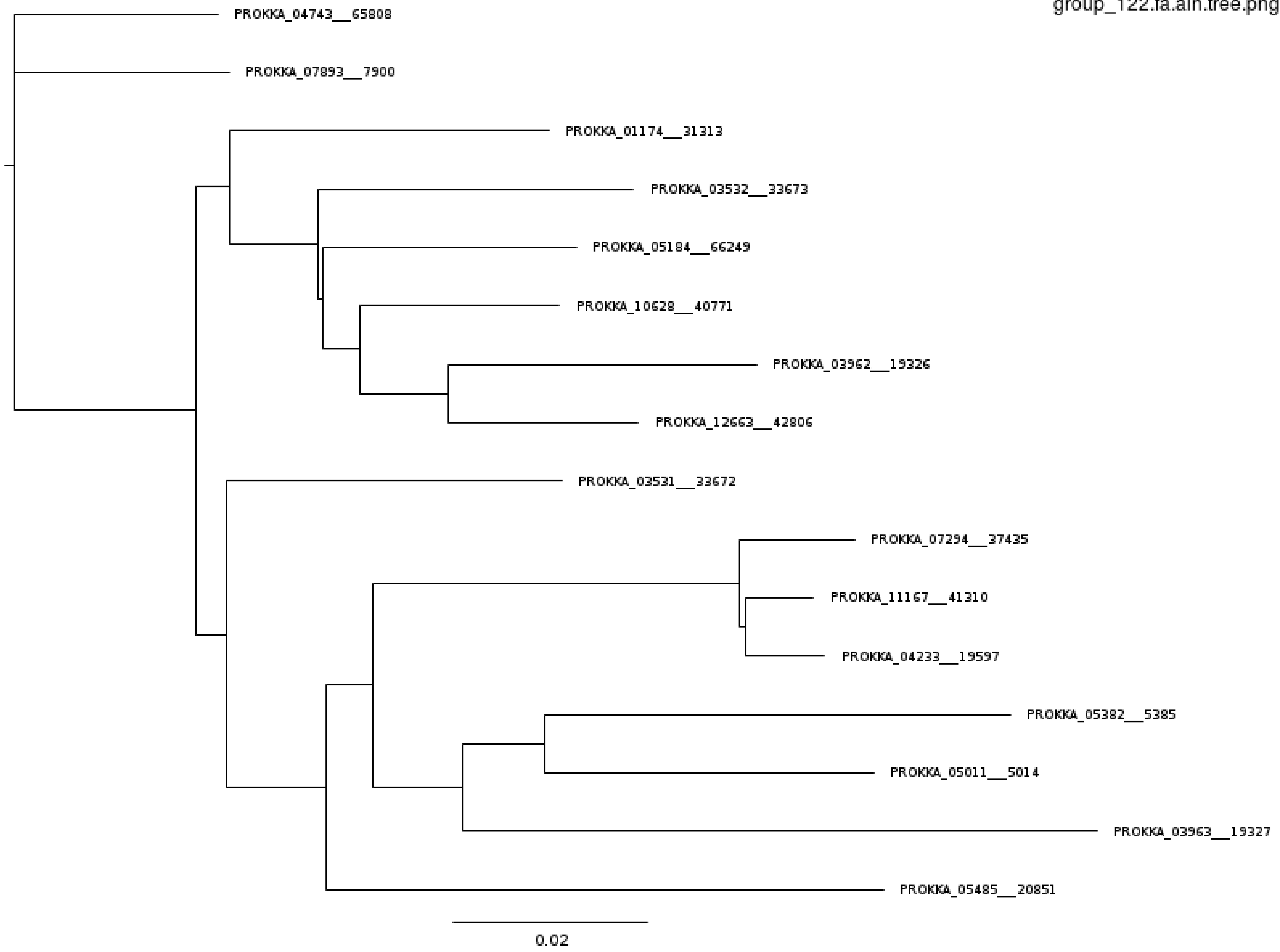

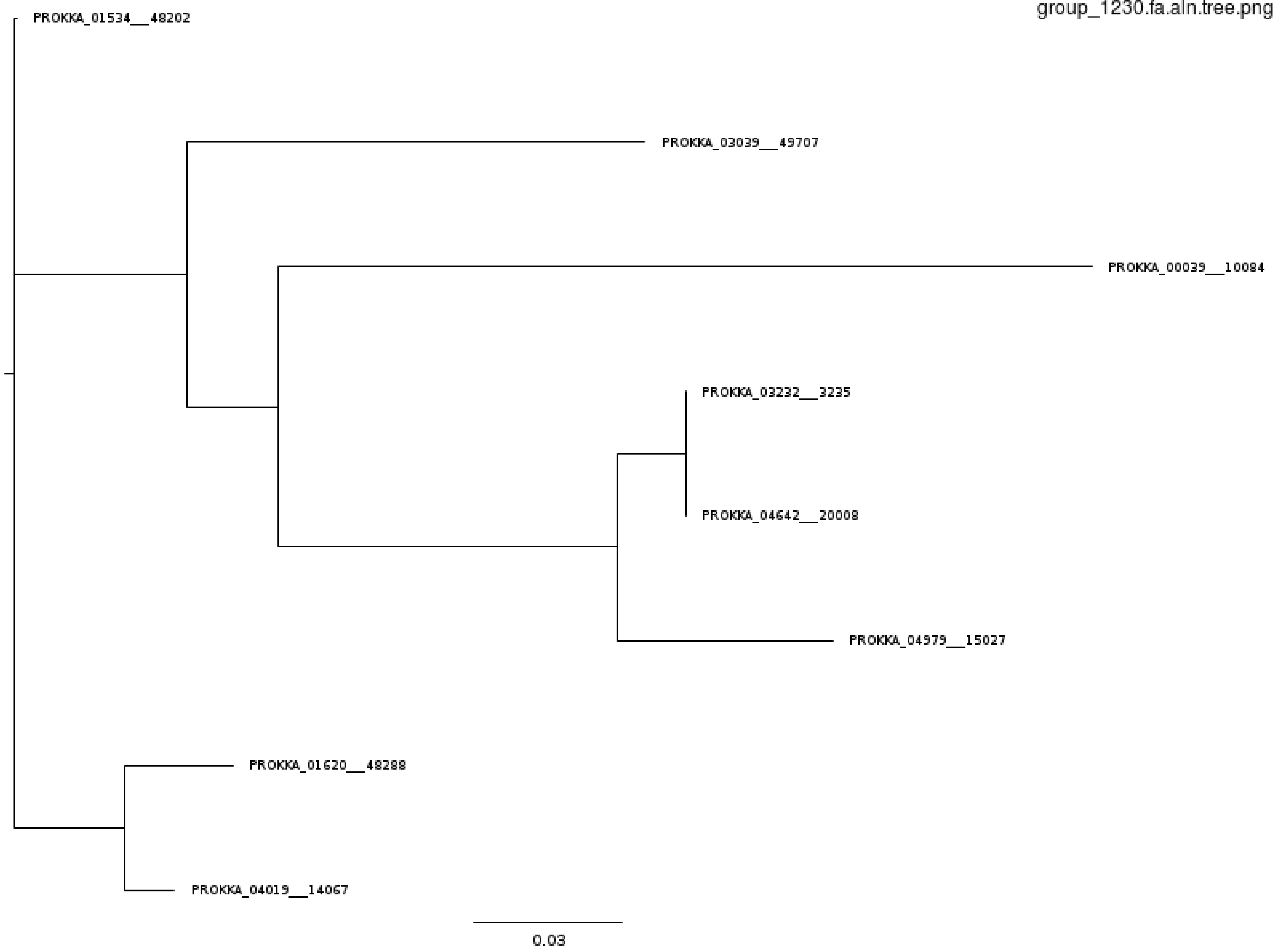
group_1230.fa.aln.tree.png
PROKKA_01534__48202
PROKKA_03039__49707
PROKKA_00039__10084
PROKKA_03232__3235
PROKKA_04642__20008
PROKKA_04979__15027
PROKKA_01620__48288
PROKKA_04019__14067
0.03

group_1231.fa.aln.tree.png

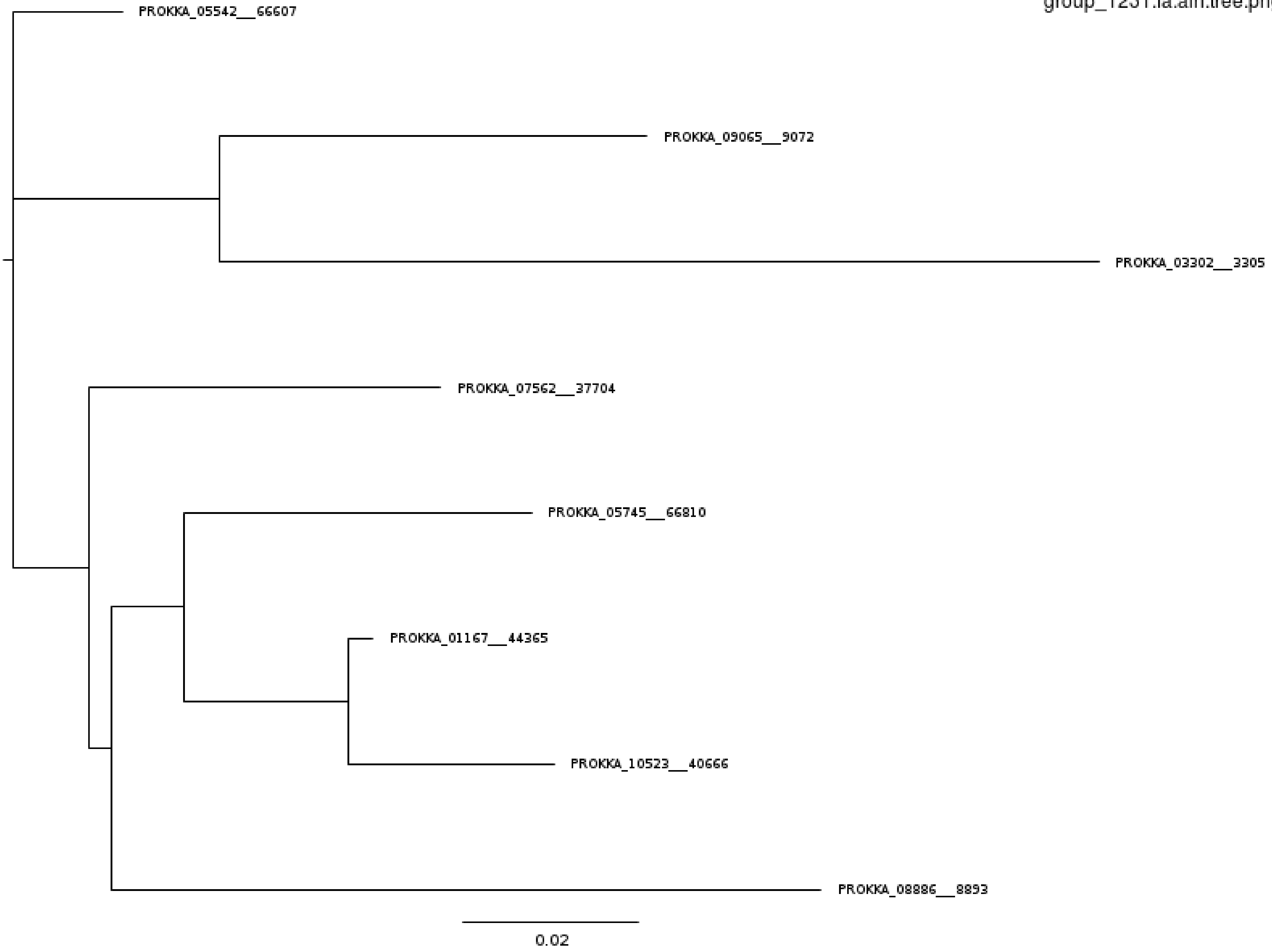

group_1233.fa.aln.tree.png

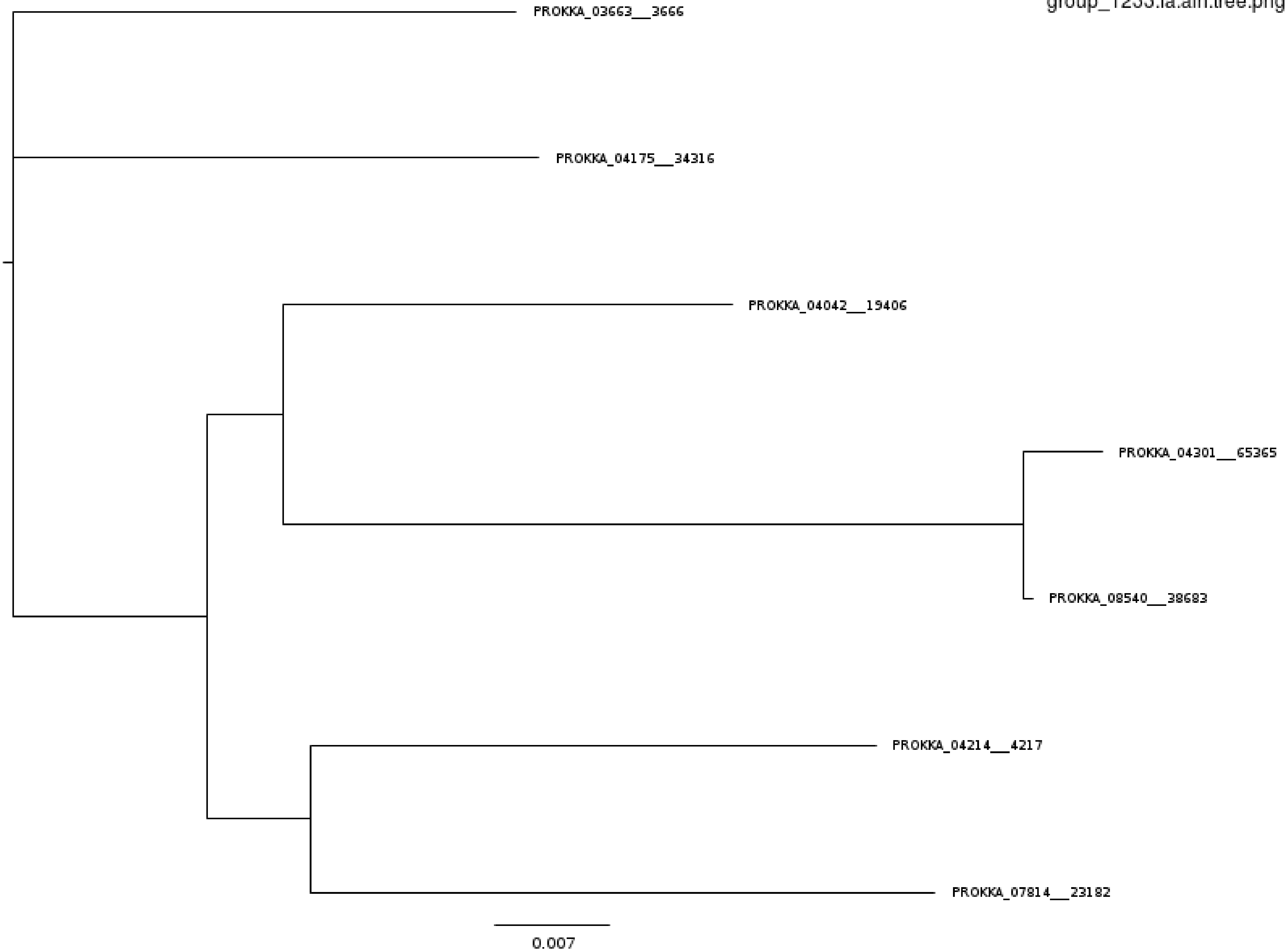

group_1237.fa.aln.tree.png

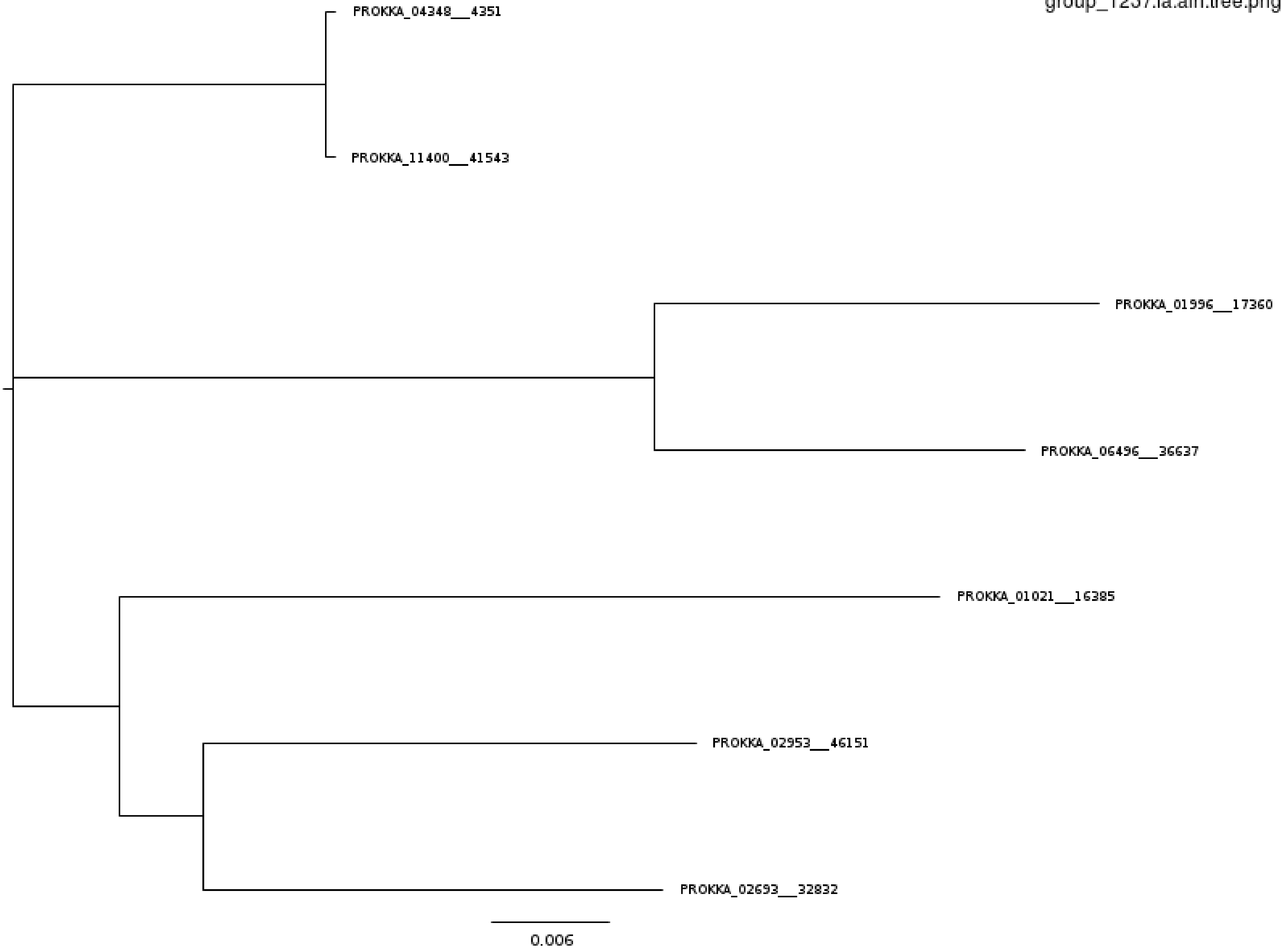

group_1238.fa.aln.tree.png

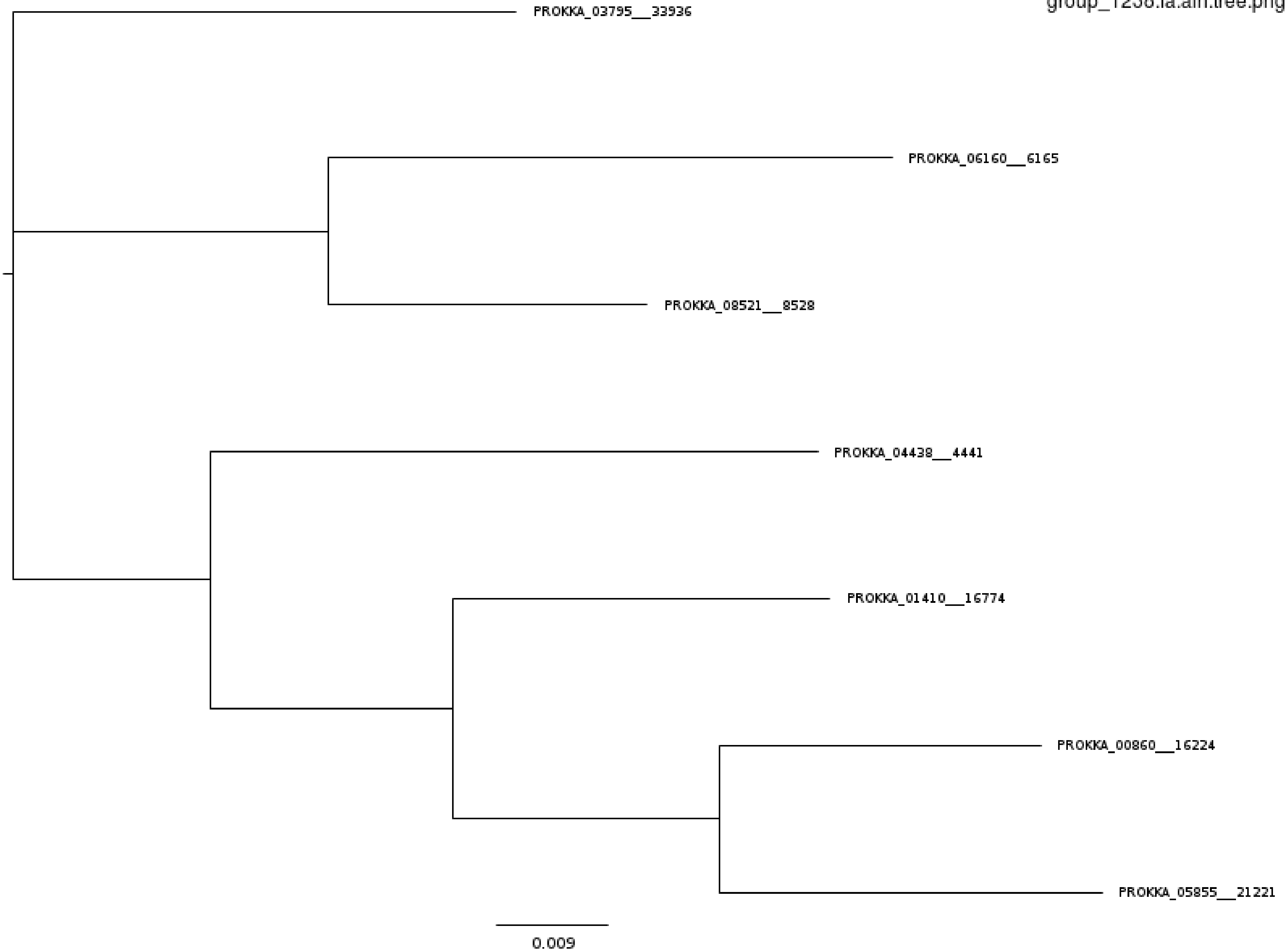

group_123.fa.aln.tree.png

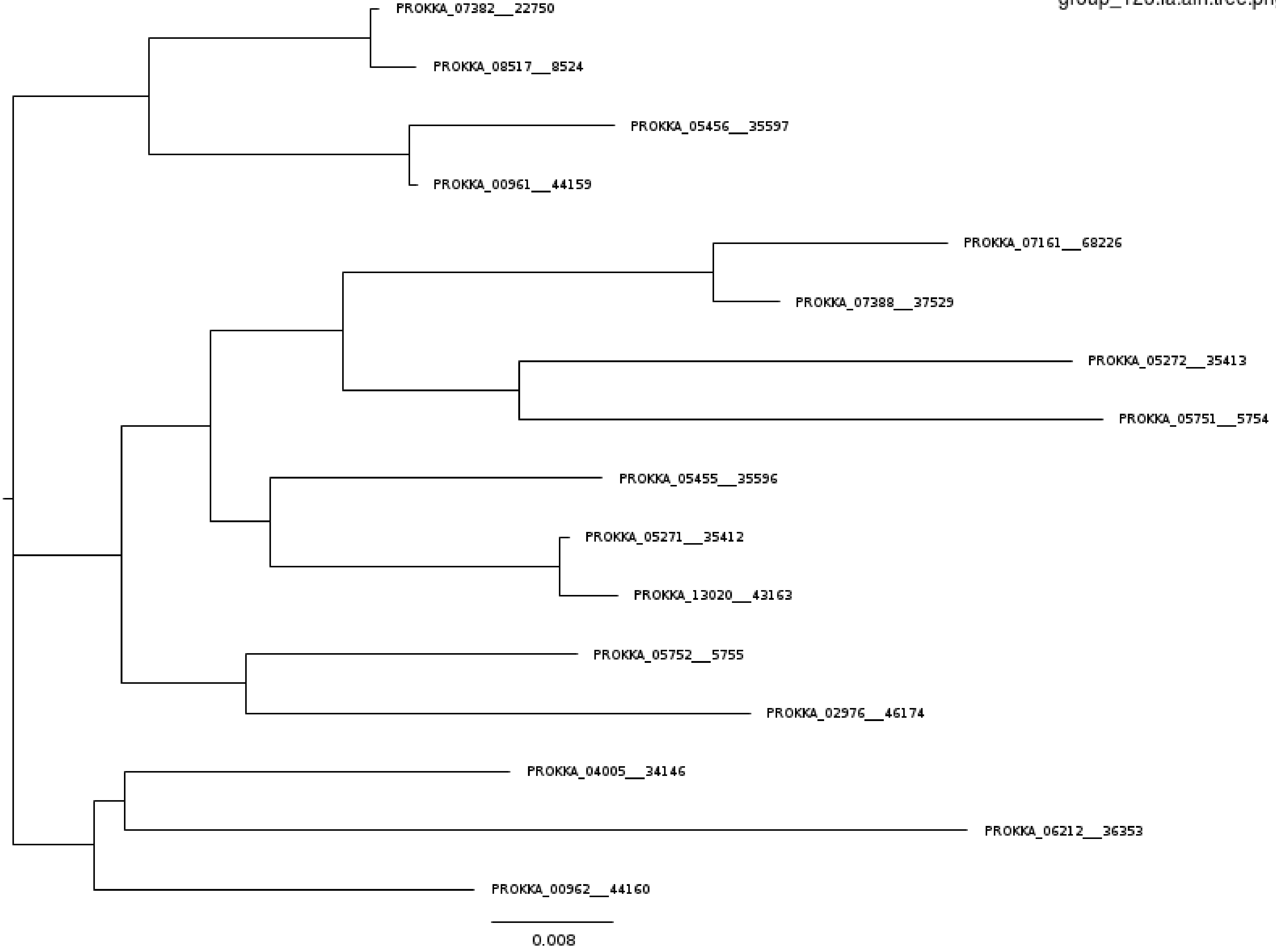

group_1240.fa.aln.tree.png

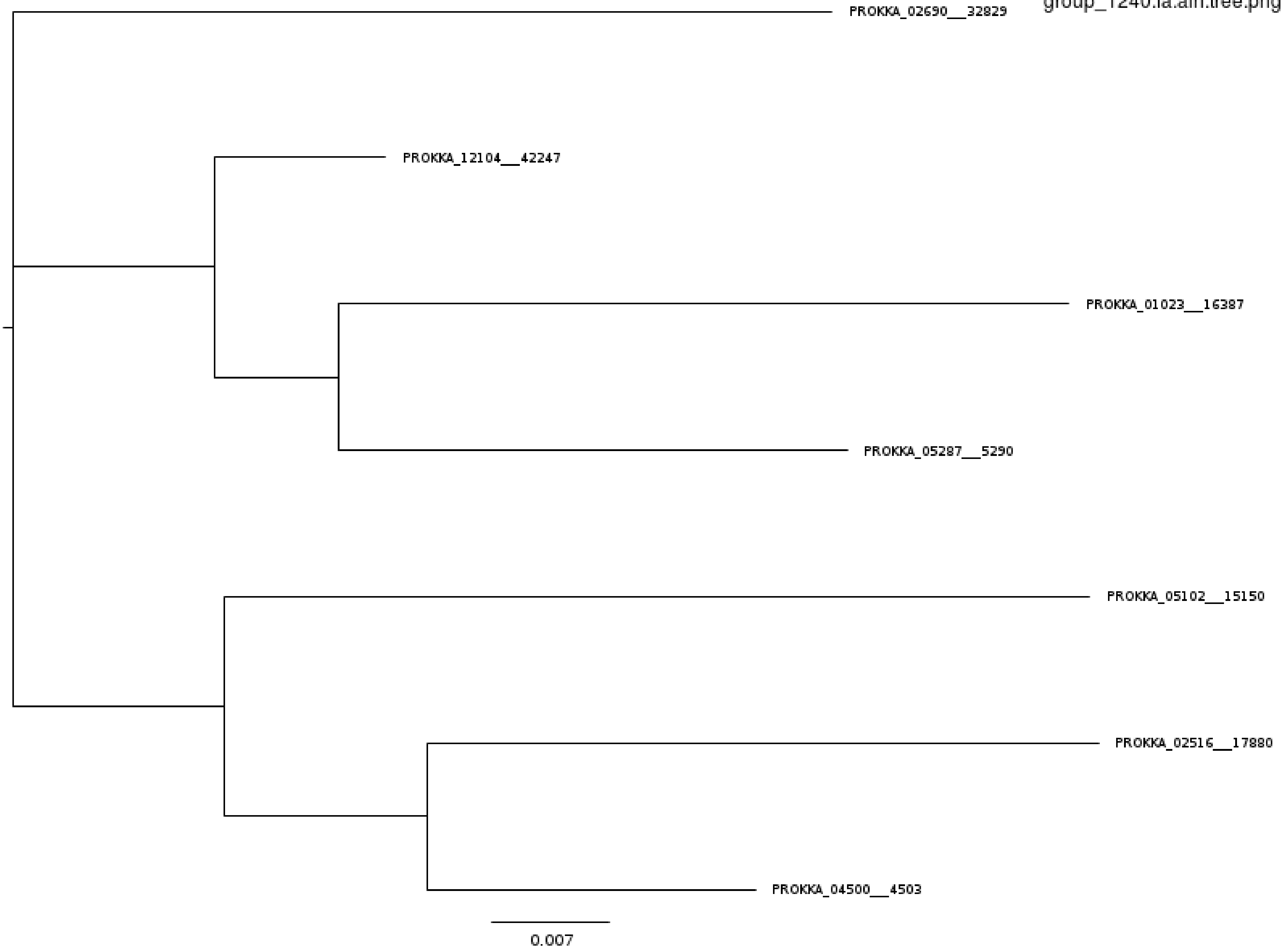

group_1247.fa.aln.tree.png

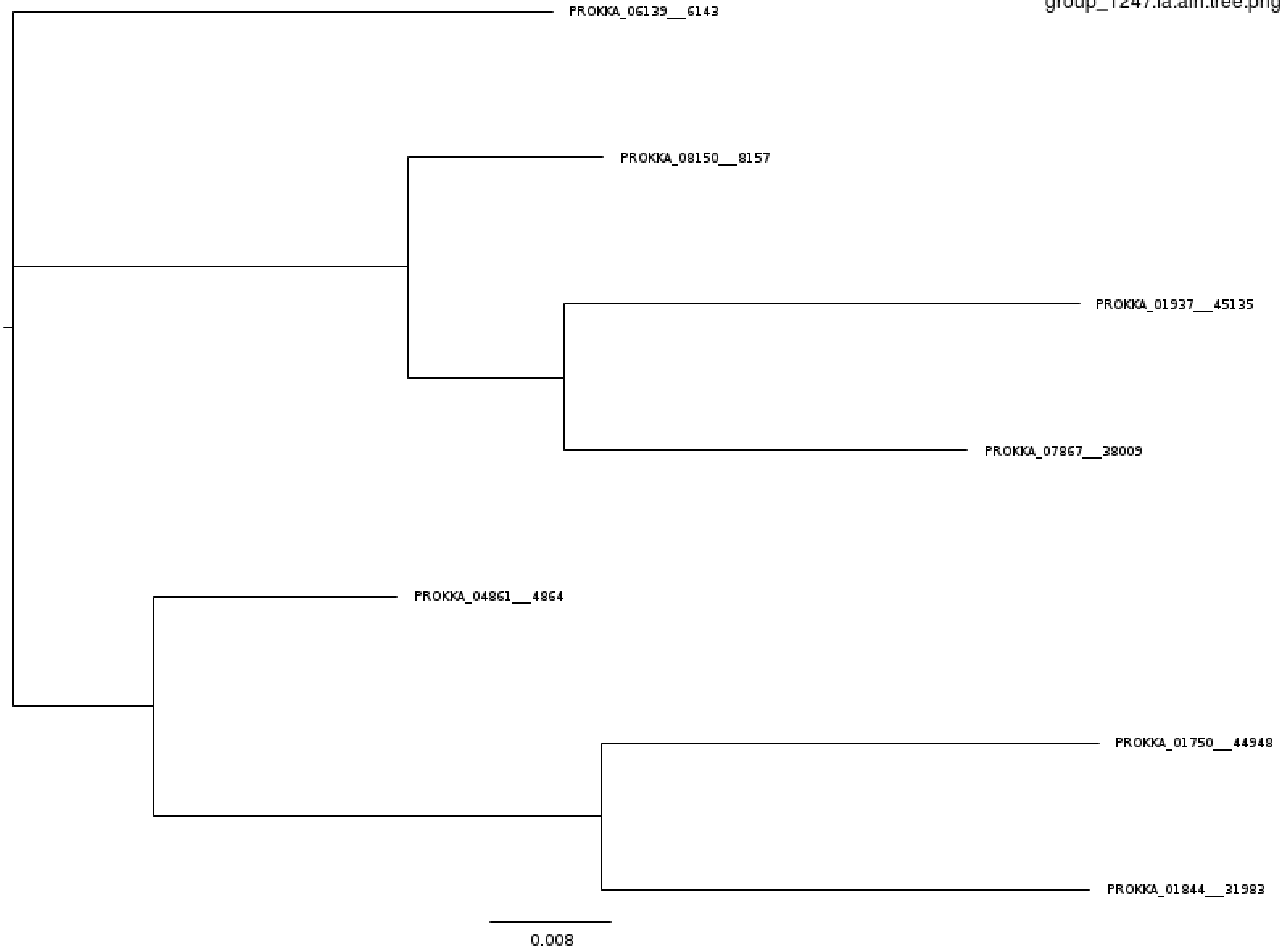

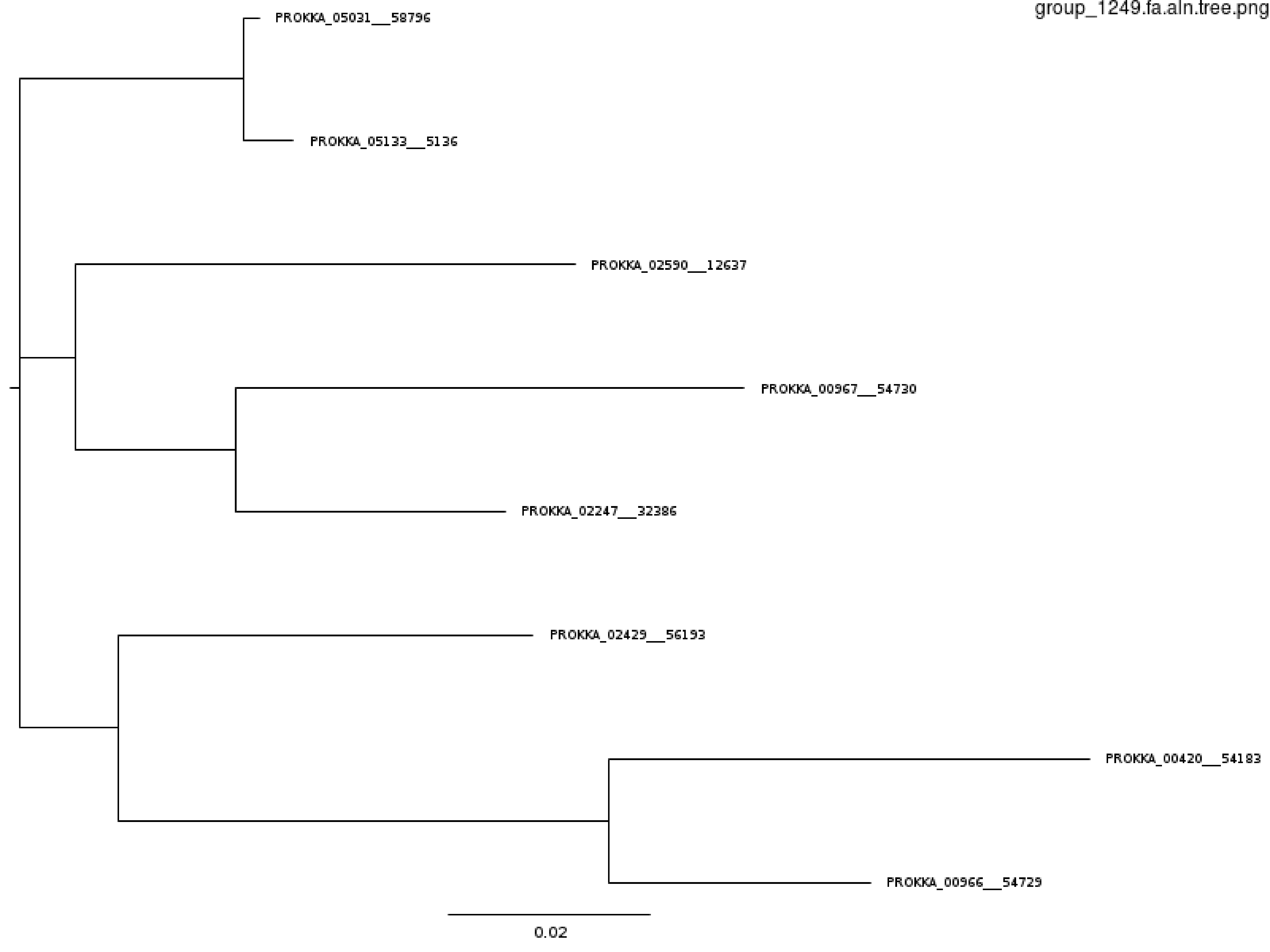
group_1249.fa.aln.tree.png
PROKKA_05031___58796
PROKKA_05133___5136
PROKKA_02590___12637
PROKKA_00967___54730
PROKKA_02247___32386
PROKKA_02429___56193
PROKKA_00420___54183
PROKKA_00966___54729
0.02

group_1250.fa.aln.tree.png

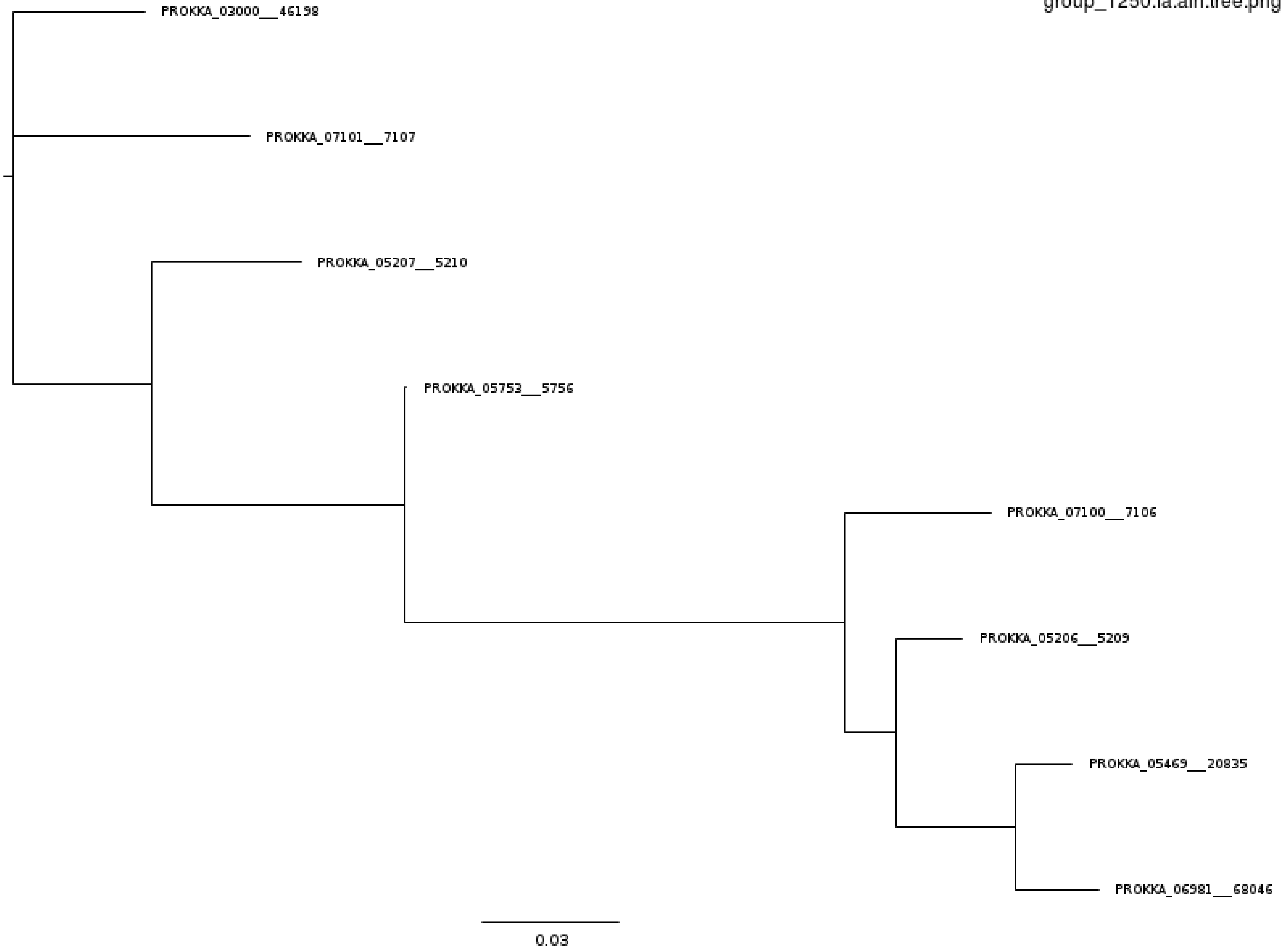

group_1252.fa.aln.tree.png

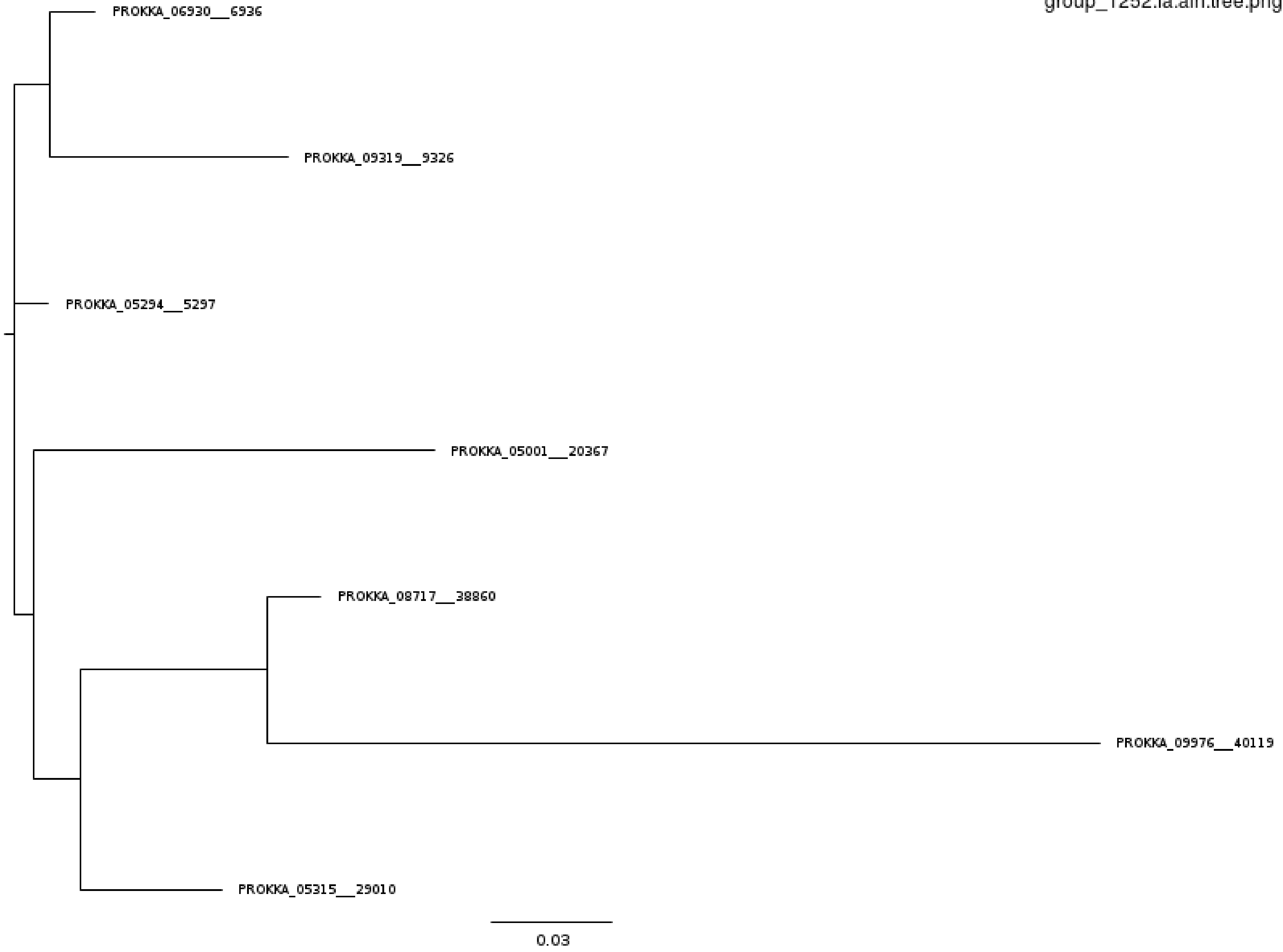

group_1253.fa.aln.tree.png

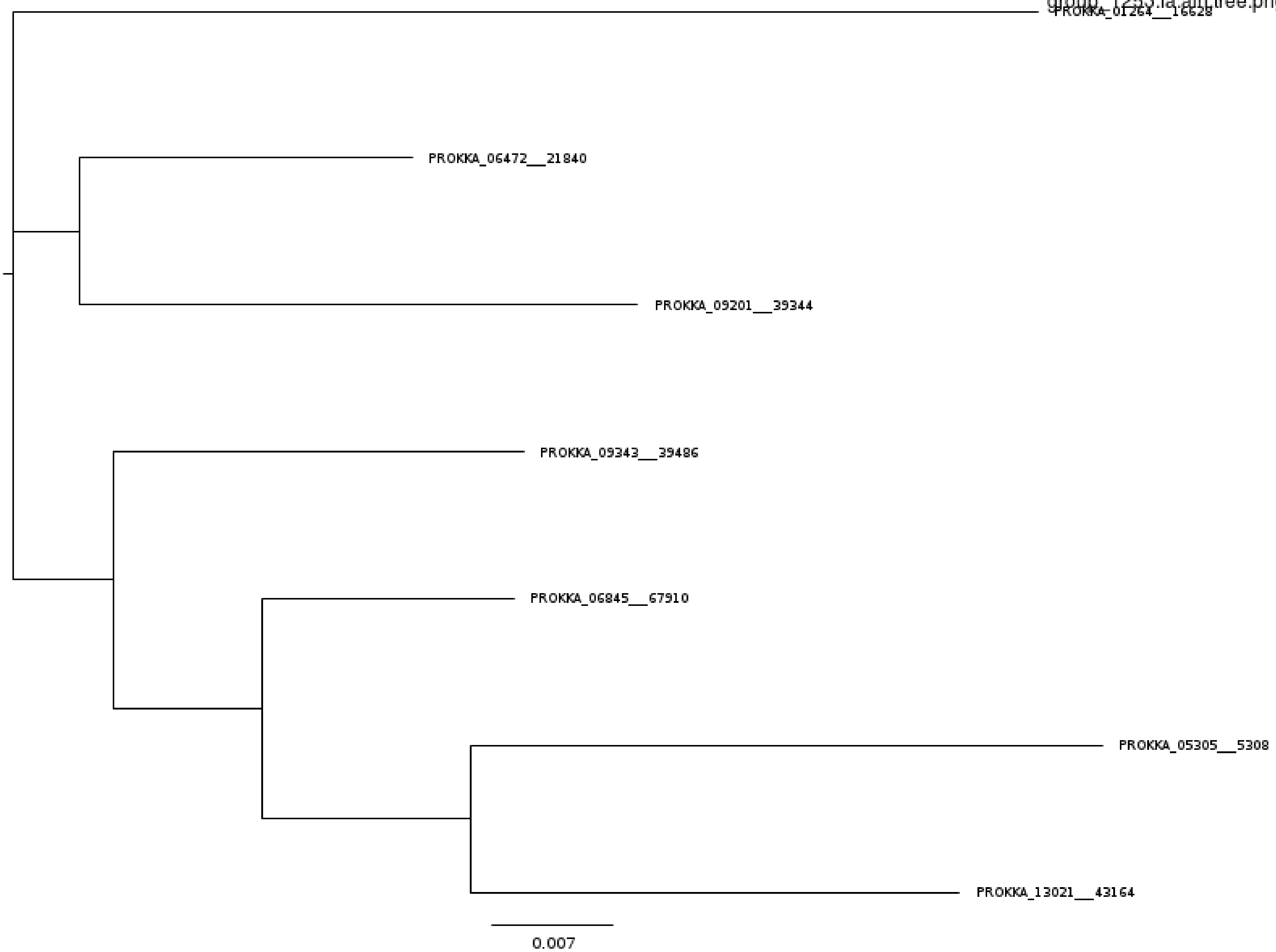

group_1255.fa.aln.tree.png

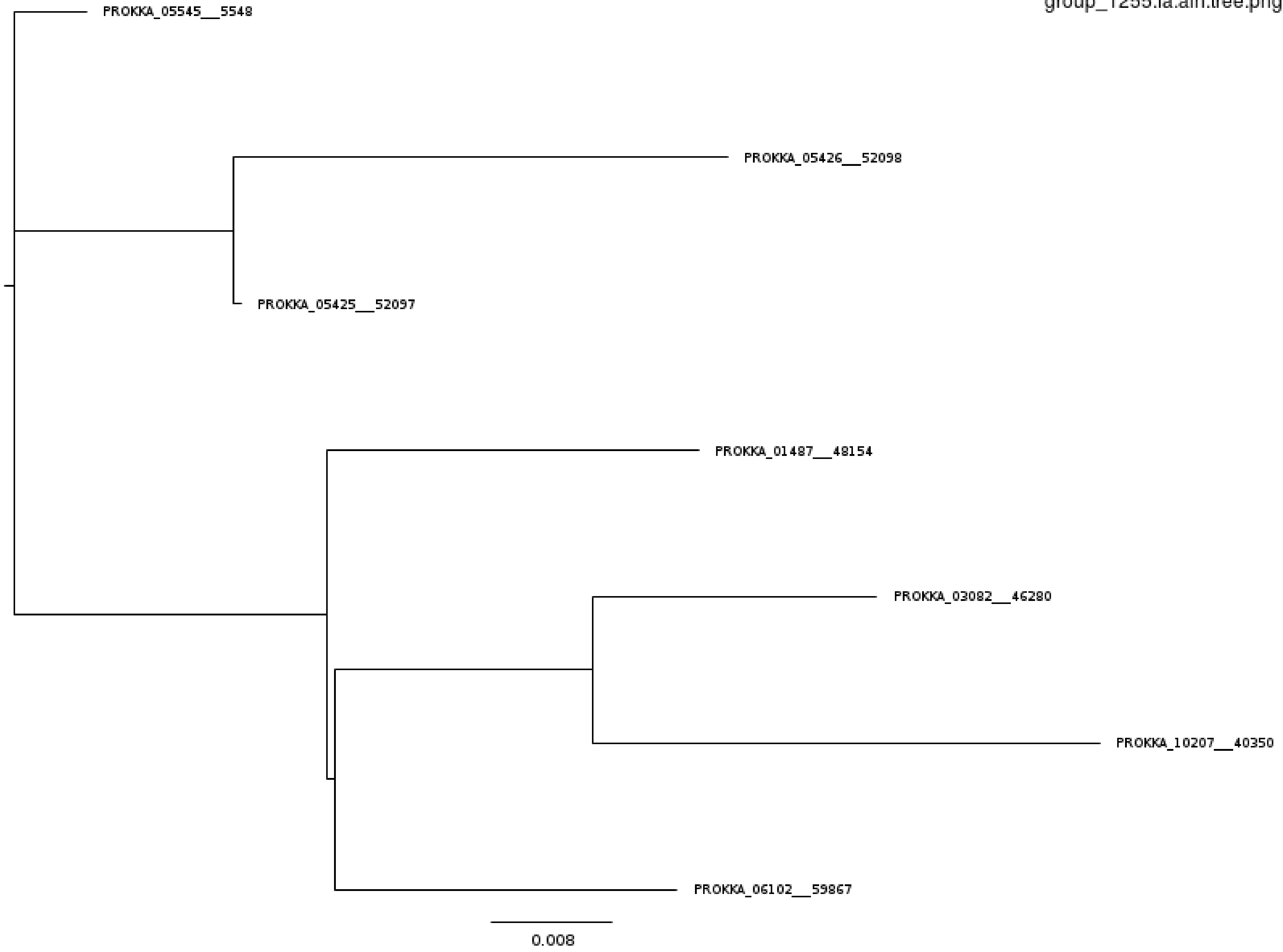

group_1257.fa.aln.tree.png

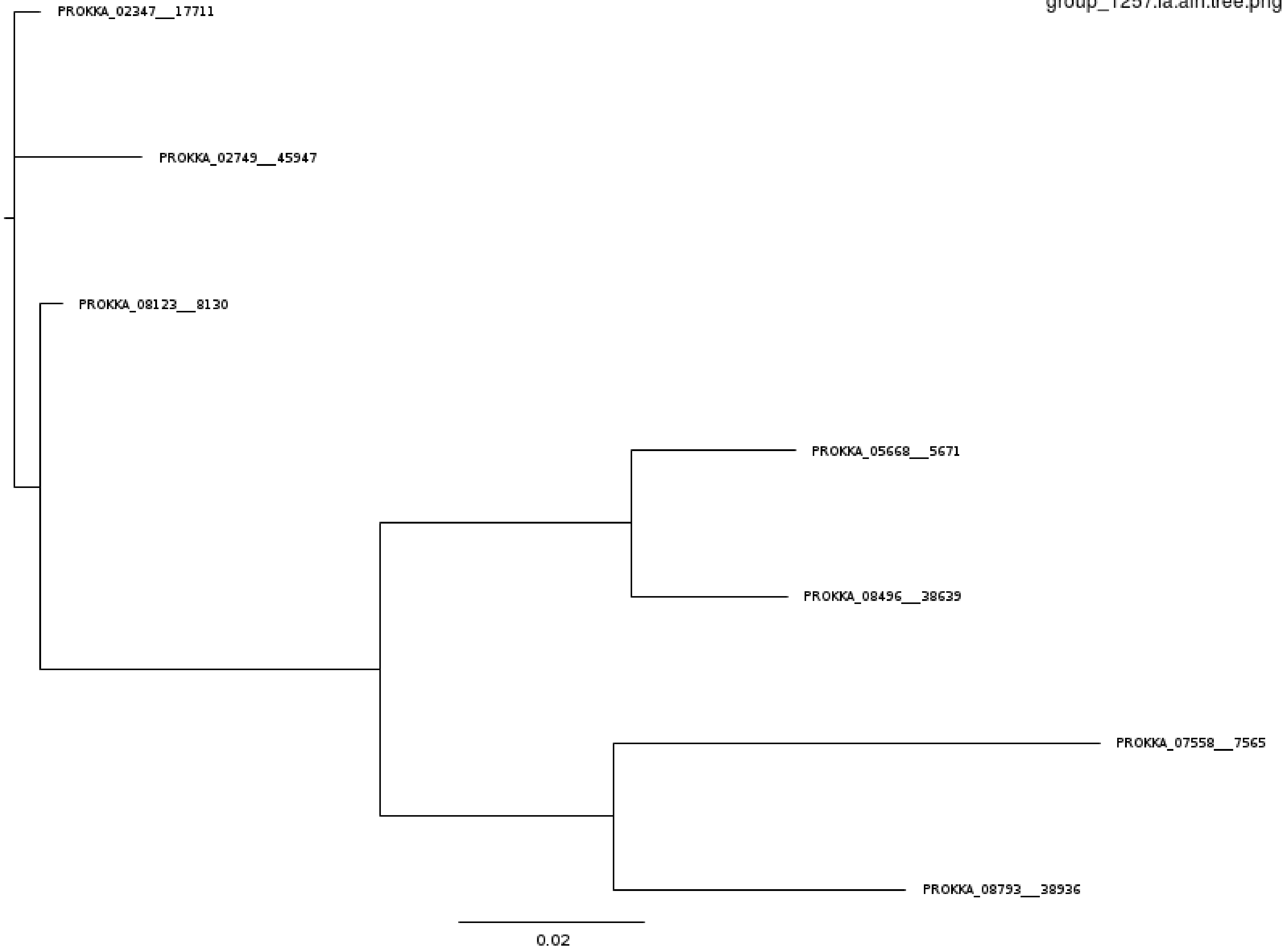

group_1259.fa.aln.tree.png

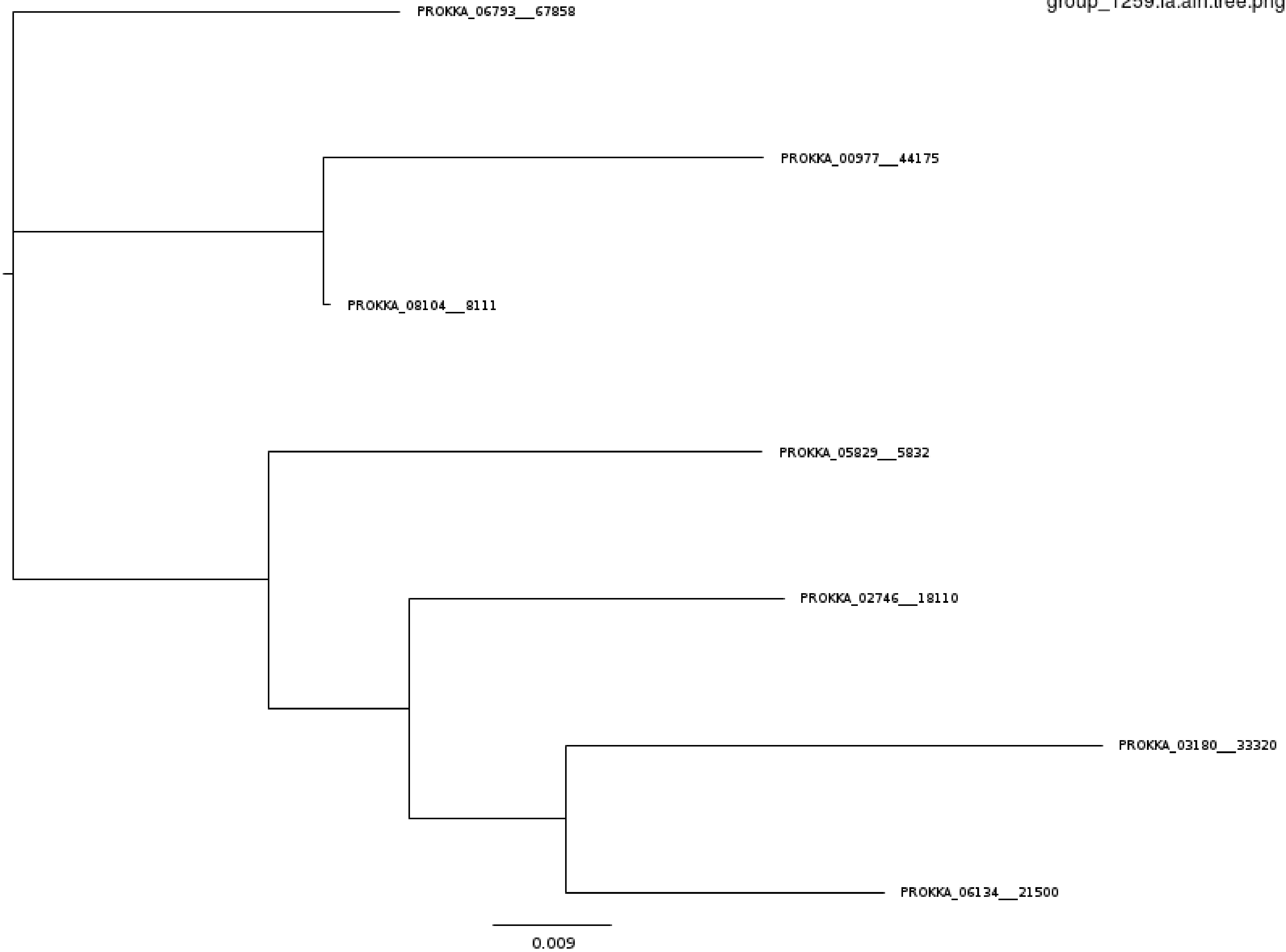

group_1261.fa.aln.tree.png

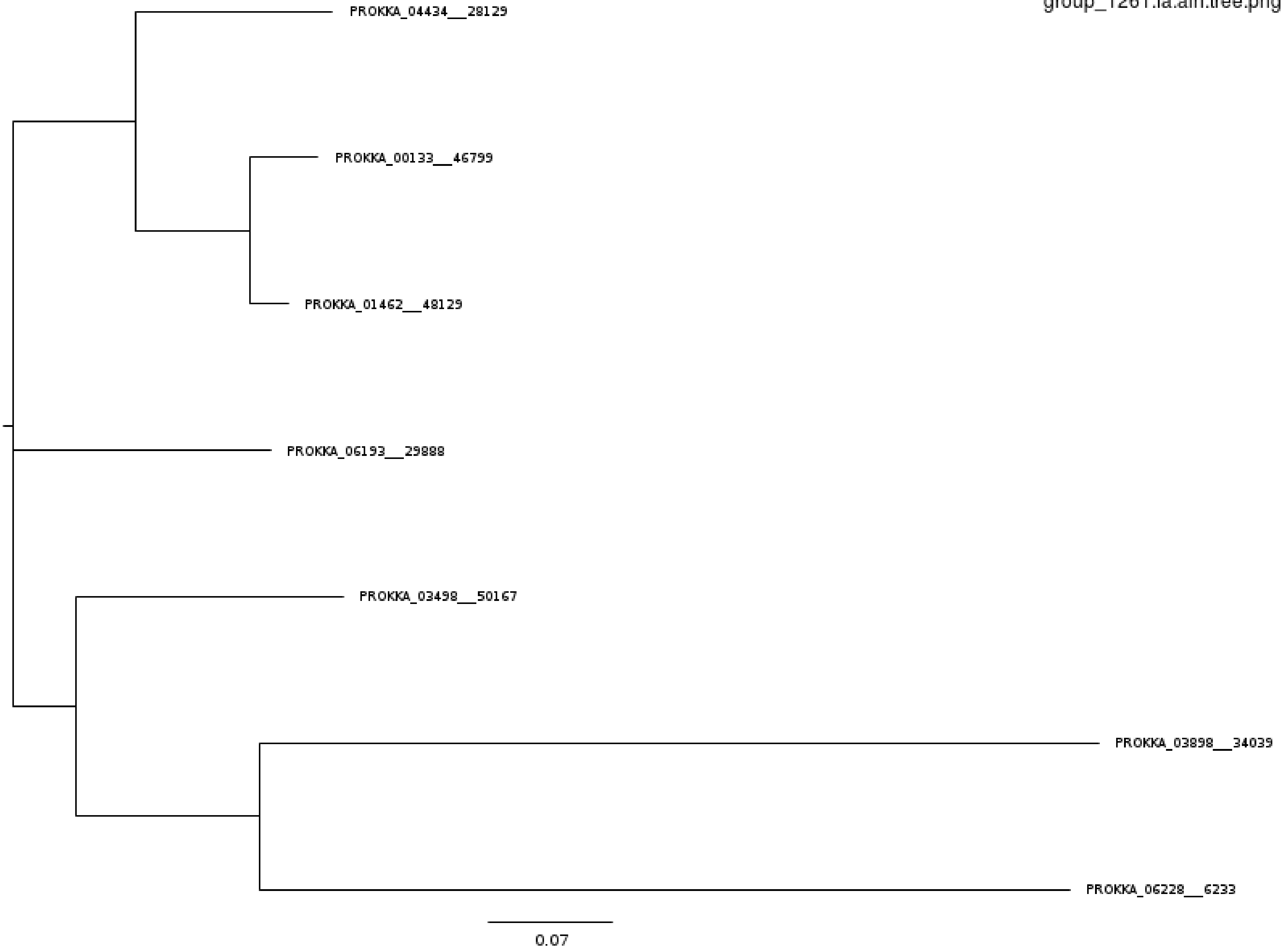

group_1263.fa.aln.tree.png

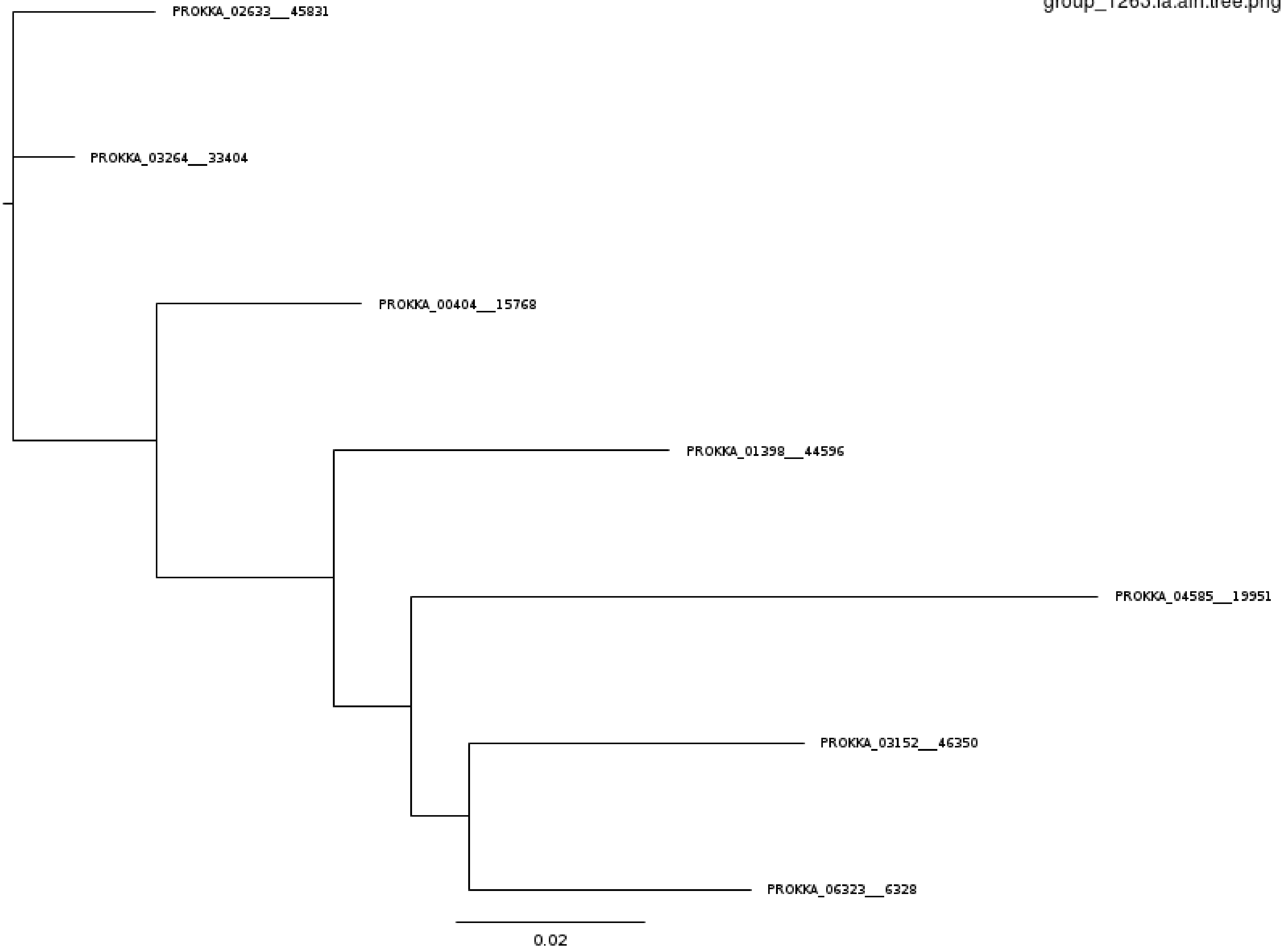

group_1264.fa.aln.tree.png

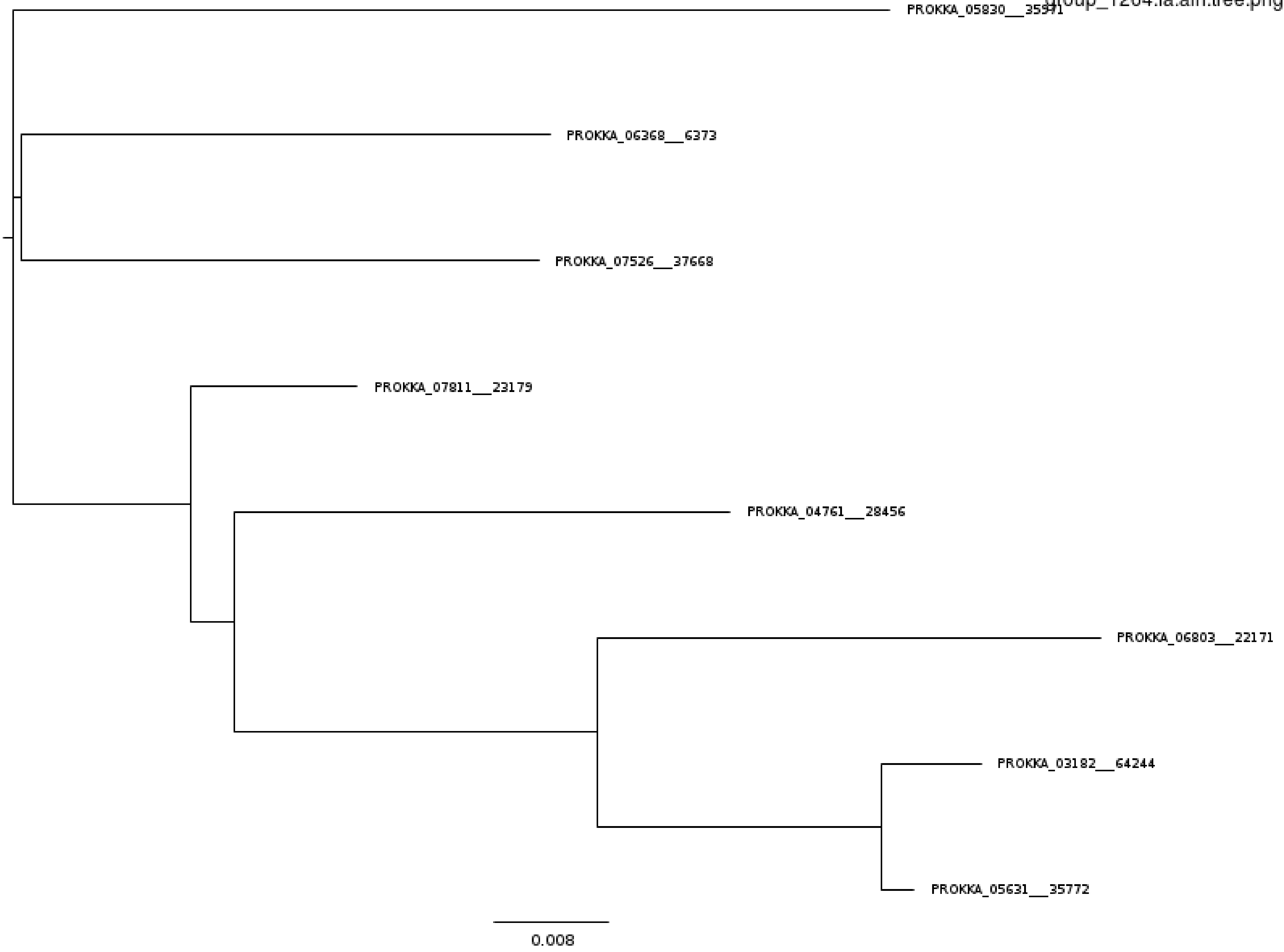

group_1265.fa.aln.tree.png

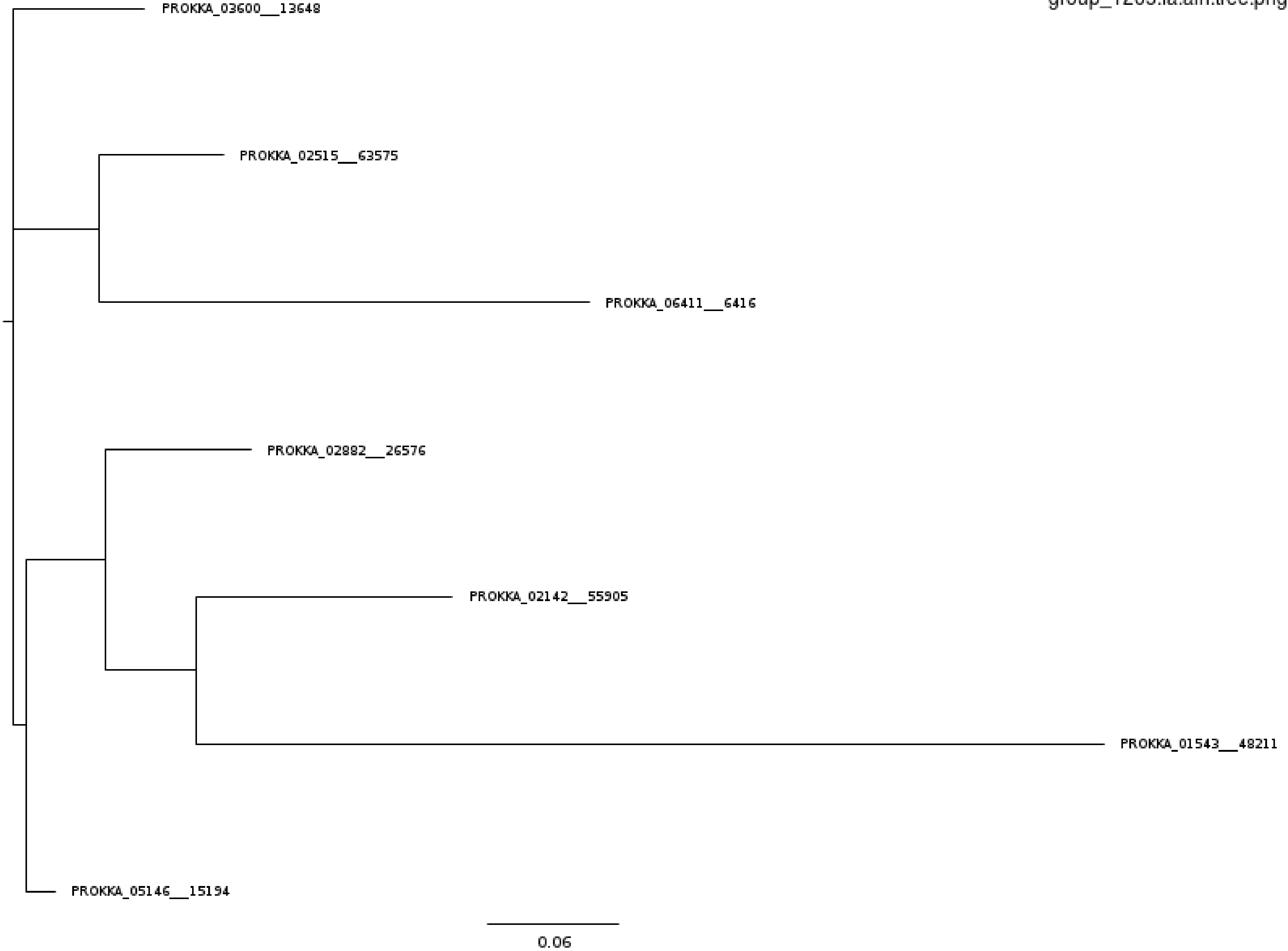

group_1266.fa.aln.tree.png

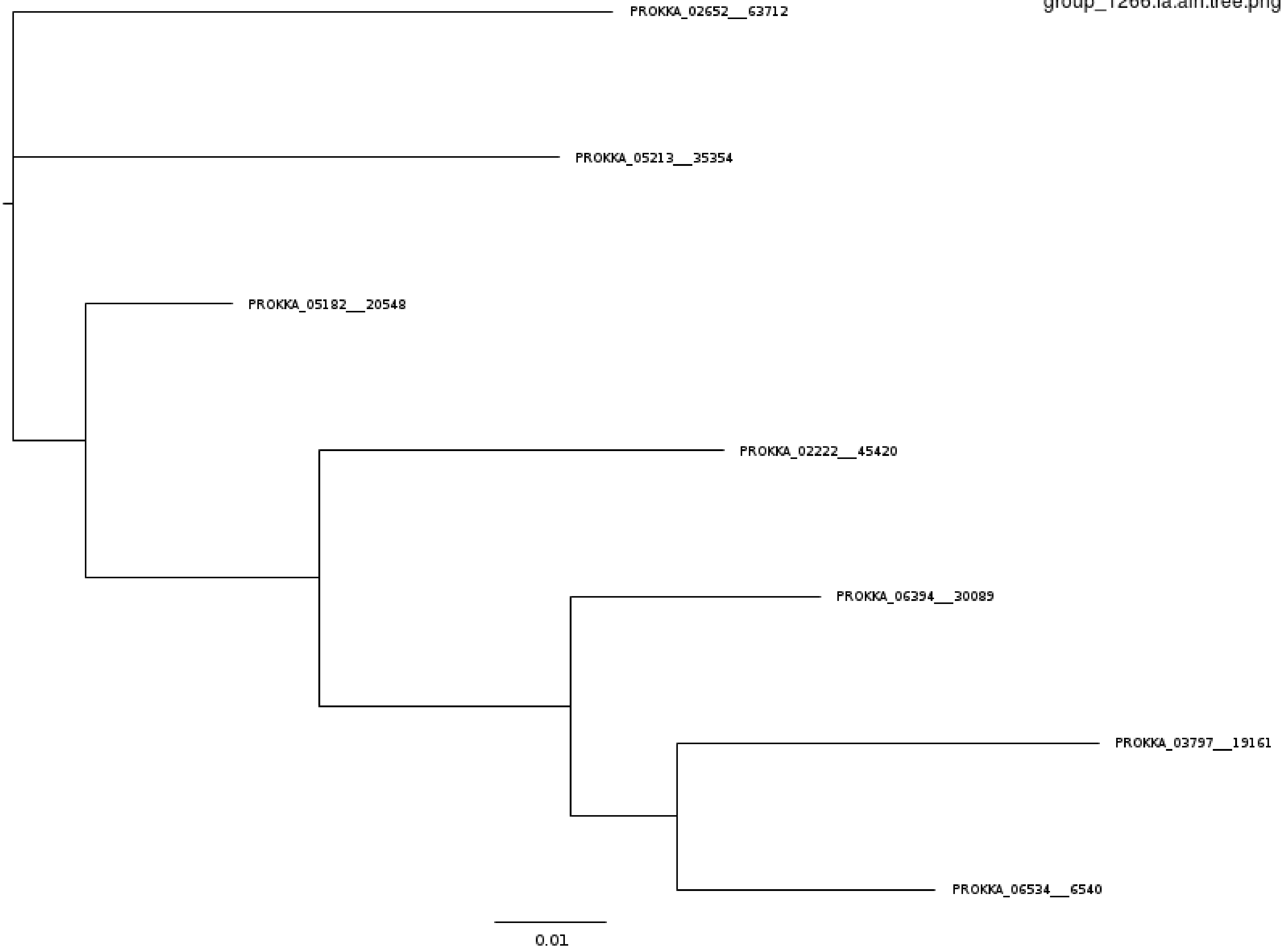

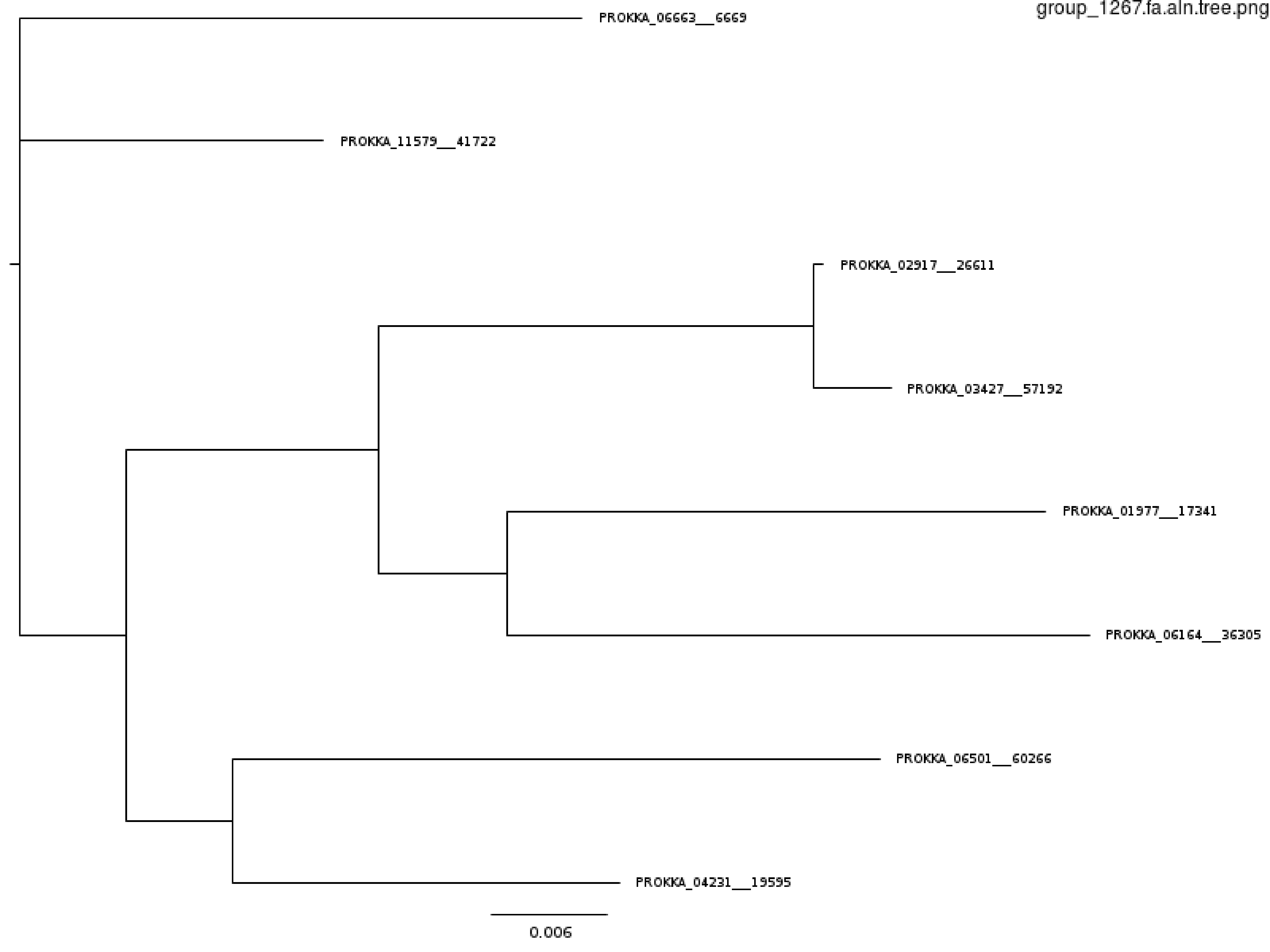

group_1267.fa.aln.tree.png
PROKKA_06663___6669
PROKKA_11579___41722
PROKKA_02917___26611
PROKKA_03427___57192
PROKKA_01977___17341
PROKKA_06164___36305
PROKKA_06501___60266
PROKKA_04231___19595
0.006

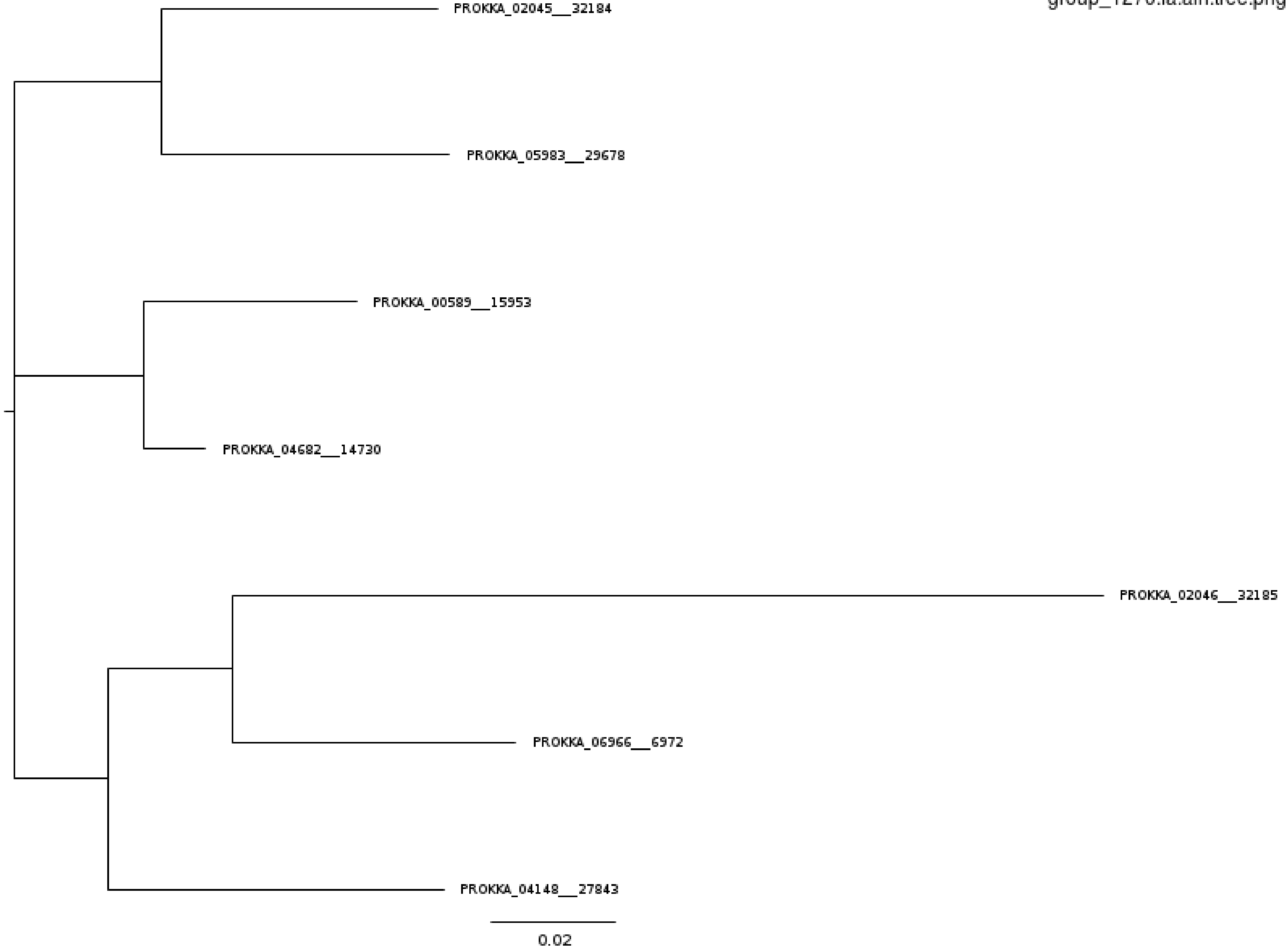
PROKKA_02045__32184
PROKKA_05983__29678
PROKKA_00589__15953
PROKKA_04682__14730
PROKKA_02046__32185
PROKKA_06966__6972
PROKKA_04148__27843
0.02

group_1276.fa.aln.tree.png

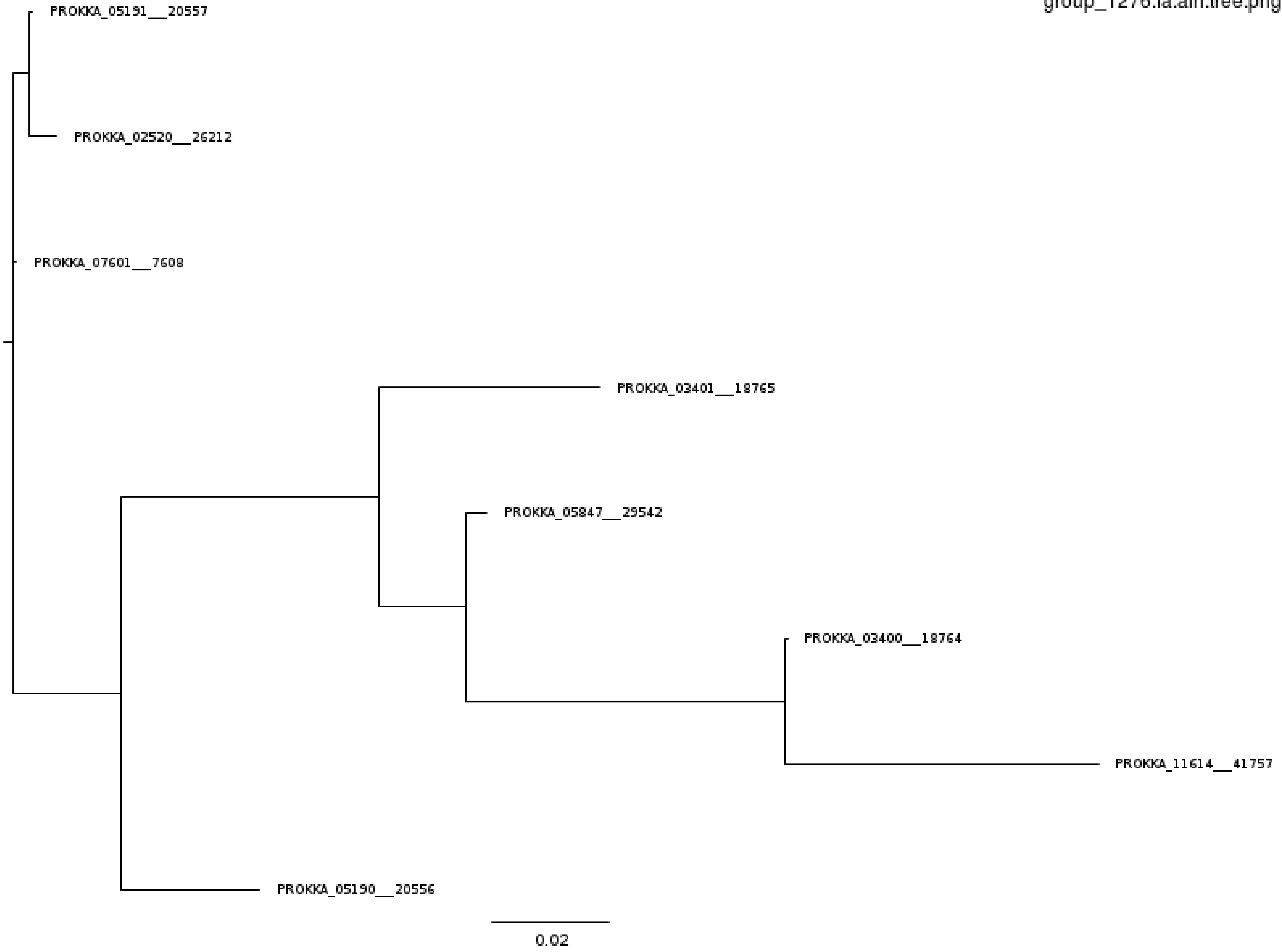

group_1277.fa.aln.tree.png

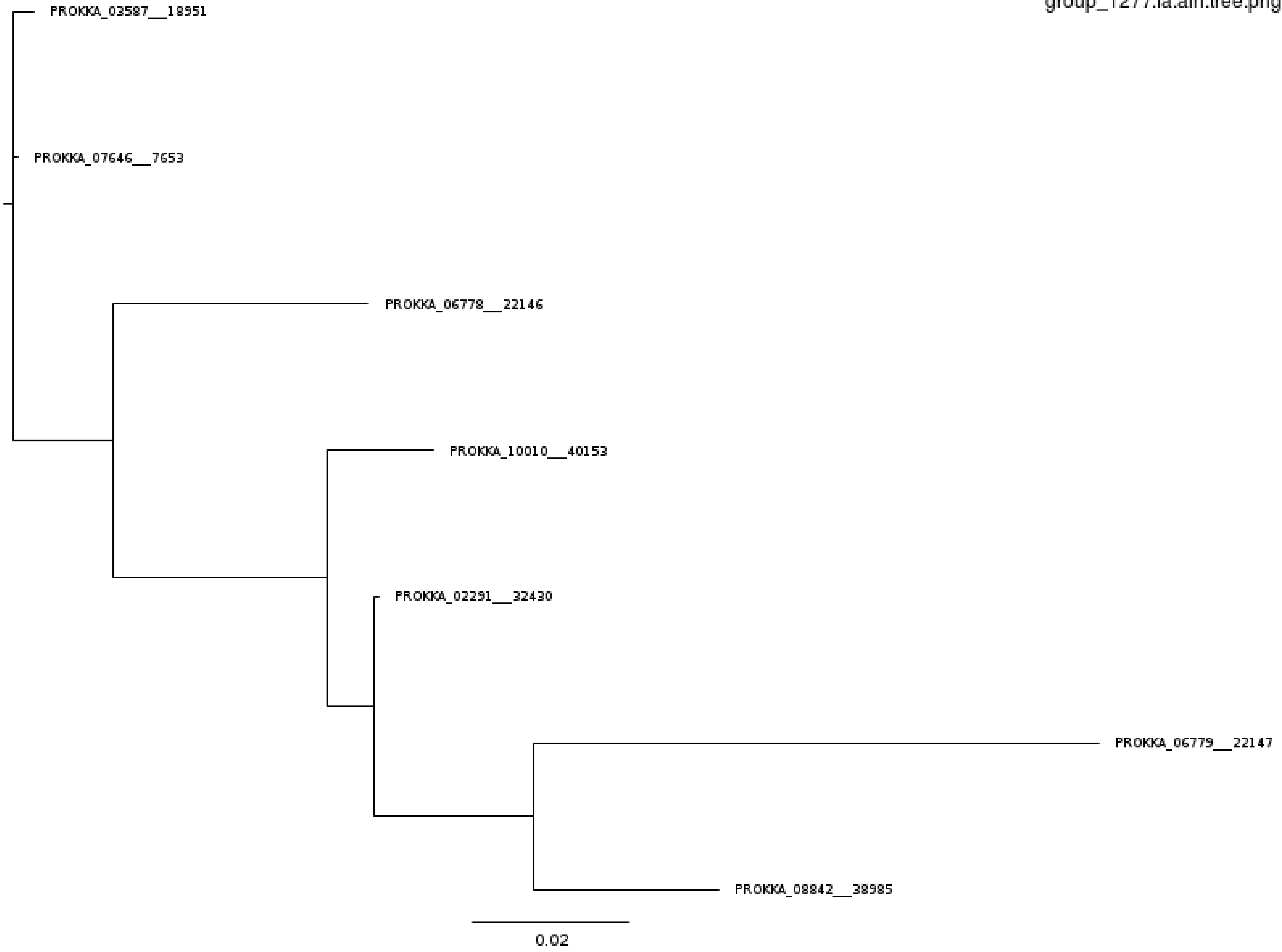

group_1278.fa.aln.tree.png

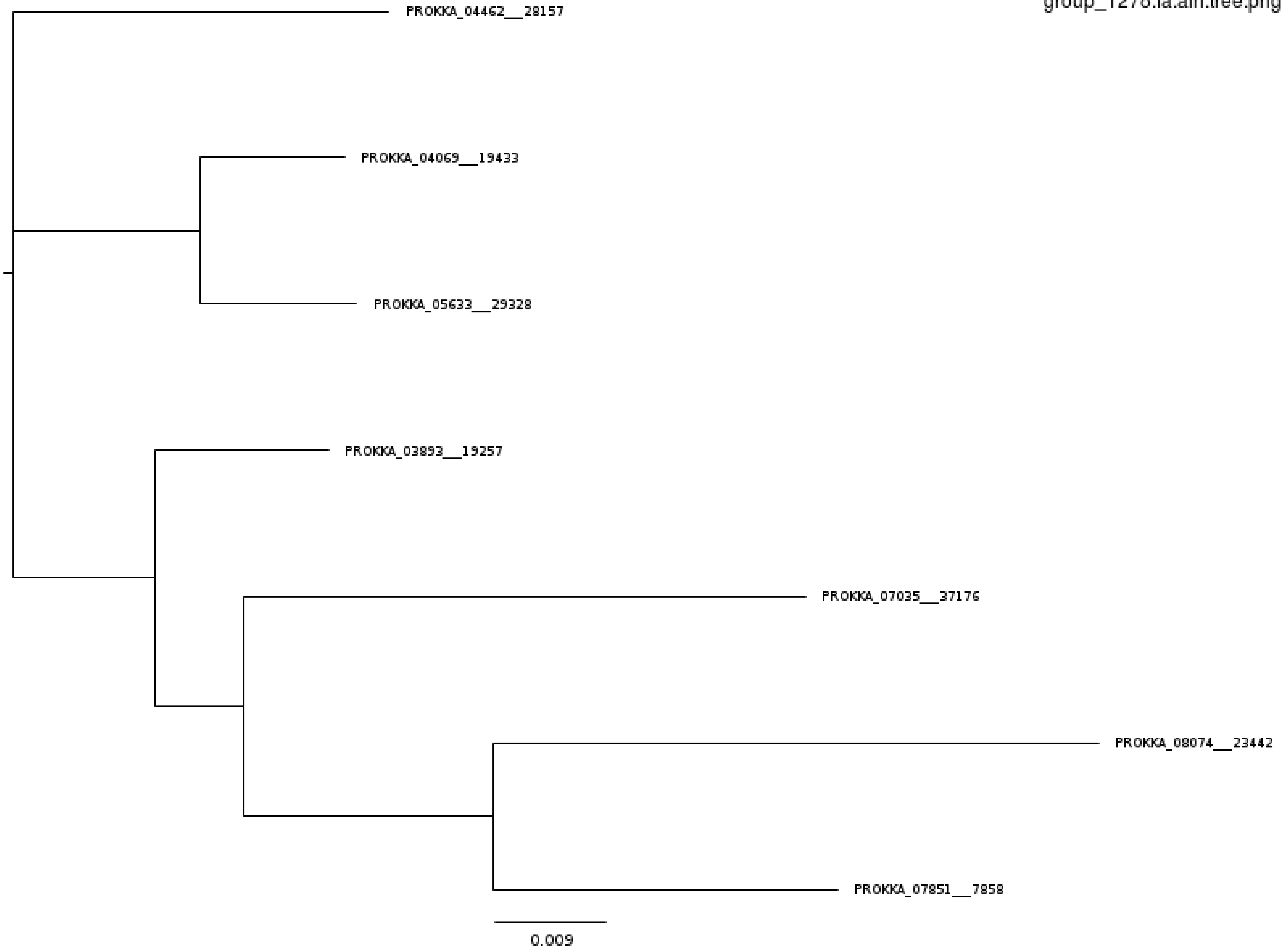

group_1280.fa.aln.tree.png

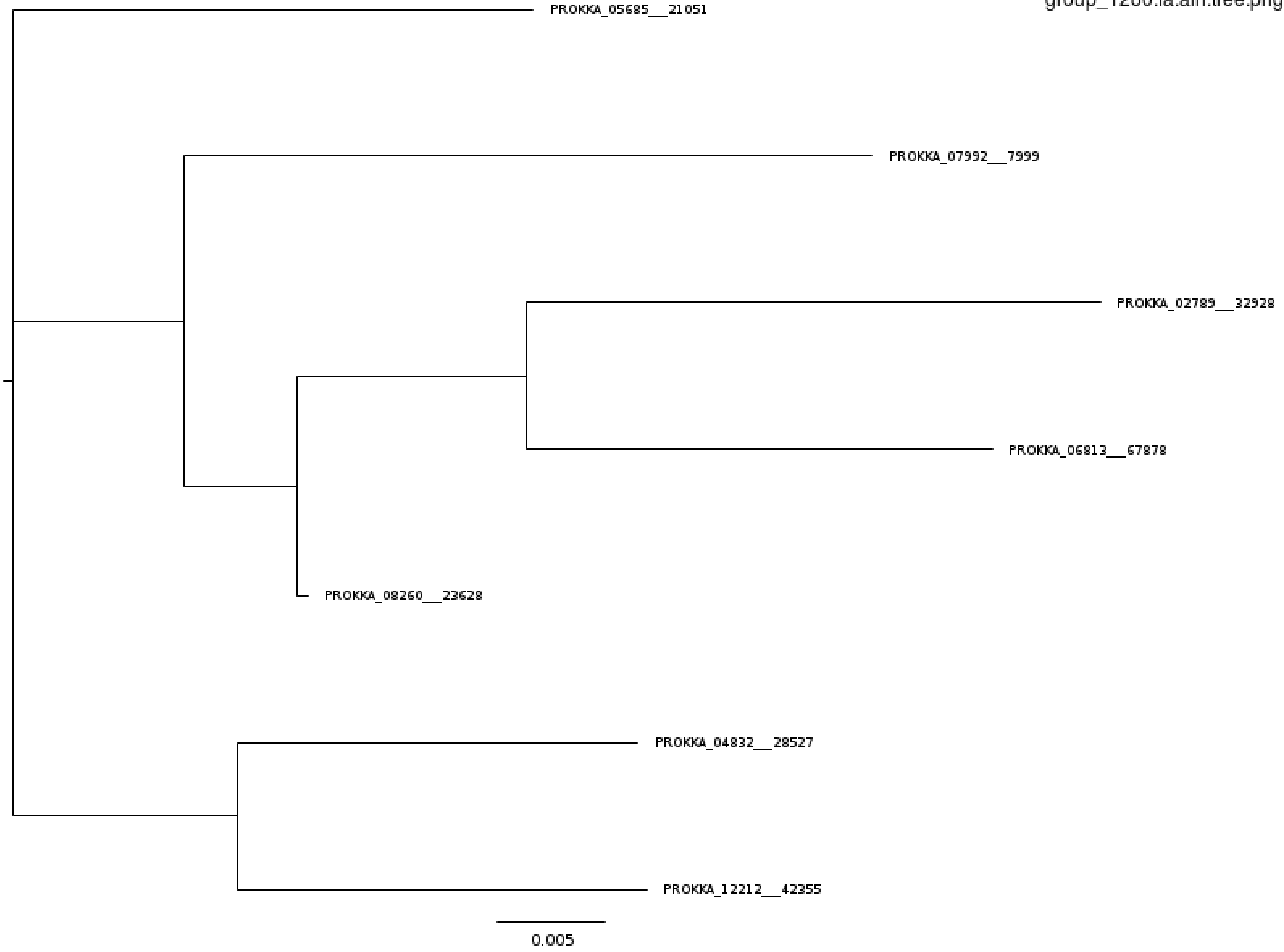

group_1283.fa.aln.tree.png

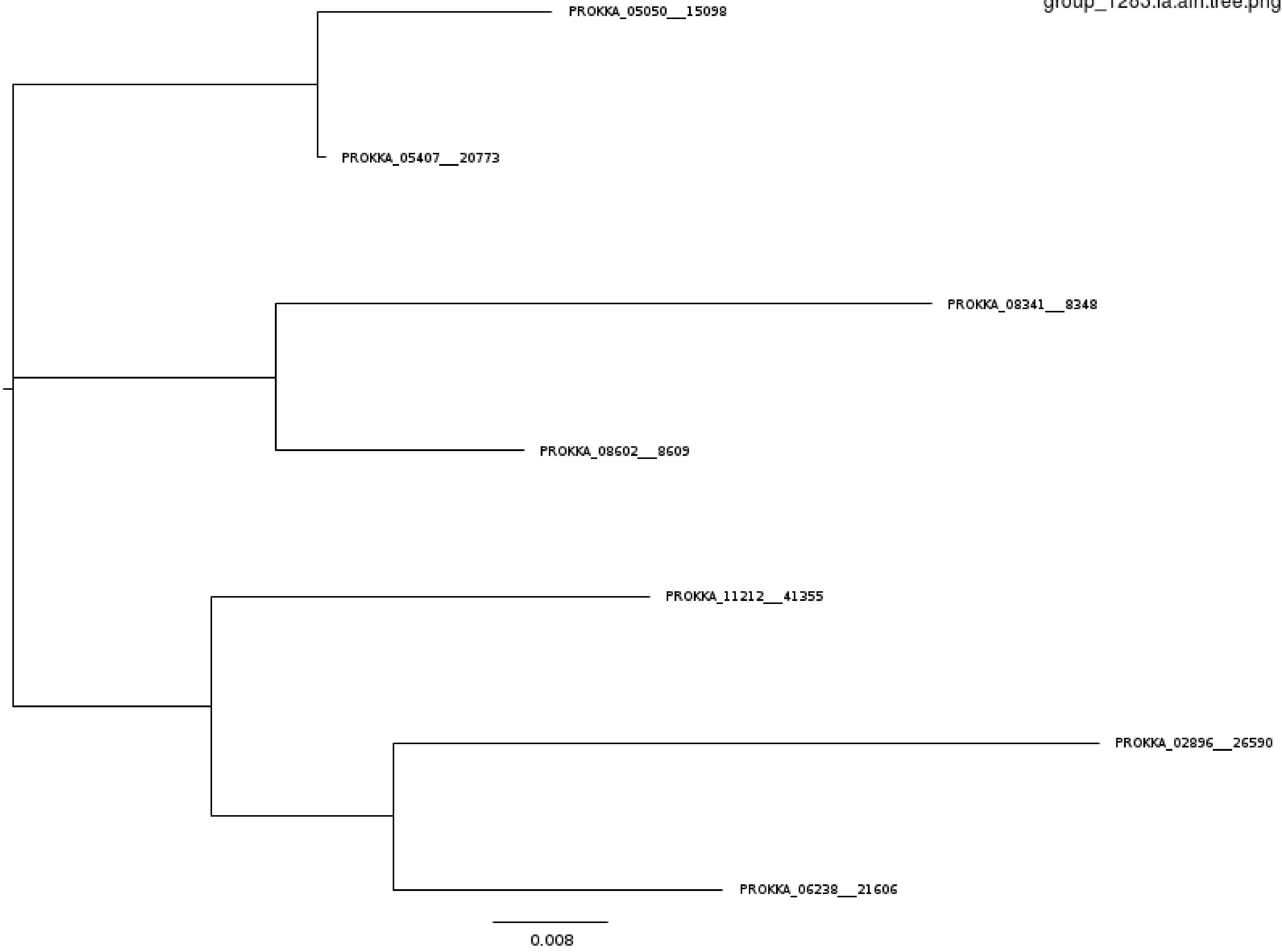

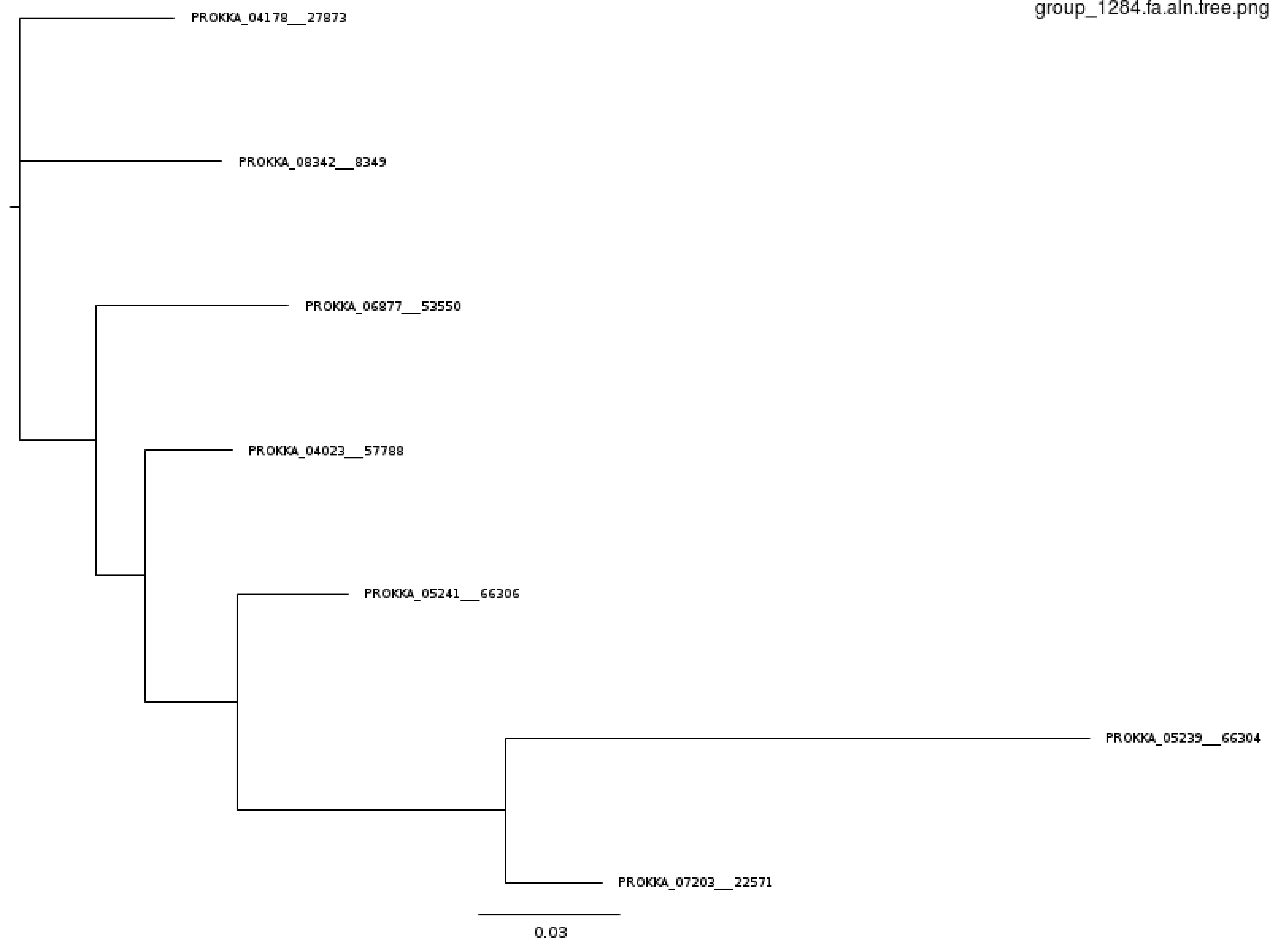
group_1284.fa.aln.tree.png
PROKKA_04178___27873
PROKKA_08342___8349
PROKKA_06877___53550
PROKKA_04023___57788
PROKKA_05241___66306
PROKKA_05239___66304
PROKKA_07203___22571
0.03

group_1286.fa.aln.tree.png

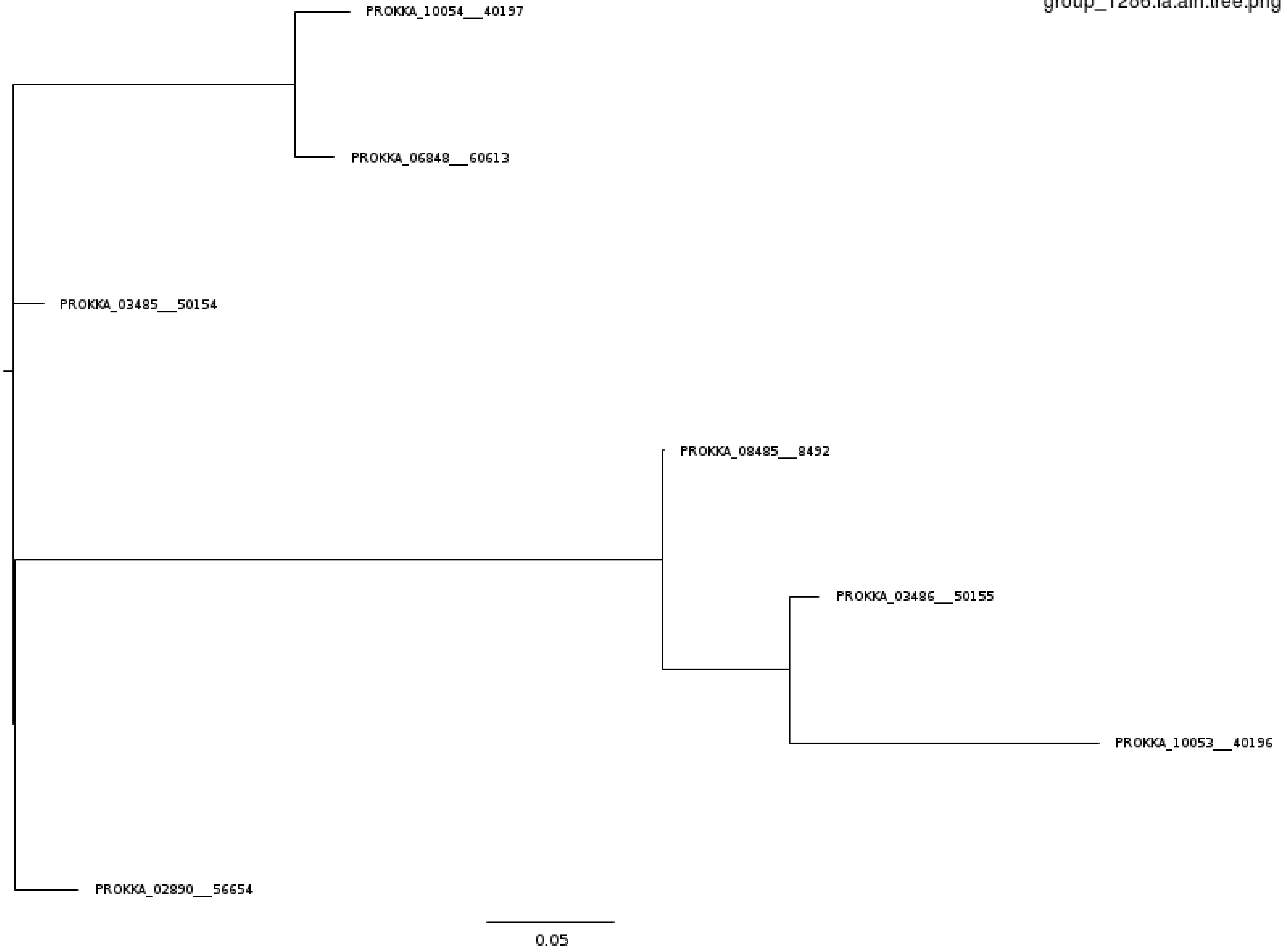

group_1289.fa.aln.tree.png

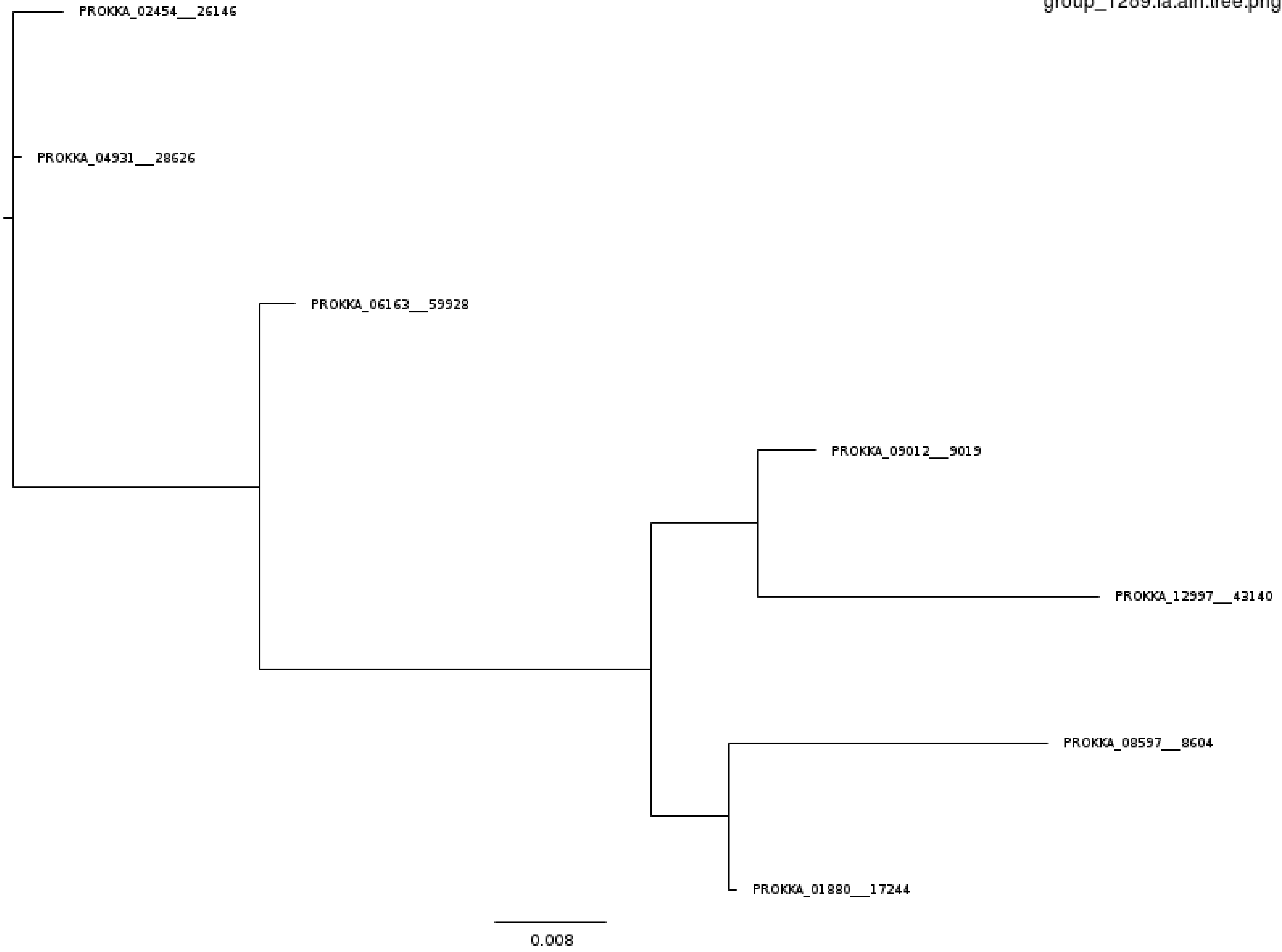

group_1290.fa.aln.tree.png

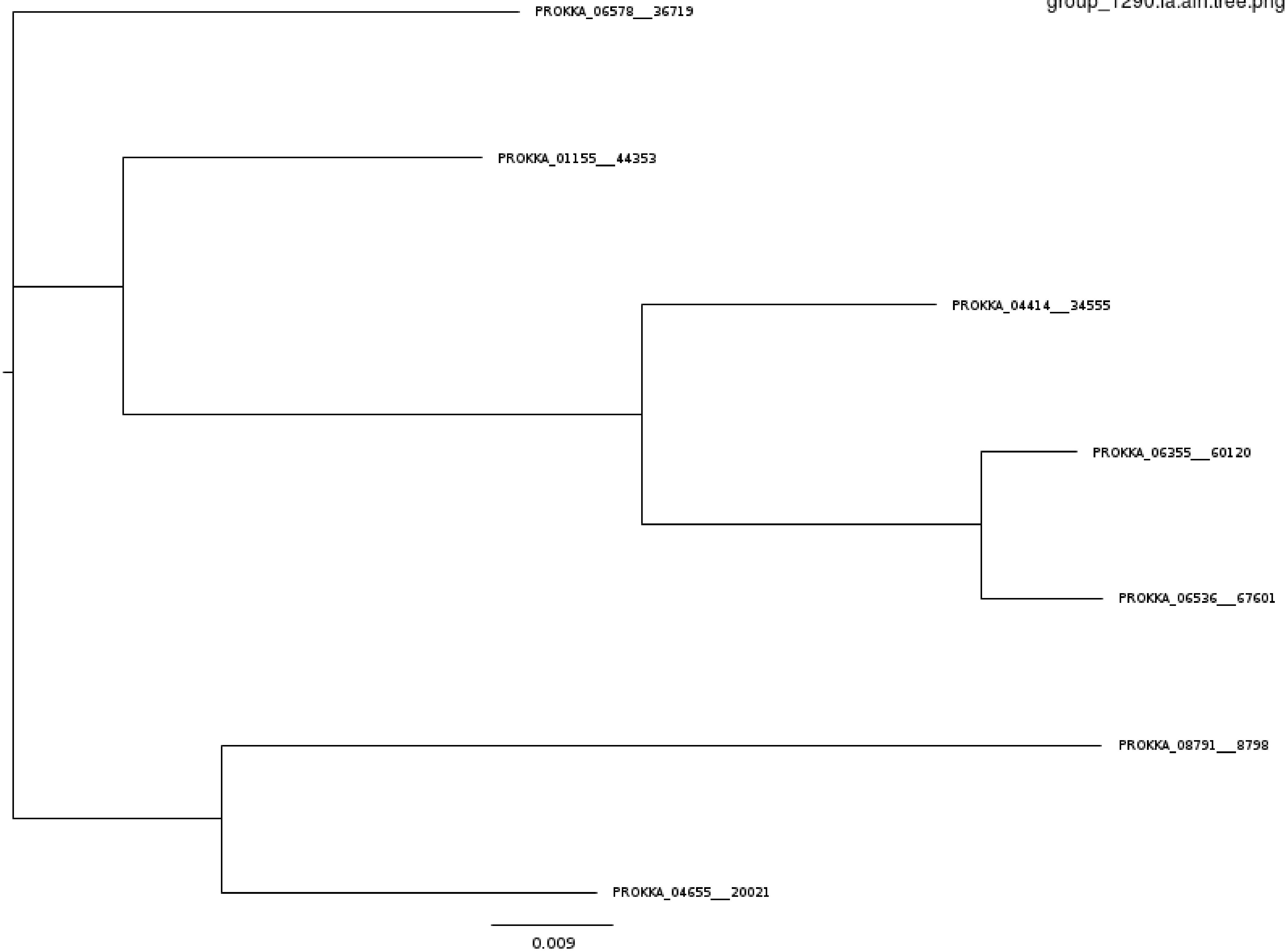

group_1294.fa.aln.tree.png

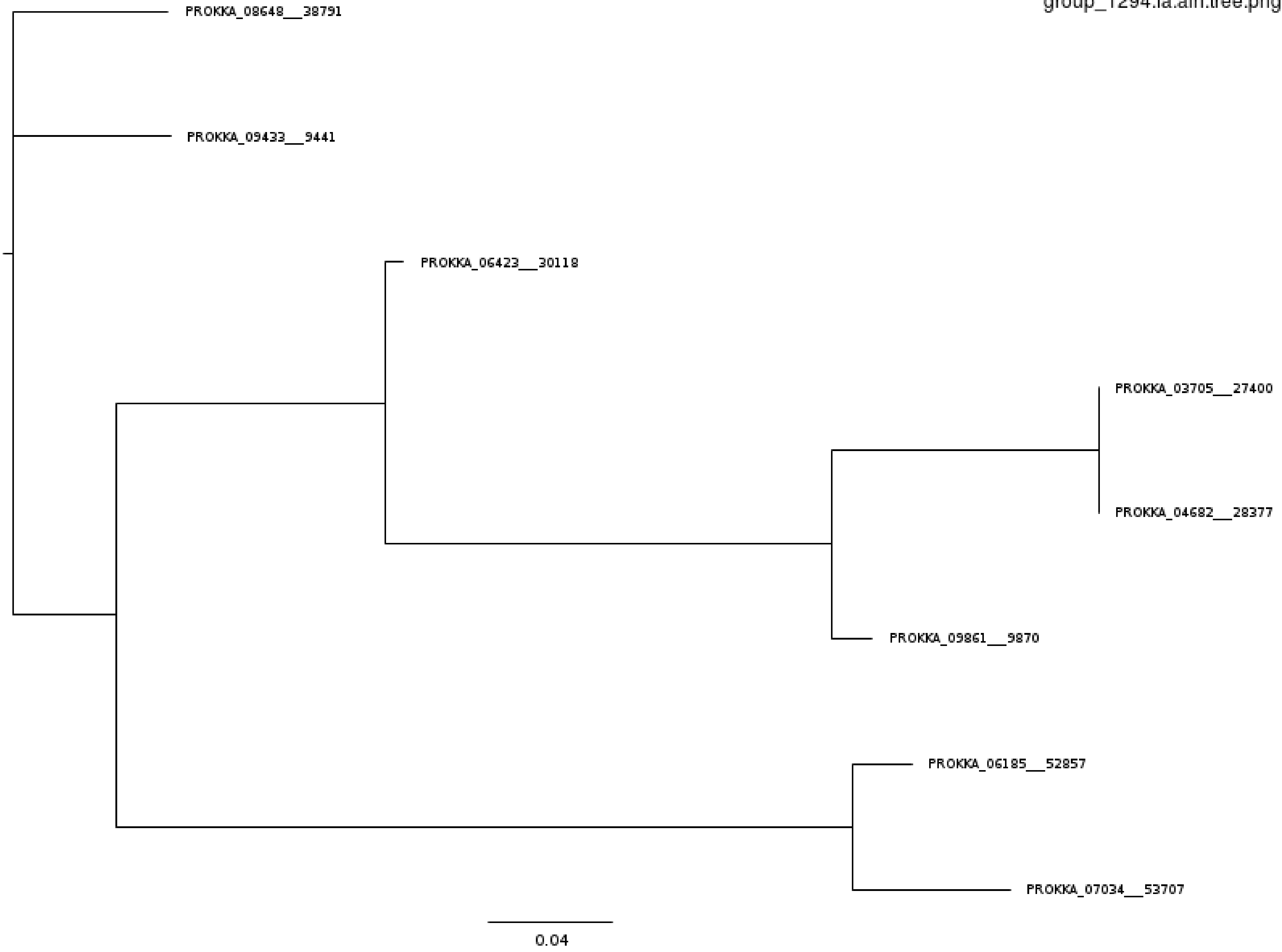

group_1297.fa.aln.tree.png

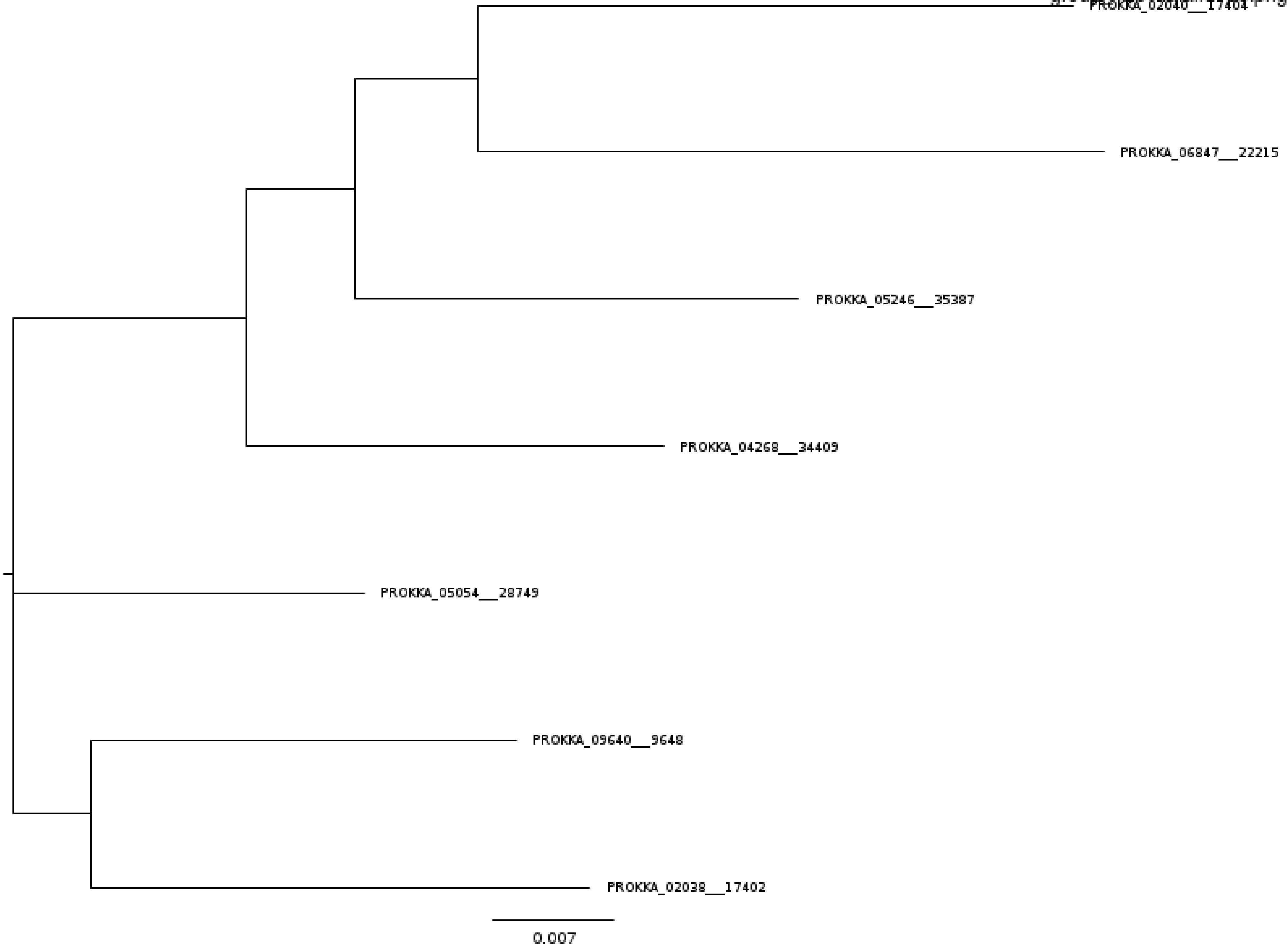

group_1298.fa.aln.tree.png

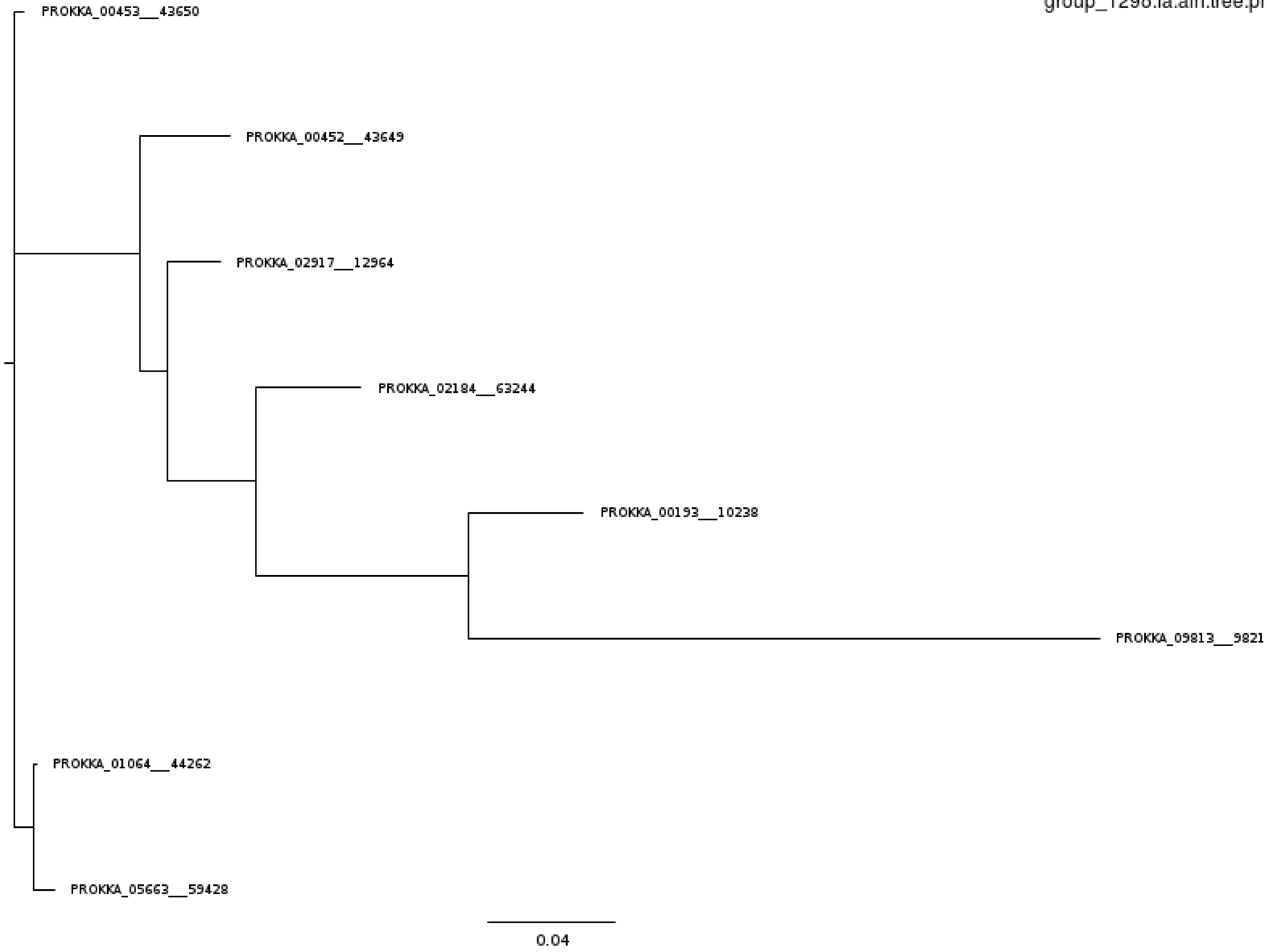

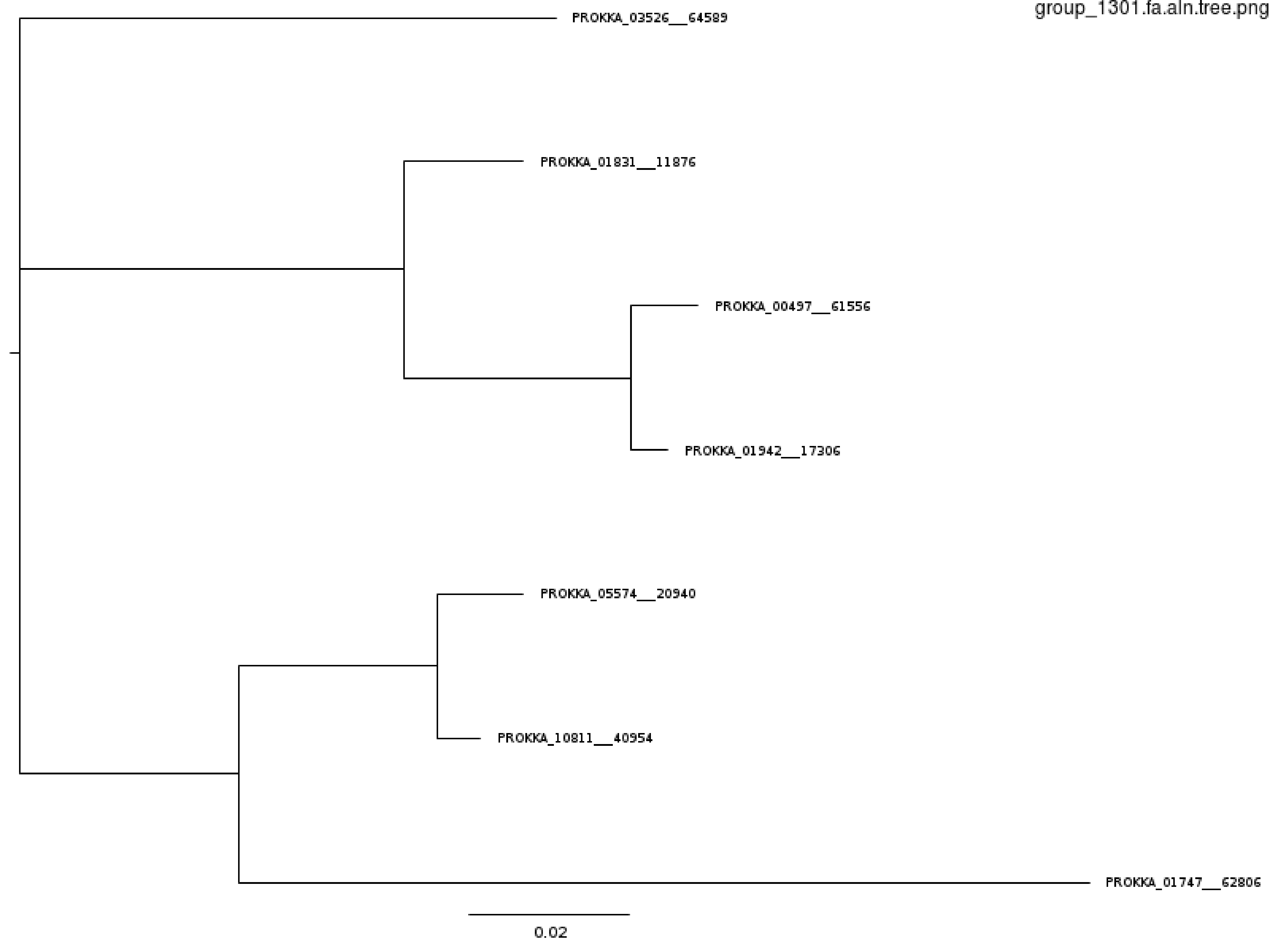
group_1301.fa.aln.tree.png
PROKKA_03526__64589
PROKKA_01831__11876
PROKKA_00497__61556
PROKKA_01942__17306
PROKKA_05574__20940
PROKKA_10811__40954
PROKKA_01747__62806
0.02

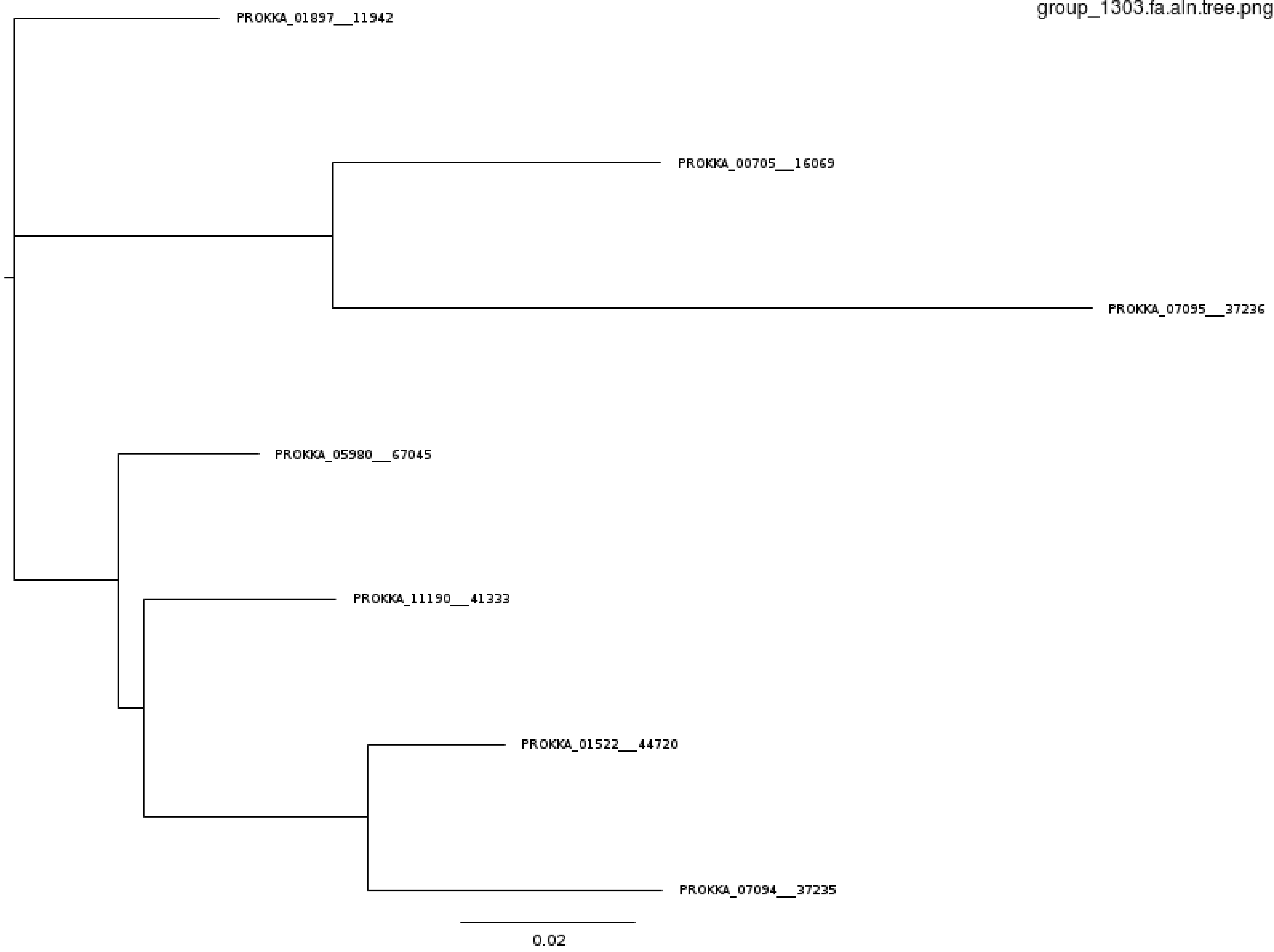
group_1303.fa.aln.tree.png
PROKKA_01897___11942
PROKKA_00705___16069
PROKKA_07095___37236
PROKKA_05980___67045
PROKKA_11190___41333
PROKKA_01522___44720
PROKKA_07094___37235
0.02

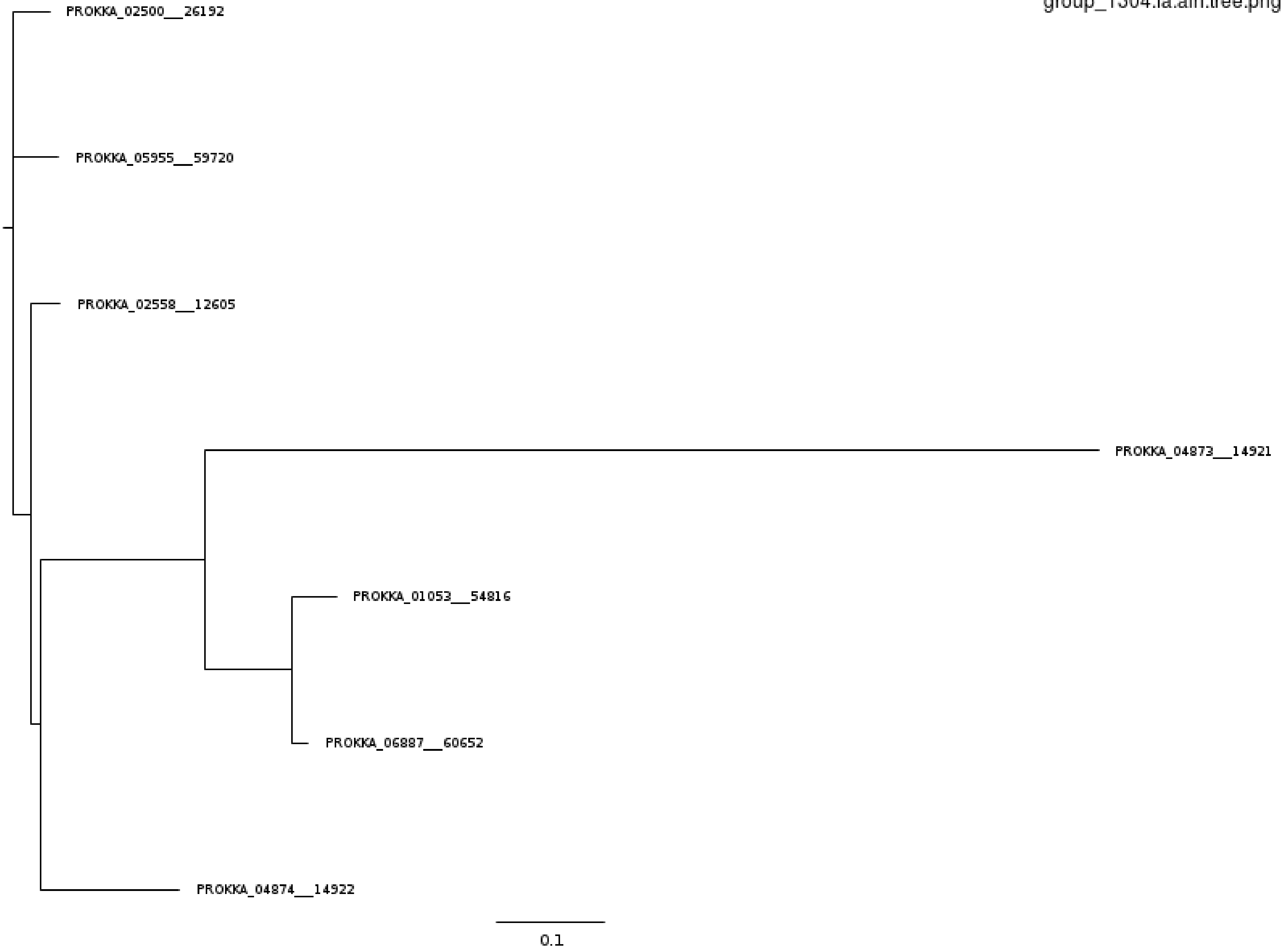
PROKKA_02500__26192
PROKKA_05955__59720
PROKKA_02558__12605
PROKKA_04873__14921
PROKKA_01053__54816
PROKKA_06887__60652
PROKKA_04874__14922
0.1

group_1306.fa.aln.tree.png

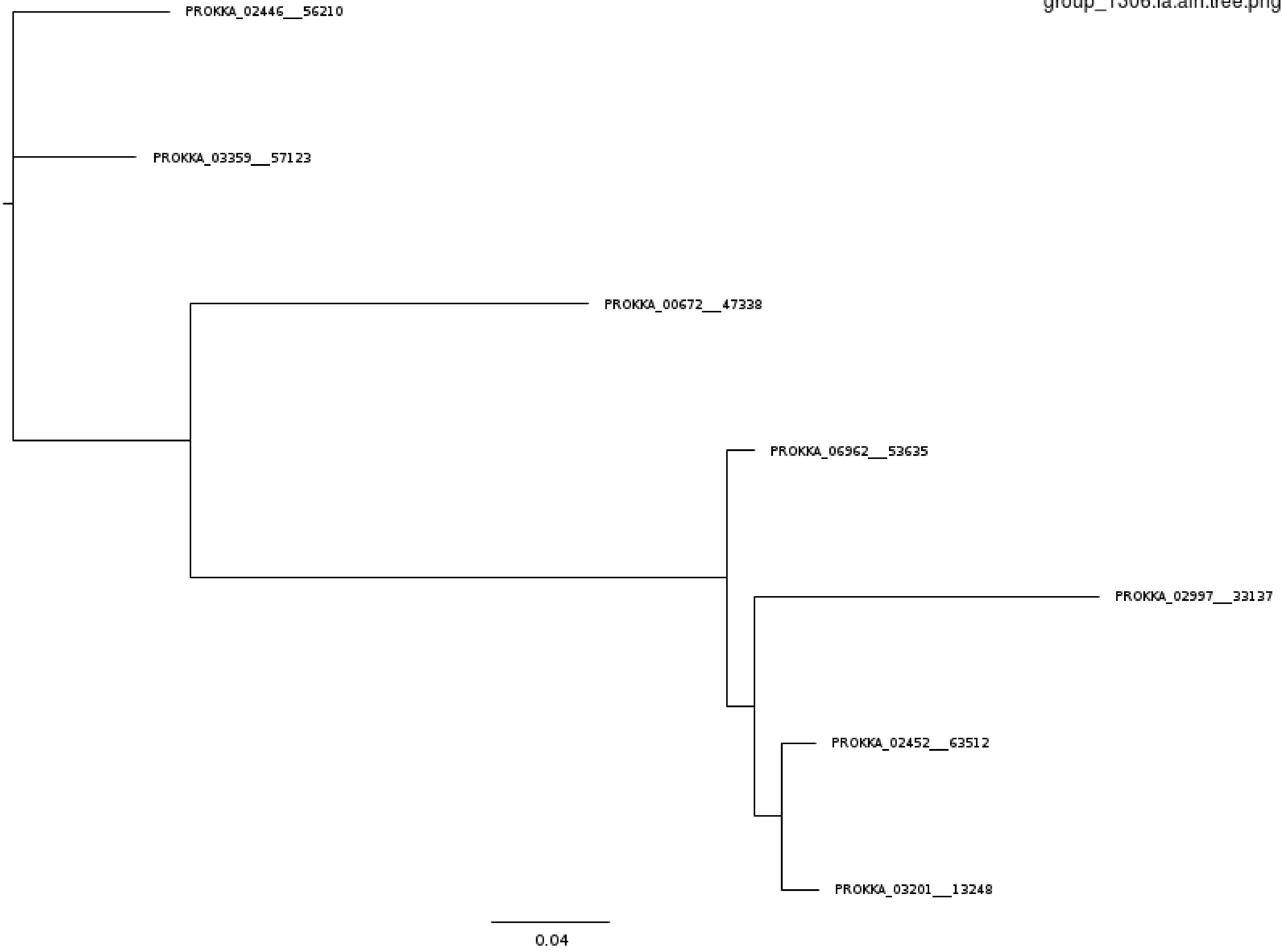

group_1310.fa.aln.tree.png

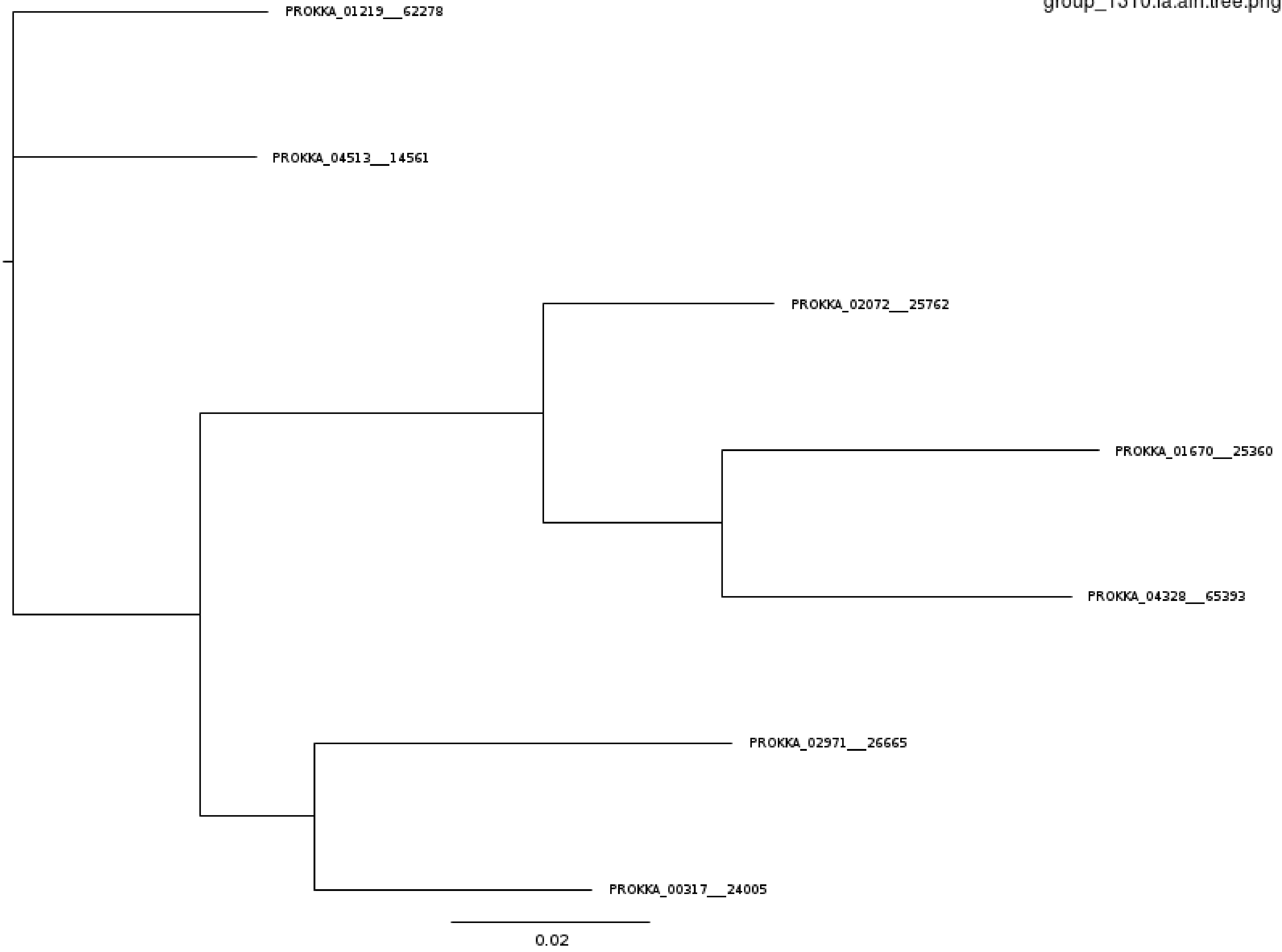

group_1312.fa.aln.tree.png

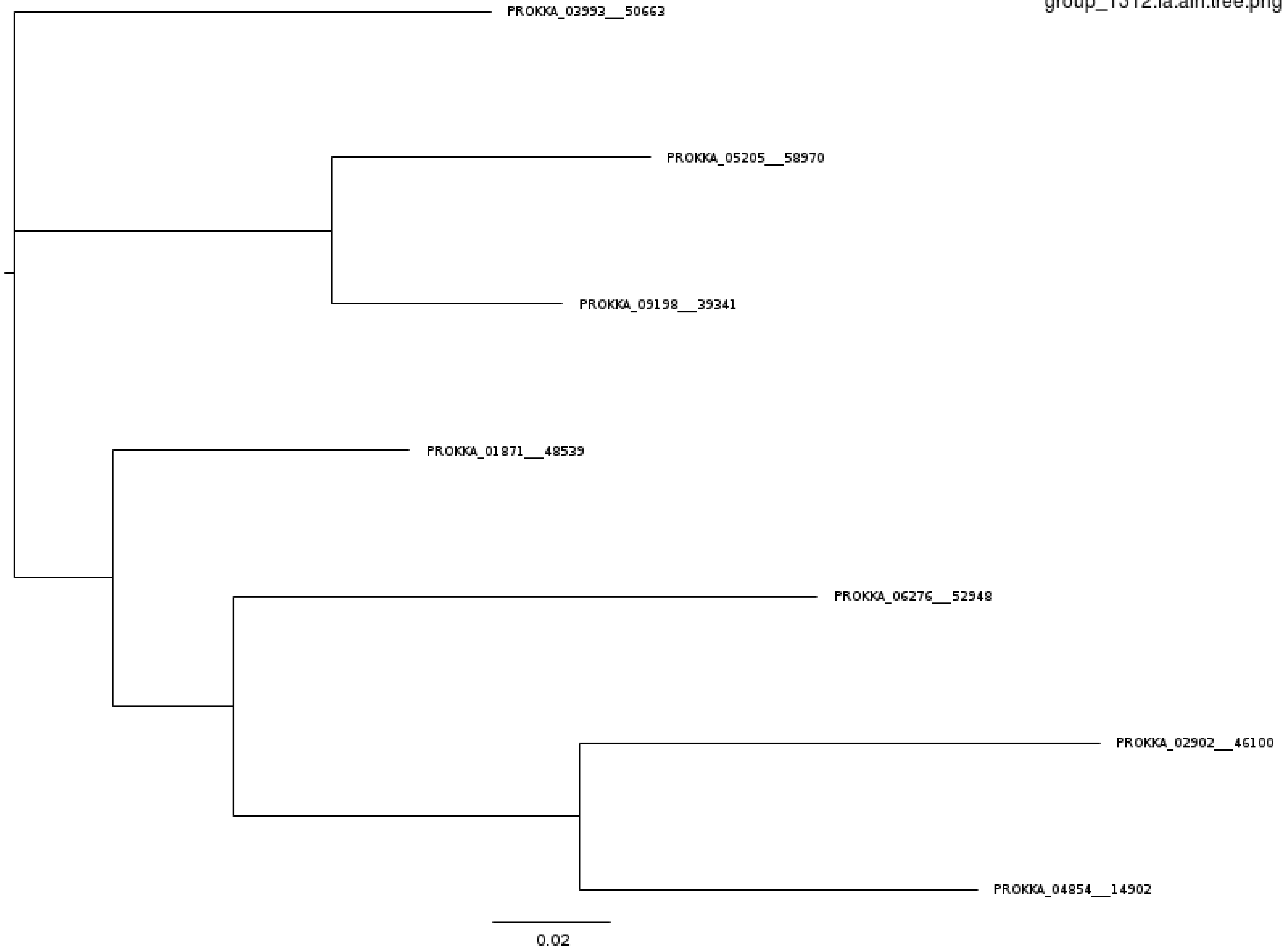

group_1314.fa.aln.tree.png

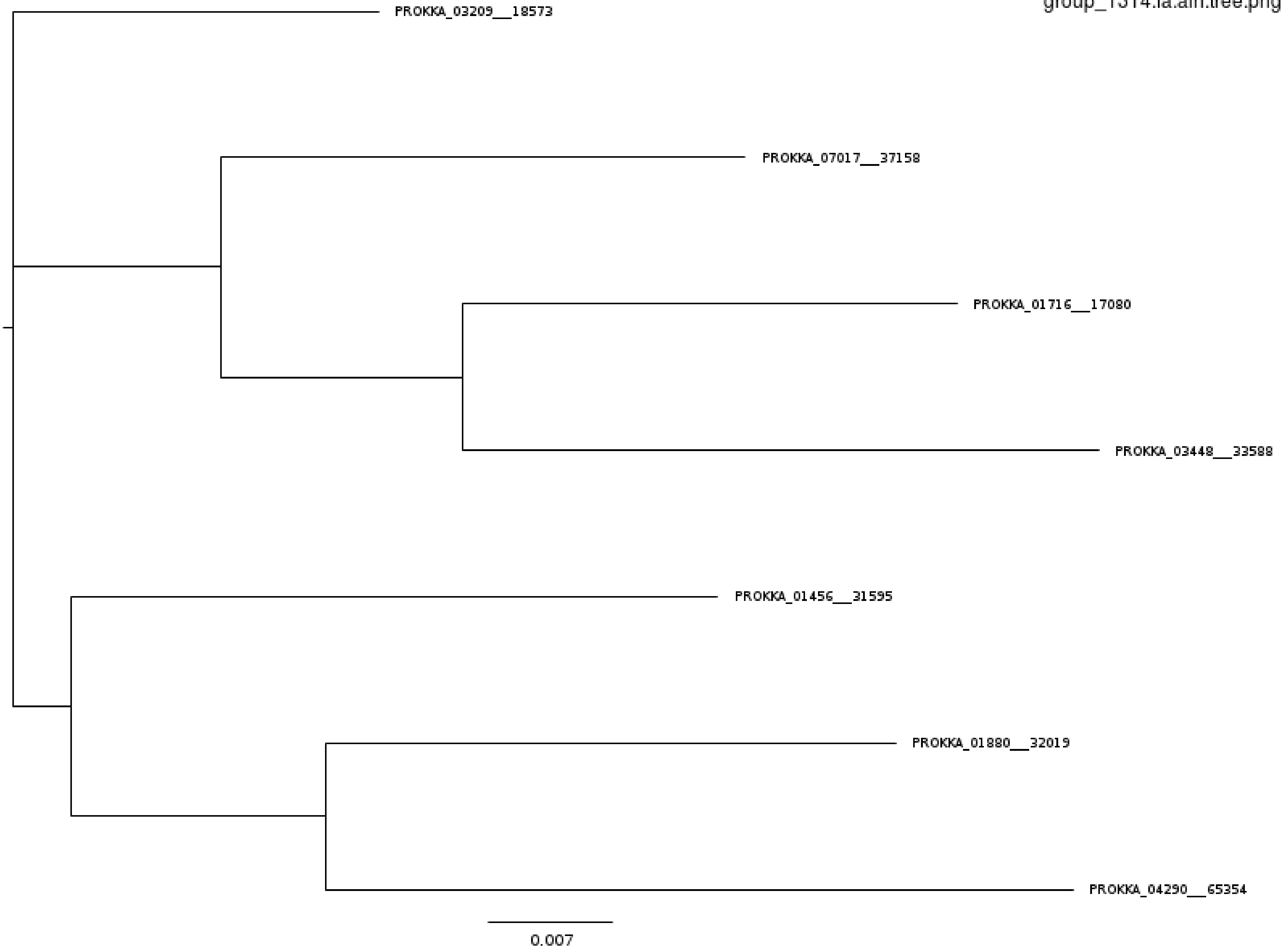

group_1320.fa.aln.tree.png

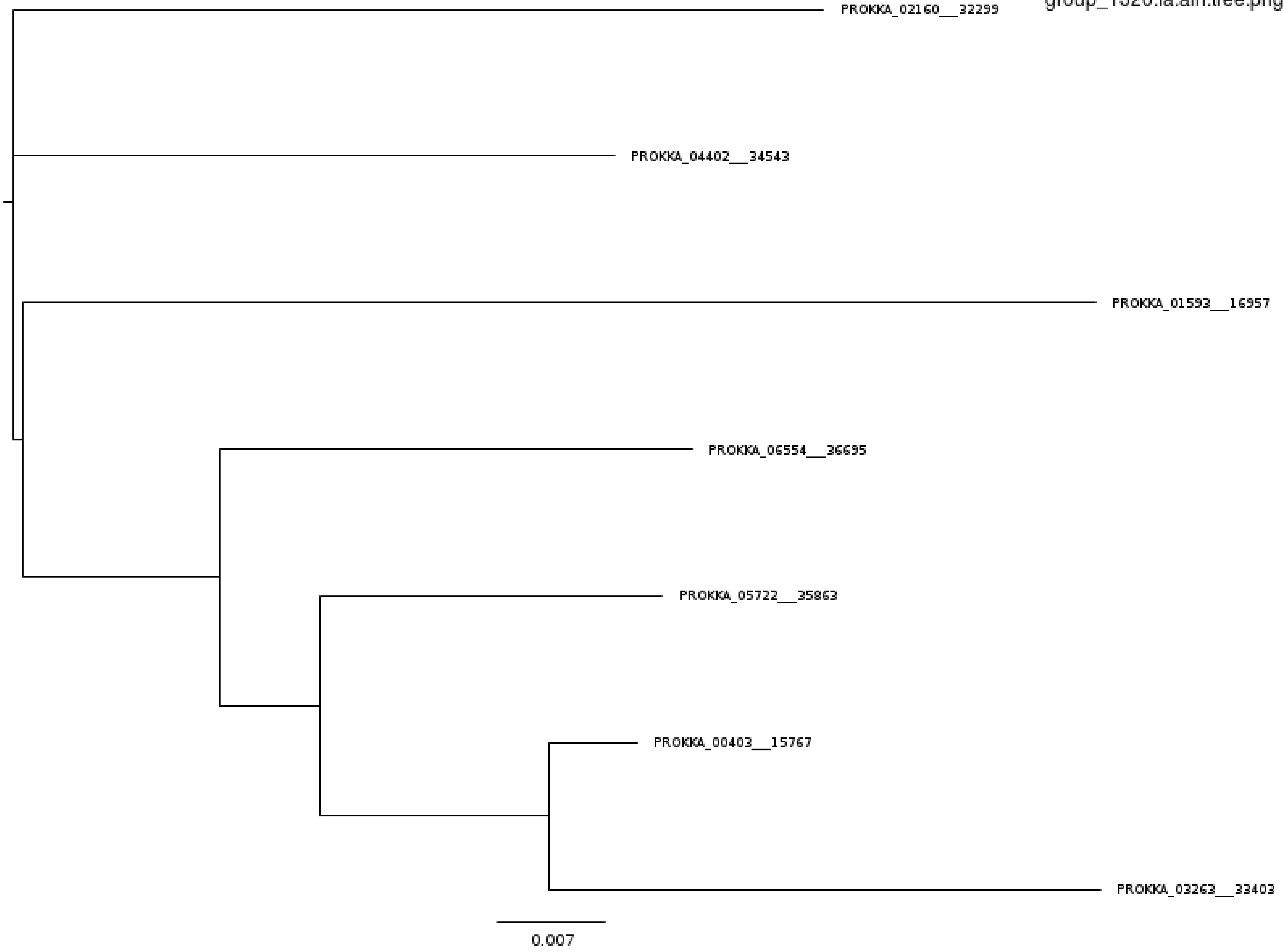

group_1324.fa.aln.tree.png

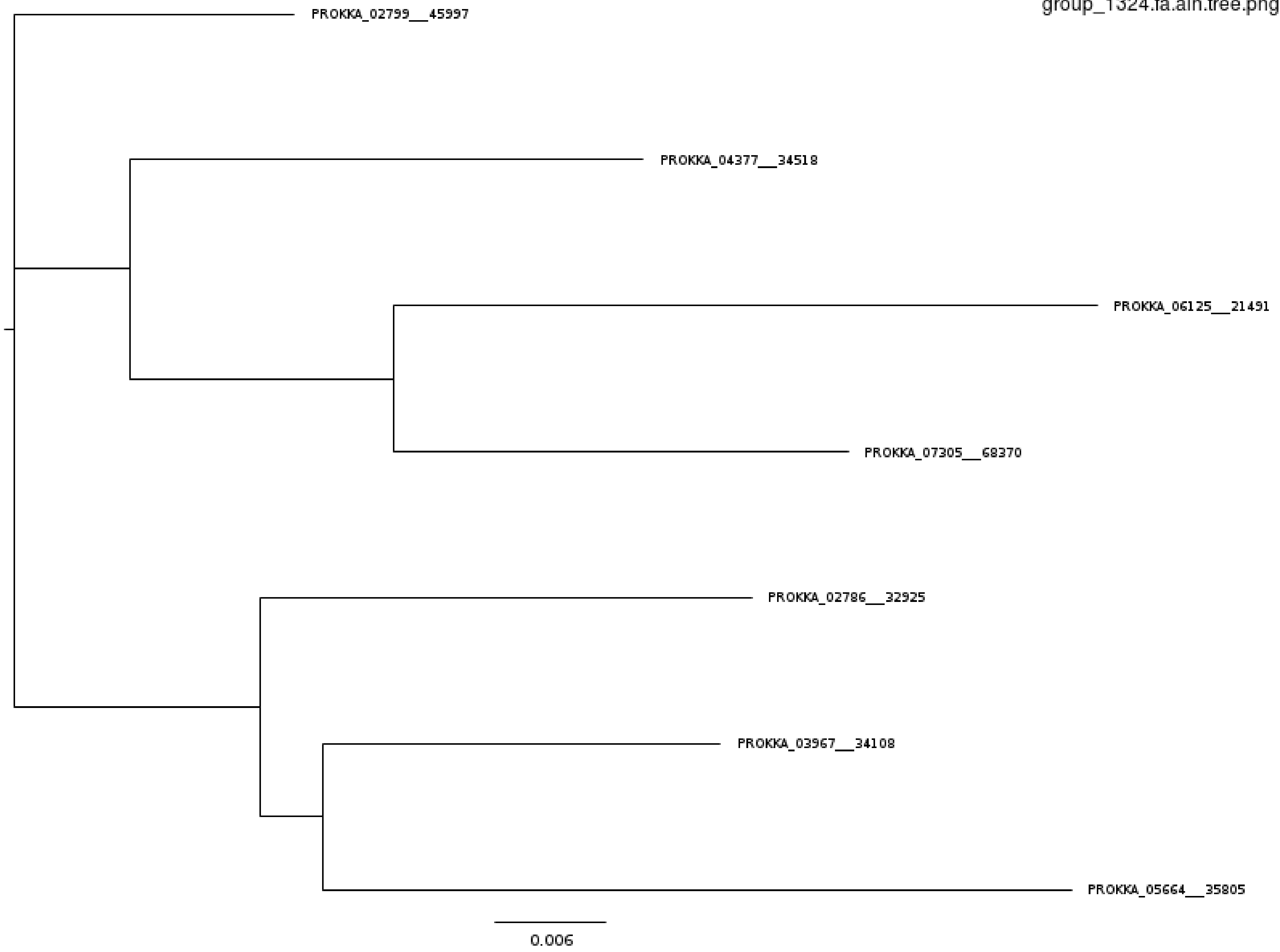

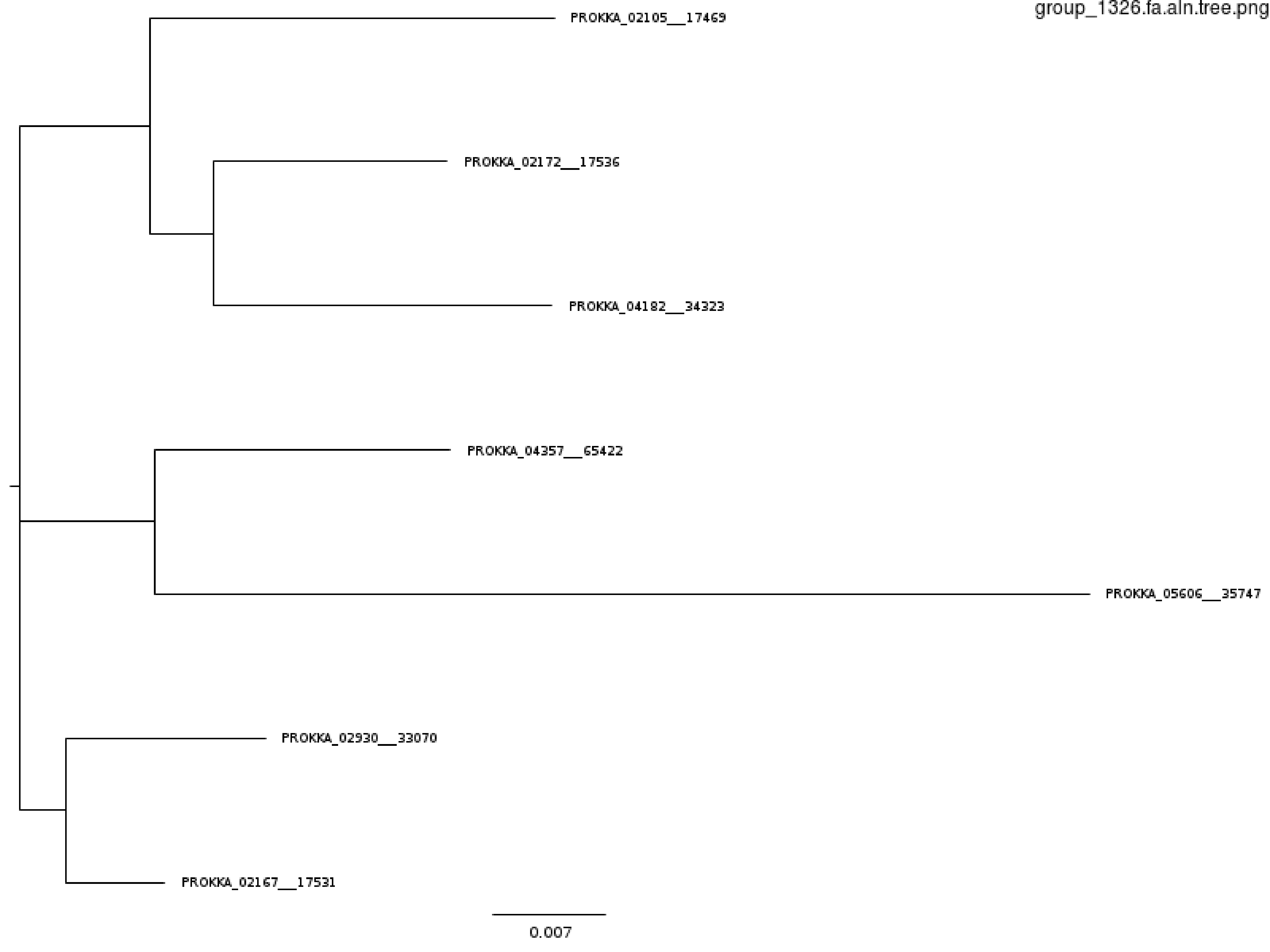
group_1326.fa.aln.tree.png
PROKKA_02105___17469
PROKKA_02172___17536
PROKKA_04182___34323
PROKKA_04357___65422
PROKKA_05606___35747
PROKKA_02930___33070
PROKKA_02167___17531
0.007

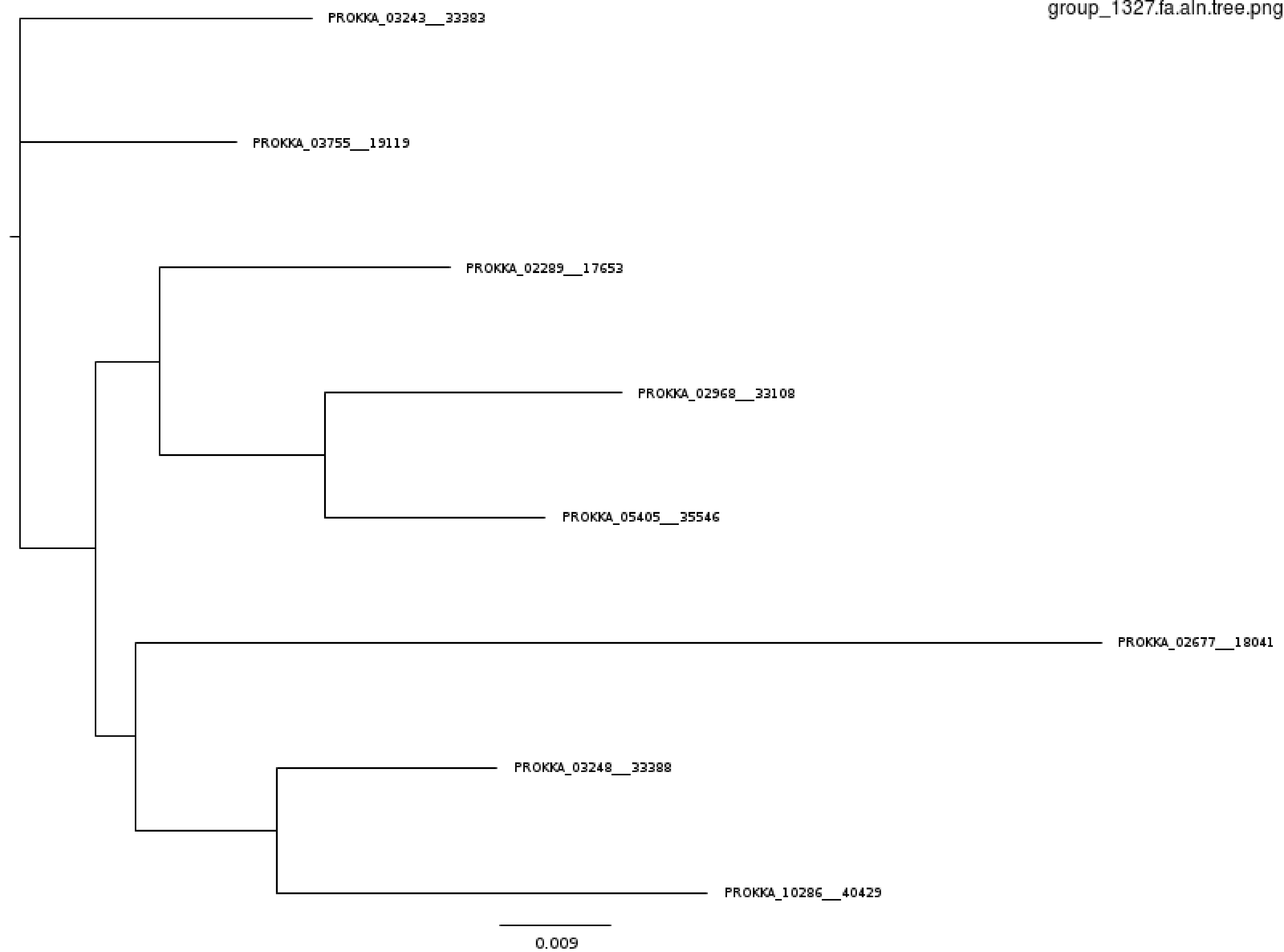
group_1327.fa.aln.tree.png
PROKKA_03243___33383
PROKKA_03755___19119
PROKKA_02289___17653
PROKKA_02968___33108
PROKKA_05405___35546
PROKKA_02677___18041
PROKKA_03248___33388
PROKKA_10286___40429
0.009

group_1330.fa.aln.tree.png

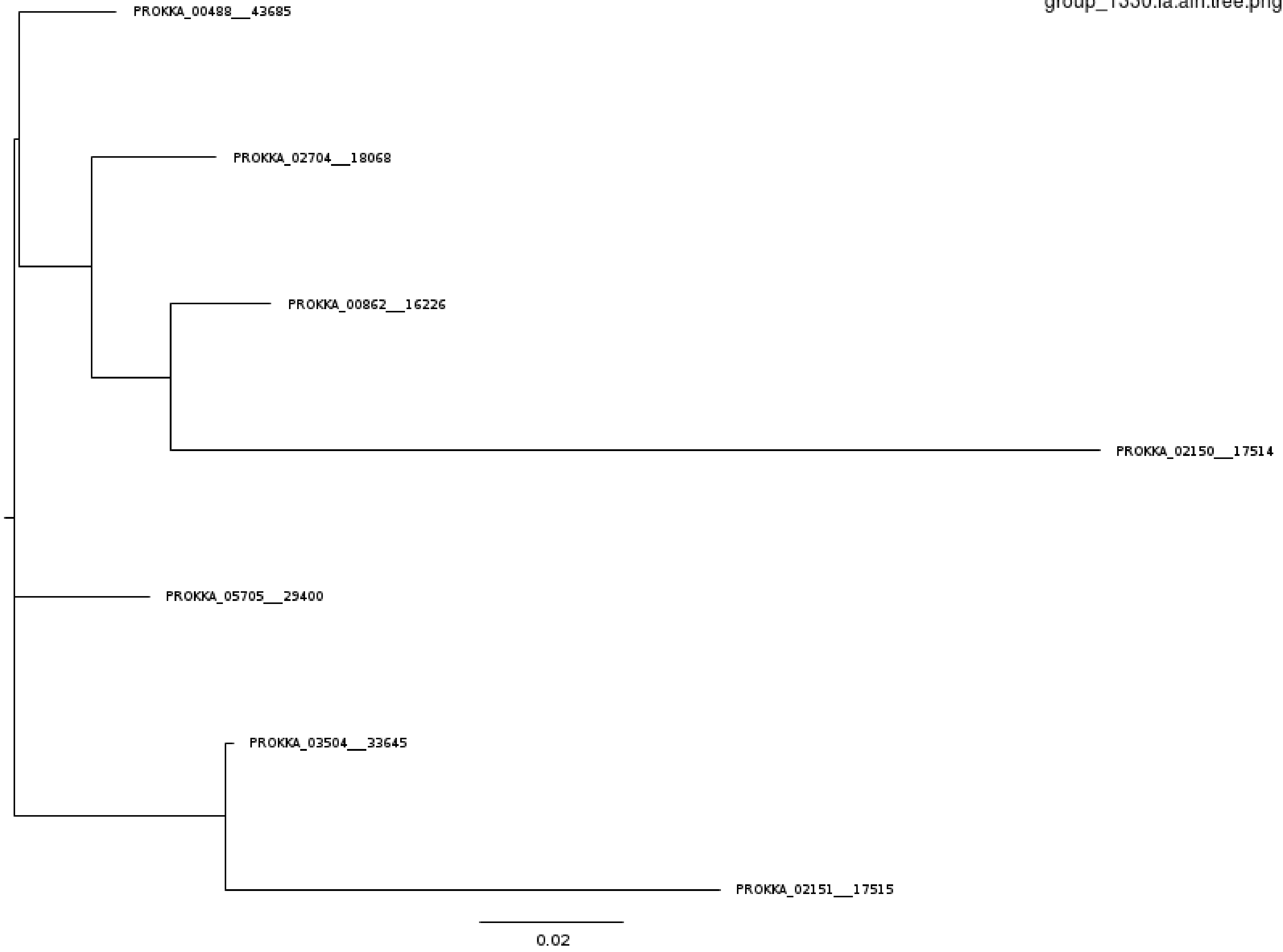

group_1331.fa.aln.tree.png

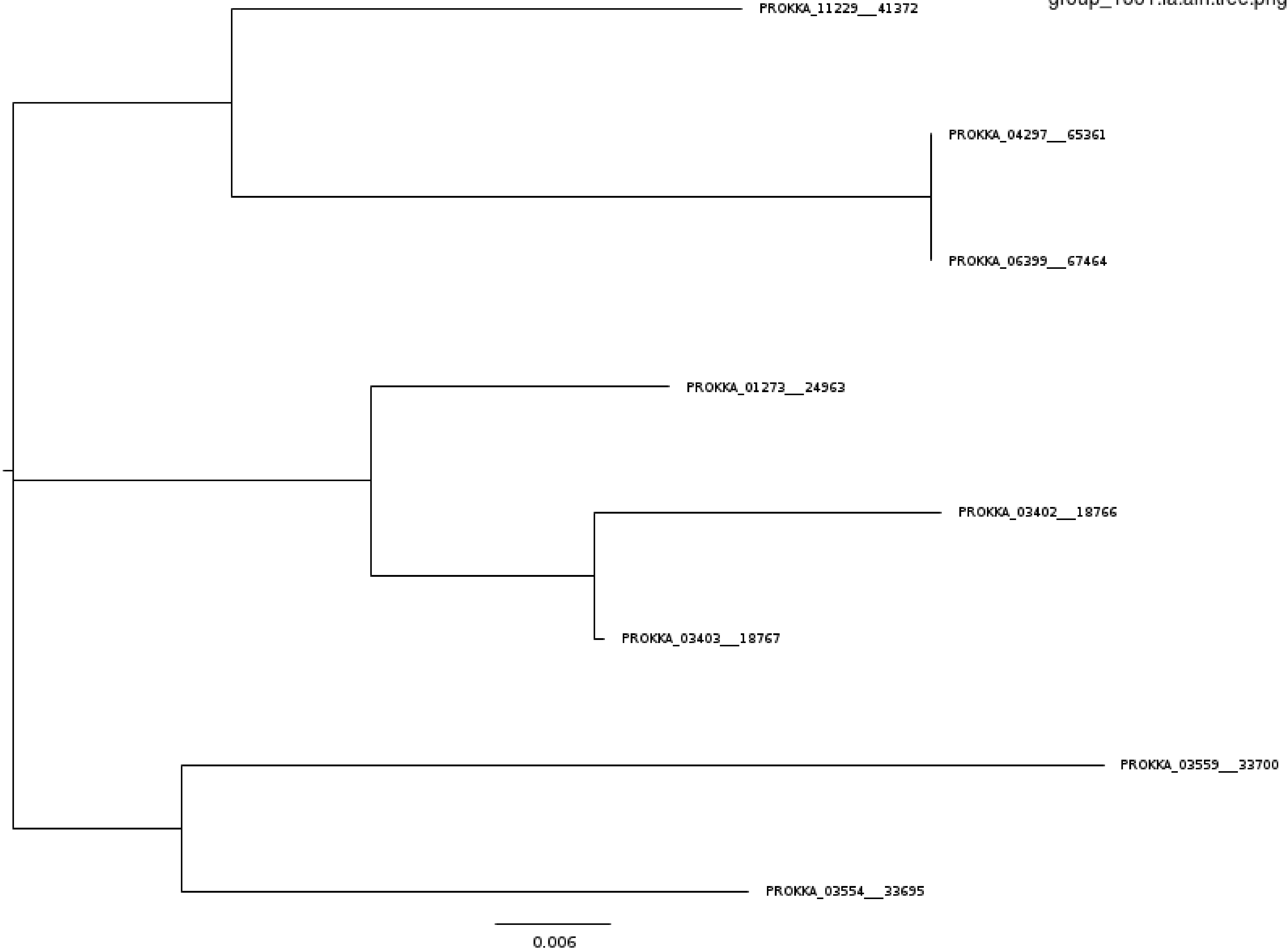

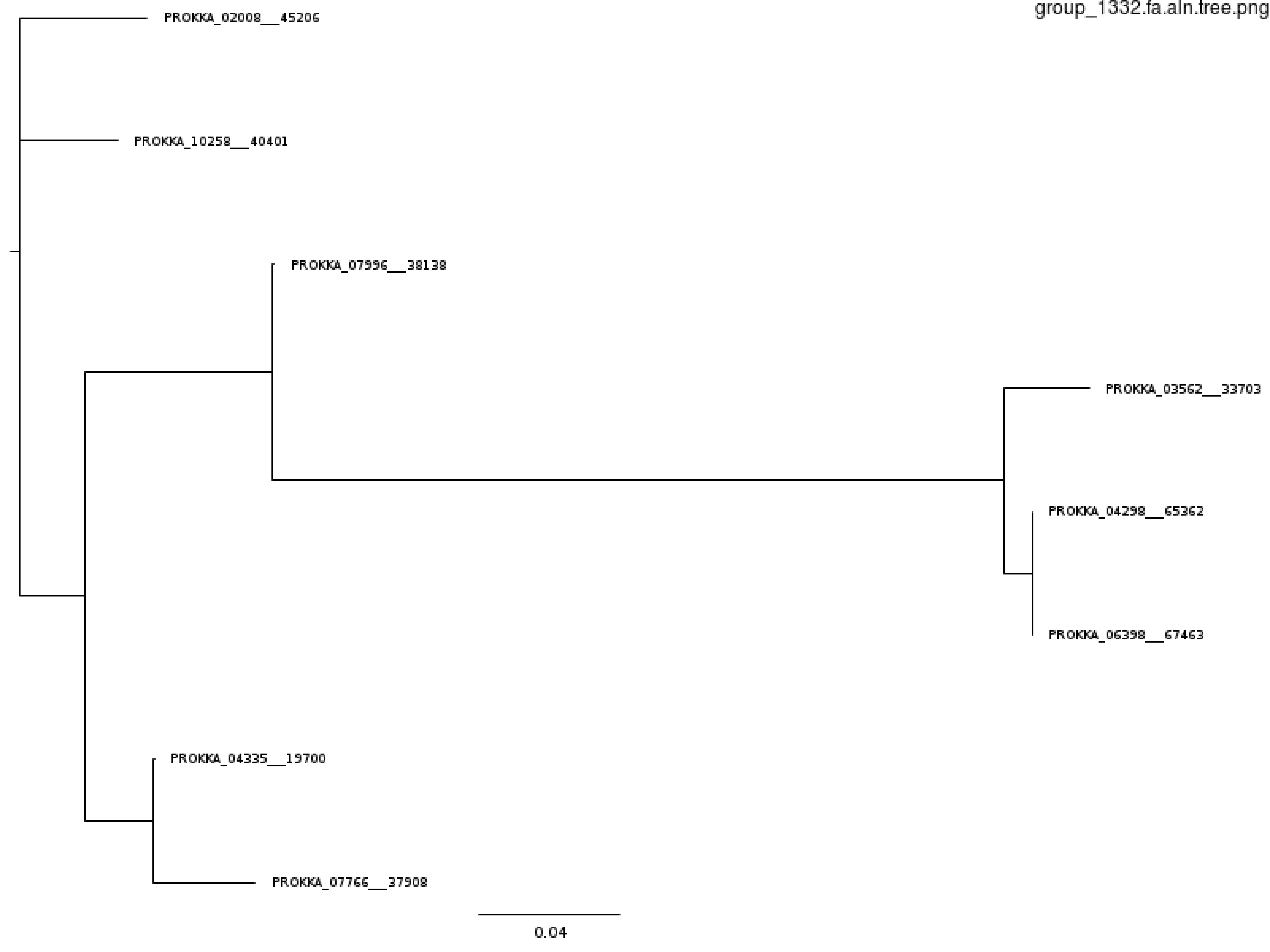

group_1332.fa.aln.tree.png
PROKKA_02008___45206
PROKKA_10258___40401
PROKKA_07996___38138
PROKKA_03562___33703
PROKKA_04298___65362
PROKKA_06398___67463
PROKKA_04335___19700
PROKKA_07766___37908
0.04

group_1333.fa.aln.tree.png

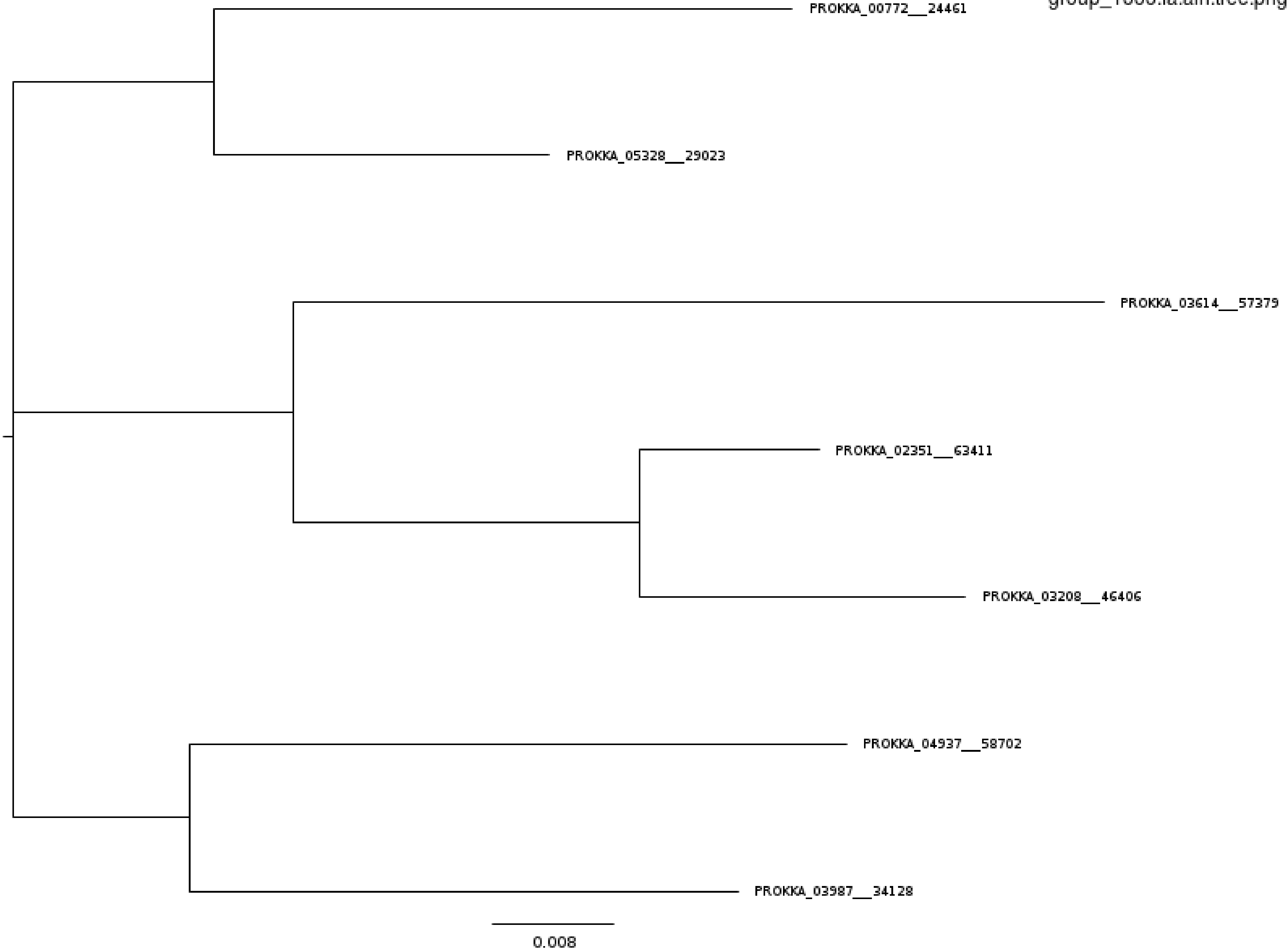

group_1336.fa.aln.tree.png

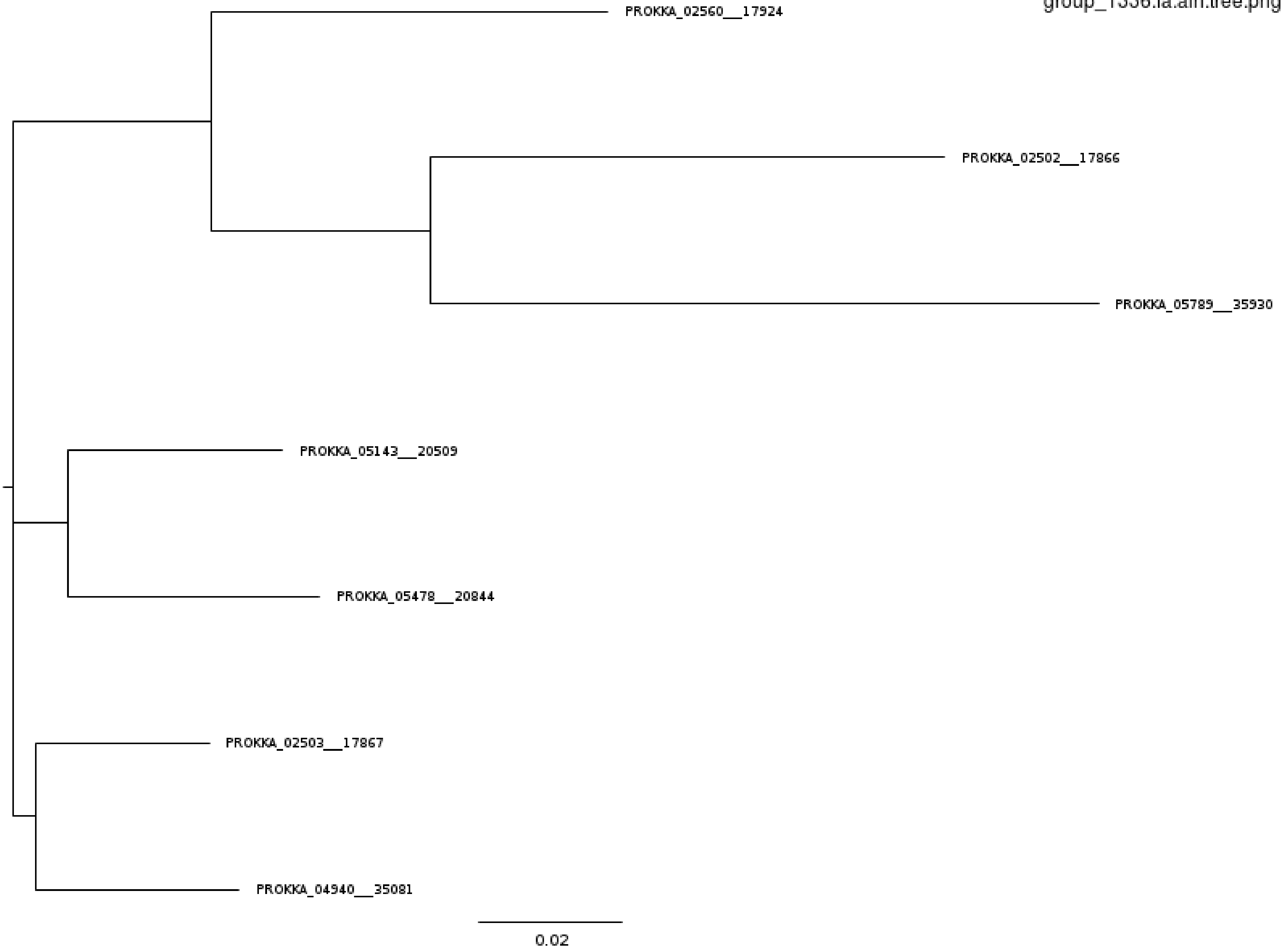

group_1338.fa.aln.tree.png

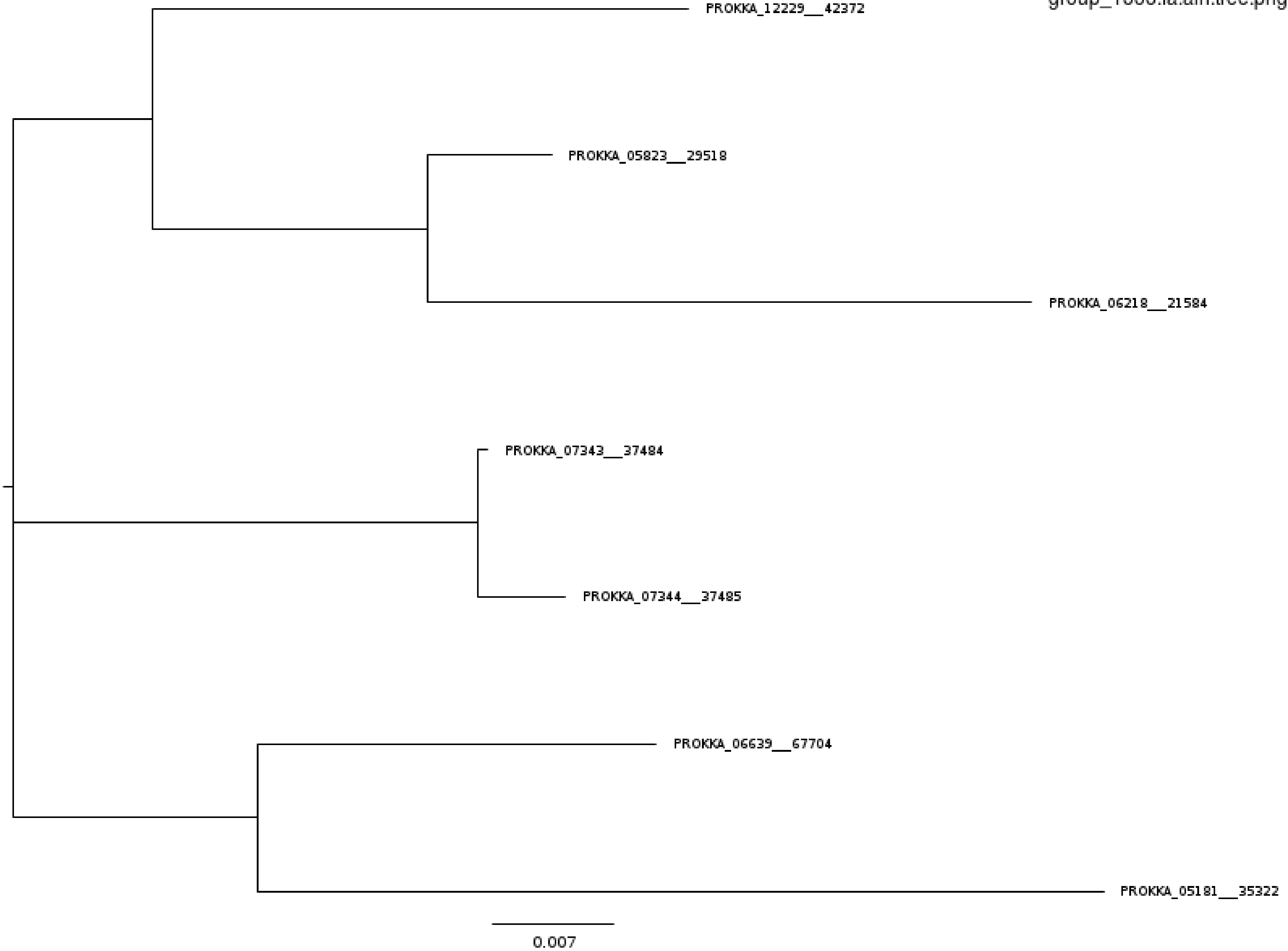

group_1339.fa.aln.tree.png

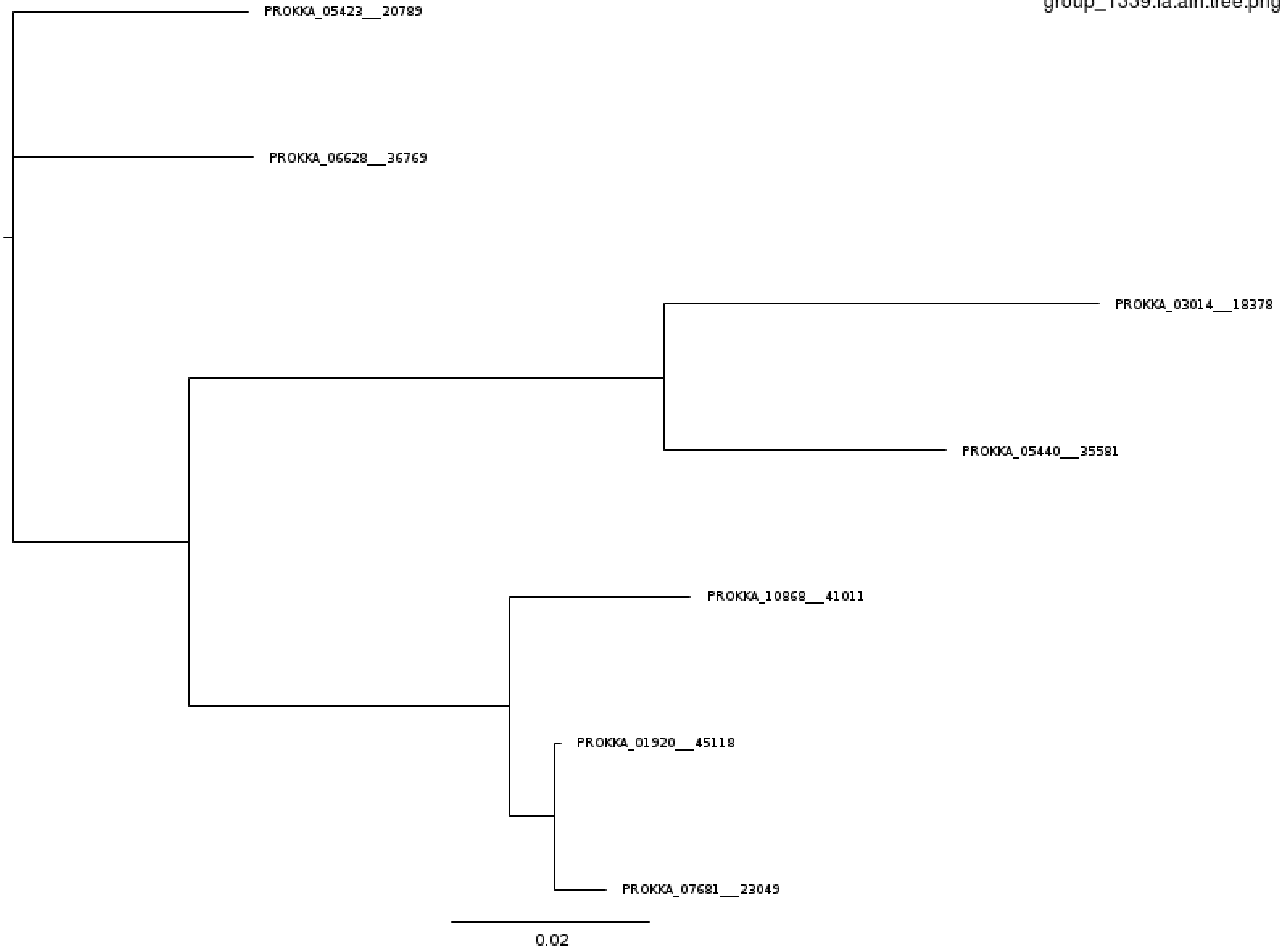

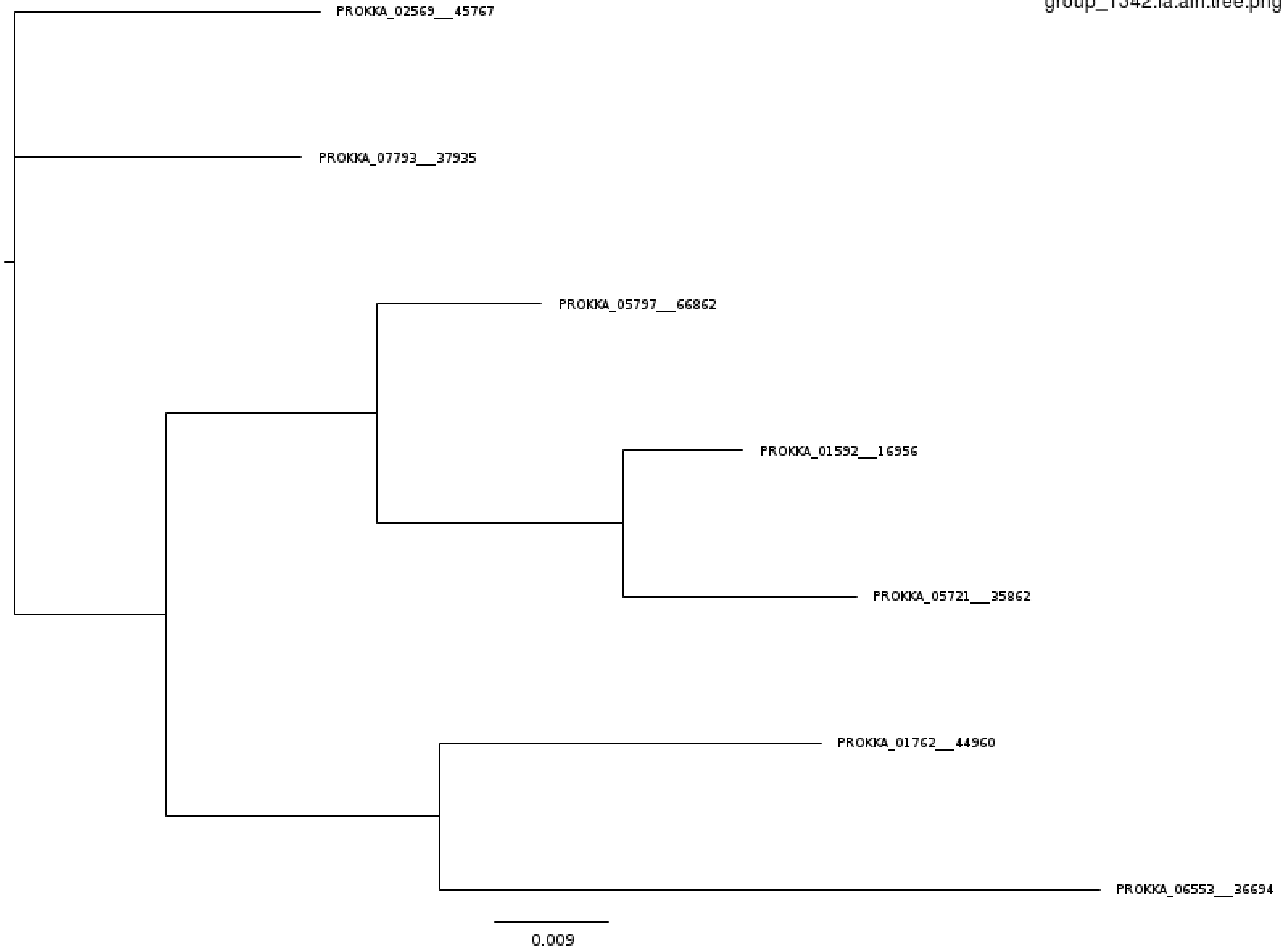
PROKKA_02569___45767
PROKKA_07793___37935
PROKKA_05797___66862
PROKKA_01592___16956
PROKKA_05721___35862
PROKKA_01762___44960
PROKKA_06553___36694
0.009

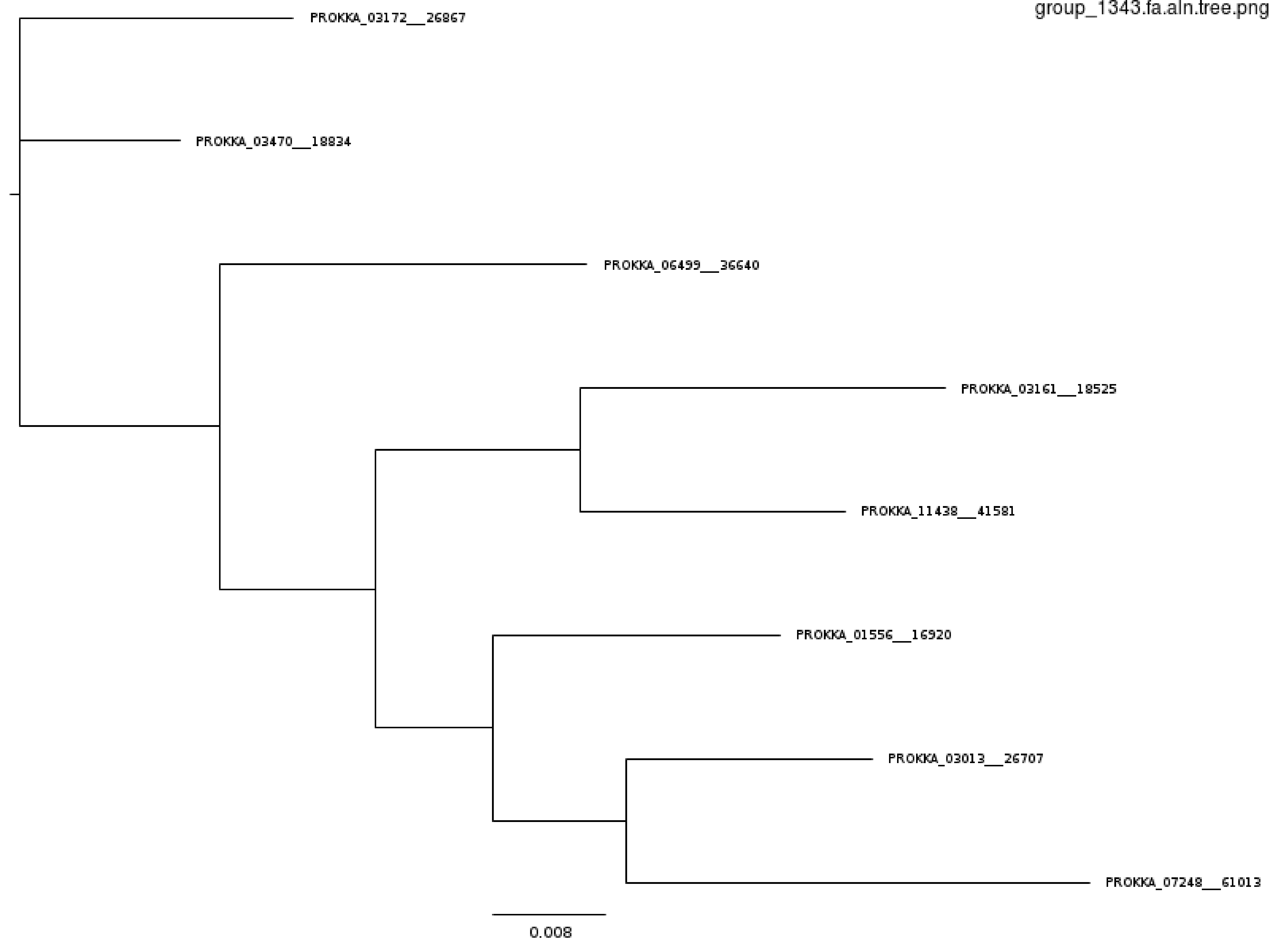
group_1343.fa.aln.tree.png
PROKKA_03172__26867
PROKKA_03470__18834
PROKKA_06499__36640
PROKKA_03161__18525
PROKKA_11438__41581
PROKKA_01556__16920
PROKKA_03013__26707
PROKKA_07248__61013
0.008

group_1345.fa.aln.tree.png

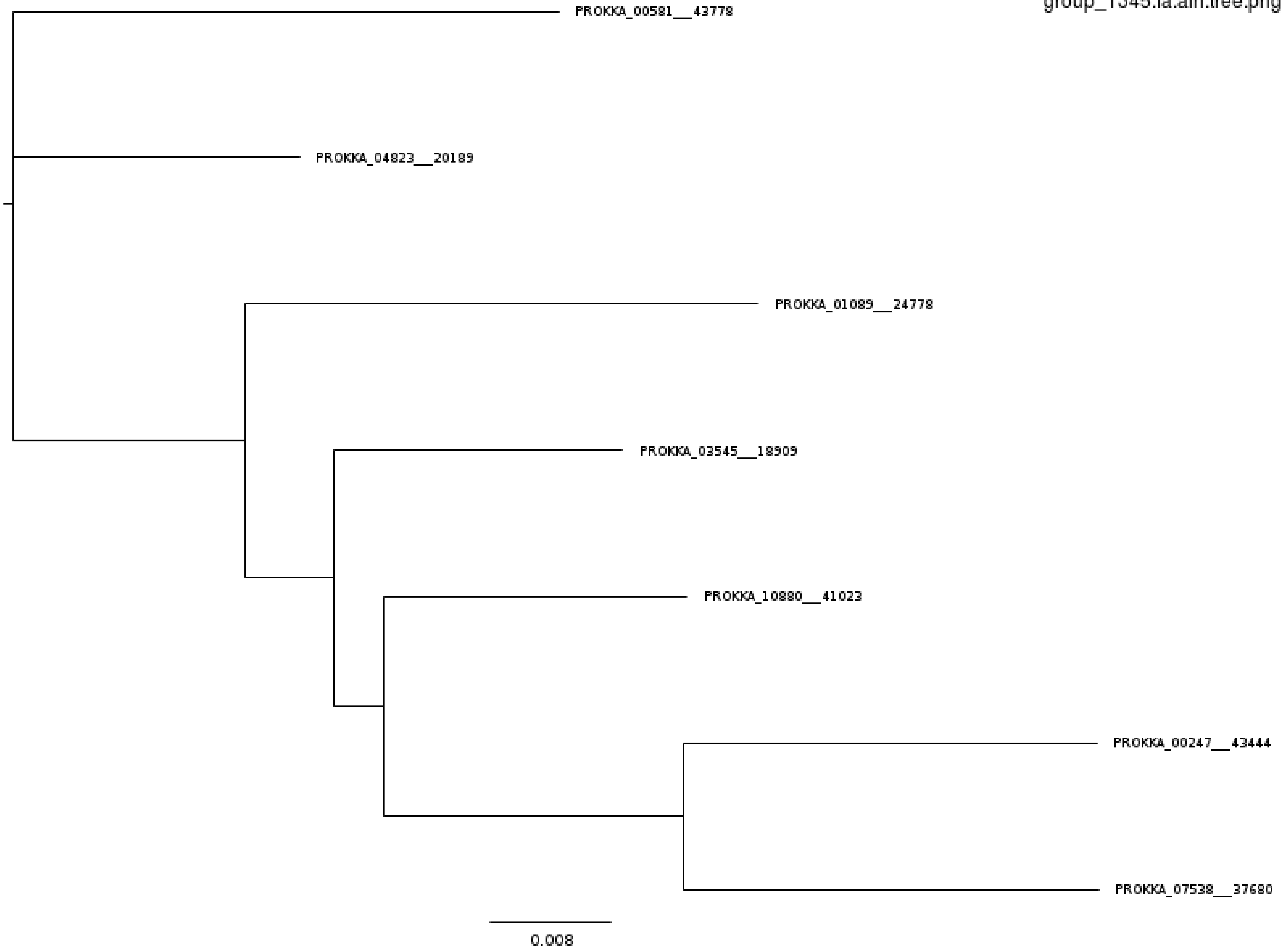

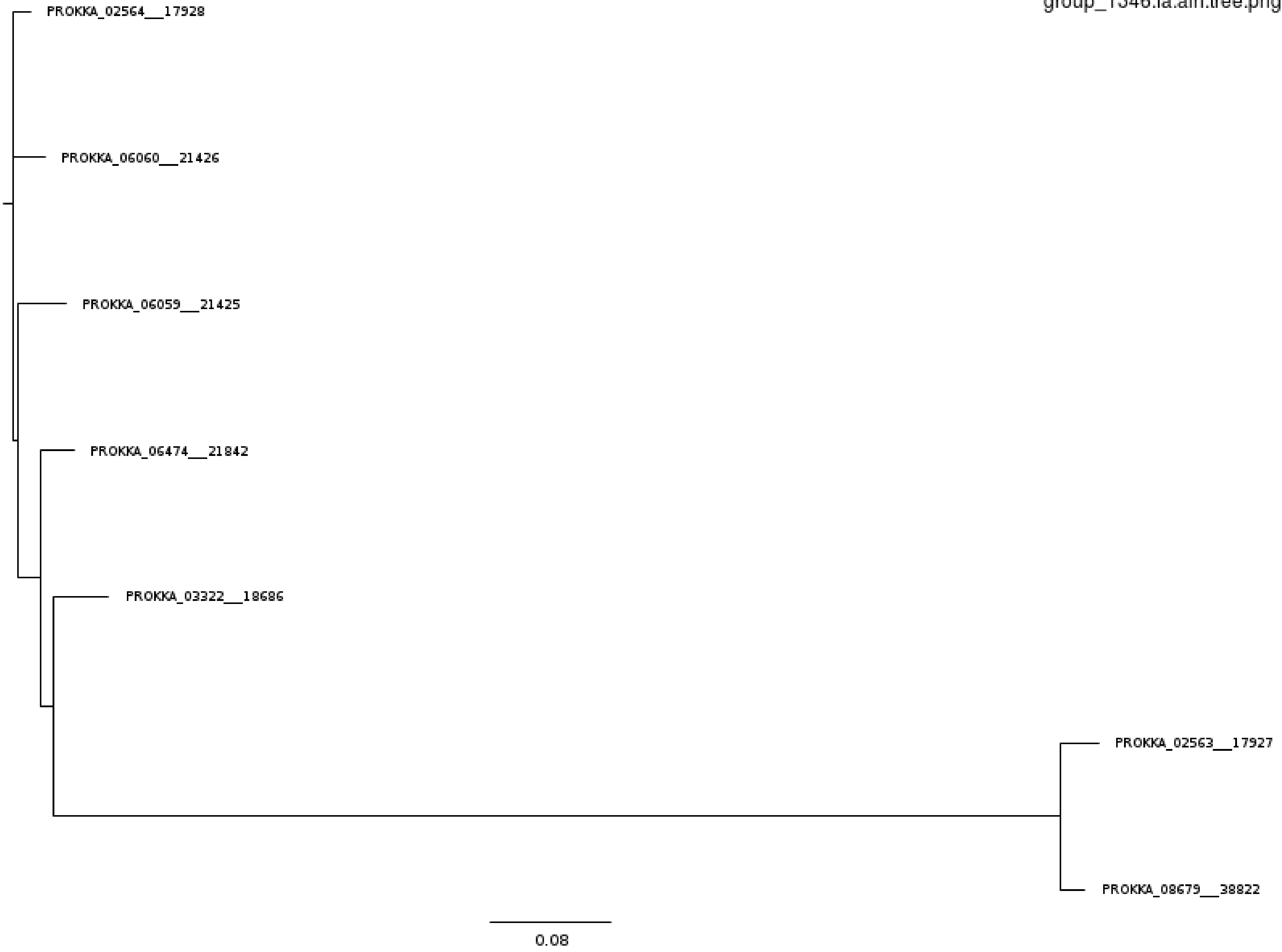
PROKKA_02564___17928
PROKKA_06060___21426
PROKKA_06059___21425
PROKKA_06474___21842
PROKKA_03322___18686
PROKKA_02563___17927
PROKKA_08679___38822
0.08

group_1347.fa.aln.tree.png

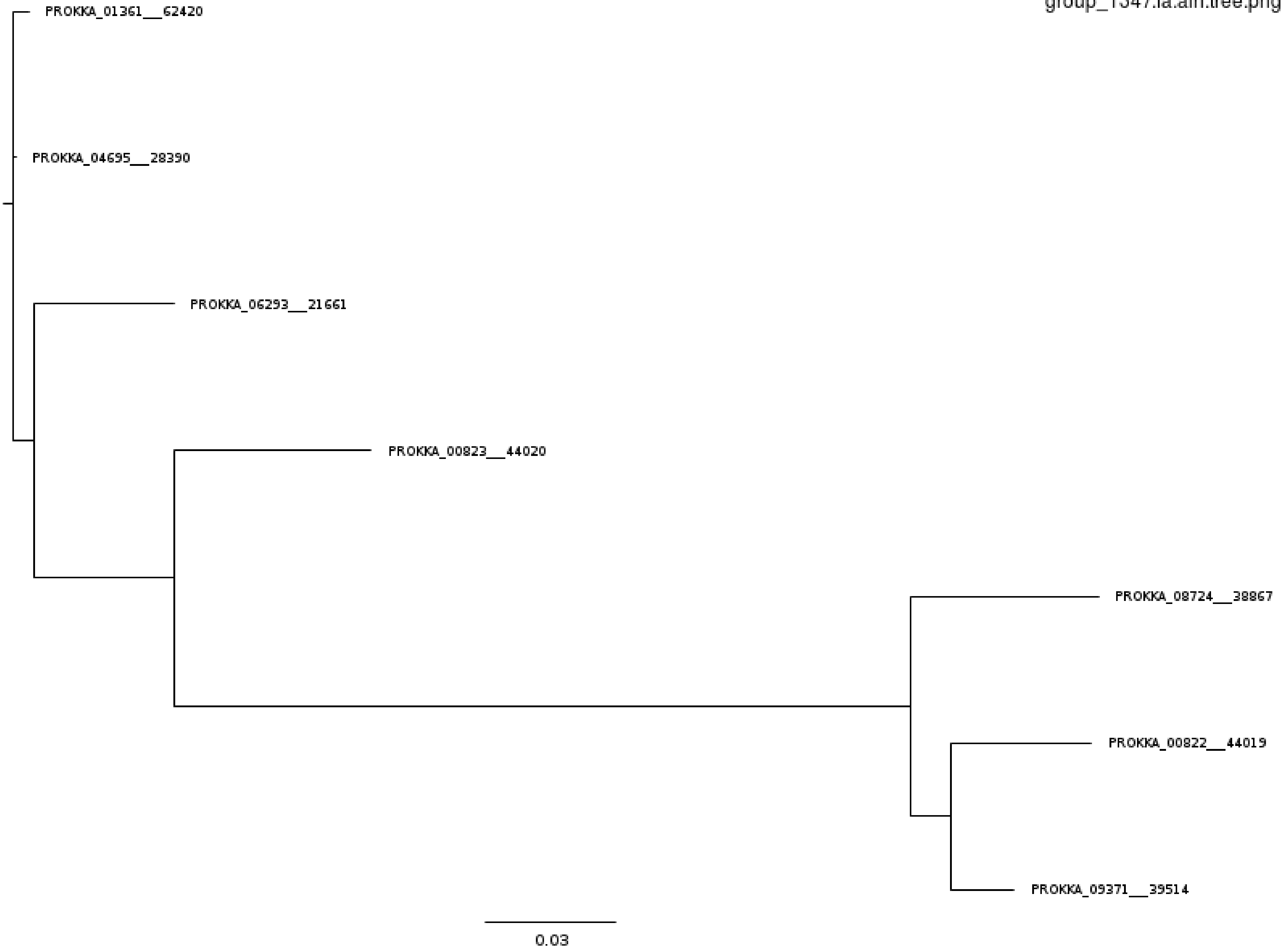

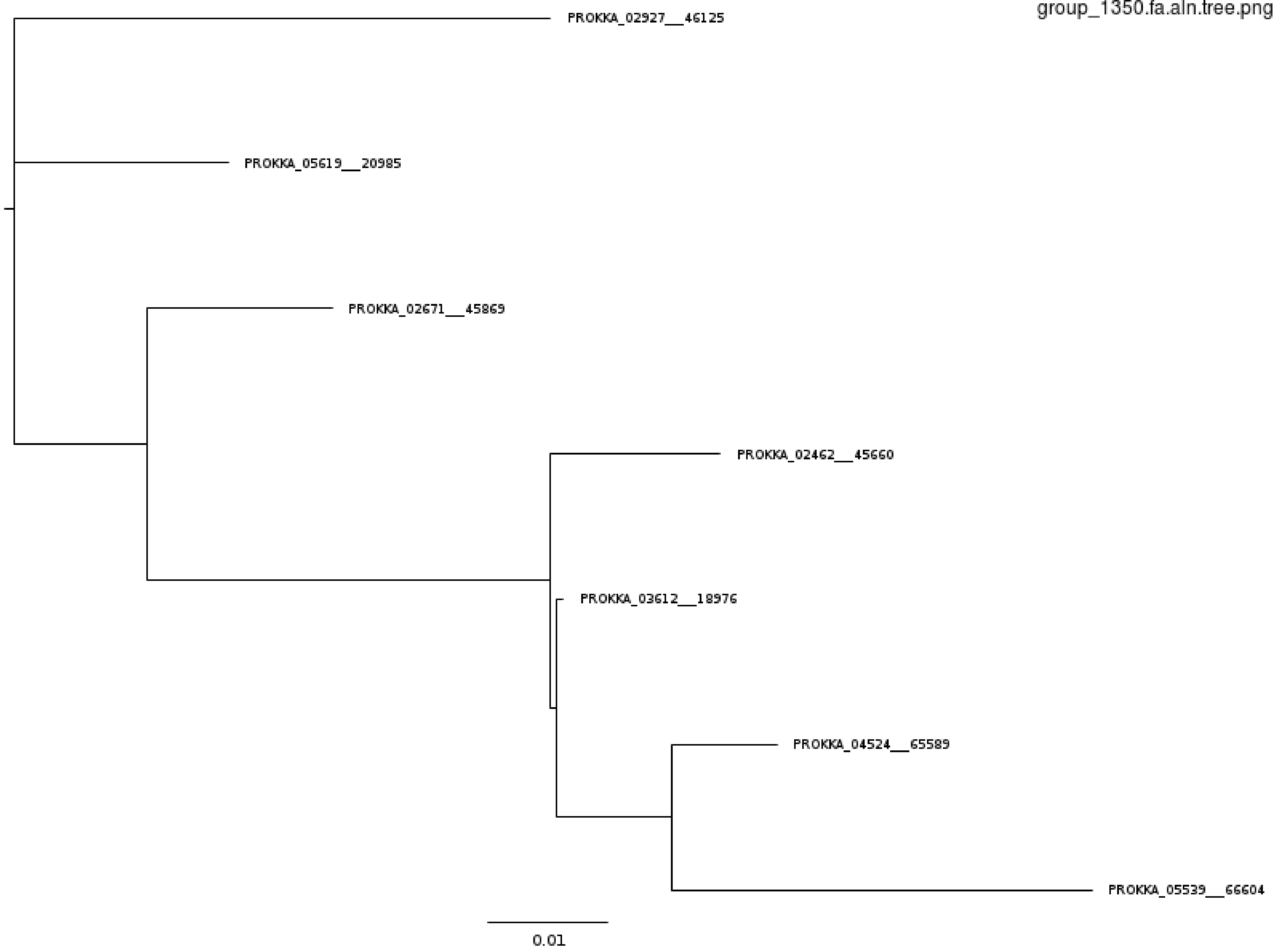
group_1350.fa.aln.tree.png
PROKKA_02927___46125
PROKKA_05619___20985
PROKKA_02671___45869
PROKKA_02462___45660
PROKKA_03612___18976
PROKKA_04524___65589
PROKKA_05539___66604
0.01

group_1352.fa.aln.tree.png

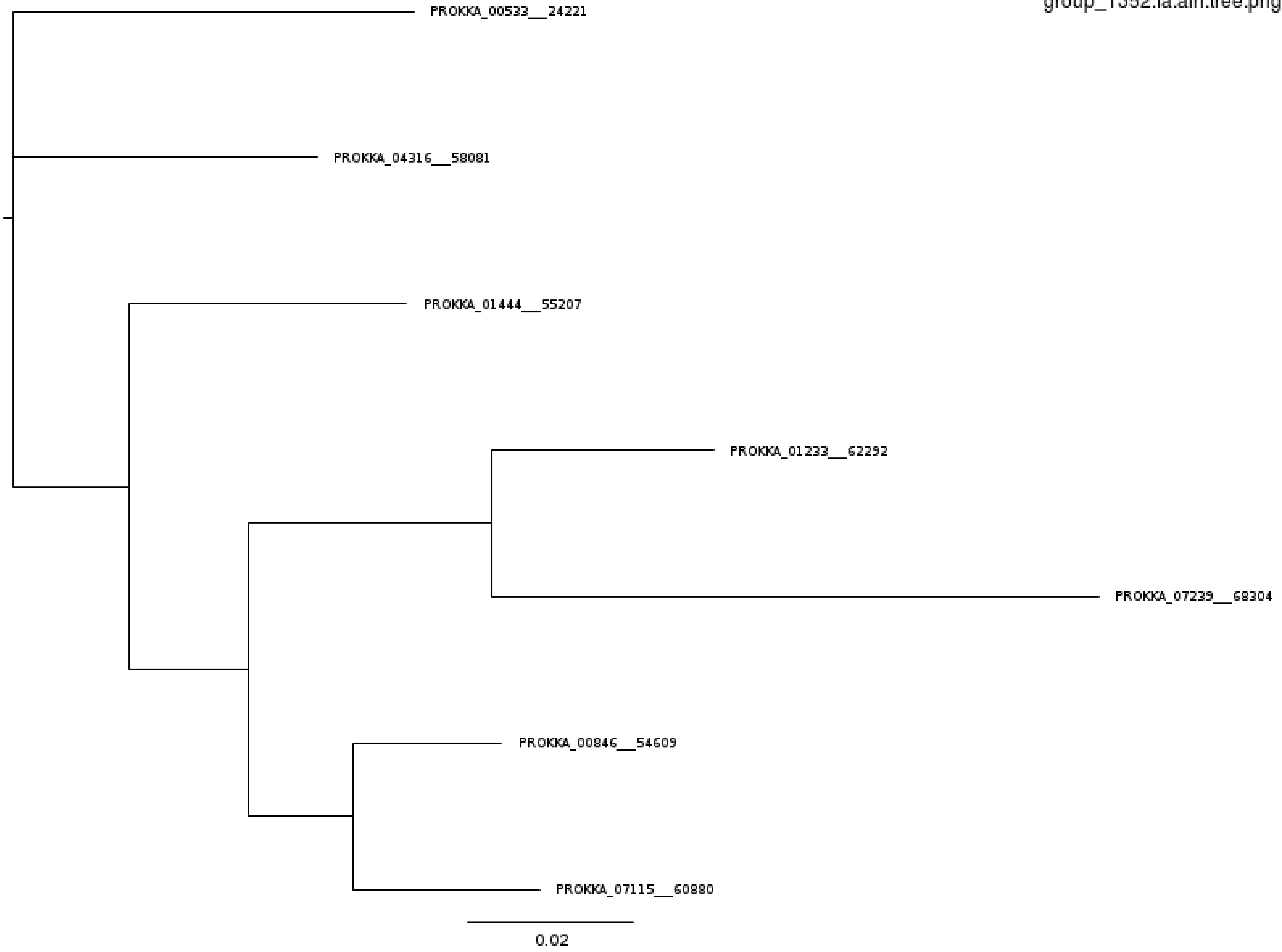

group_1355.fa.aln.tree.png

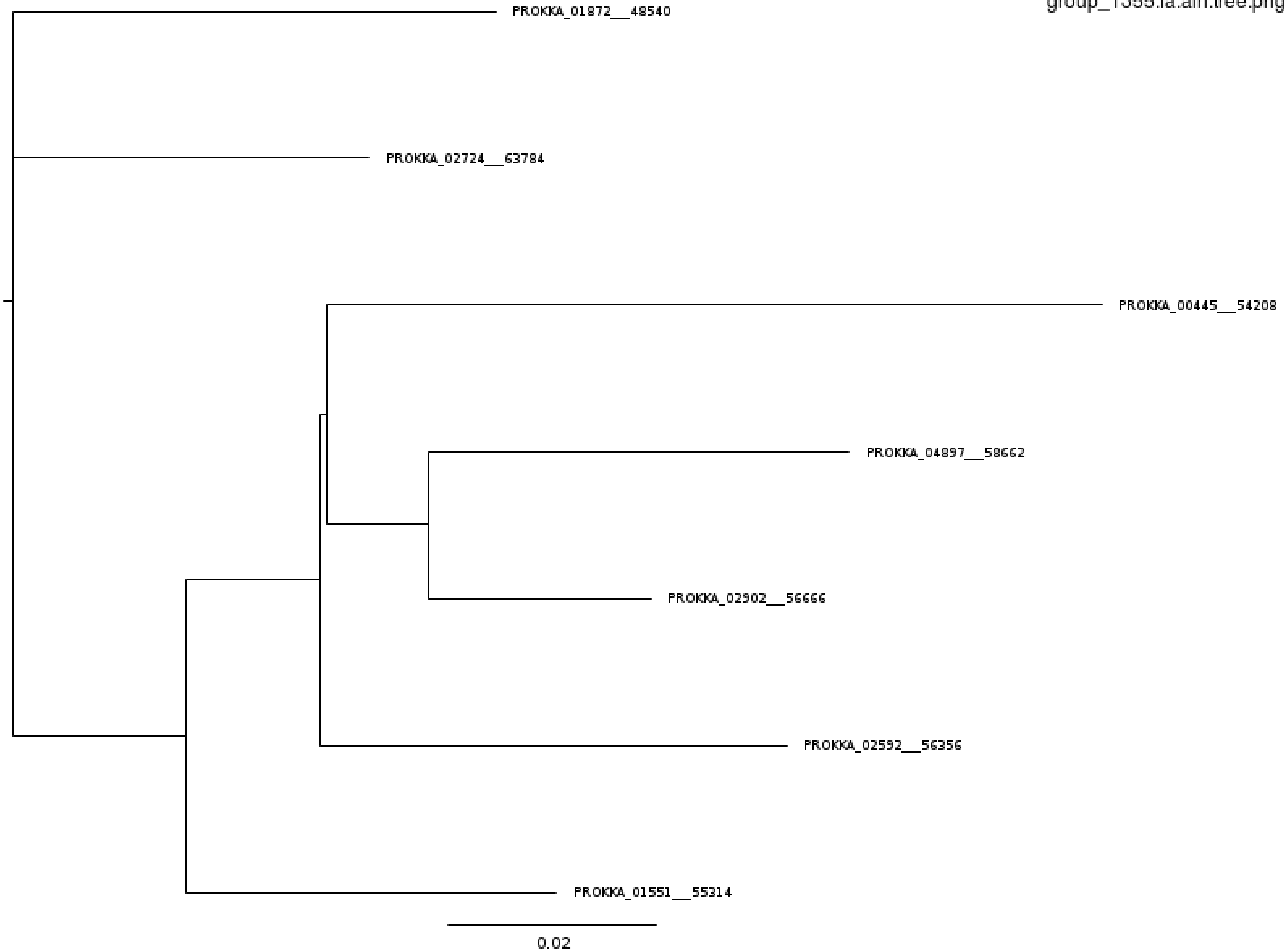

group_1357.fa.aln.tree.png

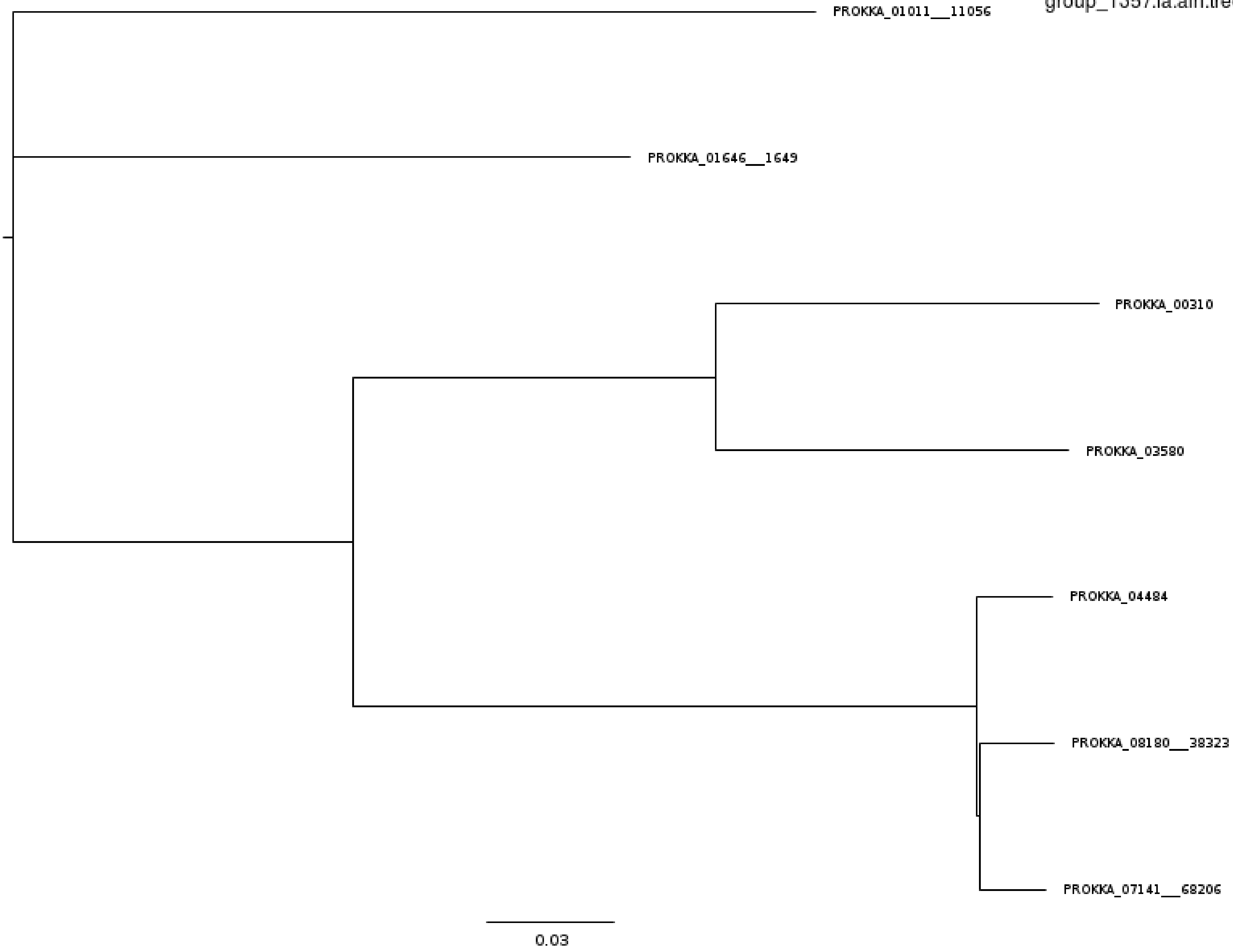

group_1358.fa.aln.tree.png

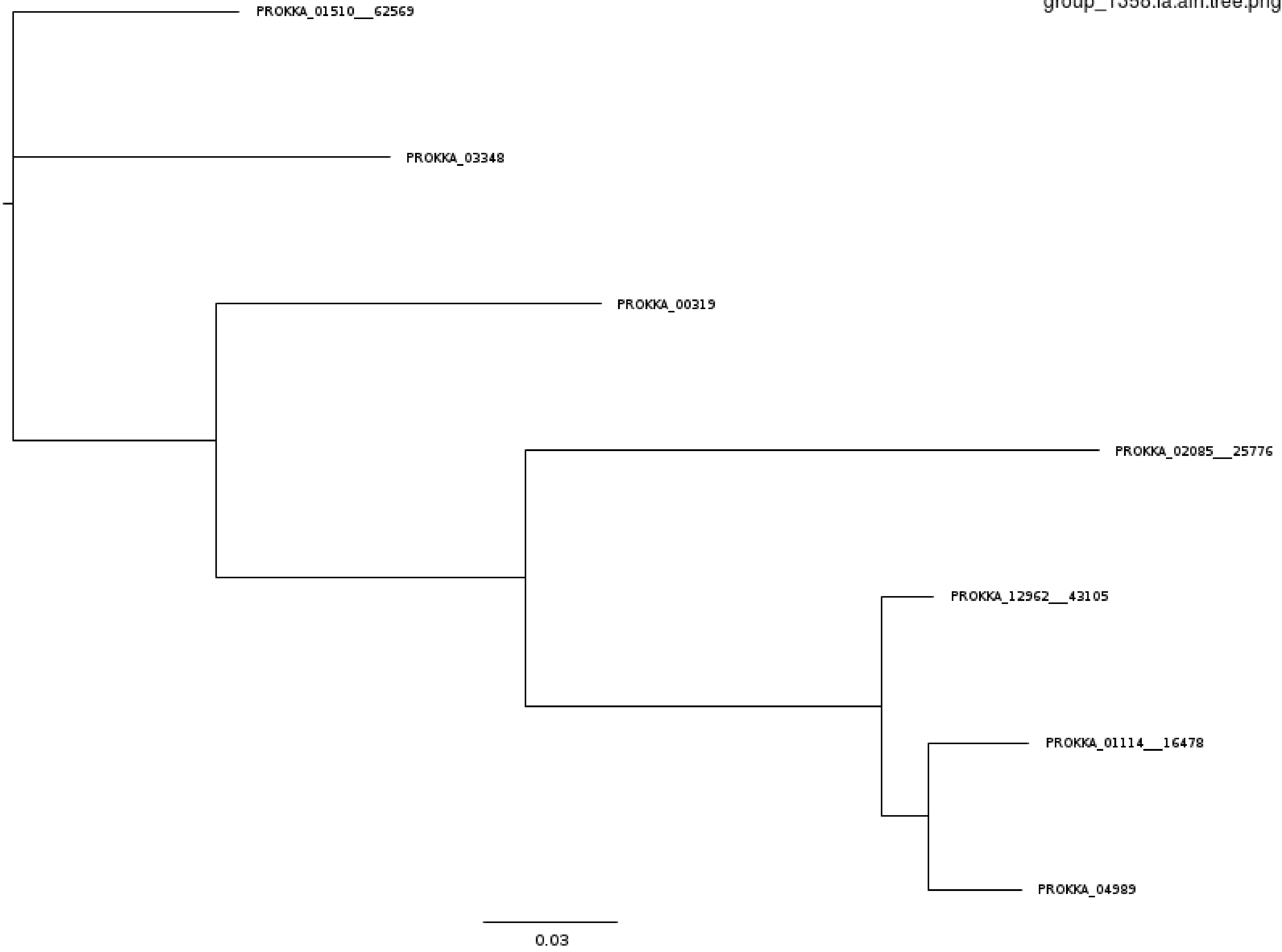

group_1361.fa.aln.tree.png

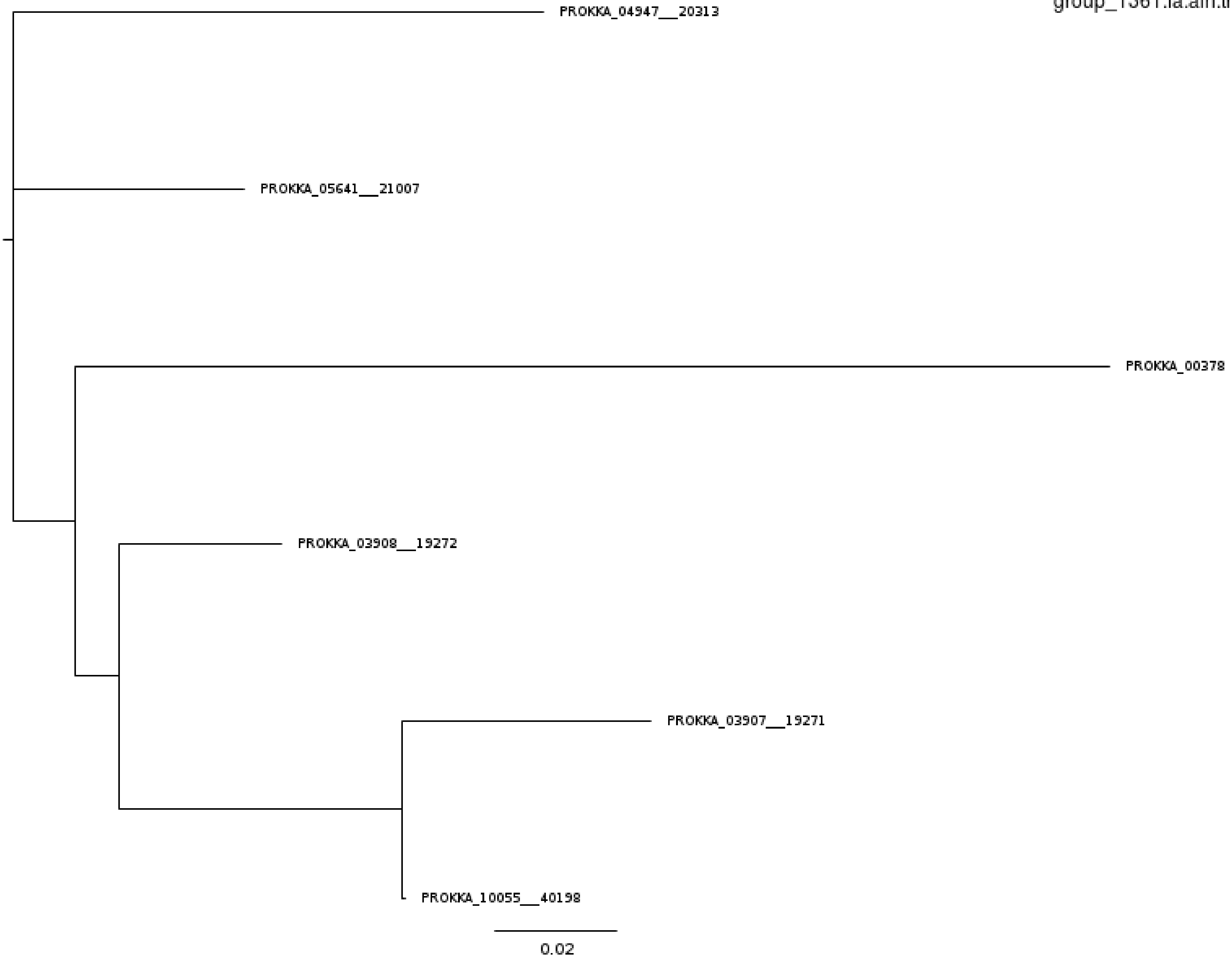

group_1369.fa.aln.tree.png

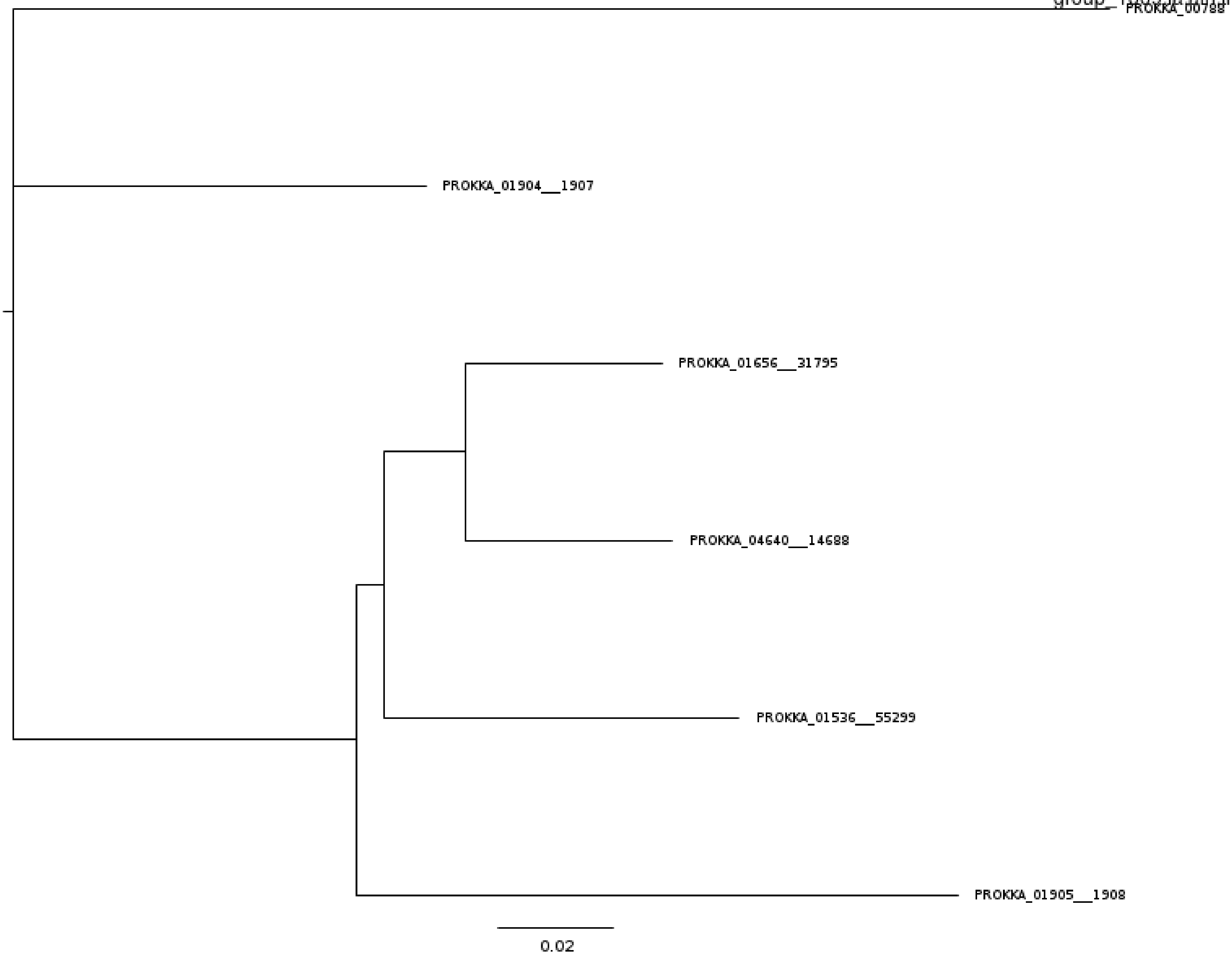

group_136.fa.aln.tree.png

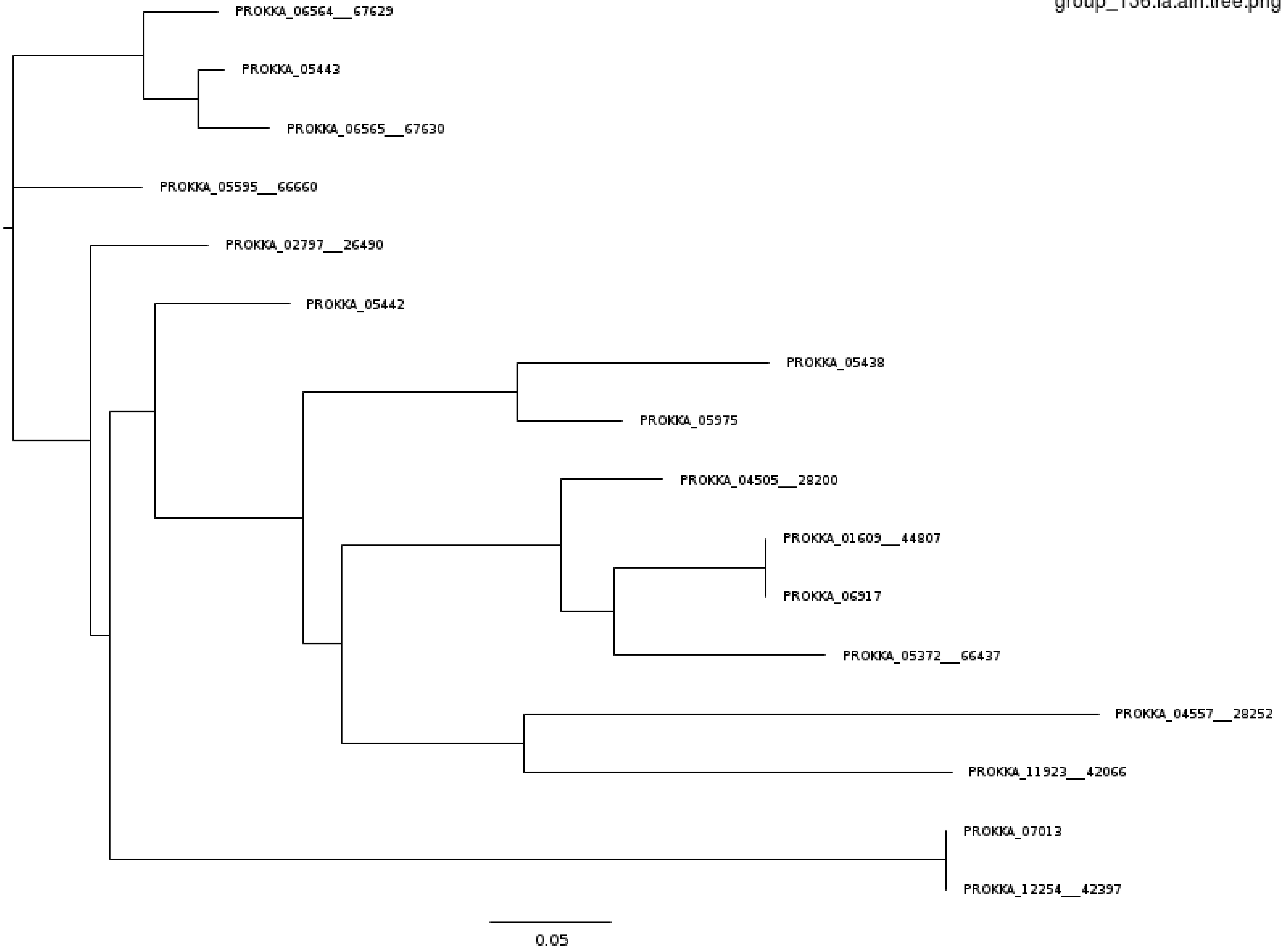

group_1371.fa.aln.tree.png

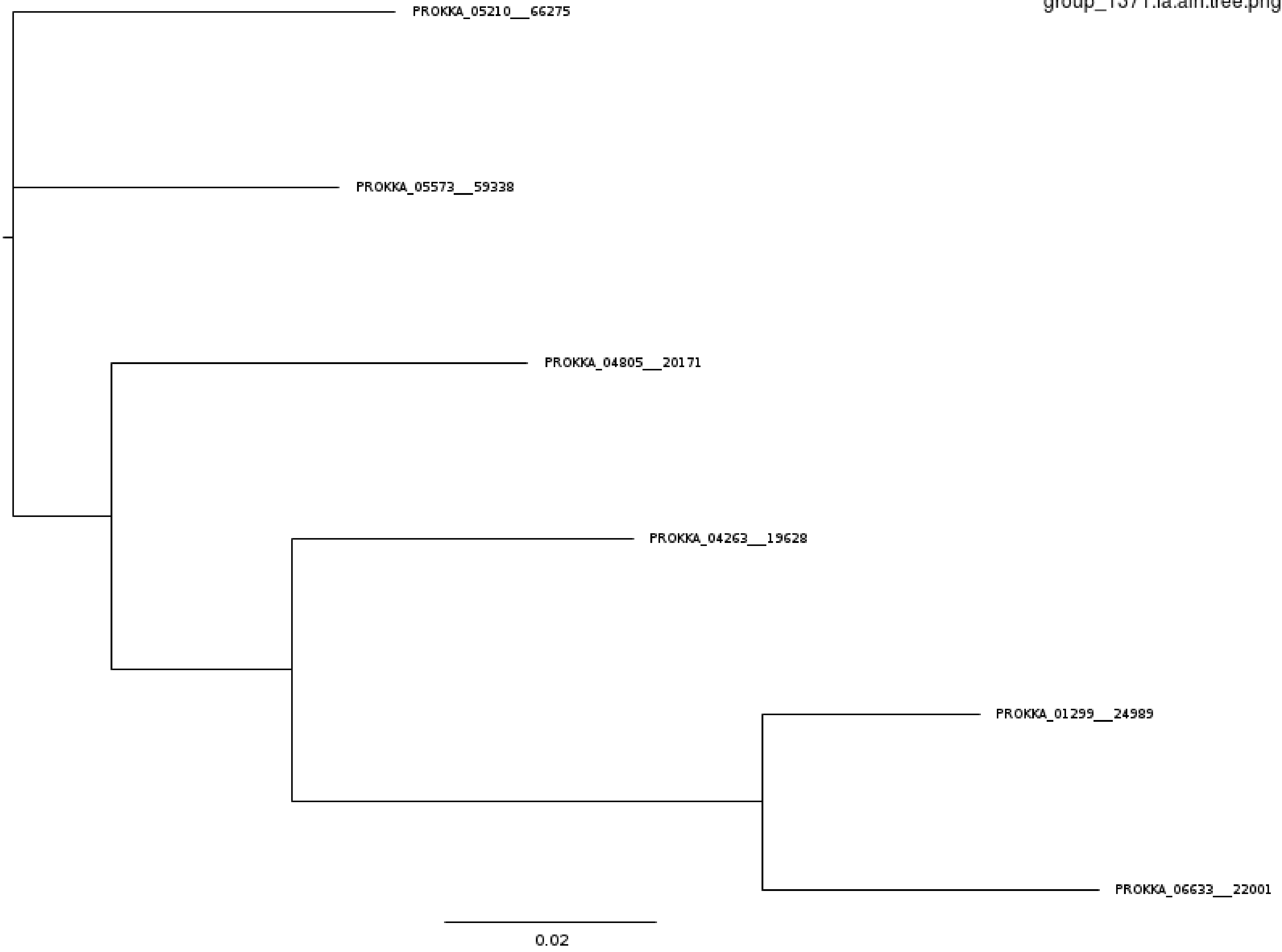

group_1373.fa.aln.tree.png

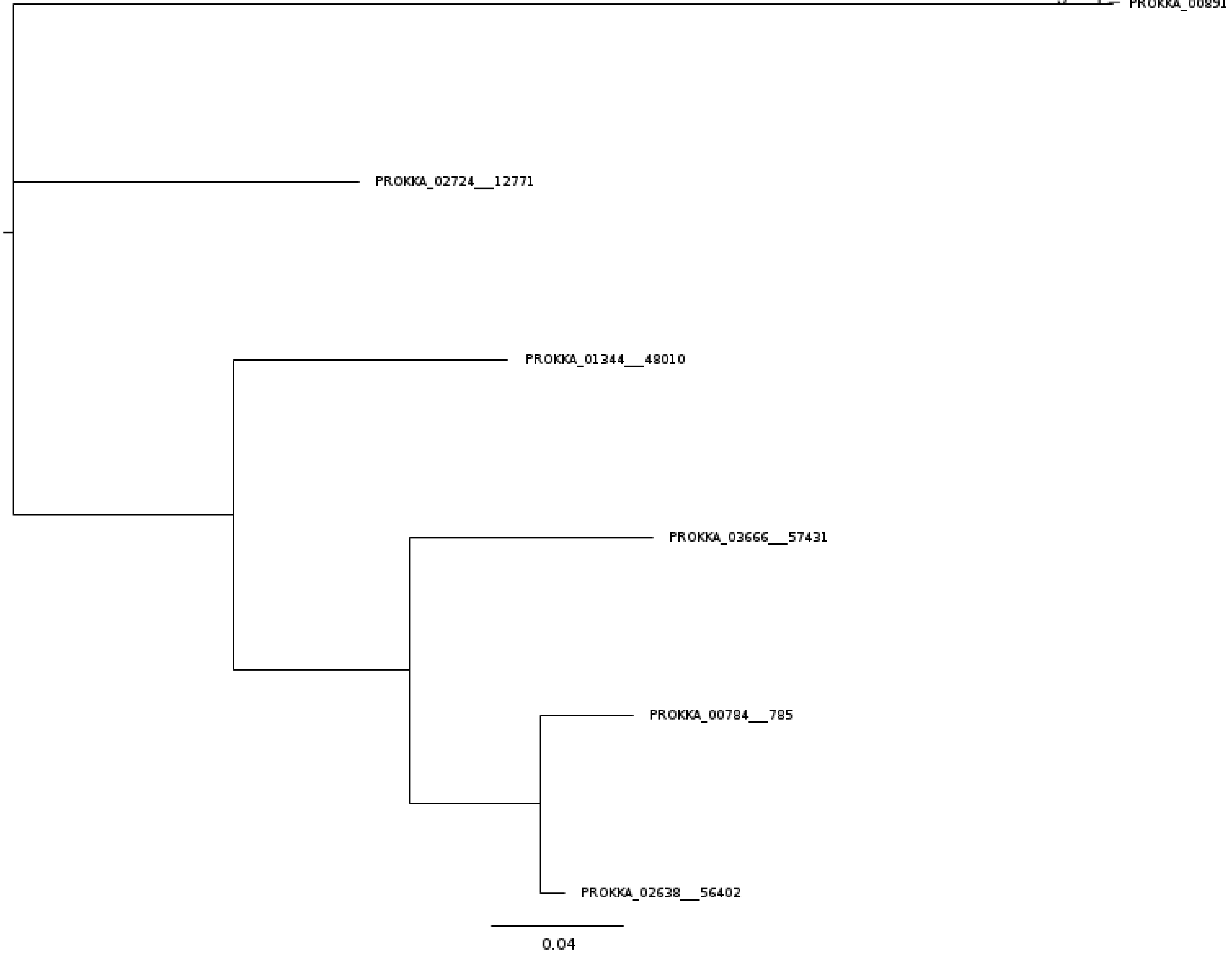

group_1374.fa.aln.tree.png

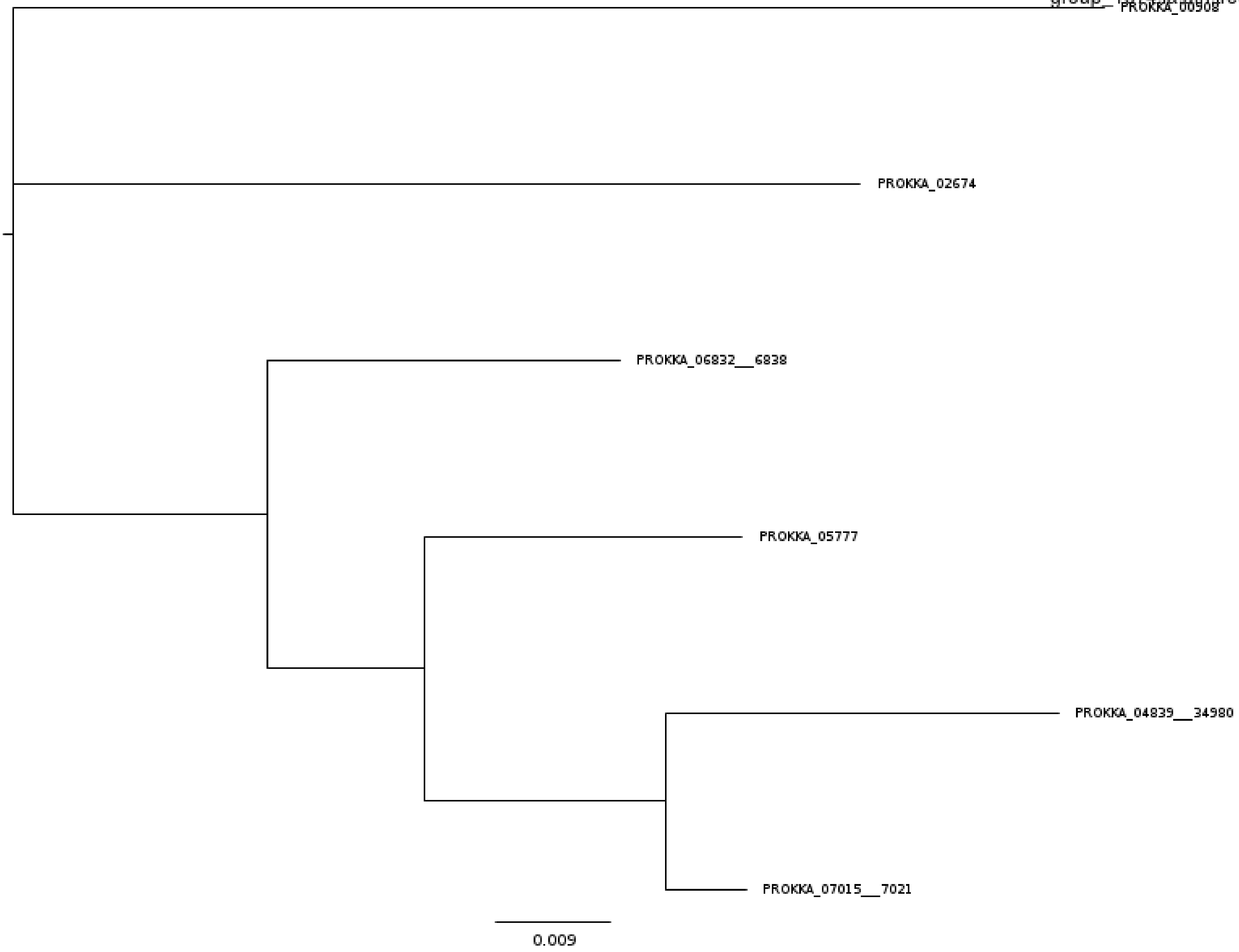

group_1376.fa.aln.tree.png

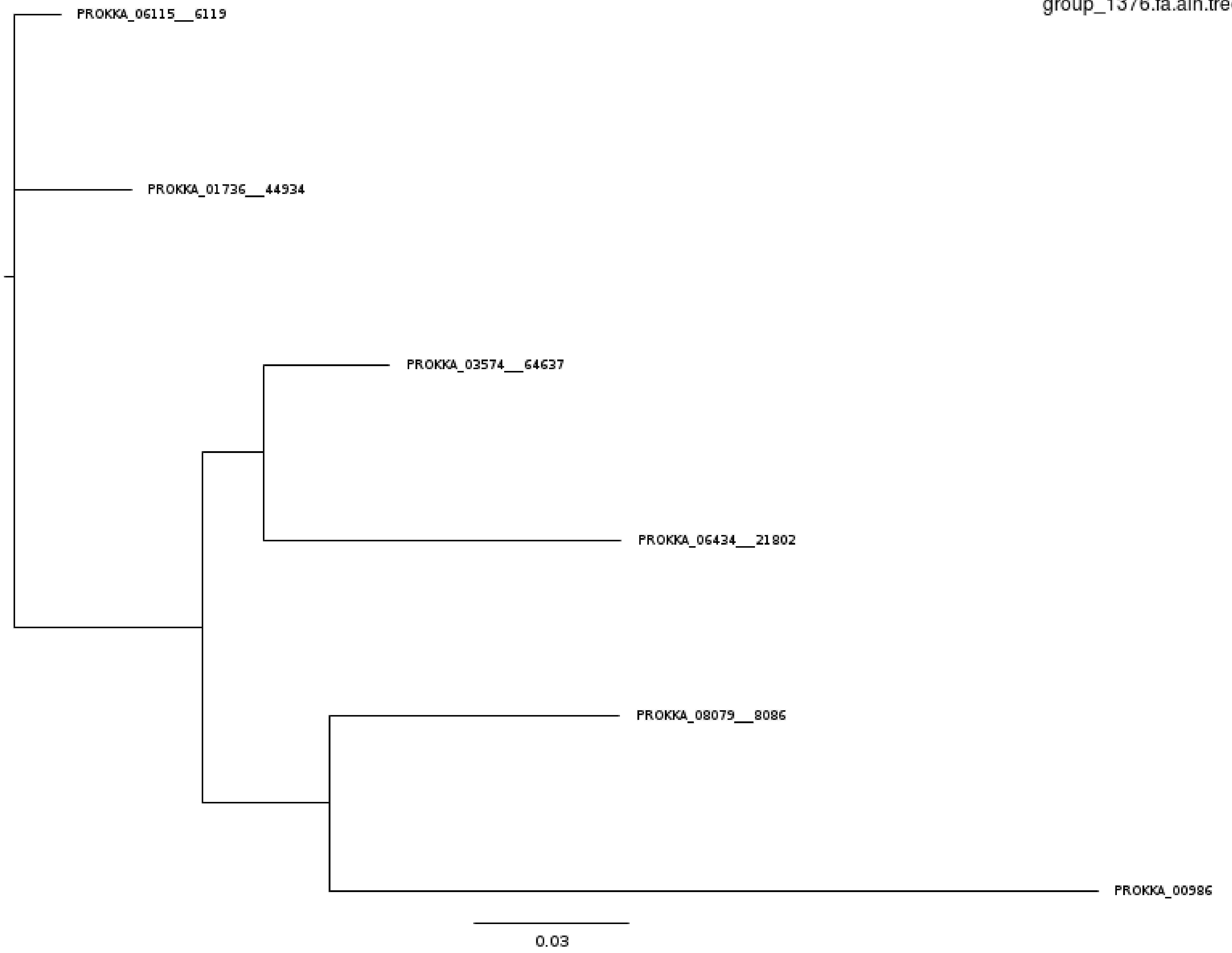

group_1378.fa.aln.tree.png

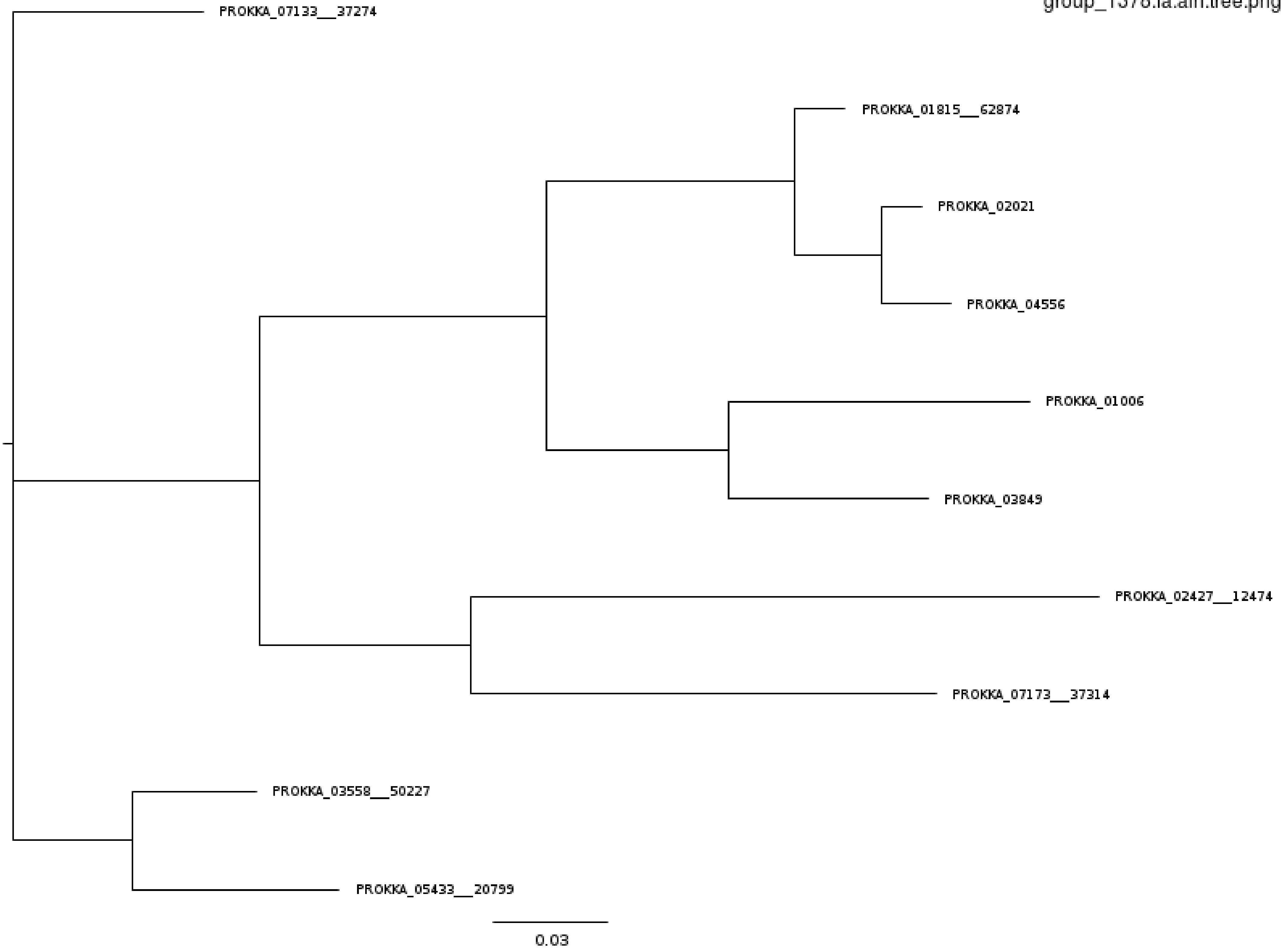

group_1381.fa.aln.tree.png

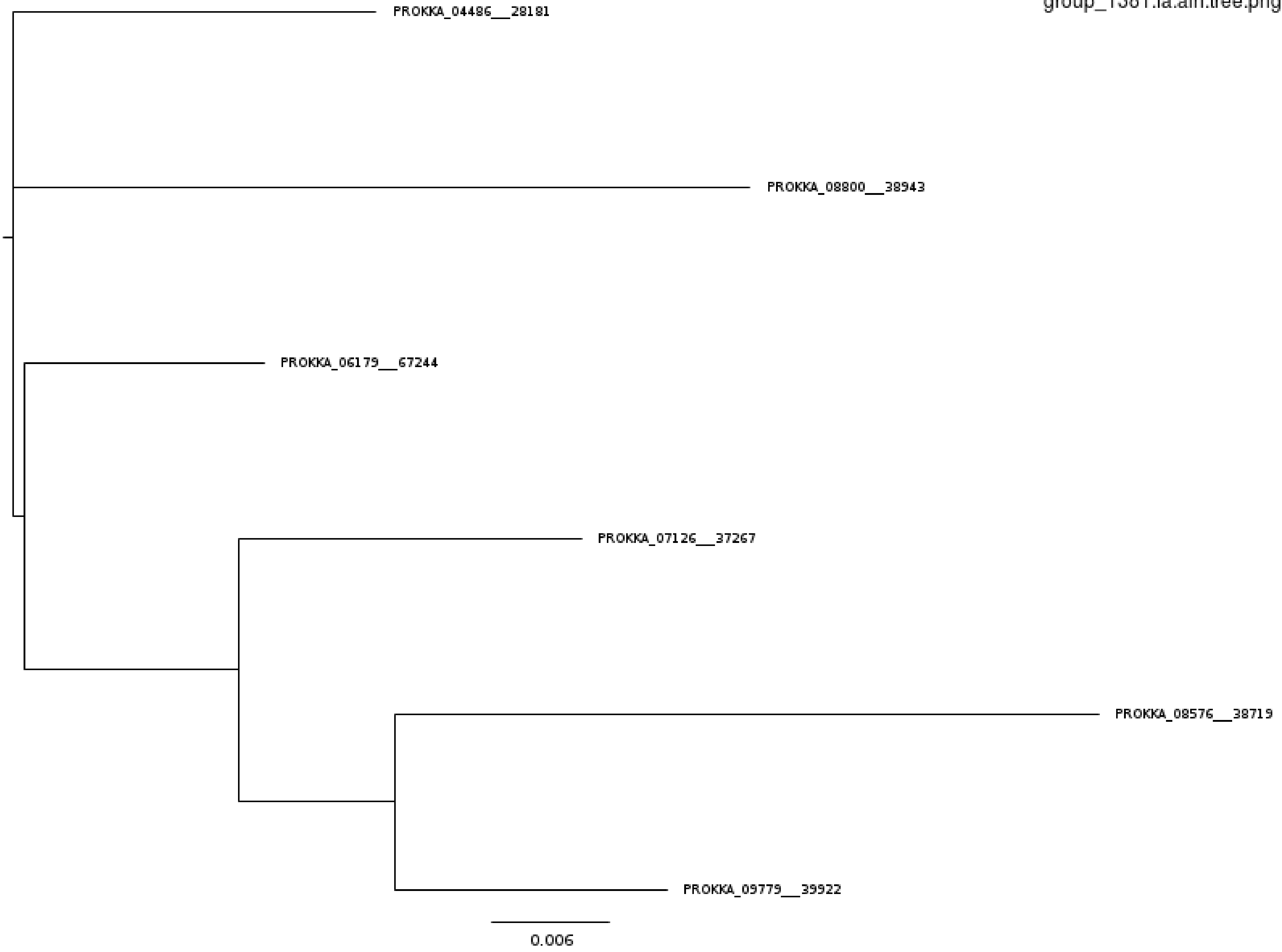

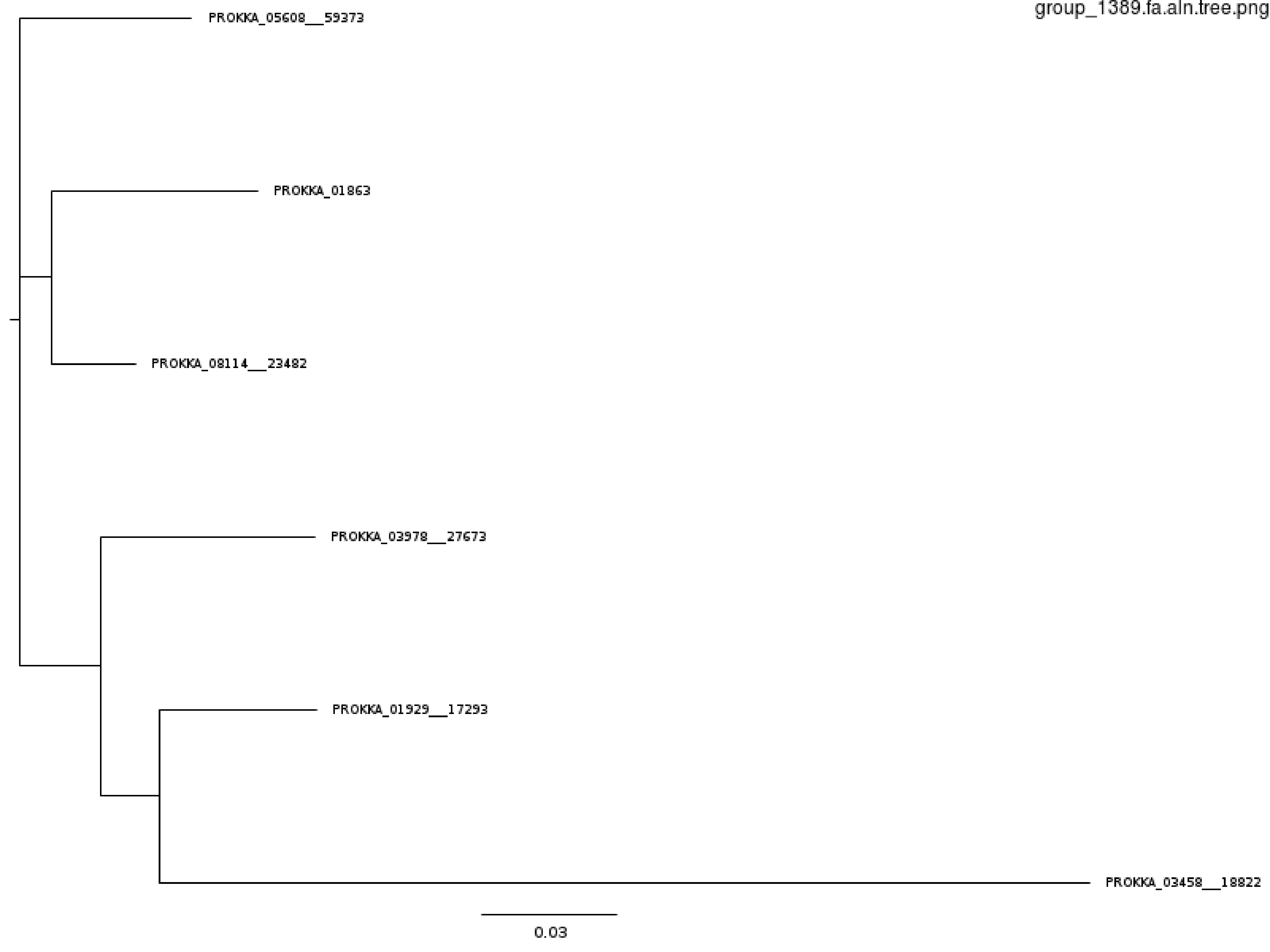
group_1389.fa.aln.tree.png
PROKKA_05608__59373
PROKKA_01863
PROKKA_08114__23482
PROKKA_03978__27673
PROKKA_01929__17293
PROKKA_03458__18822
0.03

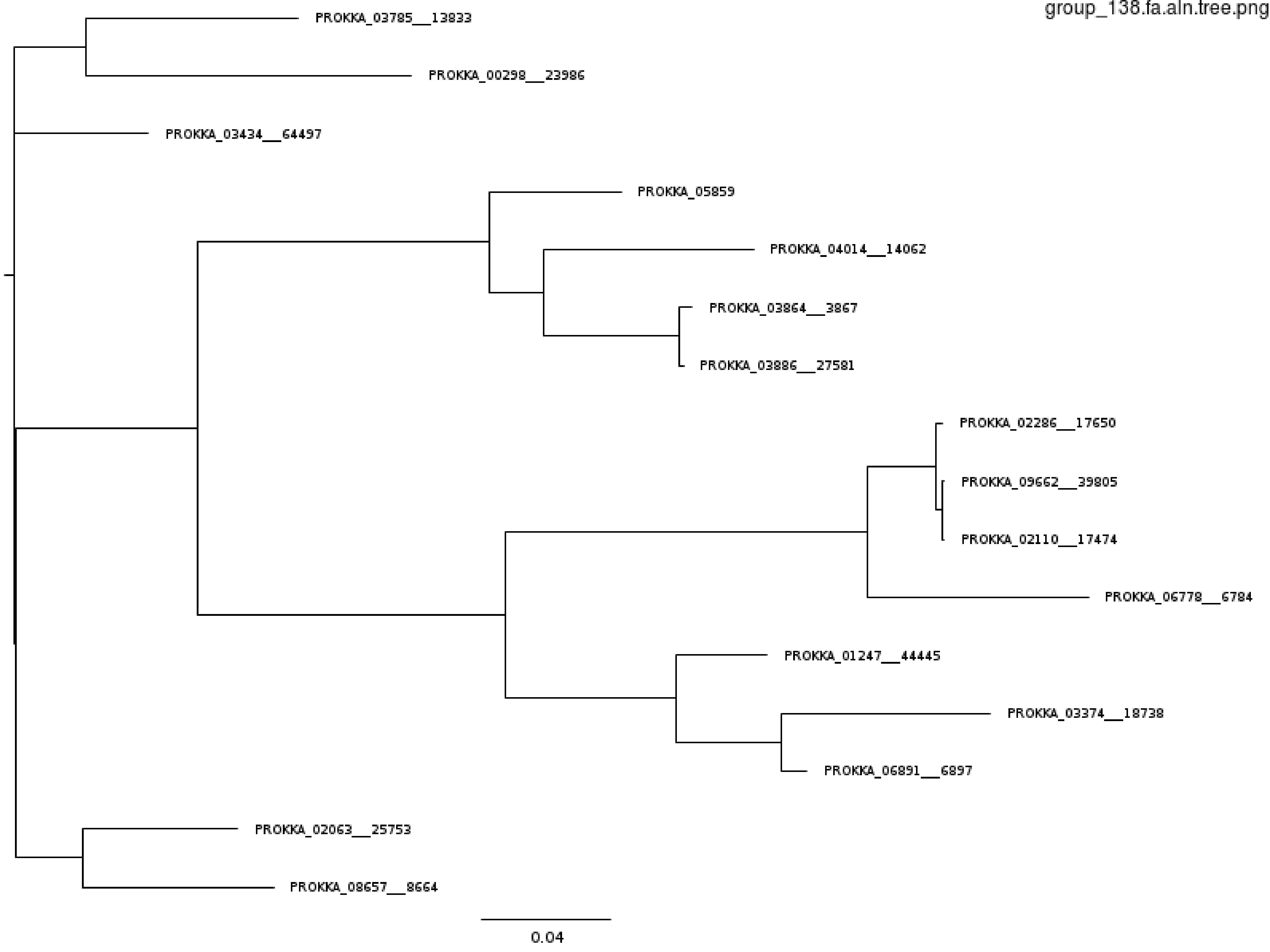
group_138.fa.aln.tree.png
PROKKA_03785___13833
PROKKA_00298___23986
PROKKA_03434___64497
PROKKA_05859
PROKKA_04014___14062
PROKKA_03864___3867
PROKKA_03886___27581
PROKKA_02286___17650
PROKKA_09662___39805
PROKKA_02110___17474
PROKKA_06778___6784
PROKKA_01247___44445
PROKKA_03374___18738
PROKKA_06891___6897
PROKKA_02063___25753
PROKKA_08657___8664
0.04

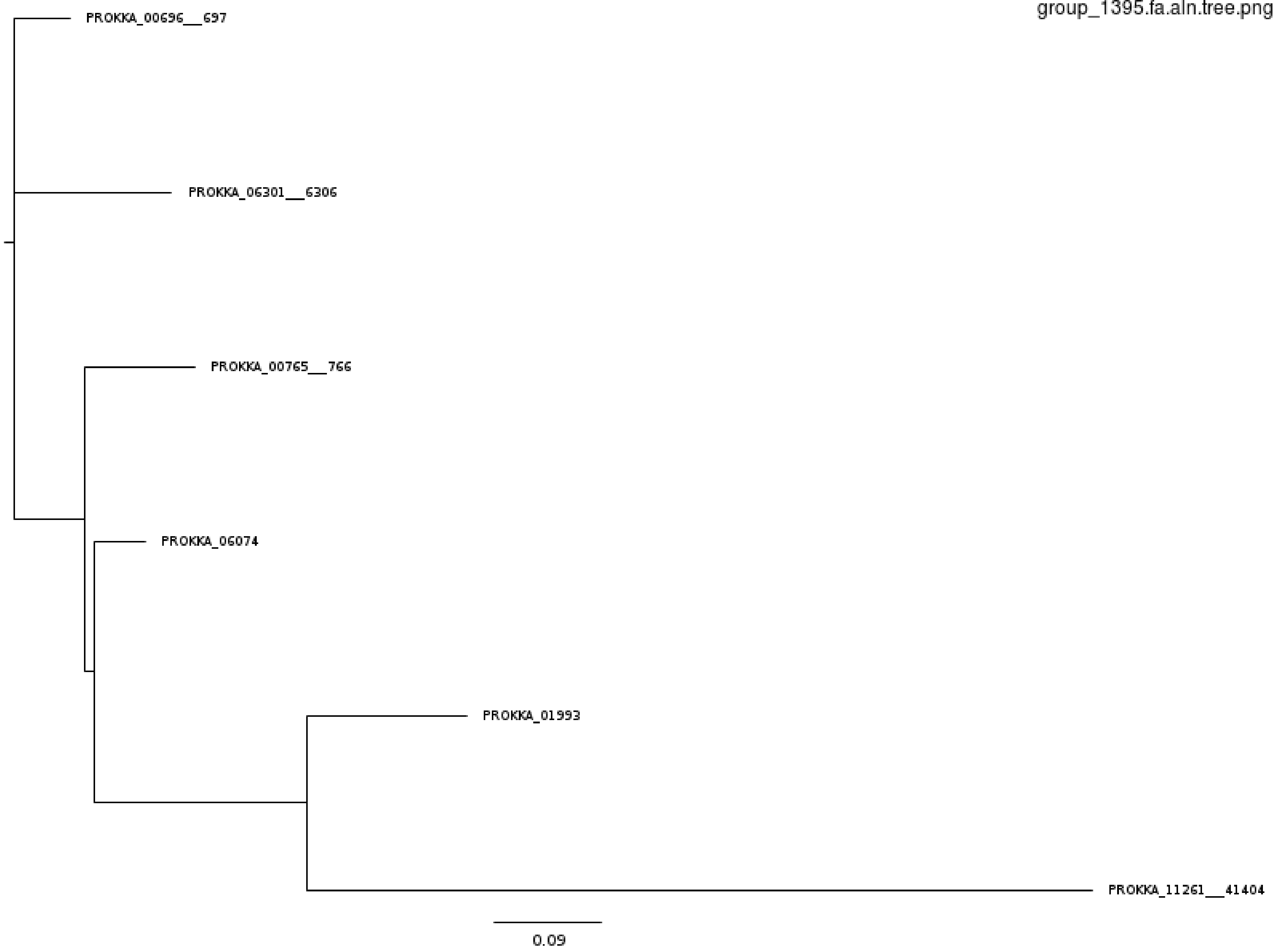
group_1395.fa.aln.tree.png
PROKKA_00696___697
PROKKA_06301___6306
PROKKA_00765___766
PROKKA_06074
PROKKA_01993
PROKKA_11261___41404
0.09

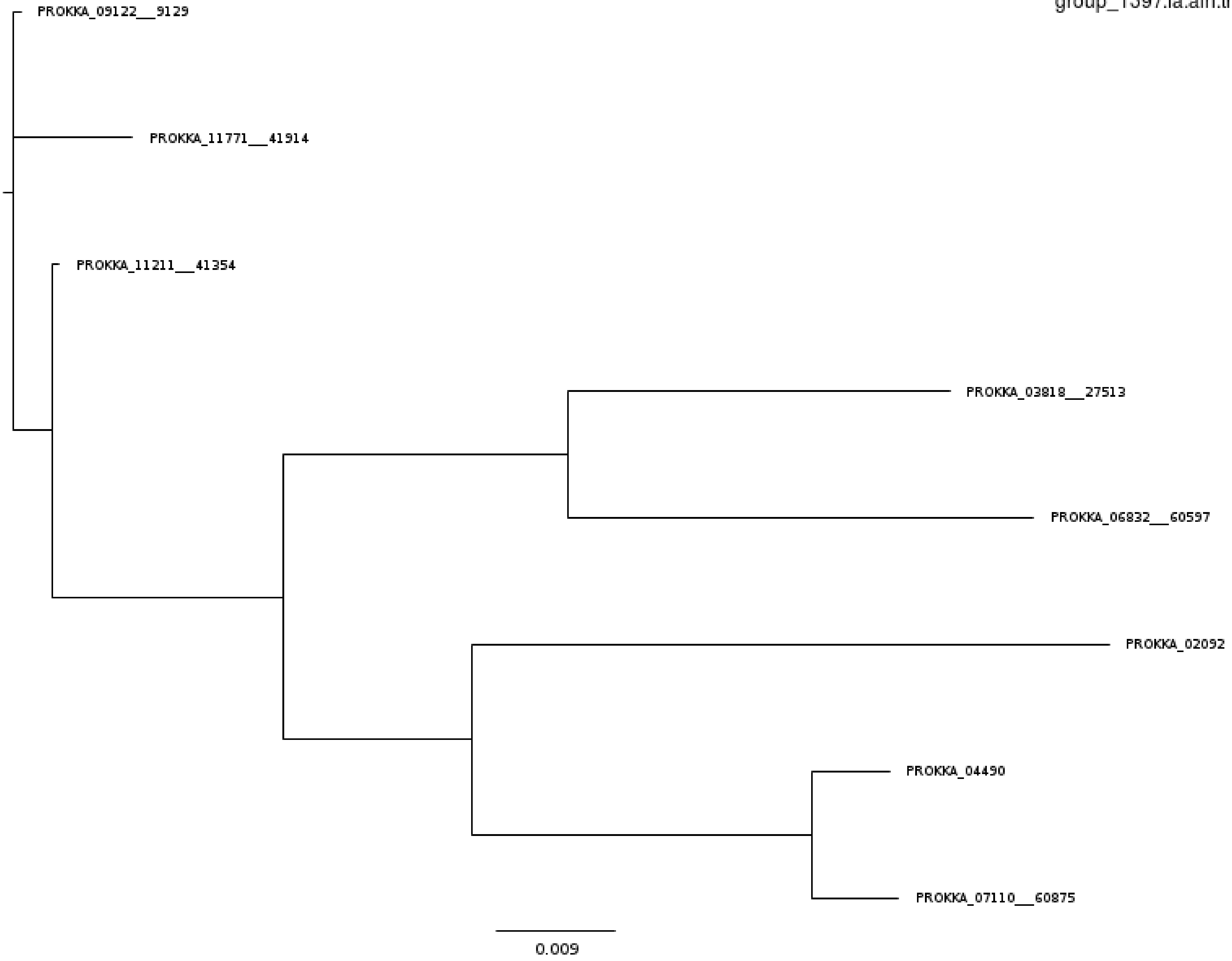
PROKKA_09122___9129
PROKKA_11771___41914
PROKKA_11211___41354
PROKKA_03818___27513
PROKKA_06832___60597
PROKKA_02092
PROKKA_04490
PROKKA_07110___60875
0.009

group_139.fa.aln.tree.png

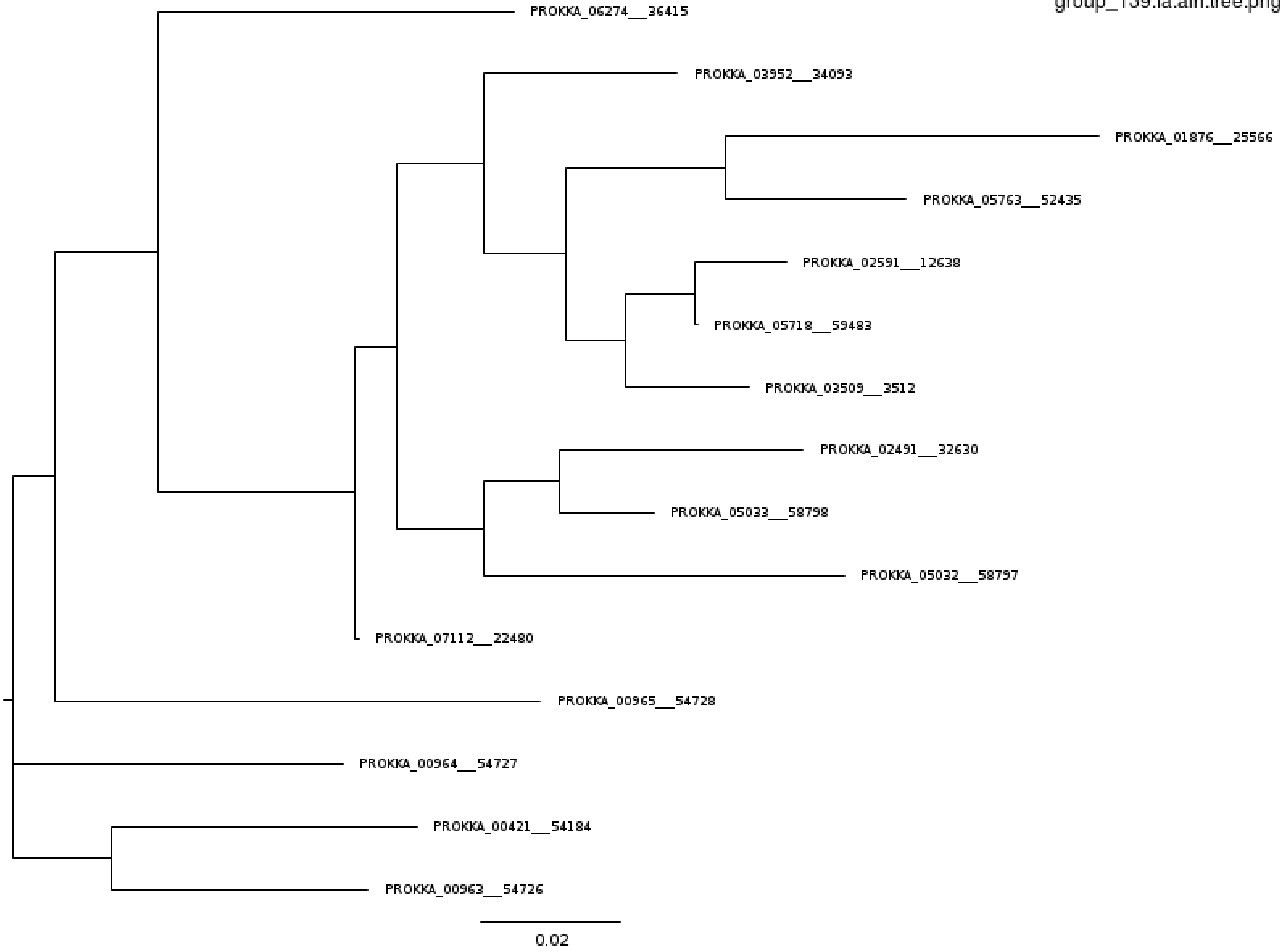

group_13.fa.aln.tree.png

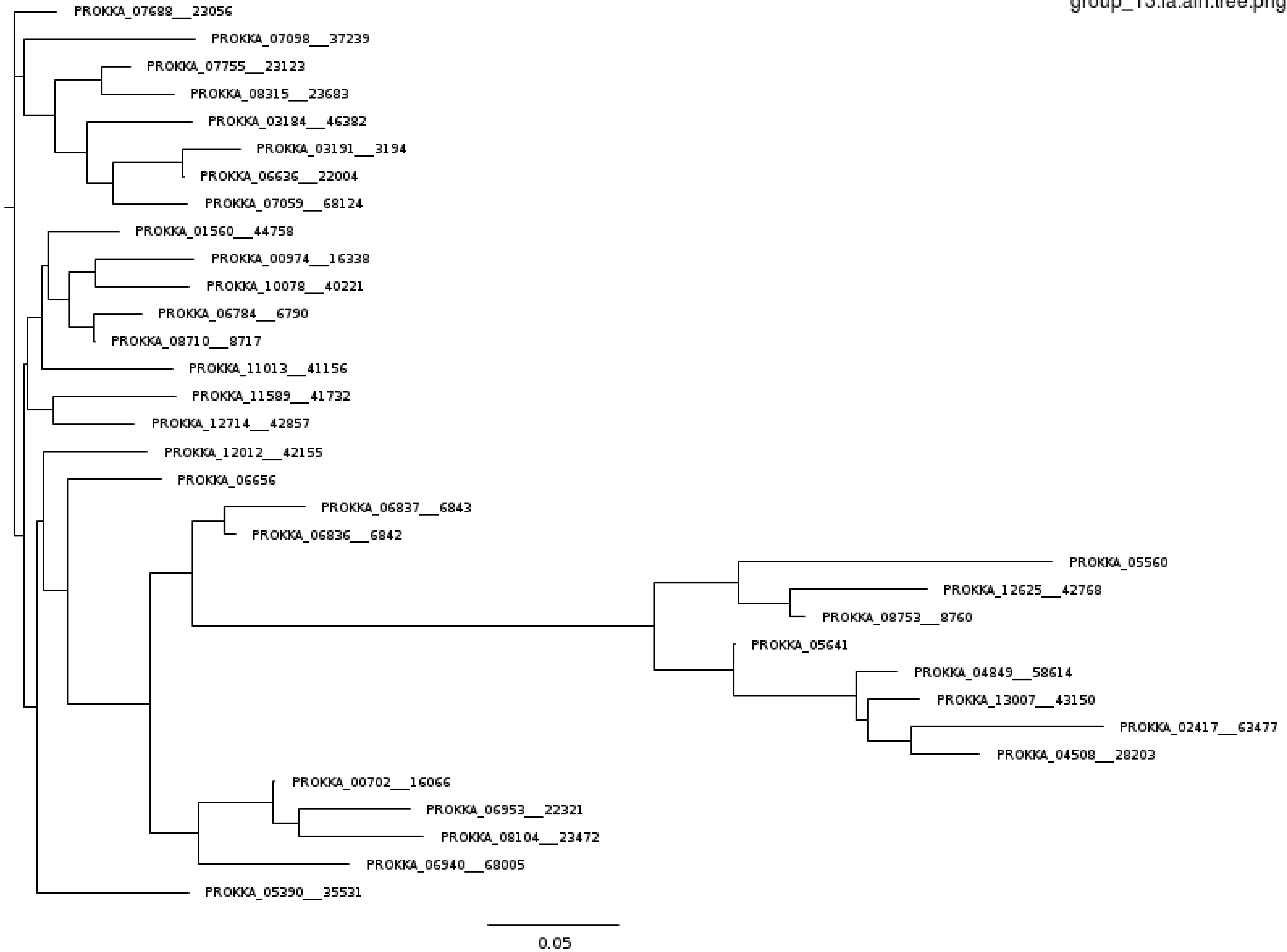

group_1400.fa.aln.tree.png

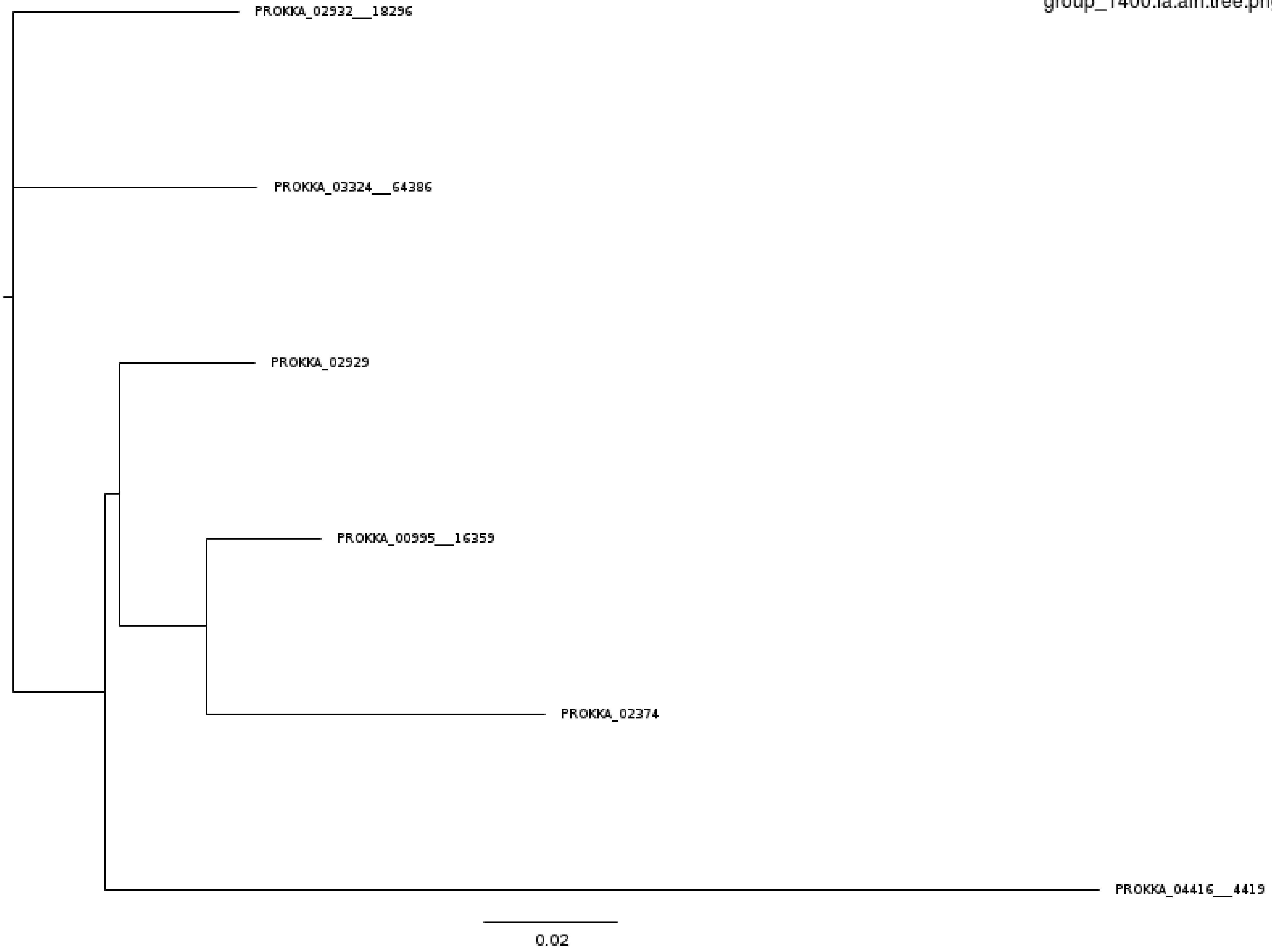

group_1402.fa.aln.tree.png

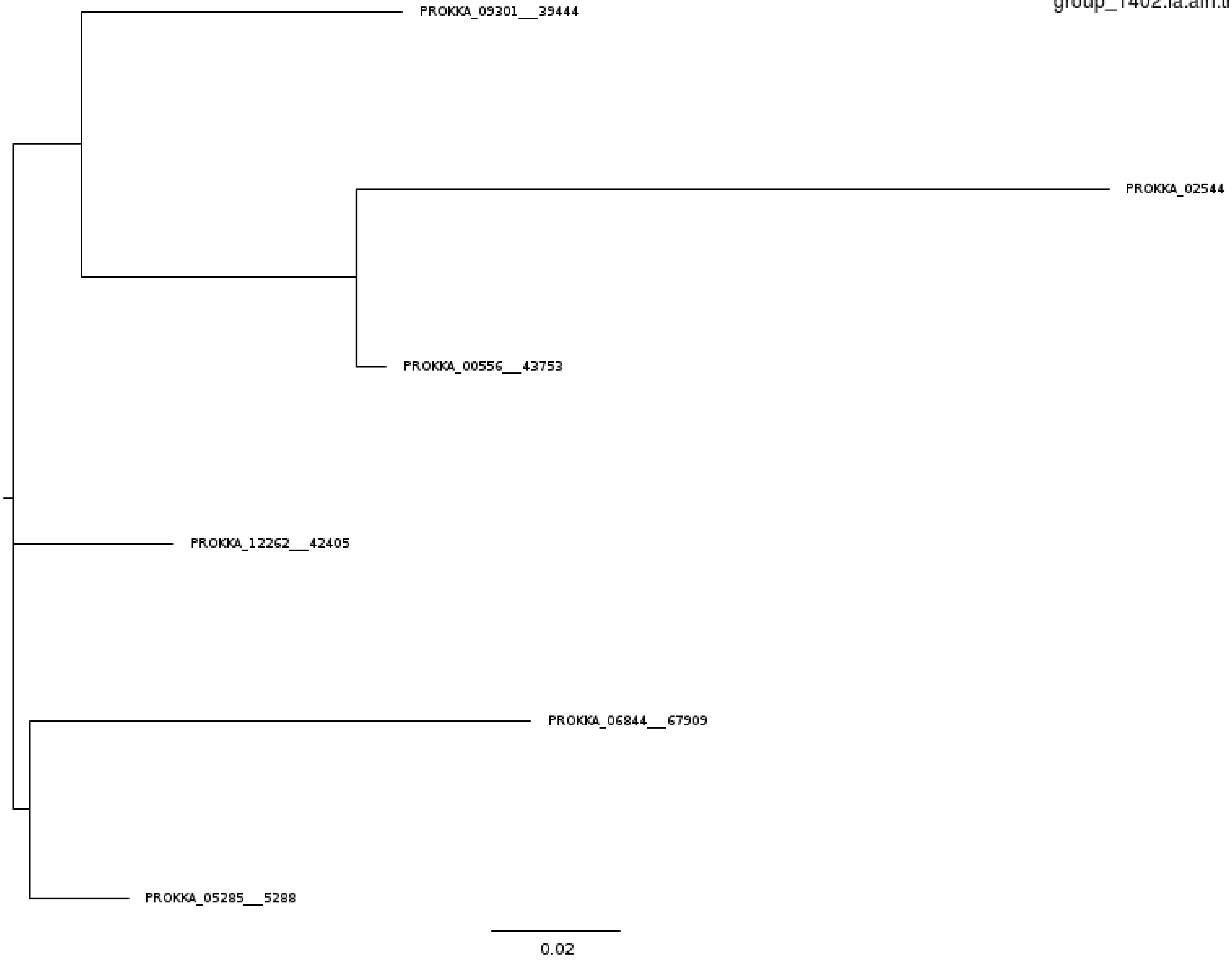

group_1403.fa.aln.tree.png

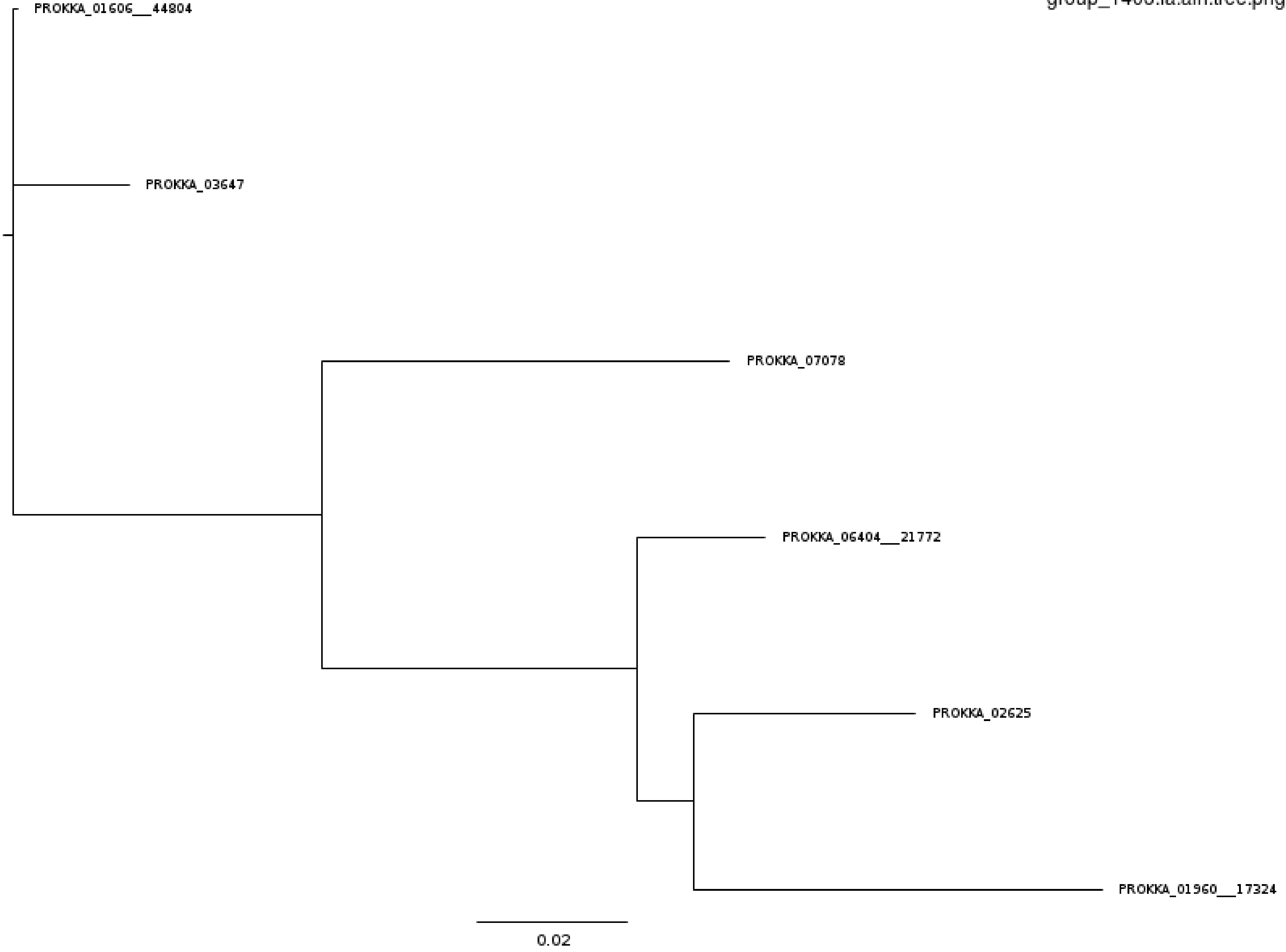

group_1409.fa.aln.tree.png

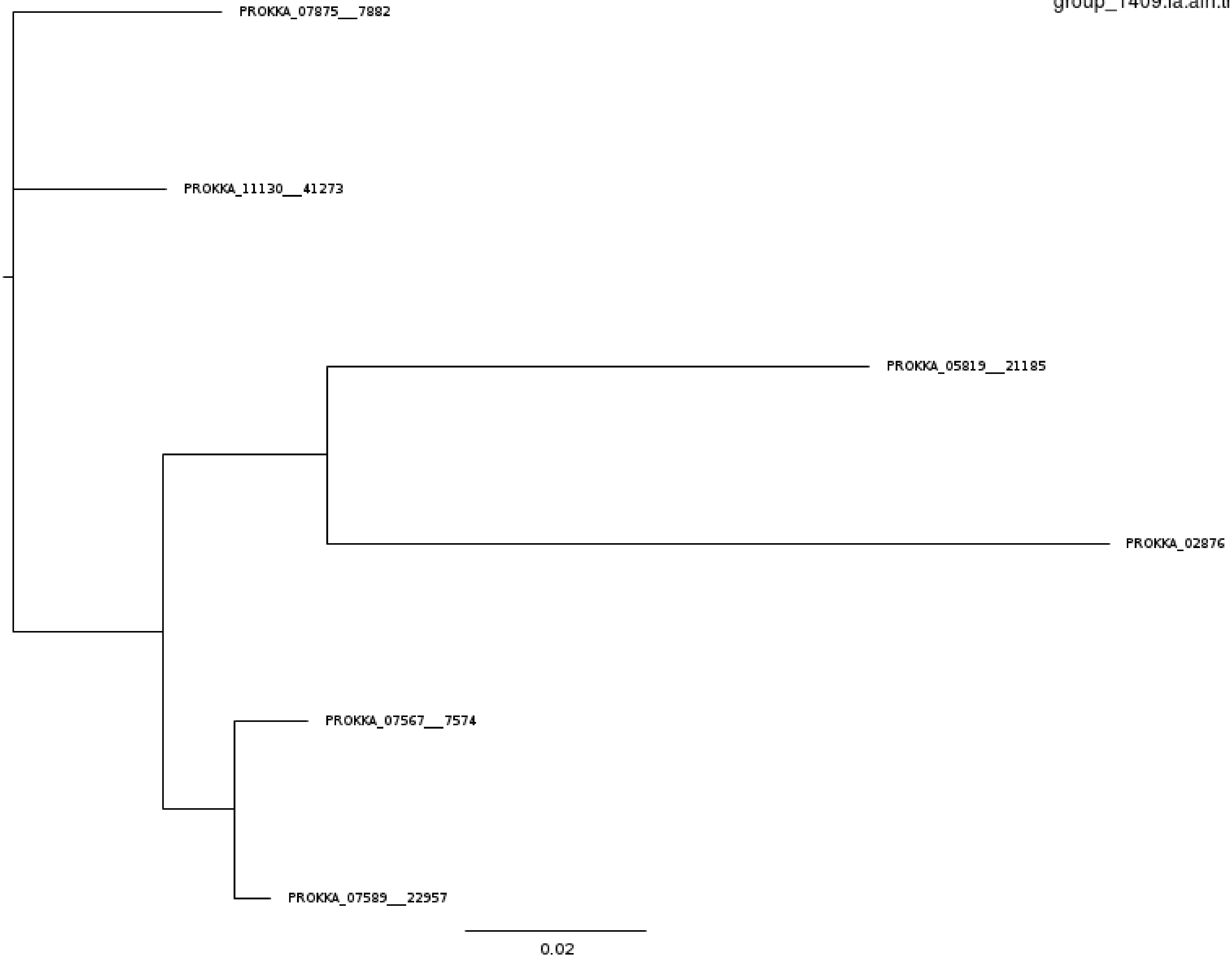

group_1418.fa.aln.tree.png

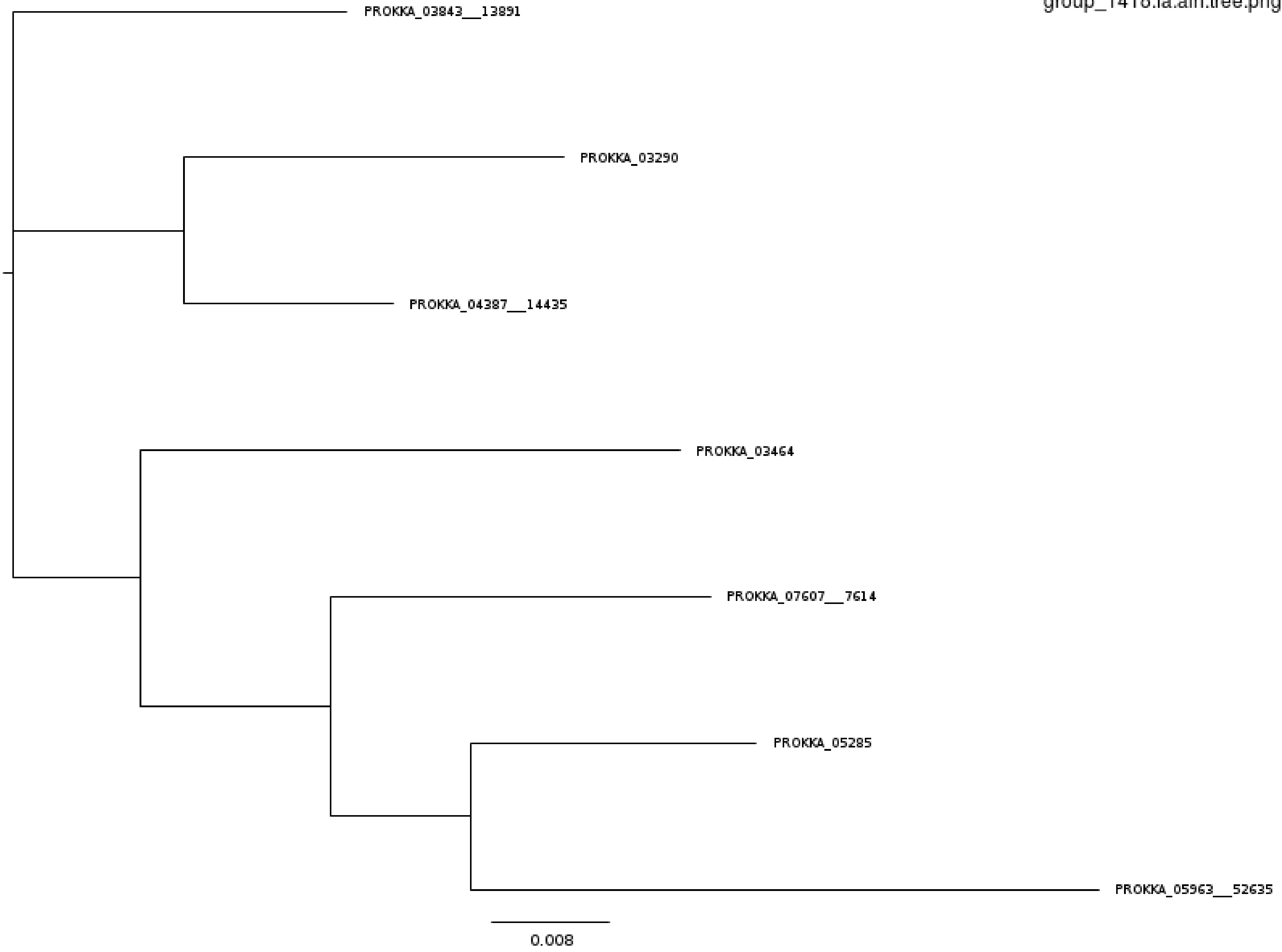

group_1420.fa.aln.tree.png

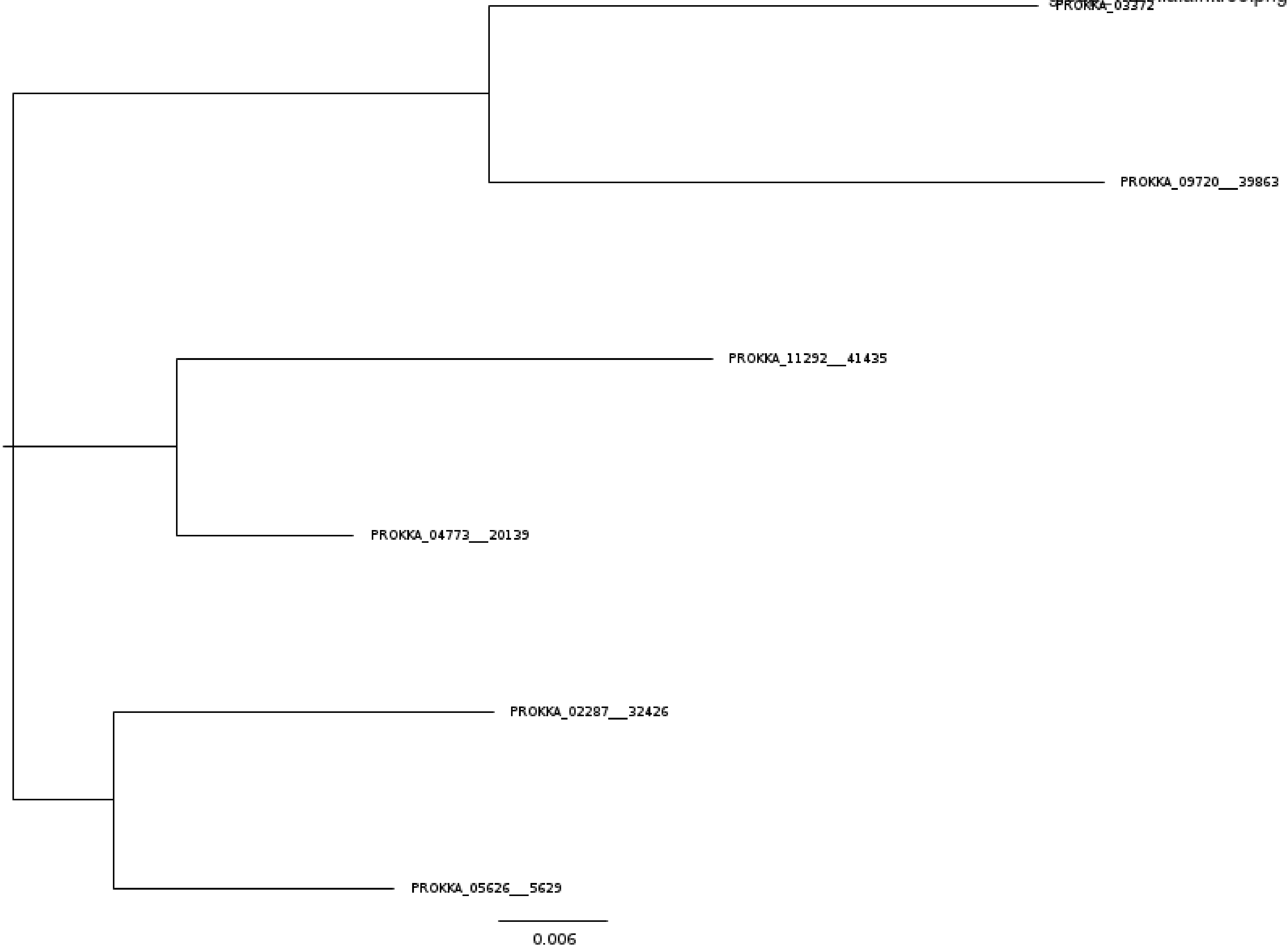

group_1421.fa.aln.tree.png

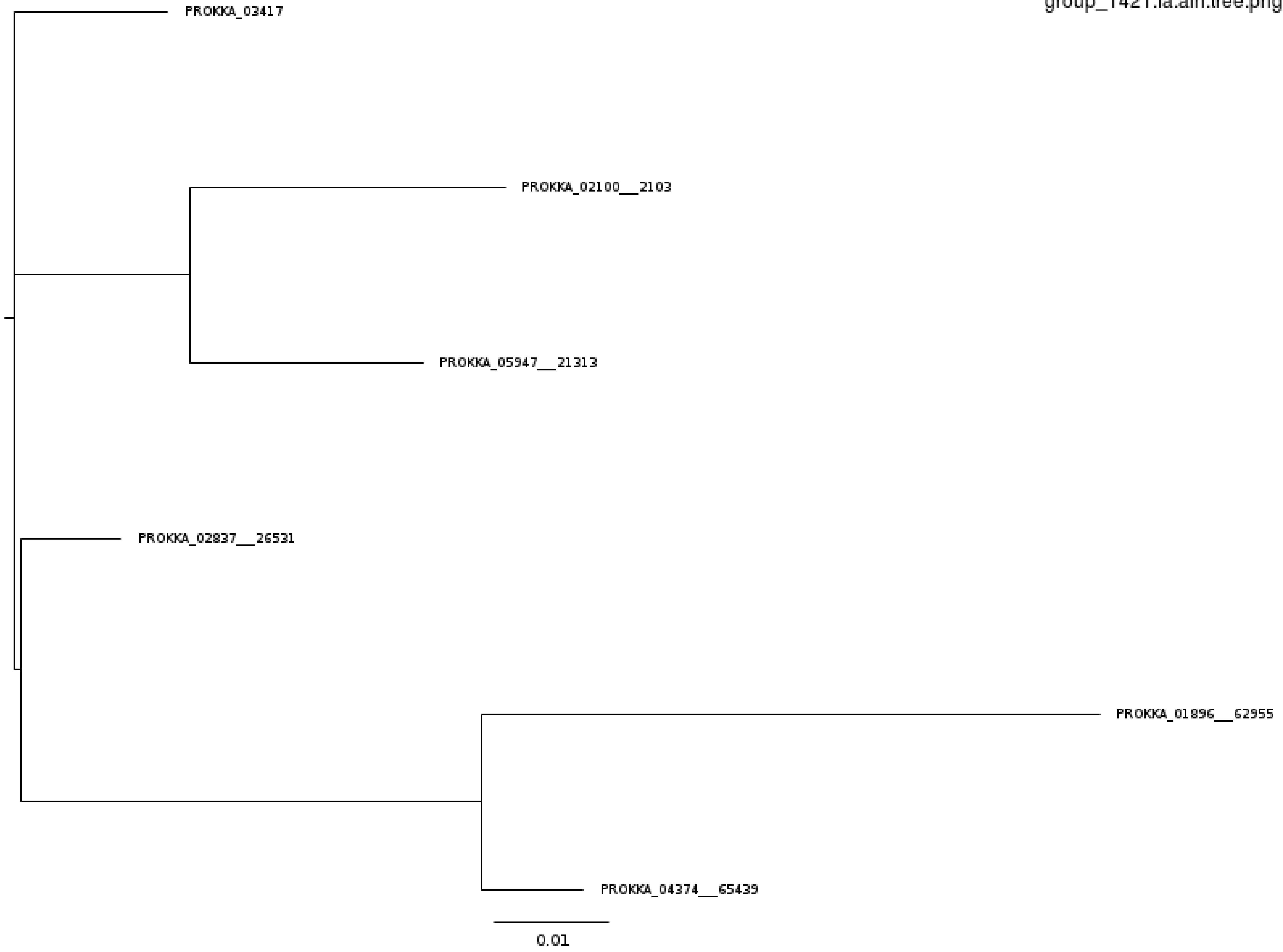

group_1422.fa.aln.tree.png

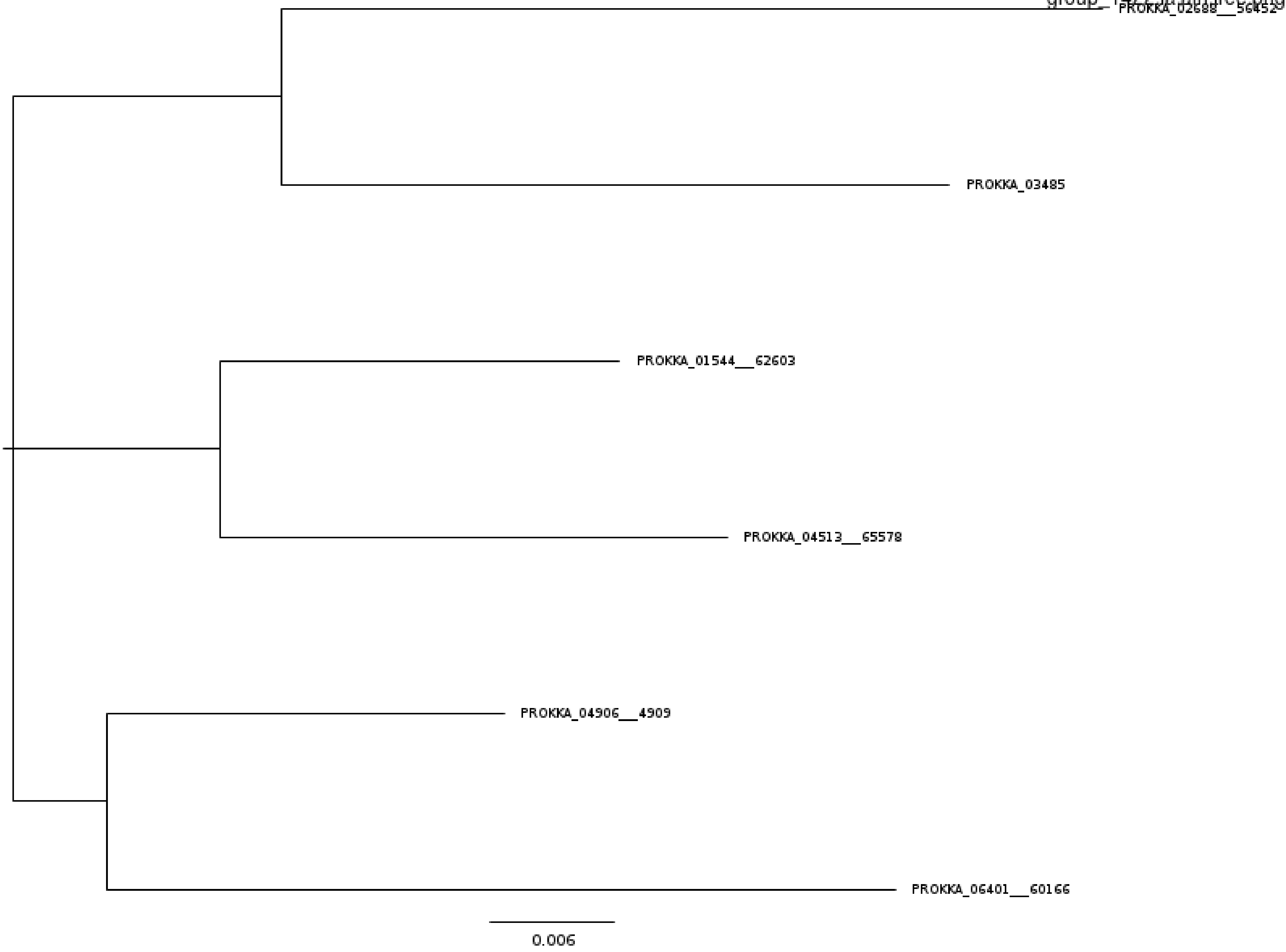

group_1423.fa.aln.tree.png

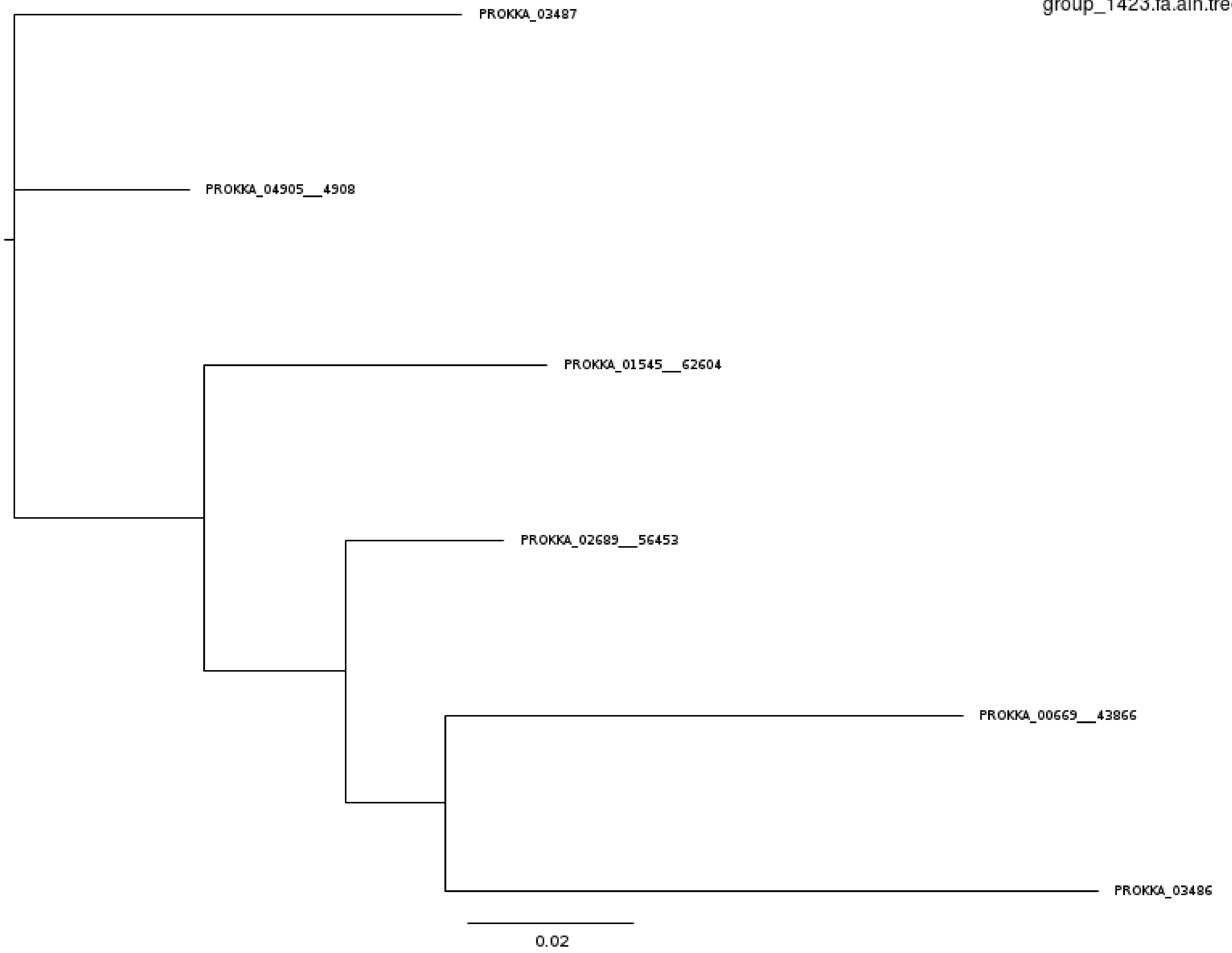

group_1424.fa.aln.tree.png

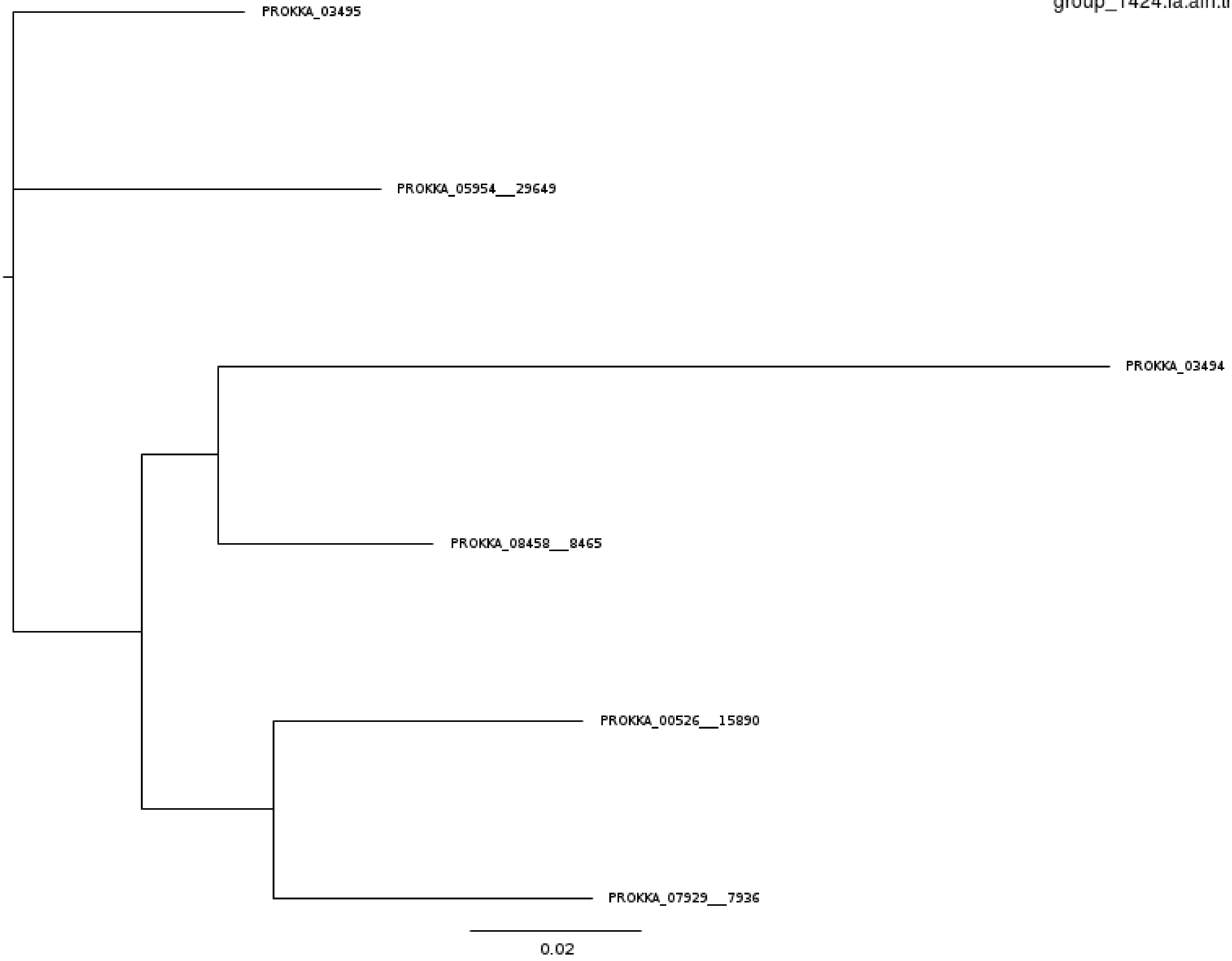

group_1426.fa.aln.tree.png

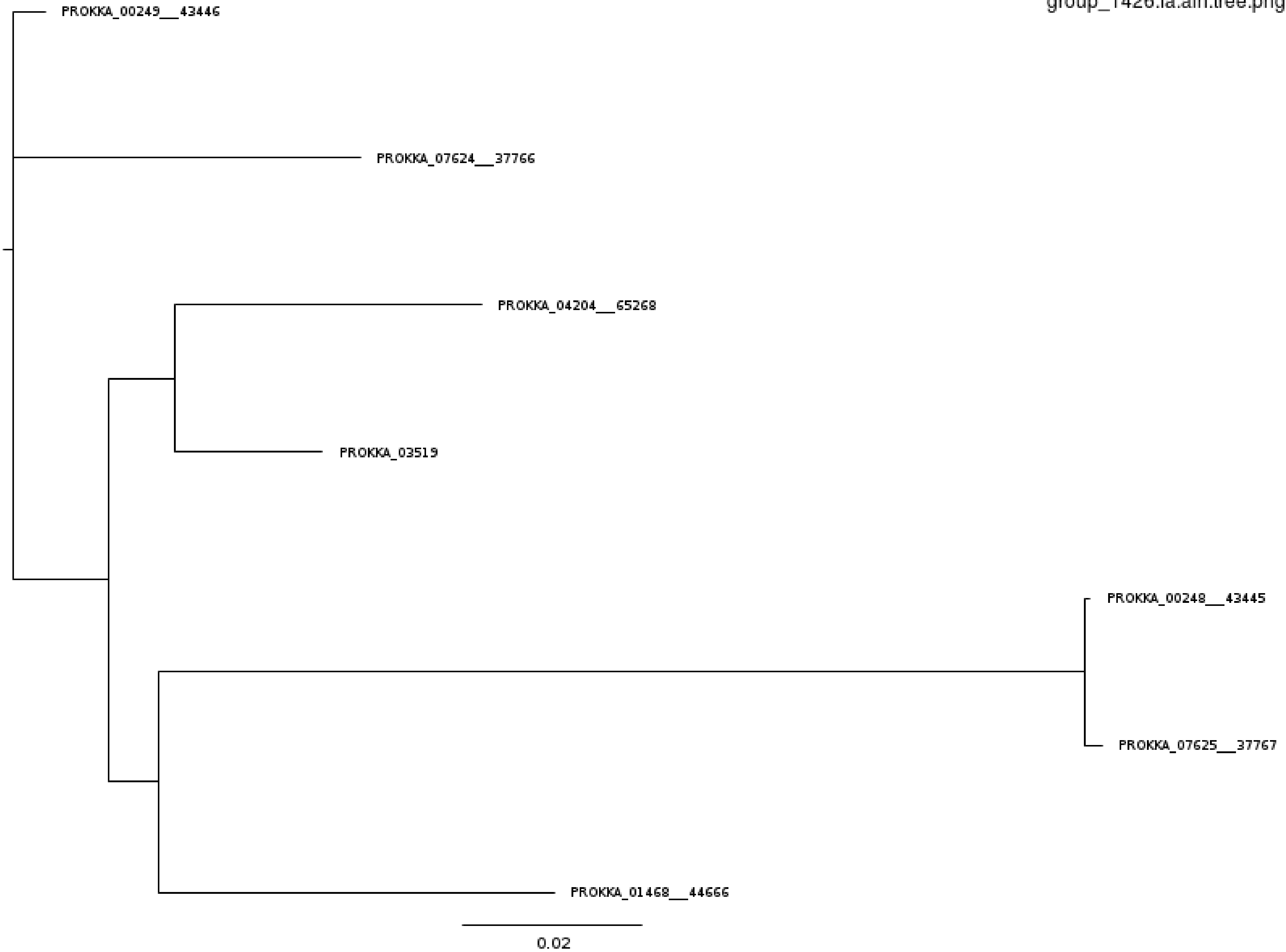

group_1428.fa.aln.tree.png

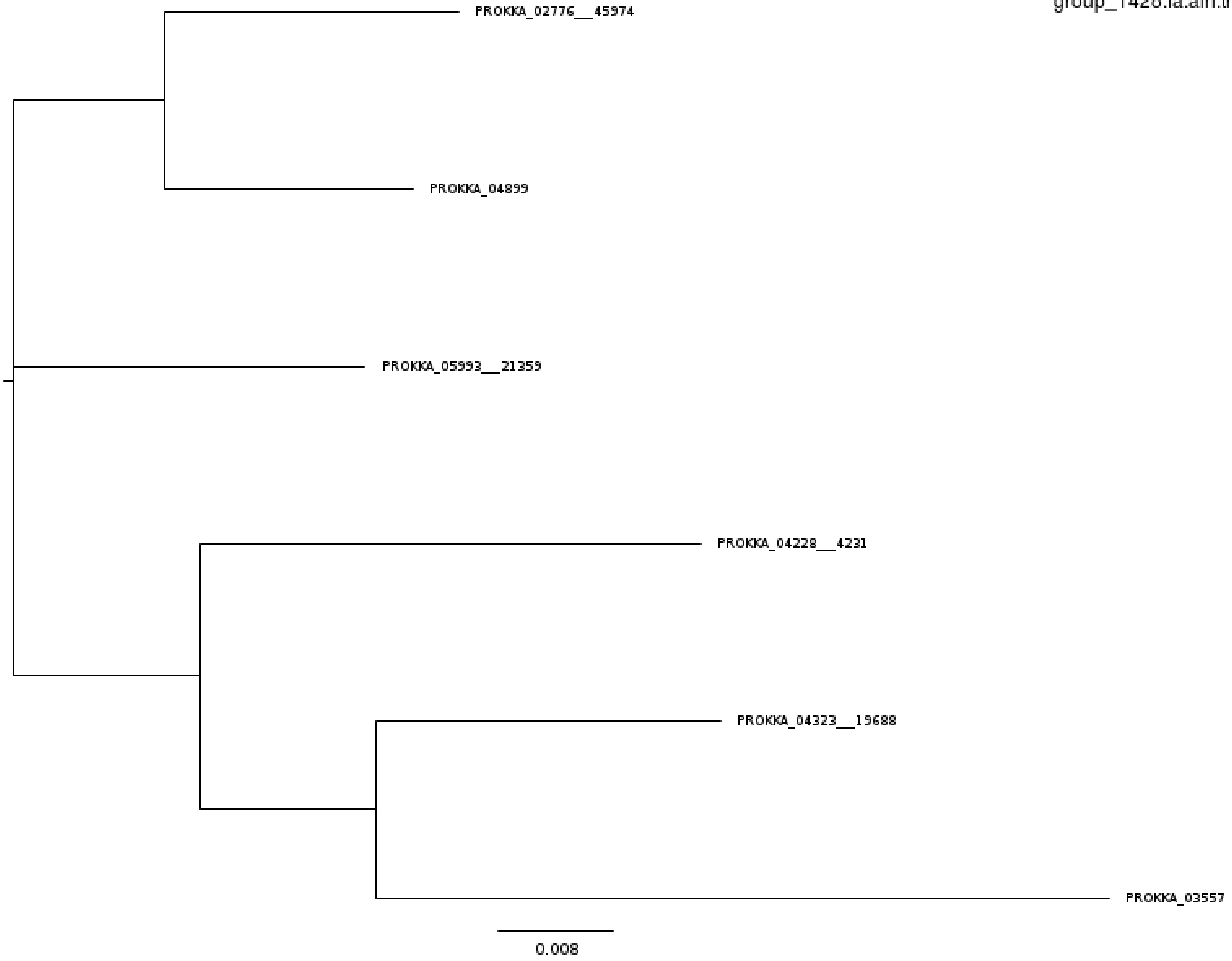

group_1430.fa.aln.tree.png

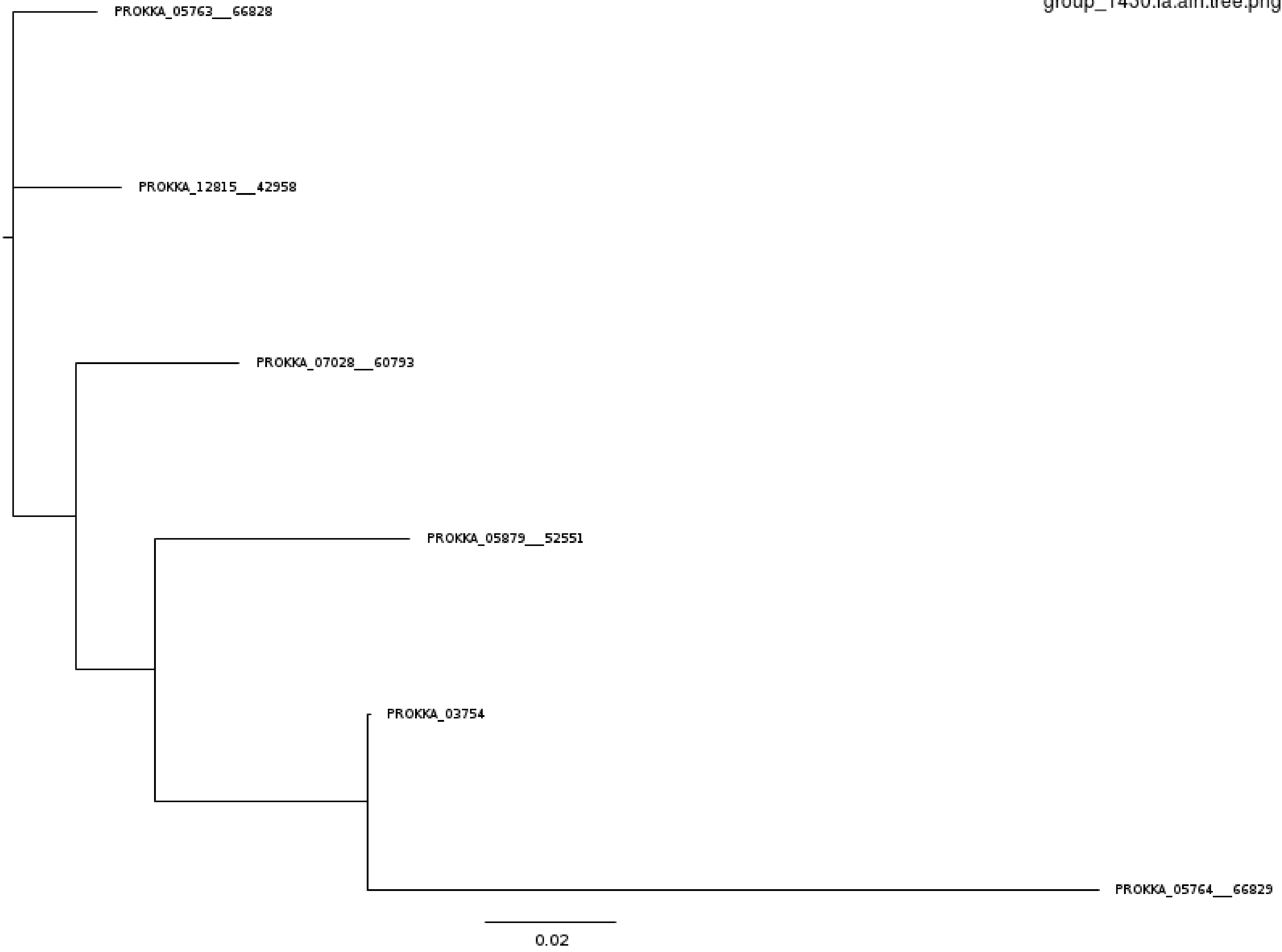

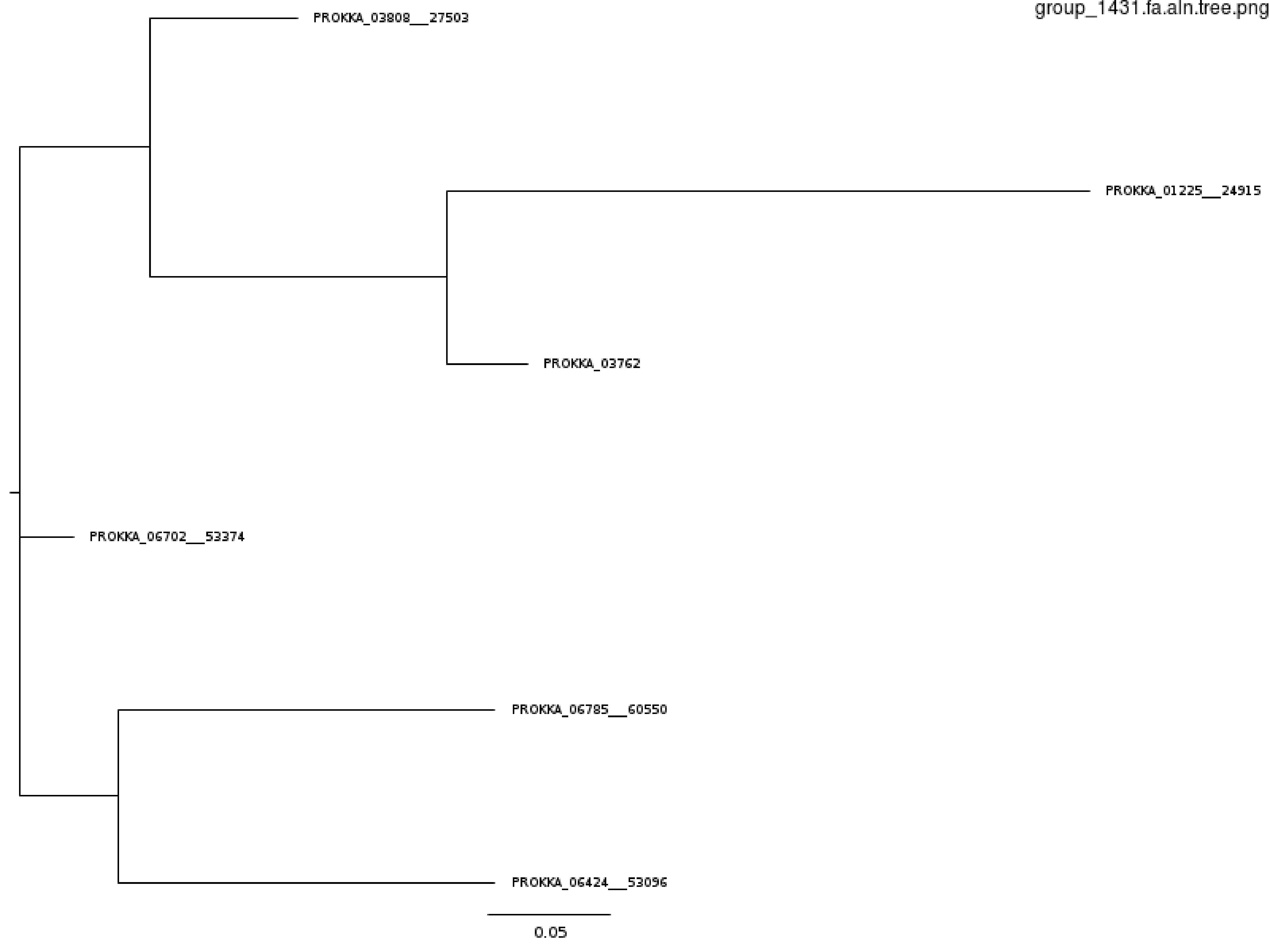
group_1431.fa.aln.tree.png
PROKKA_03808___27503
PROKKA_01225___24915
PROKKA_03762
PROKKA_06702___53374
PROKKA_06785___60550
PROKKA_06424___53096
0.05

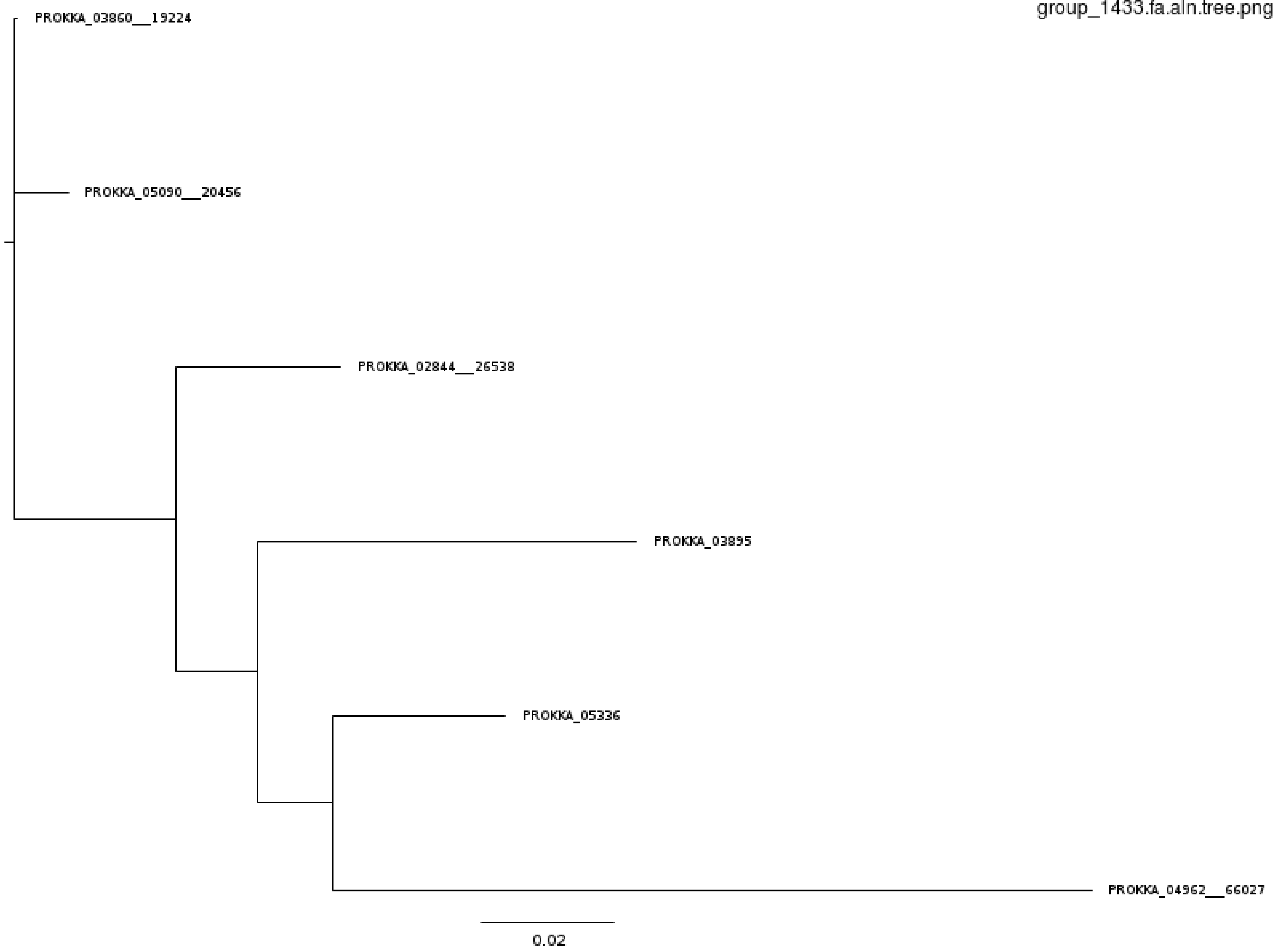
group_1433.fa.aln.tree.png
PROKKA_03860___19224
PROKKA_05090___20456
PROKKA_02844___26538
PROKKA_03895
PROKKA_05336
PROKKA_04962___66027
0.02

group_1434.fa.aln.tree.png

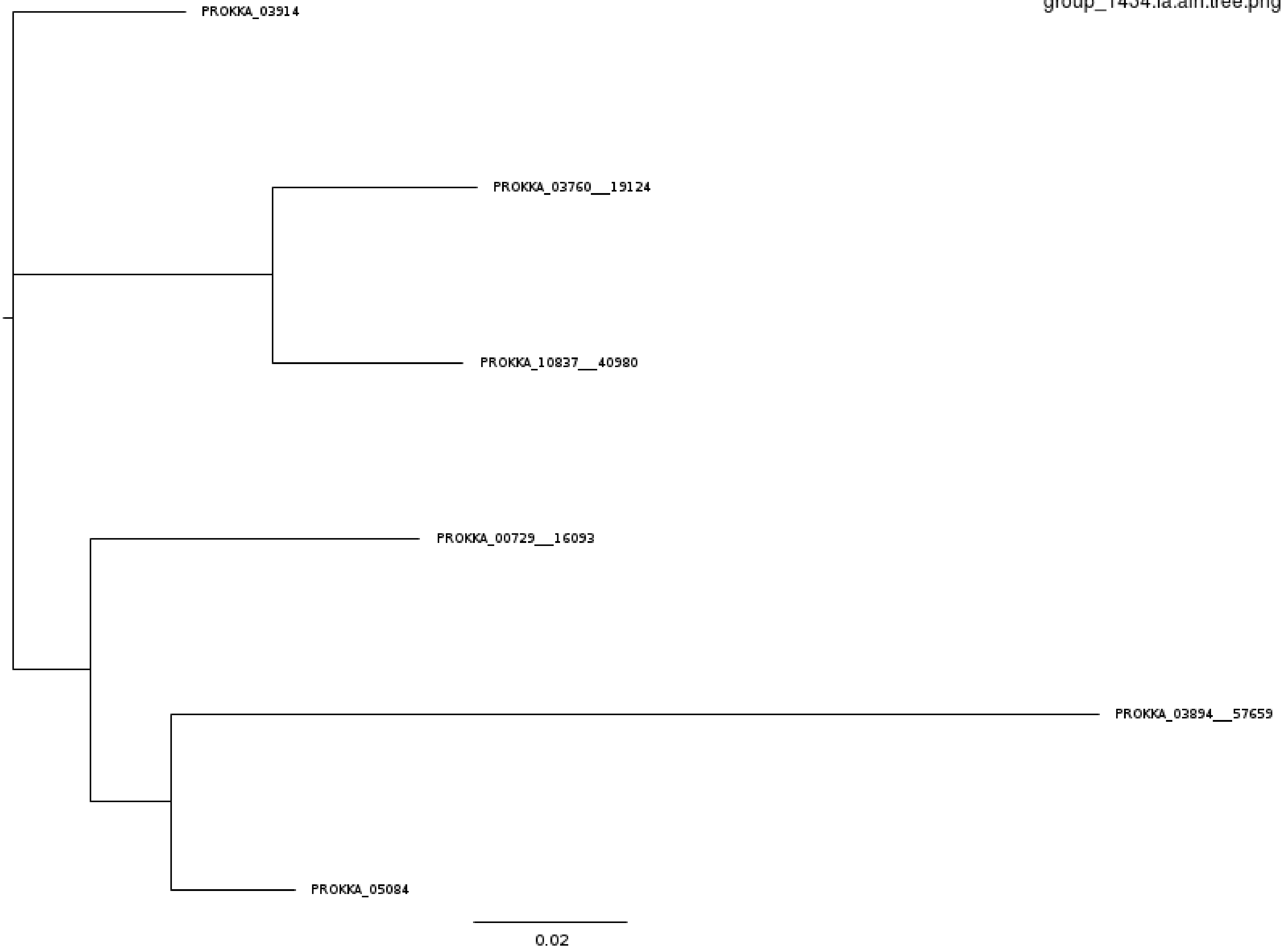

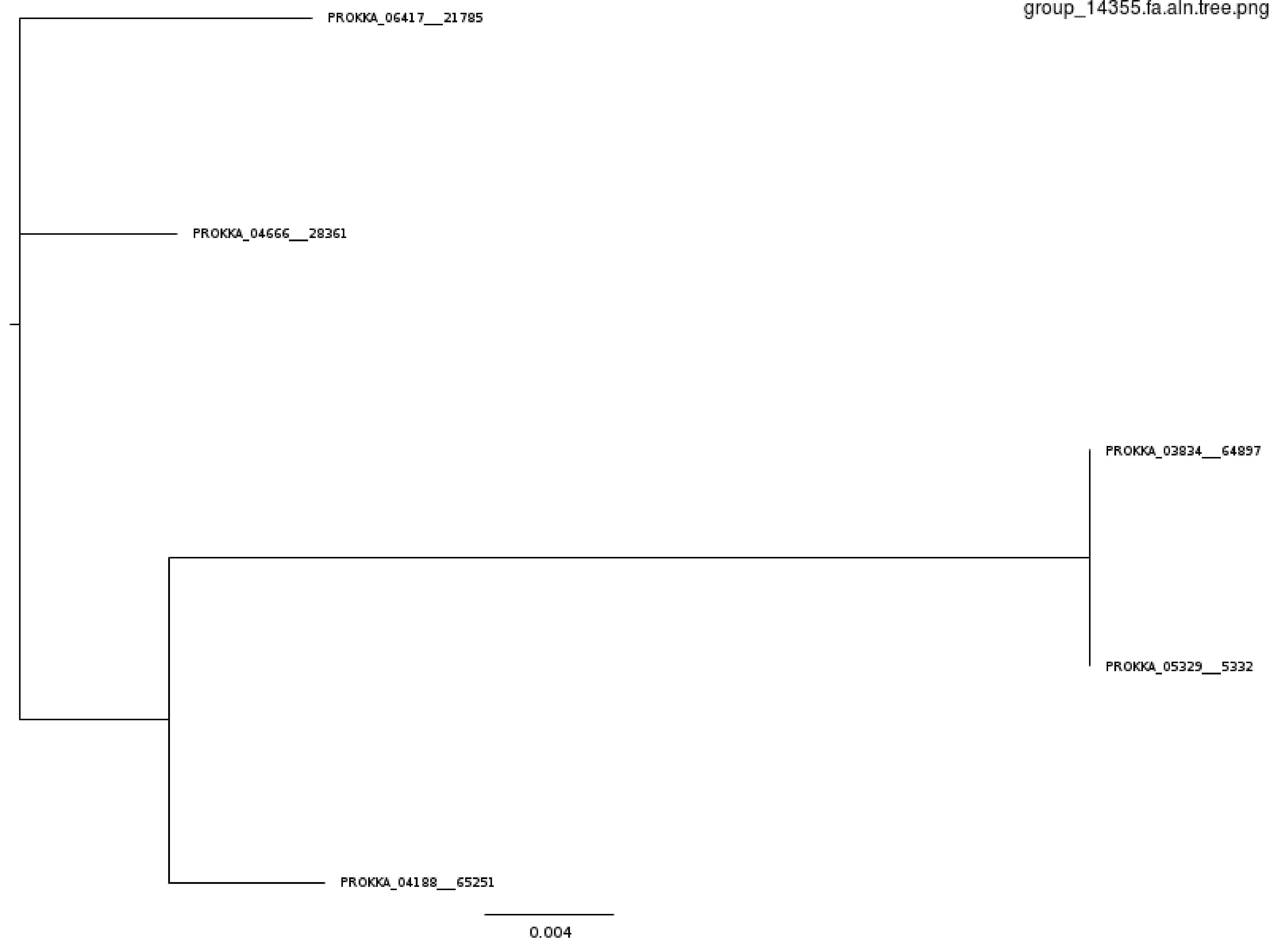
group_14355.fa.aln.tree.png
PROKKA_06417___21785
PROKKA_04666___28361
PROKKA_03834___64897
PROKKA_05329___5332
PROKKA_04188___65251
0.004

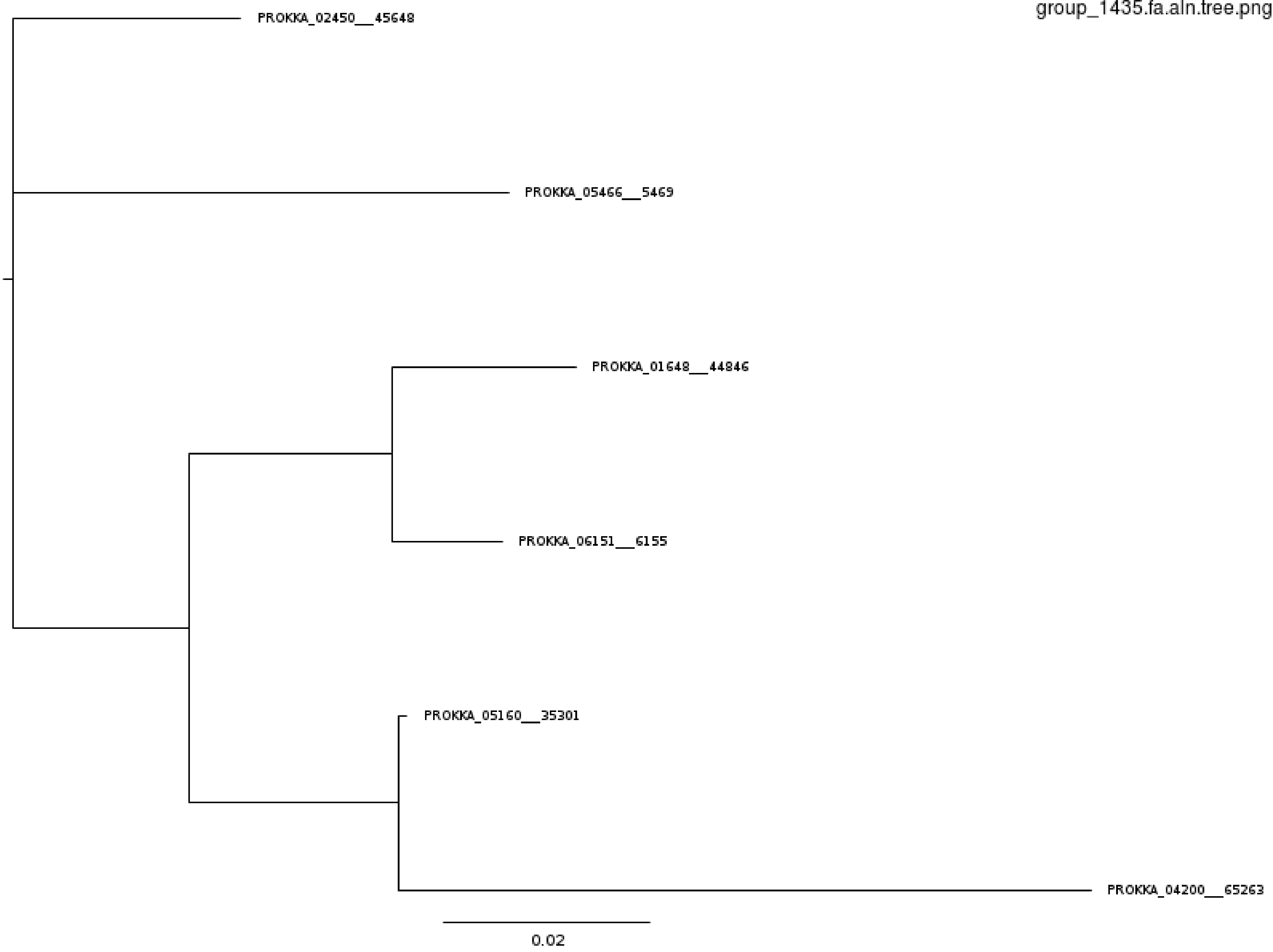
group_1435.fa.aln.tree.png
PROKKA_02450___45648
PROKKA_05466___5469
PROKKA_01648___44846
PROKKA_06151___6155
PROKKA_05160___35301
PROKKA_04200___65263
0.02

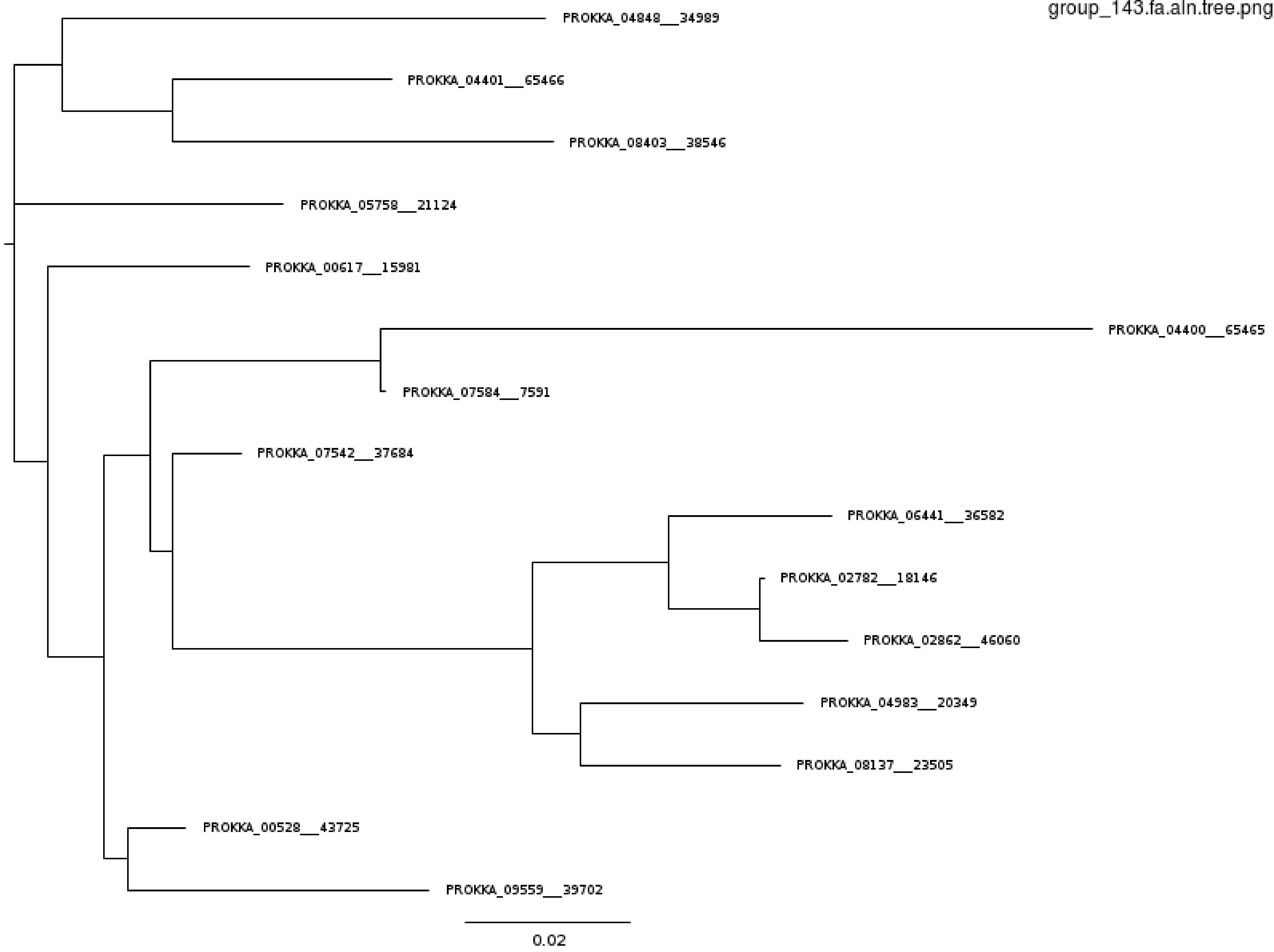

group_143.fa.aln.tree.png
PROKKA_04848___34989
PROKKA_04401___65466
PROKKA_08403___38546
PROKKA_05758___21124
PROKKA_00617___15981
PROKKA_04400___65465
PROKKA_07584___7591
PROKKA_07542___37684
PROKKA_06441___36582
PROKKA_02782___18146
PROKKA_02862___46060
PROKKA_04983___20349
PROKKA_08137___23505
PROKKA_00528___43725
PROKKA_09559___39702
0.02

group_1441.fa.aln.tree.png

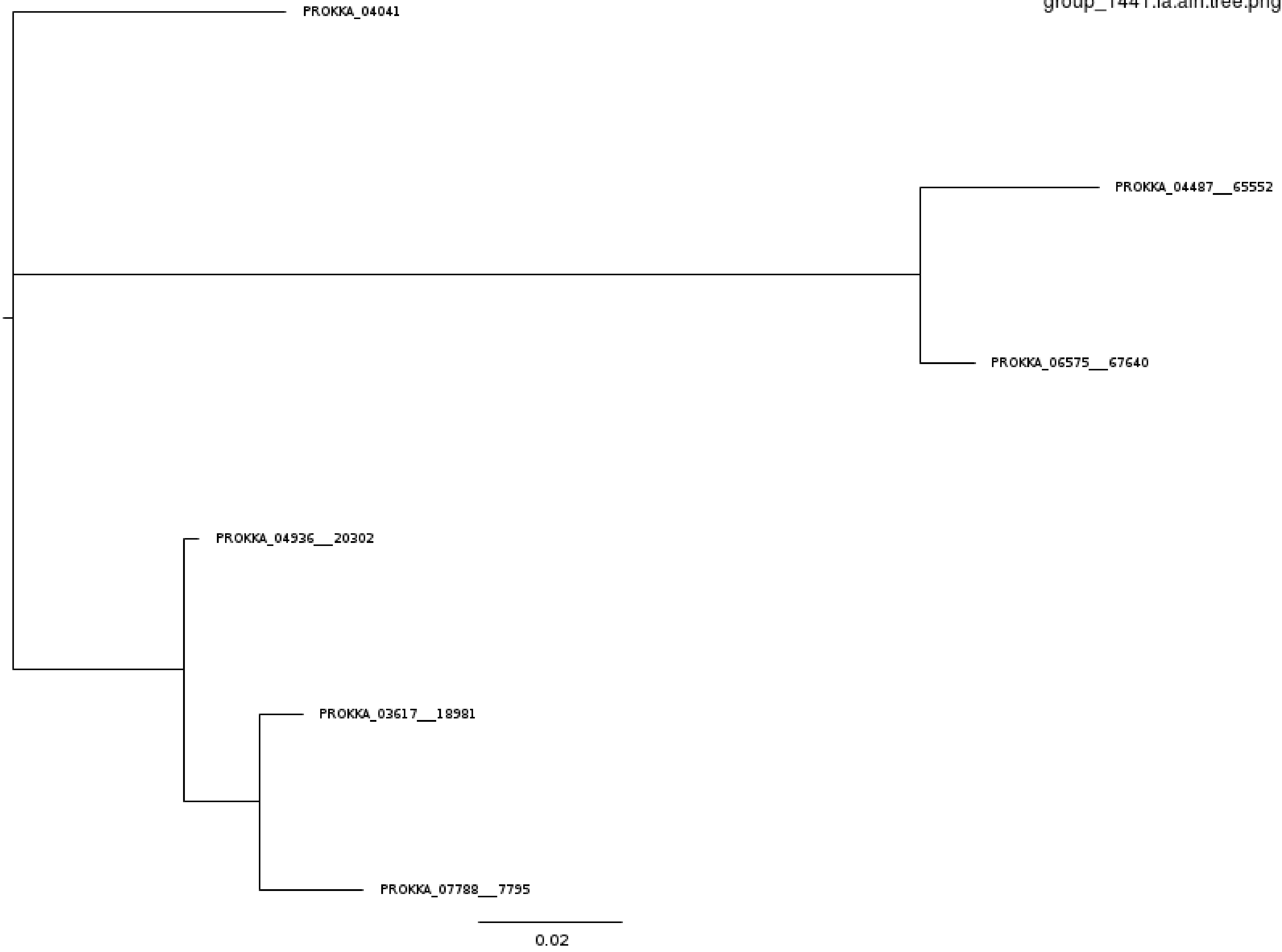

group_1446.fa.aln.tree.png

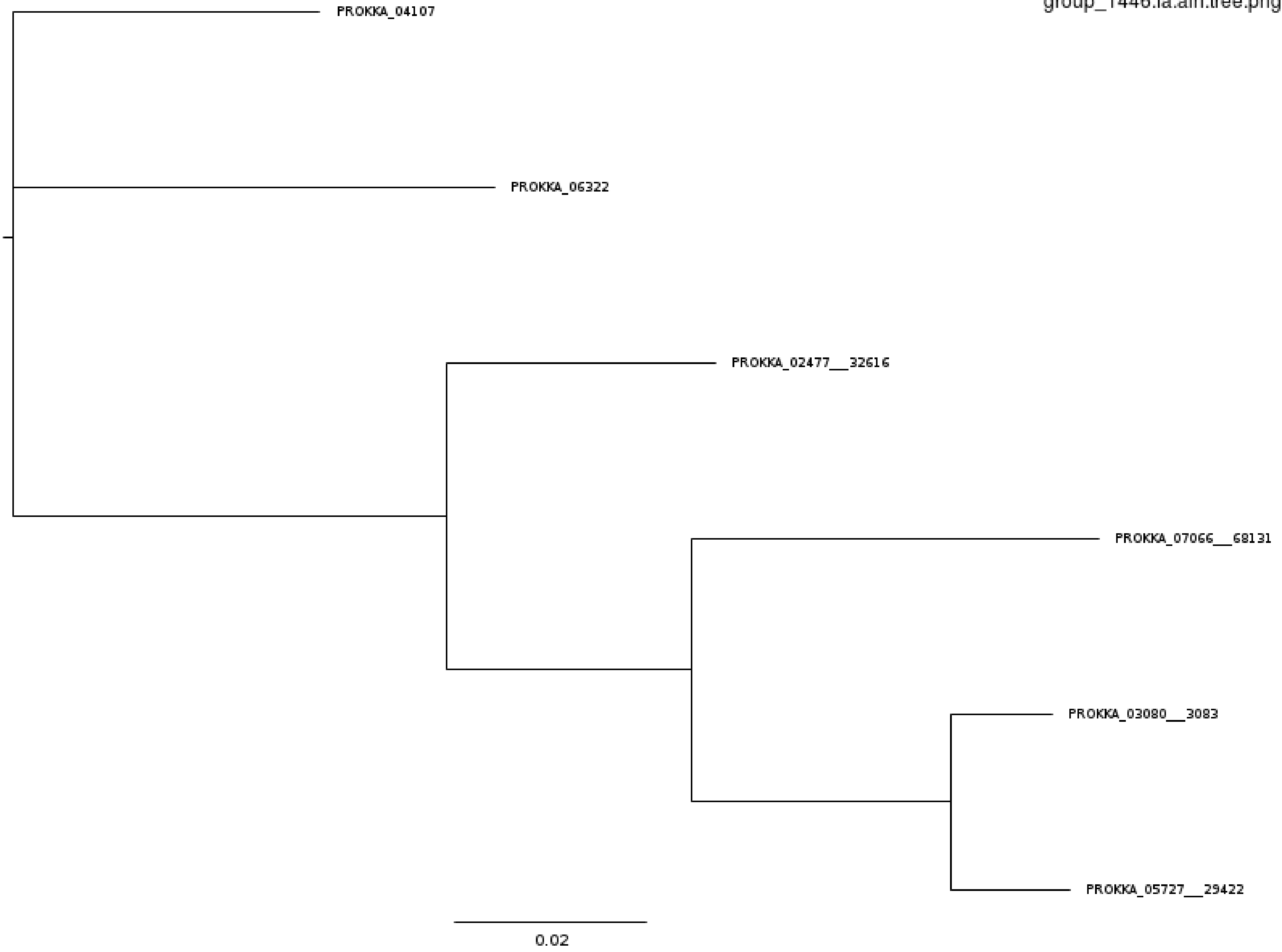

group_1447.fa.aln.tree.png

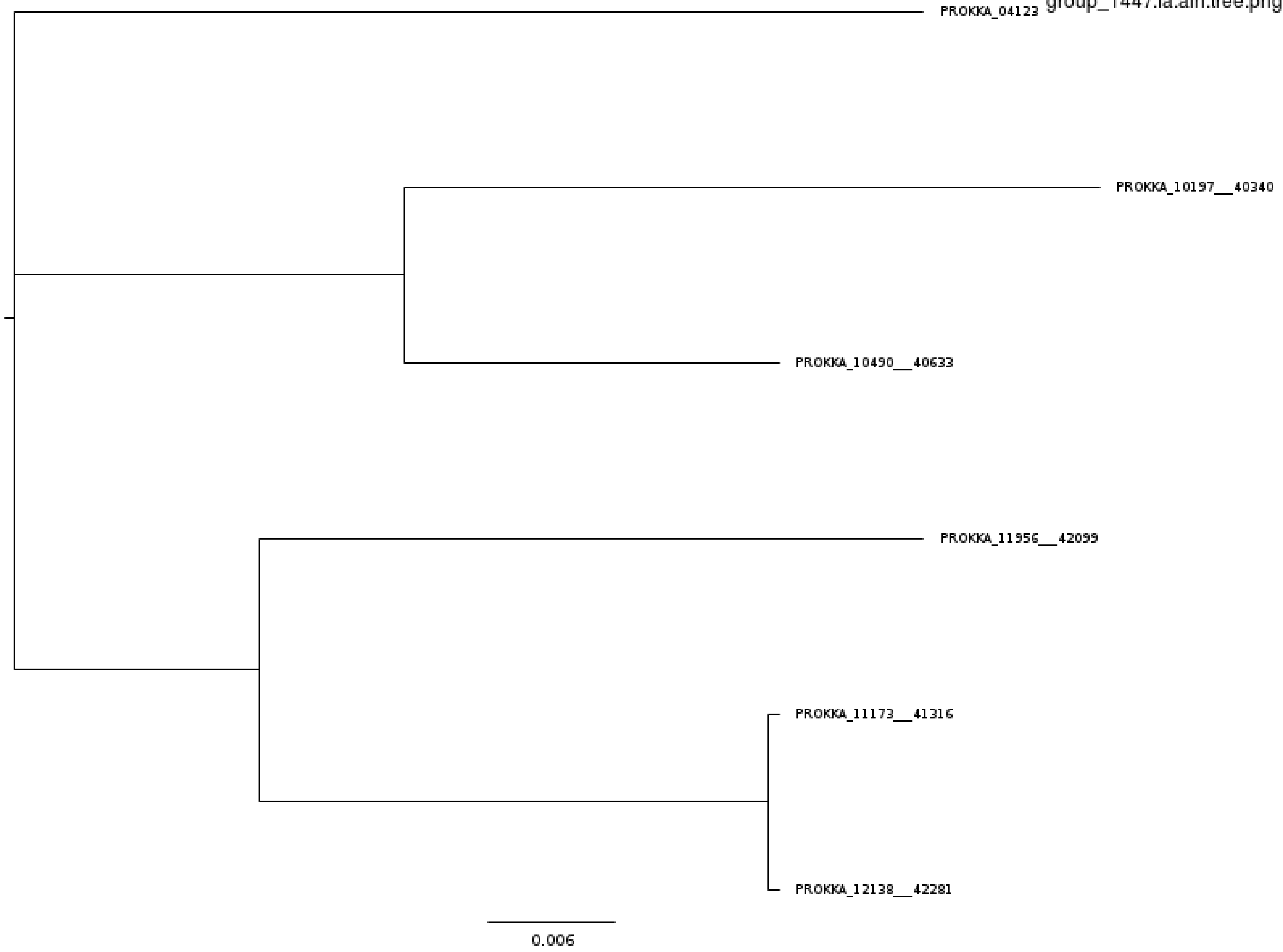

group_1448.fa.aln.tree.png

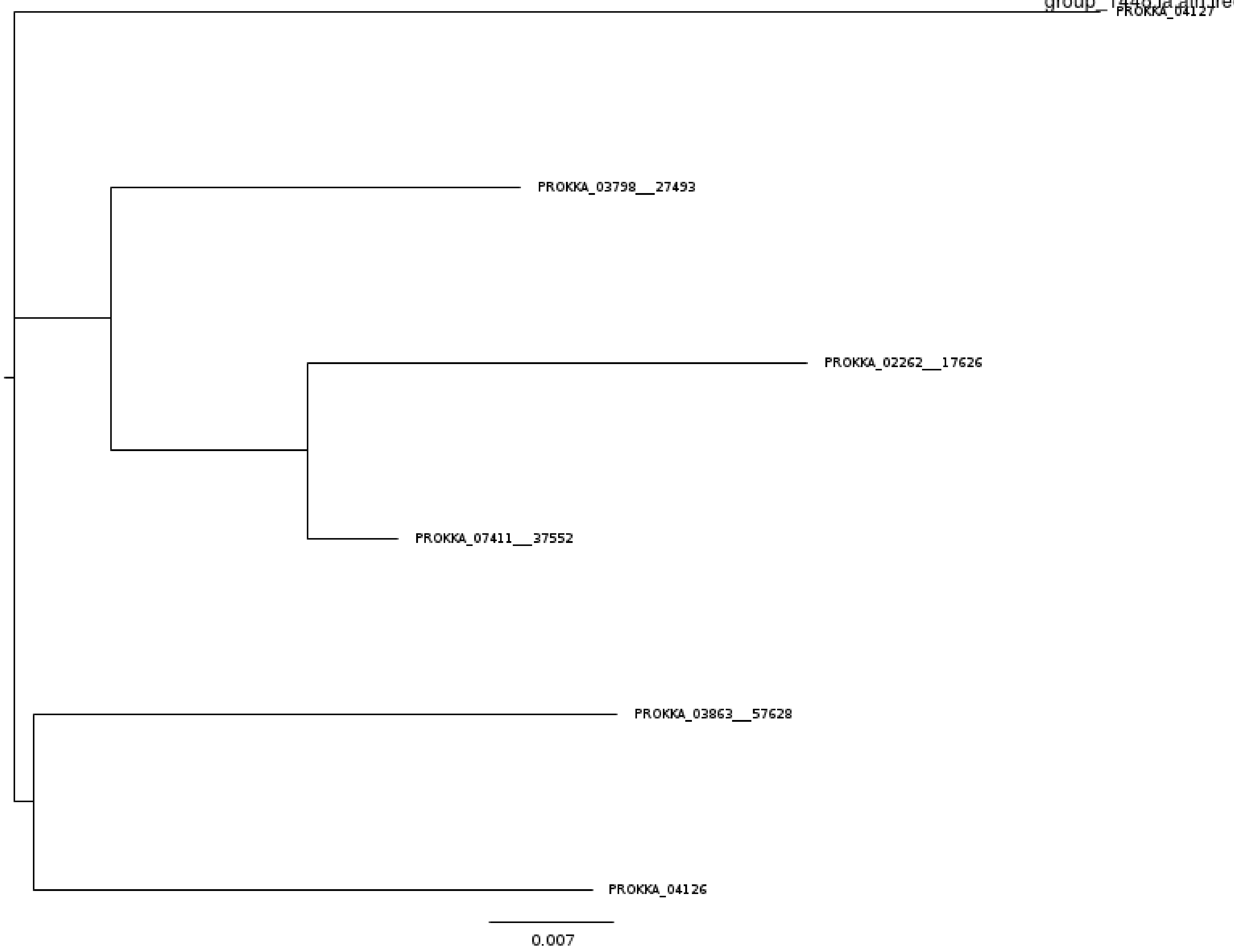

group_144.fa.aln.tree.png

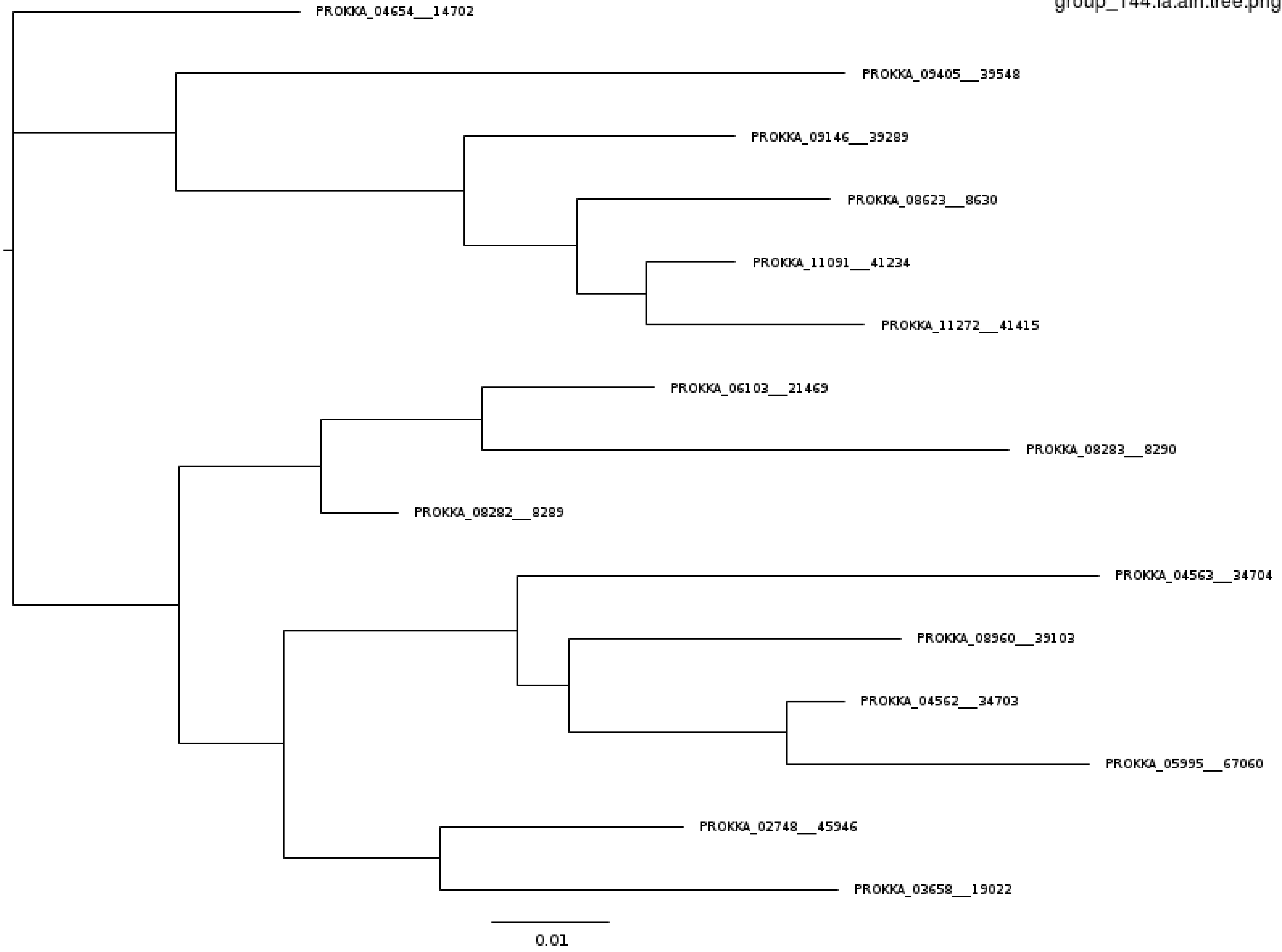

group_1450.fa.aln.tree.png

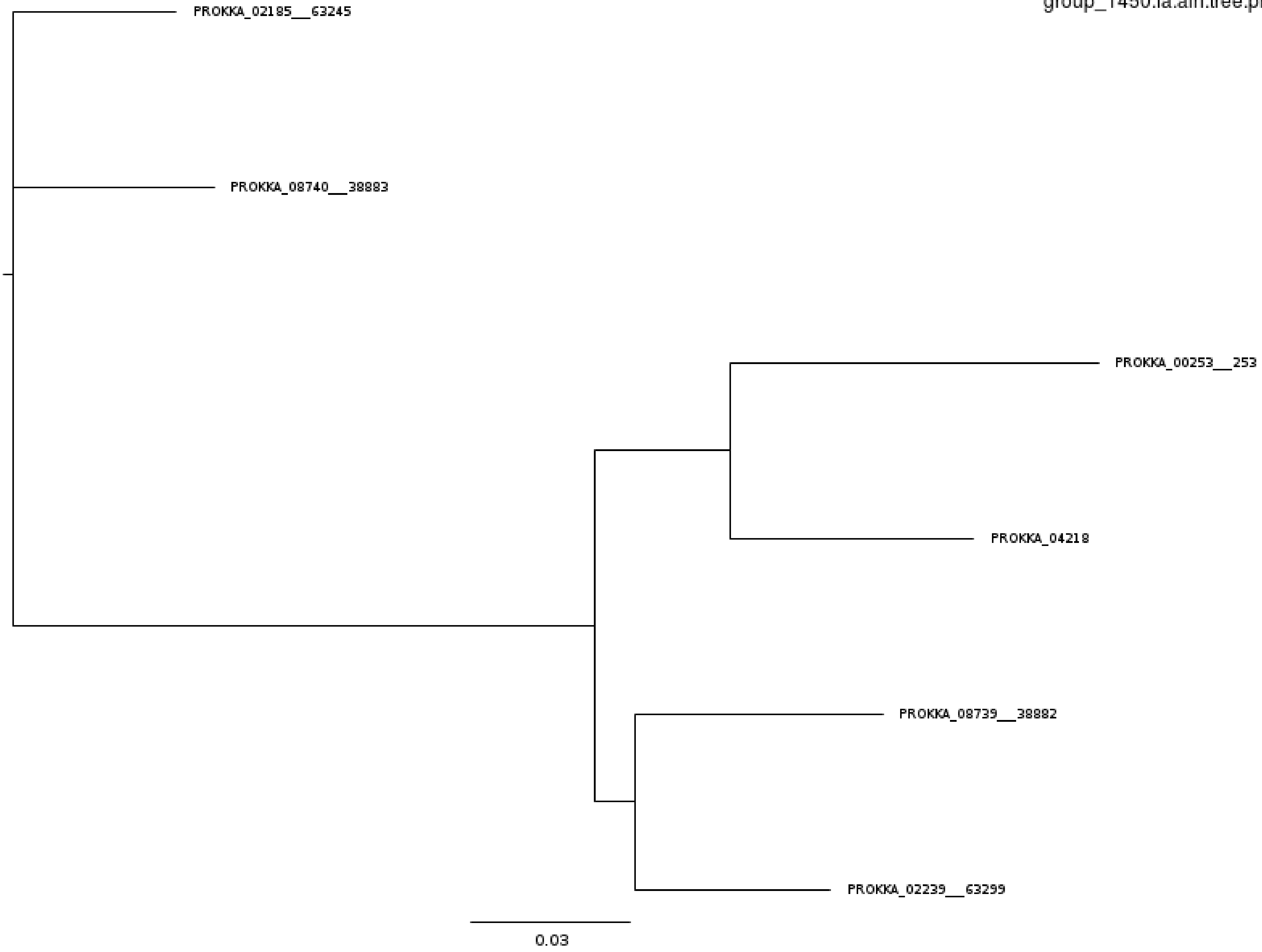

group_1453.fa.aln.tree.png

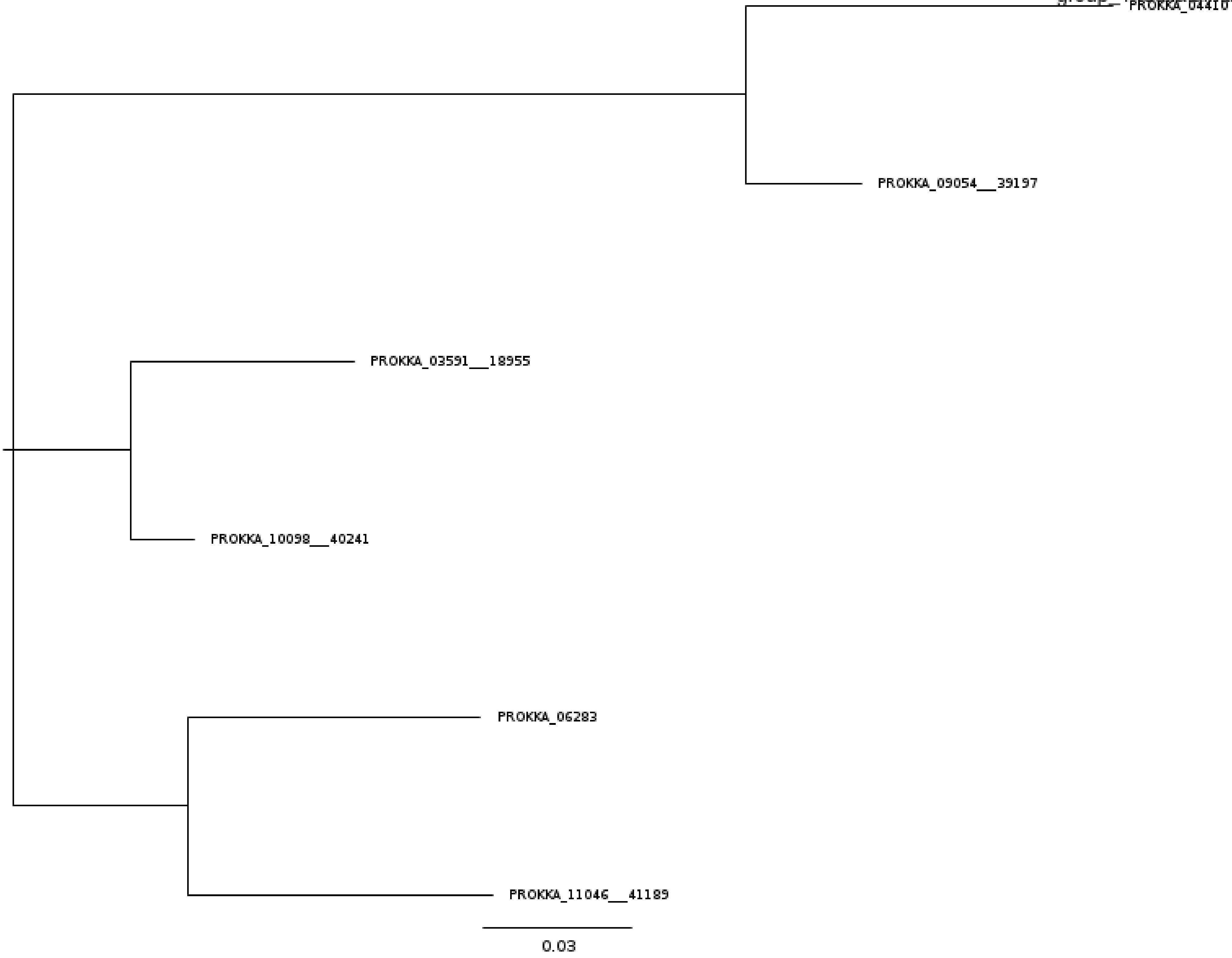

group_1455.fa.aln.tree.png

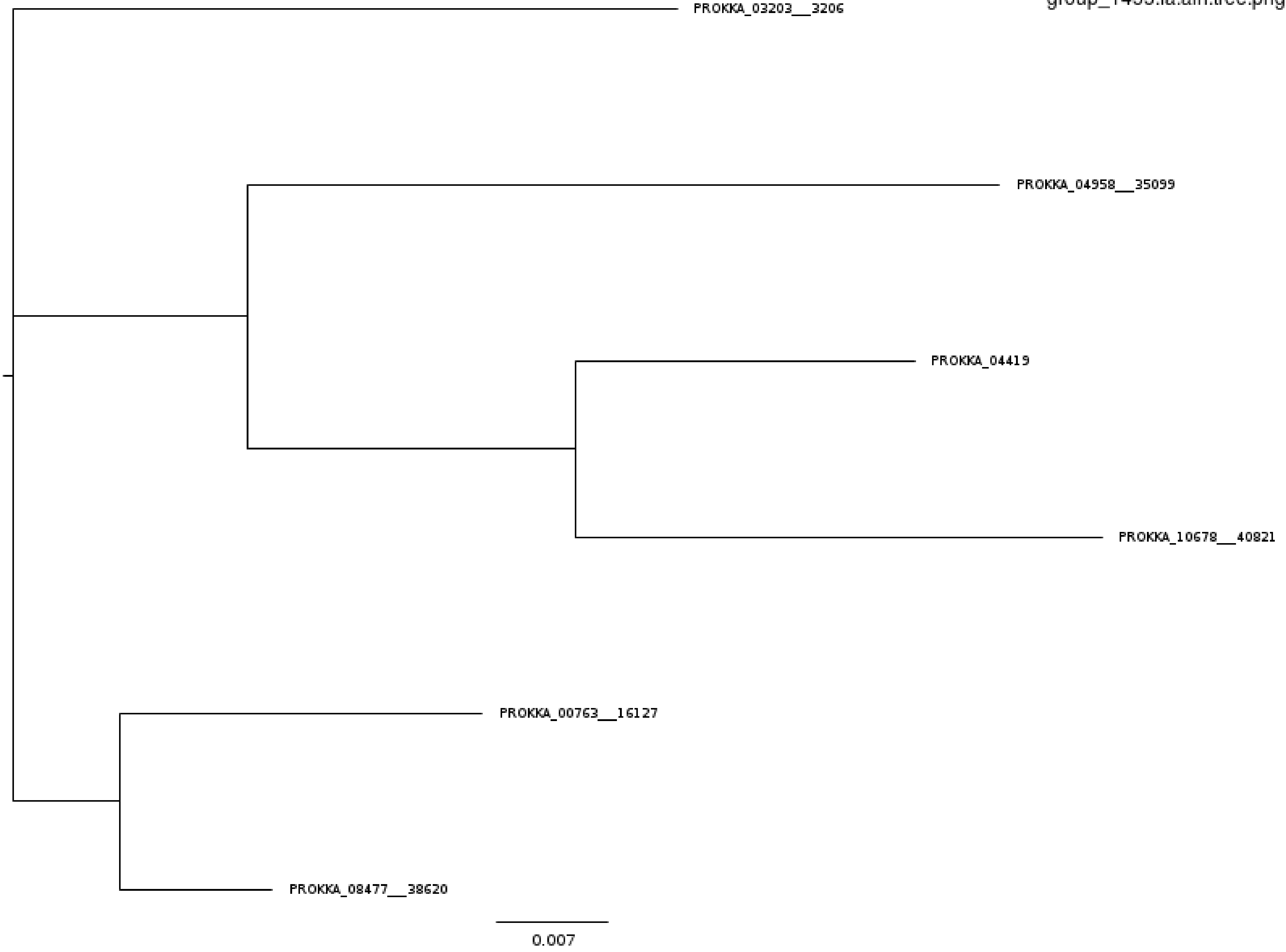

group_1457.fa.aln.tree.png

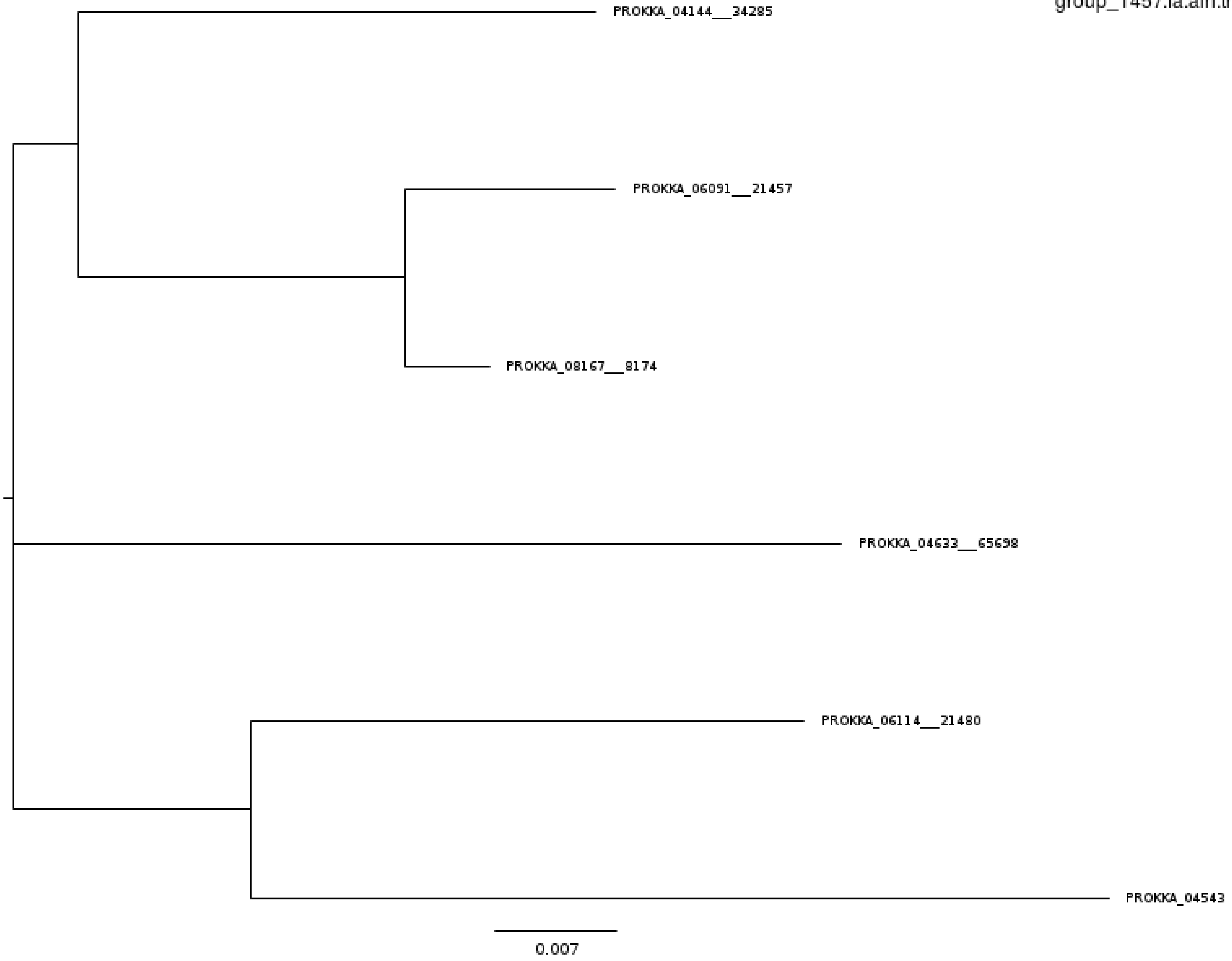

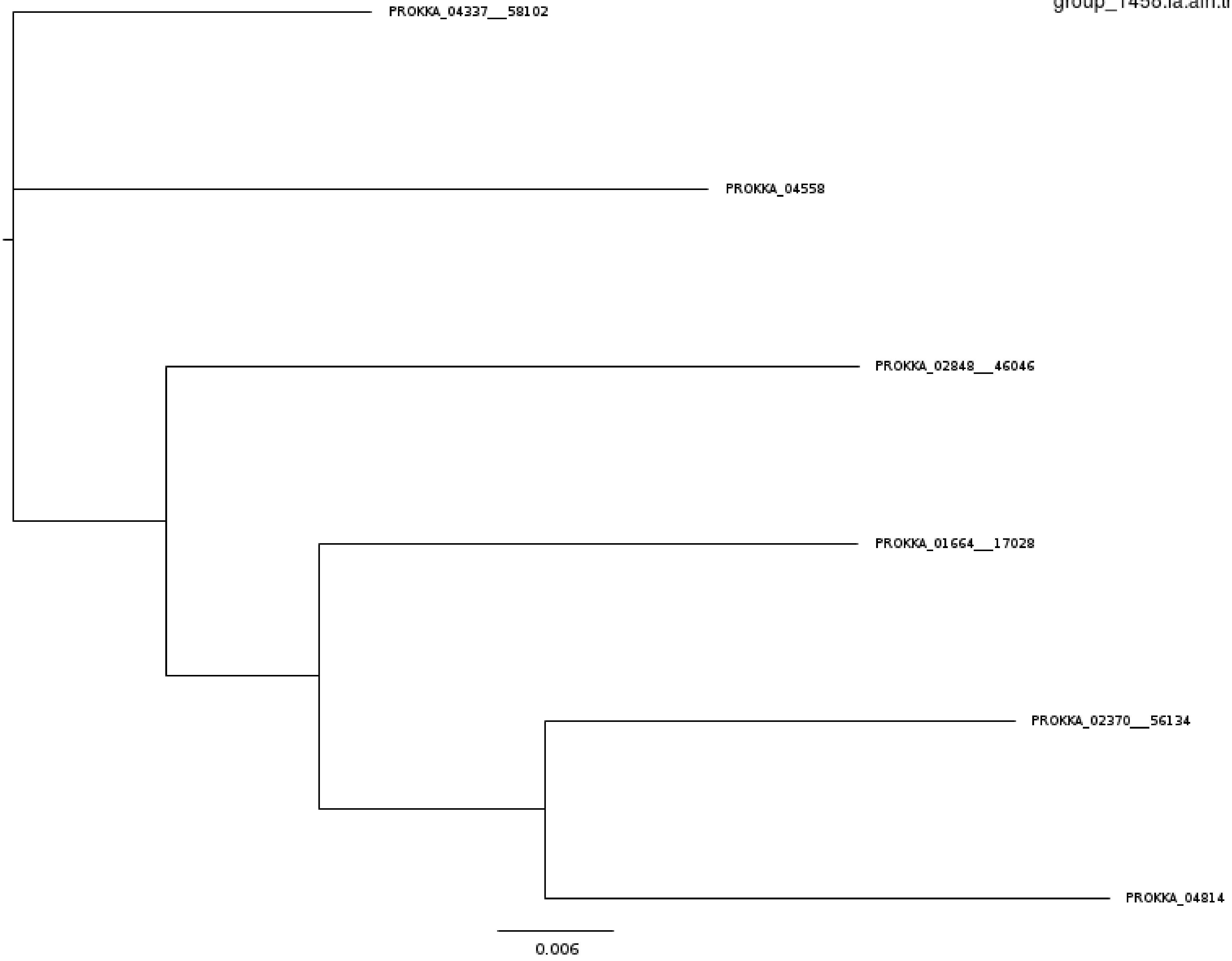
PROKKA_04337___58102
PROKKA_04558
PROKKA_02848___46046
PROKKA_01664___17028
PROKKA_02370___56134
PROKKA_04814
0.006

group_1461.fa.aln.tree.png

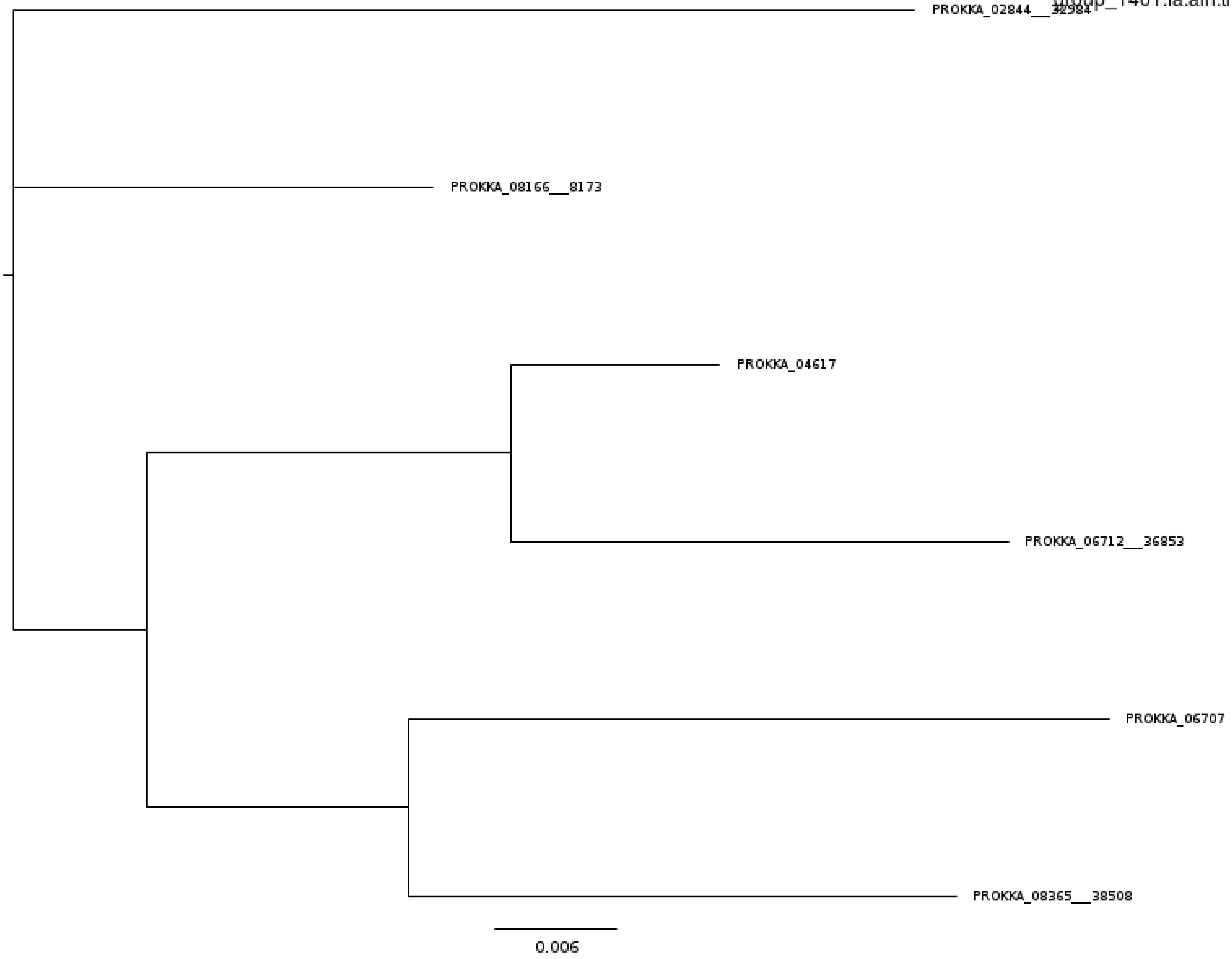

group_1465.fa.aln.tree.png

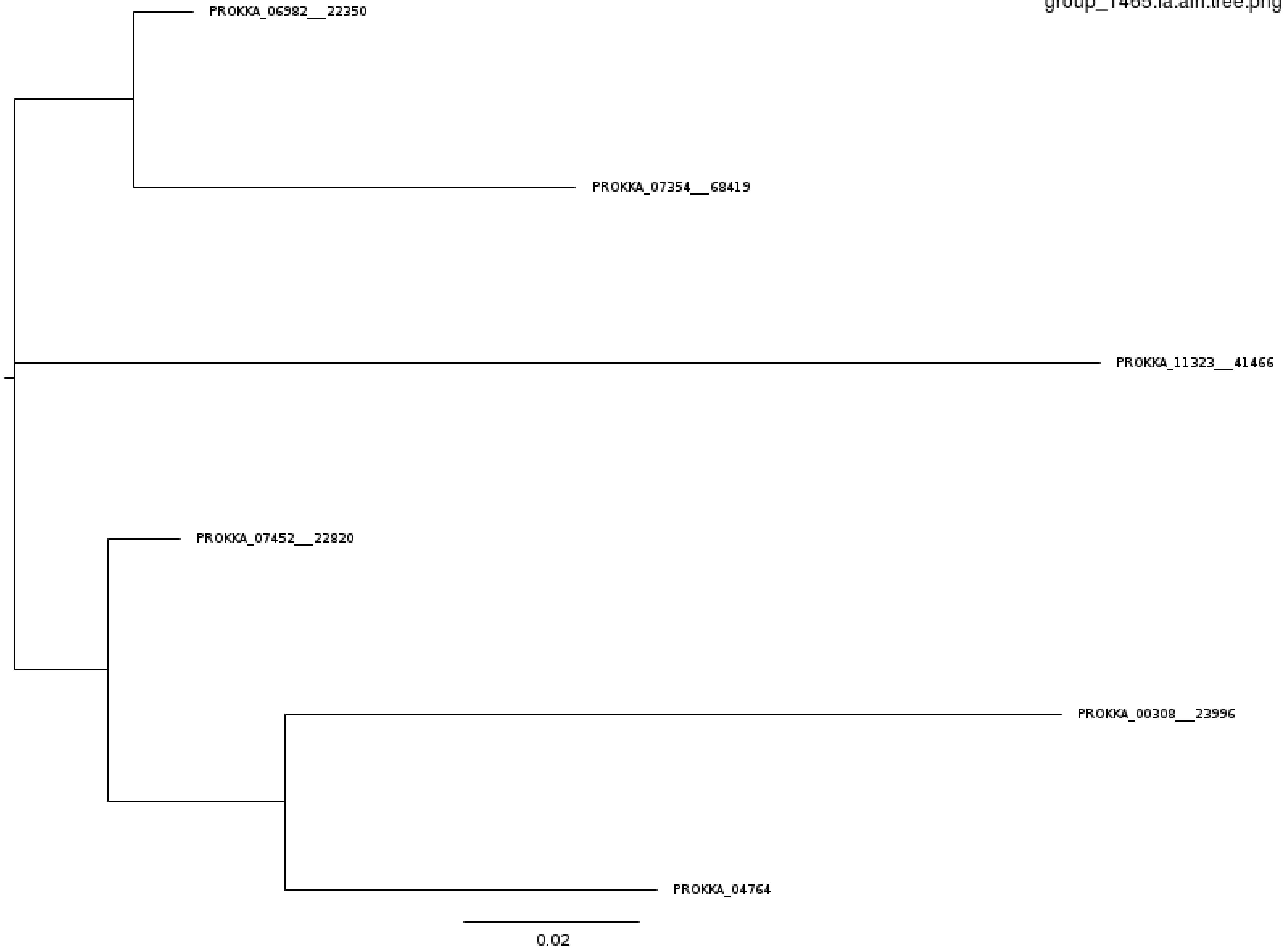

group_1467.fa.aln.tree.png

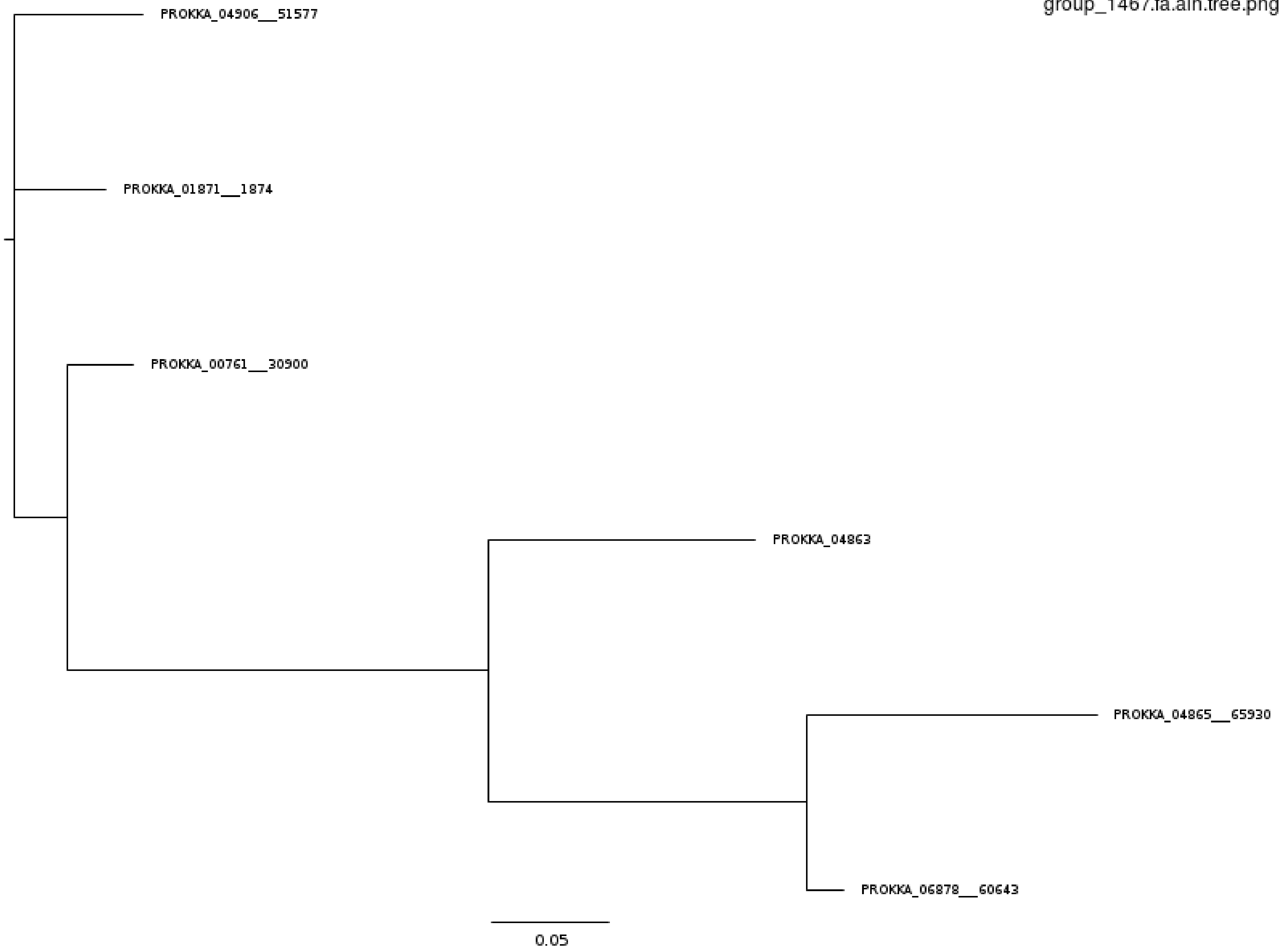

group_1468.fa.aln.tree.png

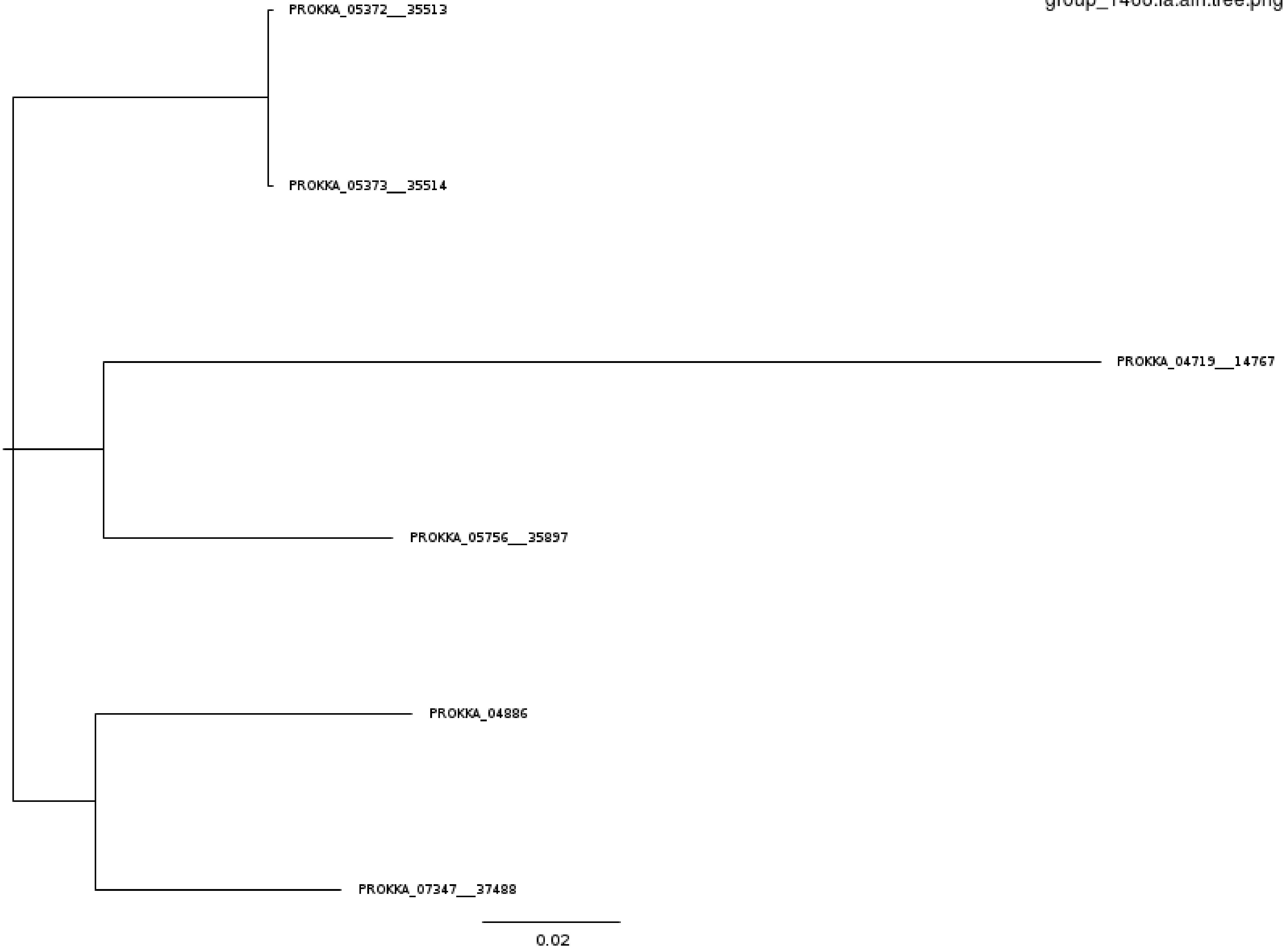

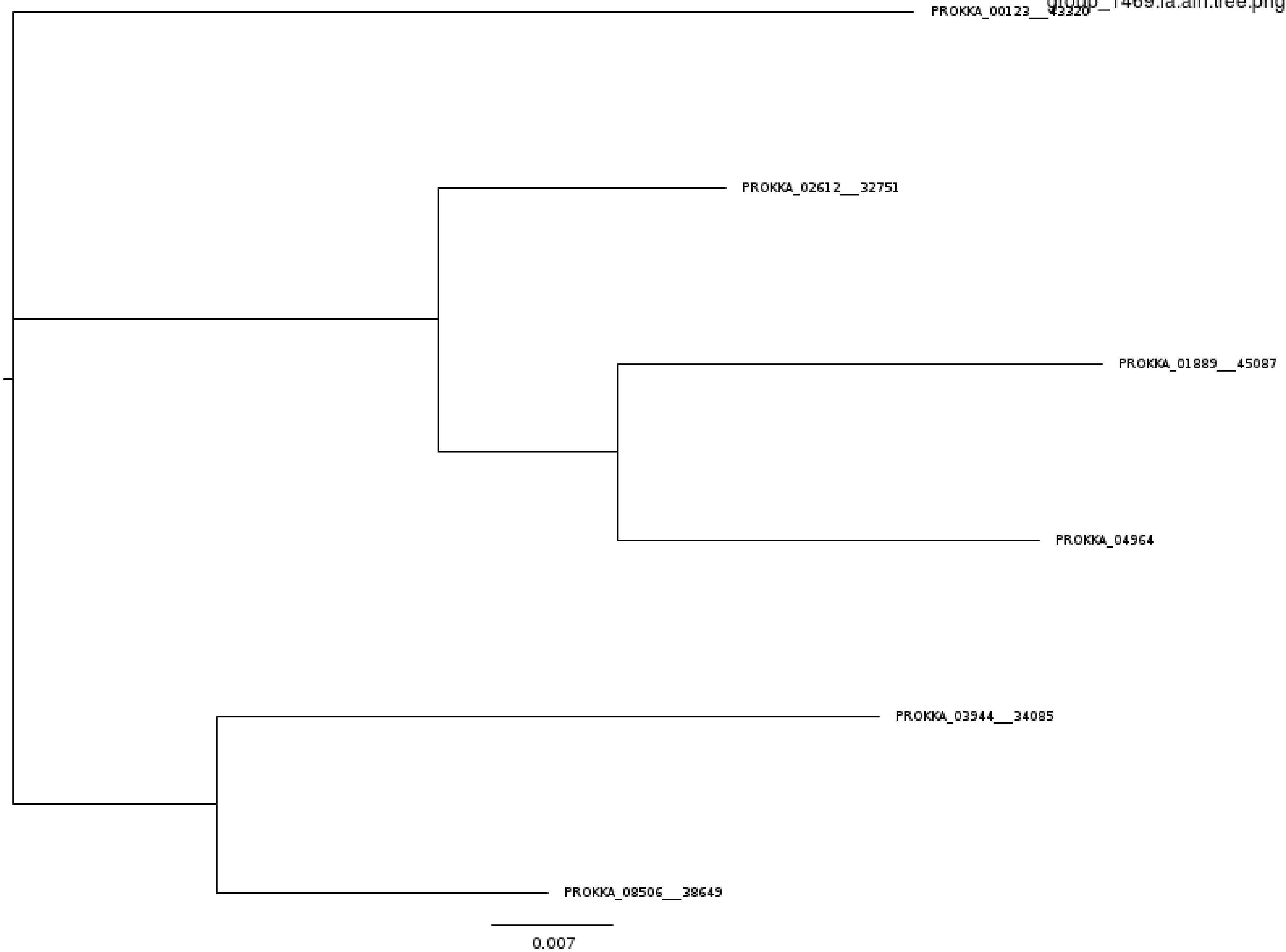
PROKKA_00123___43320
PROKKA_02612___32751
PROKKA_01889___45087
PROKKA_04964
PROKKA_03944___34085
PROKKA_08506___38649
0.007

group_1470.fa.aln.tree.png

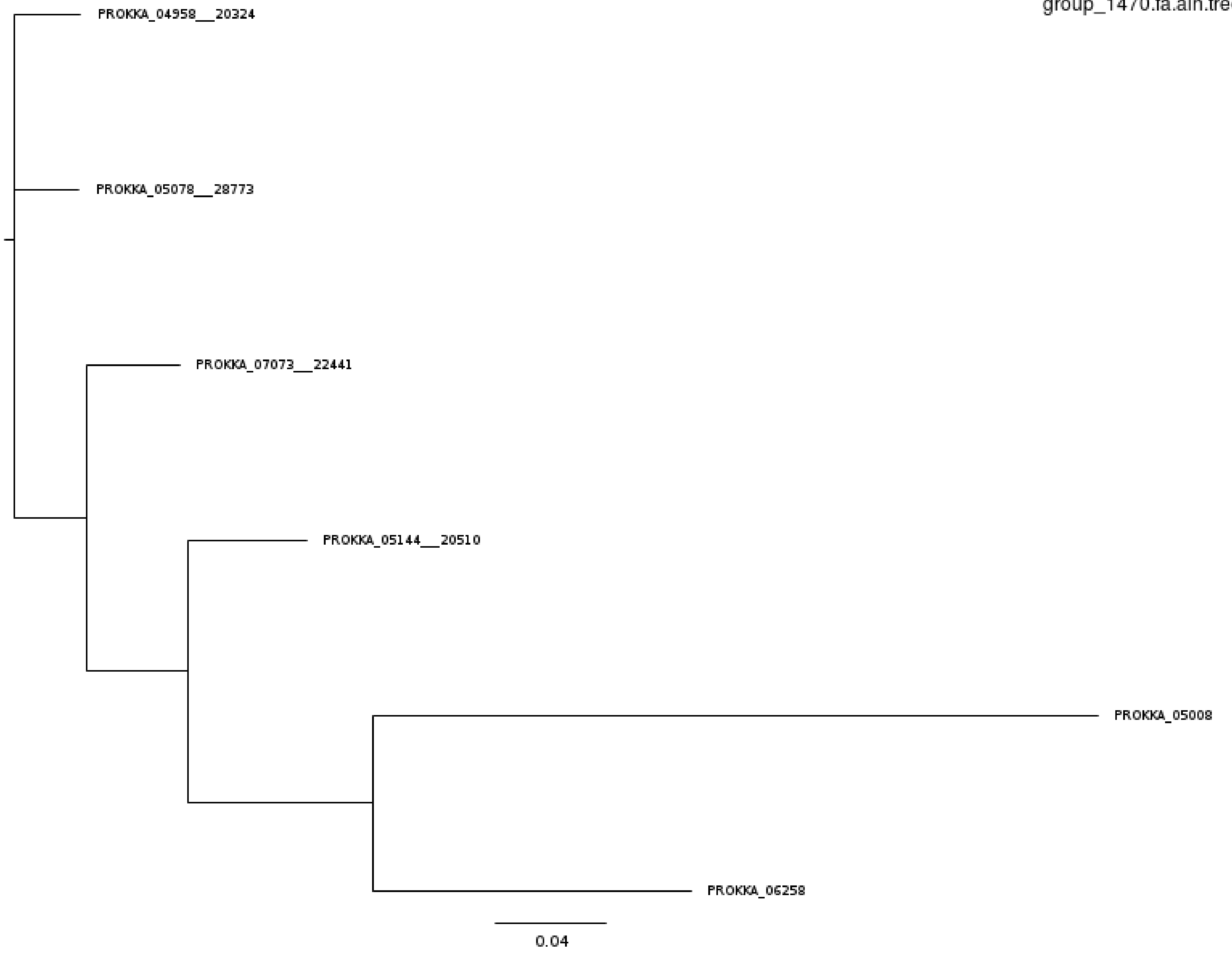

group_1471.fa.aln.tree.png

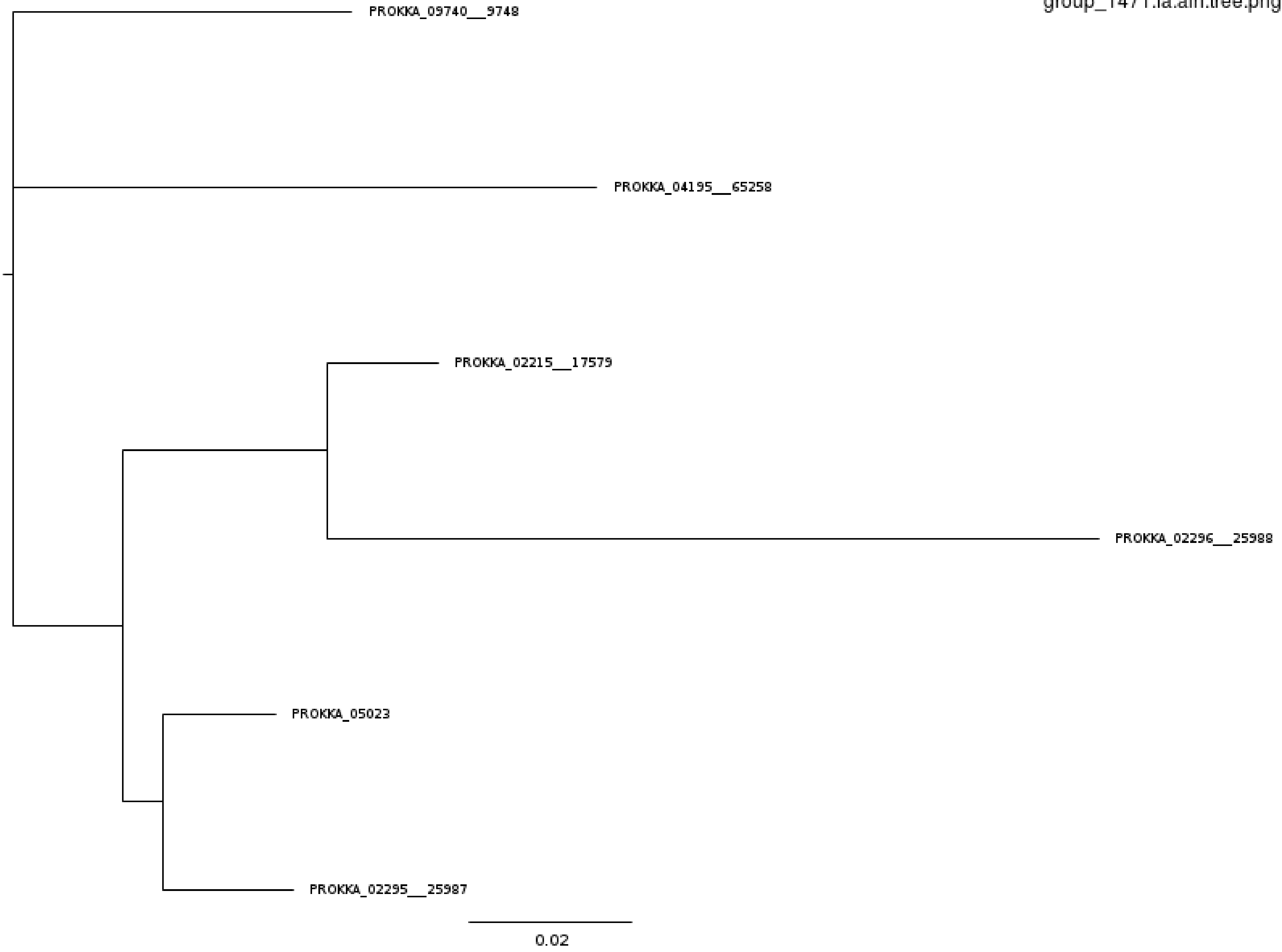

group_1472.fa.aln.tree.png

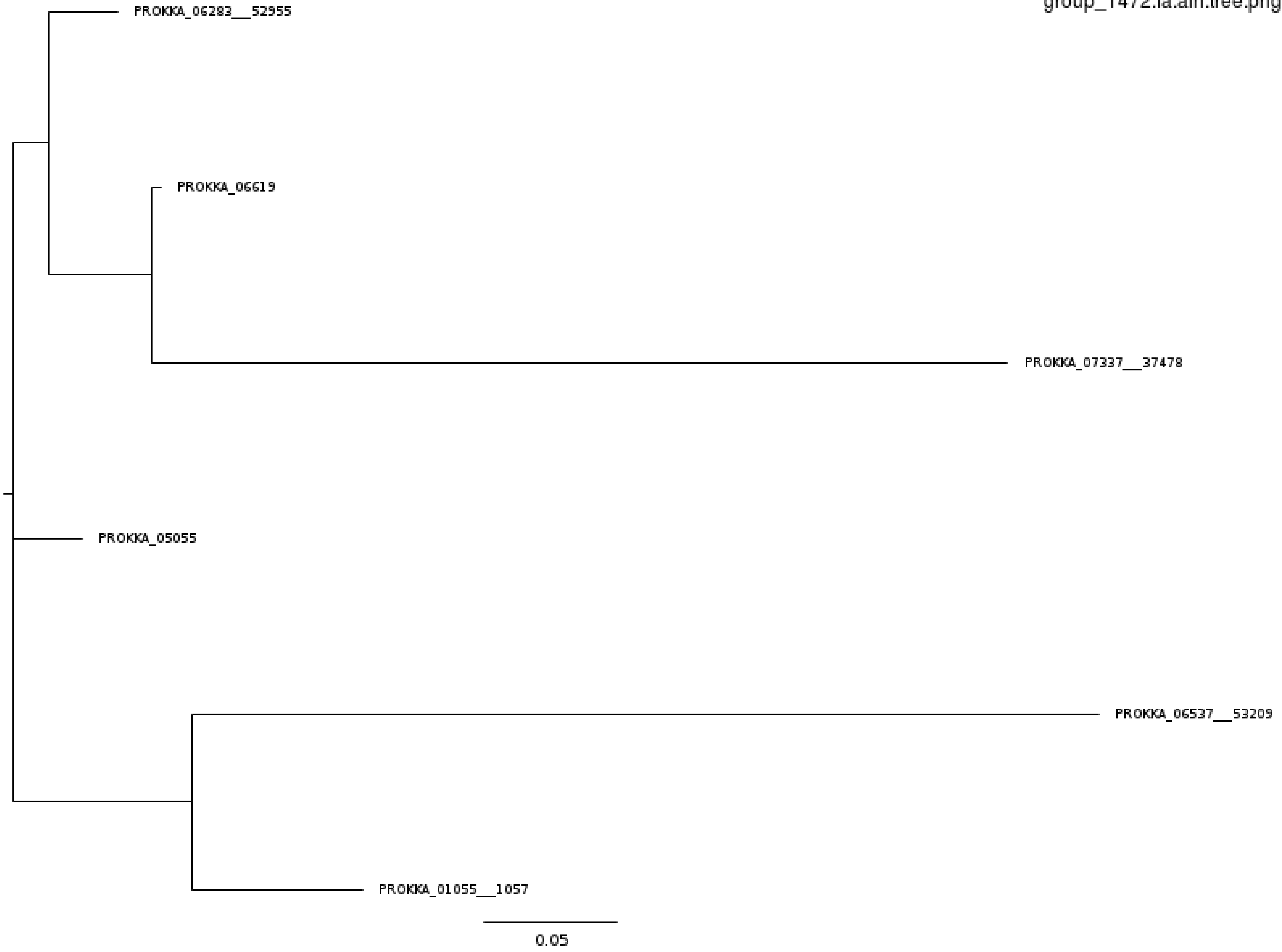

group_1476.fa.aln.tree.png

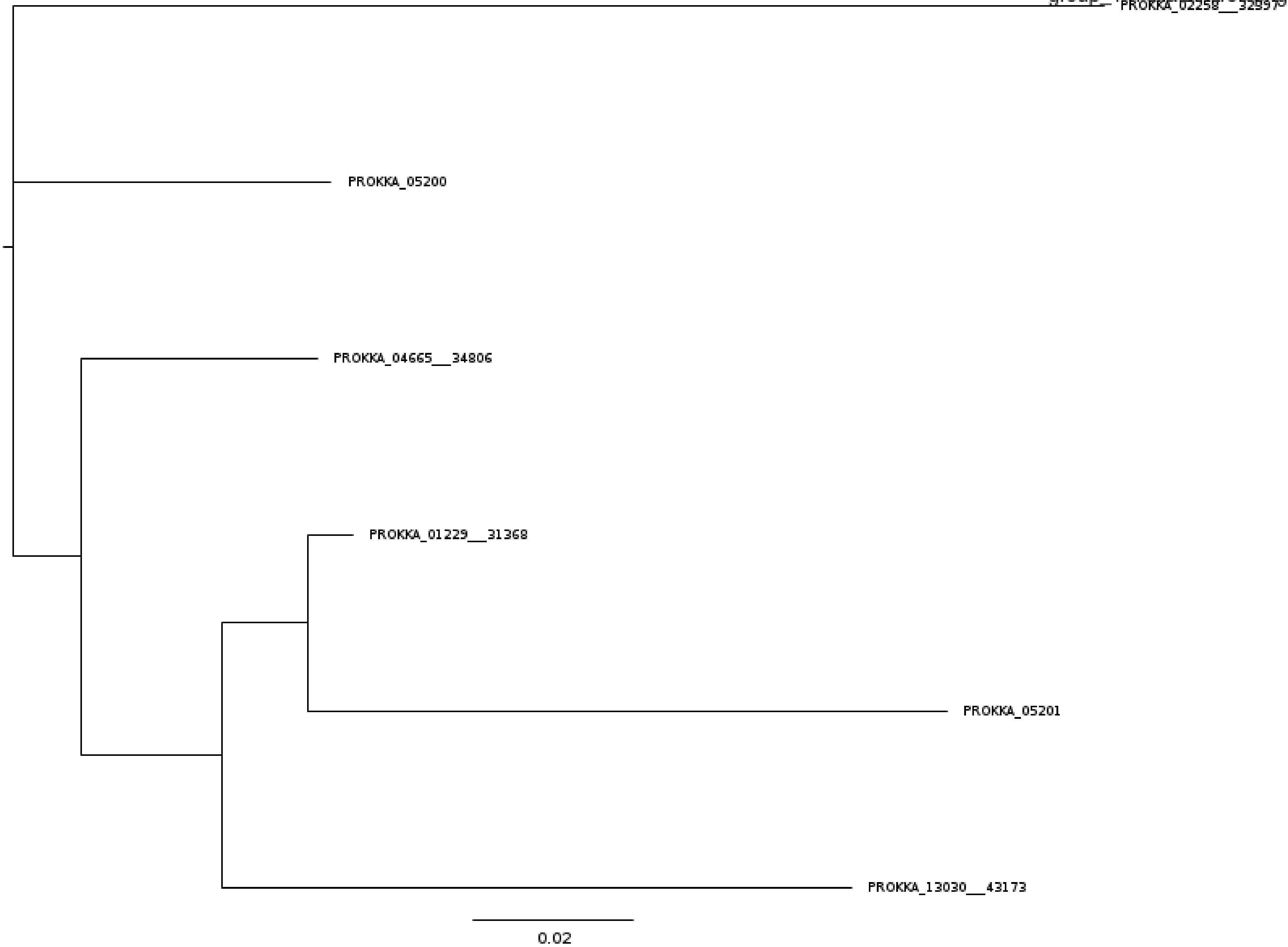

group_1477.fa.aln.tree.png

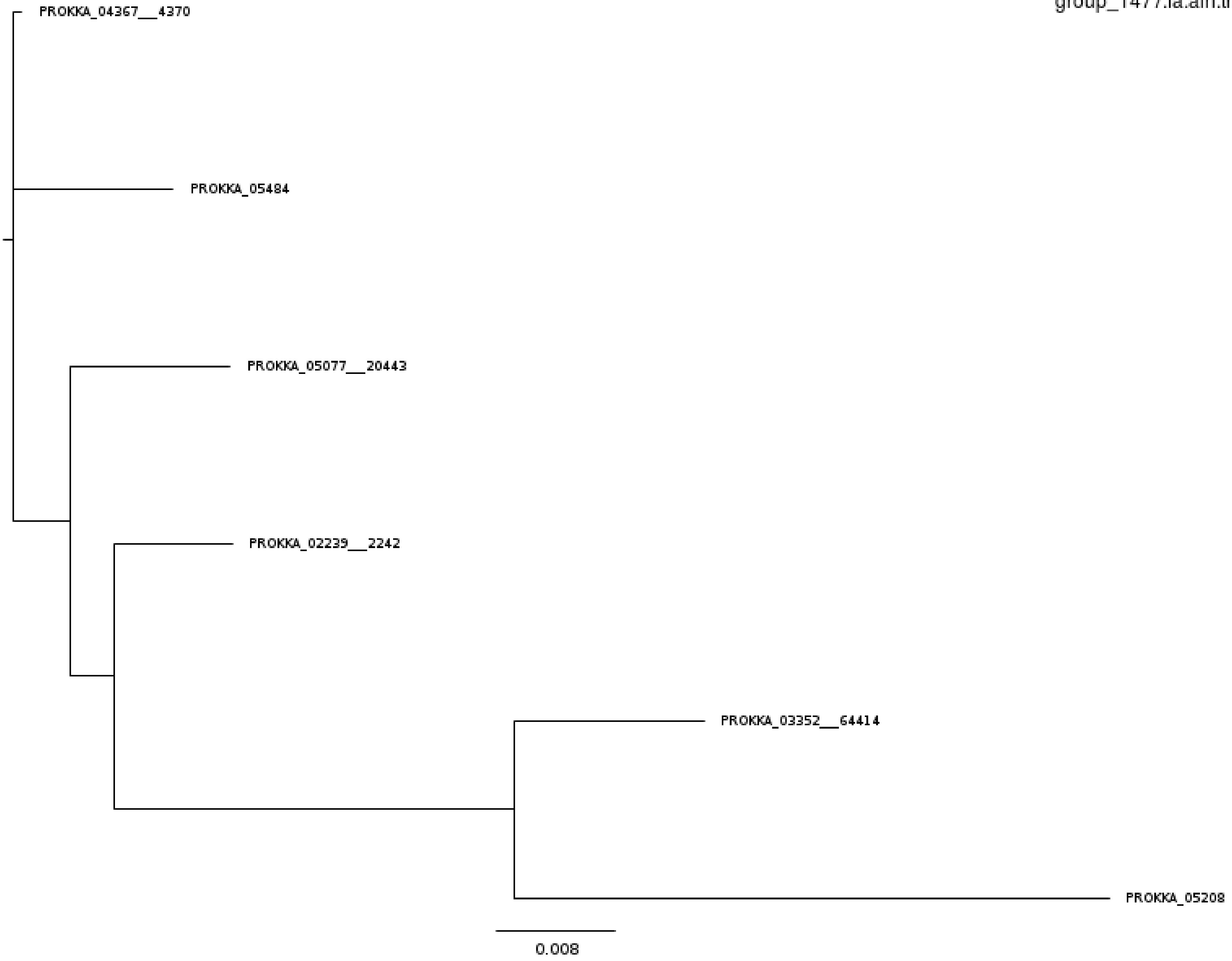

group_1480.fa.aln.tree.png

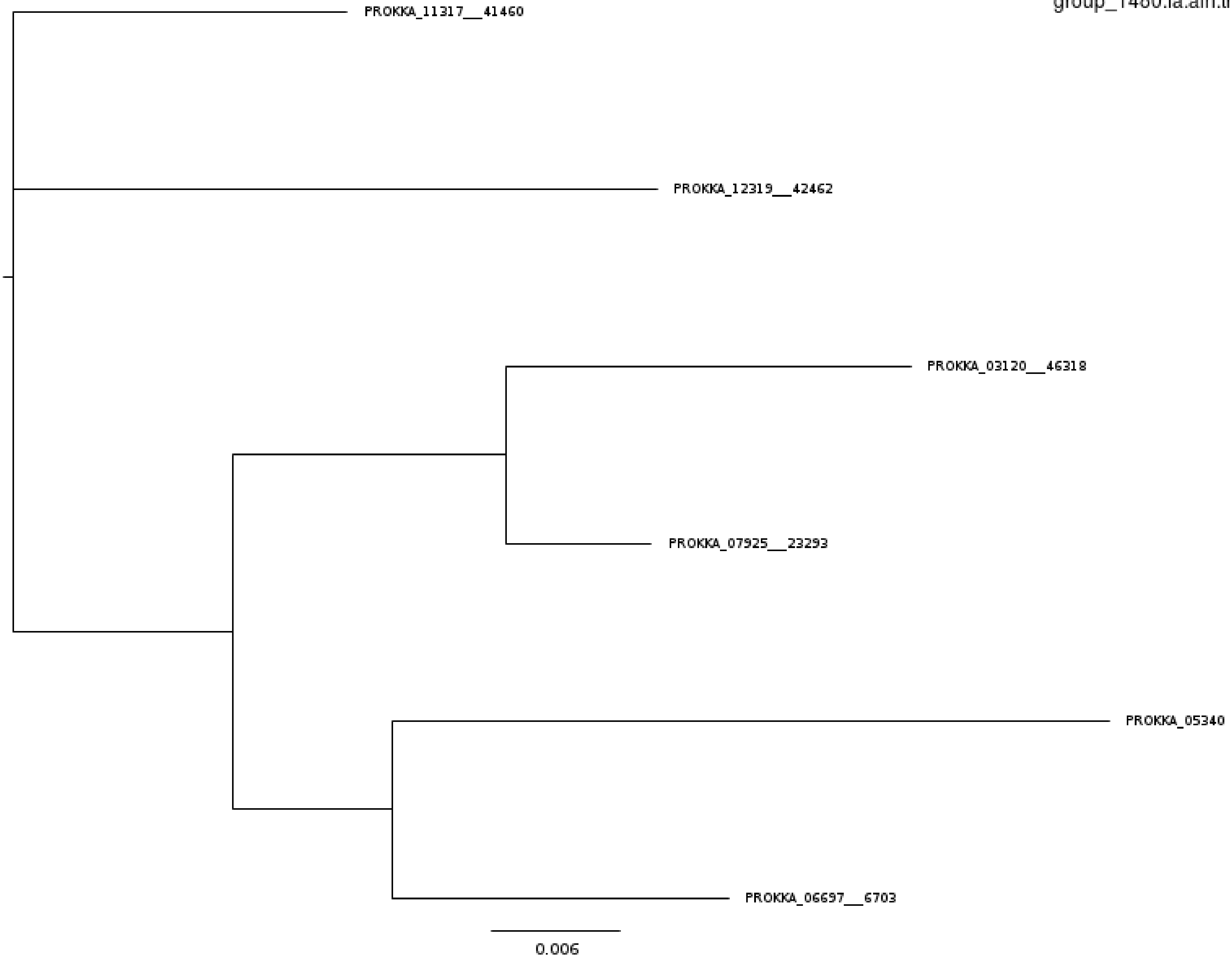

group_1481.fa.aln.tree.png

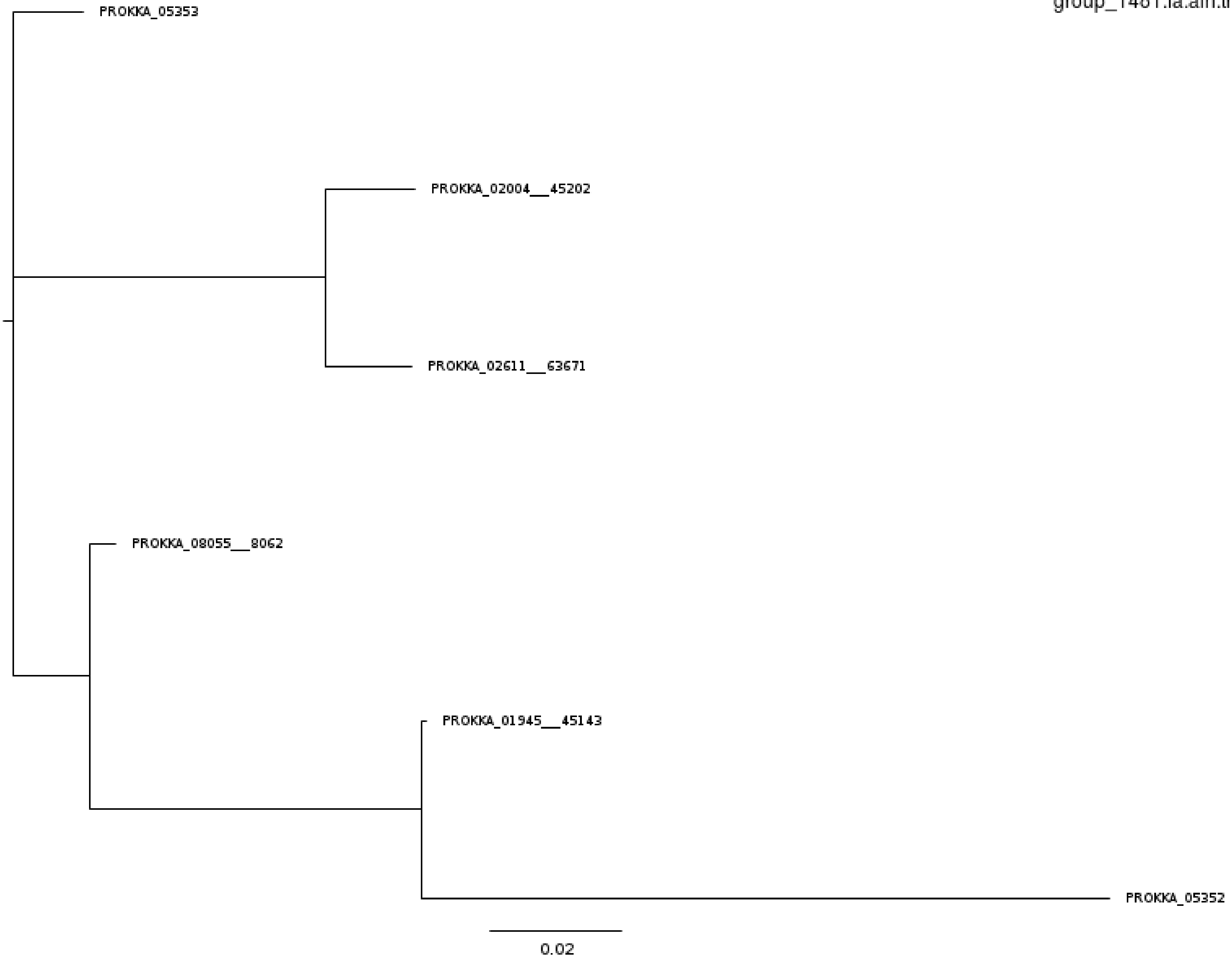

group_1490.fa.aln.tree.png

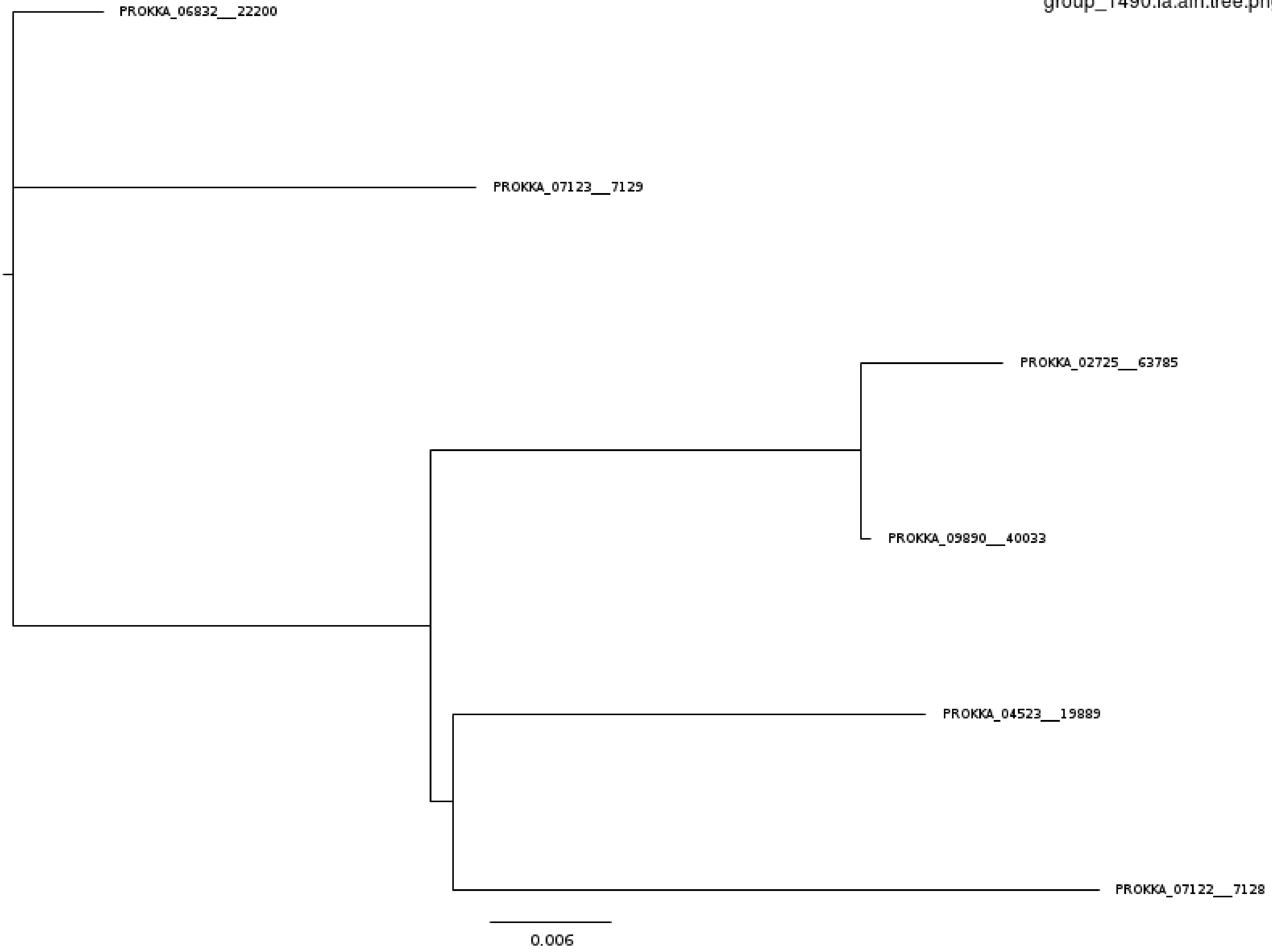

group_1492.fa.aln.tree.png

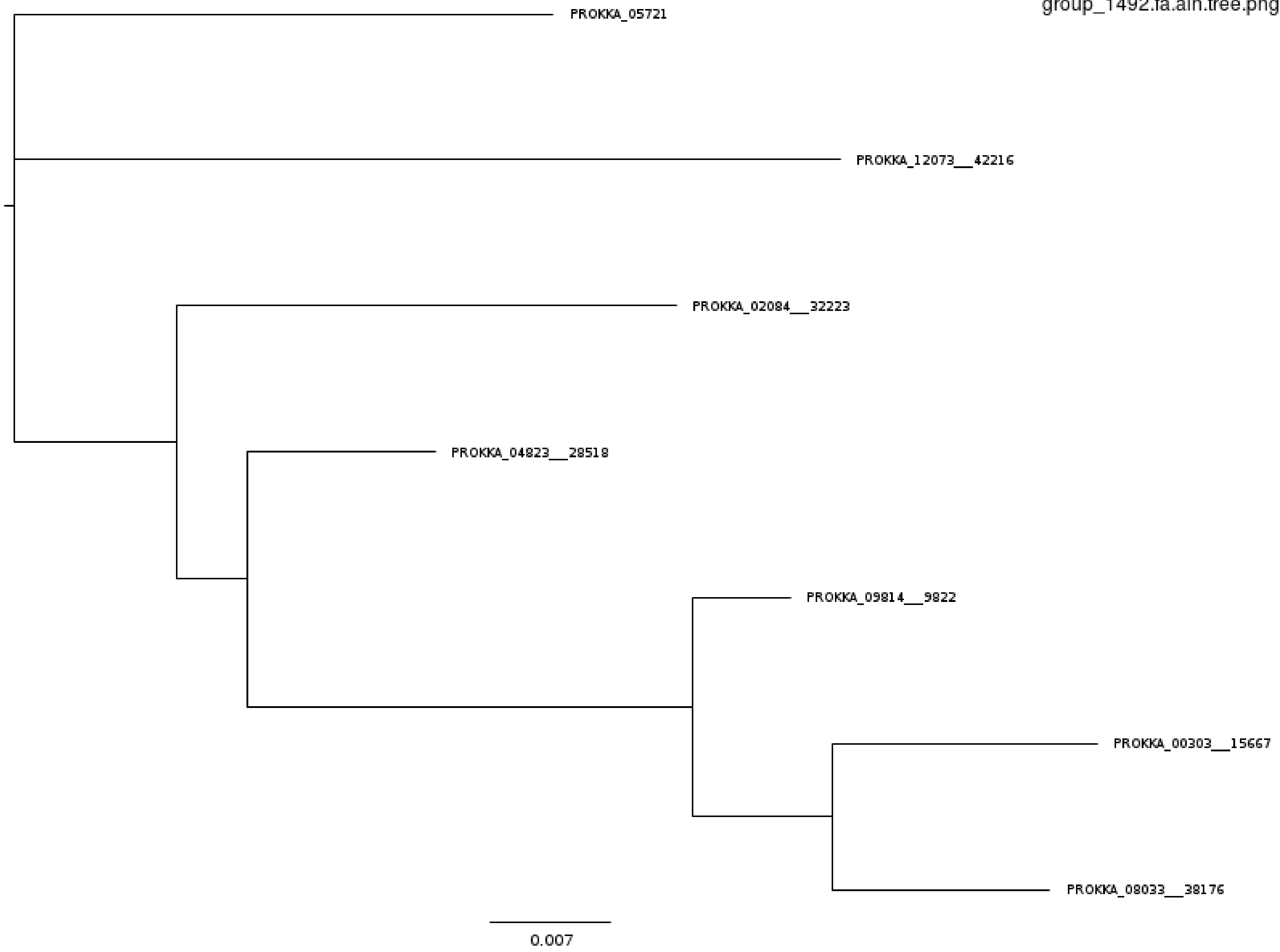

group_1497.fa.aln.tree.png

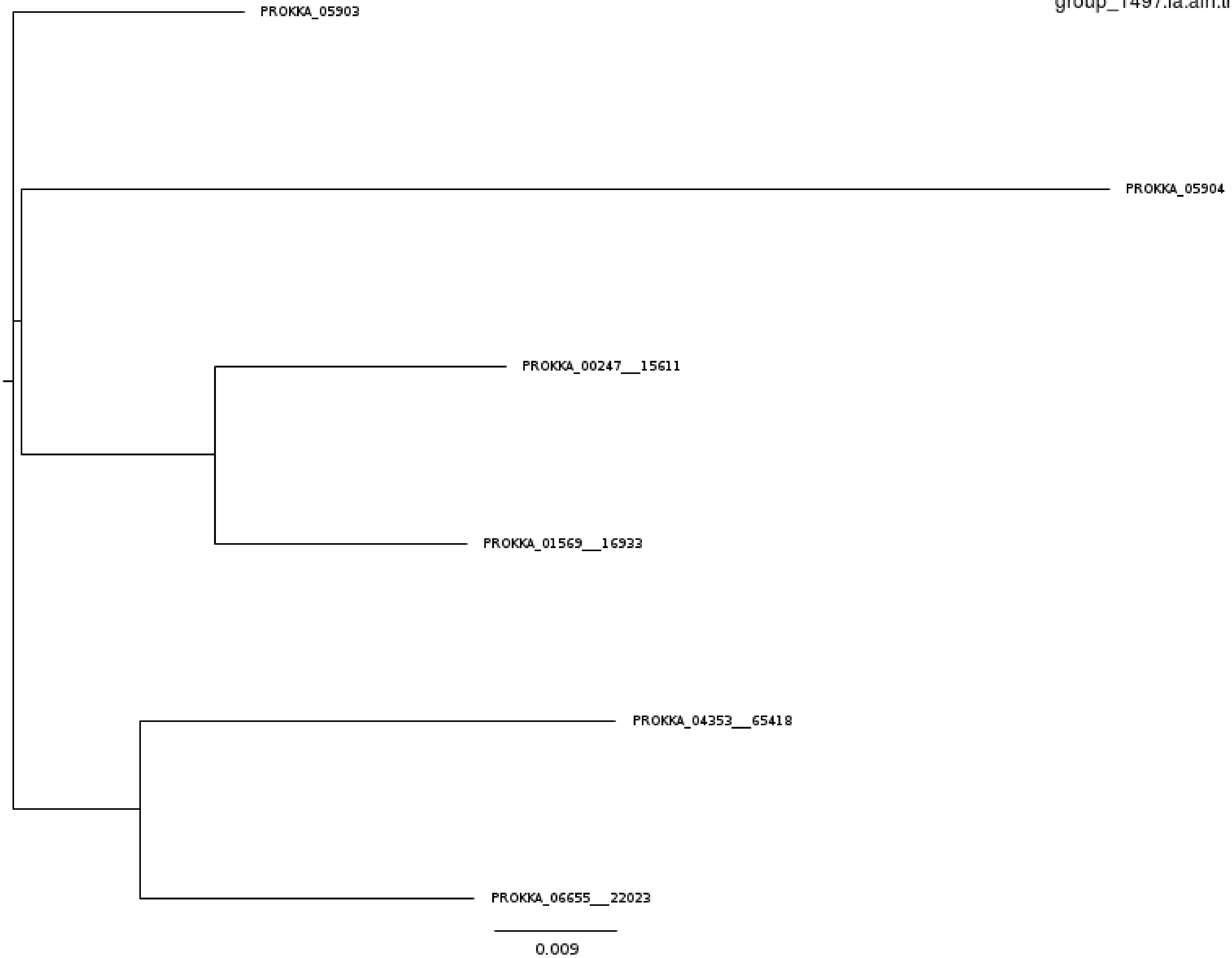

group_1498.fa.aln.tree.png

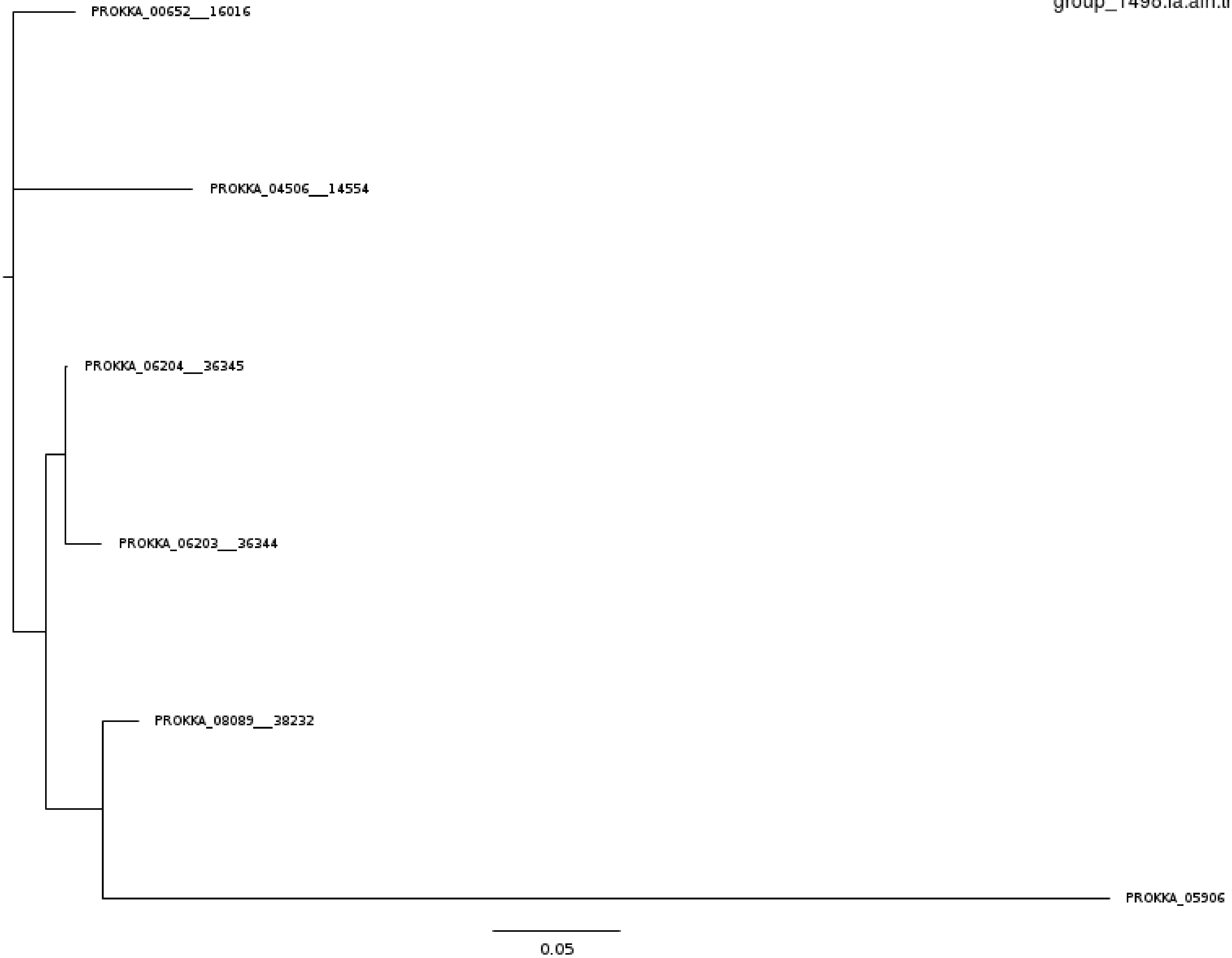

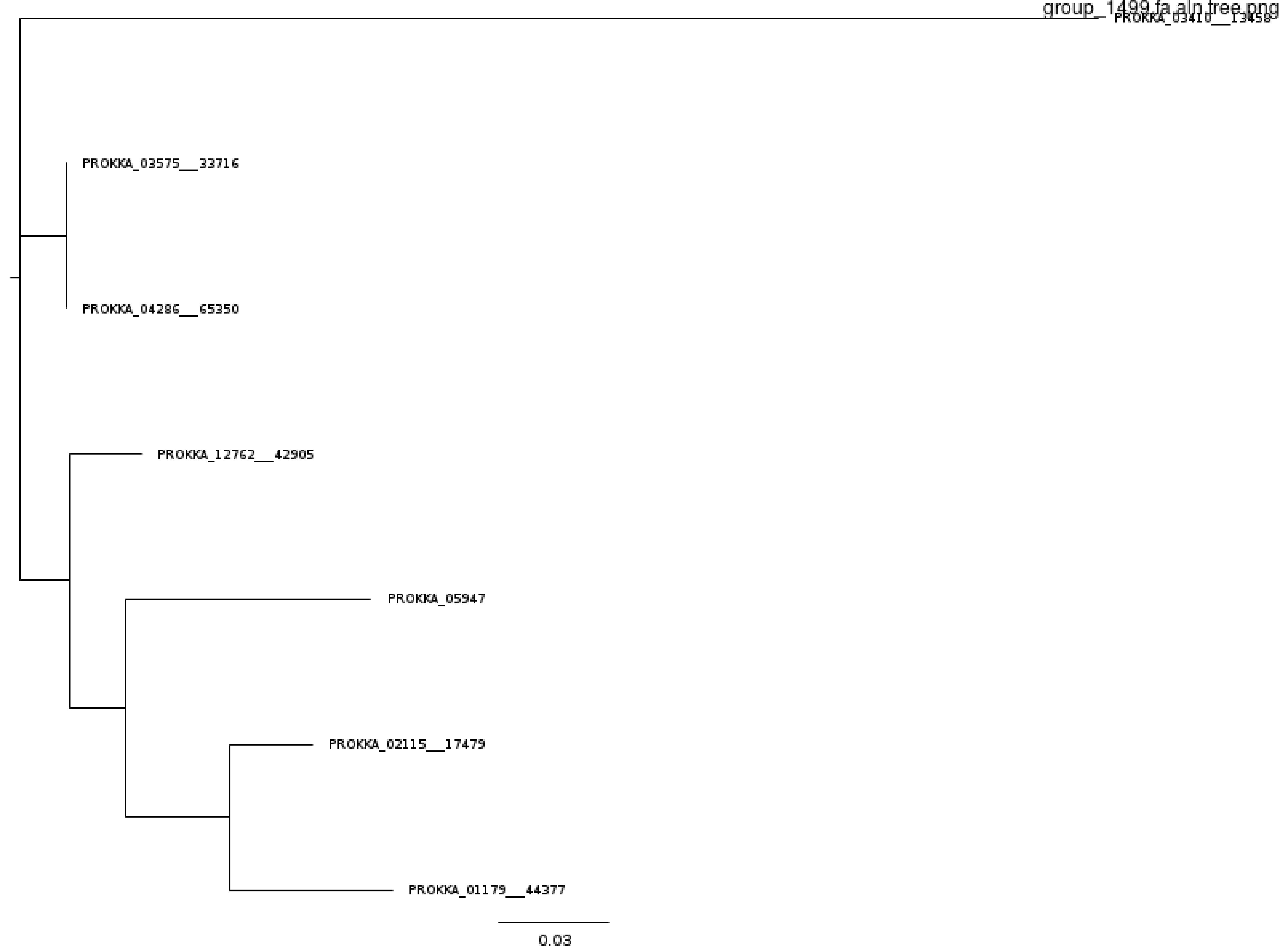
group_1499.fa.aln.tree.png
PROKKA_03410__13458
PROKKA_03575___33716
PROKKA_04286___65350
PROKKA_12762___42905
PROKKA_05947
PROKKA_02115___17479
PROKKA_01179___44377
0.03

group_14.fa.aln.tree.png

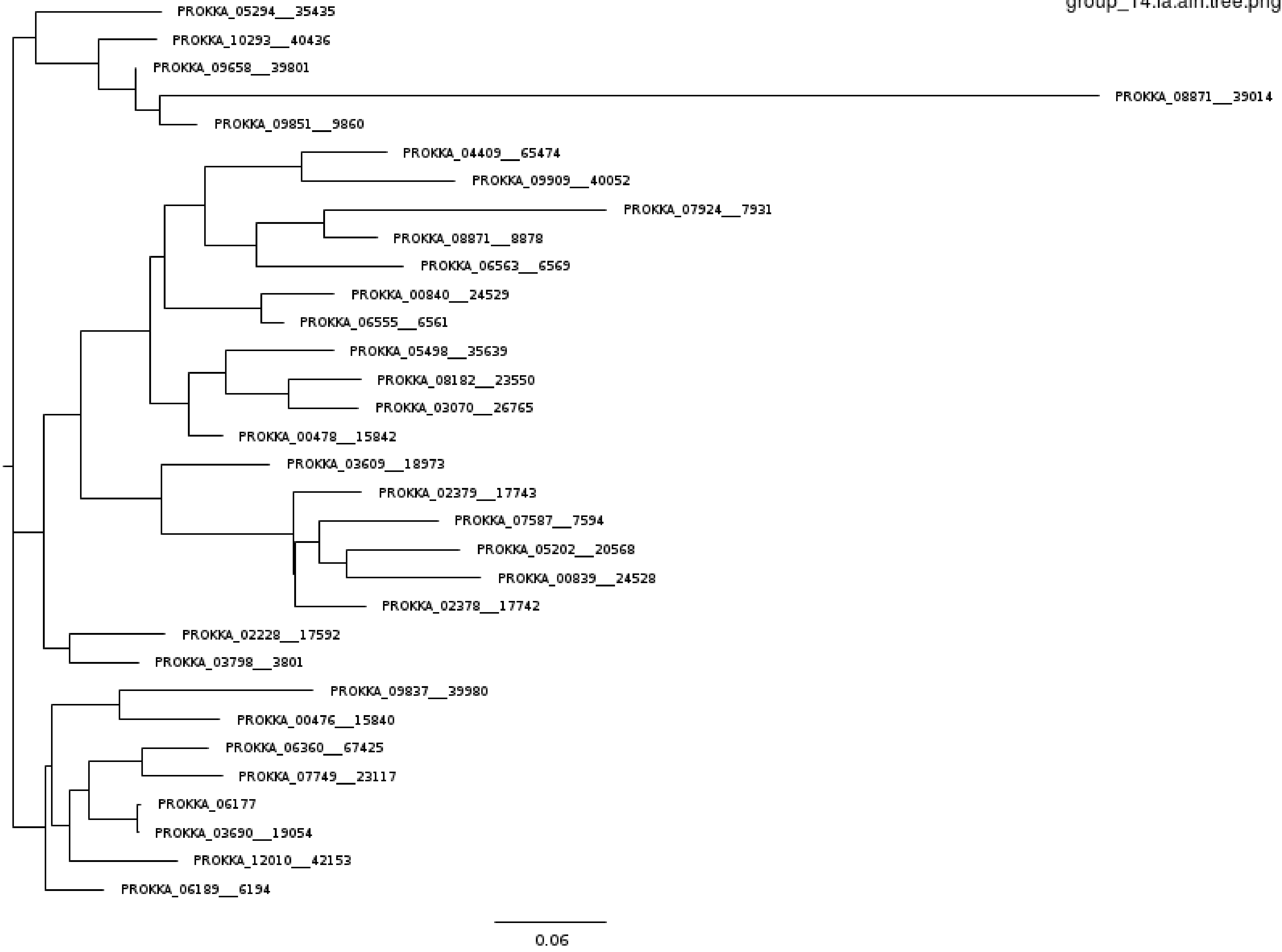

group_1500.fa.aln.tree.png

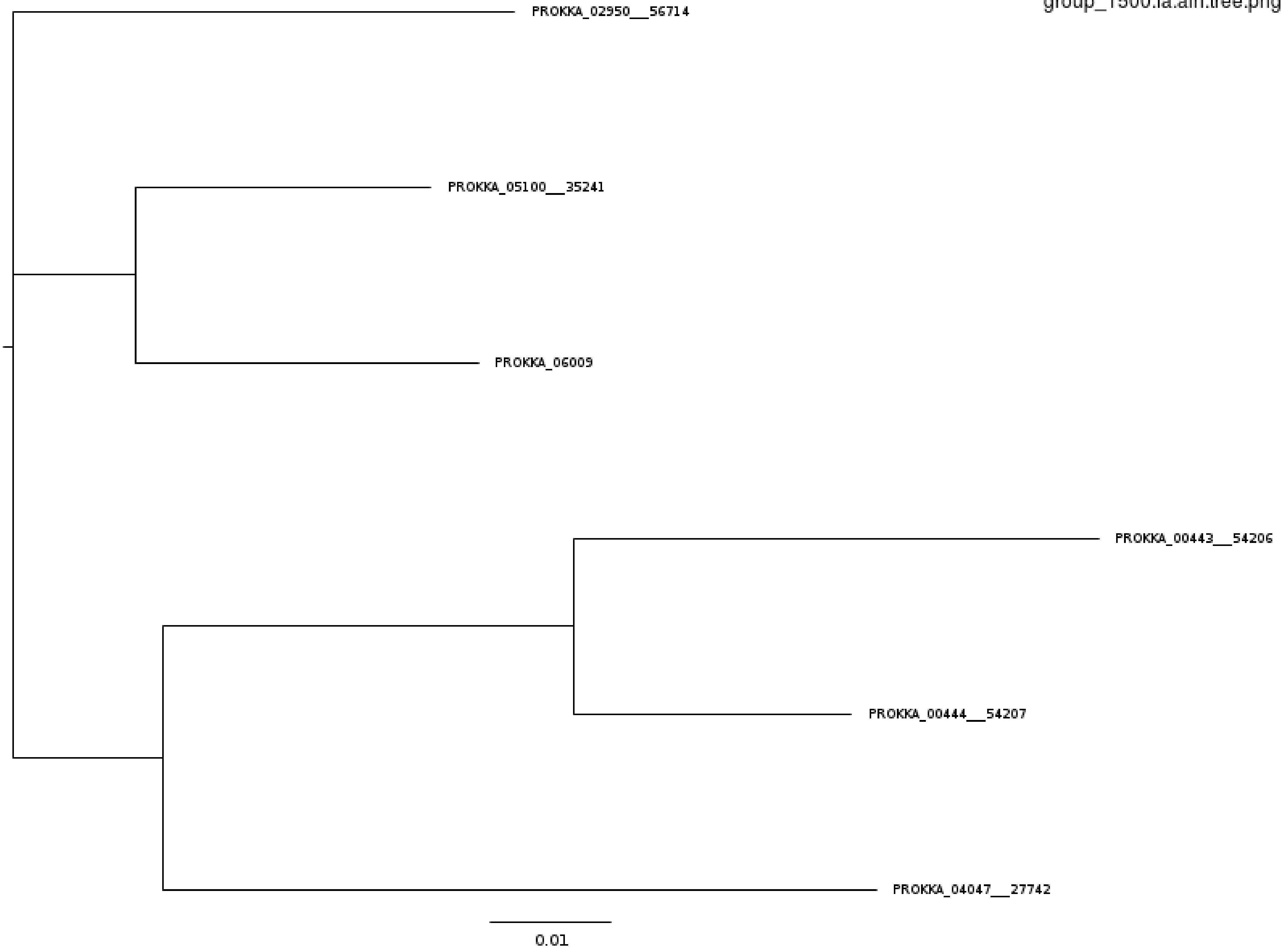

group_1503.fa.aln.tree.png

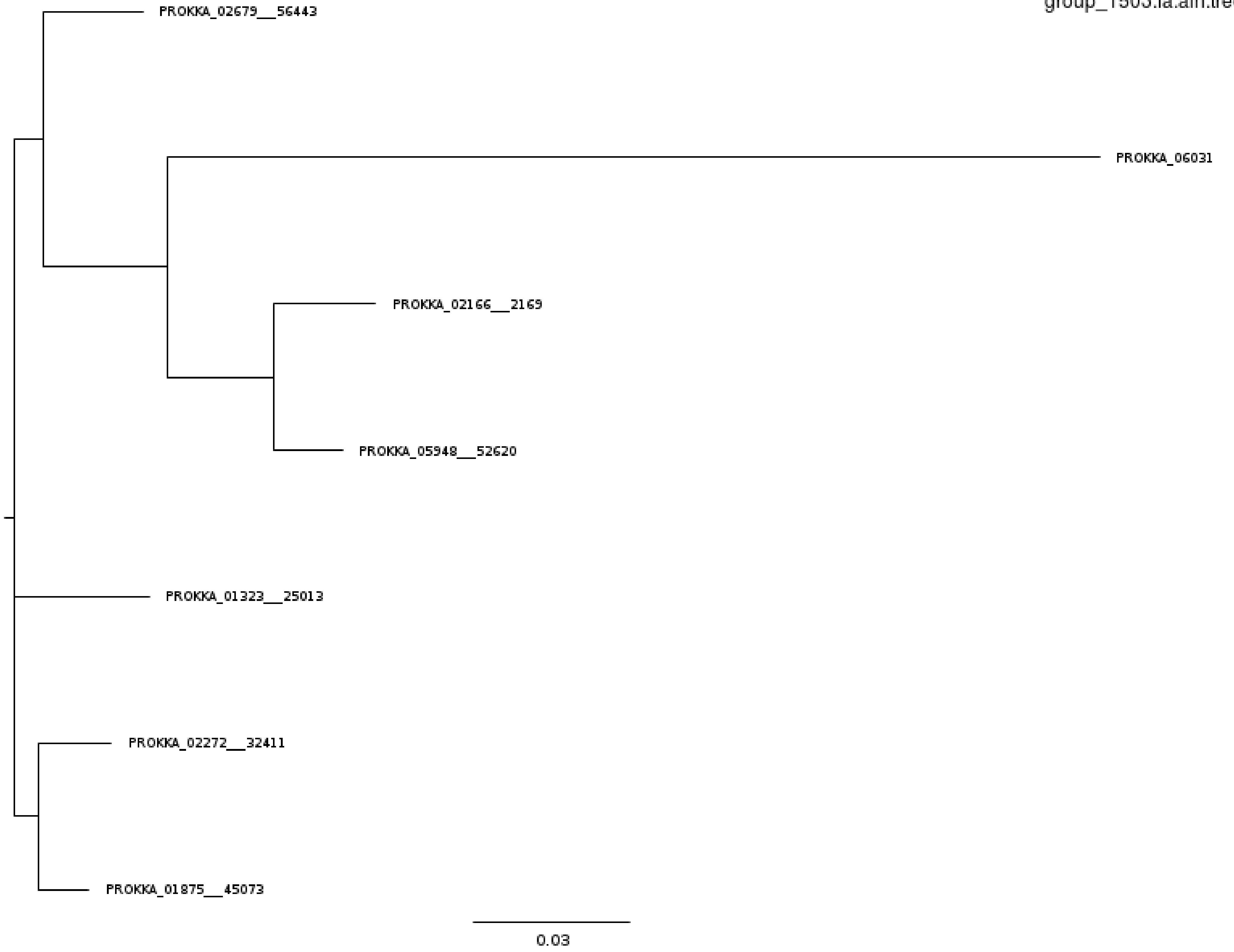

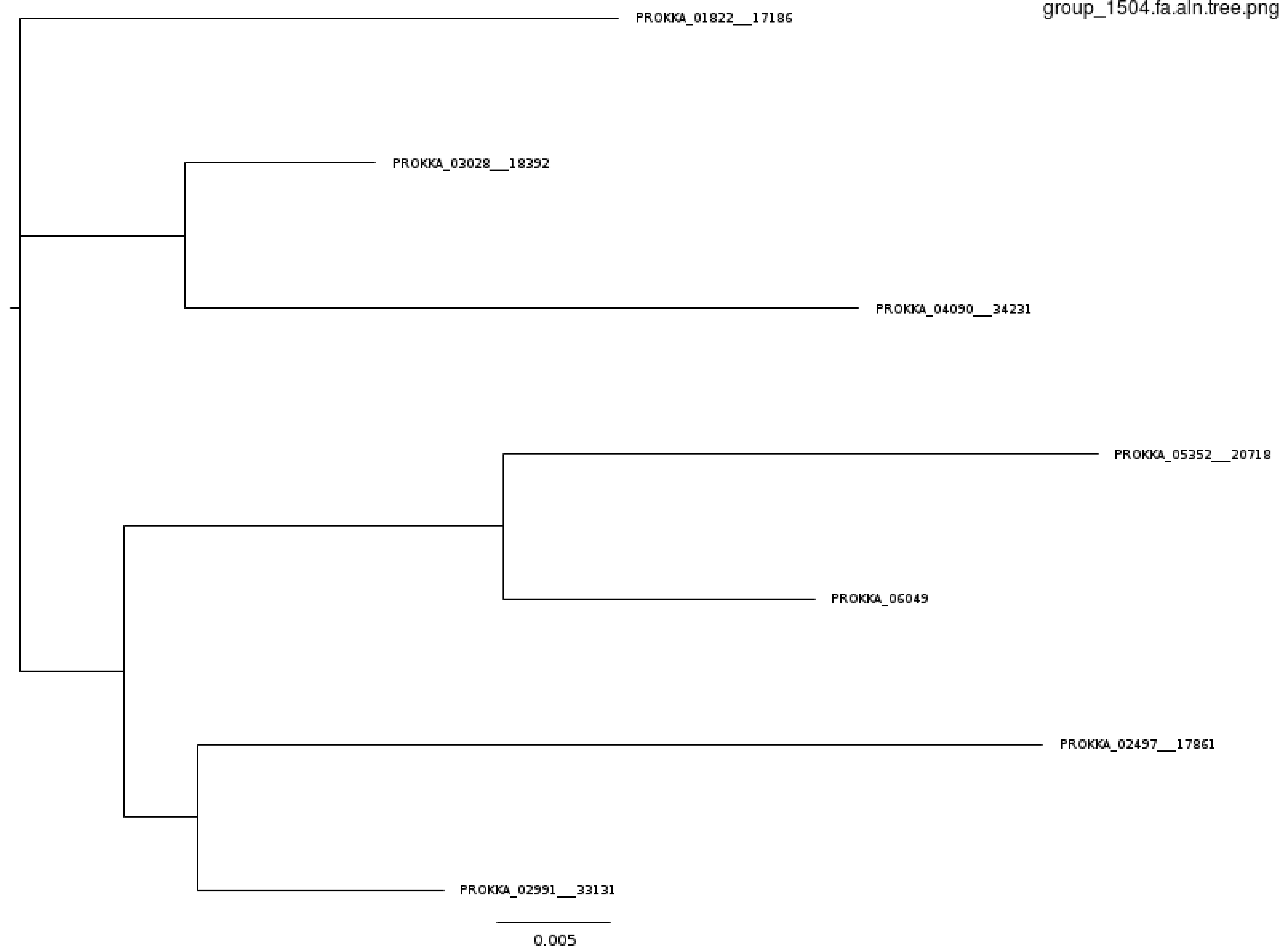
group_1504.fa.aln.tree.png
PROKKA_01822___17186
PROKKA_03028___18392
PROKKA_04090___34231
PROKKA_05352___20718
PROKKA_06049
PROKKA_02497___17861
PROKKA_02991___33131
0.005

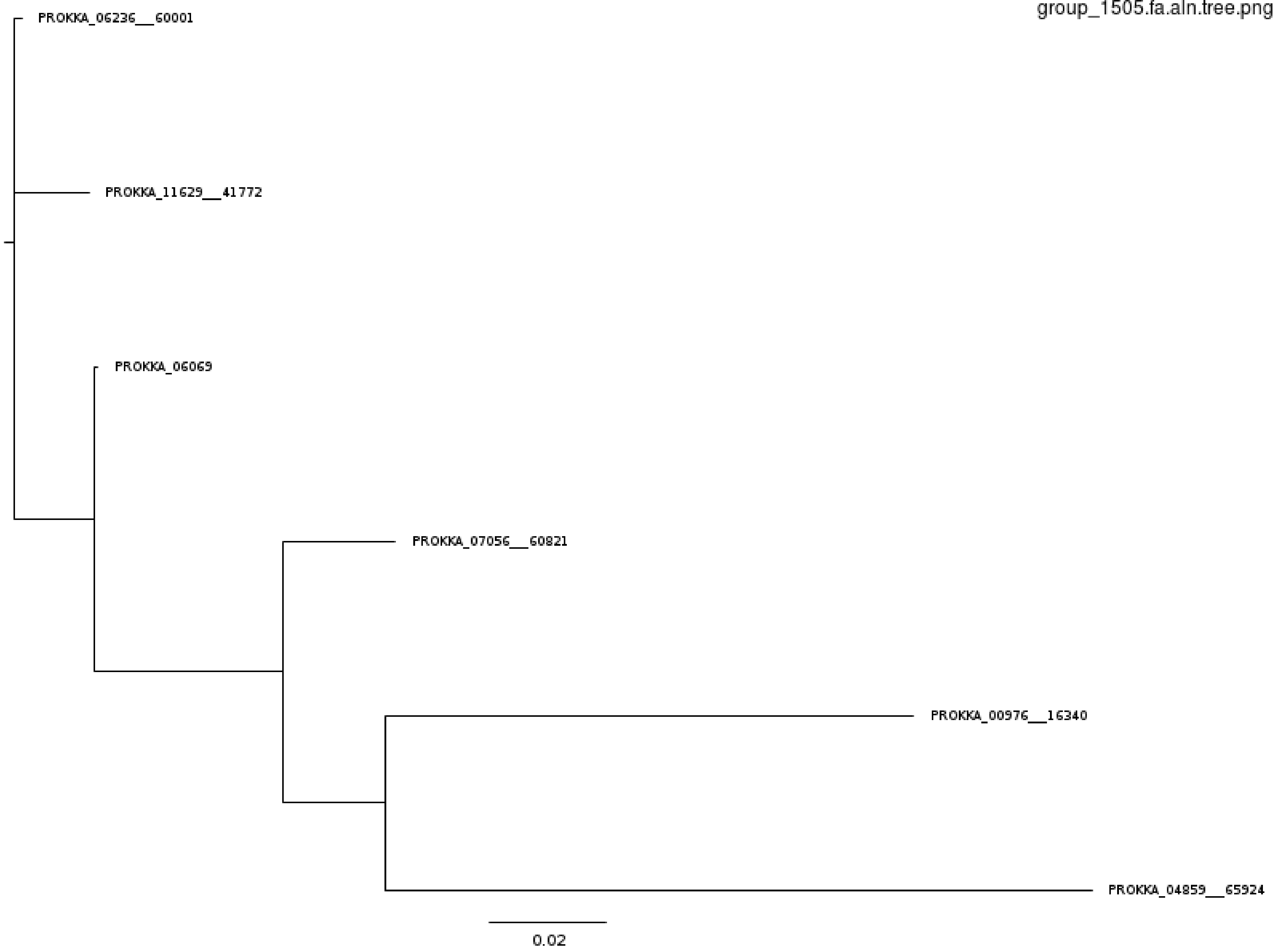
group_1505.fa.aln.tree.png
PROKKA_06236___60001
PROKKA_11629___41772
PROKKA_06069
PROKKA_07056___60821
PROKKA_00976___16340
PROKKA_04859___65924
0.02

group_1506.fa.aln.tree.png

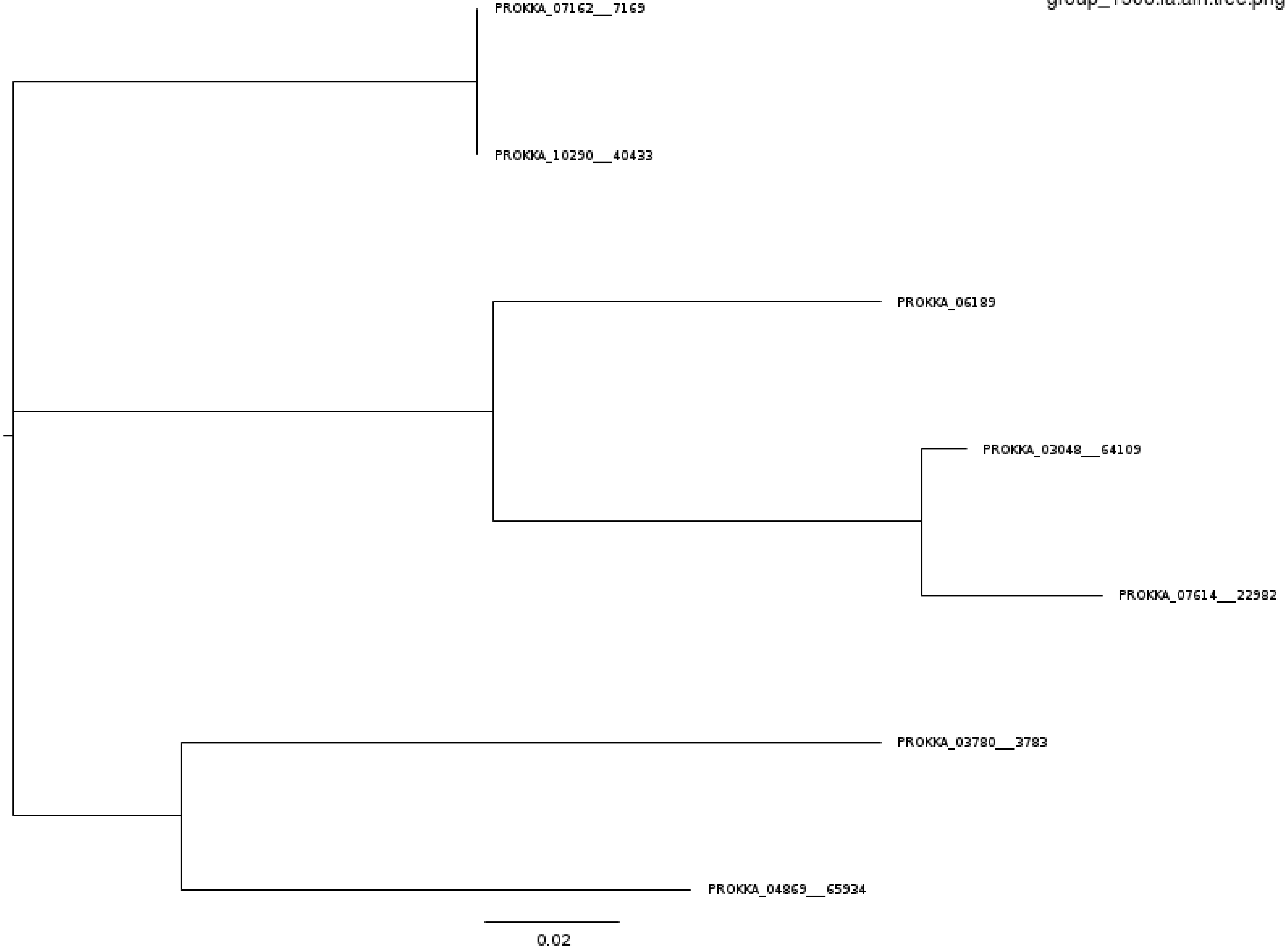

group_1507.fa.aln.tree.png

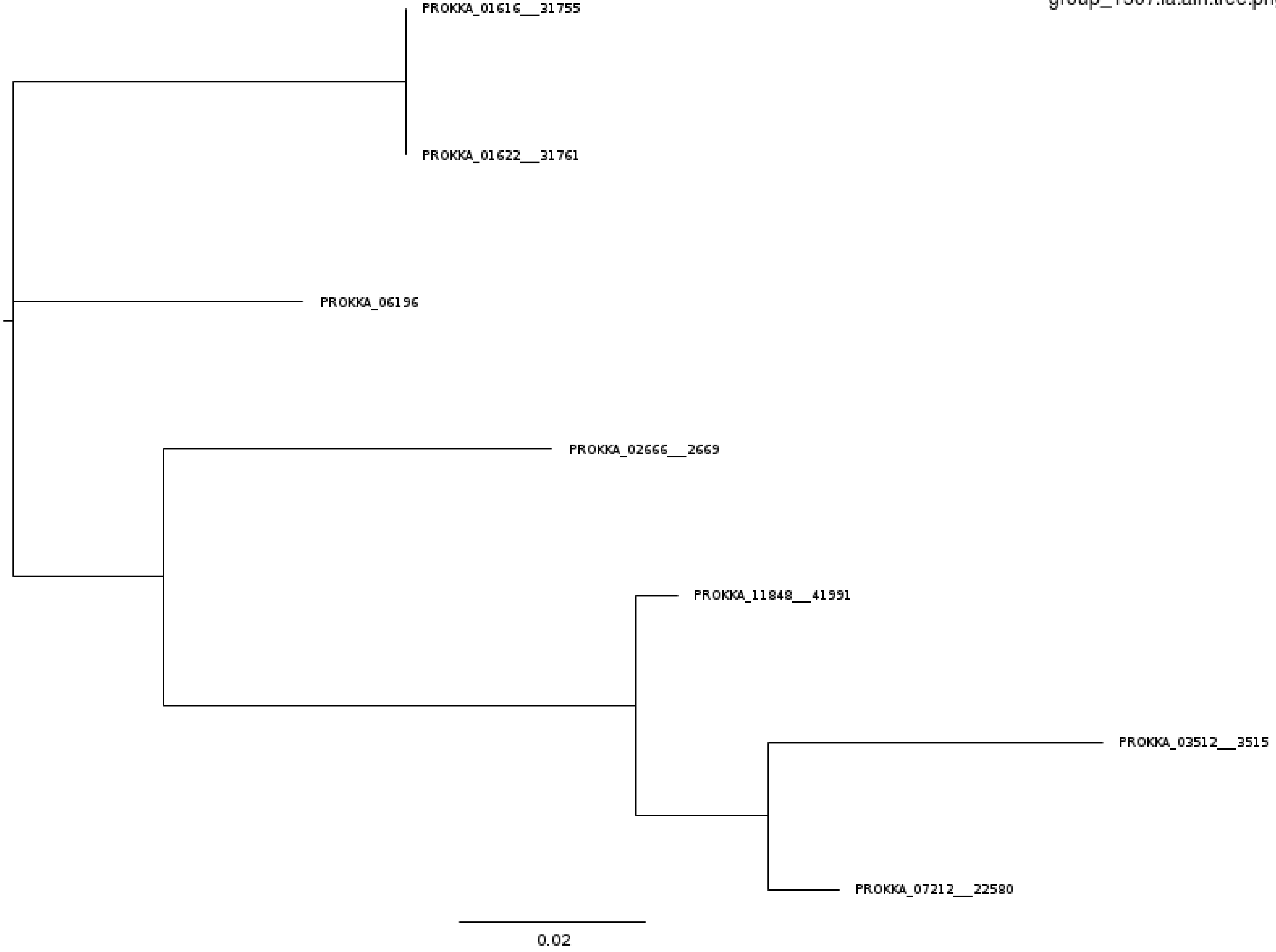

group_1509.fa.aln.tree.png

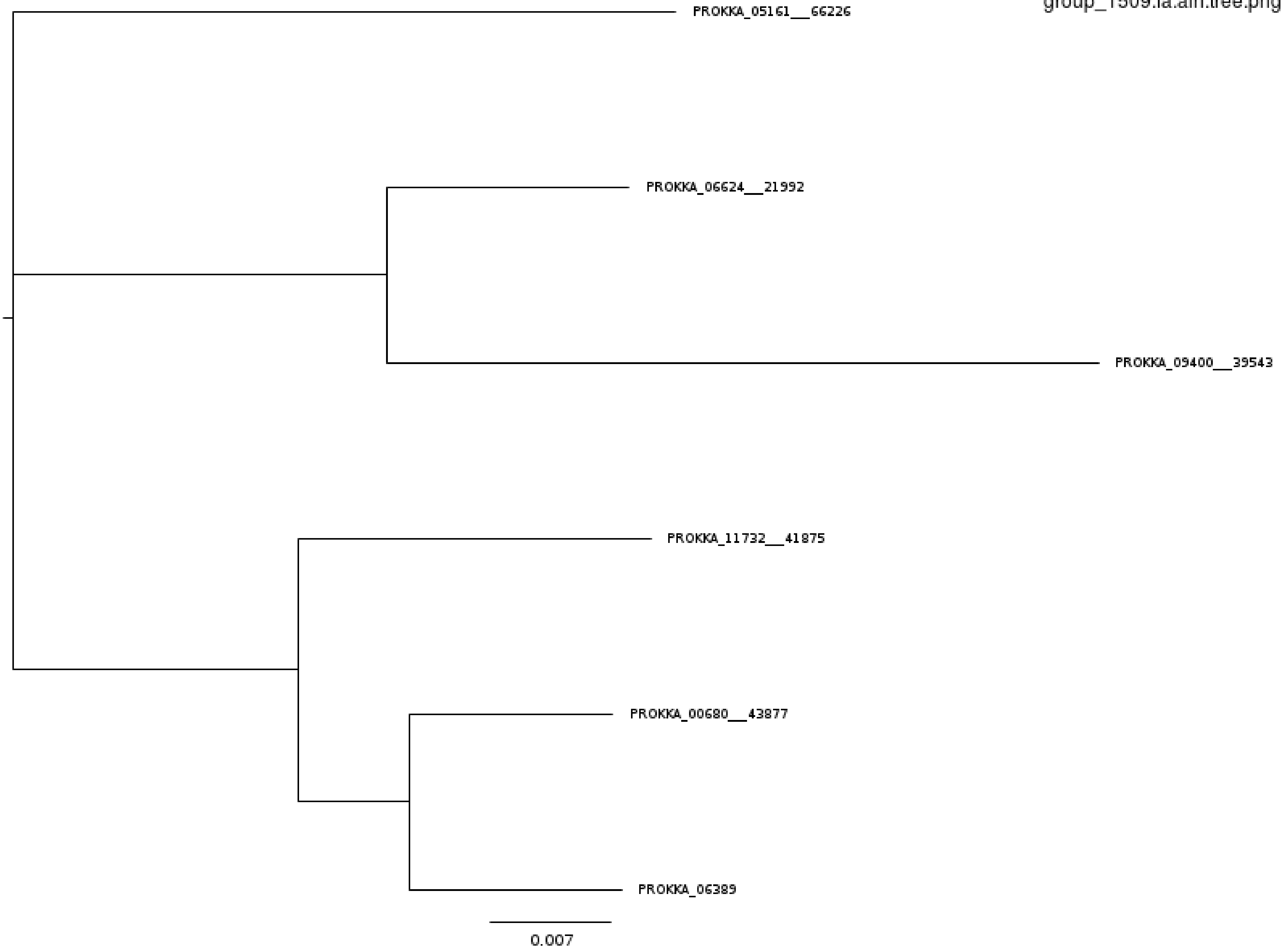

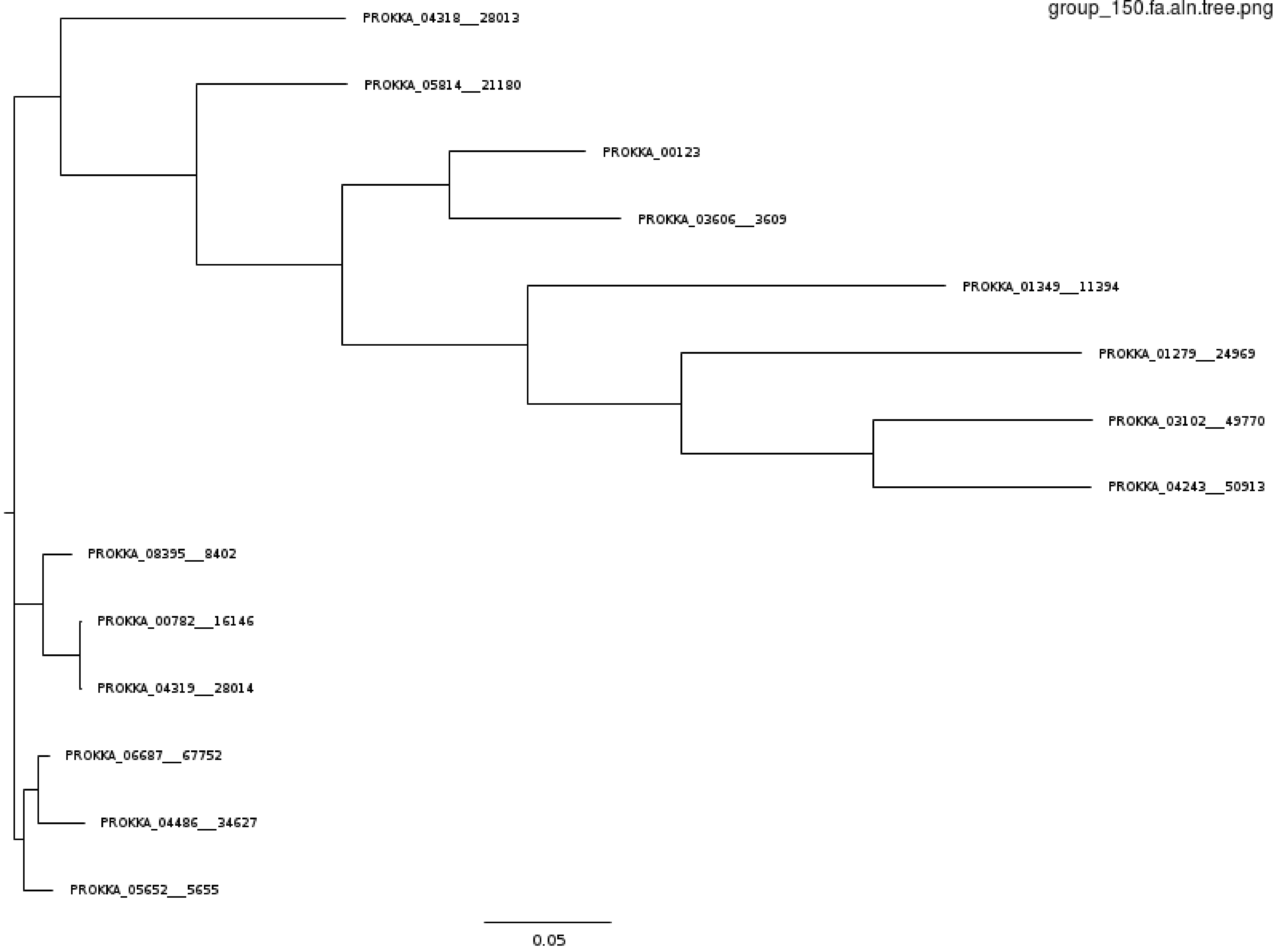

group_150.fa.aln.tree.png
PROKKA_04318___28013
PROKKA_05814___21180
PROKKA_00123
PROKKA_03606___3609
PROKKA_01349___11394
PROKKA_01279___24969
PROKKA_03102___49770
PROKKA_04243___50913
PROKKA_08395___8402
PROKKA_00782___16146
PROKKA_04319___28014
PROKKA_06687___67752
PROKKA_04486___34627
PROKKA_05652___5655
0.05

group_1511.fa.aln.tree.png

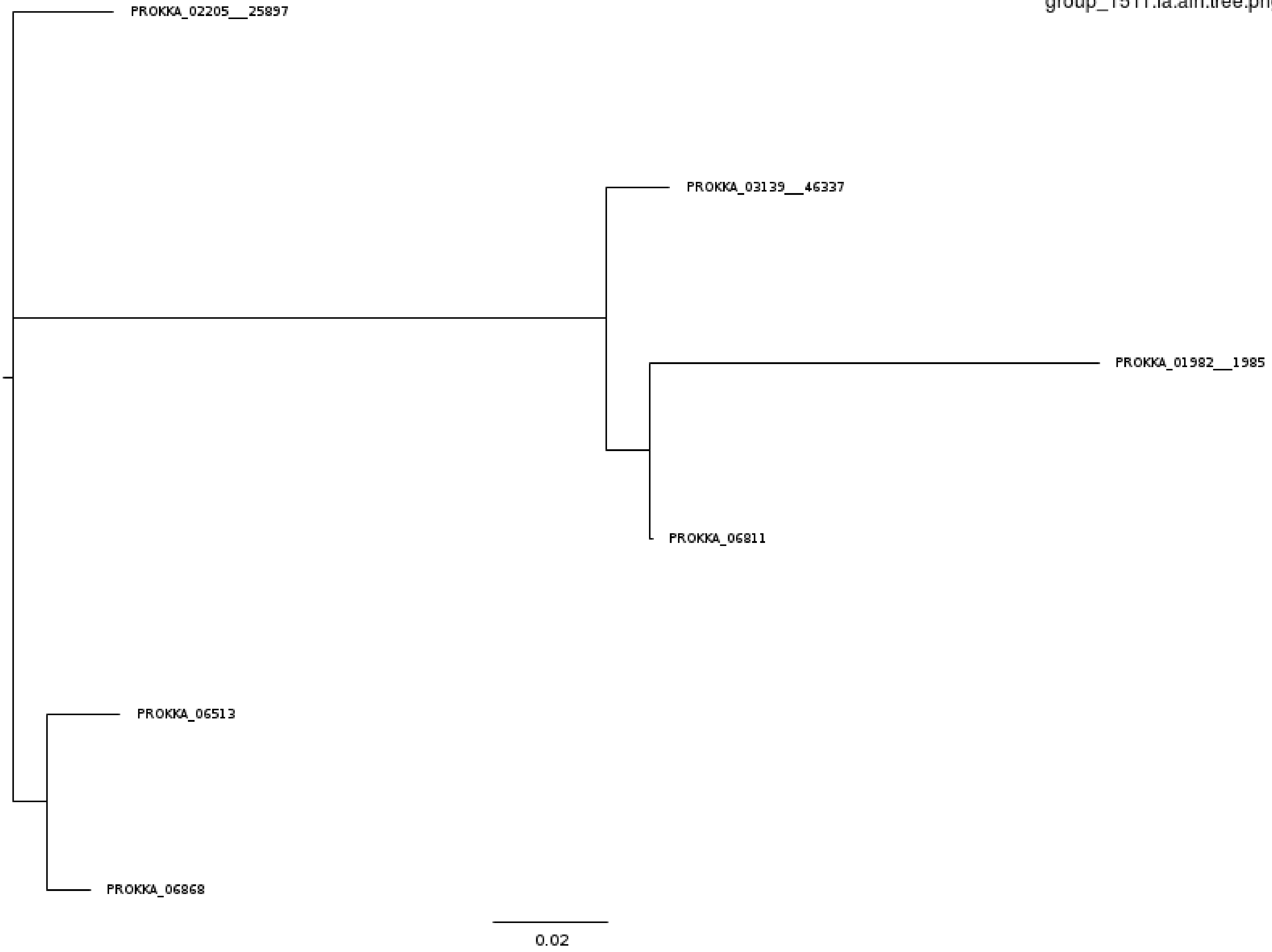

group_1514.fa.aln.tree.png

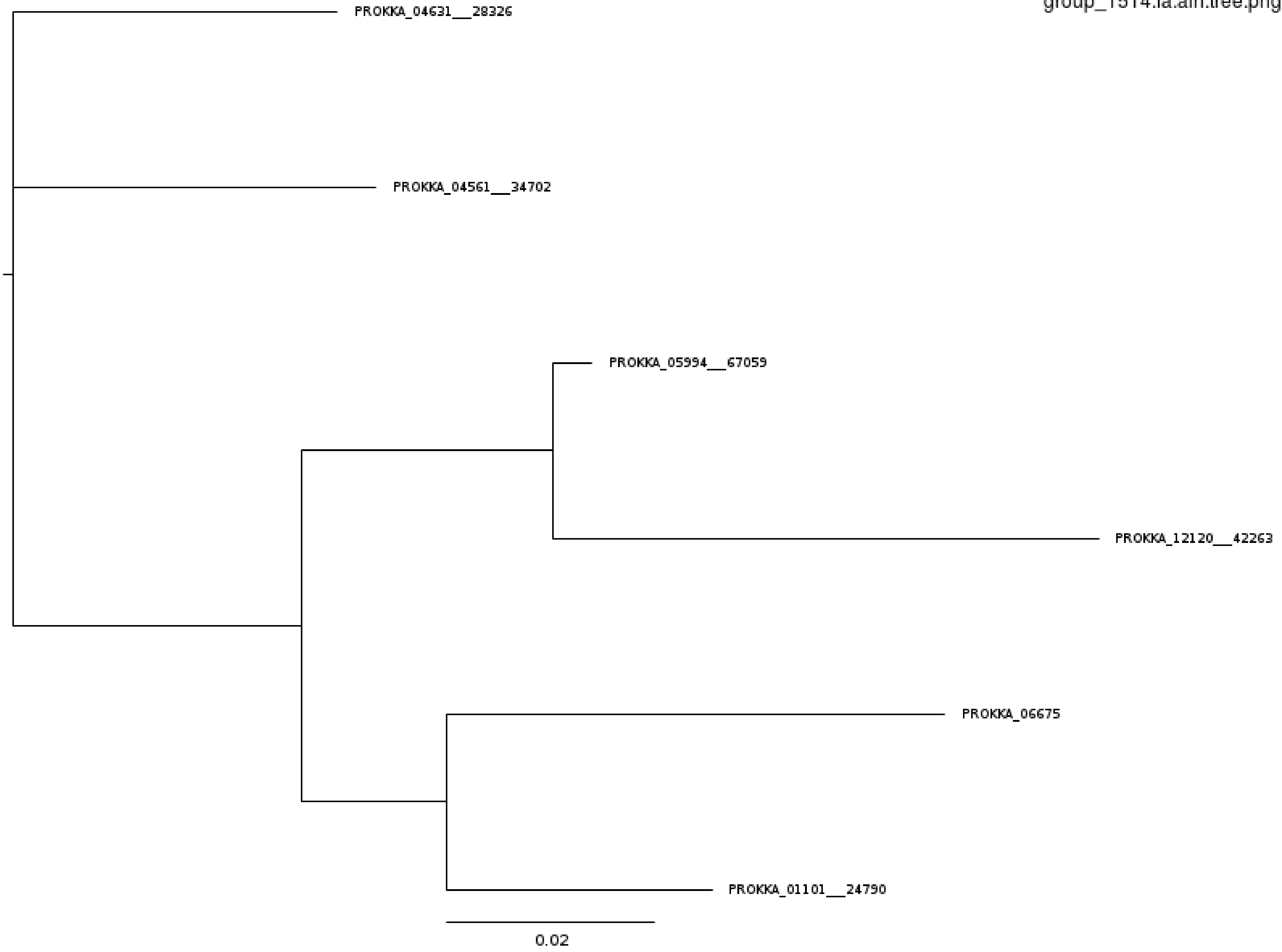

group_1515.fa.aln.tree.png

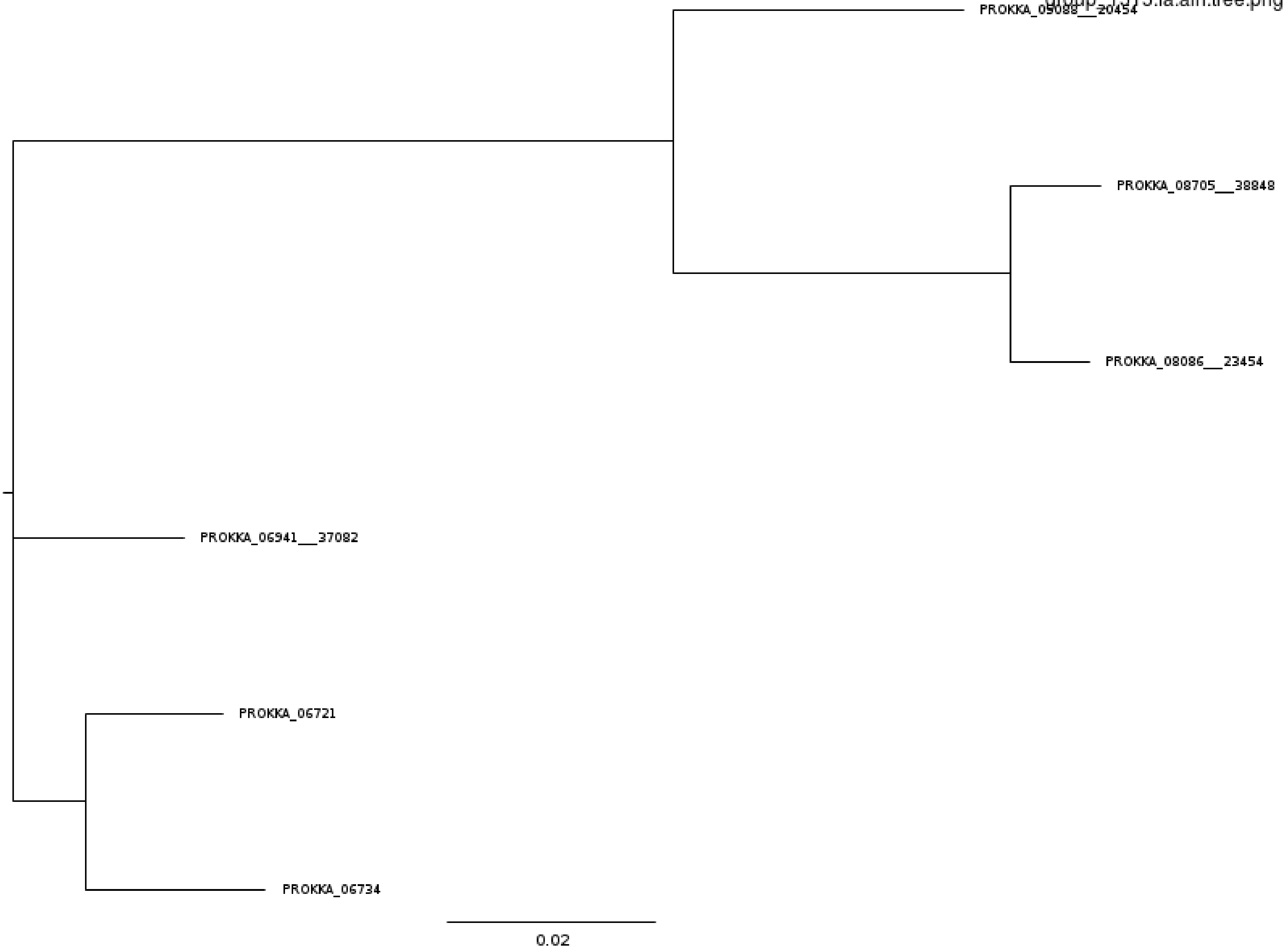

group_1516.fa.aln.tree.png

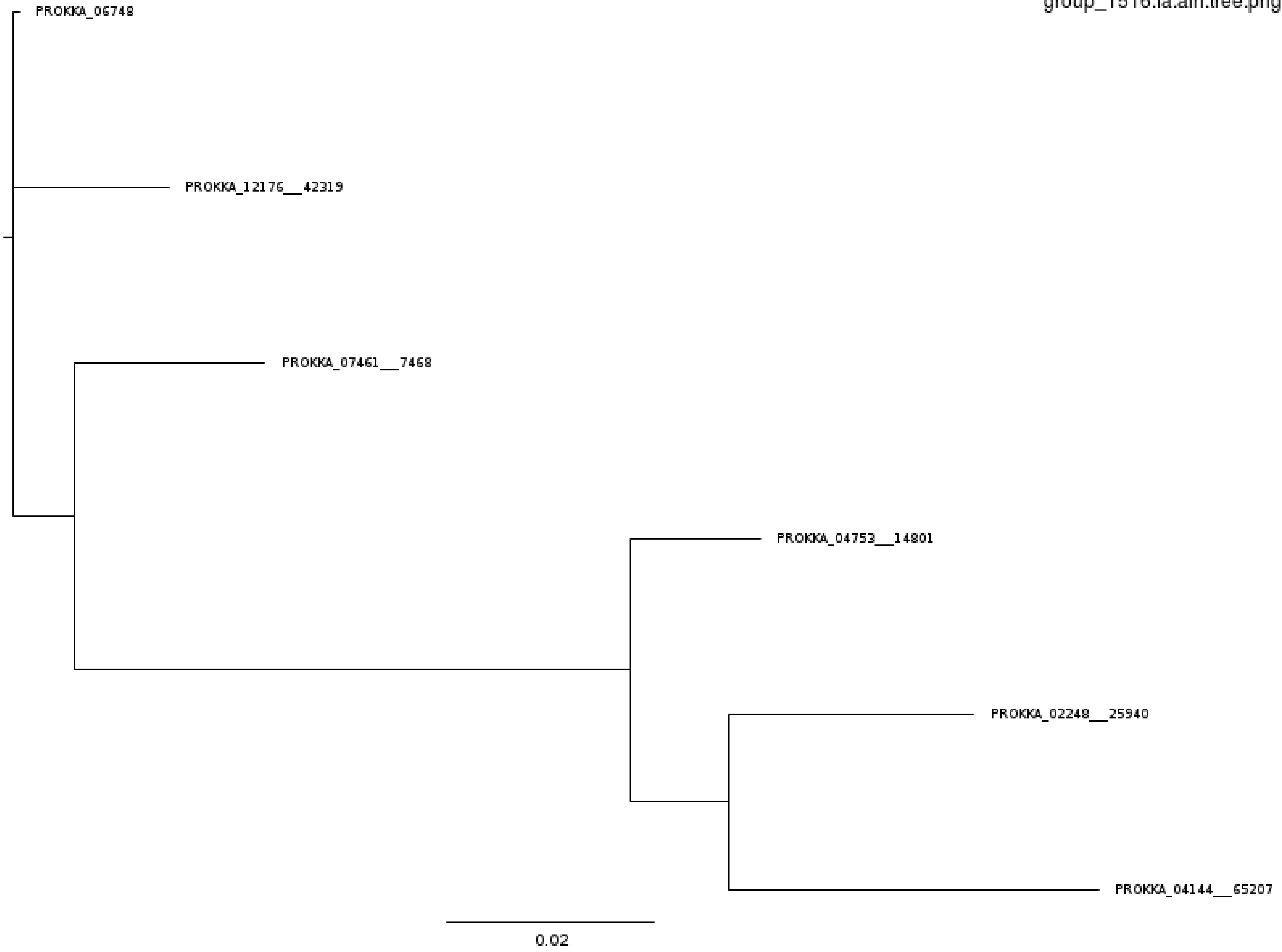

group_1520.fa.aln.tree.png

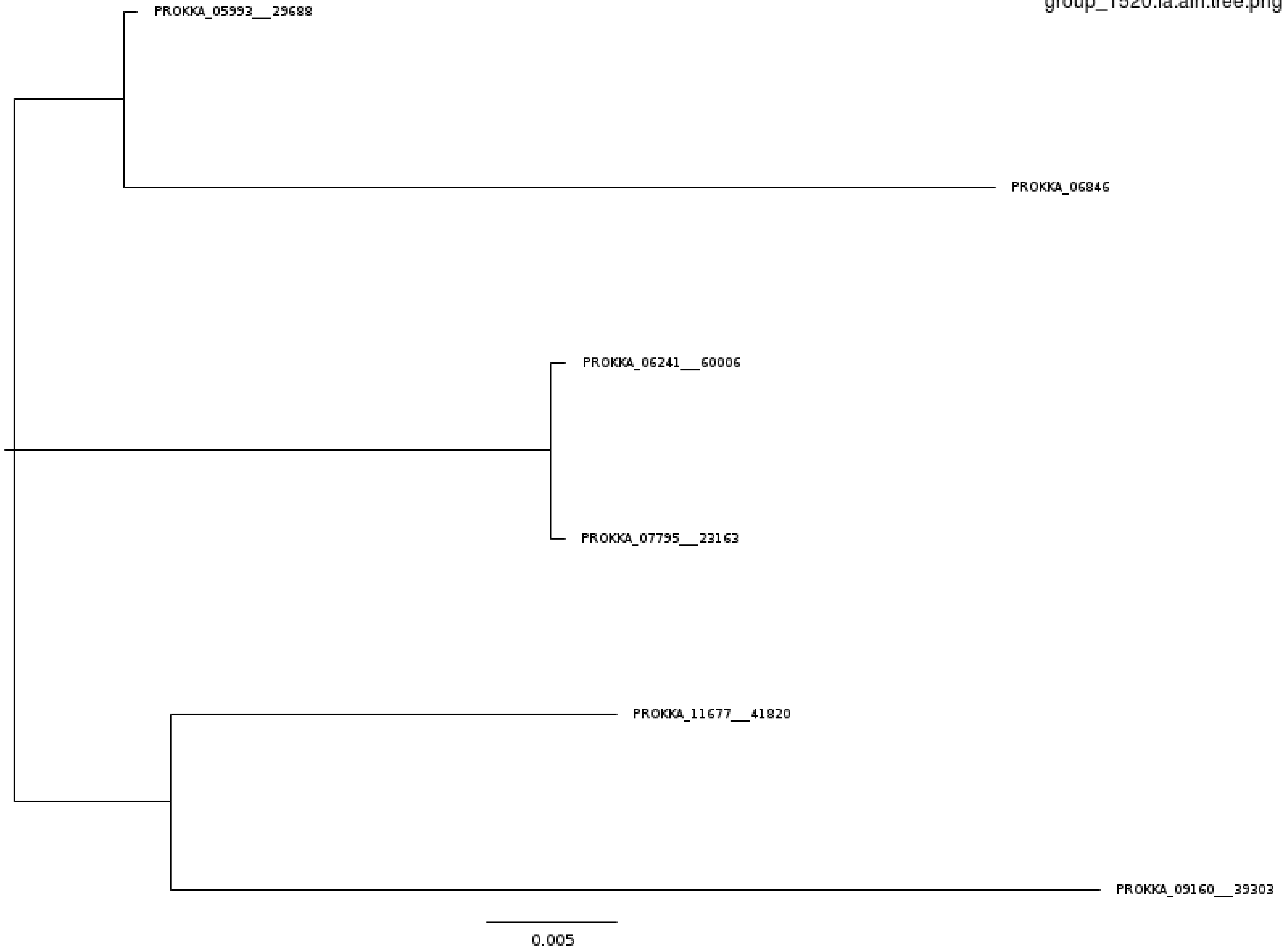

group_1524.fa.aln.tree.png

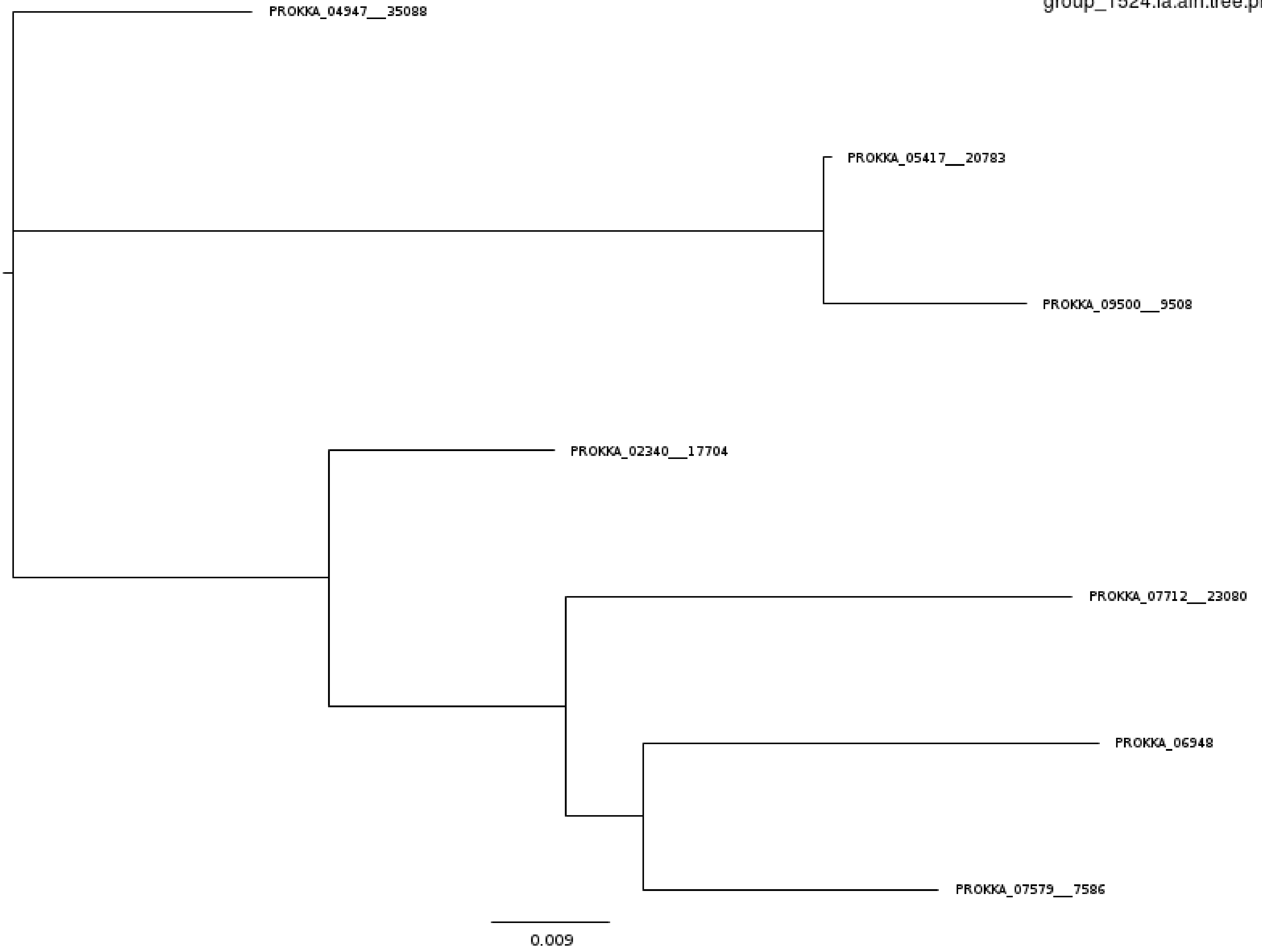

group_1525.fa.aln.tree.png

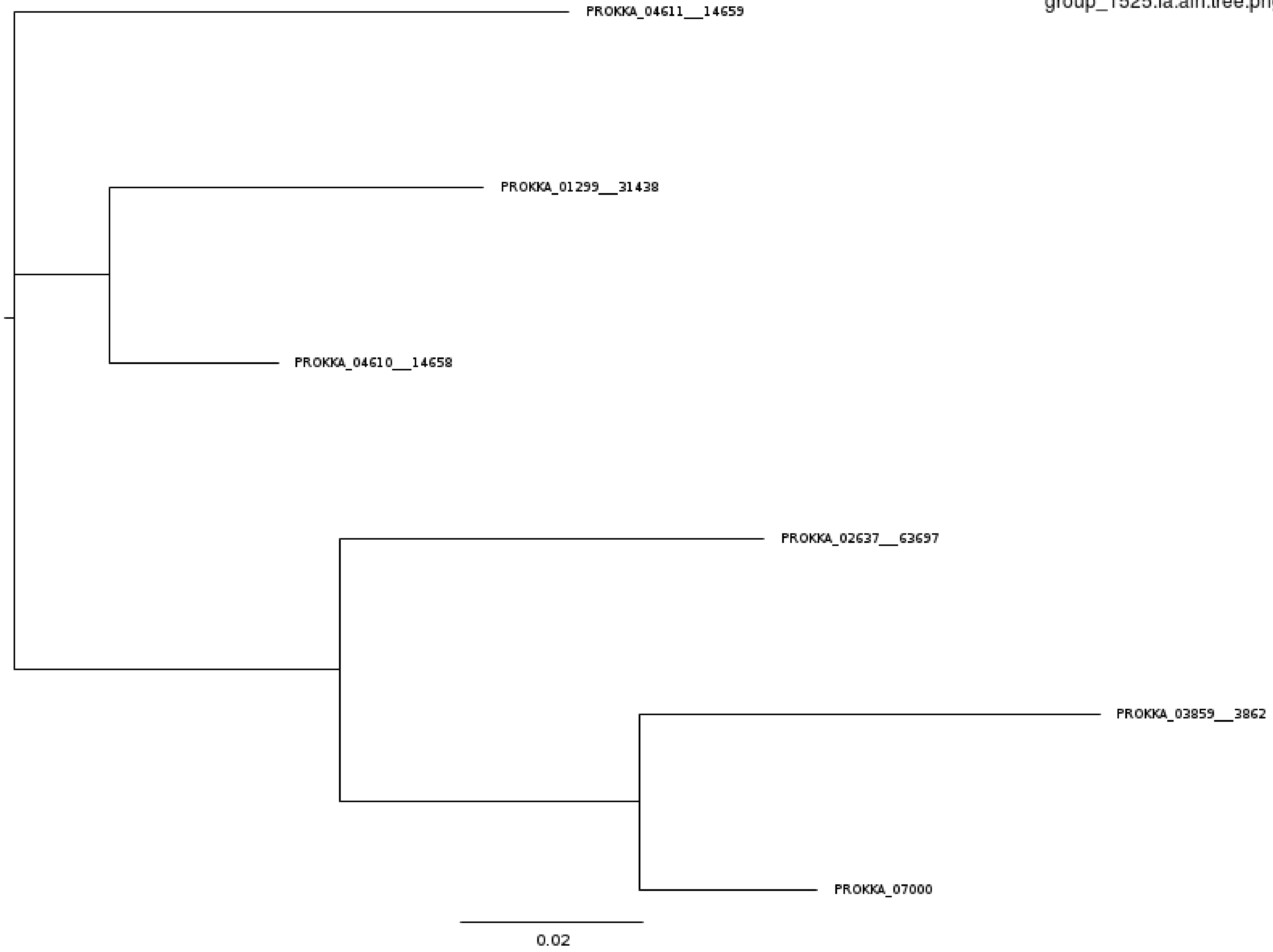

group_1526.fa.aln.tree.png

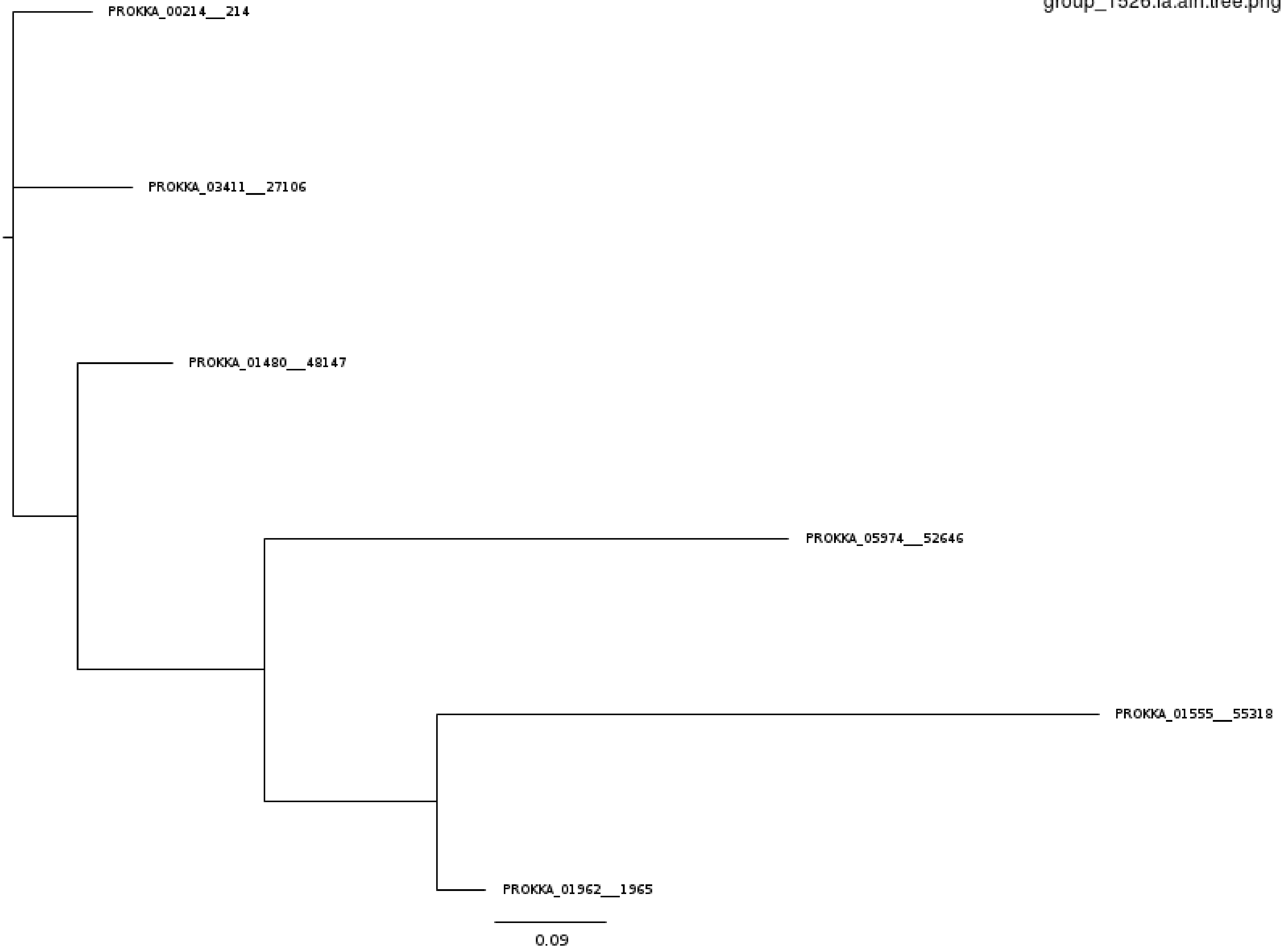

group_1528.fa.aln.tree.png

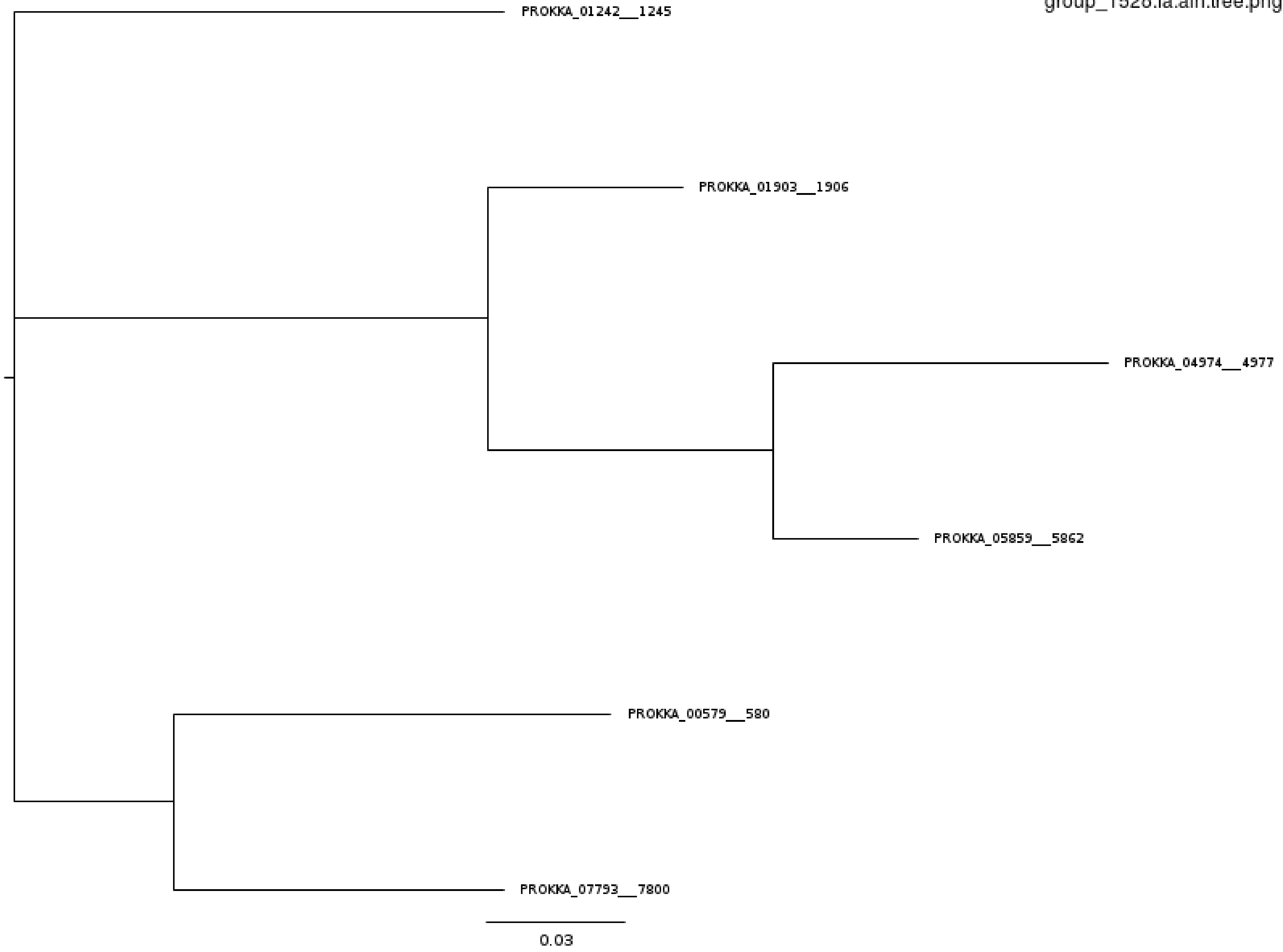

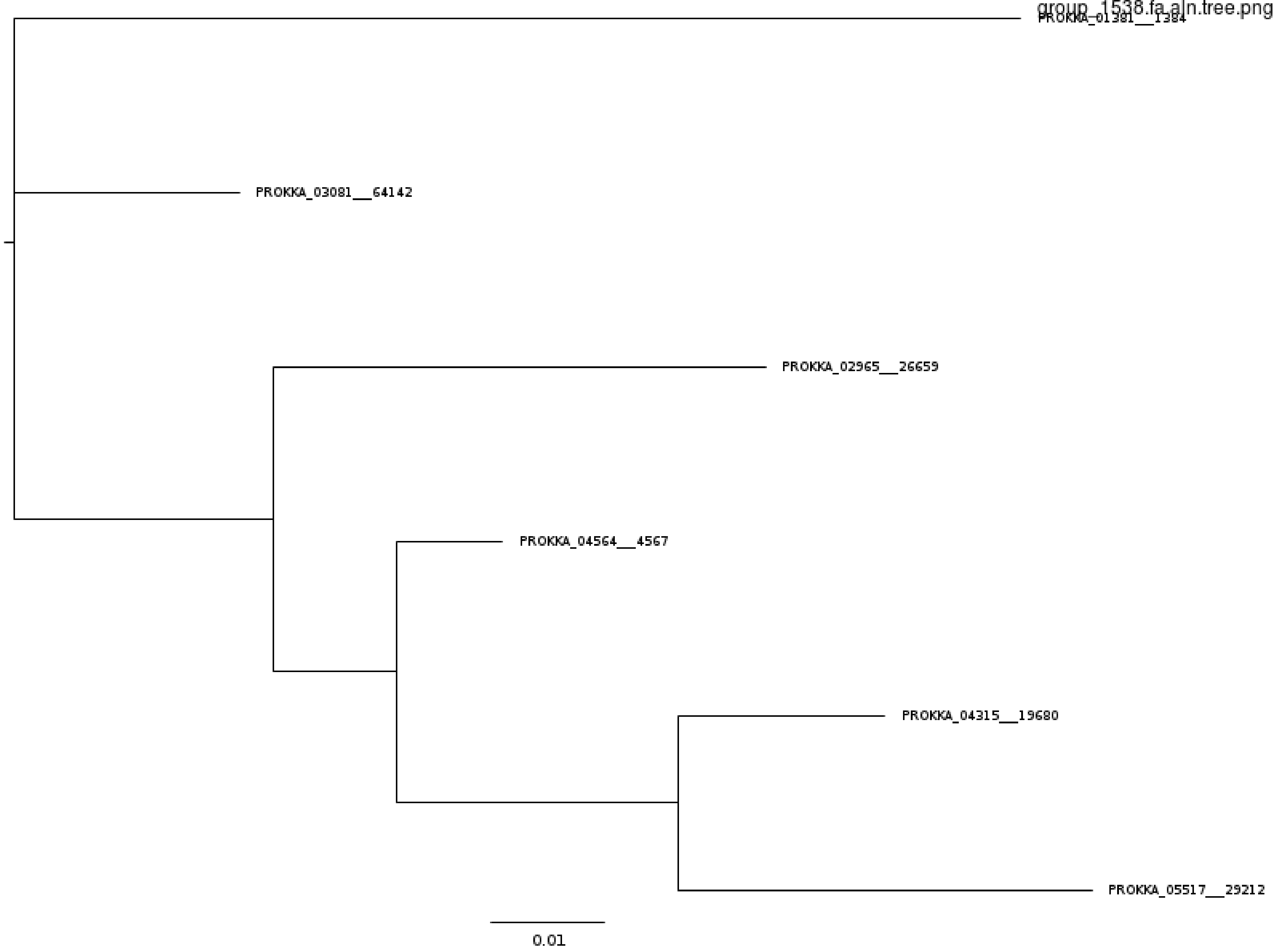
group_1538.fa.aln.tree.png
PROKKA_01381___1384
PROKKA_03081___64142
PROKKA_02965___26659
PROKKA_04564___4567
PROKKA_04315___19680
PROKKA_05517___29212
0.01

group_1541.fa.aln.tree.png

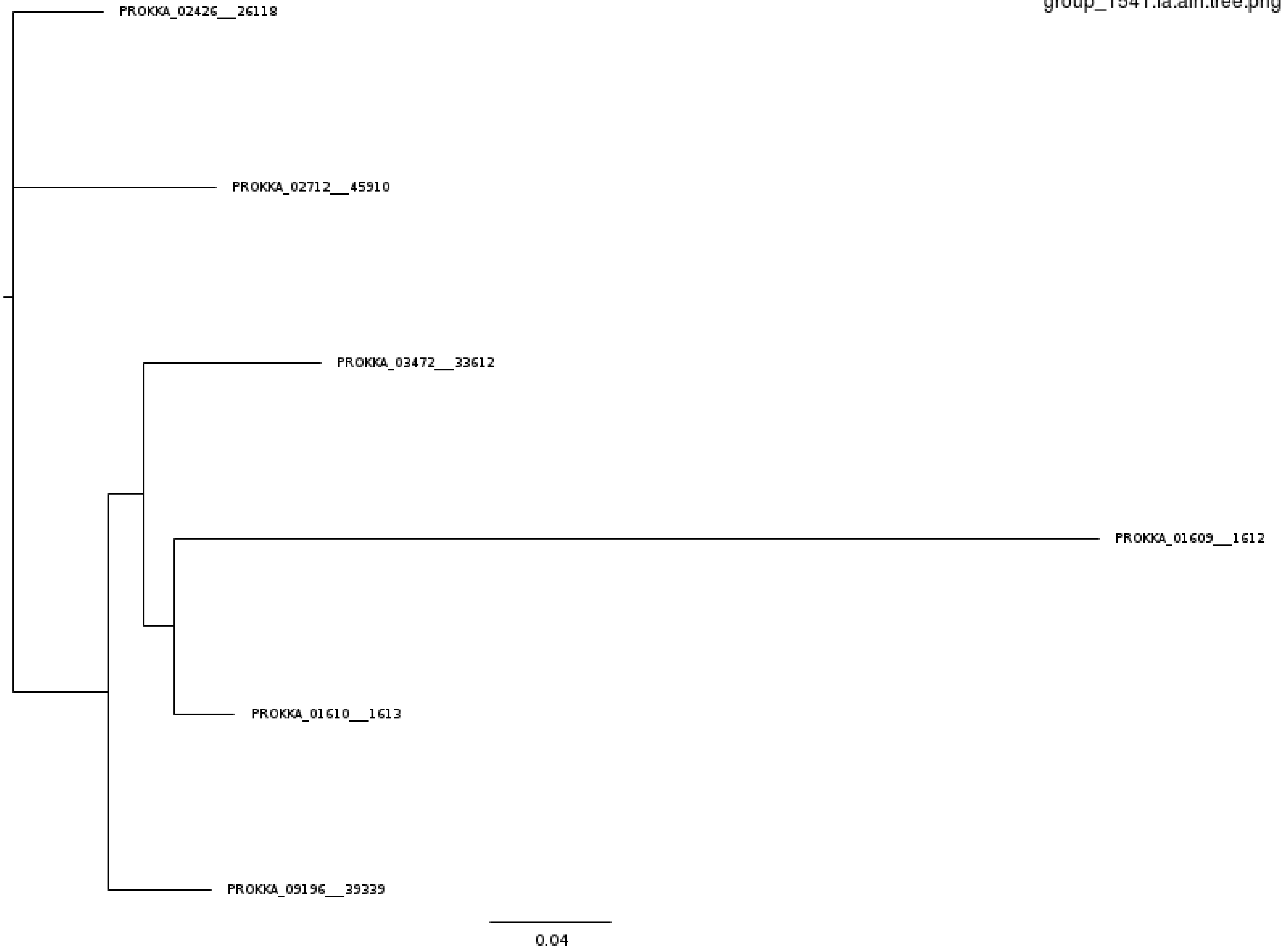

group_1544.fa.aln.tree.png

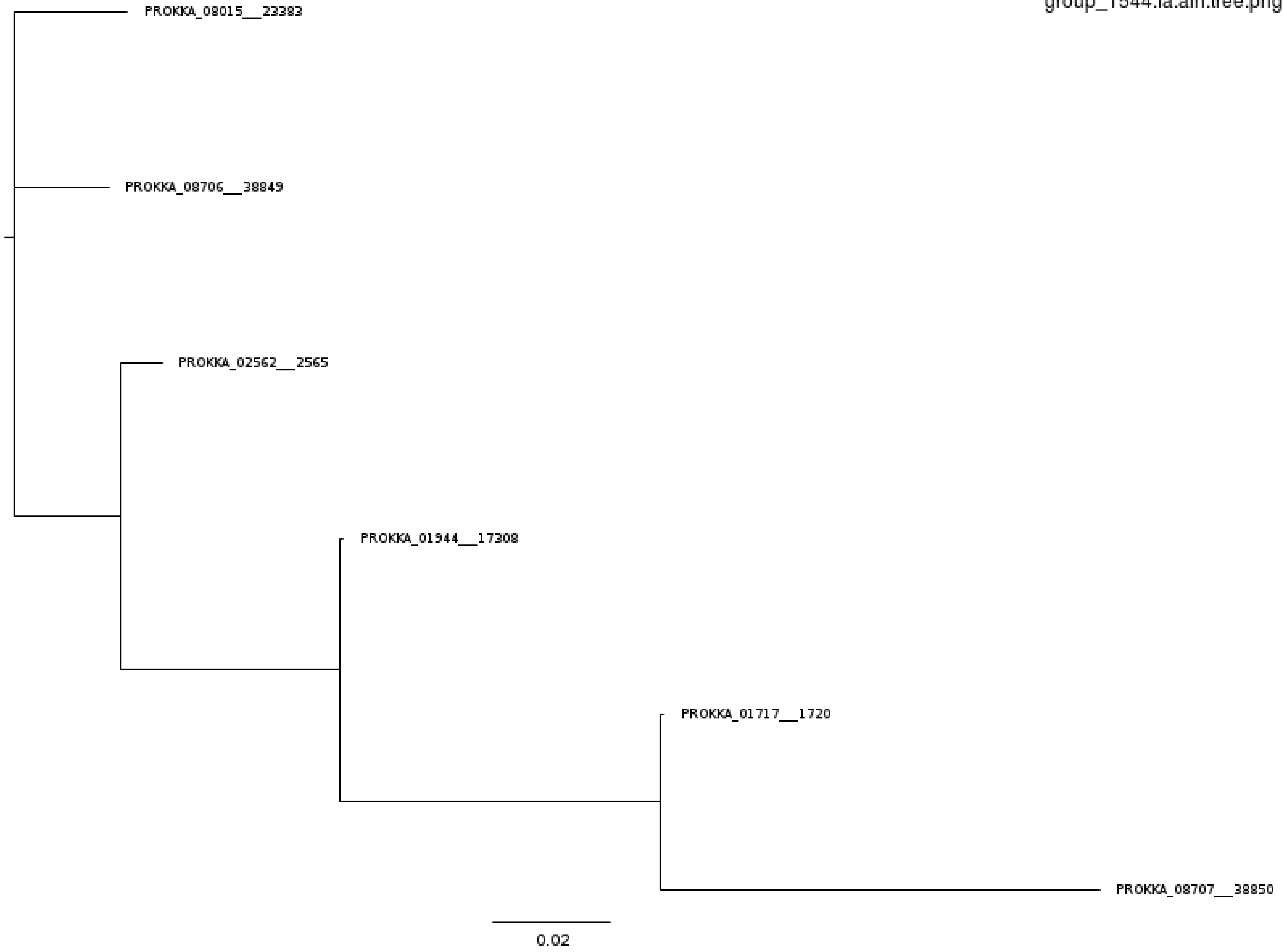

group_1546.fa.aln.tree.png

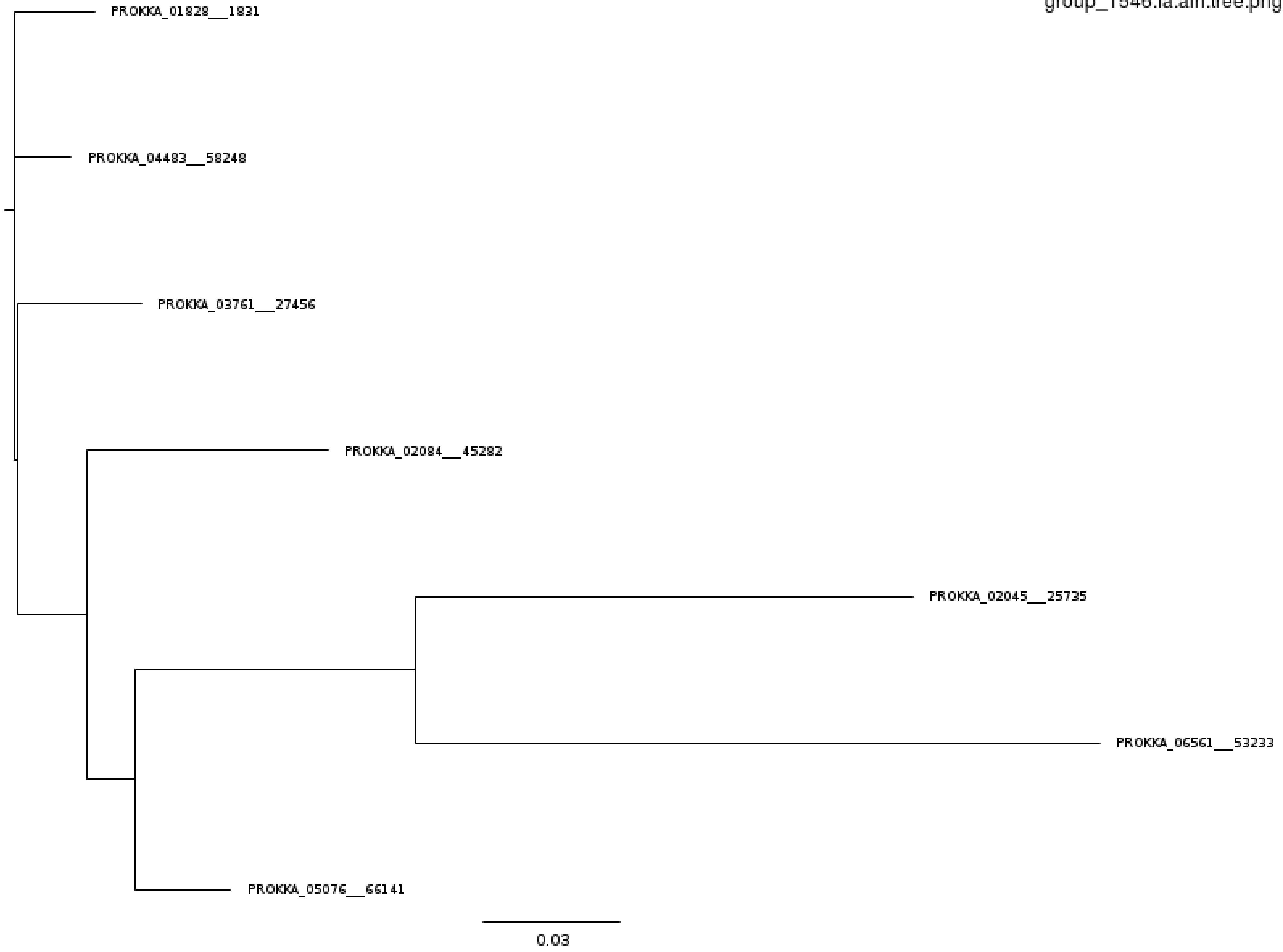

group_1547.fa.aln.tree.png

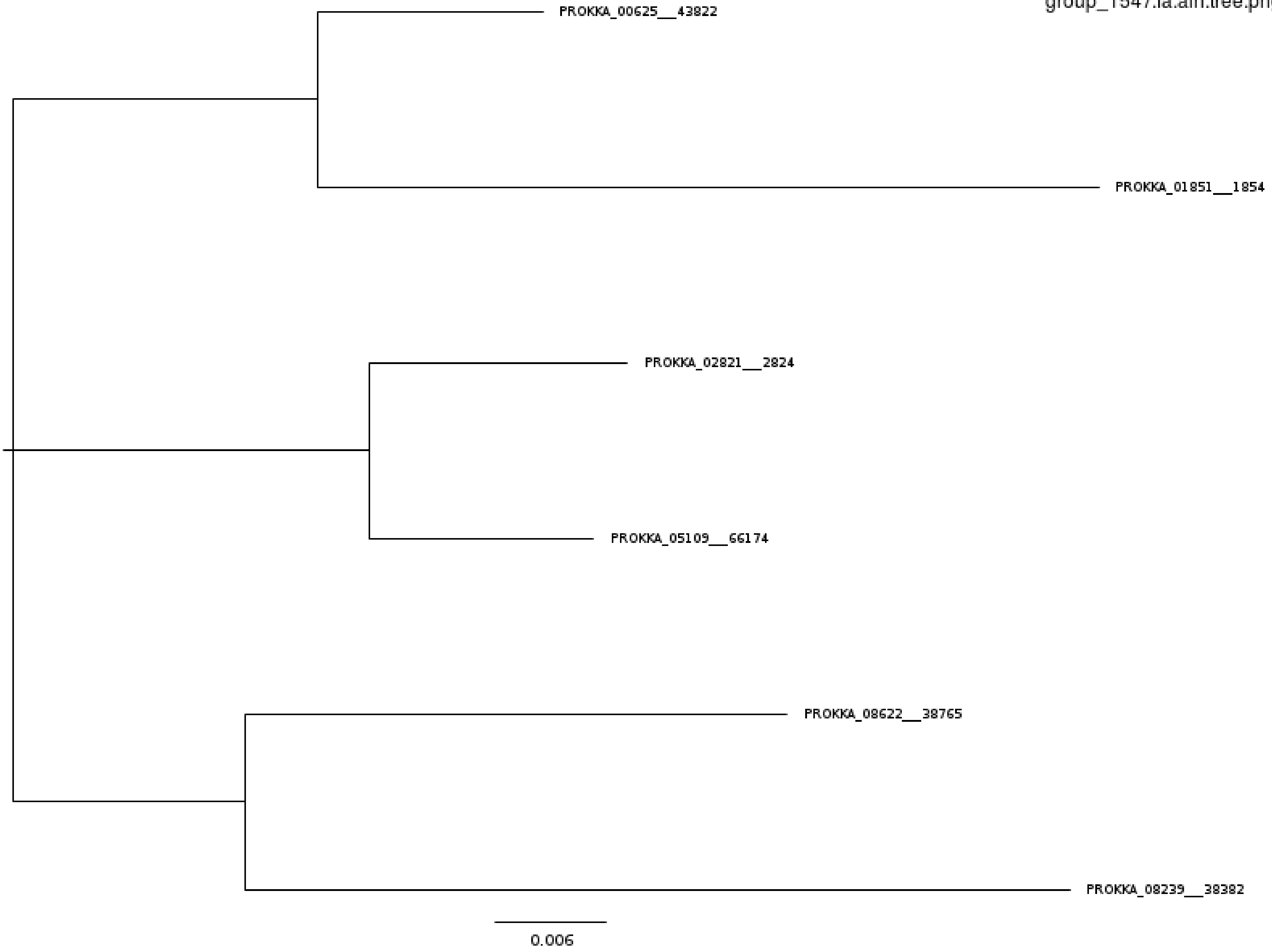

group_1550.fa.aln.tree.png

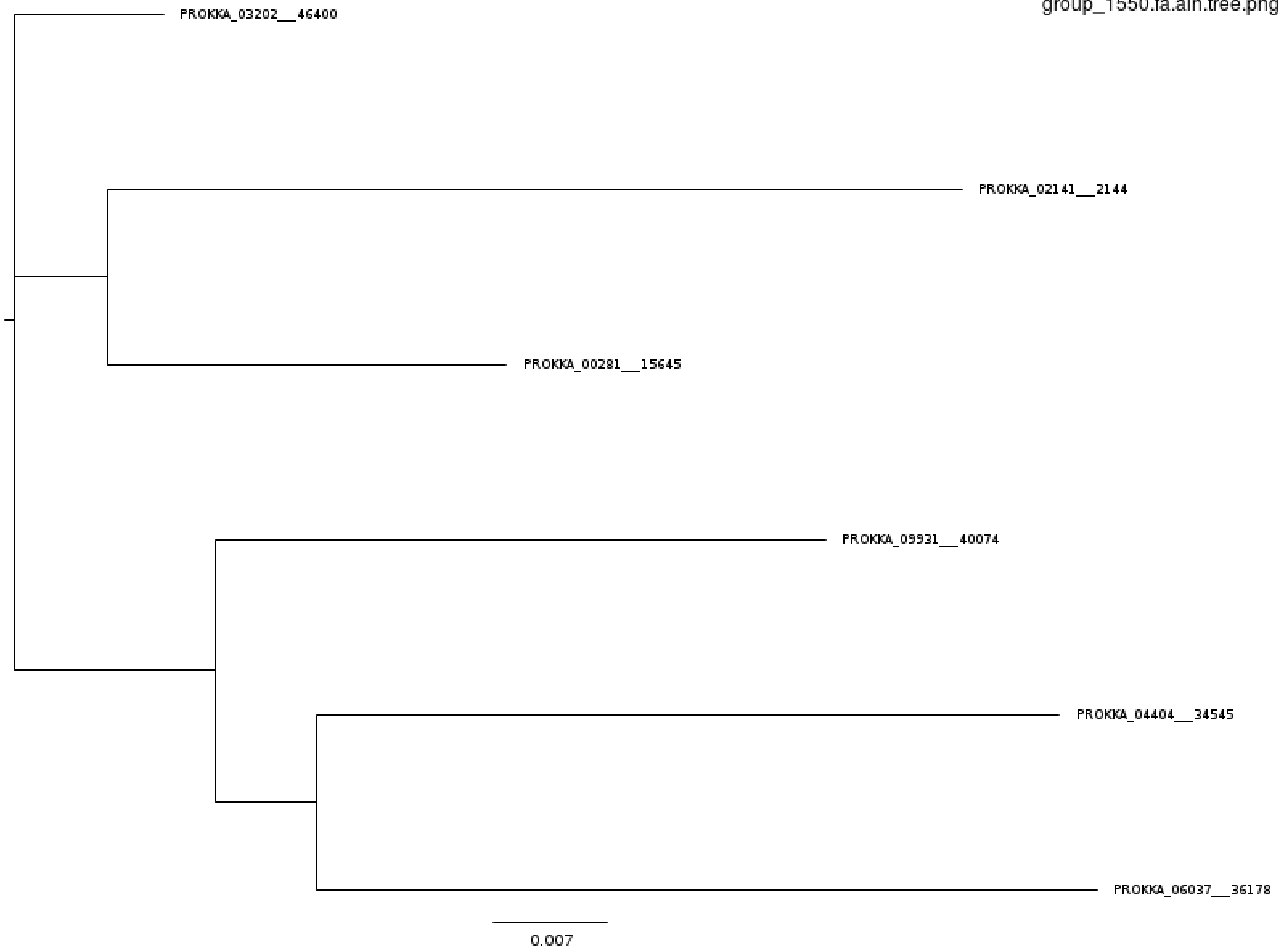

group_1553.fa.aln.tree.png

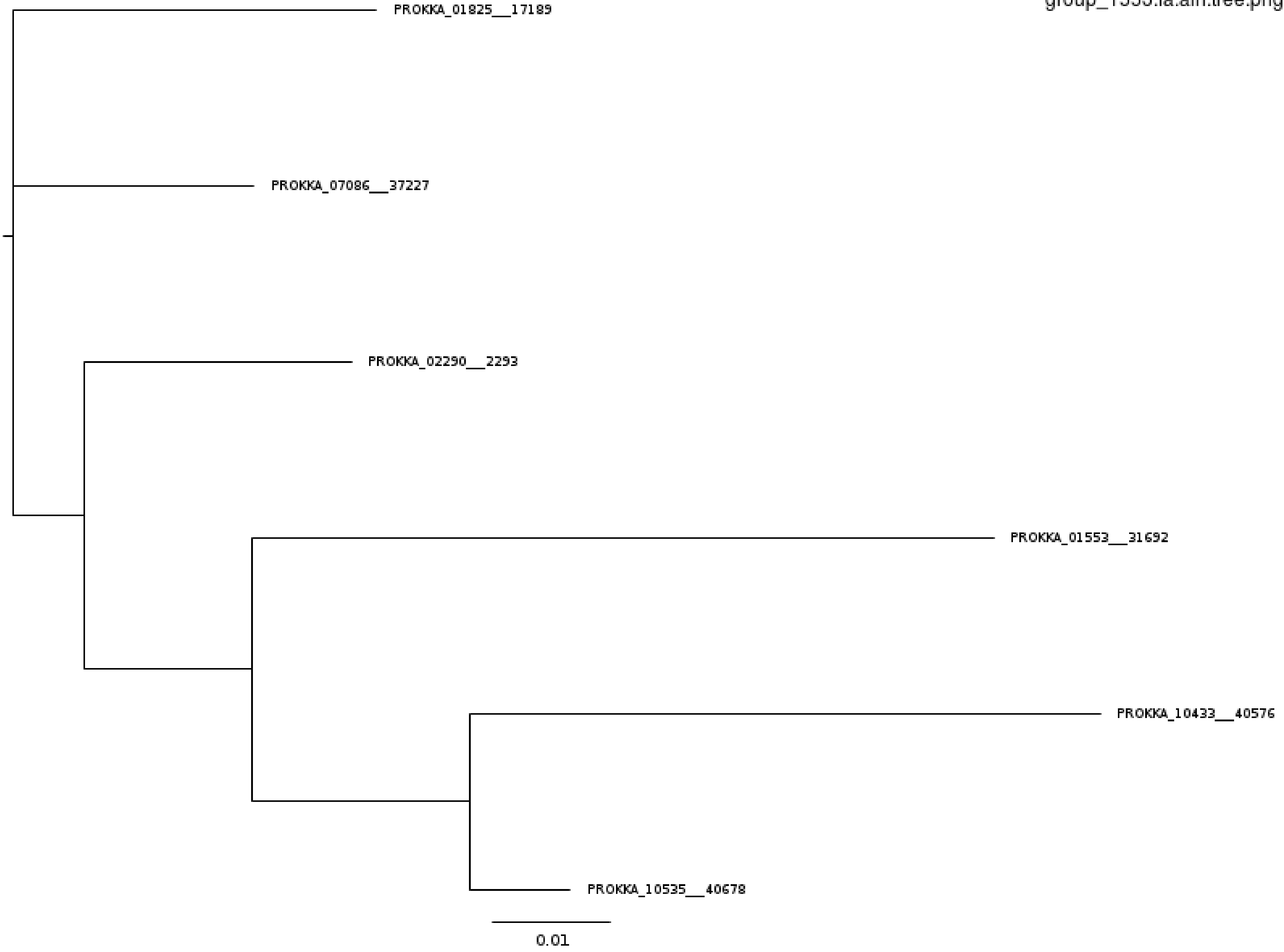

group_1554.fa.aln.tree.png

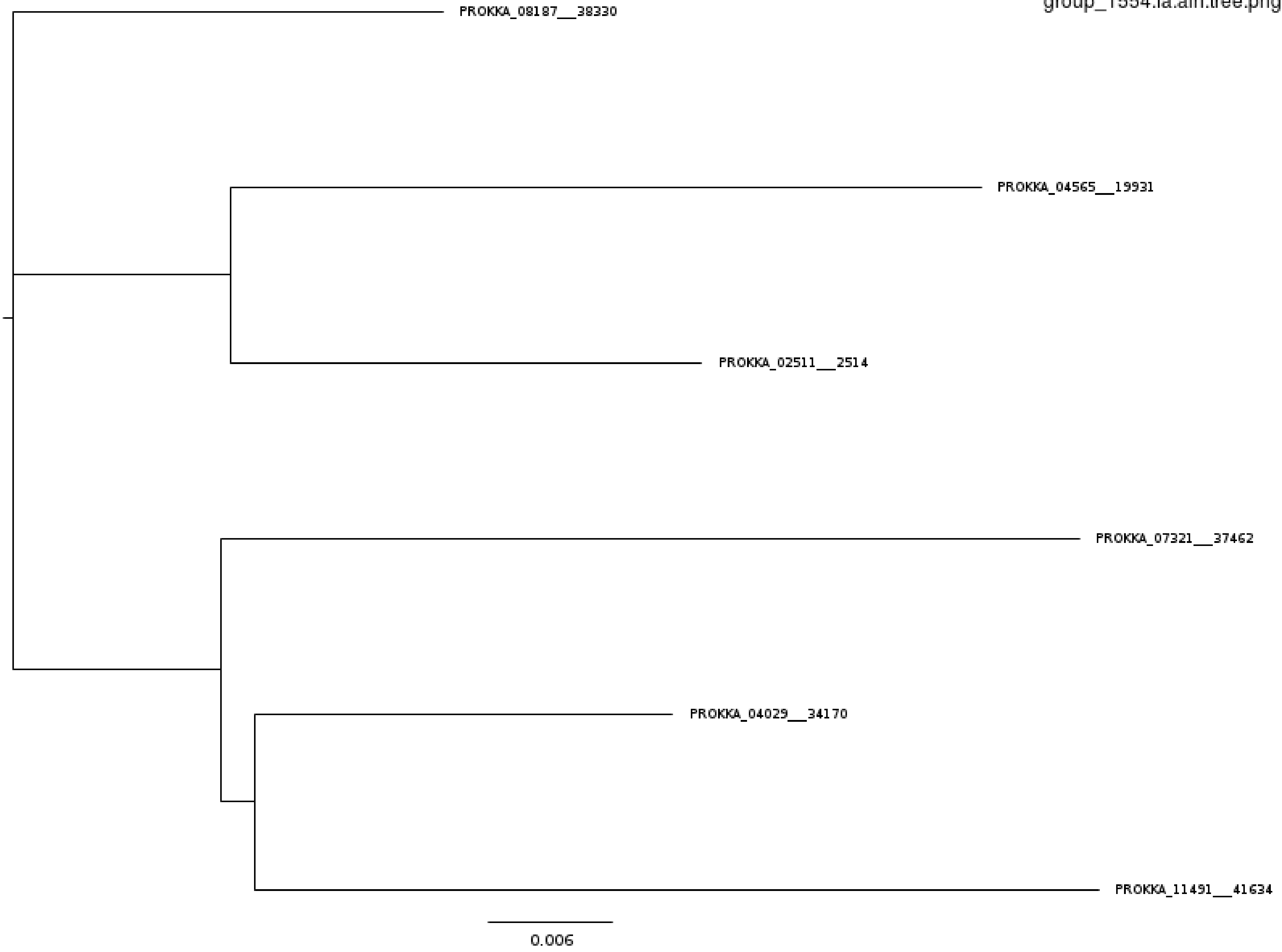

group_1559.fa.aln.tree.png

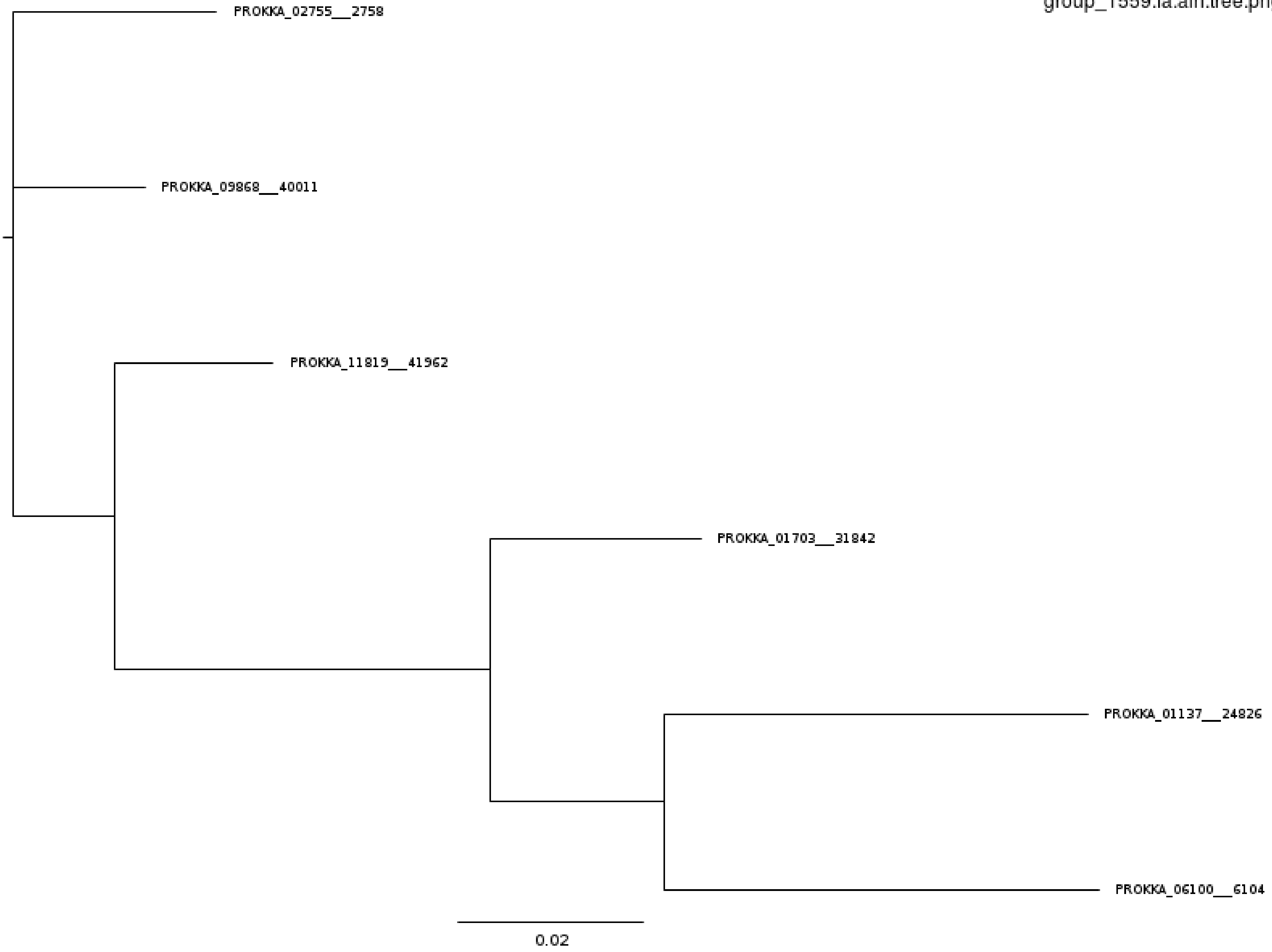

group_1562.fa.aln.tree.png

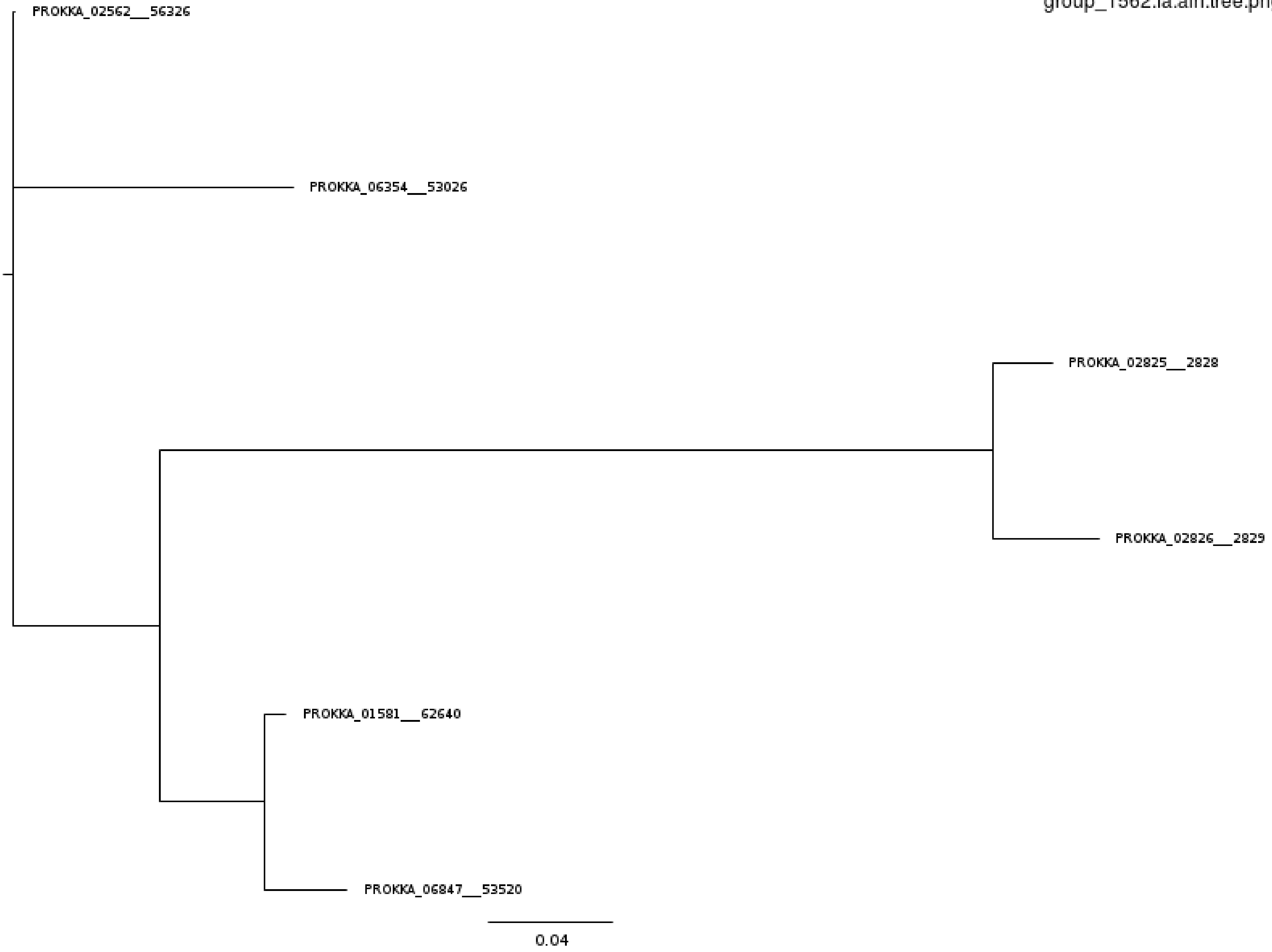

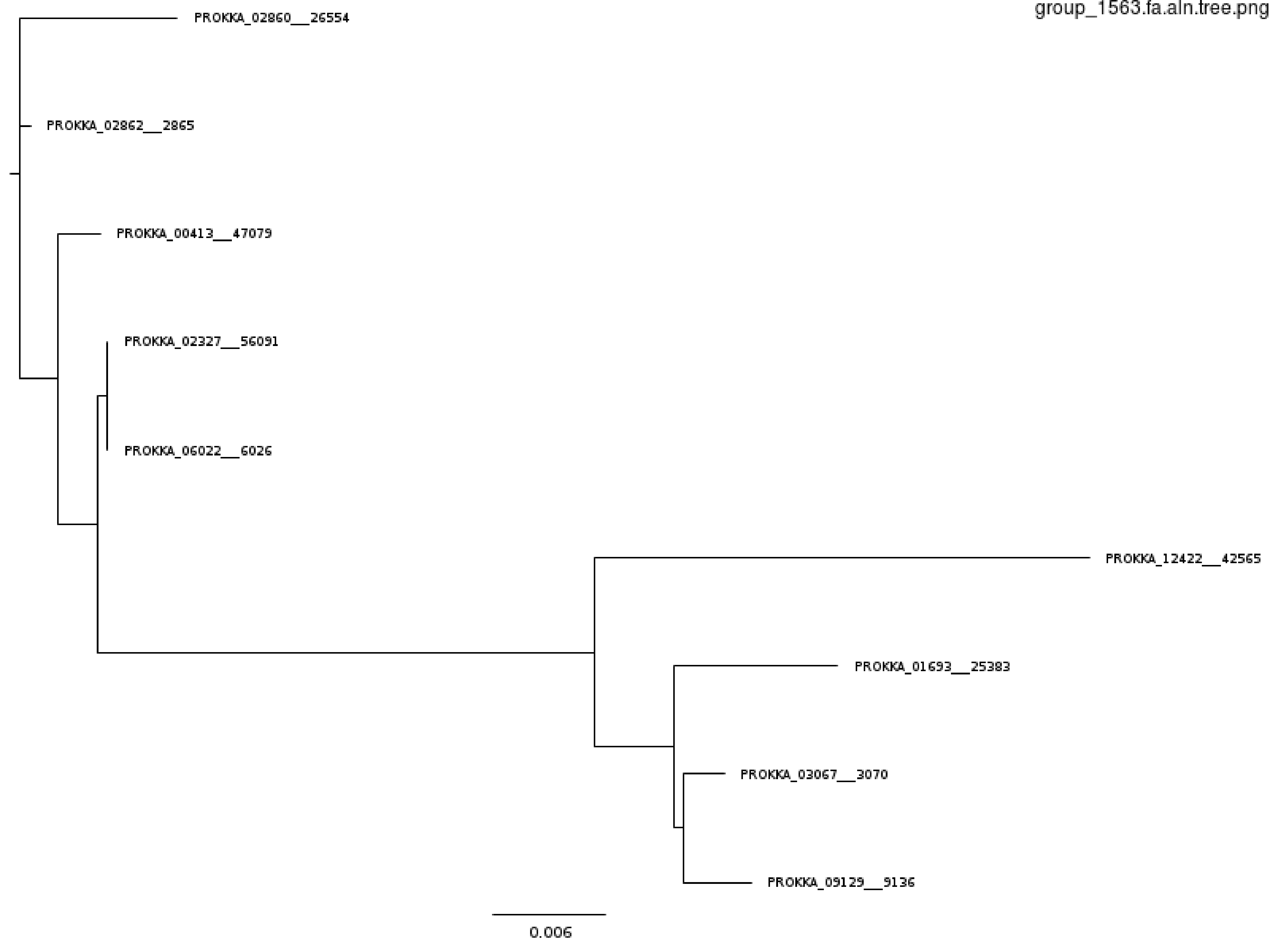
group_1563.fa.aln.tree.png
PROKKA_02860___26554
PROKKA_02862___2865
PROKKA_00413___47079
PROKKA_02327___56091
PROKKA_06022___6026
PROKKA_12422___42565
PROKKA_01693___25383
PROKKA_03067___3070
PROKKA_09129___9136
0.006

group_1565.fa.aln.tree.png

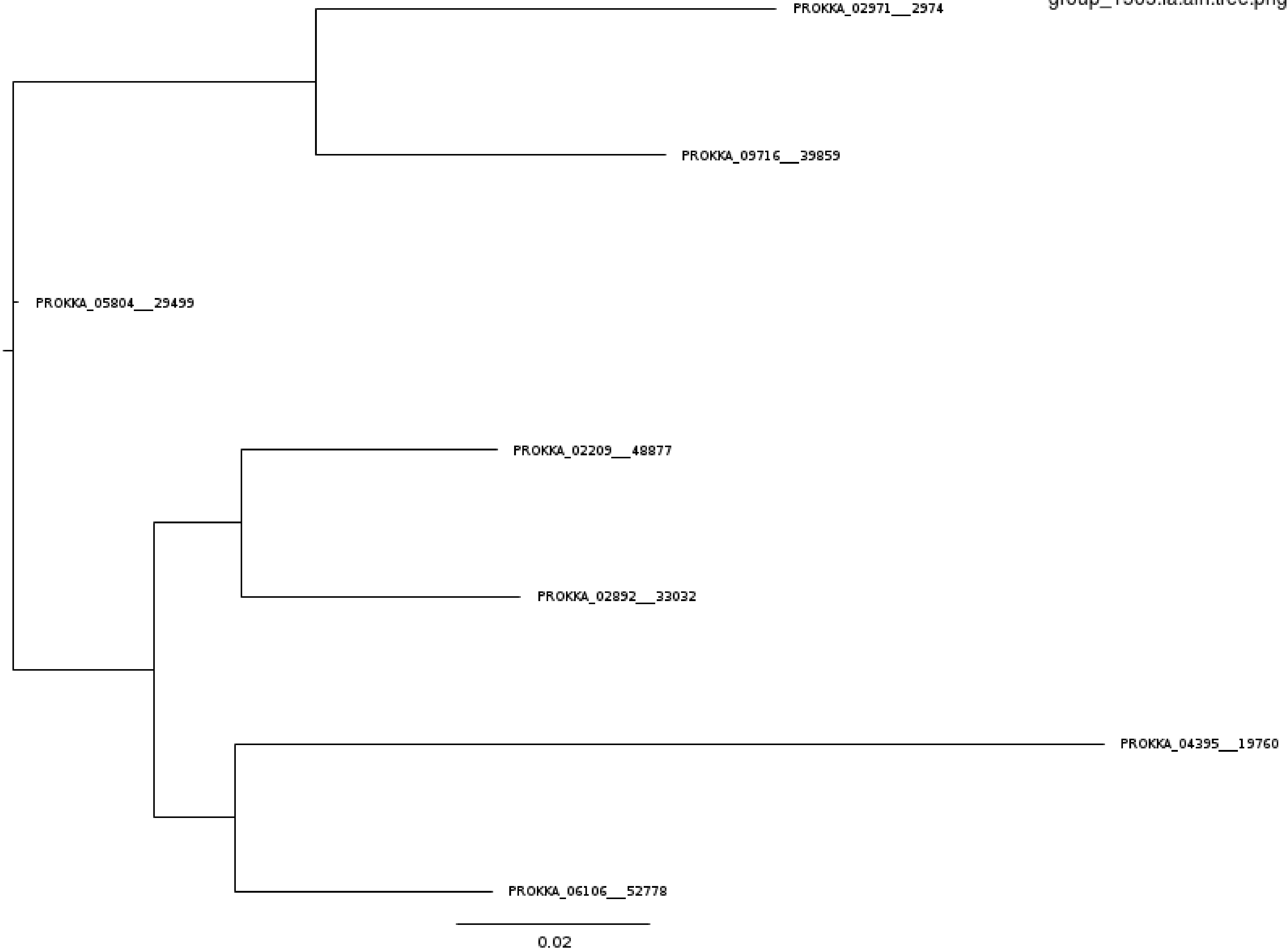

group_1568.fa.aln.tree.png

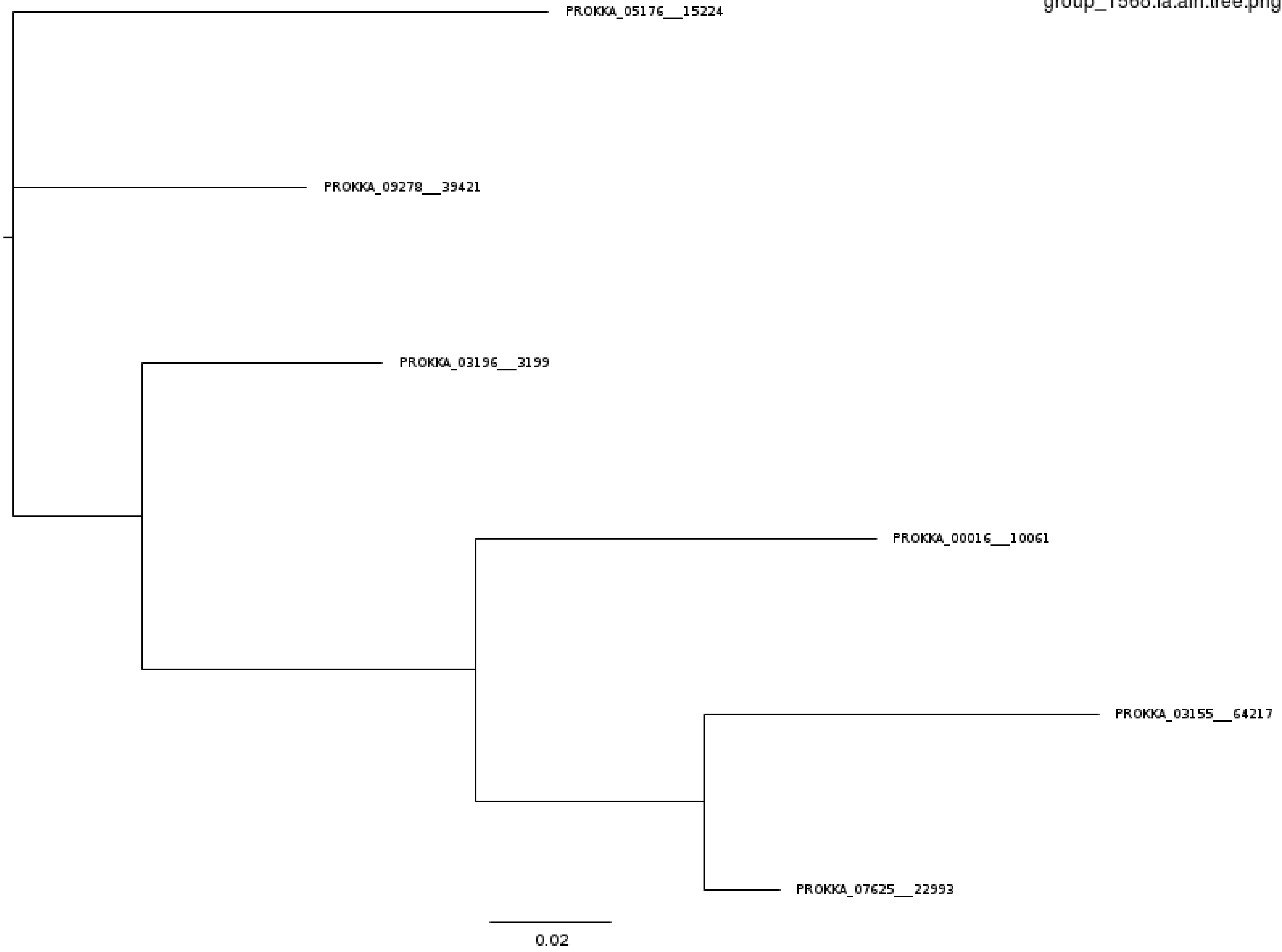

group_1569.fa.aln.tree.png

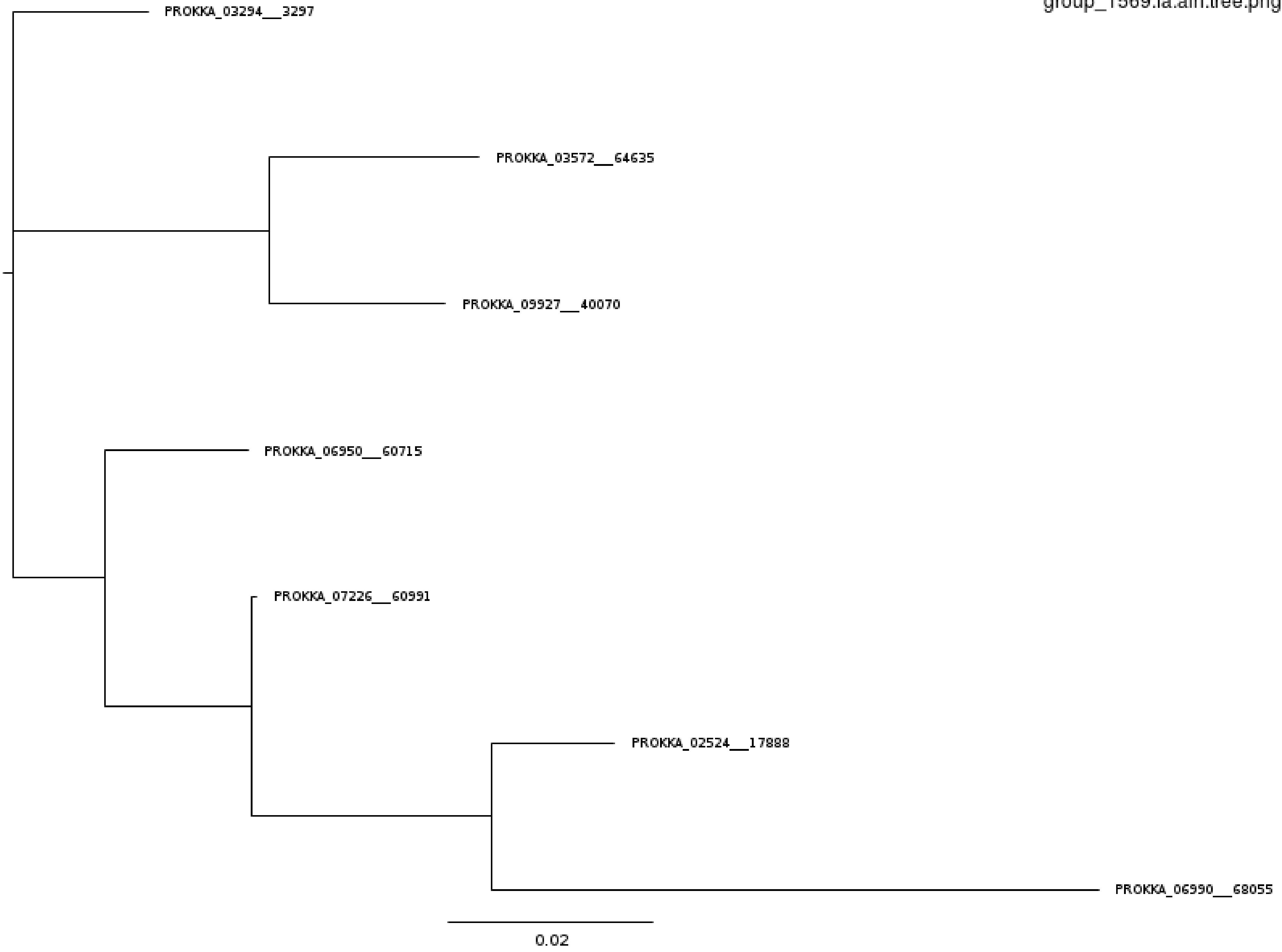

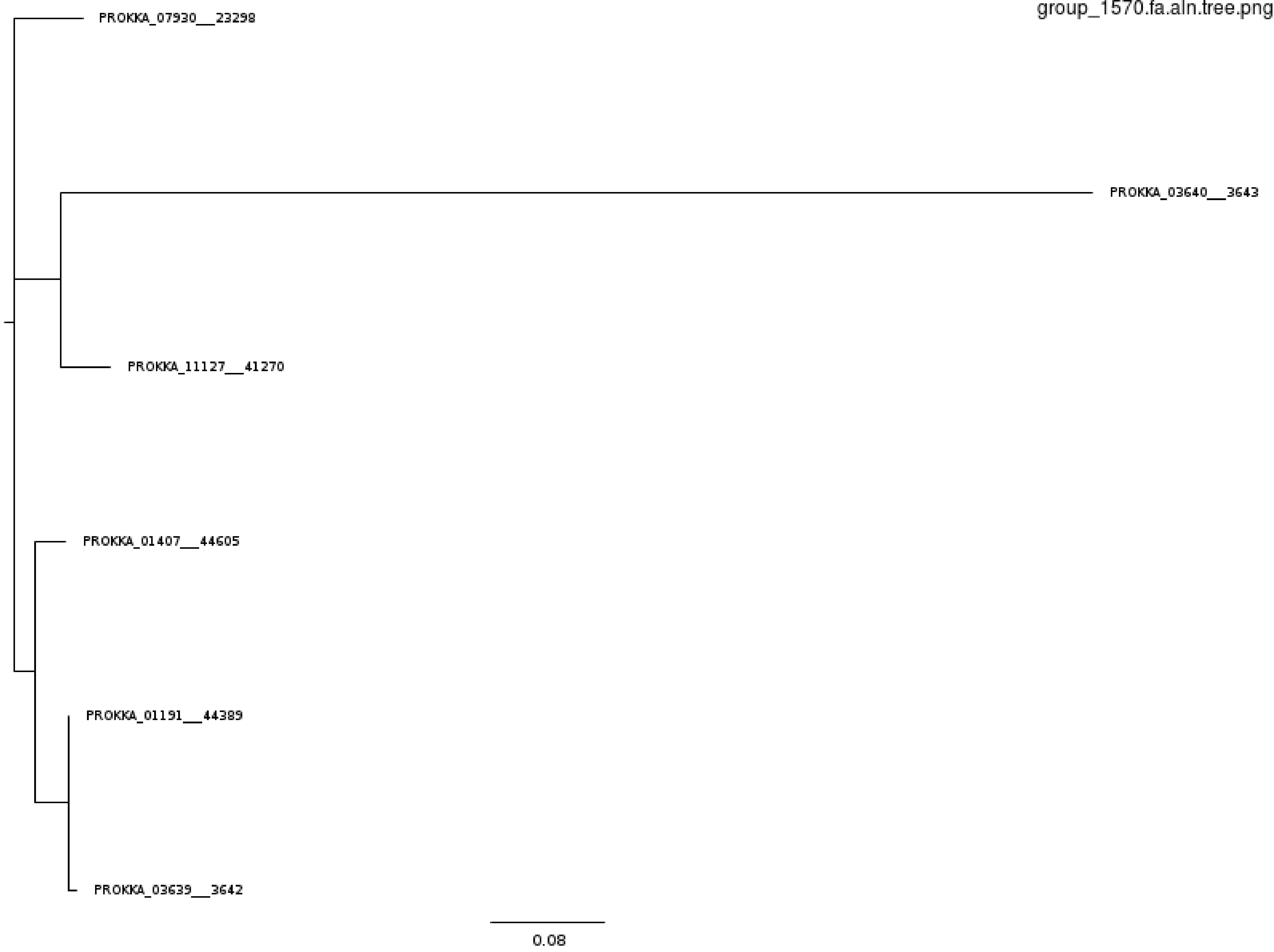
group_1570.fa.aln.tree.png
PROKKA_07930___23298
PROKKA_03640___3643
PROKKA_11127___41270
PROKKA_01407___44605
PROKKA_01191___44389
PROKKA_03639___3642
0.08

group_1571.fa.aln.tree.png

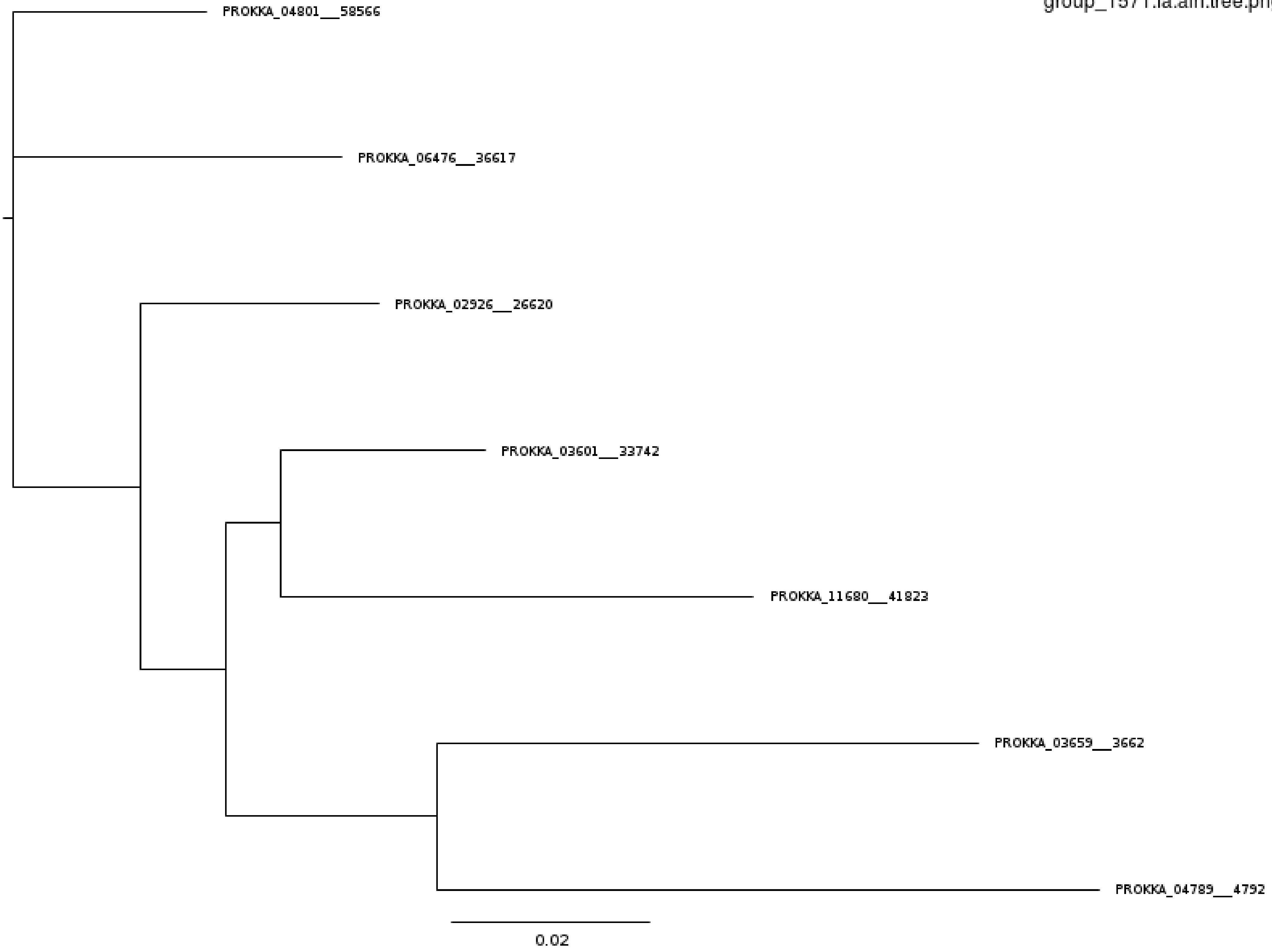

group_1572.fa.aln.tree.png

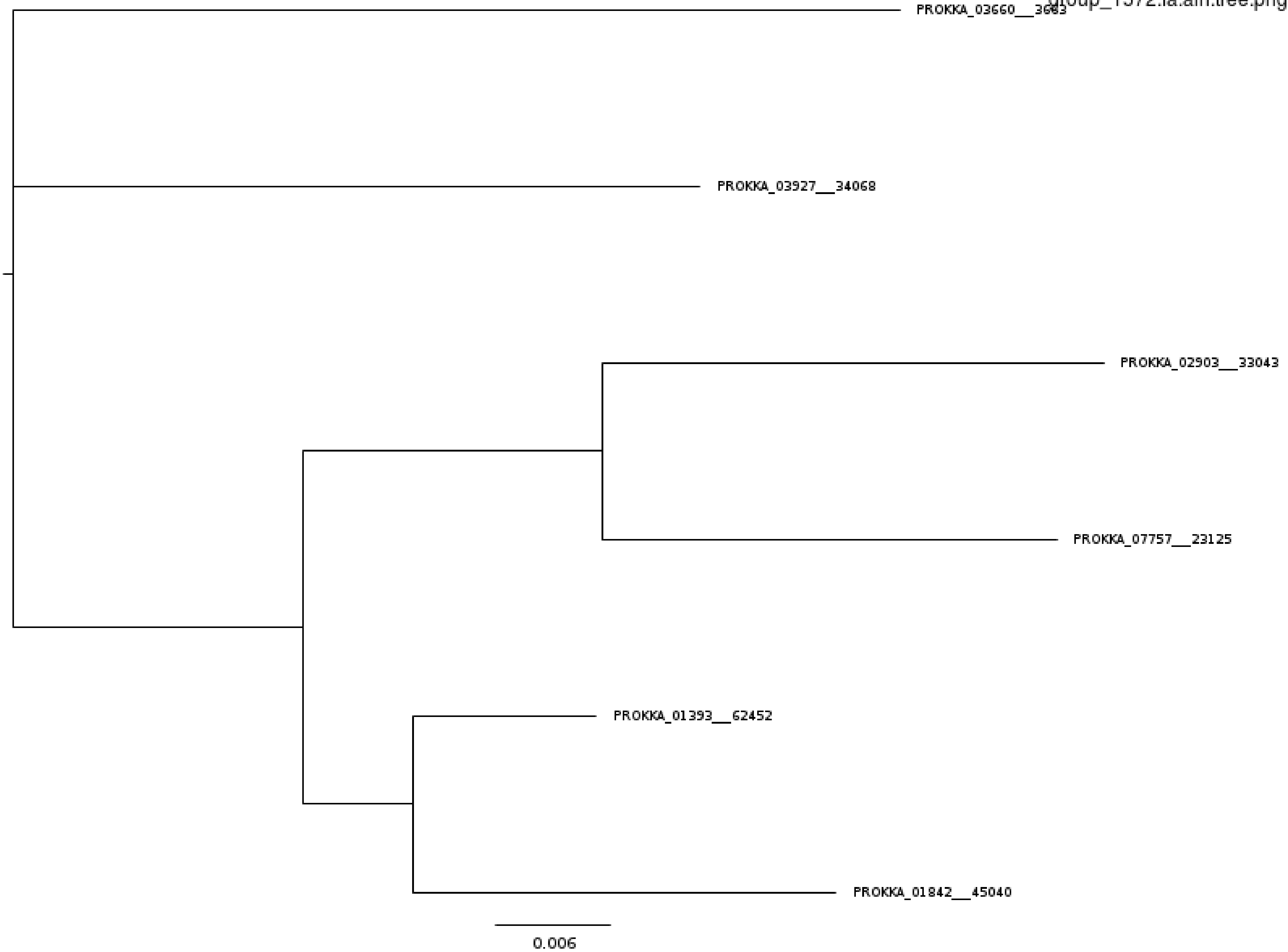

group_1573.fa.aln.tree.png

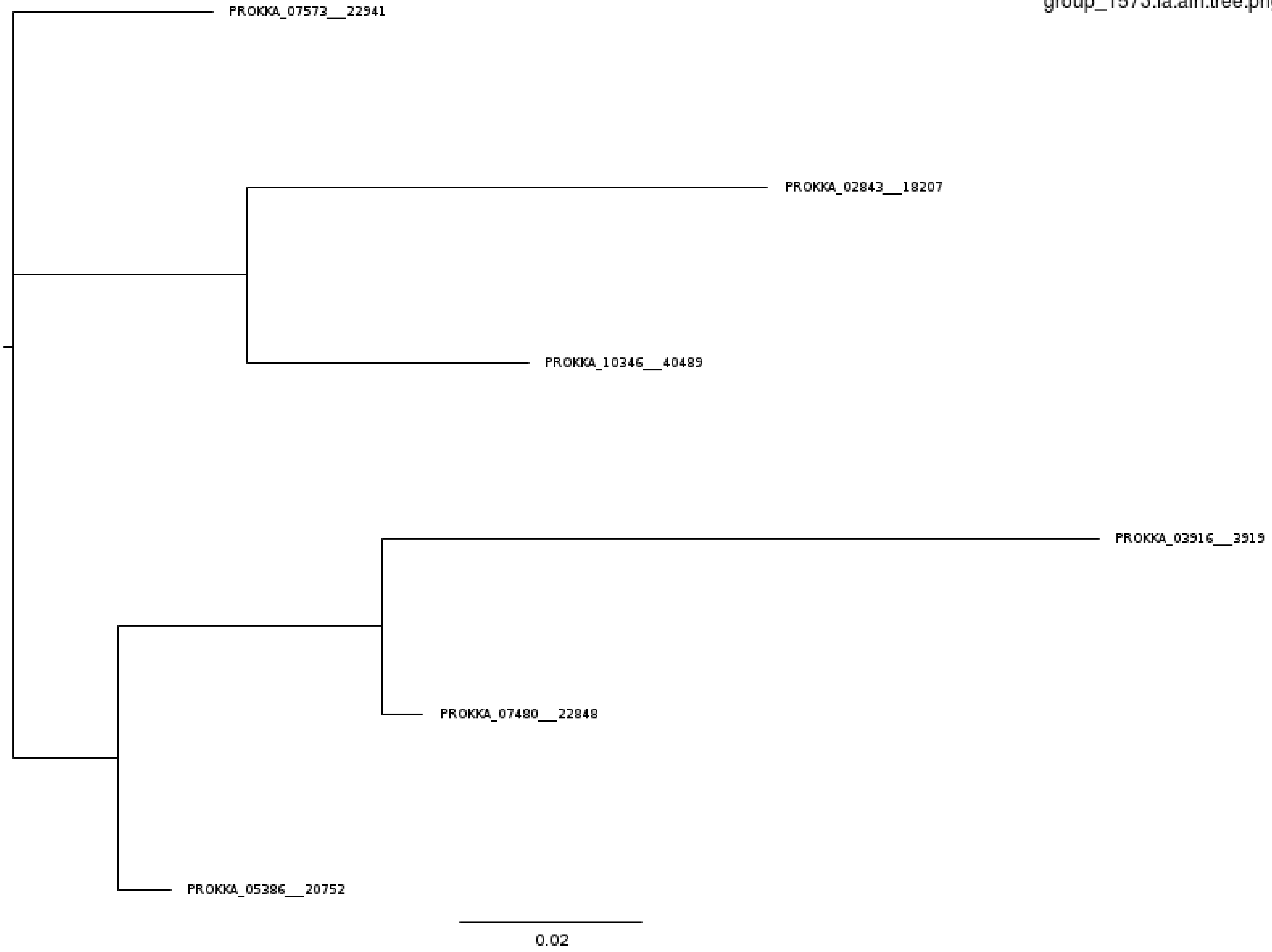

group_1574.fa.aln.tree.png

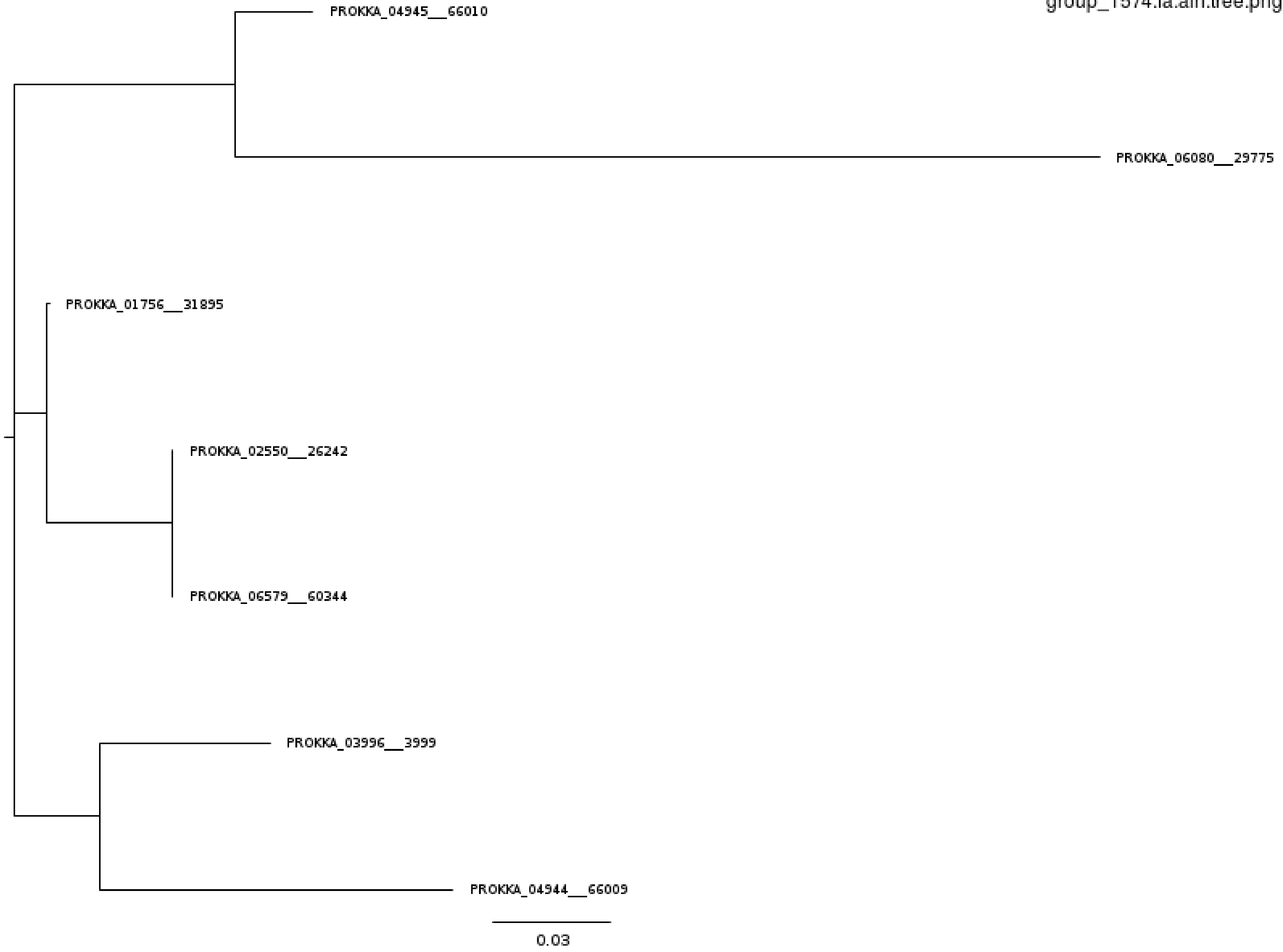

group_1575.fa.aln.tree.png

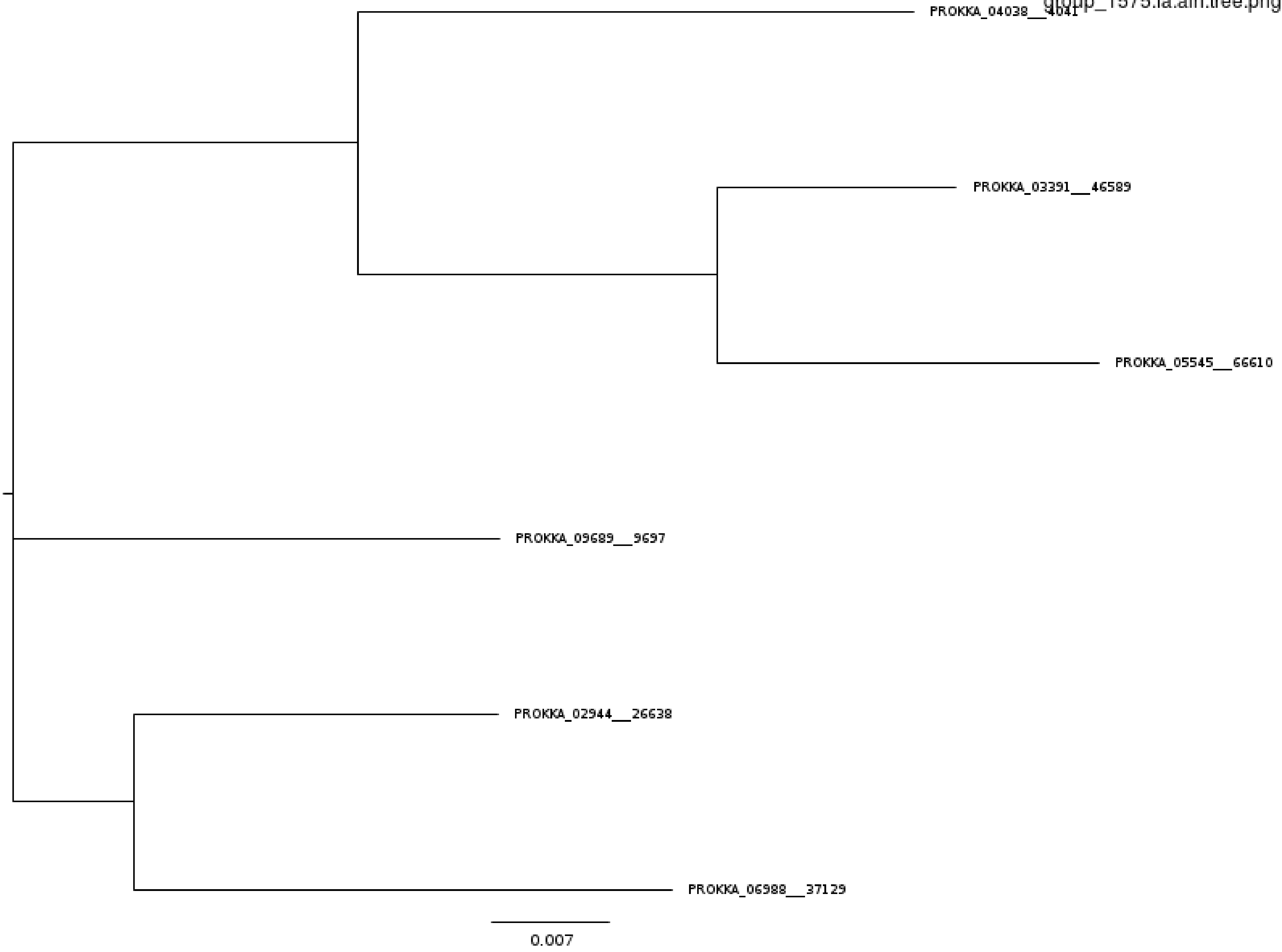

group_1578.fa.aln.tree.png

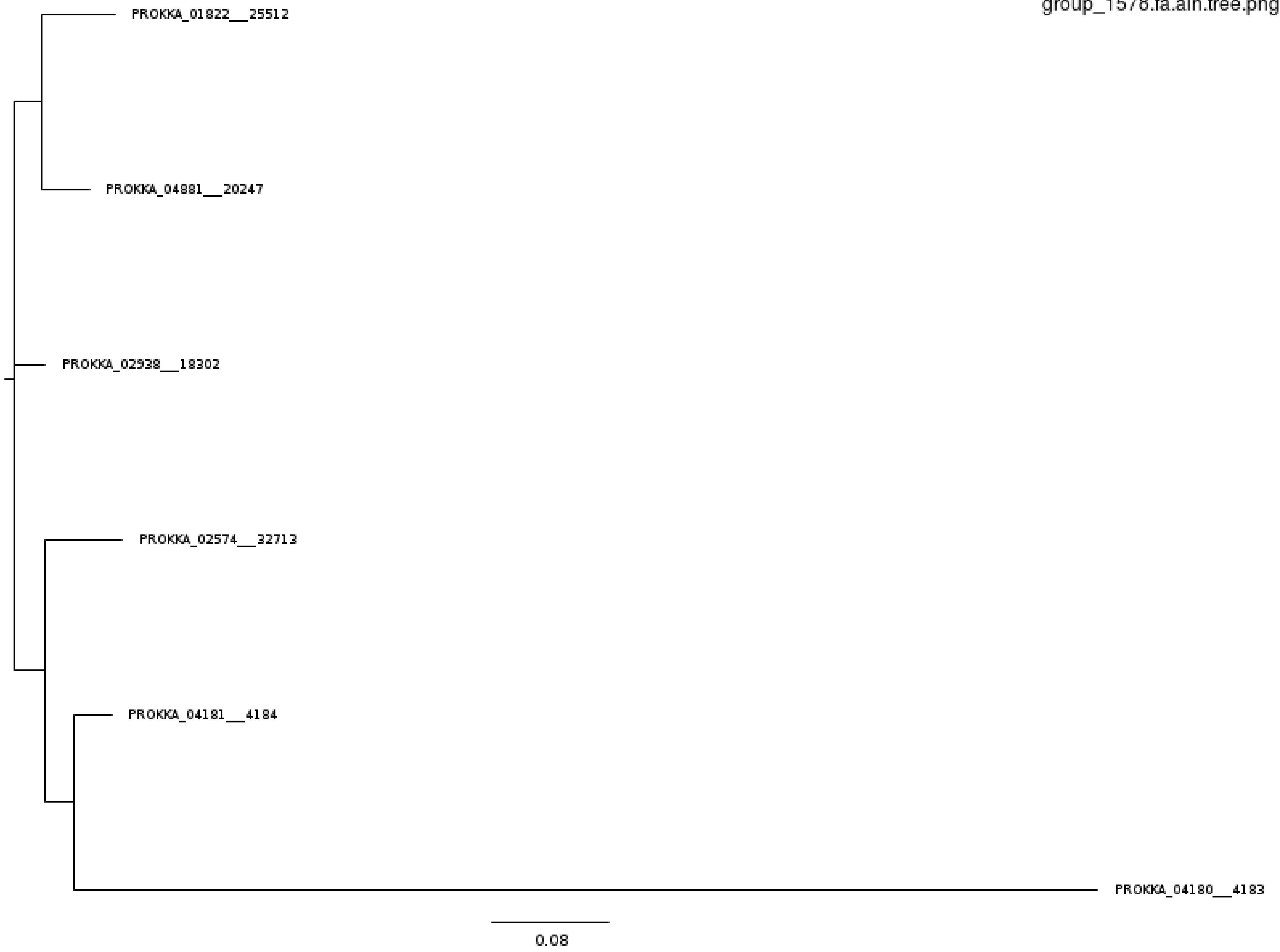

group_1584.fa.aln.tree.png

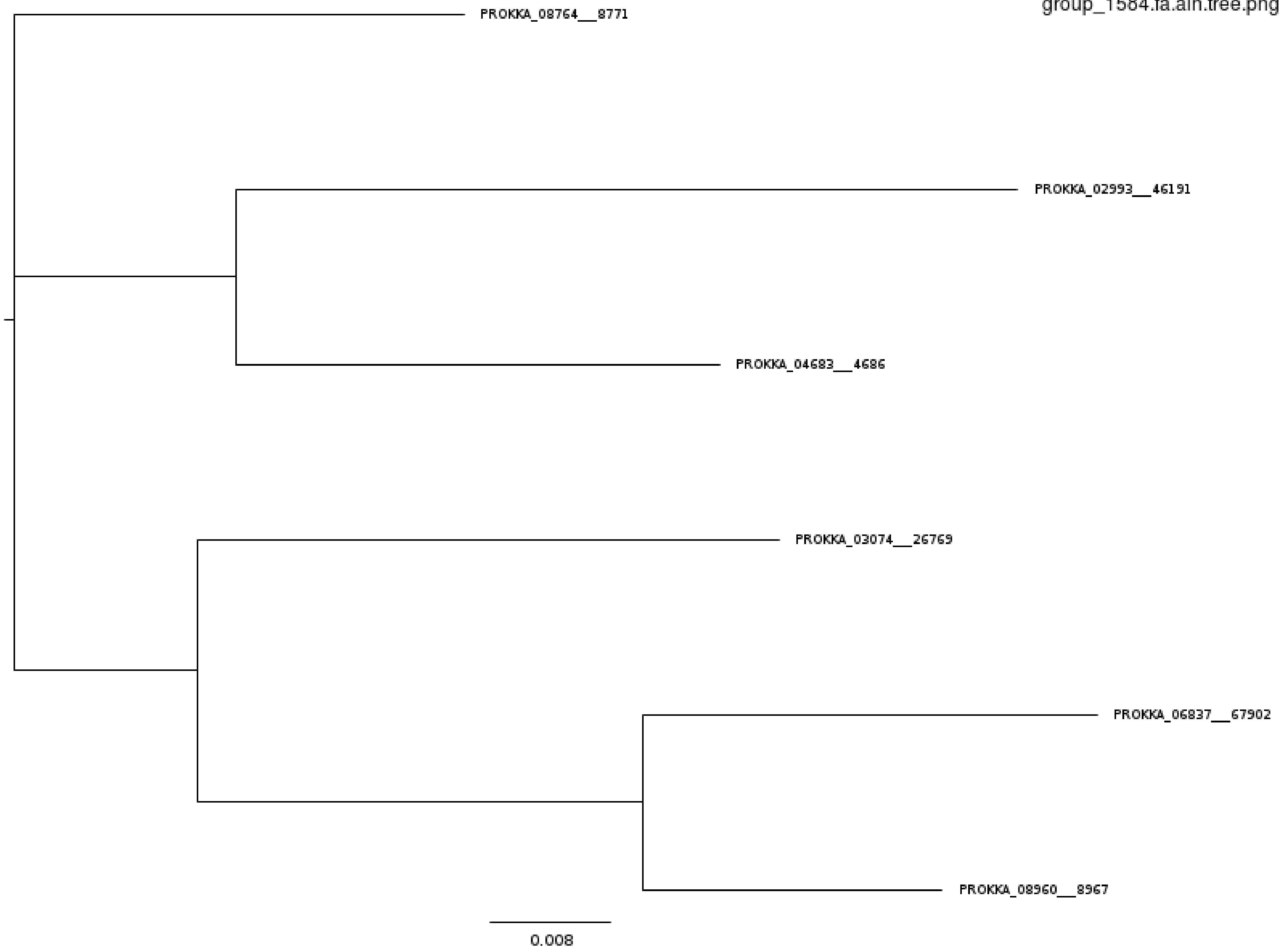

group_1587.fa.aln.tree.png

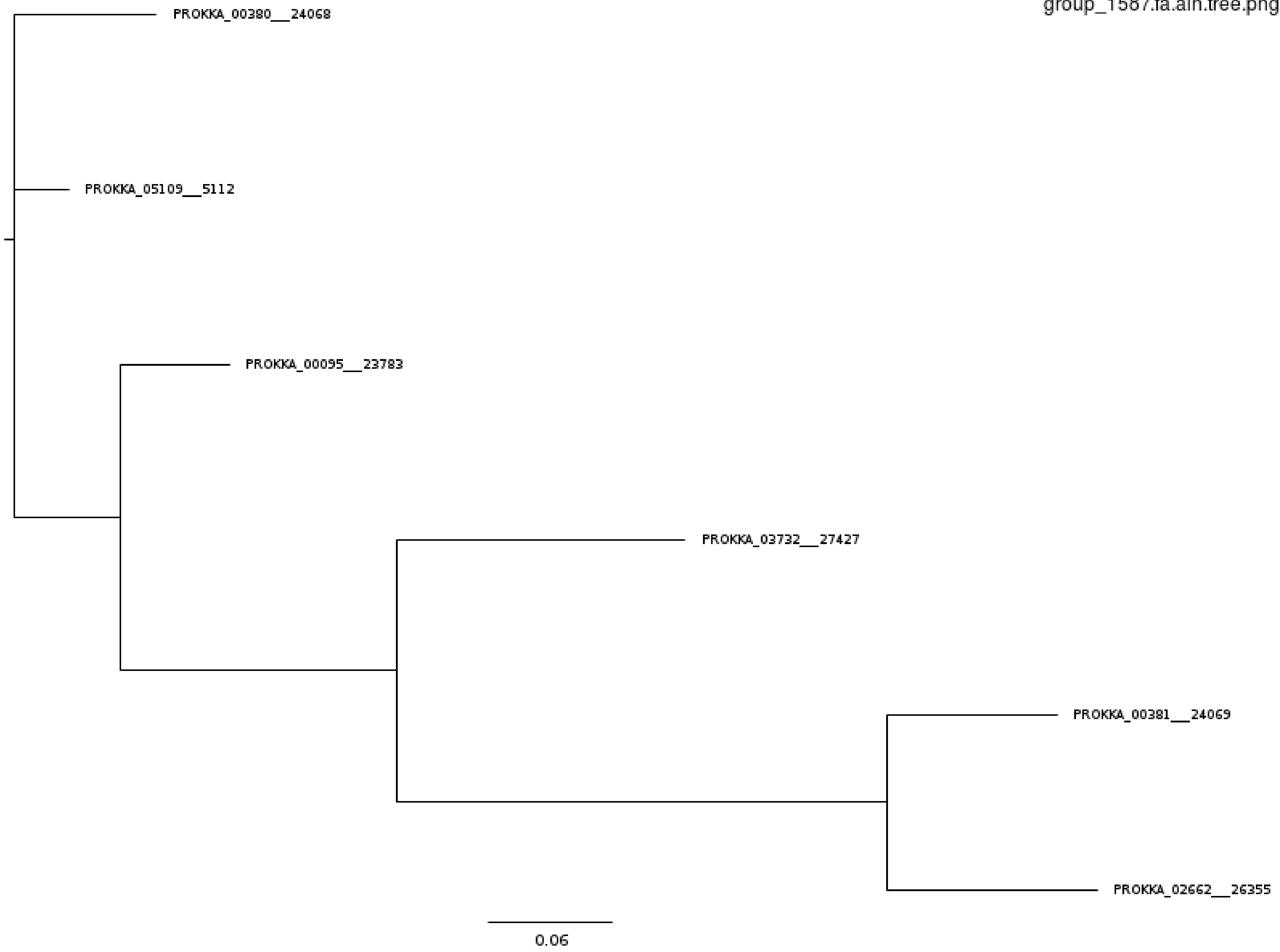

group_1589.fa.aln.tree.png

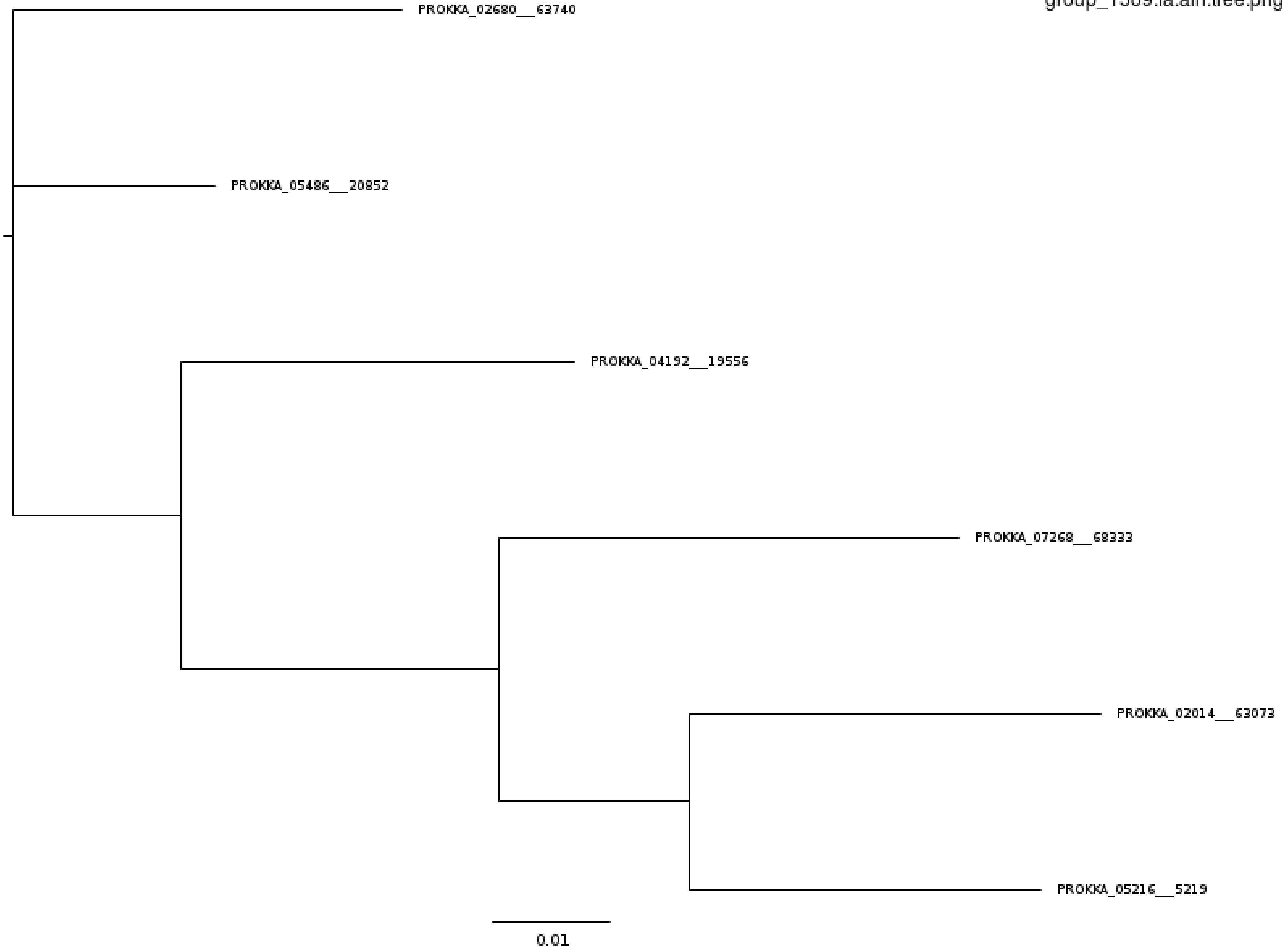

group_1593.fa.aln.tree.png

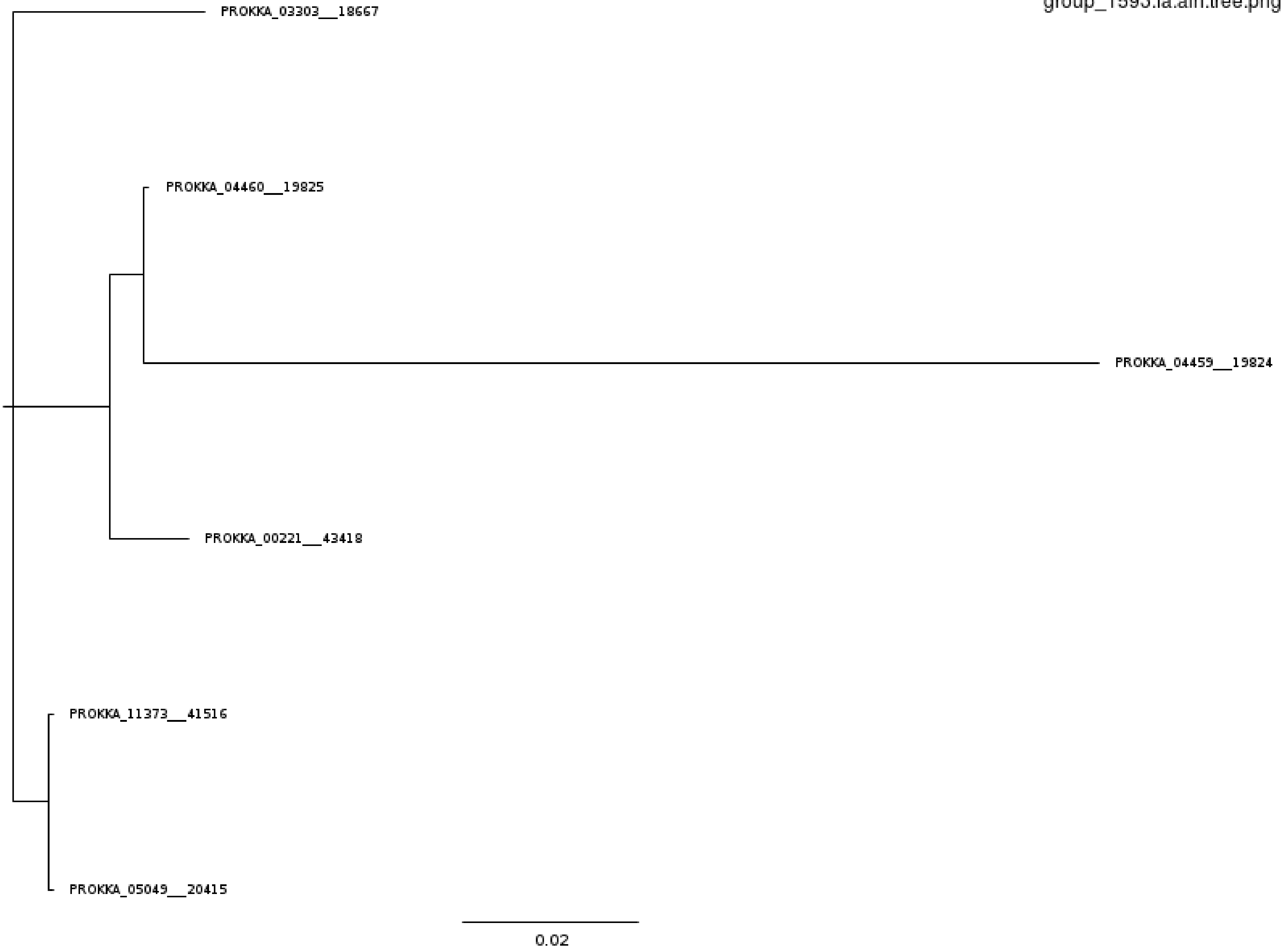

group_1597.fa.aln.tree.png

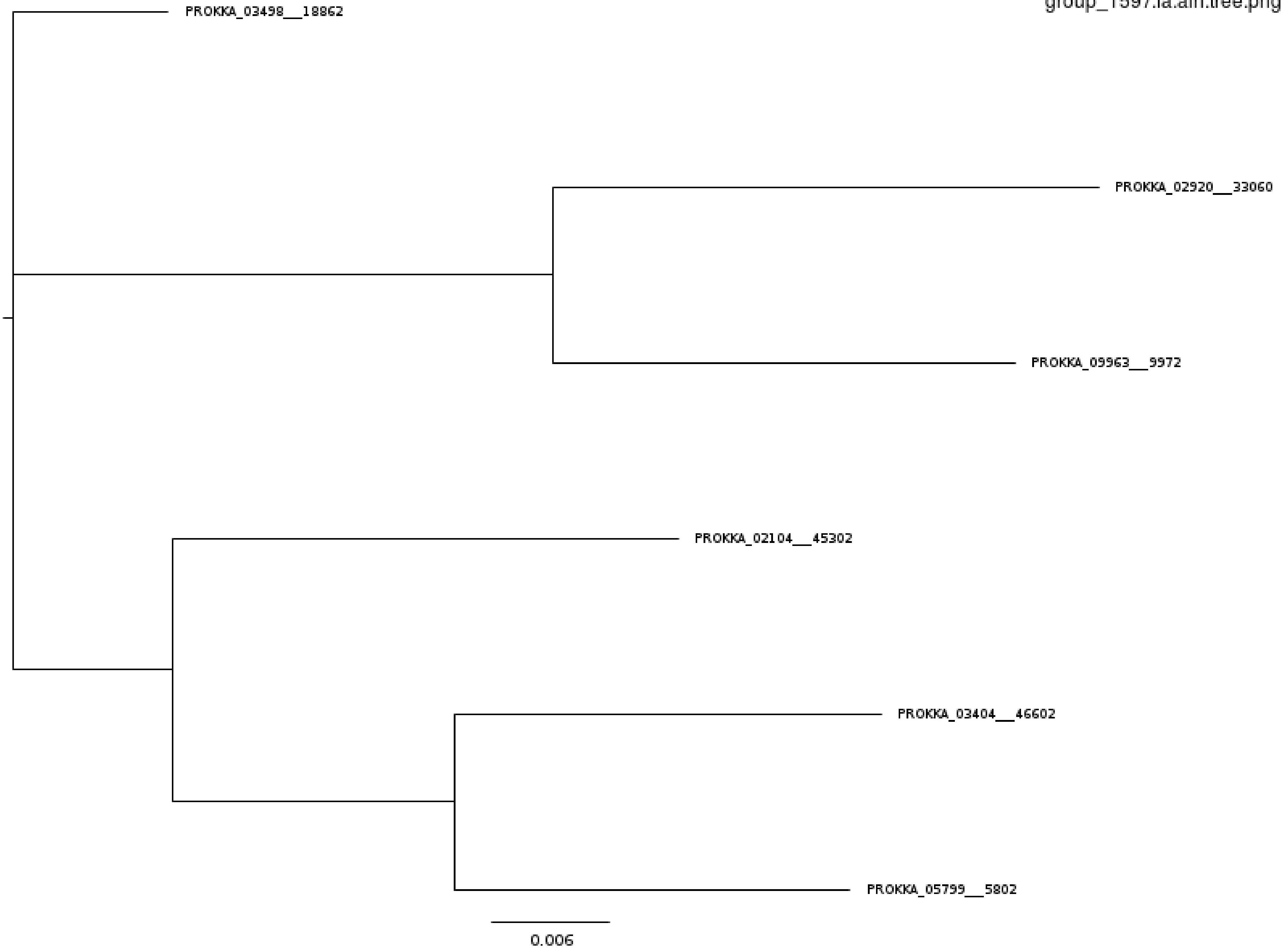

group_159.fa.aln.tree.png

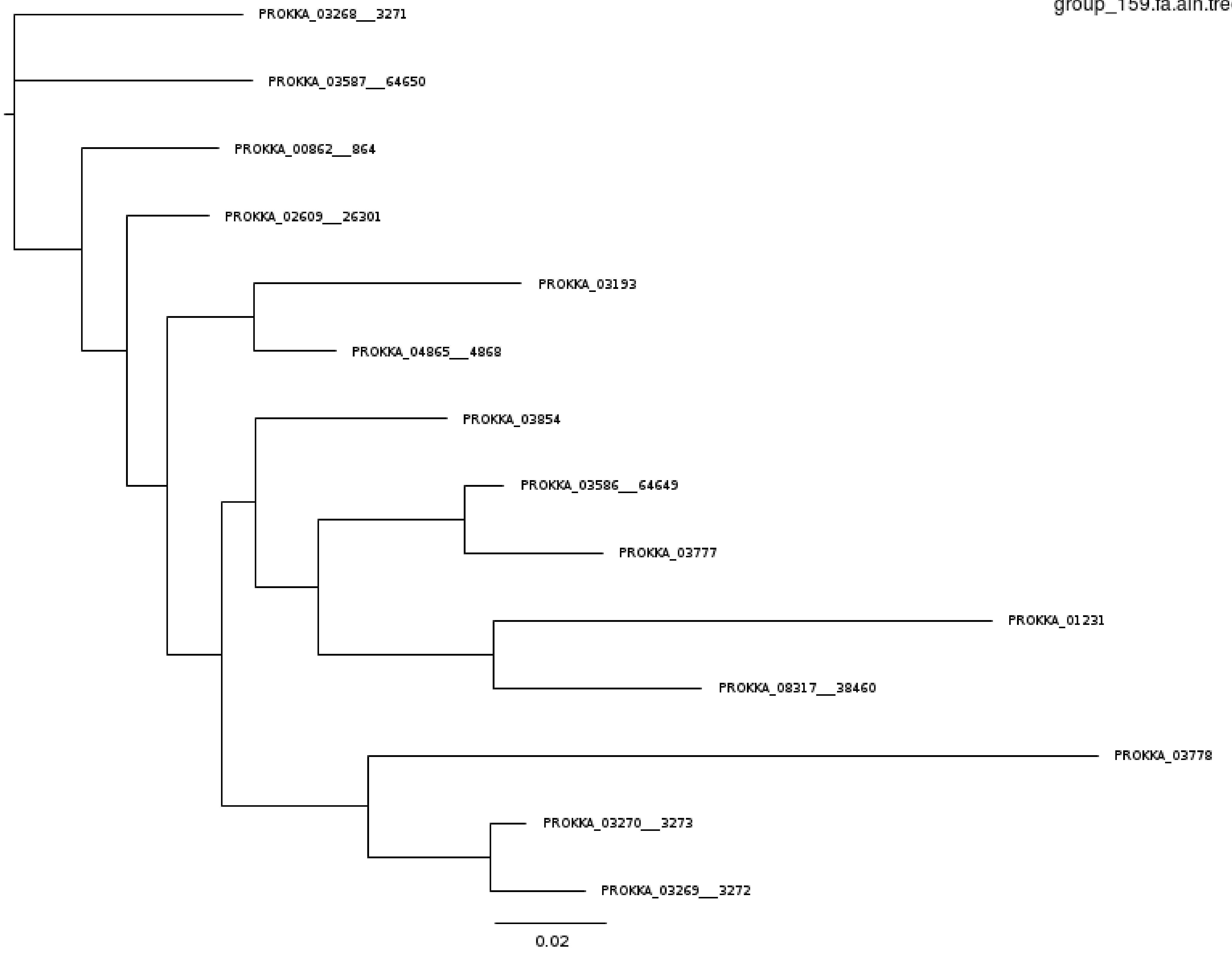

group_15.fa.aln.tree.png

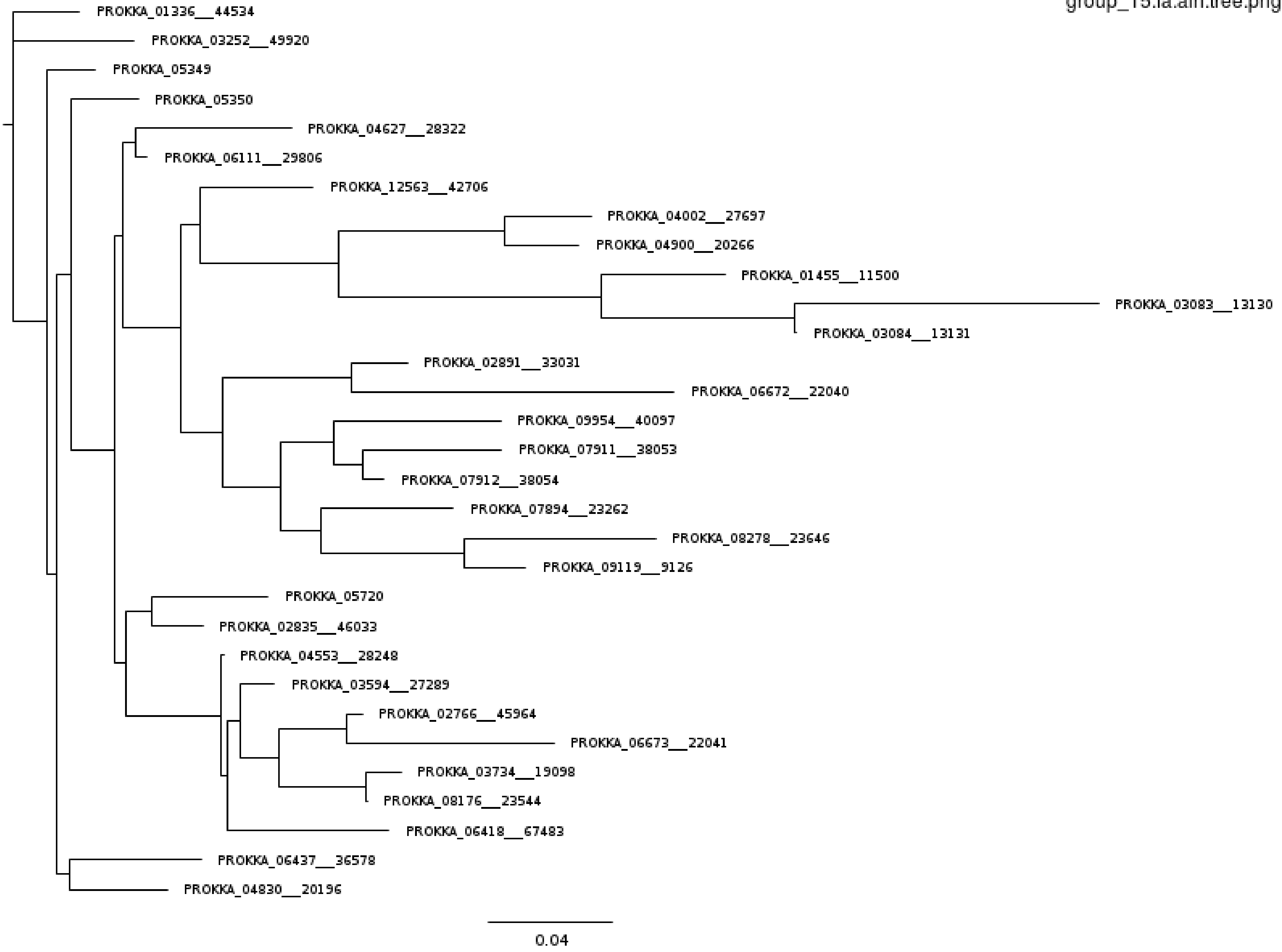

group_1603.fa.aln.tree.png

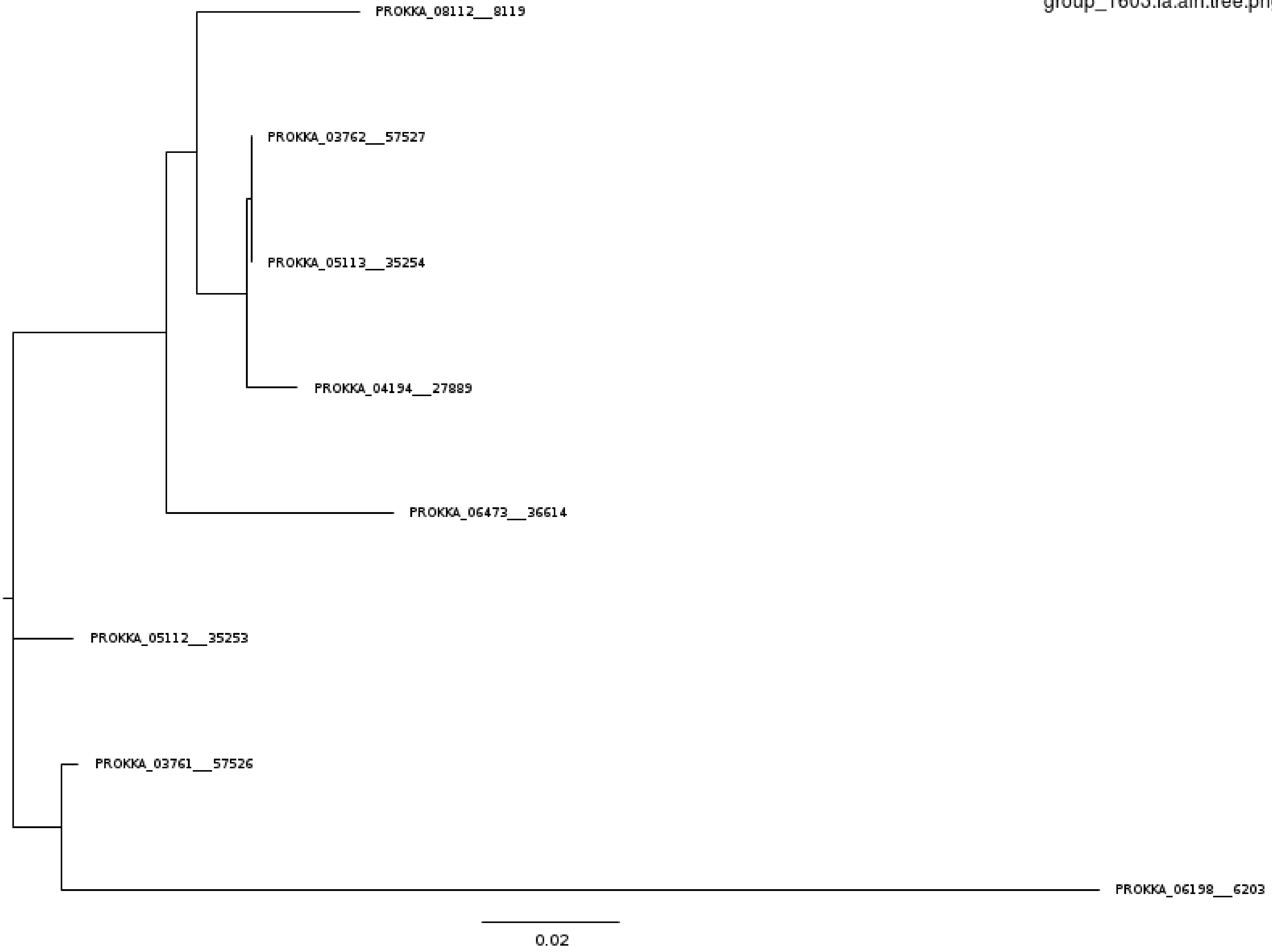

group_1606.fa.aln.tree.png

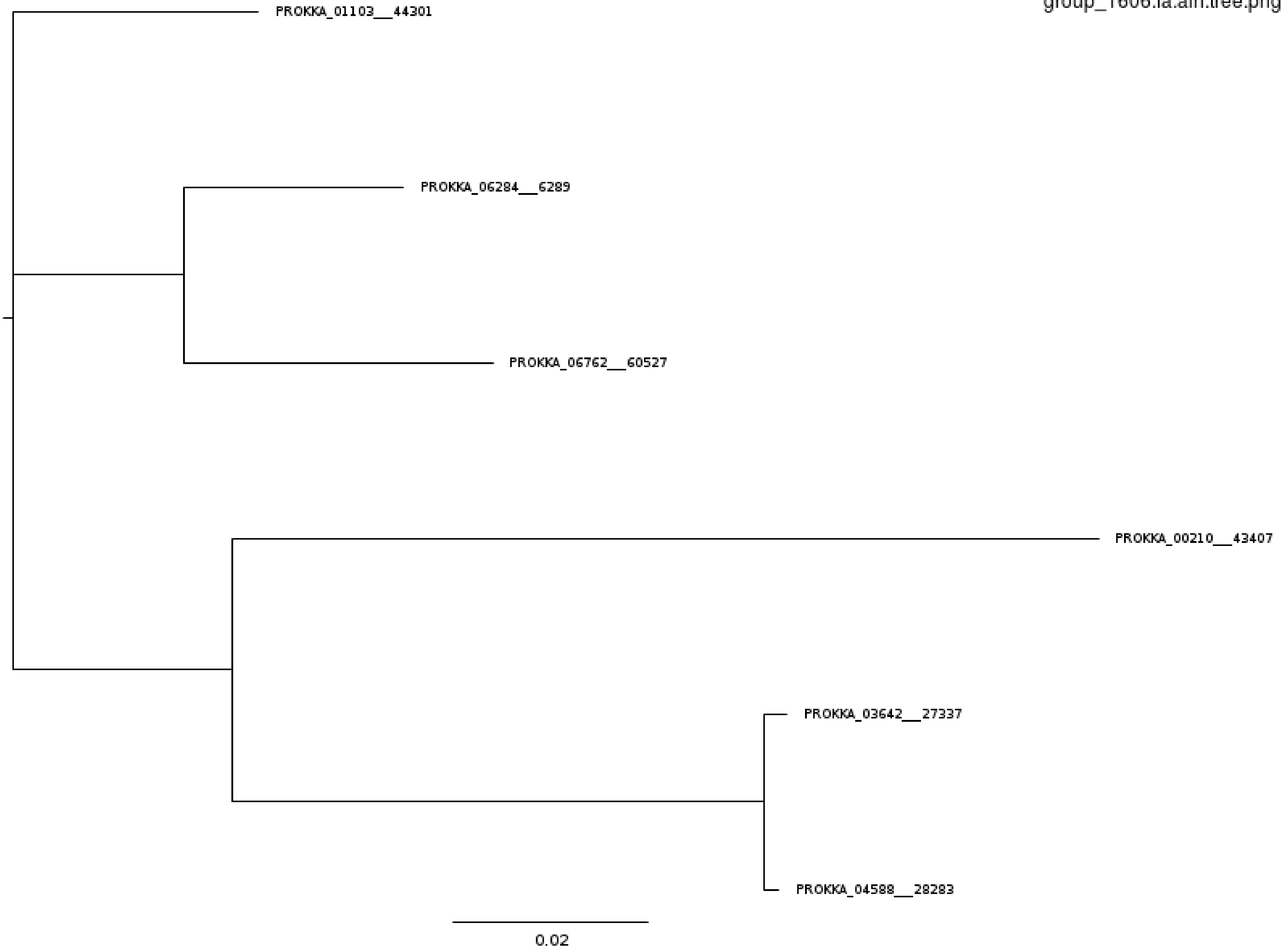

group_1612.fa.aln.tree.png

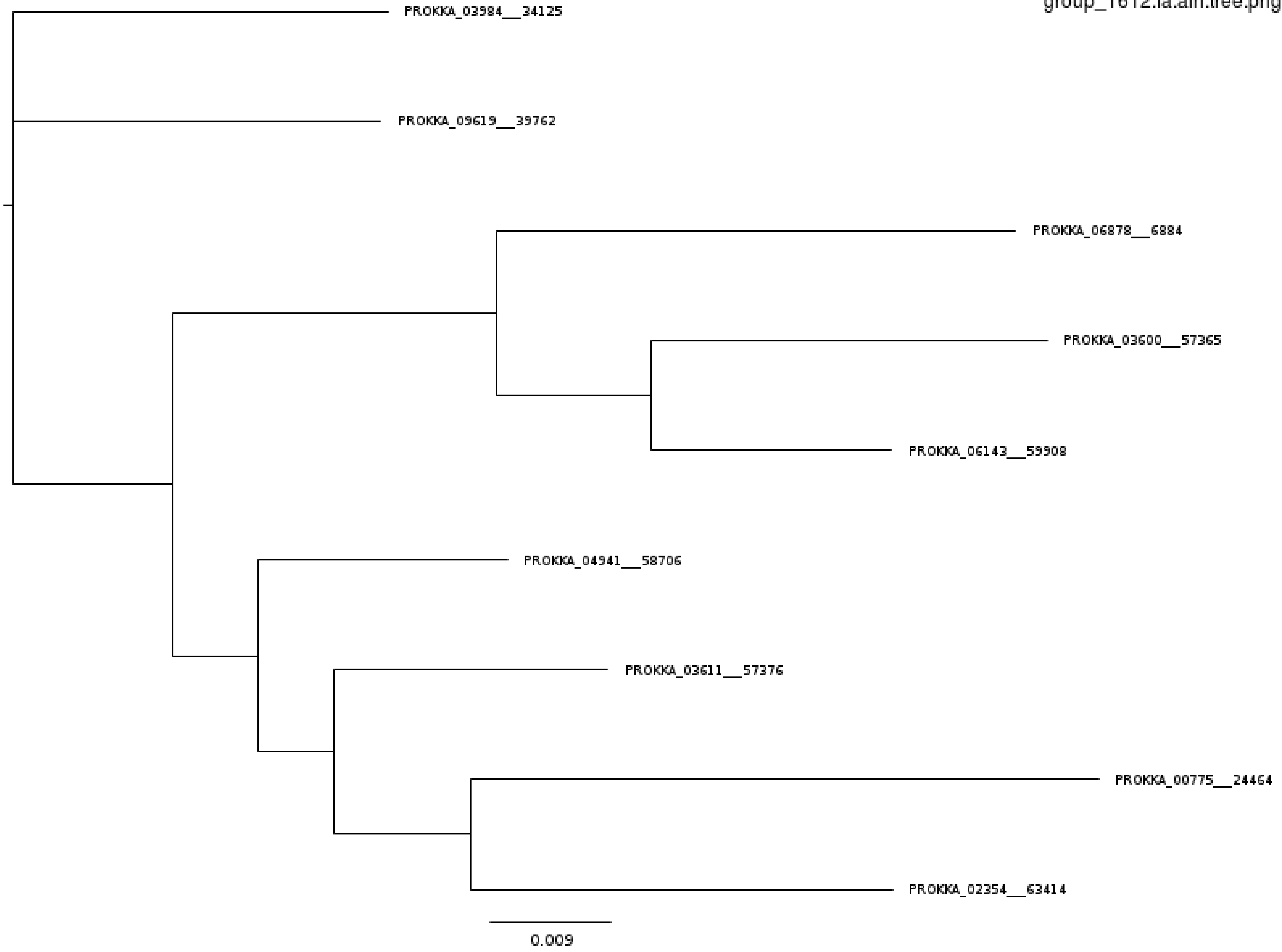

group_1615.fa.aln.tree.png

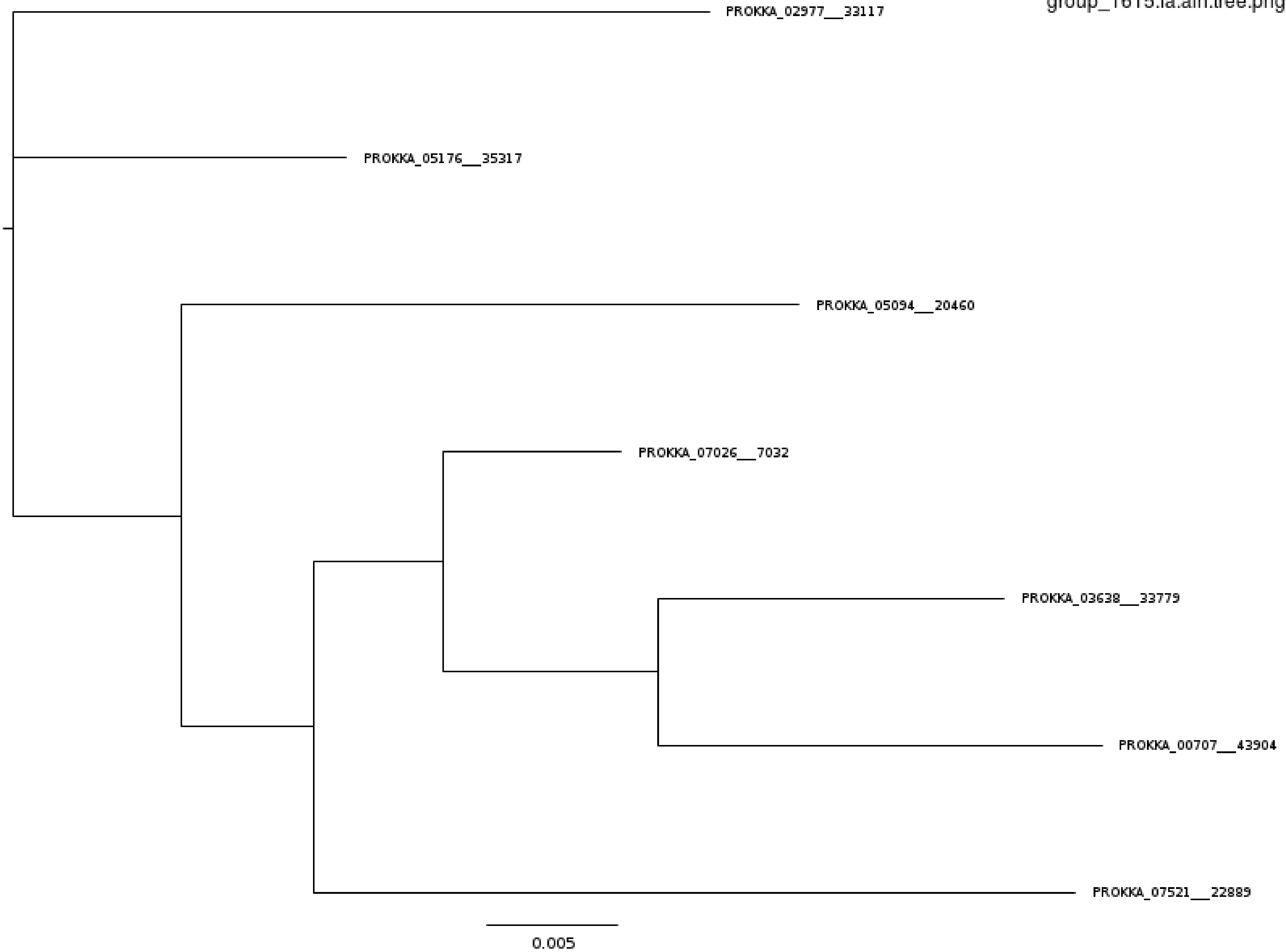

group_1617.fa.aln.tree.png

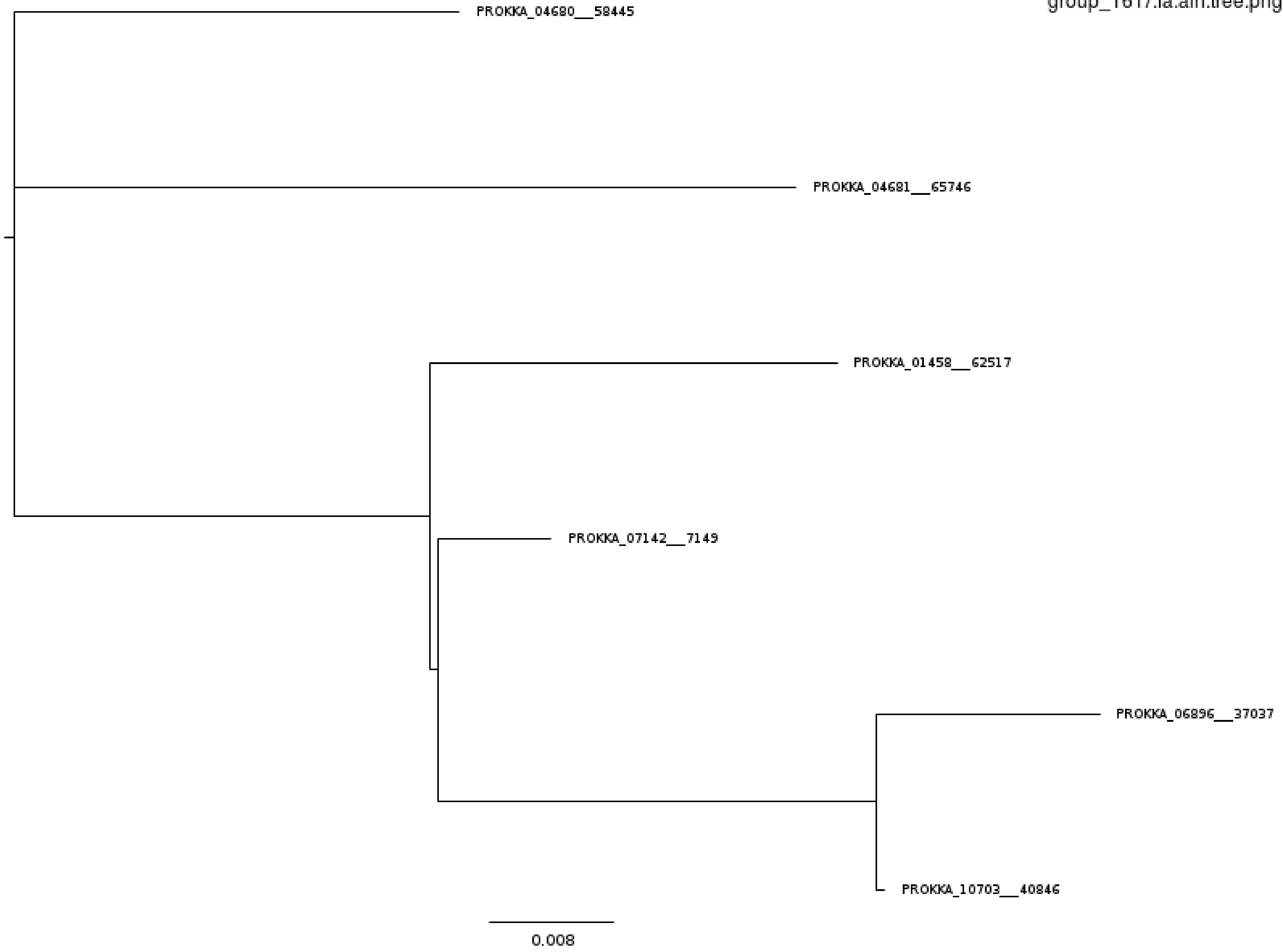

group_1618.fa.aln.tree.png

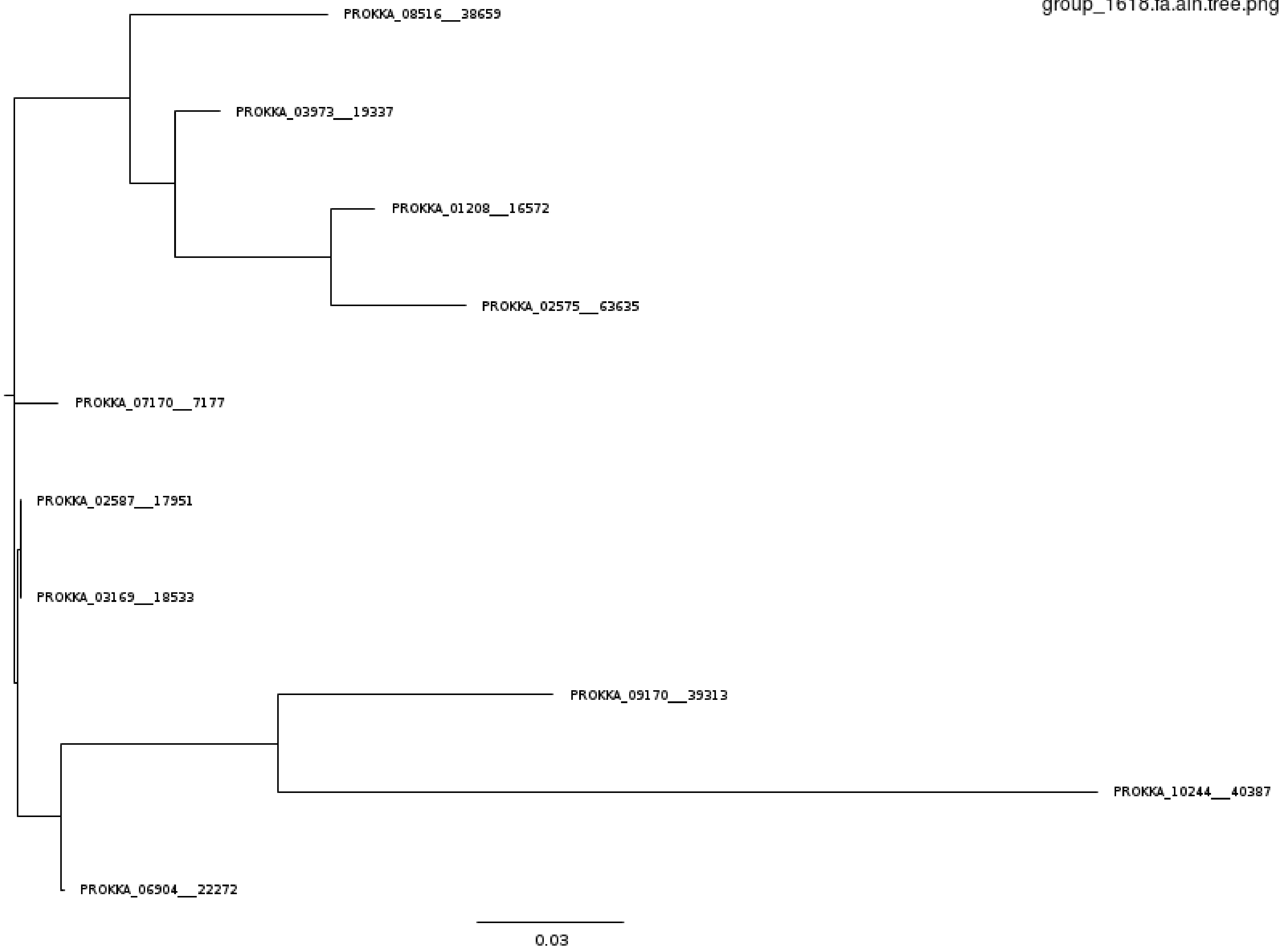

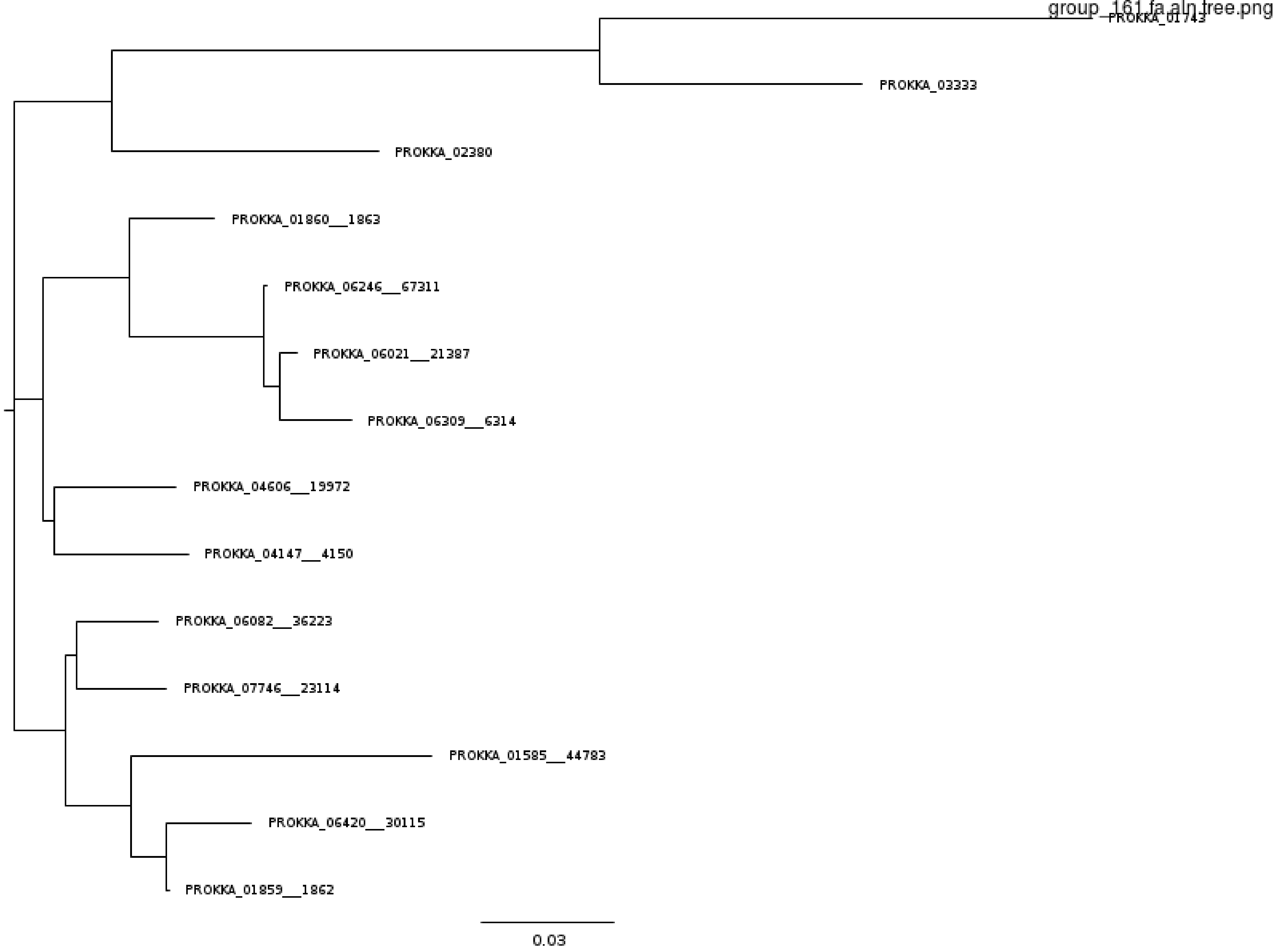
group_161.fa.aln.tree.png
PROKKA_01743
PROKKA_03333
PROKKA_02380
PROKKA_01860___1863
PROKKA_06246___67311
PROKKA_06021___21387
PROKKA_06309___6314
PROKKA_04606___19972
PROKKA_04147___4150
PROKKA_06082___36223
PROKKA_07746___23114
PROKKA_01585___44783
PROKKA_06420___30115
PROKKA_01859___1862
0.03

group_1620.fa.aln.tree.png

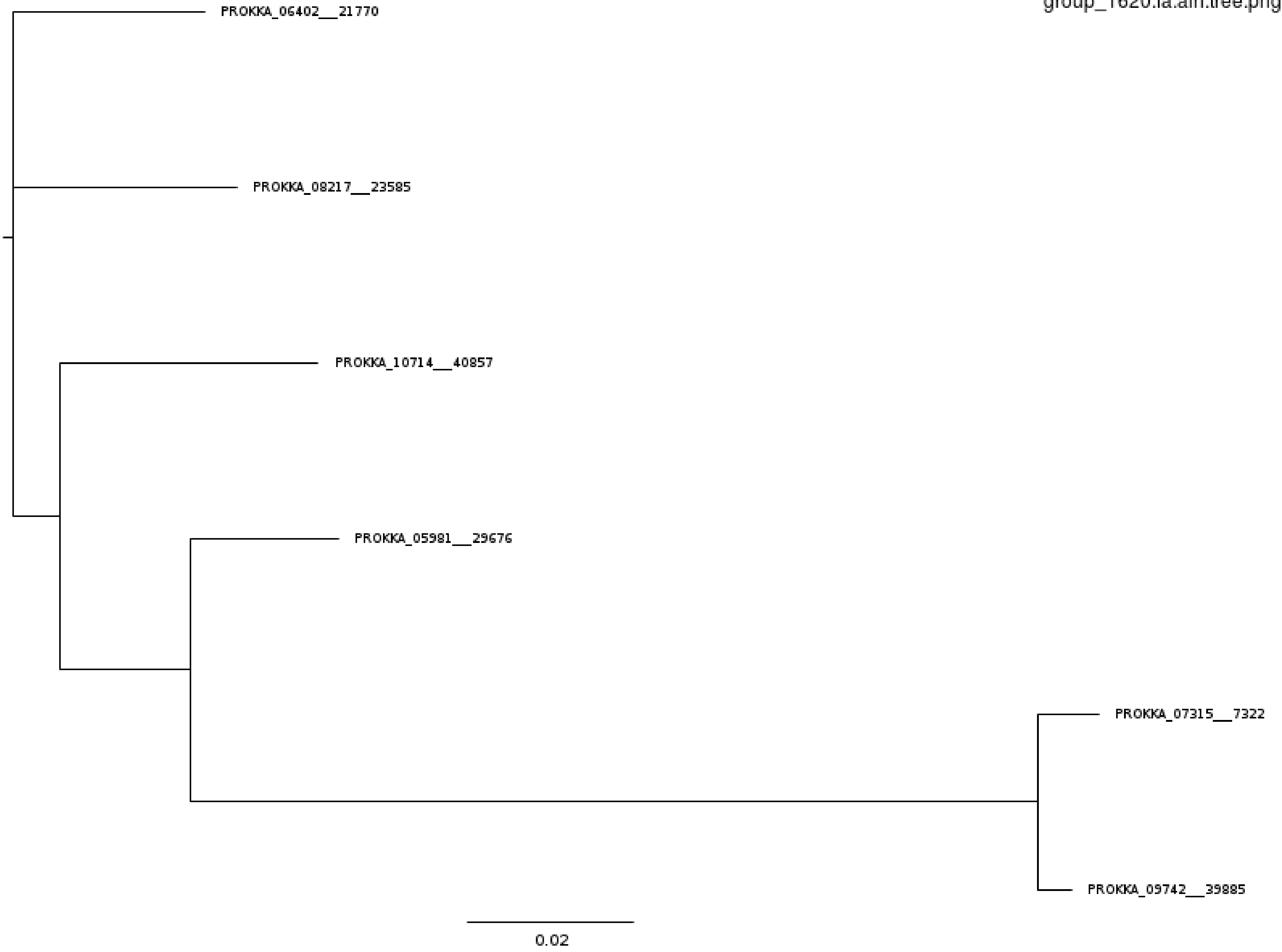

group_1621.fa.aln.tree.png

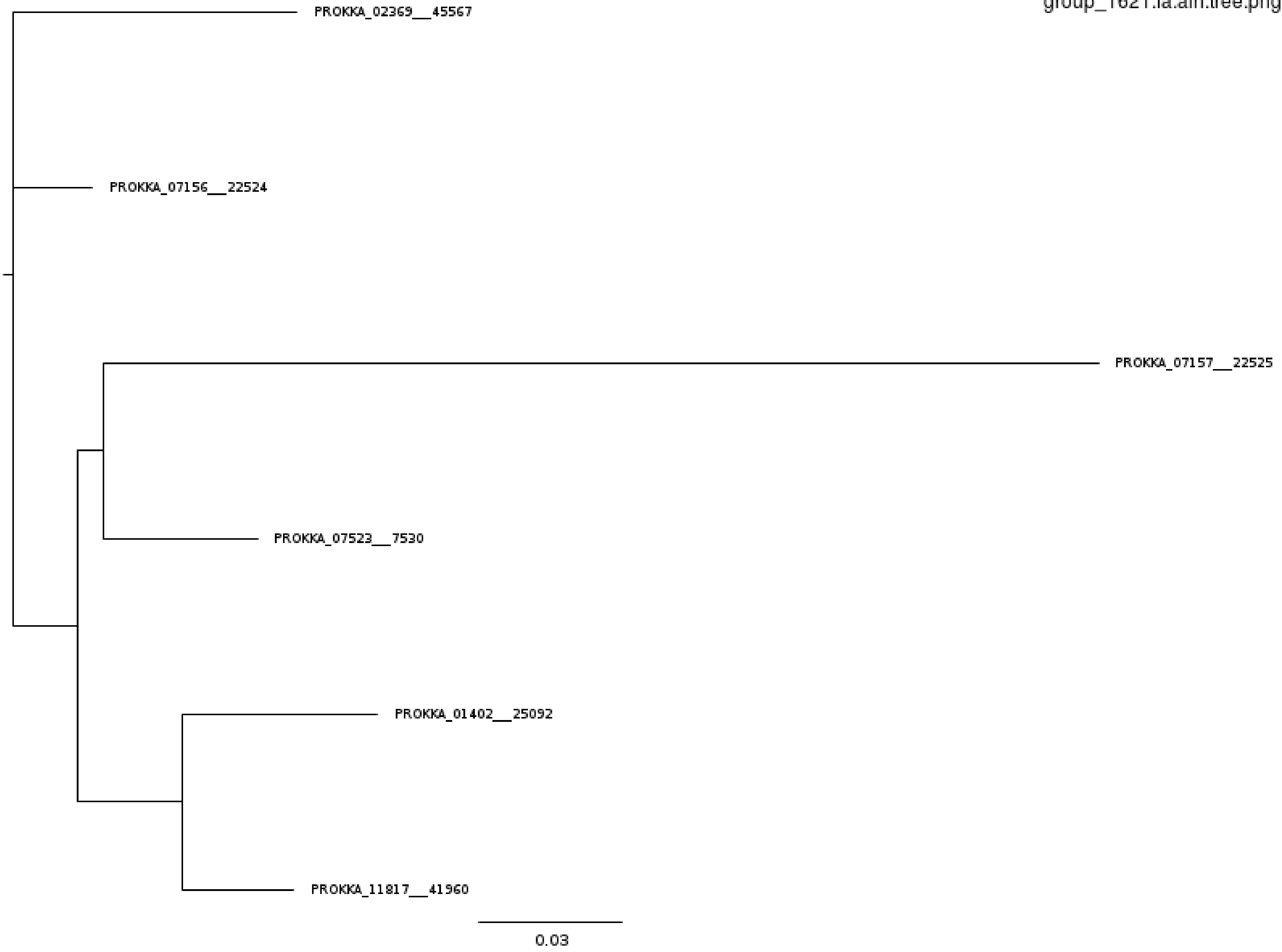

group_1624.fa.aln.tree.png

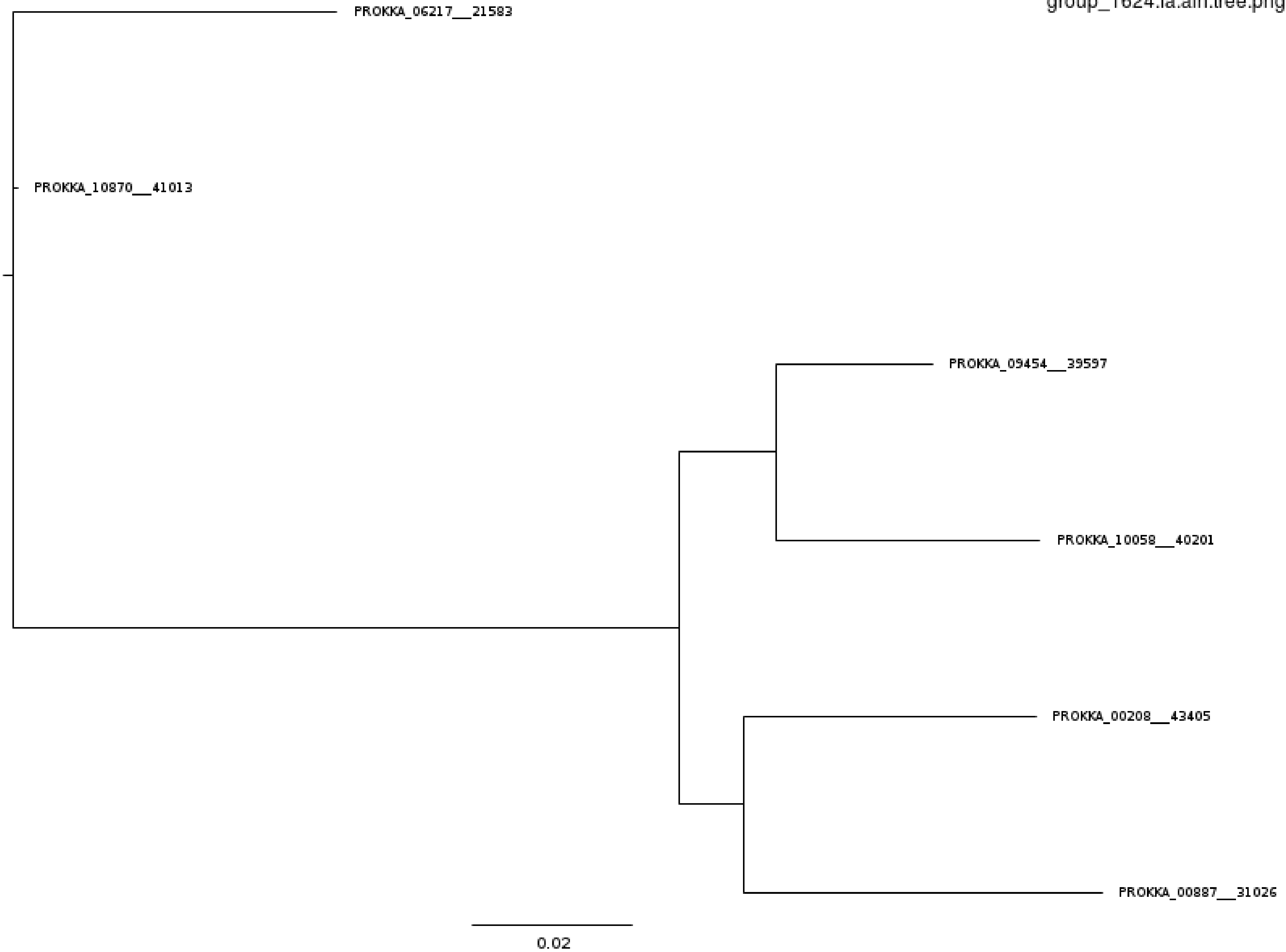

group_1628.fa.aln.tree.png

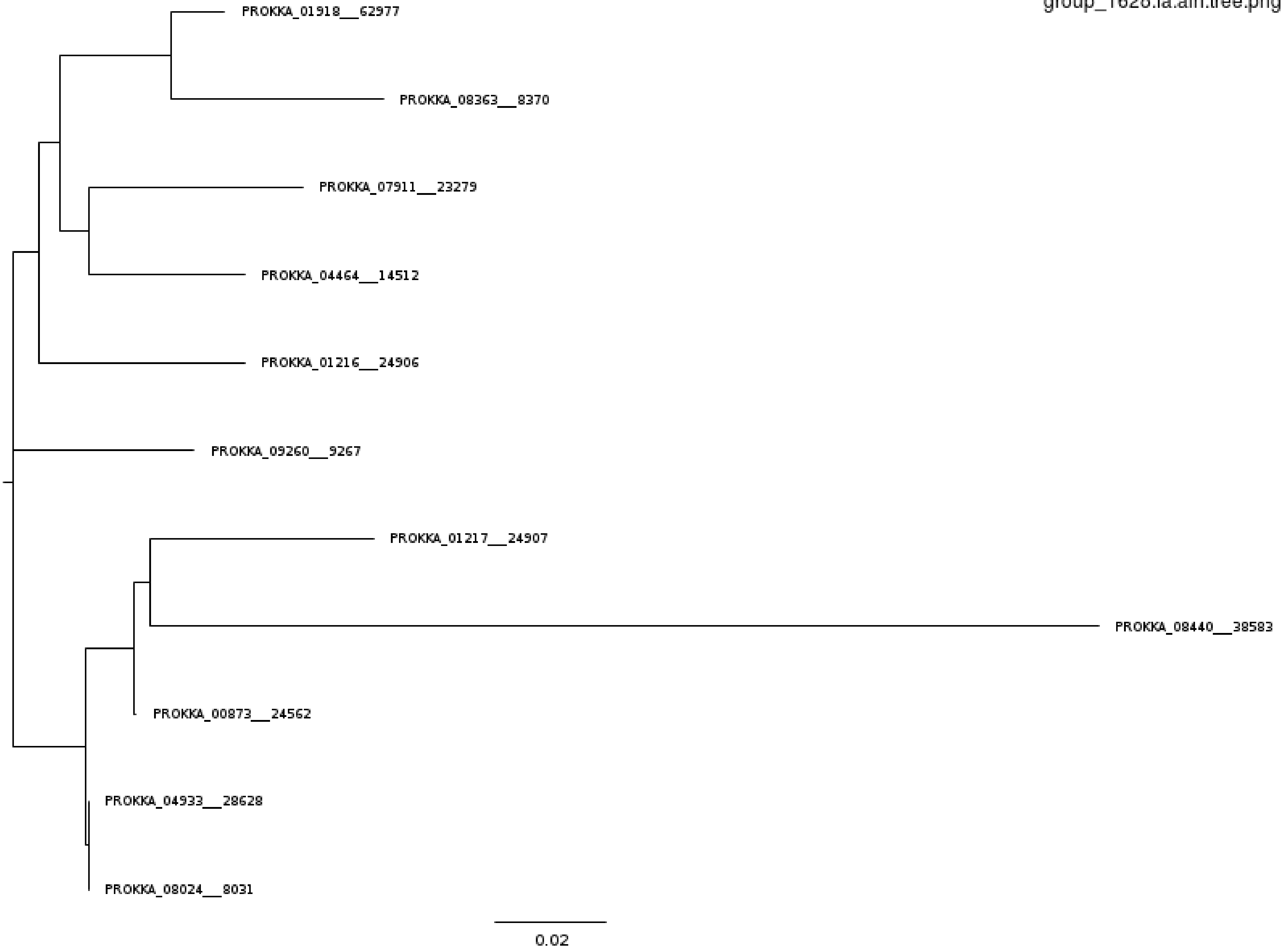

group_1631.fa.aln.tree.png

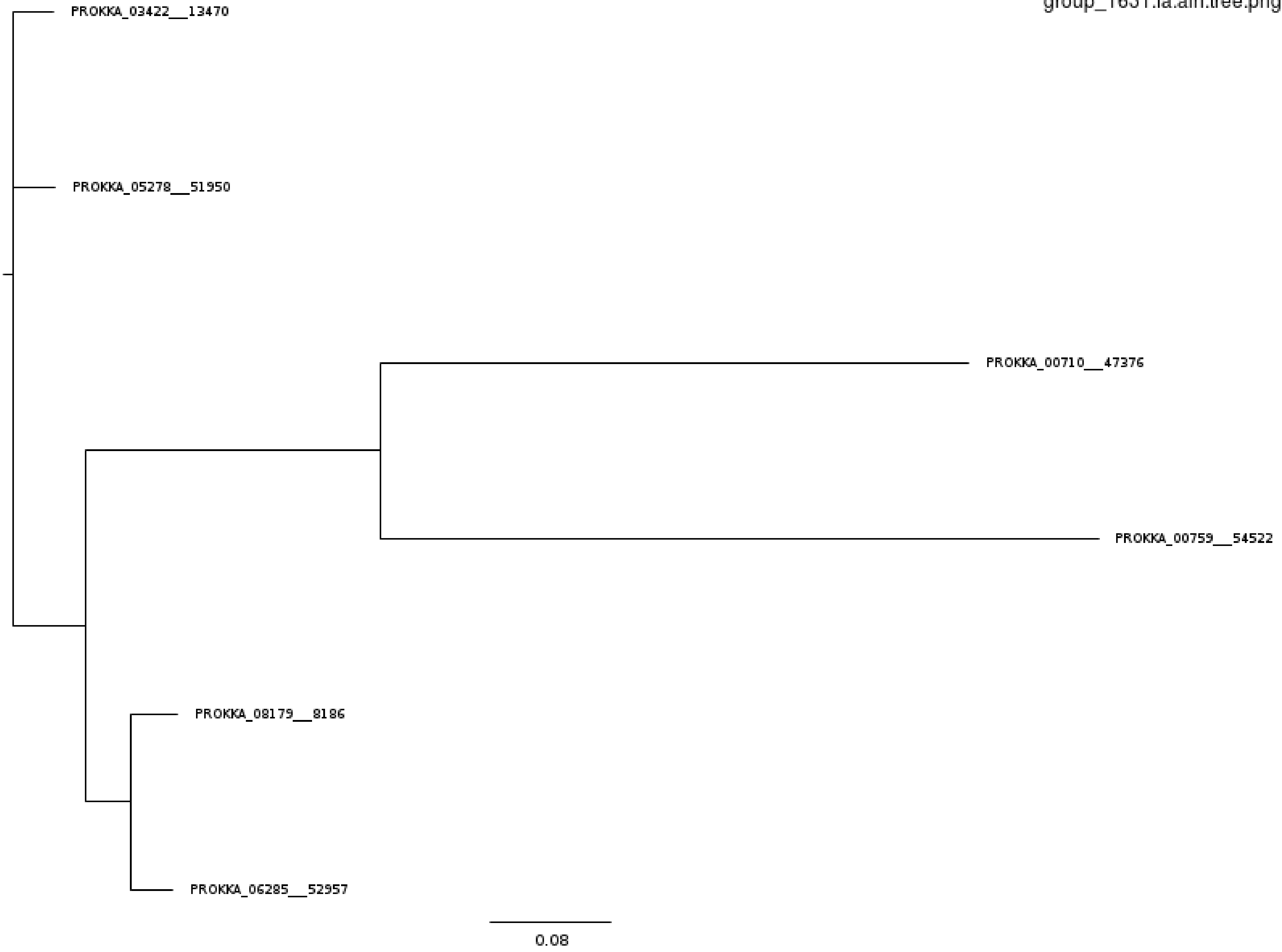

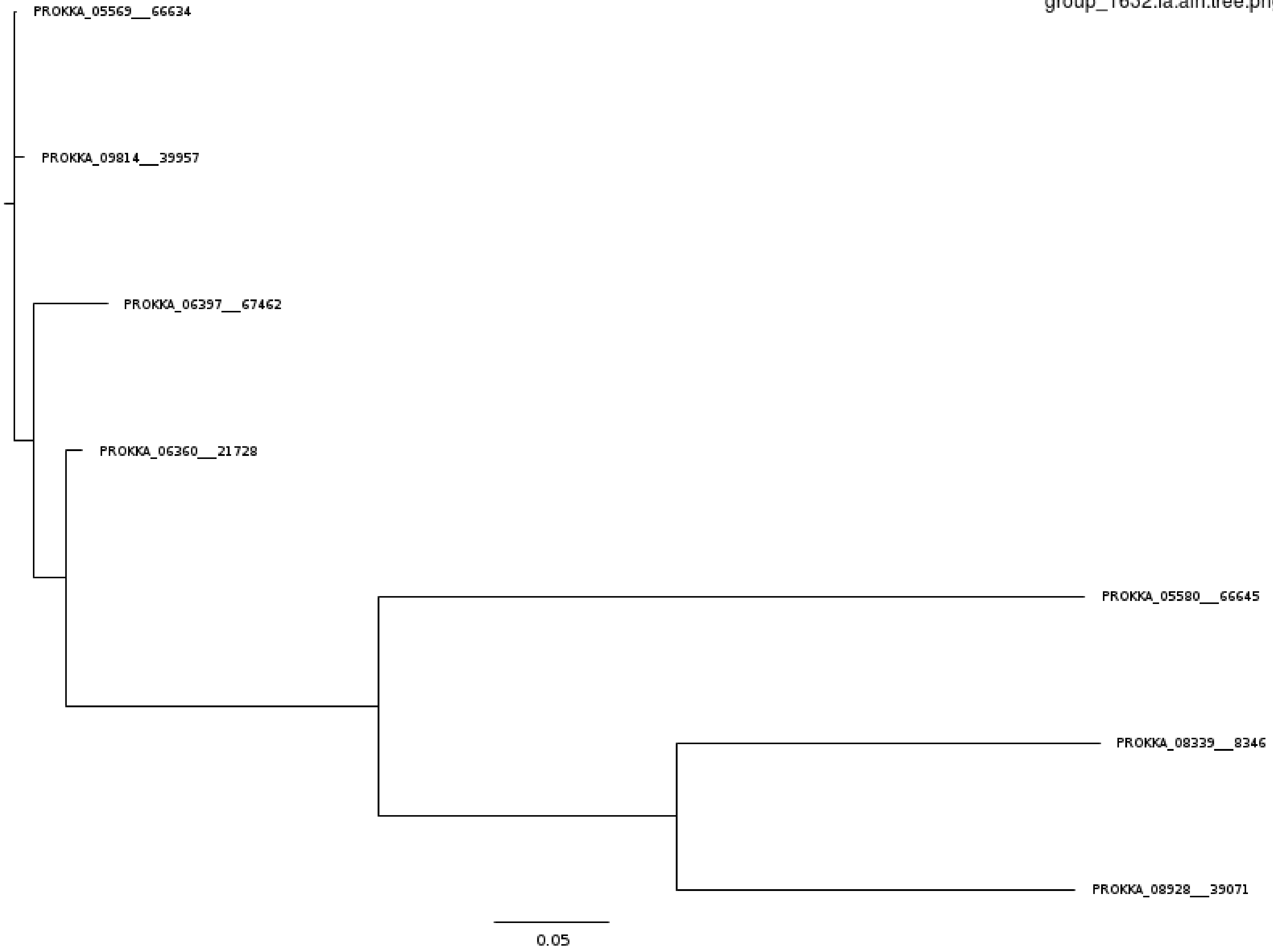
PROKKA_05569___66634
PROKKA_09814___39957
PROKKA_06397___67462
PROKKA_06360___21728
PROKKA_05580___66645
PROKKA_08339___8346
PROKKA_08928___39071
0.05

group_1634.fa.aln.tree.png

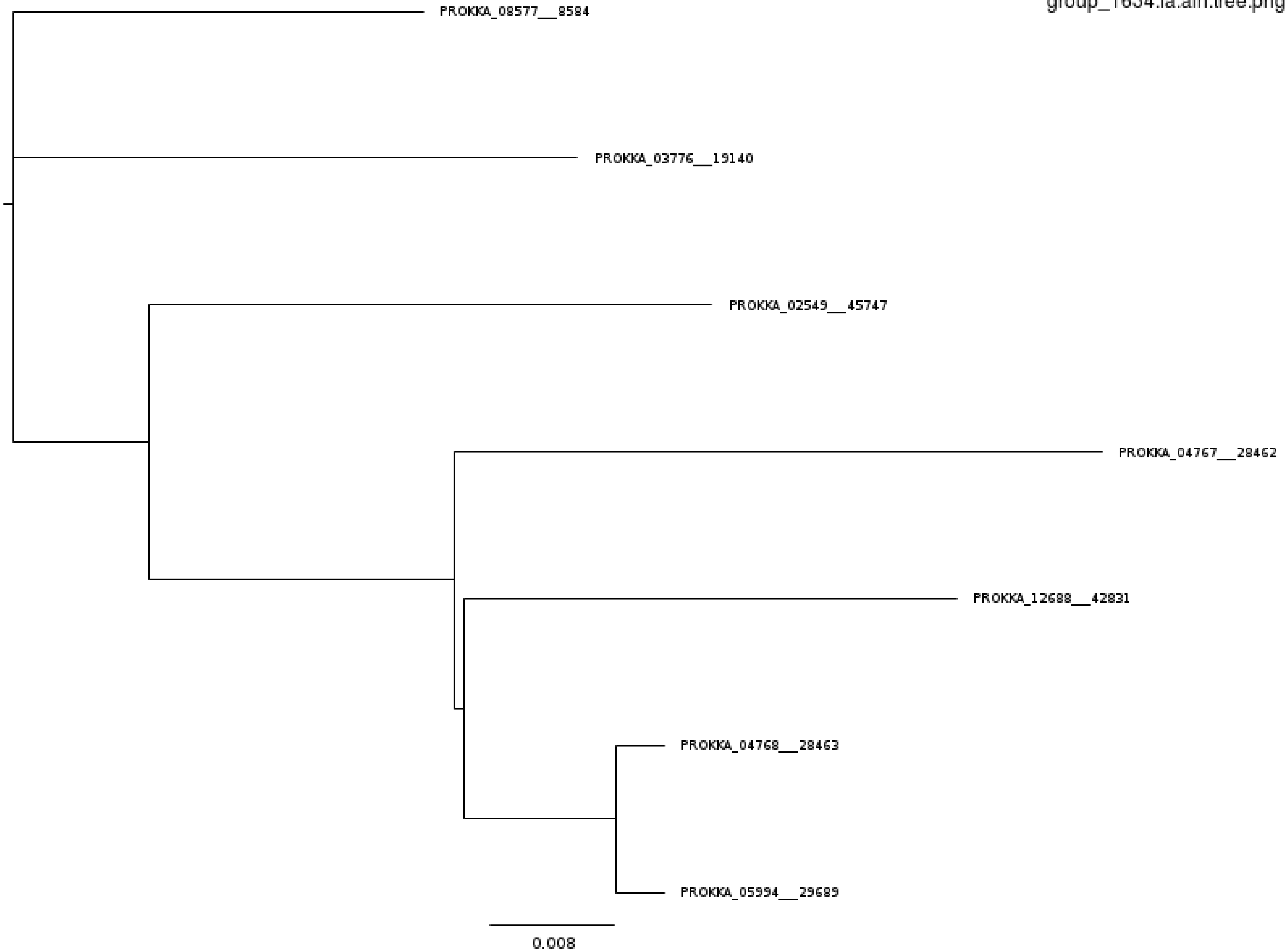

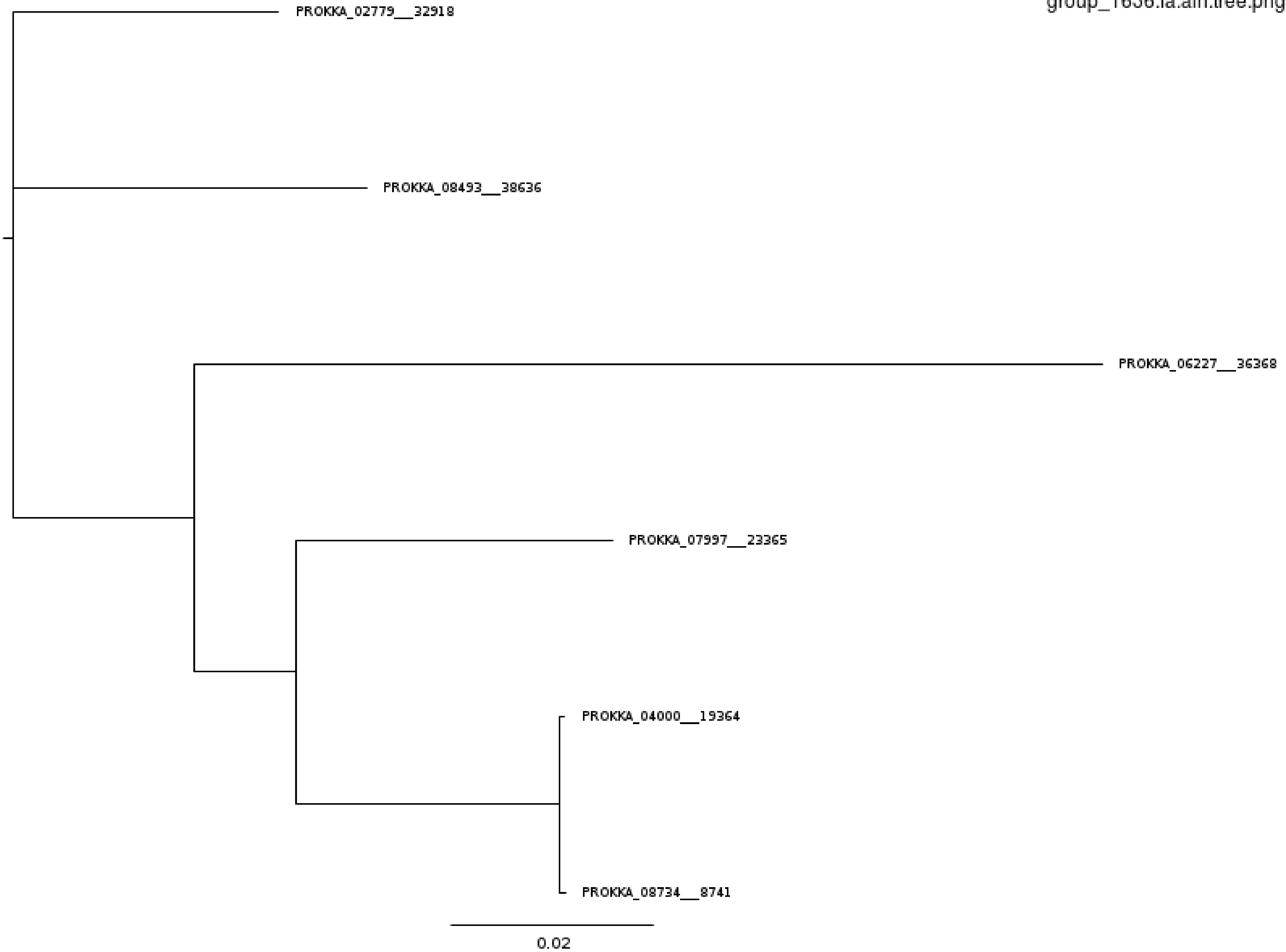
PROKKA_02779___32918
PROKKA_08493___38636
PROKKA_06227___36368
PROKKA_07997___23365
PROKKA_04000___19364
PROKKA_08734___8741
0.02

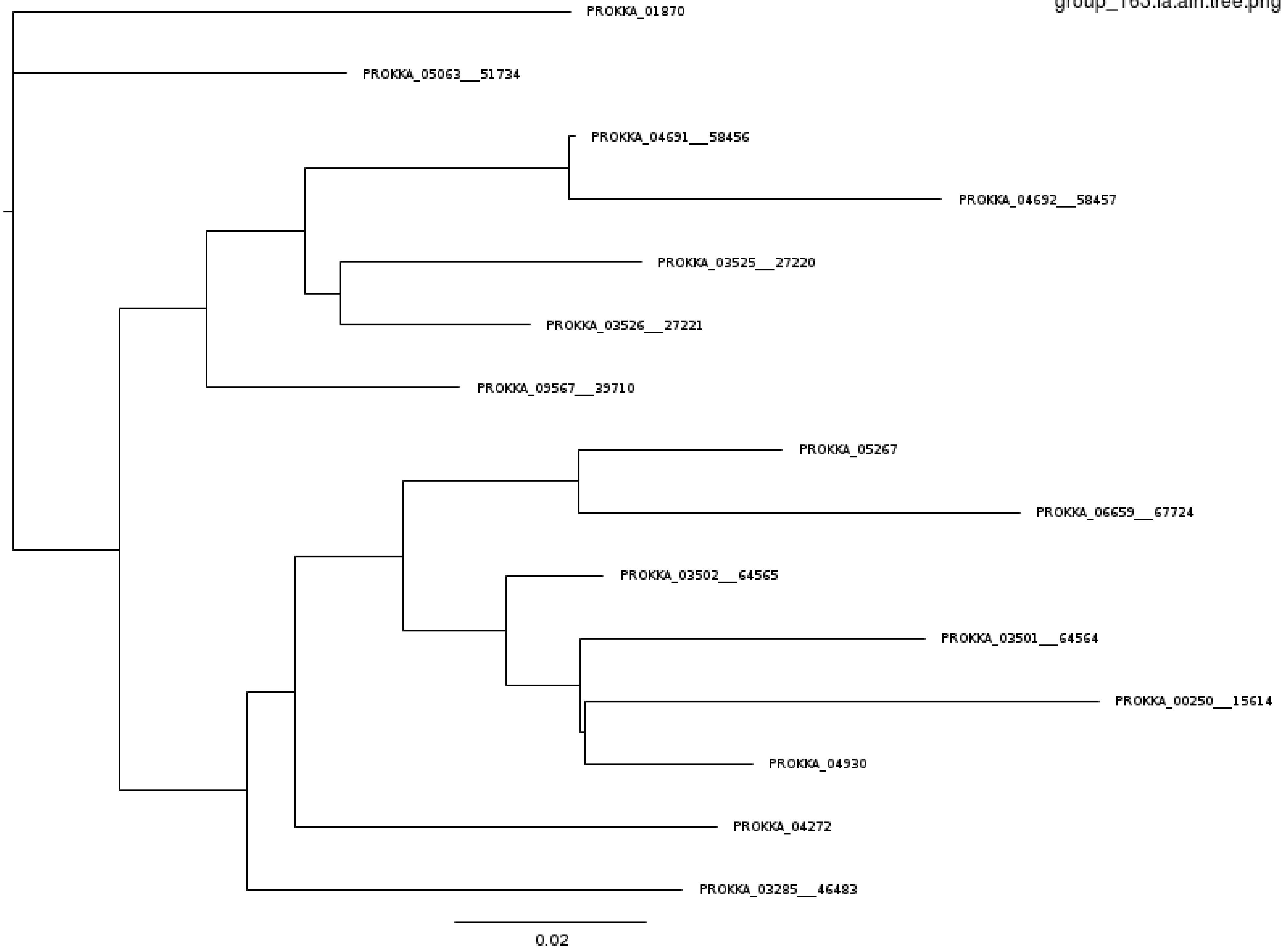
PROKKA_01870
PROKKA_05063___51734
PROKKA_04691___58456
PROKKA_04692___58457
PROKKA_03525___27220
PROKKA_03526___27221
PROKKA_09567___39710
PROKKA_05267
PROKKA_06659___67724
PROKKA_03502___64565
PROKKA_03501___64564
PROKKA_00250___15614
PROKKA_04930
PROKKA_04272
PROKKA_03285___46483
0.02

group_1640.fa.aln.tree.png

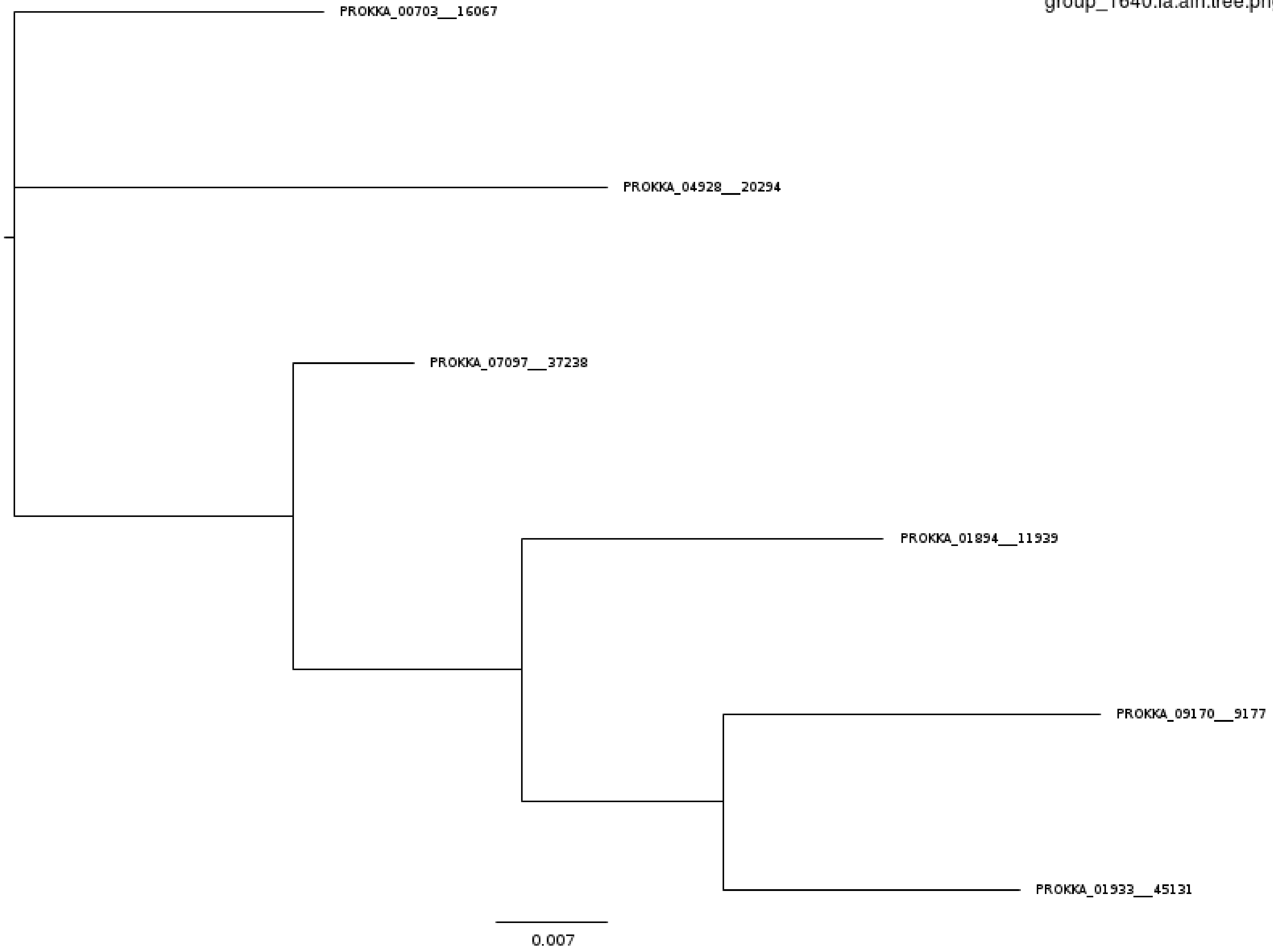

group_1644.fa.aln.tree.png

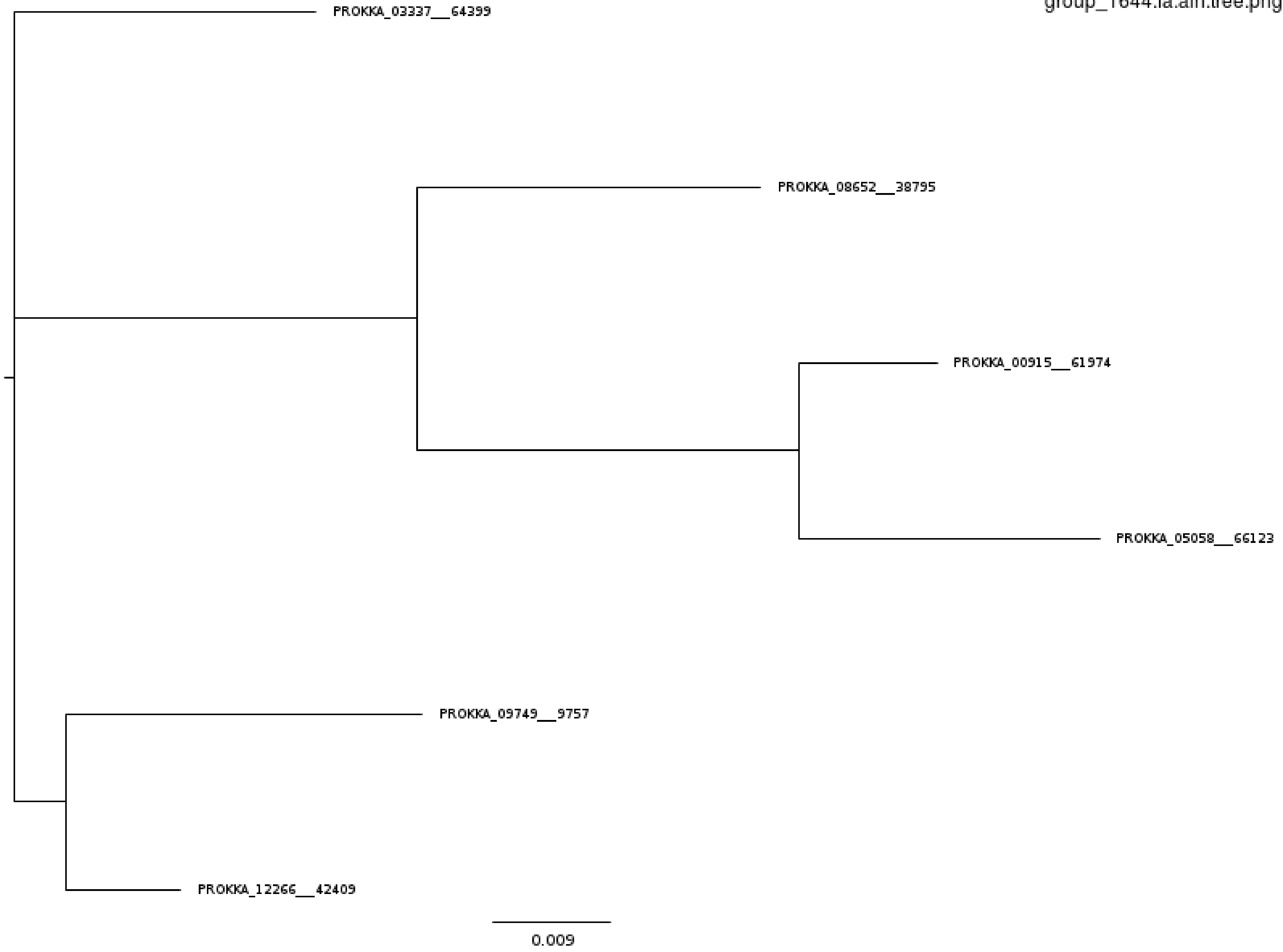

group_1645.fa.aln.tree.png

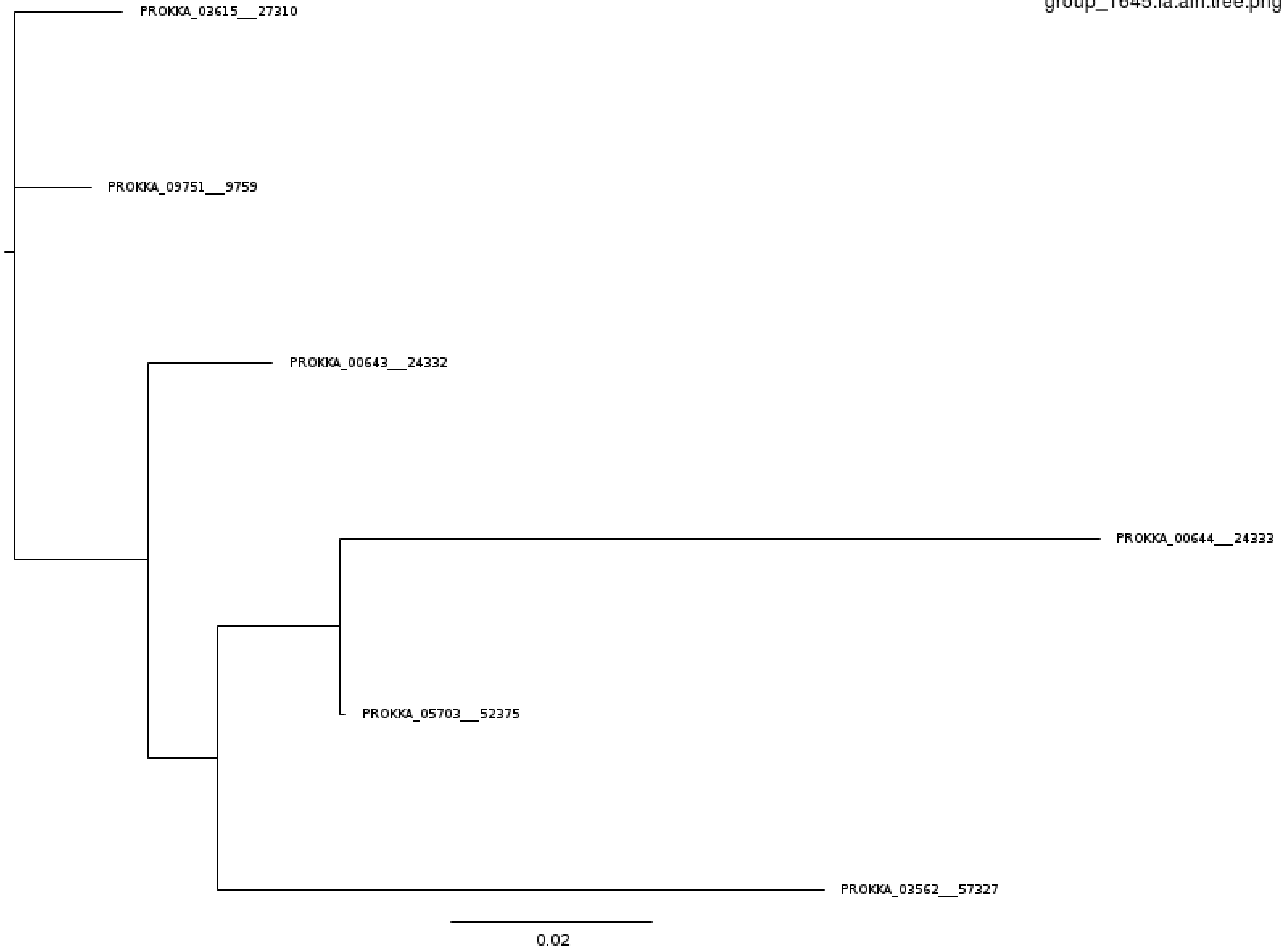

group_1648.fa.aln.tree.png

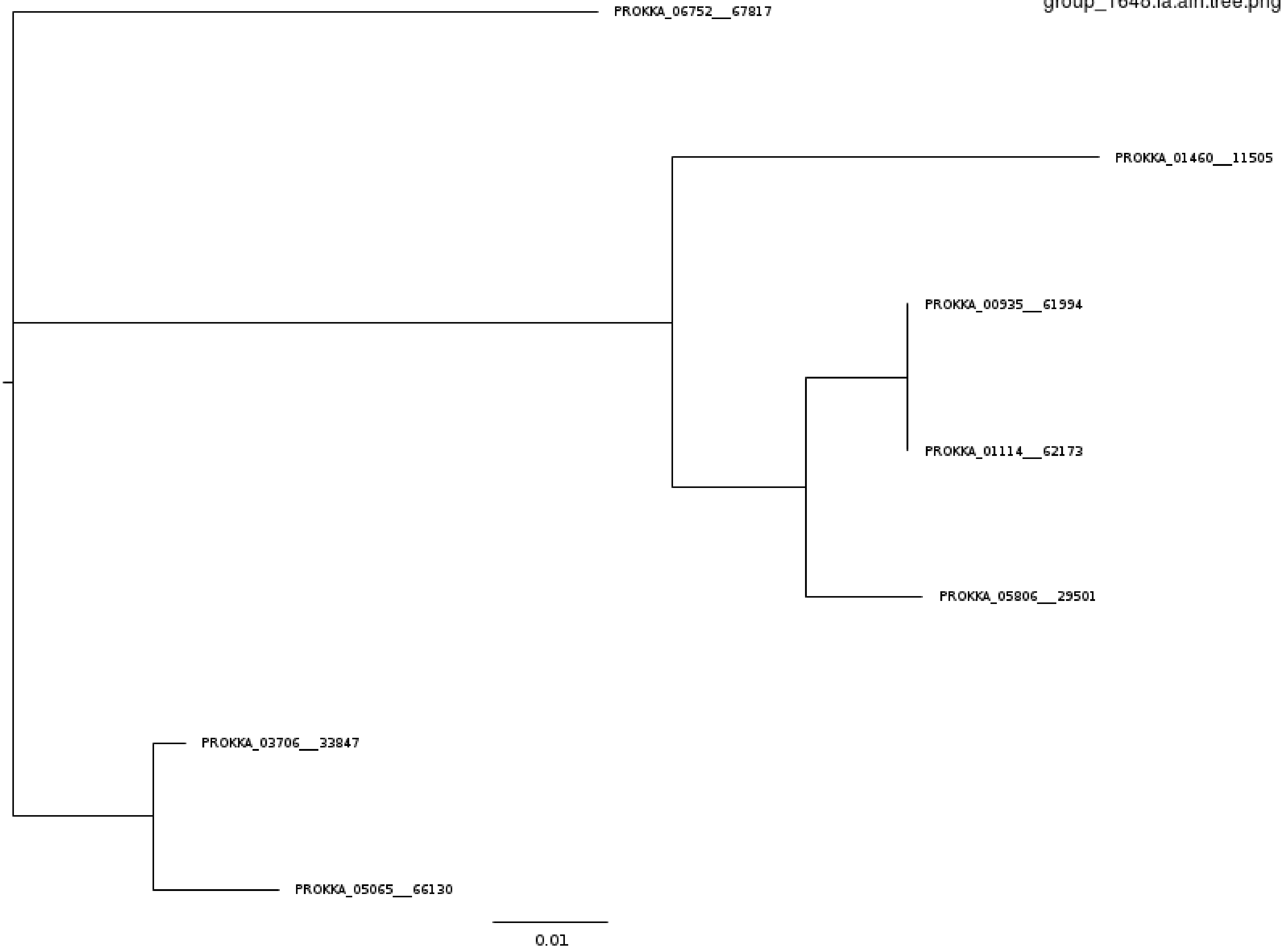

group_1649.fa.aln.tree.png

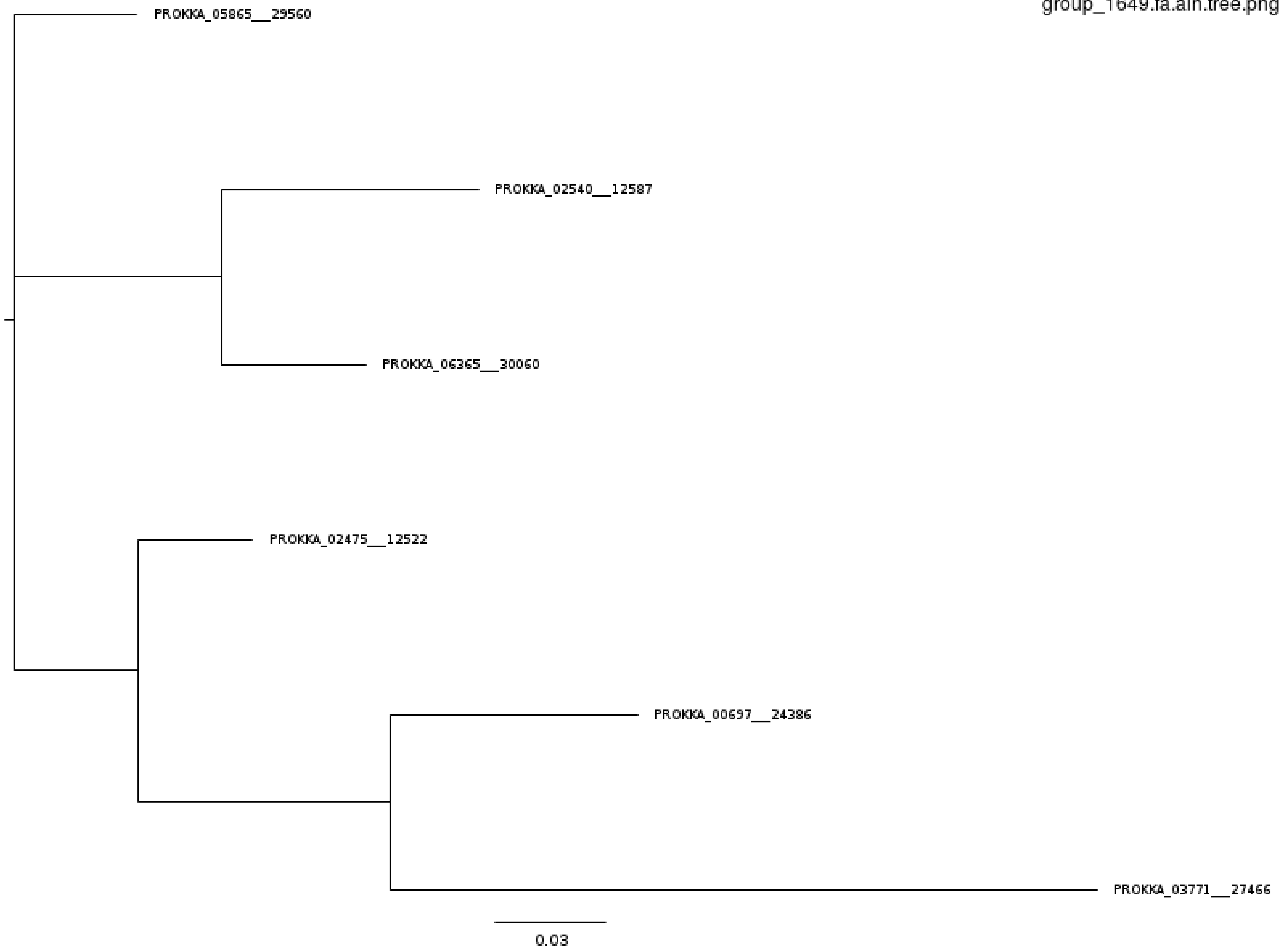

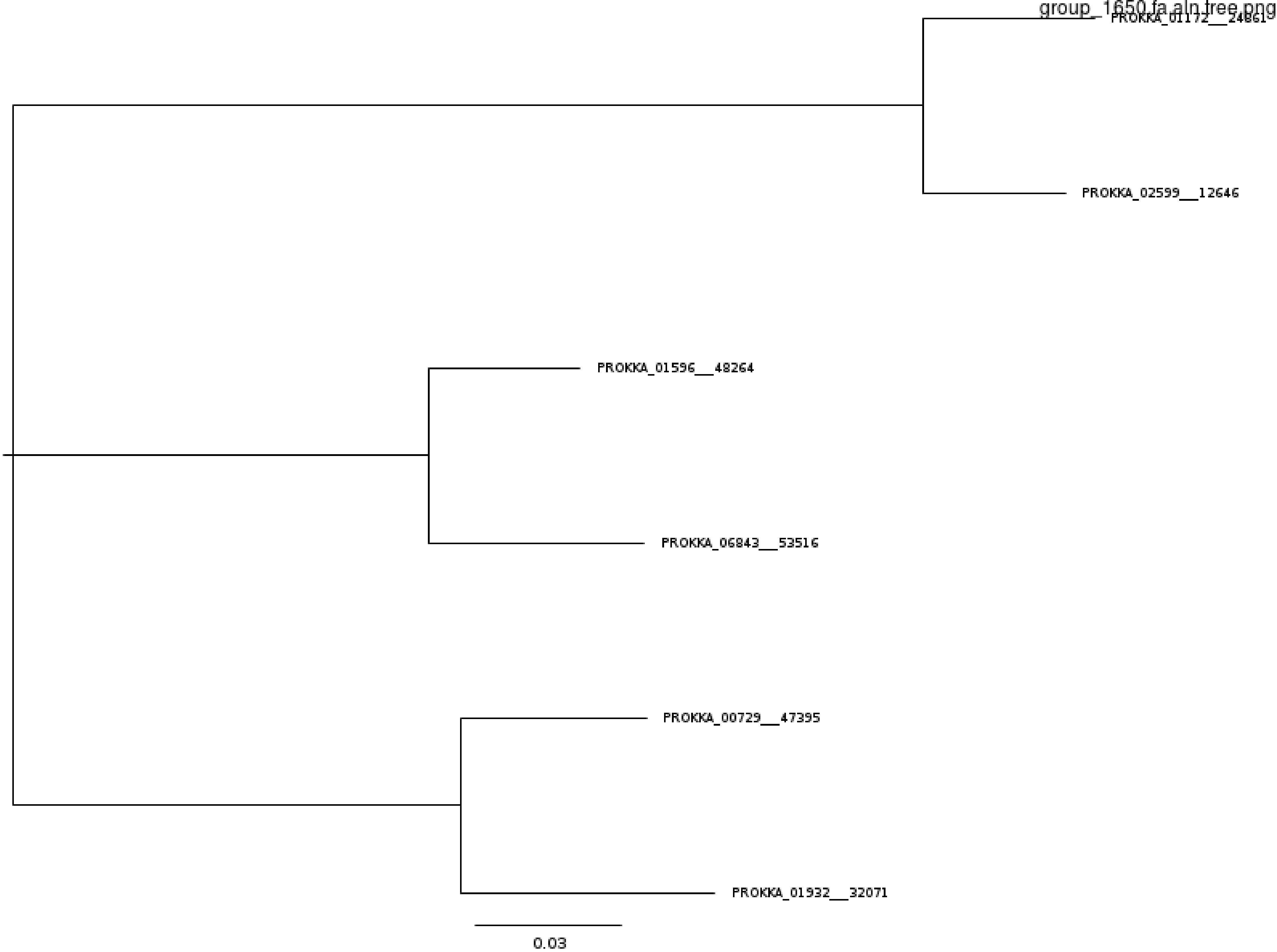
group_1650.fa.aln.tree.png
PROKKA_01172___24861
PROKKA_02599___12646
PROKKA_01596___48264
PROKKA_06843___53516
PROKKA_00729___47395
PROKKA_01932___32071
0.03

group_1653.fa.aln.tree.png

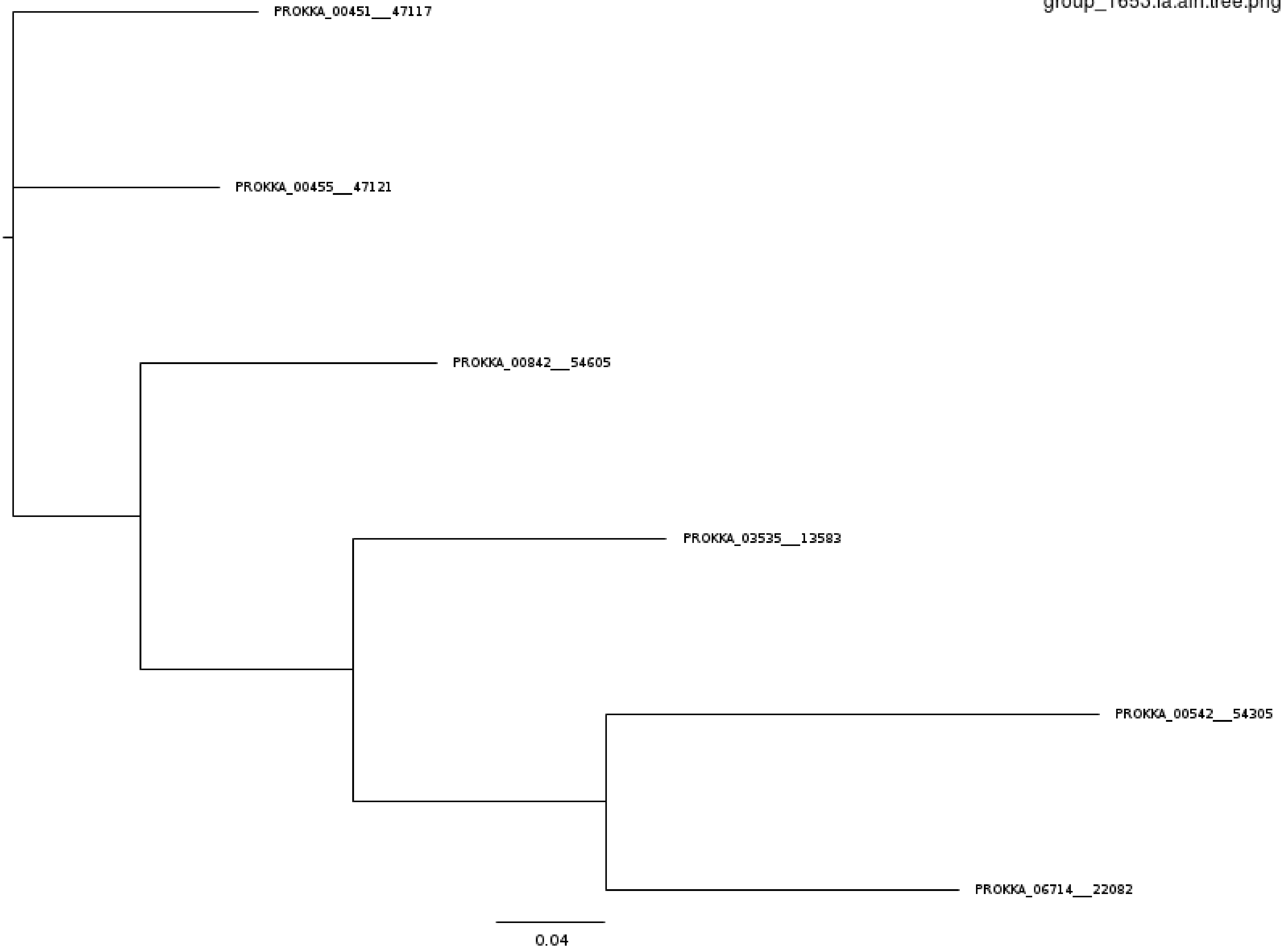

group_1654.fa.aln.tree.png

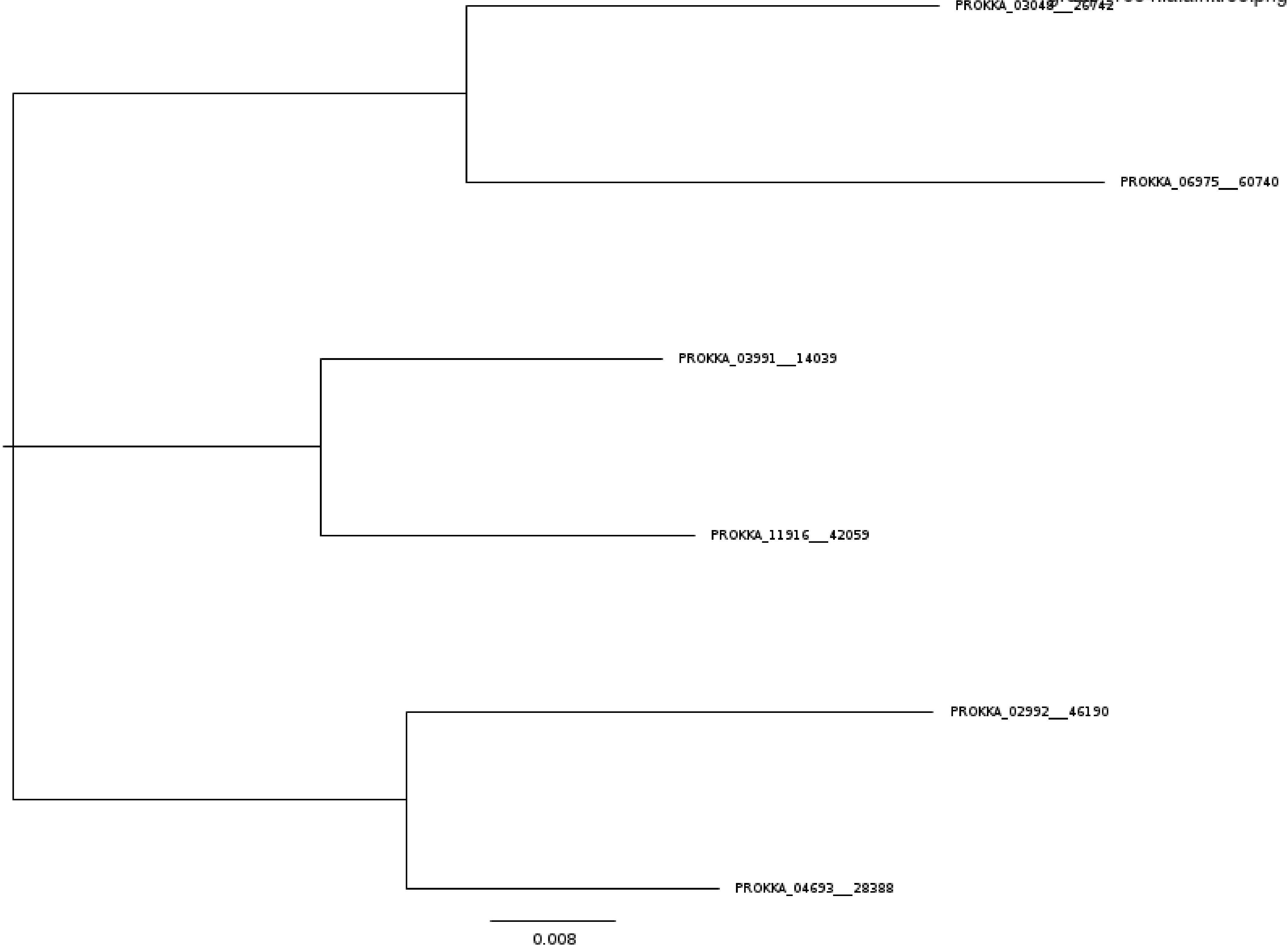

group_1656.fa.aln.tree.png

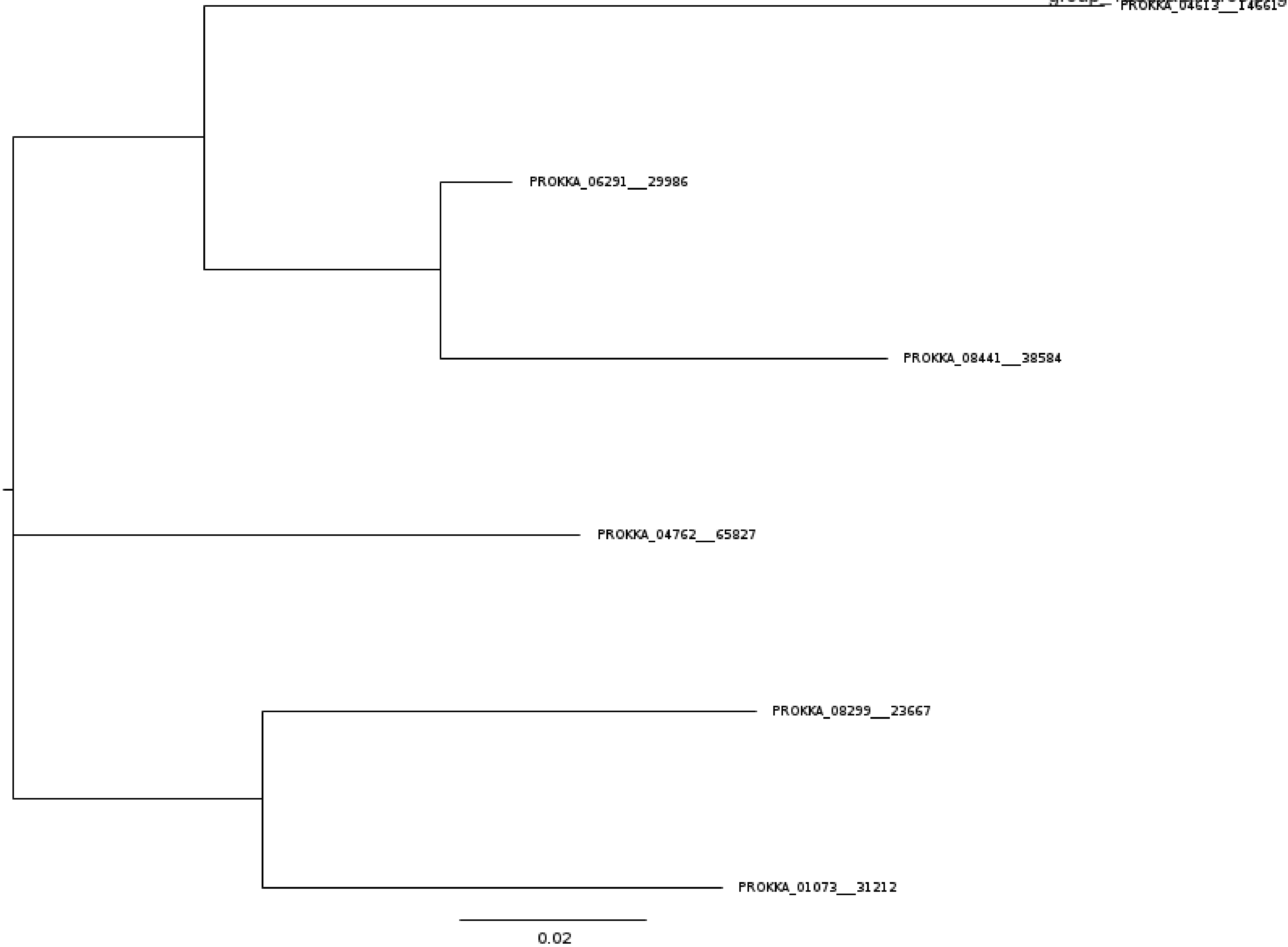

group_1657.fa.aln.tree.png

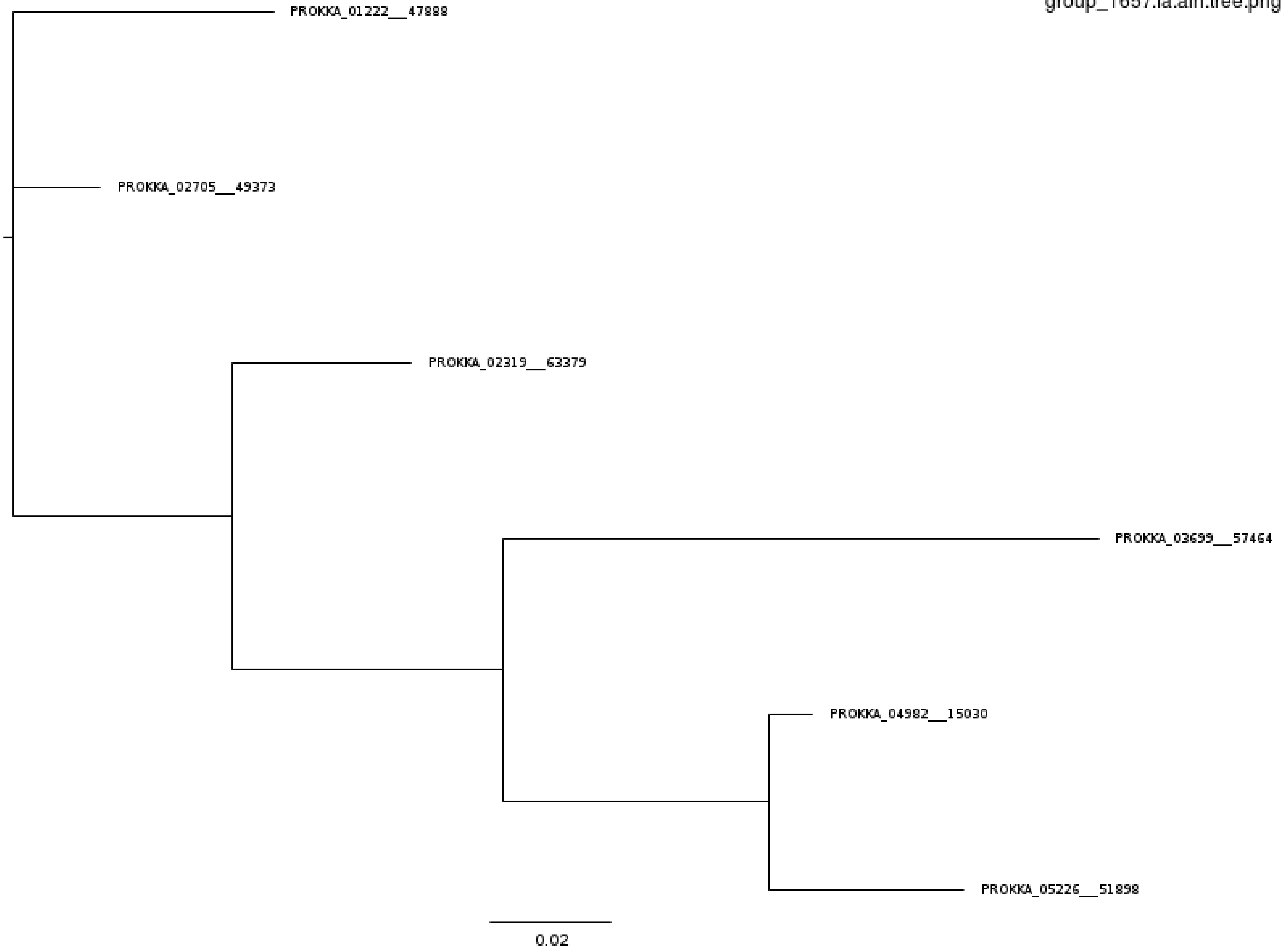

group_1658.fa.aln.tree.png

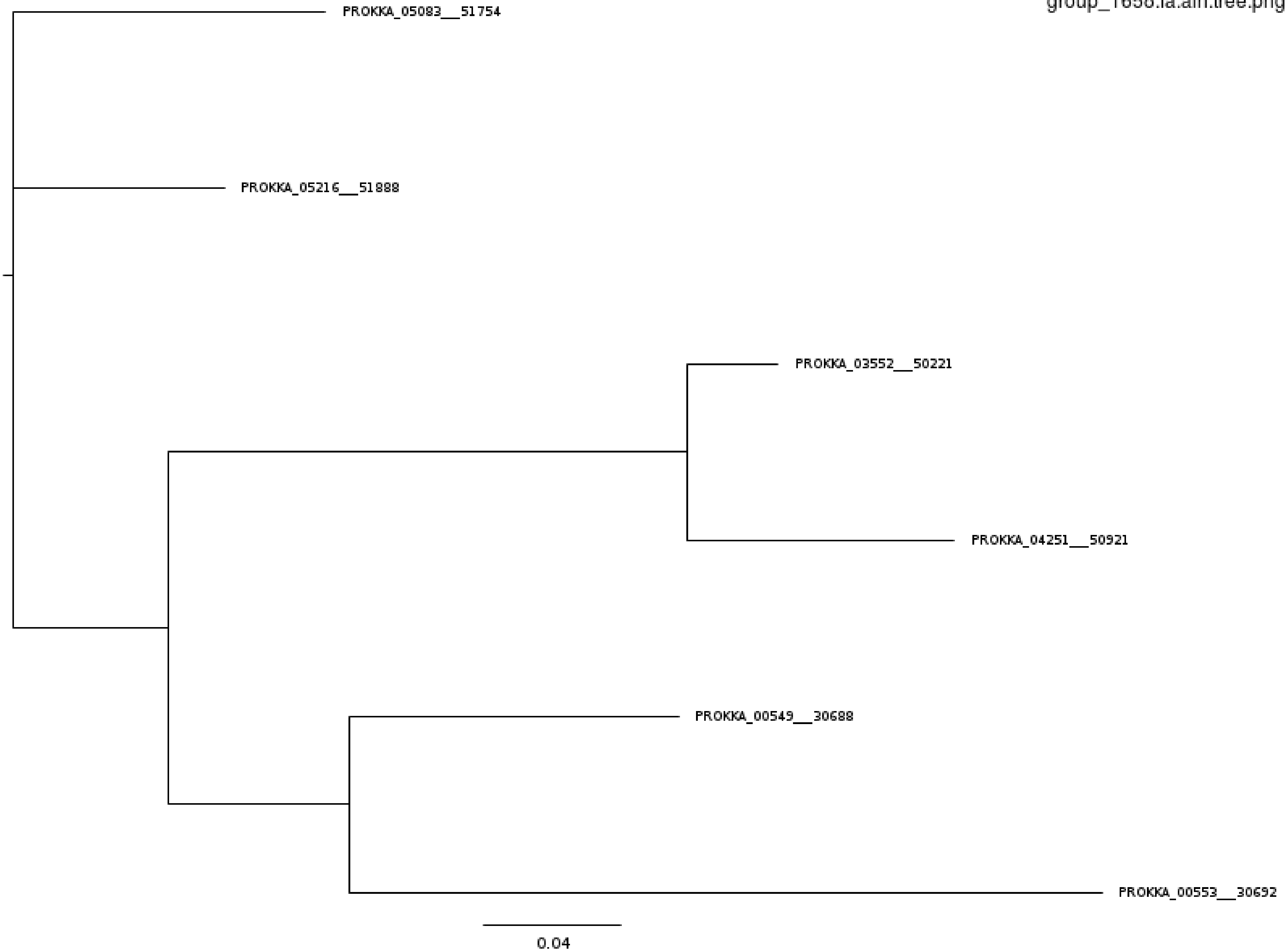

group_1659.fa.aln.tree.png

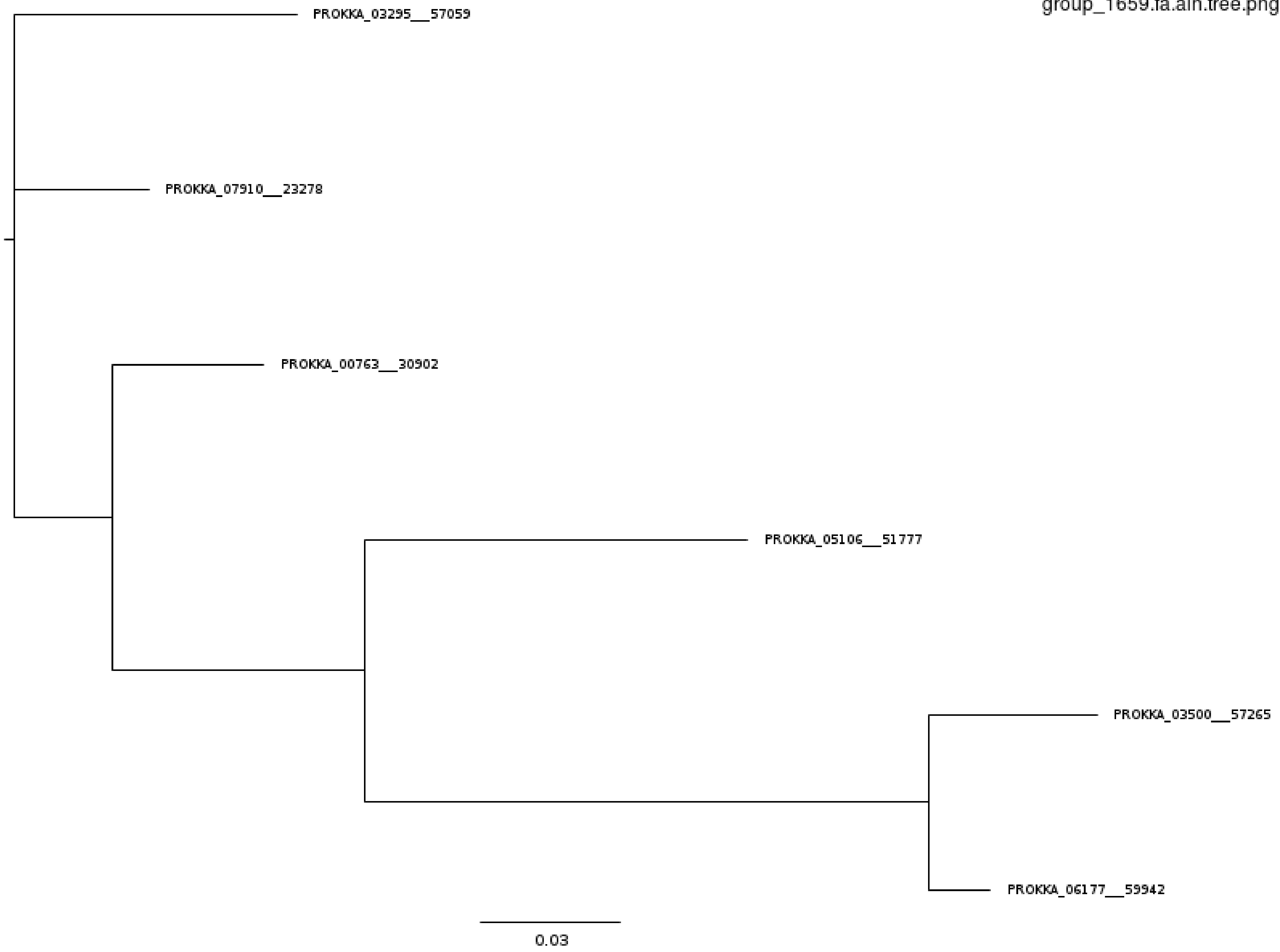

group_1660.fa.aln.tree.png

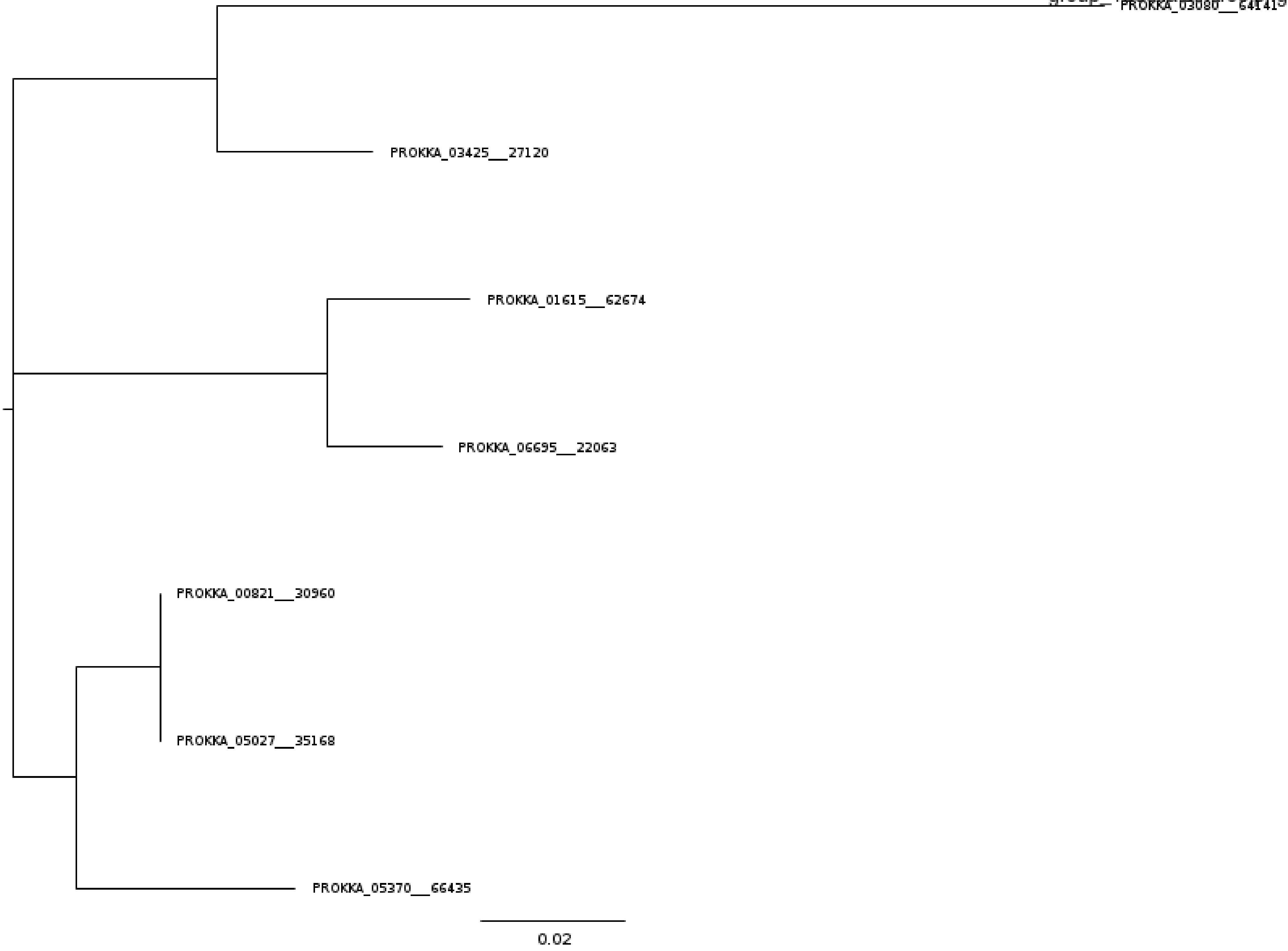

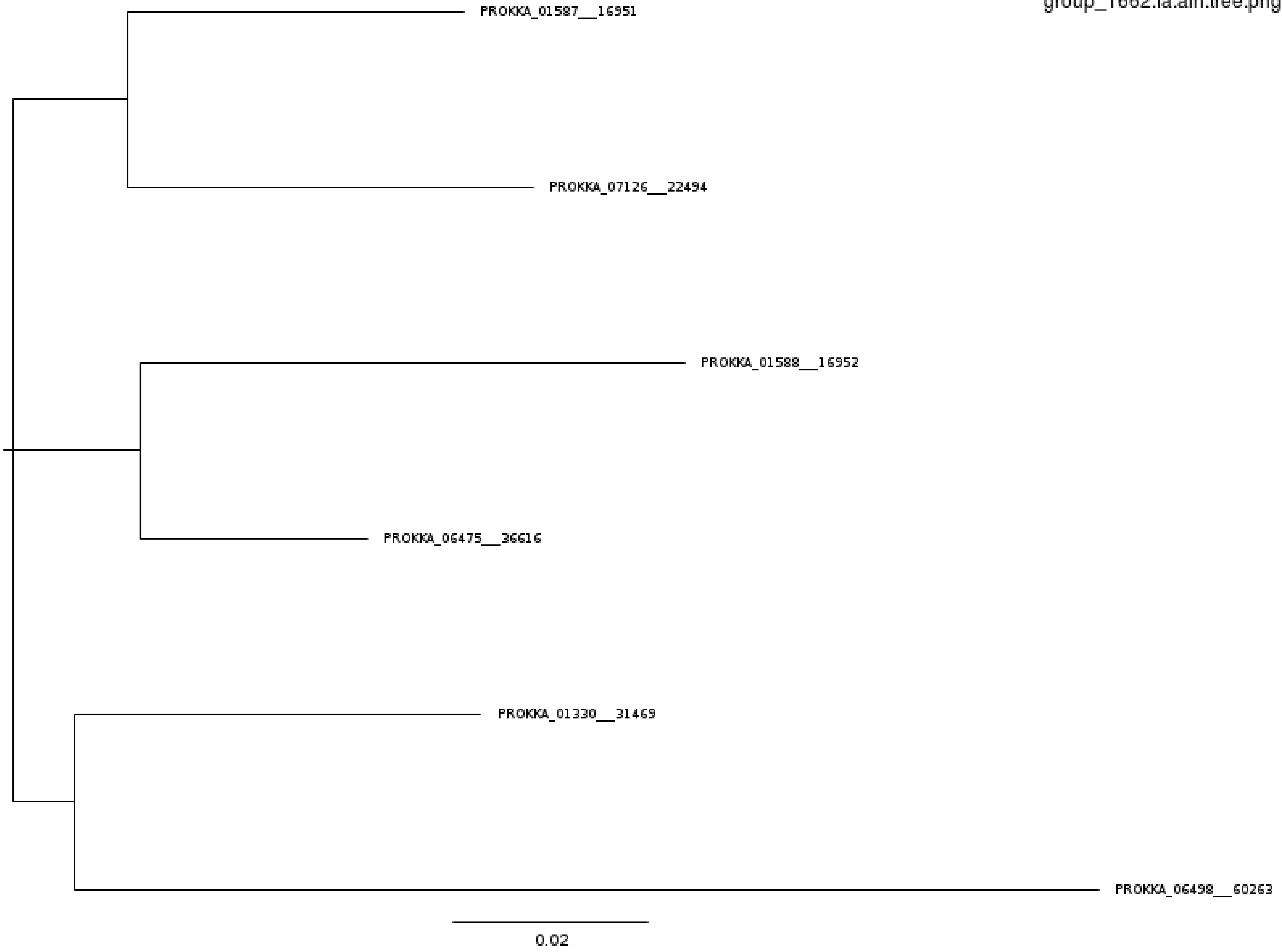
PROKKA_01587___16951
PROKKA_07126___22494
PROKKA_01588___16952
PROKKA_06475___36616
PROKKA_01330___31469
PROKKA_06498___60263
0.02

group_1663.fa.aln.tree.png

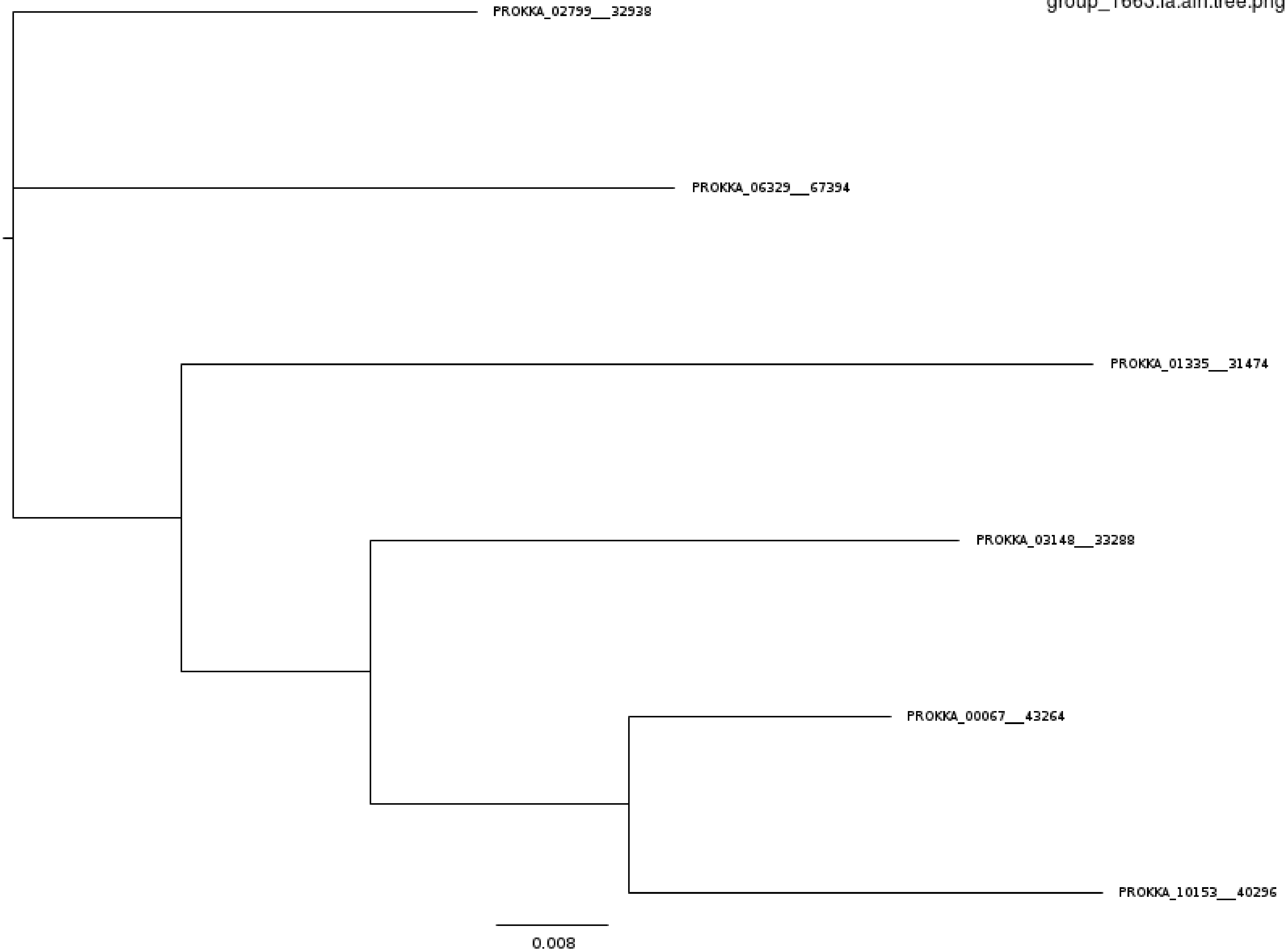

group_1669.fa.aln.tree.png

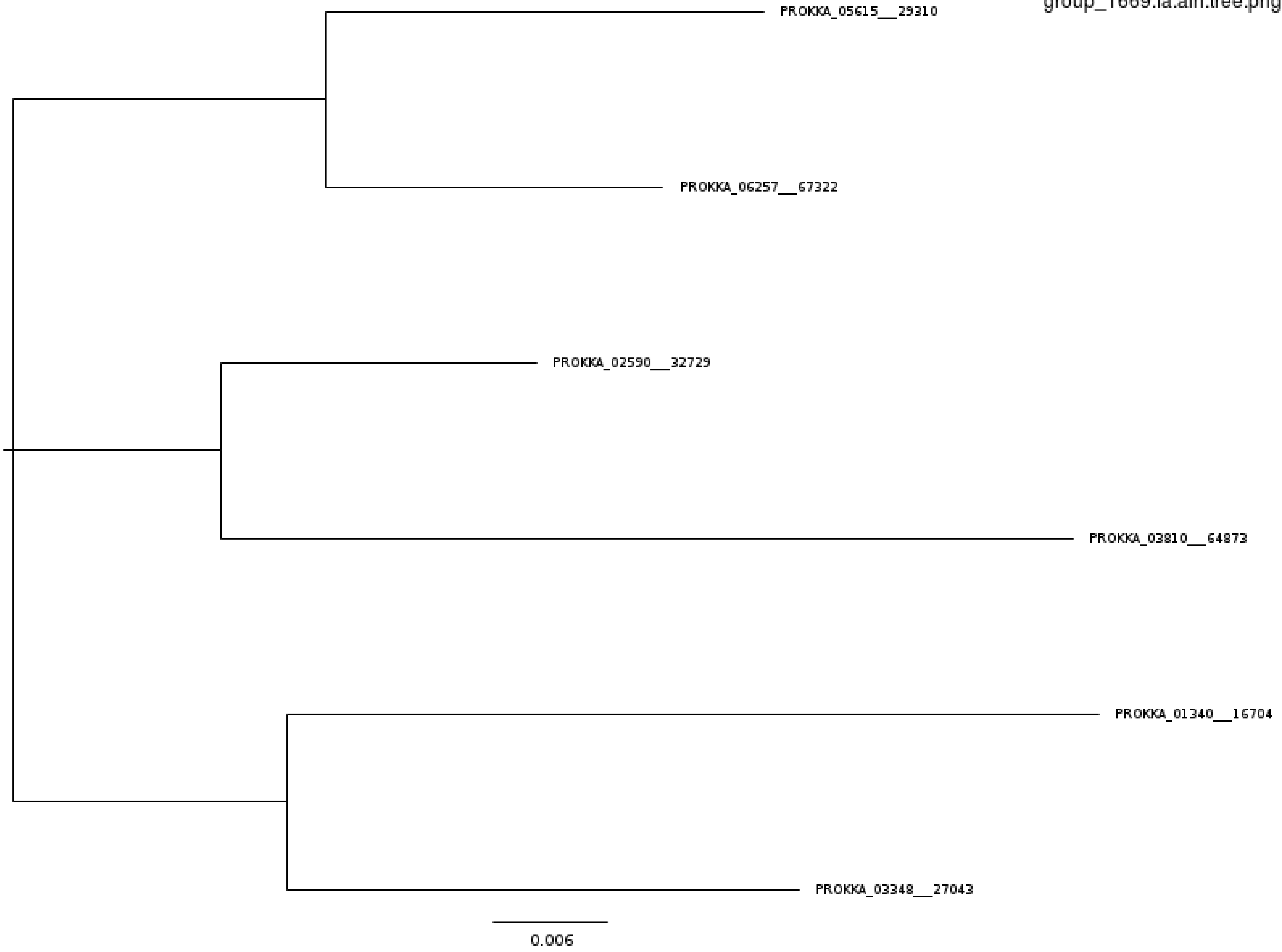

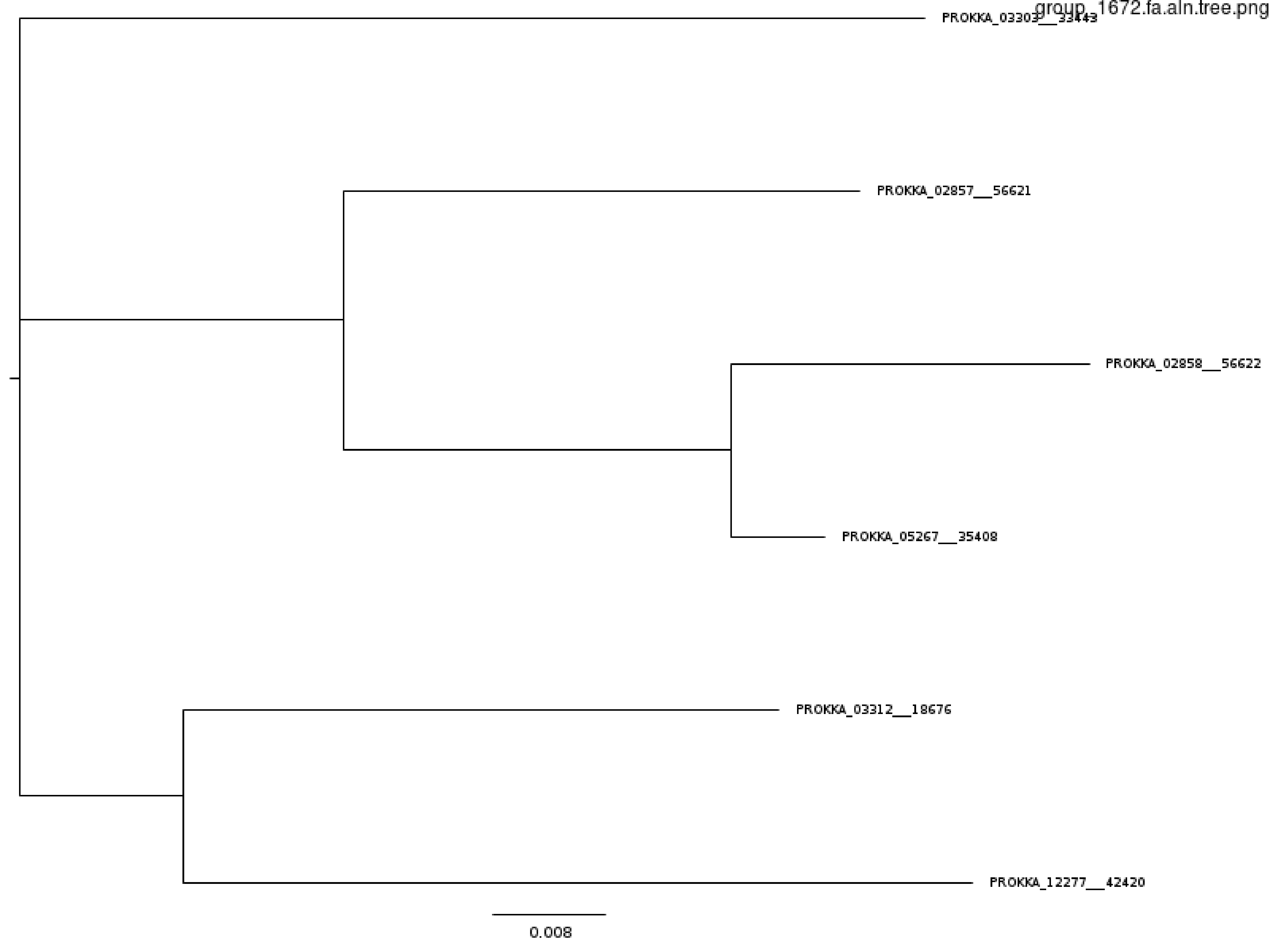

group_1672.fa.aln.tree.png
PROKKA_03303___33443
PROKKA_02857___56621
PROKKA_02858___56622
PROKKA_05267___35408
PROKKA_03312___18676
PROKKA_12277___42420
0.008

group_1673.fa.aln.tree.png

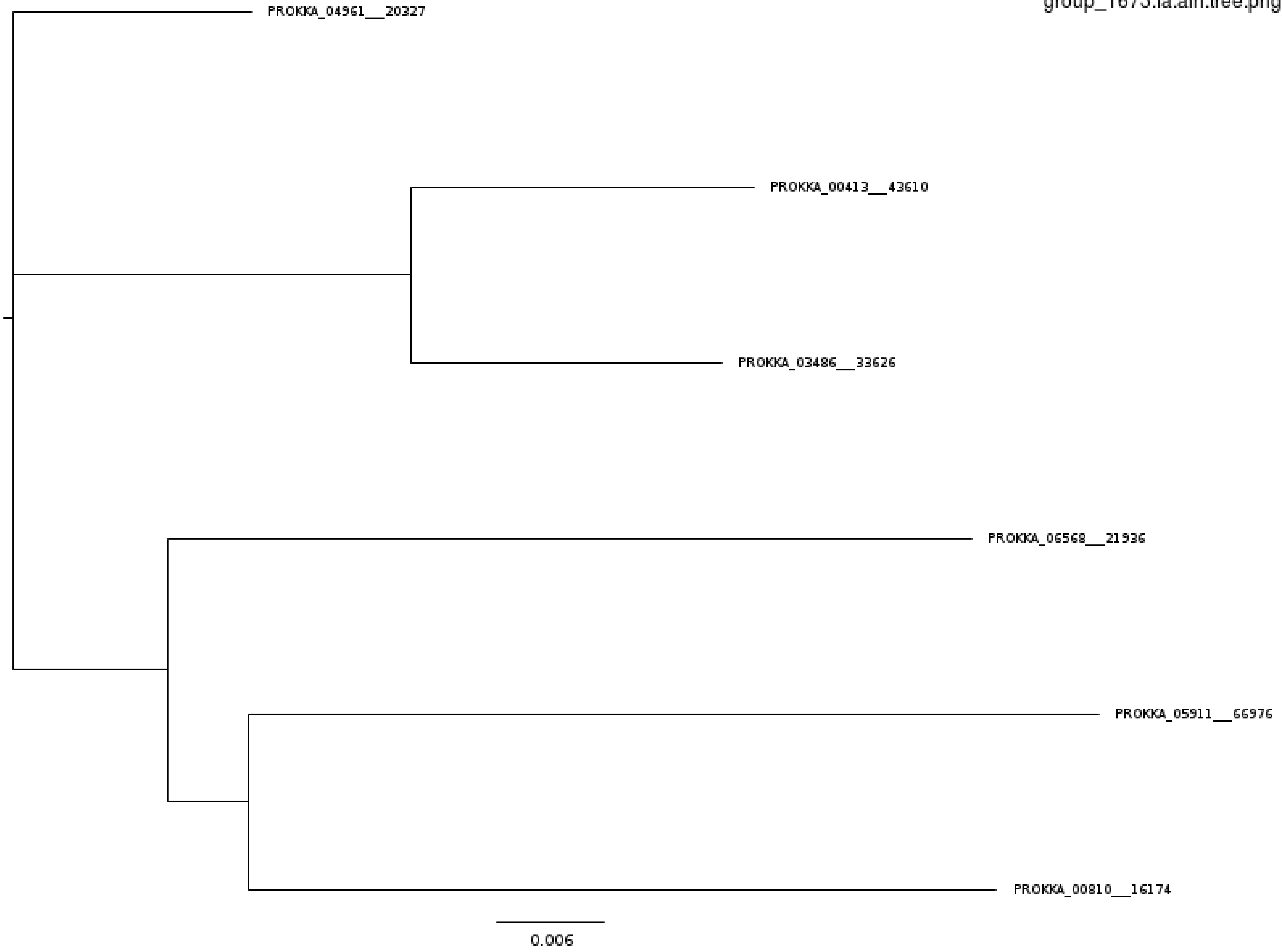

group_1674.fa.aln.tree.png

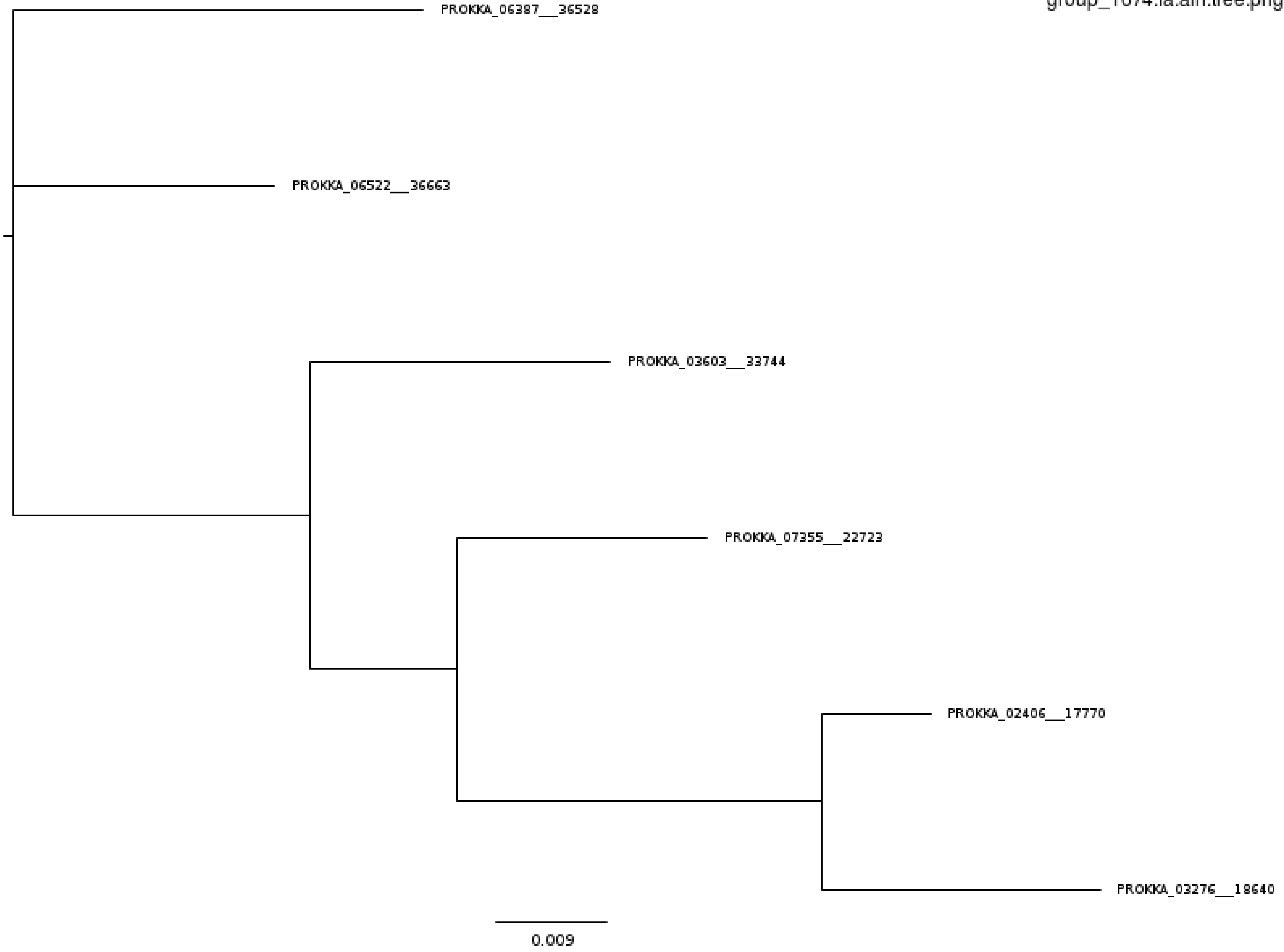

group_1677.fa.aln.tree.png

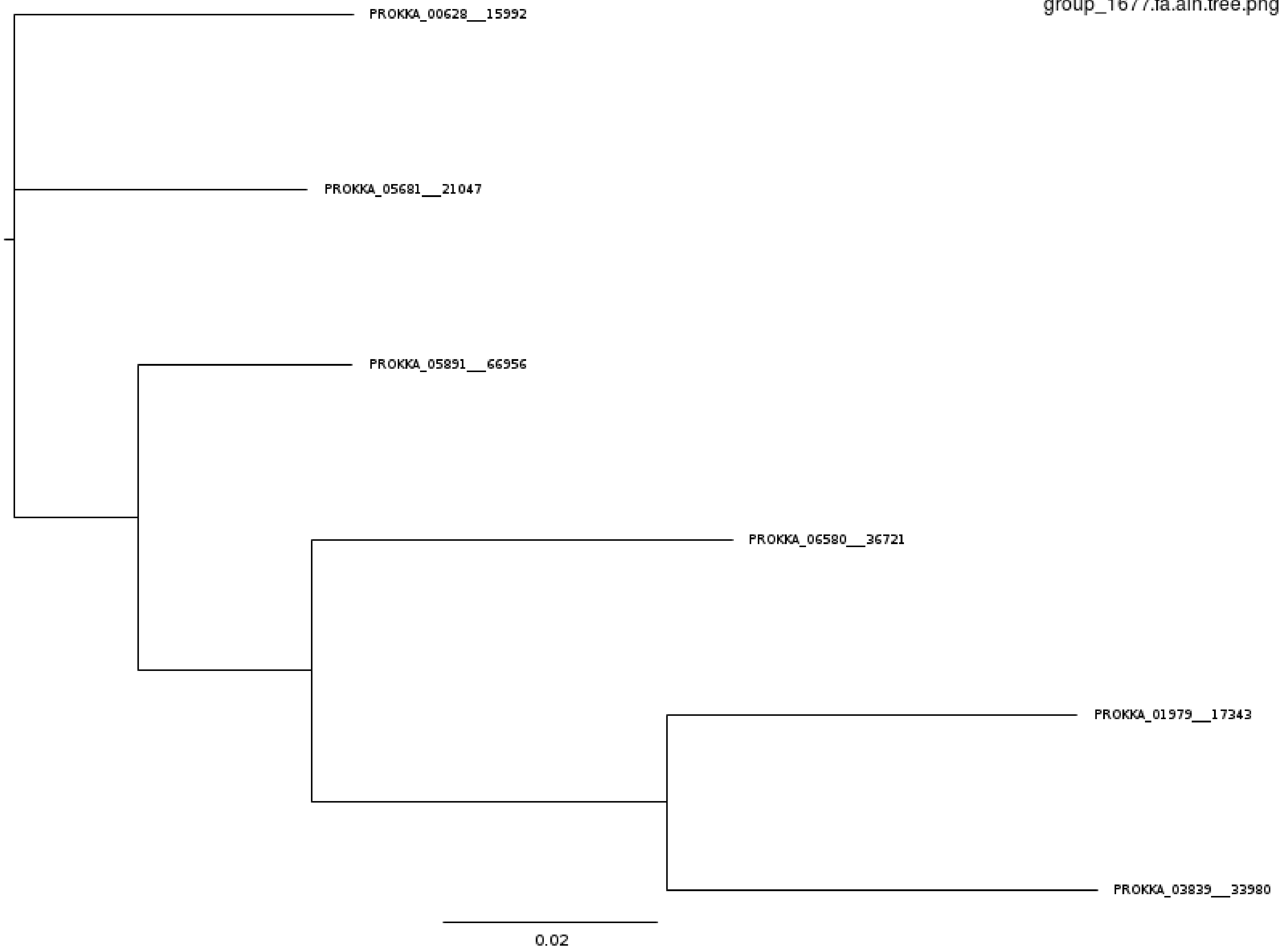

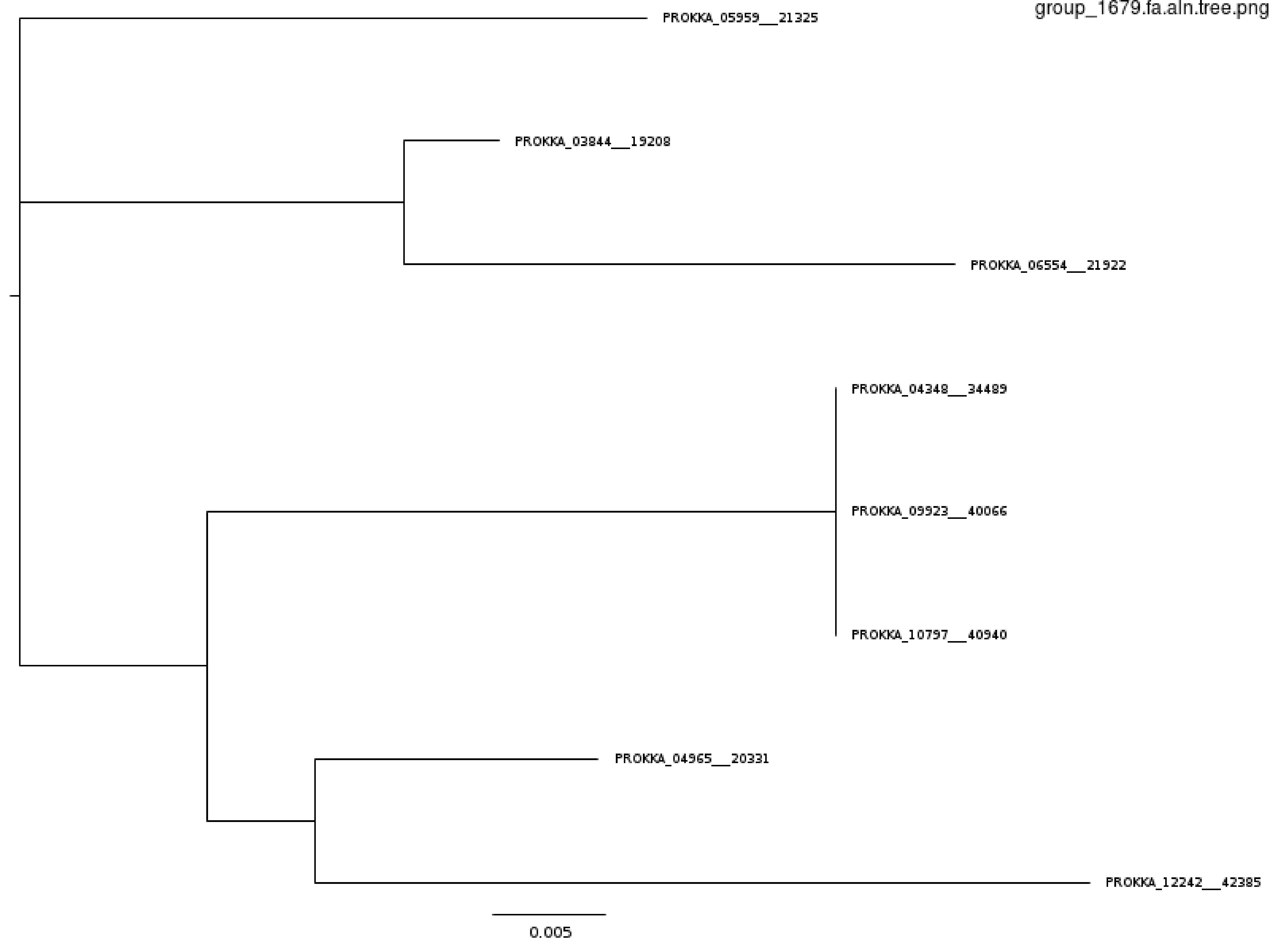
group_1679.fa.aln.tree.png
PROKKA_05959__21325
PROKKA_03844__19208
PROKKA_06554__21922
PROKKA_04348__34489
PROKKA_09923__40066
PROKKA_10797__40940
PROKKA_04965__20331
PROKKA_12242__42385
0.005

group_167.fa.aln.tree.png

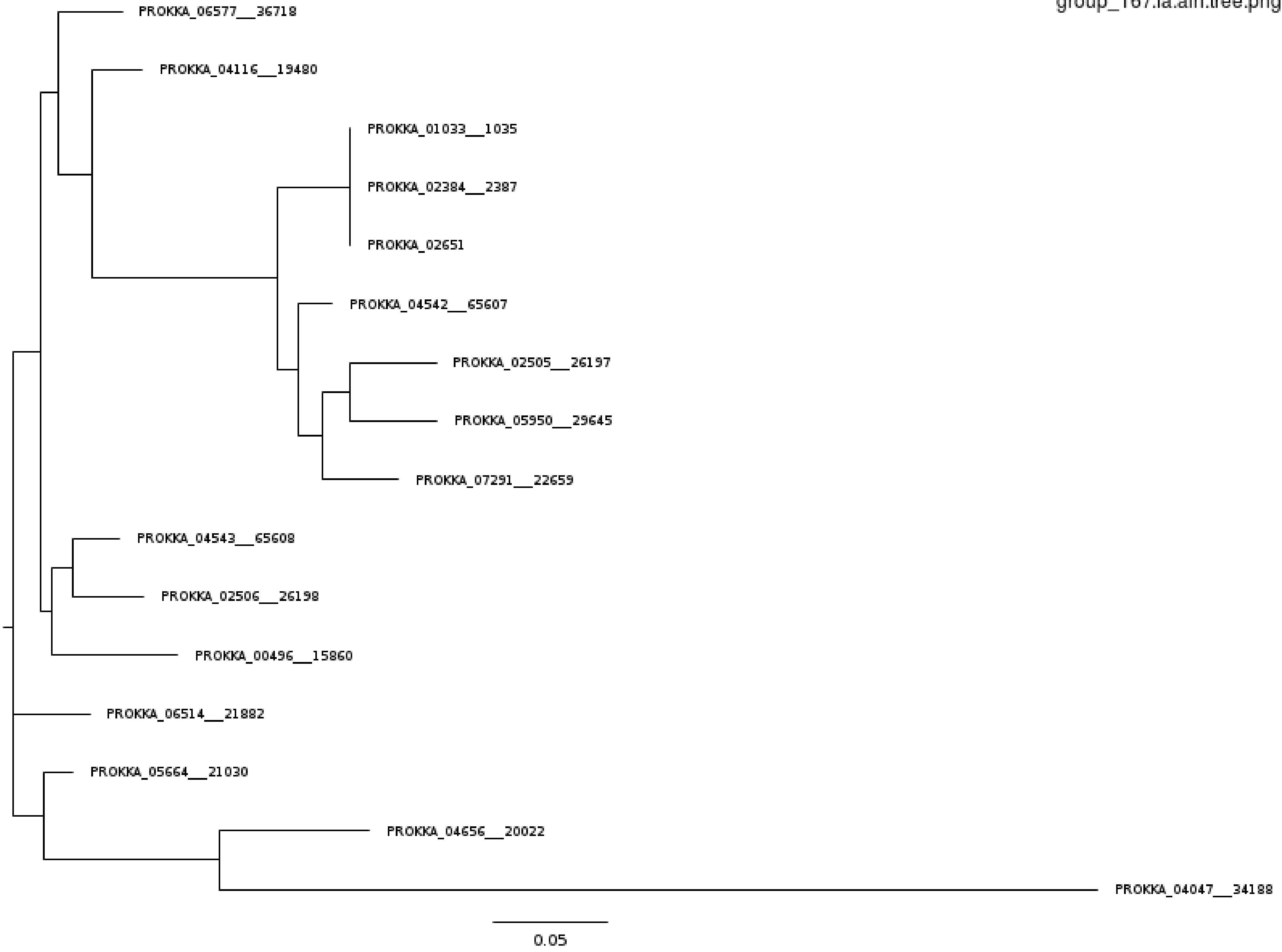

group_1681.fa.aln.tree.png

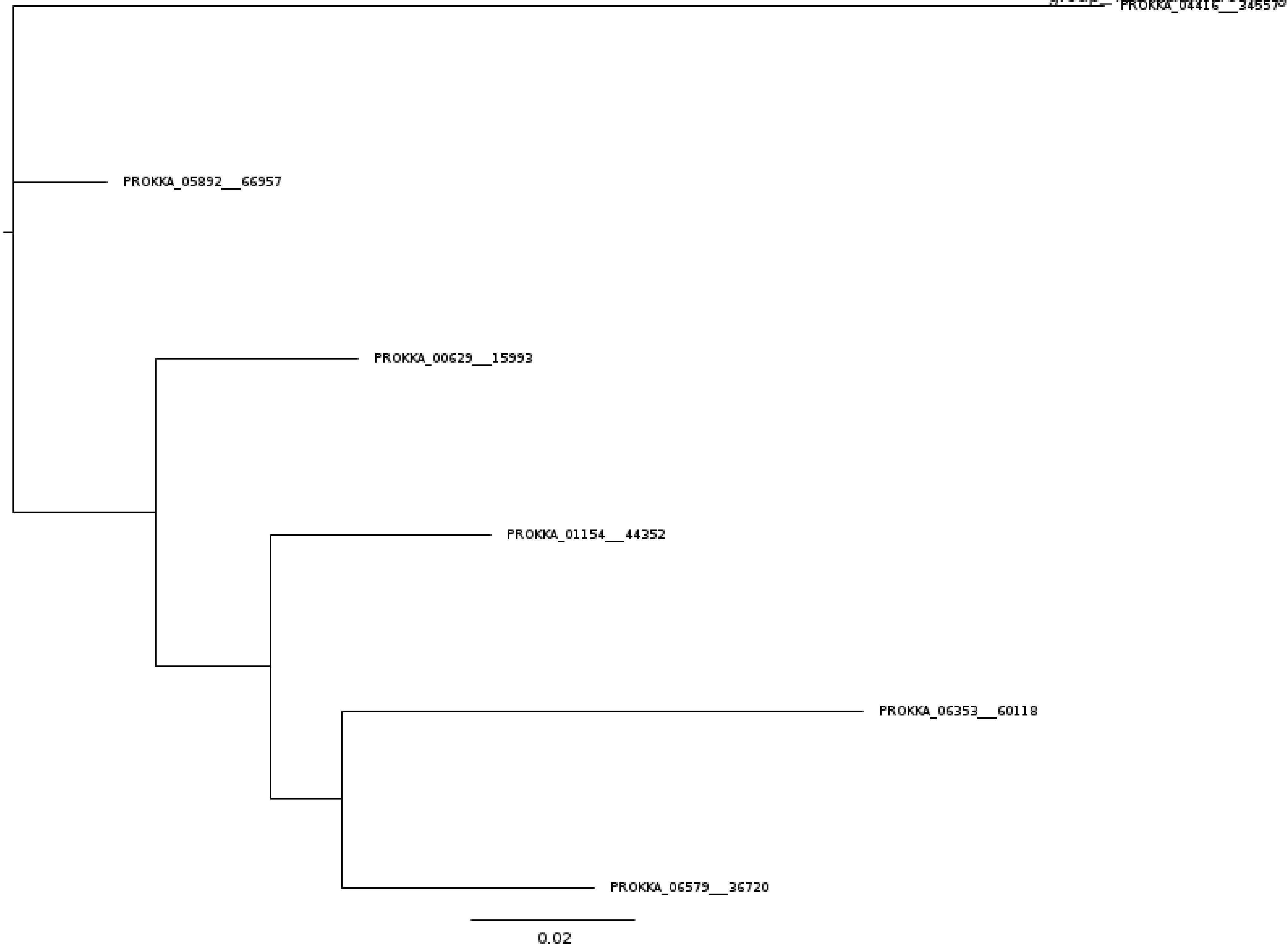

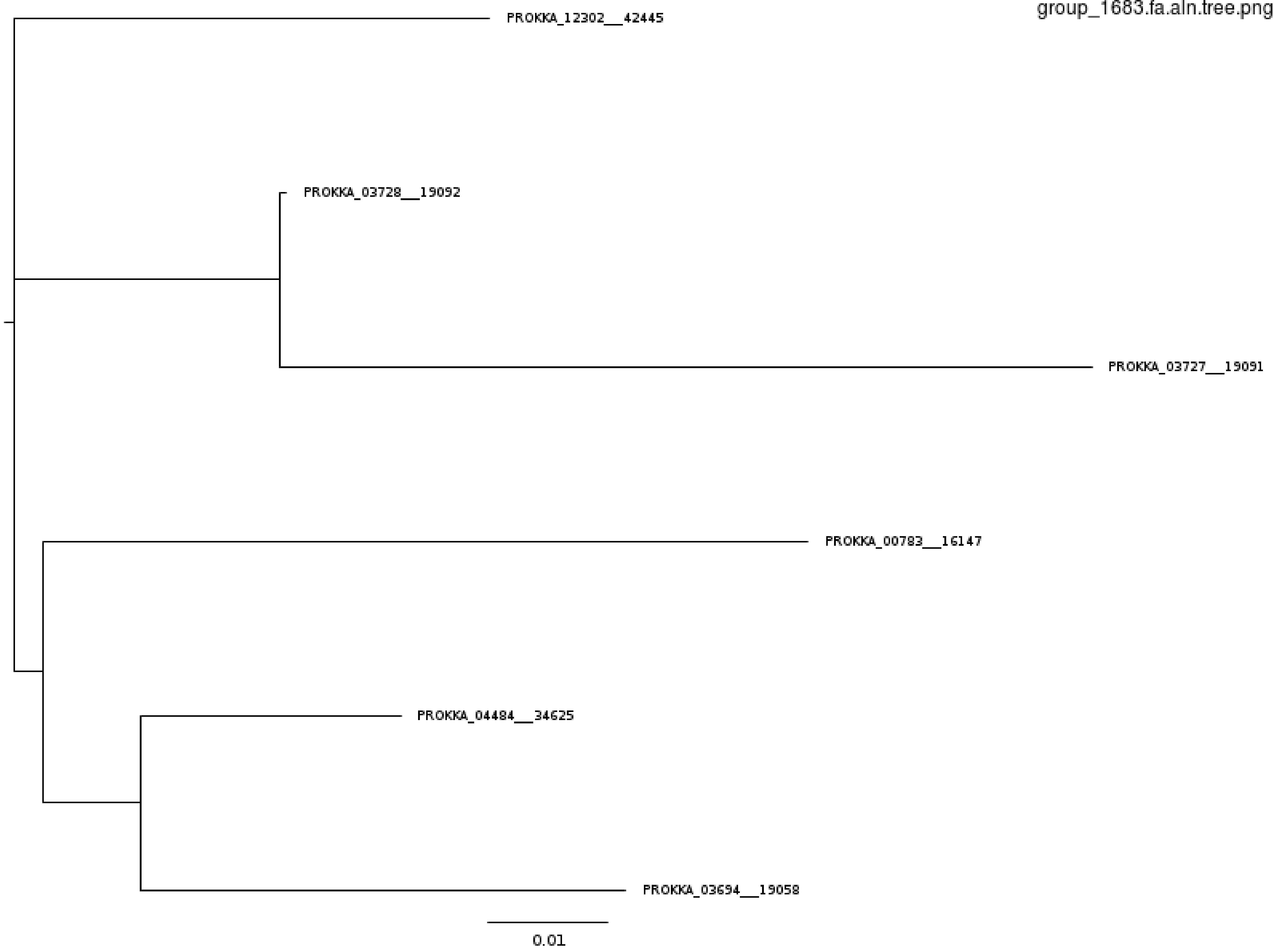
group_1683.fa.aln.tree.png
PROKKA_12302___42445
PROKKA_03728___19092
PROKKA_03727___19091
PROKKA_00783___16147
PROKKA_04484___34625
PROKKA_03694___19058
0.01

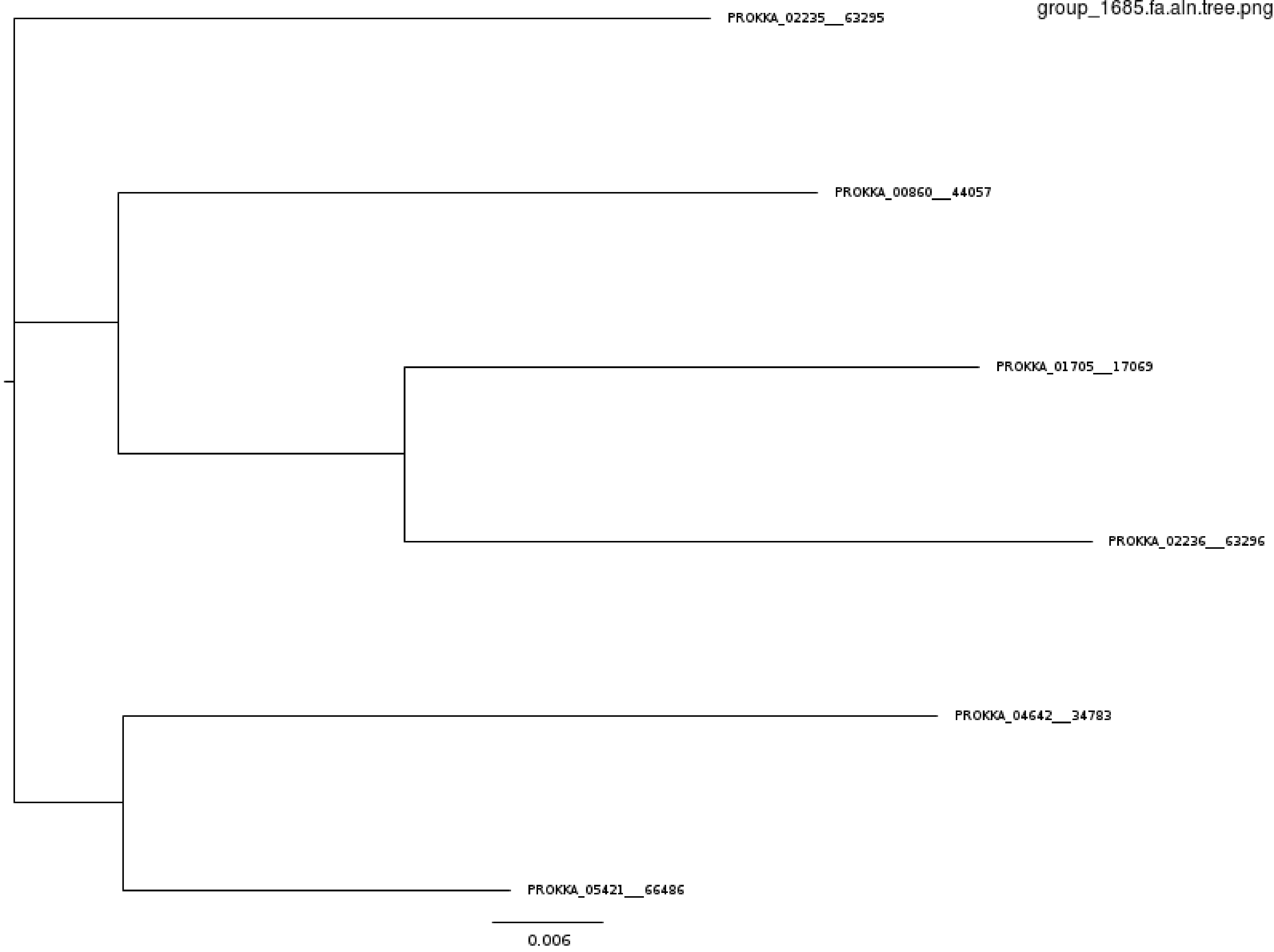
group_1685.fa.aln.tree.png
PROKKA_02235___63295
PROKKA_00860___44057
PROKKA_01705___17069
PROKKA_02236___63296
PROKKA_04642___34783
PROKKA_05421___66486
0.006

group_1686.fa.aln.tree.png

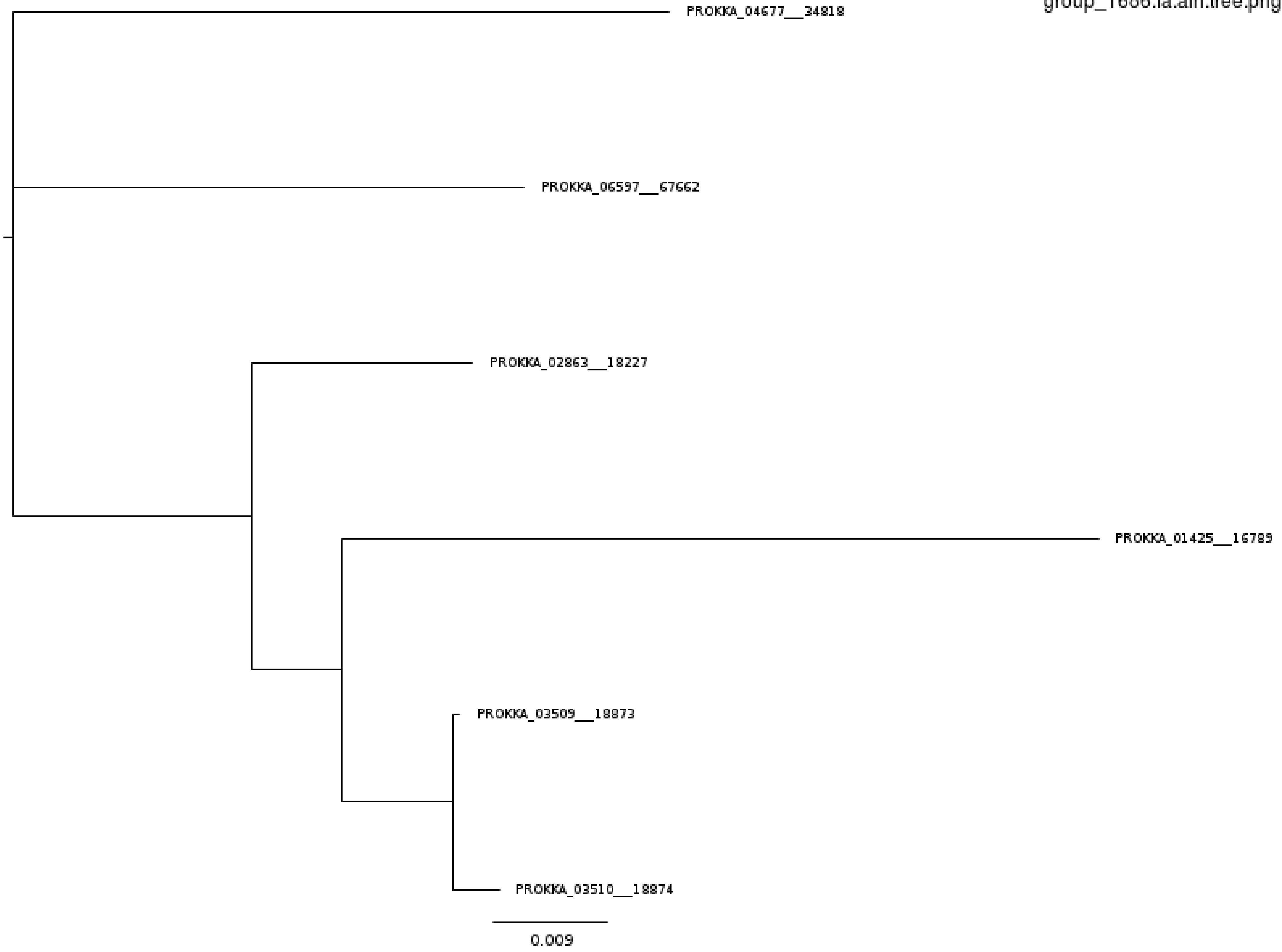

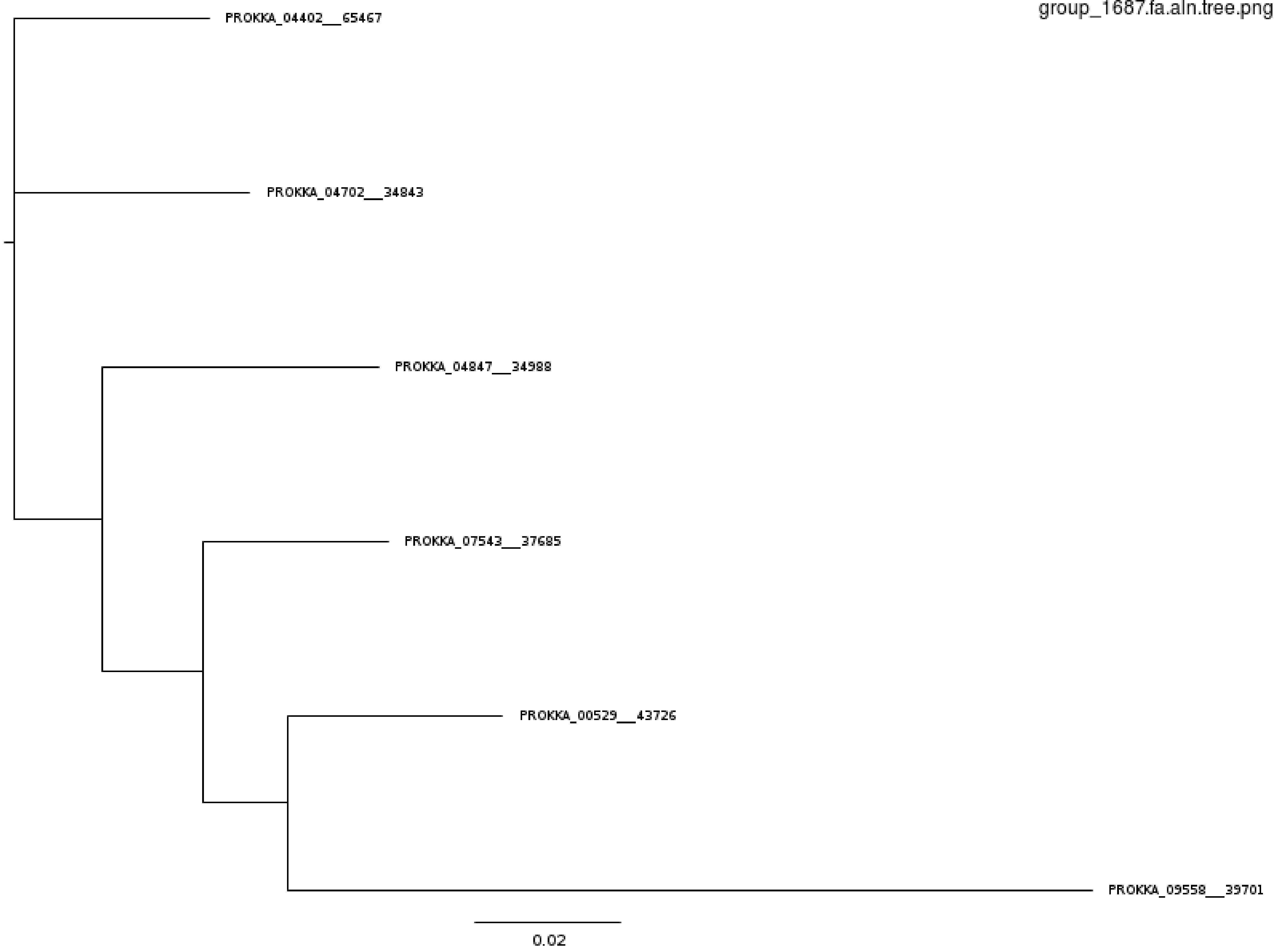
group_1687.fa.aln.tree.png
PROKKA_04402___65467
PROKKA_04702___34843
PROKKA_04847___34988
PROKKA_07543___37685
PROKKA_00529___43726
PROKKA_09558___39701
0.02

group_1689.fa.aln.tree.png

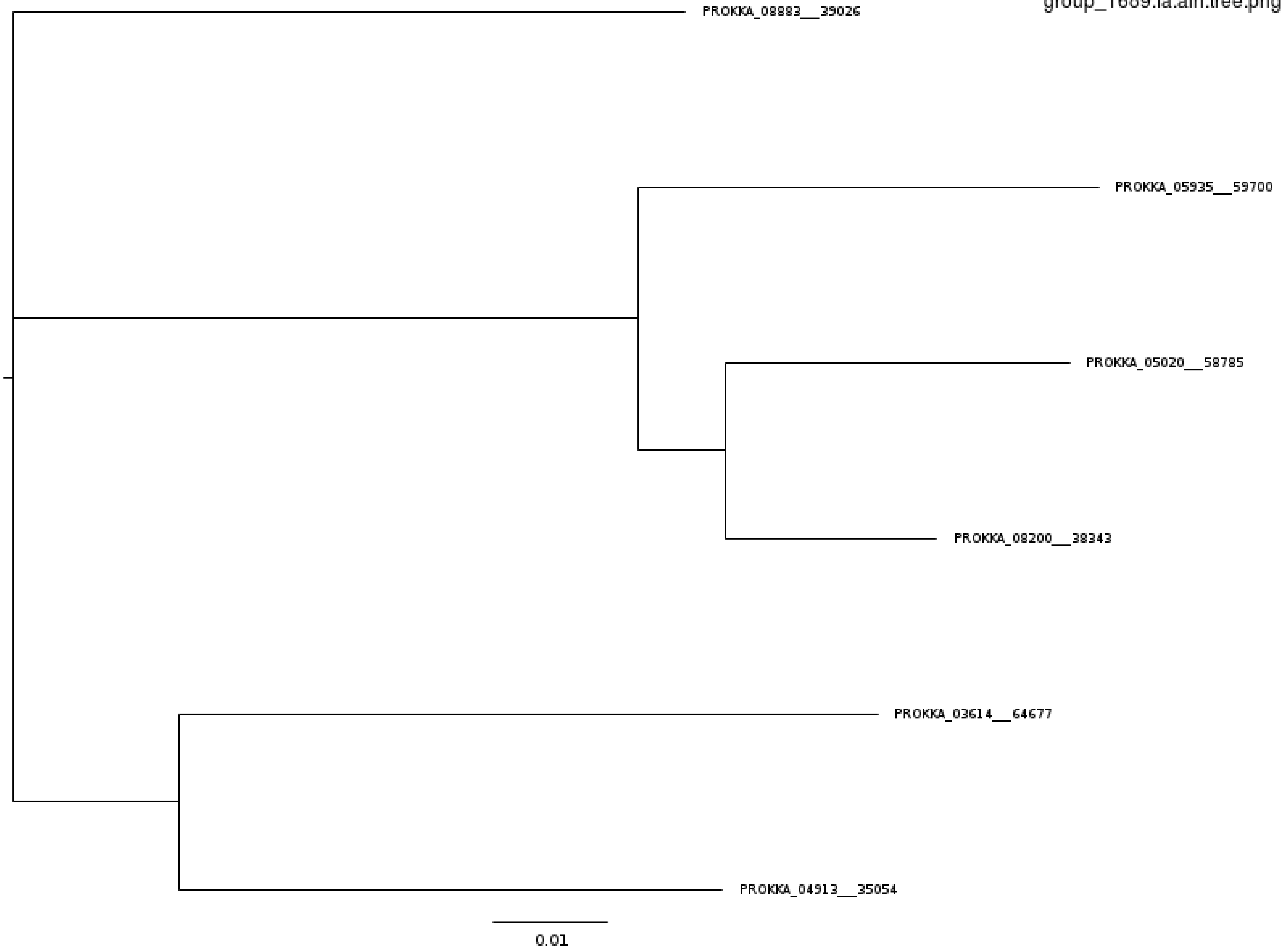

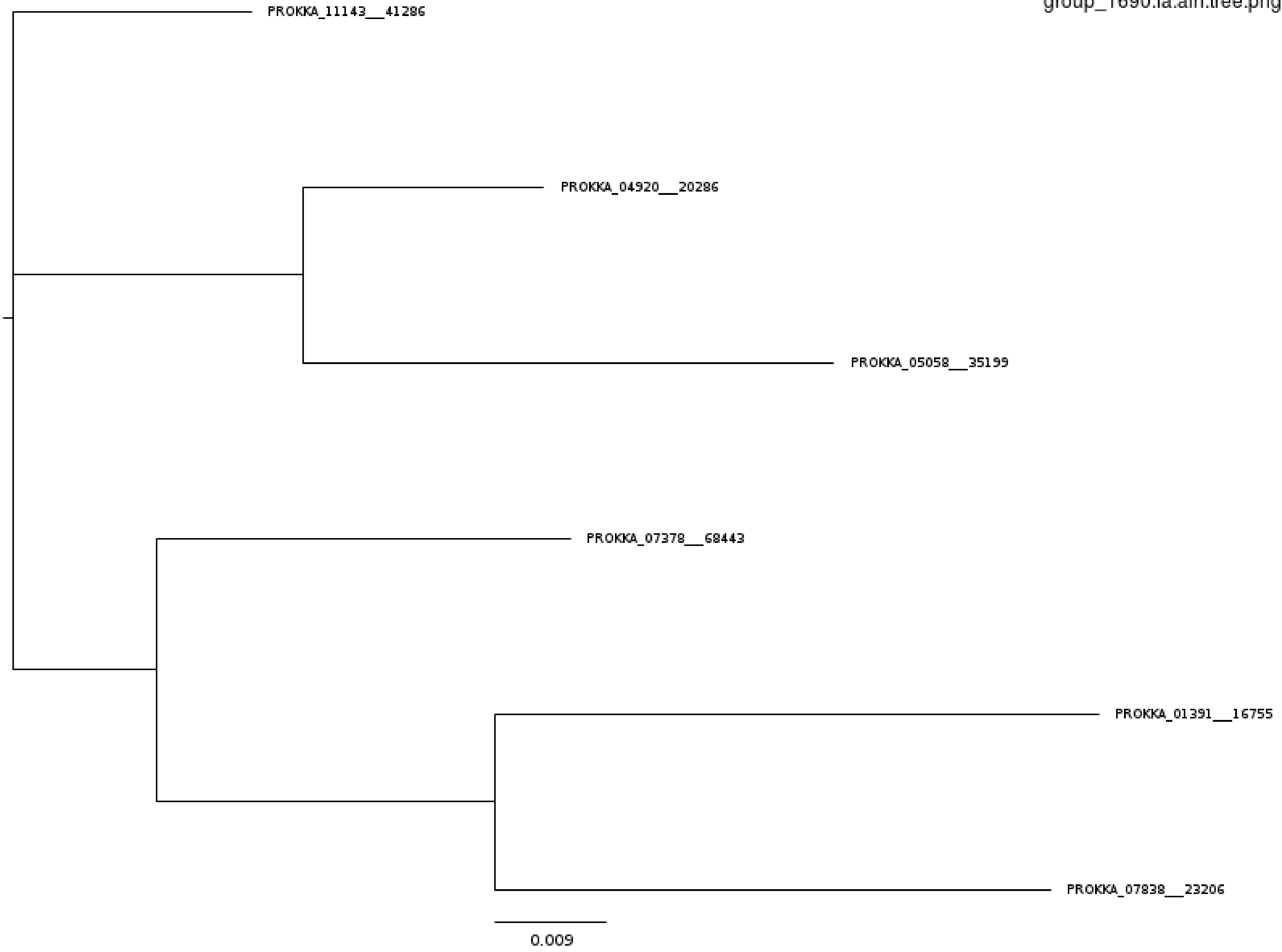
PROKKA_11143___41286
PROKKA_04920___20286
PROKKA_05058___35199
PROKKA_07378___68443
PROKKA_01391___16755
PROKKA_07838___23206
0.009

group_1692.fa.aln.tree.png

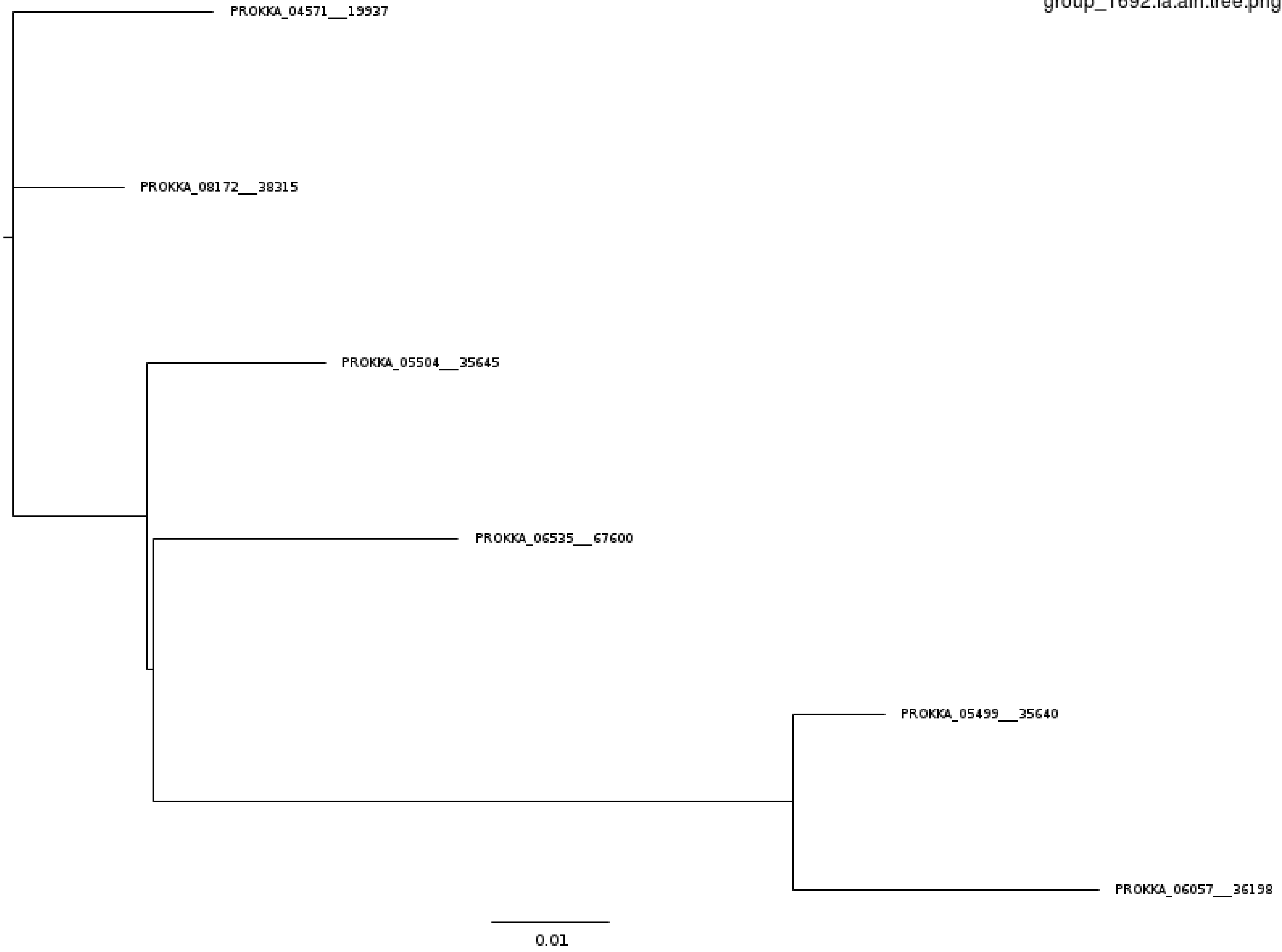

group_1697.fa.aln.tree.png

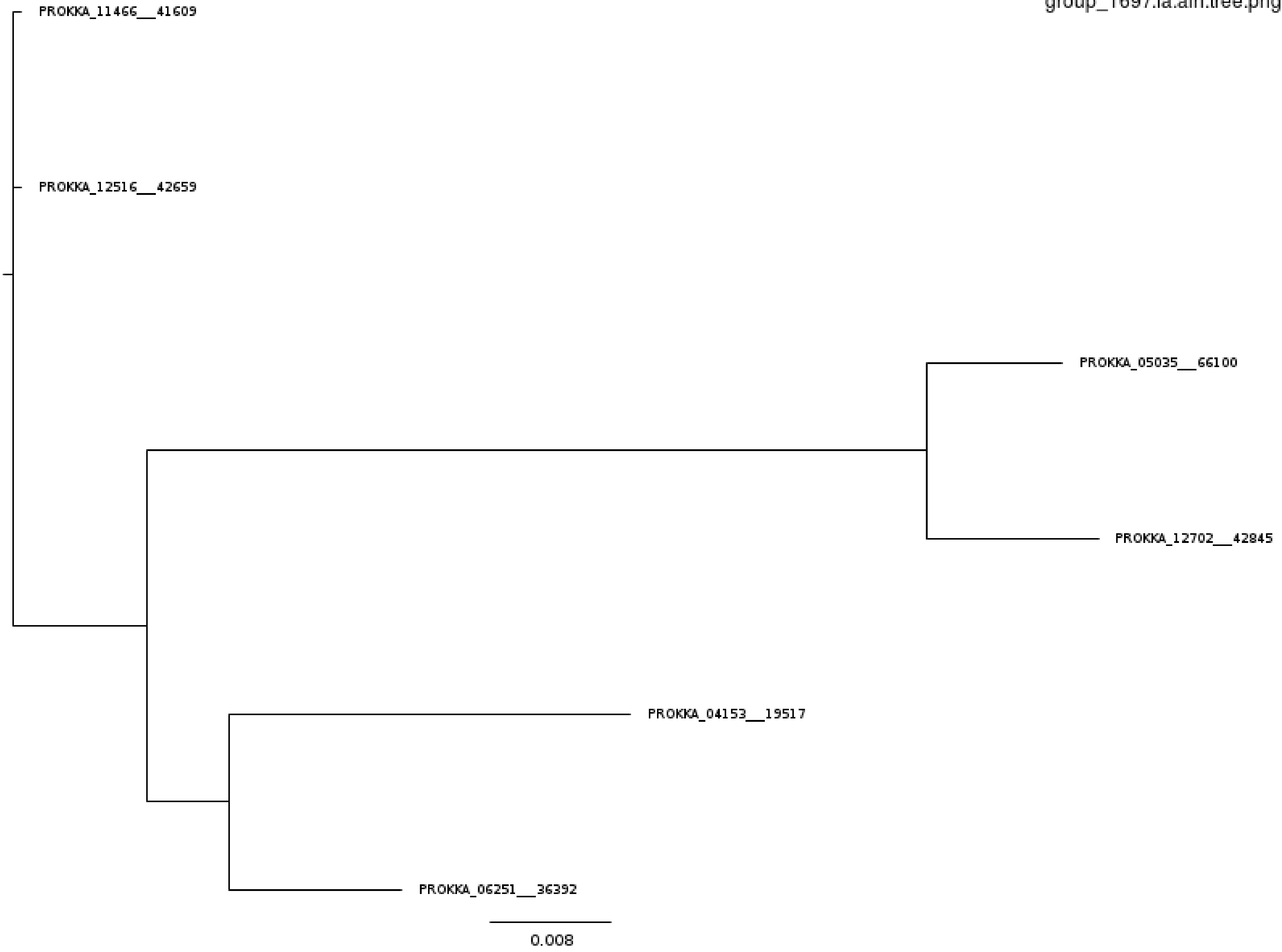

group_1698.fa.aln.tree.png

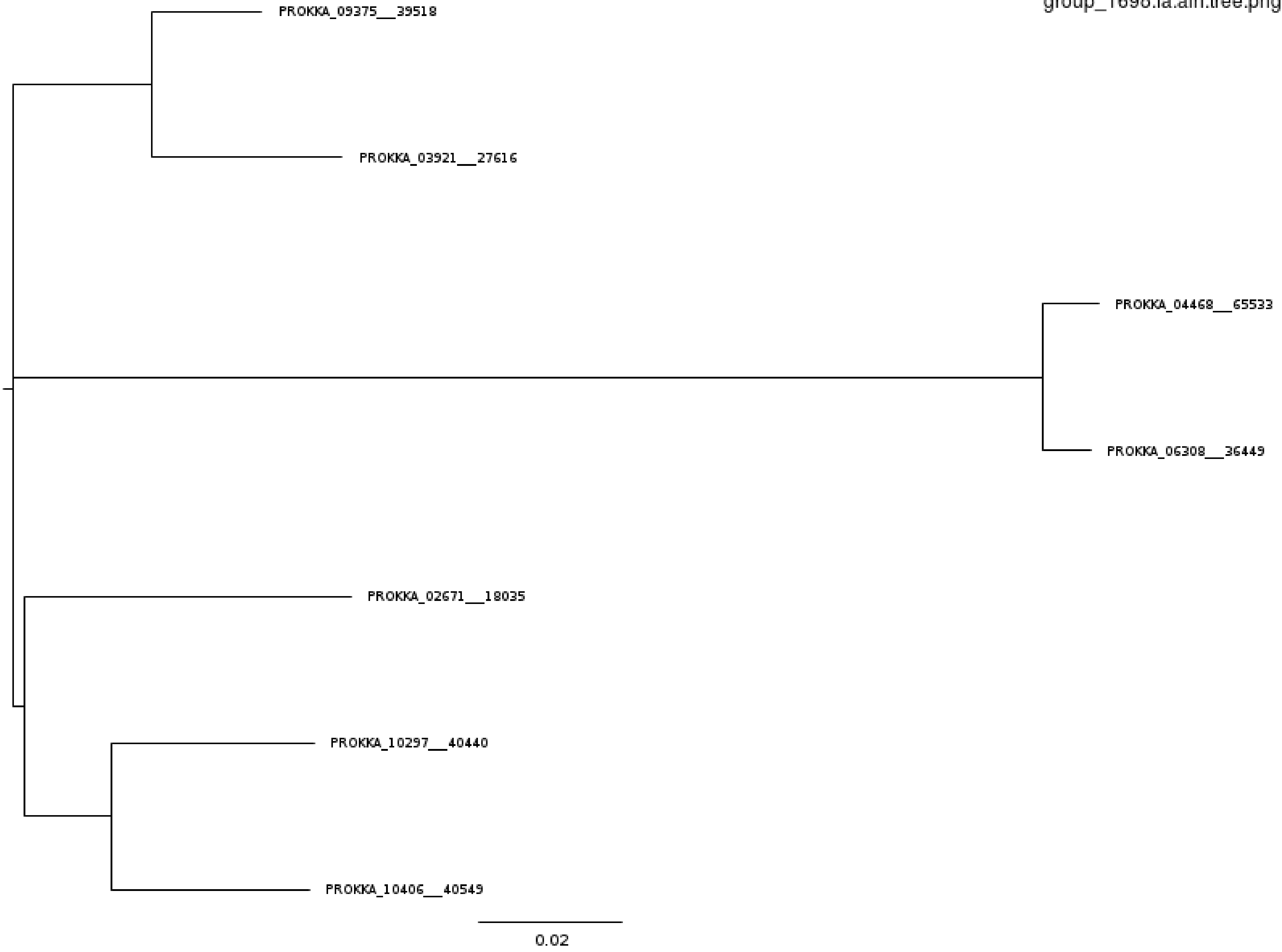

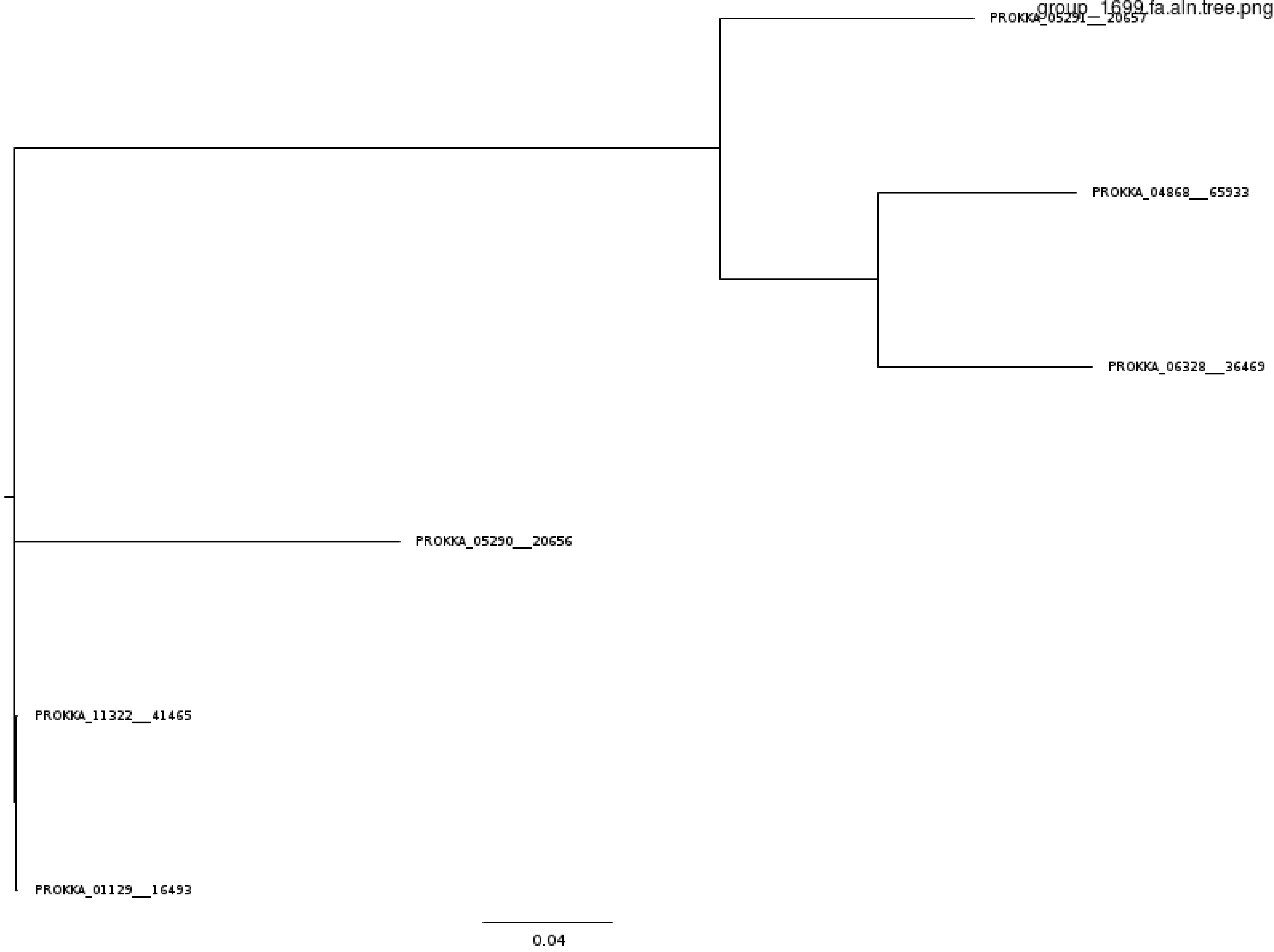
group_1699.fa.aln.tree.png
PROKKA_05291___20657
PROKKA_04868___65933
PROKKA_06328___36469
PROKKA_05290___20656
PROKKA_11322___41465
PROKKA_01129___16493
0.04

group_16.fa.aln.tree.png

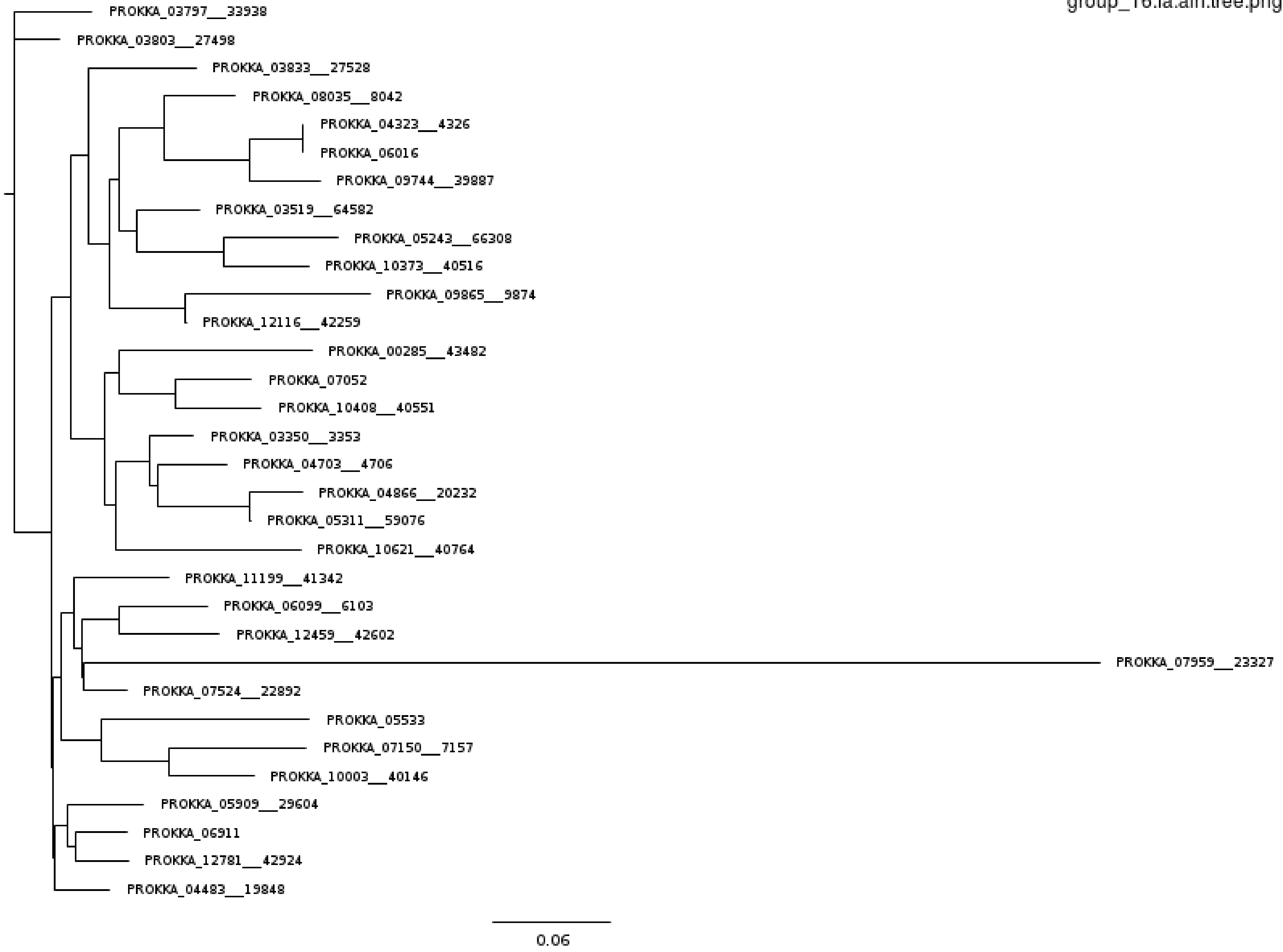

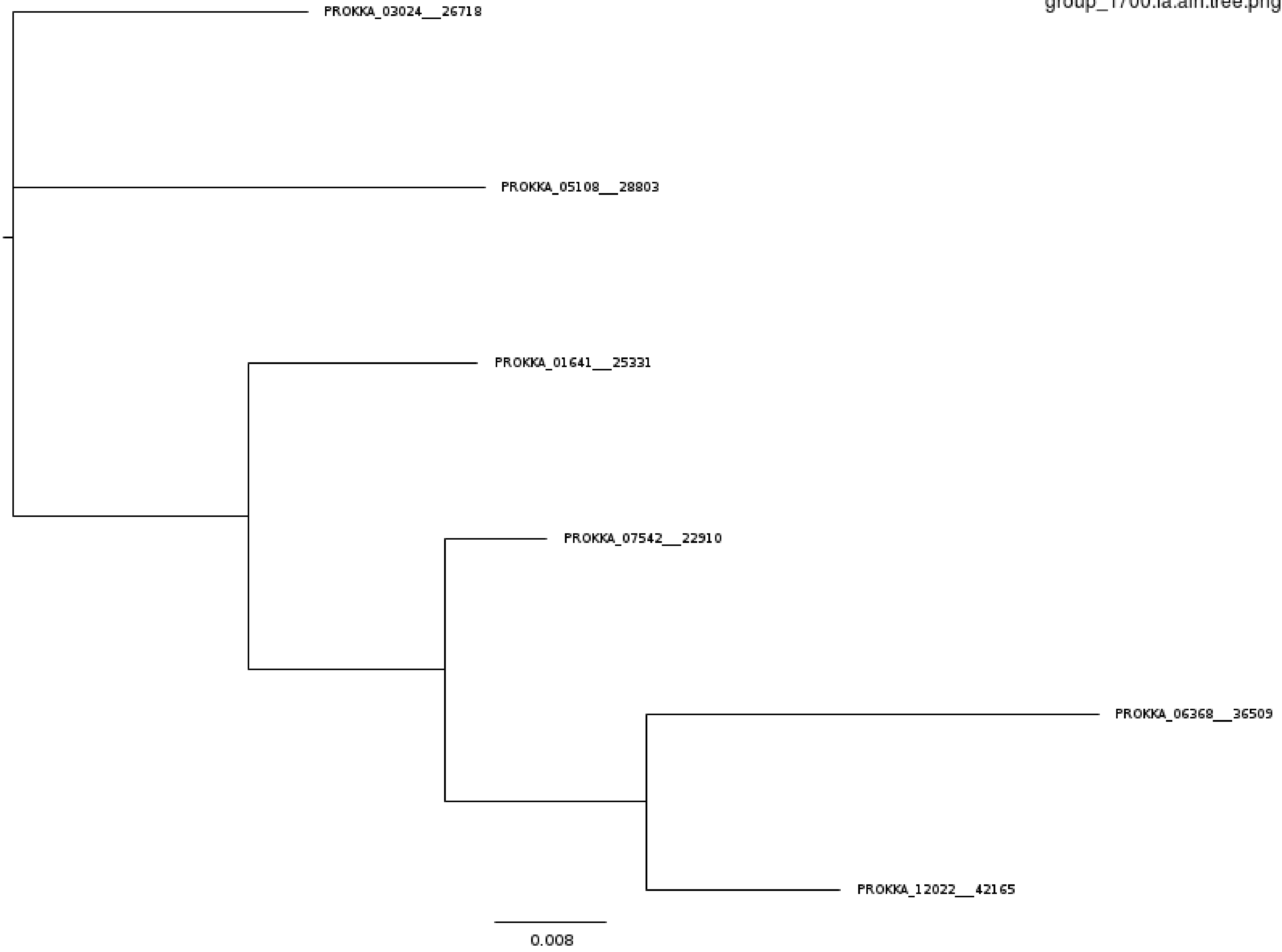
PROKKA_03024___26718
PROKKA_05108___28803
PROKKA_01641___25331
PROKKA_07542___22910
PROKKA_06368___36509
PROKKA_12022___42165
0.008

group_1701.fa.aln.tree.png

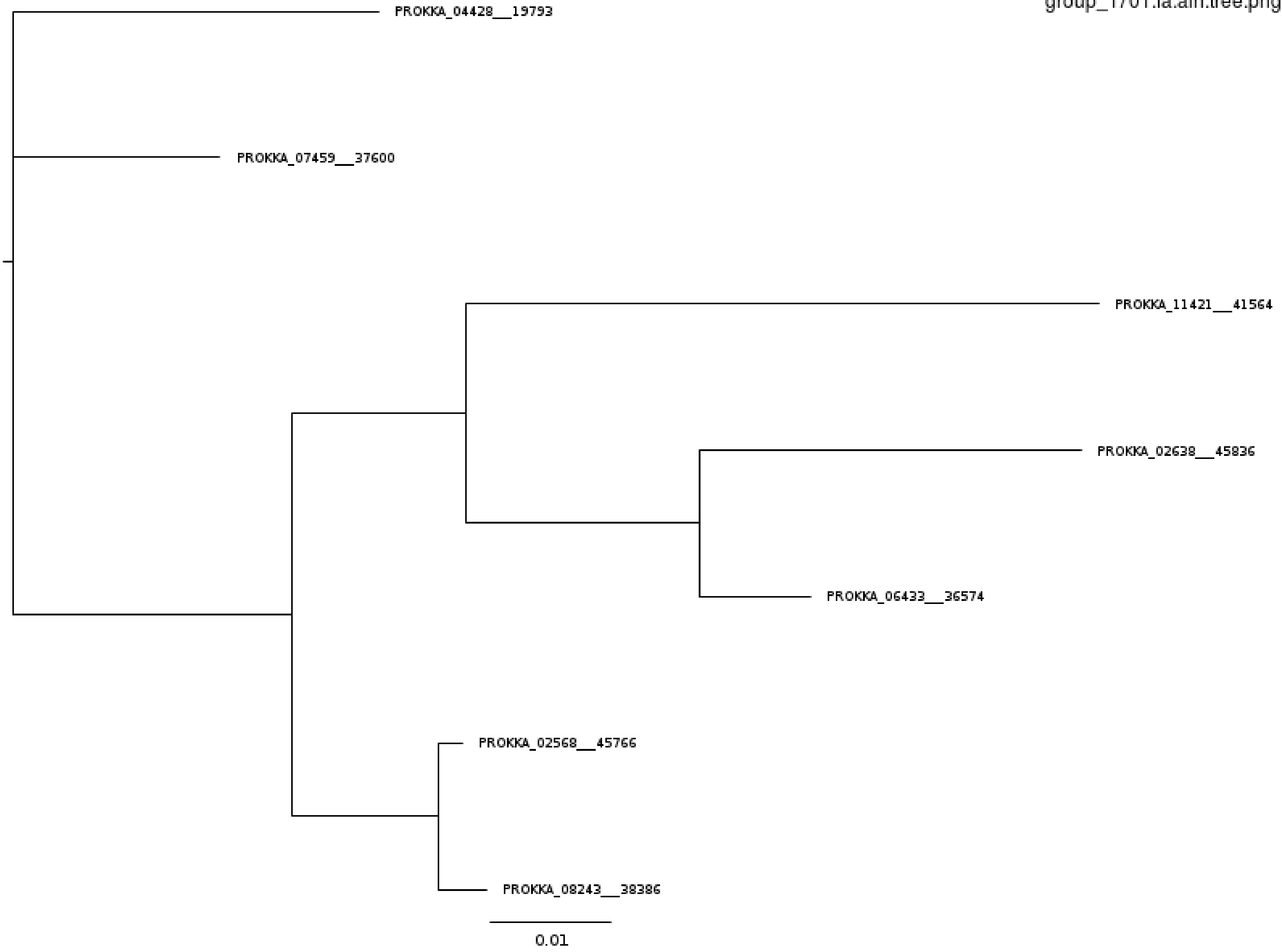

group_1702.fa.aln.tree.png

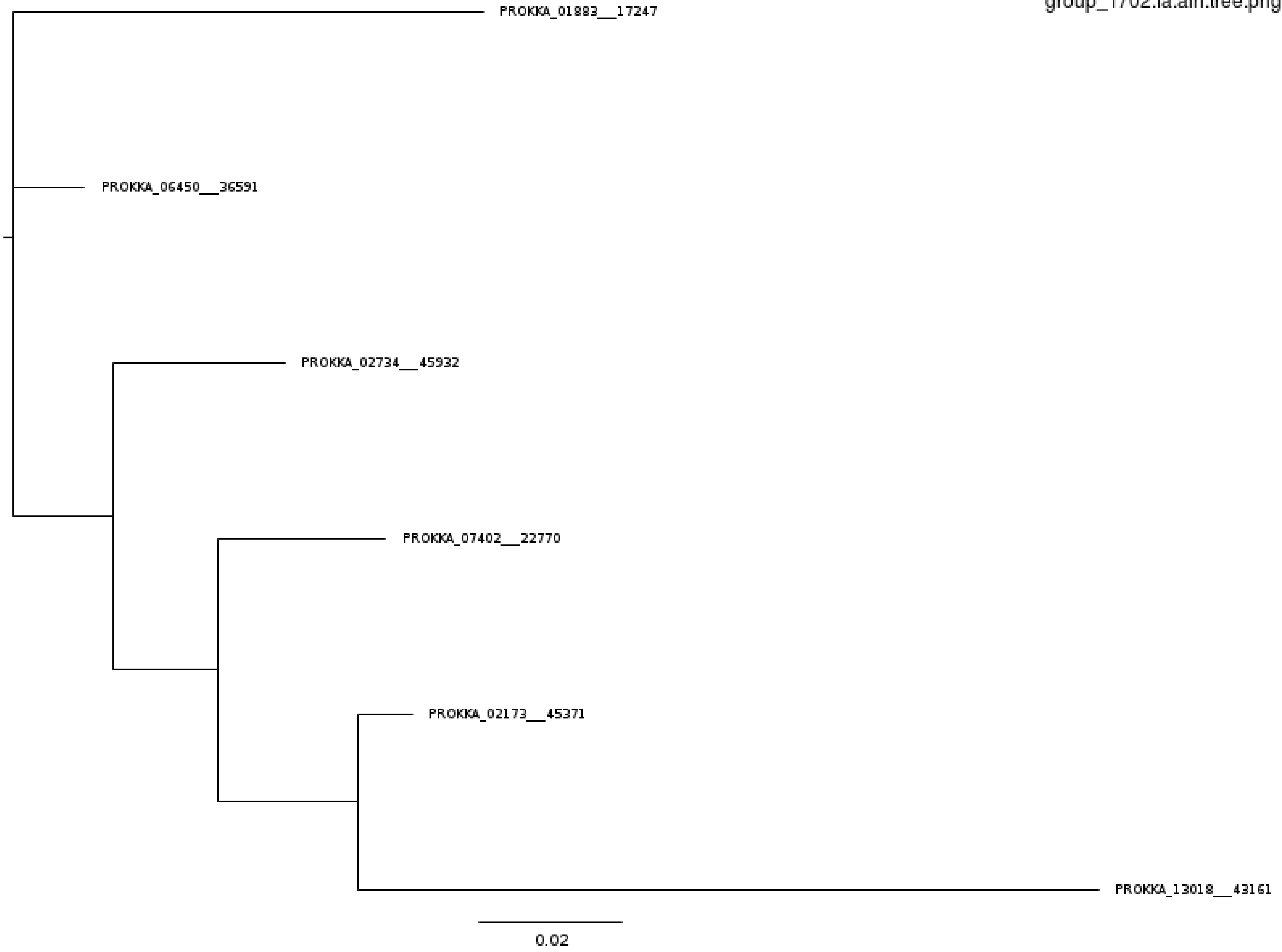

group_1703.fa.aln.tree.png

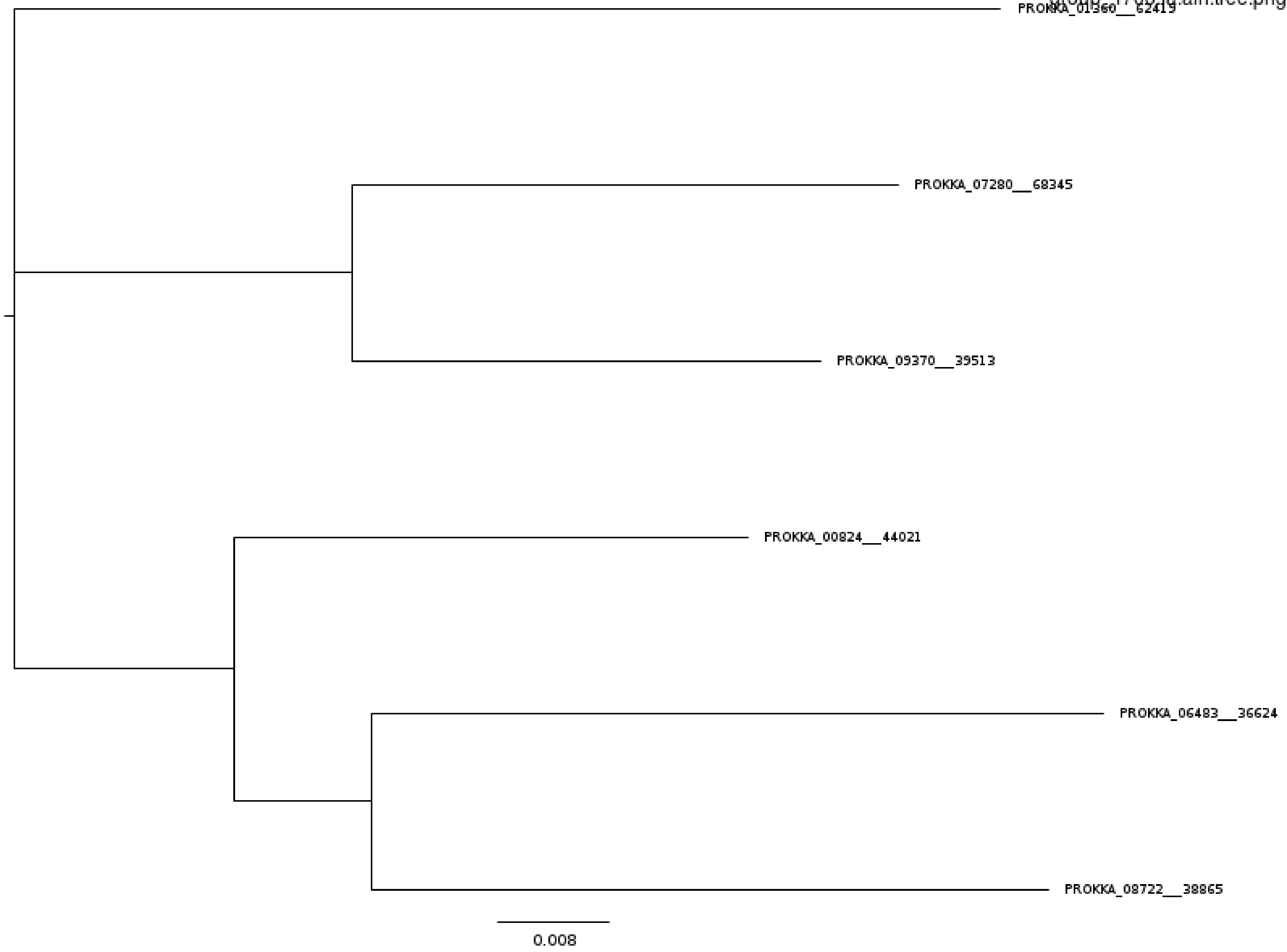

group_1704.fa.aln.tree.png

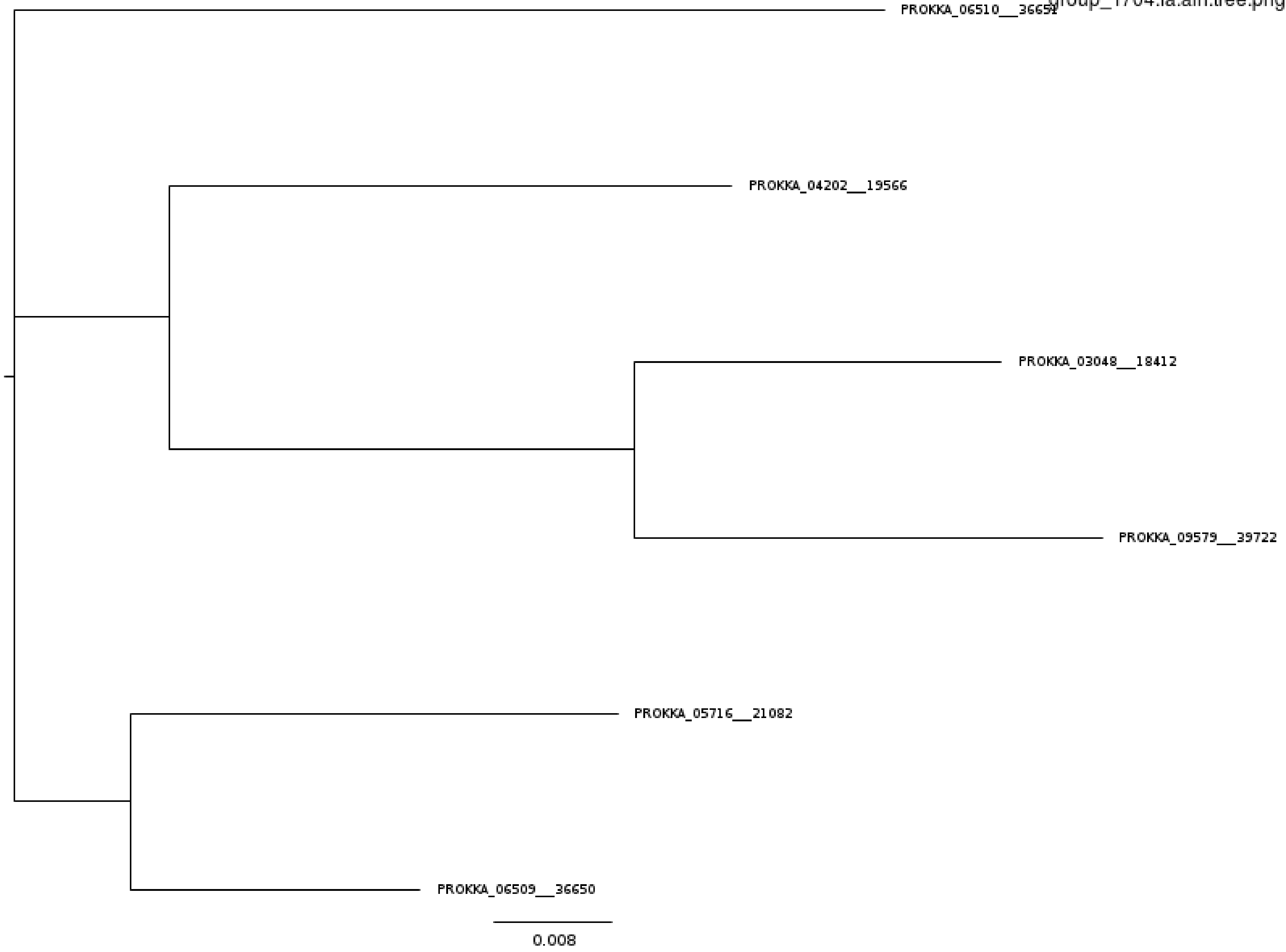

group_1705.fa.aln.tree.png

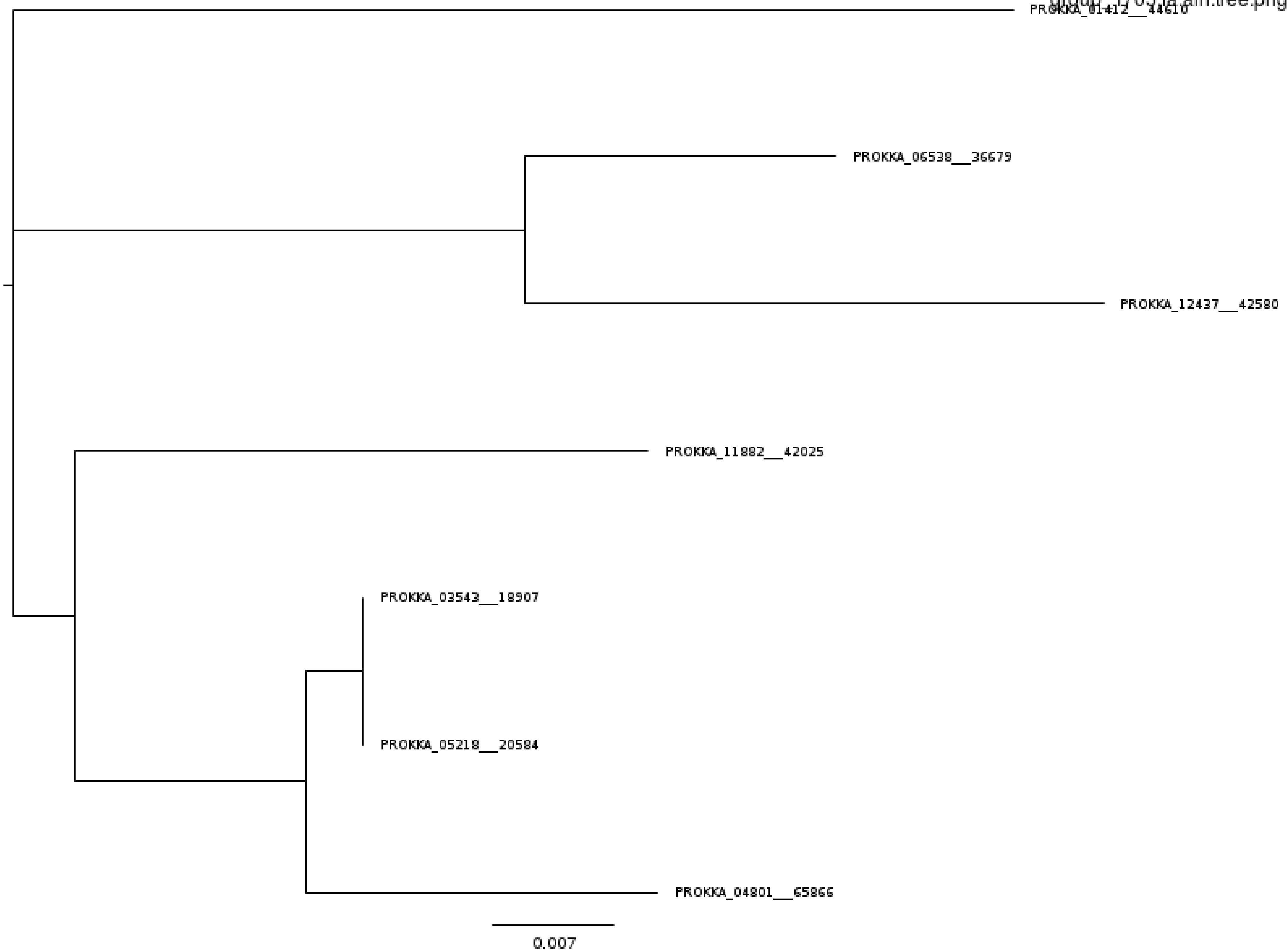

group_1708.fa.aln.tree.png

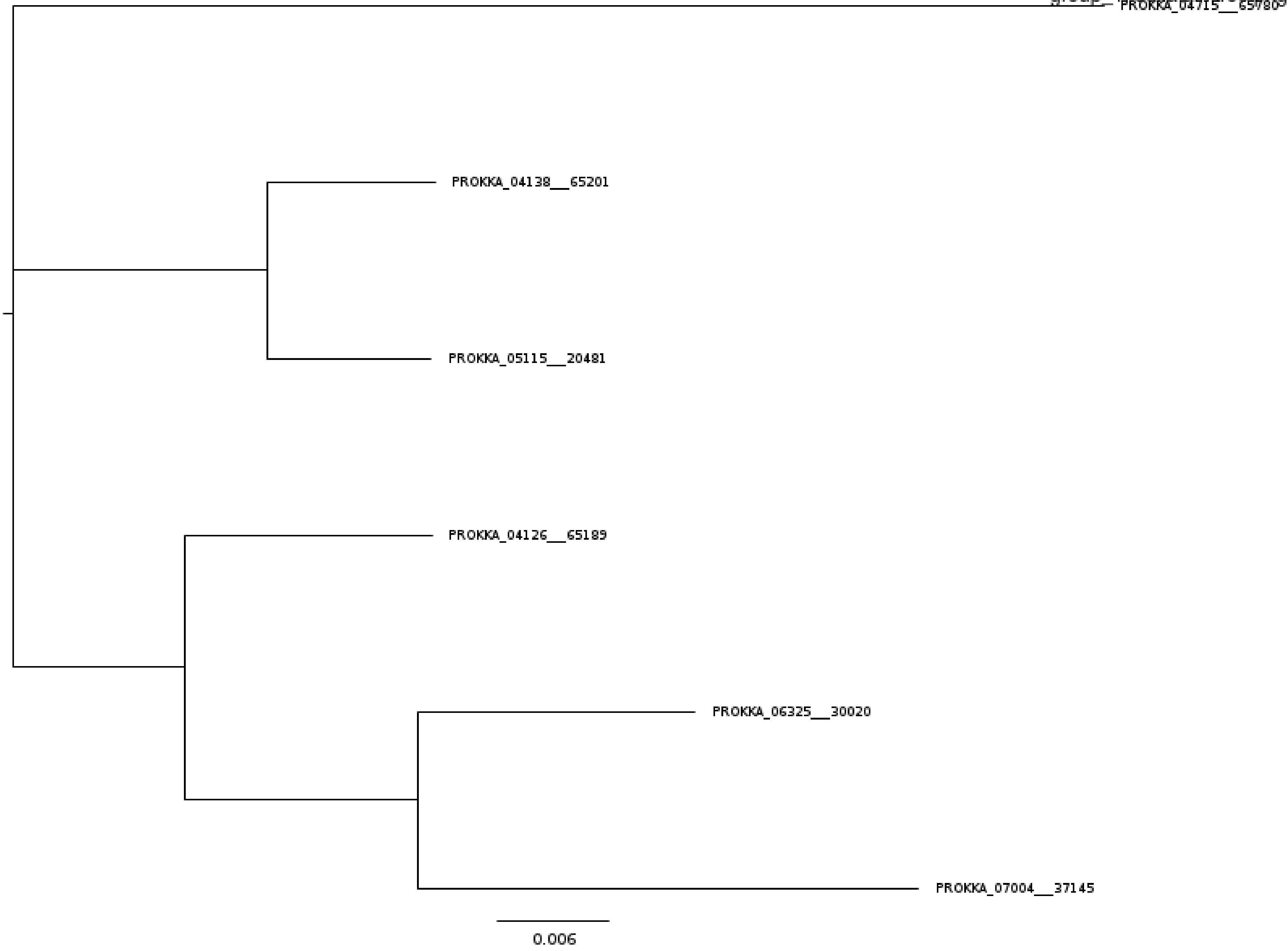

group_1710.fa.aln.tree.png

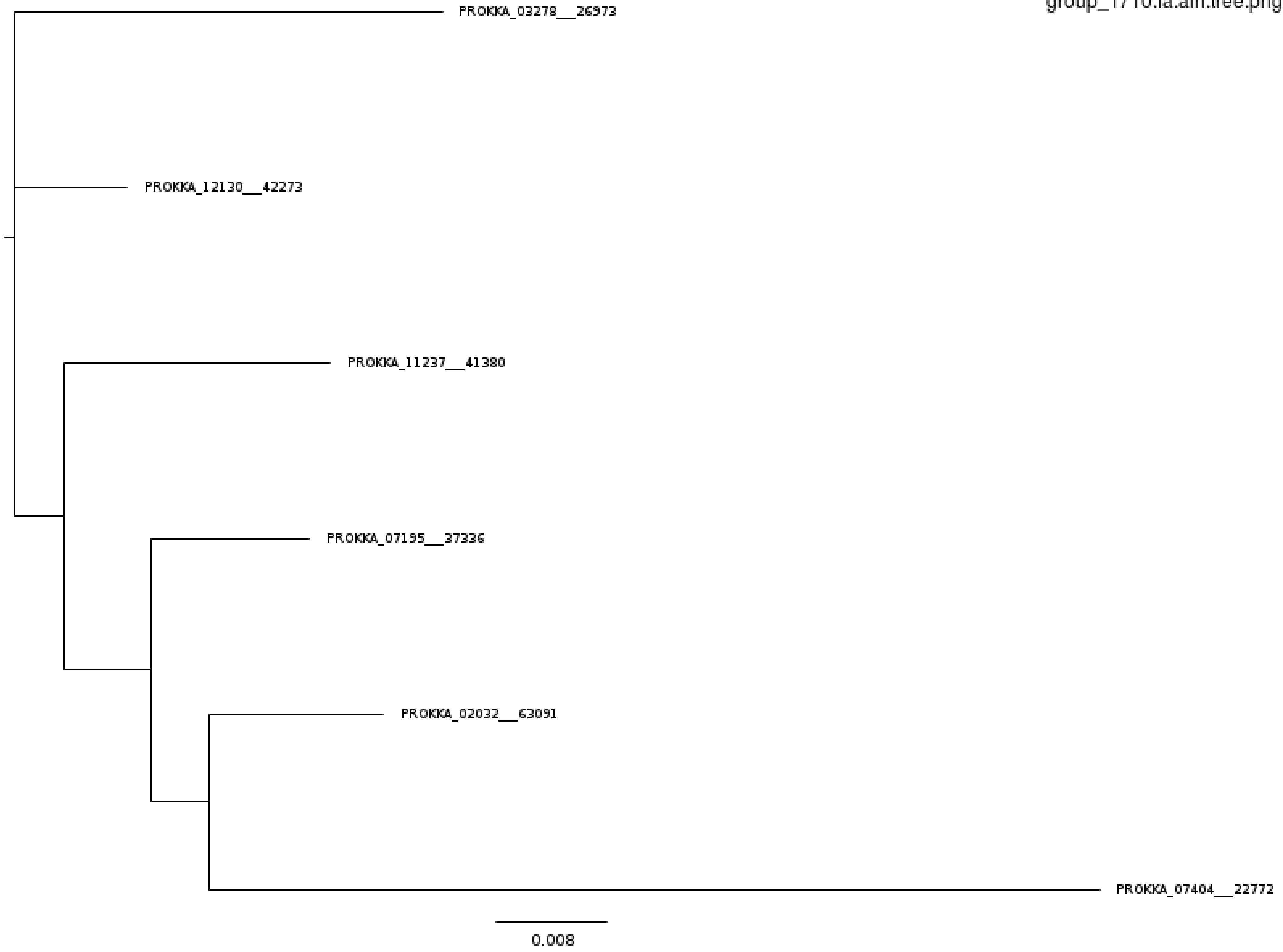

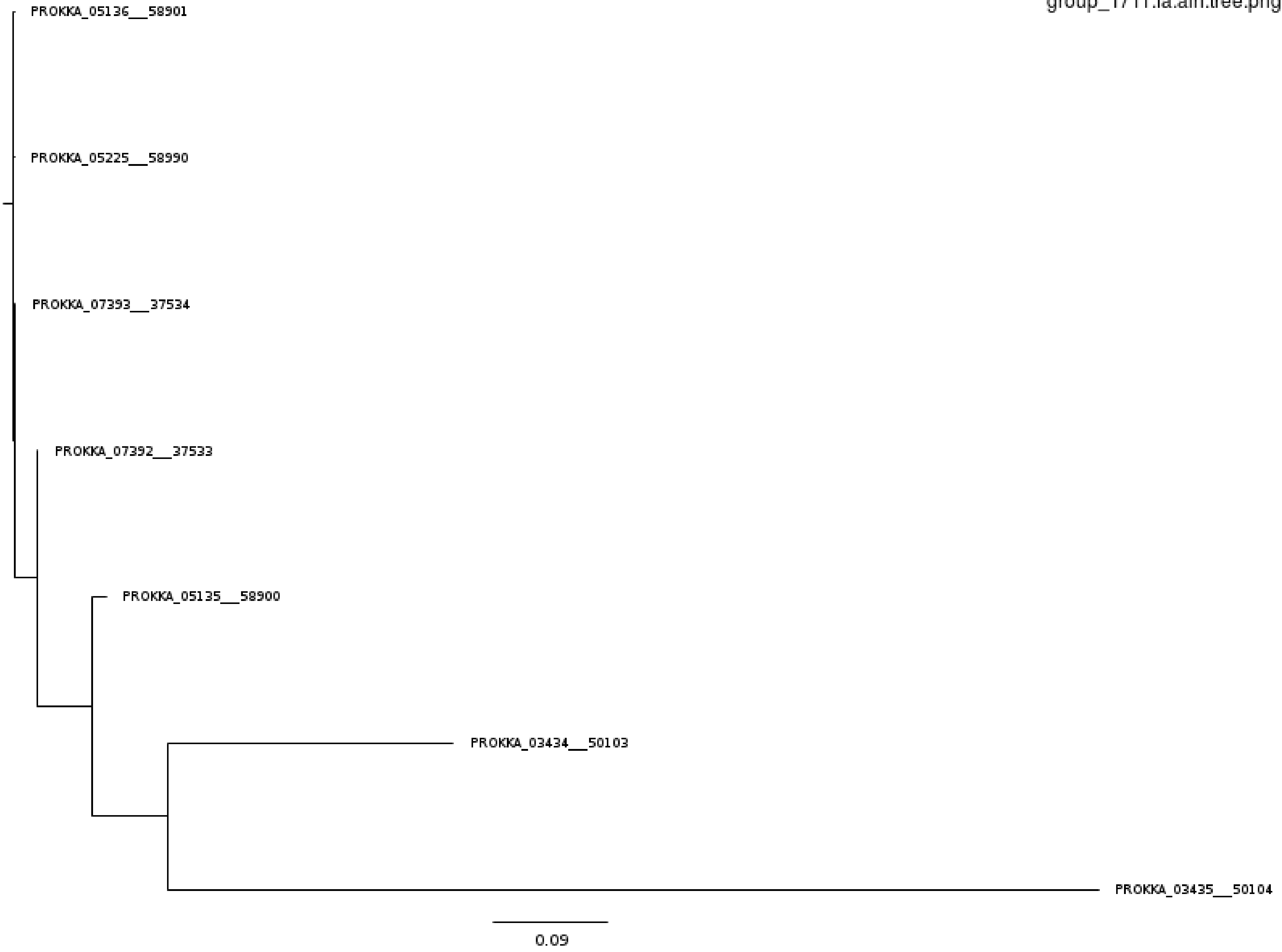
PROKKA_05136___58901
PROKKA_05225___58990
PROKKA_07393___37534
PROKKA_07392___37533
PROKKA_05135___58900
PROKKA_03434___50103
PROKKA_03435___50104
0.09

group_1712.fa.aln.tree.png

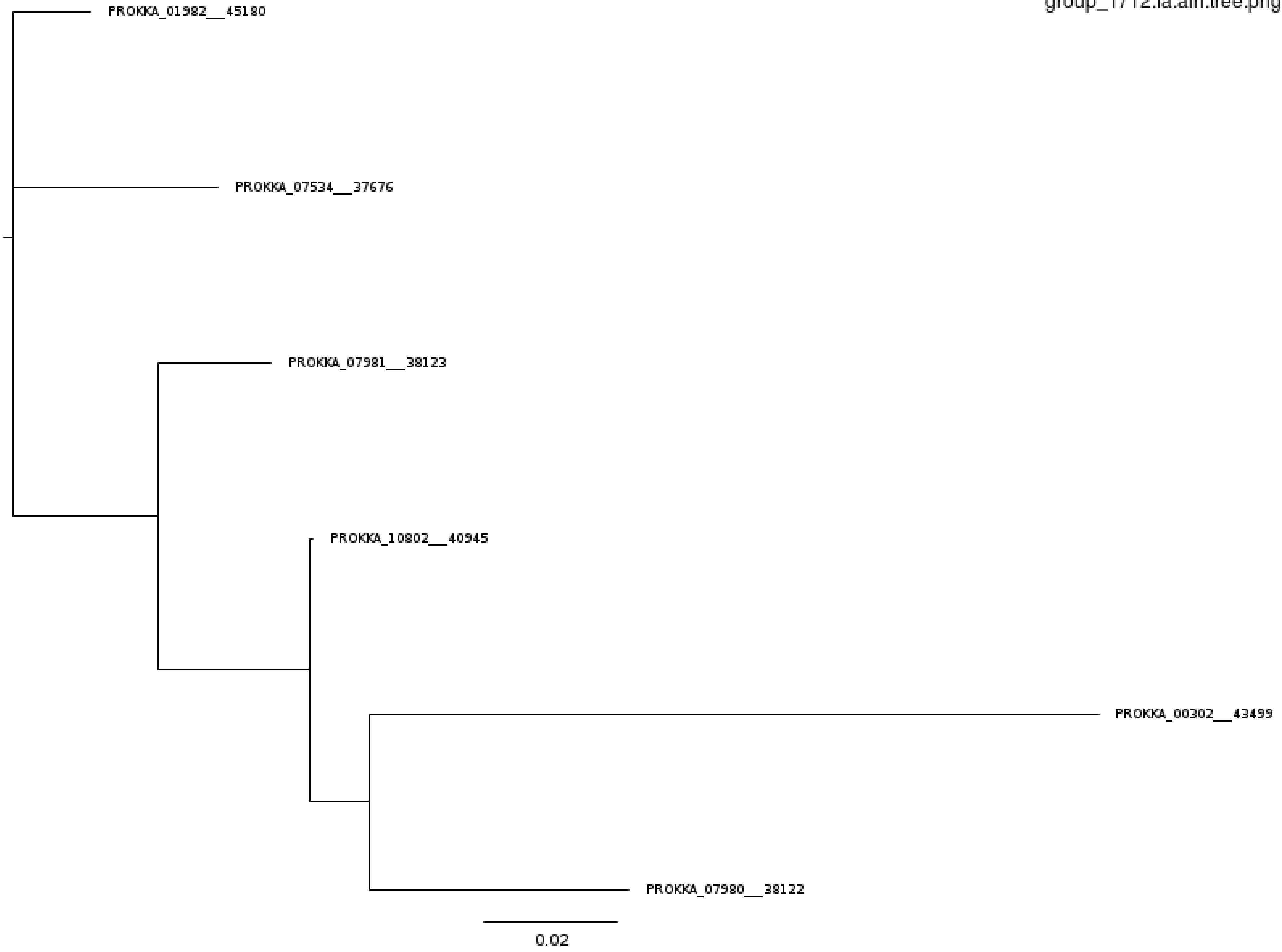

group_1714.fa.aln.tree.png

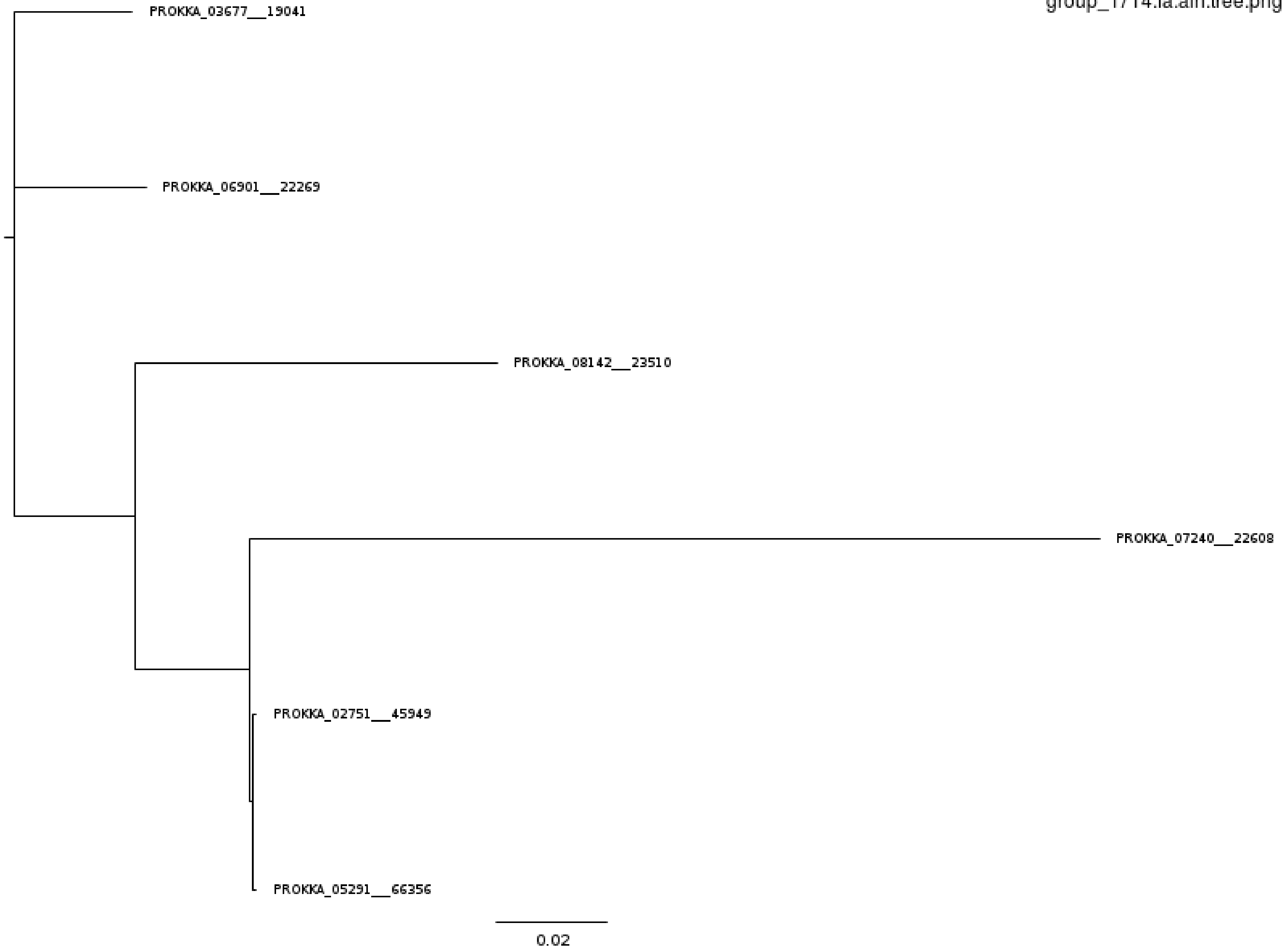

group_1715.fa.aln.tree.png

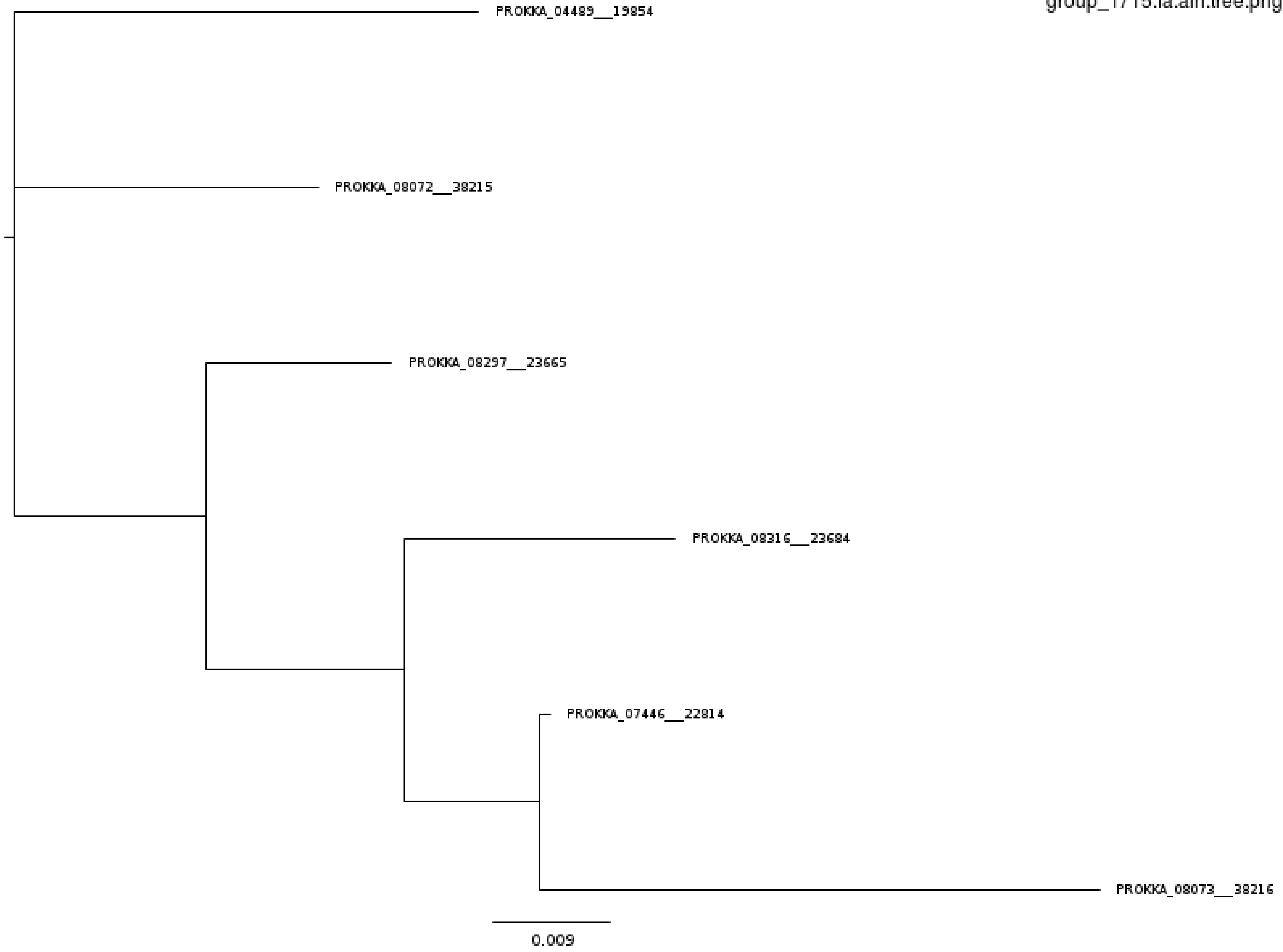

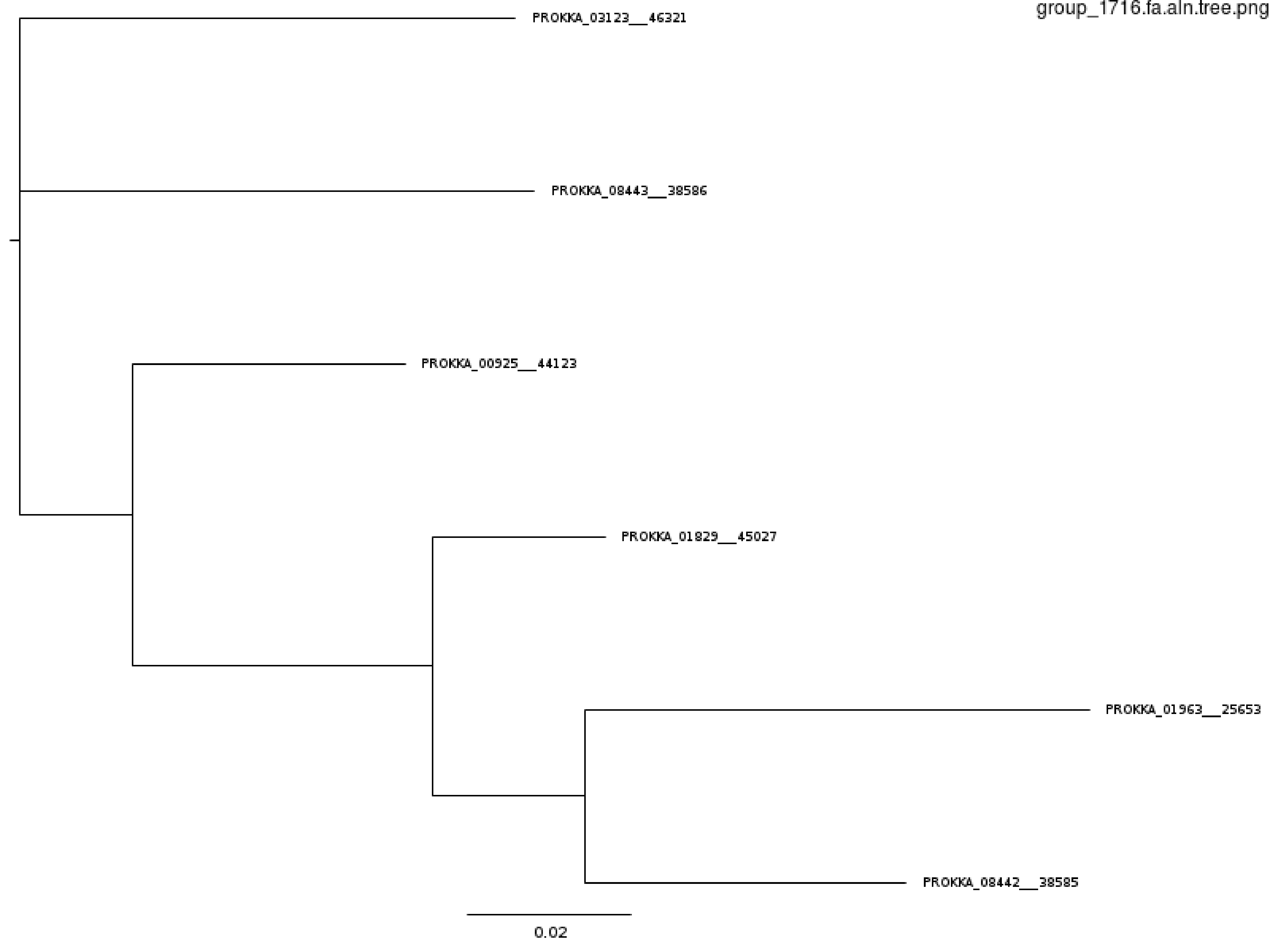
group_1716.fa.aln.tree.png
PROKKA_03123___46321
PROKKA_08443___38586
PROKKA_00925___44123
PROKKA_01829___45027
PROKKA_01963___25653
PROKKA_08442___38585
0.02

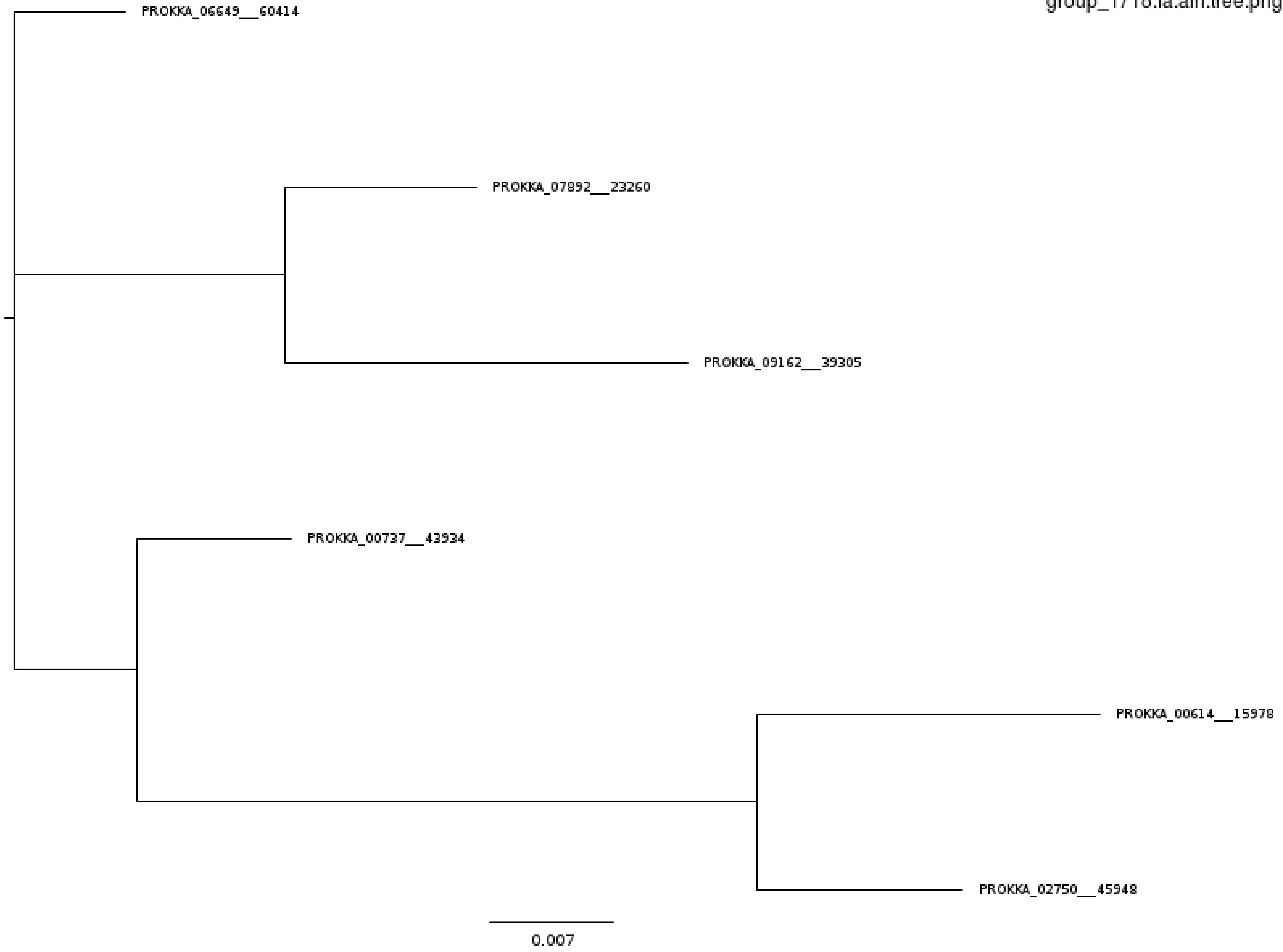
PROKKA_06649___60414
PROKKA_07892___23260
PROKKA_09162___39305
PROKKA_00737___43934
PROKKA_00614___15978
PROKKA_02750___45948
0.007

group_1719.fa.aln.tree.png

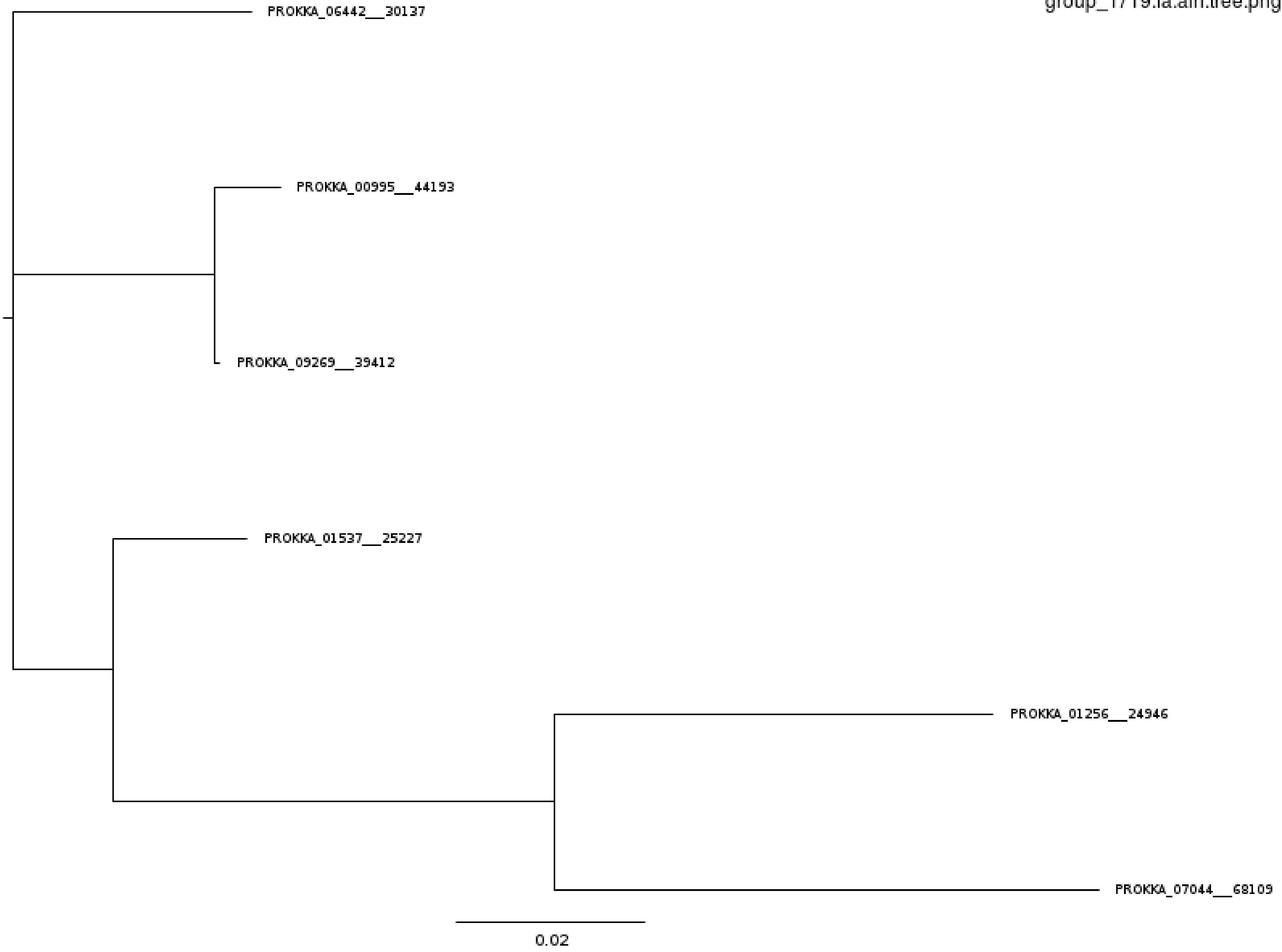

group_171.fa.aln.tree.png

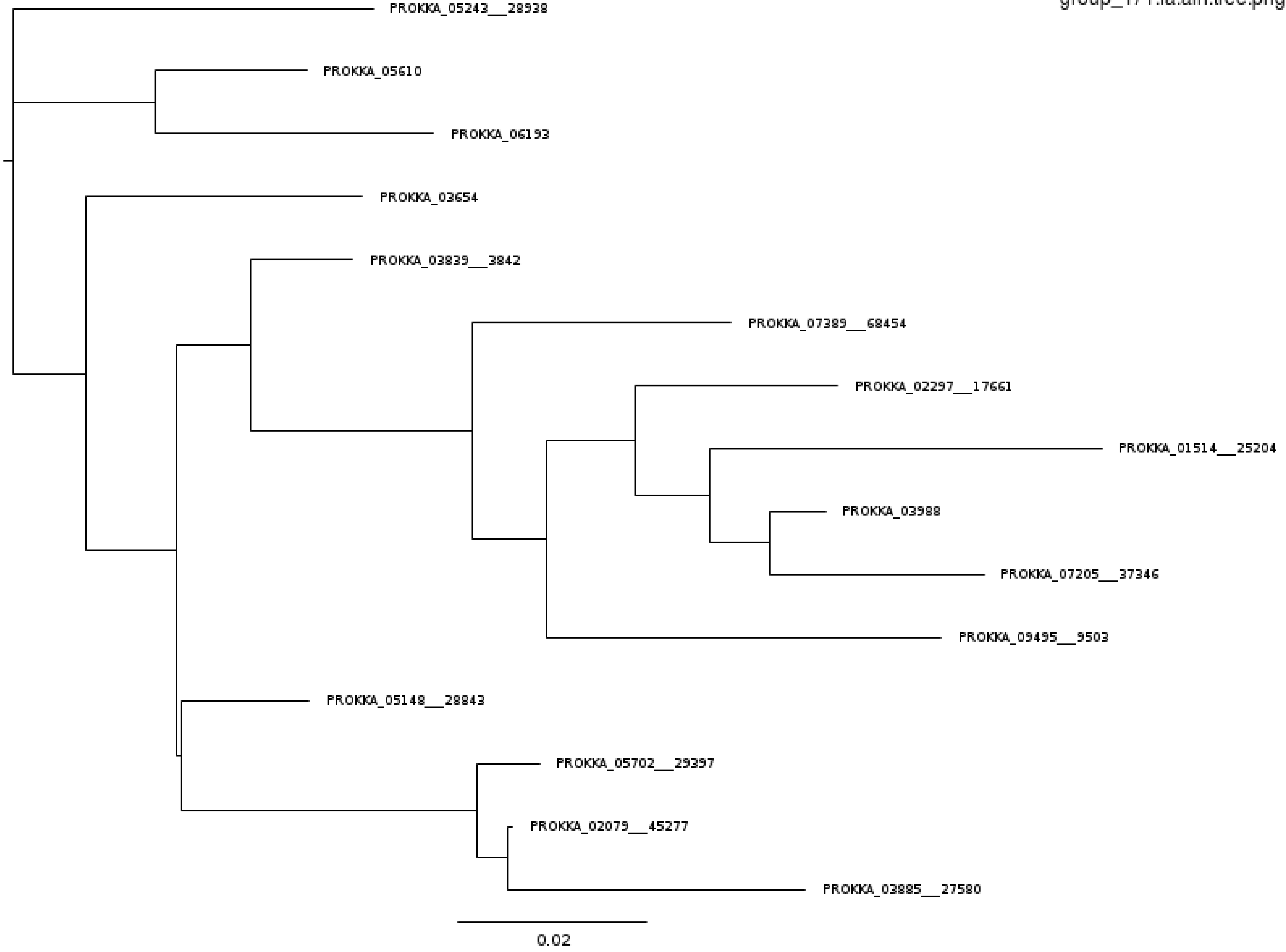

group_1722.fa.aln.tree.png

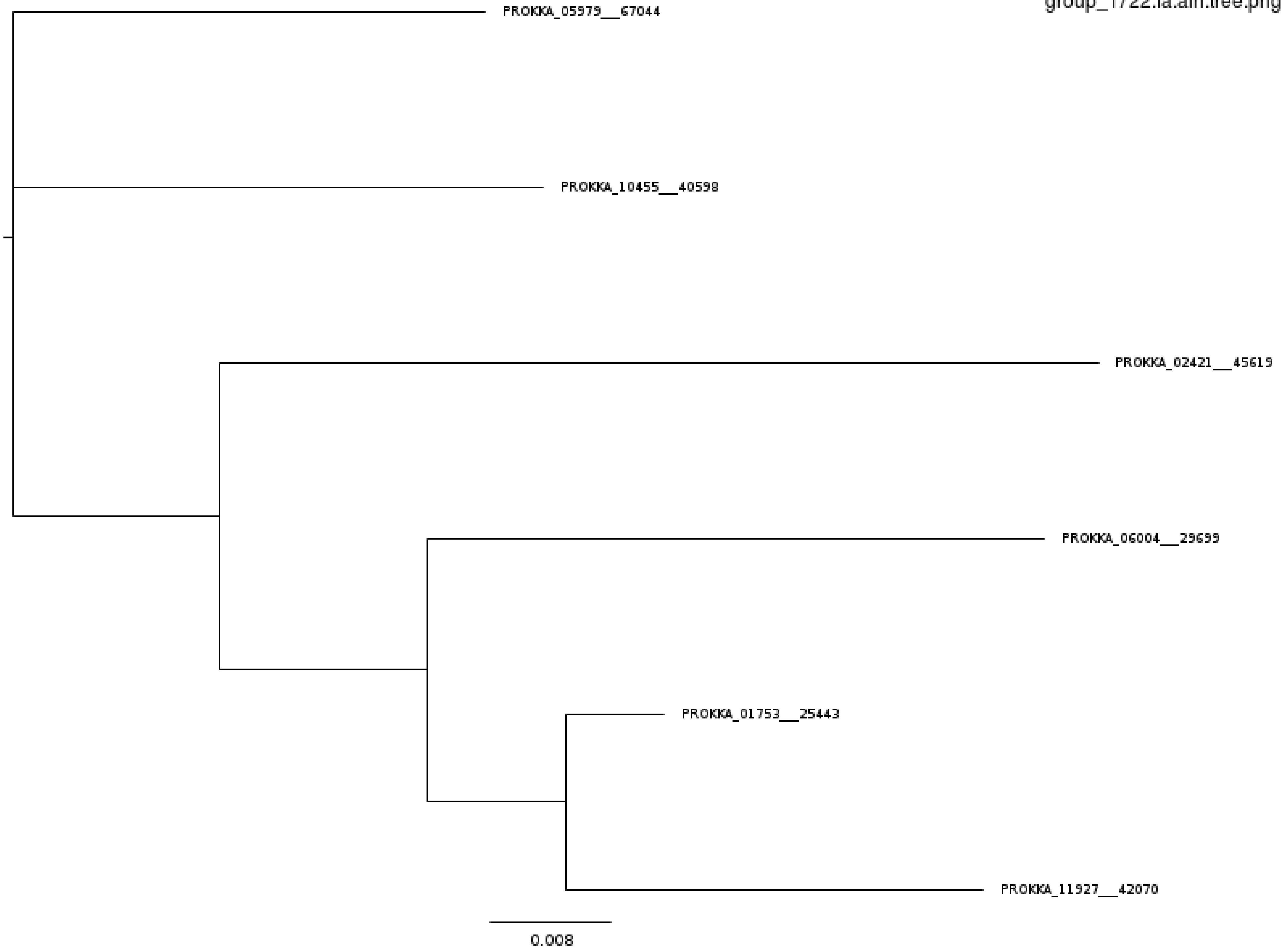

group_1723.fa.aln.tree.png

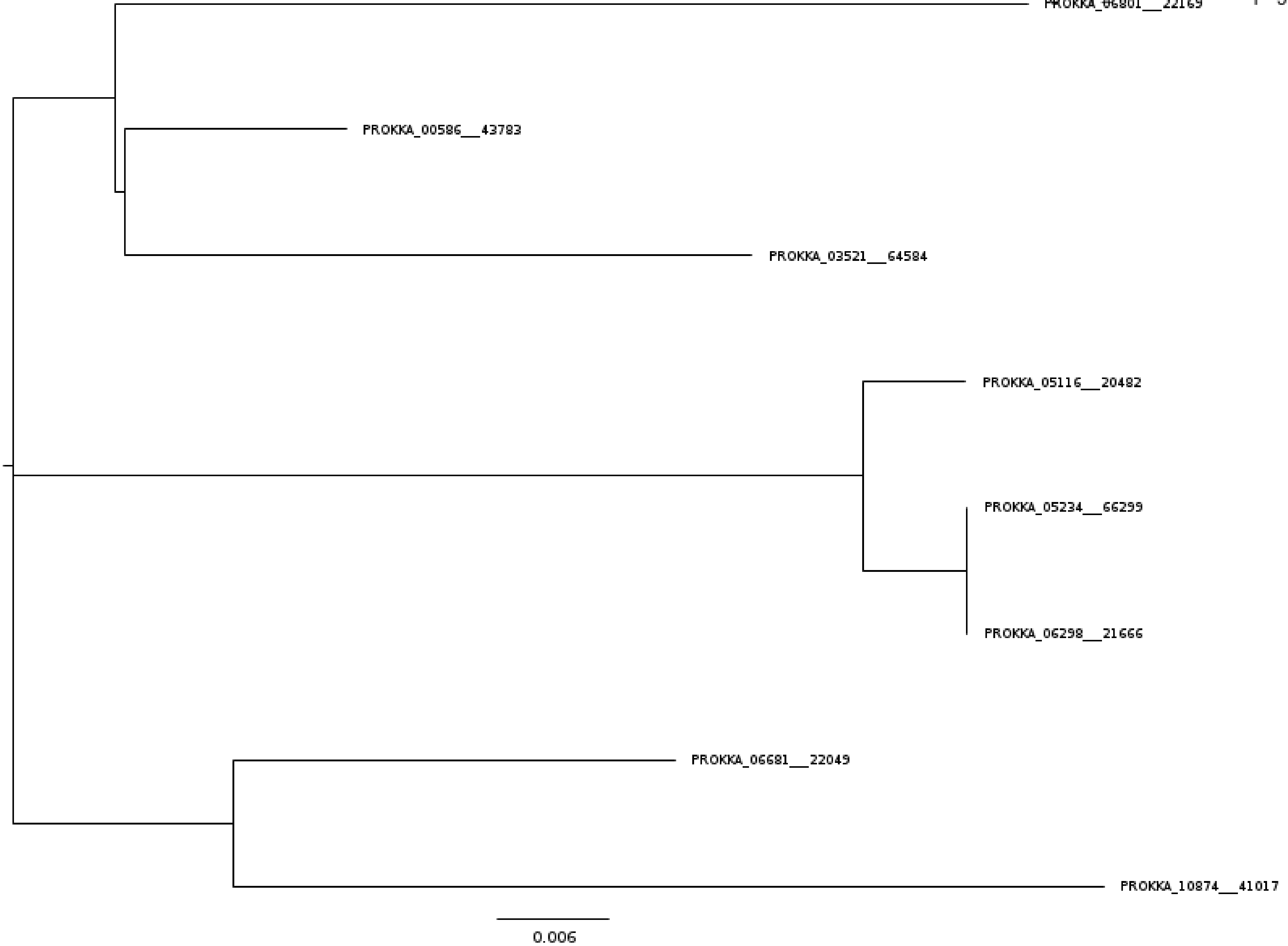

group_1726.fa.aln.tree.png

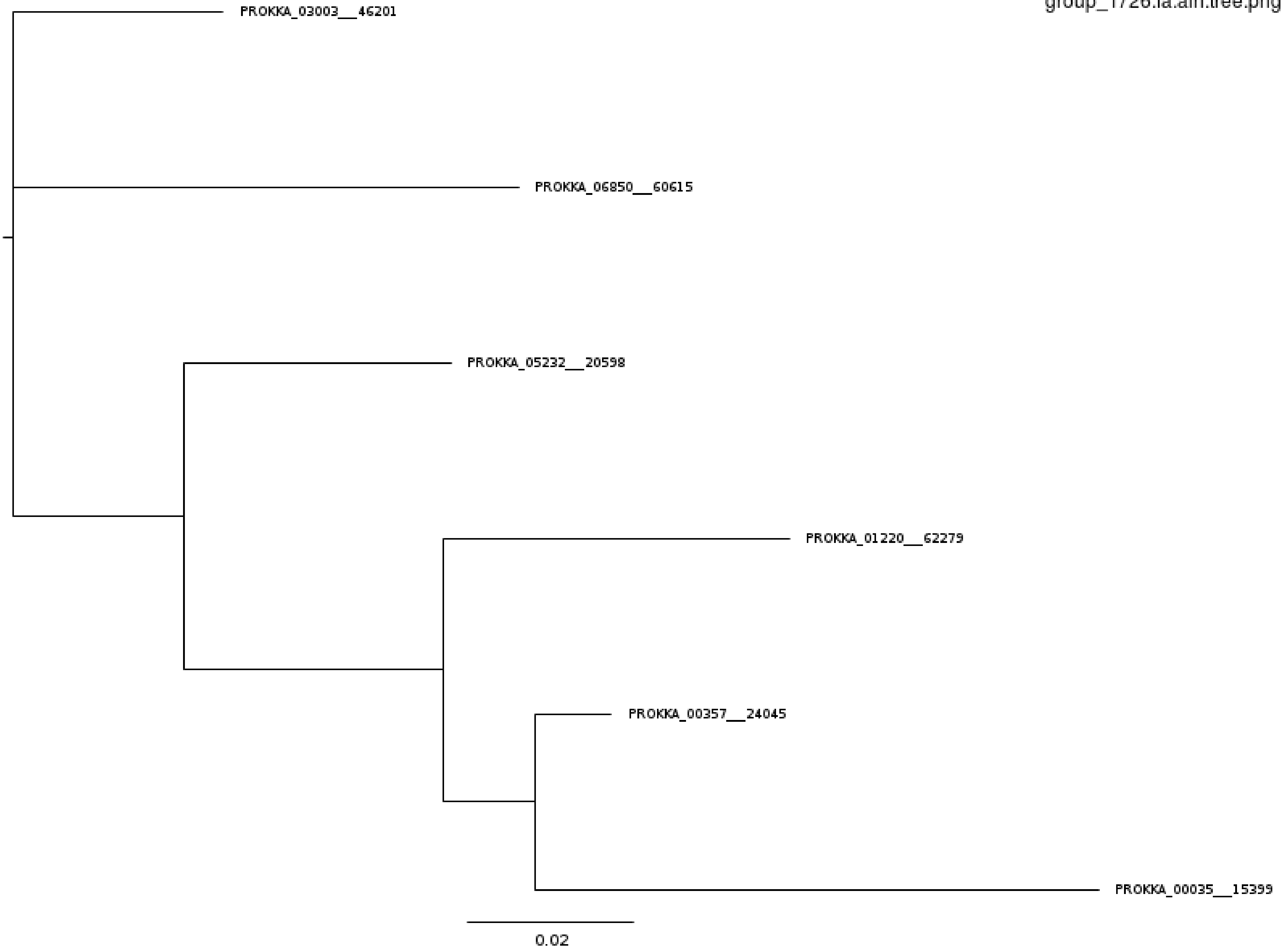

group_1727.fa.aln.tree.png

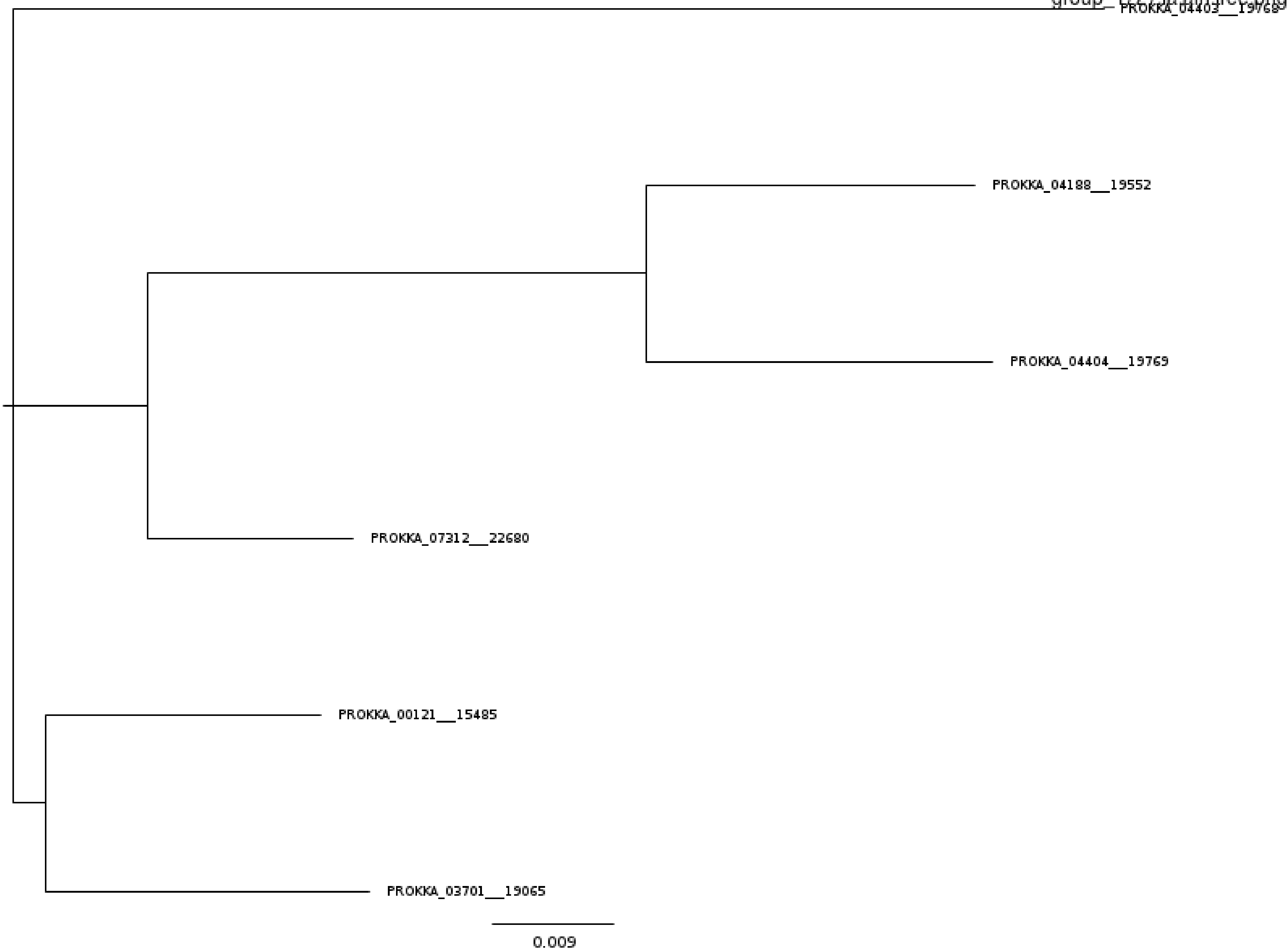

group_1729.fa.aln.tree.png

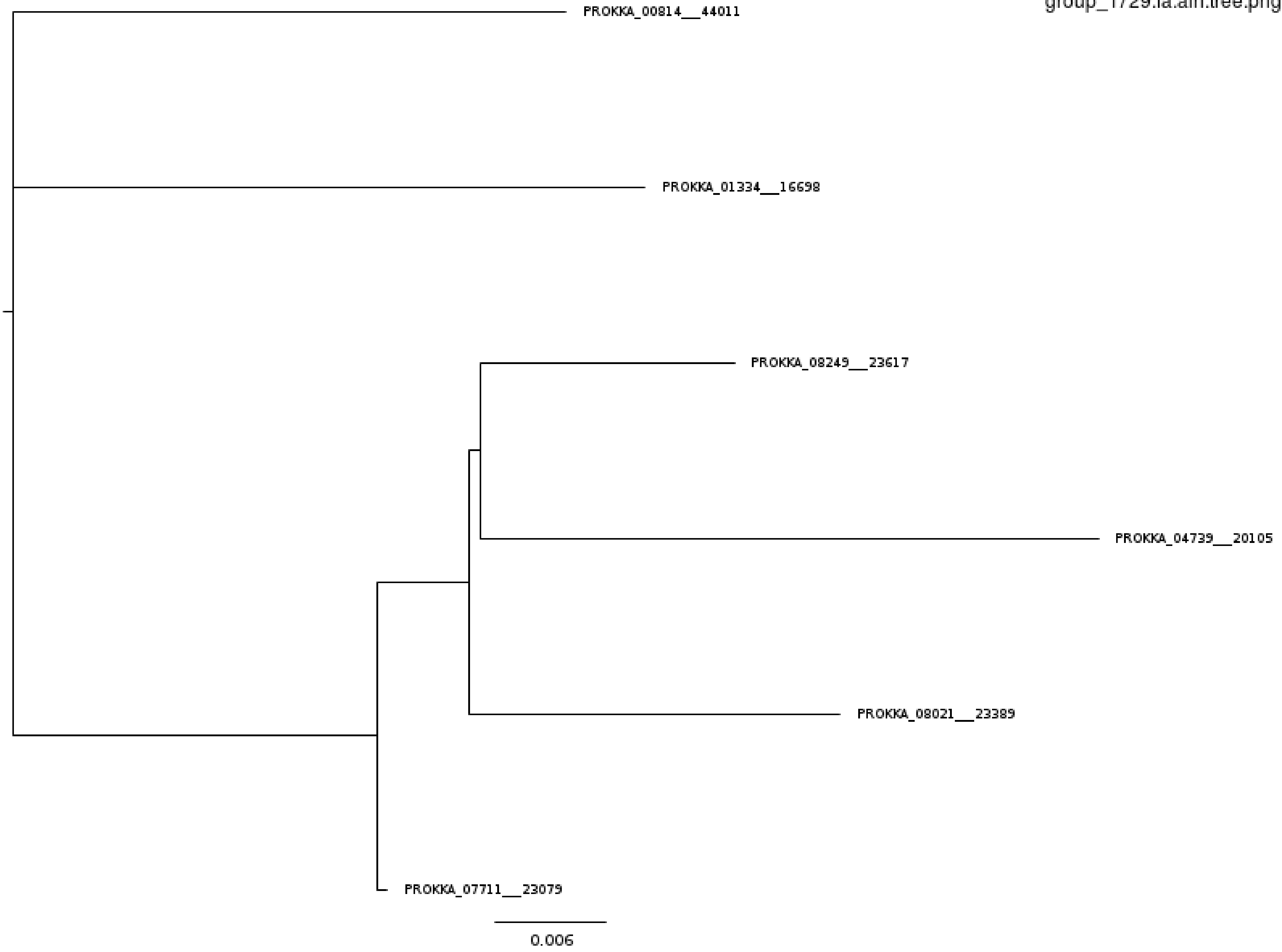

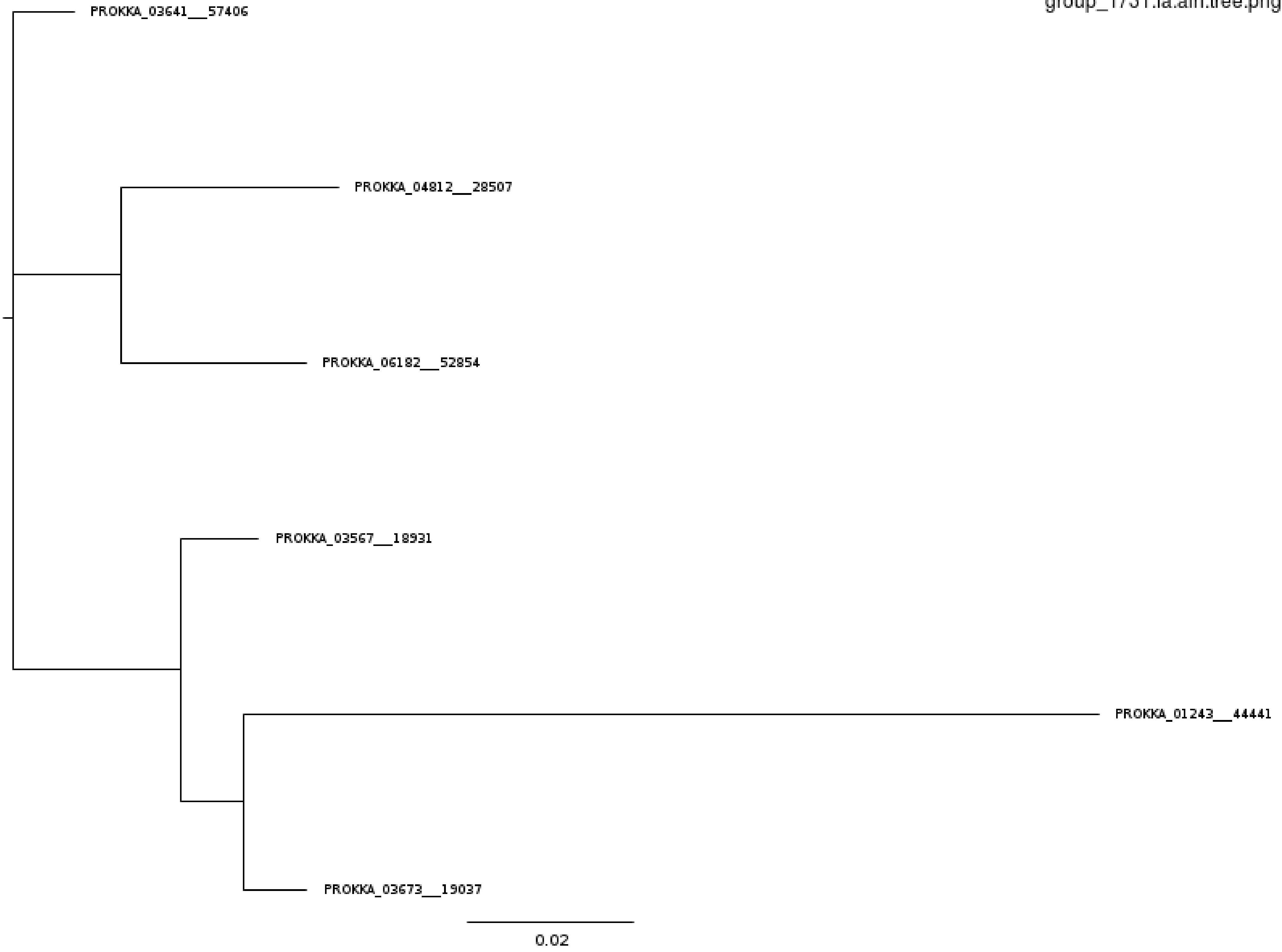
PROKKA_03641___57406
PROKKA_04812___28507
PROKKA_06182___52854
PROKKA_03567___18931
PROKKA_01243___44441
PROKKA_03673___19037
0.02

group_1733.fa.aln.tree.png

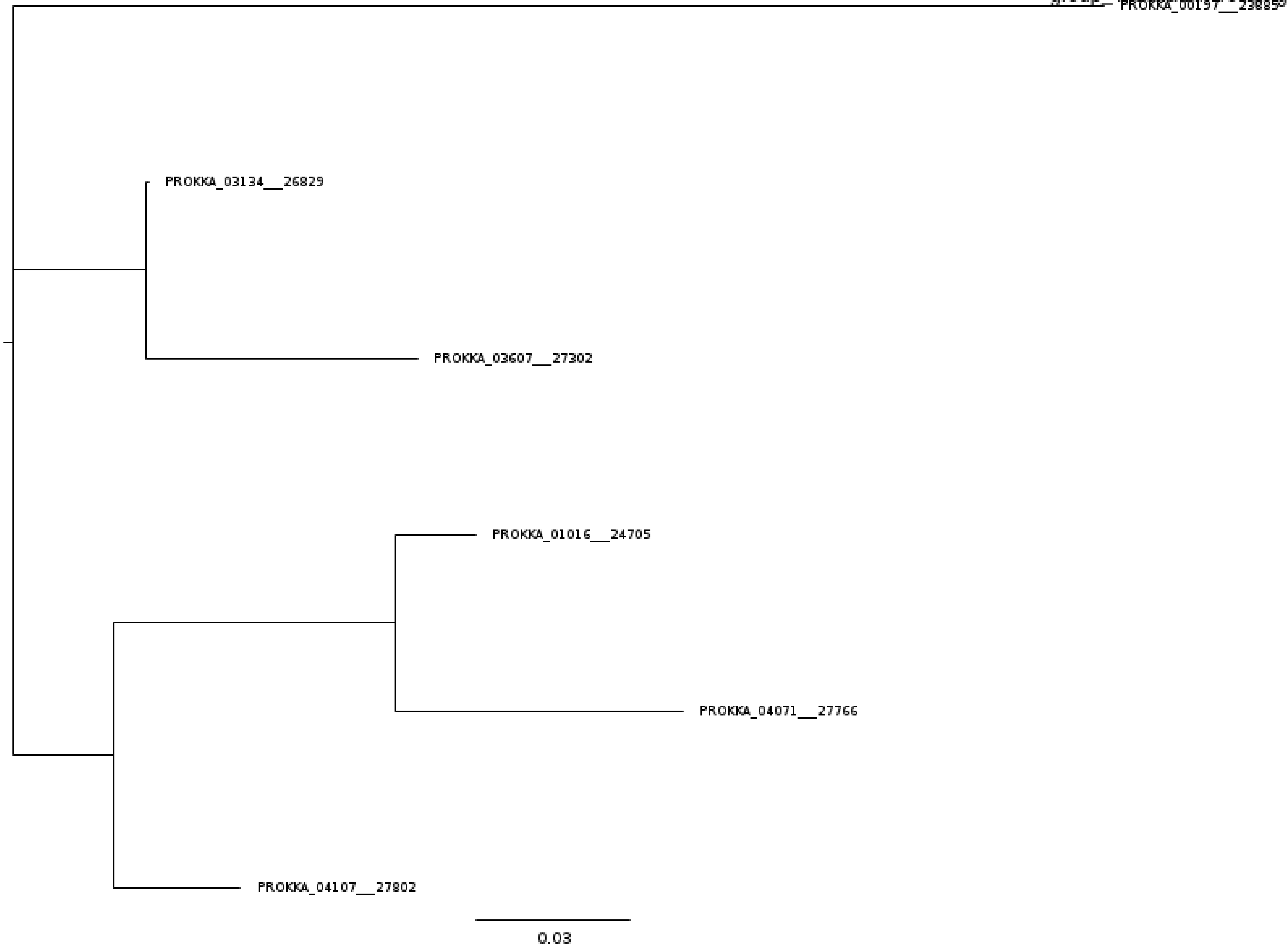

group_1738.fa.aln.tree.png

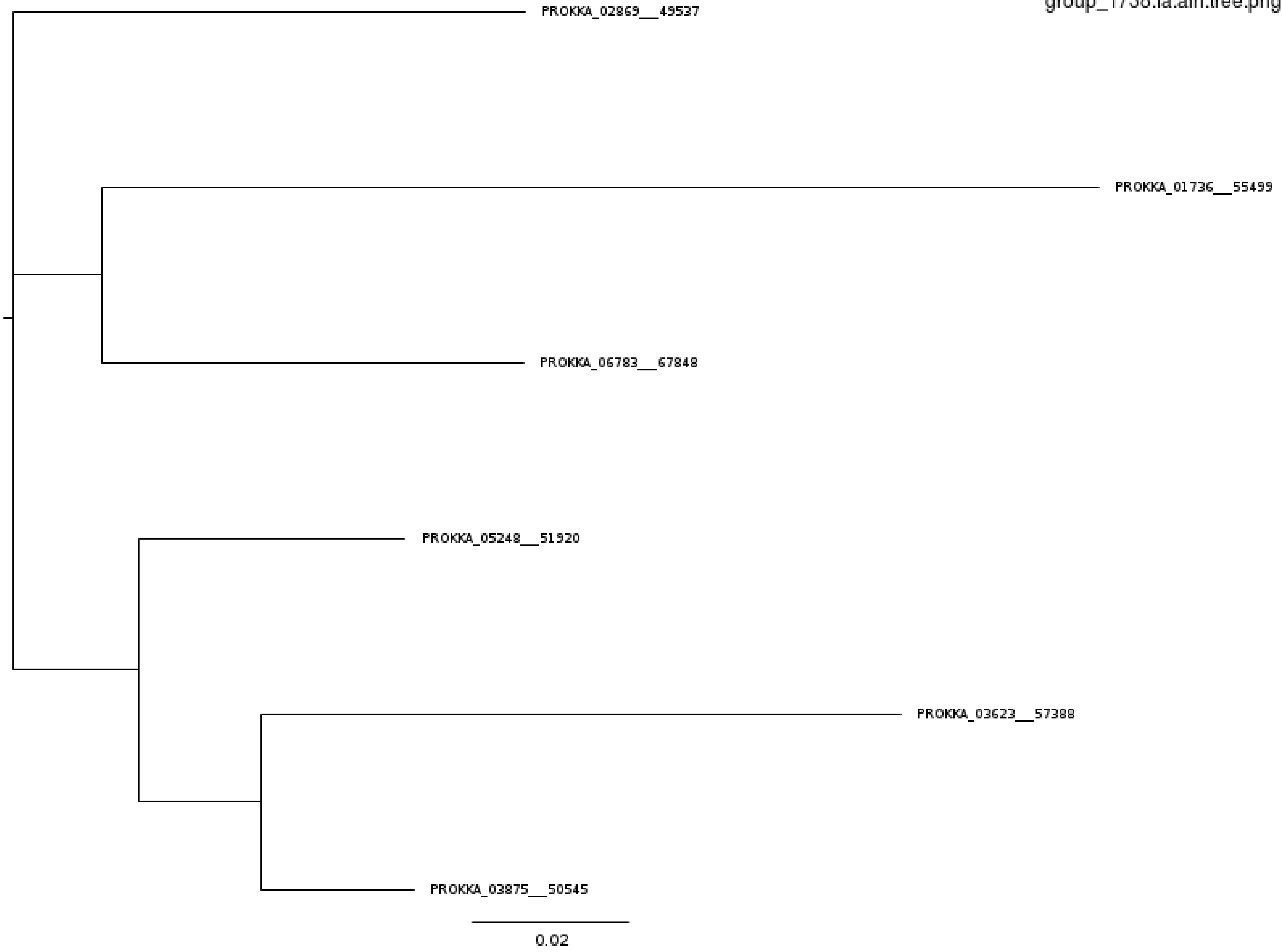

group_1739.fa.aln.tree.png

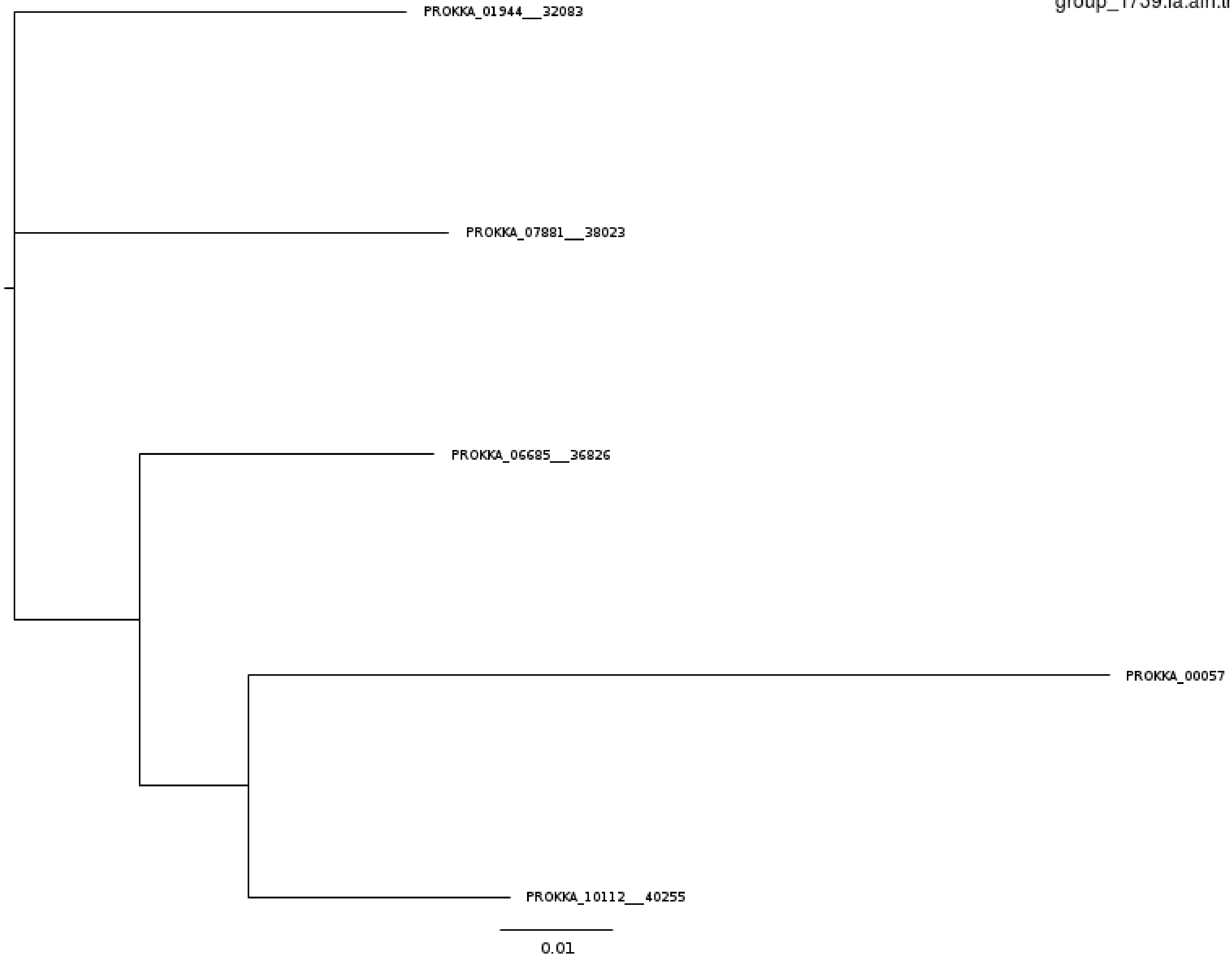

group_173.fa.aln.tree.png

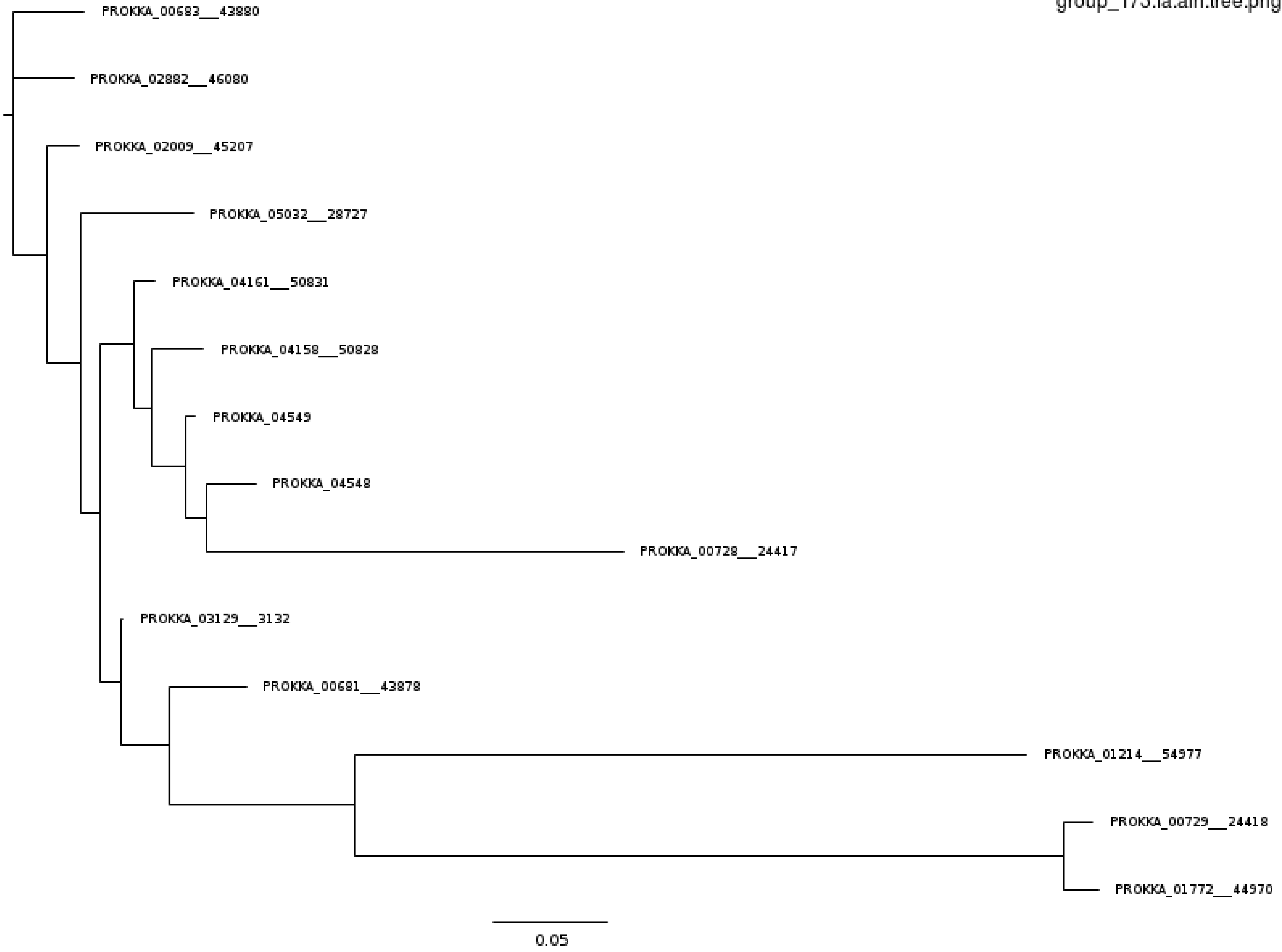

group_1740.fa.aln.tree.png

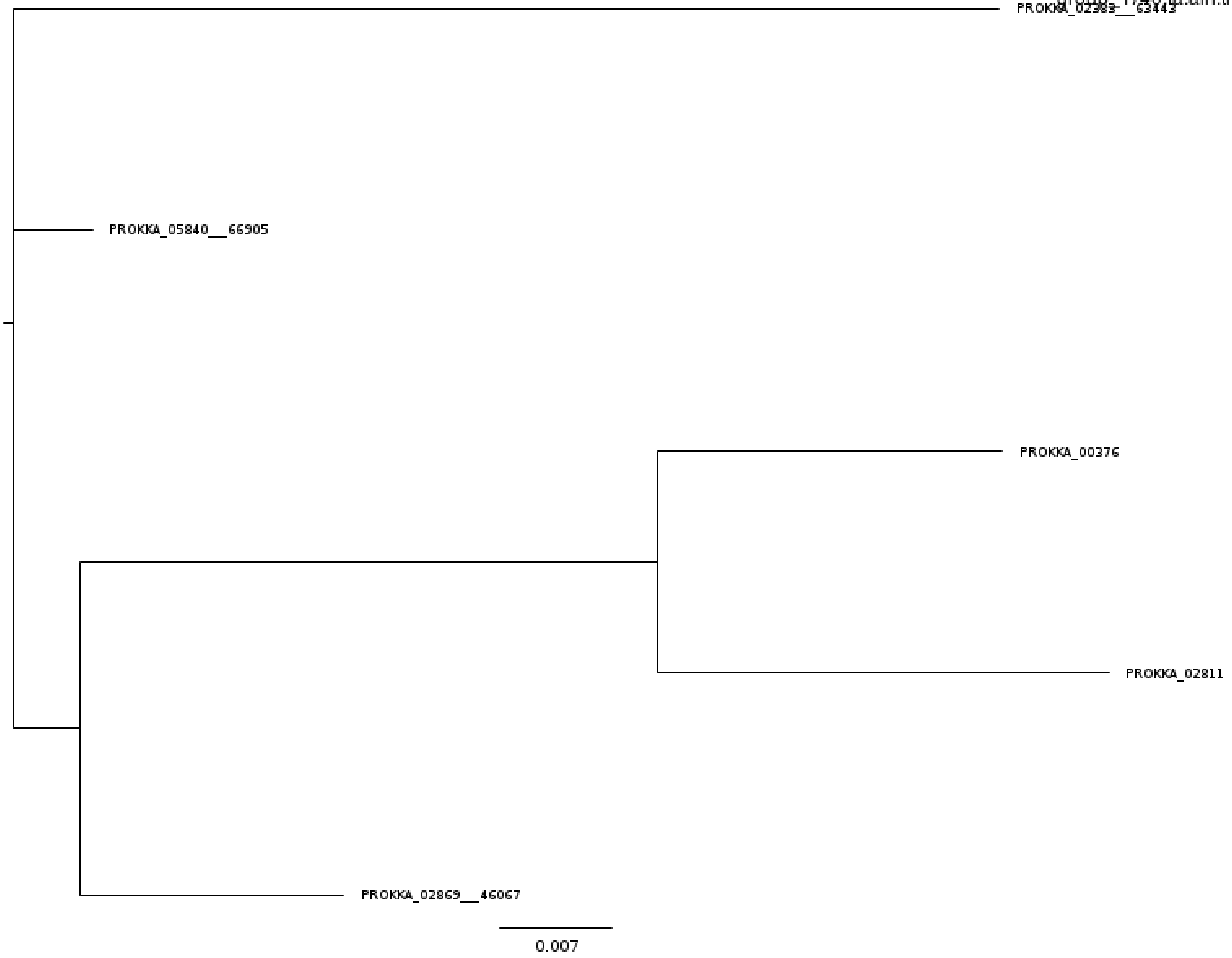

group_1742.fa.aln.tree.png

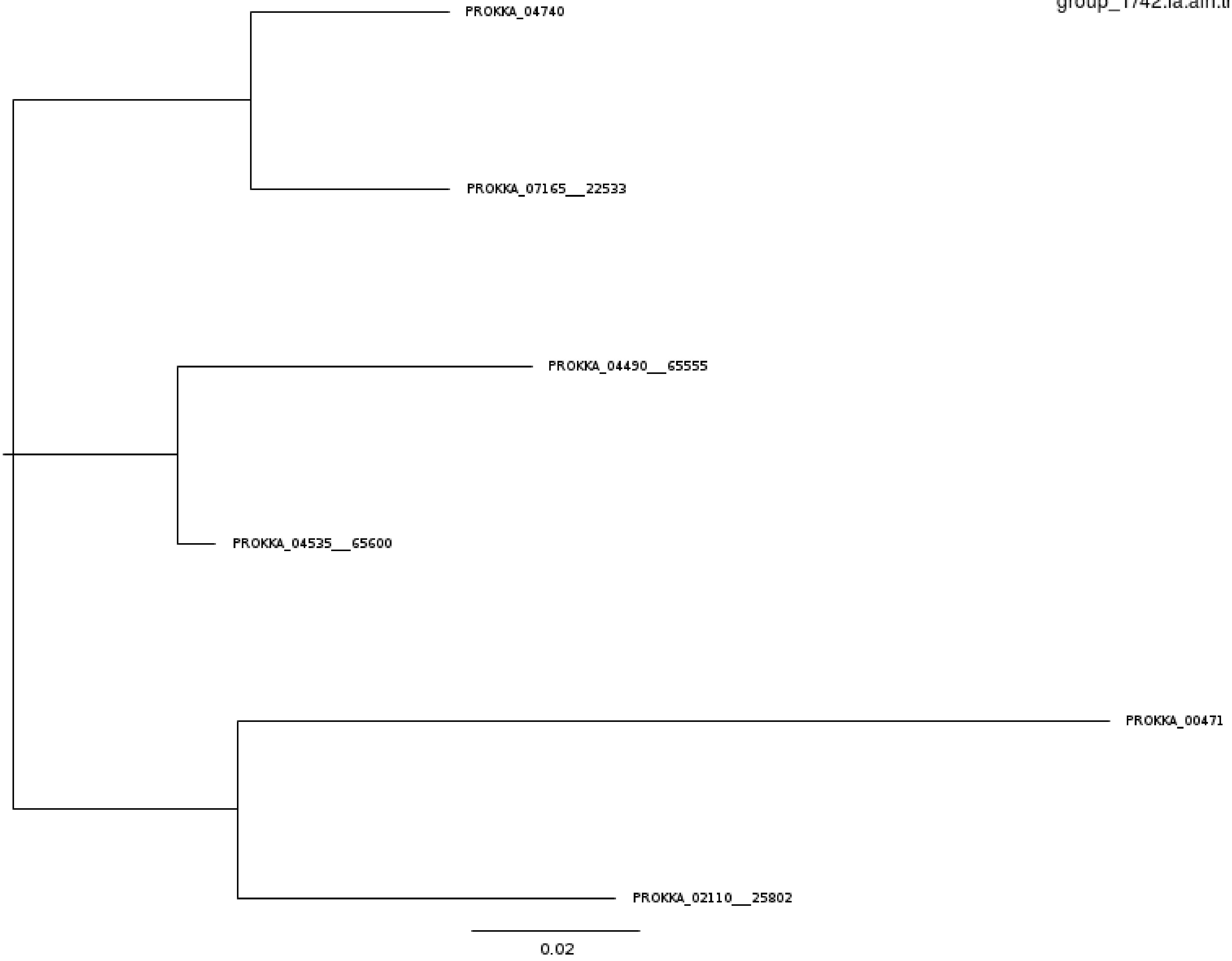

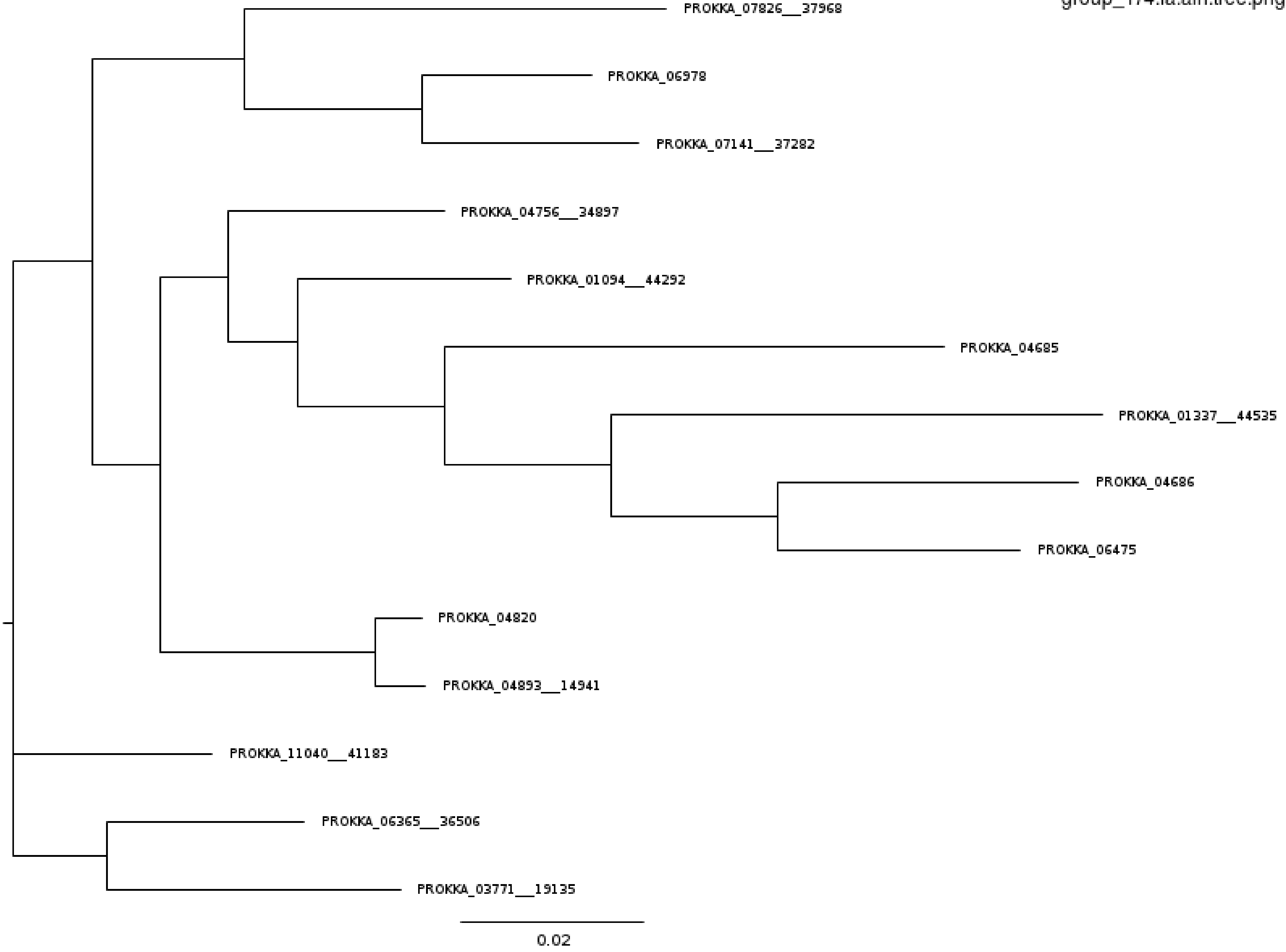
PROKKA_07826___37968
PROKKA_06978
PROKKA_07141___37282
PROKKA_04756___34897
PROKKA_01094___44292
PROKKA_04685
PROKKA_01337___44535
PROKKA_04686
PROKKA_06475
PROKKA_04820
PROKKA_04893___14941
PROKKA_11040___41183
PROKKA_06365___36506
PROKKA_03771___19135
0.02

group_1756.fa.aln.tree.png

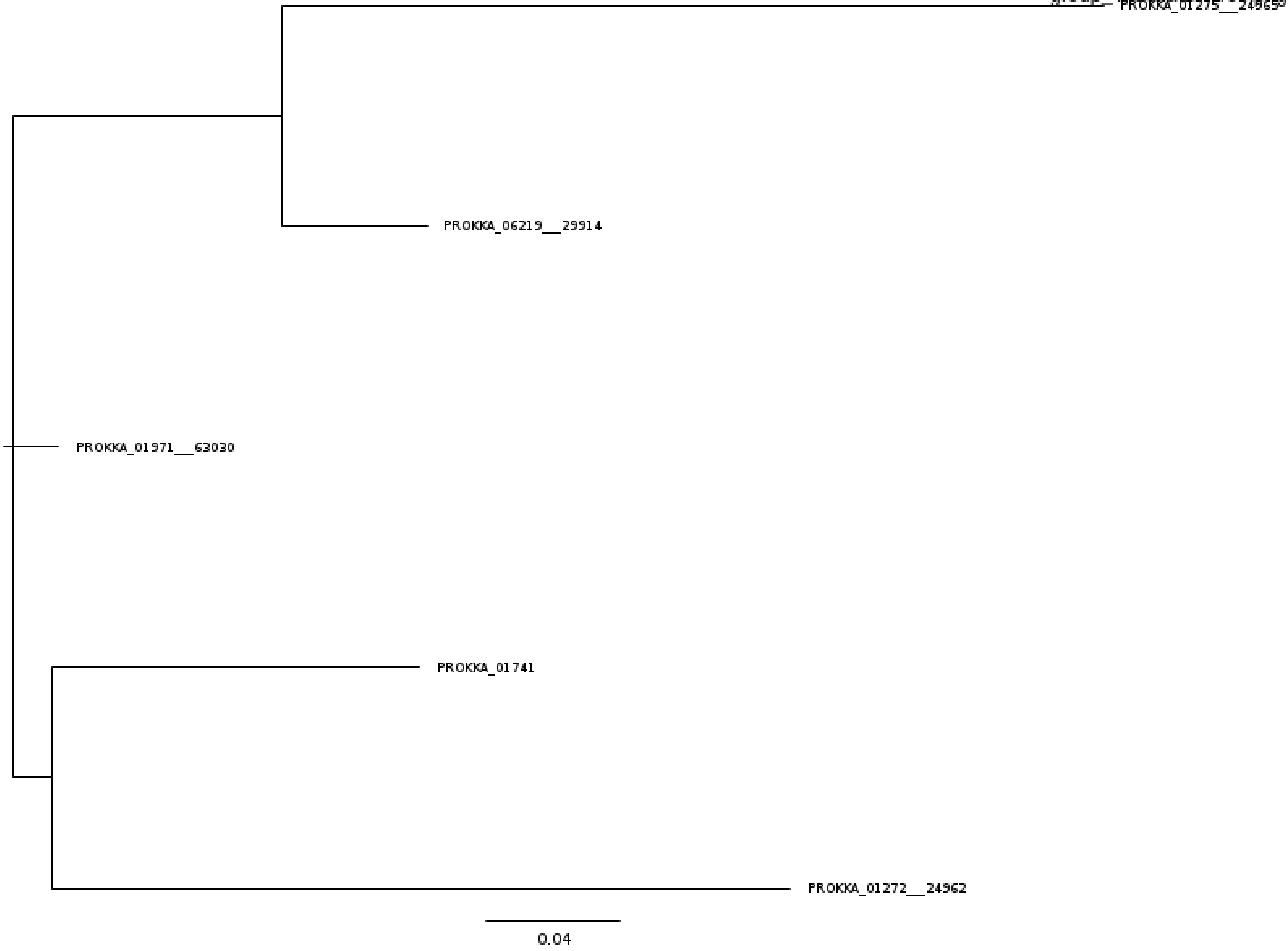

group_1759.fa.aln.tree.png

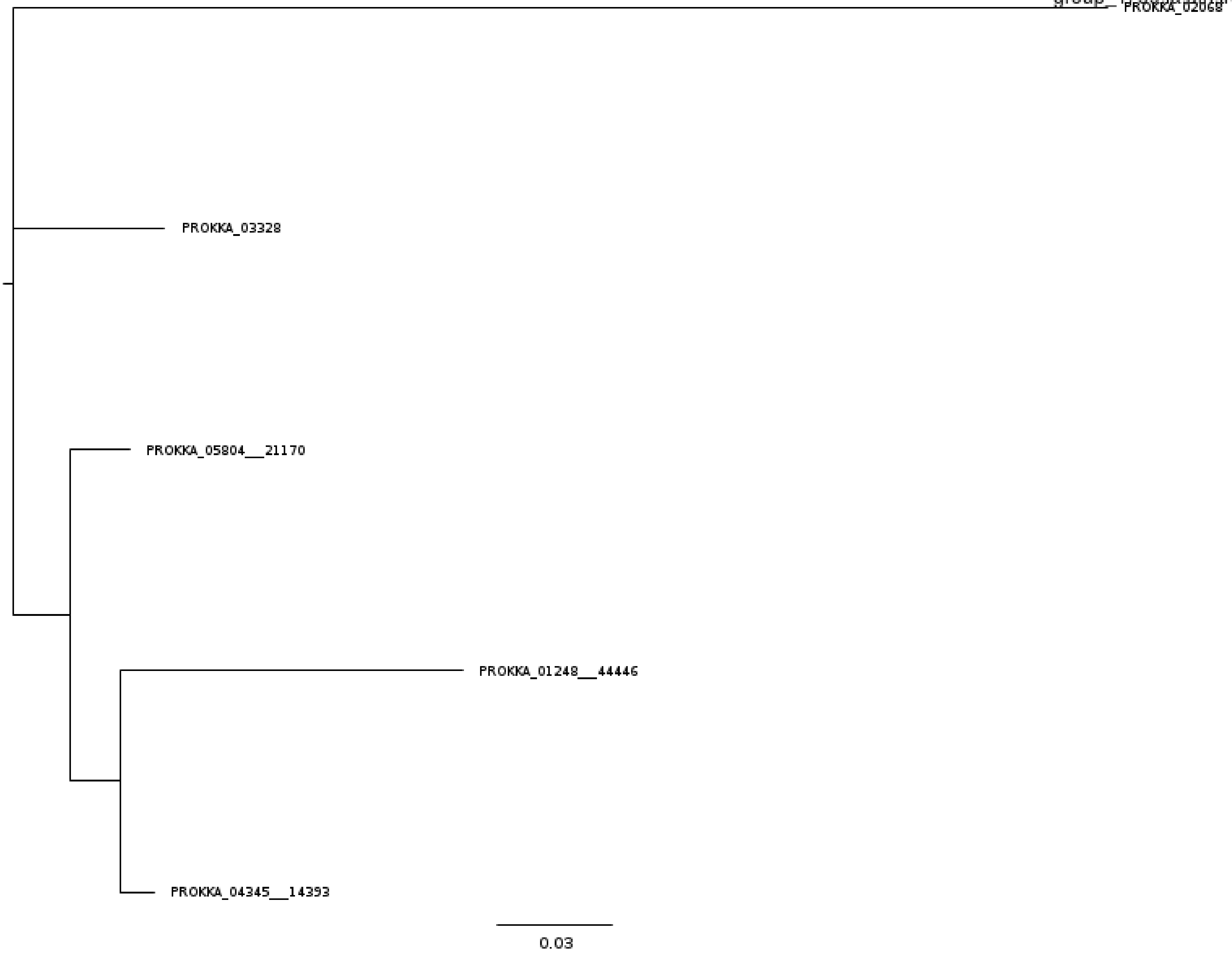

group_1761.fa.aln.tree.png

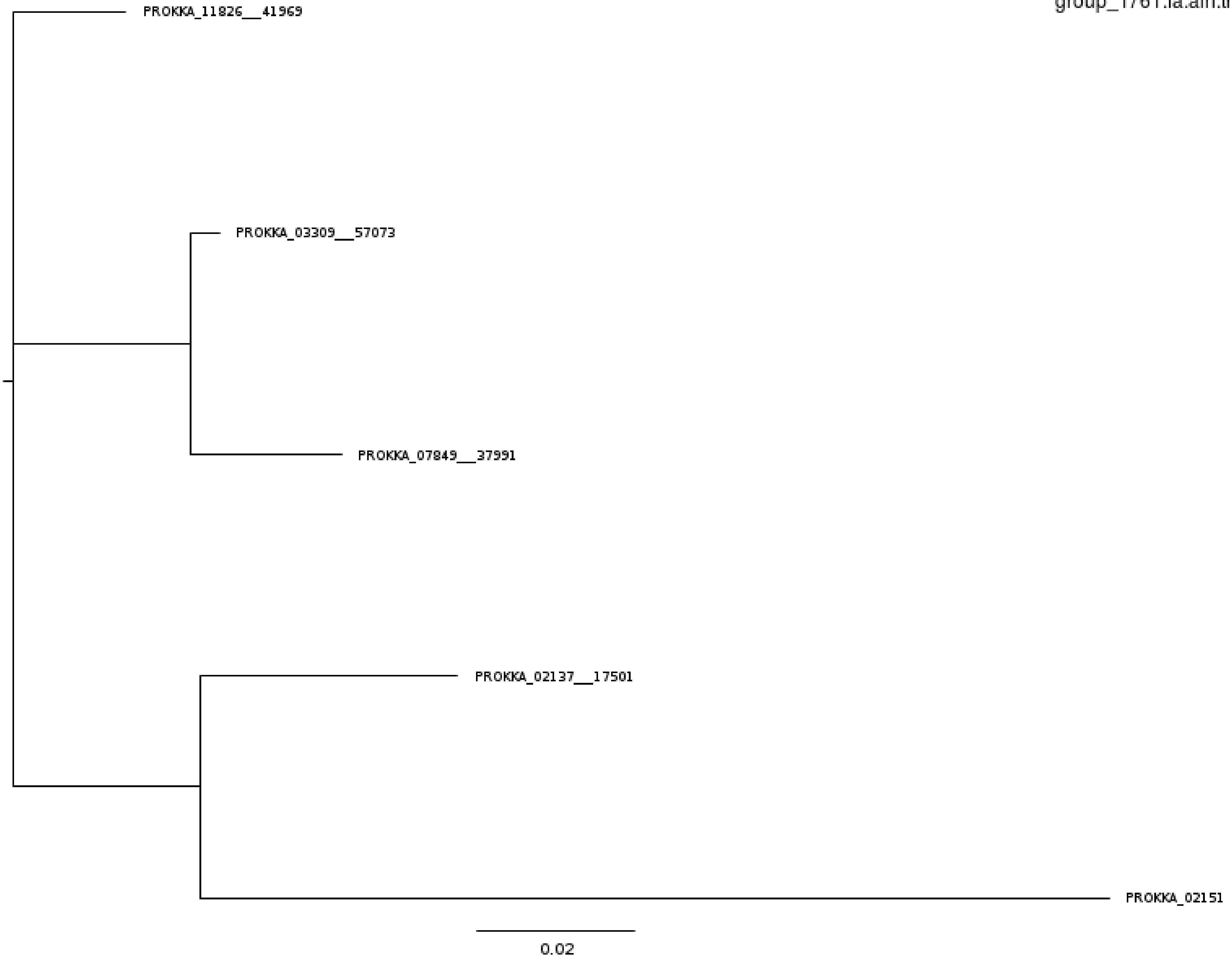

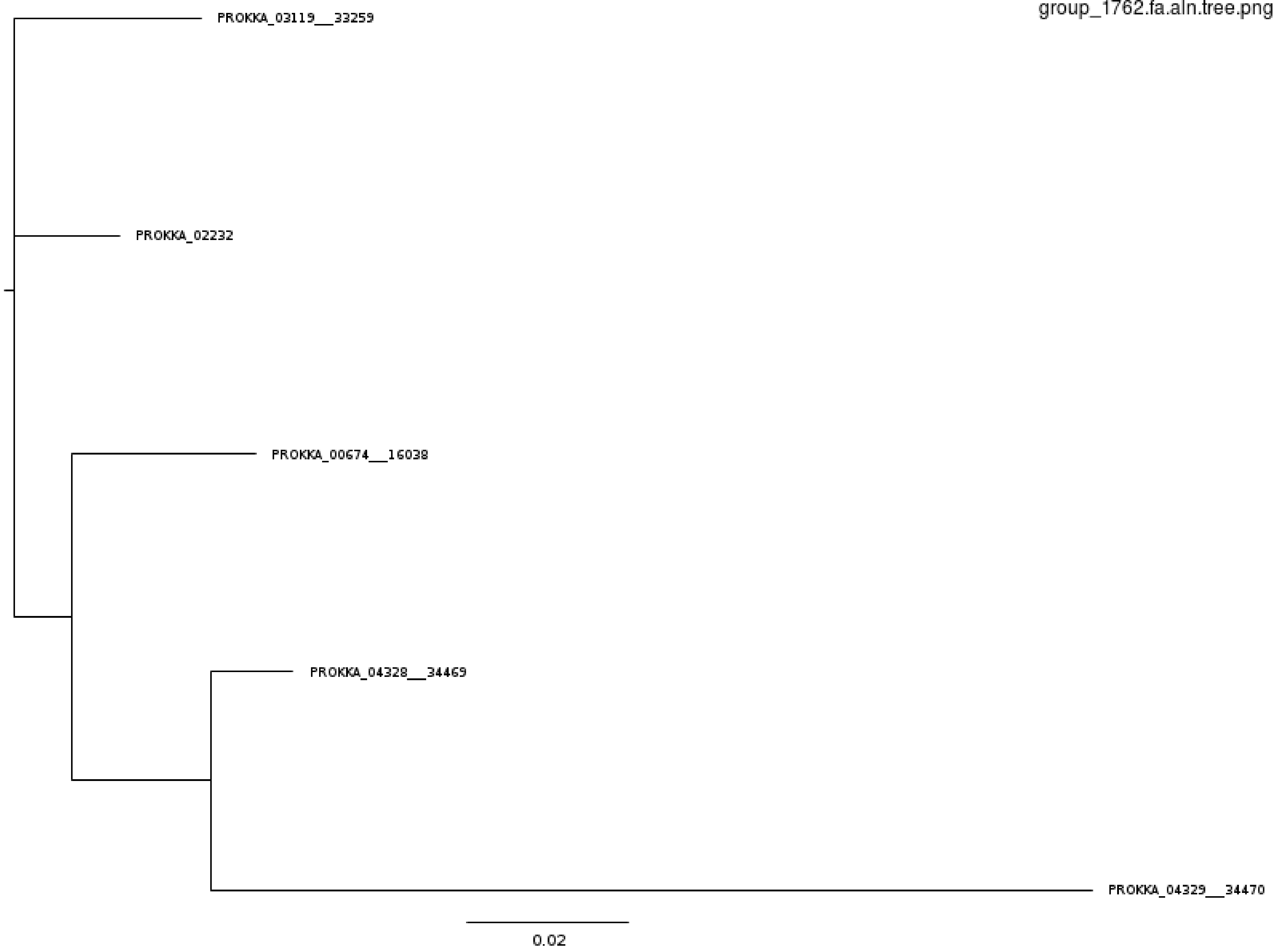
group_1762.fa.aln.tree.png
PROKKA_03119___33259
PROKKA_02232
PROKKA_00674___16038
PROKKA_04328___34469
PROKKA_04329___34470
0.02

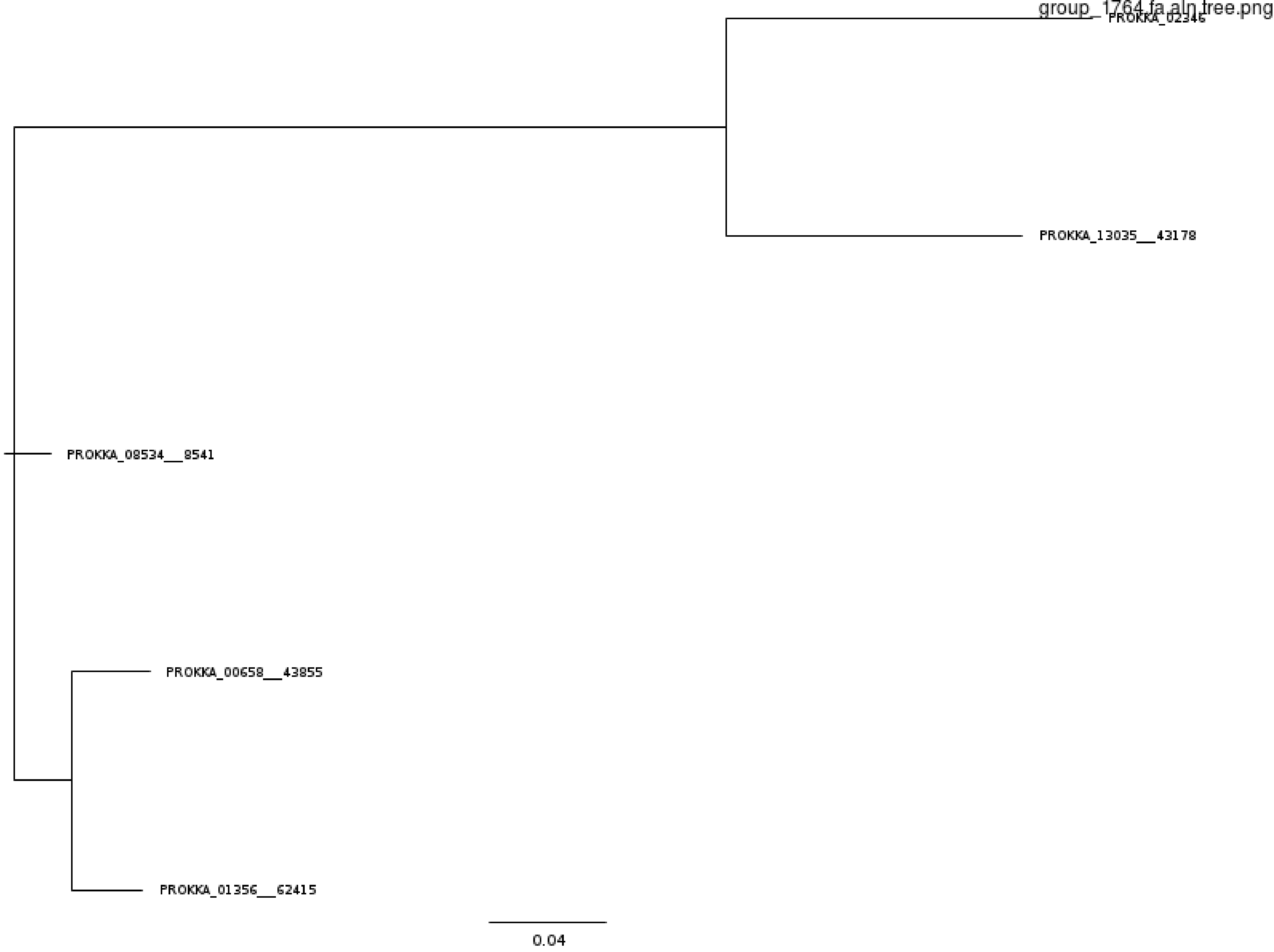
group_1764.fa.aln.tree.png
PROKKA_02346
PROKKA_13035___43178
PROKKA_08534___8541
PROKKA_00658___43855
PROKKA_01356___62415
0.04

group_1766.fa.aln.tree.png

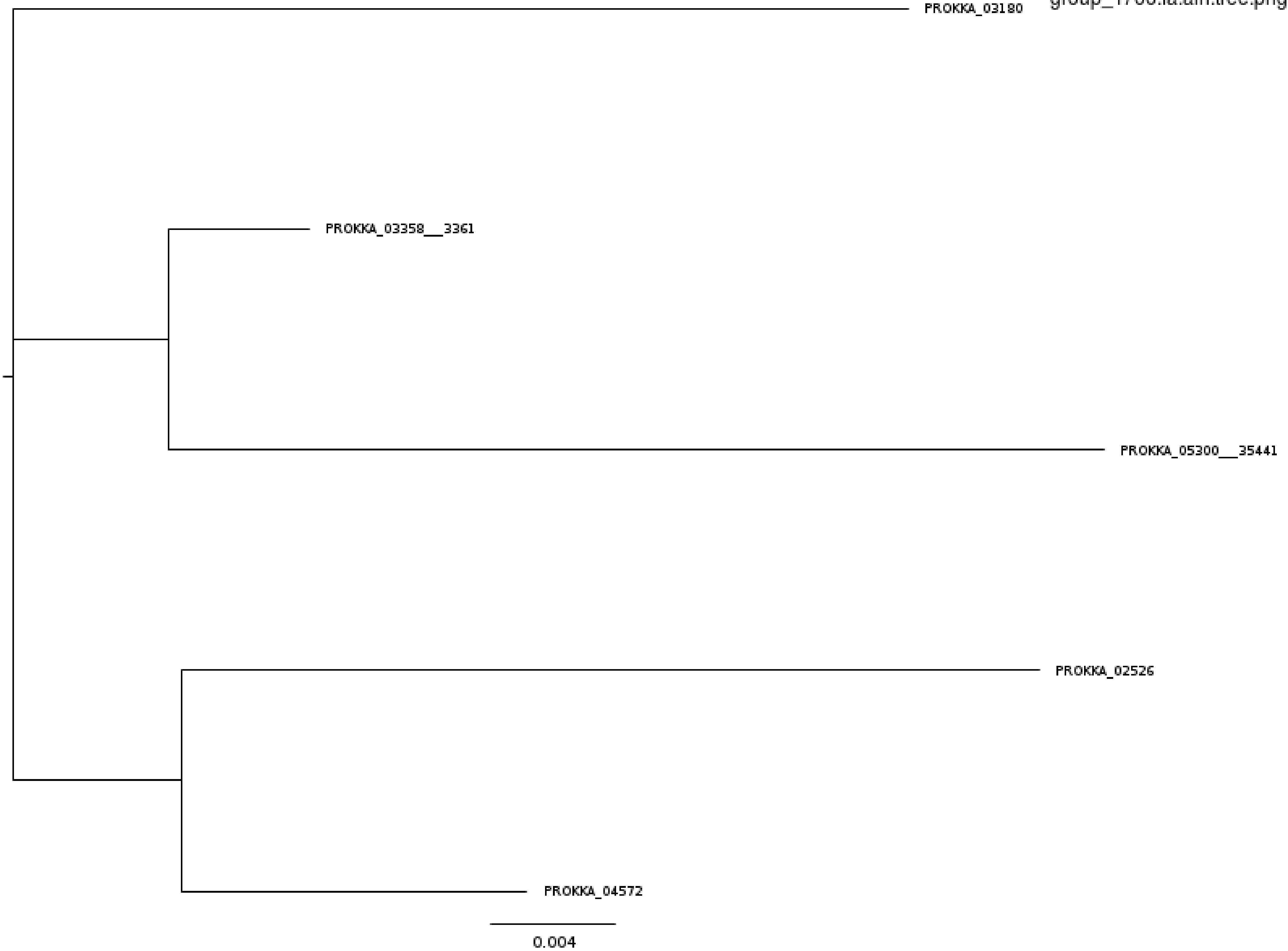

group_1767.fa.aln.tree.png

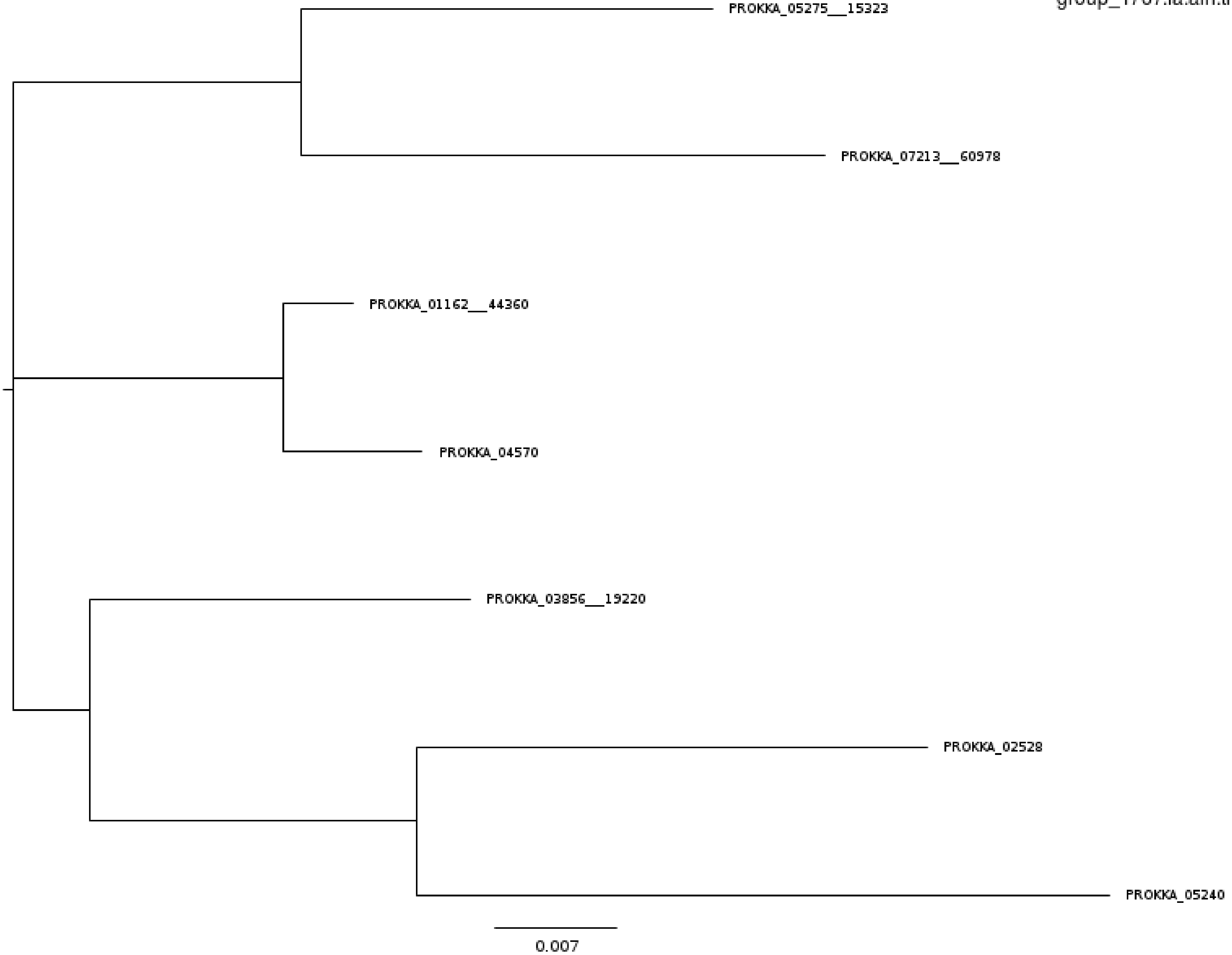

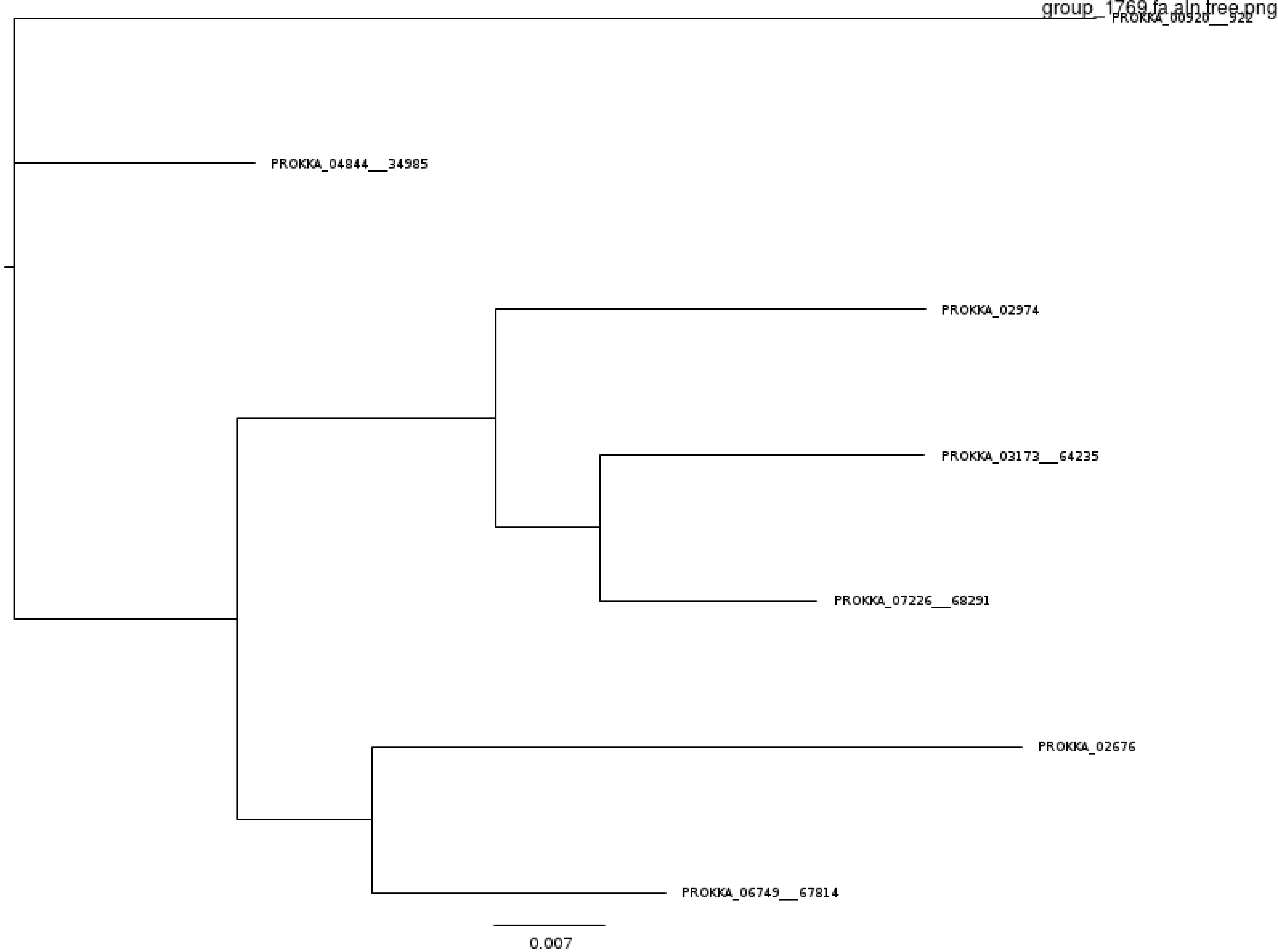

group_1769.fa.aln.tree.png
PROKKA_00920___922
PROKKA_04844___34985
PROKKA_02974
PROKKA_03173___64235
PROKKA_07226___68291
PROKKA_02676
PROKKA_06749___67814
0.007

group_1770.fa.aln.tree.png

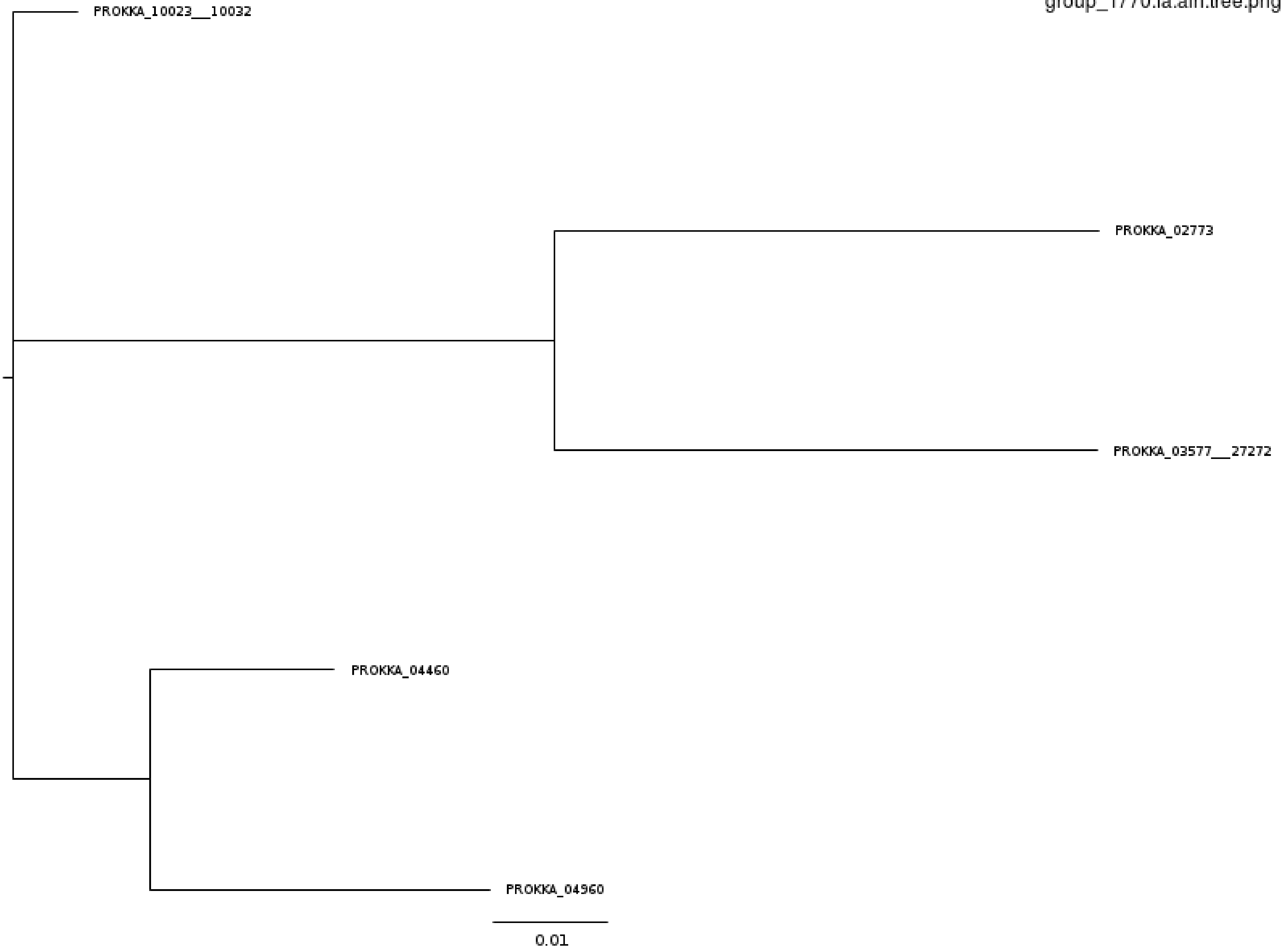

group_1773.fa.aln.tree.png

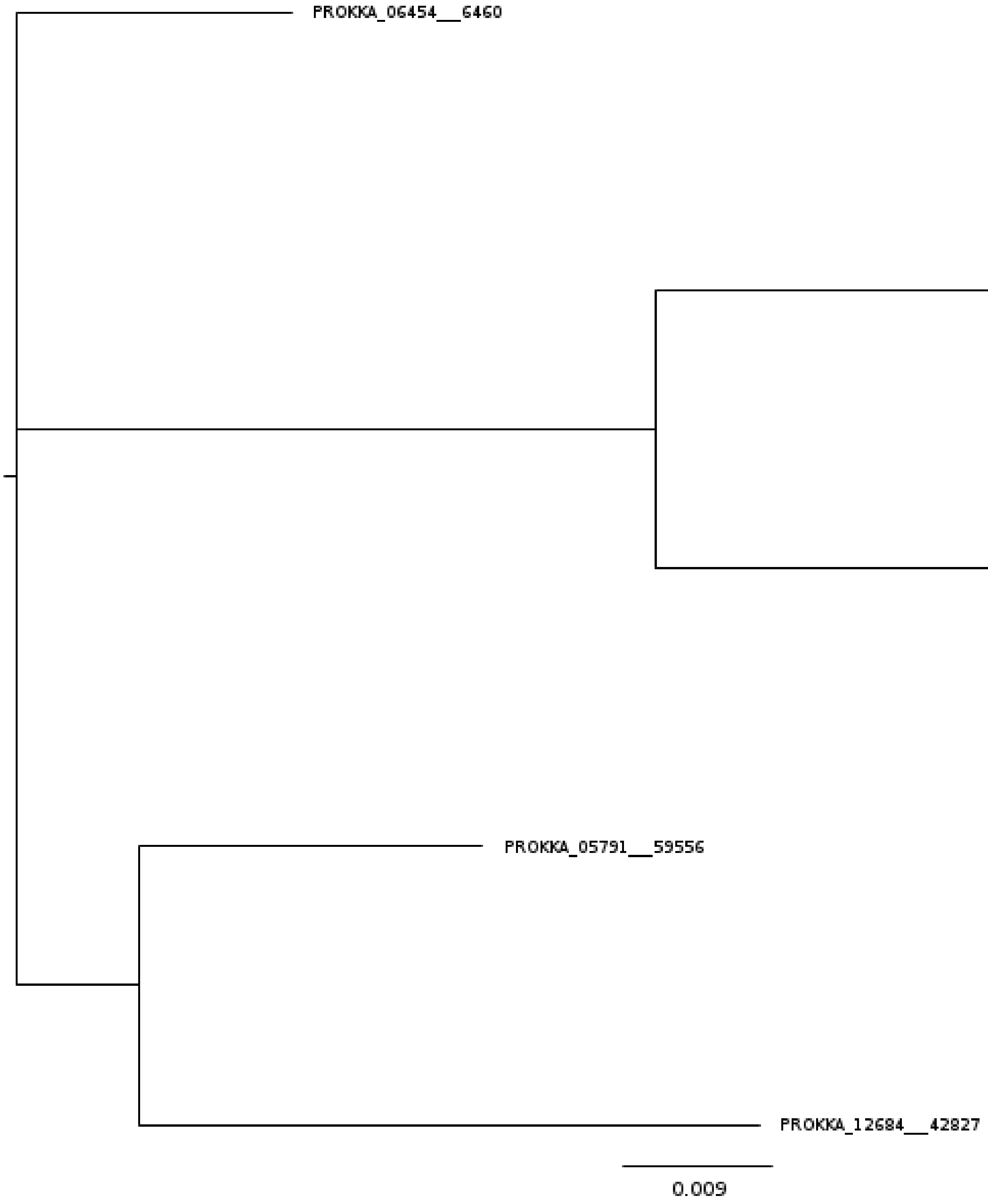

group_1774.fa.aln.tree.png

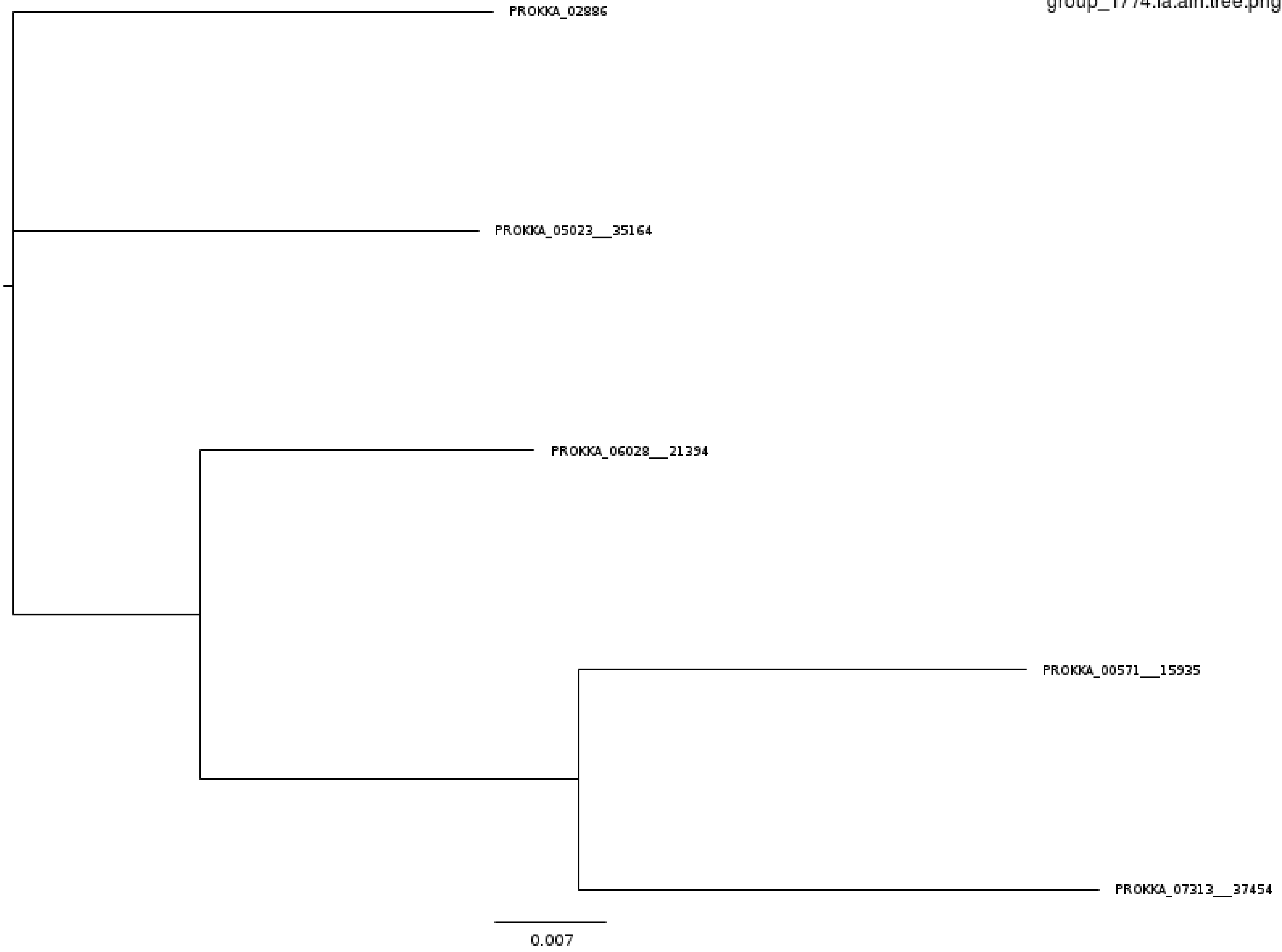

group_1776.fa.aln.tree.png

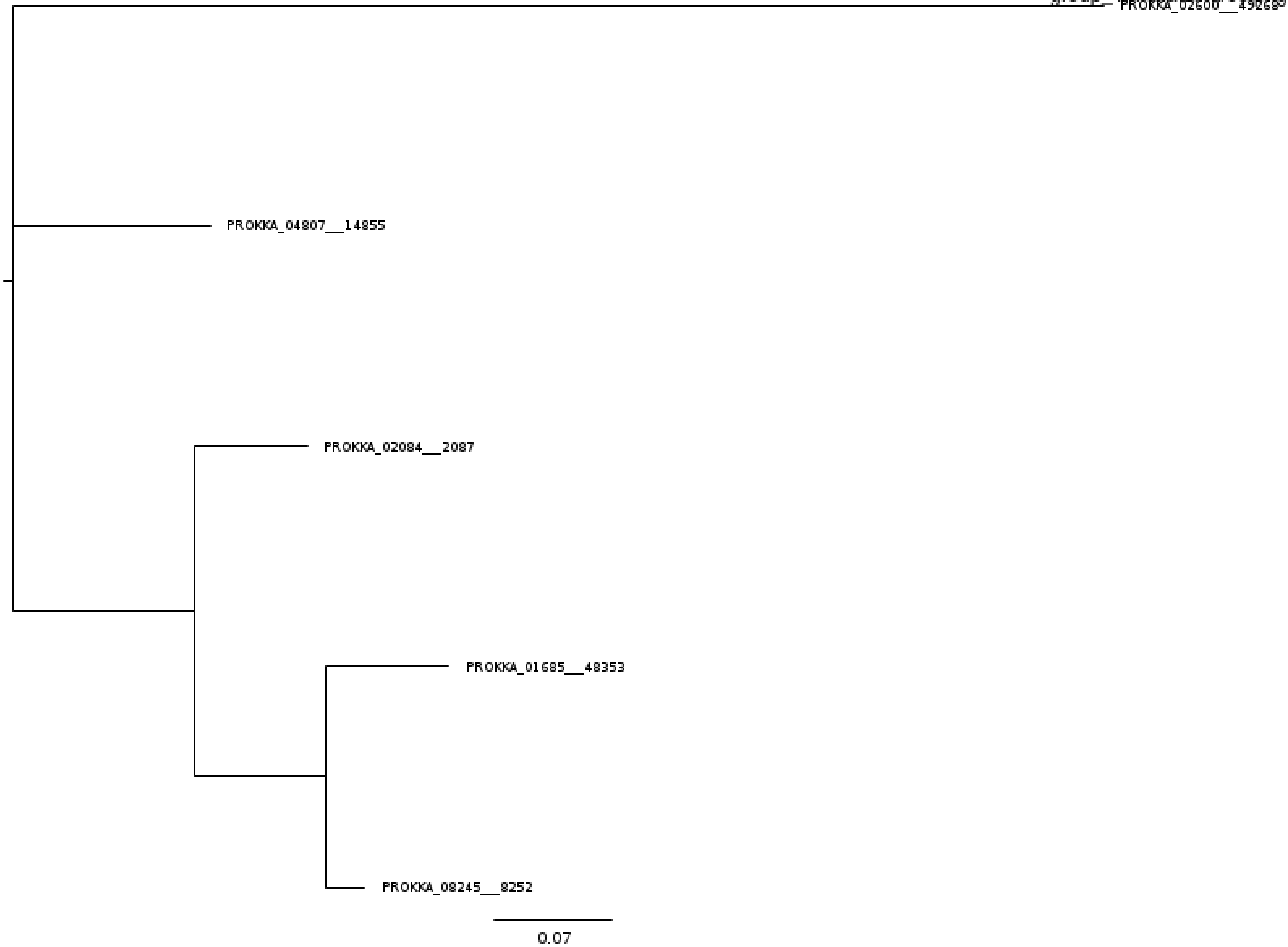

group_1778.fa.aln.tree.png

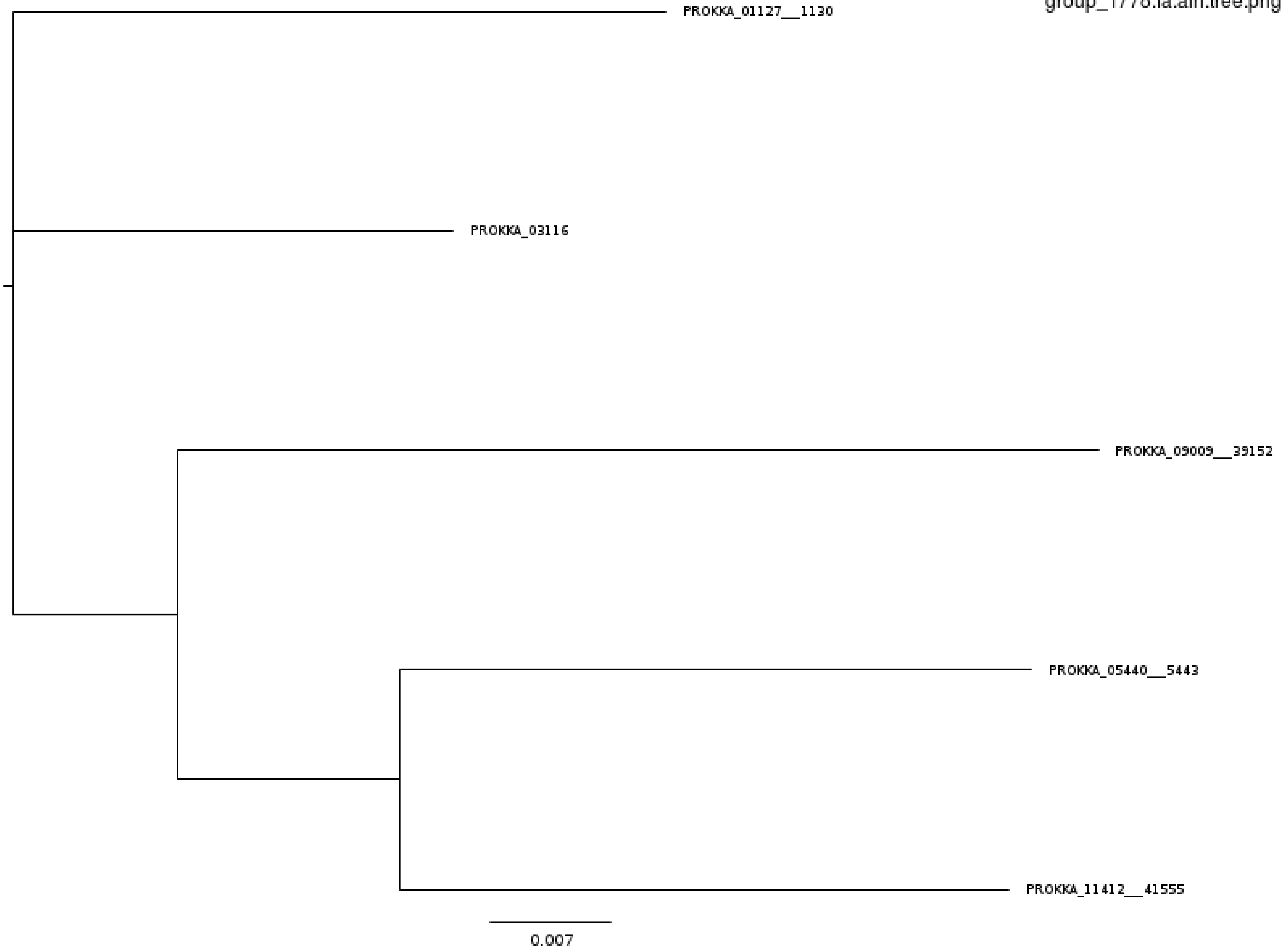

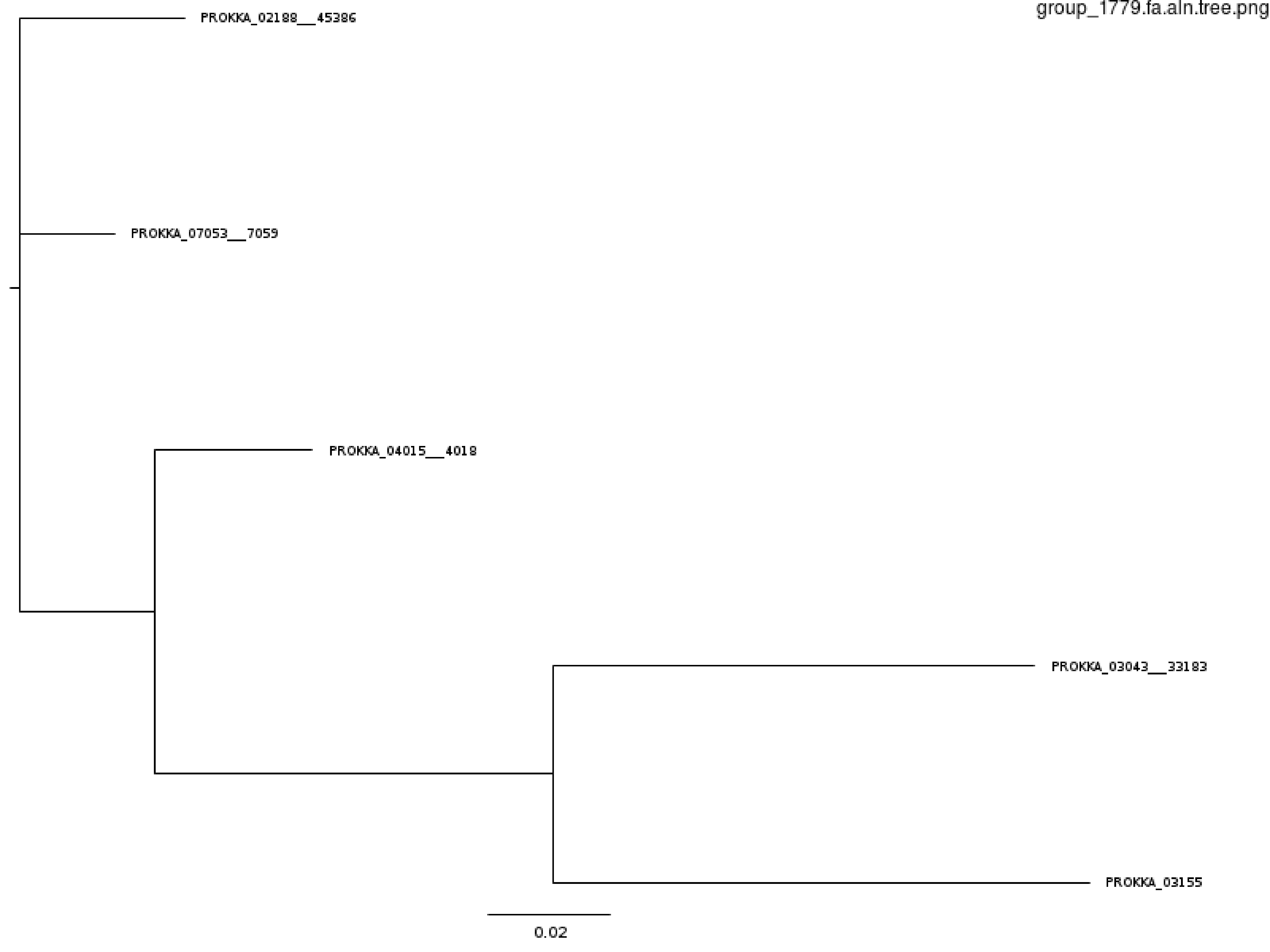
group_1779.fa.aln.tree.png
PROKKA_02188___45386
PROKKA_07053___7059
PROKKA_04015___4018
PROKKA_03043___33183
PROKKA_03155
0.02

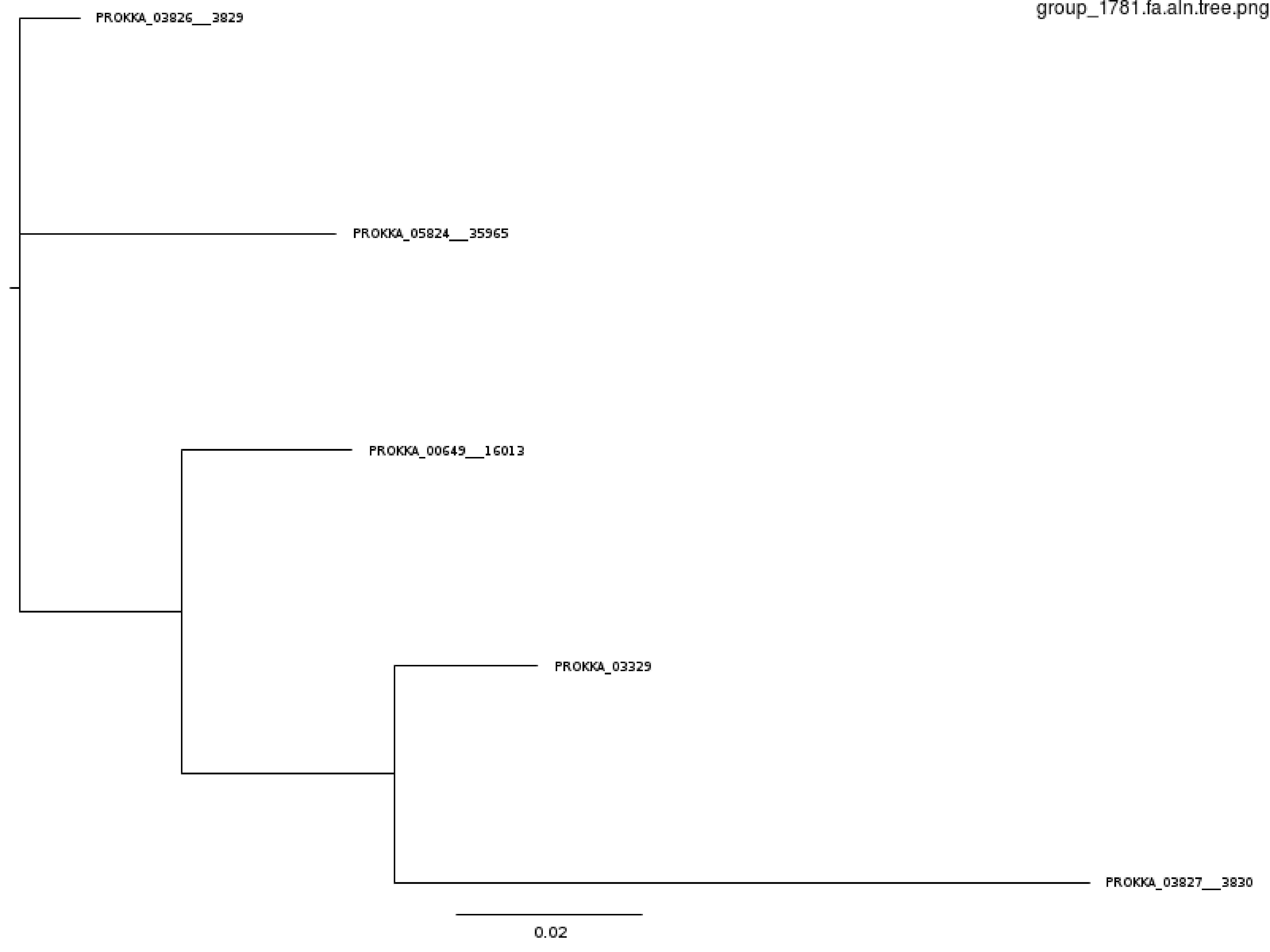
group_1781.fa.aln.tree.png
PROKKA_03826___3829
PROKKA_05824___35965
PROKKA_00649___16013
PROKKA_03329
PROKKA_03827___3830
0.02

group_1782.fa.aln.tree.png

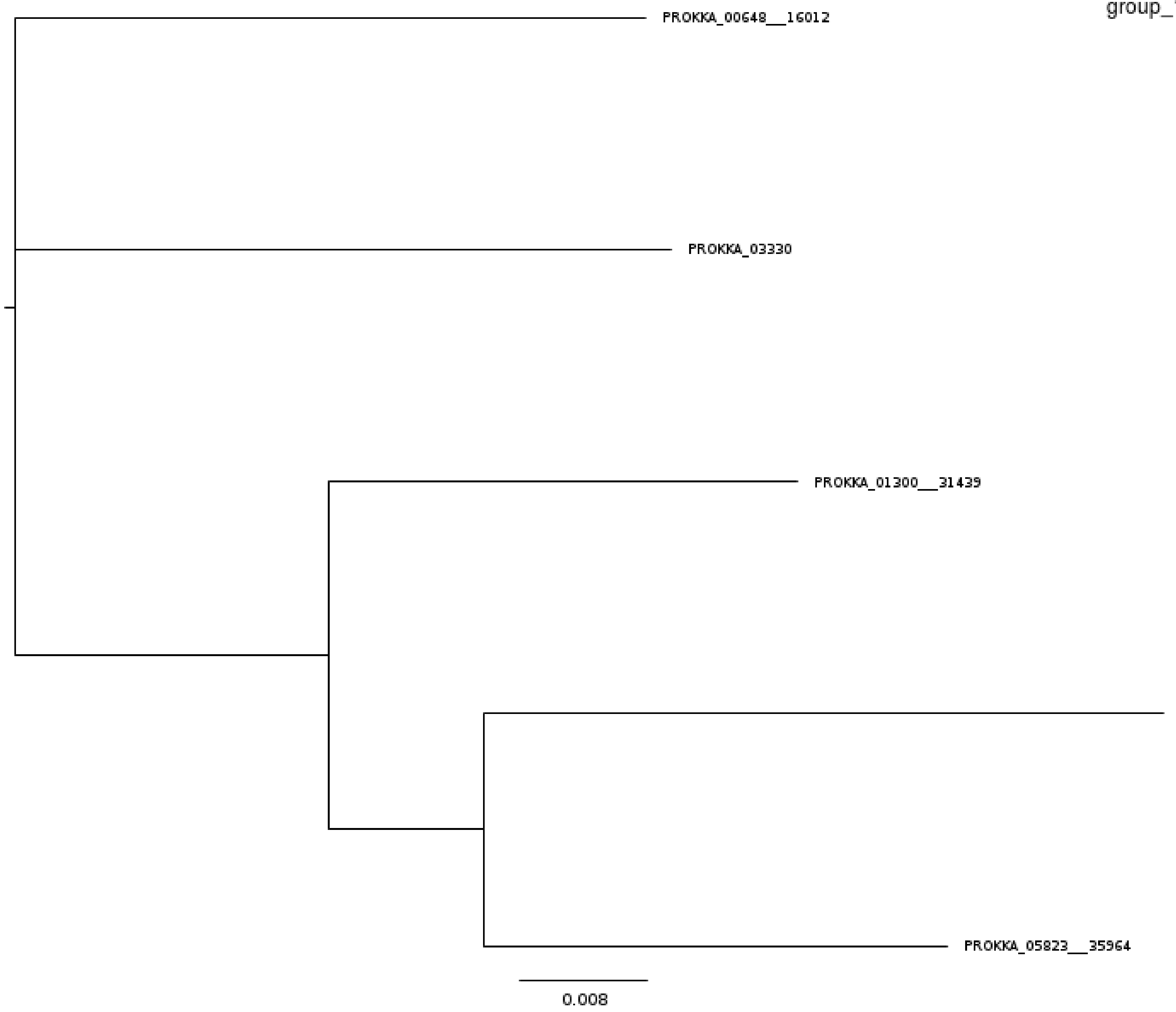

group_1783.fa.aln.tree.png

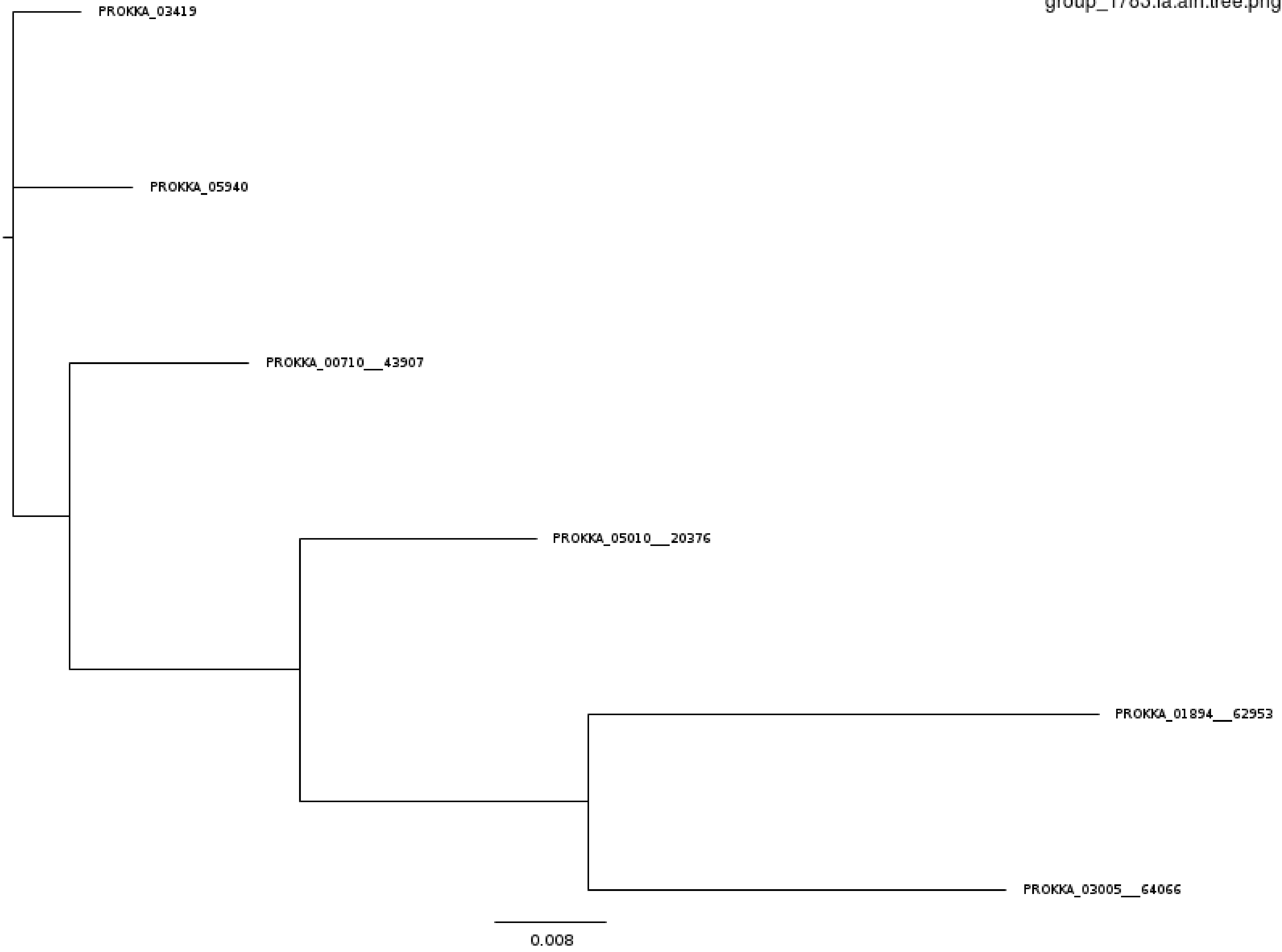

group_1784.fa.aln.tree.png

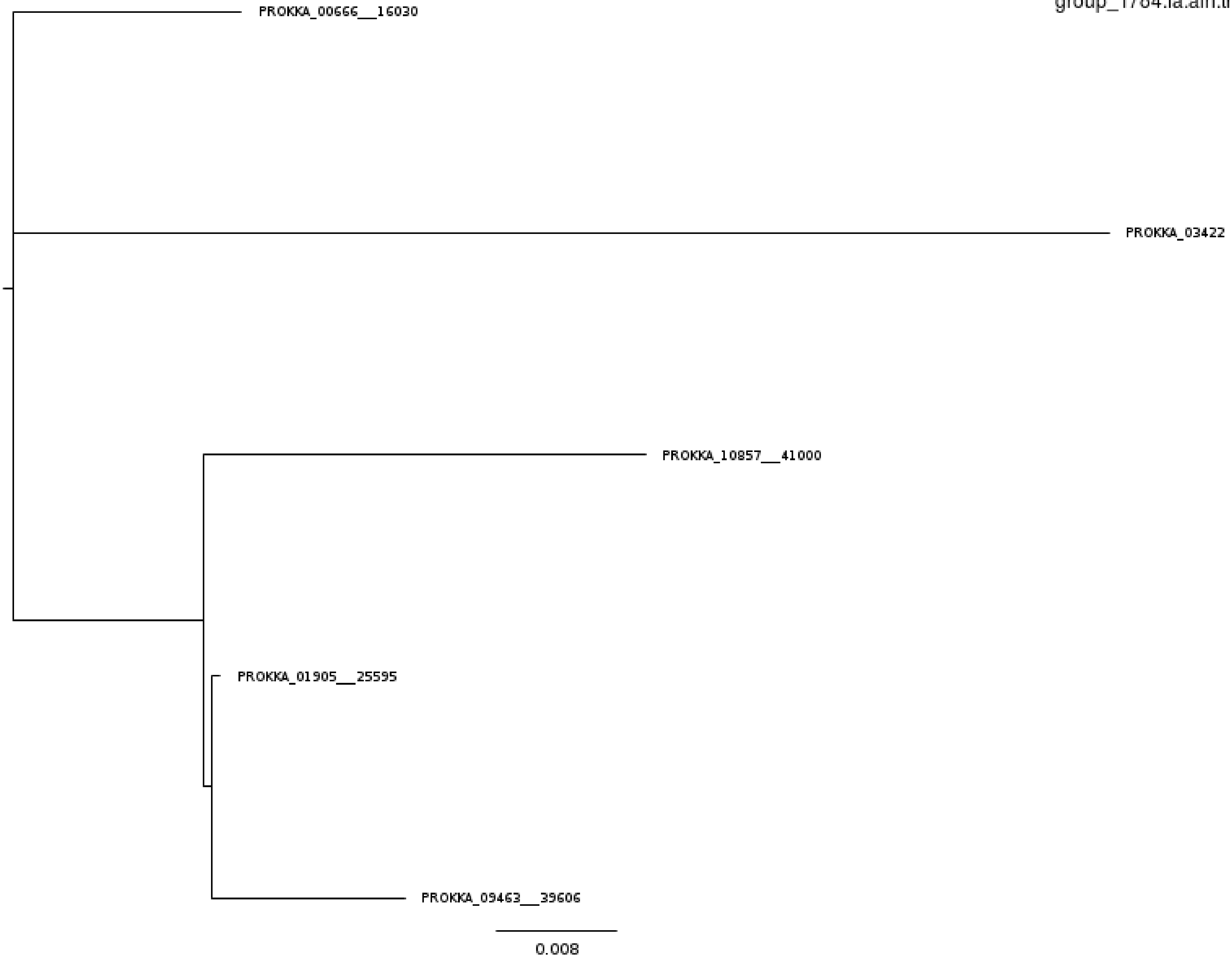

group_1789.fa.aln.tree.png

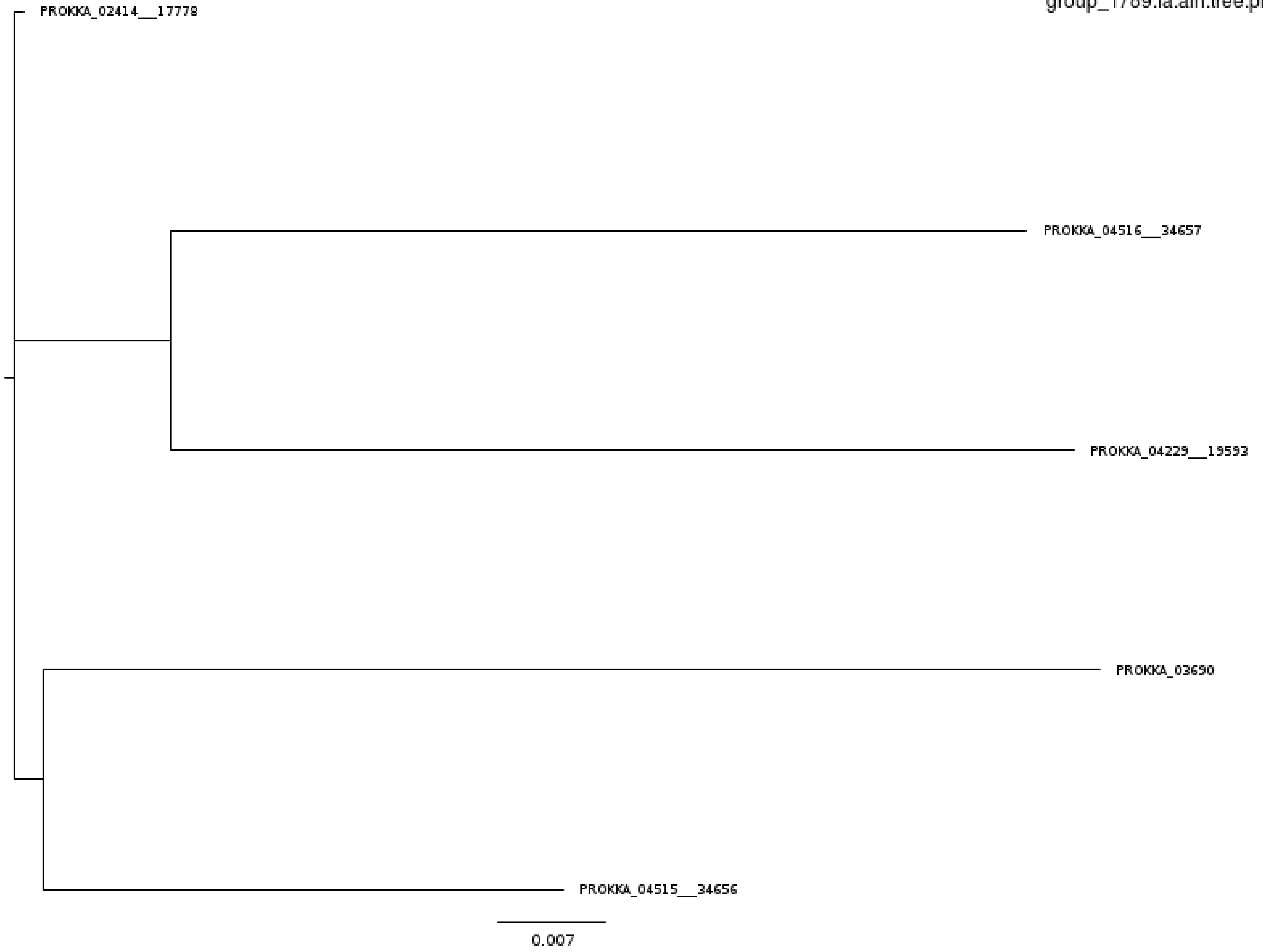

group_1792.fa.aln.tree.png

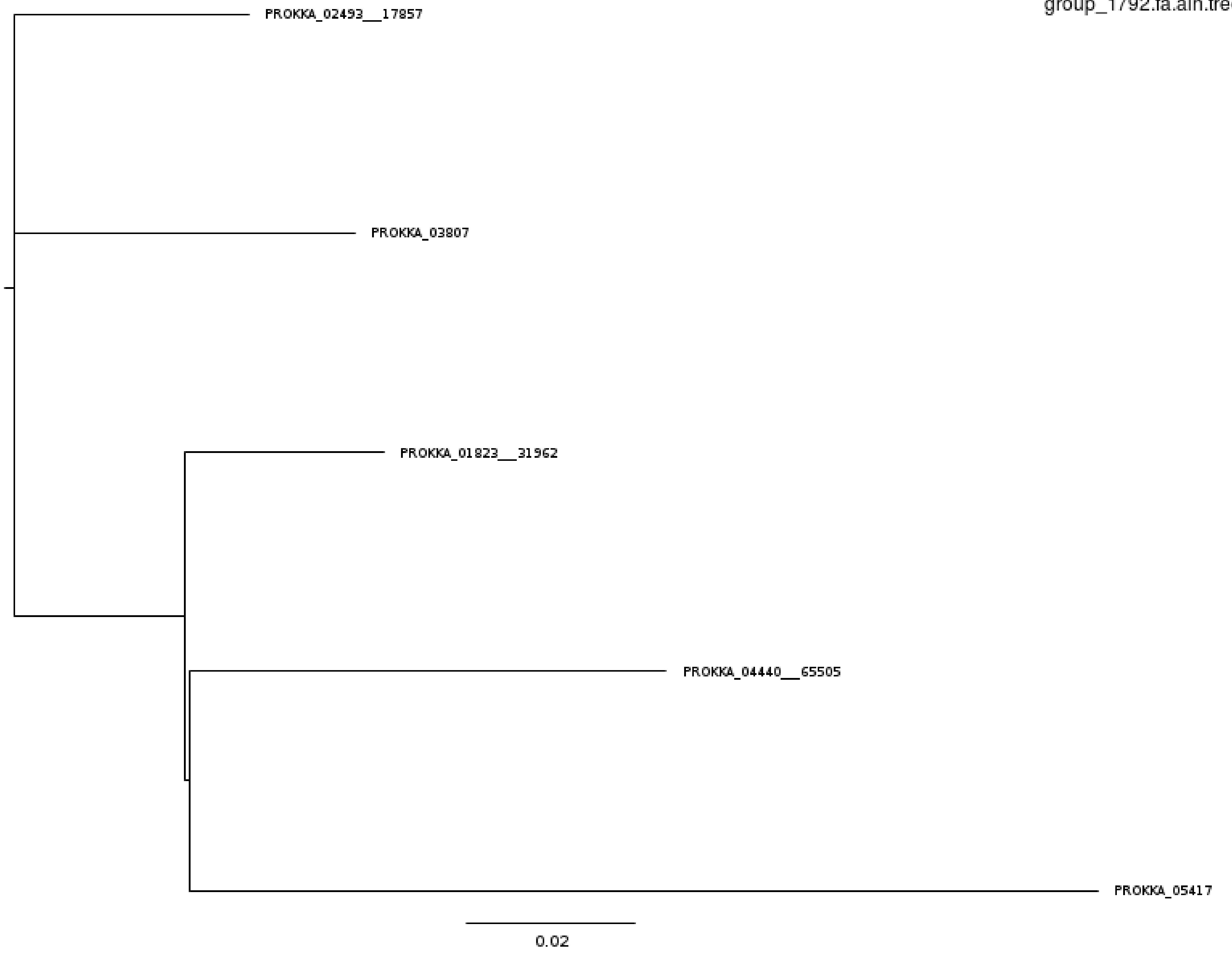

group_1793.fa.aln.tree.png

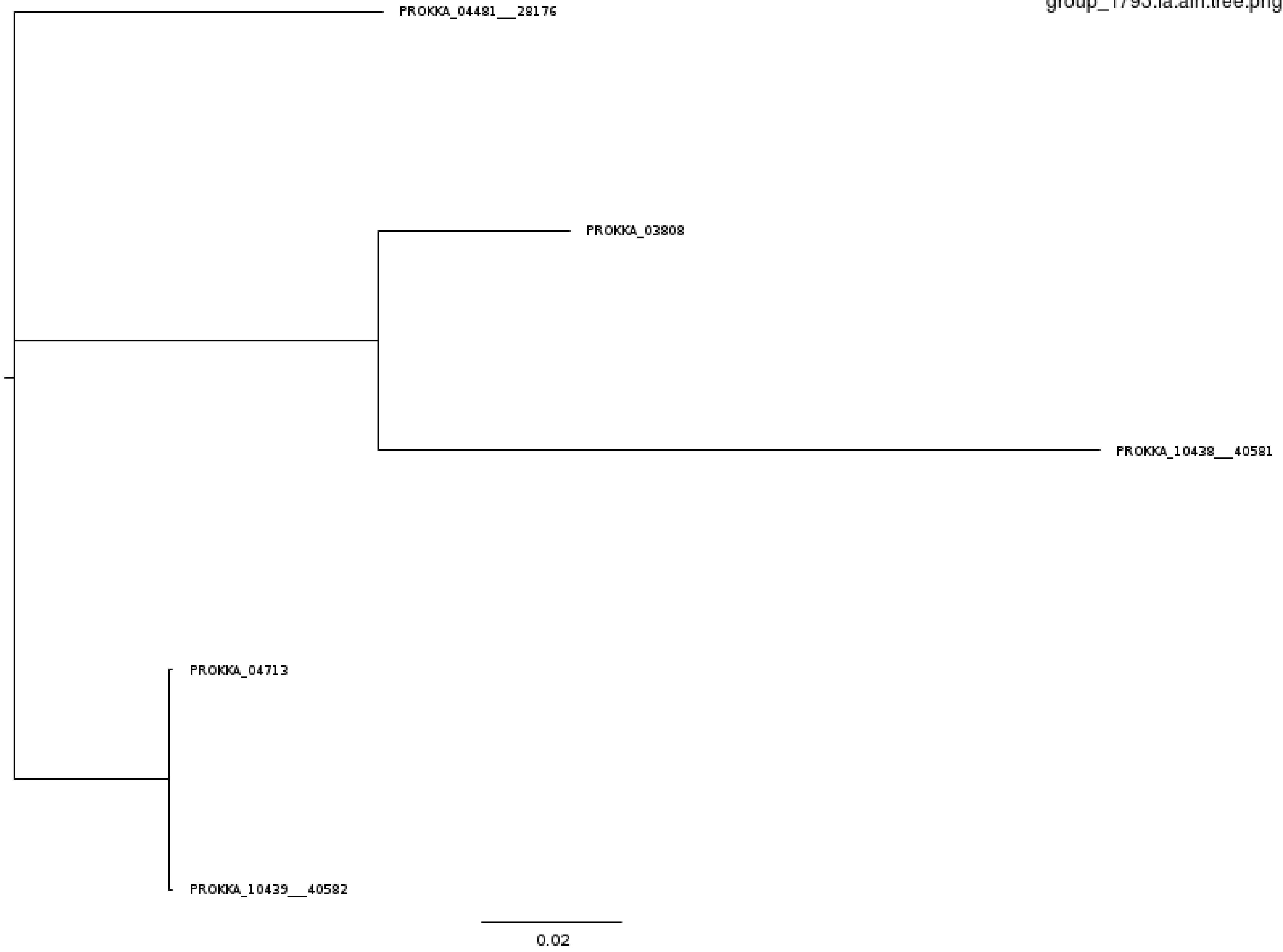

group_1795.fa.aln.tree.png

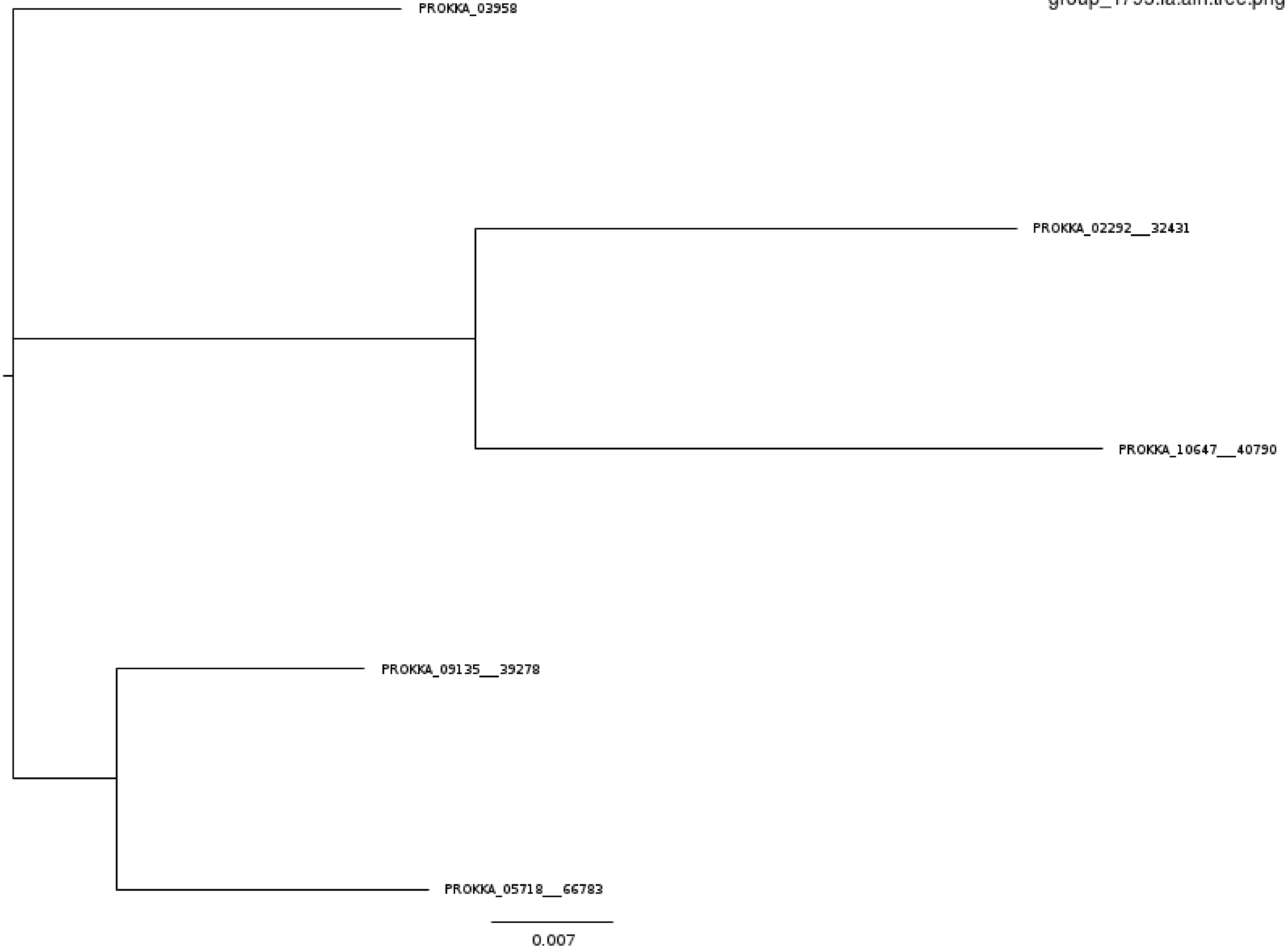

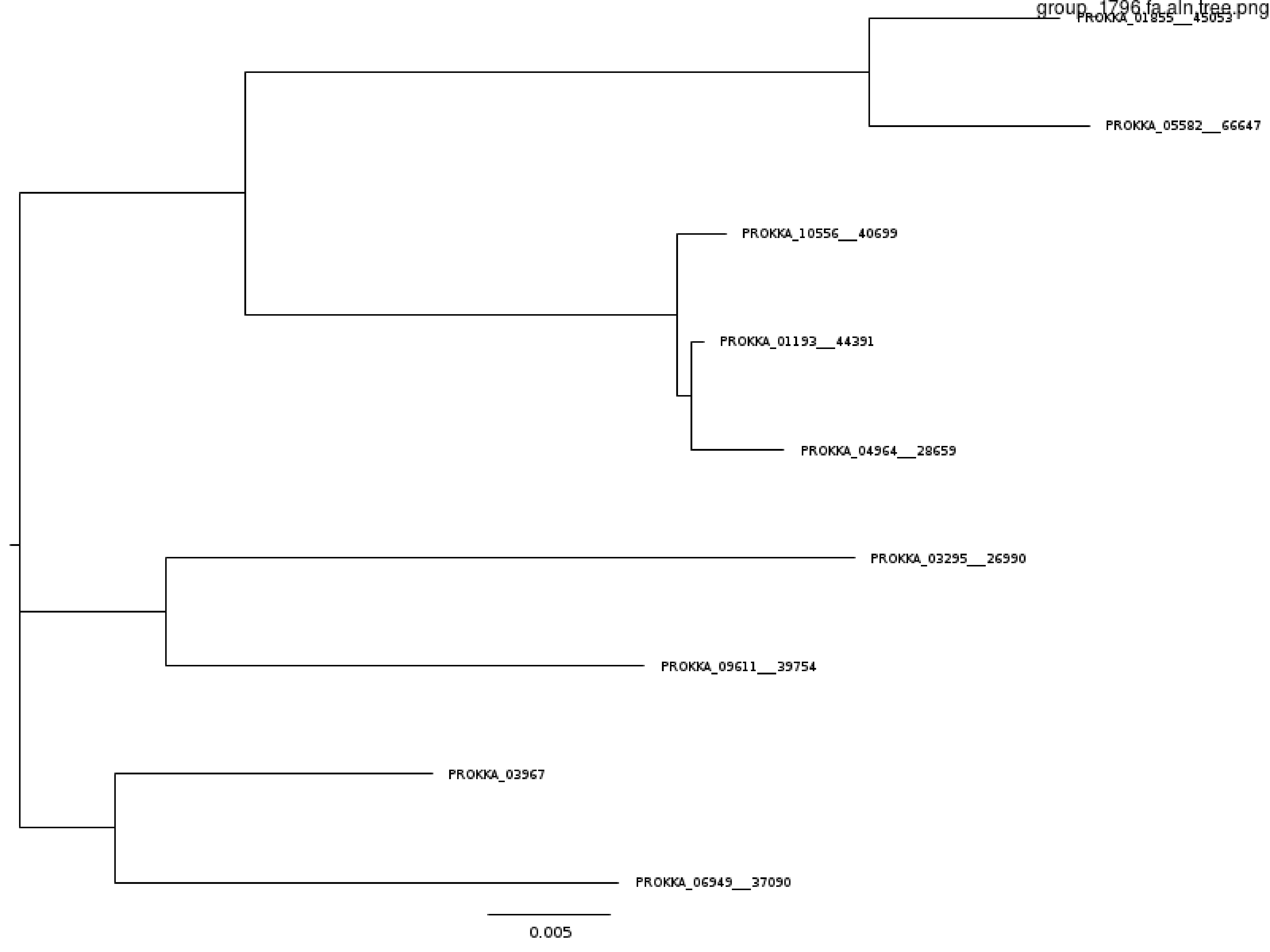
group_1796.fa.aln.tree.png
PROKKA_01855___45053
PROKKA_05582___66647
PROKKA_10556___40699
PROKKA_01193___44391
PROKKA_04964___28659
PROKKA_03295___26990
PROKKA_09611___39754
PROKKA_03967
PROKKA_06949___37090
0.005

group_1797.fa.aln.tree.png

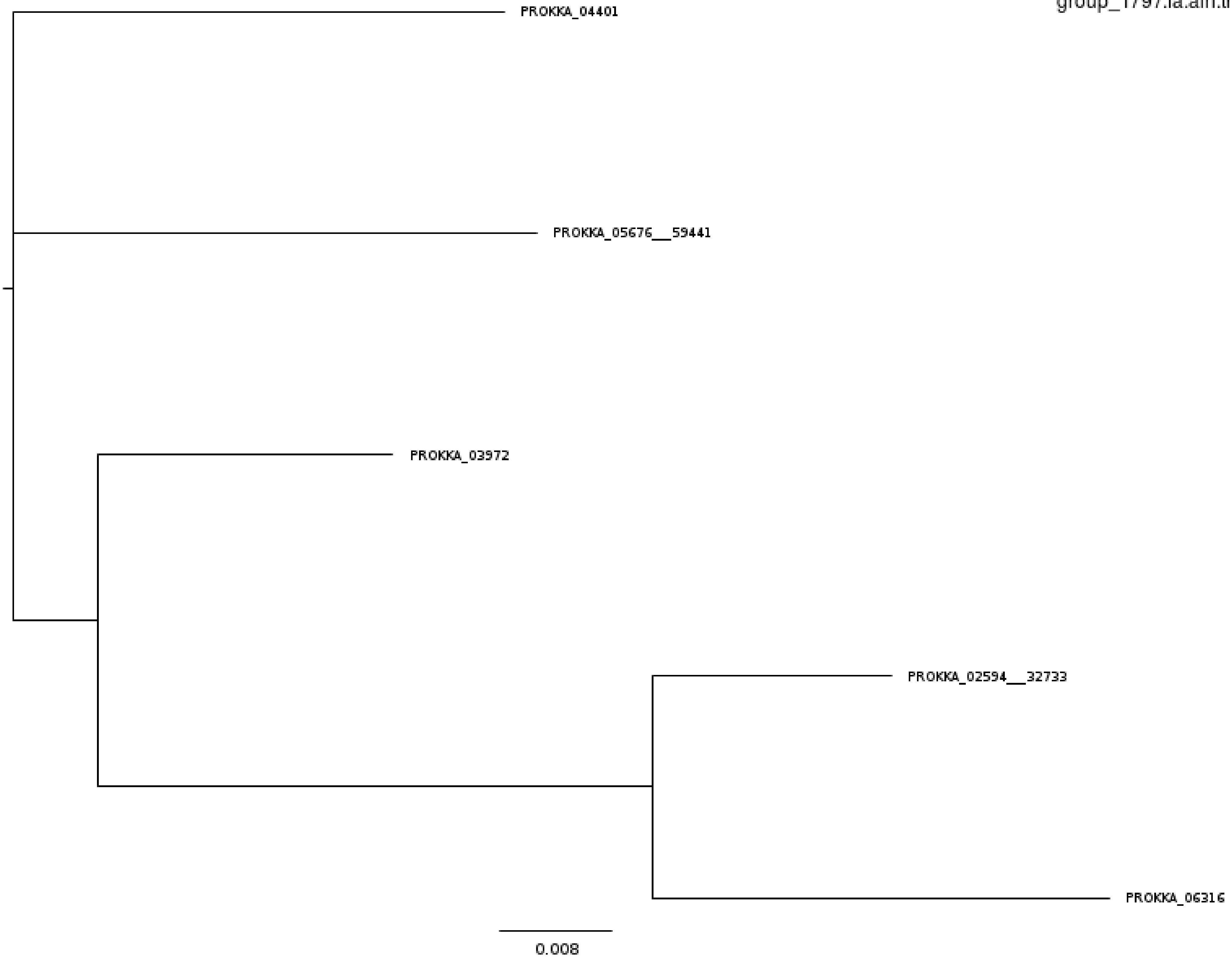

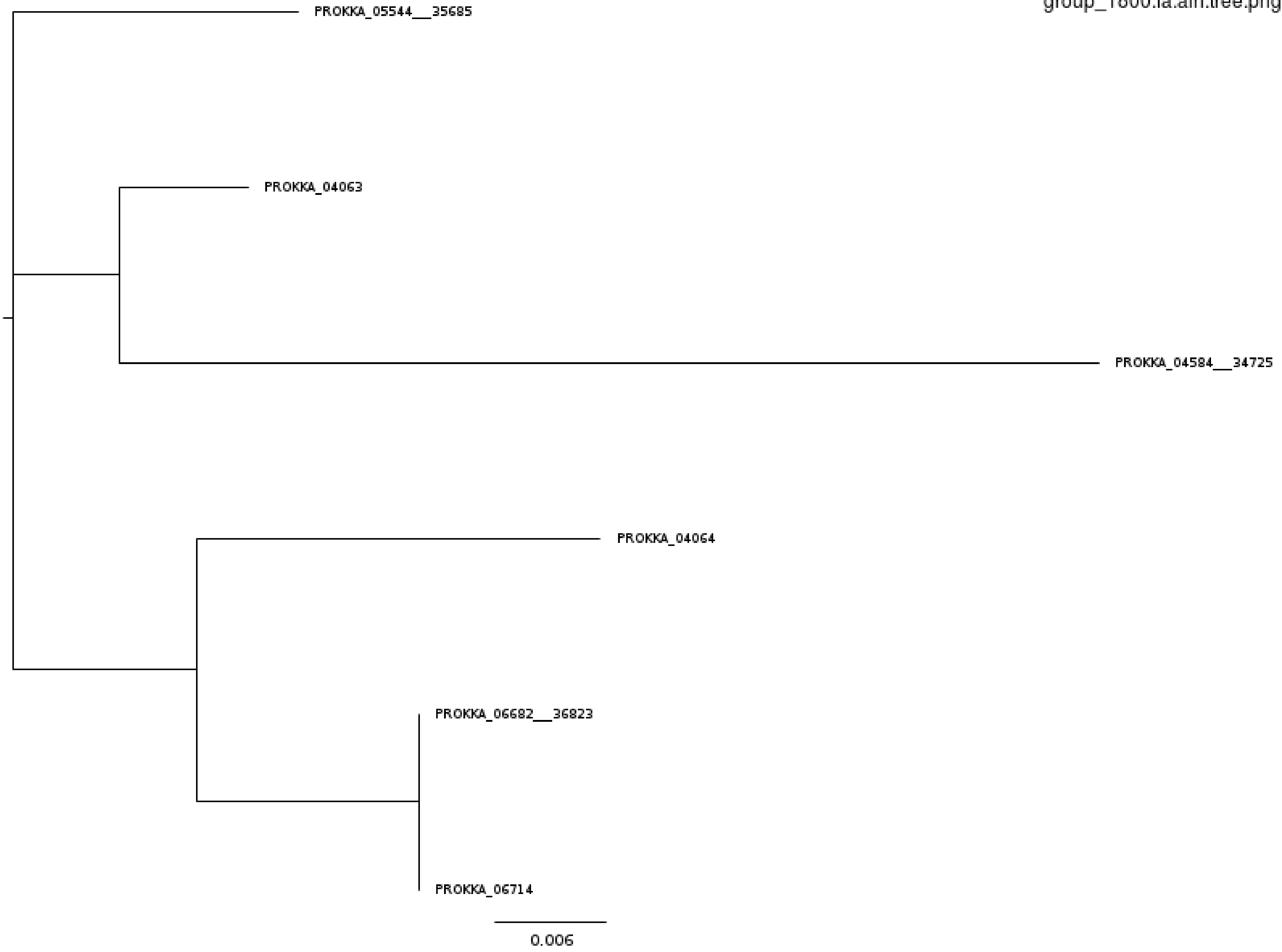
PROKKA_05544___35685
PROKKA_04063
PROKKA_04584___34725
PROKKA_04064
PROKKA_06682___36823
PROKKA_06714
0.006

group_1803.fa.aln.tree.png

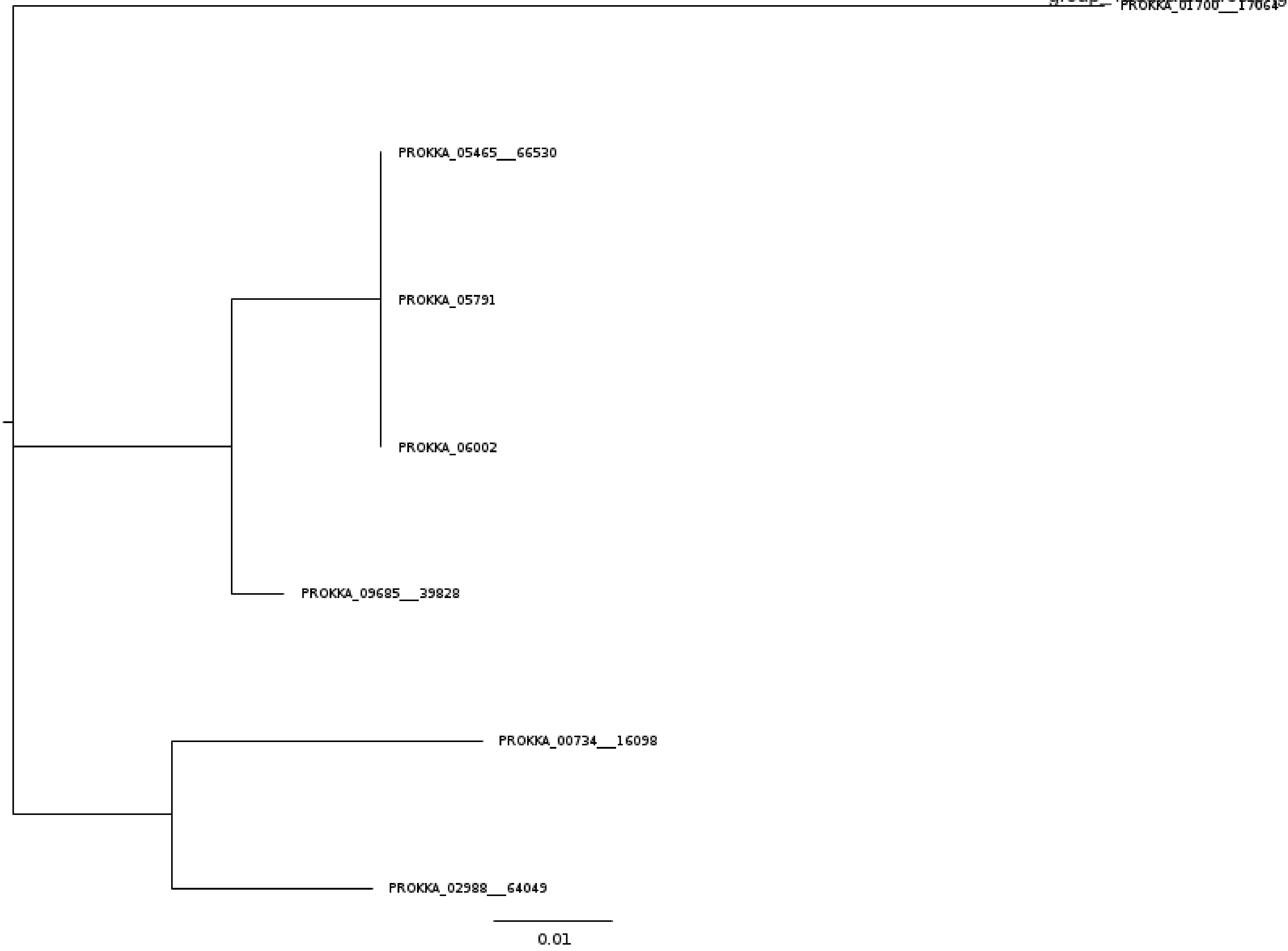

group_1808.fa.aln.tree.png

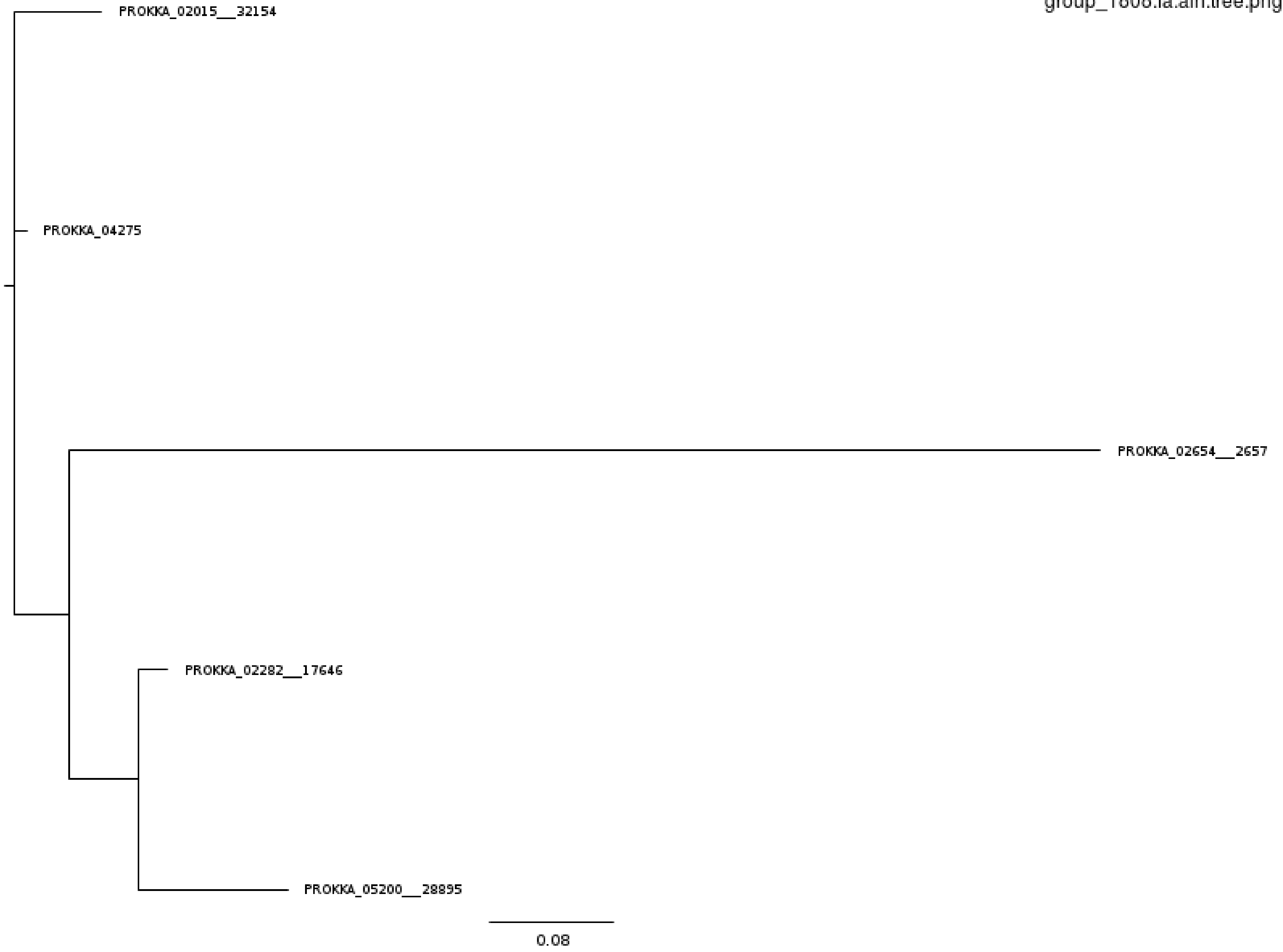

group_180.fa.aln.tree.png

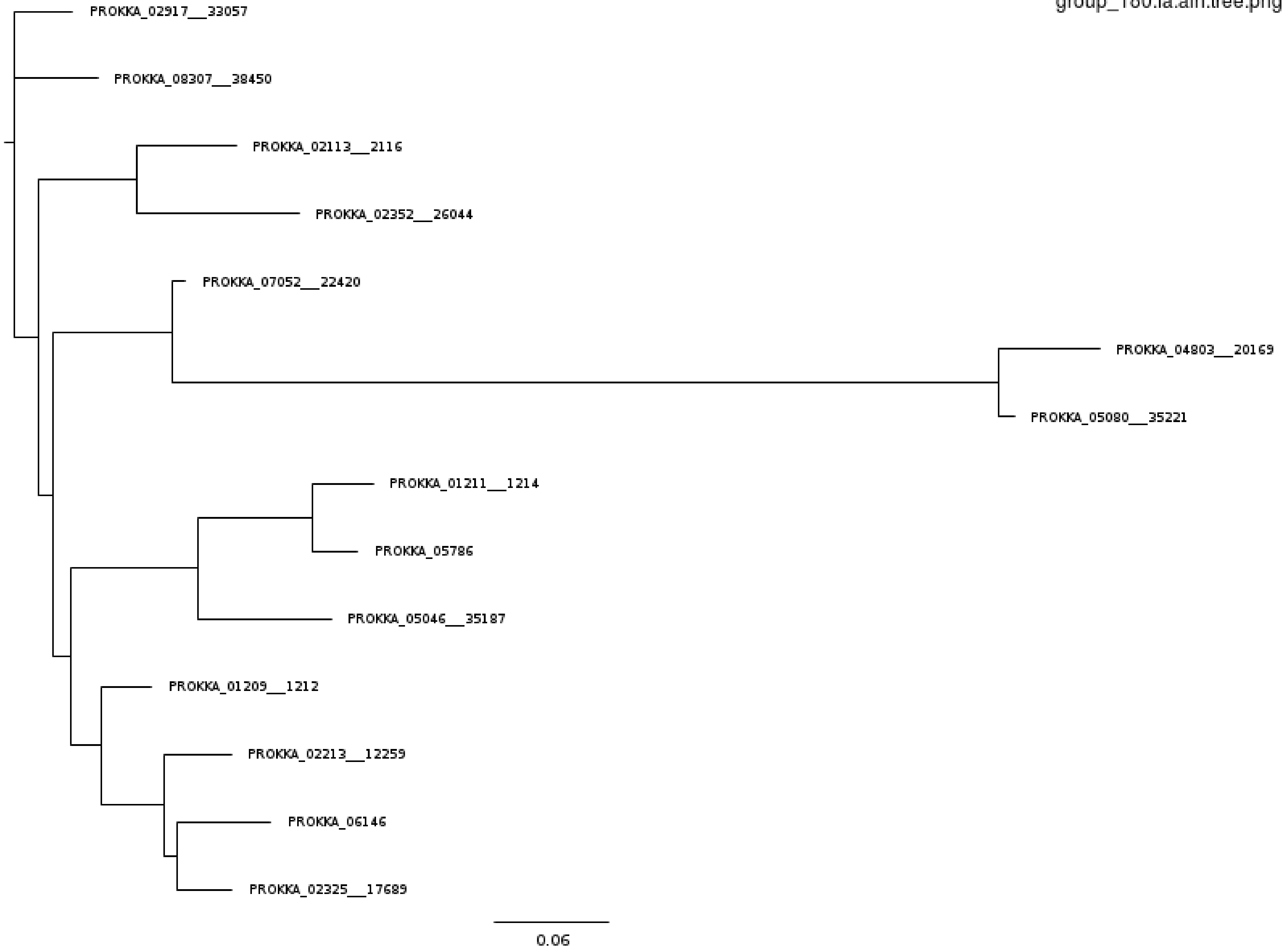

group_1810.fa.aln.tree.png

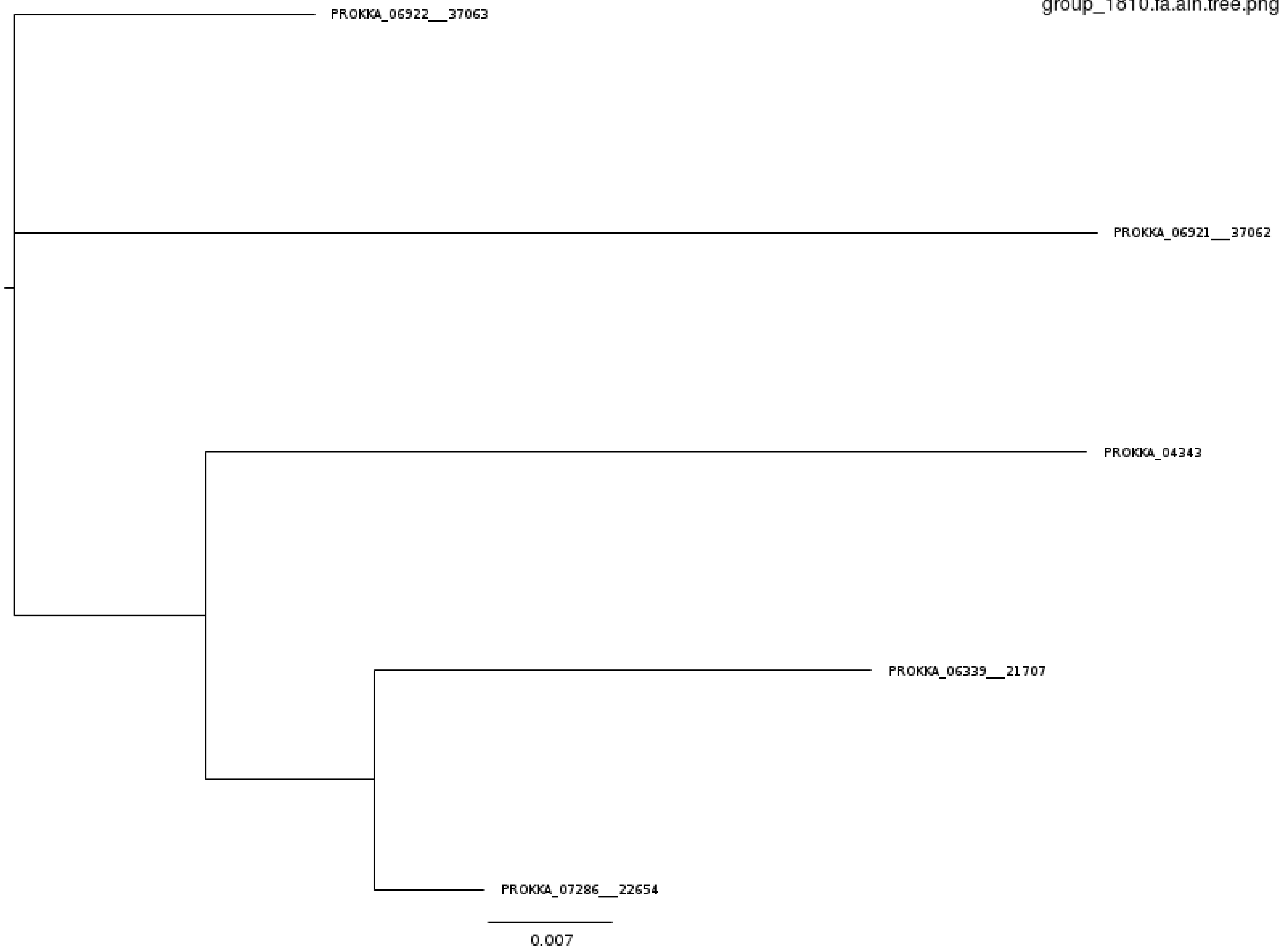

group_1811.fa.aln.tree.png

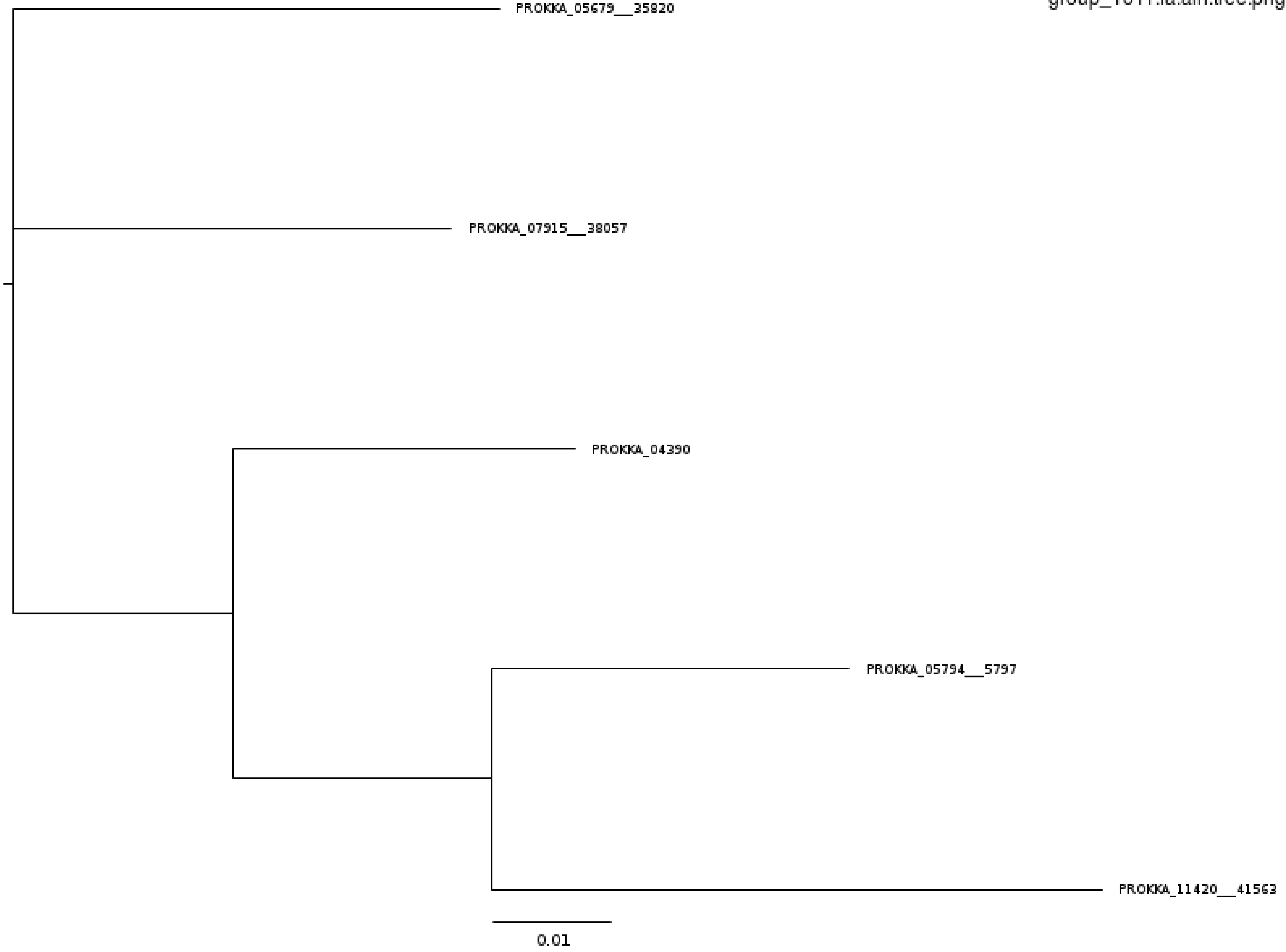

group_1813.fa.aln.tree.png

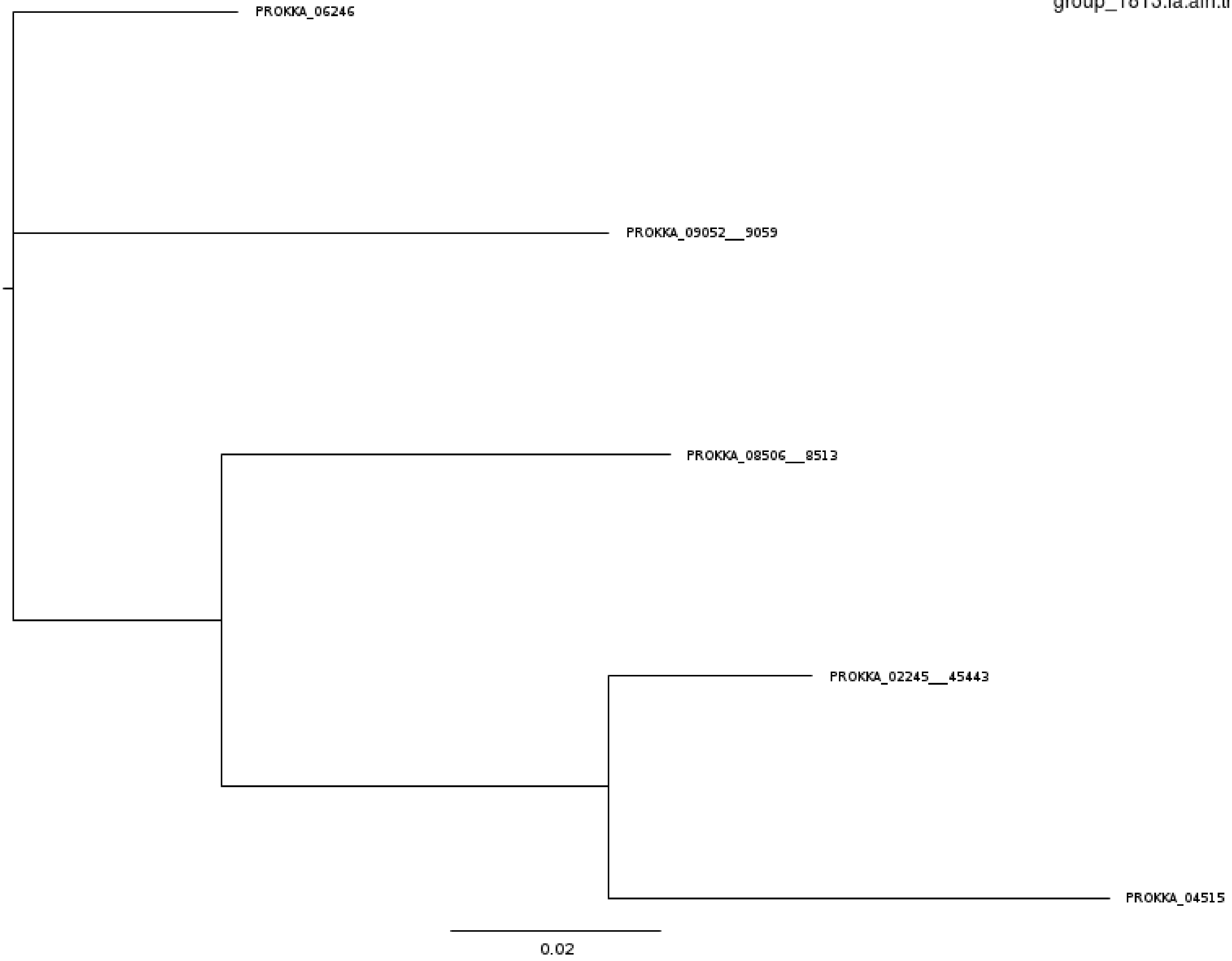

group_1815.fa.aln.tree.png

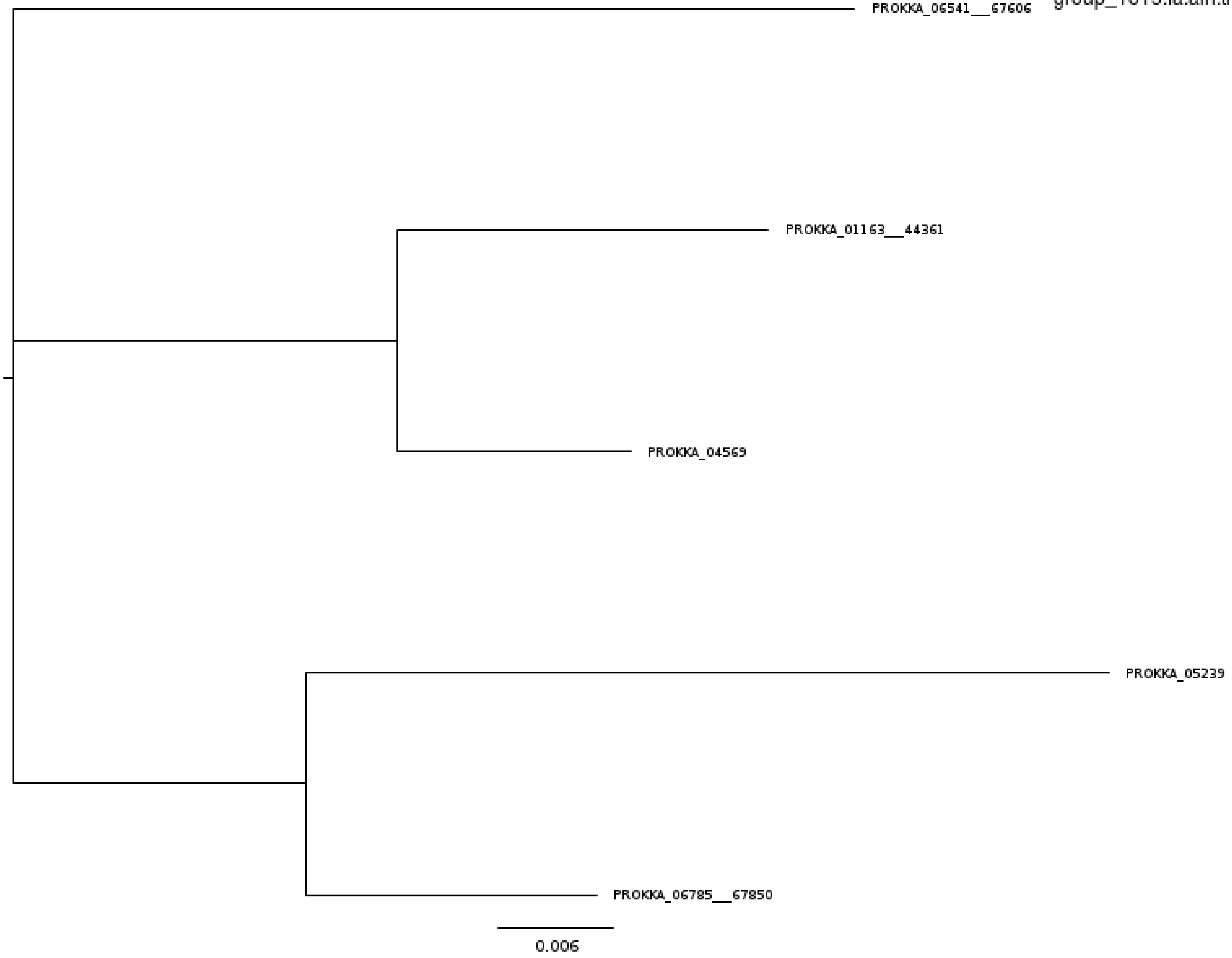

group_1817.fa.aln.tree.png

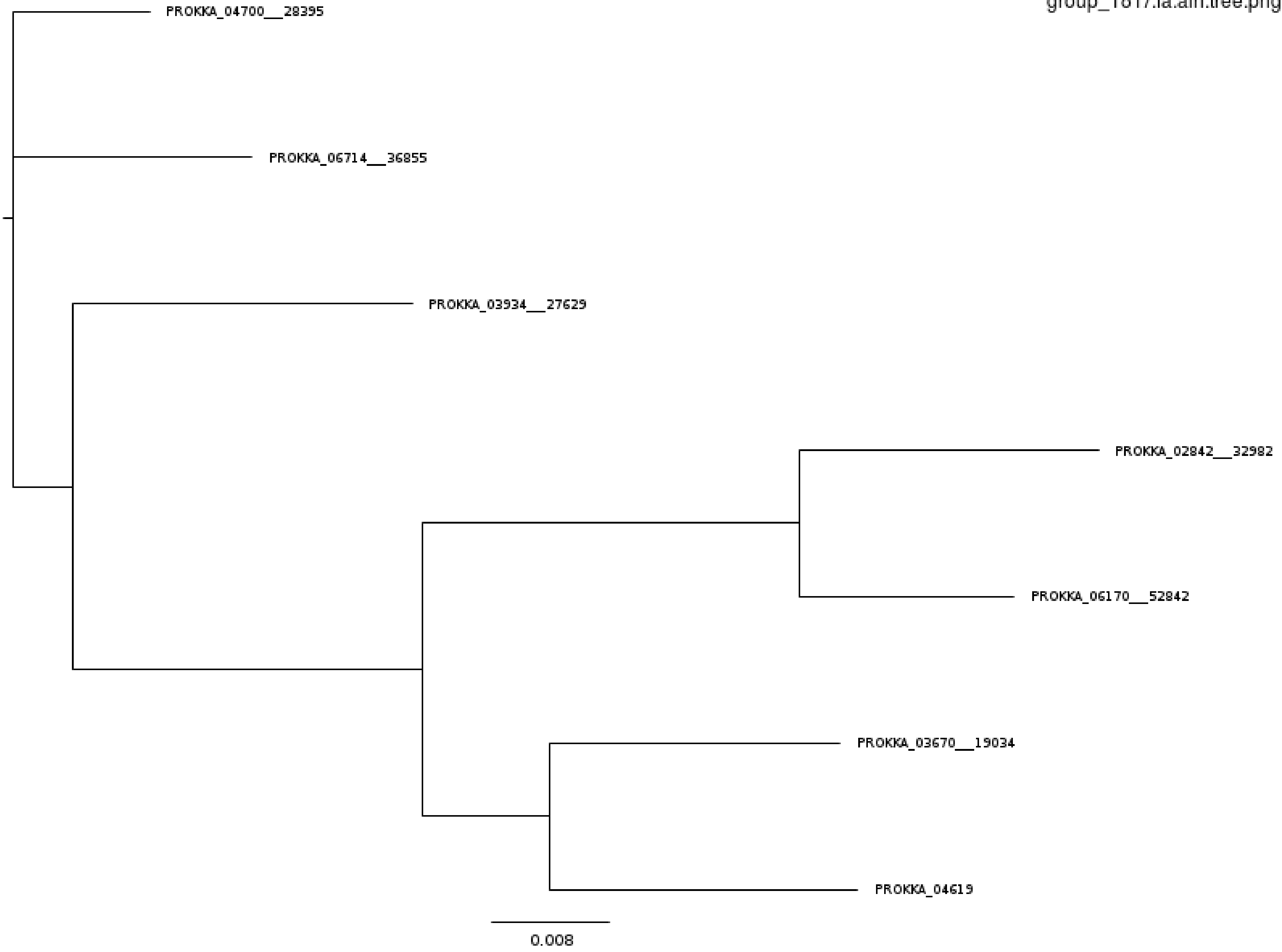

group_1818.fa.aln.tree.png

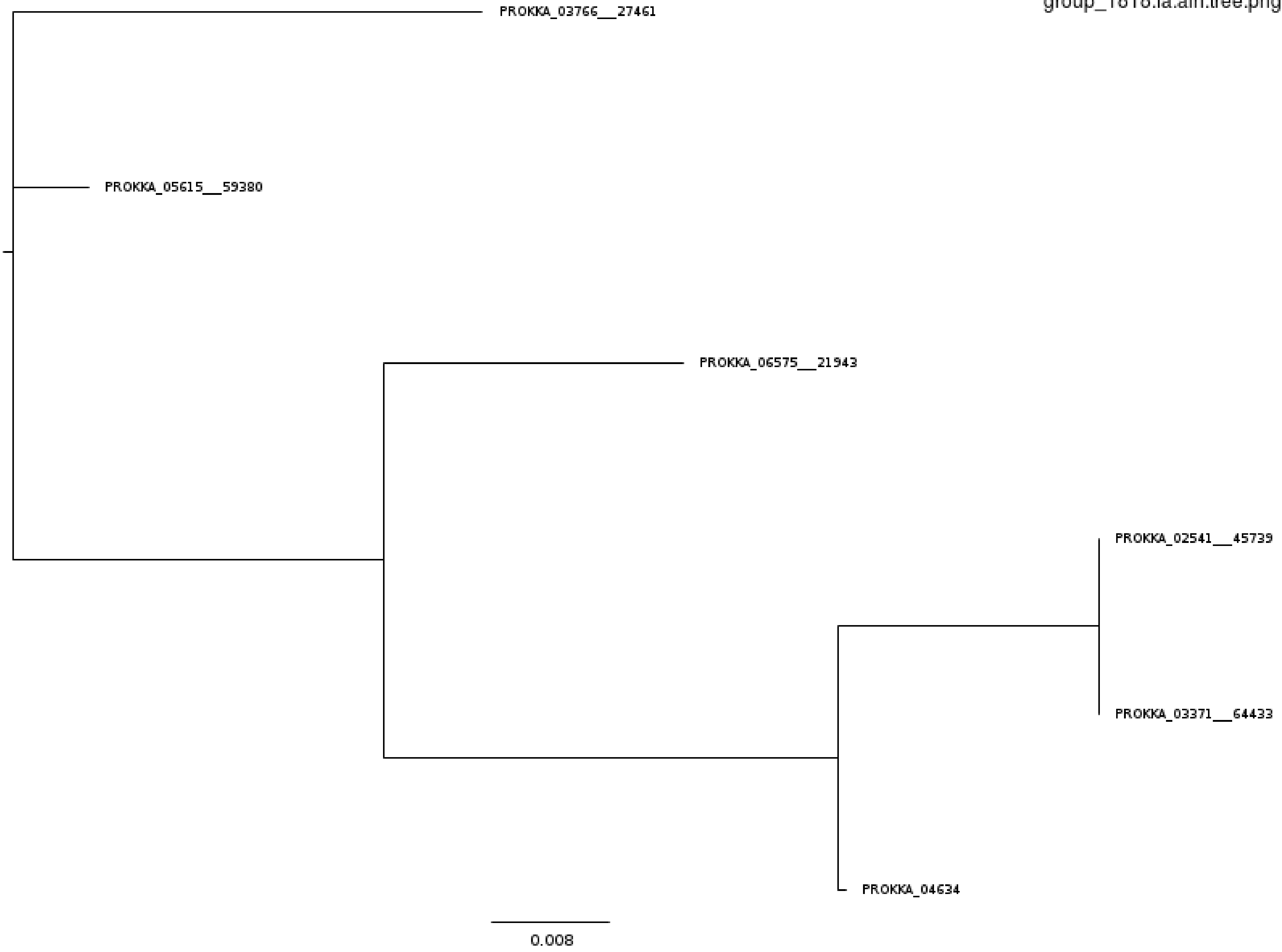

group_1819.fa.aln.tree.png

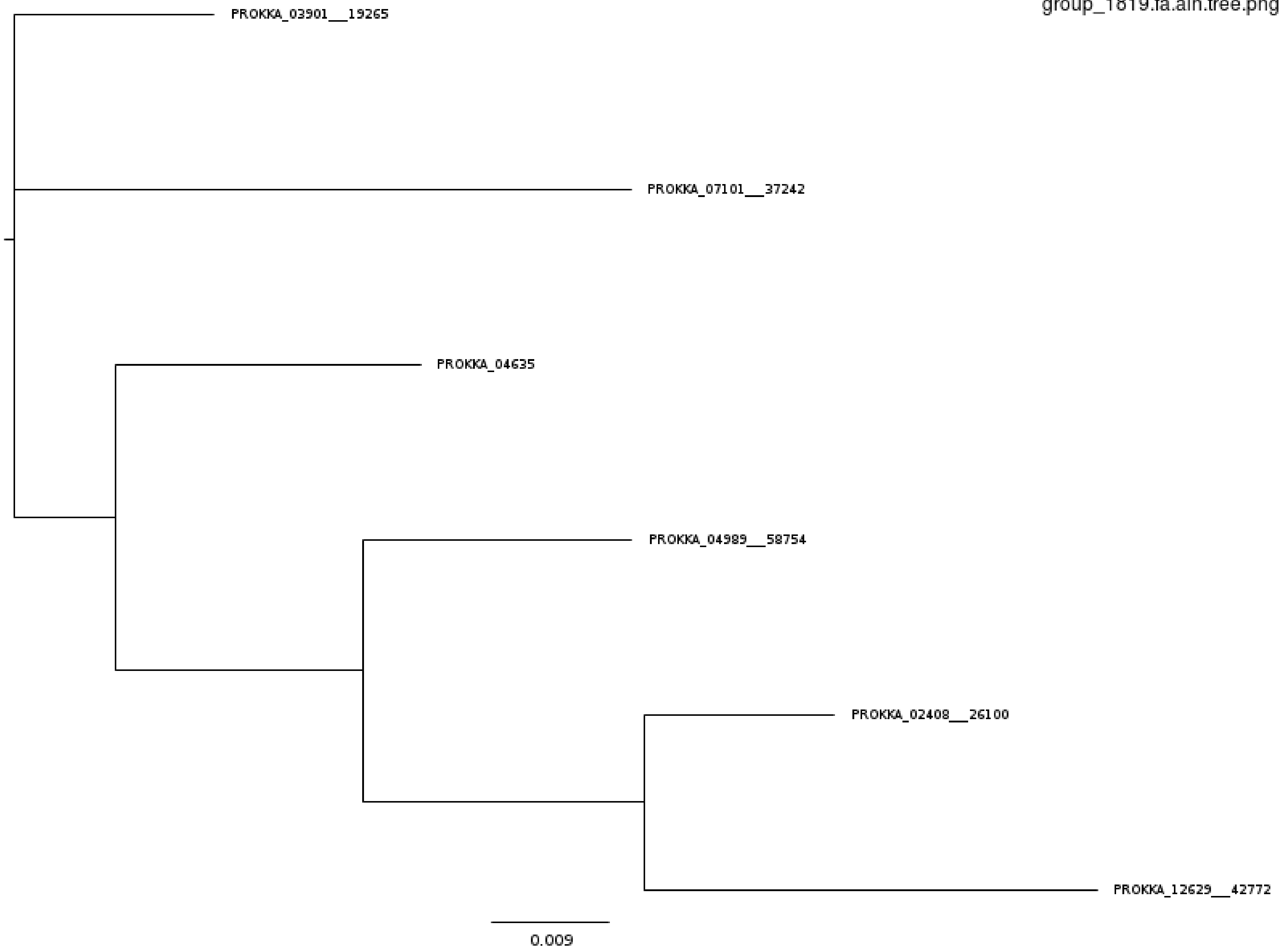

group_181.fa.aln.tree.png

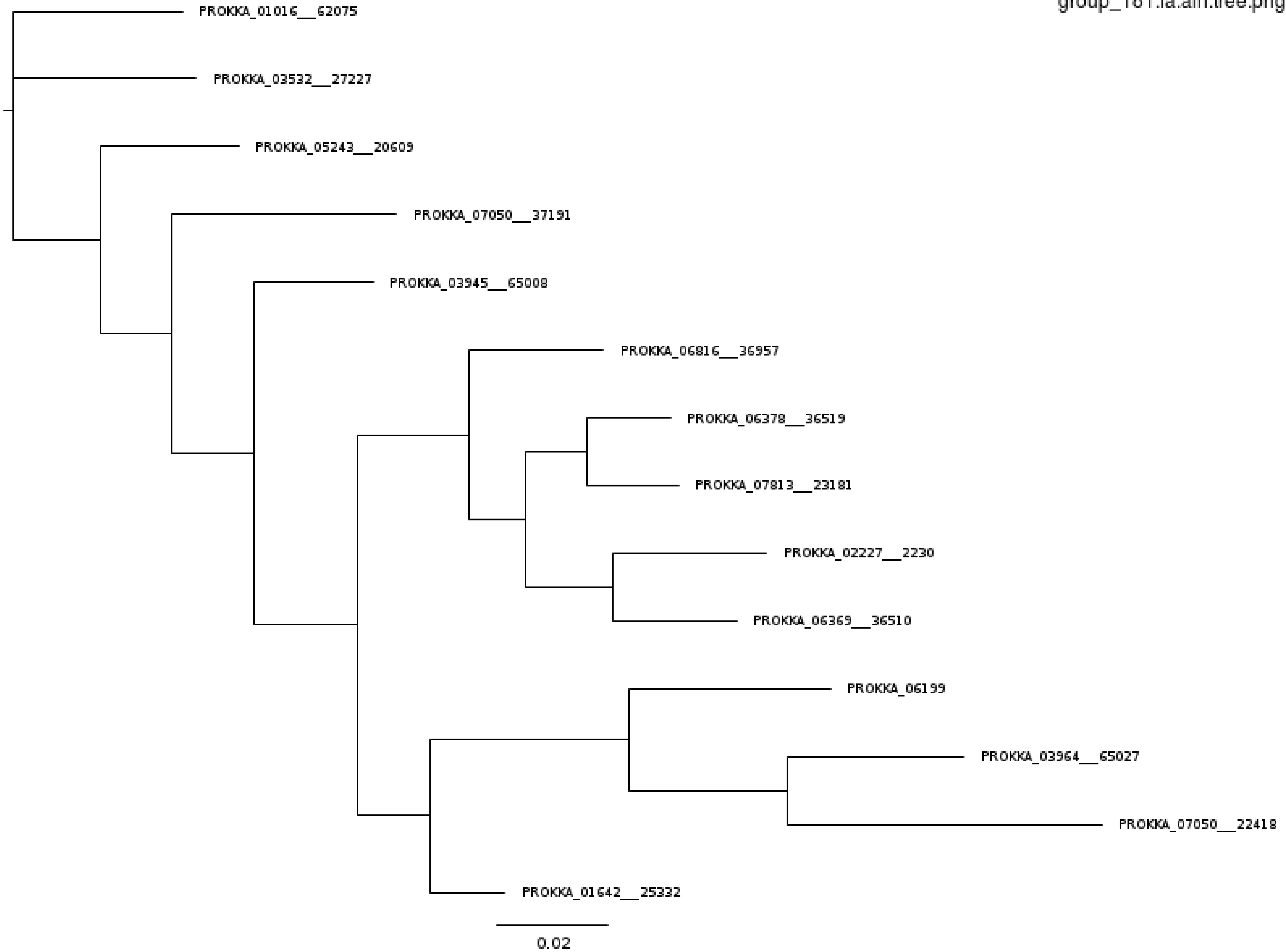

group_1820.fa.aln.tree.png

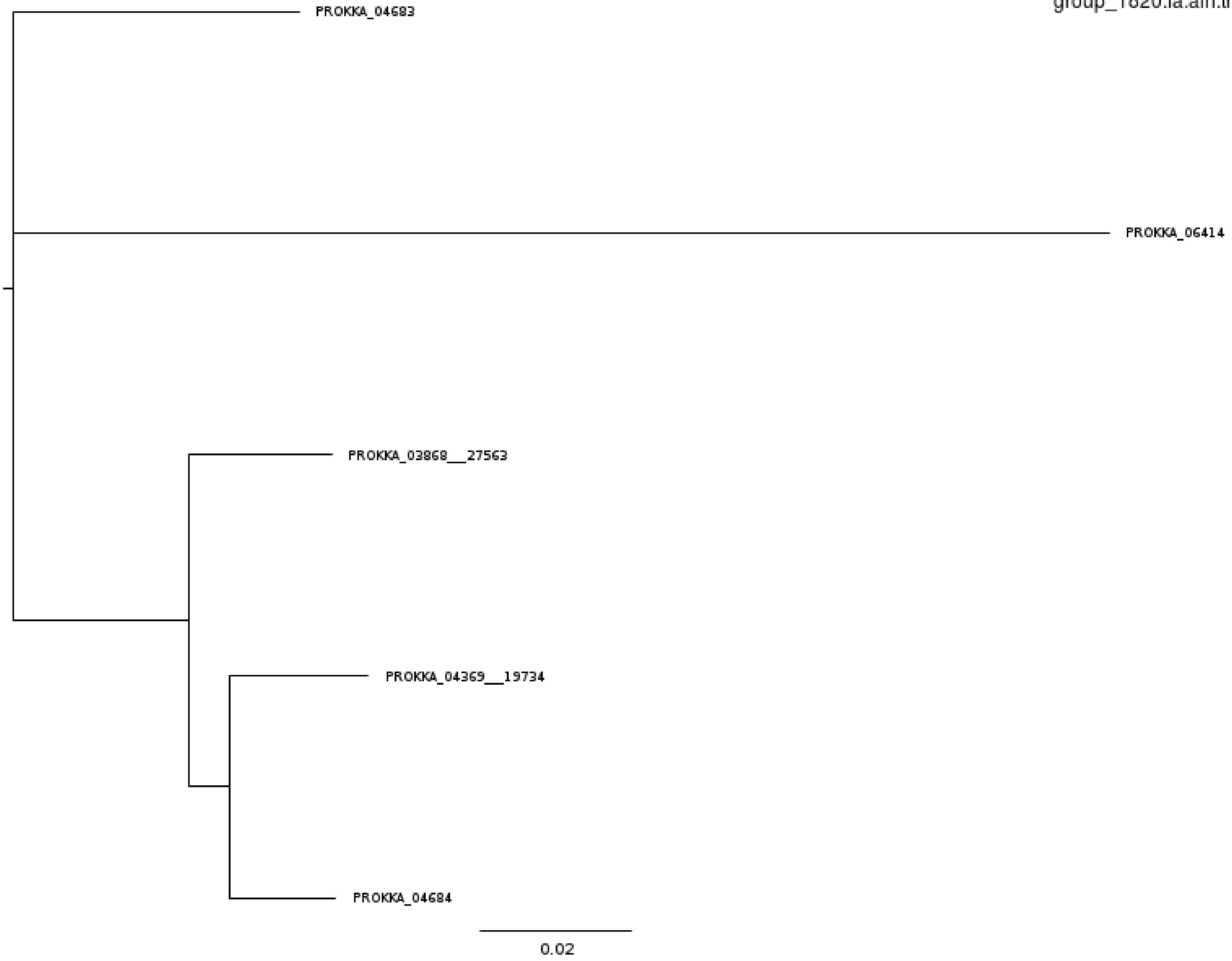

group_1821.fa.aln.tree.png

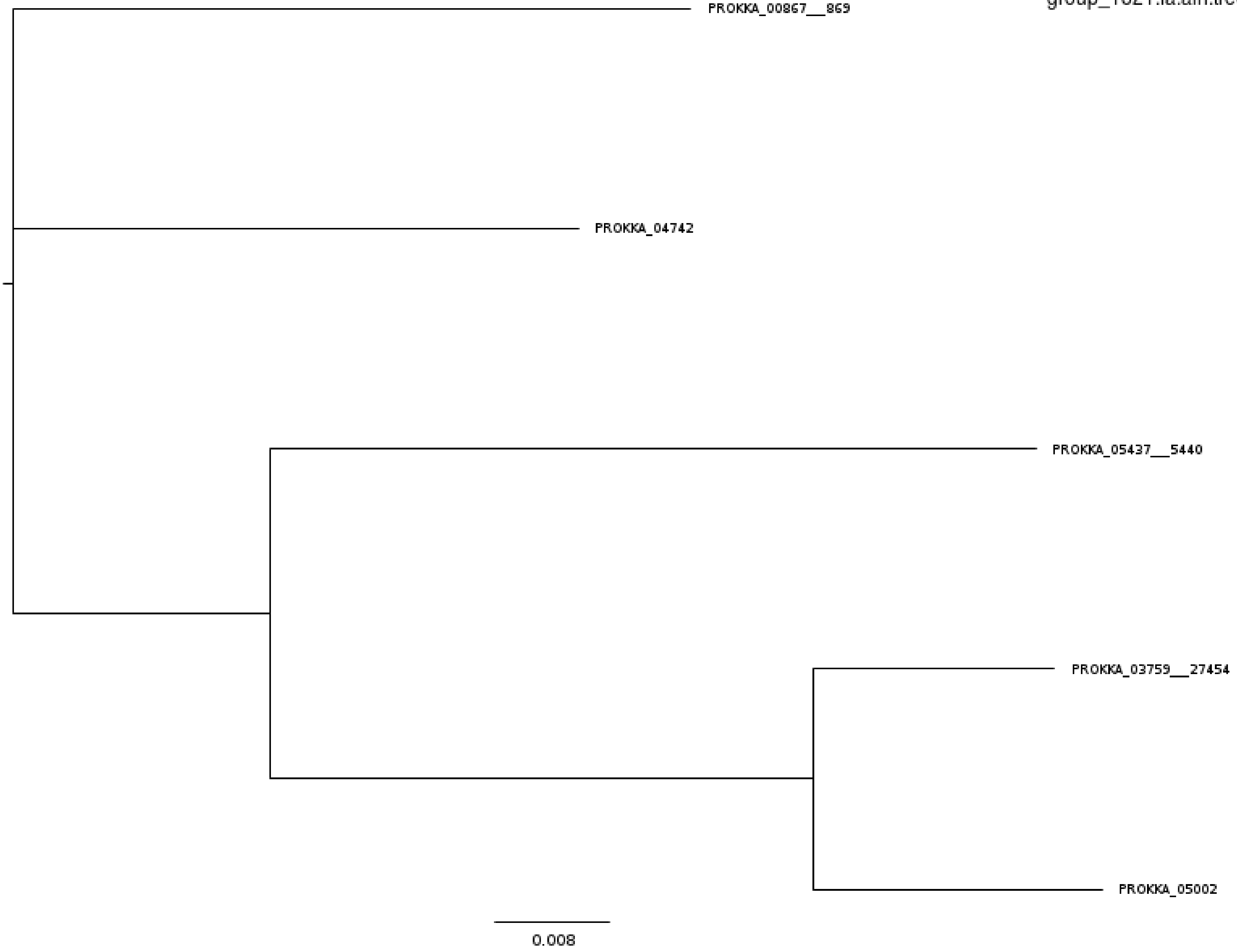

group_1824.fa.aln.tree.png

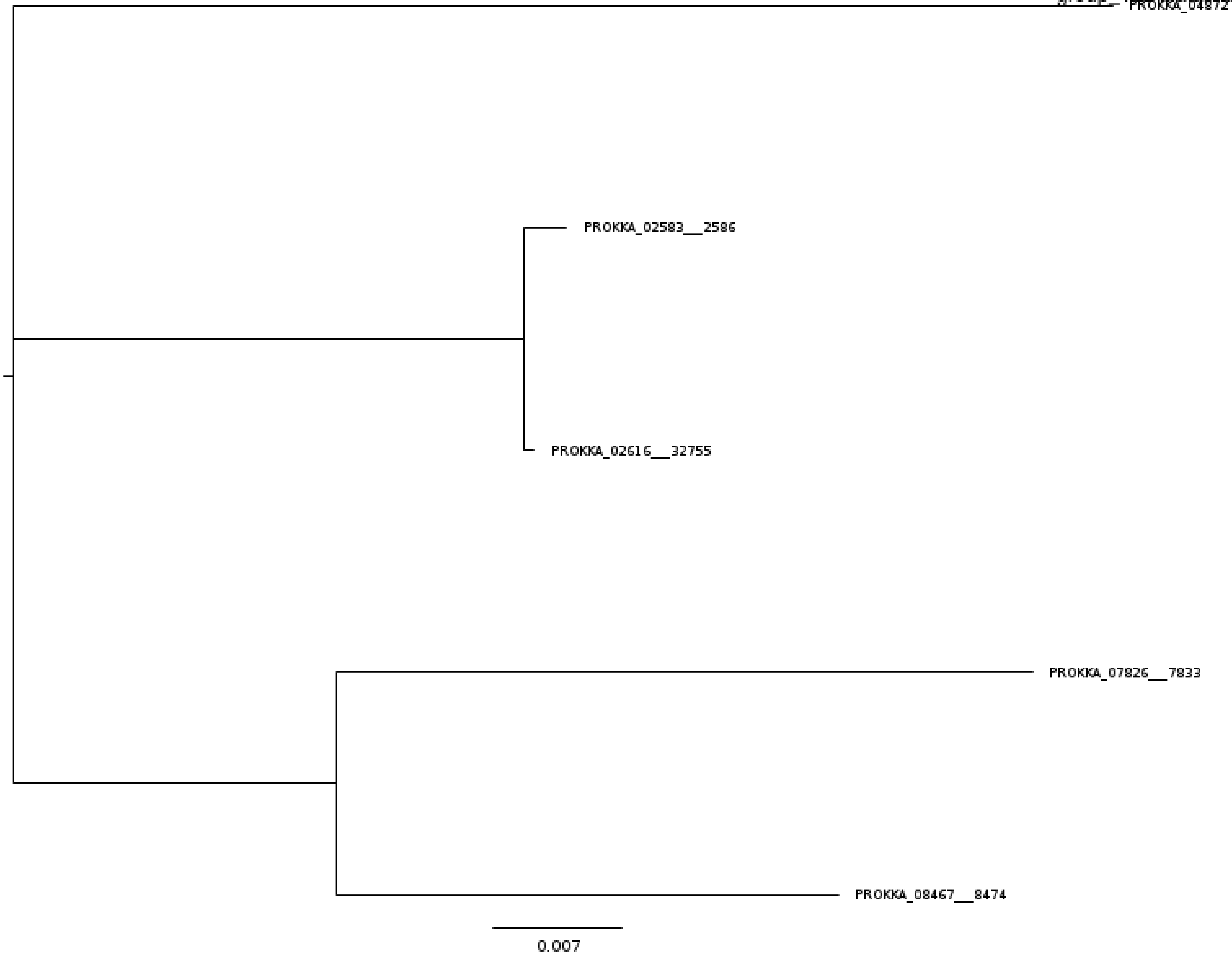

group_1825.fa.aln.tree.png

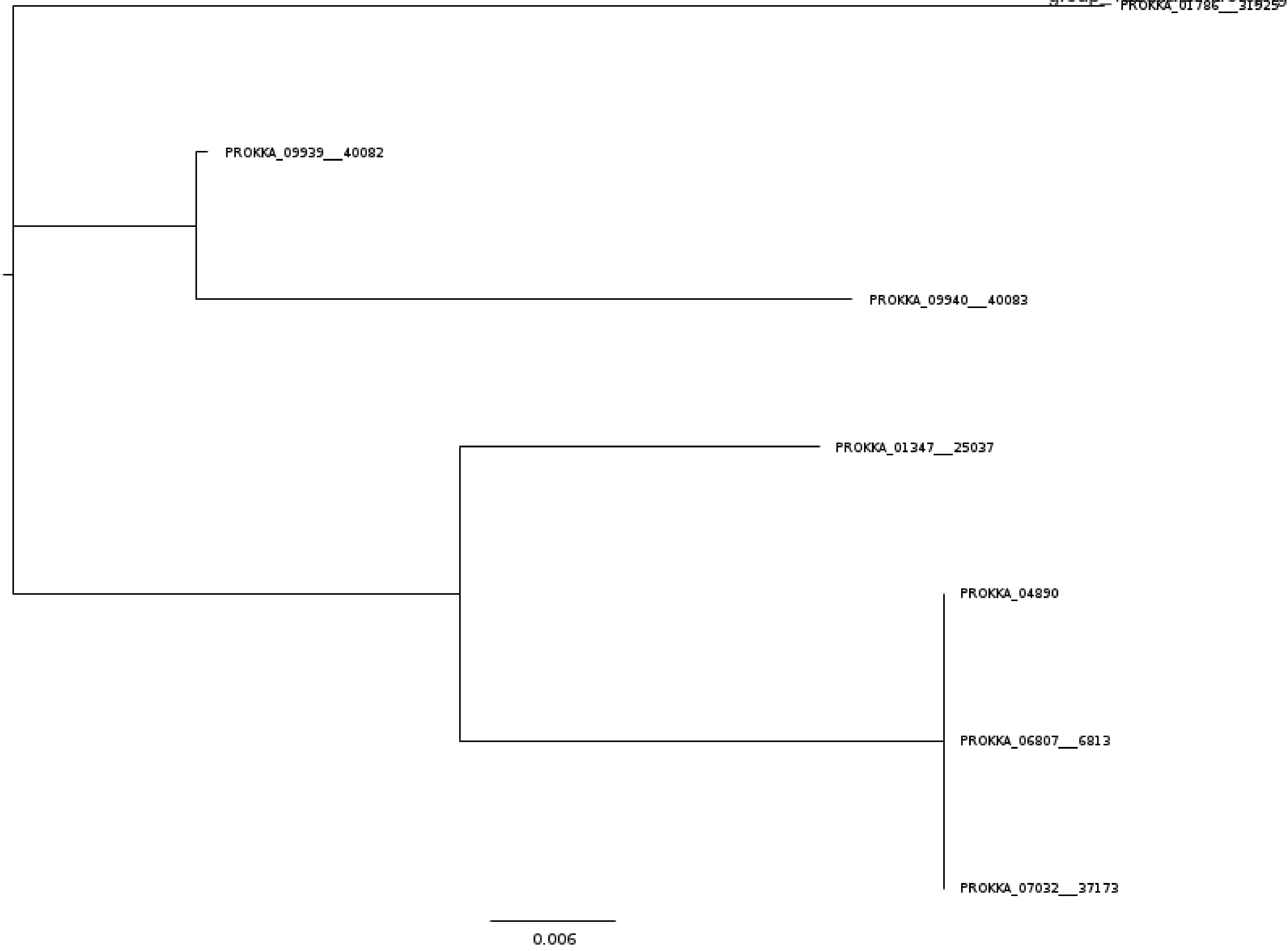

group_1828.fa.aln.tree.png

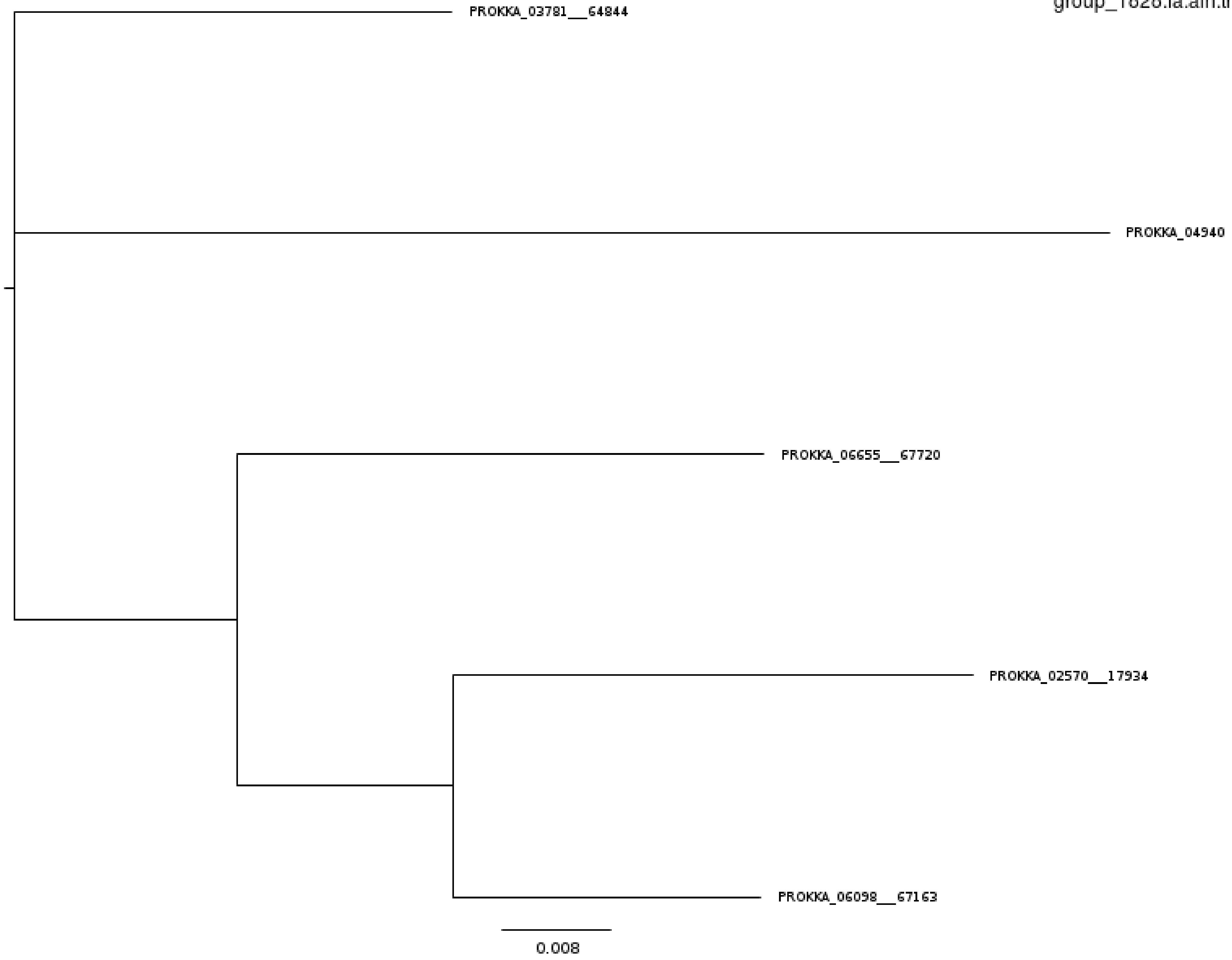

group_182.fa.aln.tree.png

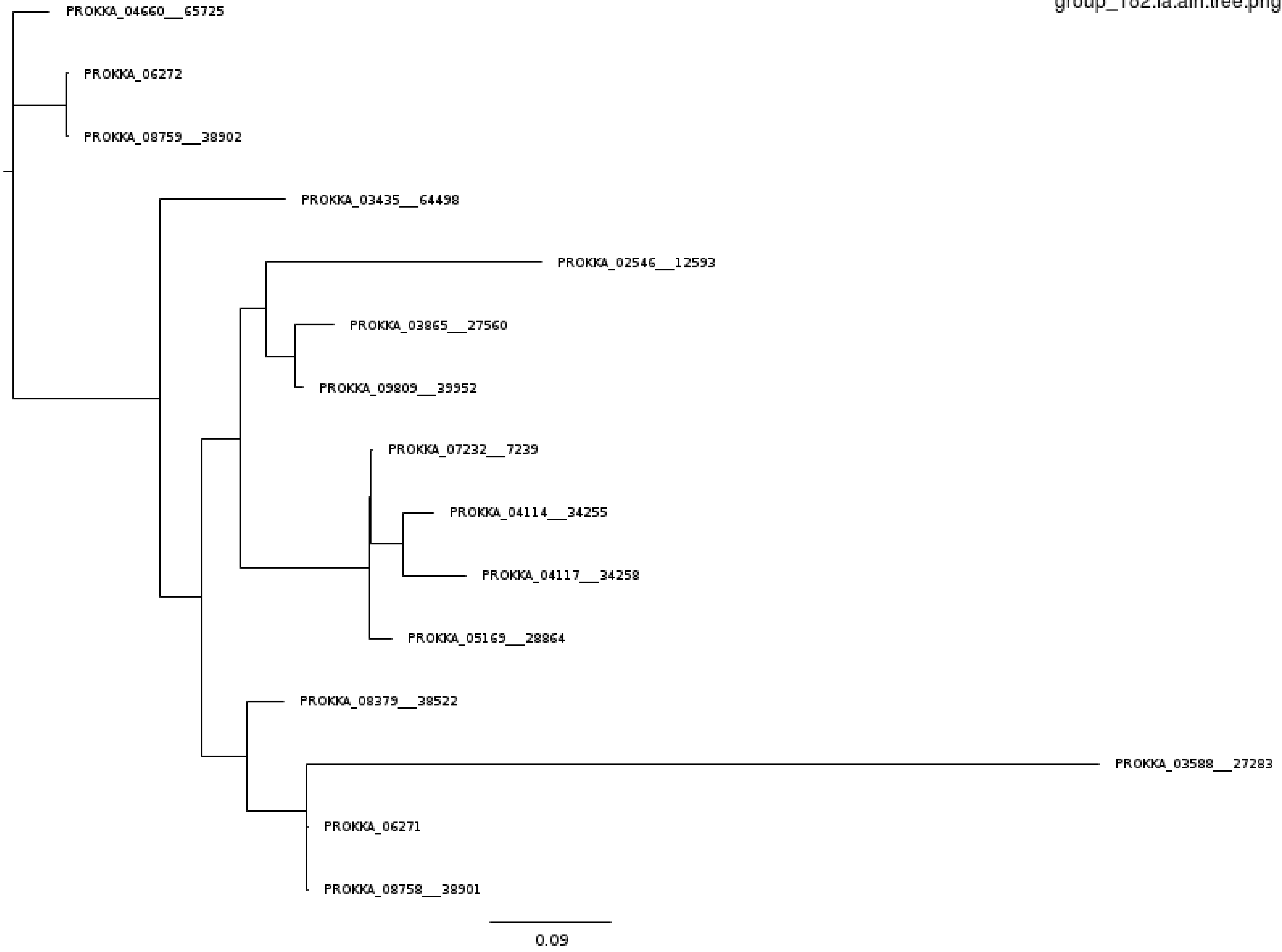

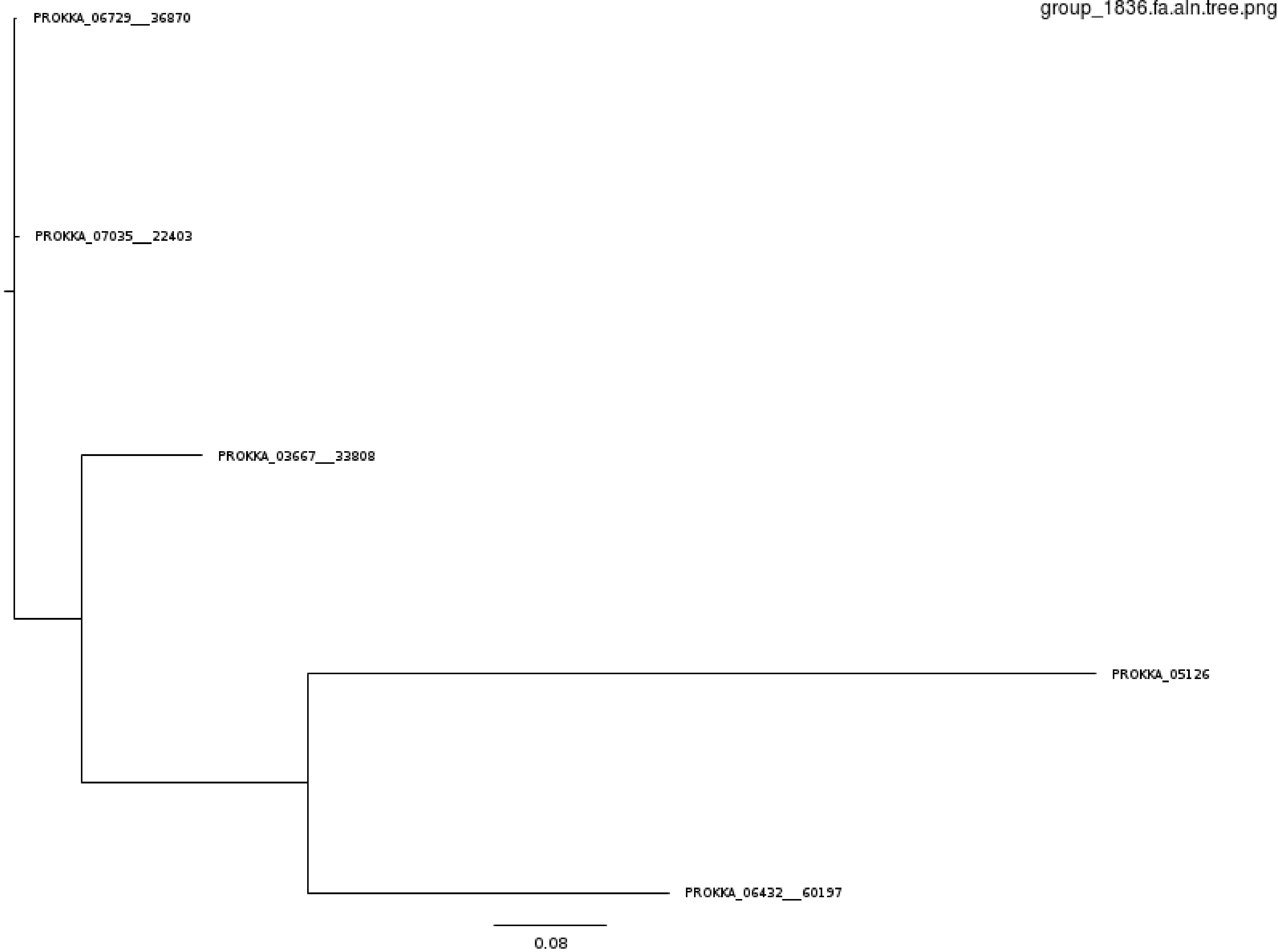
group_1836.fa.aln.tree.png
PROKKA_06729___36870
PROKKA_07035___22403
PROKKA_03667___33808
PROKKA_05126
PROKKA_06432___60197
0.08

group_1838.fa.aln.tree.png

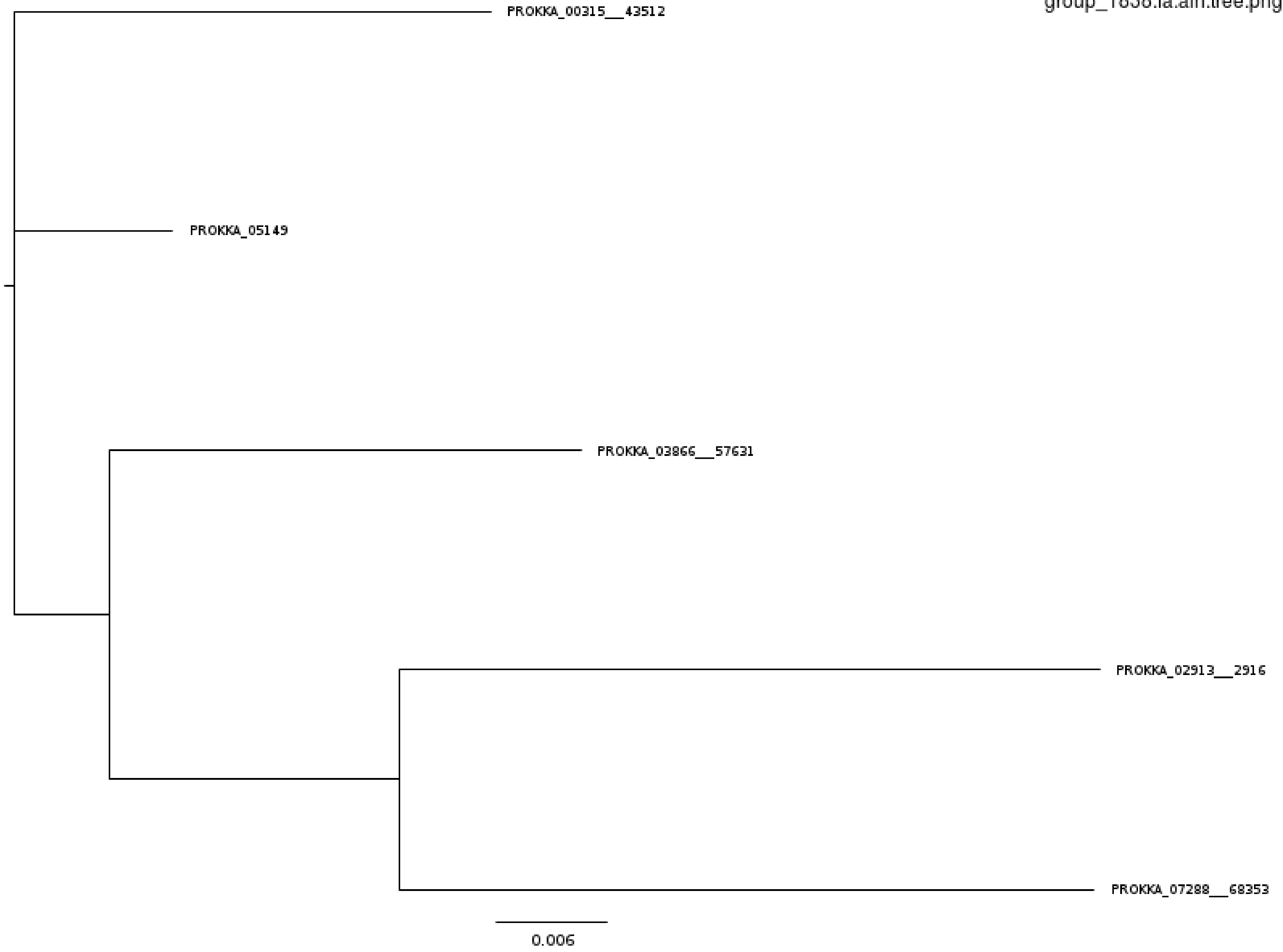

group_1840.fa.aln.tree.png

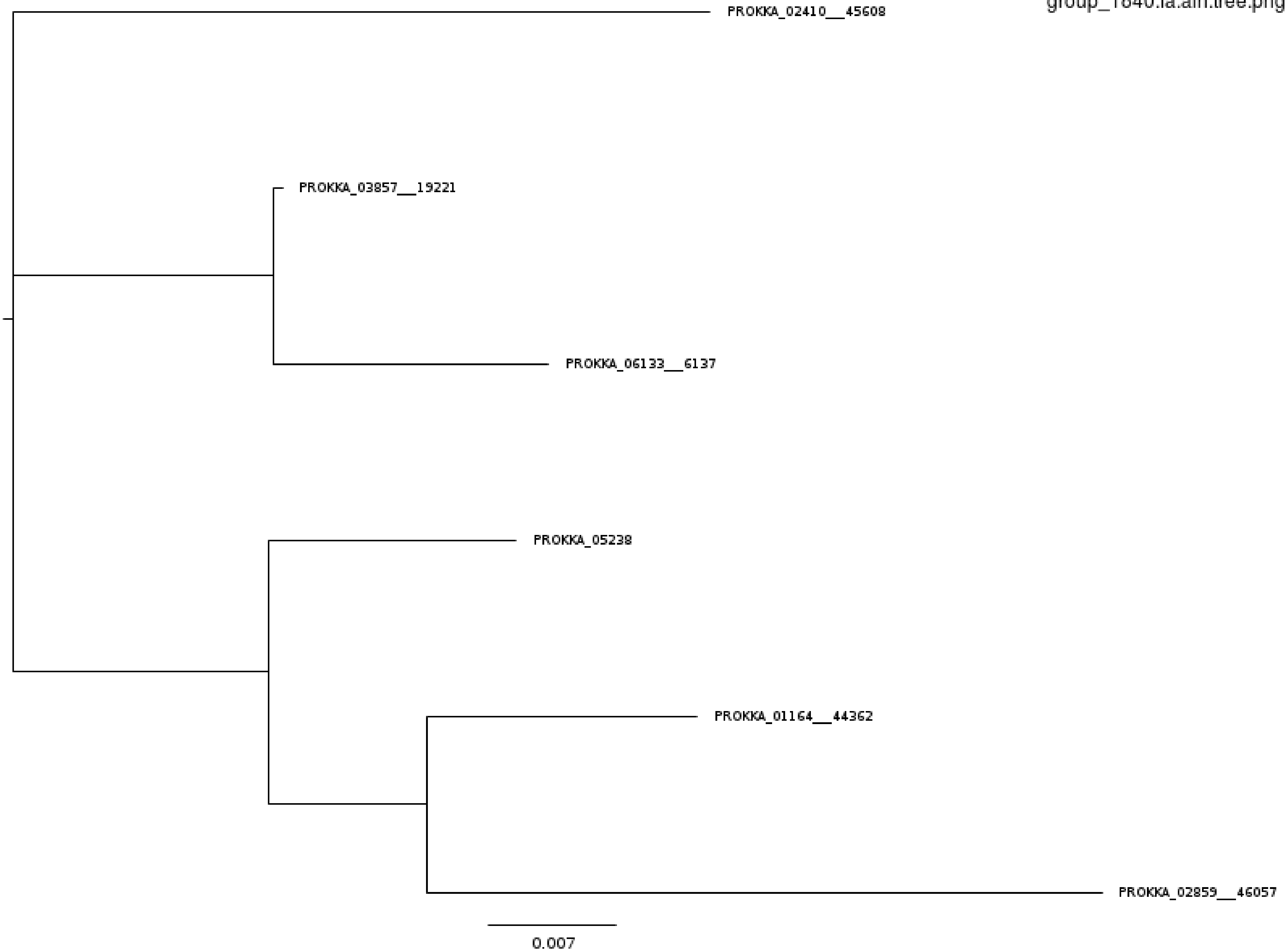

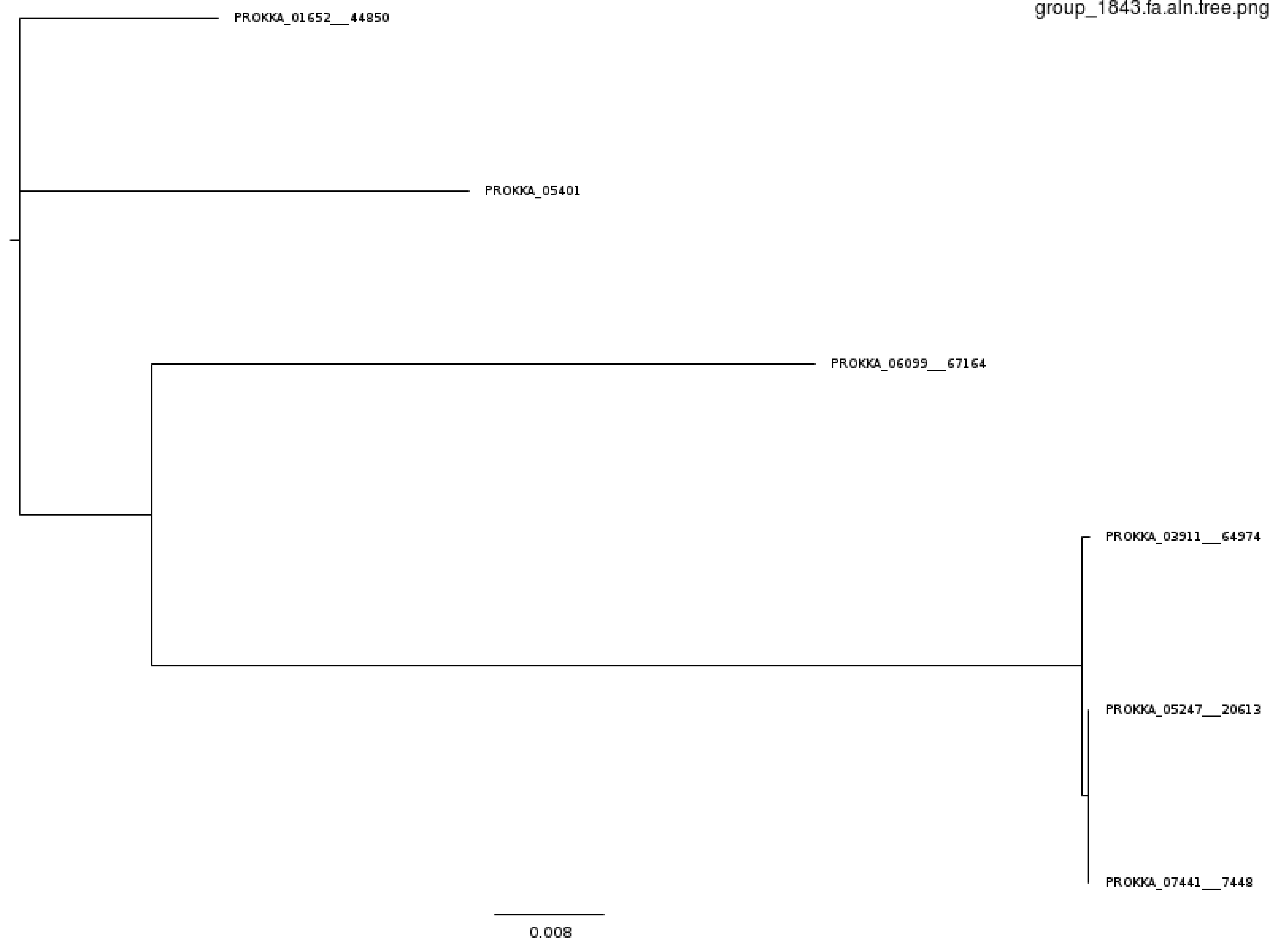
group_1843.fa.aln.tree.png
PROKKA_01652___44850
PROKKA_05401
PROKKA_06099___67164
PROKKA_03911___64974
PROKKA_05247___20613
PROKKA_07441___7448
0.008

group_1844.fa.aln.tree.png

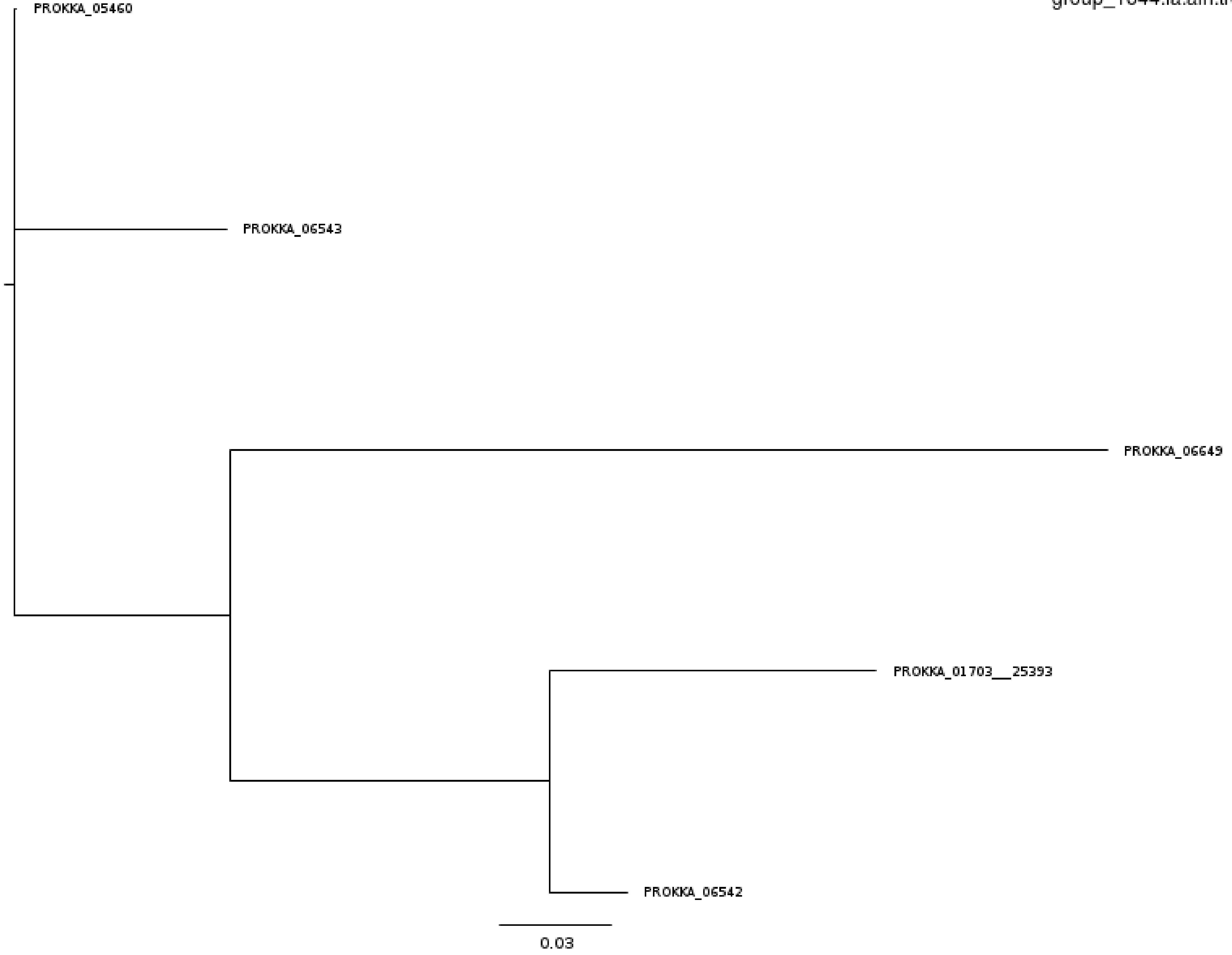

group_1845.fa.aln.tree.png

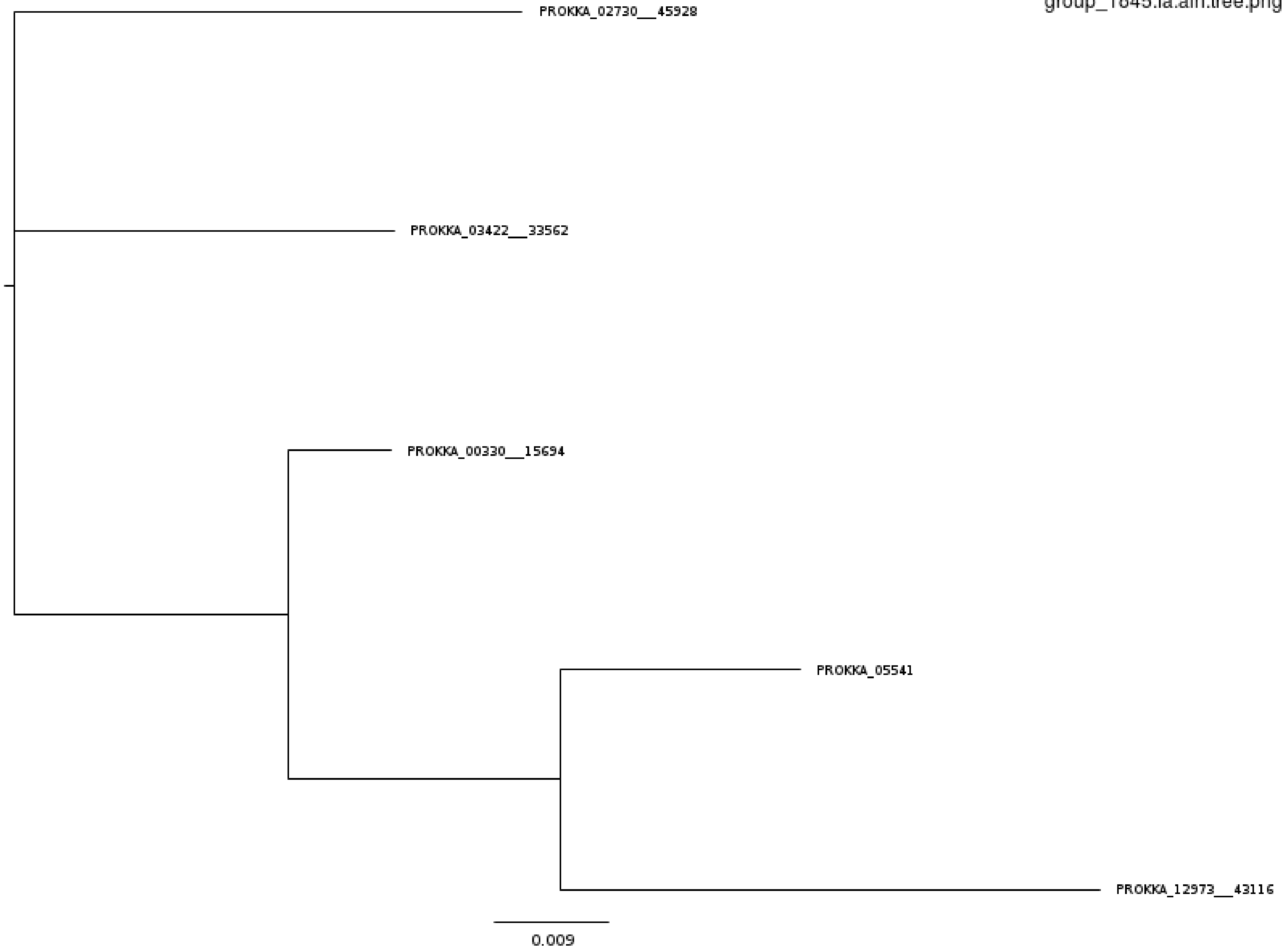

group_1847.fa.aln.tree.png

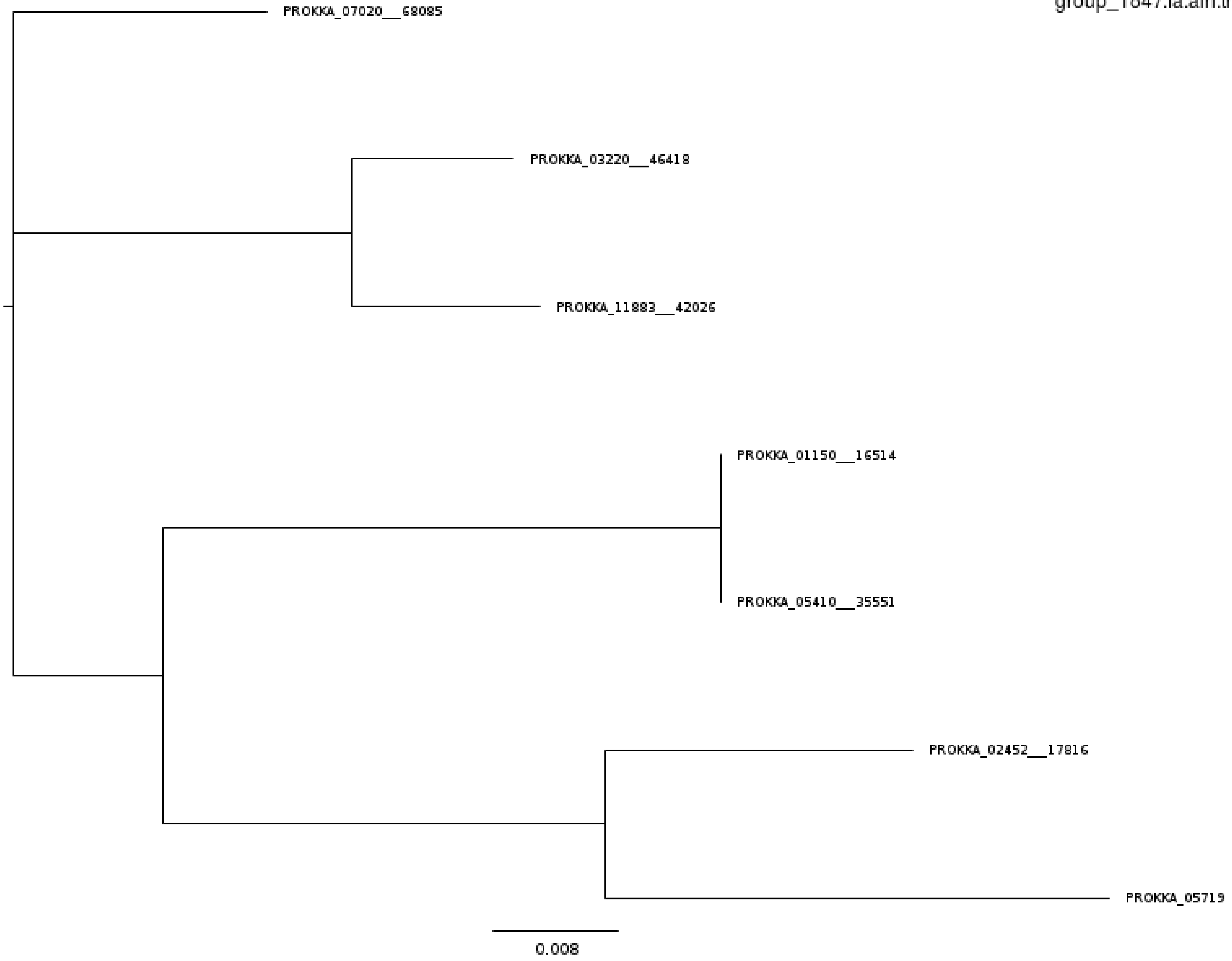

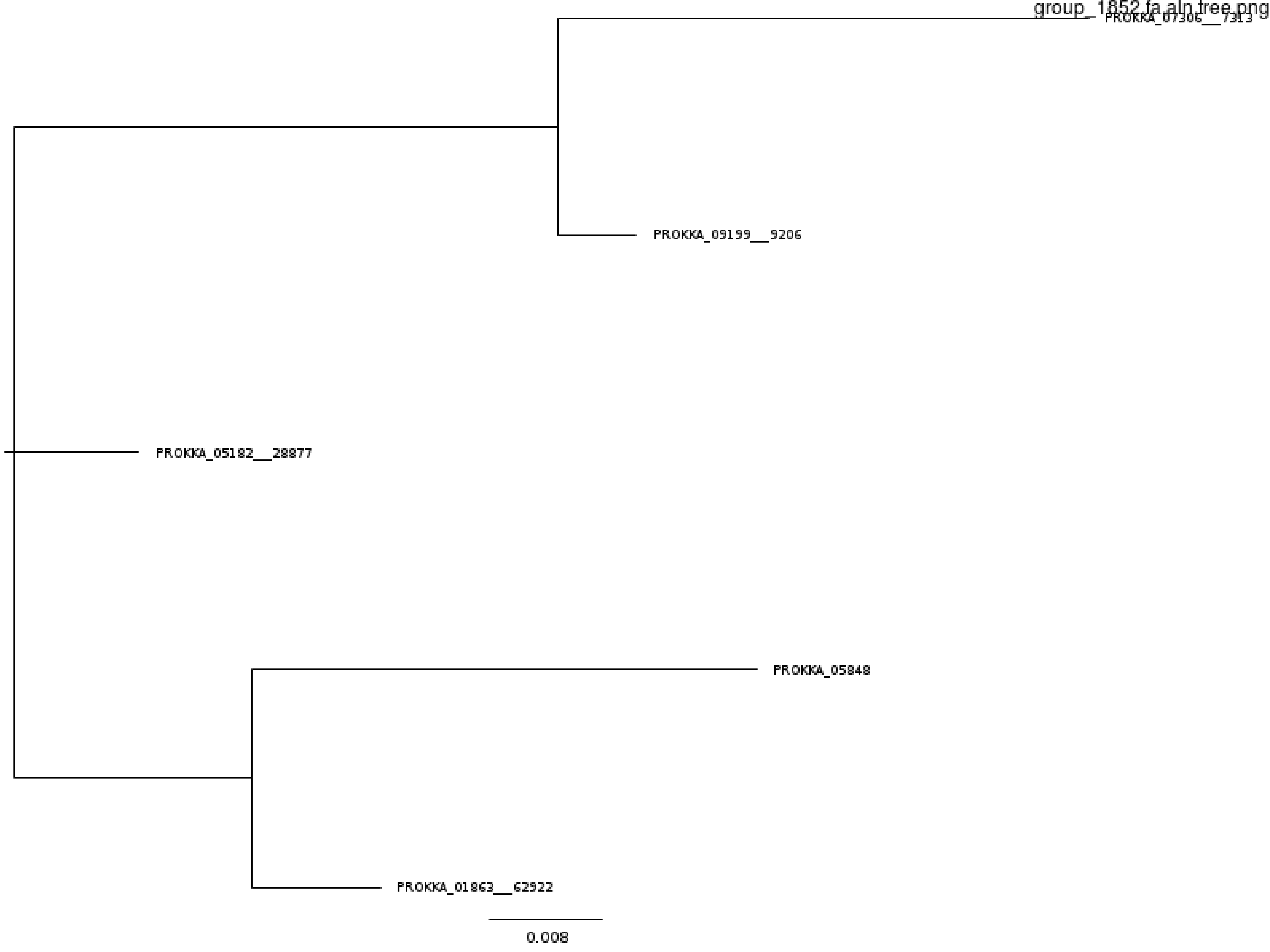

group_1852.fa.aln.tree.png
PROKKA_07306___7313
PROKKA_09199___9206
PROKKA_05182___28877
PROKKA_05848
PROKKA_01863___62922
0.008

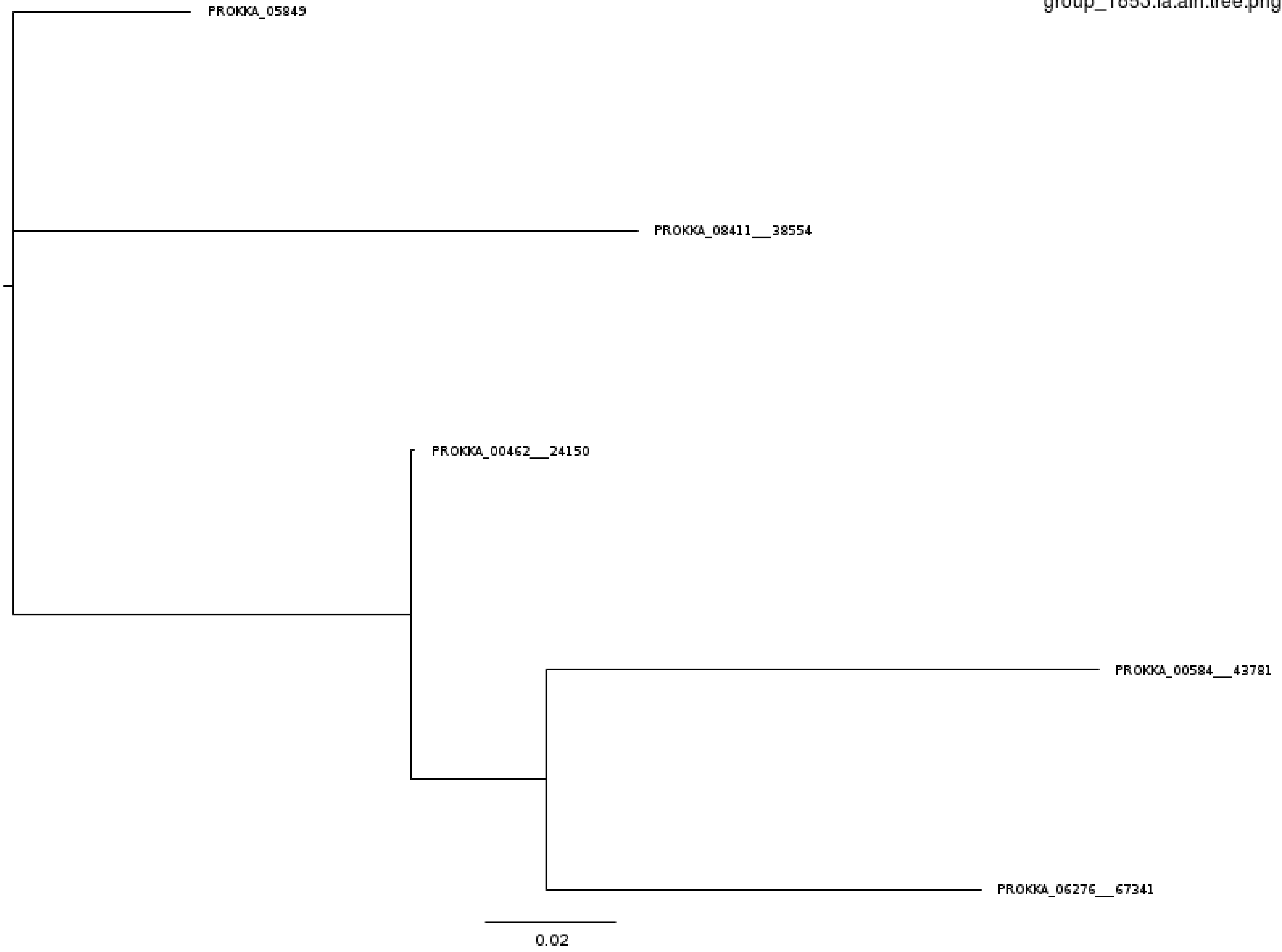
PROKKA_05849
PROKKA_08411___38554
PROKKA_00462___24150
PROKKA_00584___43781
PROKKA_06276___67341
0.02

group_1854.fa.aln.tree.png

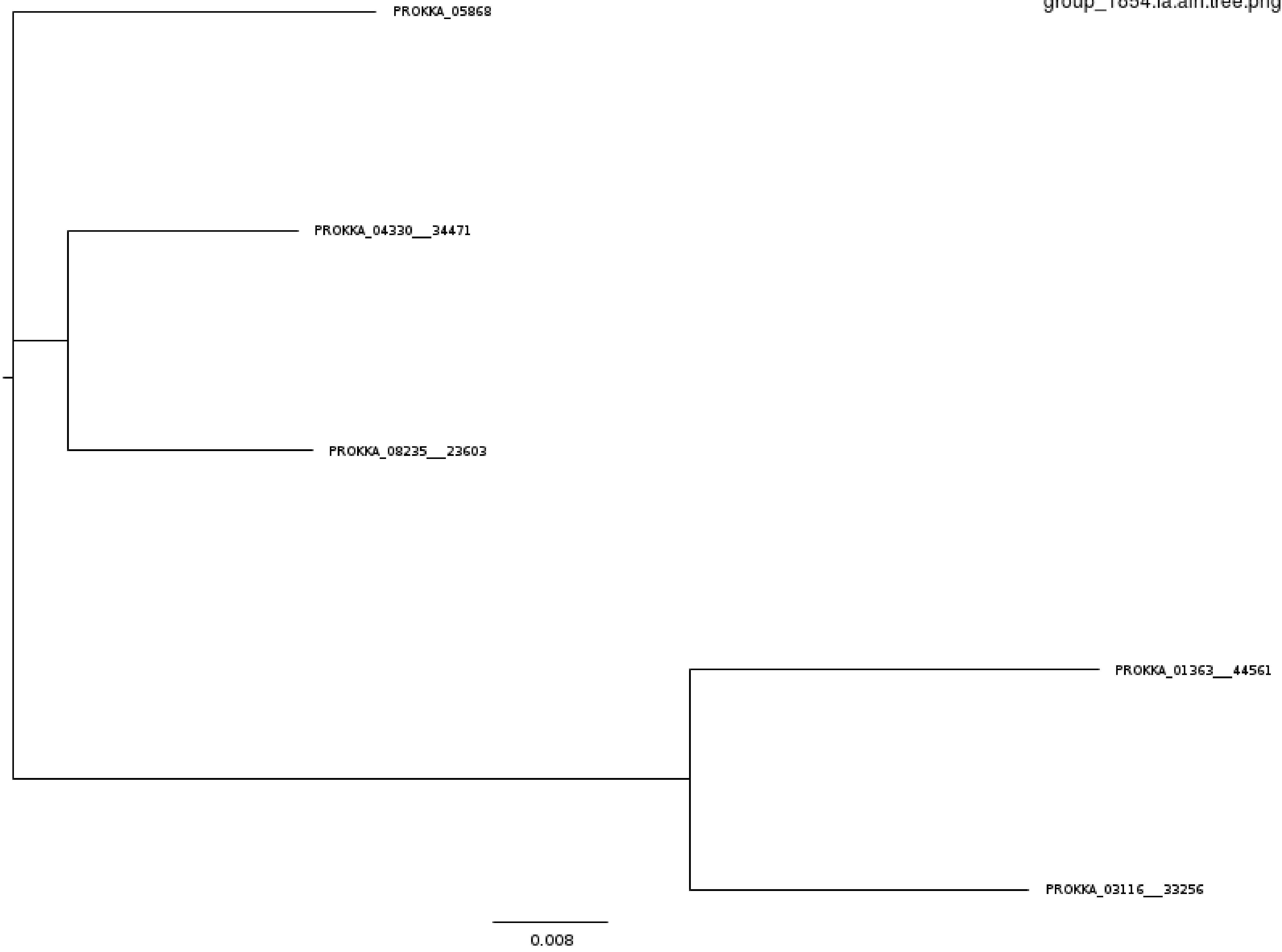

group_1856.fa.aln.tree.png

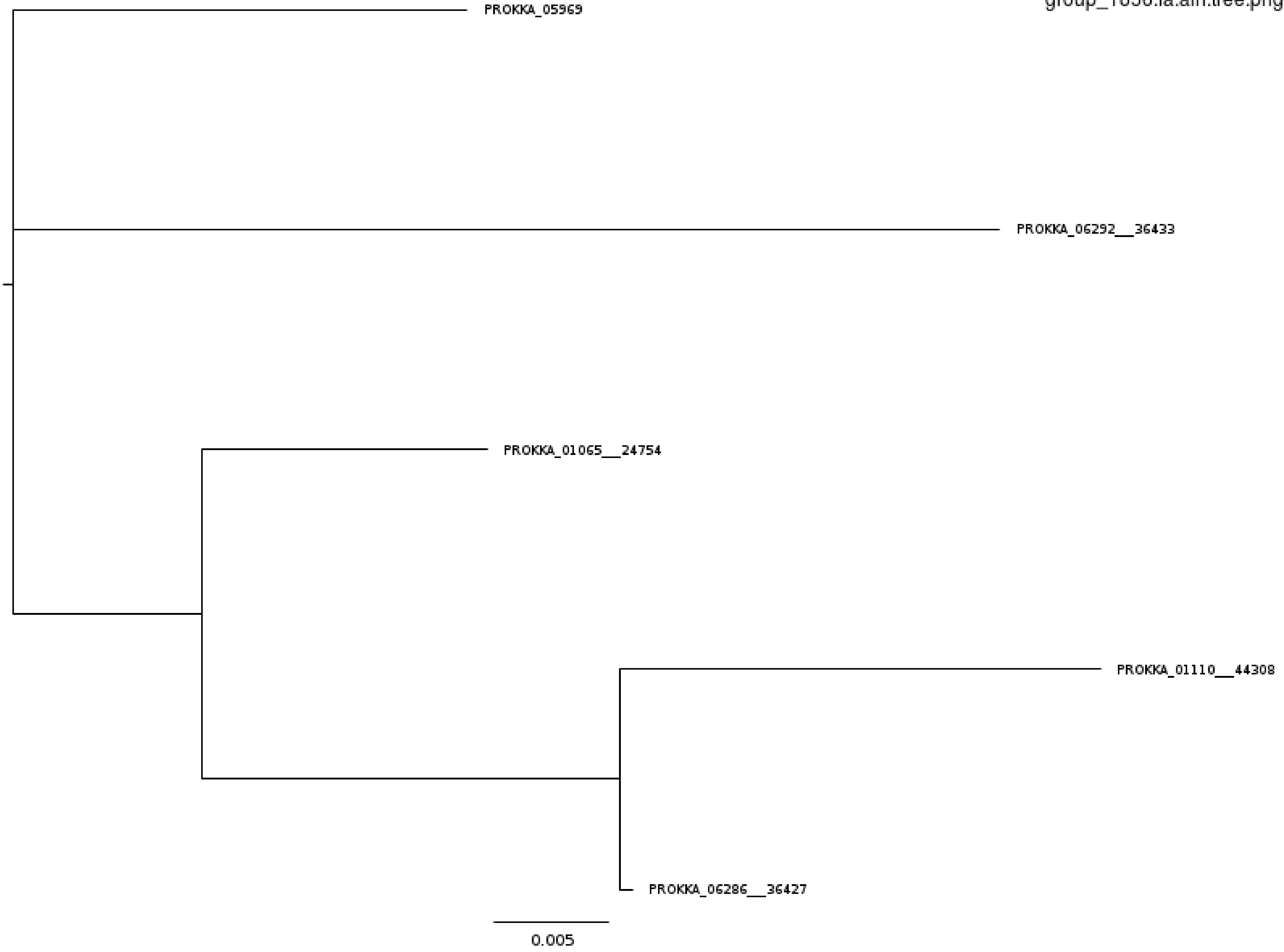

group_1857.fa.aln.tree.png

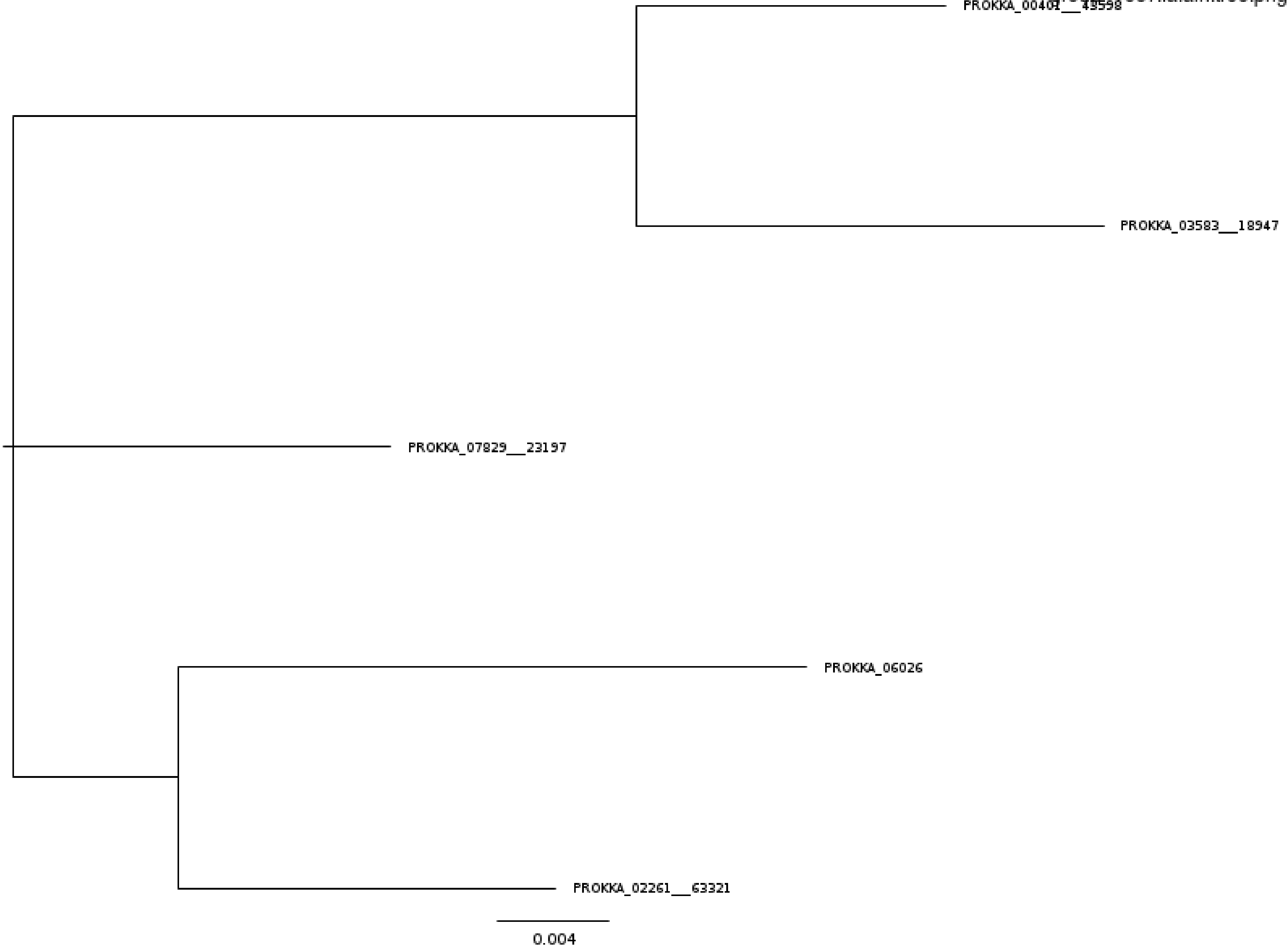

group_1858.fa.aln.tree.png

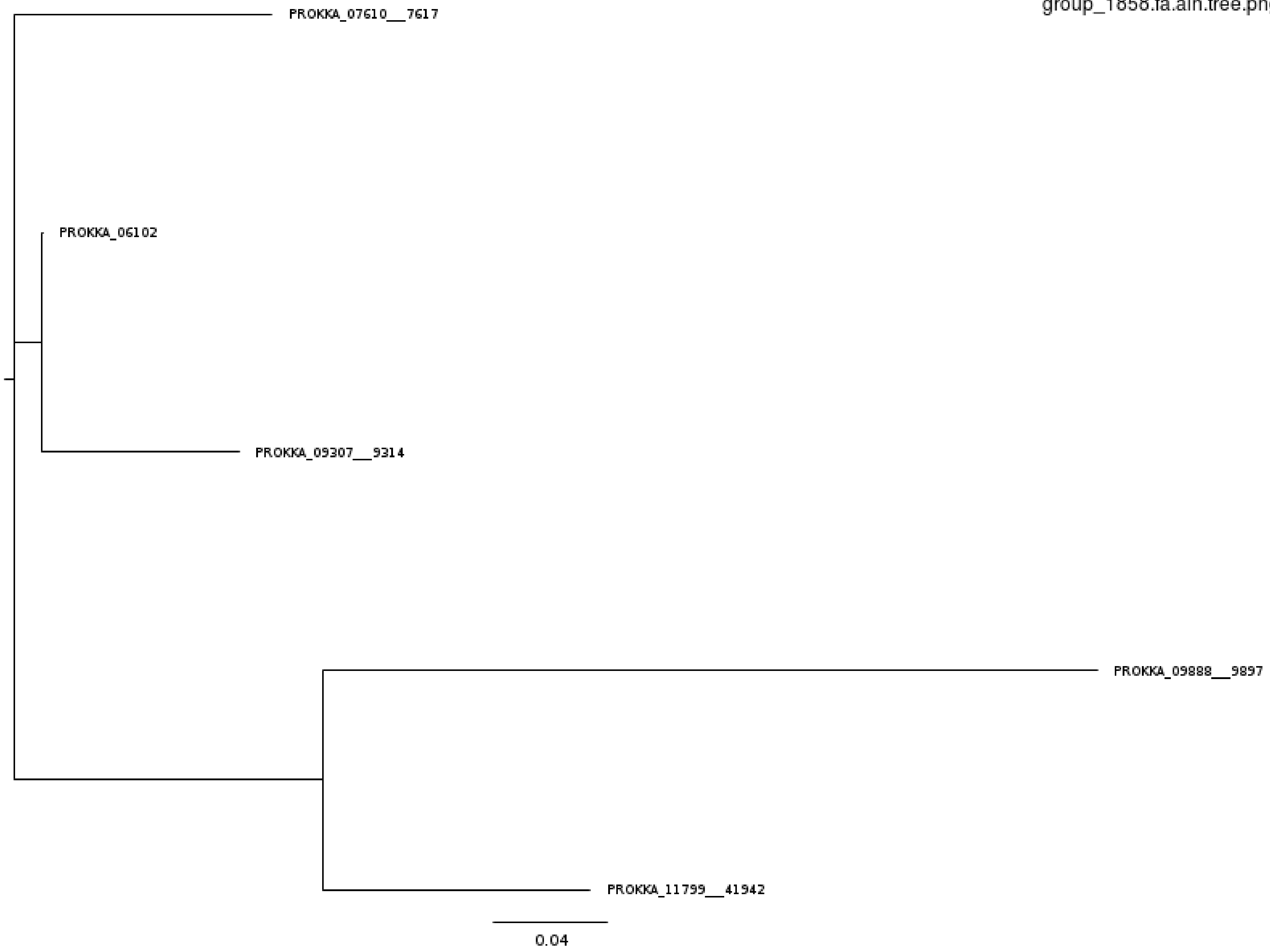

group_185.fa.aln.tree.png

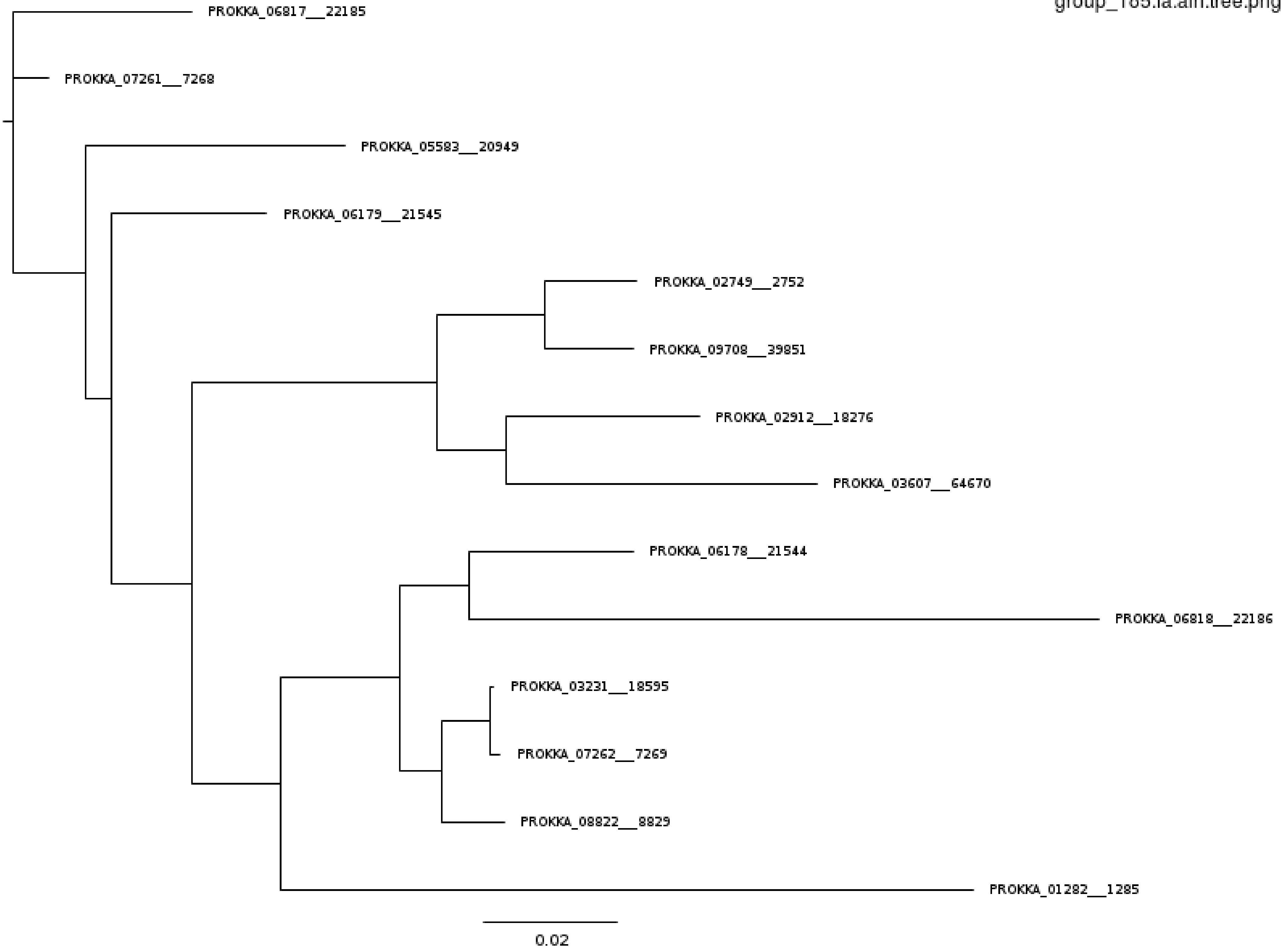

group_1860.fa.aln.tree.png

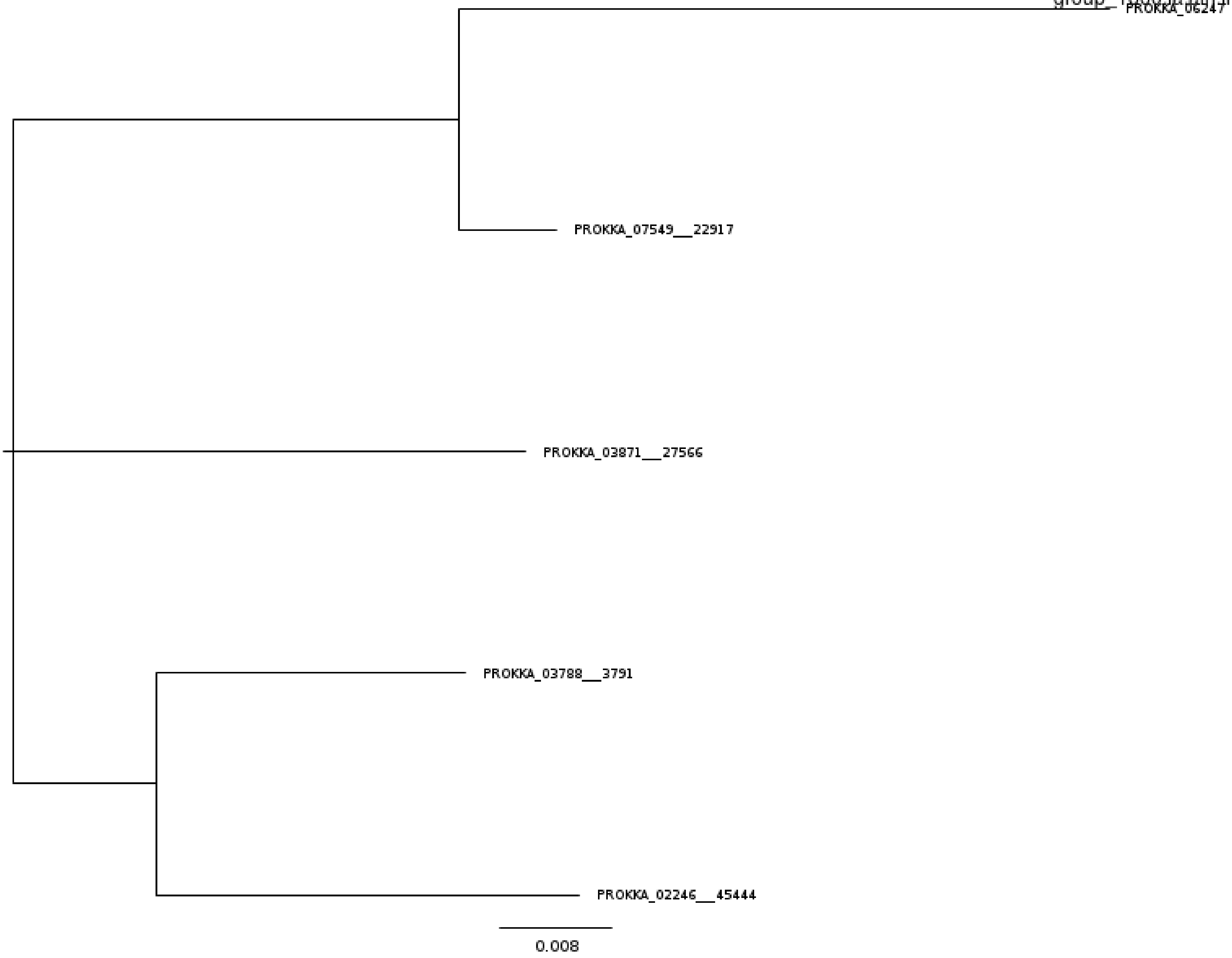

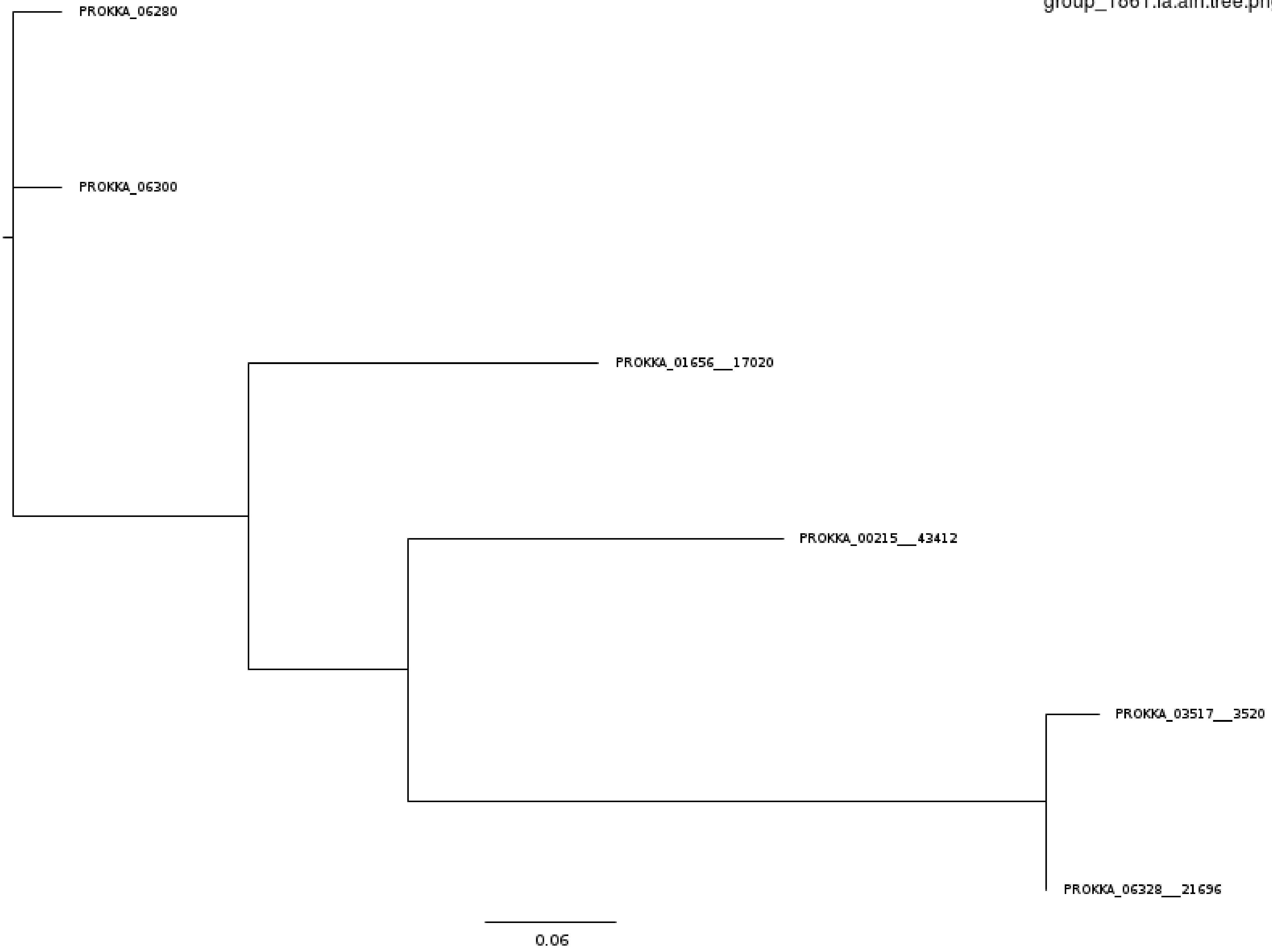
PROKKA_06280
PROKKA_06300
PROKKA_01656__17020
PROKKA_00215__43412
PROKKA_03517__3520
PROKKA_06328__21696
0.06

group_1862.fa.aln.tree.png

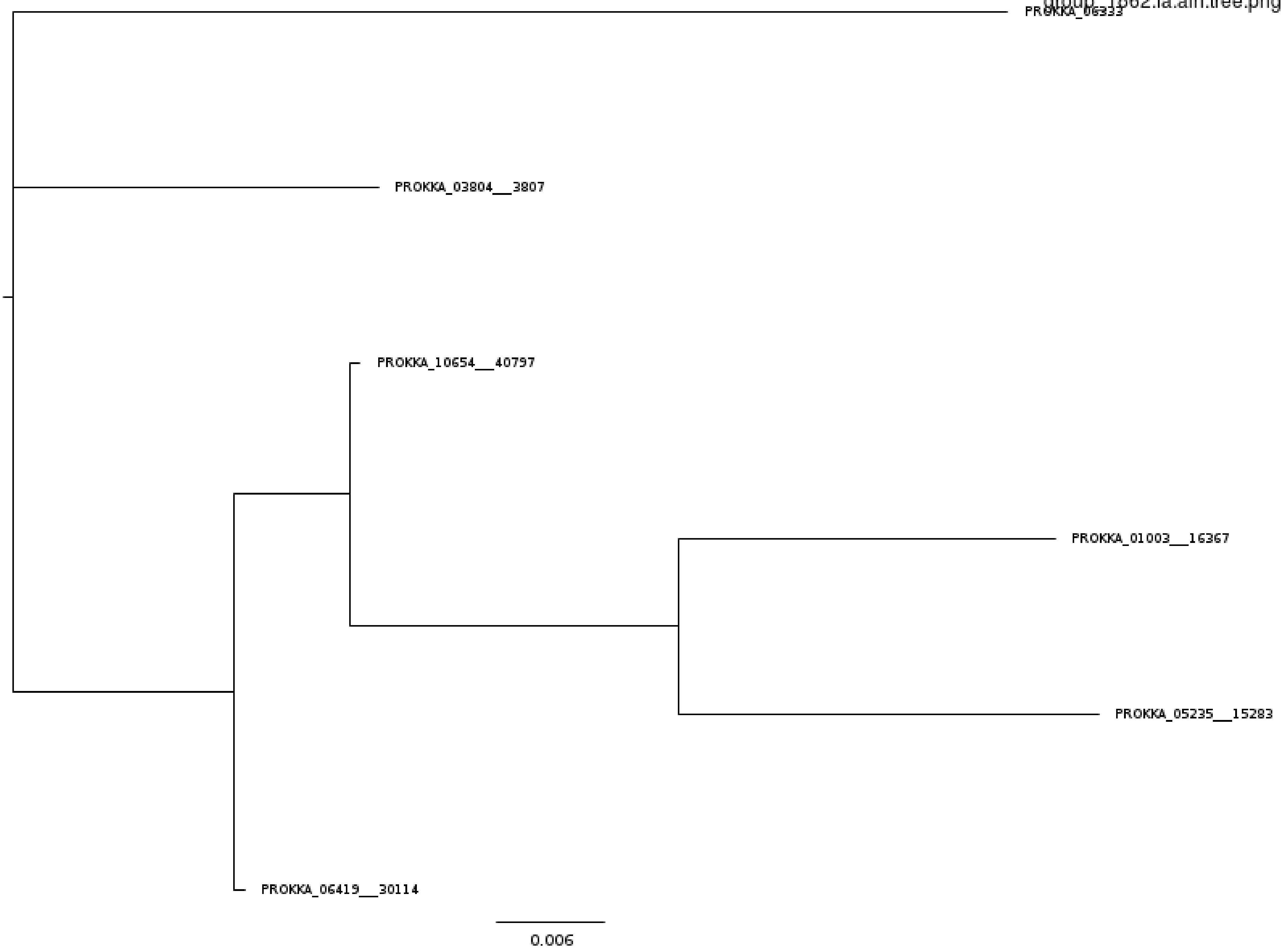

group_1867.fa.aln.tree.png

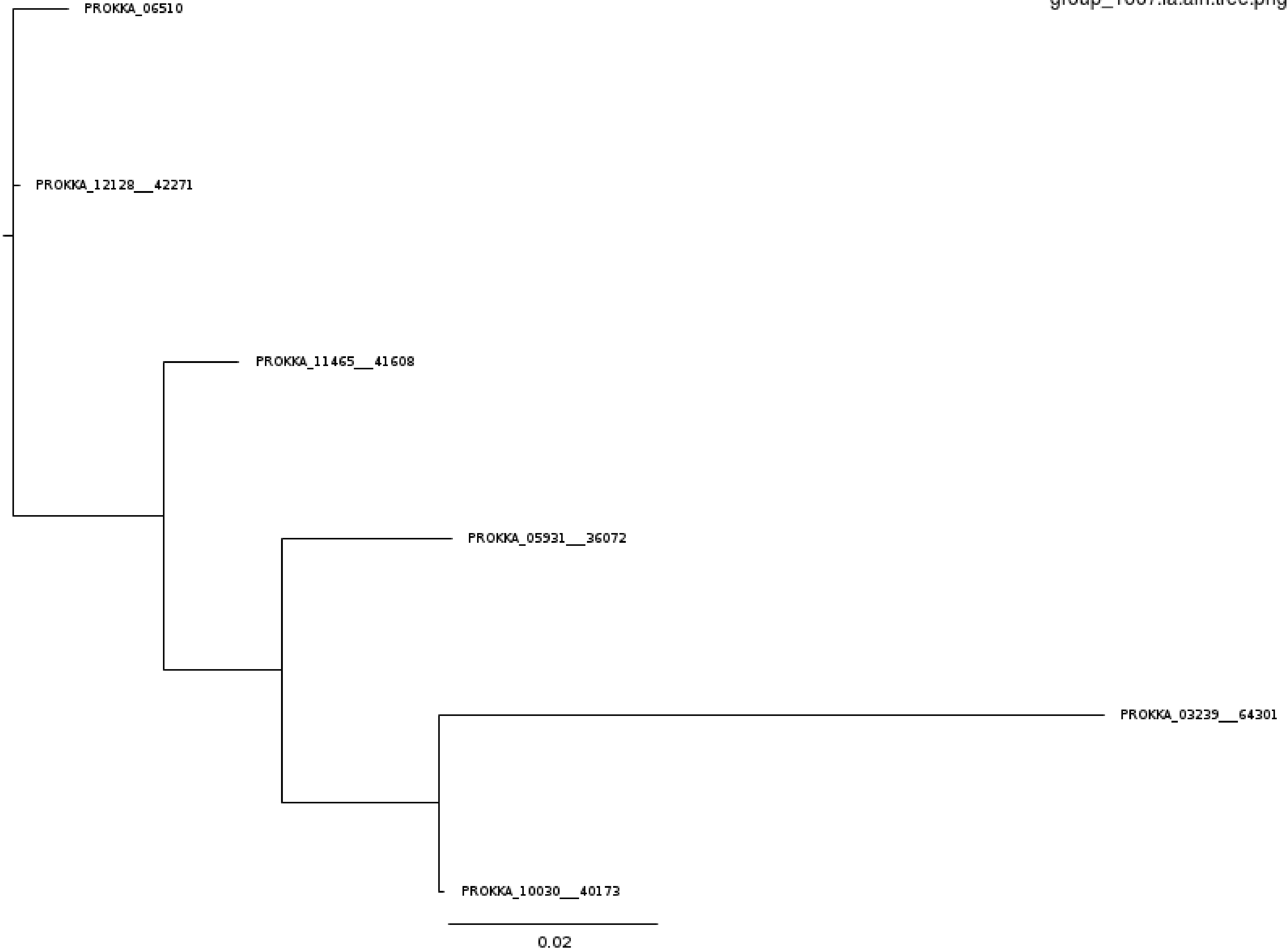

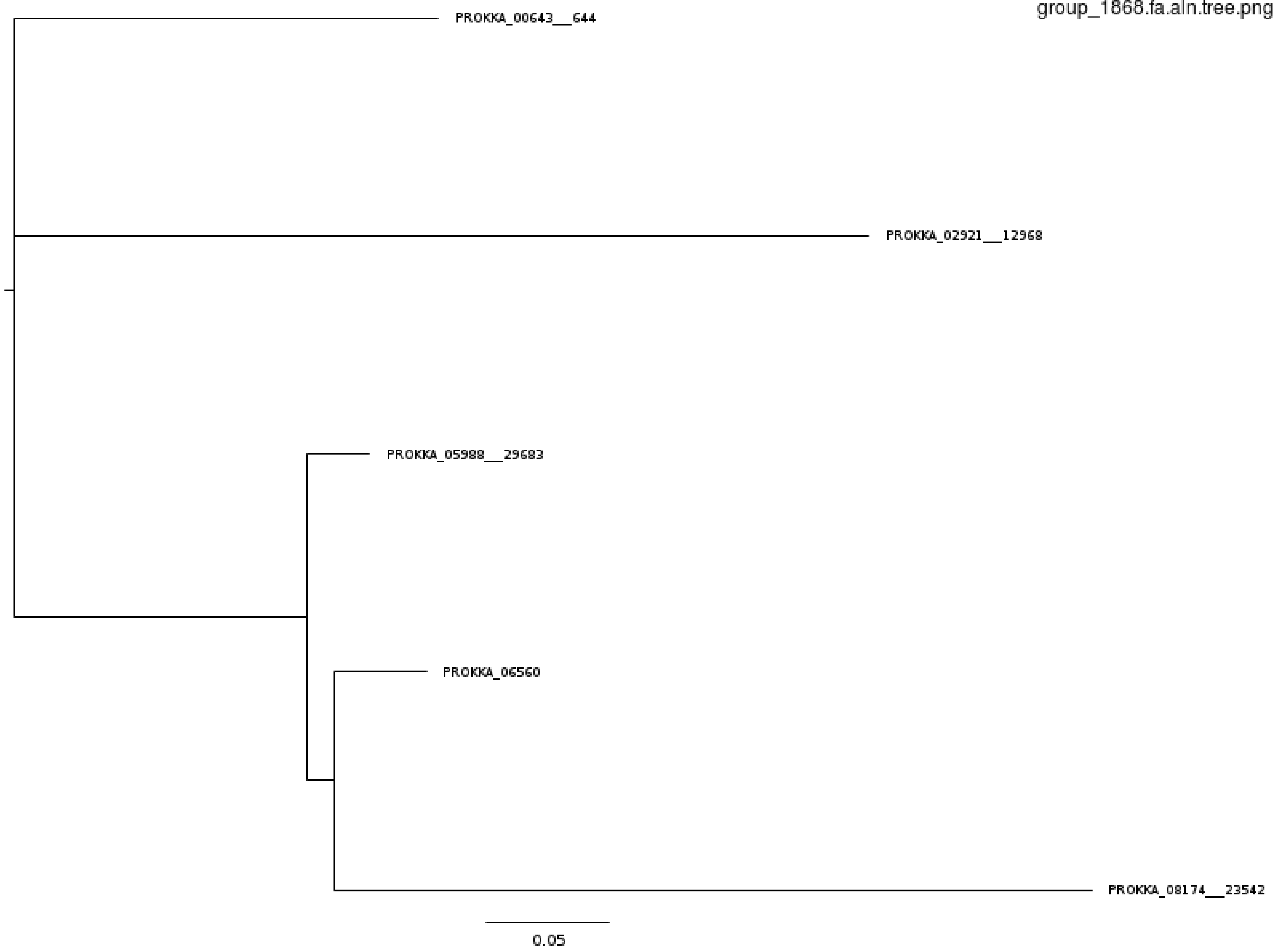
group_1868.fa.aln.tree.png
PROKKA_00643___644
PROKKA_02921___12968
PROKKA_05988___29683
PROKKA_06560
PROKKA_08174___23542
0.05

group_1871.fa.aln.tree.png

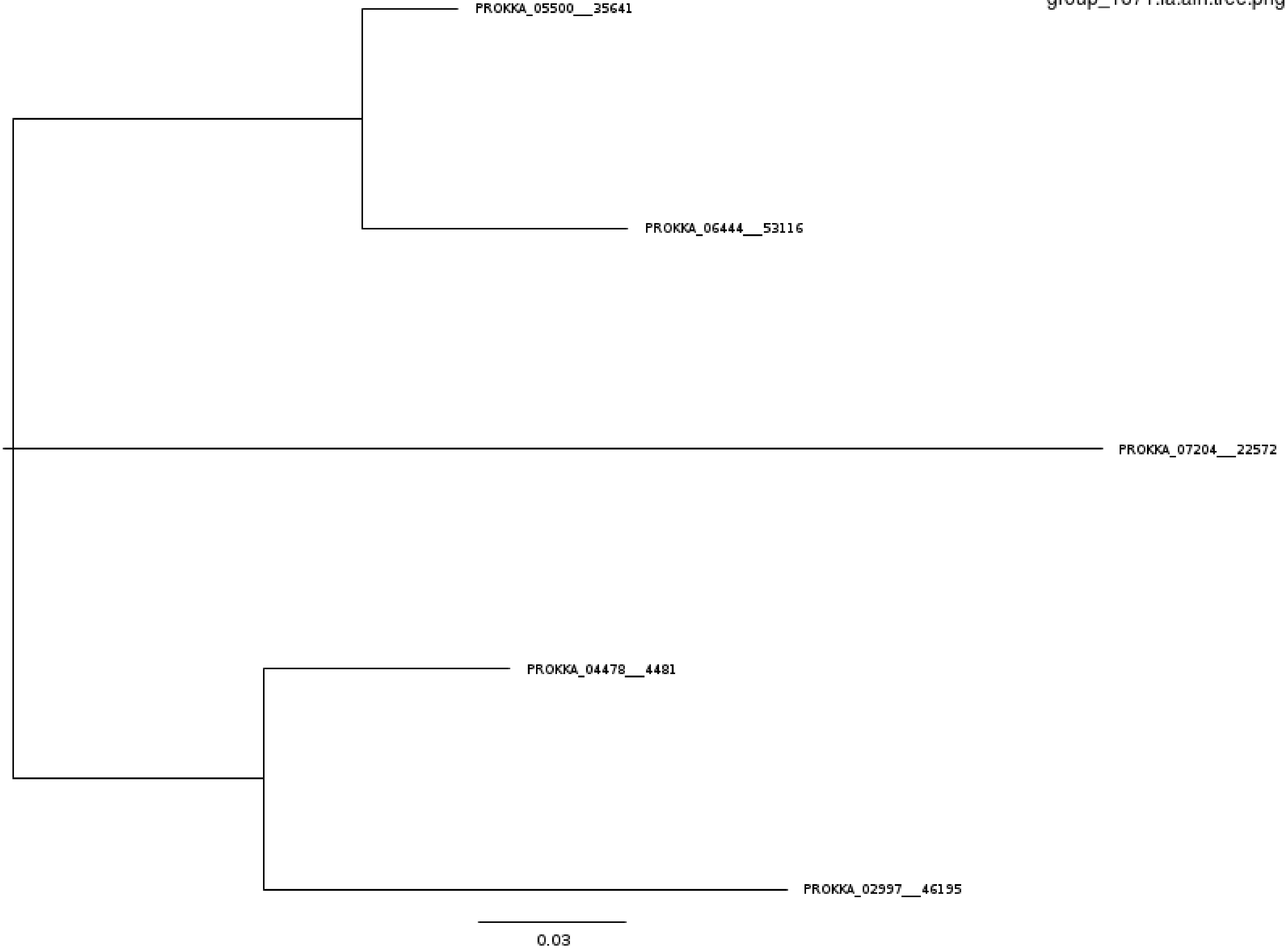

group_1875.fa.aln.tree.png

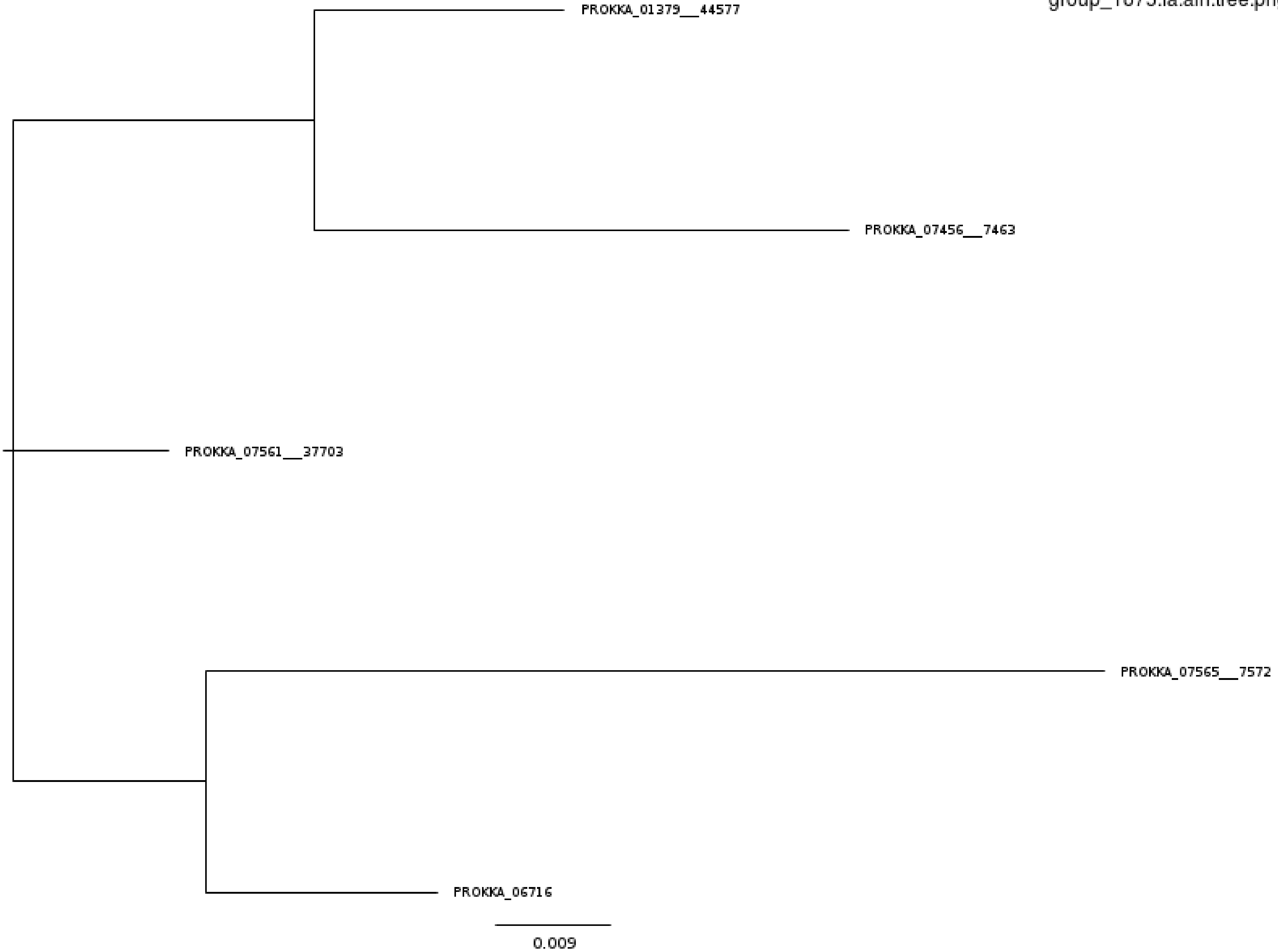

group_1877.fa.aln.tree.png

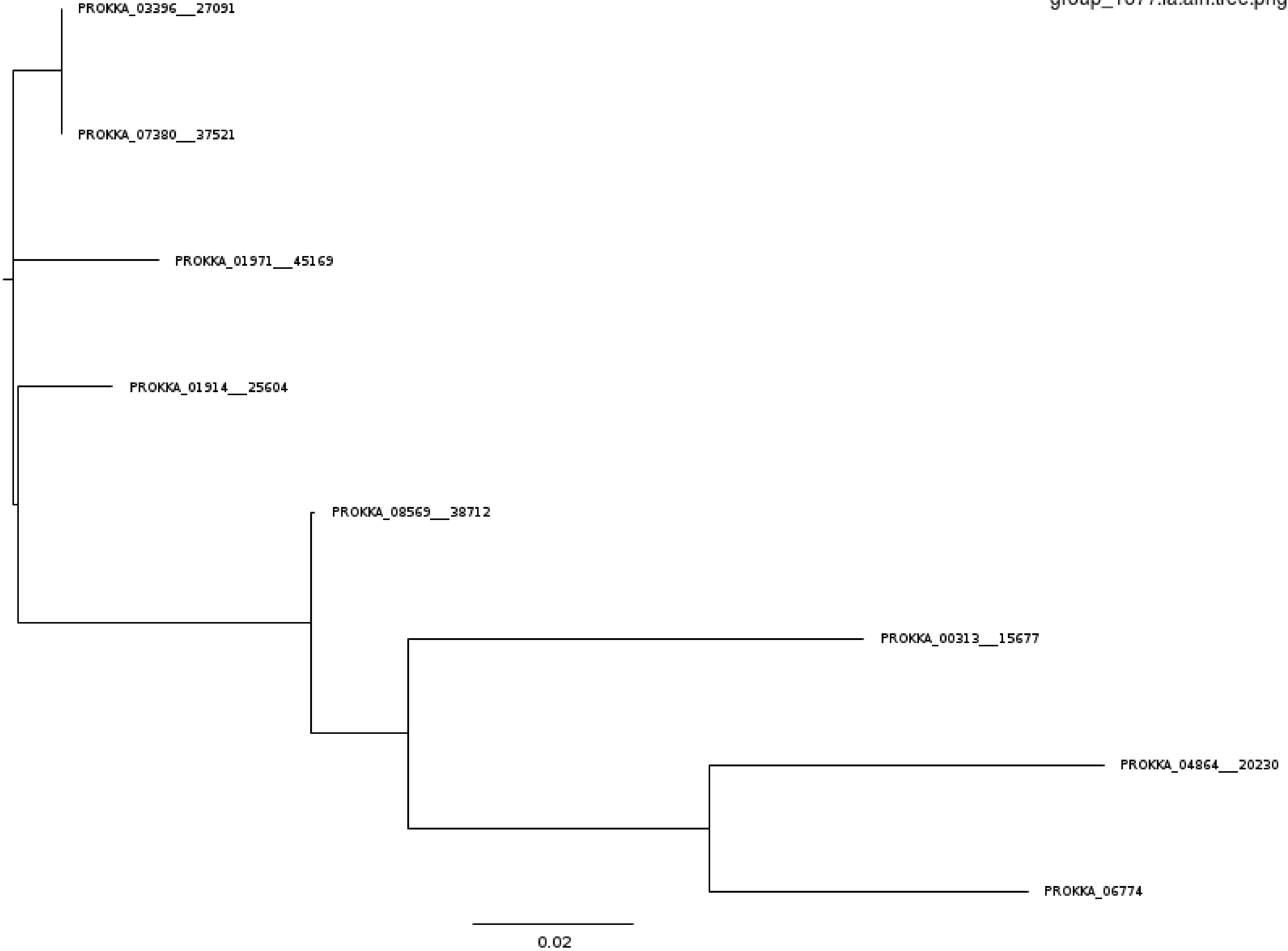

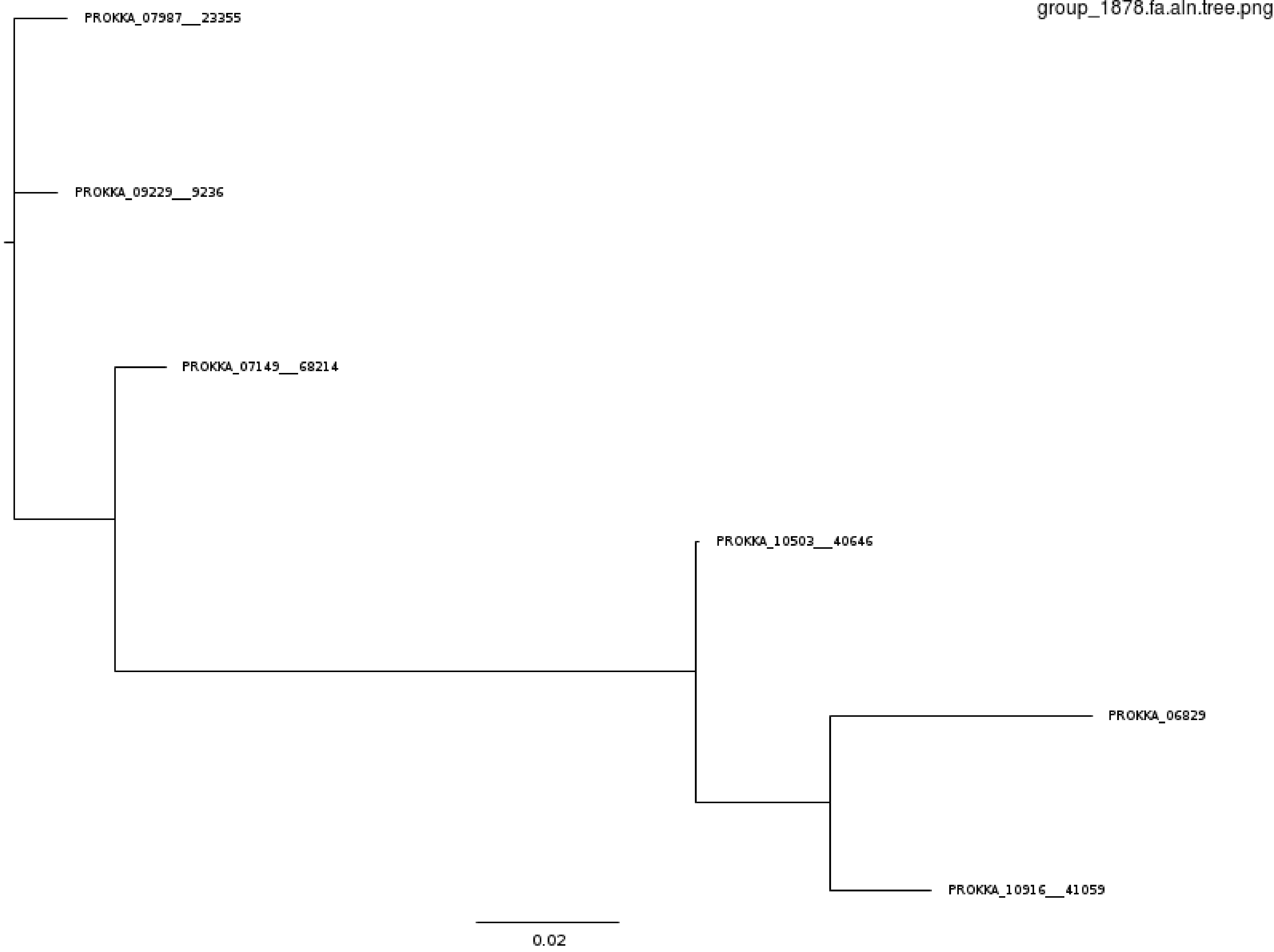
group_1878.fa.aln.tree.png
PROKKA_07987___23355
PROKKA_09229___9236
PROKKA_07149___68214
PROKKA_10503___40646
PROKKA_06829
PROKKA_10916___41059
0.02

group_1881.fa.aln.tree.png

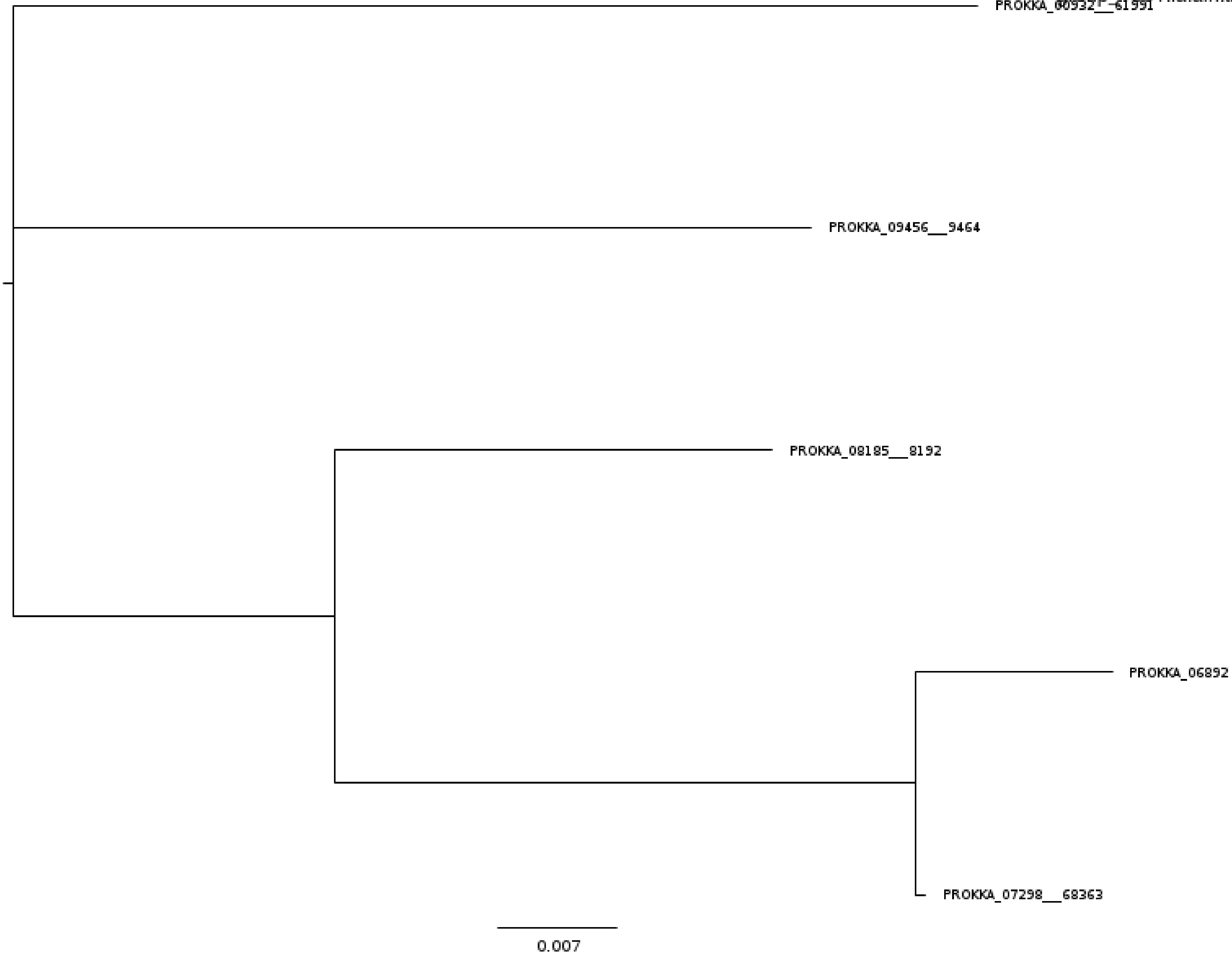

group_1882.fa.aln.tree.png

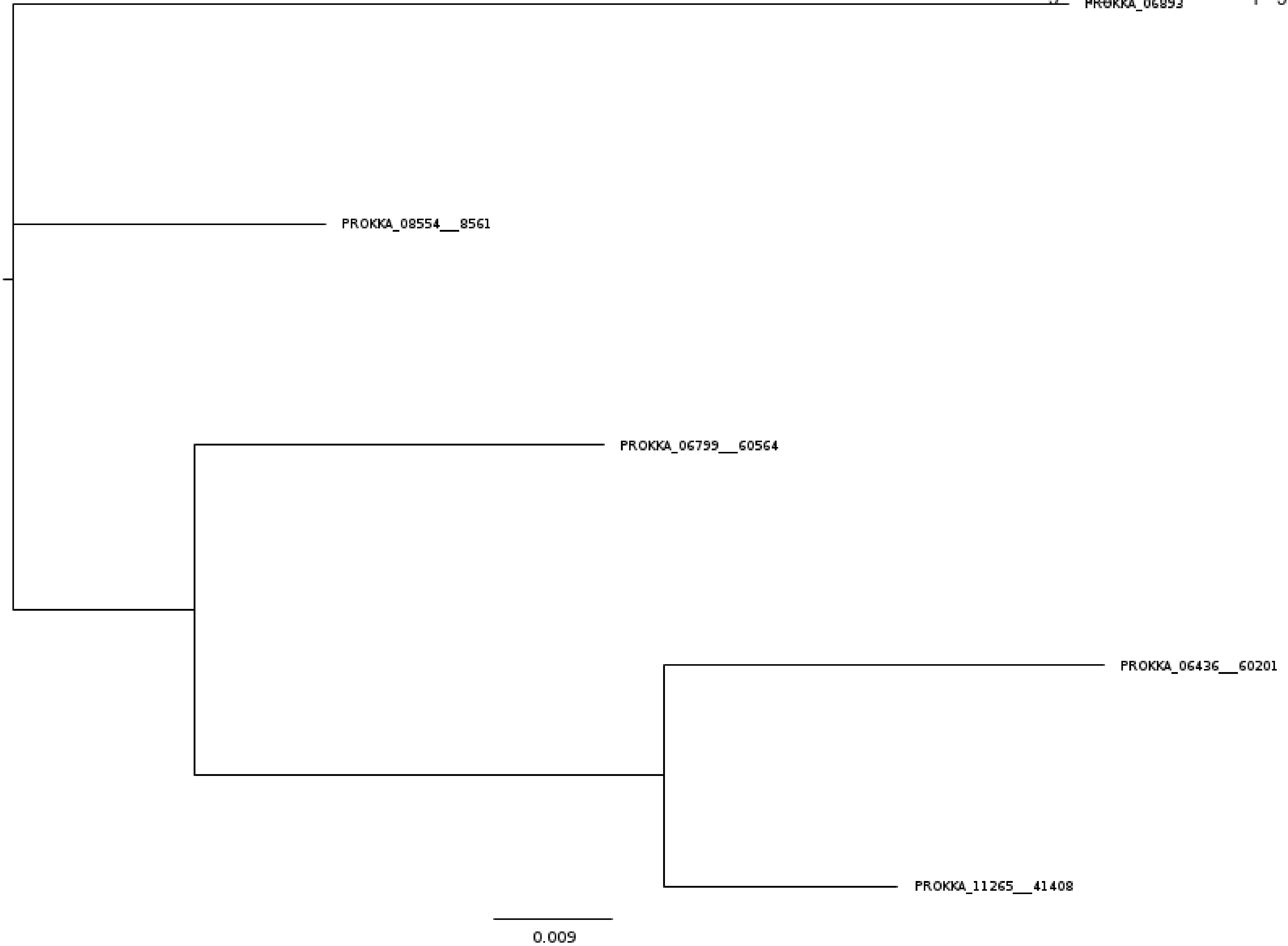

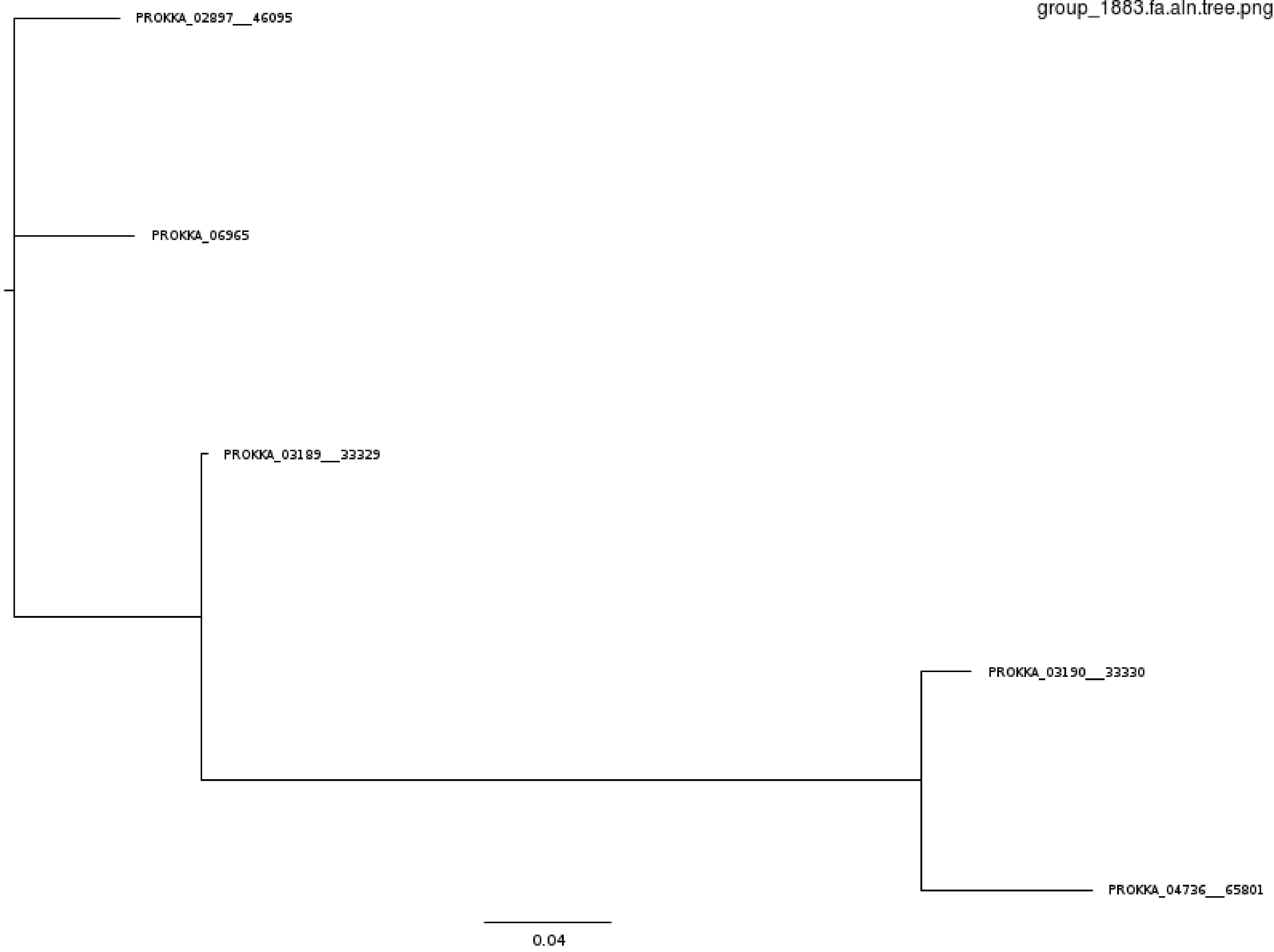
group_1883.fa.aln.tree.png
PROKKA_02897___46095
PROKKA_06965
PROKKA_03189___33329
PROKKA_03190___33330
PROKKA_04736___65801
0.04

group_1889.fa.aln.tree.png

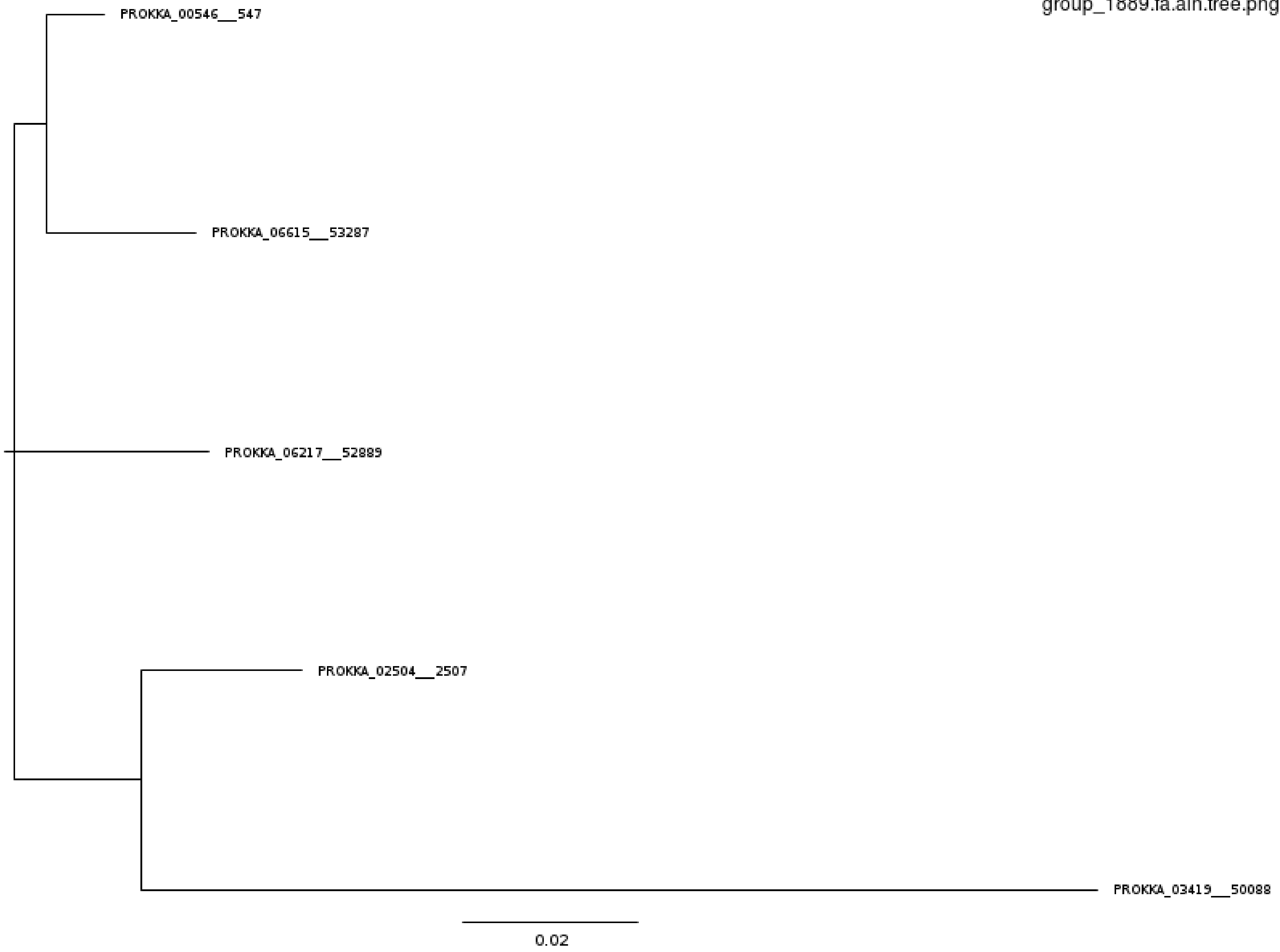

group_188.fa.aln.tree.png

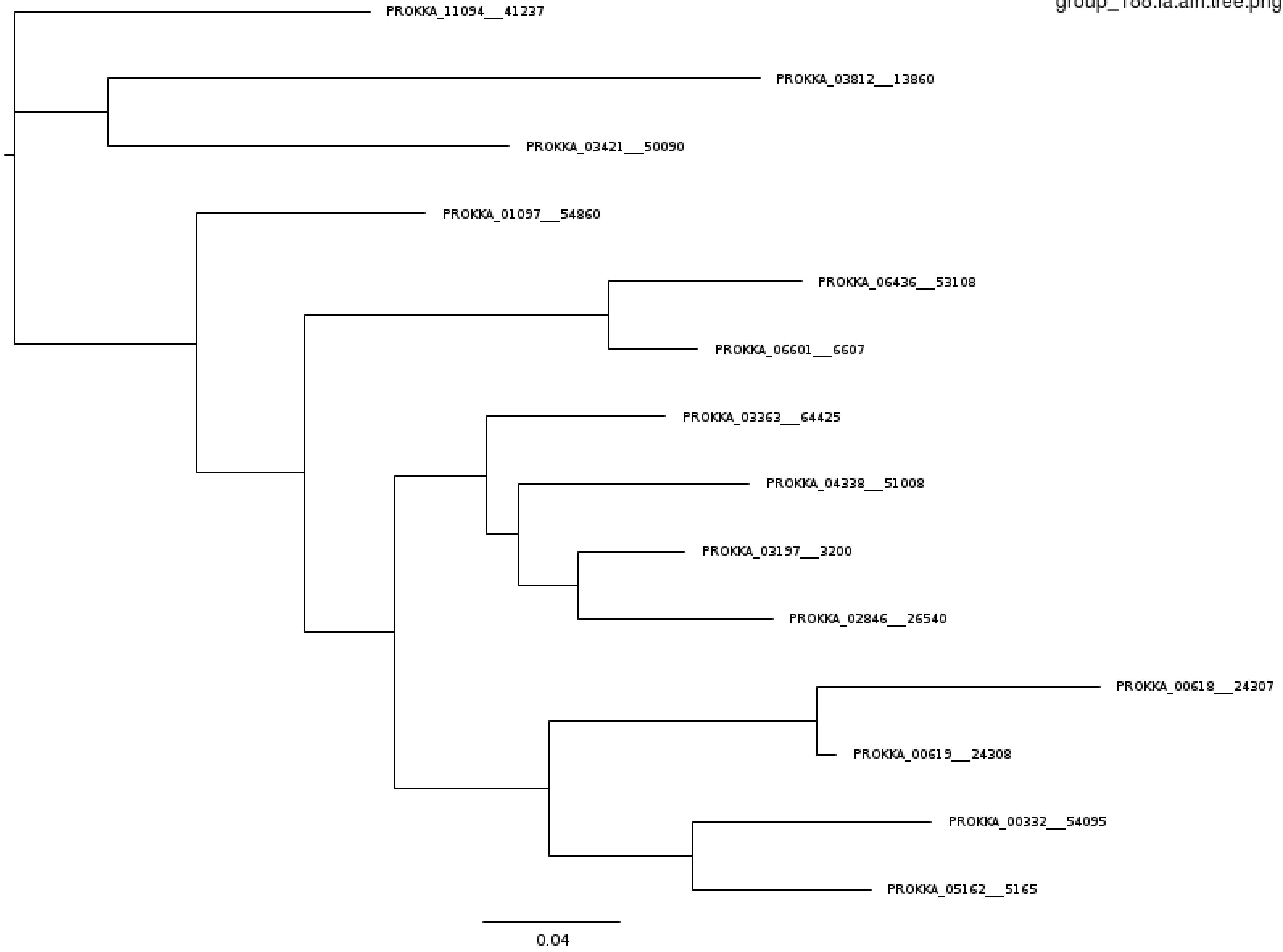

group_1893.fa.aln.tree.png

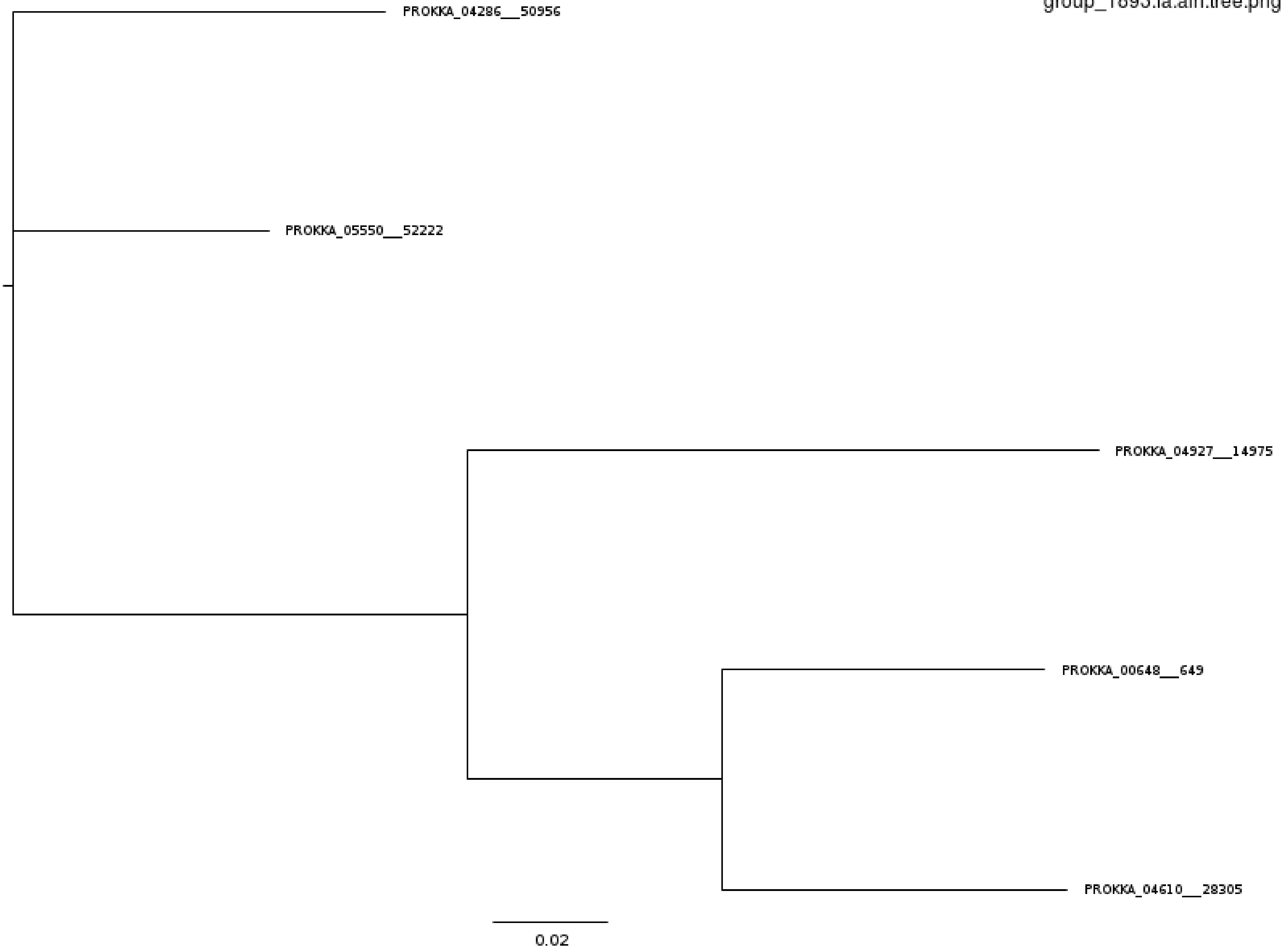

group_1895.fa.aln.tree.png

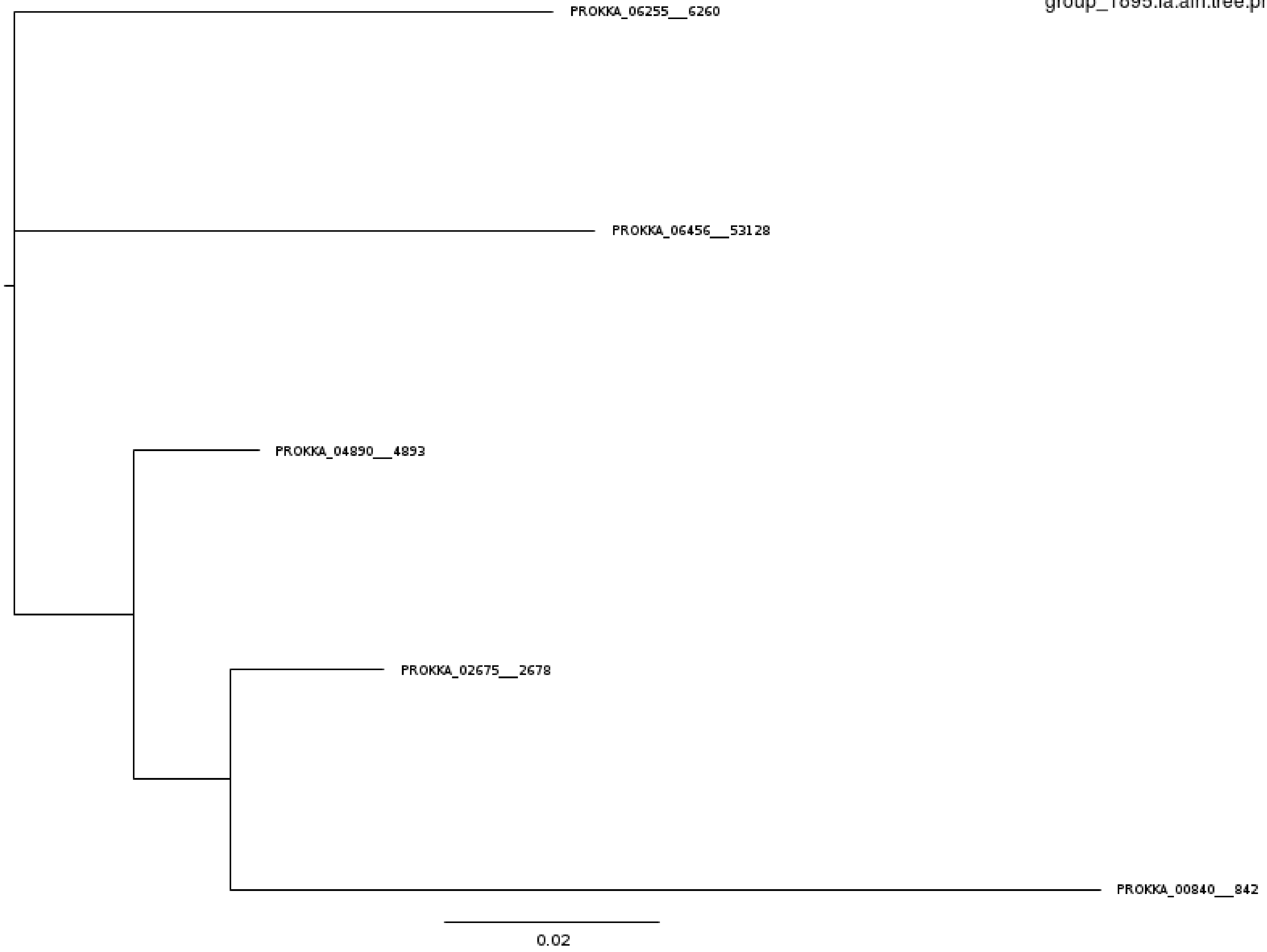

group_1897.fa.aln.tree.png

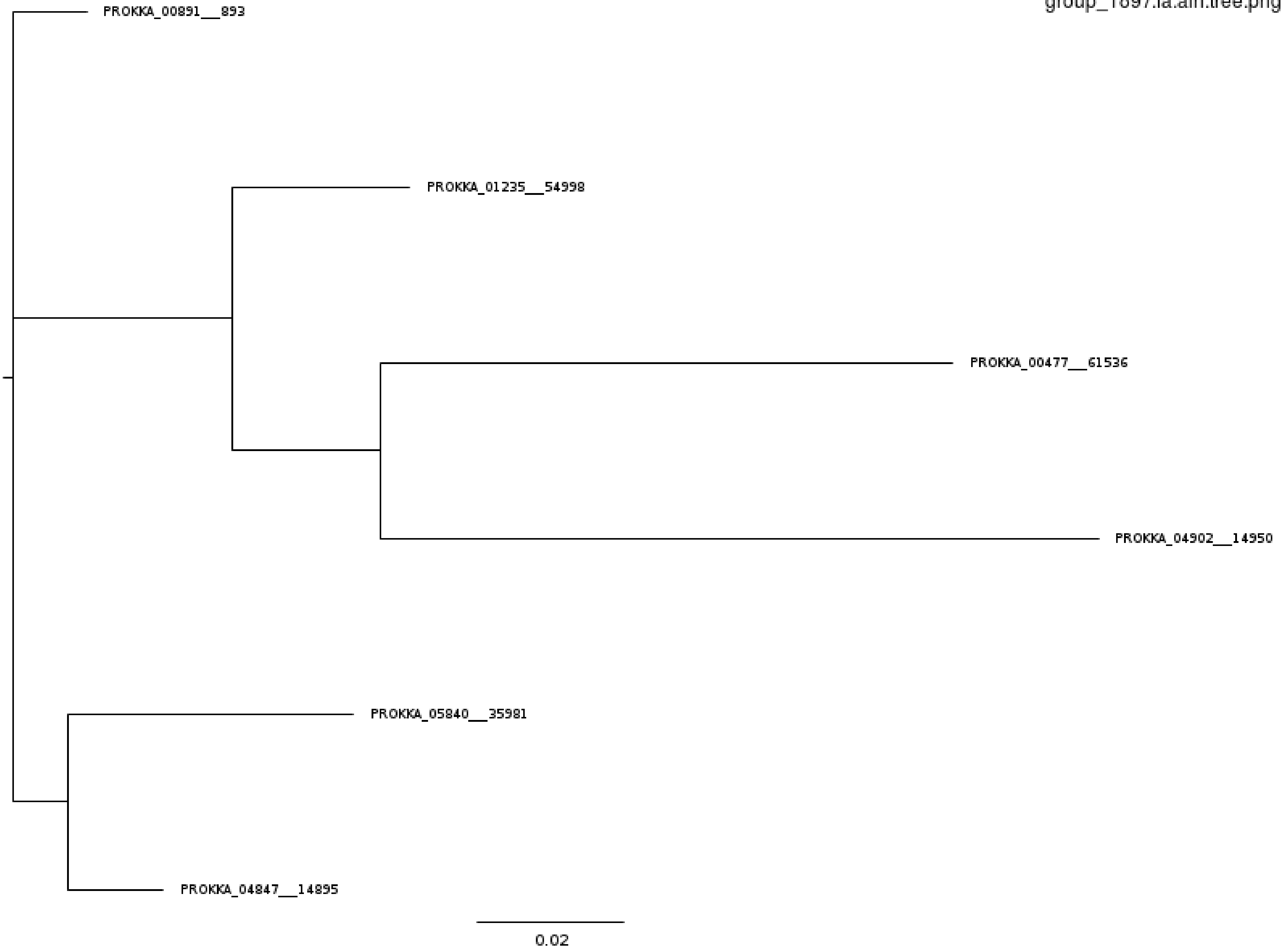

group_189.fa.aln.tree.png

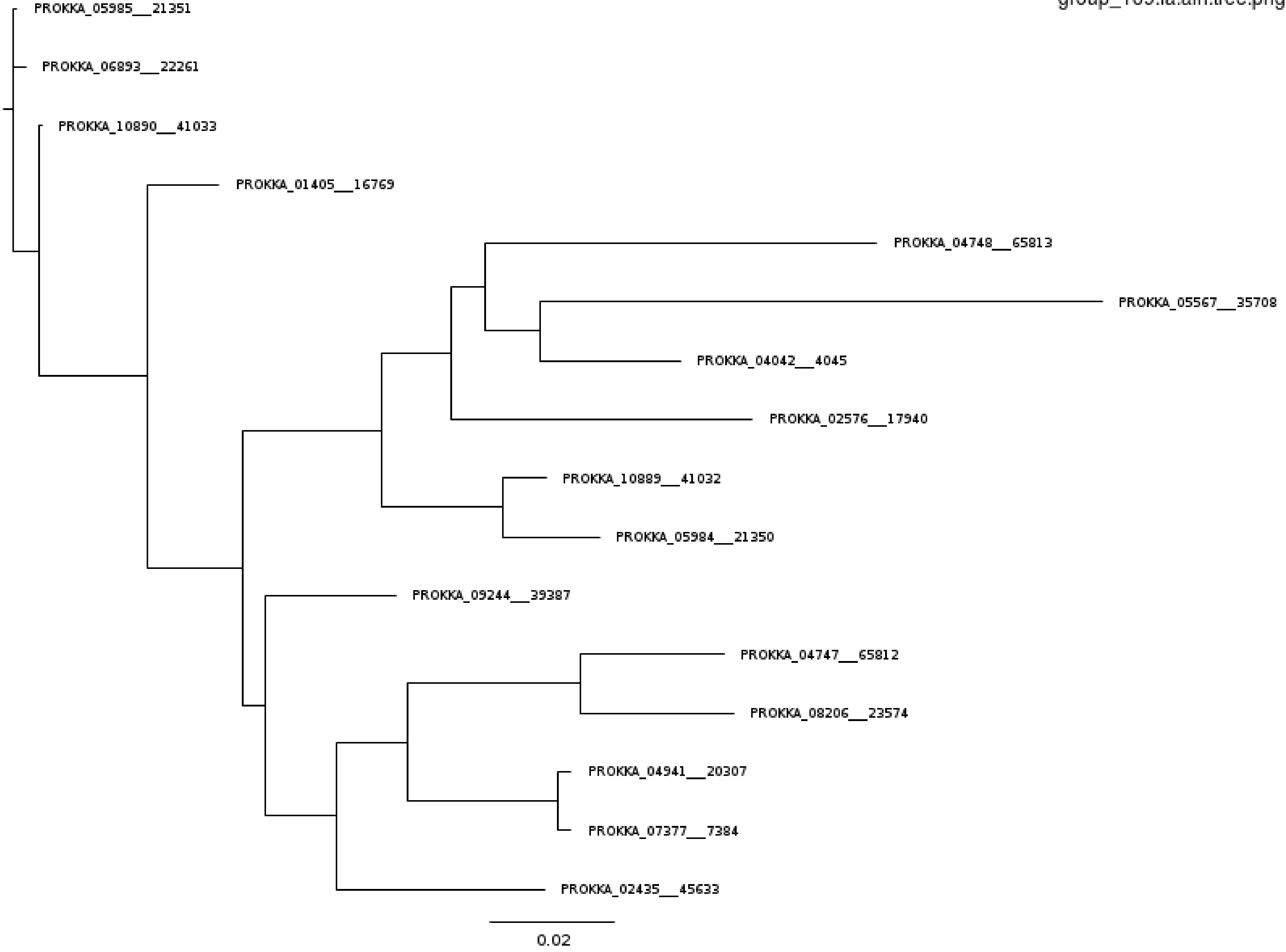

group_1902.fa.aln.tree.png

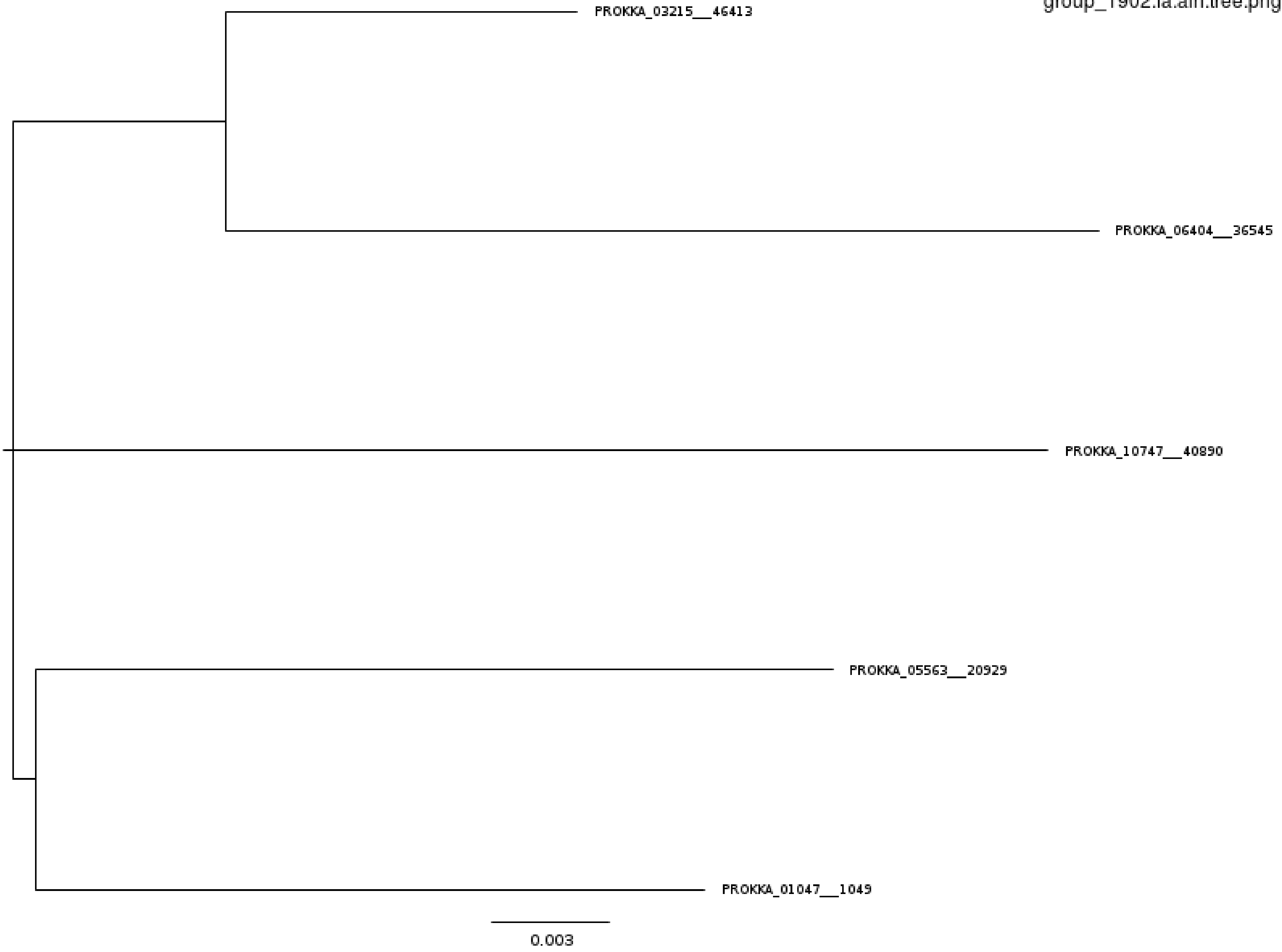

group_1904.fa.aln.tree.png

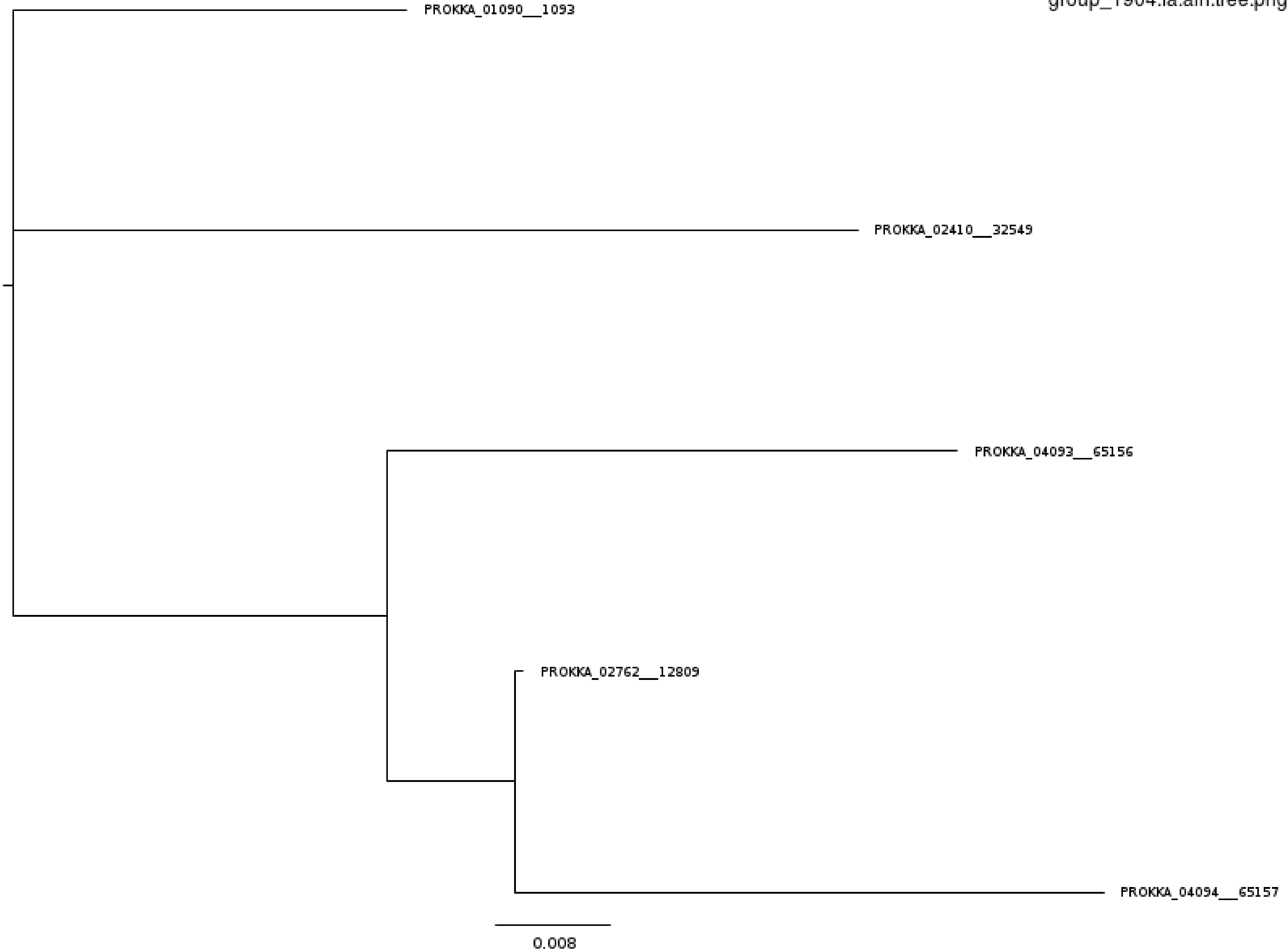

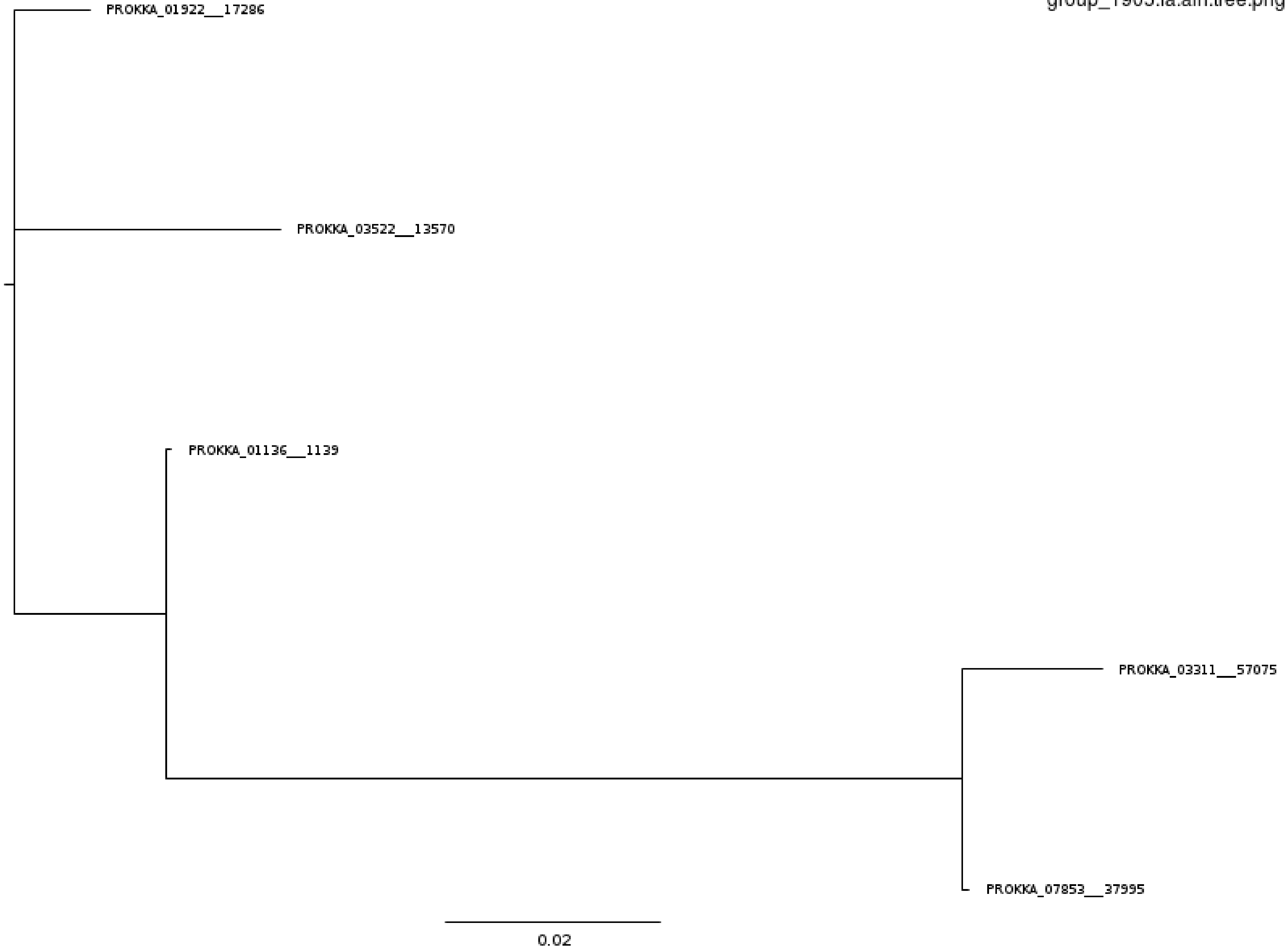
PROKKA_01922___17286
PROKKA_03522___13570
PROKKA_01136___1139
PROKKA_03311___57075
PROKKA_07853___37995
0.02

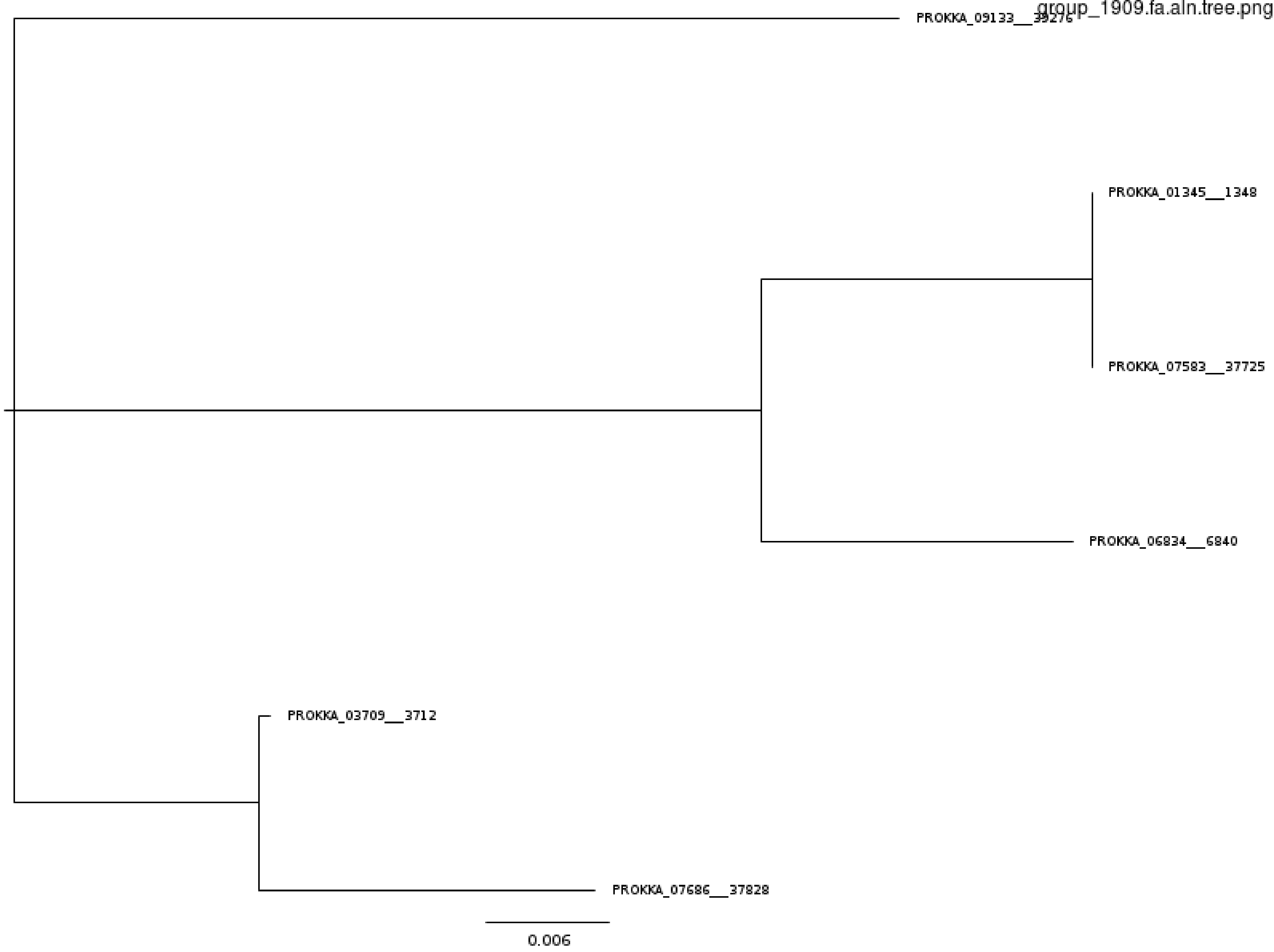
group_1909.fa.aln.tree.png
PROKKA_09133__39276
PROKKA_01345__1348
PROKKA_07583__37725
PROKKA_06834__6840
PROKKA_03709__3712
PROKKA_07686__37828
0.006

group_190.fa.aln.tree.png

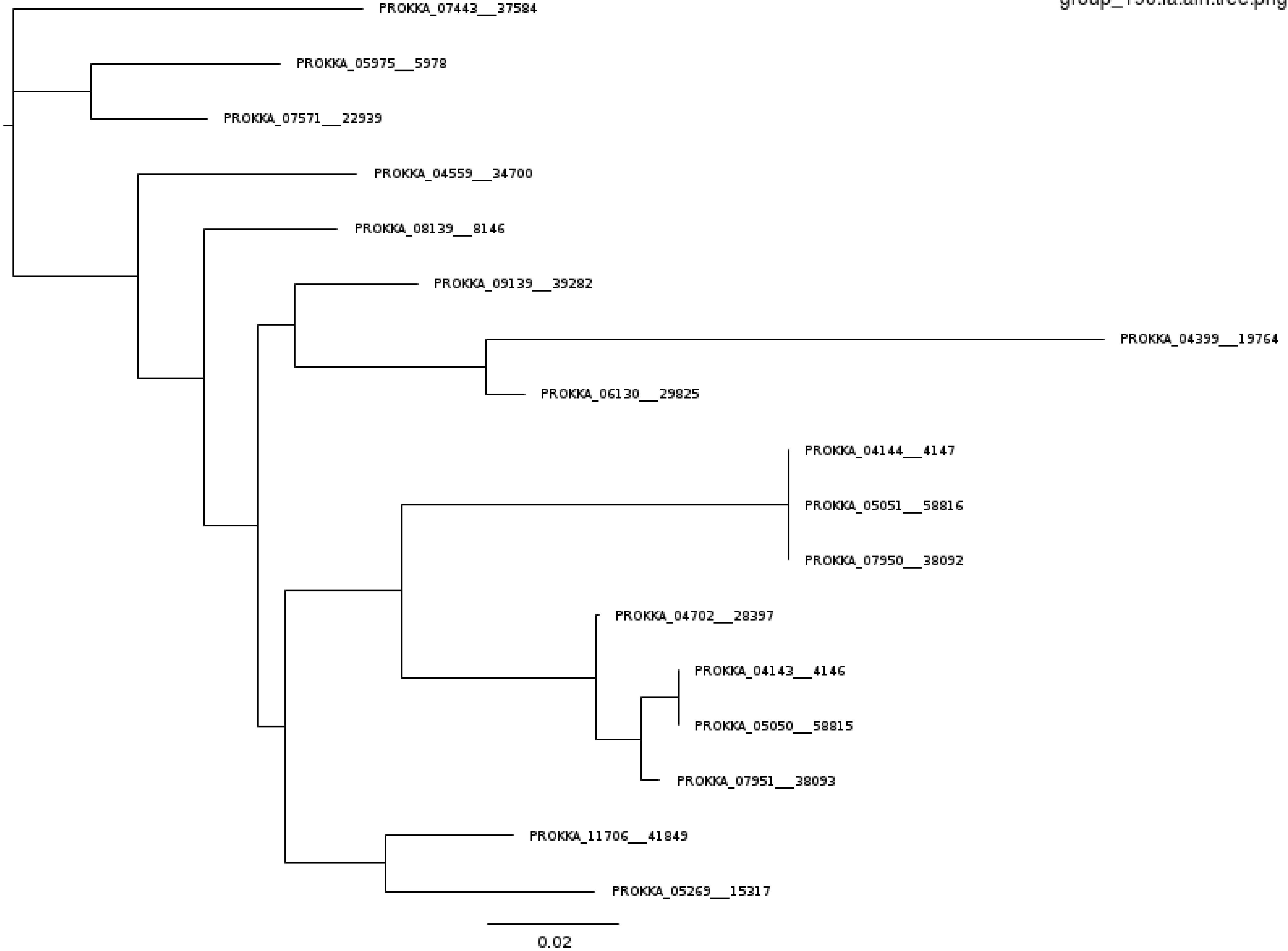

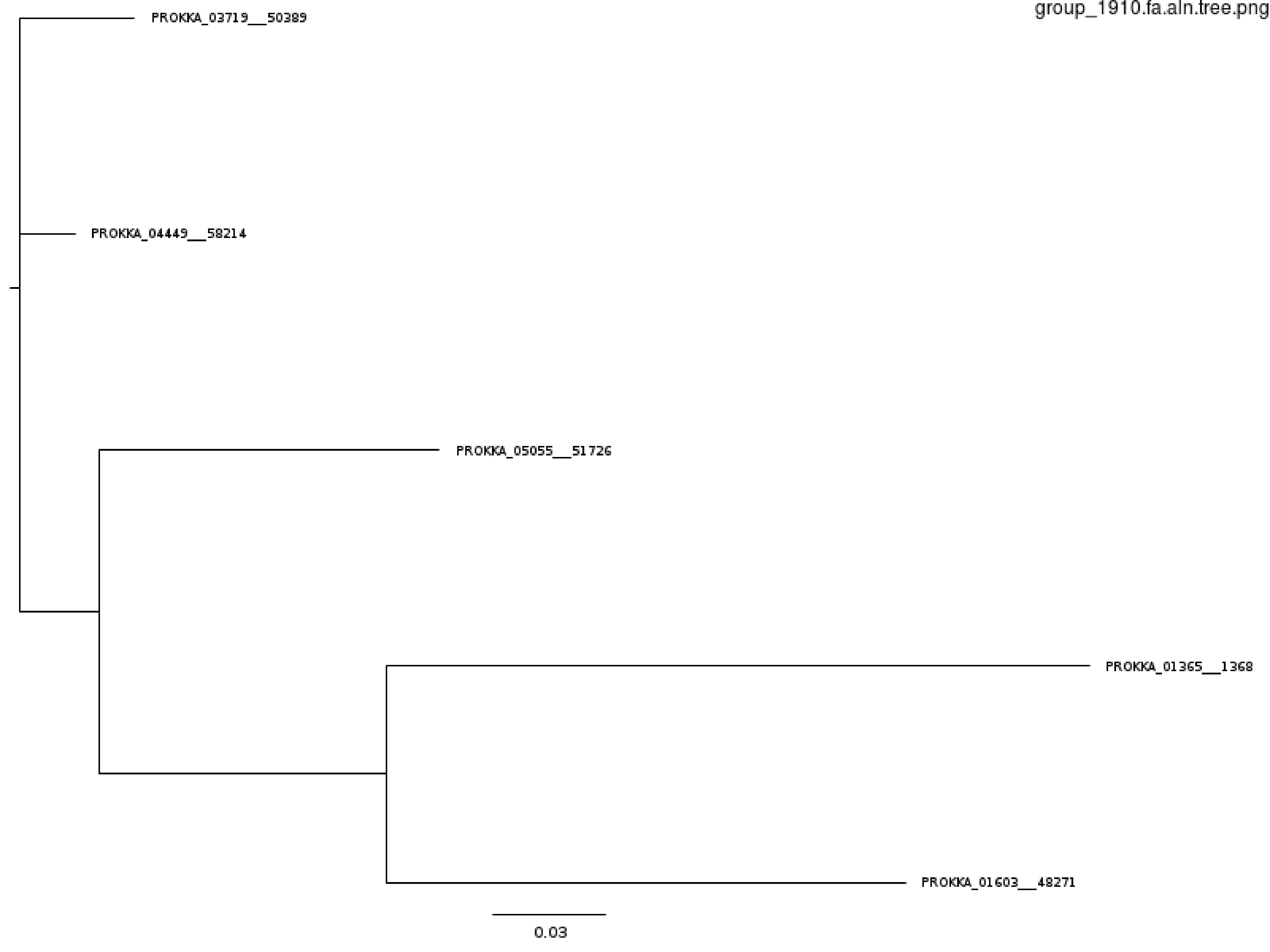
group_1910.fa.aln.tree.png
PROKKA_03719___50389
PROKKA_04449___58214
PROKKA_05055___51726
PROKKA_01365___1368
PROKKA_01603___48271
0.03

group_1911.fa.aln.tree.png

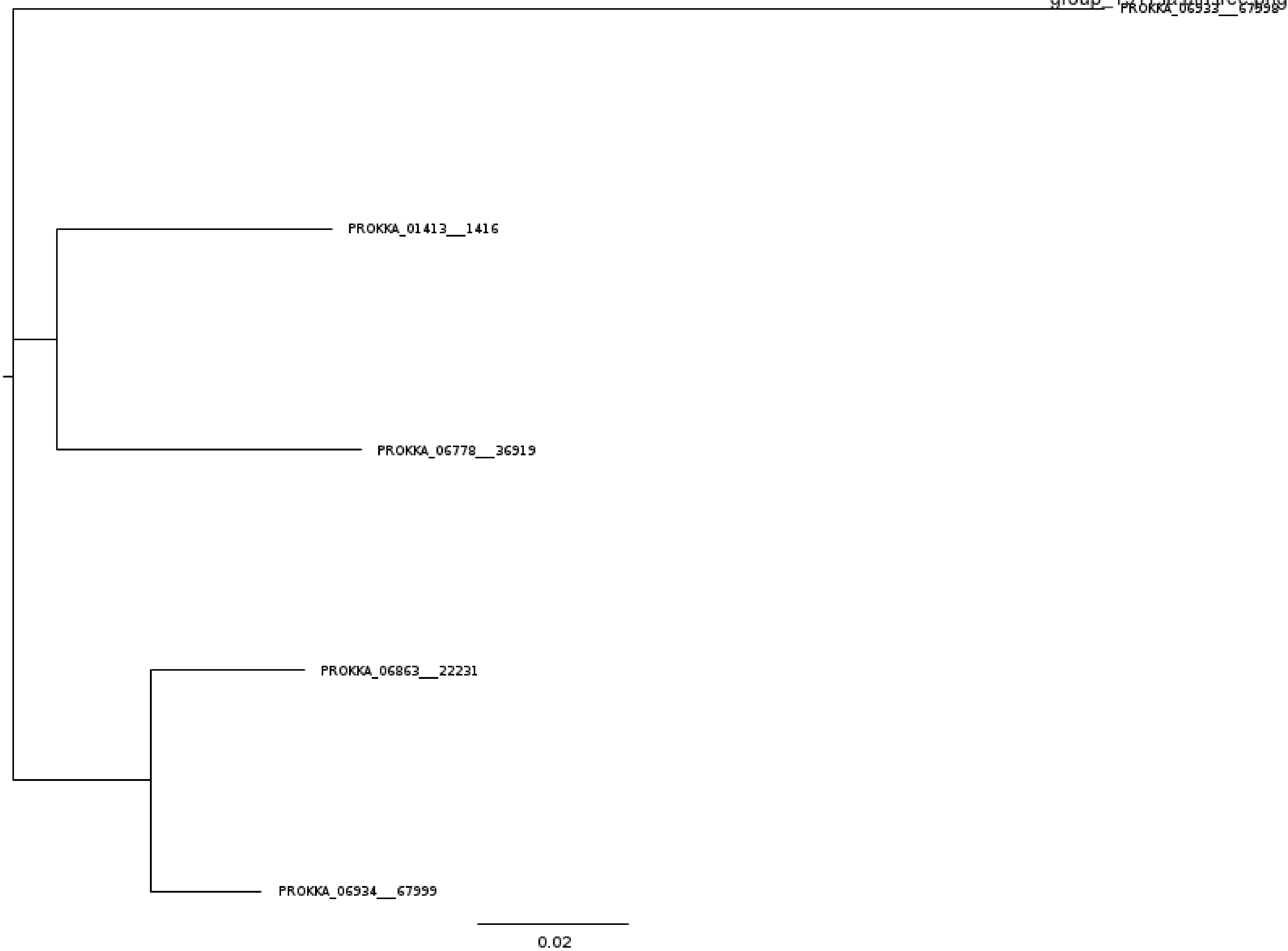

group_1912.fa.aln.tree.png

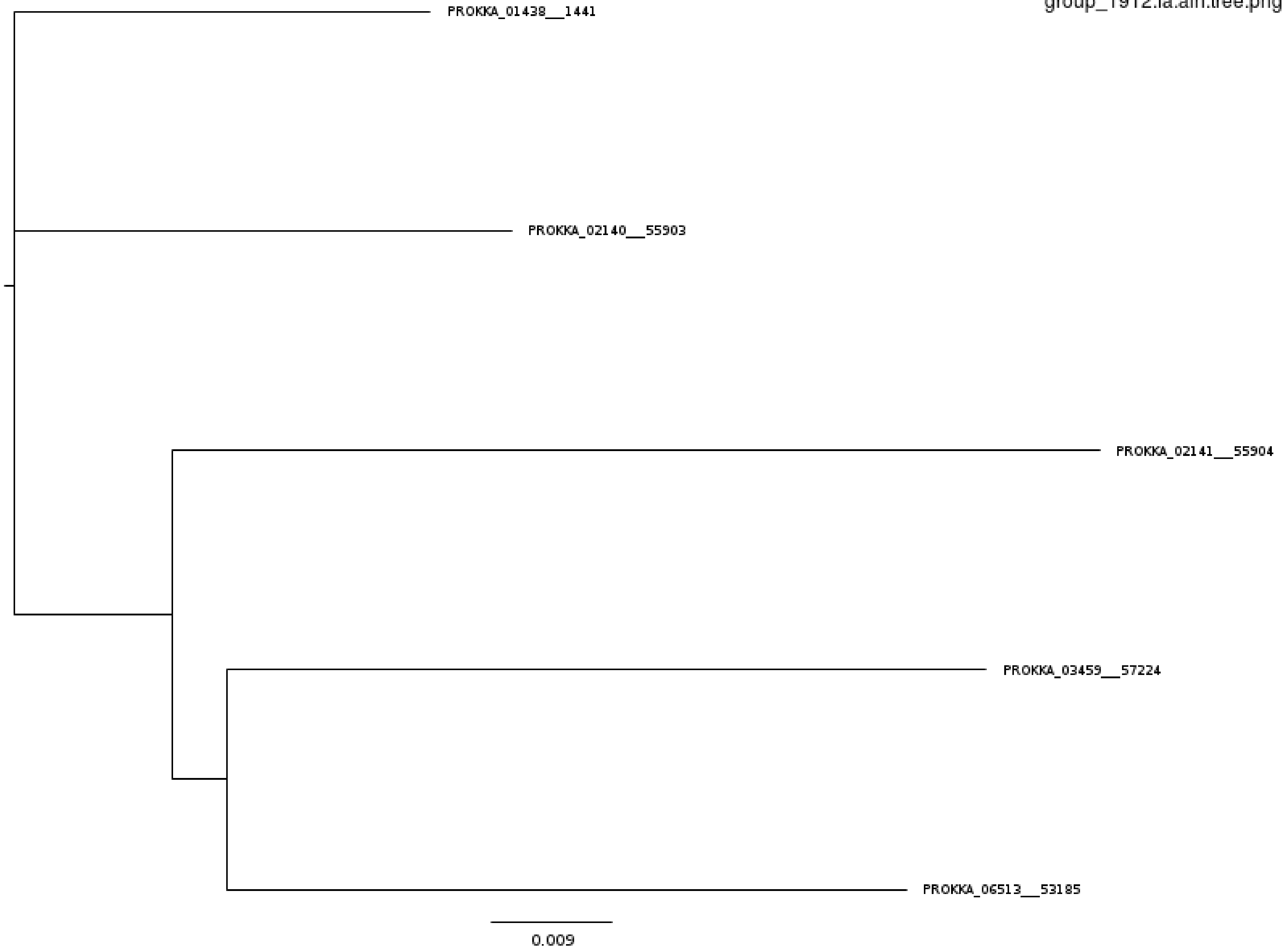

group_1914.fa.aln.tree.png

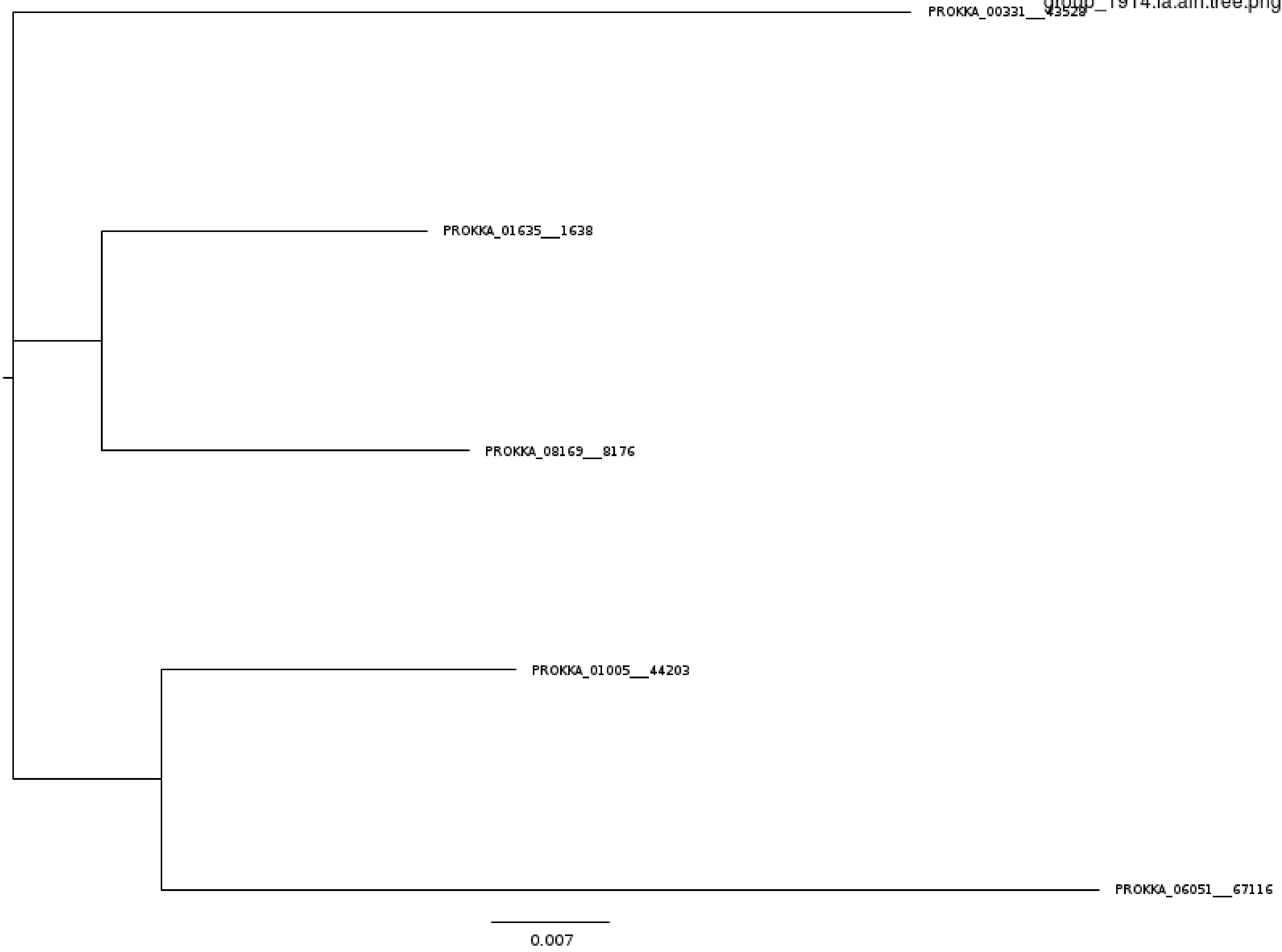

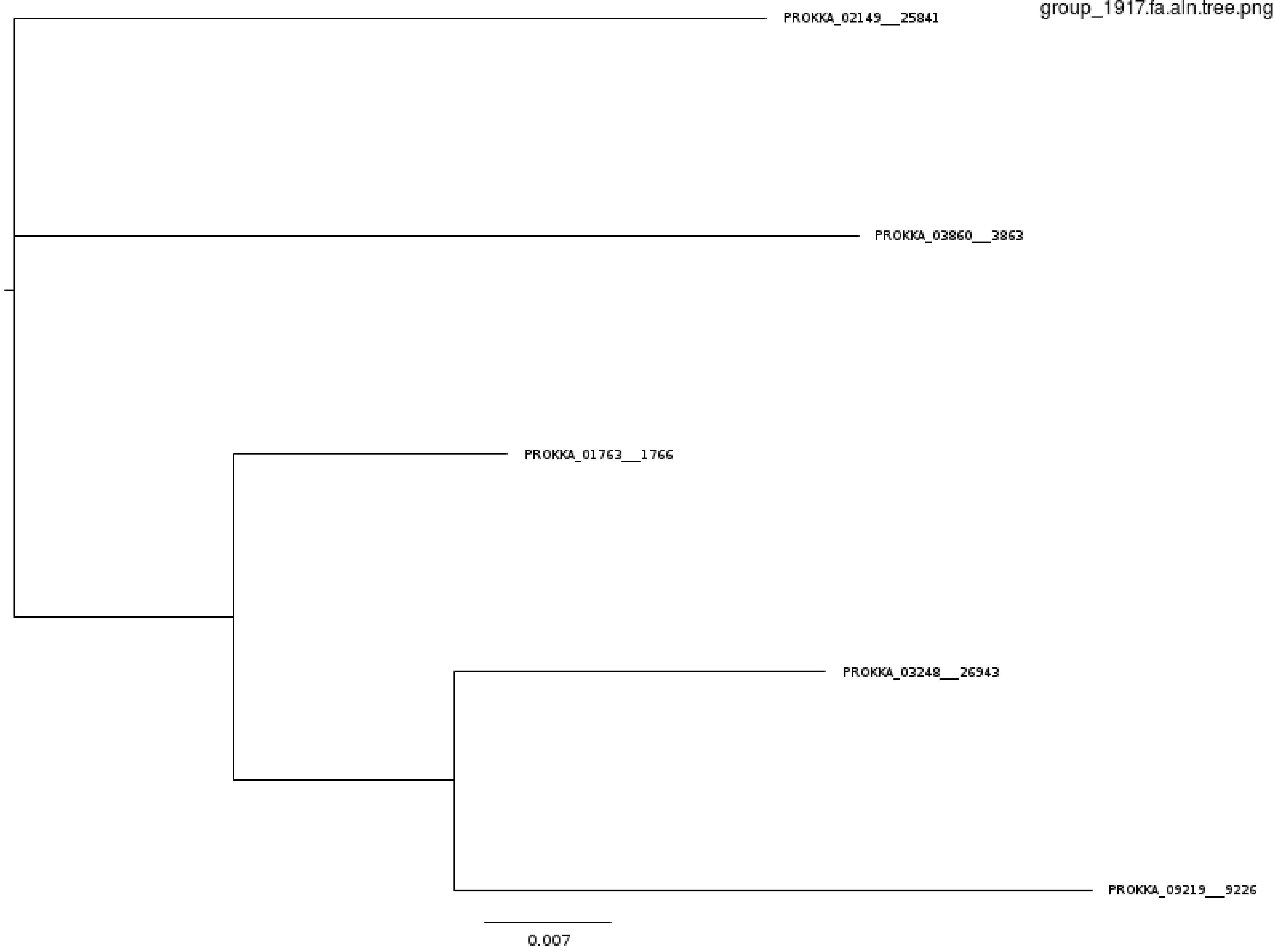
group_1917.fa.aln.tree.png
PROKKA_02149___25841
PROKKA_03860___3863
PROKKA_01763___1766
PROKKA_03248___26943
PROKKA_09219___9226
0.007

group_1919.fa.aln.tree.png

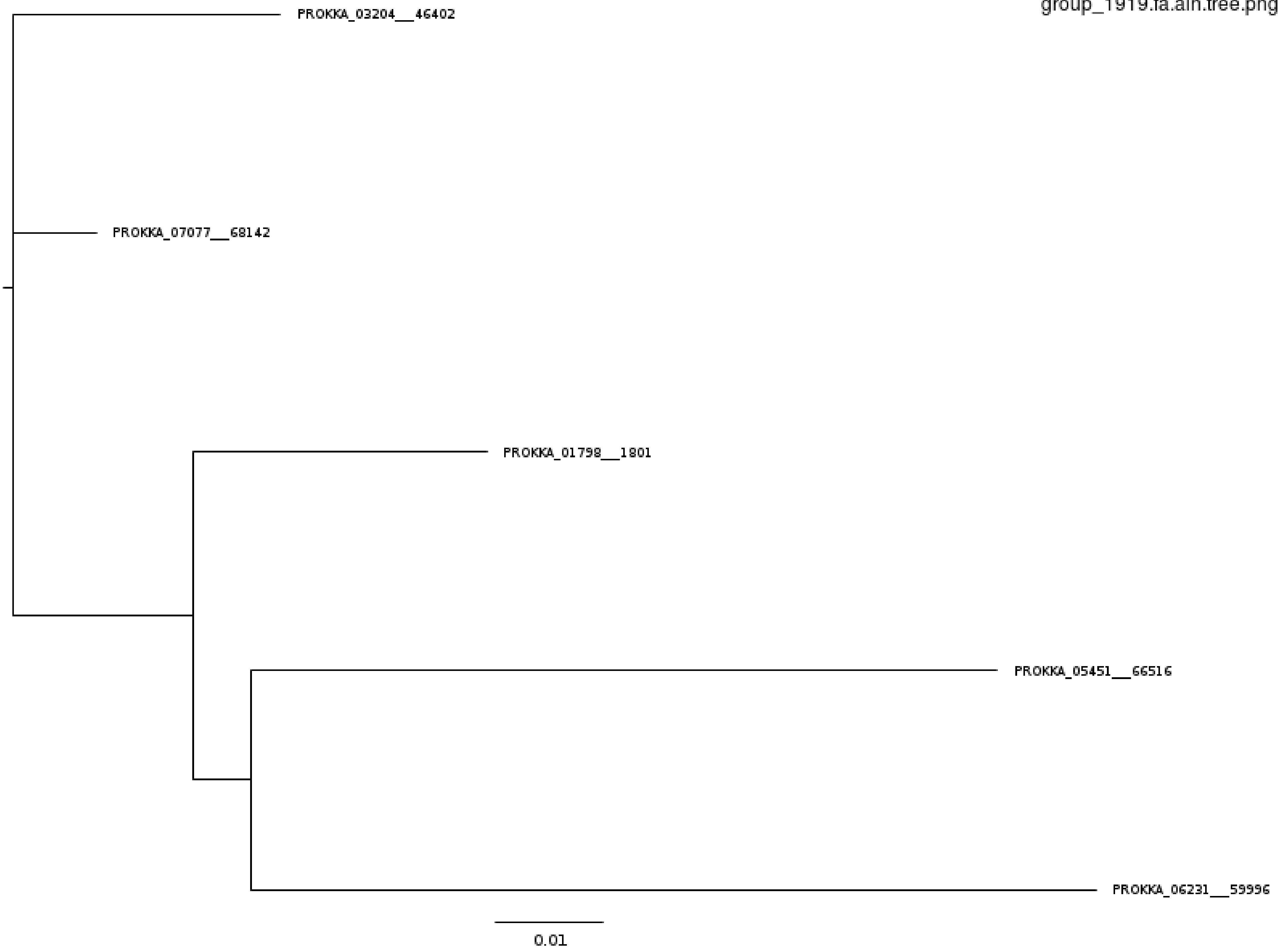

group_191.fa.aln.tree.png

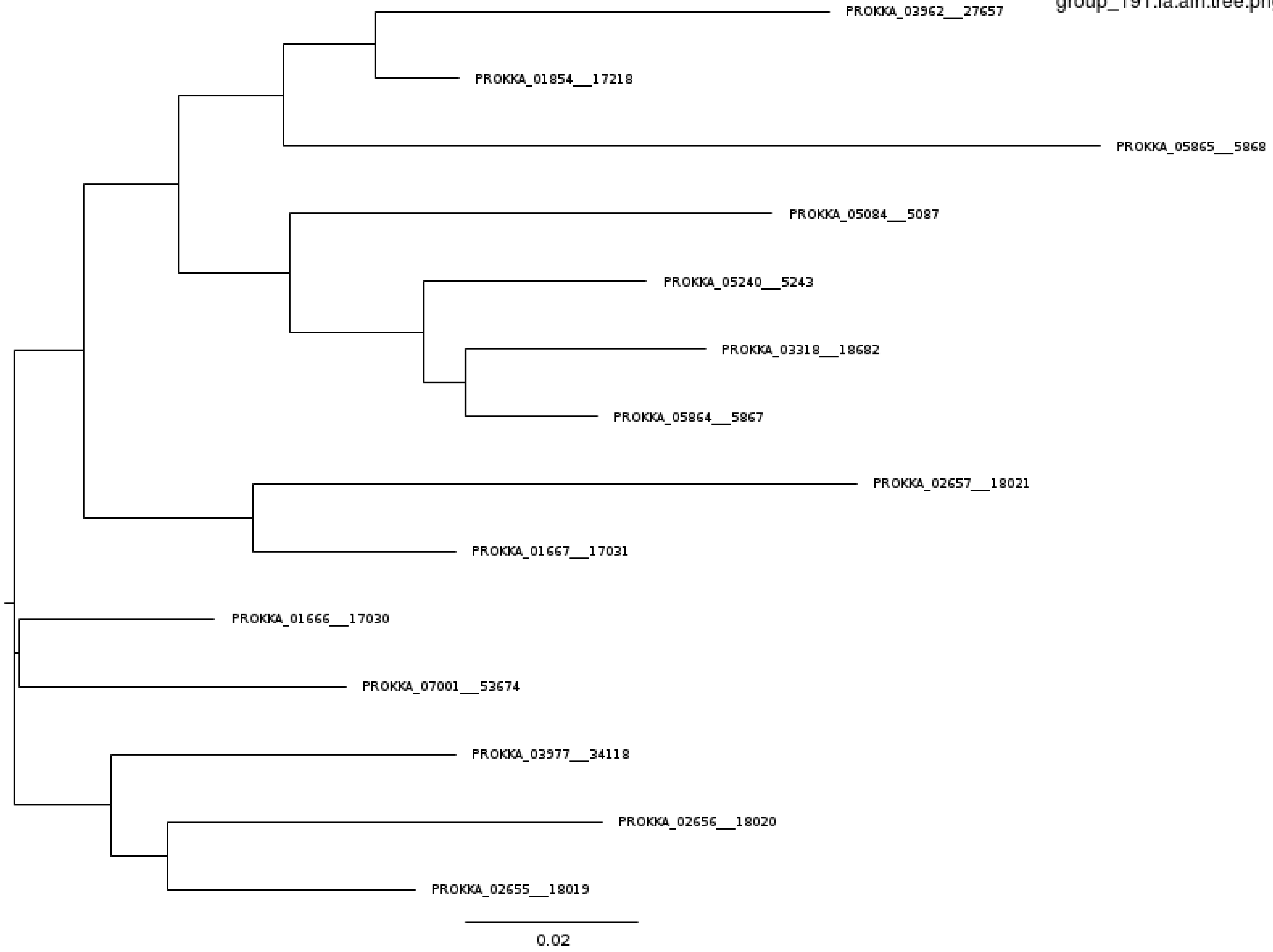

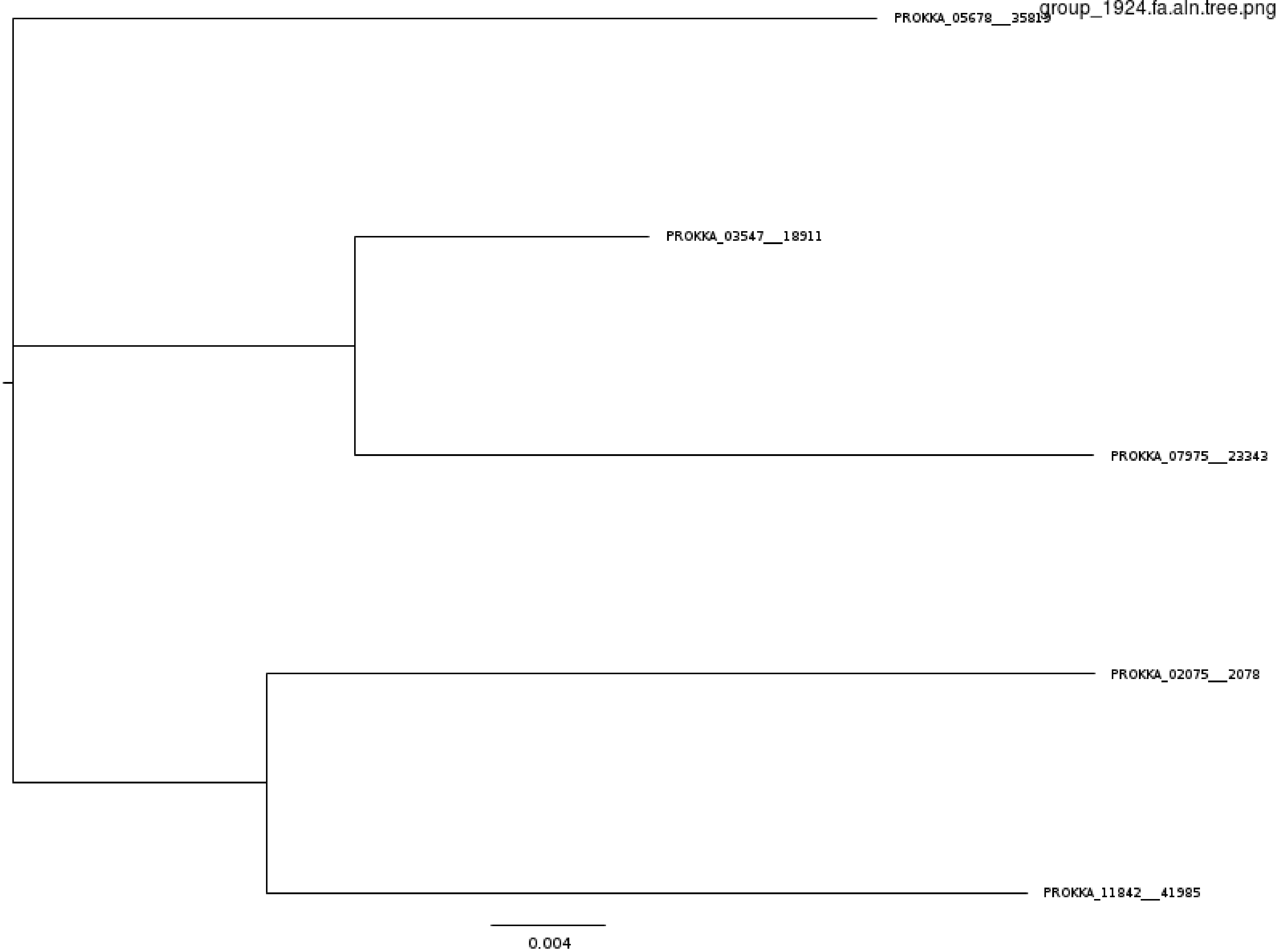
group_1924.fa.aln.tree.png
PROKKA_05678__35819
PROKKA_03547__18911
PROKKA_07975__23343
PROKKA_02075__2078
PROKKA_11842__41985
0.004

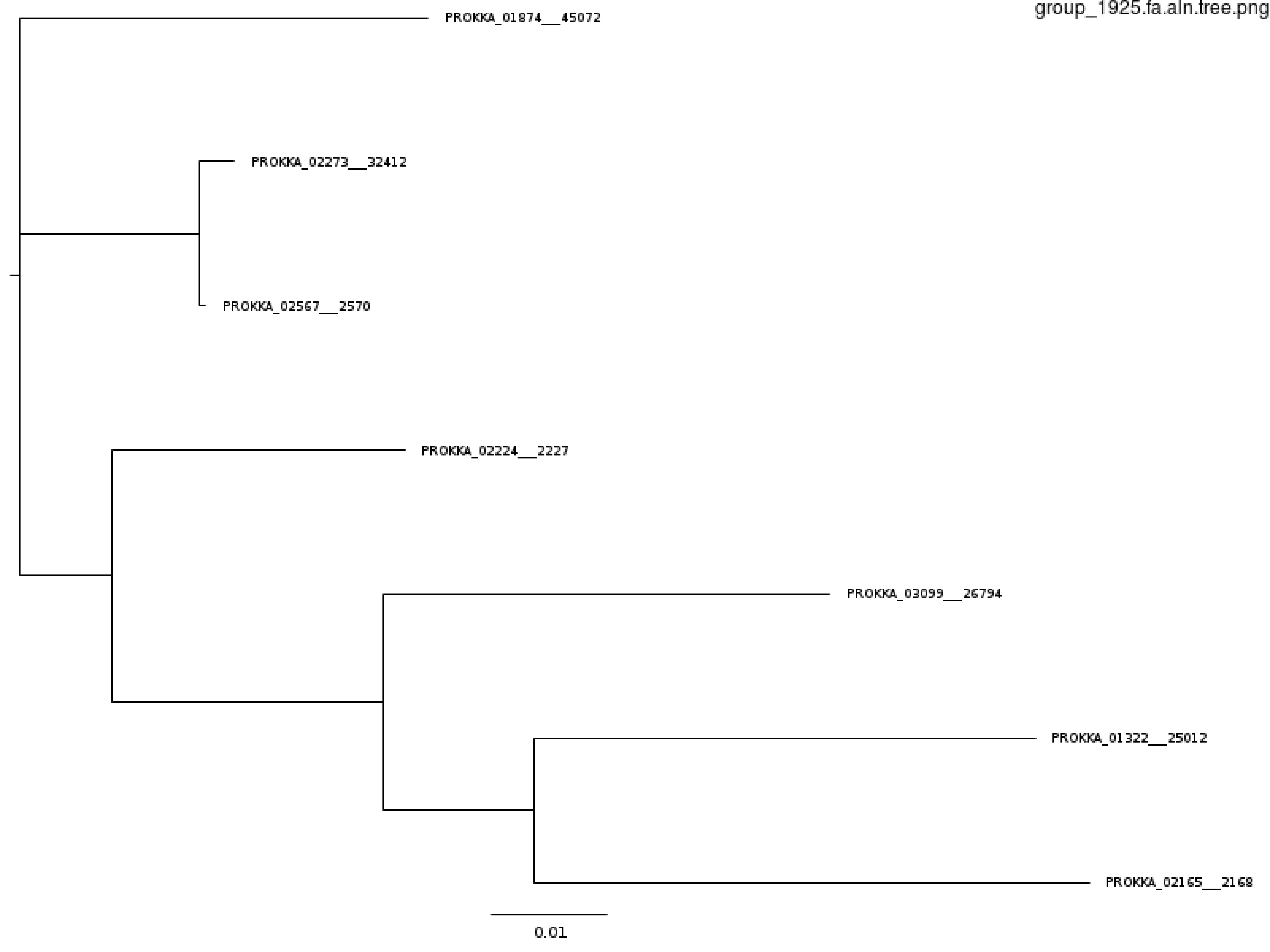
group_1925.fa.aln.tree.png
PROKKA_01874___45072
PROKKA_02273___32412
PROKKA_02567___2570
PROKKA_02224___2227
PROKKA_03099___26794
PROKKA_01322___25012
PROKKA_02165___2168
0.01

group_1928.fa.aln.tree.png

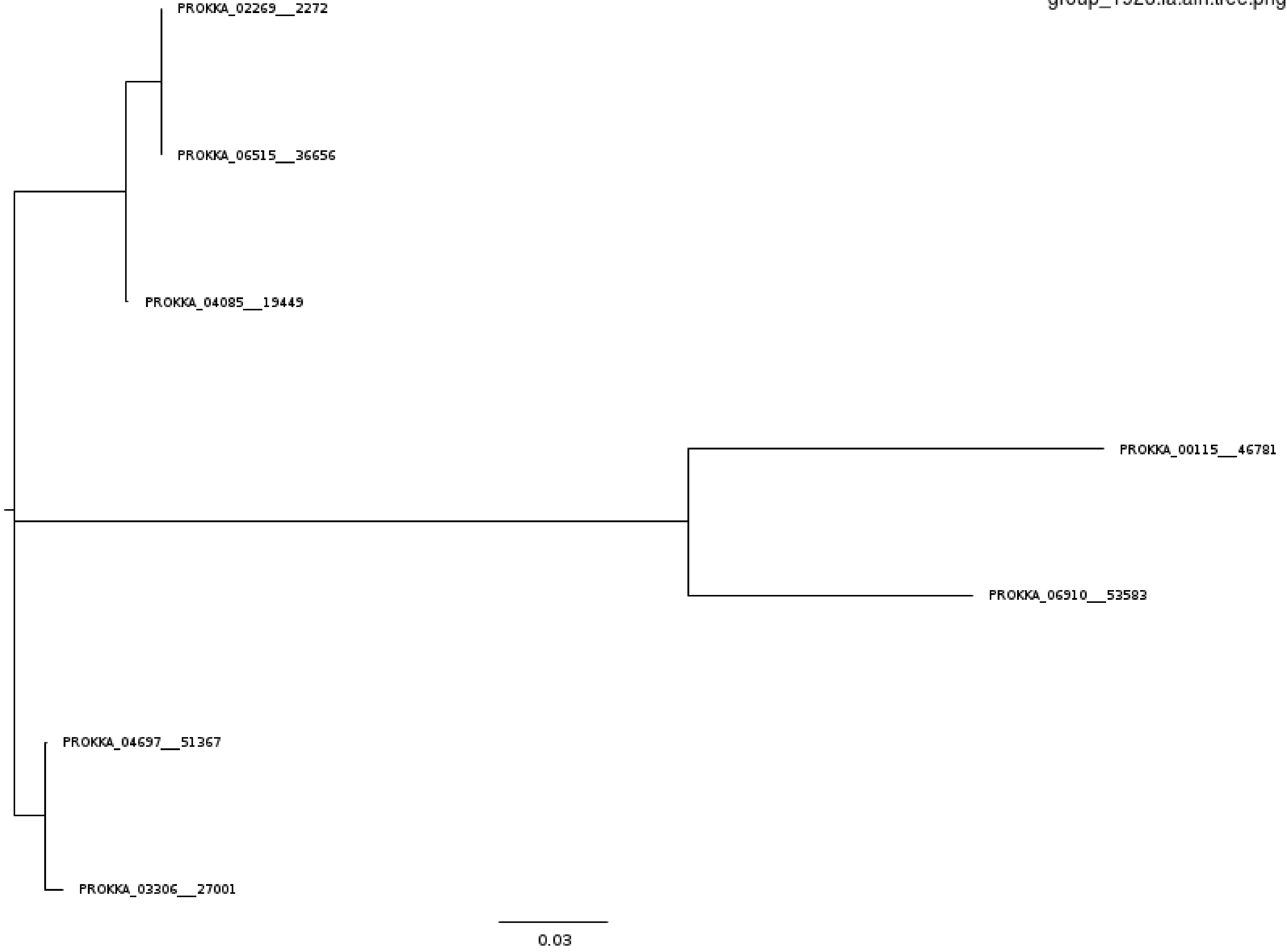

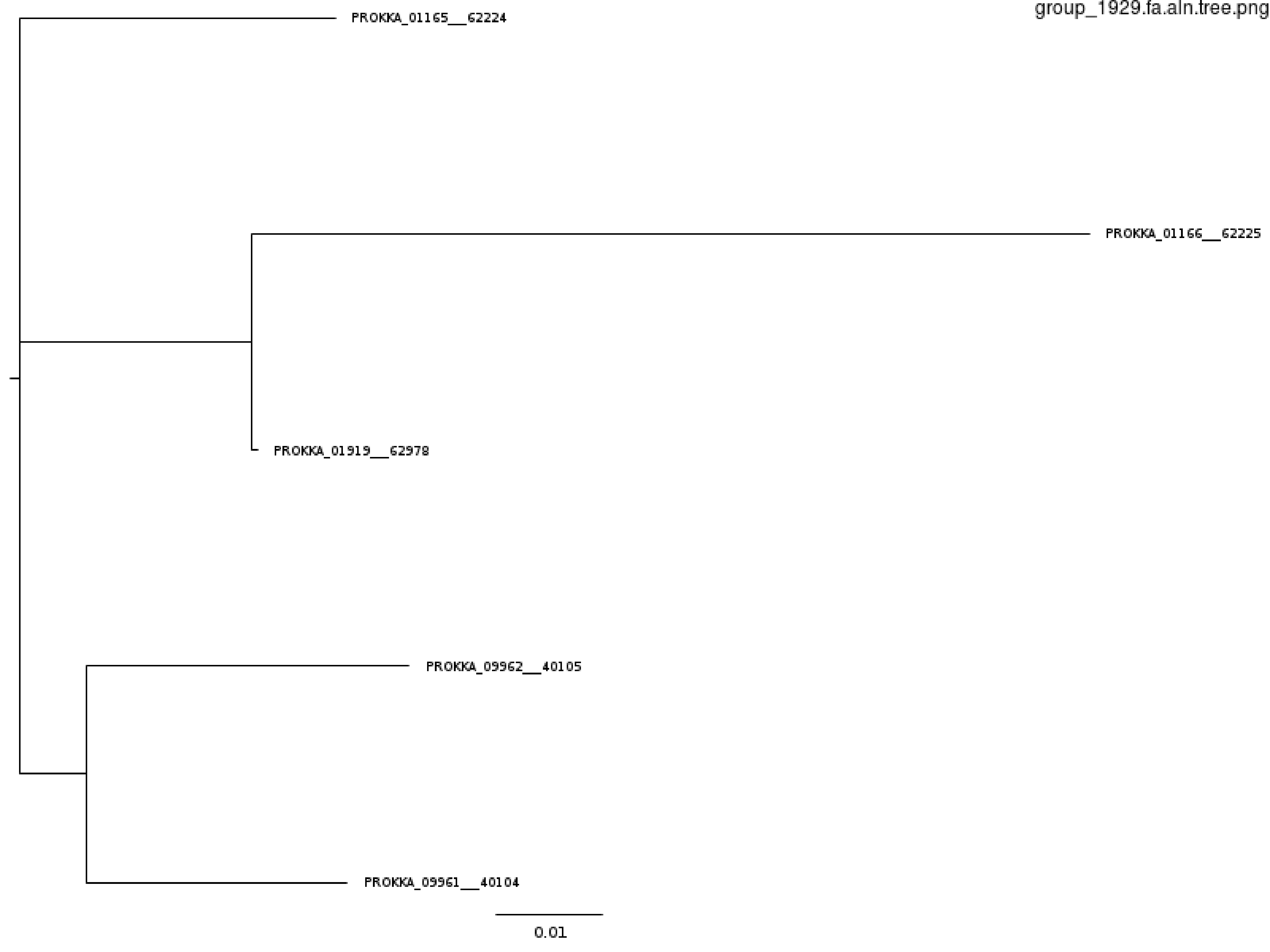
group_1929.fa.aln.tree.png
PROKKA_01165___62224
PROKKA_01166___62225
PROKKA_01919___62978
PROKKA_09962___40105
PROKKA_09961___40104
0.01

group_1931.fa.aln.tree.png

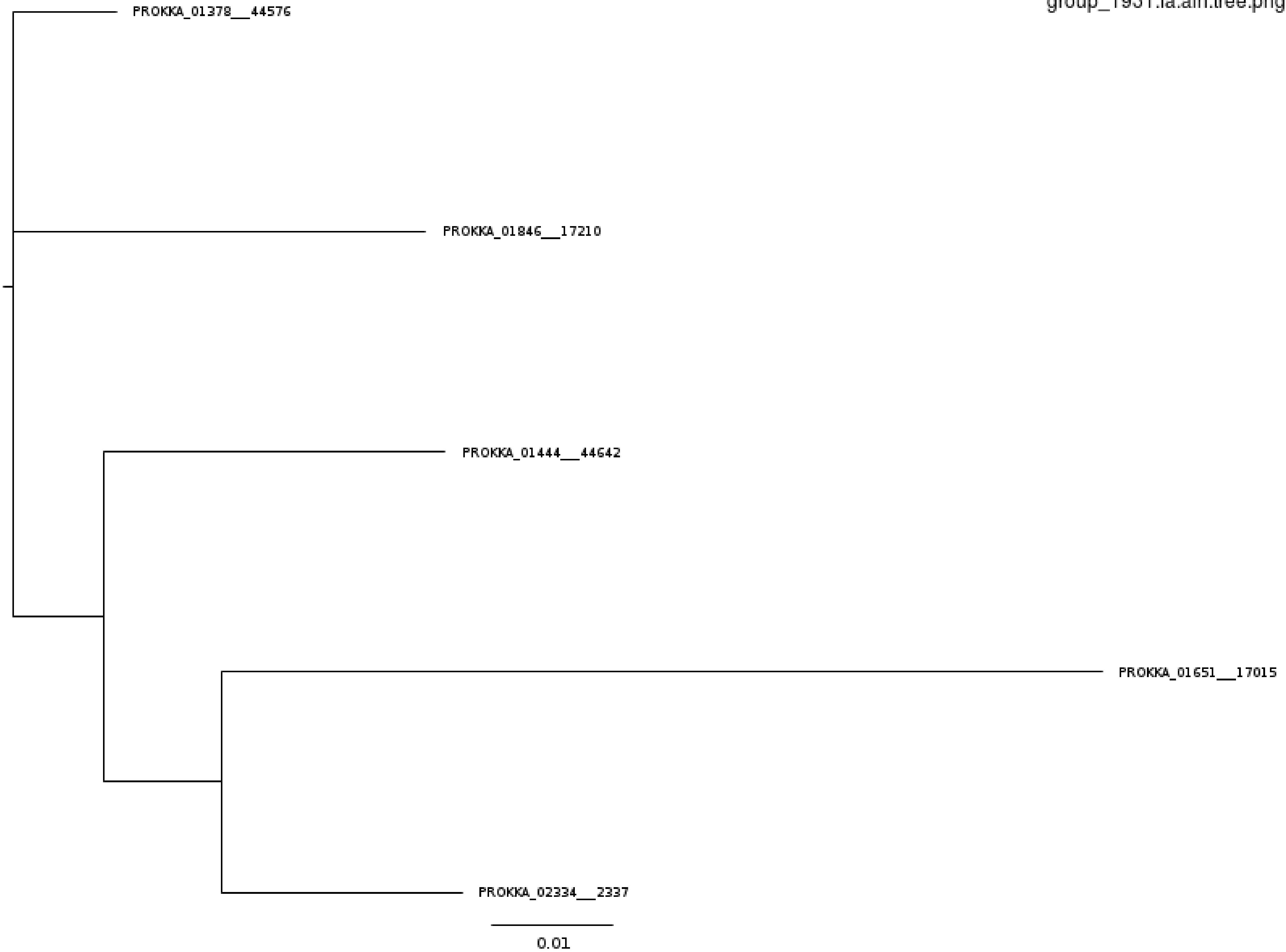

group_1938.fa.aln.tree.png

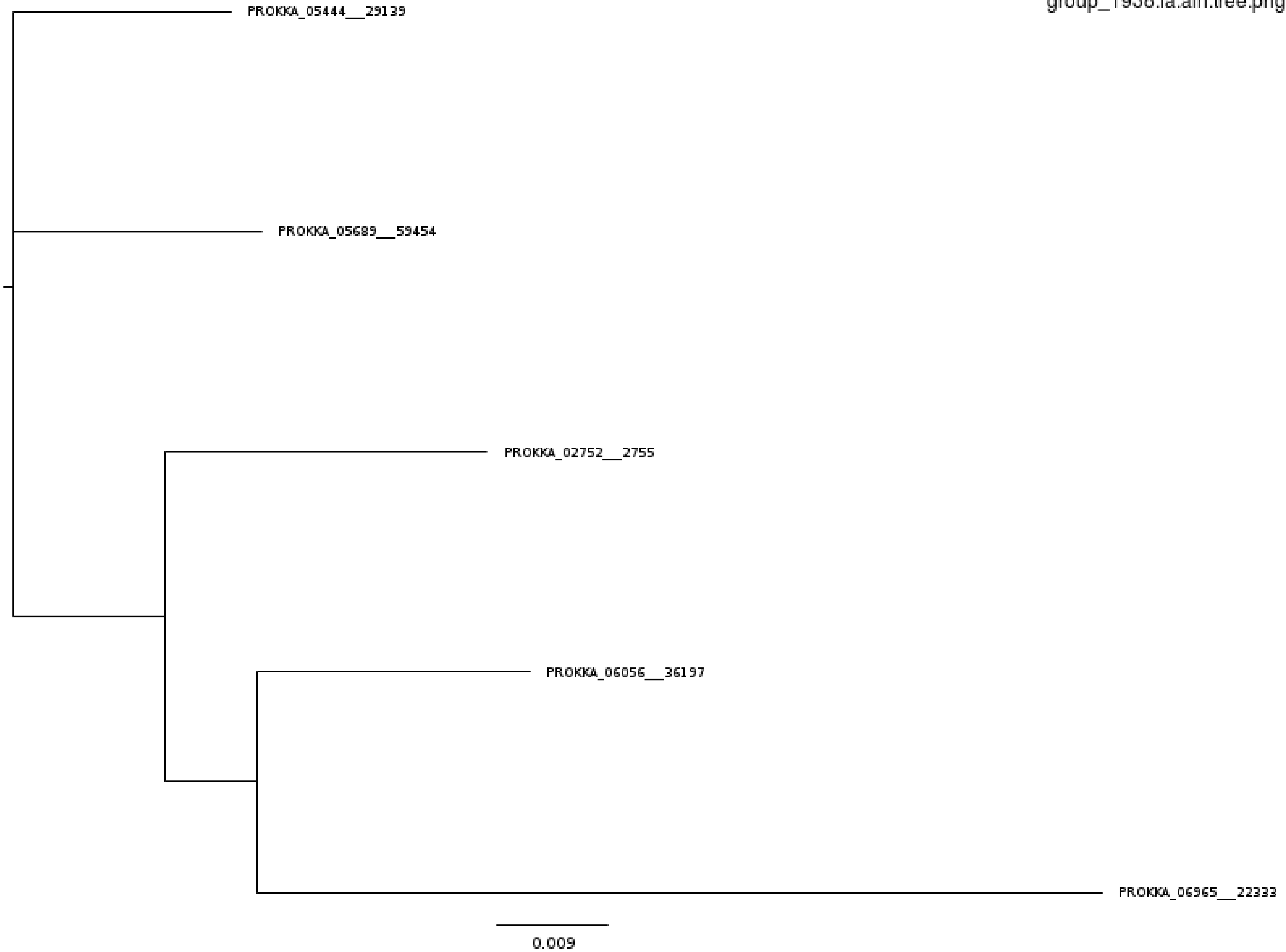

group_1939.fa.aln.tree.png

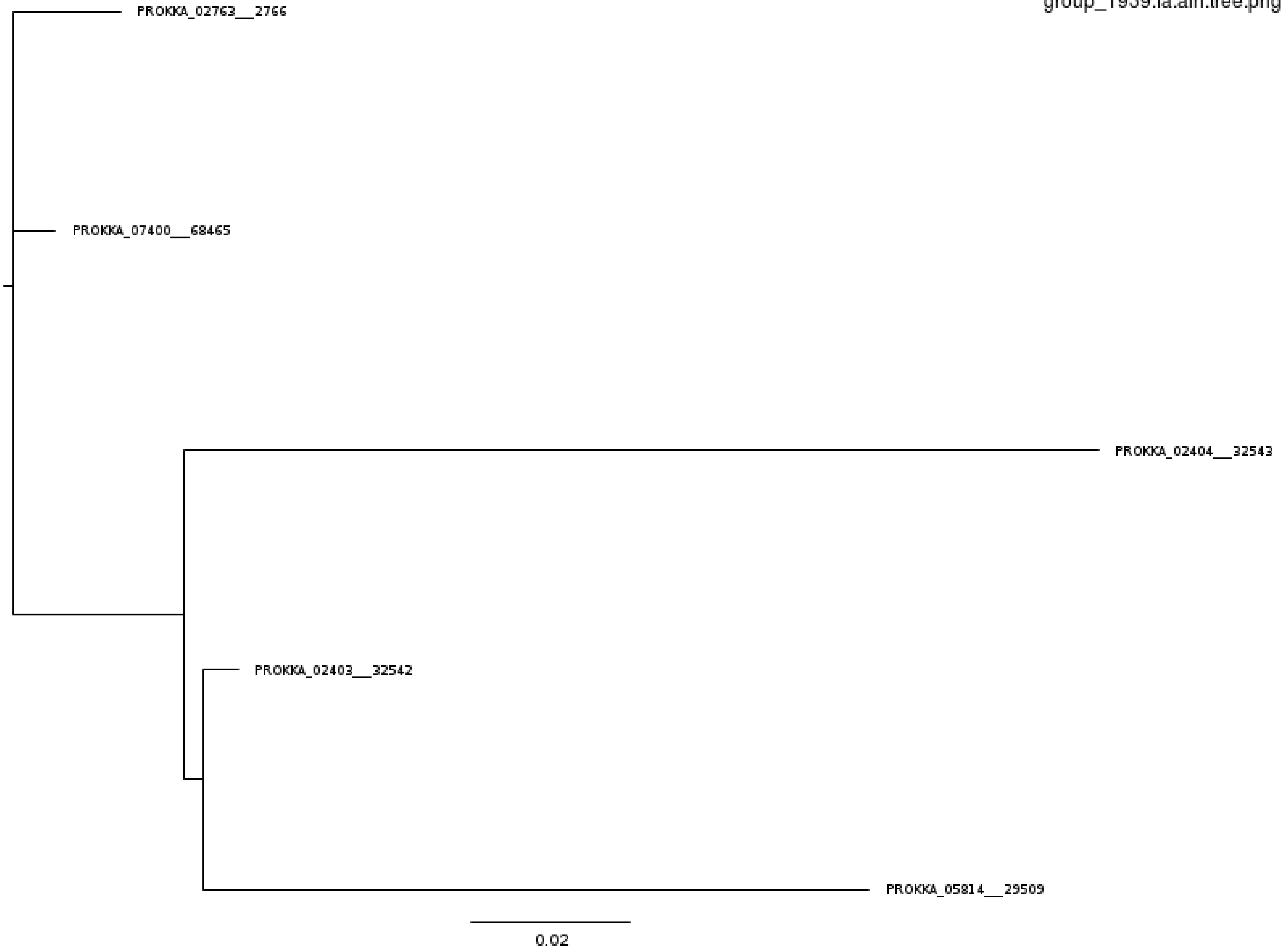

group_1940.fa.aln.tree.png

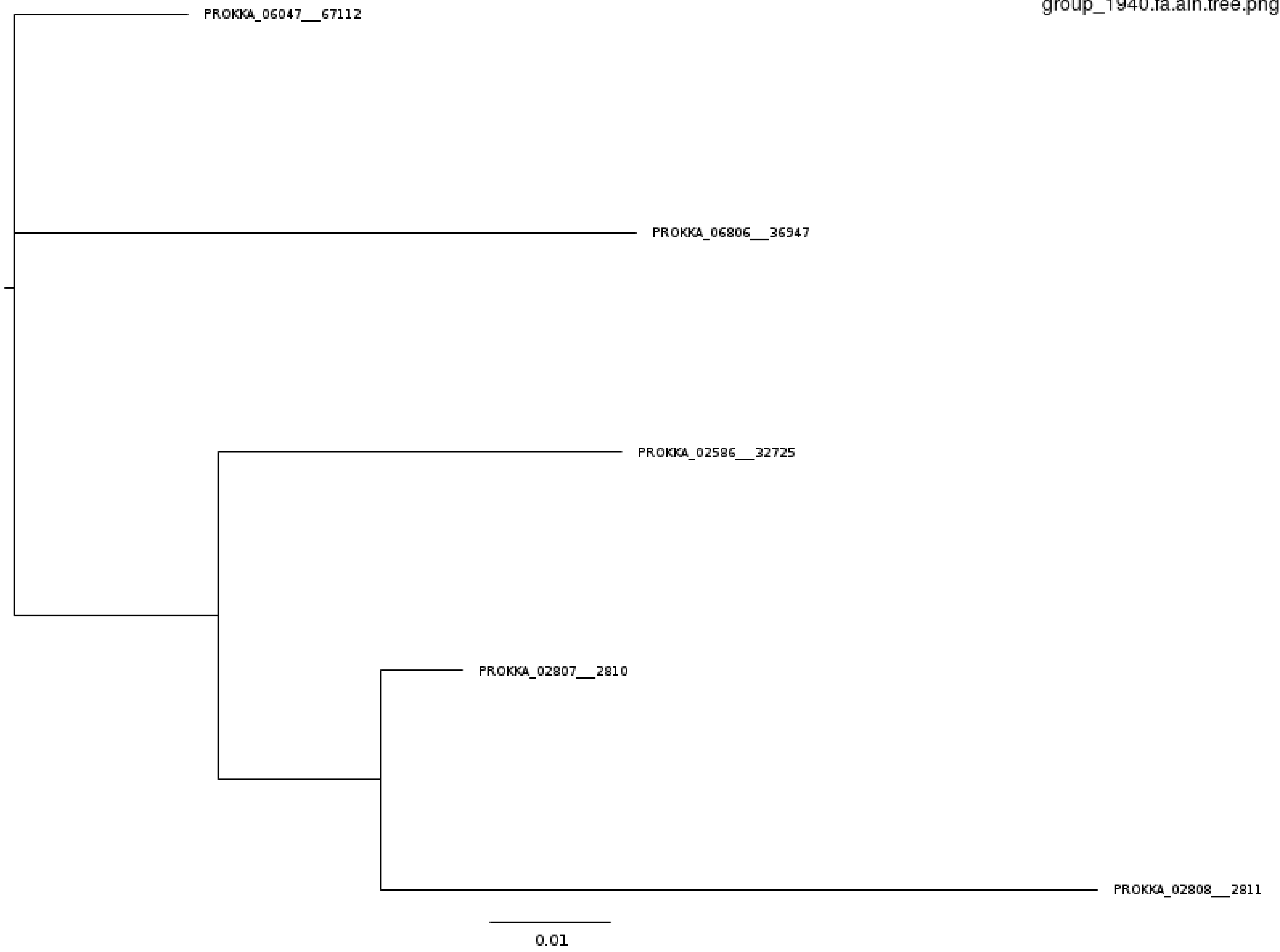

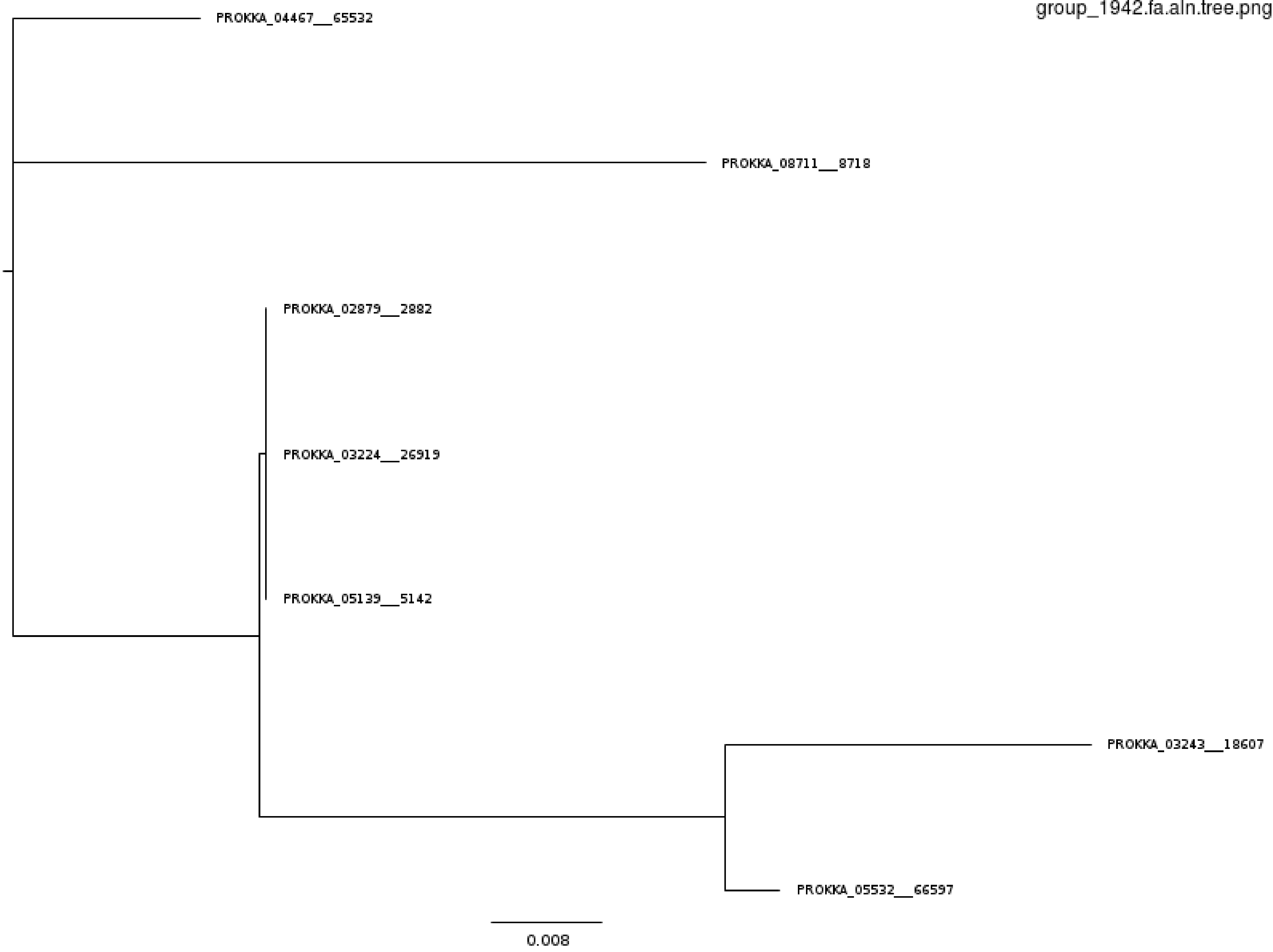
group_1942.fa.aln.tree.png
PROKKA_04467__65532
PROKKA_08711__8718
PROKKA_02879__2882
PROKKA_03224__26919
PROKKA_05139__5142
PROKKA_03243__18607
PROKKA_05532__66597
0.008

group_1943.fa.aln.tree.png

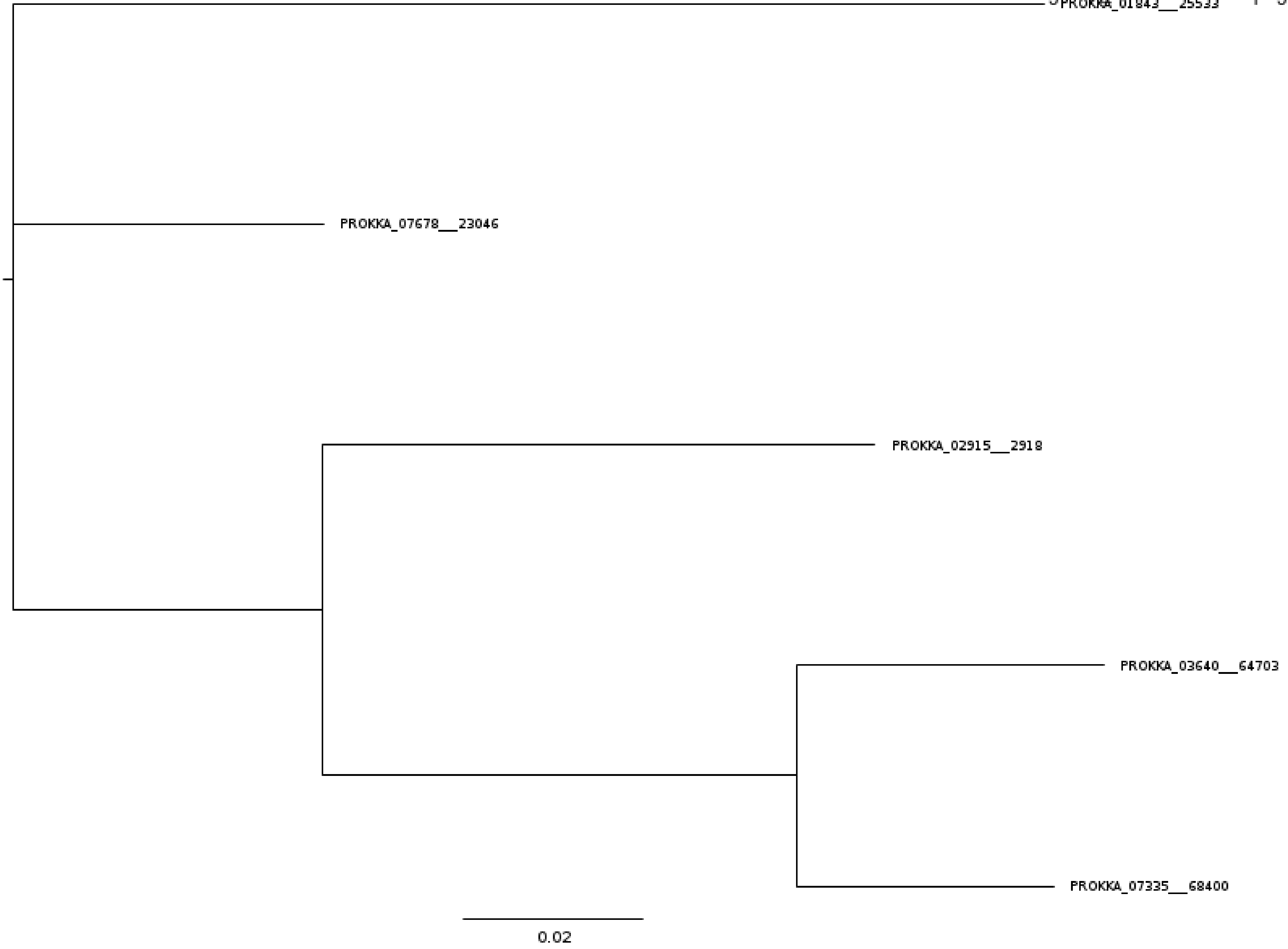

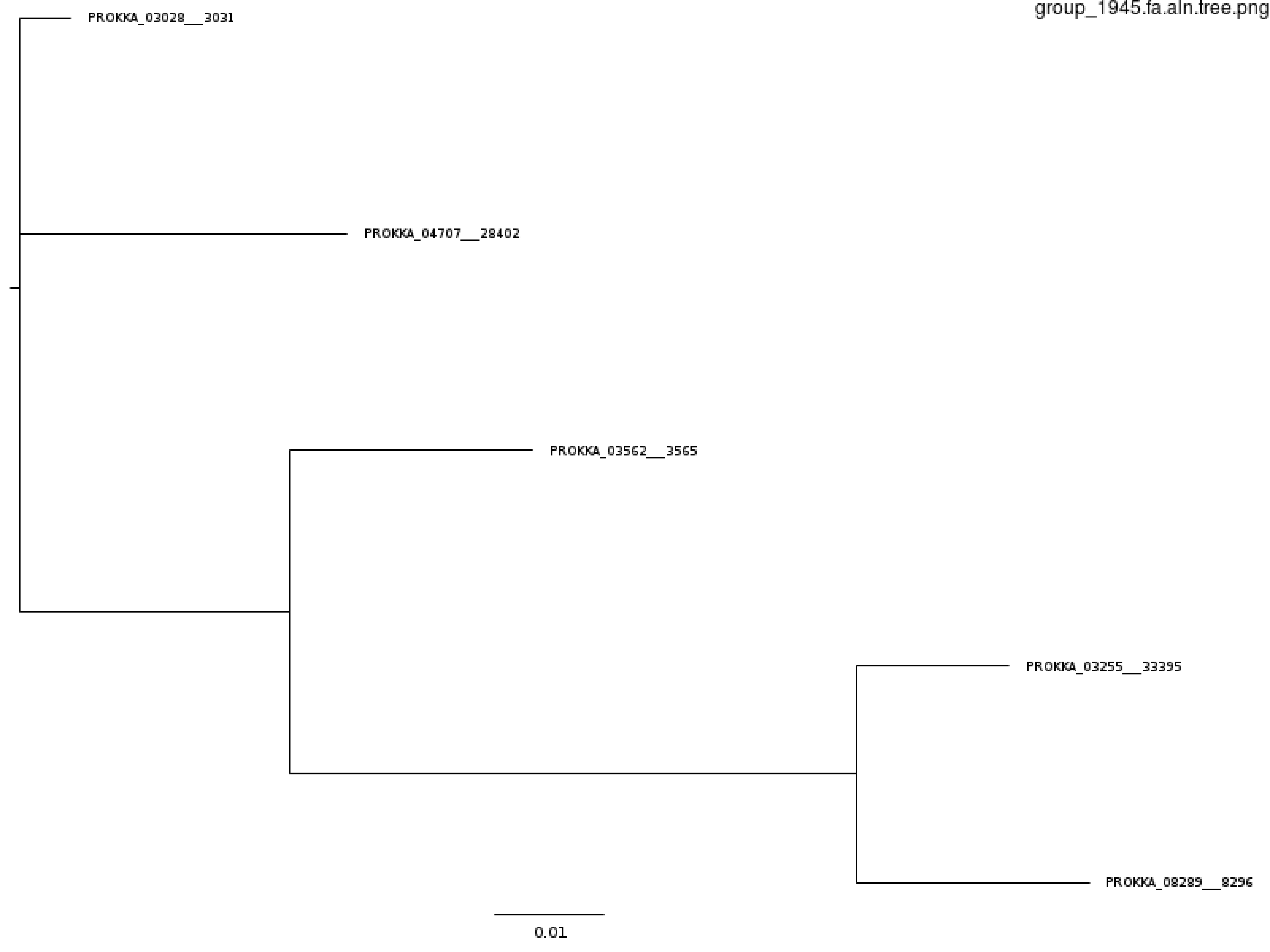
group_1945.fa.aln.tree.png
PROKKA_03028___3031
PROKKA_04707___28402
PROKKA_03562___3565
PROKKA_03255___33395
PROKKA_08289___8296
0.01

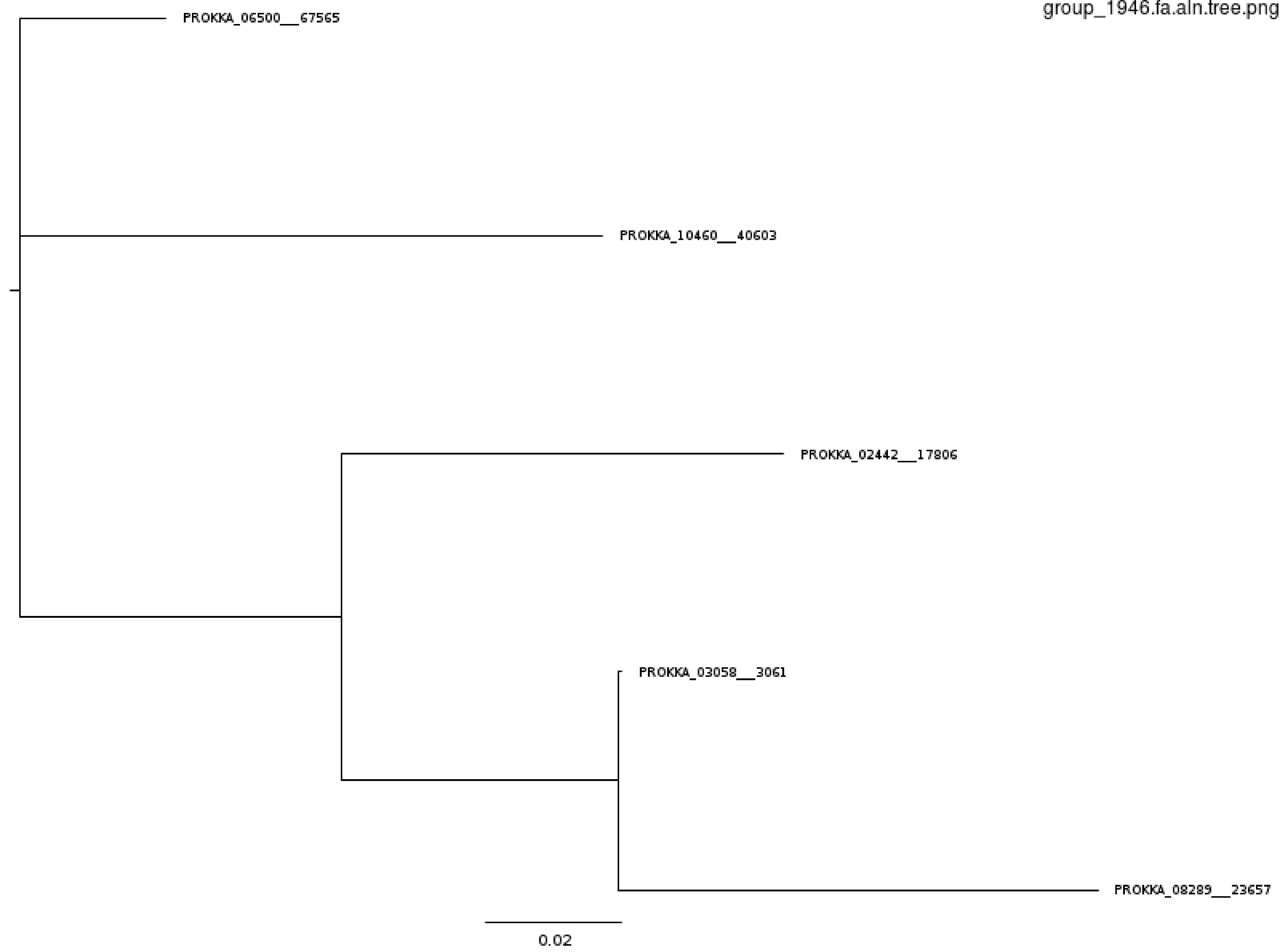
group_1946.fa.aln.tree.png
PROKKA_06500___67565
PROKKA_10460___40603
PROKKA_02442___17806
PROKKA_03058___3061
PROKKA_08289___23657
0.02

group_1947.fa.aln.tree.png

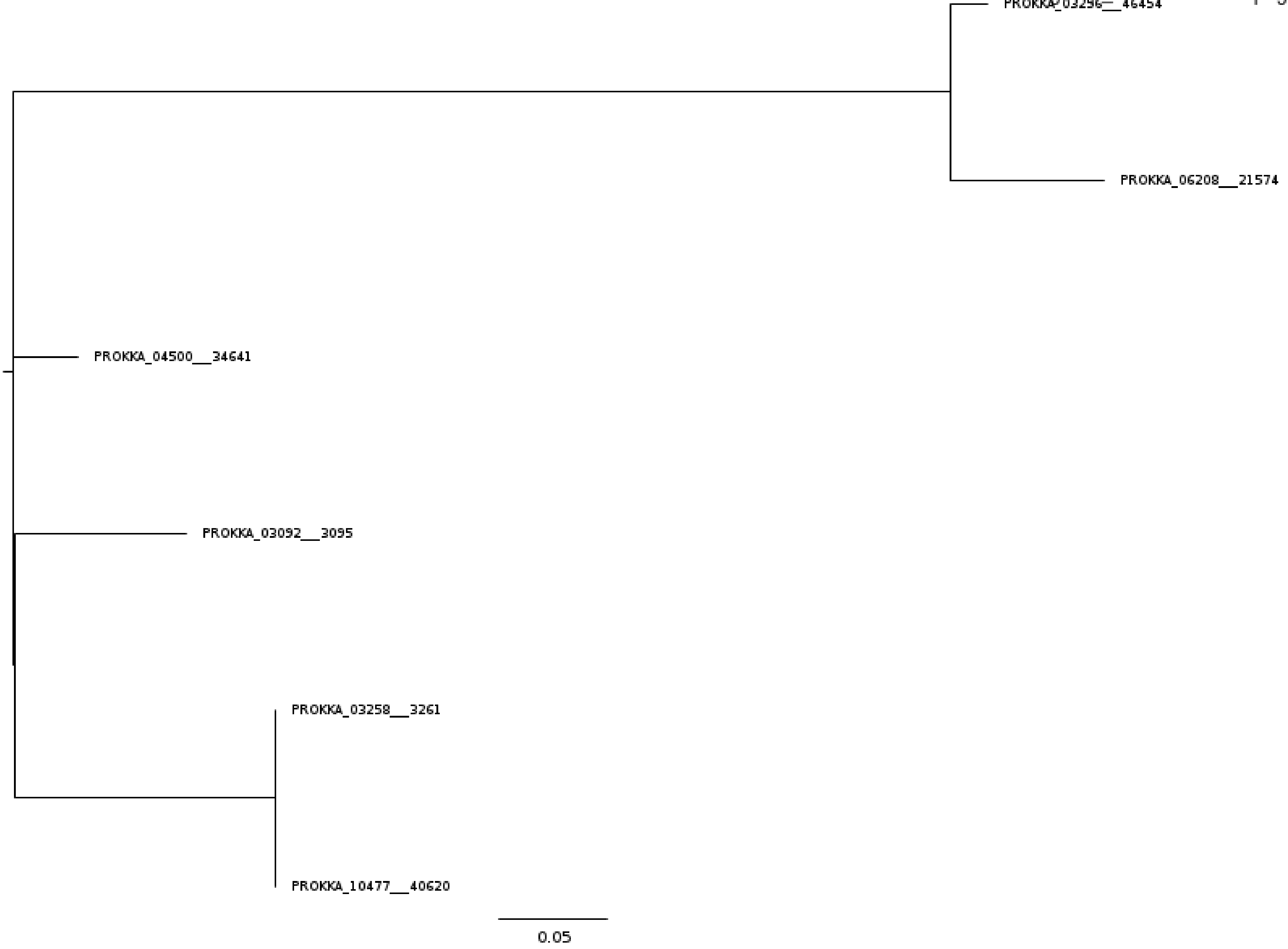

group_1948.fa.aln.tree.png

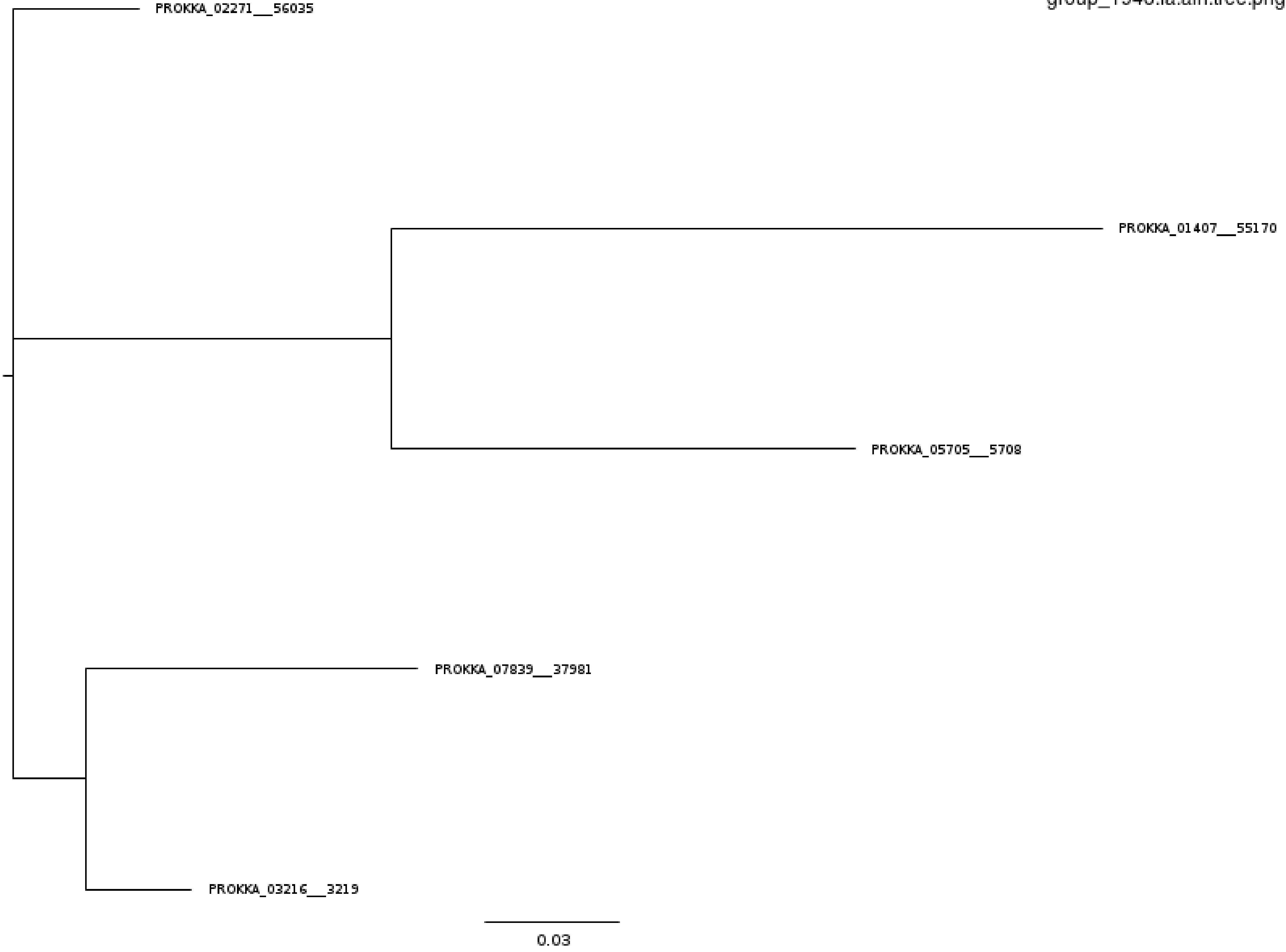

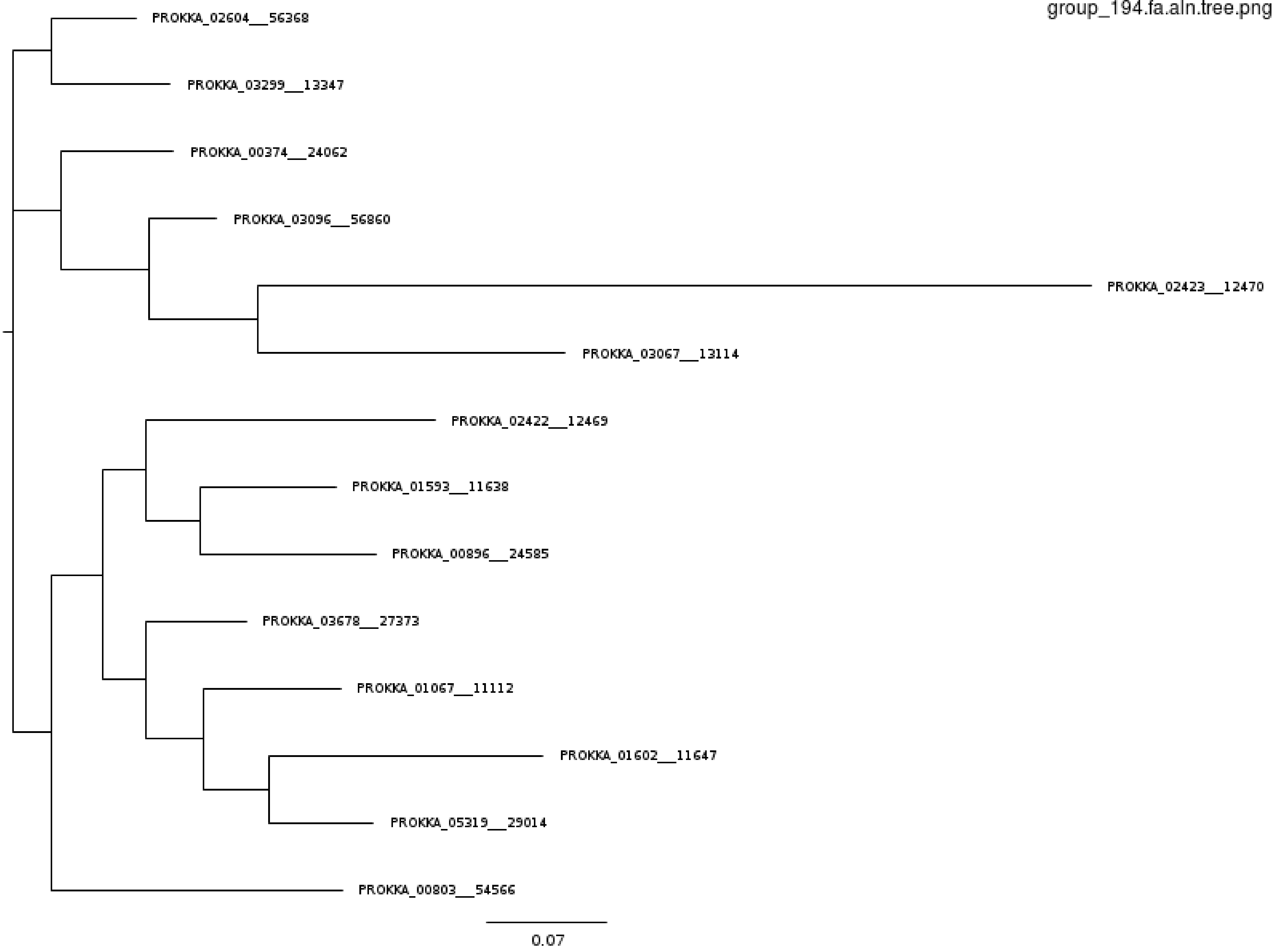
group_194.fa.aln.tree.png
PROKKA_02604___56368
PROKKA_03299___13347
PROKKA_00374___24062
PROKKA_03096___56860
PROKKA_02423___12470
PROKKA_03067___13114
PROKKA_02422___12469
PROKKA_01593___11638
PROKKA_00896___24585
PROKKA_03678___27373
PROKKA_01067___11112
PROKKA_01602___11647
PROKKA_05319___29014
PROKKA_00803___54566
0.07

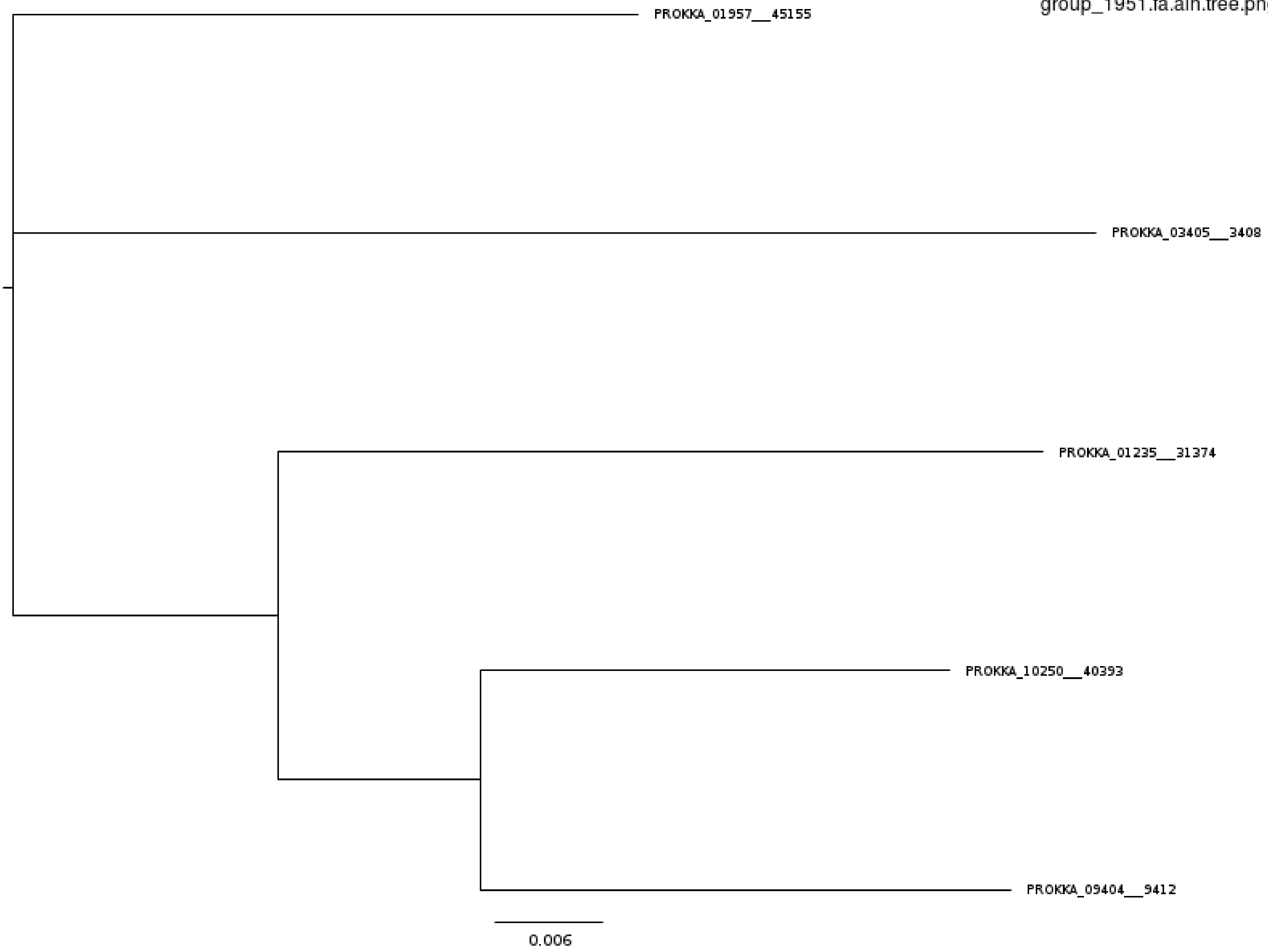
PROKKA_01957___45155
PROKKA_03405___3408
PROKKA_01235___31374
PROKKA_10250___40393
PROKKA_09404___9412
0.006

group_1953.fa.aln.tree.png

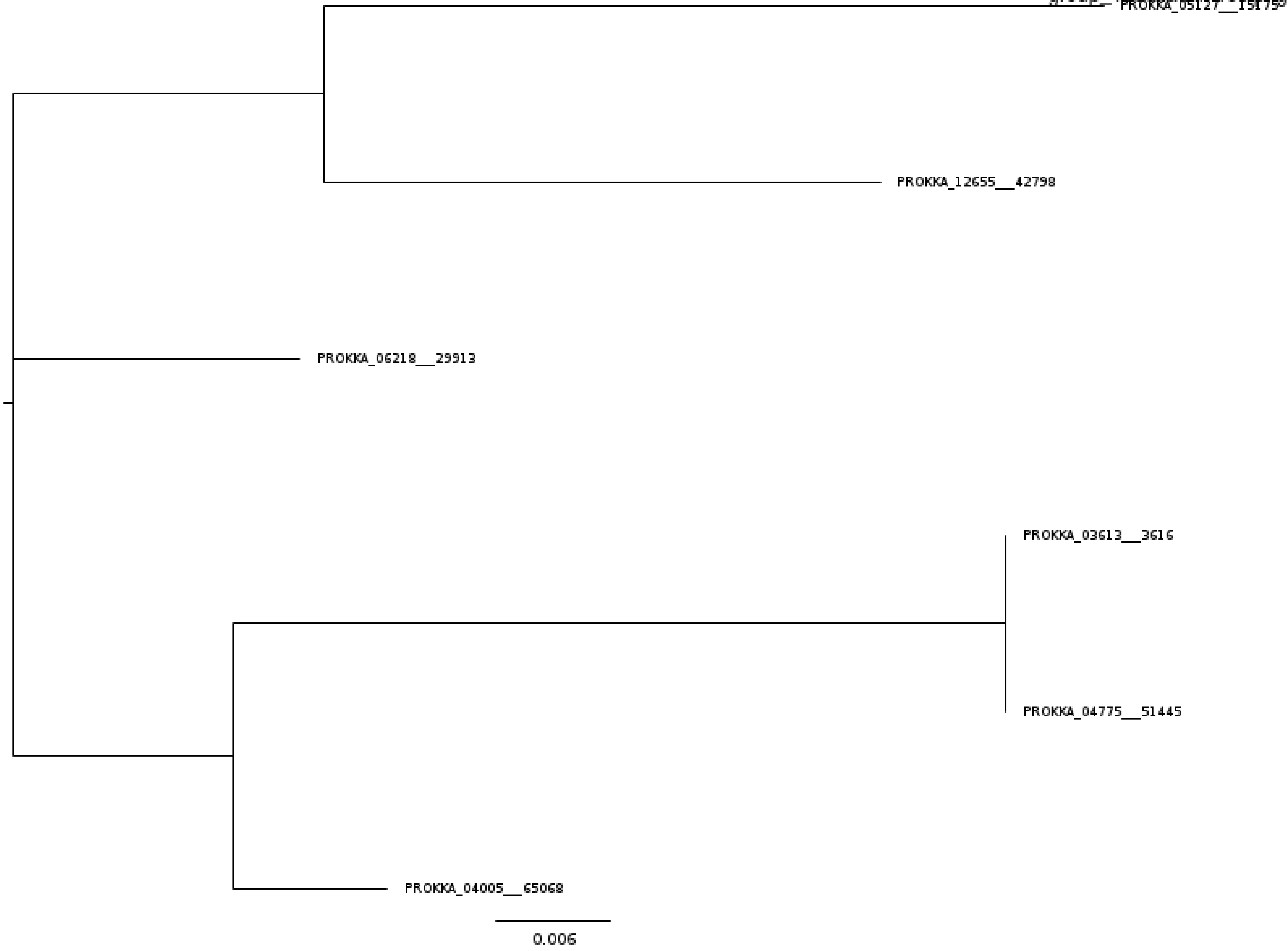

group_1958.fa.aln.tree.png

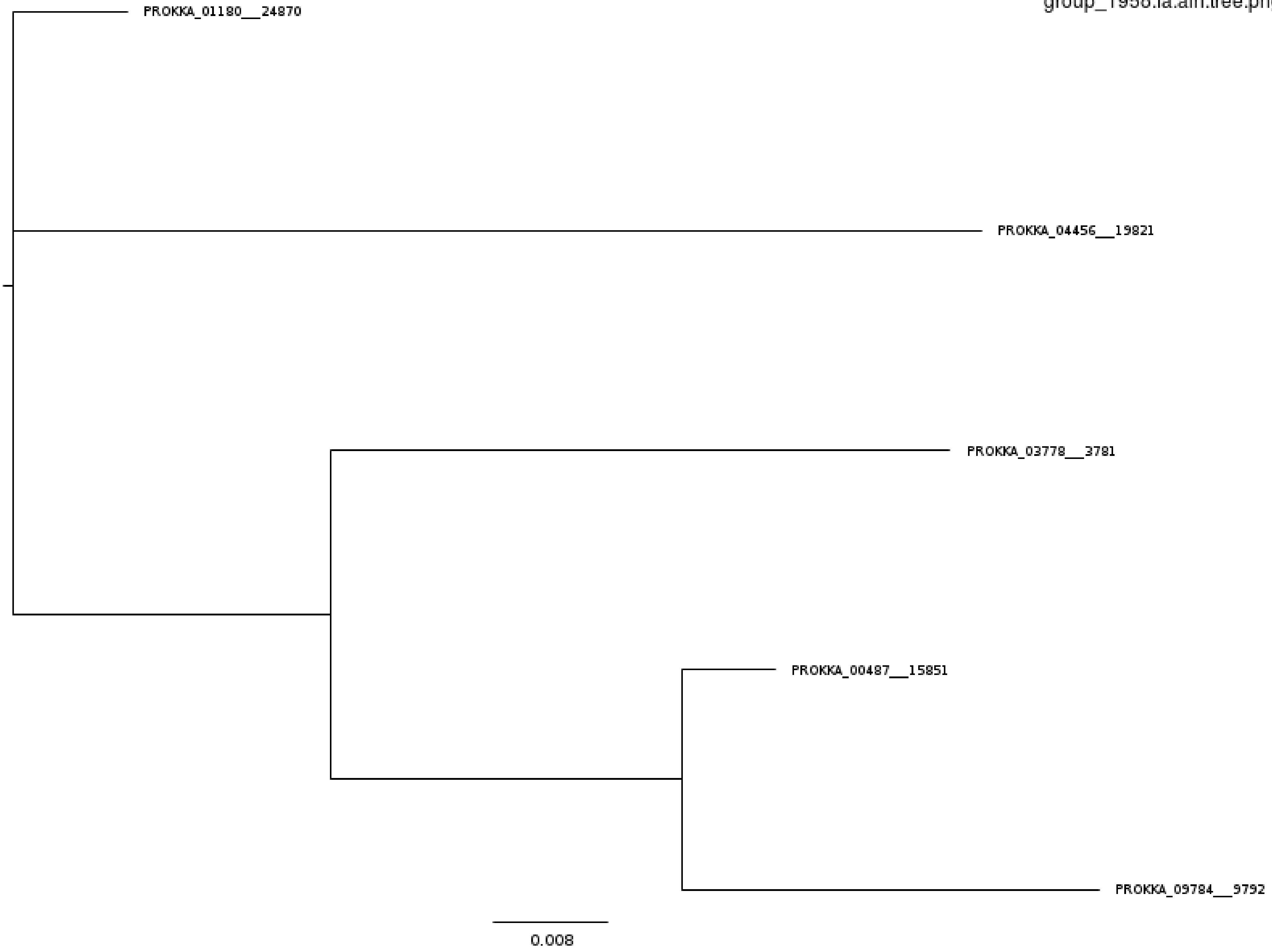

group_1962.fa.aln.tree.png

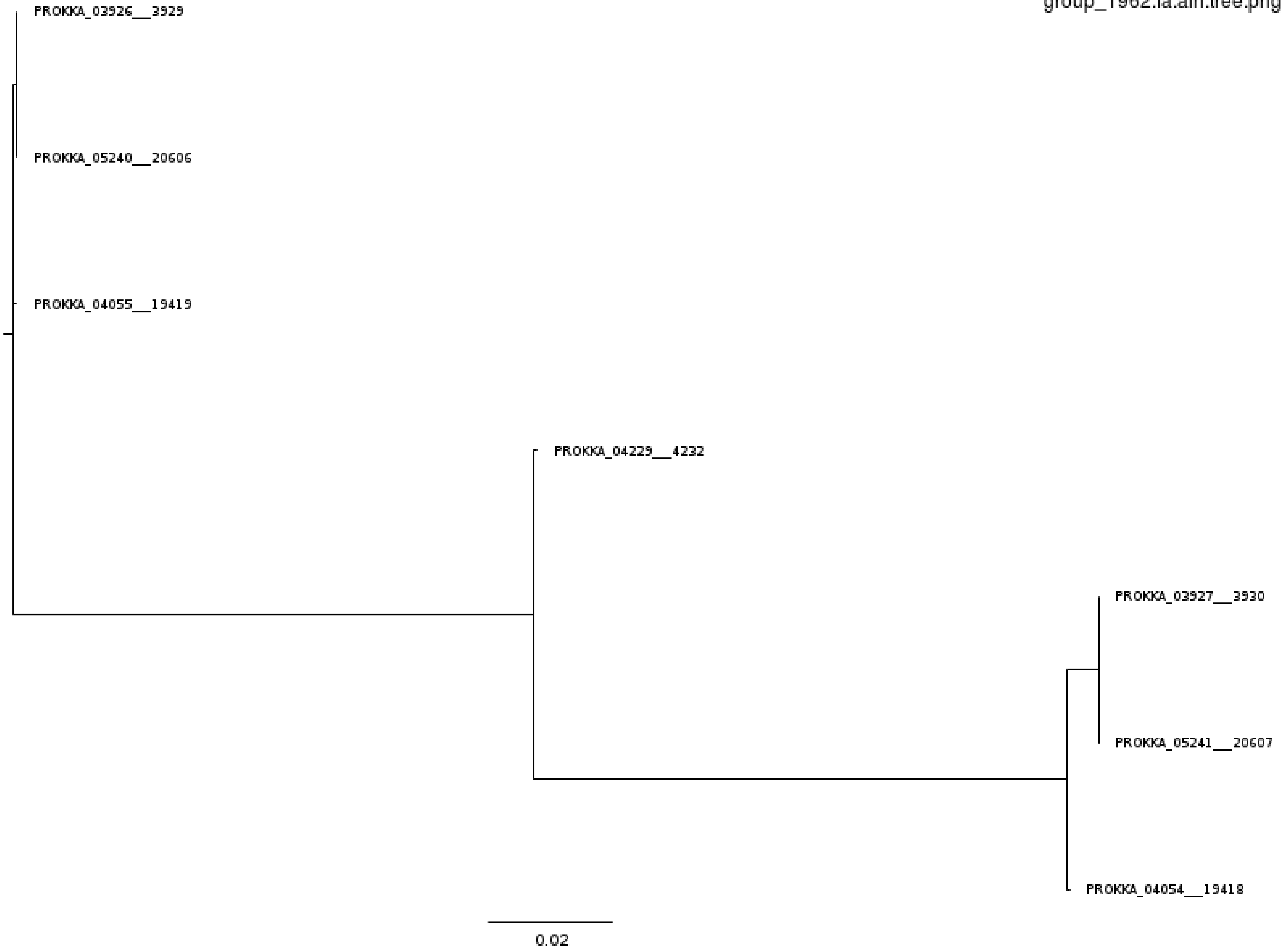

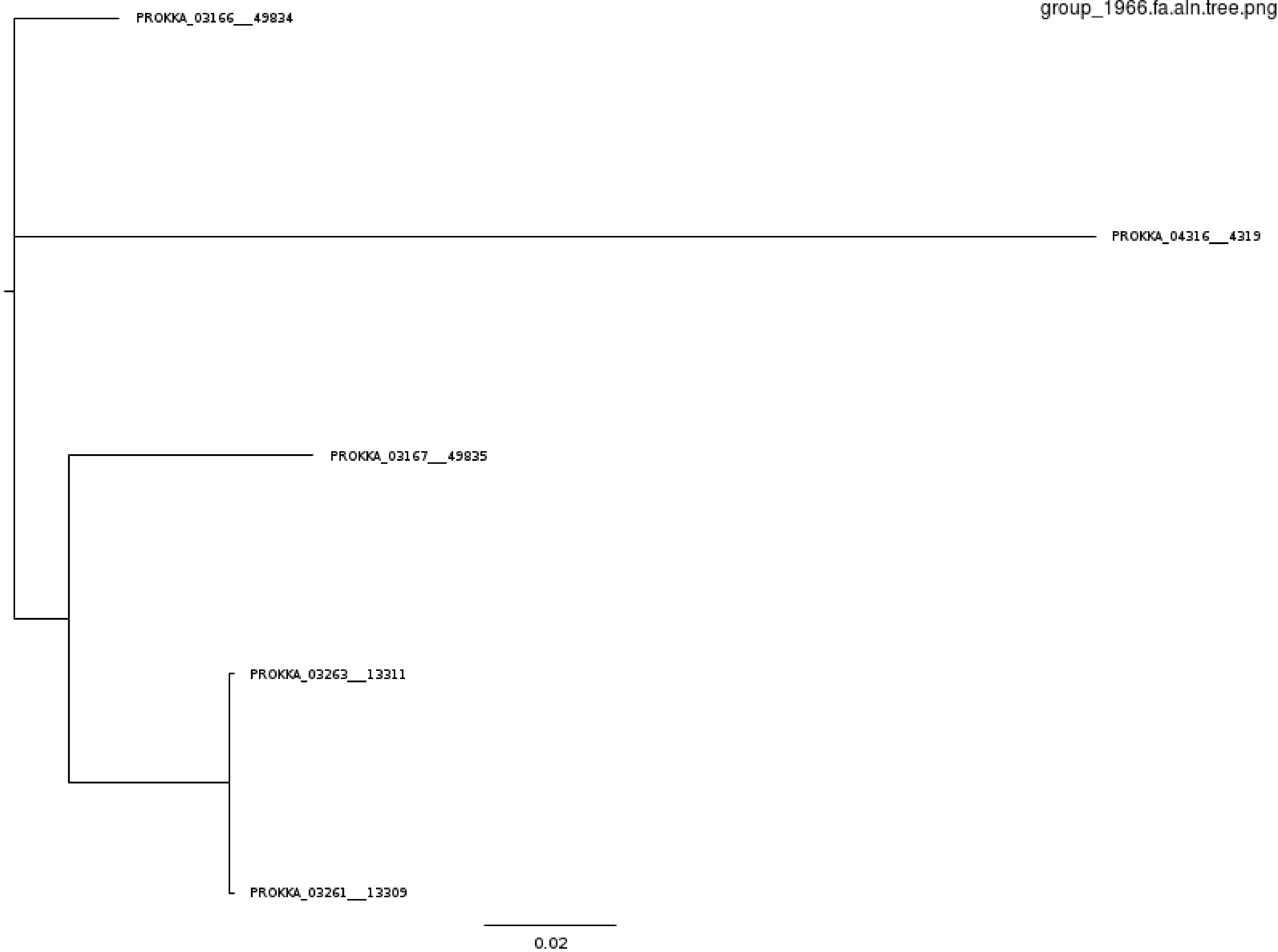
group_1966.fa.aln.tree.png
PROKKA_03166__49834
PROKKA_04316__4319
PROKKA_03167__49835
PROKKA_03263__13311
PROKKA_03261__13309
0.02

group_1973.fa.aln.tree.png

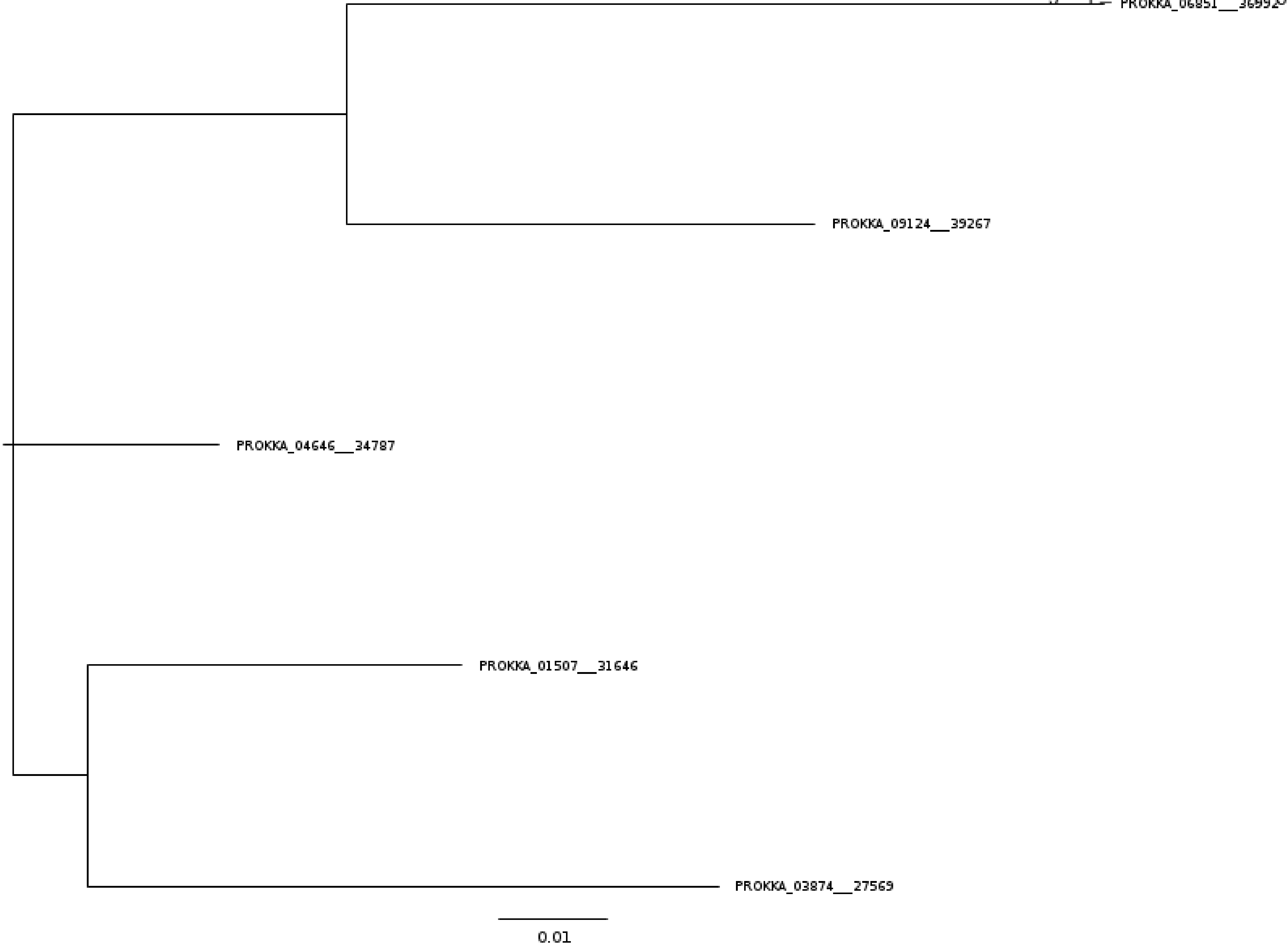

group_1974.fa.aln.tree.png

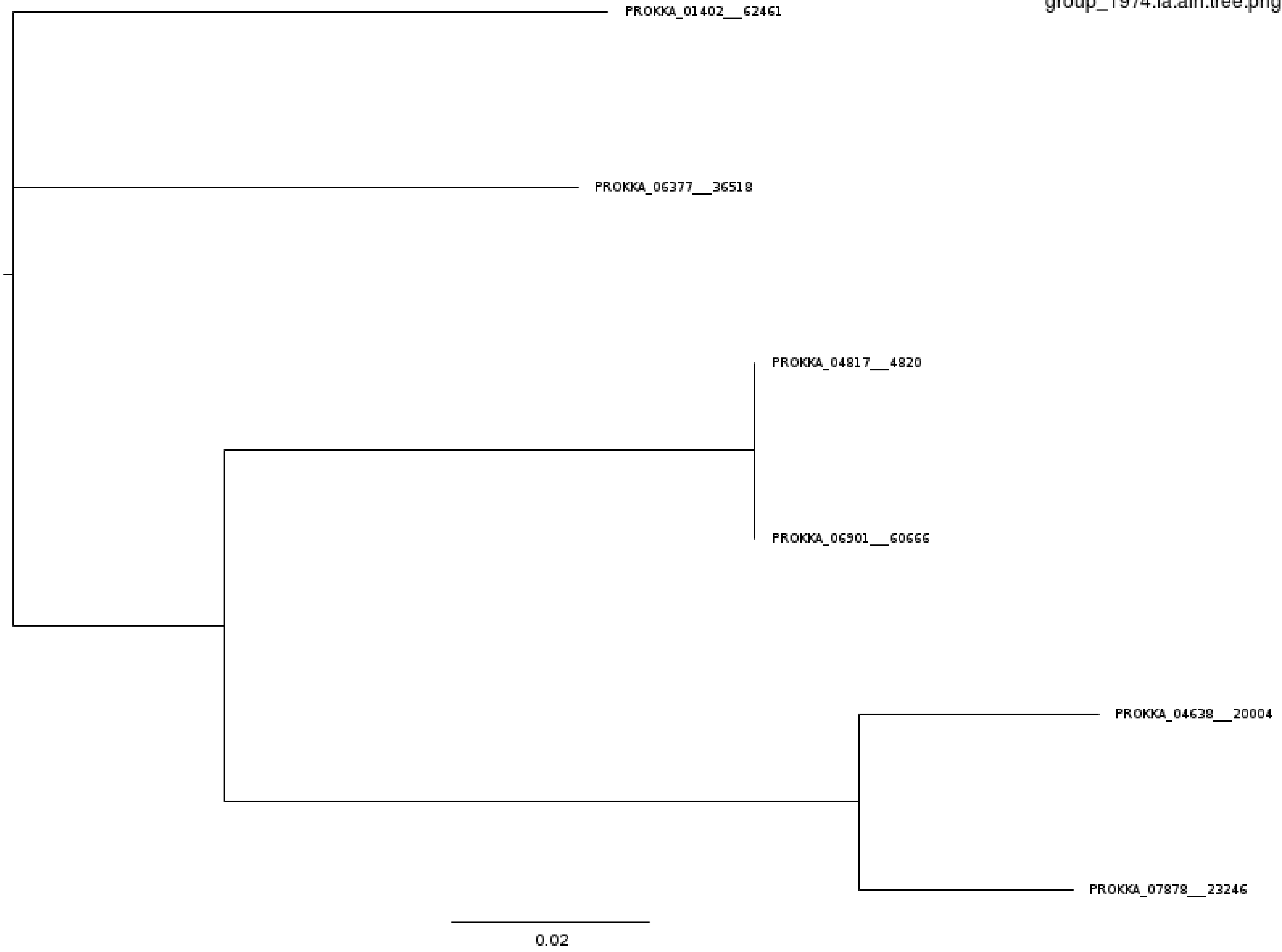

group_1976.fa.aln.tree.png

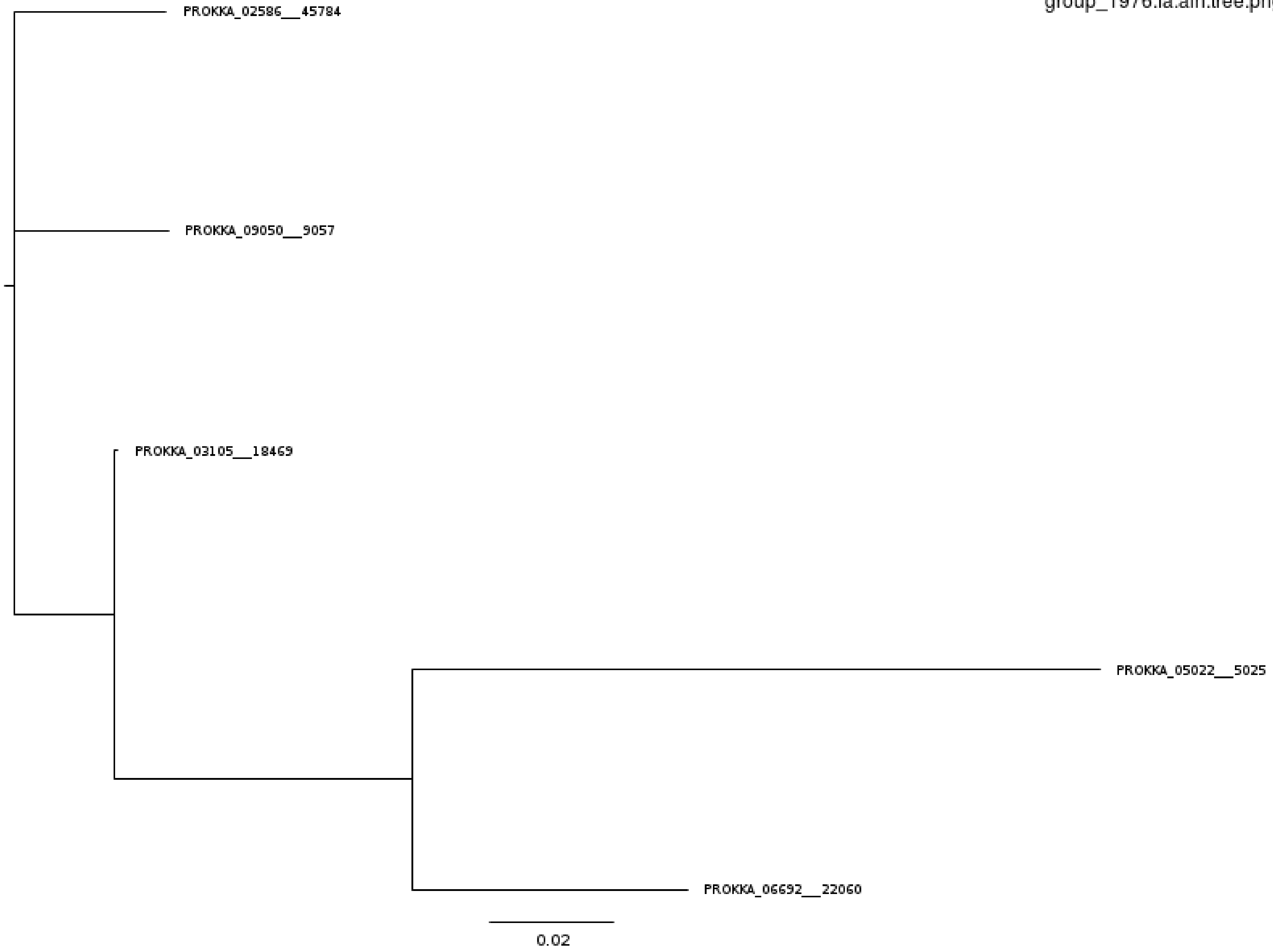

group_1977.fa.aln.tree.png

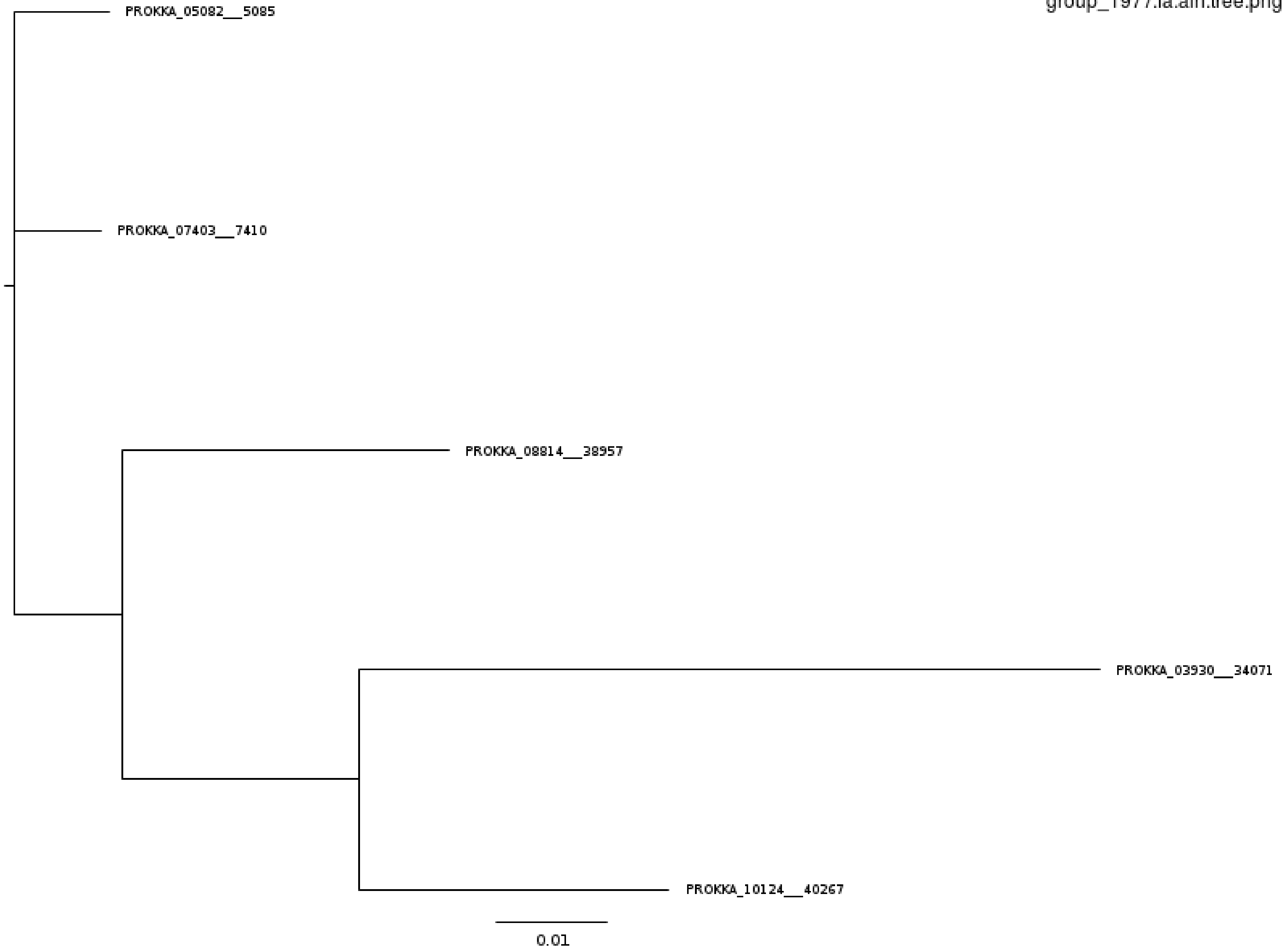

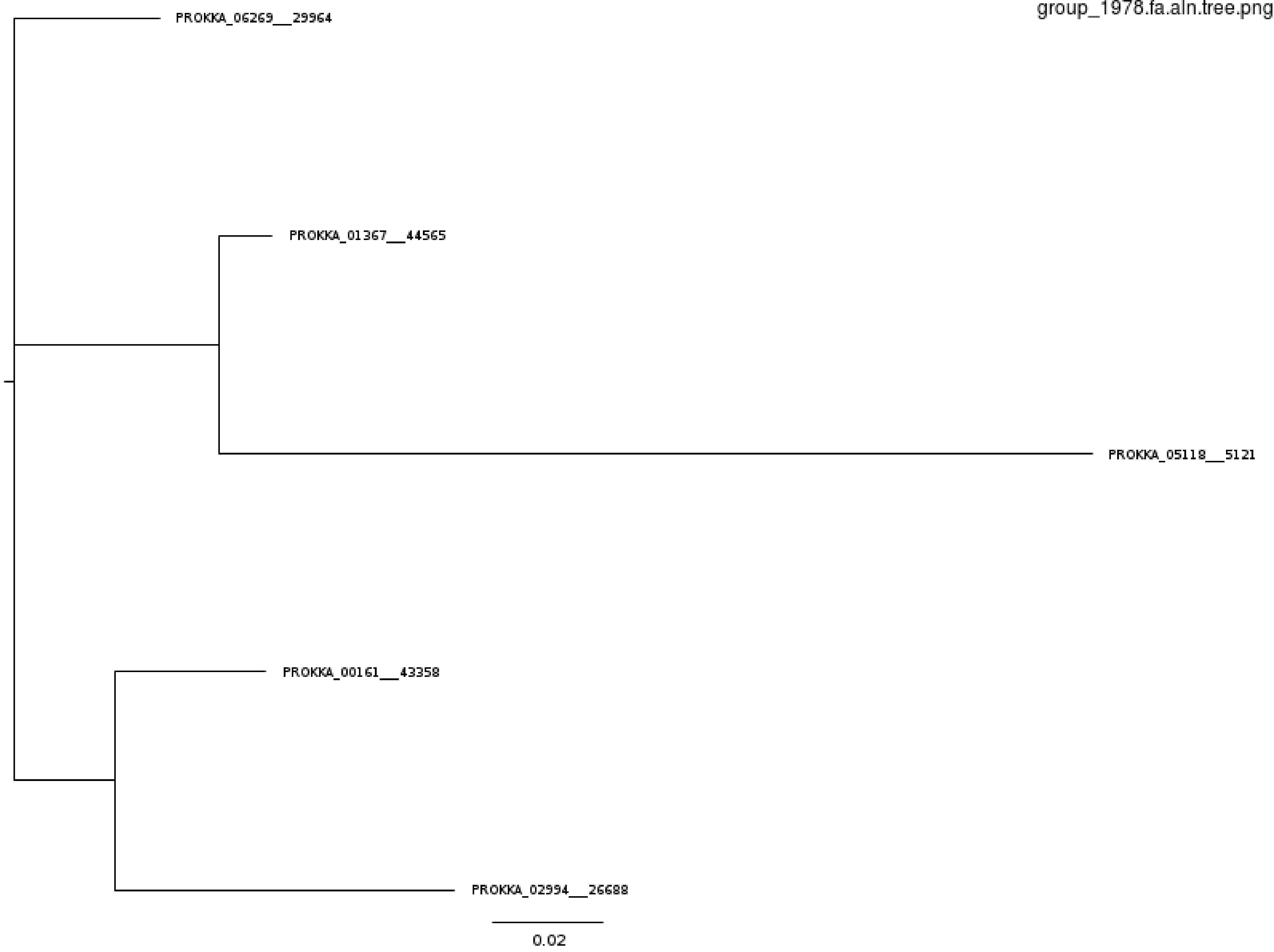
group_1978.fa.aln.tree.png
PROKKA_06269__29964
PROKKA_01367__44565
PROKKA_05118__5121
PROKKA_00161__43358
PROKKA_02994__26688
0.02

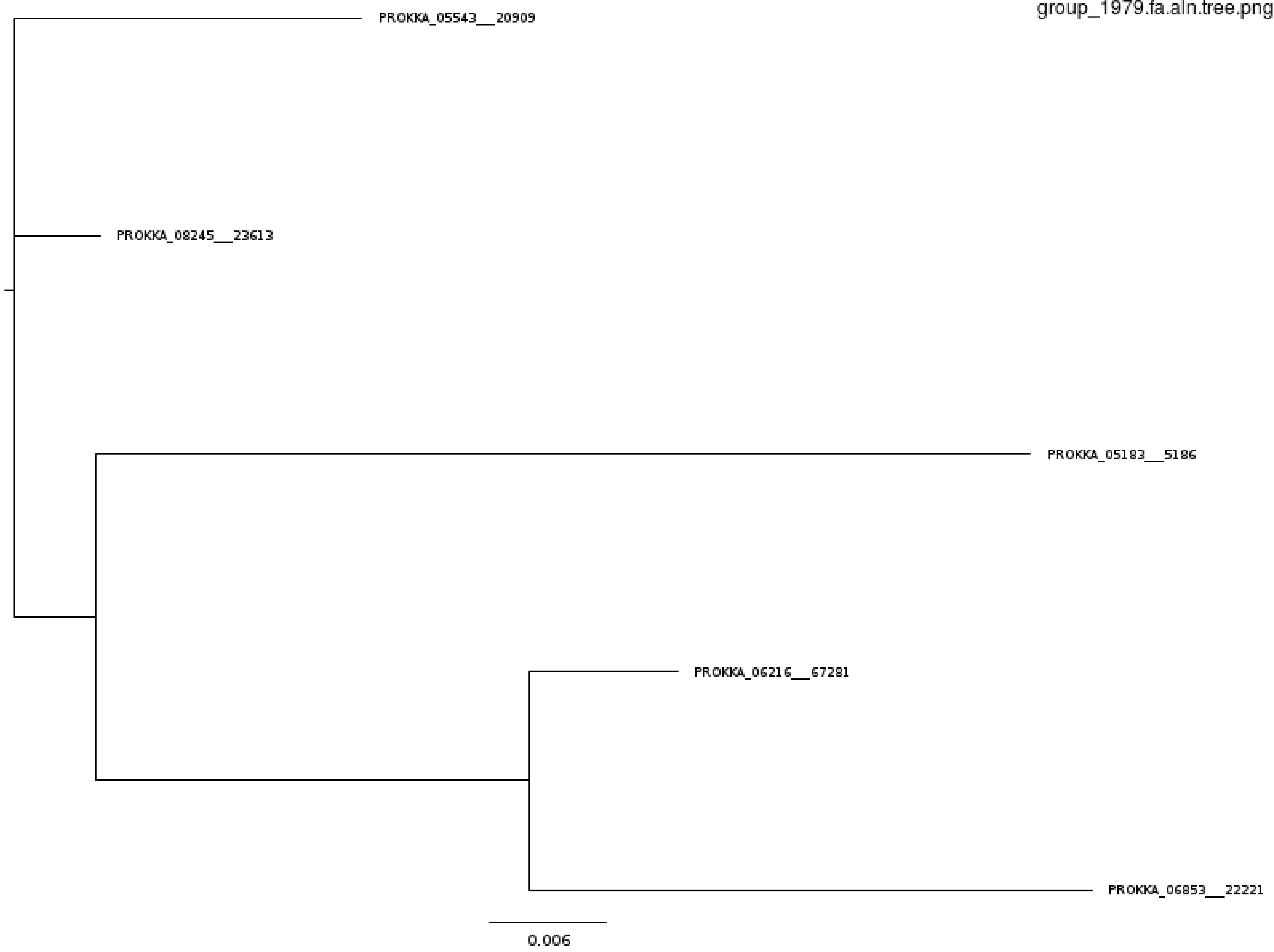
group_1979.fa.aln.tree.png
PROKKA_05543__20909
PROKKA_08245__23613
PROKKA_05183__5186
PROKKA_06216__67281
PROKKA_06853__22221
0.006

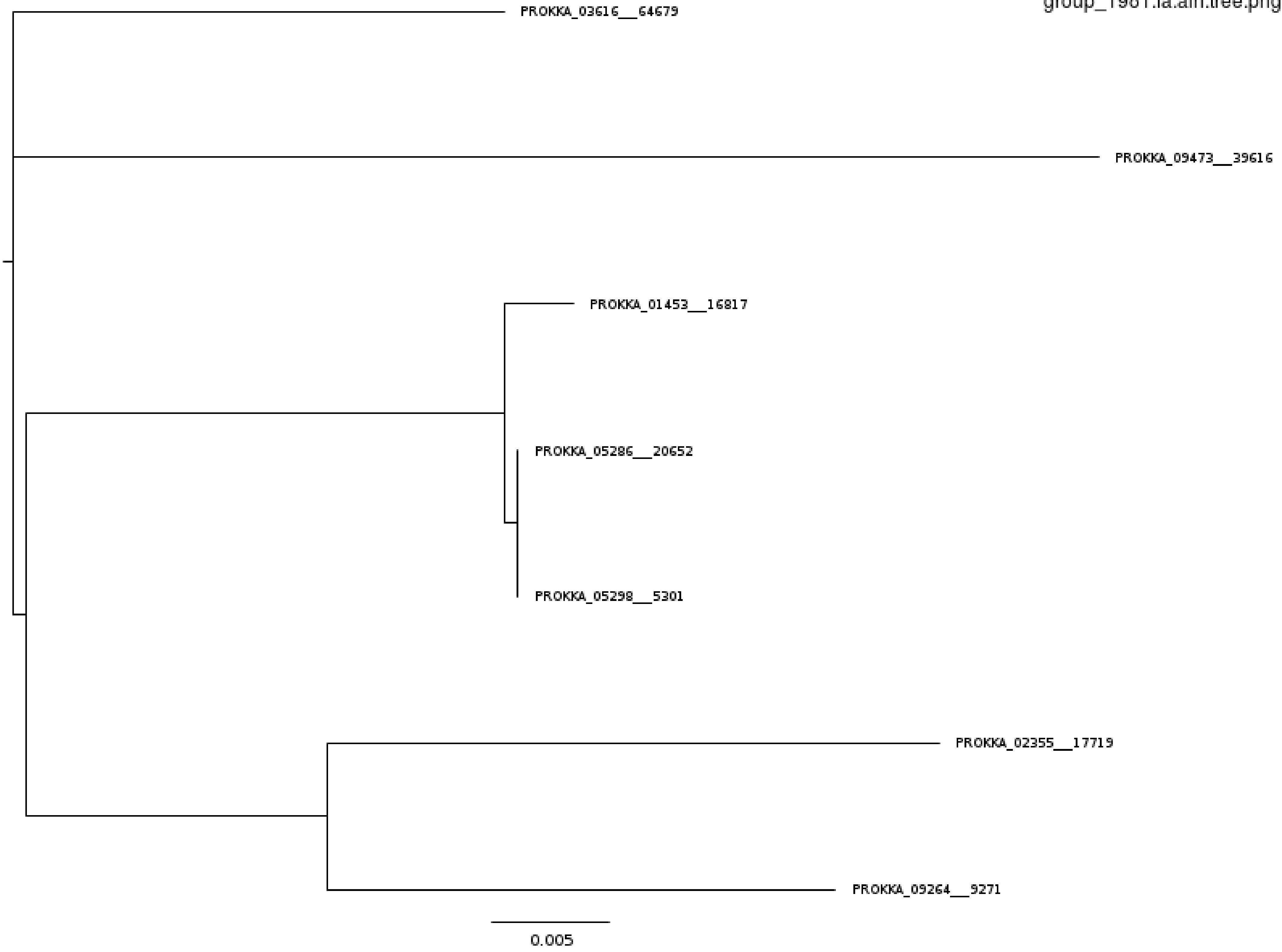
PROKKA_03616___64679
PROKKA_09473___39616
PROKKA_01453___16817
PROKKA_05286___20652
PROKKA_05298___5301
PROKKA_02355___17719
PROKKA_09264___9271
0.005

group_1982.fa.aln.tree.png

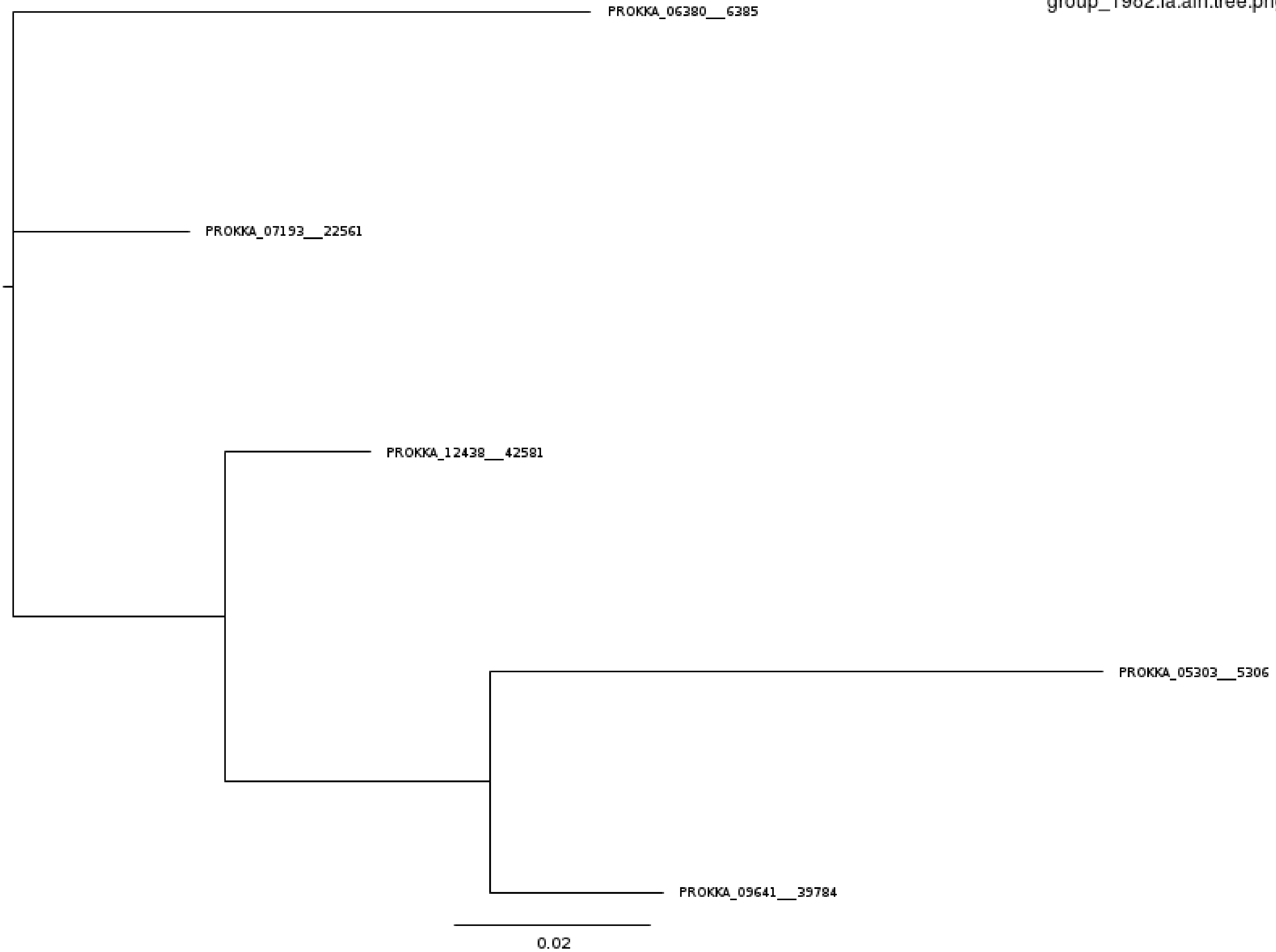

group_1983.fa.aln.tree.png

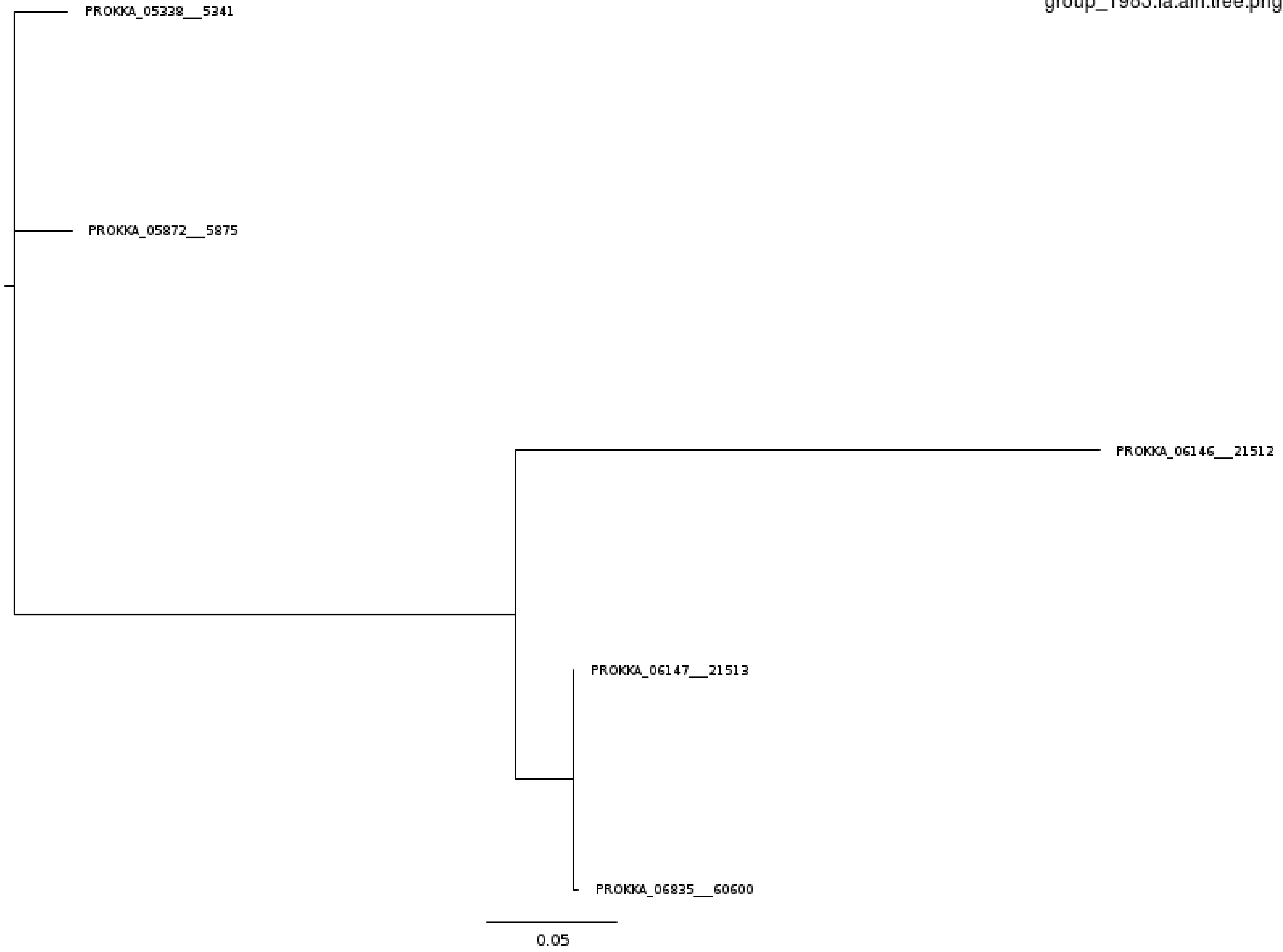

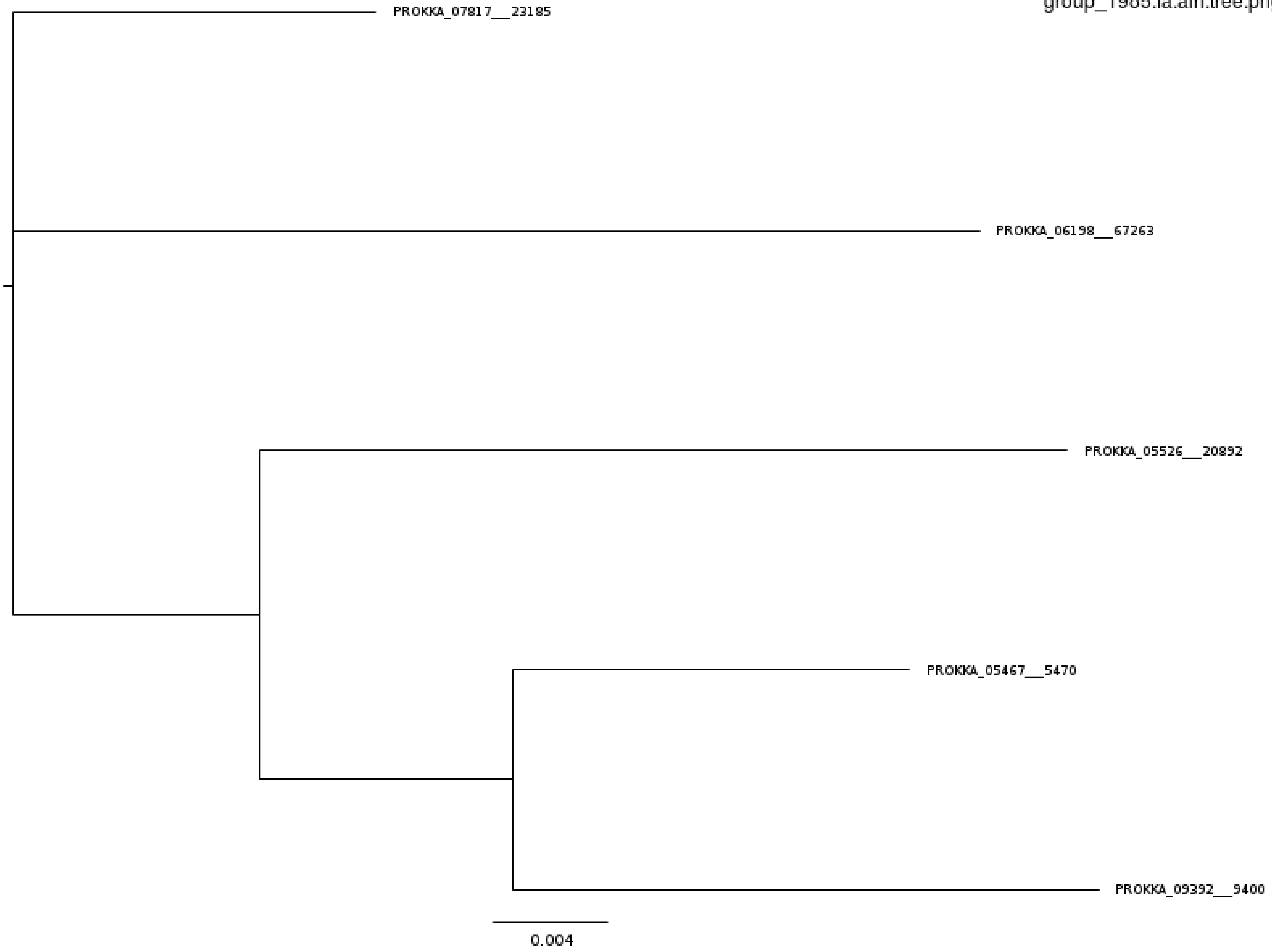
PROKKA_07817___23185
PROKKA_06198___67263
PROKKA_05526___20892
PROKKA_05467___5470
PROKKA_09392___9400
0.004

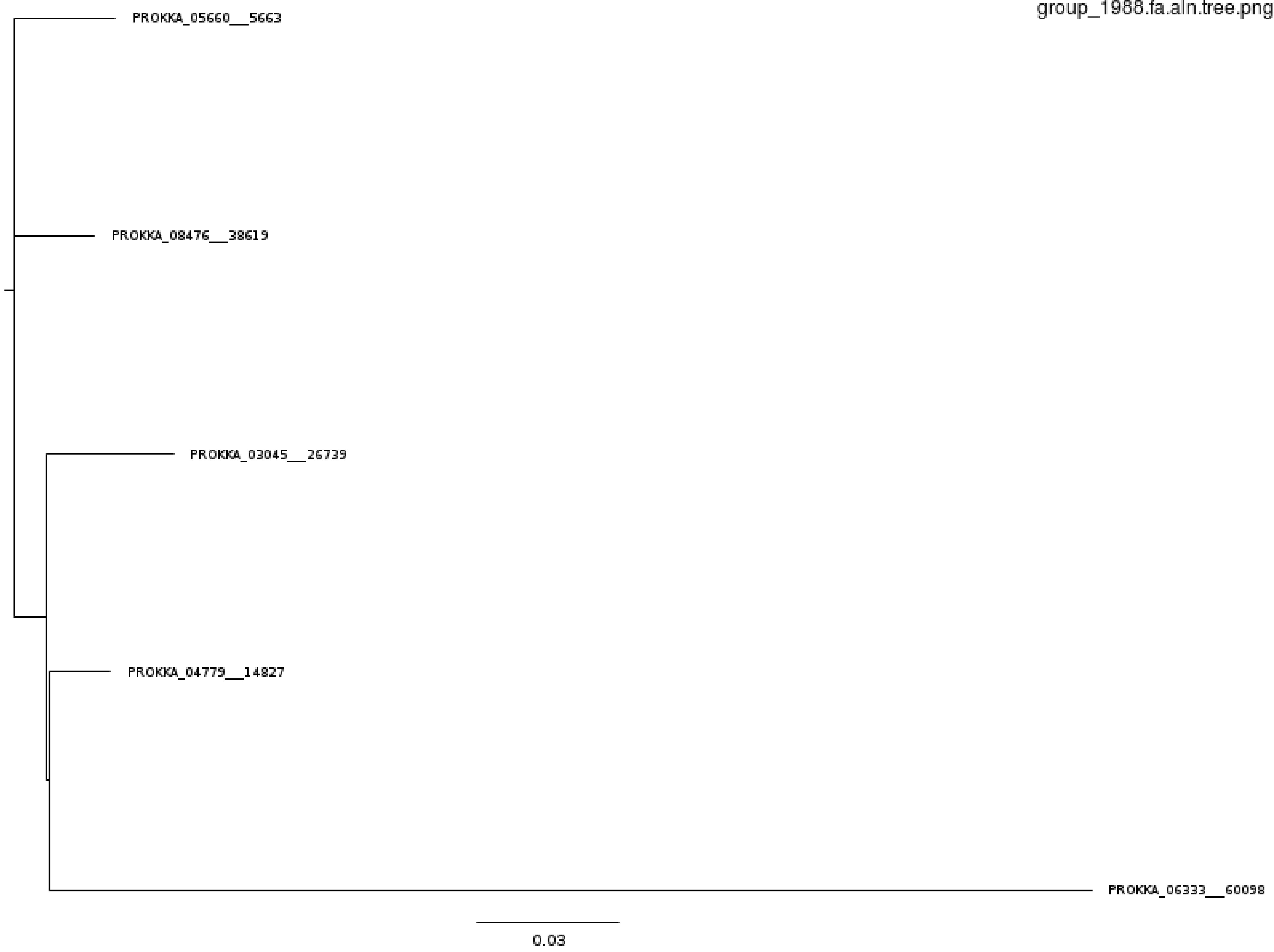
group_1988.fa.aln.tree.png
PROKKA_05660___5663
PROKKA_08476___38619
PROKKA_03045___26739
PROKKA_04779___14827
PROKKA_06333___60098
0.03

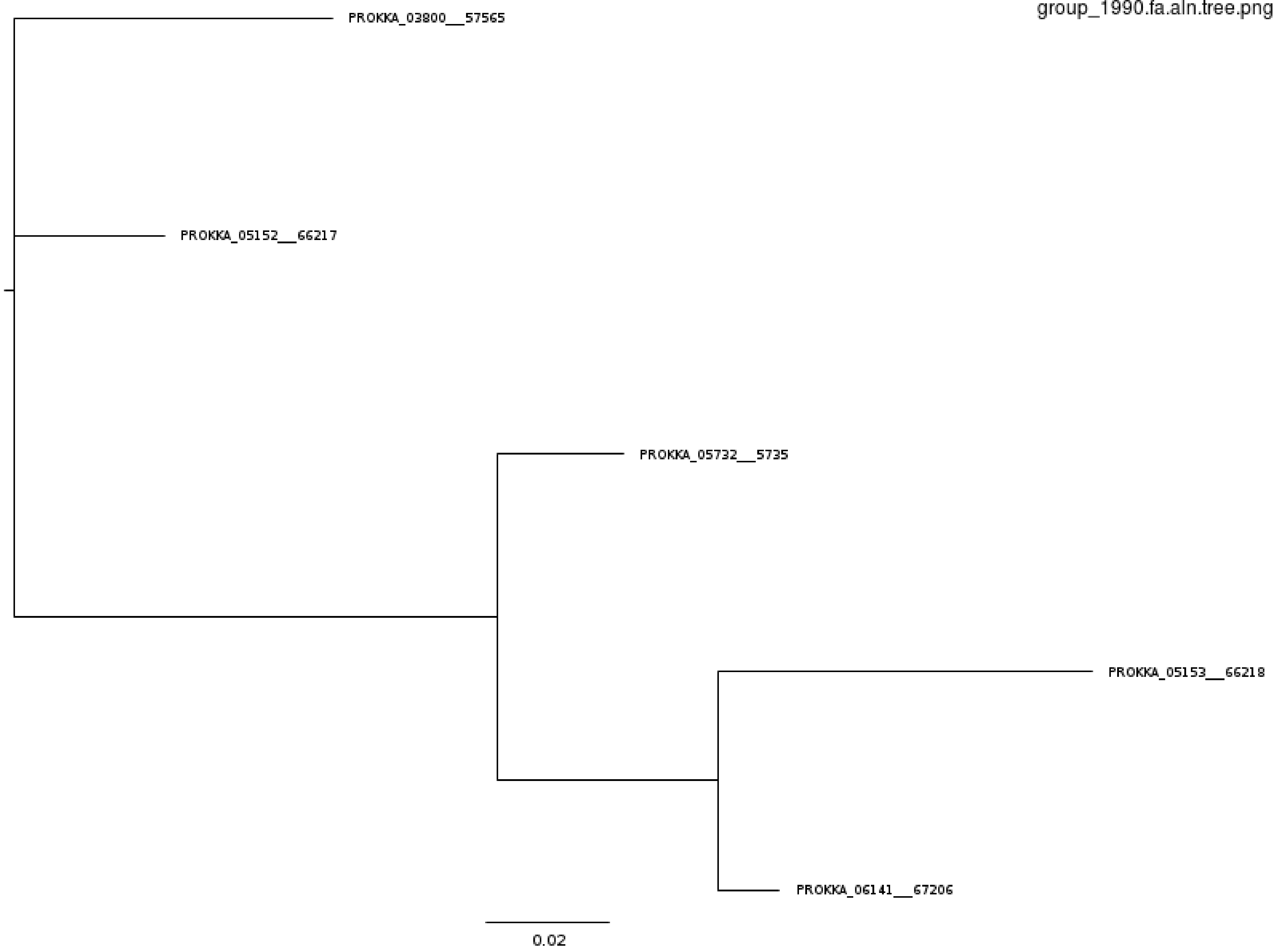
group_1990.fa.aln.tree.png
PROKKA_03800___57565
PROKKA_05152___66217
PROKKA_05732___5735
PROKKA_05153___66218
PROKKA_06141___67206
0.02

group_1994.fa.aln.tree.png

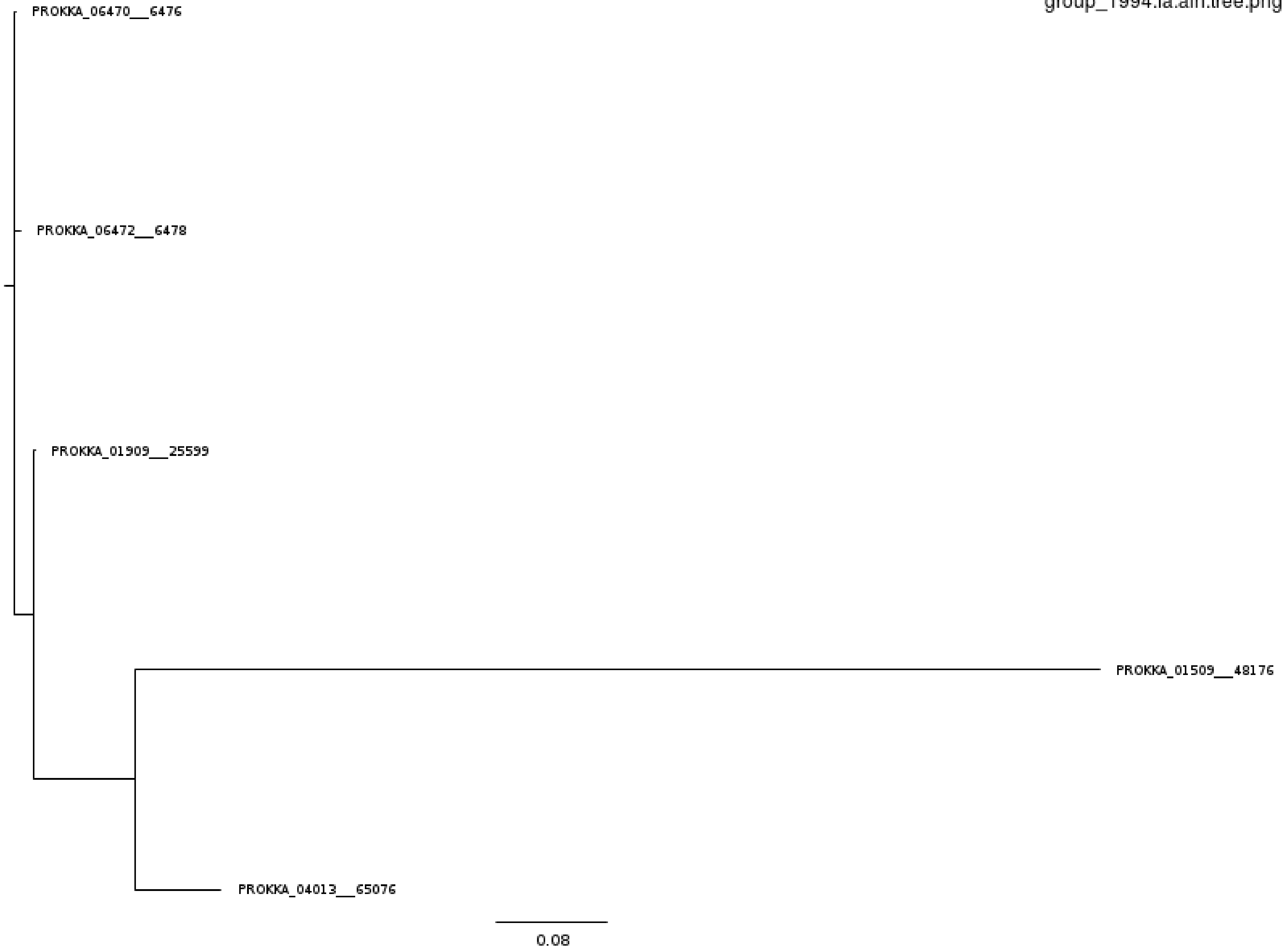

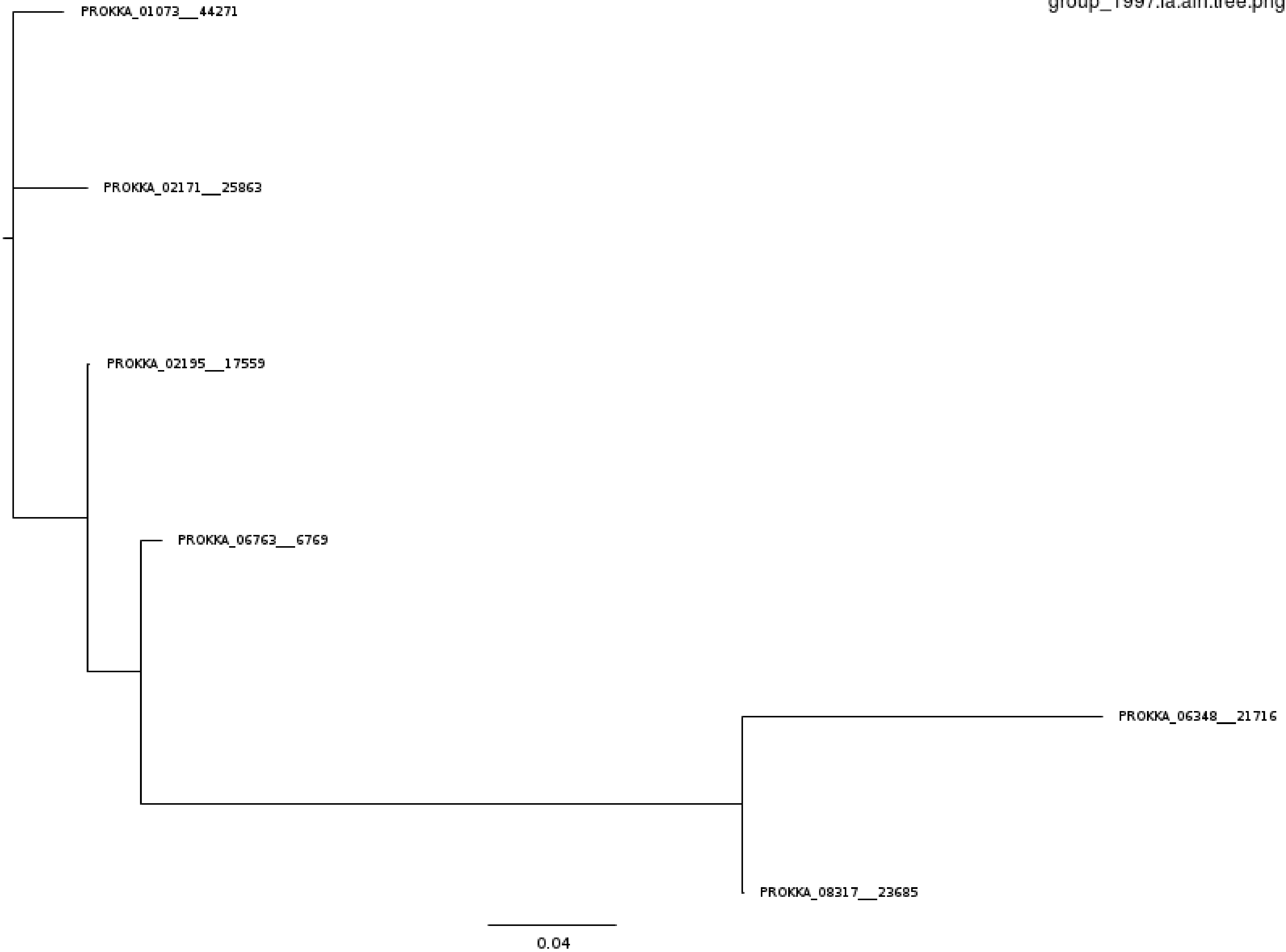
PROKKA_01073___44271
PROKKA_02171___25863
PROKKA_02195___17559
PROKKA_06763___6769
PROKKA_06348___21716
PROKKA_08317___23685
0.04

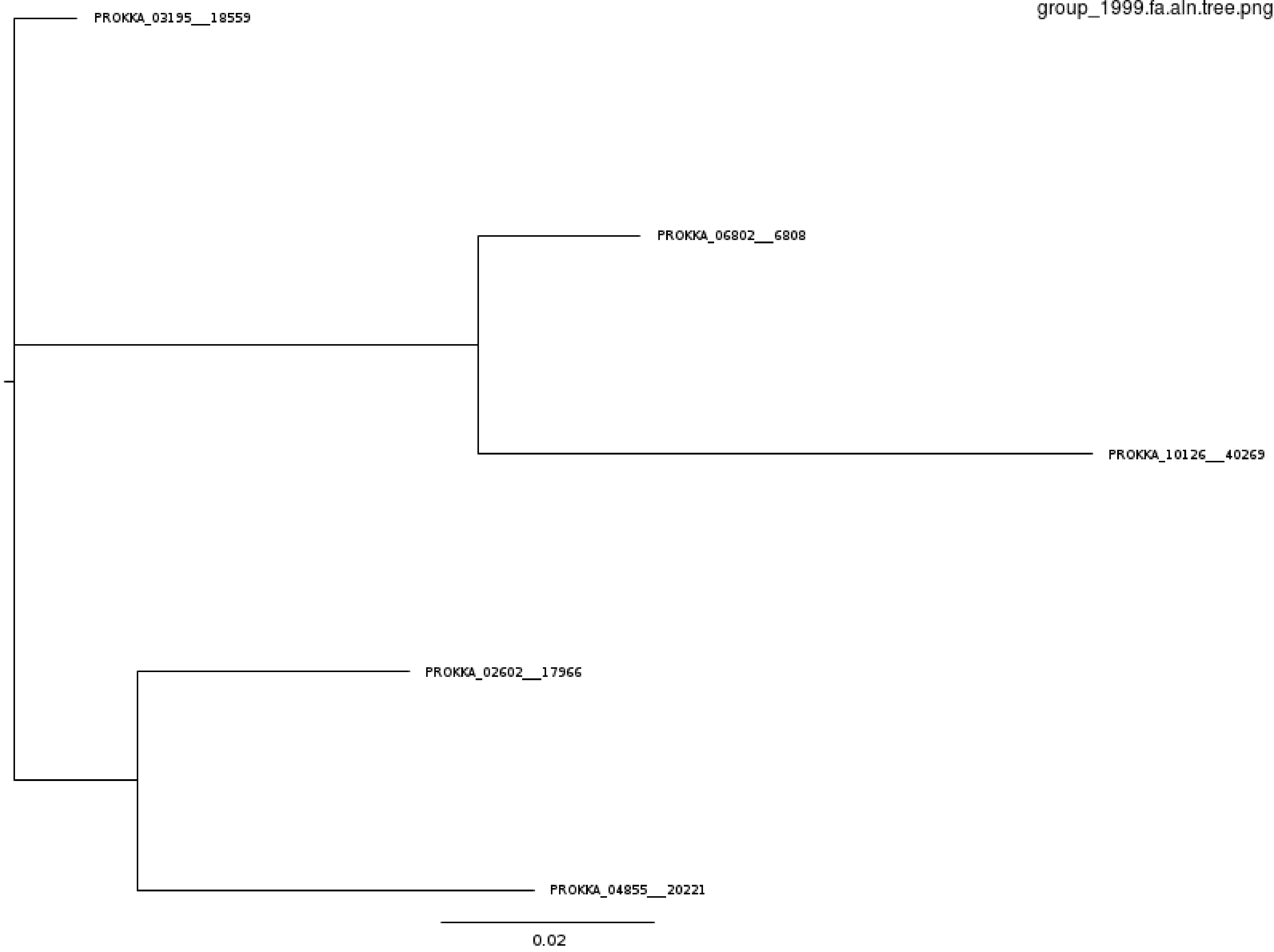
group_1999.fa.aln.tree.png
PROKKA_03195__18559
PROKKA_06802__6808
PROKKA_10126__40269
PROKKA_02602__17966
PROKKA_04855__20221
0.02

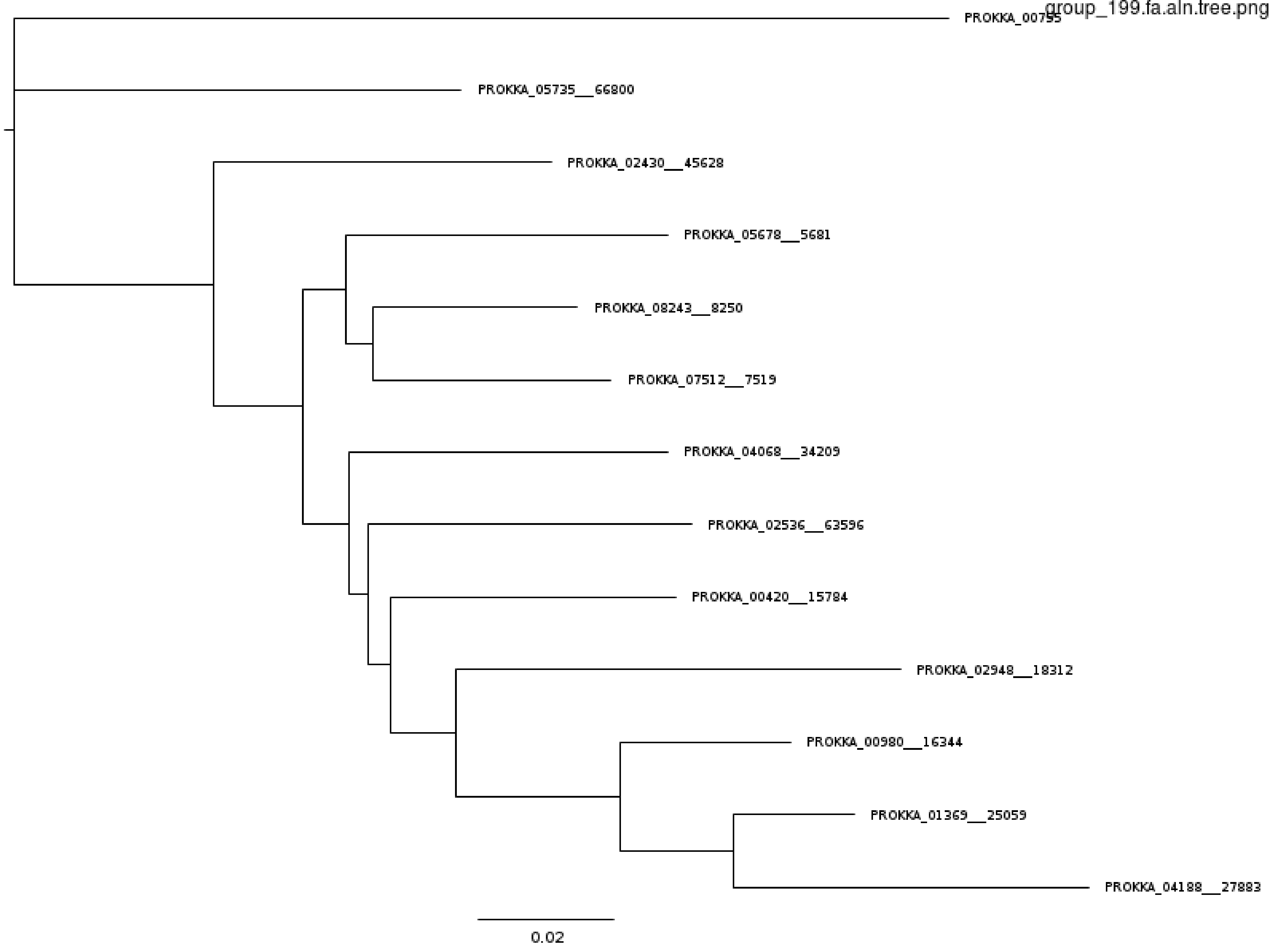
group_199.fa.aln.tree.png
PROKKA_00755
PROKKA_05735___66800
PROKKA_02430___45628
PROKKA_05678___5681
PROKKA_08243___8250
PROKKA_07512___7519
PROKKA_04068___34209
PROKKA_02536___63596
PROKKA_00420___15784
PROKKA_02948___18312
PROKKA_00980___16344
PROKKA_01369___25059
PROKKA_04188___27883
0.02

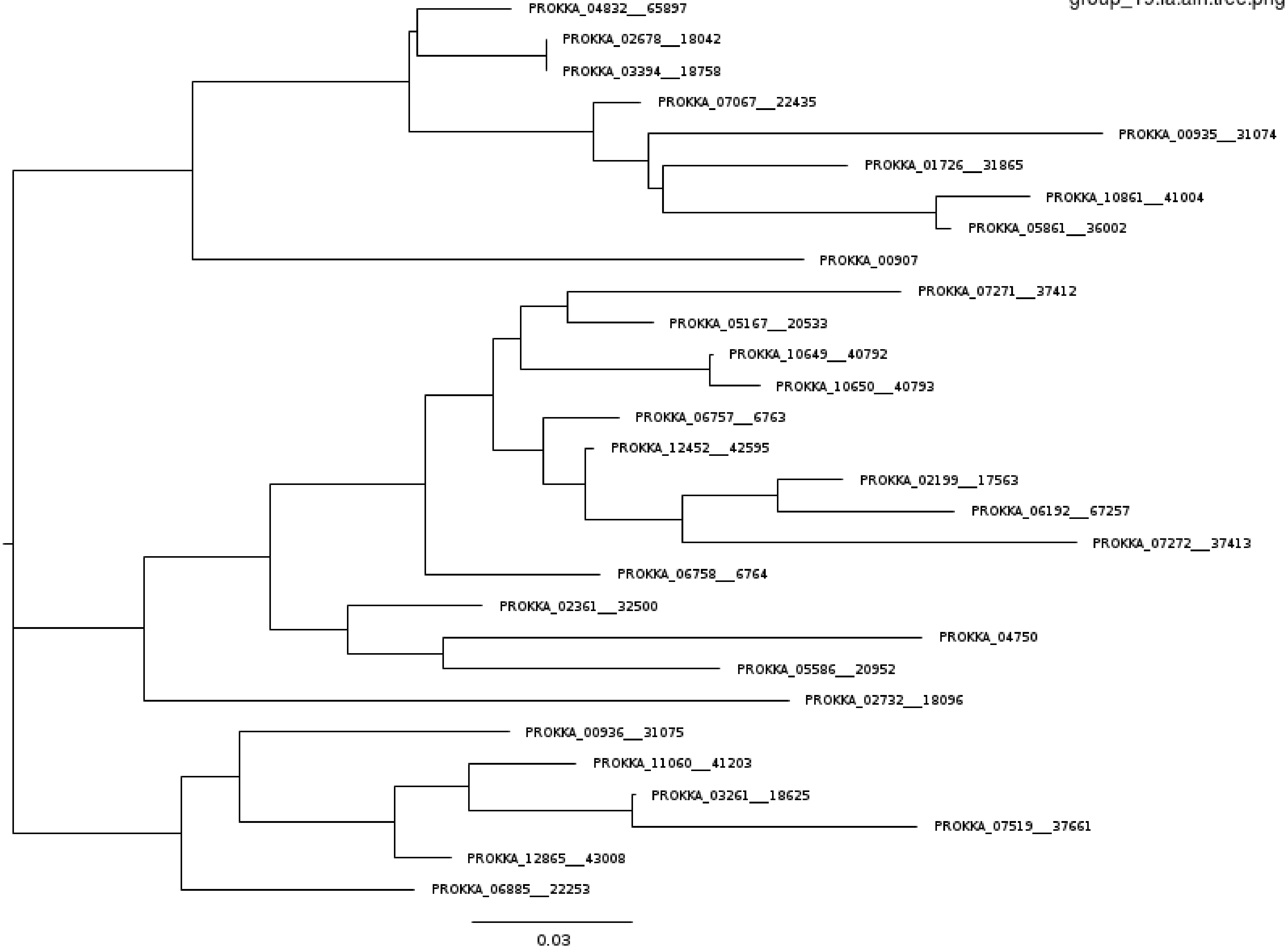
PROKKA_04832___65897
PROKKA_02678___18042
PROKKA_03394___18758
PROKKA_07067___22435
PROKKA_00935___31074
PROKKA_01726___31865
PROKKA_10861___41004
PROKKA_05861___36002
PROKKA_00907
PROKKA_07271___37412
PROKKA_05167___20533
PROKKA_10649___40792
PROKKA_10650___40793
PROKKA_06757___6763
PROKKA_12452___42595
PROKKA_02199___17563
PROKKA_06192___67257
PROKKA_07272___37413
PROKKA_06758___6764
PROKKA_02361___32500
PROKKA_04750
PROKKA_05586___20952
PROKKA_02732___18096
PROKKA_00936___31075
PROKKA_11060___41203
PROKKA_03261___18625
PROKKA_07519___37661
PROKKA_12865___43008
PROKKA_06885___22253
0.03

group_1.fa.aln.tree.png

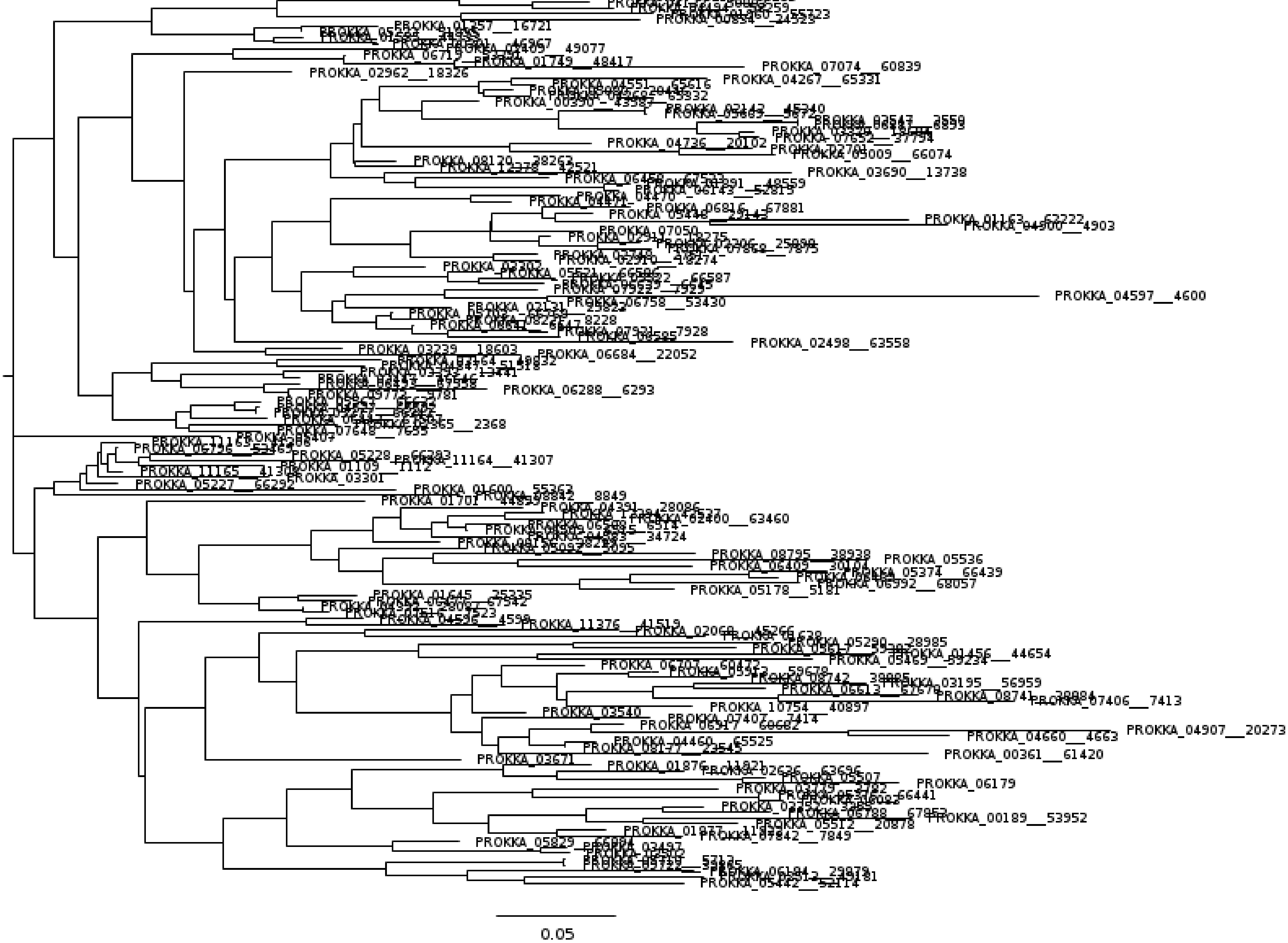

group_2000.fa.aln.tree.png

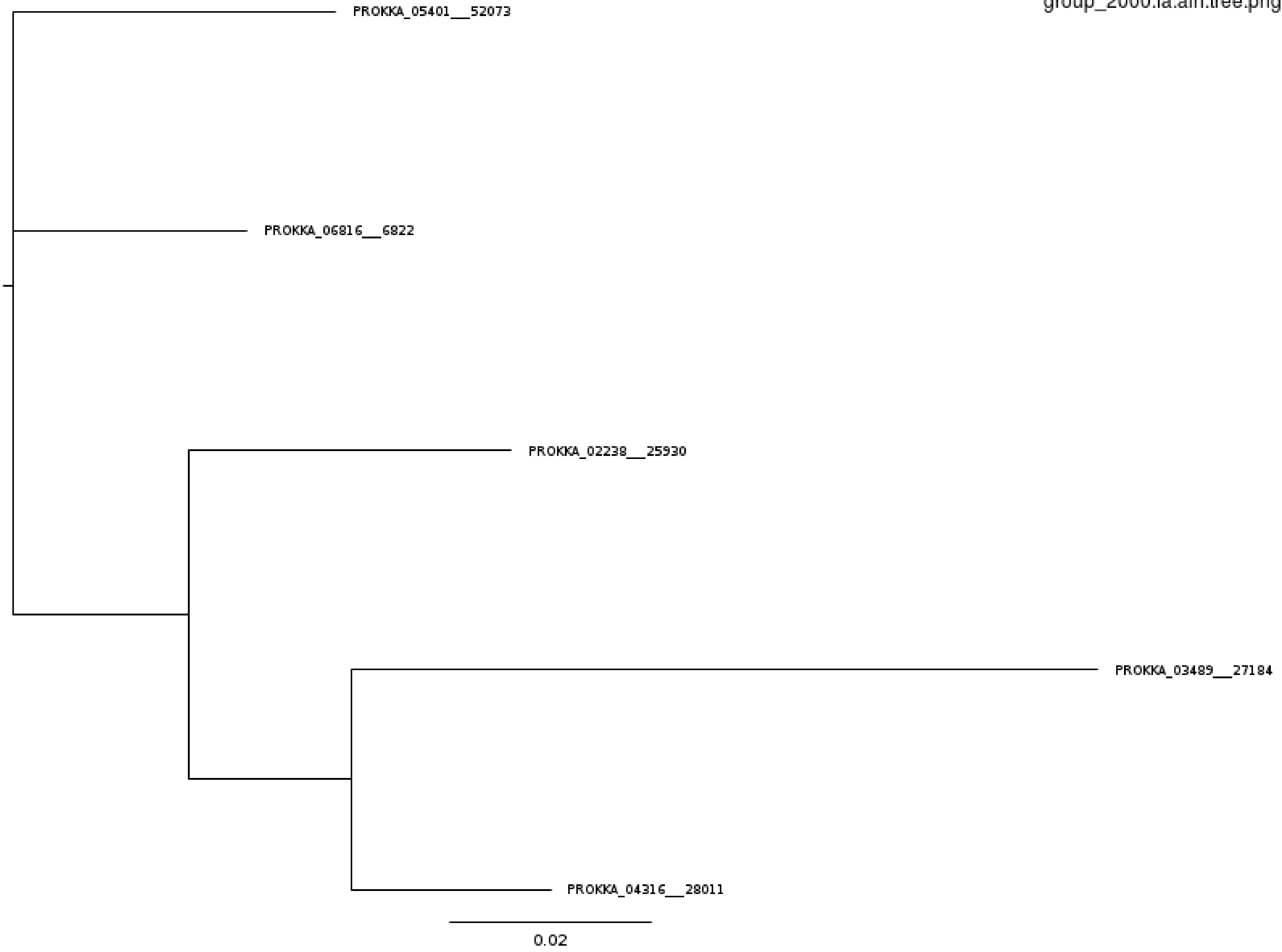

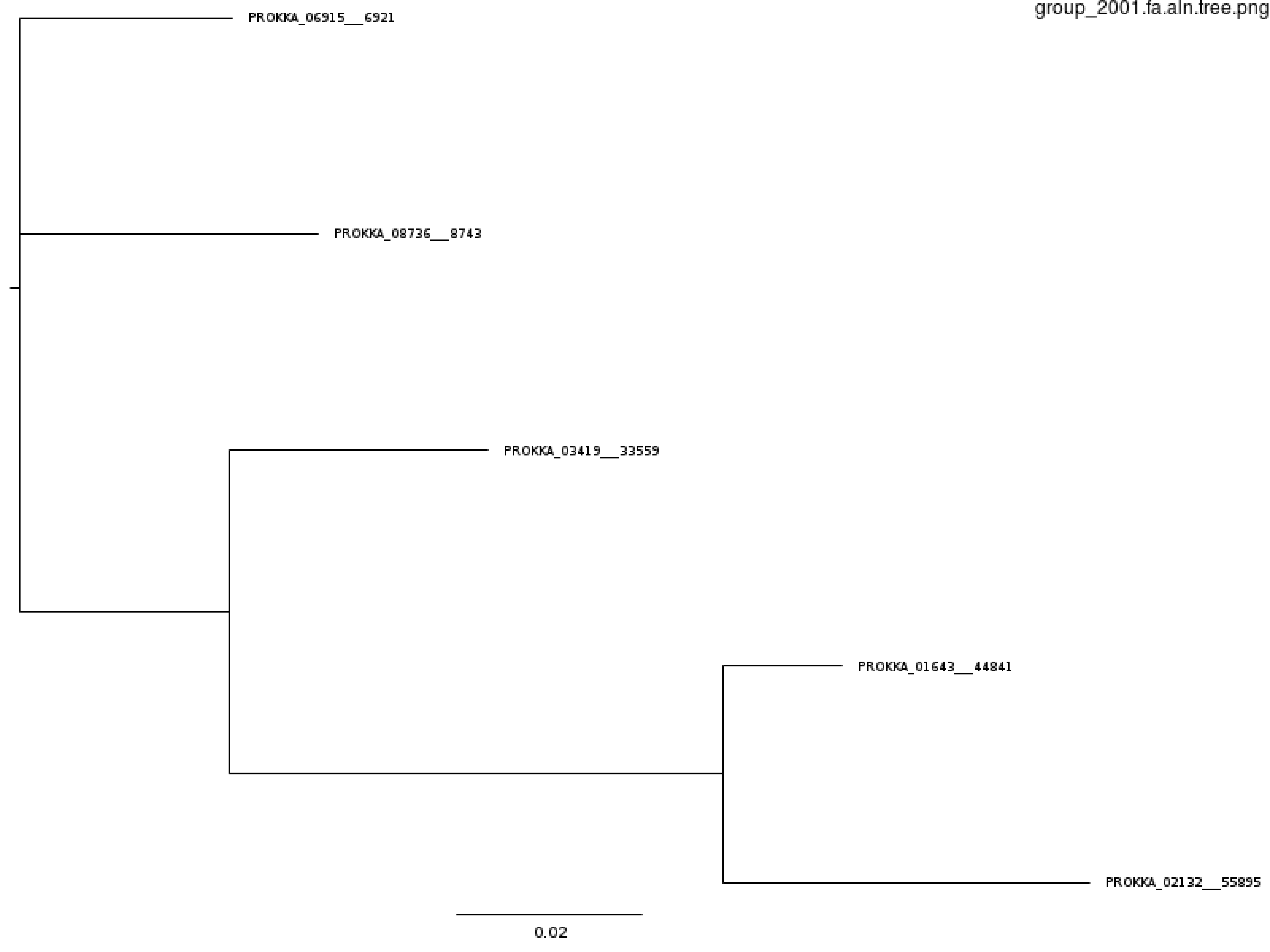
group_2001.fa.aln.tree.png
PROKKA_06915___6921
PROKKA_08736___8743
PROKKA_03419___33559
PROKKA_01643___44841
PROKKA_02132___55895
0.02

group_2002.fa.aln.tree.png

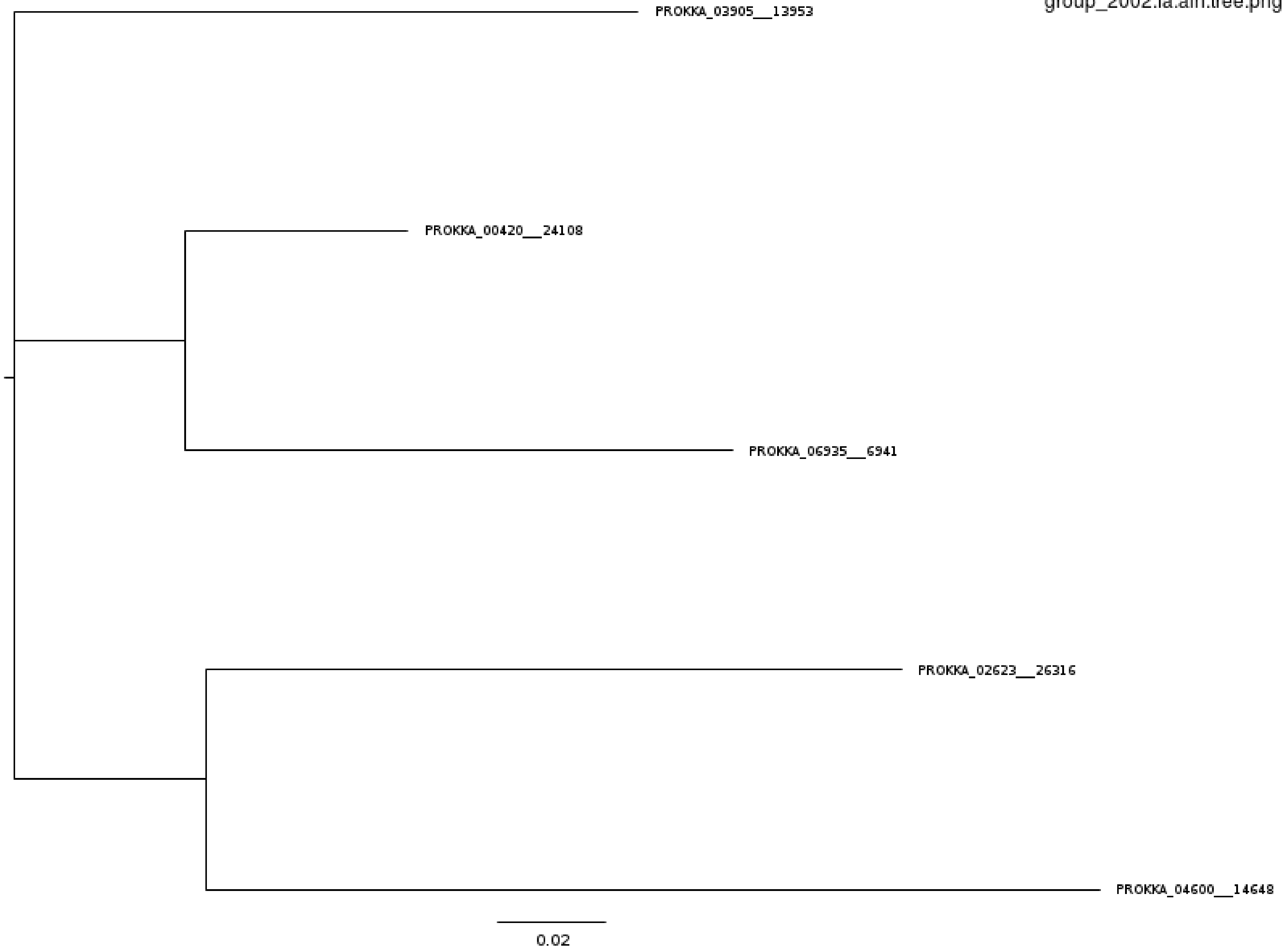

group_2003.fa.aln.tree.png

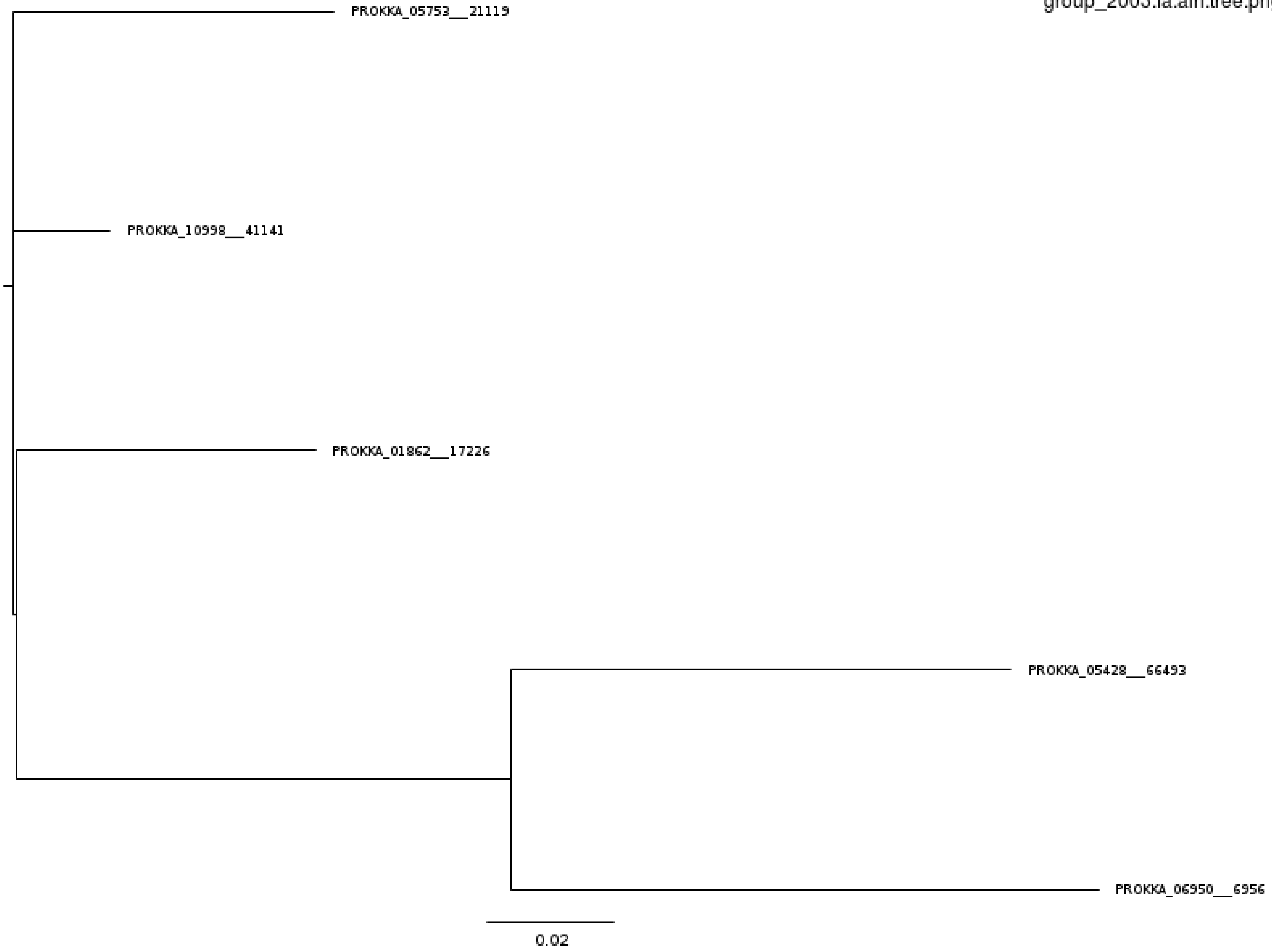

group_2005.fa.aln.tree.png

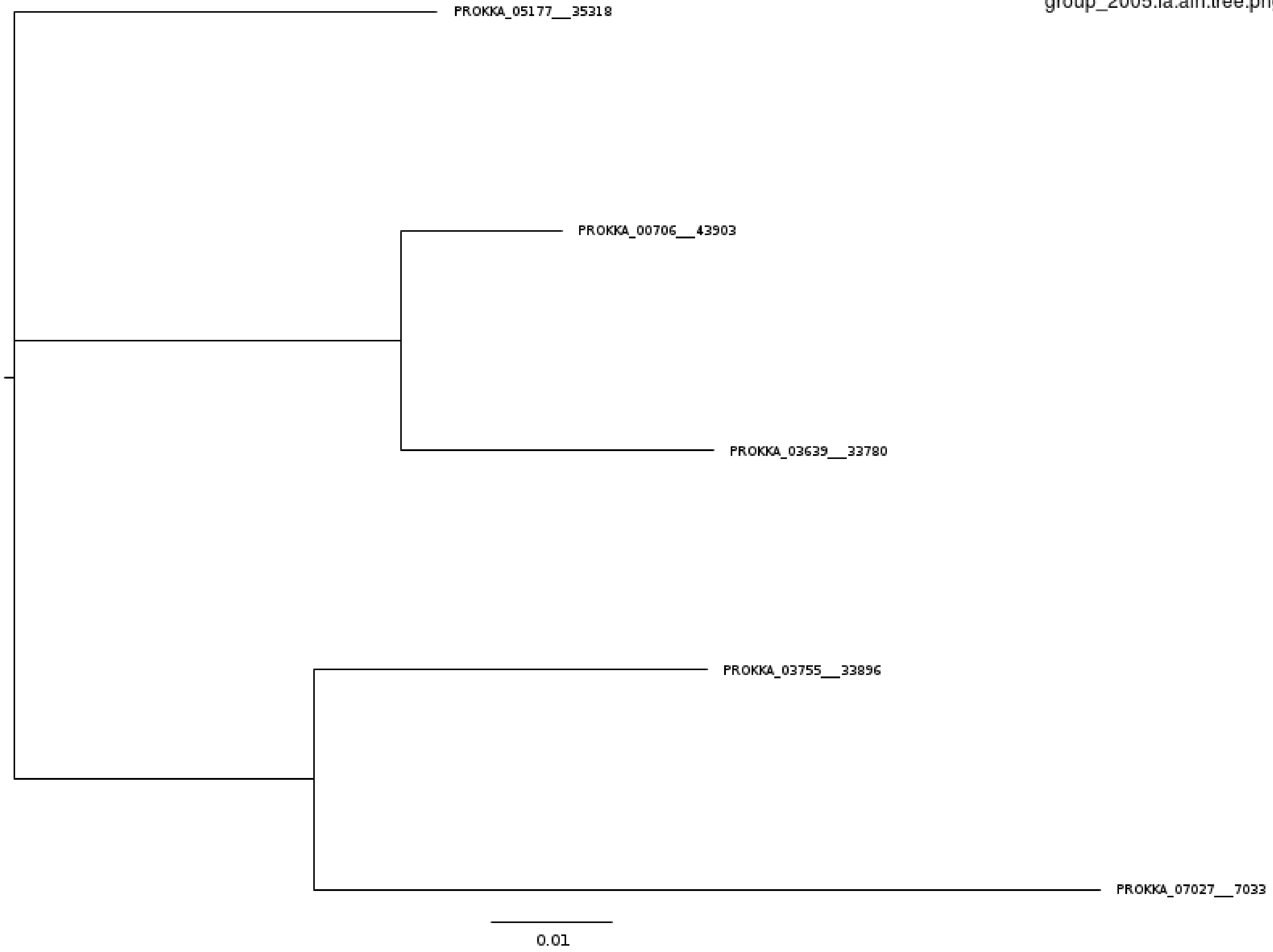

group_2007.fa.aln.tree.png

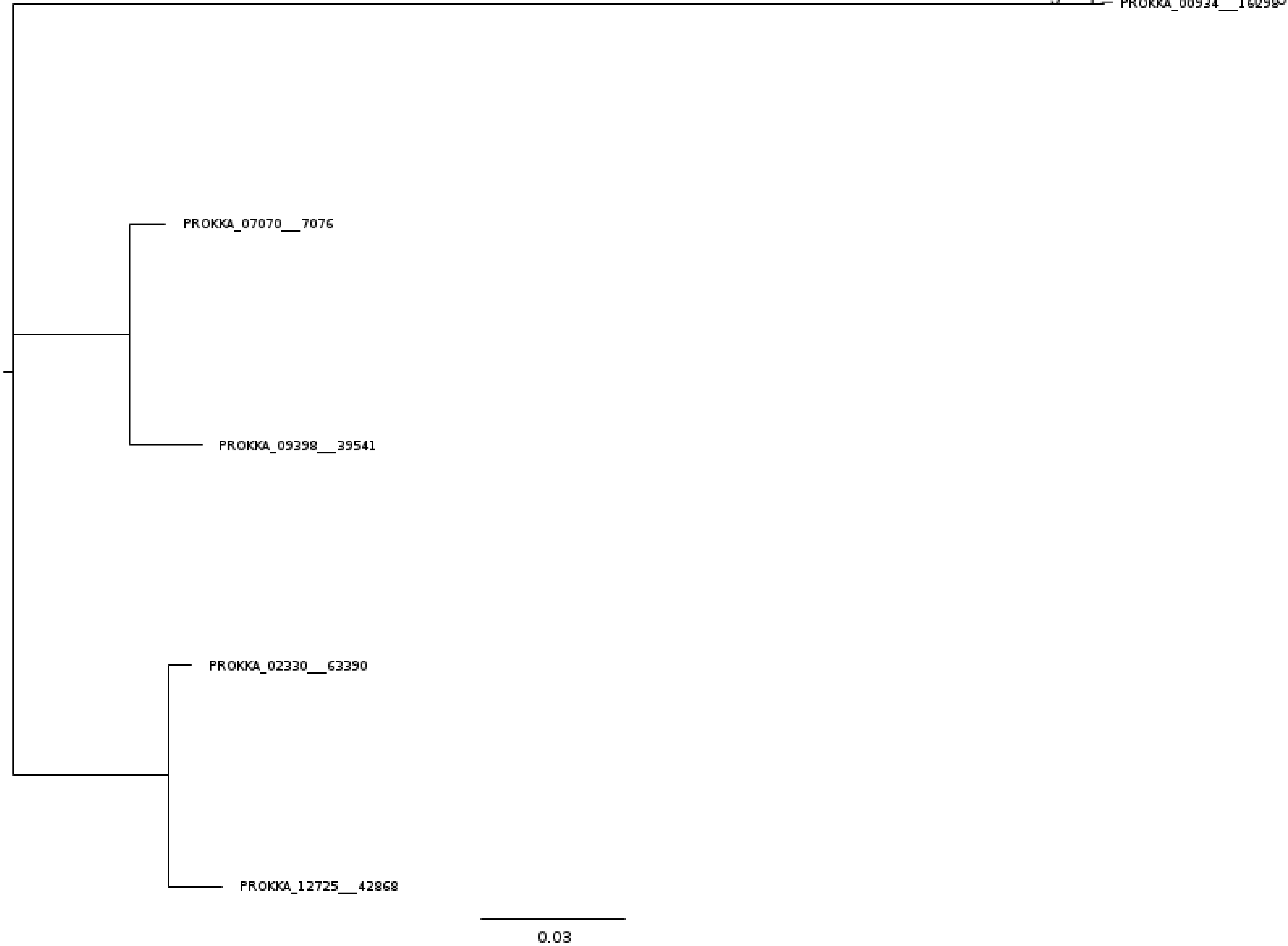

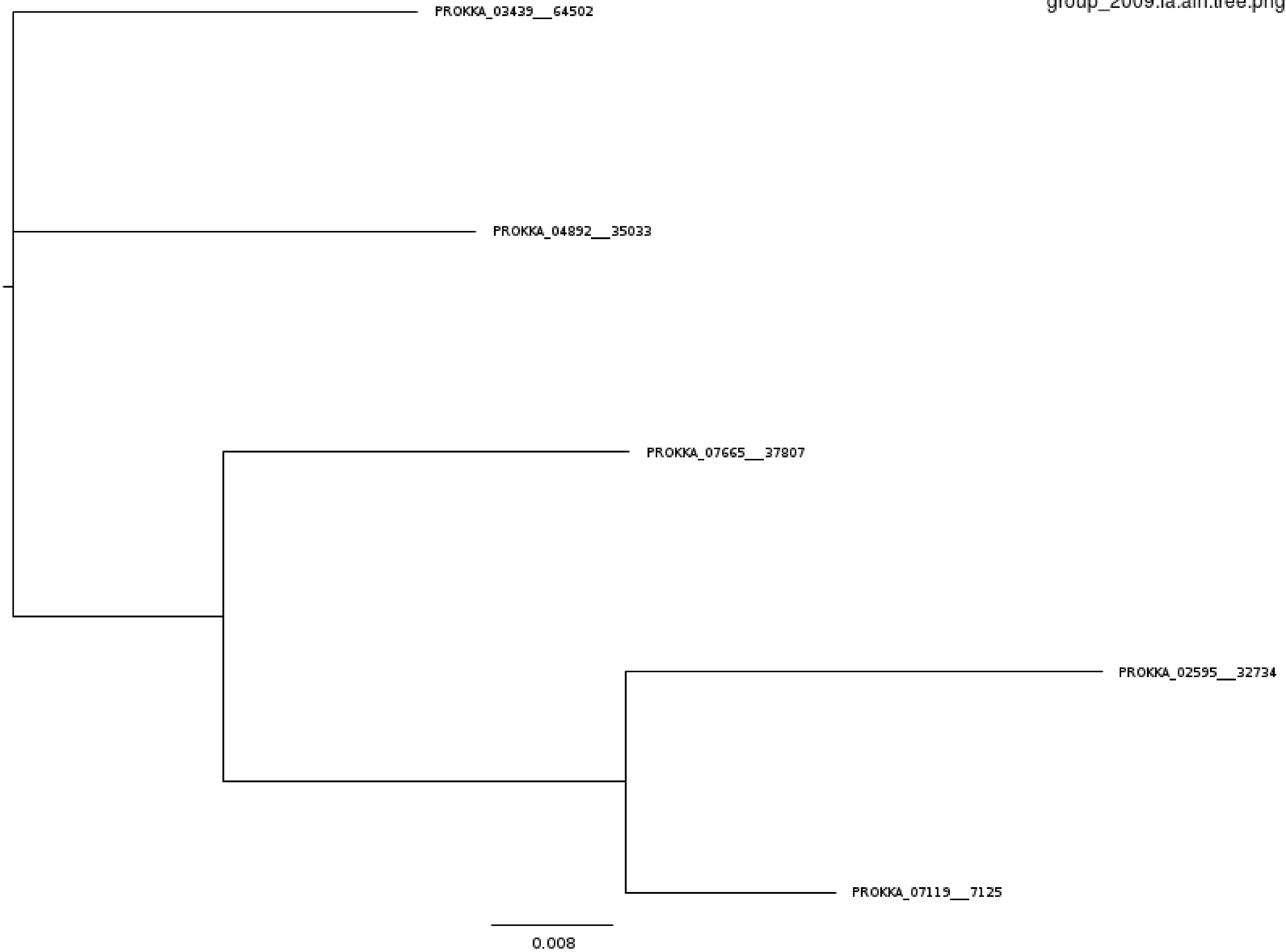
PROKKA_03439___64502
PROKKA_04892___35033
PROKKA_07665___37807
PROKKA_02595___32734
PROKKA_07119___7125
0.008

group_2011.fa.aln.tree.png

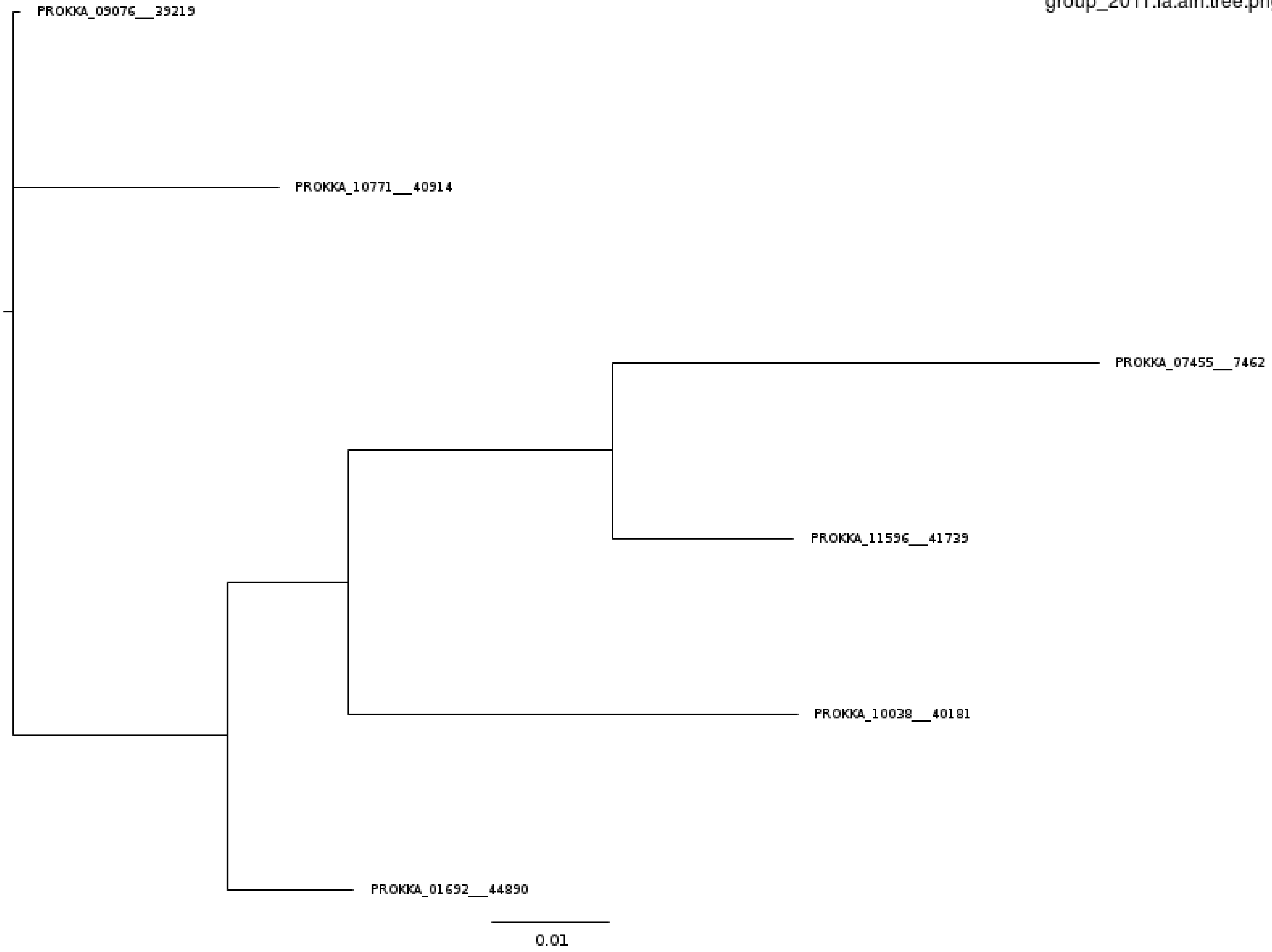

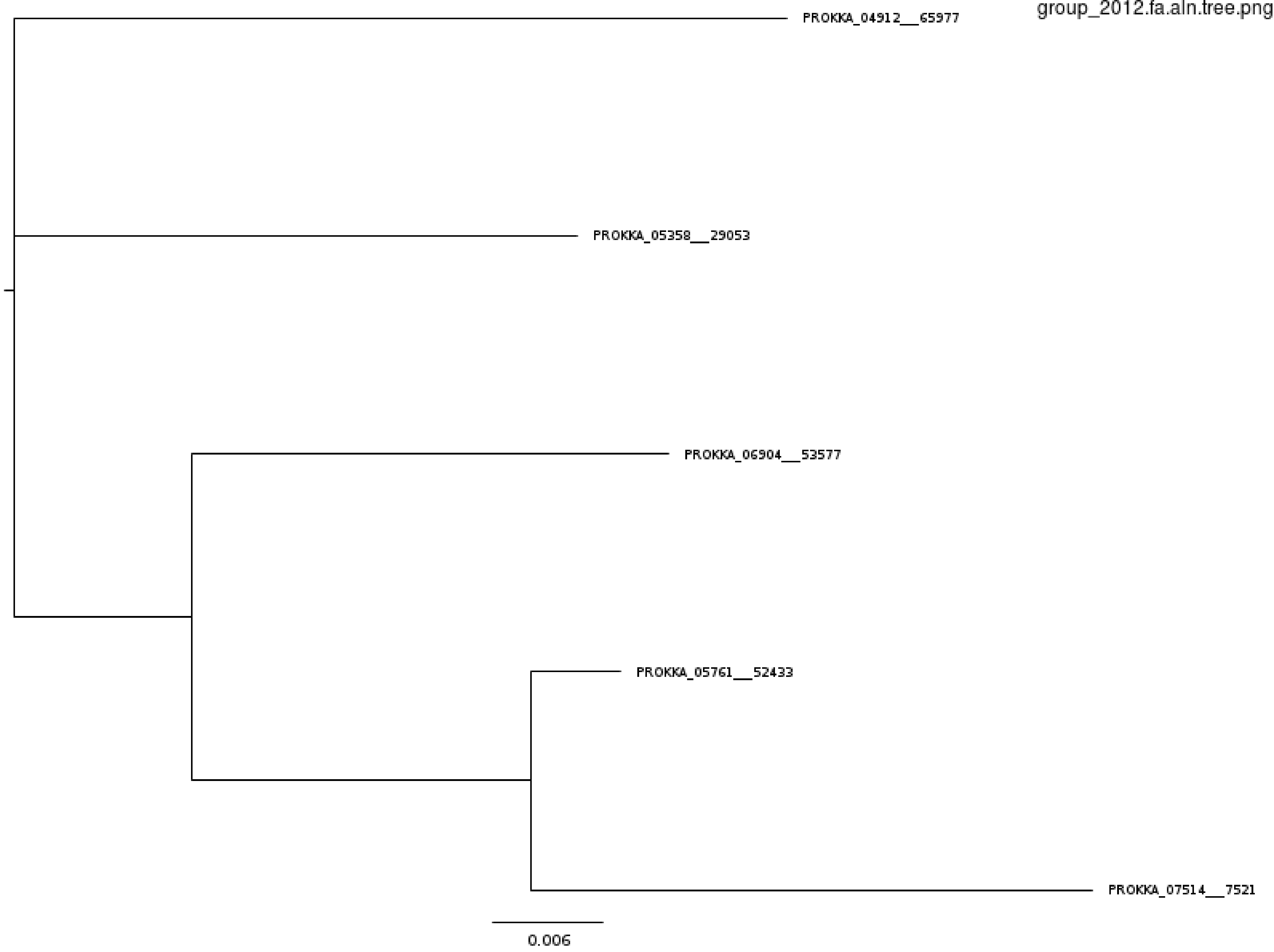
group_2012.fa.aln.tree.png
PROKKA_04912___65977
PROKKA_05358___29053
PROKKA_06904___53577
PROKKA_05761___52433
PROKKA_07514___7521
0.006

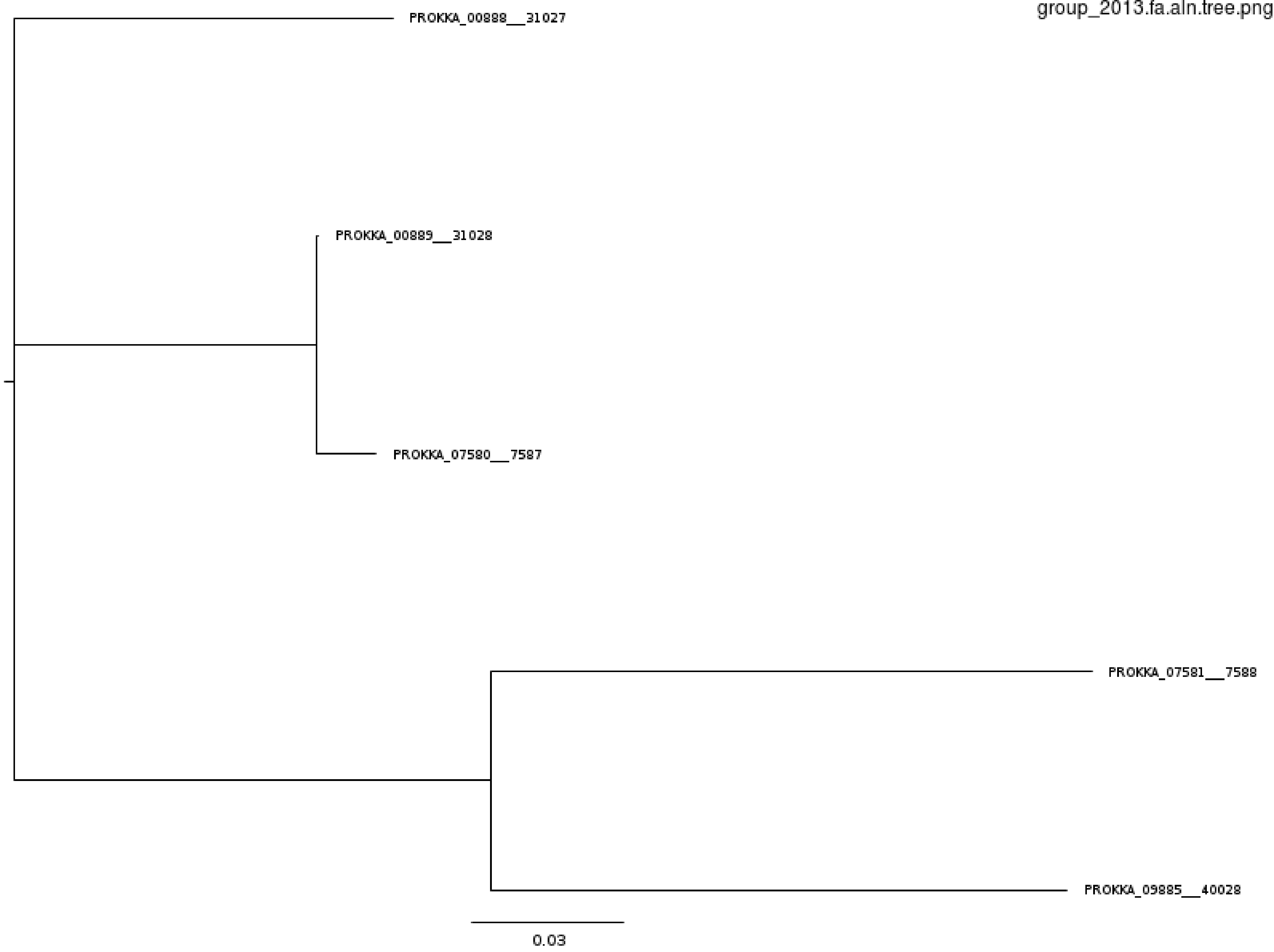
group_2013.fa.aln.tree.png
PROKKA_00888__31027
PROKKA_00889__31028
PROKKA_07580__7587
PROKKA_07581__7588
PROKKA_09885__40028
0.03

group_2015.fa.aln.tree.png

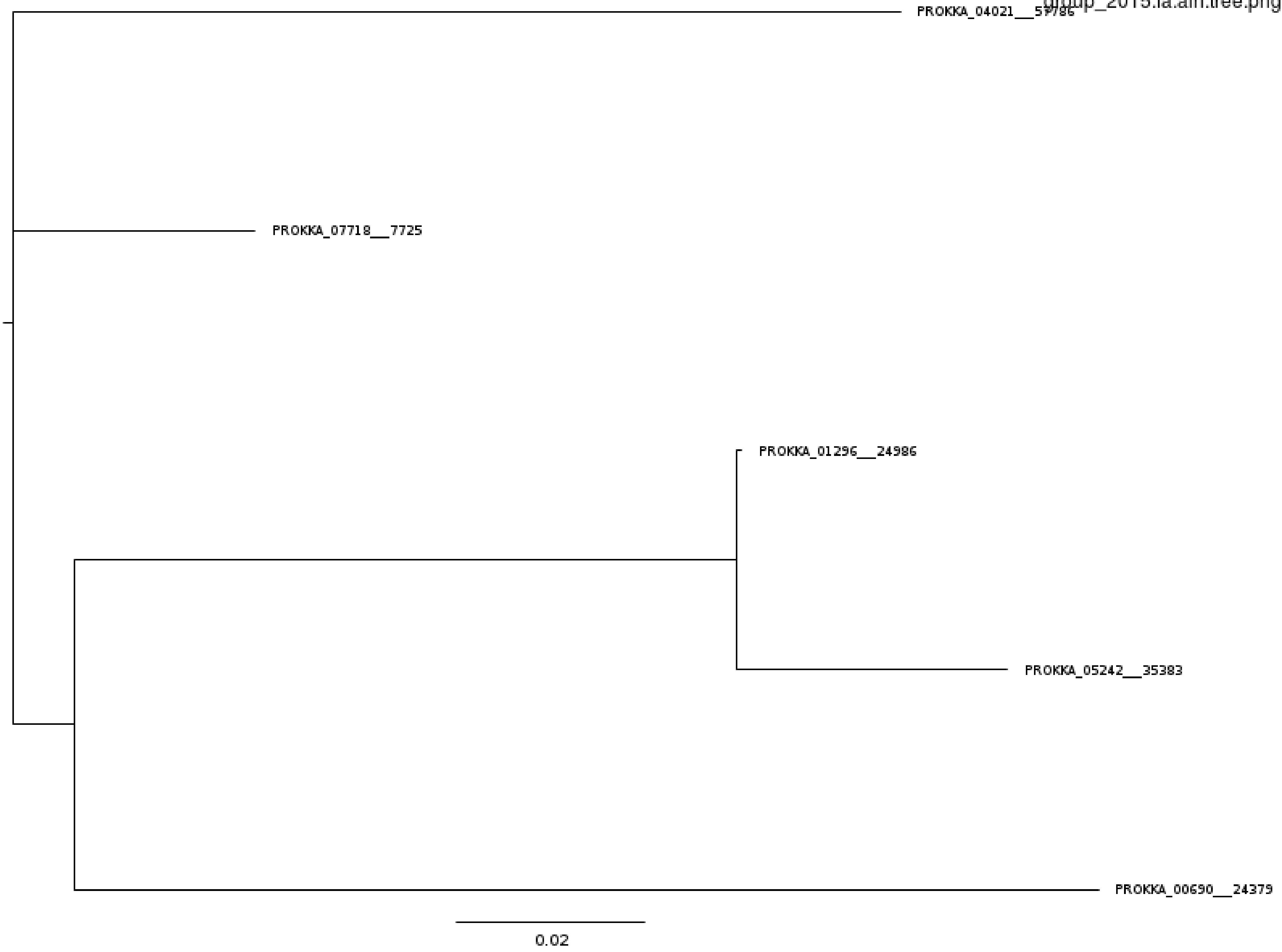

group_2017.fa.aln.tree.png

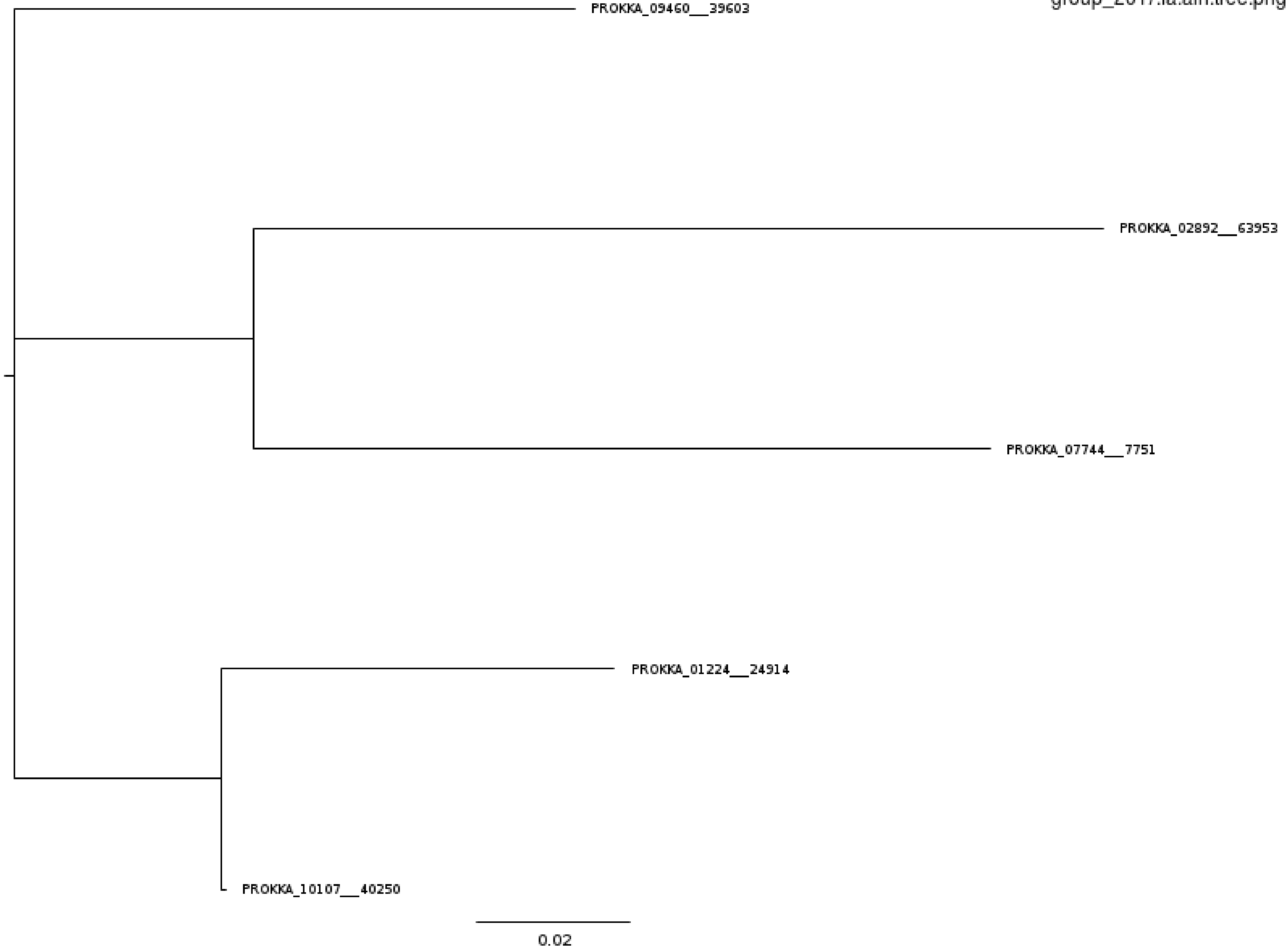

group_2018.fa.aln.tree.png

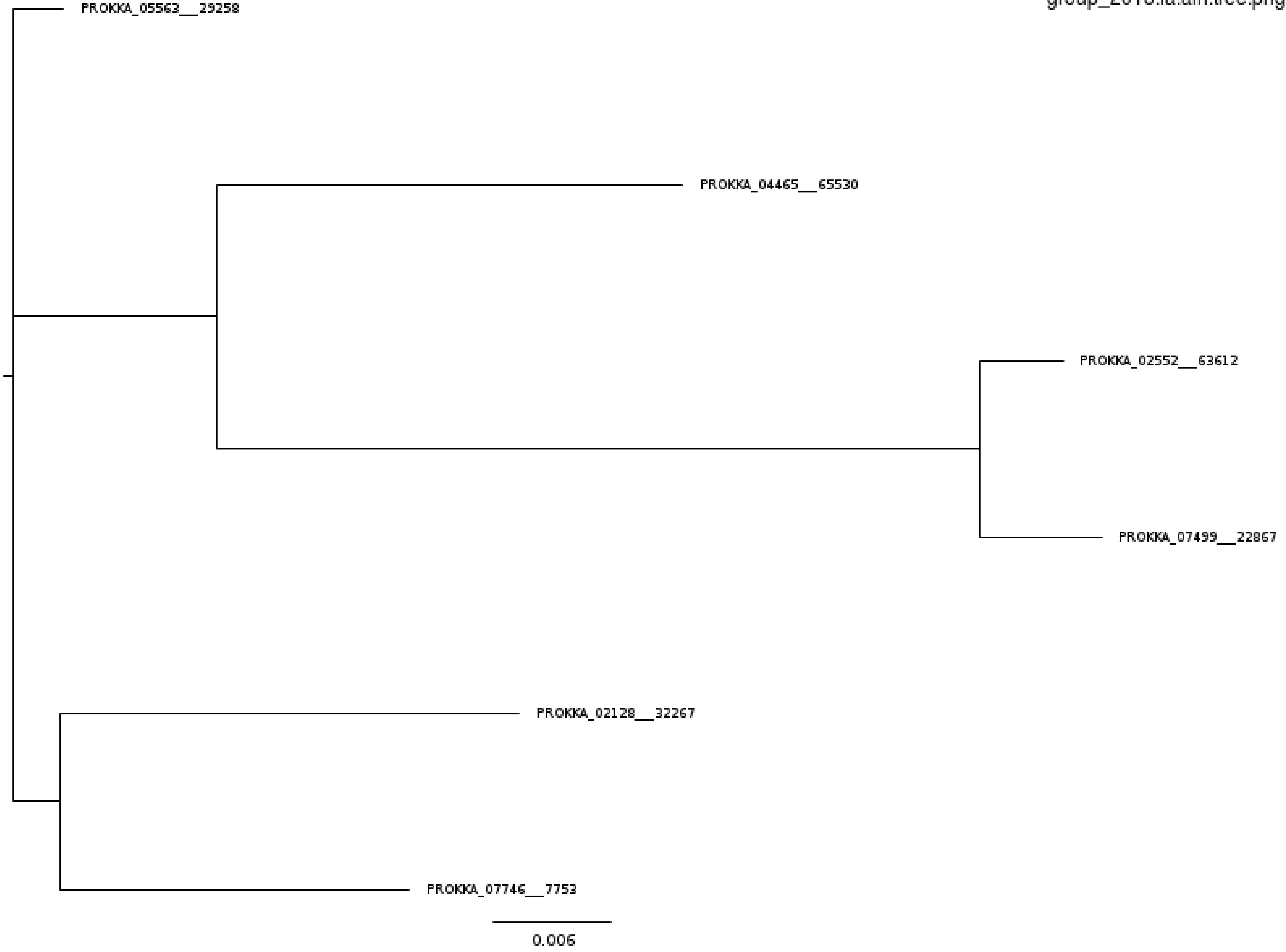

group_2019.fa.aln.tree.png

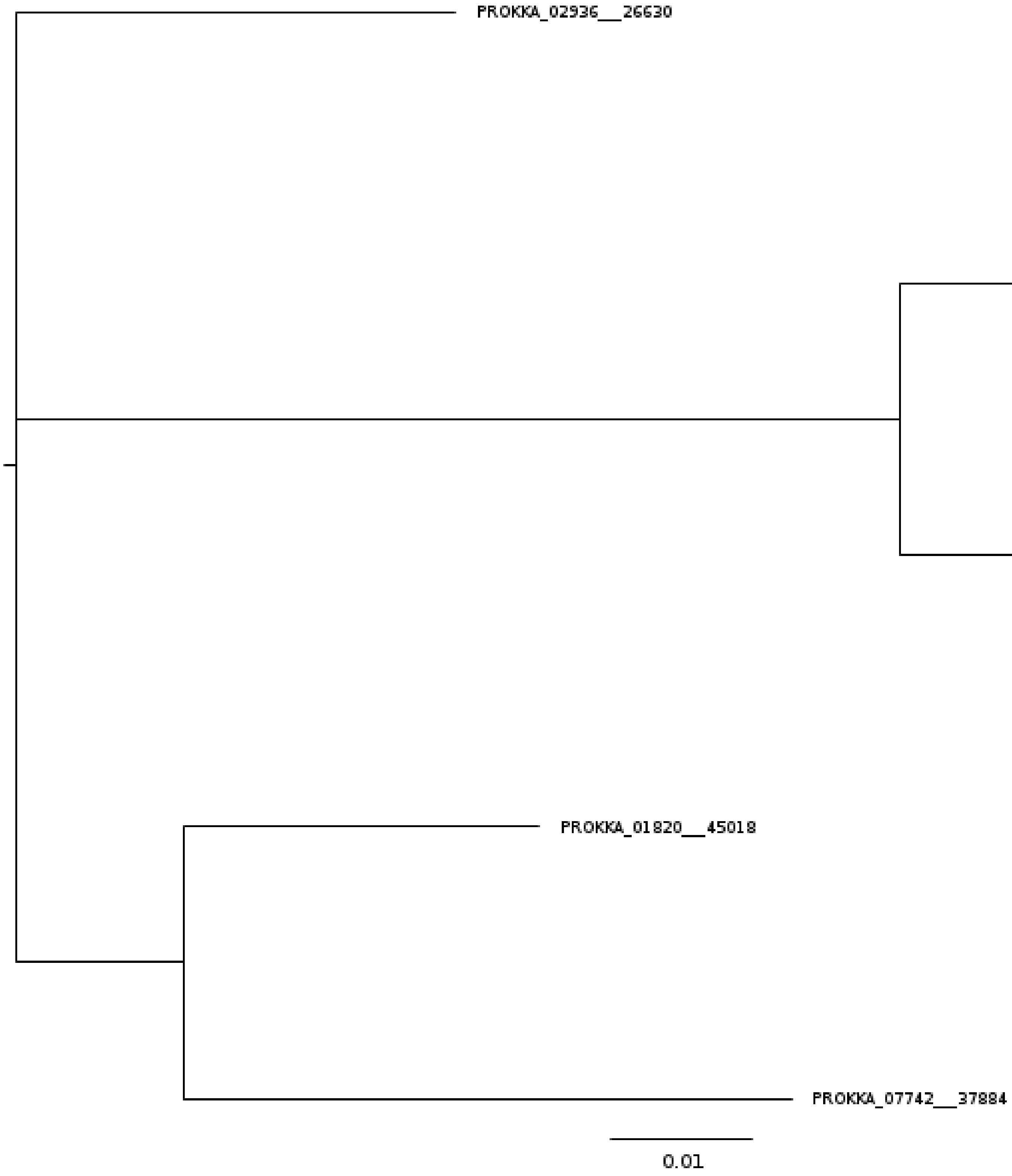

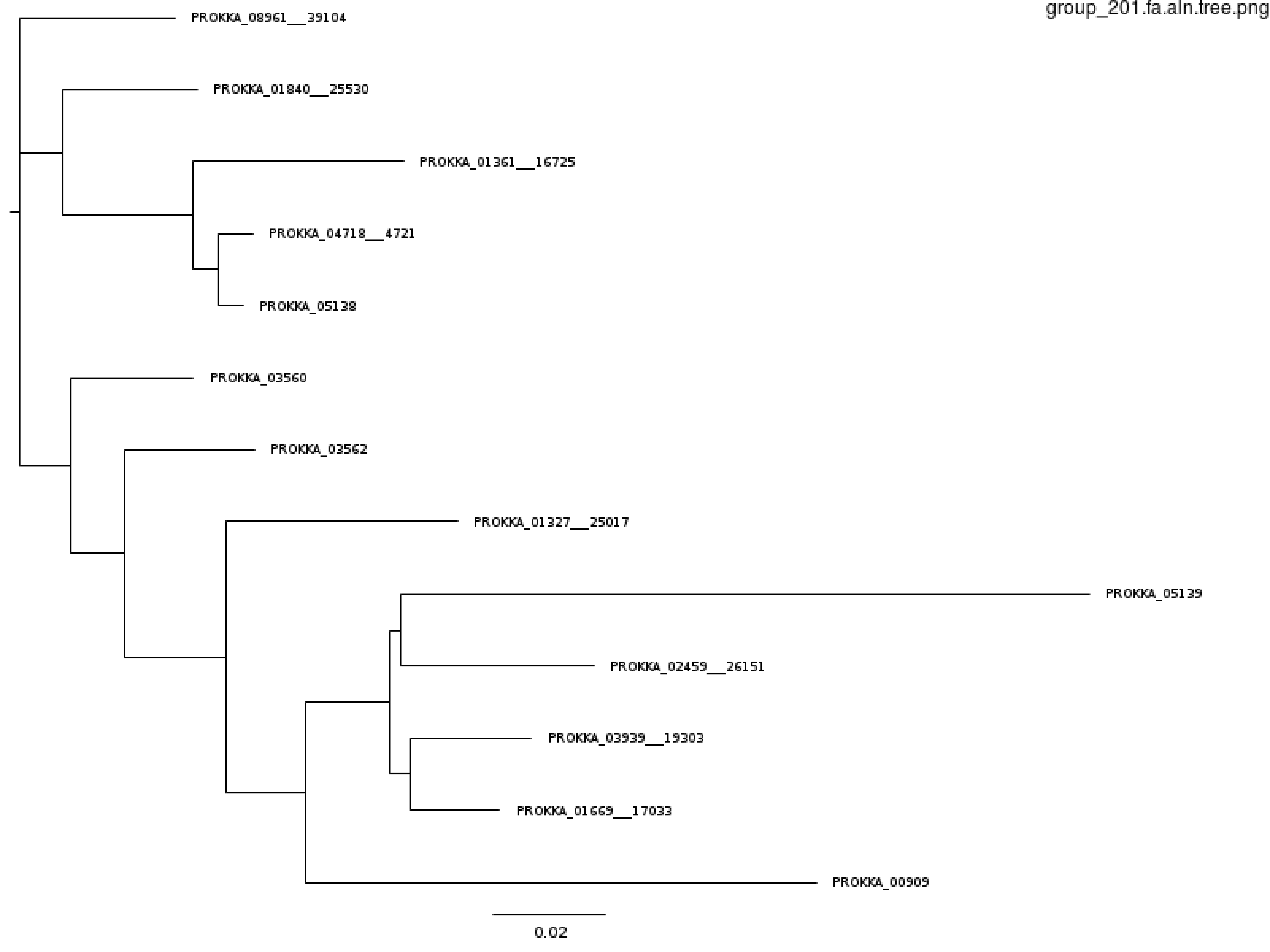
group_201.fa.aln.tree.png
PROKKA_08961___39104
PROKKA_01840___25530
PROKKA_01361___16725
PROKKA_04718___4721
PROKKA_05138
PROKKA_03560
PROKKA_03562
PROKKA_01327___25017
PROKKA_05139
PROKKA_02459___26151
PROKKA_03939___19303
PROKKA_01669___17033
PROKKA_00909
0.02

group_2020.fa.aln.tree.png

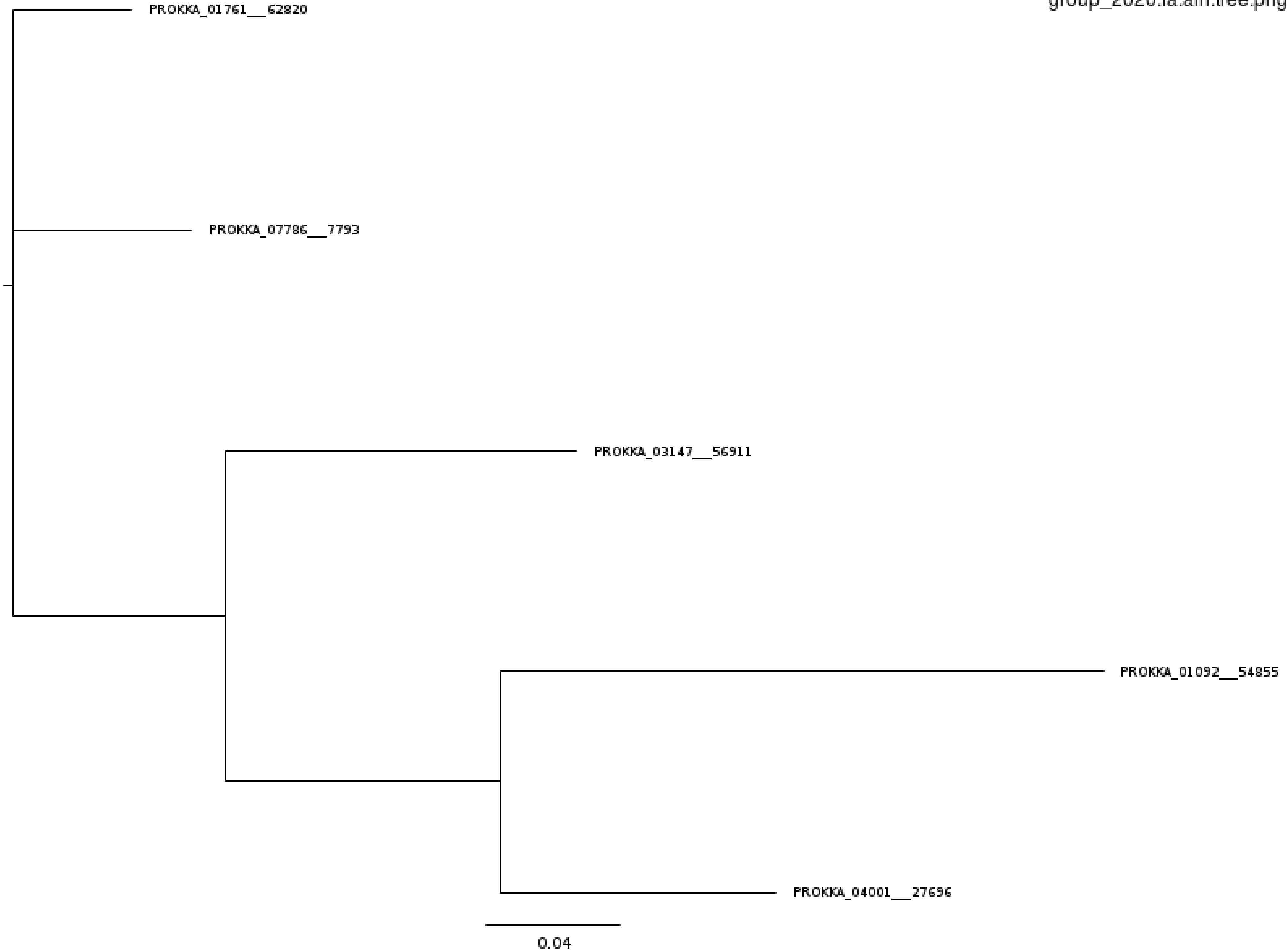

group_2021.fa.aln.tree.png

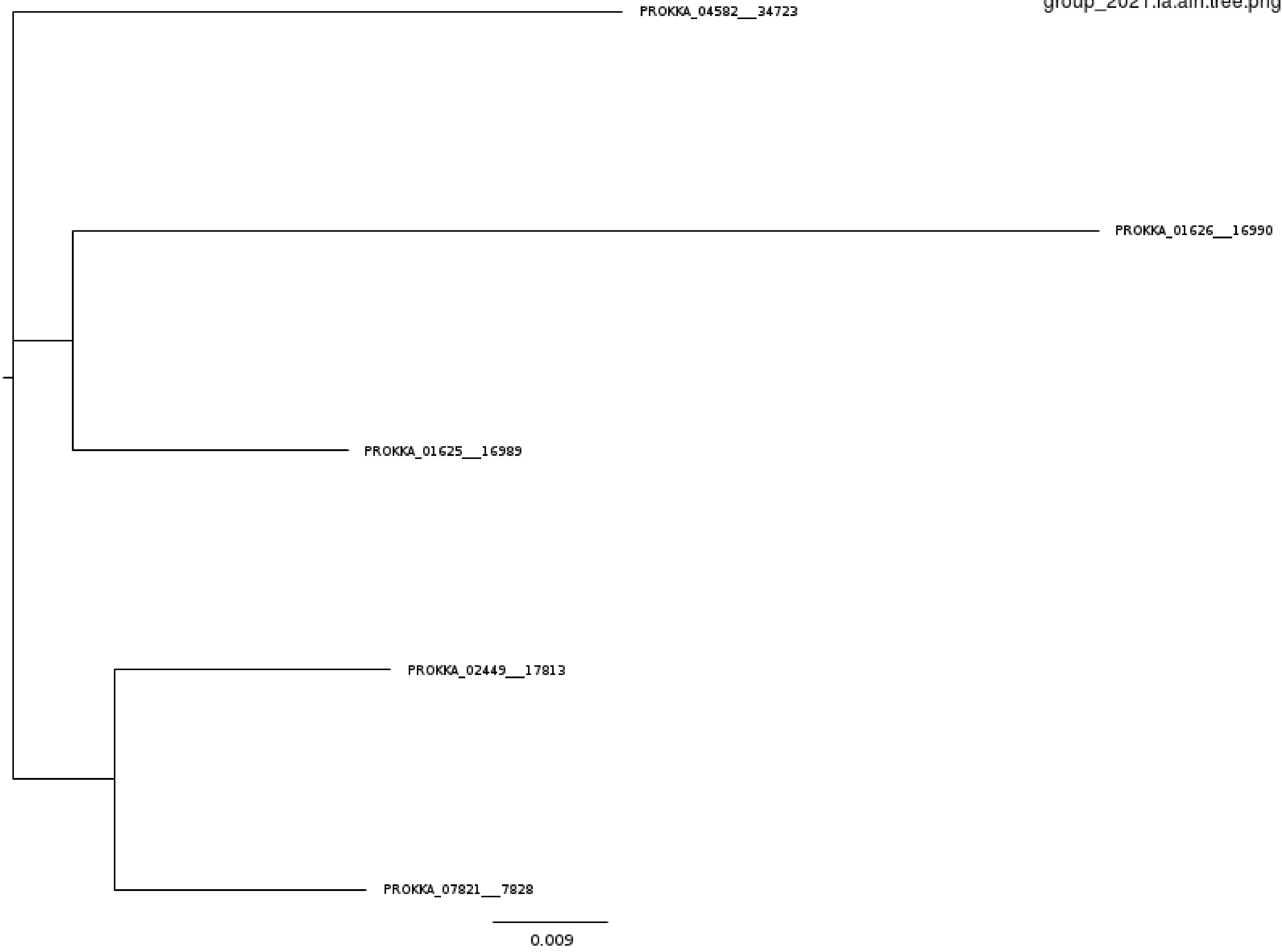

group_2022.fa.aln.tree.png

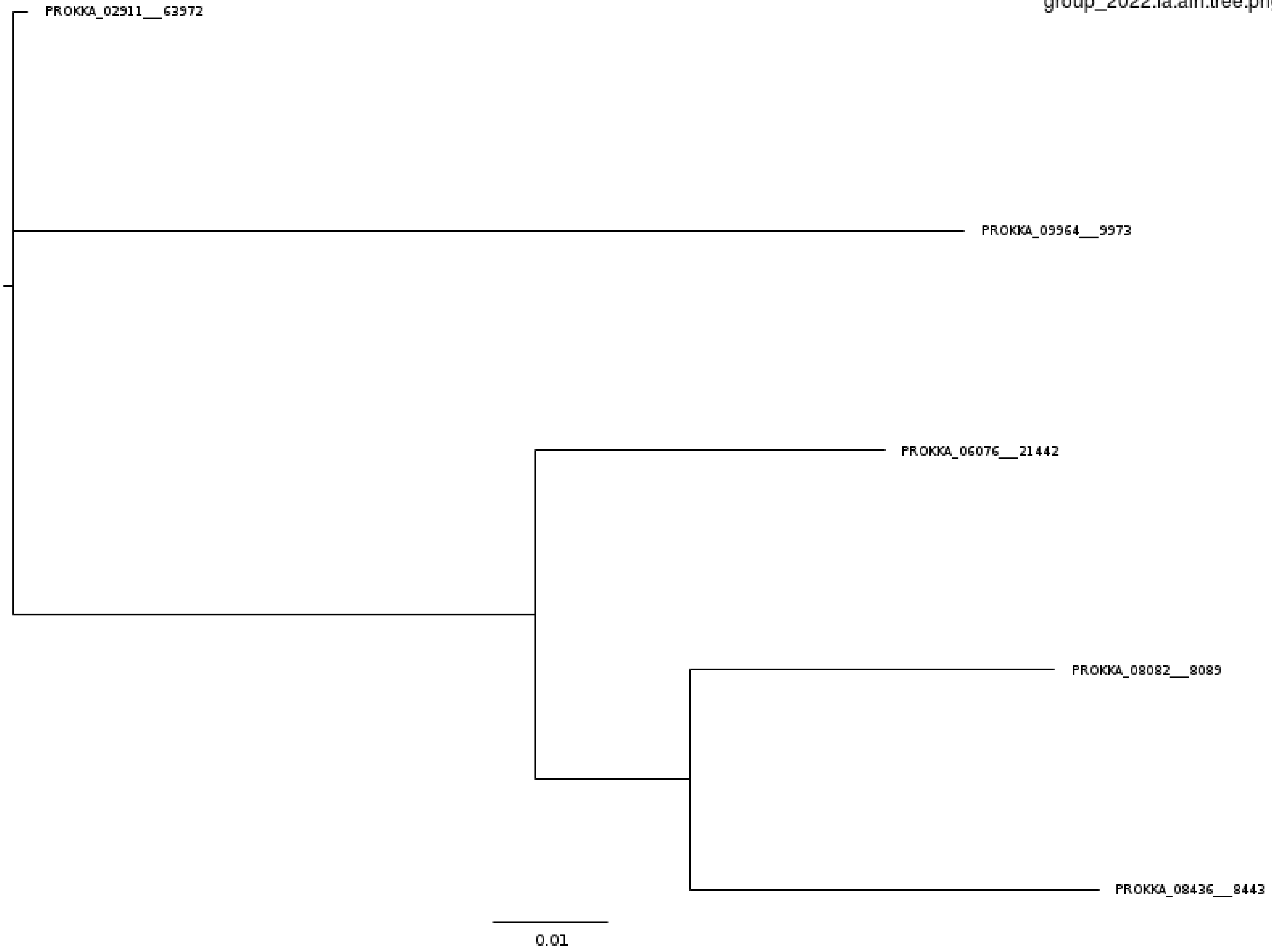

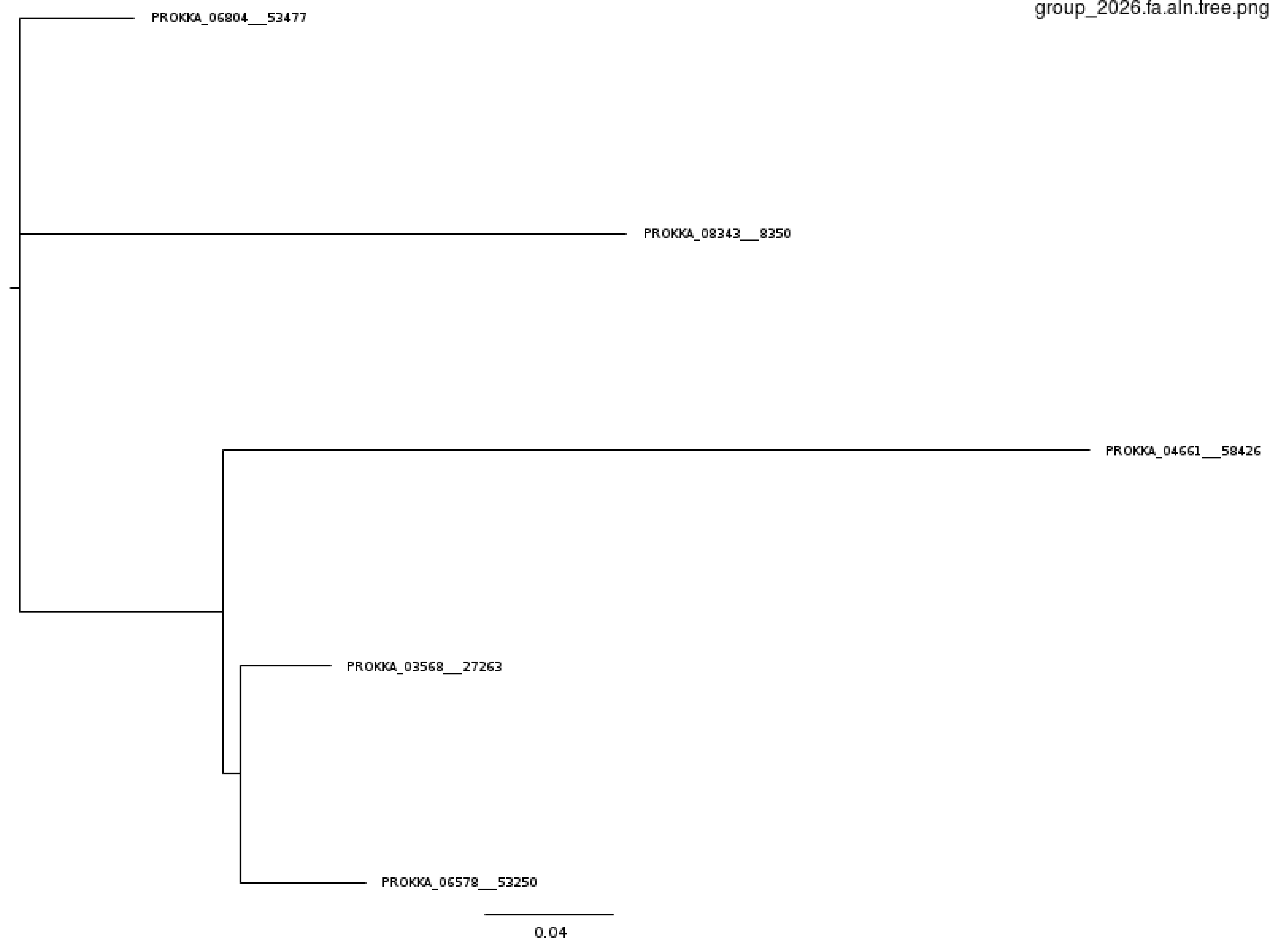
group_2026.fa.aln.tree.png
PROKKA_06804___53477
PROKKA_08343___8350
PROKKA_04661___58426
PROKKA_03568___27263
PROKKA_06578___53250
0.04

group_2027.fa.aln.tree.png

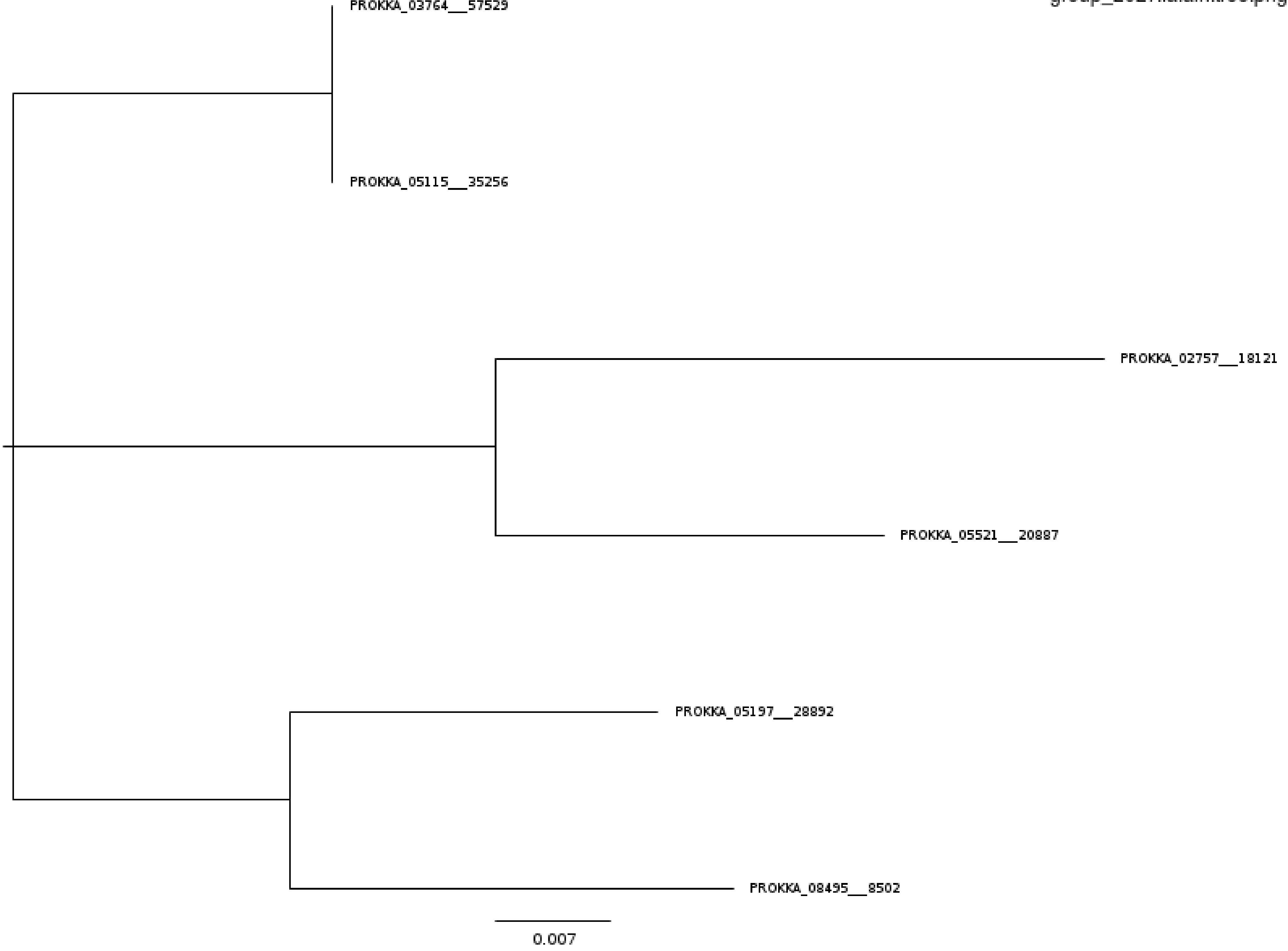

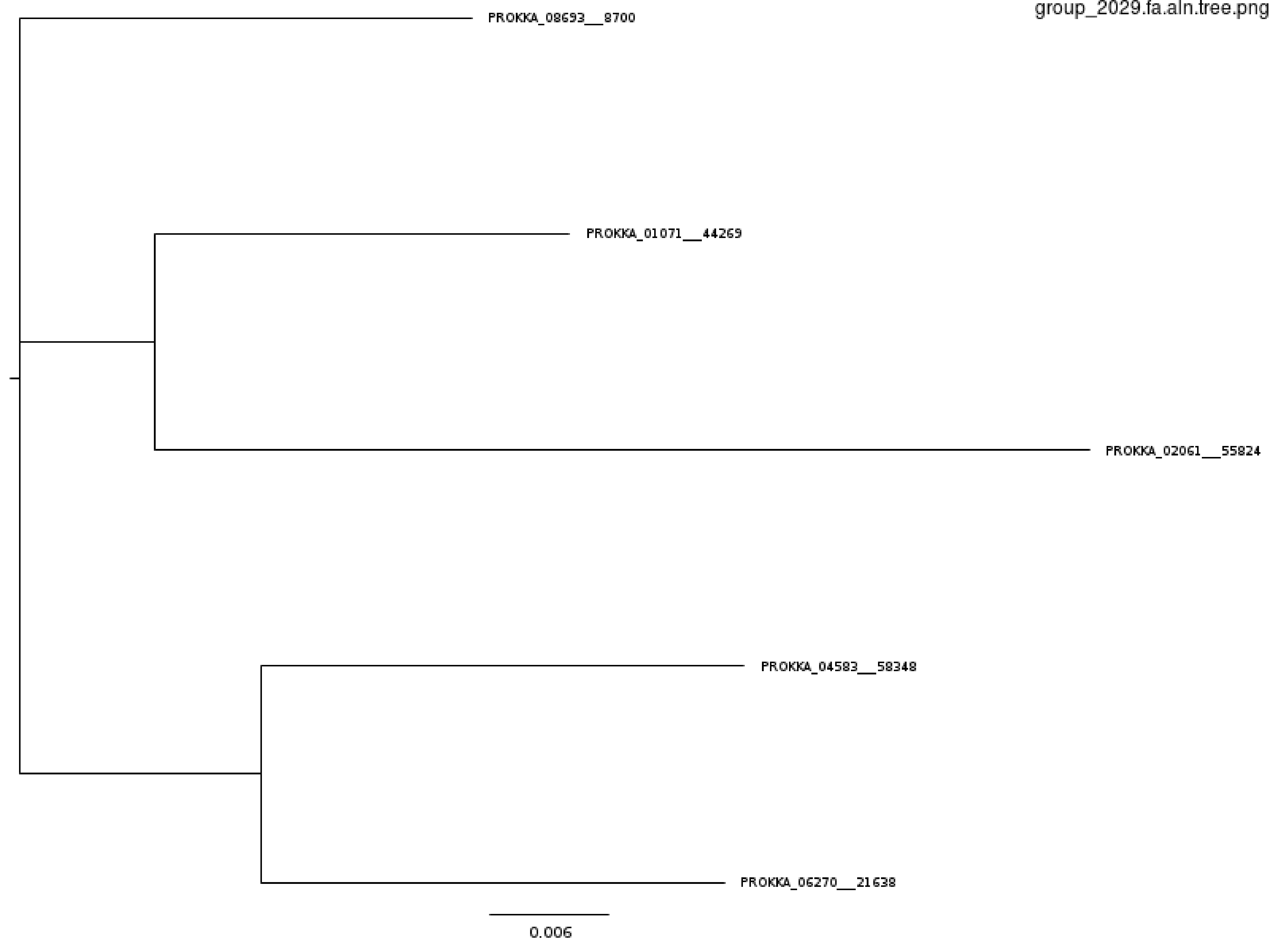
group_2029.fa.aln.tree.png
PROKKA_08693___8700
PROKKA_01071___44269
PROKKA_02061___55824
PROKKA_04583___58348
PROKKA_06270___21638
0.006

group_2034.fa.aln.tree.png

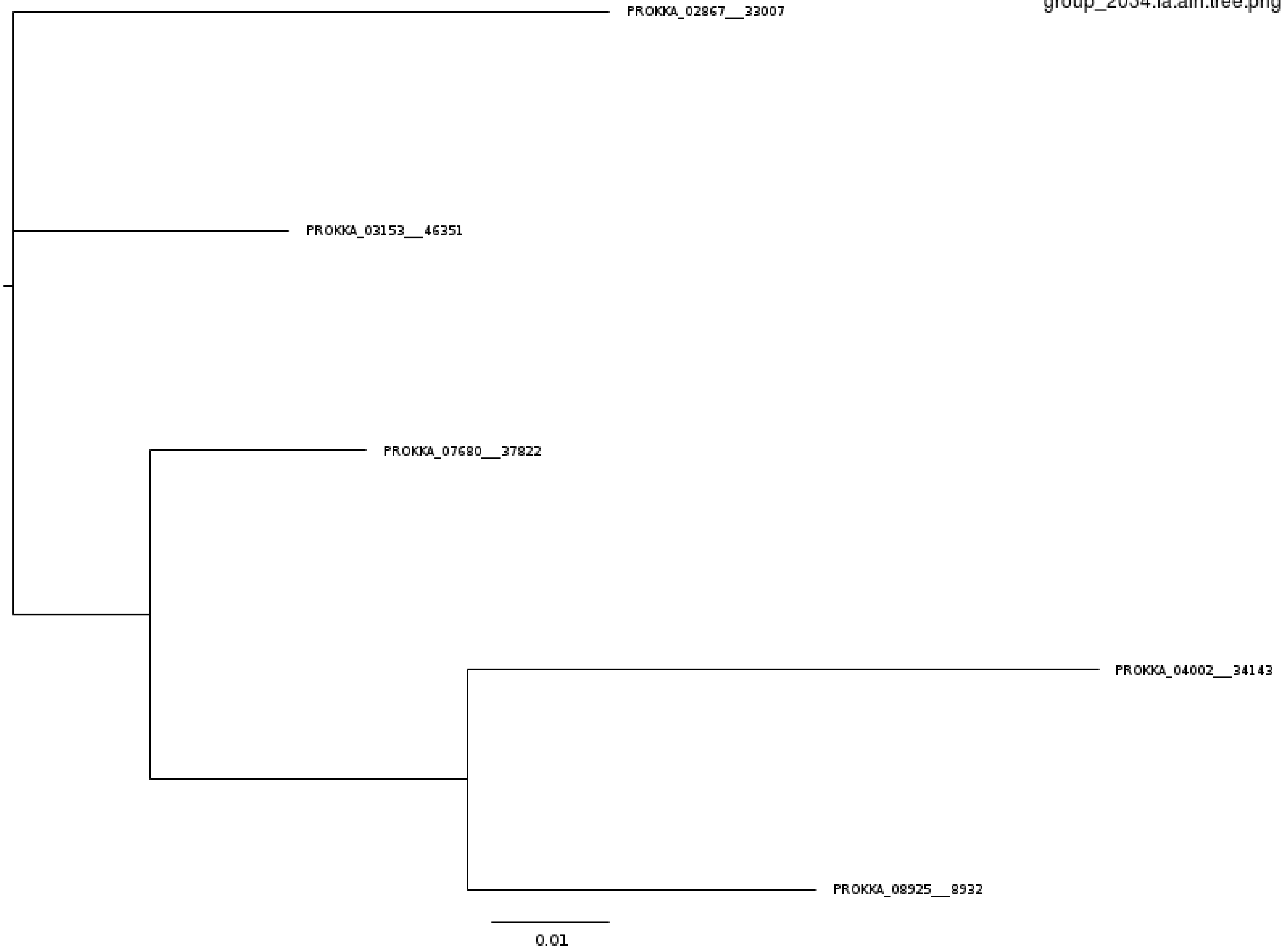

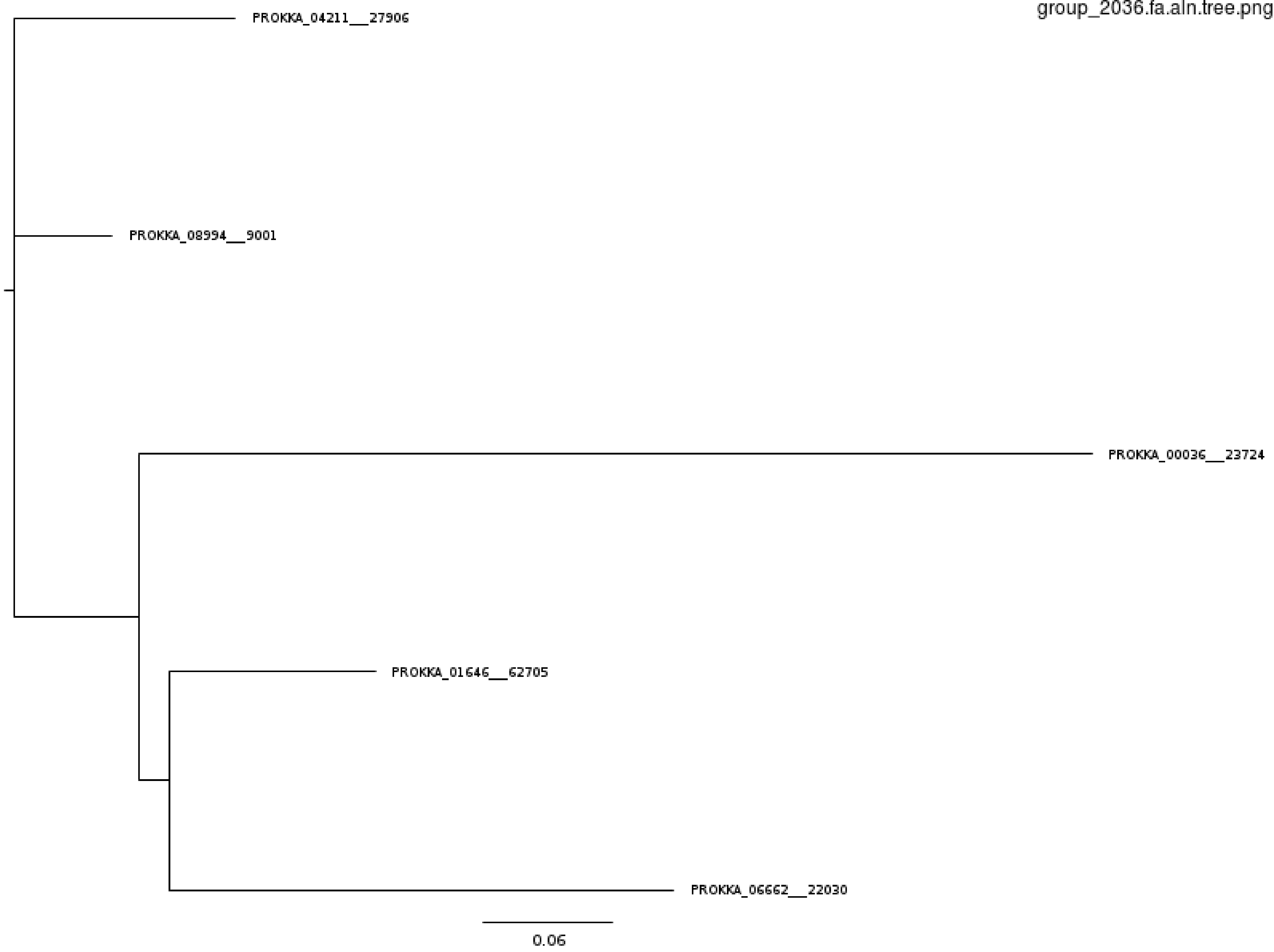
group_2036.fa.aln.tree.png
PROKKA_04211__27906
PROKKA_08994__9001
PROKKA_00036__23724
PROKKA_01646__62705
PROKKA_06662__22030
0.06

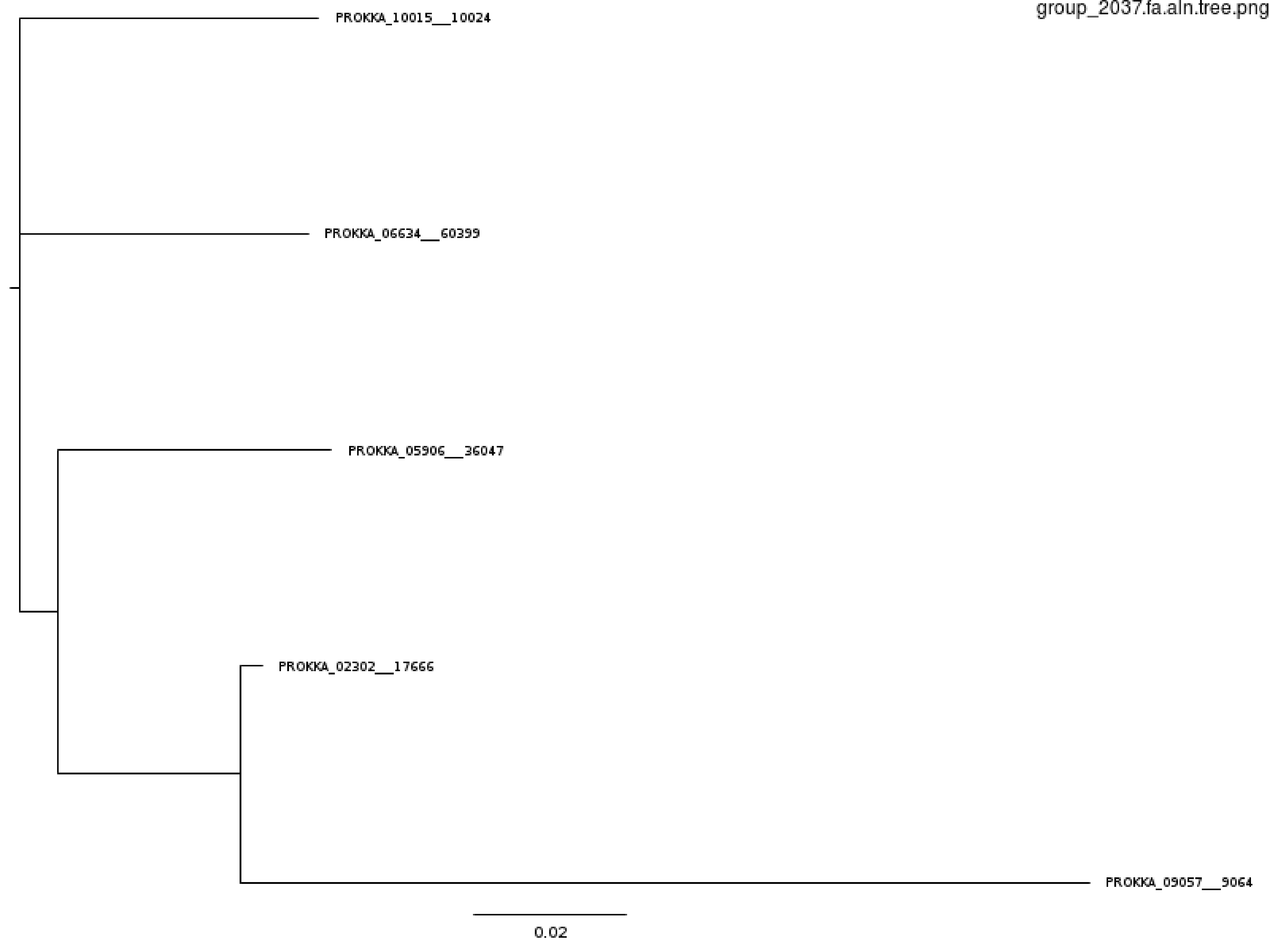
group_2037.fa.aln.tree.png
PROKKA_10015___10024
PROKKA_06634___60399
PROKKA_05906___36047
PROKKA_02302___17666
PROKKA_09057___9064
0.02

group_2038.fa.aln.tree.png

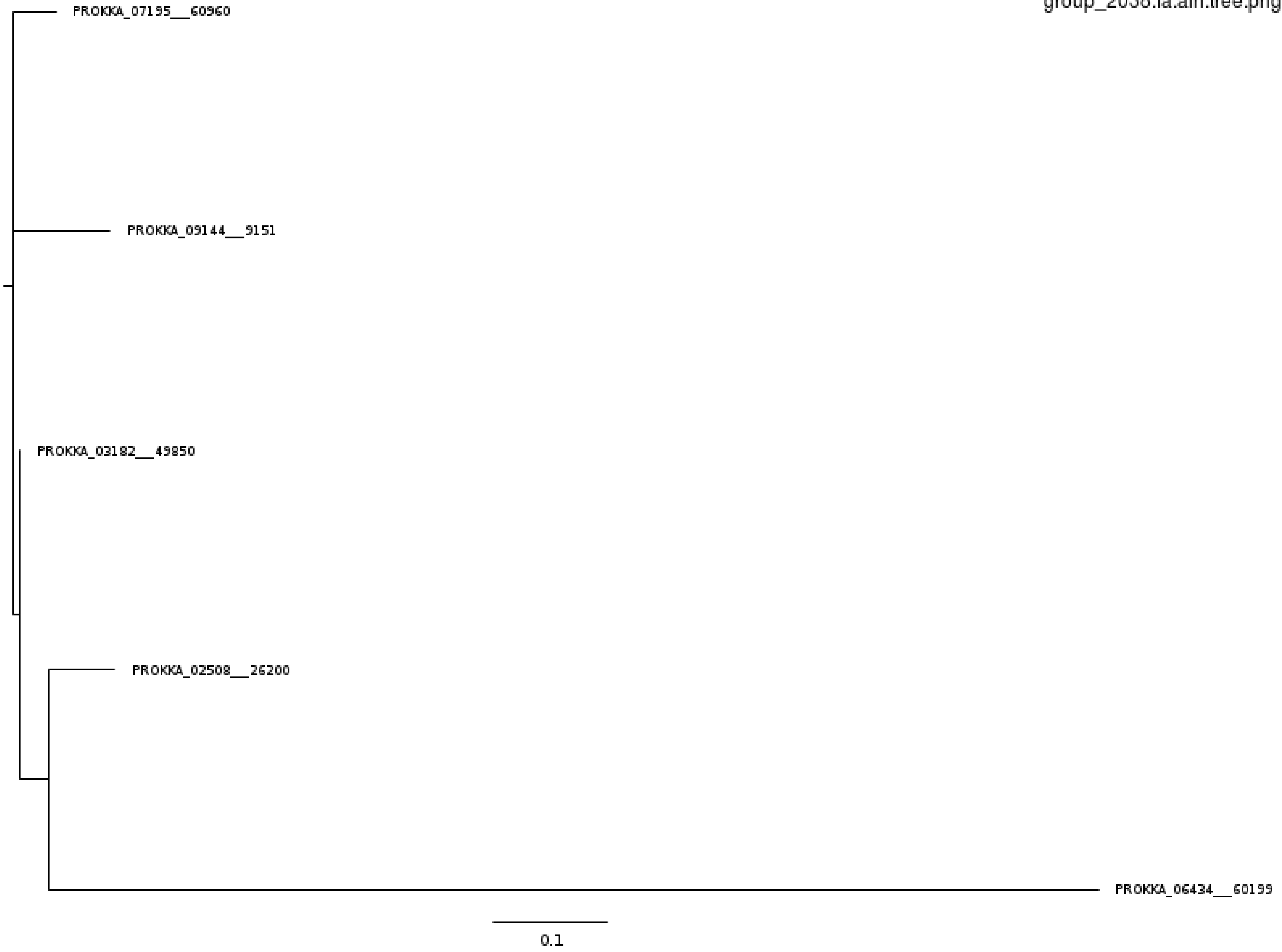

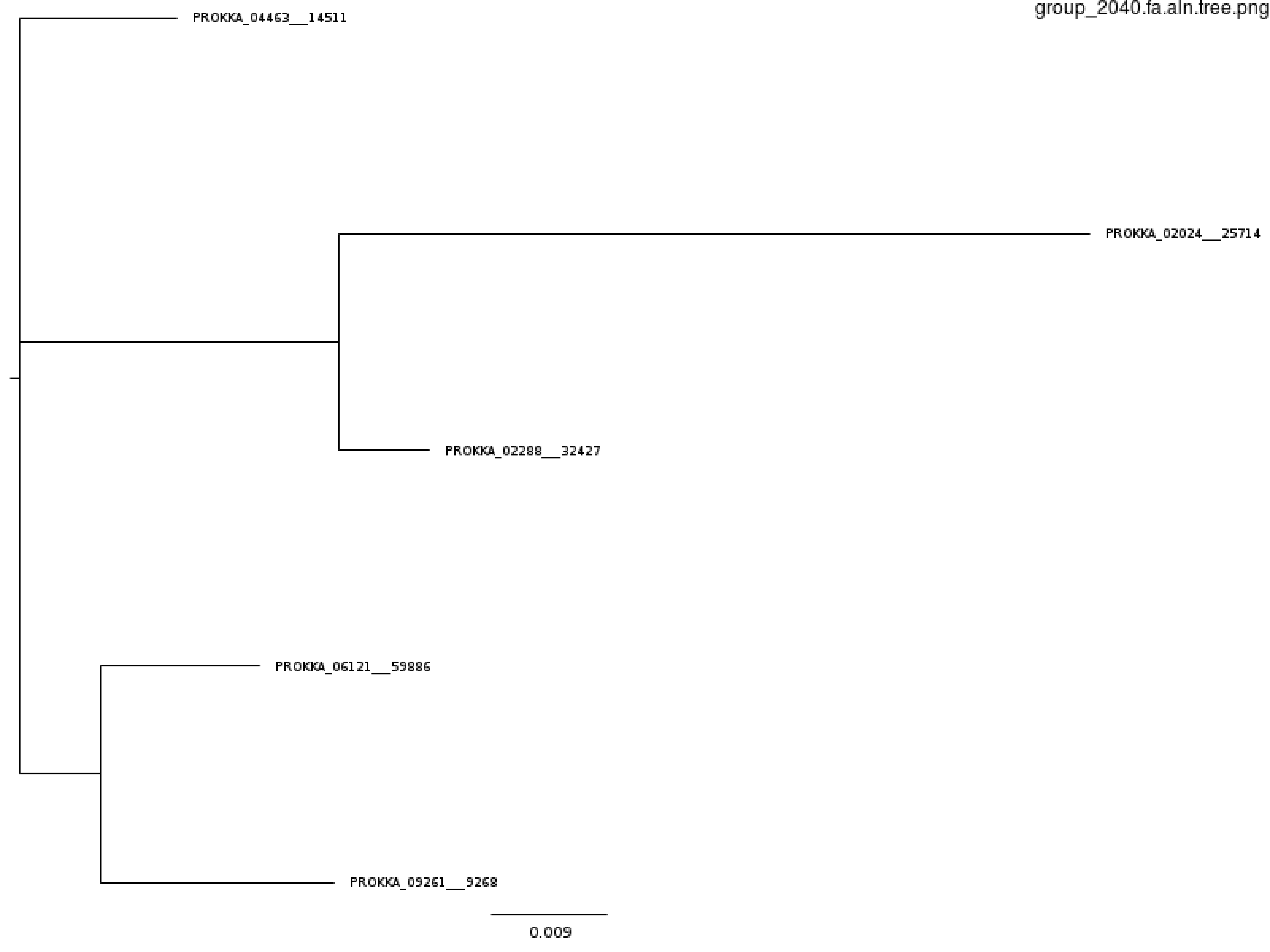
group_2040.fa.aln.tree.png
PROKKA_04463___14511
PROKKA_02024___25714
PROKKA_02288___32427
PROKKA_06121___59886
PROKKA_09261___9268
0.009

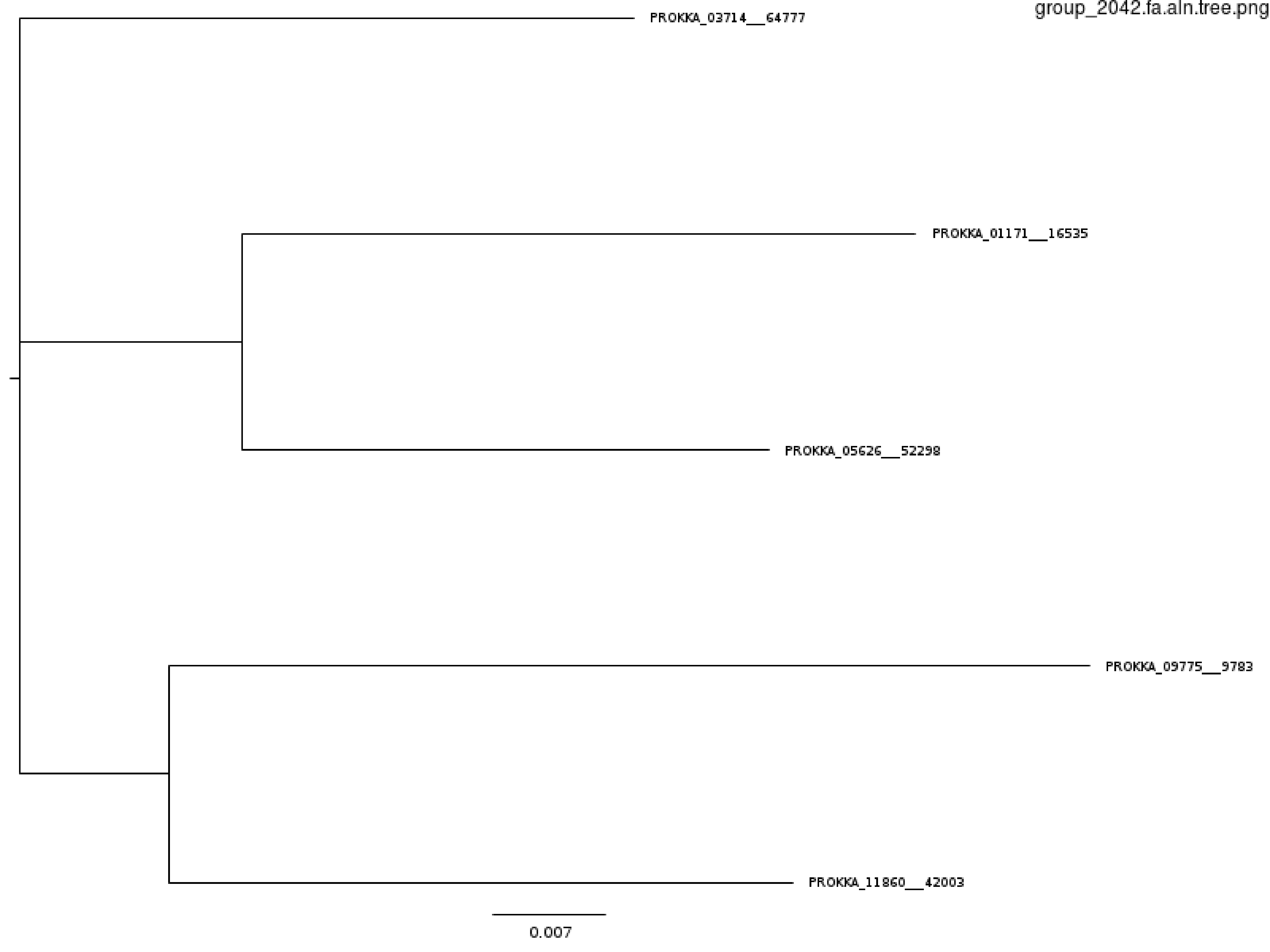
group_2042.fa.aln.tree.png
PROKKA_03714___64777
PROKKA_01171___16535
PROKKA_05626___52298
PROKKA_09775___9783
PROKKA_11860___42003
0.007

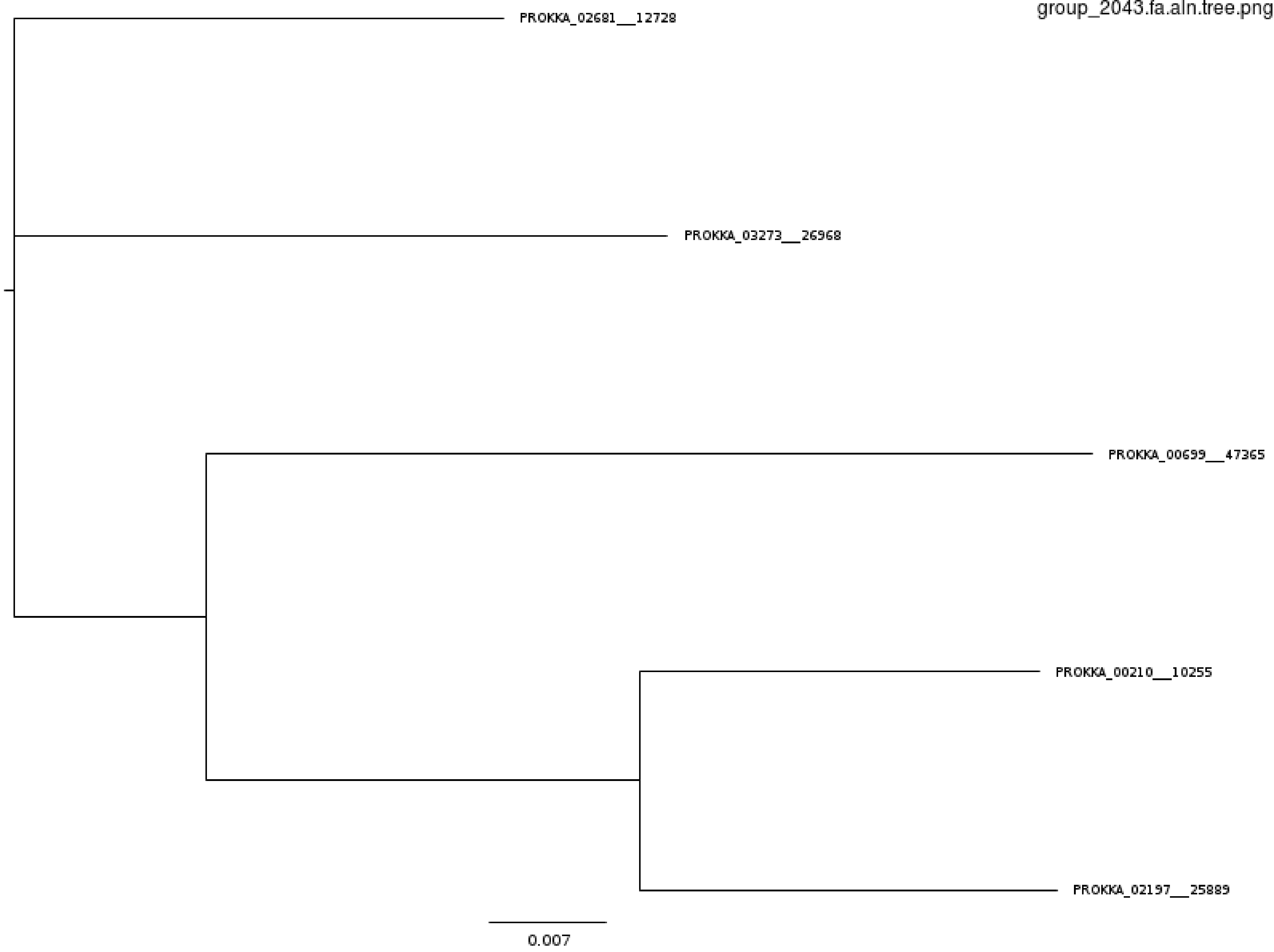
group_2043.fa.aln.tree.png
PROKKA_02681___12728
PROKKA_03273___26968
PROKKA_00699___47365
PROKKA_00210___10255
PROKKA_02197___25889
0.007

group_2046.fa.aln.tree.png

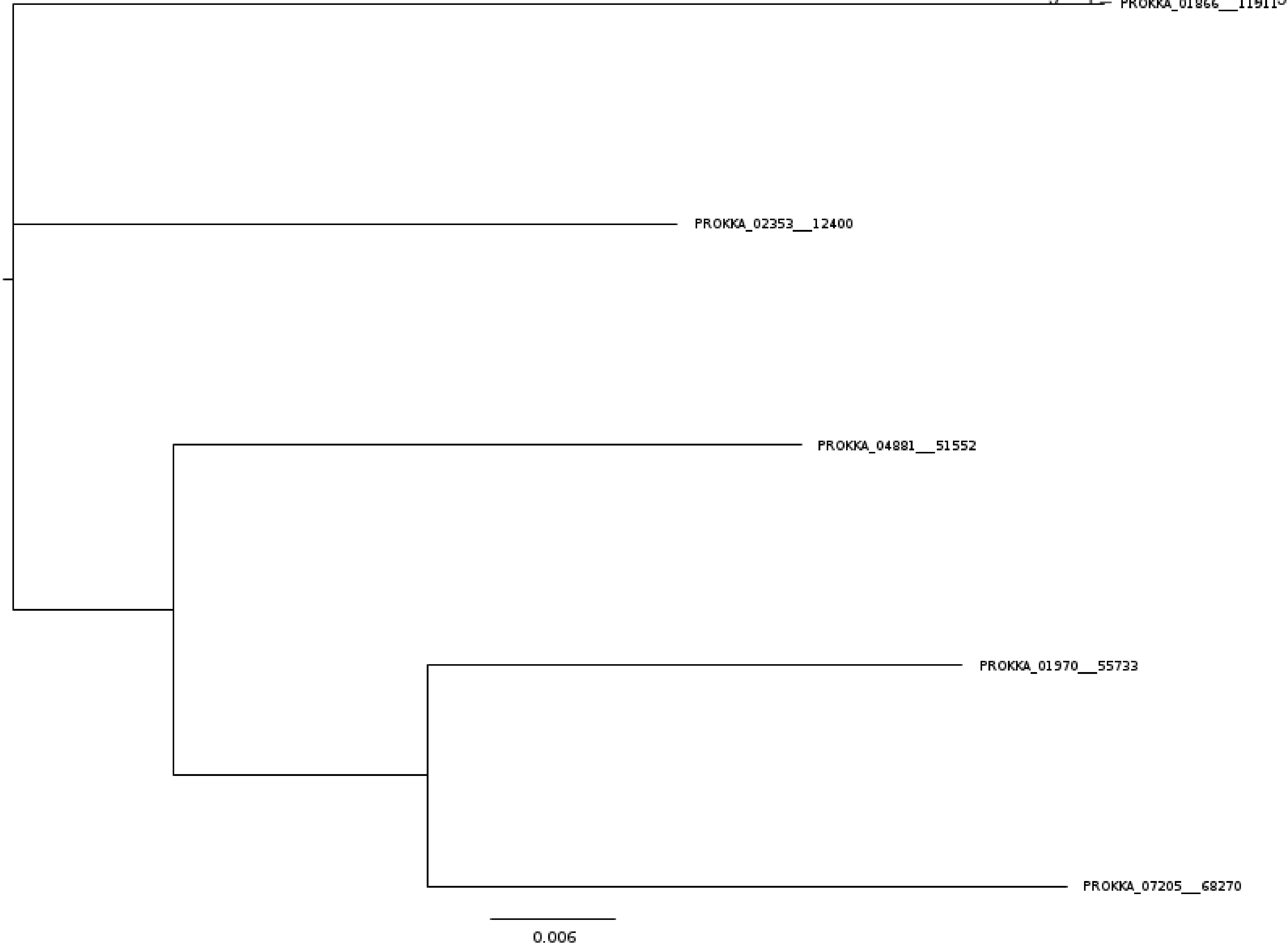

group_2047.fa.aln.tree.png

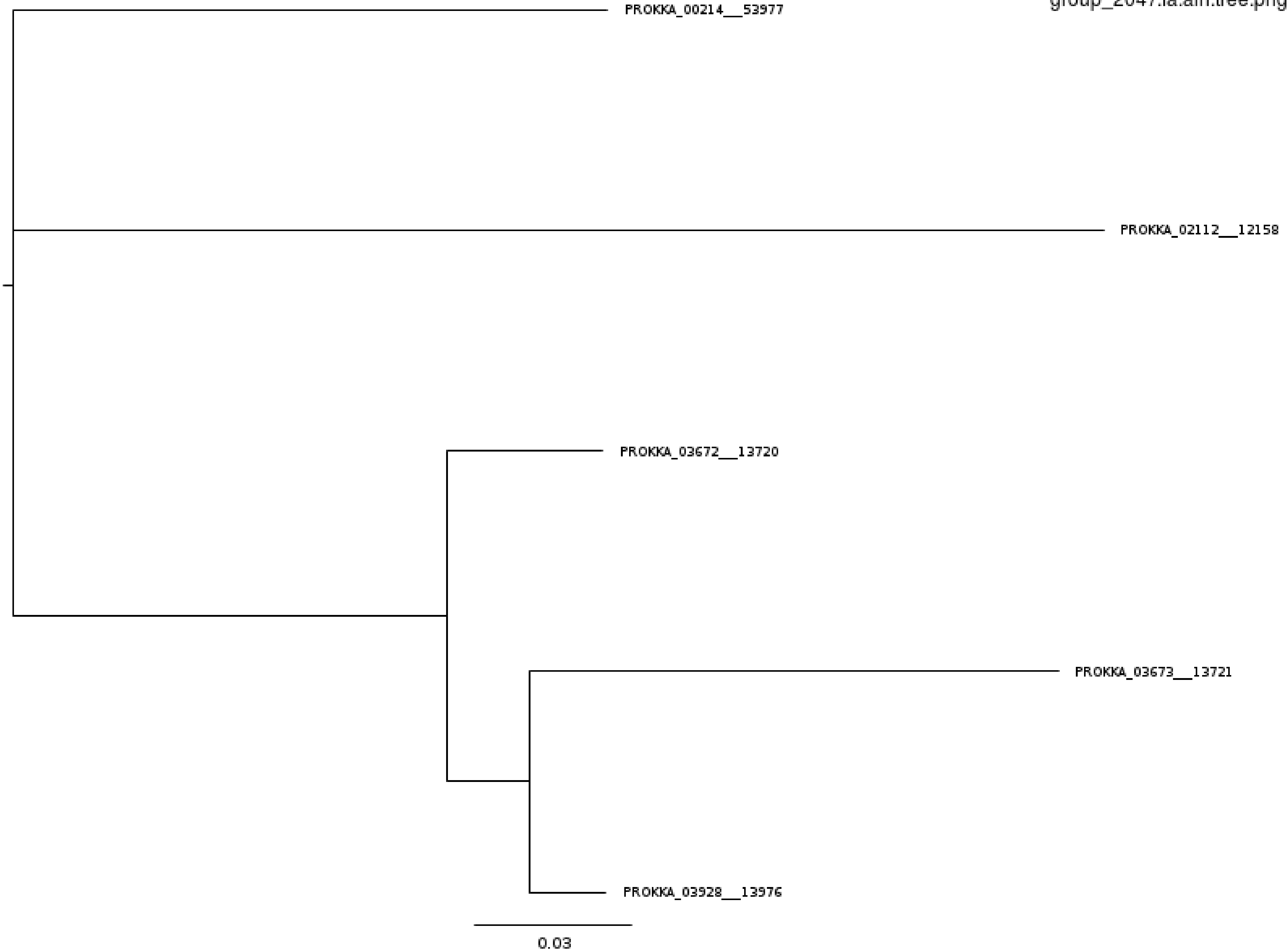

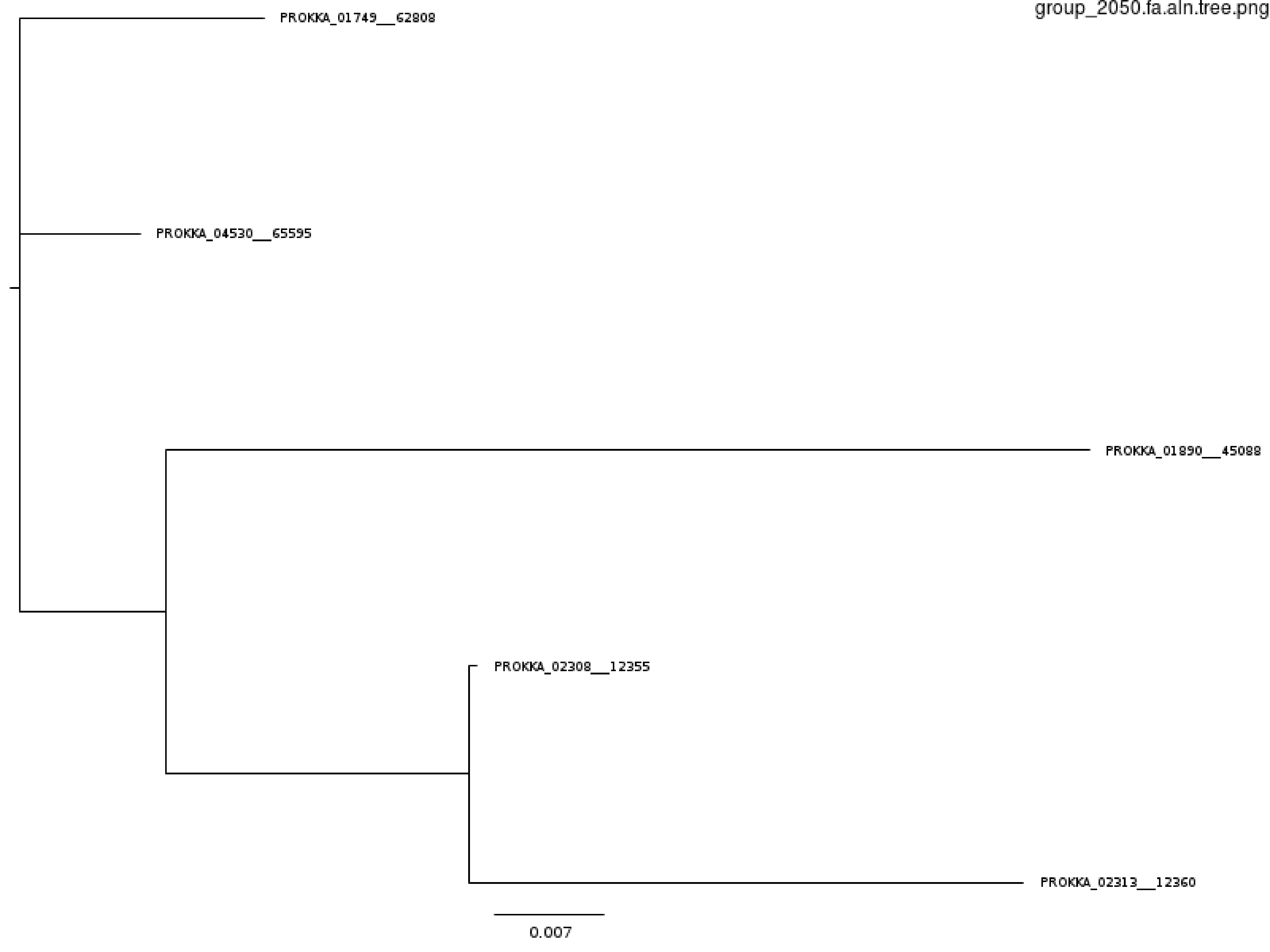
group_2050.fa.aln.tree.png
PROKKA_01749___62808
PROKKA_04530___65595
PROKKA_01890___45088
PROKKA_02308___12355
PROKKA_02313___12360
0.007

group_2055.fa.aln.tree.png

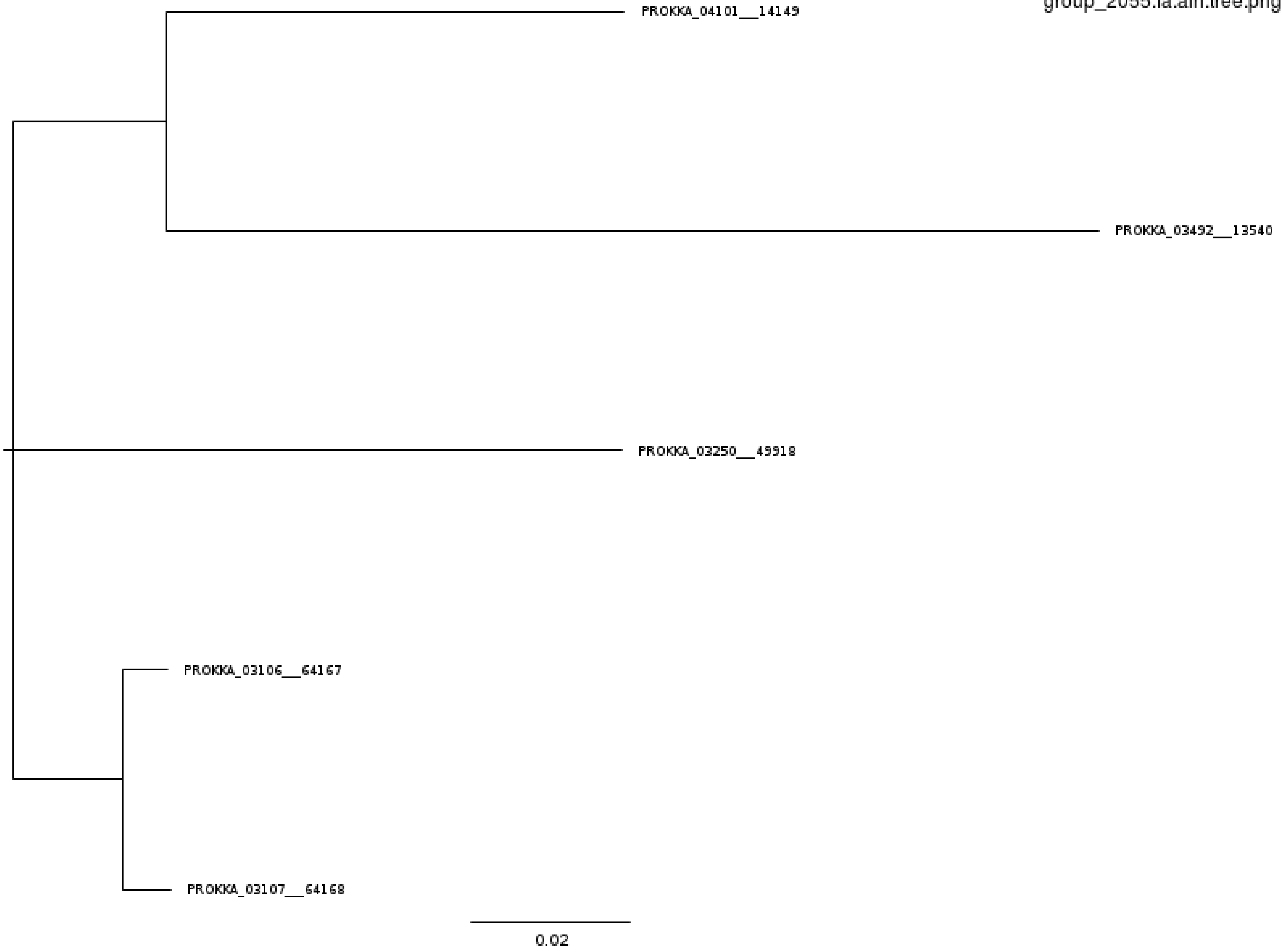

group_2056.fa.aln.tree.png

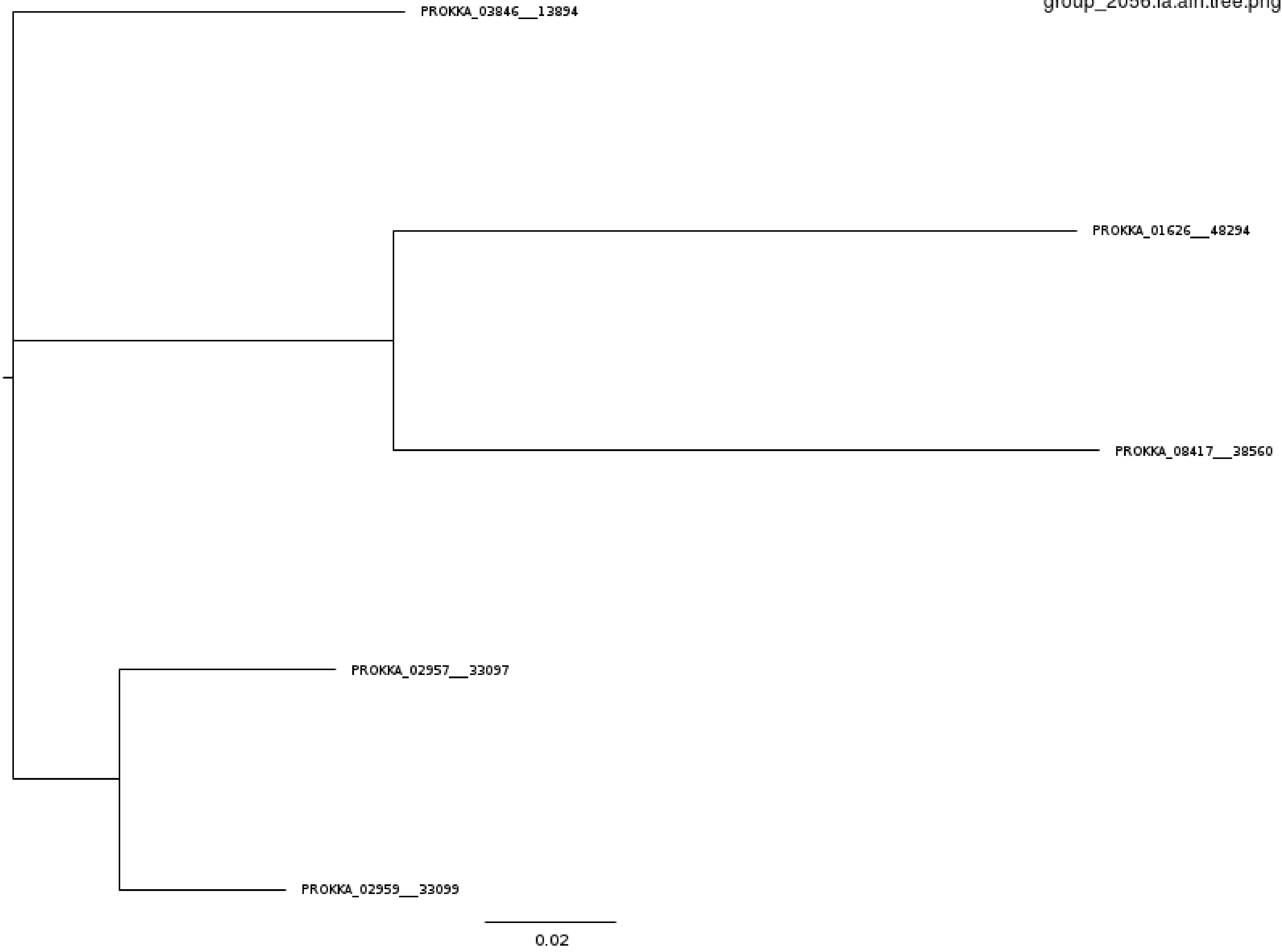

group_2057.fa.aln.tree.png

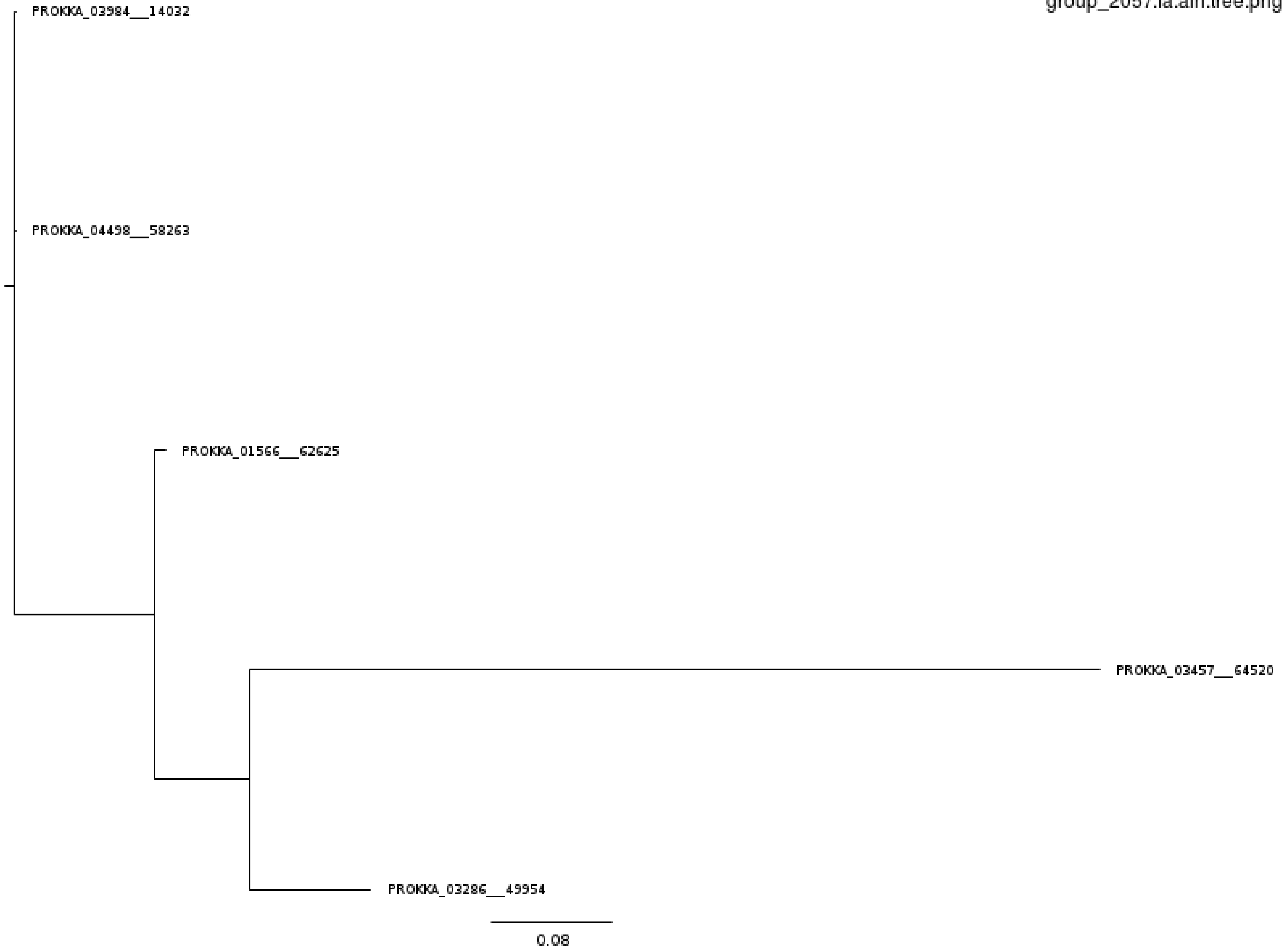

group_2061.fa.aln.tree.png

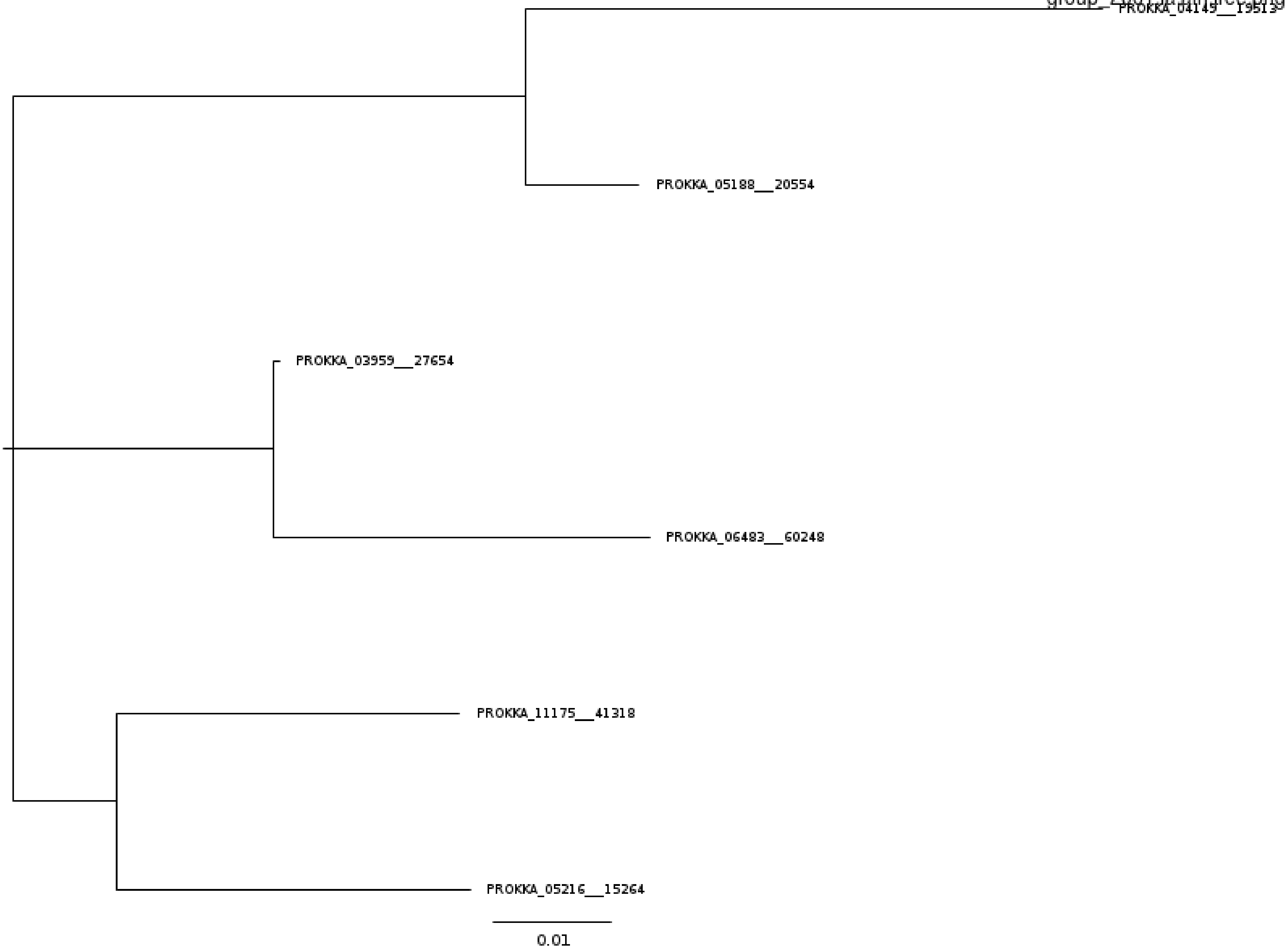

group_2063.fa.aln.tree.png

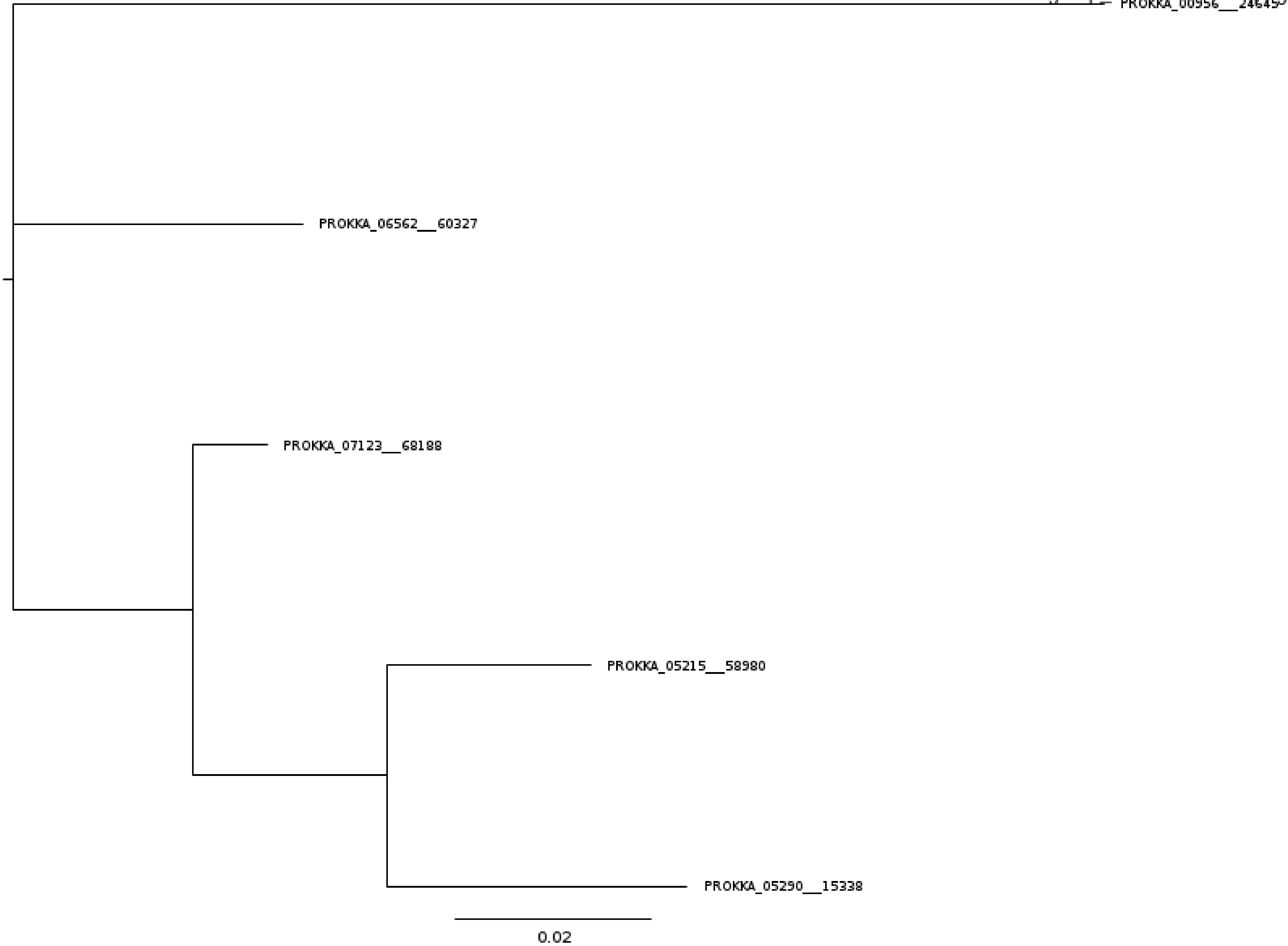

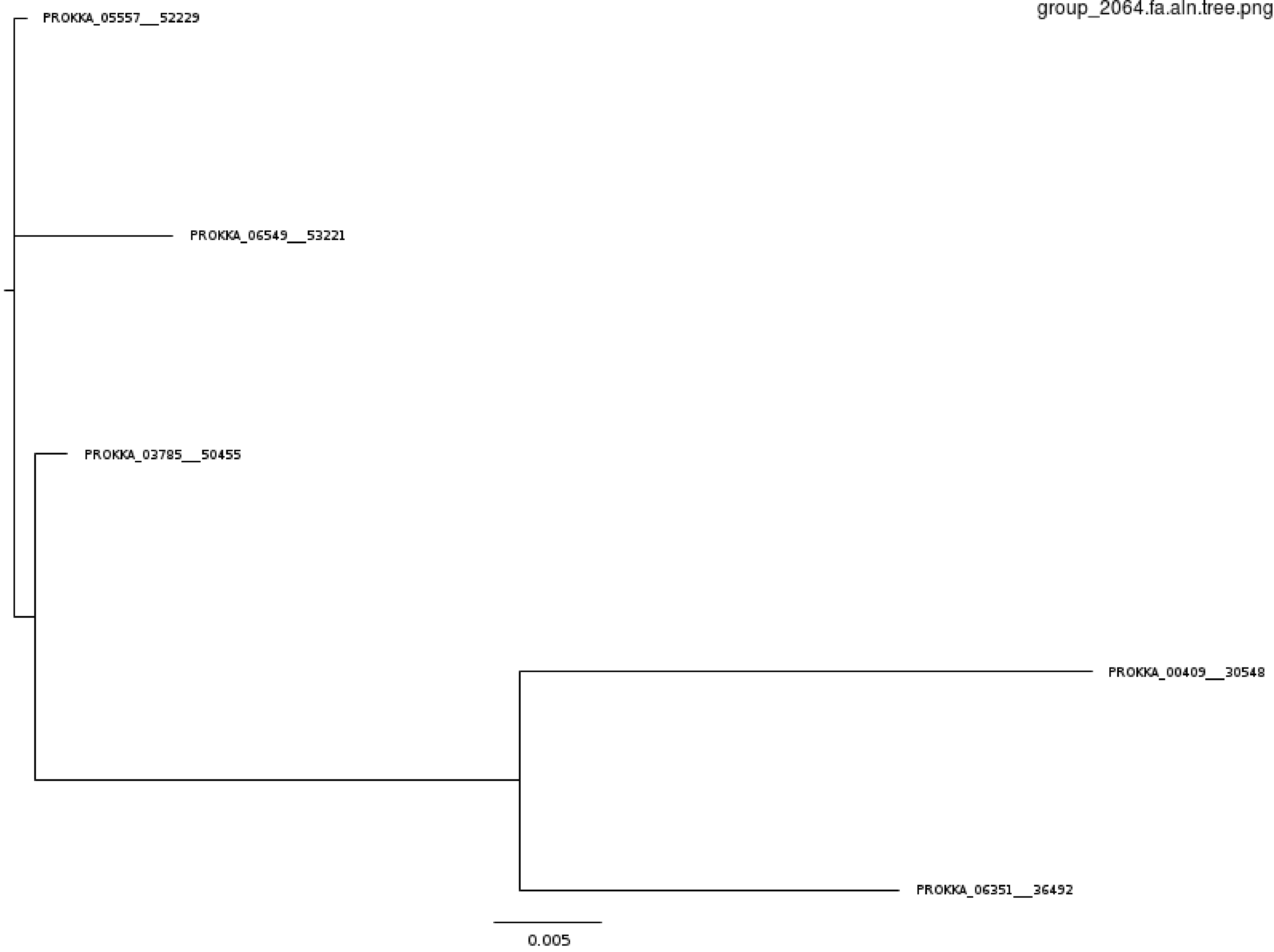

group_2064.fa.aln.tree.png
PROKKA_05557___52229
PROKKA_06549___53221
PROKKA_03785___50455
PROKKA_00409___30548
PROKKA_06351___36492
0.005

group_2065.fa.aln.tree.png

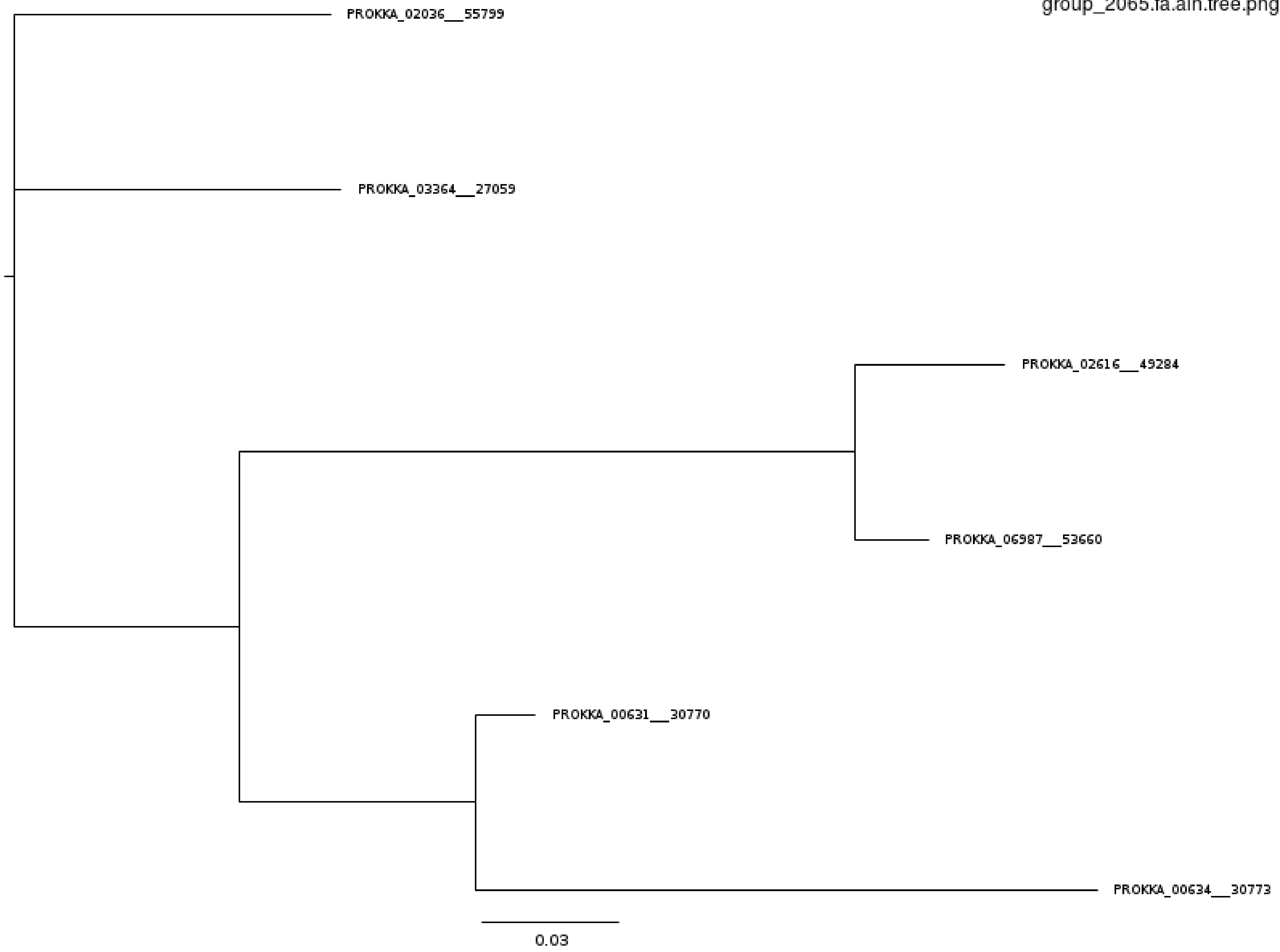

group_2066.fa.aln.tree.png

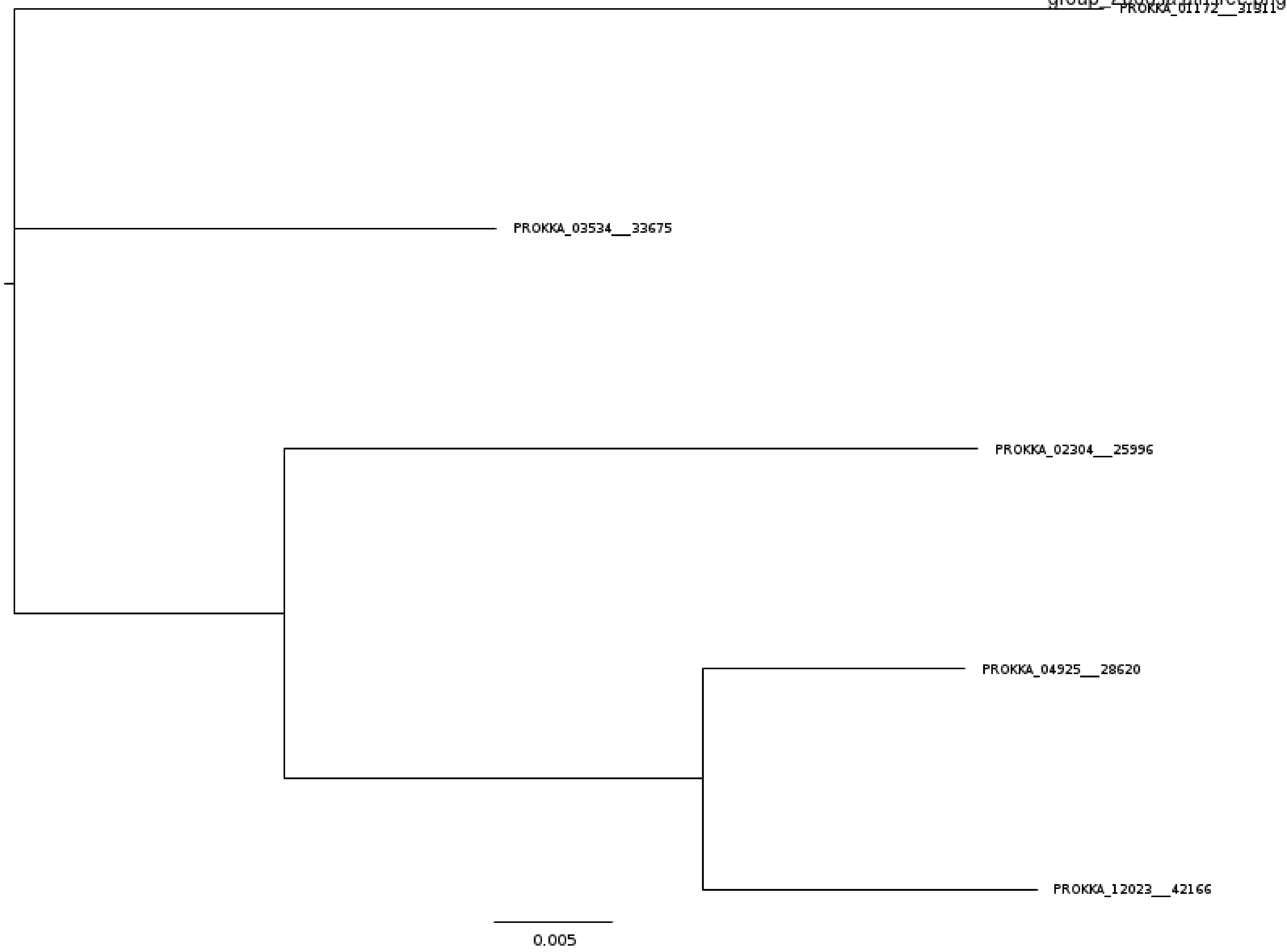

group_2067.fa.aln.tree.png

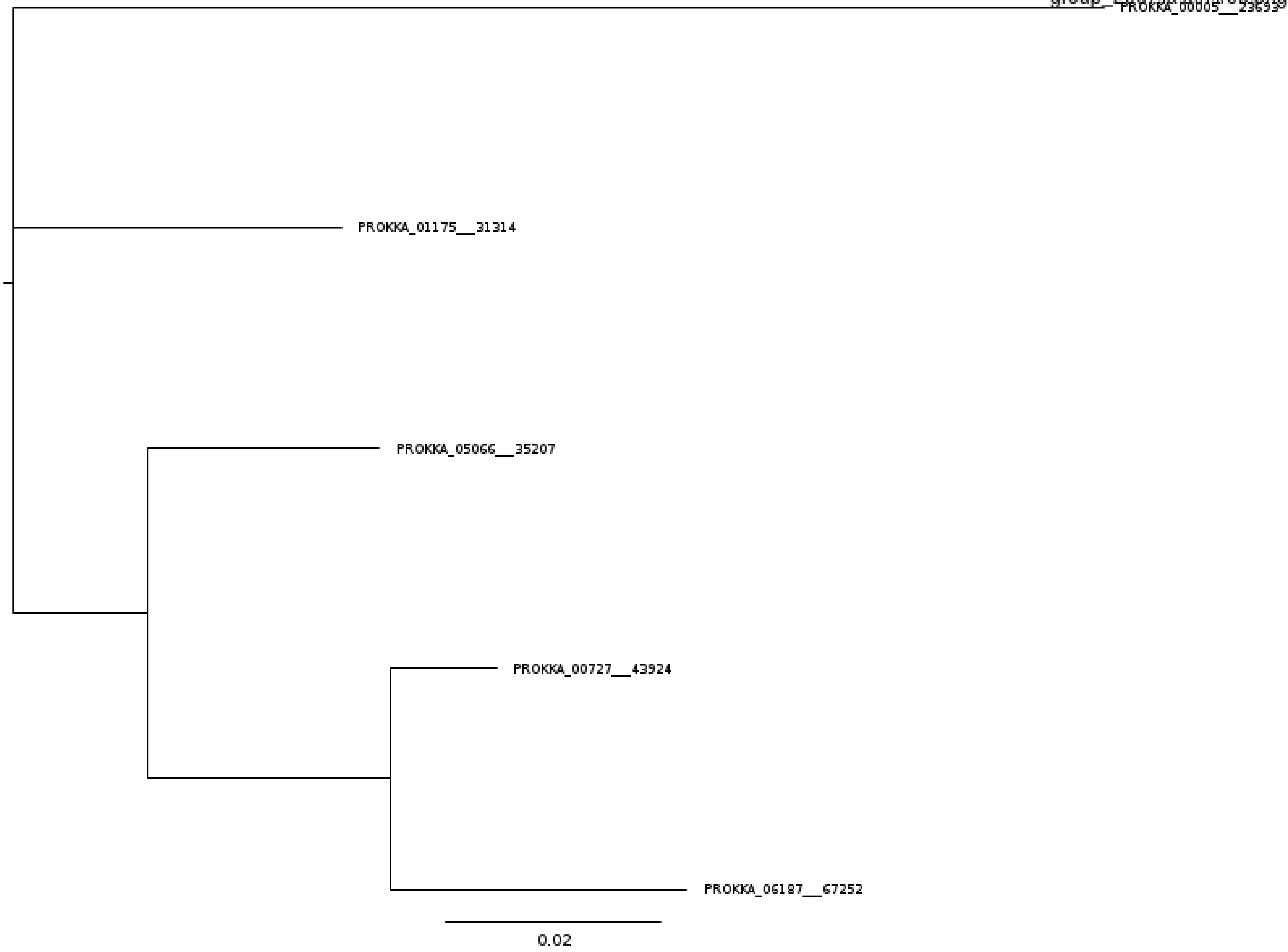

group_2069.fa.aln.tree.png

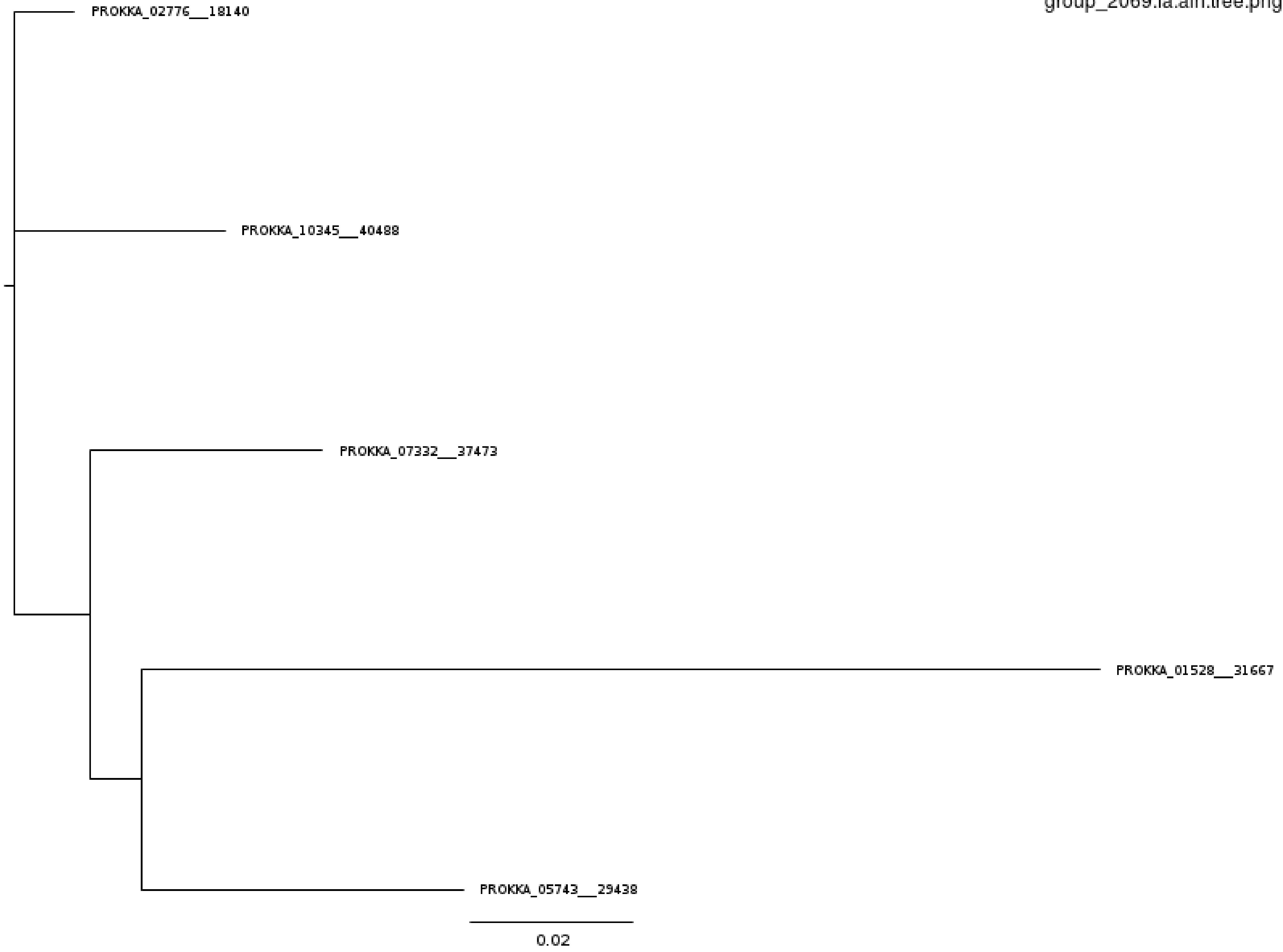

group_2070.fa.aln.tree.png

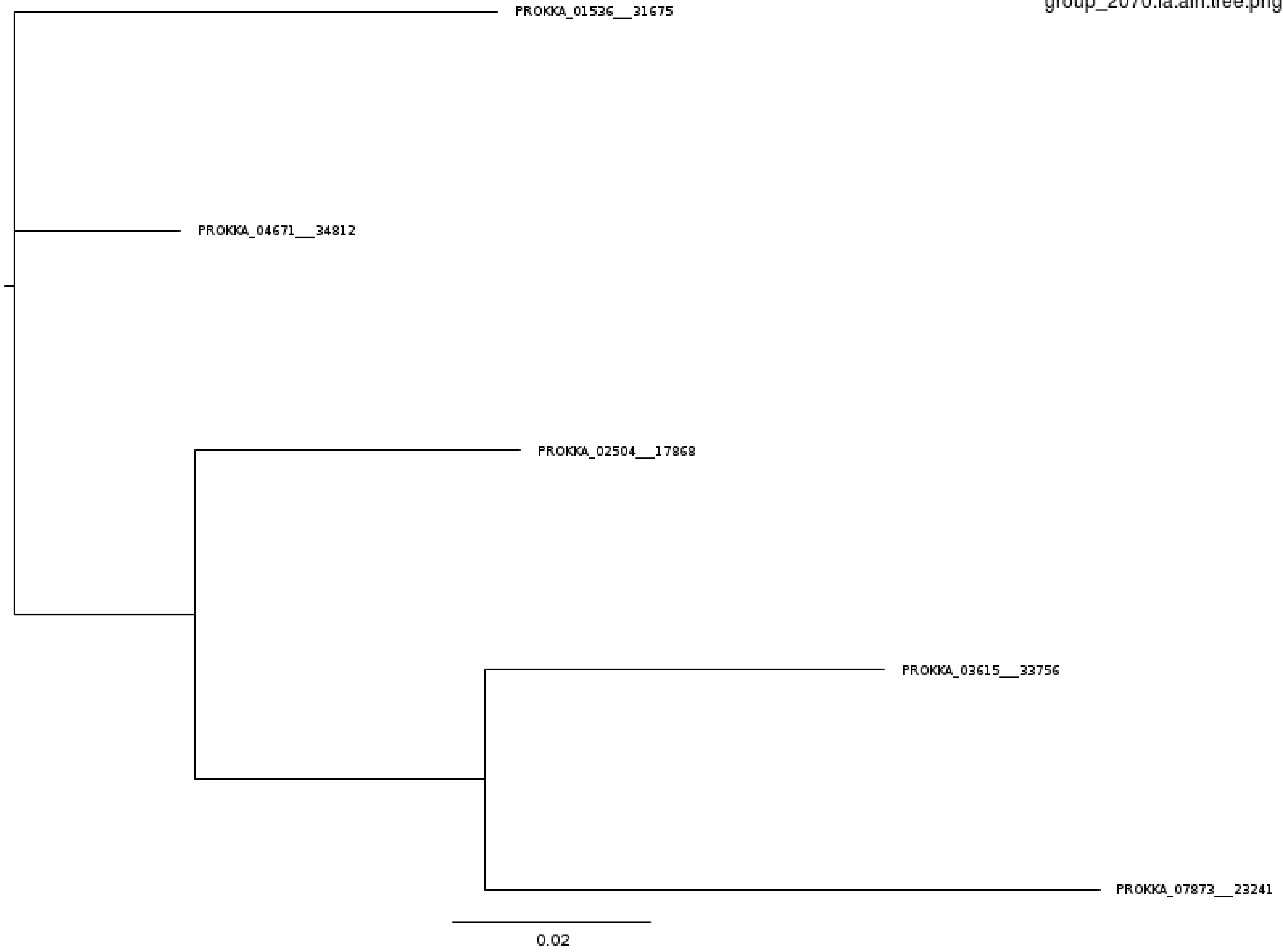

group_2071.fa.aln.tree.png

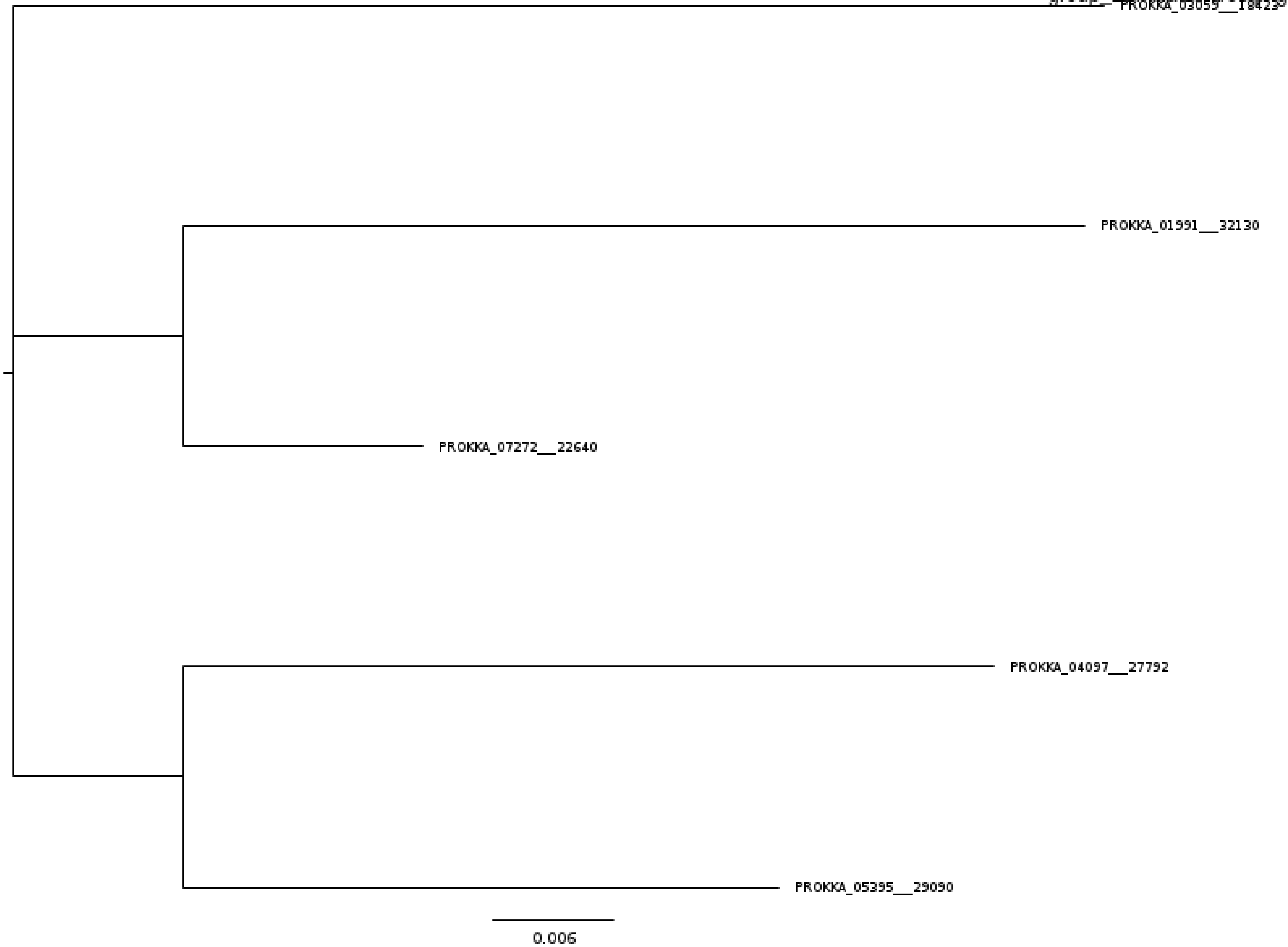

group_2074.fa.aln.tree.png

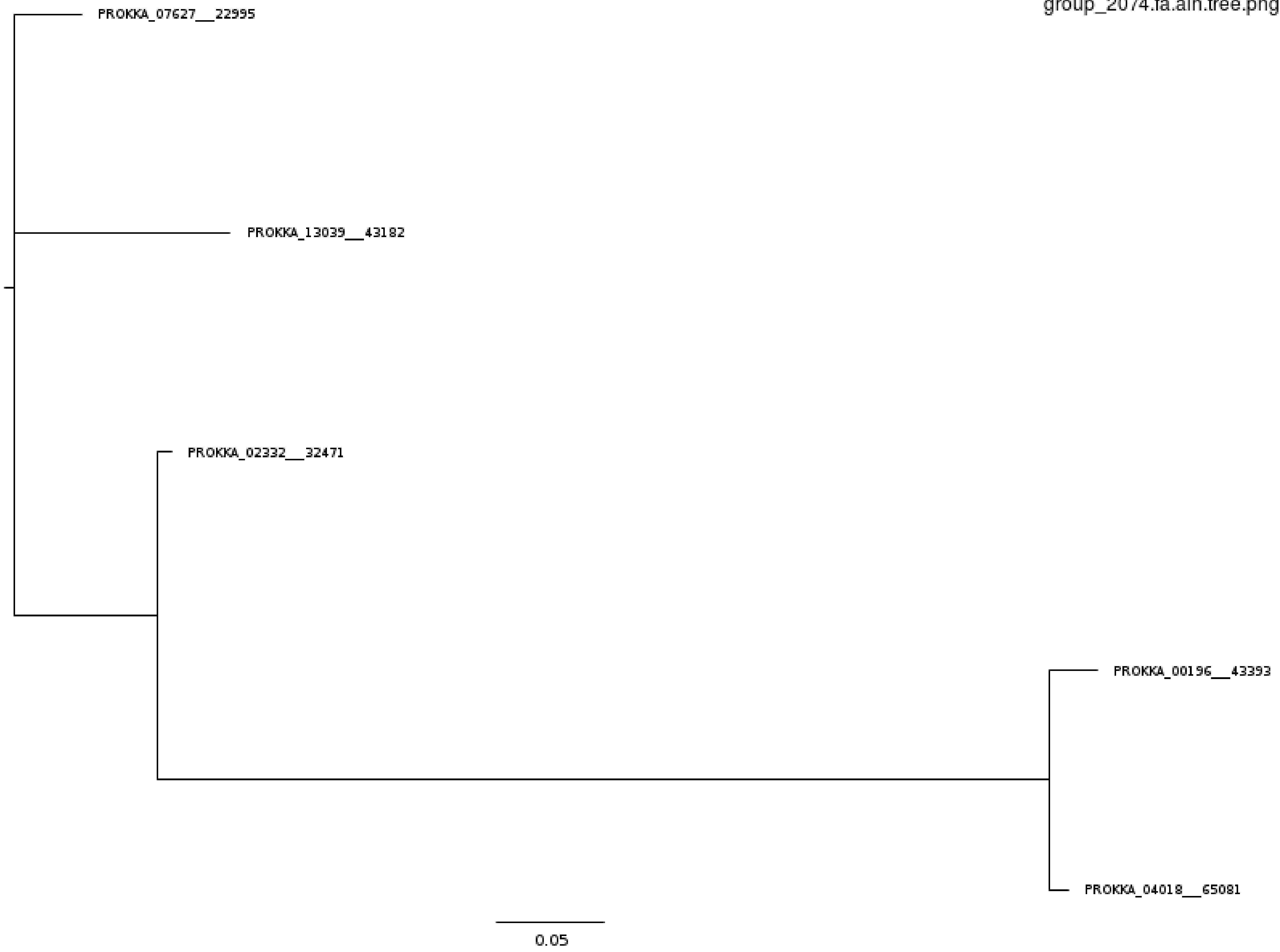

group_2075.fa.aln.tree.png

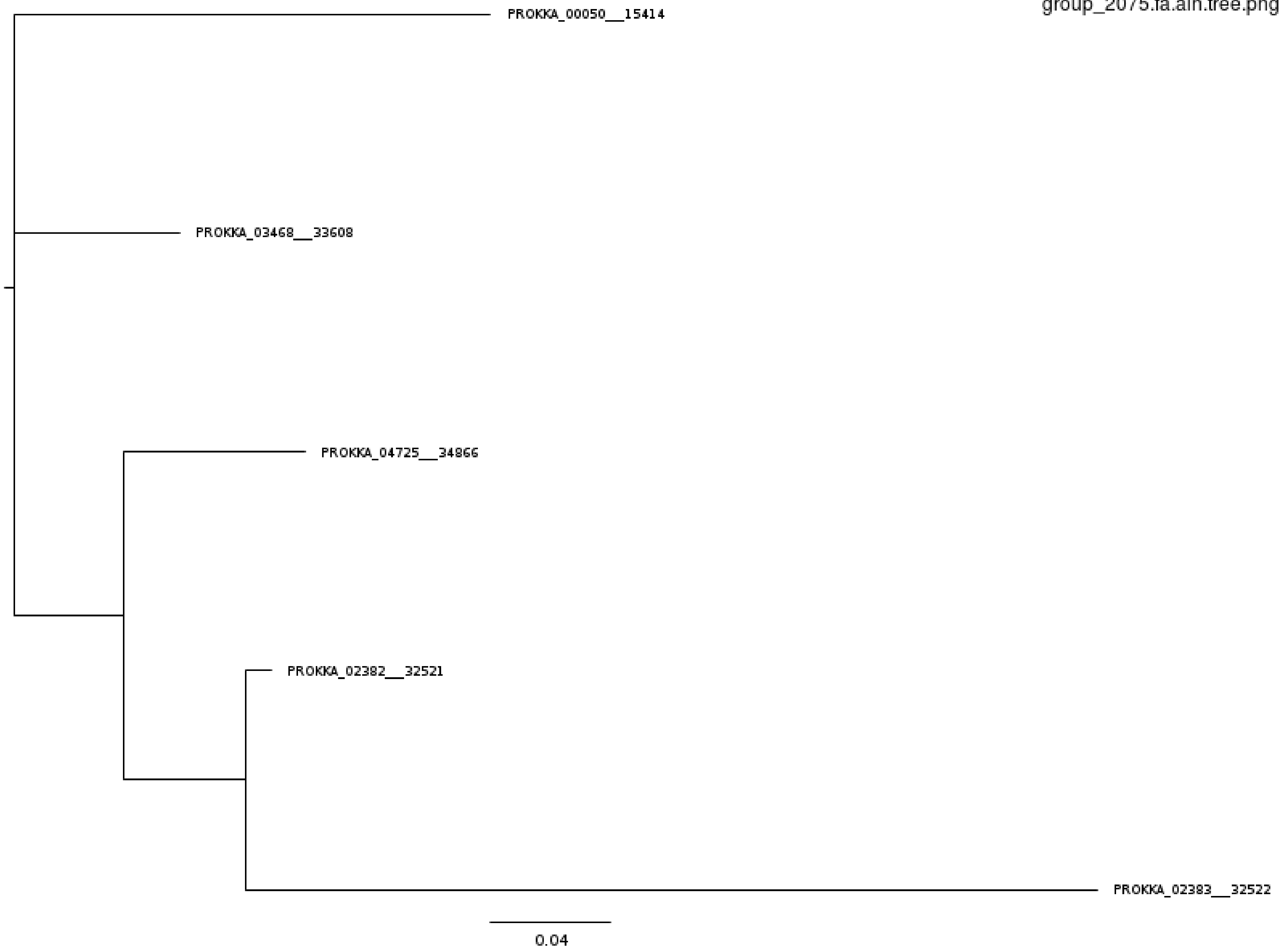

group_2077.fa.aln.tree.png

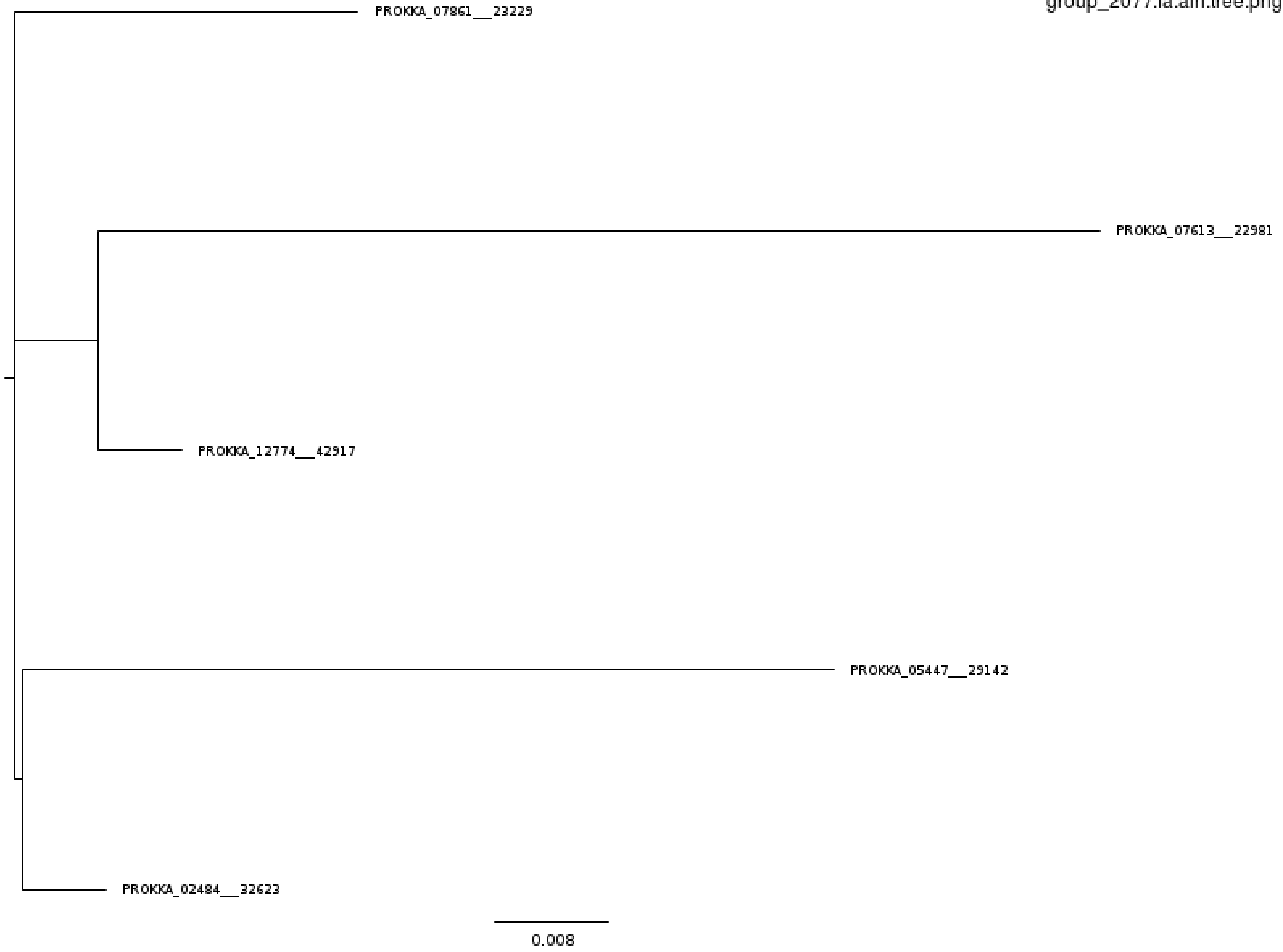

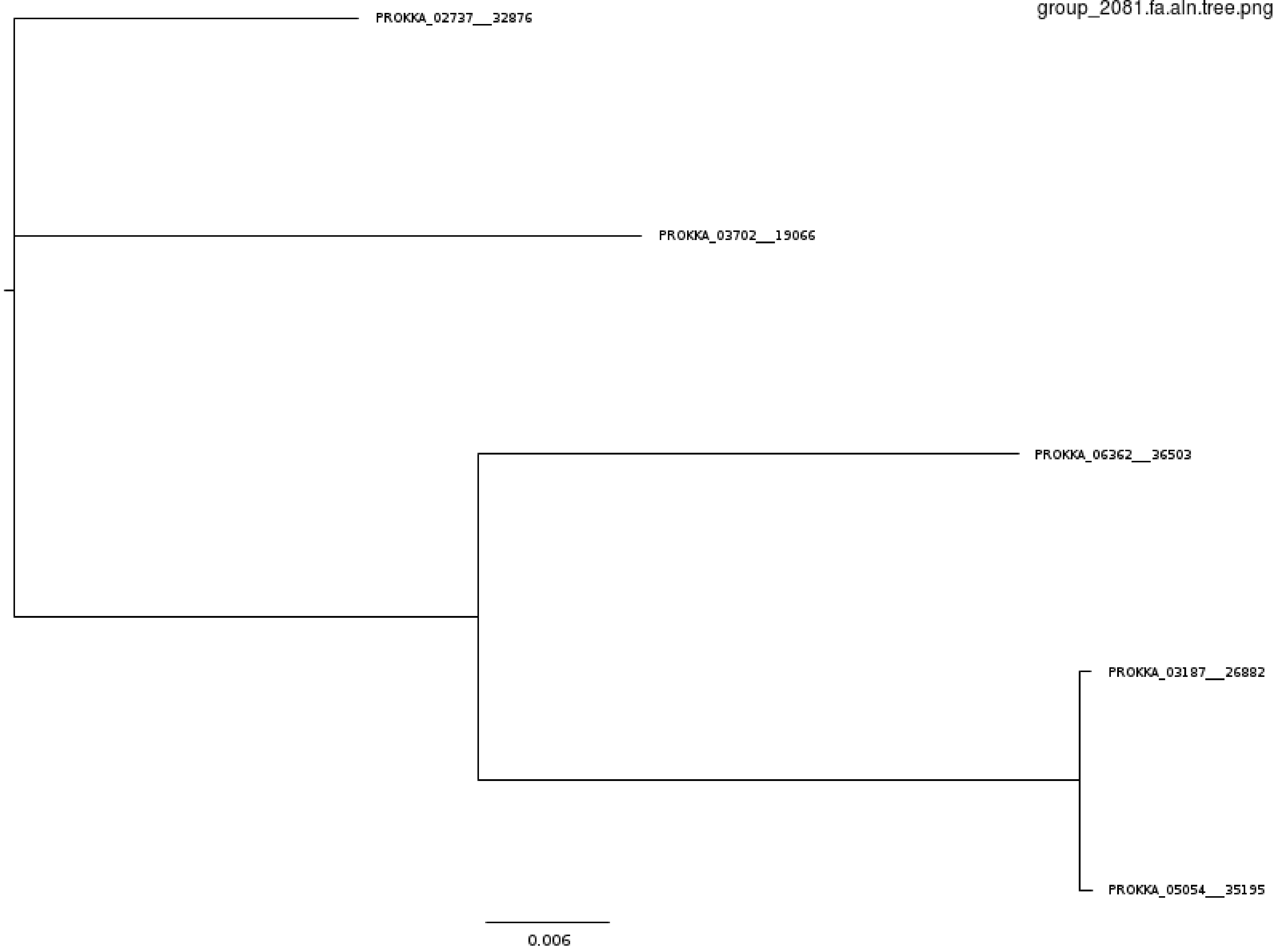
group_2081.fa.aln.tree.png
PROKKA_02737___32876
PROKKA_03702___19066
PROKKA_06362___36503
PROKKA_03187___26882
PROKKA_05054___35195
0.006

group_2083.fa.aln.tree.png

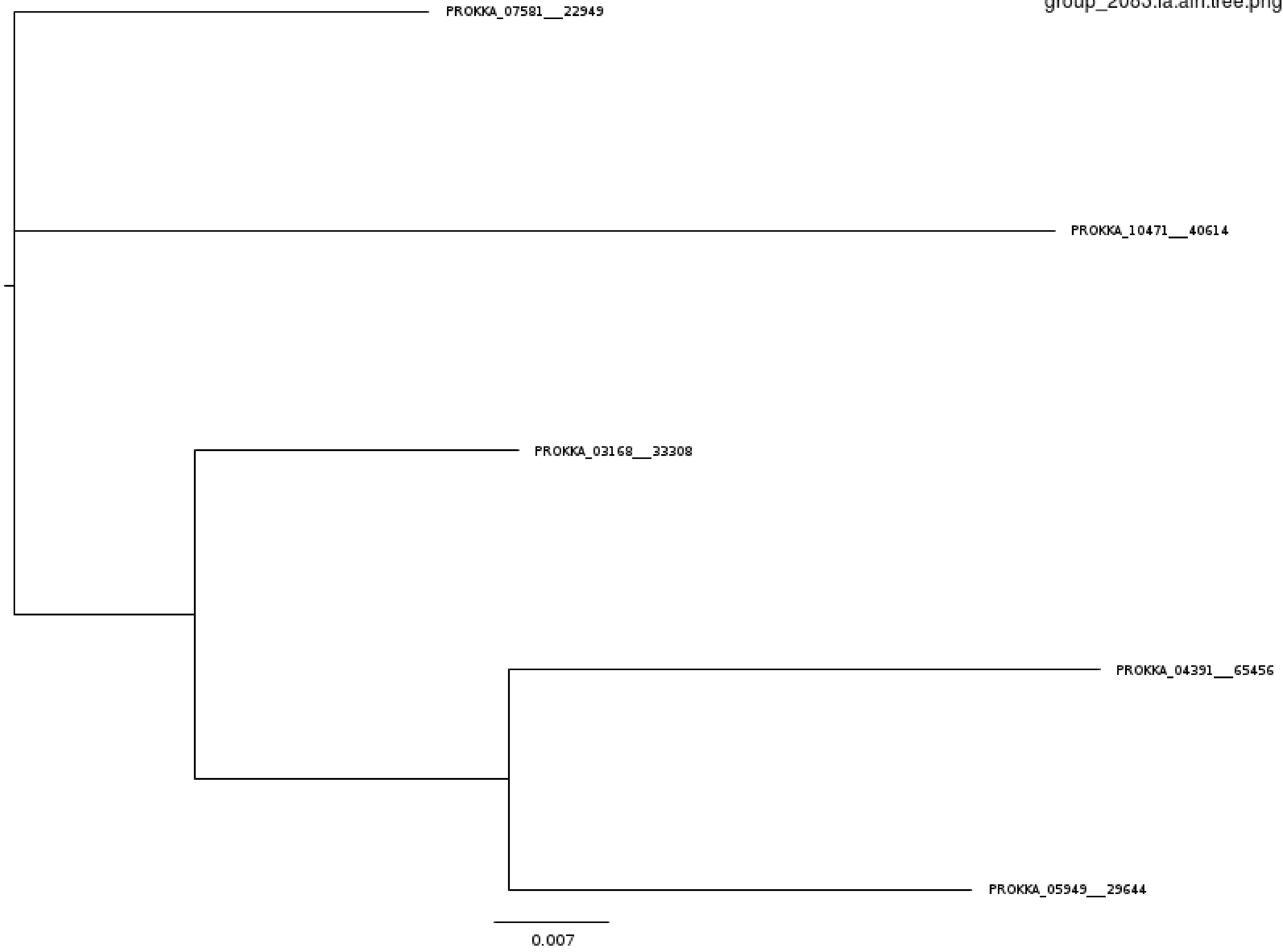

group_2085.fa.aln.tree.png

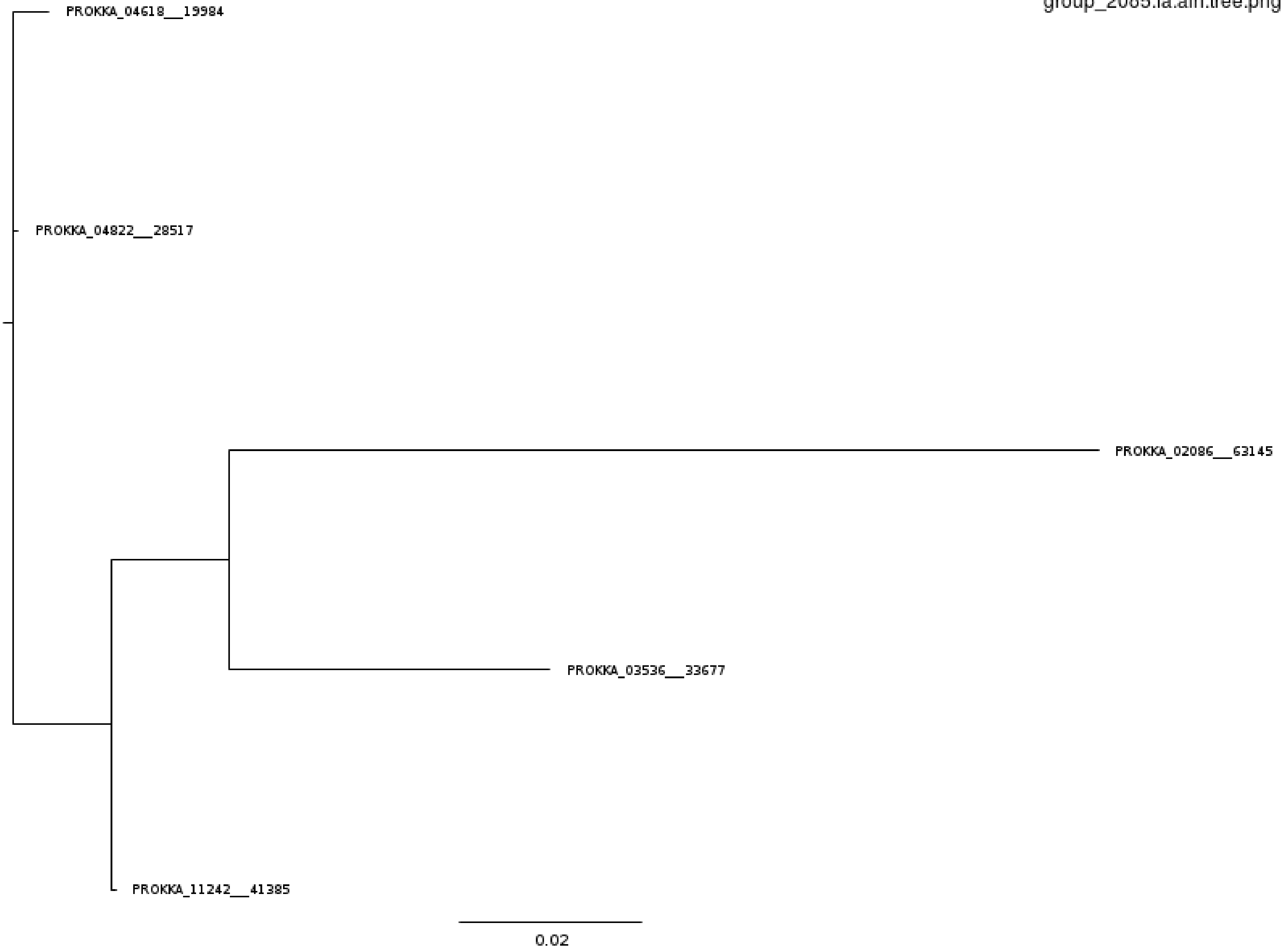

group_2086.fa.aln.tree.png

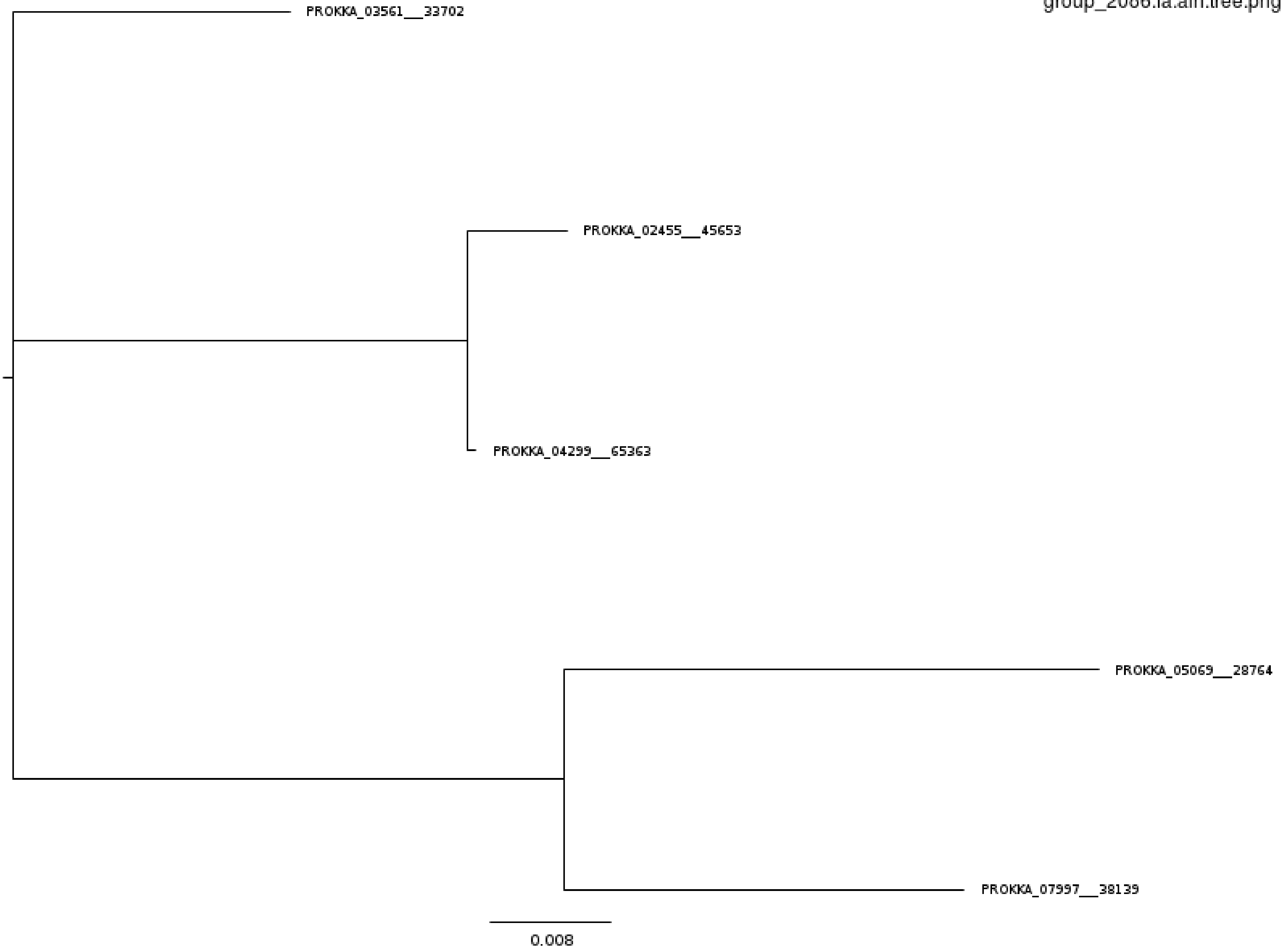

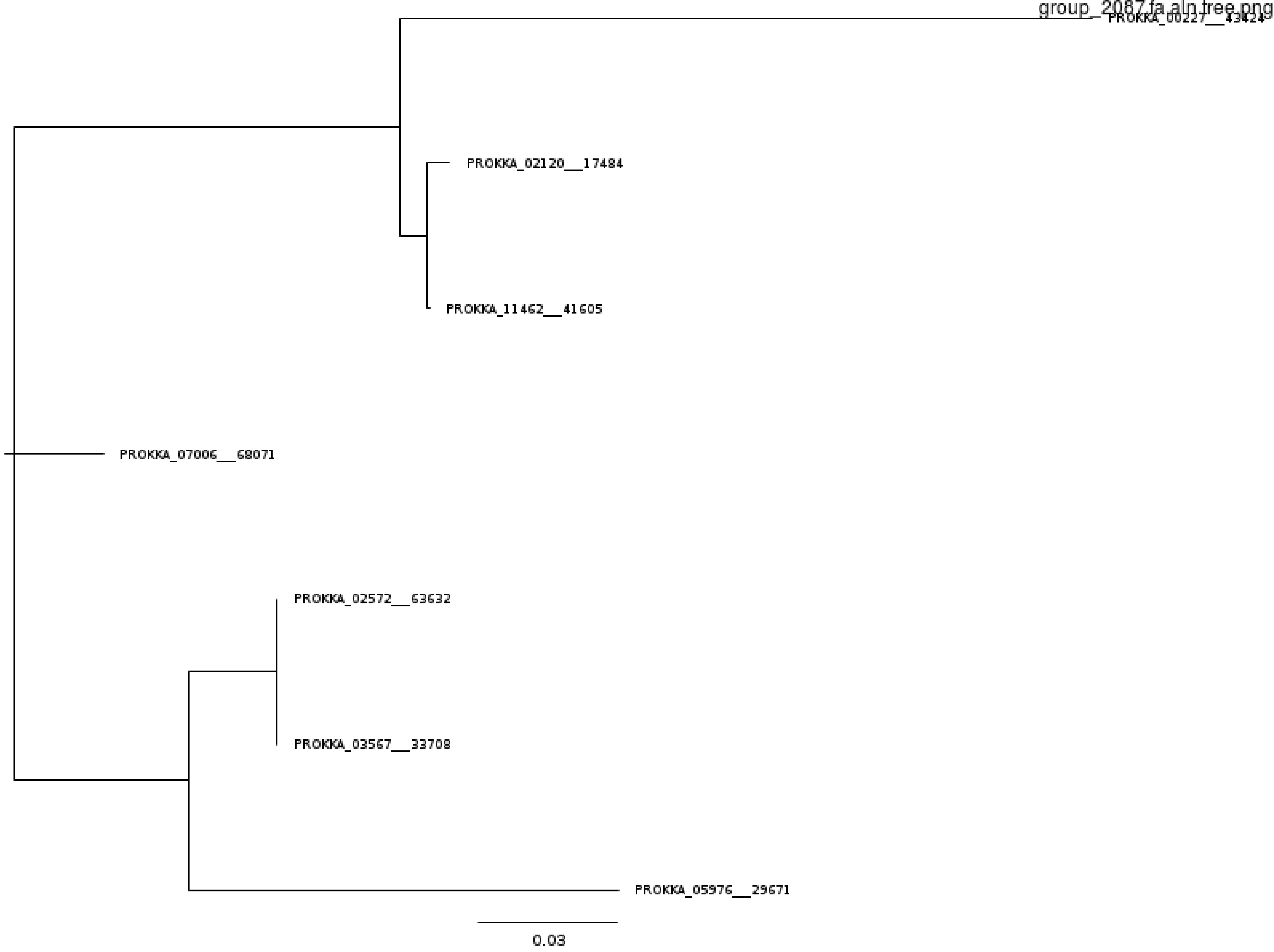

group_2087.fa.aln.tree.png
PROKKA_00227__43424
PROKKA_02120__17484
PROKKA_11462__41605
PROKKA_07006__68071
PROKKA_02572__63632
PROKKA_03567__33708
PROKKA_05976__29671
0.03

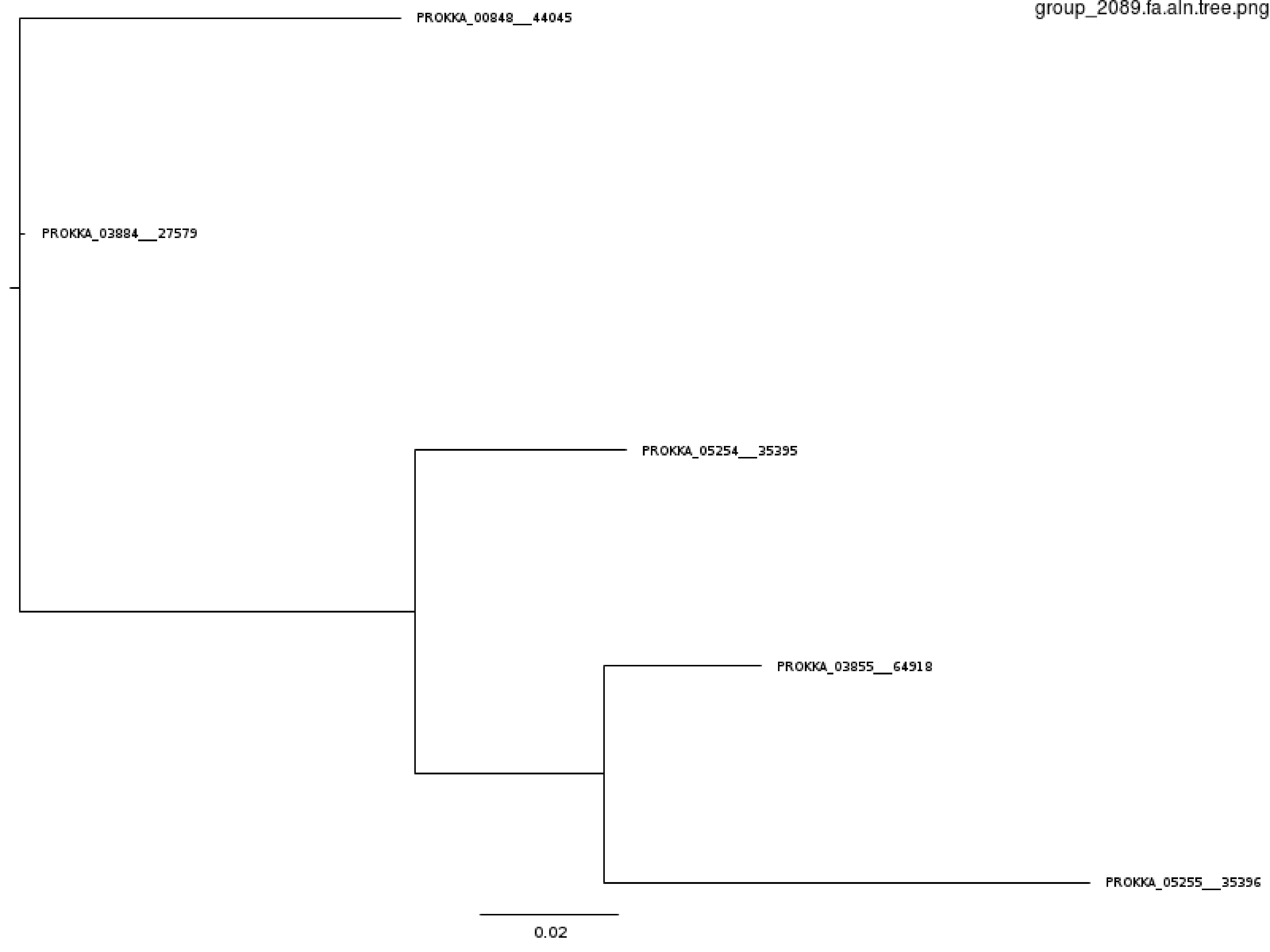
group_2089.fa.aln.tree.png
PROKKA_00848___44045
PROKKA_03884___27579
PROKKA_05254___35395
PROKKA_03855___64918
PROKKA_05255___35396
0.02

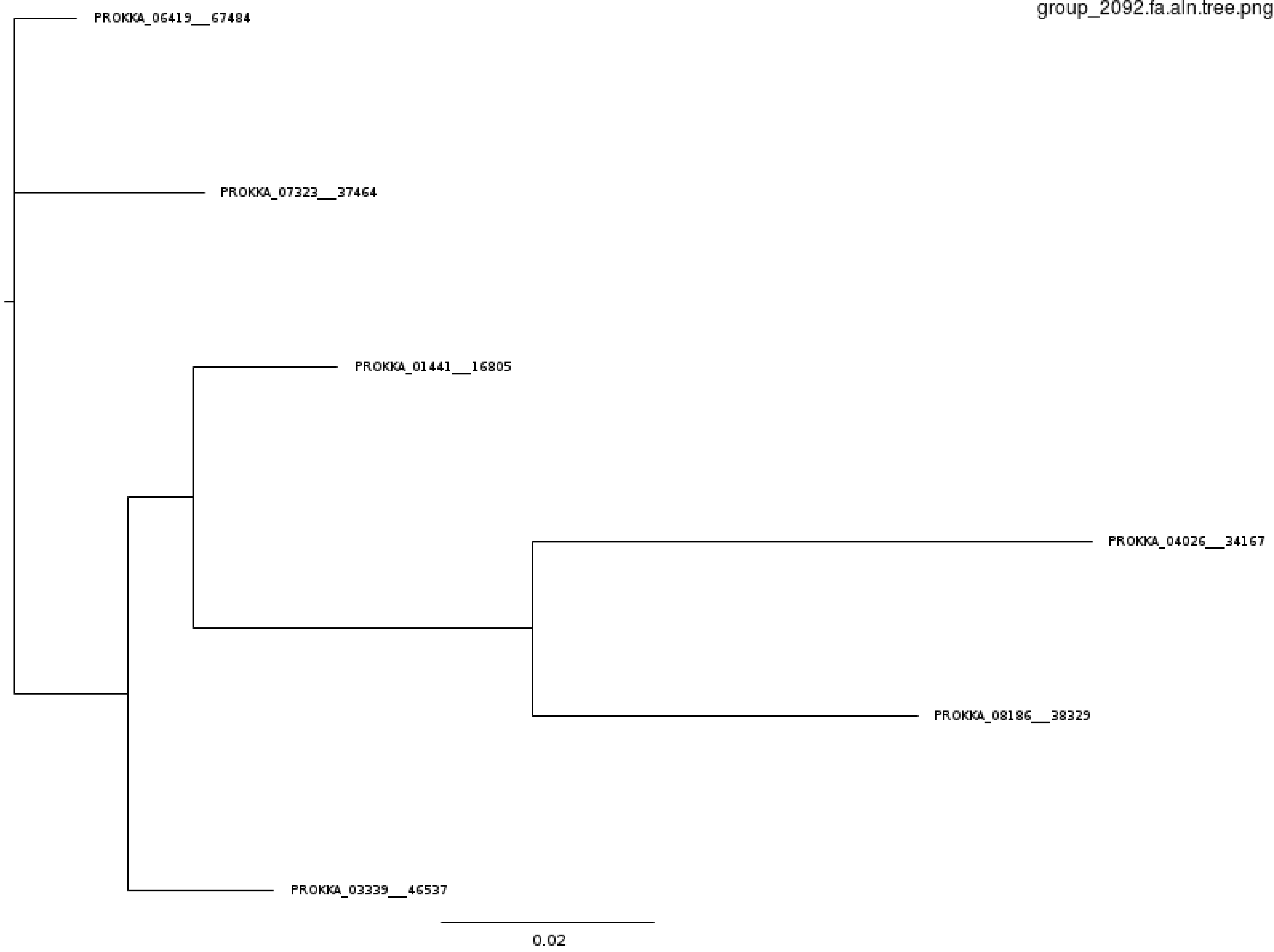
group_2092.fa.aln.tree.png
PROKKA_06419___67484
PROKKA_07323___37464
PROKKA_01441___16805
PROKKA_04026___34167
PROKKA_08186___38329
PROKKA_03339___46537
0.02

group_2095.fa.aln.tree.png

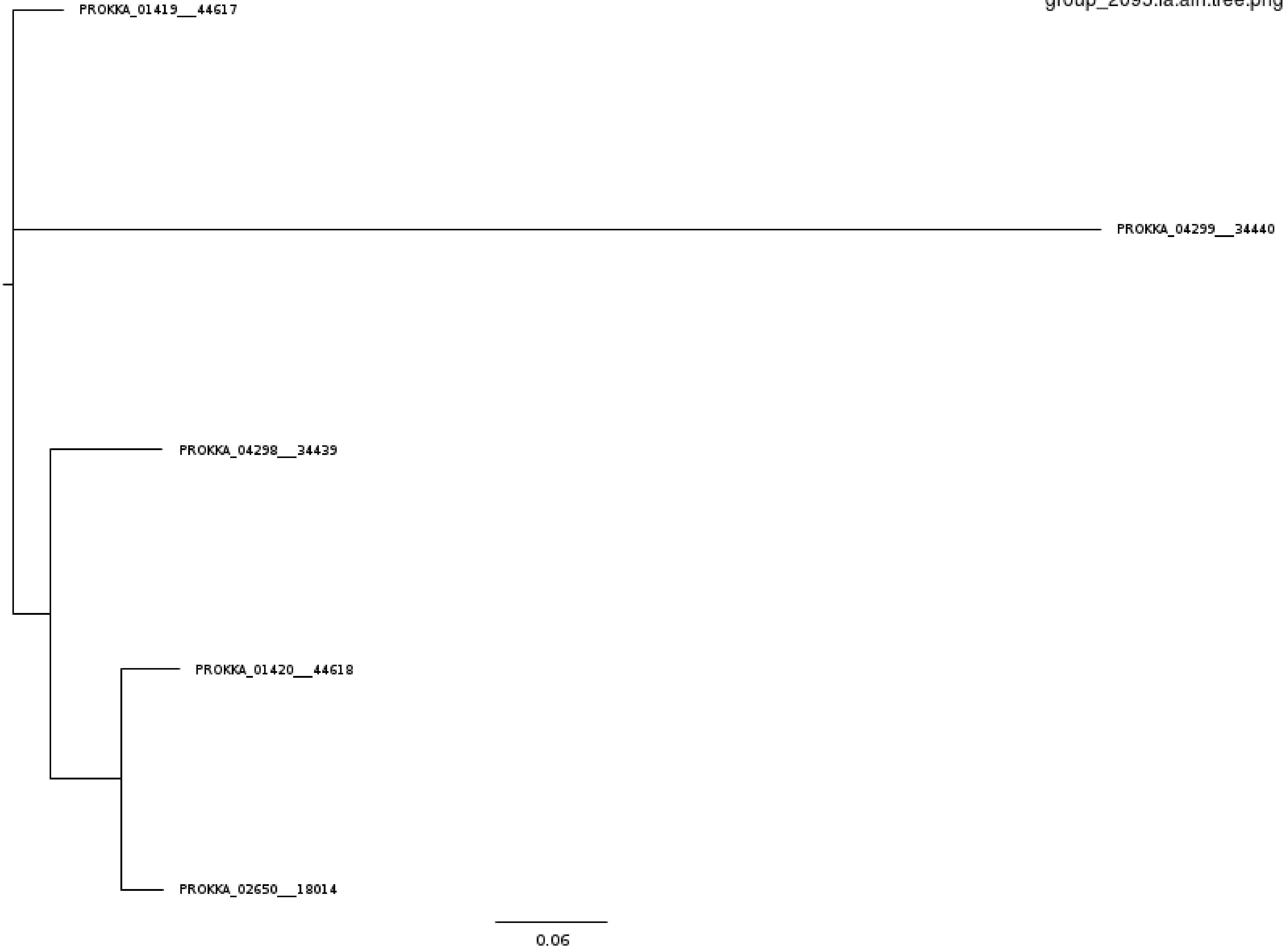

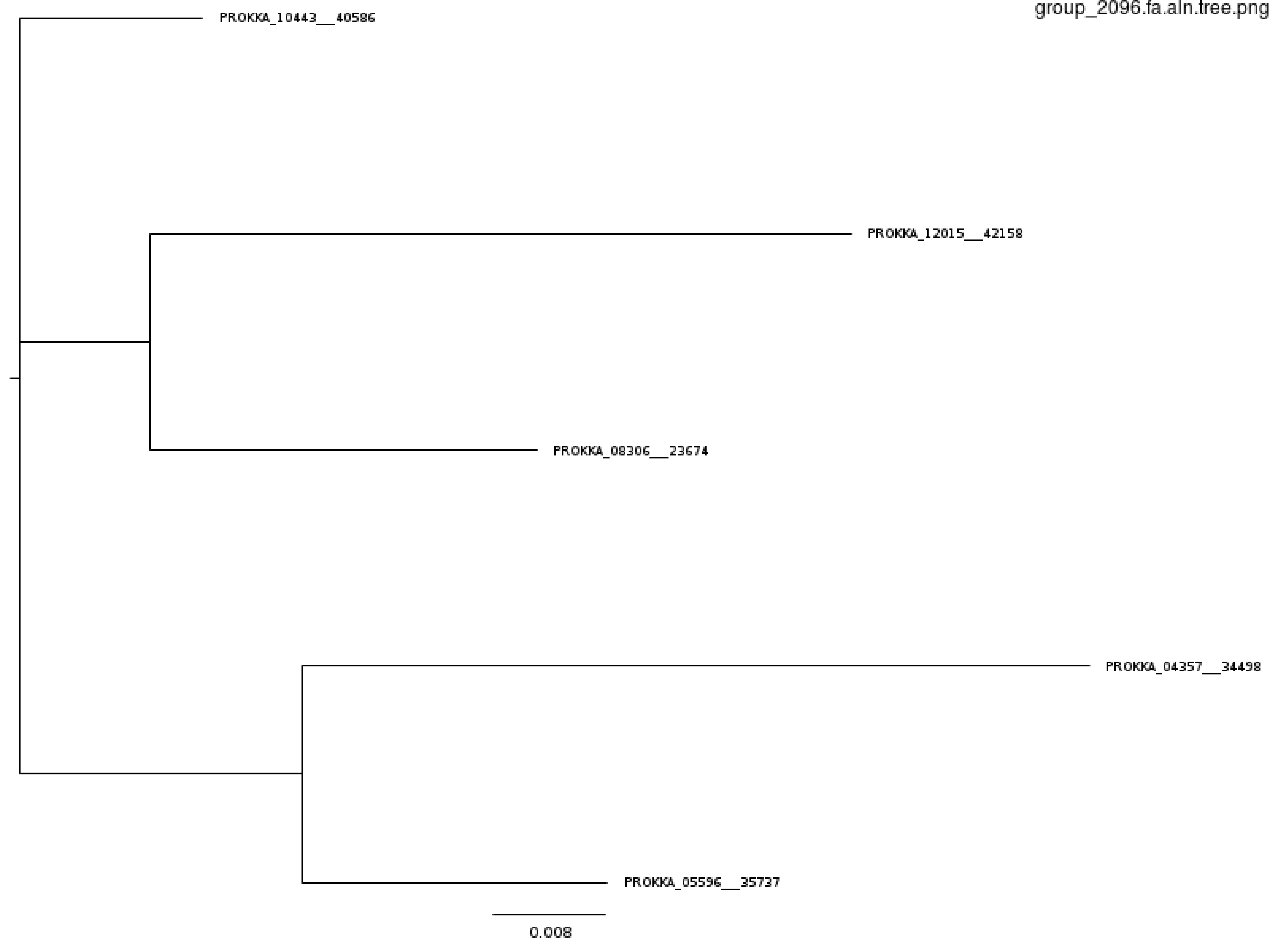
group_2096.fa.aln.tree.png
PROKKA_10443__40586
PROKKA_12015__42158
PROKKA_08306__23674
PROKKA_04357__34498
PROKKA_05596__35737
0.008

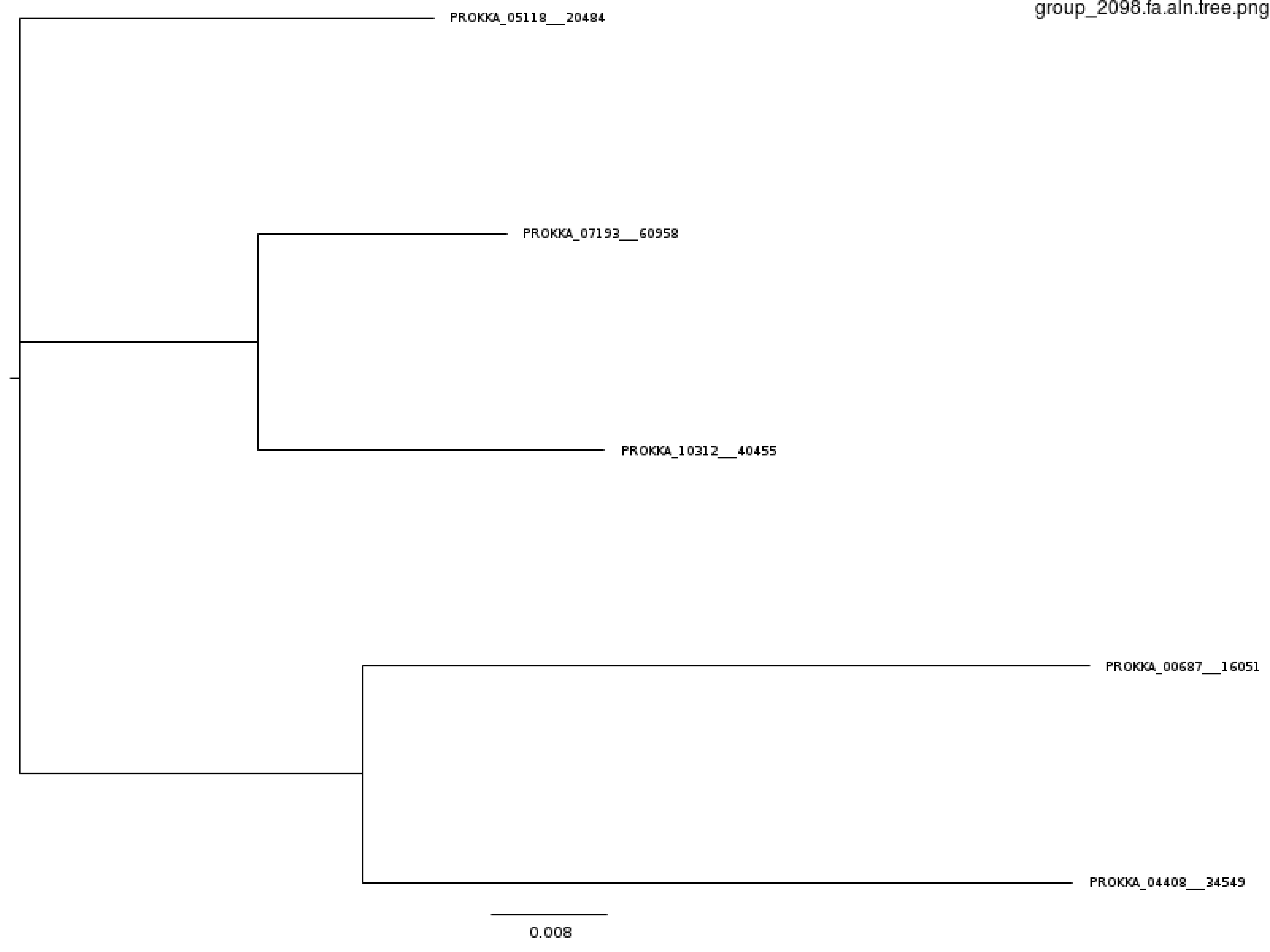
group_2098.fa.aln.tree.png
PROKKA_05118___20484
PROKKA_07193___60958
PROKKA_10312___40455
PROKKA_00687___16051
PROKKA_04408___34549
0.008

group_2099.fa.aln.tree.png

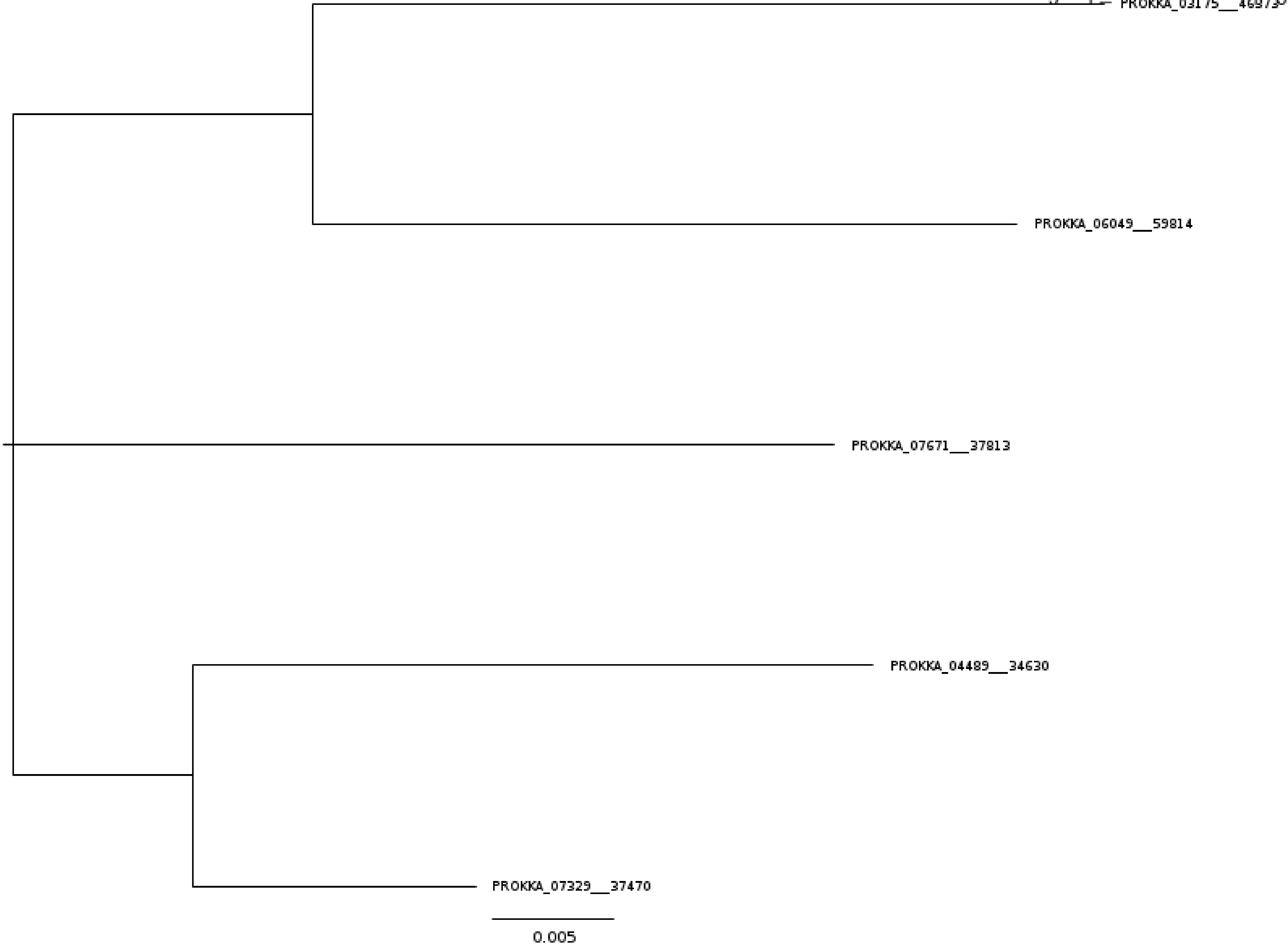

group_2100.fa.aln.tree.png

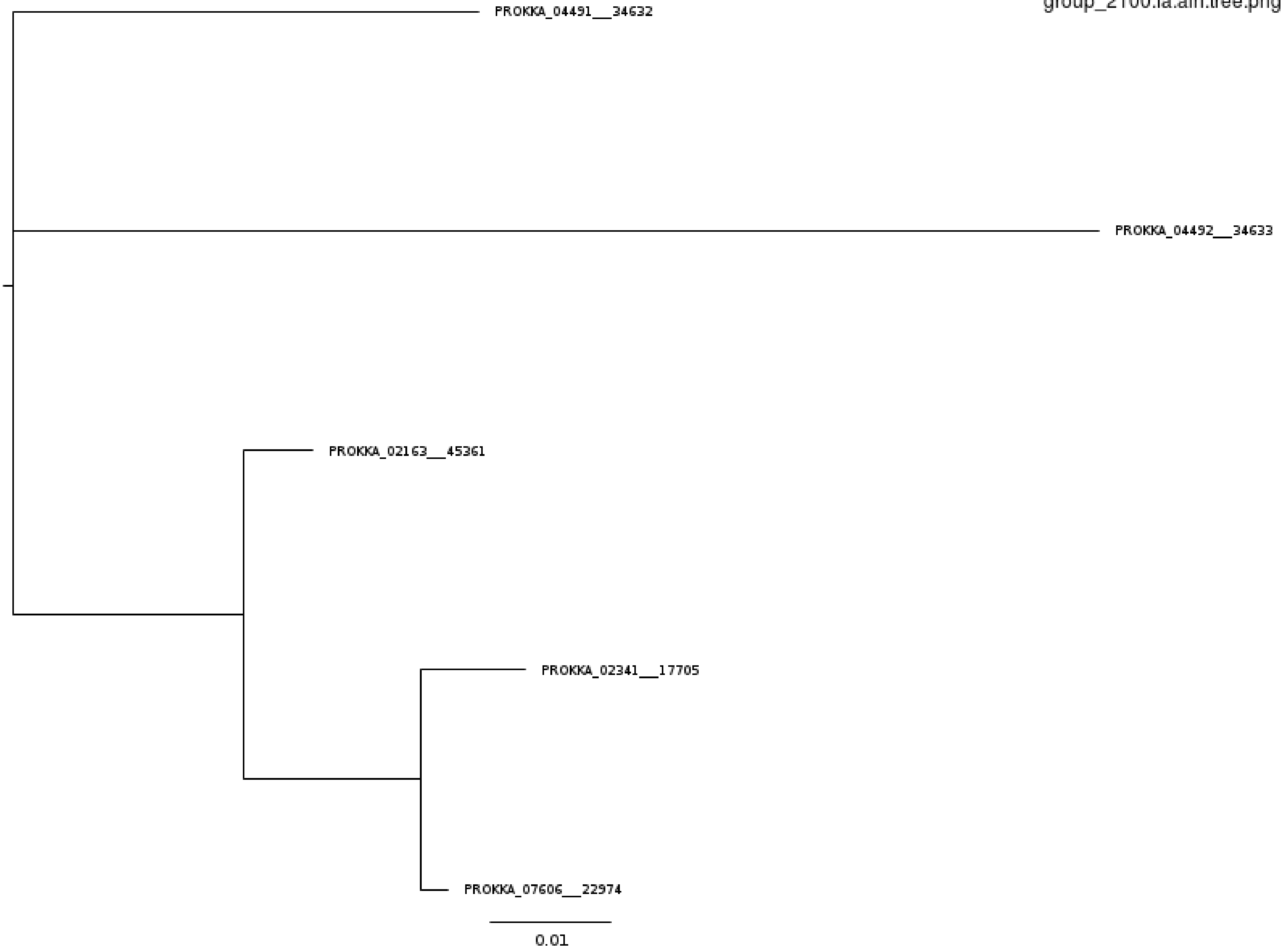

group_2104.fa.aln.tree.png

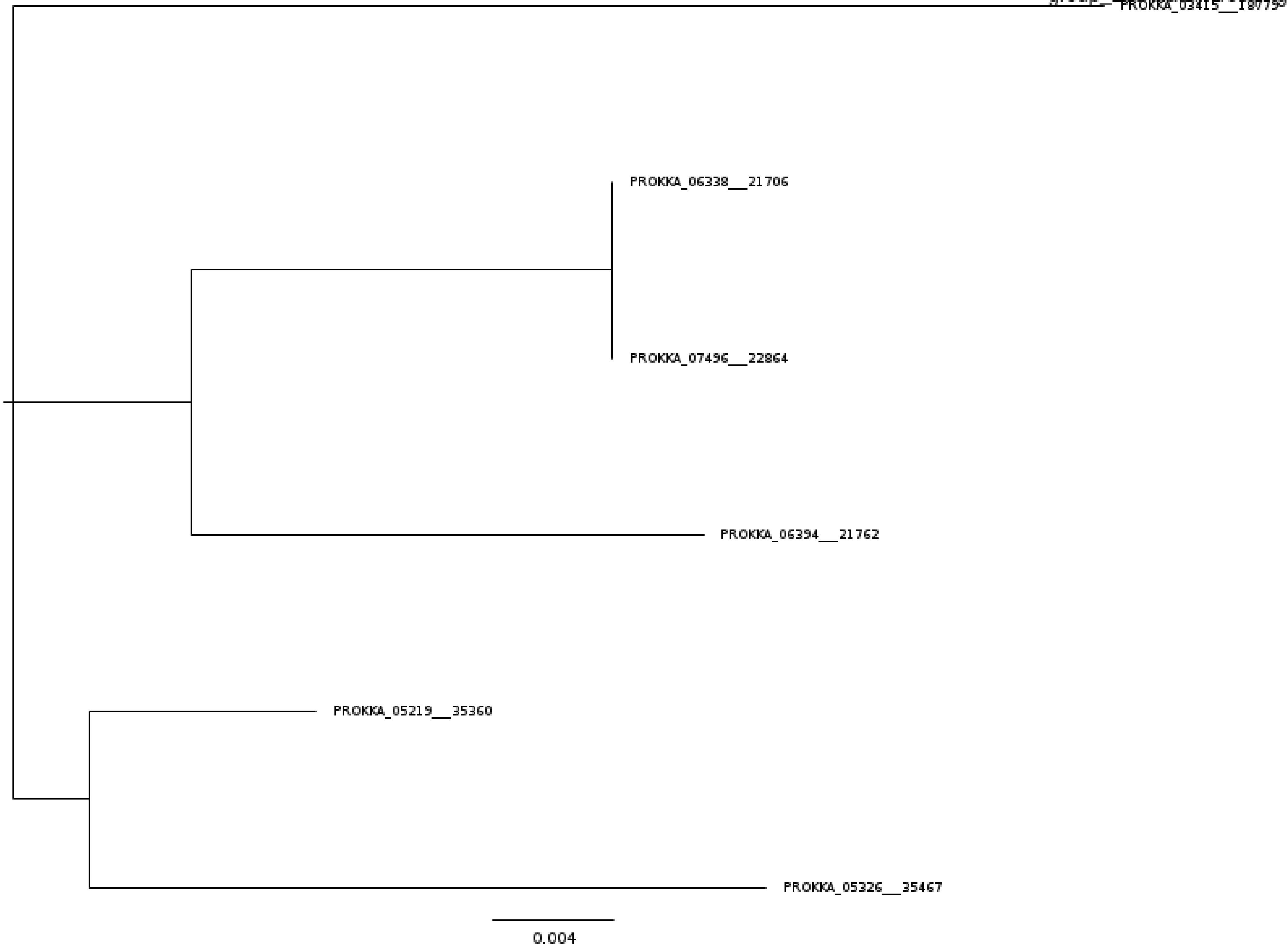

group_2105.fa.aln.tree.png

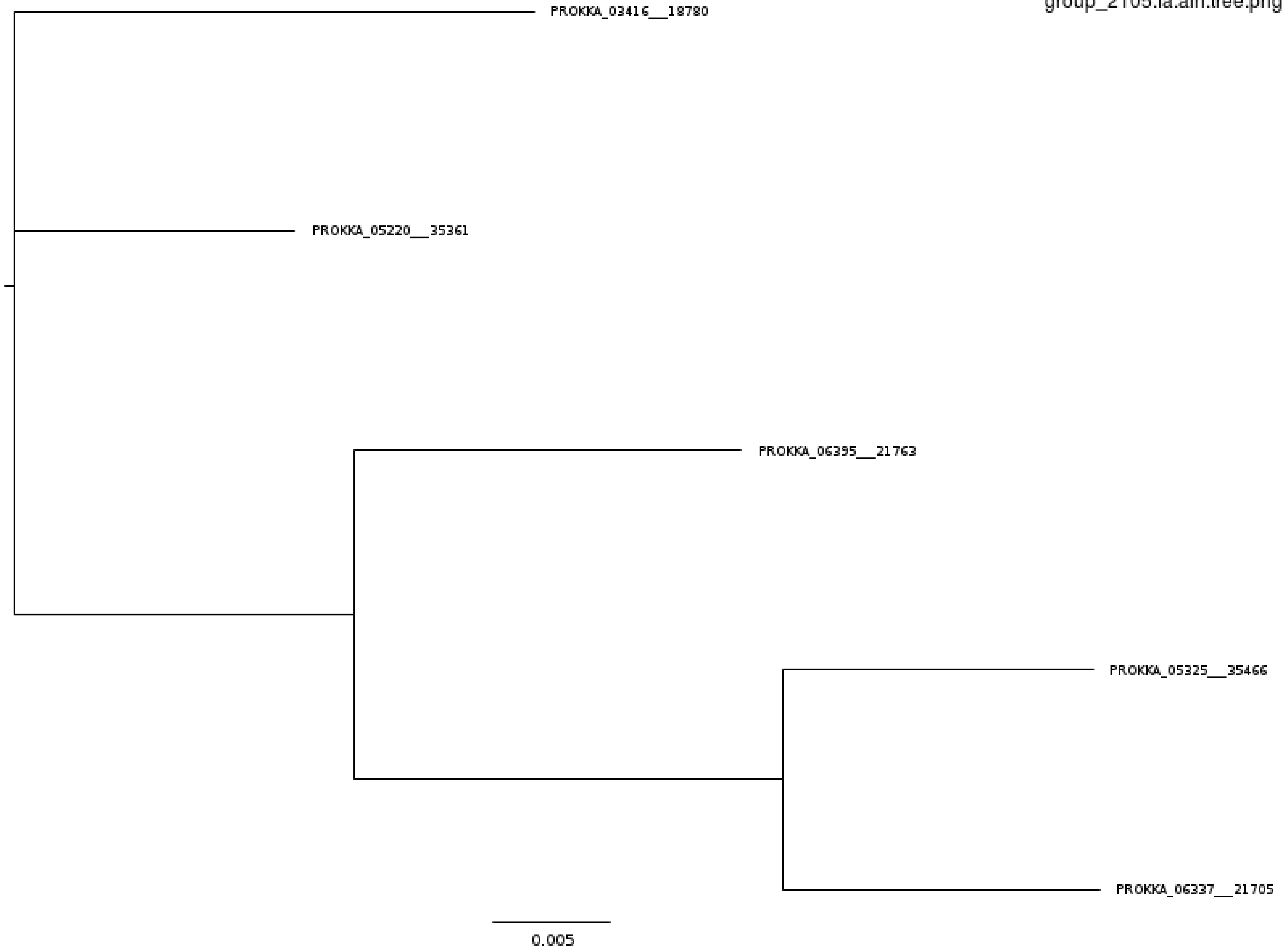

group_2107.fa.aln.tree.png

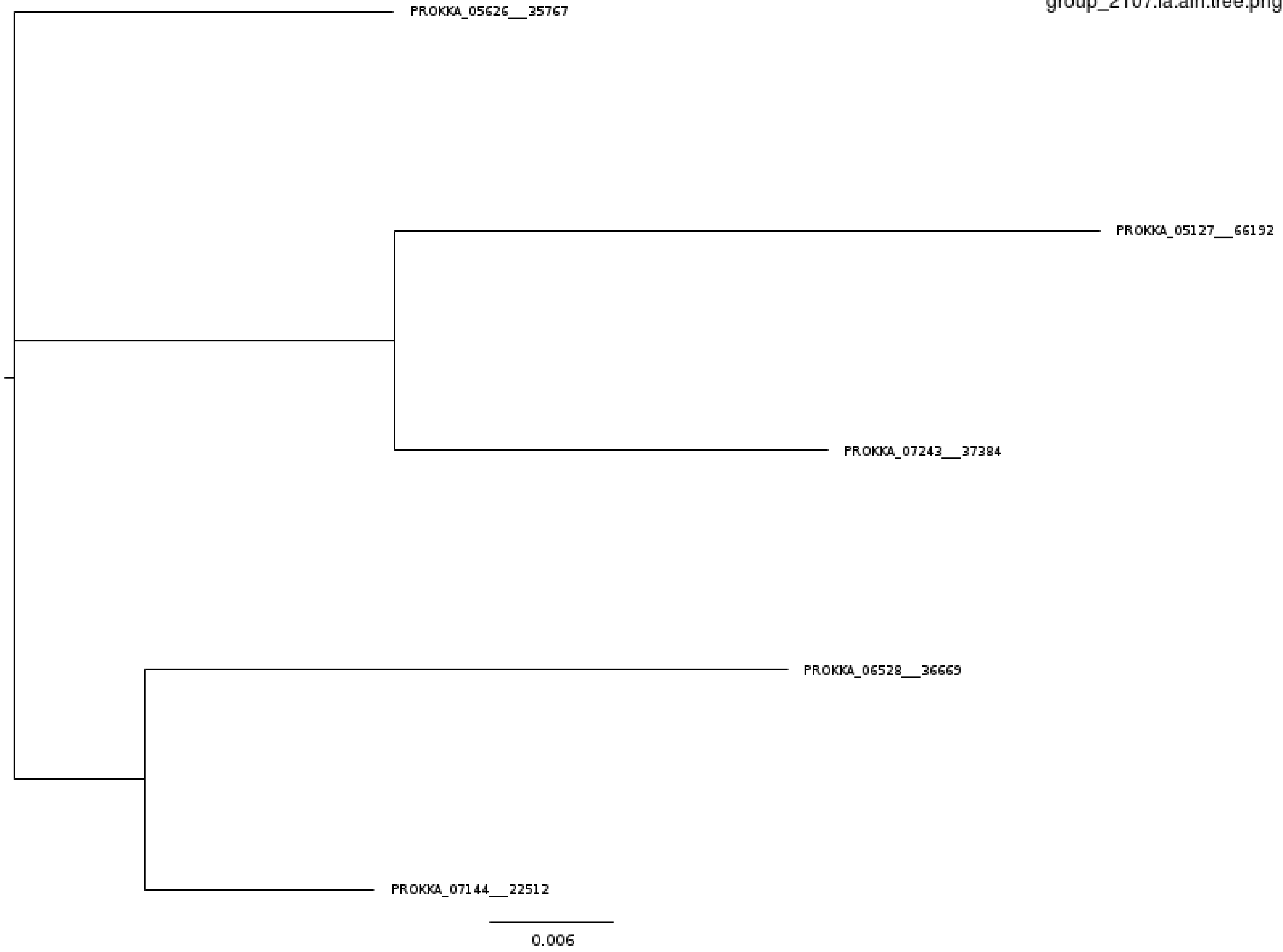

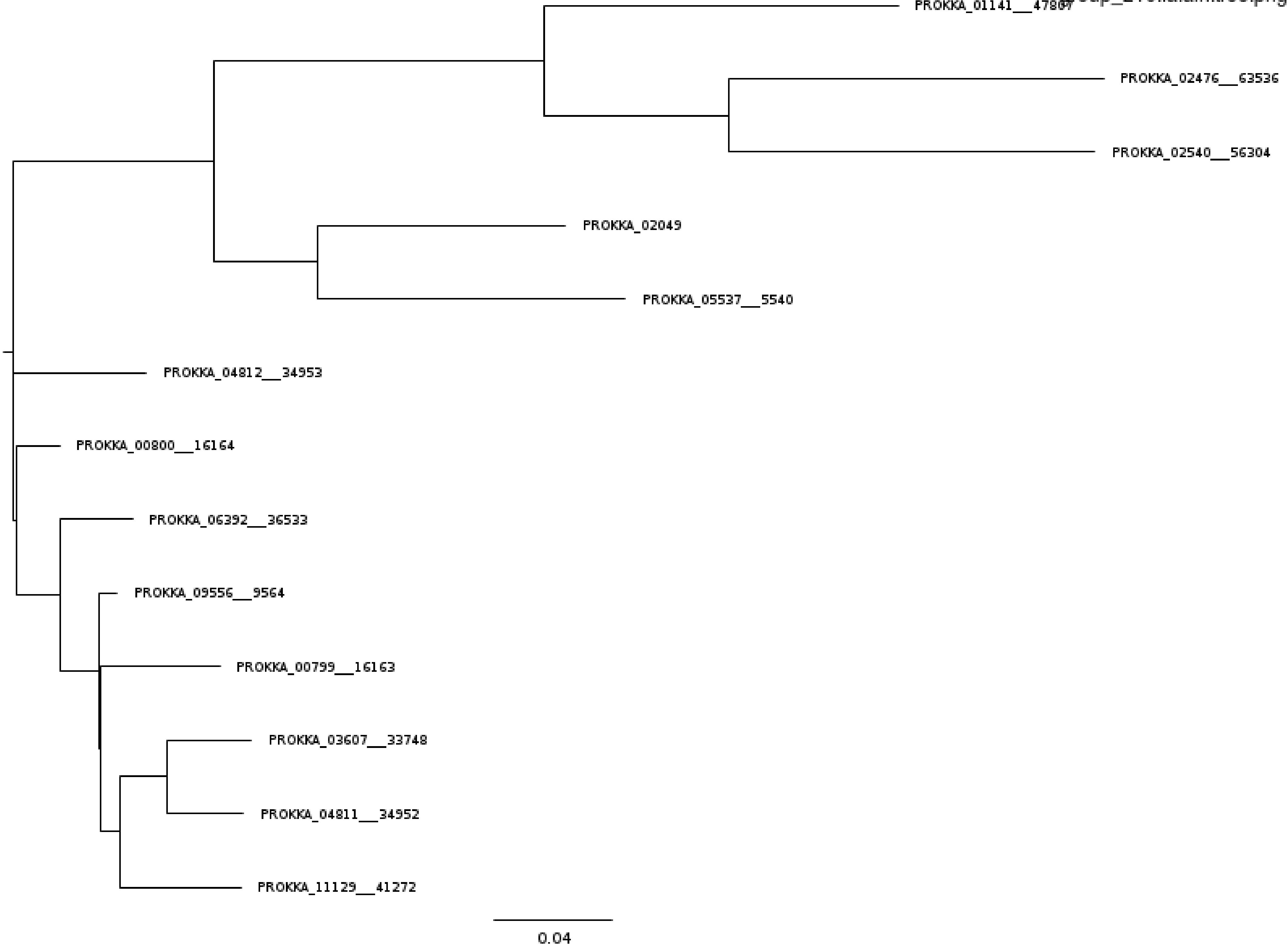
PROKKA_01141___47867
PROKKA_02476___63536
PROKKA_02540___56304
PROKKA_02049
PROKKA_05537___5540
PROKKA_04812___34953
PROKKA_00800___16164
PROKKA_06392___36533
PROKKA_09556___9564
PROKKA_00799___16163
PROKKA_03607___33748
PROKKA_04811___34952
PROKKA_11129___41272
0.04

group_2111.fa.aln.tree.png

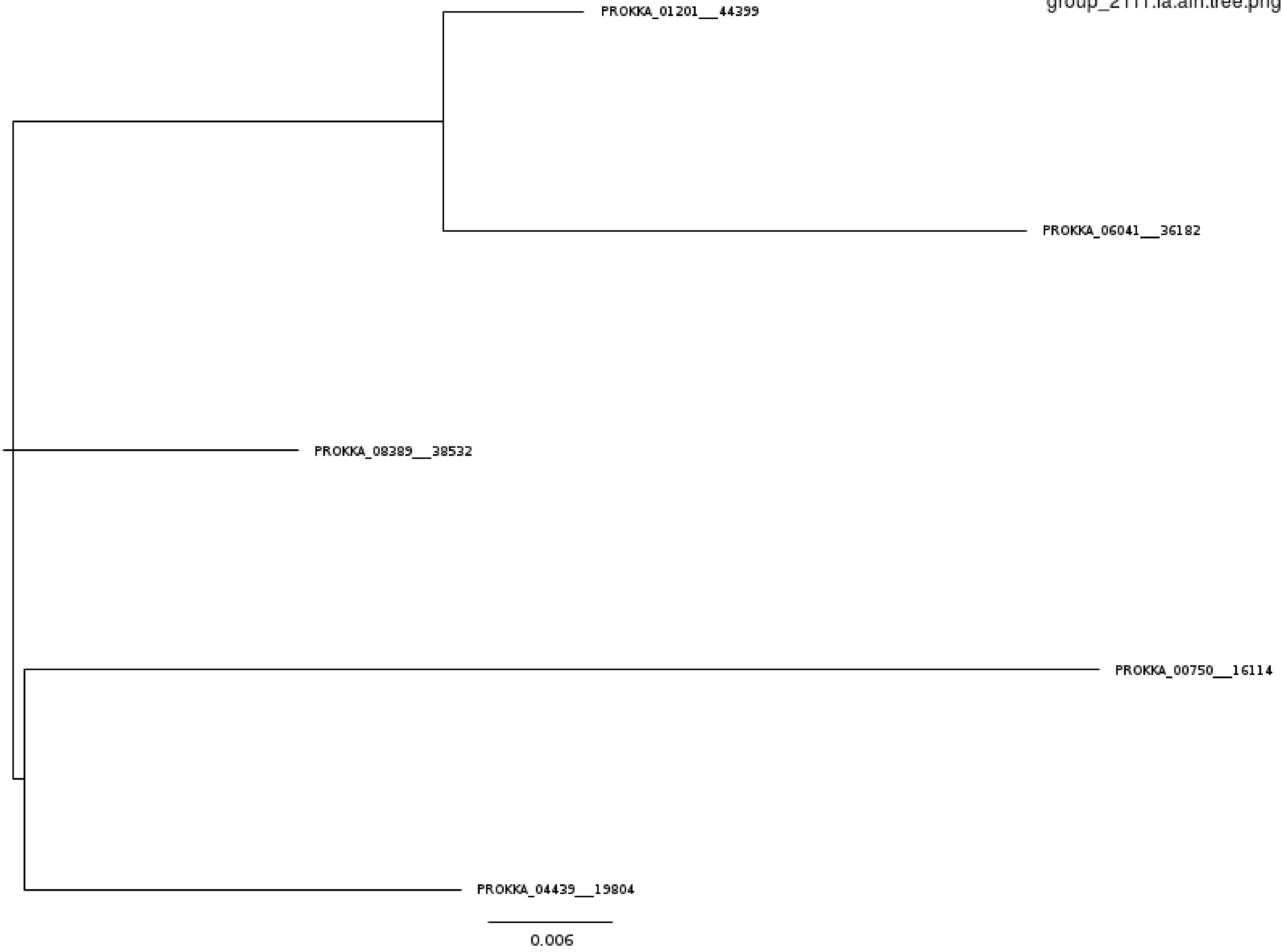

group_2112.fa.aln.tree.png

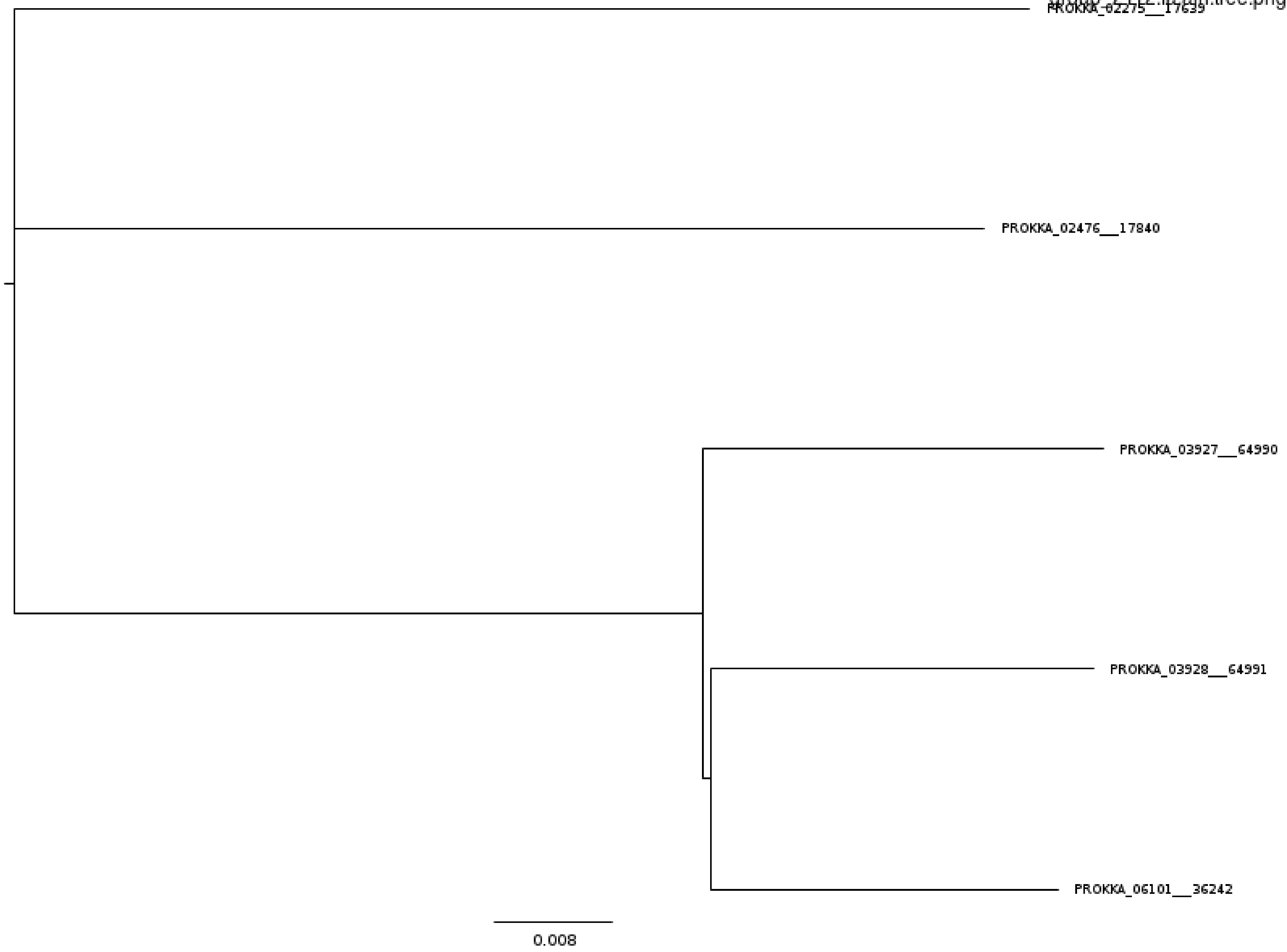

group_2114.fa.aln.tree.png

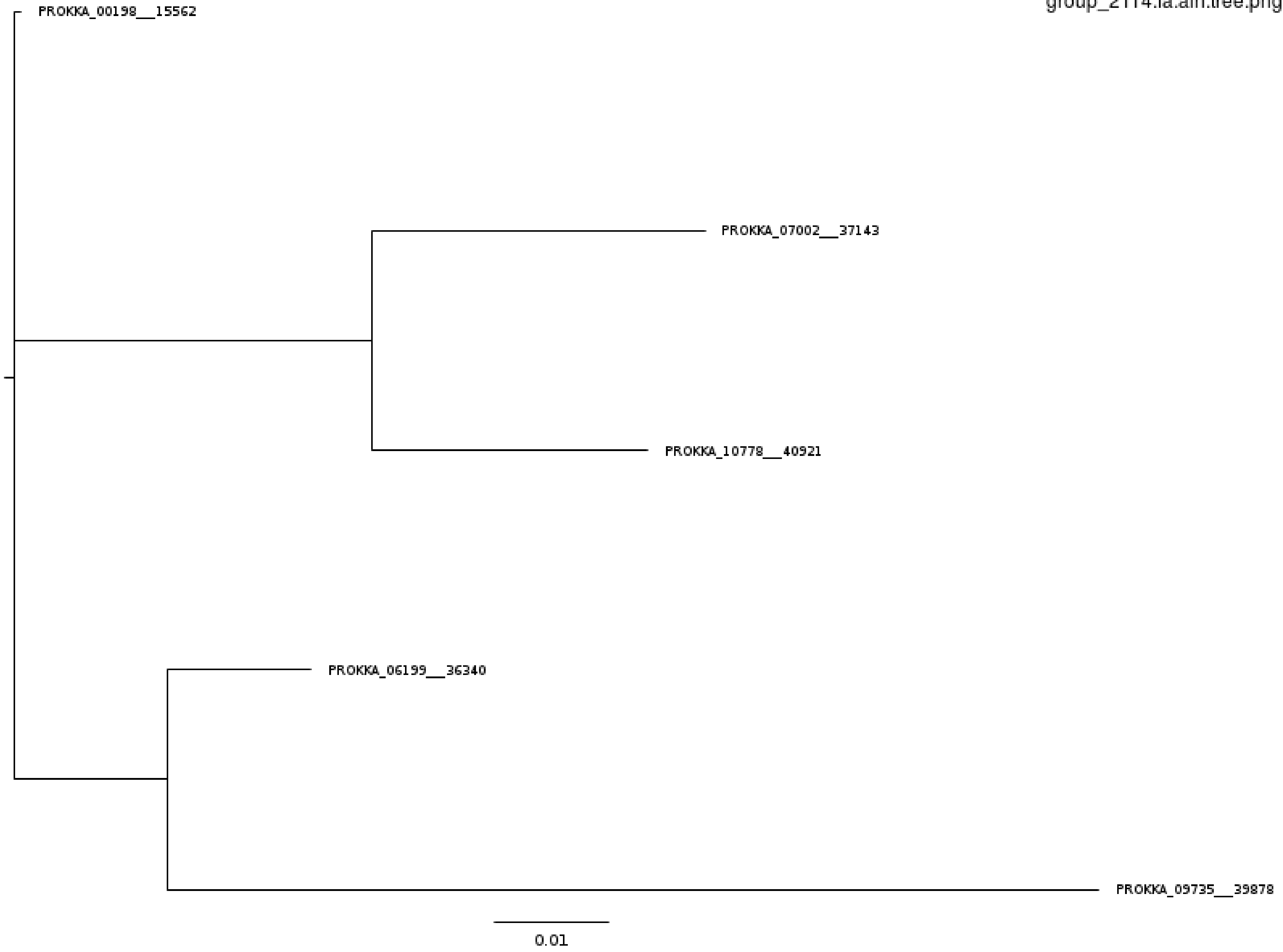

group_2116.fa.aln.tree.png

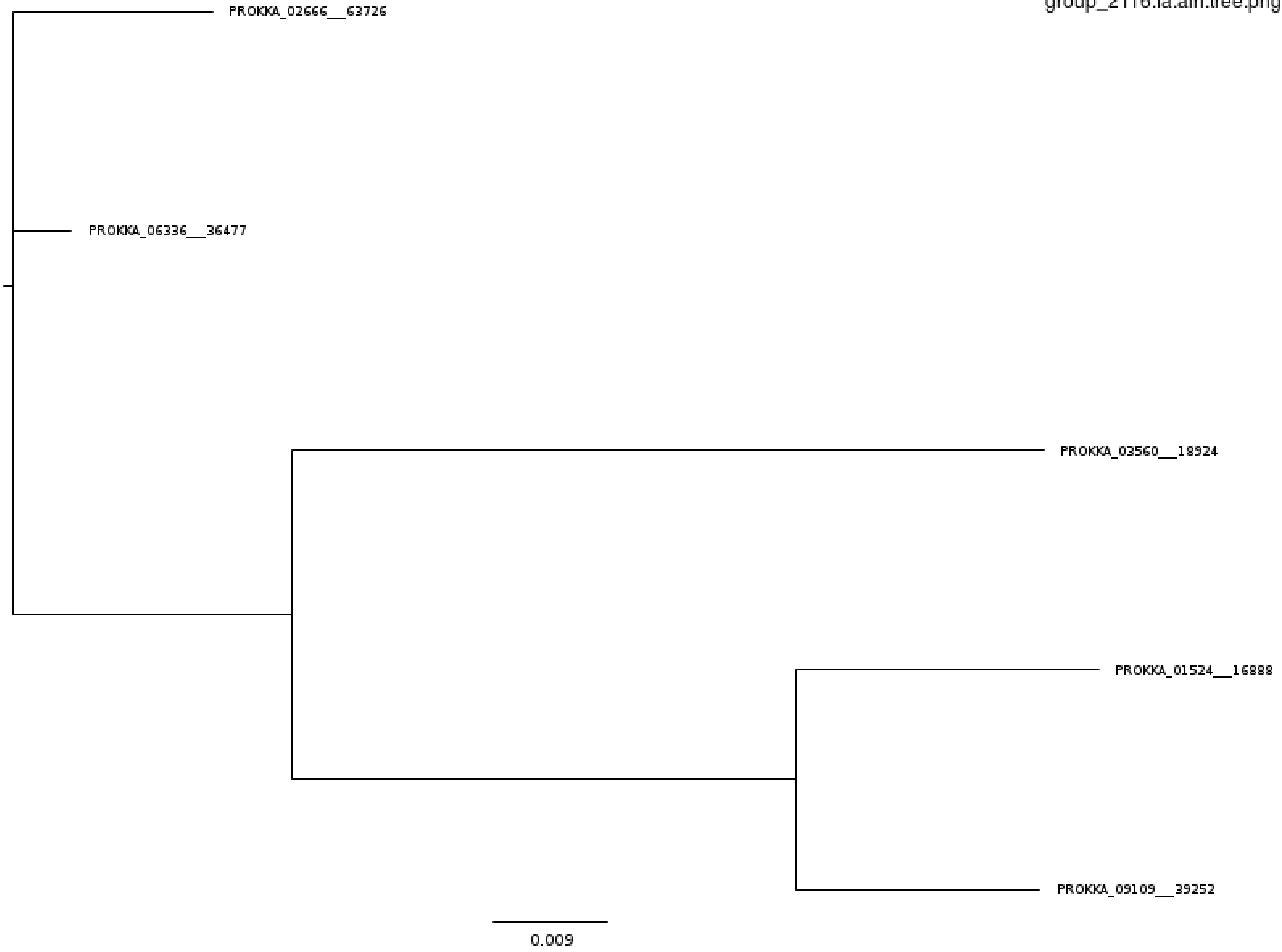

group_2121.fa.aln.tree.png

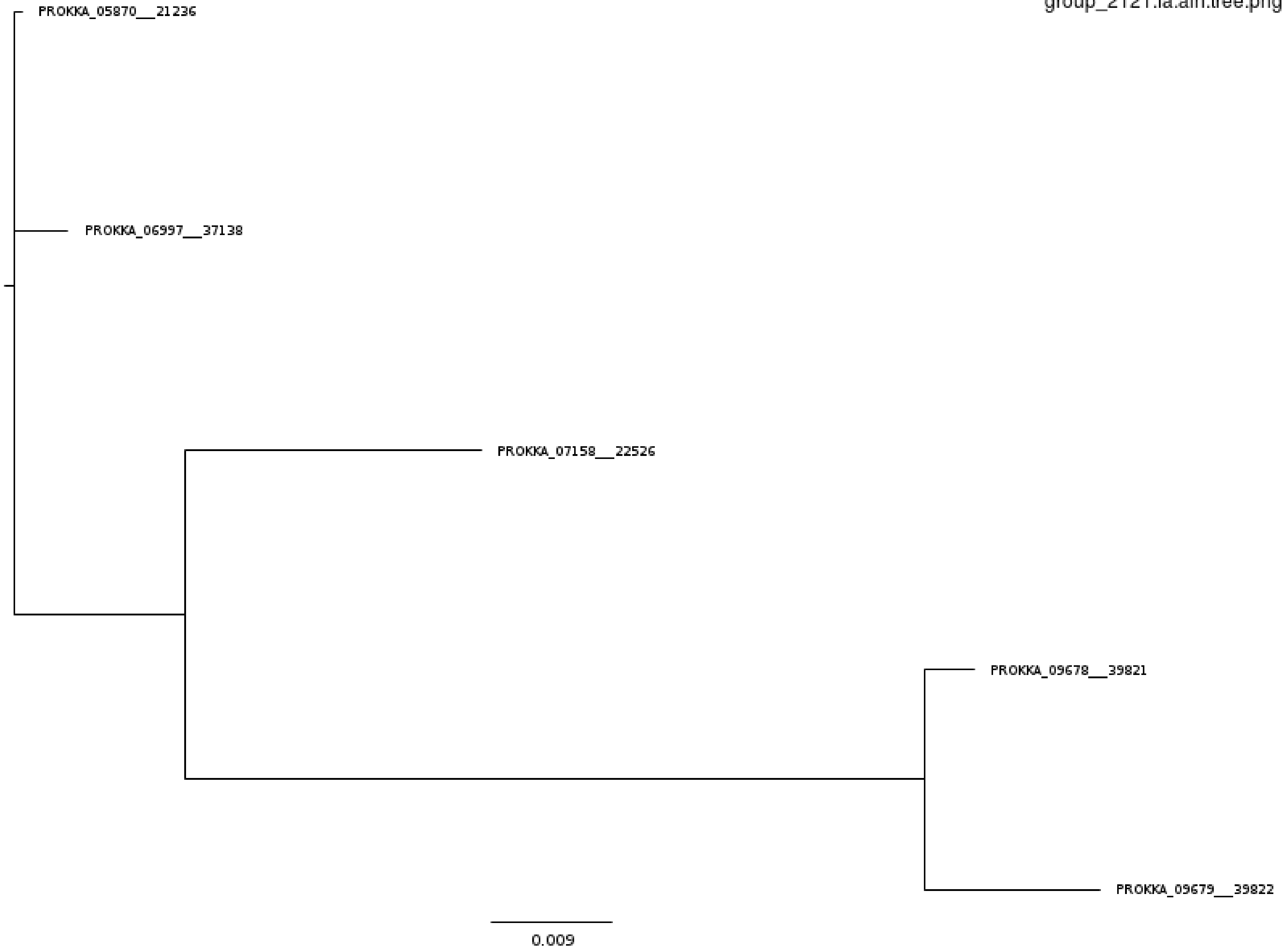

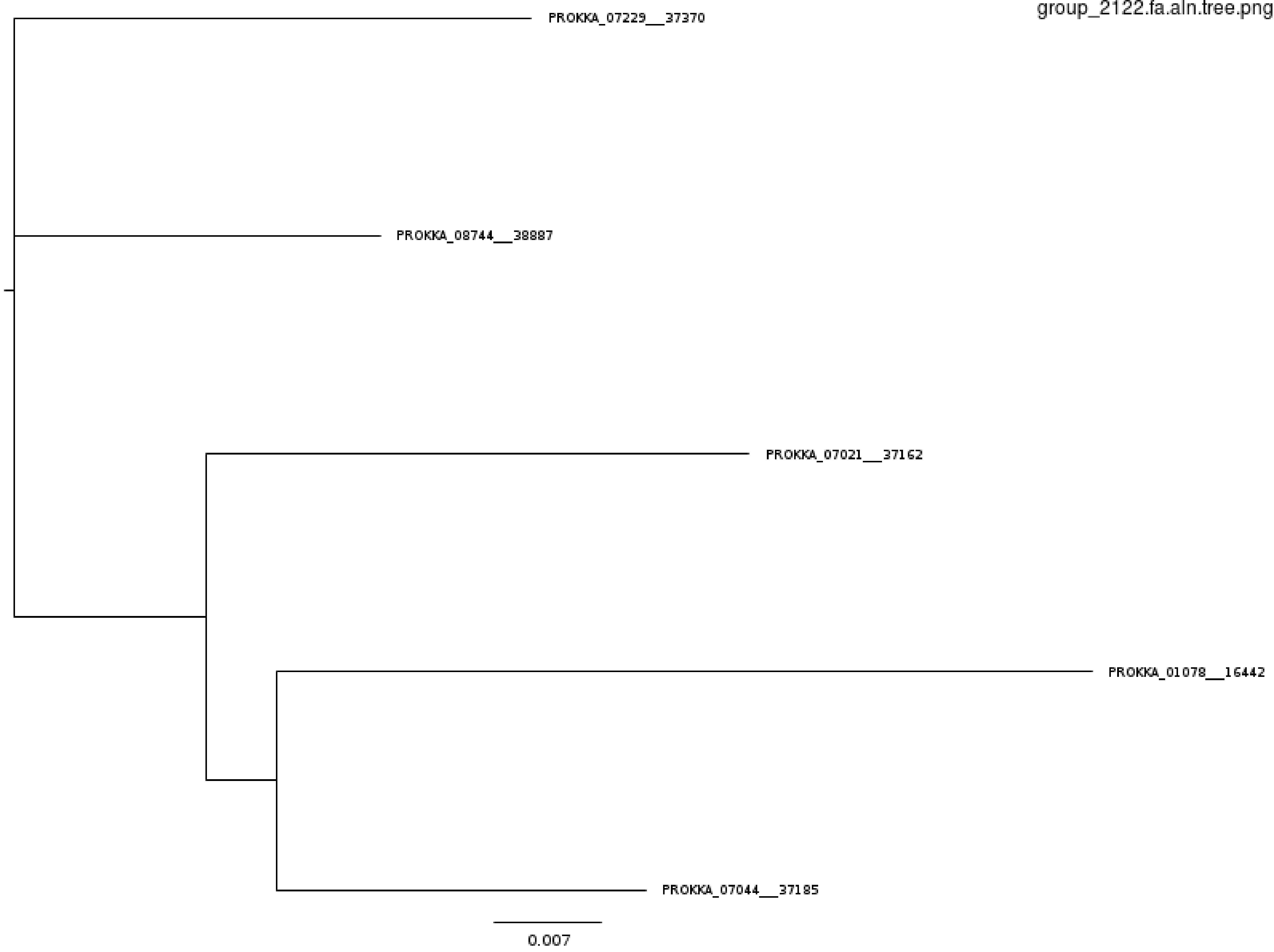
group_2122.fa.aln.tree.png
PROKKA_07229___37370
PROKKA_08744___38887
PROKKA_07021___37162
PROKKA_01078___16442
PROKKA_07044___37185
0.007

group_2124.fa.aln.tree.png

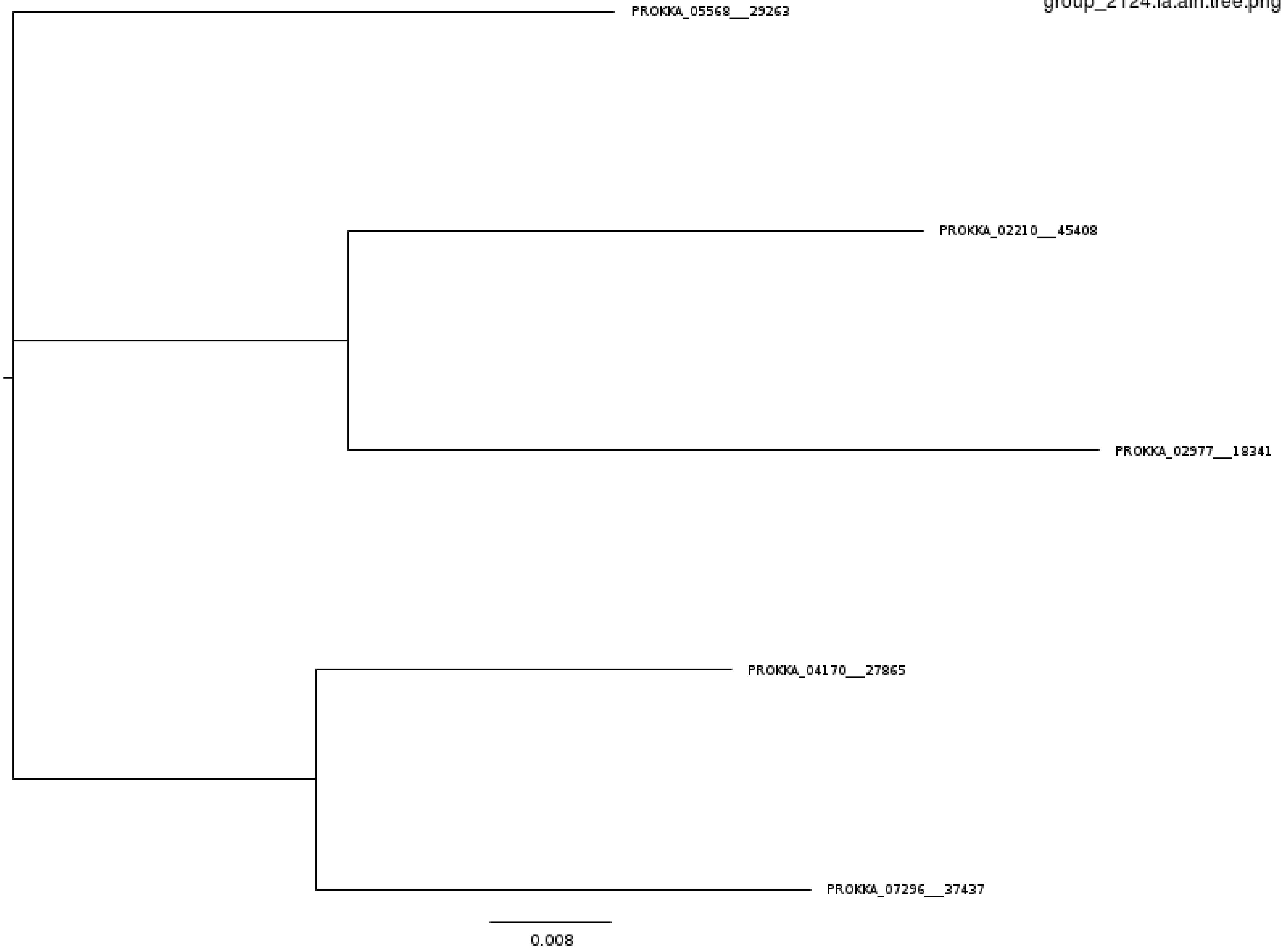

group_2125.fa.aln.tree.png

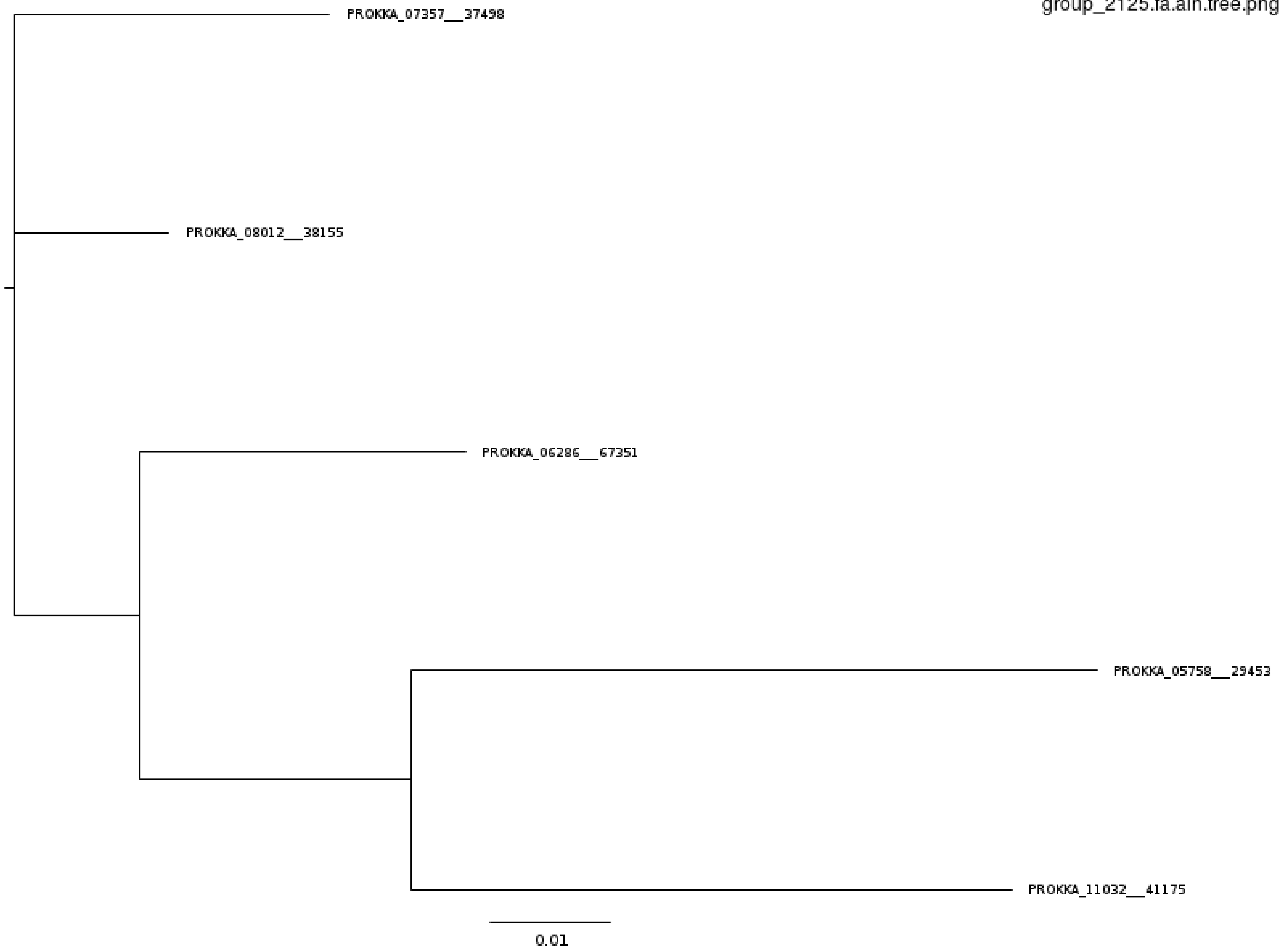

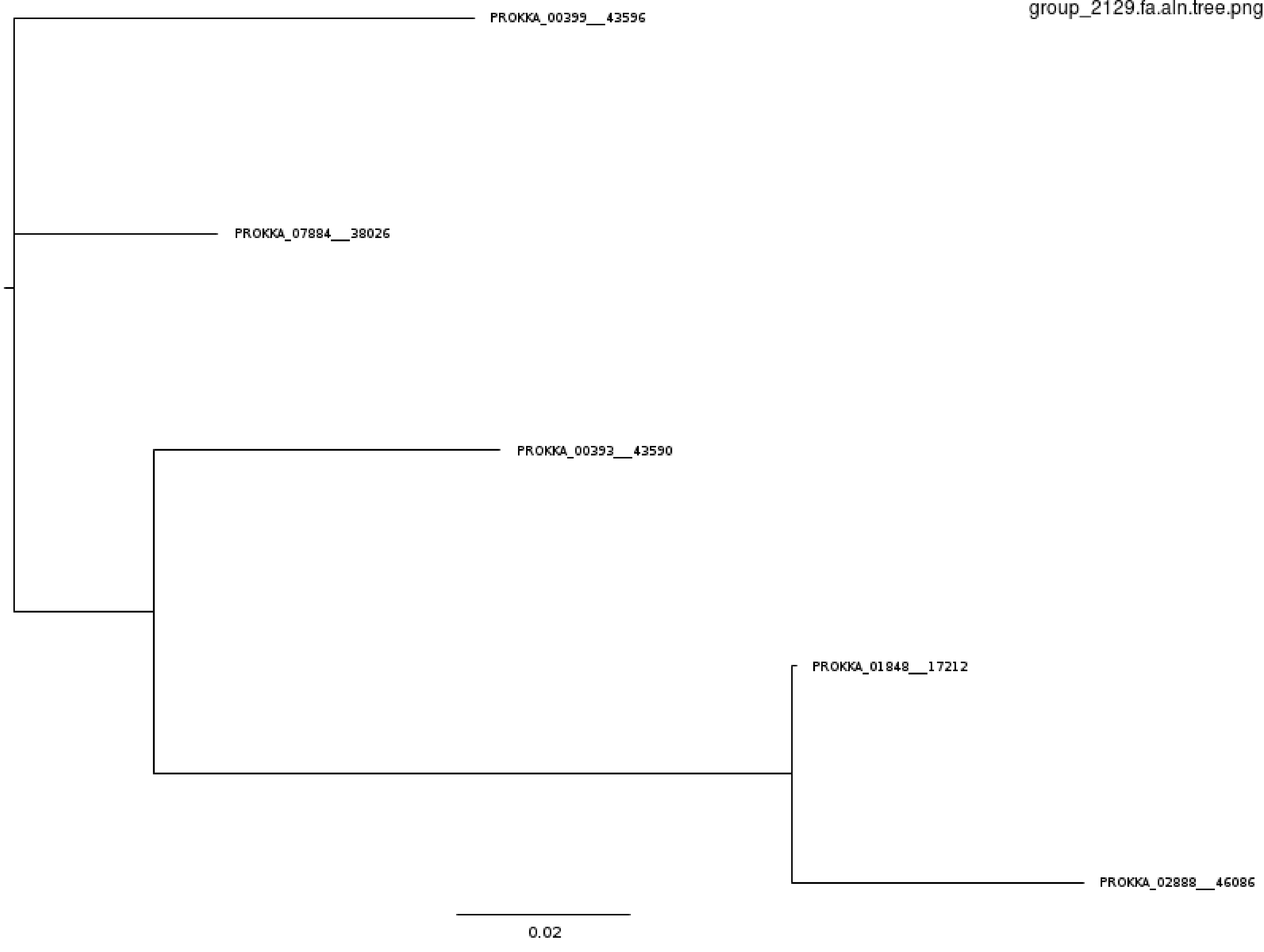
group_2129.fa.aln.tree.png
PROKKA_00399___43596
PROKKA_07884___38026
PROKKA_00393___43590
PROKKA_01848___17212
PROKKA_02888___46086
0.02

group_2132.fa.aln.tree.png

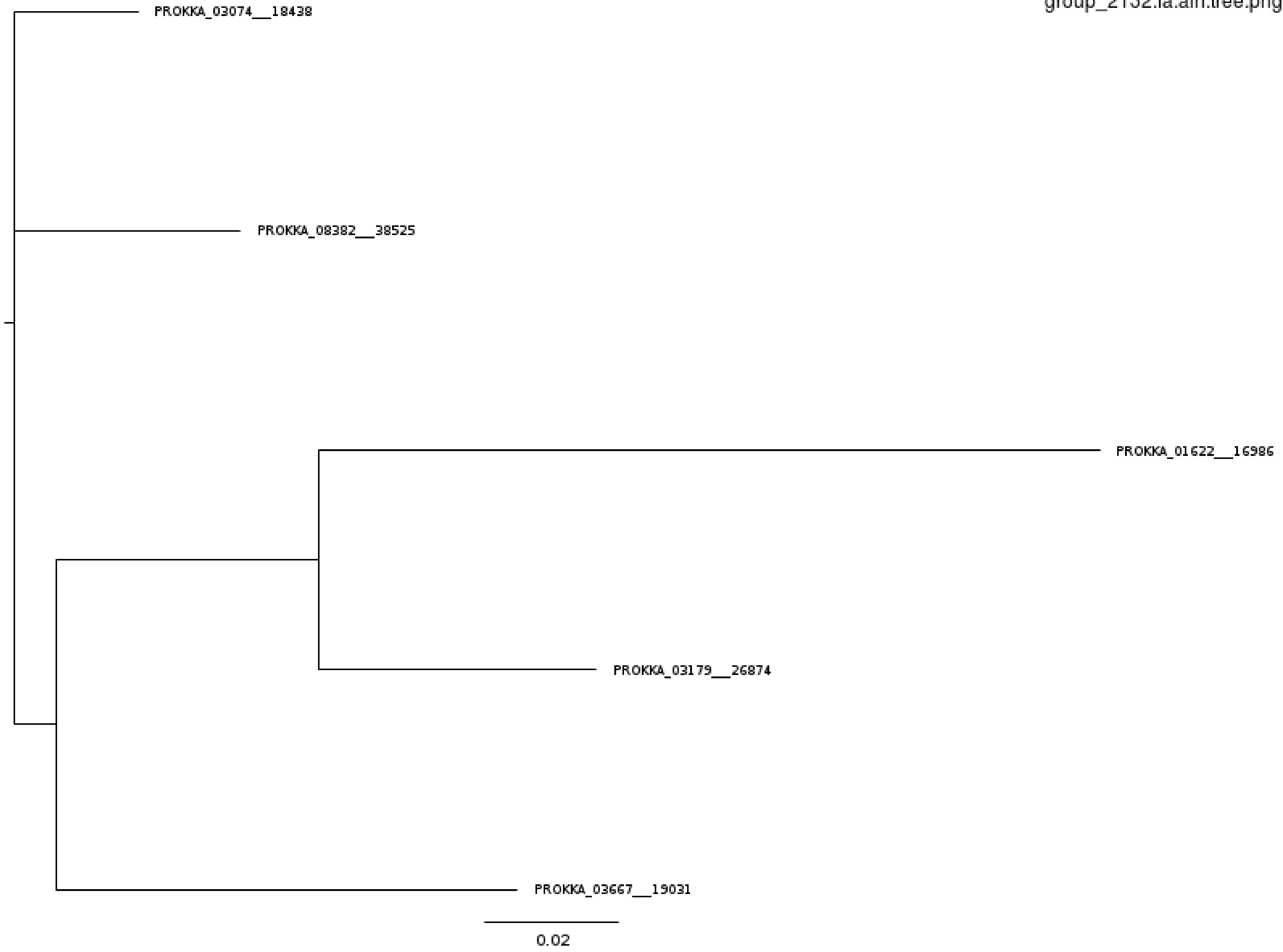

group_2135.fa.aln.tree.png

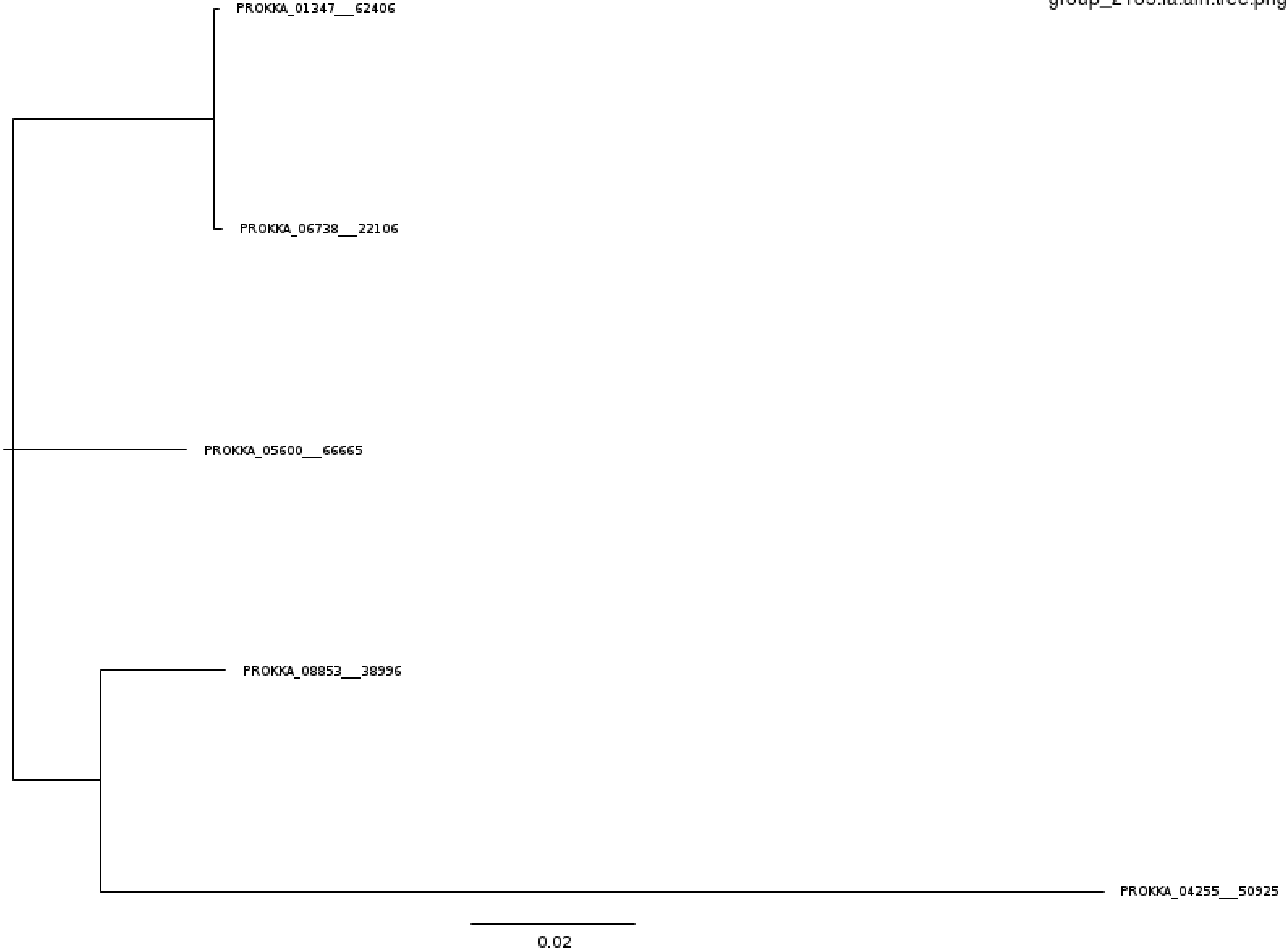

group_2137.fa.aln.tree.png

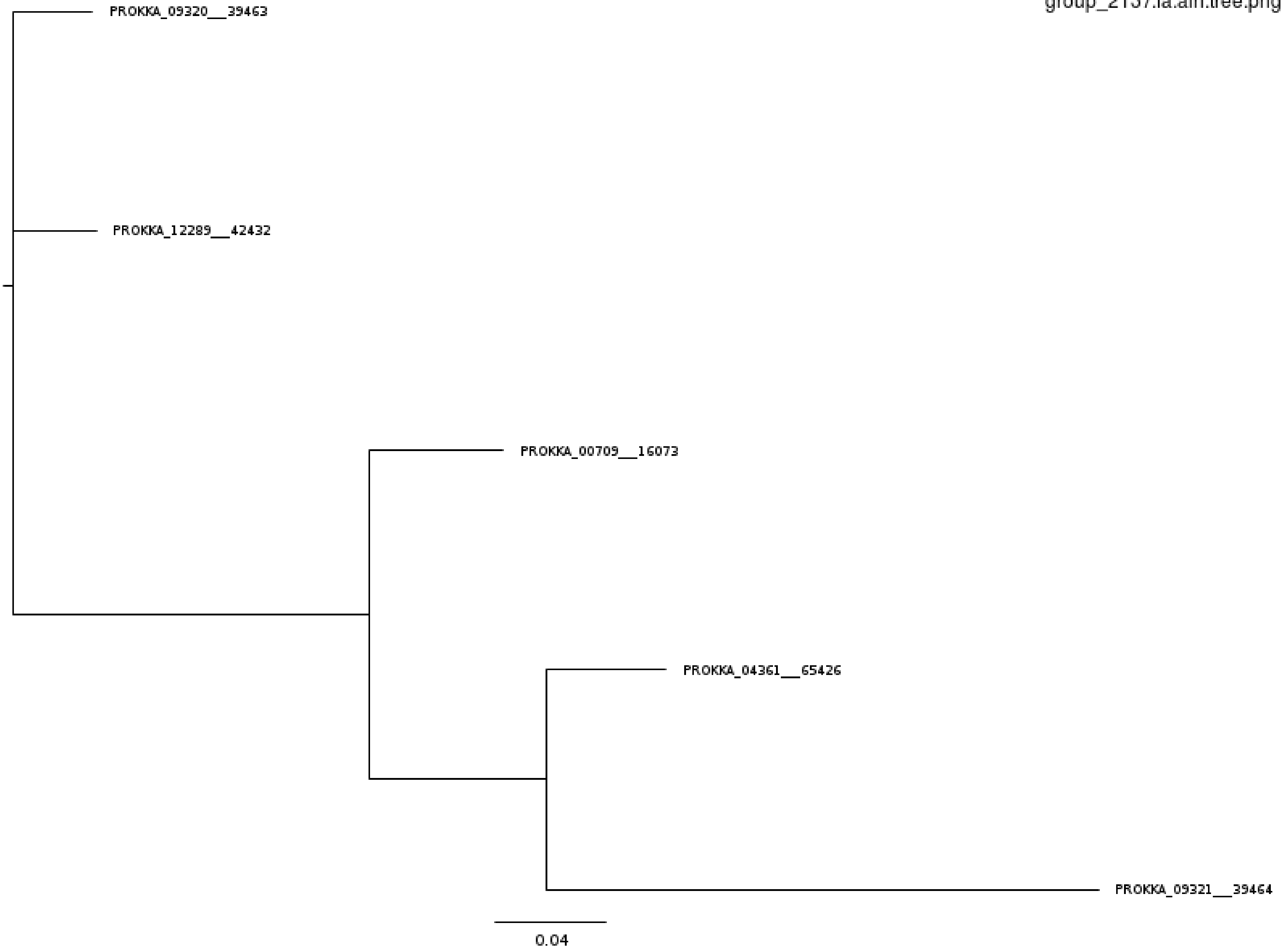

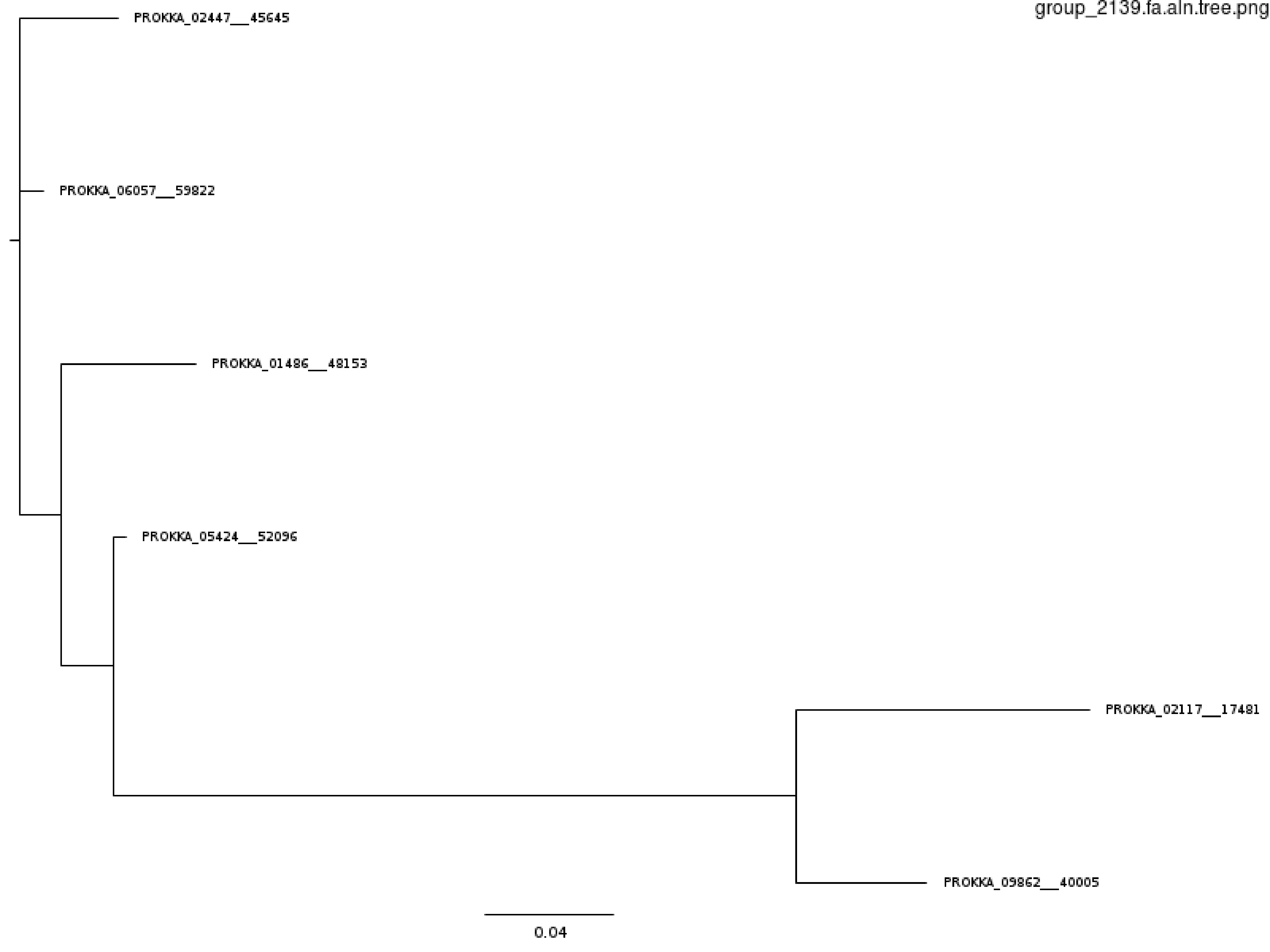
group_2139.fa.aln.tree.png
PROKKA_02447___45645
PROKKA_06057___59822
PROKKA_01486___48153
PROKKA_05424___52096
PROKKA_02117___17481
PROKKA_09862___40005
0.04

group_2140.fa.aln.tree.png

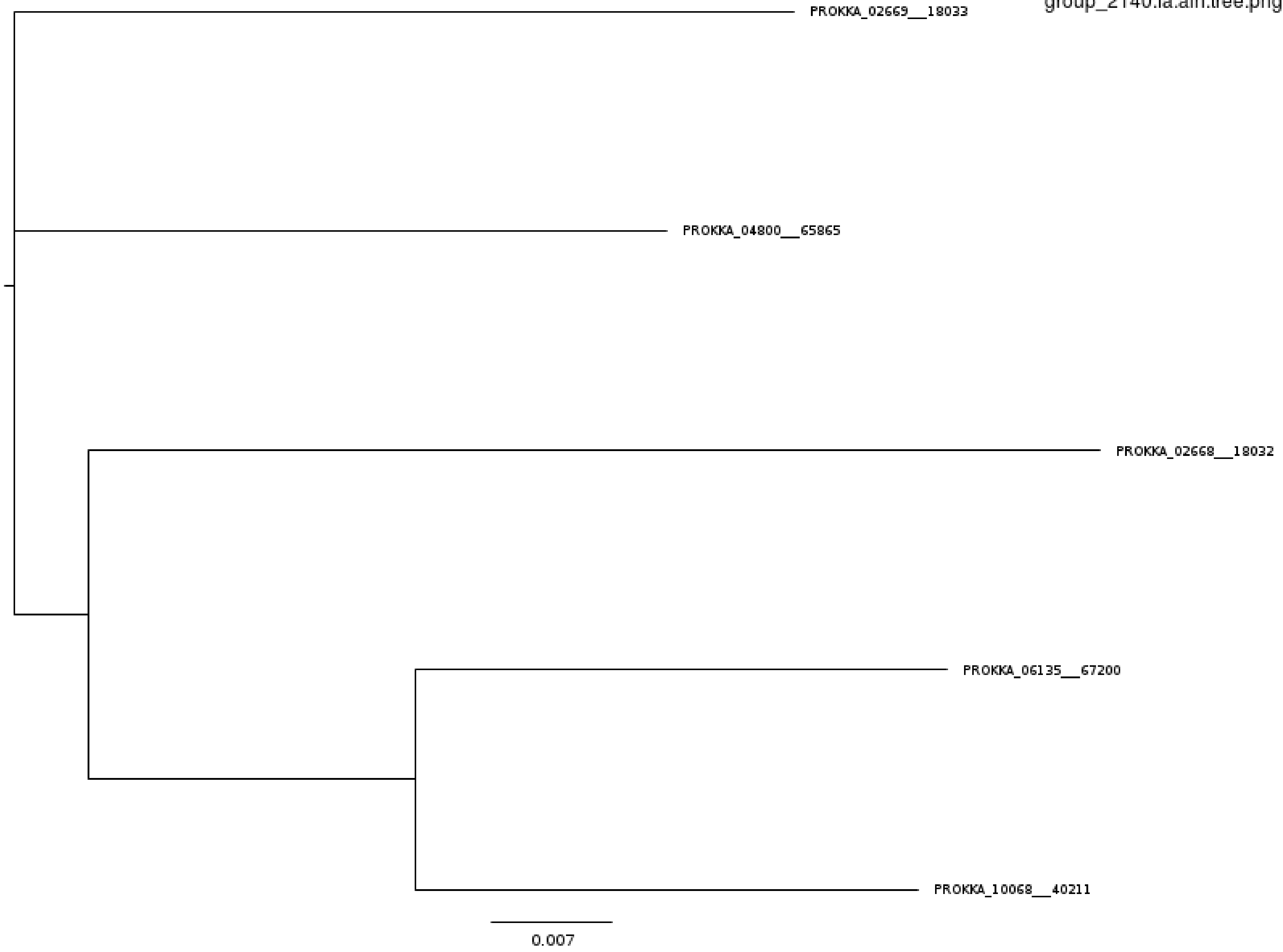

group_2141.fa.aln.tree.png

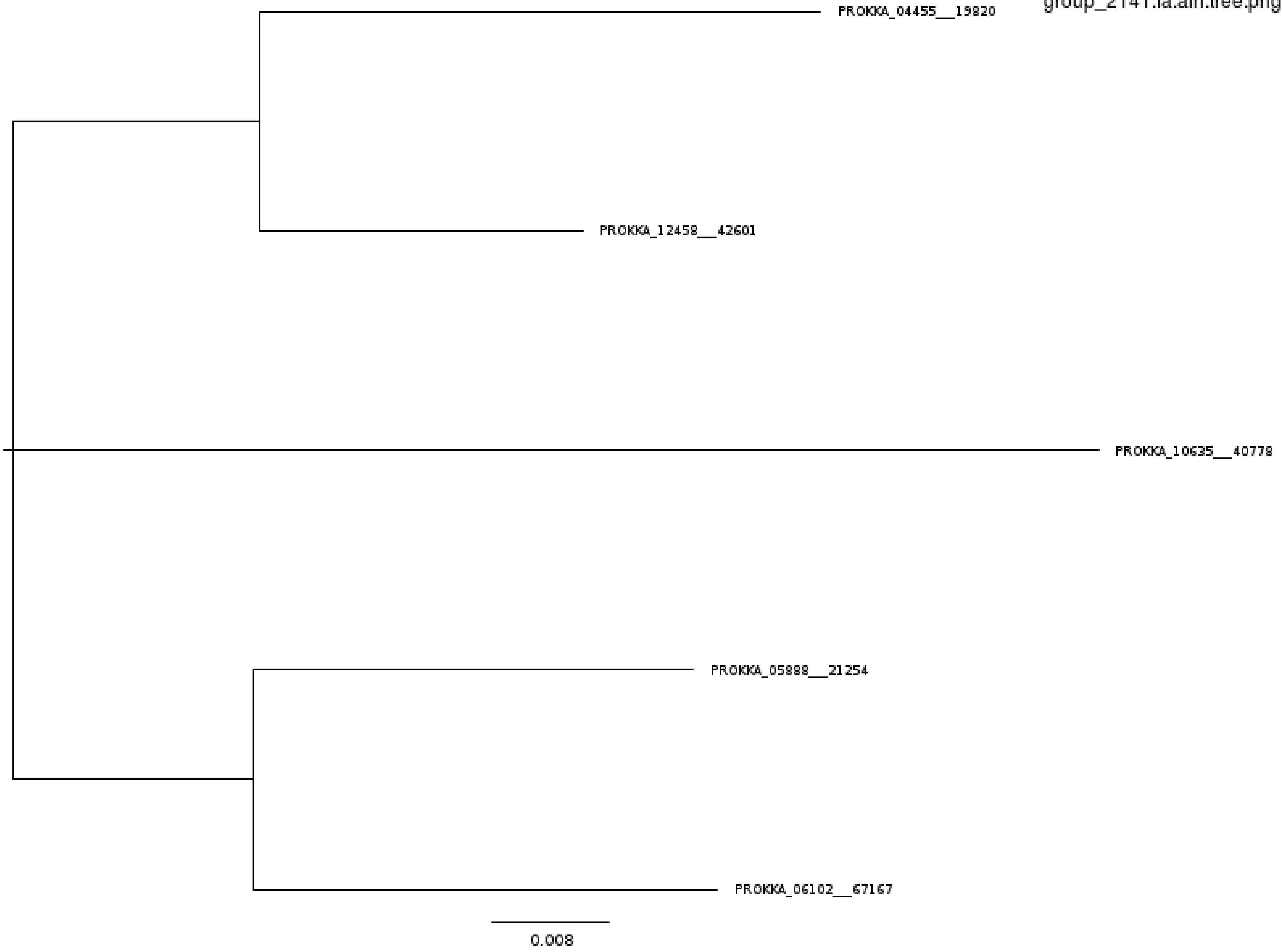

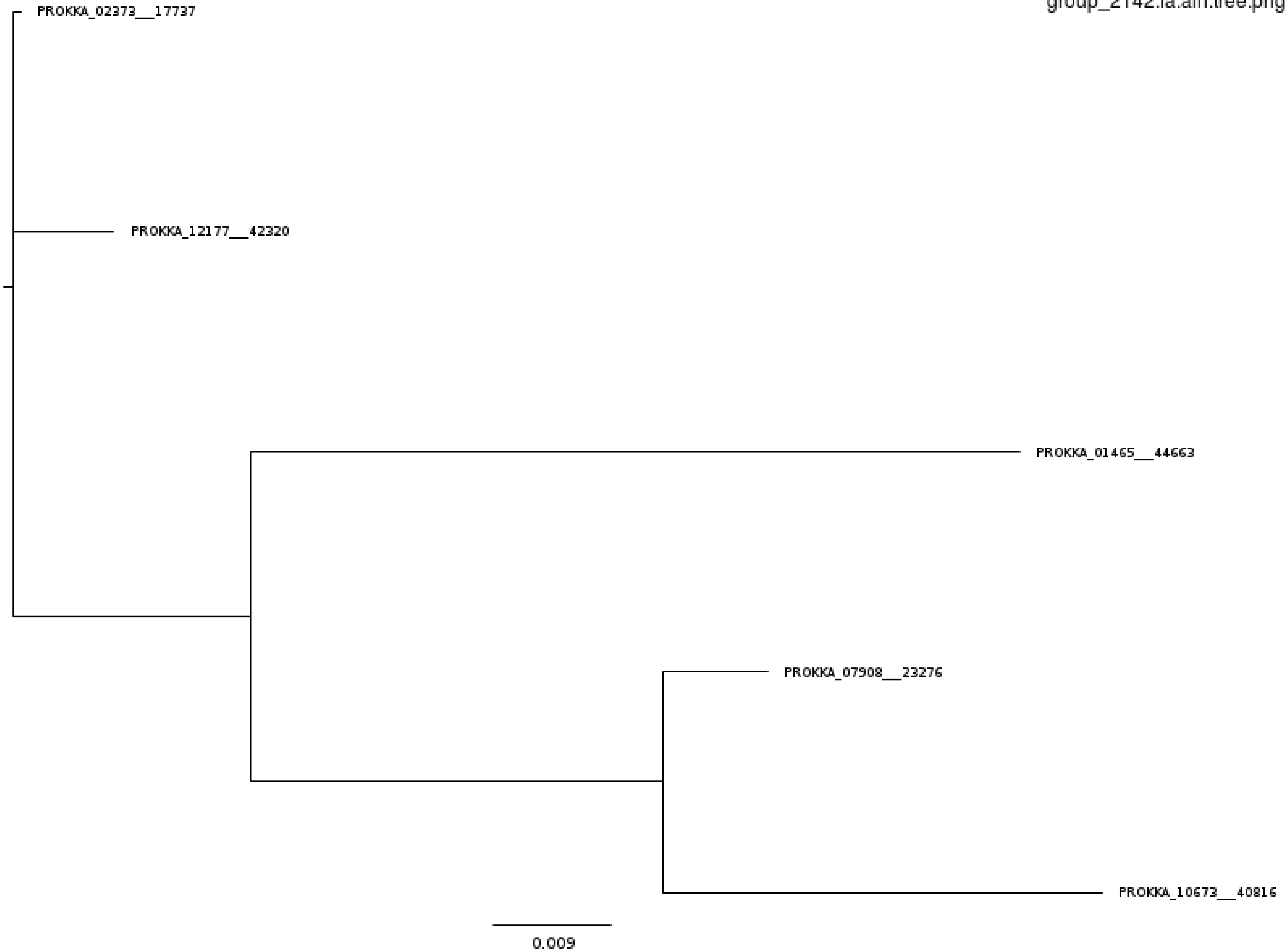
PROKKA_02373___17737
PROKKA_12177___42320
PROKKA_01465___44663
PROKKA_07908___23276
PROKKA_10673___40816
0.009

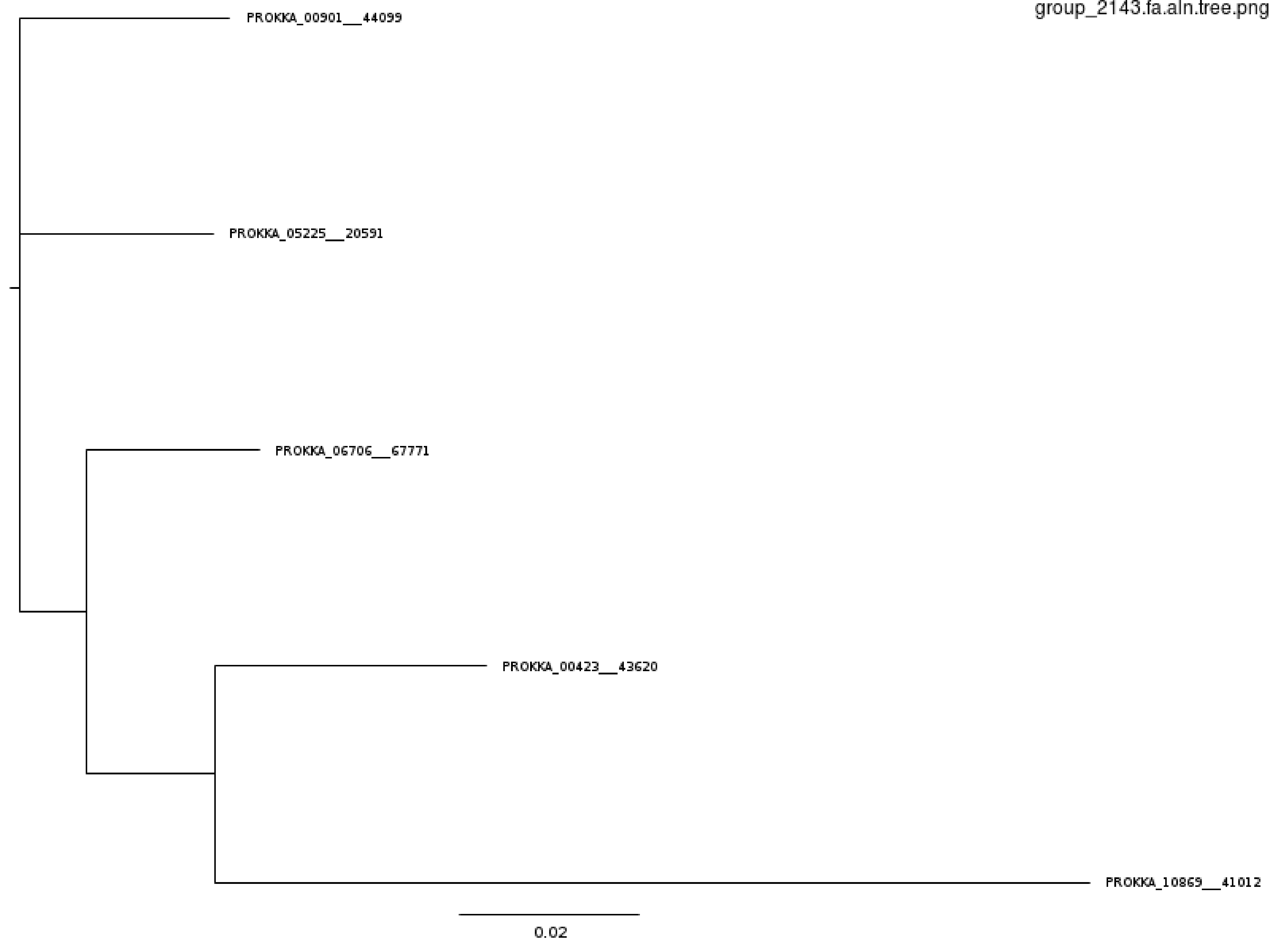
group_2143.fa.aln.tree.png
PROKKA_00901___44099
PROKKA_05225___20591
PROKKA_06706___67771
PROKKA_00423___43620
PROKKA_10869___41012
0.02

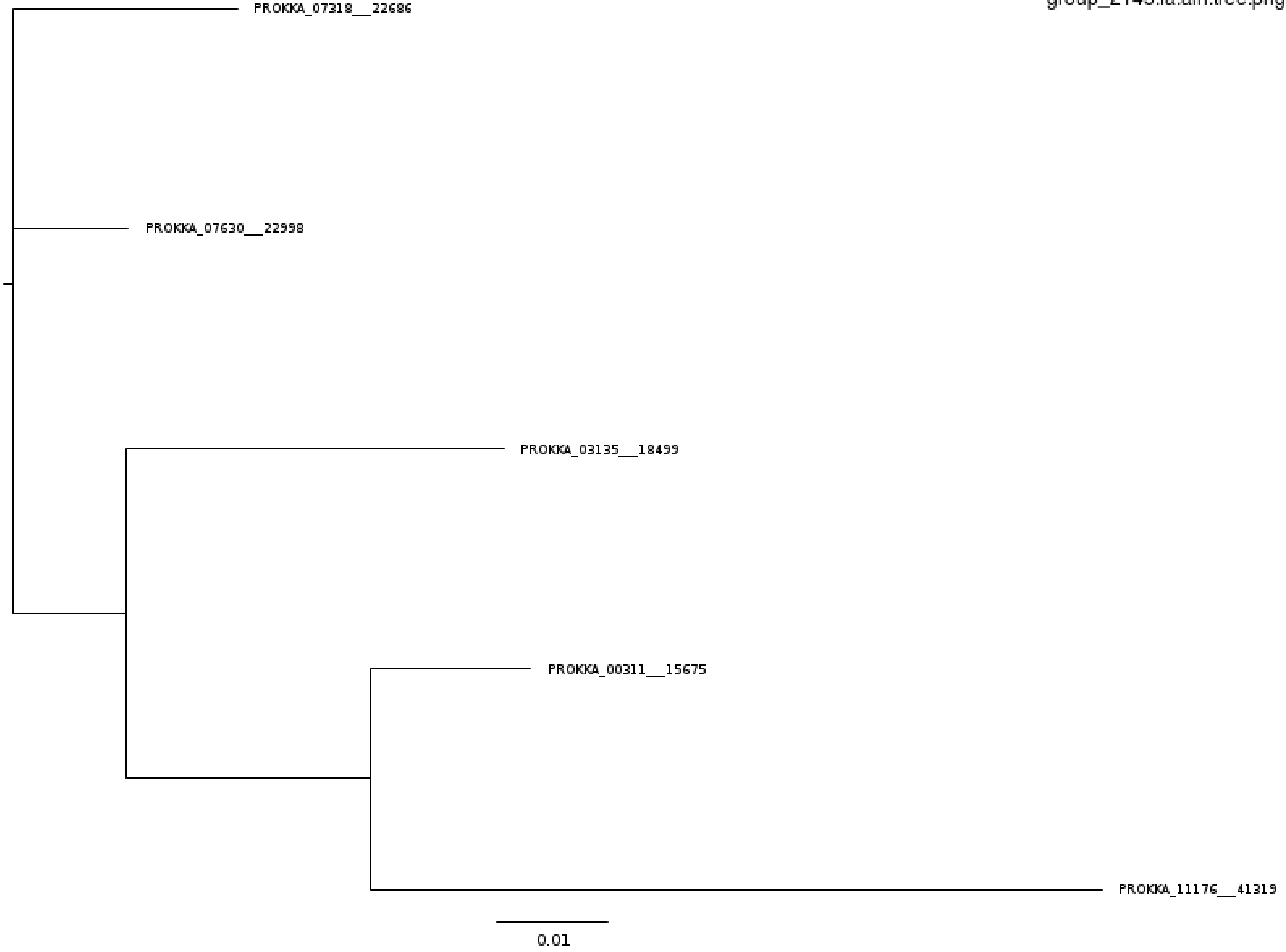
PROKKA_07318___22686
PROKKA_07630___22998
PROKKA_03135___18499
PROKKA_00311___15675
PROKKA_11176___41319
0.01

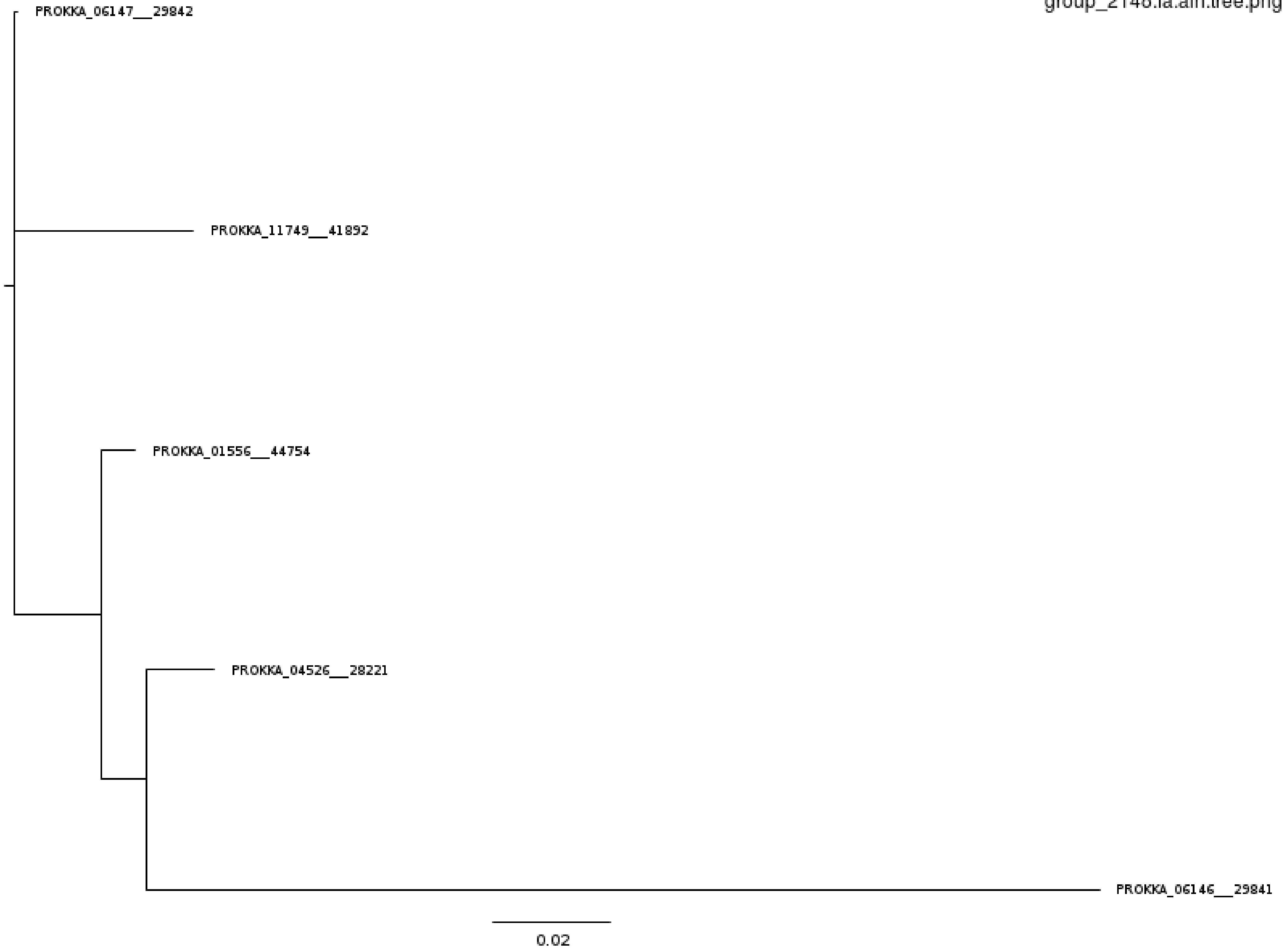
PROKKA_06147__29842
PROKKA_11749__41892
PROKKA_01556__44754
PROKKA_04526__28221
PROKKA_06146__29841
0.02

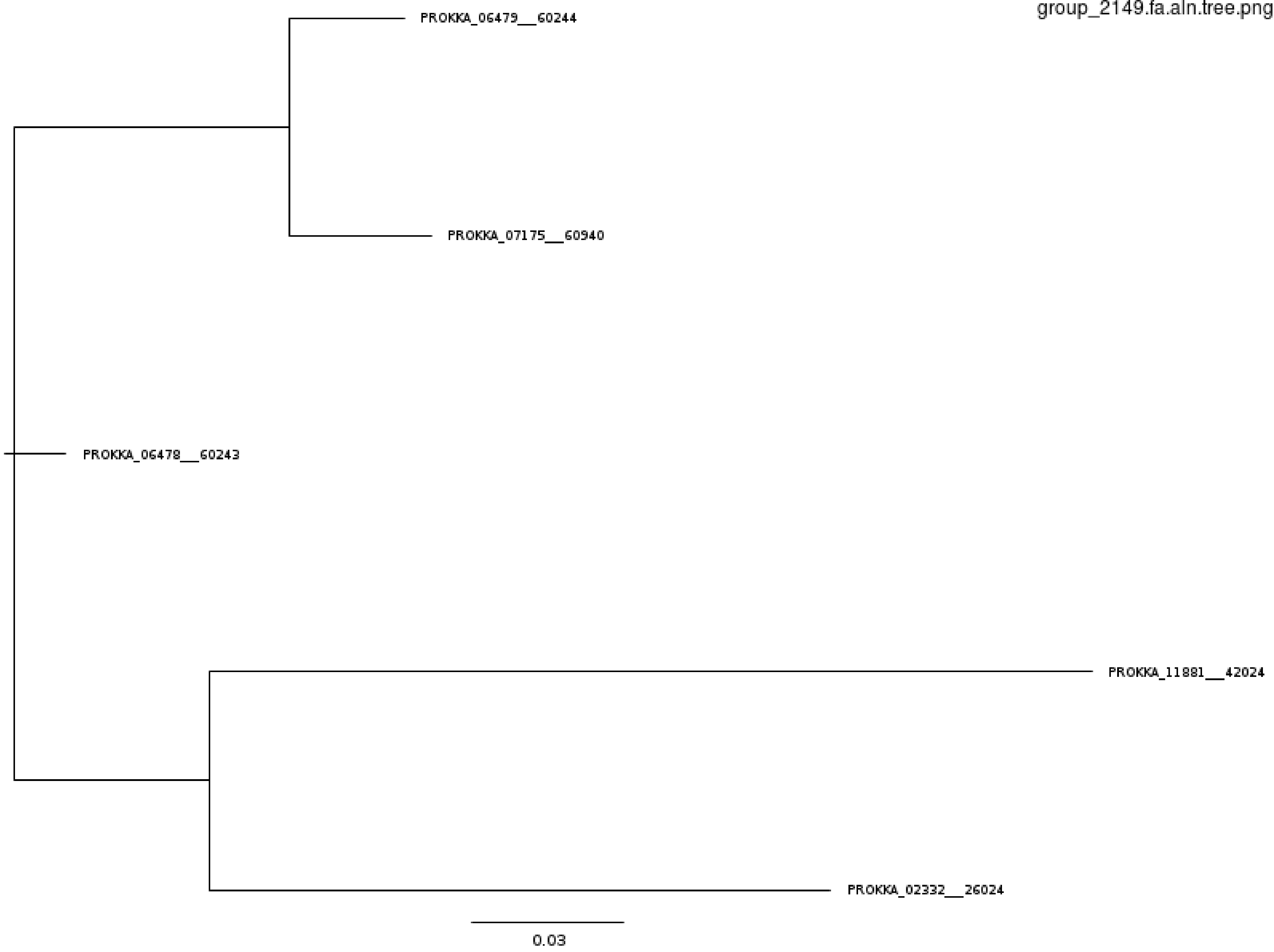
group_2149.fa.aln.tree.png
PROKKA_06479___60244
PROKKA_07175___60940
PROKKA_06478___60243
PROKKA_11881___42024
PROKKA_02332___26024
0.03

group_214.fa.aln.tree.png

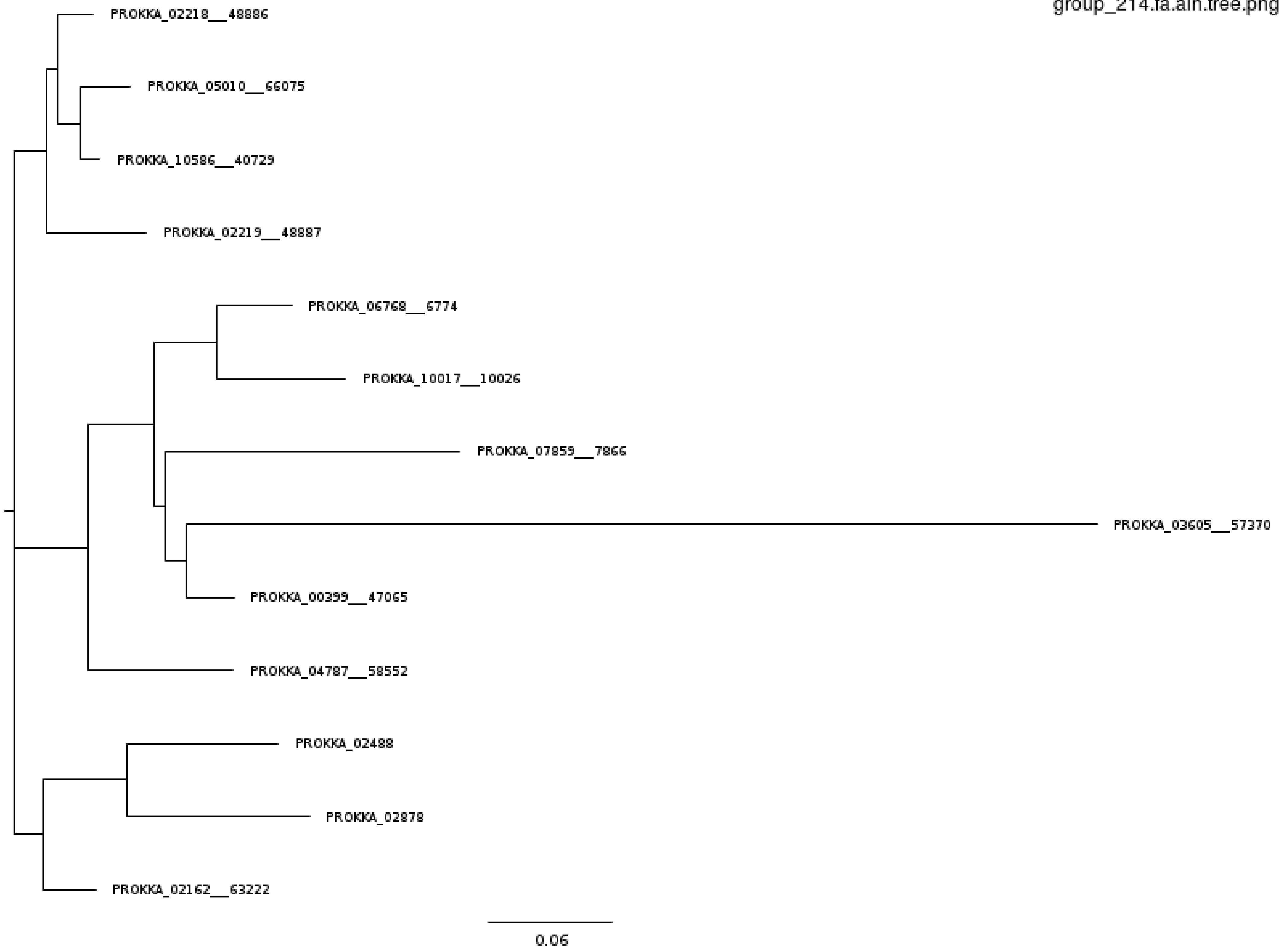

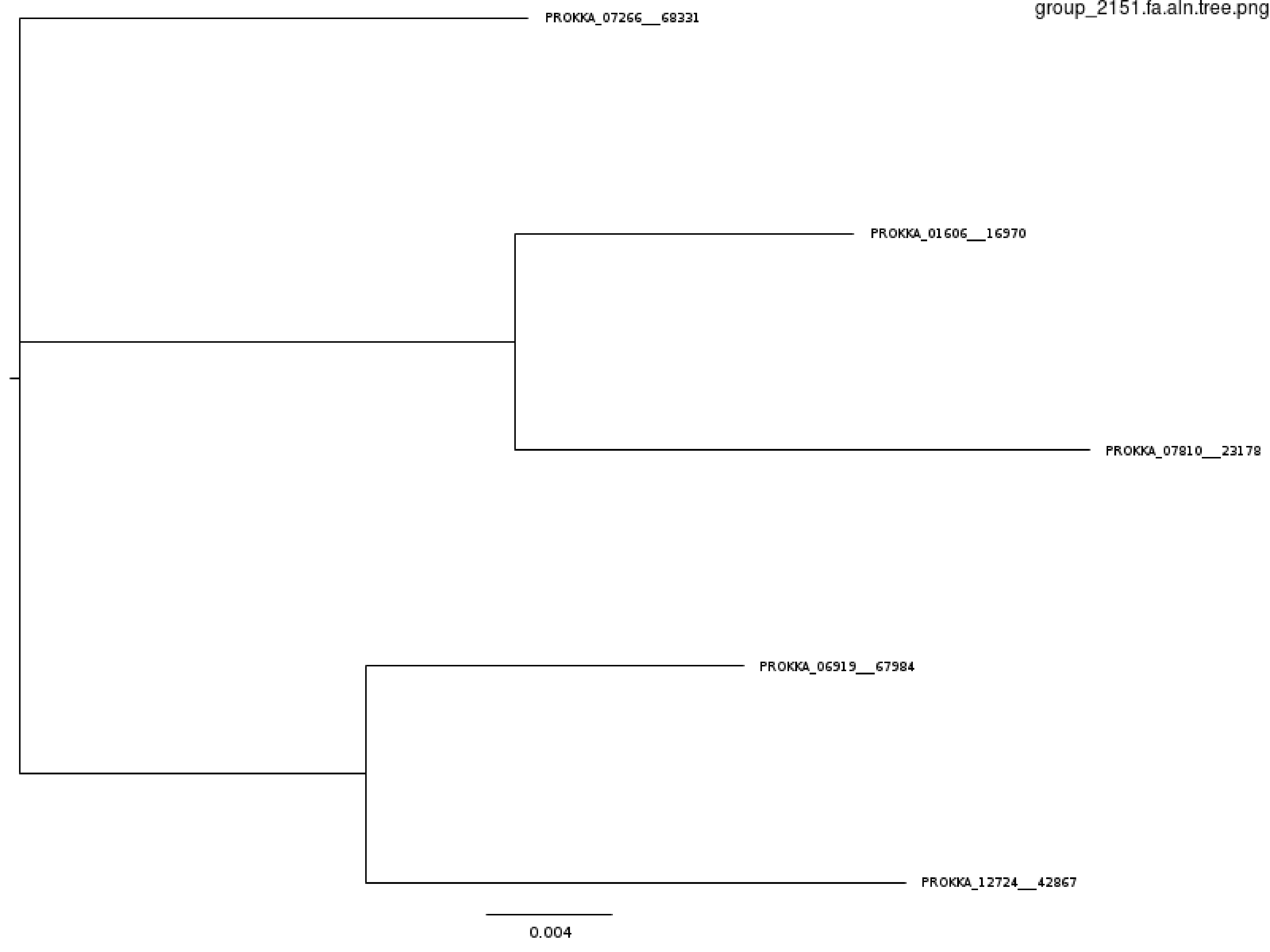

group_2151.fa.aln.tree.png
PROKKA_07266__68331
PROKKA_01606__16970
PROKKA_07810__23178
PROKKA_06919__67984
PROKKA_12724__42867
0.004

group_2152.fa.aln.tree.png

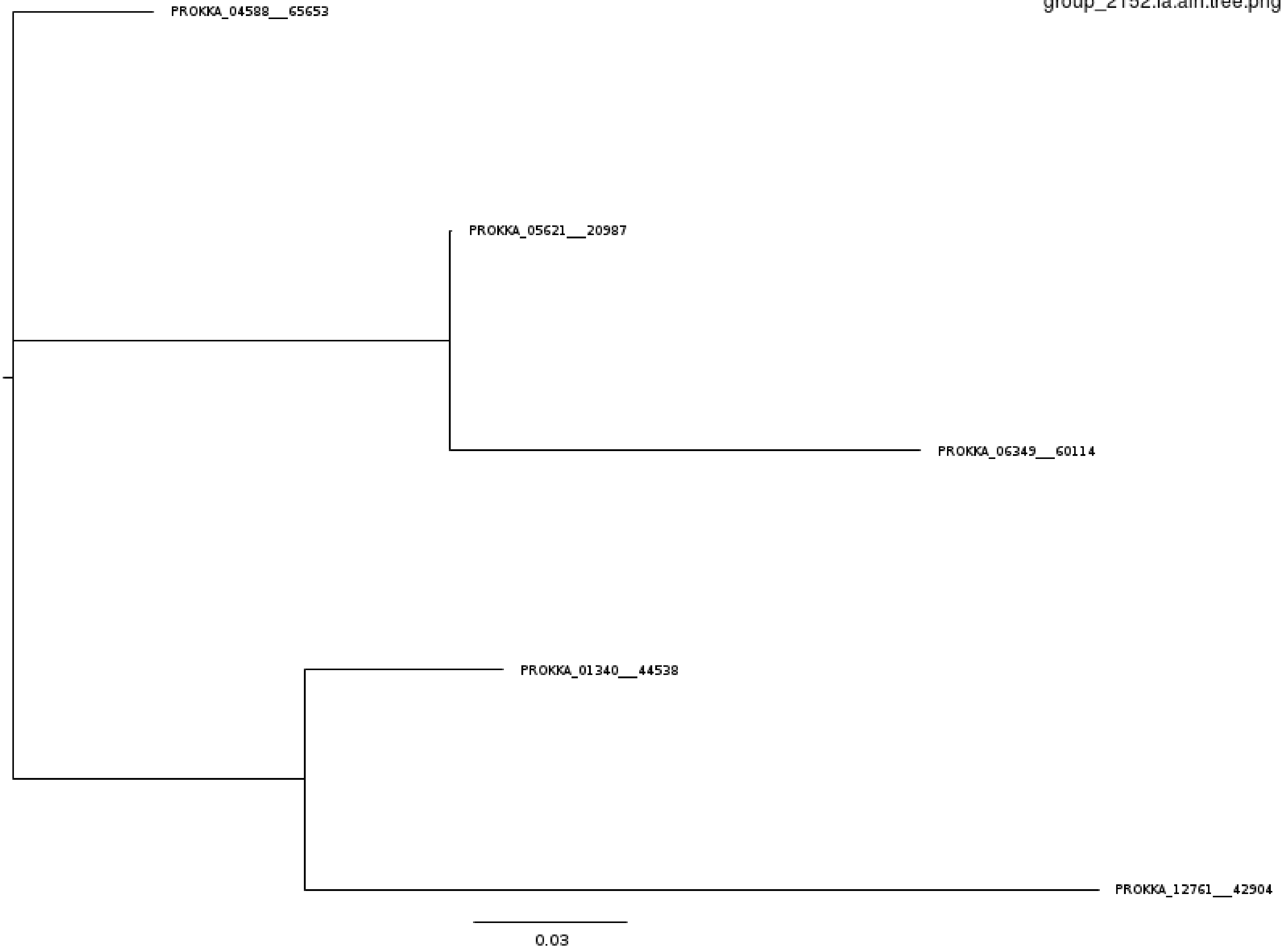

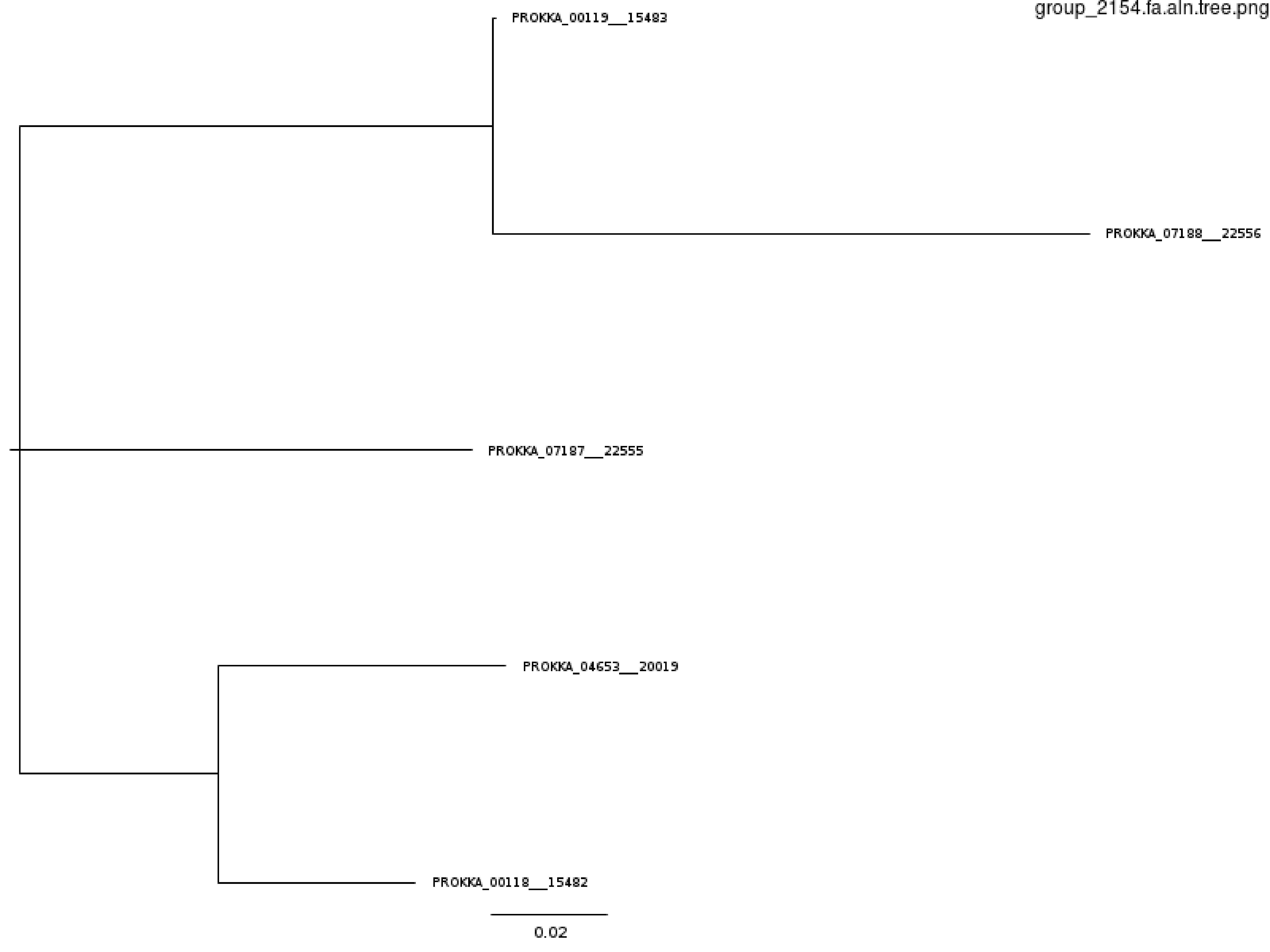
group_2154.fa.aln.tree.png
PROKKA_00119__15483
PROKKA_07188__22556
PROKKA_07187__22555
PROKKA_04653__20019
PROKKA_00118__15482
0.02

group_2155.fa.aln.tree.png

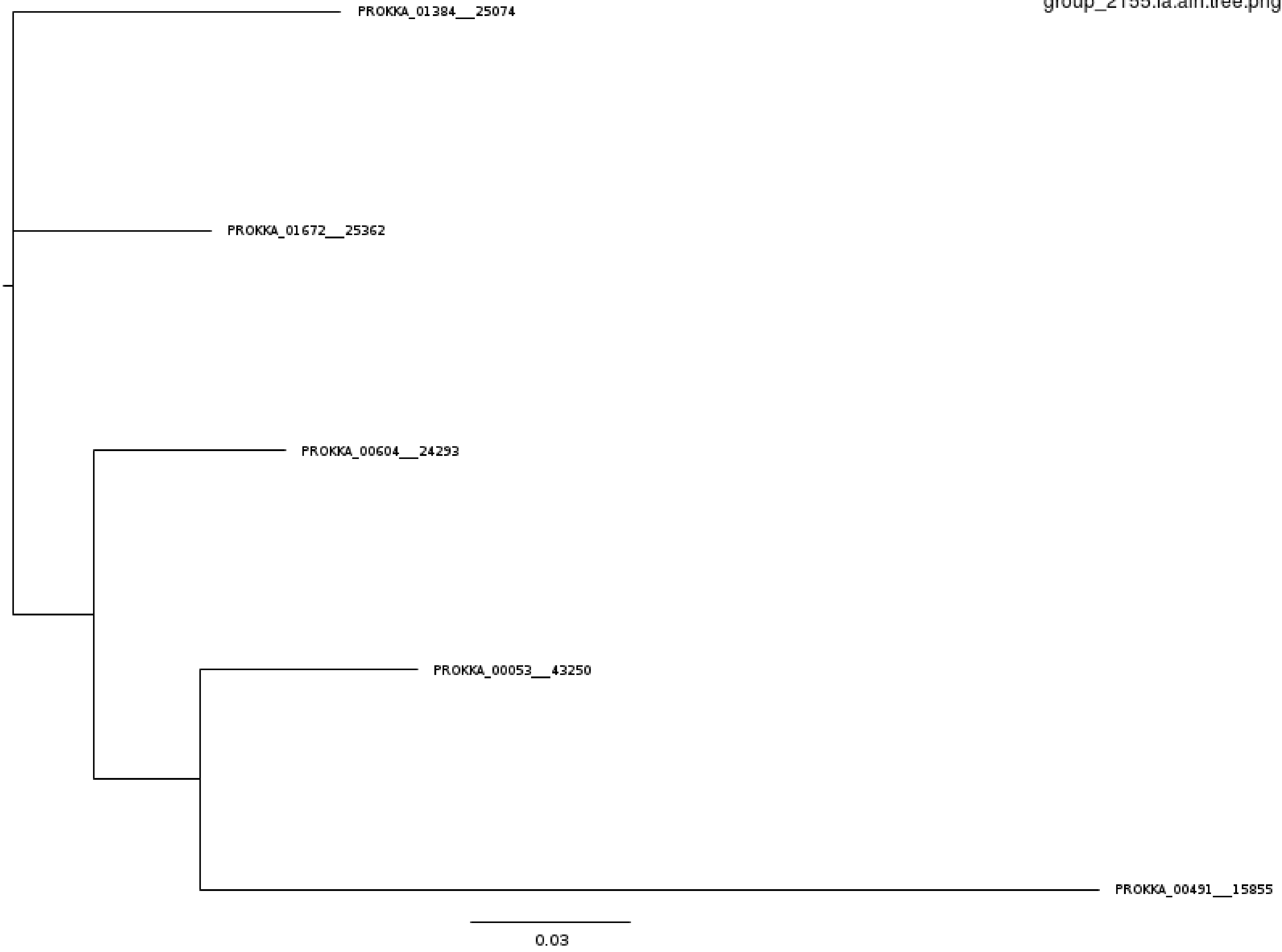

group_2156.fa.aln.tree.png

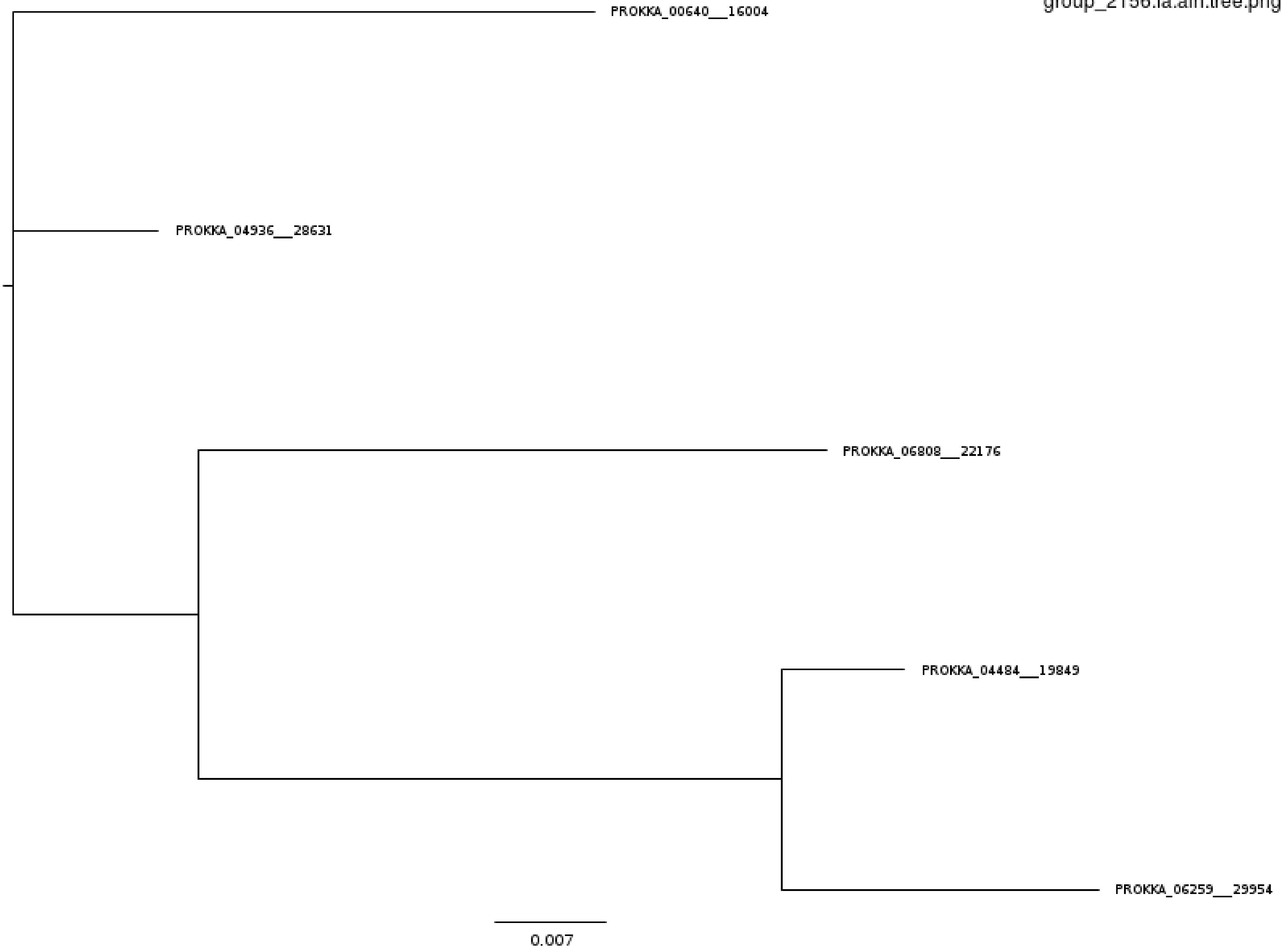

group_2157.fa.aln.tree.png

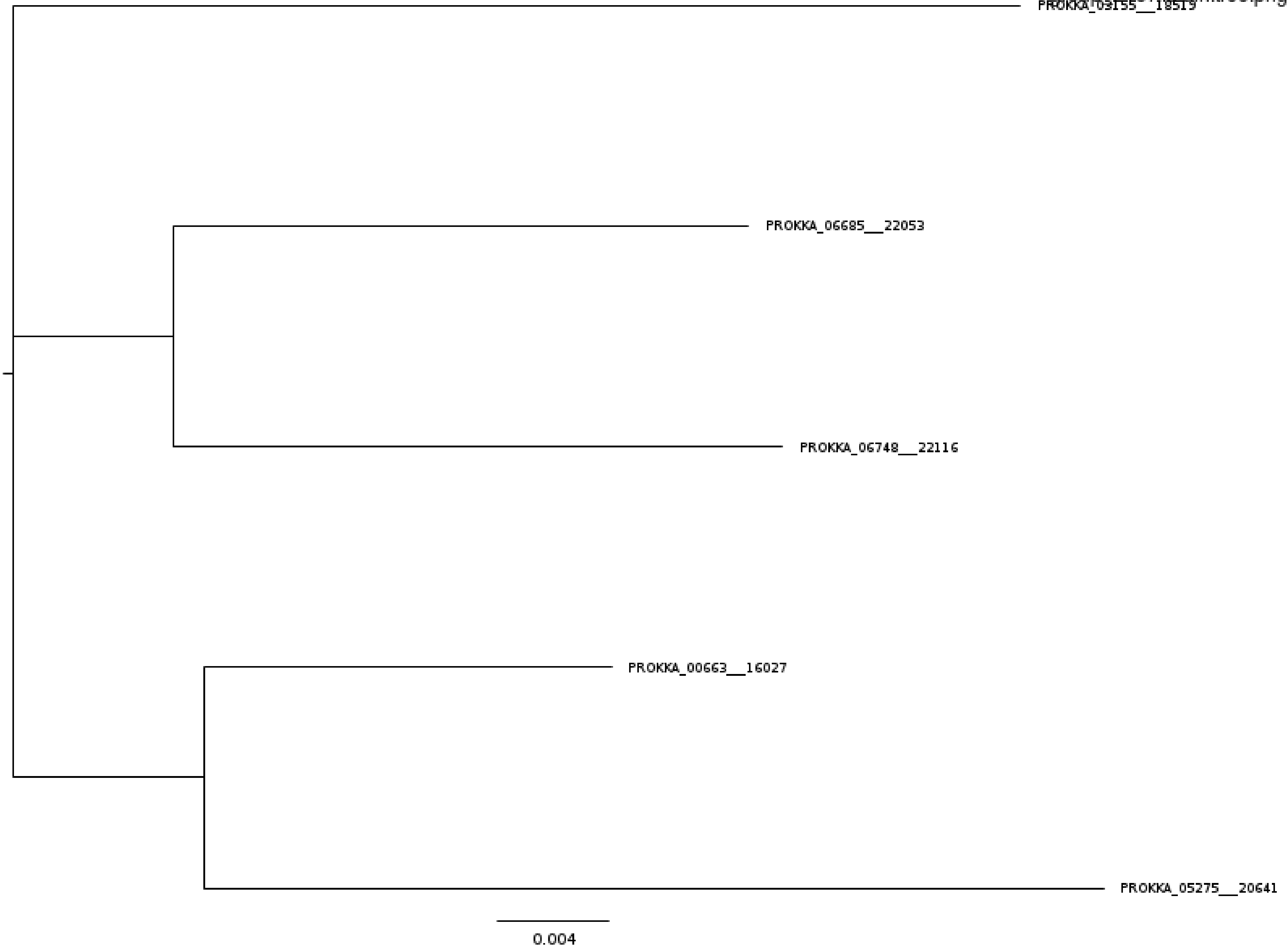

group_2158.fa.aln.tree.png

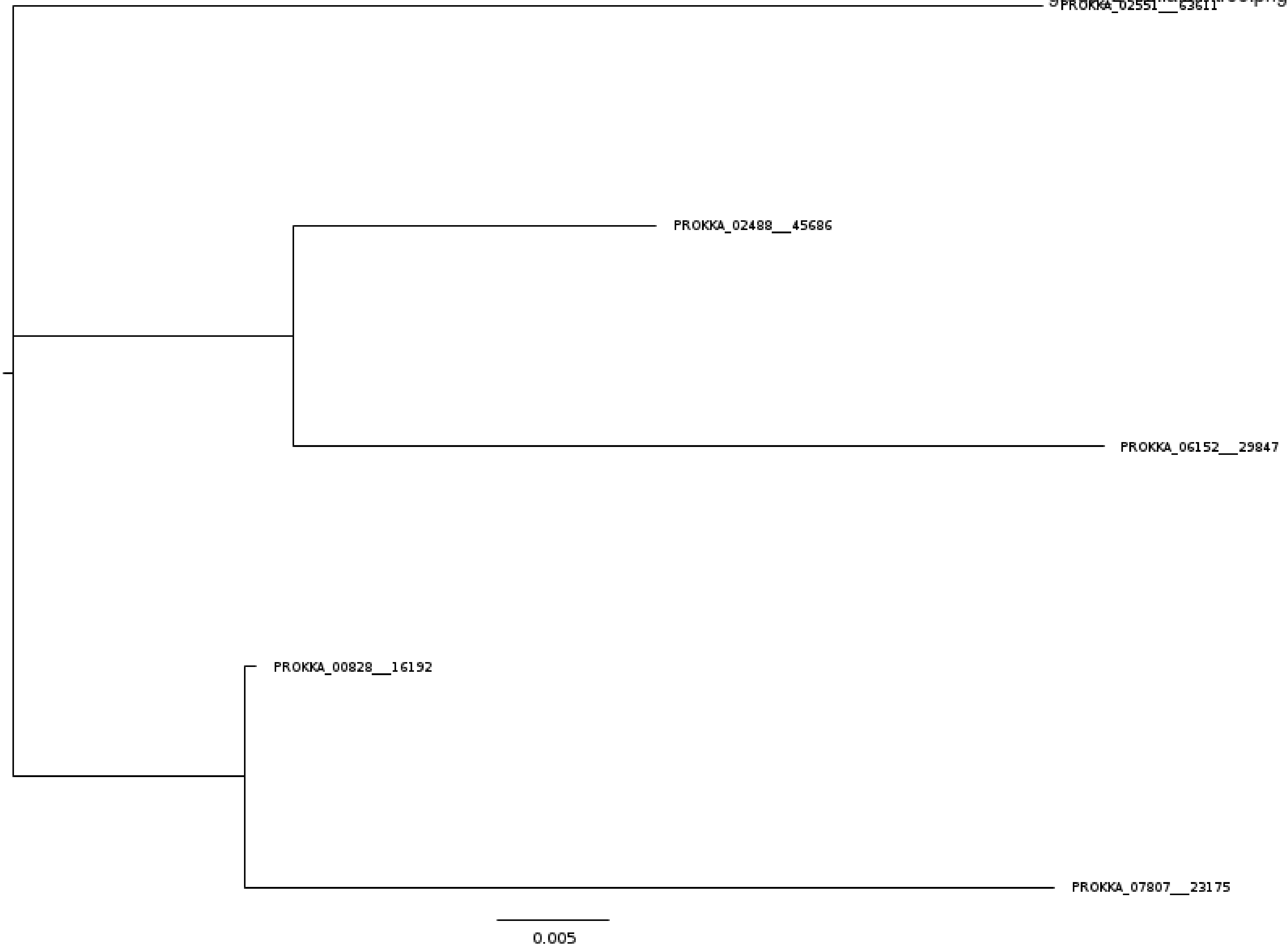

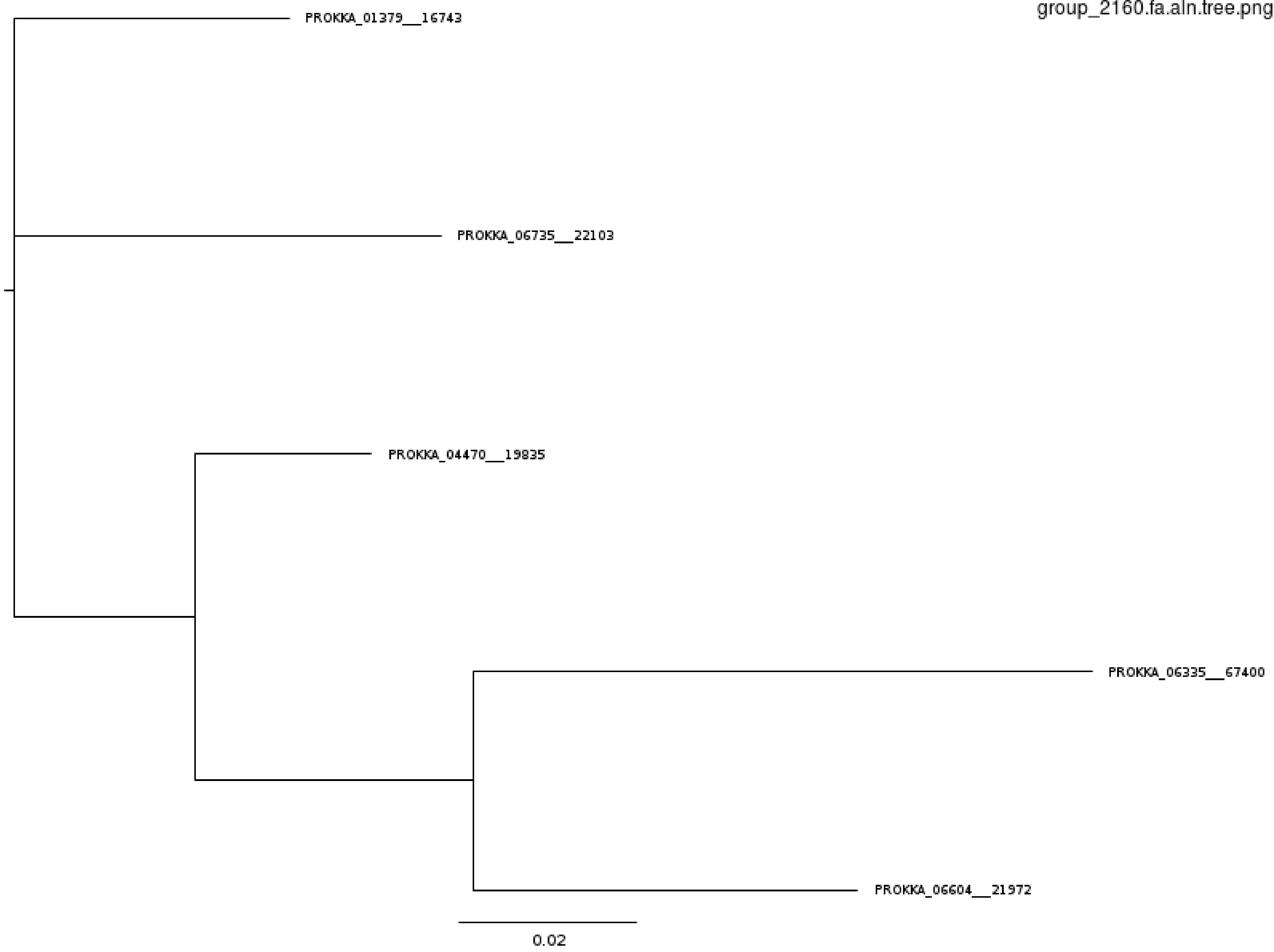
group_2160.fa.aln.tree.png
PROKKA_01379___16743
PROKKA_06735___22103
PROKKA_04470___19835
PROKKA_06335___67400
PROKKA_06604___21972
0.02

group_2161.fa.aln.tree.png

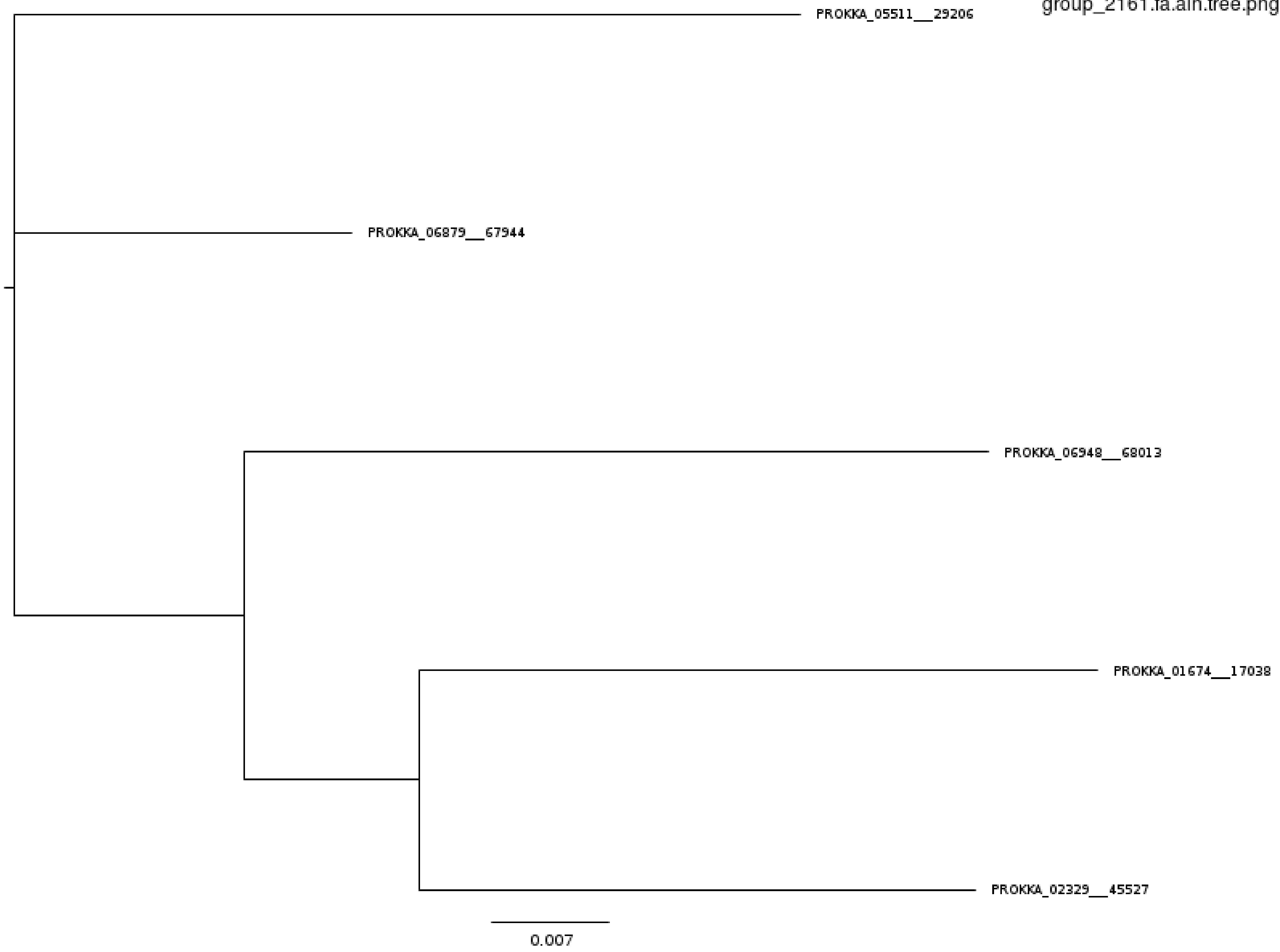

group_2165.fa.aln.tree.png

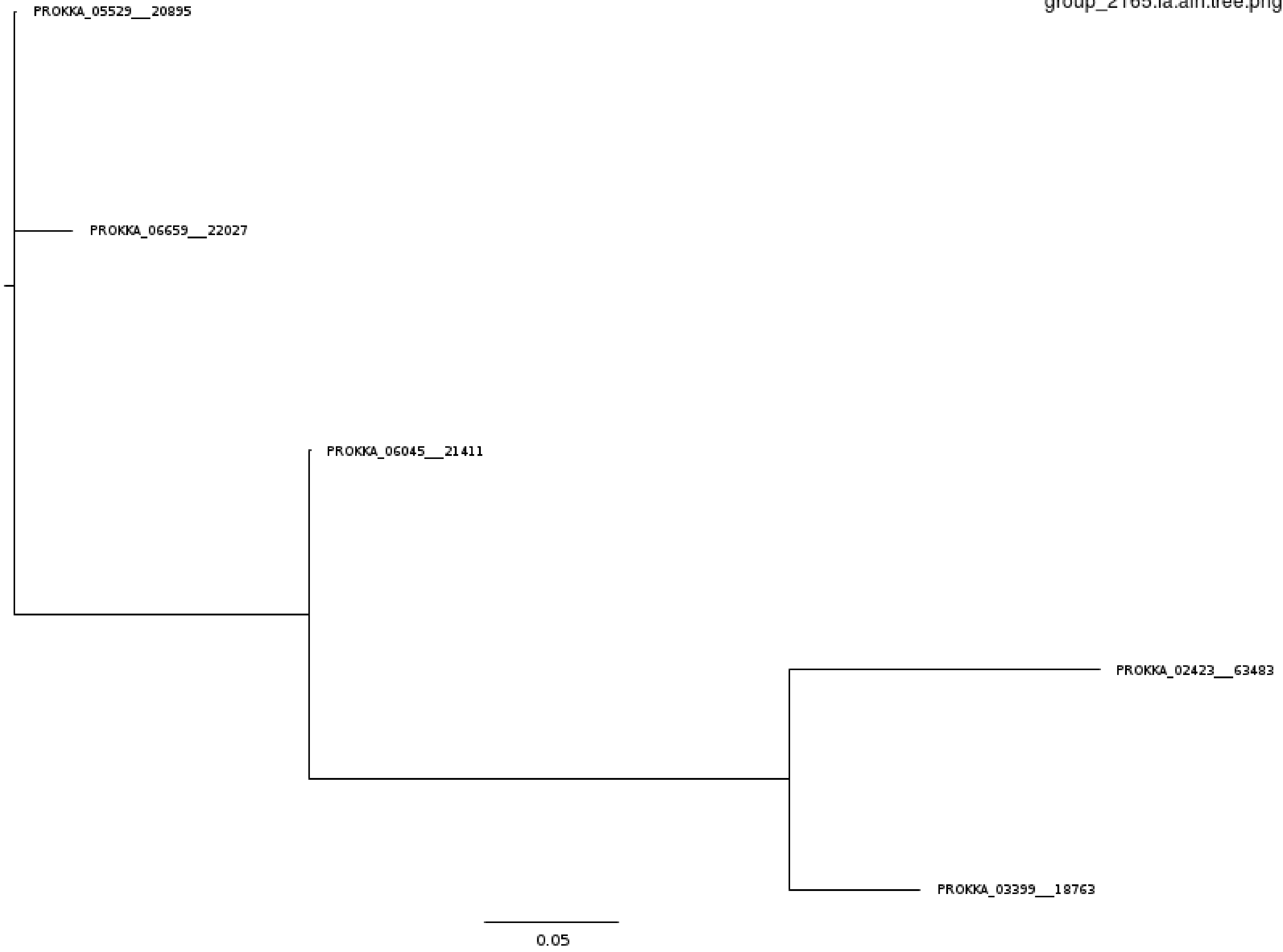

group_2170.fa.aln.tree.png

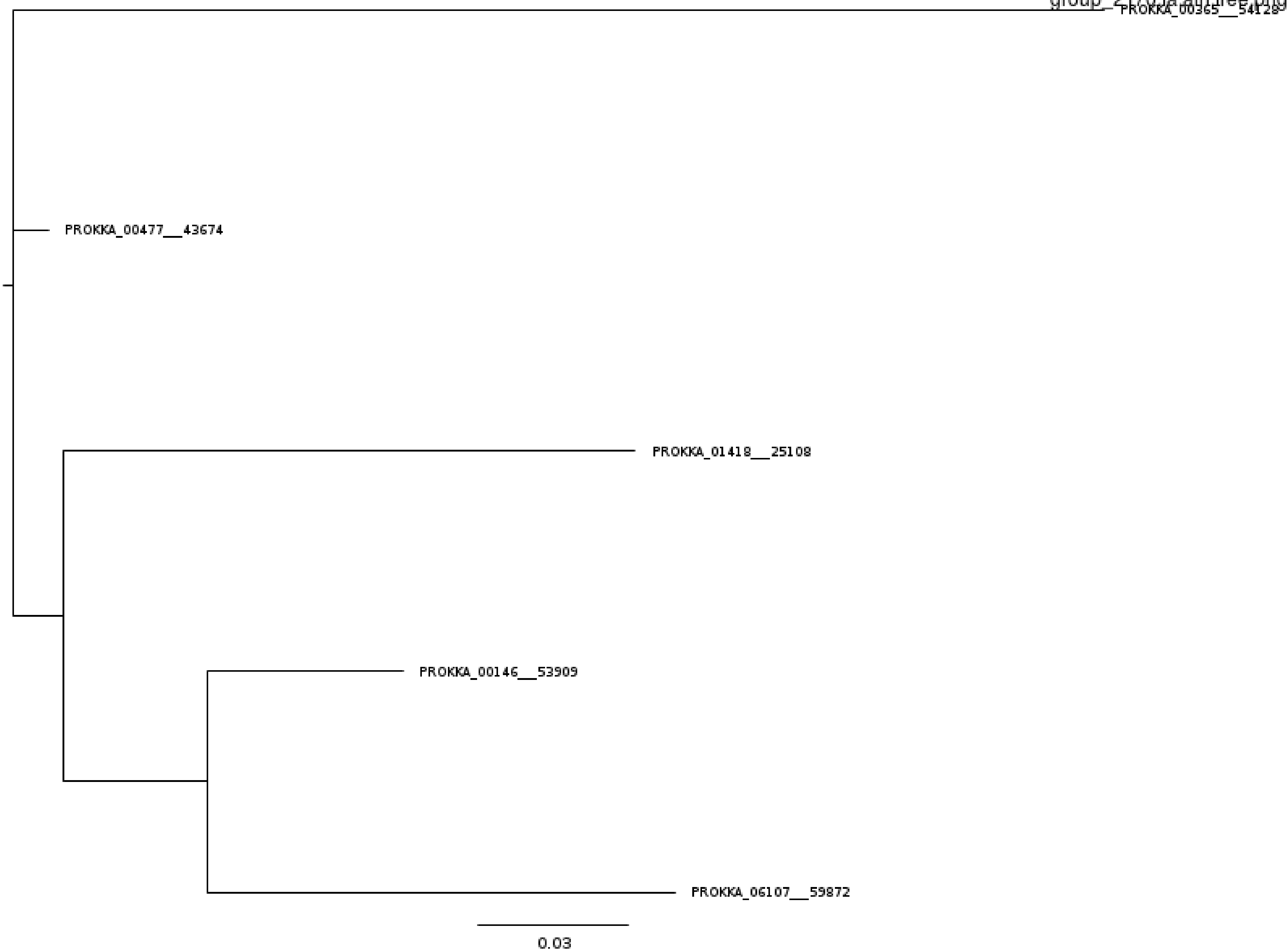

group_2173.fa.aln.tree.png

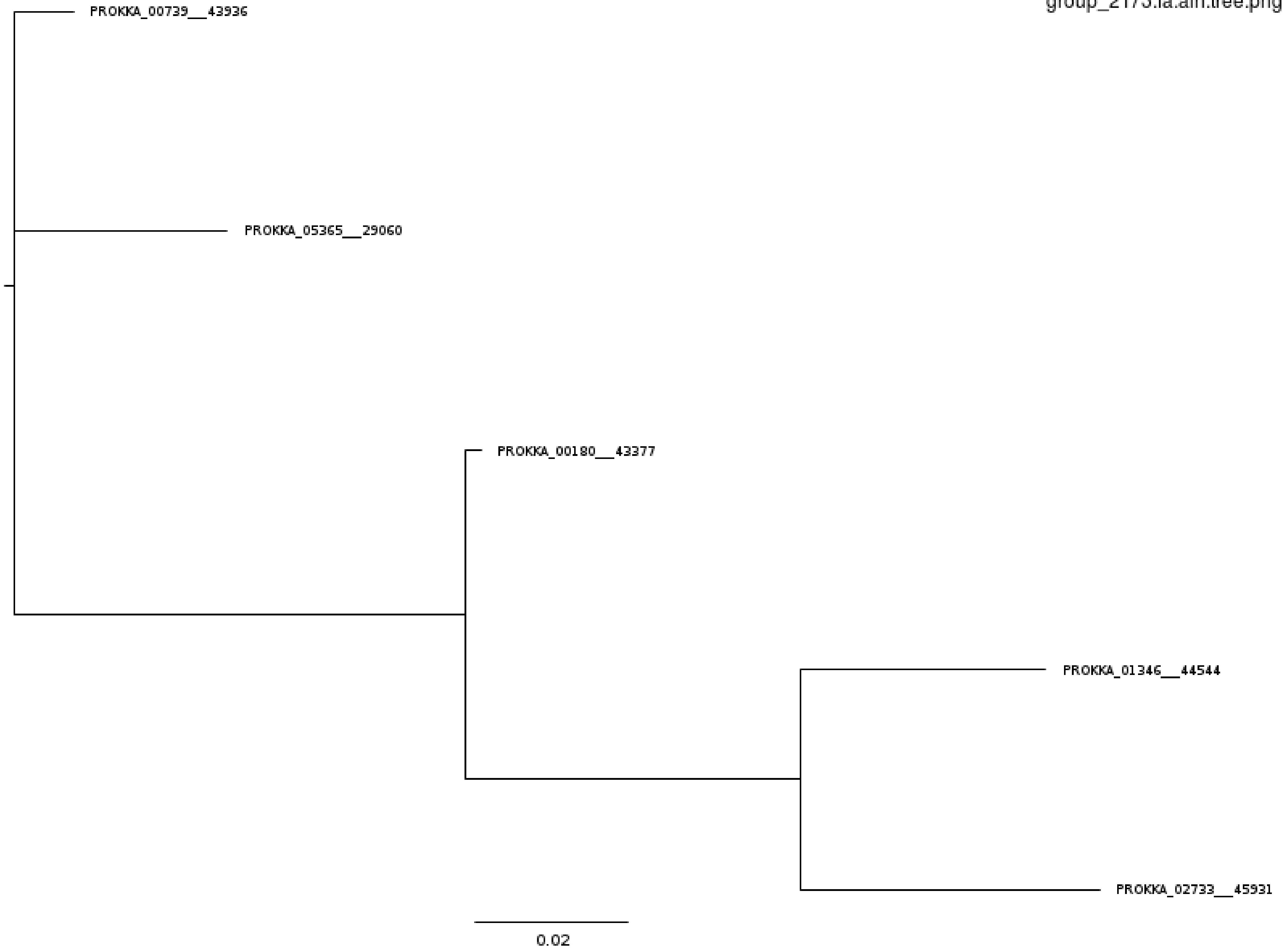

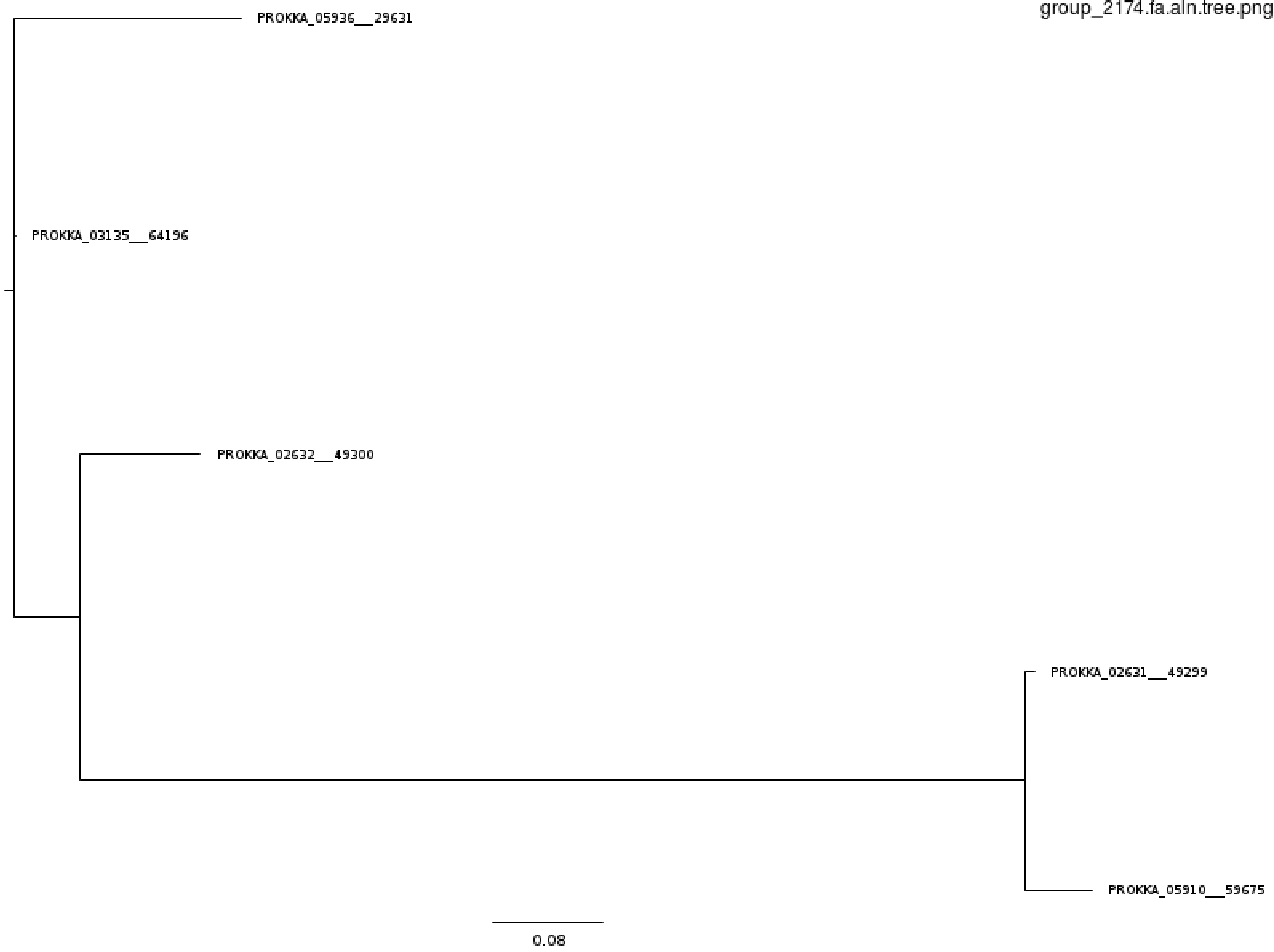
group_2174.fa.aln.tree.png
PROKKA_05936___29631
PROKKA_03135___64196
PROKKA_02632___49300
PROKKA_02631___49299
PROKKA_05910___59675
0.08

group_2175.fa.aln.tree.png

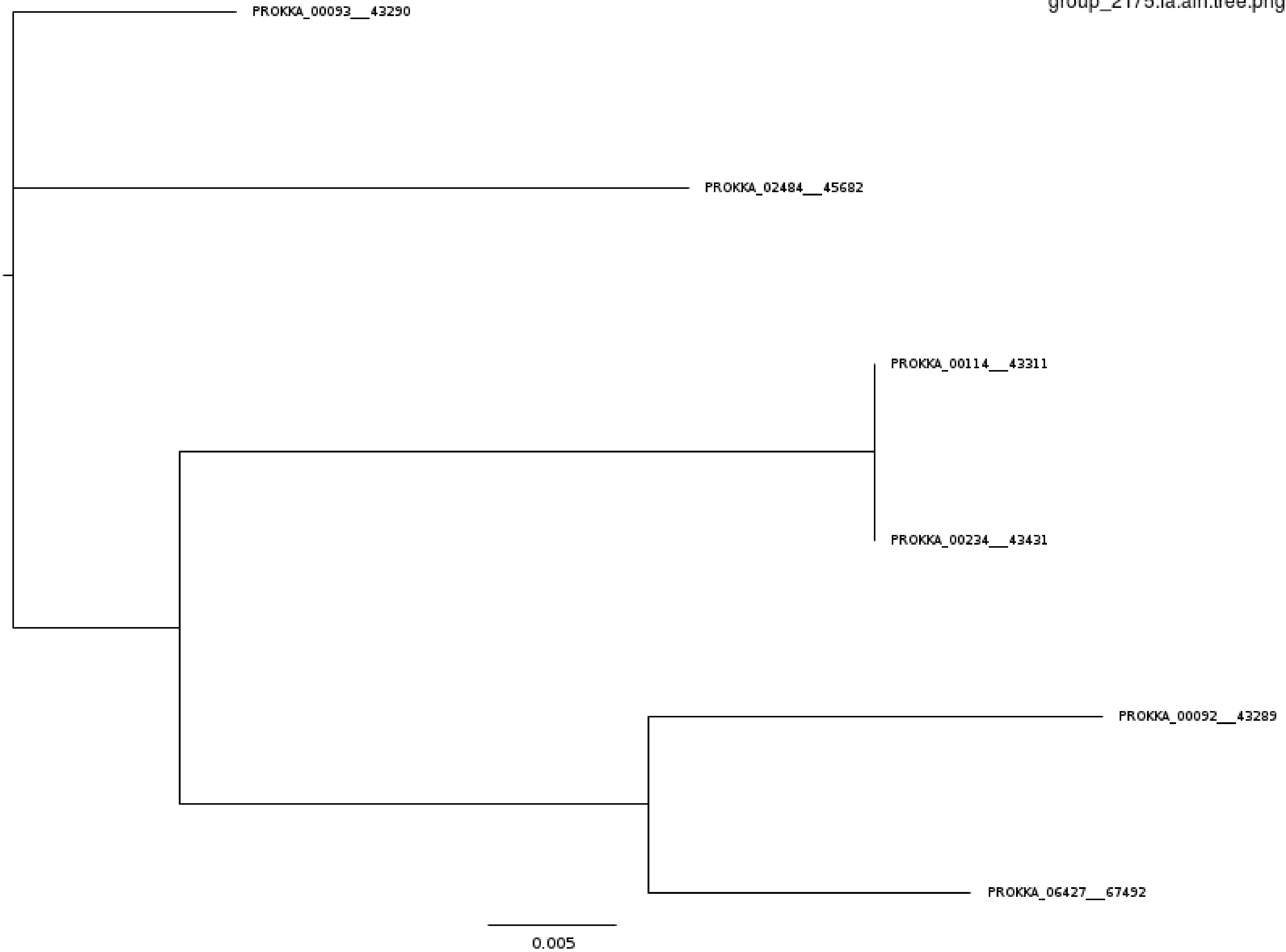

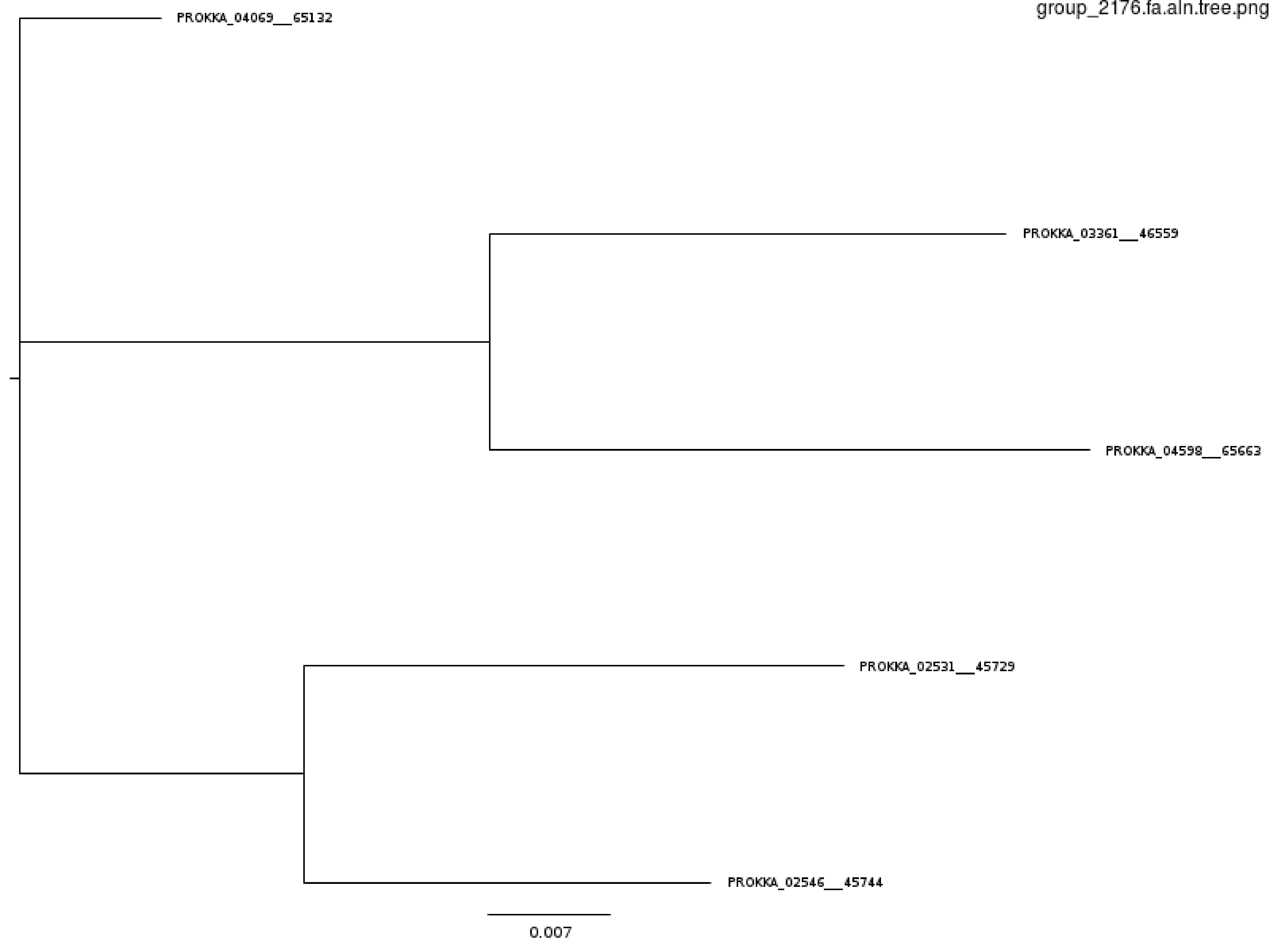
group_2176.fa.aln.tree.png
PROKKA_04069___65132
PROKKA_03361___46559
PROKKA_04598___65663
PROKKA_02531___45729
PROKKA_02546___45744
0.007

group_2177.fa.aln.tree.png

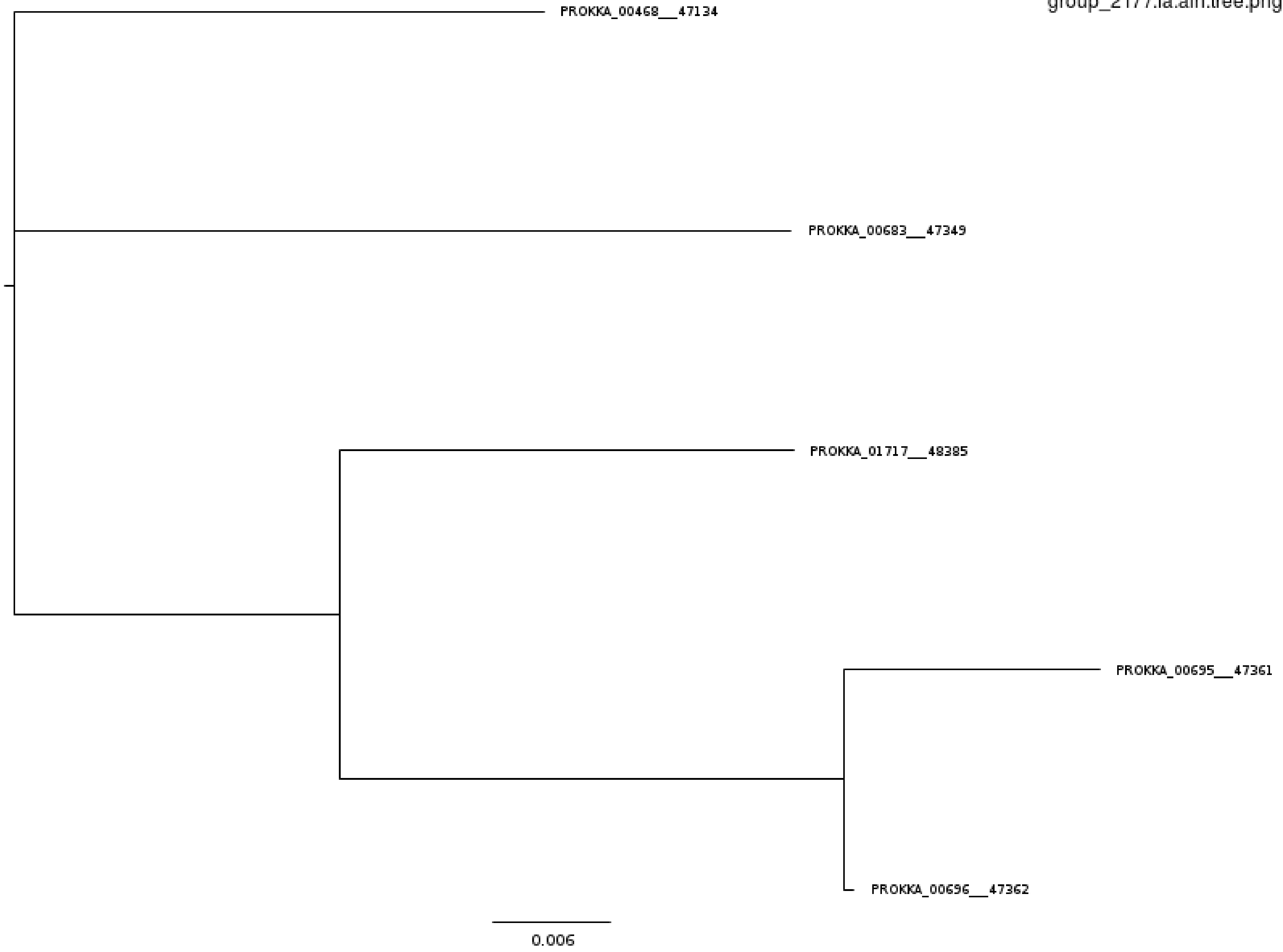

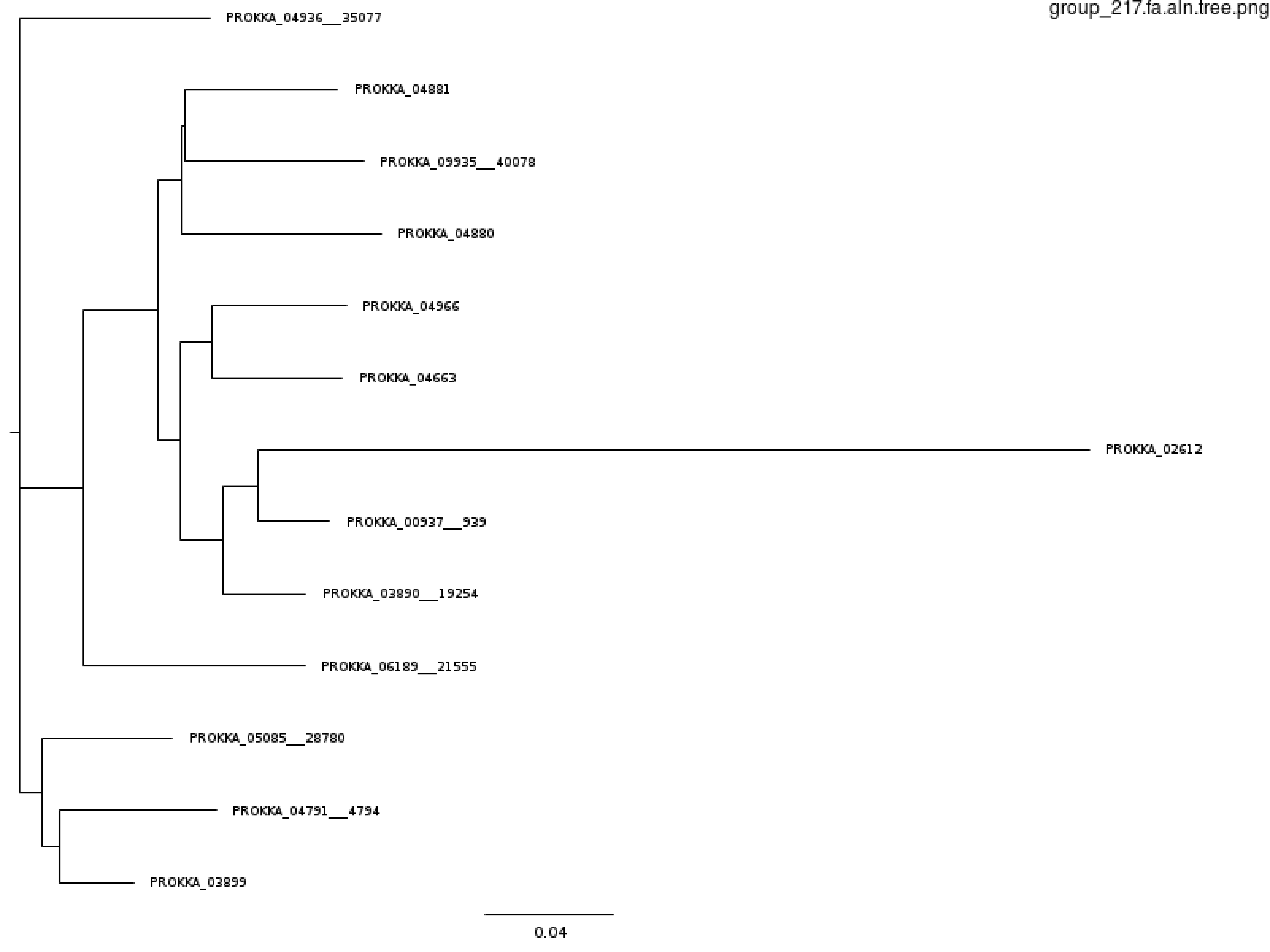
group_217.fa.aln.tree.png
PROKKA_04936___35077
PROKKA_04881
PROKKA_09935___40078
PROKKA_04880
PROKKA_04966
PROKKA_04663
PROKKA_02612
PROKKA_00937___939
PROKKA_03890___19254
PROKKA_06189___21555
PROKKA_05085___28780
PROKKA_04791___4794
PROKKA_03899
0.04

group_2181.fa.aln.tree.png

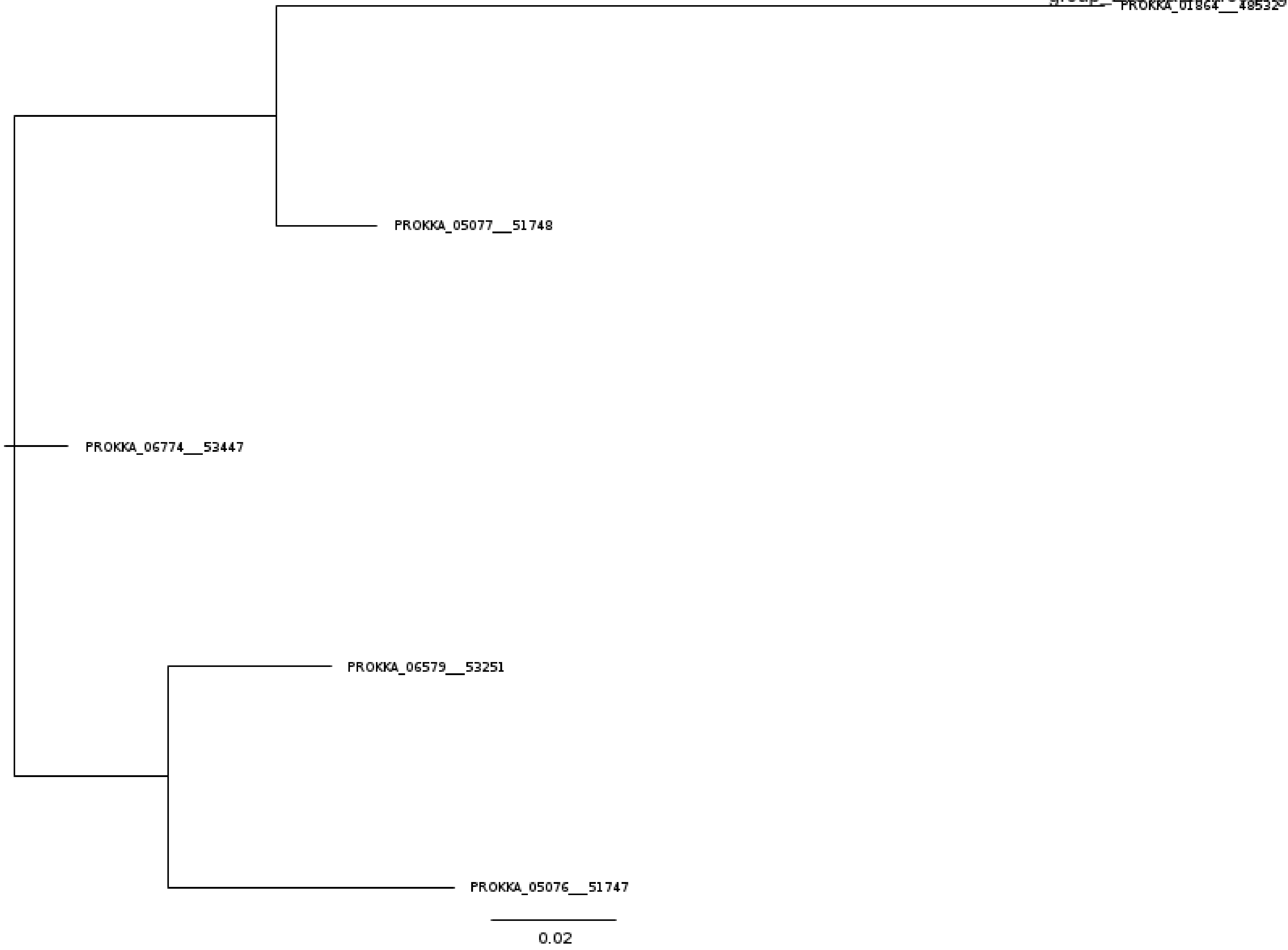

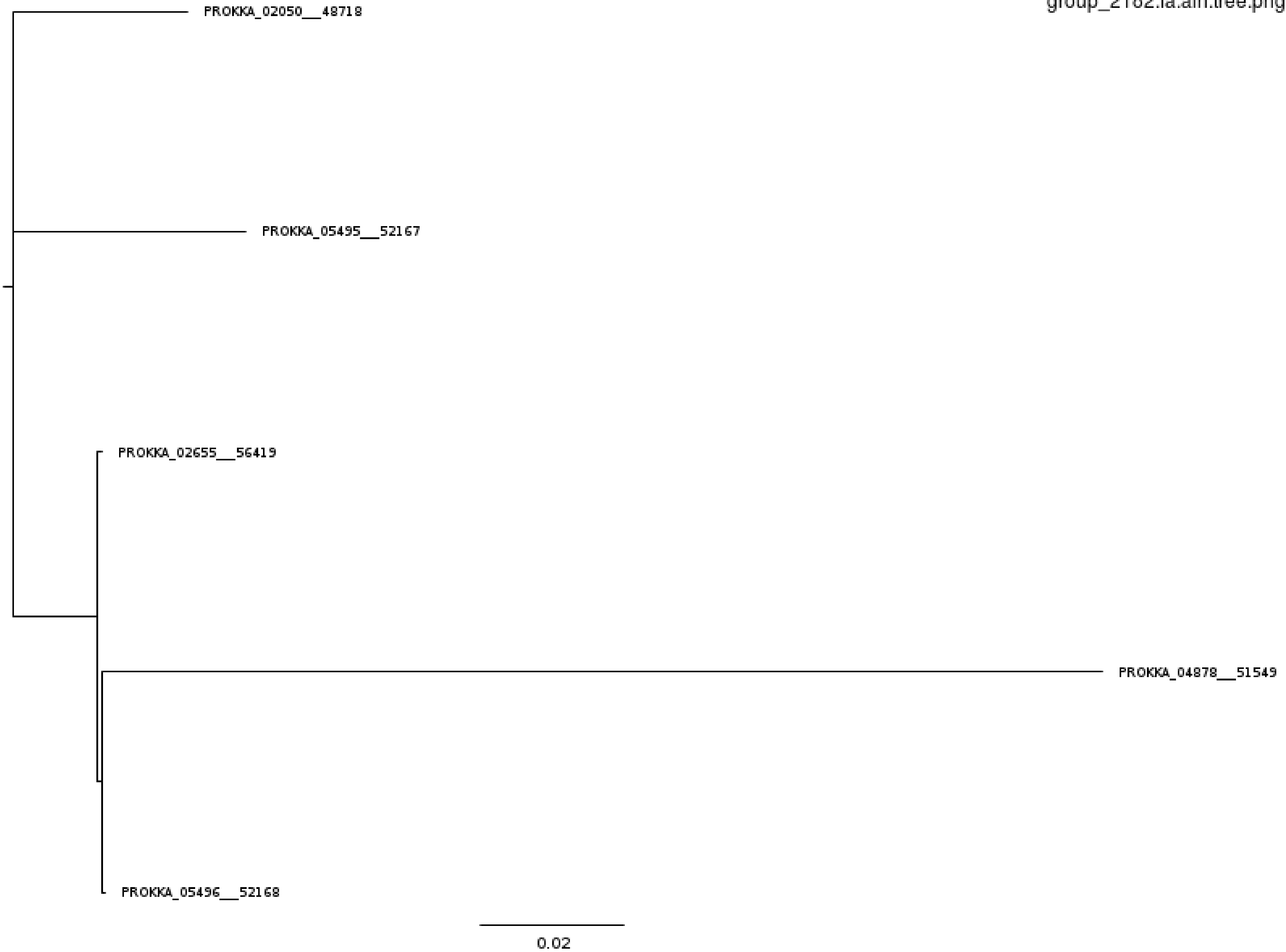
PROKKA_02050__48718
PROKKA_05495__52167
PROKKA_02655__56419
PROKKA_04878__51549
PROKKA_05496__52168
0.02

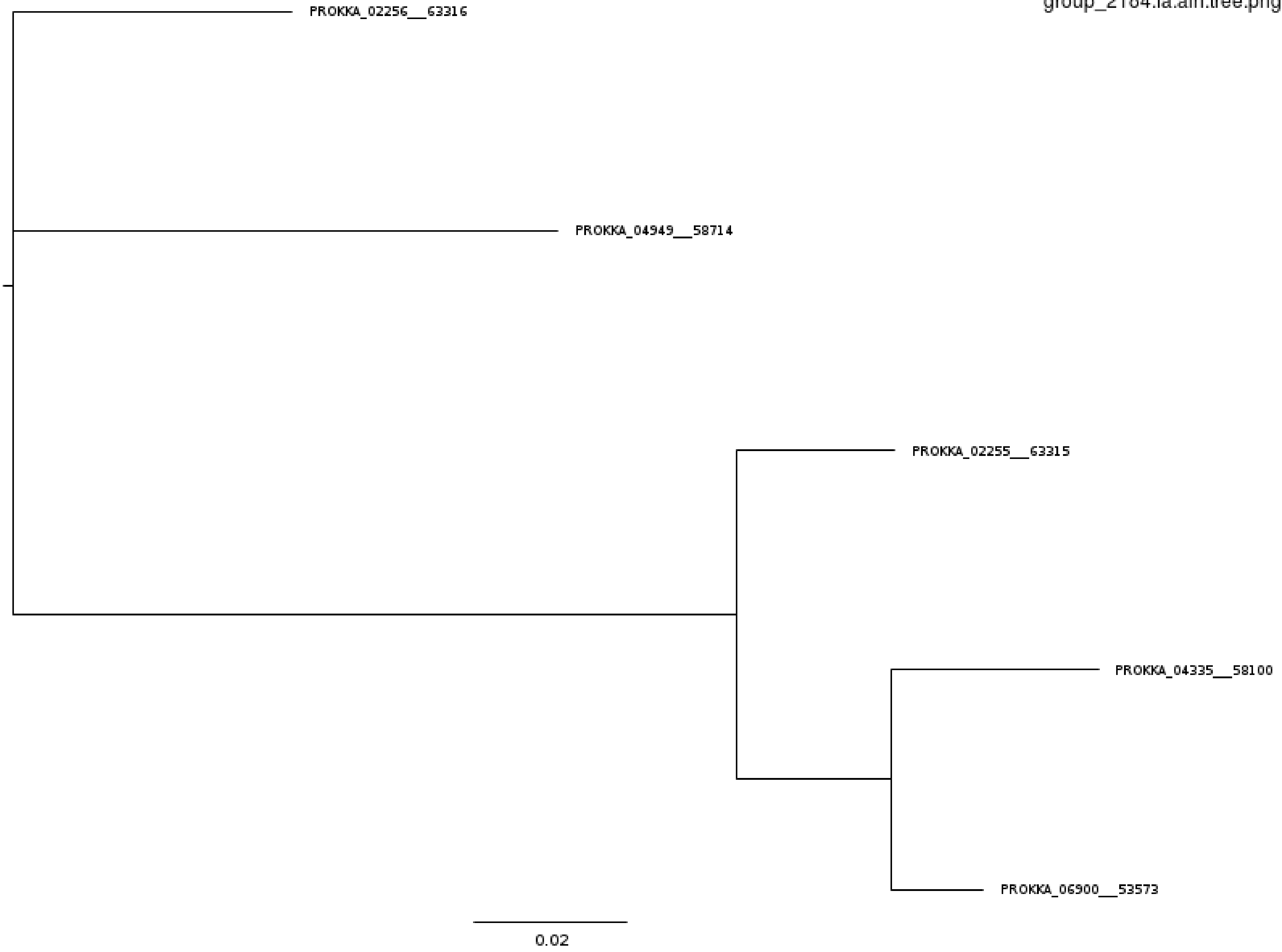
PROKKA_02256___63316
PROKKA_04949___58714
PROKKA_02255___63315
PROKKA_04335___58100
PROKKA_06900___53573
0.02

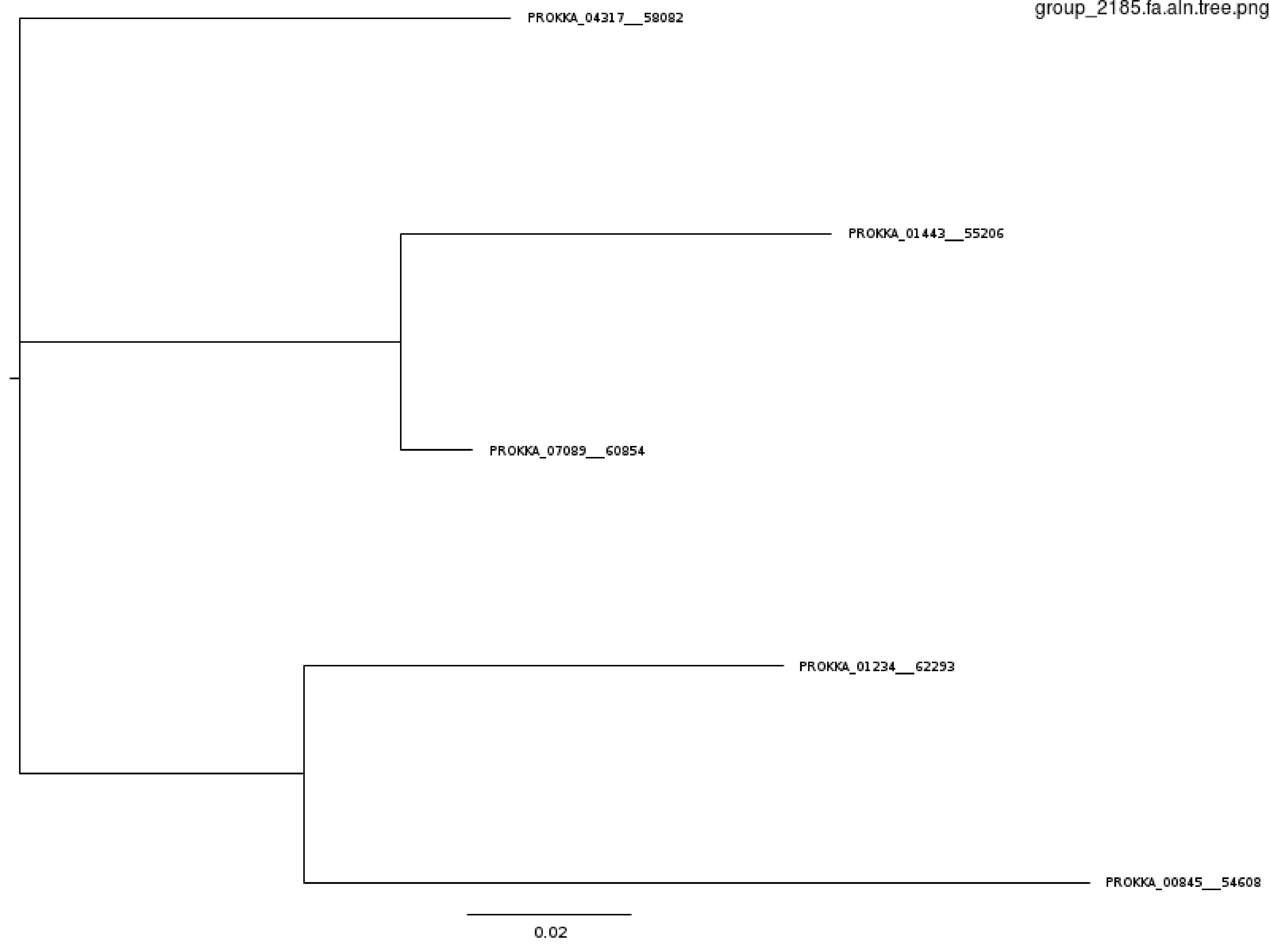
group_2185.fa.aln.tree.png
PROKKA_04317___58082
PROKKA_01443___55206
PROKKA_07089___60854
PROKKA_01234___62293
PROKKA_00845___54608
0.02

group_2193.fa.aln.tree.png

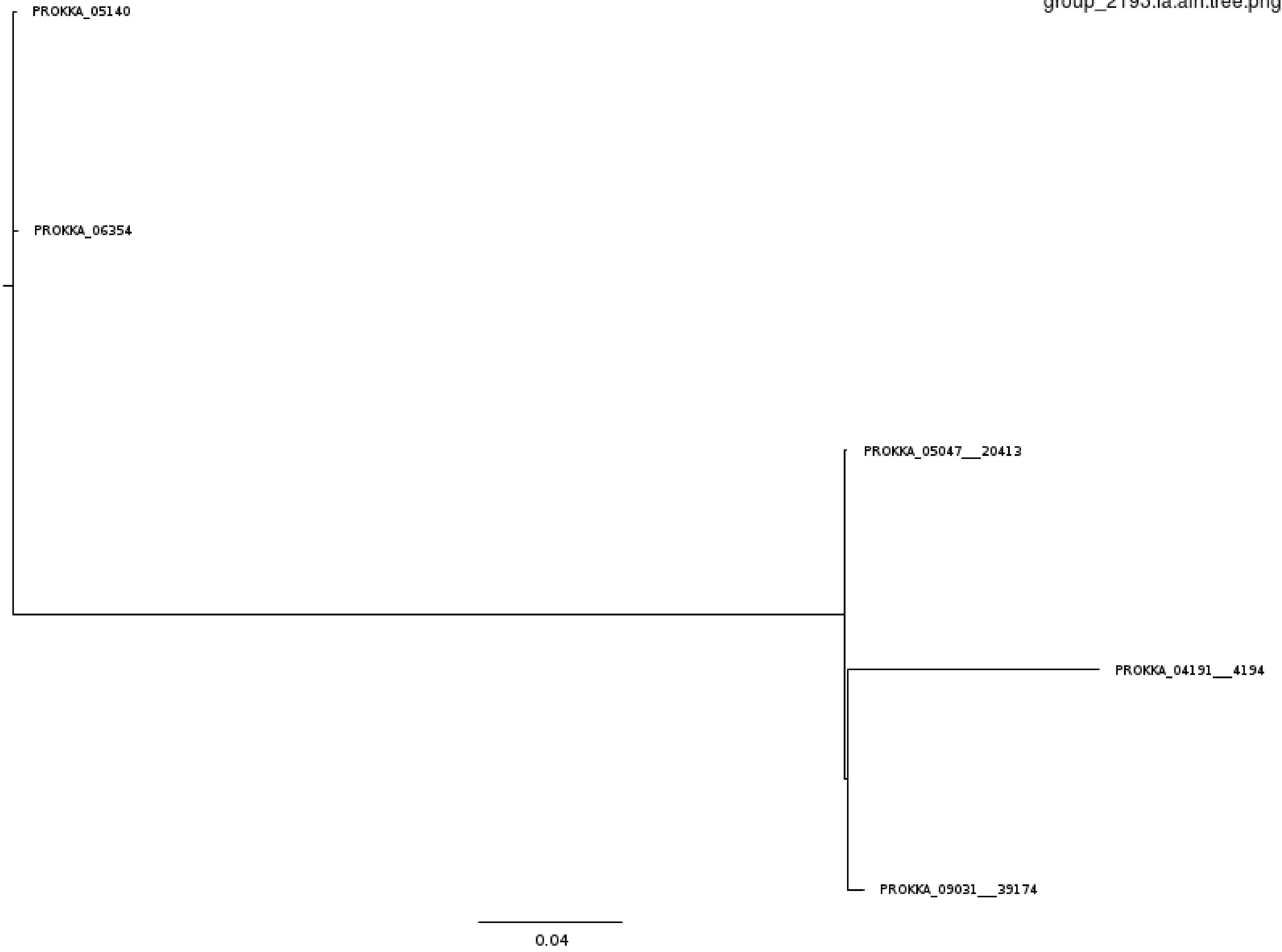

group_21.fa.aln.tree.png

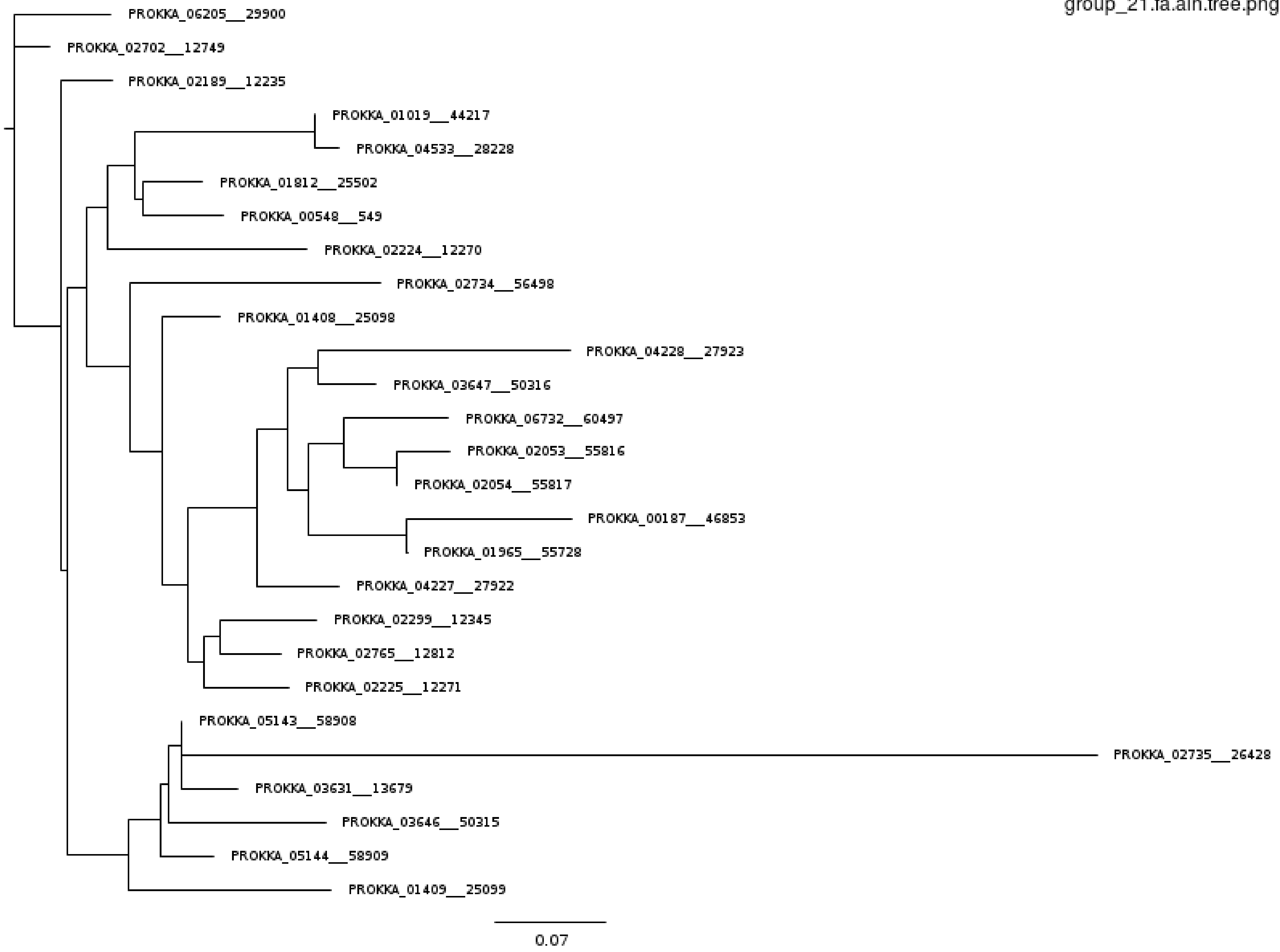

group_2200.fa.aln.tree.png

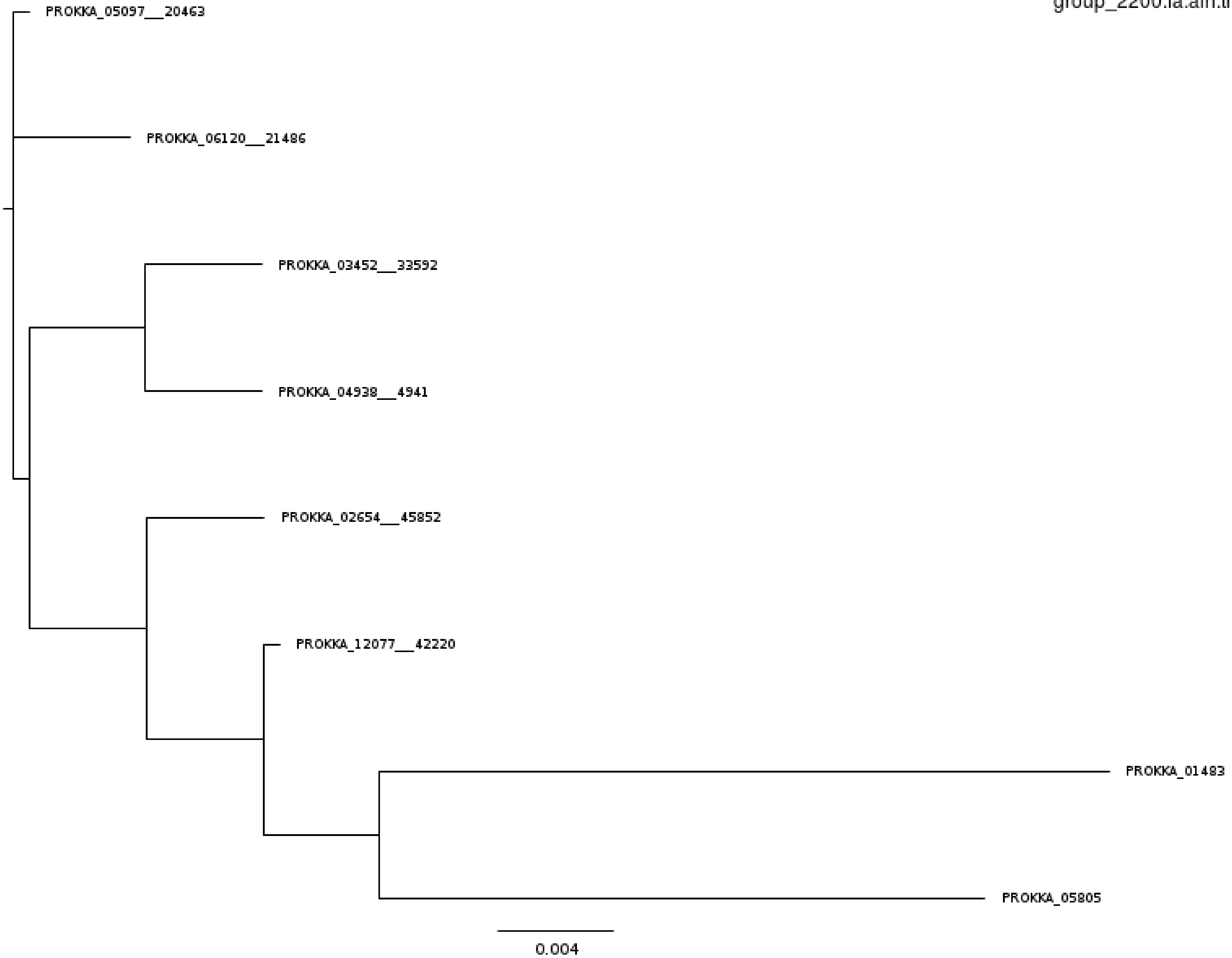

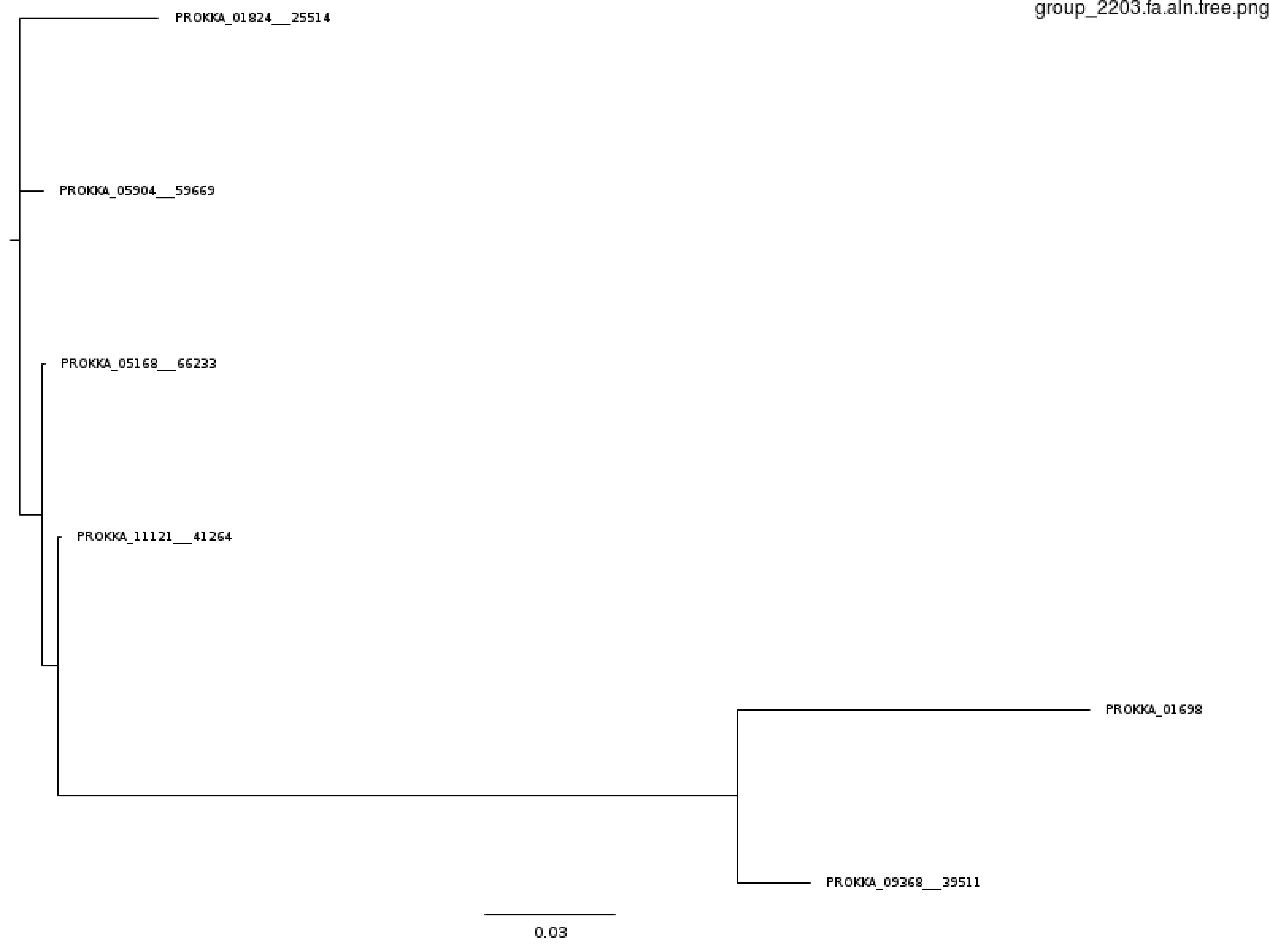
group_2203.fa.aln.tree.png
PROKKA_01824___25514
PROKKA_05904___59669
PROKKA_05168___66233
PROKKA_11121___41264
PROKKA_01698
PROKKA_09368___39511
0.03

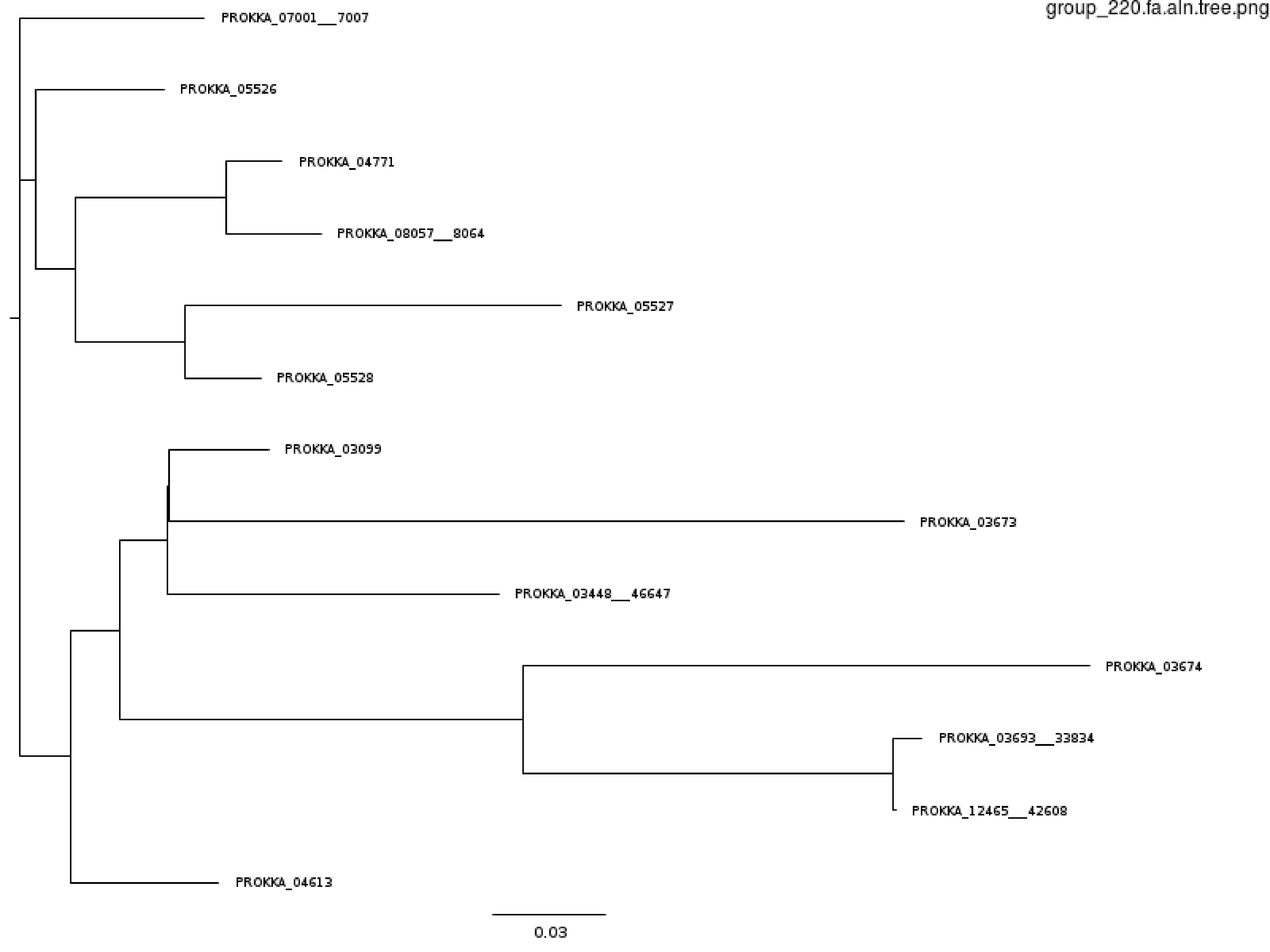
group_220.fa.aln.tree.png
PROKKA_07001___7007
PROKKA_05526
PROKKA_04771
PROKKA_08057___8064
PROKKA_05527
PROKKA_05528
PROKKA_03099
PROKKA_03673
PROKKA_03448___46647
PROKKA_03674
PROKKA_03693___33834
PROKKA_12465___42608
PROKKA_04613
0.03

group_2237.fa.aln.tree.png

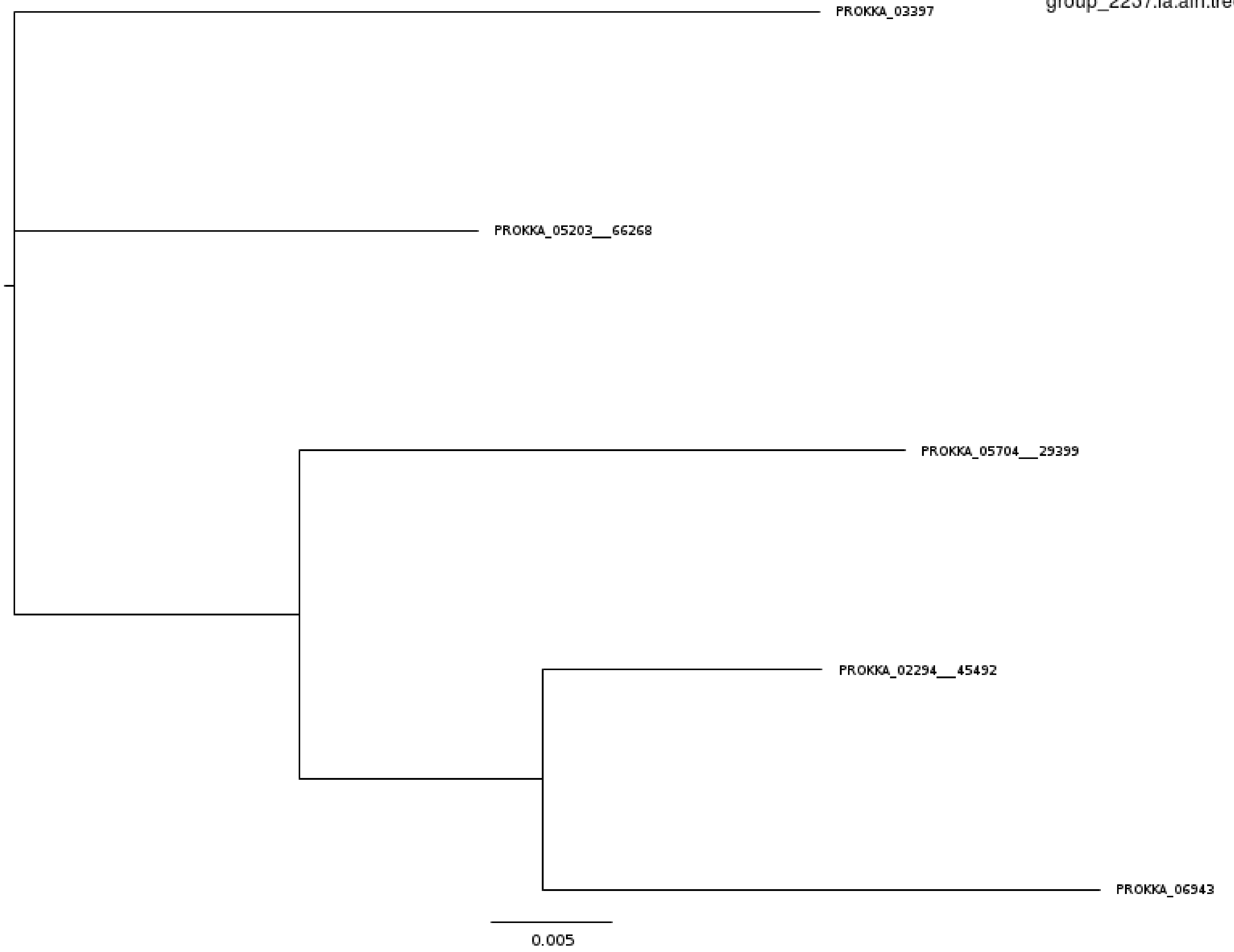

group_2240.fa.aln.tree.png

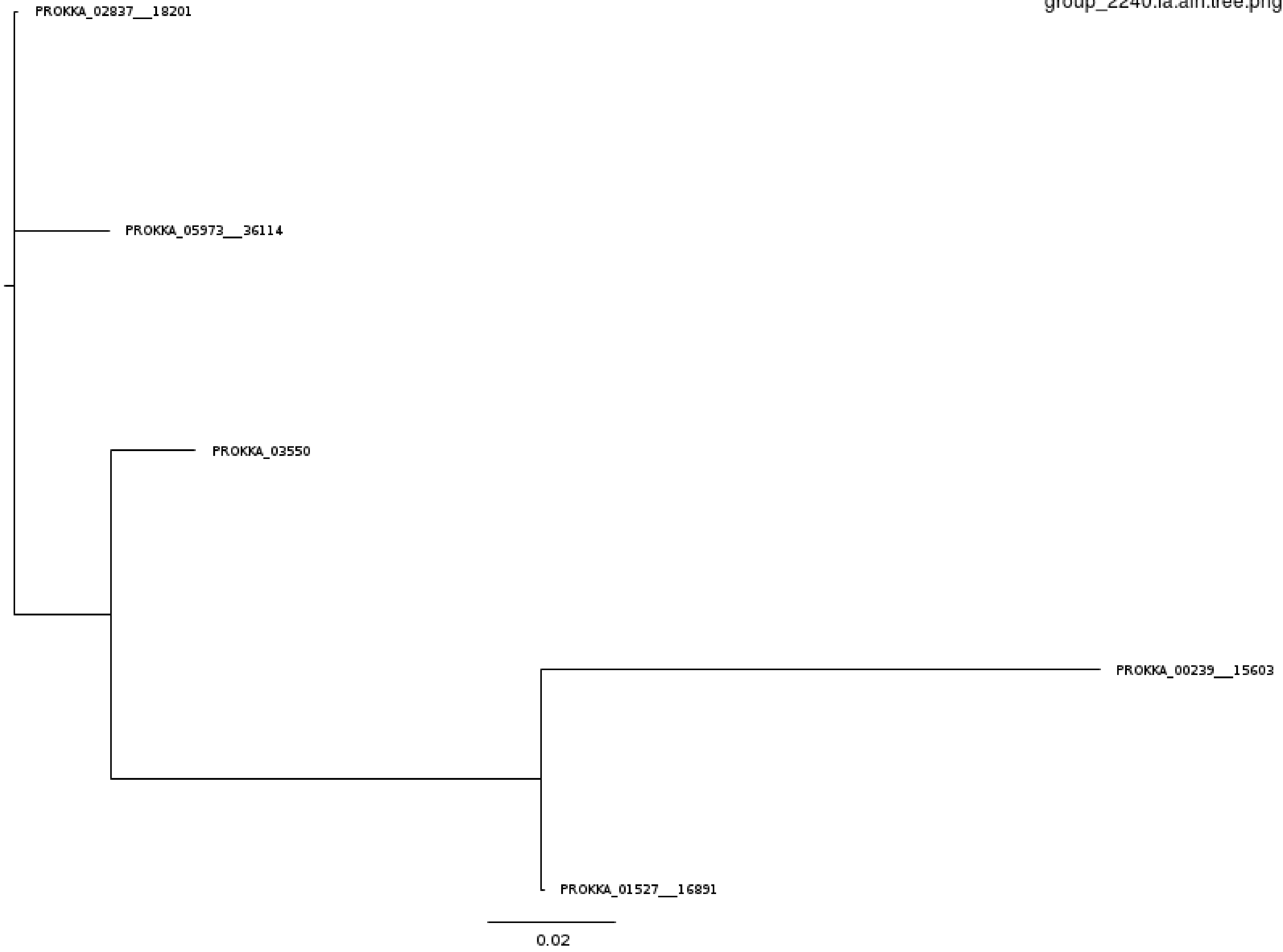

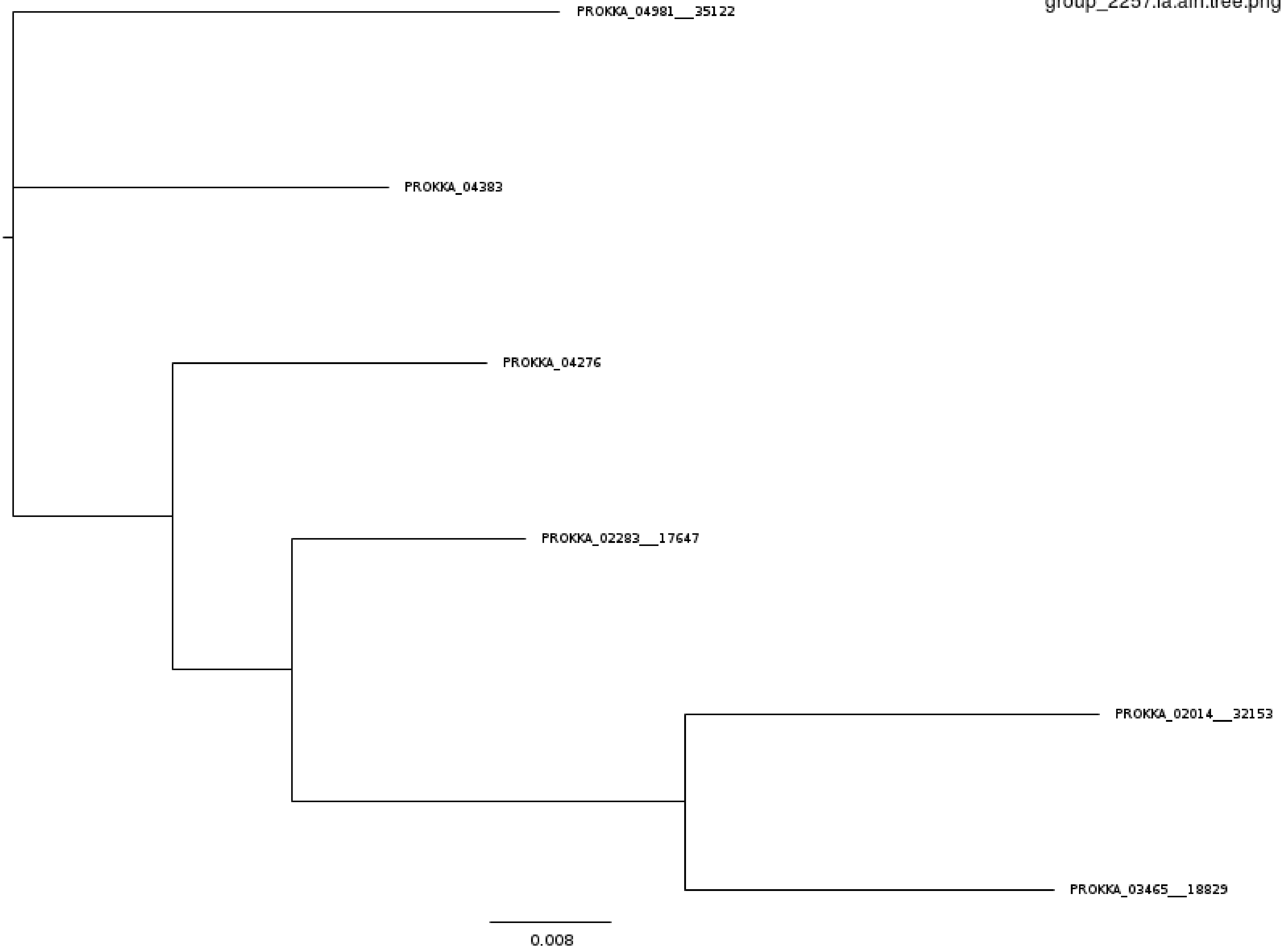
PROKKA_04981___35122
PROKKA_04383
PROKKA_04276
PROKKA_02283___17647
PROKKA_02014___32153
PROKKA_03465___18829
0.008

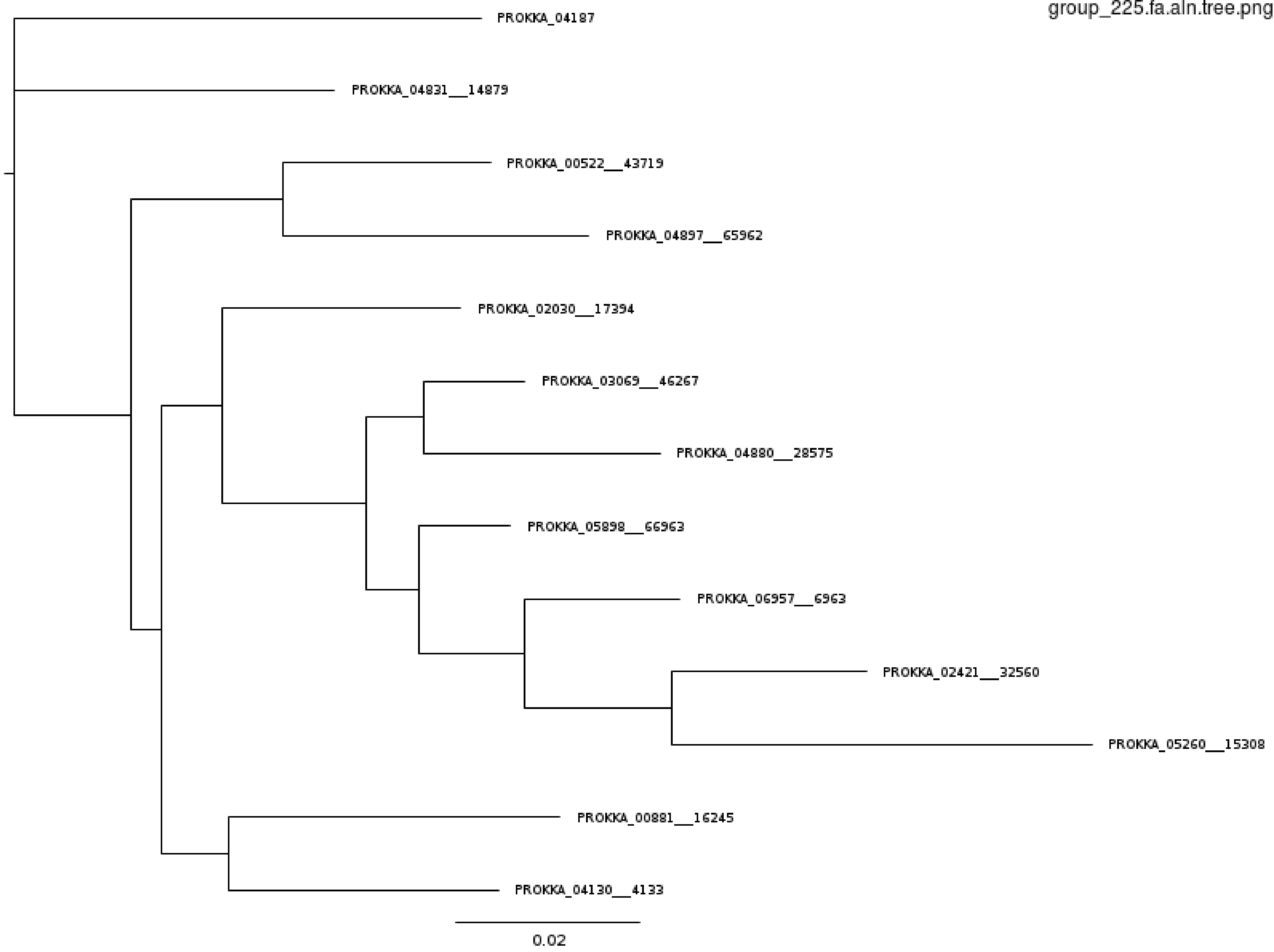
group_225.fa.aln.tree.png
PROKKA_04187
PROKKA_04831___14879
PROKKA_00522___43719
PROKKA_04897___65962
PROKKA_02030___17394
PROKKA_03069___46267
PROKKA_04880___28575
PROKKA_05898___66963
PROKKA_06957___6963
PROKKA_02421___32560
PROKKA_05260___15308
PROKKA_00881___16245
PROKKA_04130___4133
0.02

group_2264.fa.aln.tree.png

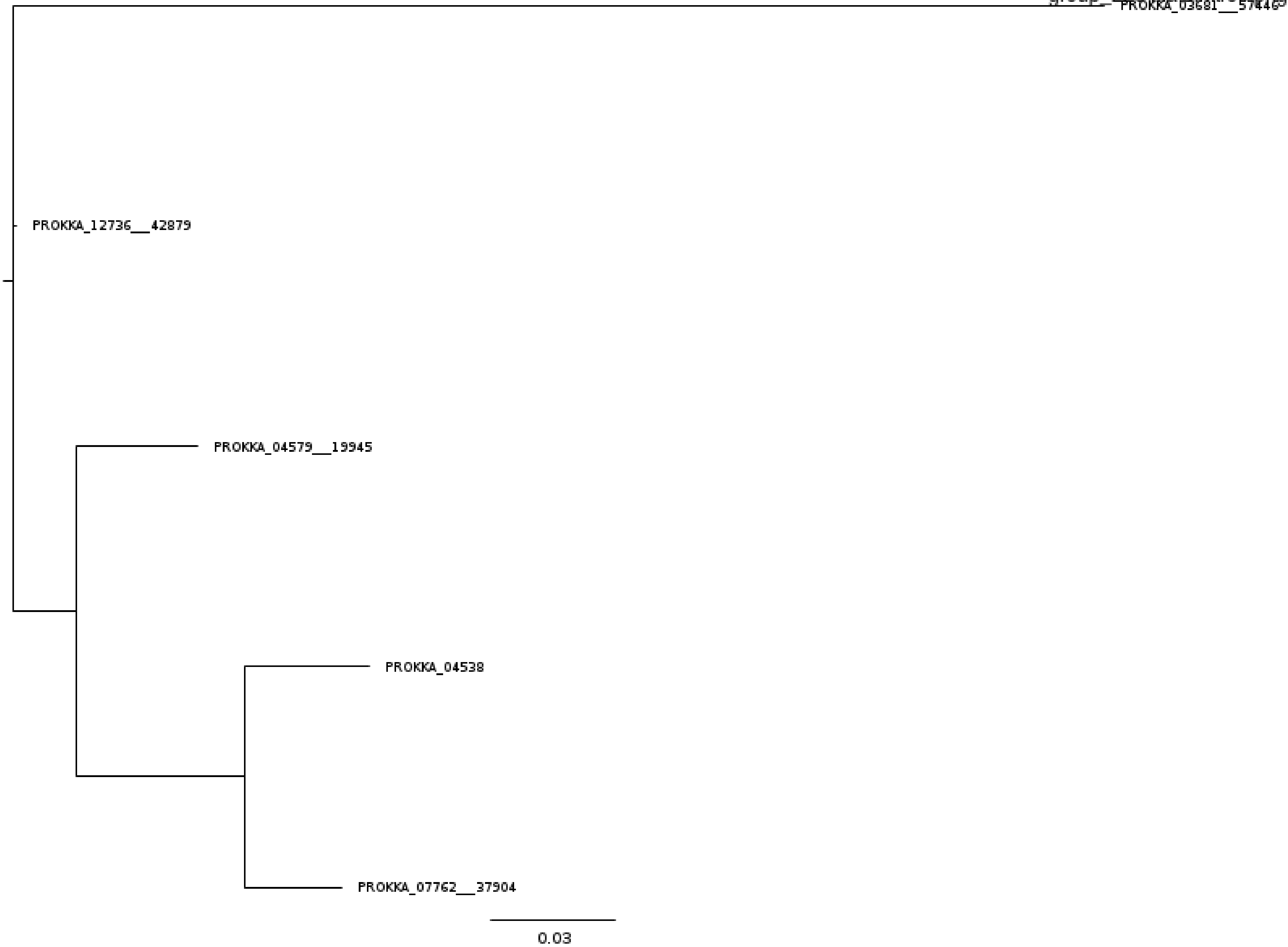

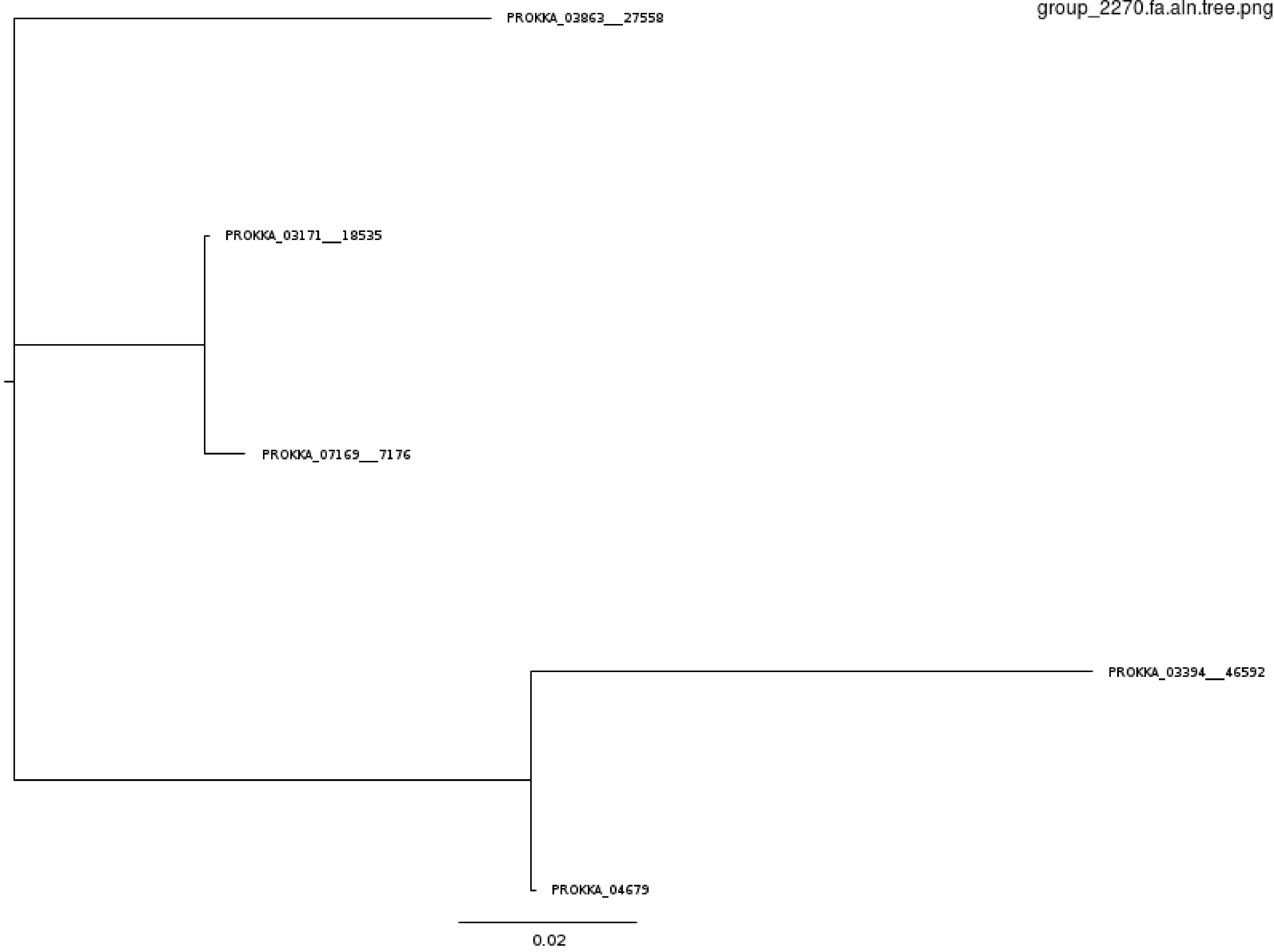
group_2270.fa.aln.tree.png
PROKKA_03863___27558
PROKKA_03171___18535
PROKKA_07169___7176
PROKKA_03394___46592
PROKKA_04679
0.02

group_2281.fa.aln.tree.png

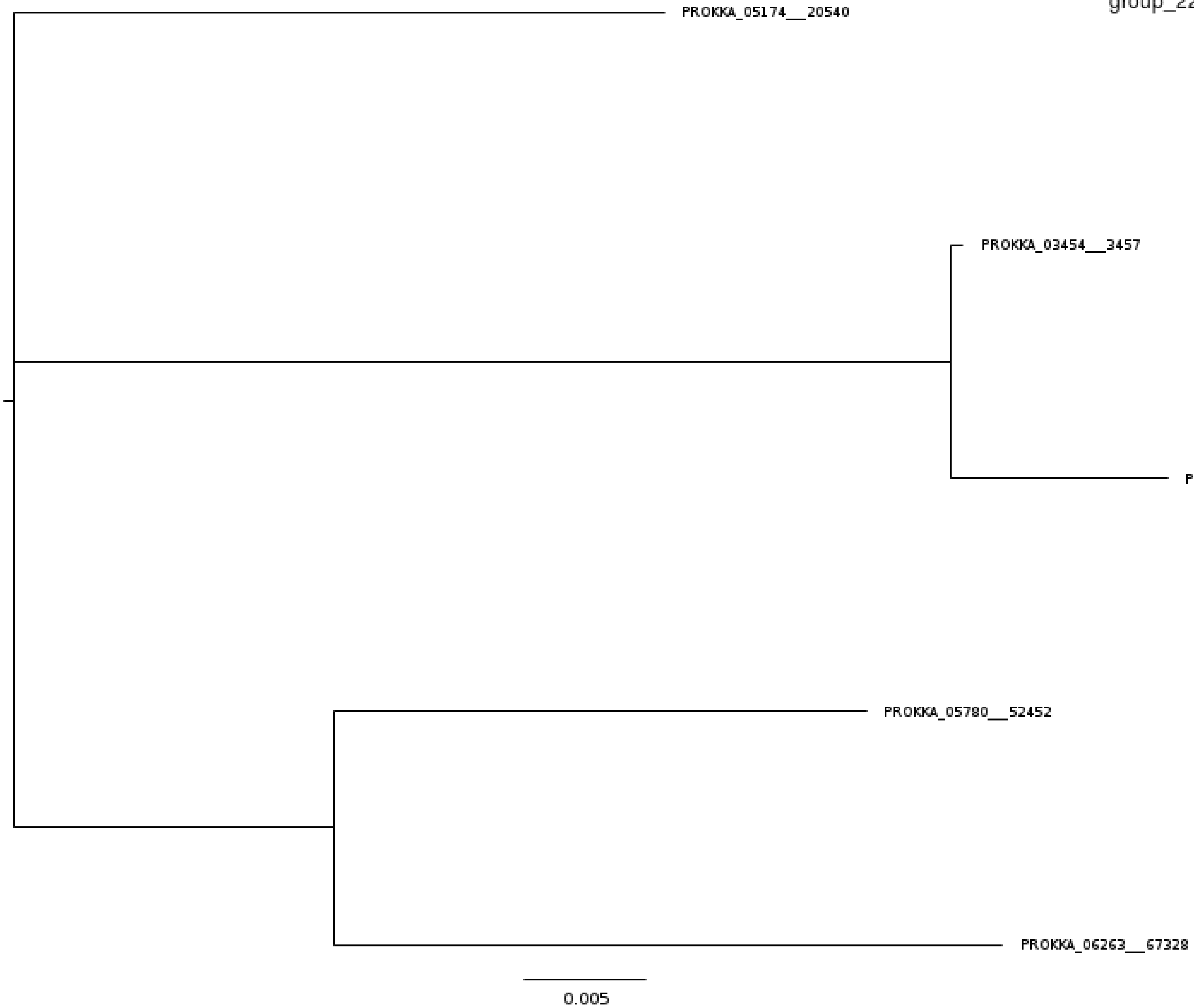

group_2285.fa.aln.tree.png

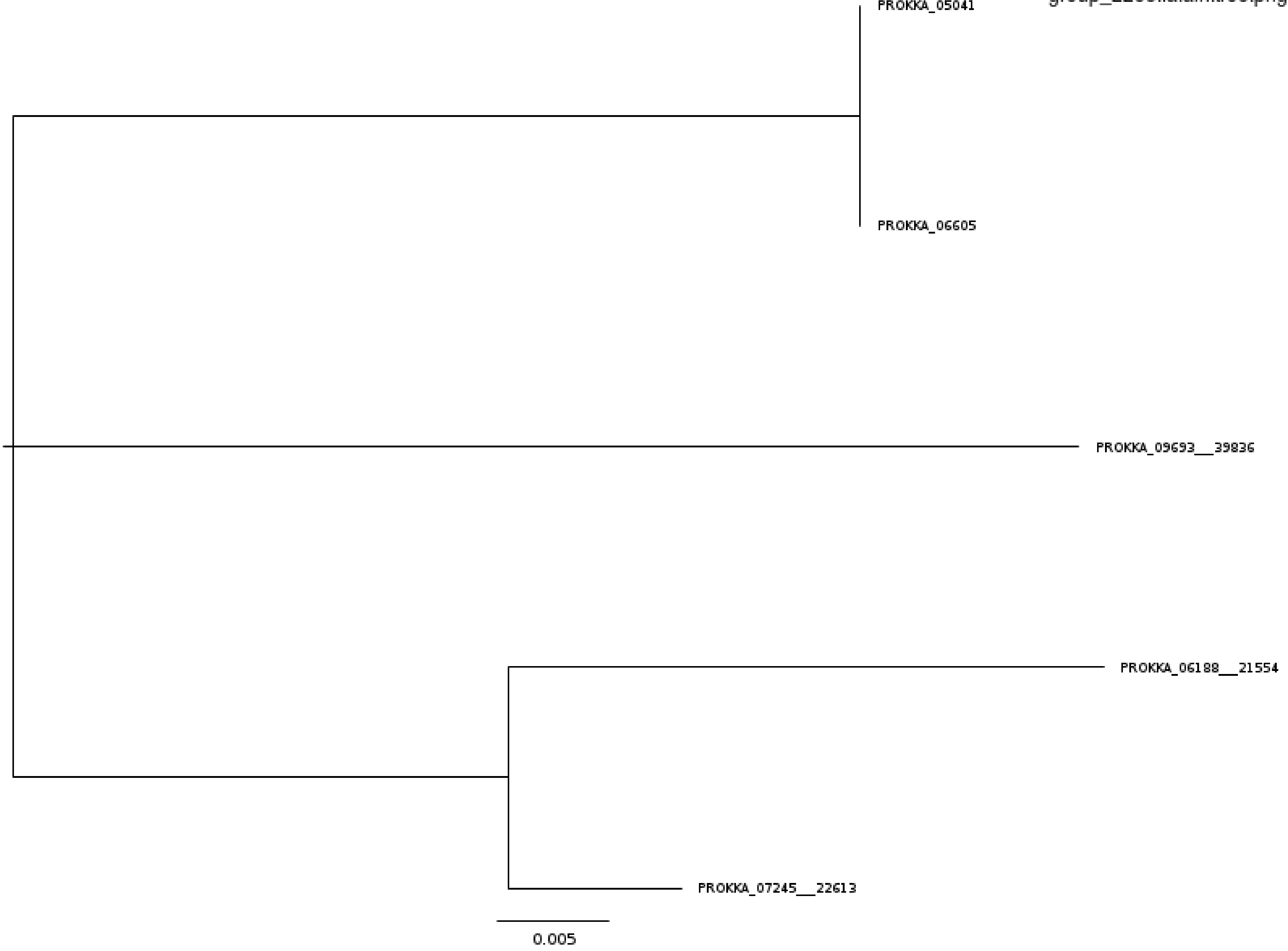

group_2286.fa.aln.tree.png

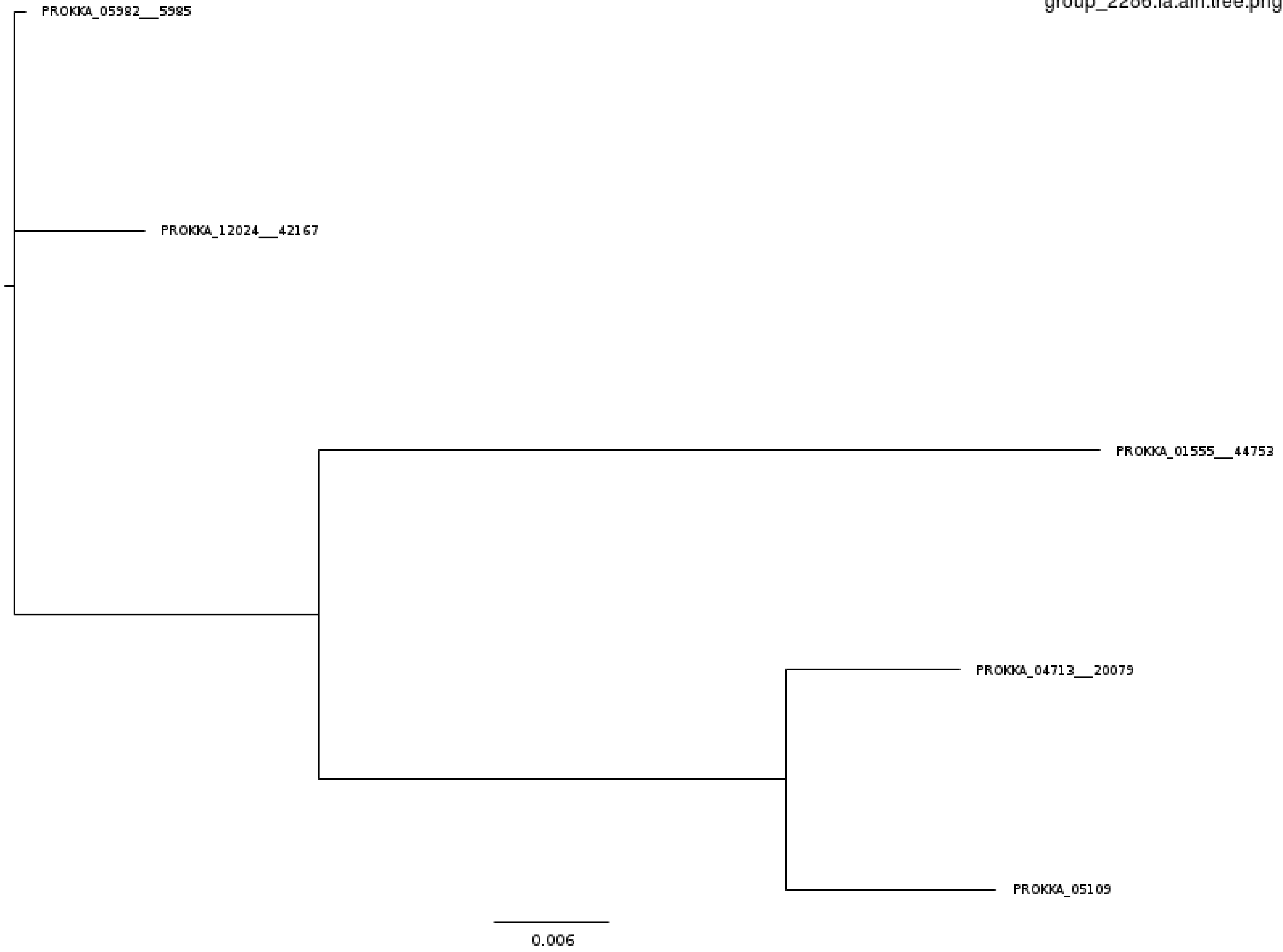

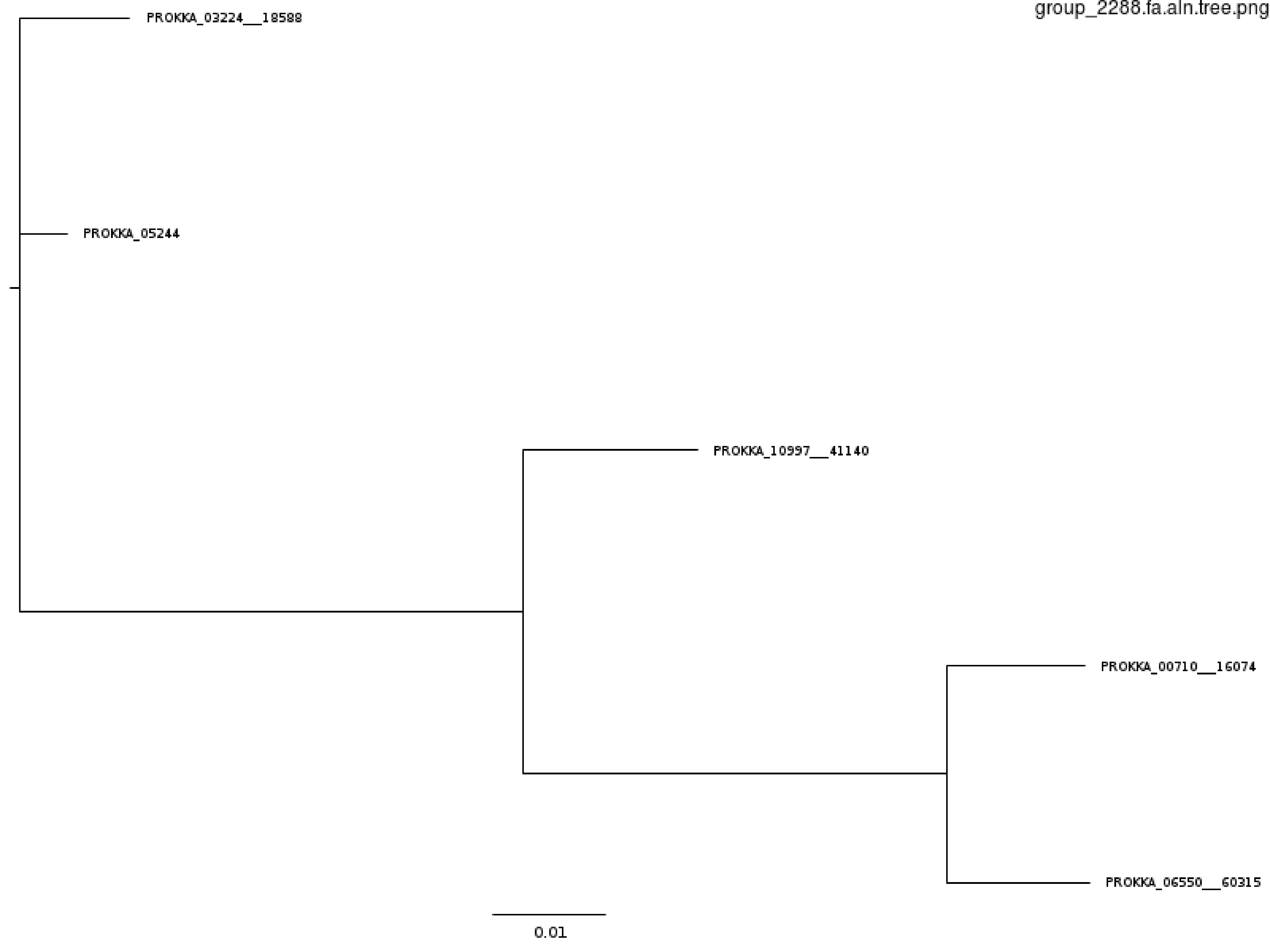
group_2288.fa.aln.tree.png
PROKKA_03224__18588
PROKKA_05244
PROKKA_10997__41140
PROKKA_00710__16074
PROKKA_06550__60315
0.01

group_2293.fa.aln.tree.png

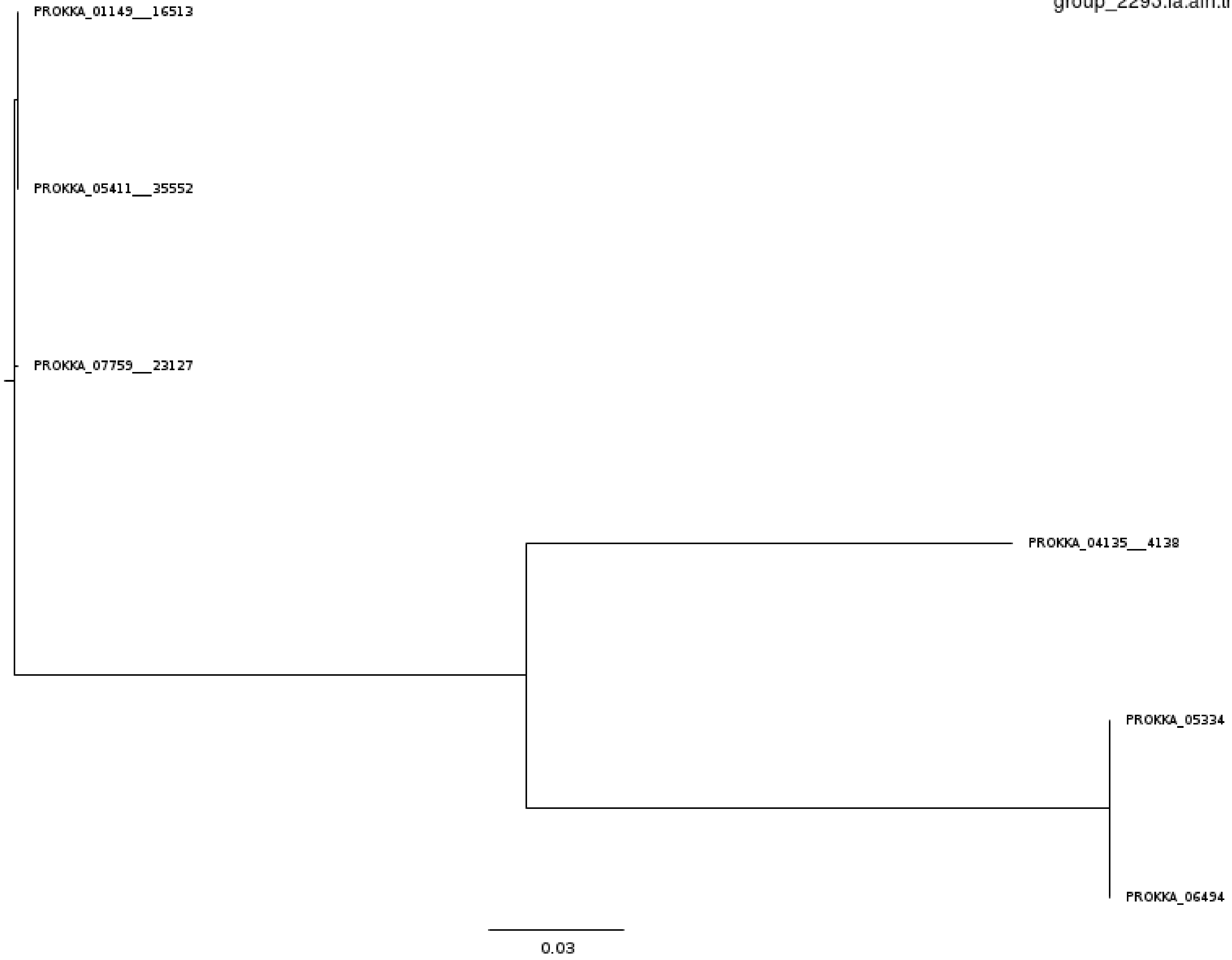

group_2297.fa.aln.tree.png

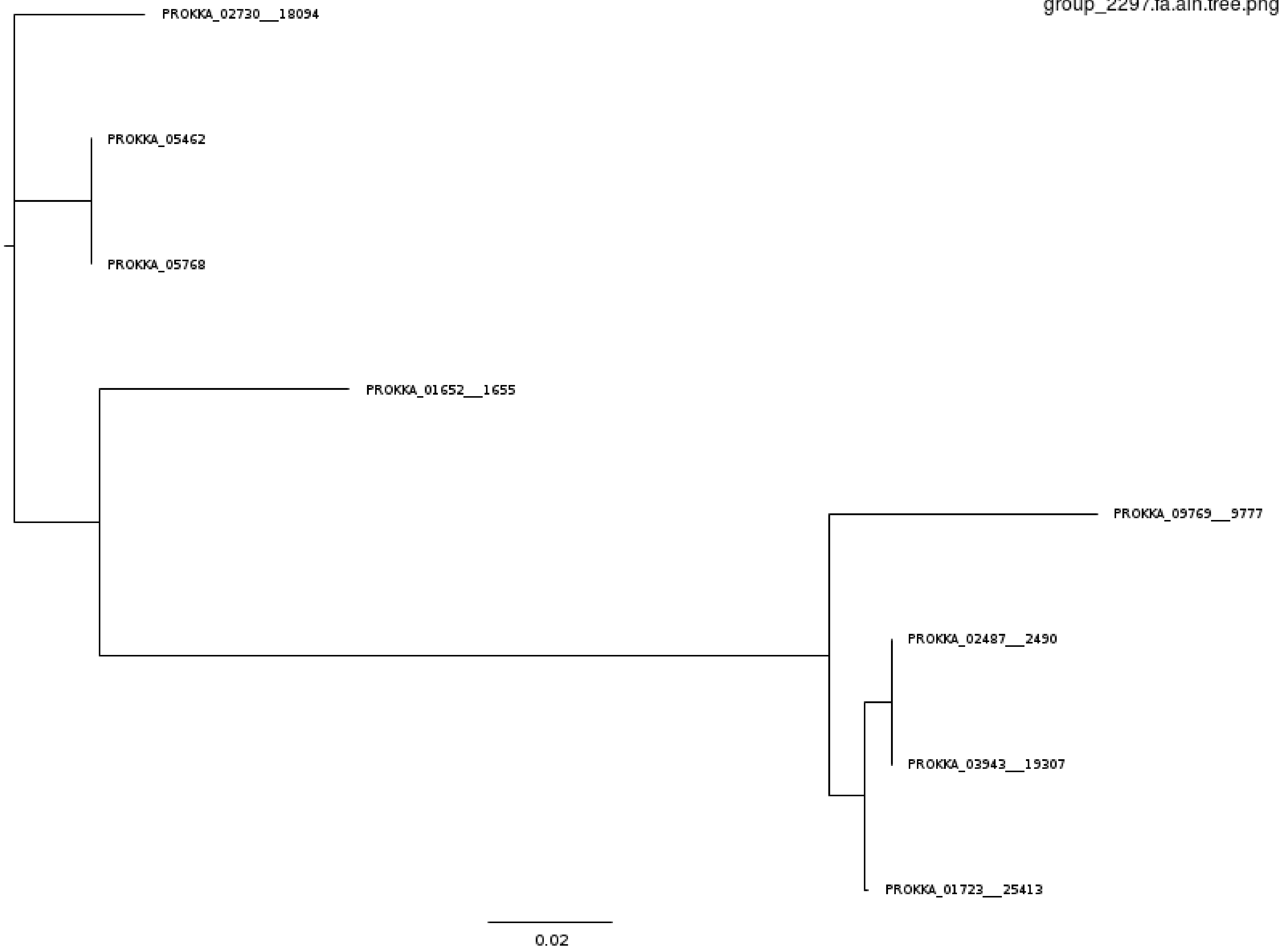

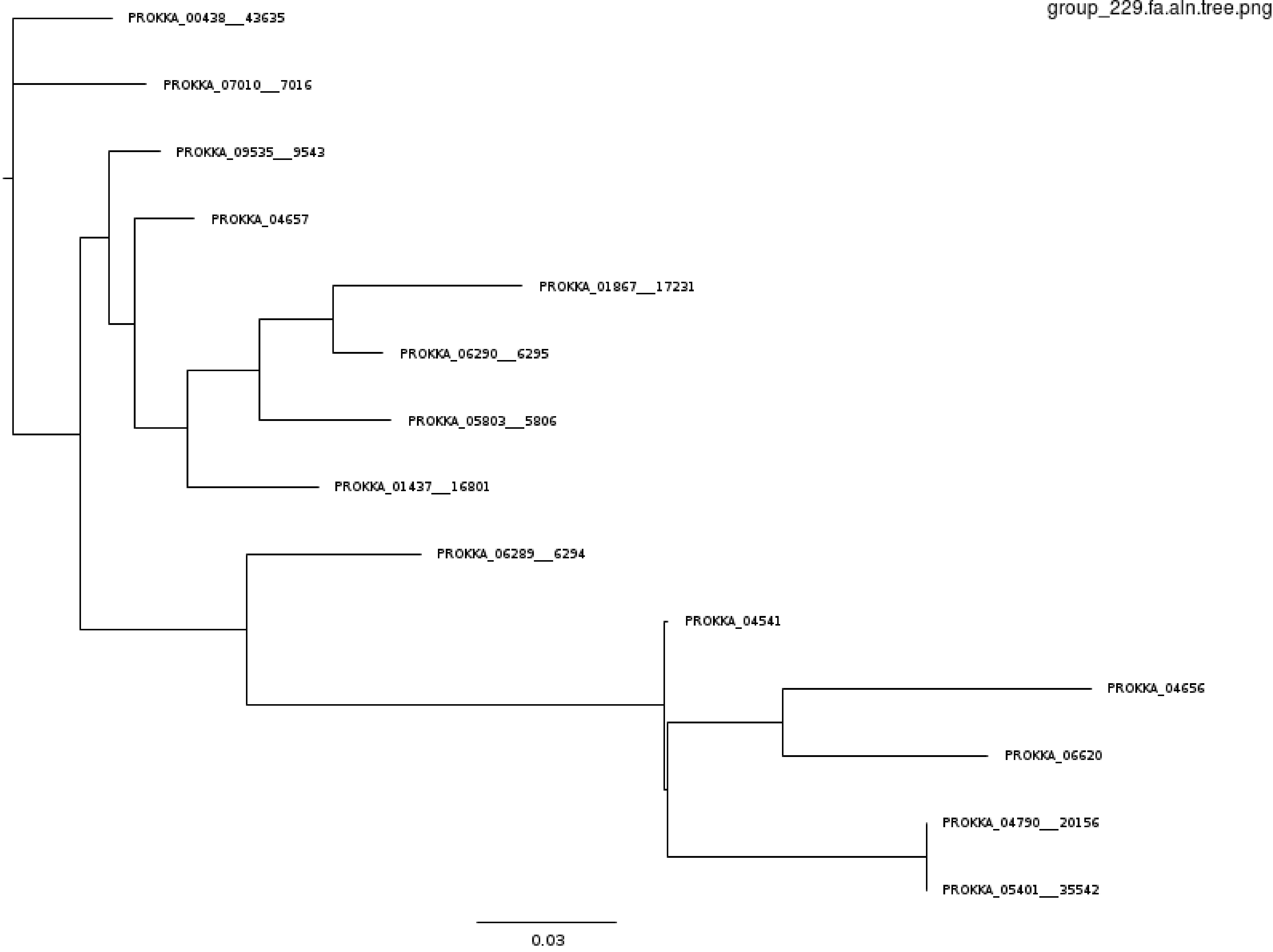

group_229.fa.aln.tree.png
PROKKA_00438___43635
PROKKA_07010___7016
PROKKA_09535___9543
PROKKA_04657
PROKKA_01867___17231
PROKKA_06290___6295
PROKKA_05803___5806
PROKKA_01437___16801
PROKKA_06289___6294
PROKKA_04541
PROKKA_04656
PROKKA_06620
PROKKA_04790___20156
PROKKA_05401___35542
0.03

group_2301.fa.aln.tree.png

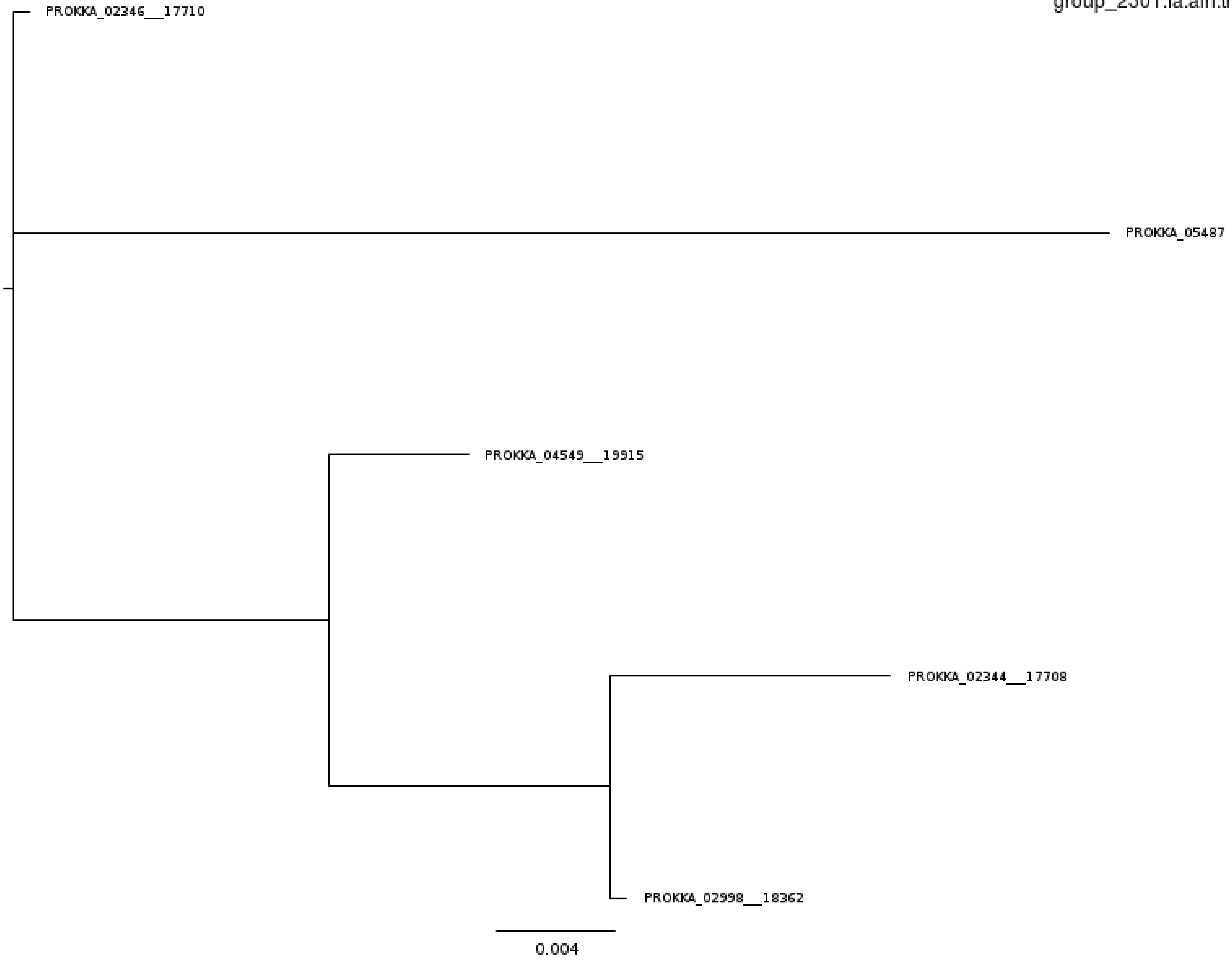

group_2306.fa.aln.tree.png

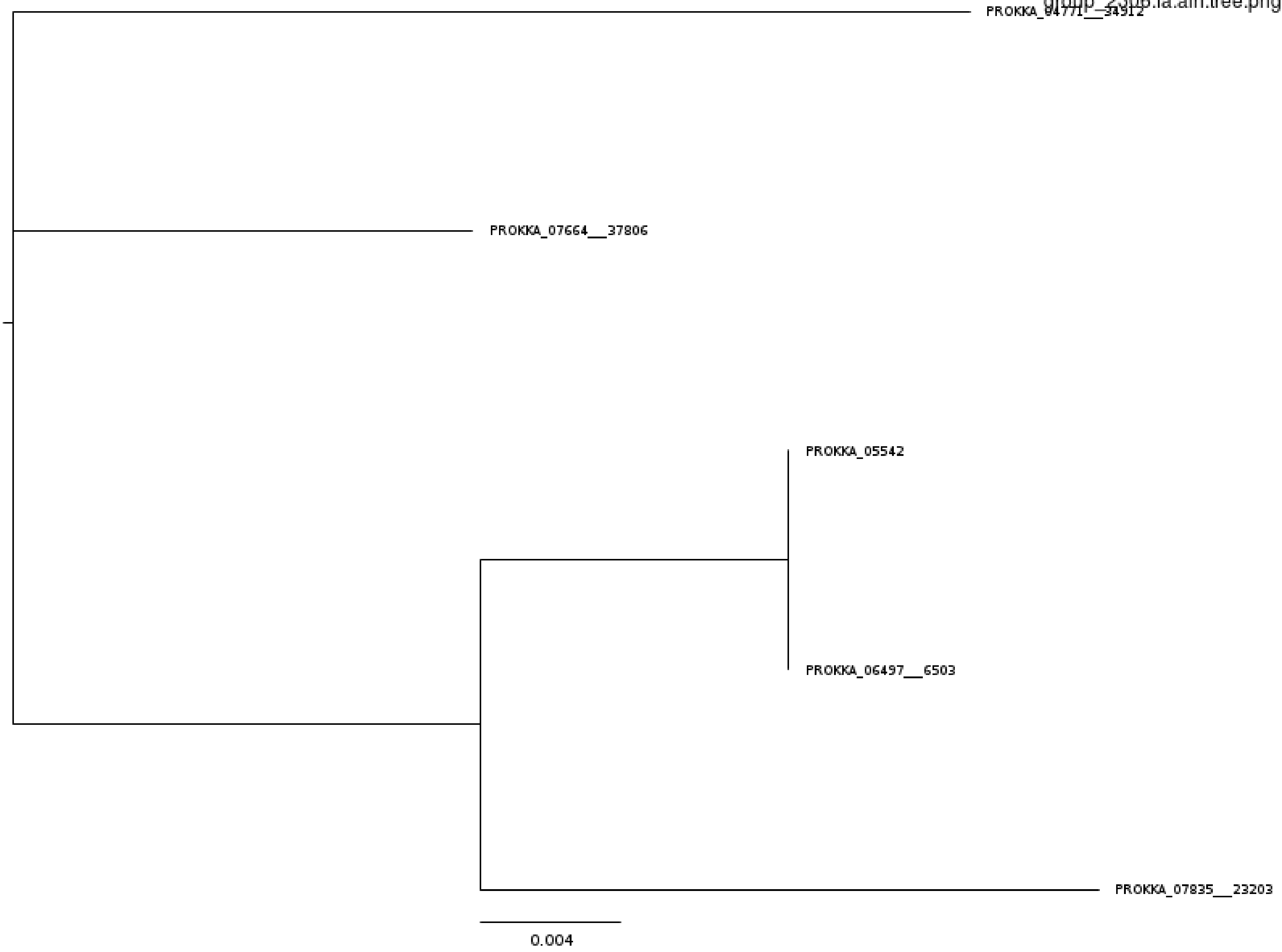

group_2311.fa.aln.tree.png

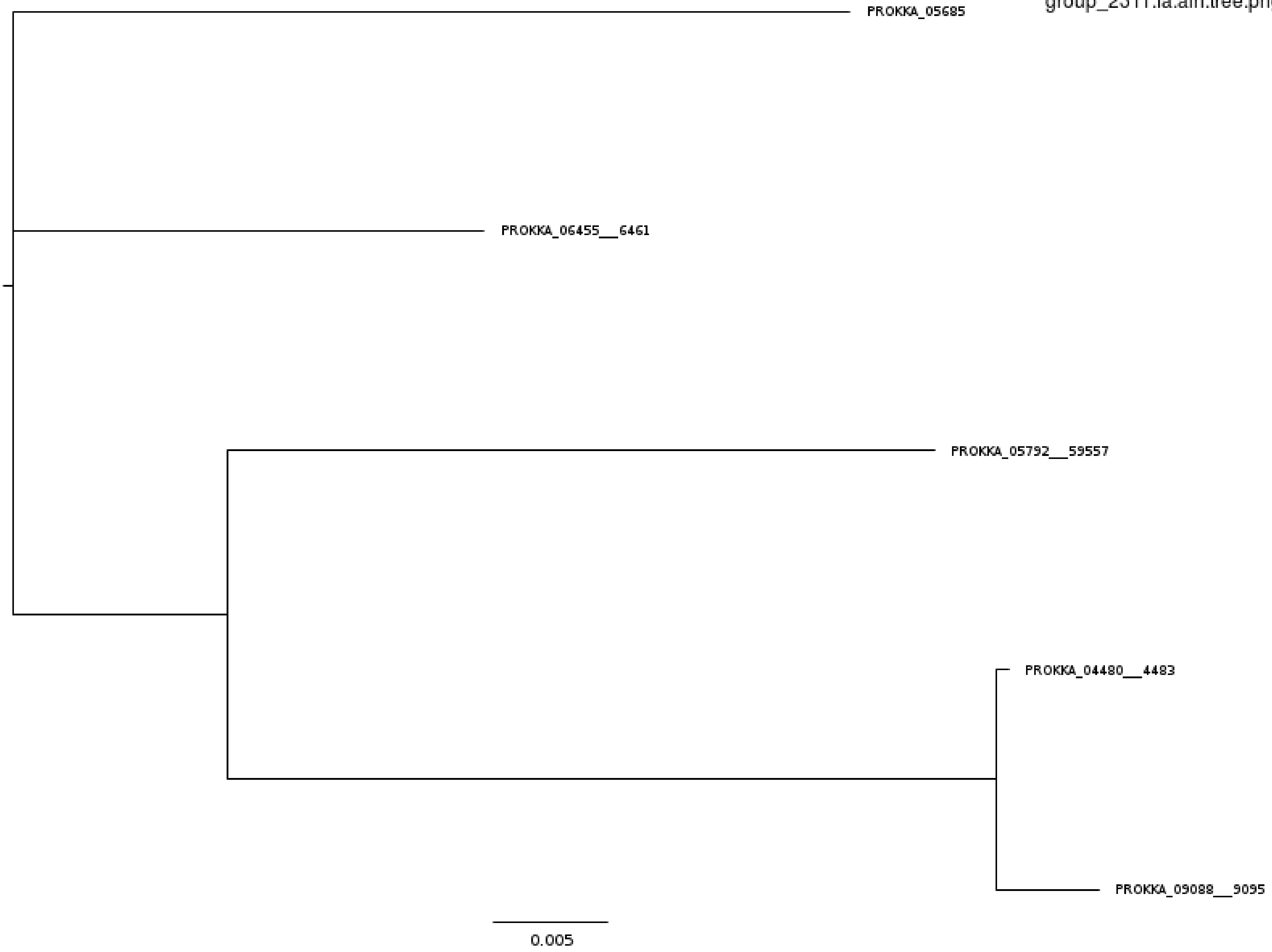

group_2312.fa.aln.tree.png

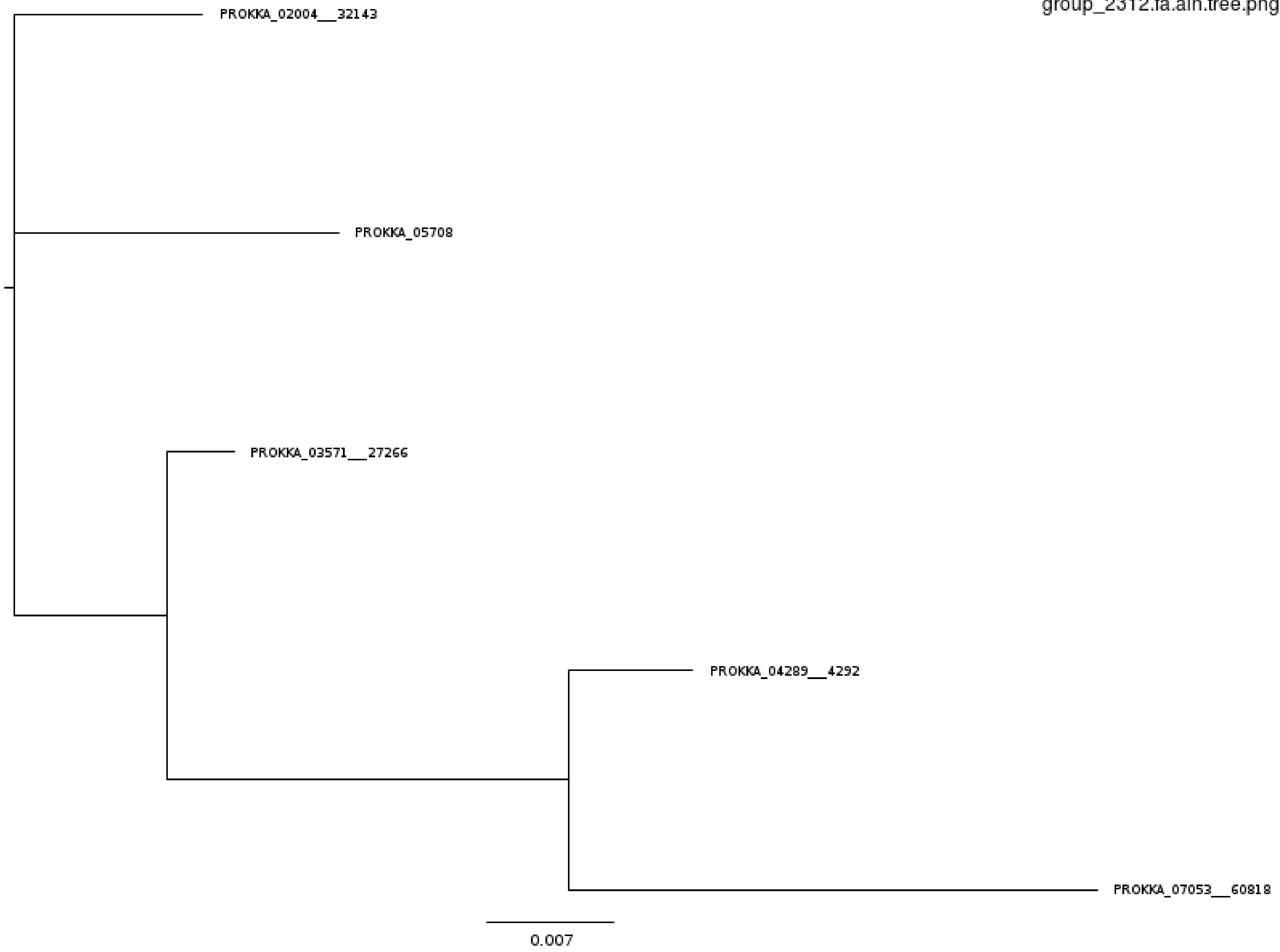

group_2316.fa.aln.tree.png

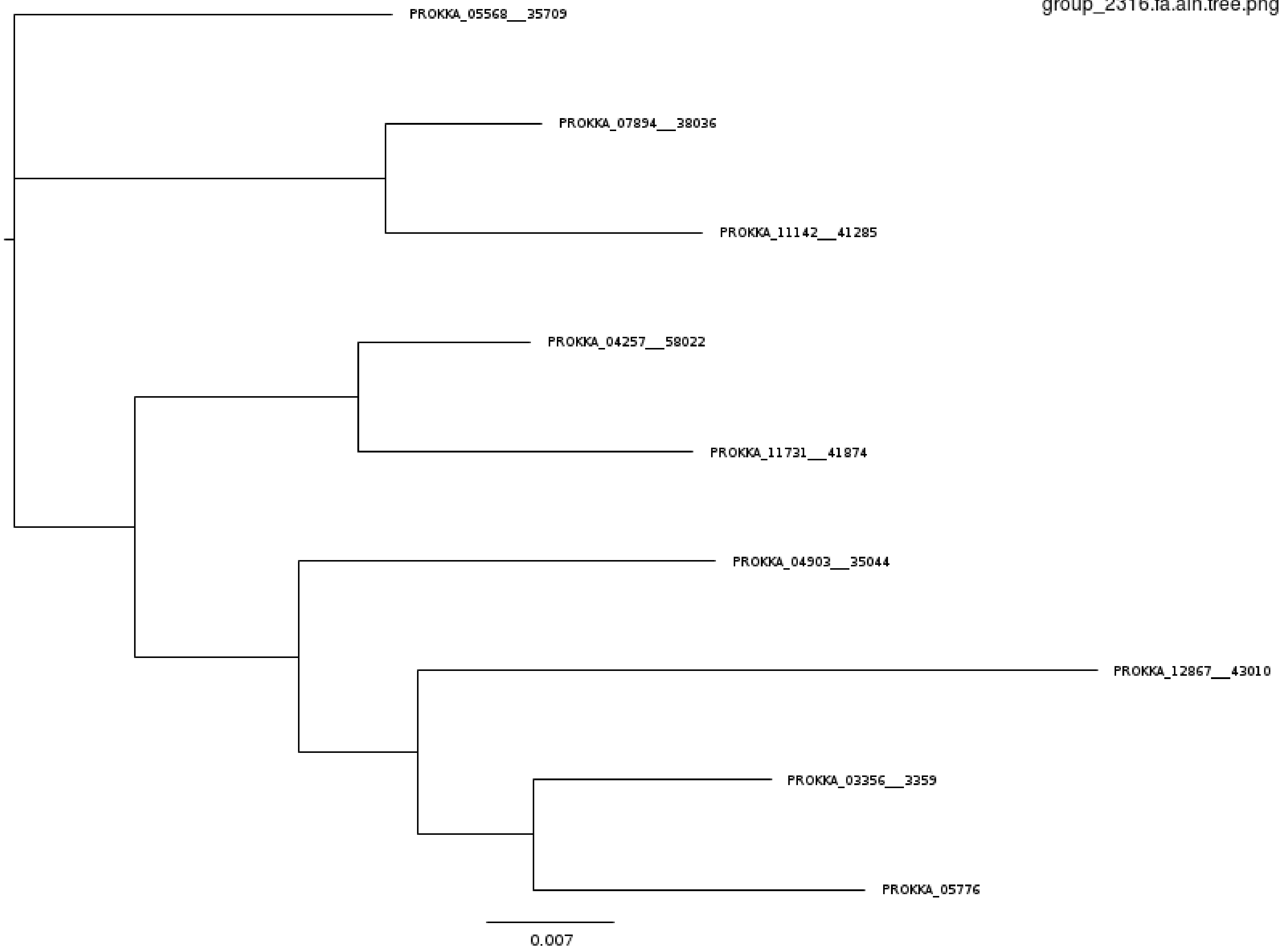

group_2318.fa.aln.tree.png

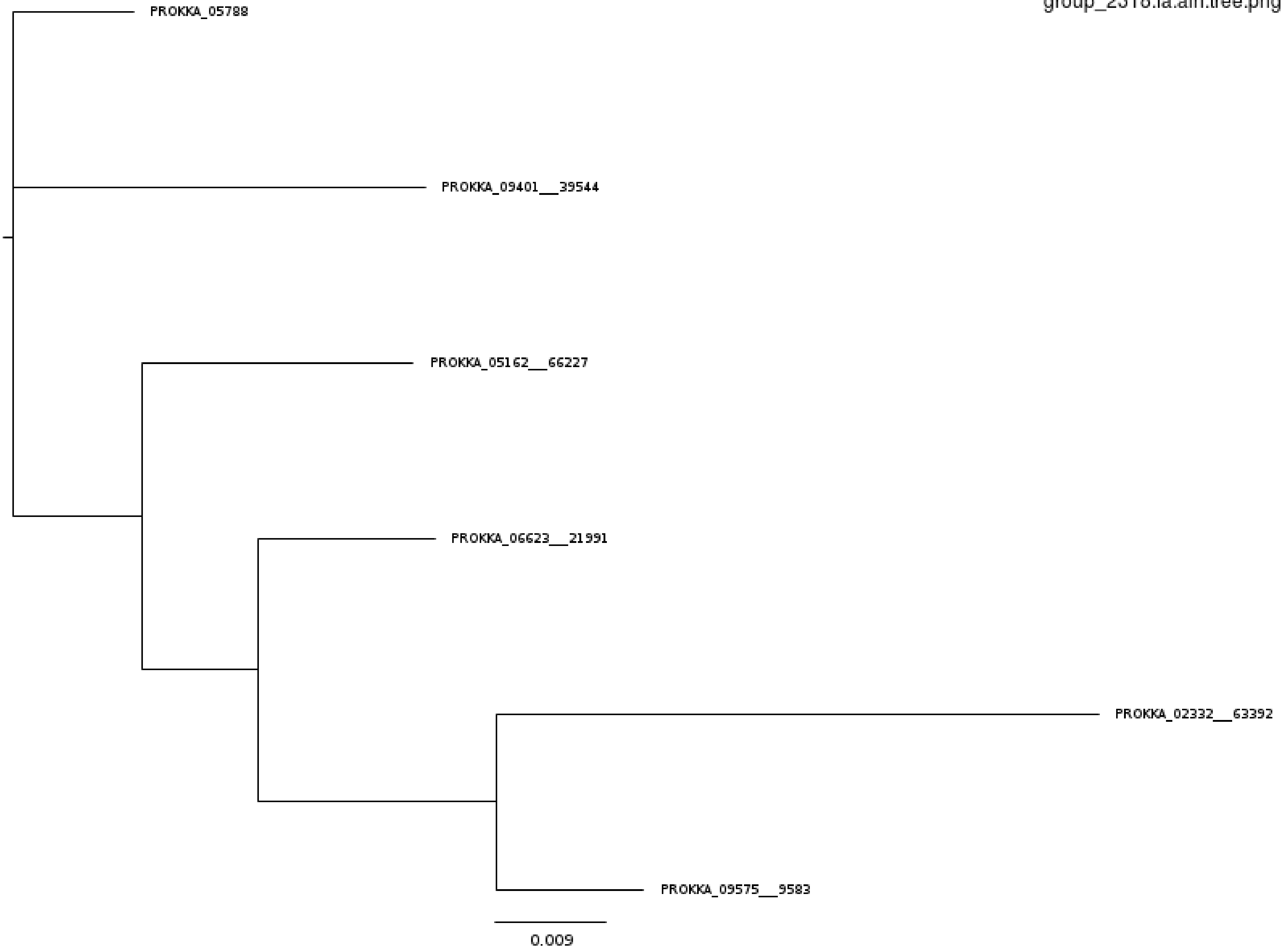

group_231.fa.aln.tree.png

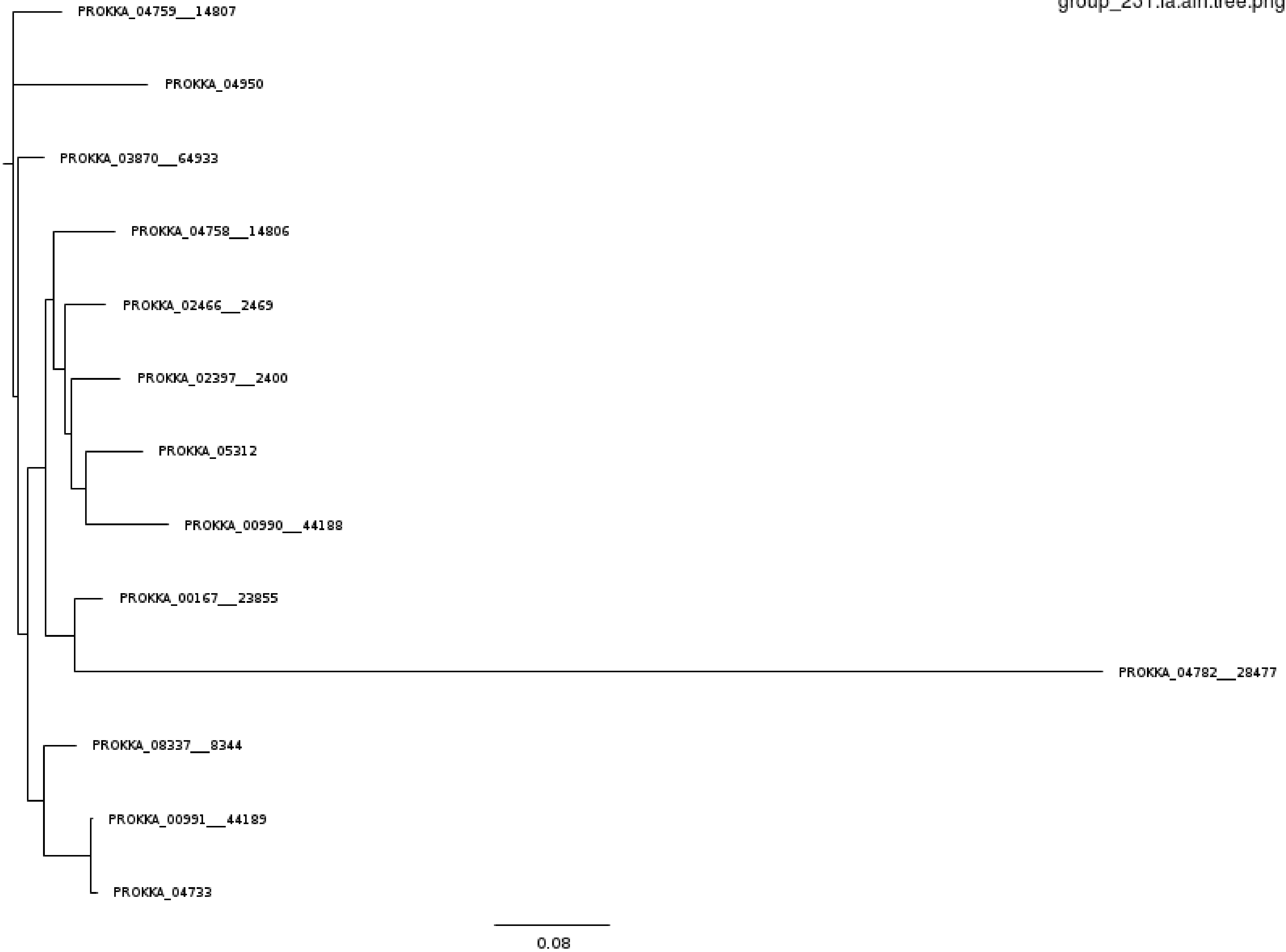

group_2326.fa.aln.tree.png

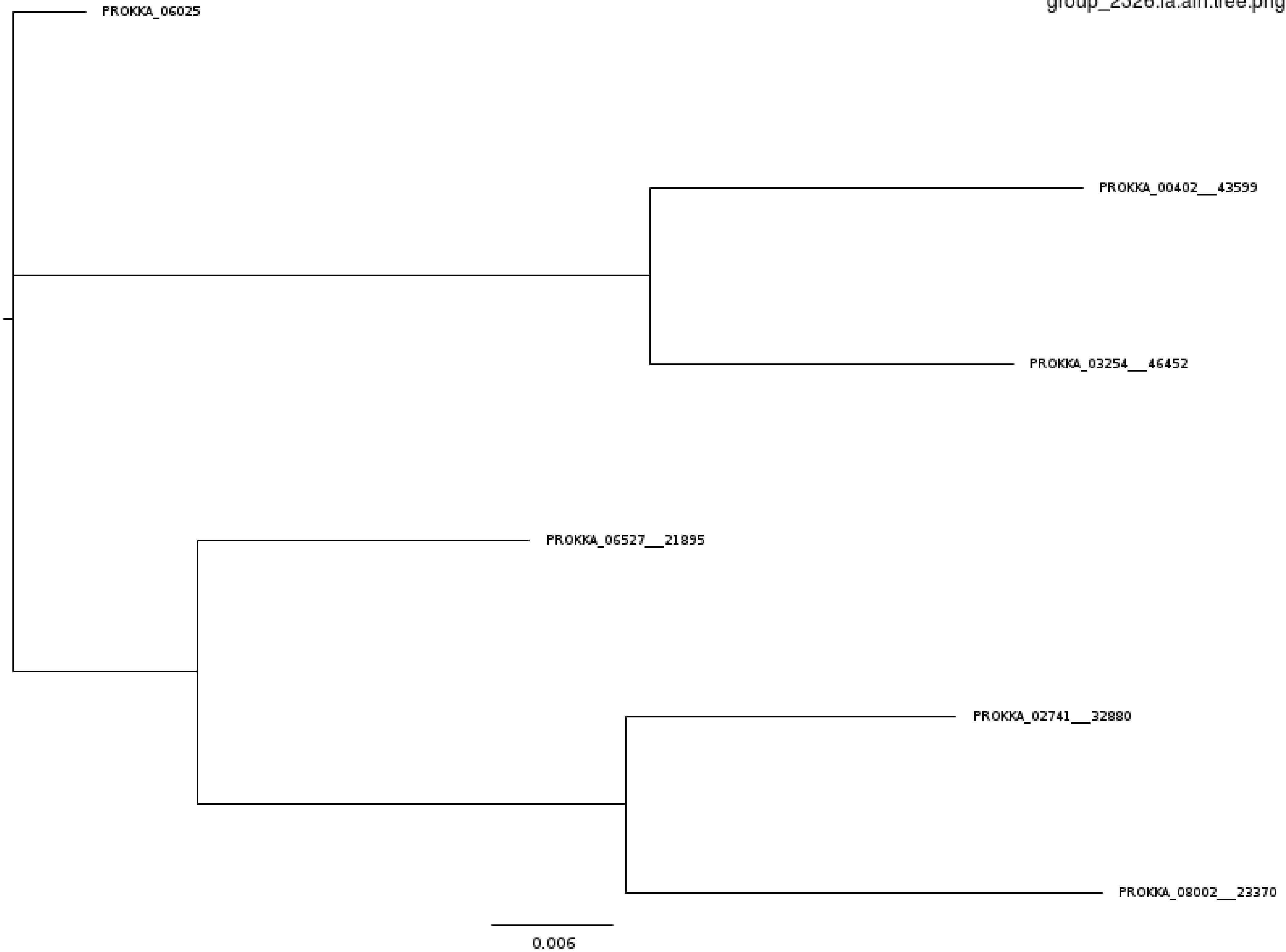

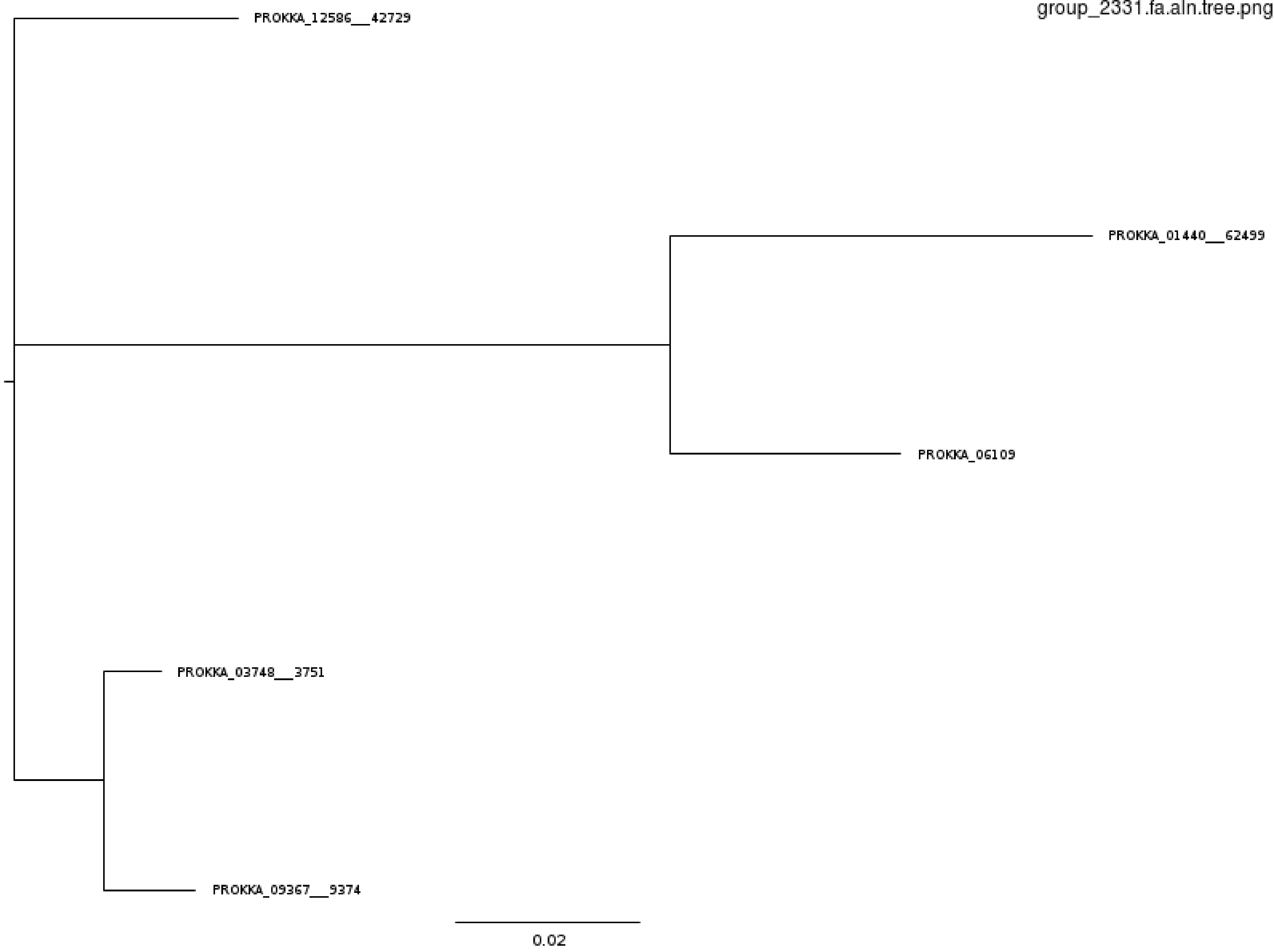
group_2331.fa.aln.tree.png
PROKKA_12586___42729
PROKKA_01440___62499
PROKKA_06109
PROKKA_03748___3751
PROKKA_09367___9374
0.02

group_2332.fa.aln.tree.png

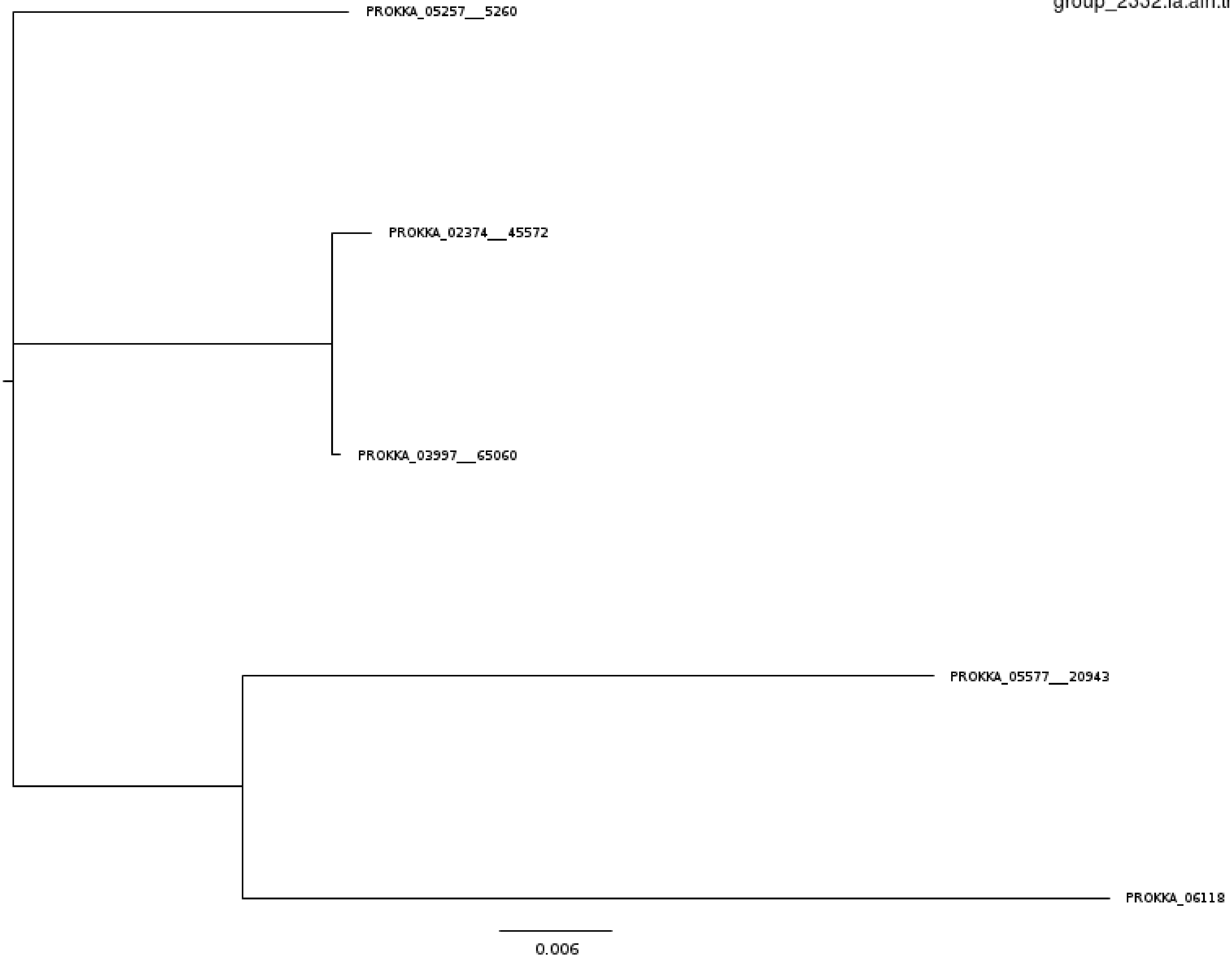

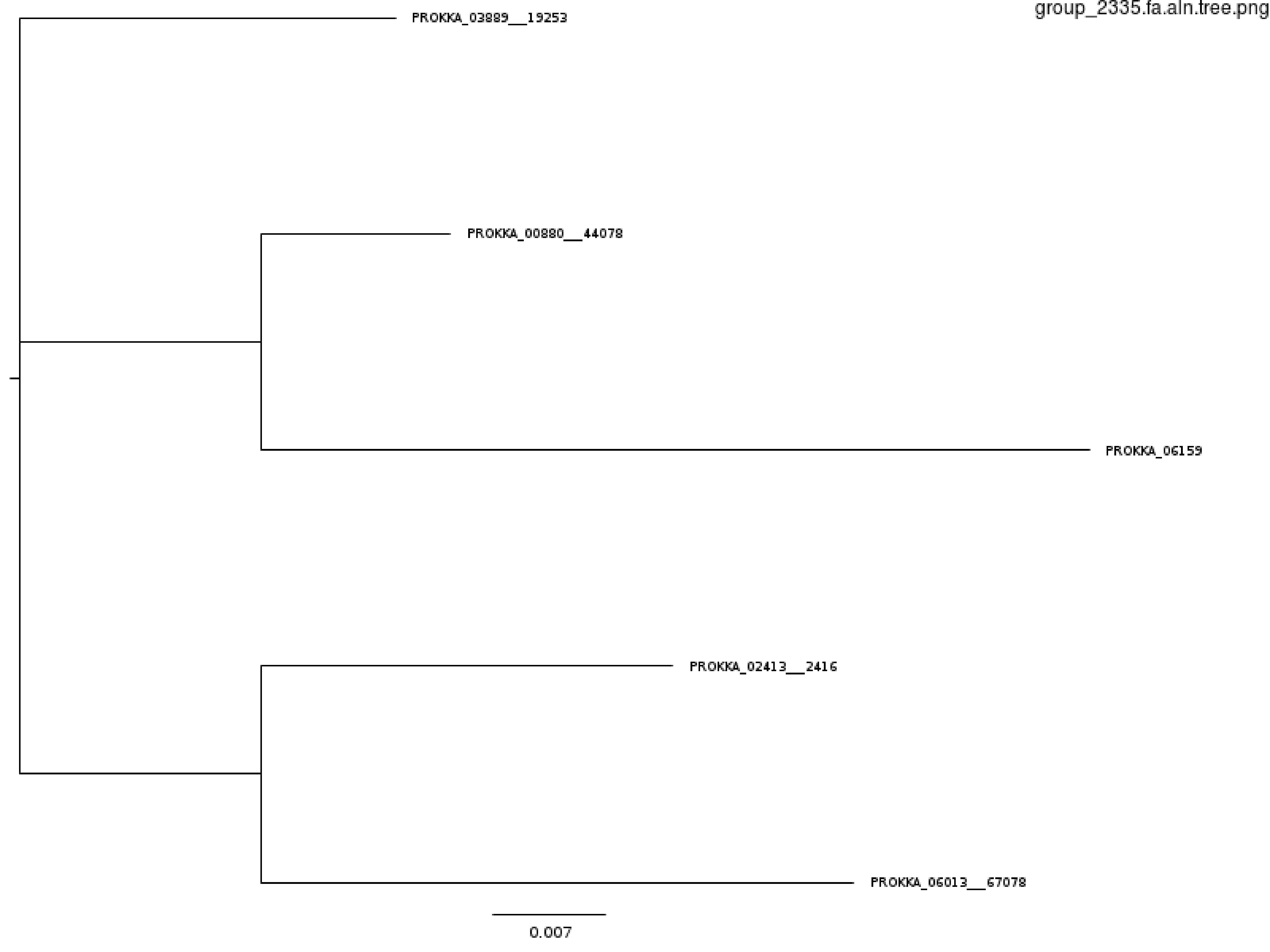
group_2335.fa.aln.tree.png
PROKKA_03889___19253
PROKKA_00880___44078
PROKKA_06159
PROKKA_02413___2416
PROKKA_06013___67078
0.007

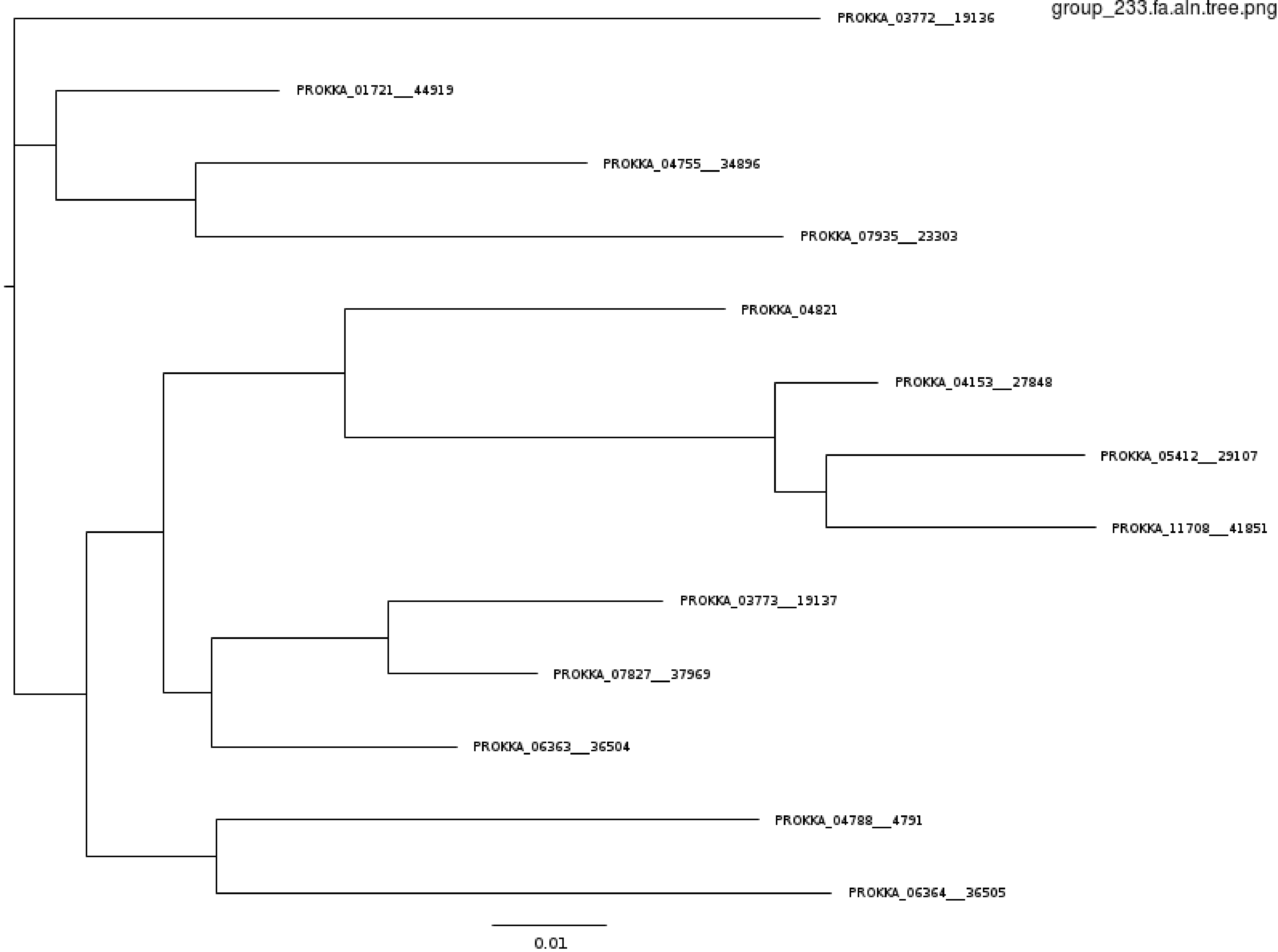
group_233.fa.aln.tree.png
PROKKA_03772___19136
PROKKA_01721___44919
PROKKA_04755___34896
PROKKA_07935___23303
PROKKA_04821
PROKKA_04153___27848
PROKKA_05412___29107
PROKKA_11708___41851
PROKKA_03773___19137
PROKKA_07827___37969
PROKKA_06363___36504
PROKKA_04788___4791
PROKKA_06364___36505
0.01

group_23542.fa.aln.tree.png

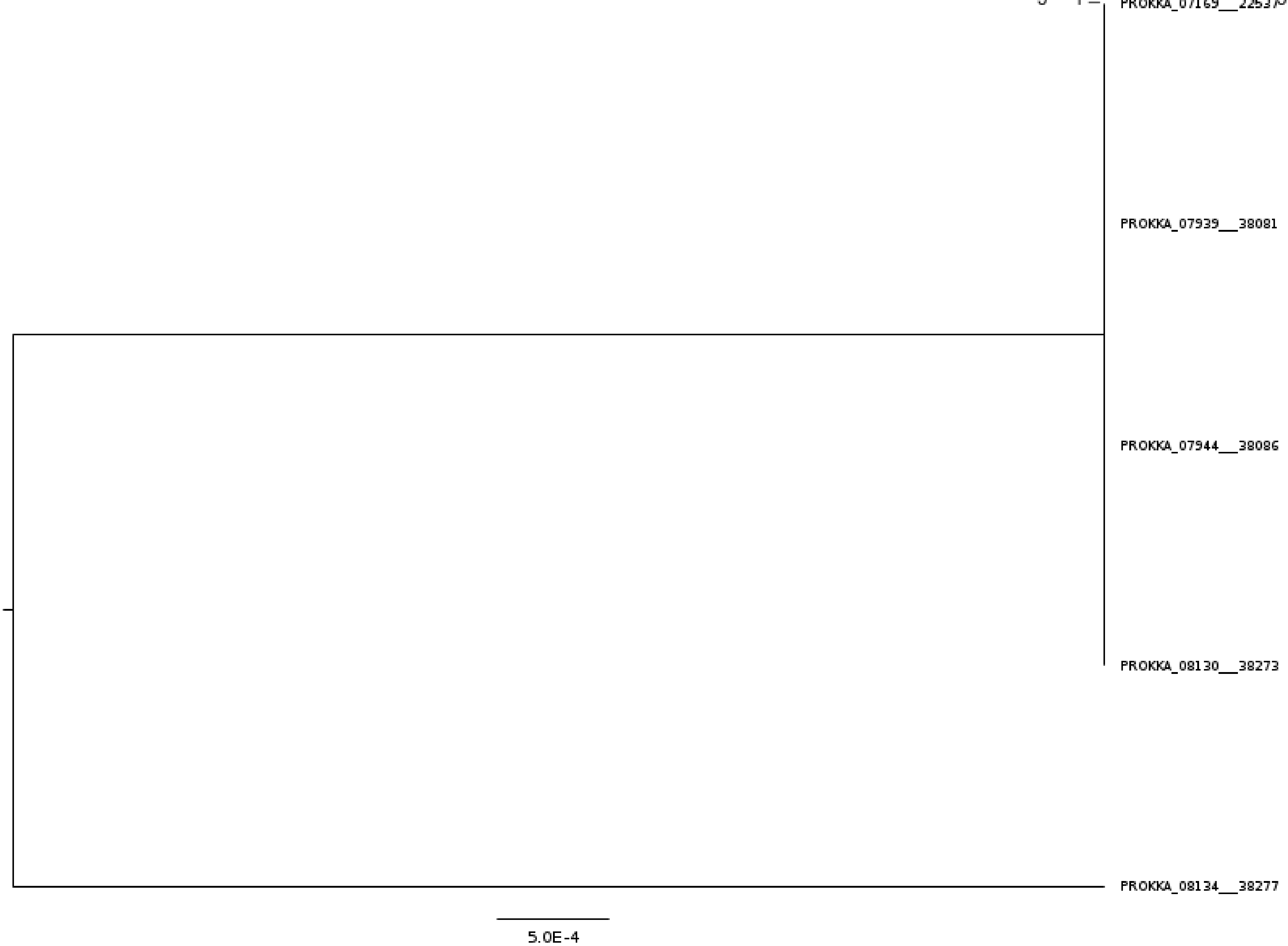

group_23552.fa.aln.tree.png

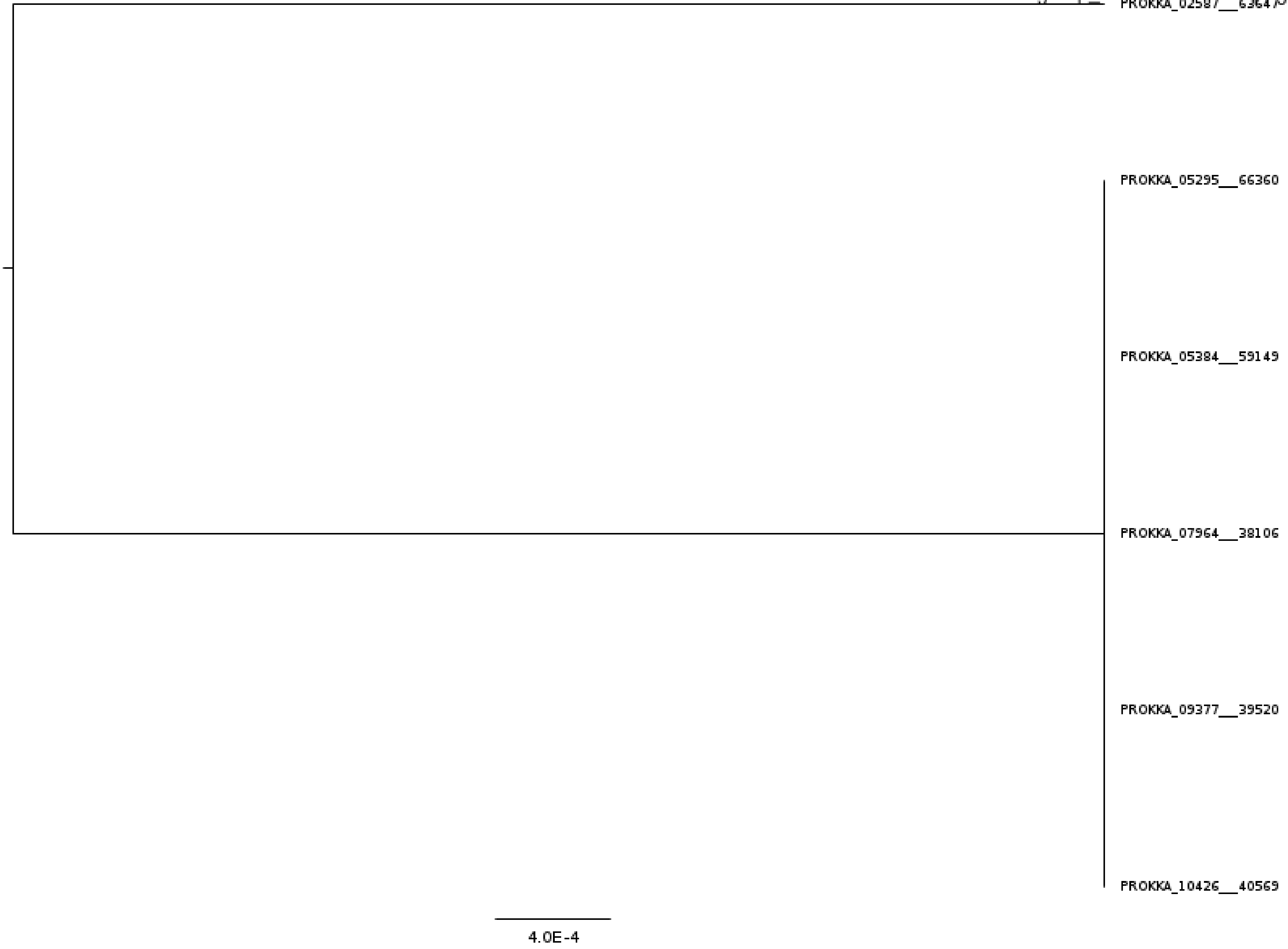

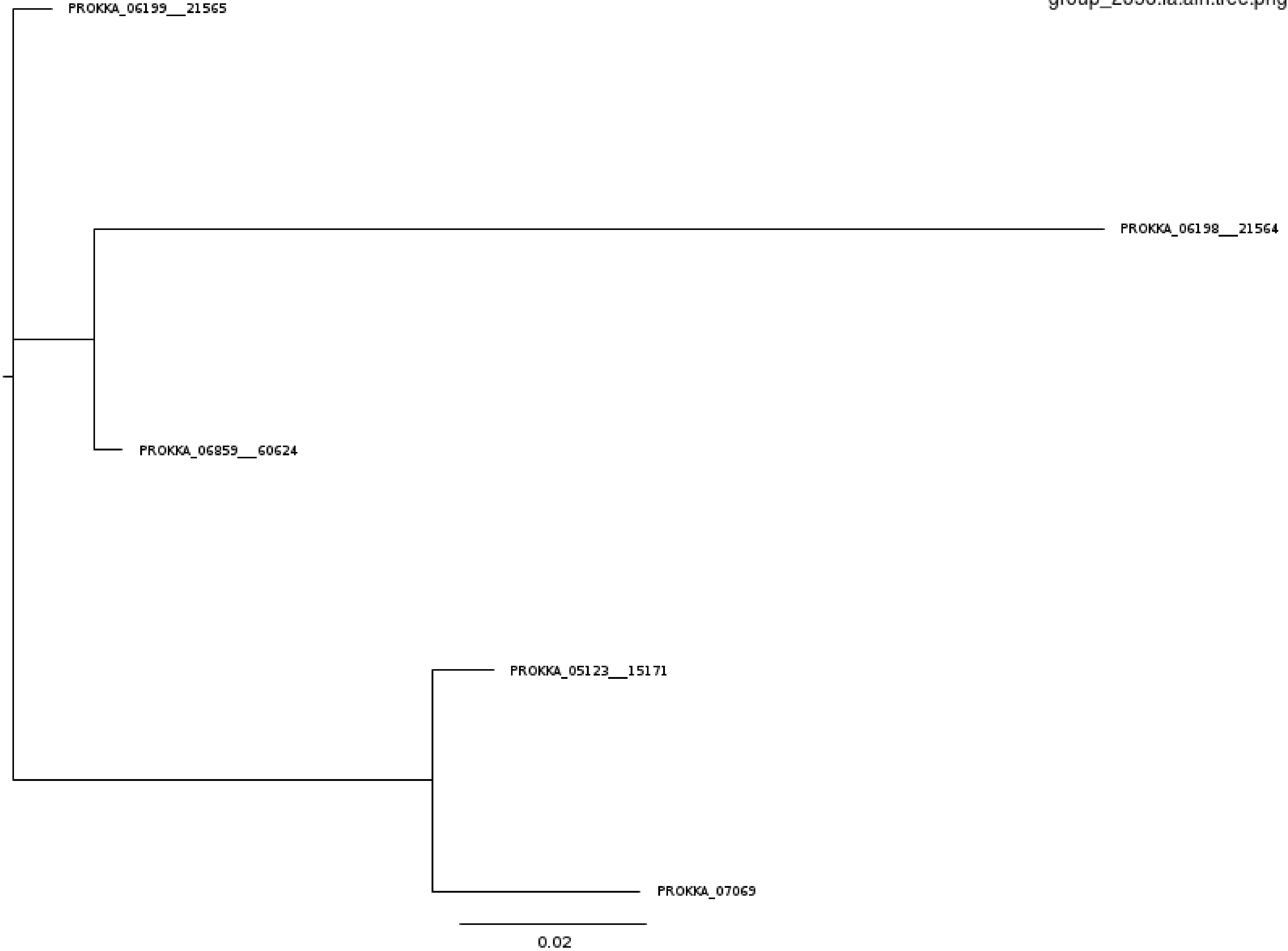
PROKKA_06199___21565
PROKKA_06198___21564
PROKKA_06859___60624
PROKKA_05123___15171
PROKKA_07069
0.02

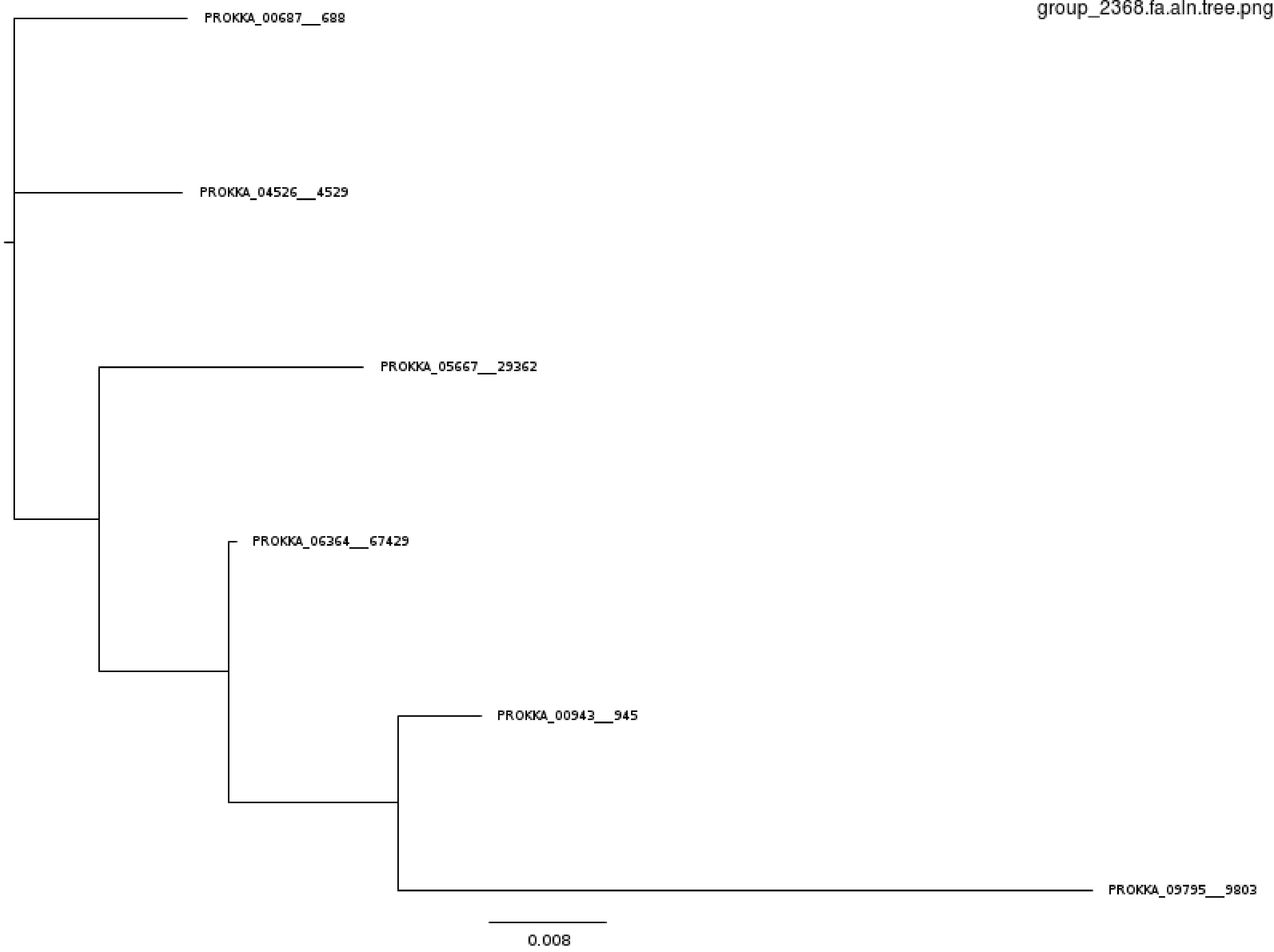
group_2368.fa.aln.tree.png
PROKKA_00687___688
PROKKA_04526___4529
PROKKA_05667___29362
PROKKA_06364___67429
PROKKA_00943___945
PROKKA_09795___9803
0.008

group_2372.fa.aln.tree.png

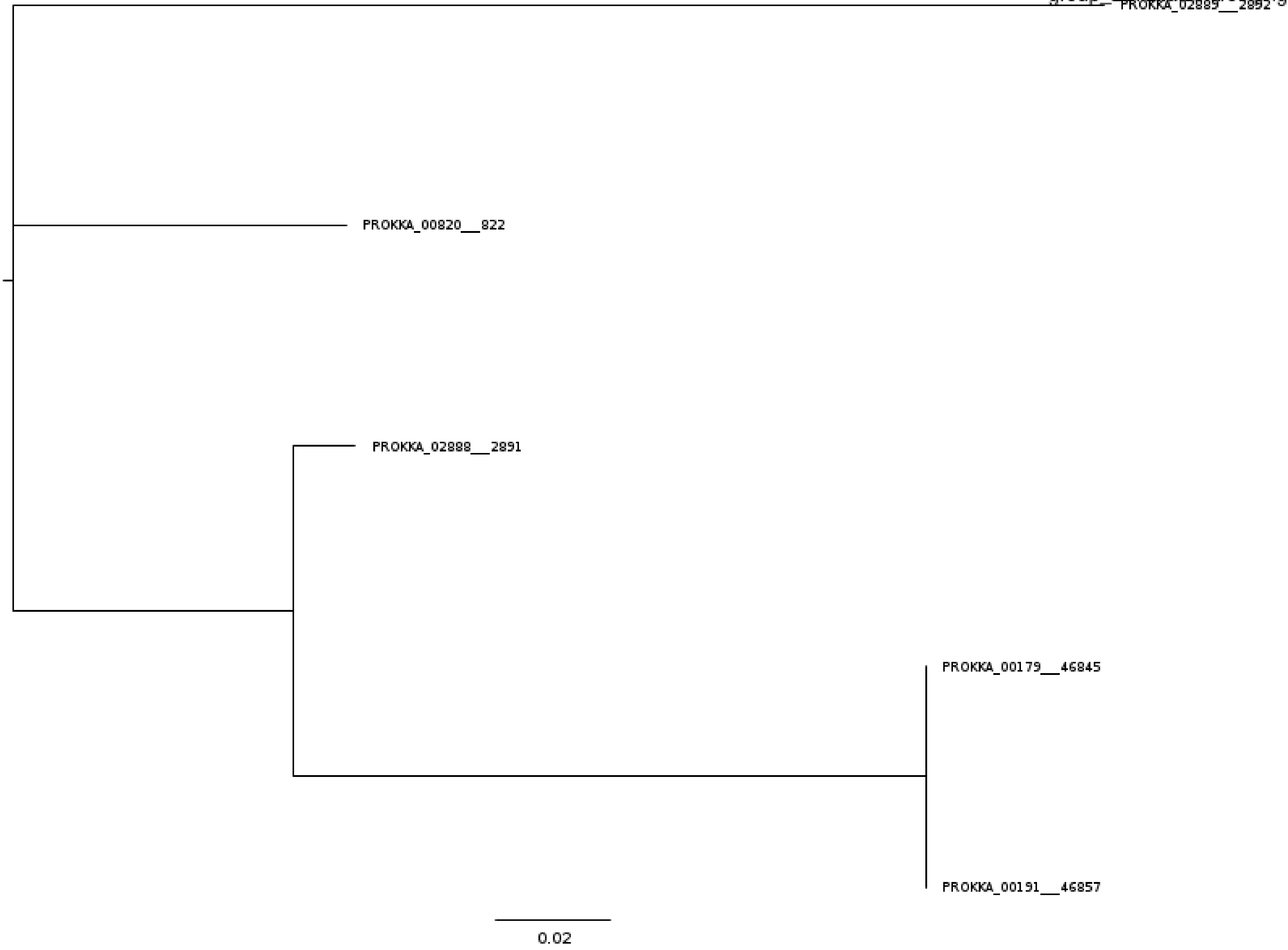

group_2389.fa.aln.tree.png

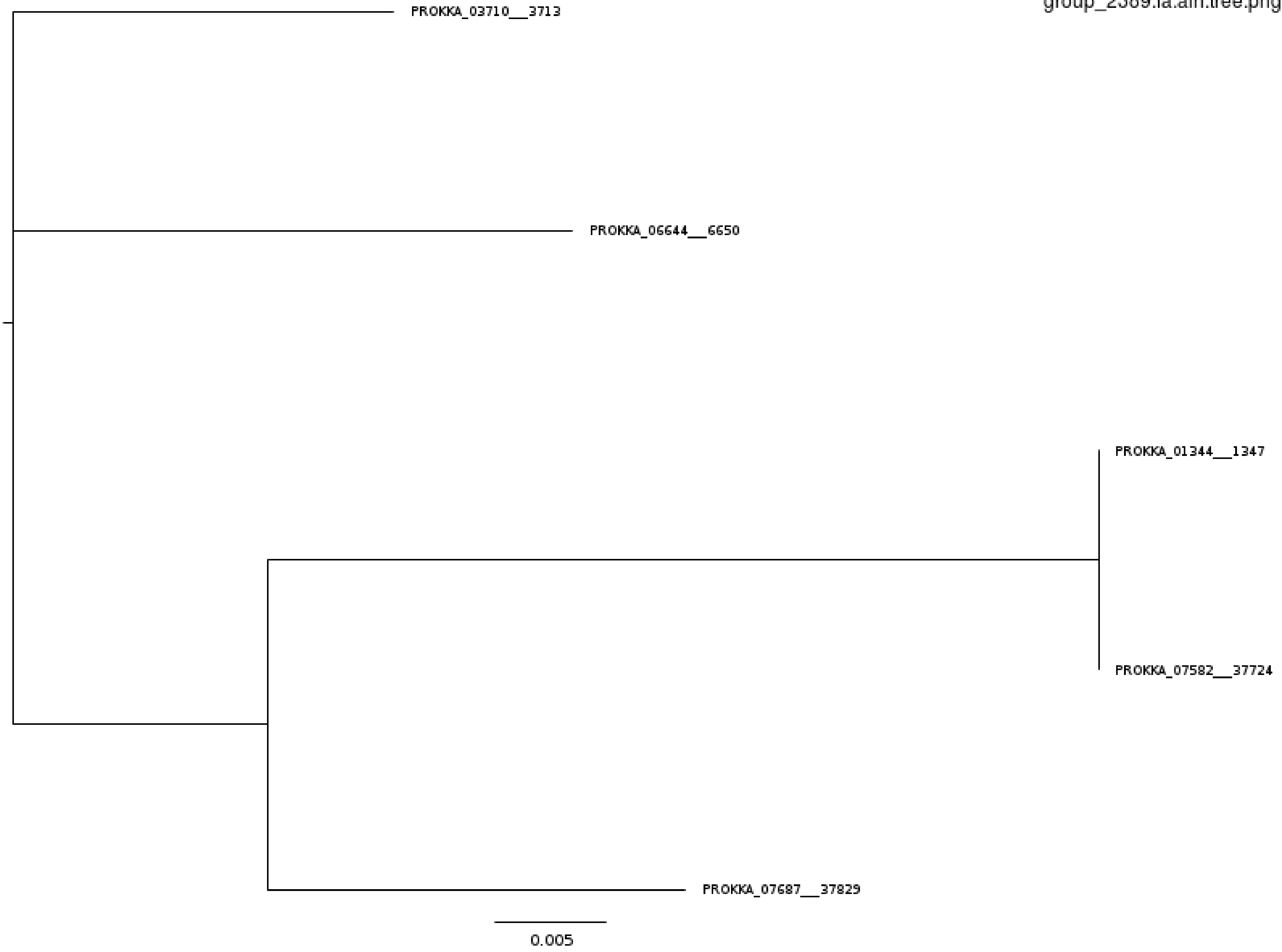

group_2396.fa.aln.tree.png

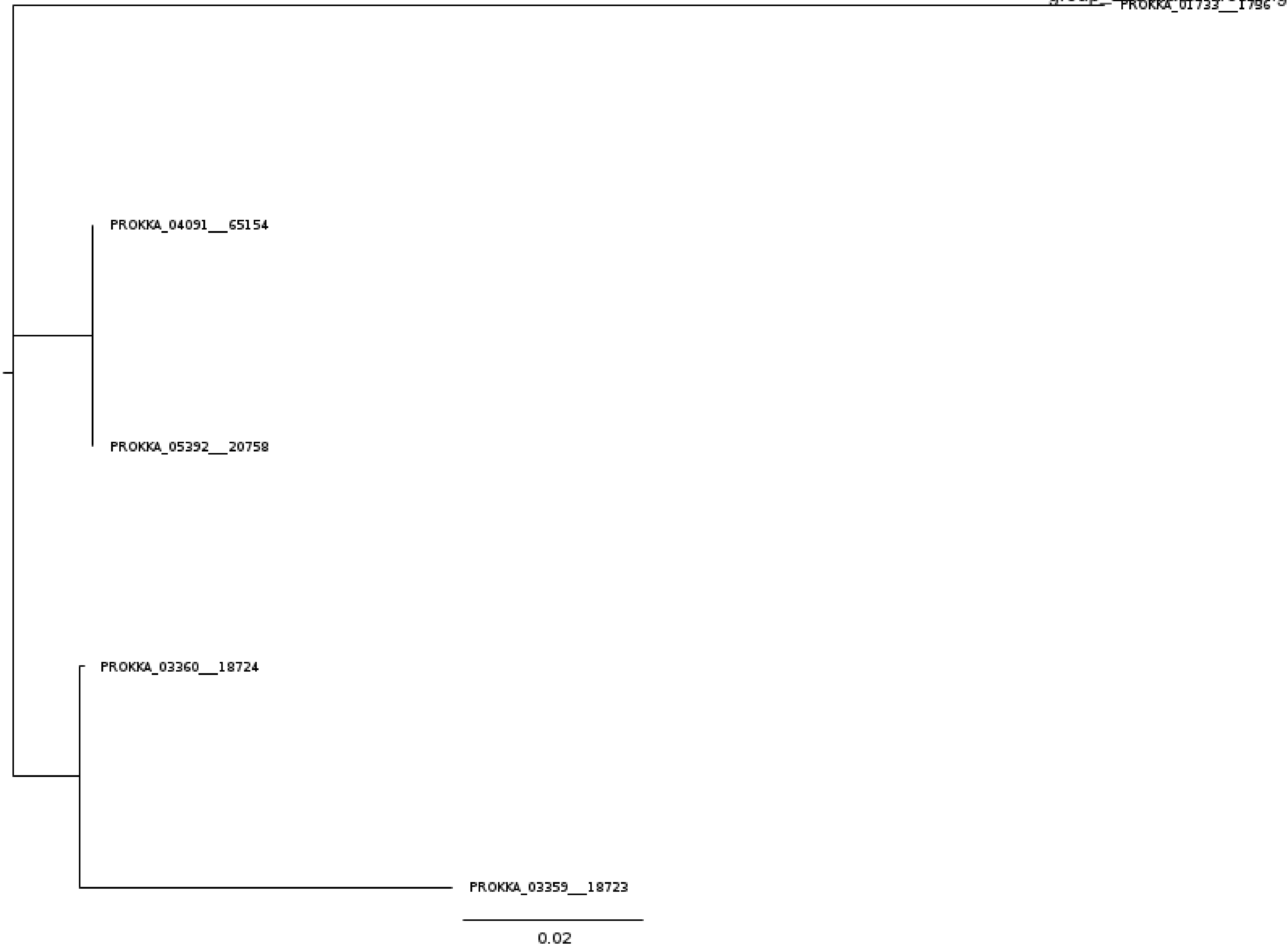

group_241.fa.aln.tree.png

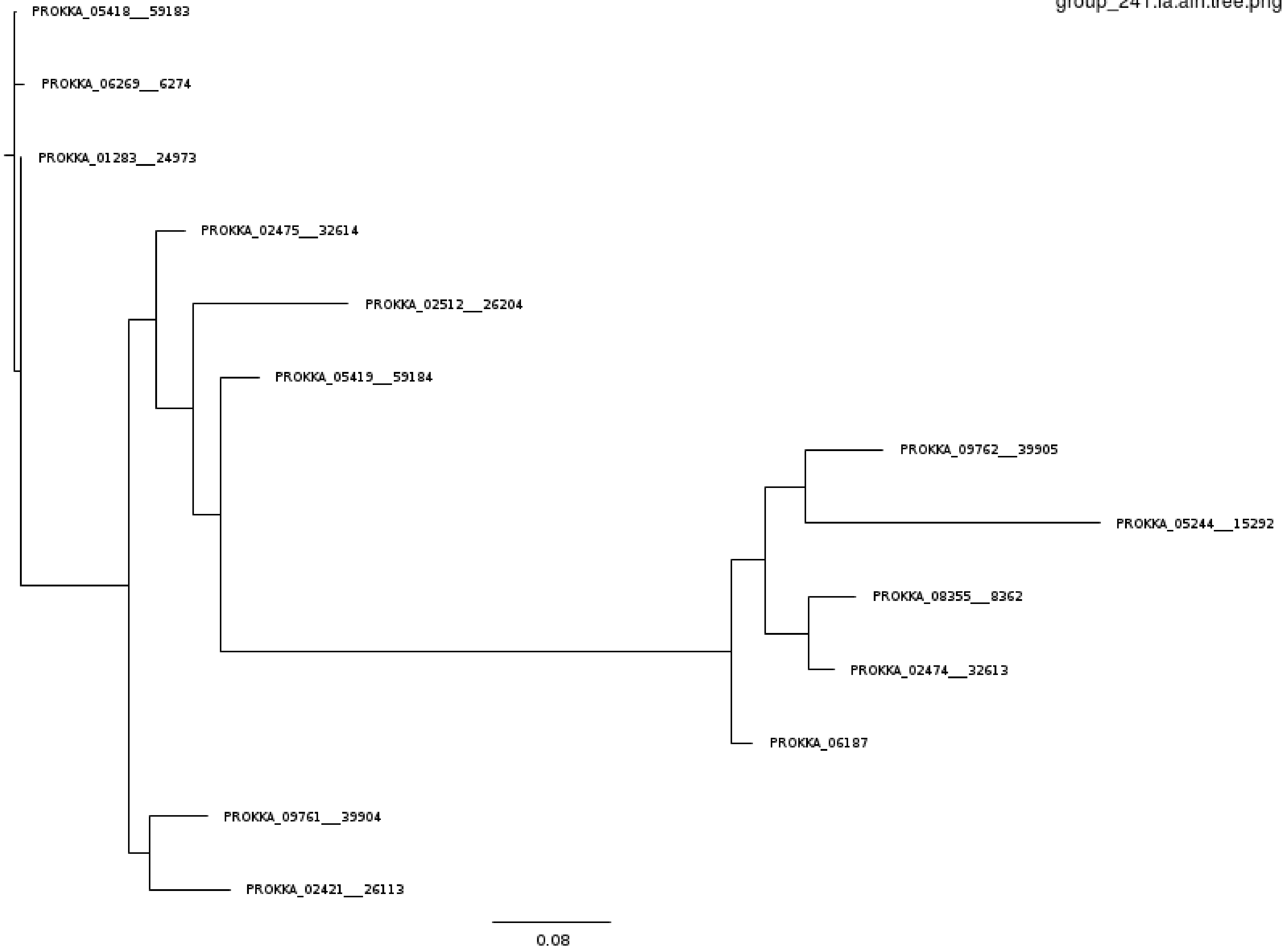

group_2436.fa.aln.tree.png

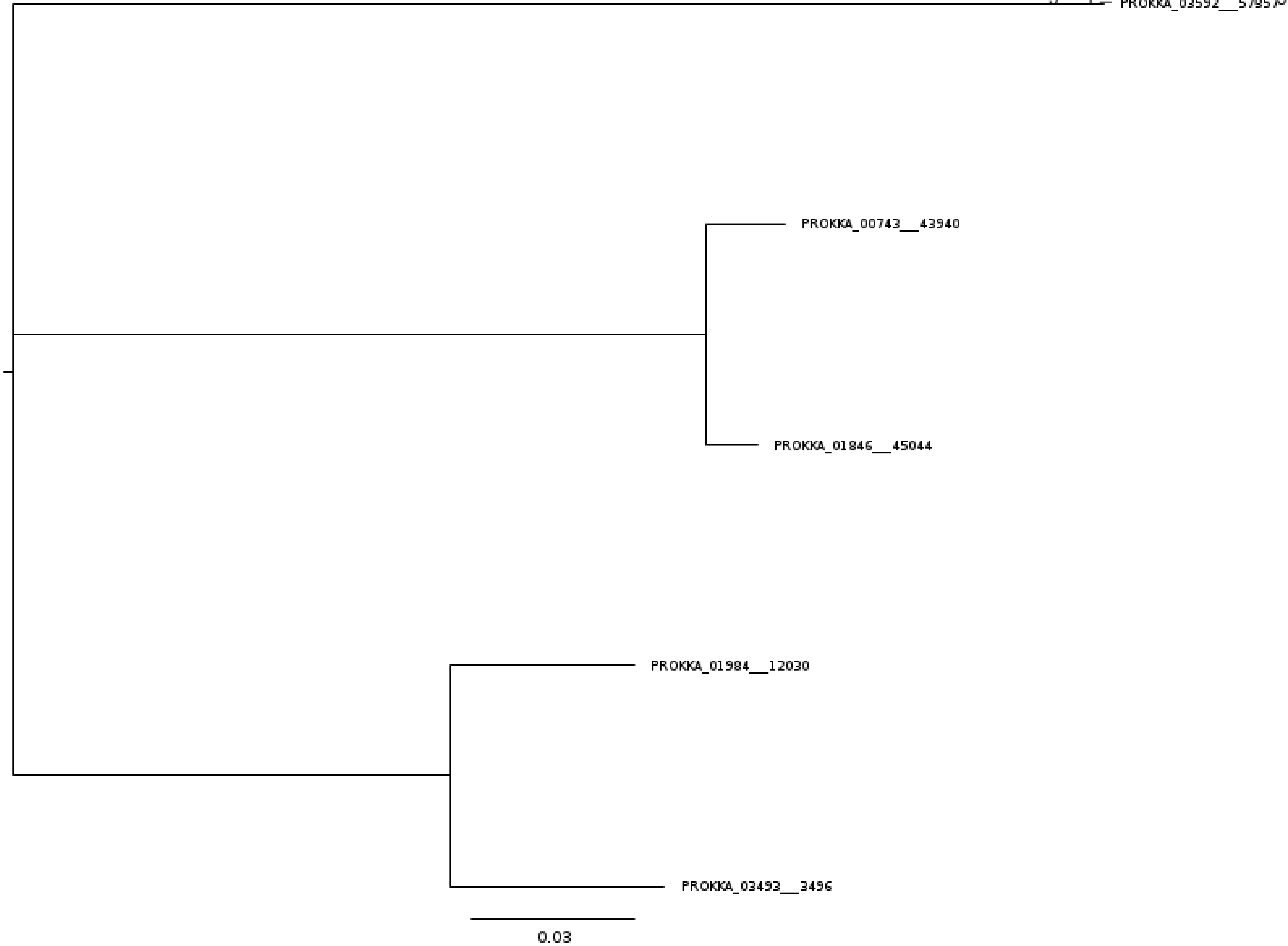

group_2440.fa.aln.tree.png

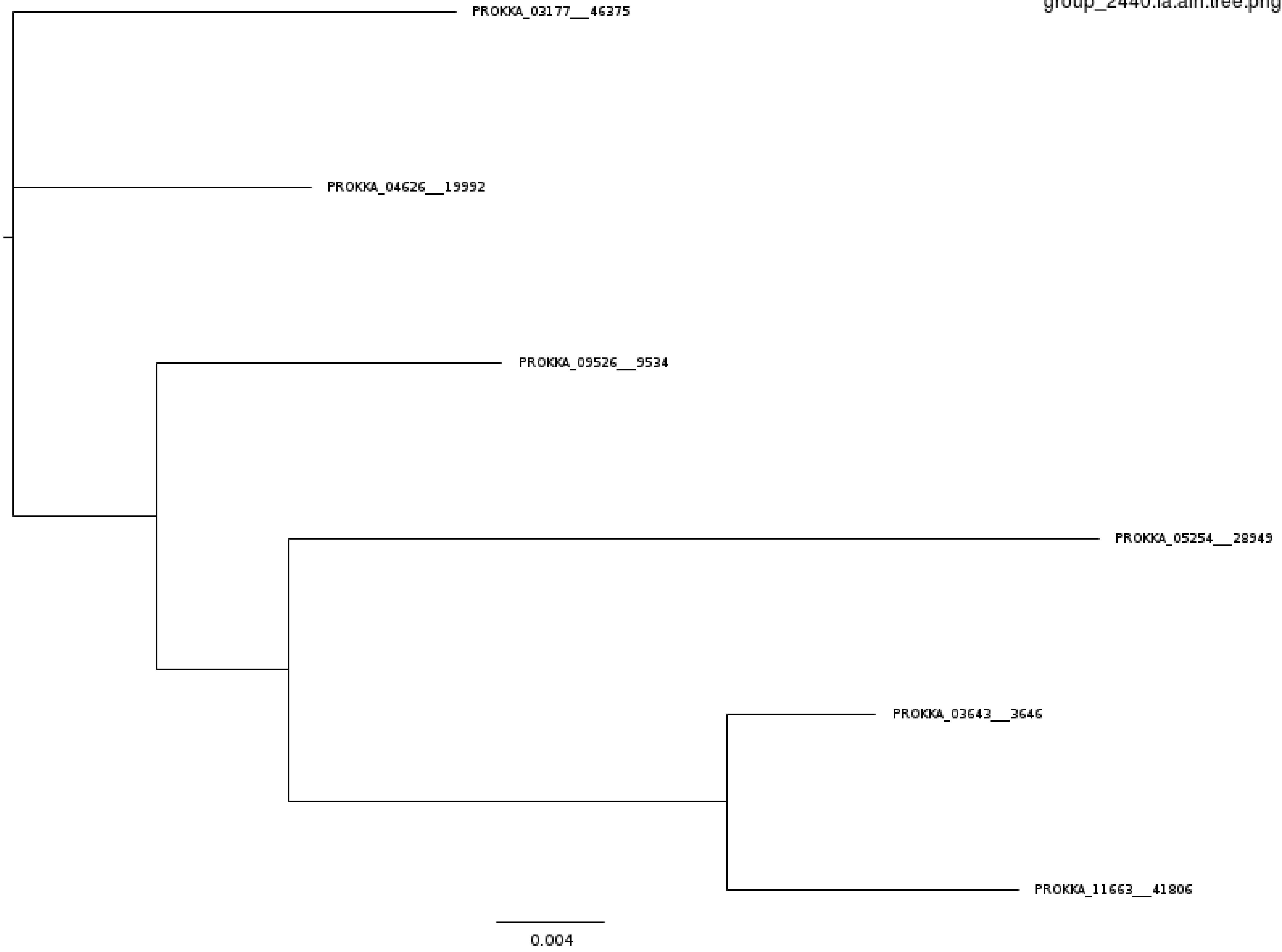

group_2448.fa.aln.tree.png

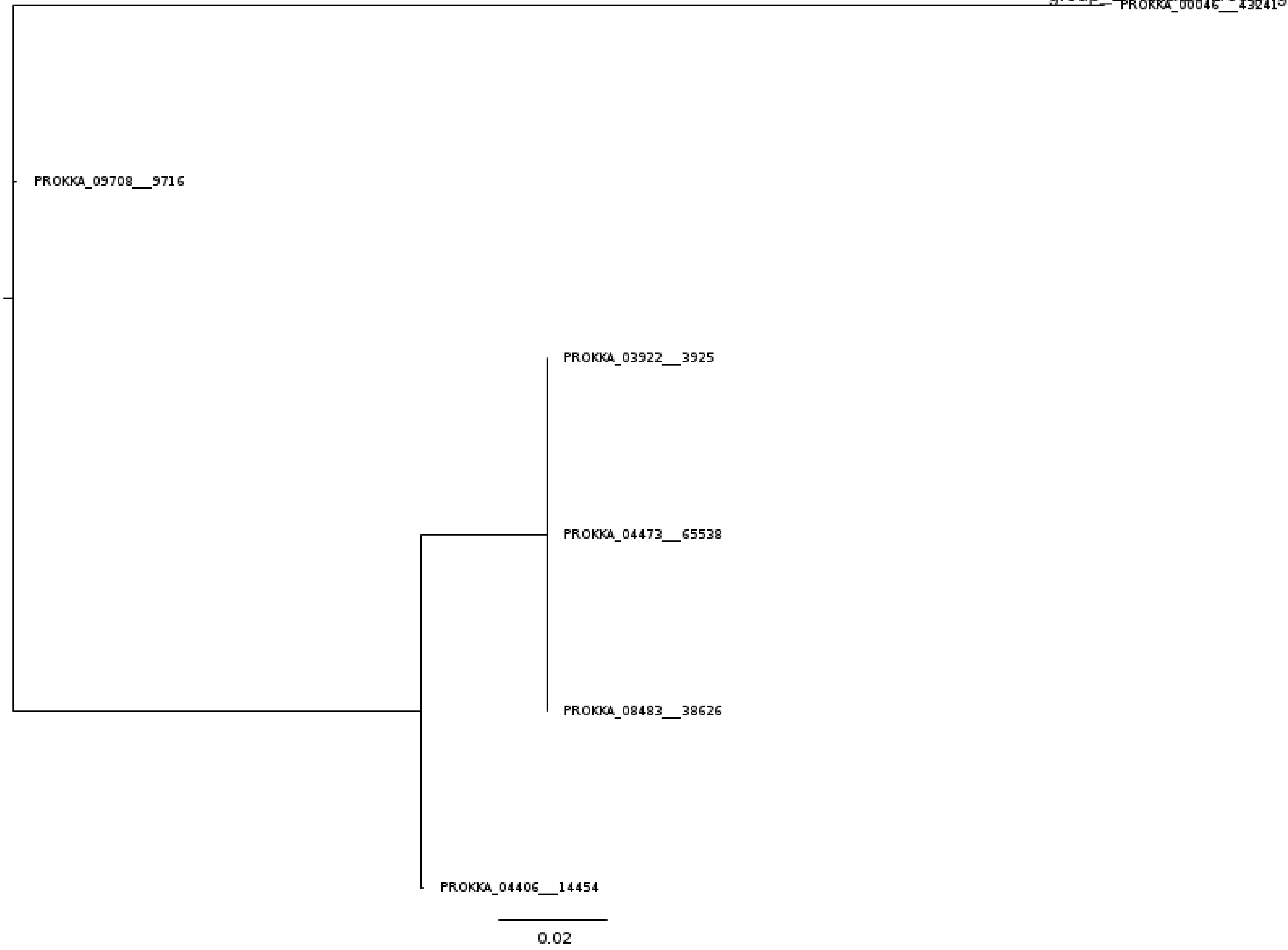

group_244.fa.aln.tree.png

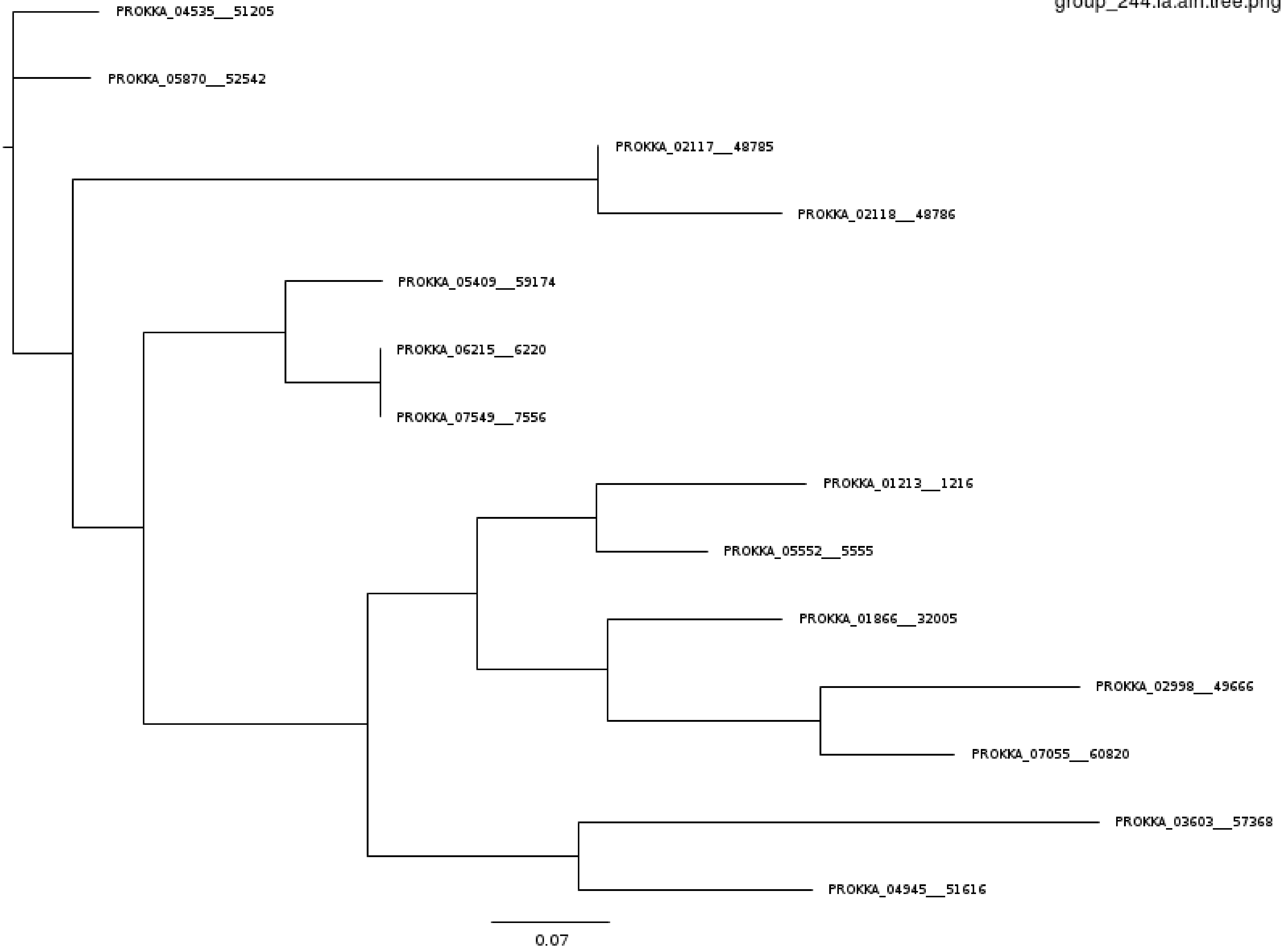

group_2463.fa.aln.tree.png

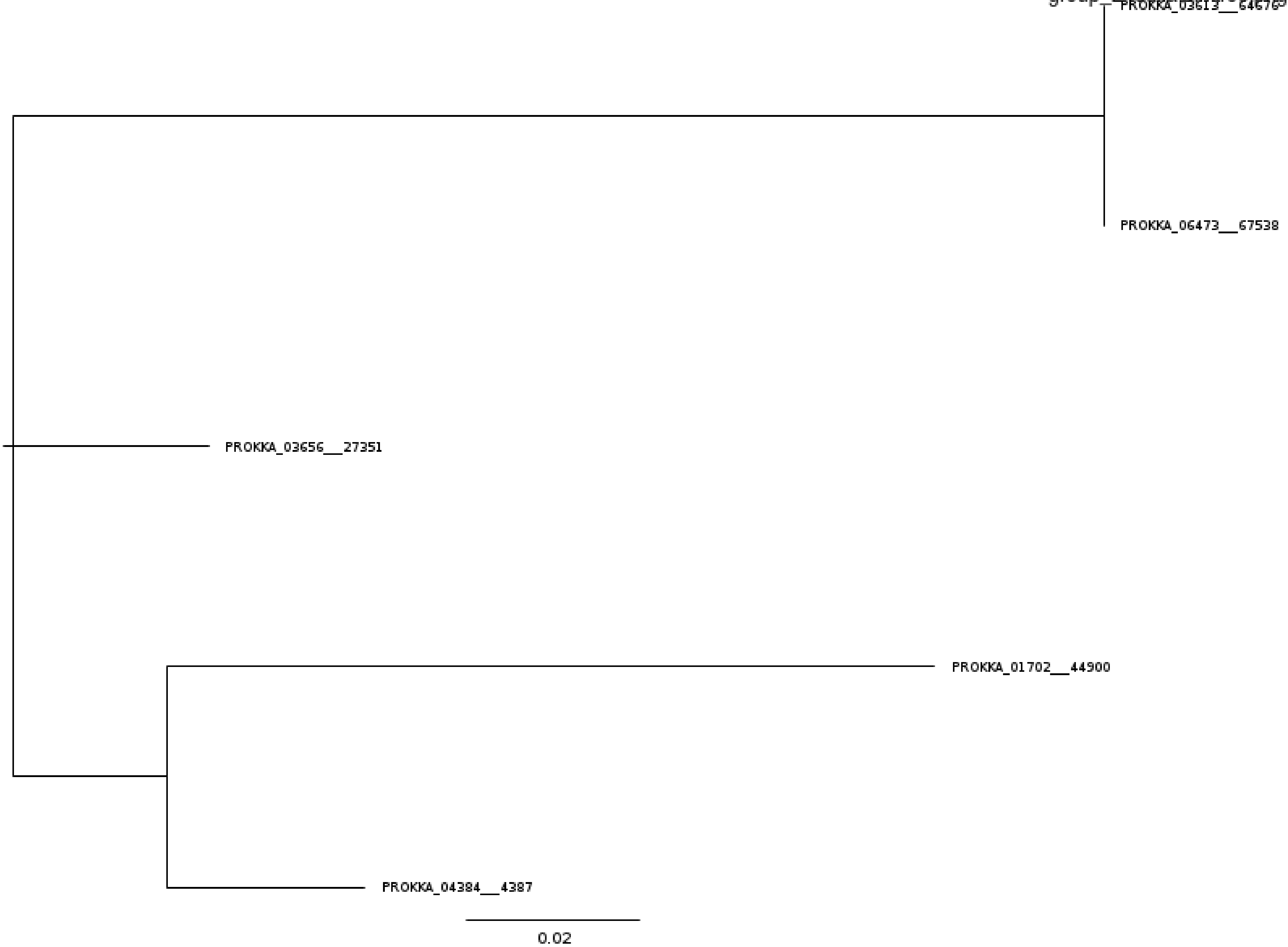

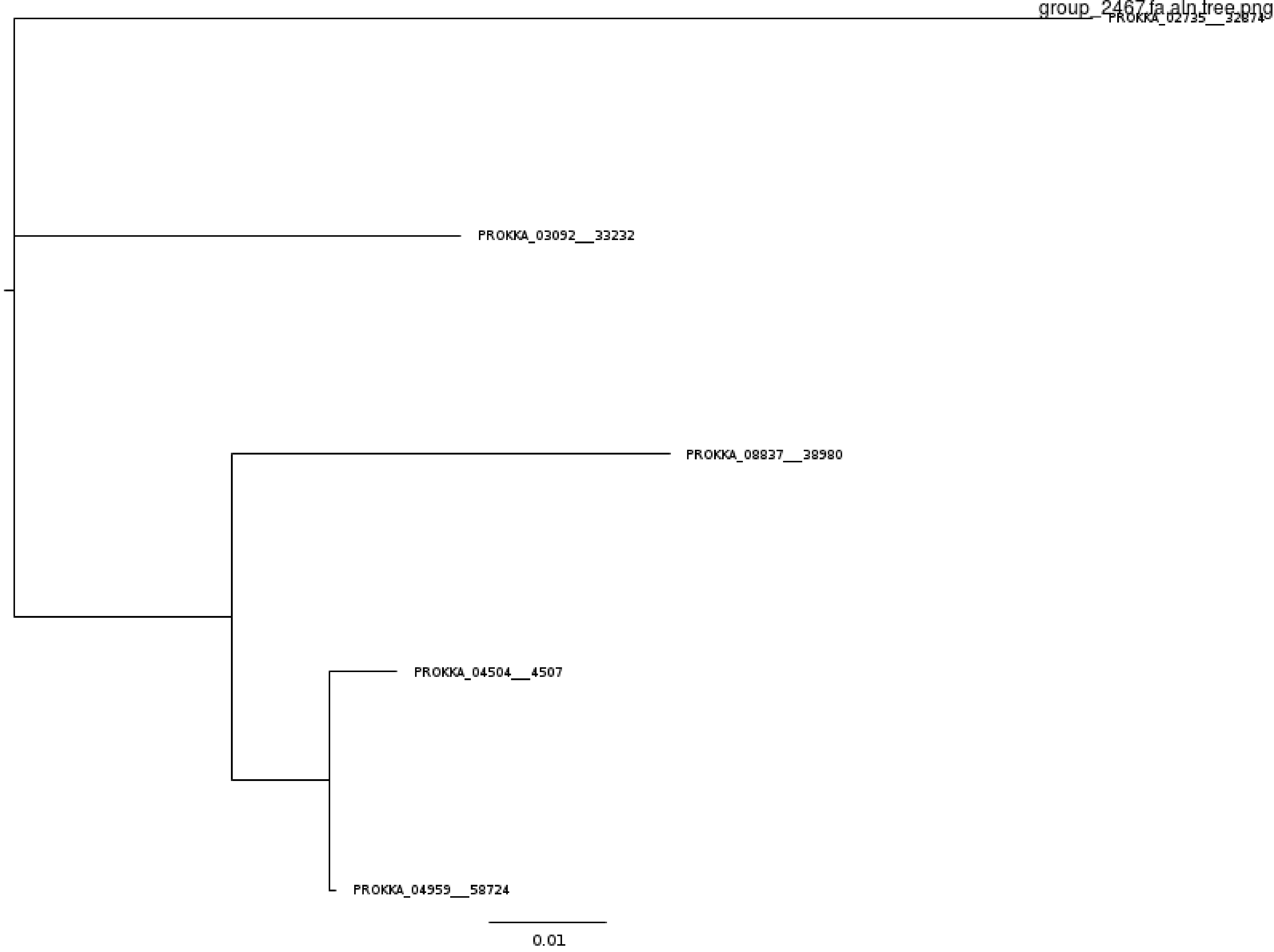

group_2467.fa.aln.tree.png
PROKKA_02735___32874
PROKKA_03092___33232
PROKKA_08837___38980
PROKKA_04504___4507
PROKKA_04959___58724
0.01

group_246.fa.aln.tree.png

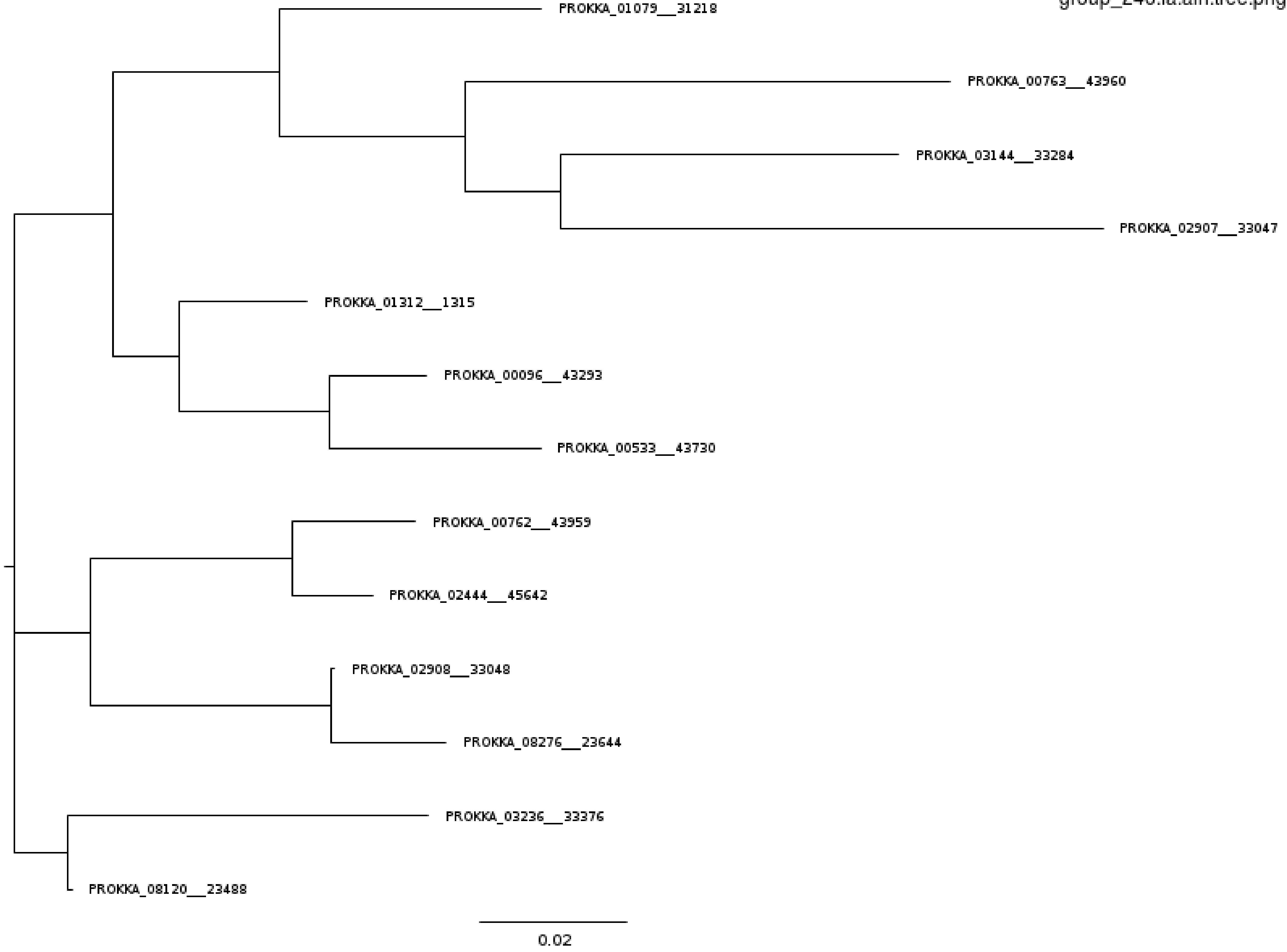

group_248.fa.aln.tree.png

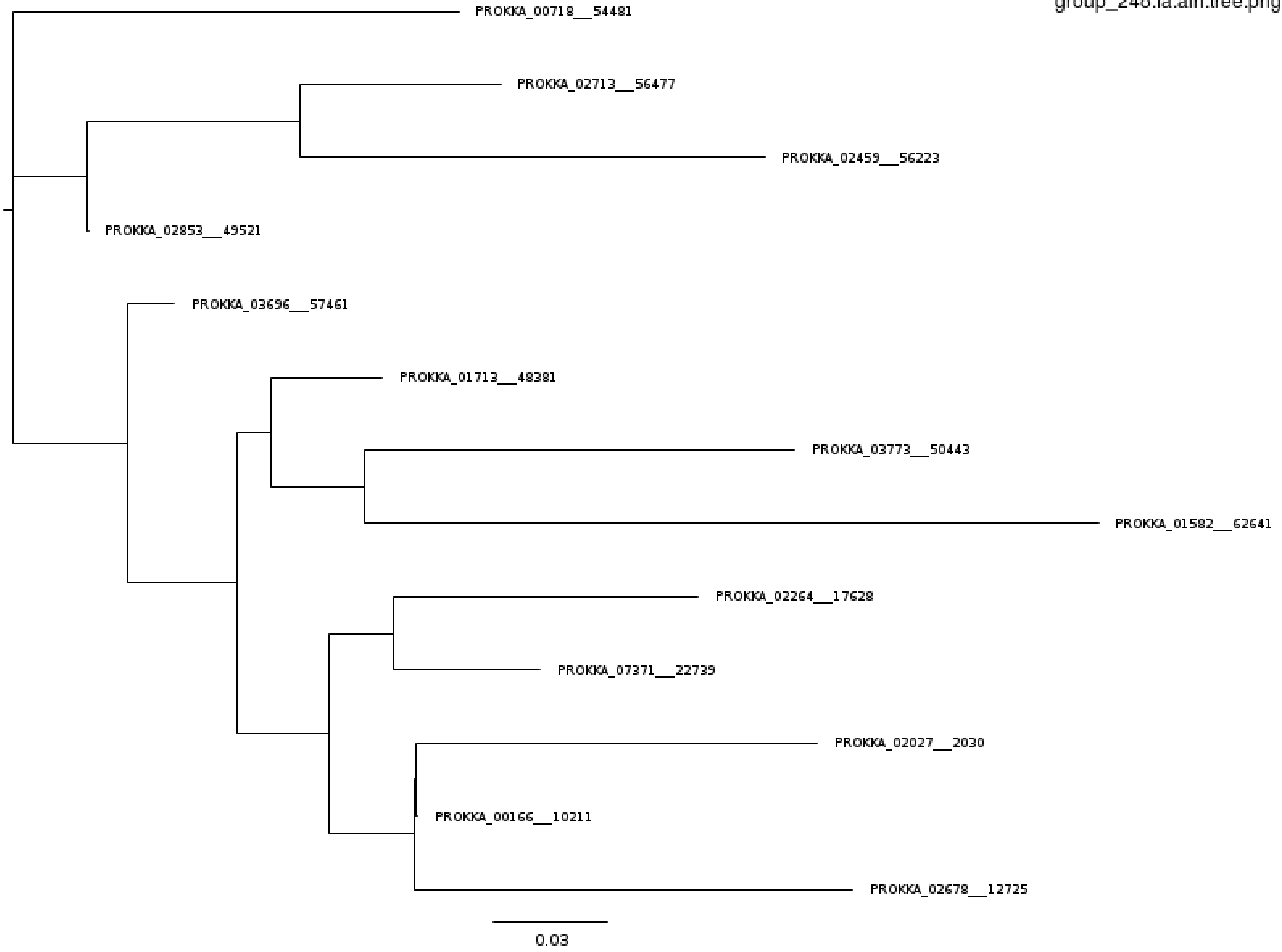

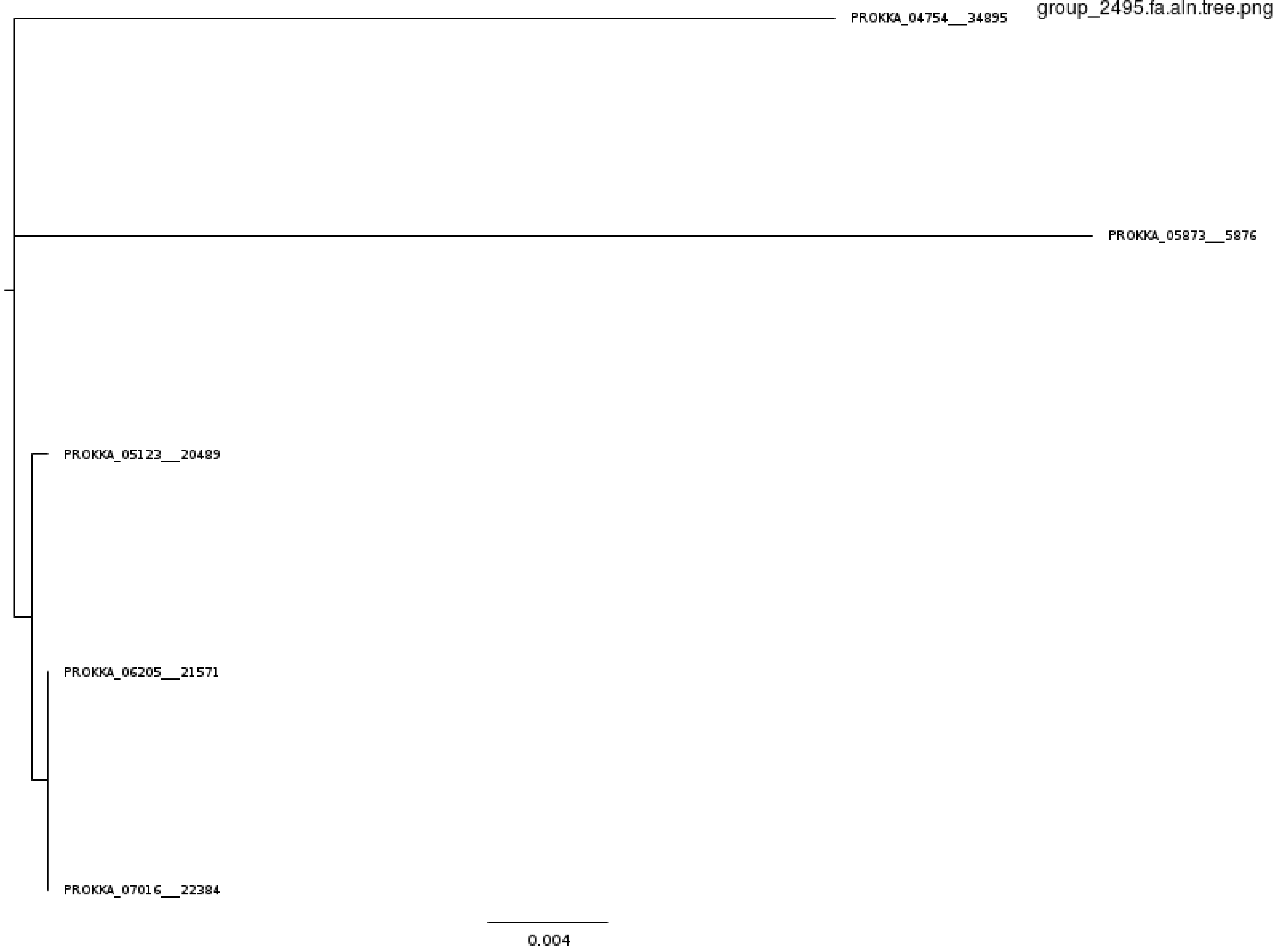
group_2495.fa.aln.tree.png
PROKKA_04754___34895
PROKKA_05873___5876
PROKKA_05123___20489
PROKKA_06205___21571
PROKKA_07016___22384
0.004

group_24.fa.aln.tree.png

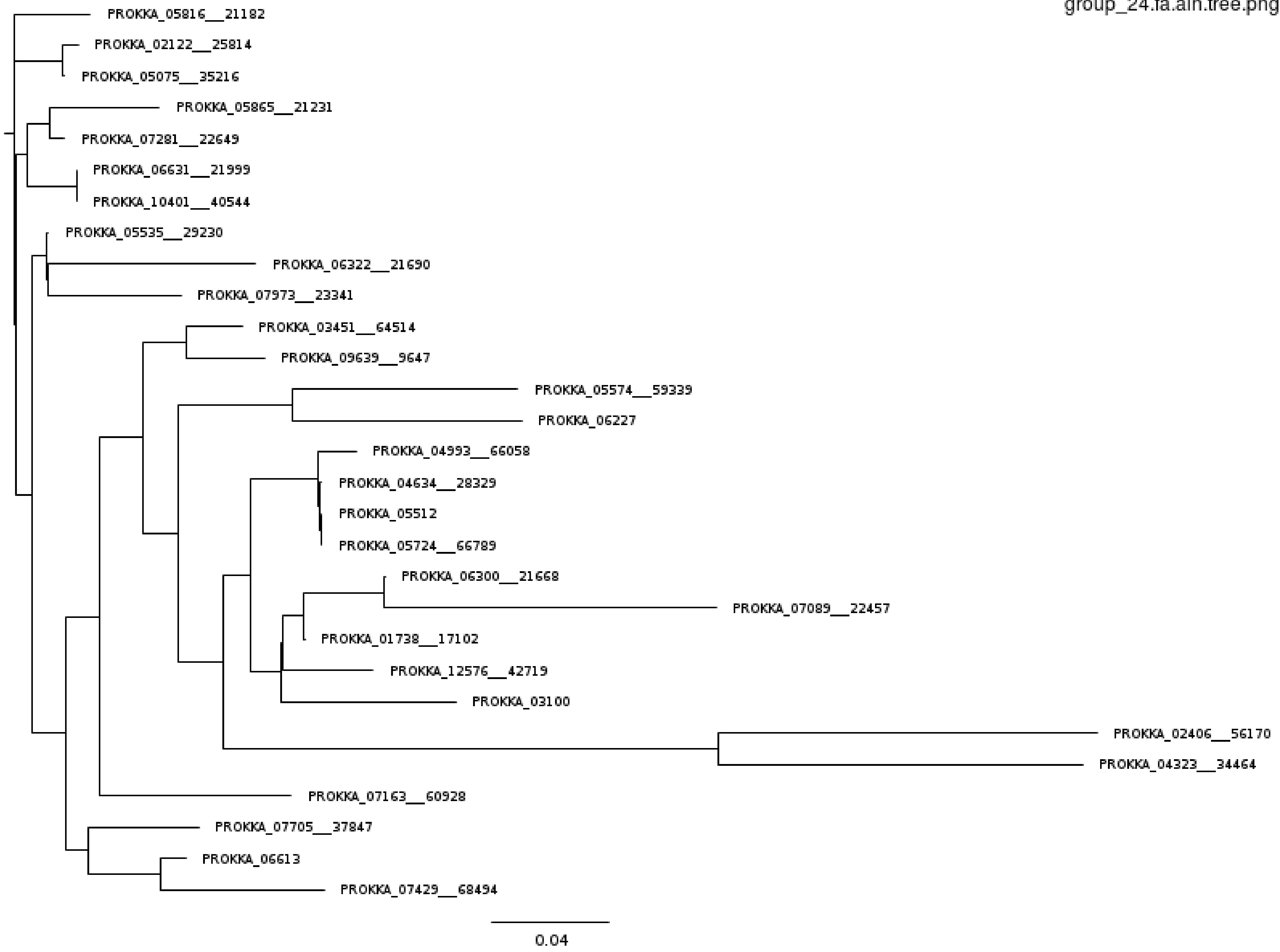

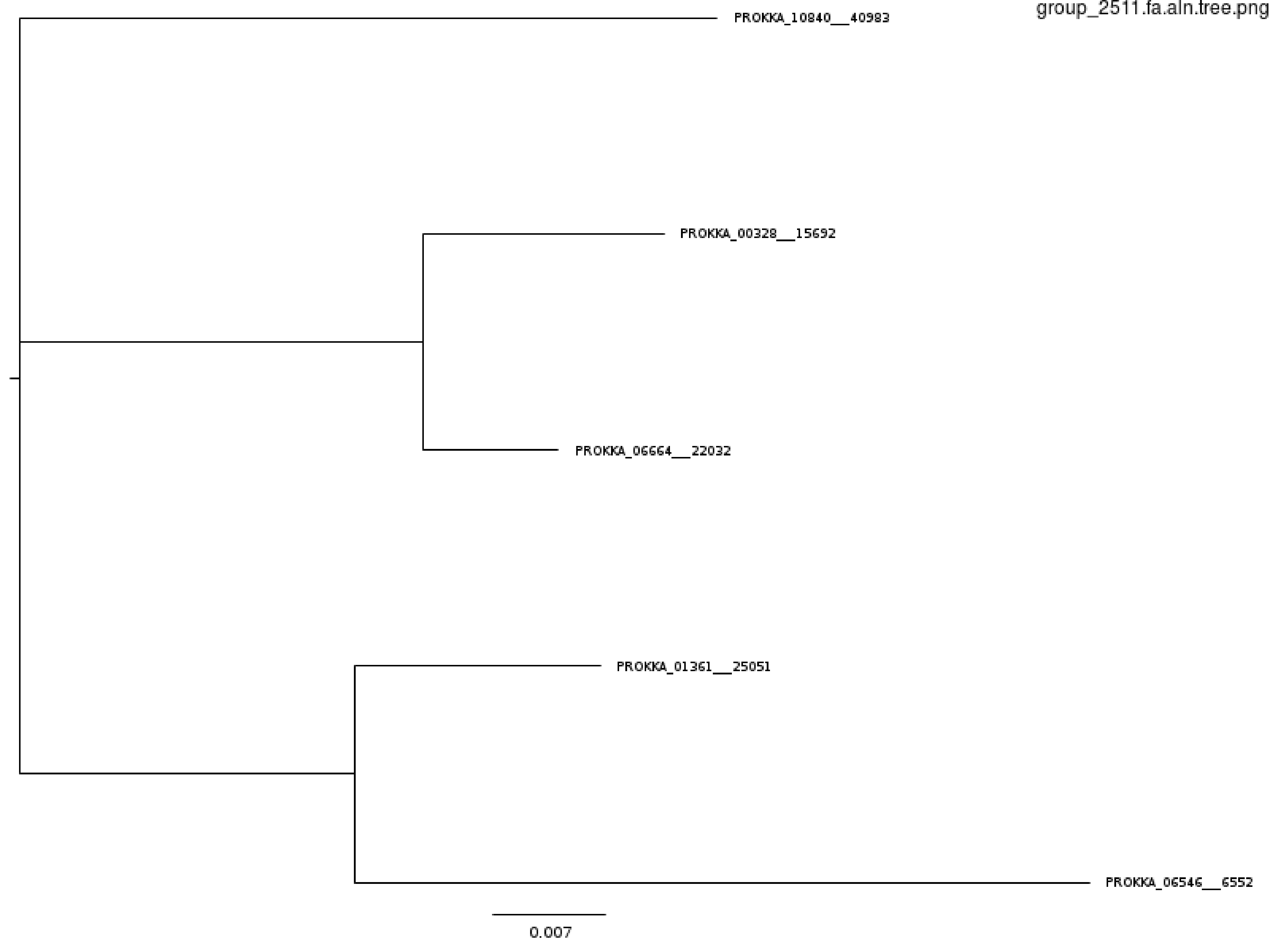

group_2511.fa.aln.tree.png
PROKKA_10840__40983
PROKKA_00328__15692
PROKKA_06664__22032
PROKKA_01361__25051
PROKKA_06546__6552
0.007

group_2514.fa.aln.tree.png

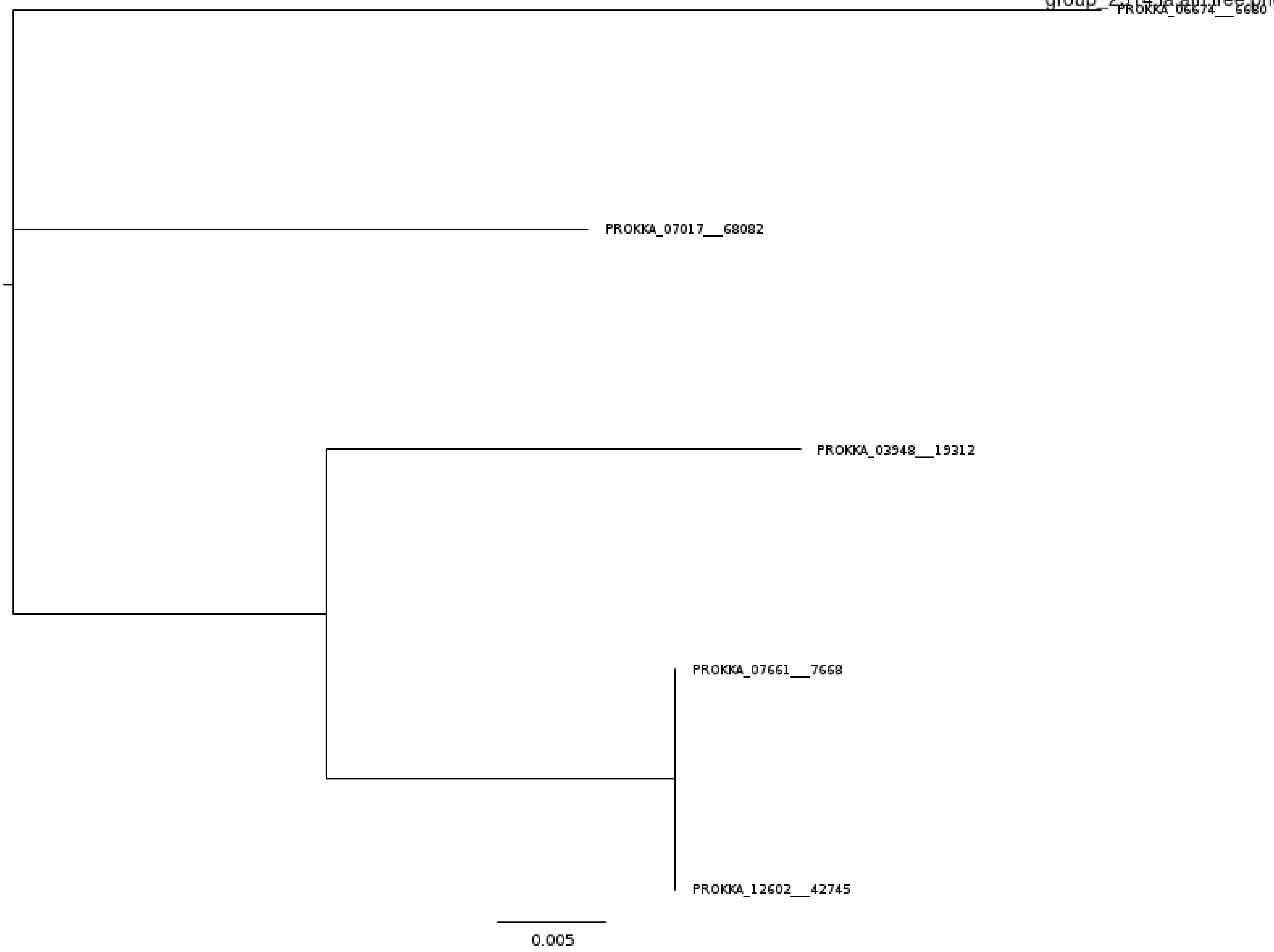

group_2519.fa.aln.tree.png

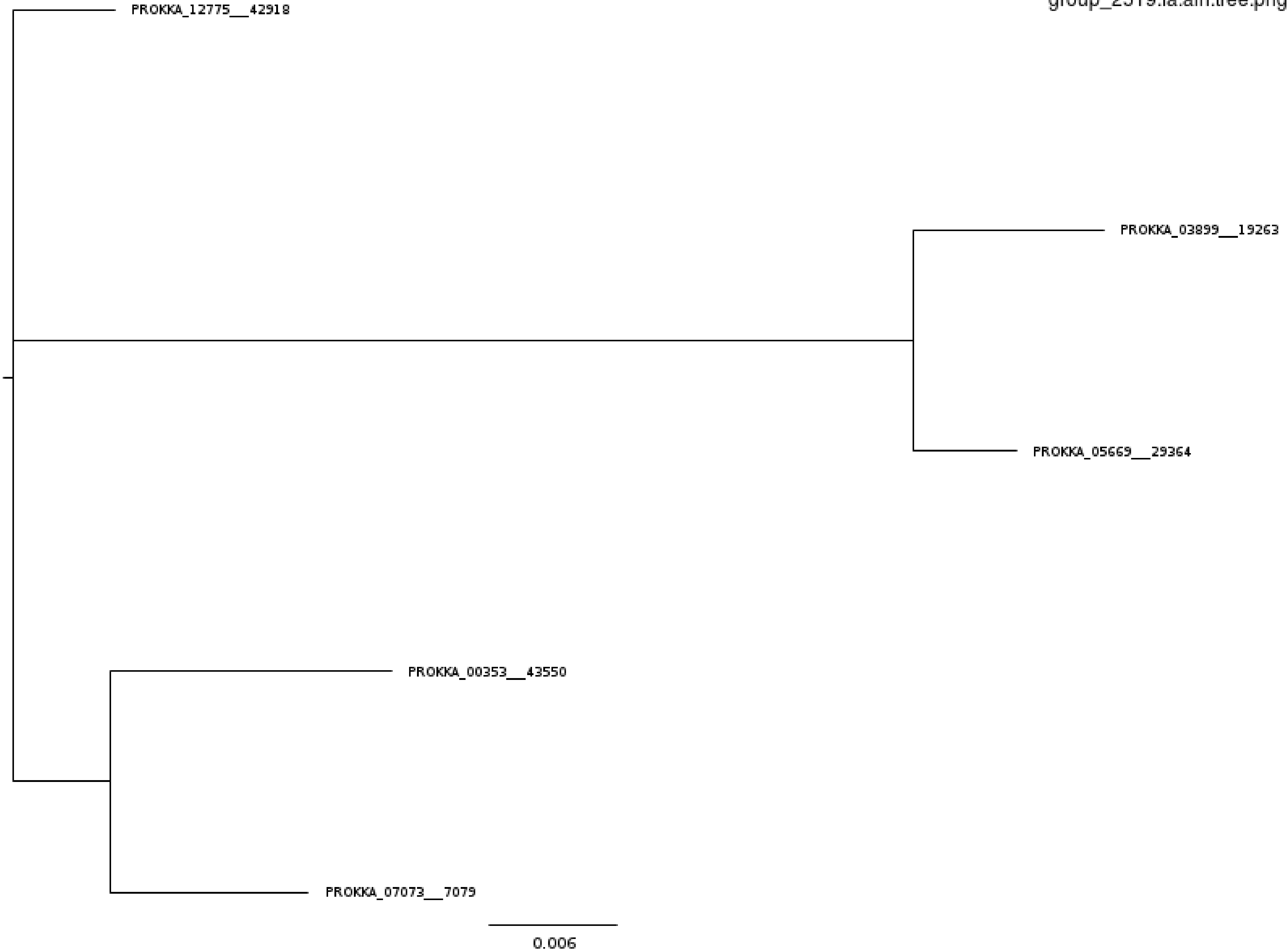

group_252.fa.aln.tree.png

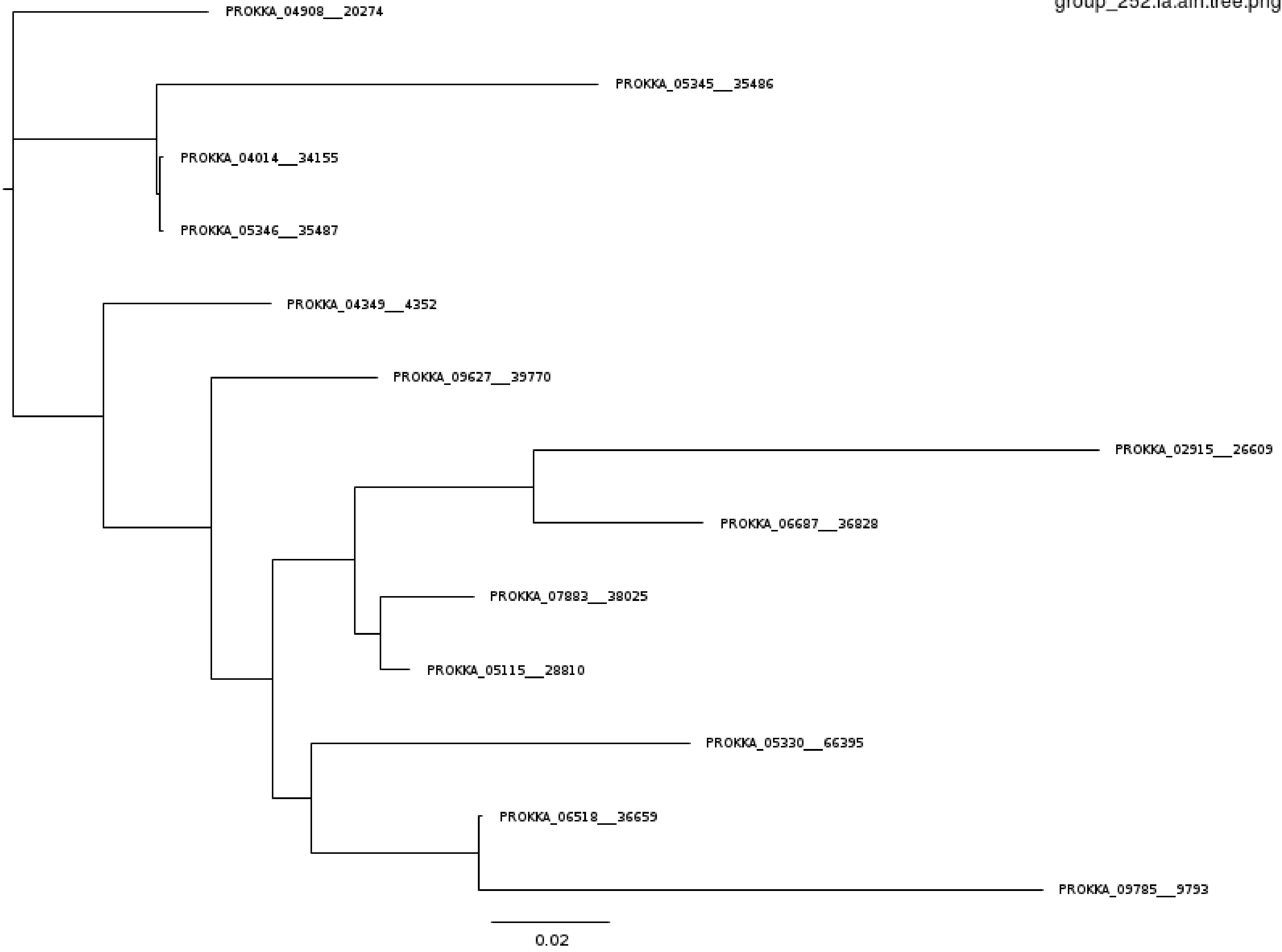

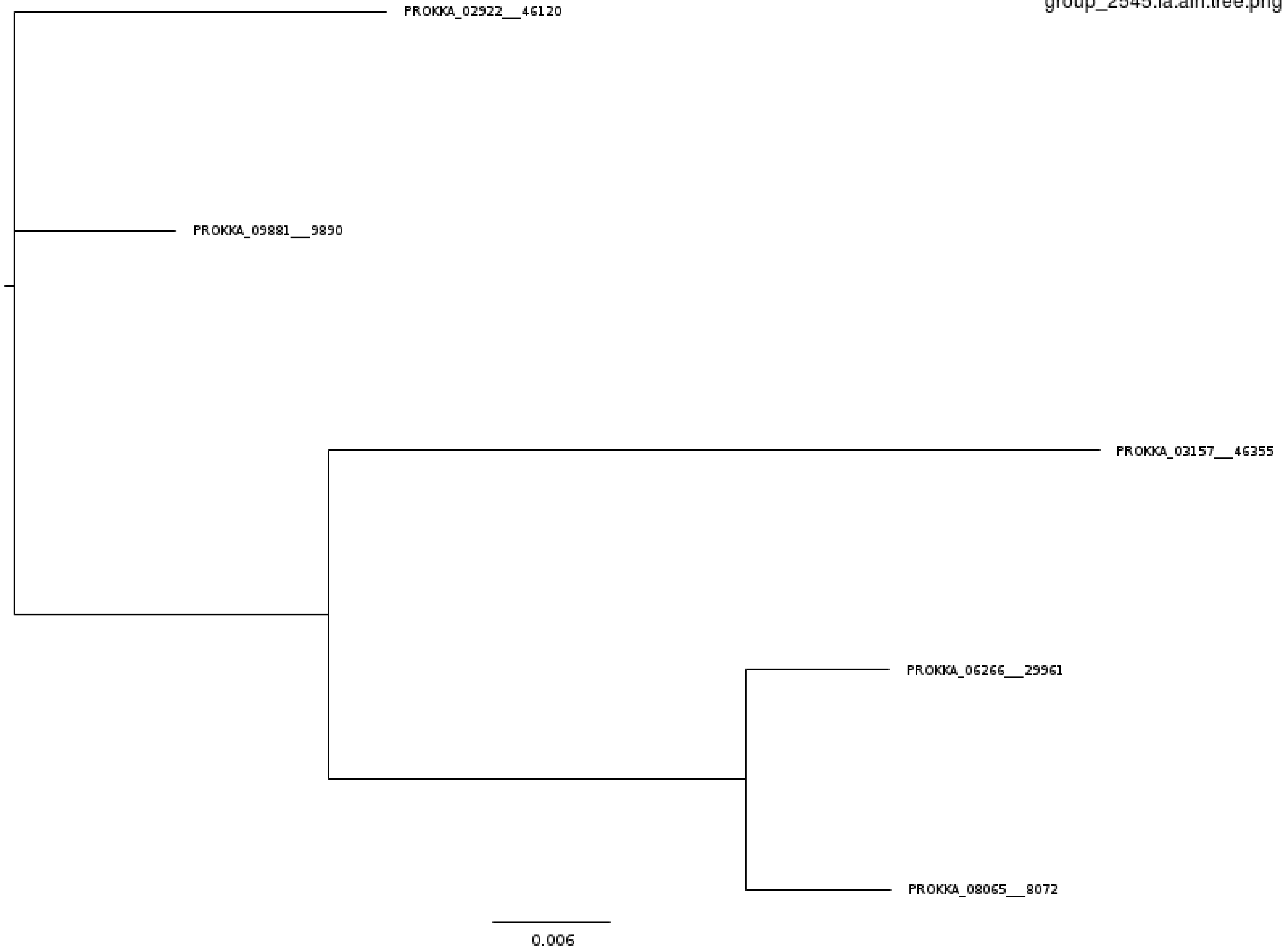
PROKKA_02922___46120
PROKKA_09881___9890
PROKKA_03157___46355
PROKKA_06266___29961
PROKKA_08065___8072
0.006

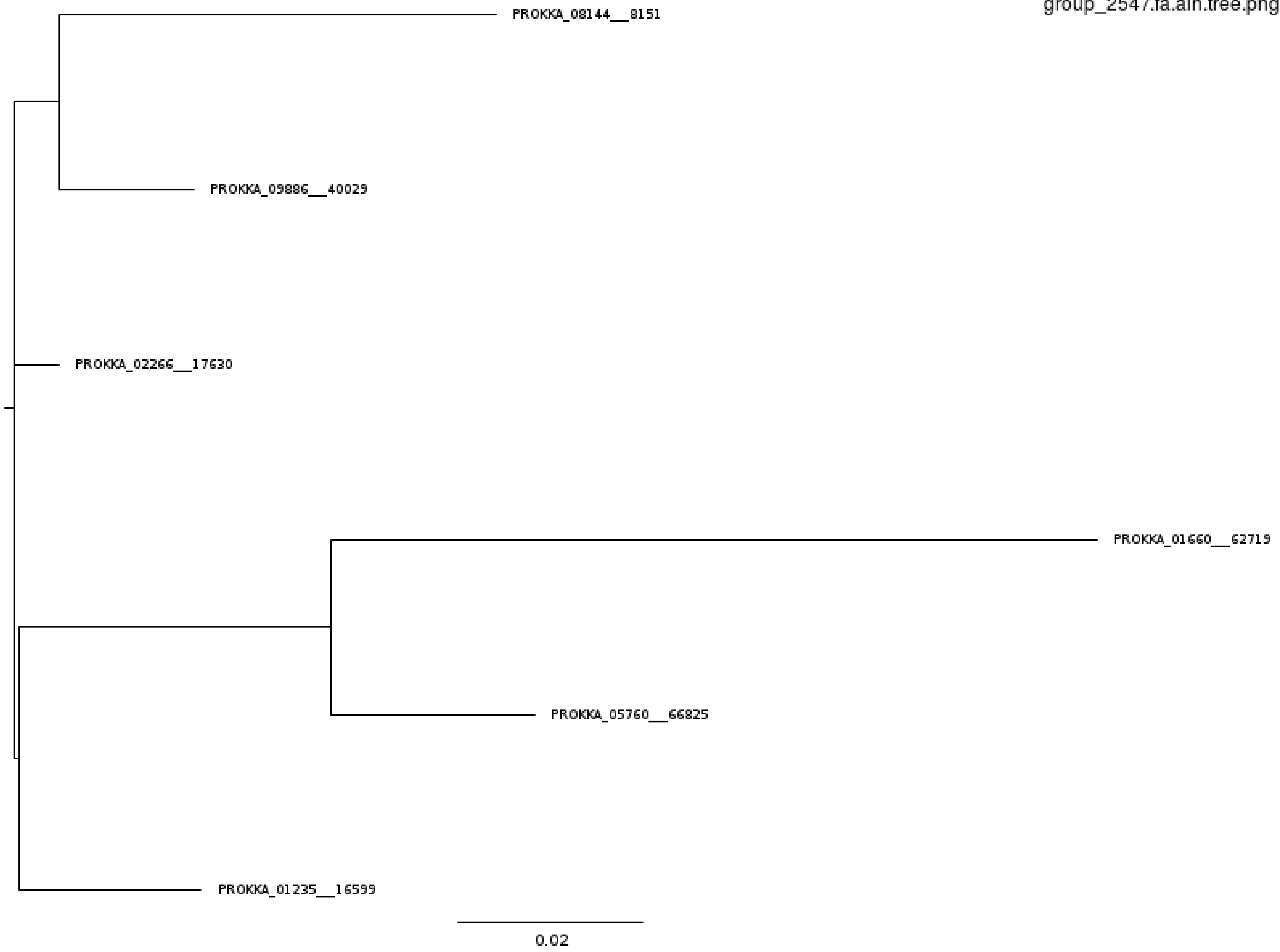
PROKKA_08144___8151
PROKKA_09886___40029
PROKKA_02266___17630
PROKKA_01660___62719
PROKKA_05760___66825
PROKKA_01235___16599
0.02

group_254.fa.aln.tree.png

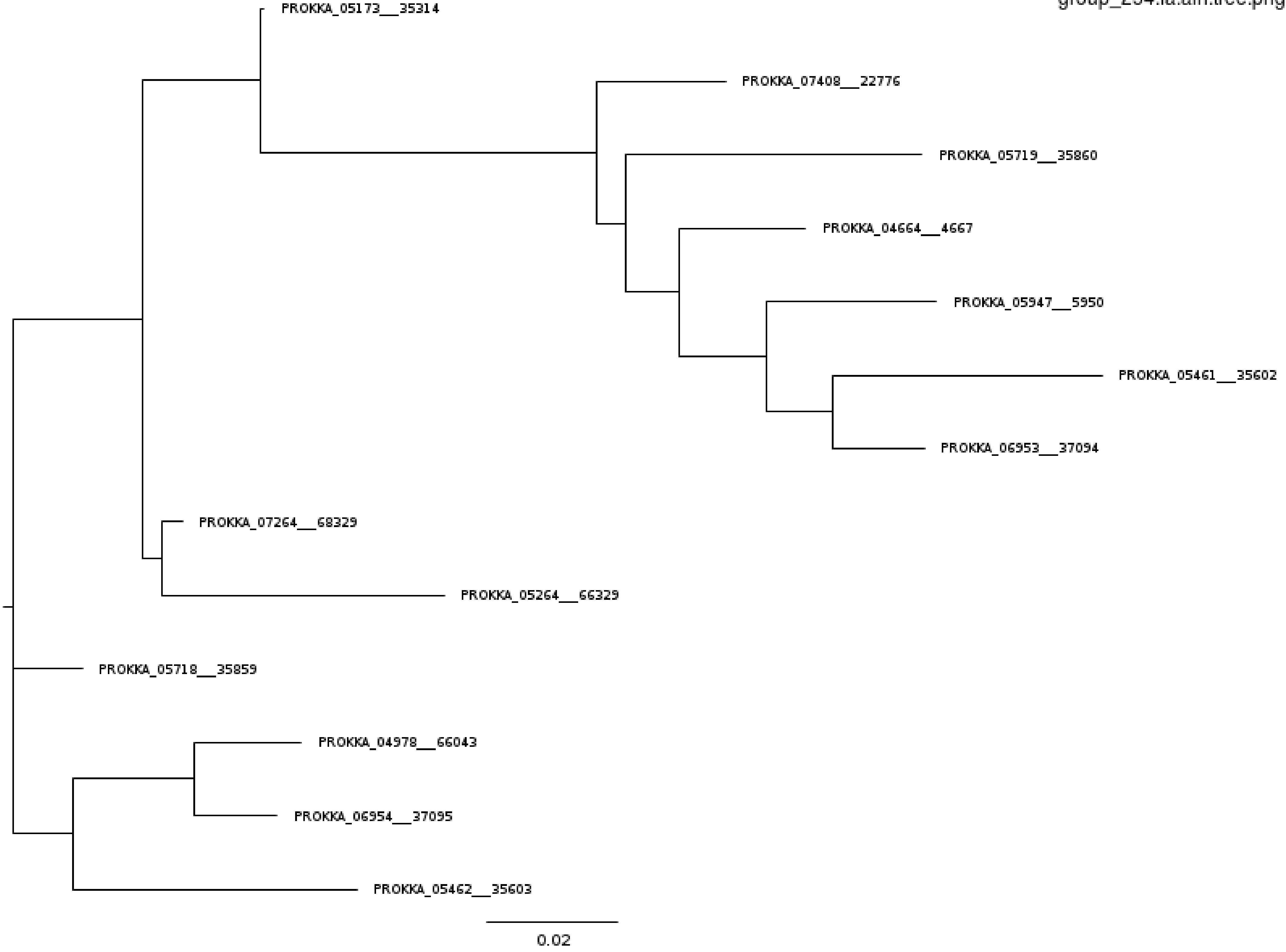

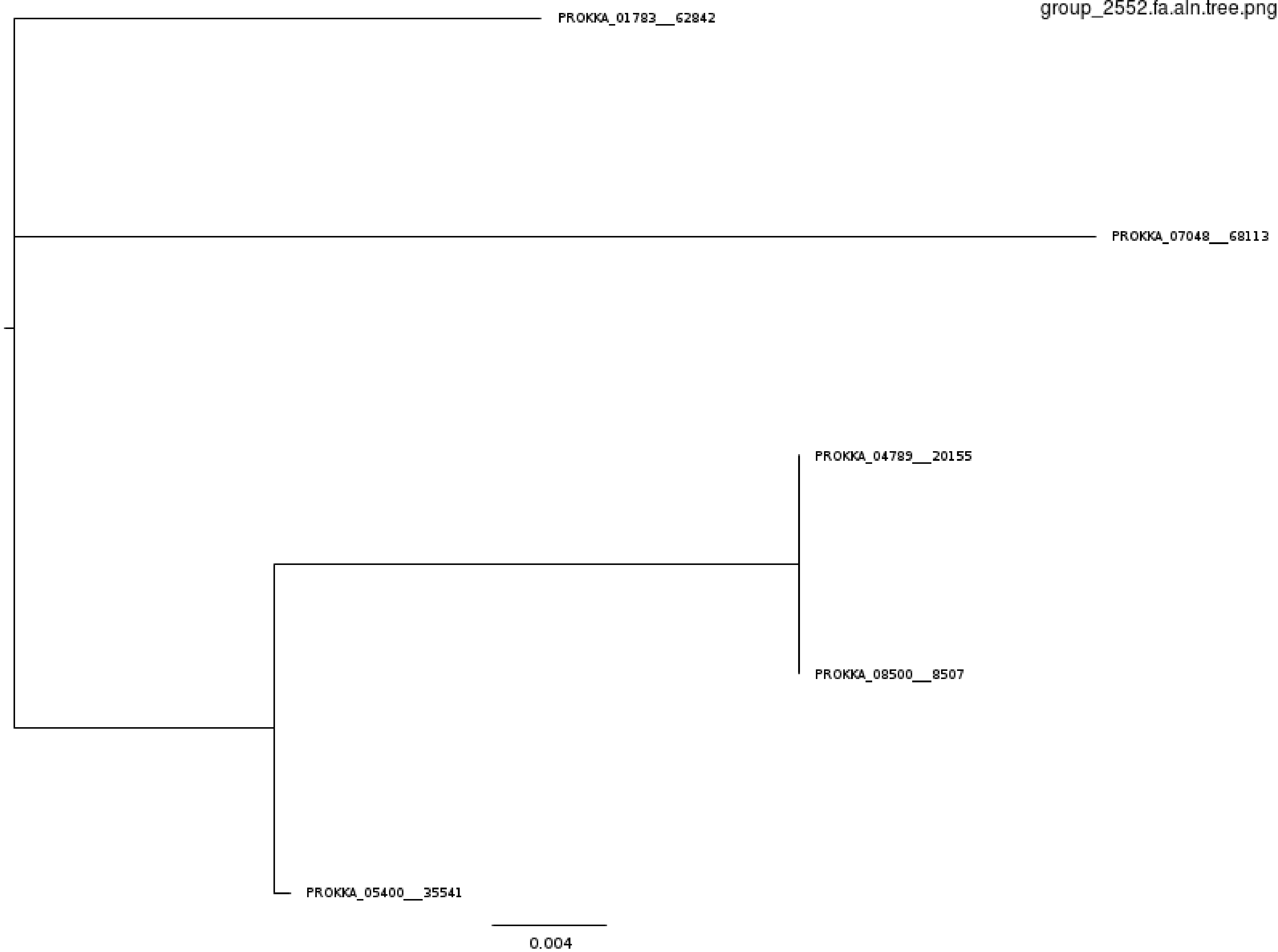
group_2552.fa.aln.tree.png
PROKKA_01783___62842
PROKKA_07048___68113
PROKKA_04789___20155
PROKKA_08500___8507
PROKKA_05400___35541
0.004

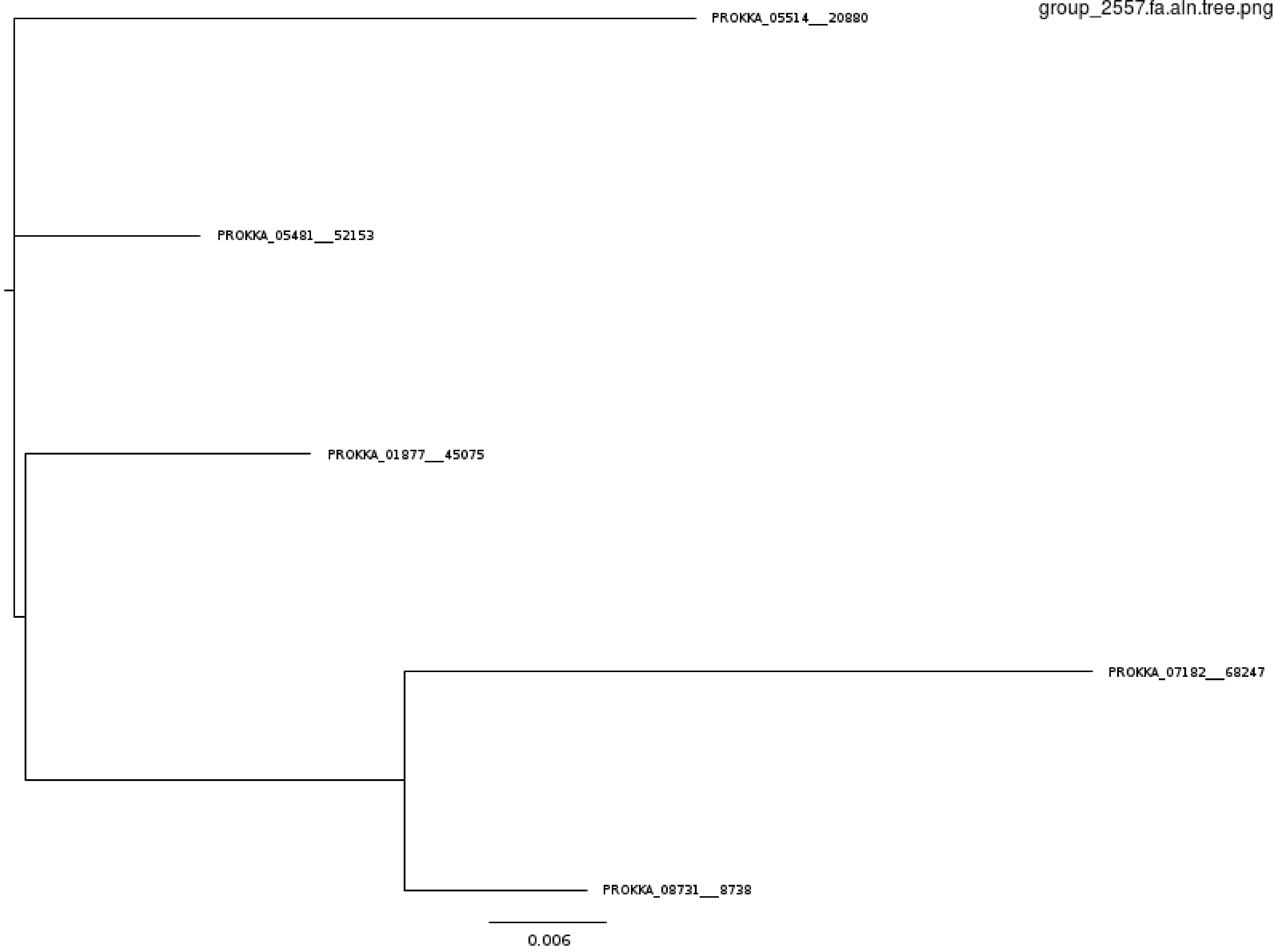
group_2557.fa.aln.tree.png
PROKKA_05514___20880
PROKKA_05481___52153
PROKKA_01877___45075
PROKKA_07182___68247
PROKKA_08731___8738
0.006

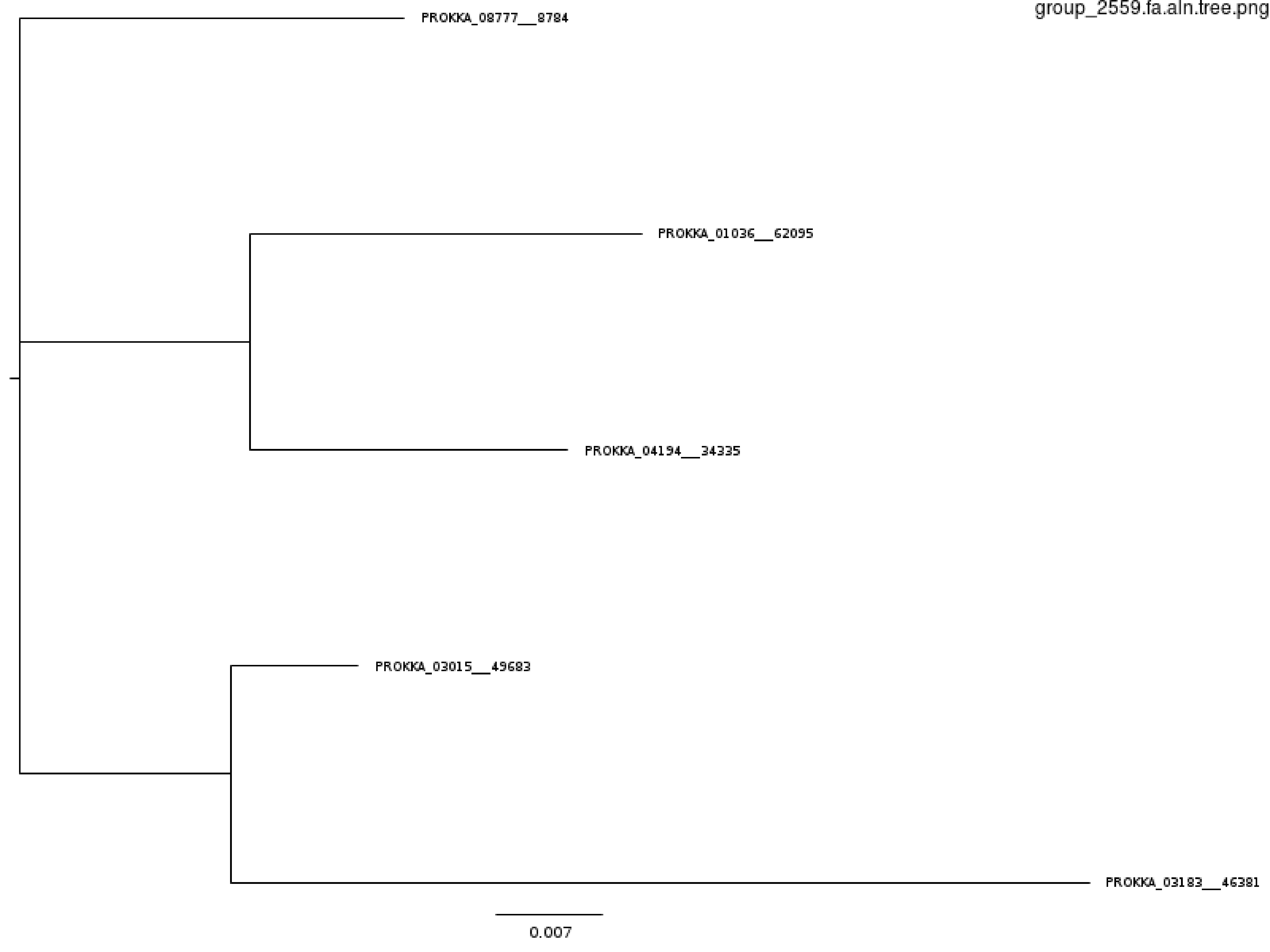
group_2559.fa.aln.tree.png
PROKKA_08777___8784
PROKKA_01036___62095
PROKKA_04194___34335
PROKKA_03015___49683
PROKKA_03183___46381
0.007

group_2561.fa.aln.tree.png

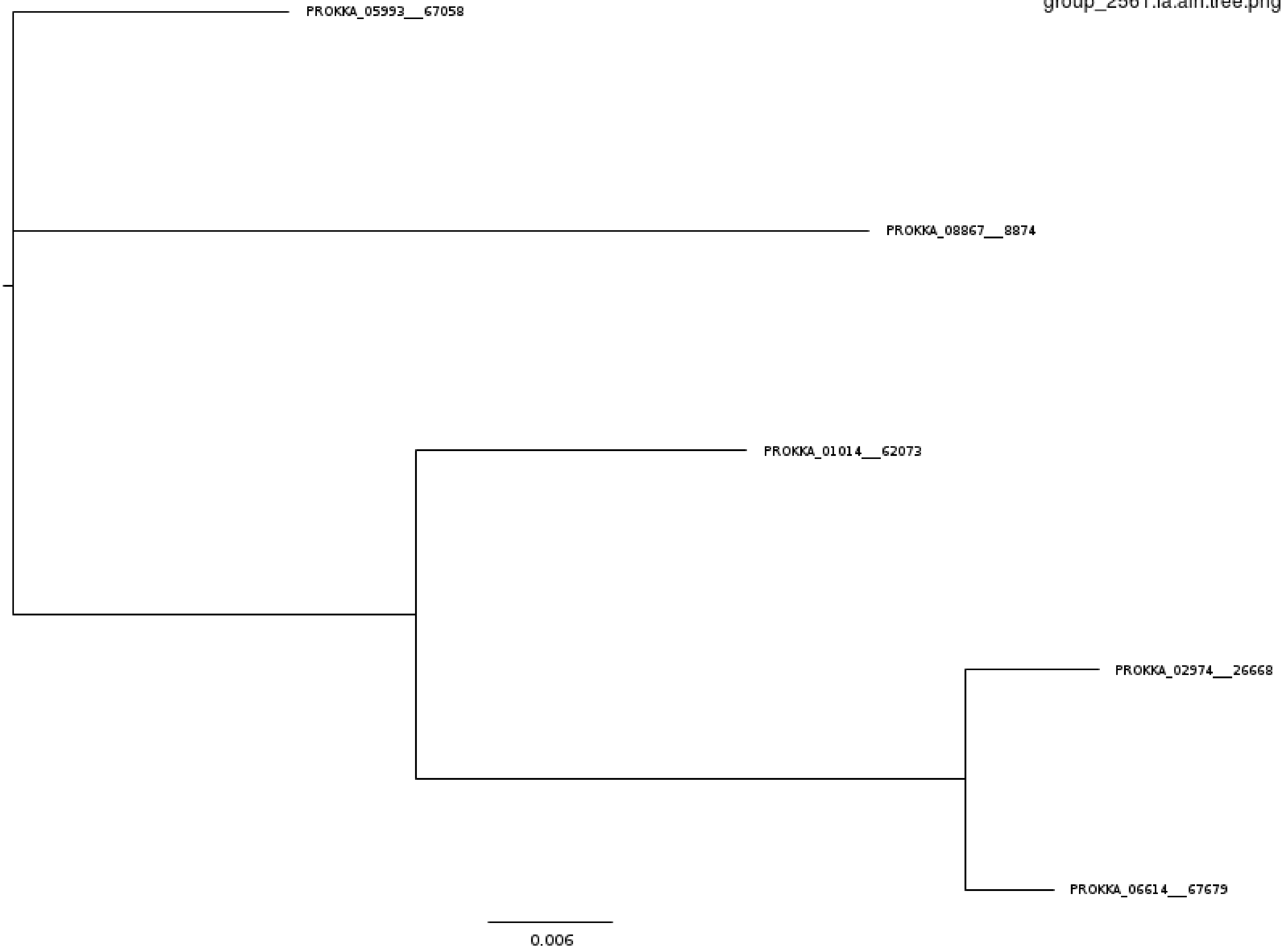

group_2562.fa.aln.tree.png

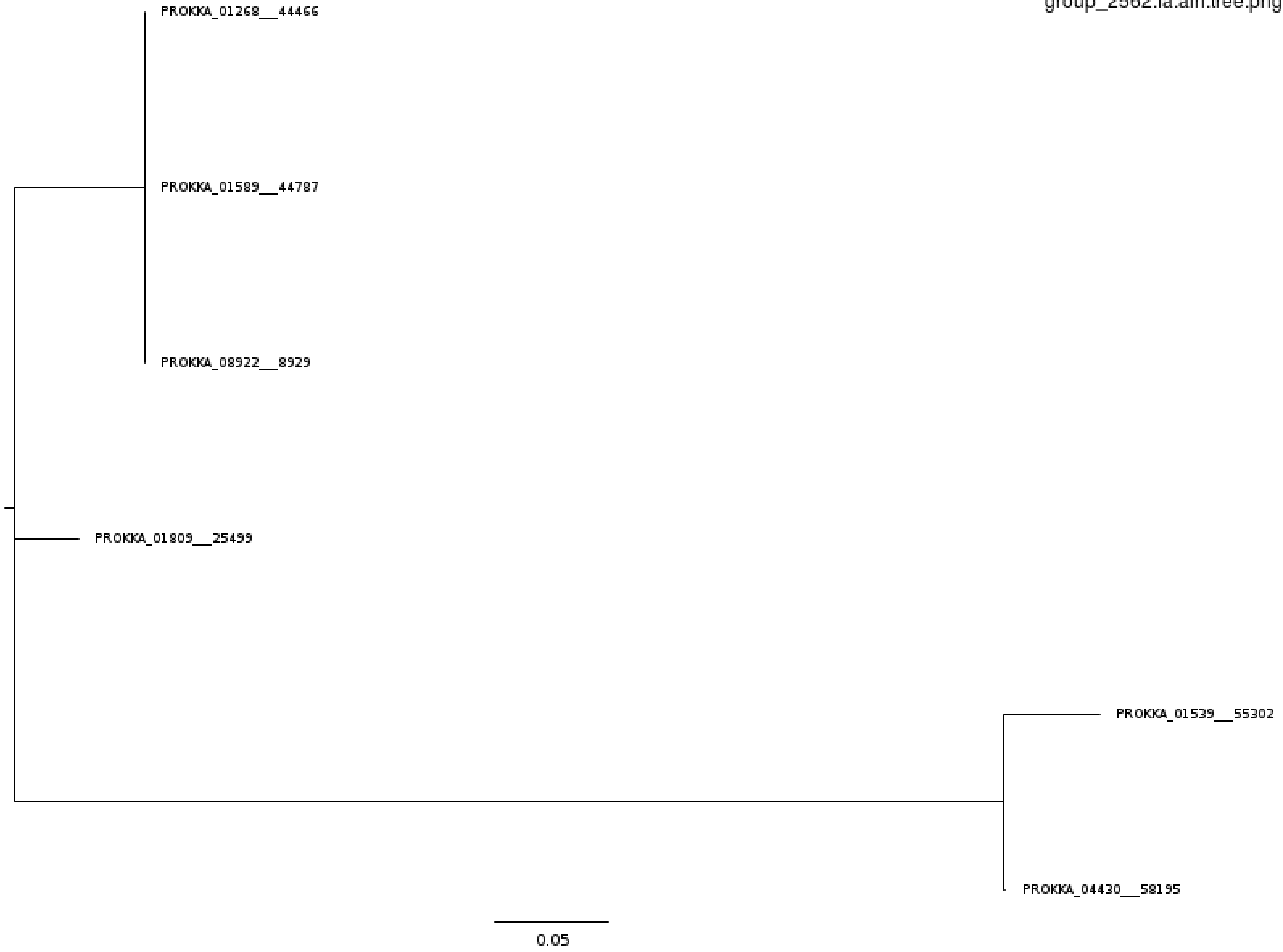

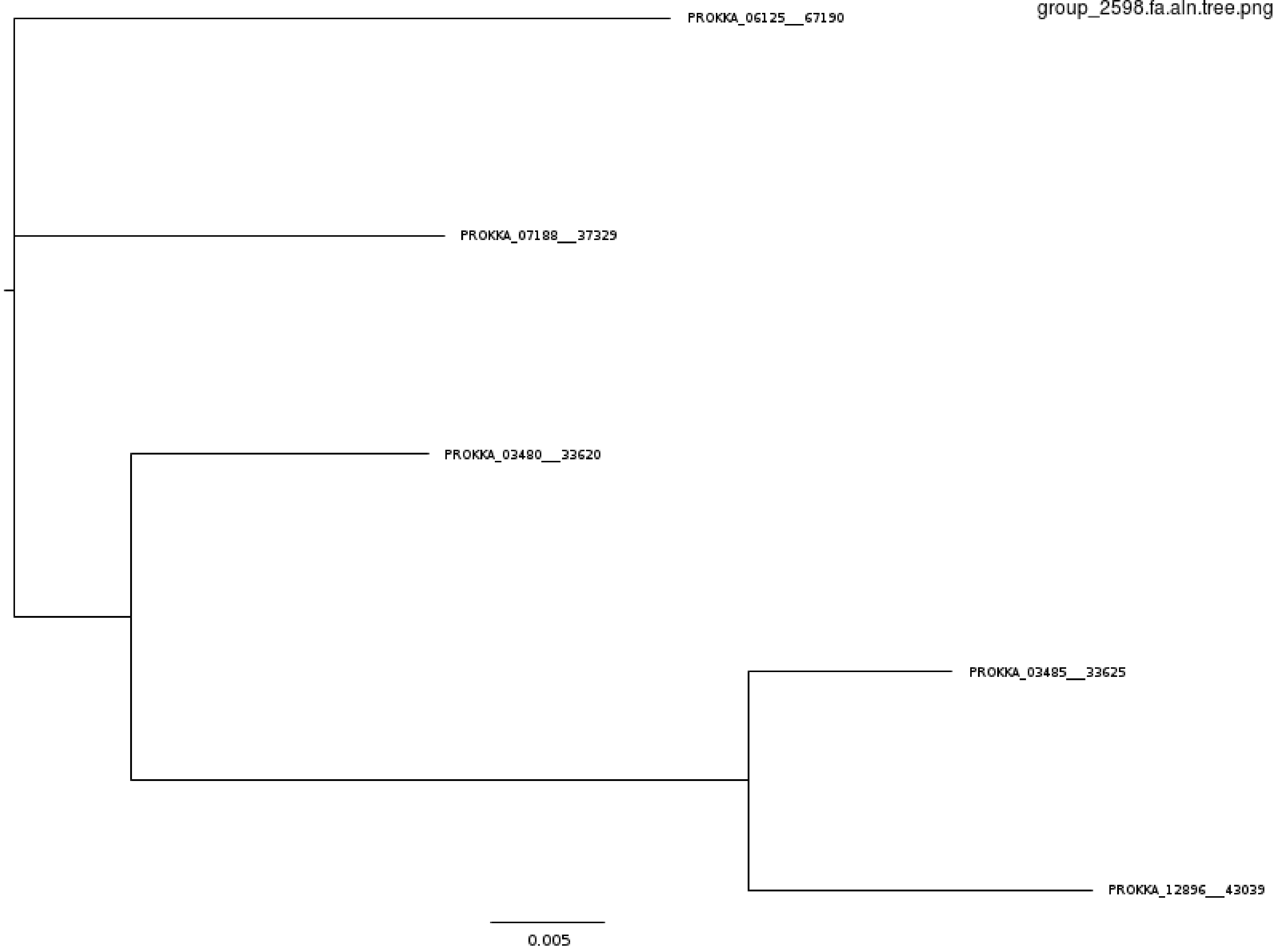
group_2598.fa.aln.tree.png
PROKKA_06125___67190
PROKKA_07188___37329
PROKKA_03480___33620
PROKKA_03485___33625
PROKKA_12896___43039
0.005

group_259.fa.aln.tree.png

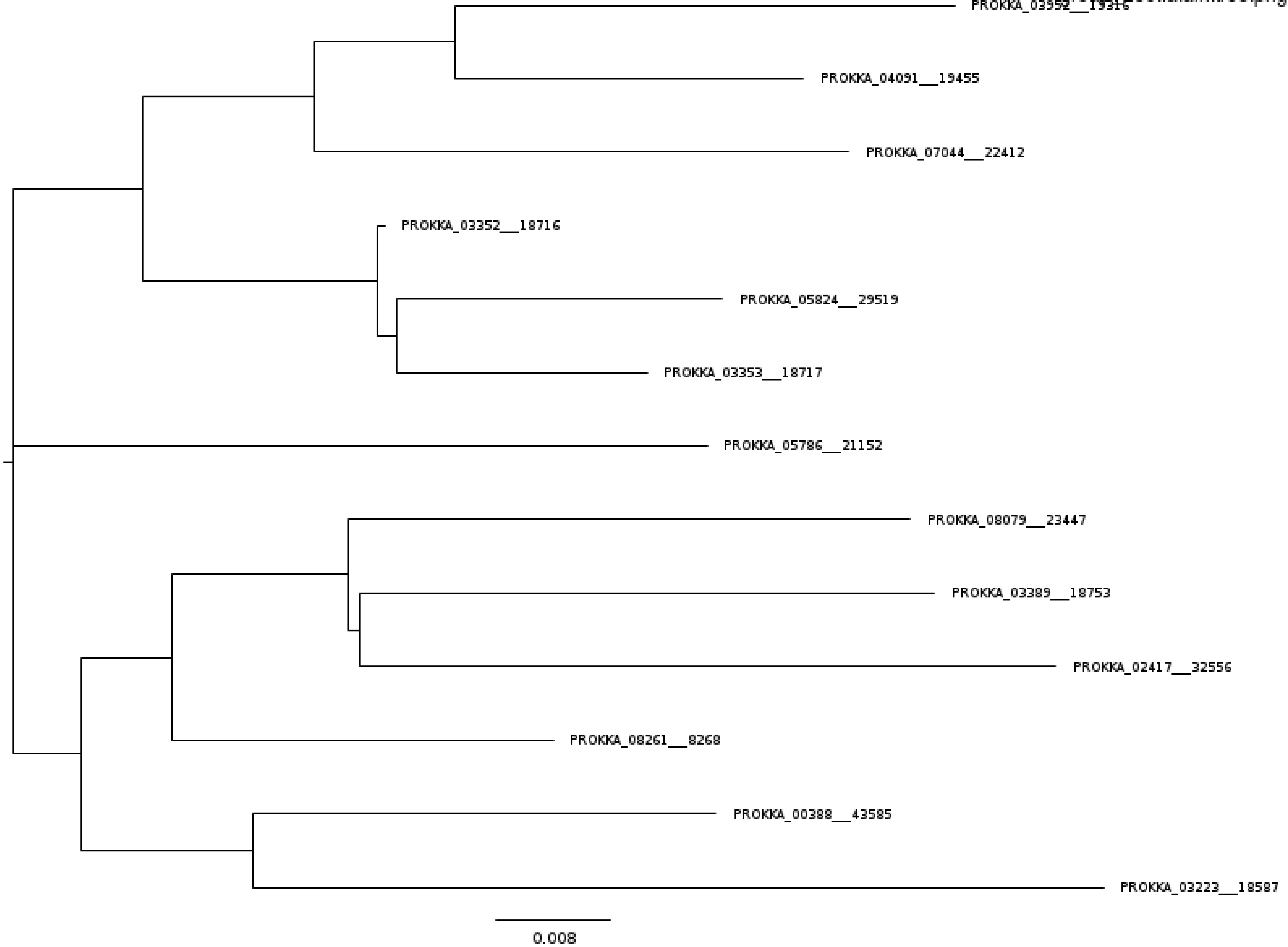

group_2607.fa.aln.tree.png

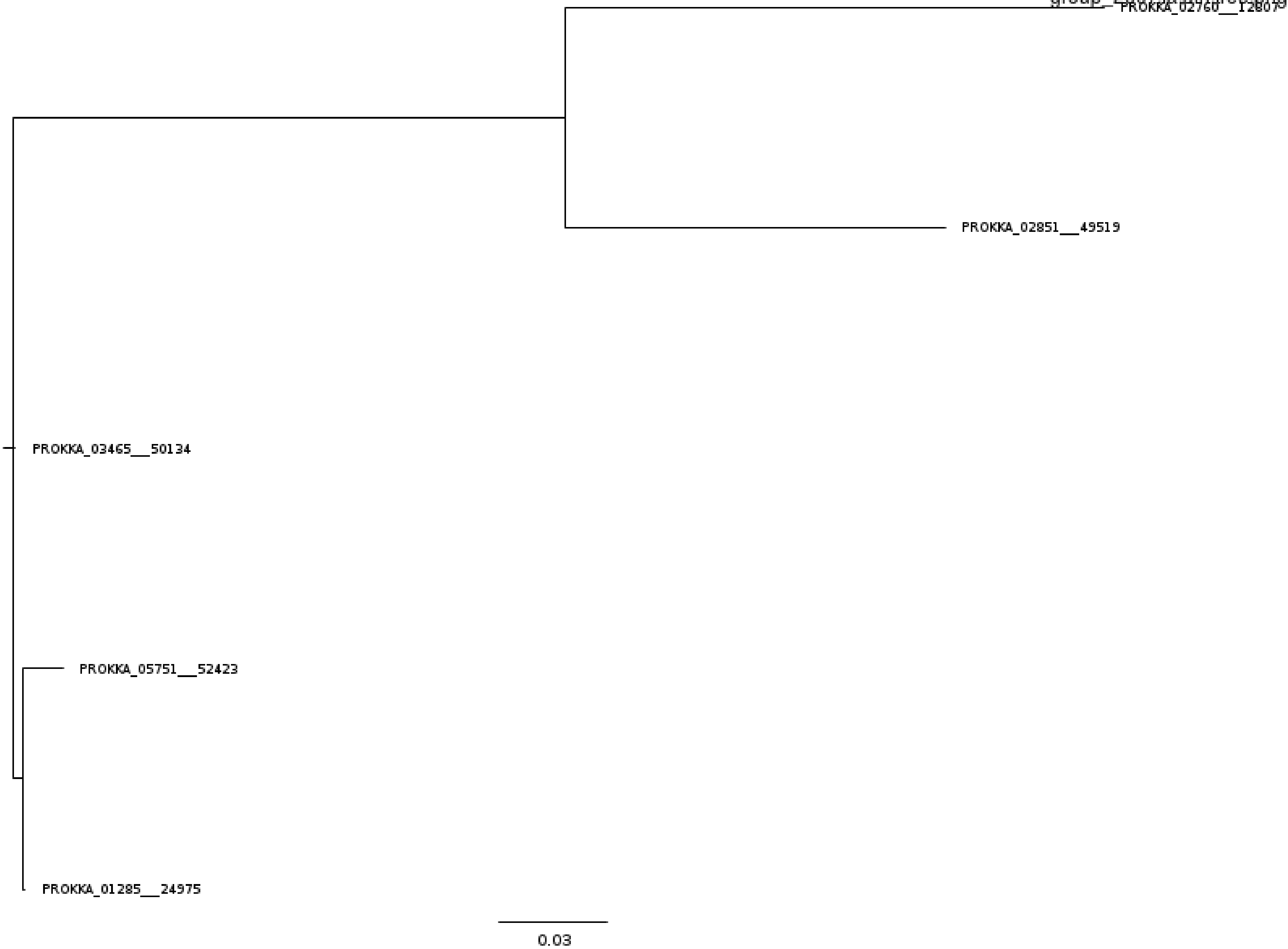

group_2621.fa.aln.tree.png

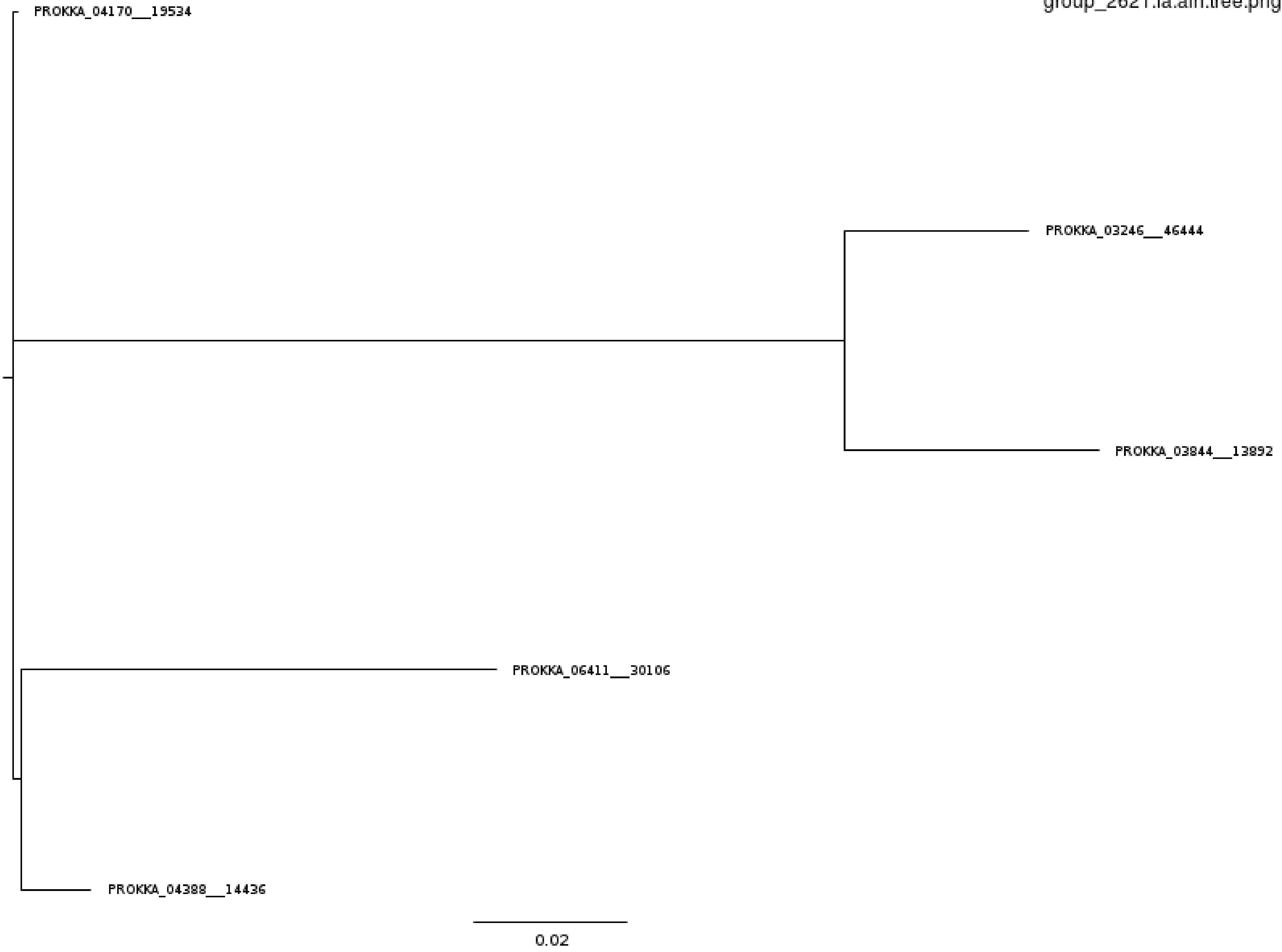

group_2630.fa.aln.tree.png

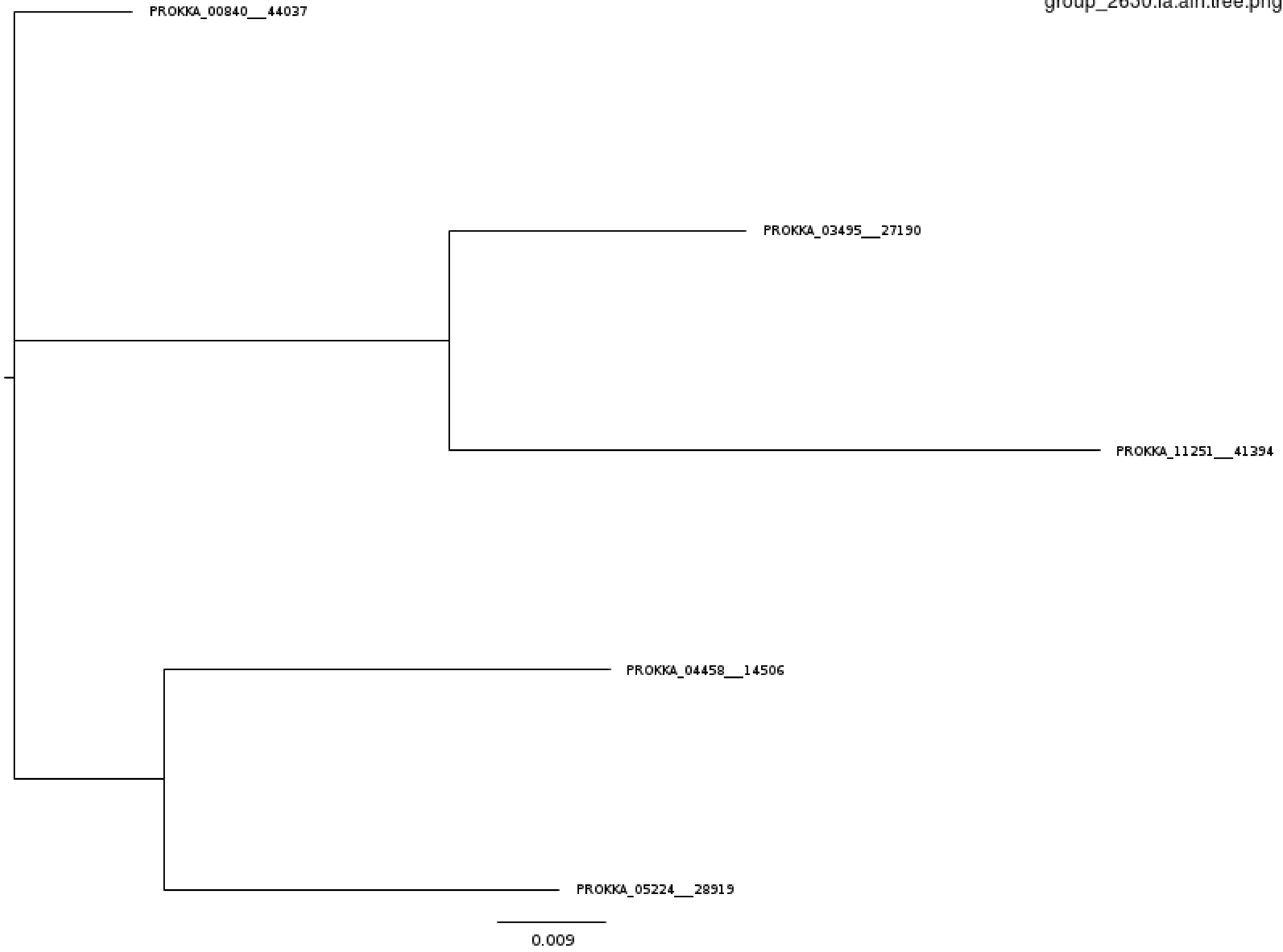

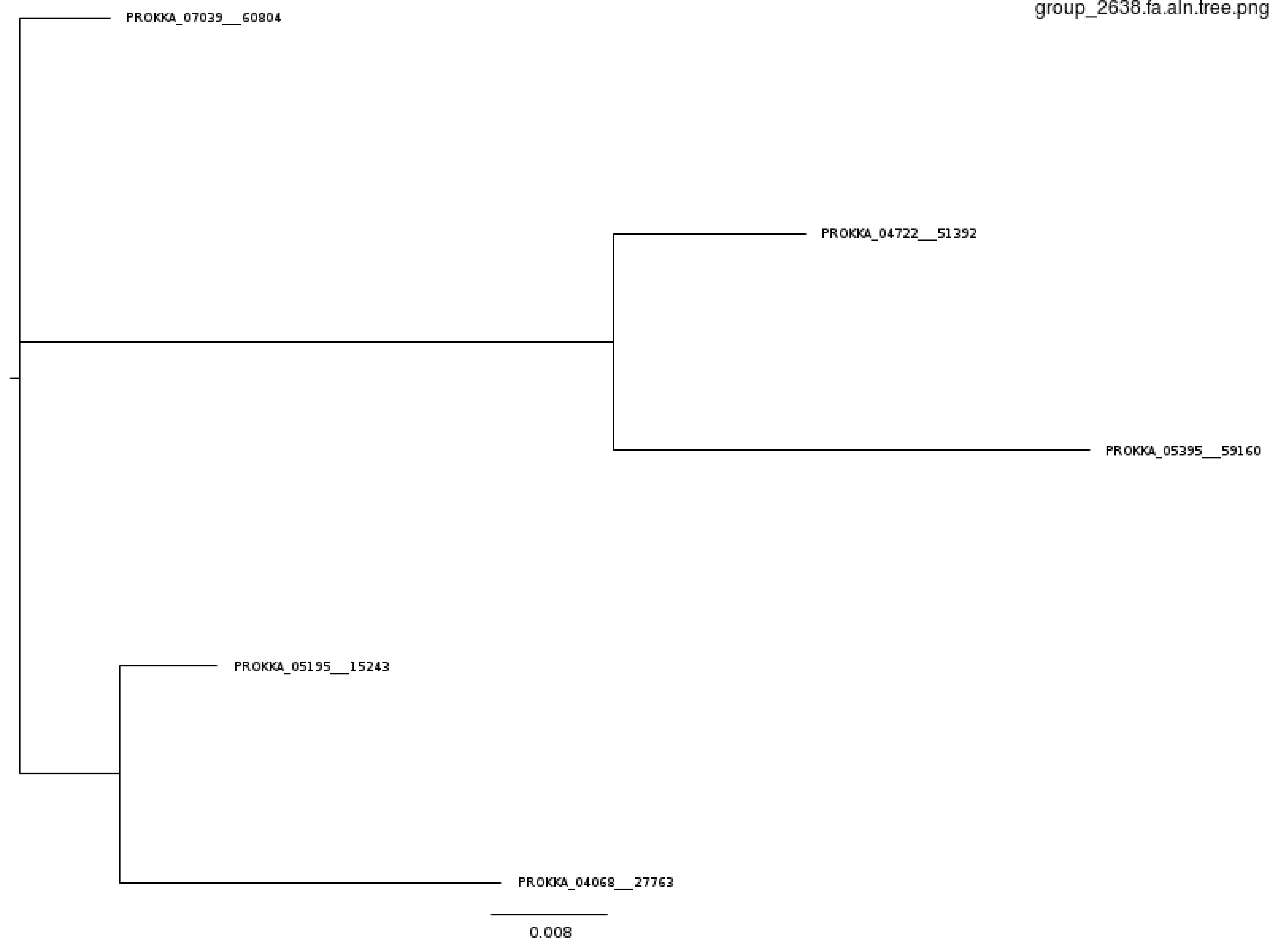
group_2638.fa.aln.tree.png
PROKKA_07039___60804
PROKKA_04722___51392
PROKKA_05395___59160
PROKKA_05195___15243
PROKKA_04068___27763
0.008

group_2677.fa.aln.tree.png

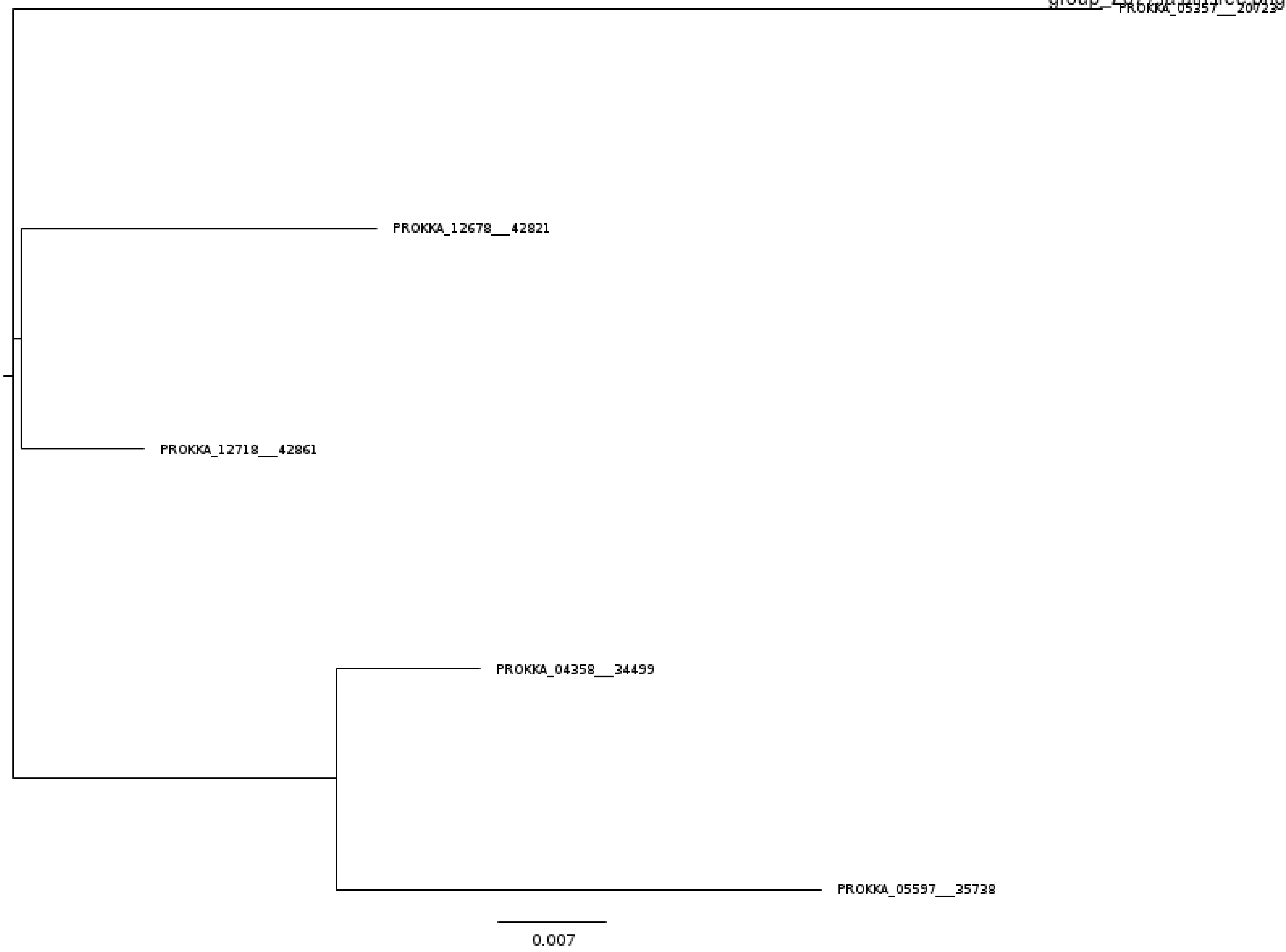

group_2713.fa.aln.tree.png

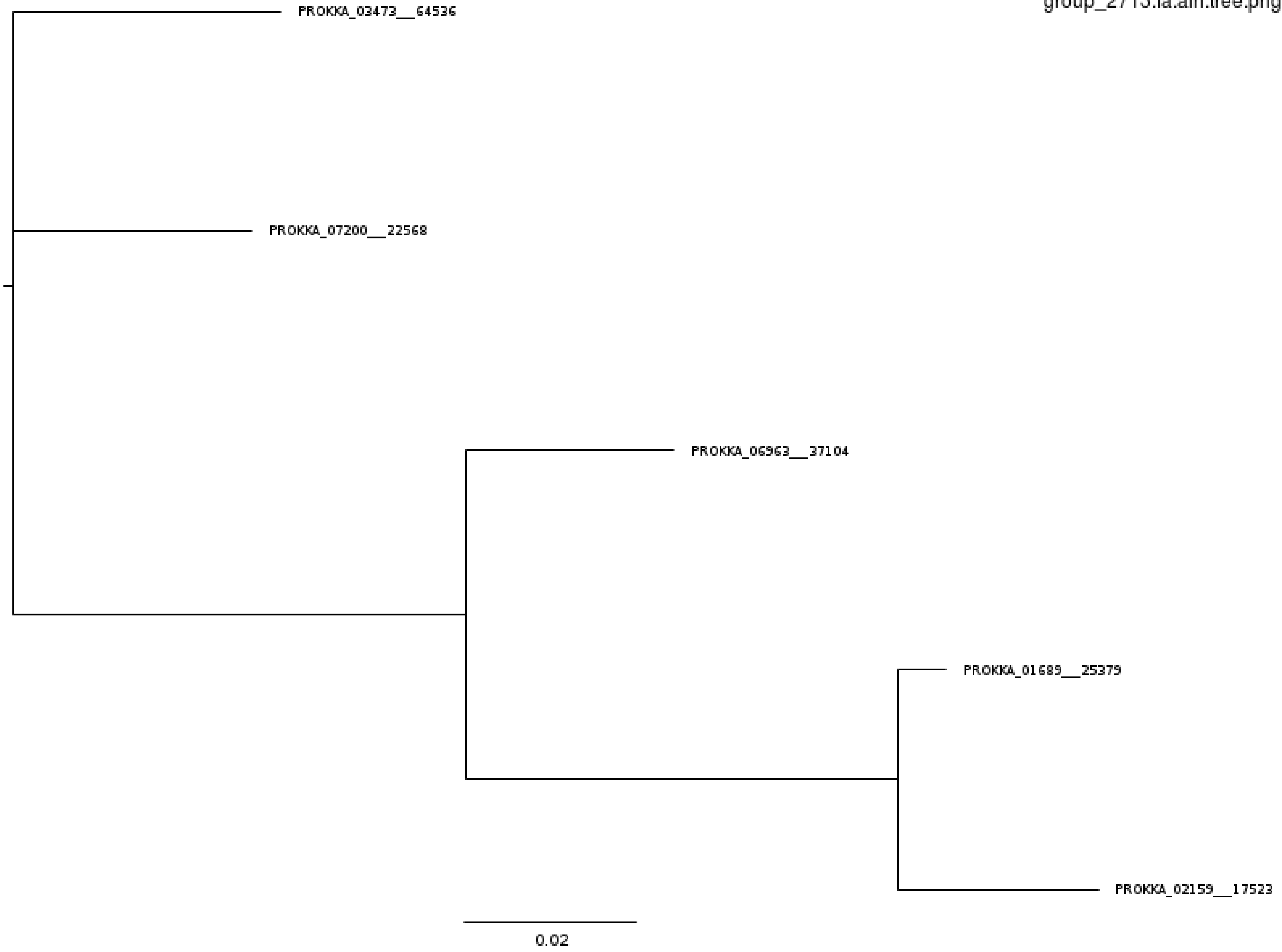

group_2715.fa.aln.tree.png

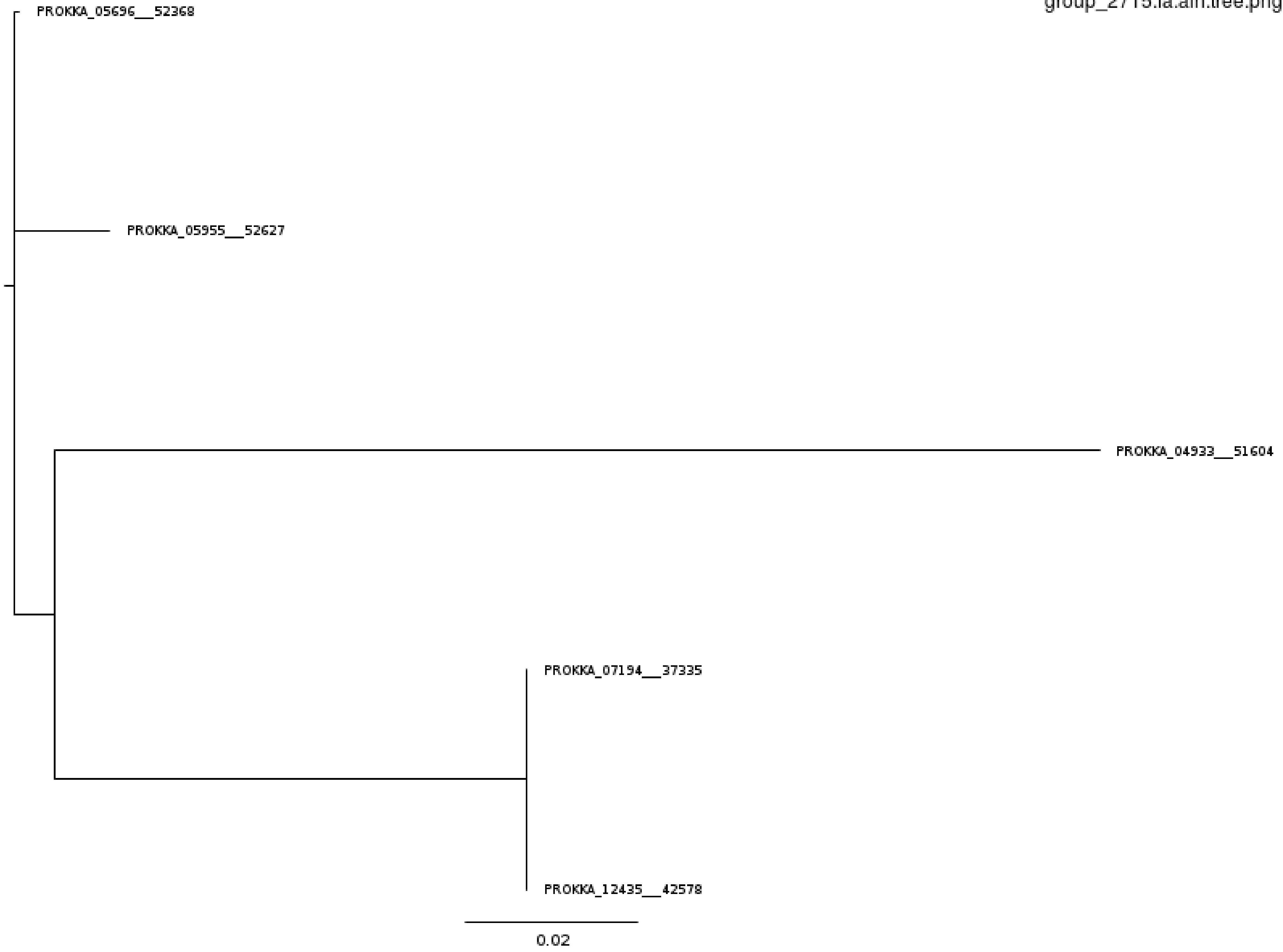

group_2727.fa.aln.tree.png

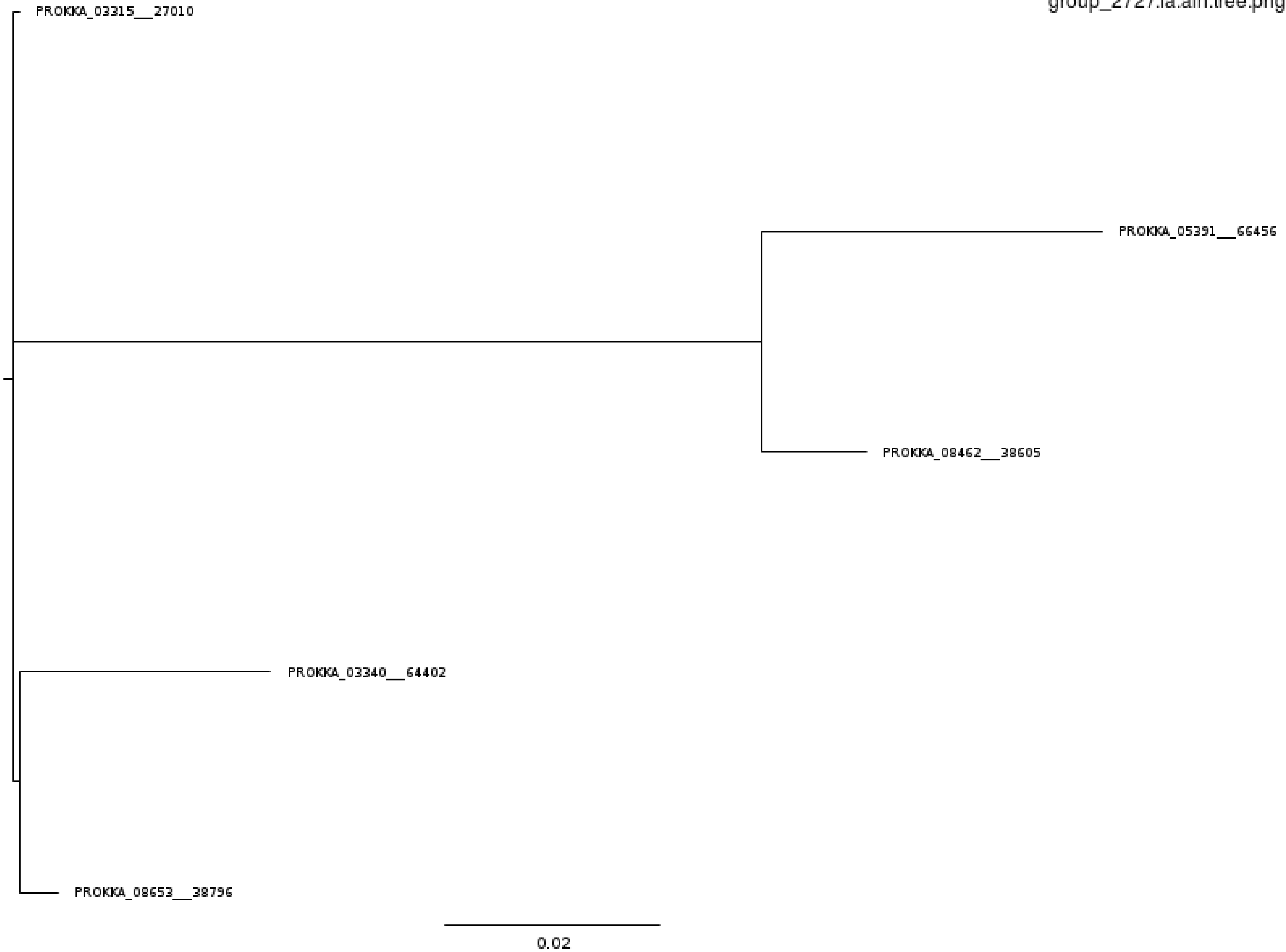

group_2735.fa.aln.tree.png

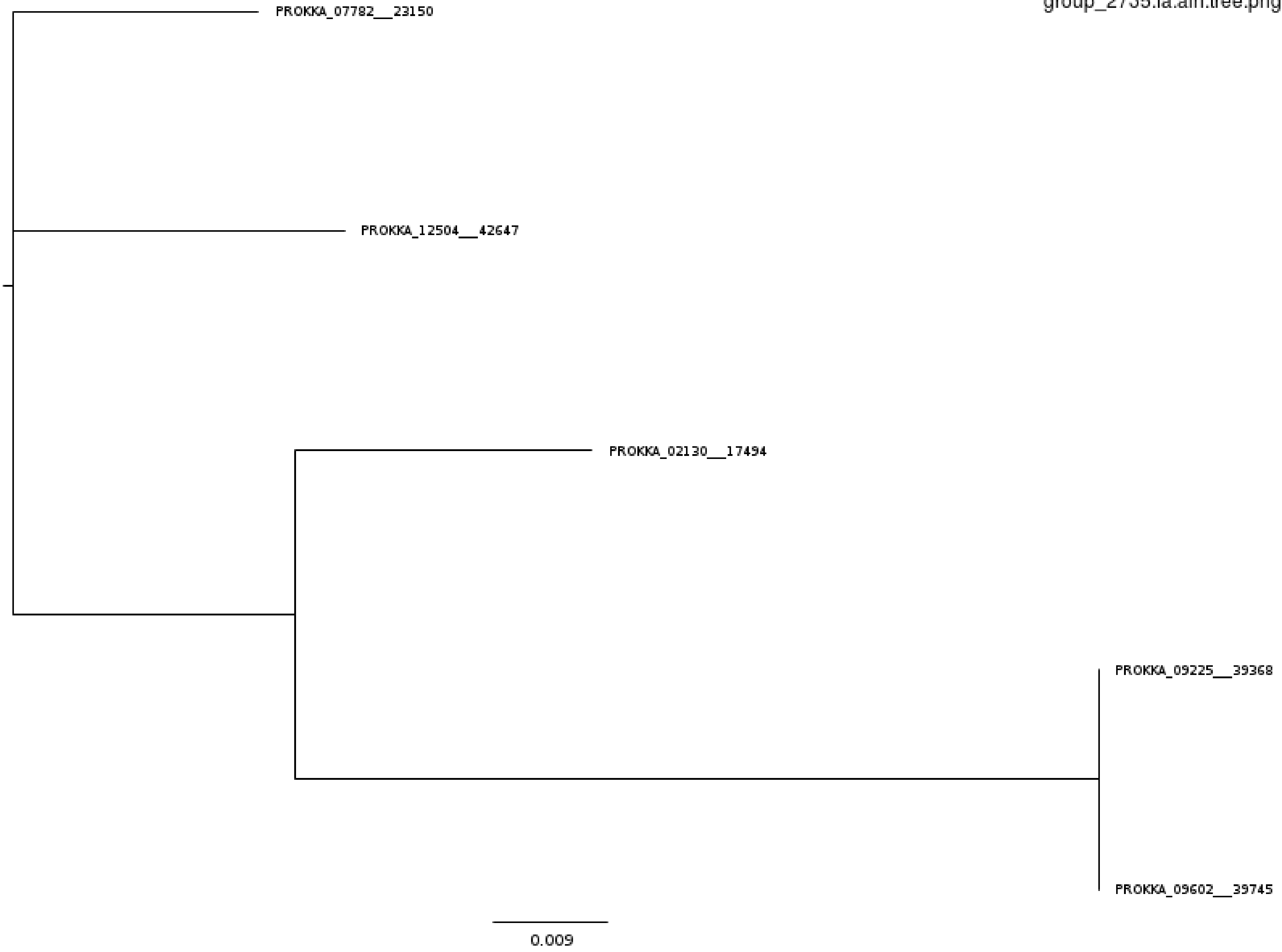

group_2753.fa.aln.tree.png

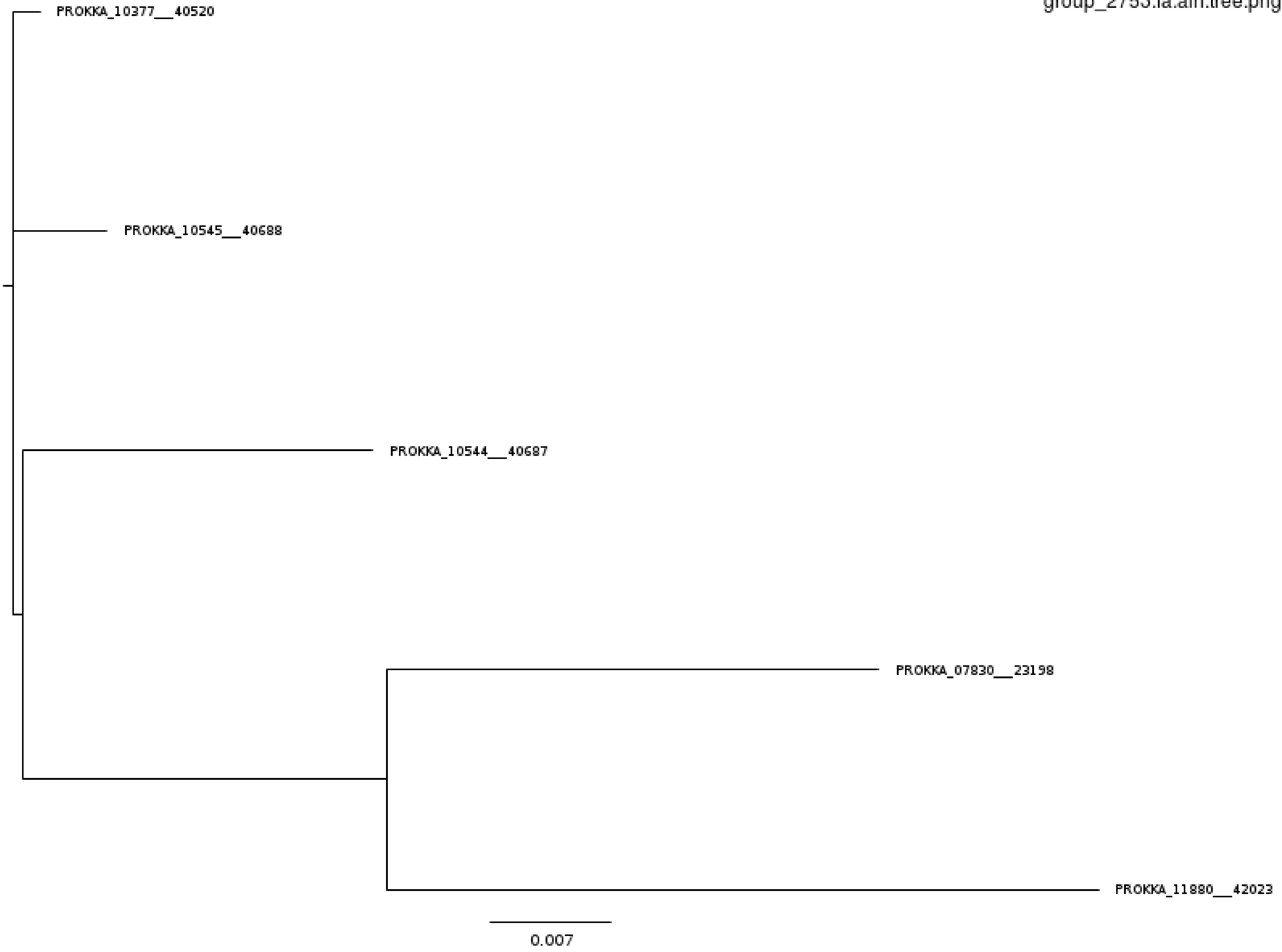

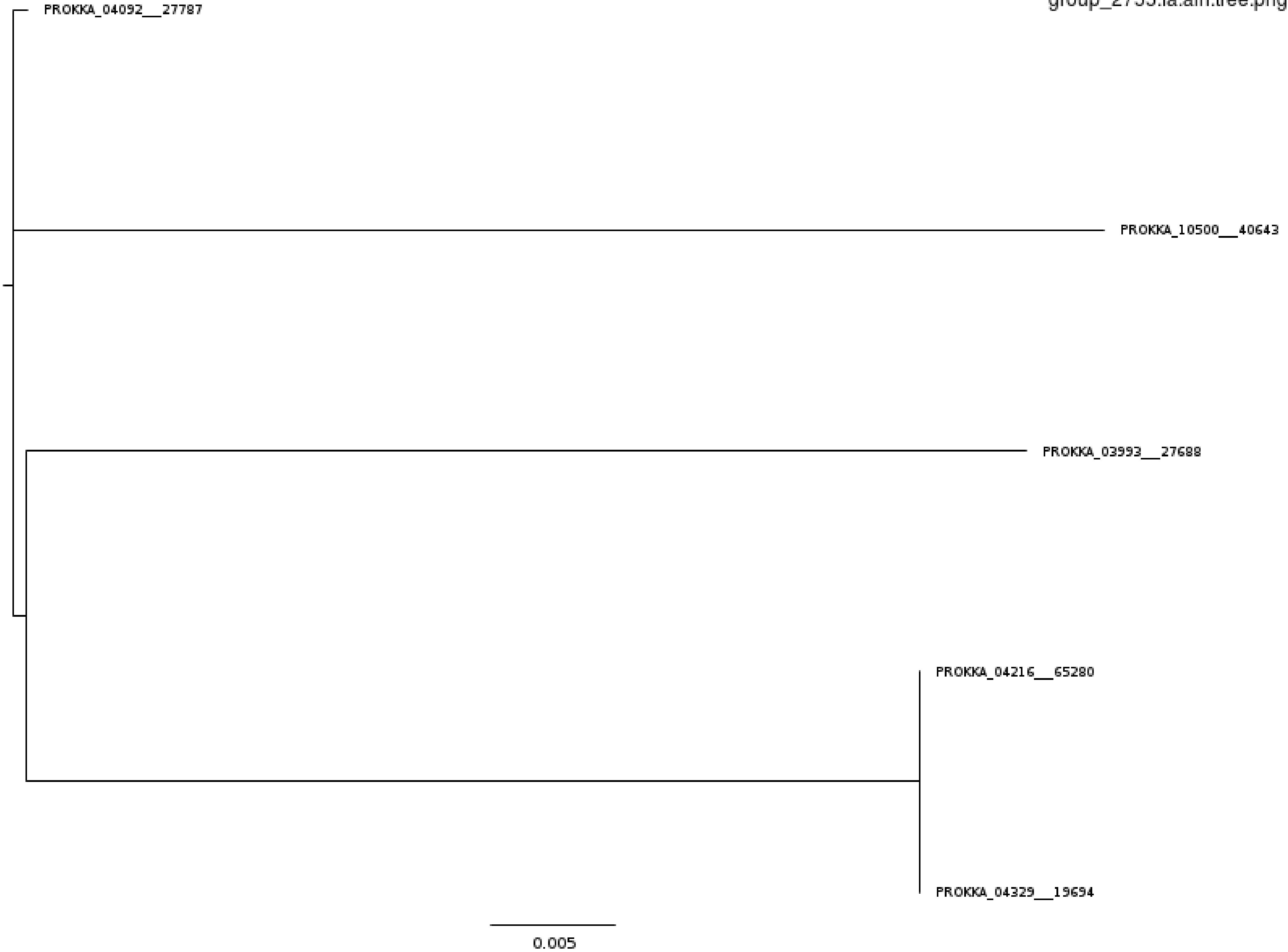
PROKKA_04092__27787
PROKKA_10500__40643
PROKKA_03993__27688
PROKKA_04216__65280
PROKKA_04329__19694
0.005

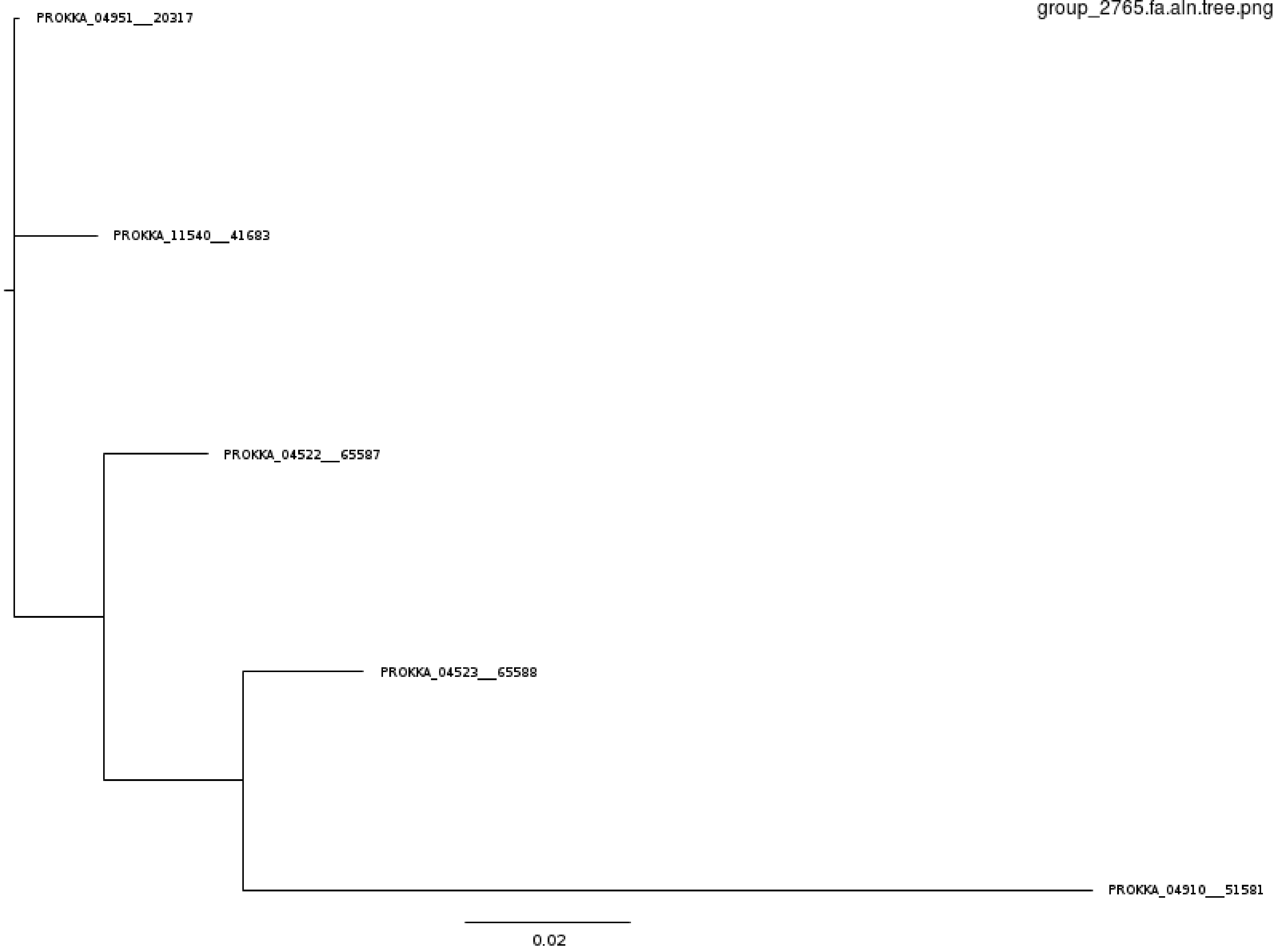
group_2765.fa.aln.tree.png
PROKKA_04951___20317
PROKKA_11540___41683
PROKKA_04522___65587
PROKKA_04523___65588
PROKKA_04910___51581
0.02

group_2767.fa.aln.tree.png

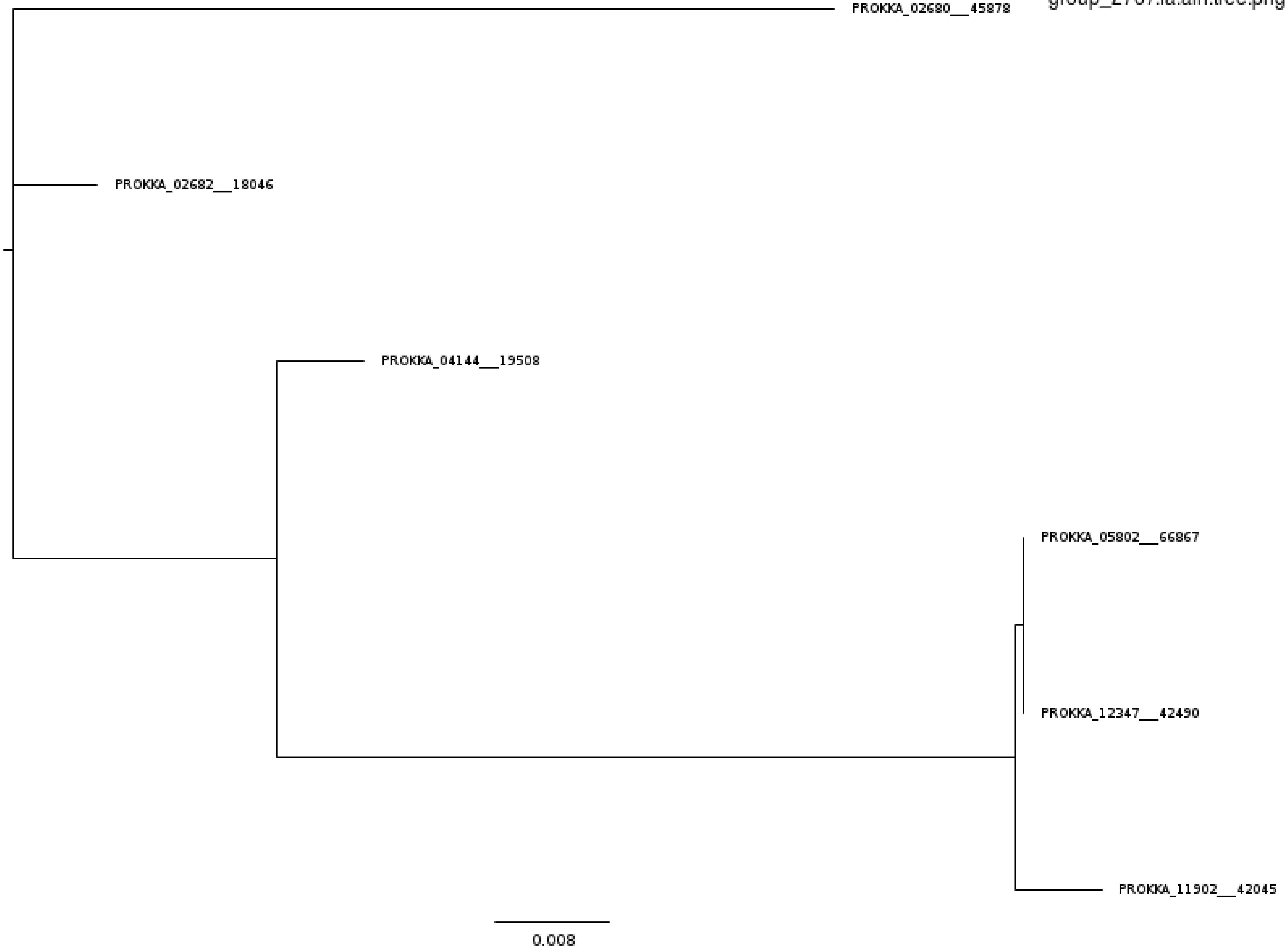

group_2773.fa.aln.tree.png

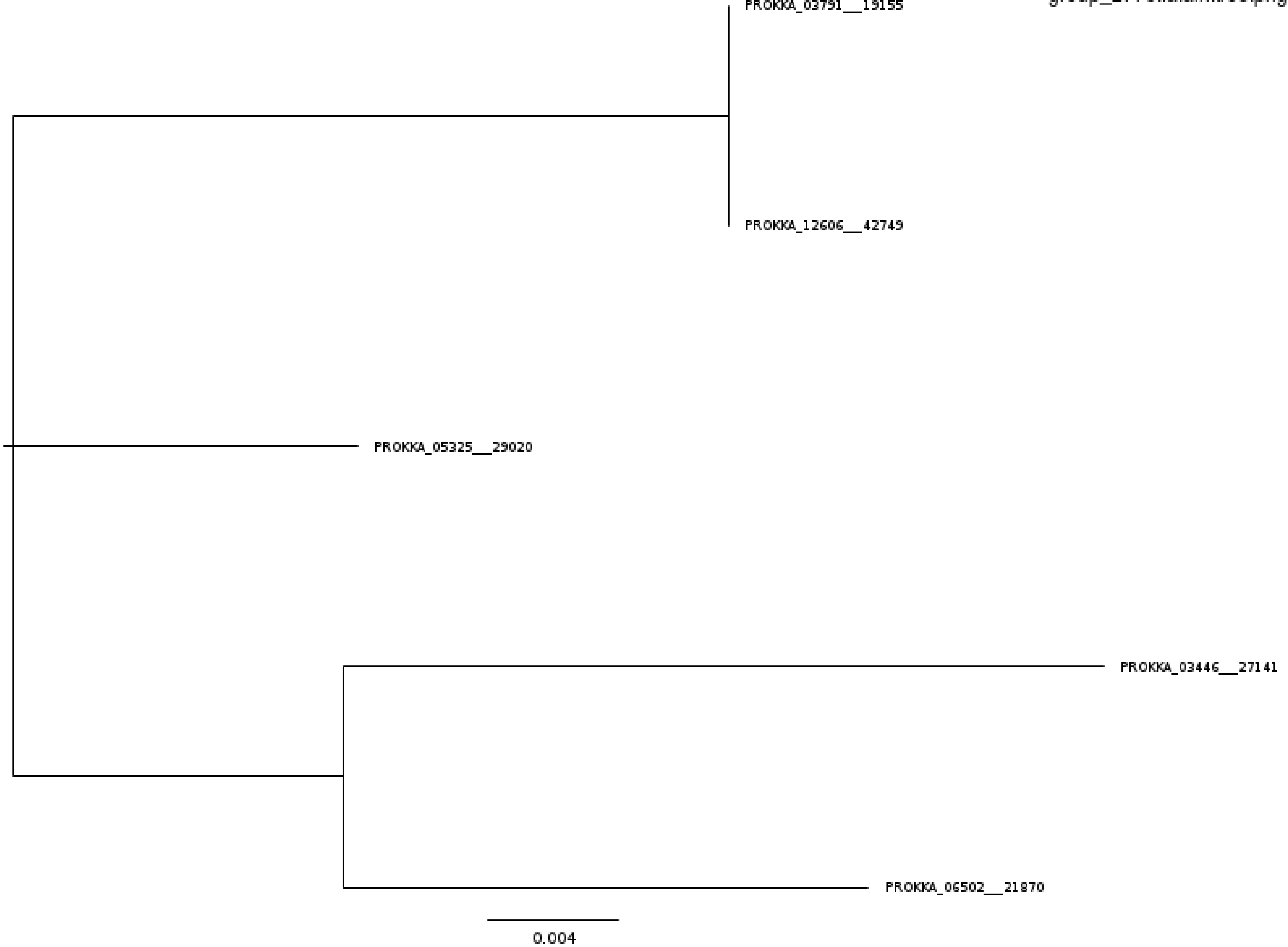

group_2778.fa.aln.tree.png

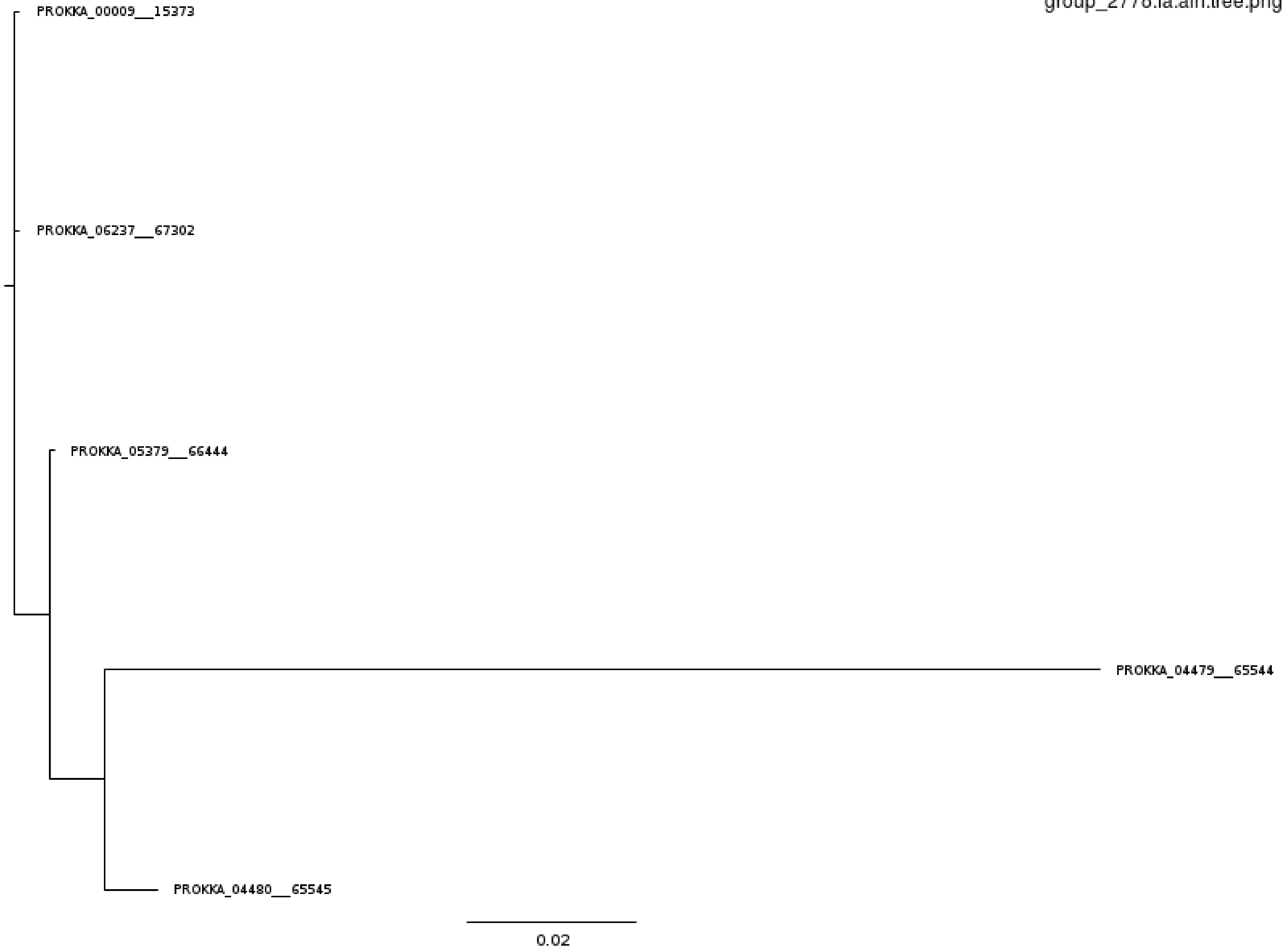

group_279.fa.aln.tree.png

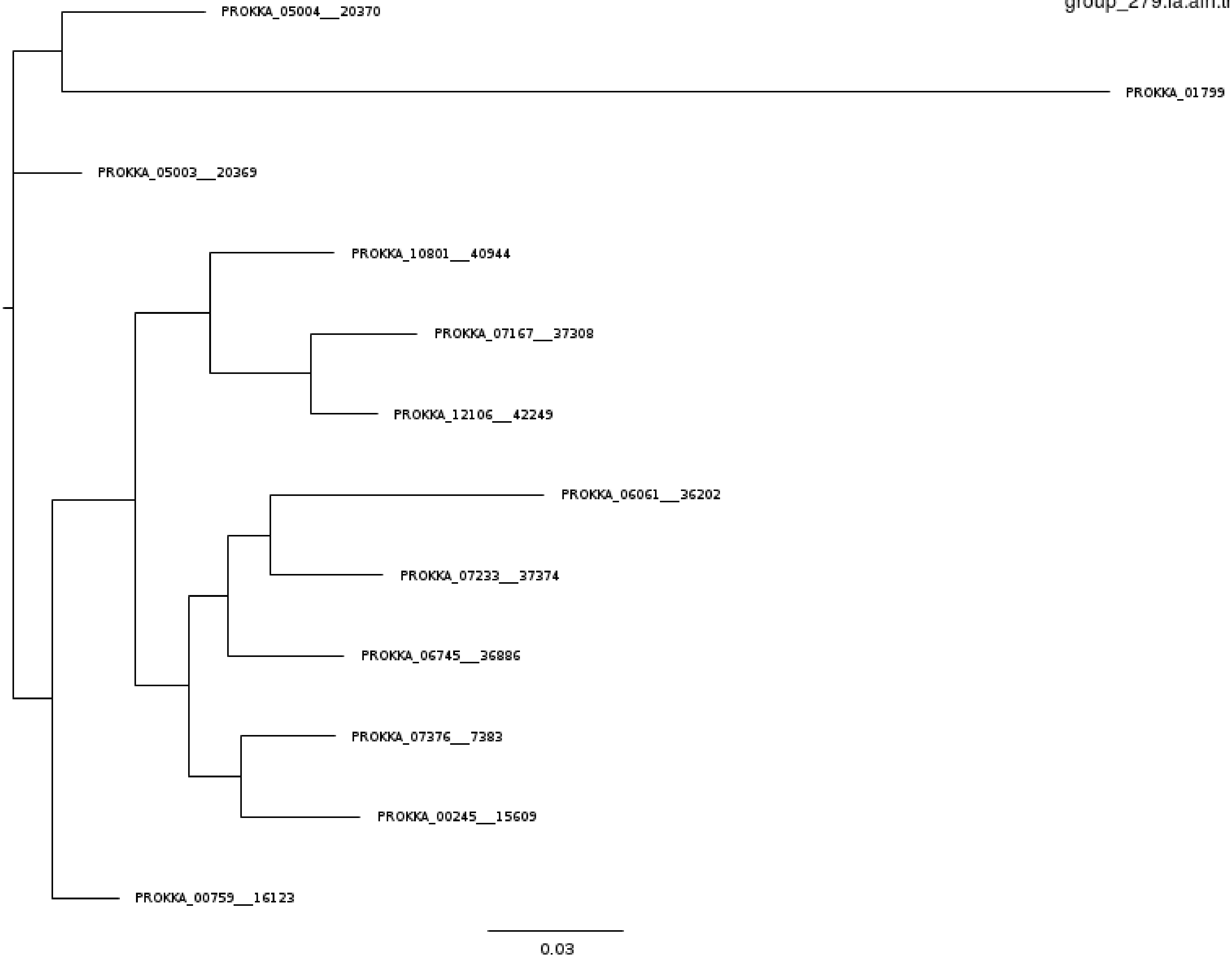

group_2805.fa.aln.tree.png

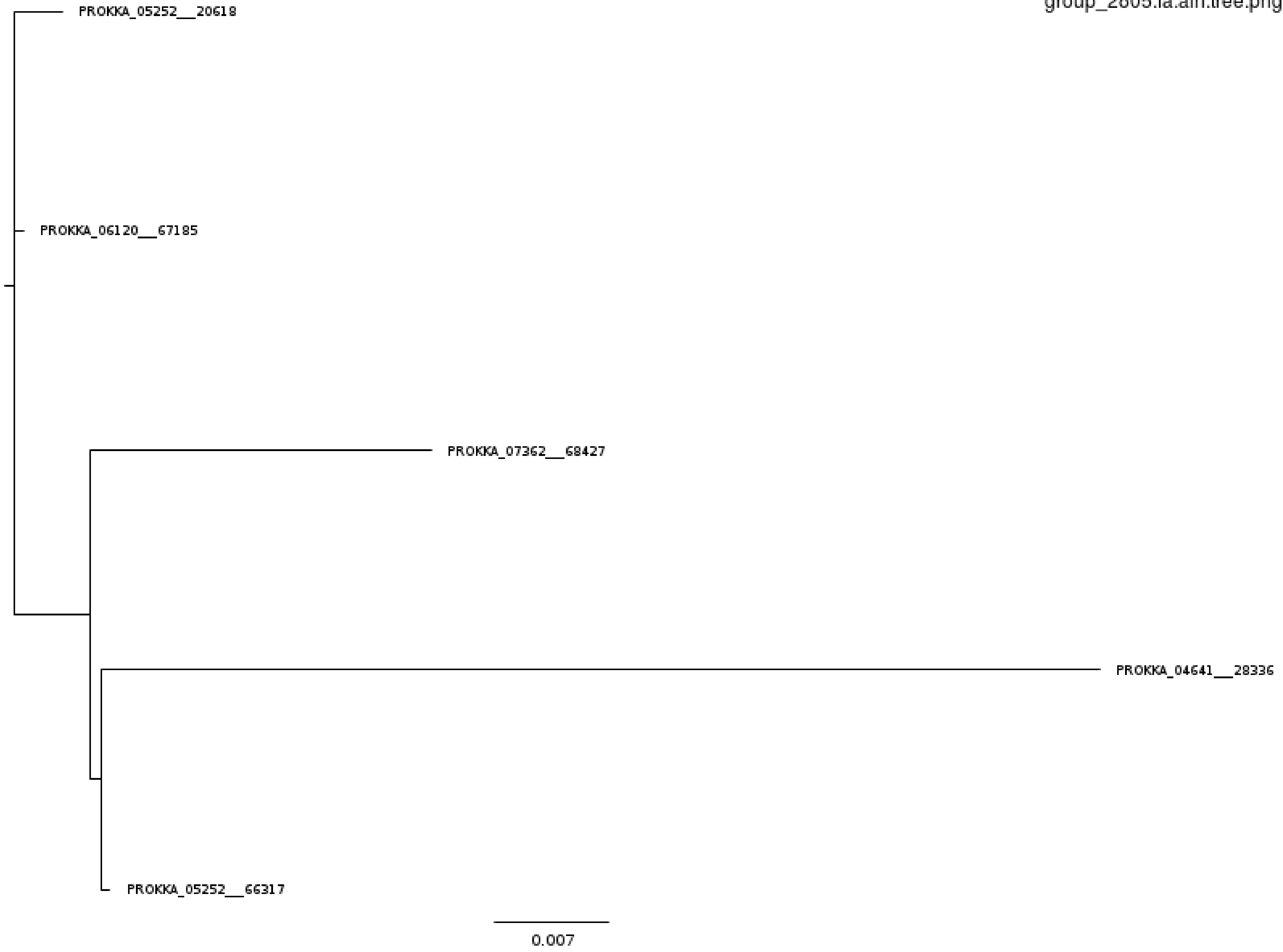

group_282.fa.aln.tree.png

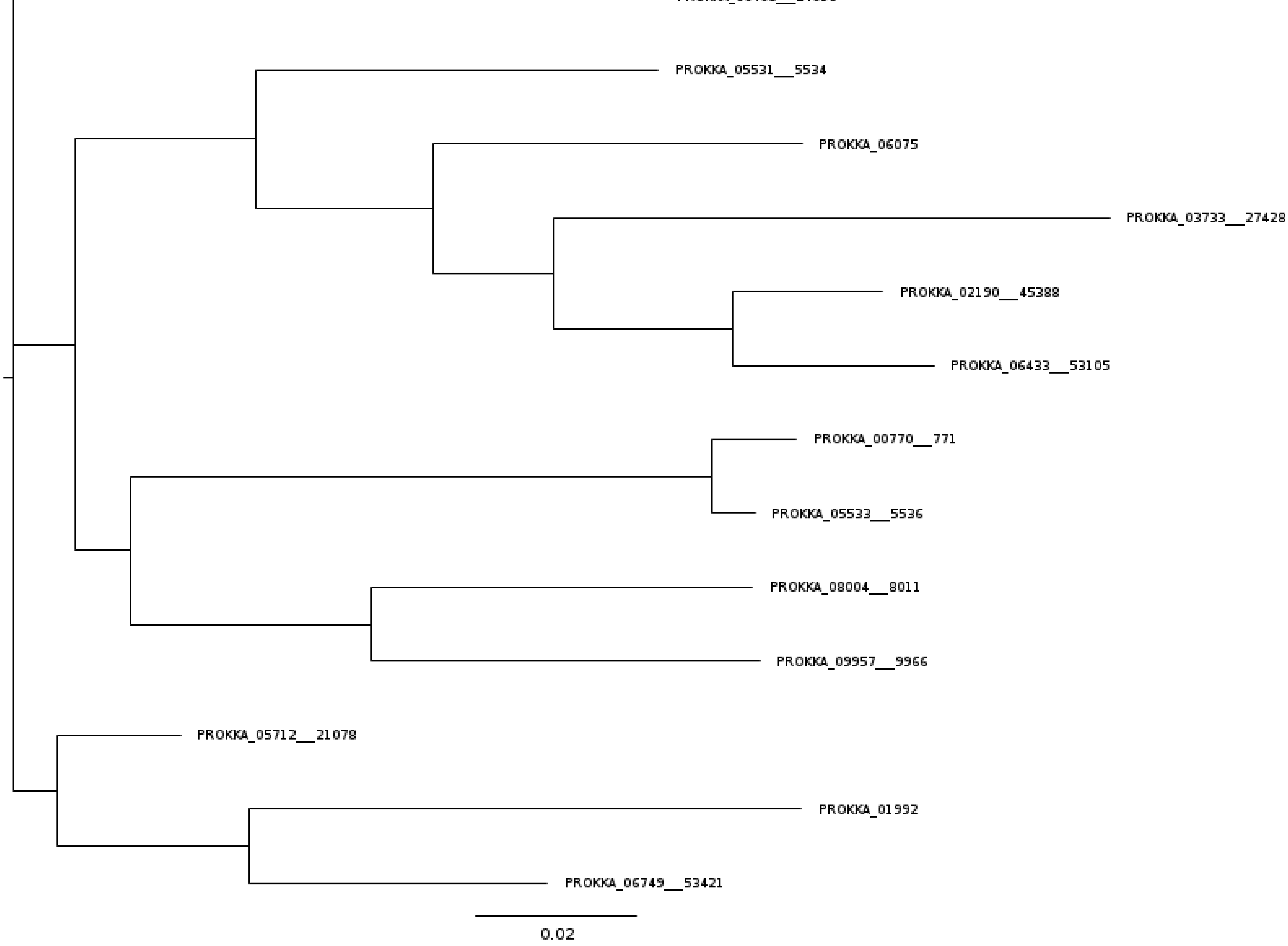

group_2849.fa.aln.tree.png

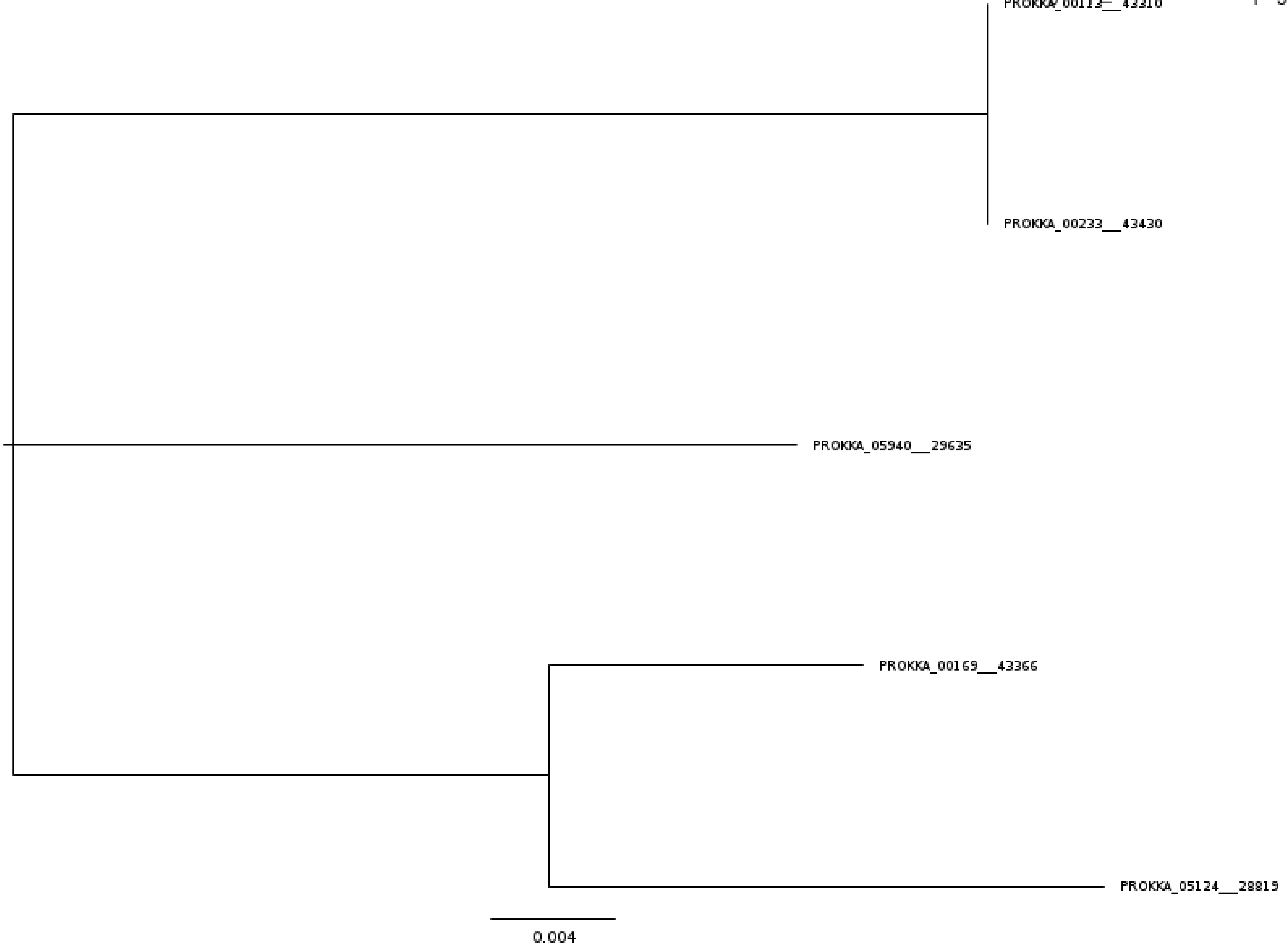

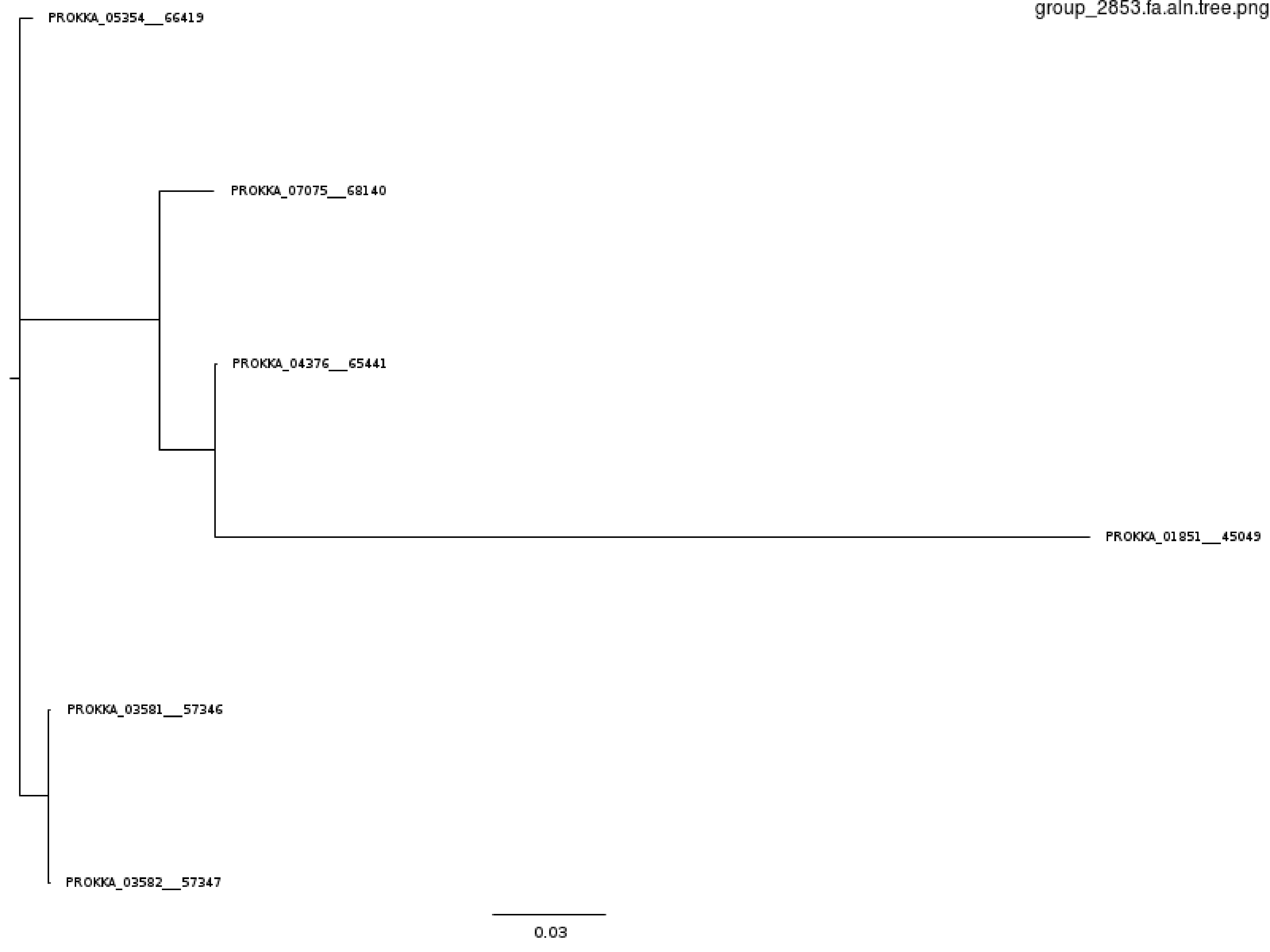
group_2853.fa.aln.tree.png
PROKKA_05354___66419
PROKKA_07075___68140
PROKKA_04376___65441
PROKKA_01851___45049
PROKKA_03581___57346
PROKKA_03582___57347
0.03

group_2855.fa.aln.tree.png

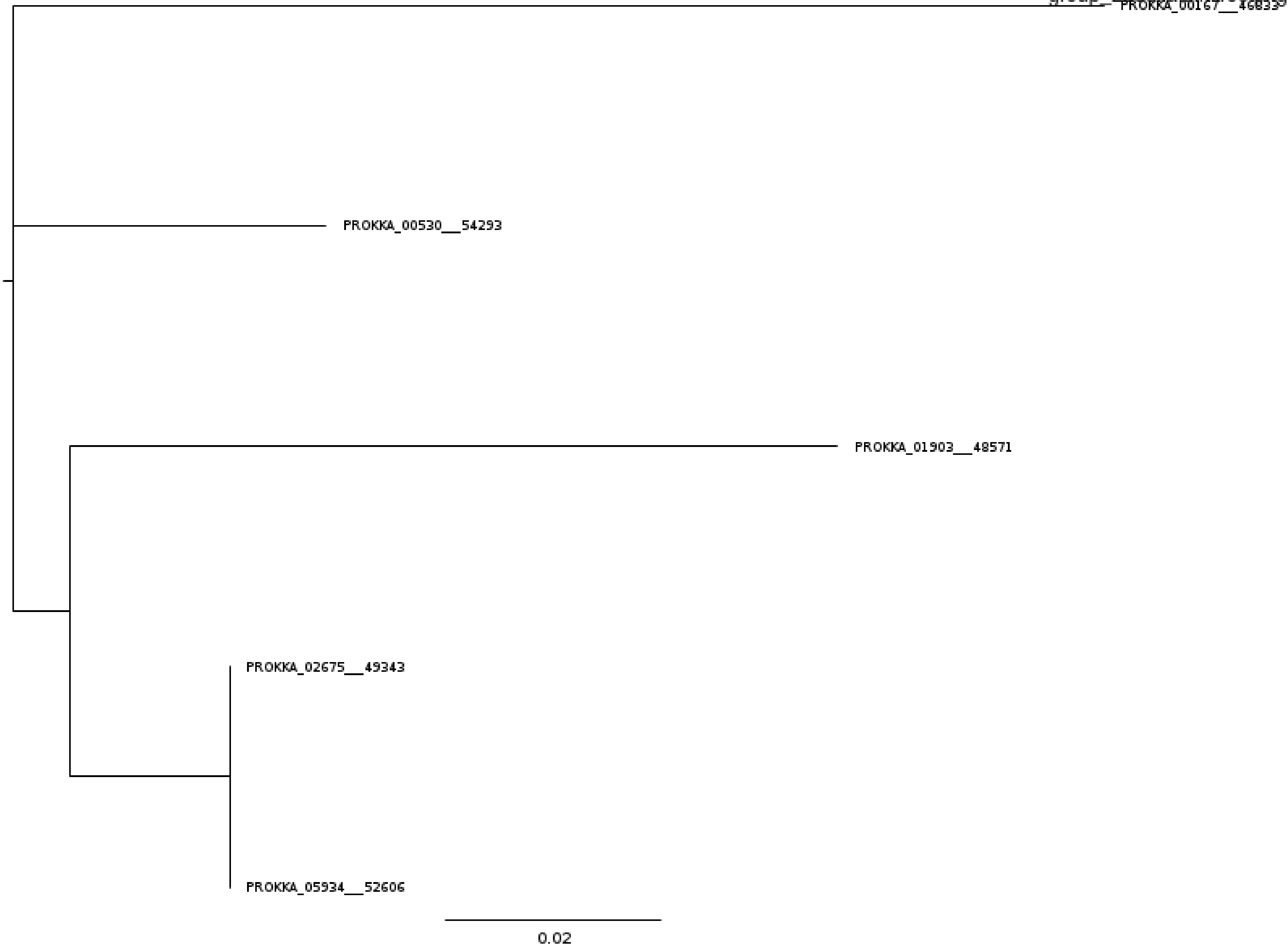

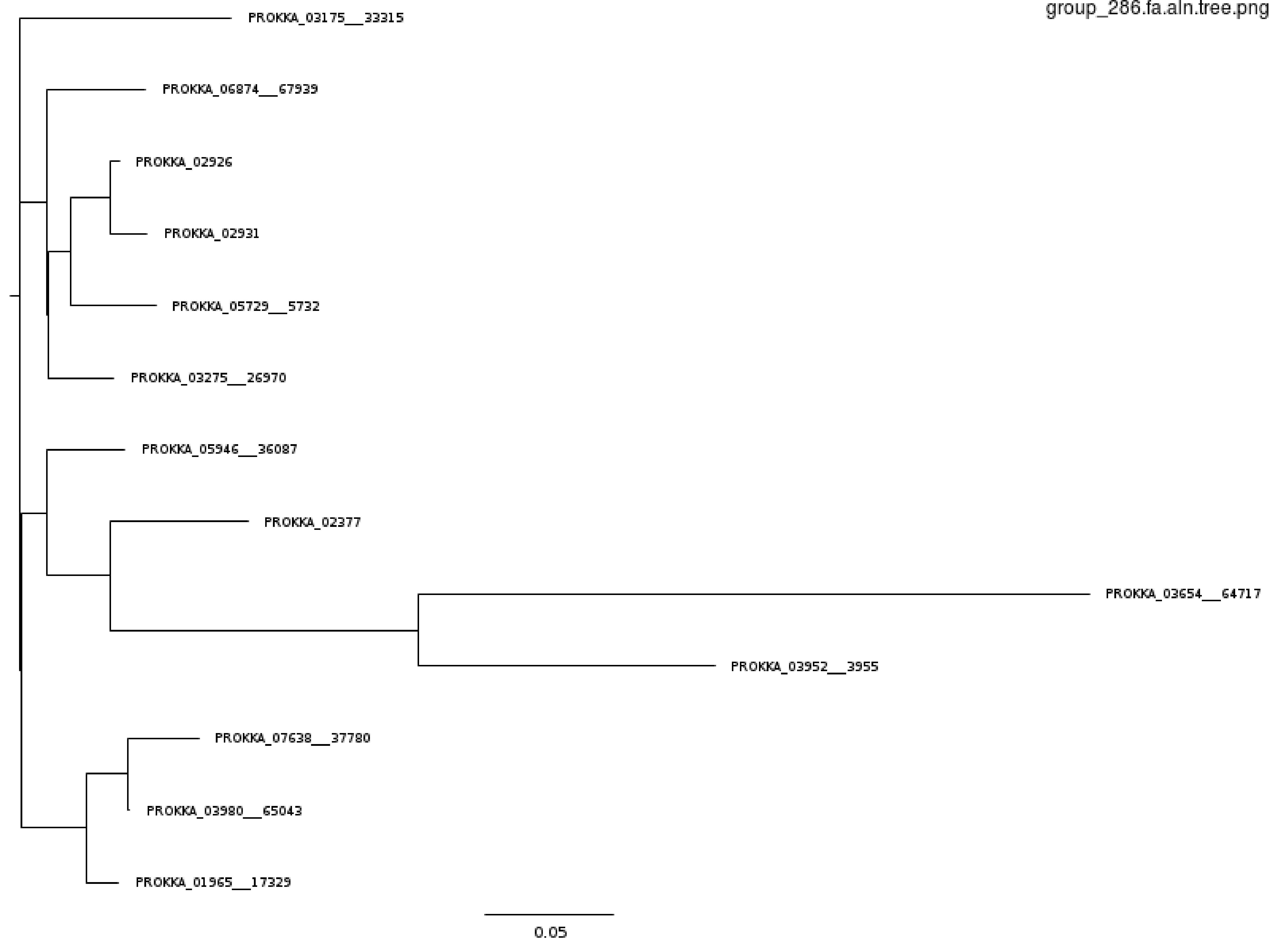
group_286.fa.aln.tree.png
PROKKA_03175___33315
PROKKA_06874___67939
PROKKA_02926
PROKKA_02931
PROKKA_05729___5732
PROKKA_03275___26970
PROKKA_05946___36087
PROKKA_02377
PROKKA_03654___64717
PROKKA_03952___3955
PROKKA_07638___37780
PROKKA_03980___65043
PROKKA_01965___17329
0.05

group_287.fa.aln.tree.png

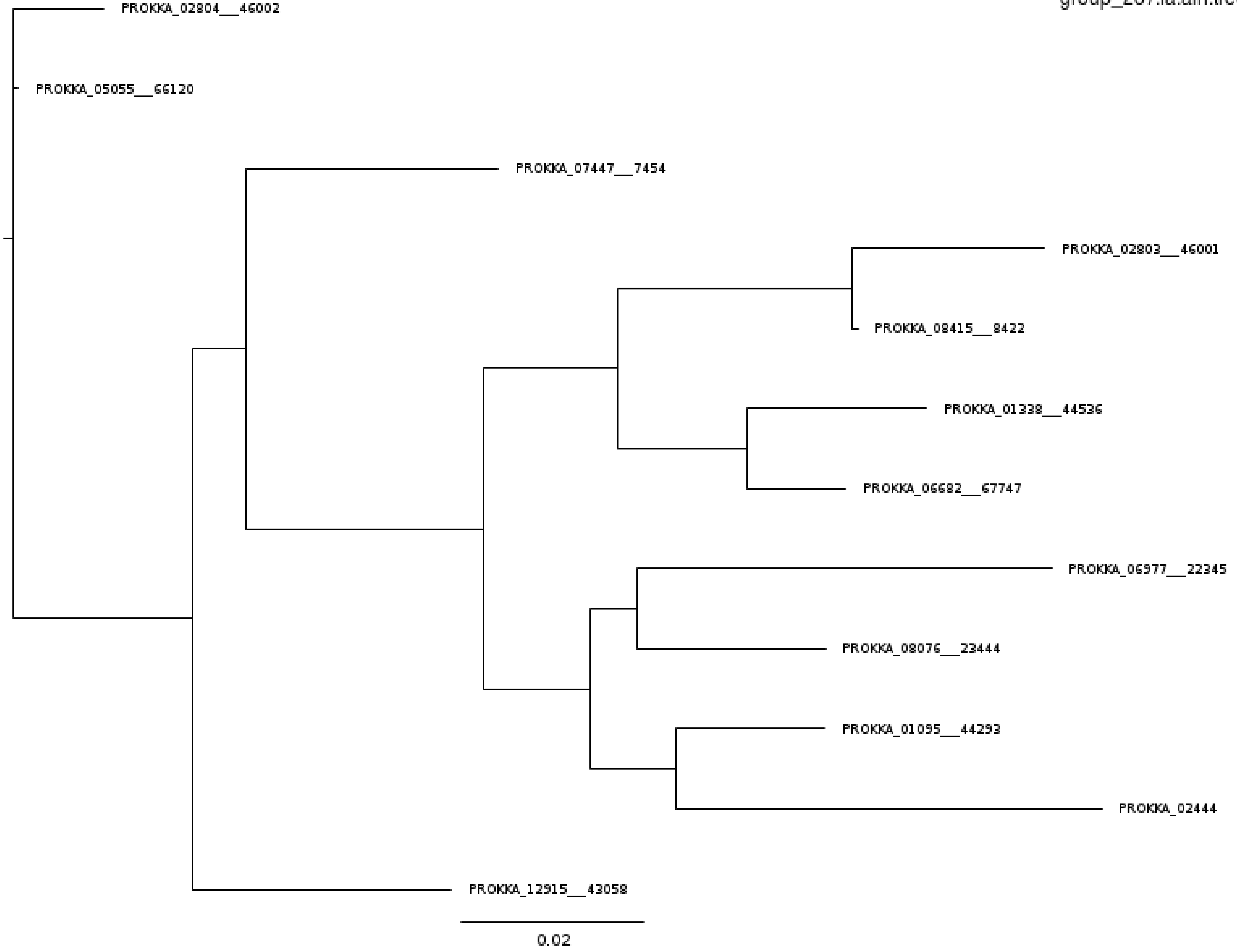

group_290.fa.aln.tree.png

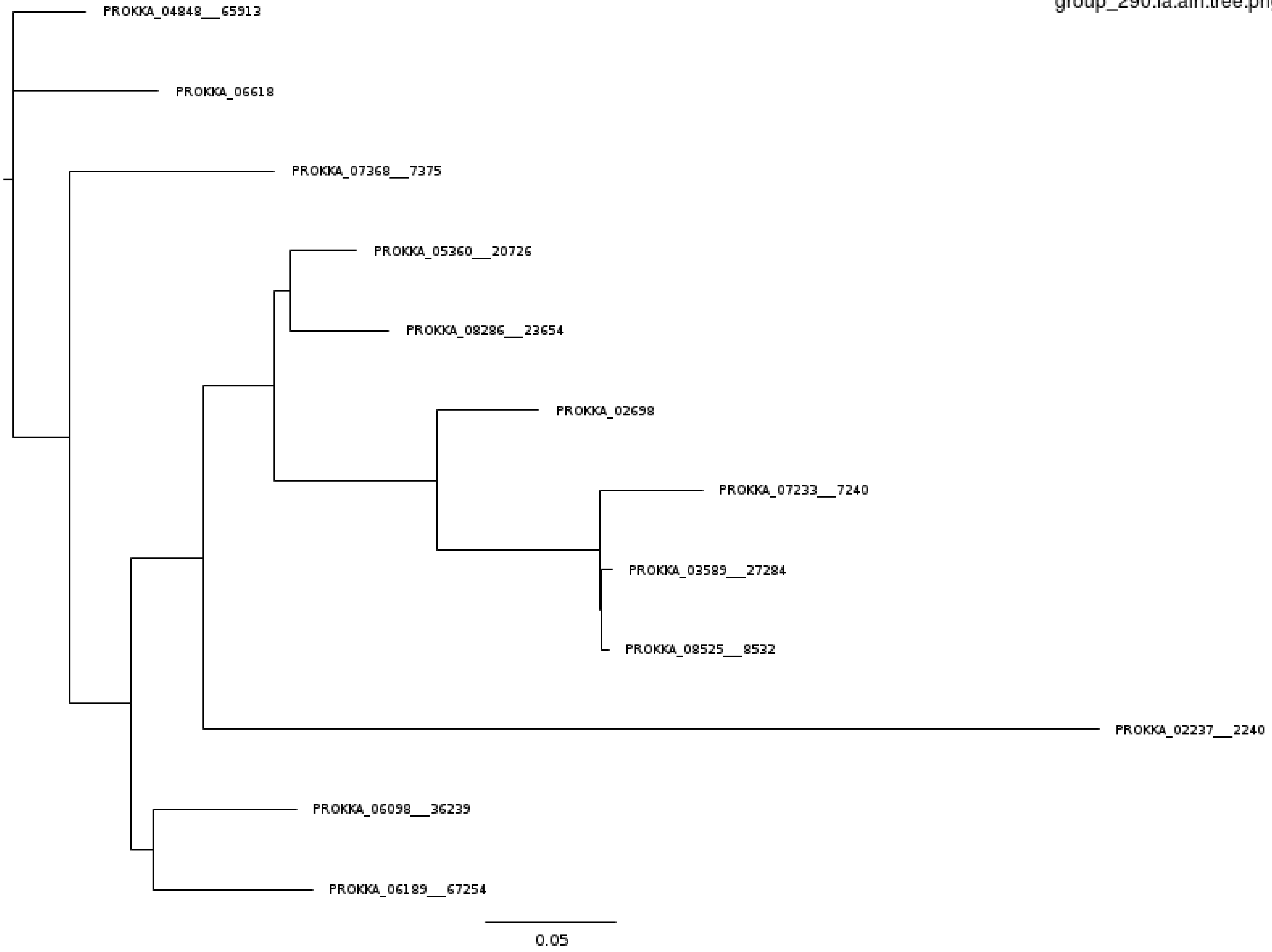

group_293.fa.aln.tree.png

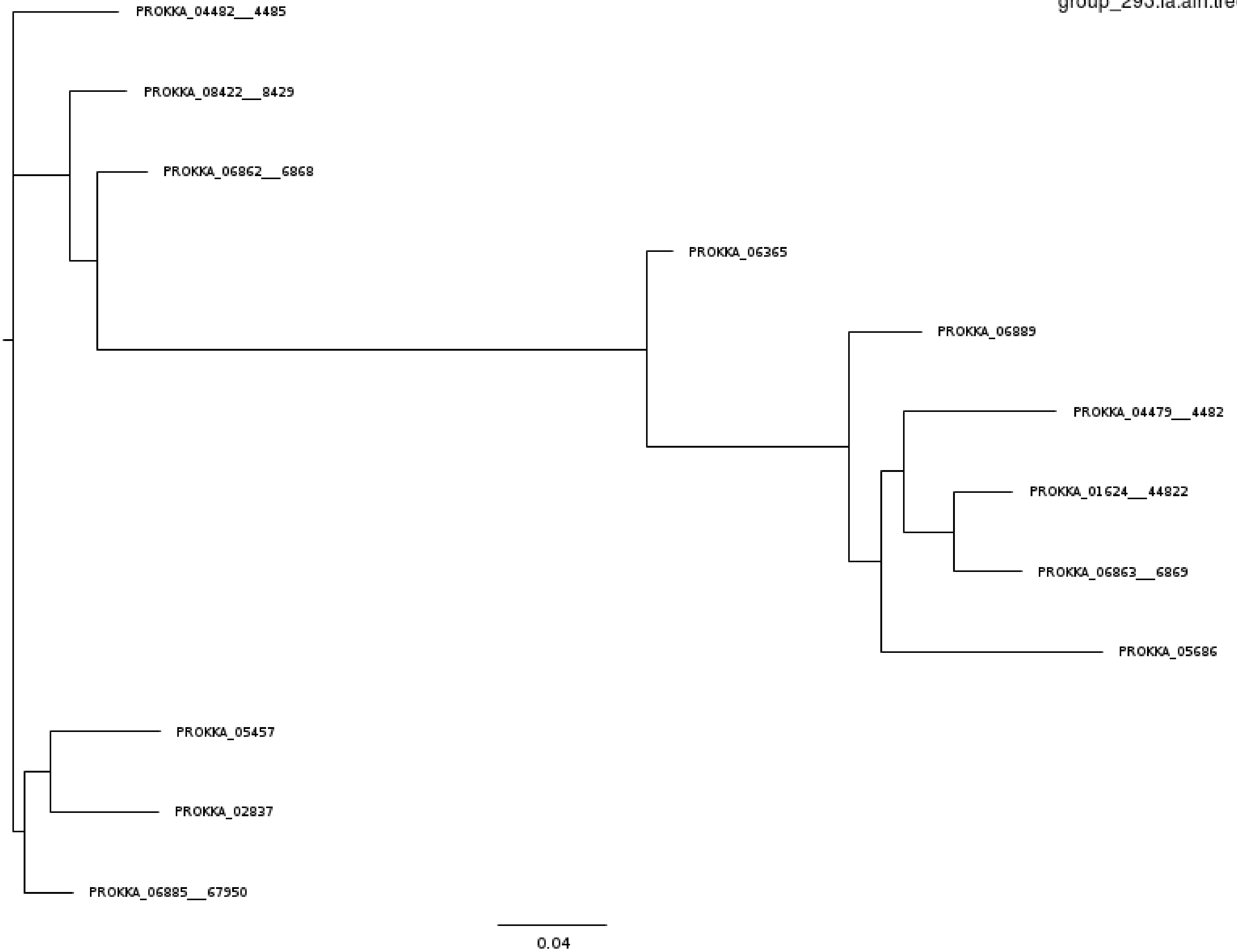

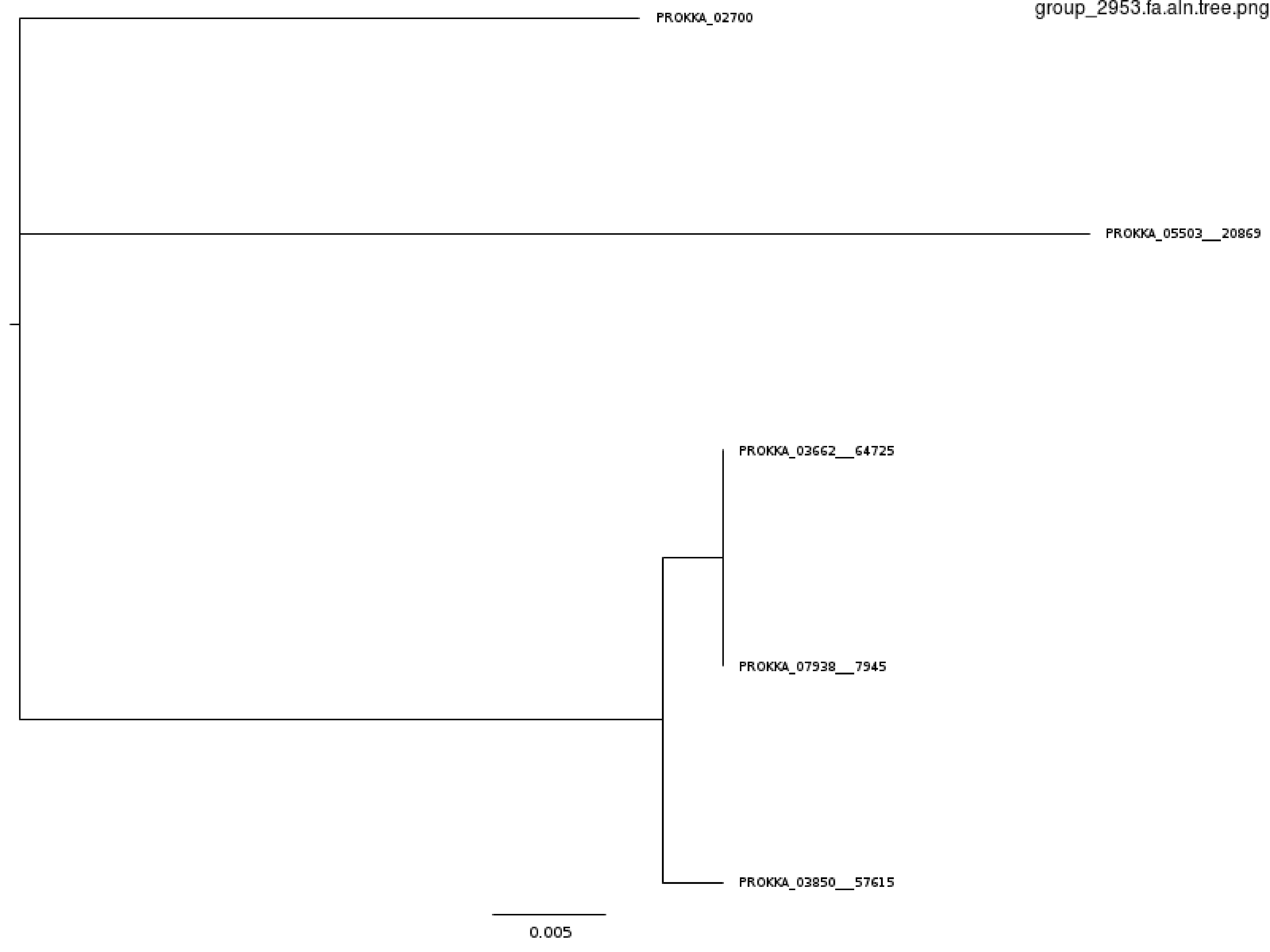
group_2953.fa.aln.tree.png
PROKKA_02700
PROKKA_05503___20869
PROKKA_03662___64725
PROKKA_07938___7945
PROKKA_03850___57615
0.005

group_296.fa.aln.tree.png

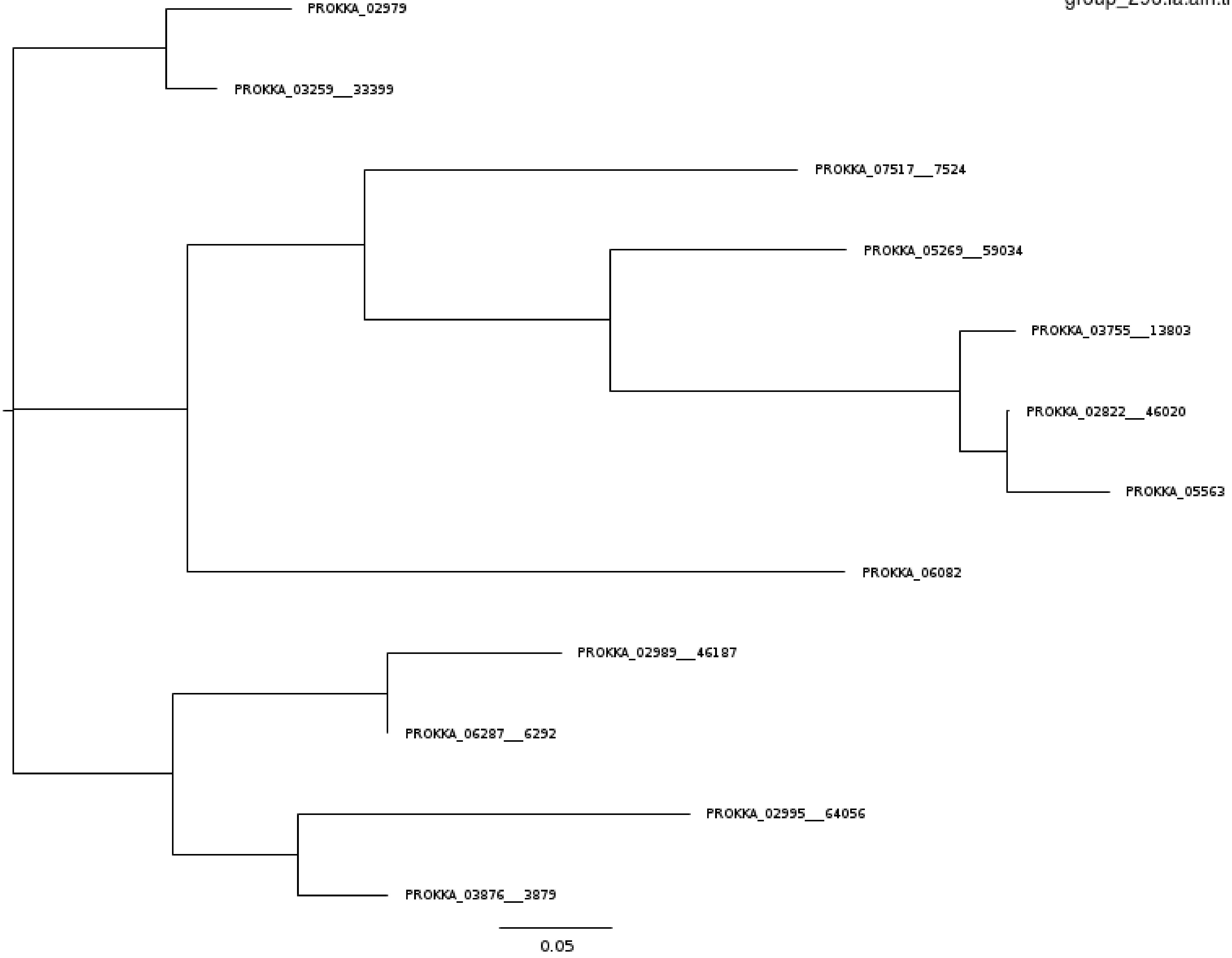

group_2994.fa.aln.tree.png

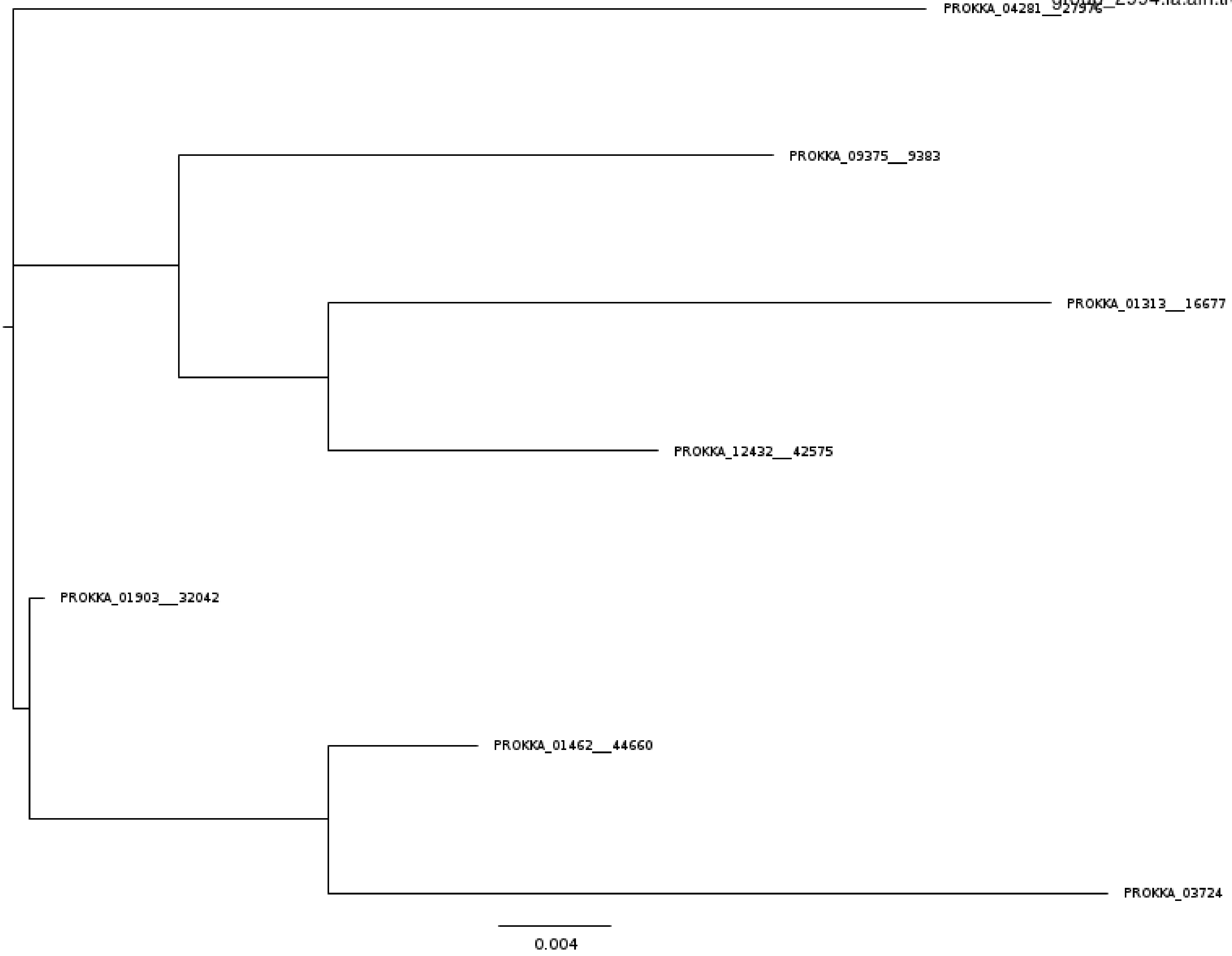

group_299.fa.aln.tree.png

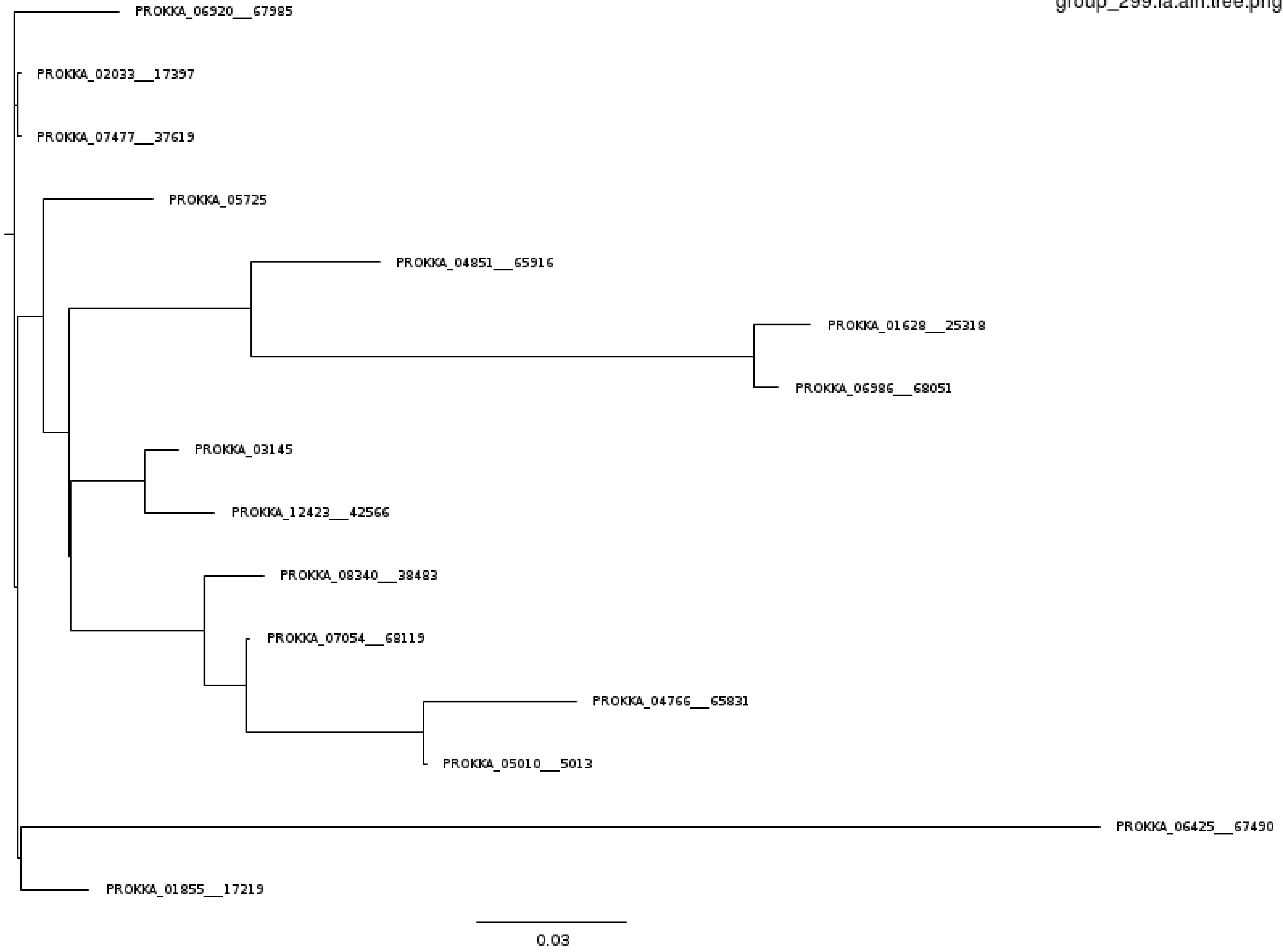

group_301.fa.aln.tree.png

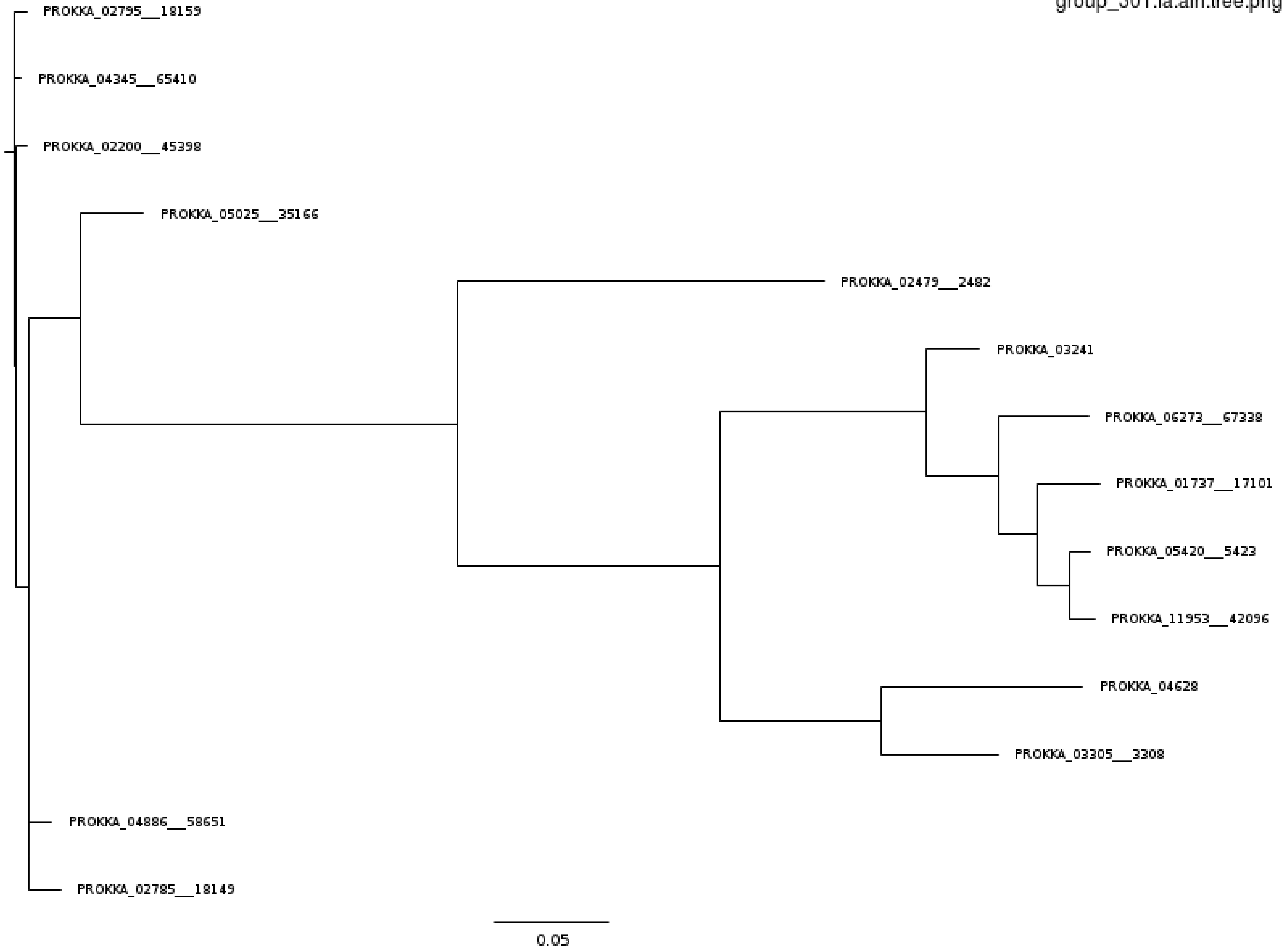

group_3030.fa.aln.tree.png

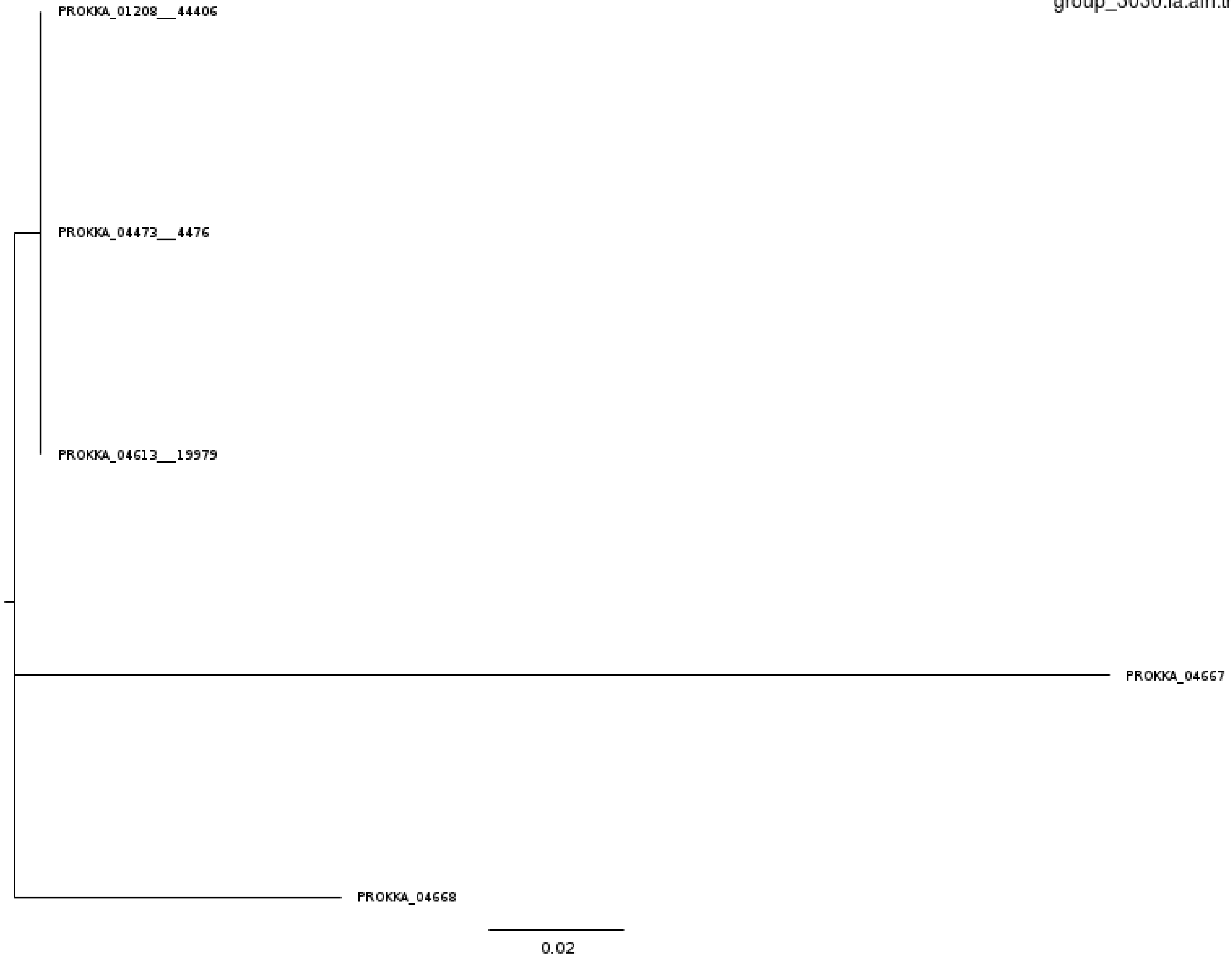

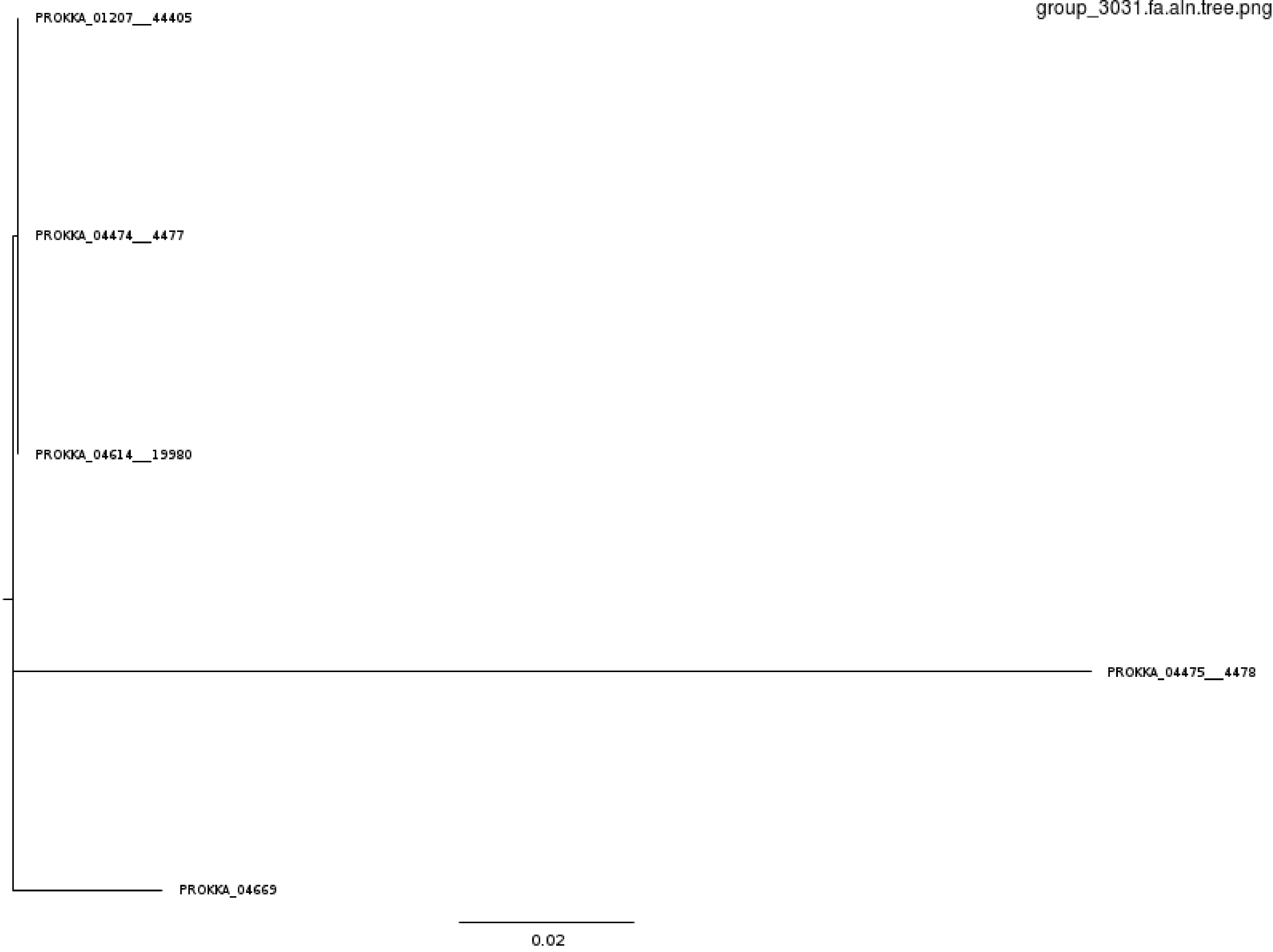
group_3031.fa.aln.tree.png
PROKKA_01207___44405
PROKKA_04474___4477
PROKKA_04614___19980
PROKKA_04475___4478
PROKKA_04669
0.02

group_3048.fa.aln.tree.png

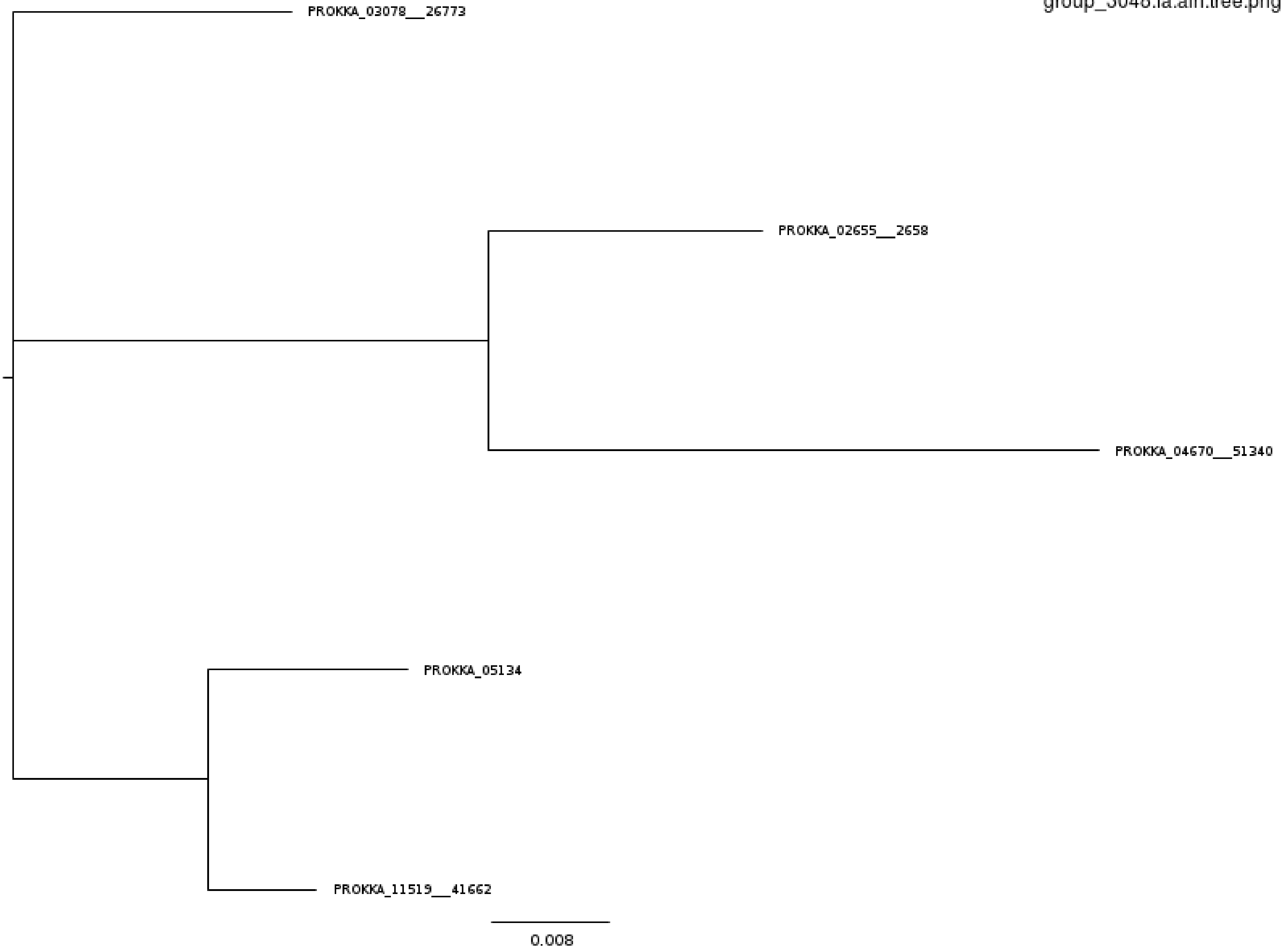

group_3064.fa.aln.tree.png

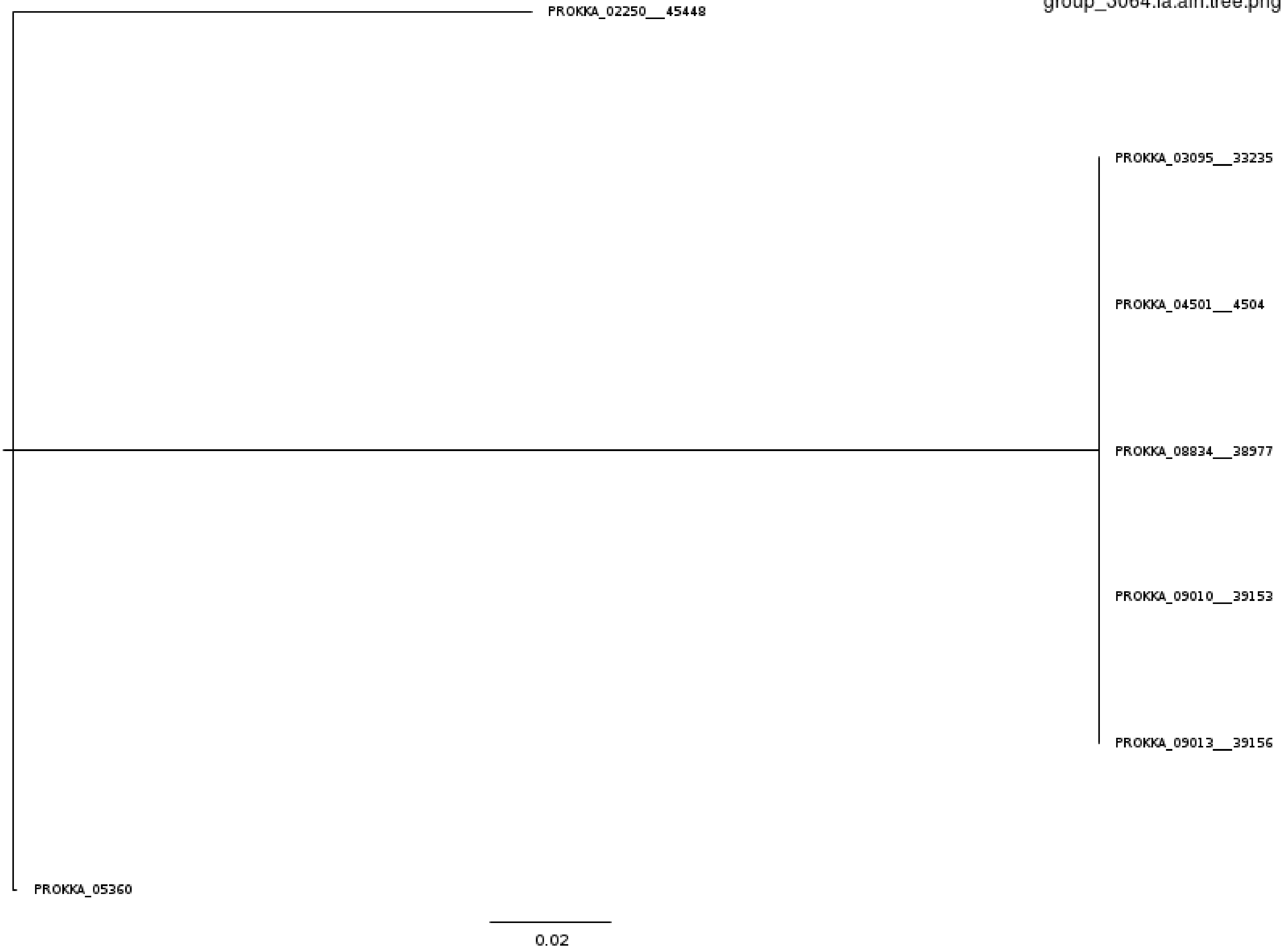

group_306.fa.aln.tree.png

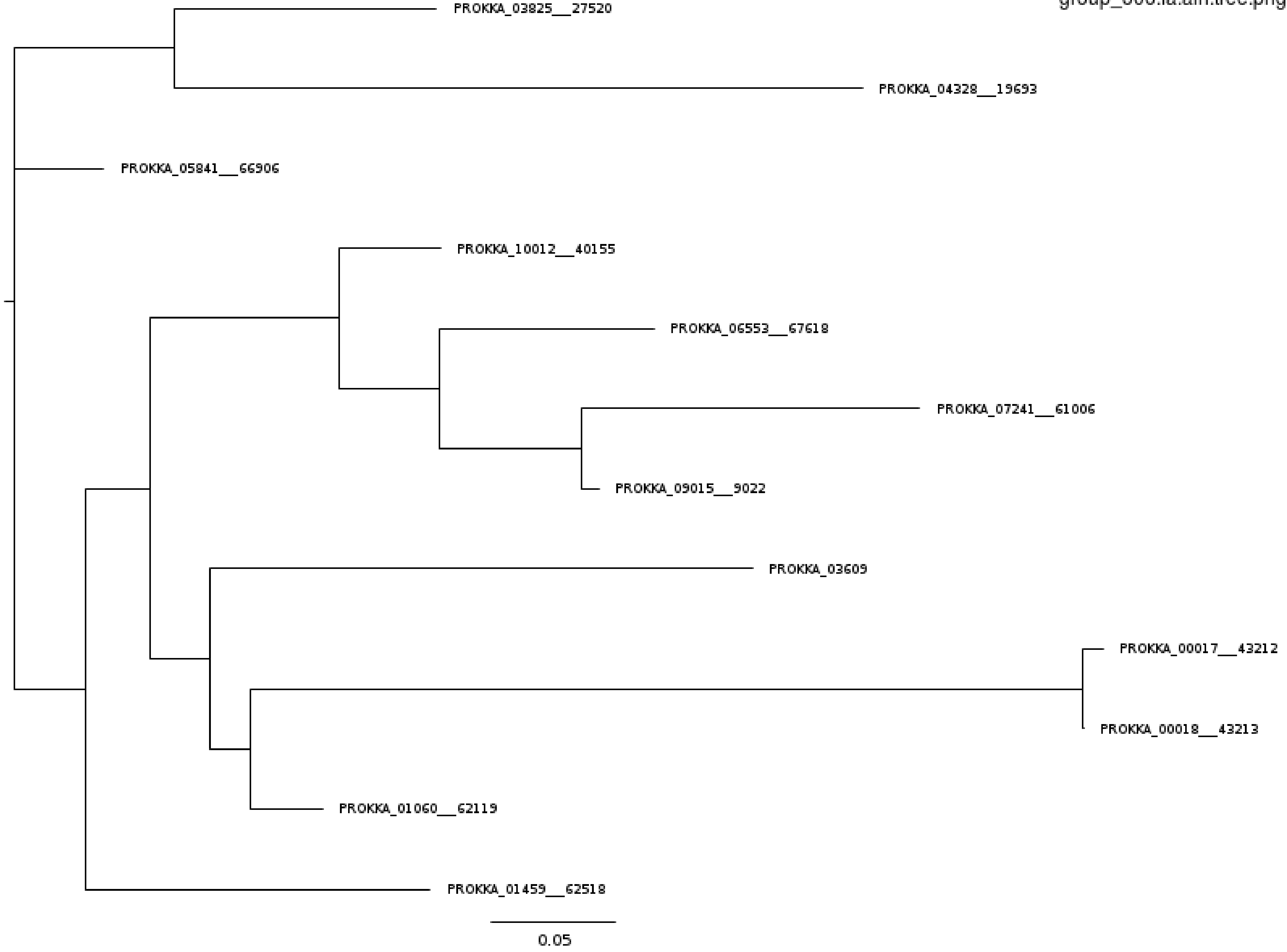

group_307.fa.aln.tree.png

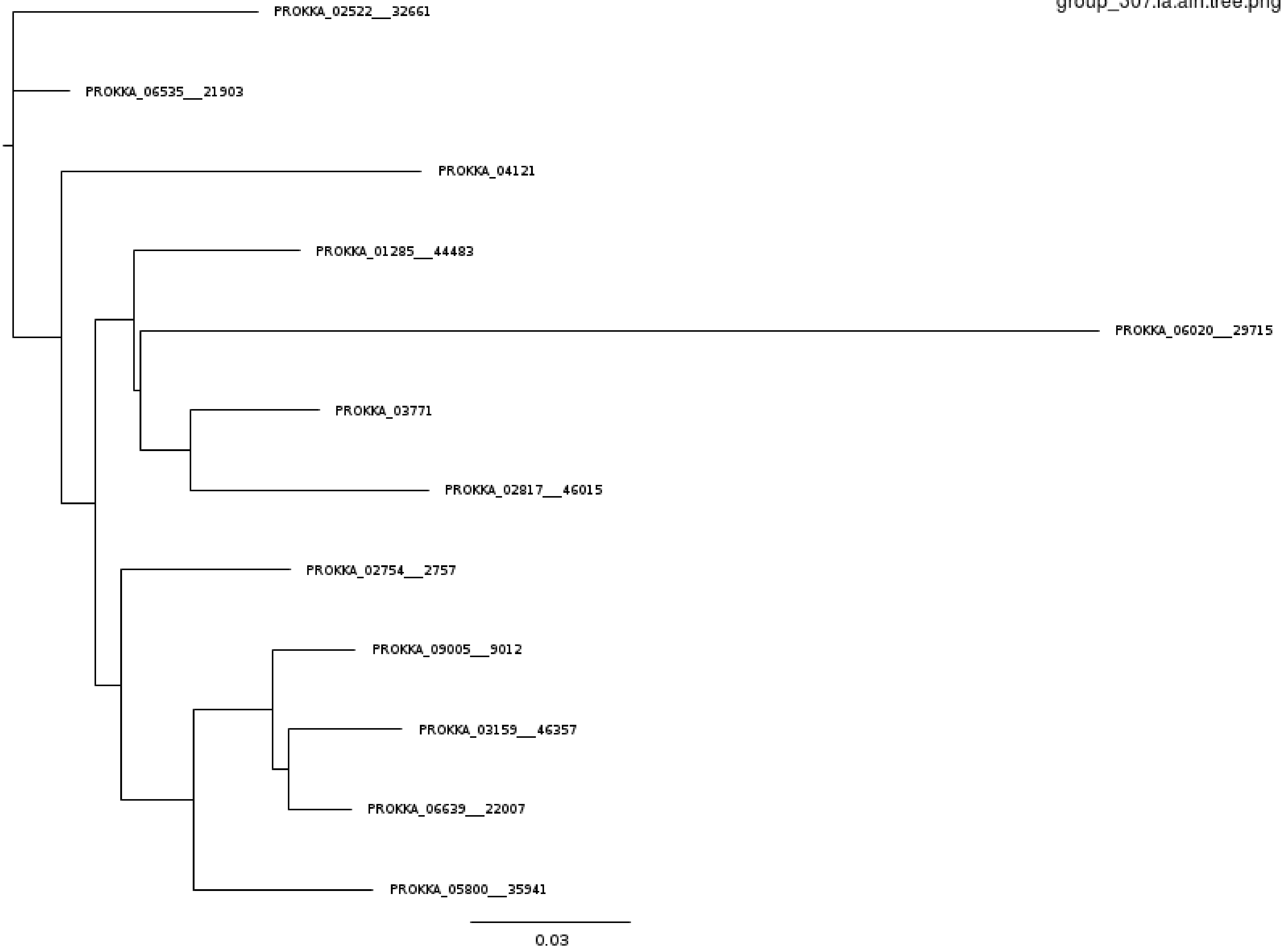

group_314.fa.aln.tree.png

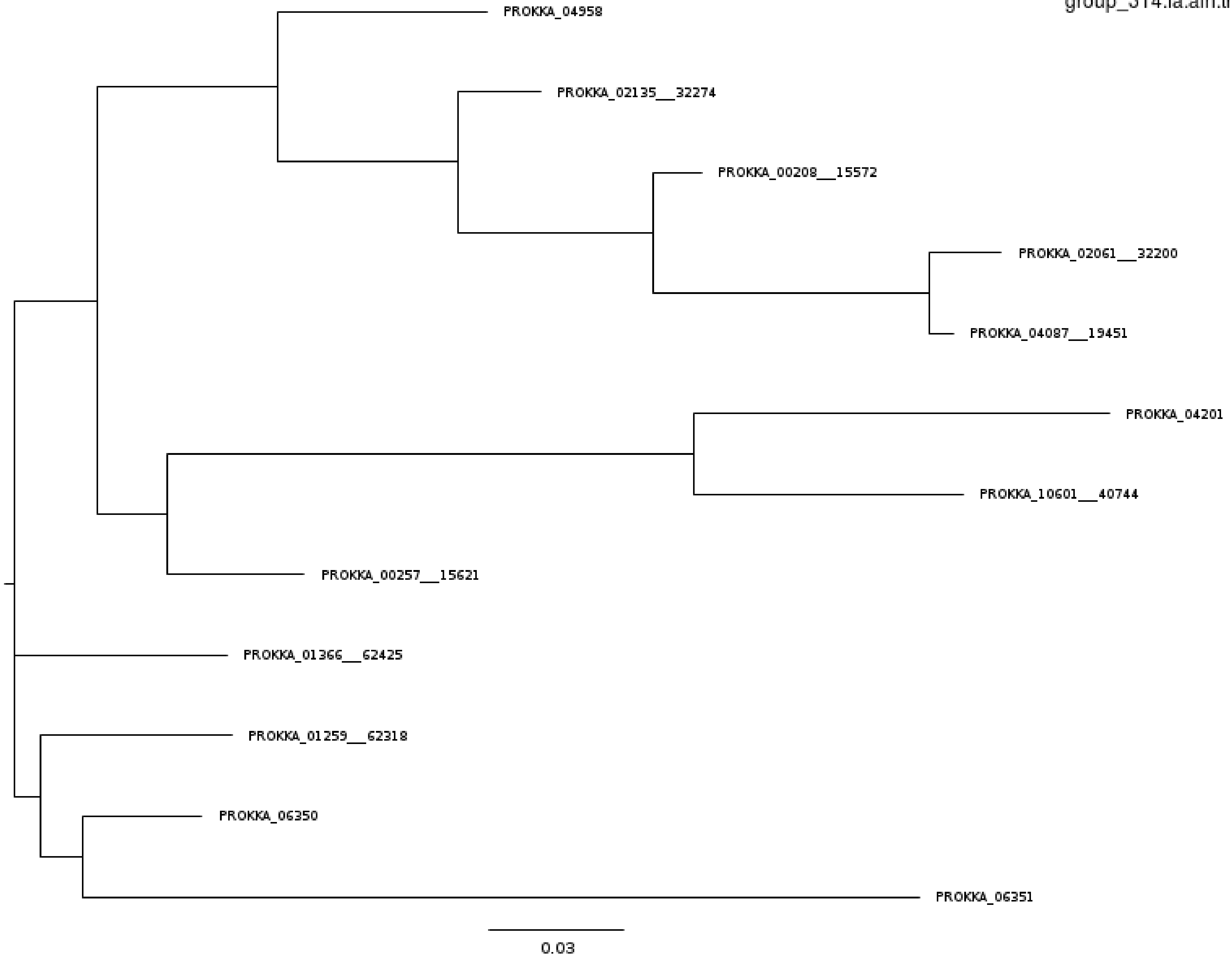

group_315.fa.aln.tree.png

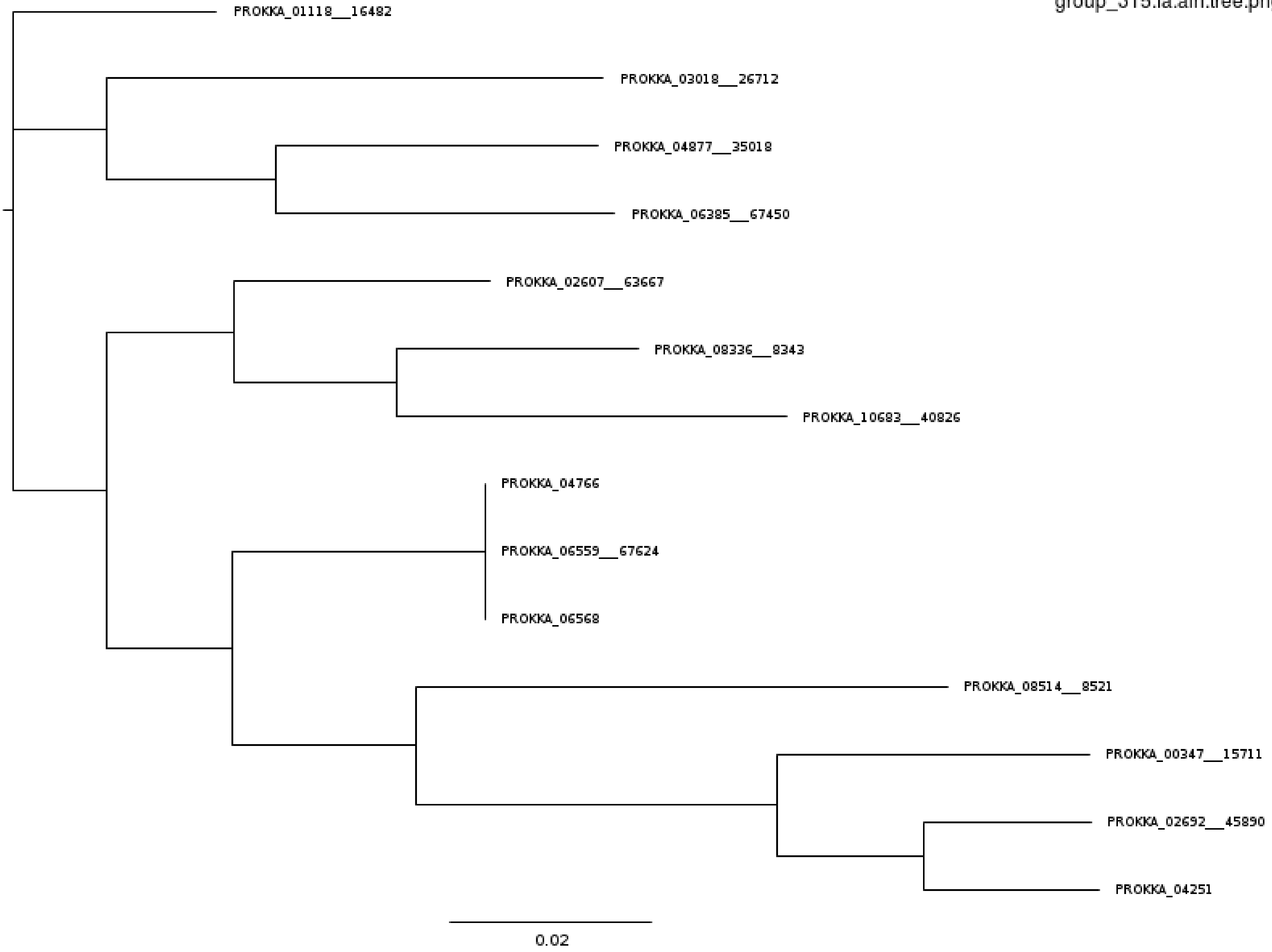

group_3197.fa.aln.tree.png

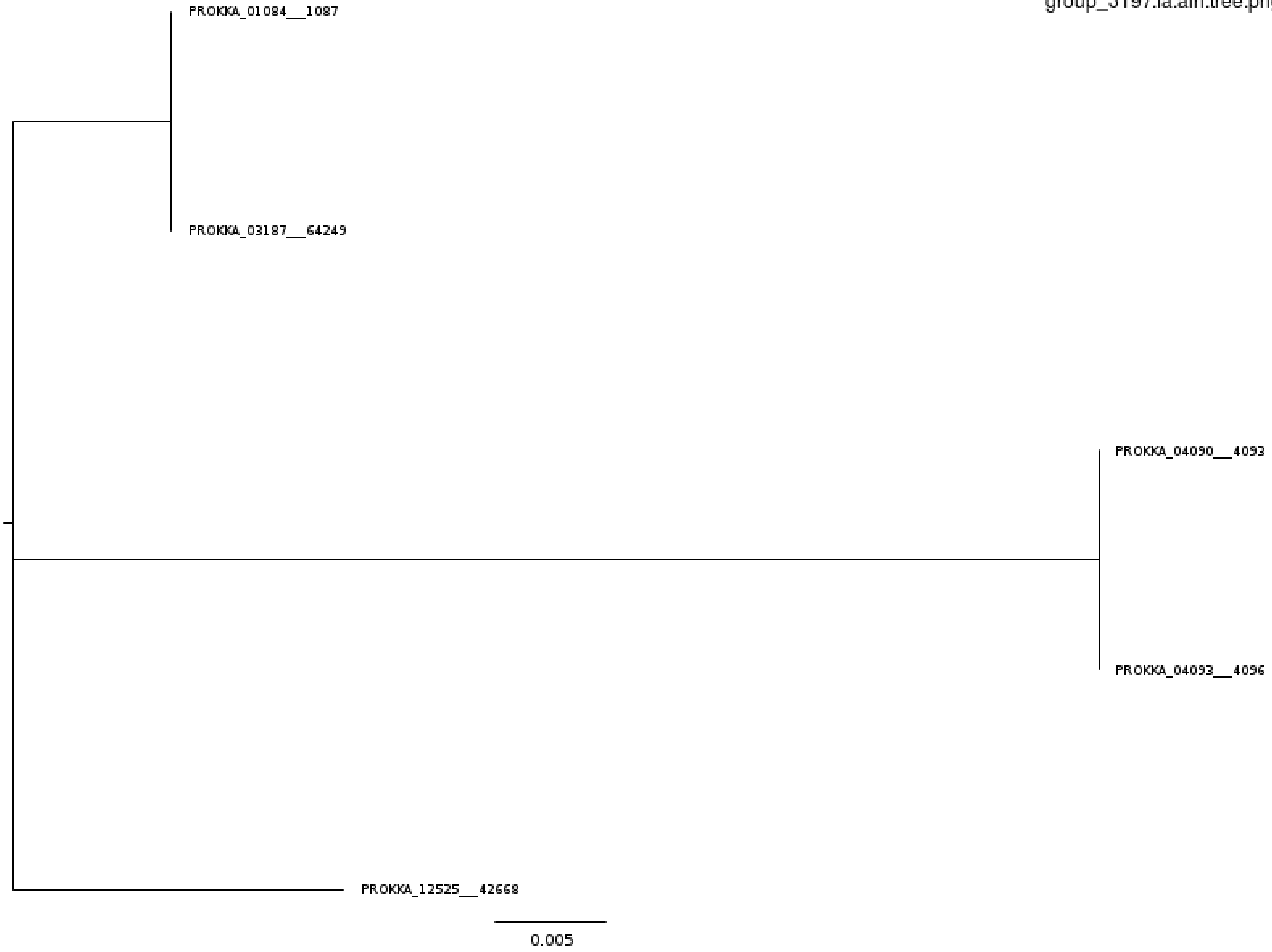

group_323.fa.aln.tree.png

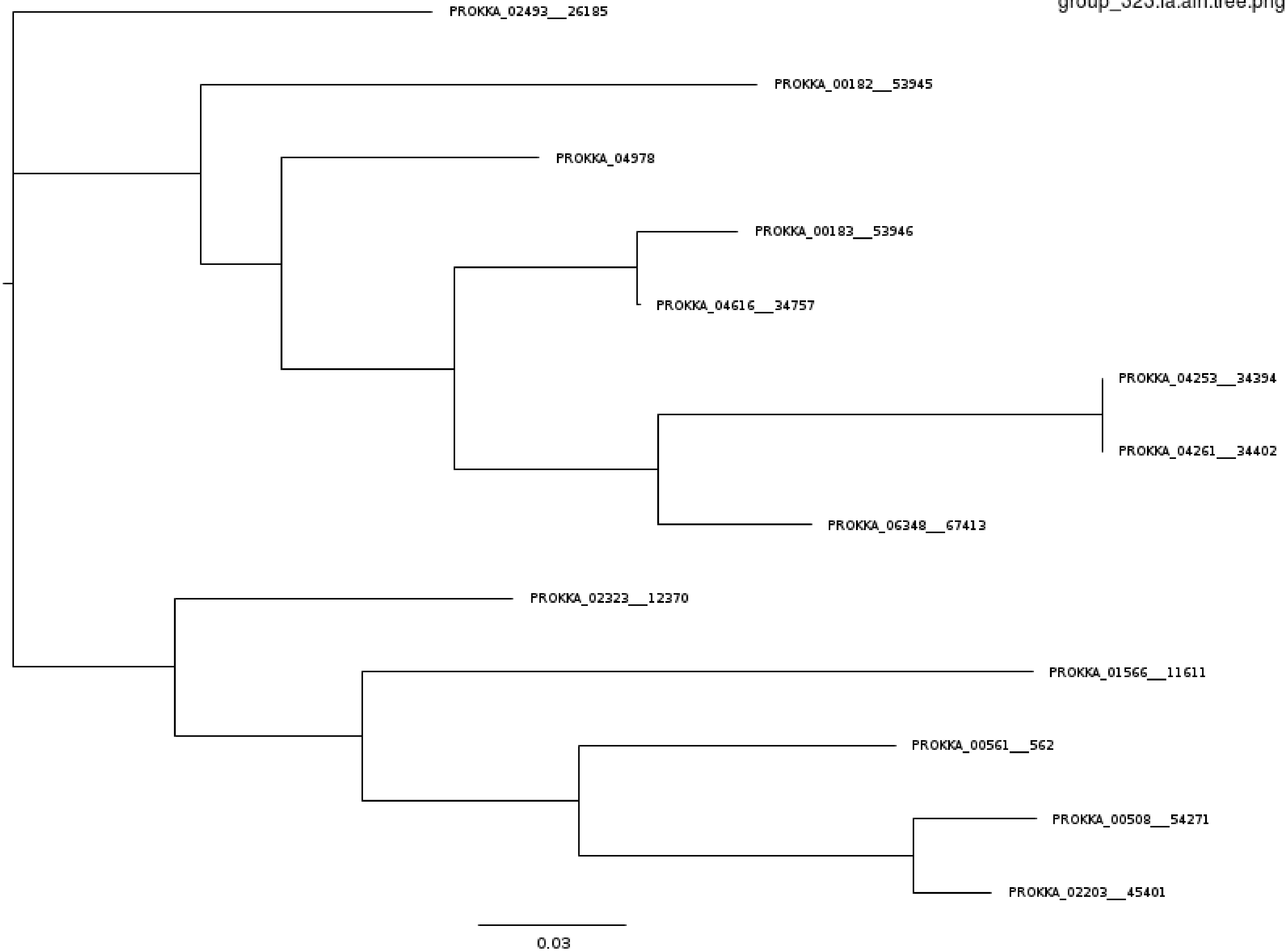

group_324.fa.aln.tree.png

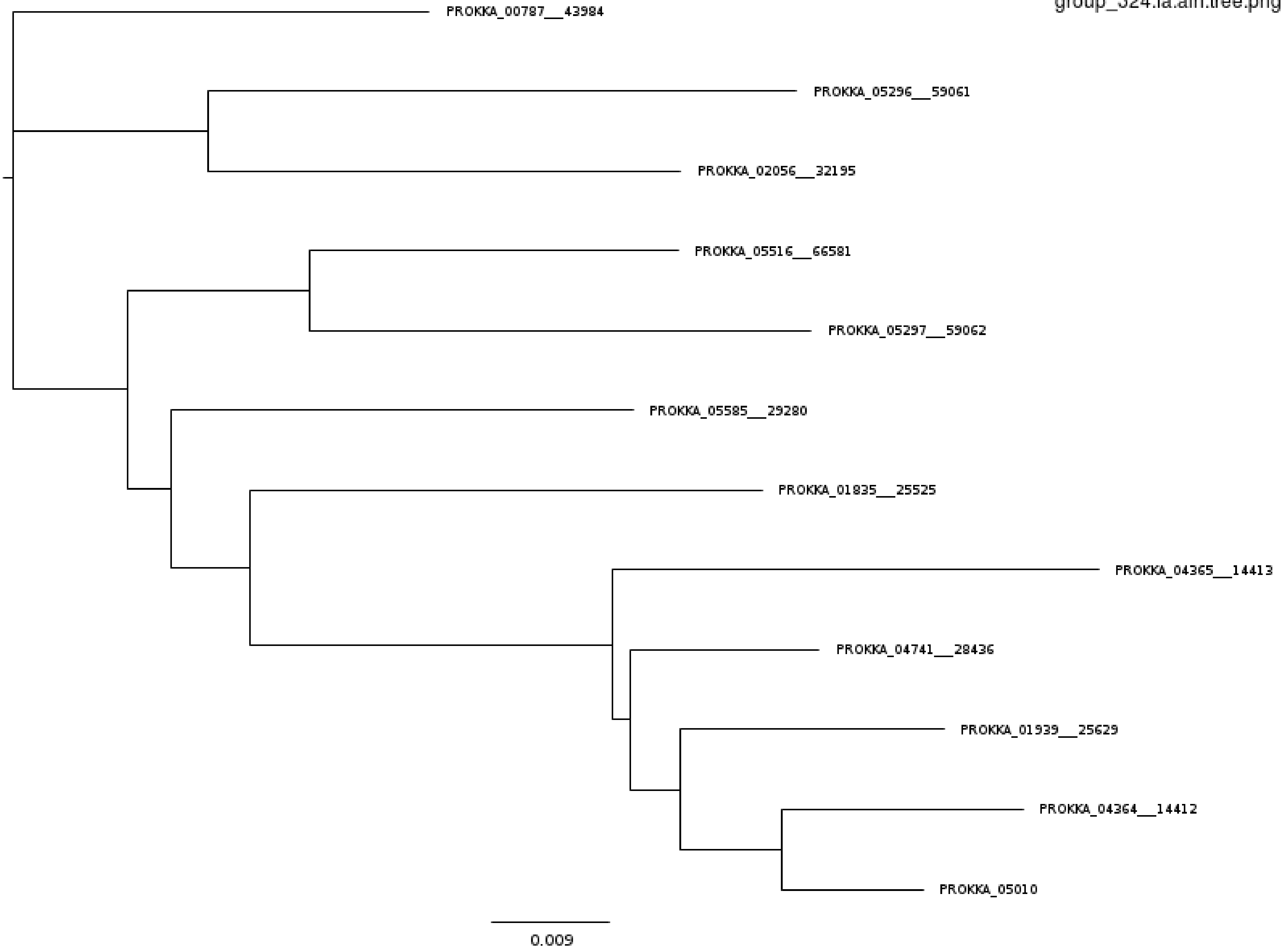

group_3255.fa.aln.tree.png

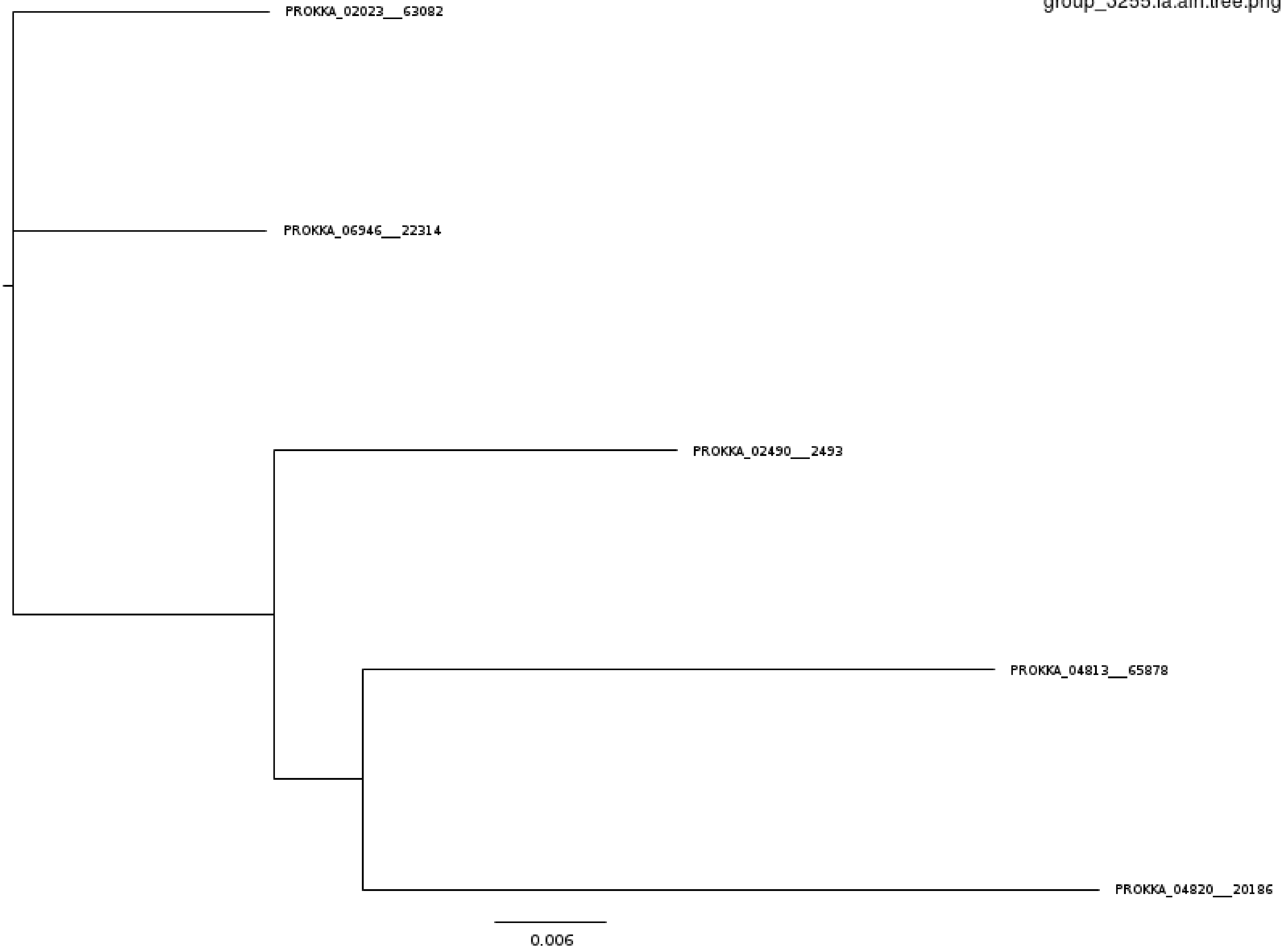

group_327.fa.aln.tree.png

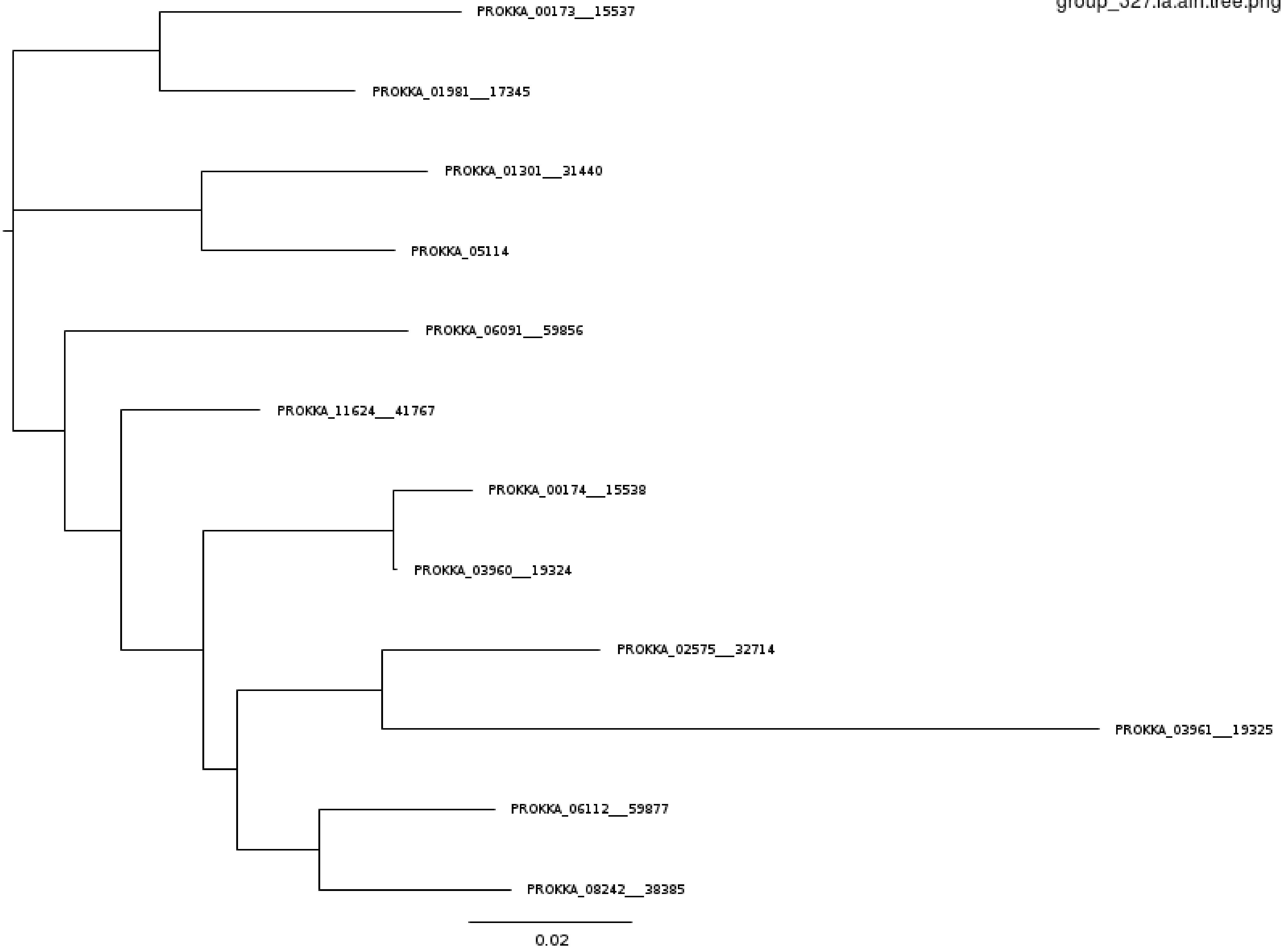

group_329.fa.aln.tree.png

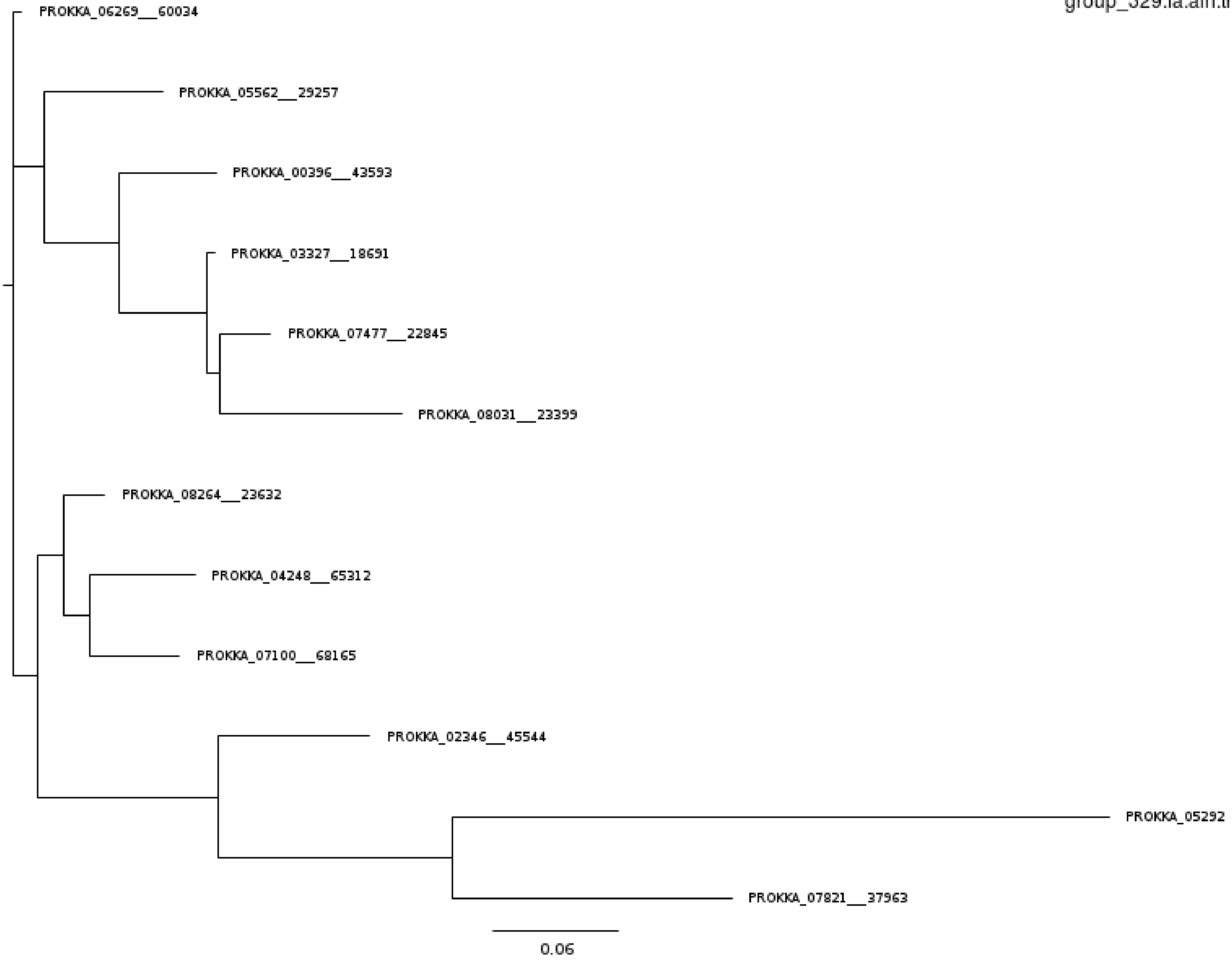

group_330.fa.aln.tree.png

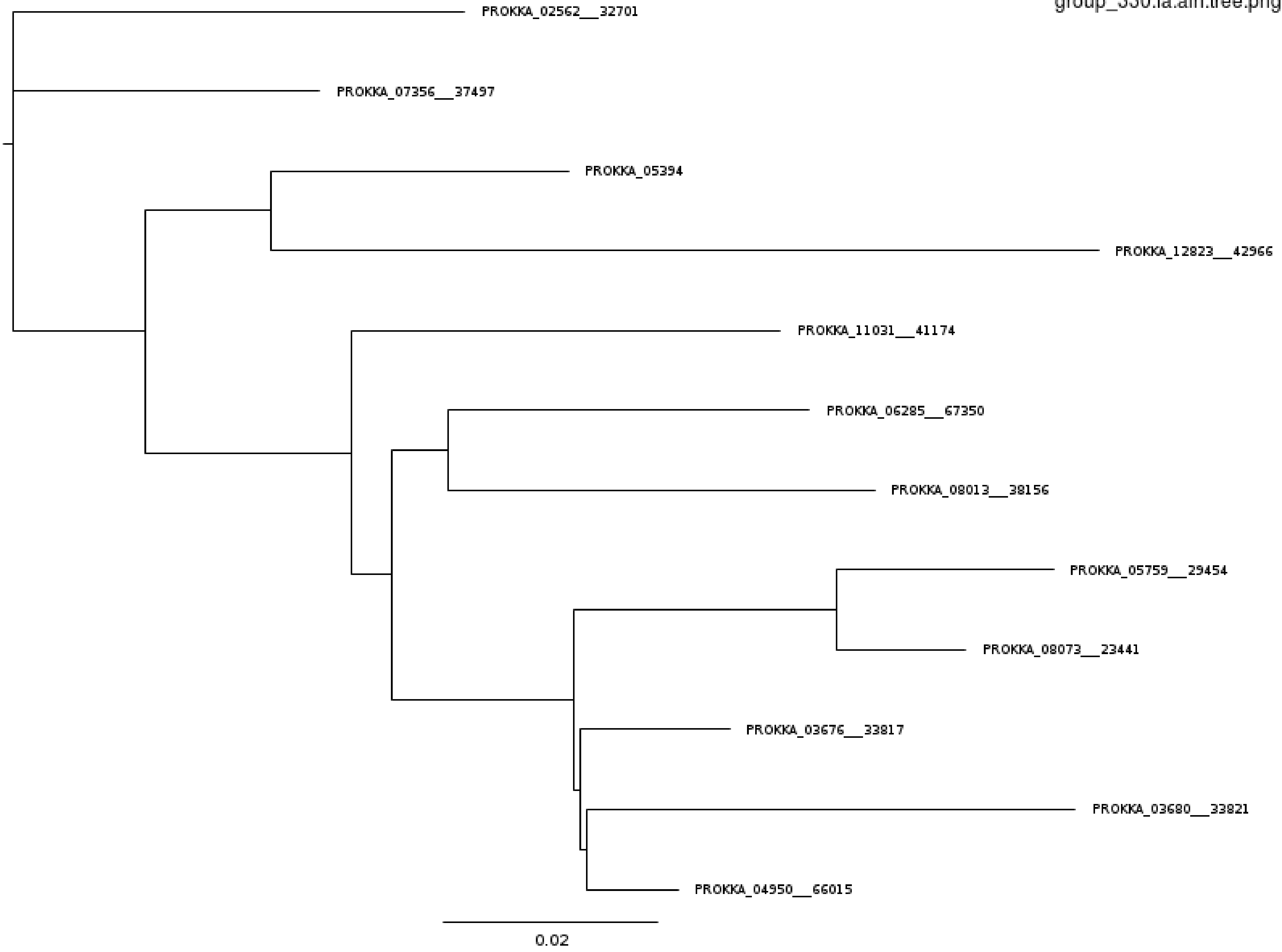

group_3320.fa.aln.tree.png

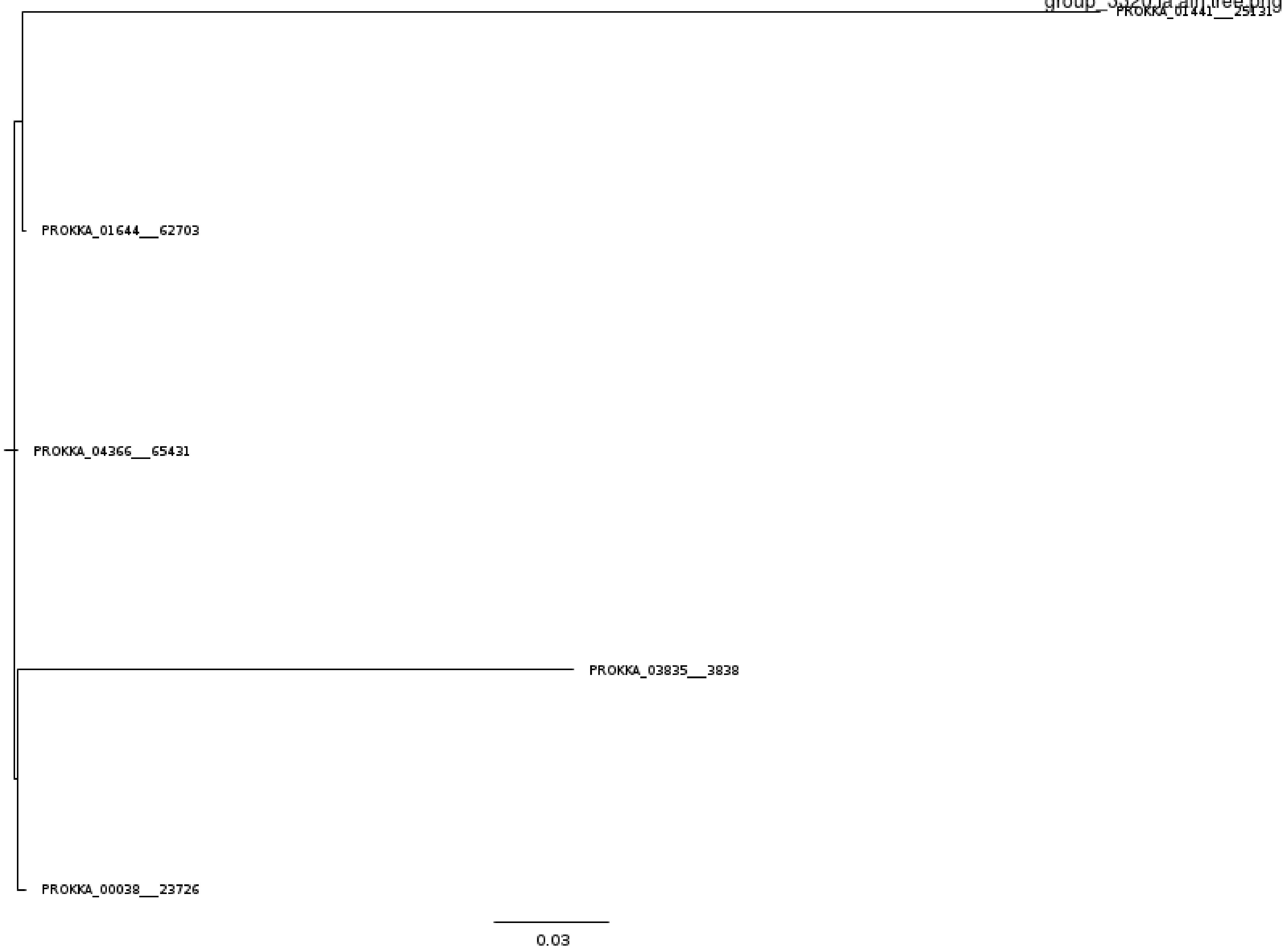

group_335.fa.aln.tree.png

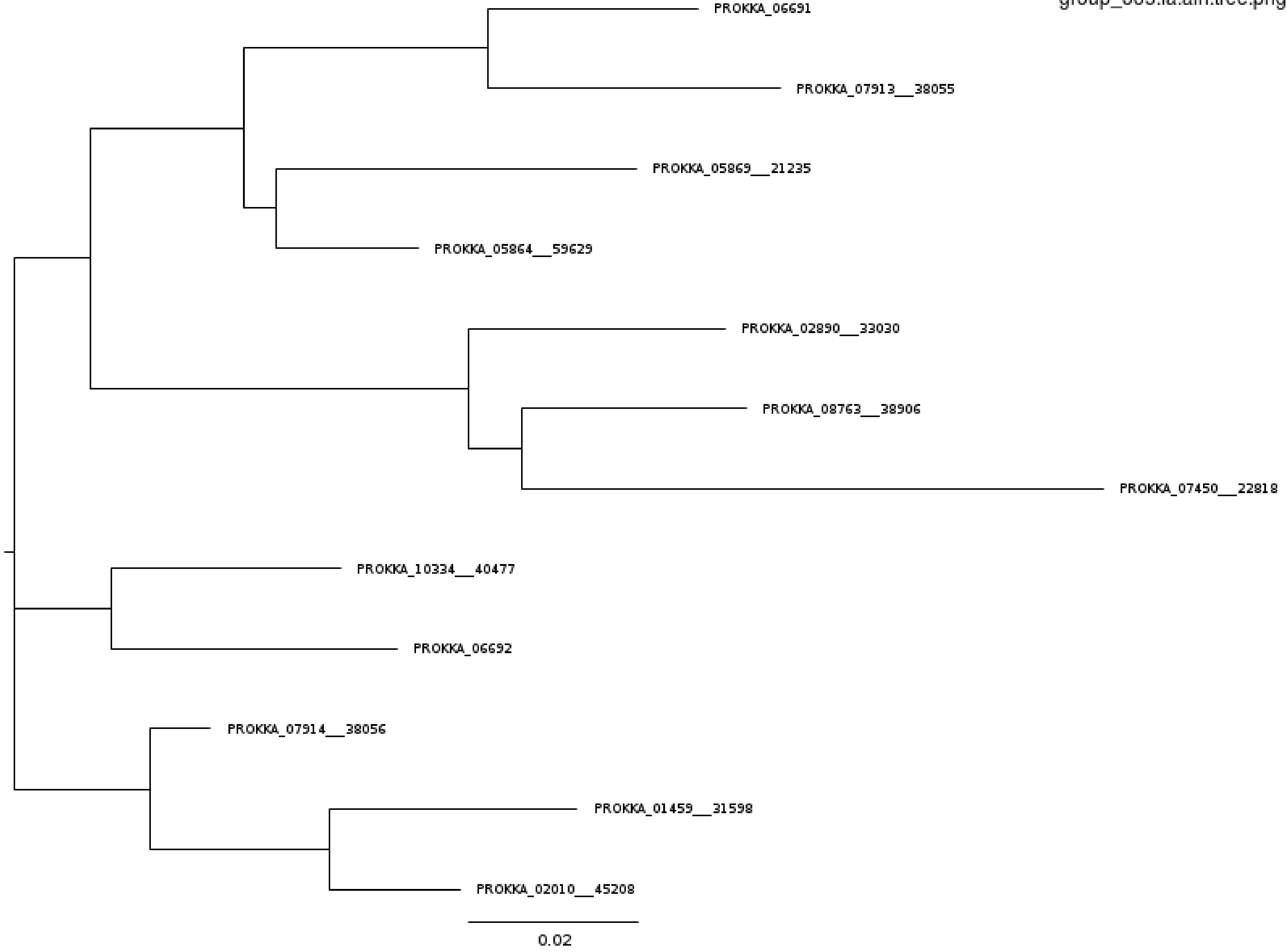

group_341.fa.aln.tree.png

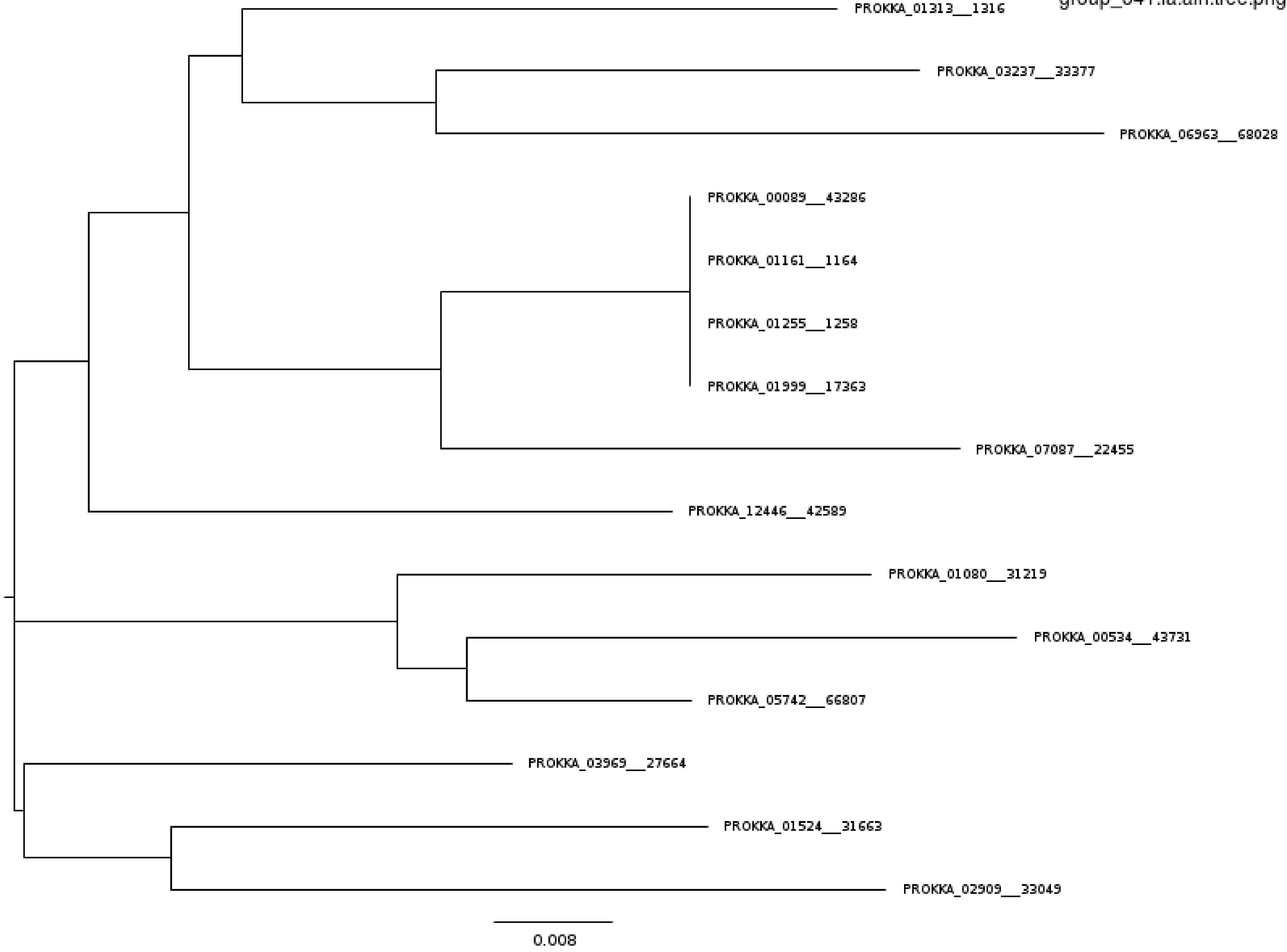

group_3430.fa.aln.tree.png

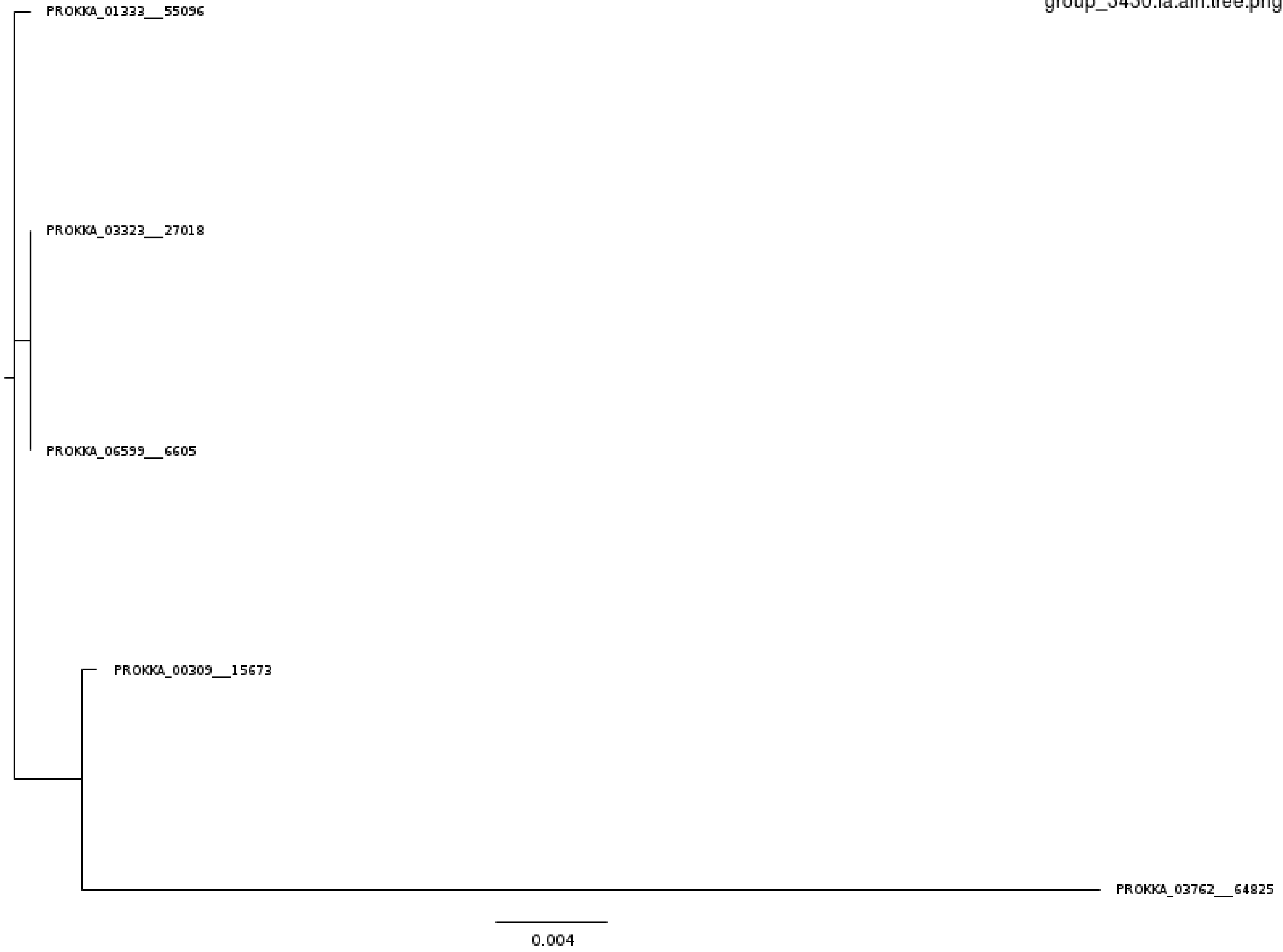

group_346.fa.aln.tree.png

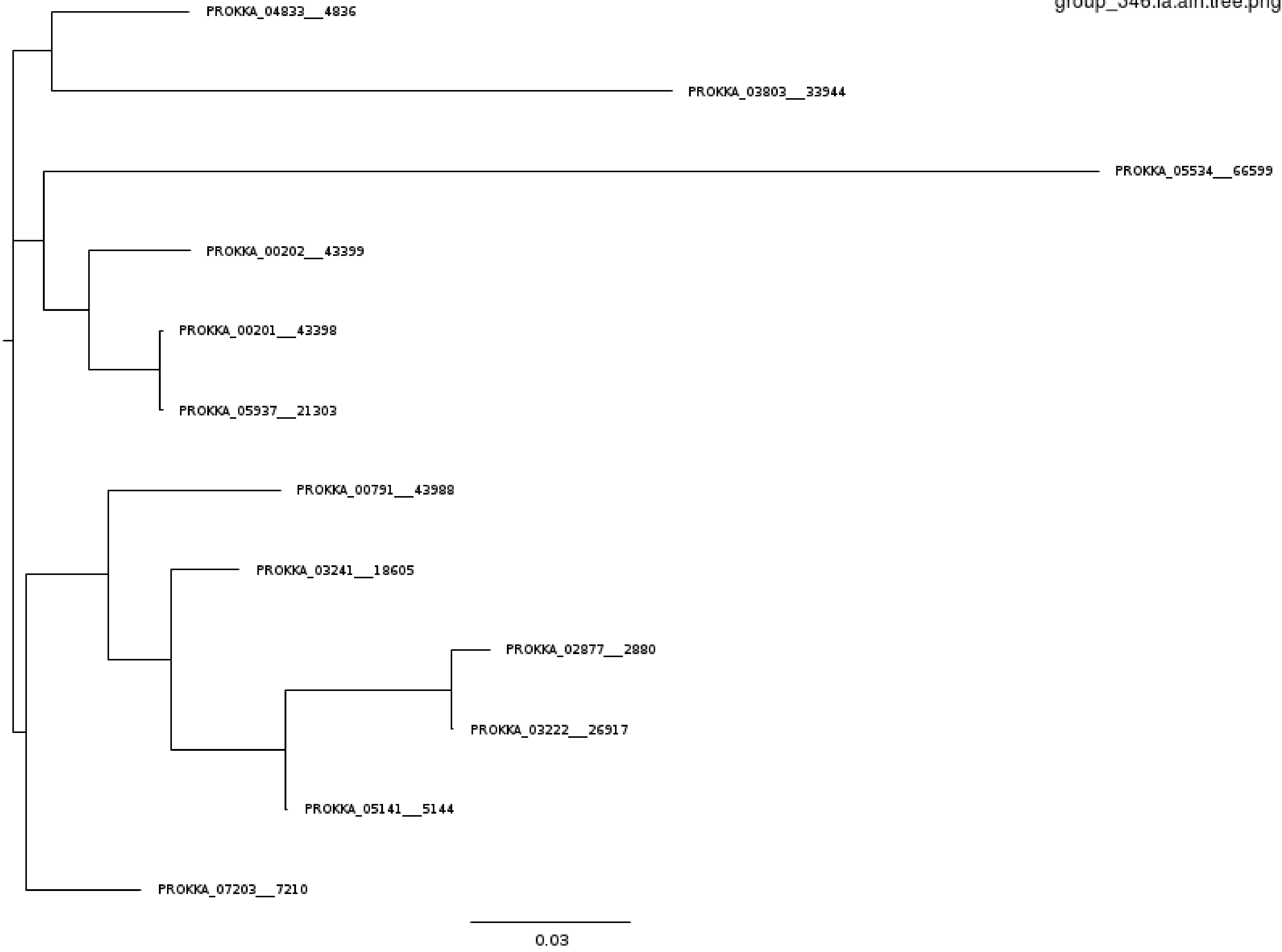

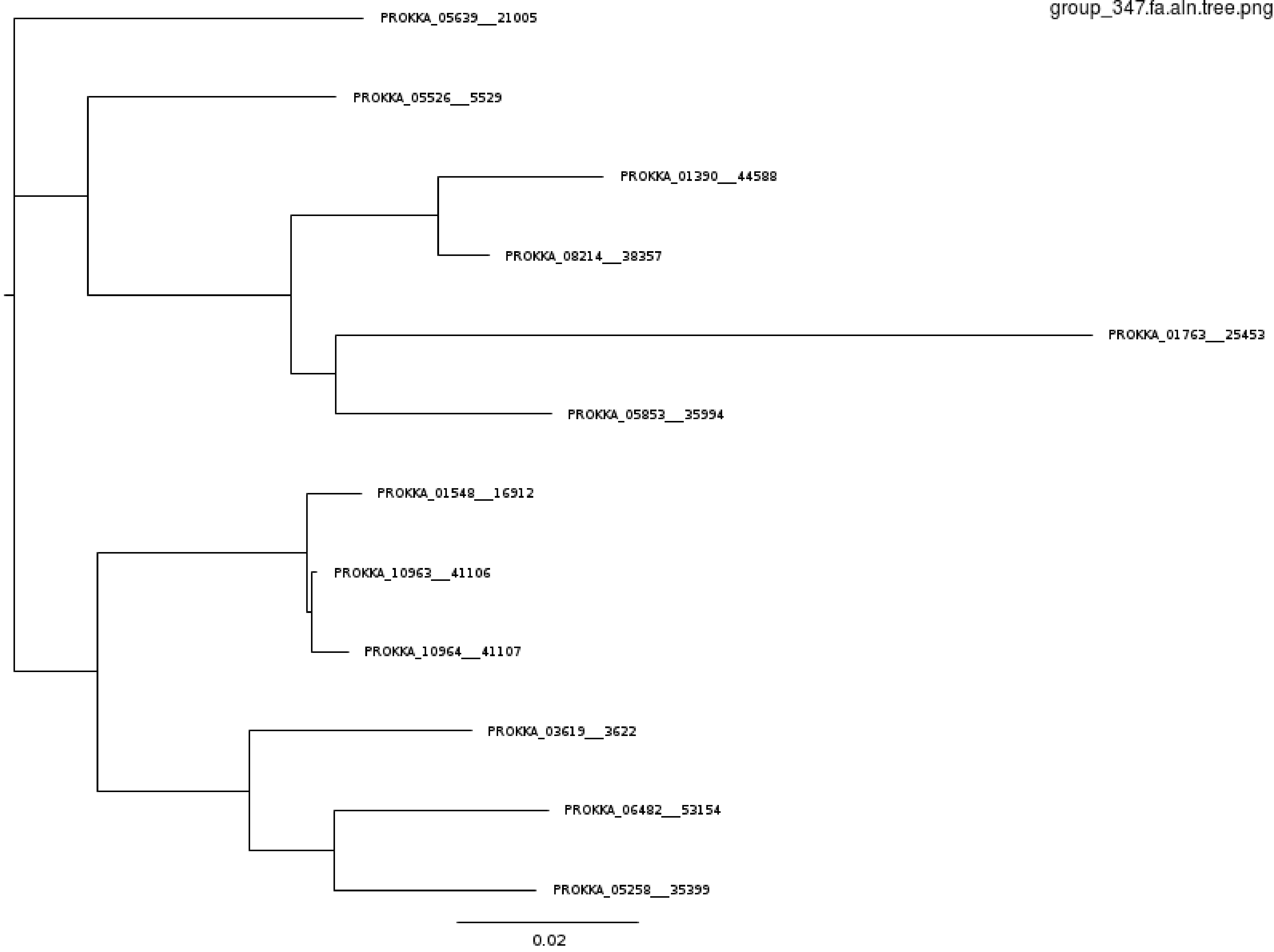
group_347.fa.aln.tree.png
PROKKA_05639___21005
PROKKA_05526___5529
PROKKA_01390___44588
PROKKA_08214___38357
PROKKA_01763___25453
PROKKA_05853___35994
PROKKA_01548___16912
PROKKA_10963___41106
PROKKA_10964___41107
PROKKA_03619___3622
PROKKA_06482___53154
PROKKA_05258___35399
0.02

group_3515.fa.aln.tree.png

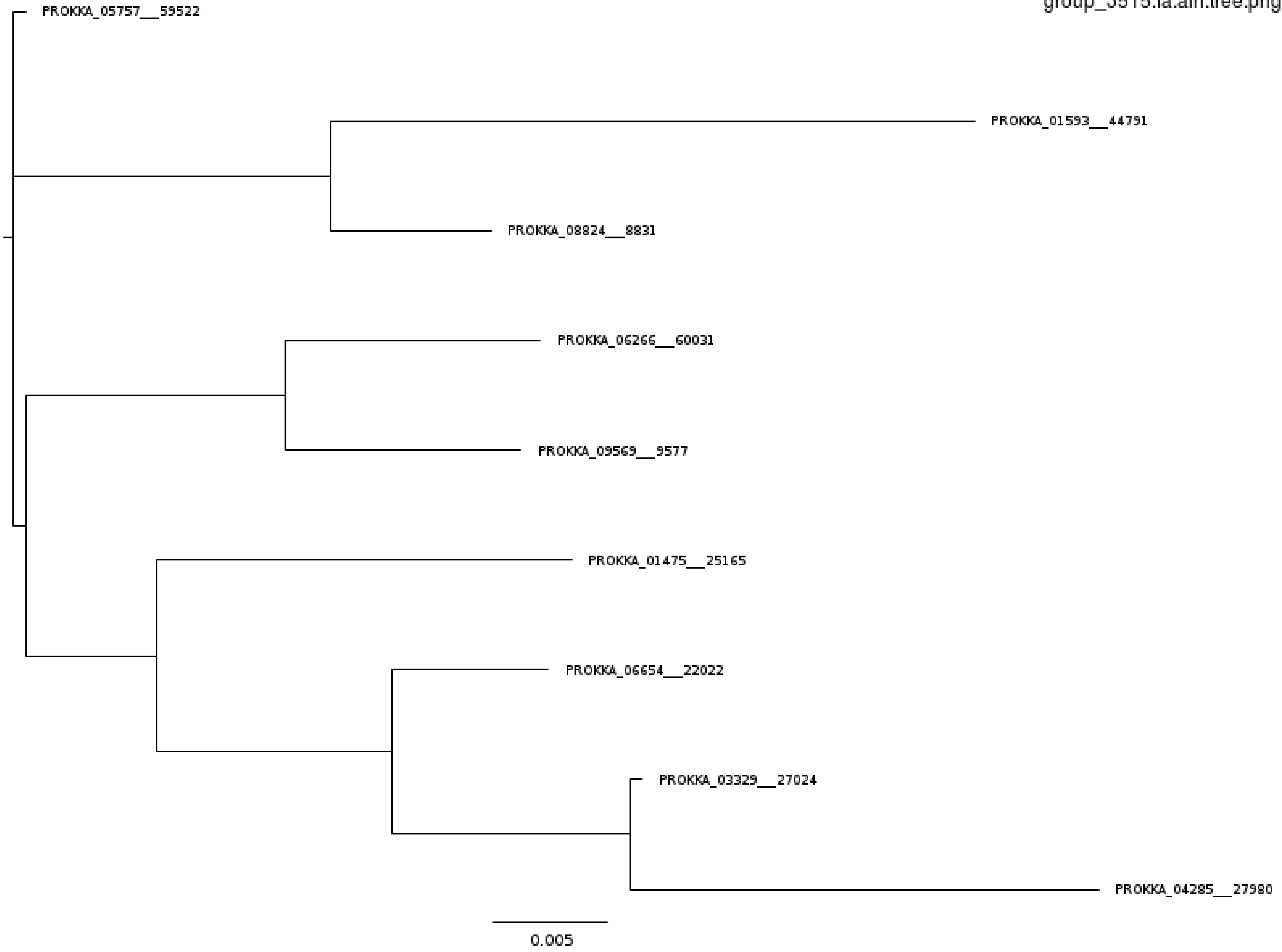

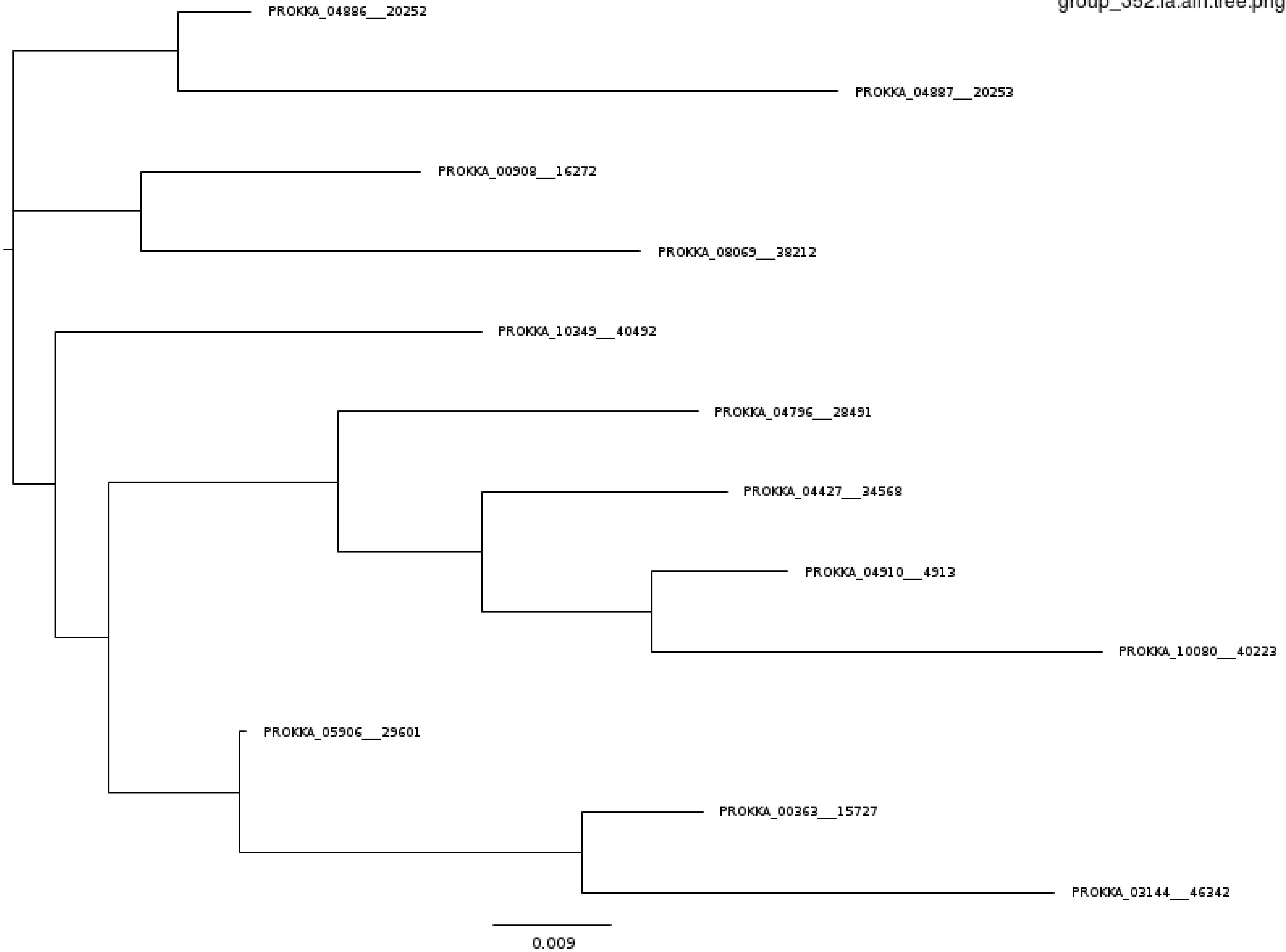
PROKKA_04886___20252
PROKKA_04887___20253
PROKKA_00908___16272
PROKKA_08069___38212
PROKKA_10349___40492
PROKKA_04796___28491
PROKKA_04427___34568
PROKKA_04910___4913
PROKKA_10080___40223
PROKKA_05906___29601
PROKKA_00363___15727
PROKKA_03144___46342
0.009

group_357.fa.aln.tree.png

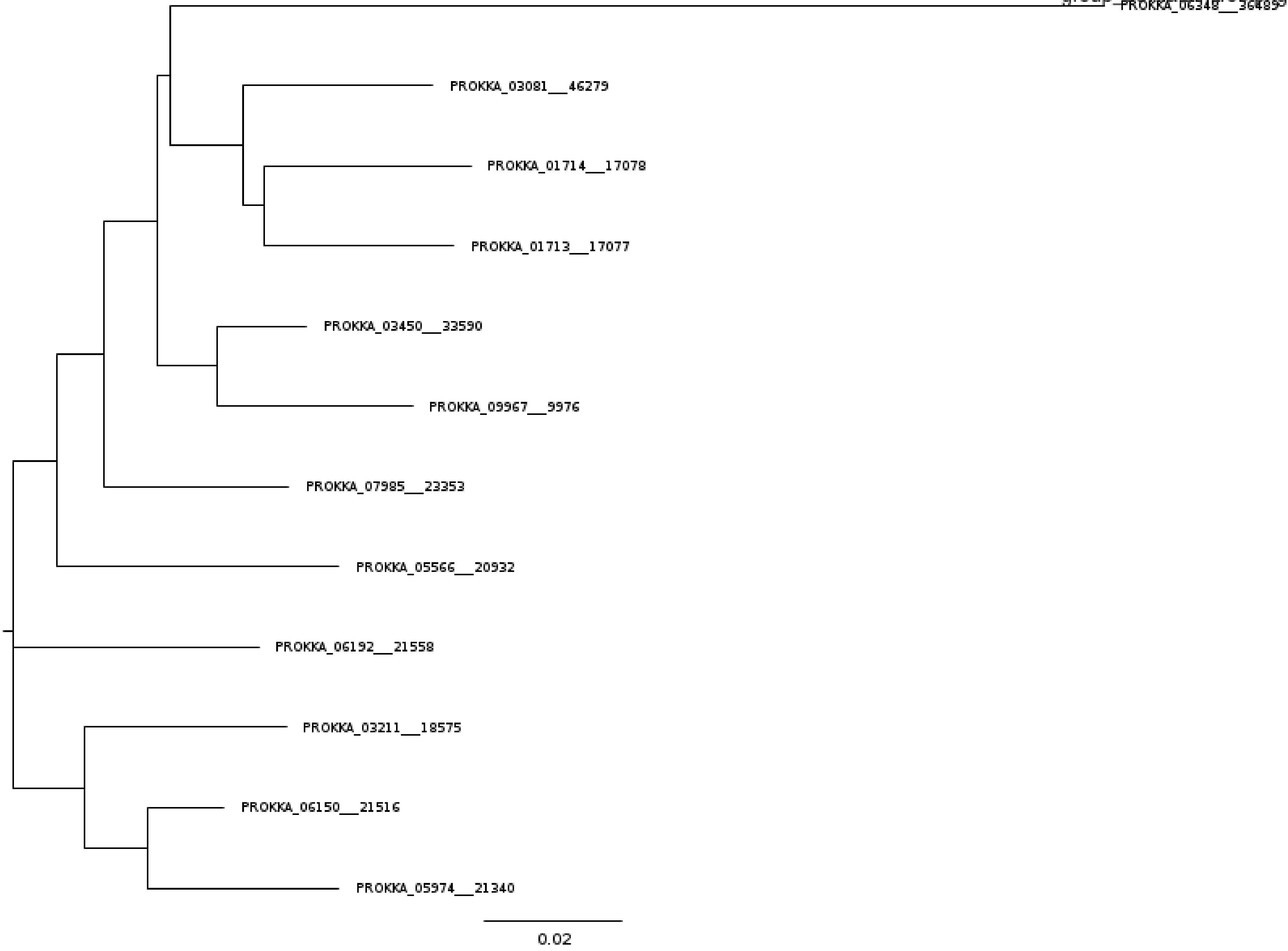

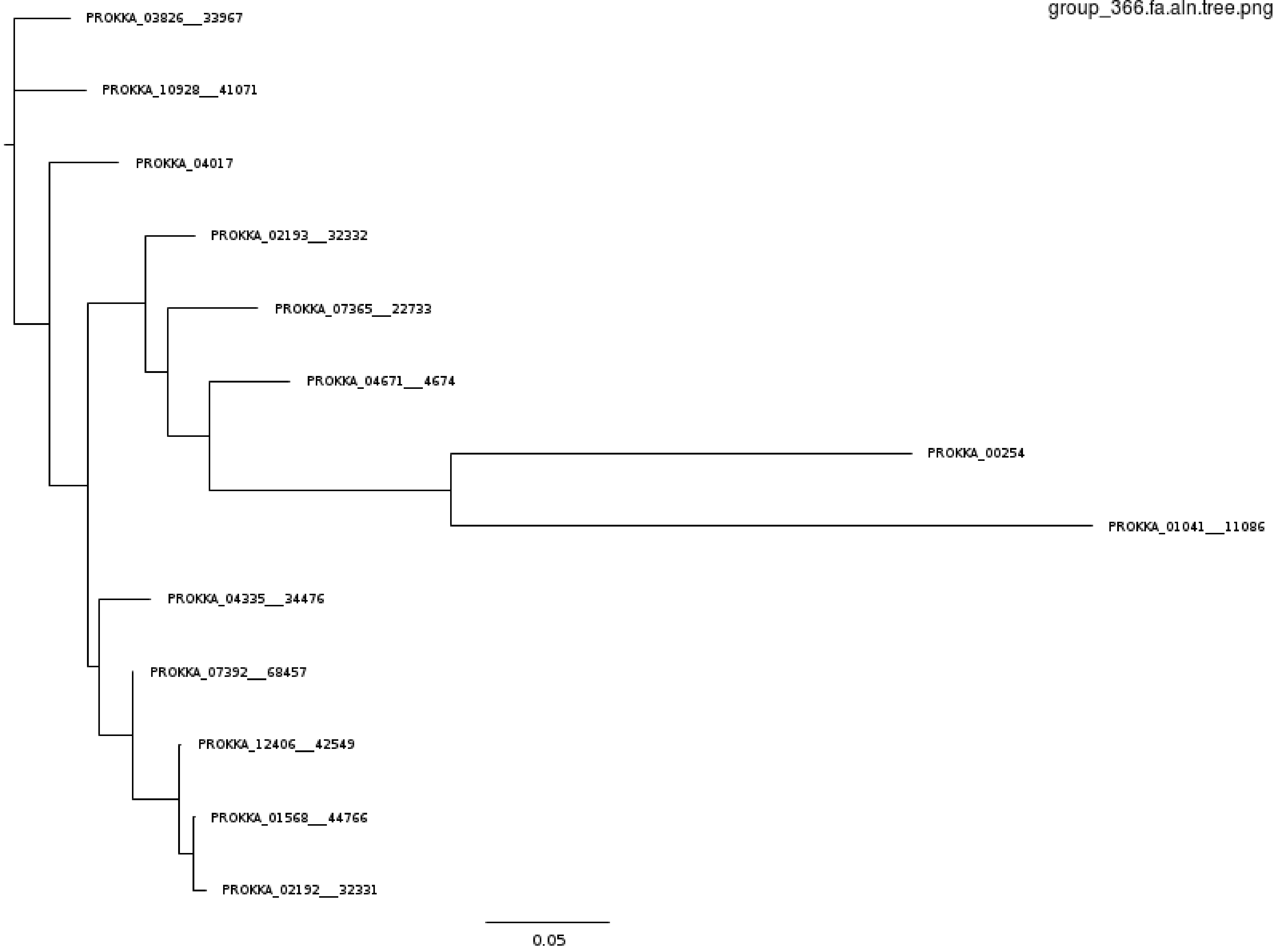
group_366.fa.aln.tree.png
PROKKA_03826___33967
PROKKA_10928___41071
PROKKA_04017
PROKKA_02193___32332
PROKKA_07365___22733
PROKKA_04671___4674
PROKKA_00254
PROKKA_01041___11086
PROKKA_04335___34476
PROKKA_07392___68457
PROKKA_12406___42549
PROKKA_01568___44766
PROKKA_02192___32331
0.05

group_372.fa.aln.tree.png

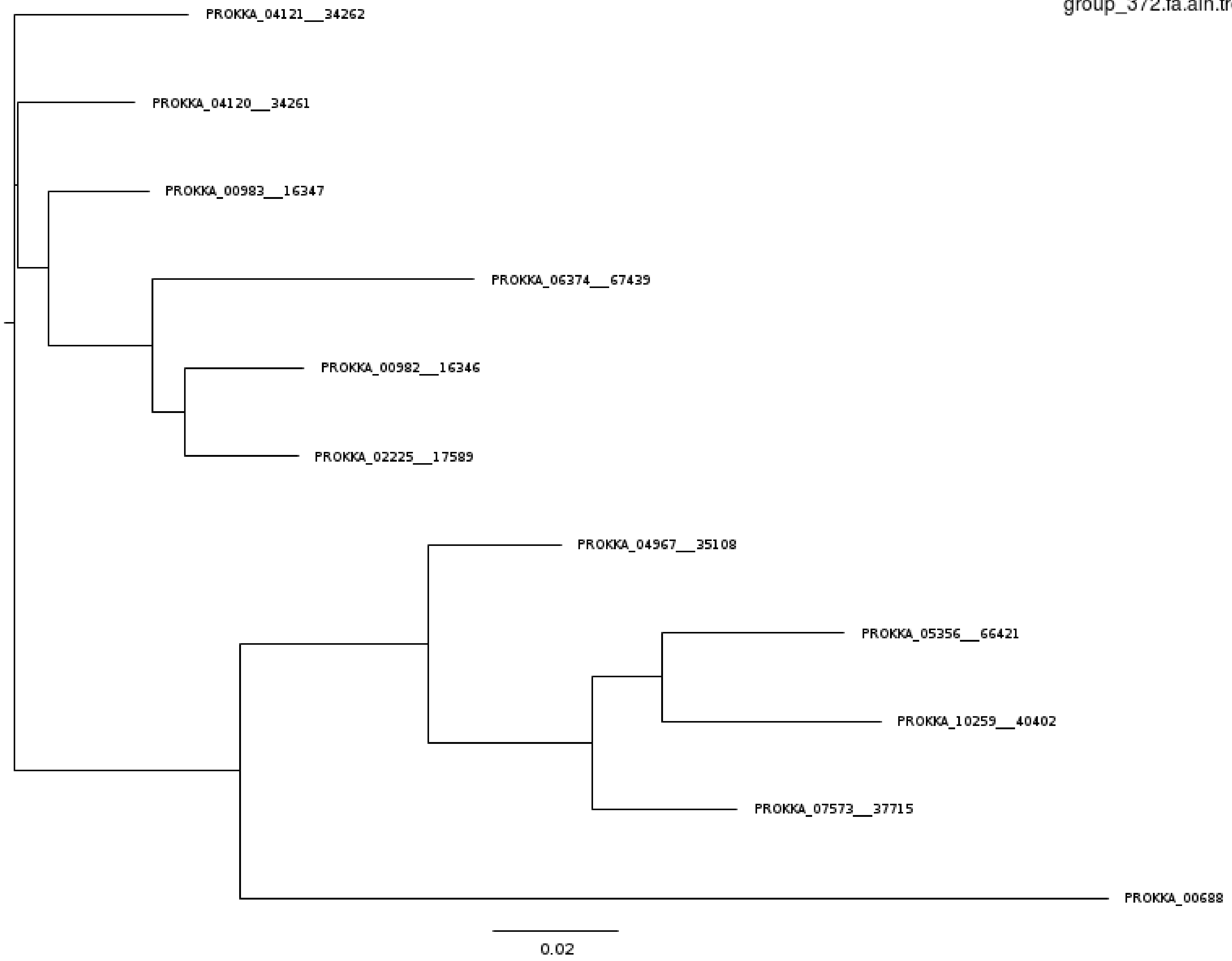

group_3856.fa.aln.tree.png

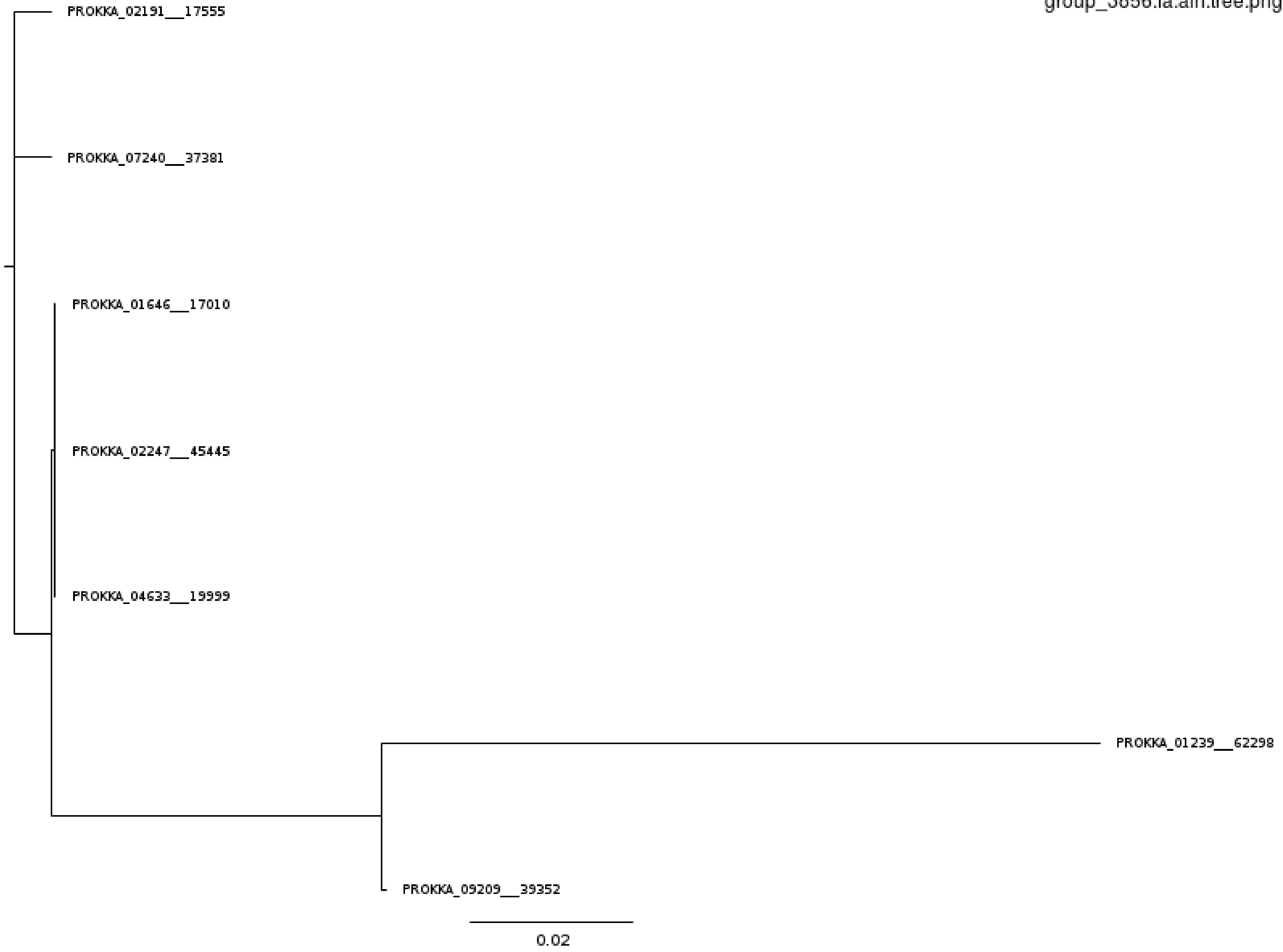

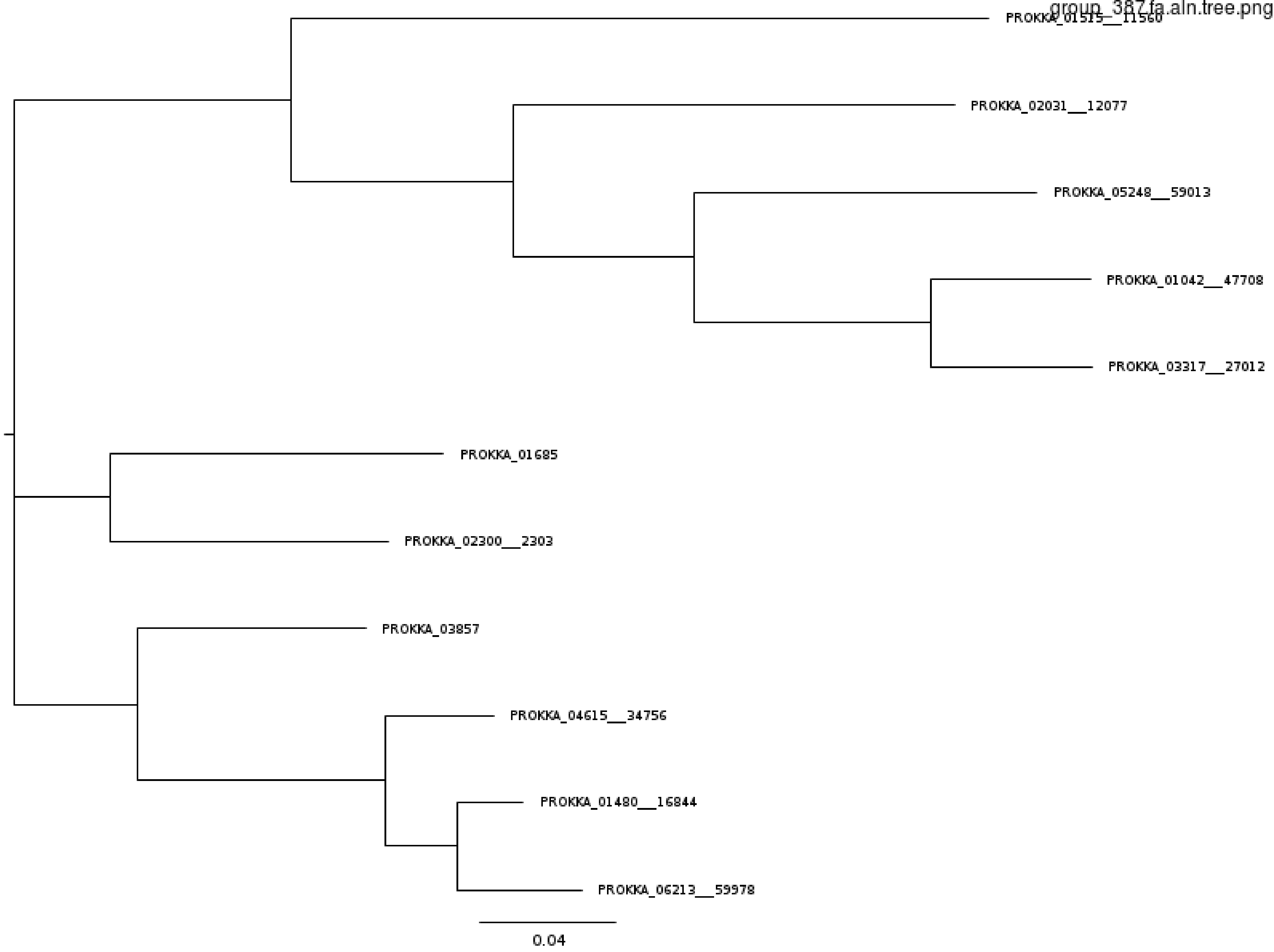
group_387.fa.aln.tree.png
PROKKA_01515___11560
PROKKA_02031___12077
PROKKA_05248___59013
PROKKA_01042___47708
PROKKA_03317___27012
PROKKA_01685
PROKKA_02300___2303
PROKKA_03857
PROKKA_04615___34756
PROKKA_01480___16844
PROKKA_06213___59978
0.04

group_389.fa.aln.tree.png

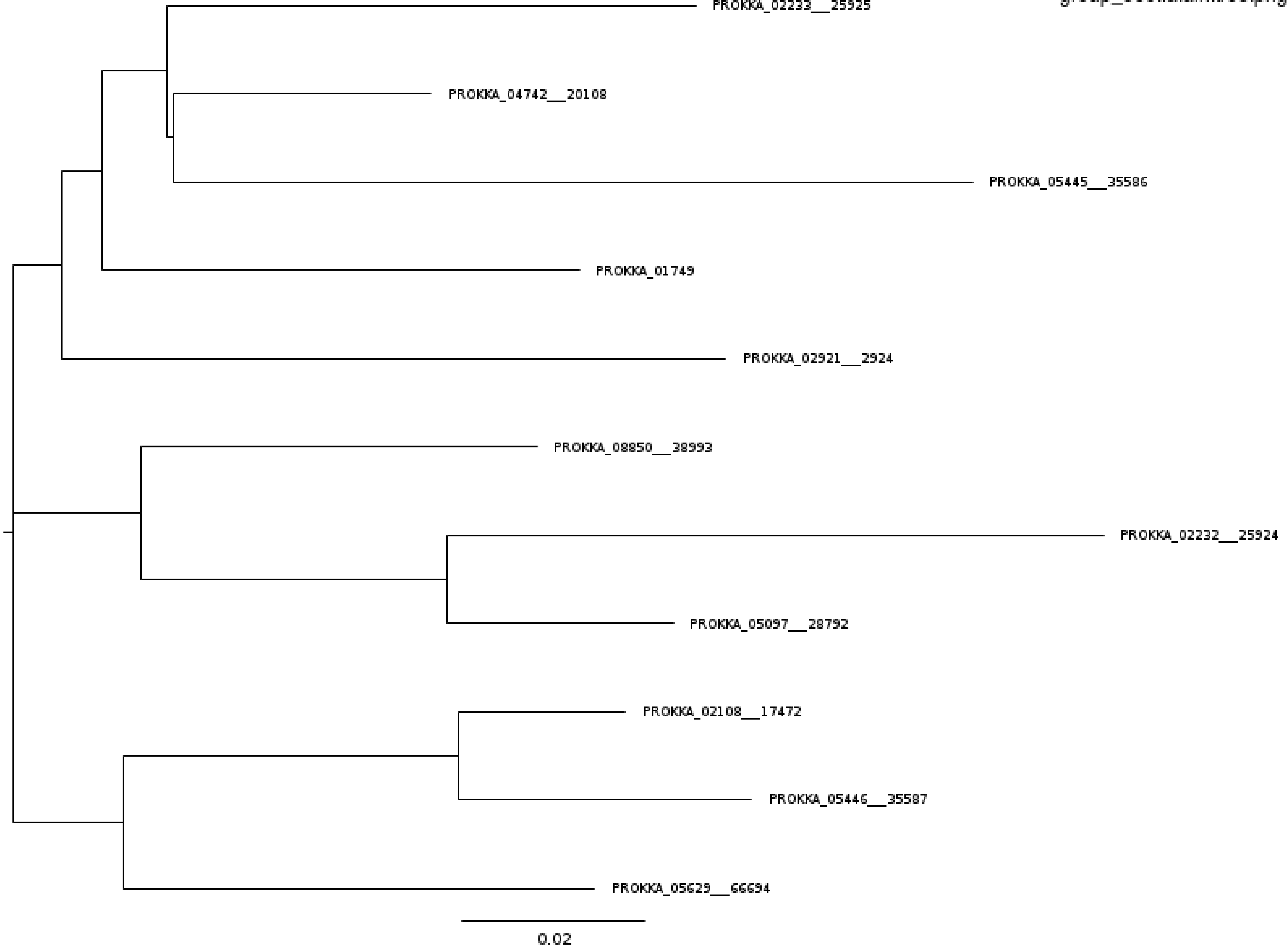

group_3928.fa.aln.tree.png

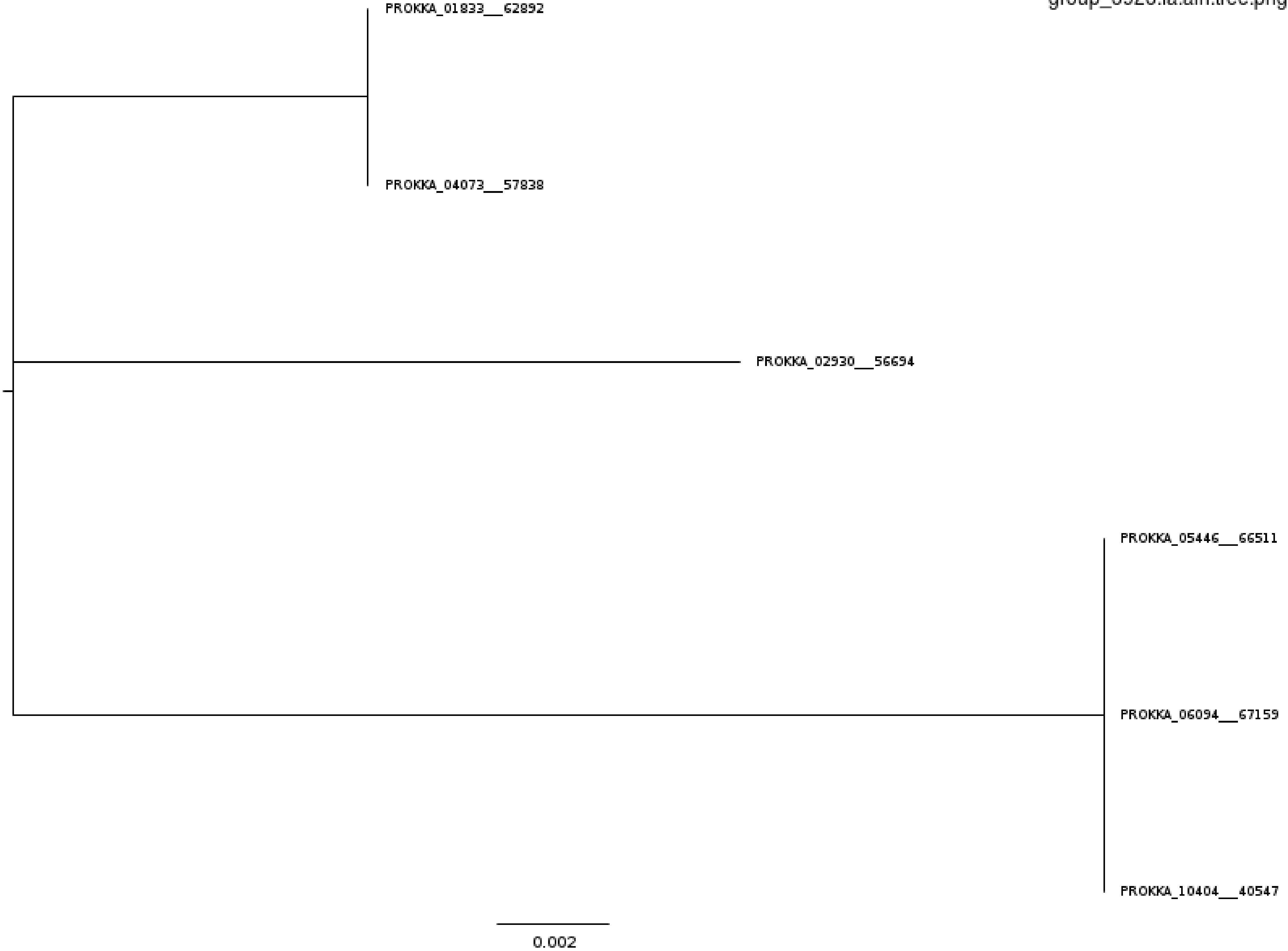

group_393.fa.aln.tree.png

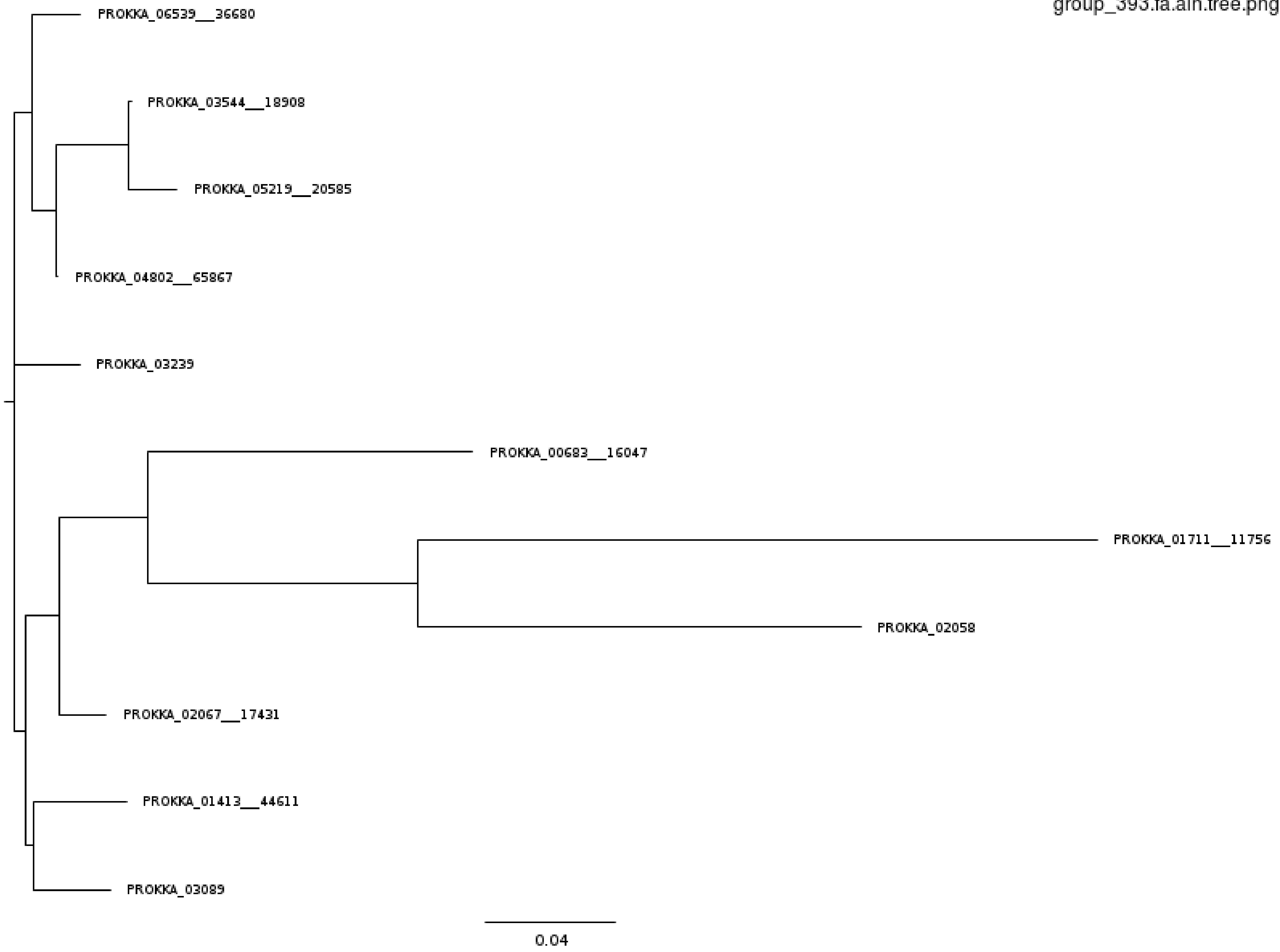

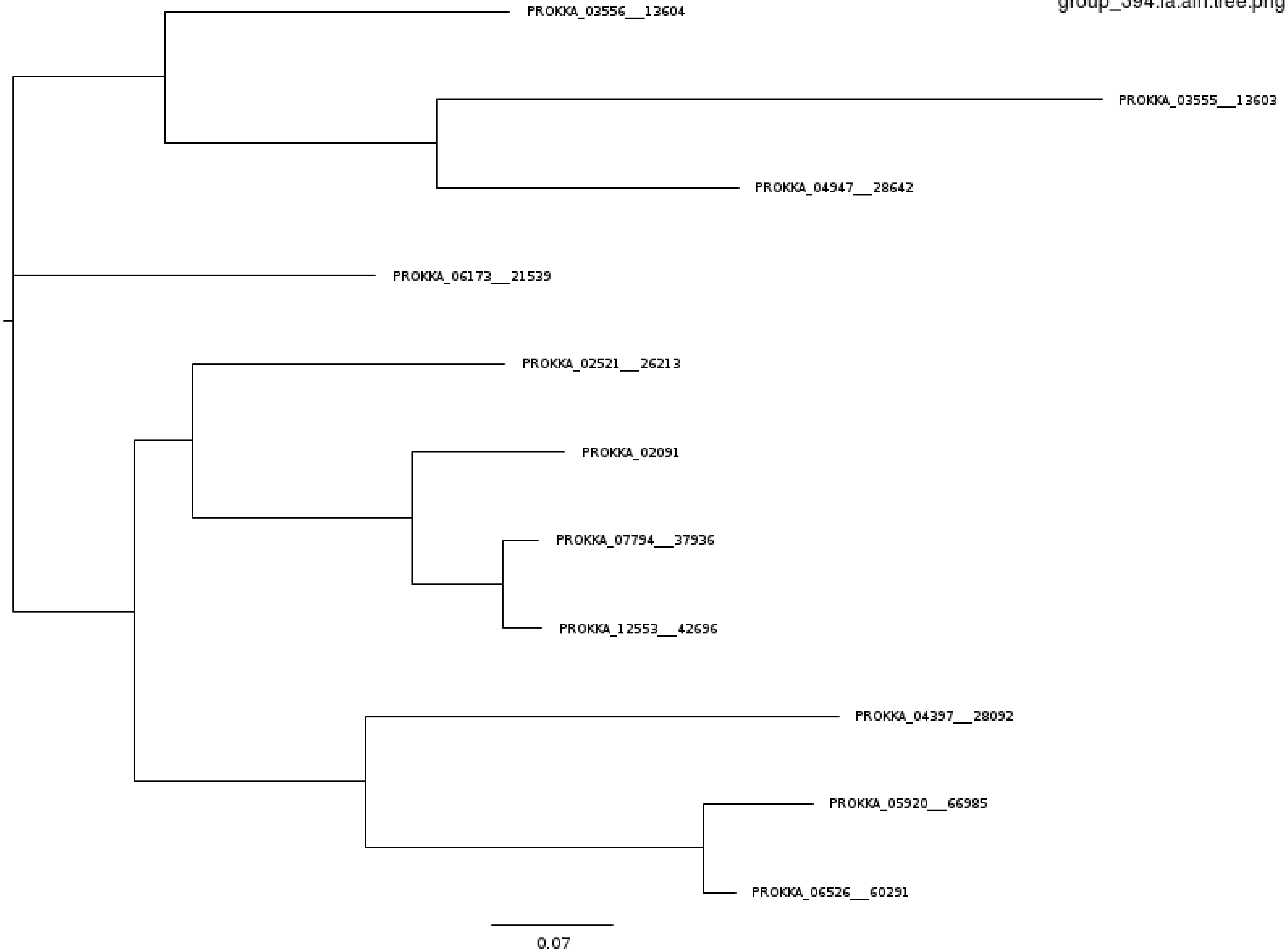
PROKKA_03556___13604
PROKKA_03555___13603
PROKKA_04947___28642
PROKKA_06173___21539
PROKKA_02521___26213
PROKKA_02091
PROKKA_07794___37936
PROKKA_12553___42696
PROKKA_04397___28092
PROKKA_05920___66985
PROKKA_06526___60291
0.07

group_395.fa.aln.tree.png

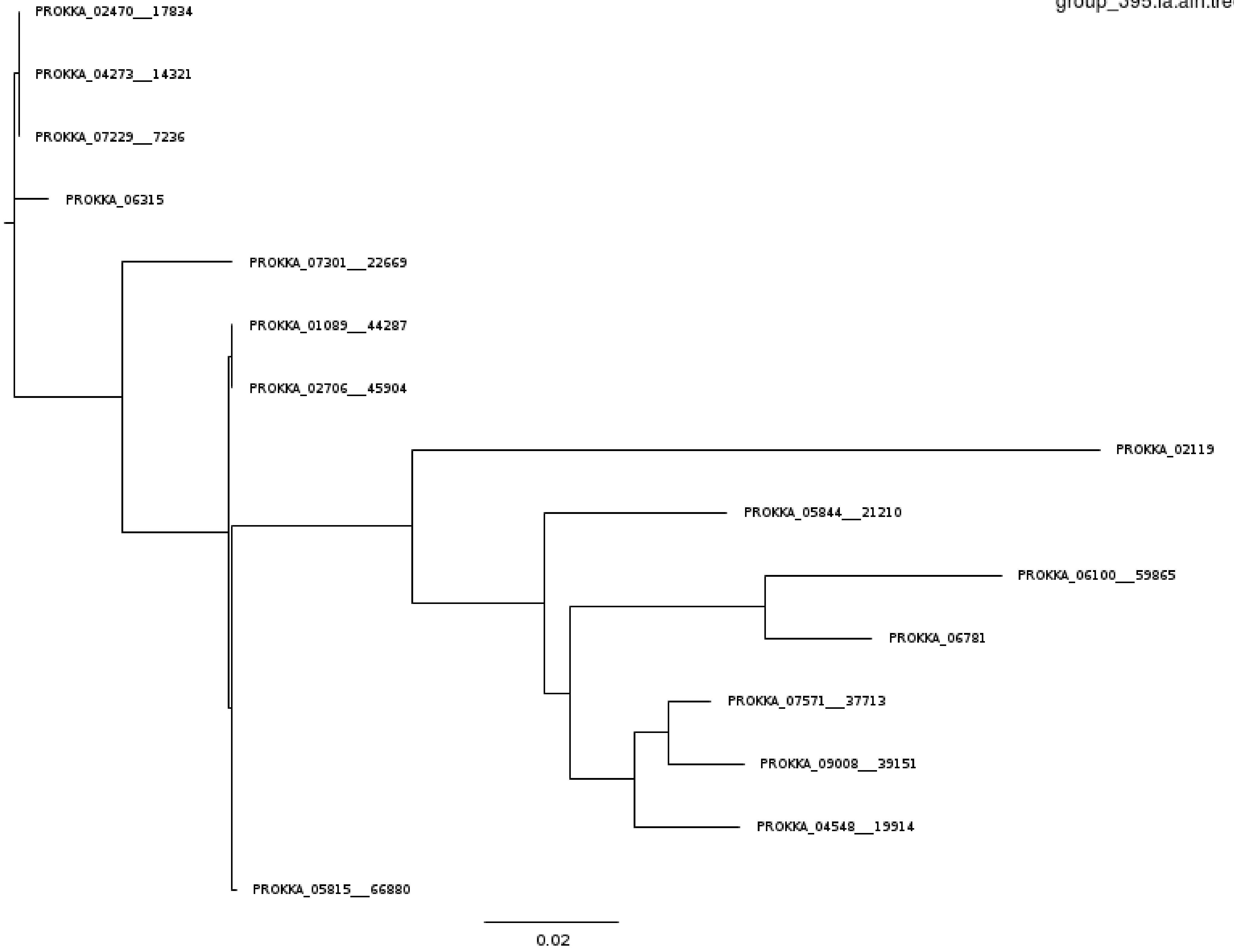

group_396.fa.aln.tree.png

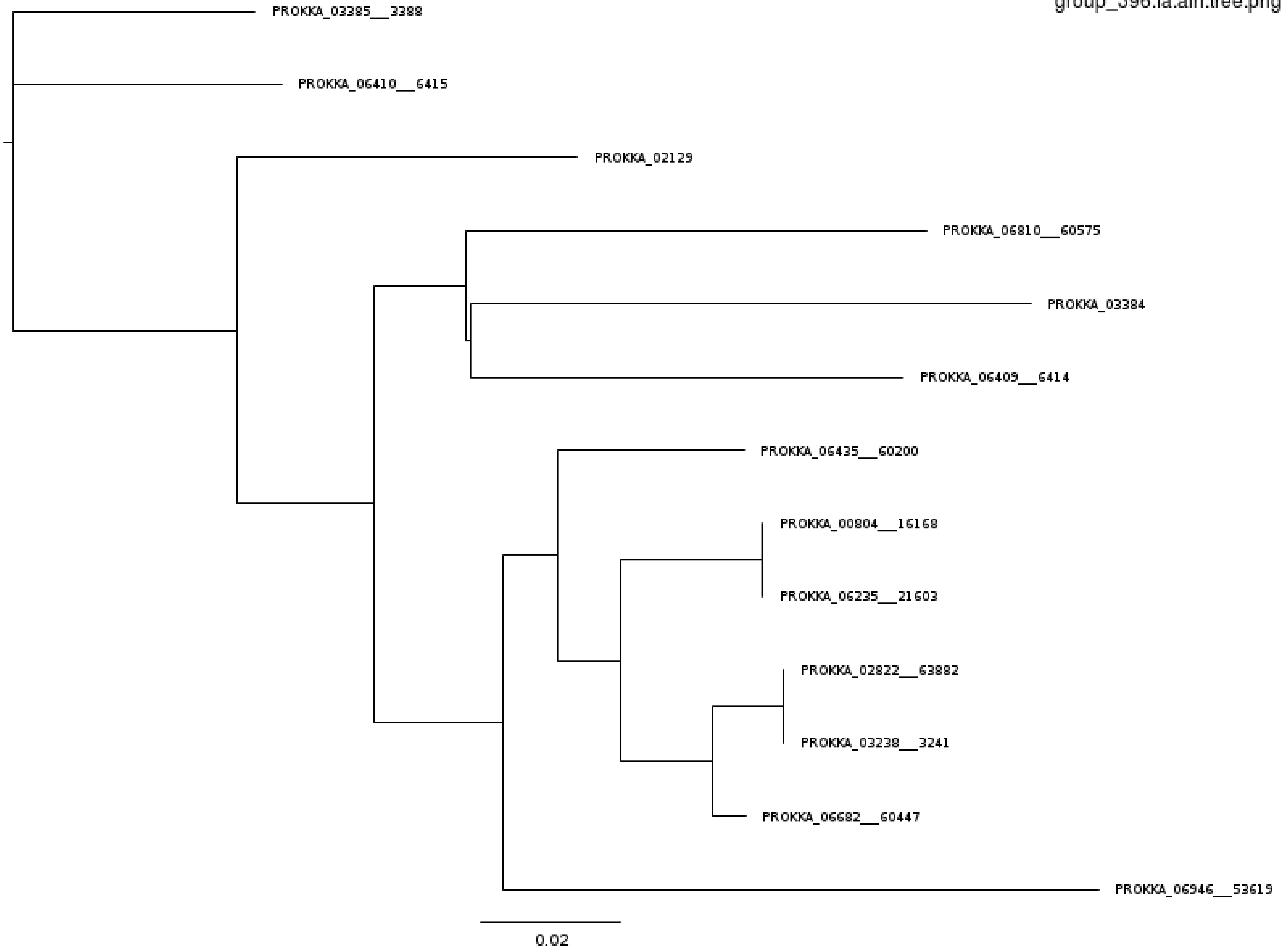

group_397.fa.aln.tree.png

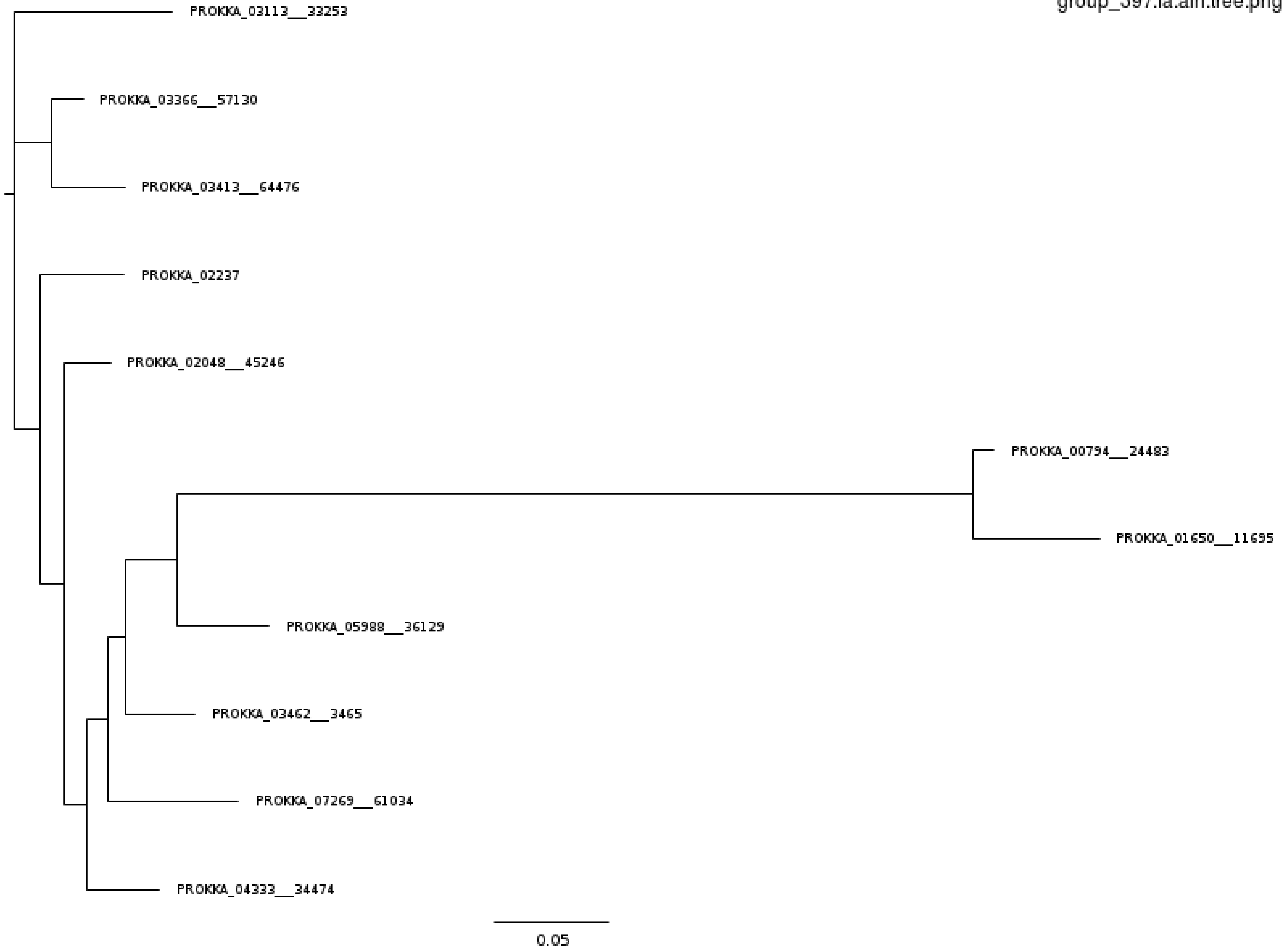

group_398.fa.aln.tree.png

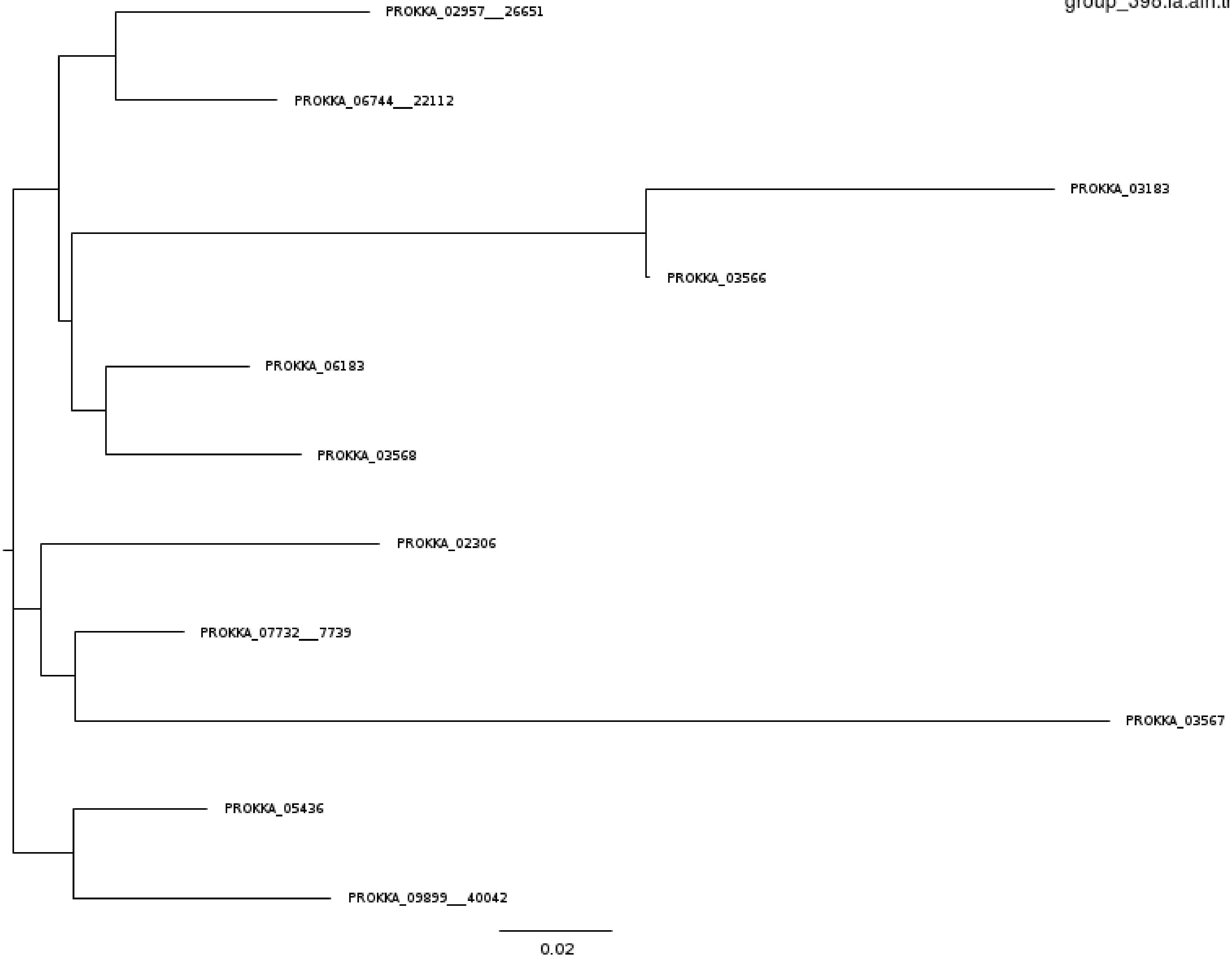

group_3.fa.aln.tree.png

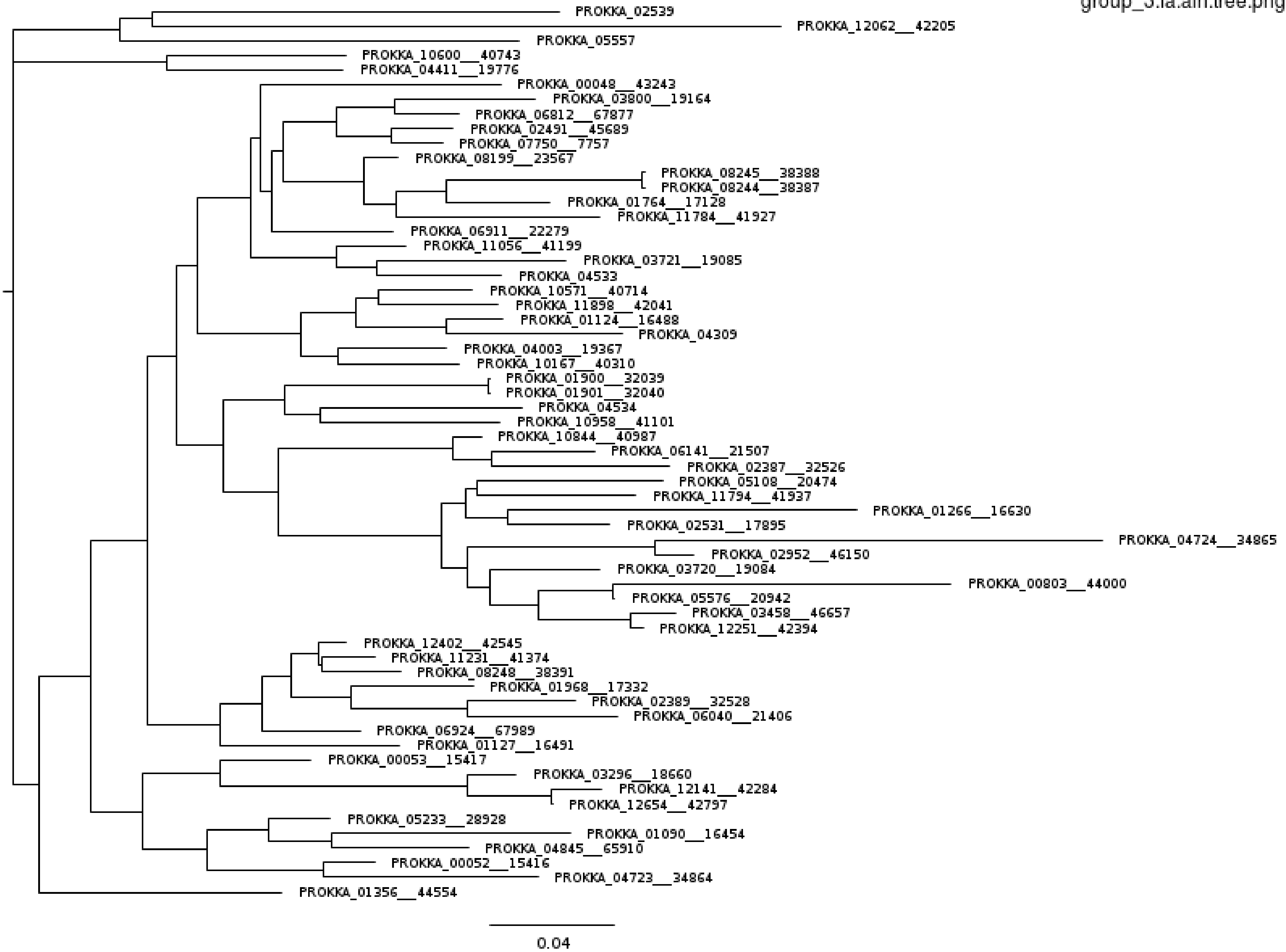

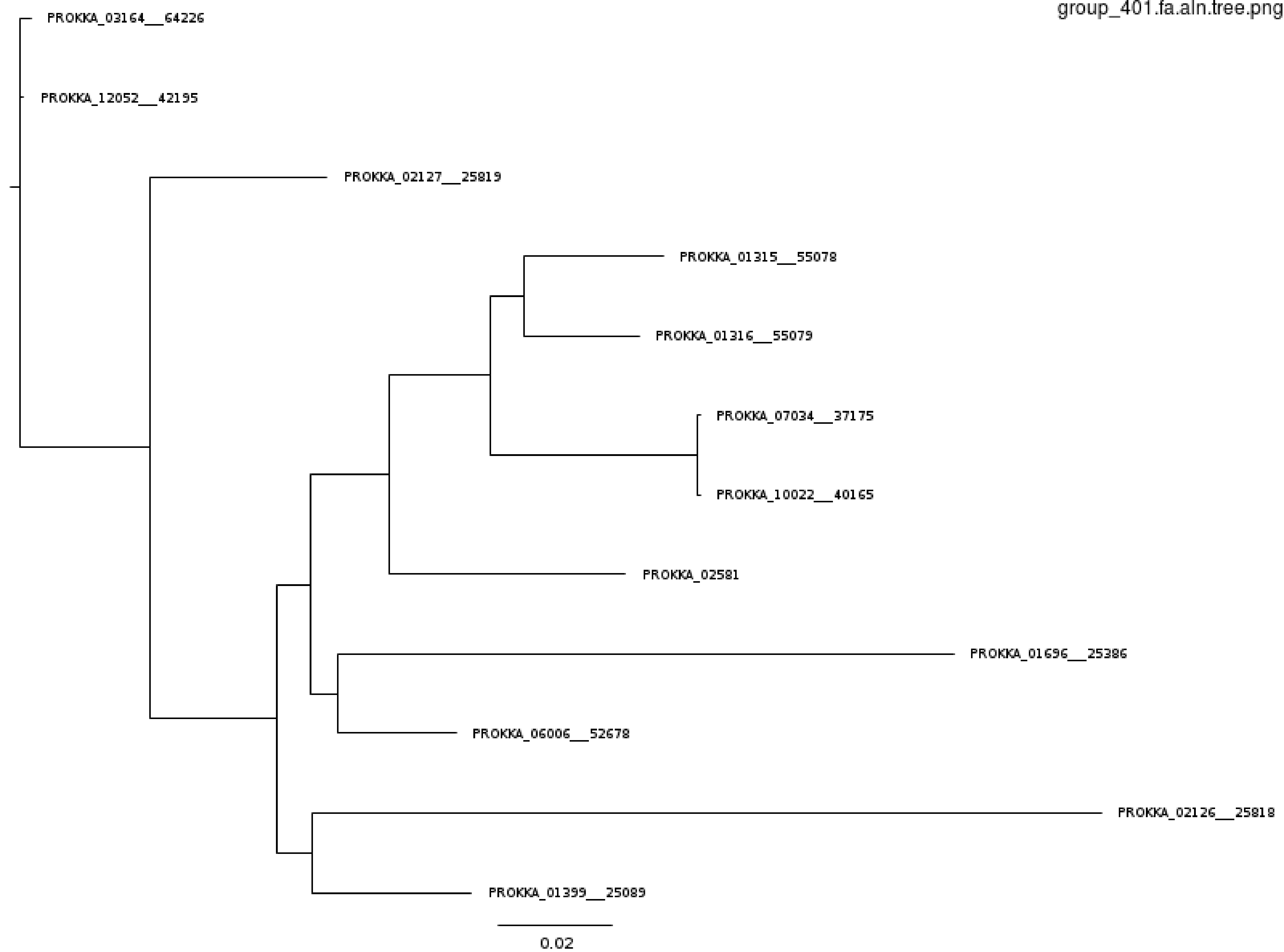
group_401.fa.aln.tree.png
PROKKA_03164___64226
PROKKA_12052___42195
PROKKA_02127___25819
PROKKA_01315___55078
PROKKA_01316___55079
PROKKA_07034___37175
PROKKA_10022___40165
PROKKA_02581
PROKKA_01696___25386
PROKKA_06006___52678
PROKKA_02126___25818
PROKKA_01399___25089
0.02

group_4023.fa.aln.tree.png

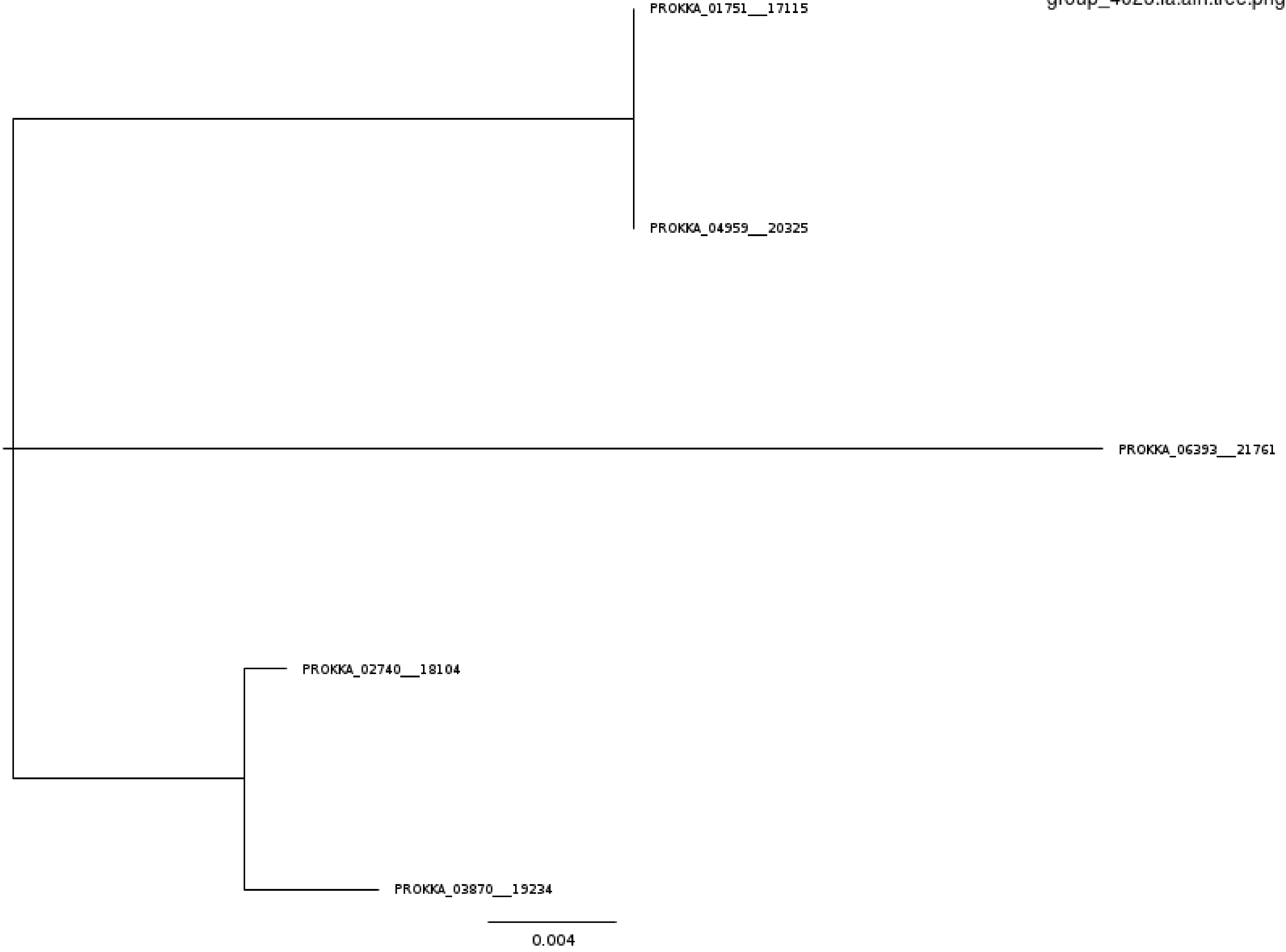

group_4065.fa.aln.tree.png

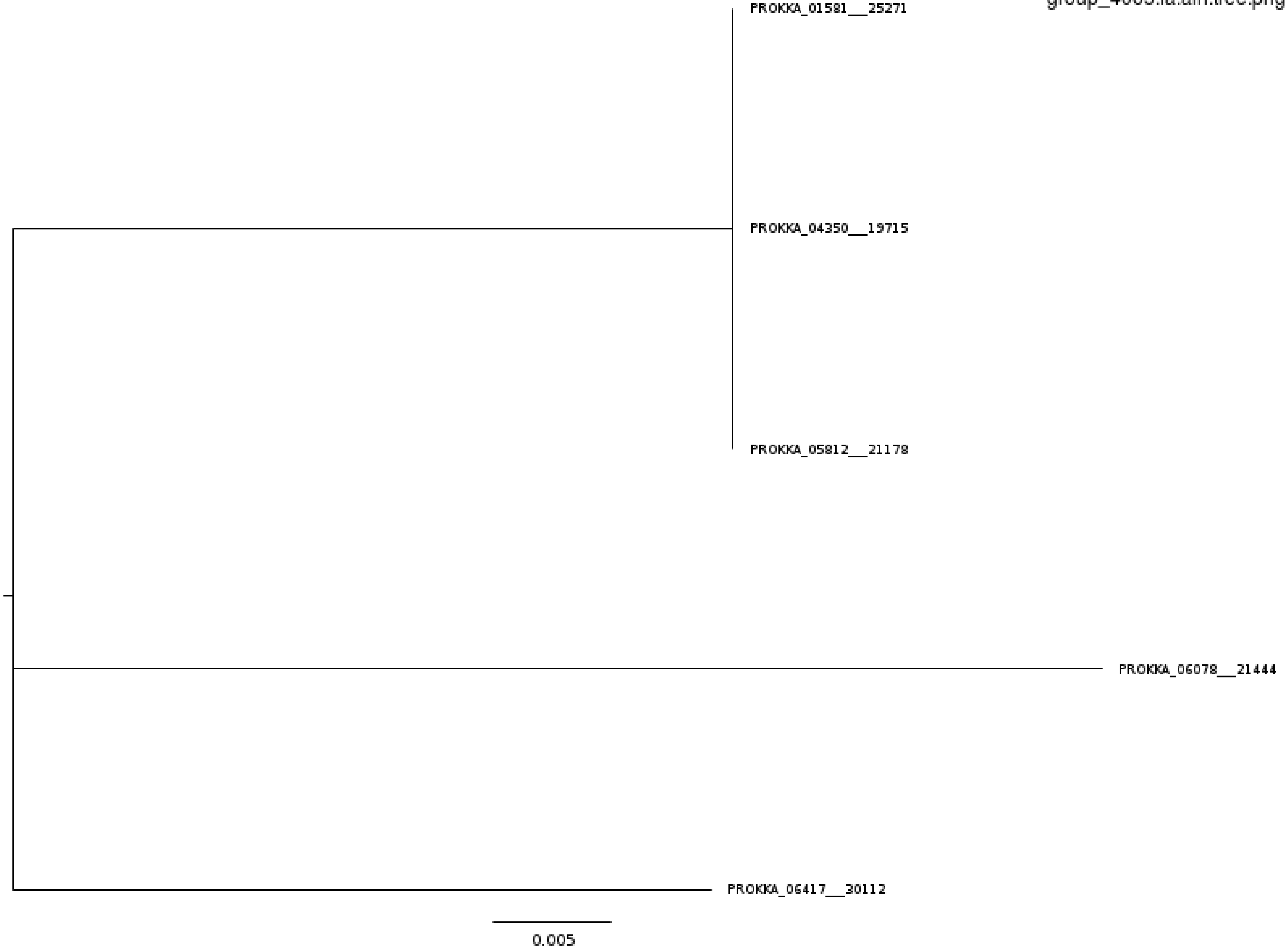

group_407.fa.aln.tree.png

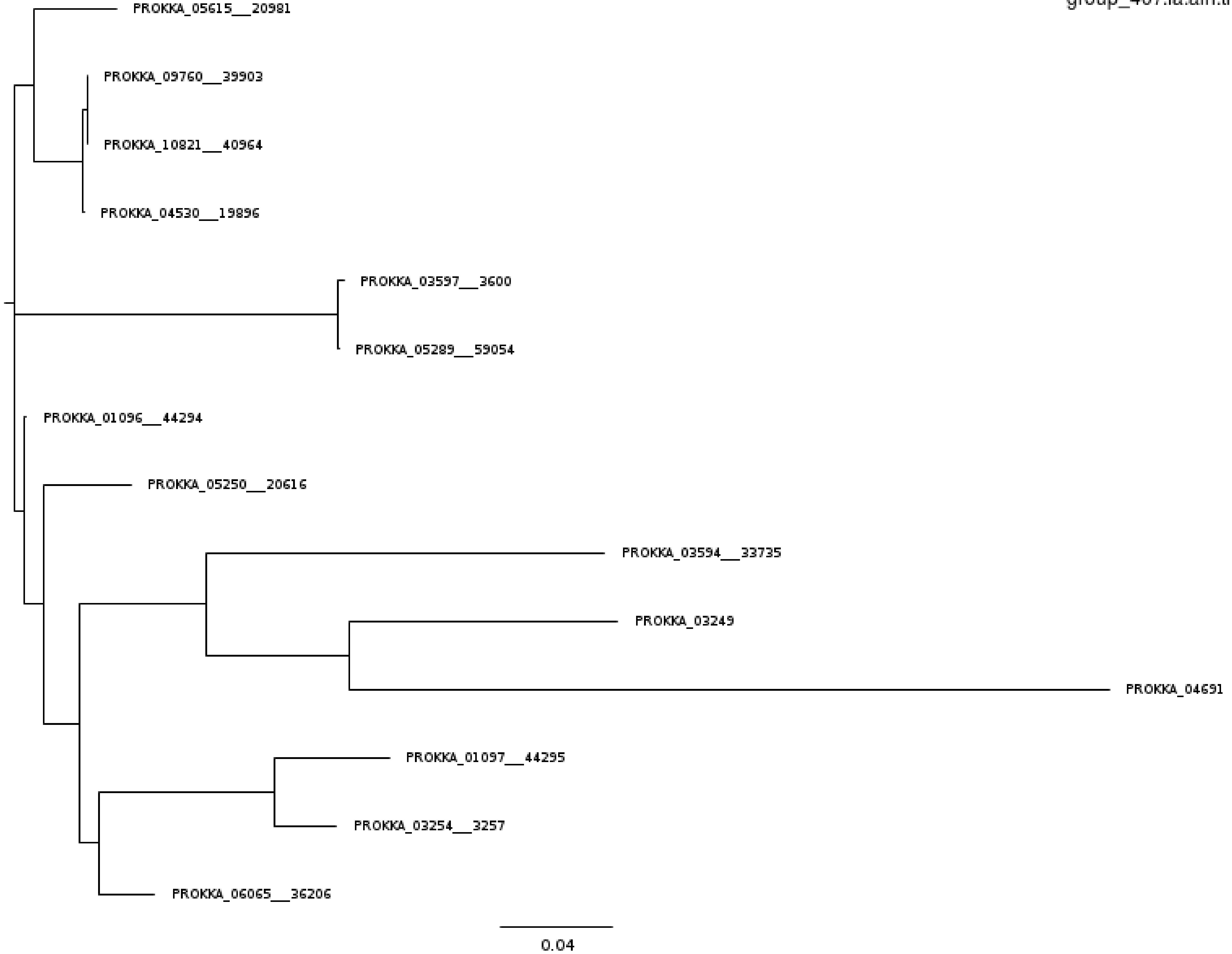

group_417.fa.aln.tree.png

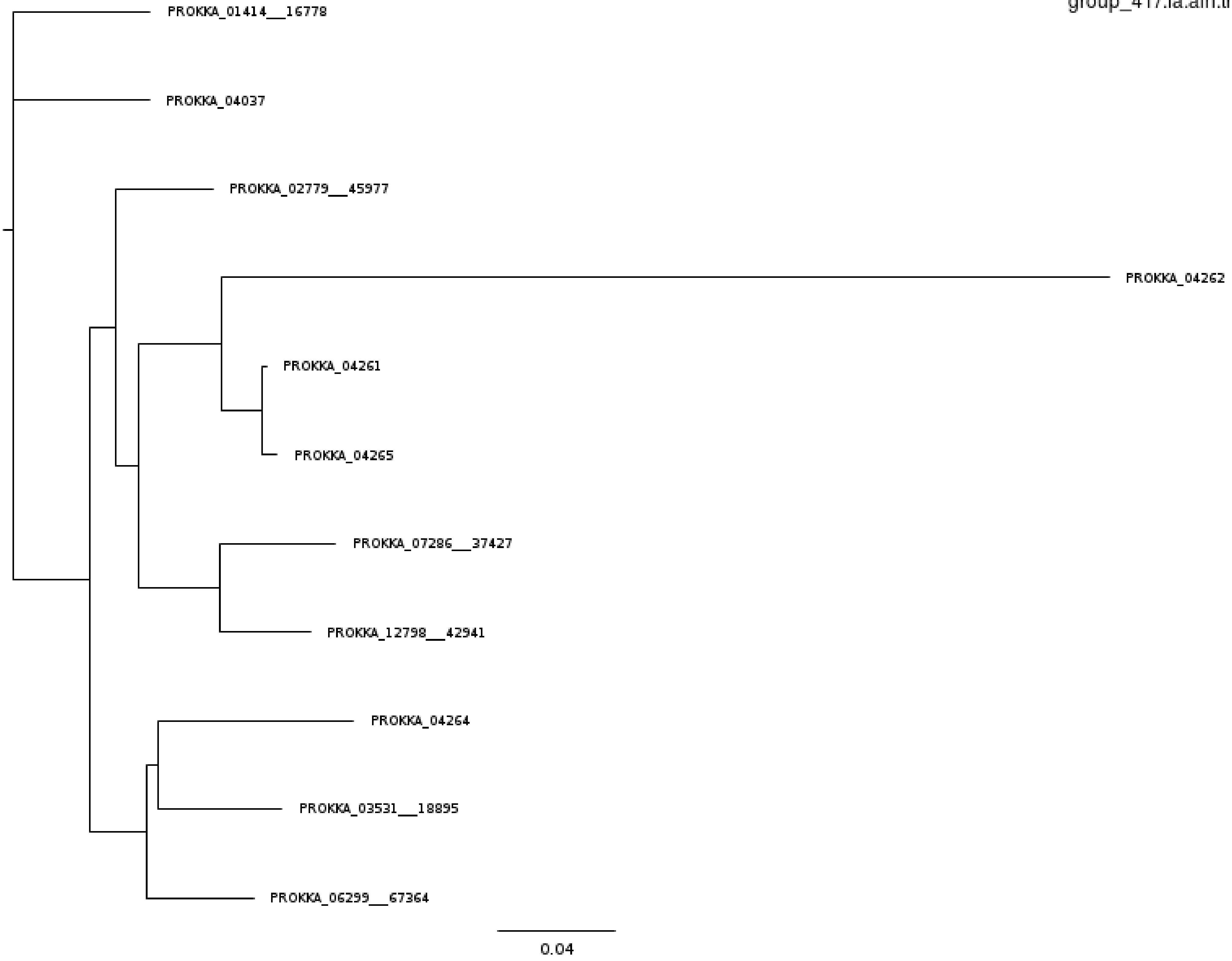

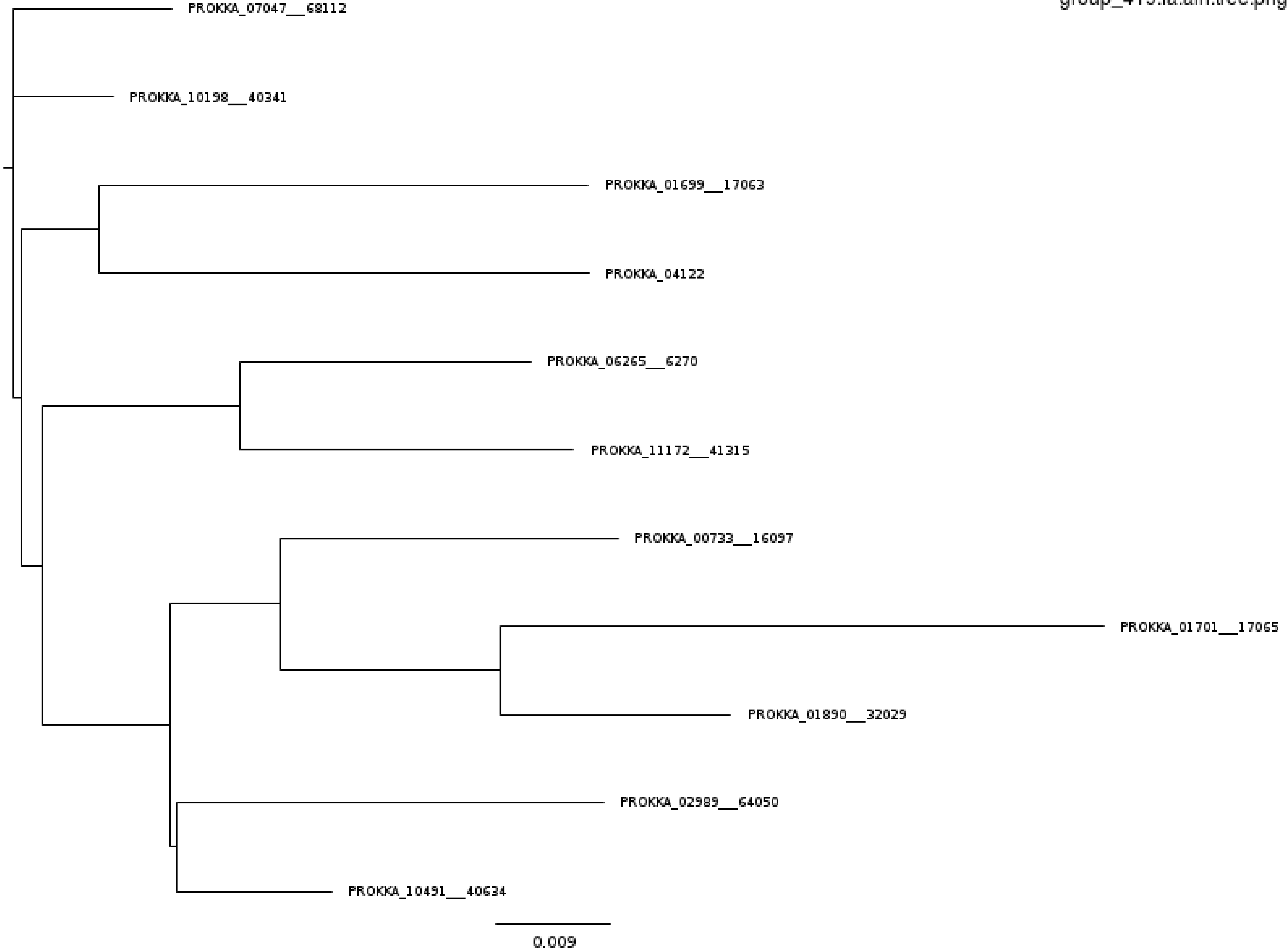

PROKKA_07047___68112
PROKKA_10198___40341
PROKKA_01699___17063
PROKKA_04122
PROKKA_06265___6270
PROKKA_11172___41315
PROKKA_00733___16097
PROKKA_01701___17065
PROKKA_01890___32029
PROKKA_02989___64050
PROKKA_10491___40634
0.009

group_426.fa.aln.tree.png

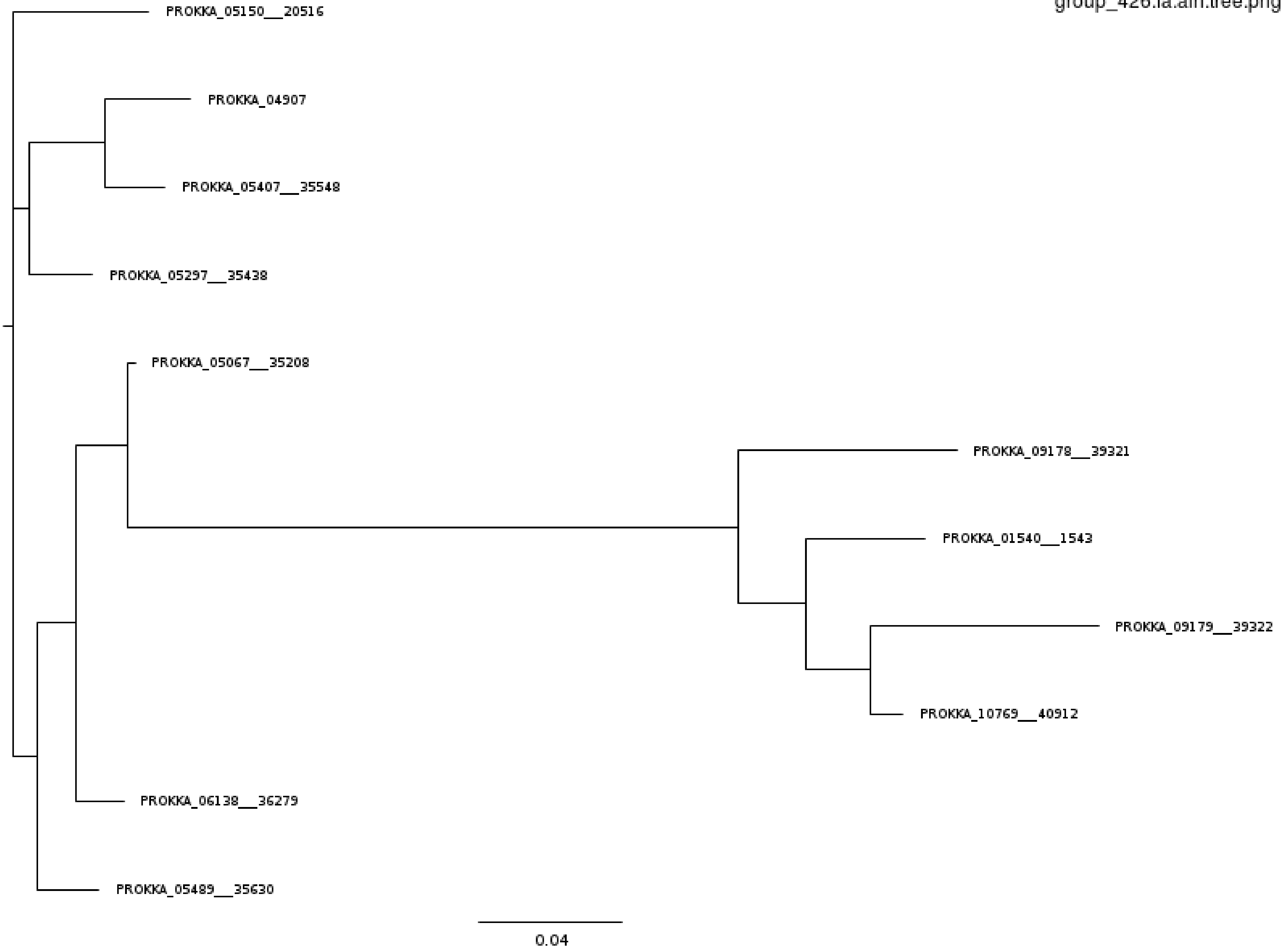

group_427.fa.aln.tree.png

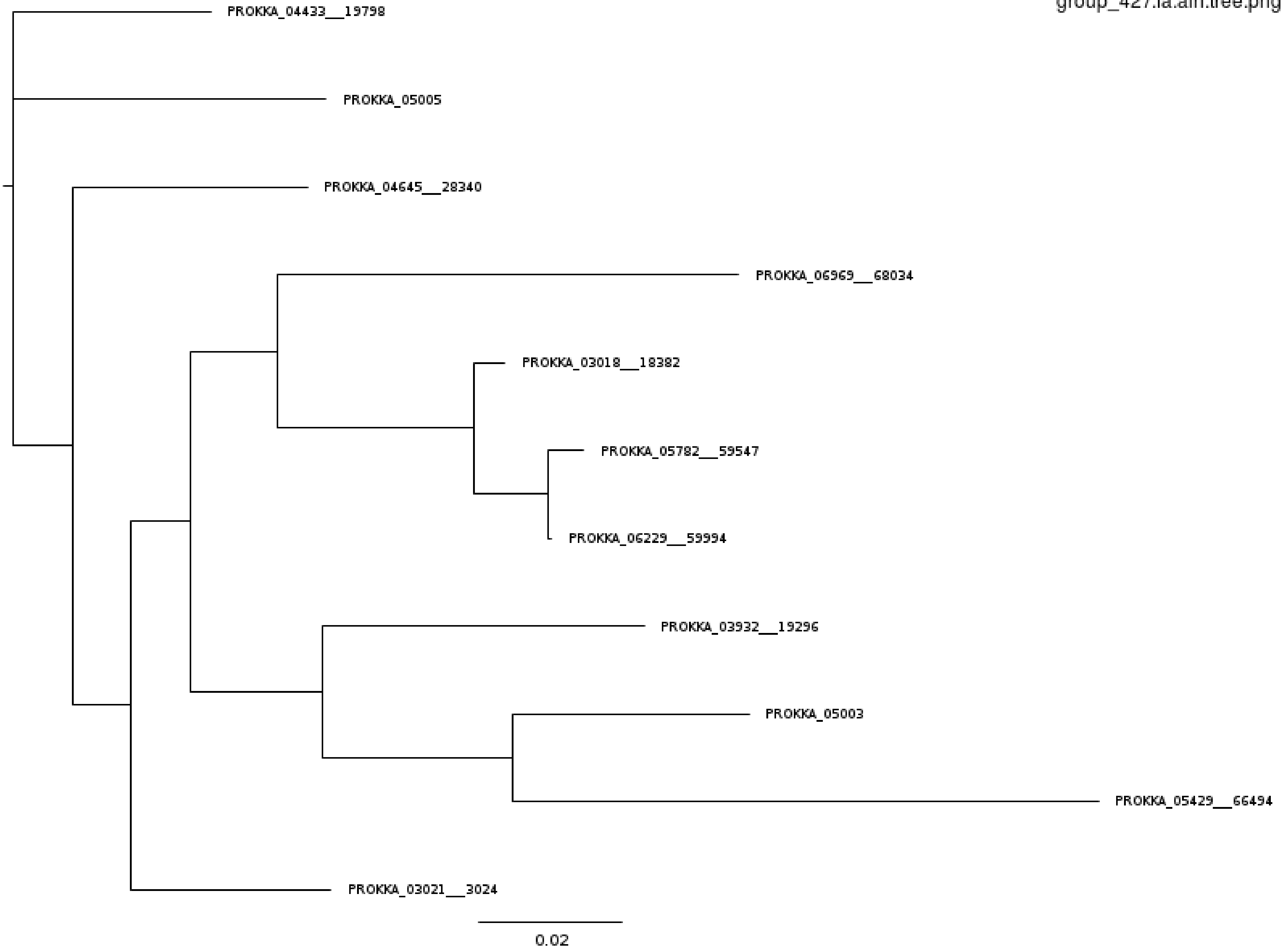

group_428.fa.aln.tree.png

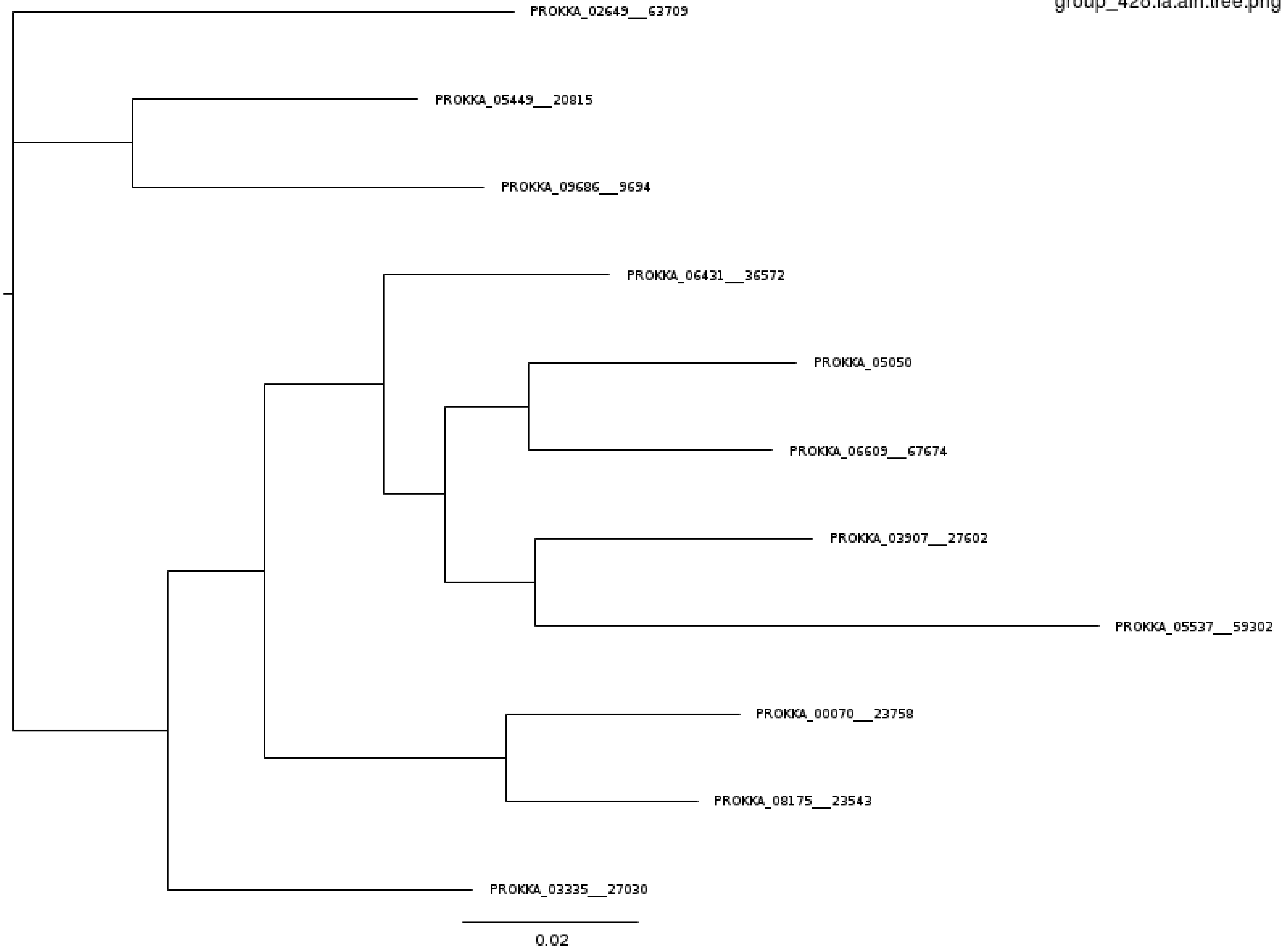

group_42.fa.aln.tree.png

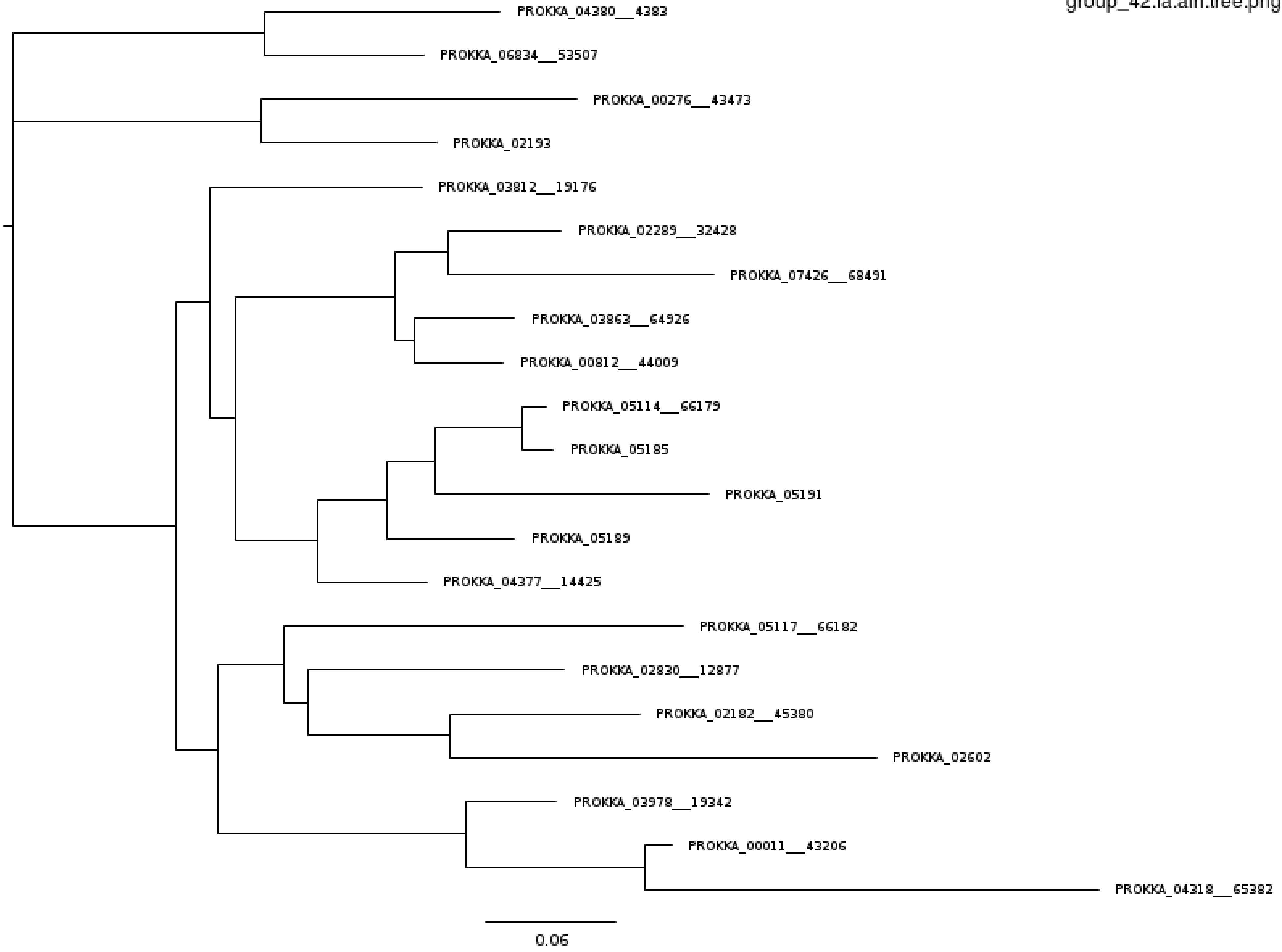

group_430.fa.aln.tree.png

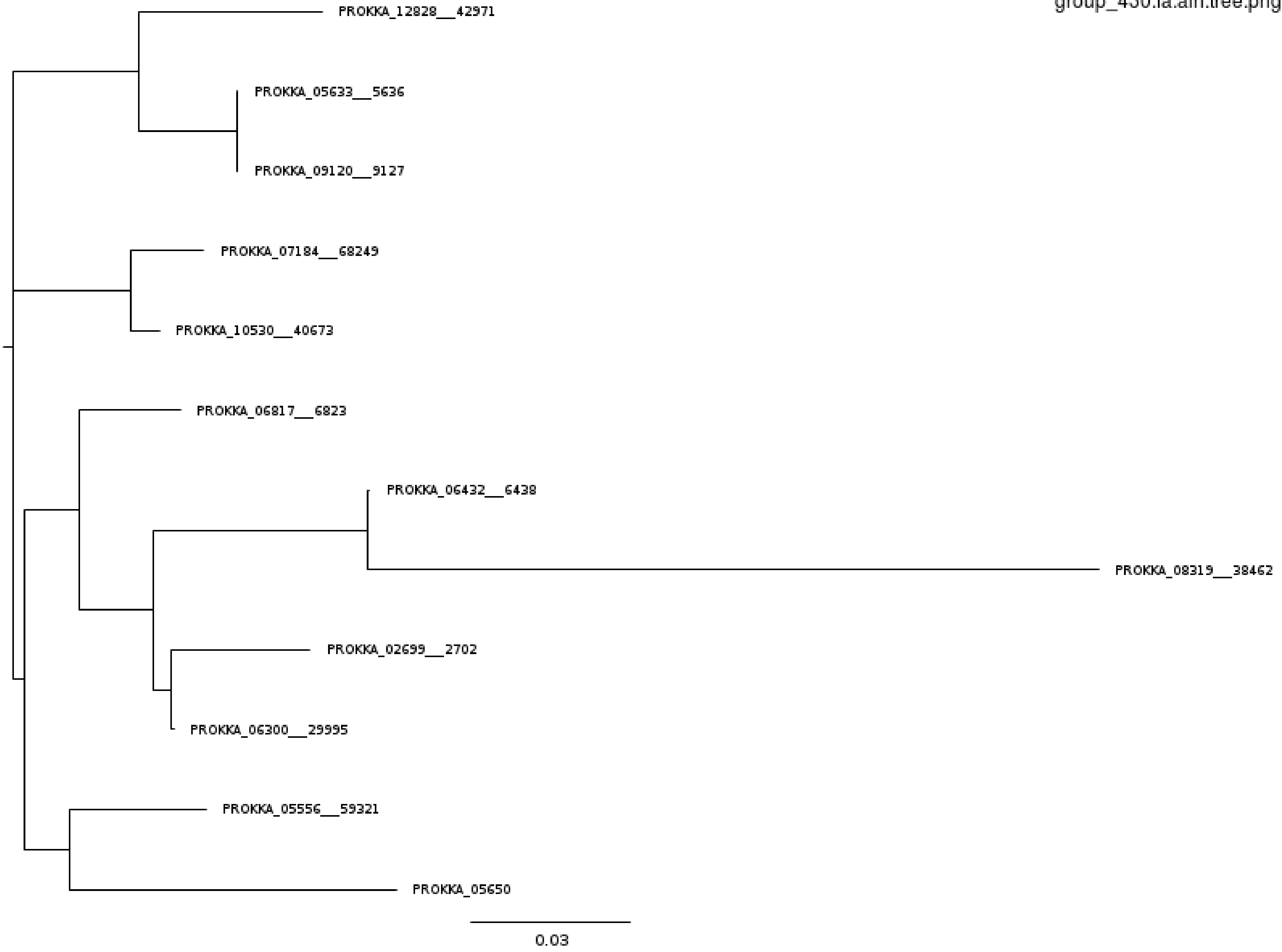

group_434.fa.aln.tree.png

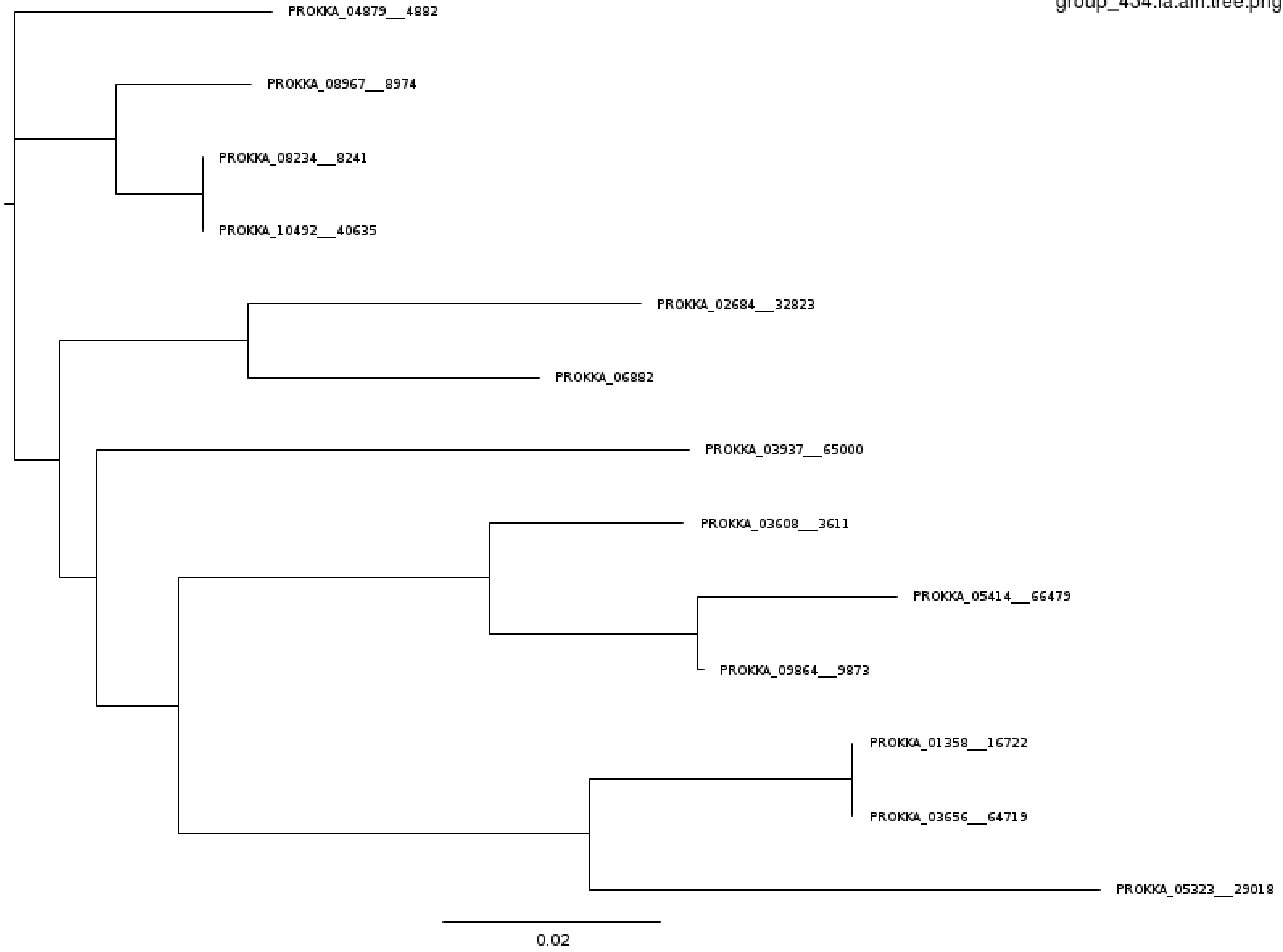

group_437.fa.aln.tree.png

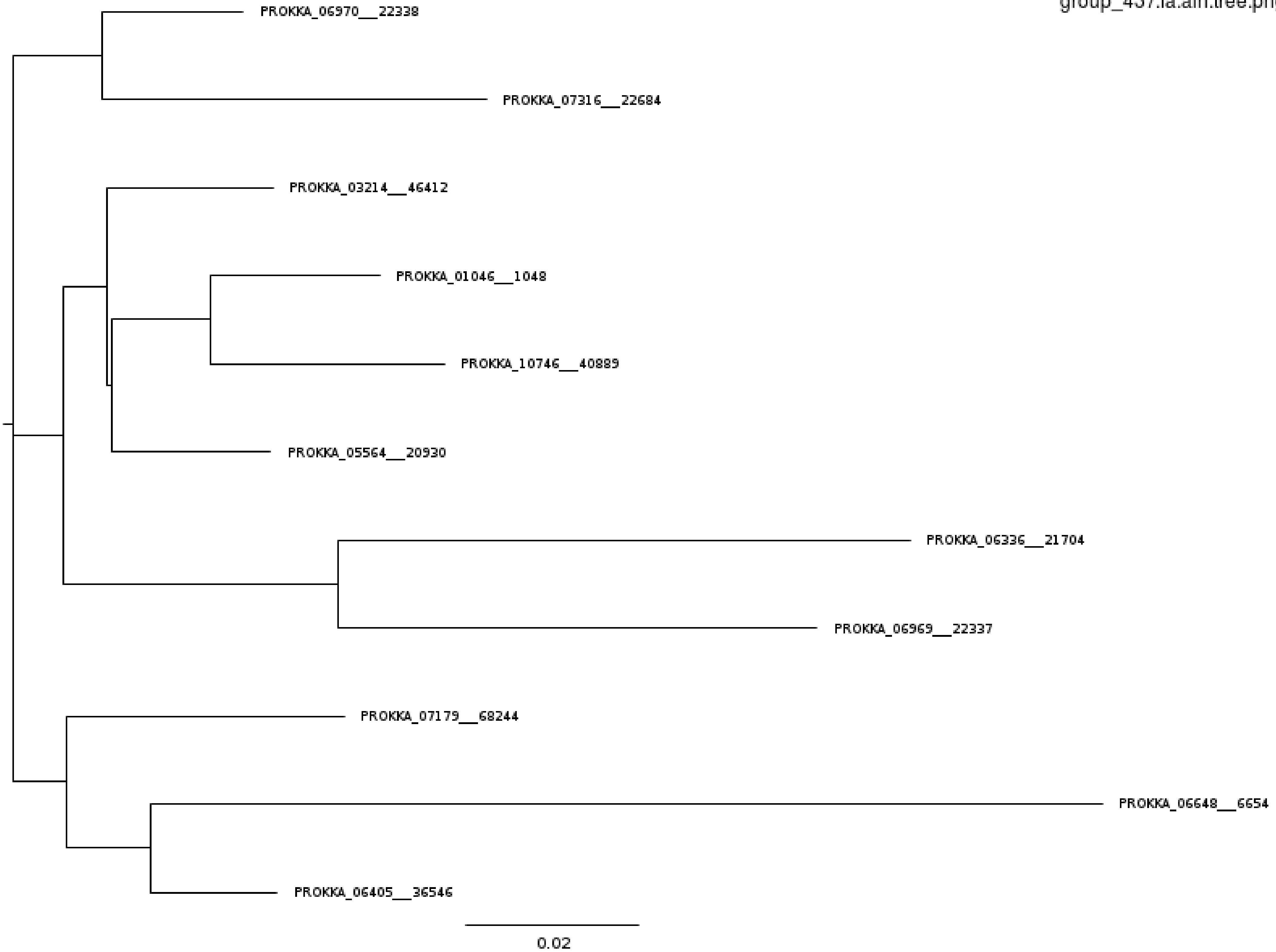

group_43.fa.aln.tree.png

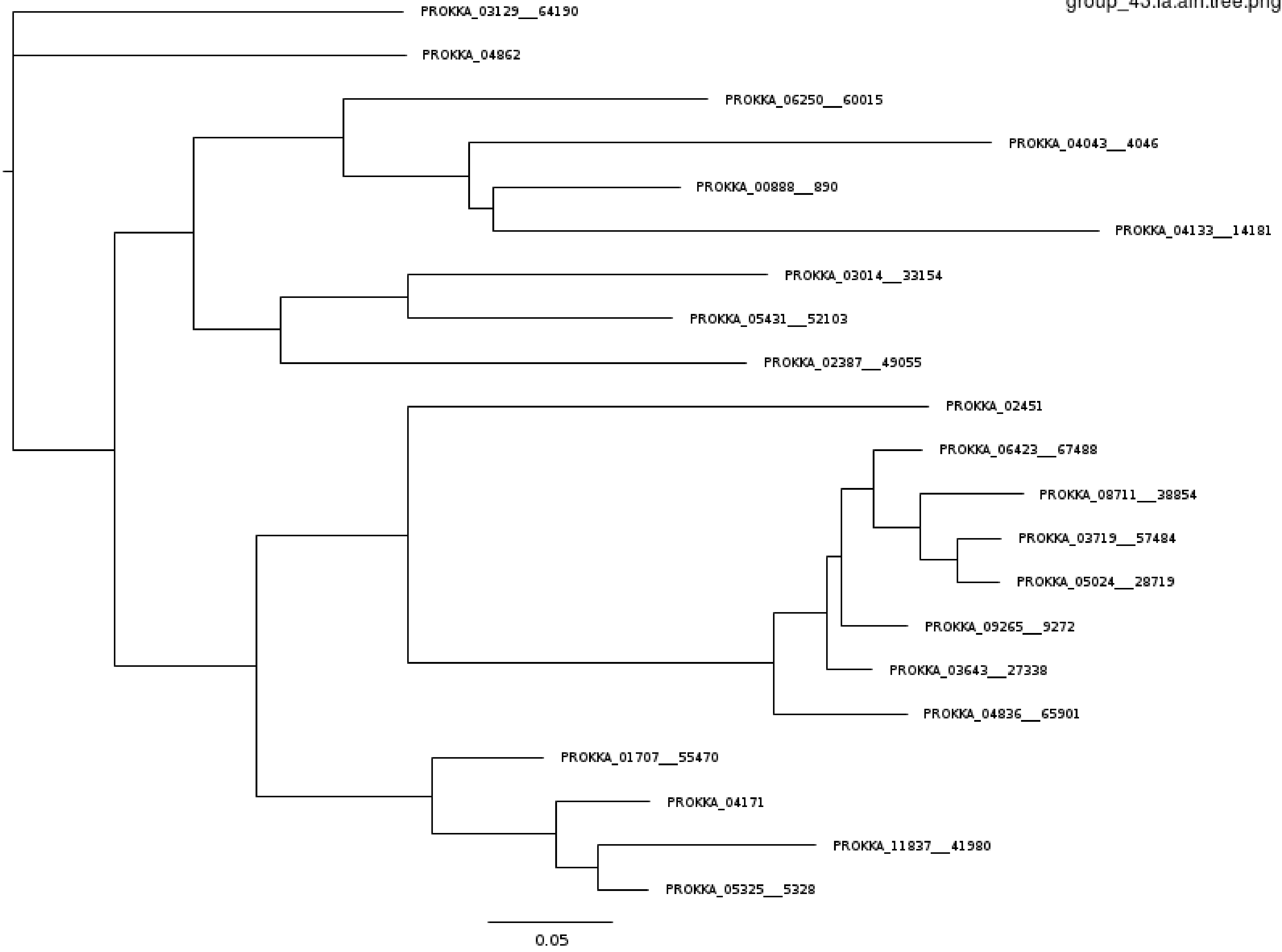

group_441.fa.aln.tree.png

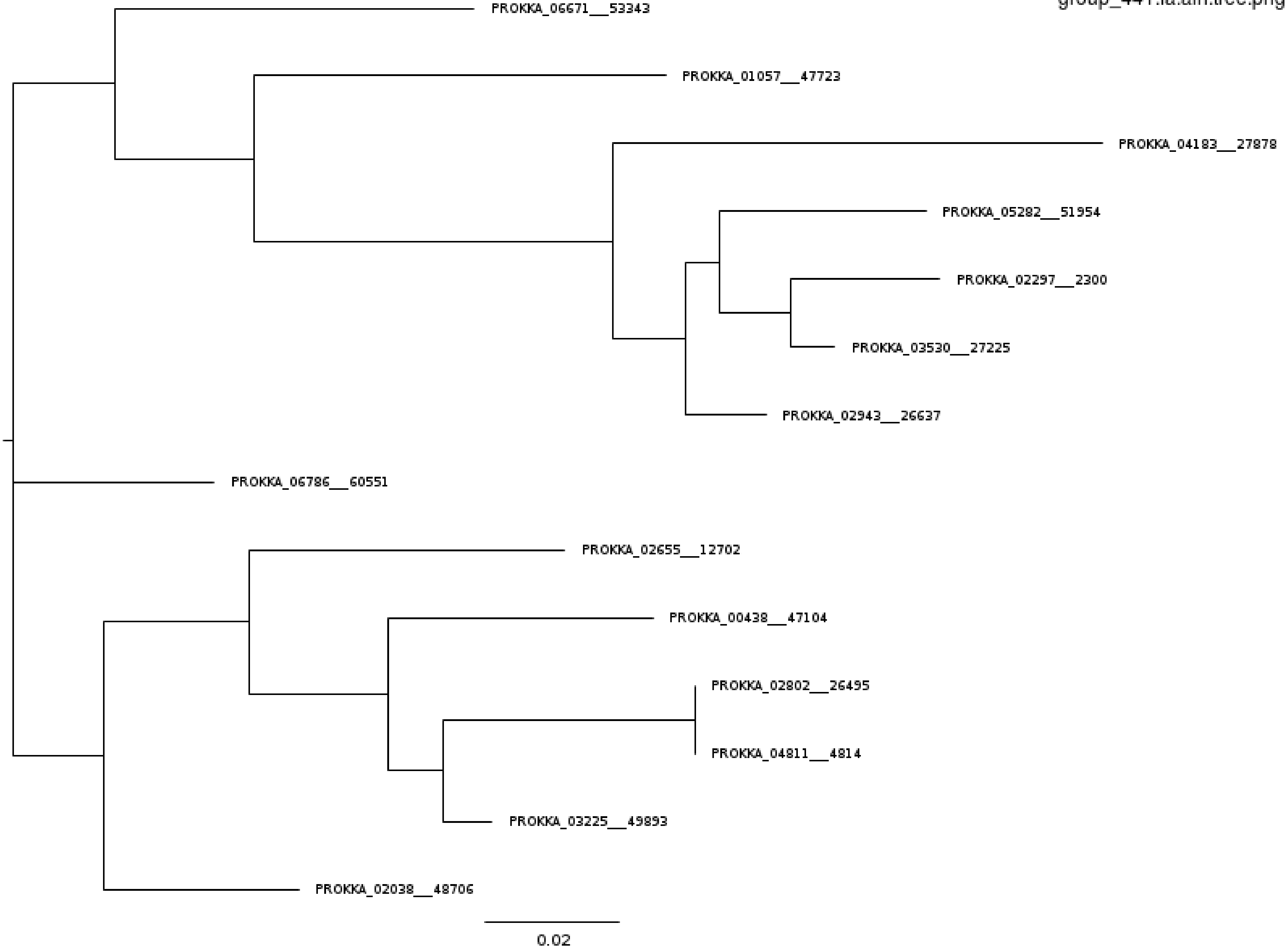

group_44272.fa.aln.tree.png

PROKKA_02581___17945

PROKKA_03888___64951

PROKKA_04383___65448

PROKKA_05353___35494

PROKKA_06010___21376

PROKKA_06013

PROKKA_06230___59995

PROKKA_06575___36716

PROKKA_06919___37060

PROKKA_07451___7458

group_44273.fa.aln.tree.png

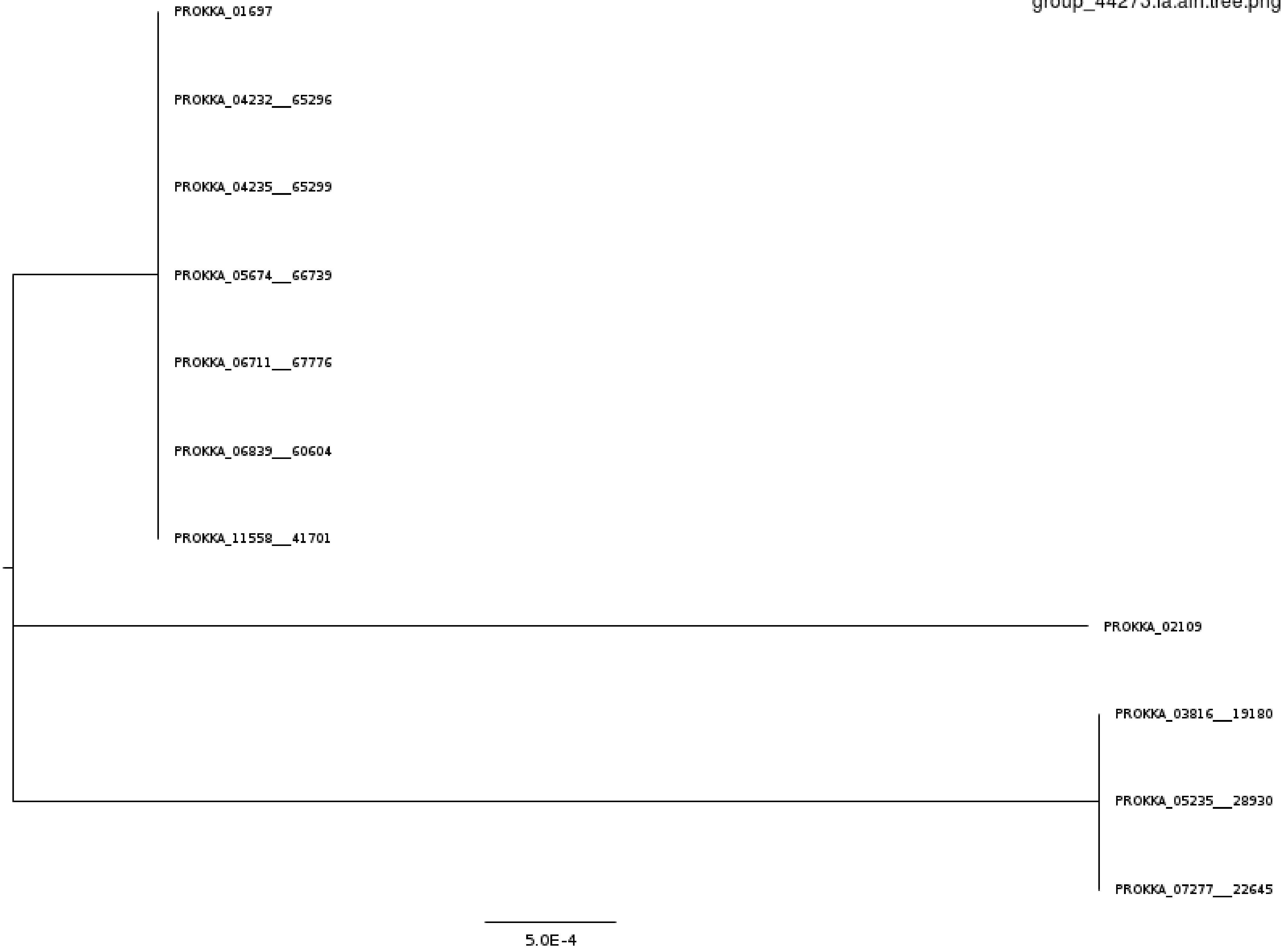

group_447.fa.aln.tree.png

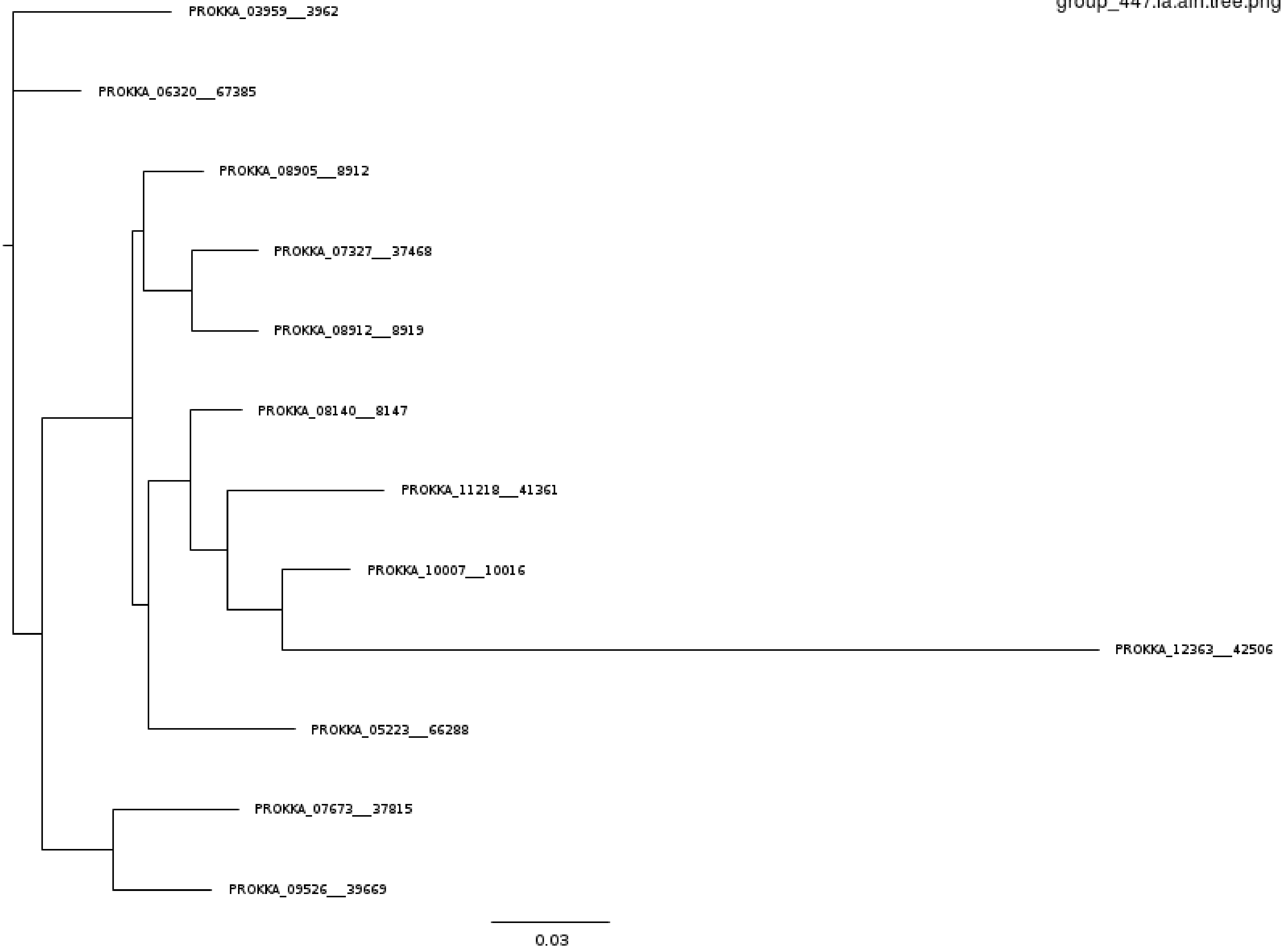

group_449.fa.aln.tree.png

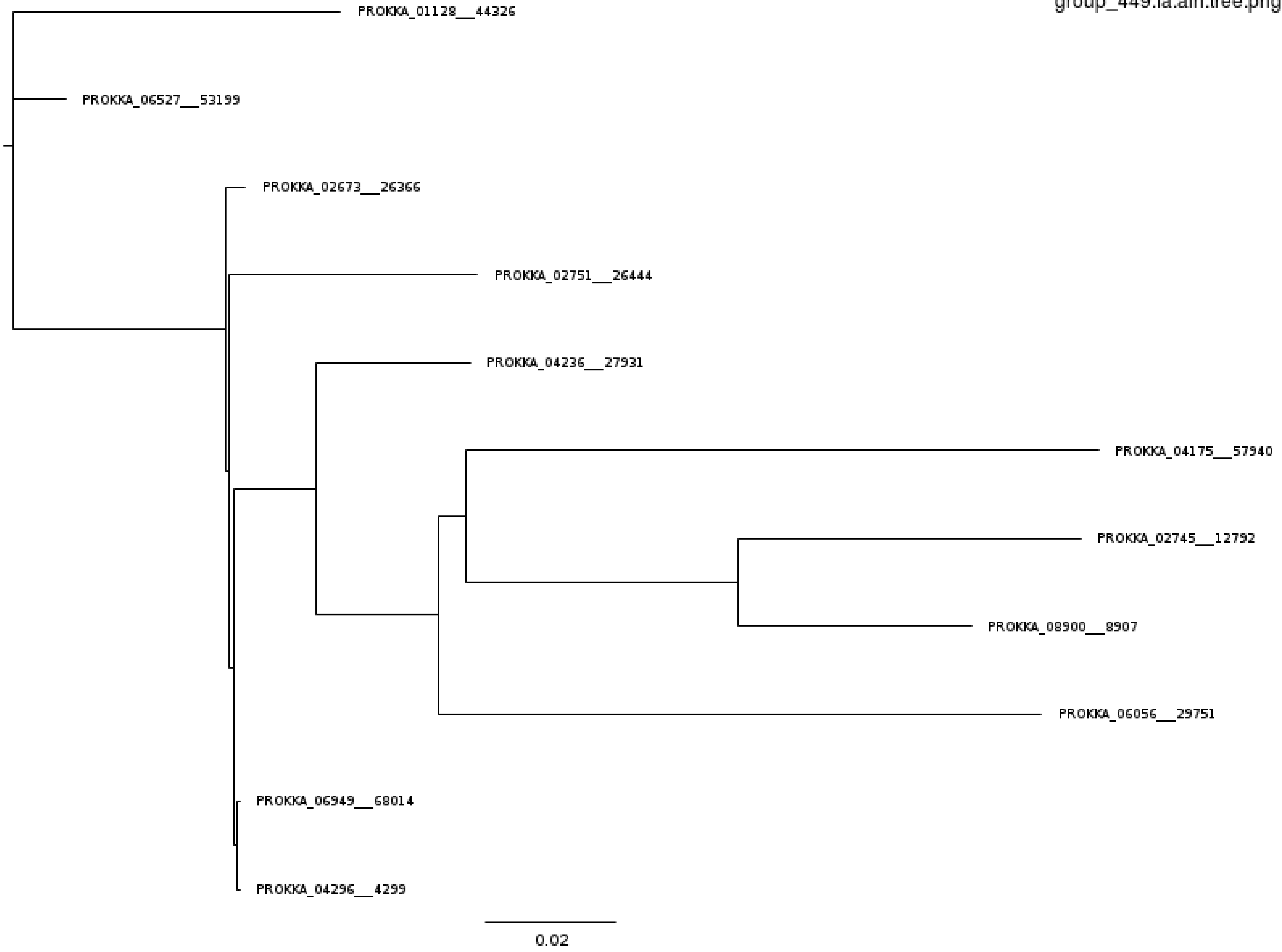

group_451.fa.aln.tree.png

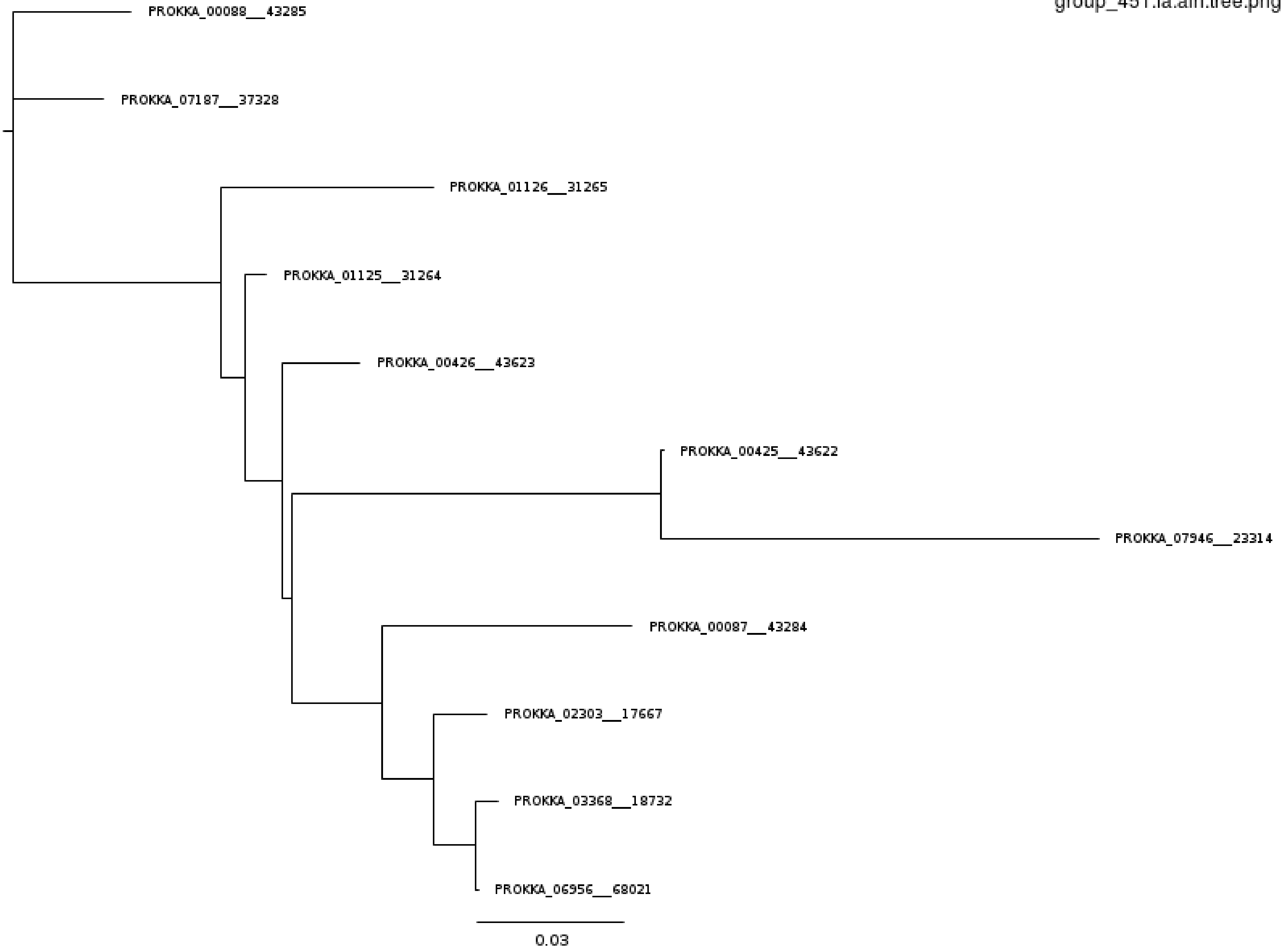

group_455.fa.aln.tree.png

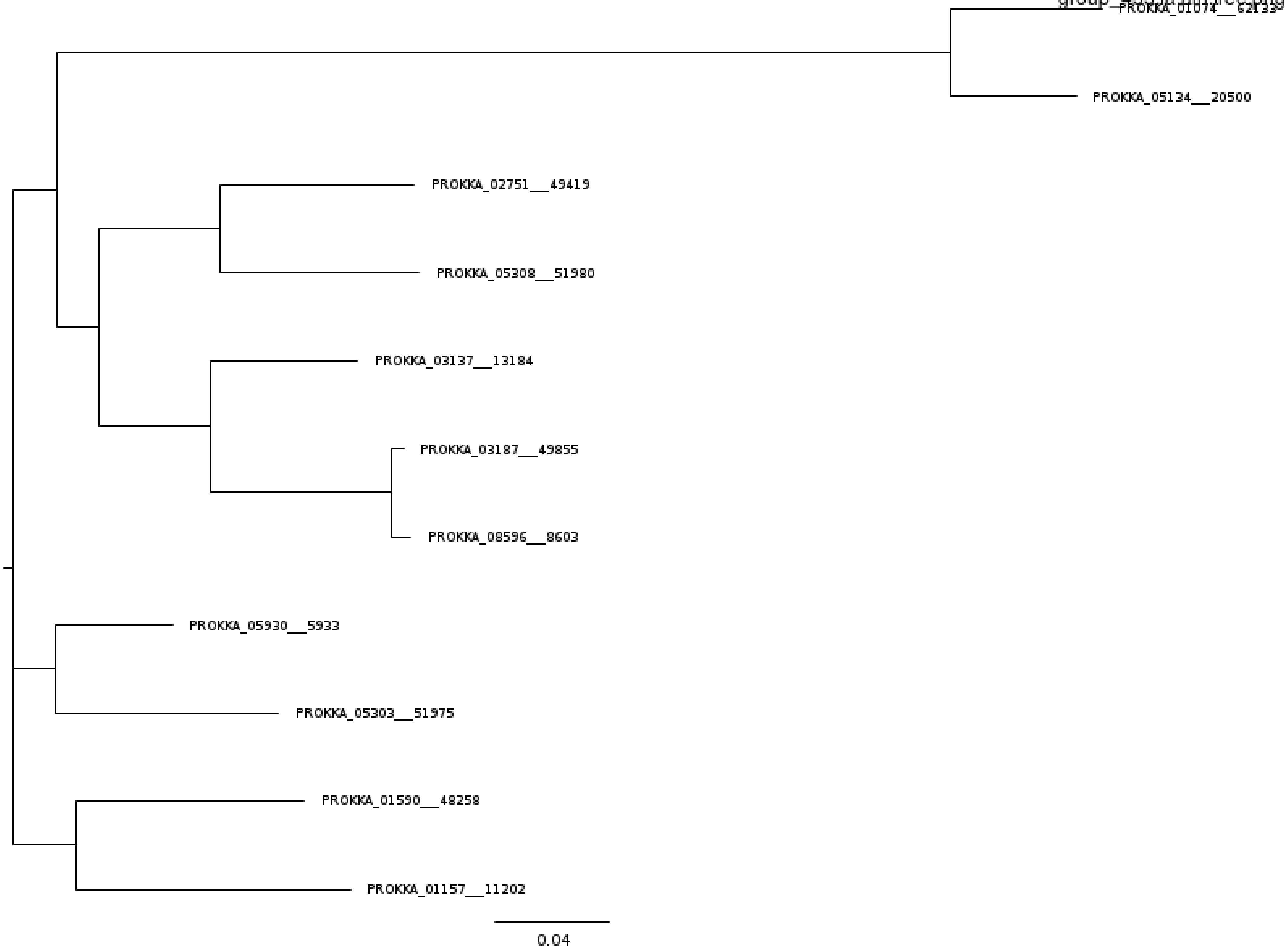

group_456.fa.aln.tree.png

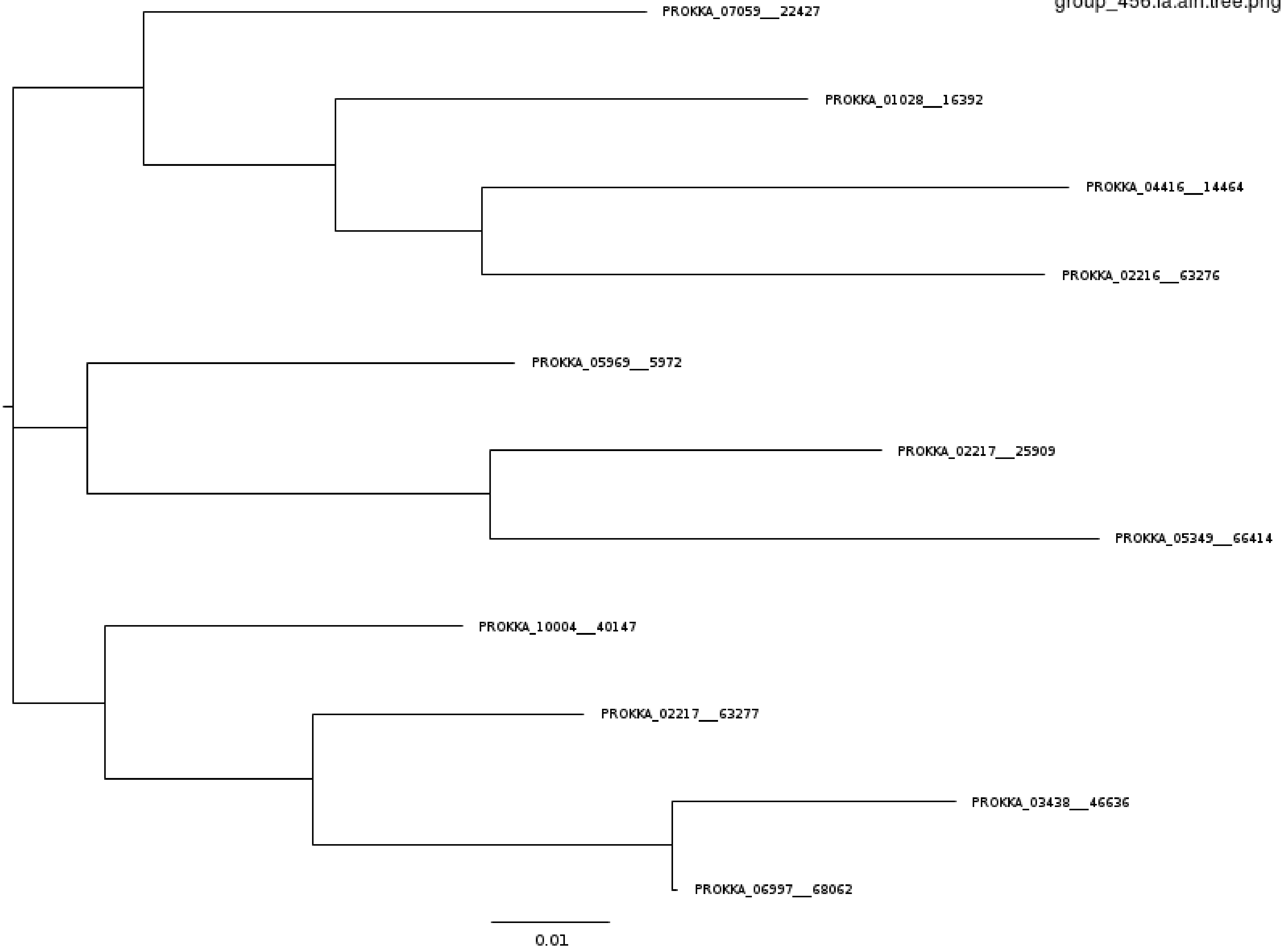

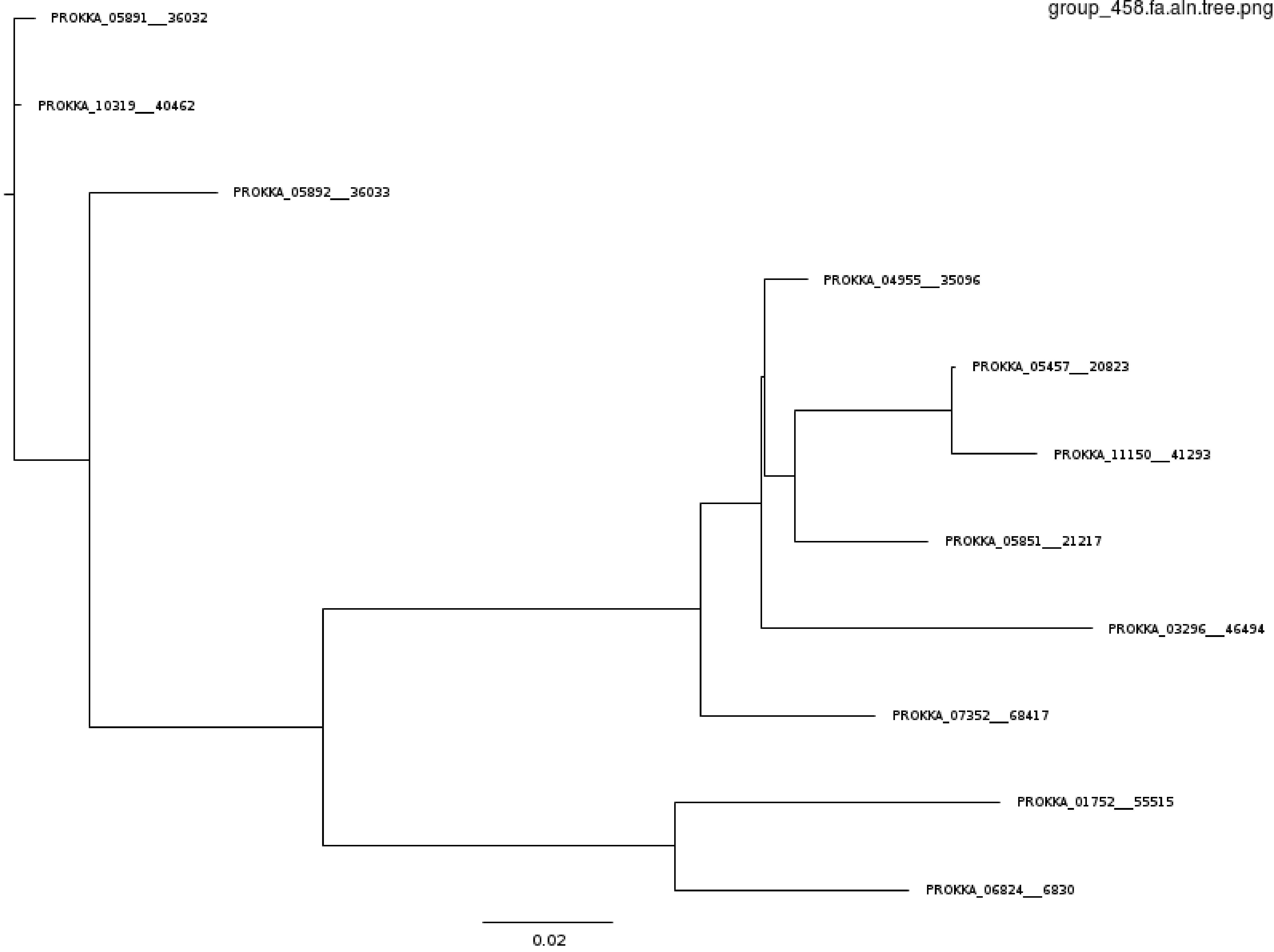

group_458.fa.aln.tree.png
PROKKA_05891___36032
PROKKA_10319___40462
PROKKA_05892___36033
PROKKA_04955___35096
PROKKA_05457___20823
PROKKA_11150___41293
PROKKA_05851___21217
PROKKA_03296___46494
PROKKA_07352___68417
PROKKA_01752___55515
PROKKA_06824___6830
0.02

group_4597.fa.aln.tree.png

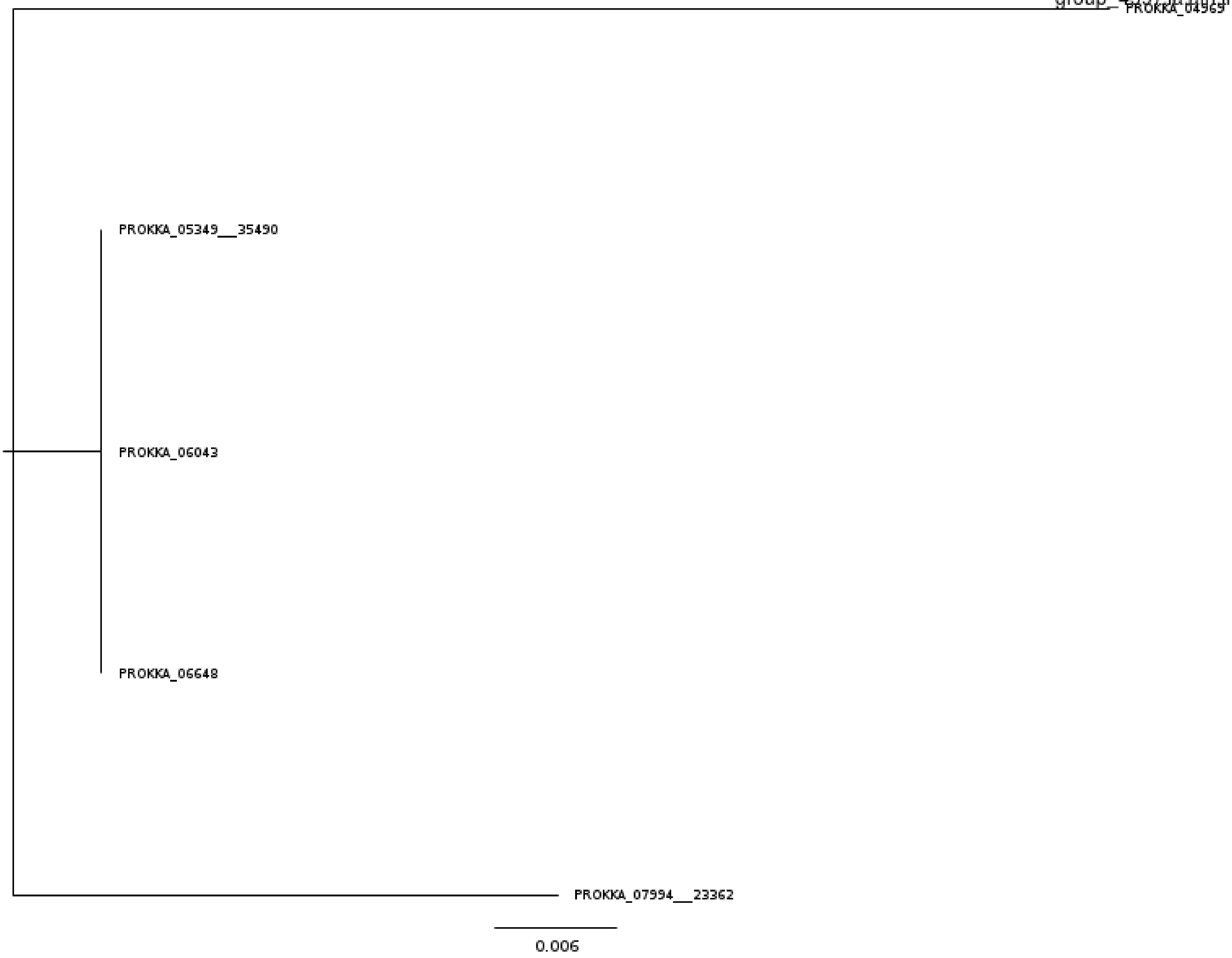

group_461.fa.aln.tree.png

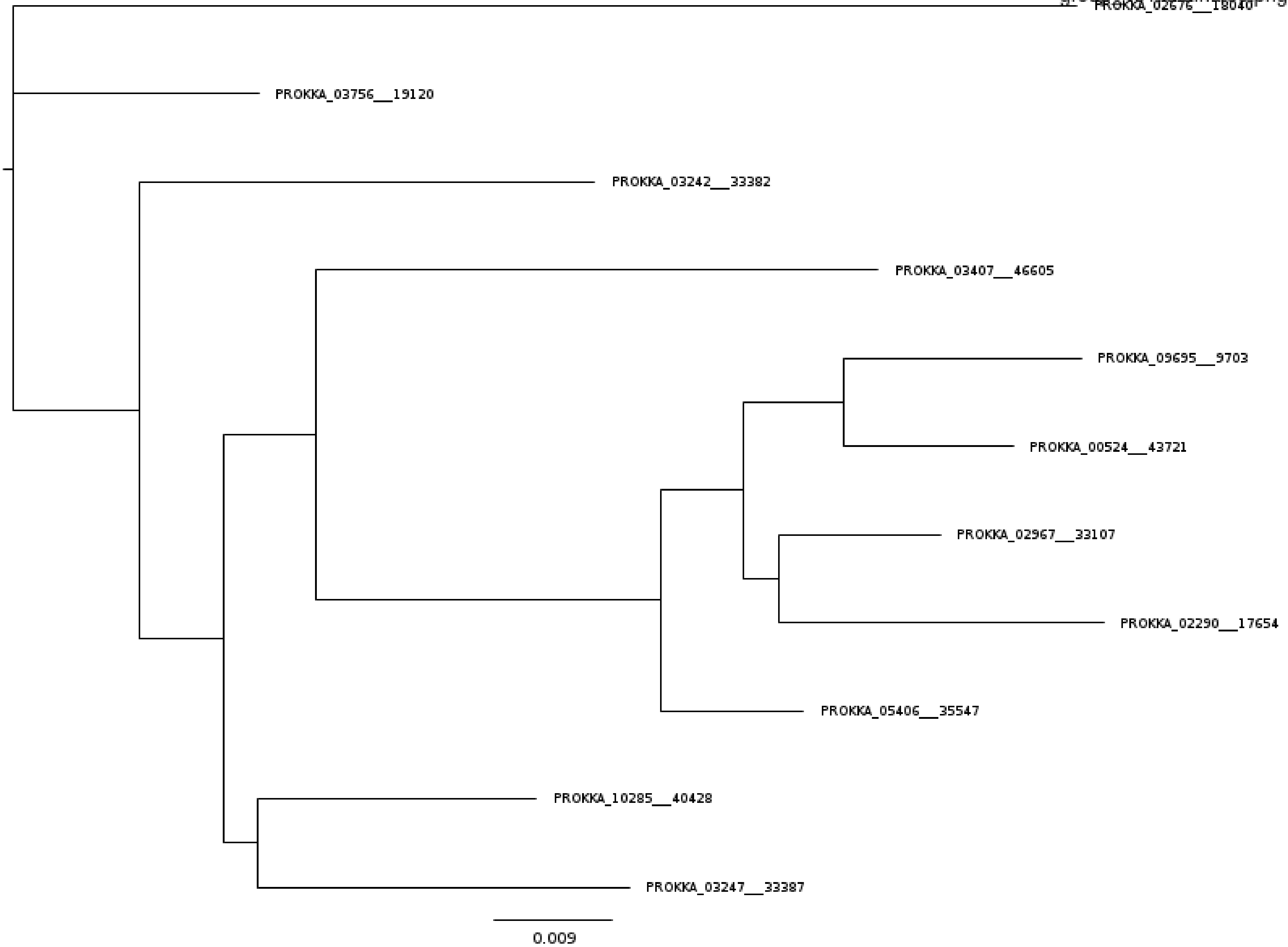

group_463.fa.aln.tree.png

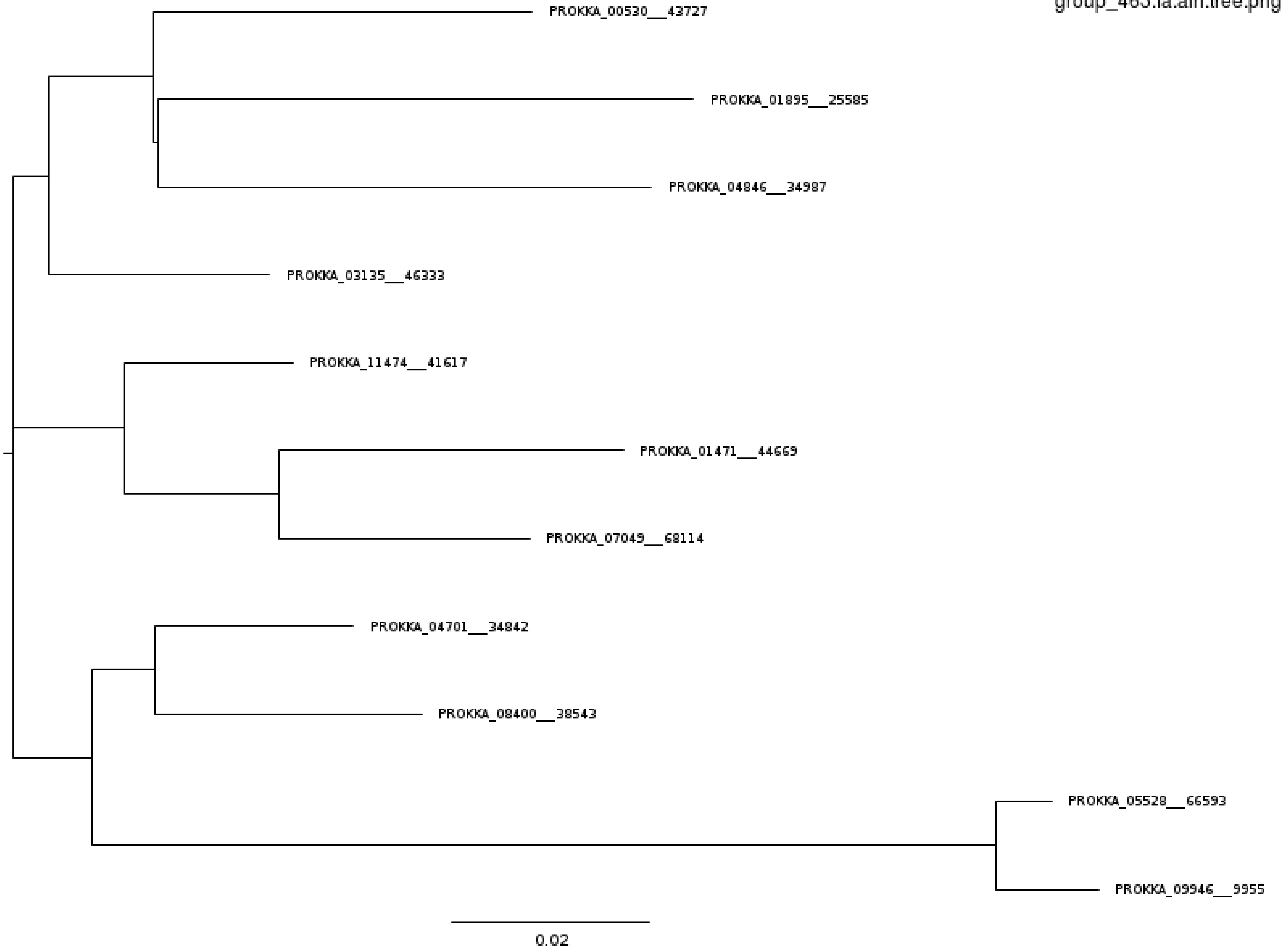

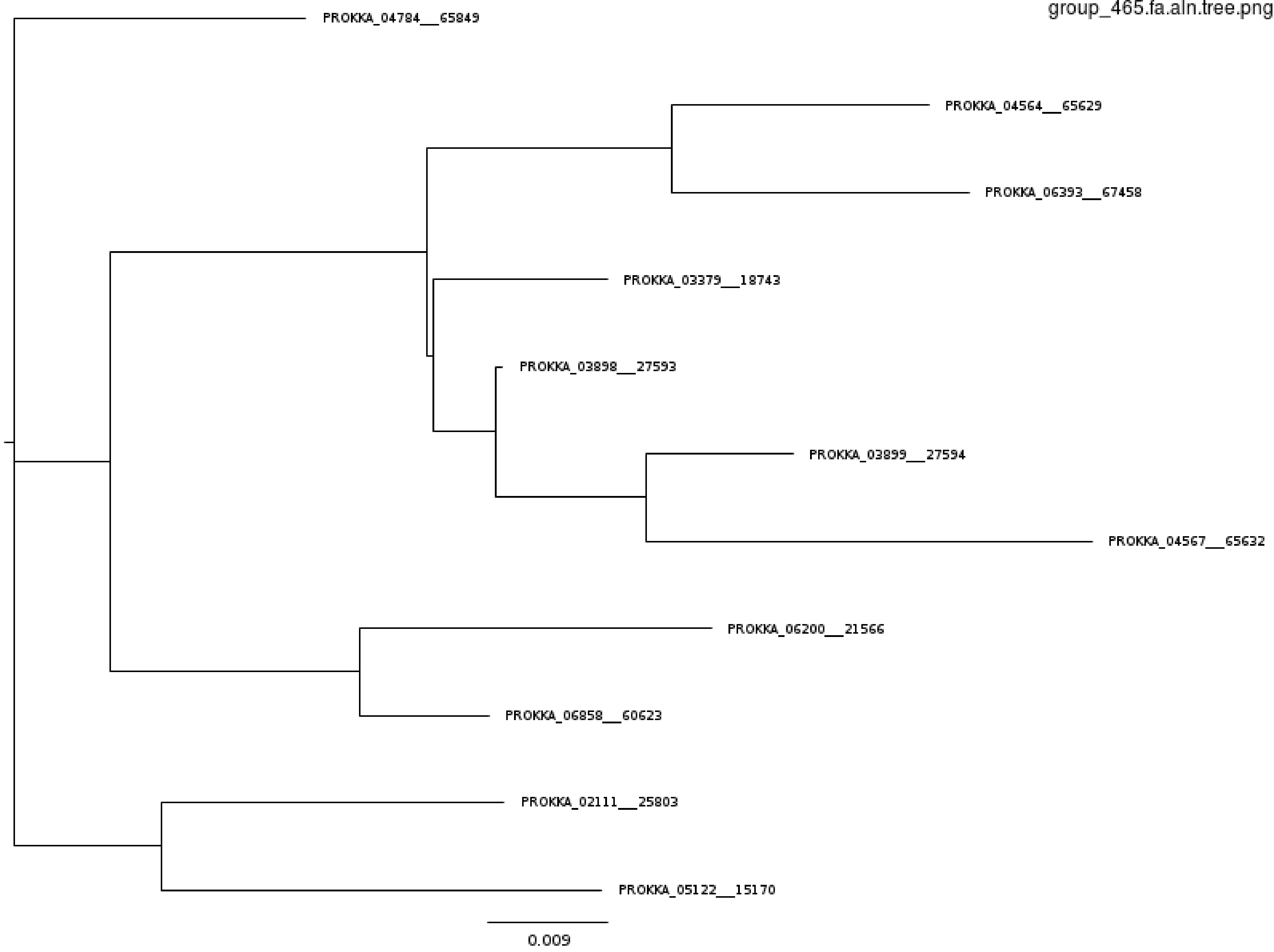
group_465.fa.aln.tree.png
PROKKA_04784___65849
PROKKA_04564___65629
PROKKA_06393___67458
PROKKA_03379___18743
PROKKA_03898___27593
PROKKA_03899___27594
PROKKA_04567___65632
PROKKA_06200___21566
PROKKA_06858___60623
PROKKA_02111___25803
PROKKA_05122___15170
0.009

group_4696.fa.aln.tree.png

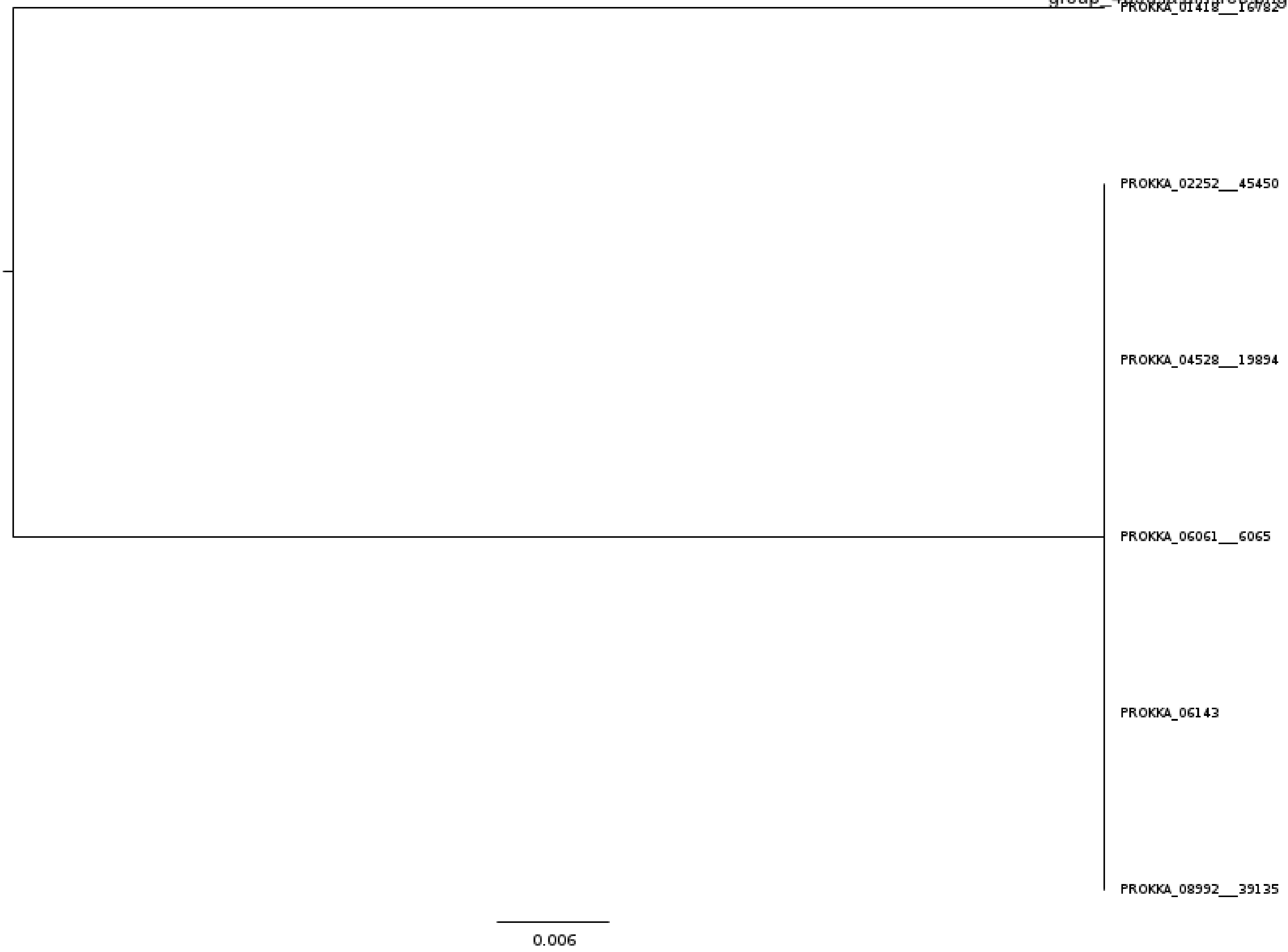

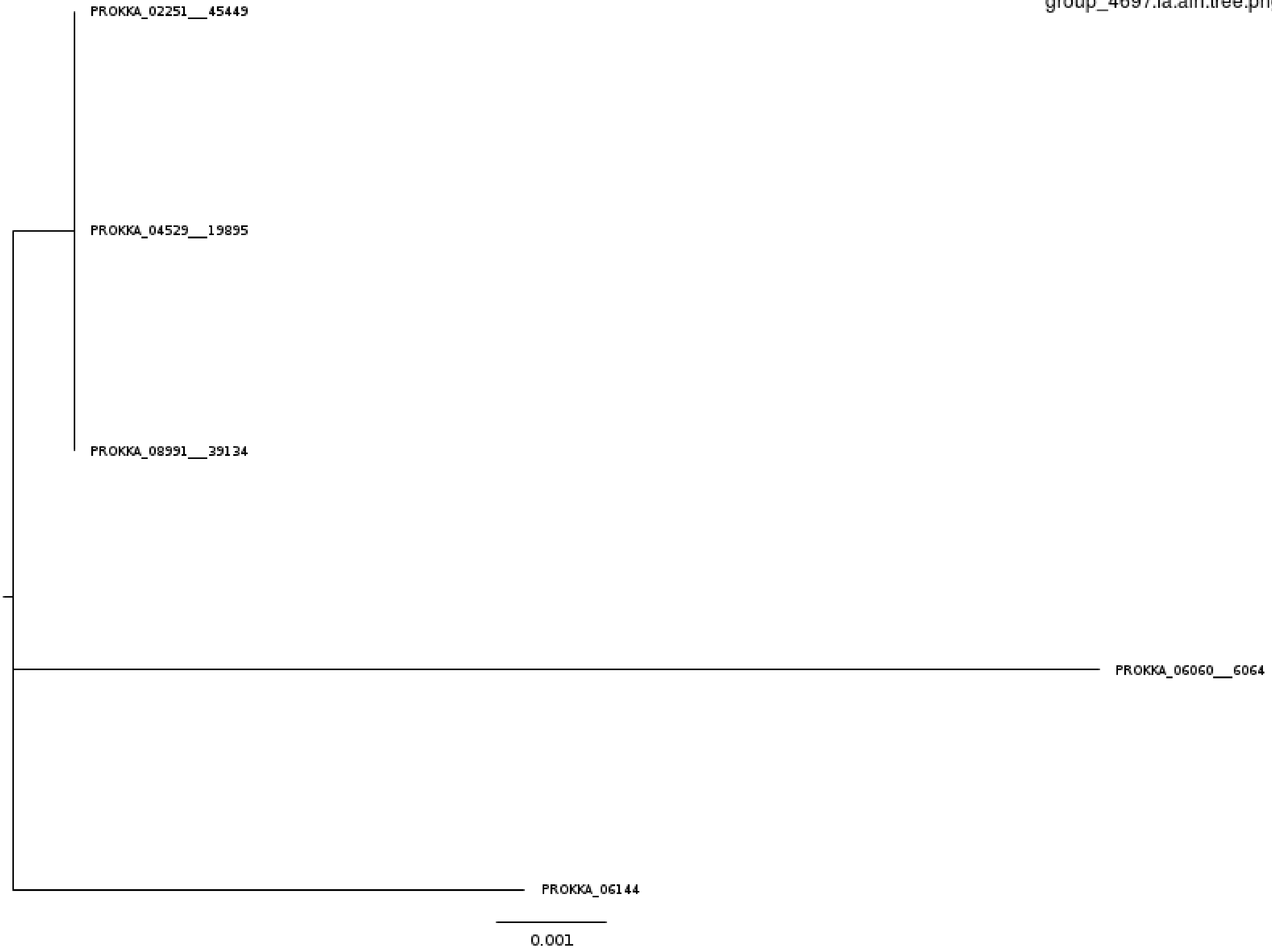
PROKKA_02251___45449
PROKKA_04529___19895
PROKKA_08991___39134
PROKKA_06060___6064
PROKKA_06144
0.001

group_474.fa.aln.tree.png

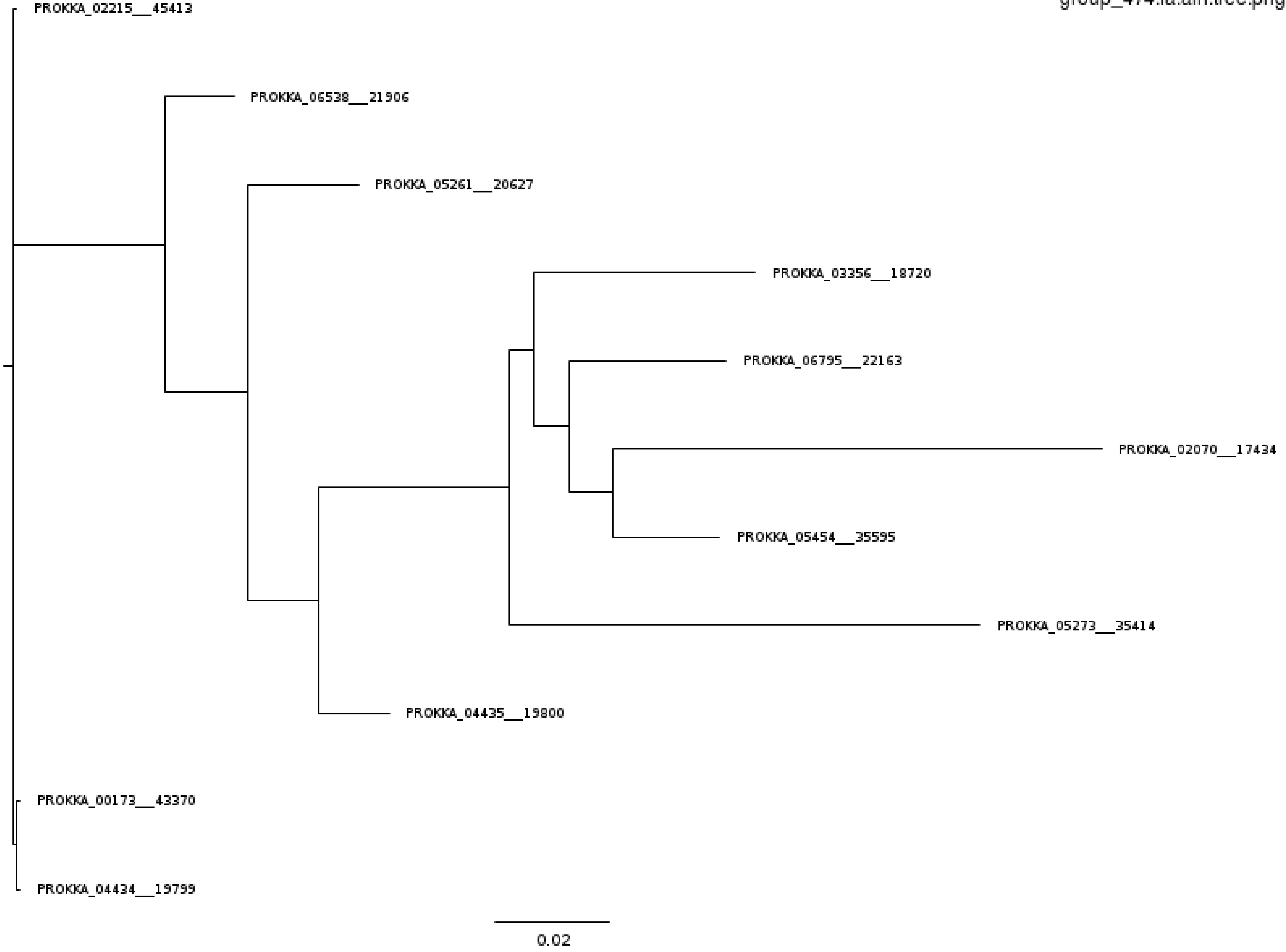

group_4753.fa.aln.tree.png

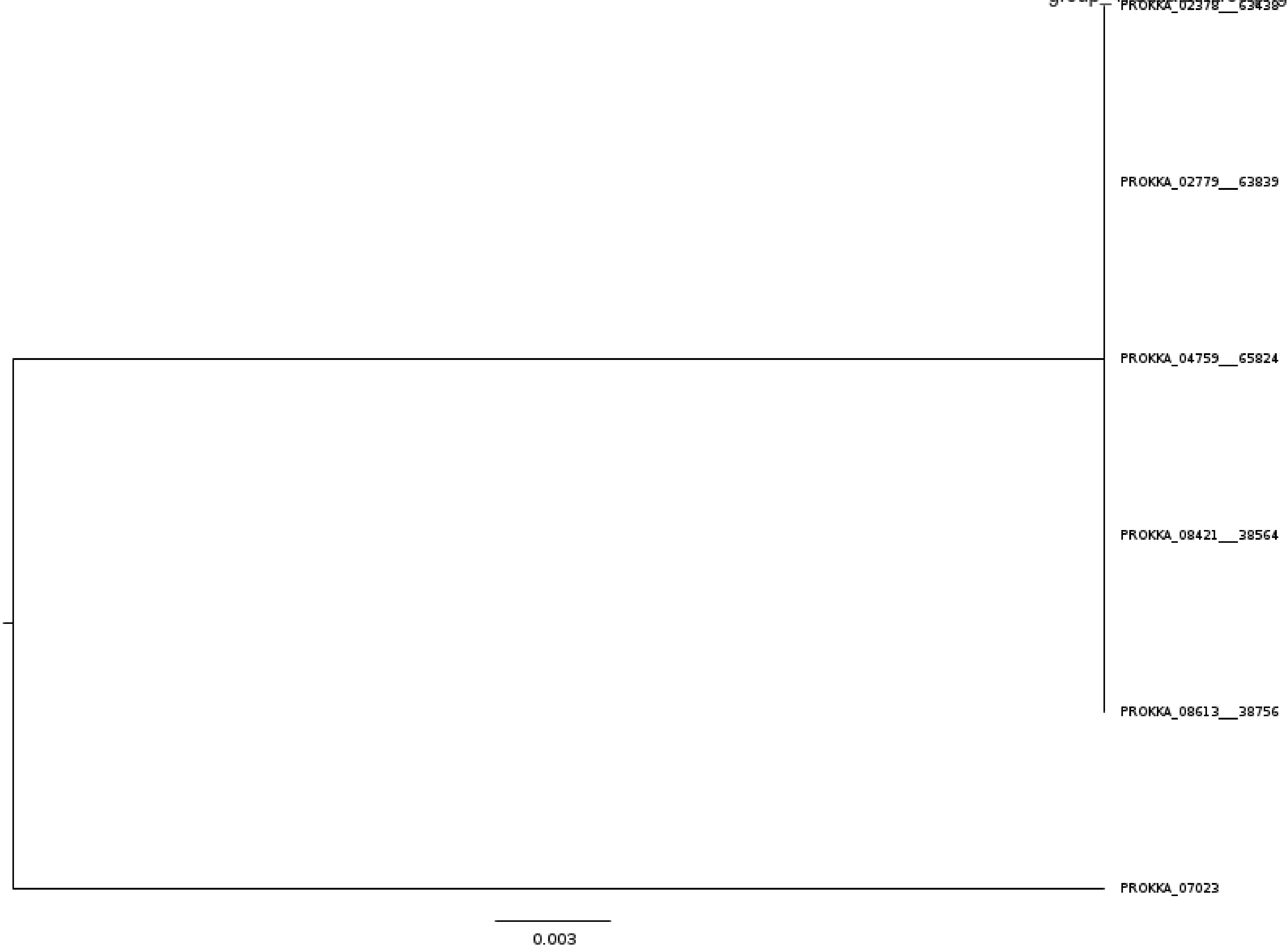

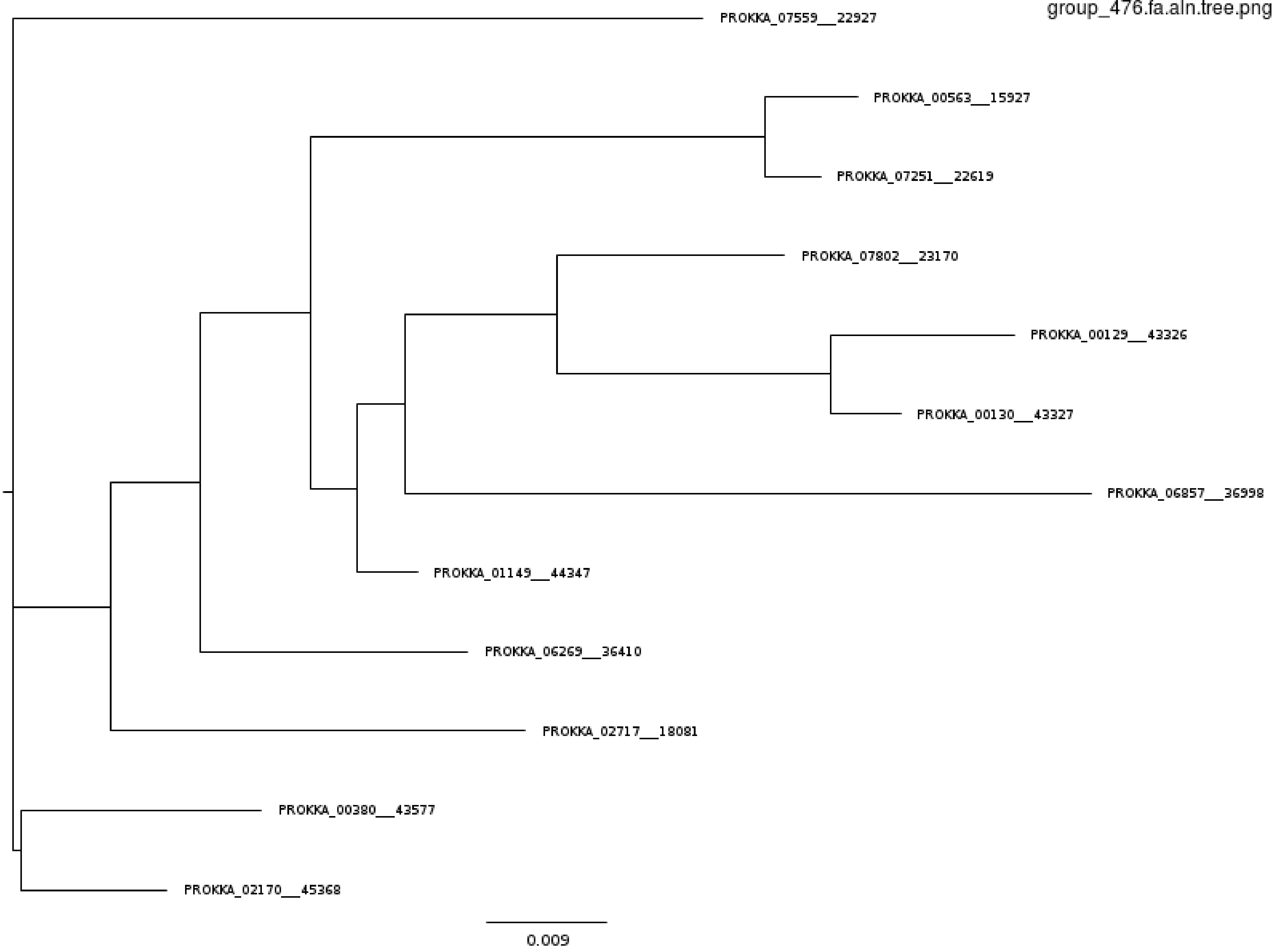
group_476.fa.aln.tree.png
PROKKA_07559___22927
PROKKA_00563___15927
PROKKA_07251___22619
PROKKA_07802___23170
PROKKA_00129___43326
PROKKA_00130___43327
PROKKA_06857___36998
PROKKA_01149___44347
PROKKA_06269___36410
PROKKA_02717___18081
PROKKA_00380___43577
PROKKA_02170___45368
0.009

group_47.fa.aln.tree.png

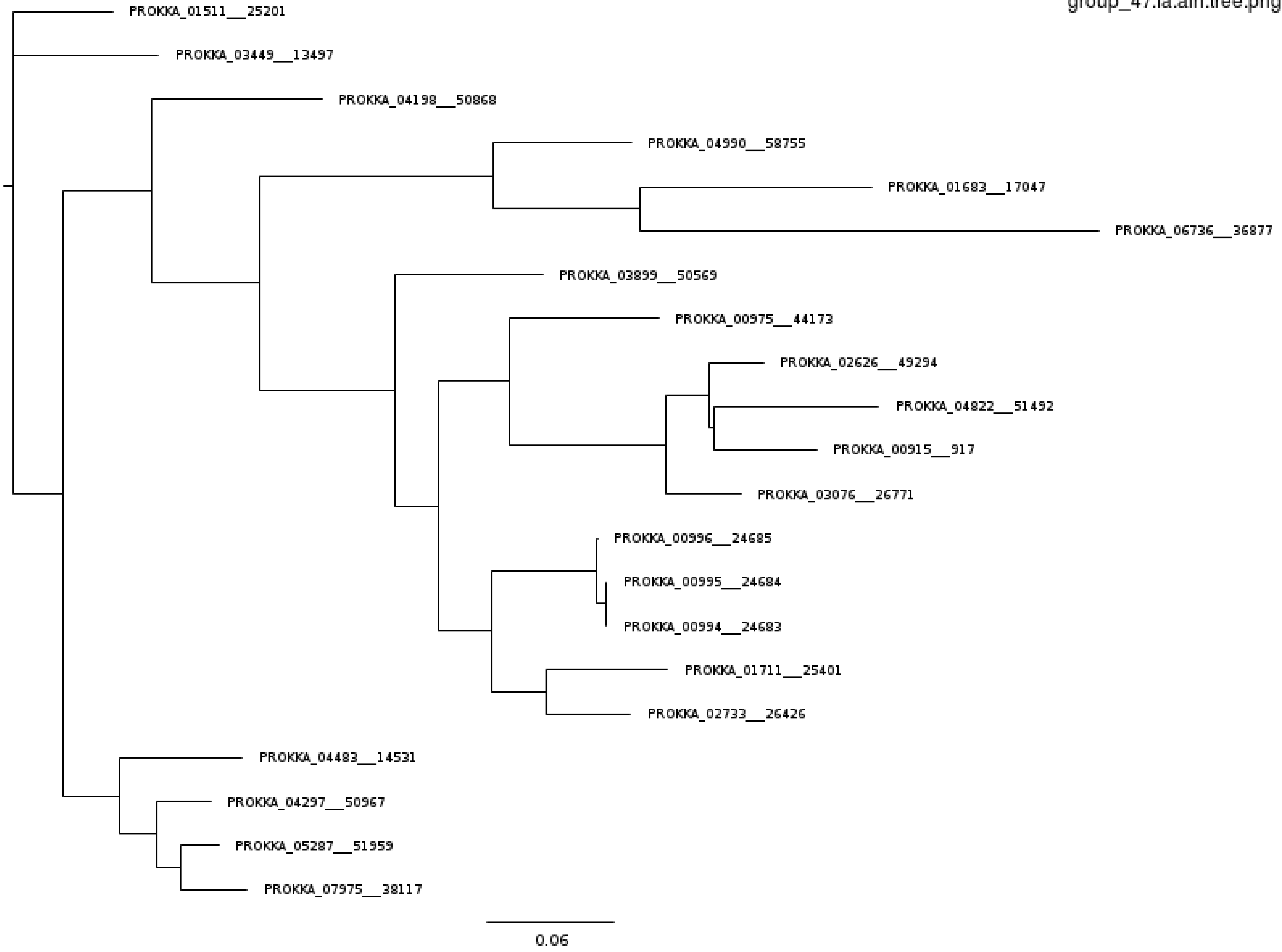

group_486.fa.aln.tree.png

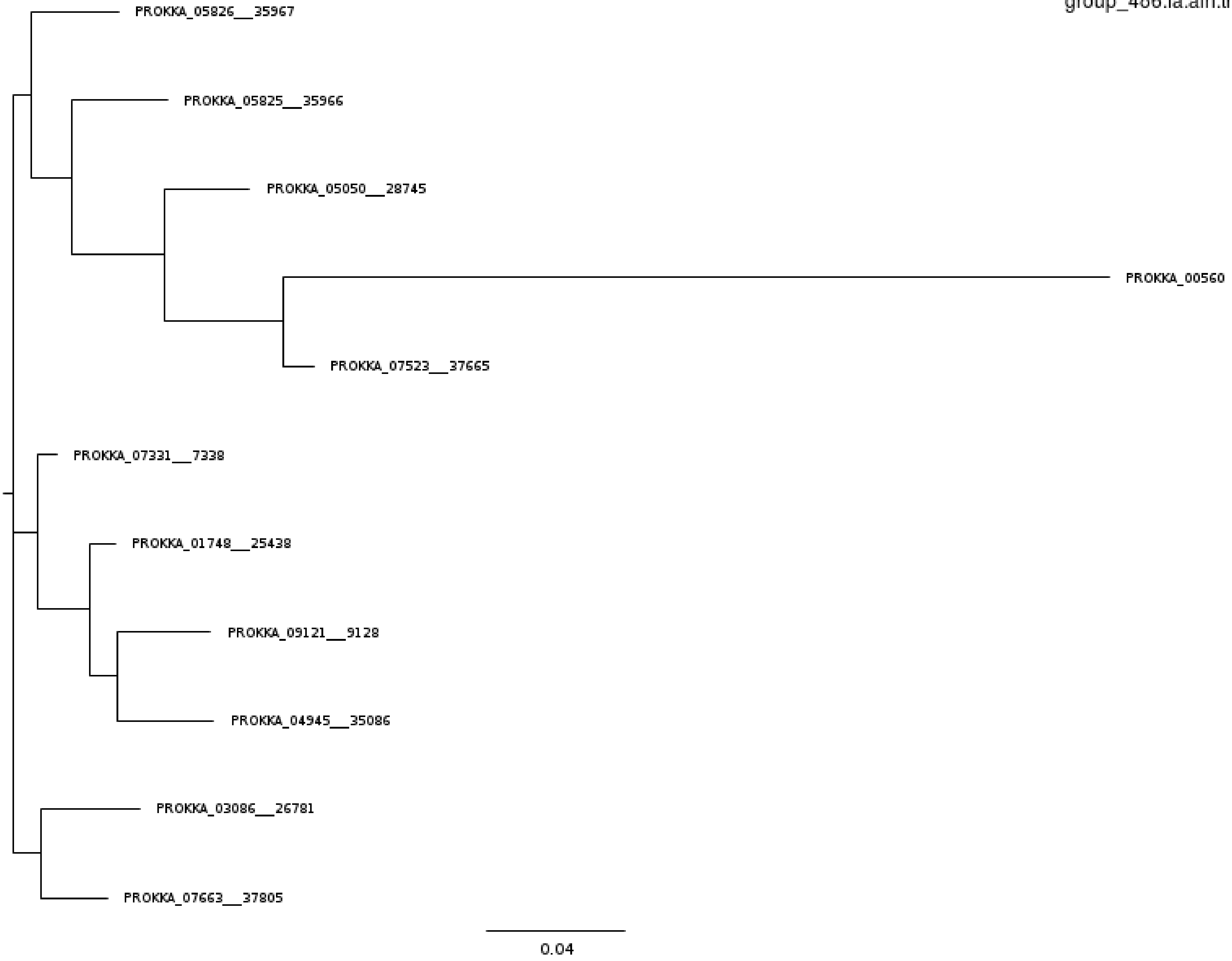

group_491.fa.aln.tree.png

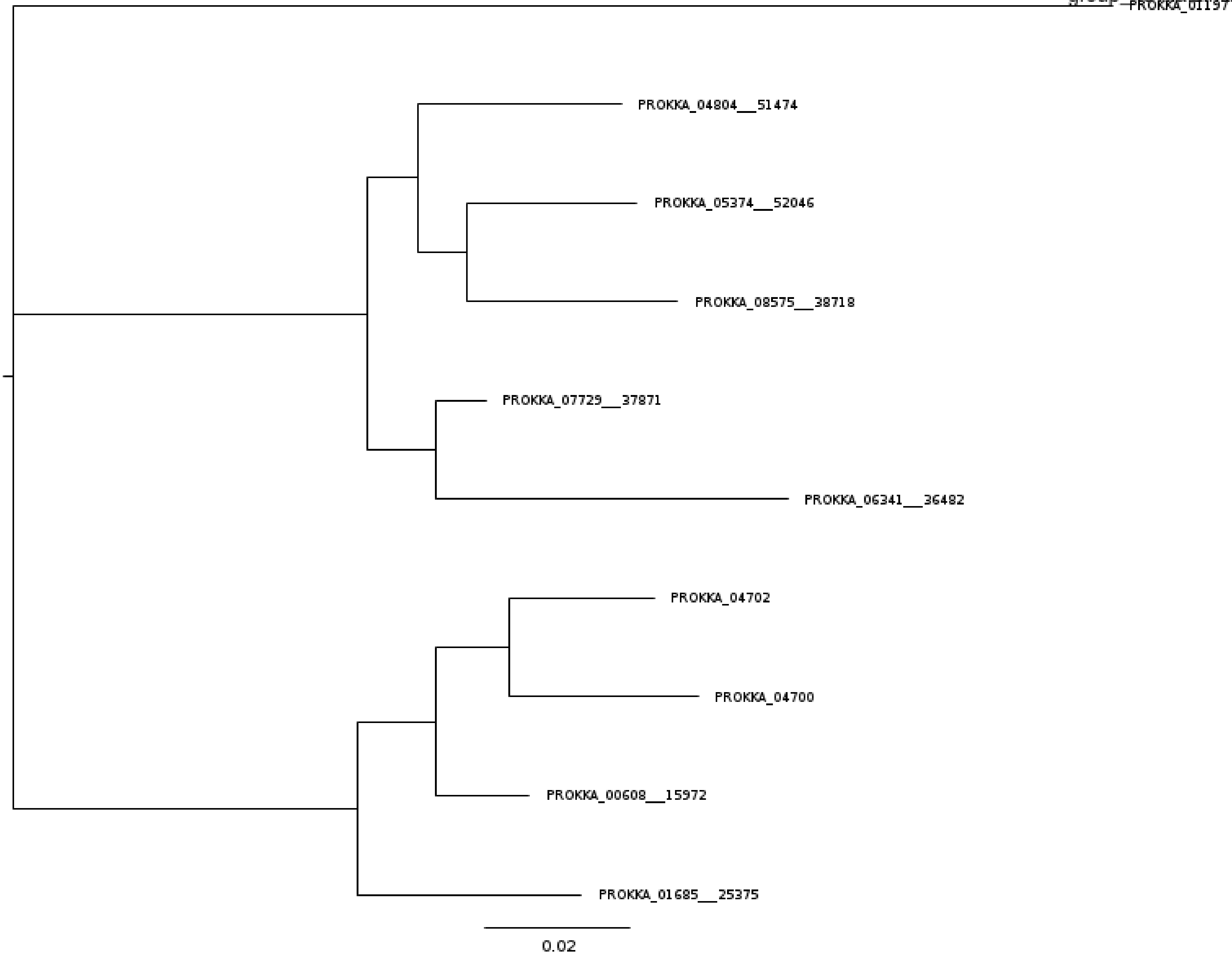

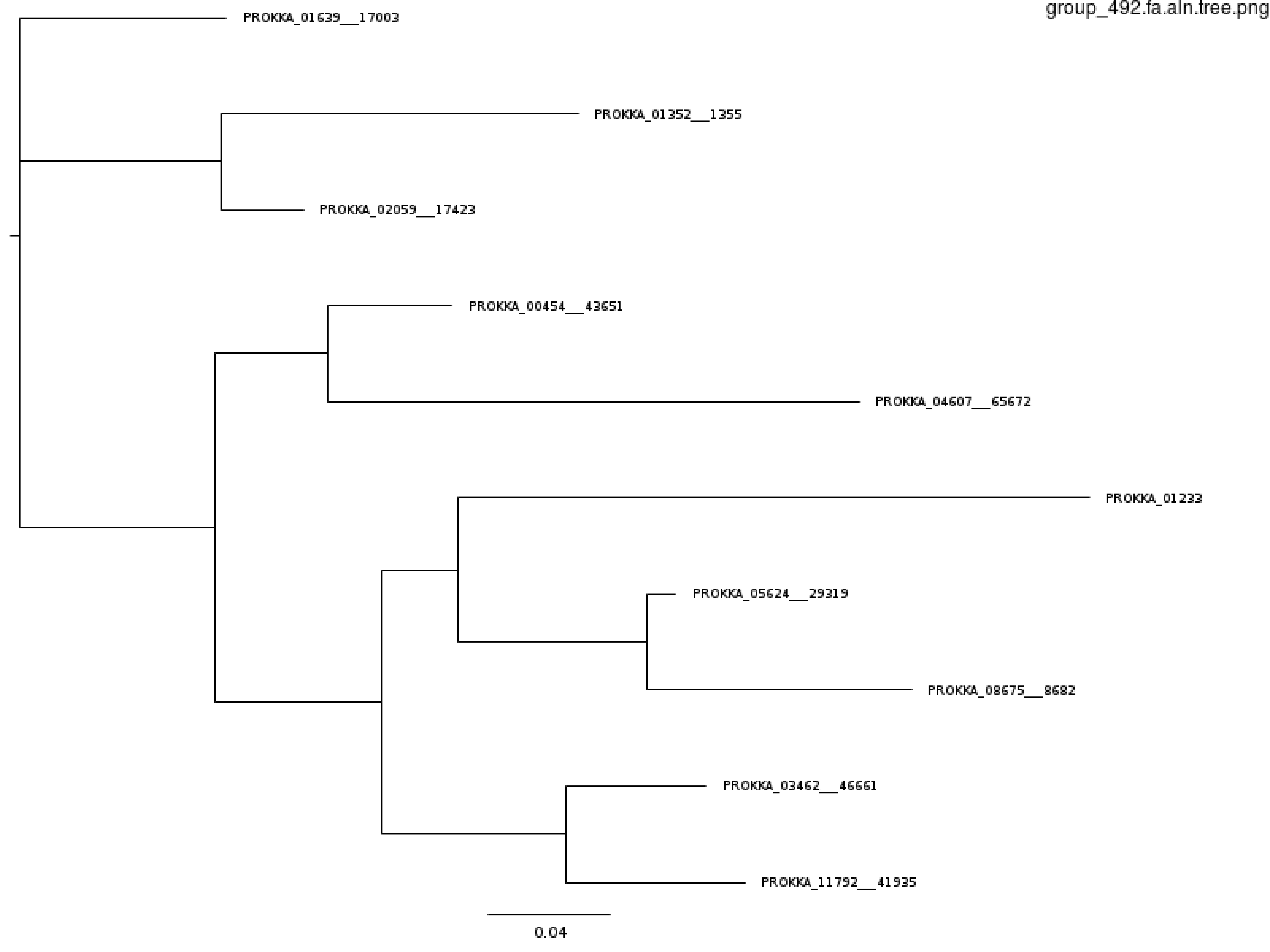
group_492.fa.aln.tree.png
PROKKA_01639___17003
PROKKA_01352___1355
PROKKA_02059___17423
PROKKA_00454___43651
PROKKA_04607___65672
PROKKA_01233
PROKKA_05624___29319
PROKKA_08675___8682
PROKKA_03462___46661
PROKKA_11792___41935
0.04

group_494.fa.aln.tree.png

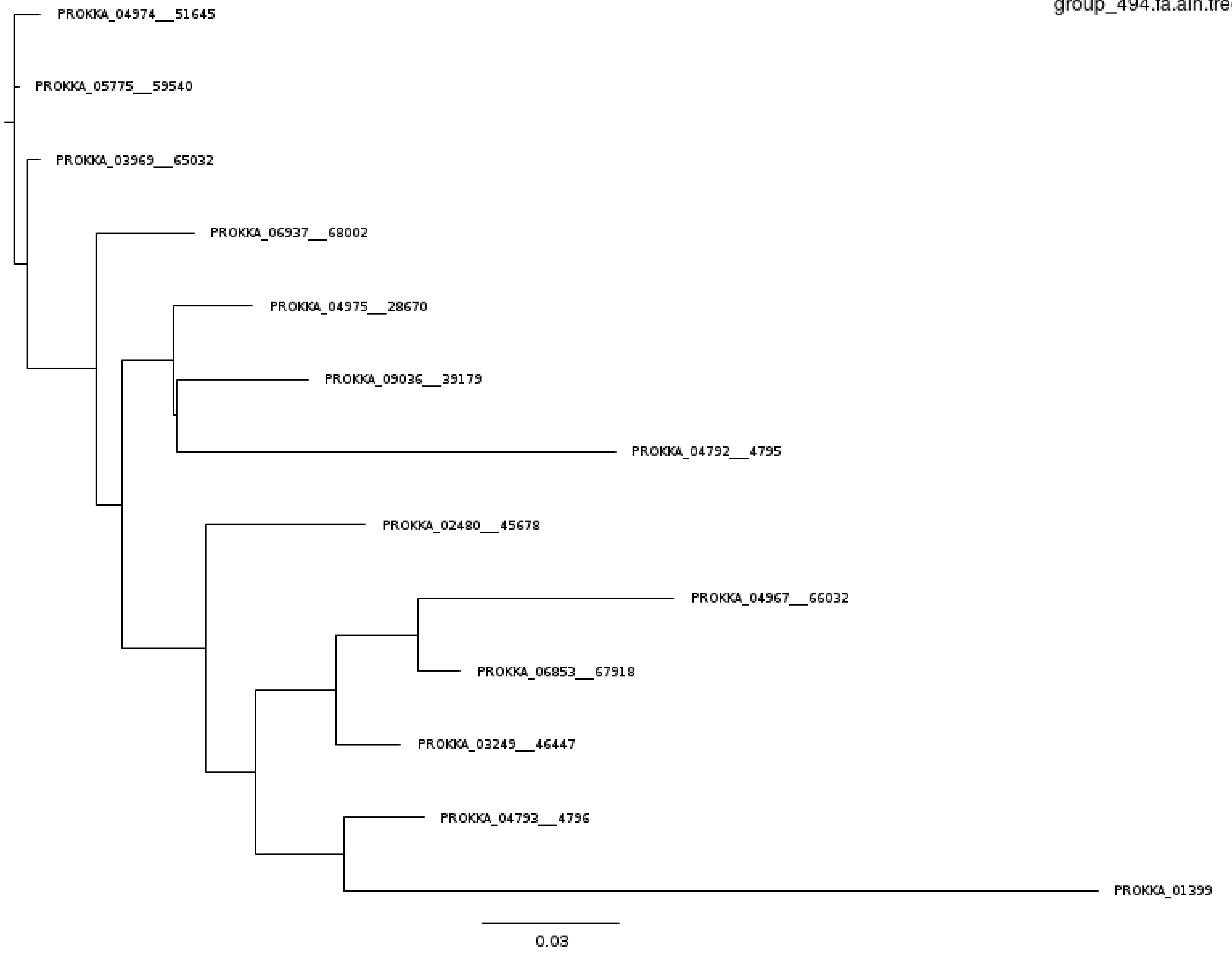

group_498.fa.aln.tree.png

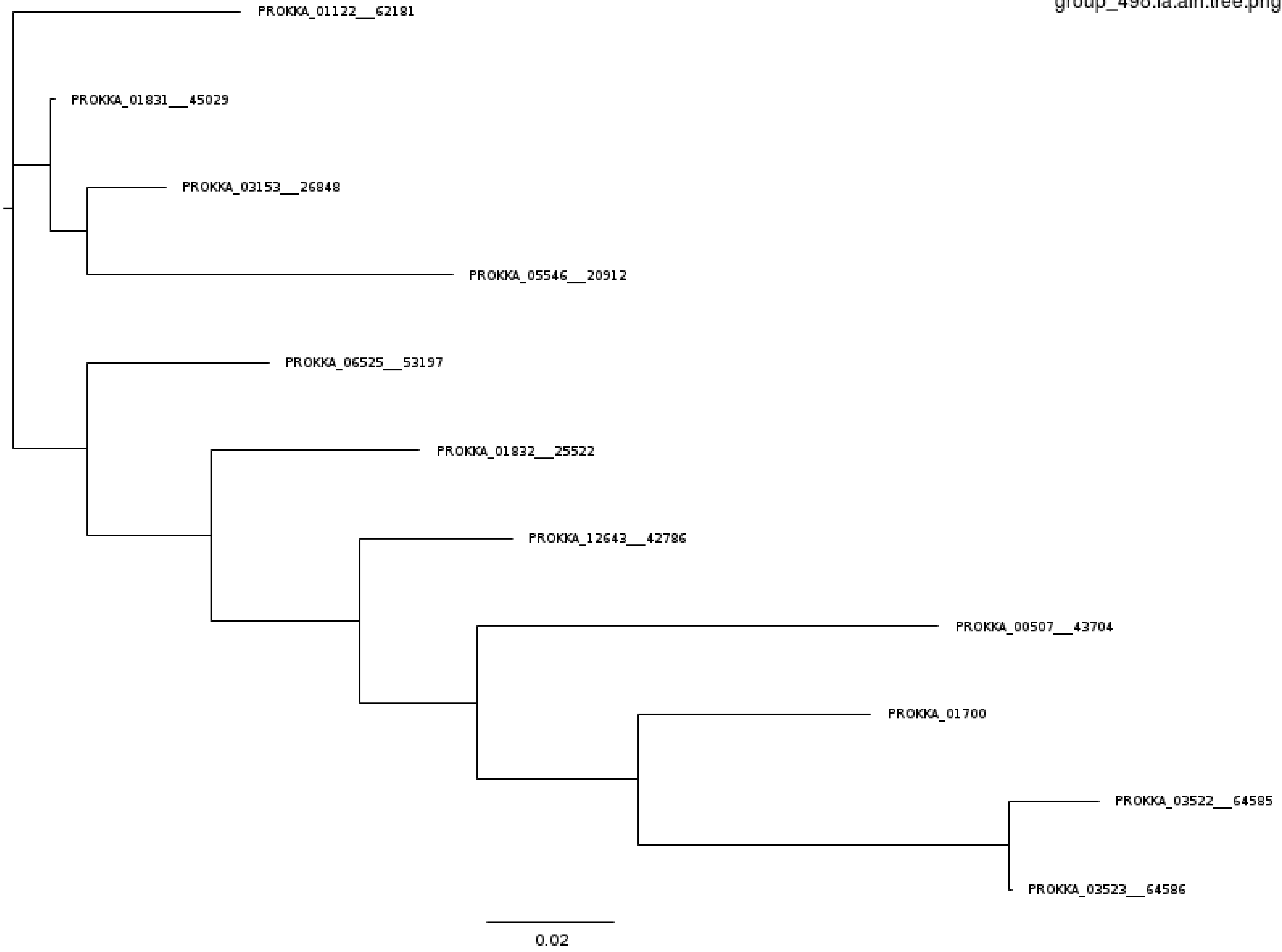

group_511.fa.aln.tree.png

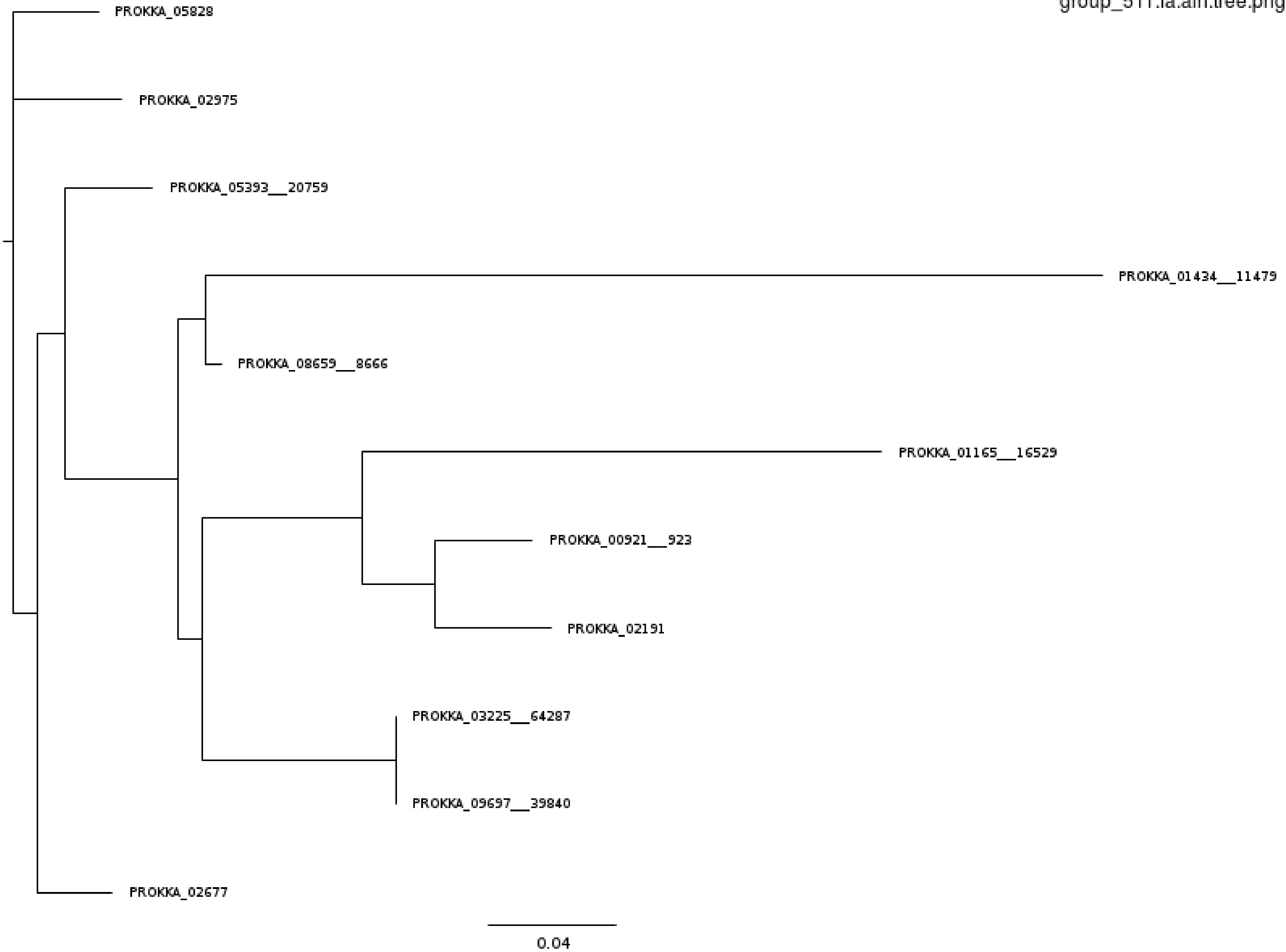

group_515.fa.aln.tree.png

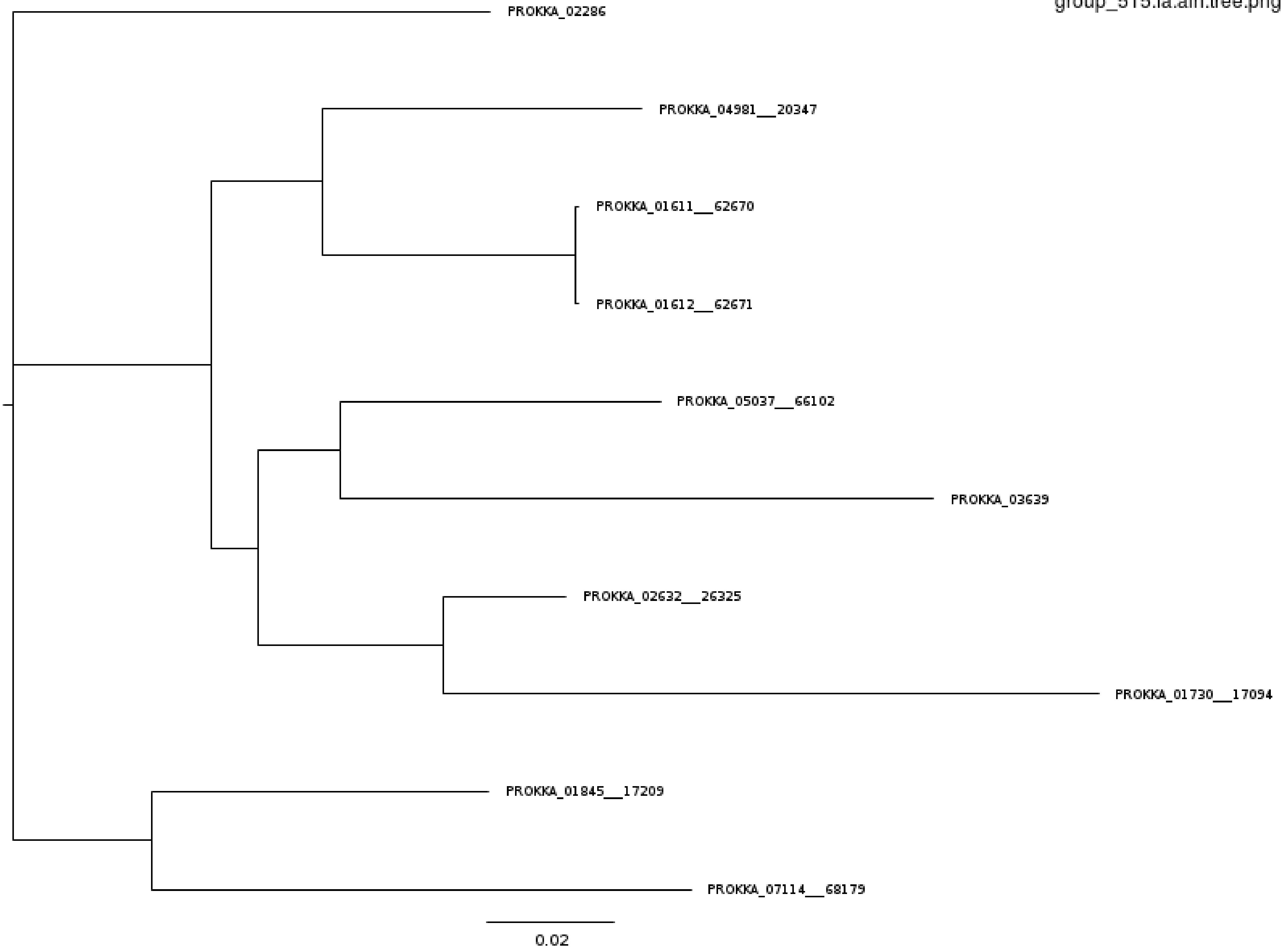

group_517.fa.aln.tree.png

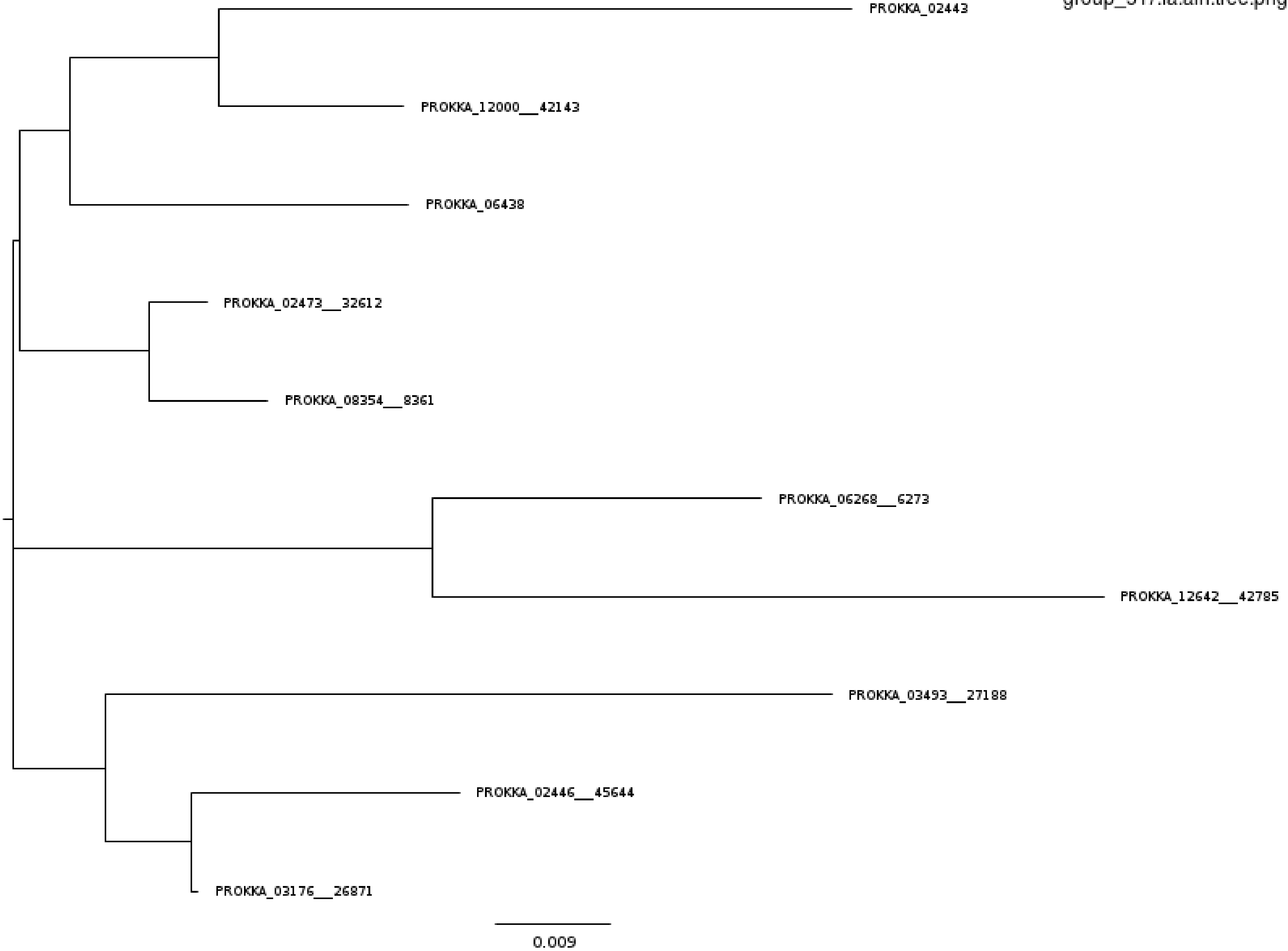

group_519.fa.aln.tree.png

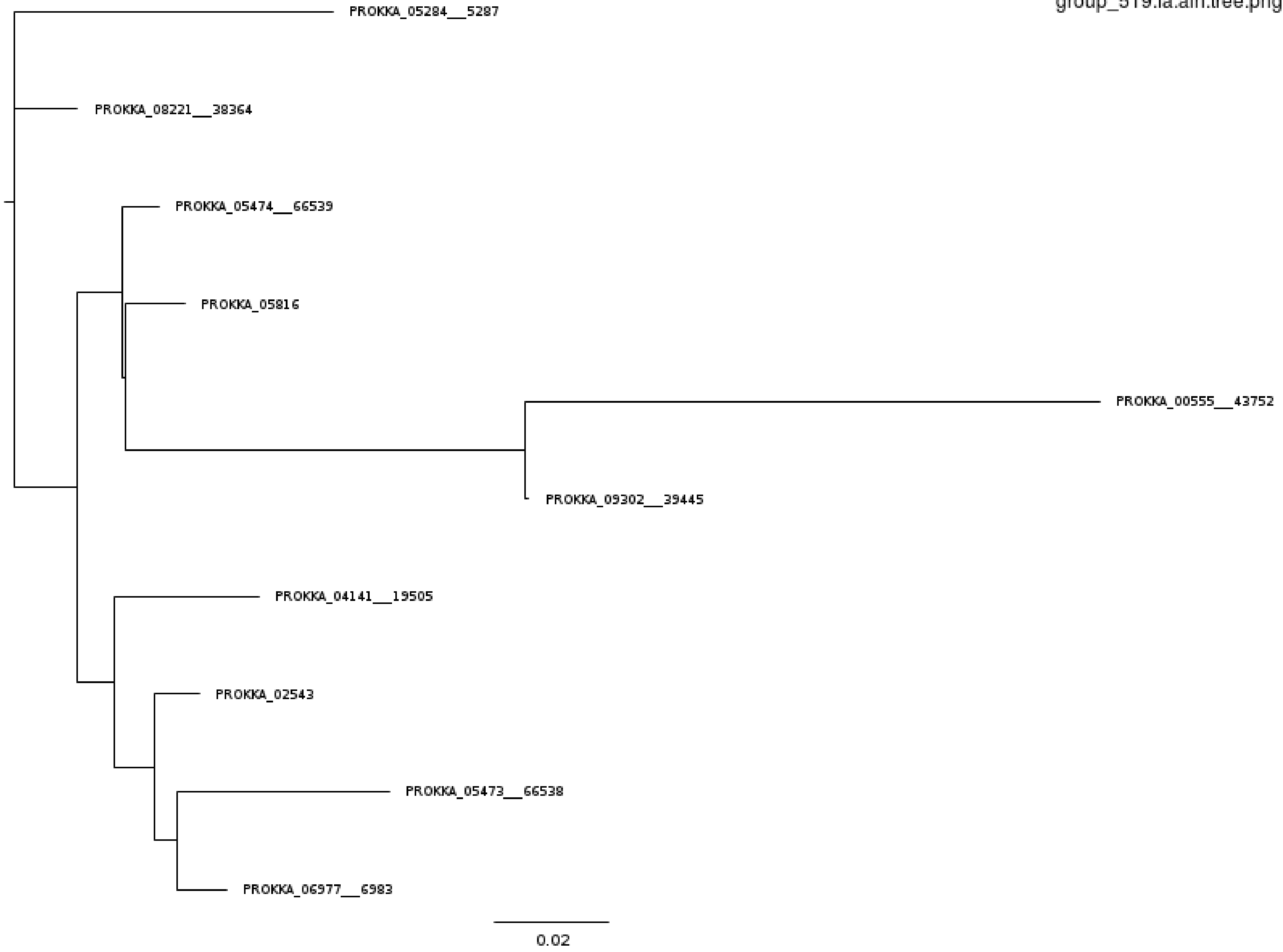

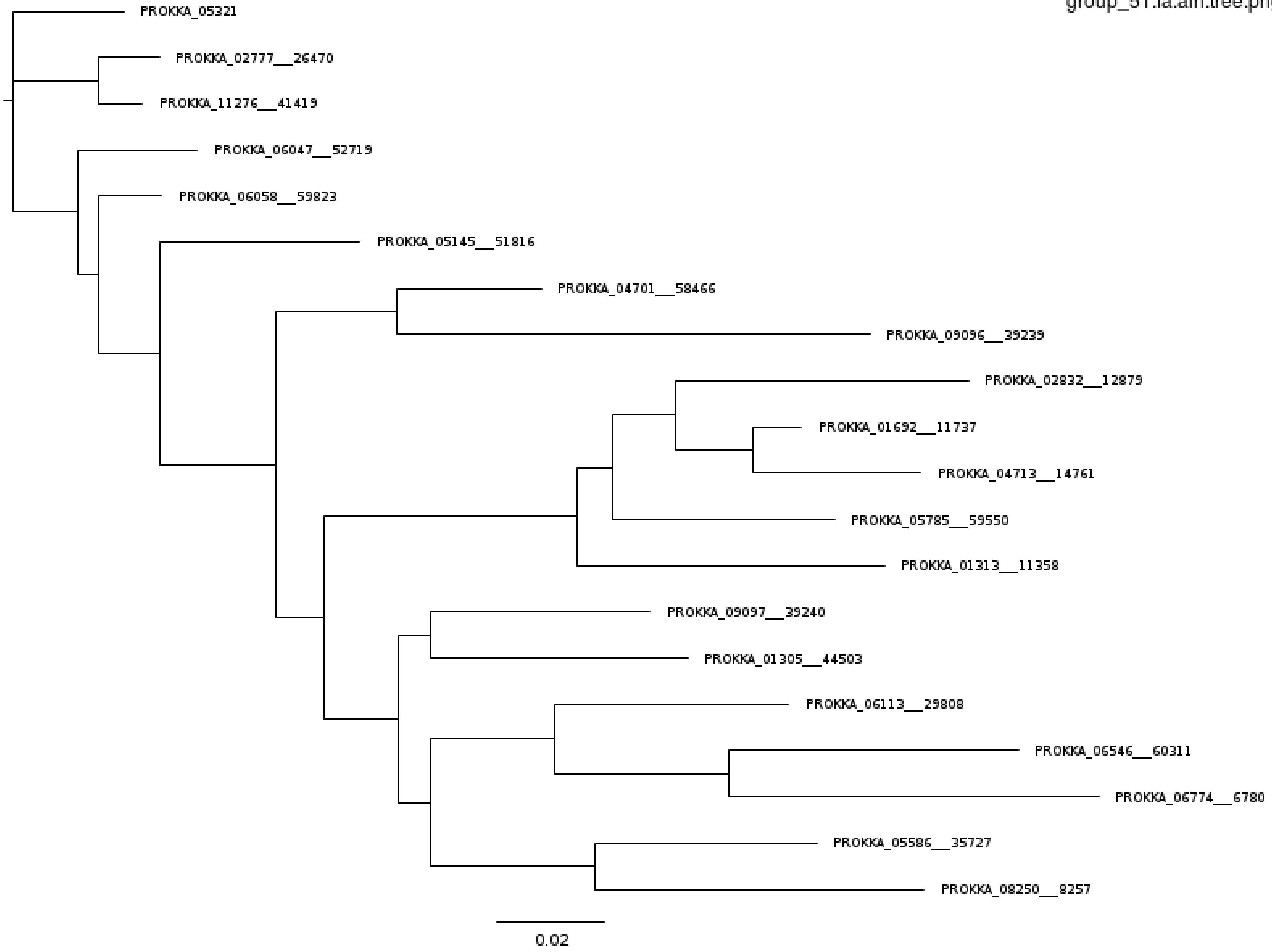
PROKKA_05321
PROKKA_02777__26470
PROKKA_11276__41419
PROKKA_06047__52719
PROKKA_06058__59823
PROKKA_05145__51816
PROKKA_04701__58466
PROKKA_09096__39239
PROKKA_02832__12879
PROKKA_01692__11737
PROKKA_04713__14761
PROKKA_05785__59550
PROKKA_01313__11358
PROKKA_09097__39240
PROKKA_01305__44503
PROKKA_06113__29808
PROKKA_06546__60311
PROKKA_06774__6780
PROKKA_05586__35727
PROKKA_08250__8257
0.02

group_522.fa.aln.tree.png

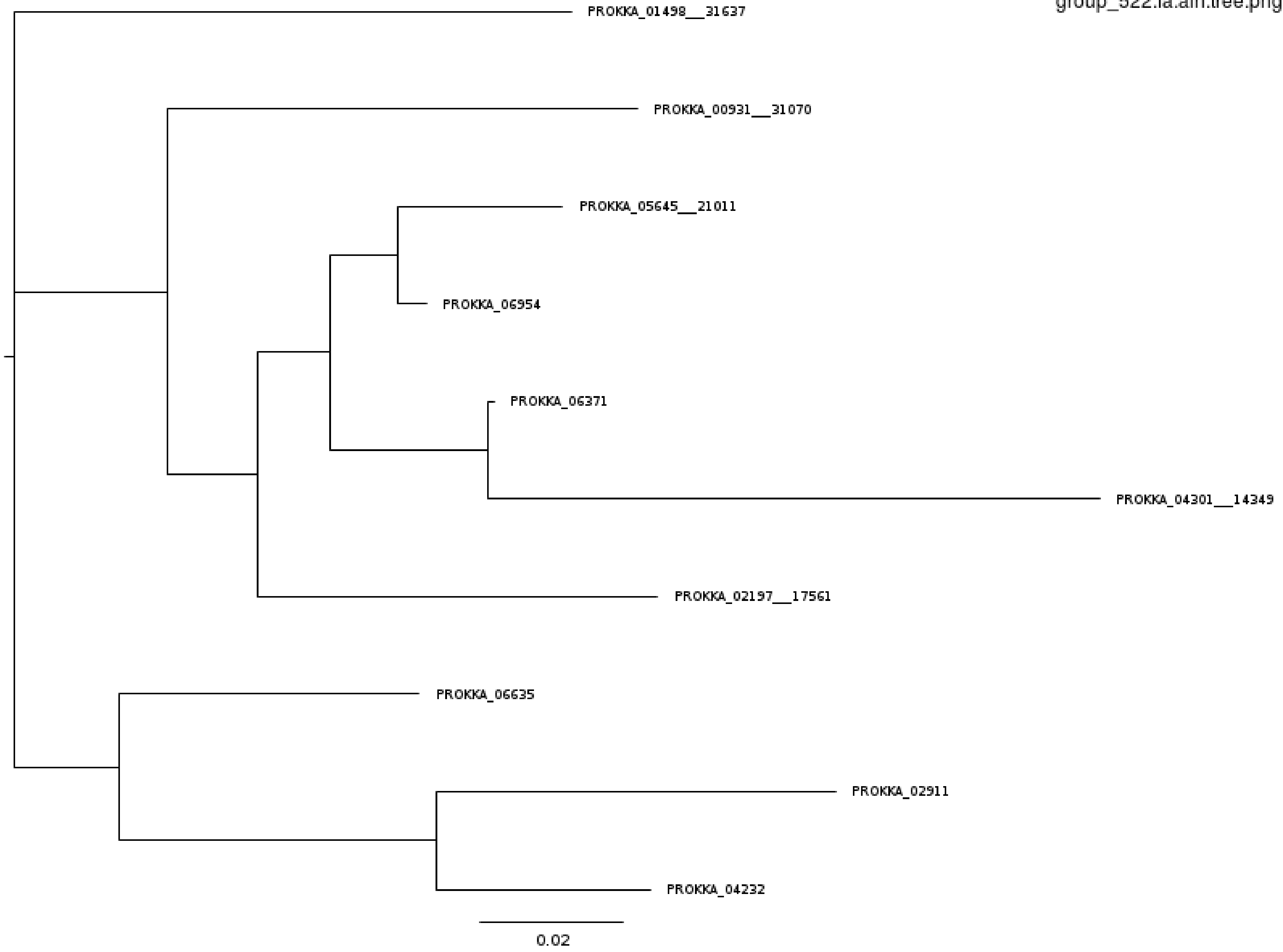

group_524.fa.aln.tree.png

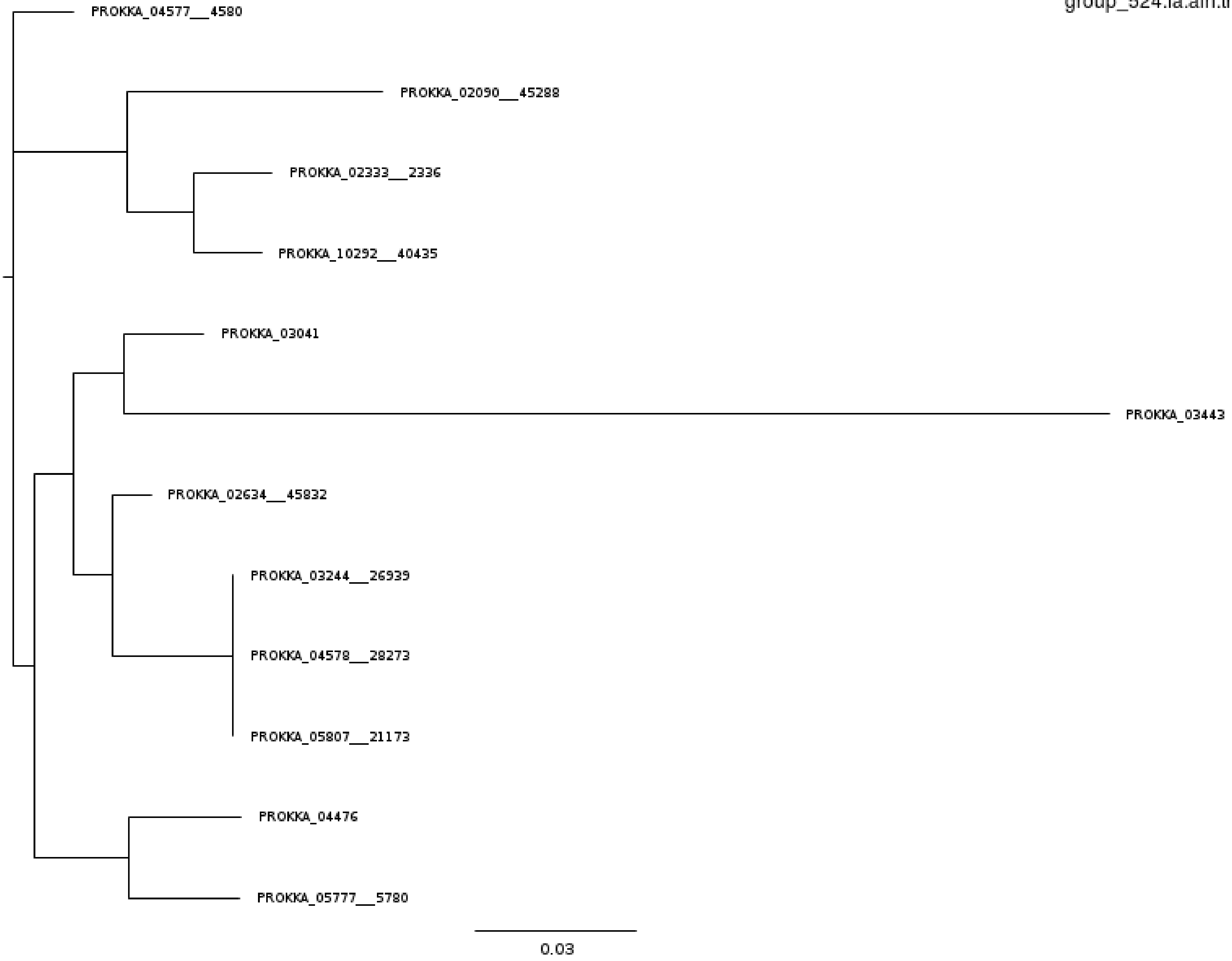

group_52.fa.aln.tree.png

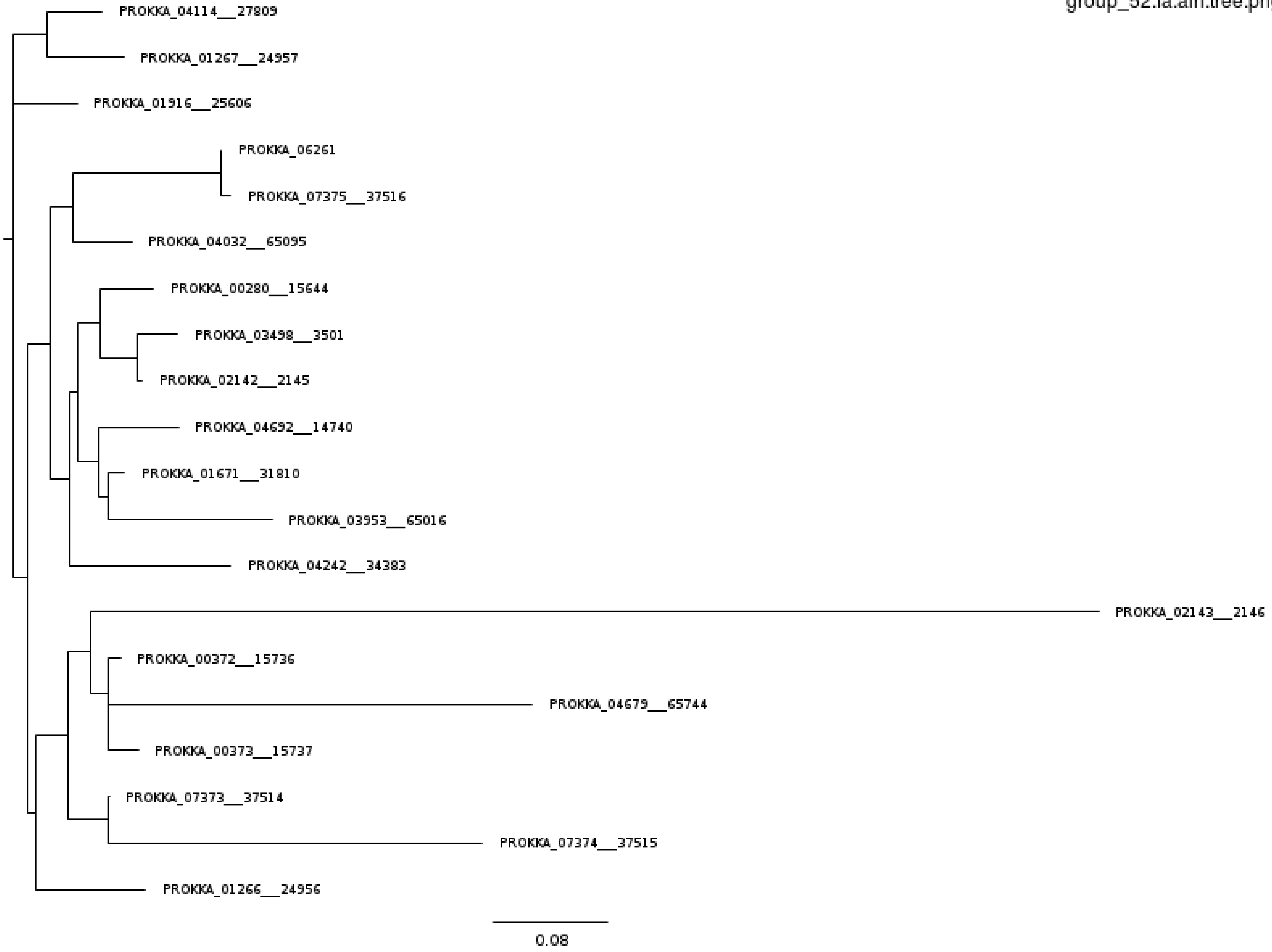

group_530.fa.aln.tree.png

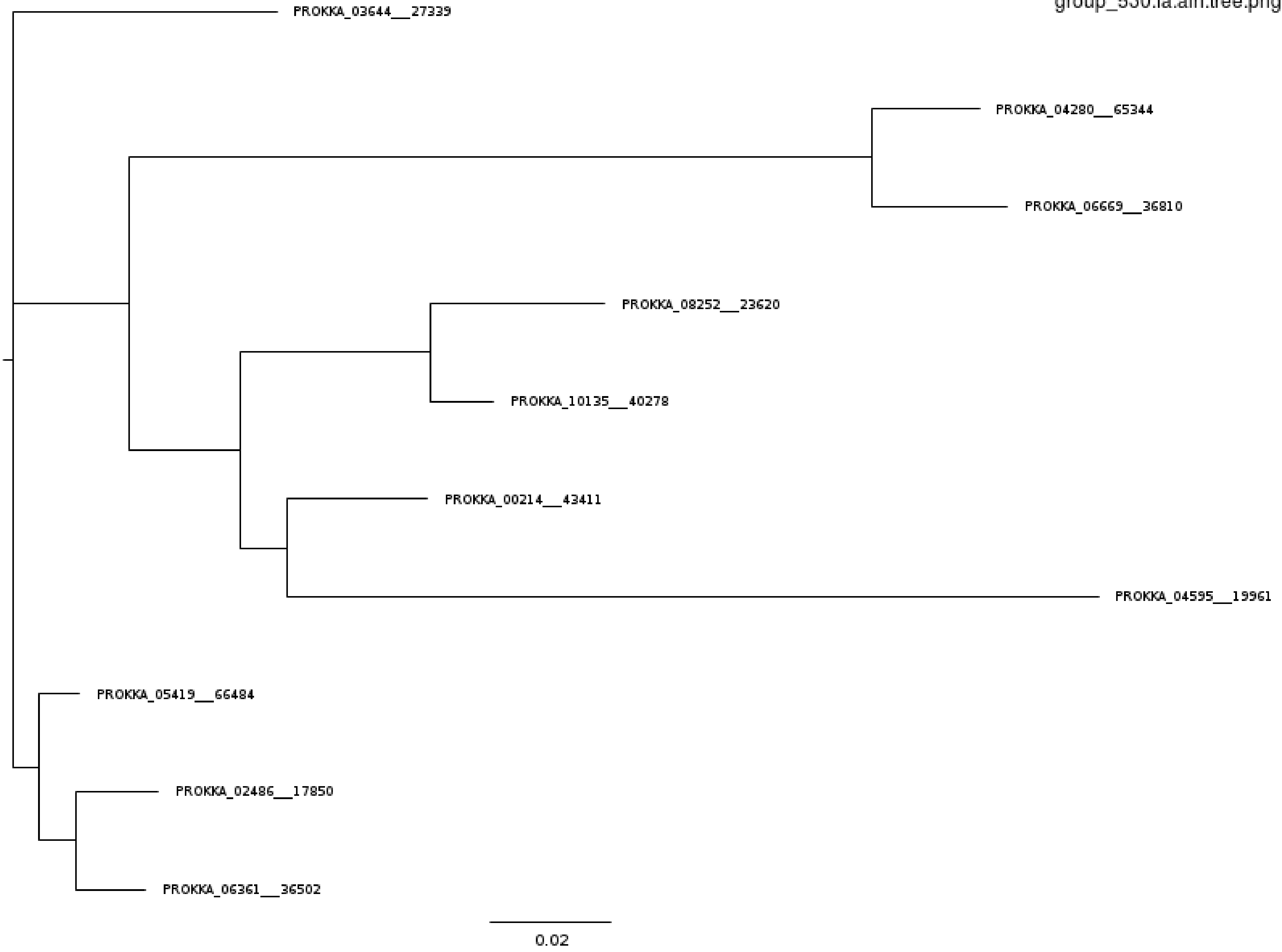

group_533.fa.aln.tree.png

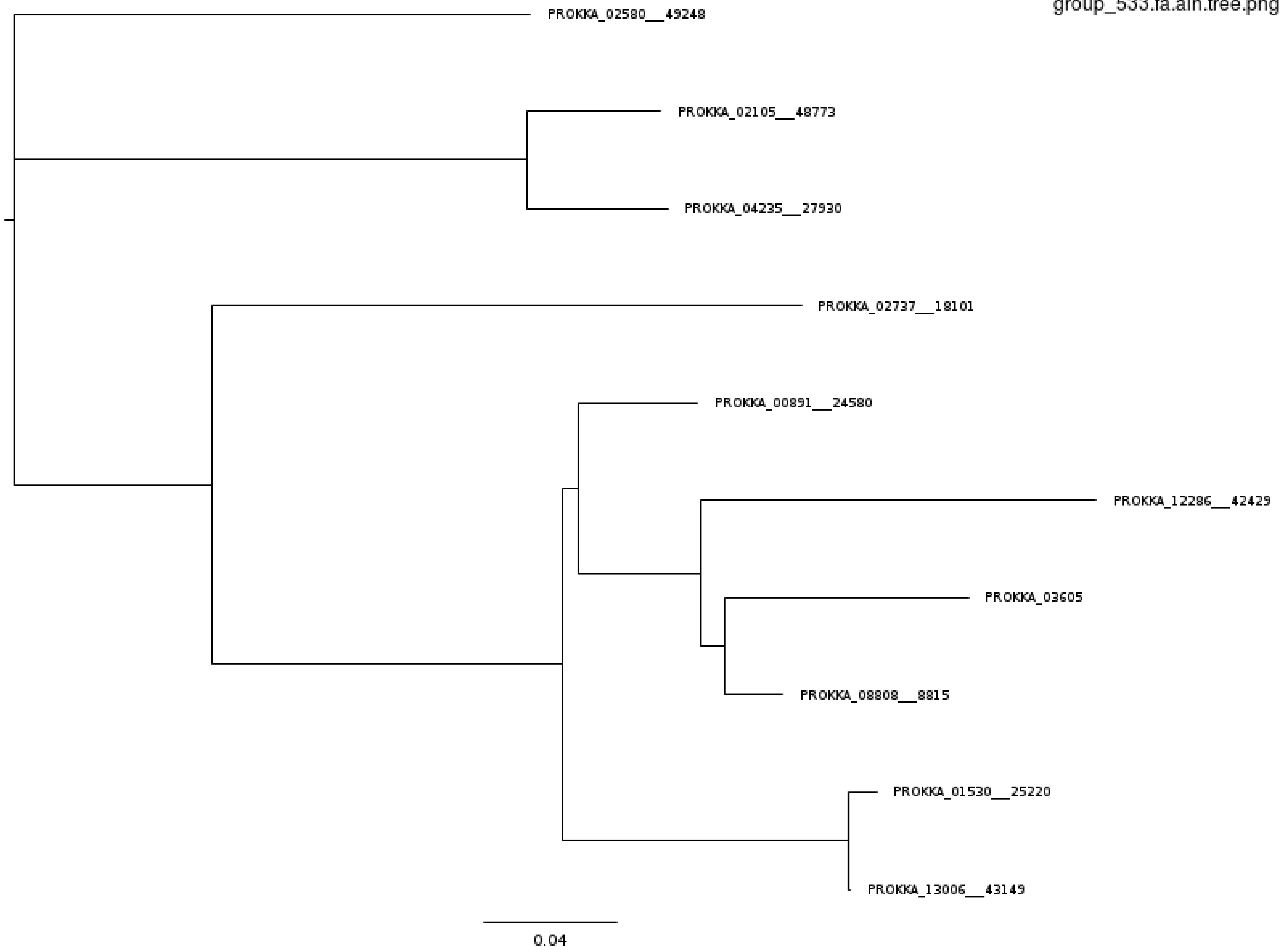

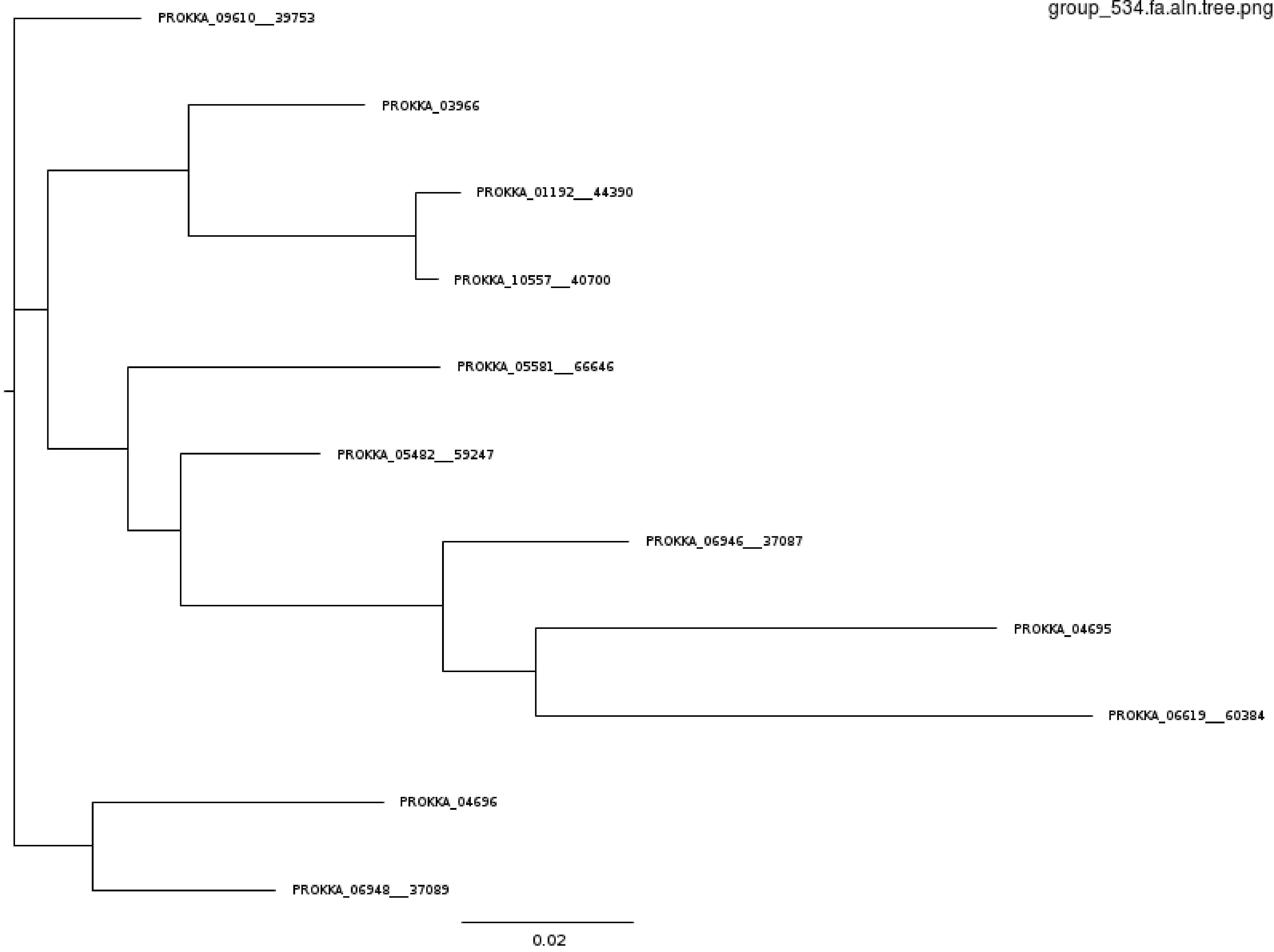
group_534.fa.aln.tree.png
PROKKA_09610___39753
PROKKA_03966
PROKKA_01192___44390
PROKKA_10557___40700
PROKKA_05581___66646
PROKKA_05482___59247
PROKKA_06946___37087
PROKKA_04695
PROKKA_06619___60384
PROKKA_04696
PROKKA_06948___37089
0.02

group_537.fa.aln.tree.png

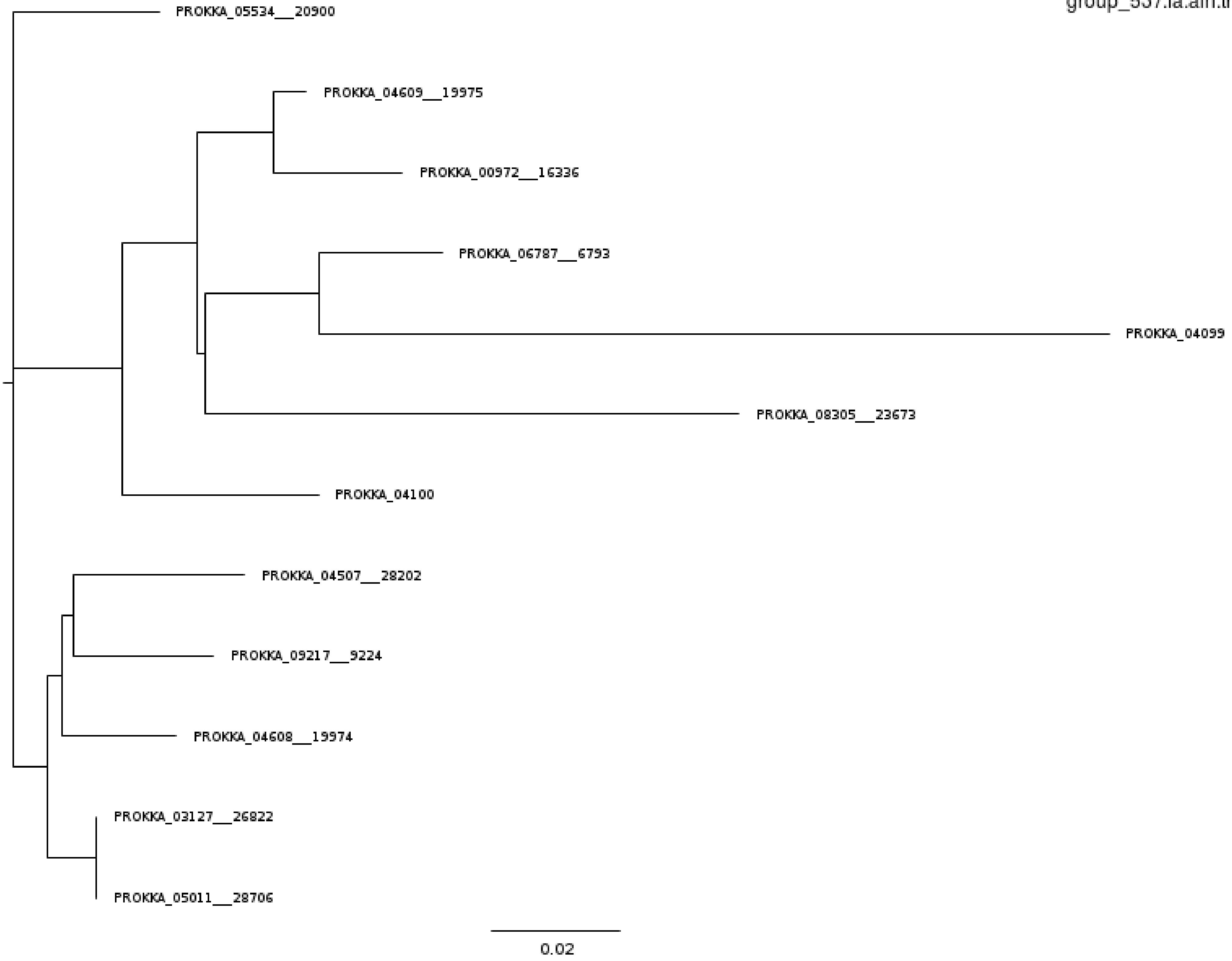

group_538.fa.aln.tree.png

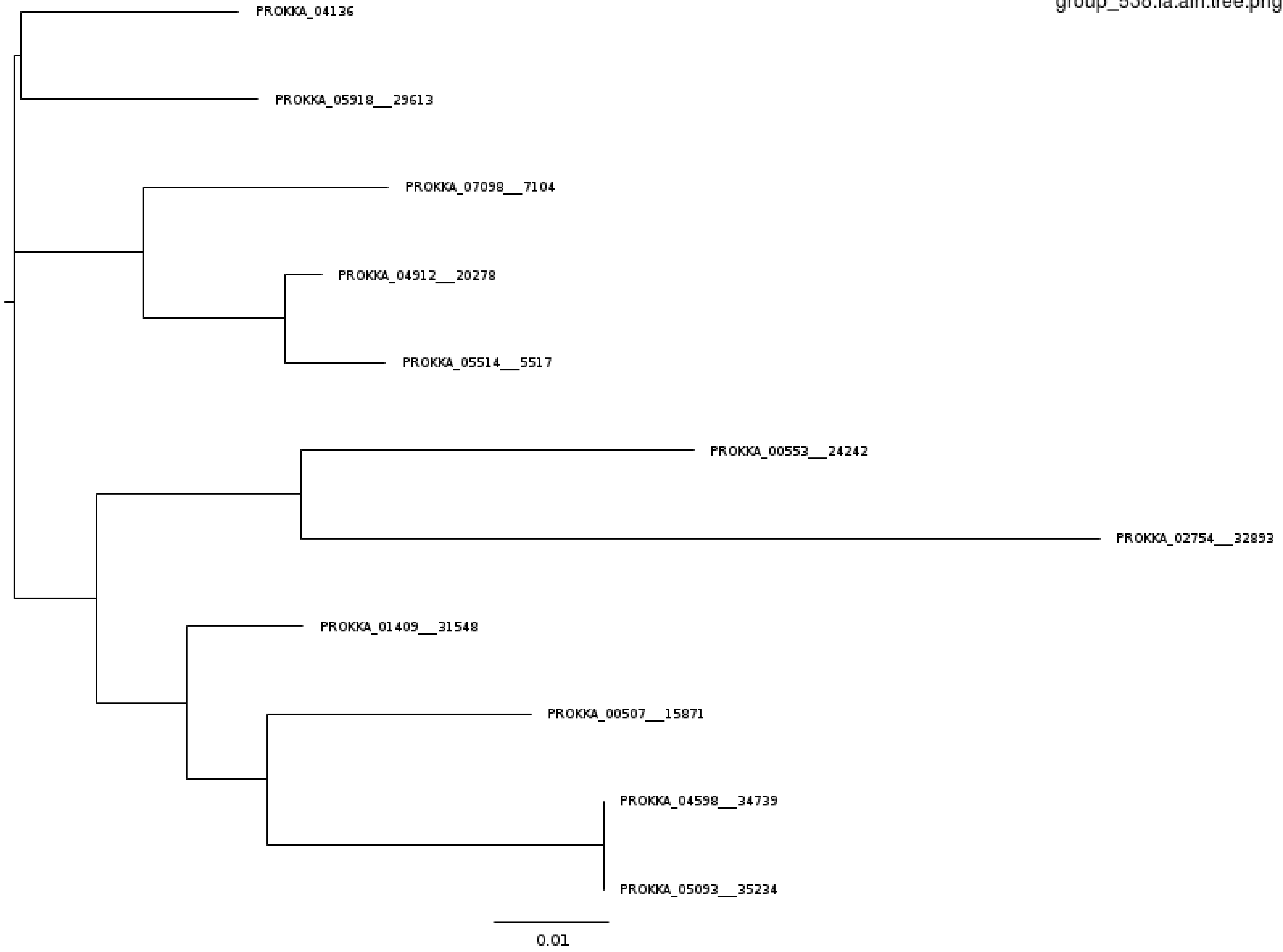

group_547.fa.aln.tree.png

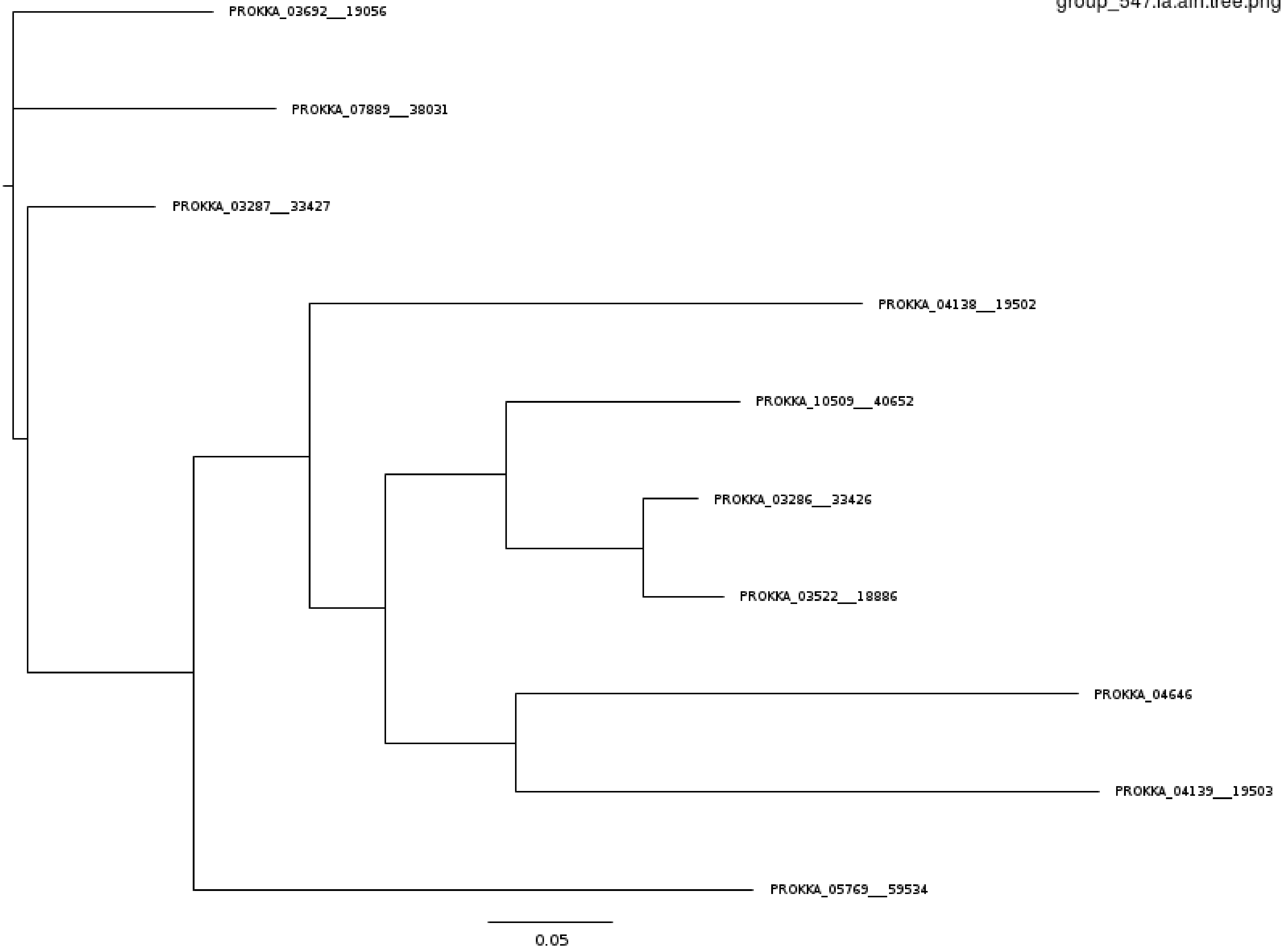

group_54.fa.aln.tree.png

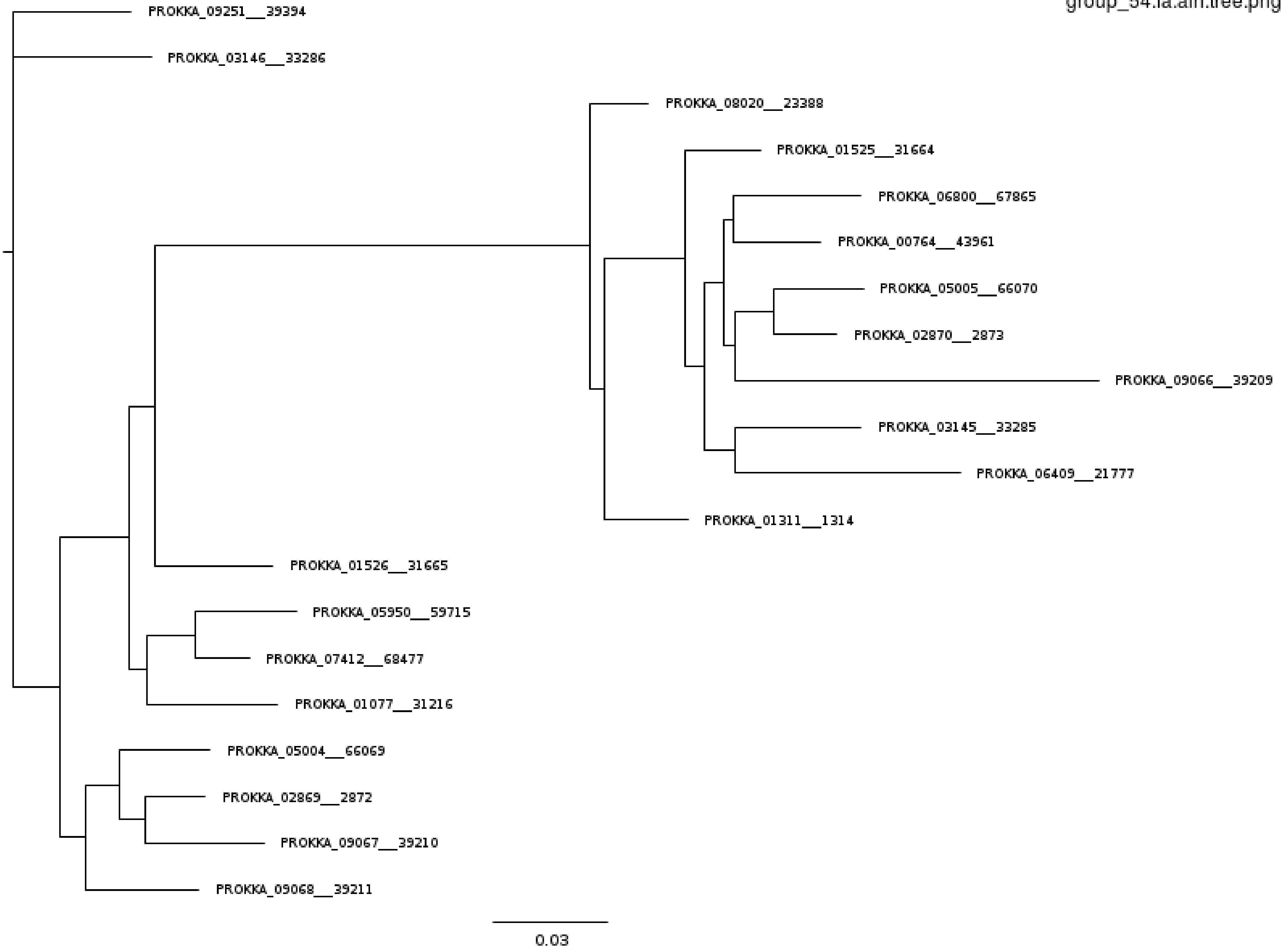

group_551.fa.aln.tree.png

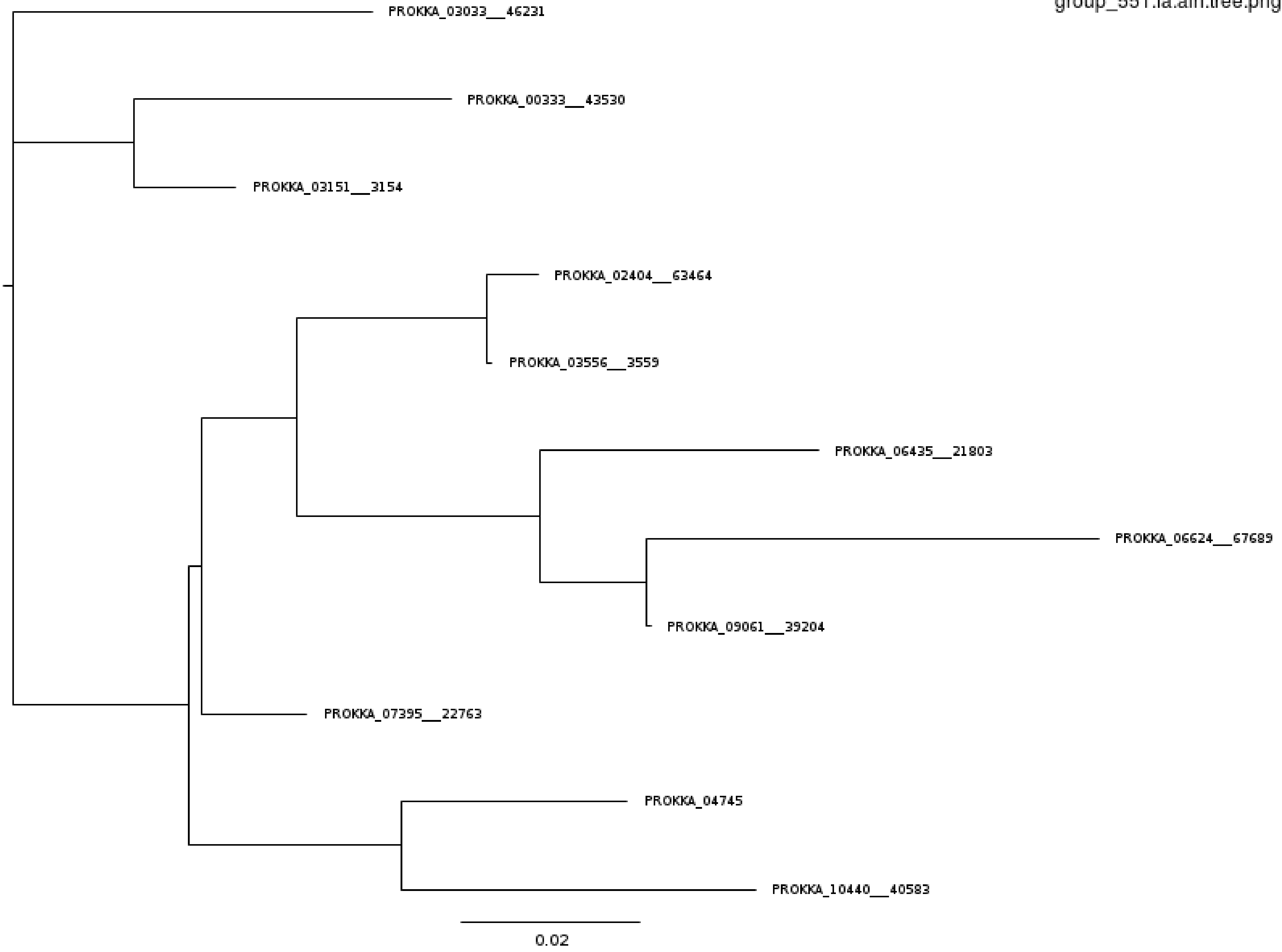

group_554.fa.aln.tree.png

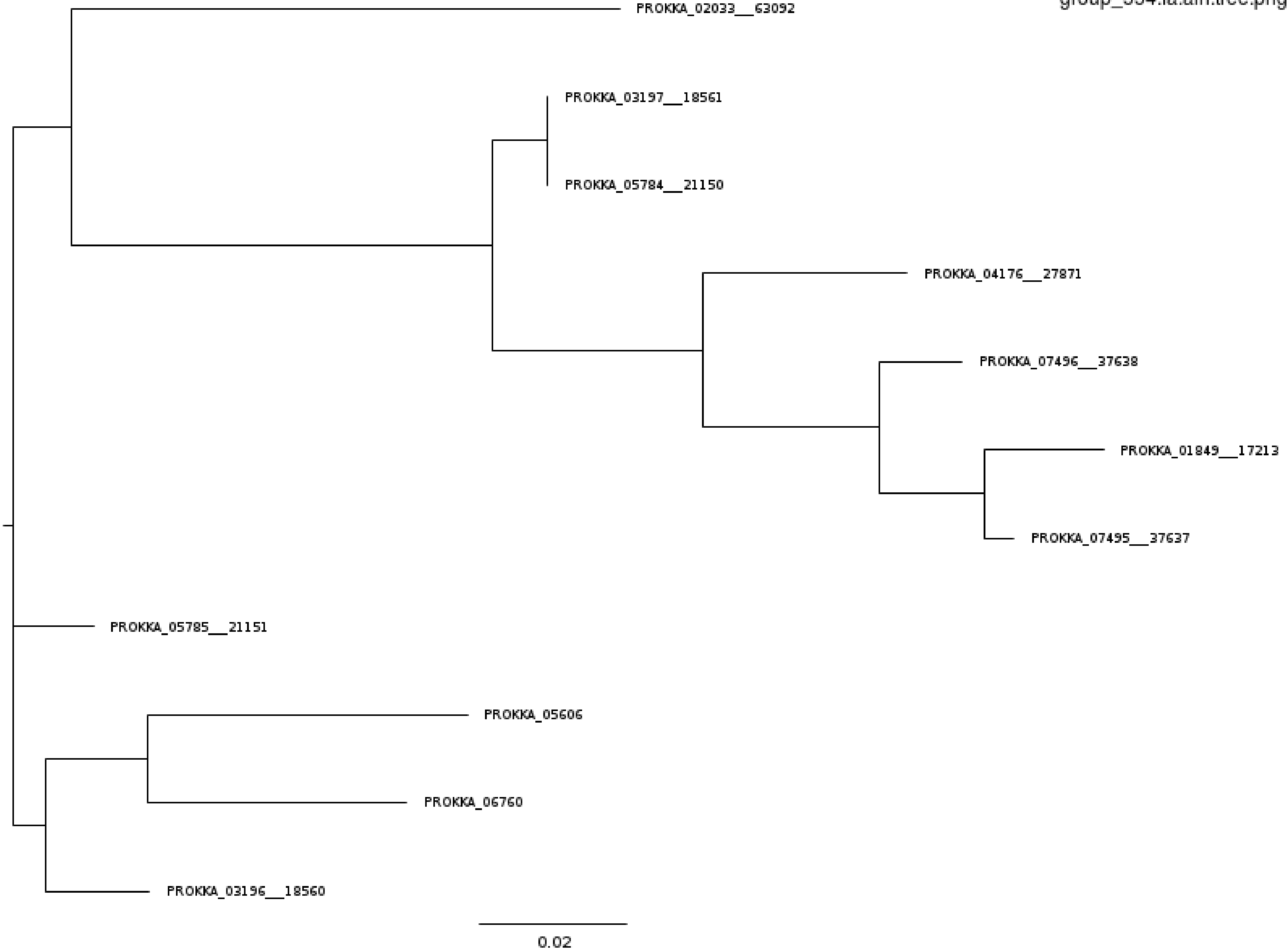

group_560.fa.aln.tree.png

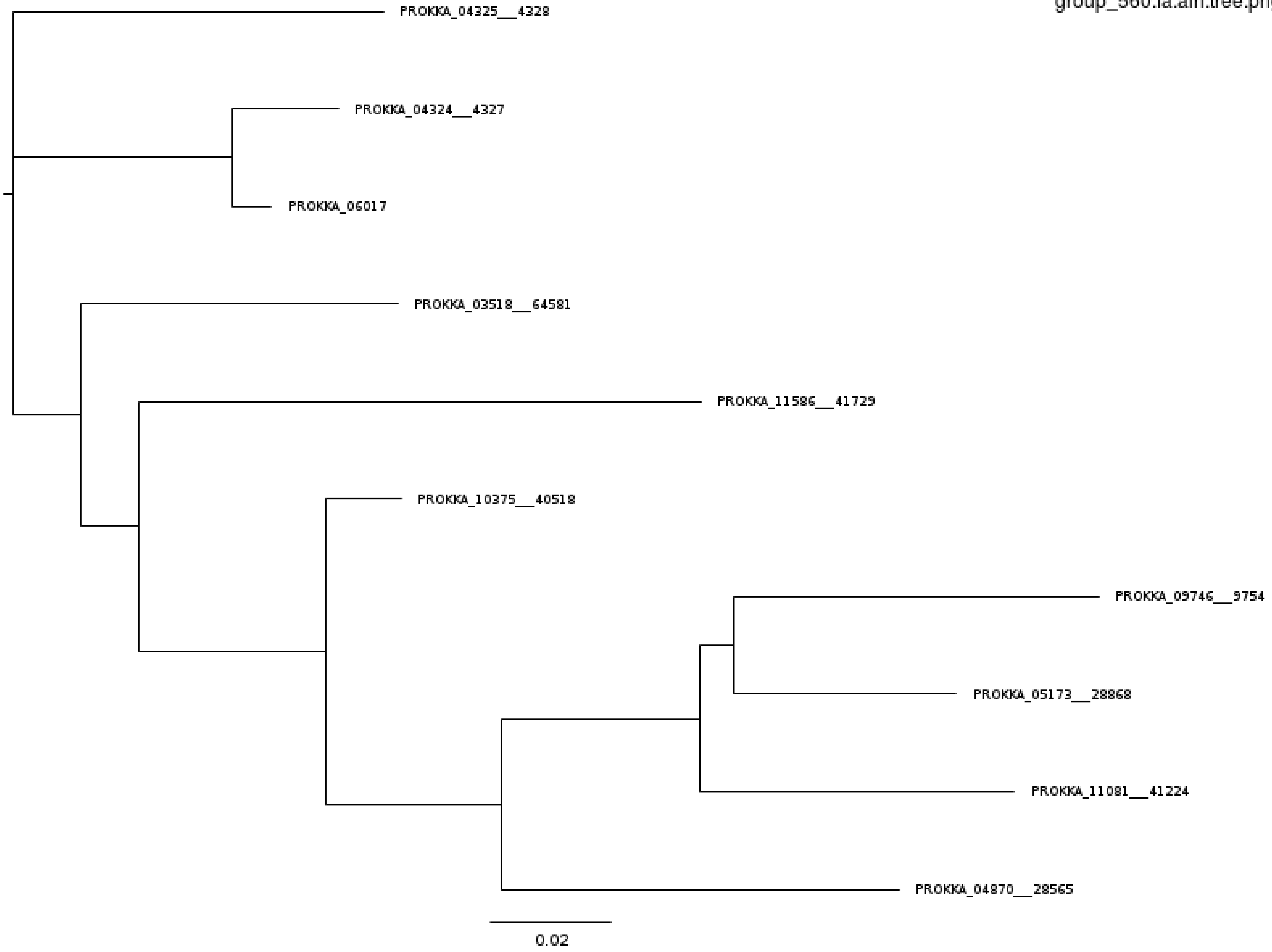

group_562.fa.aln.tree.png

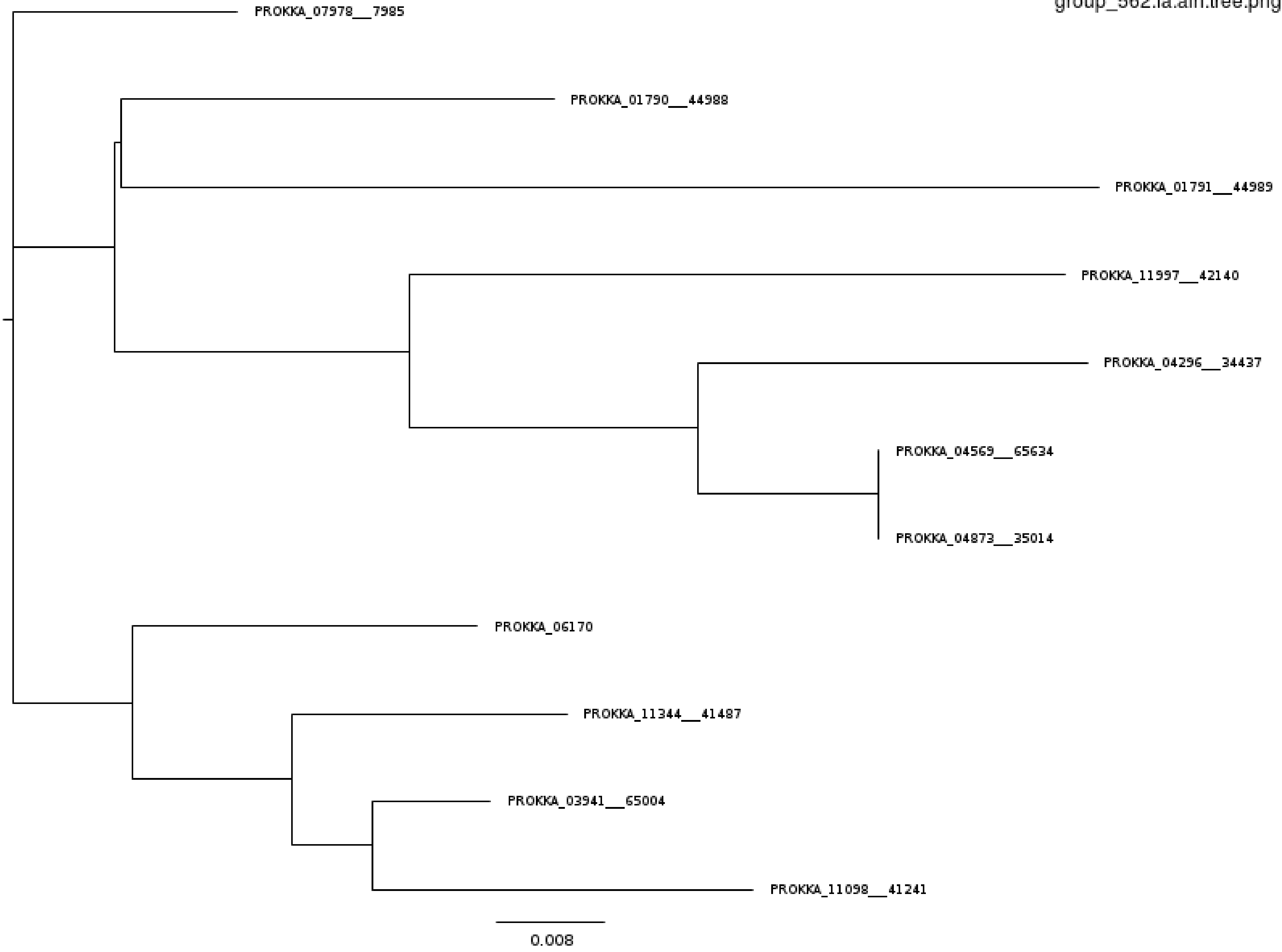

group_564.fa.aln.tree.png

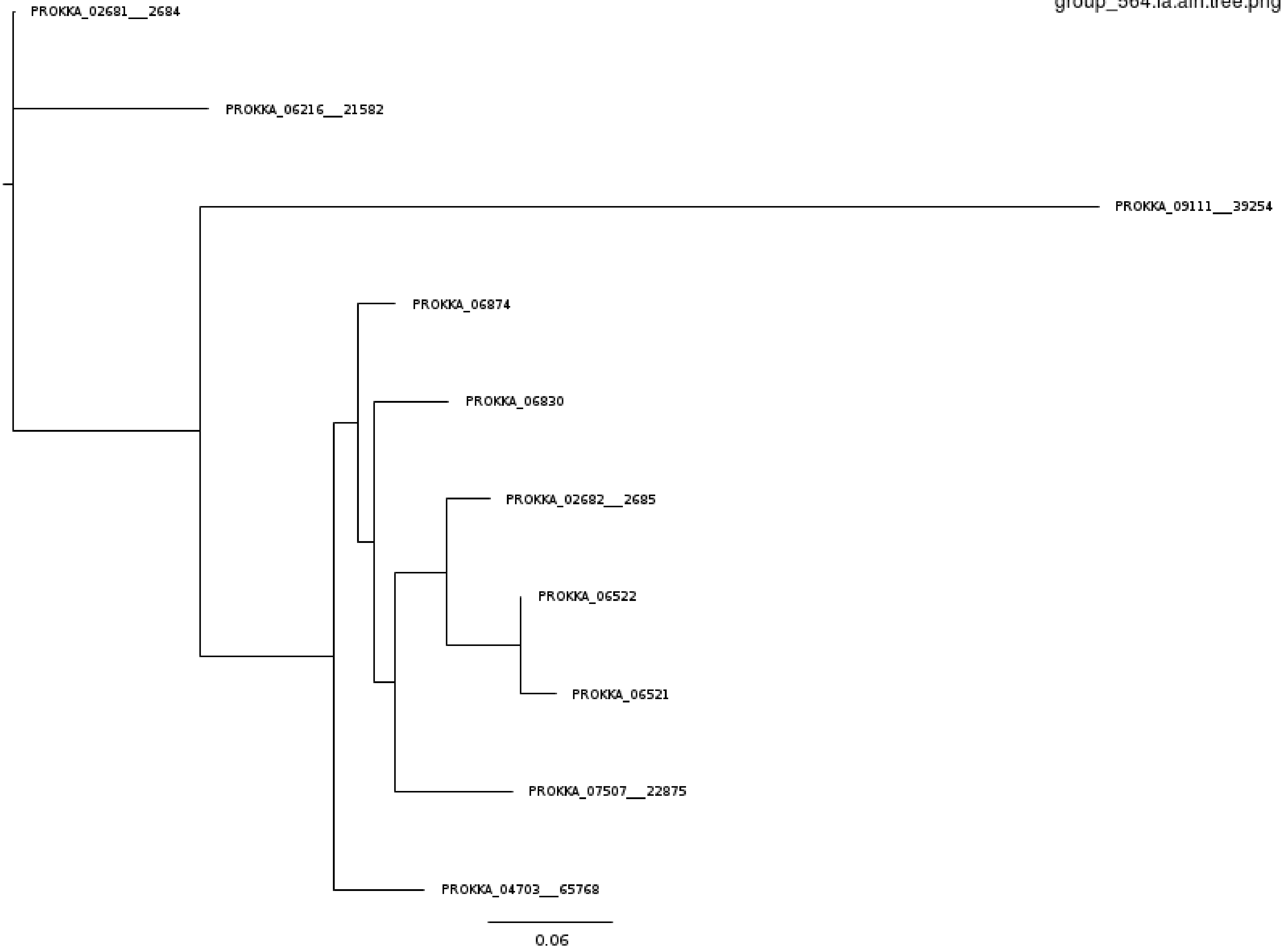

group_566.fa.aln.tree.png

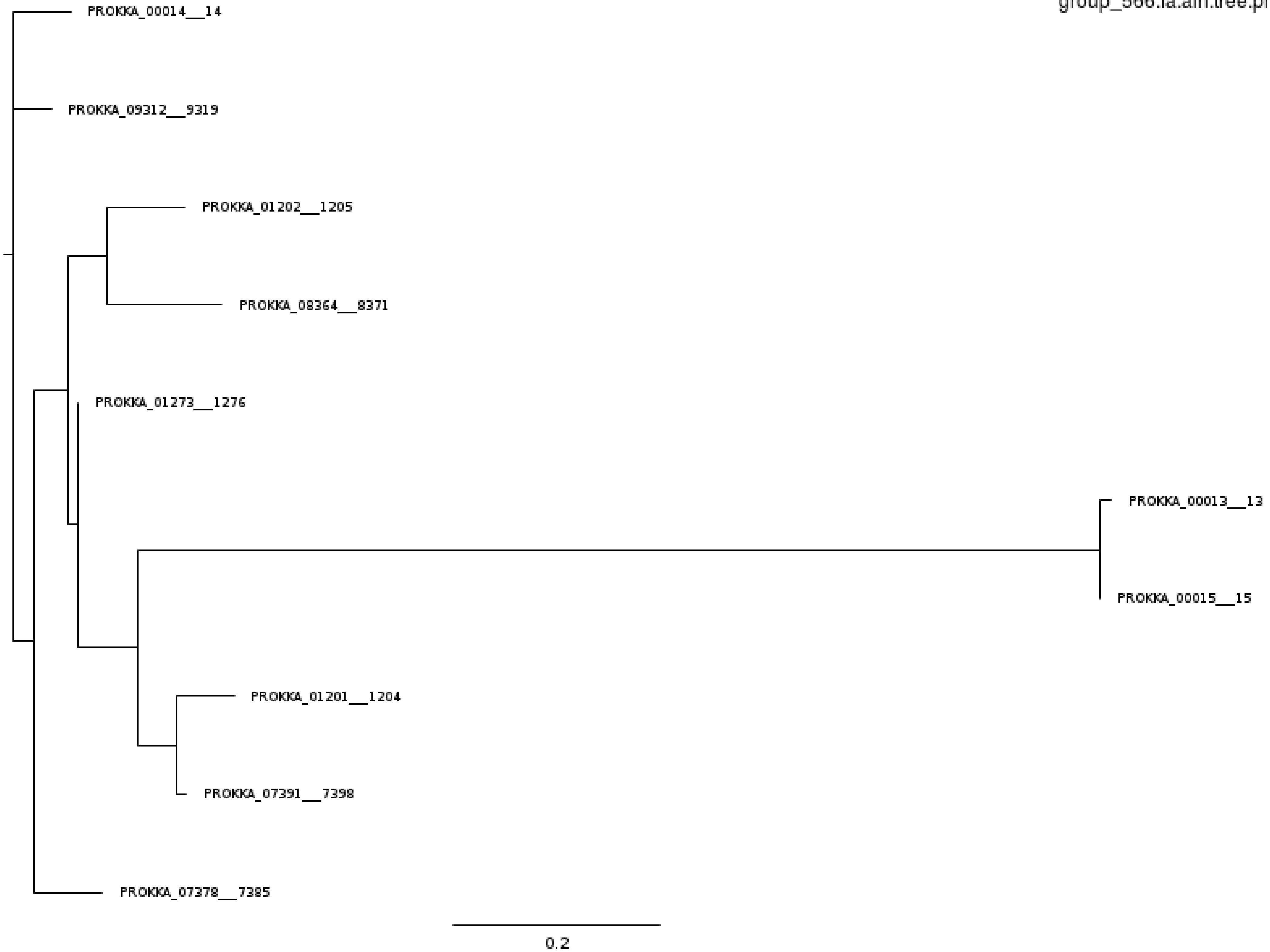

group_567.fa.aln.tree.png

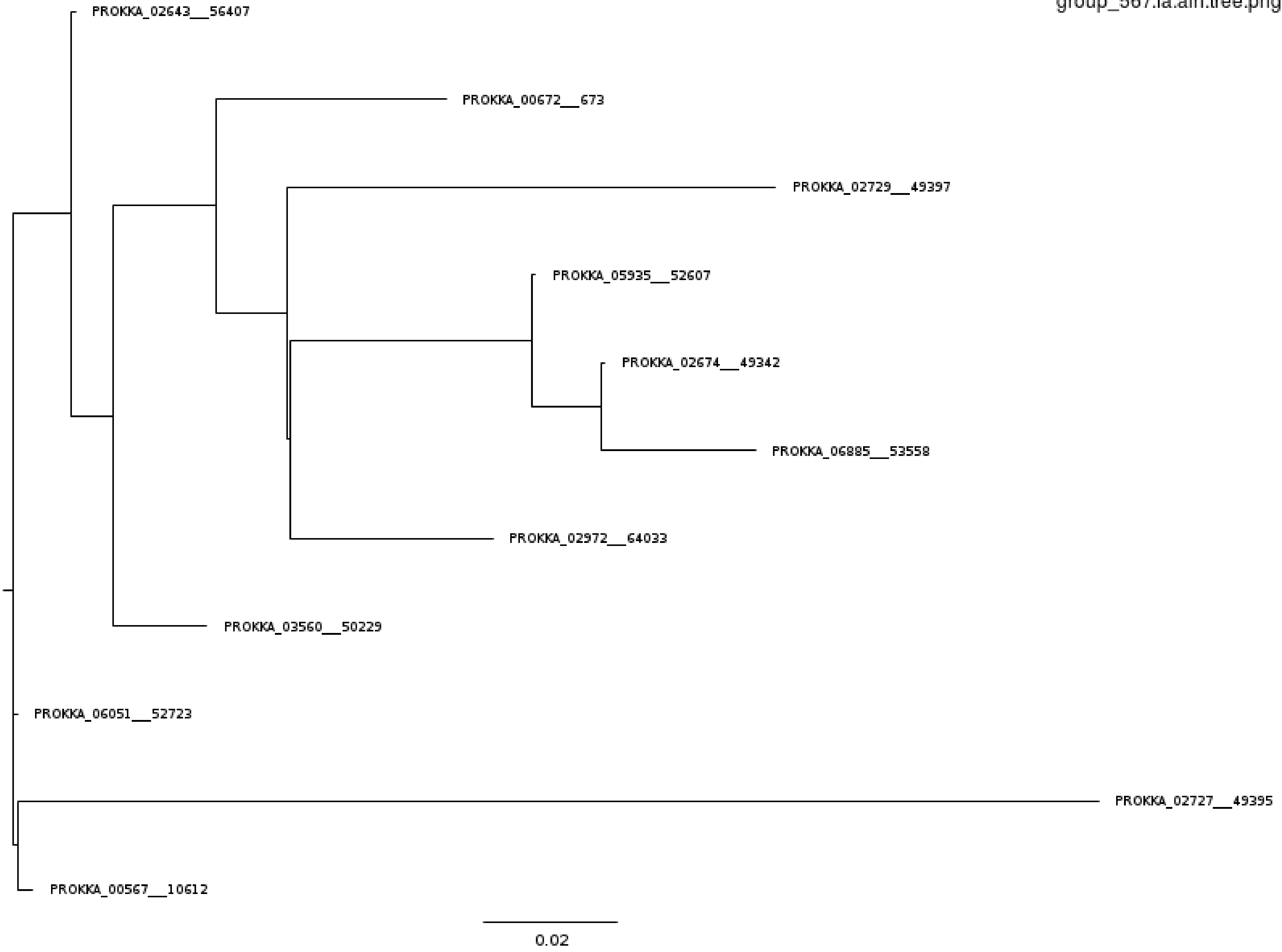

group_569.fa.aln.tree.png

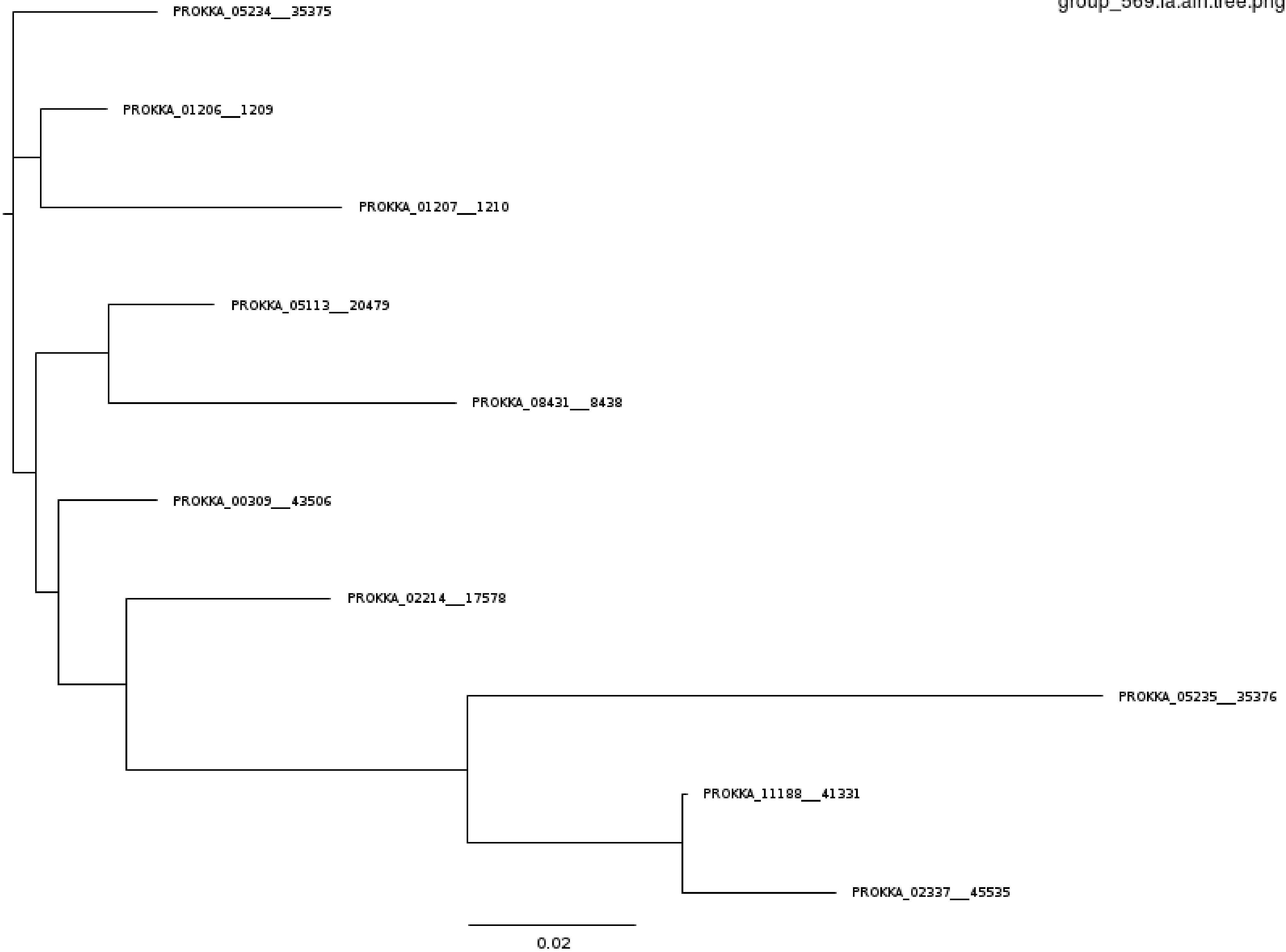

group_570.fa.aln.tree.png

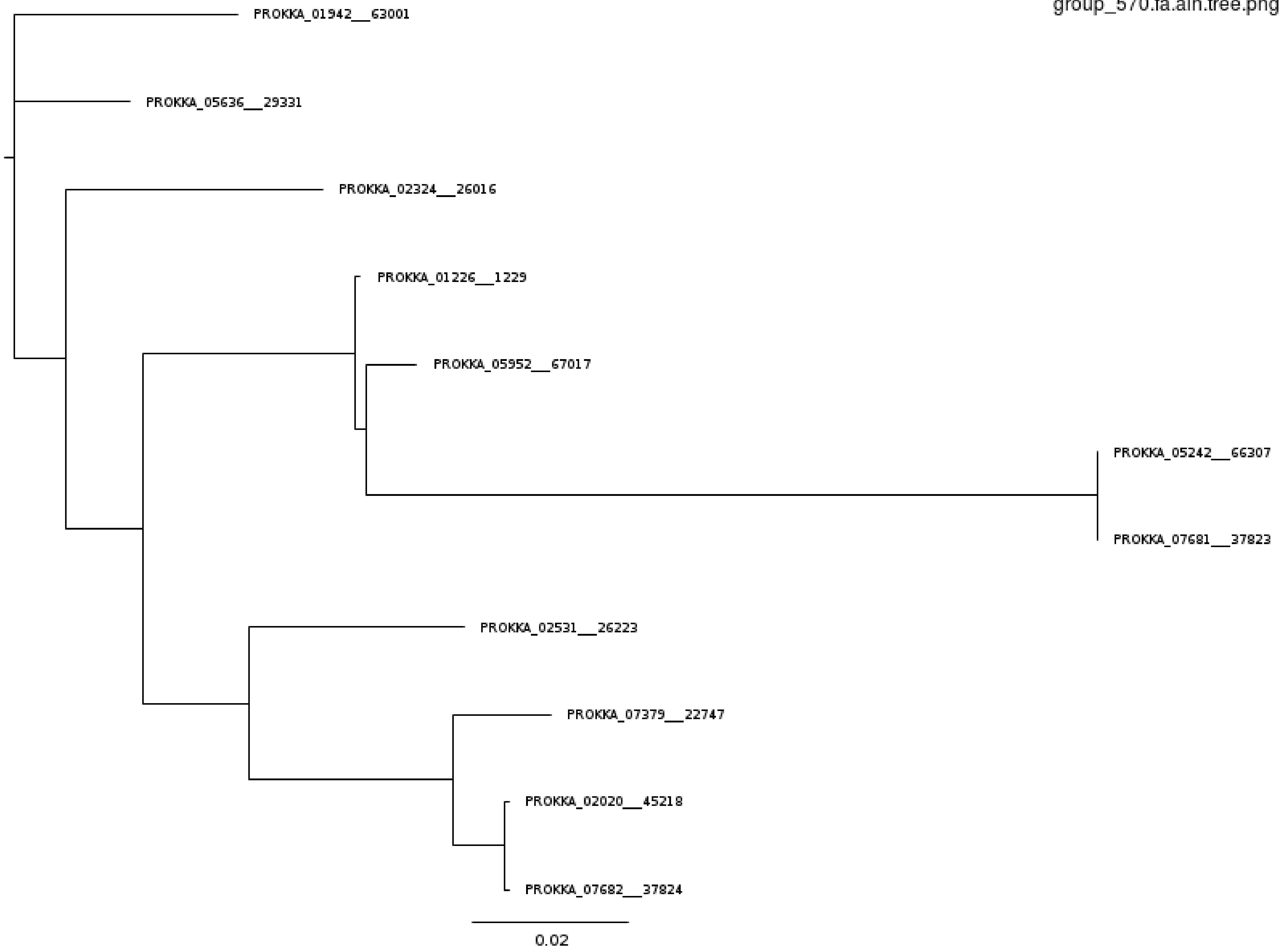

group_573.fa.aln.tree.png

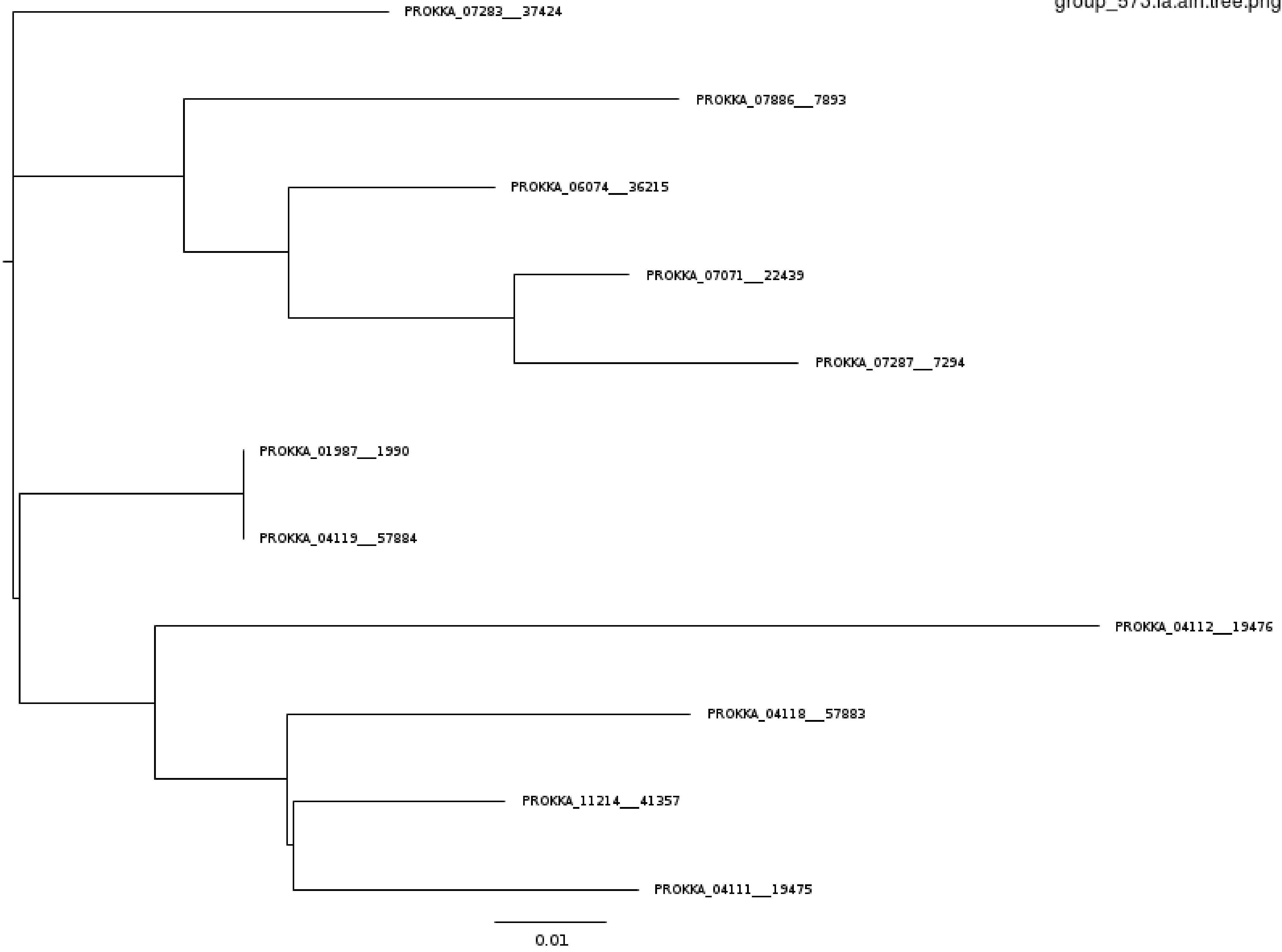

group_576.fa.aln.tree.png

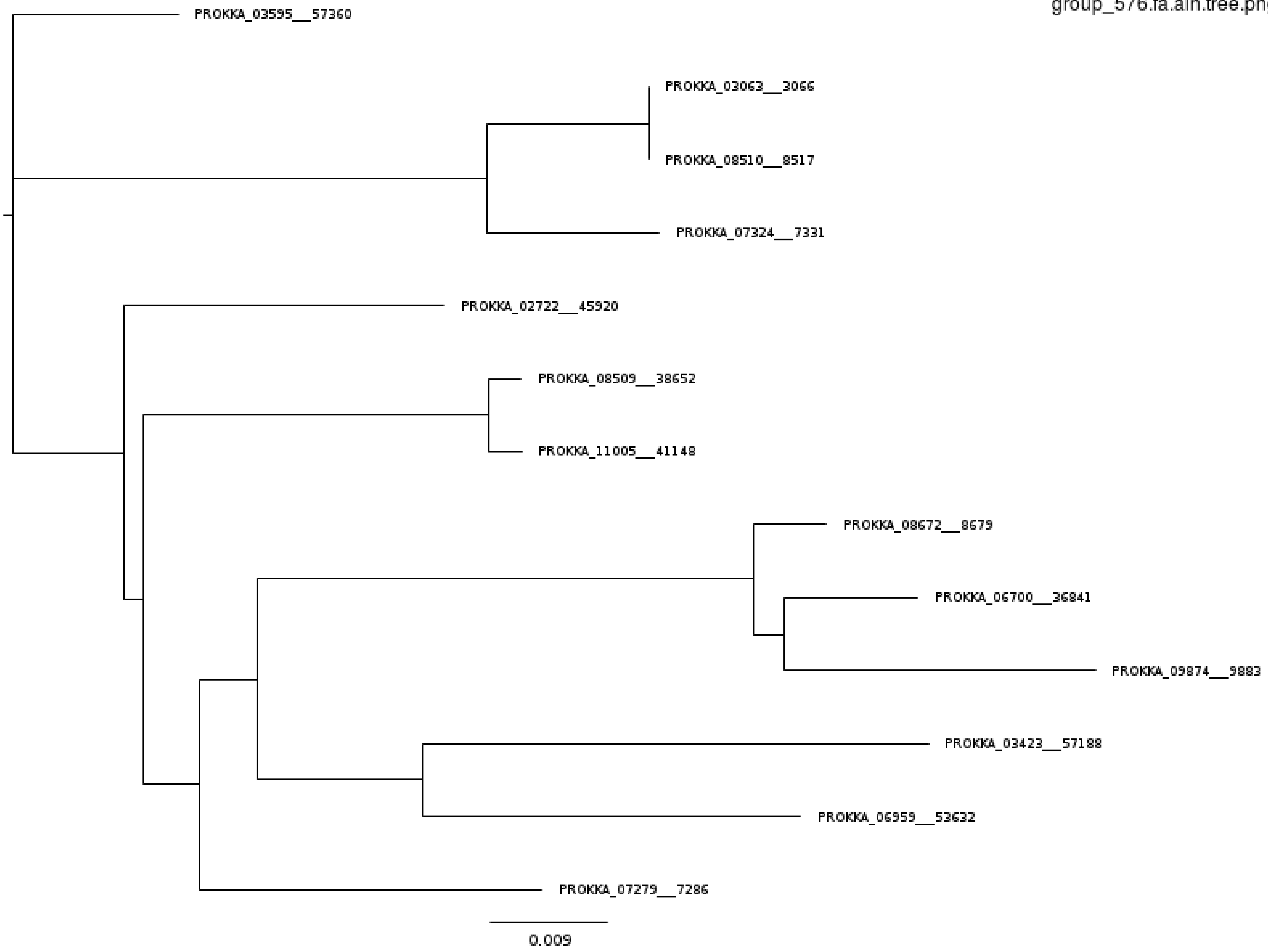

group_579.fa.aln.tree.png

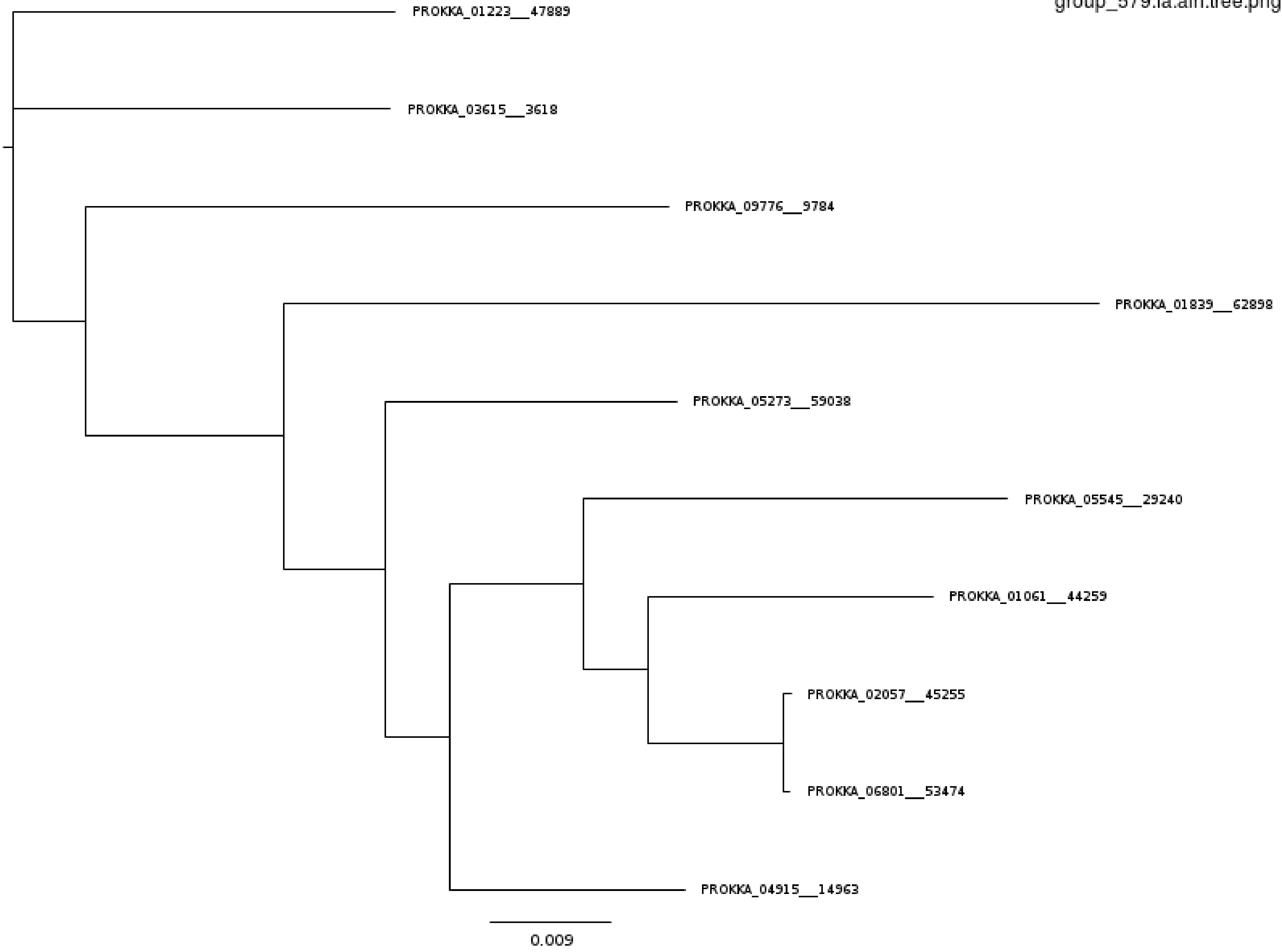

group_583.fa.aln.tree.png

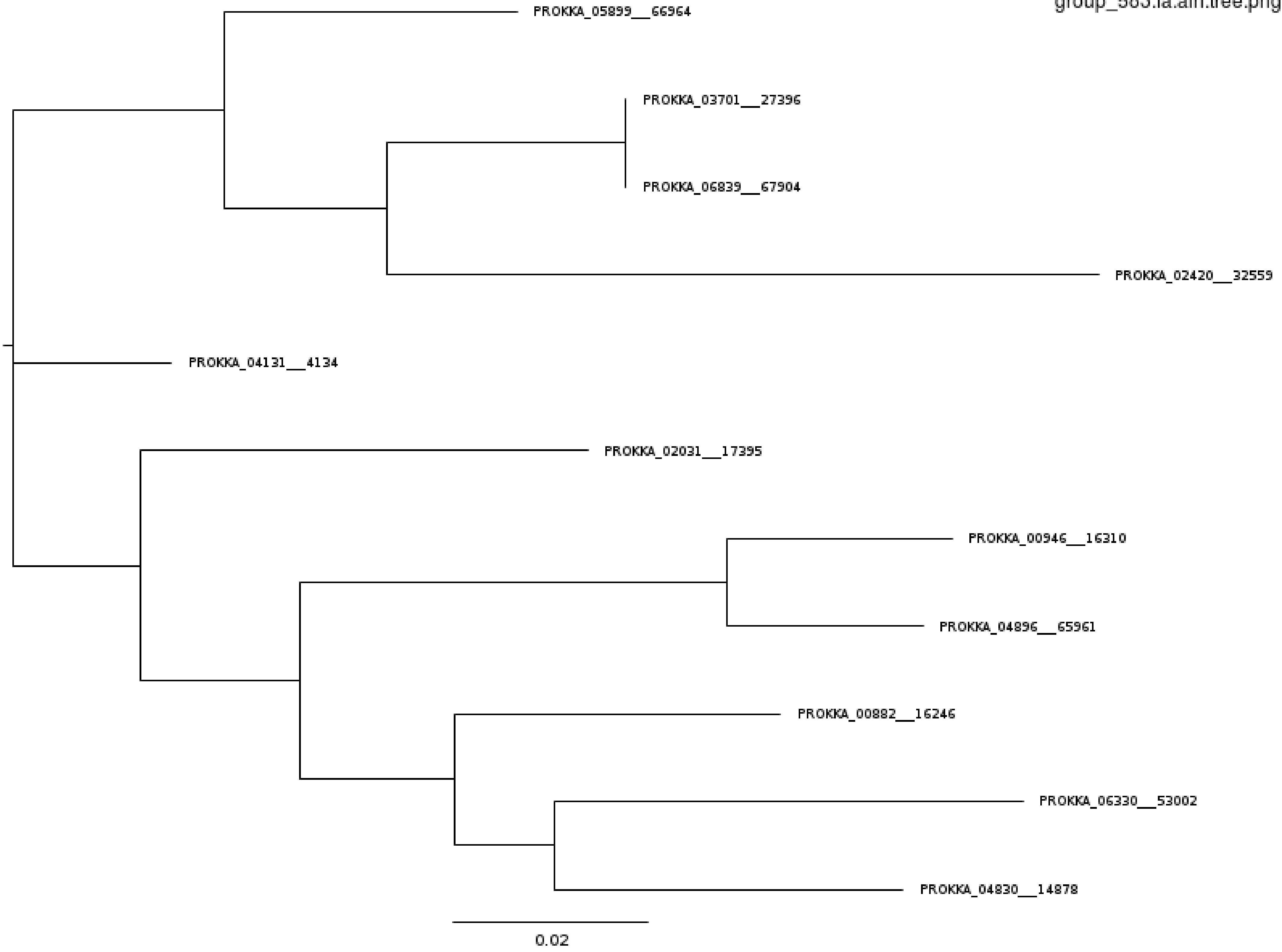

group_584.fa.aln.tree.png

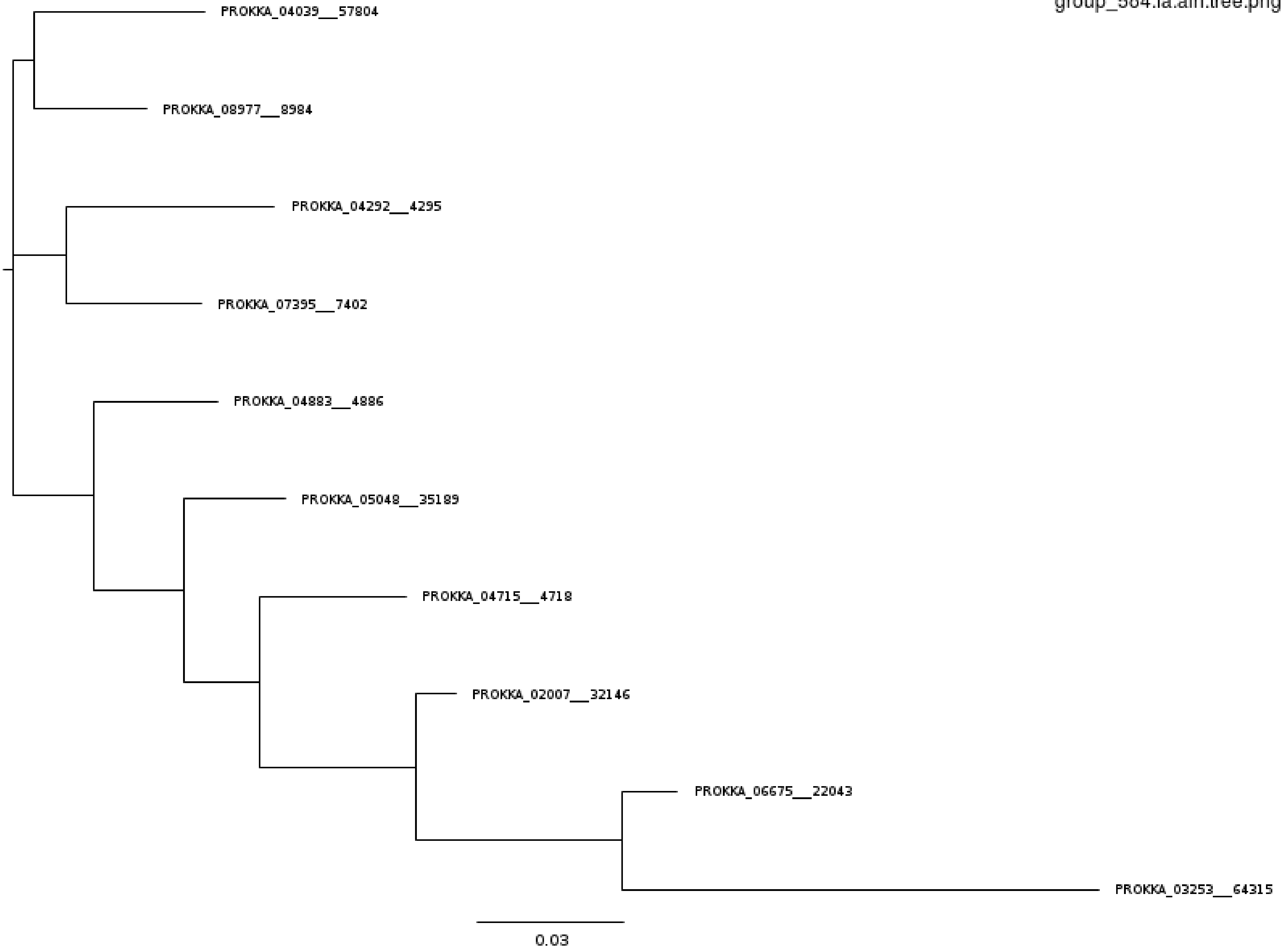

group_585.fa.aln.tree.png

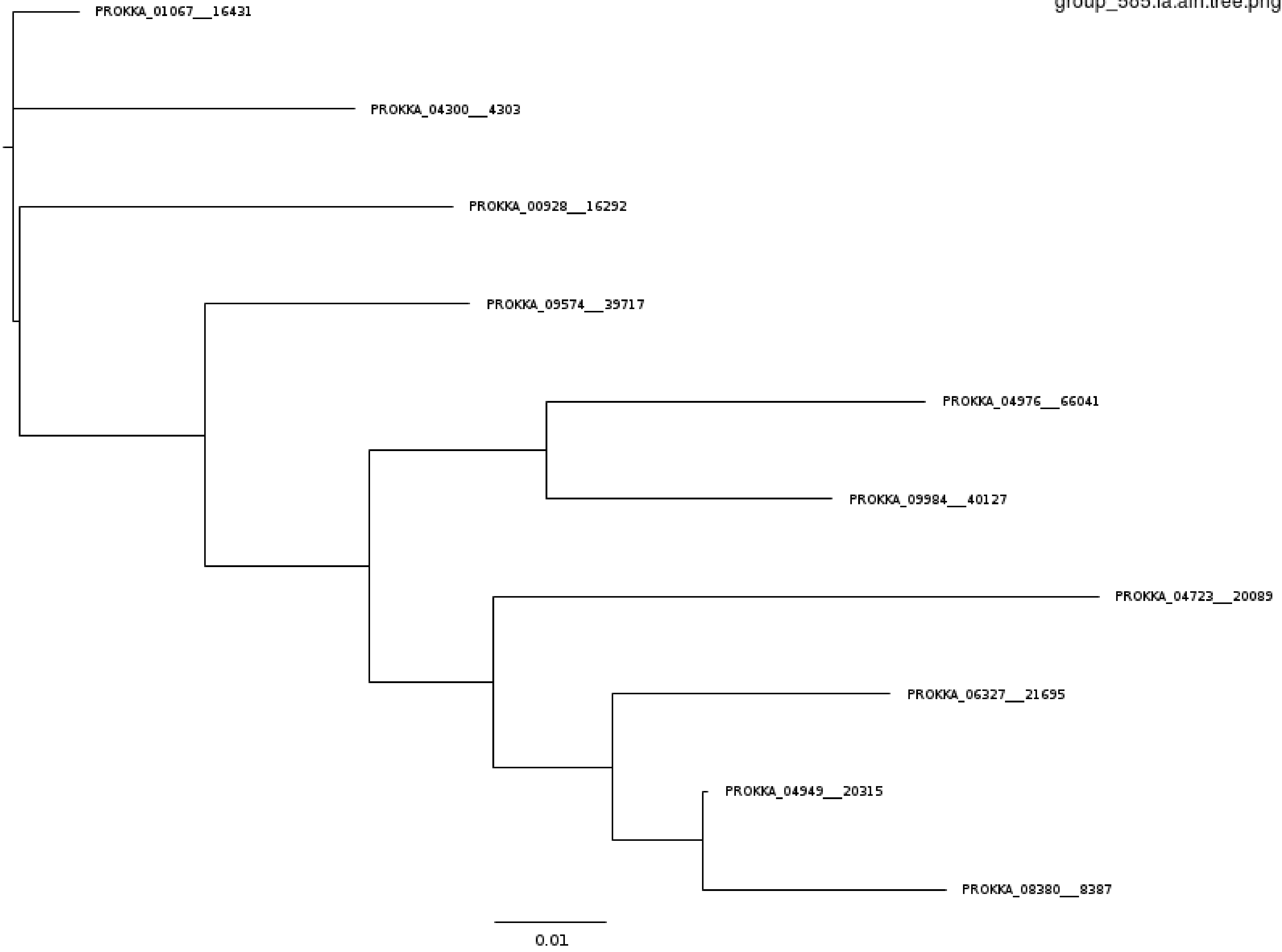

group_5878.fa.aln.tree.png

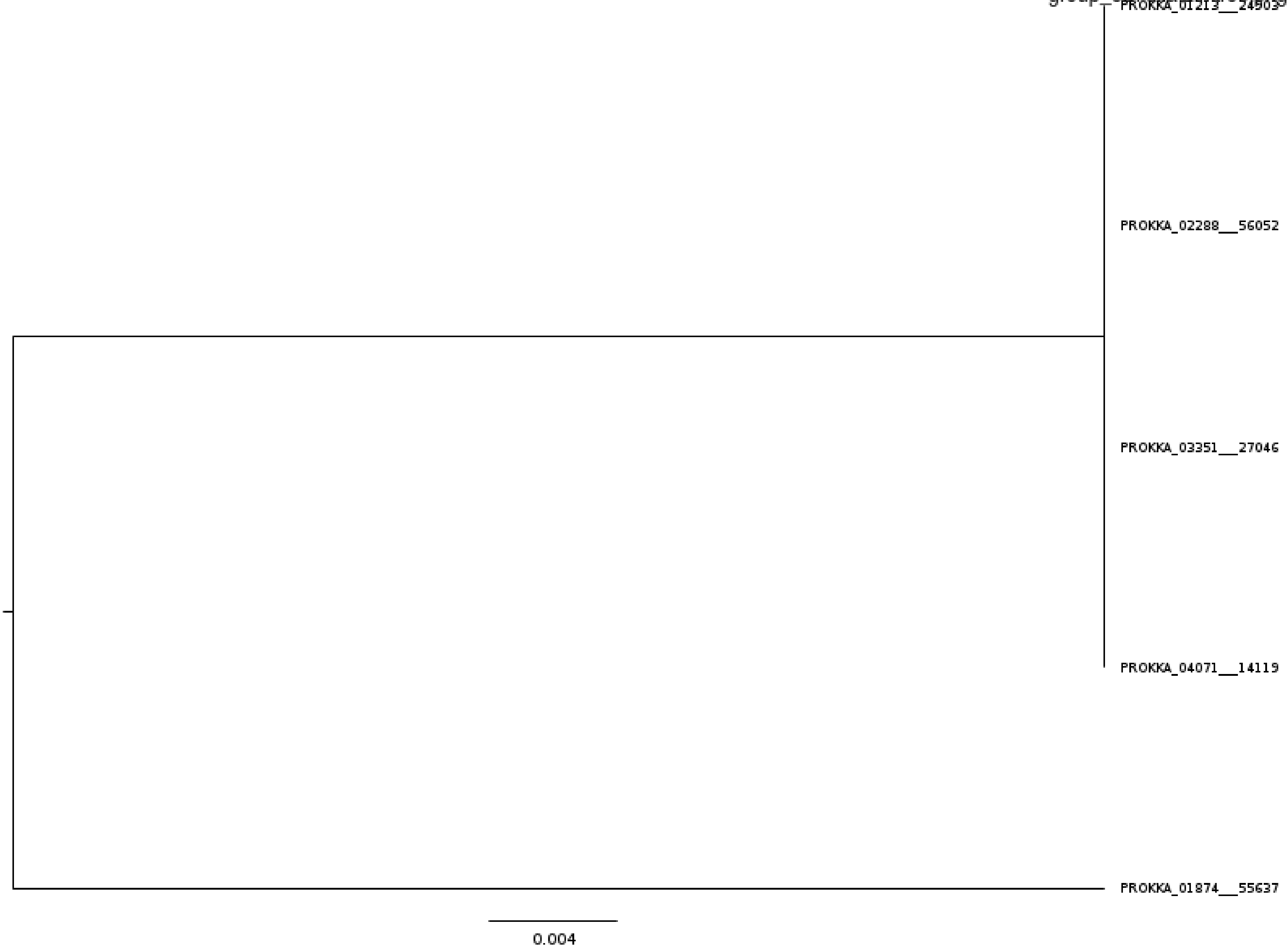

group_588.fa.aln.tree.png

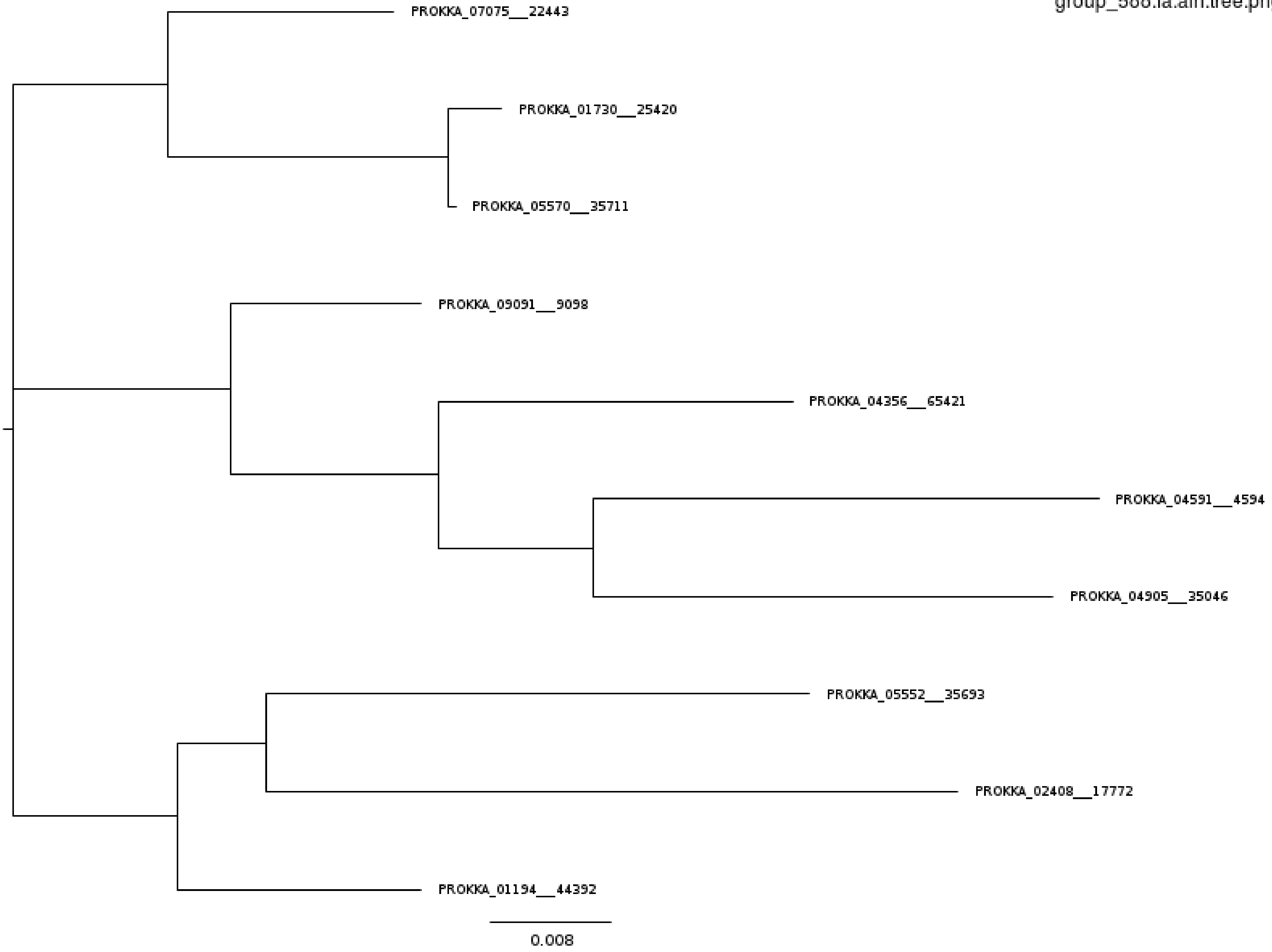

group_589.fa.aln.tree.png

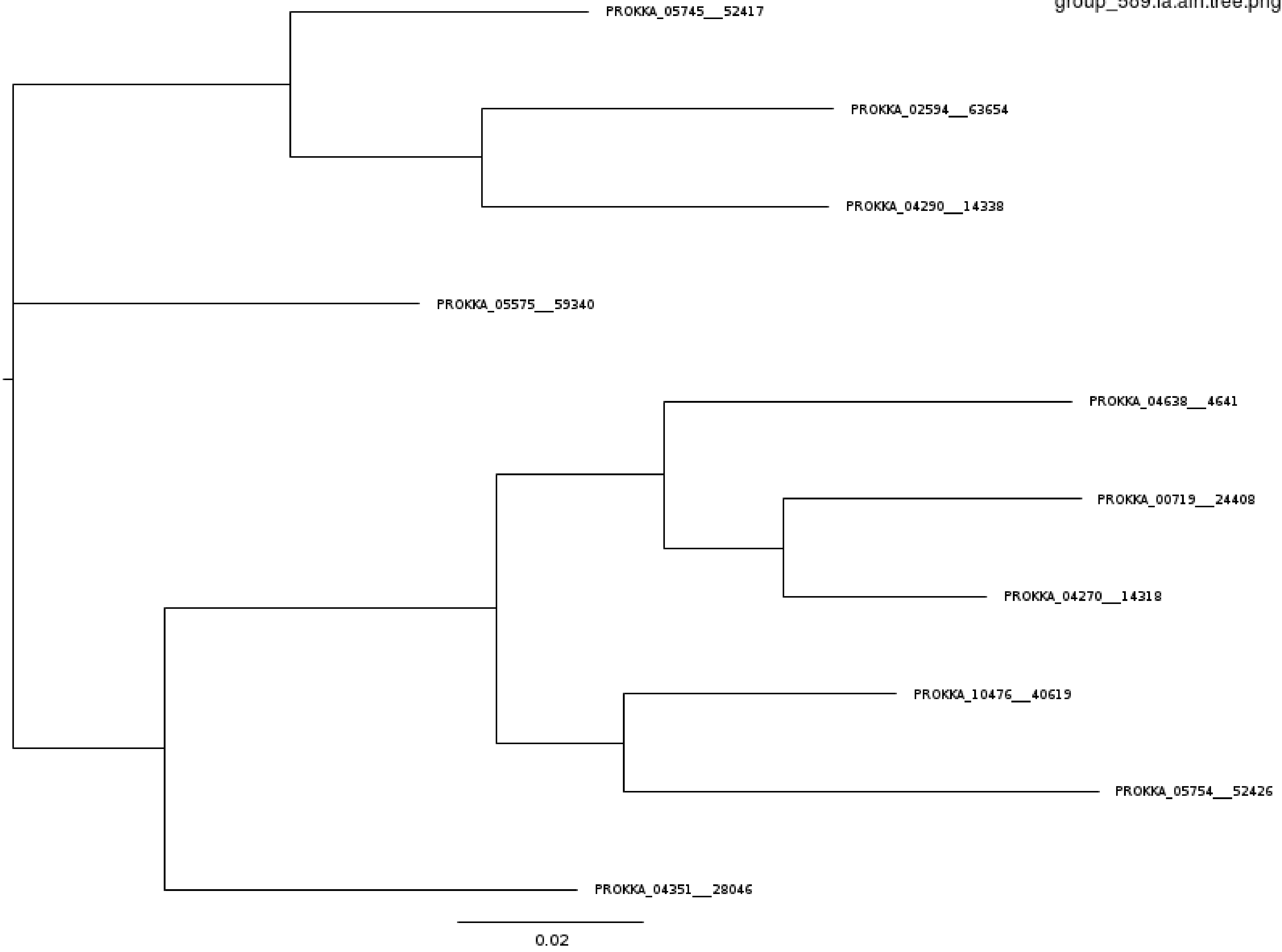

group_591.fa.aln.tree.png

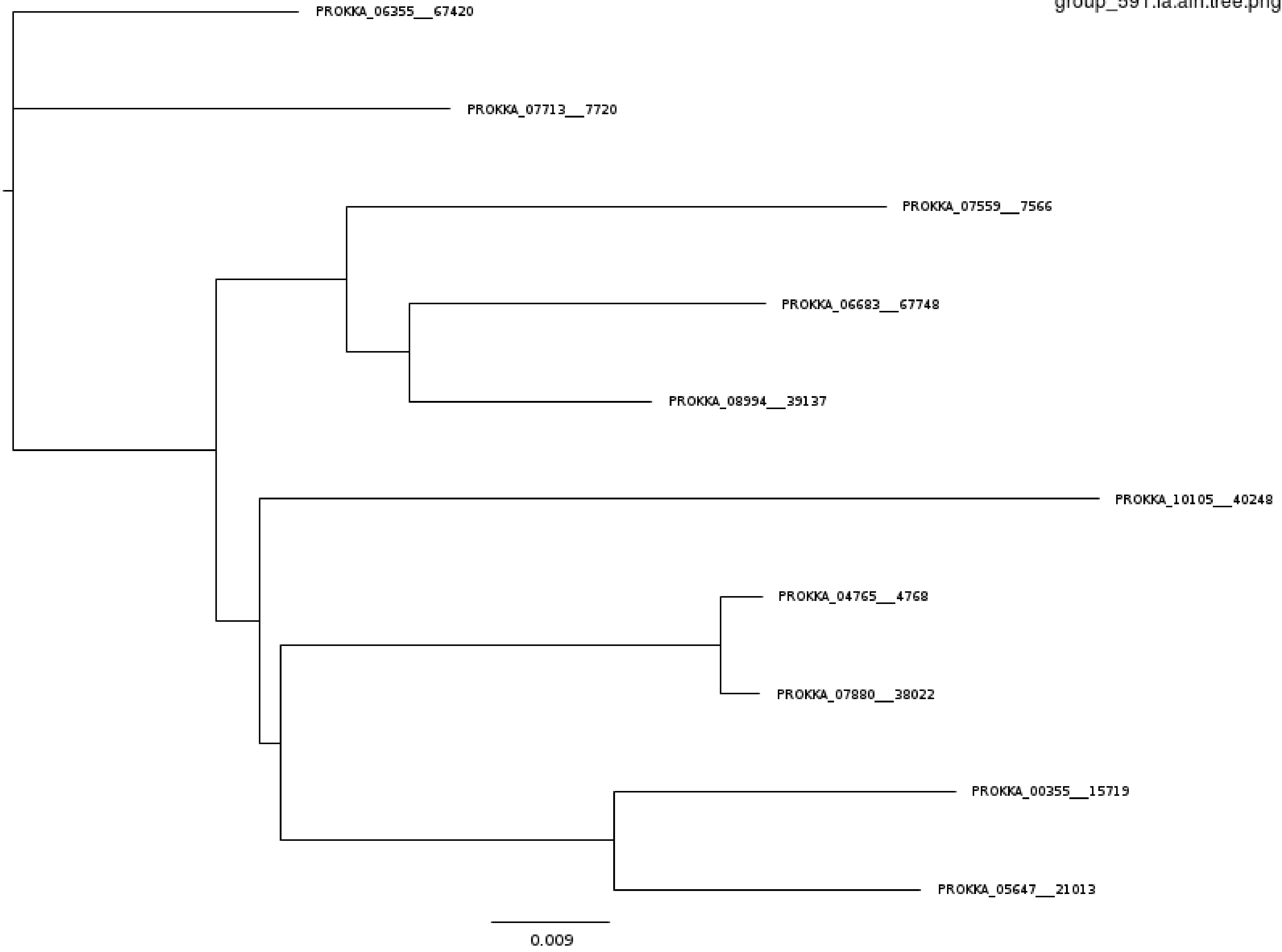

group_602.fa.aln.tree.png

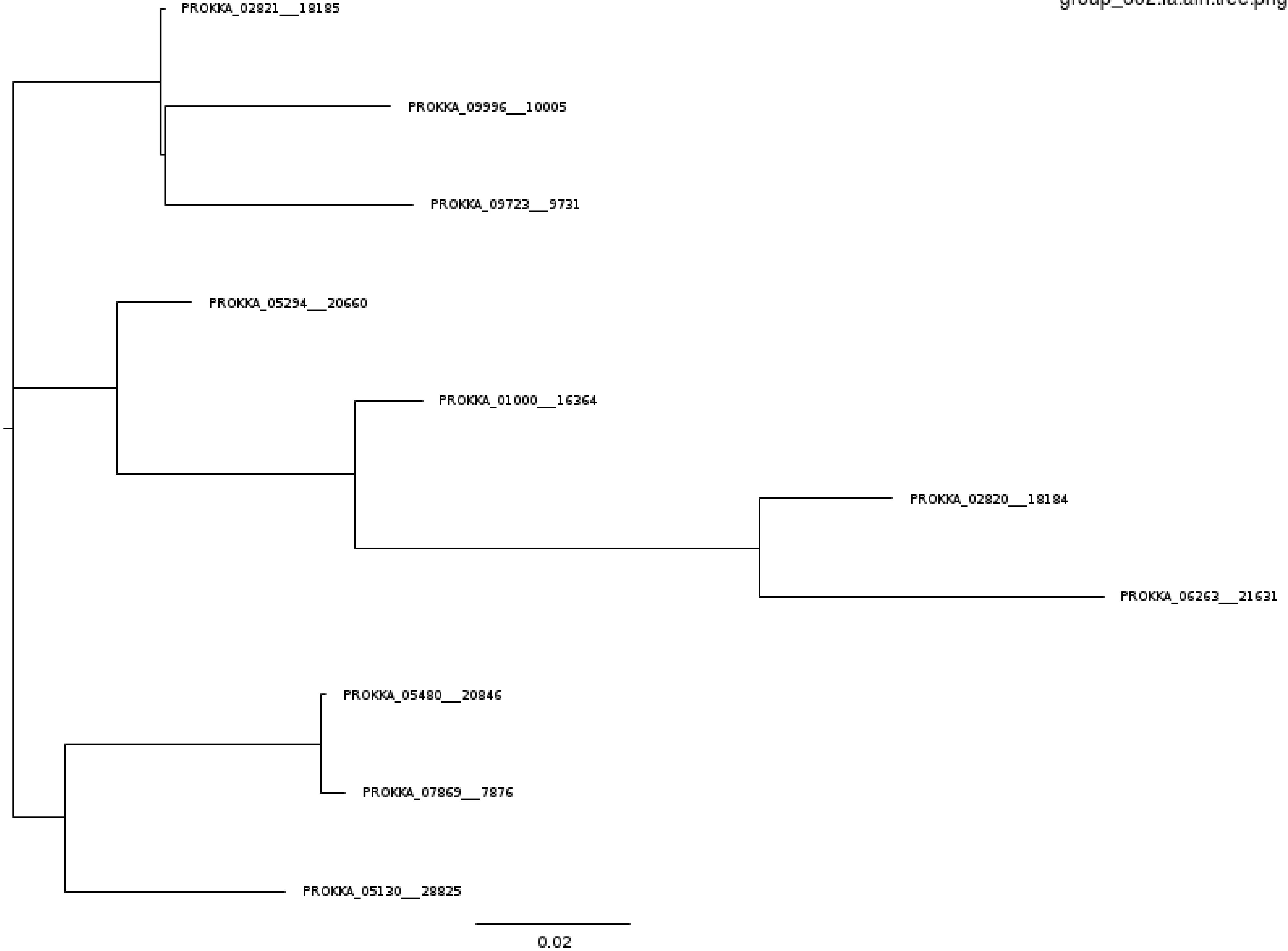

group_604.fa.aln.tree.png

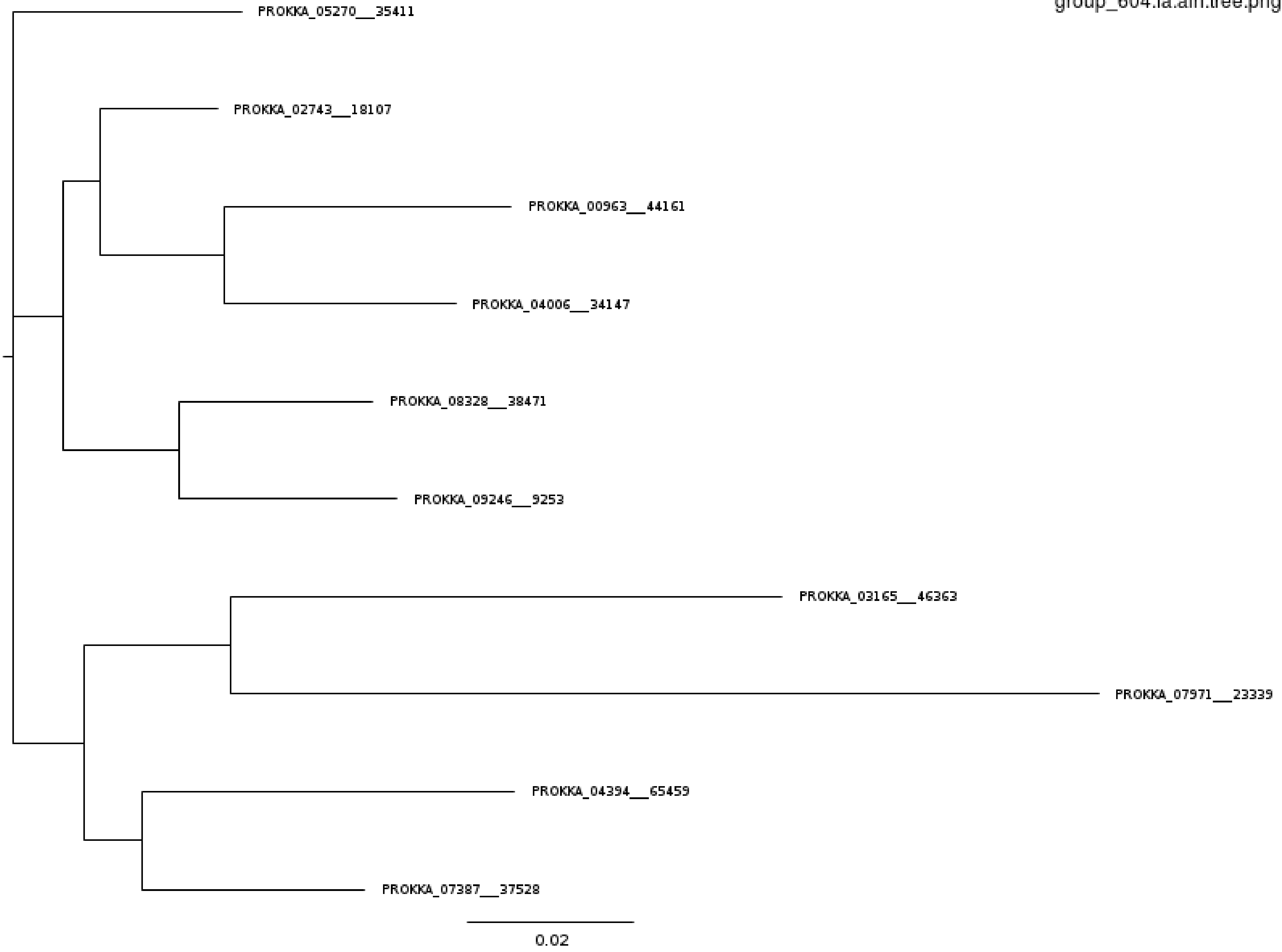

group_611.fa.aln.tree.png

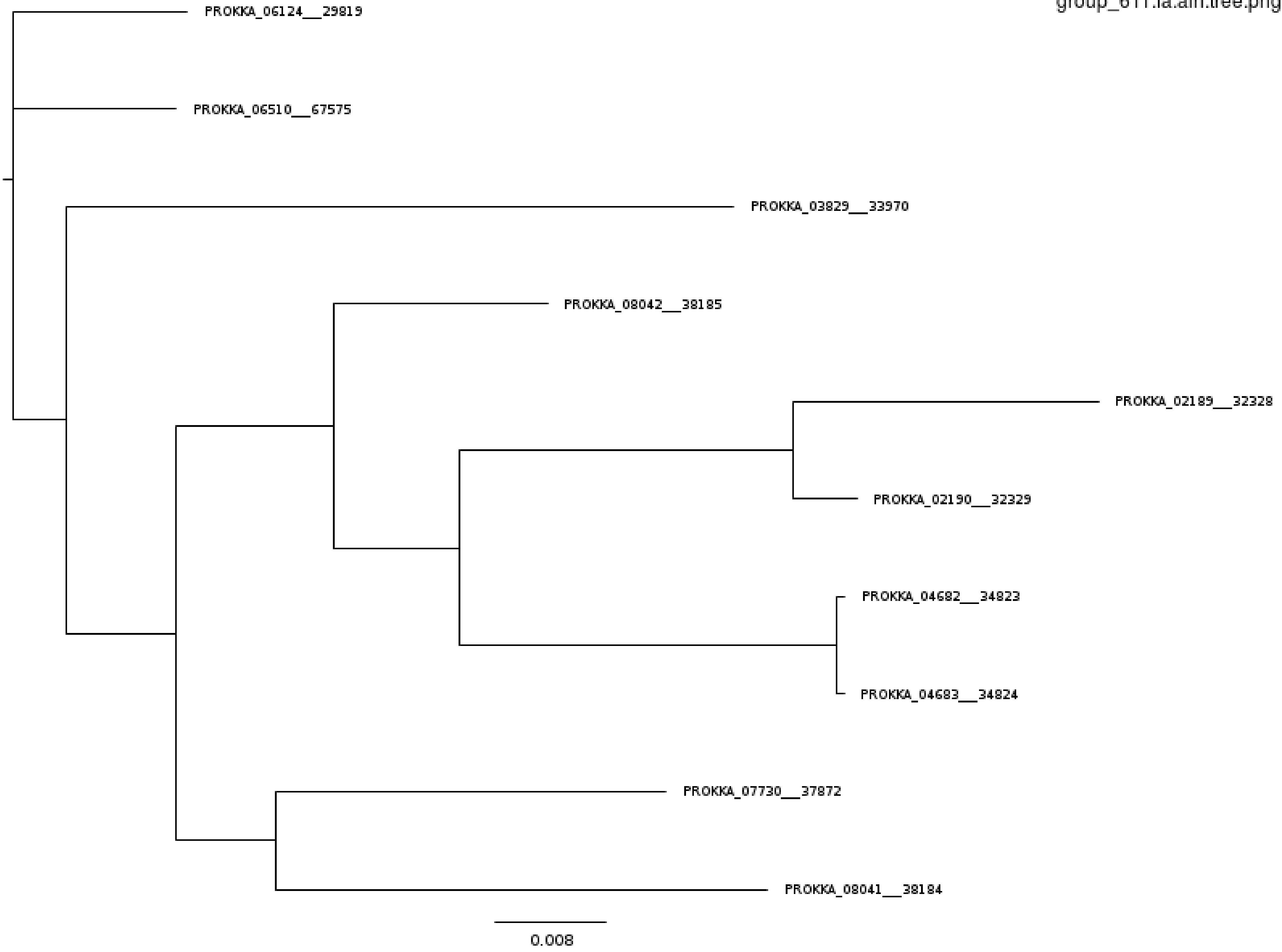

group_612.fa.aln.tree.png

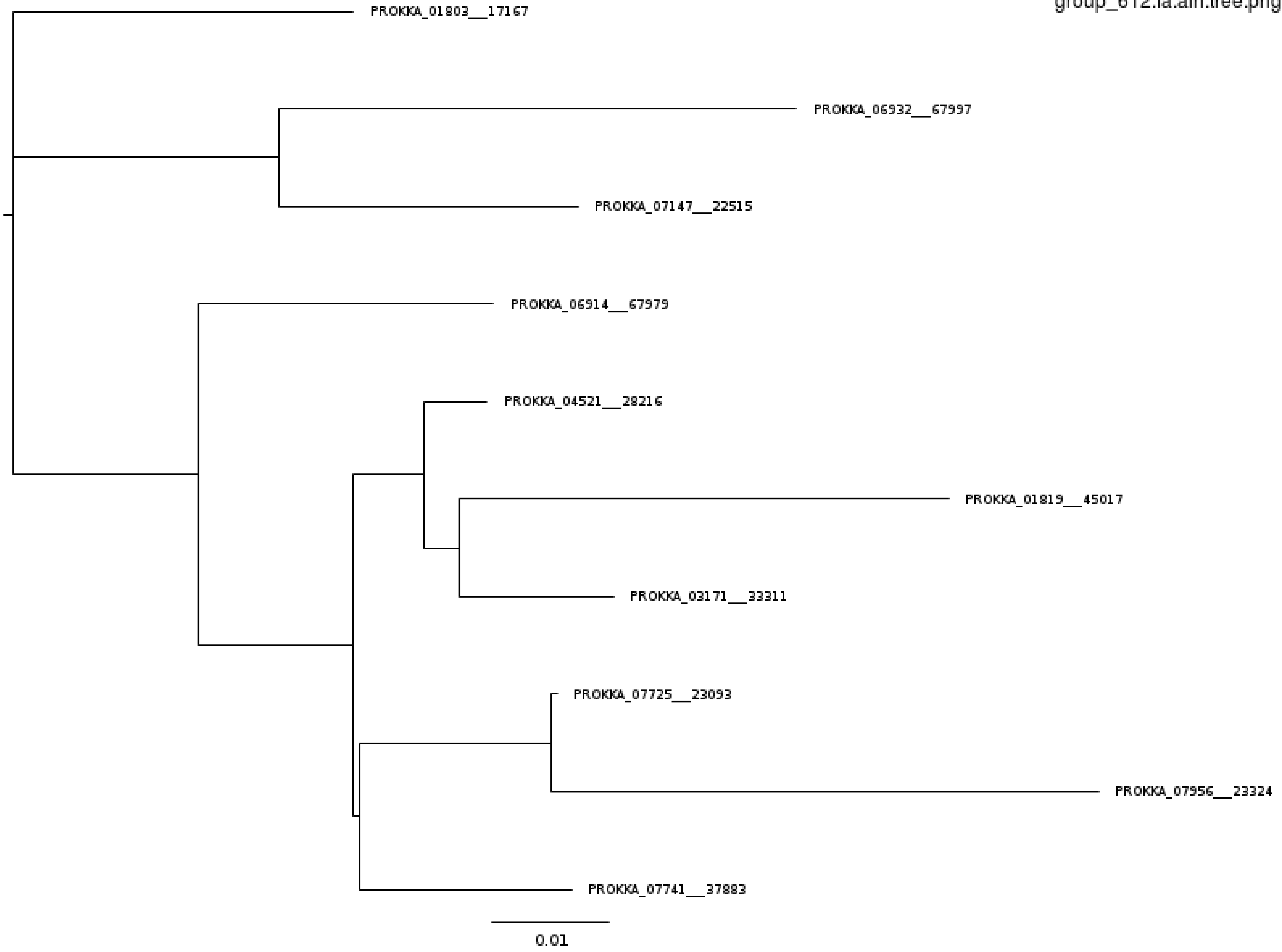

group_613.fa.aln.tree.png

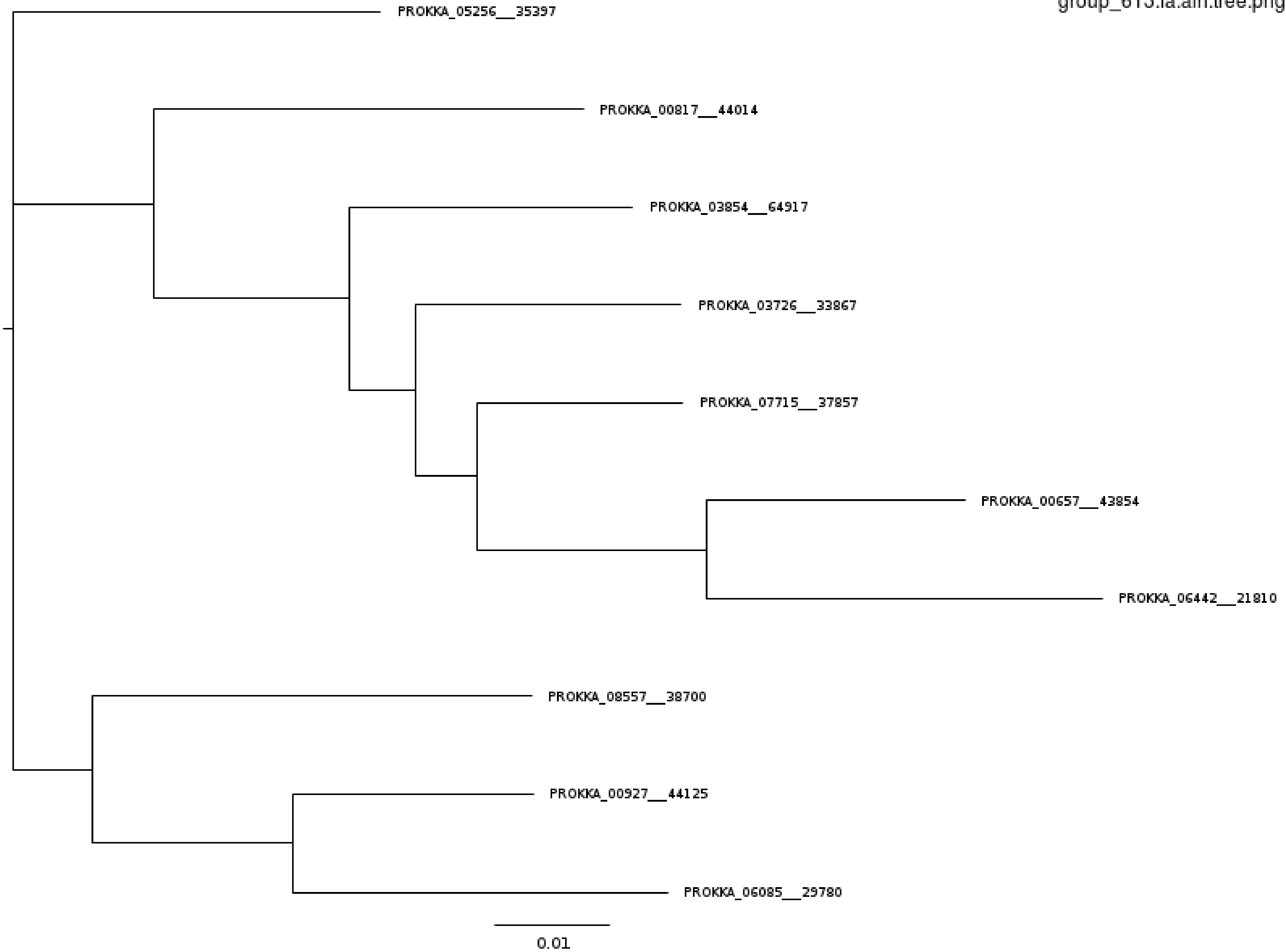

group_615.fa.aln.tree.png

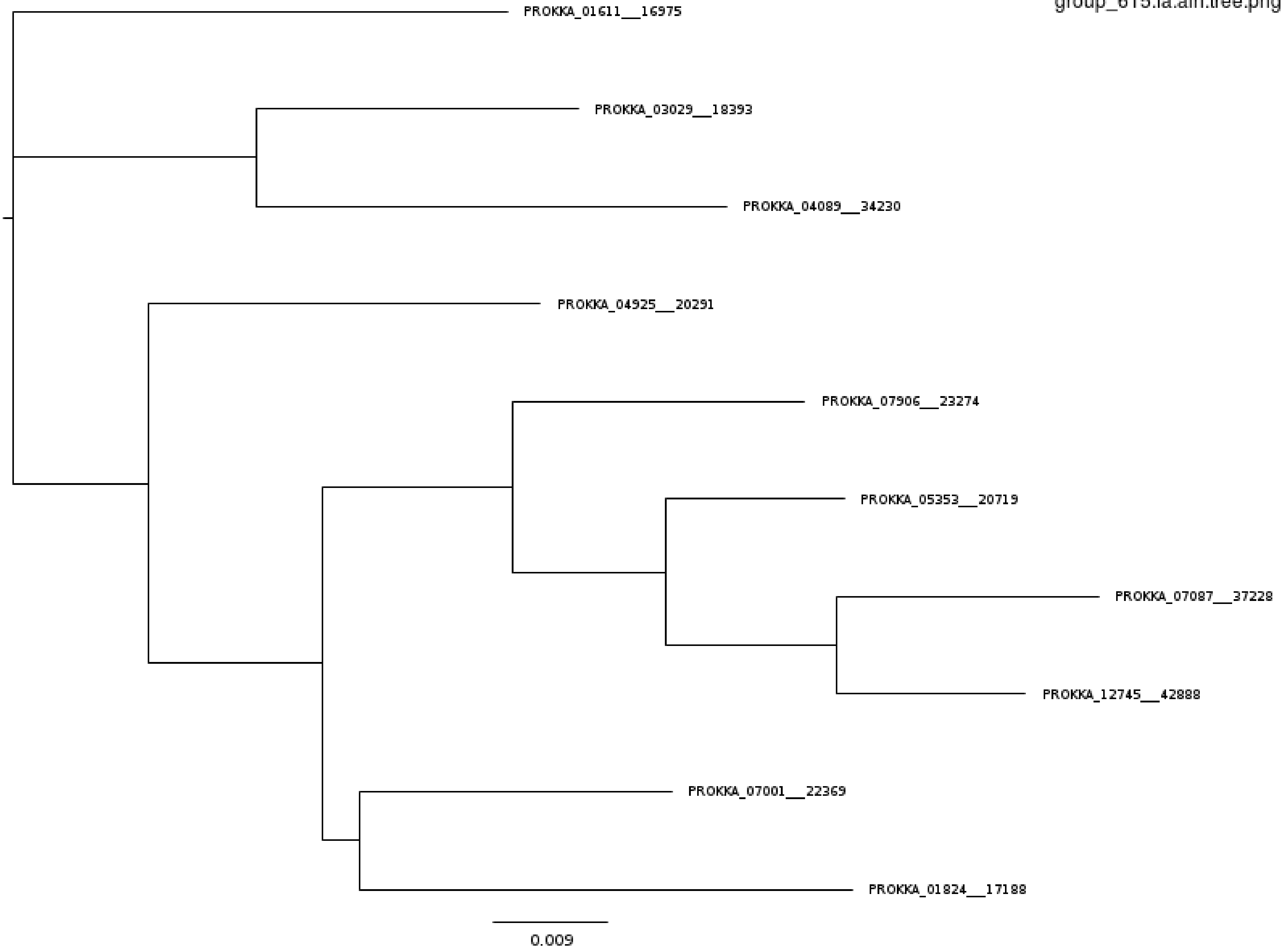

group_616.fa.aln.tree.png

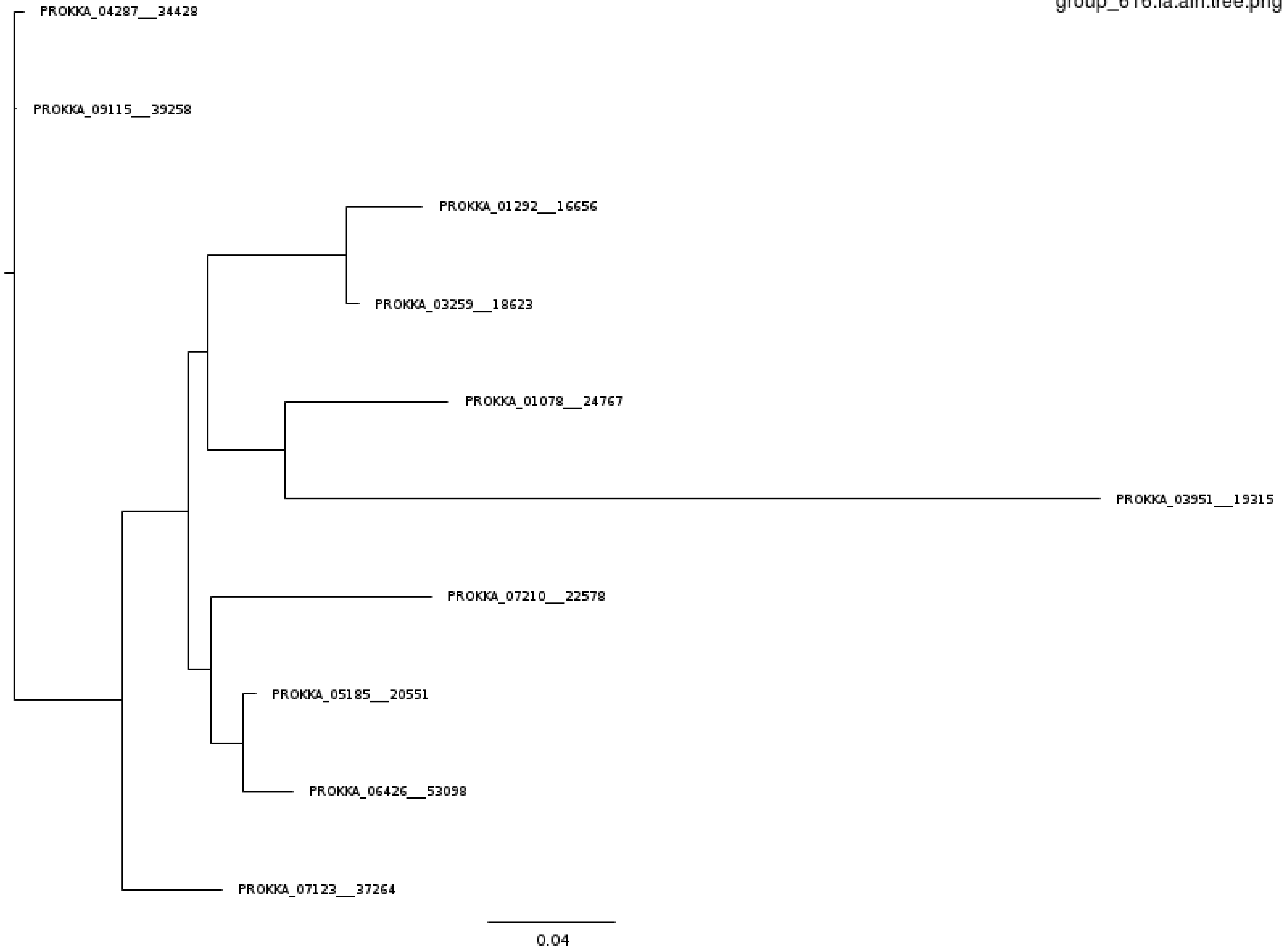

group_622.fa.aln.tree.png

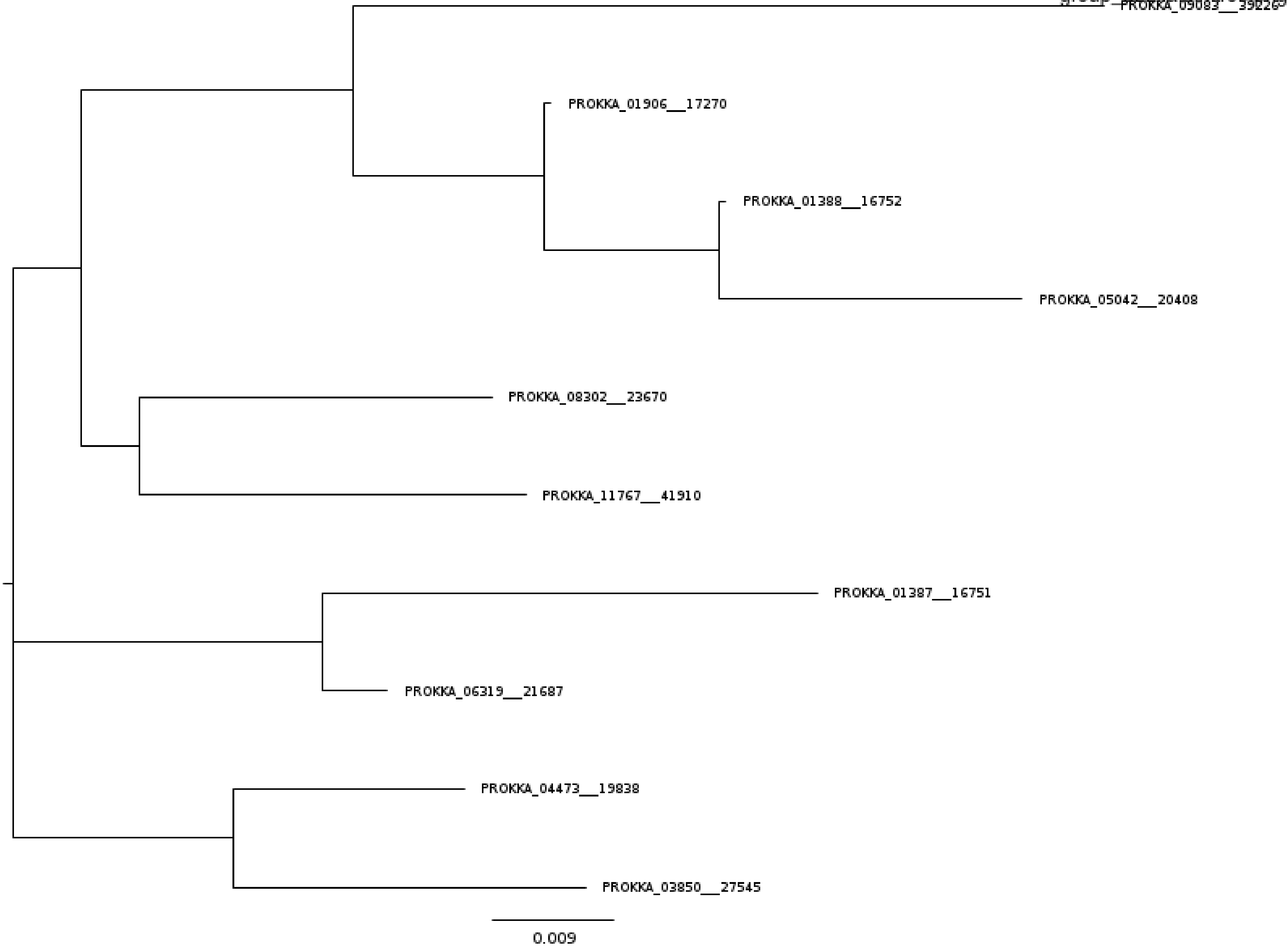

group_624.fa.aln.tree.png

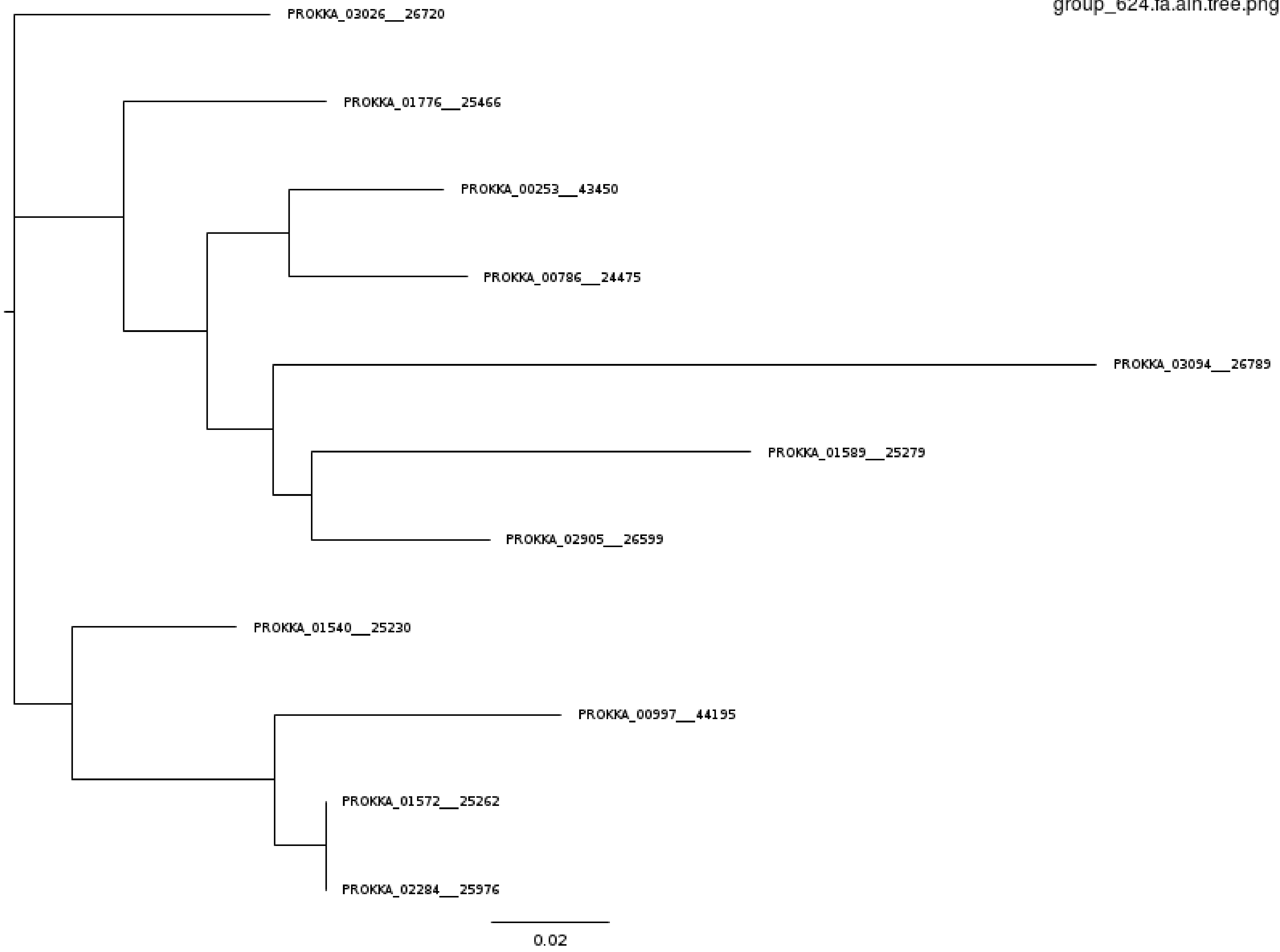

group_629.fa.aln.tree.png

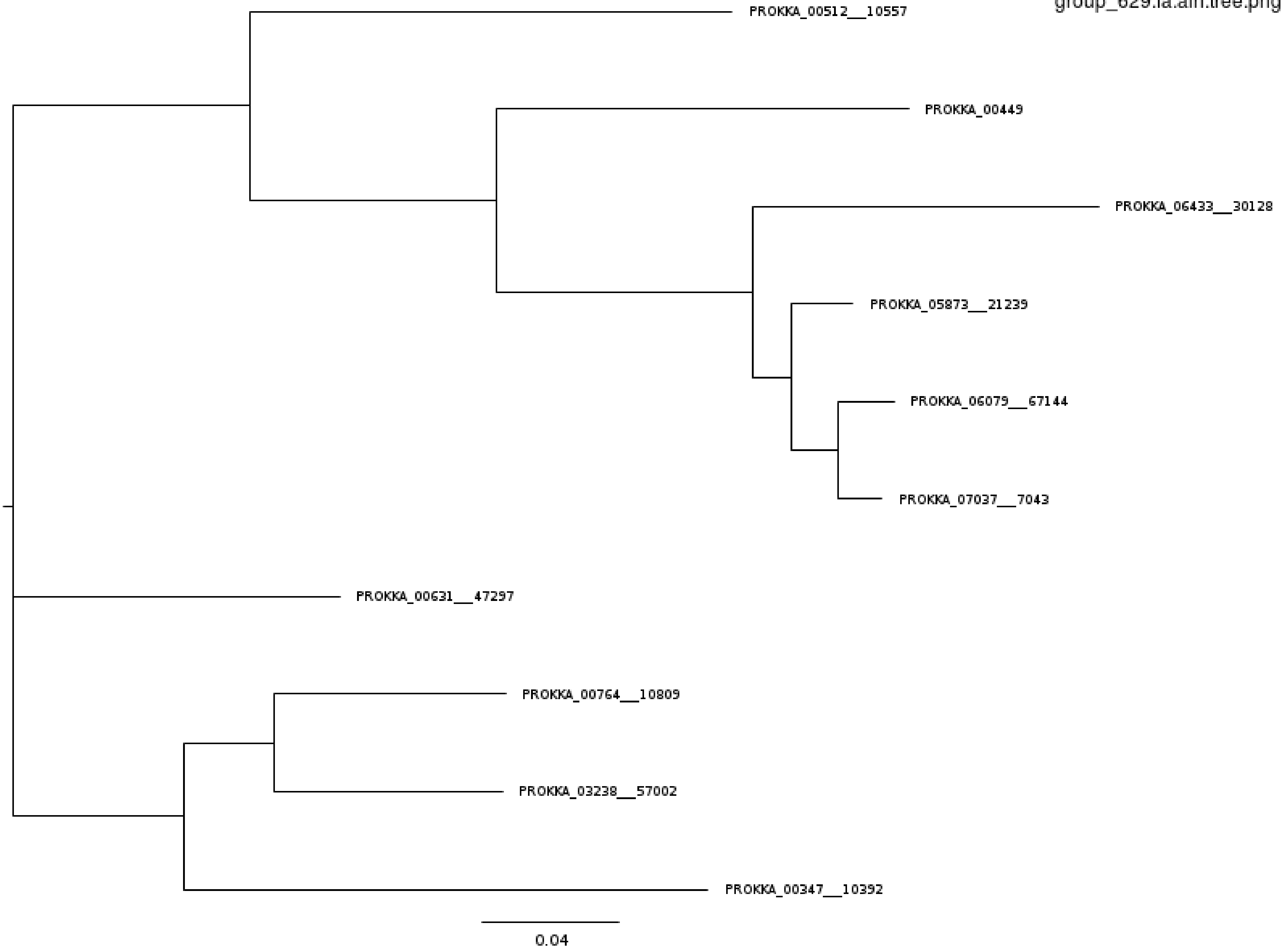

group_62.fa.aln.tree.png

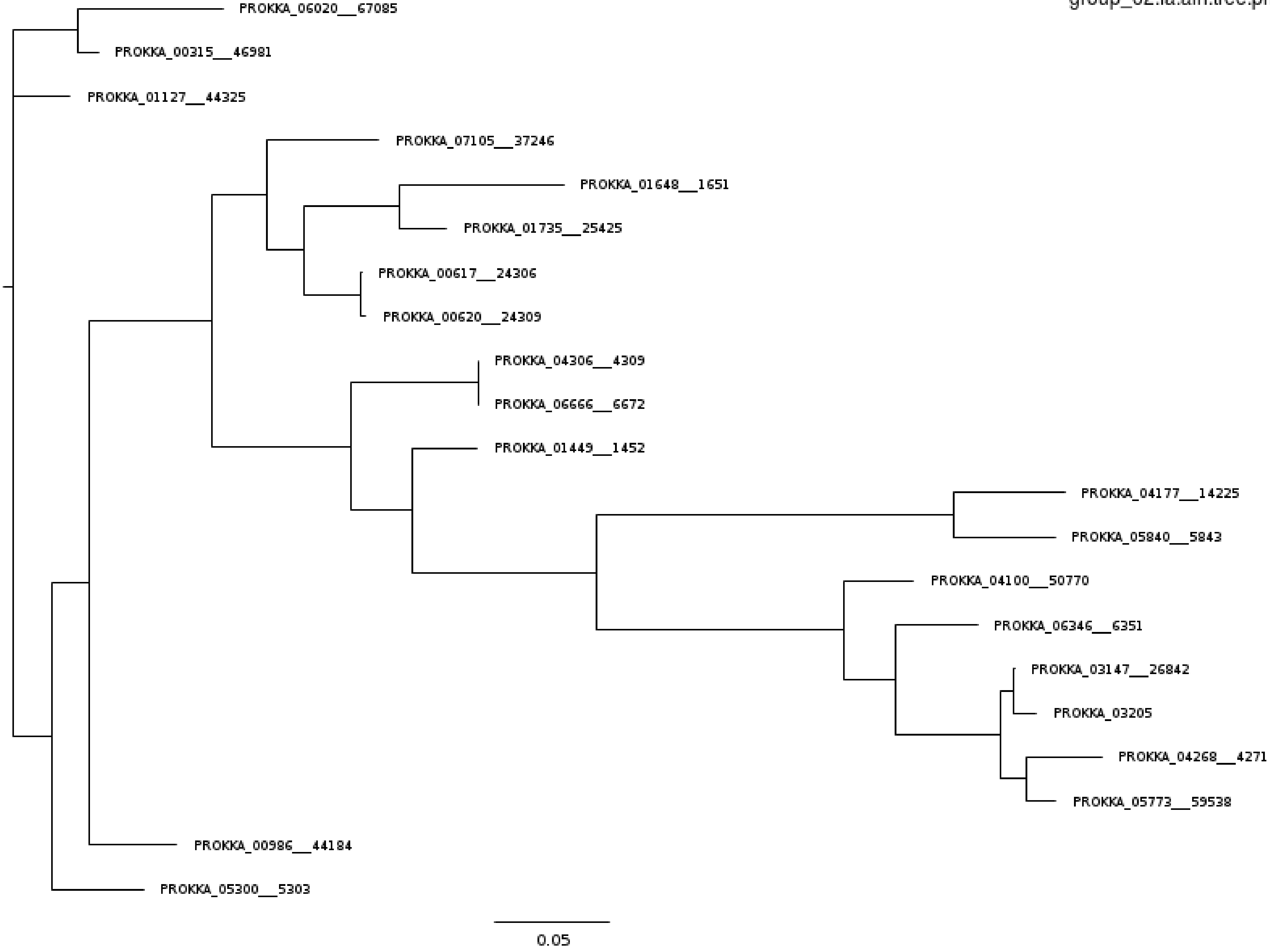

group_6356.fa.aln.tree.png

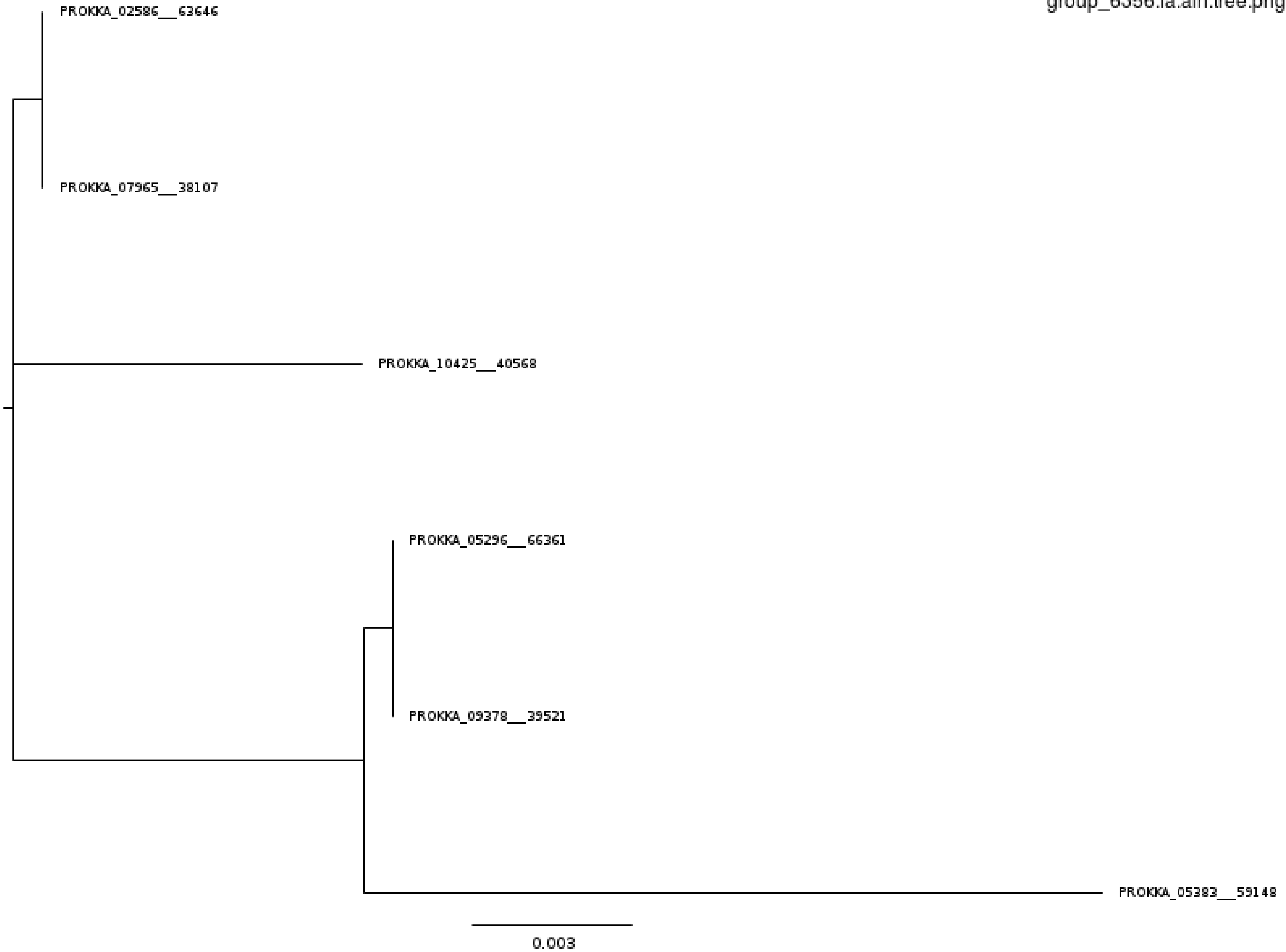

group_636.fa.aln.tree.png

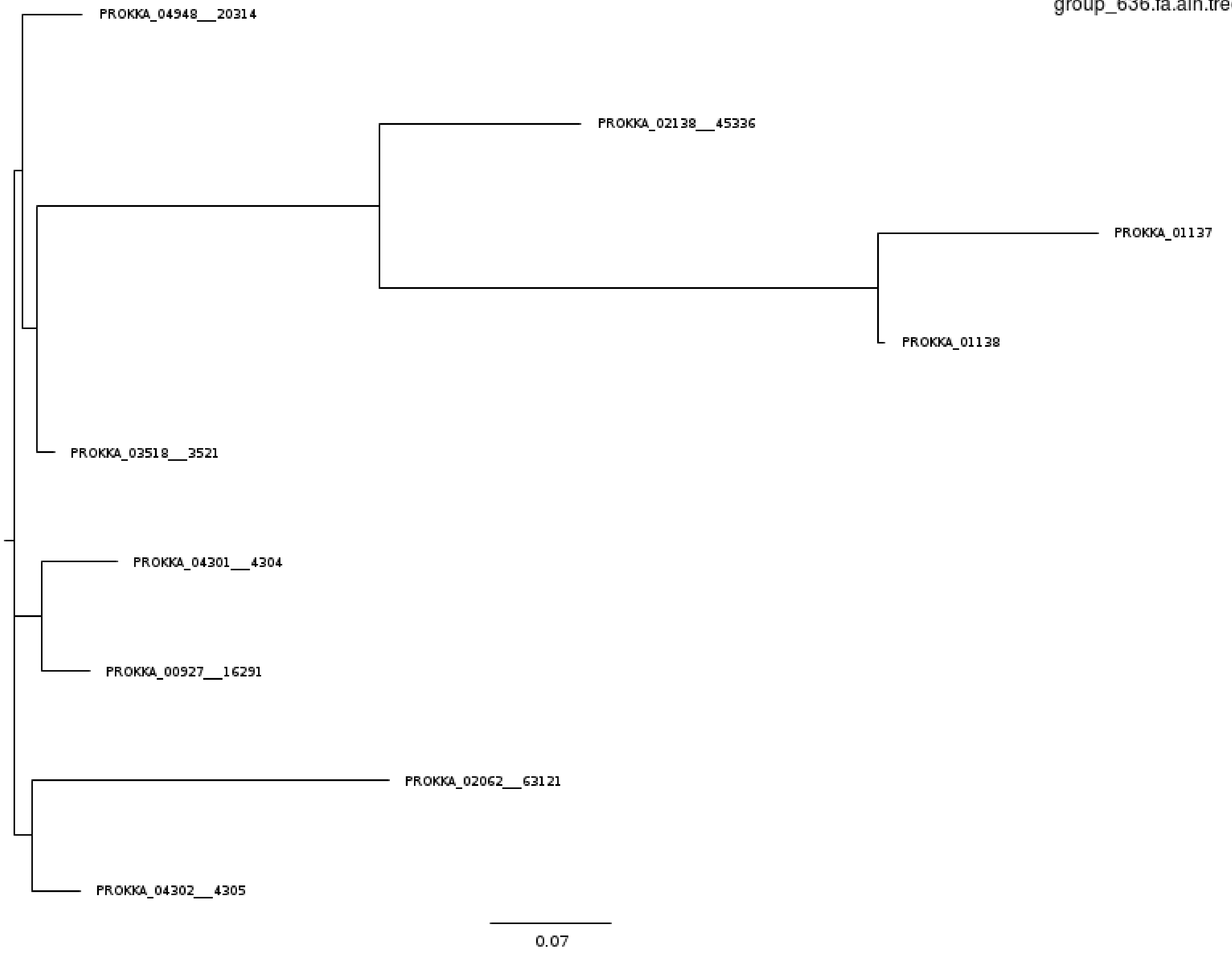

group_637.fa.aln.tree.png

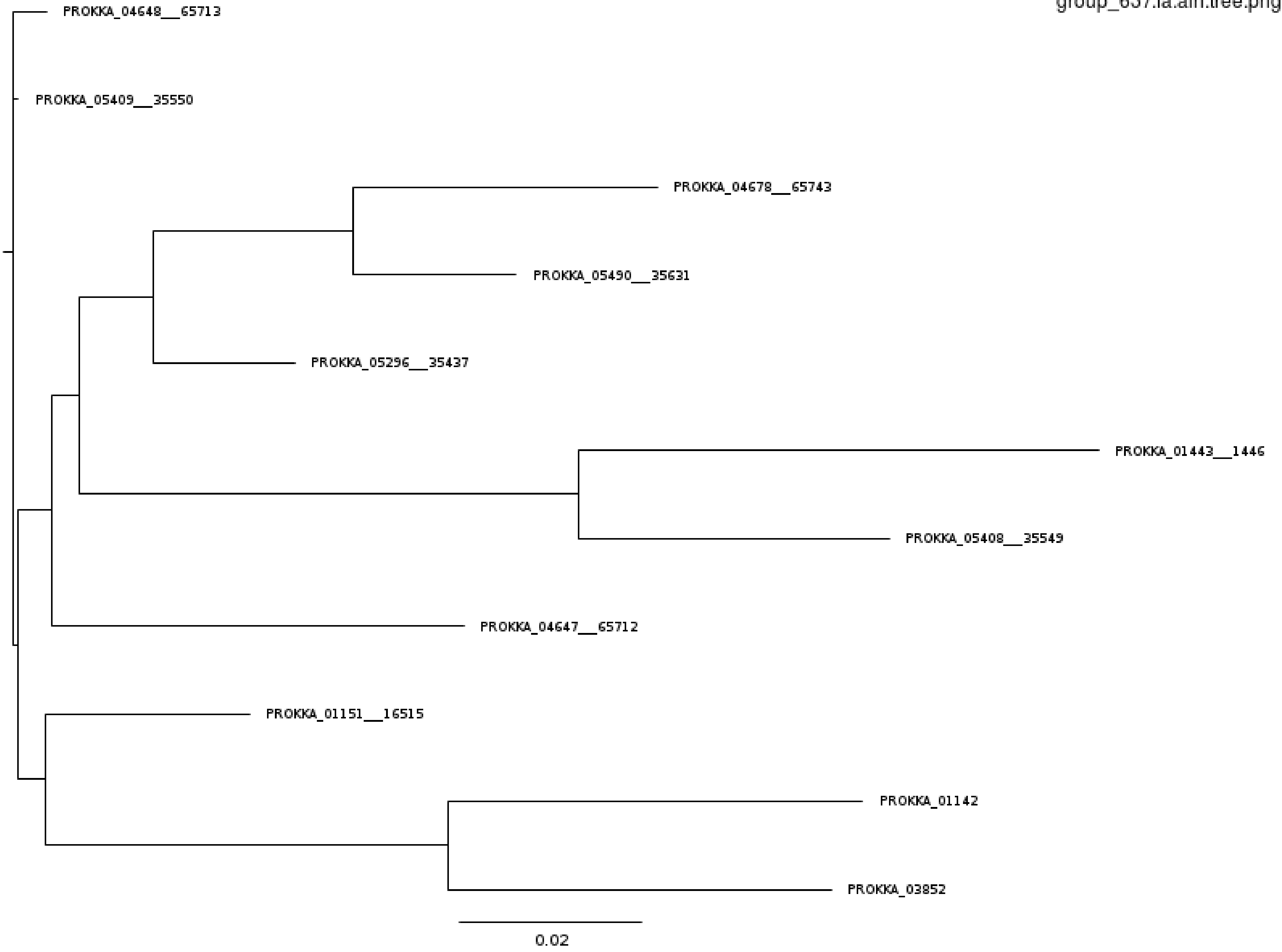

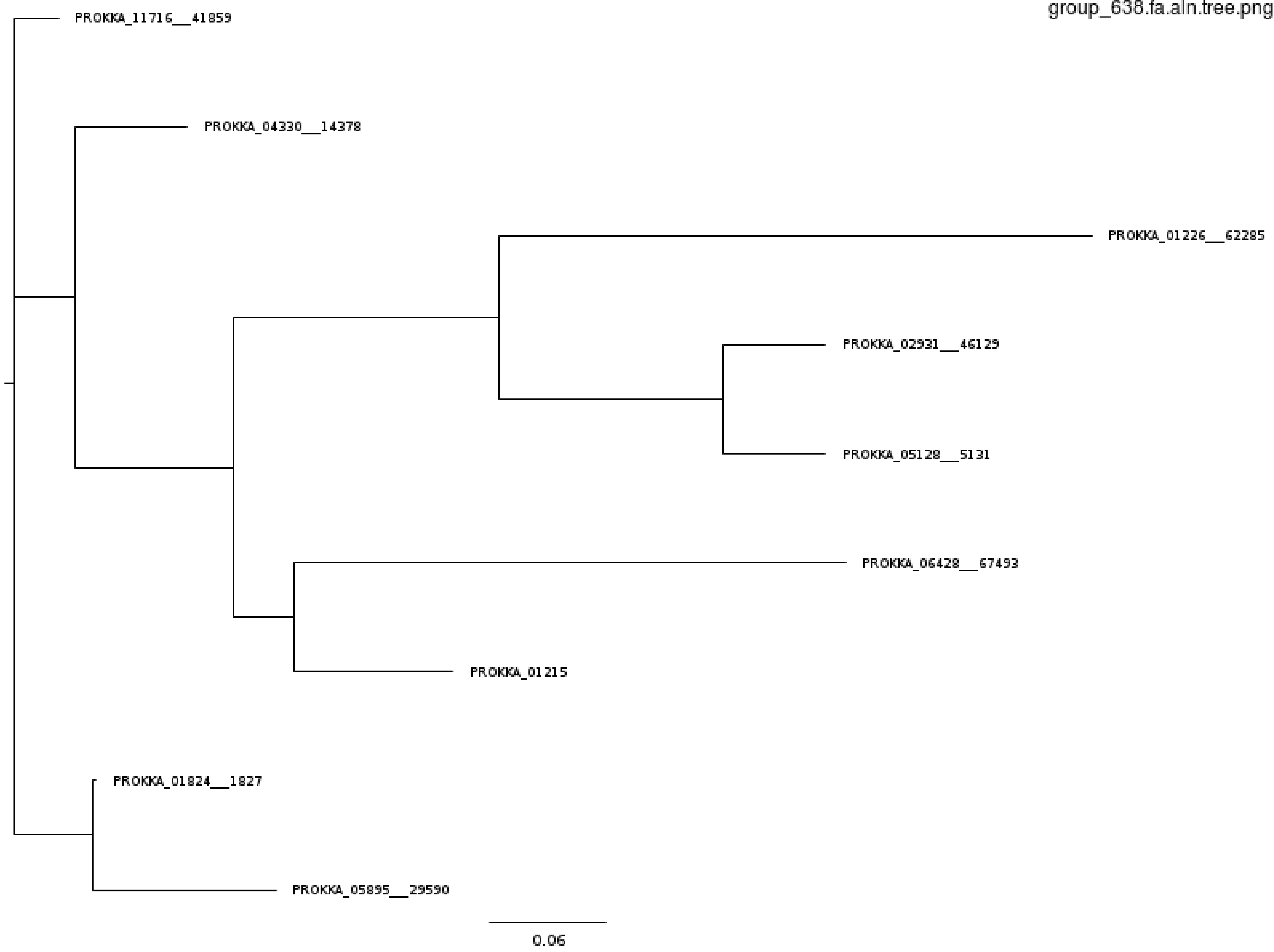
group_638.fa.aln.tree.png
PROKKA_11716___41859
PROKKA_04330___14378
PROKKA_01226___62285
PROKKA_02931___46129
PROKKA_05128___5131
PROKKA_06428___67493
PROKKA_01215
PROKKA_01824___1827
PROKKA_05895___29590
0.06

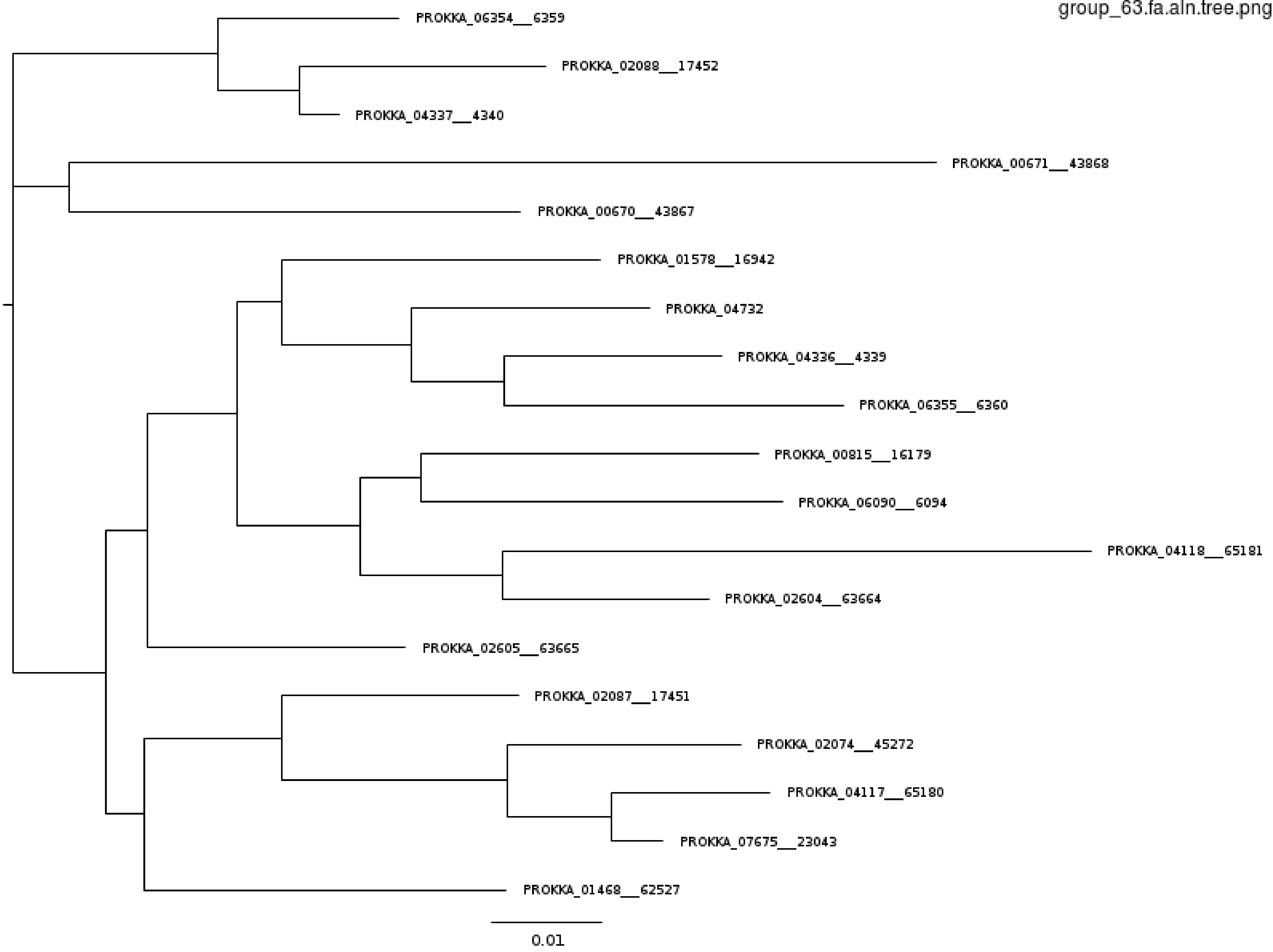
group_63.fa.aln.tree.png
PROKKA_06354___6359
PROKKA_02088___17452
PROKKA_04337___4340
PROKKA_00671___43868
PROKKA_00670___43867
PROKKA_01578___16942
PROKKA_04732
PROKKA_04336___4339
PROKKA_06355___6360
PROKKA_00815___16179
PROKKA_06090___6094
PROKKA_04118___65181
PROKKA_02604___63664
PROKKA_02605___63665
PROKKA_02087___17451
PROKKA_02074___45272
PROKKA_04117___65180
PROKKA_07675___23043
PROKKA_01468___62527
0.01

group_640.fa.aln.tree.png

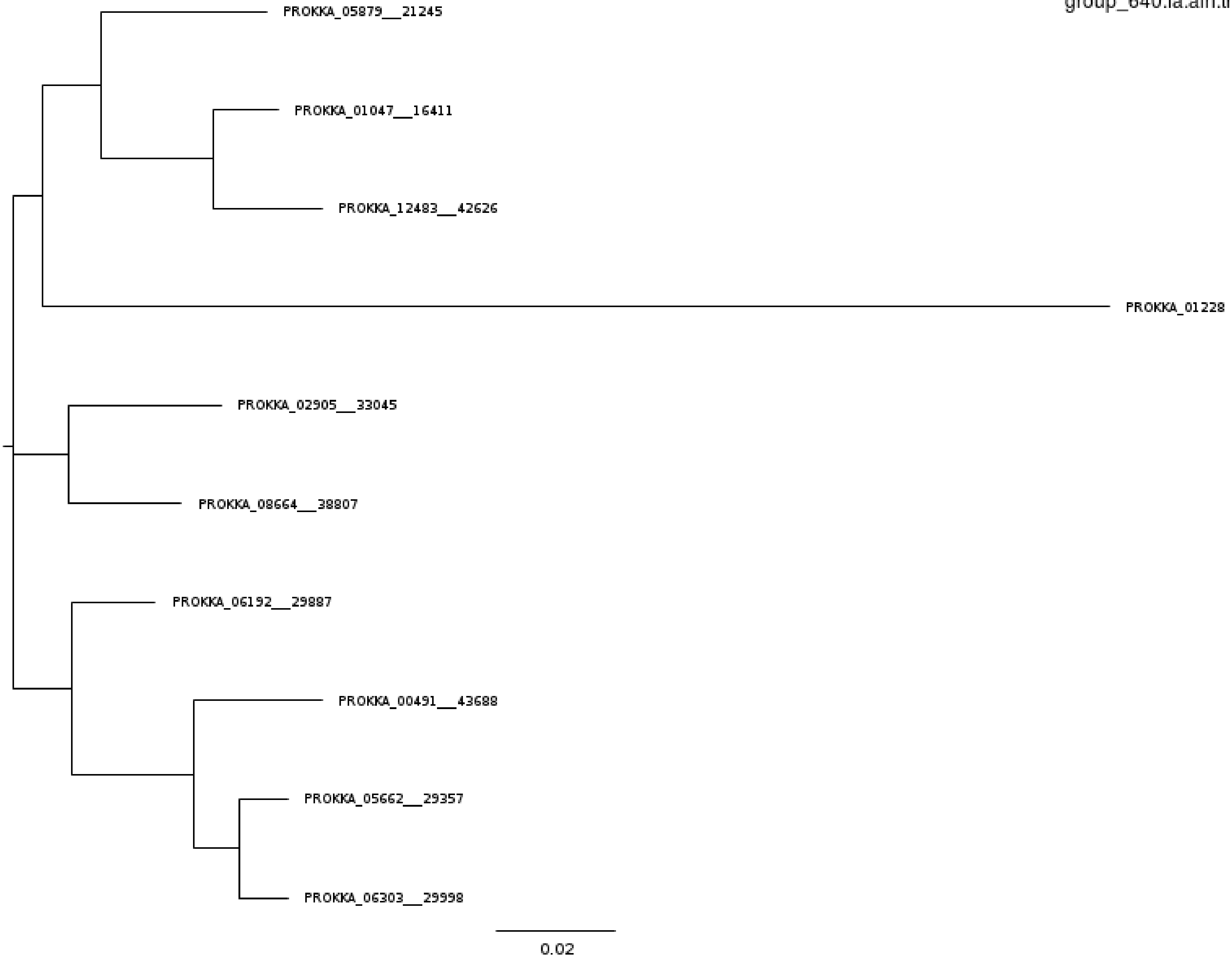

group_641.fa.aln.tree.png

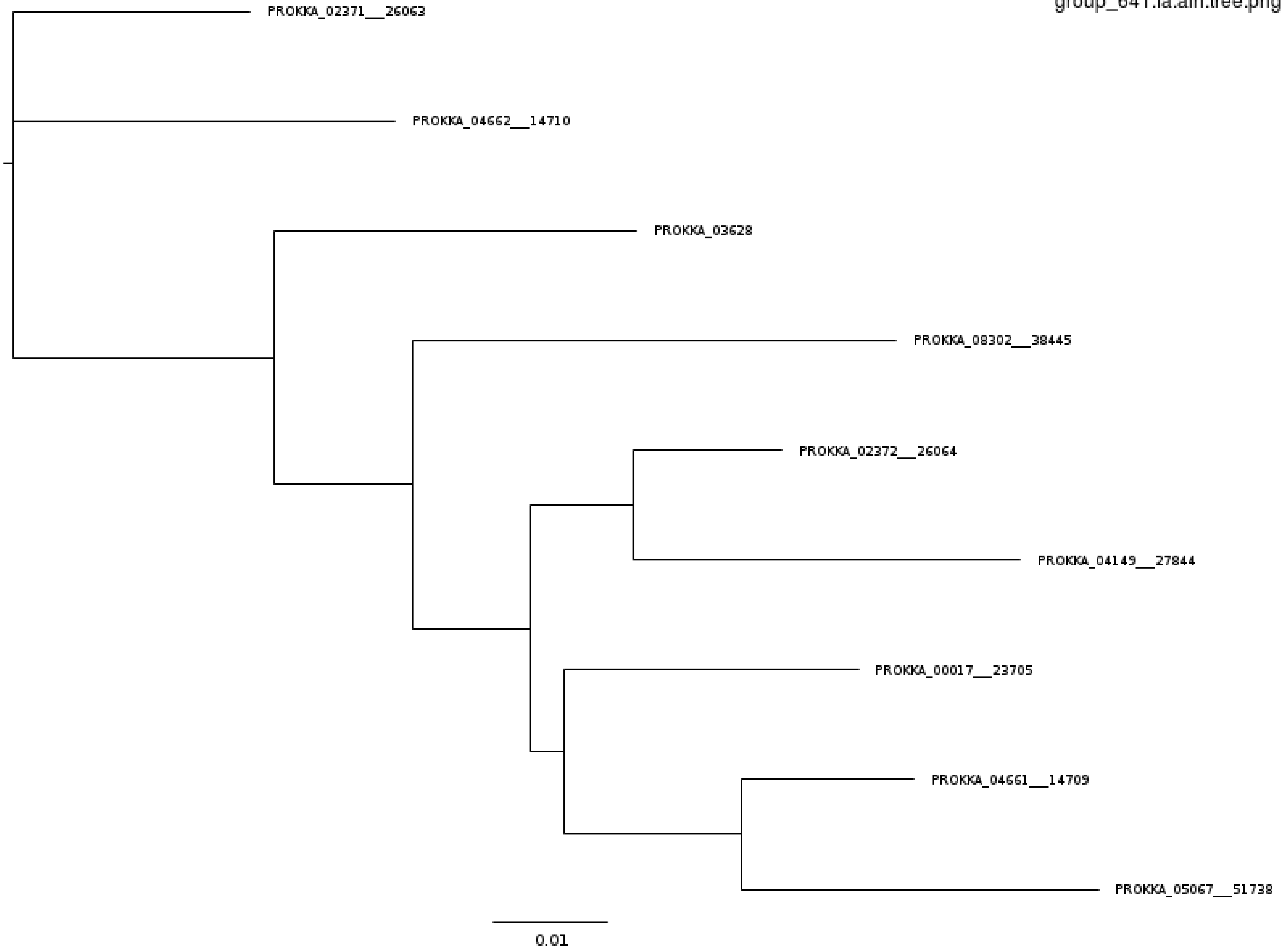

group_642.fa.aln.tree.png

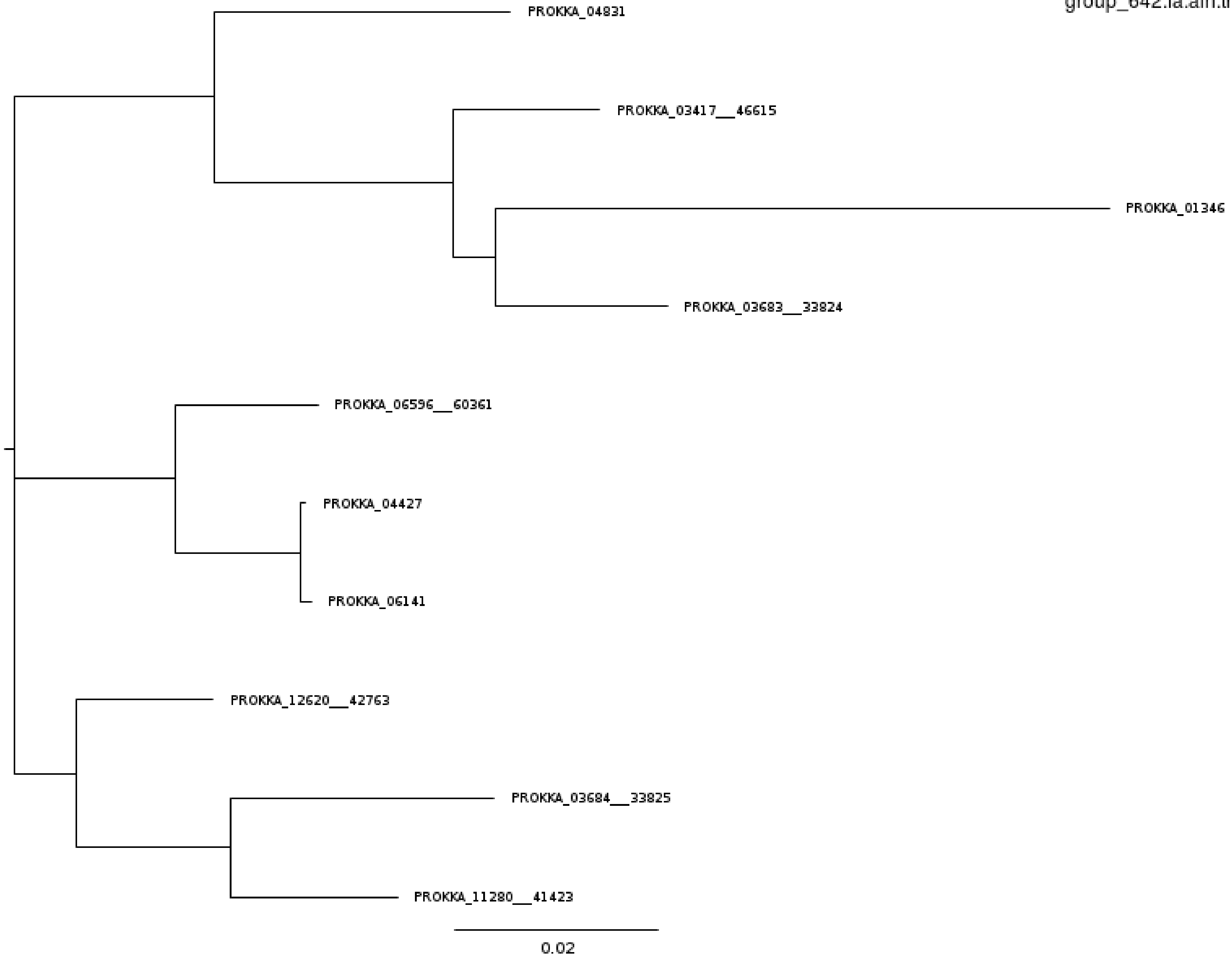

group_6504.fa.aln.tree.png

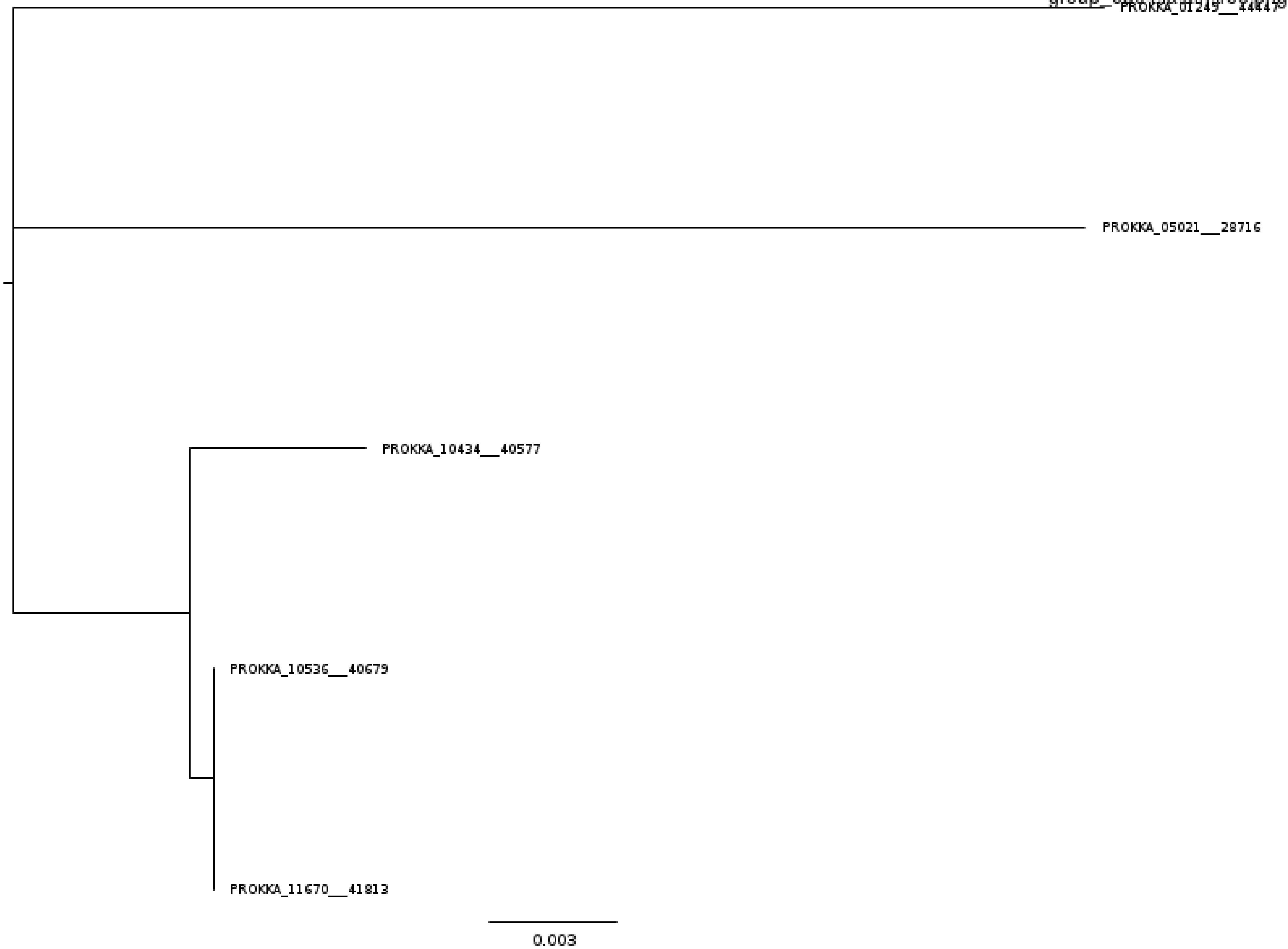

group_651.fa.aln.tree.png

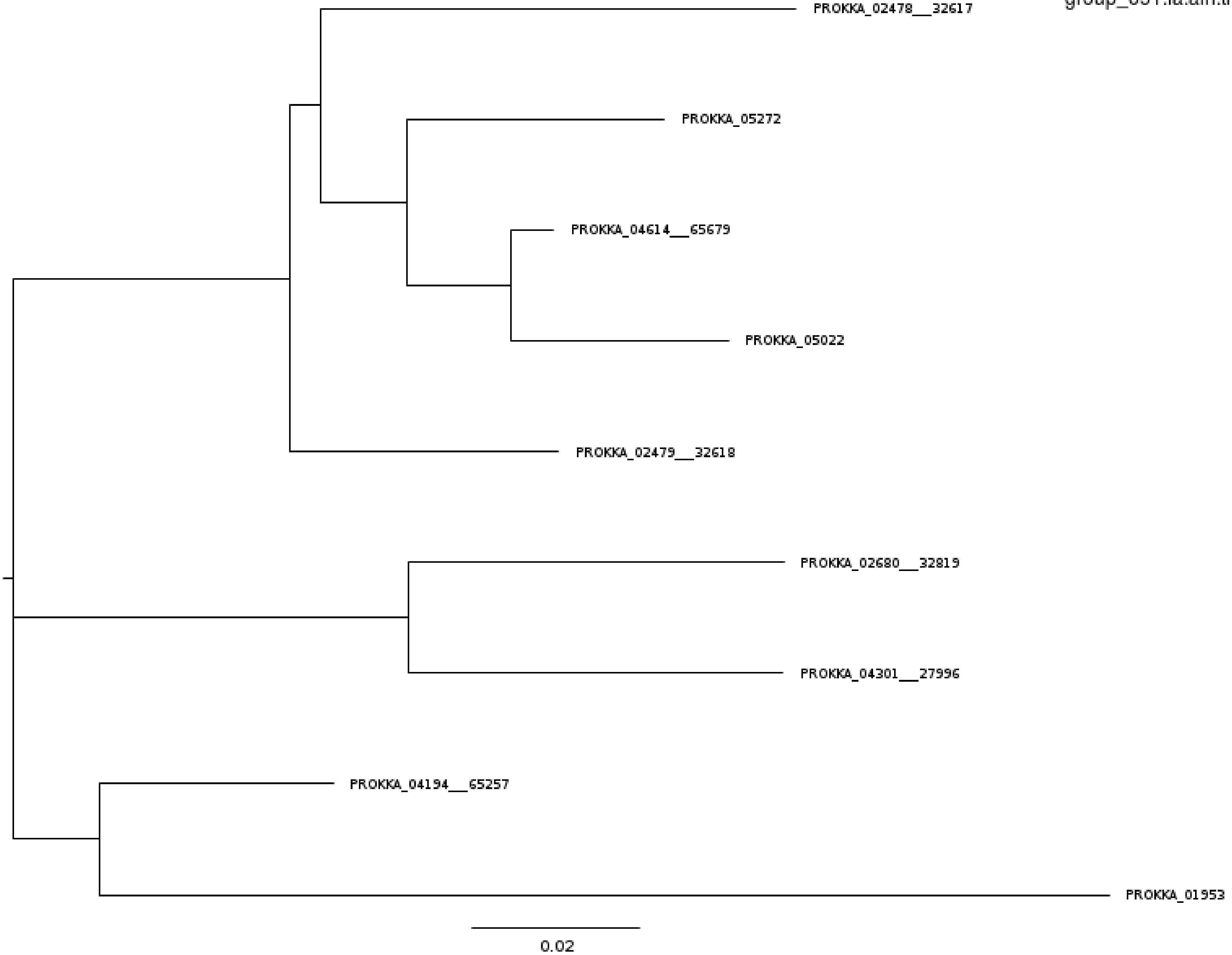

group_654.fa.aln.tree.png

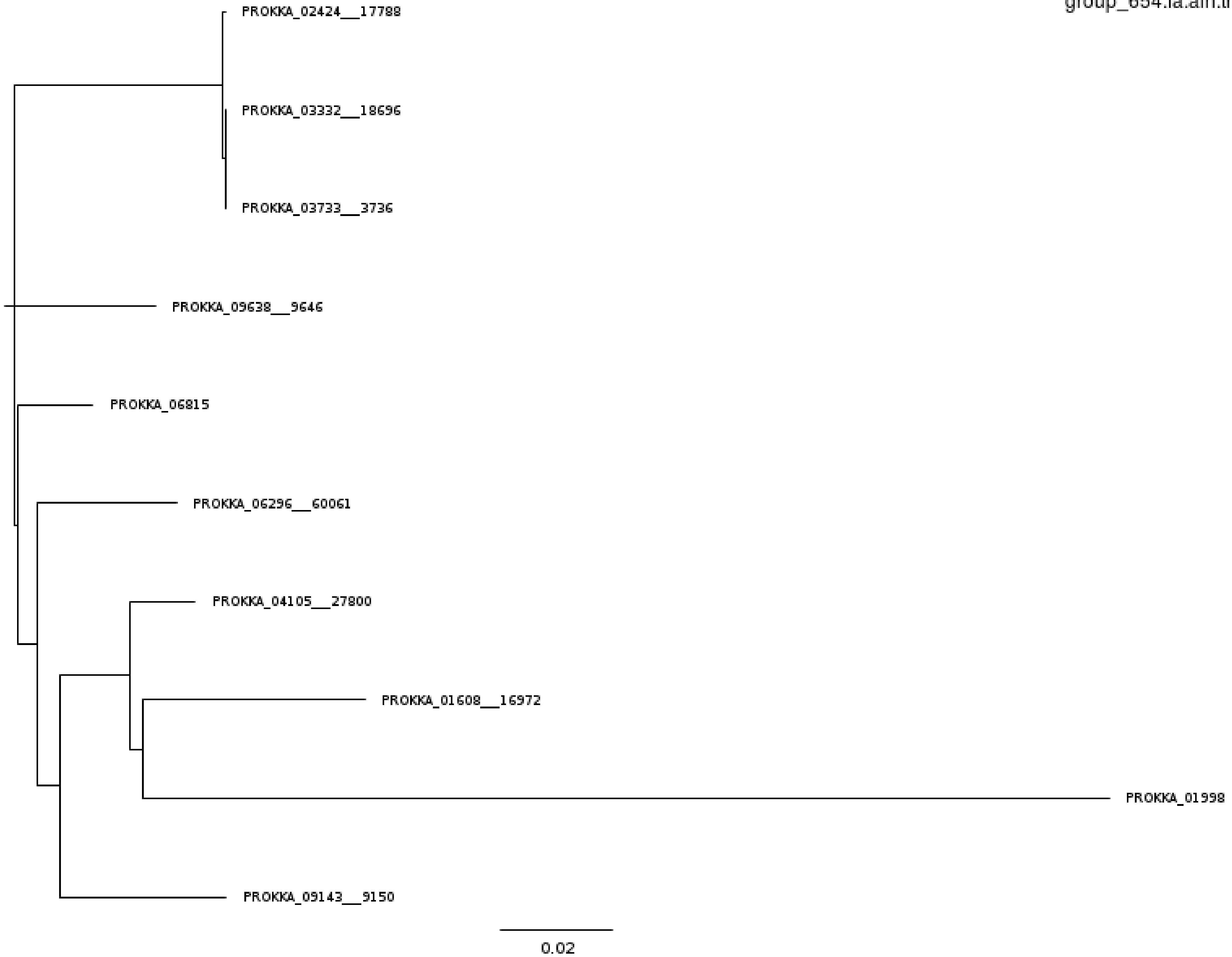

group_6551.fa.aln.tree.png

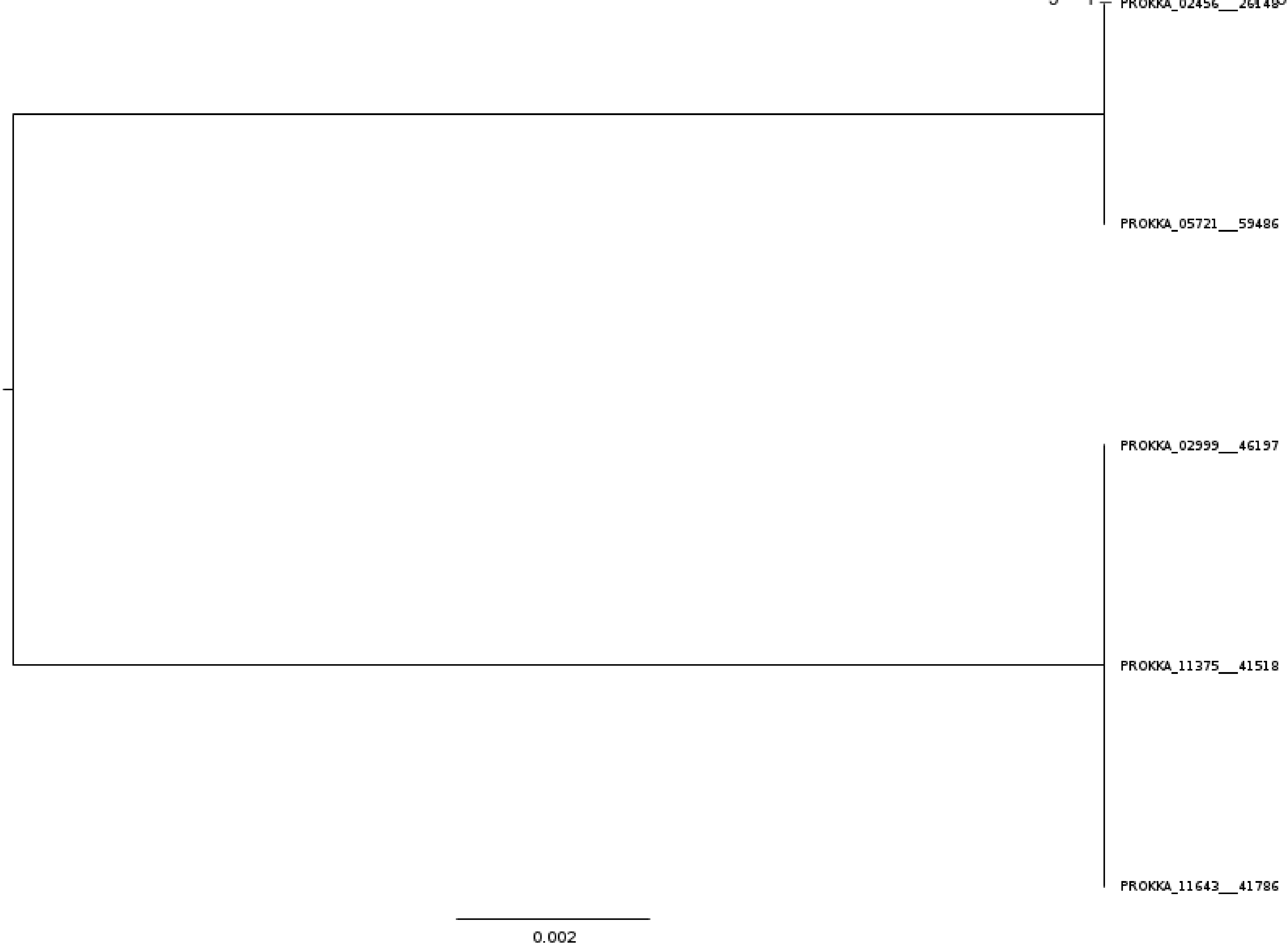

group_655.fa.aln.tree.png

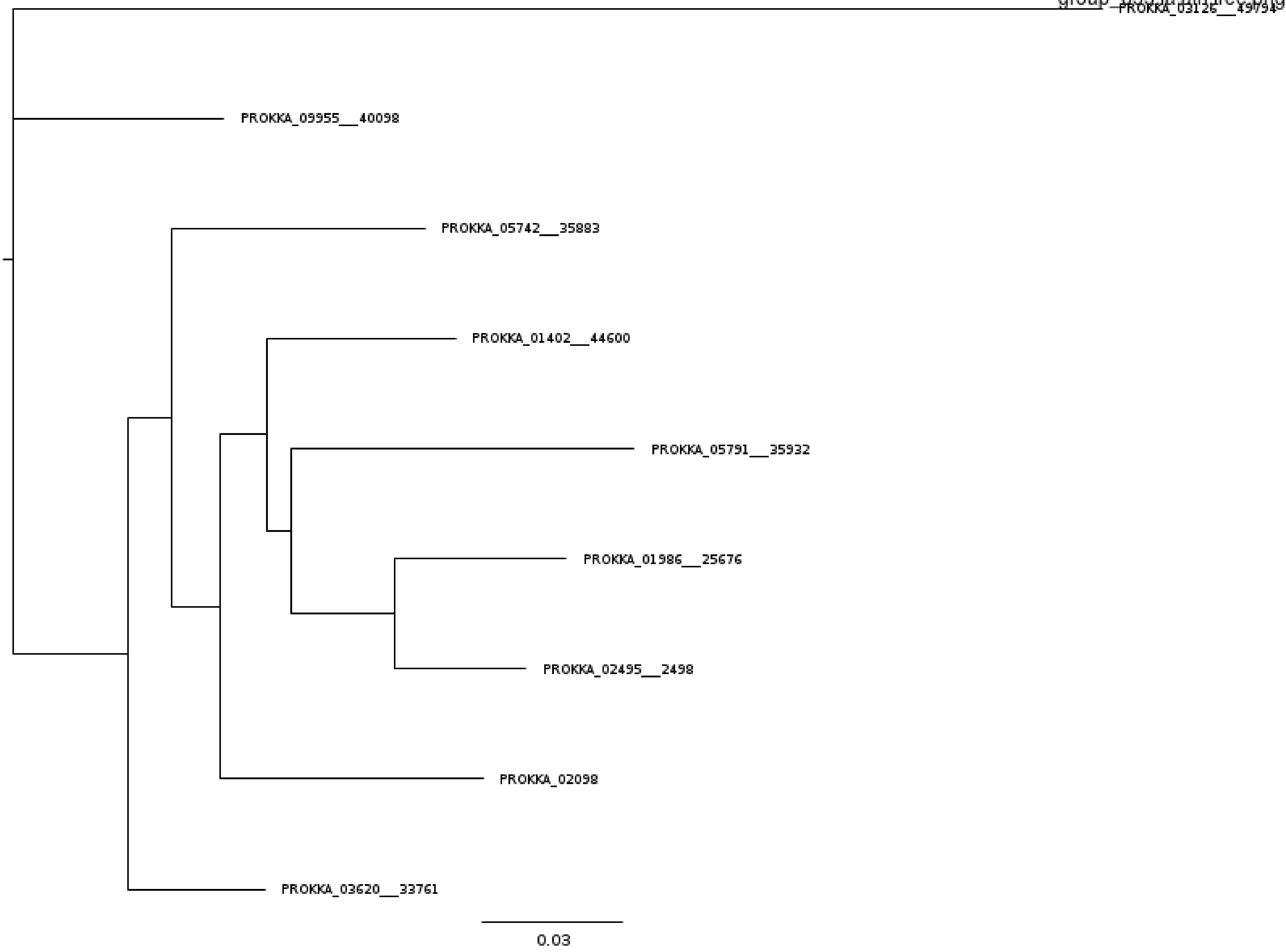

group_659.fa.aln.tree.png

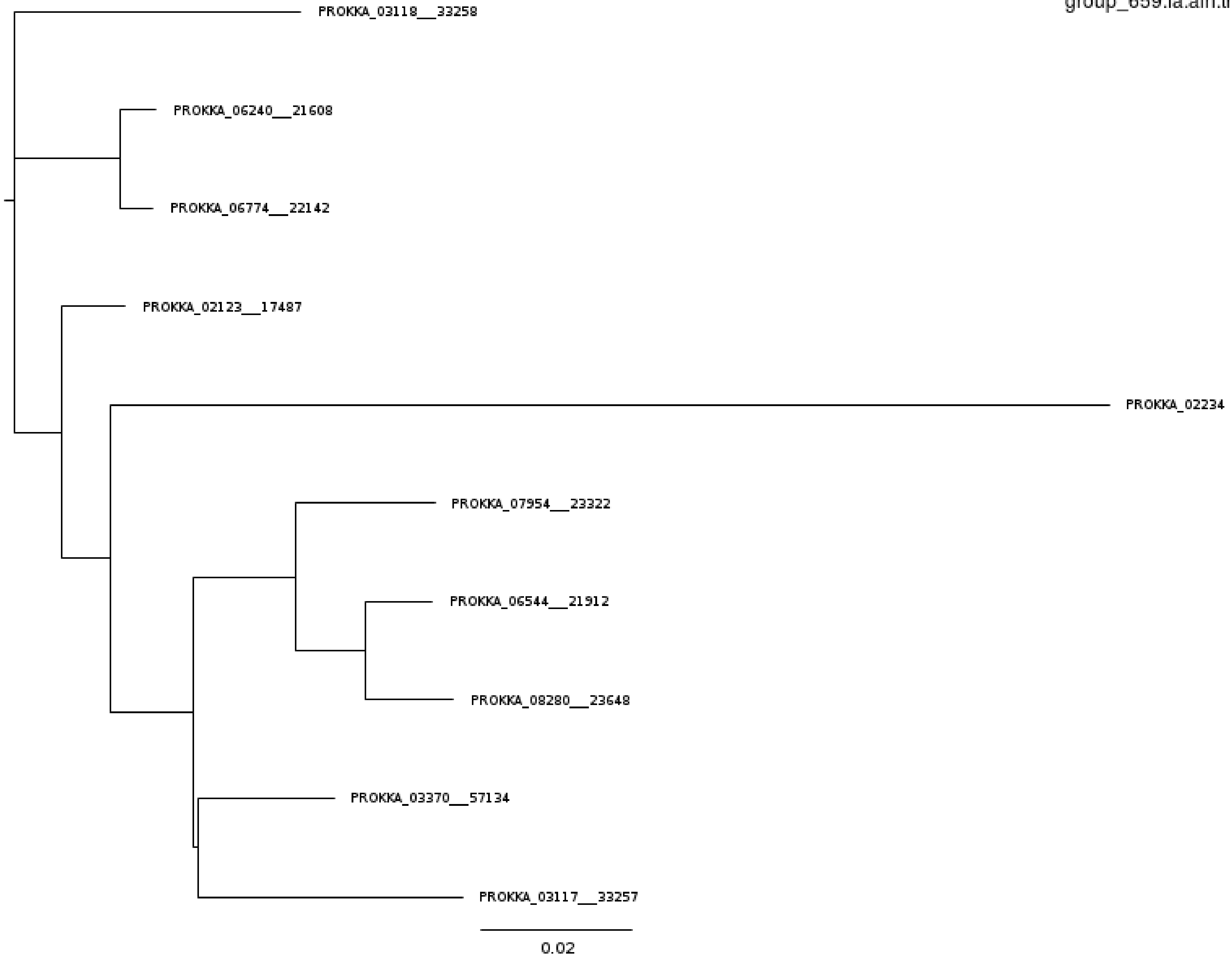

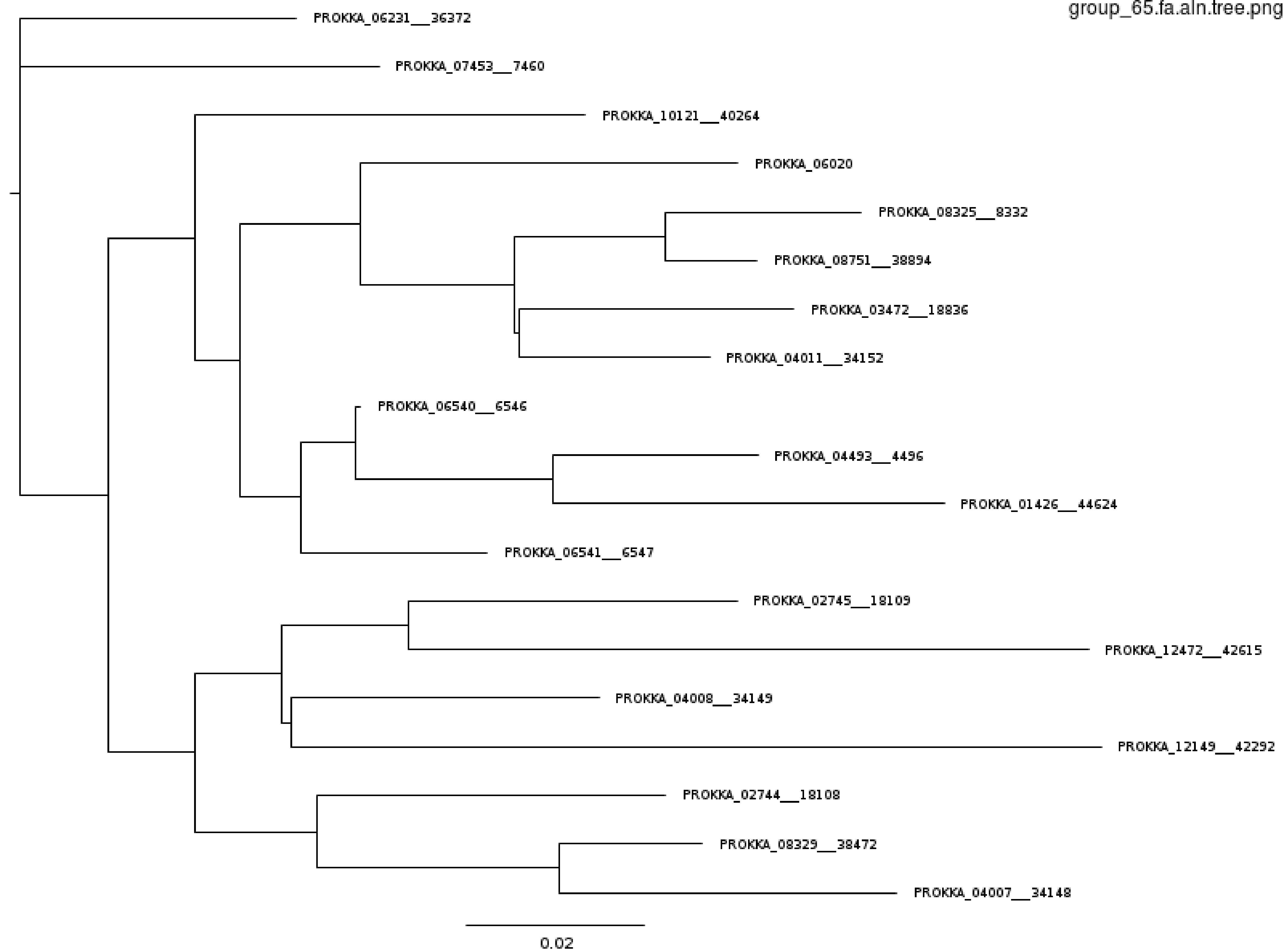

group_65.fa.aln.tree.png
PROKKA_06231___36372
PROKKA_07453___7460
PROKKA_10121___40264
PROKKA_06020
PROKKA_08325___8332
PROKKA_08751___38894
PROKKA_03472___18836
PROKKA_04011___34152
PROKKA_06540___6546
PROKKA_04493___4496
PROKKA_01426___44624
PROKKA_06541___6547
PROKKA_02745___18109
PROKKA_12472___42615
PROKKA_04008___34149
PROKKA_12149___42292
PROKKA_02744___18108
PROKKA_08329___38472
PROKKA_04007___34148
0.02

group_661.fa.aln.tree.png

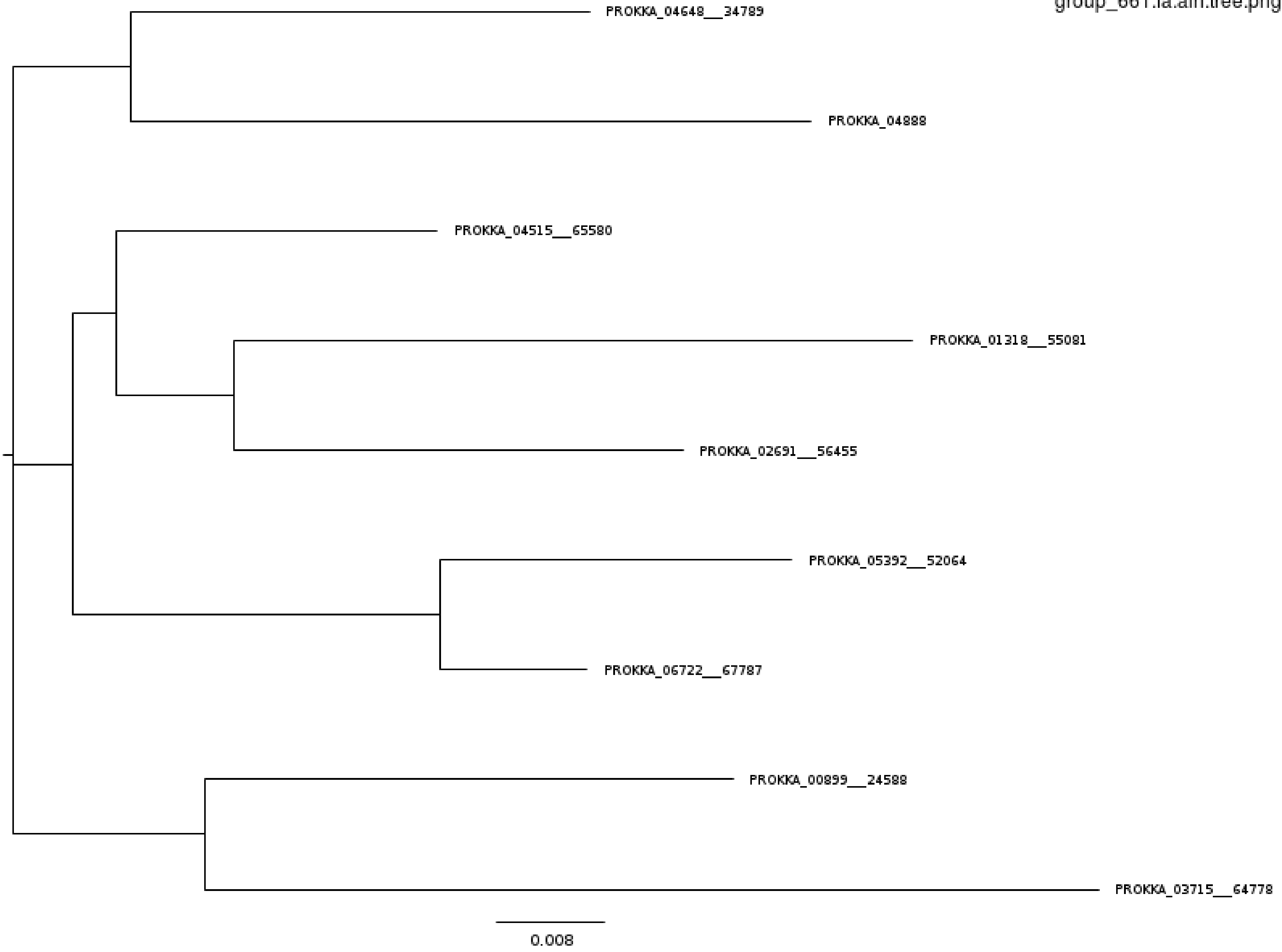

group_662.fa.aln.tree.png

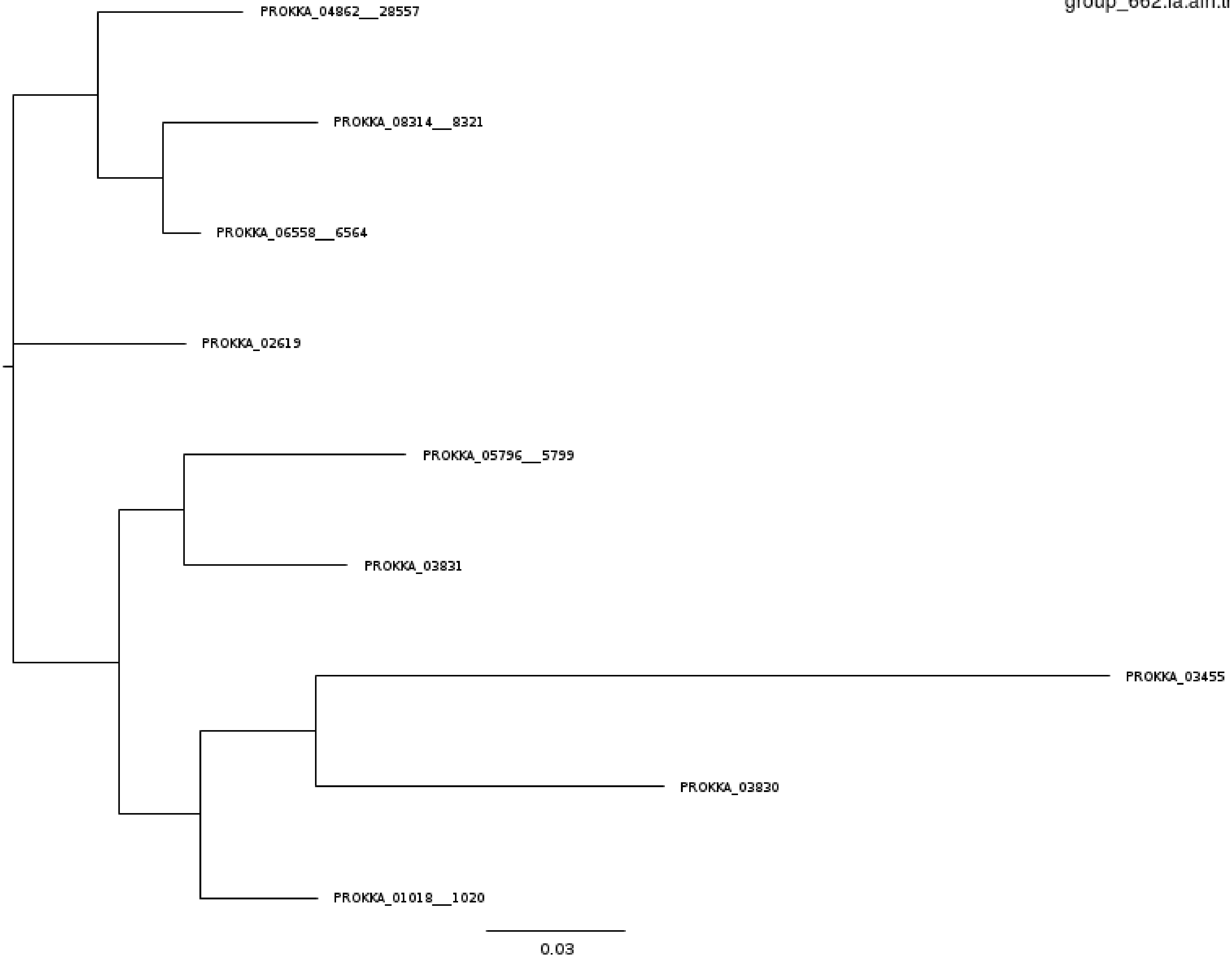

group_664.fa.aln.tree.png

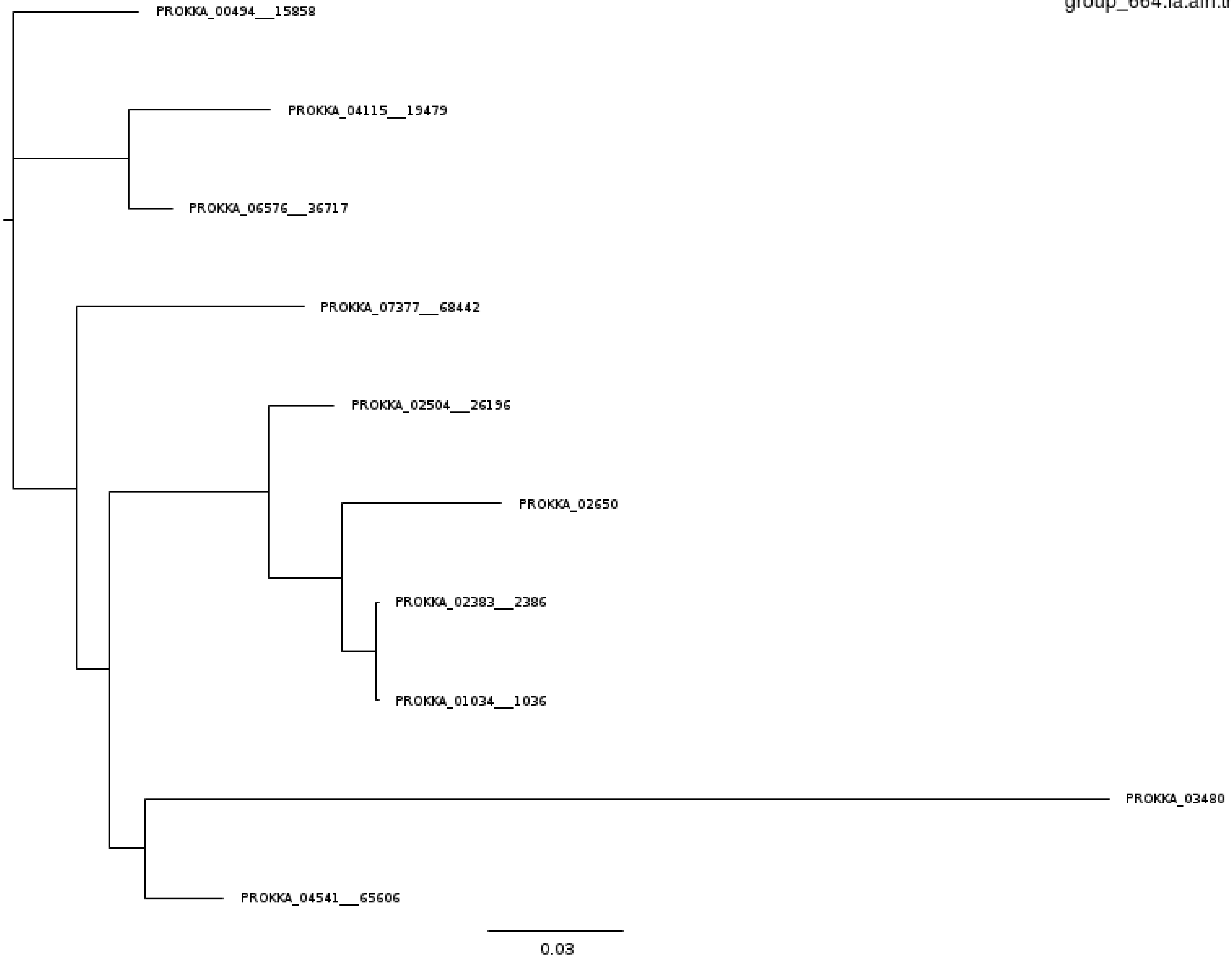

group_665.fa.aln.tree.png

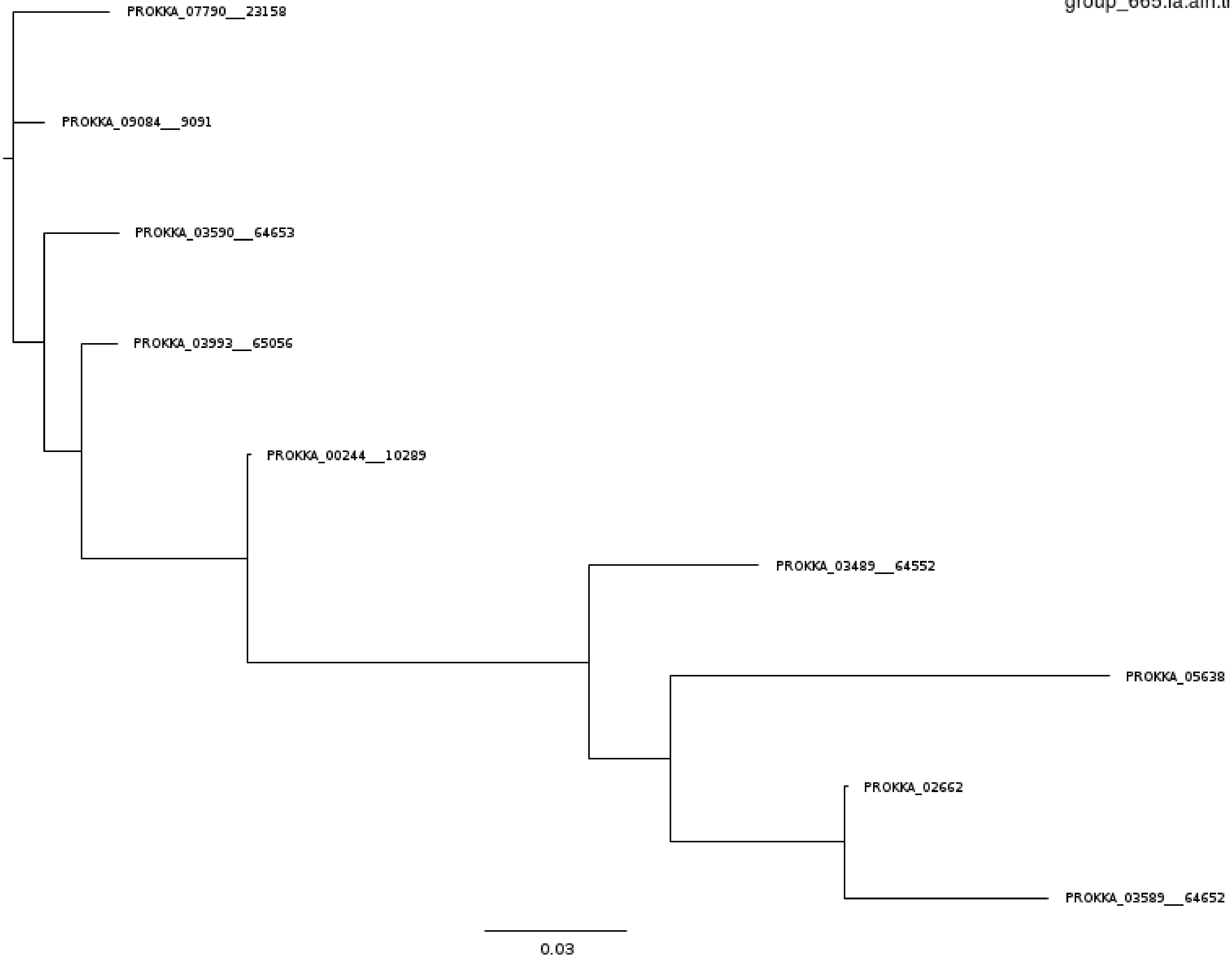

group_666.fa.aln.tree.png

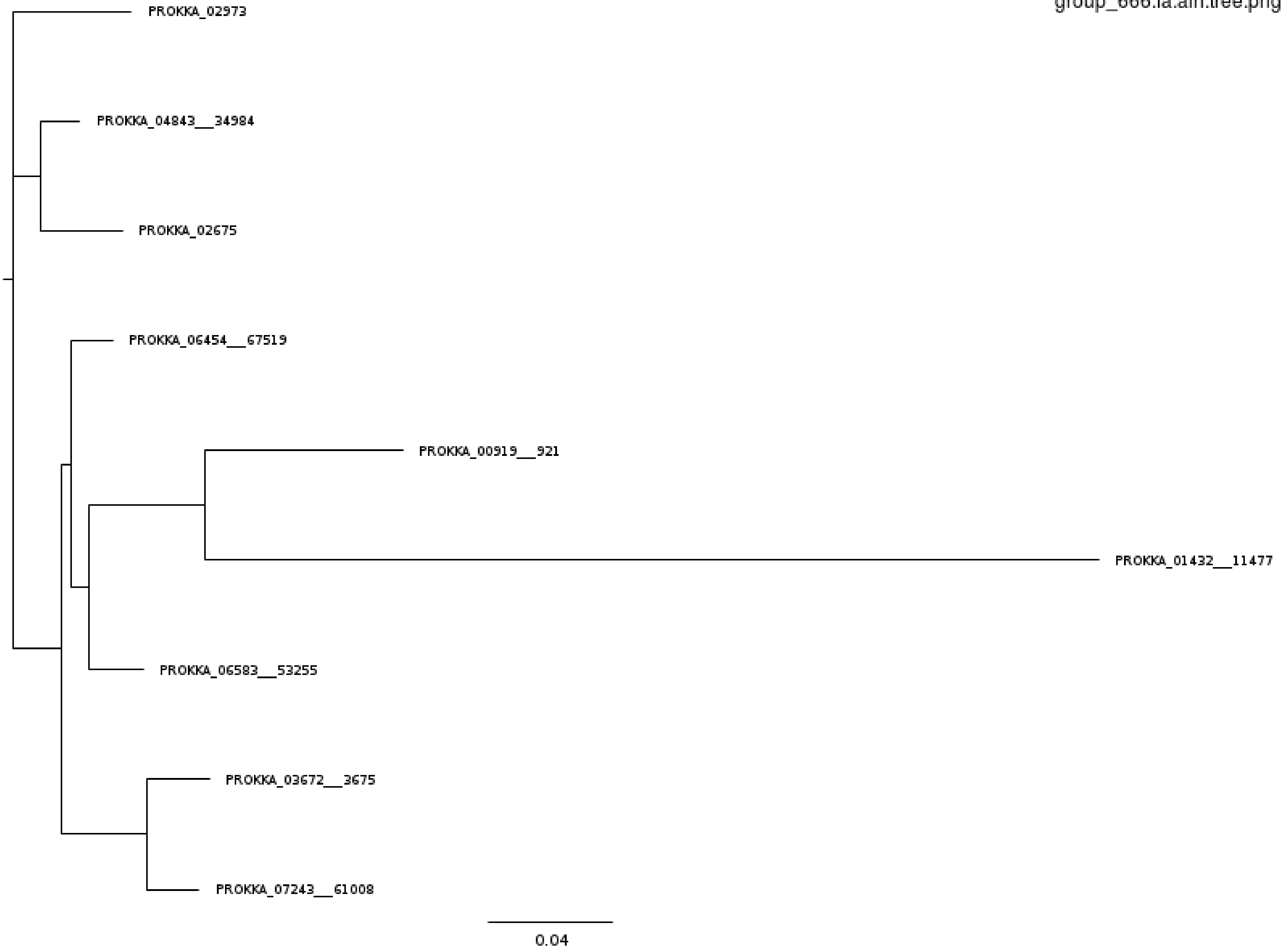

group_670.fa.aln.tree.png

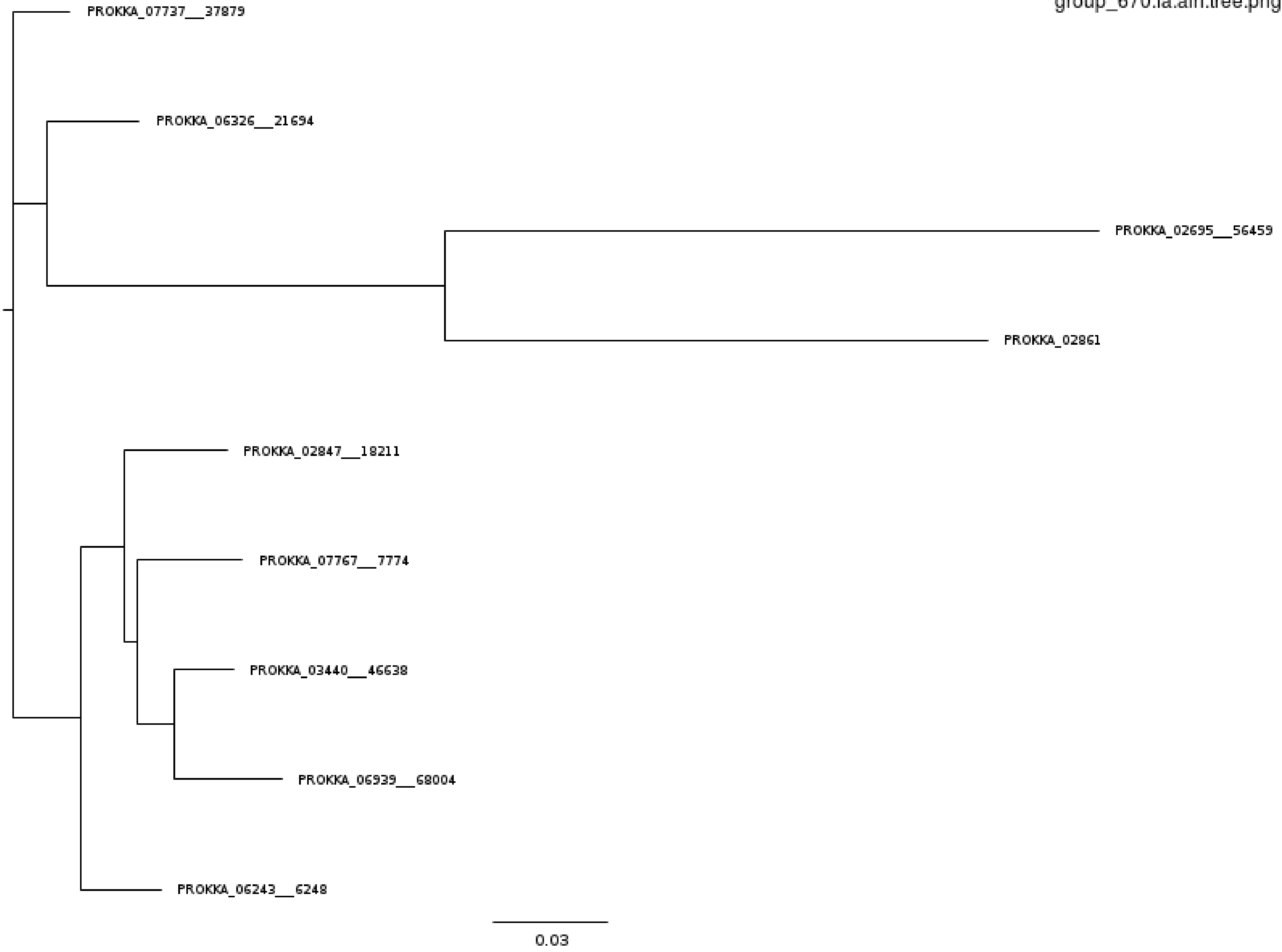

group_671.fa.aln.tree.png

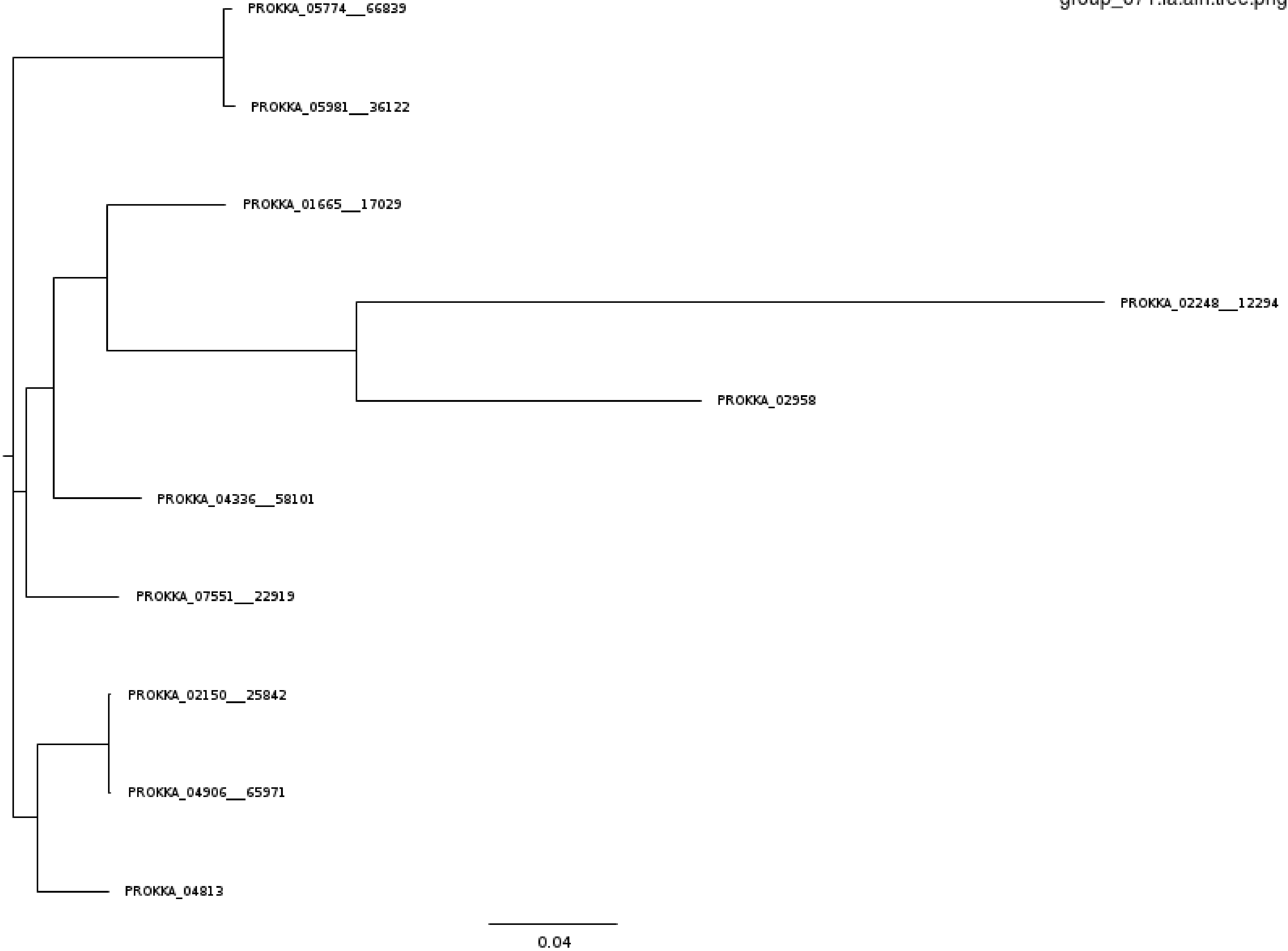

group_672.fa.aln.tree.png

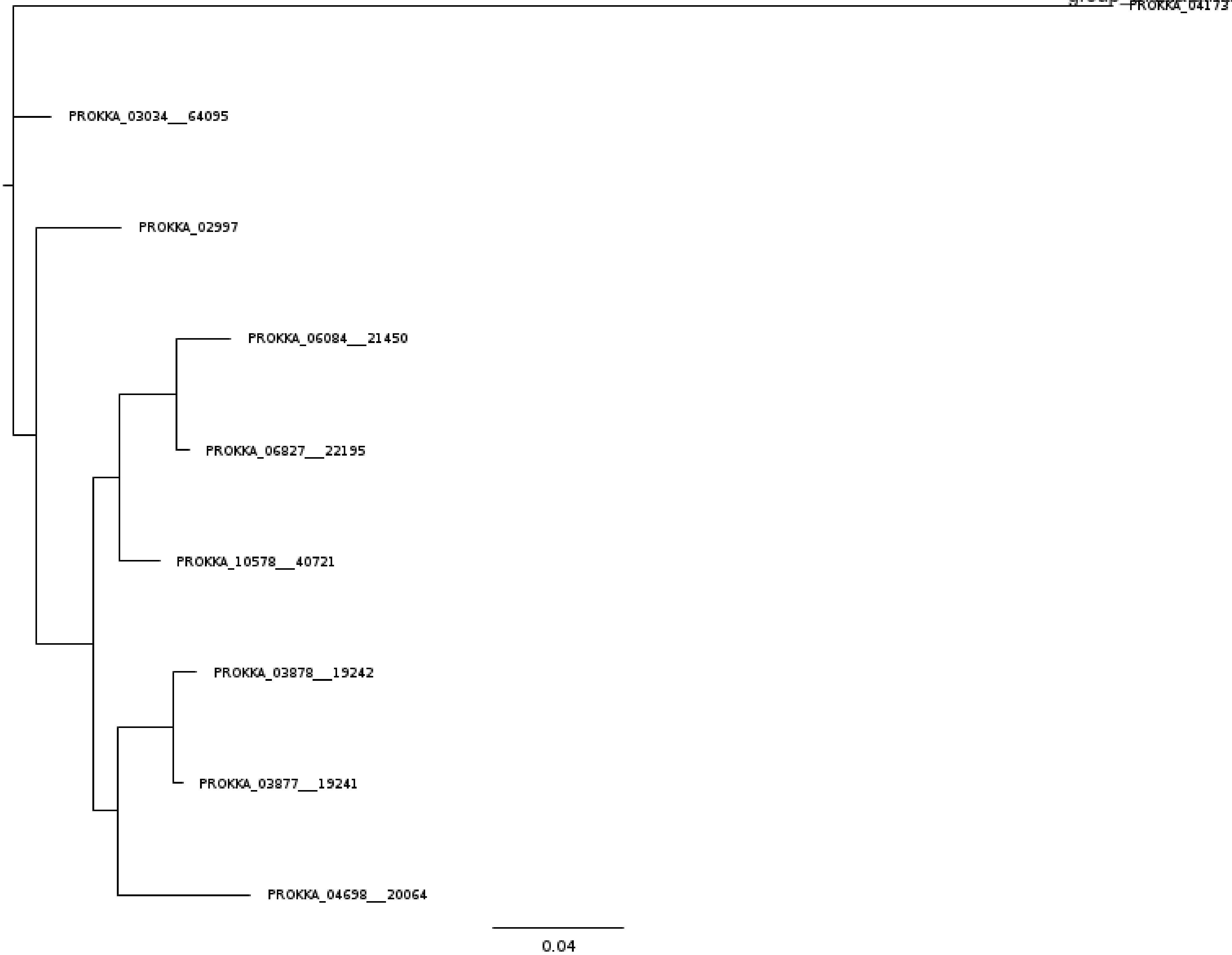

group_673.fa.aln.tree.png

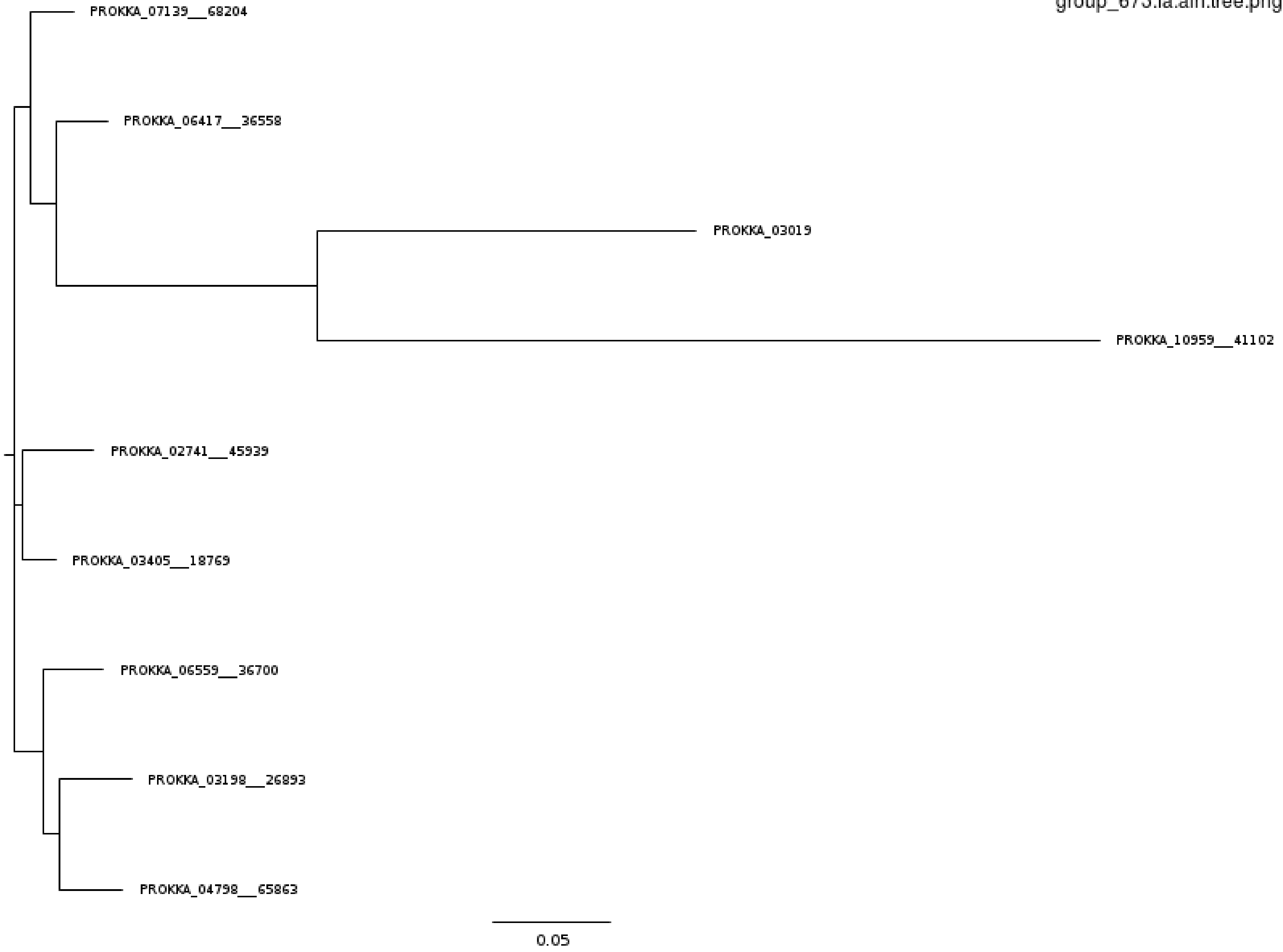

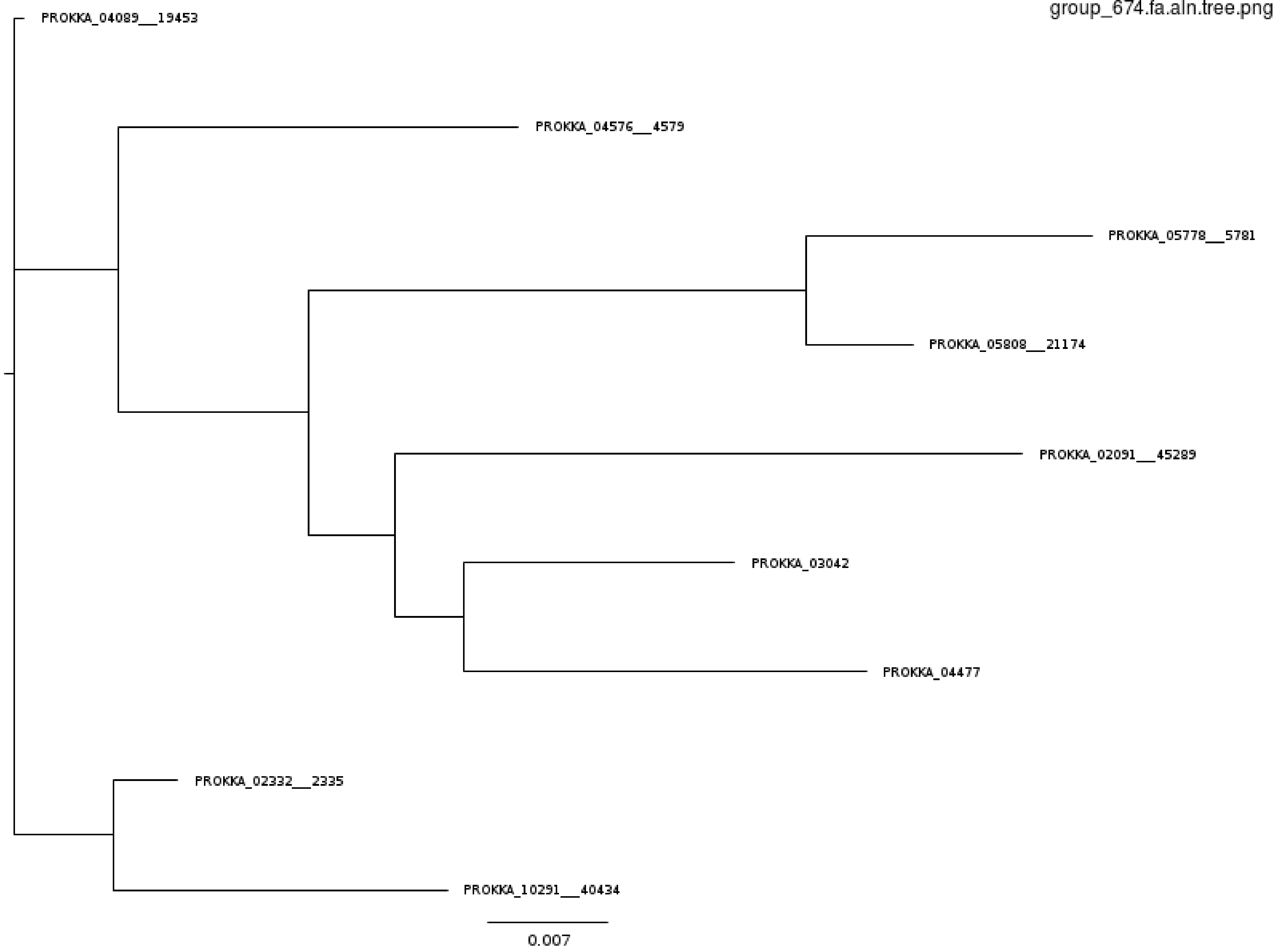
group_674.fa.aln.tree.png
PROKKA_04089___19453
PROKKA_04576___4579
PROKKA_05778___5781
PROKKA_05808___21174
PROKKA_02091___45289
PROKKA_03042
PROKKA_04477
PROKKA_02332___2335
PROKKA_10291___40434
0.007

group_677.fa.aln.tree.png

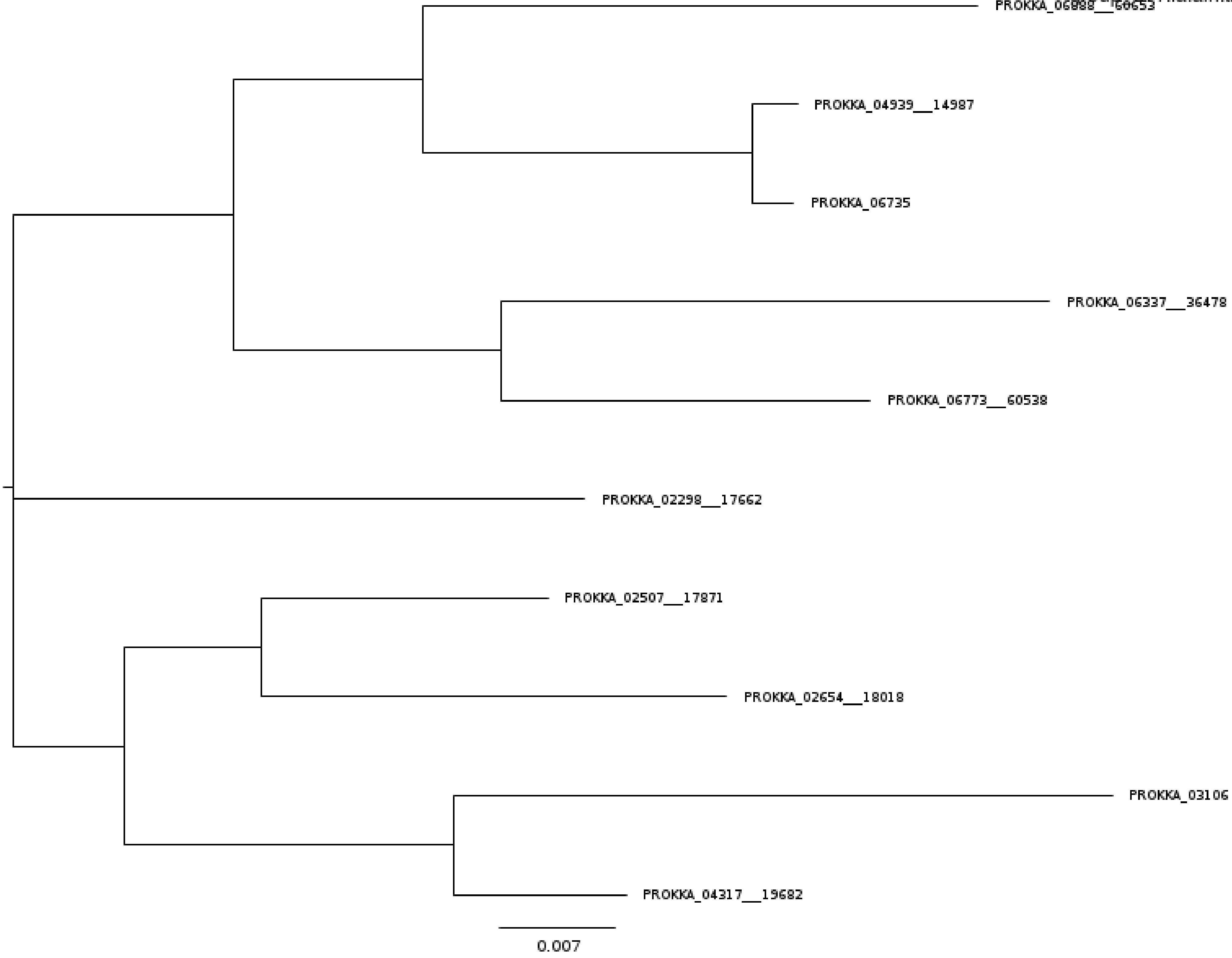

group_685.fa.aln.tree.png

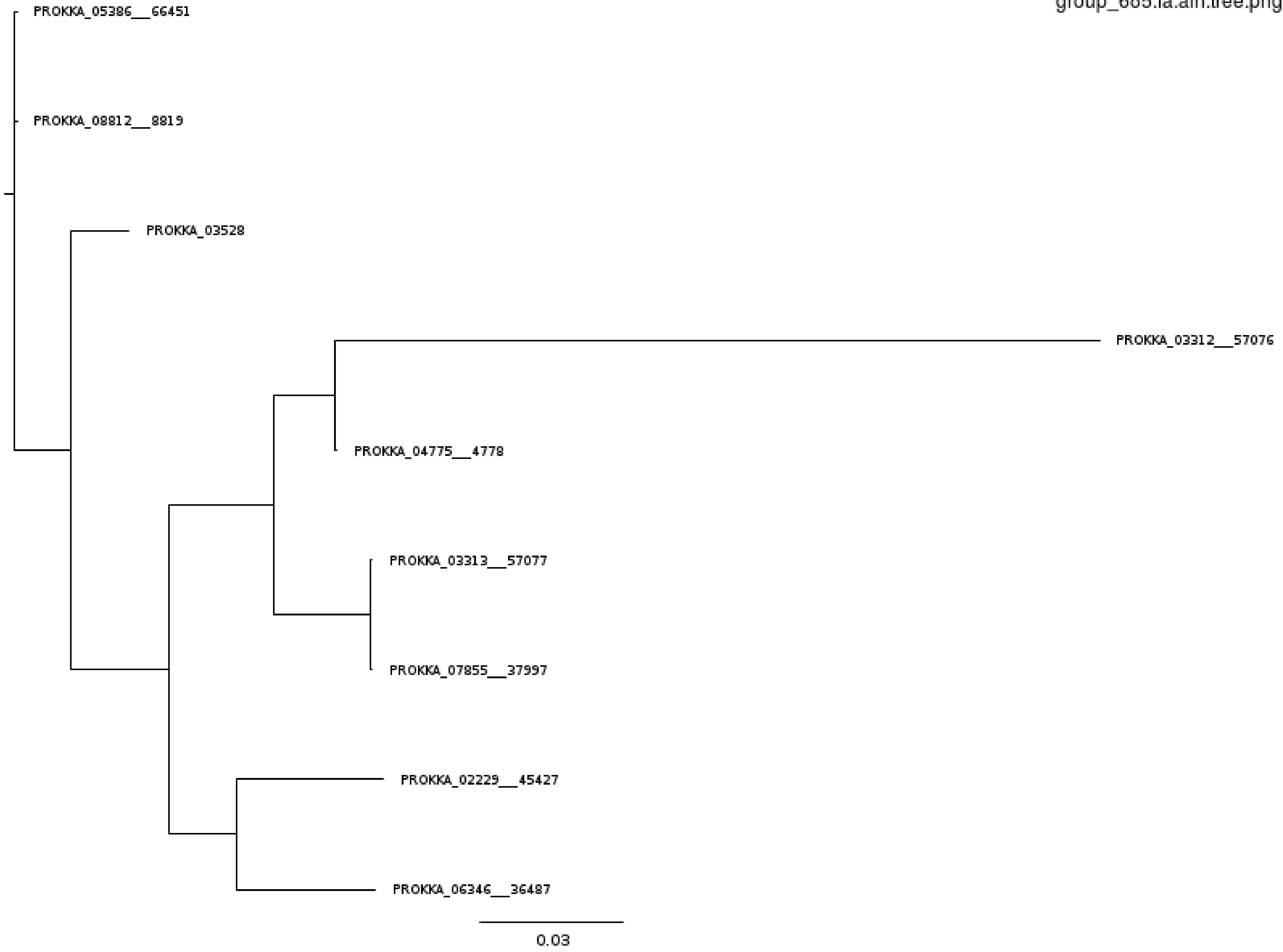

group_687.fa.aln.tree.png

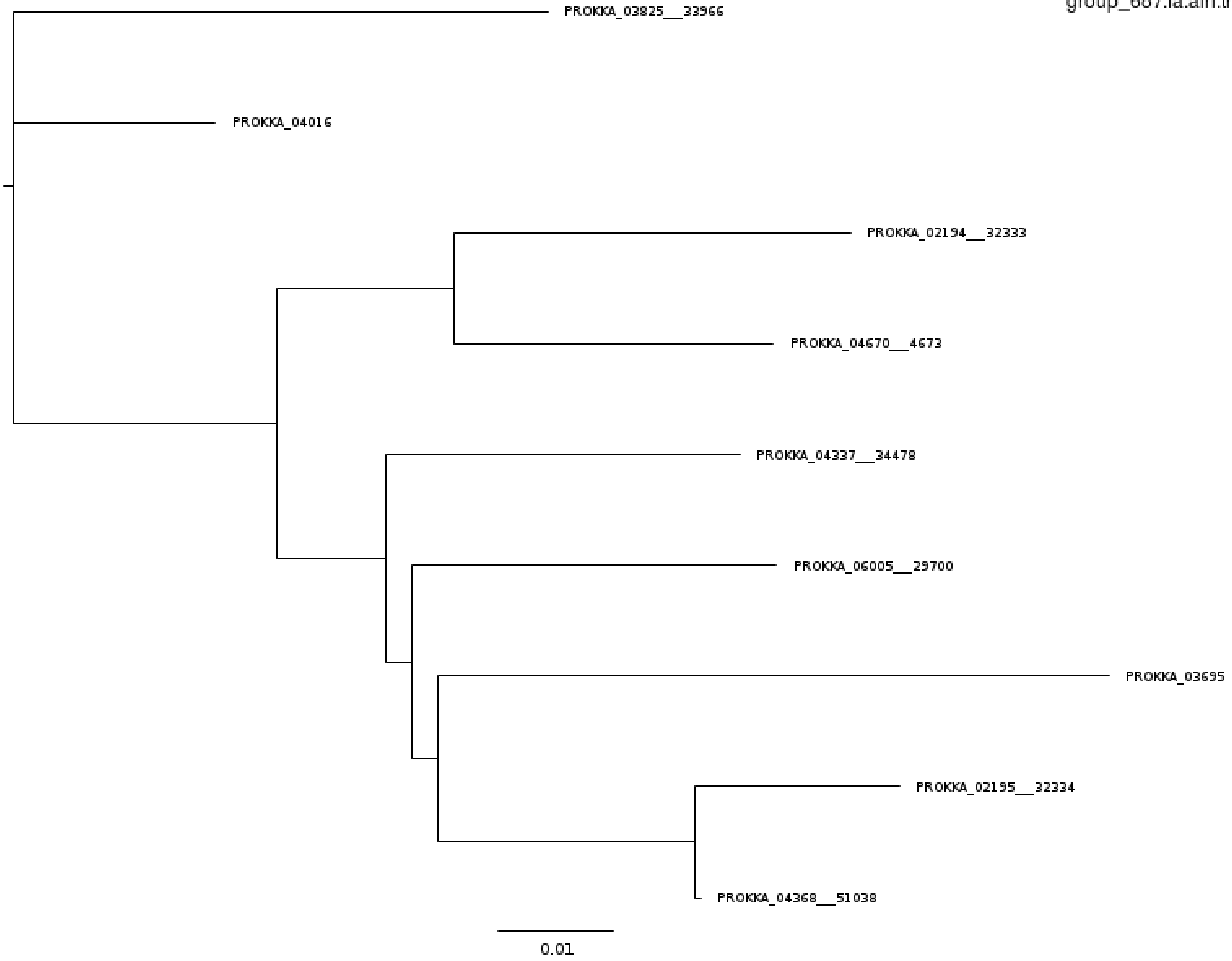

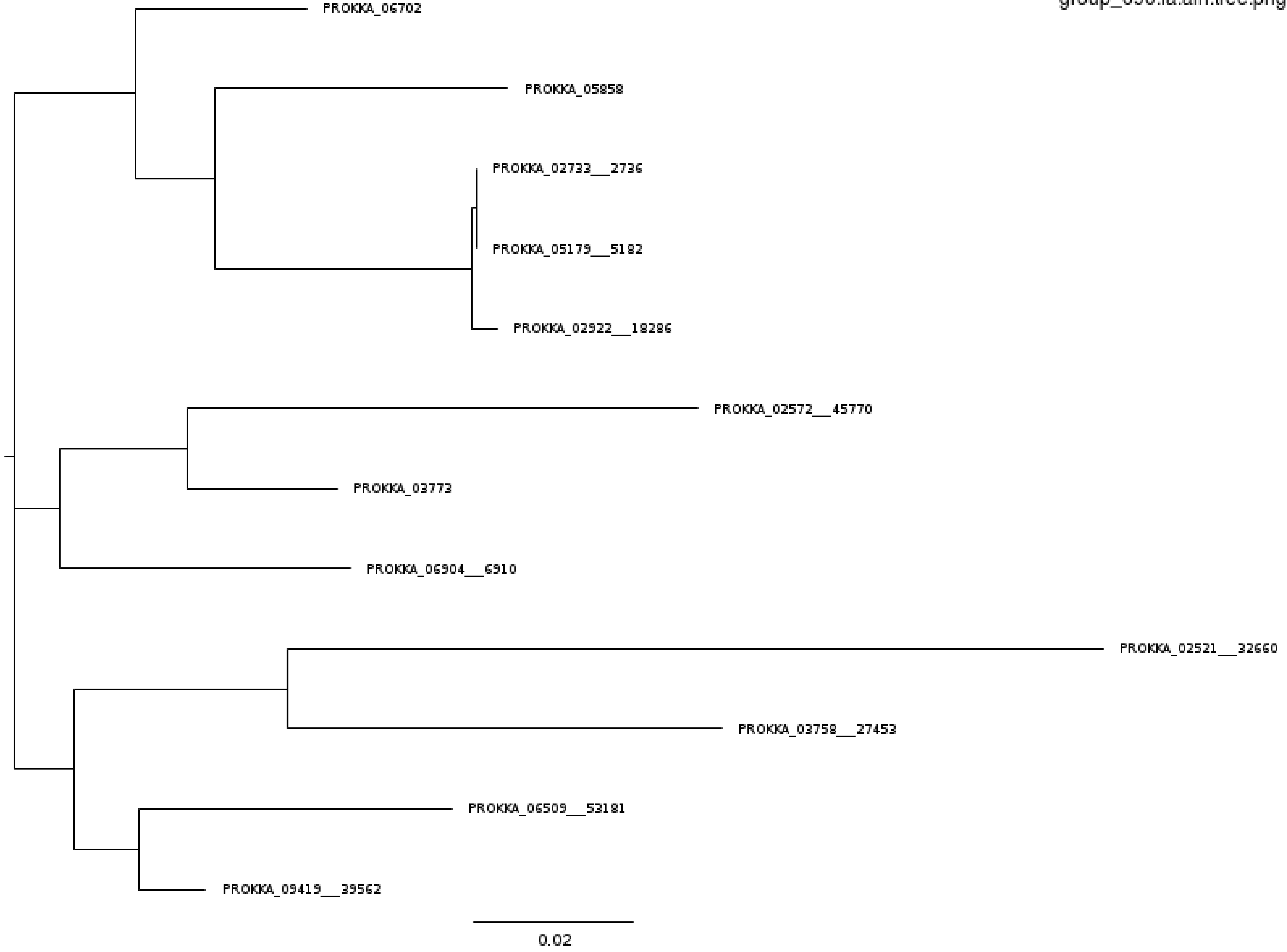
PROKKA_06702
PROKKA_05858
PROKKA_02733___2736
PROKKA_05179___5182
PROKKA_02922___18286
PROKKA_02572___45770
PROKKA_03773
PROKKA_06904___6910
PROKKA_02521___32660
PROKKA_03758___27453
PROKKA_06509___53181
PROKKA_09419___39562
0.02

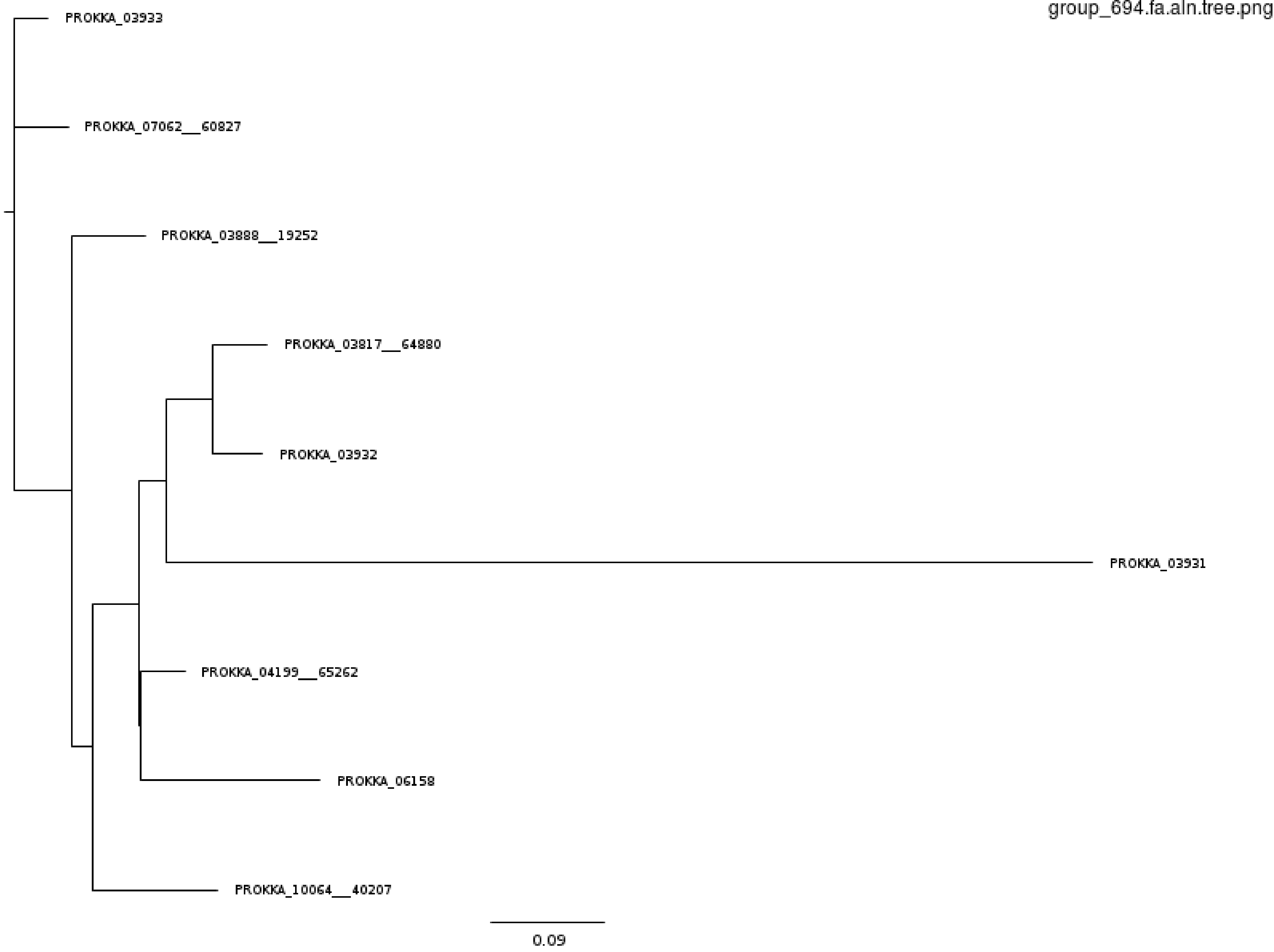
group_694.fa.aln.tree.png
PROKKA_03933
PROKKA_07062___60827
PROKKA_03888___19252
PROKKA_03817___64880
PROKKA_03932
PROKKA_03931
PROKKA_04199___65262
PROKKA_06158
PROKKA_10064___40207
0.09

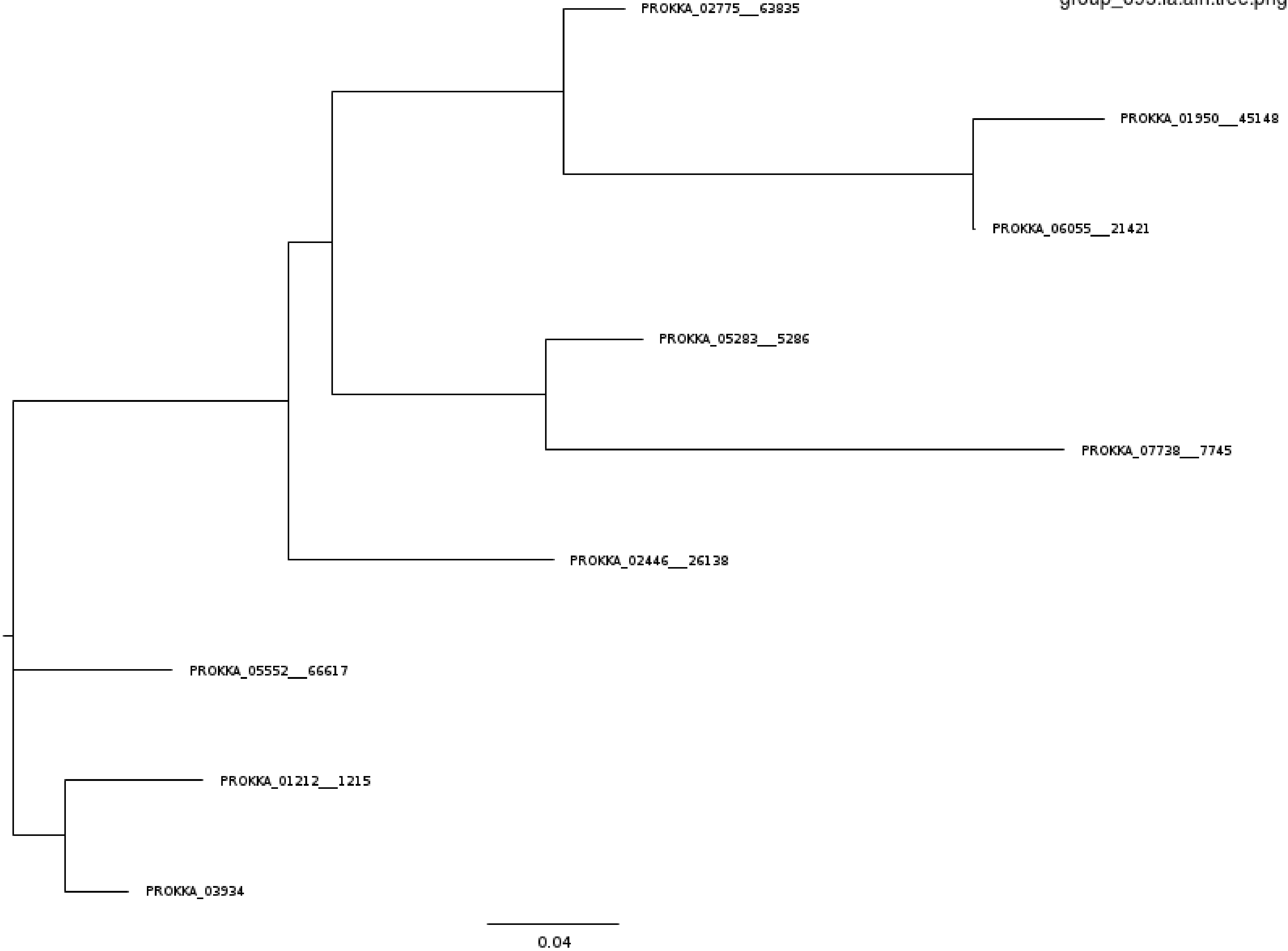
PROKKA_02775___63835
PROKKA_01950___45148
PROKKA_06055___21421
PROKKA_05283___5286
PROKKA_07738___7745
PROKKA_02446___26138
PROKKA_05552___66617
PROKKA_01212___1215
PROKKA_03934
0.04

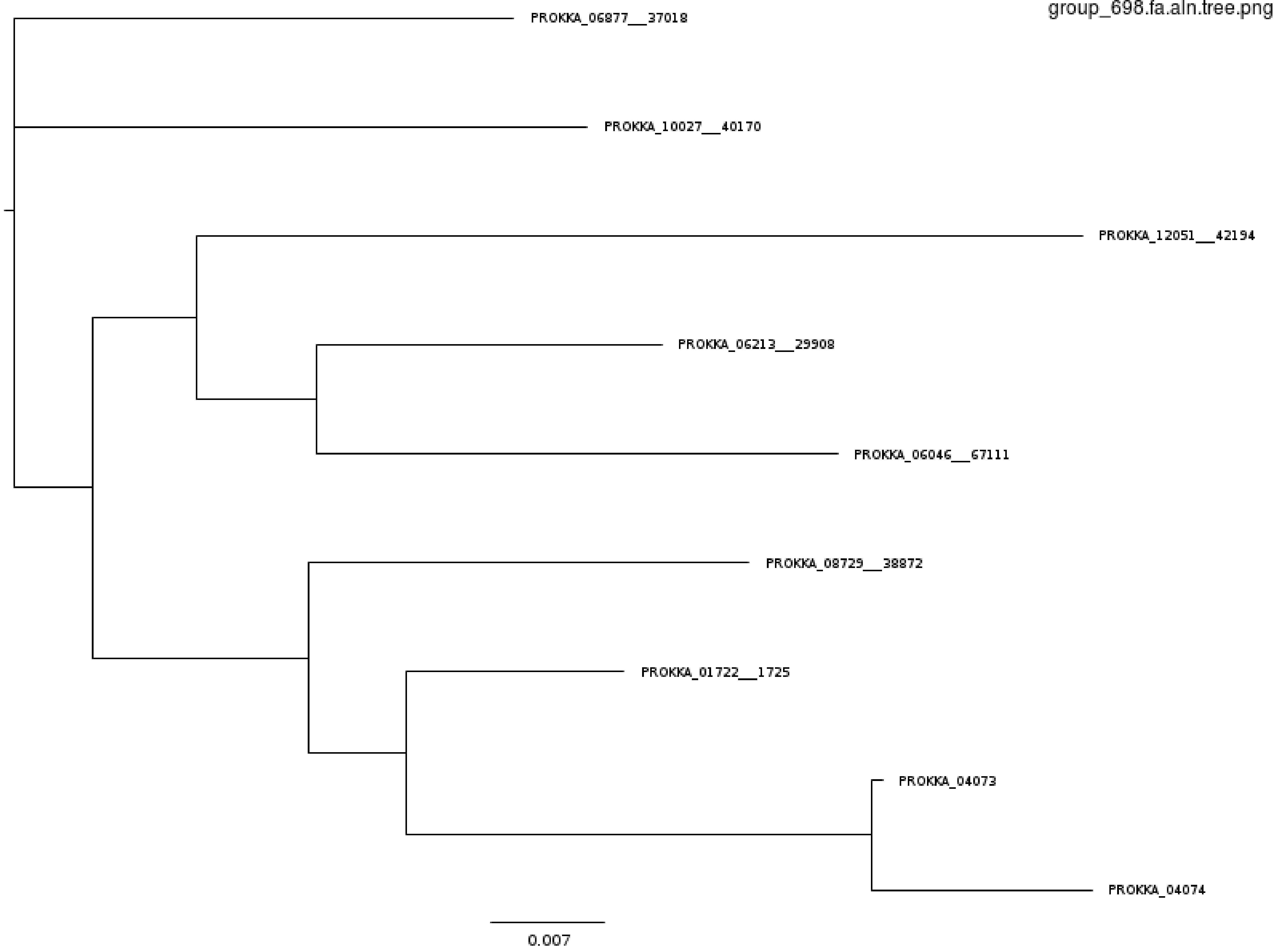

group_698.fa.aln.tree.png
PROKKA_06877___37018
PROKKA_10027___40170
PROKKA_12051___42194
PROKKA_06213___29908
PROKKA_06046___67111
PROKKA_08729___38872
PROKKA_01722___1725
PROKKA_04073
PROKKA_04074
0.007

group_699.fa.aln.tree.png

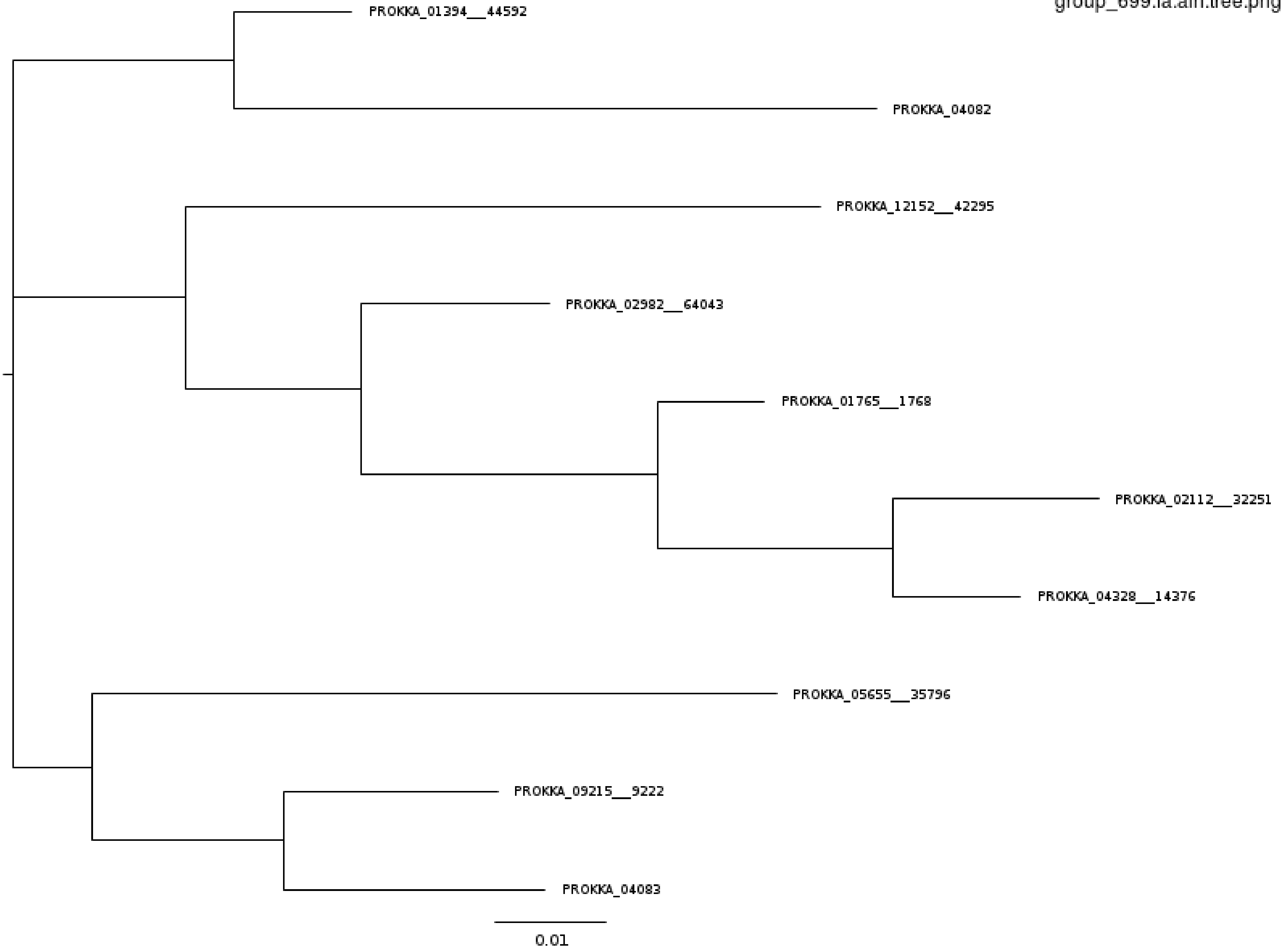

group_69.fa.aln.tree.png

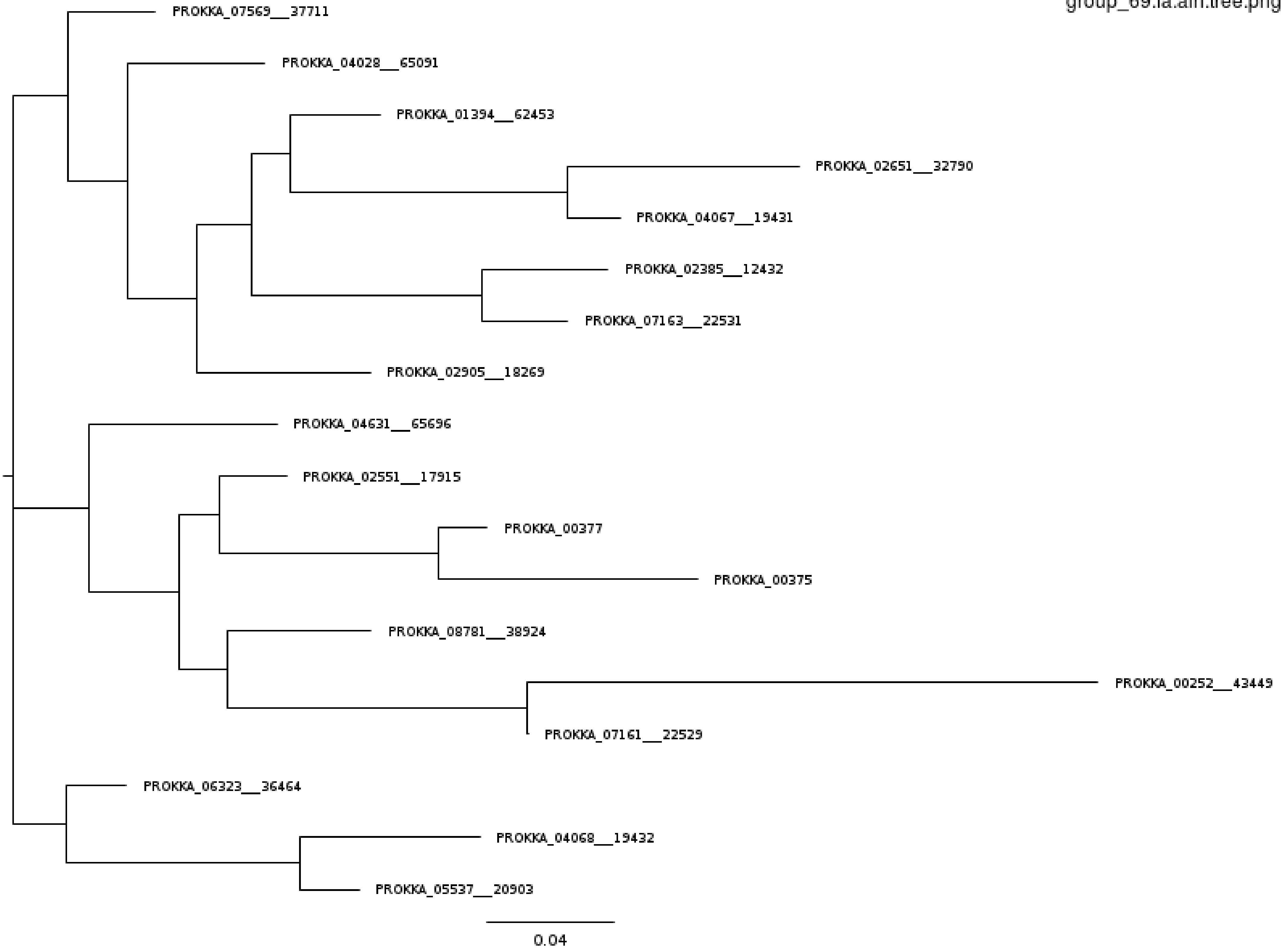

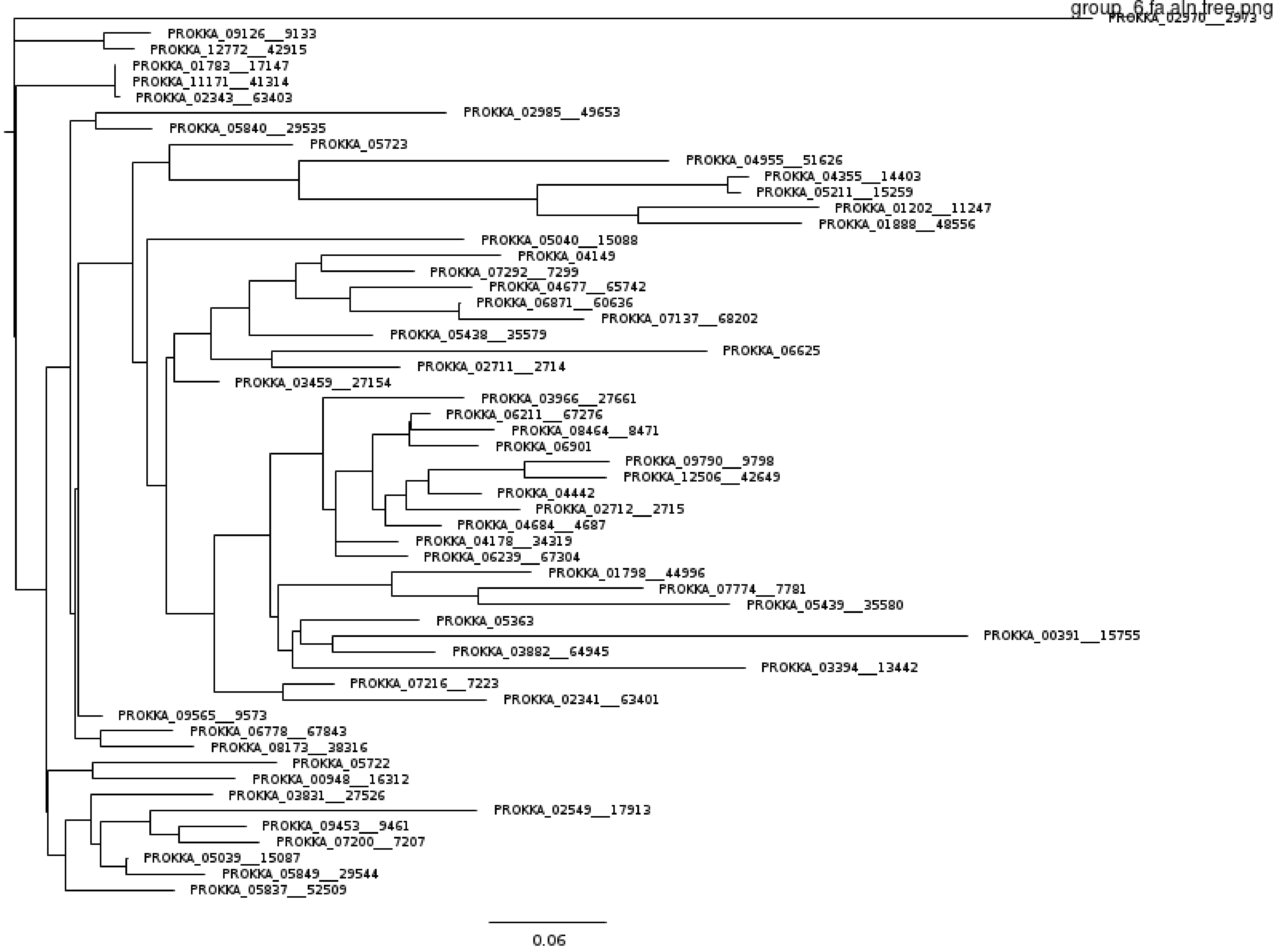
group_6.fa.aln.tree.png
PROKKA_02970___2973
PROKKA_09126___9133
PROKKA_12772___42915
PROKKA_01783___17147
PROKKA_11171___41314
PROKKA_02343___63403
PROKKA_02985___49653
PROKKA_05840___29535
PROKKA_05723
PROKKA_04955___51626
PROKKA_04355___14403
PROKKA_05211___15259
PROKKA_01202___11247
PROKKA_01888___48556
PROKKA_05040___15088
PROKKA_04149
PROKKA_07292___7299
PROKKA_04677___65742
PROKKA_06871___60636
PROKKA_07137___68202
PROKKA_05438___35579
PROKKA_06625
PROKKA_02711___2714
PROKKA_03459___27154
PROKKA_03966___27661
PROKKA_06211___67276
PROKKA_08464___8471
PROKKA_06901
PROKKA_09790___9798
PROKKA_12506___42649
PROKKA_04442
PROKKA_02712___2715
PROKKA_04684___4687
PROKKA_04178___34319
PROKKA_06239___67304
PROKKA_01798___44996
PROKKA_07774___7781
PROKKA_05439___35580
PROKKA_05363
PROKKA_00391___15755
PROKKA_03882___64945
PROKKA_03394___13442
PROKKA_07216___7223
PROKKA_02341___63401
PROKKA_09565___9573
PROKKA_06778___67843
PROKKA_08173___38316
PROKKA_05722
PROKKA_00948___16312
PROKKA_03831___27526
PROKKA_02549___17913
PROKKA_09453___9461
PROKKA_07200___7207
PROKKA_05039___15087
PROKKA_05849___29544
PROKKA_05837___52509
0.06

group_709.fa.aln.tree.png

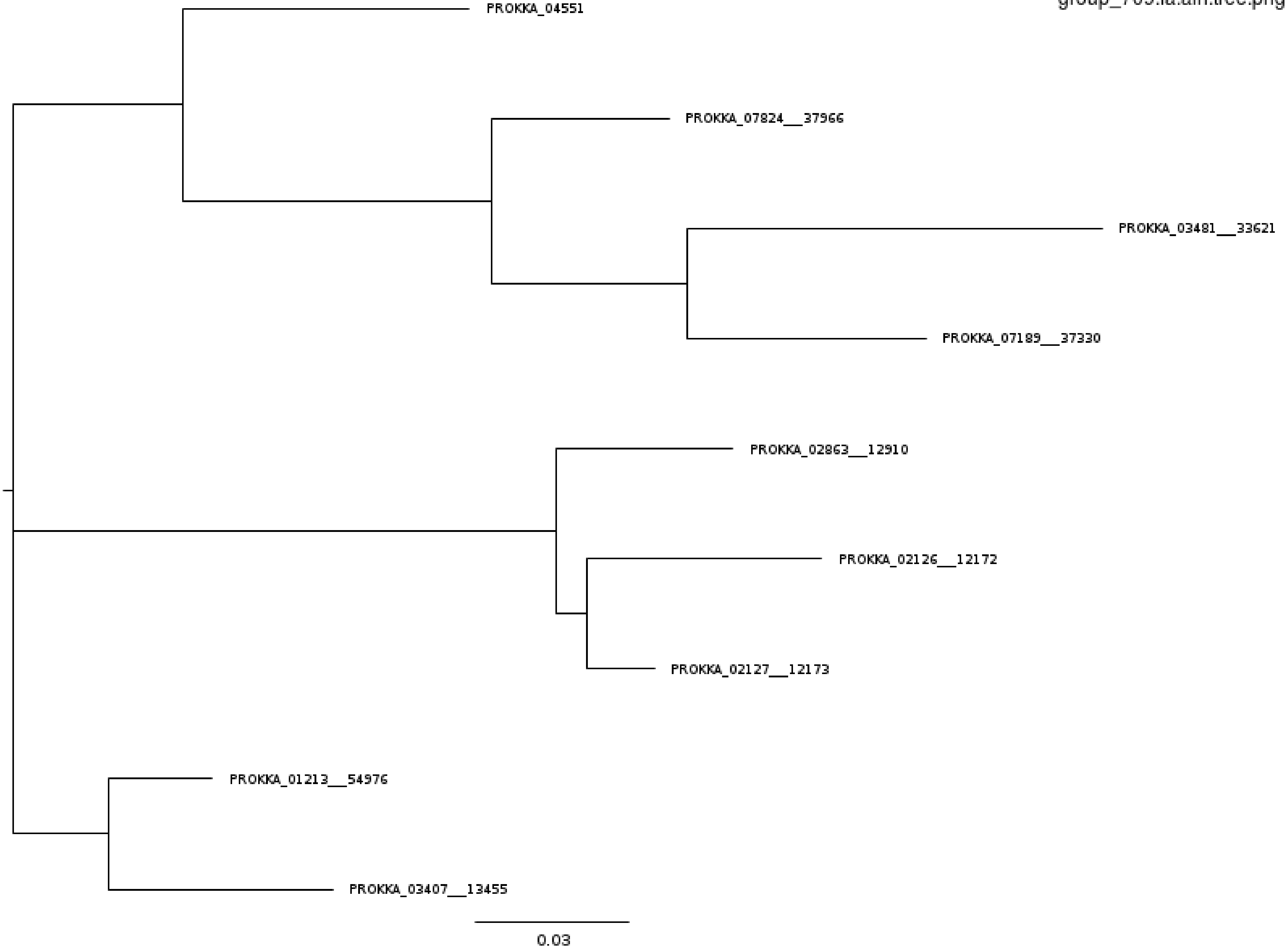

group_70.fa.aln.tree.png

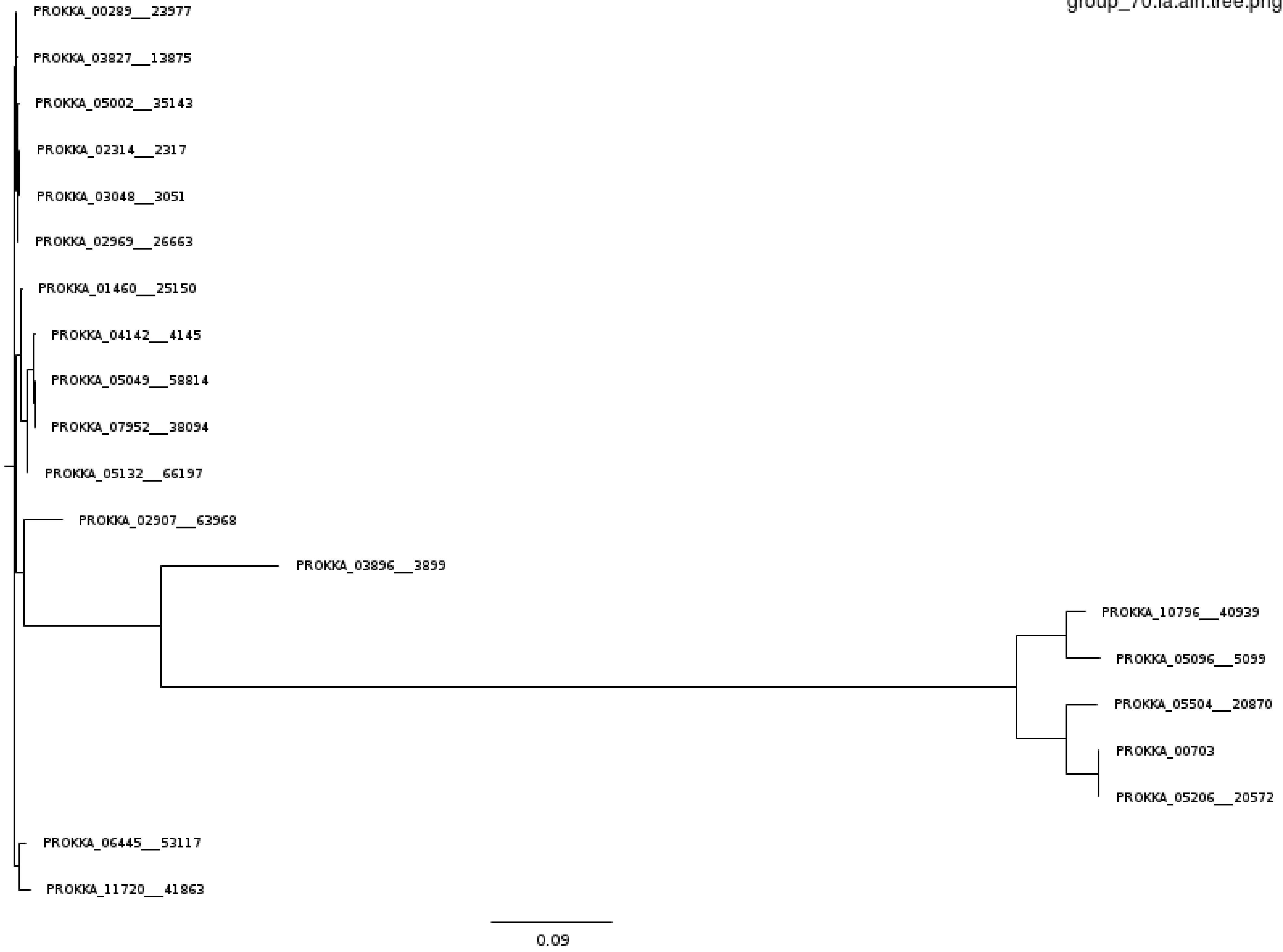

group_717.fa.aln.tree.png

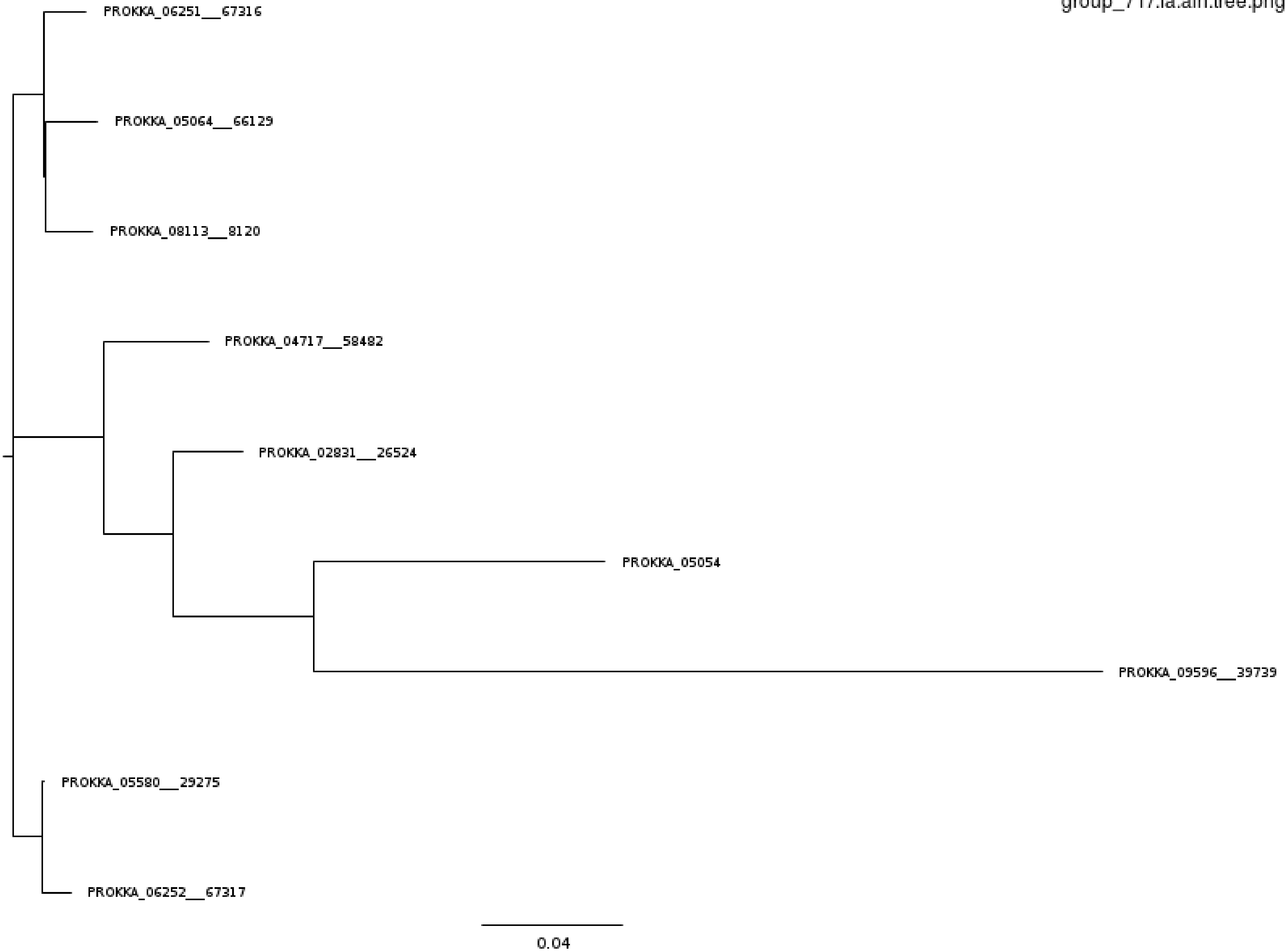

group_718.fa.aln.tree.png

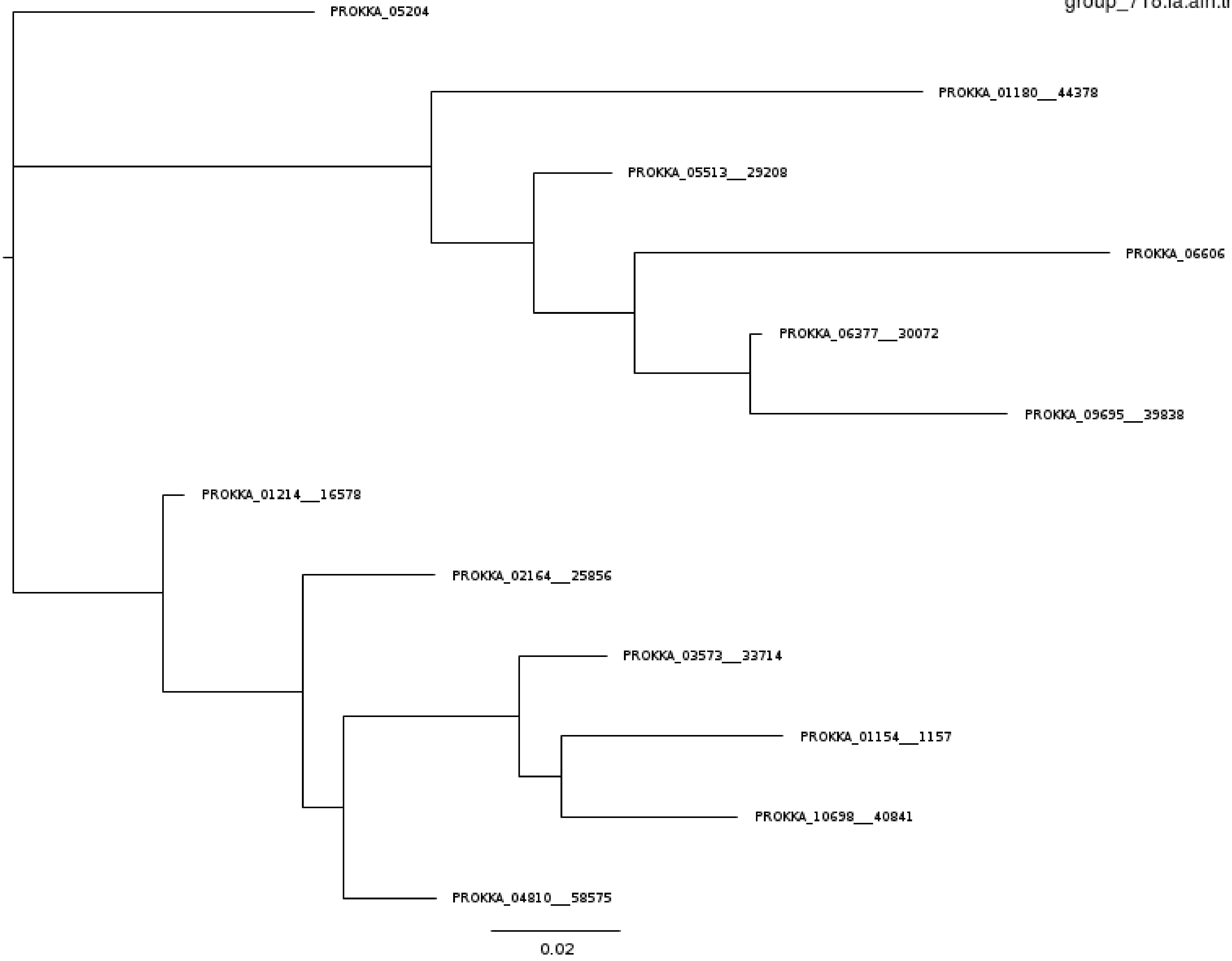

group_720.fa.aln.tree.png

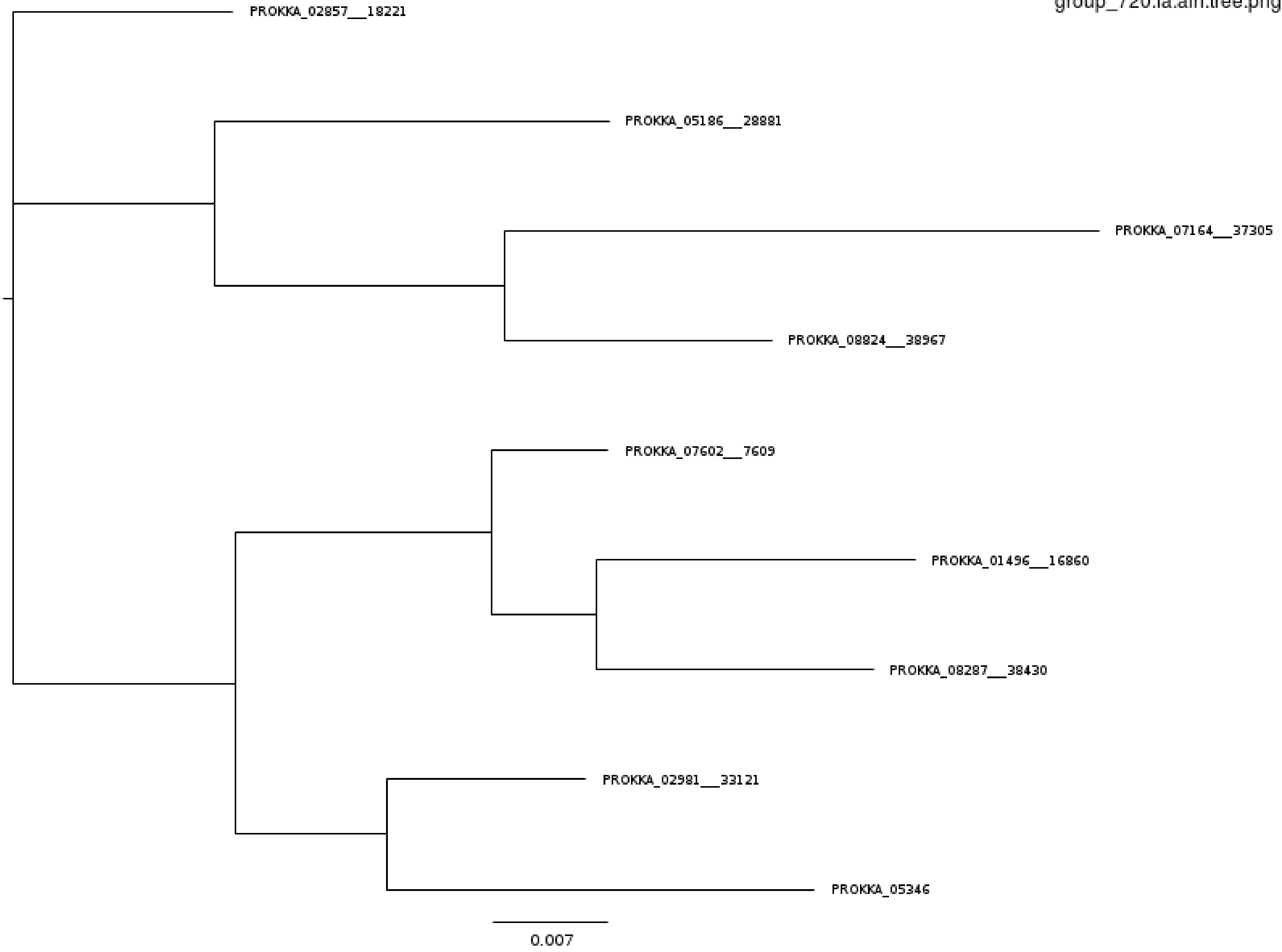

group_721.fa.aln.tree.png

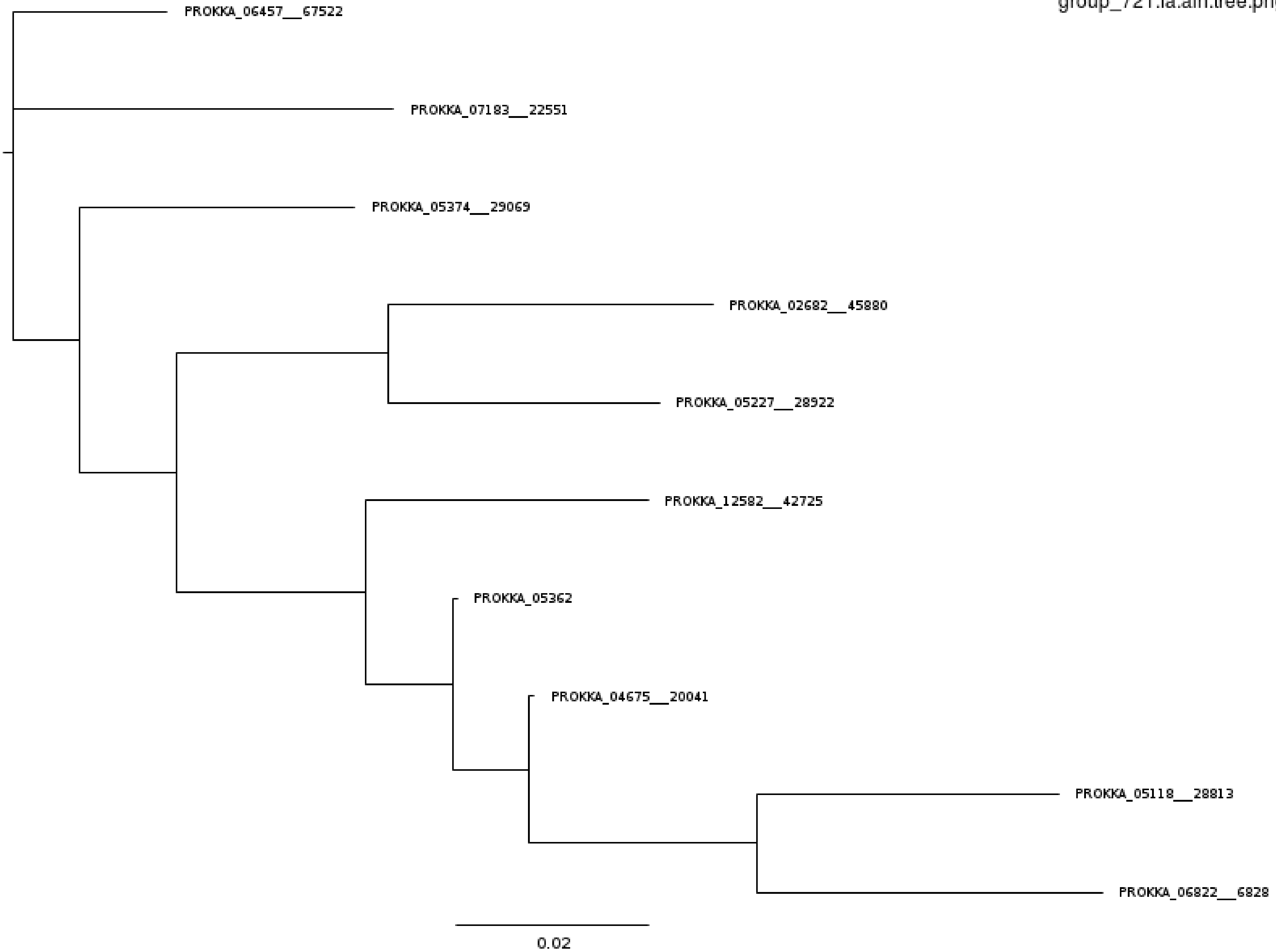

group_727.fa.aln.tree.png

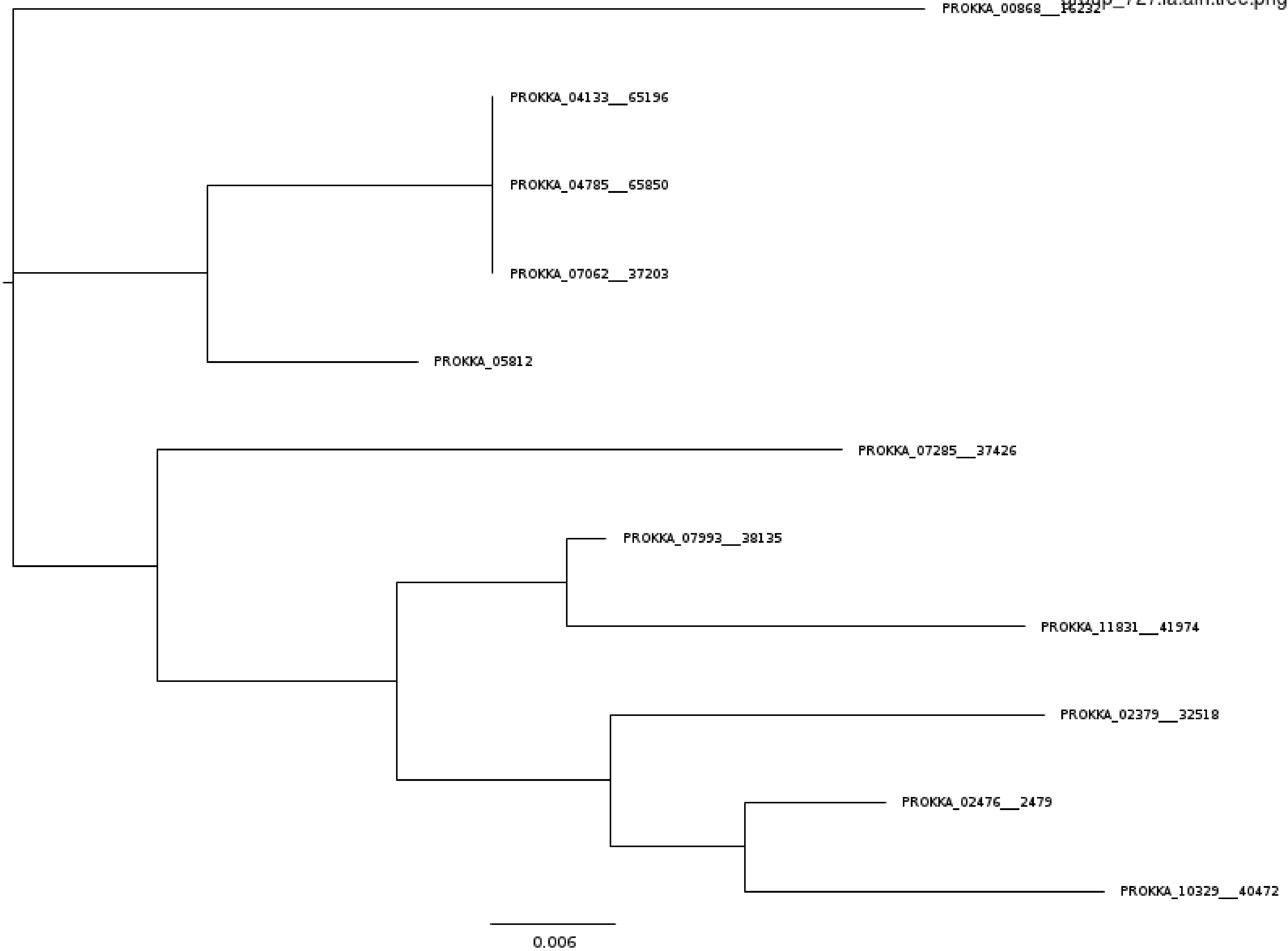

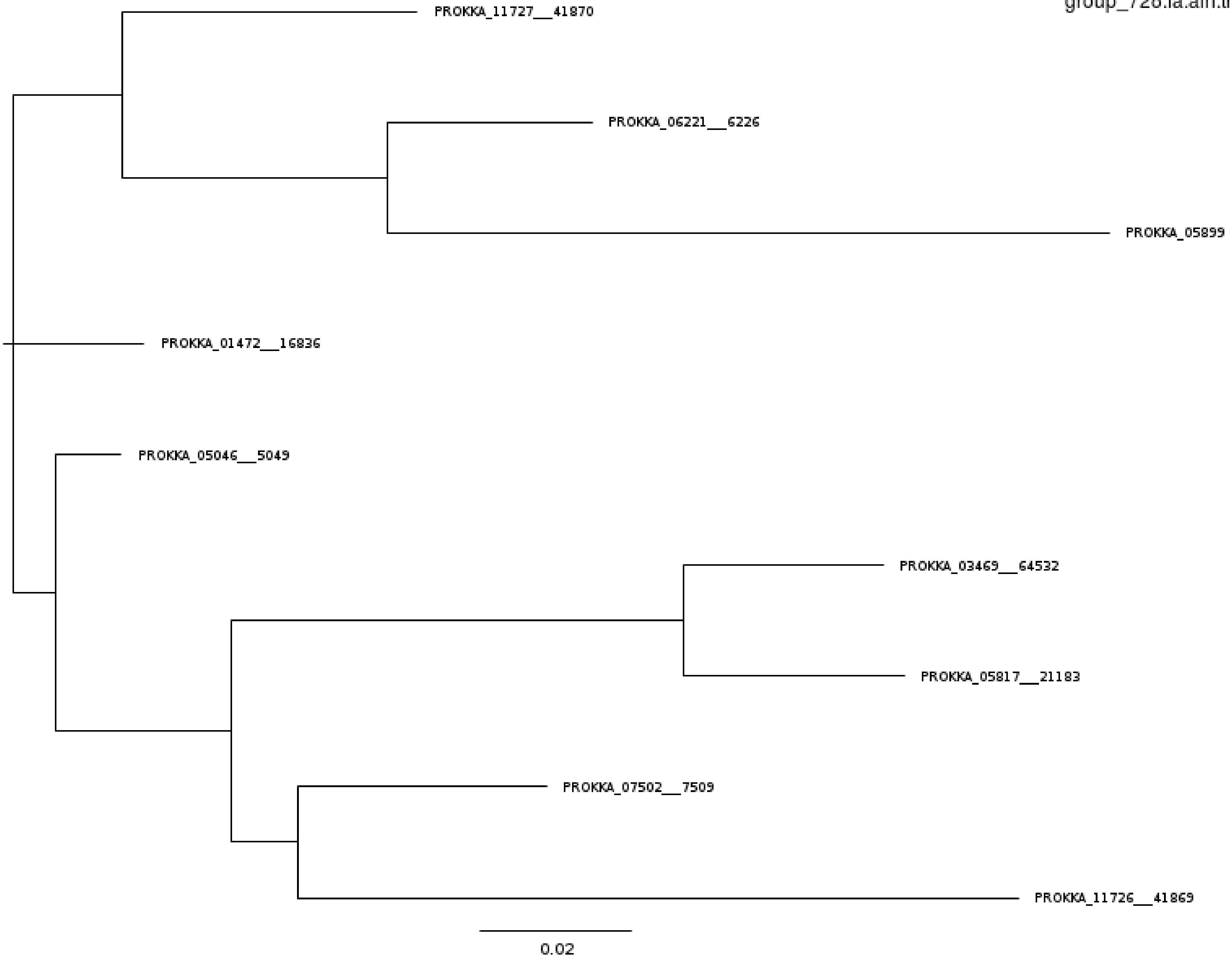
PROKKA_11727___41870
PROKKA_06221___6226
PROKKA_05899
PROKKA_01472___16836
PROKKA_05046___5049
PROKKA_03469___64532
PROKKA_05817___21183
PROKKA_07502___7509
PROKKA_11726___41869
0.02

group_741.fa.aln.tree.png

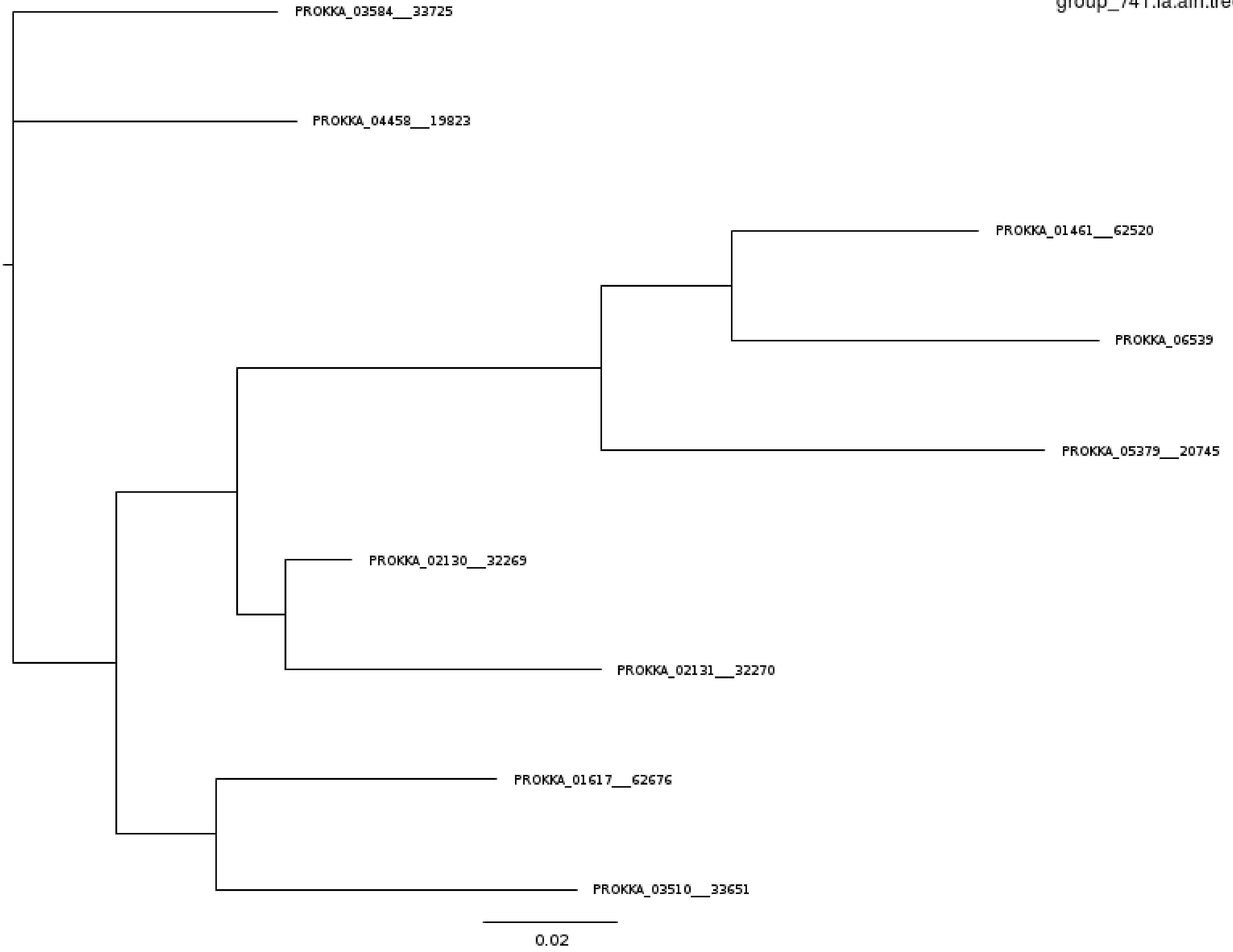

group_743.fa.aln.tree.png

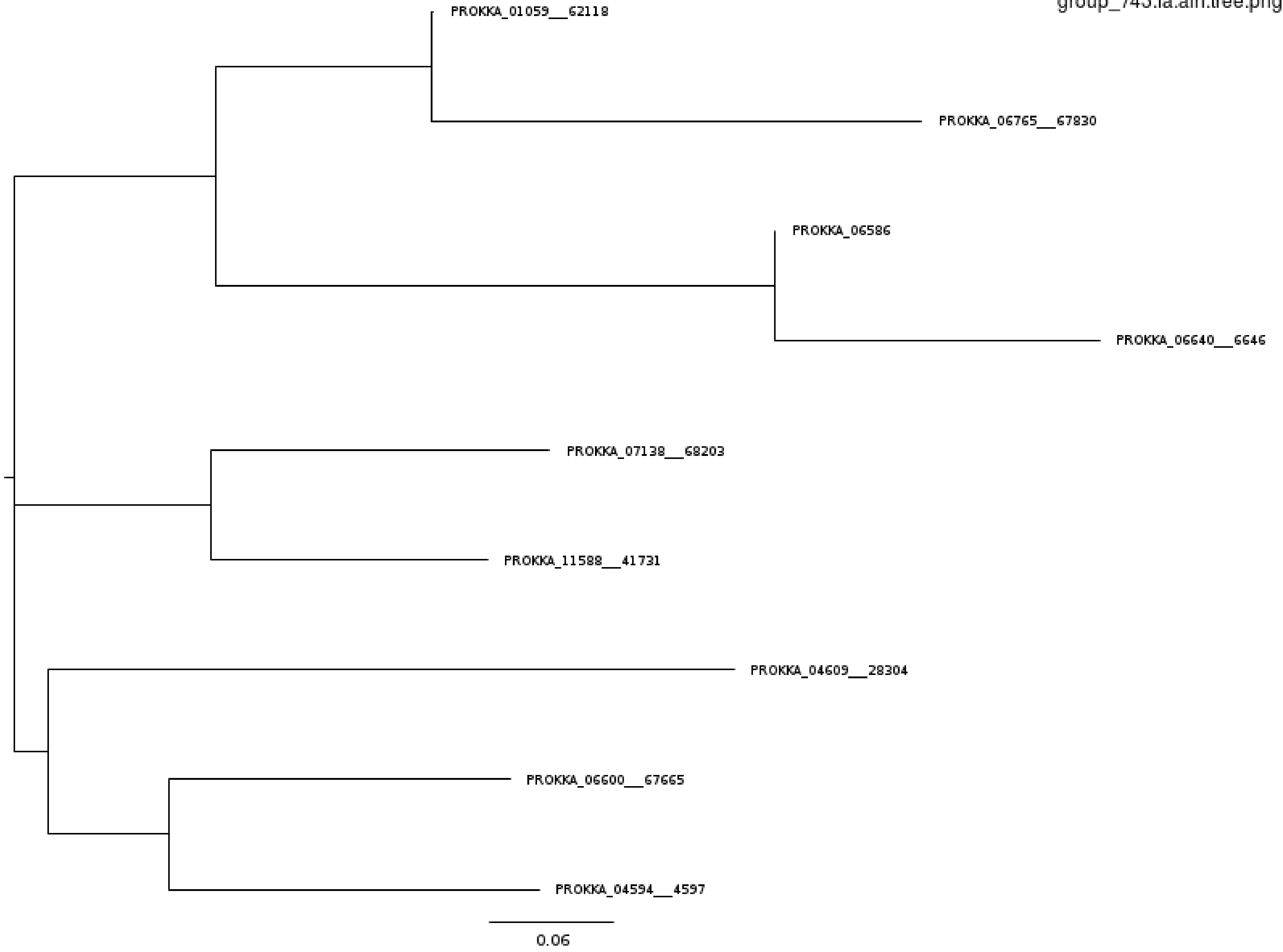

group_745.fa.aln.tree.png

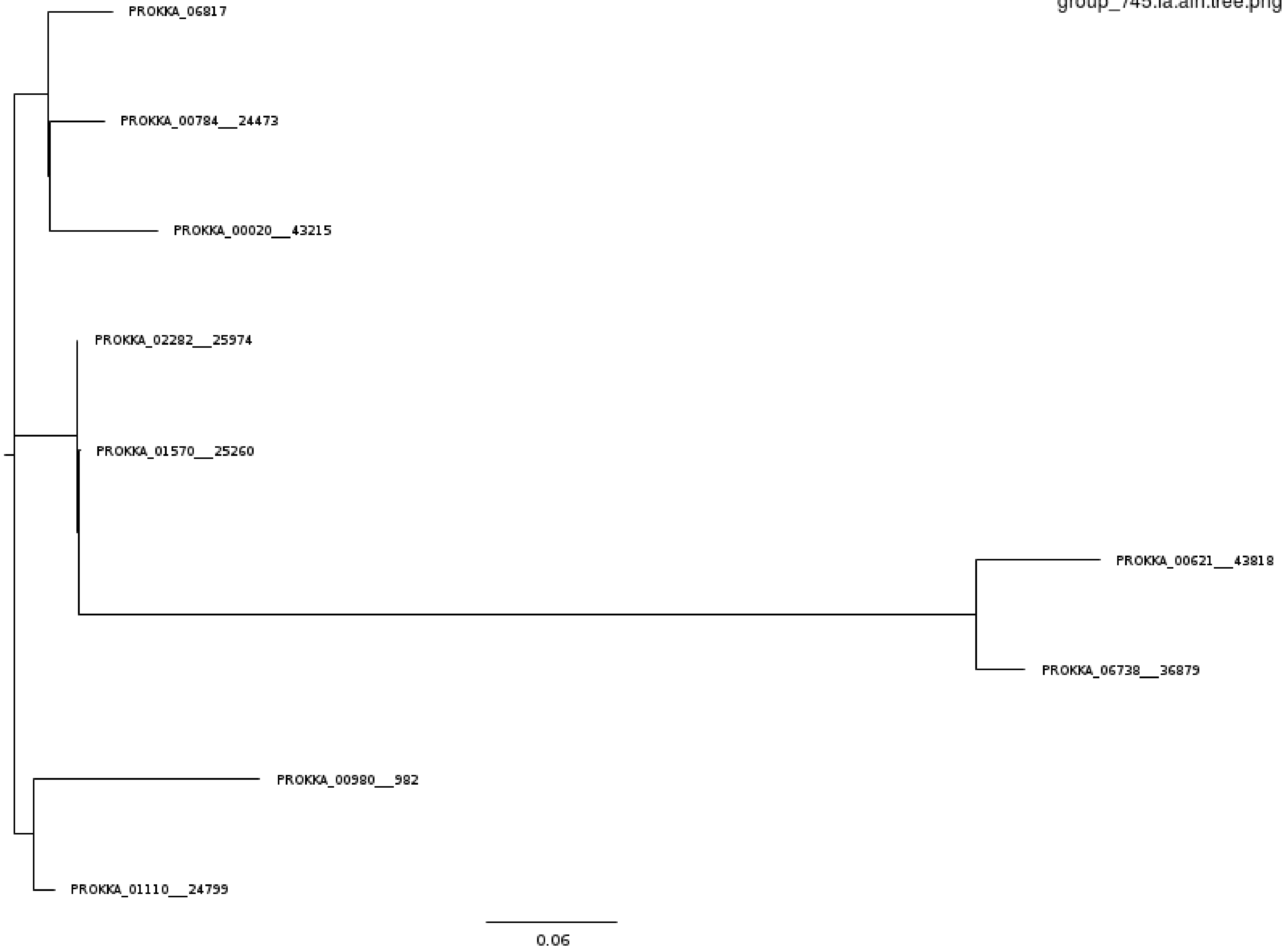

group_747.fa.aln.tree.png

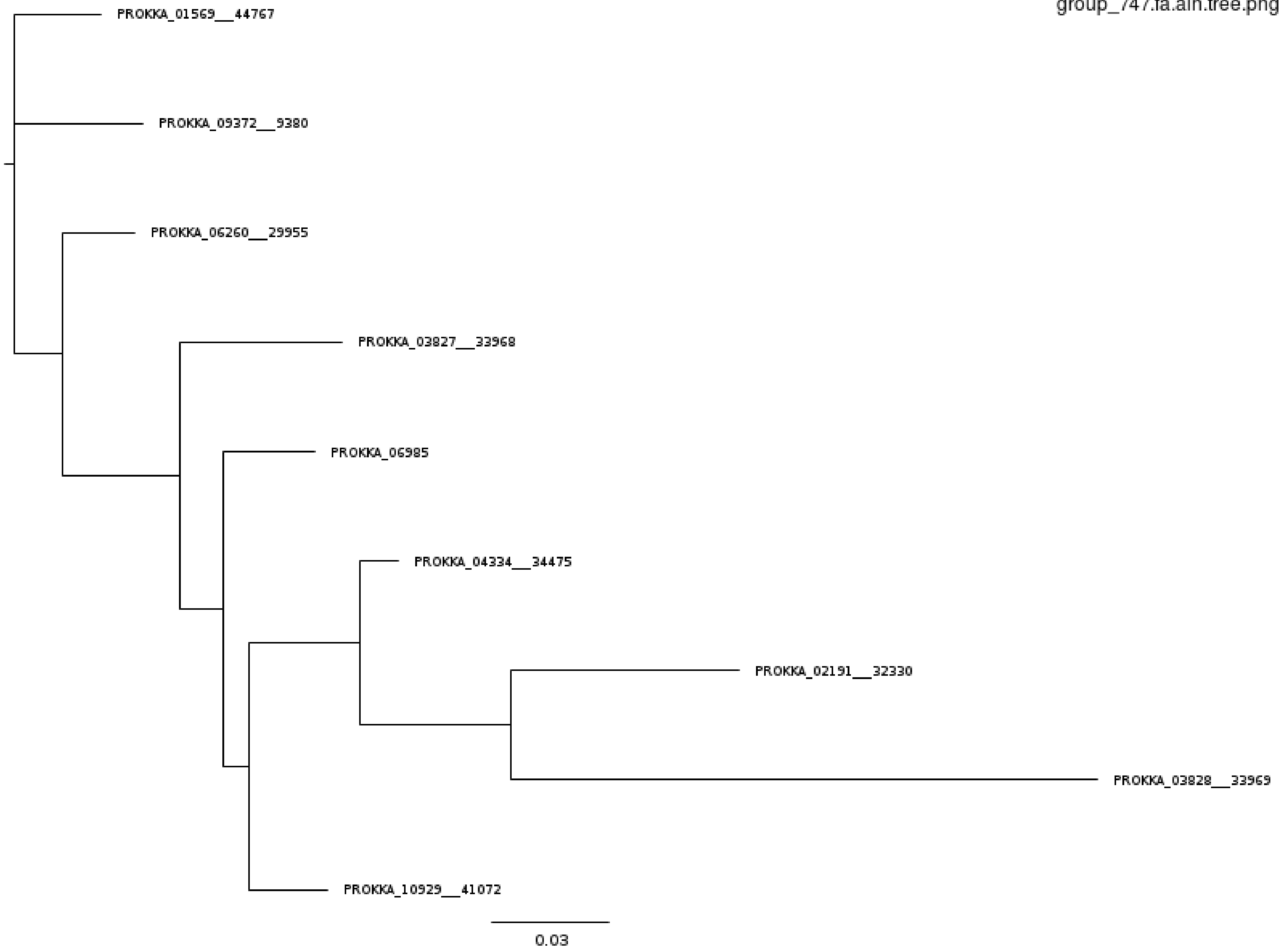

group_752.fa.aln.tree.png

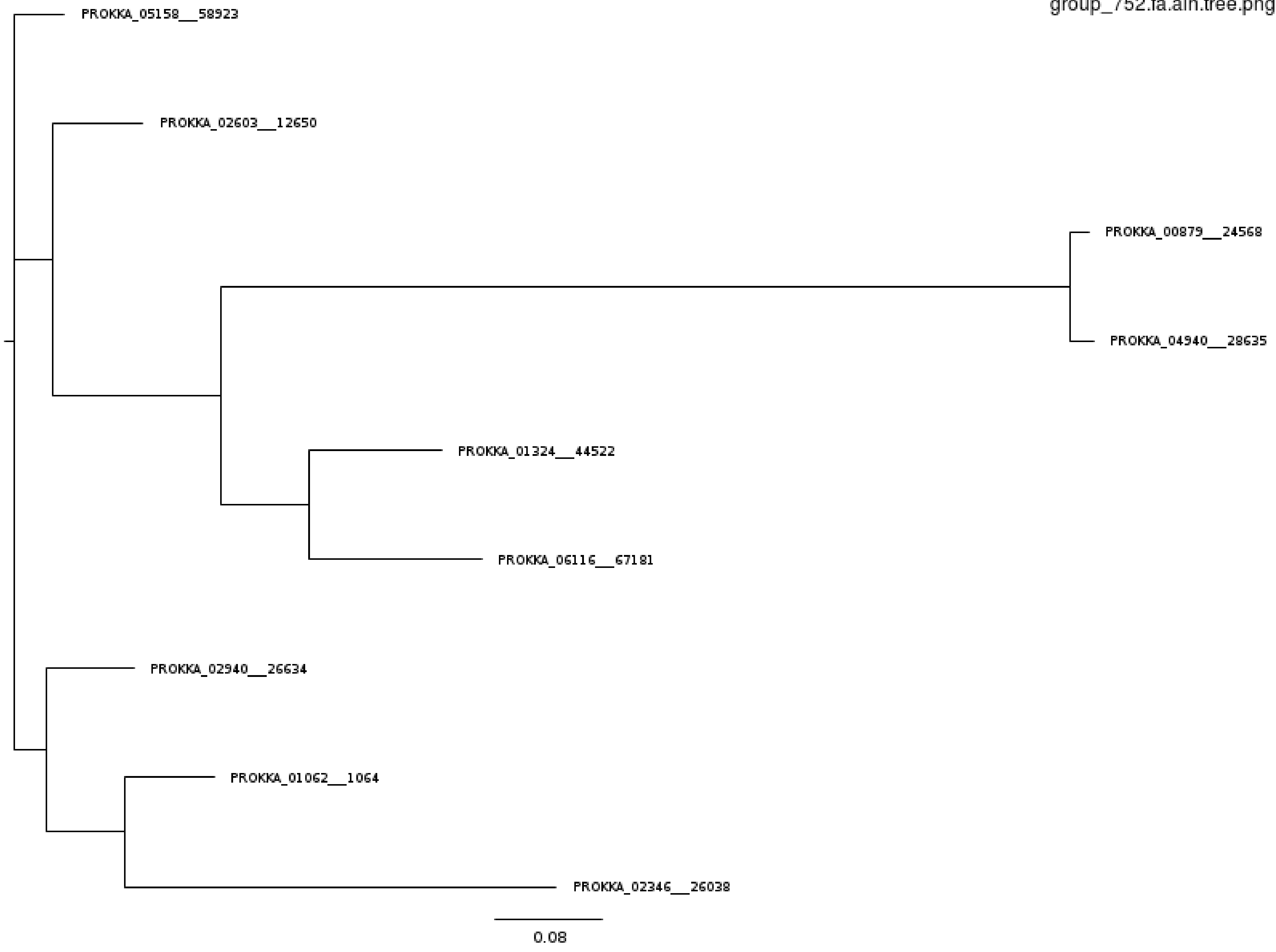

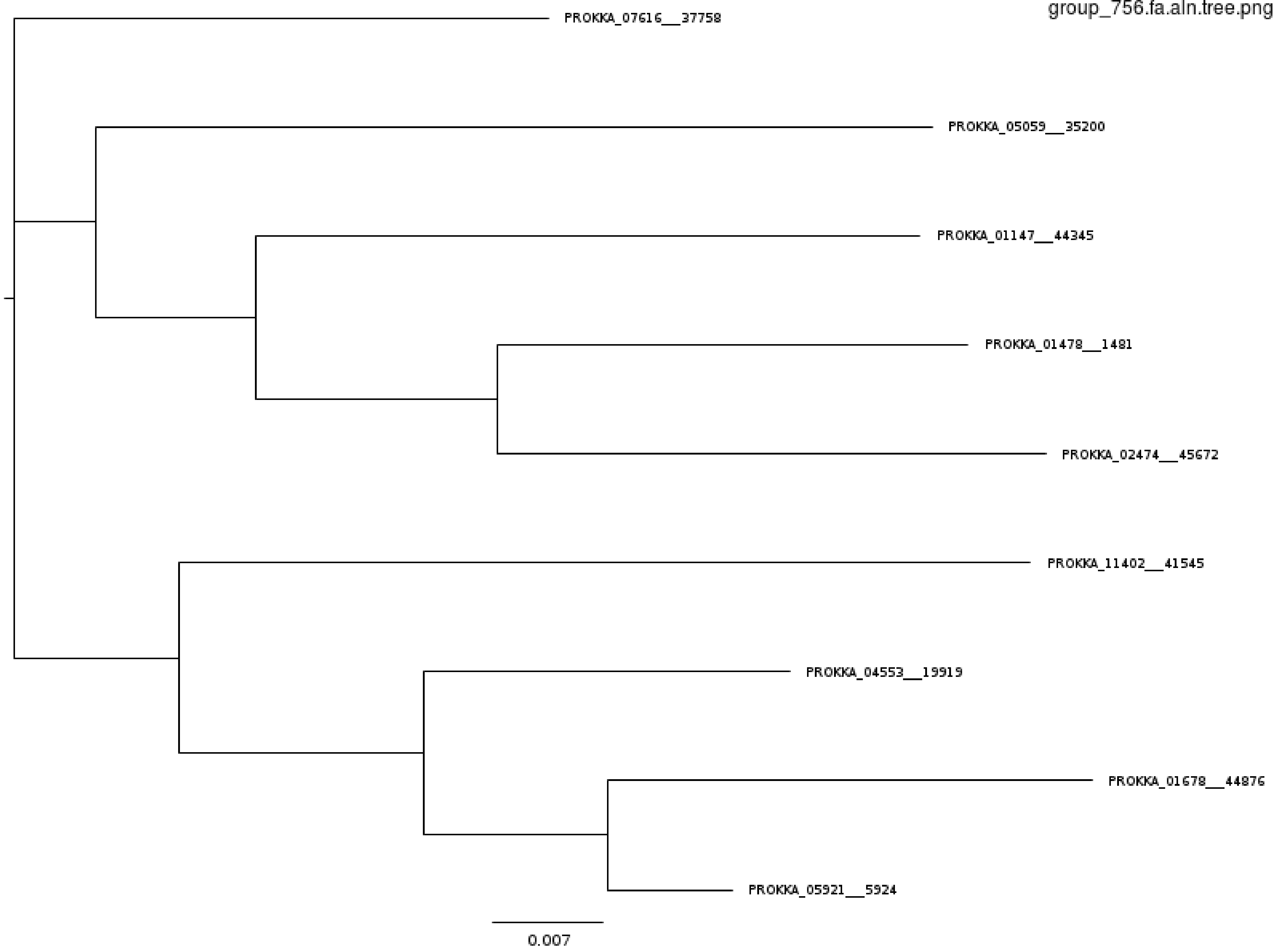

group_756.fa.aln.tree.png
PROKKA_07616__37758
PROKKA_05059__35200
PROKKA_01147__44345
PROKKA_01478__1481
PROKKA_02474__45672
PROKKA_11402__41545
PROKKA_04553__19919
PROKKA_01678__44876
PROKKA_05921__5924
0.007

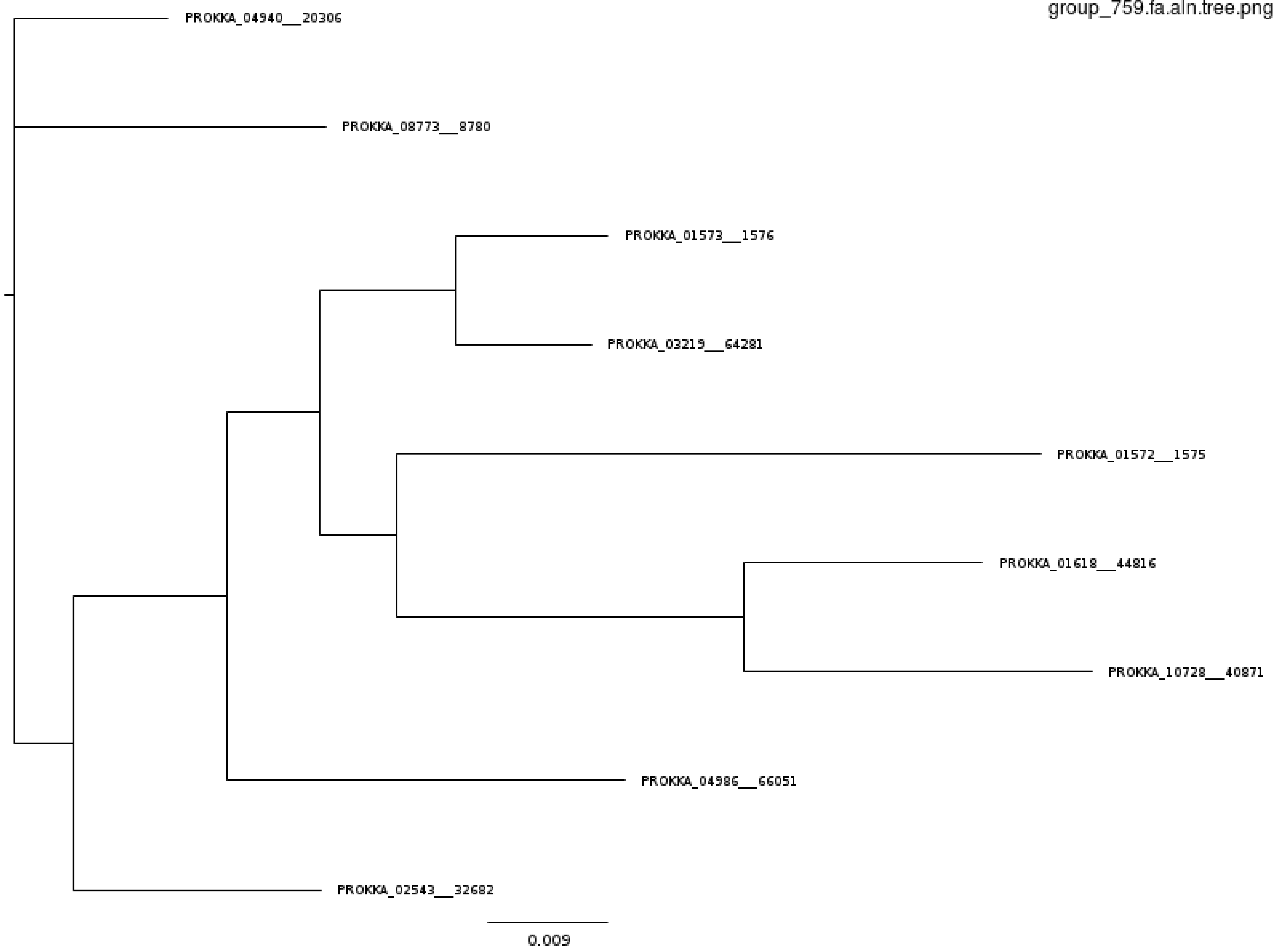
group_759.fa.aln.tree.png
PROKKA_04940___20306
PROKKA_08773___8780
PROKKA_01573___1576
PROKKA_03219___64281
PROKKA_01572___1575
PROKKA_01618___44816
PROKKA_10728___40871
PROKKA_04986___66051
PROKKA_02543___32682
0.009

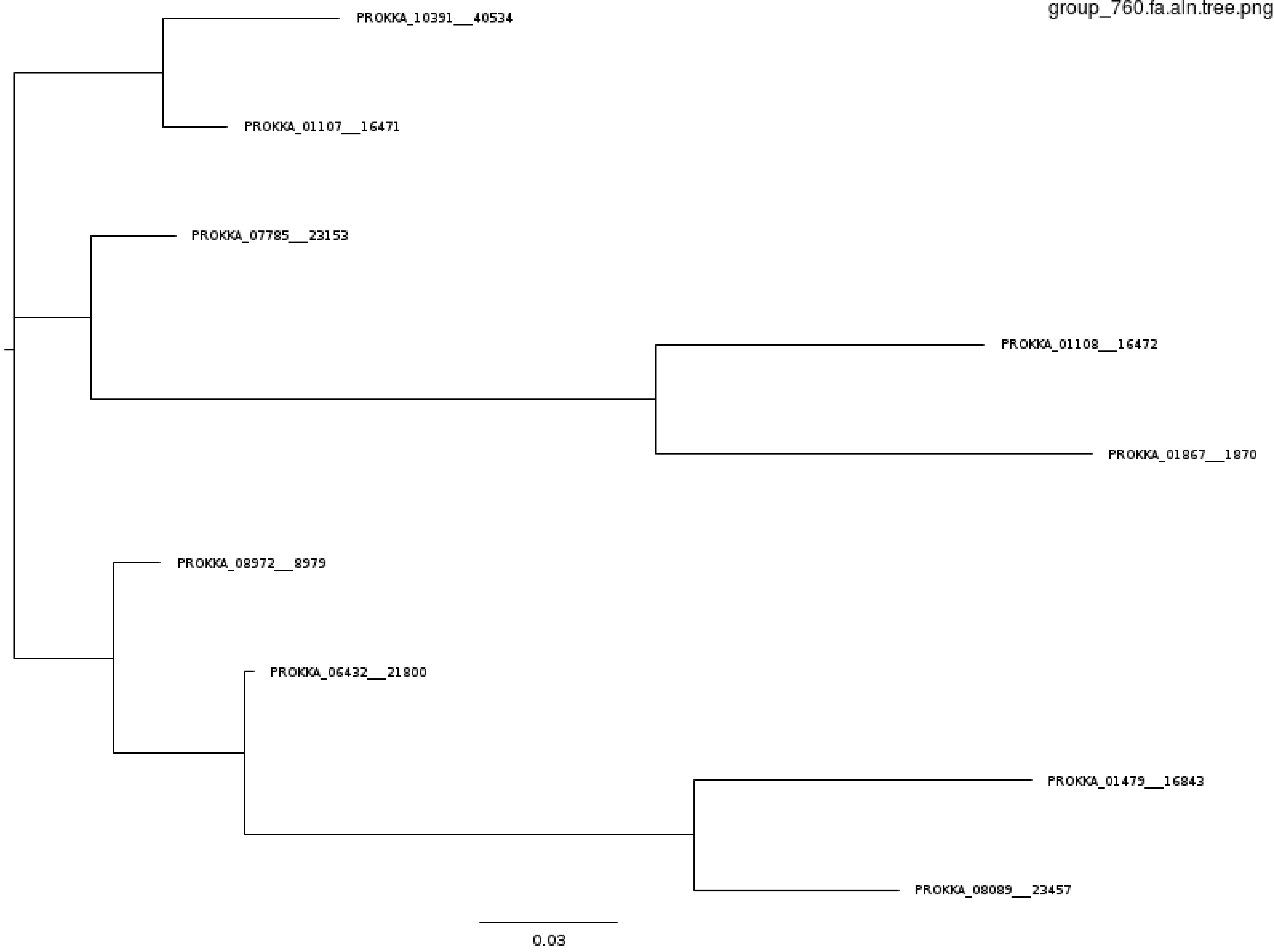
group_760.fa.aln.tree.png
PROKKA_10391___40534
PROKKA_01107___16471
PROKKA_07785___23153
PROKKA_01108___16472
PROKKA_01867___1870
PROKKA_08972___8979
PROKKA_06432___21800
PROKKA_01479___16843
PROKKA_08089___23457
0.03

group_762.fa.aln.tree.png

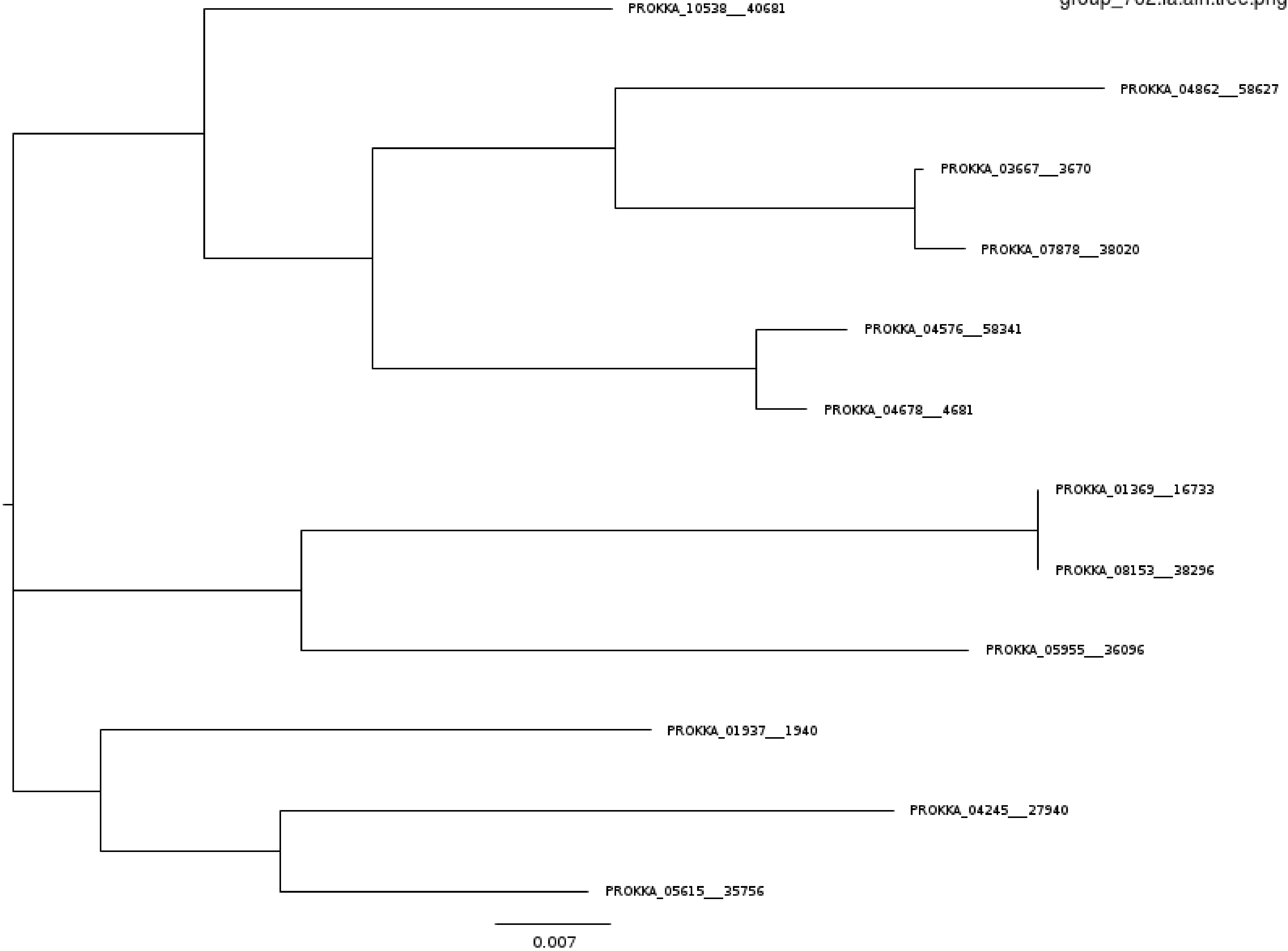

group_764.fa.aln.tree.png

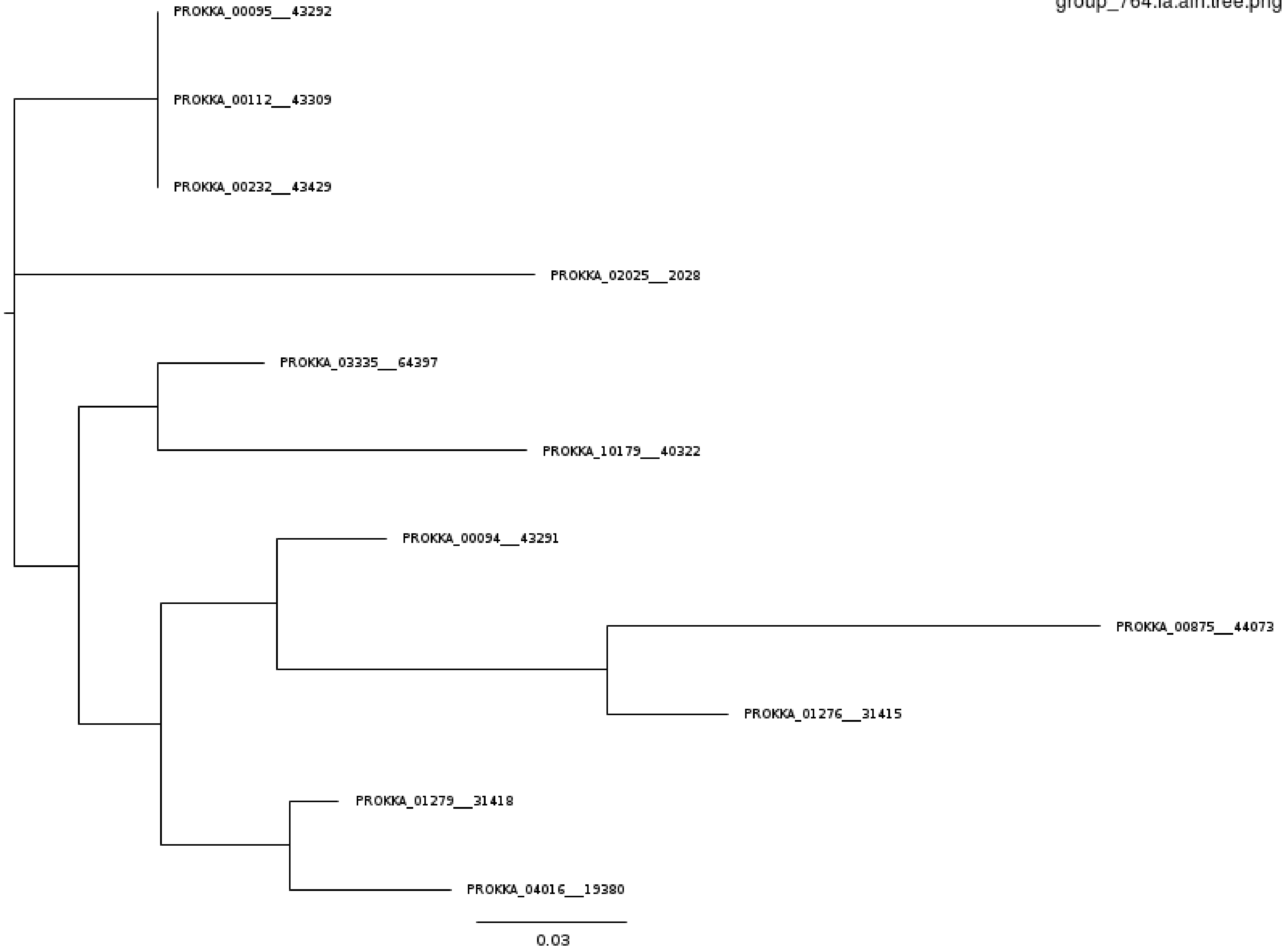

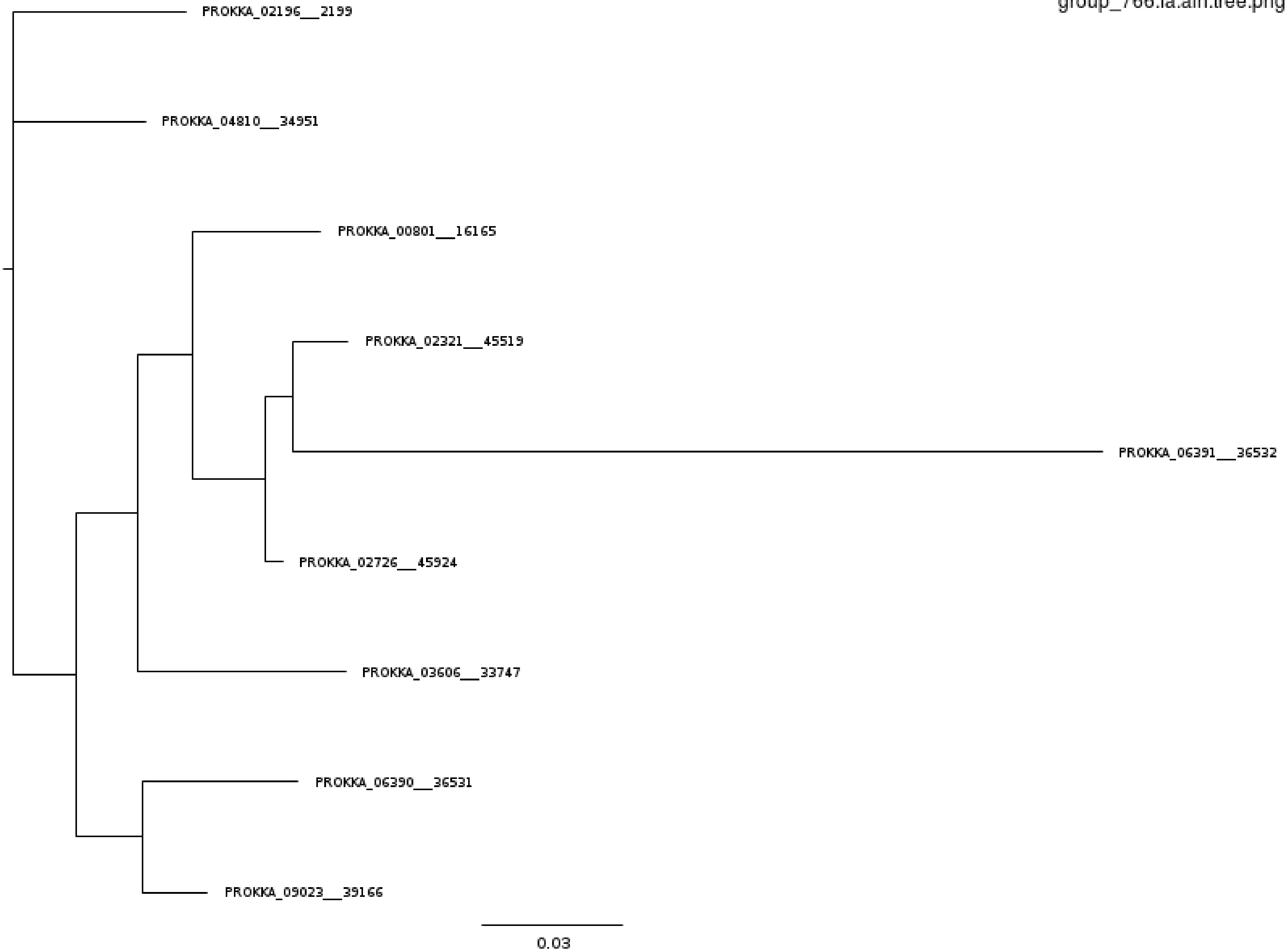
PROKKA_02196___2199
PROKKA_04810___34951
PROKKA_00801___16165
PROKKA_02321___45519
PROKKA_06391___36532
PROKKA_02726___45924
PROKKA_03606___33747
PROKKA_06390___36531
PROKKA_09023___39166
0.03

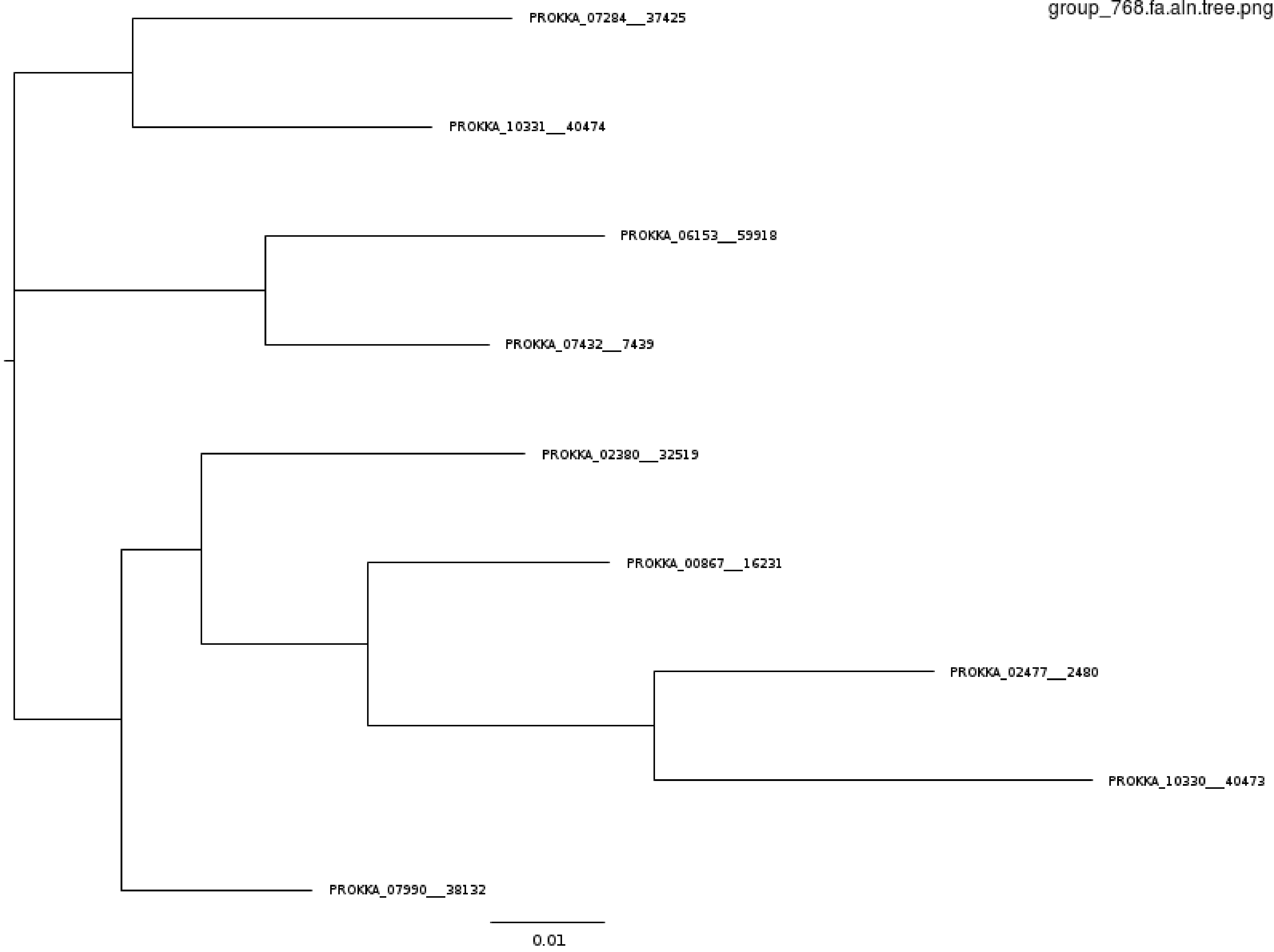
group_768.fa.aln.tree.png
PROKKA_07284___37425
PROKKA_10331___40474
PROKKA_06153___59918
PROKKA_07432___7439
PROKKA_02380___32519
PROKKA_00867___16231
PROKKA_02477___2480
PROKKA_10330___40473
PROKKA_07990___38132
0.01

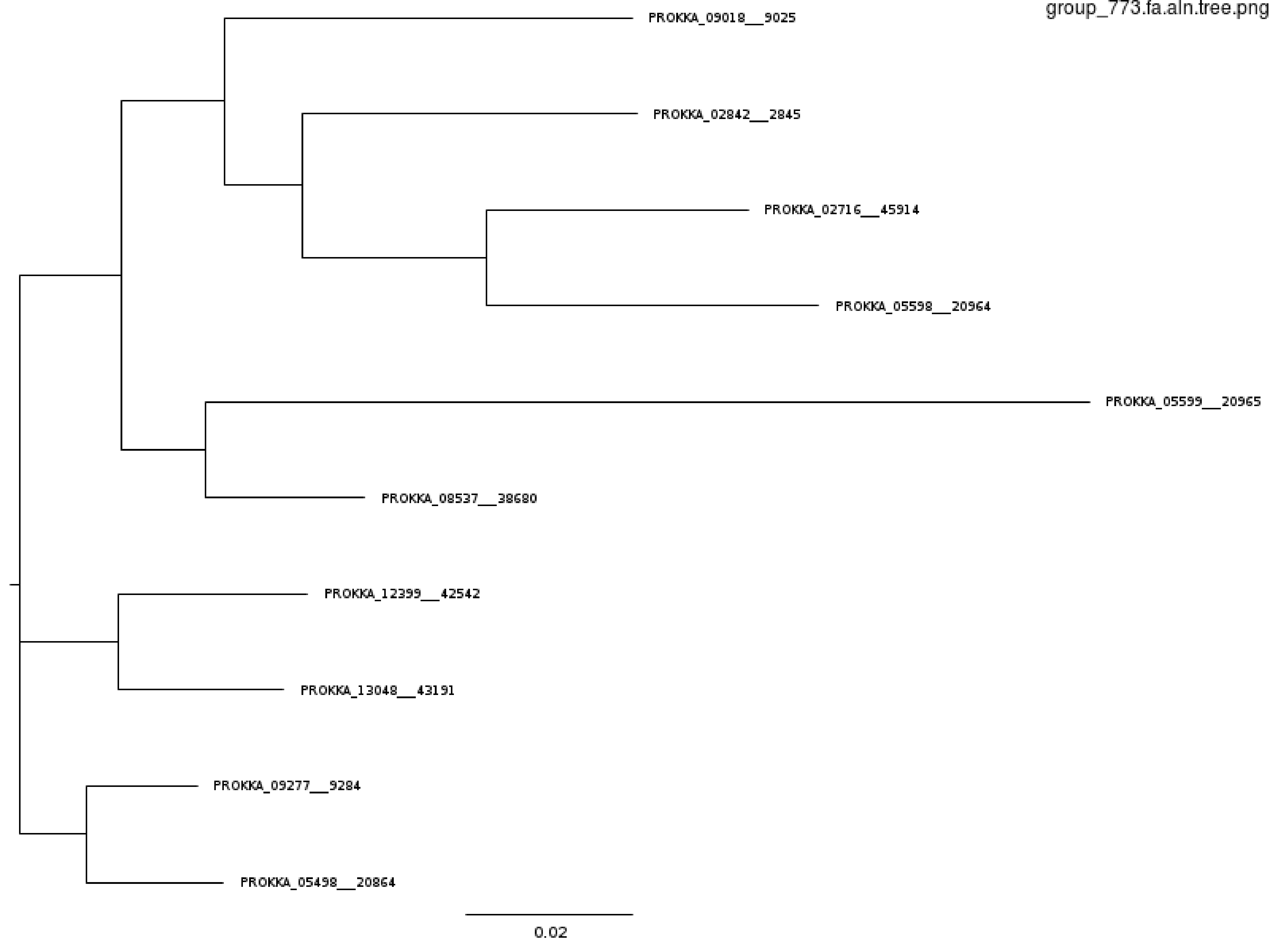
group_773.fa.aln.tree.png
PROKKA_09018___9025
PROKKA_02842___2845
PROKKA_02716___45914
PROKKA_05598___20964
PROKKA_05599___20965
PROKKA_08537___38680
PROKKA_12399___42542
PROKKA_13048___43191
PROKKA_09277___9284
PROKKA_05498___20864
0.02

group_775.fa.aln.tree.png

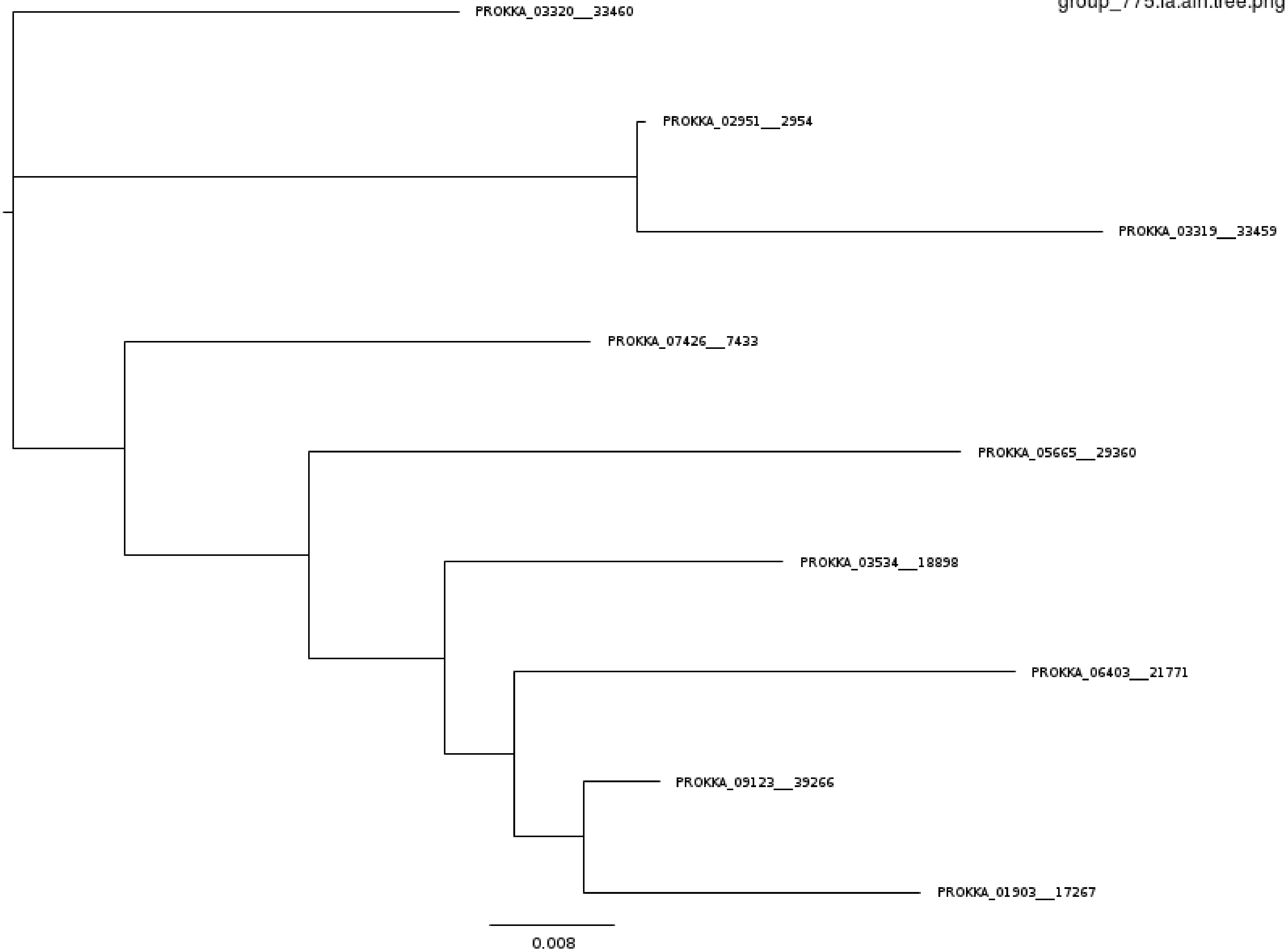

group_776.fa.aln.tree.png

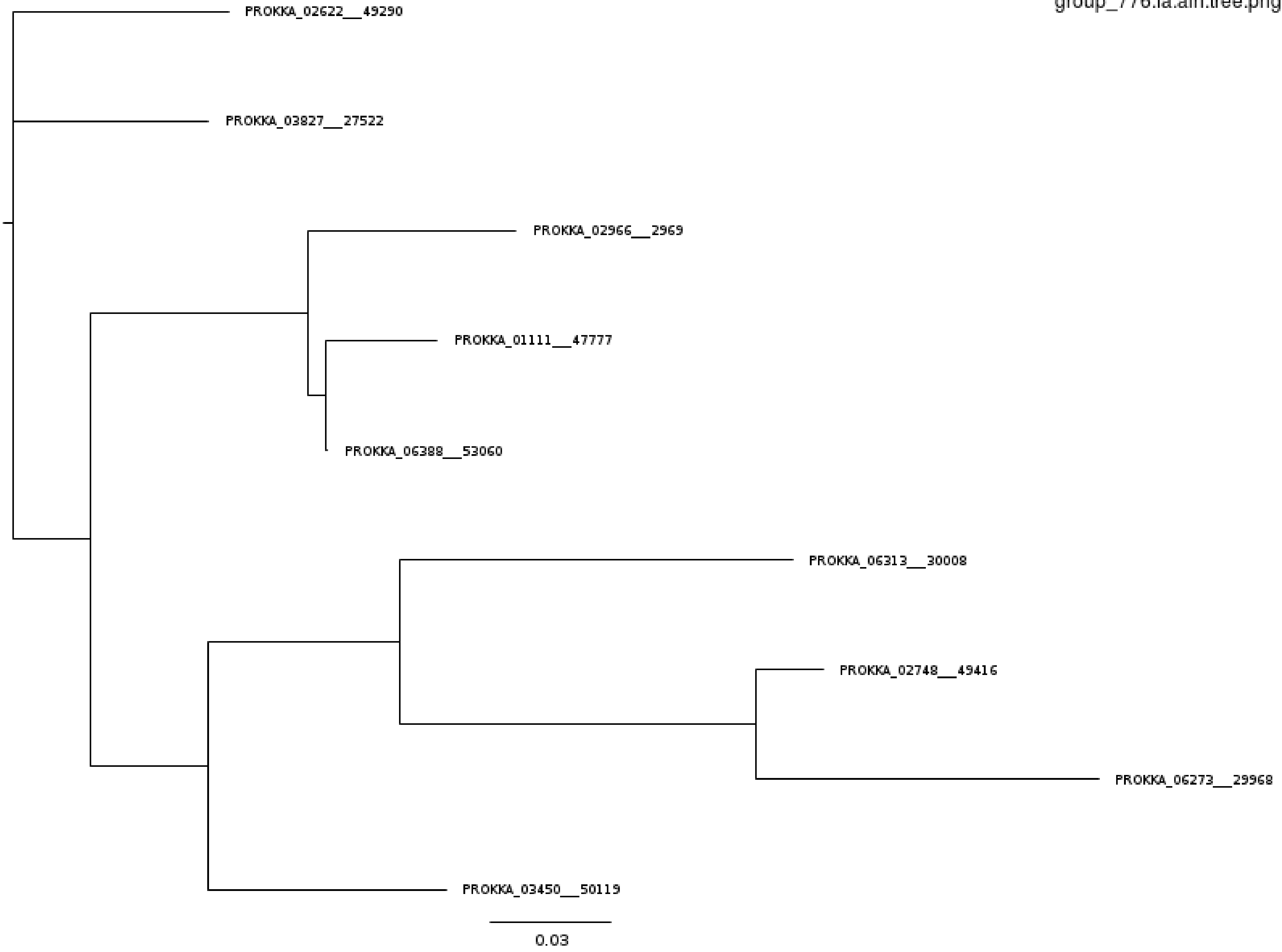

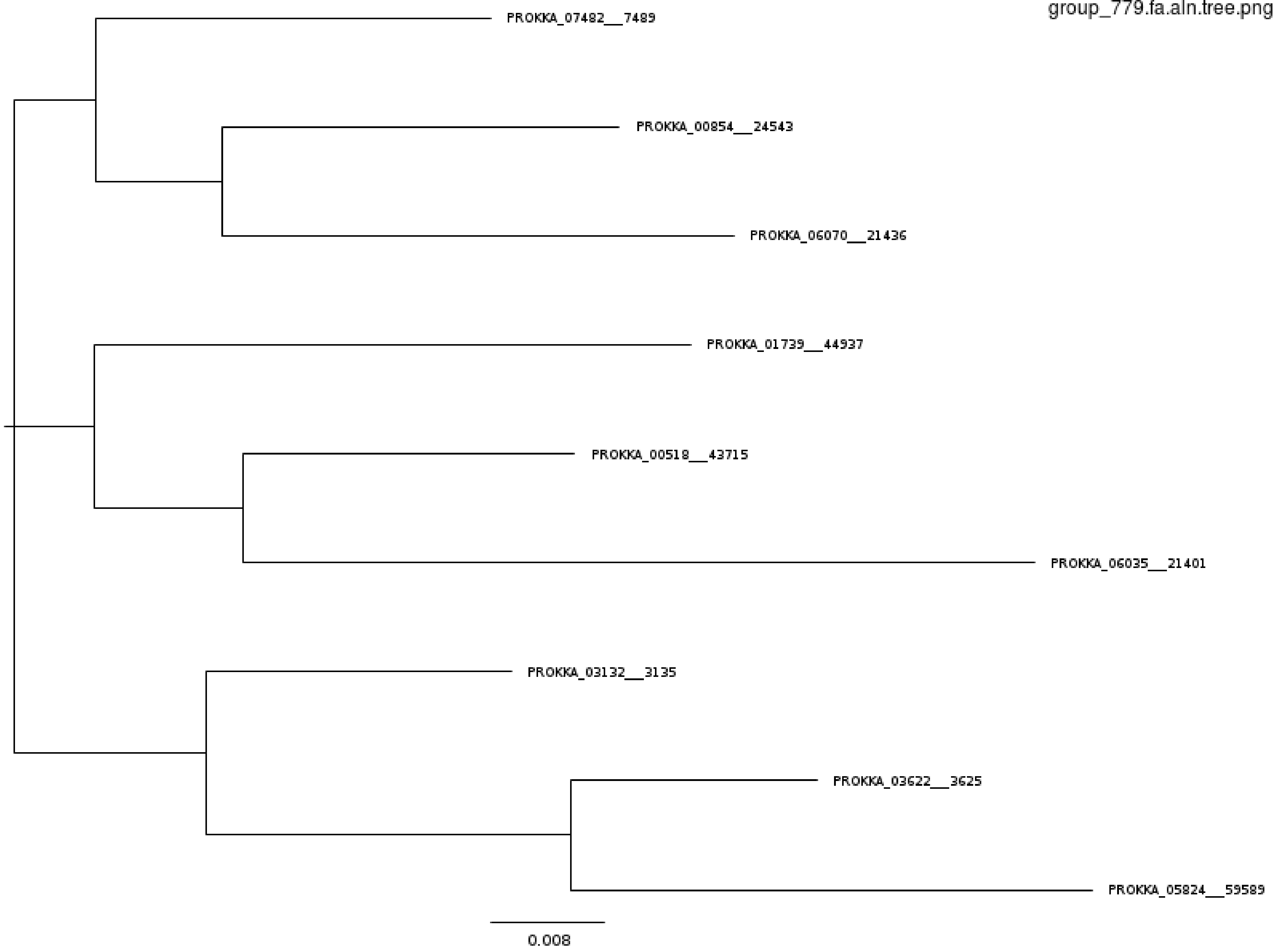
group_779.fa.aln.tree.png
PROKKA_07482___7489
PROKKA_00854___24543
PROKKA_06070___21436
PROKKA_01739___44937
PROKKA_00518___43715
PROKKA_06035___21401
PROKKA_03132___3135
PROKKA_03622___3625
PROKKA_05824___59589
0.008

group_77.fa.aln.tree.png

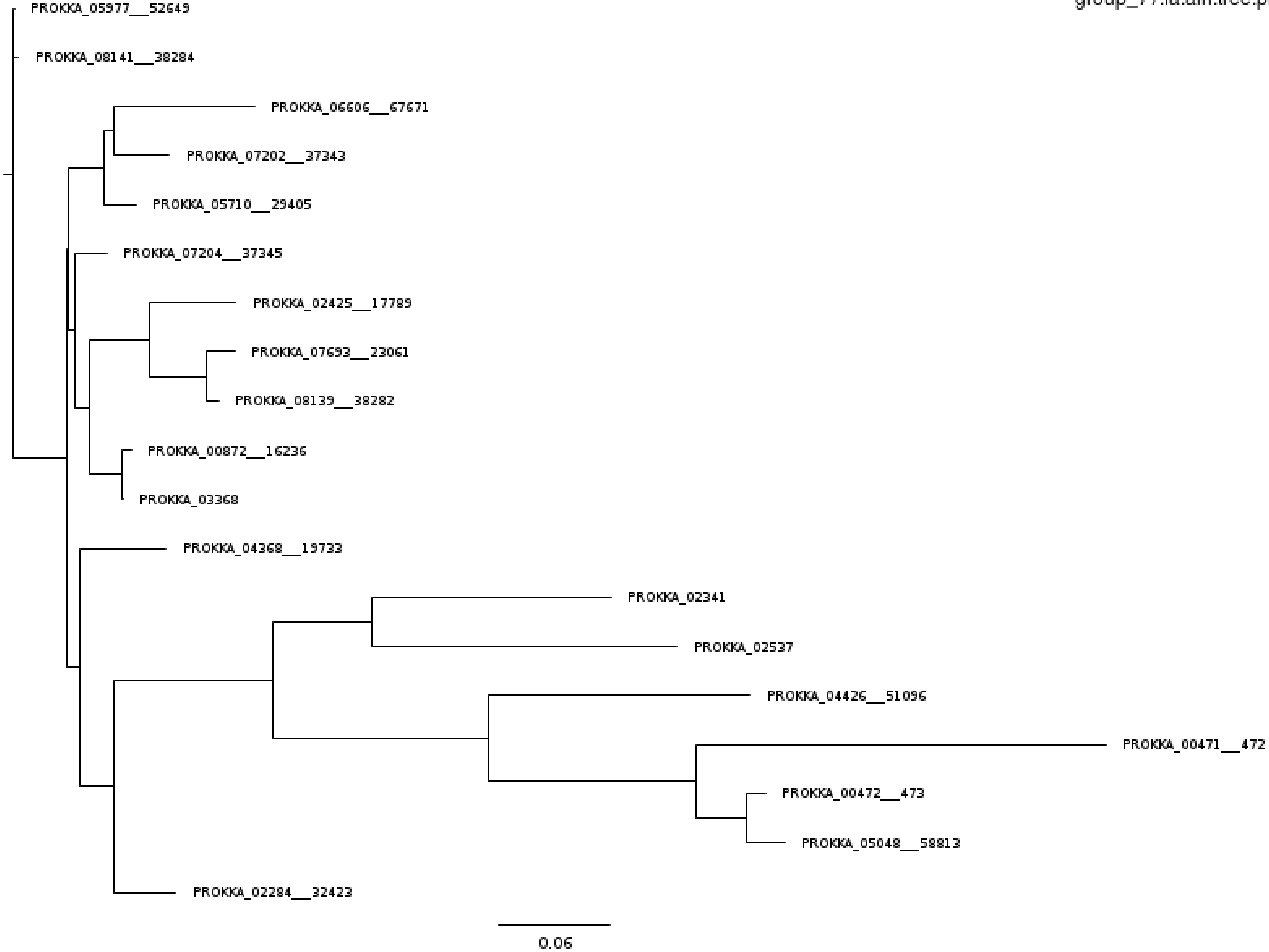

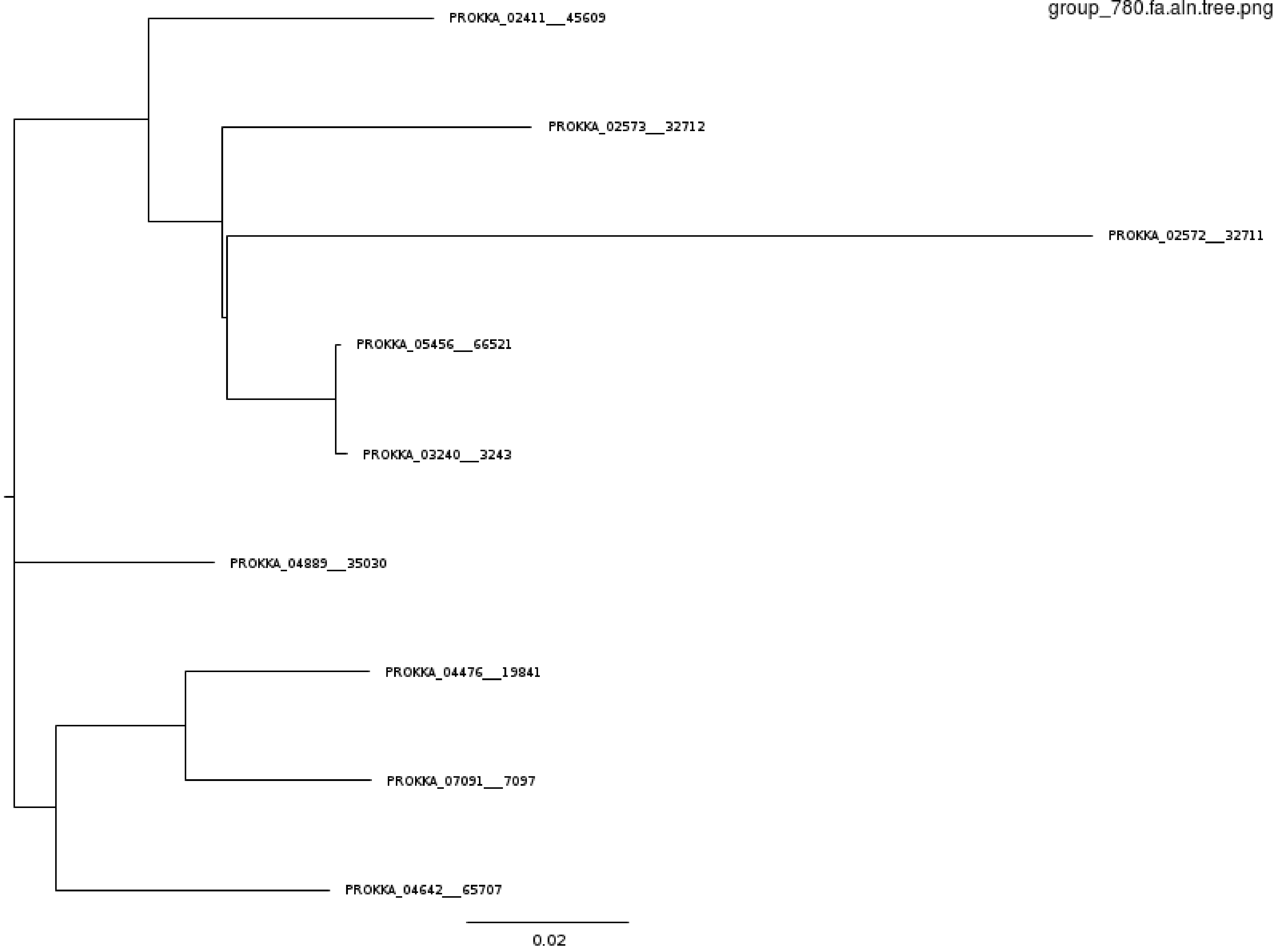
group_780.fa.aln.tree.png
PROKKA_02411___45609
PROKKA_02573___32712
PROKKA_02572___32711
PROKKA_05456___66521
PROKKA_03240___3243
PROKKA_04889___35030
PROKKA_04476___19841
PROKKA_07091___7097
PROKKA_04642___65707
0.02

group_794.fa.aln.tree.png

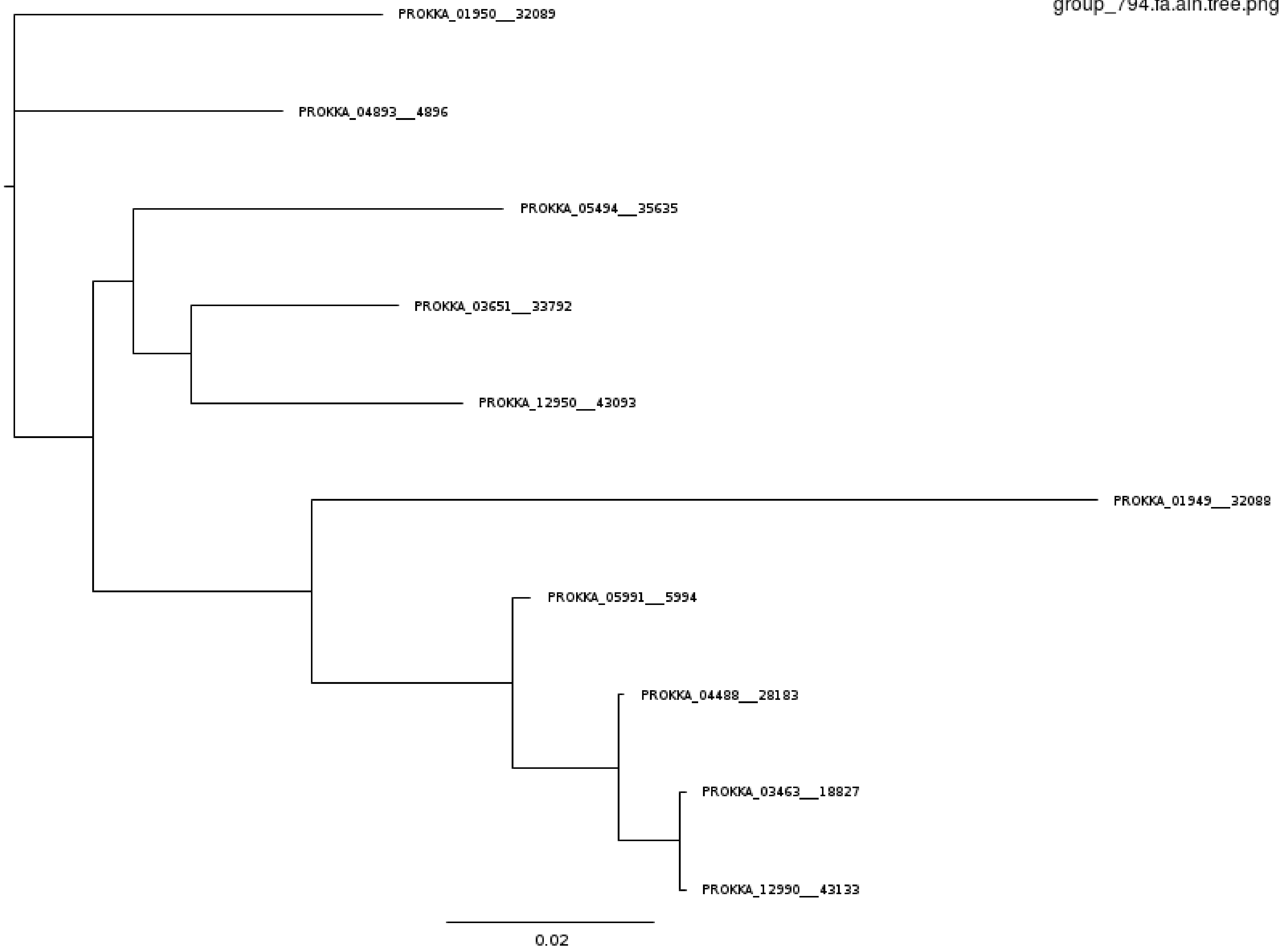

group_795.fa.aln.tree.png

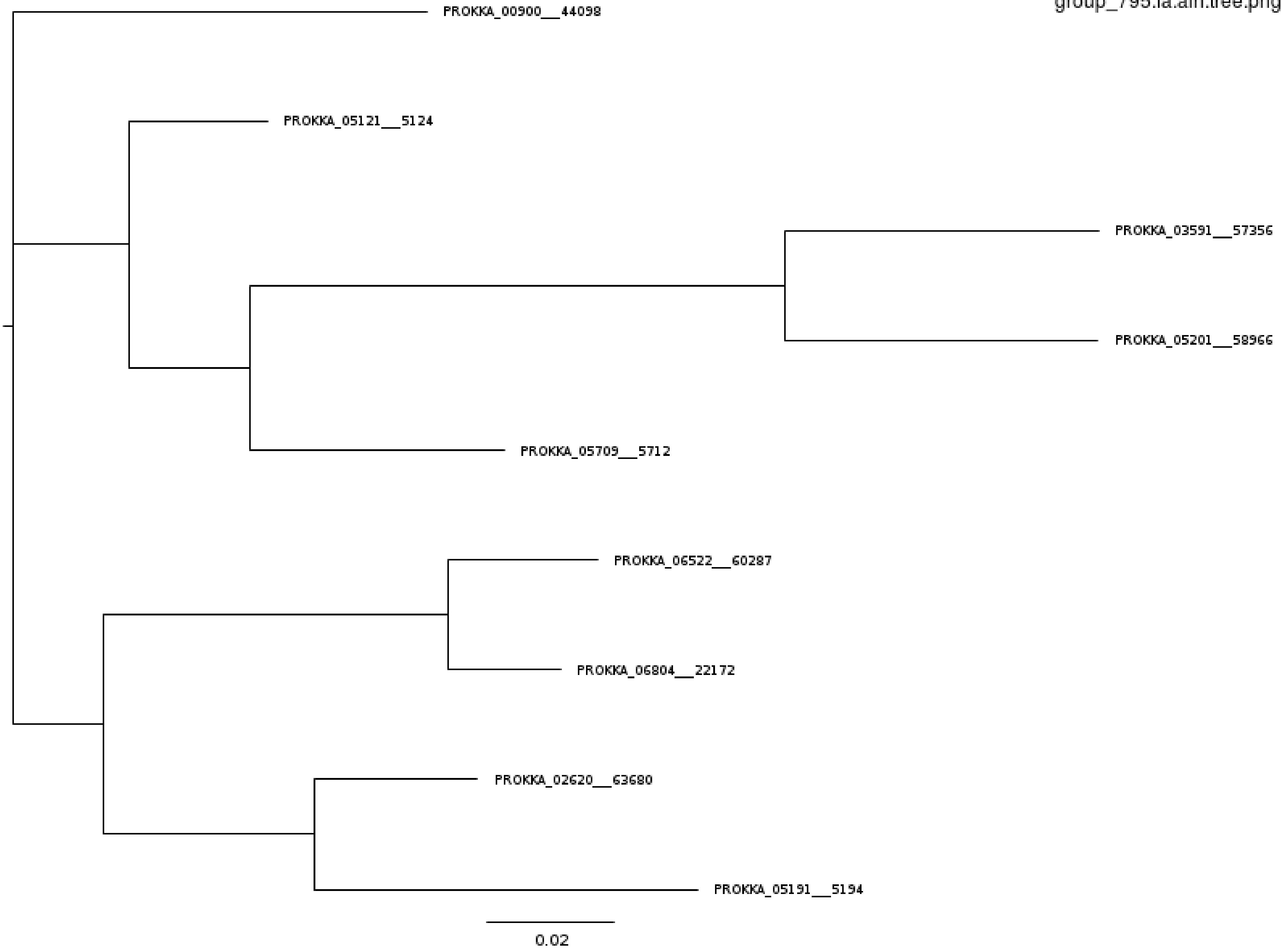

group_797.fa.aln.tree.png

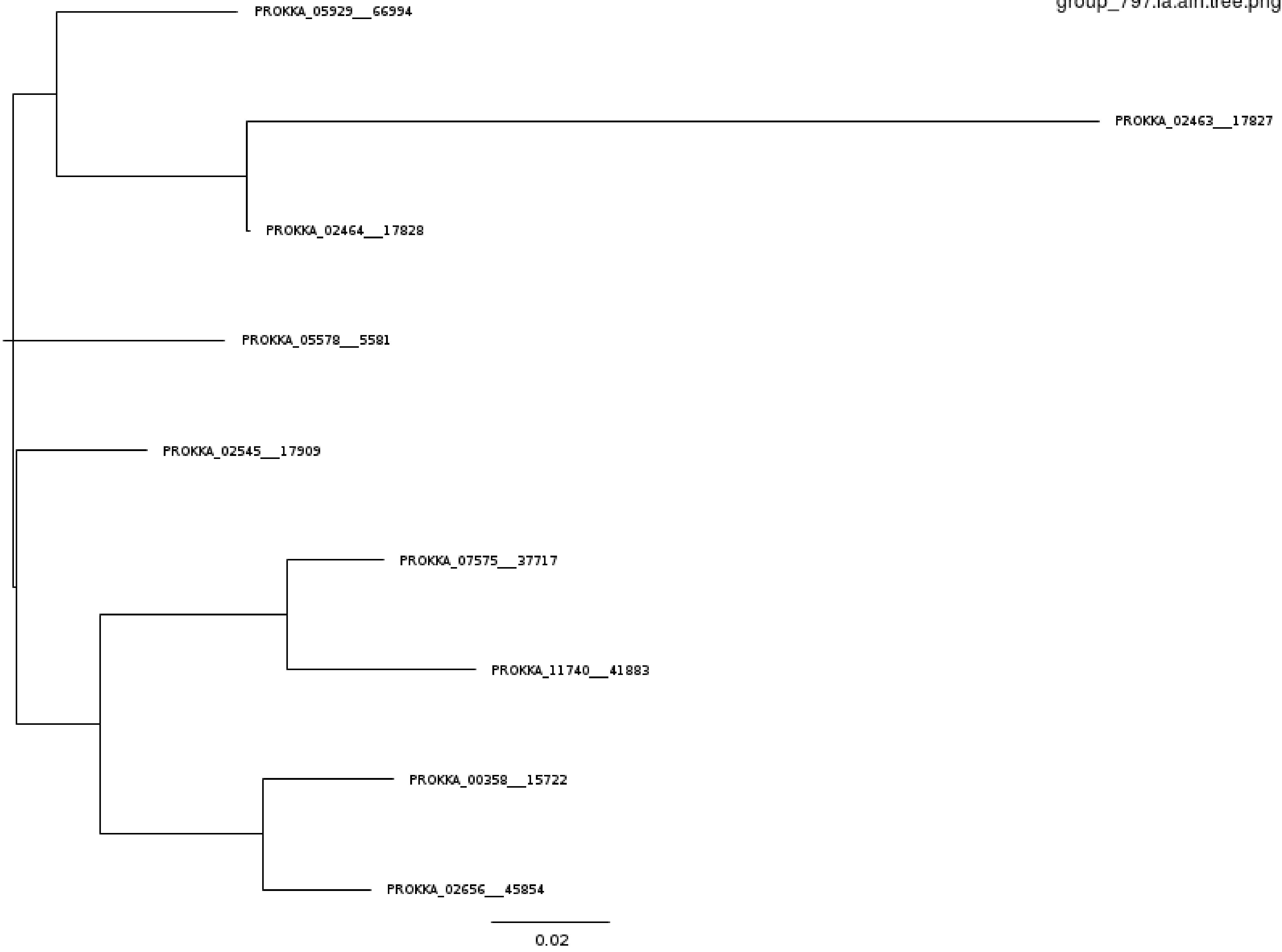

group_799.fa.aln.tree.png

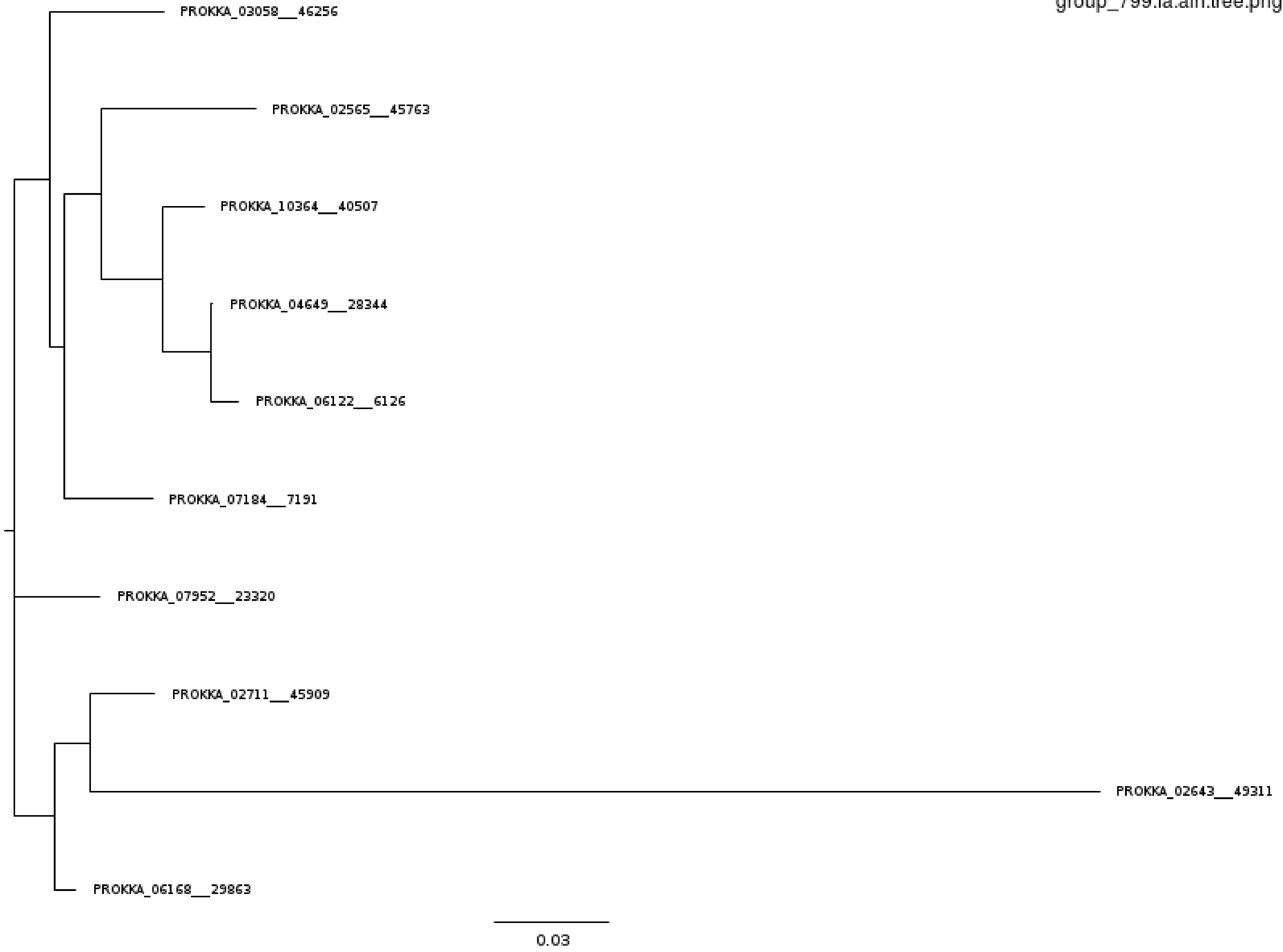

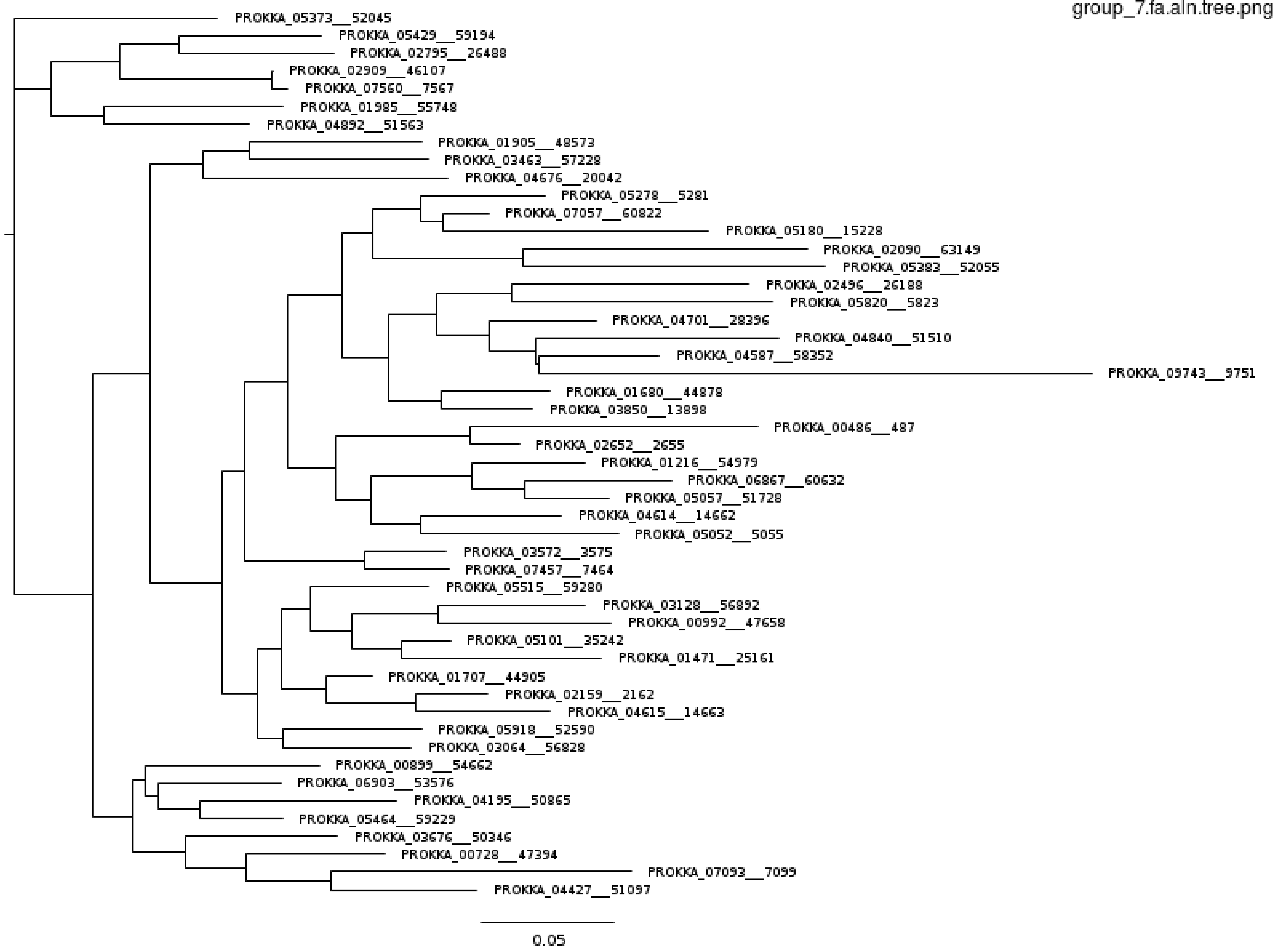
group_7.fa.aln.tree.png
PROKKA_05373___52045
PROKKA_05429___59194
PROKKA_02795___26488
PROKKA_02909___46107
PROKKA_07560___7567
PROKKA_01985___55748
PROKKA_04892___51563
PROKKA_01905___48573
PROKKA_03463___57228
PROKKA_04676___20042
PROKKA_05278___5281
PROKKA_07057___60822
PROKKA_05180___15228
PROKKA_02090___63149
PROKKA_05383___52055
PROKKA_02496___26188
PROKKA_05820___5823
PROKKA_04701___28396
PROKKA_04840___51510
PROKKA_04587___58352
PROKKA_09743___9751
PROKKA_01680___44878
PROKKA_03850___13898
PROKKA_00486___487
PROKKA_02652___2655
PROKKA_01216___54979
PROKKA_06867___60632
PROKKA_05057___51728
PROKKA_04614___14662
PROKKA_05052___5055
PROKKA_03572___3575
PROKKA_07457___7464
PROKKA_05515___59280
PROKKA_03128___56892
PROKKA_00992___47658
PROKKA_05101___35242
PROKKA_01471___25161
PROKKA_01707___44905
PROKKA_02159___2162
PROKKA_04615___14663
PROKKA_05918___52590
PROKKA_03064___56828
PROKKA_00899___54662
PROKKA_06903___53576
PROKKA_04195___50865
PROKKA_05464___59229
PROKKA_03676___50346
PROKKA_00728___47394
PROKKA_07093___7099
PROKKA_04427___51097
0.05

group_800.fa.aln.tree.png

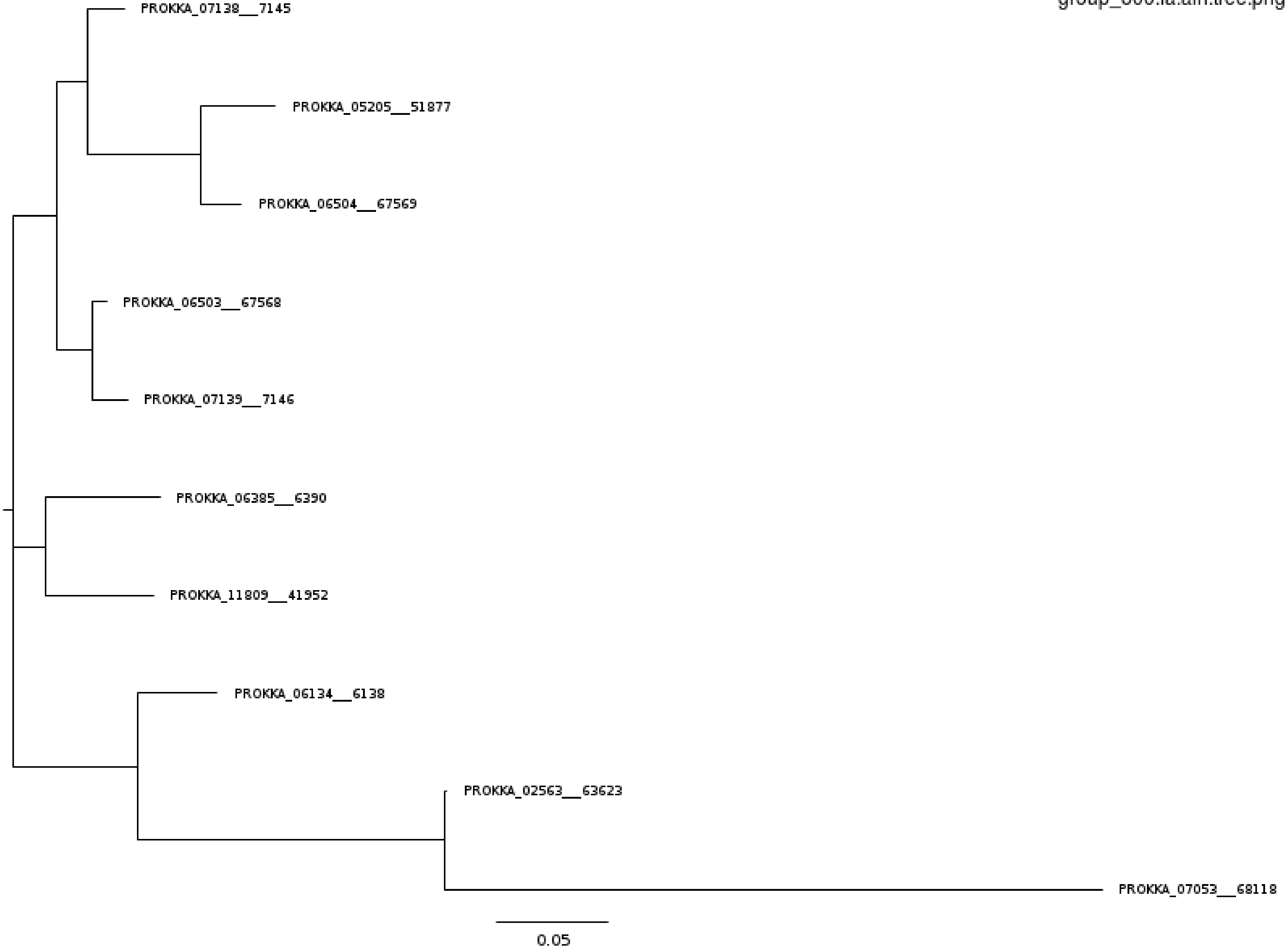

group_801.fa.aln.tree.png

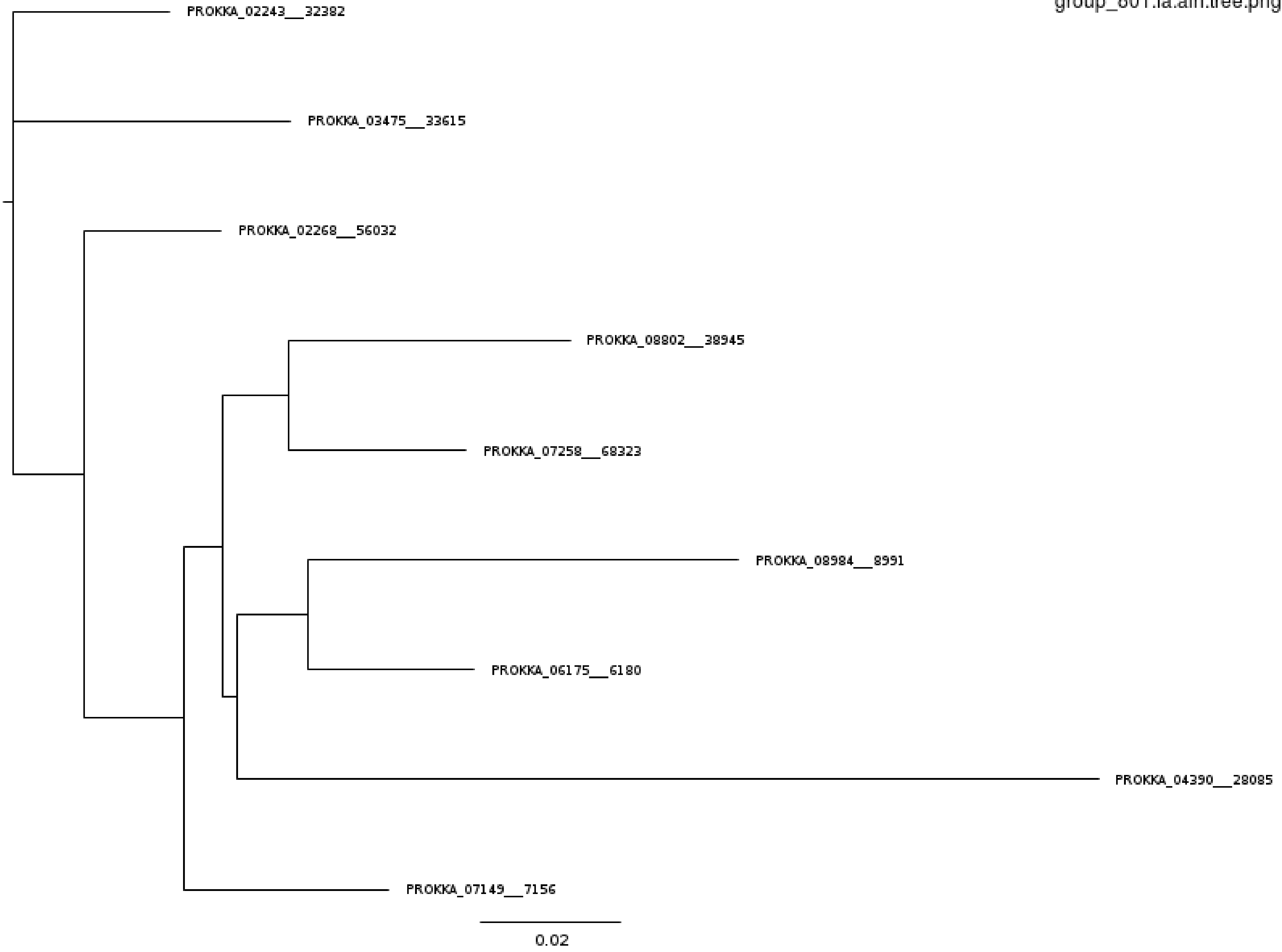

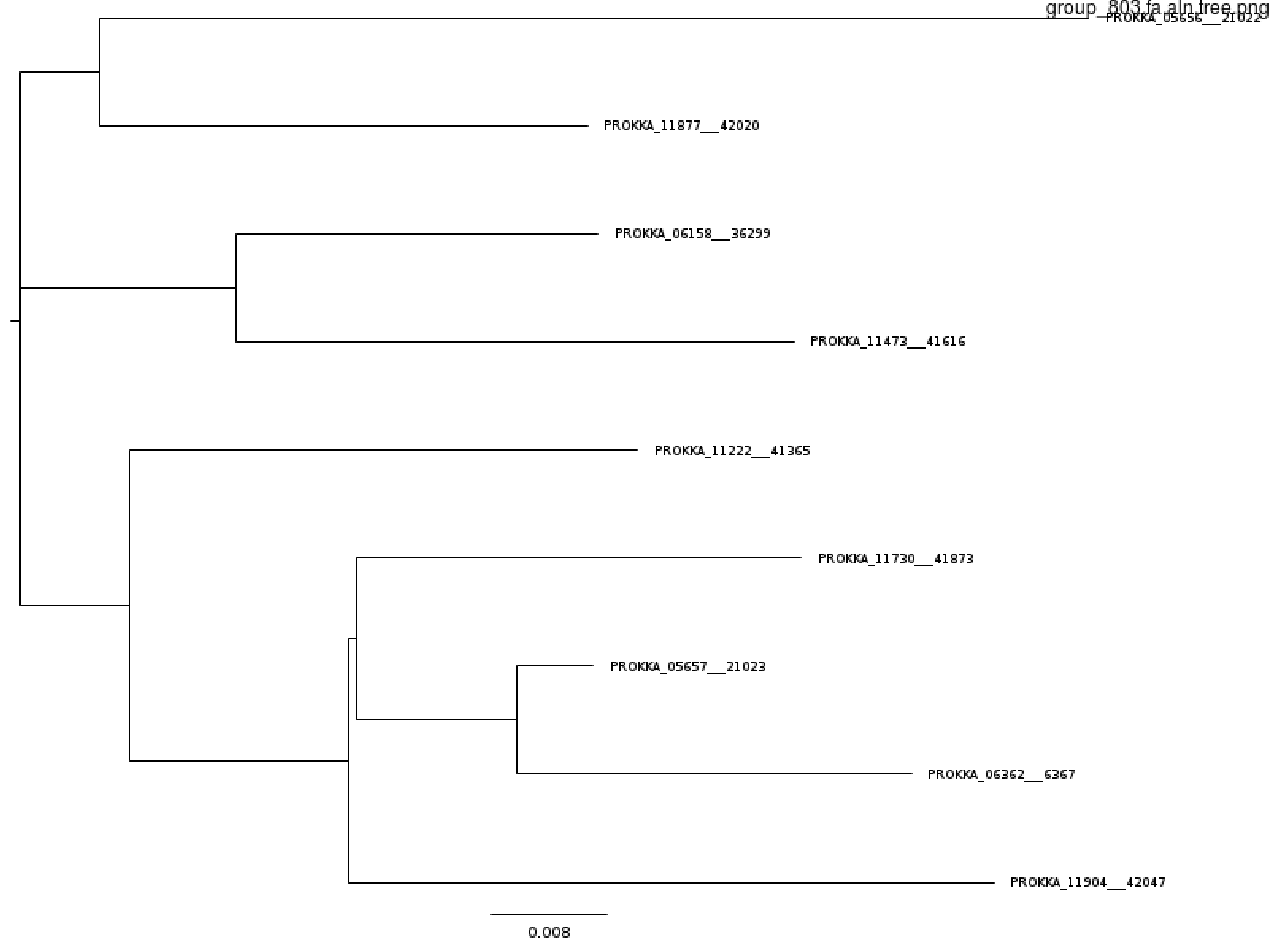
group_803.fa.aln.tree.png
PROKKA_05656___21022
PROKKA_11877___42020
PROKKA_06158___36299
PROKKA_11473___41616
PROKKA_11222___41365
PROKKA_11730___41873
PROKKA_05657___21023
PROKKA_06362___6367
PROKKA_11904___42047
0.008

group_807.fa.aln.tree.png

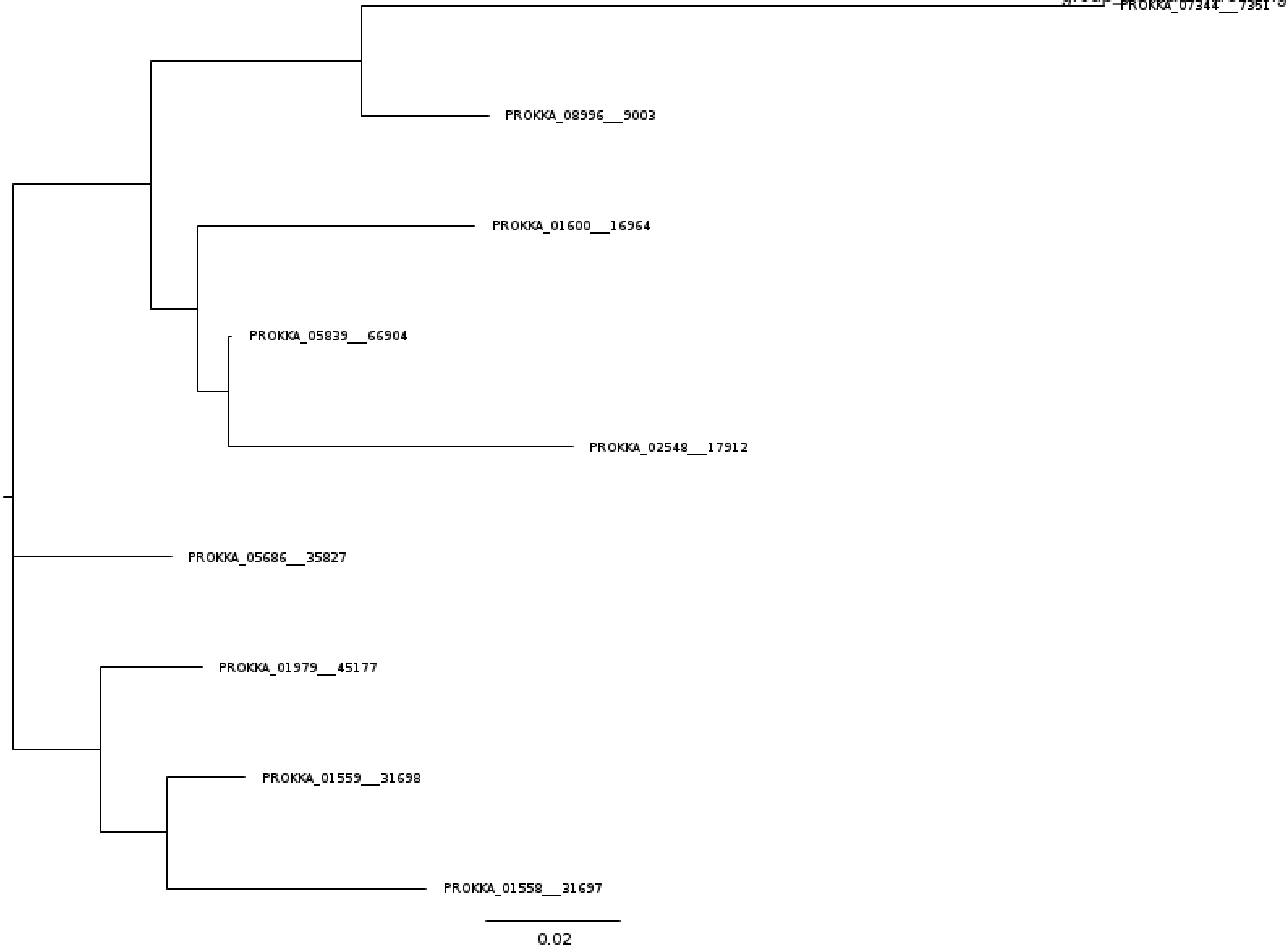

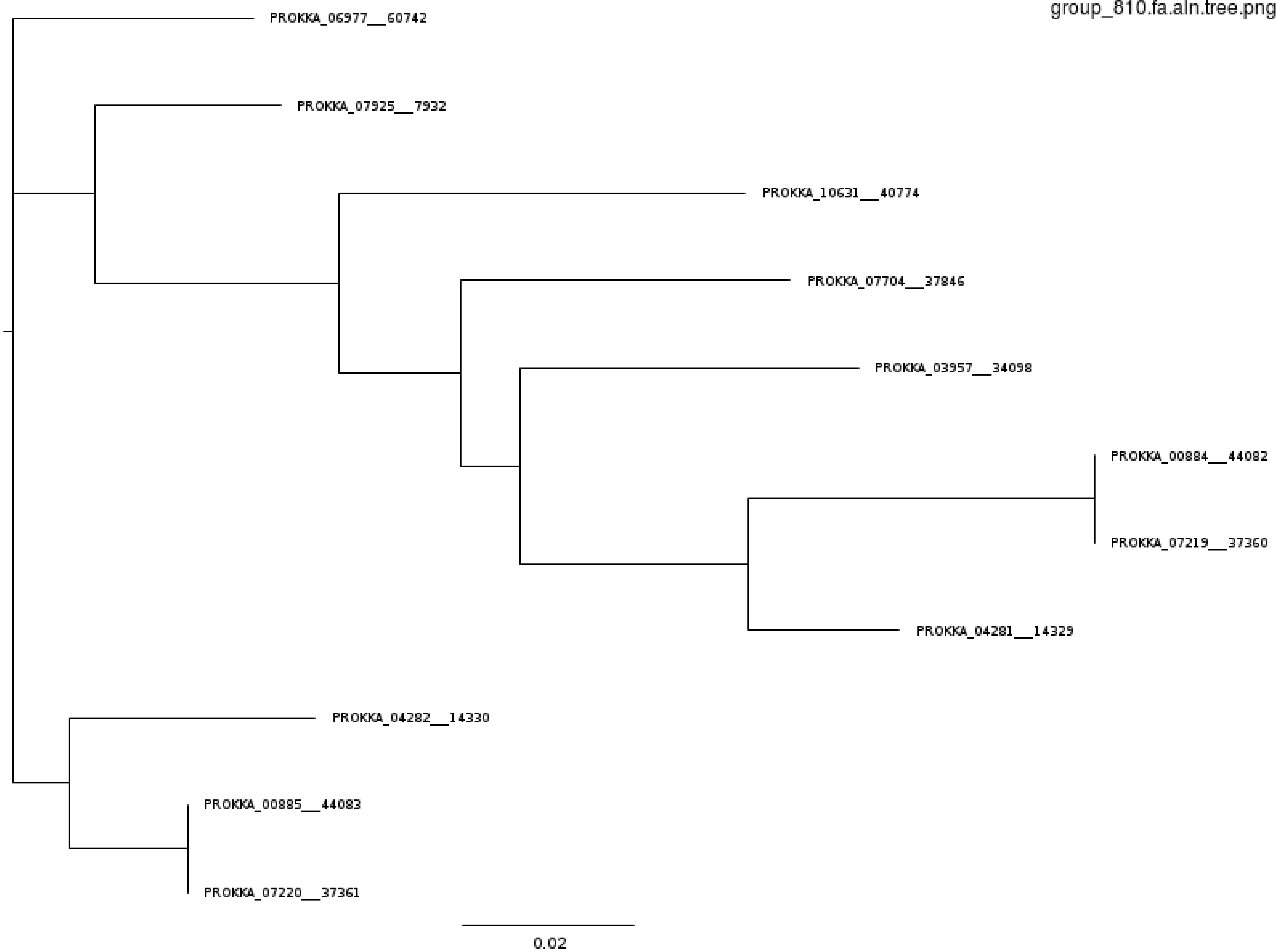
group_810.fa.aln.tree.png
PROKKA_06977___60742
PROKKA_07925___7932
PROKKA_10631___40774
PROKKA_07704___37846
PROKKA_03957___34098
PROKKA_00884___44082
PROKKA_07219___37360
PROKKA_04281___14329
PROKKA_04282___14330
PROKKA_00885___44083
PROKKA_07220___37361
0.02

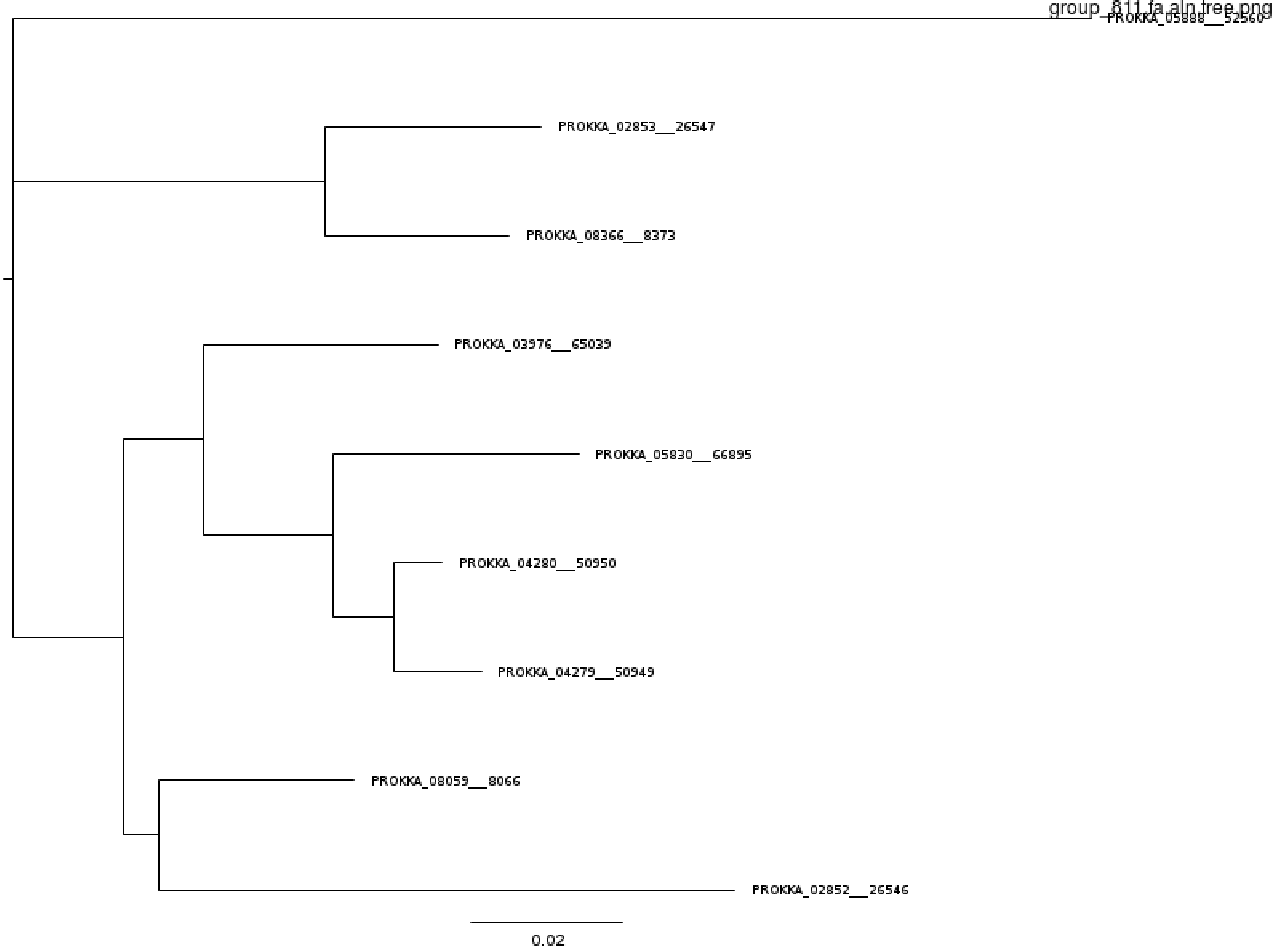
group_811.fa.aln.tree.png
PROKKA_05888___52560
PROKKA_02853___26547
PROKKA_08366___8373
PROKKA_03976___65039
PROKKA_05830___66895
PROKKA_04280___50950
PROKKA_04279___50949
PROKKA_08059___8066
PROKKA_02852___26546
0.02

group_812.fa.aln.tree.png

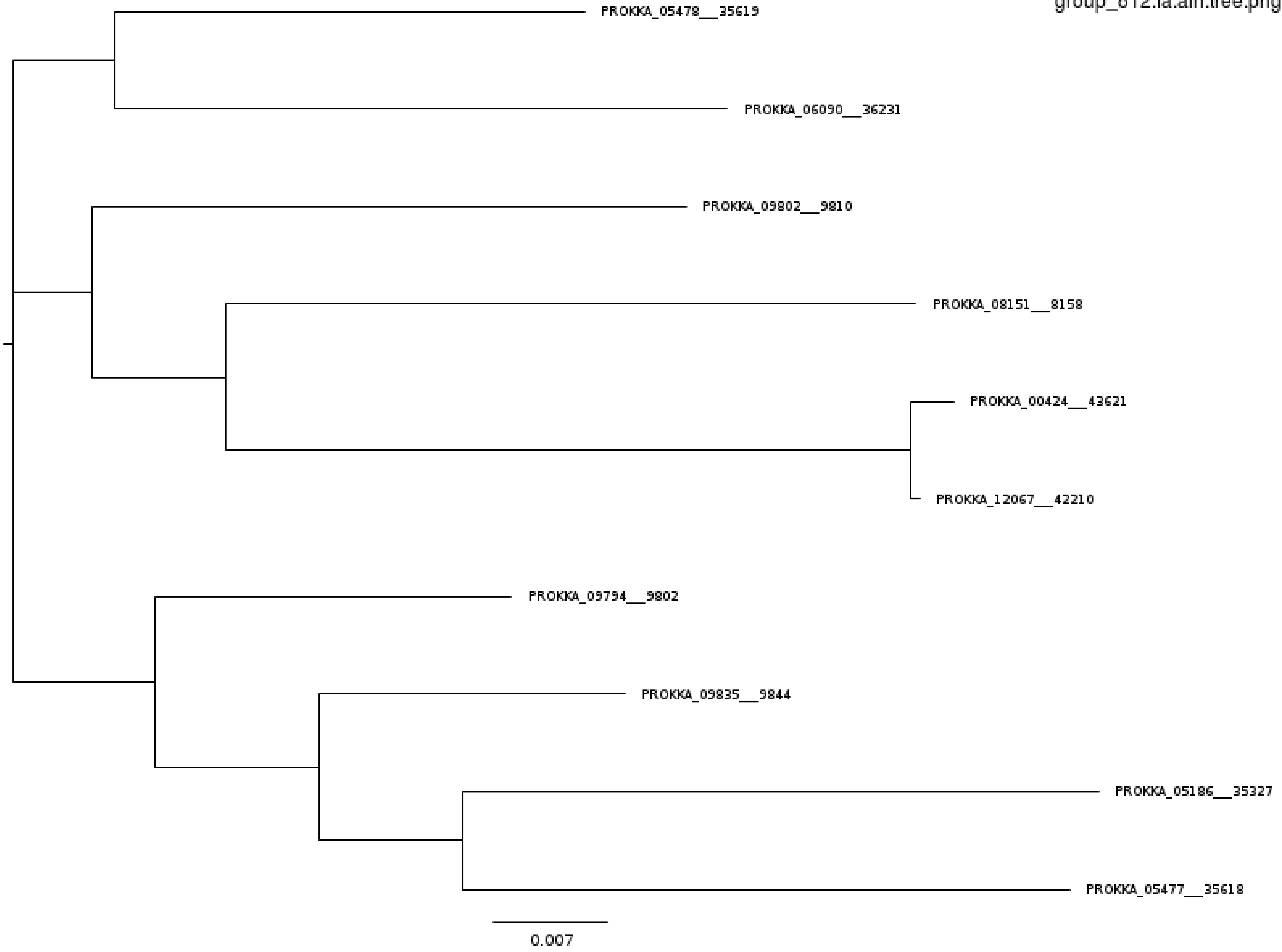

group_814.fa.aln.tree.png

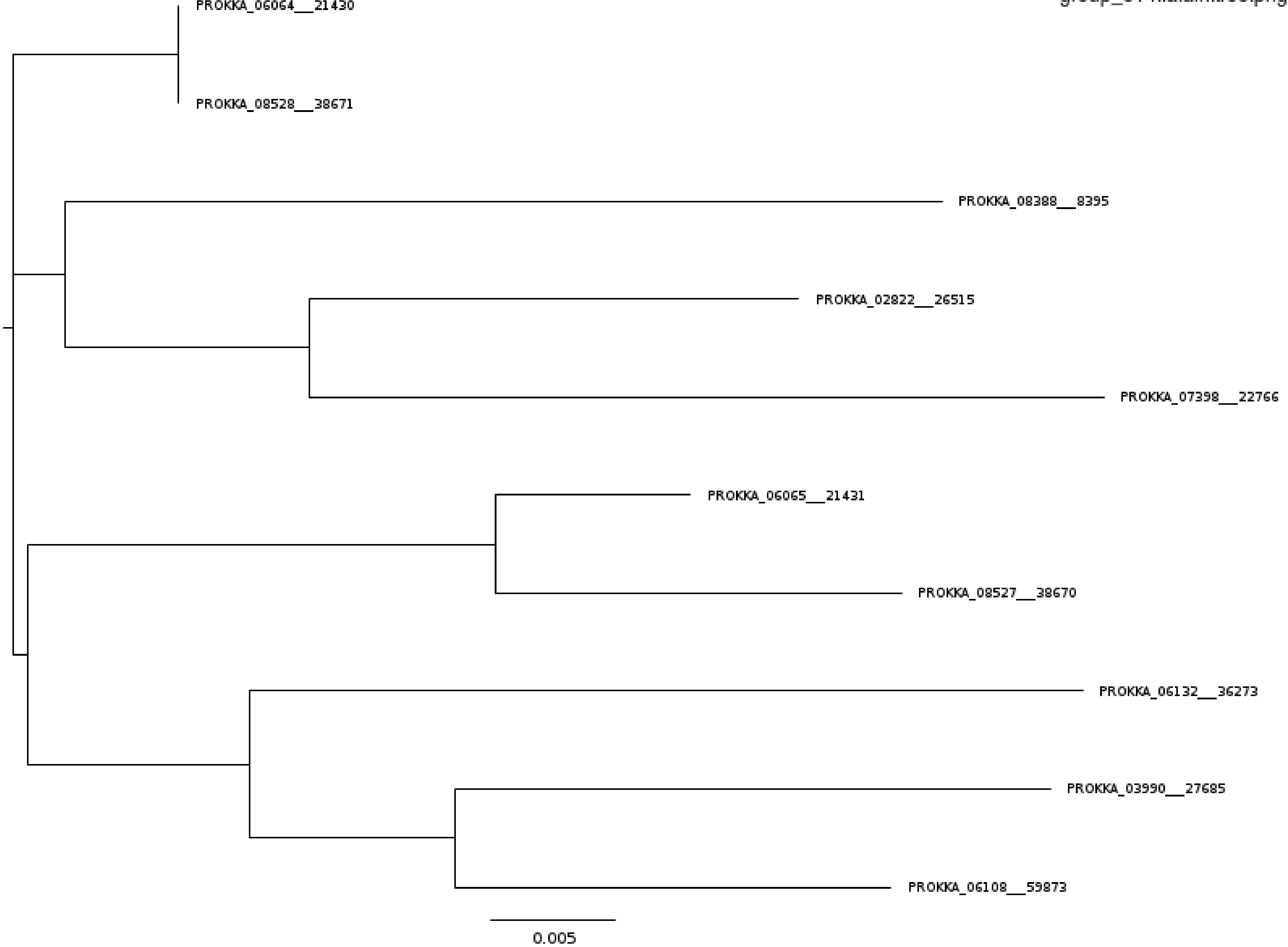

group_819.fa.aln.tree.png

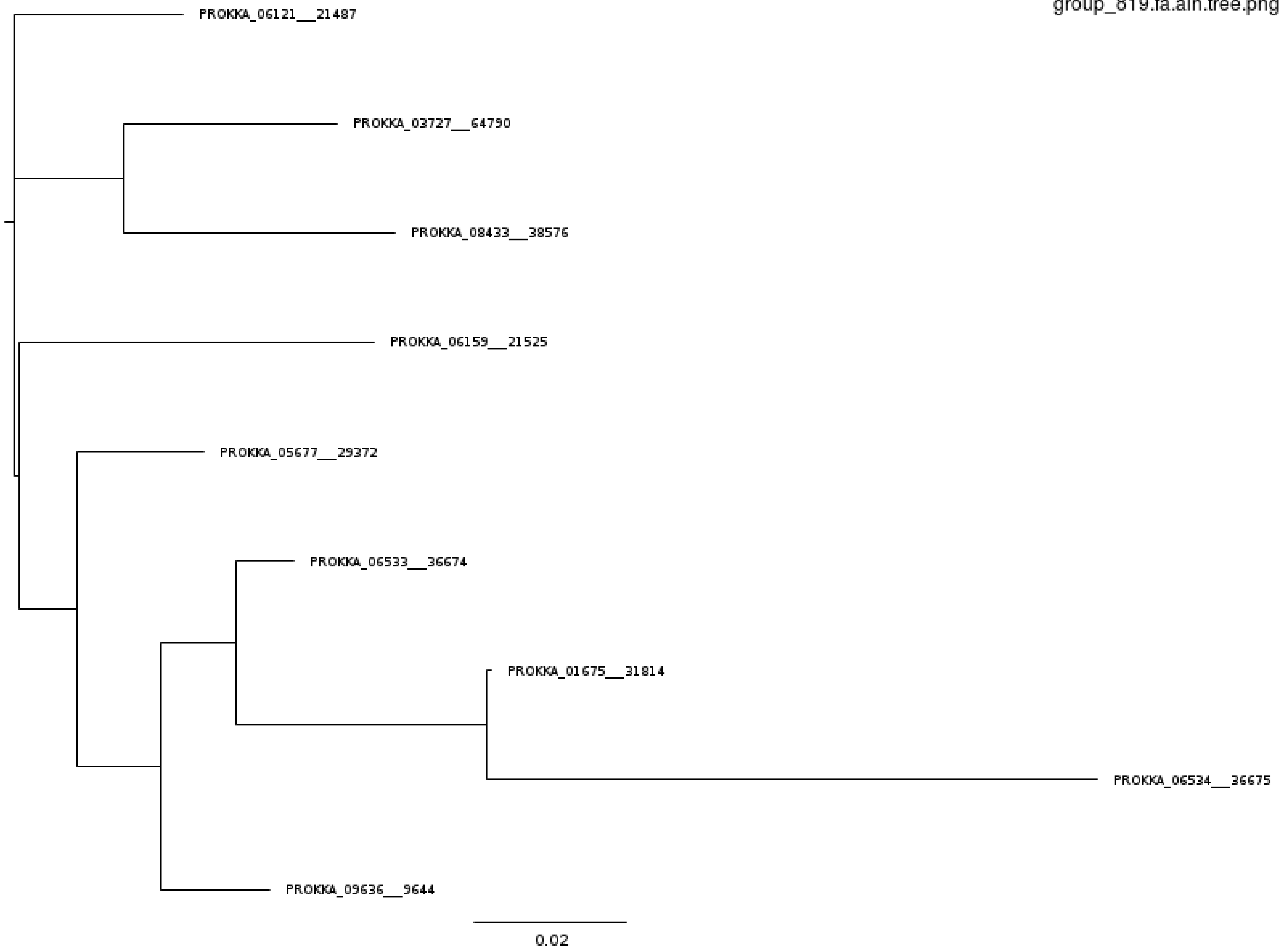

group_820.fa.aln.tree.png

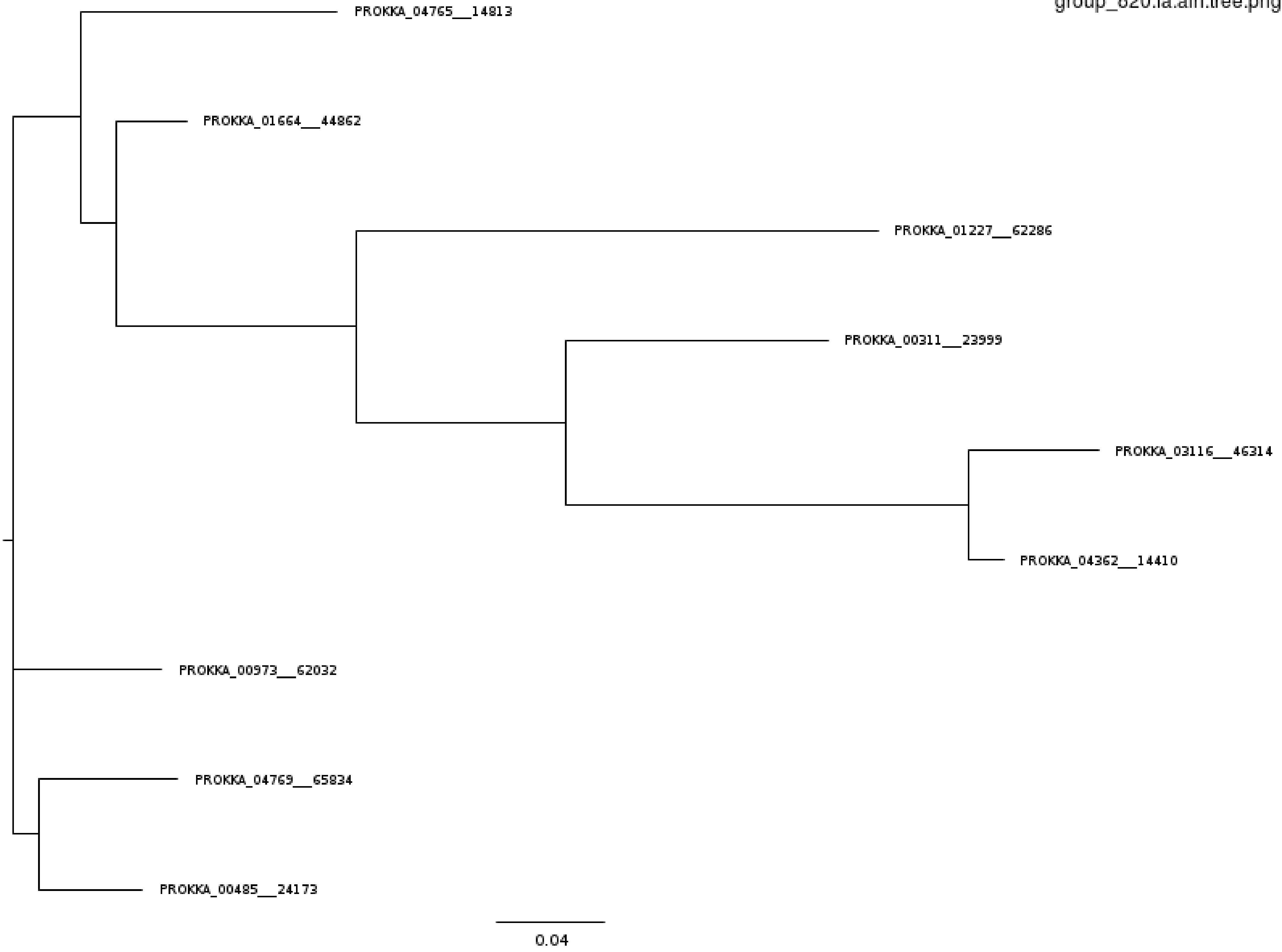

group_821.fa.aln.tree.png

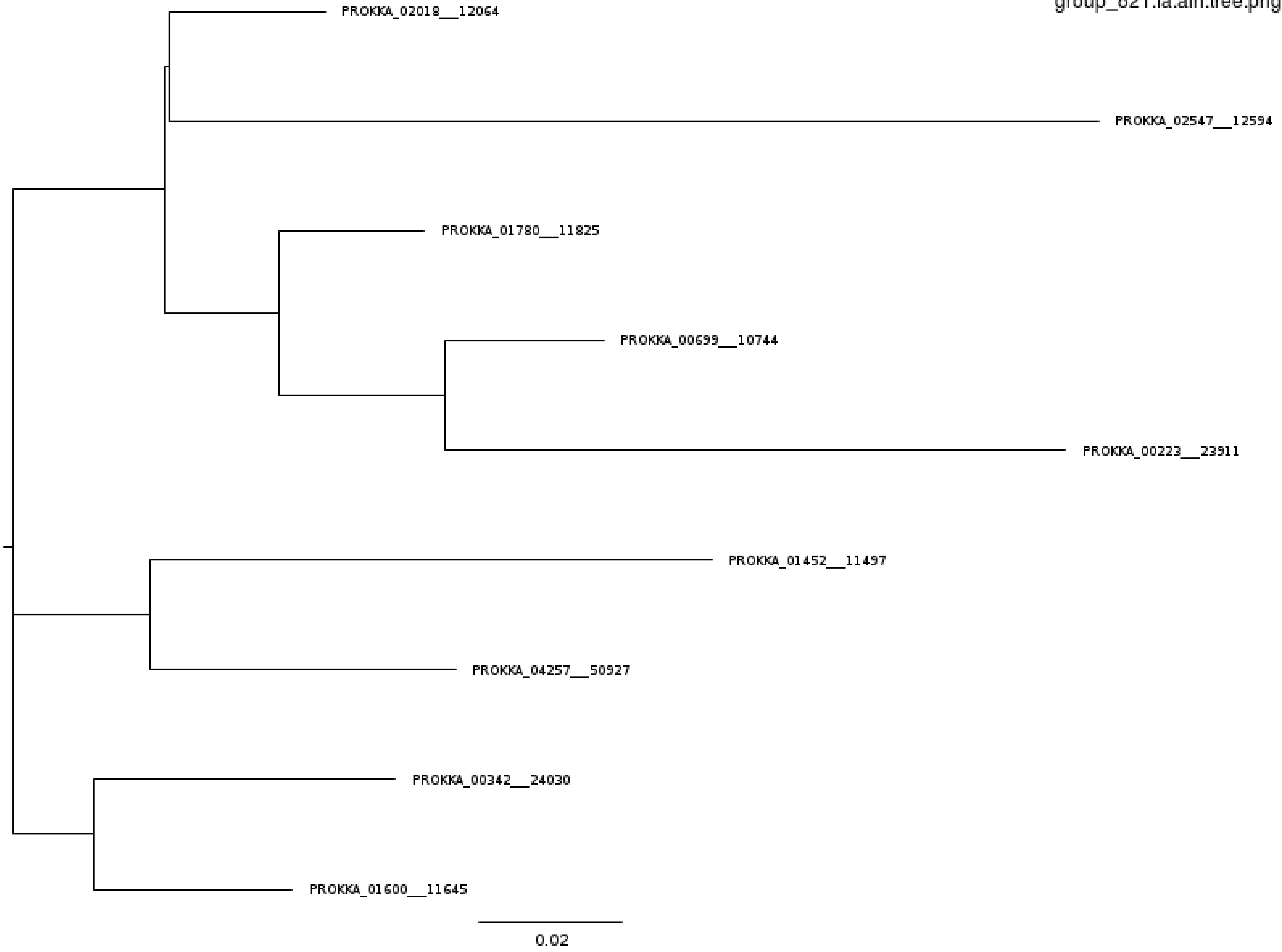

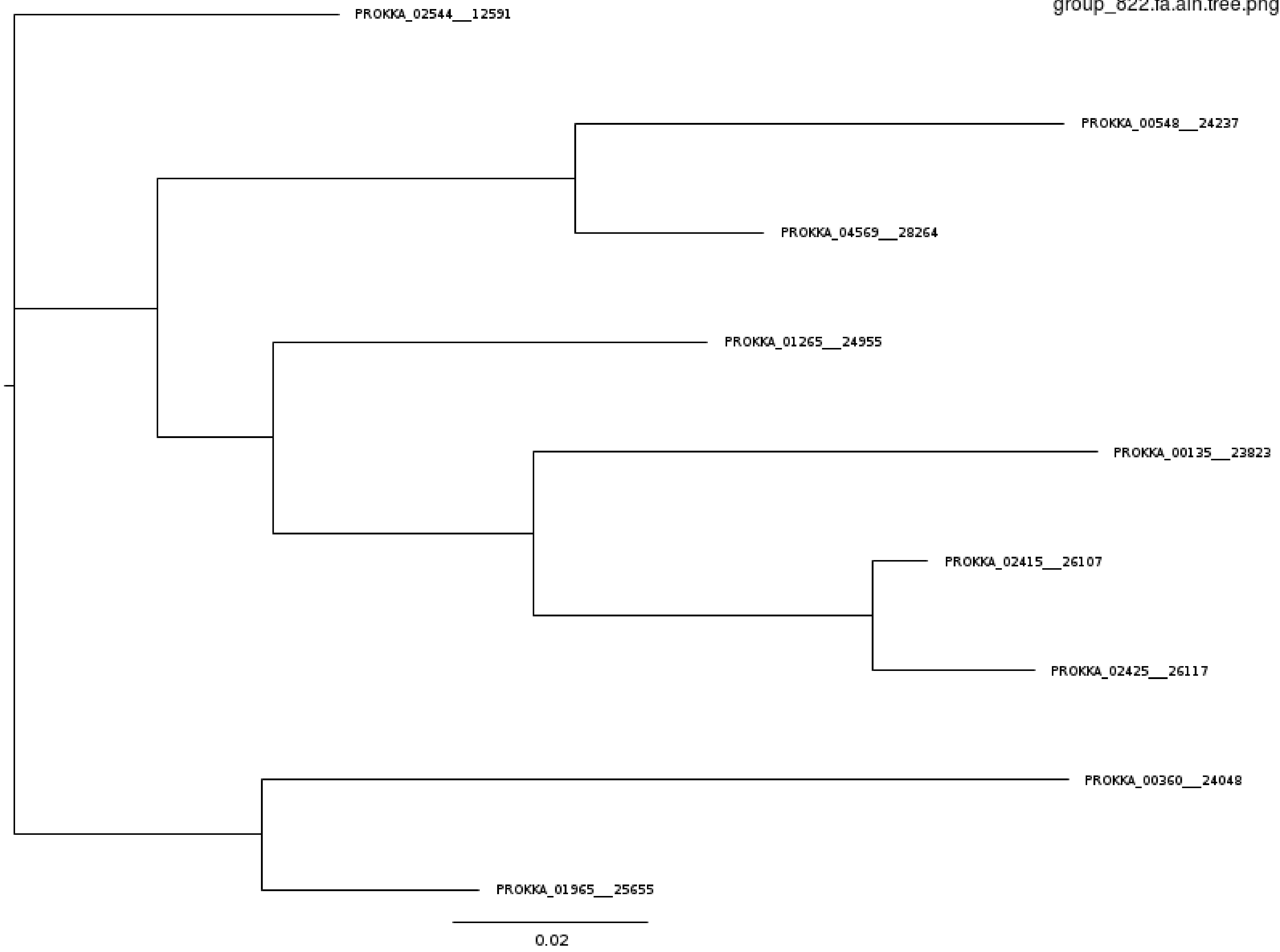
PROKKA_02544___12591
PROKKA_00548___24237
PROKKA_04569___28264
PROKKA_01265___24955
PROKKA_00135___23823
PROKKA_02415___26107
PROKKA_02425___26117
PROKKA_00360___24048
PROKKA_01965___25655
0.02

group_824.fa.aln.tree.png

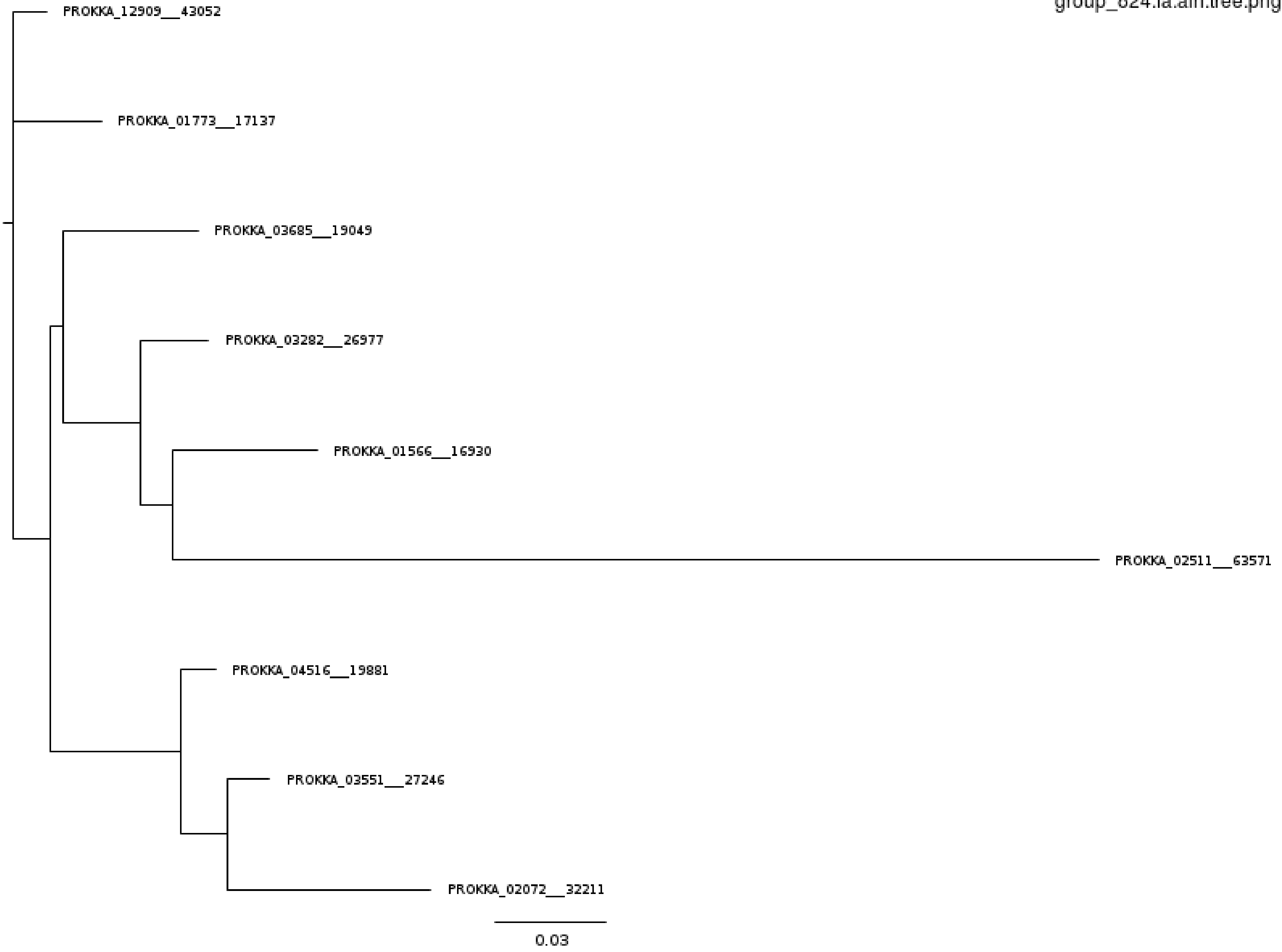

group_828.fa.aln.tree.png

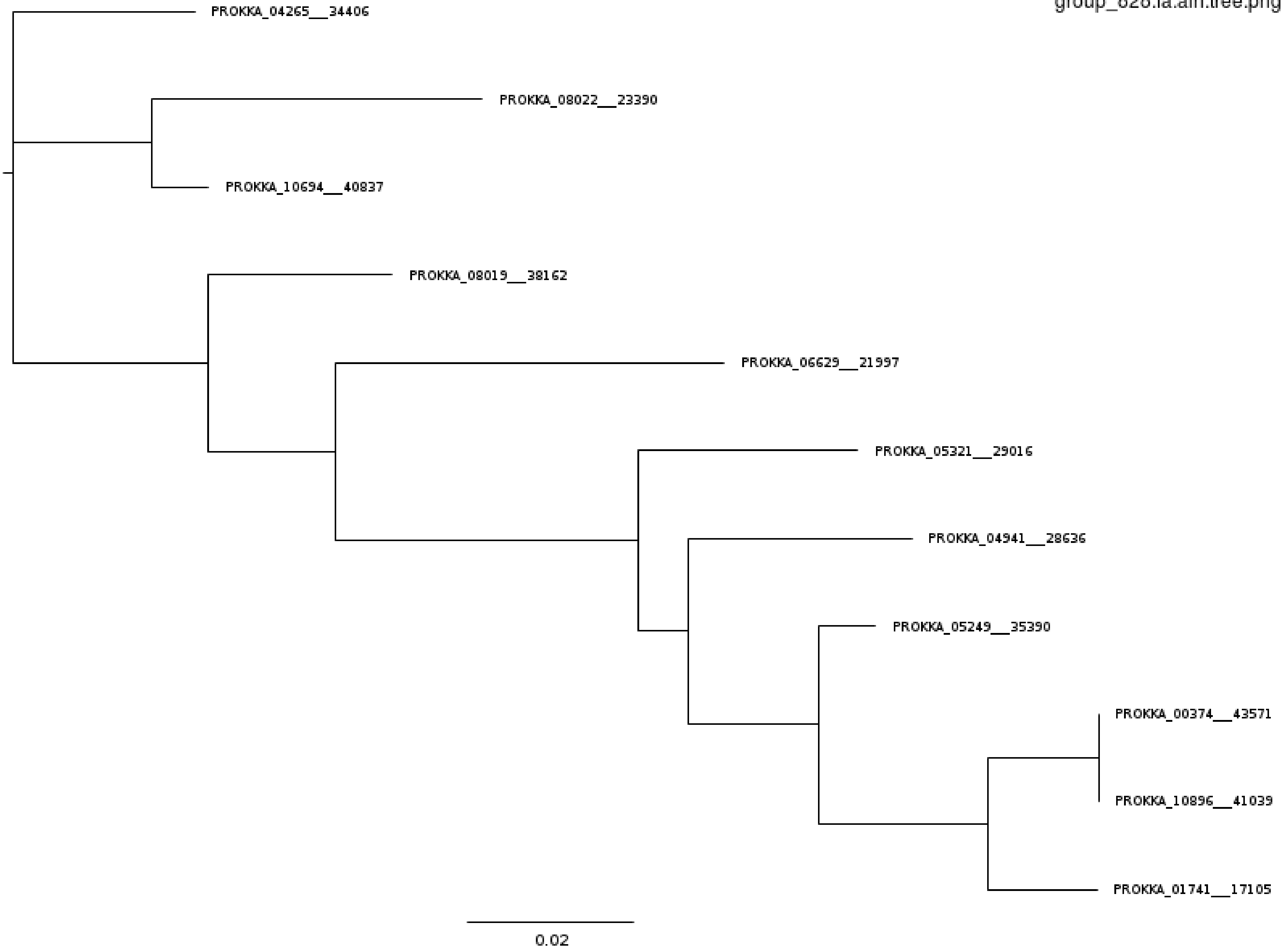

group_82.fa.aln.tree.png

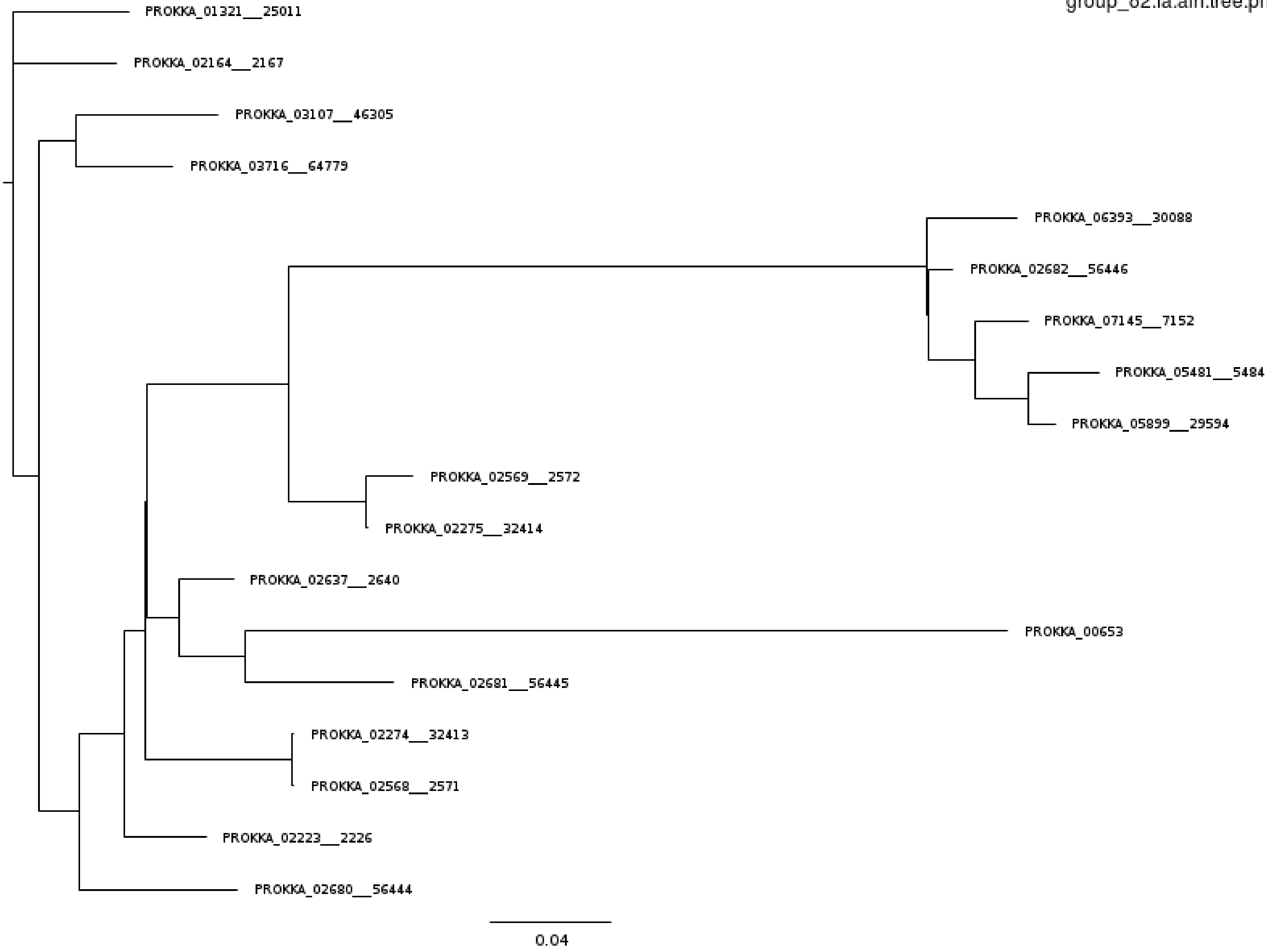

group_830.fa.aln.tree.png

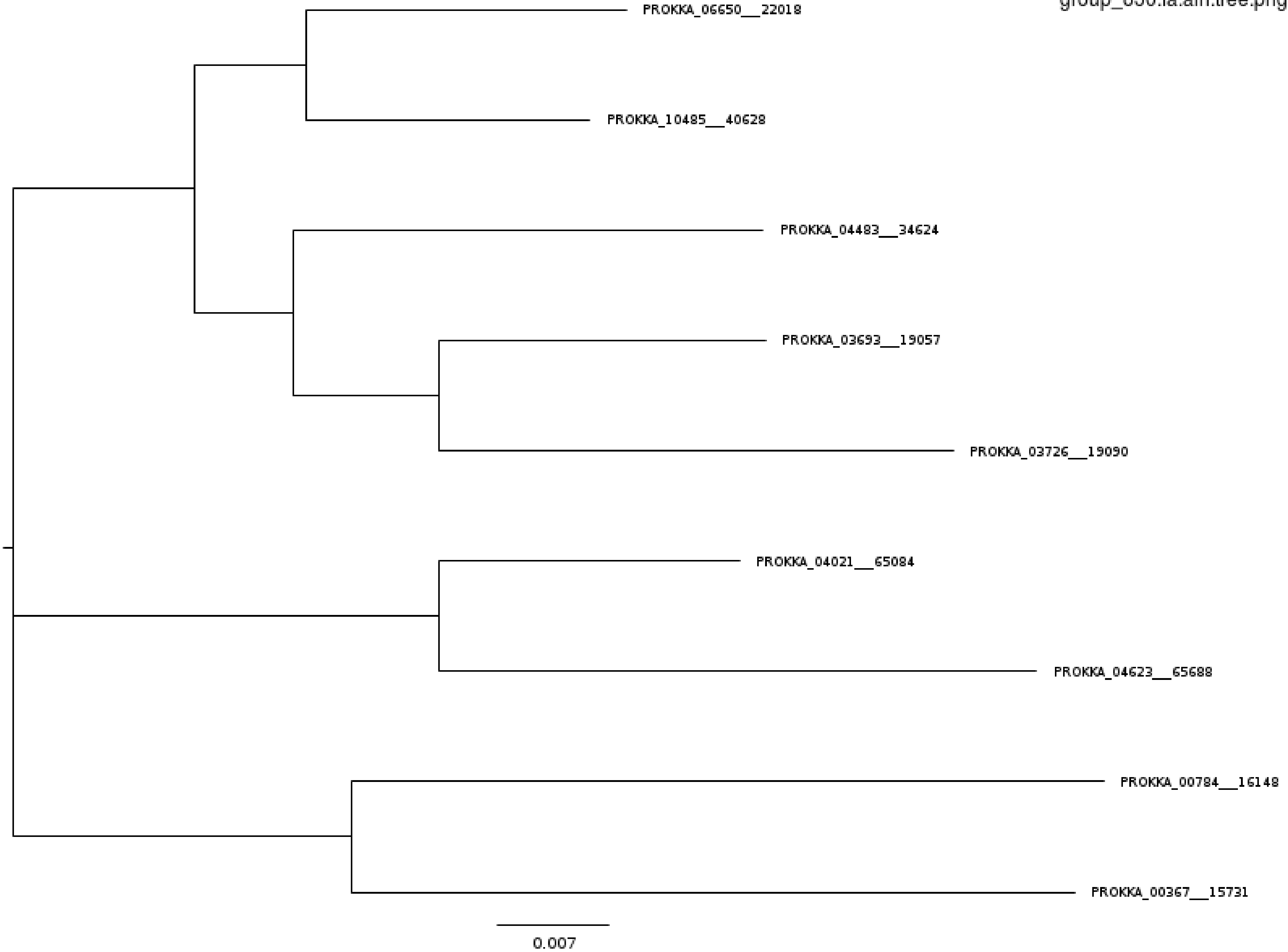

group_831.fa.aln.tree.png

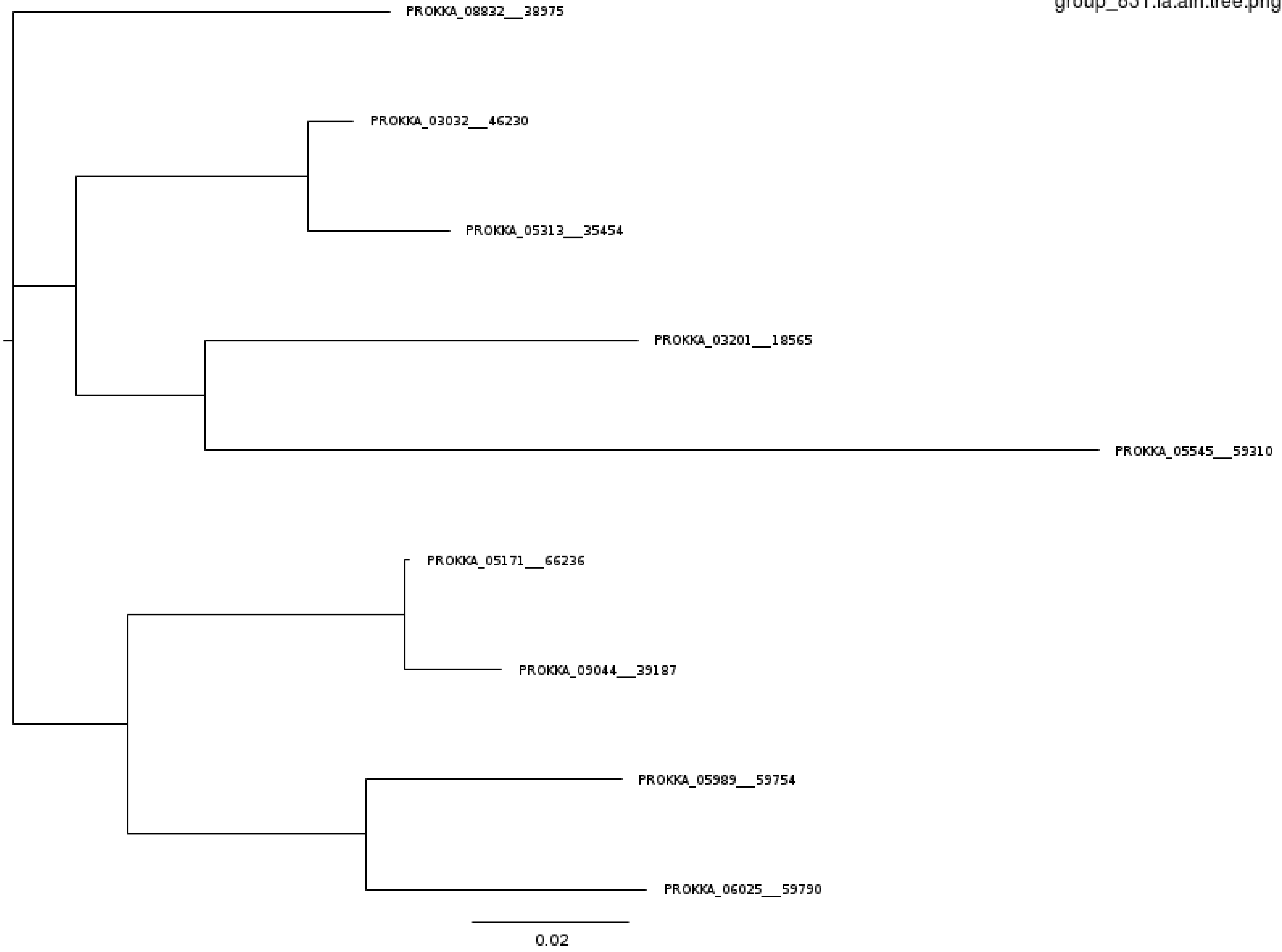

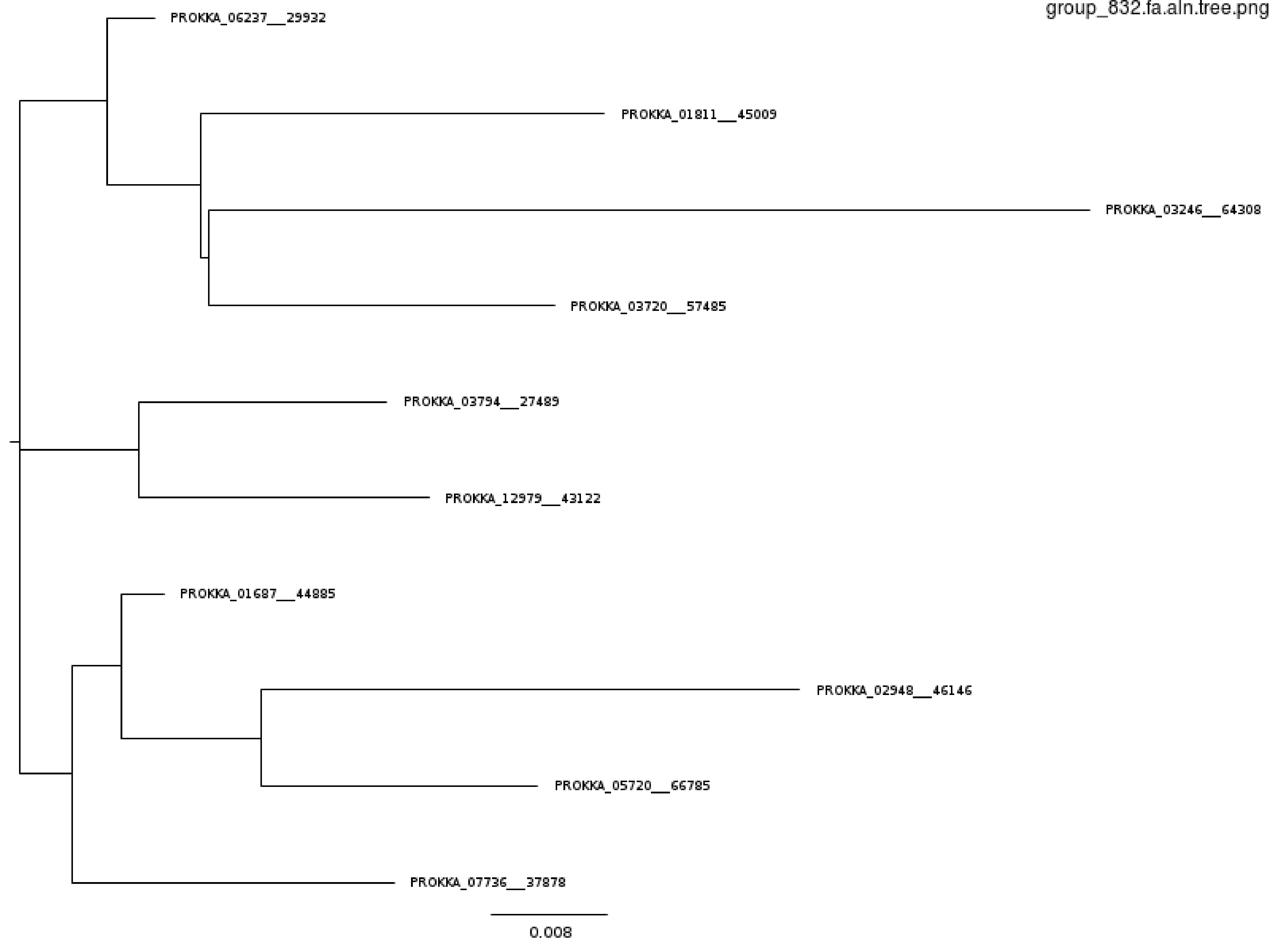
group_832.fa.aln.tree.png
PROKKA_06237___29932
PROKKA_01811___45009
PROKKA_03246___64308
PROKKA_03720___57485
PROKKA_03794___27489
PROKKA_12979___43122
PROKKA_01687___44885
PROKKA_02948___46146
PROKKA_05720___66785
PROKKA_07736___37878
0.008

group_834.fa.aln.tree.png

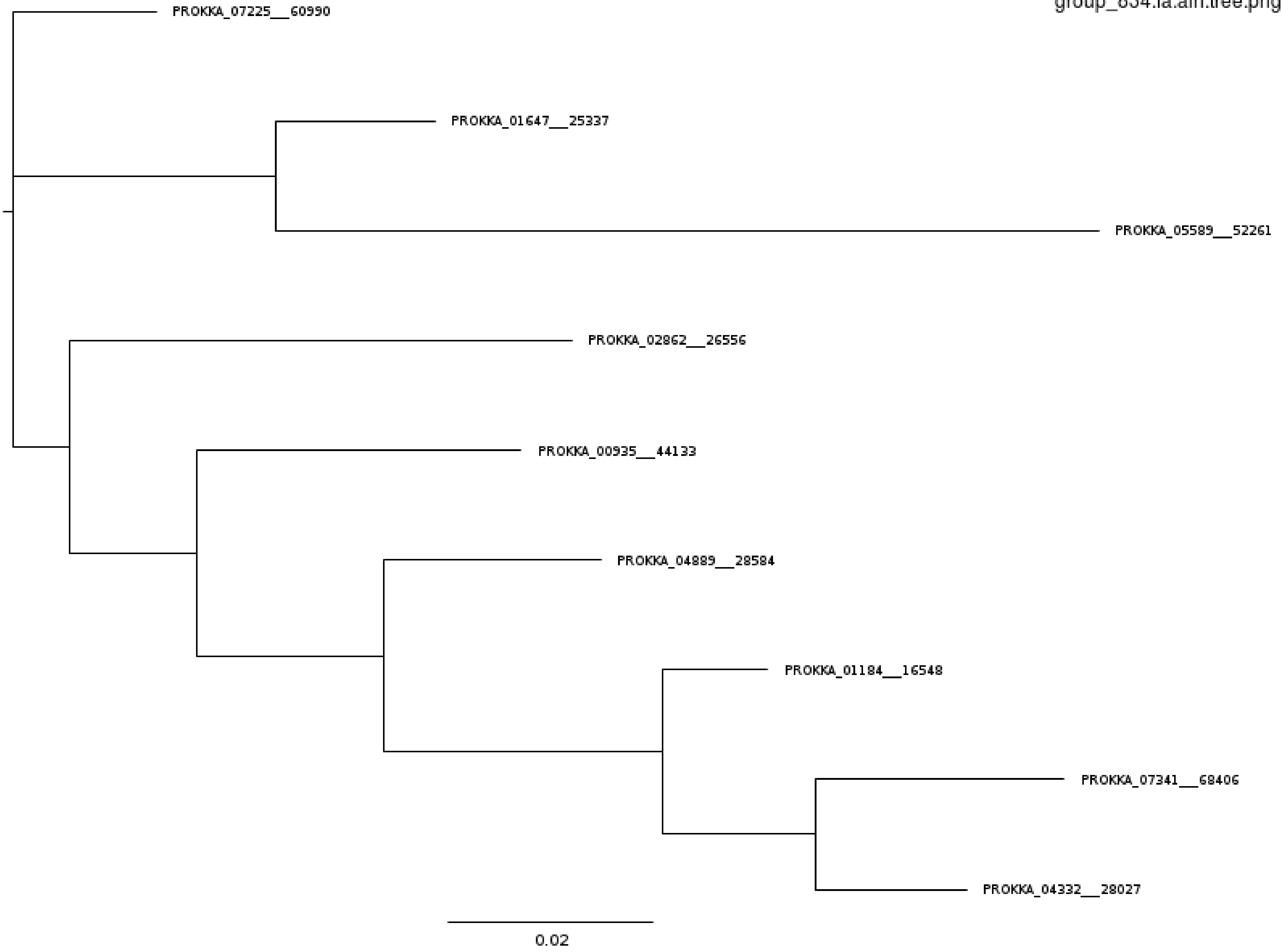

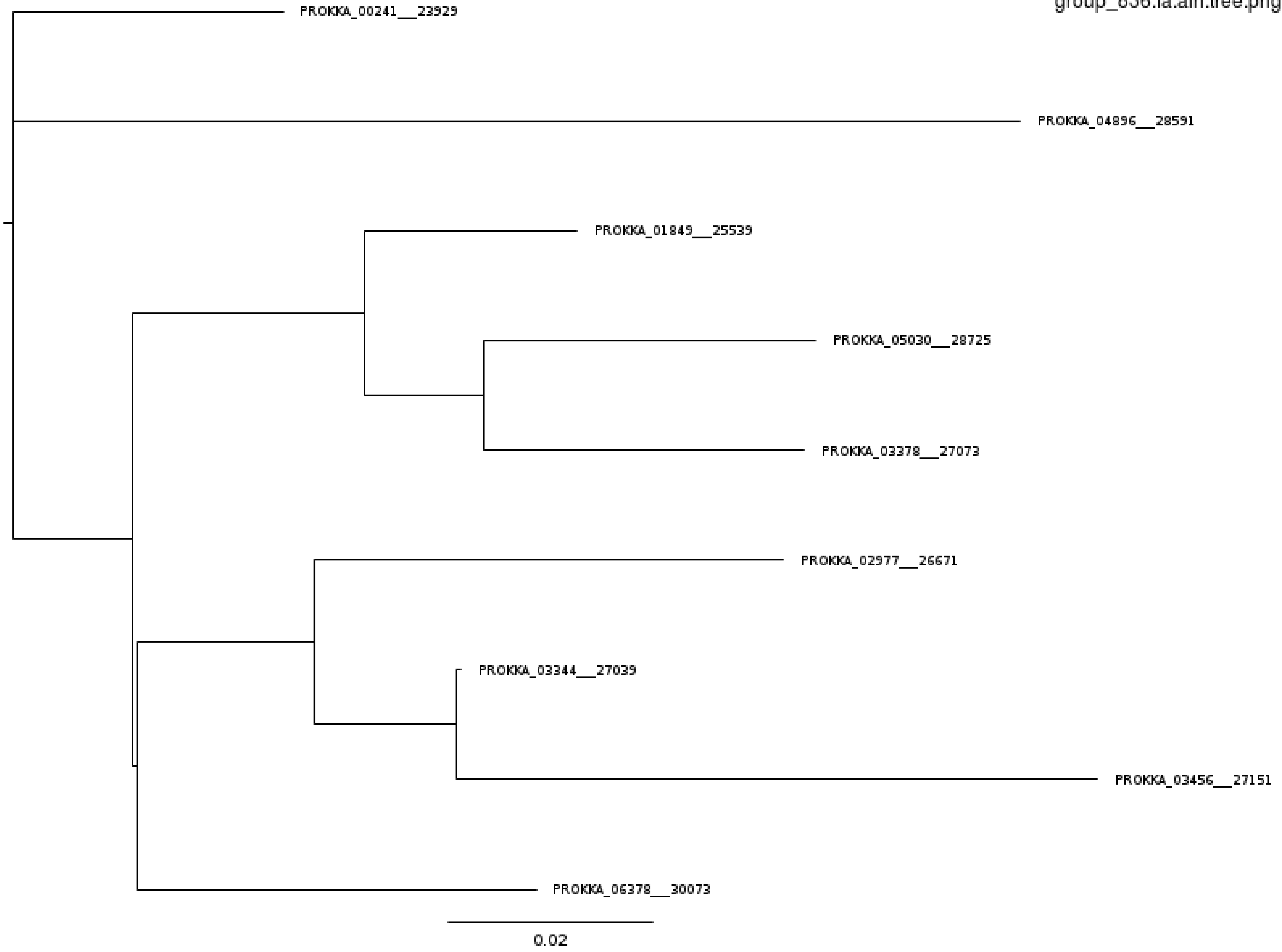
PROKKA_00241___23929
PROKKA_04896___28591
PROKKA_01849___25539
PROKKA_05030___28725
PROKKA_03378___27073
PROKKA_02977___26671
PROKKA_03344___27039
PROKKA_03456___27151
PROKKA_06378___30073
0.02

group_842.fa.aln.tree.png

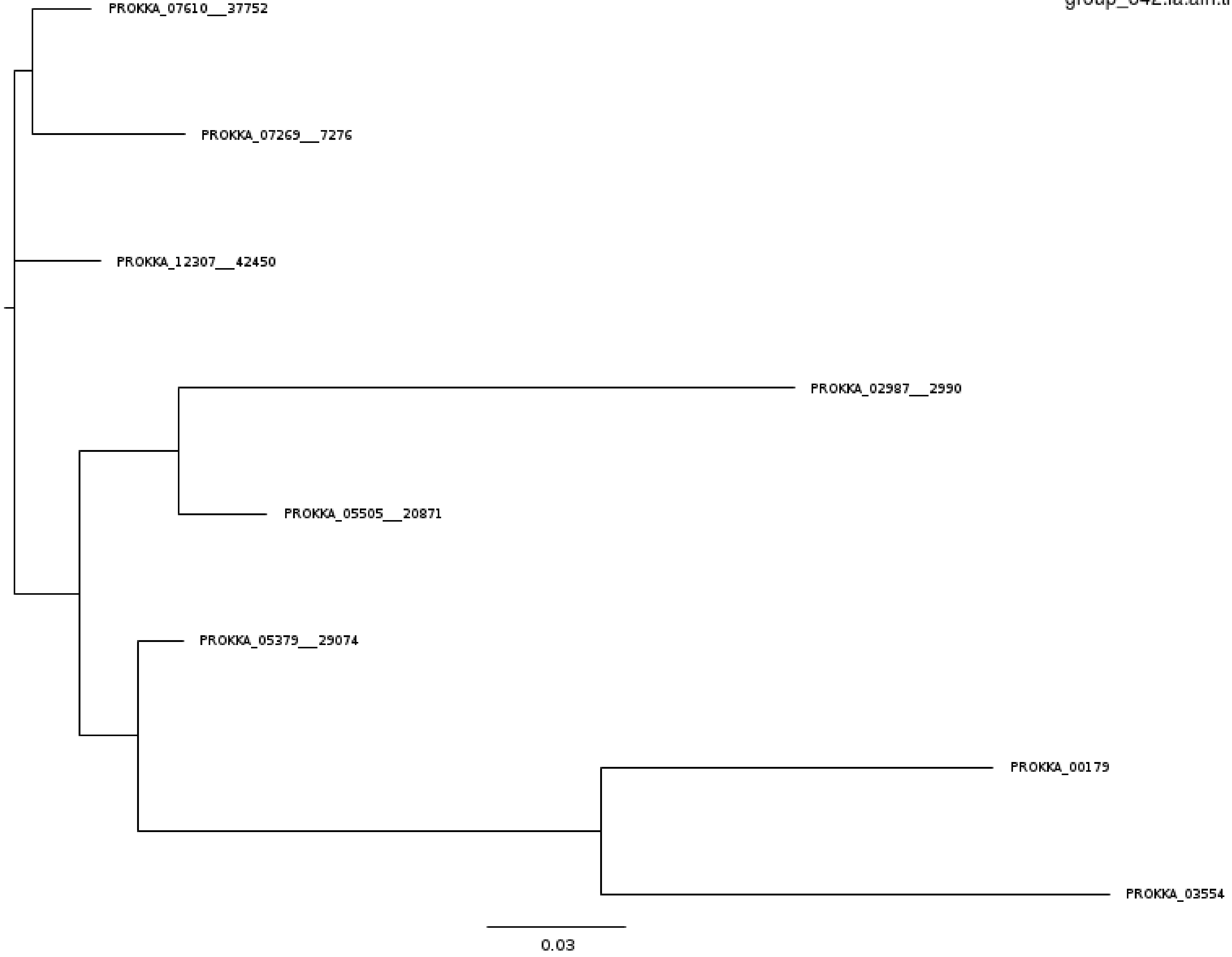

group_845.fa.aln.tree.png

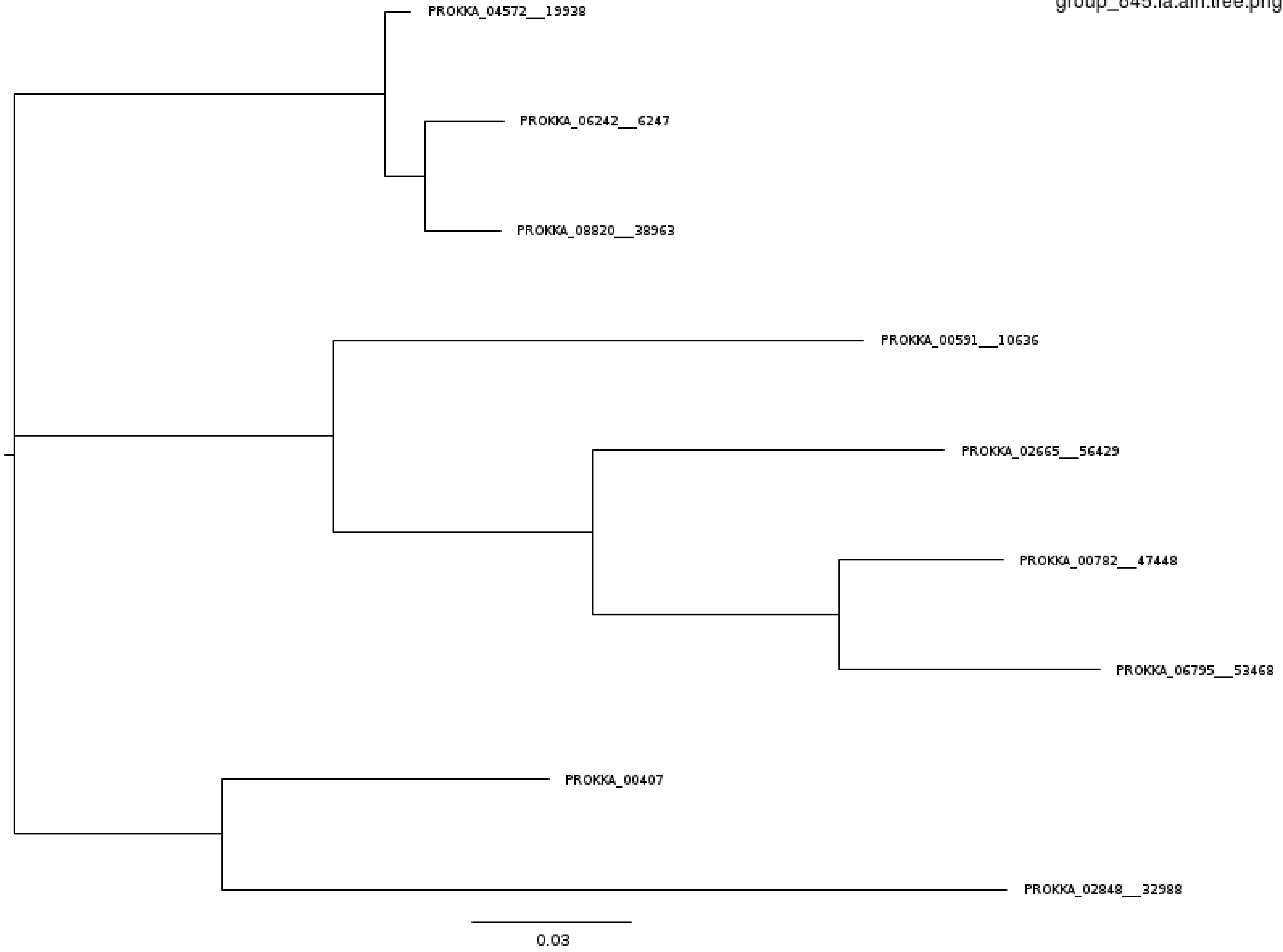

group_853.fa.aln.tree.png

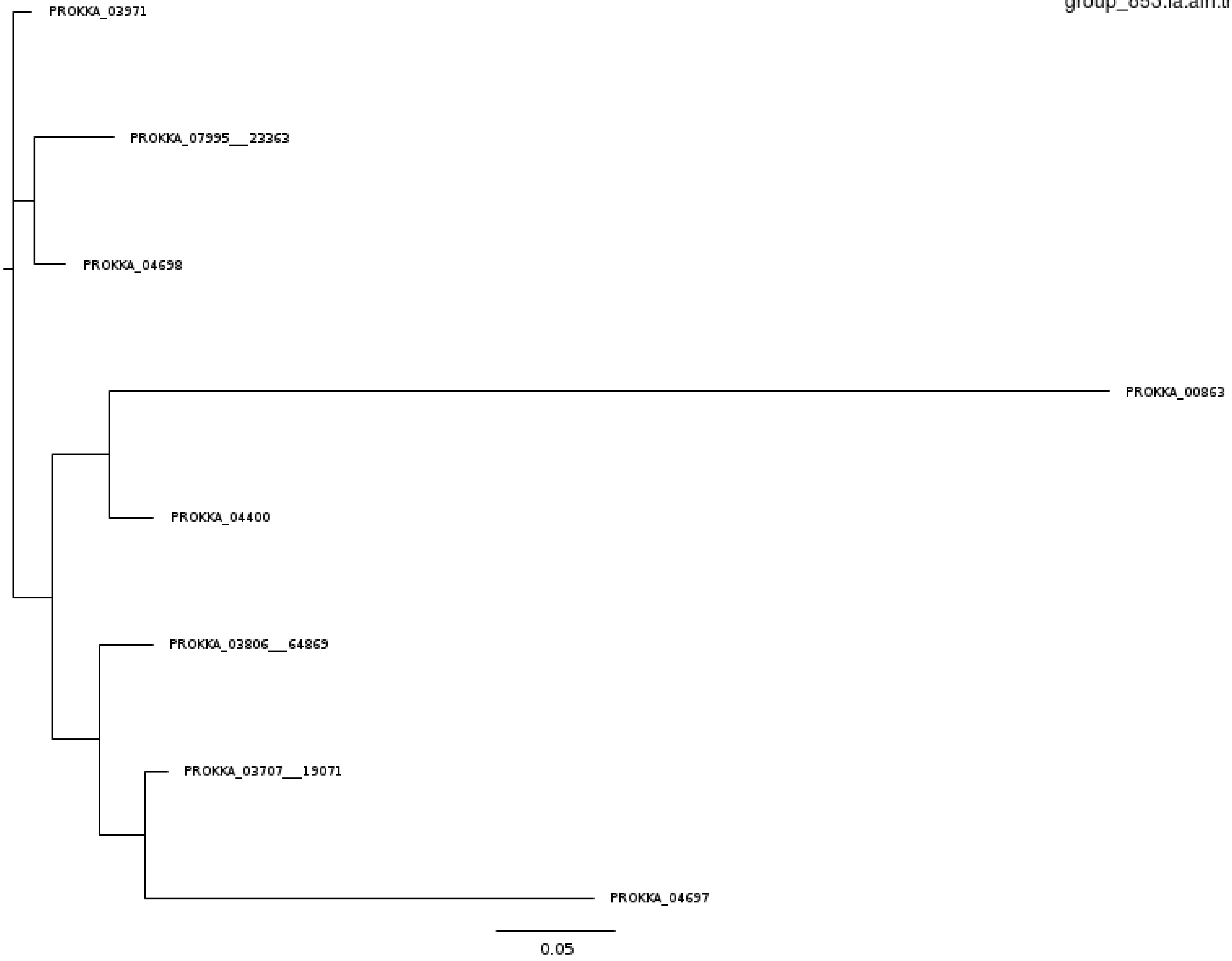

group_857.fa.aln.tree.png

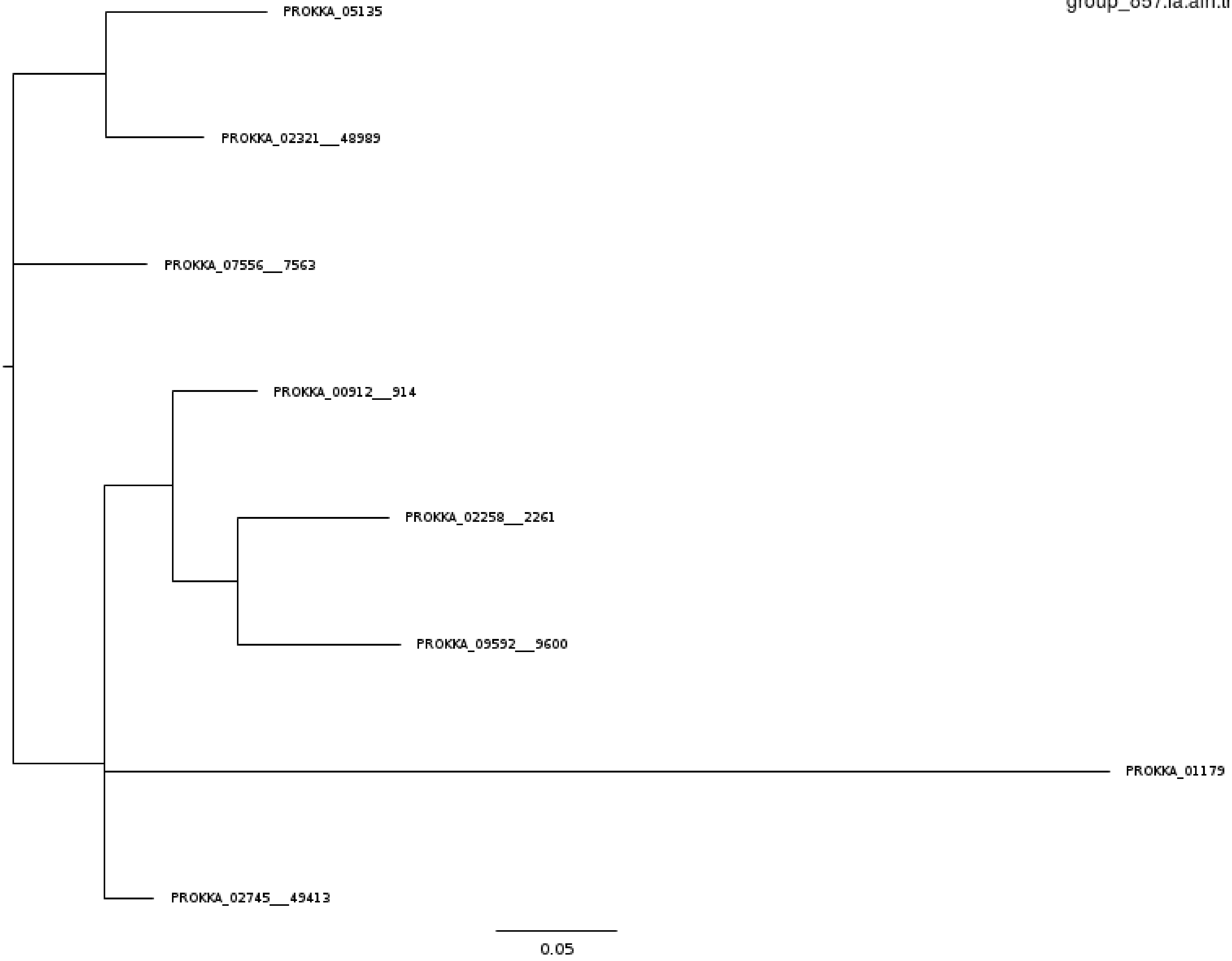

group_860.fa.aln.tree.png

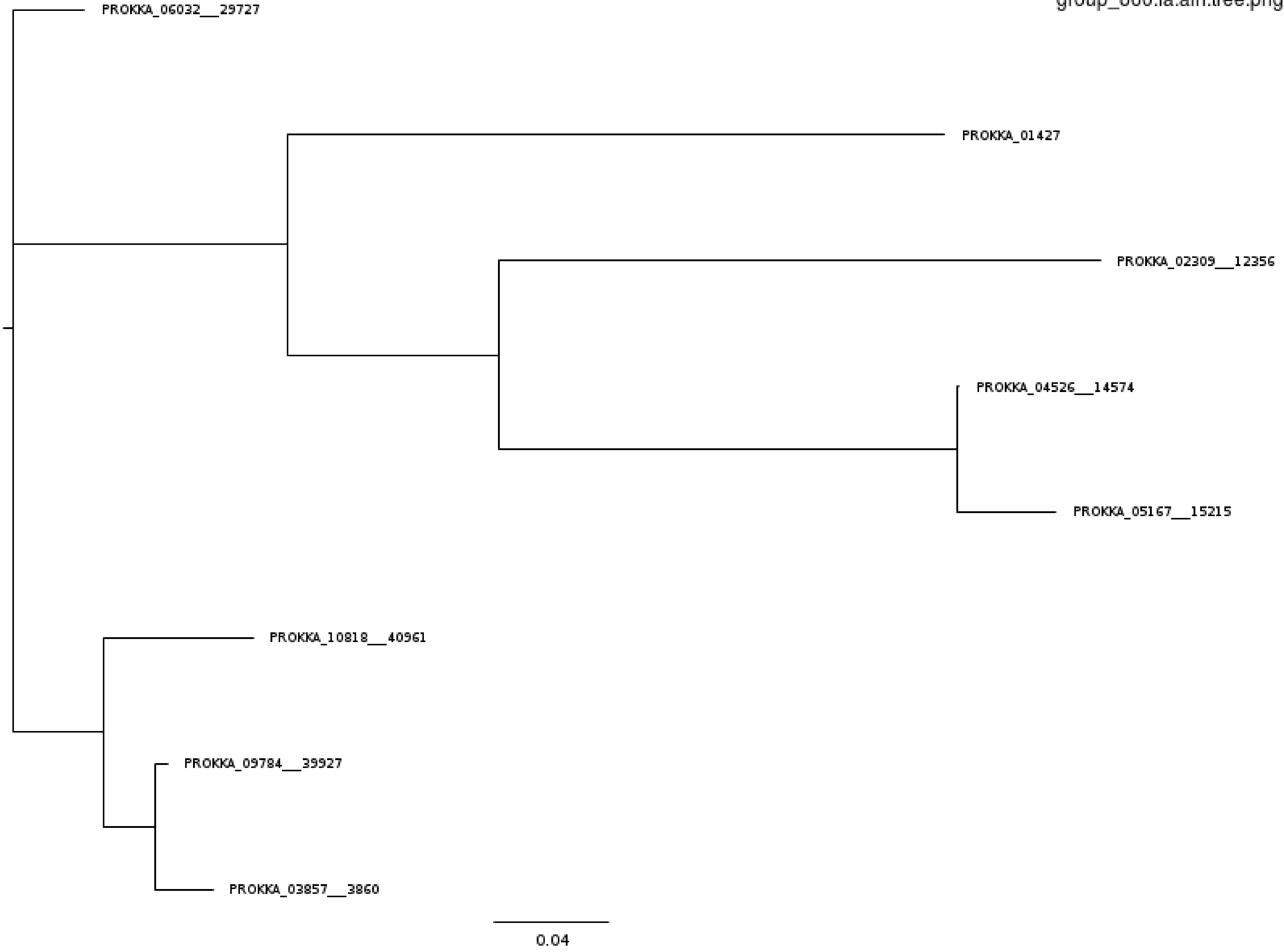

group_870.fa.aln.tree.png

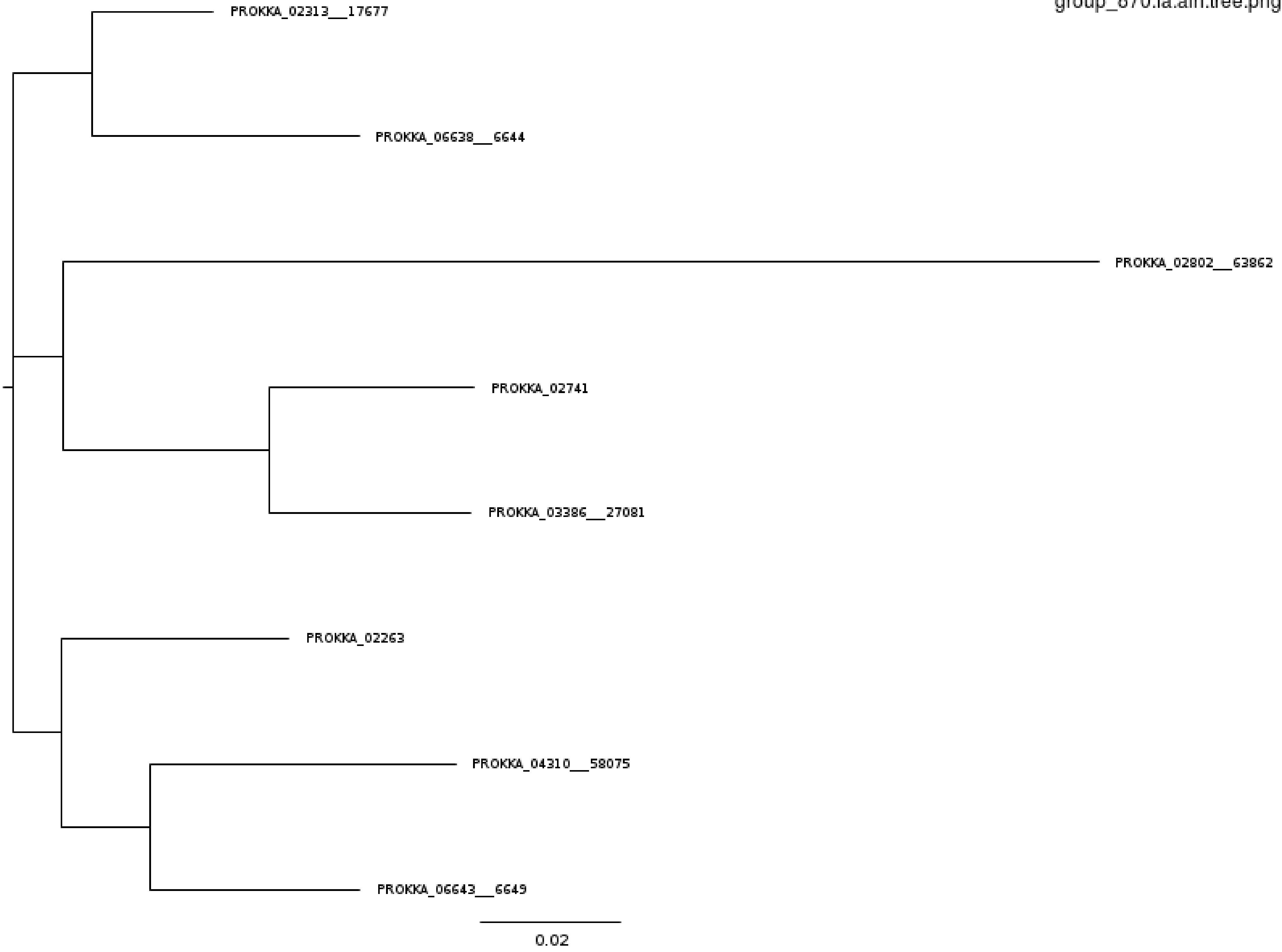

group_871.fa.aln.tree.png

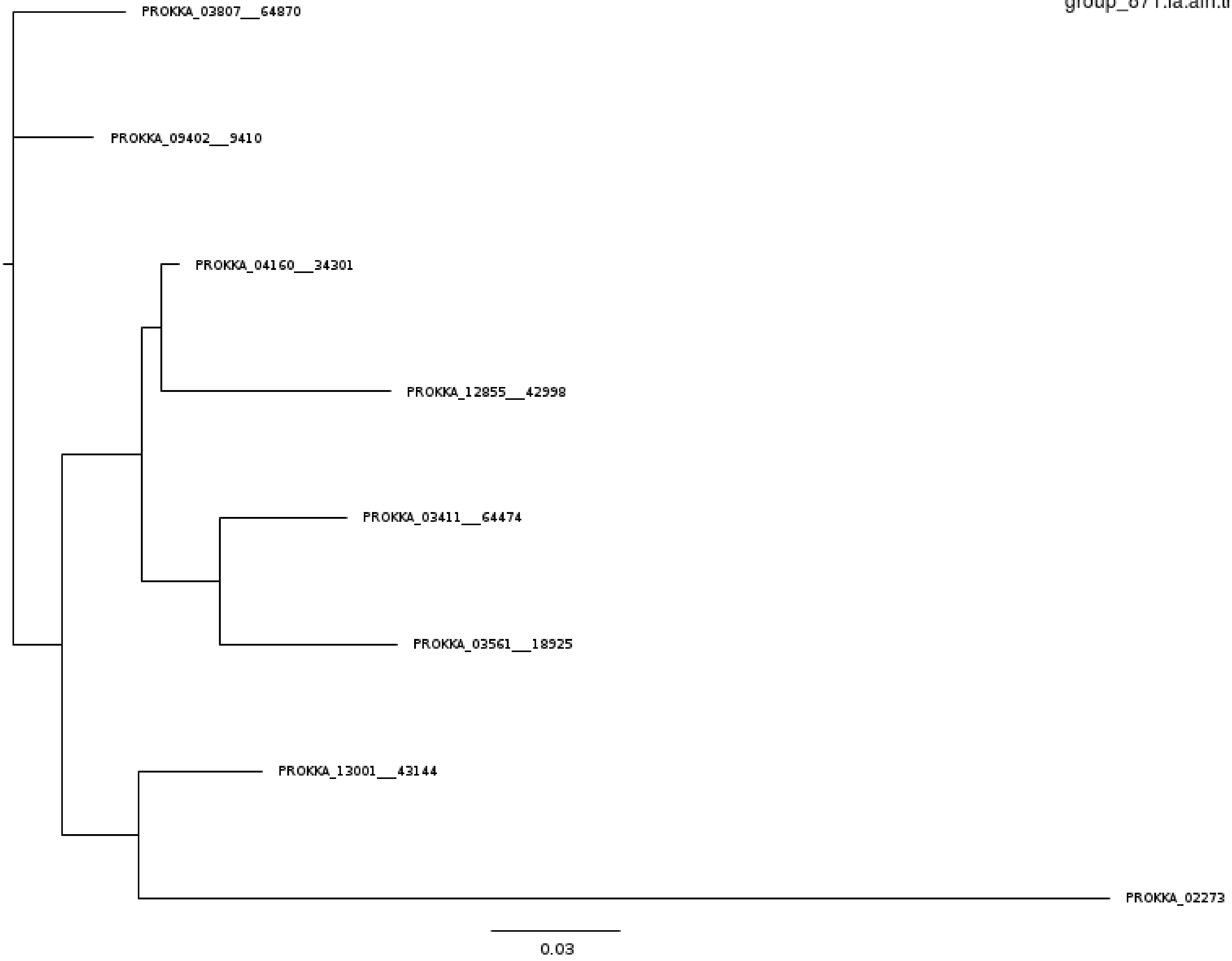

group_872.fa.aln.tree.png

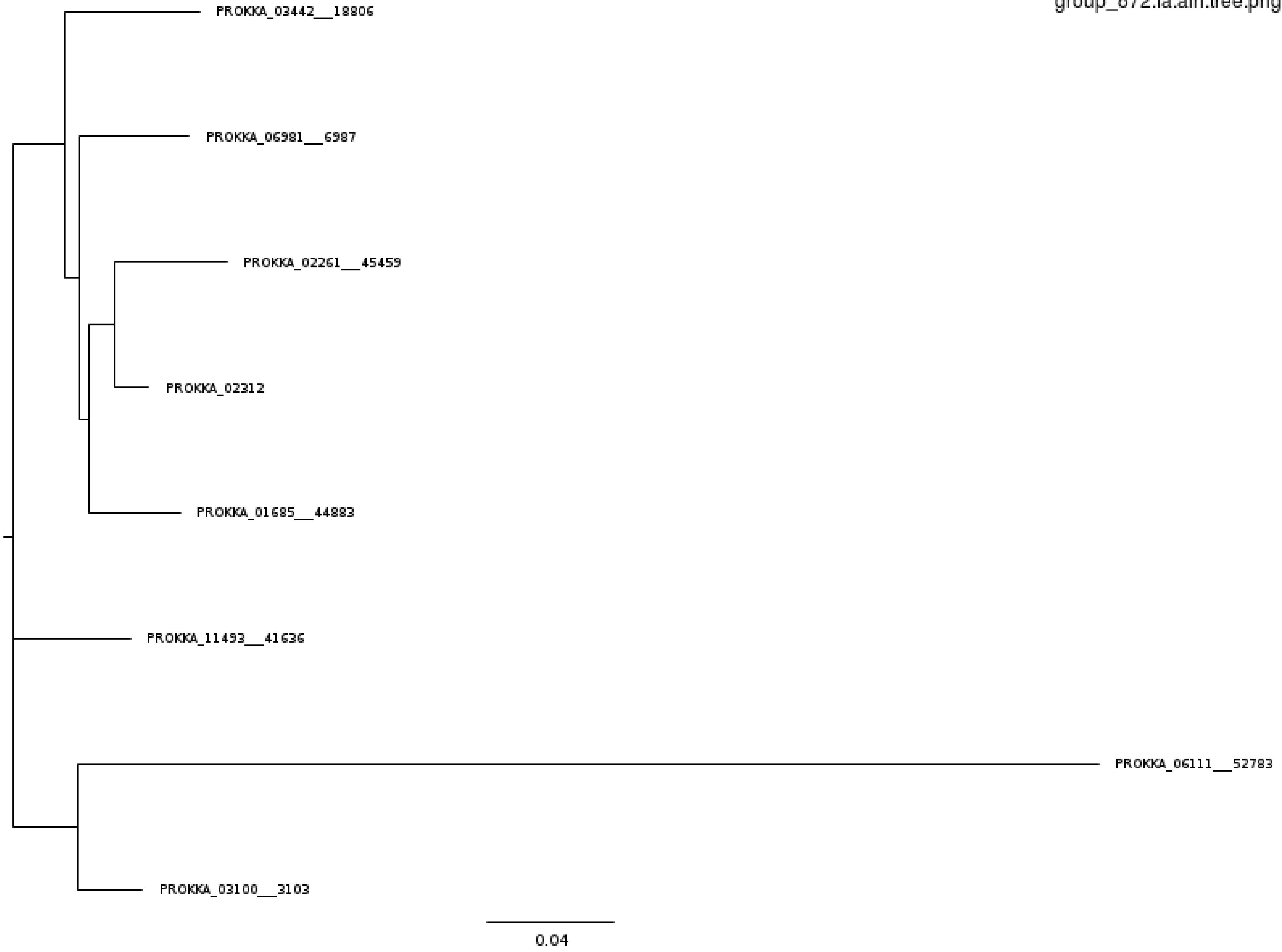

group_873.fa.aln.tree.png

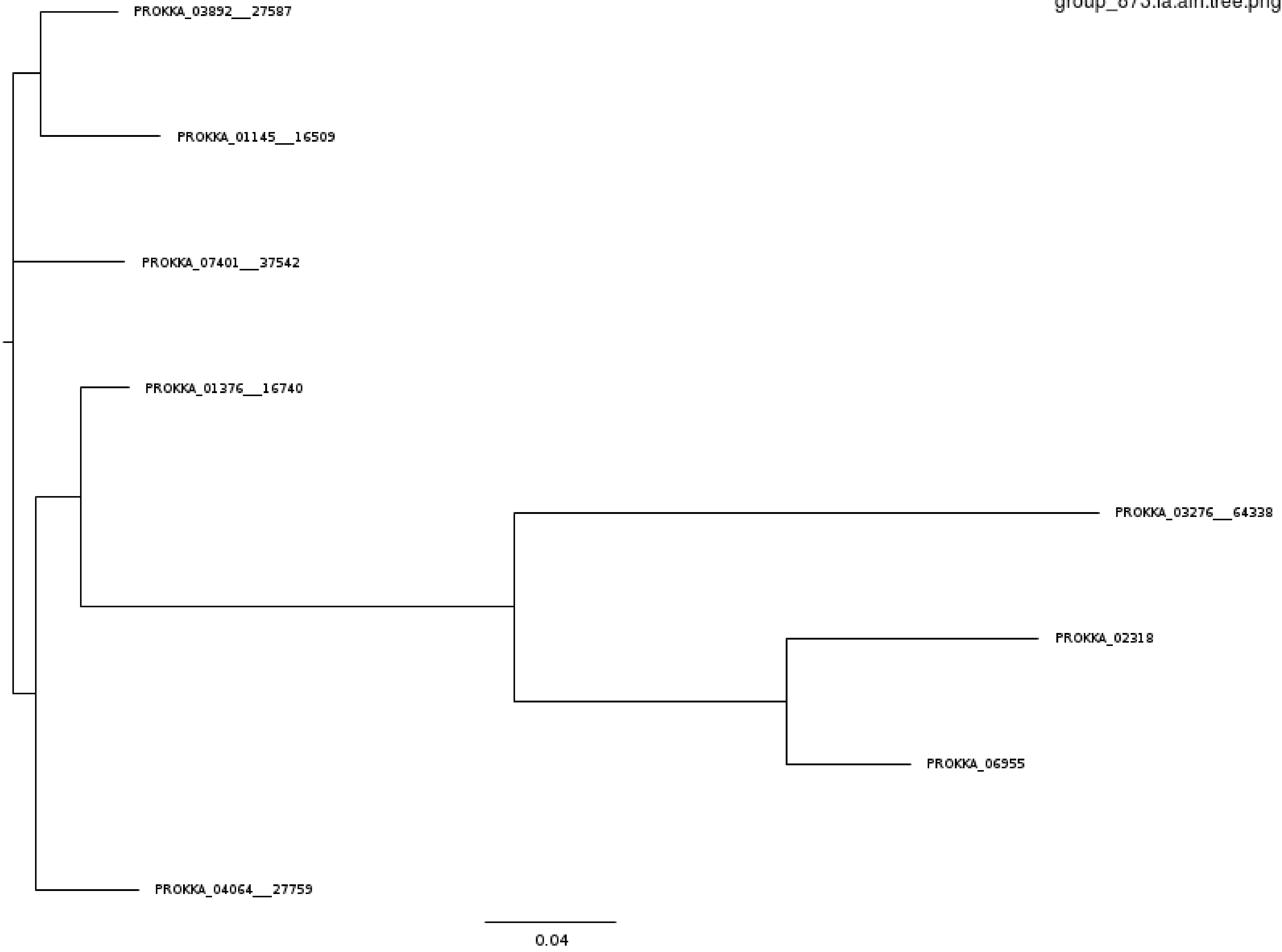

group_874.fa.aln.tree.png

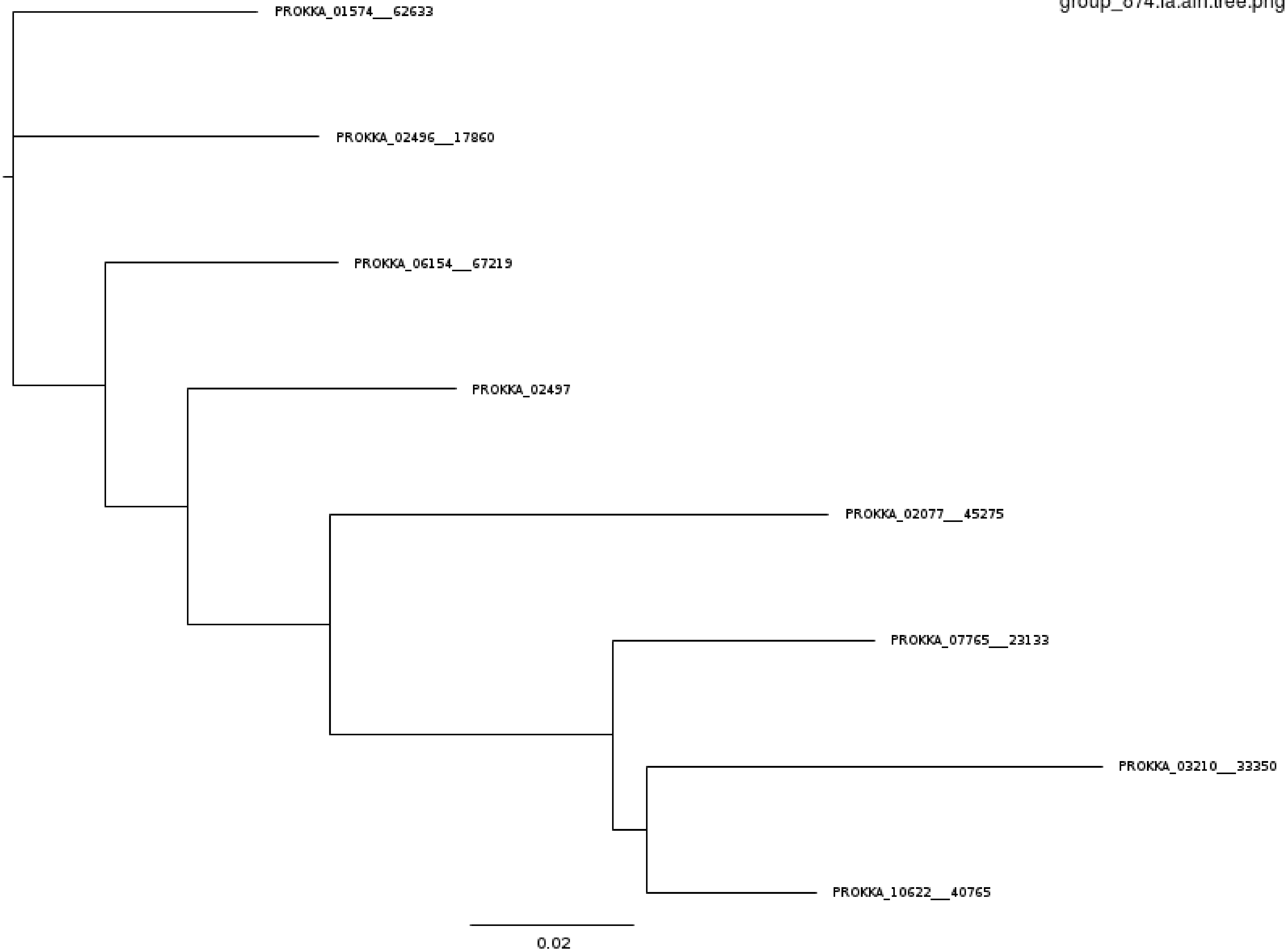

group_875.fa.aln.tree.png

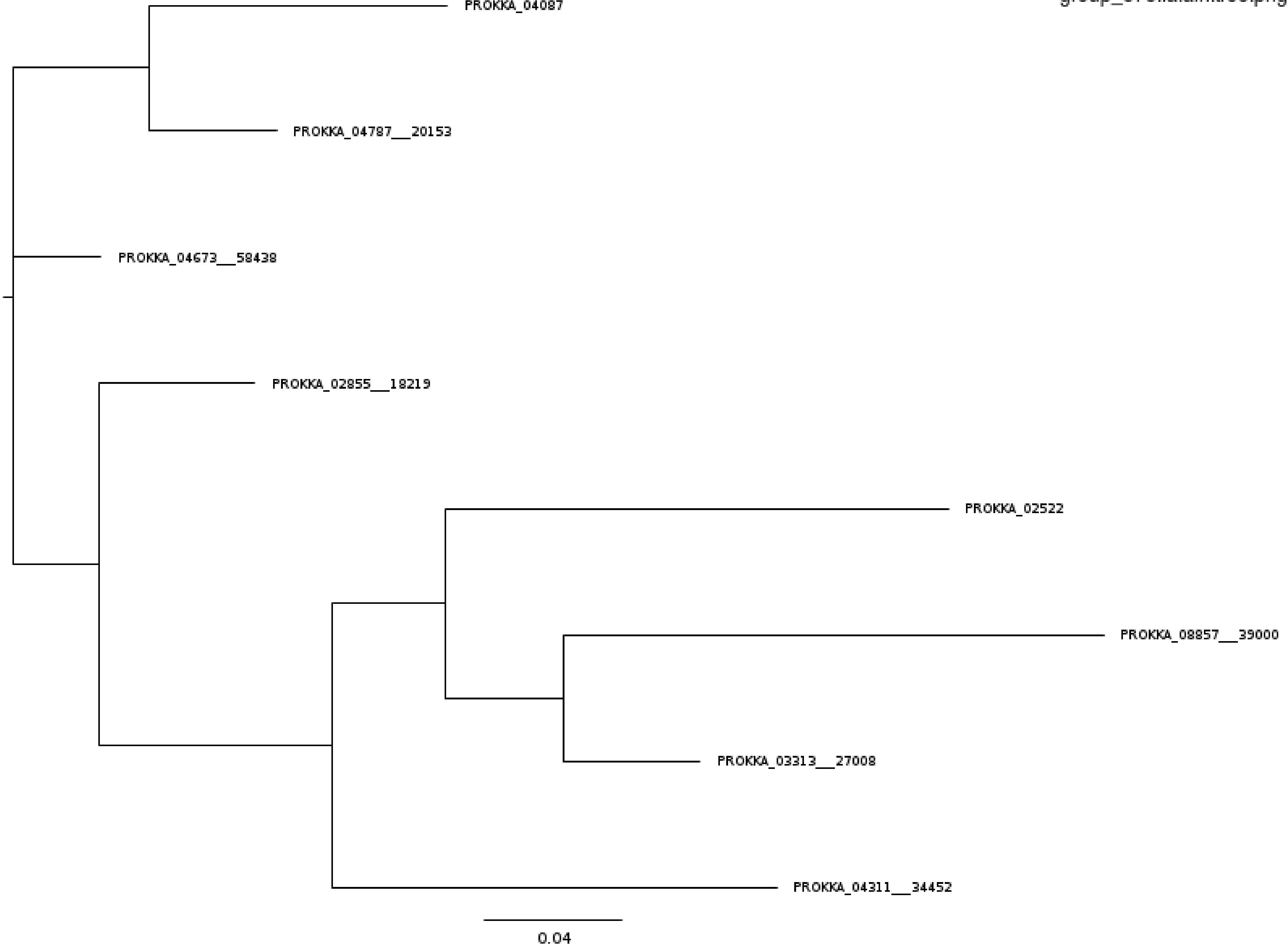

group_876.fa.aln.tree.png

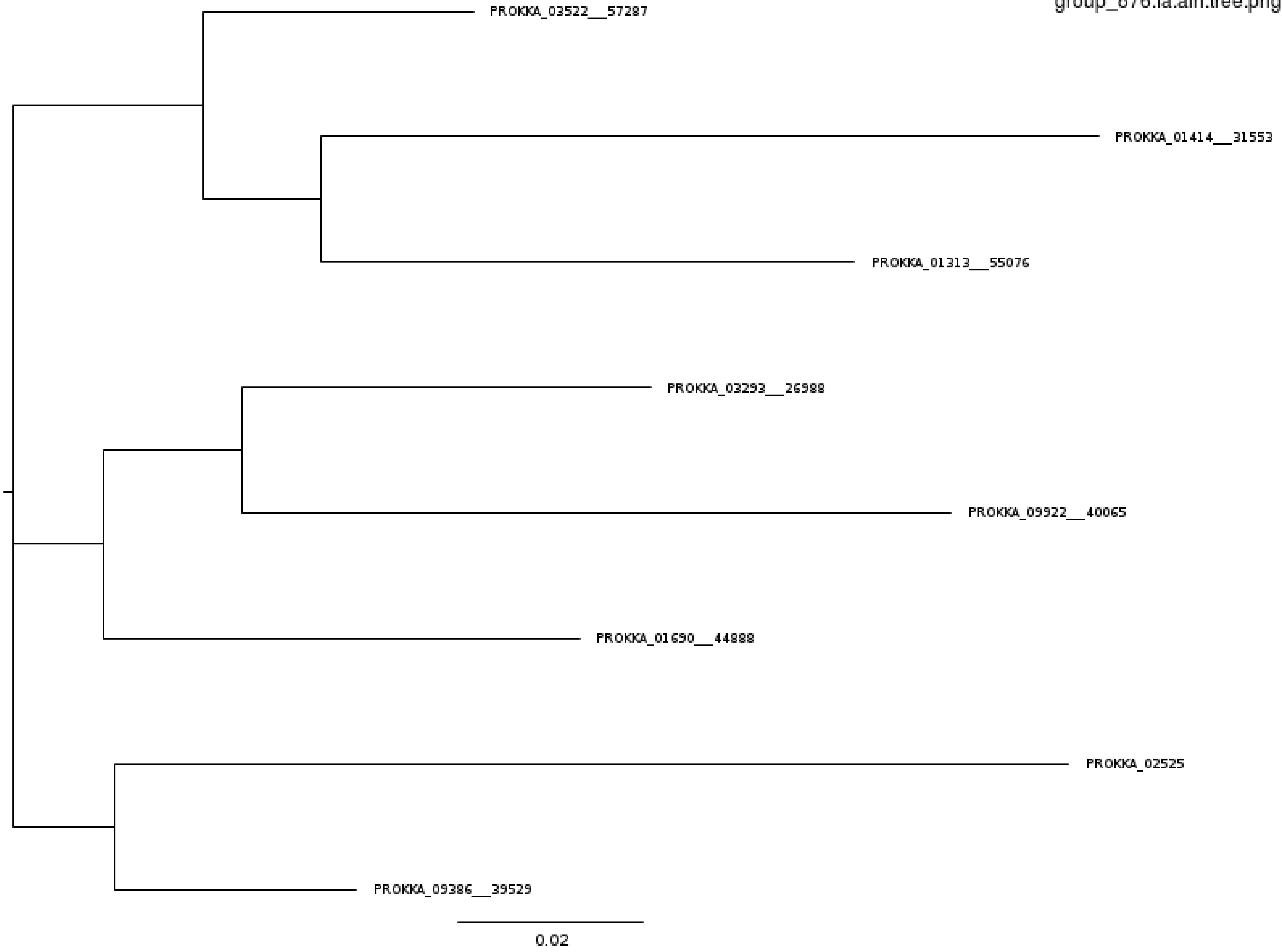

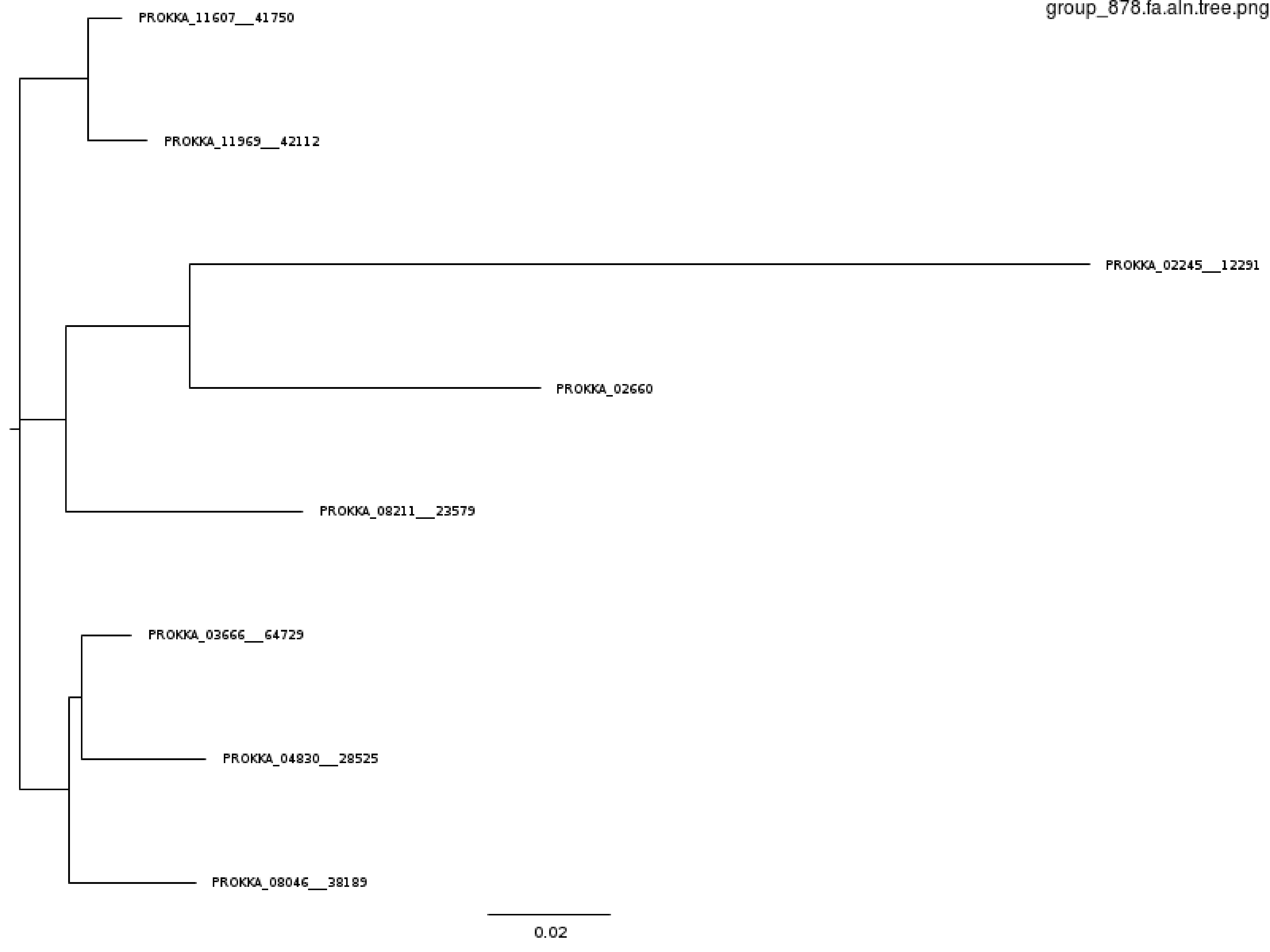
group_878.fa.aln.tree.png
PROKKA_11607___41750
PROKKA_11969___42112
PROKKA_02245___12291
PROKKA_02660
PROKKA_08211___23579
PROKKA_03666___64729
PROKKA_04830___28525
PROKKA_08046___38189
0.02

group_87.fa.aln.tree.png

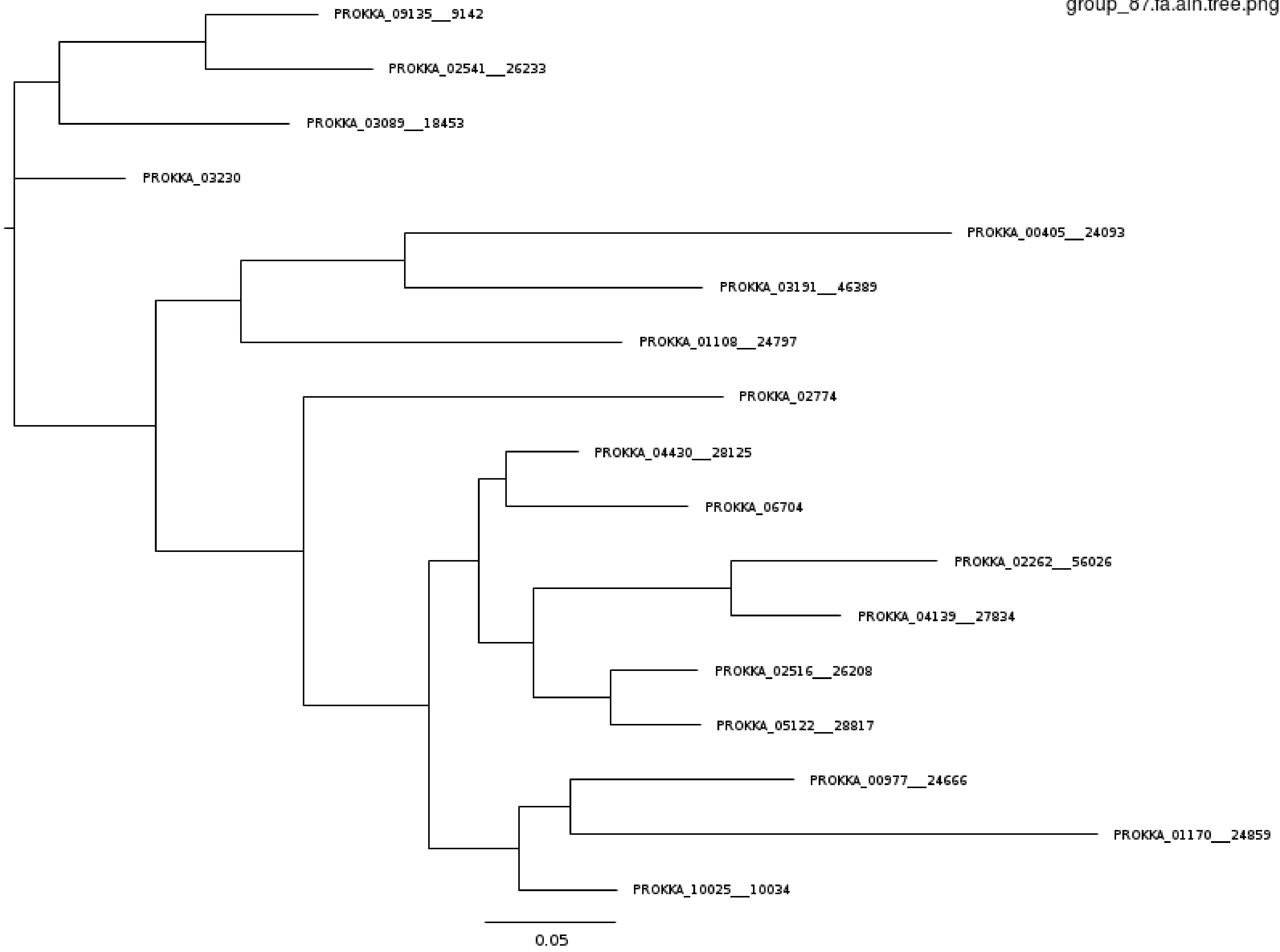

group_881.fa.aln.tree.png

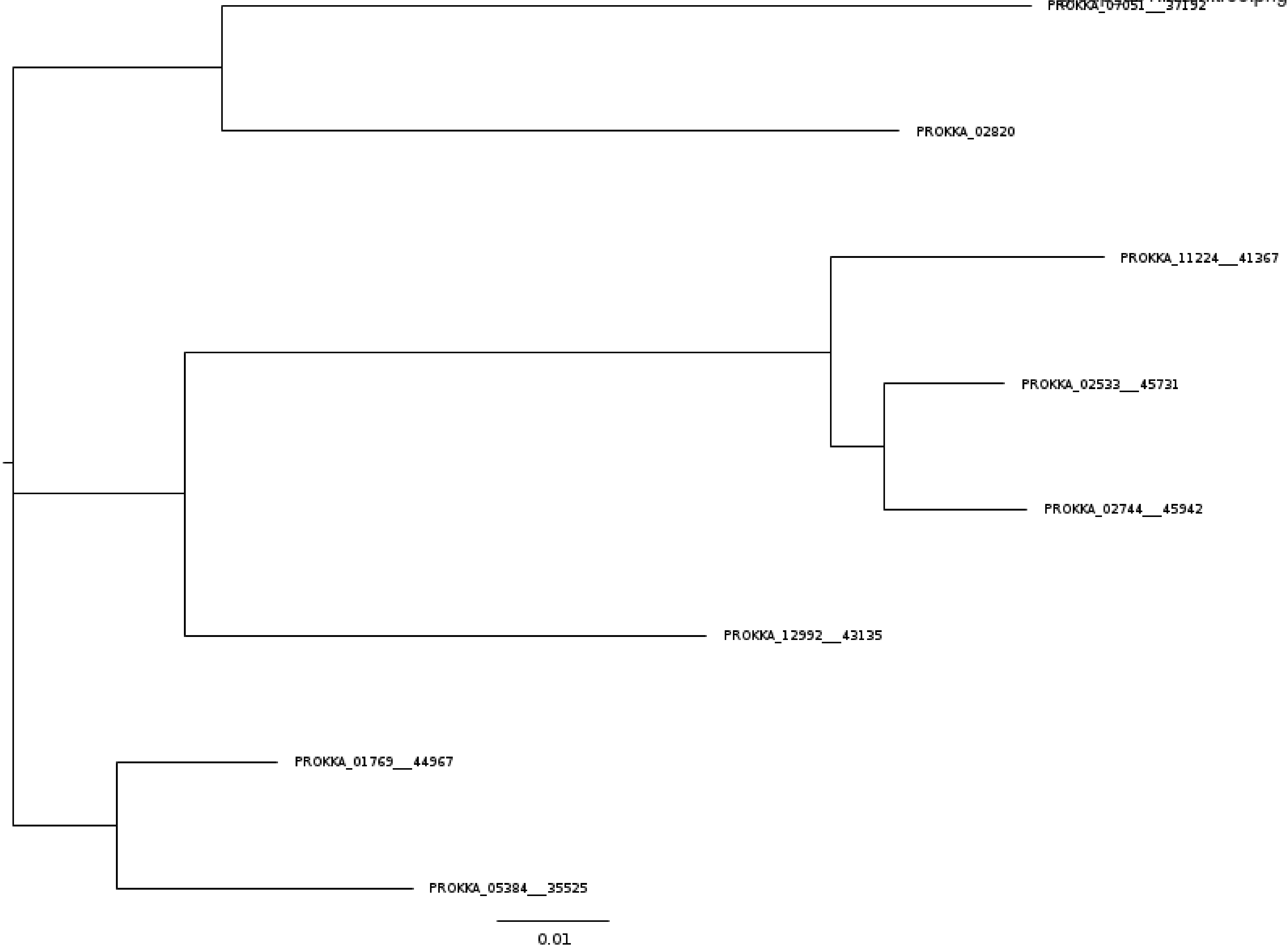

group_882.fa.aln.tree.png

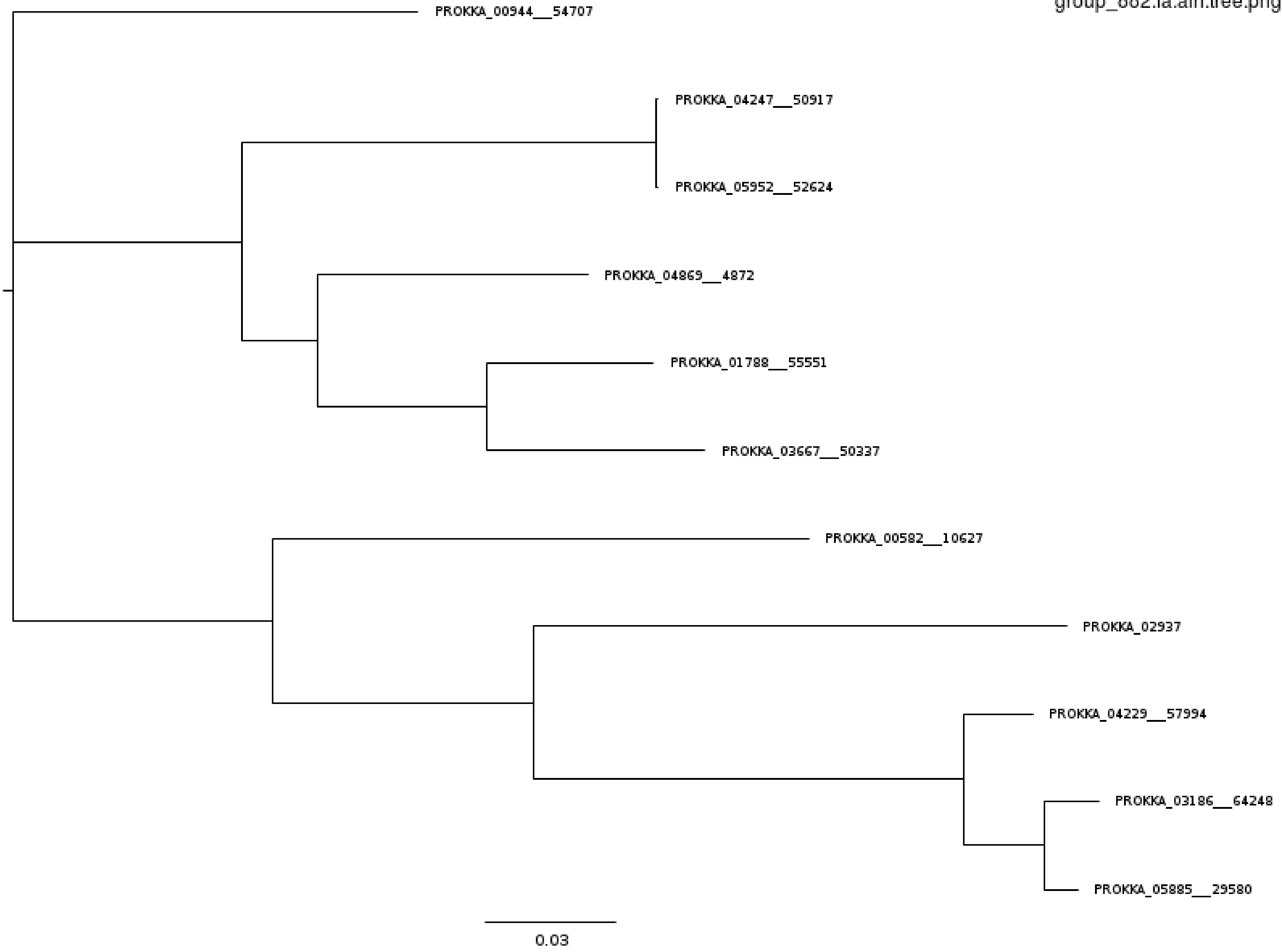

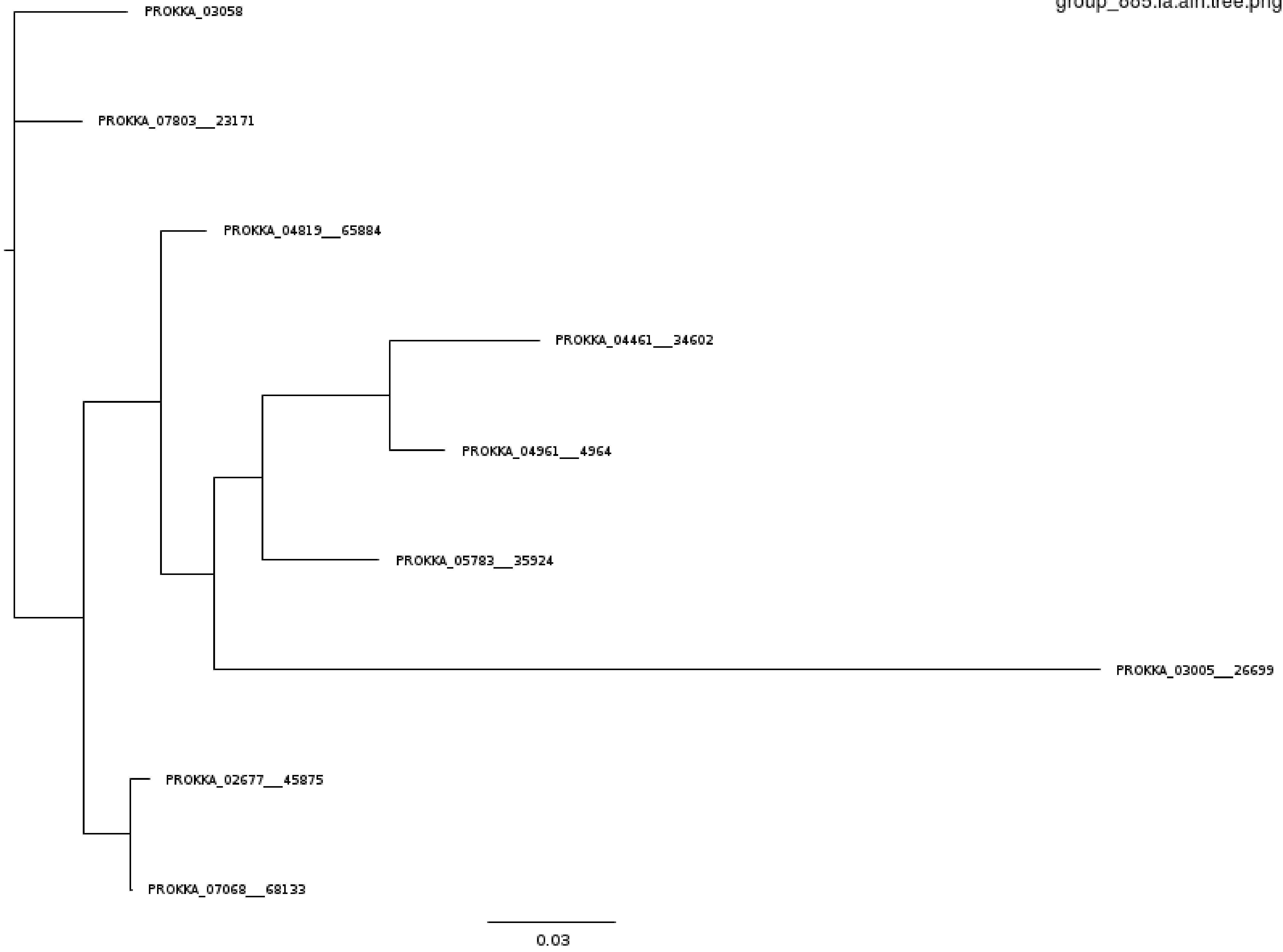
PROKKA_03058
PROKKA_07803___23171
PROKKA_04819___65884
PROKKA_04461___34602
PROKKA_04961___4964
PROKKA_05783___35924
PROKKA_03005___26699
PROKKA_02677___45875
PROKKA_07068___68133
0.03

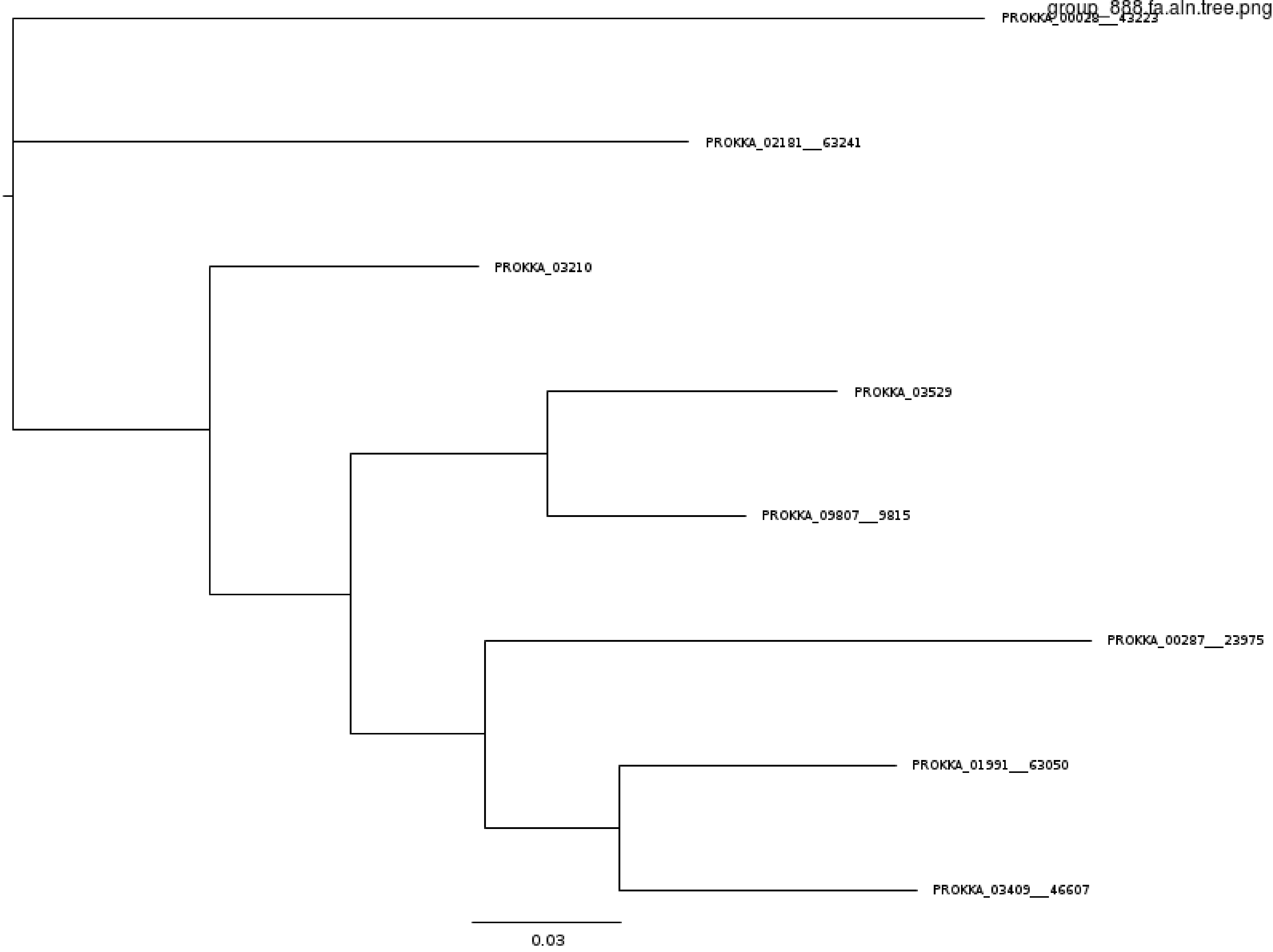
group_888.fa.aln.tree.png
PROKKA_00028___43223
PROKKA_02181___63241
PROKKA_03210
PROKKA_03529
PROKKA_09807___9815
PROKKA_00287___23975
PROKKA_01991___63050
PROKKA_03409___46607
0.03

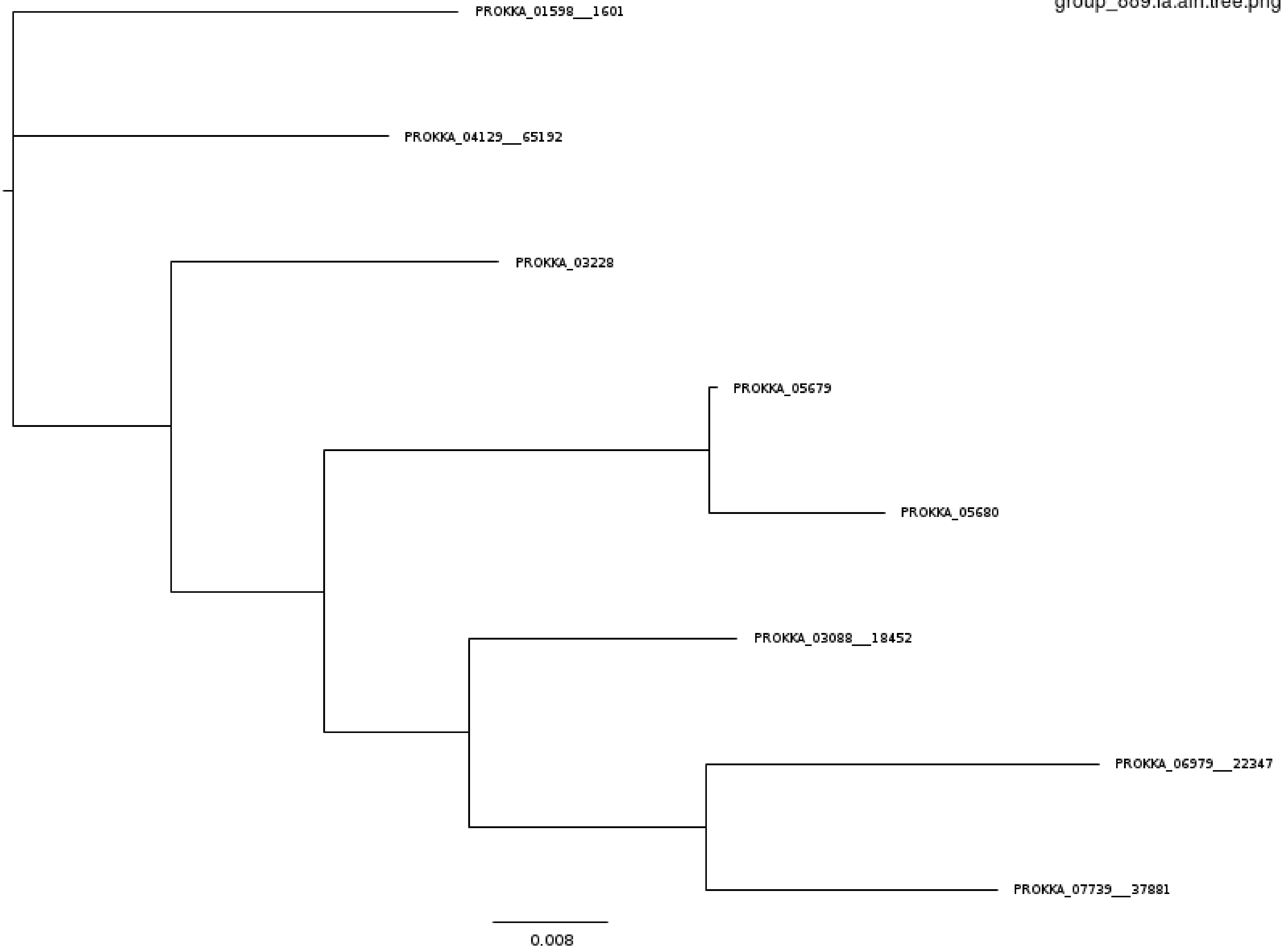
PROKKA_01598___1601
PROKKA_04129___65192
PROKKA_03228
PROKKA_05679
PROKKA_05680
PROKKA_03088___18452
PROKKA_06979___22347
PROKKA_07739___37881
0.008

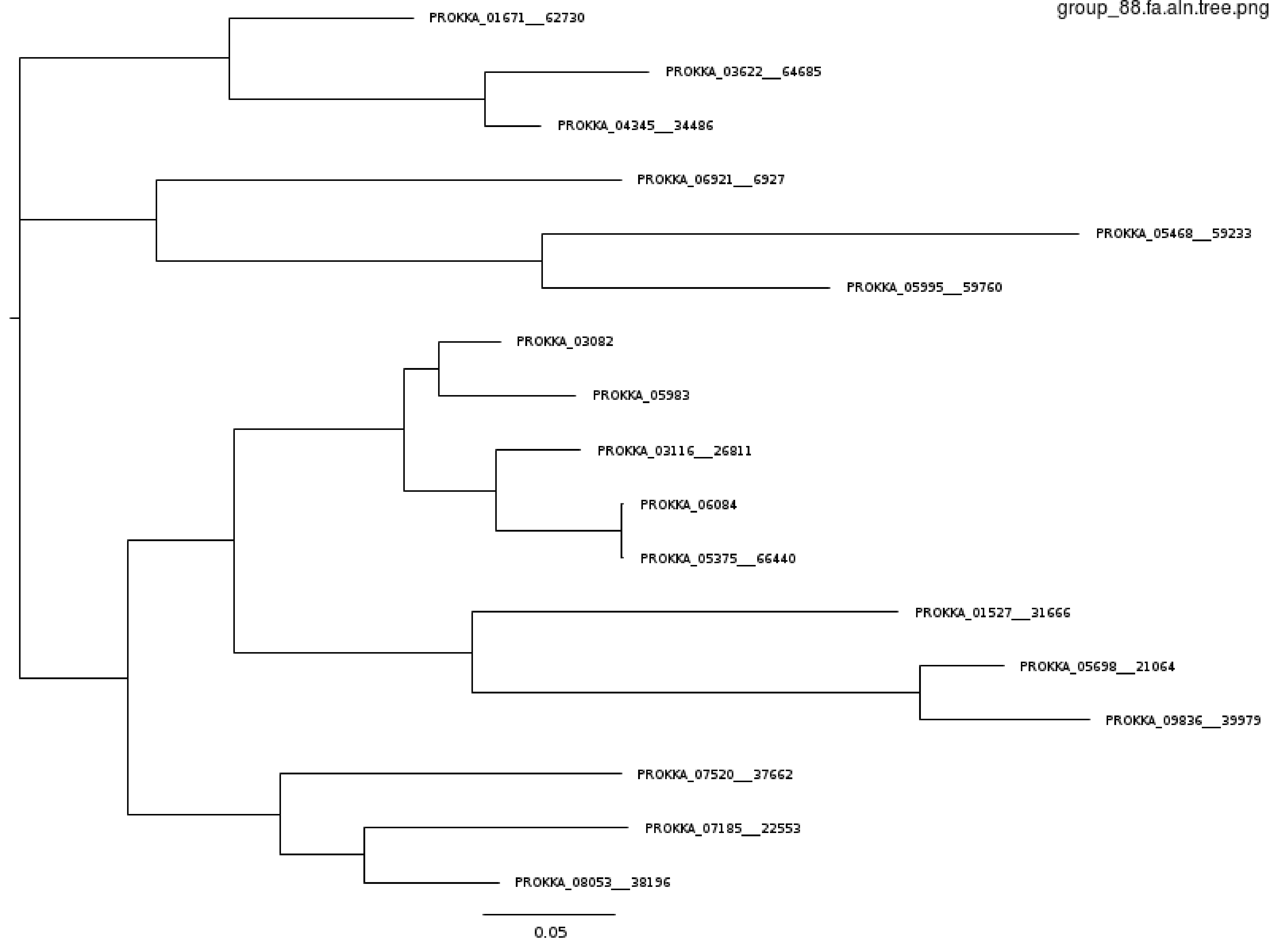
group_88.fa.aln.tree.png
PROKKA_01671___62730
PROKKA_03622___64685
PROKKA_04345___34486
PROKKA_06921___6927
PROKKA_05468___59233
PROKKA_05995___59760
PROKKA_03082
PROKKA_05983
PROKKA_03116___26811
PROKKA_06084
PROKKA_05375___66440
PROKKA_01527___31666
PROKKA_05698___21064
PROKKA_09836___39979
PROKKA_07520___37662
PROKKA_07185___22553
PROKKA_08053___38196
0.05

group_895.fa.aln.tree.png

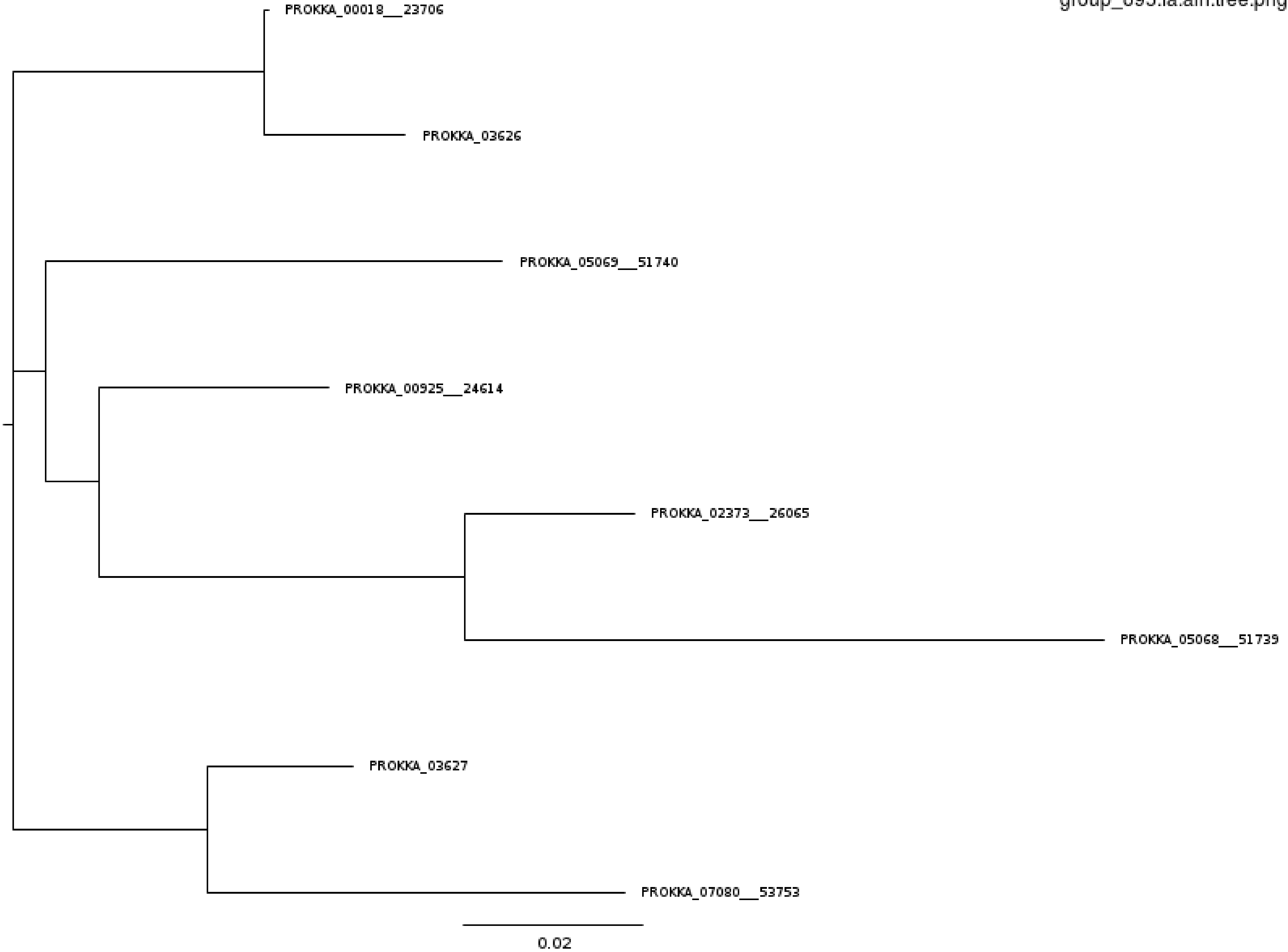

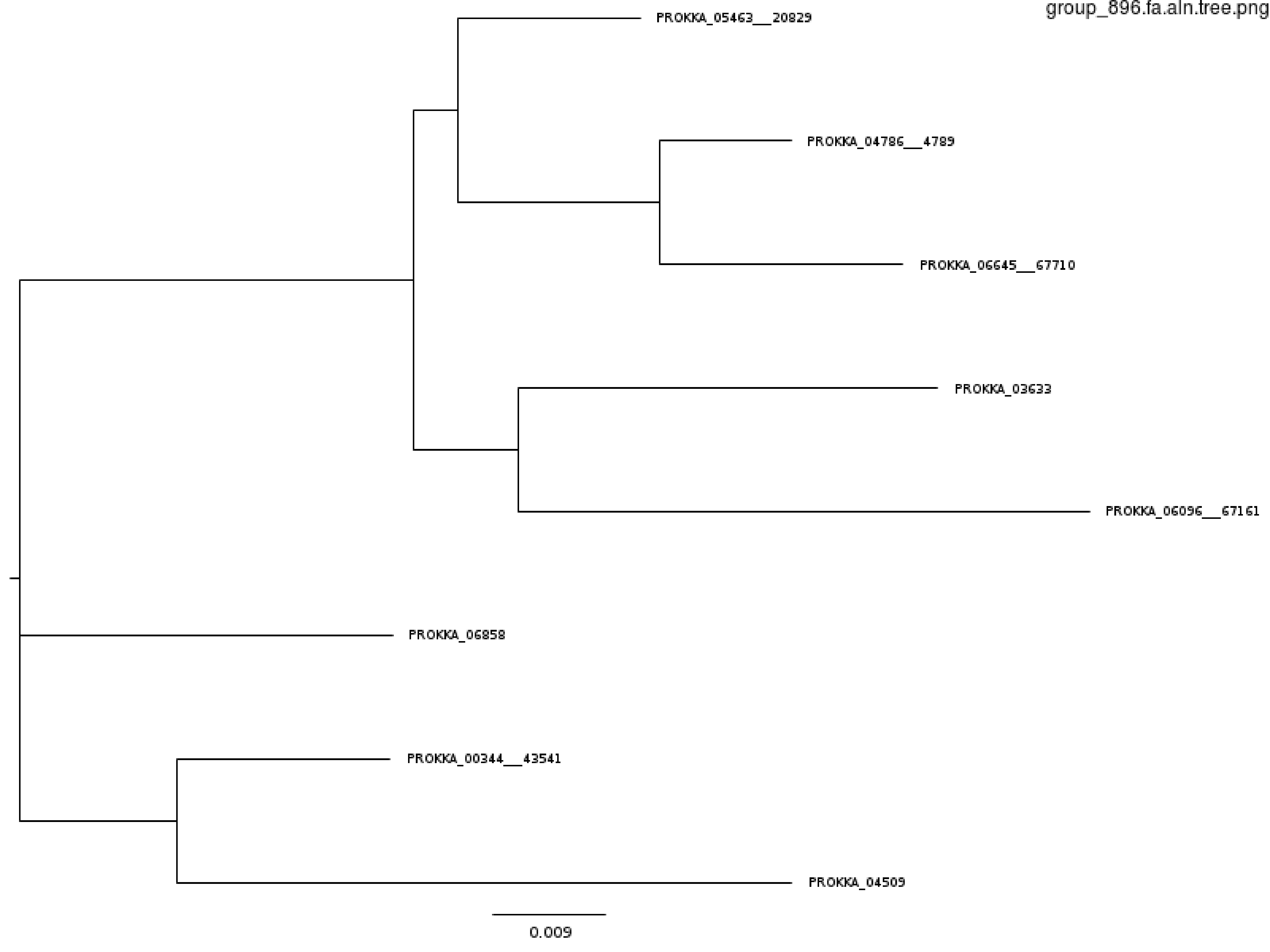
group_896.fa.aln.tree.png
PROKKA_05463___20829
PROKKA_04786___4789
PROKKA_06645___67710
PROKKA_03633
PROKKA_06096___67161
PROKKA_06858
PROKKA_00344___43541
PROKKA_04509
0.009

group_897.fa.aln.tree.png

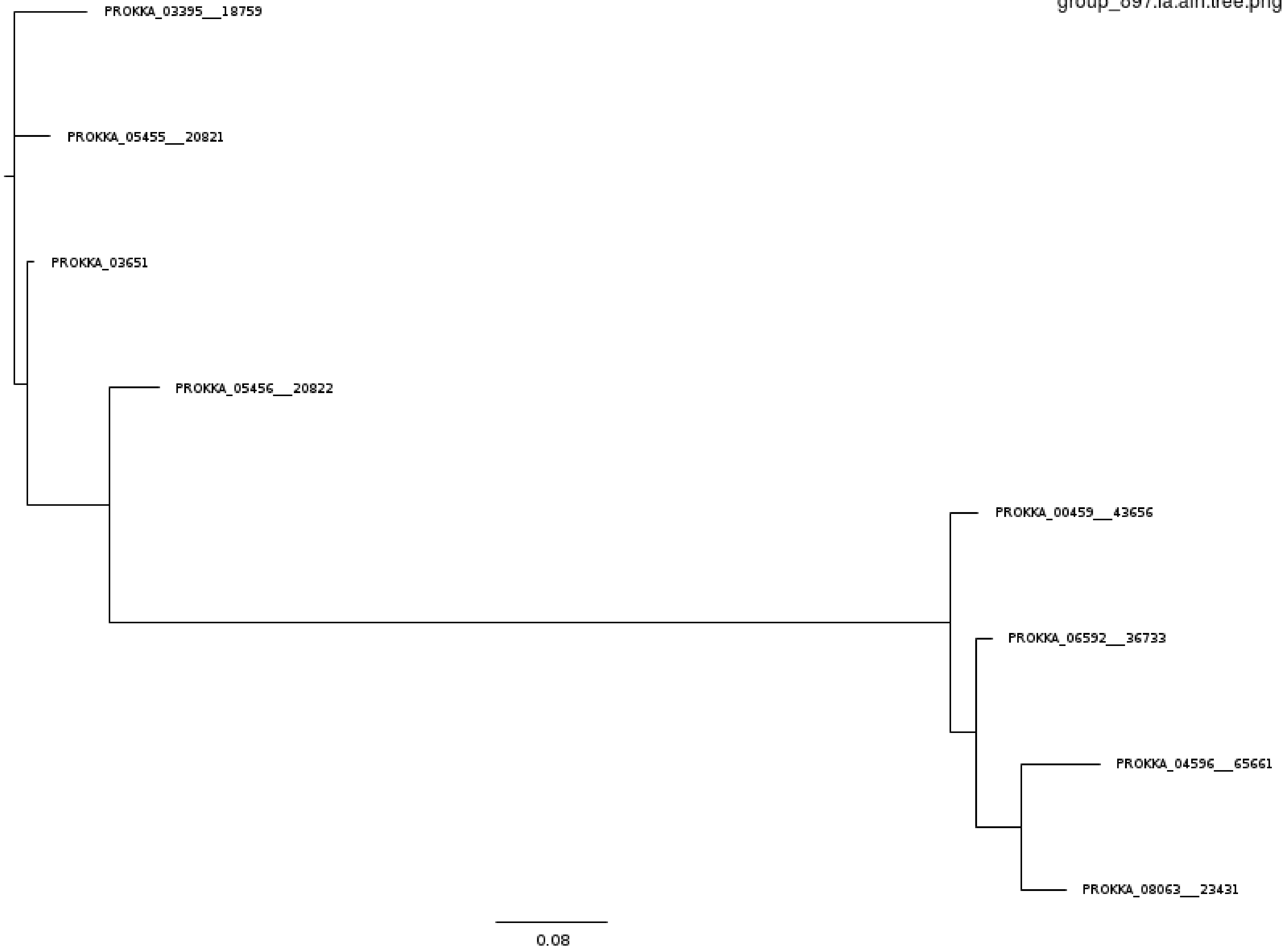

group_89.fa.aln.tree.png

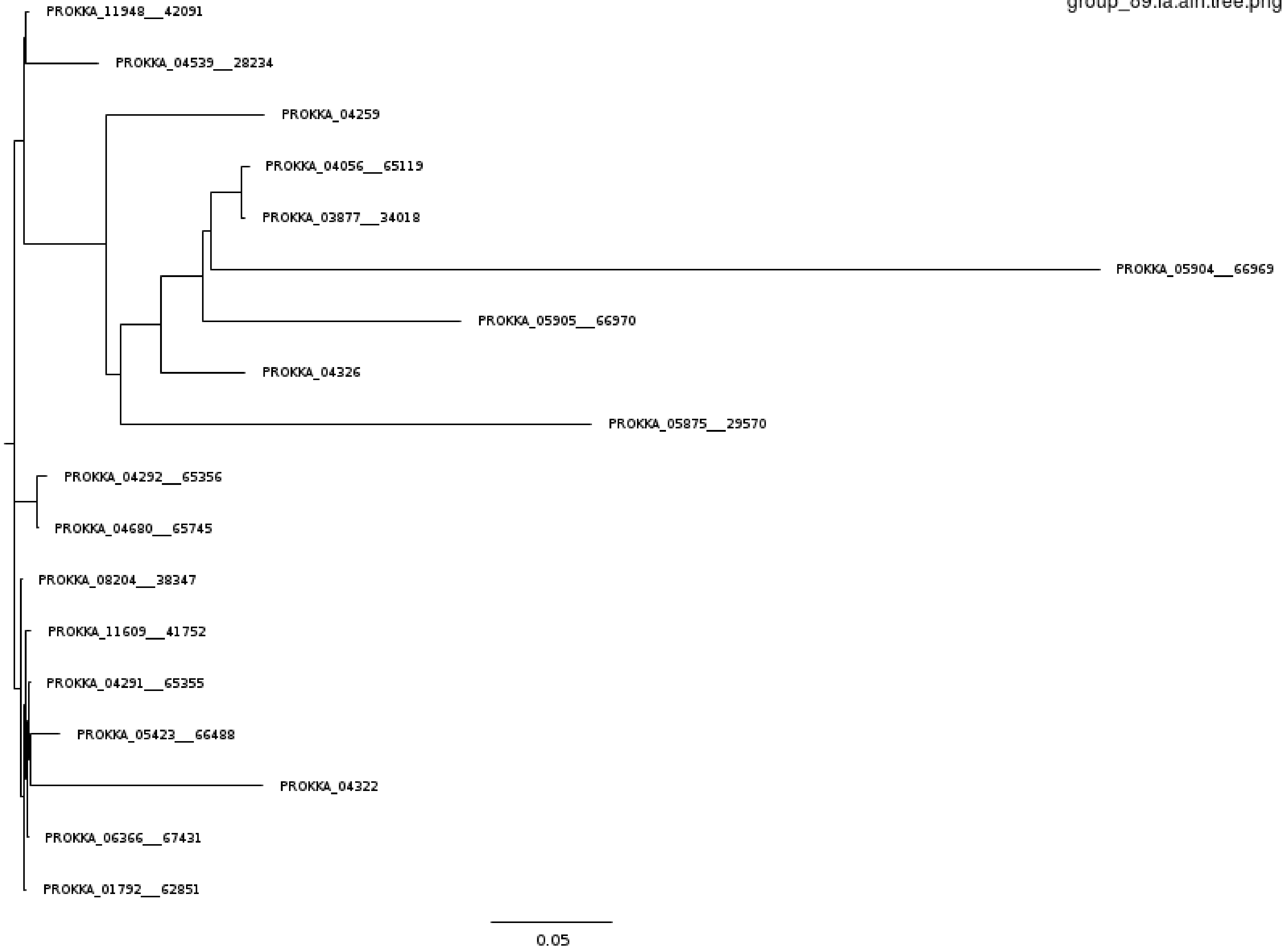

group_8.fa.aln.tree.png

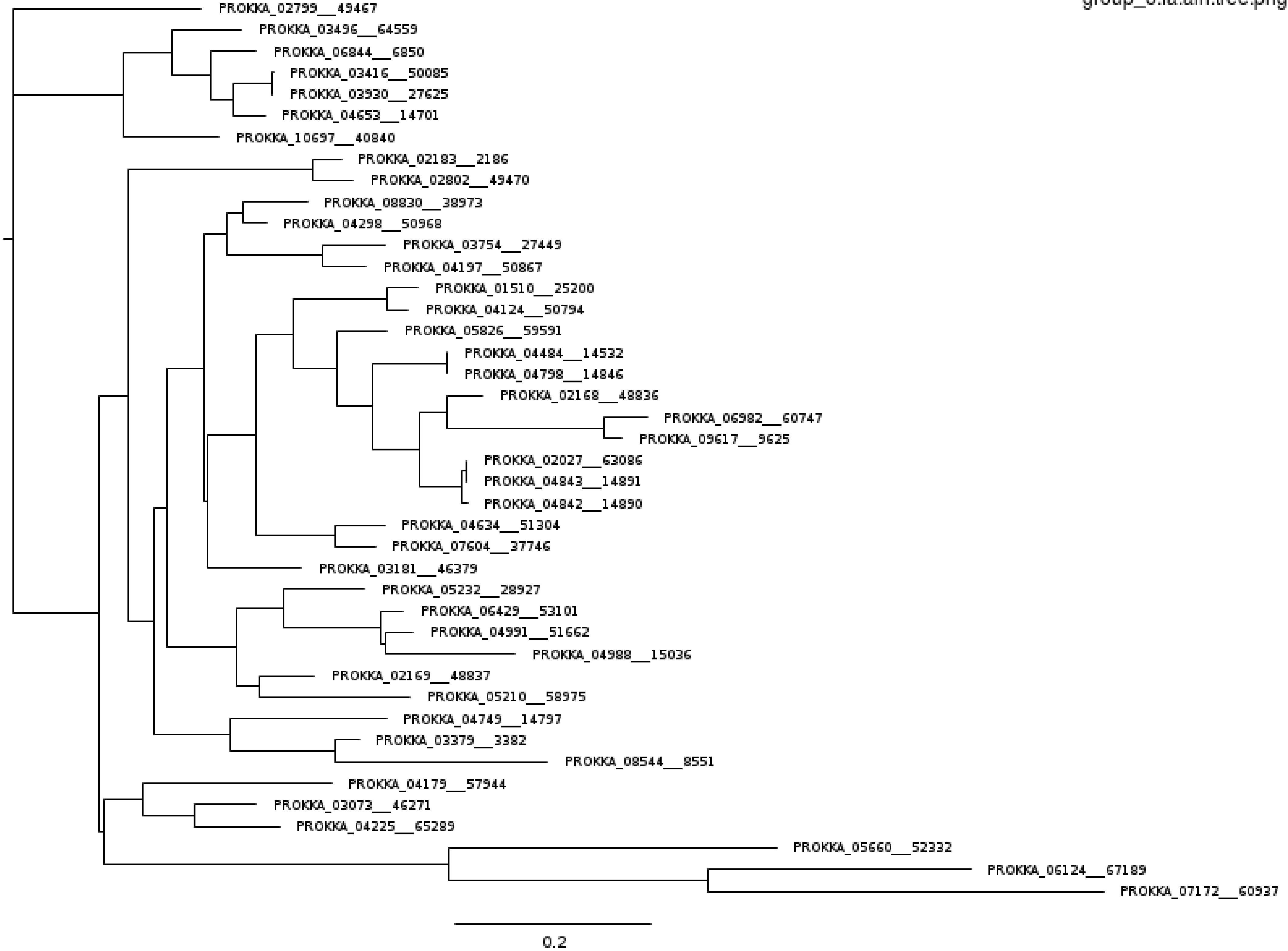

group_908.fa.aln.tree.png

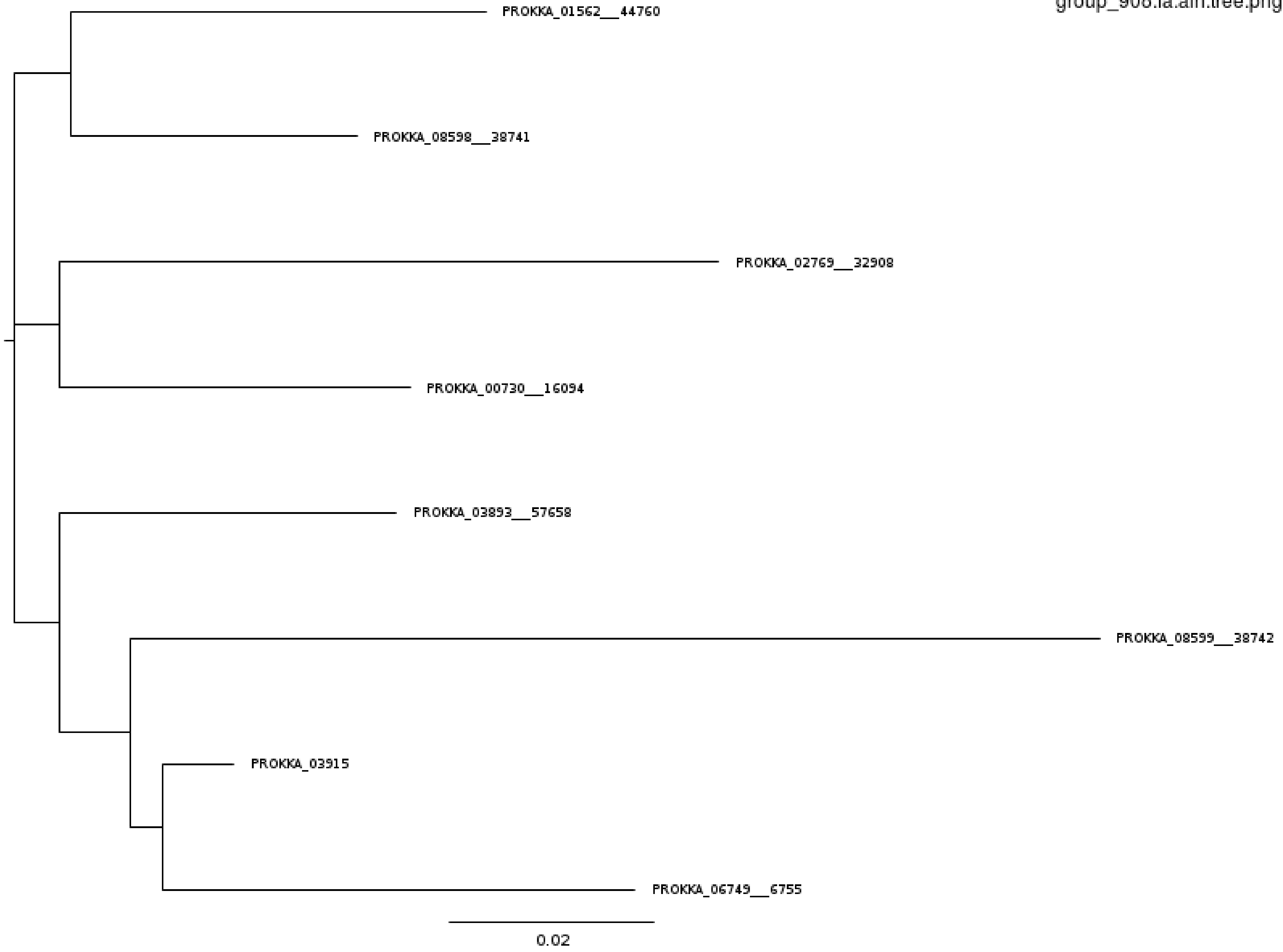

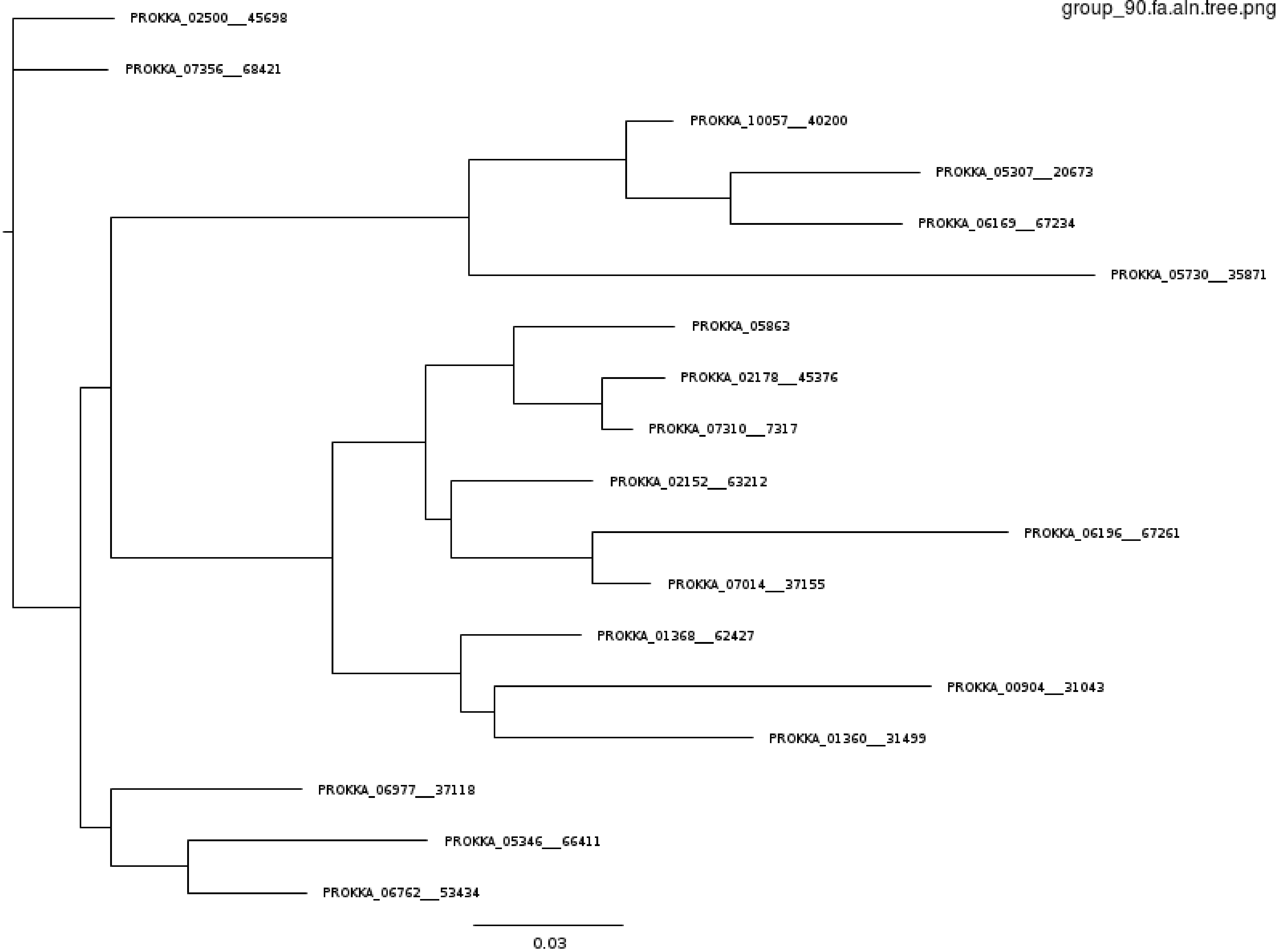

group_90.fa.aln.tree.png
PROKKA_02500__45698
PROKKA_07356__68421
PROKKA_10057__40200
PROKKA_05307__20673
PROKKA_06169__67234
PROKKA_05730__35871
PROKKA_05863
PROKKA_02178__45376
PROKKA_07310__7317
PROKKA_02152__63212
PROKKA_06196__67261
PROKKA_07014__37155
PROKKA_01368__62427
PROKKA_00904__31043
PROKKA_01360__31499
PROKKA_06977__37118
PROKKA_05346__66411
PROKKA_06762__53434
0.03

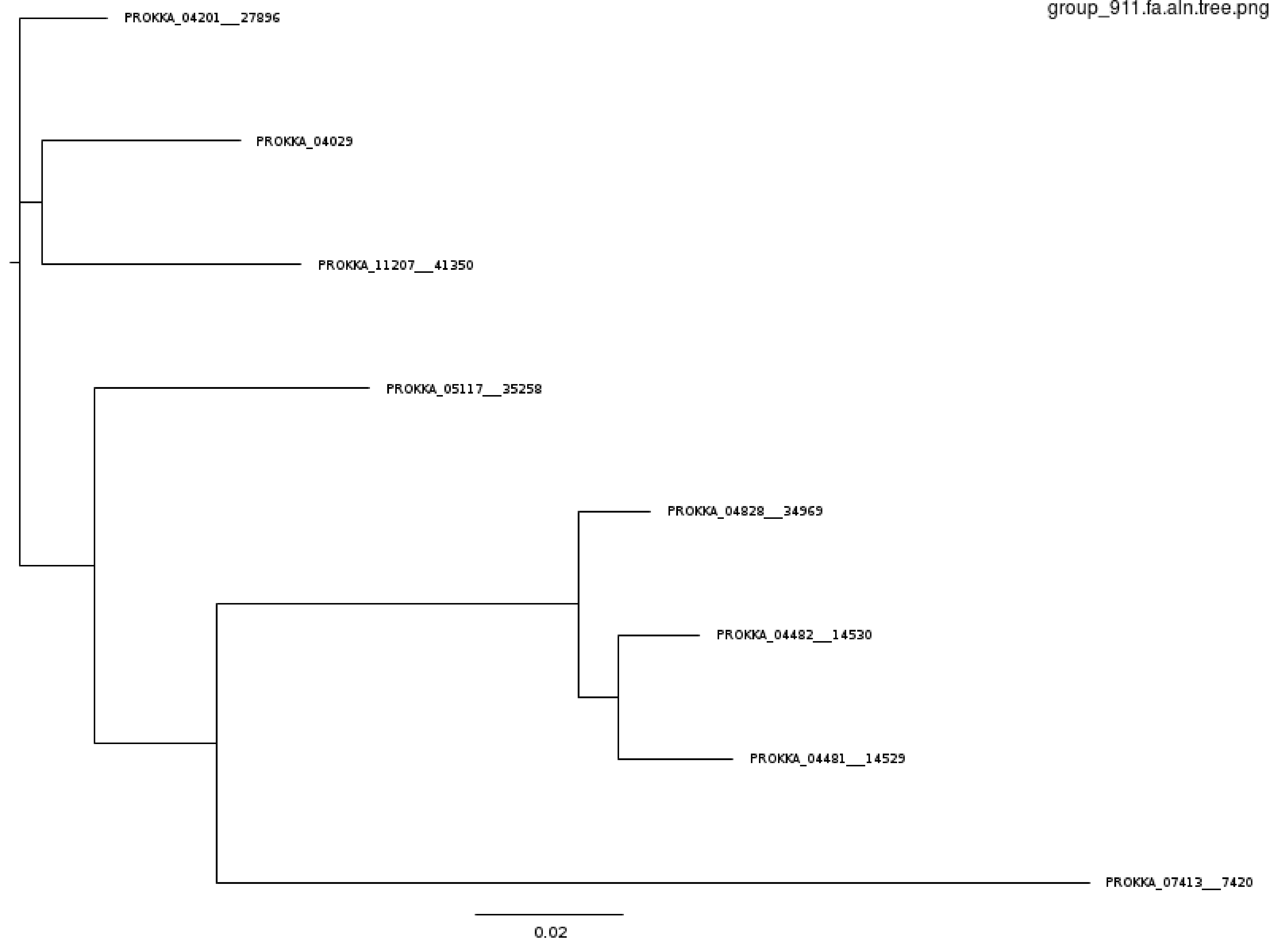
group_911.fa.aln.tree.png
PROKKA_04201___27896
PROKKA_04029
PROKKA_11207___41350
PROKKA_05117___35258
PROKKA_04828___34969
PROKKA_04482___14530
PROKKA_04481___14529
PROKKA_07413___7420
0.02

group_913.fa.aln.tree.png

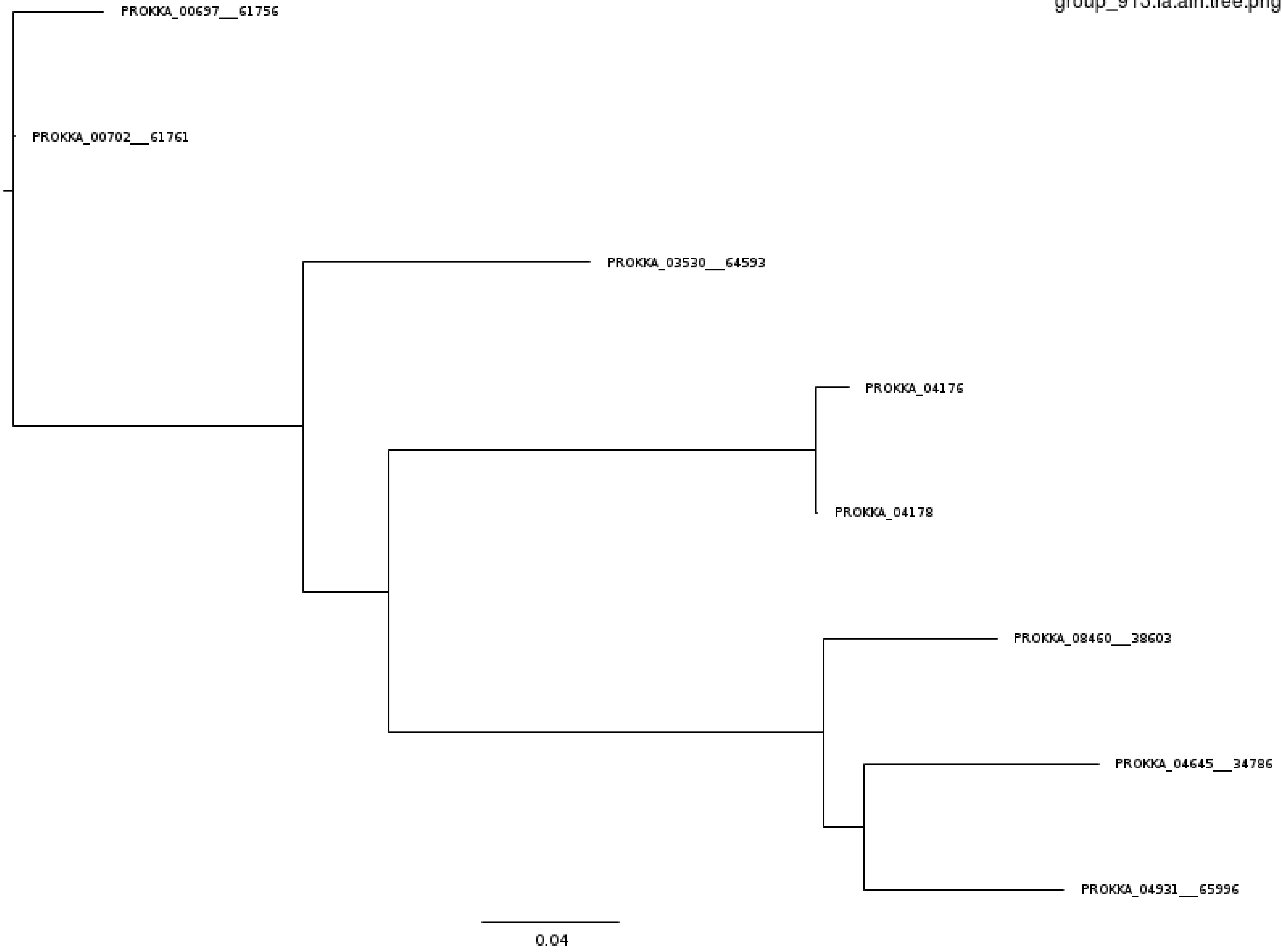

group_914.fa.aln.tree.png

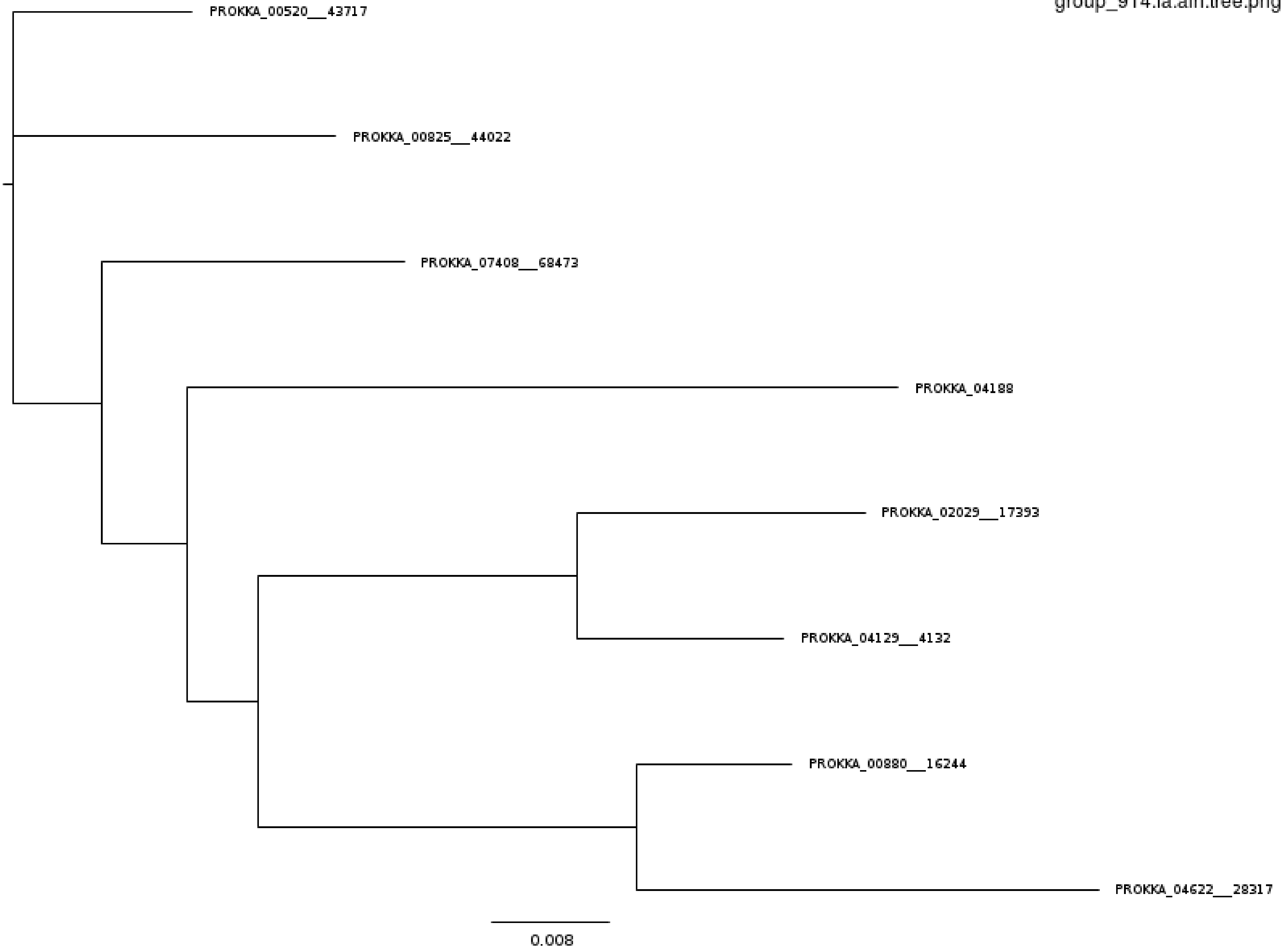

group_917.fa.aln.tree.png

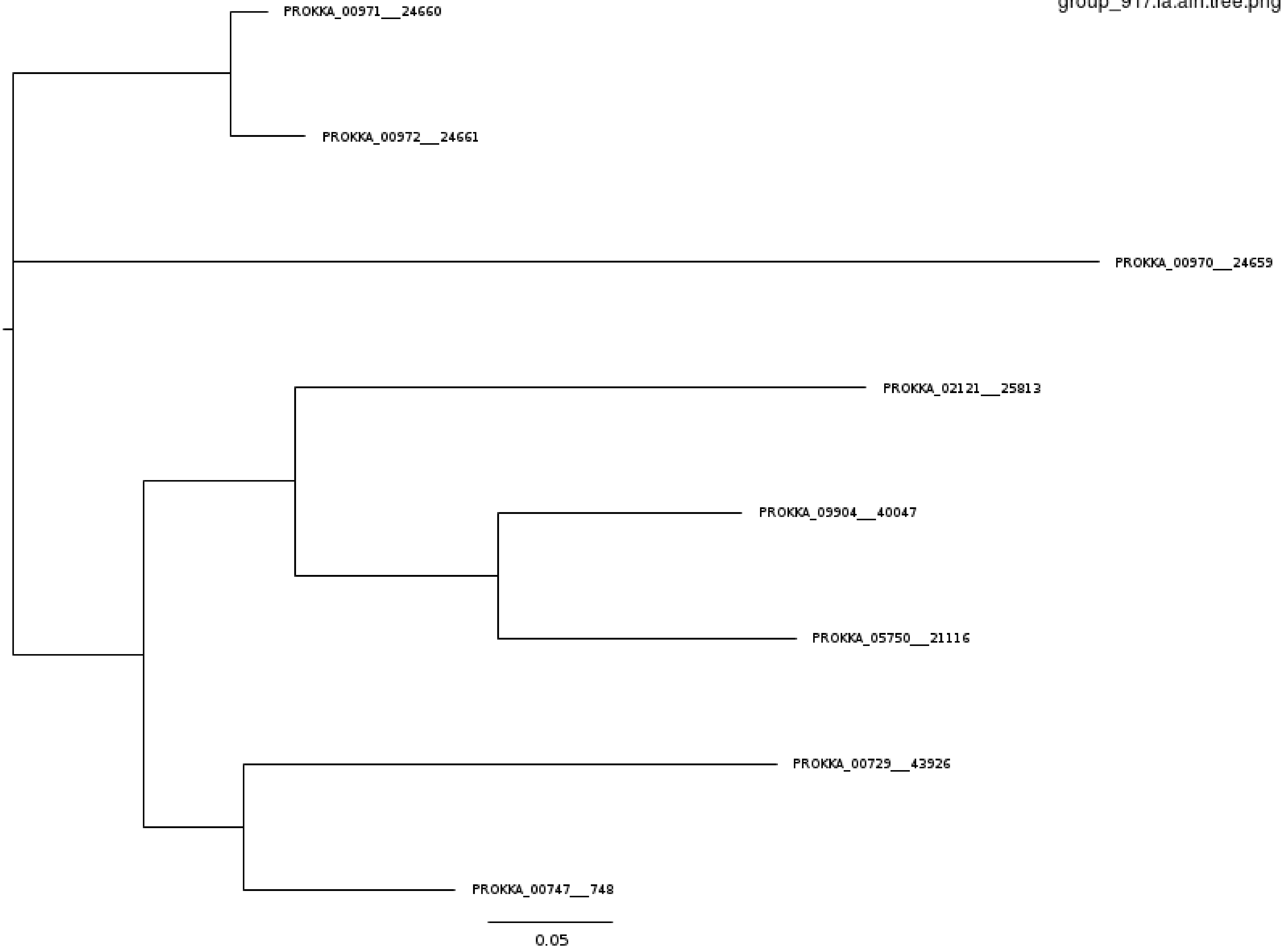

group_919.fa.aln.tree.png

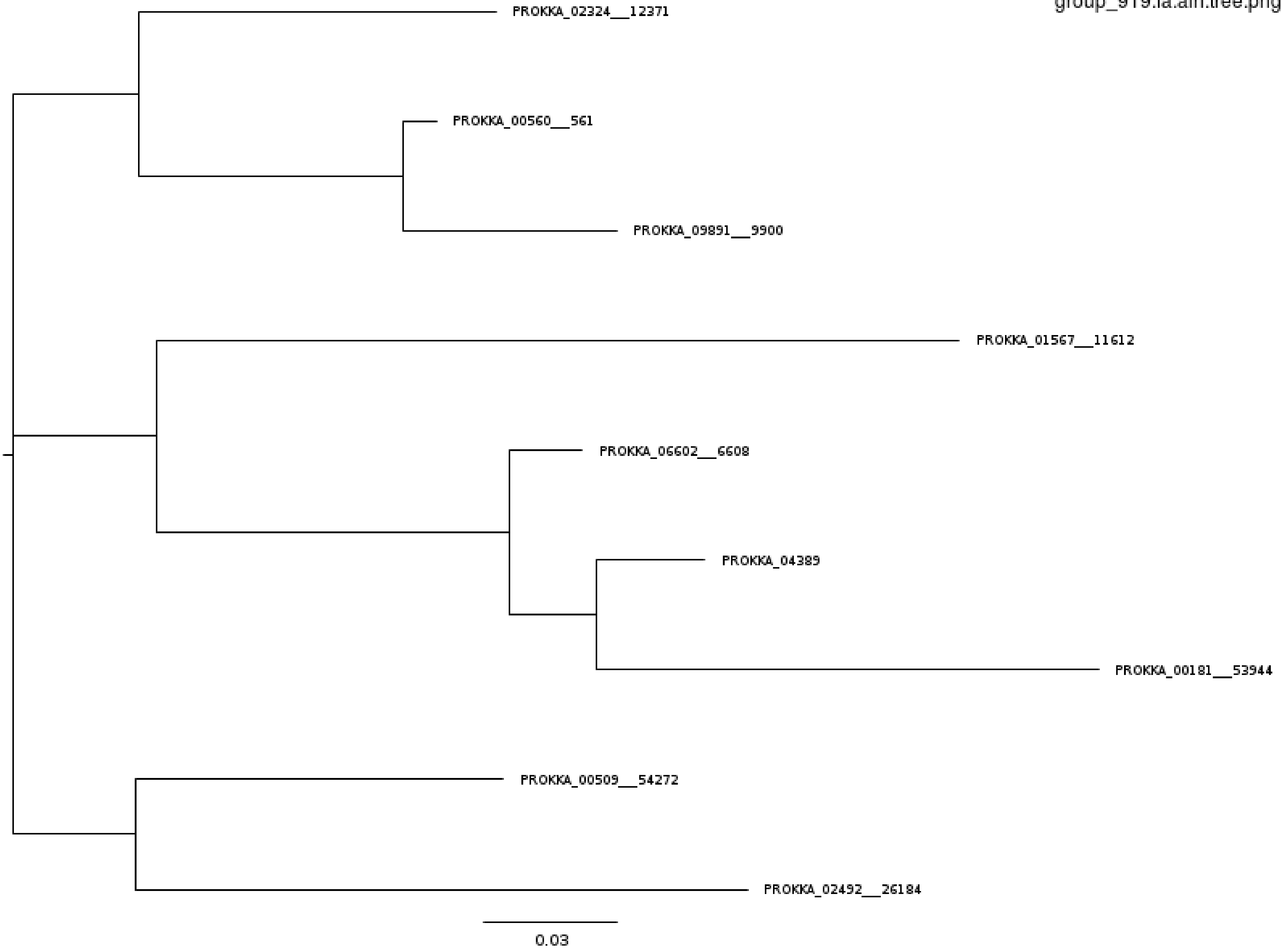

group_920.fa.aln.tree.png

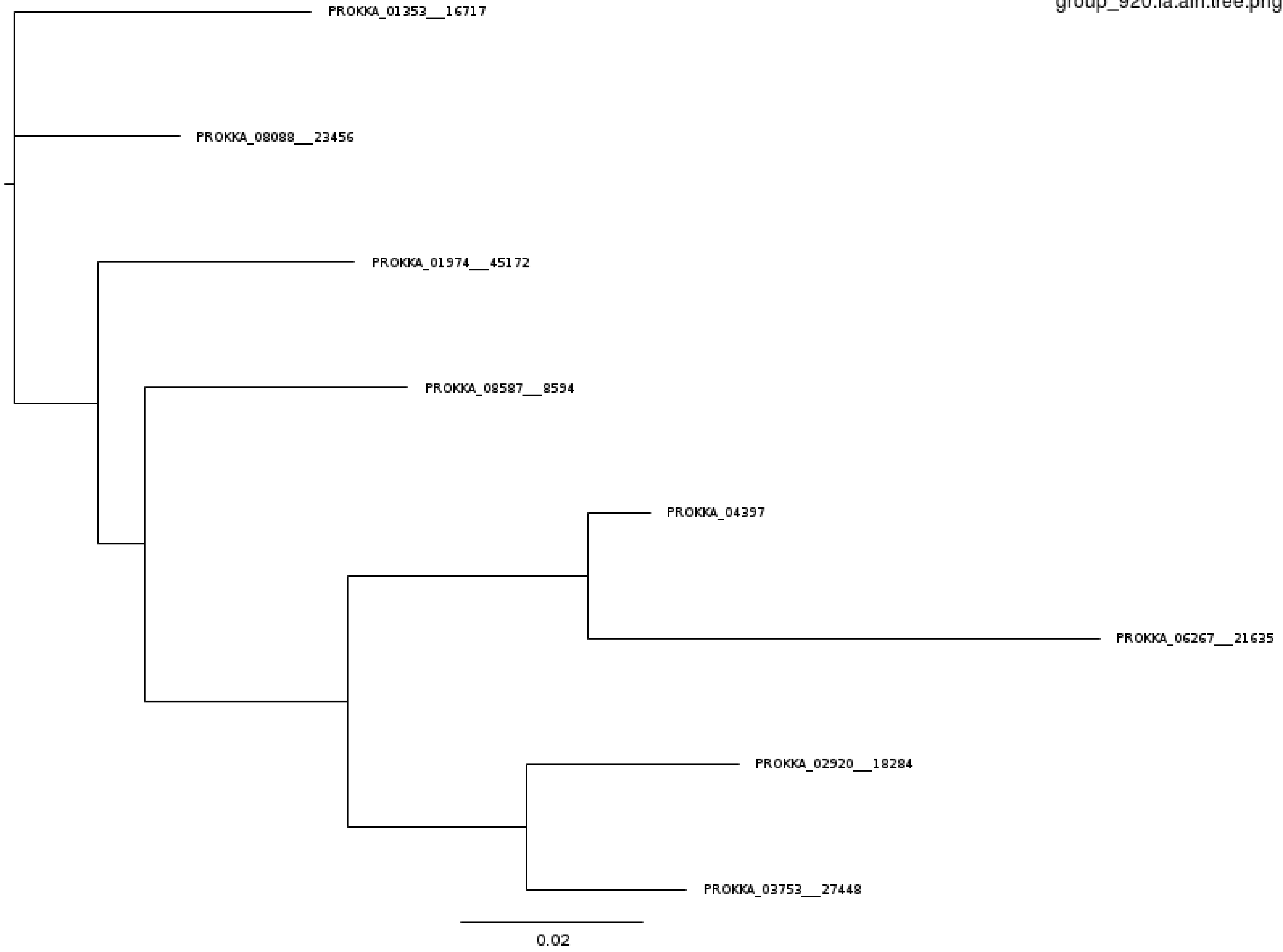

group_921.fa.aln.tree.png

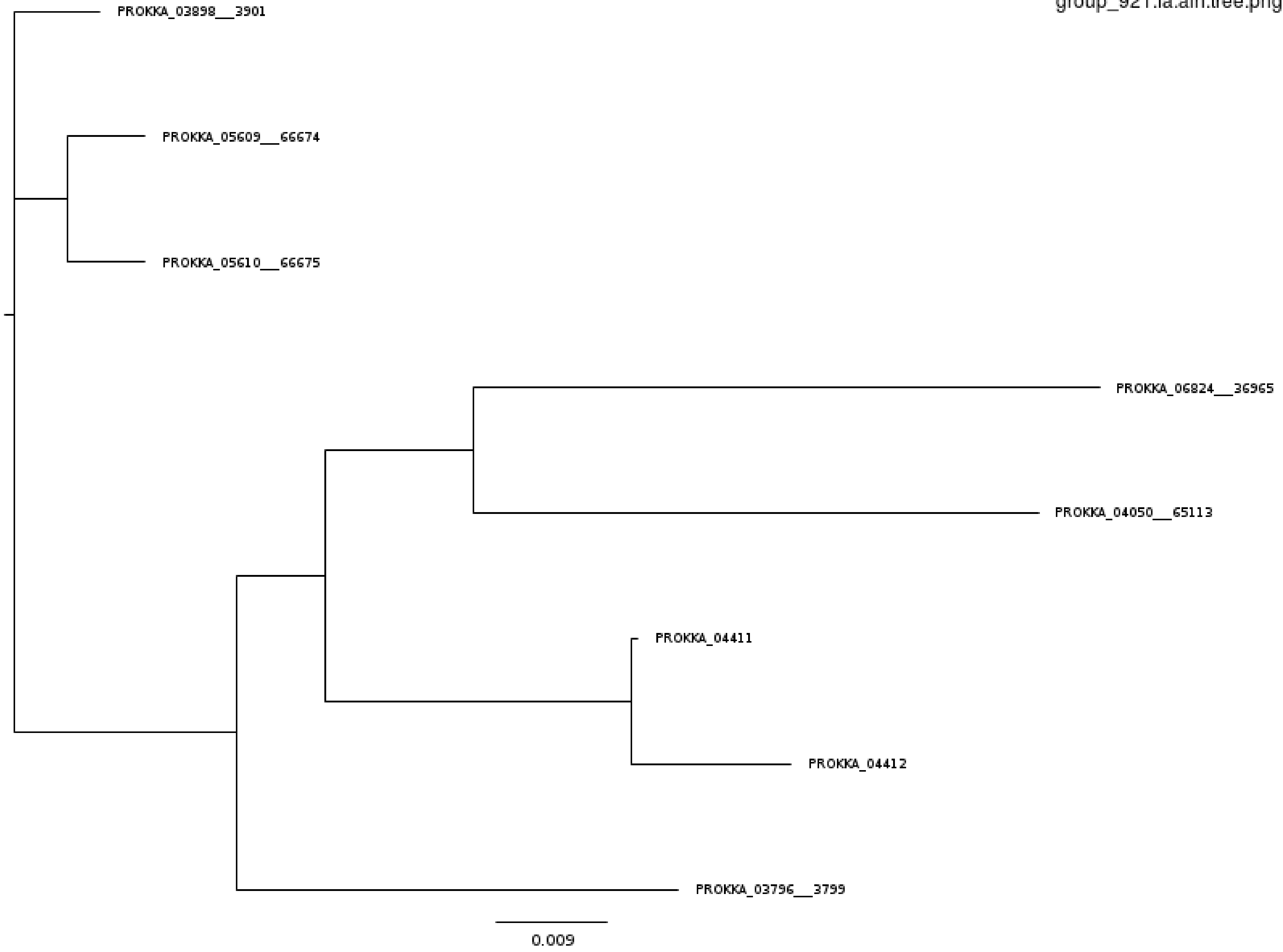

group_925.fa.aln.tree.png

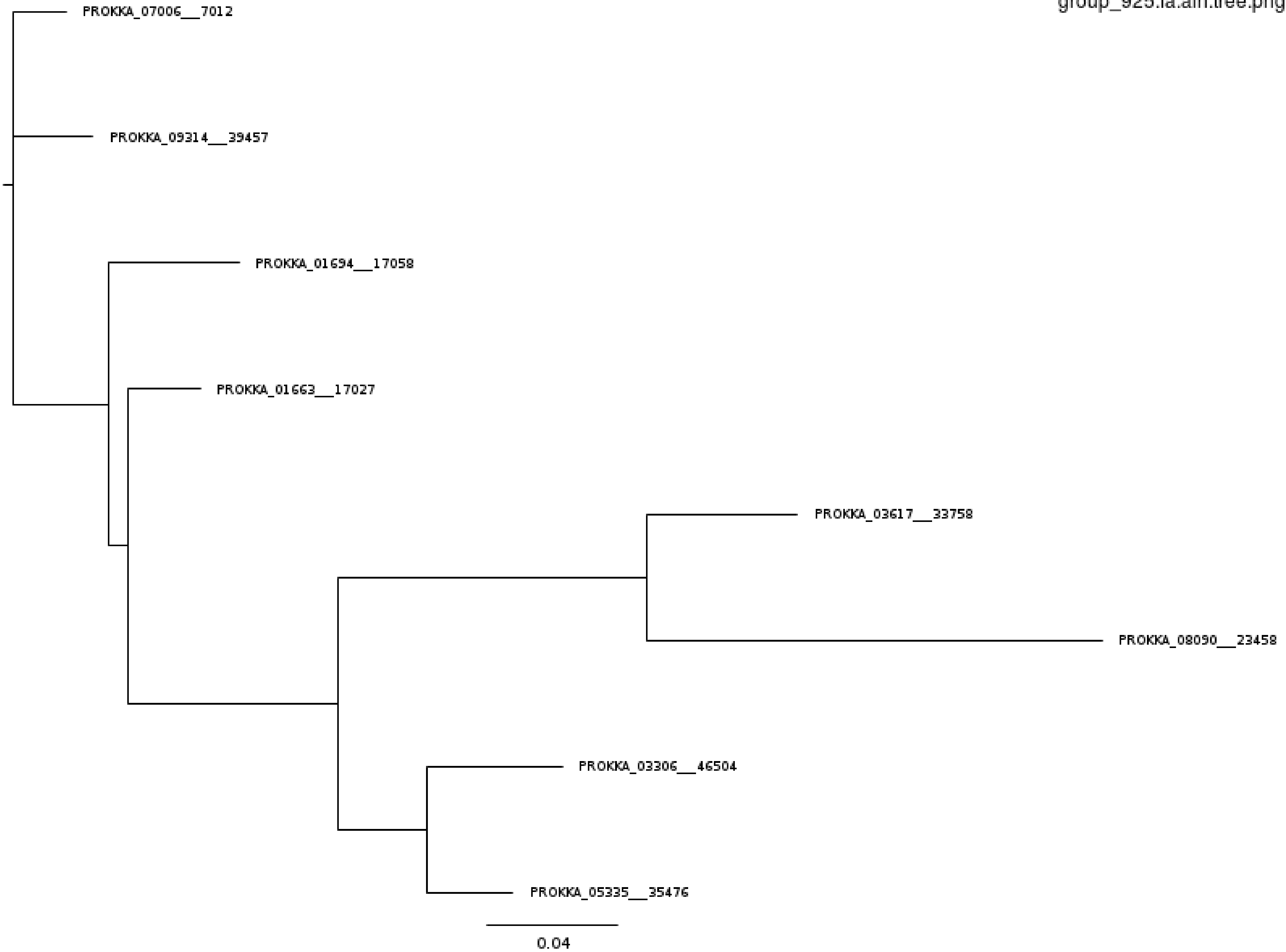

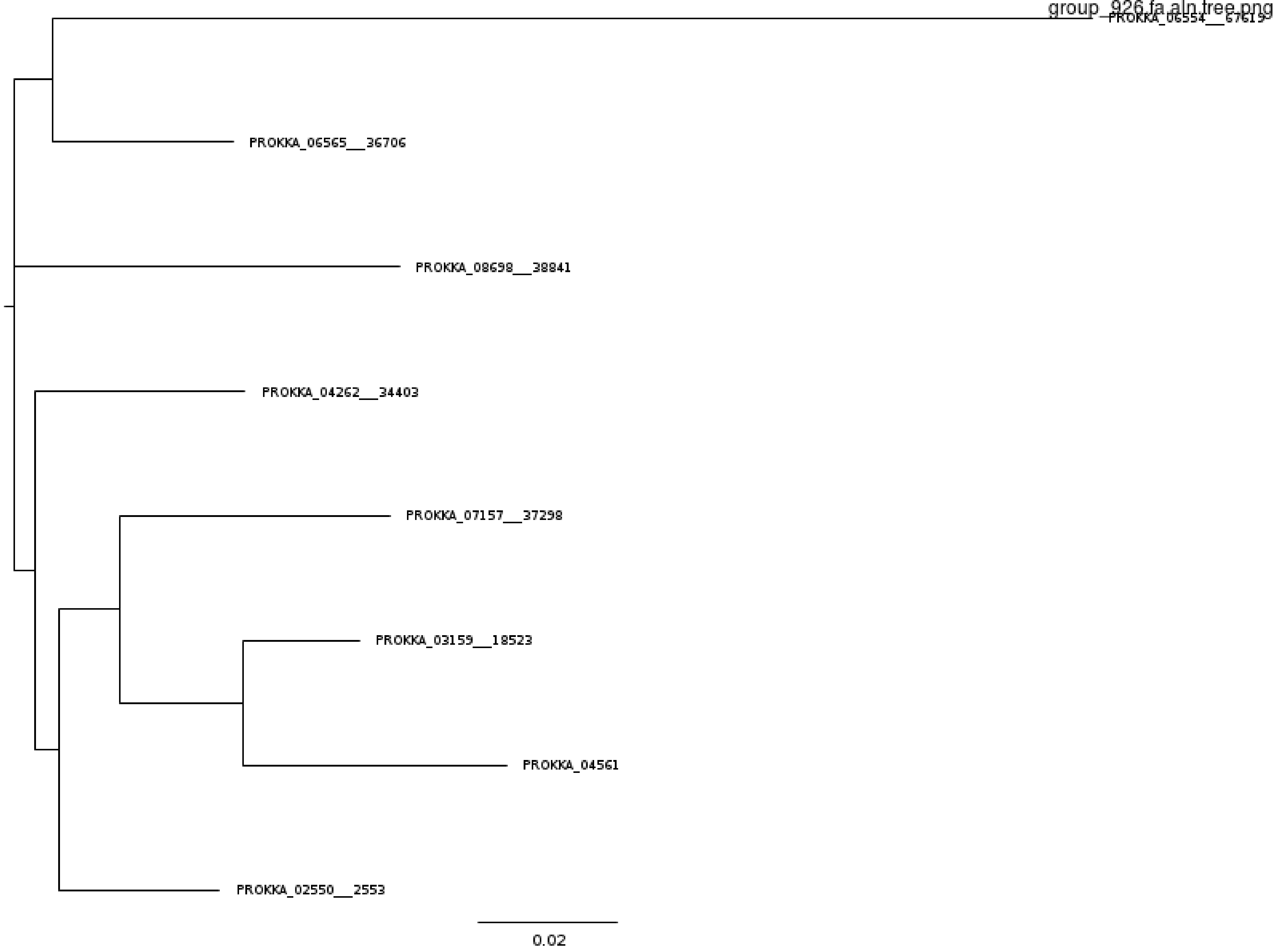
group_926.fa.aln.tree.png
PROKKA_06554___67619
PROKKA_06565___36706
PROKKA_08698___38841
PROKKA_04262___34403
PROKKA_07157___37298
PROKKA_03159___18523
PROKKA_04561
PROKKA_02550___2553
0.02

group_931.fa.aln.tree.png

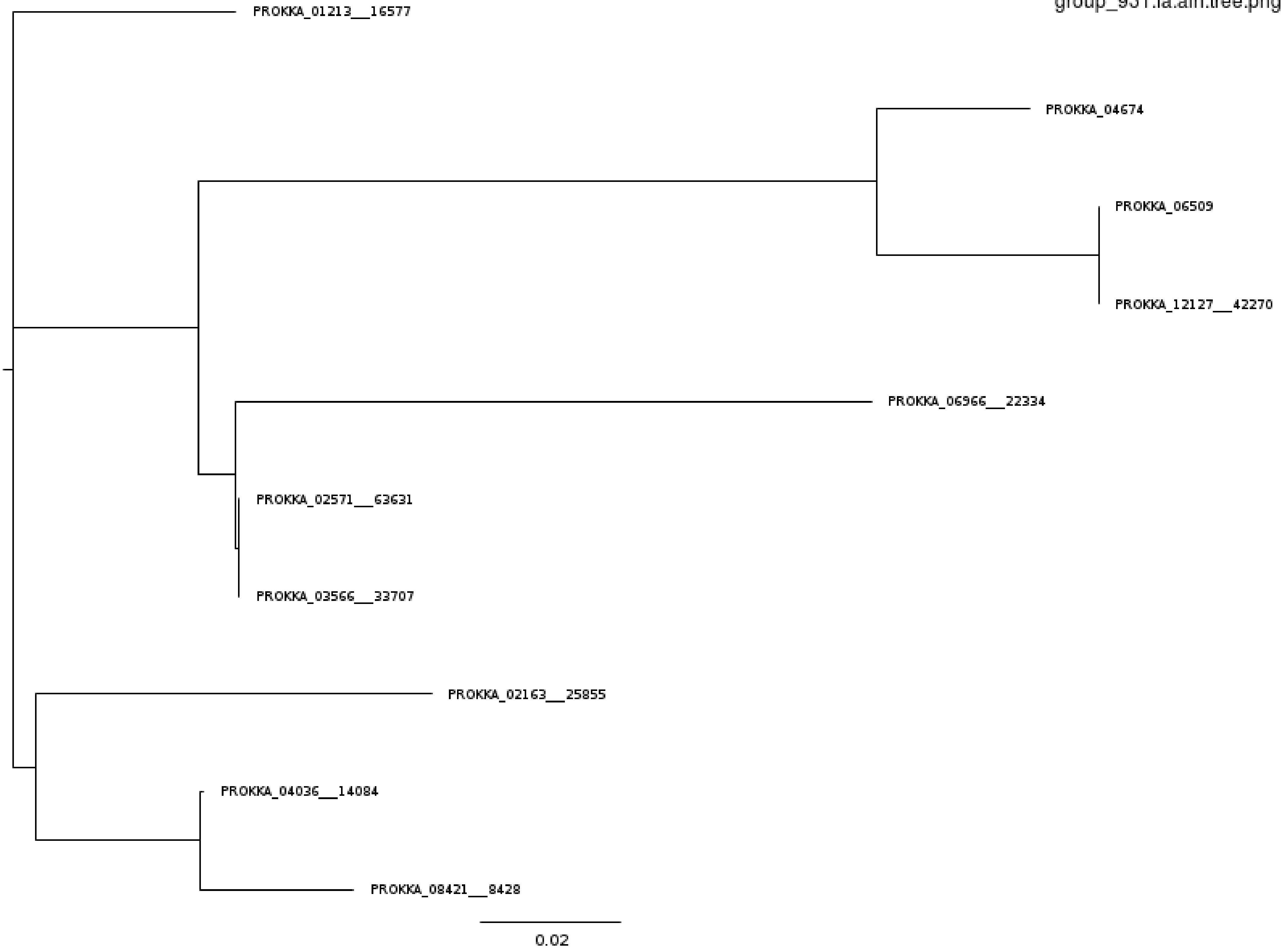

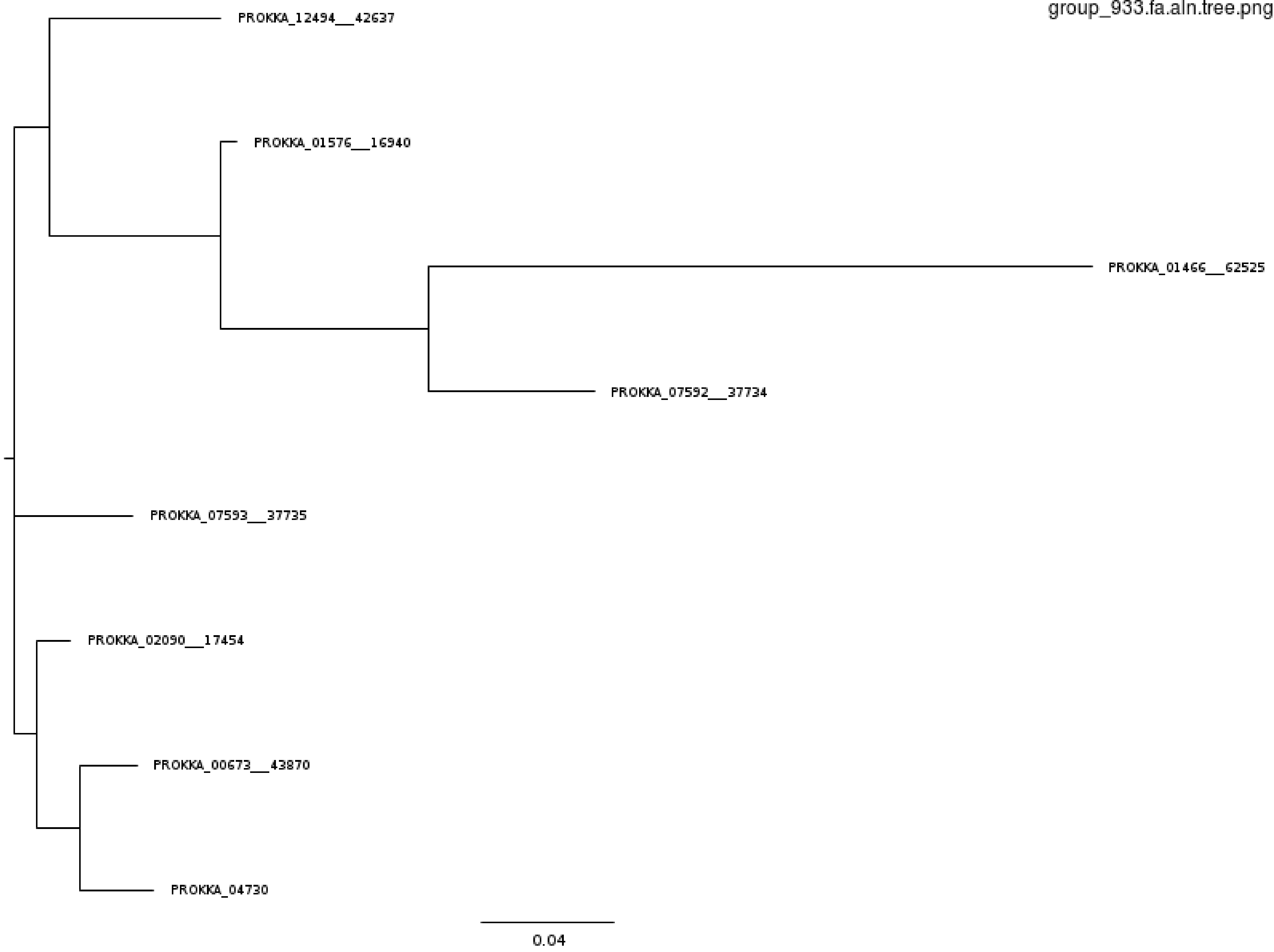
group_933.fa.aln.tree.png
PROKKA_12494___42637
PROKKA_01576___16940
PROKKA_01466___62525
PROKKA_07592___37734
PROKKA_07593___37735
PROKKA_02090___17454
PROKKA_00673___43870
PROKKA_04730
0.04

group_934.fa.aln.tree.png

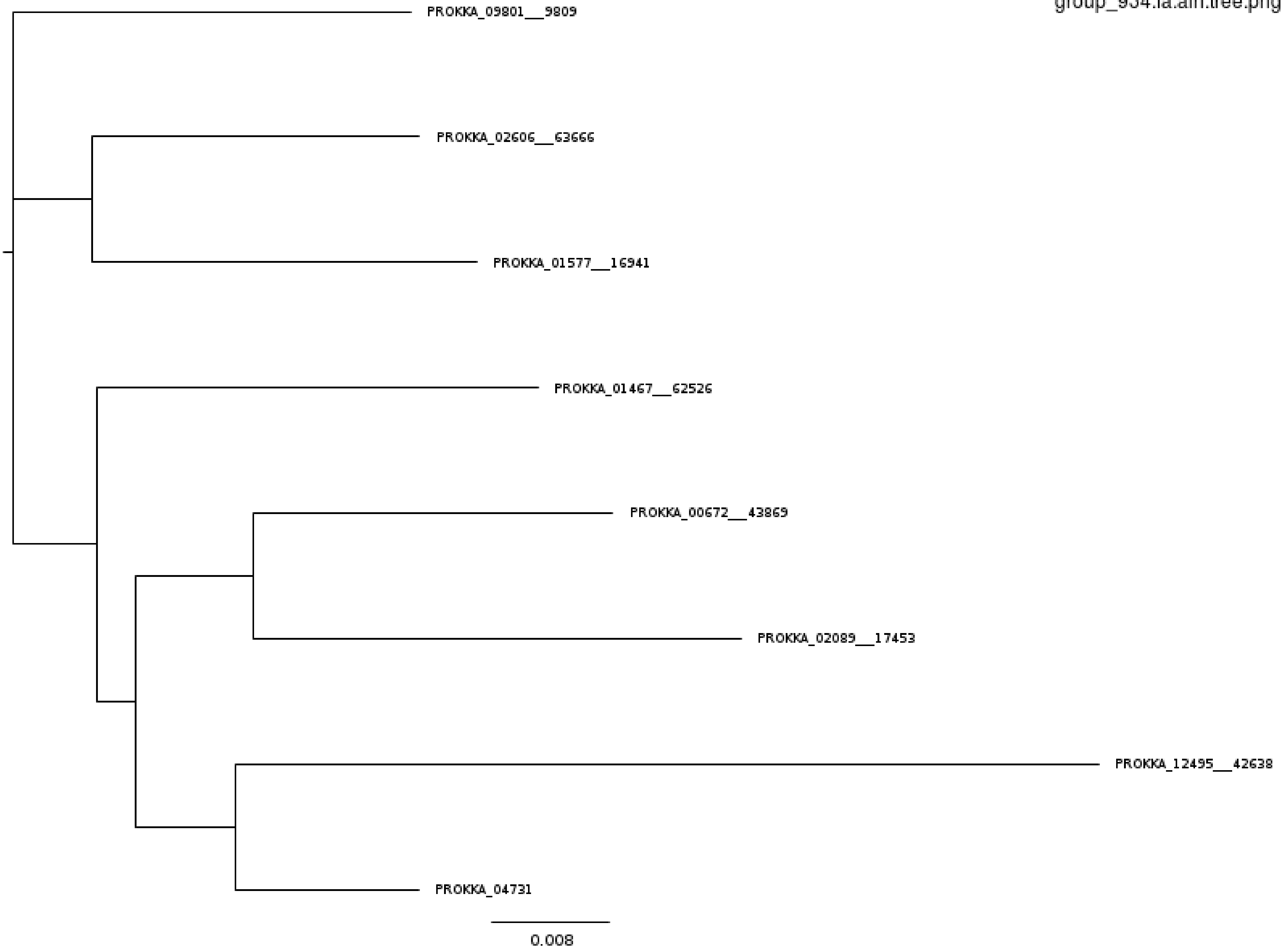

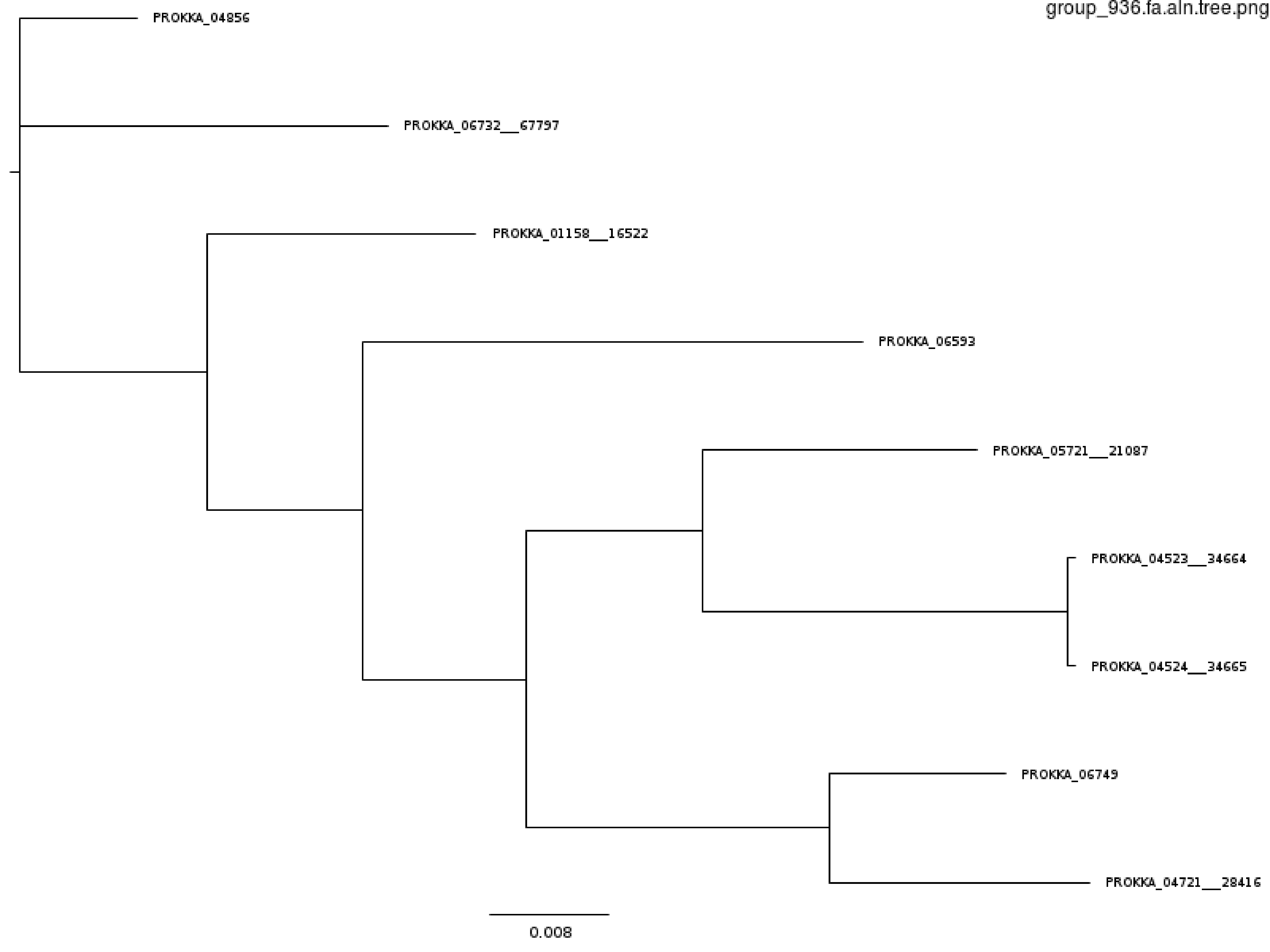

group_936.fa.aln.tree.png
PROKKA_04856
PROKKA_06732___67797
PROKKA_01158___16522
PROKKA_06593
PROKKA_05721___21087
PROKKA_04523___34664
PROKKA_04524___34665
PROKKA_06749
PROKKA_04721___28416
0.008

group_937.fa.aln.tree.png

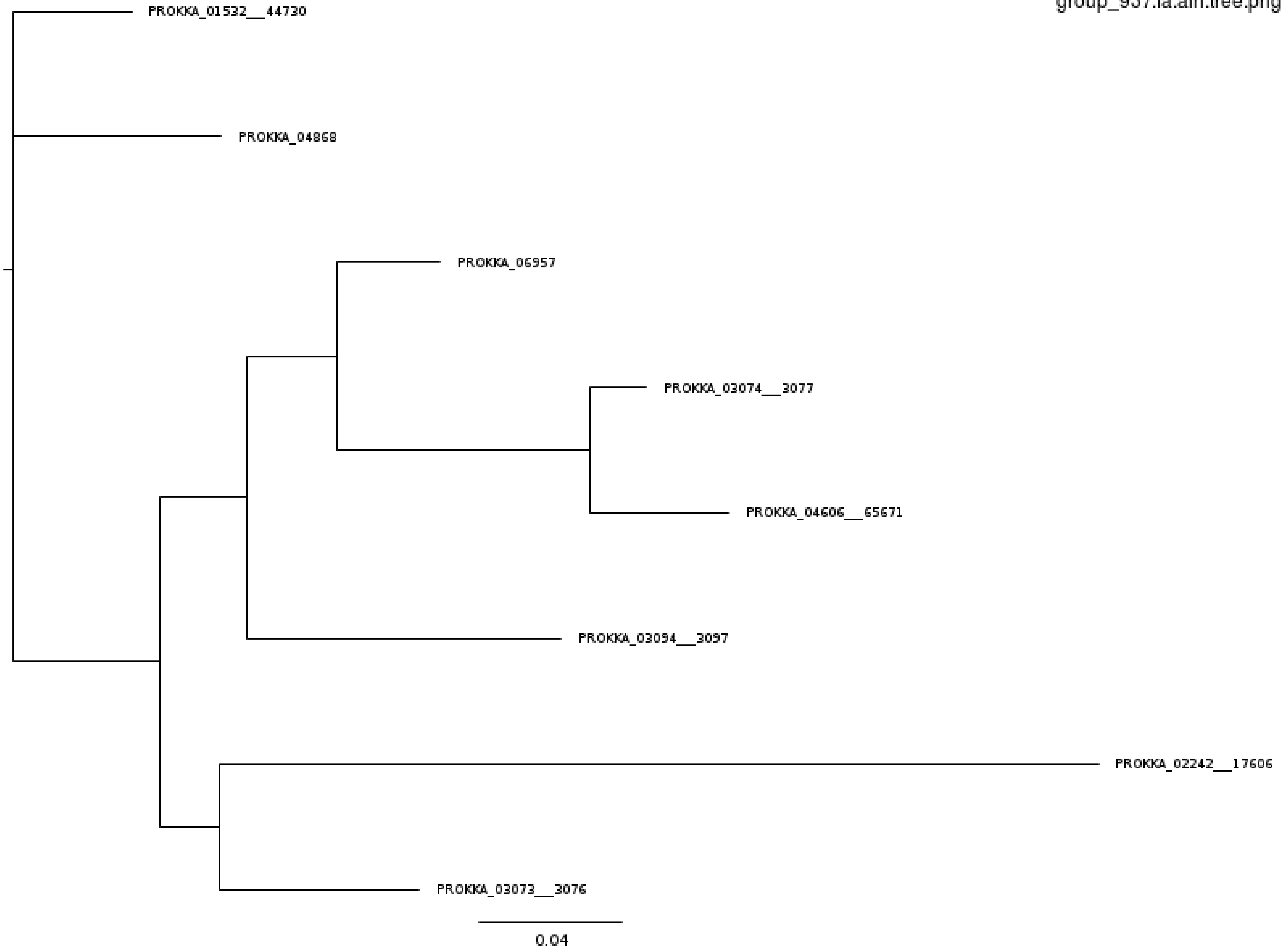

group_938.fa.aln.tree.png

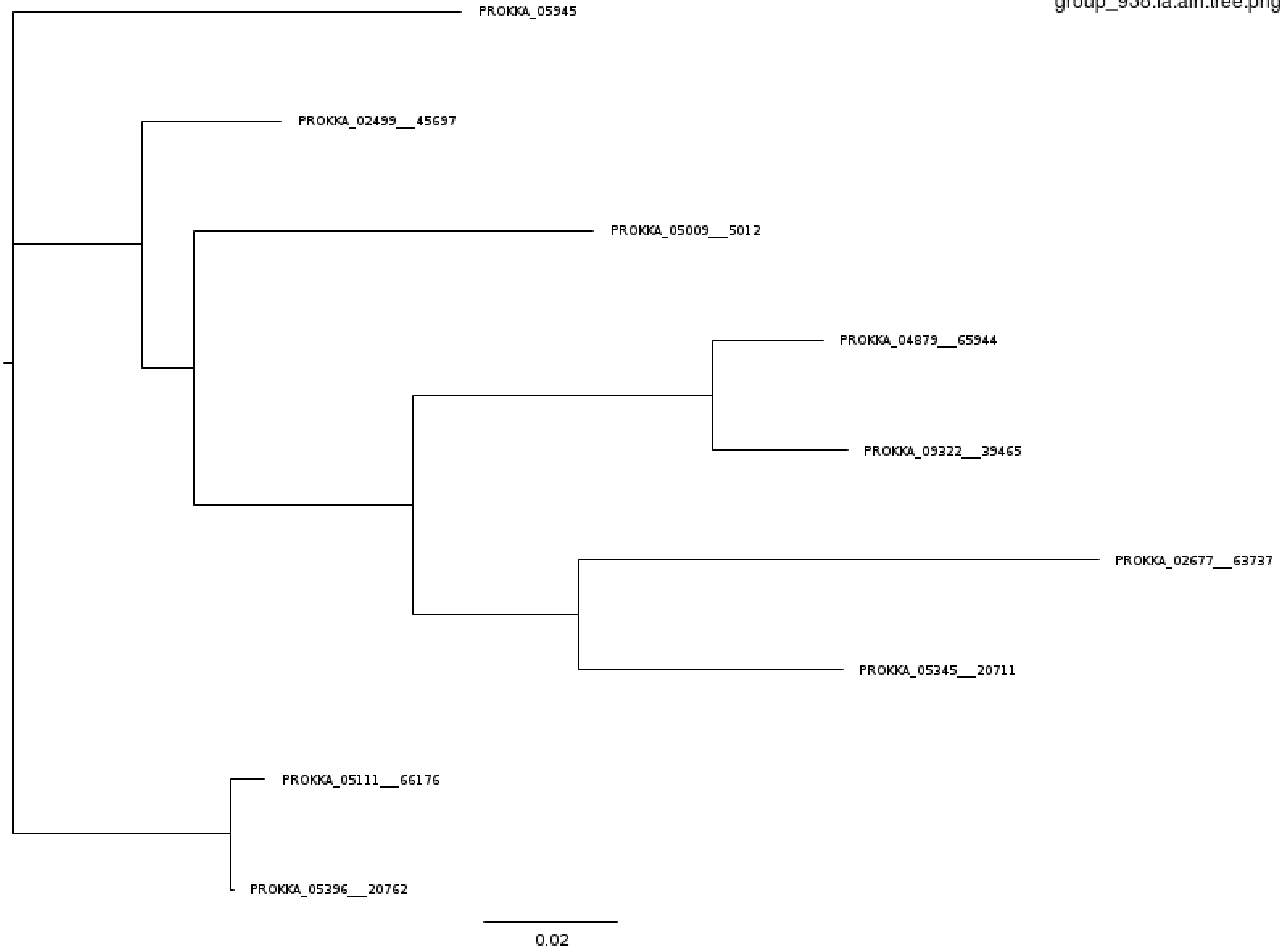

group_939.fa.aln.tree.png

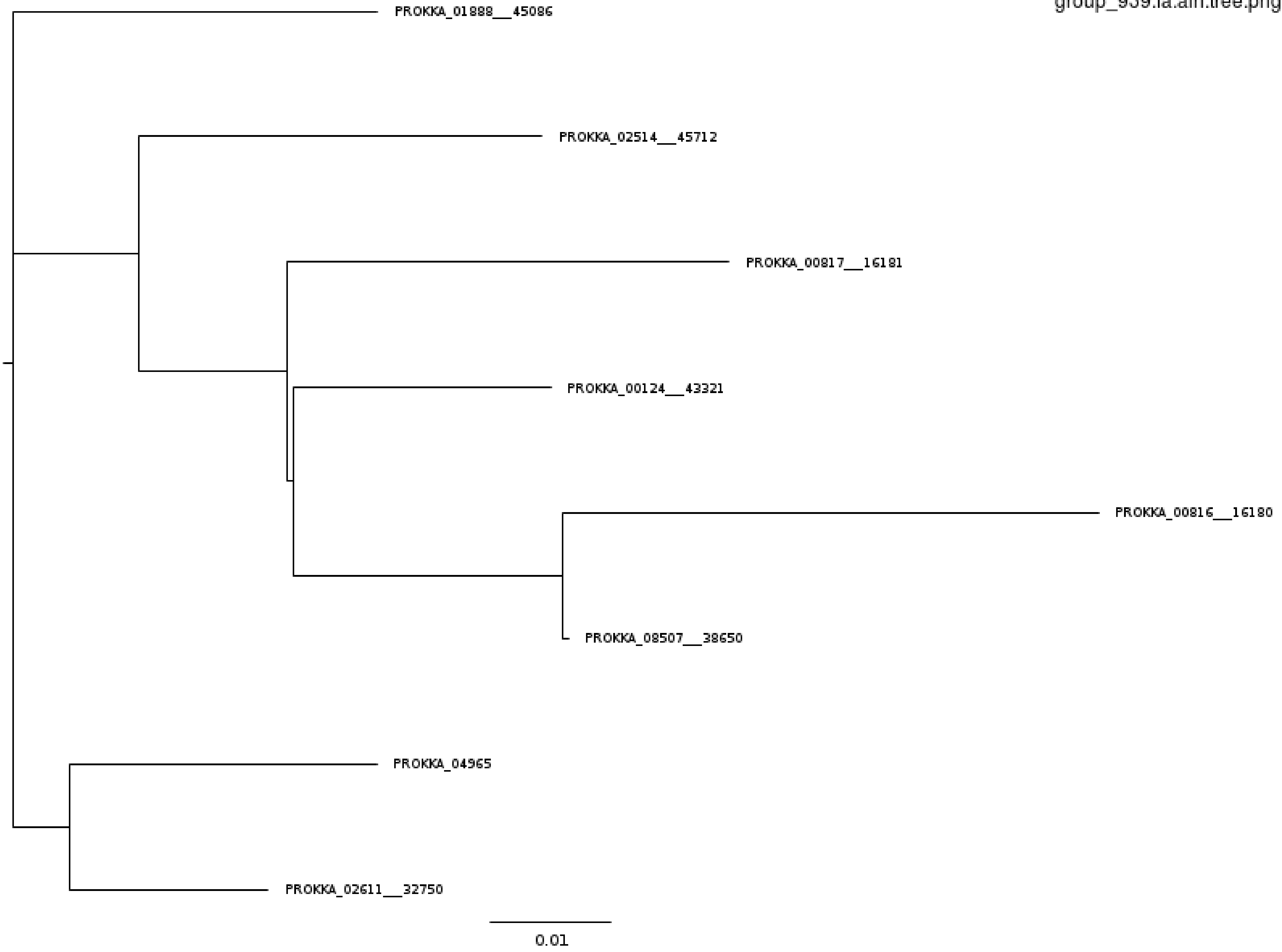

group_93.fa.aln.tree.png

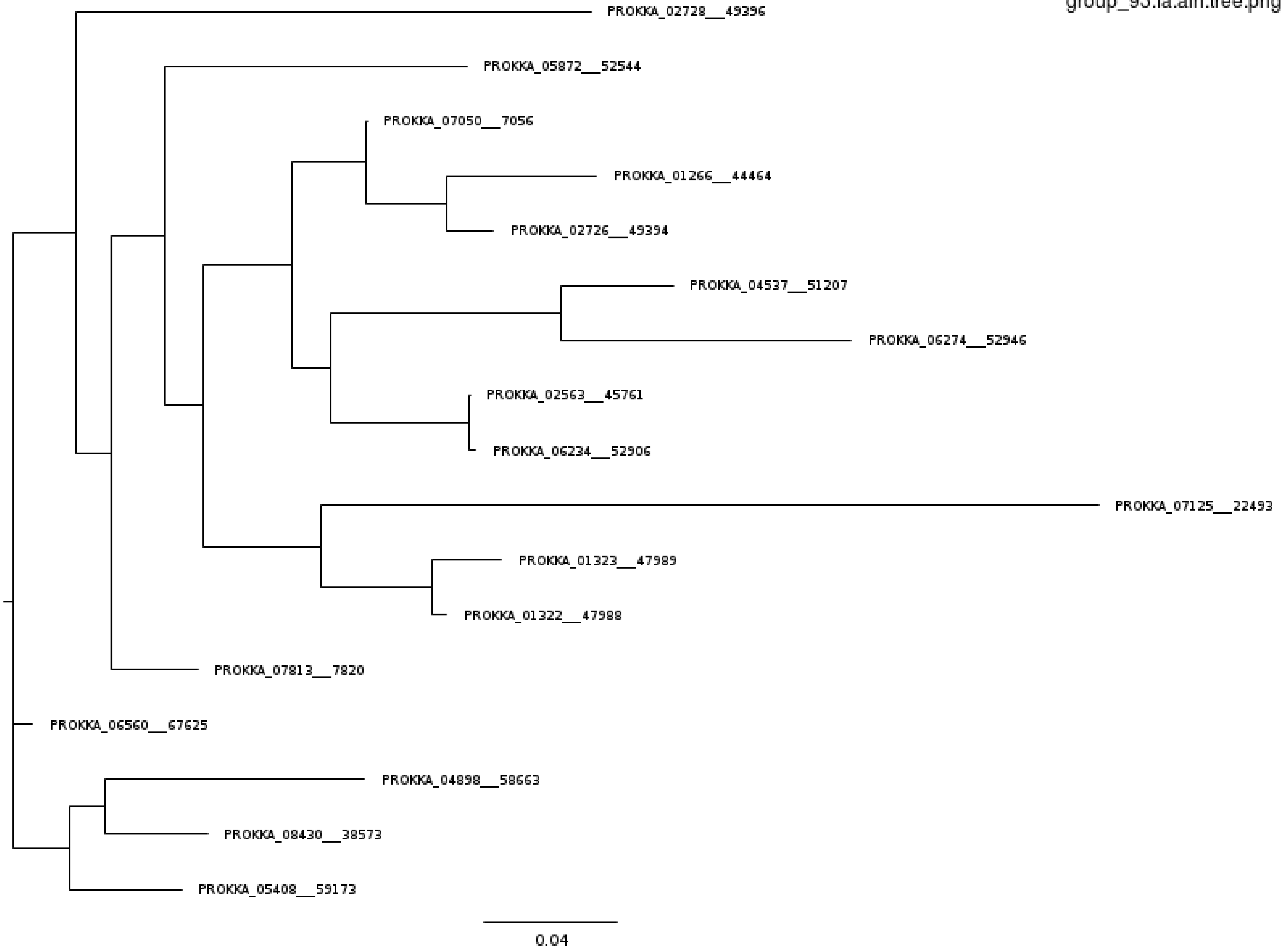

group_940.fa.aln.tree.png

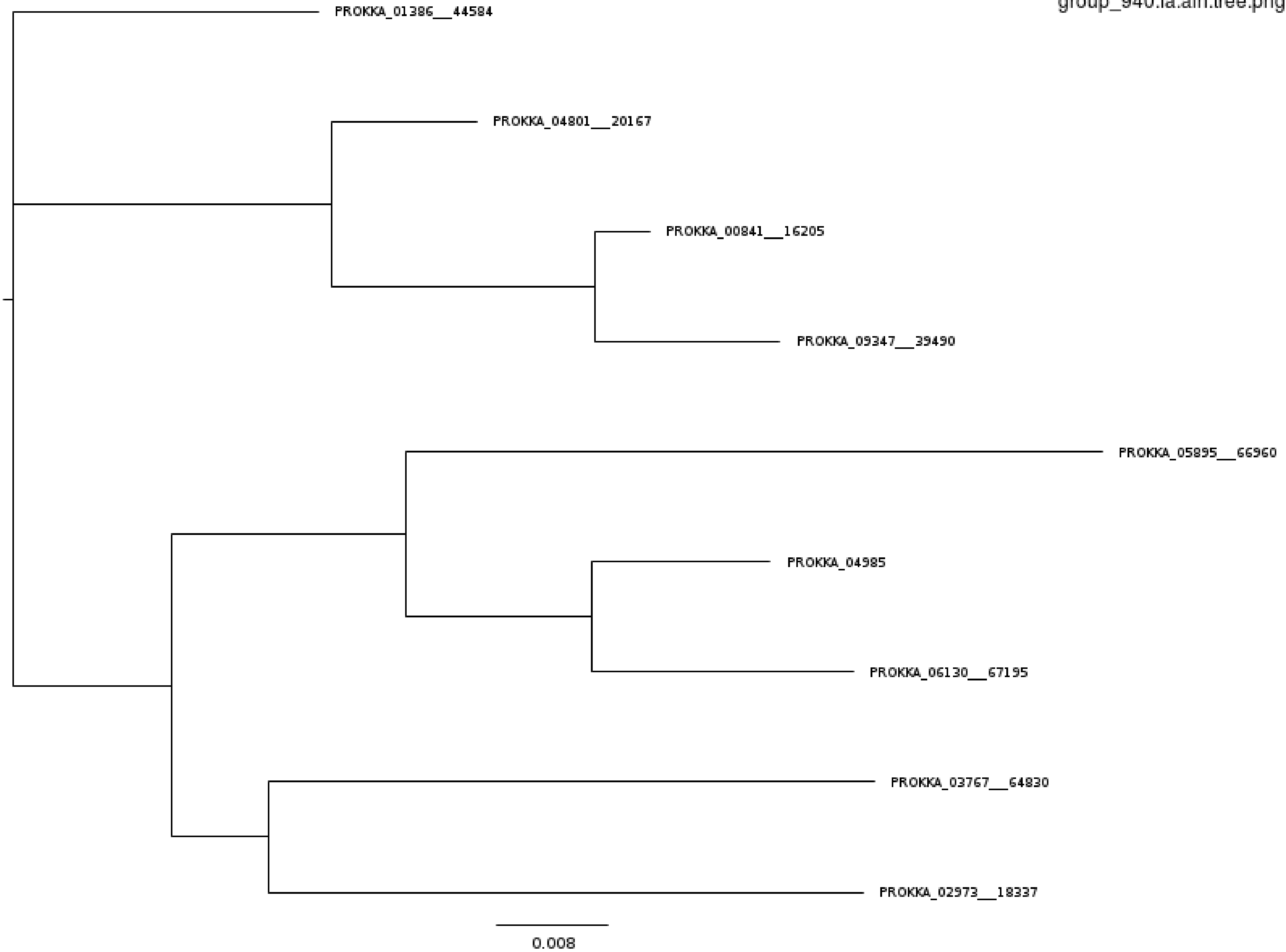

group_942.fa.aln.tree.png

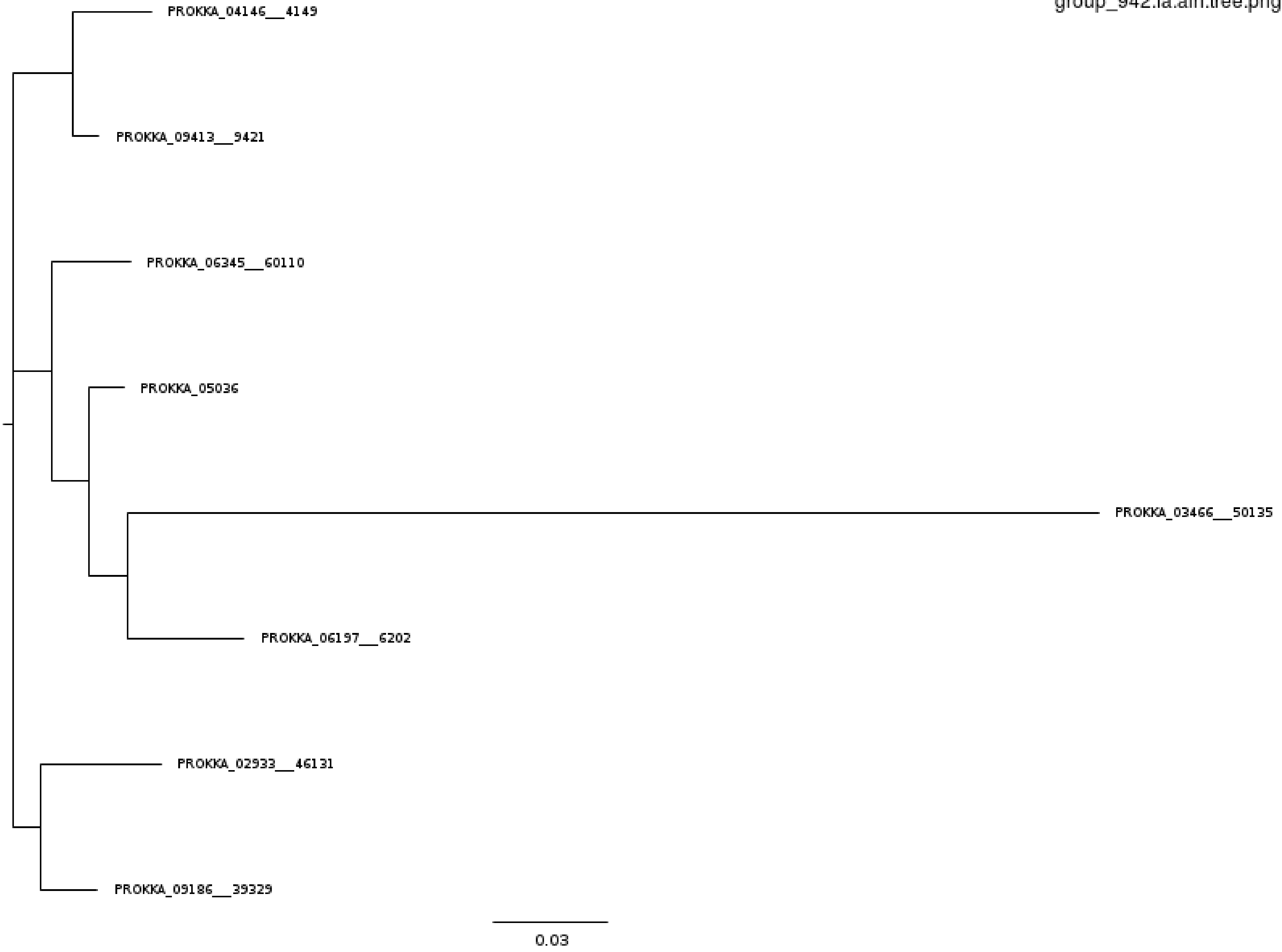

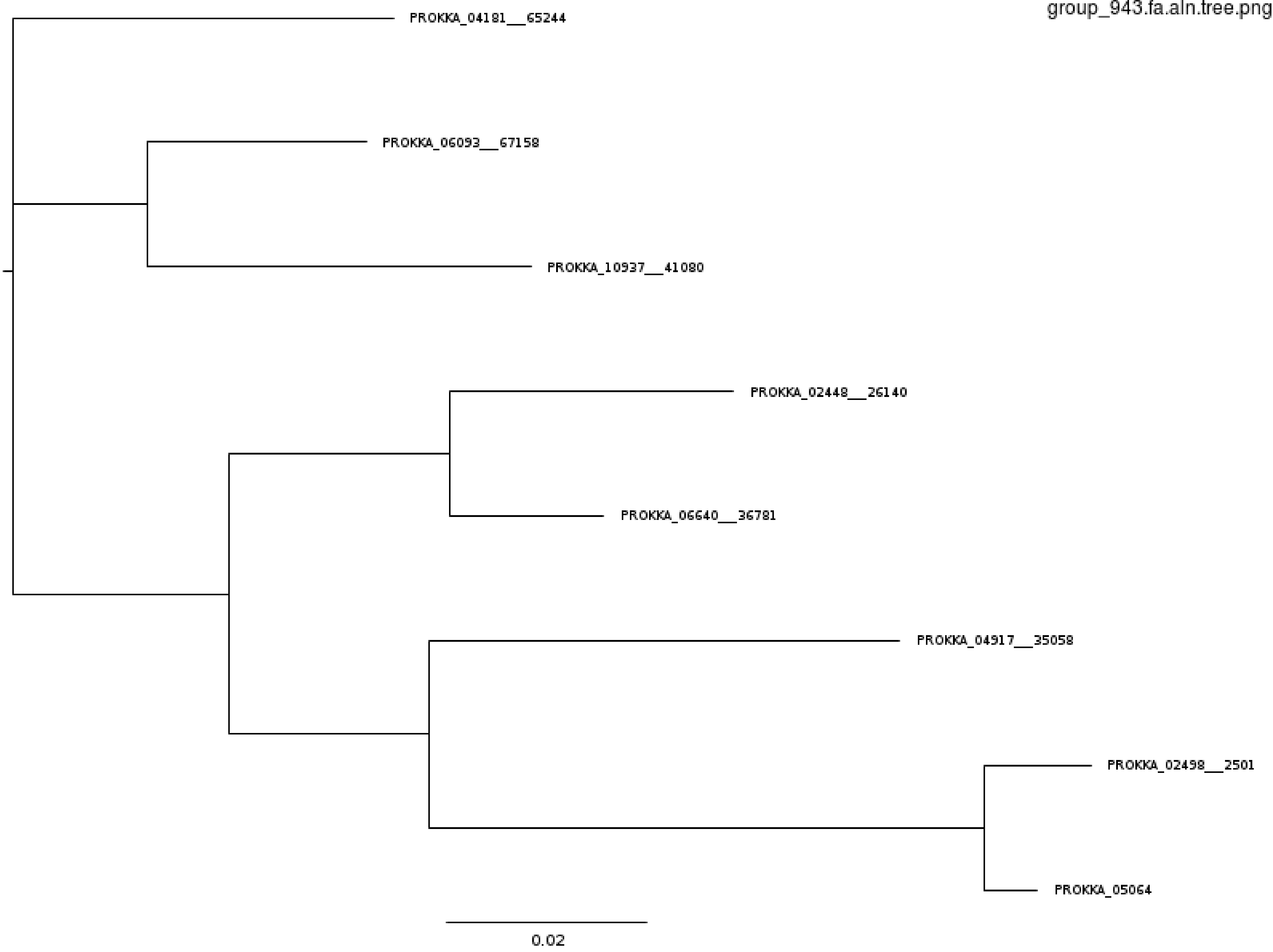
group_943.fa.aln.tree.png
PROKKA_04181___65244
PROKKA_06093___67158
PROKKA_10937___41080
PROKKA_02448___26140
PROKKA_06640___36781
PROKKA_04917___35058
PROKKA_02498___2501
PROKKA_05064
0.02

group_944.fa.aln.tree.png

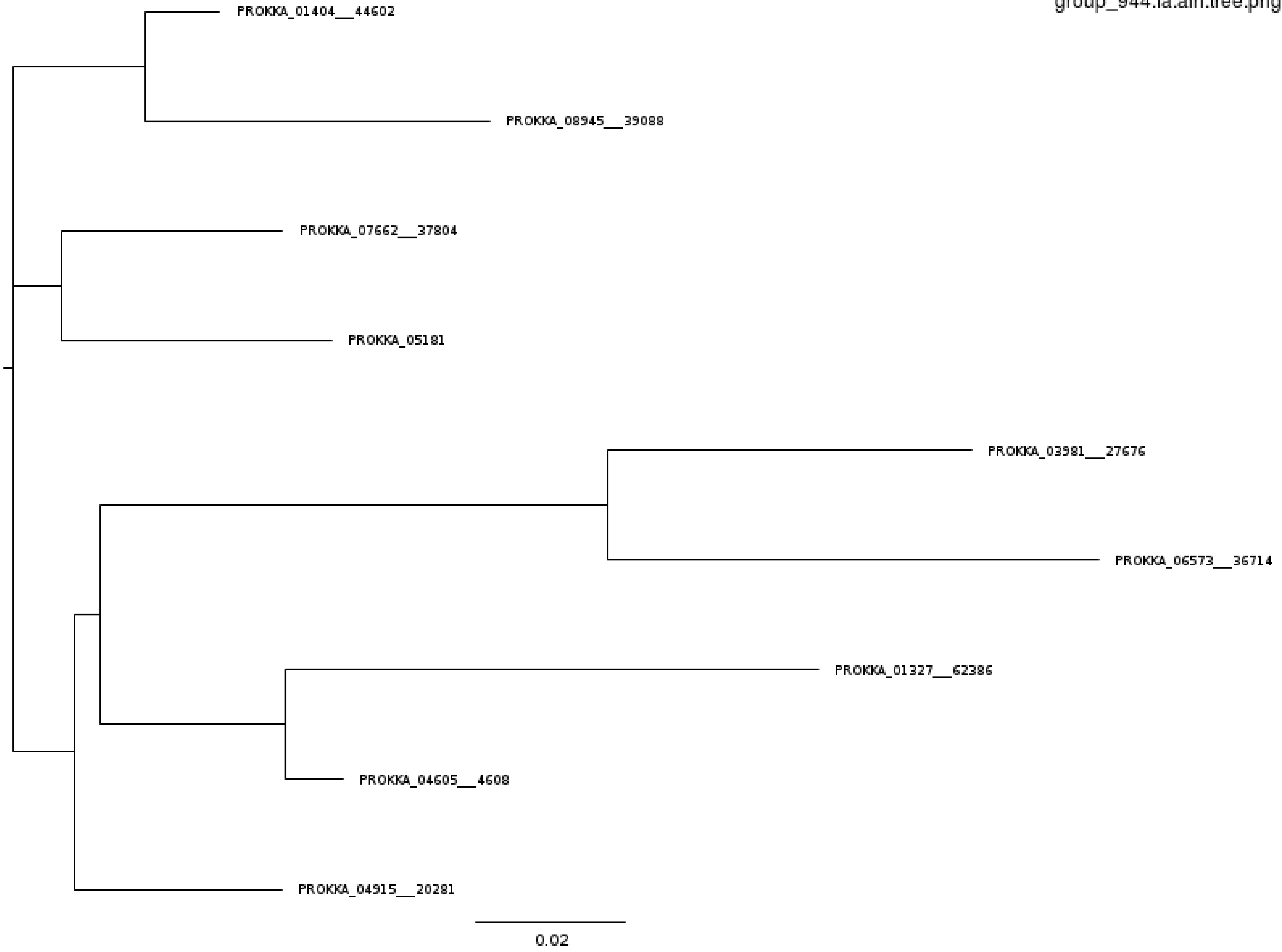

group_948.fa.aln.tree.png

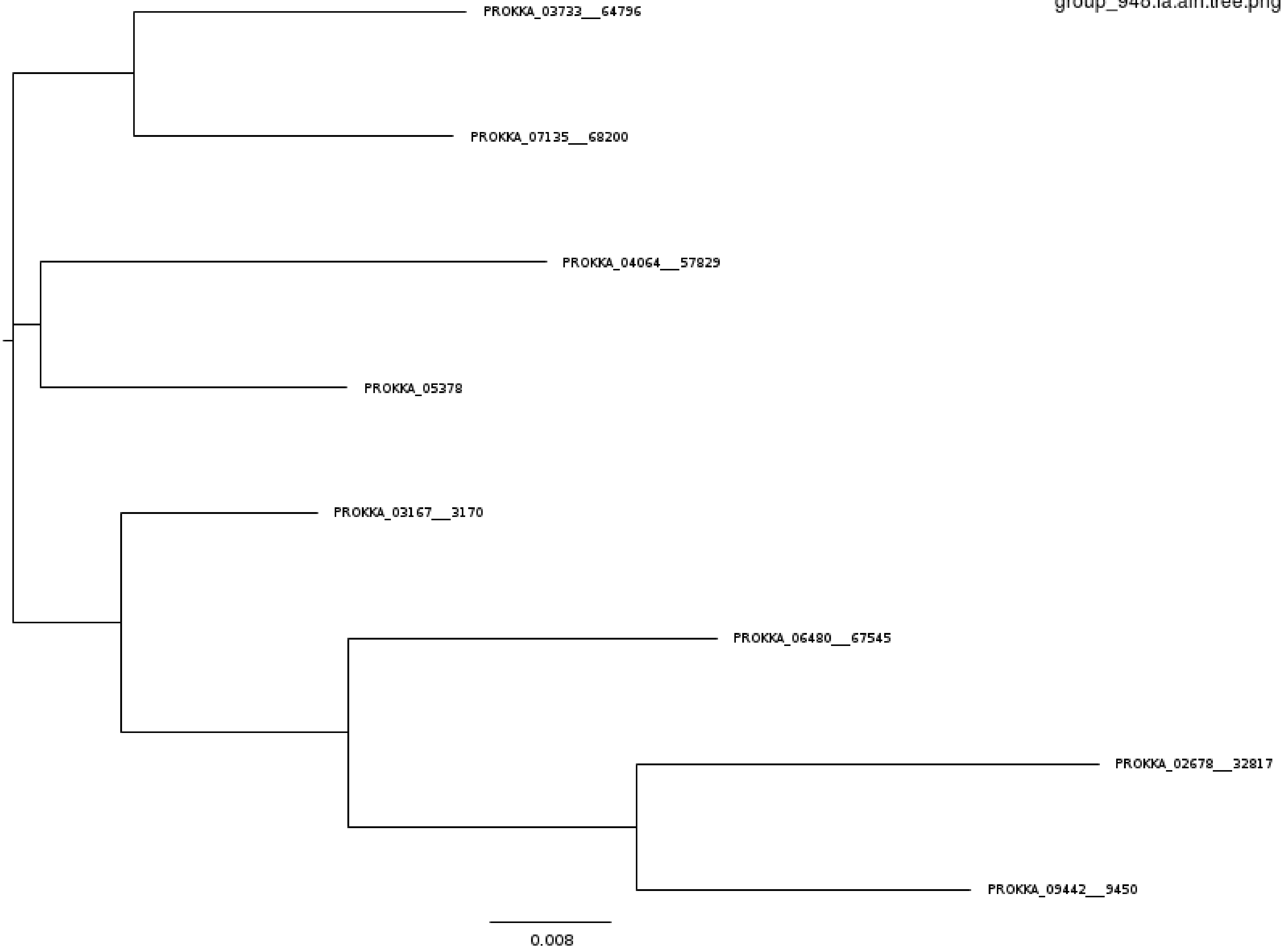

group_949.fa.aln.tree.png

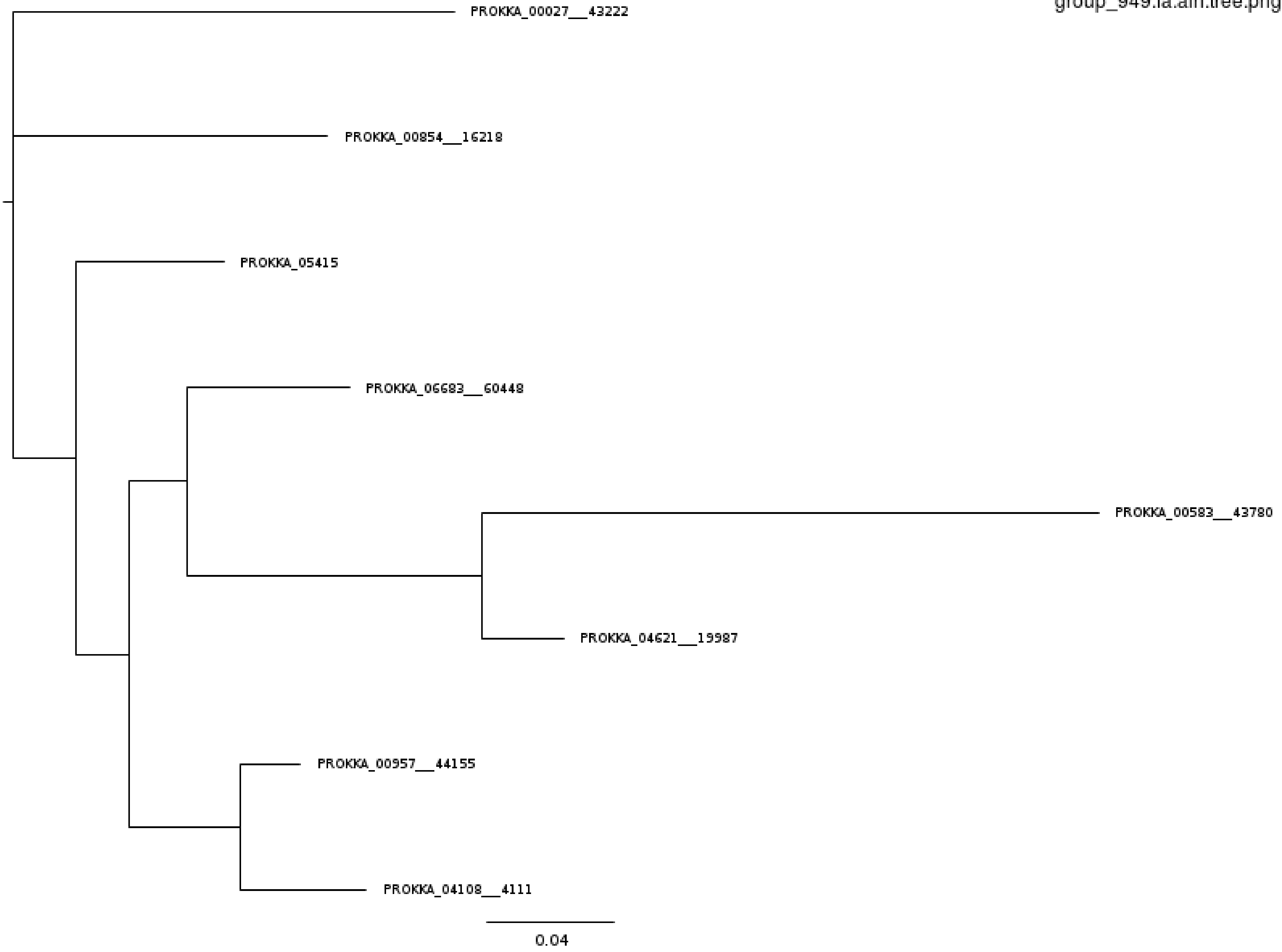

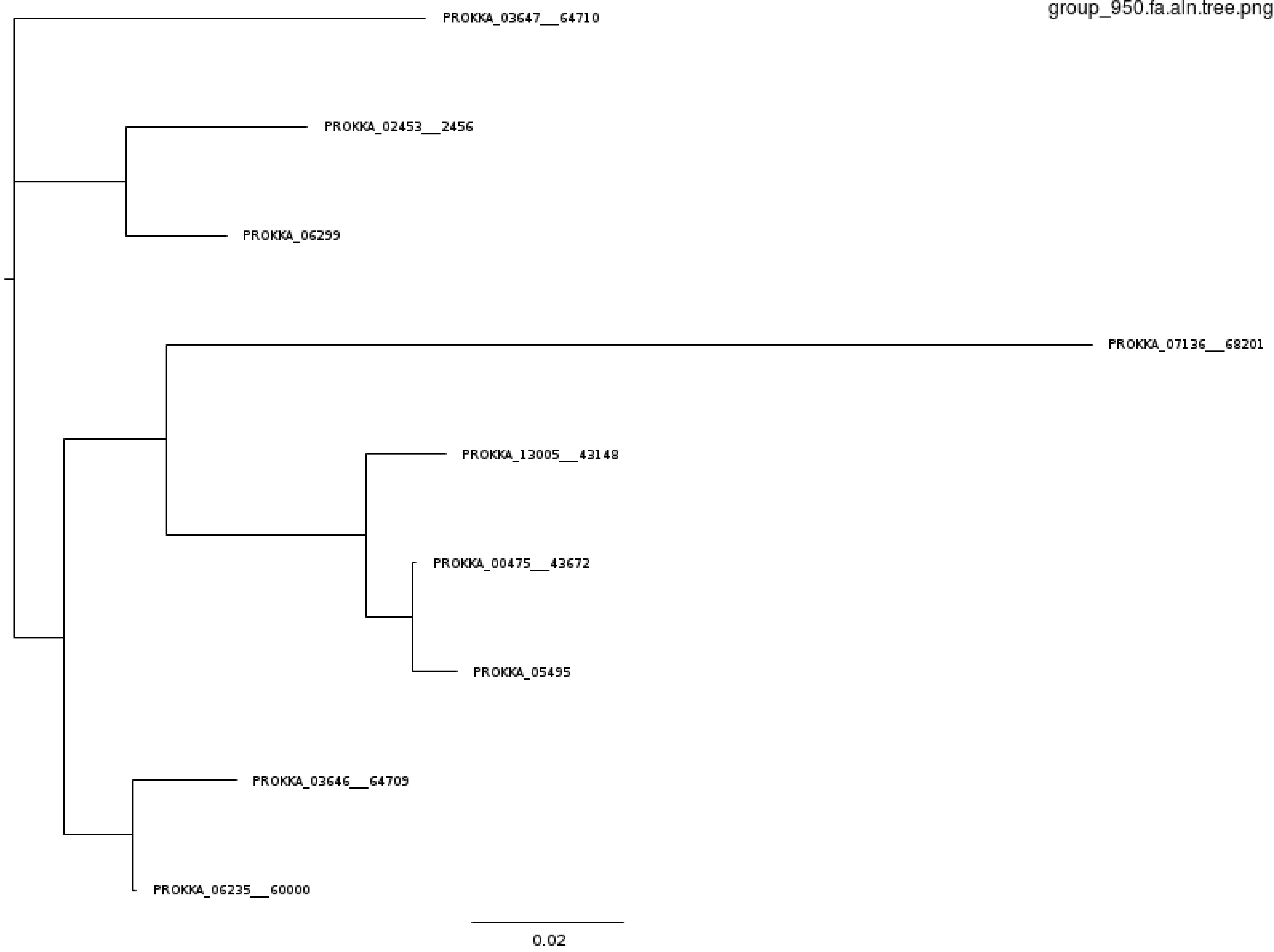
group_950.fa.aln.tree.png
PROKKA_03647___64710
PROKKA_02453___2456
PROKKA_06299
PROKKA_07136___68201
PROKKA_13005___43148
PROKKA_00475___43672
PROKKA_05495
PROKKA_03646___64709
PROKKA_06235___60000
0.02

group_951.fa.aln.tree.png

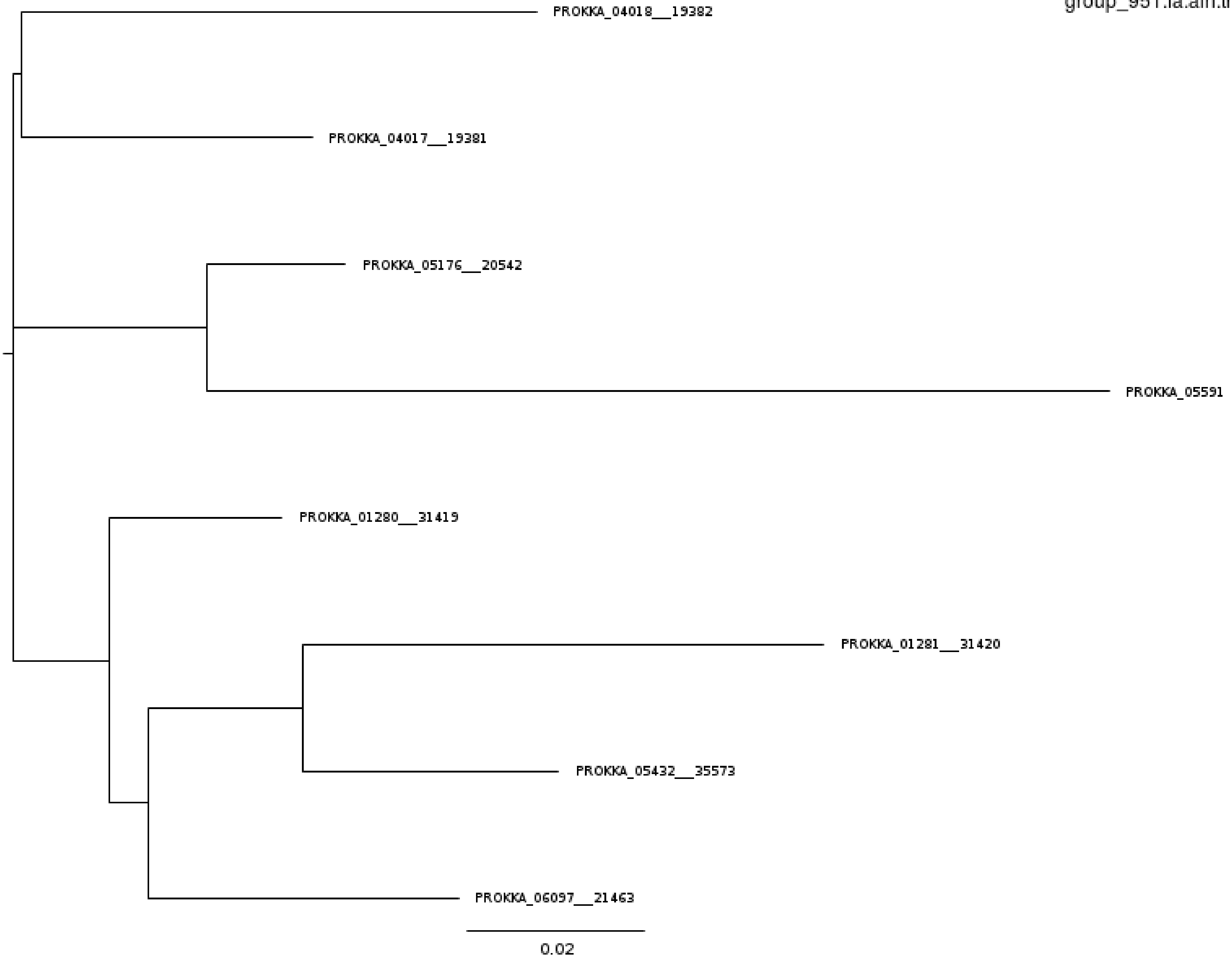

group_952.fa.aln.tree.png

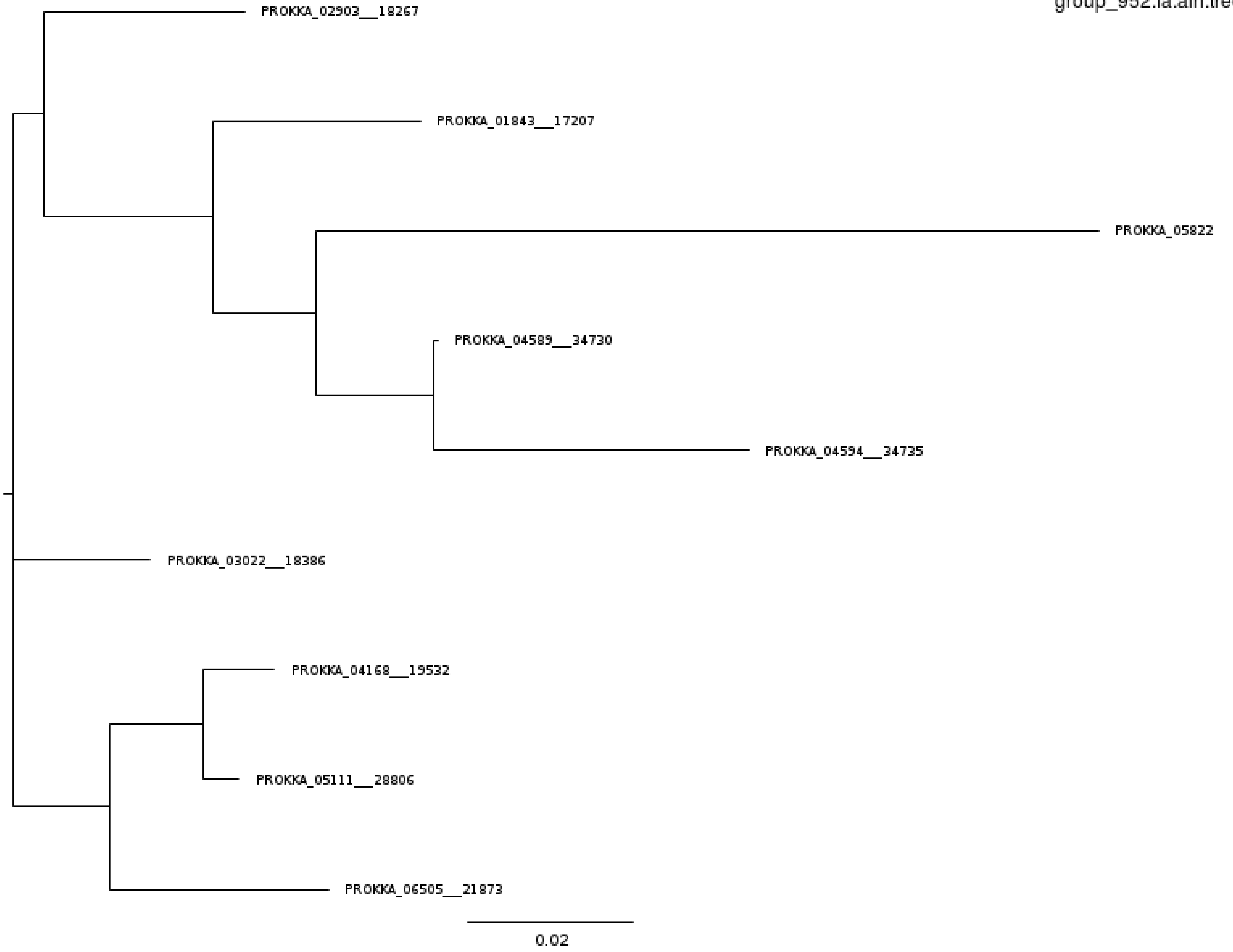

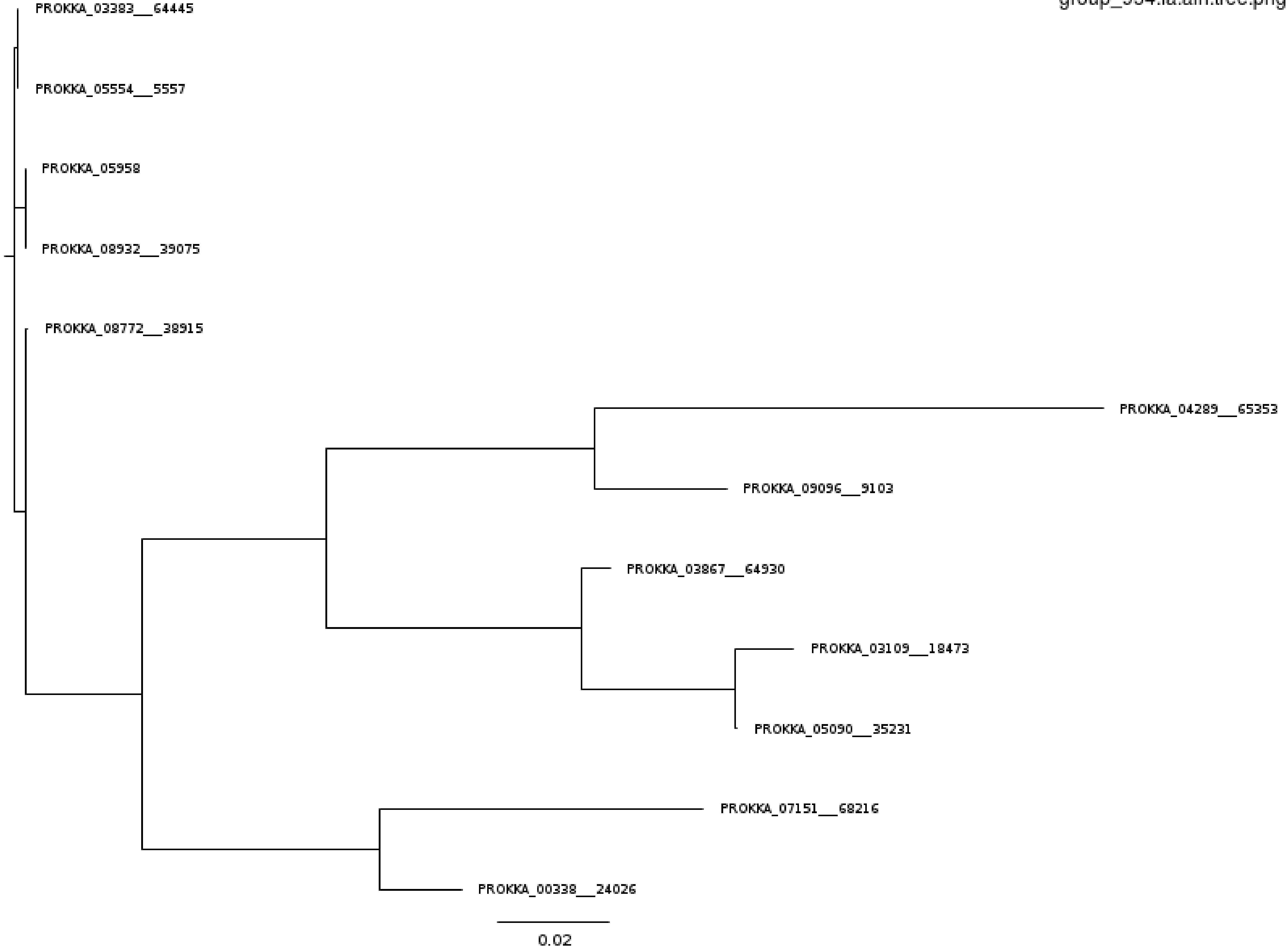
PROKKA_03383___64445
PROKKA_05554___5557
PROKKA_05958
PROKKA_08932___39075
PROKKA_08772___38915
PROKKA_04289___65353
PROKKA_09096___9103
PROKKA_03867___64930
PROKKA_03109___18473
PROKKA_05090___35231
PROKKA_07151___68216
PROKKA_00338___24026
0.02

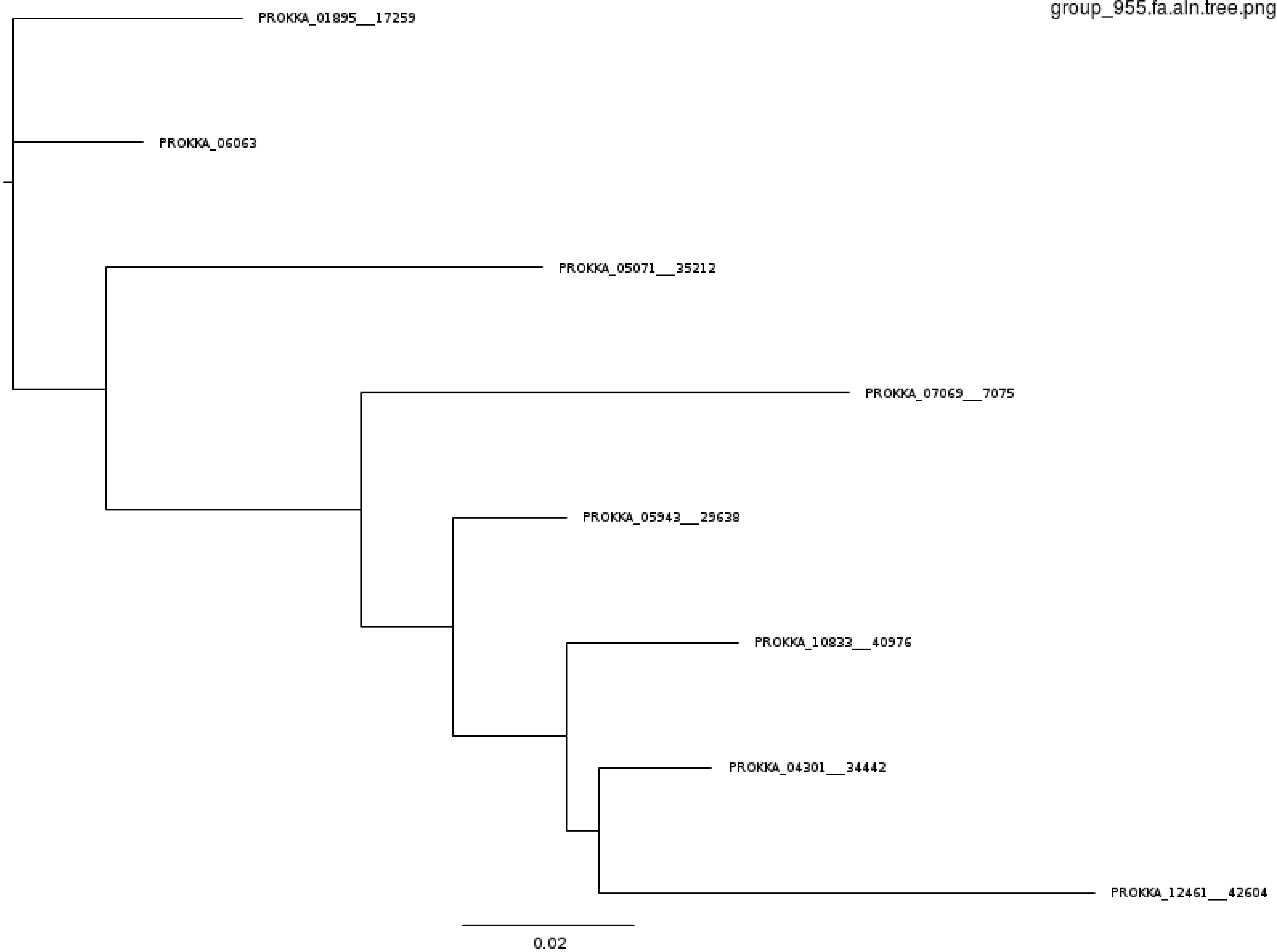

group_955.fa.aln.tree.png
PROKKA_01895___17259
PROKKA_06063
PROKKA_05071___35212
PROKKA_07069___7075
PROKKA_05943___29638
PROKKA_10833___40976
PROKKA_04301___34442
PROKKA_12461___42604
0.02

group_956.fa.aln.tree.png

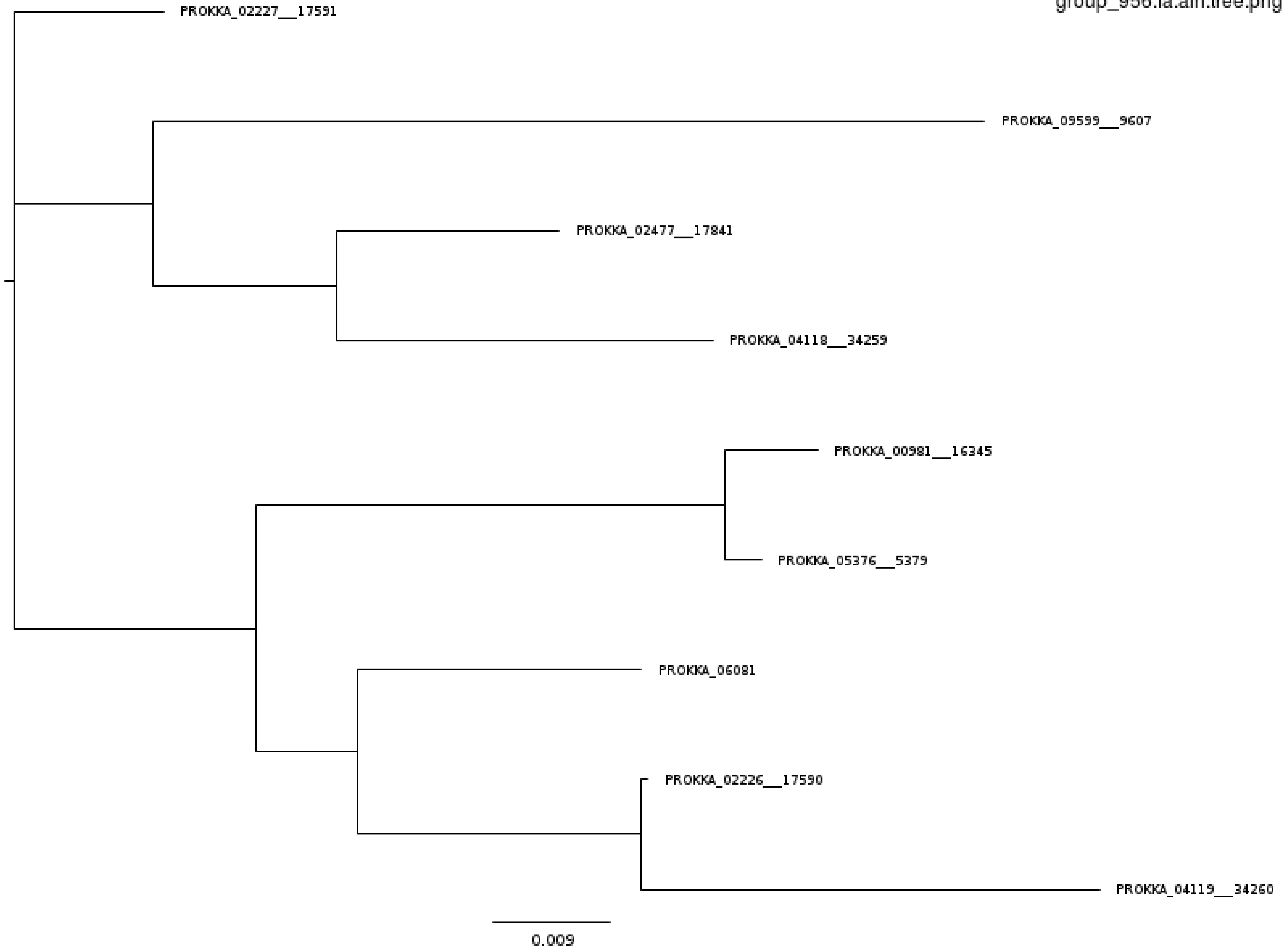

group_957.fa.aln.tree.png

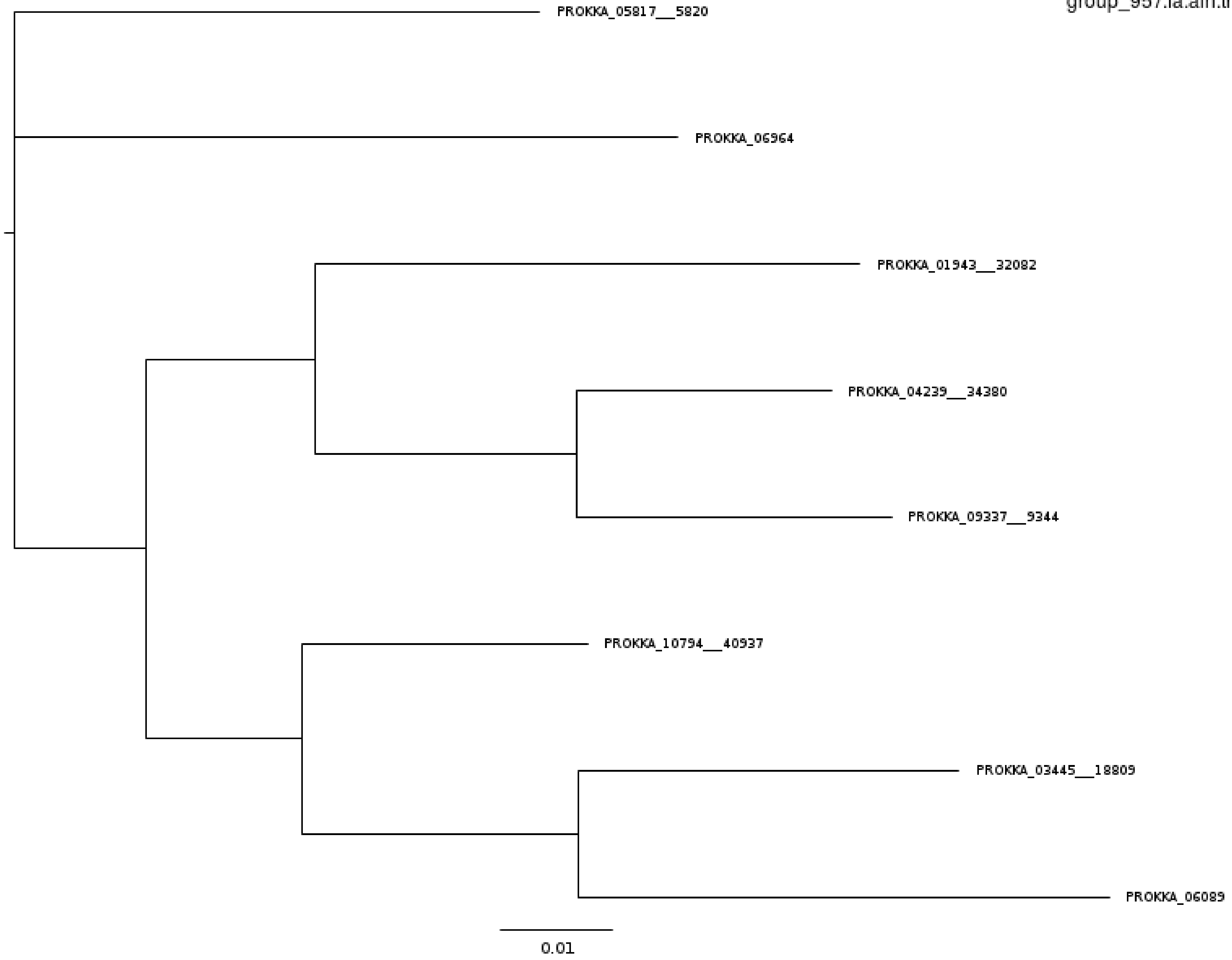

group_963.fa.aln.tree.png

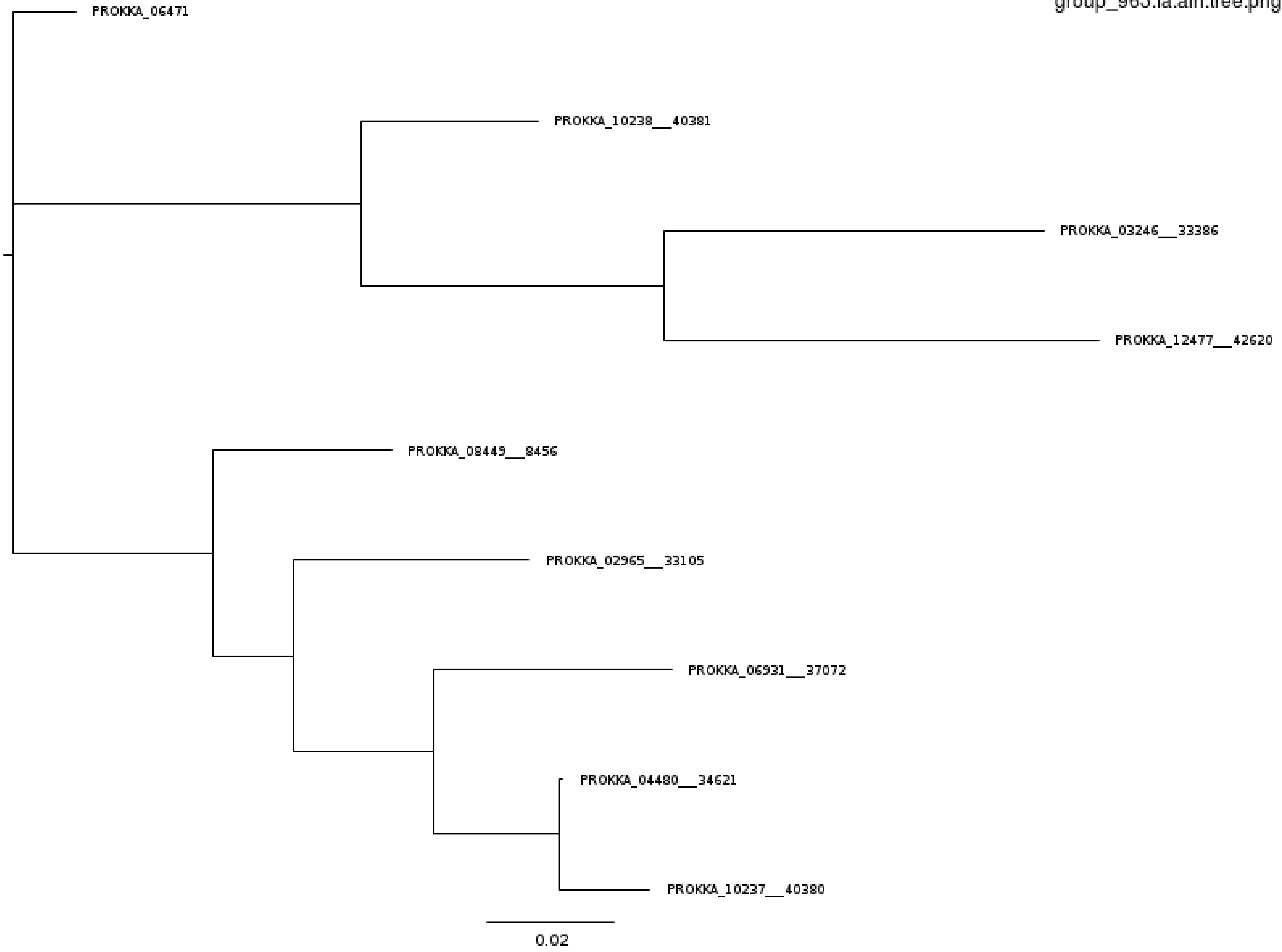

group_969.fa.aln.tree.png

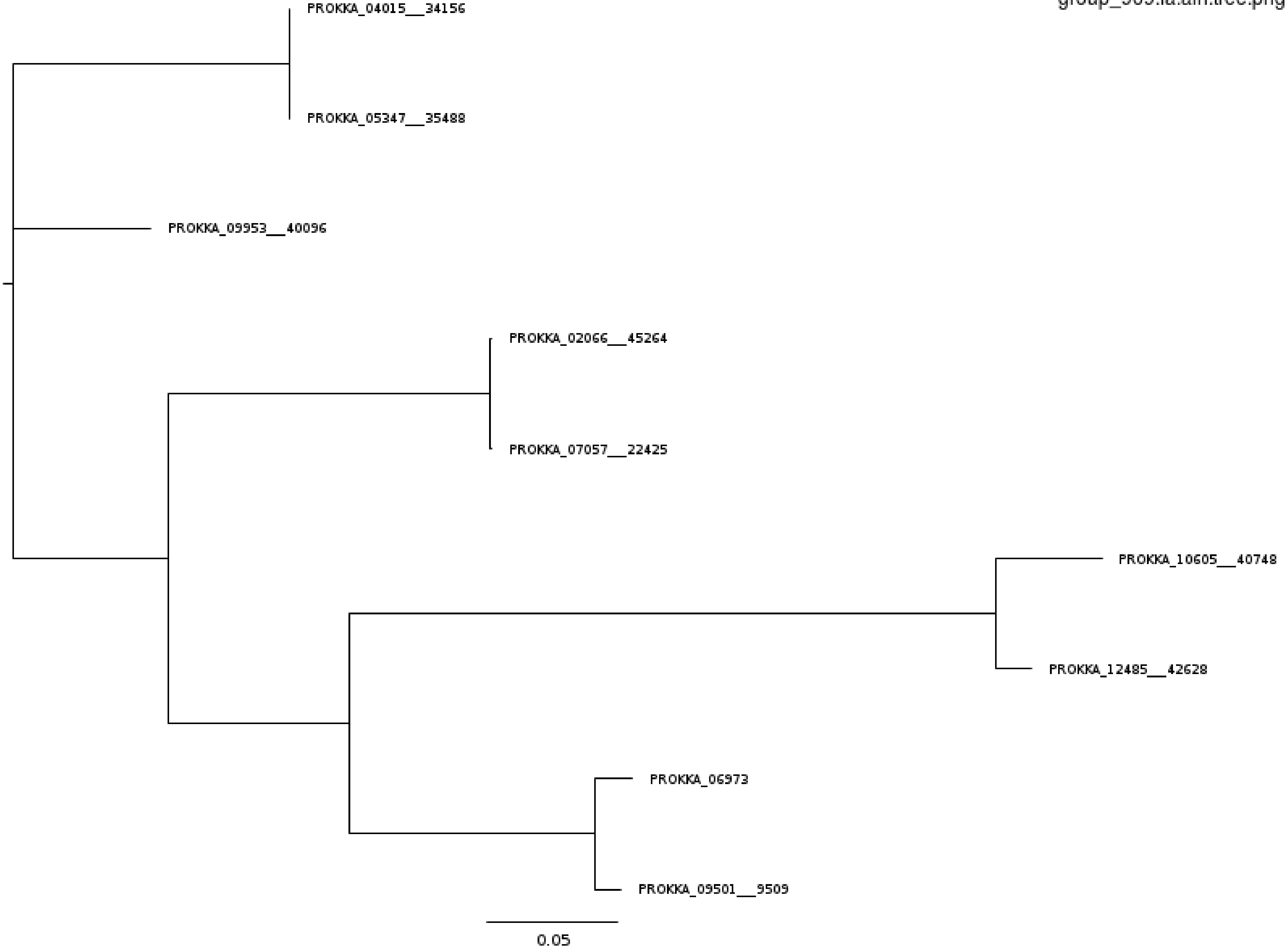

group_970.fa.aln.tree.png

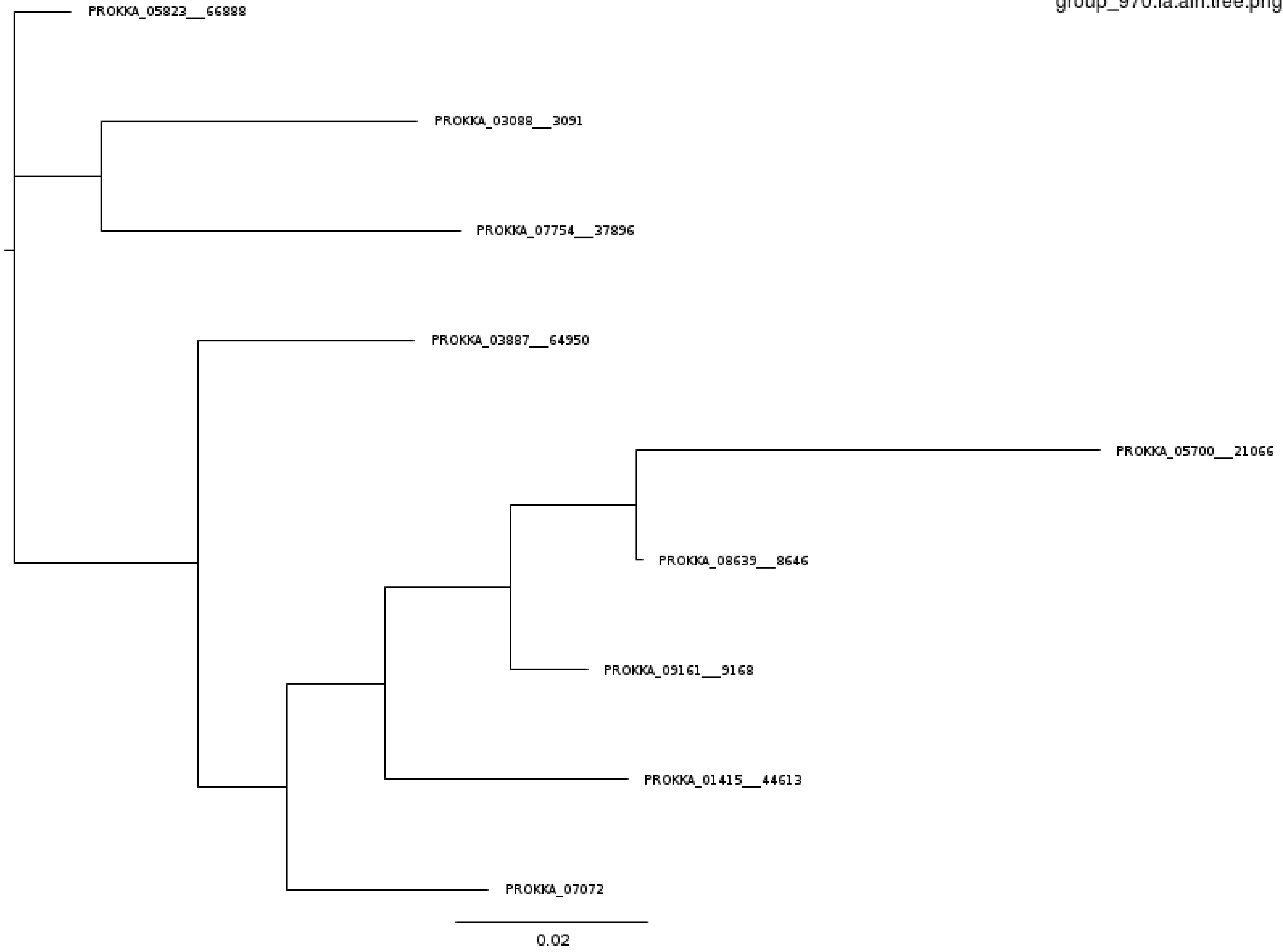

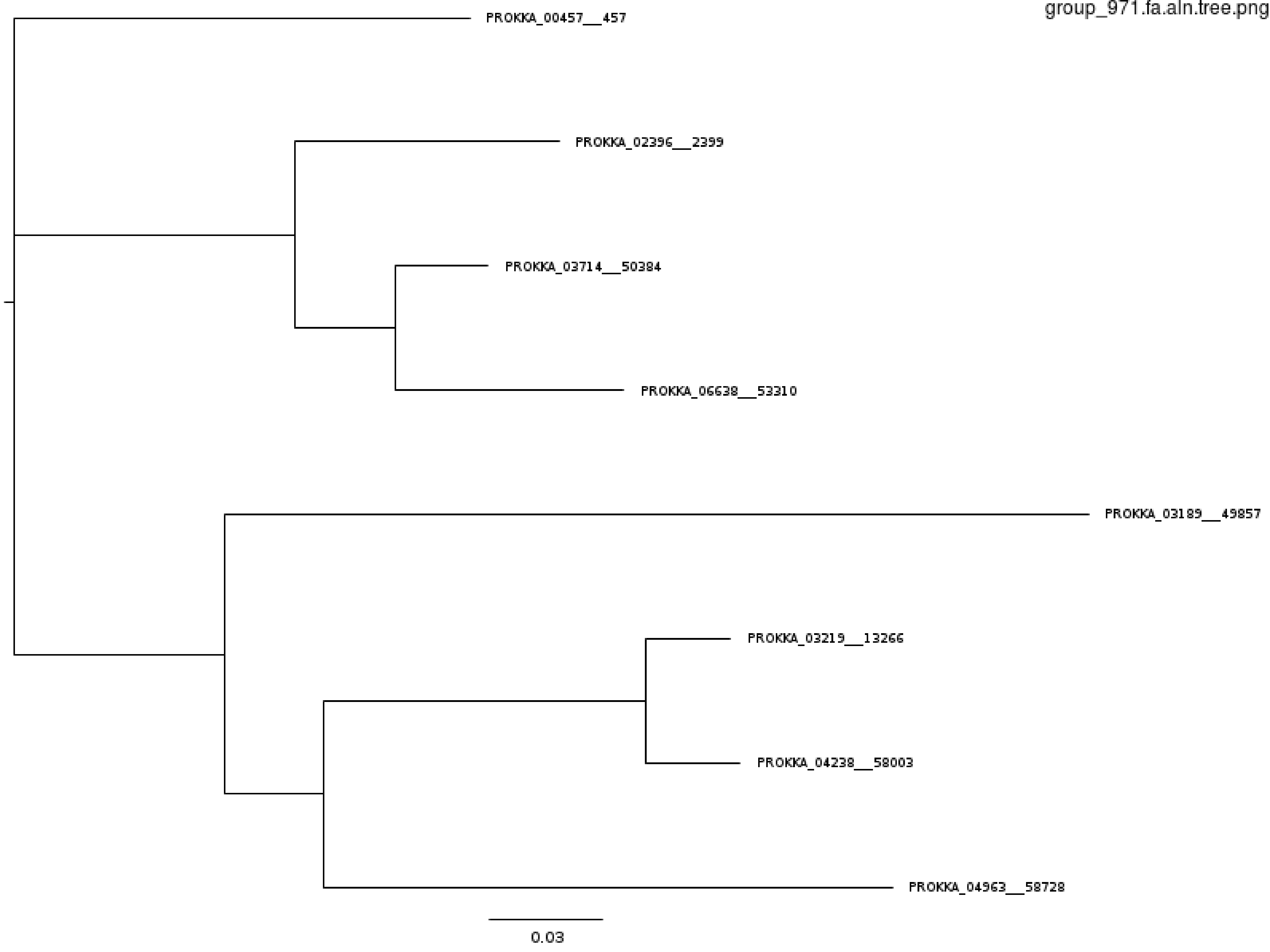
group_971.fa.aln.tree.png
PROKKA_00457___457
PROKKA_02396___2399
PROKKA_03714___50384
PROKKA_06638___53310
PROKKA_03189___49857
PROKKA_03219___13266
PROKKA_04238___58003
PROKKA_04963___58728
0.03

group_972.fa.aln.tree.png

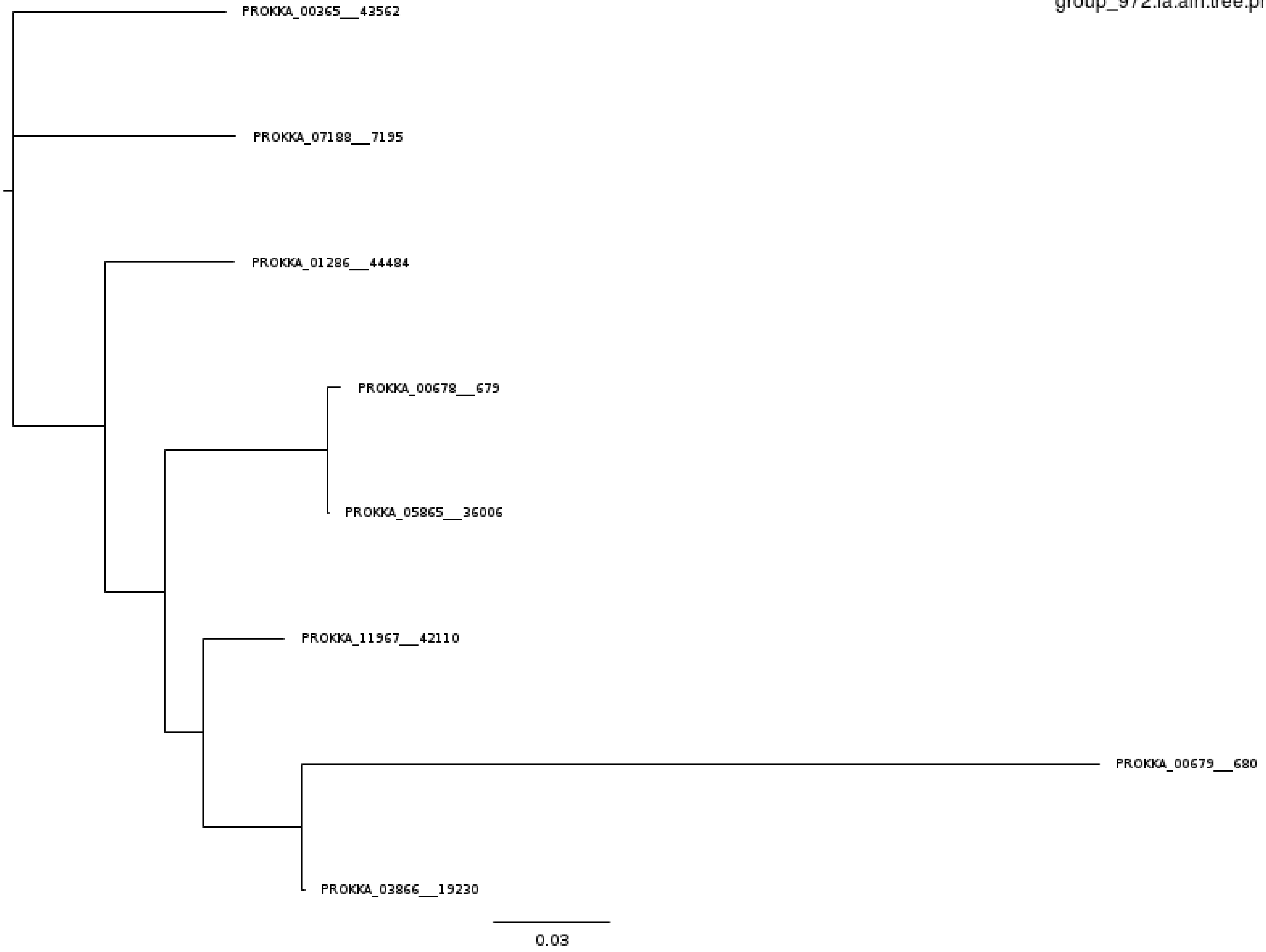

group_973.fa.aln.tree.png

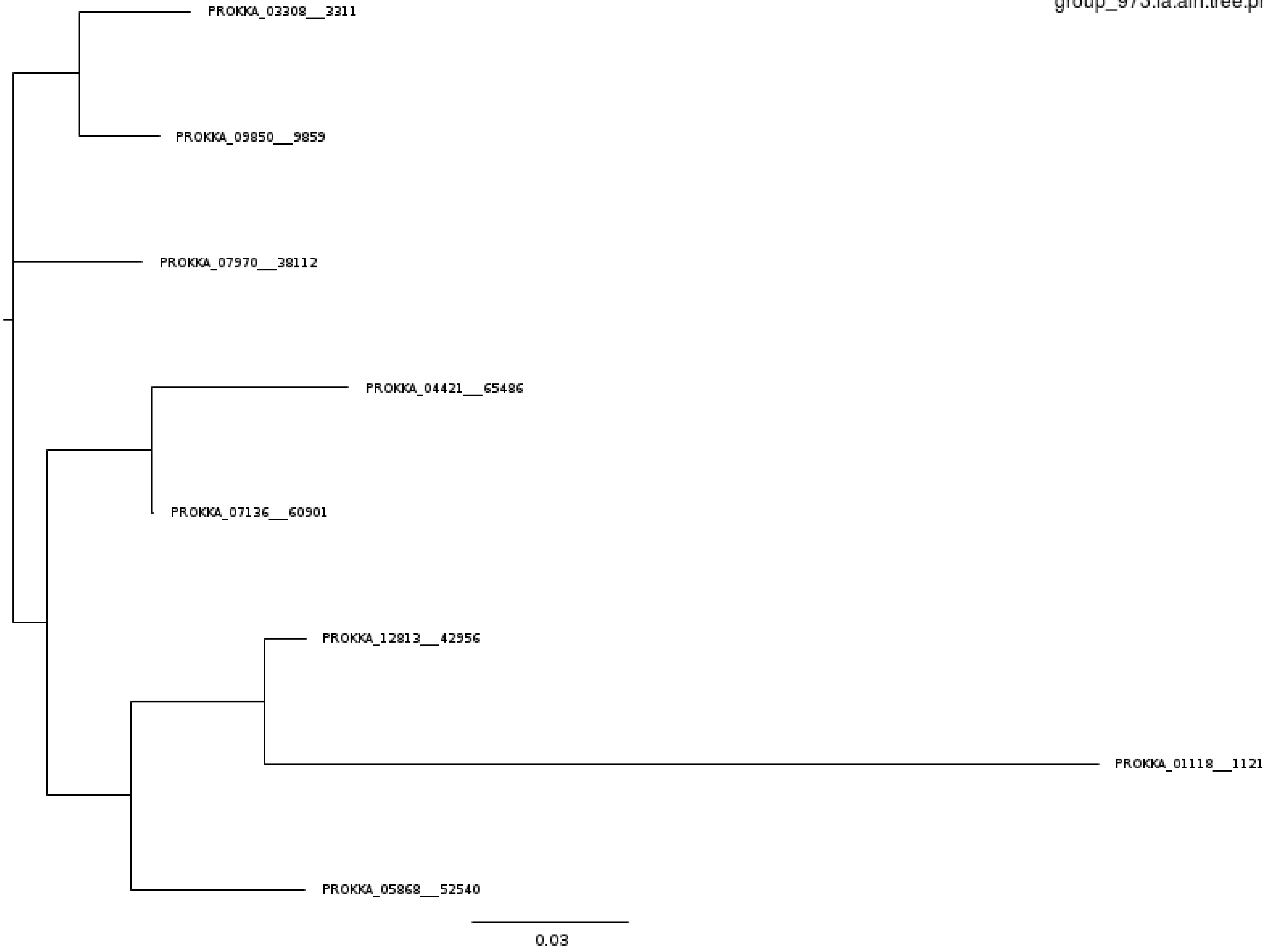

group_987.fa.aln.tree.png

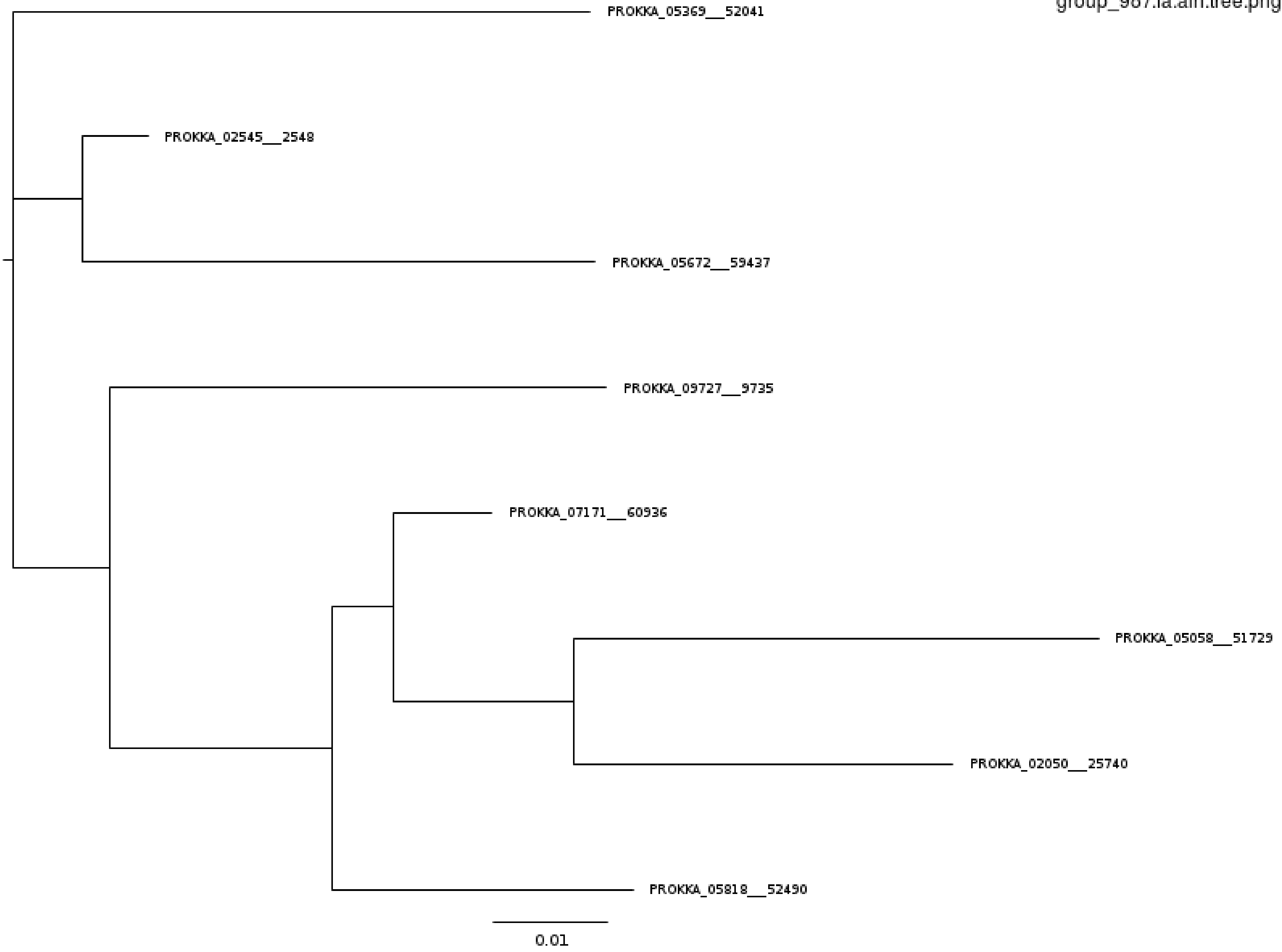

group_990.fa.aln.tree.png

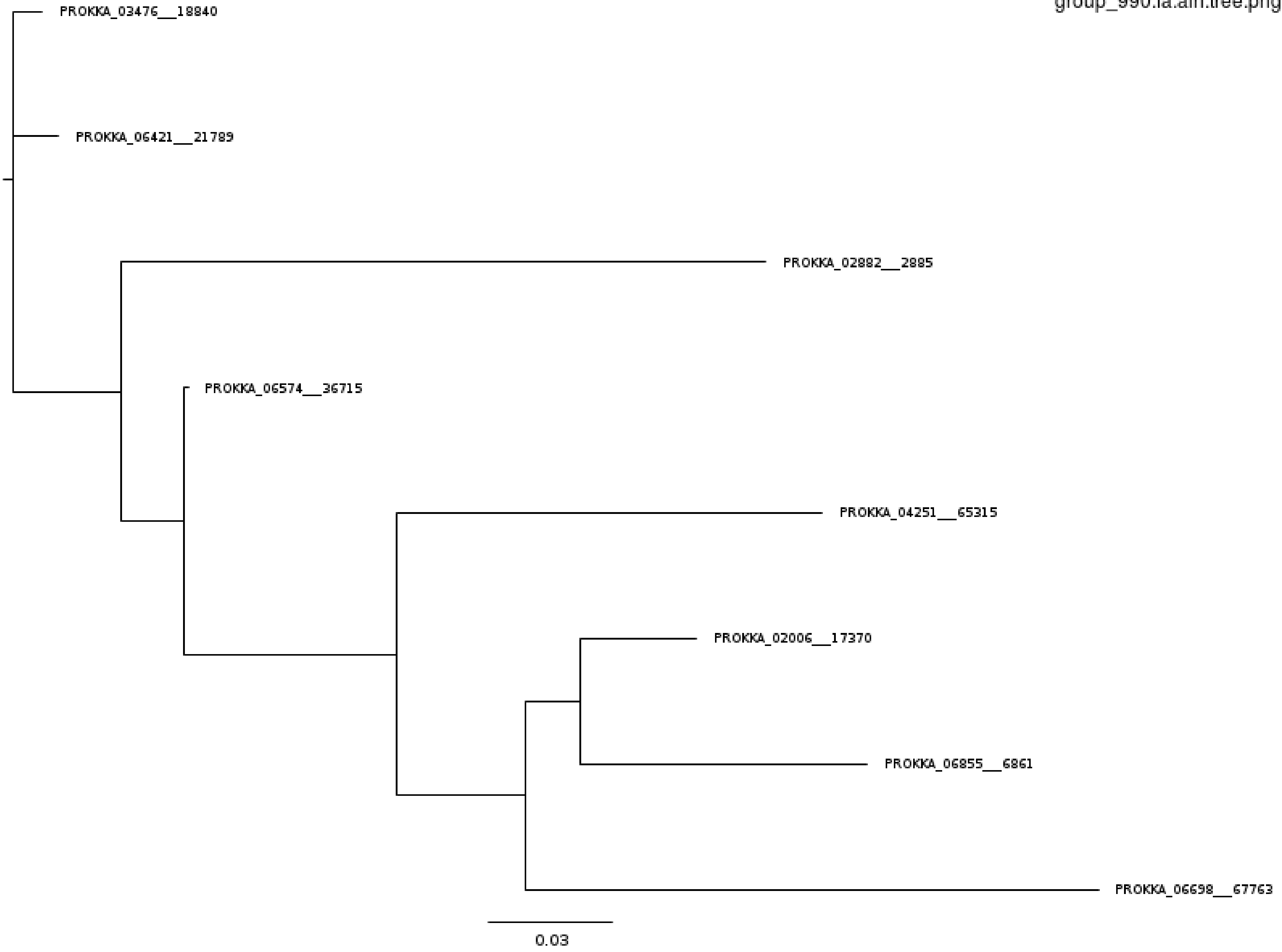

group_991.fa.aln.tree.png

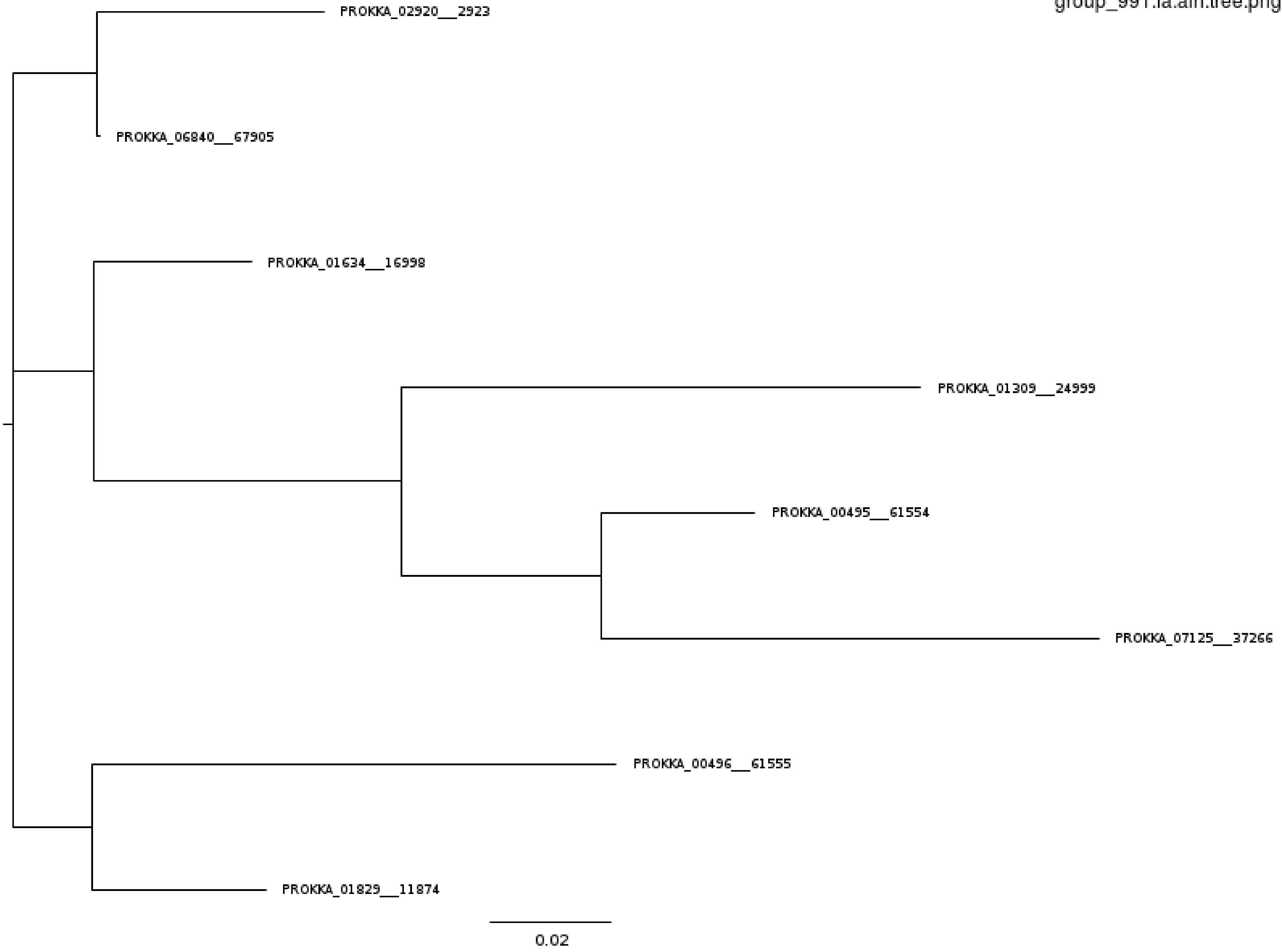

group_992.fa.aln.tree.png

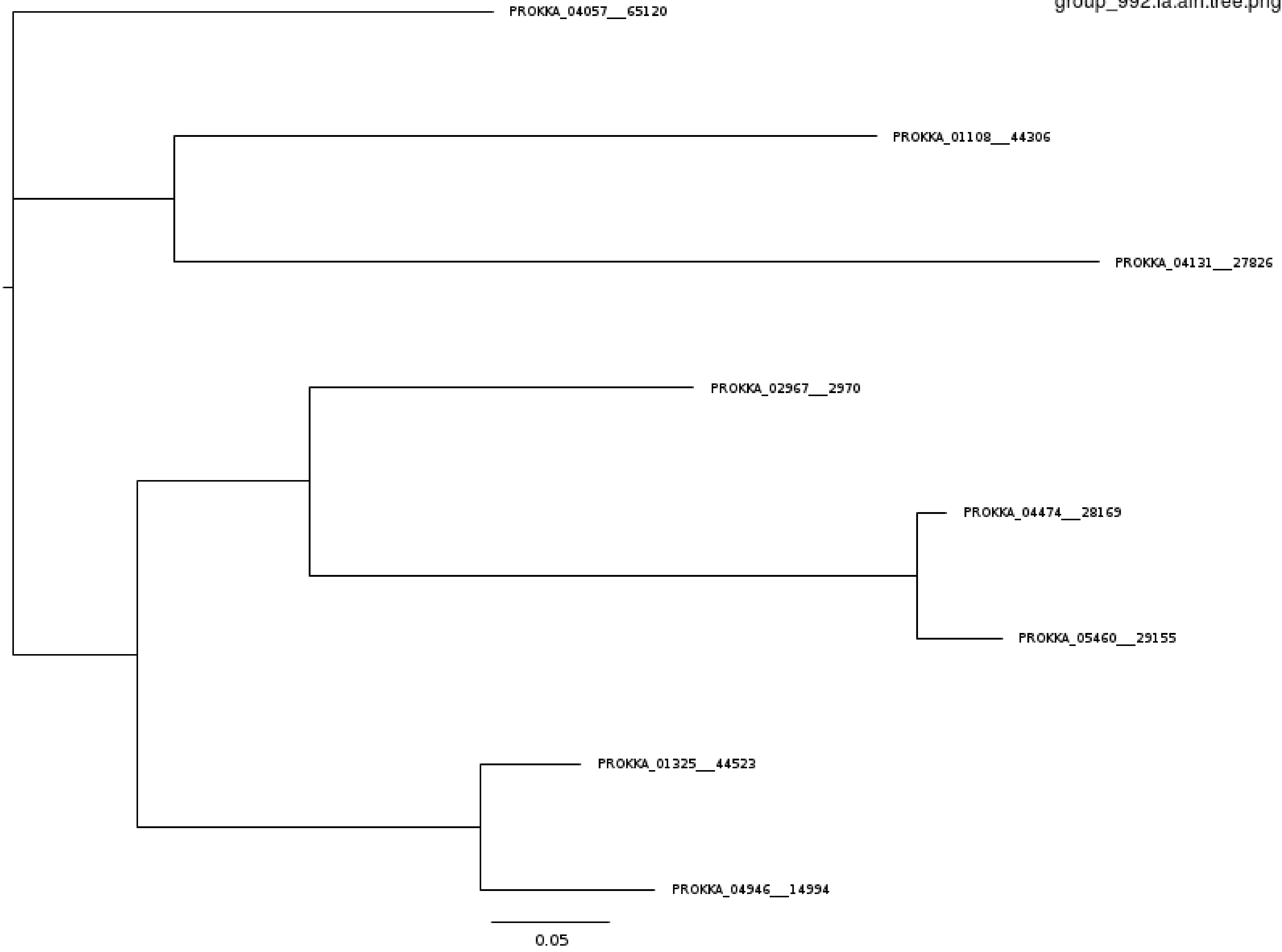

group_993.fa.aln.tree.png

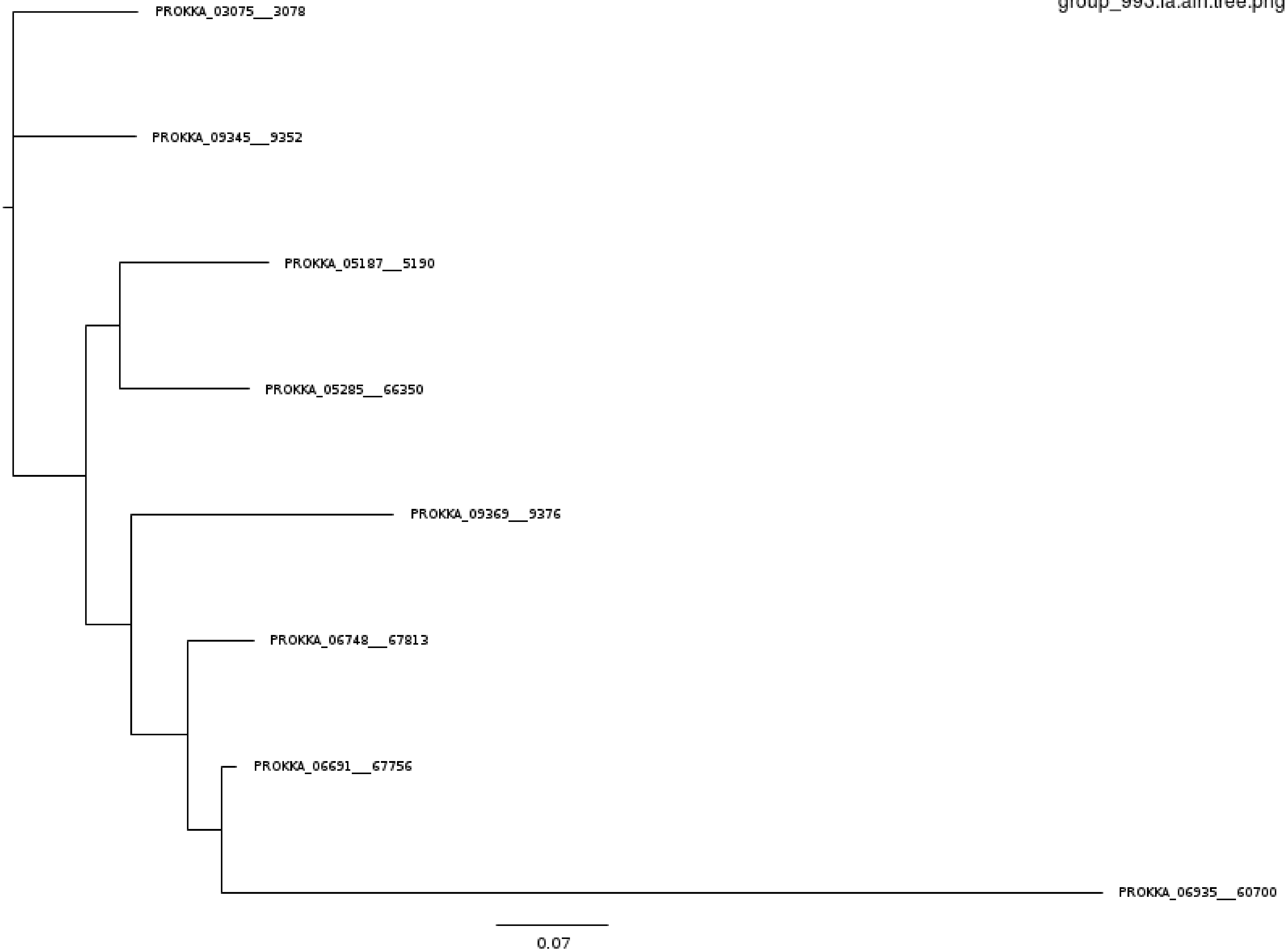

group_994.fa.aln.tree.png

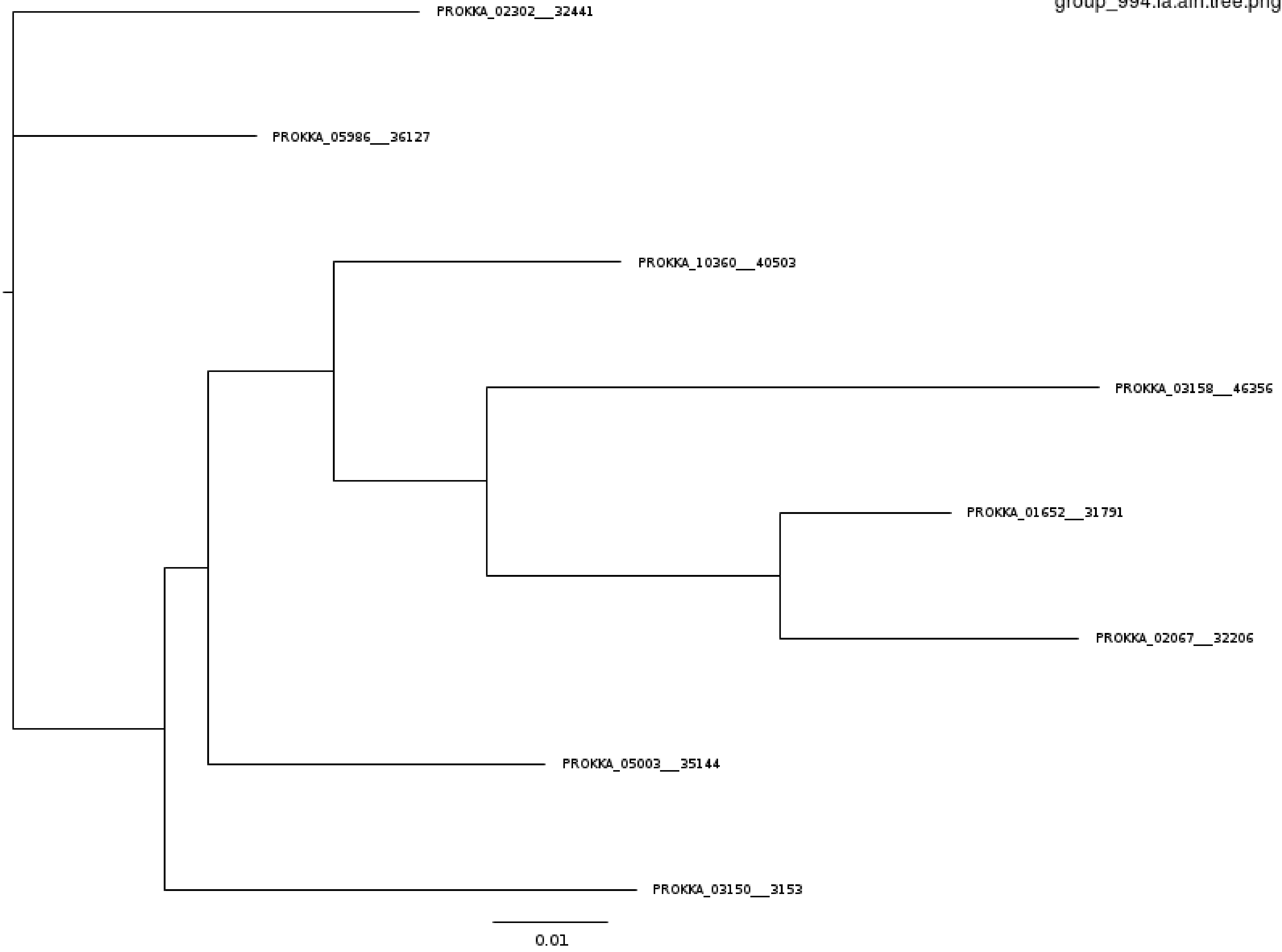

group_997.fa.aln.tree.png

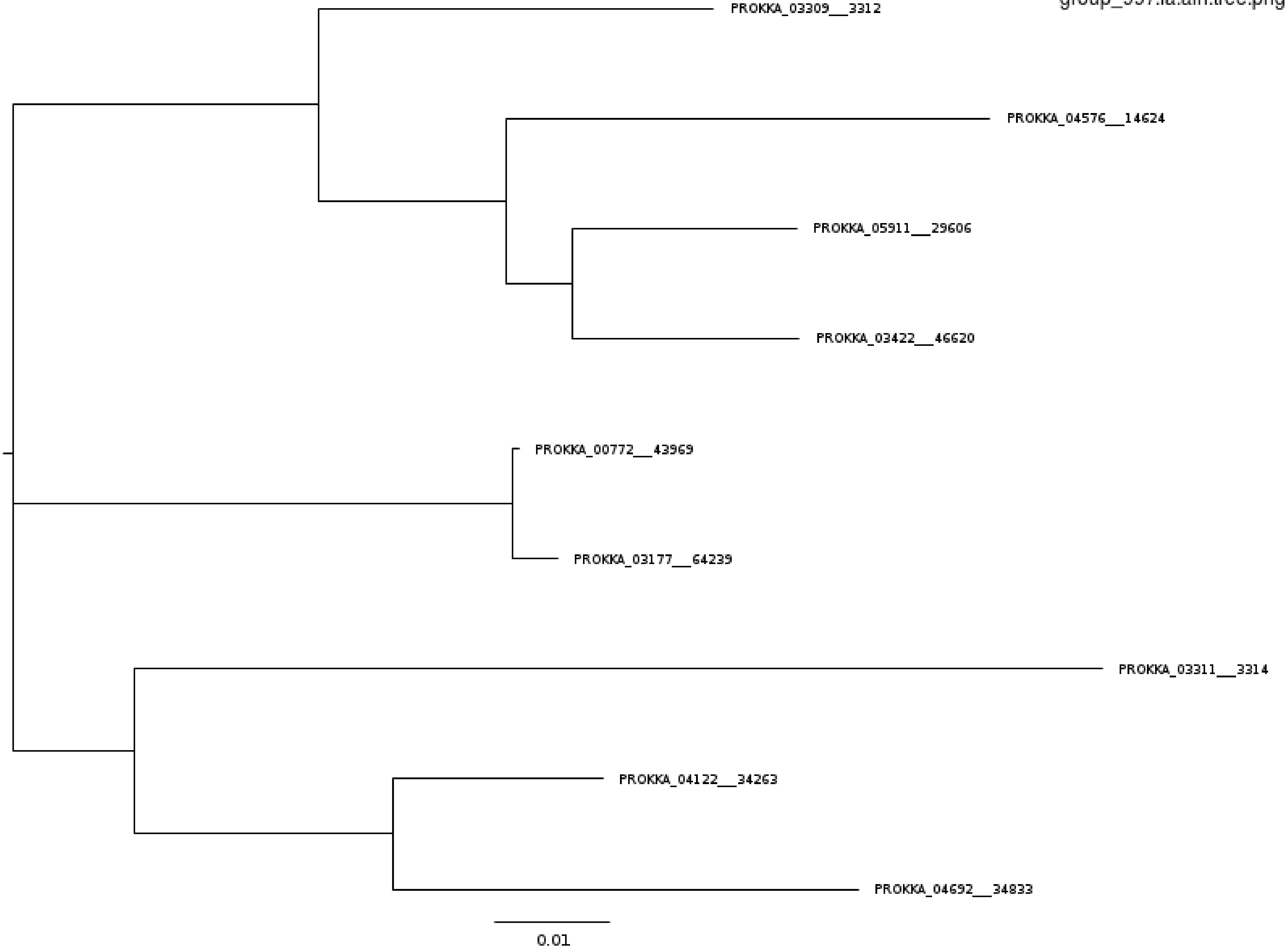

group_9.fa.aln.tree.png

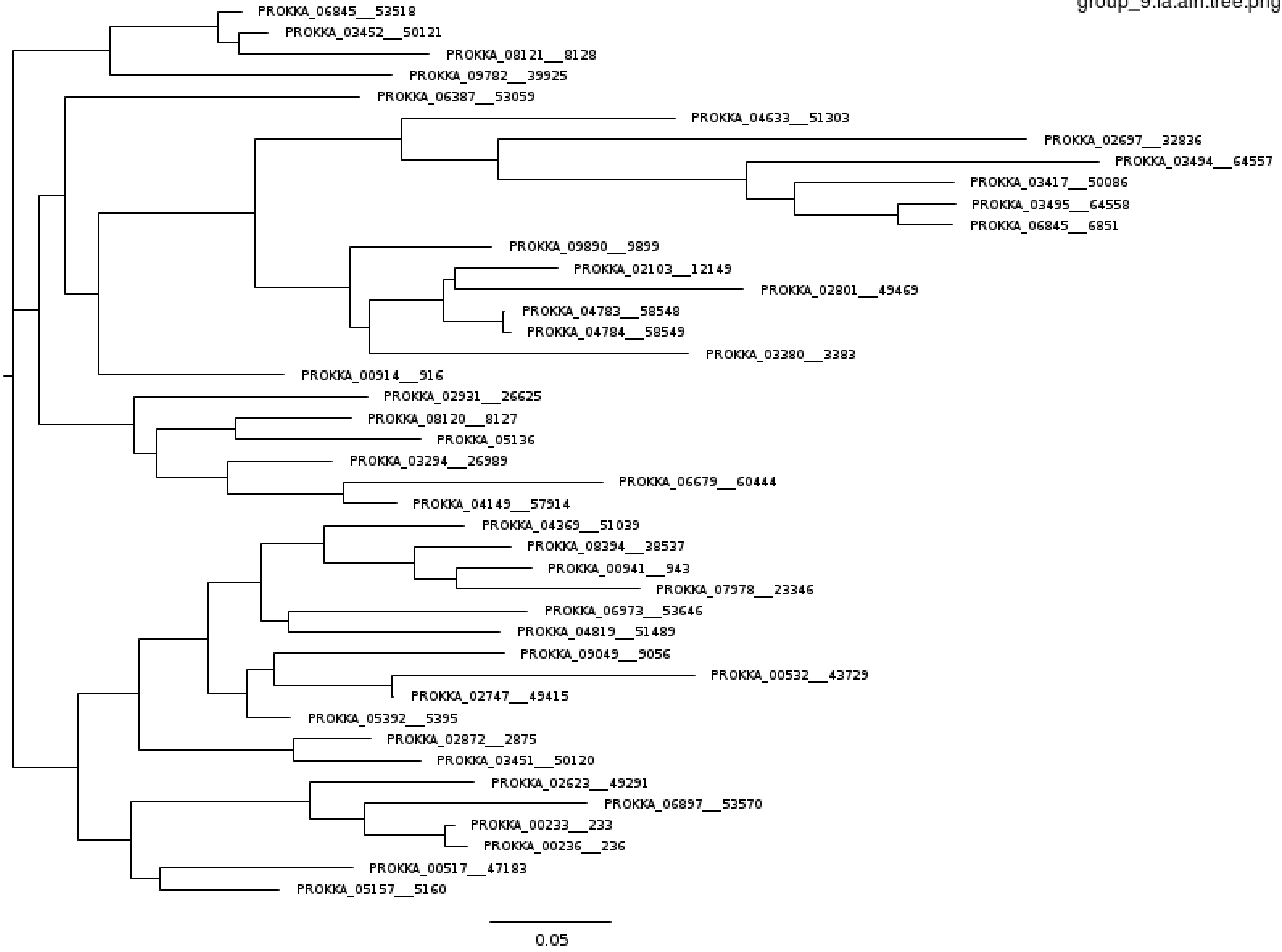

grpE_1.fa.aln.tree.png

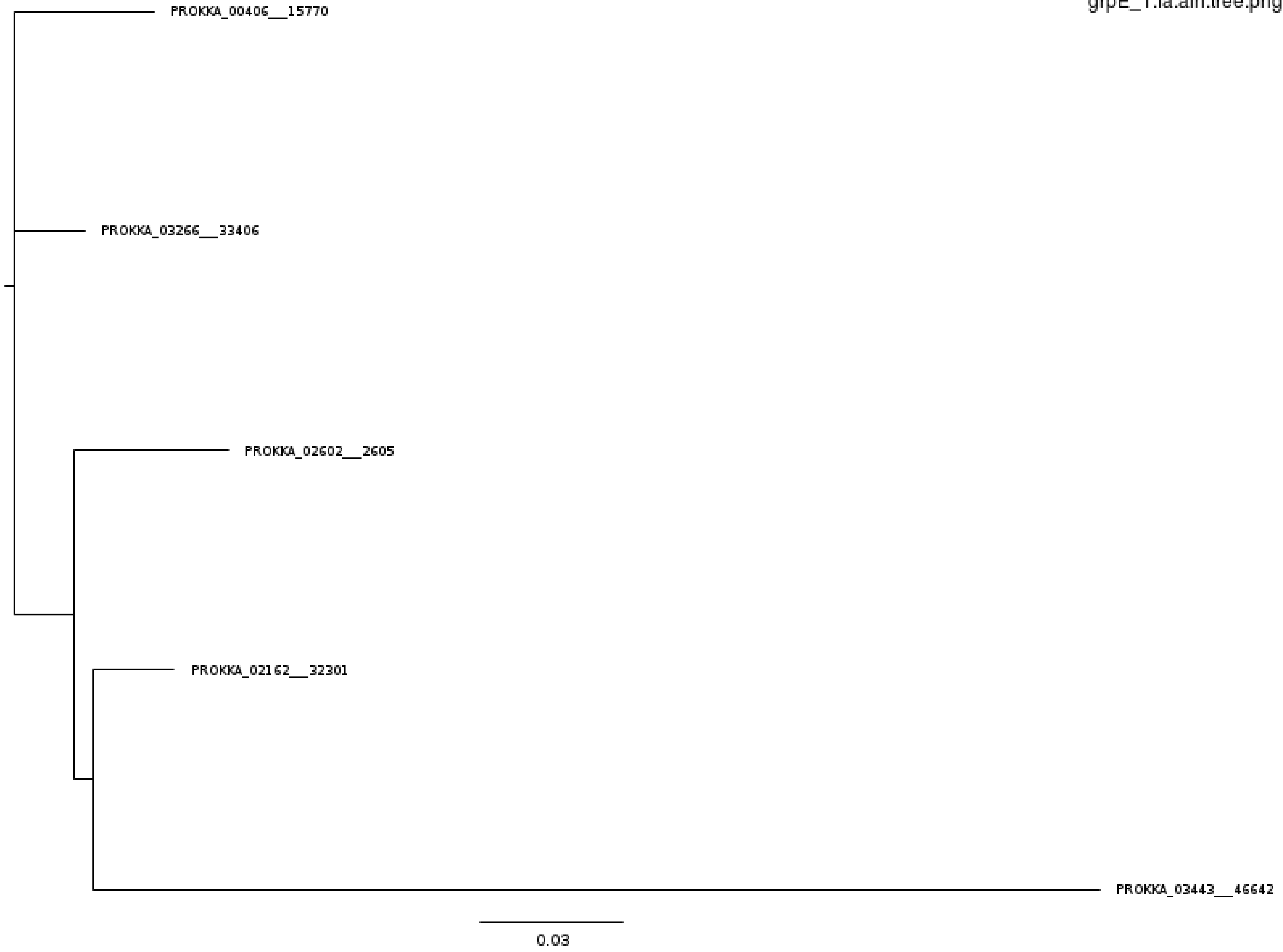

grxC_1.fa.aln.tree.png

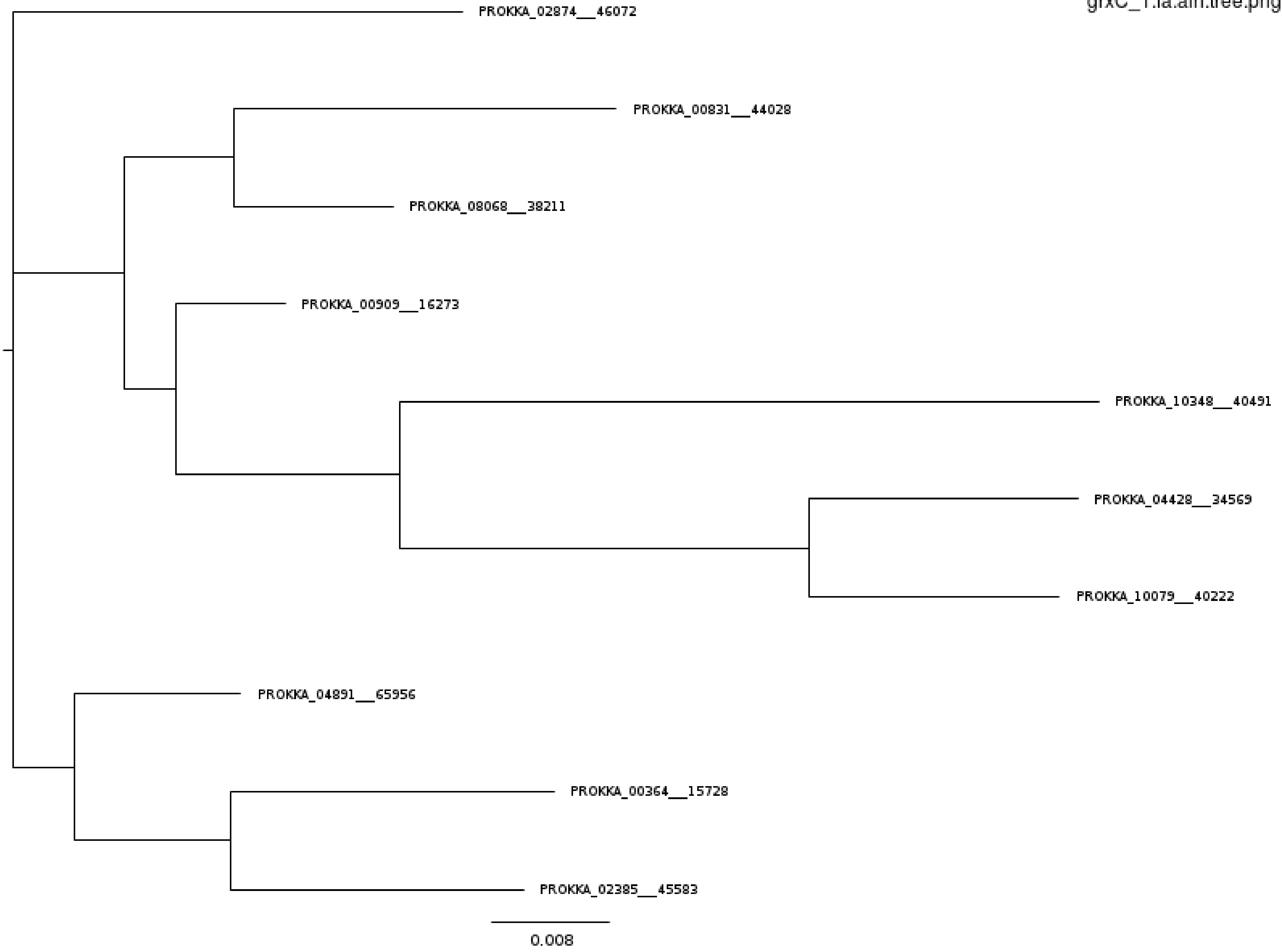

gshA_2.fa.aln.tree.png

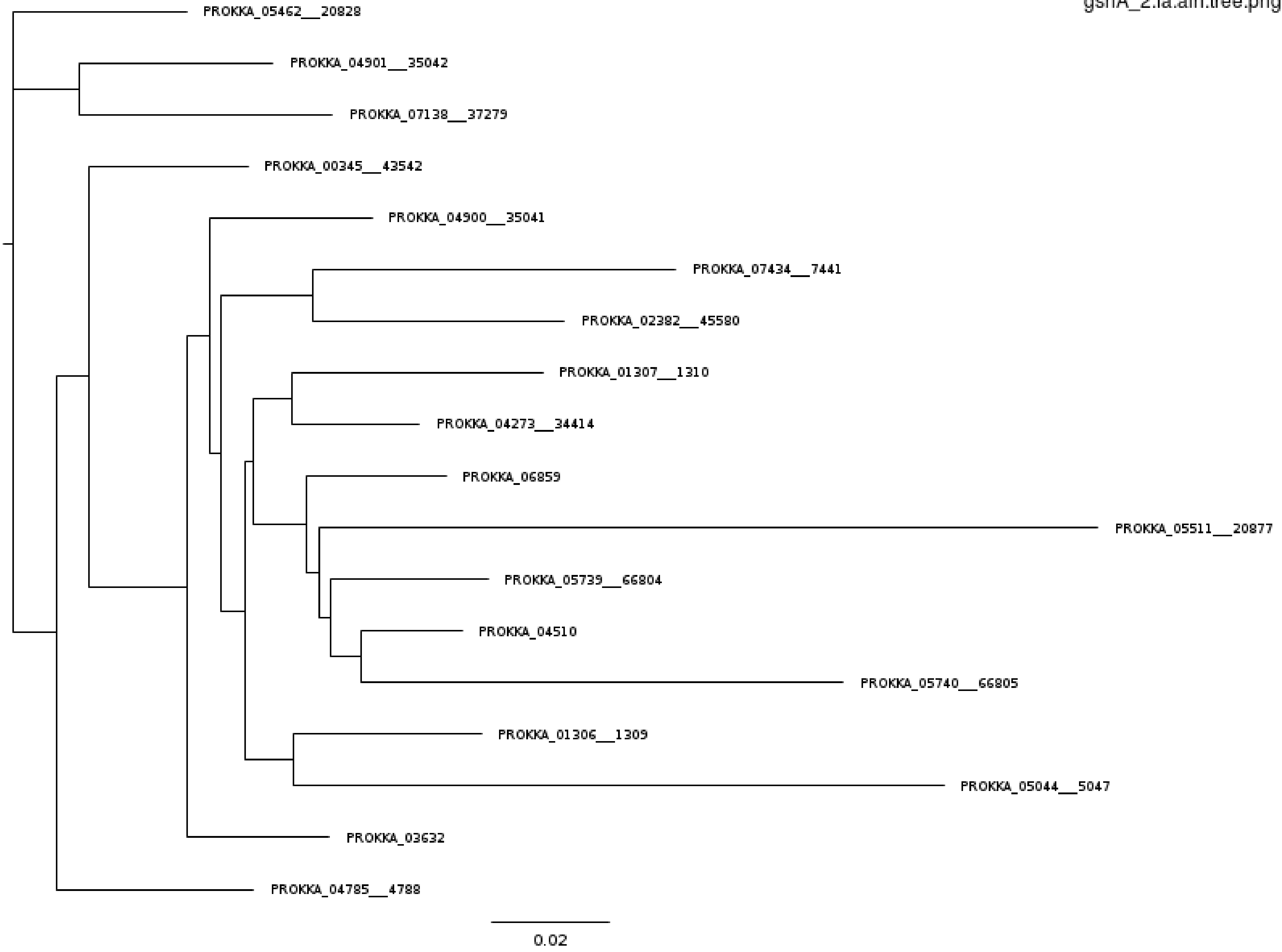

gsiD_1.fa.aln.tree.png

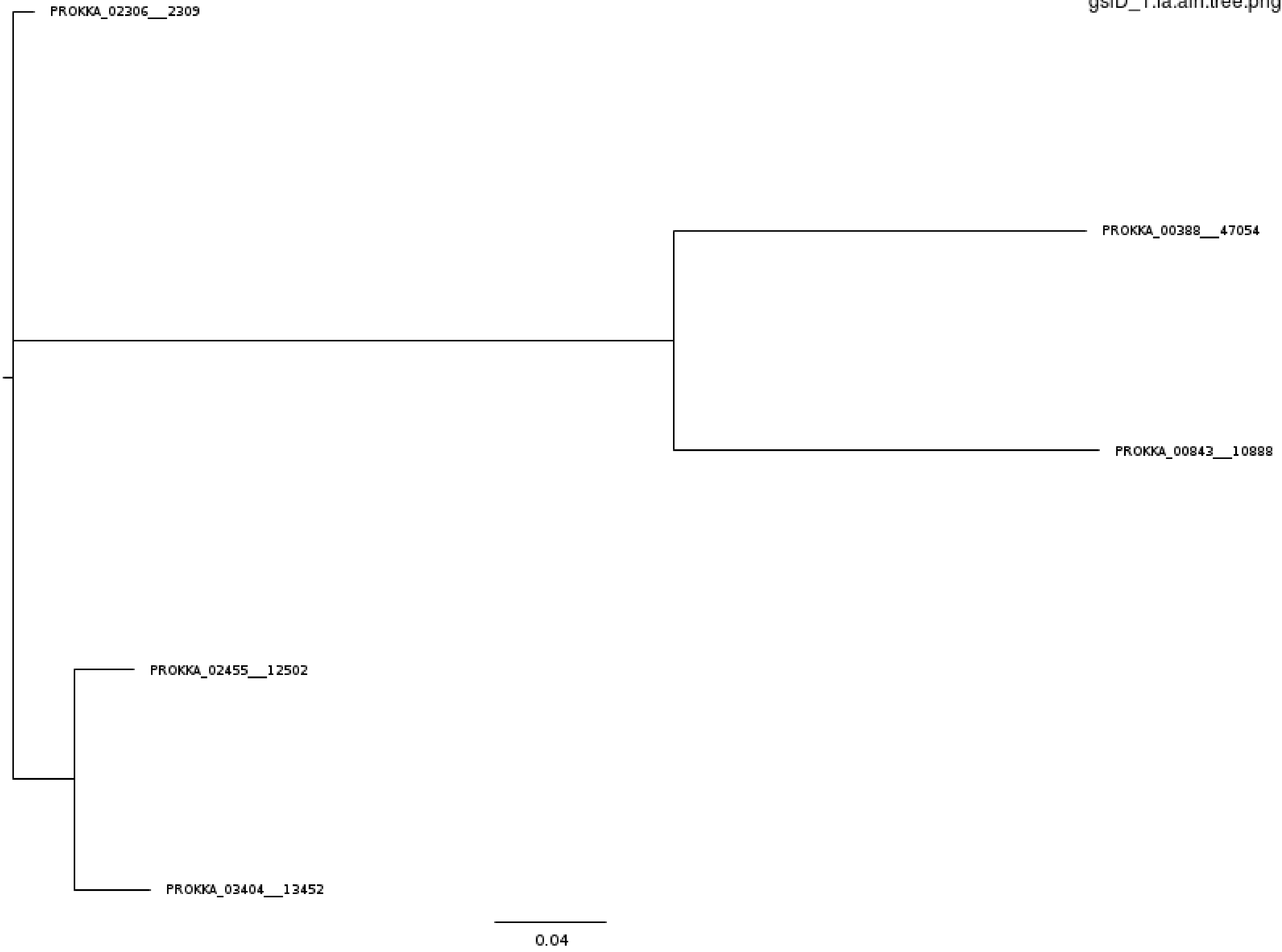

gspE.fa.aln.tree.png

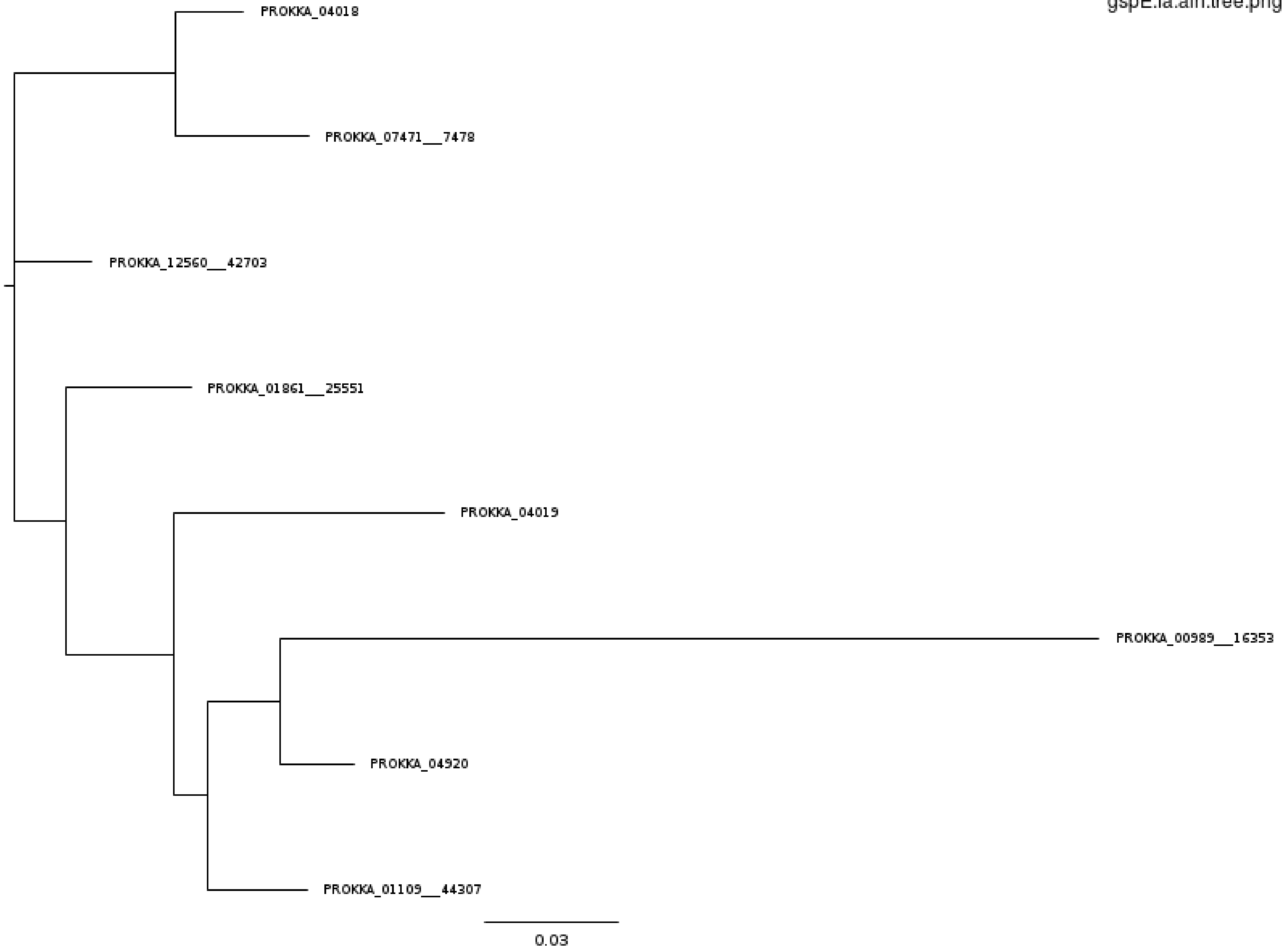

guaB_1.fa.aln.tree.png

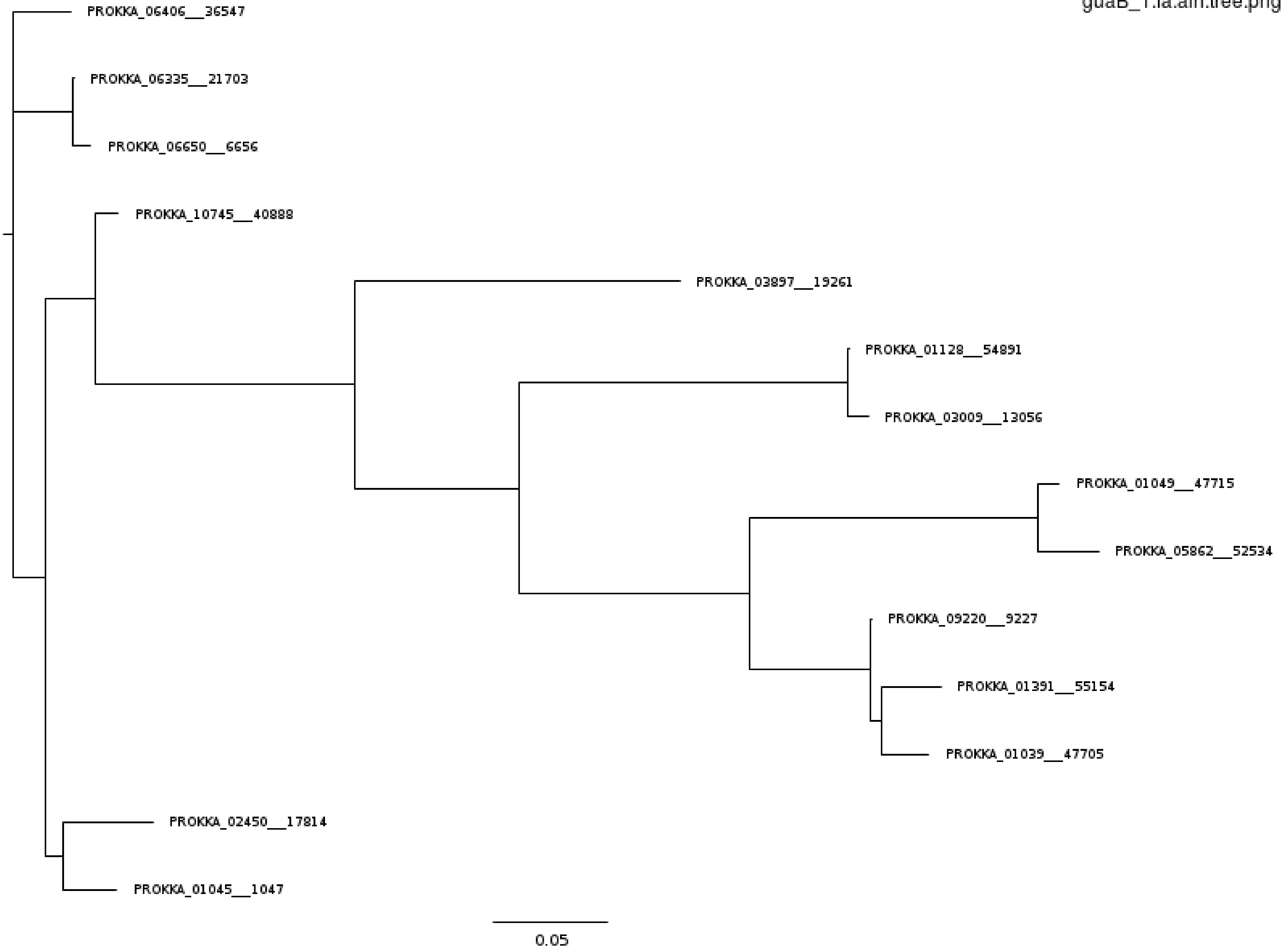

guaB_2.fa.aln.tree.png

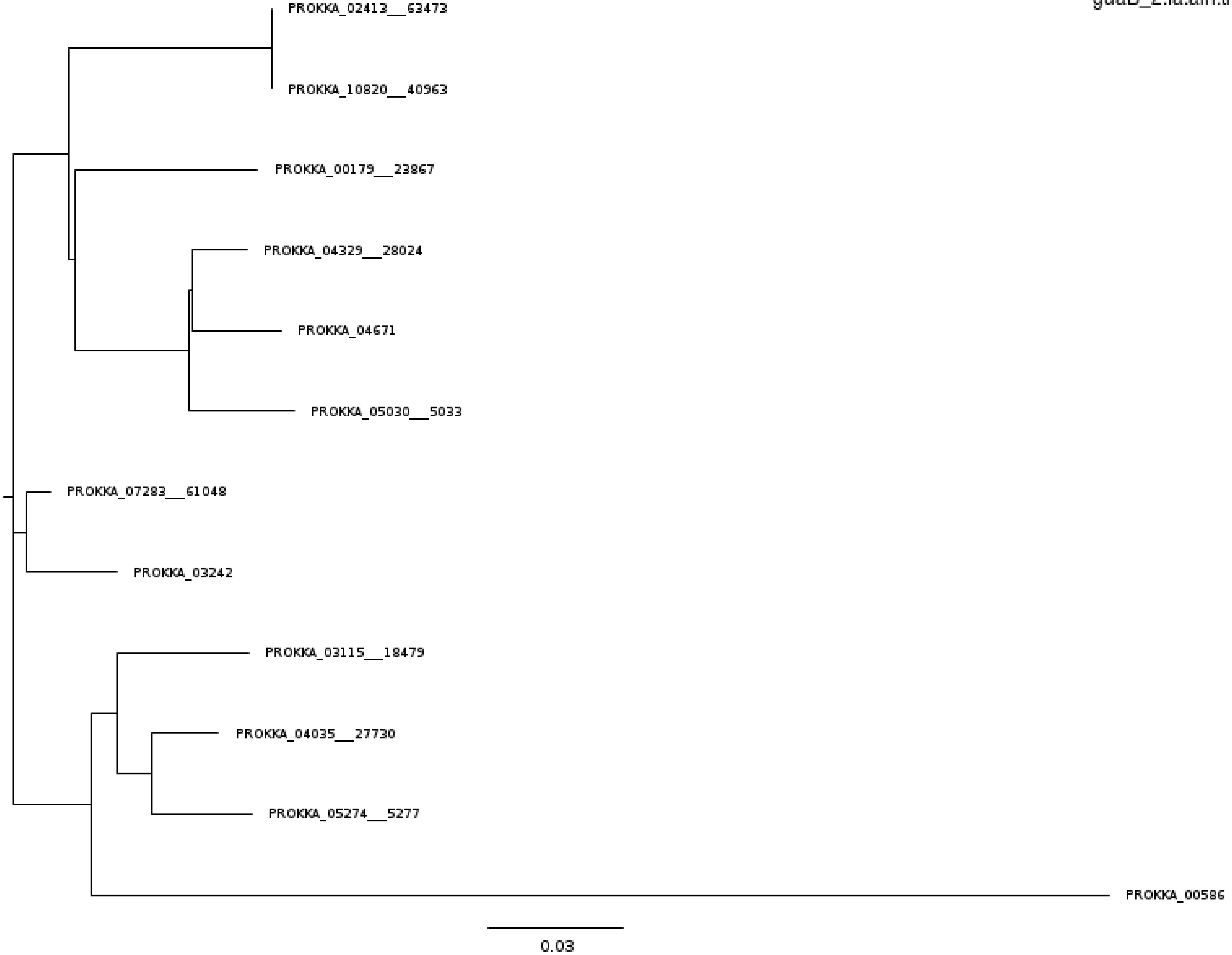

guaB_3.fa.aln.tree.png

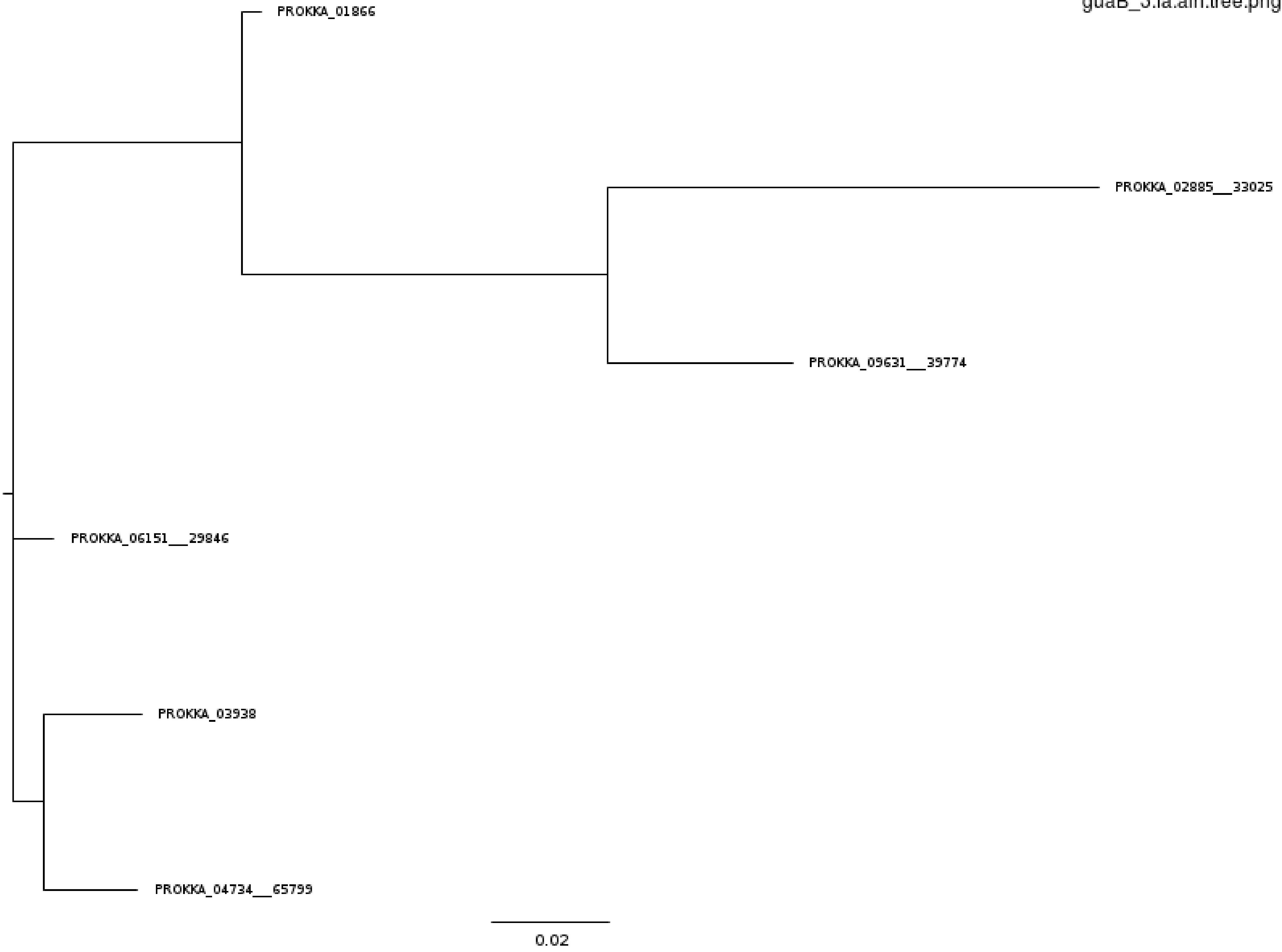

gyrA_1.fa.aln.tree.png

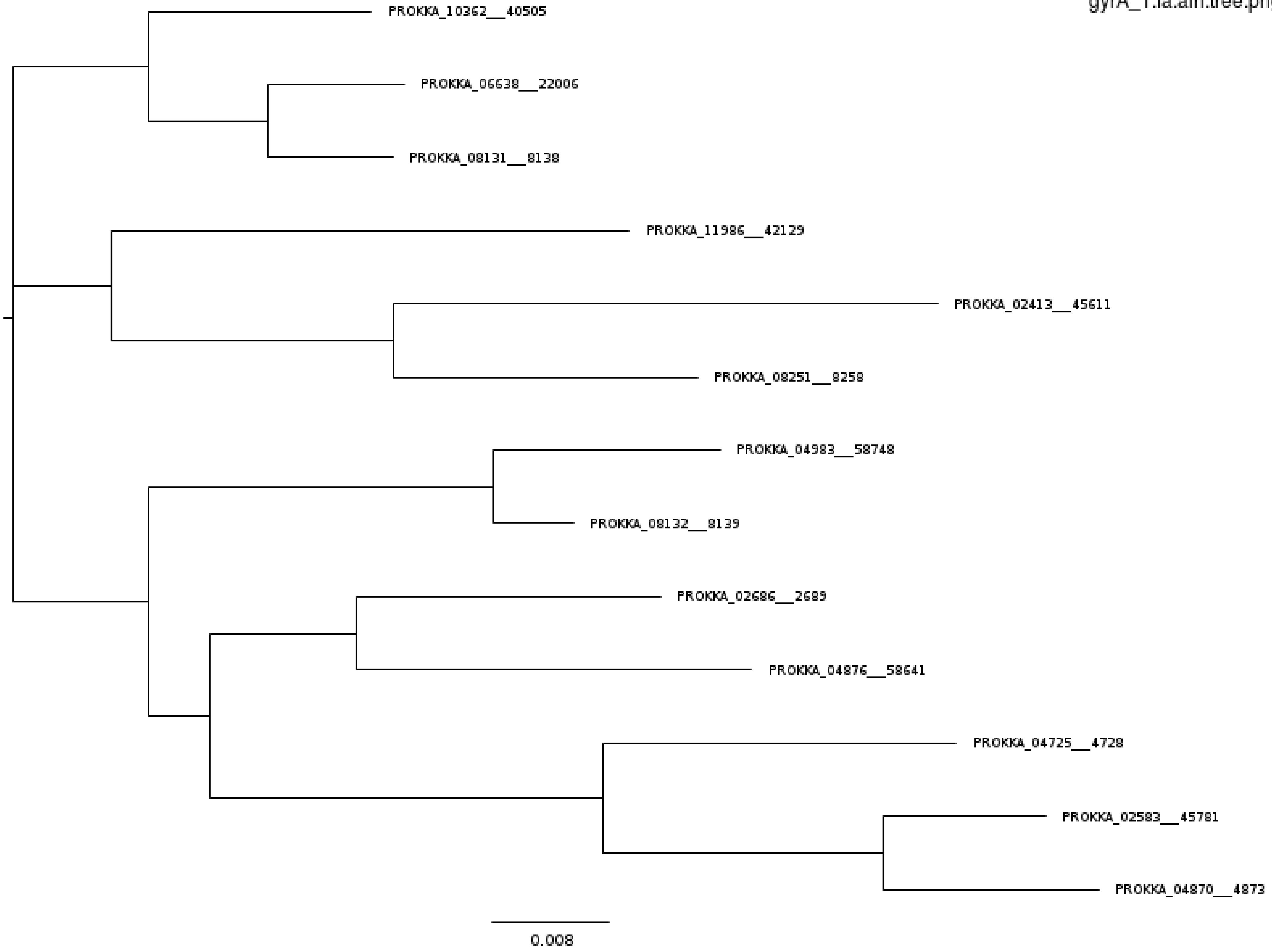

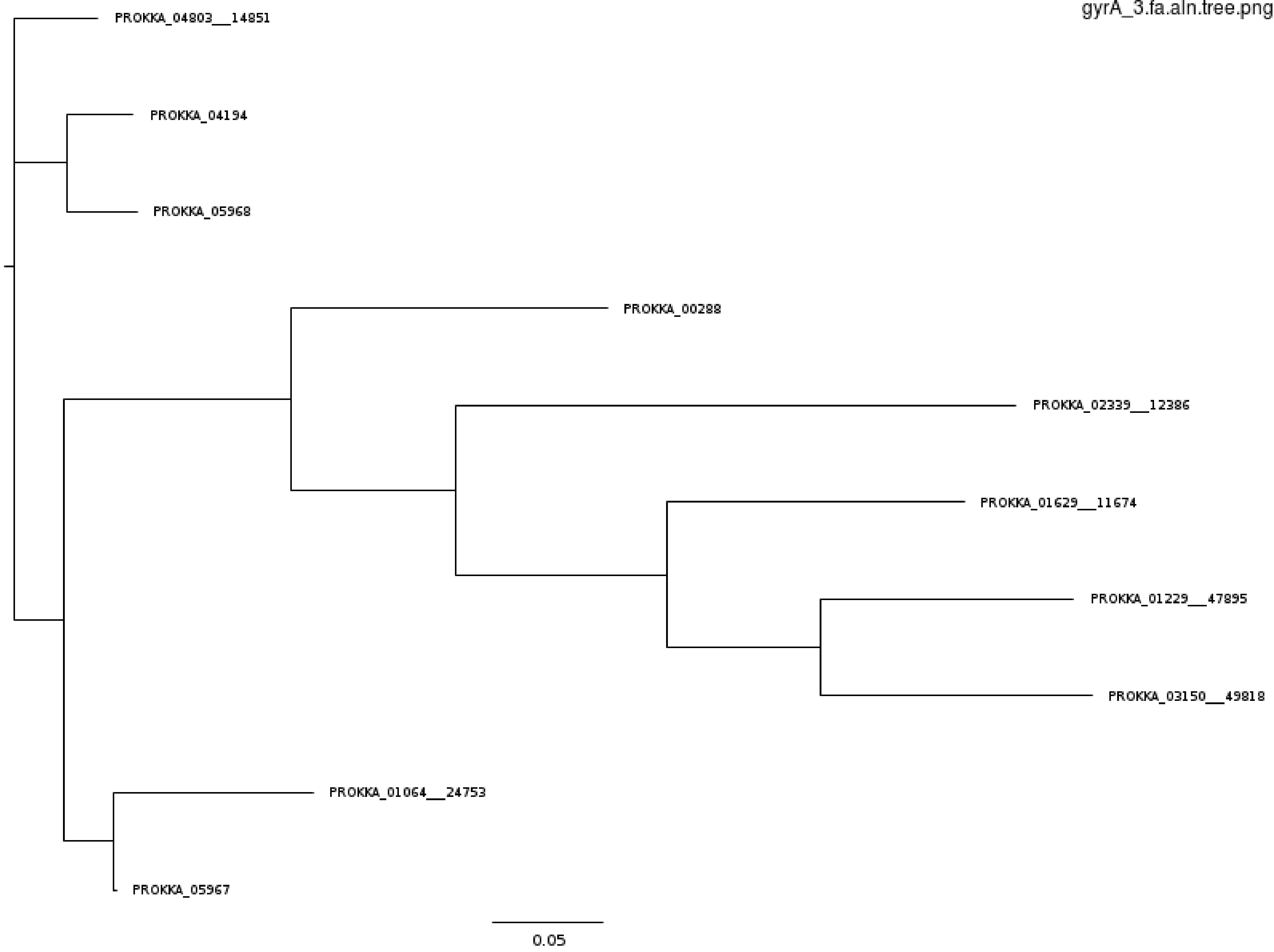
gyrA_3.fa.aln.tree.png
PROKKA_04803___14851
PROKKA_04194
PROKKA_05968
PROKKA_00288
PROKKA_02339___12386
PROKKA_01629___11674
PROKKA_01229___47895
PROKKA_03150___49818
PROKKA_01064___24753
PROKKA_05967
0.05

gyrB_2.fa.aln.tree.png

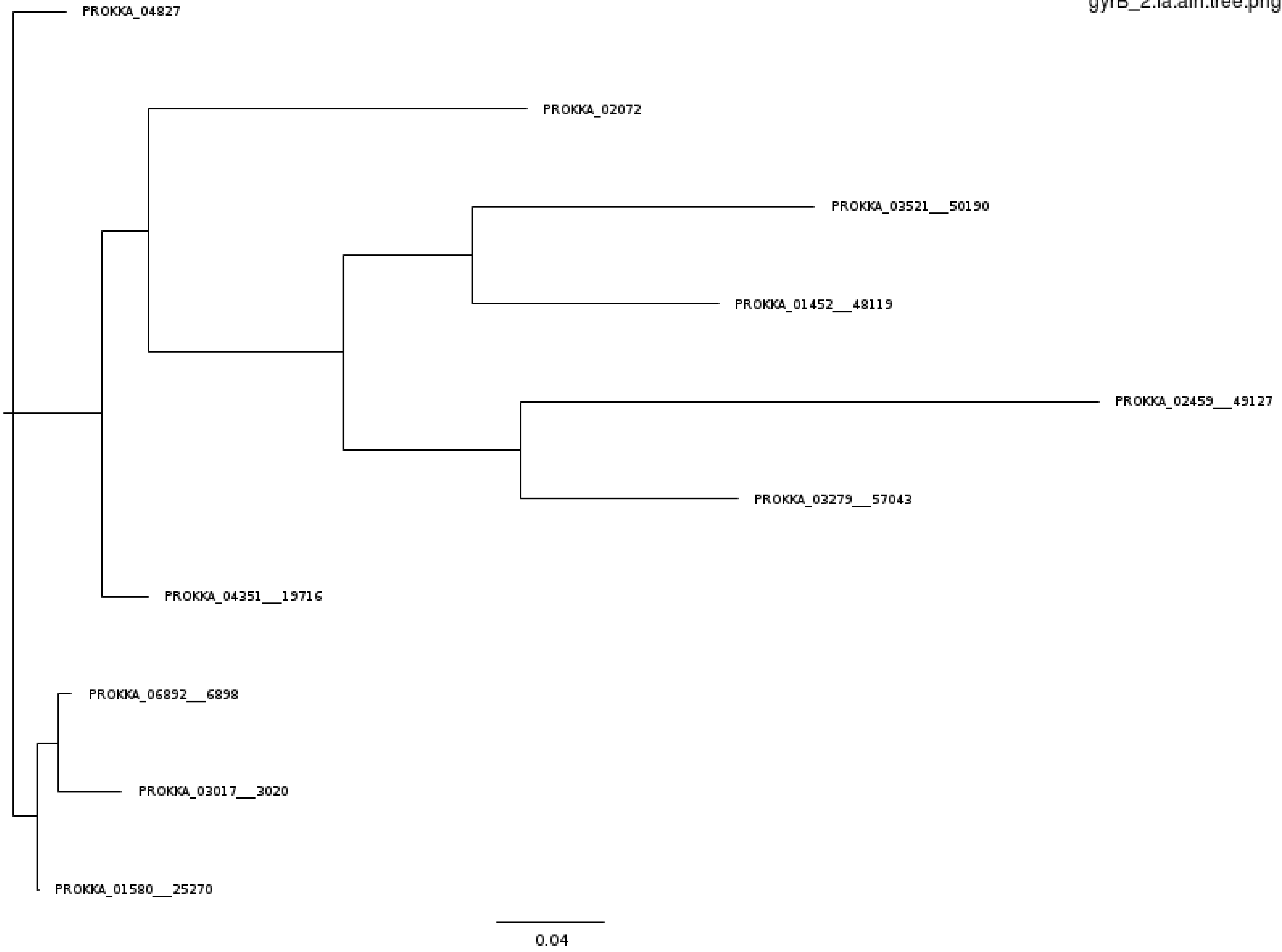

hacB_3.fa.aln.tree.png

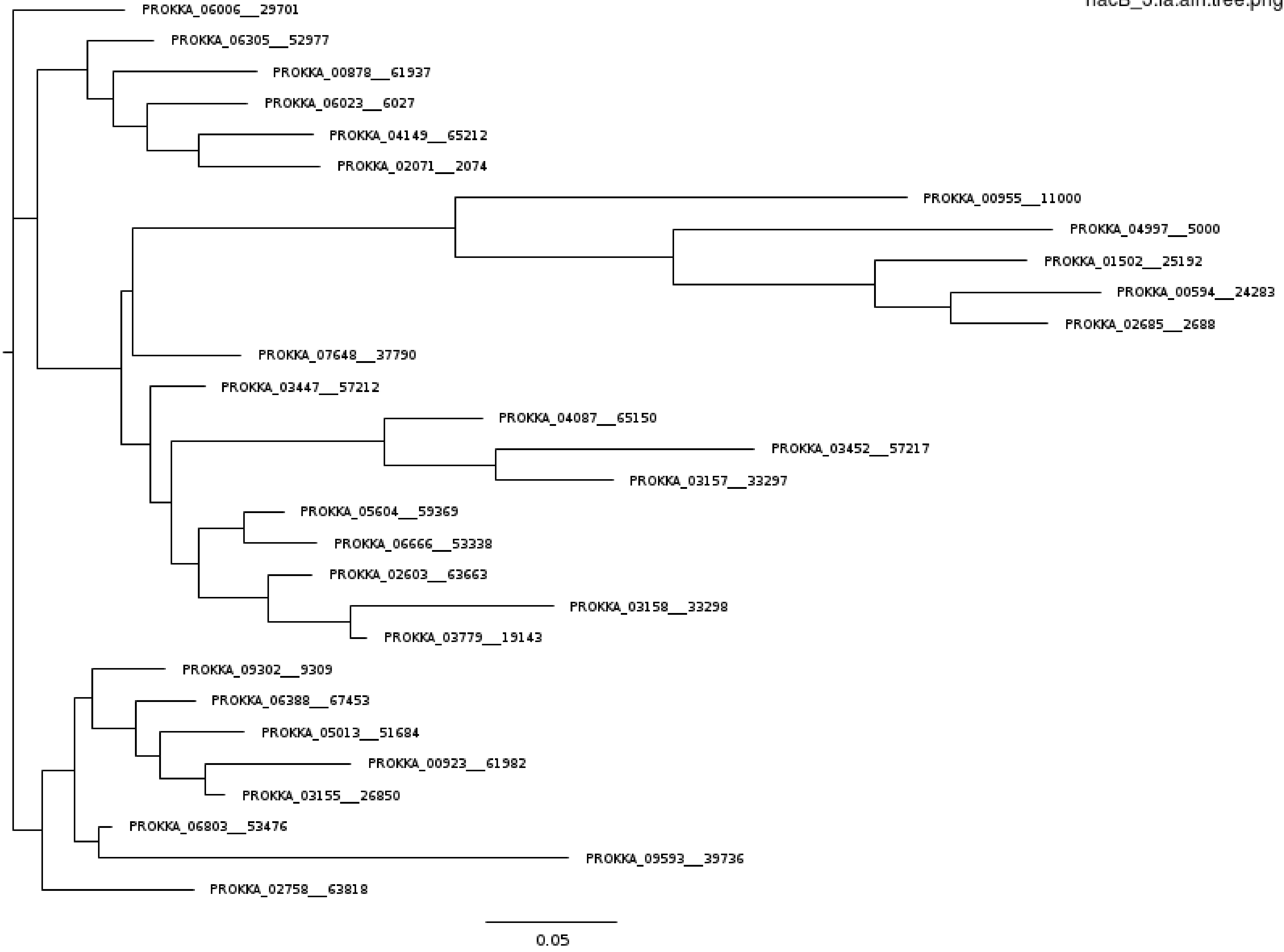

hbpA_2.fa.aln.tree.png

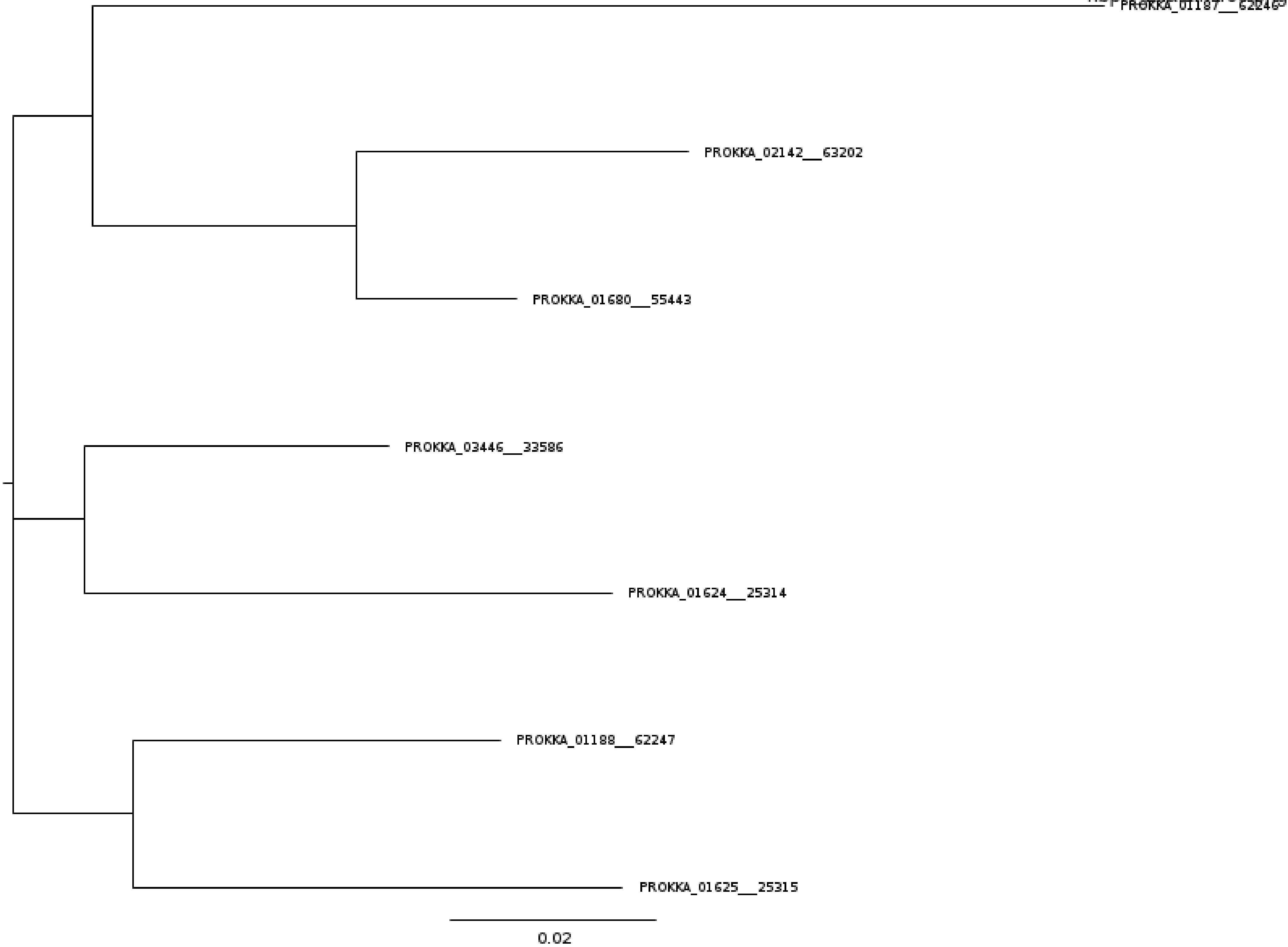

hcp_2.fa.aln.tree.png

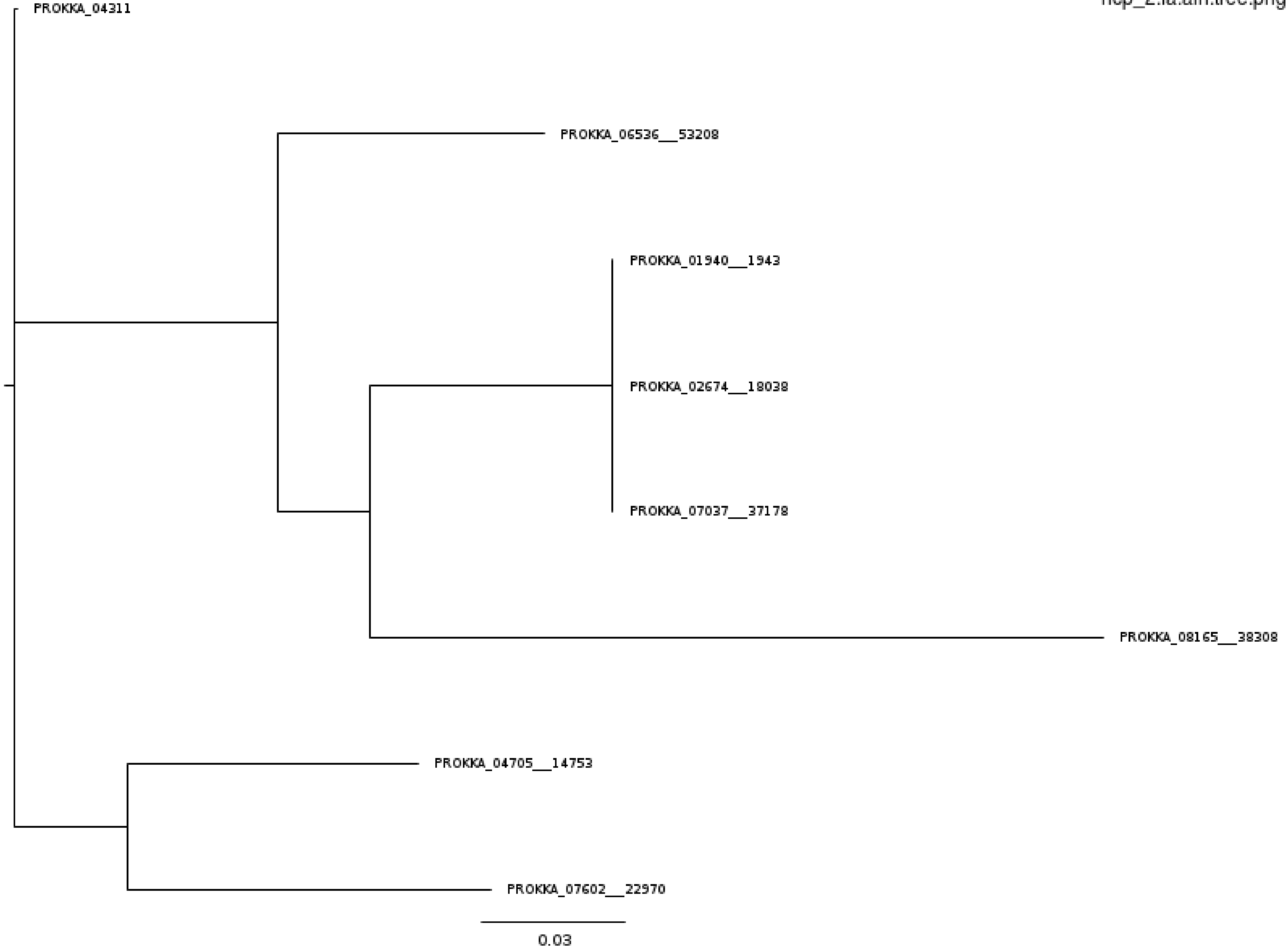

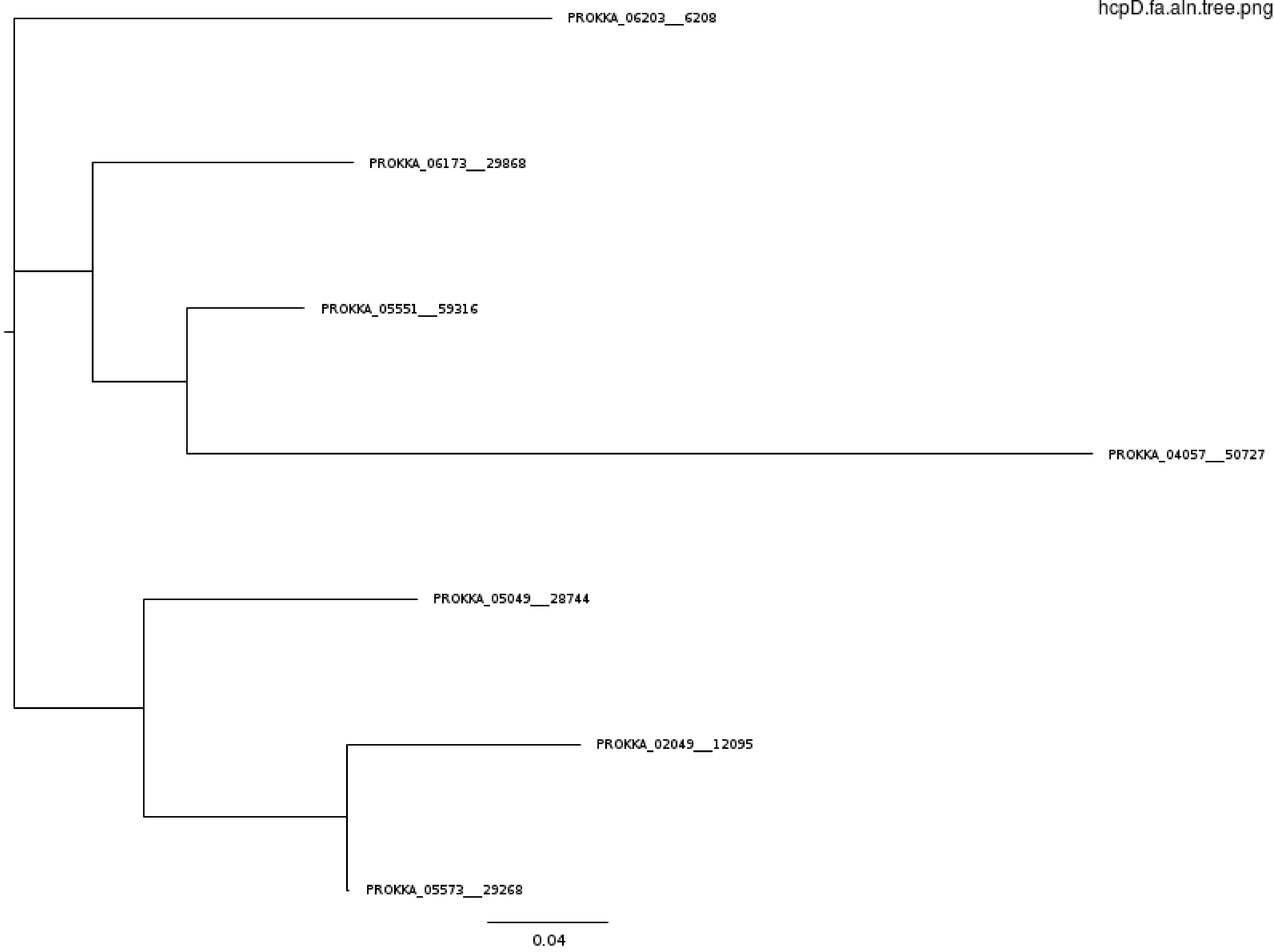
hcpD.fa.aln.tree.png
PROKKA_06203___6208
PROKKA_06173___29868
PROKKA_05551___59316
PROKKA_04057___50727
PROKKA_05049___28744
PROKKA_02049___12095
PROKKA_05573___29268
0.04

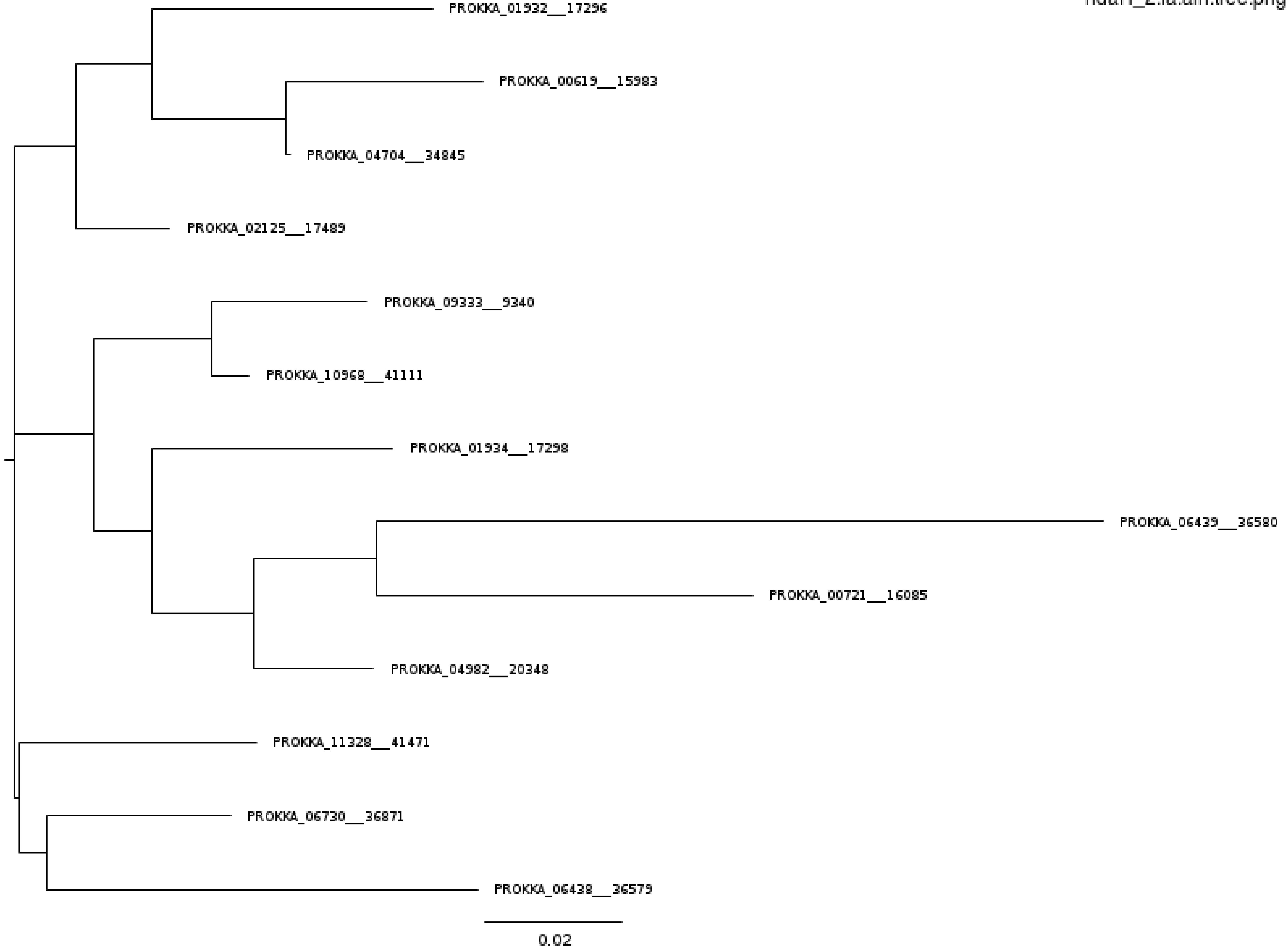
PROKKA_01932___17296
PROKKA_00619___15983
PROKKA_04704___34845
PROKKA_02125___17489
PROKKA_09333___9340
PROKKA_10968___41111
PROKKA_01934___17298
PROKKA_06439___36580
PROKKA_00721___16085
PROKKA_04982___20348
PROKKA_11328___41471
PROKKA_06730___36871
PROKKA_06438___36579
0.02

hemB_2.fa.aln.tree.png

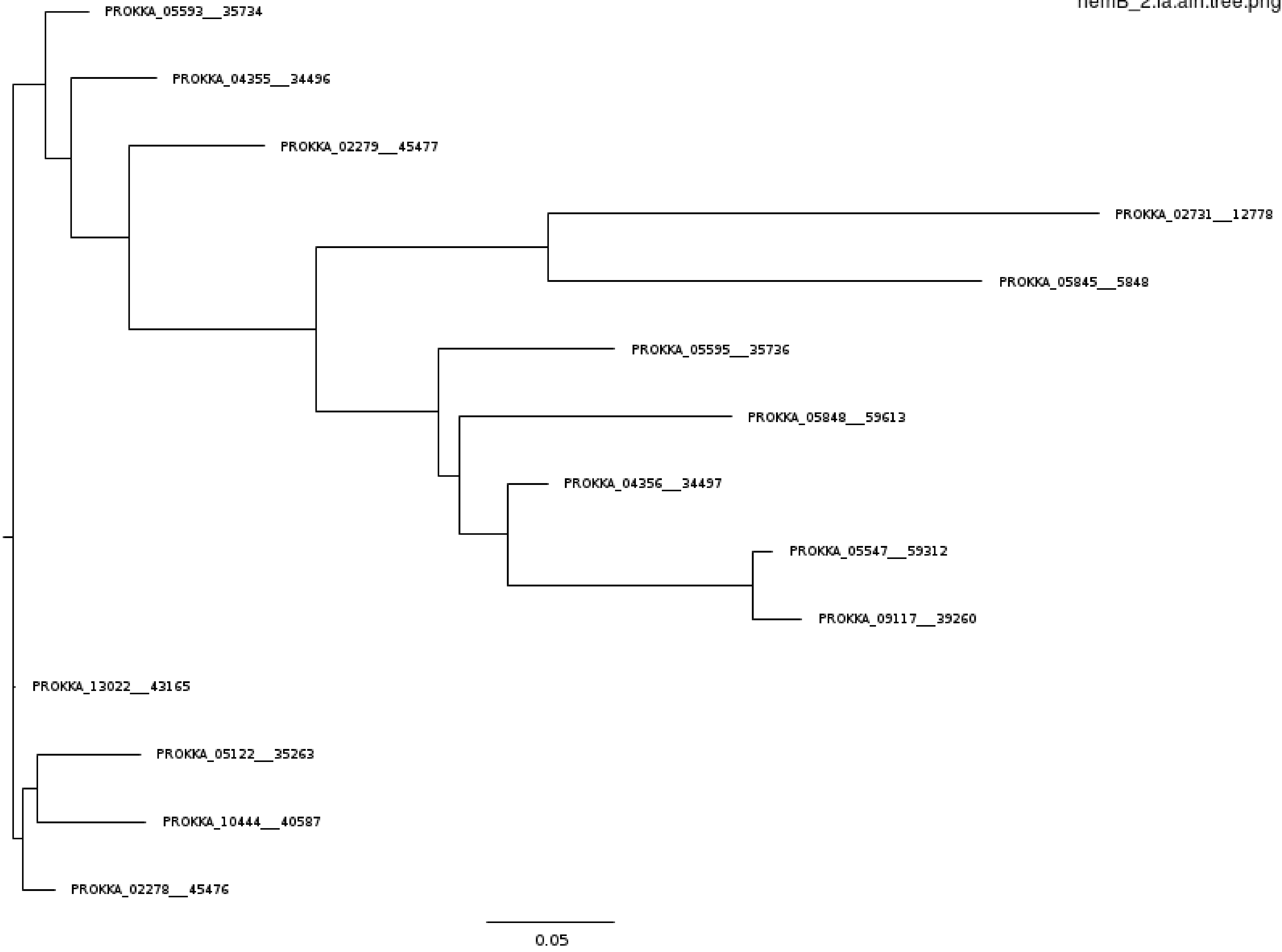

hemC_2.fa.aln.tree.png

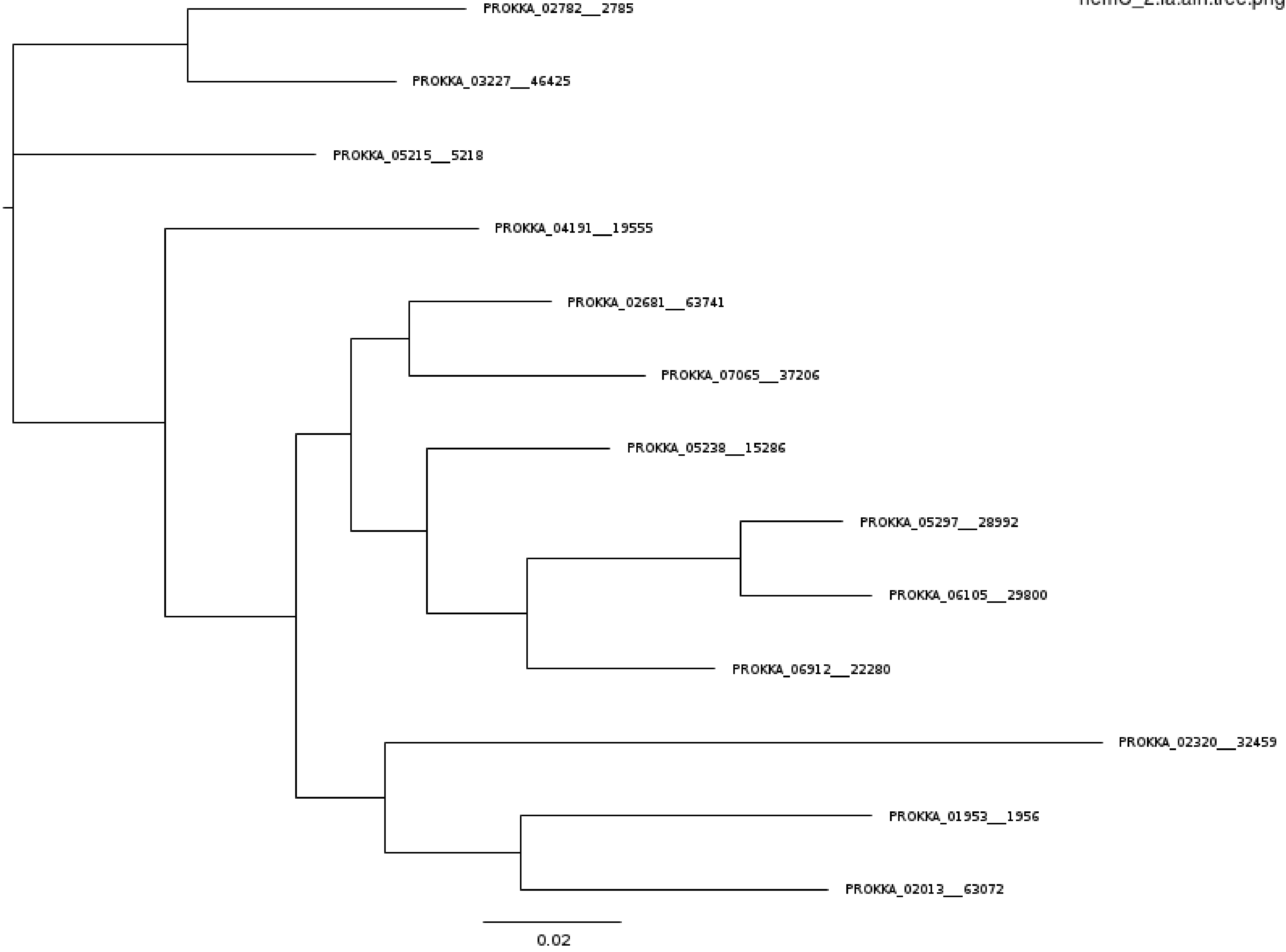

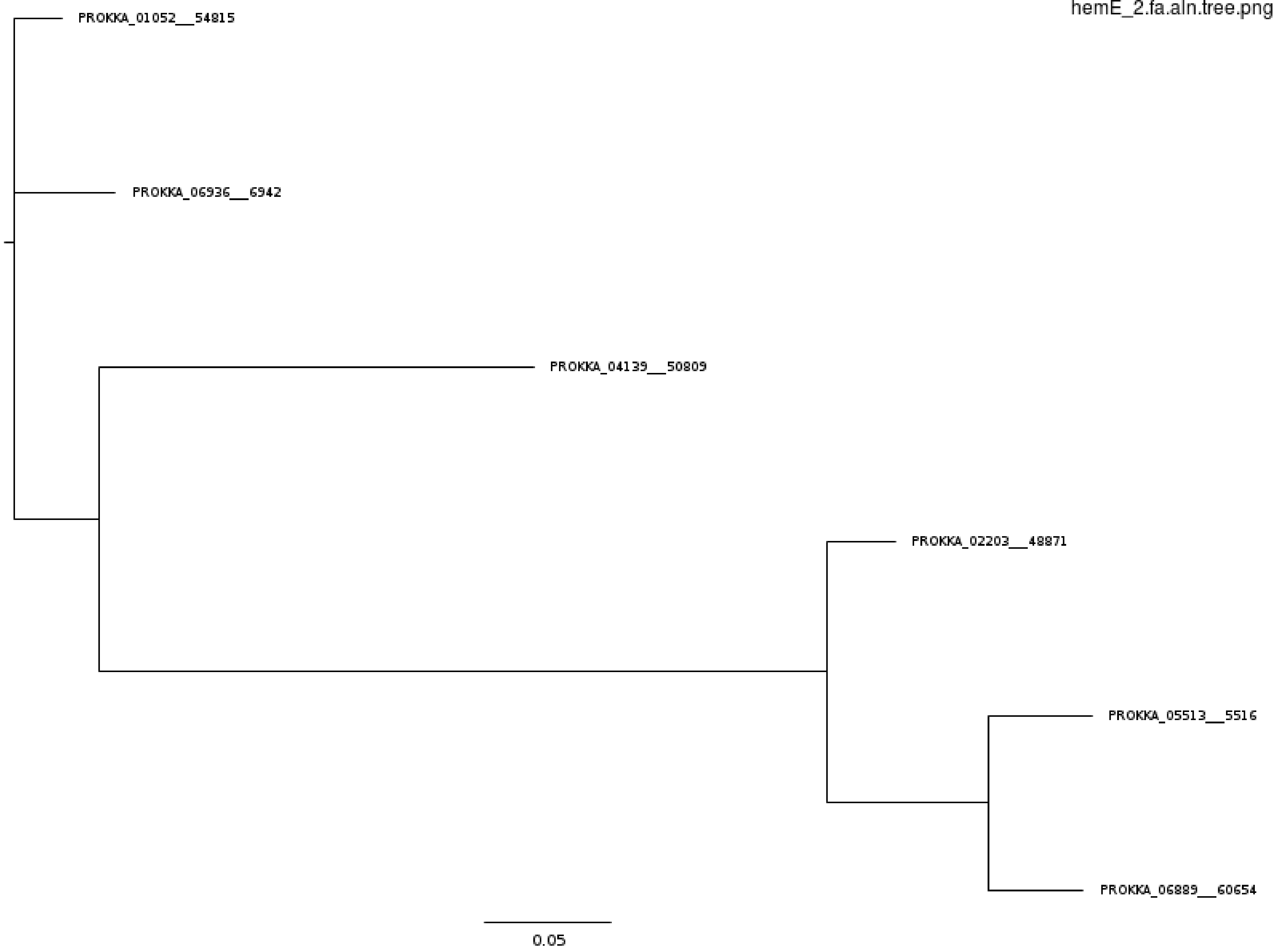
hemE_2.fa.aln.tree.png
PROKKA_01052__54815
PROKKA_06936__6942
PROKKA_04139__50809
PROKKA_02203__48871
PROKKA_05513__5516
PROKKA_06889__60654
0.05

hemH_4.fa.aln.tree.png

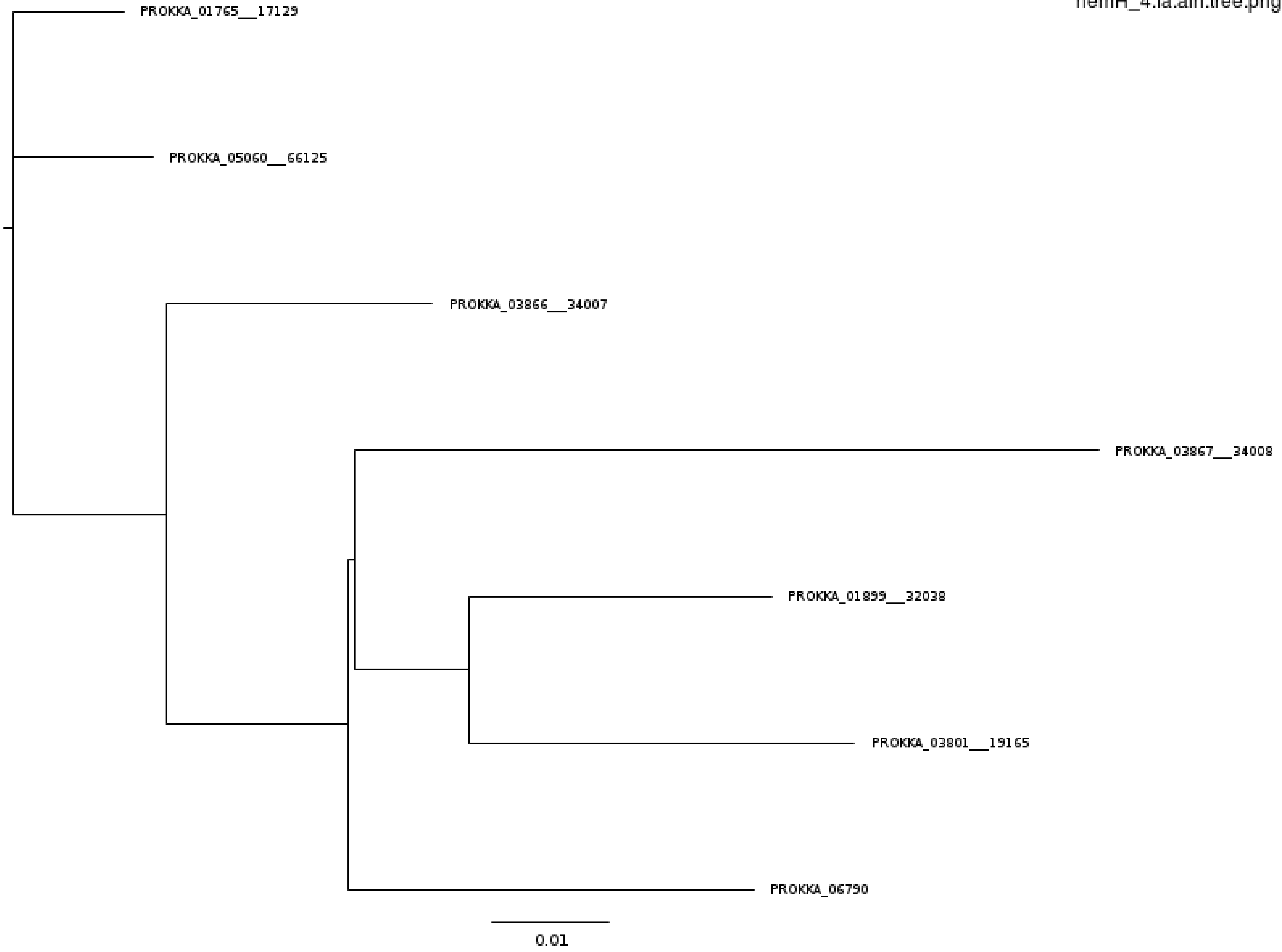

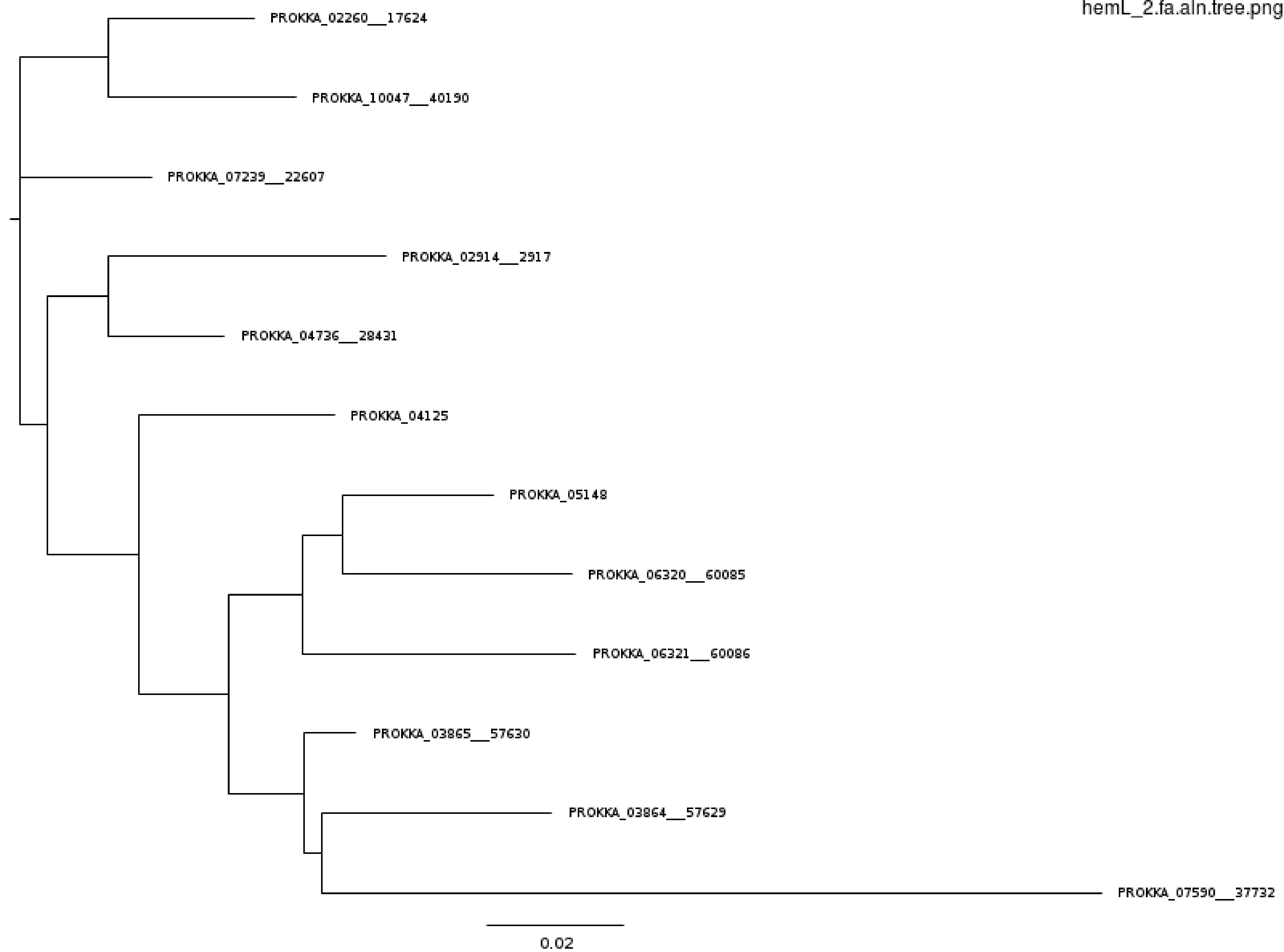
hemL_2.fa.aln.tree.png
PROKKA_02260__17624
PROKKA_10047__40190
PROKKA_07239__22607
PROKKA_02914__2917
PROKKA_04736__28431
PROKKA_04125
PROKKA_05148
PROKKA_06320__60085
PROKKA_06321__60086
PROKKA_03865__57630
PROKKA_03864__57629
PROKKA_07590__37732
0.02

hemN_3.fa.aln.tree.png

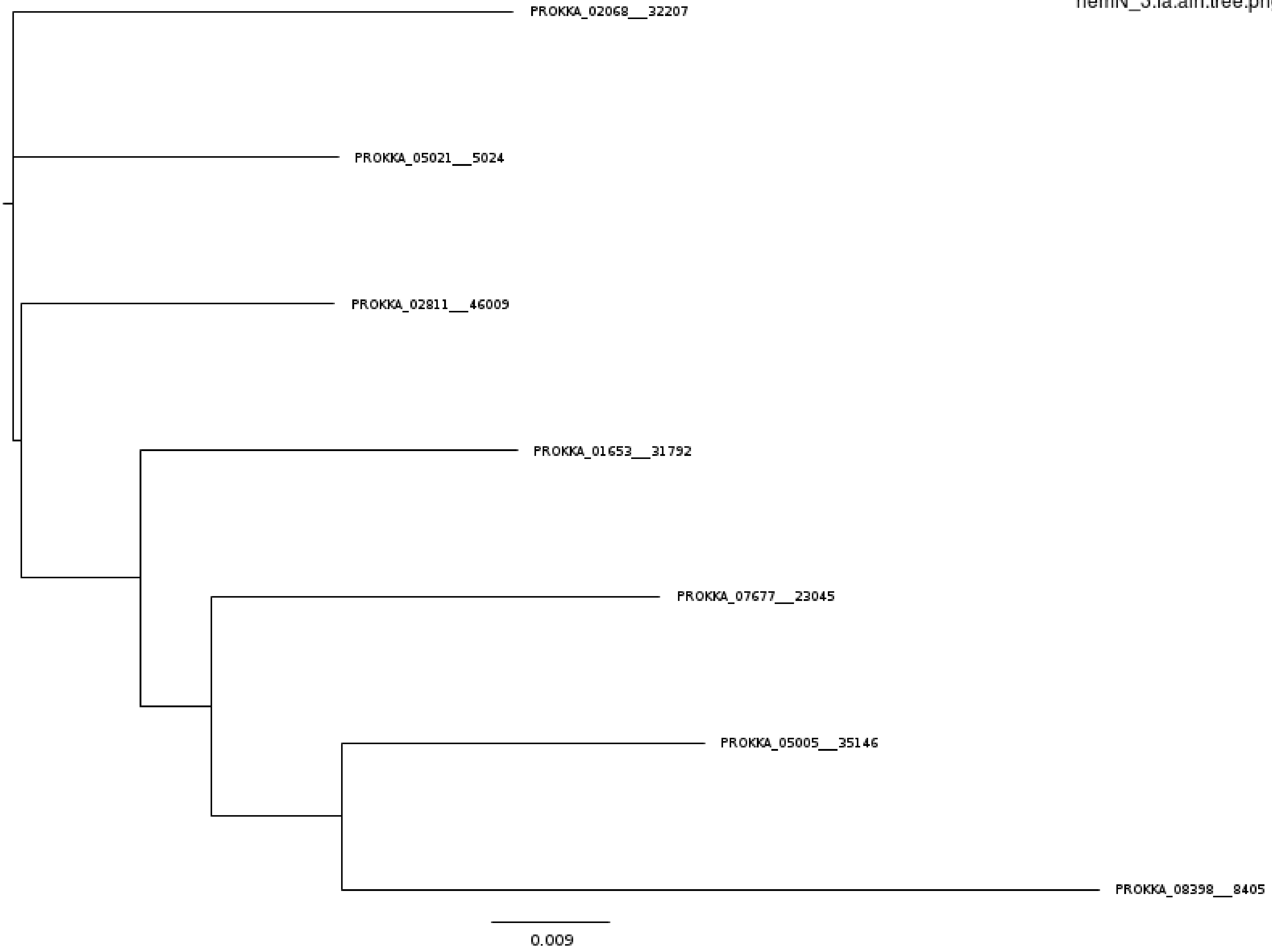

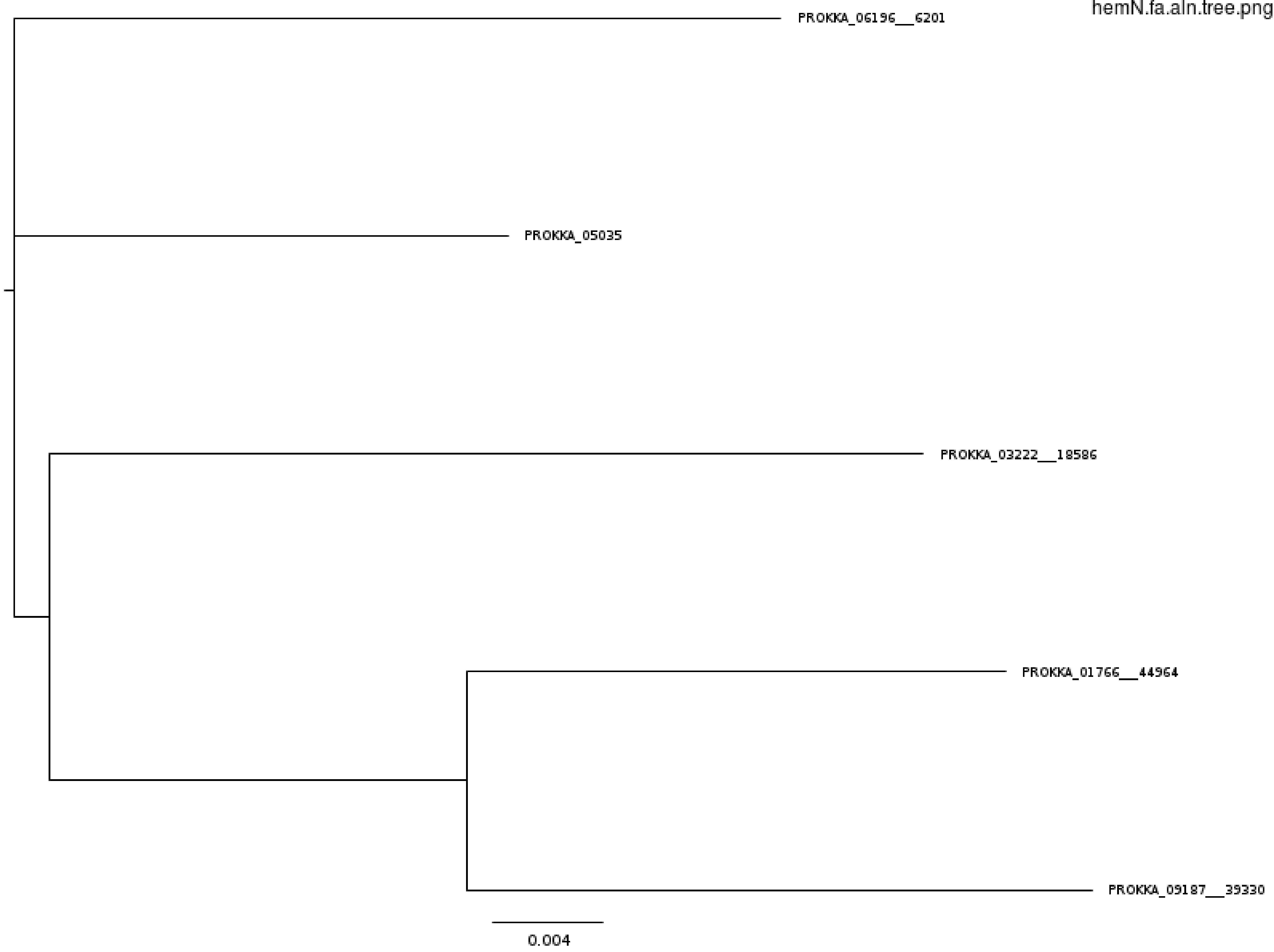
hemN.fa.aln.tree.png
PROKKA_06196___6201
PROKKA_05035
PROKKA_03222___18586
PROKKA_01766___44964
PROKKA_09187___39330
0.004

hflC_1.fa.aln.tree.png

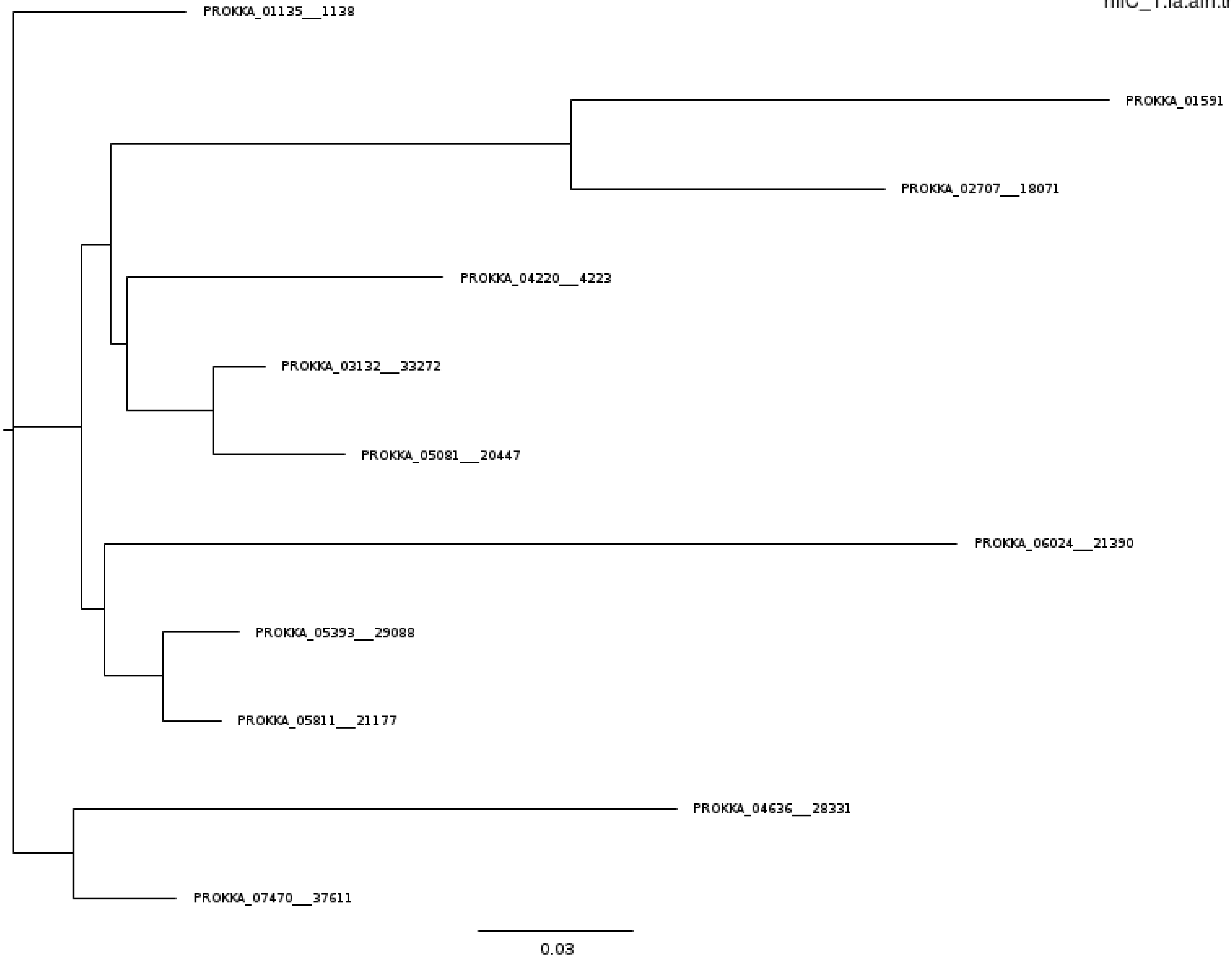

hflC_2.fa.aln.tree.png

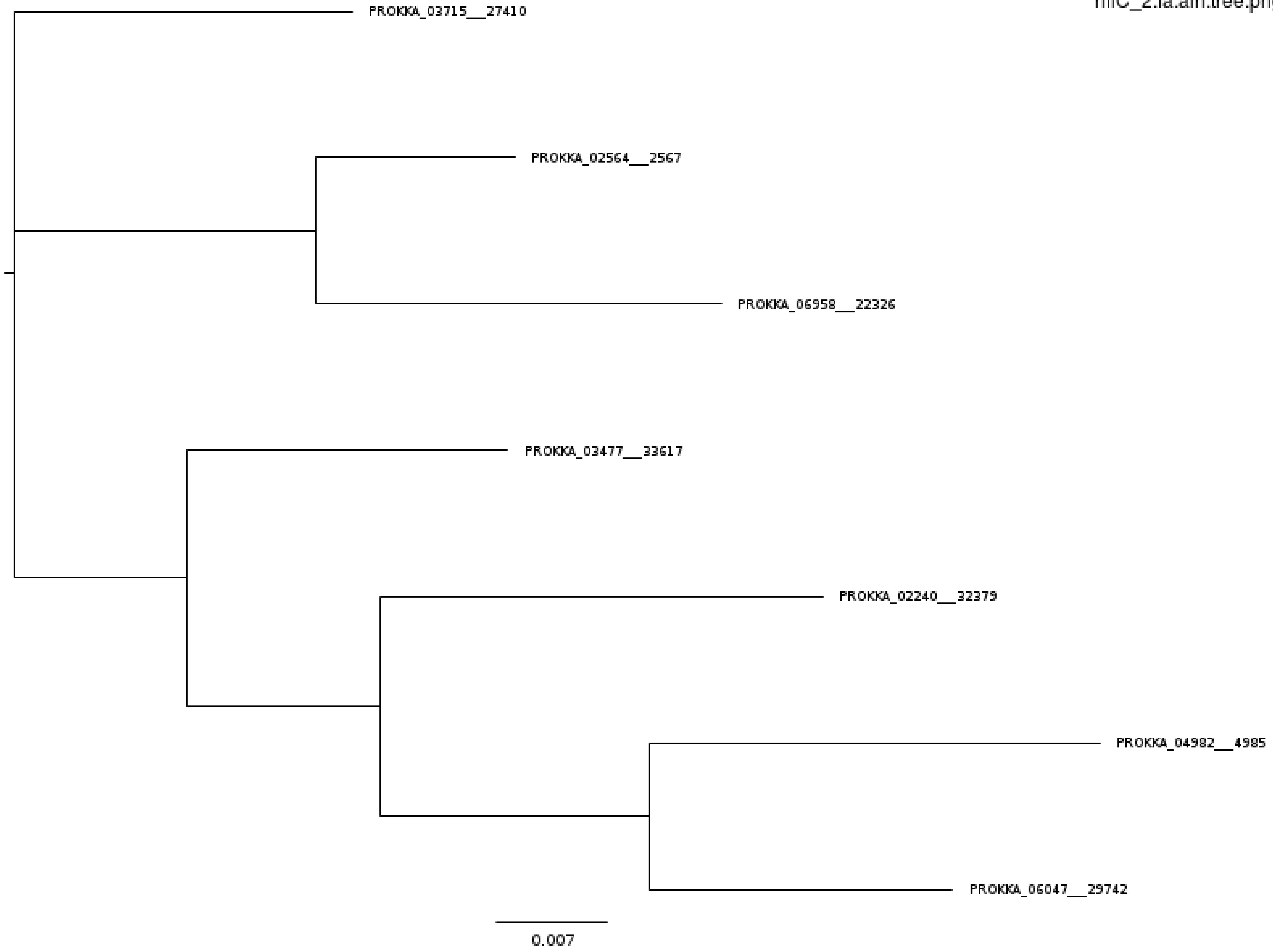

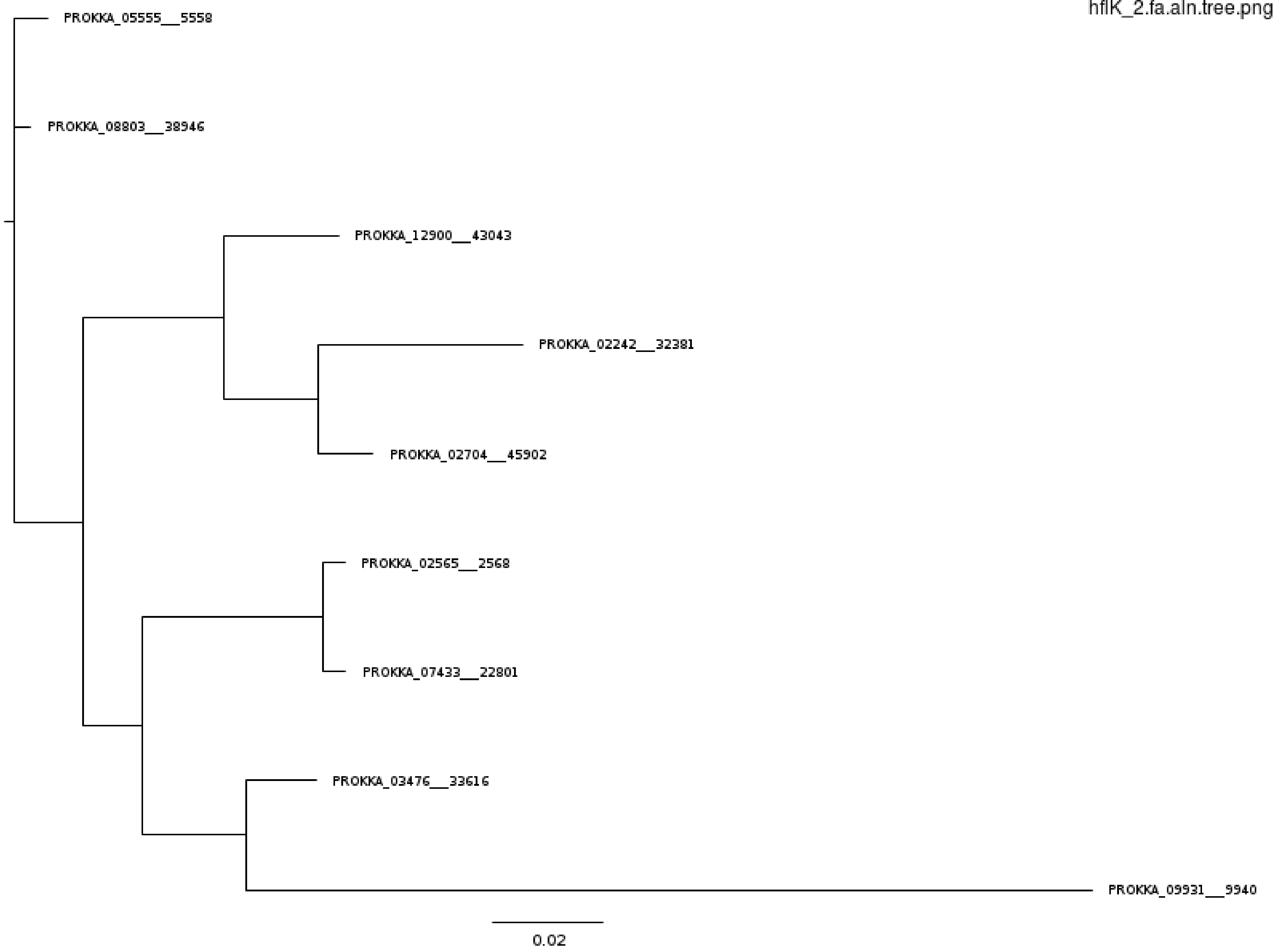
hflK_2.fa.aln.tree.png
PROKKA_05555___5558
PROKKA_08803___38946
PROKKA_12900___43043
PROKKA_02242___32381
PROKKA_02704___45902
PROKKA_02565___2568
PROKKA_07433___22801
PROKKA_03476___33616
PROKKA_09931___9940
0.02

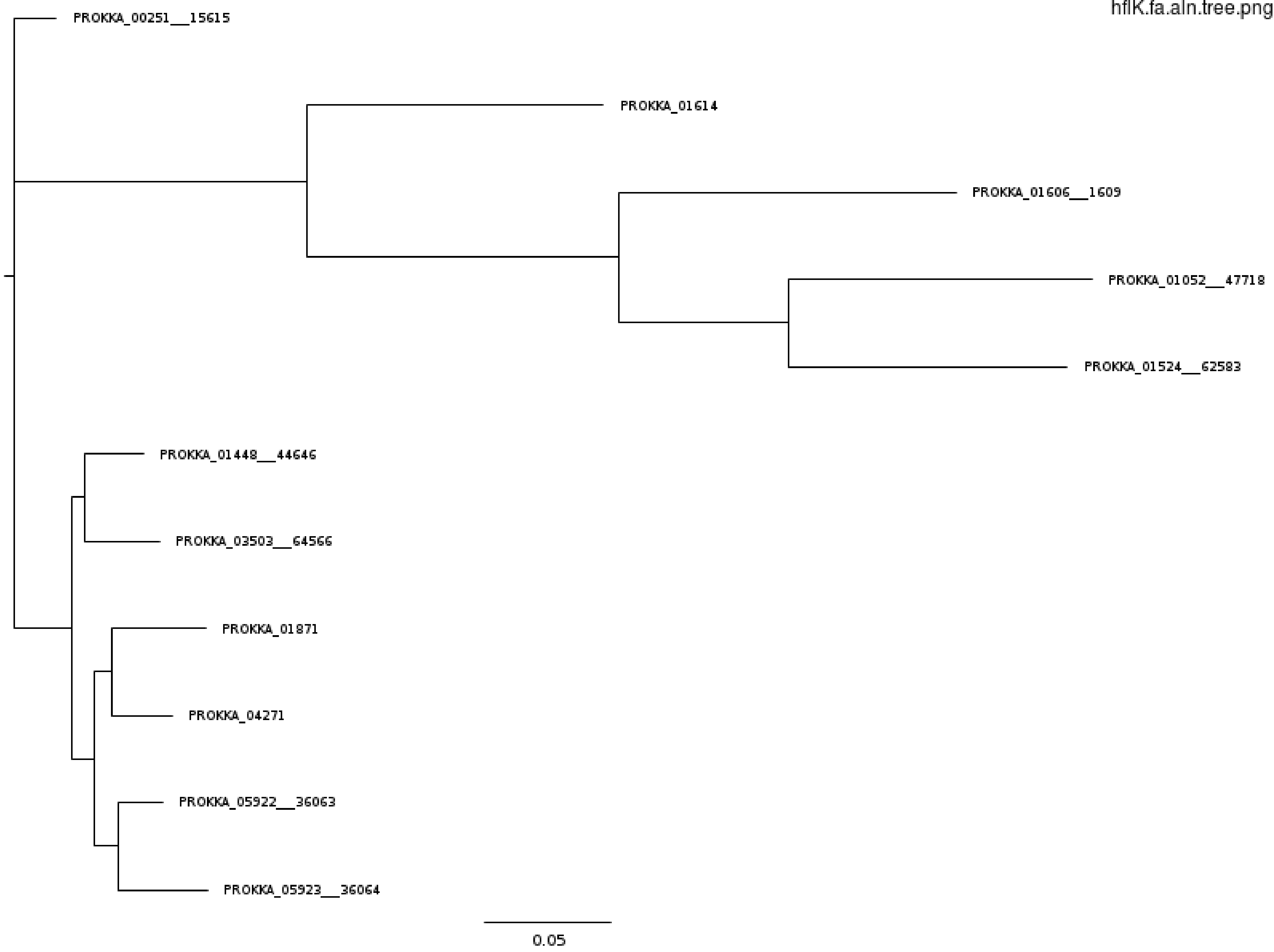
hflK.fa.aln.tree.png
PROKKA_00251___15615
PROKKA_01614
PROKKA_01606___1609
PROKKA_01052___47718
PROKKA_01524___62583
PROKKA_01448___44646
PROKKA_03503___64566
PROKKA_01871
PROKKA_04271
PROKKA_05922___36063
PROKKA_05923___36064
0.05

hfq_3.fa.aln.tree.png

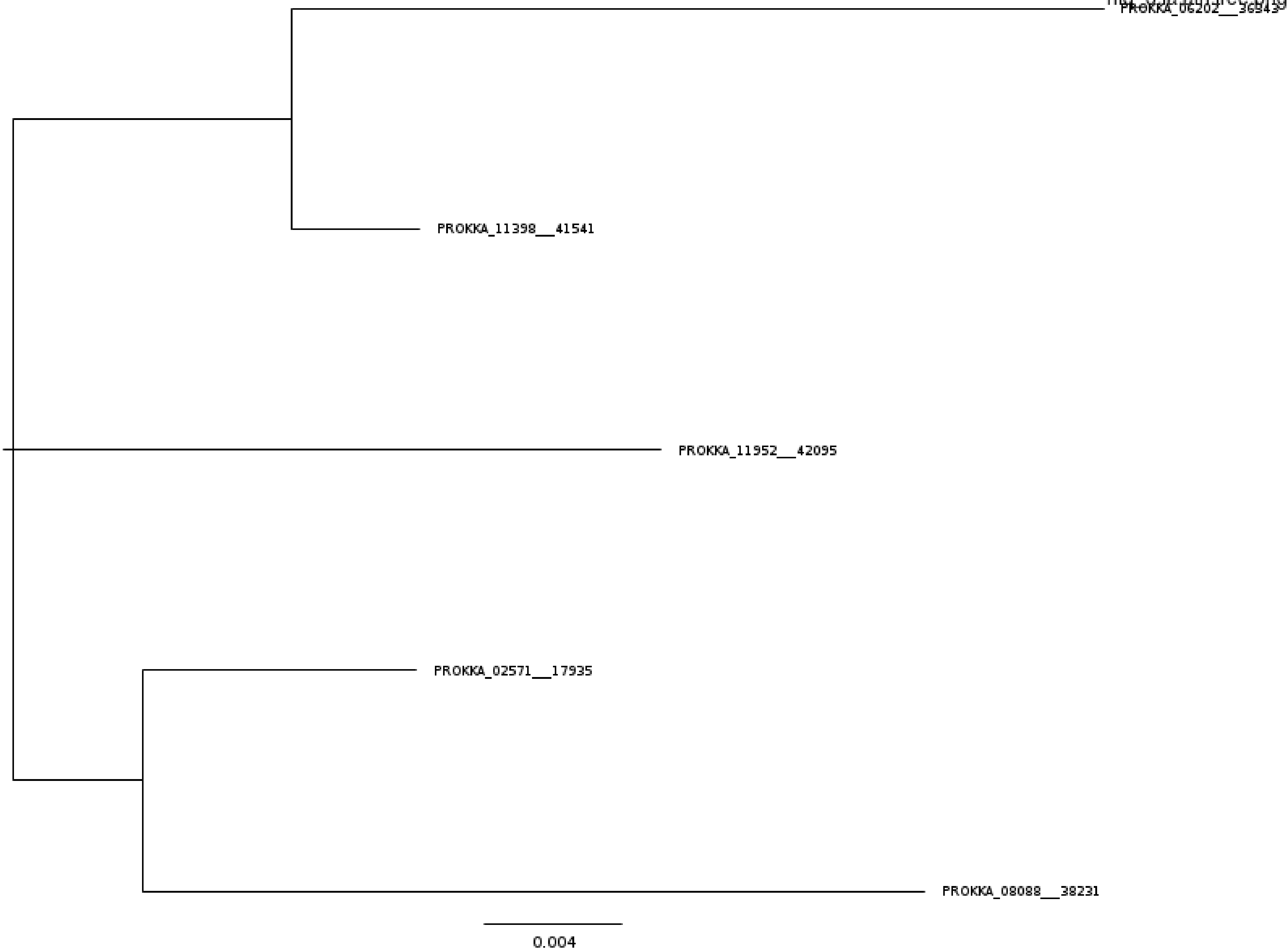

hicB_3.fa.aln.tree.png

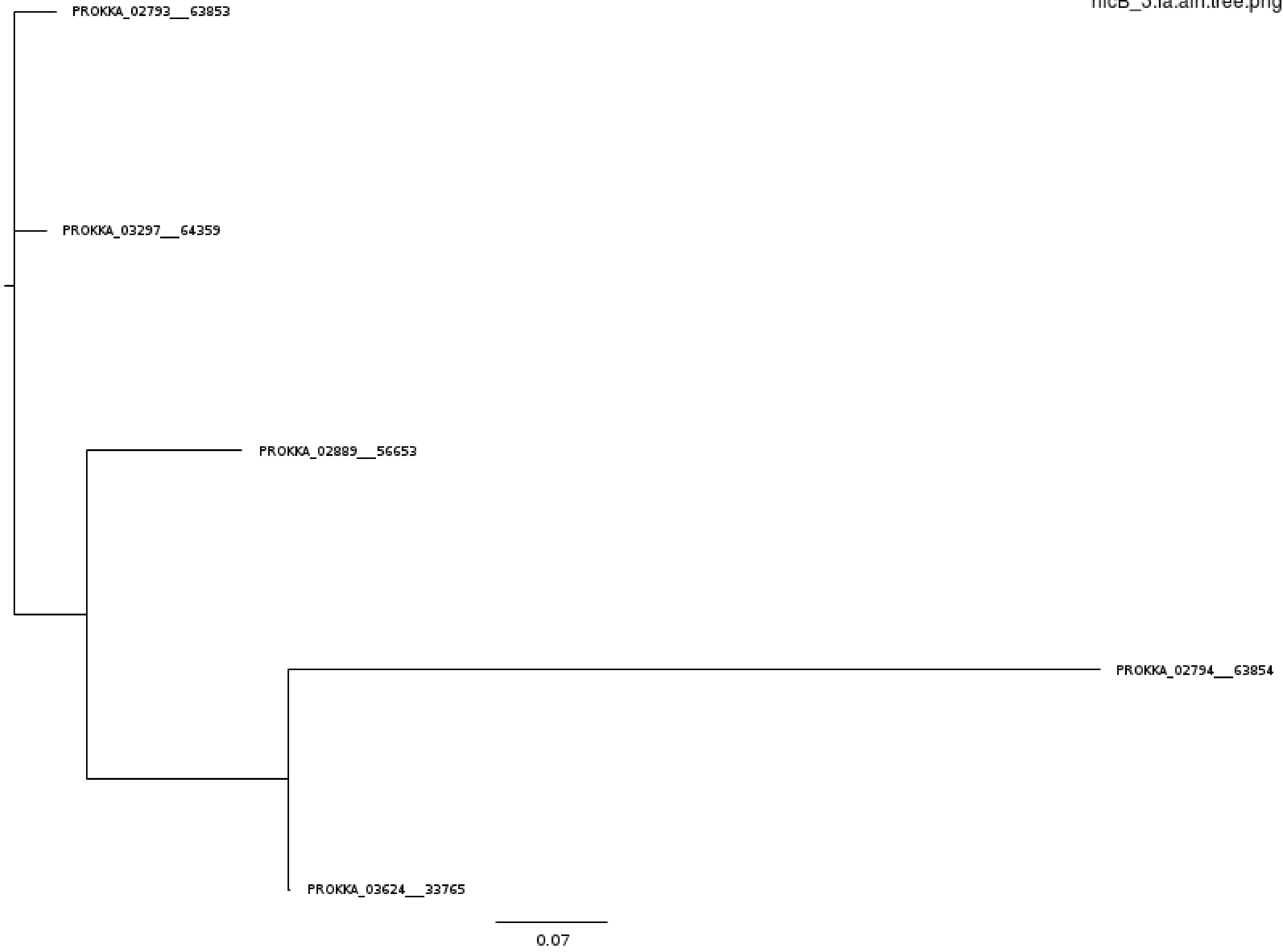

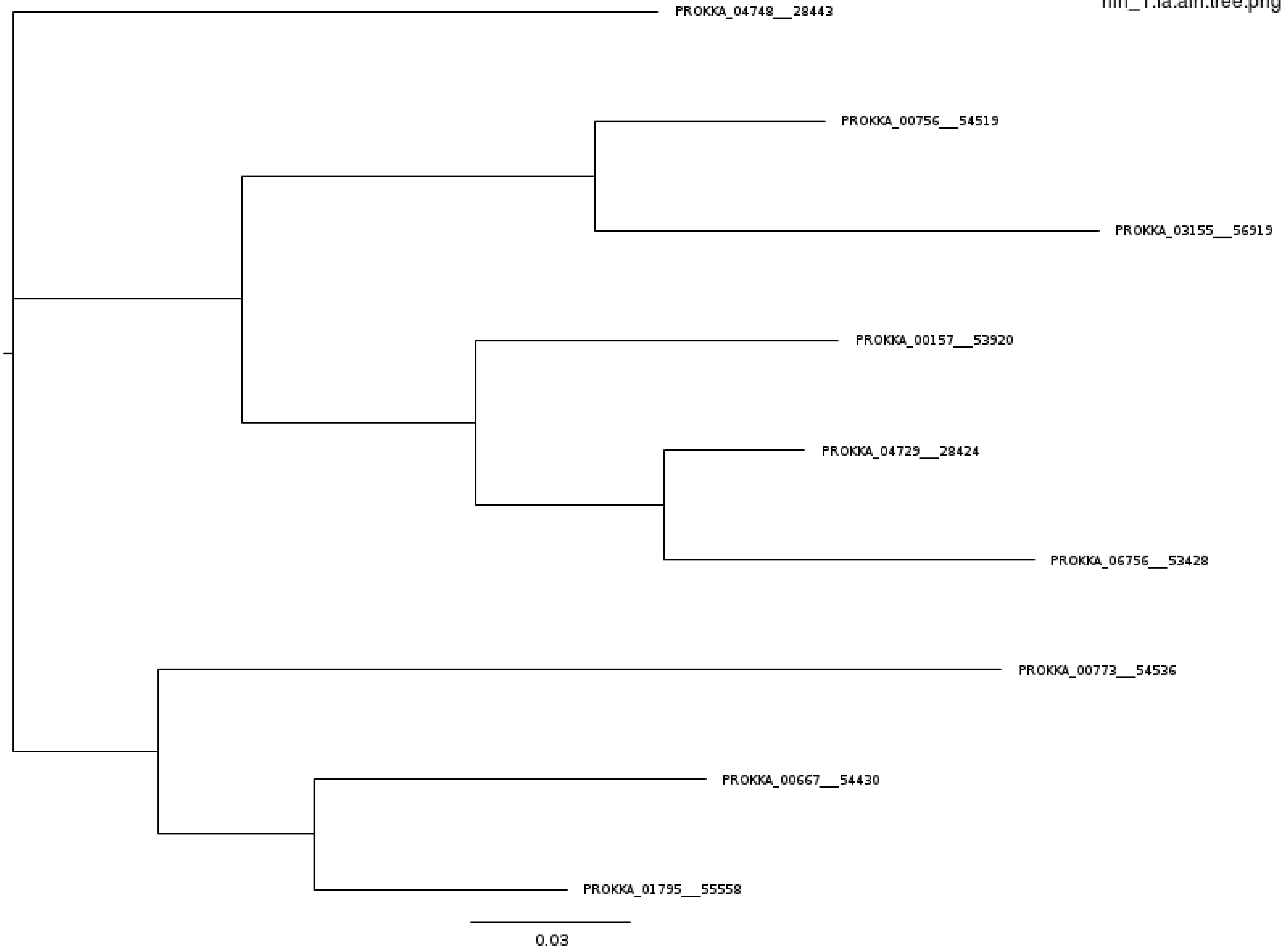
PROKKA_04748___28443
PROKKA_00756___54519
PROKKA_03155___56919
PROKKA_00157___53920
PROKKA_04729___28424
PROKKA_06756___53428
PROKKA_00773___54536
PROKKA_00667___54430
PROKKA_01795___55558
0.03

hin_2.fa.aln.tree.png

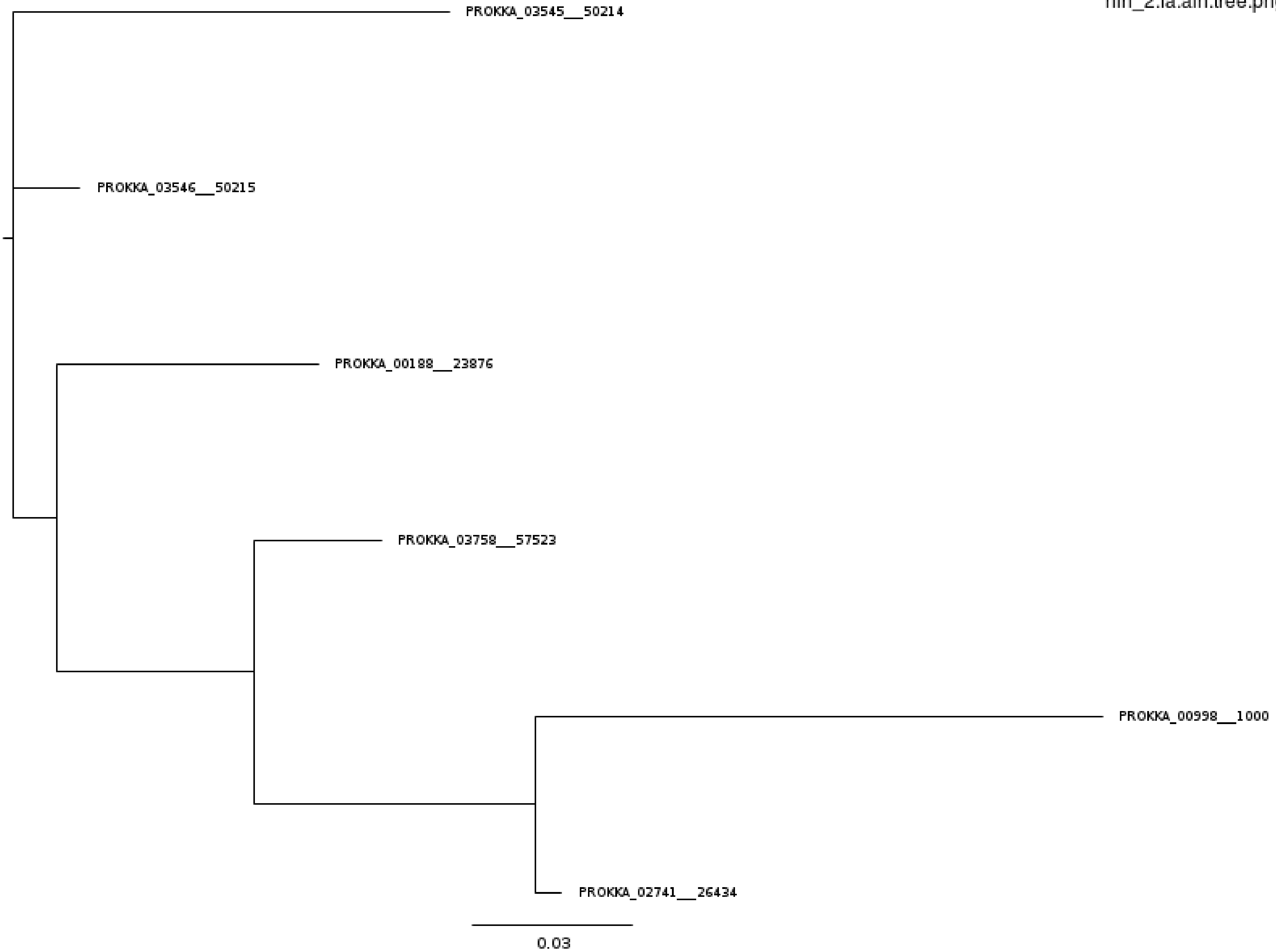

hisA.fa.aln.tree.png

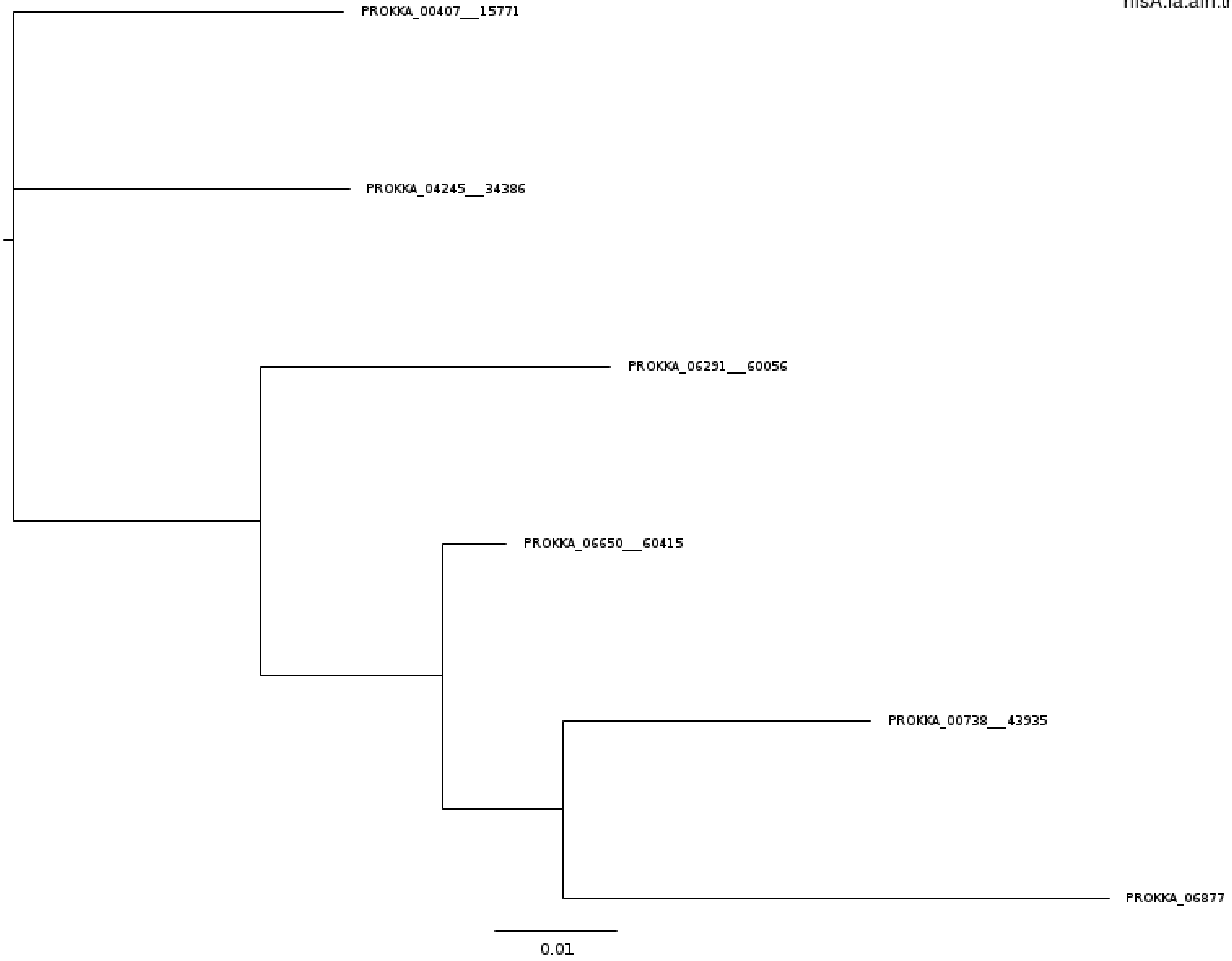

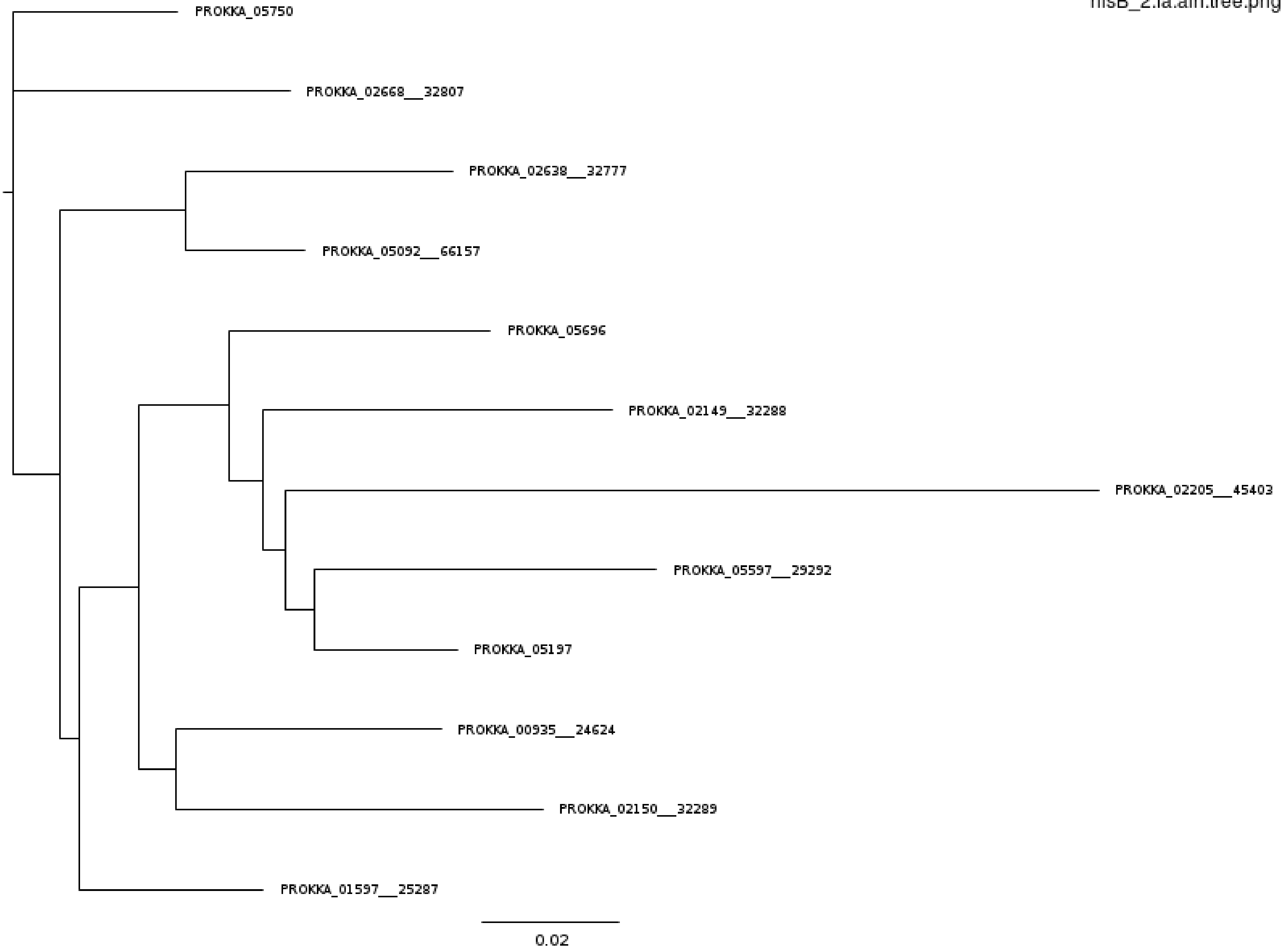
PROKKA_05750
PROKKA_02668___32807
PROKKA_02638___32777
PROKKA_05092___66157
PROKKA_05696
PROKKA_02149___32288
PROKKA_02205___45403
PROKKA_05597___29292
PROKKA_05197
PROKKA_00935___24624
PROKKA_02150___32289
PROKKA_01597___25287
0.02

hisC2_1.fa.aln.tree.png

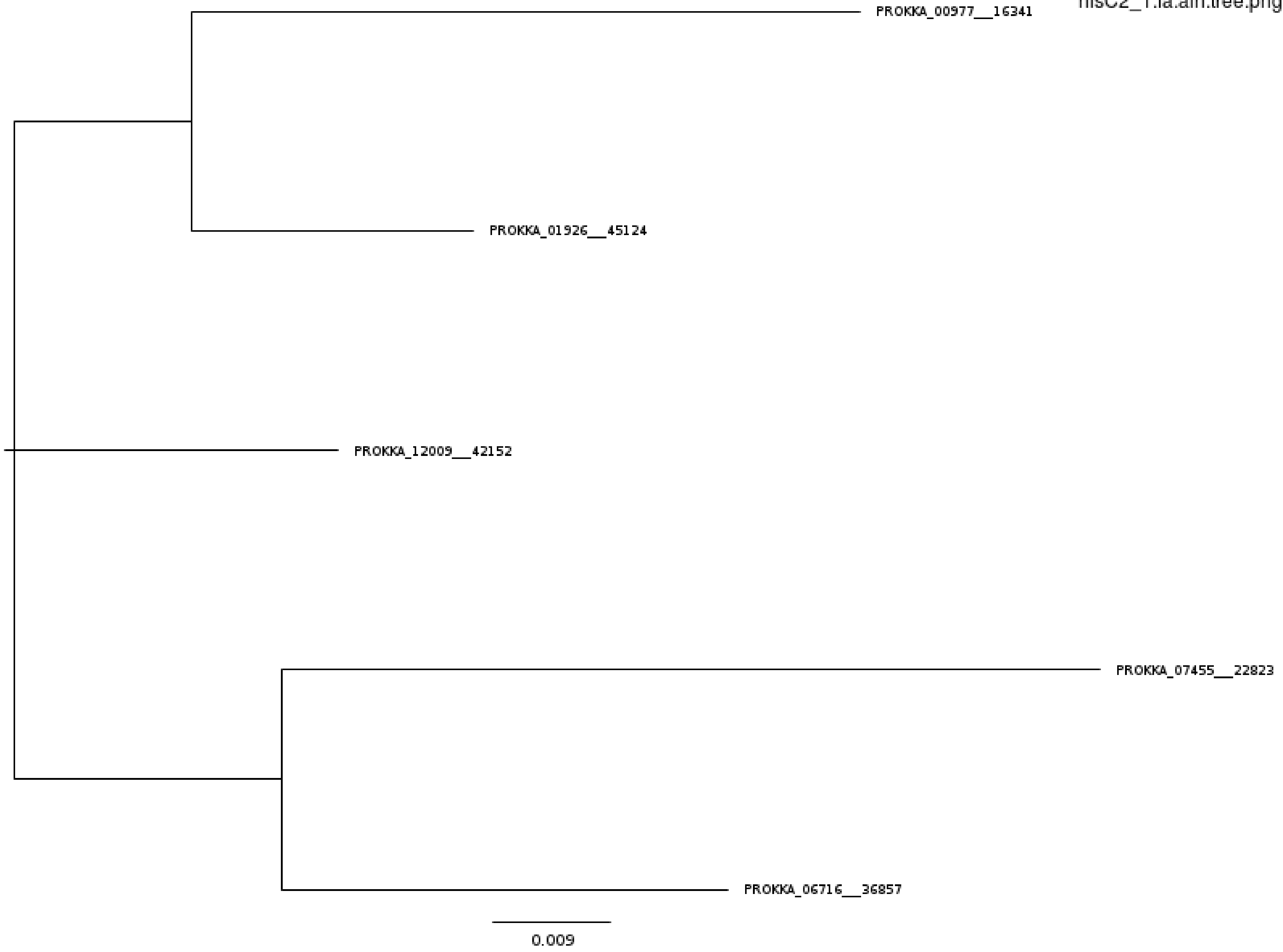

hisD_2.fa.aln.tree.png

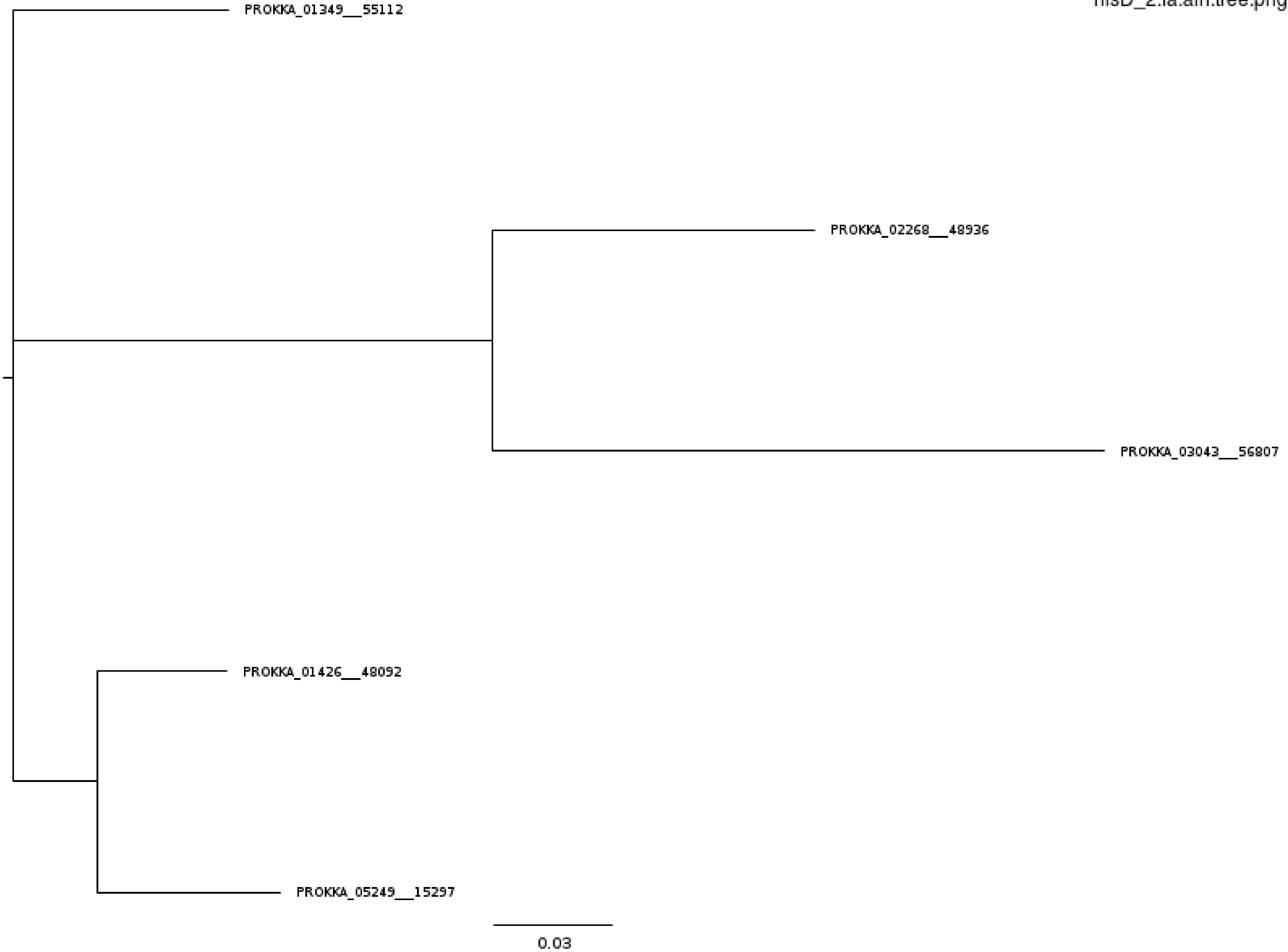

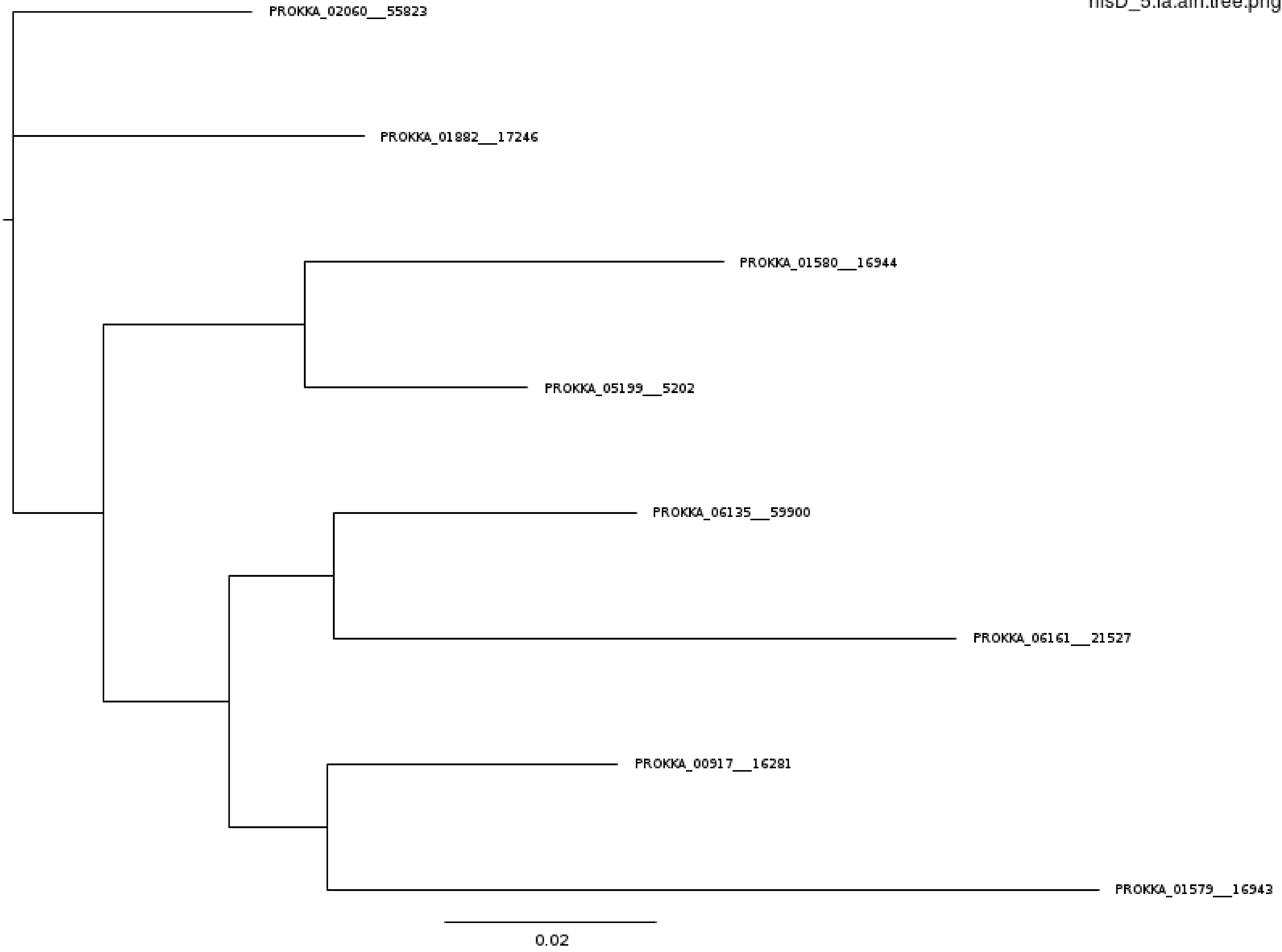
PROKKA_02060__55823
PROKKA_01882__17246
PROKKA_01580__16944
PROKKA_05199__5202
PROKKA_06135__59900
PROKKA_06161__21527
PROKKA_00917__16281
PROKKA_01579__16943
0.02

hisF_2.fa.aln.tree.png

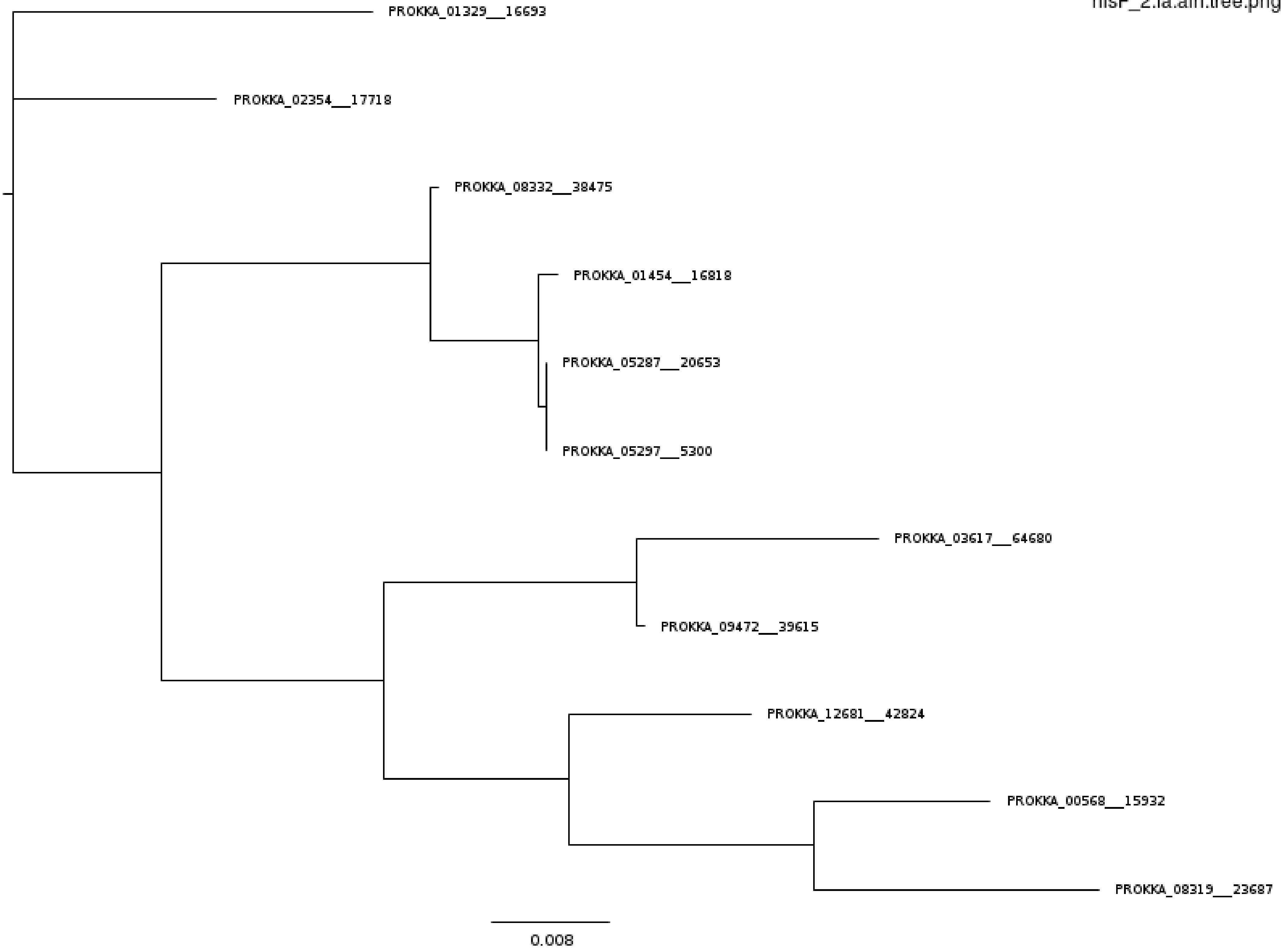

hisG_2.fa.aln.tree.png

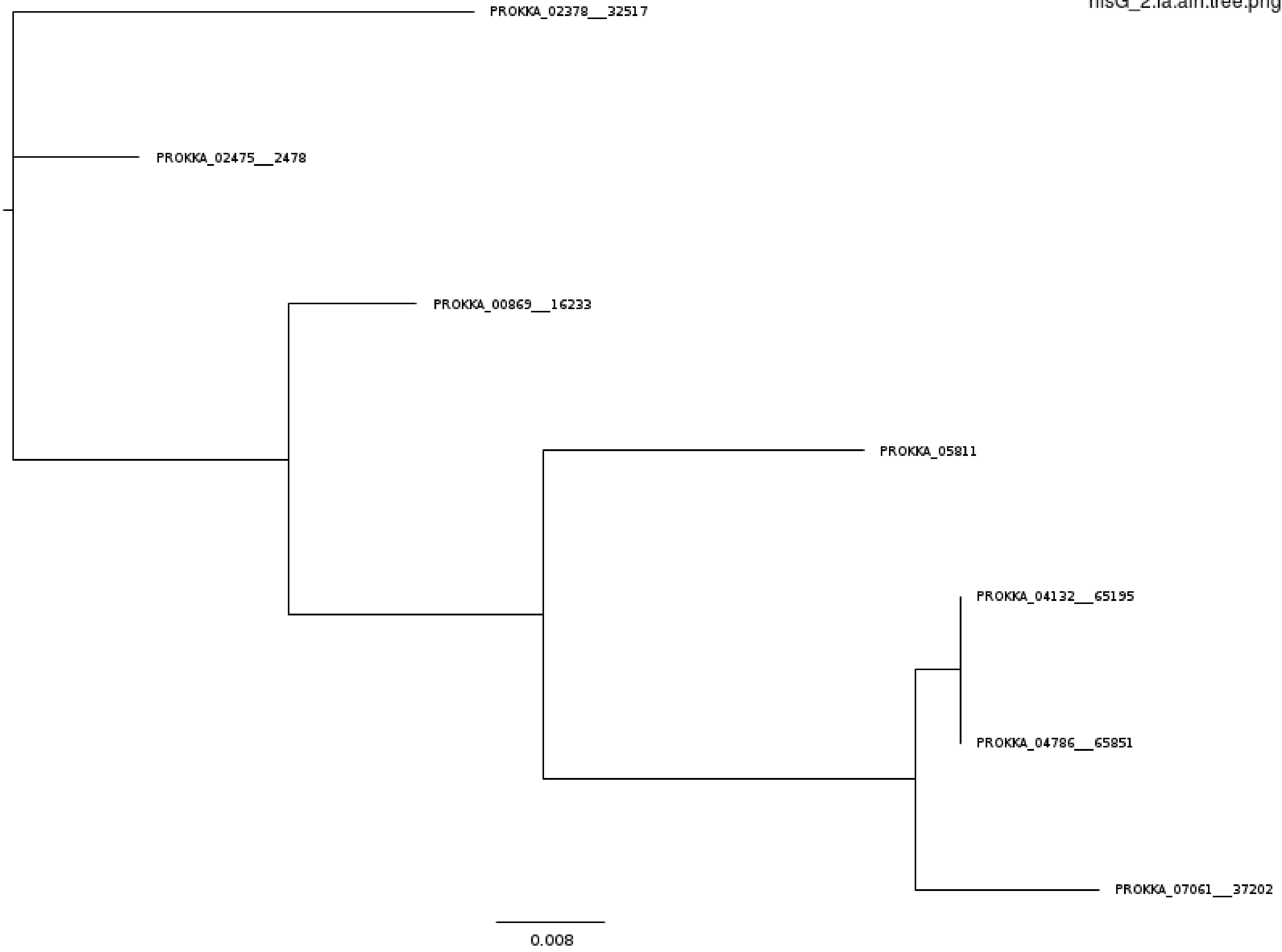

hisH1_2.fa.aln.tree.png

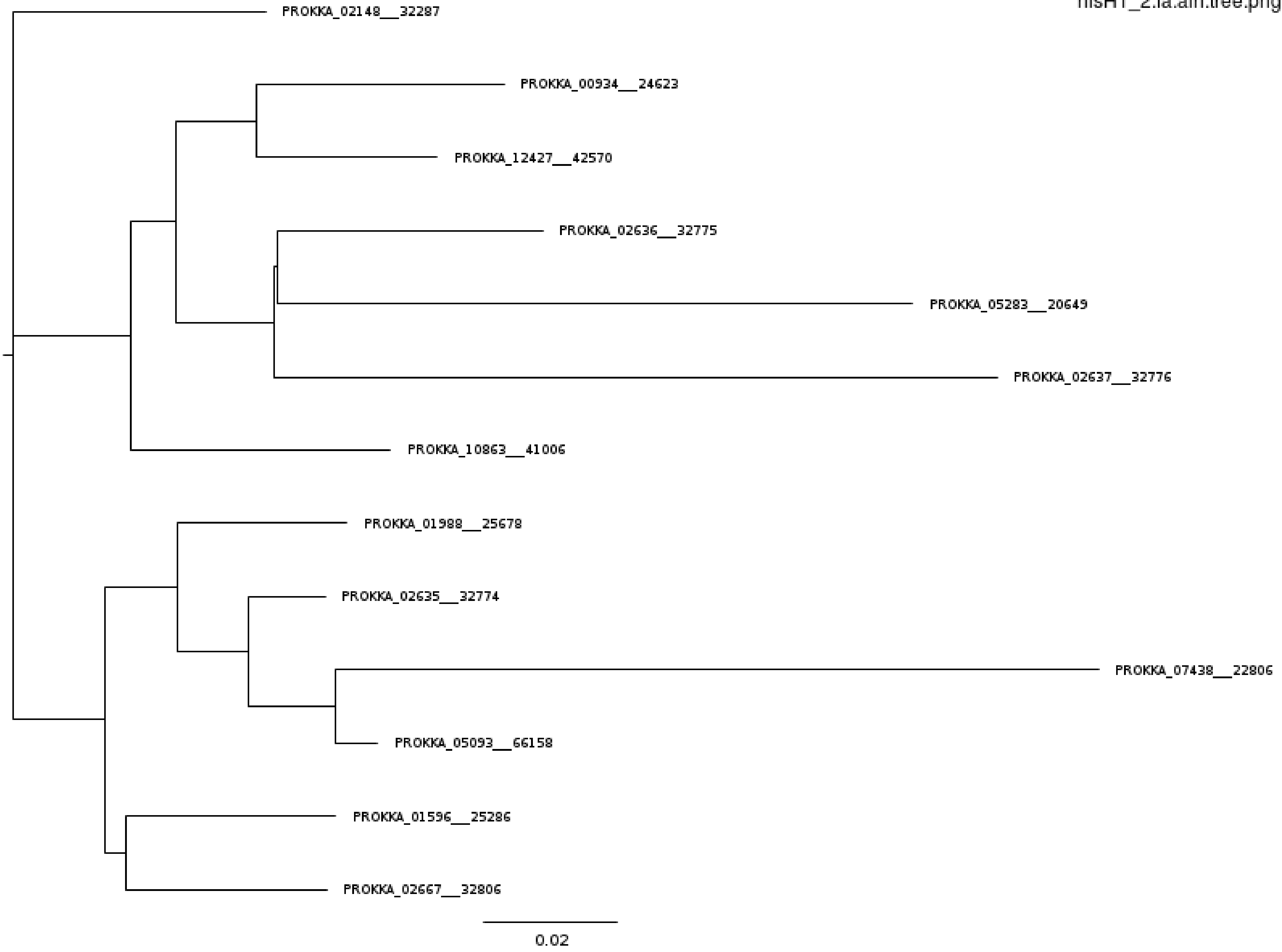

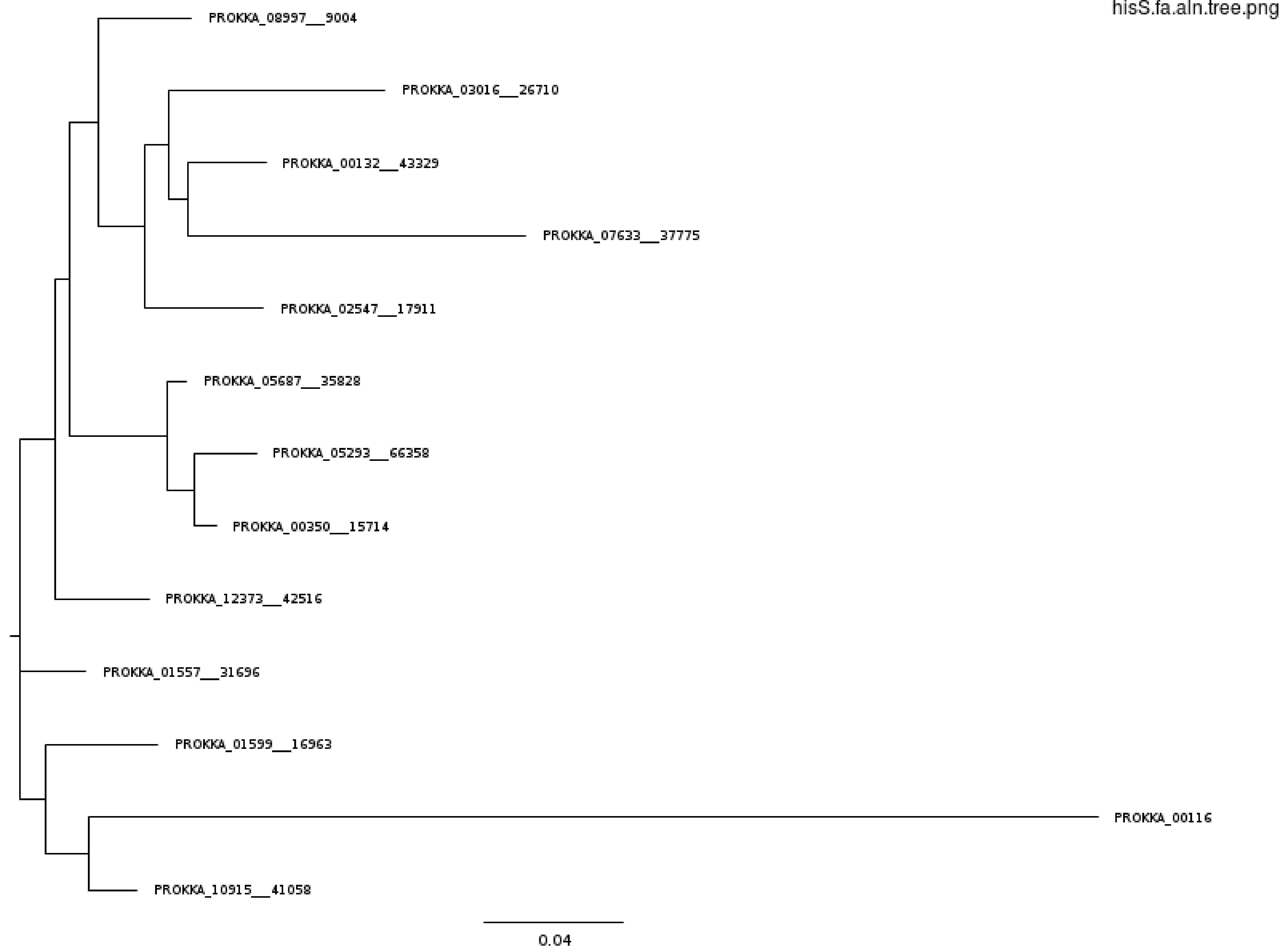

hisS.fa.aln.tree.png
PROKKA_08997___9004
PROKKA_03016___26710
PROKKA_00132___43329
PROKKA_07633___37775
PROKKA_02547___17911
PROKKA_05687___35828
PROKKA_05293___66358
PROKKA_00350___15714
PROKKA_12373___42516
PROKKA_01557___31696
PROKKA_01599___16963
PROKKA_00116
PROKKA_10915___41058
0.04

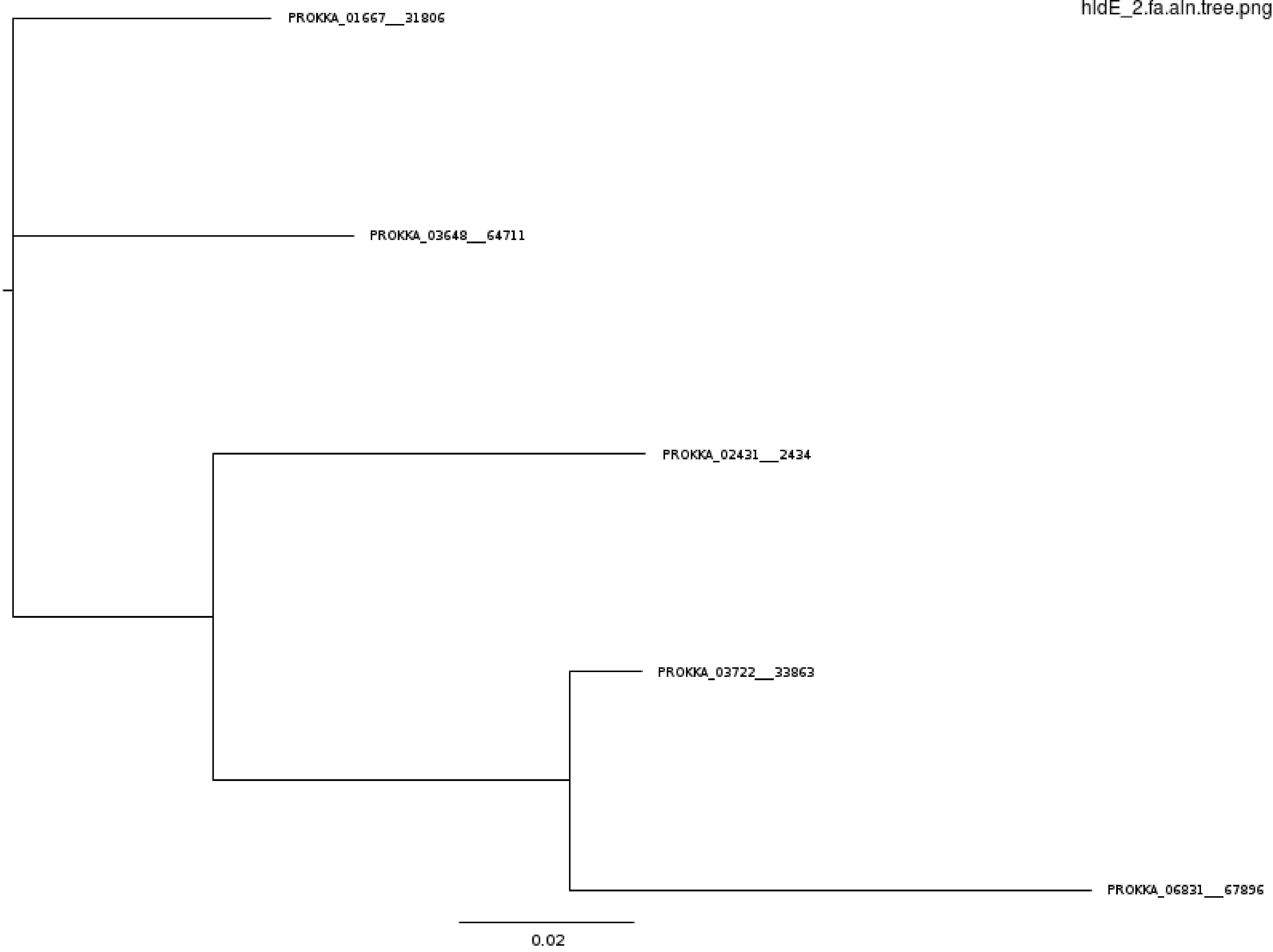
hldE_2.fa.aln.tree.png
PROKKA_01667___31806
PROKKA_03648___64711
PROKKA_02431___2434
PROKKA_03722___33863
PROKKA_06831___67896
0.02

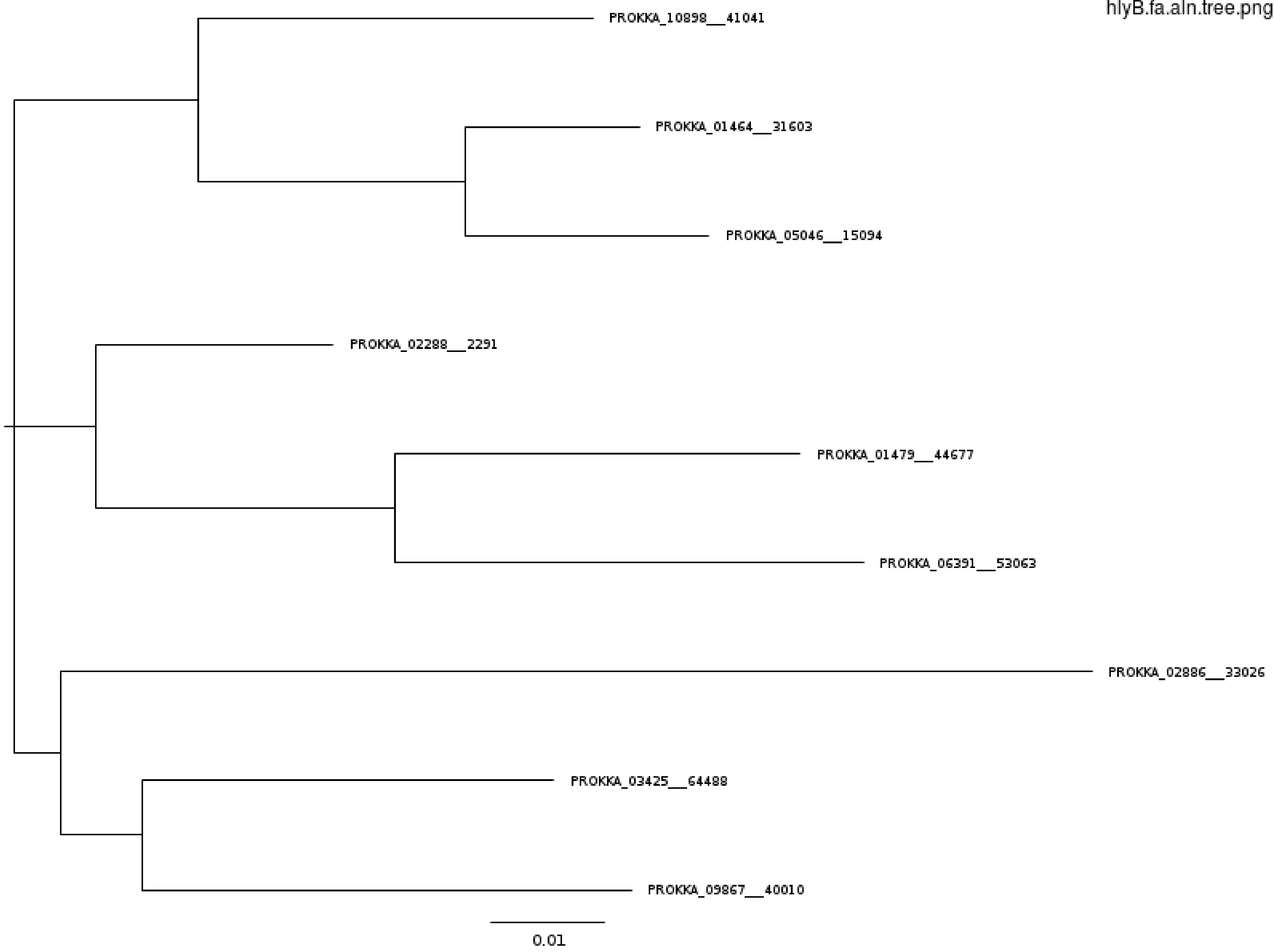
hlyB.fa.aln.tree.png
PROKKA_10898___41041
PROKKA_01464___31603
PROKKA_05046___15094
PROKKA_02288___2291
PROKKA_01479___44677
PROKKA_06391___53063
PROKKA_02886___33026
PROKKA_03425___64488
PROKKA_09867___40010
0.01

hlyD_1.fa.aln.tree.png

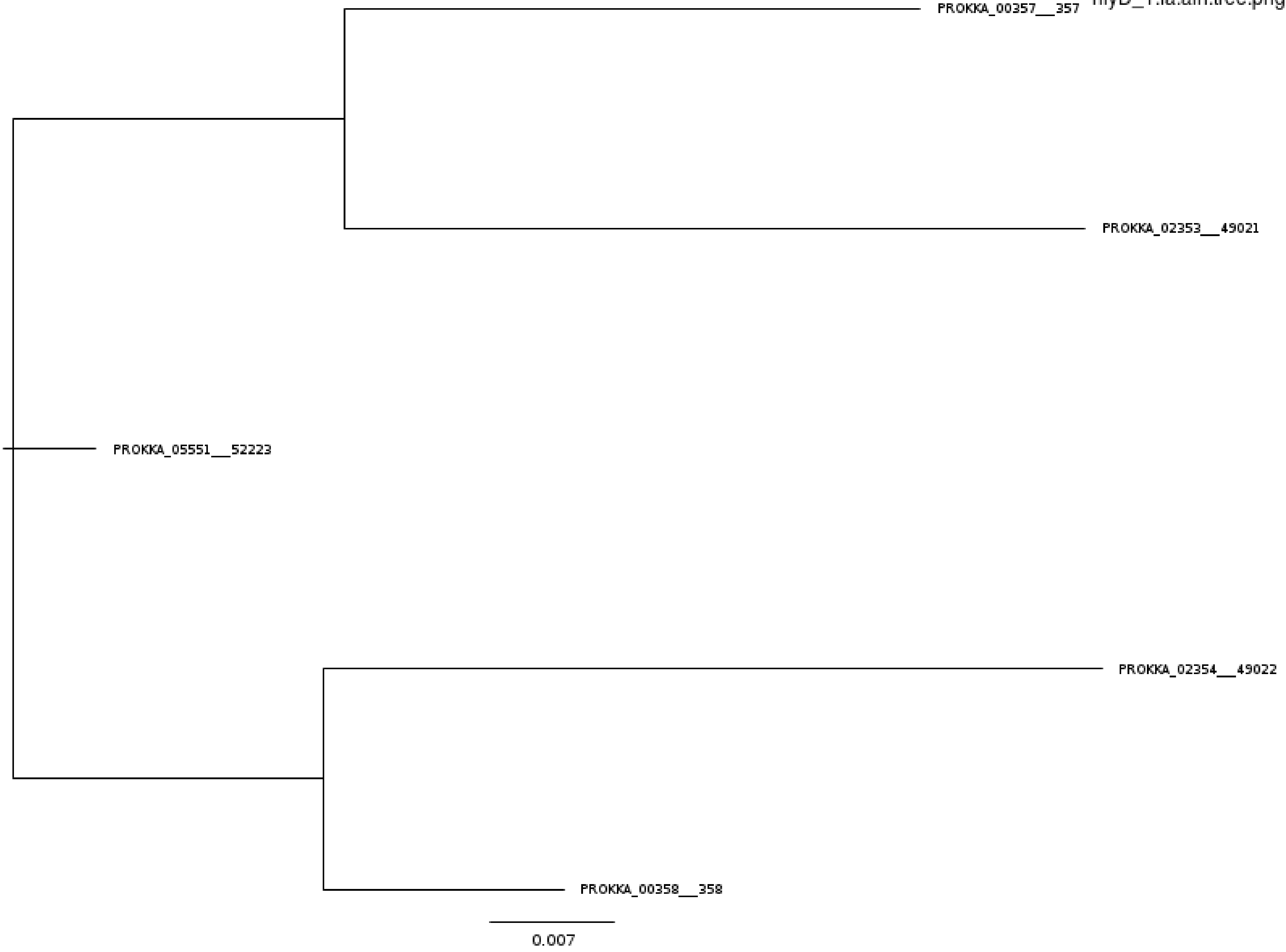

hmp.fa.aln.tree.png

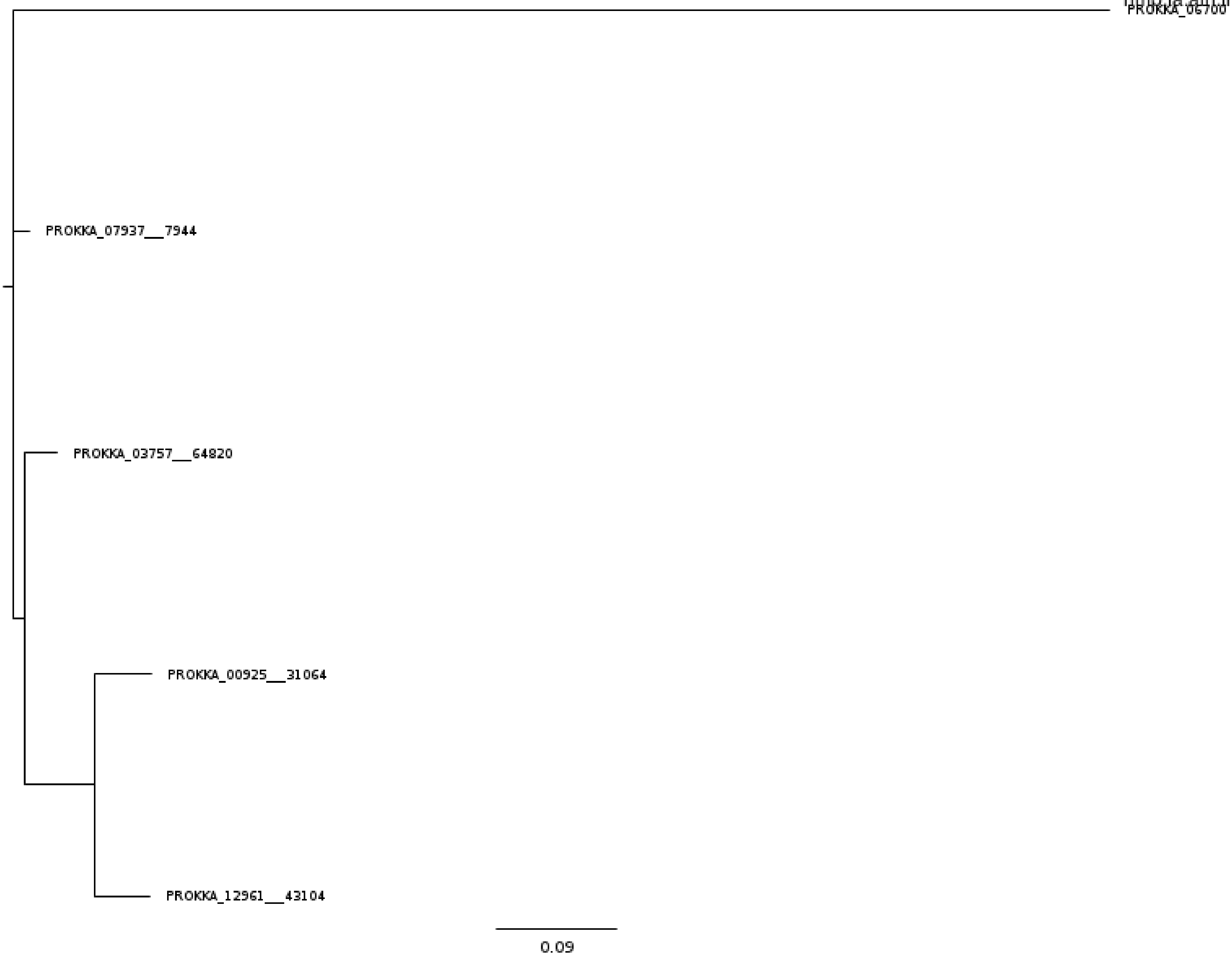

holA.fa.aln.tree.png

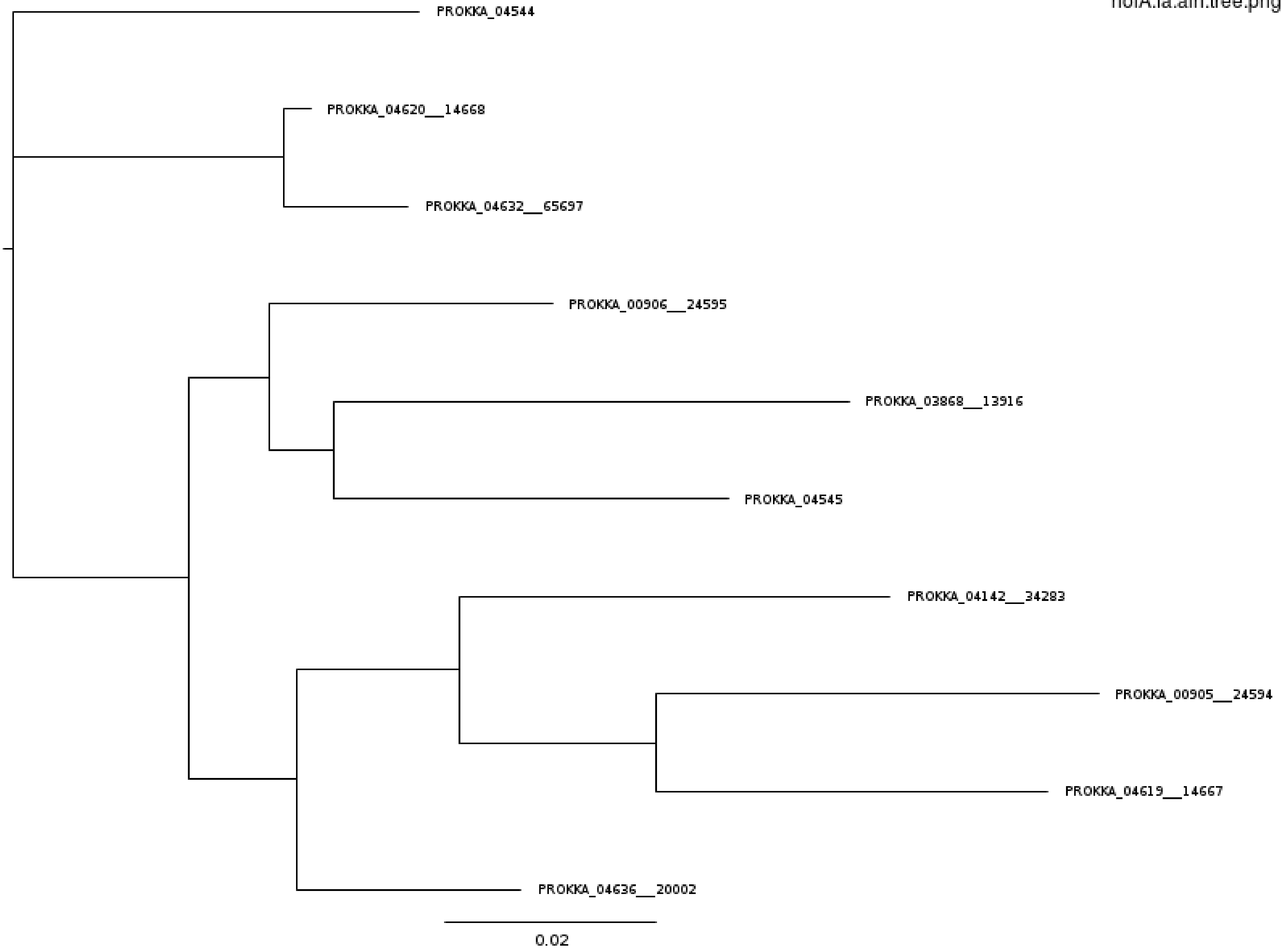

holB_1.fa.aln.tree.png

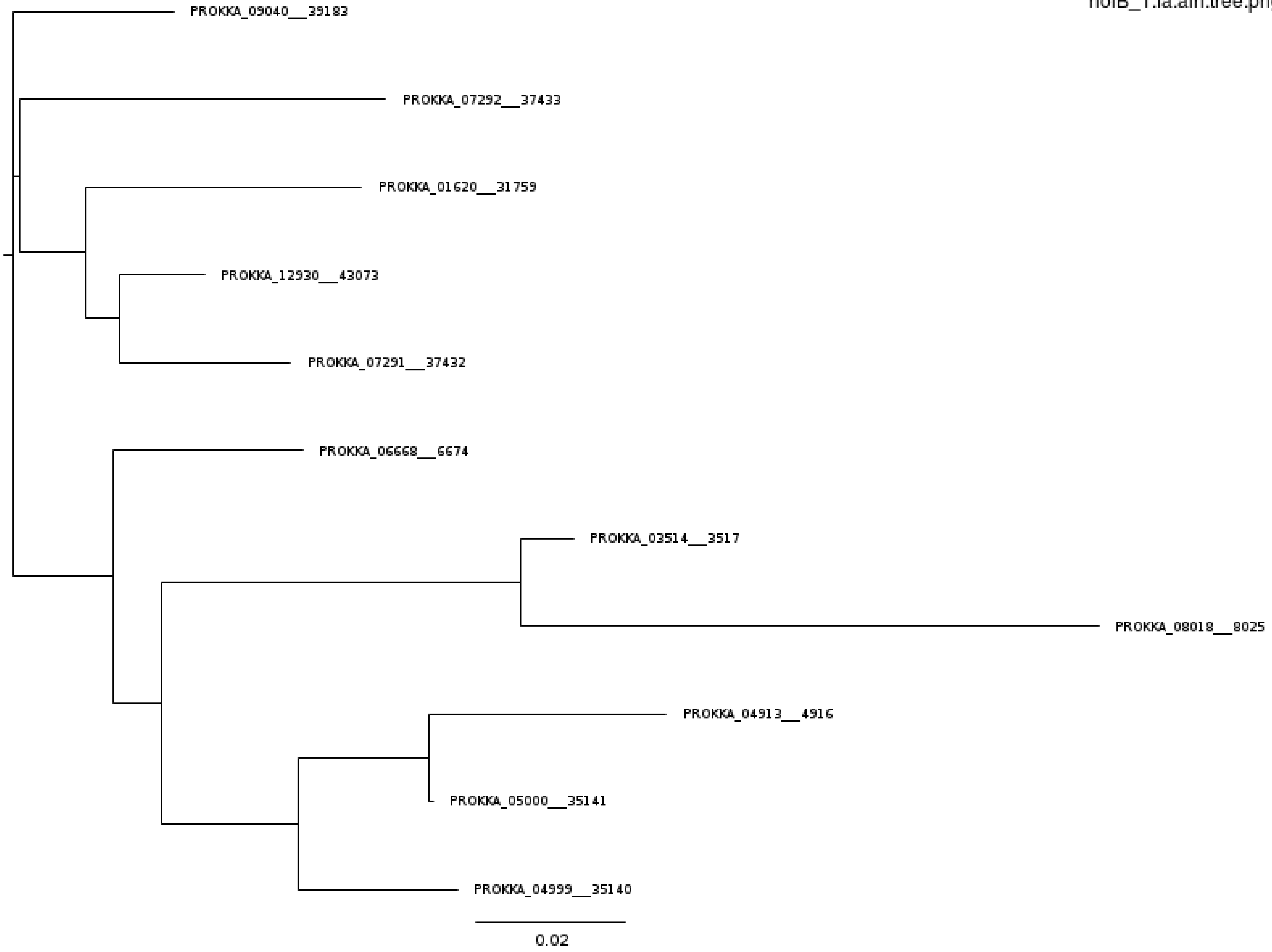

holC_4.fa.aln.tree.png

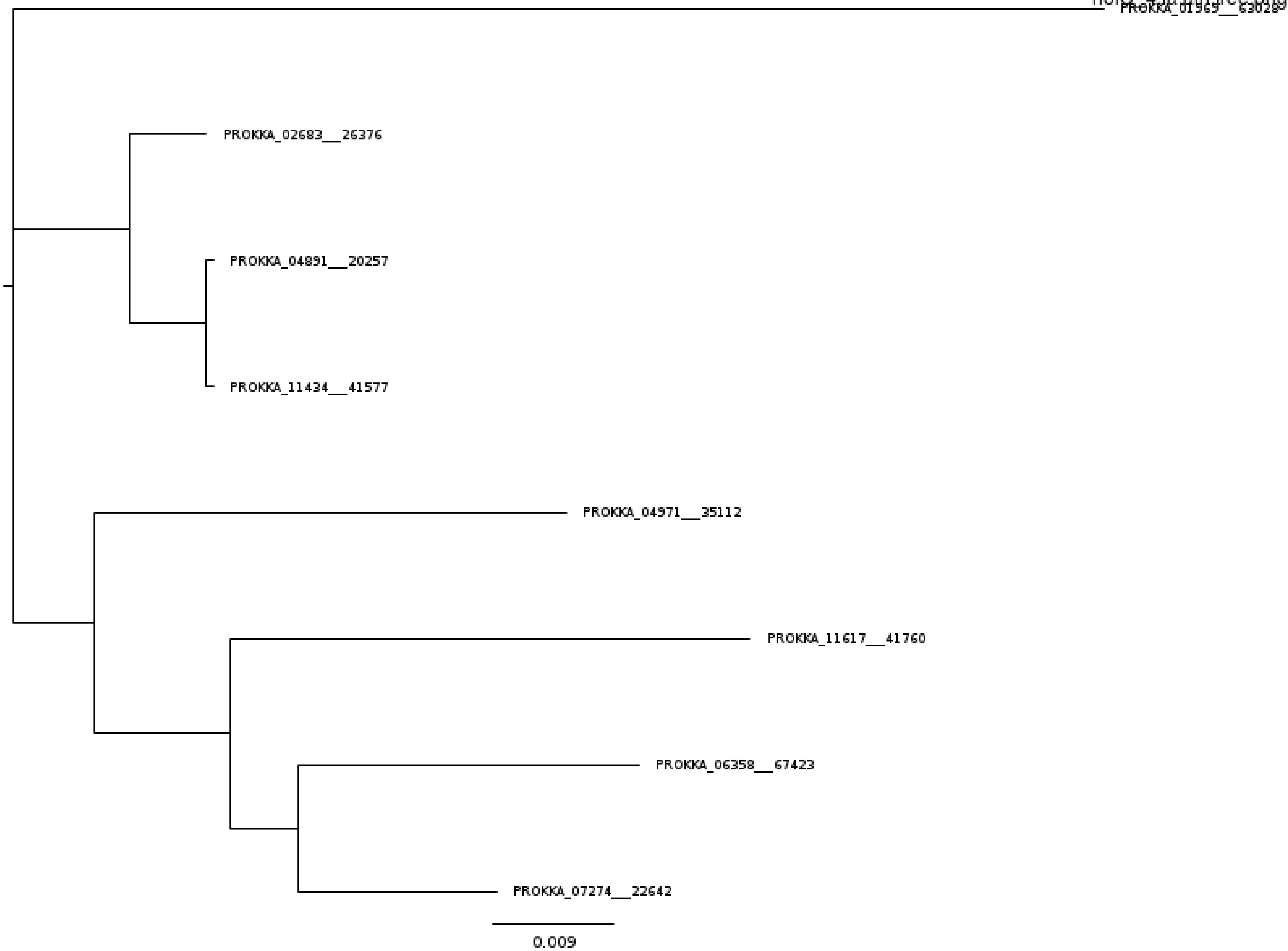

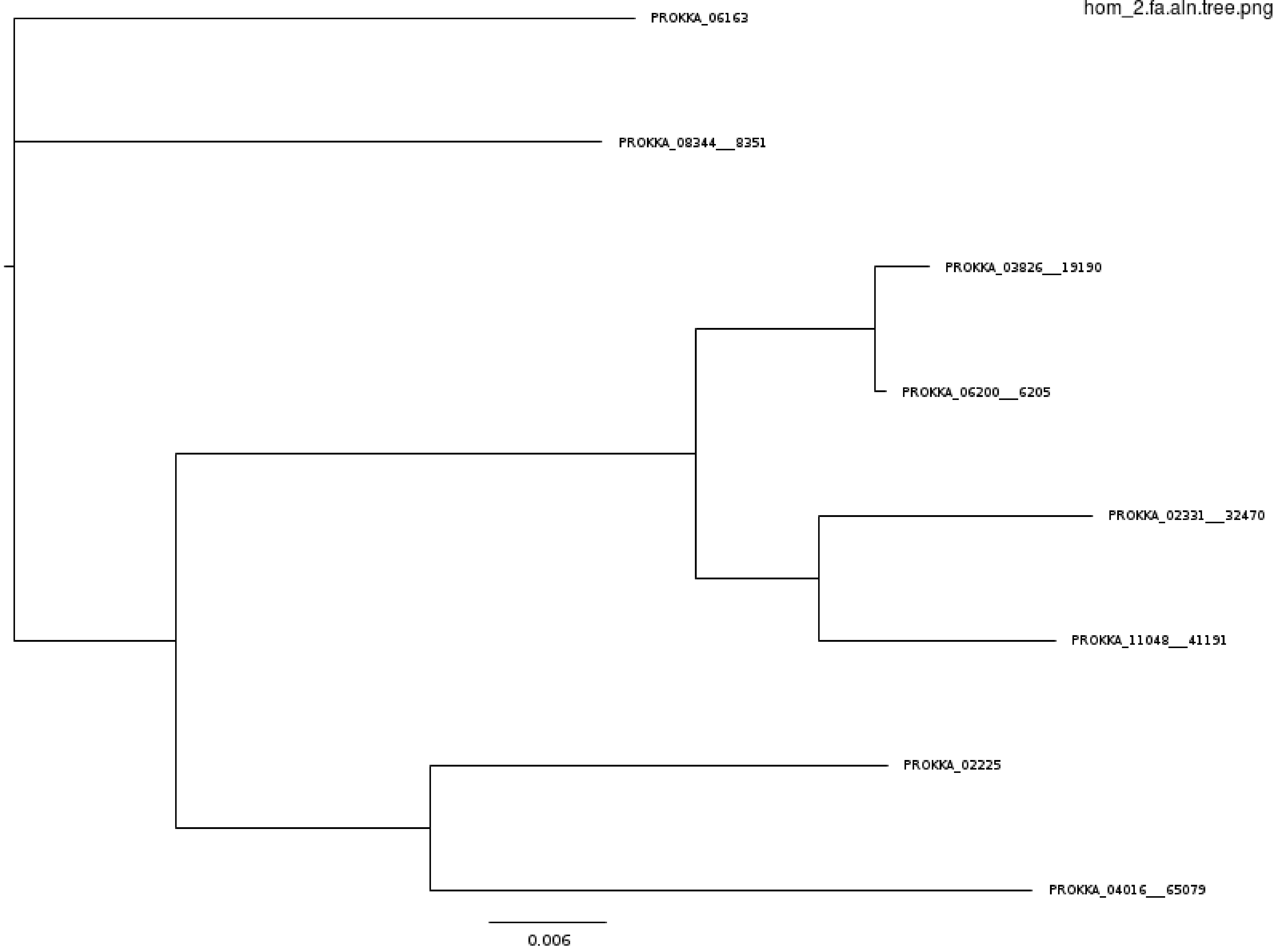
hom_2.fa.aln.tree.png
PROKKA_06163
PROKKA_08344__8351
PROKKA_03826__19190
PROKKA_06200__6205
PROKKA_02331__32470
PROKKA_11048__41191
PROKKA_02225
PROKKA_04016__65079
0.006

hoxS_3.fa.aln.tree.png

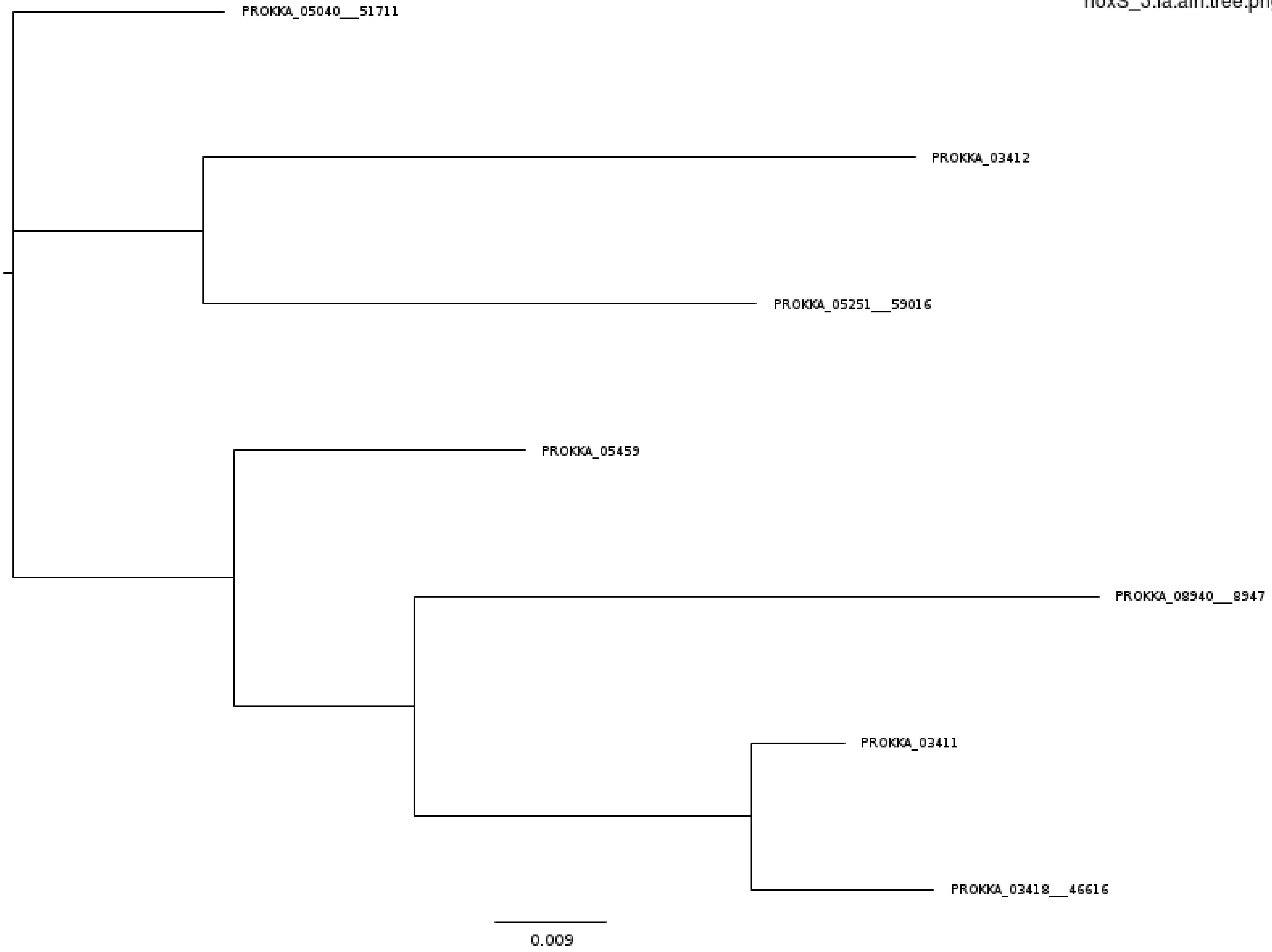

hpf_1.fa.aln.tree.png

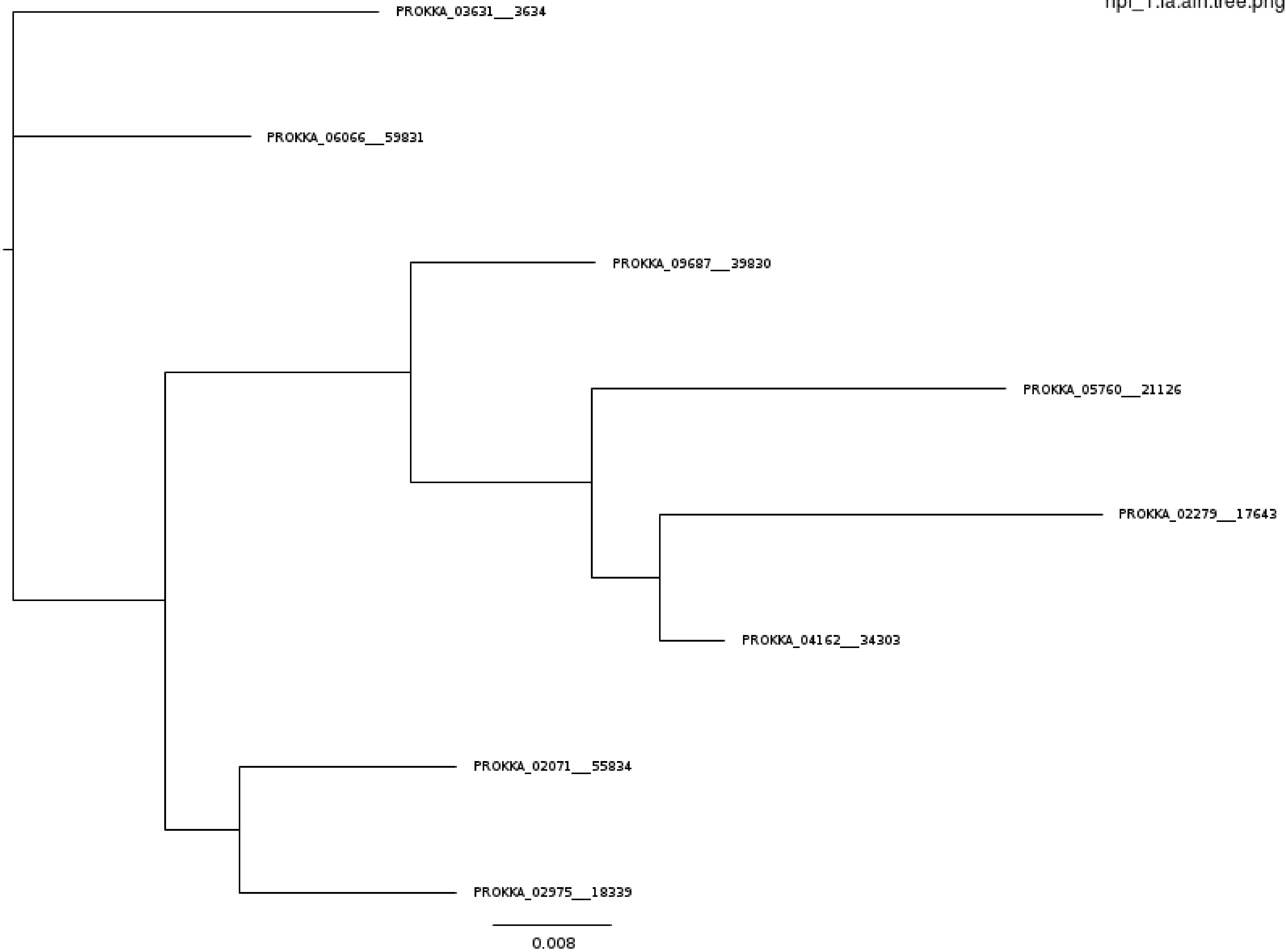

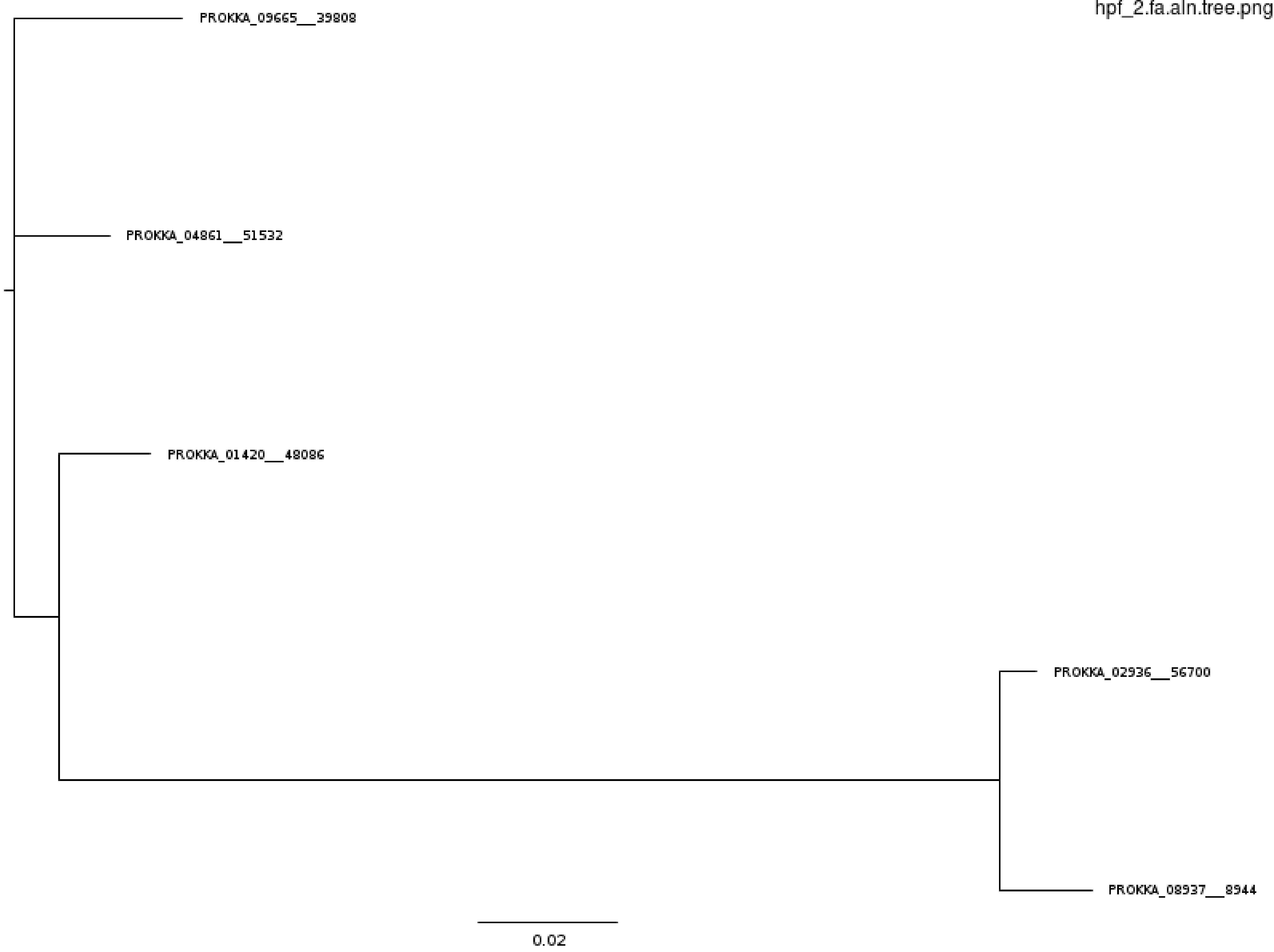
hpf_2.fa.aln.tree.png
PROKKA_09665___39808
PROKKA_04861___51532
PROKKA_01420___48086
PROKKA_02936___56700
PROKKA_08937___8944
0.02

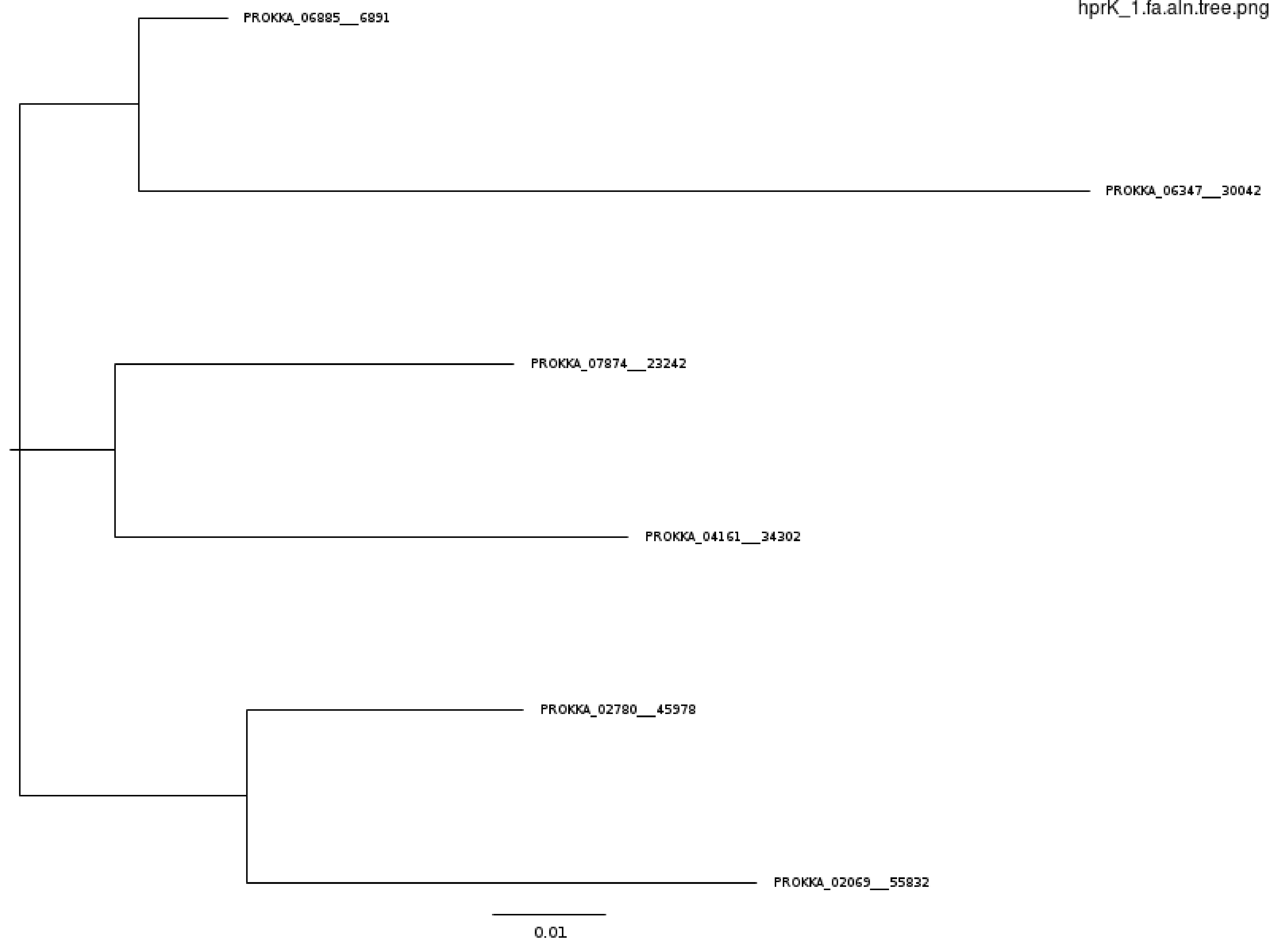
hprK_1.fa.aln.tree.png
PROKKA_06885___6891
PROKKA_06347___30042
PROKKA_07874___23242
PROKKA_04161___34302
PROKKA_02780___45978
PROKKA_02069___55832
0.01

hpt_1.fa.aln.tree.png

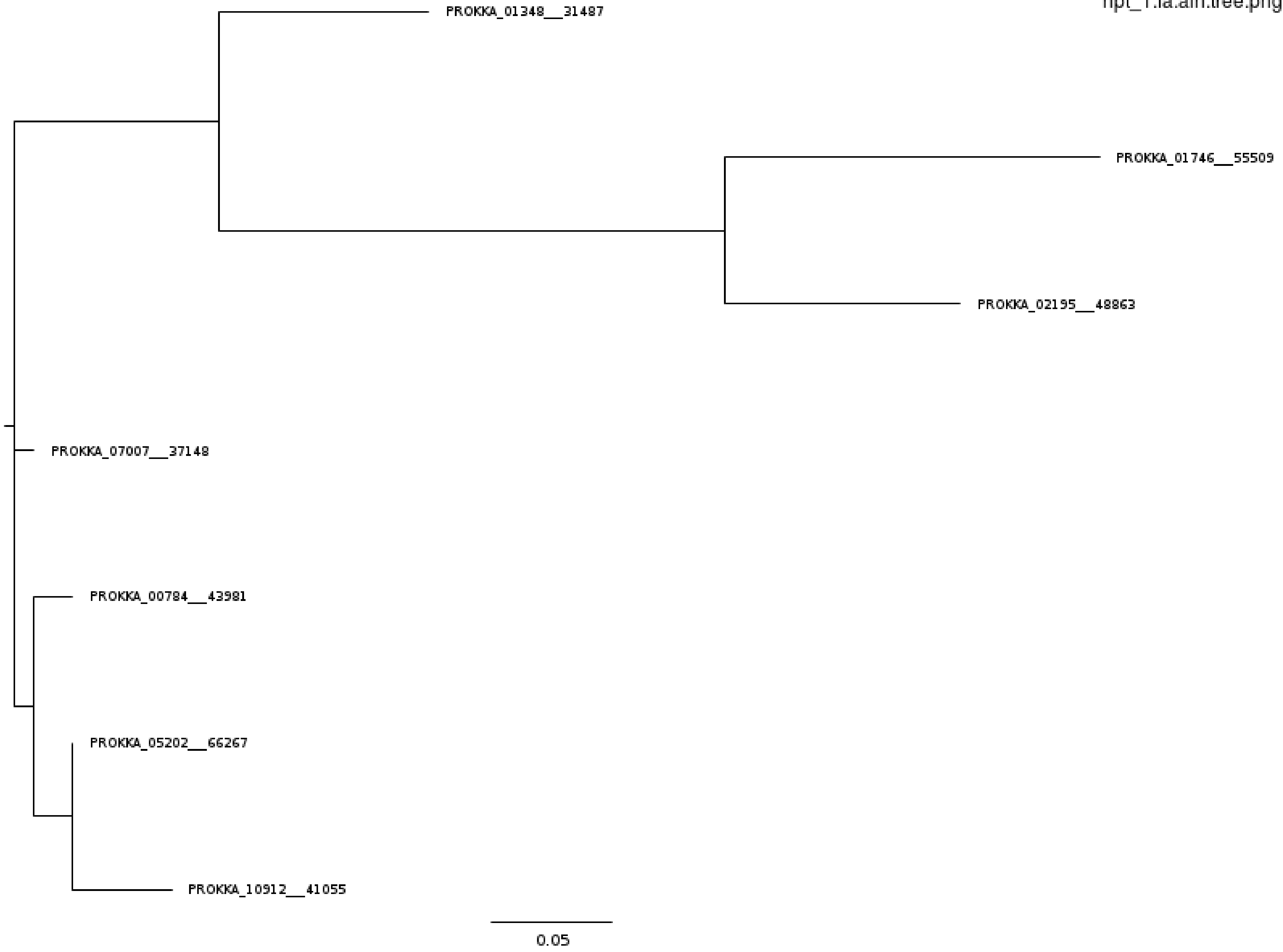

hrcA_3.fa.aln.tree.png

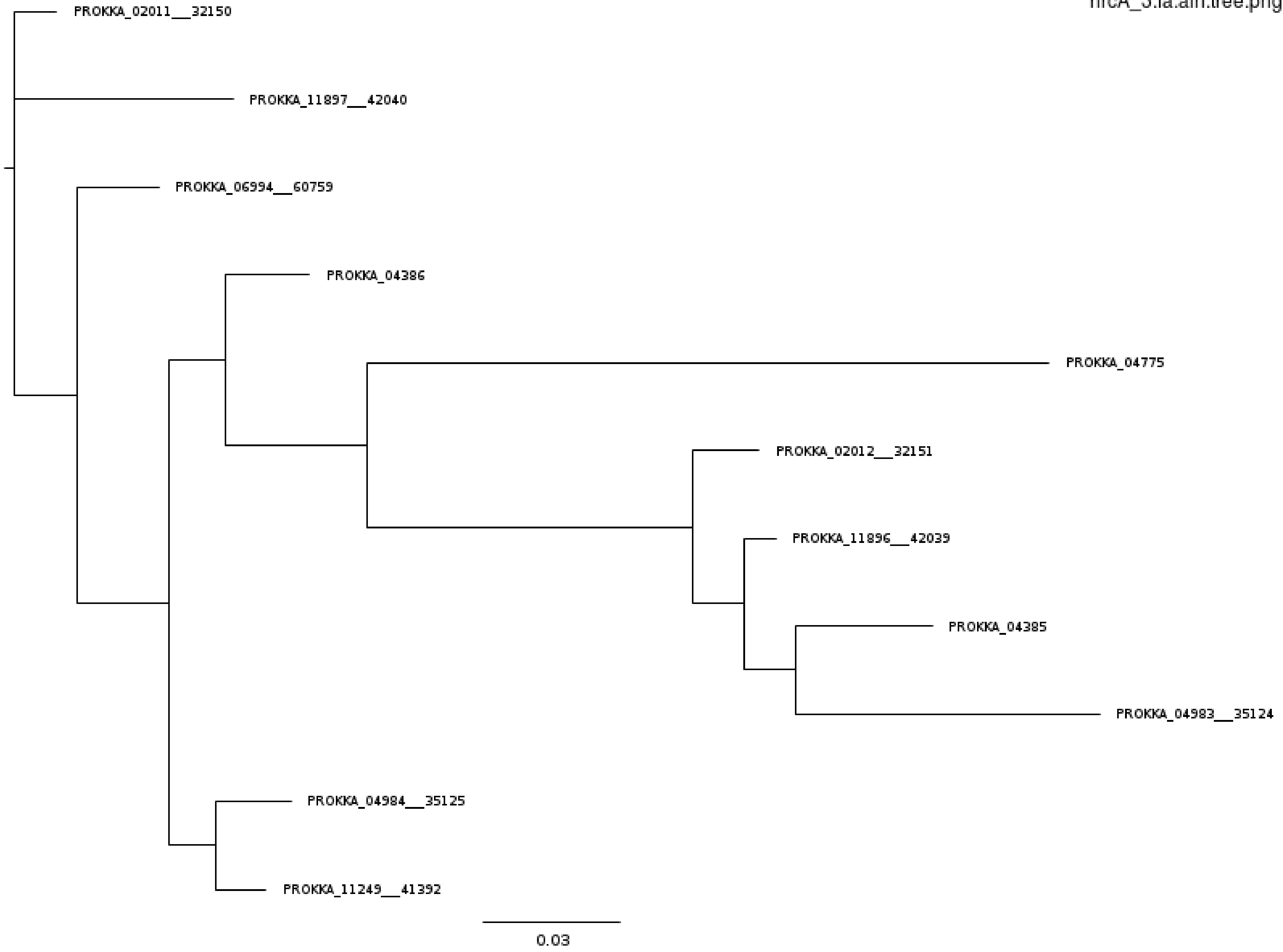

hrpB_6.fa.aln.tree.png

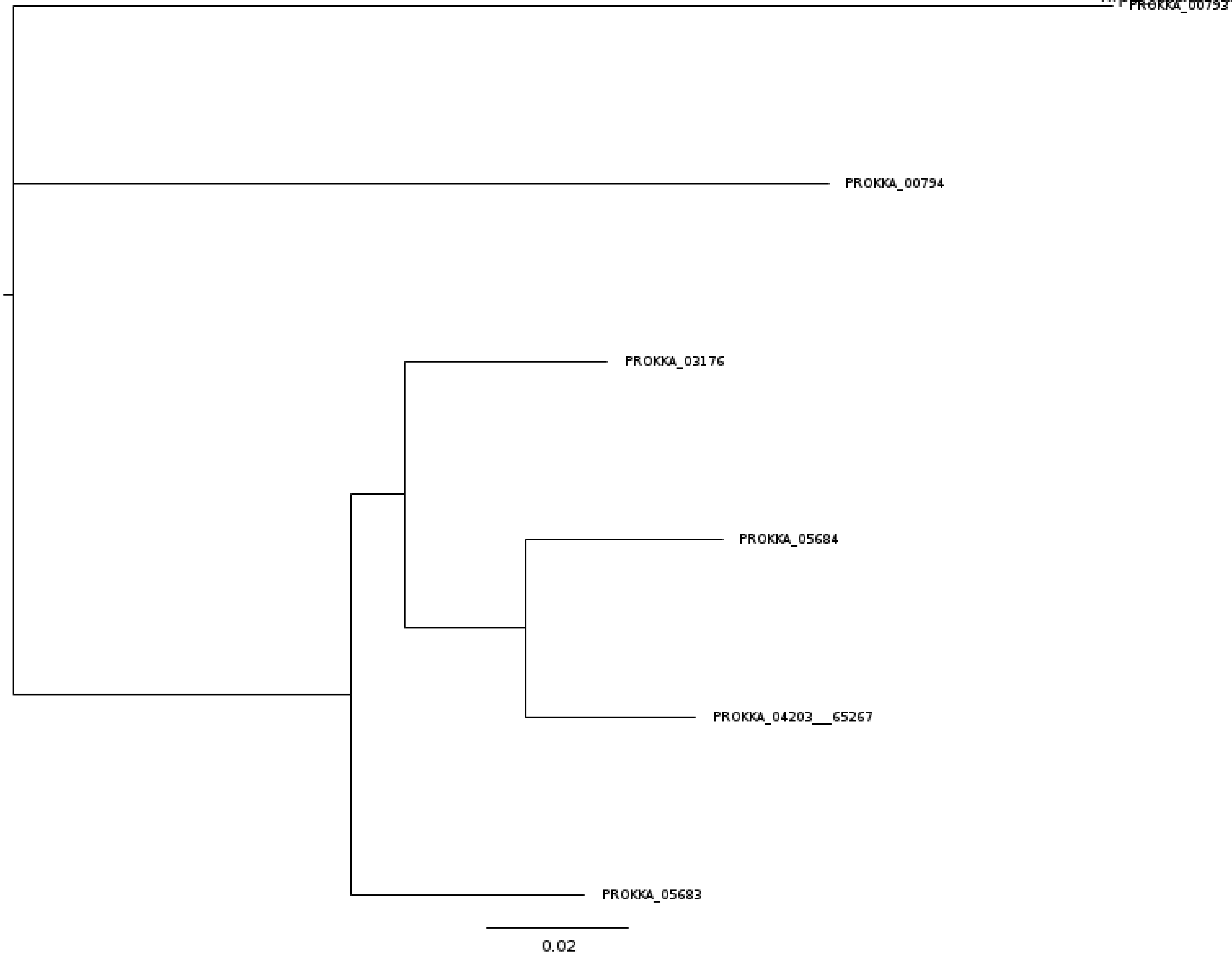

hscA_1.fa.aln.tree.png

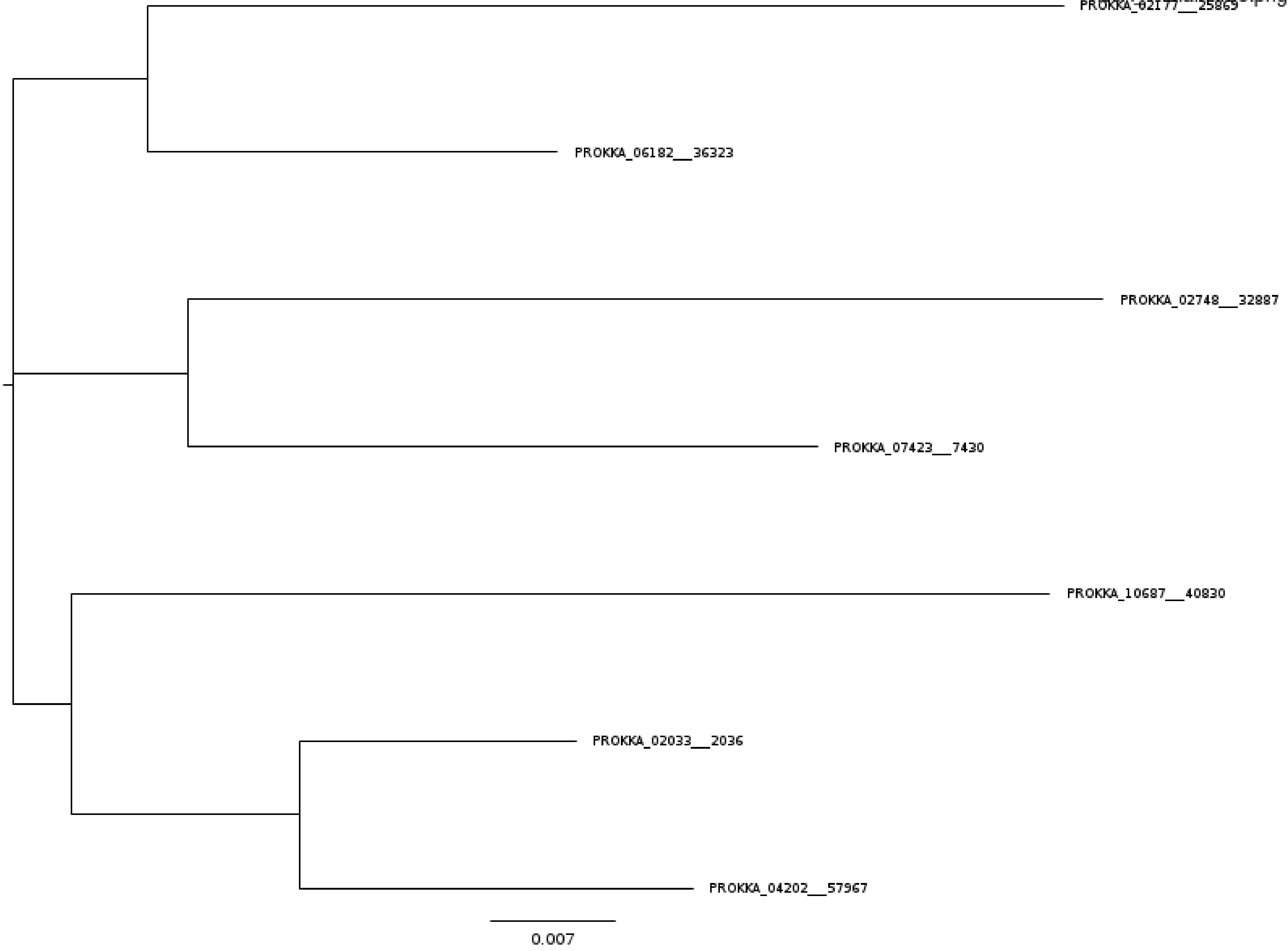

hscB_2.fa.aln.tree.png

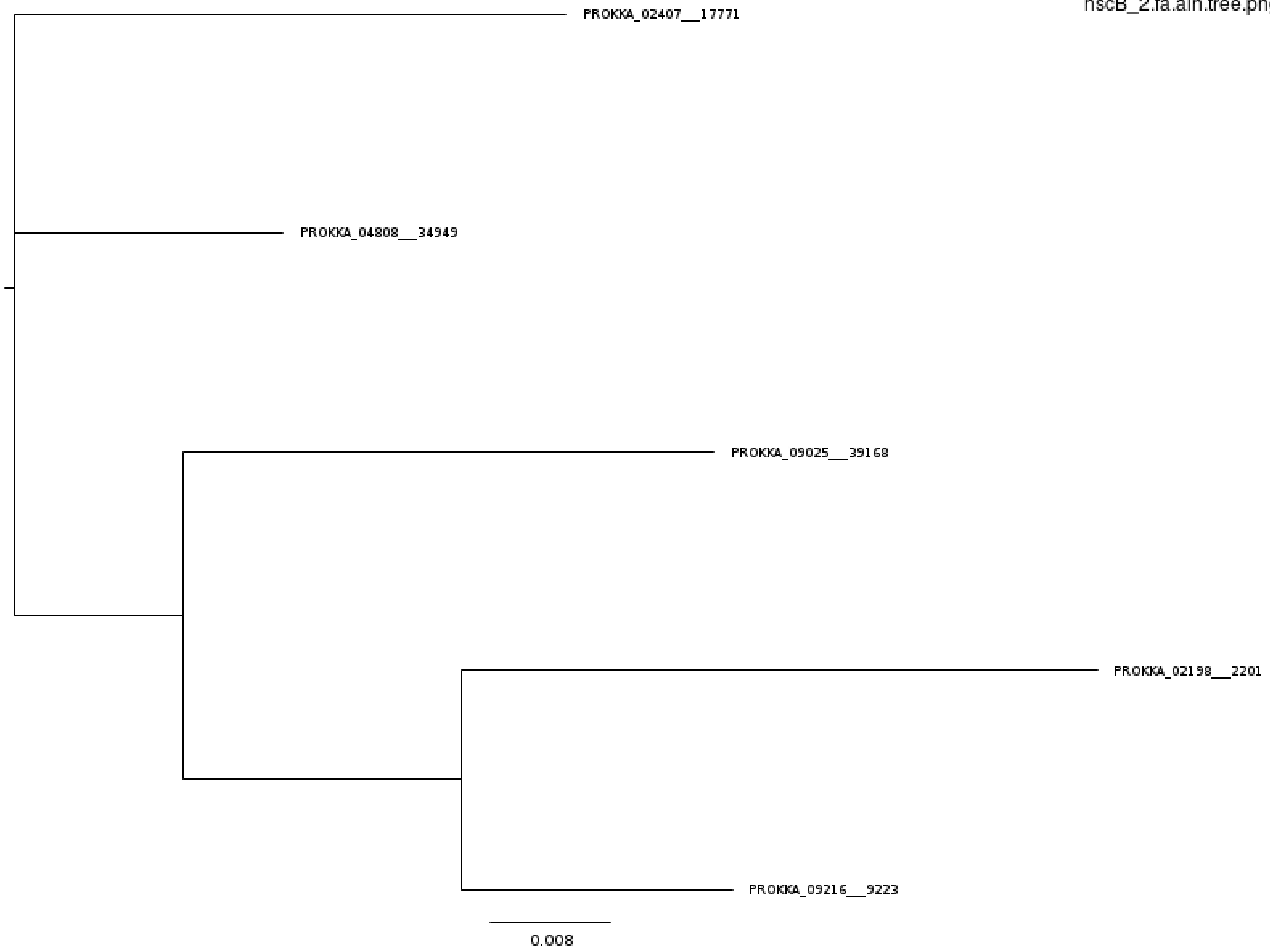

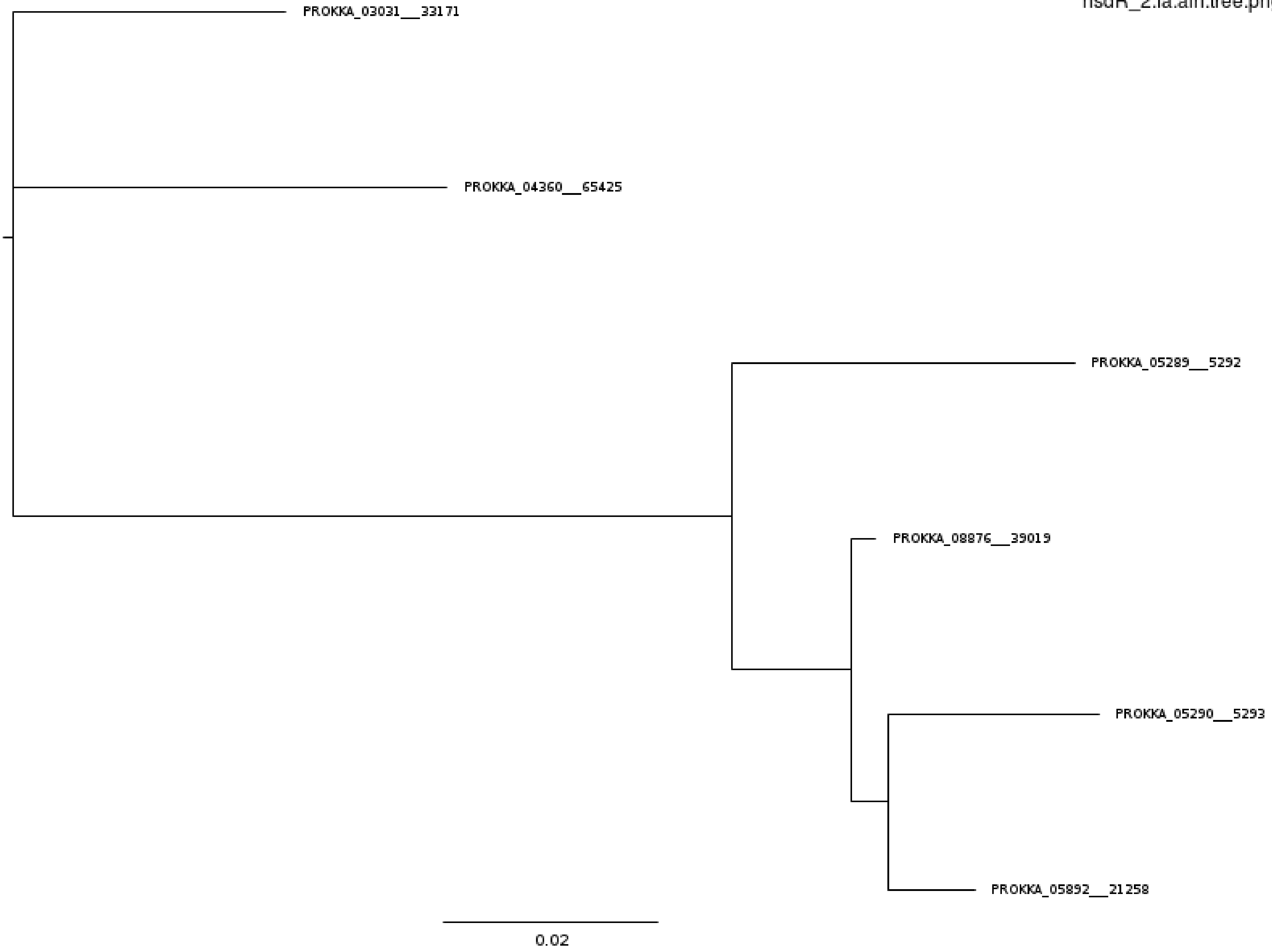
PROKKA_03031___33171
PROKKA_04360___65425
PROKKA_05289___5292
PROKKA_08876___39019
PROKKA_05290___5293
PROKKA_05892___21258
0.02

hslR.fa.aln.tree.png

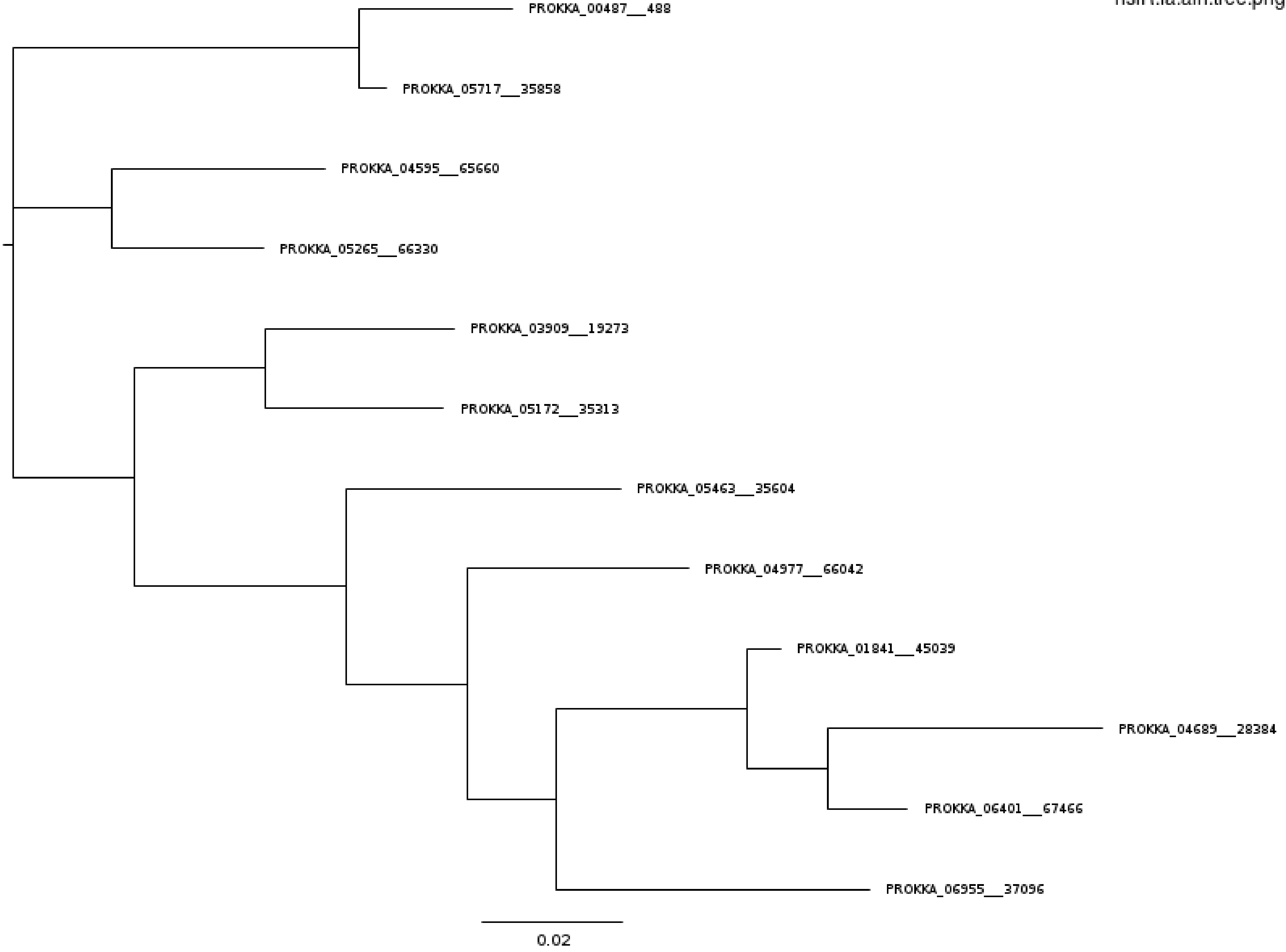

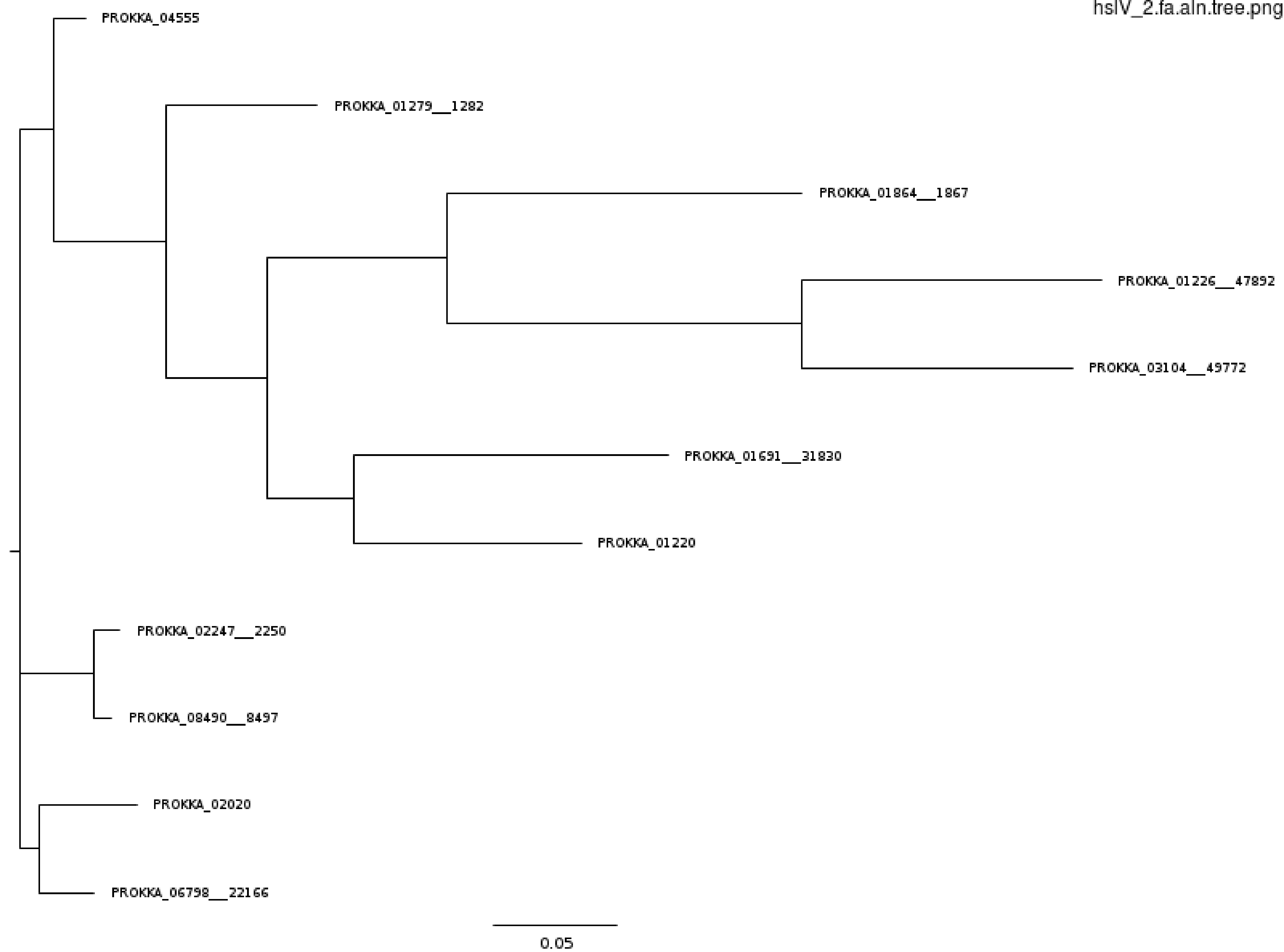

hslV_2.fa.aln.tree.png
PROKKA_04555
PROKKA_01279___1282
PROKKA_01864___1867
PROKKA_01226___47892
PROKKA_03104___49772
PROKKA_01691___31830
PROKKA_01220
PROKKA_02247___2250
PROKKA_08490___8497
PROKKA_02020
PROKKA_06798___22166
0.05

htpG_1.fa.aln.tree.png

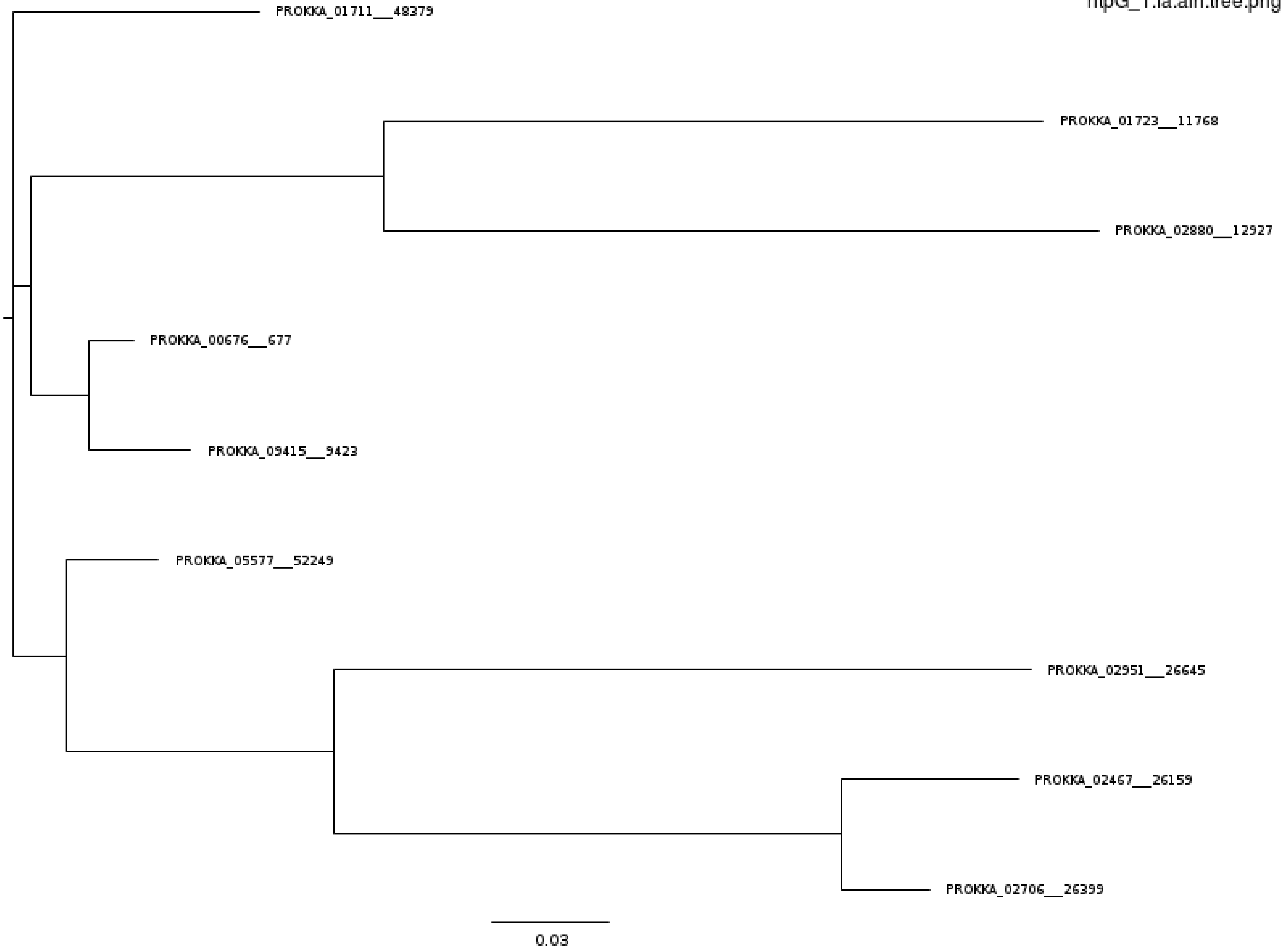

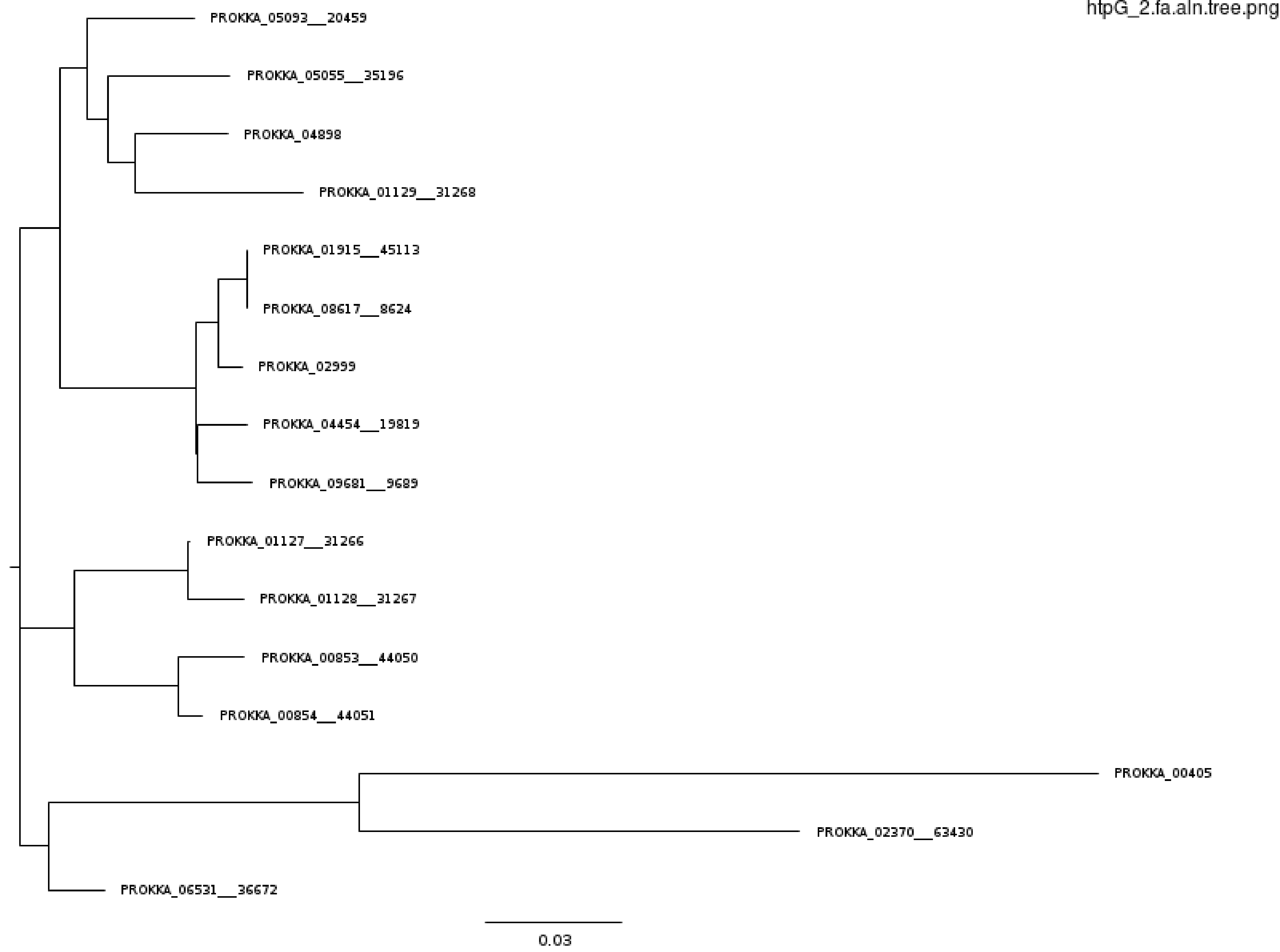

htpG_2.fa.aln.tree.png
PROKKA_05093___20459
PROKKA_05055___35196
PROKKA_04898
PROKKA_01129___31268
PROKKA_01915___45113
PROKKA_08617___8624
PROKKA_02999
PROKKA_04454___19819
PROKKA_09681___9689
PROKKA_01127___31266
PROKKA_01128___31267
PROKKA_00853___44050
PROKKA_00854___44051
PROKKA_00405
PROKKA_02370___63430
PROKKA_06531___36672
0.03

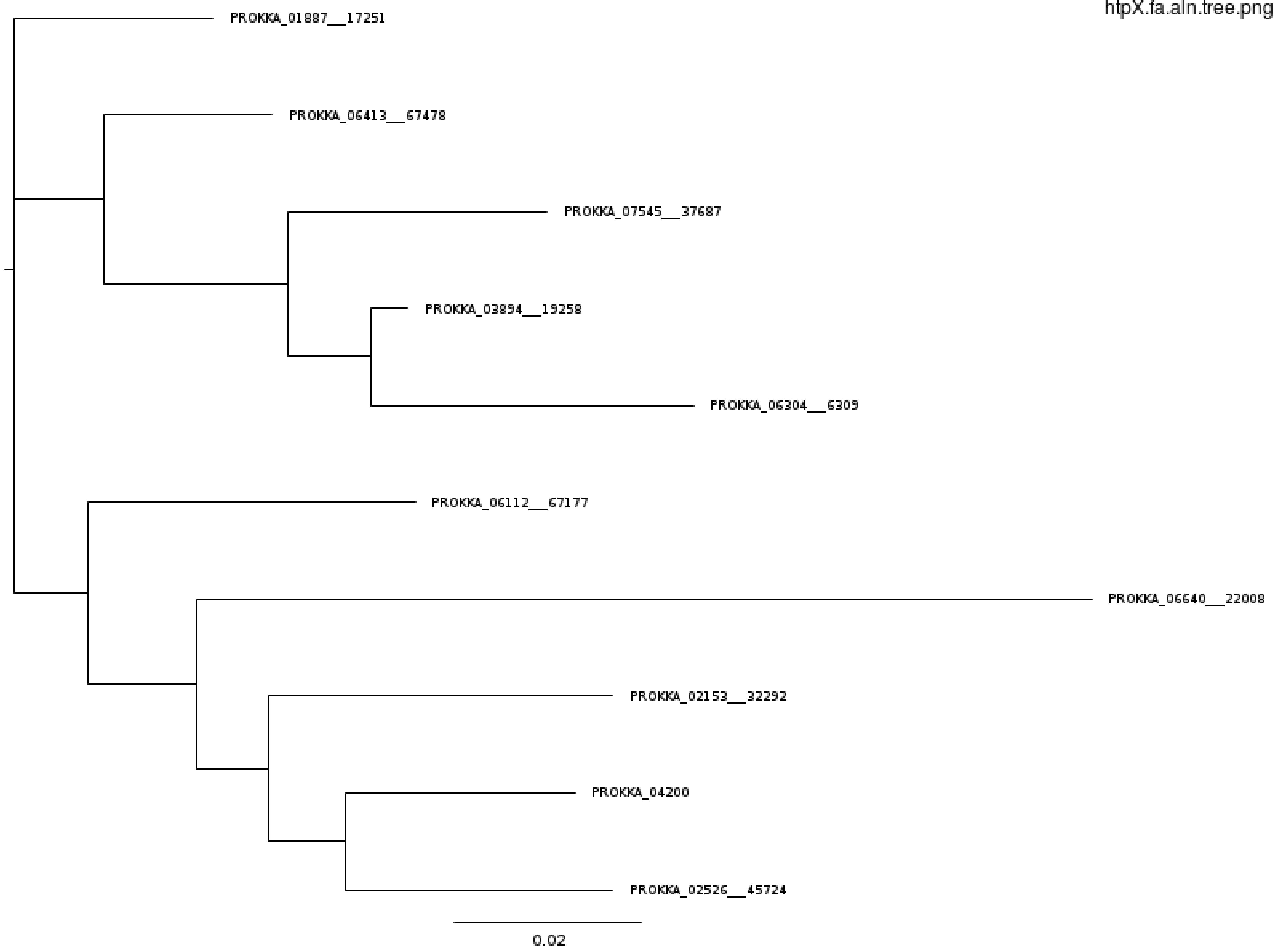

htpX.fa.aln.tree.png
PROKKA_01887___17251
PROKKA_06413___67478
PROKKA_07545___37687
PROKKA_03894___19258
PROKKA_06304___6309
PROKKA_06112___67177
PROKKA_06640___22008
PROKKA_02153___32292
PROKKA_04200
PROKKA_02526___45724
0.02

htrA_3.fa.aln.tree.png

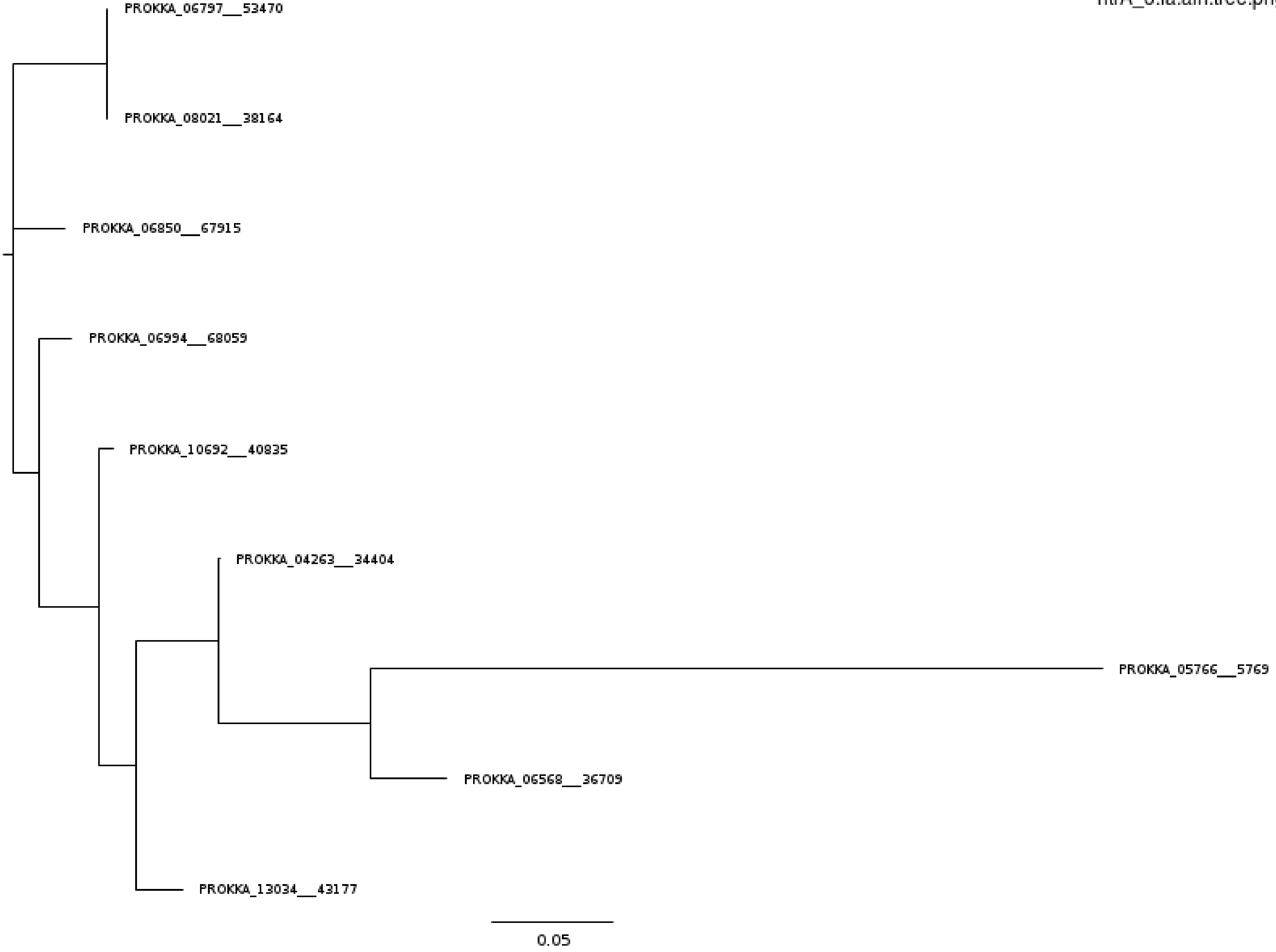

htrB_2.fa.aln.tree.png

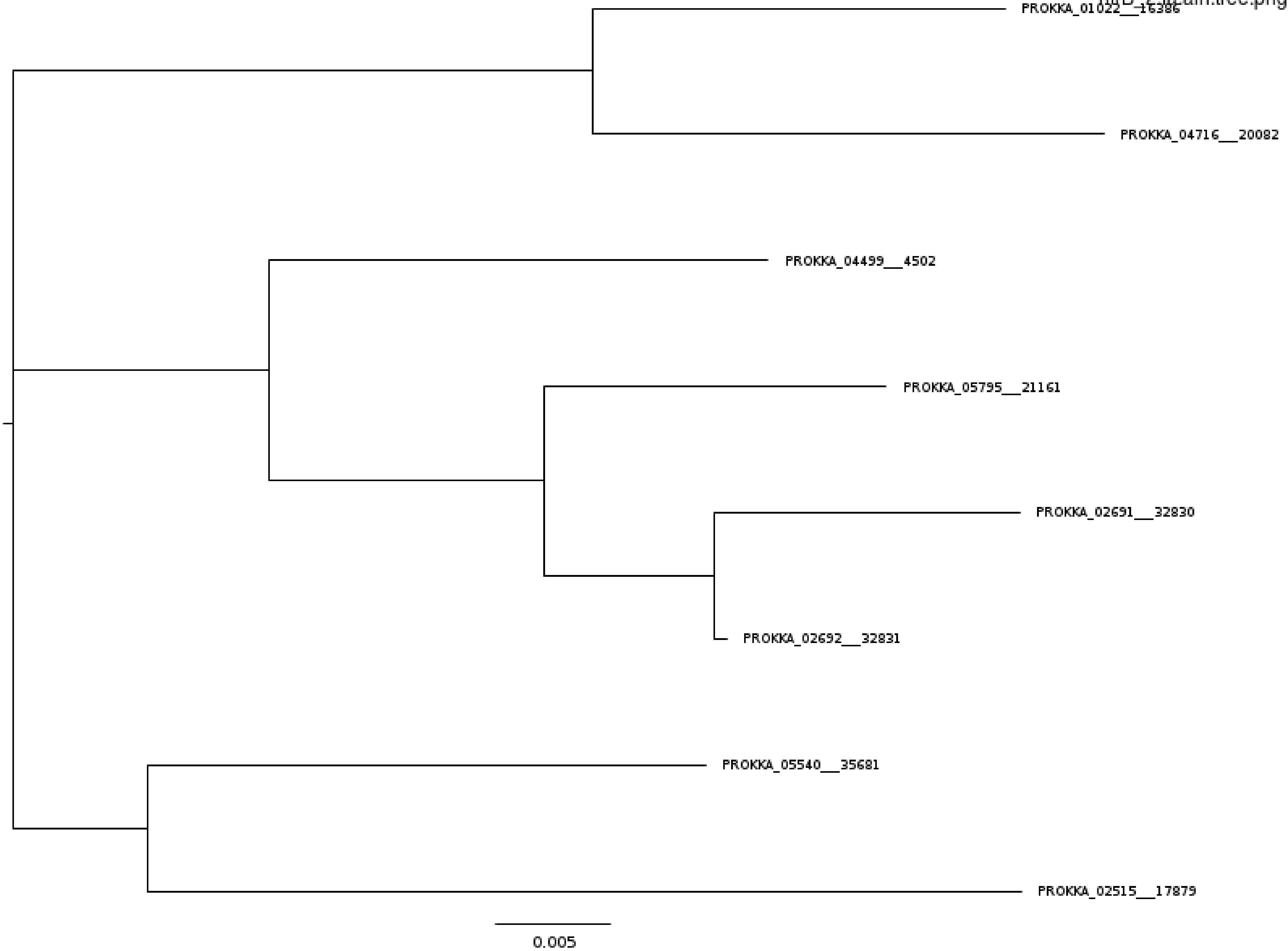

hupR1_2.fa.aln.tree.png

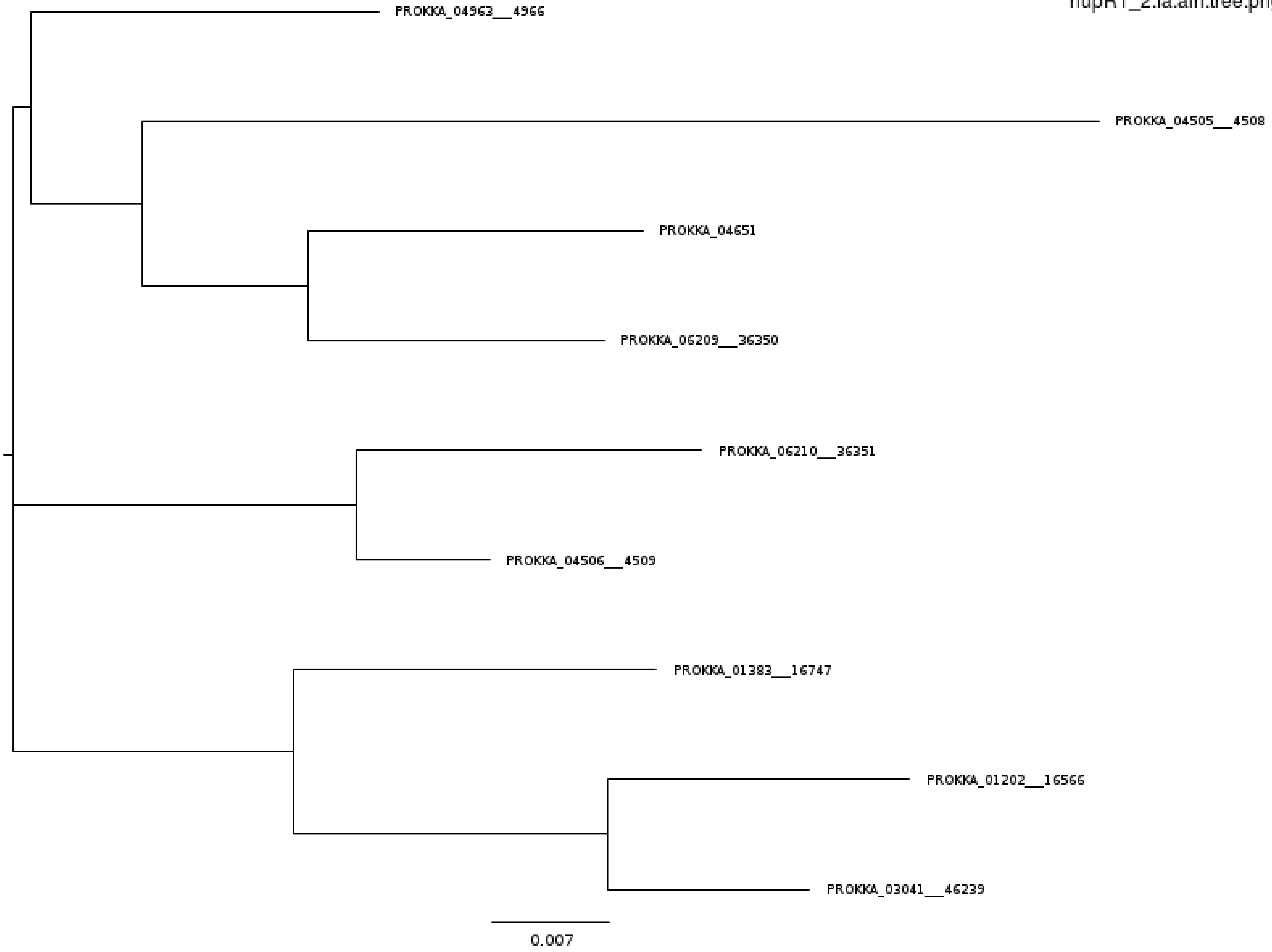

hupR1.fa.aln.tree.png

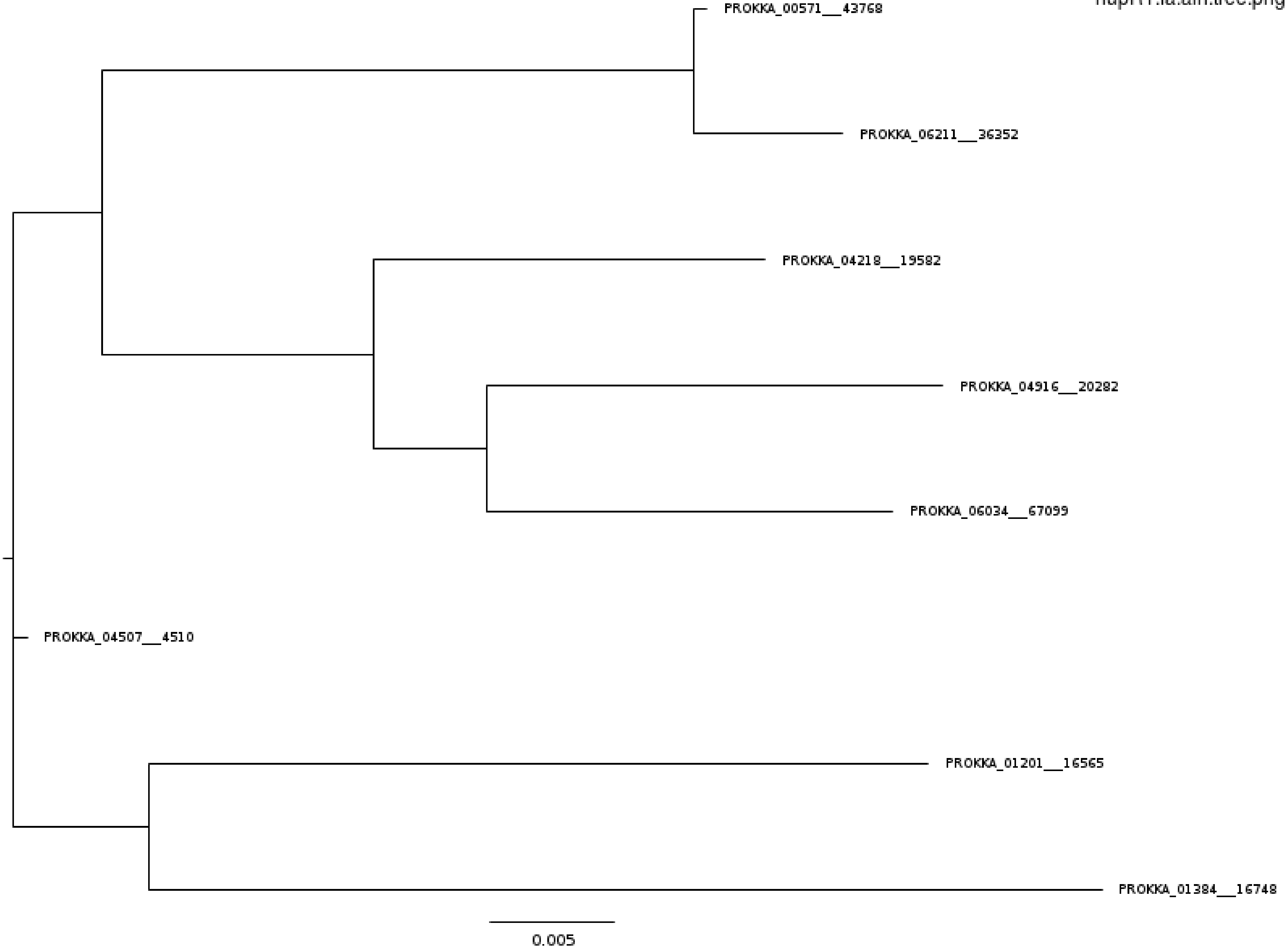

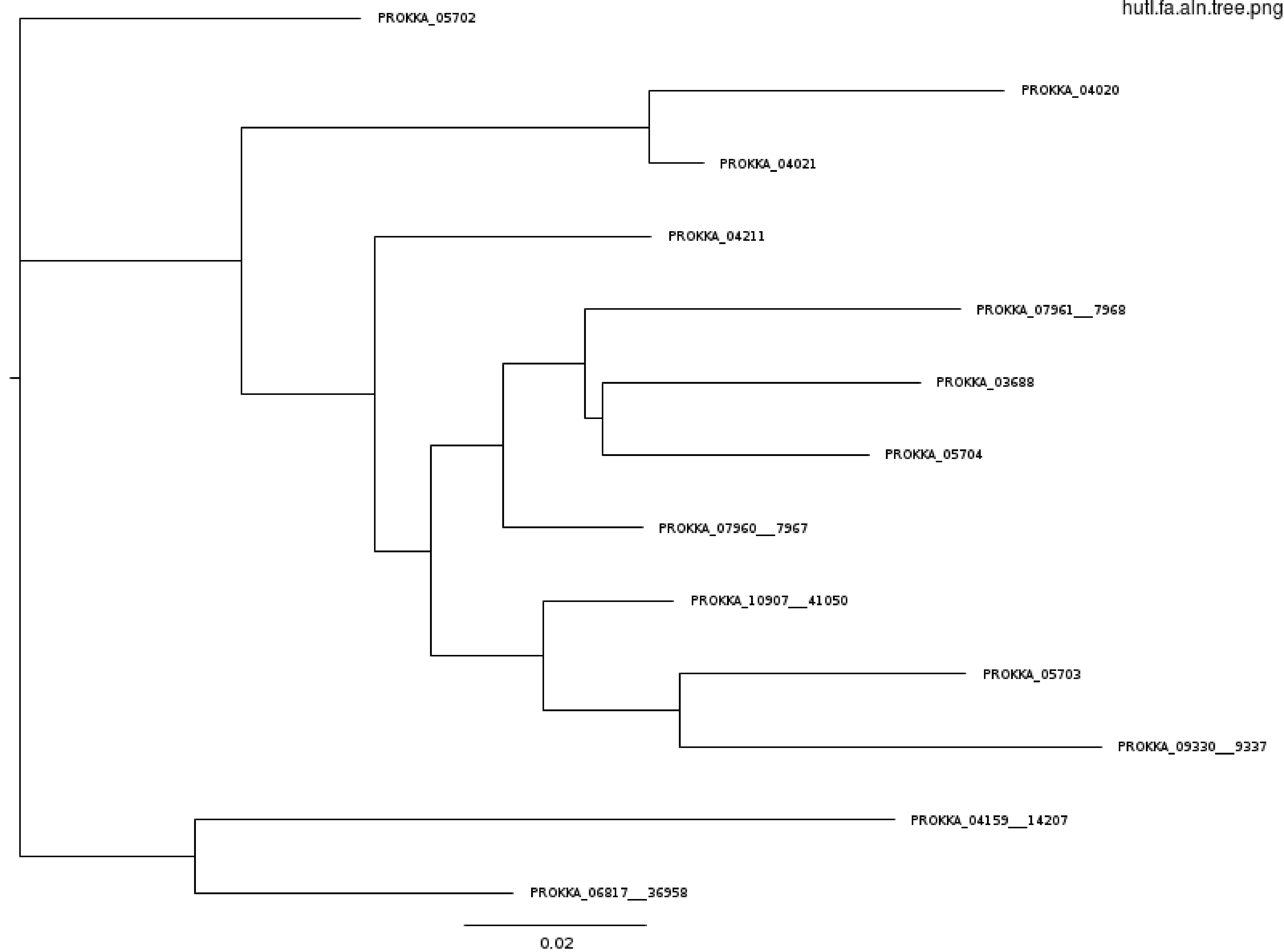
hutI.fa.aln.tree.png
PROKKA_05702
PROKKA_04020
PROKKA_04021
PROKKA_04211
PROKKA_07961__7968
PROKKA_03688
PROKKA_05704
PROKKA_07960__7967
PROKKA_10907__41050
PROKKA_05703
PROKKA_09330__9337
PROKKA_04159__14207
PROKKA_06817__36958
0.02

hyaB.fa.aln.tree.png

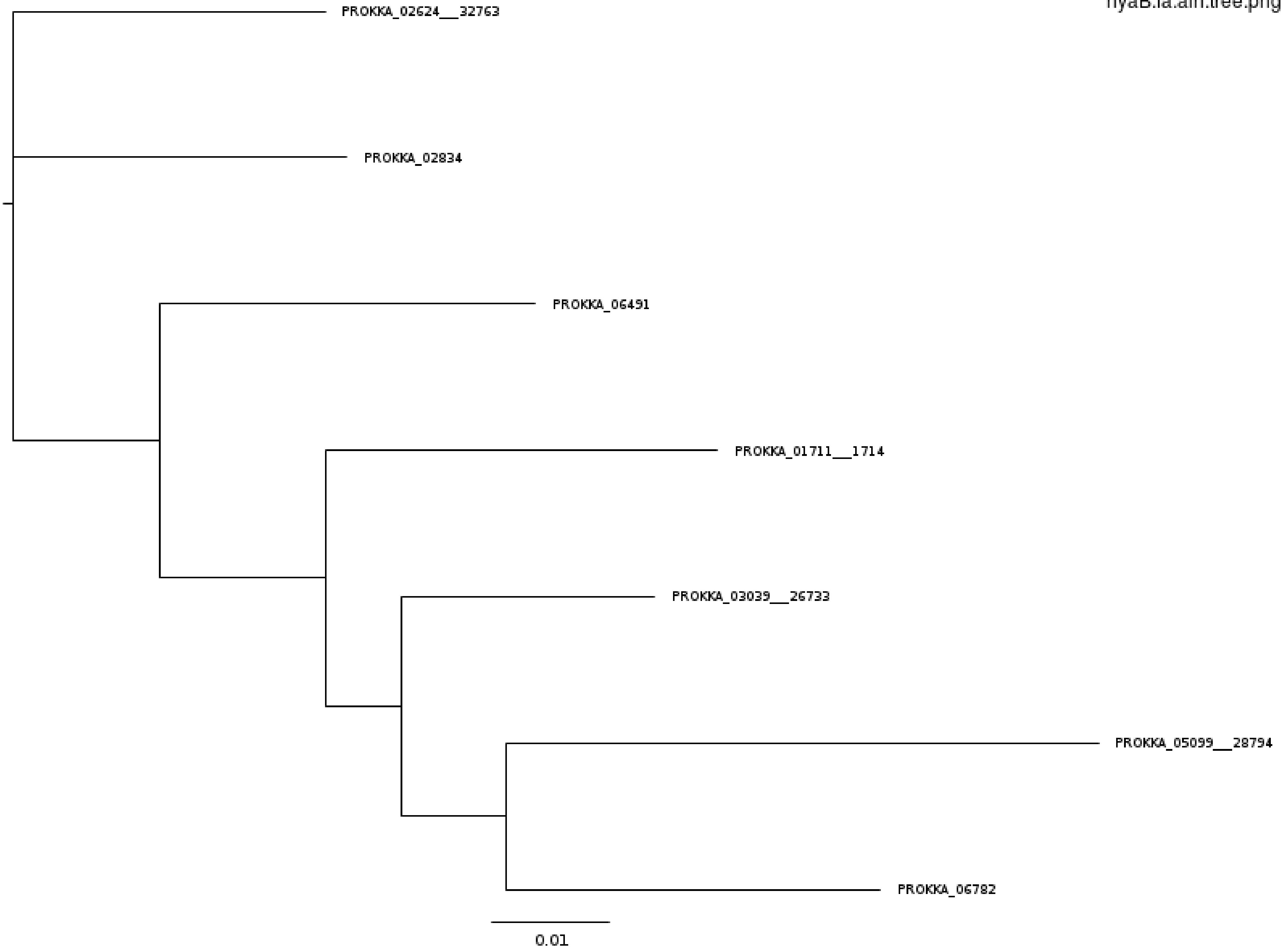

hypA.fa.aln.tree.png

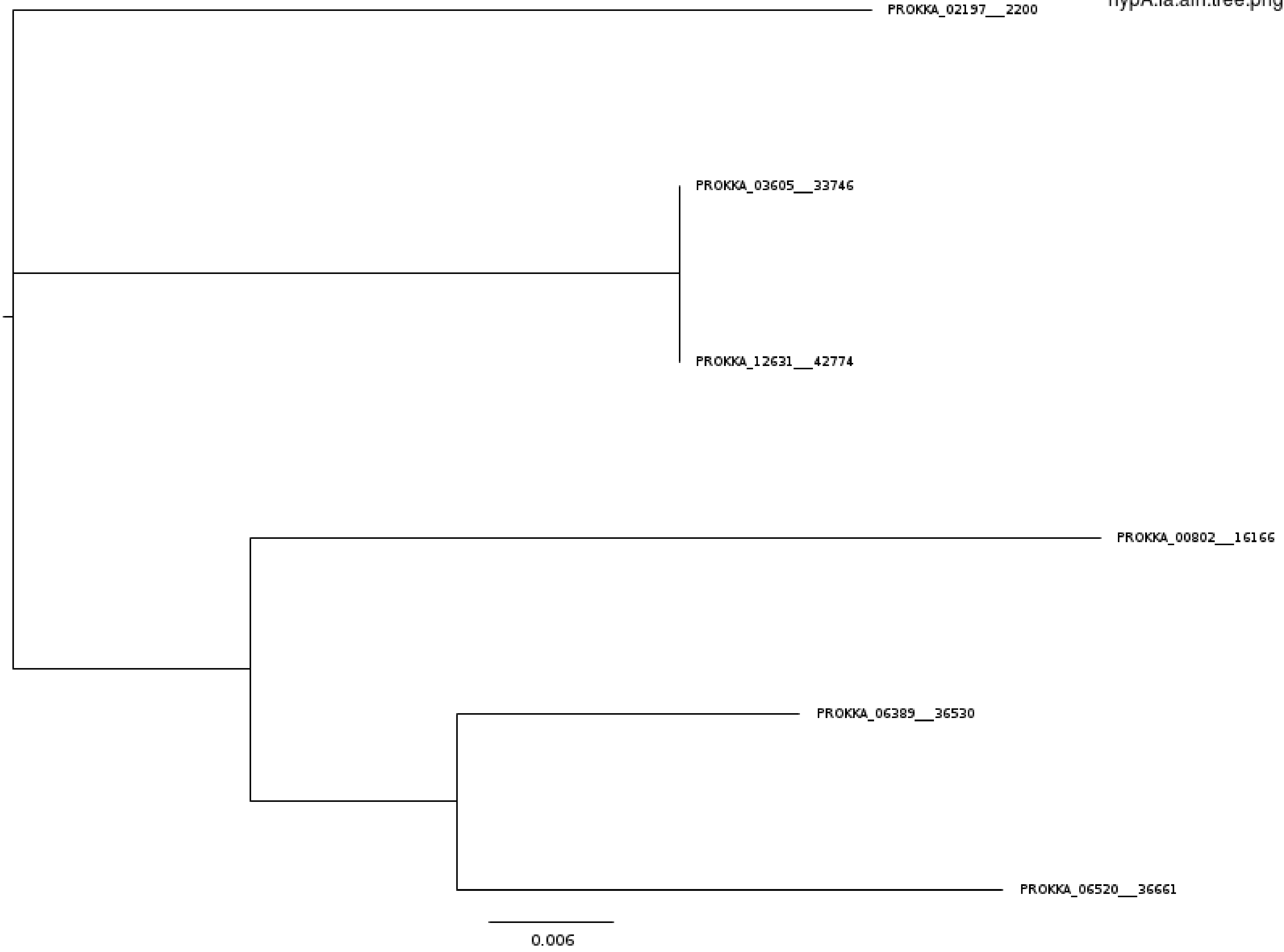

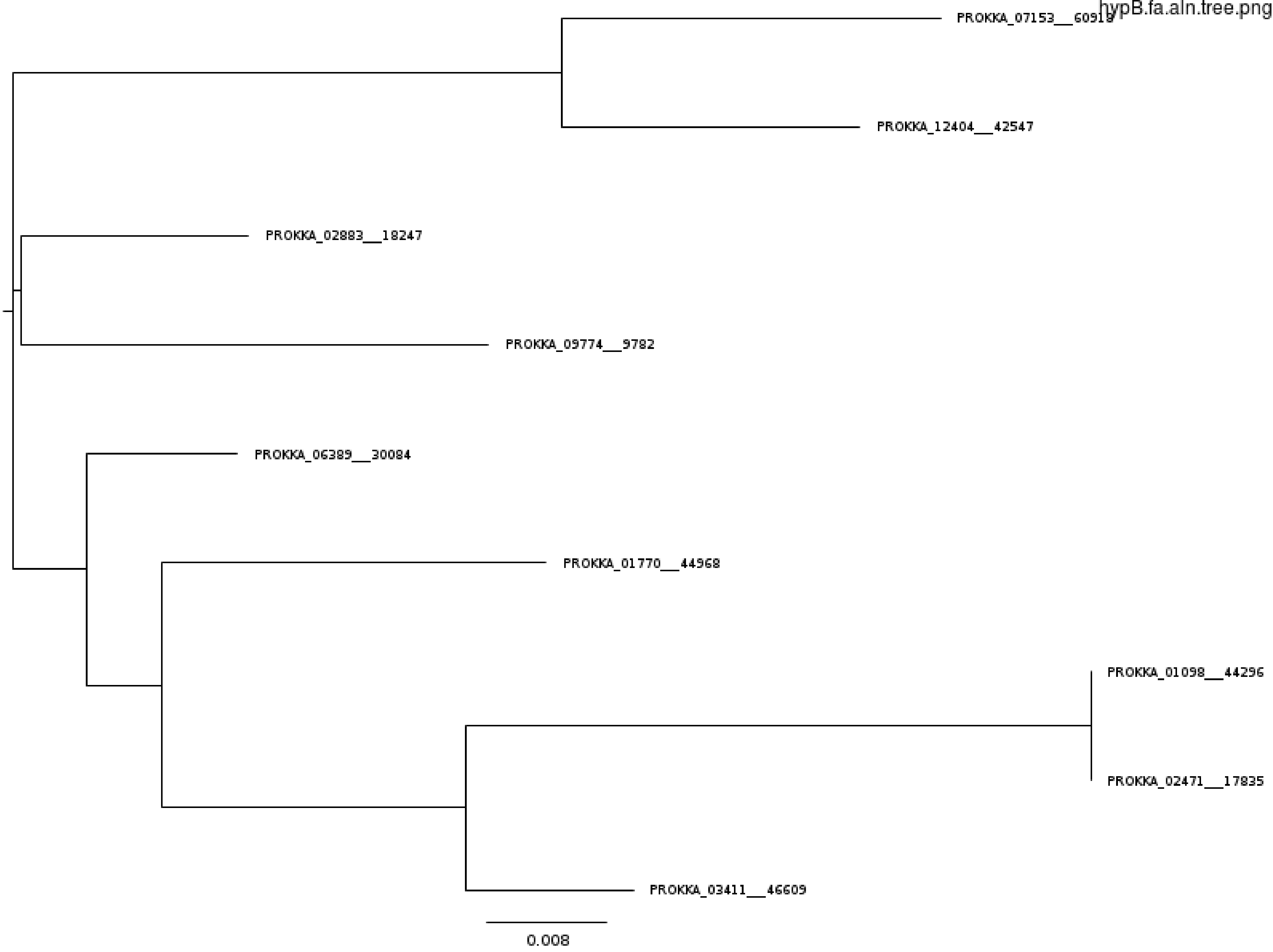
hypB.fa.aln.tree.png
PROKKA_07153___60919
PROKKA_12404___42547
PROKKA_02883___18247
PROKKA_09774___9782
PROKKA_06389___30084
PROKKA_01770___44968
PROKKA_01098___44296
PROKKA_02471___17835
PROKKA_03411___46609
0.008

hypC_2.fa.aln.tree.png

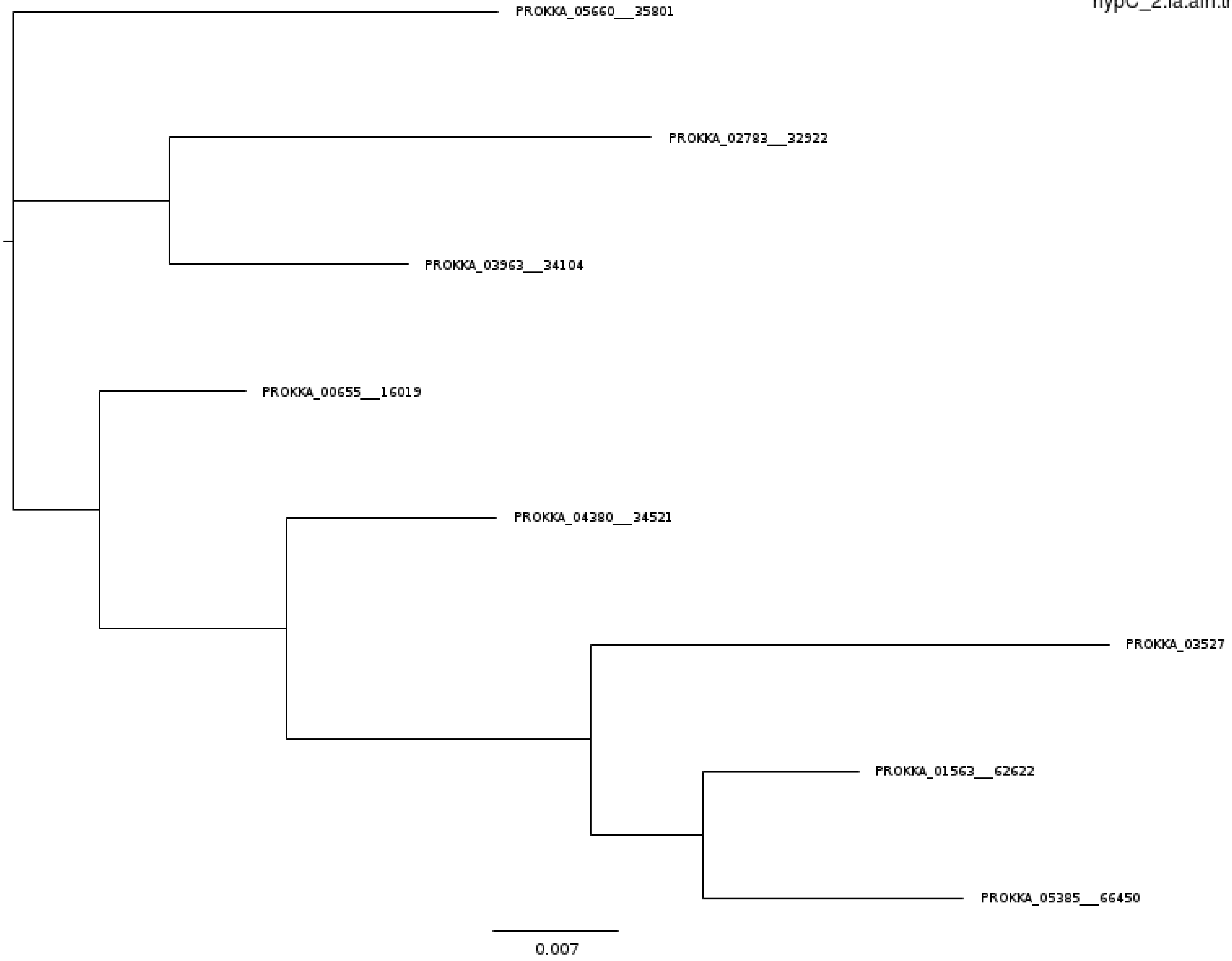

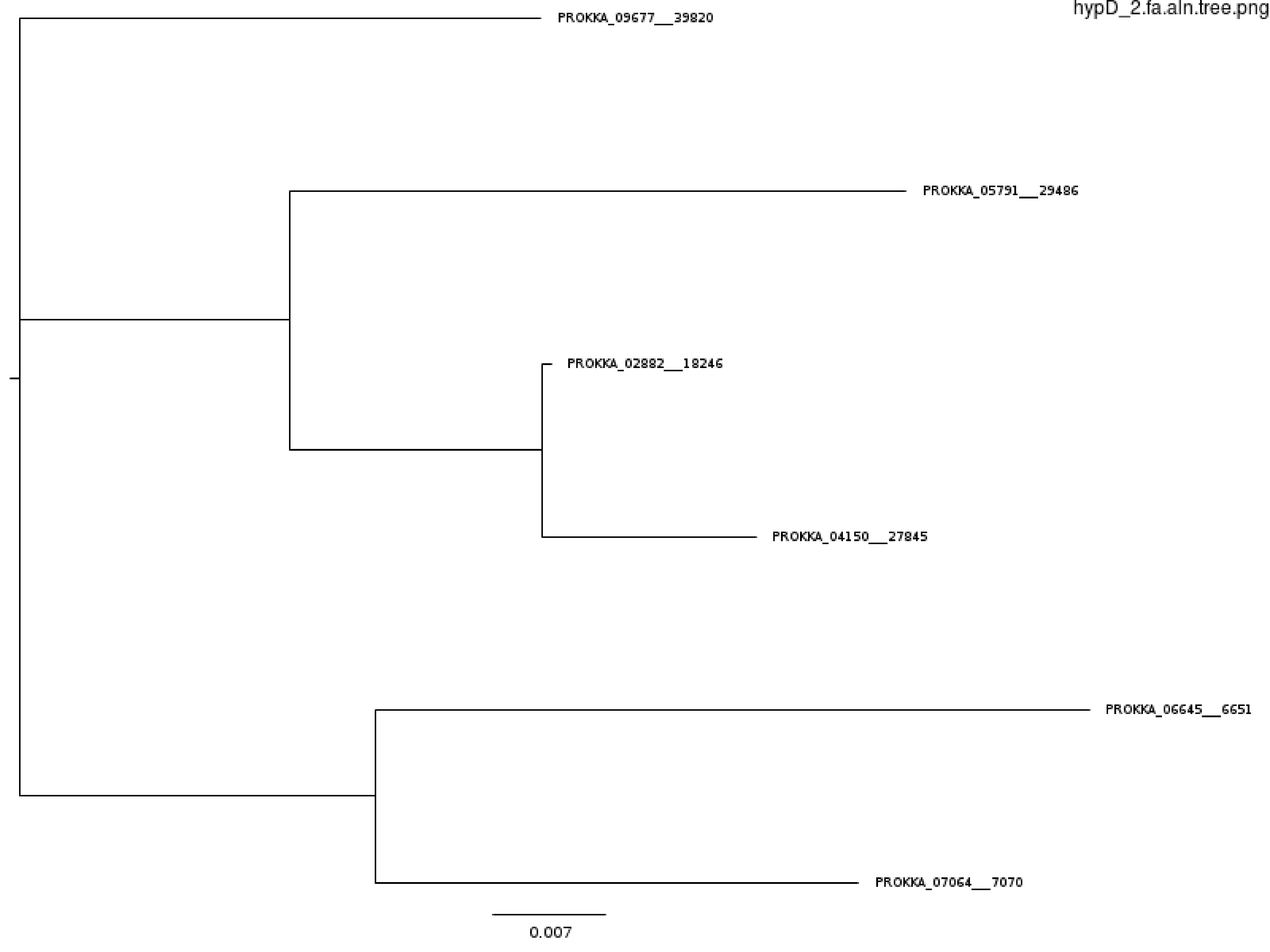
hypD_2.fa.aln.tree.png
PROKKA_09677___39820
PROKKA_05791___29486
PROKKA_02882___18246
PROKKA_04150___27845
PROKKA_06645___6651
PROKKA_07064___7070
0.007

hypE_1.fa.aln.tree.png

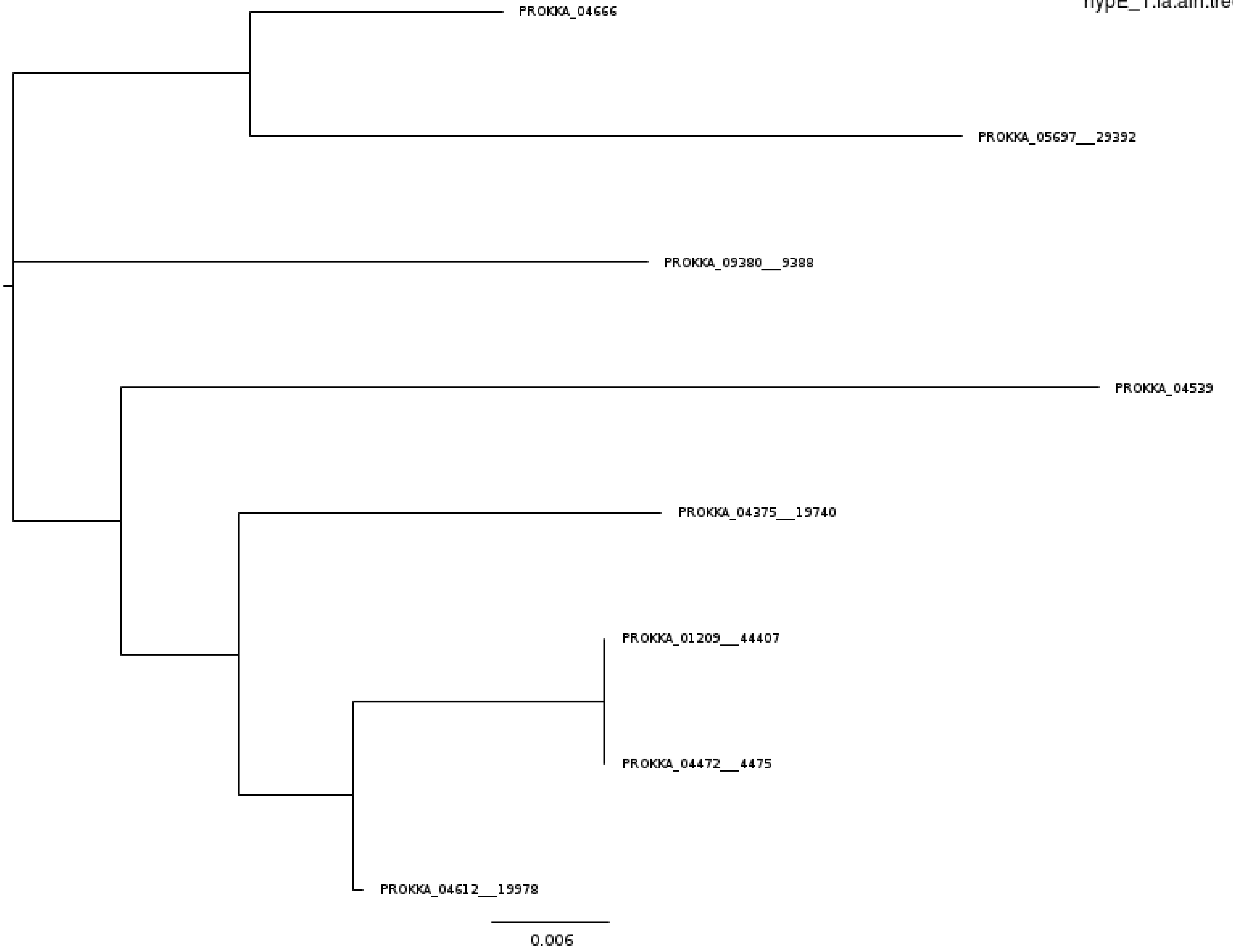

hypF_1.fa.aln.tree.png

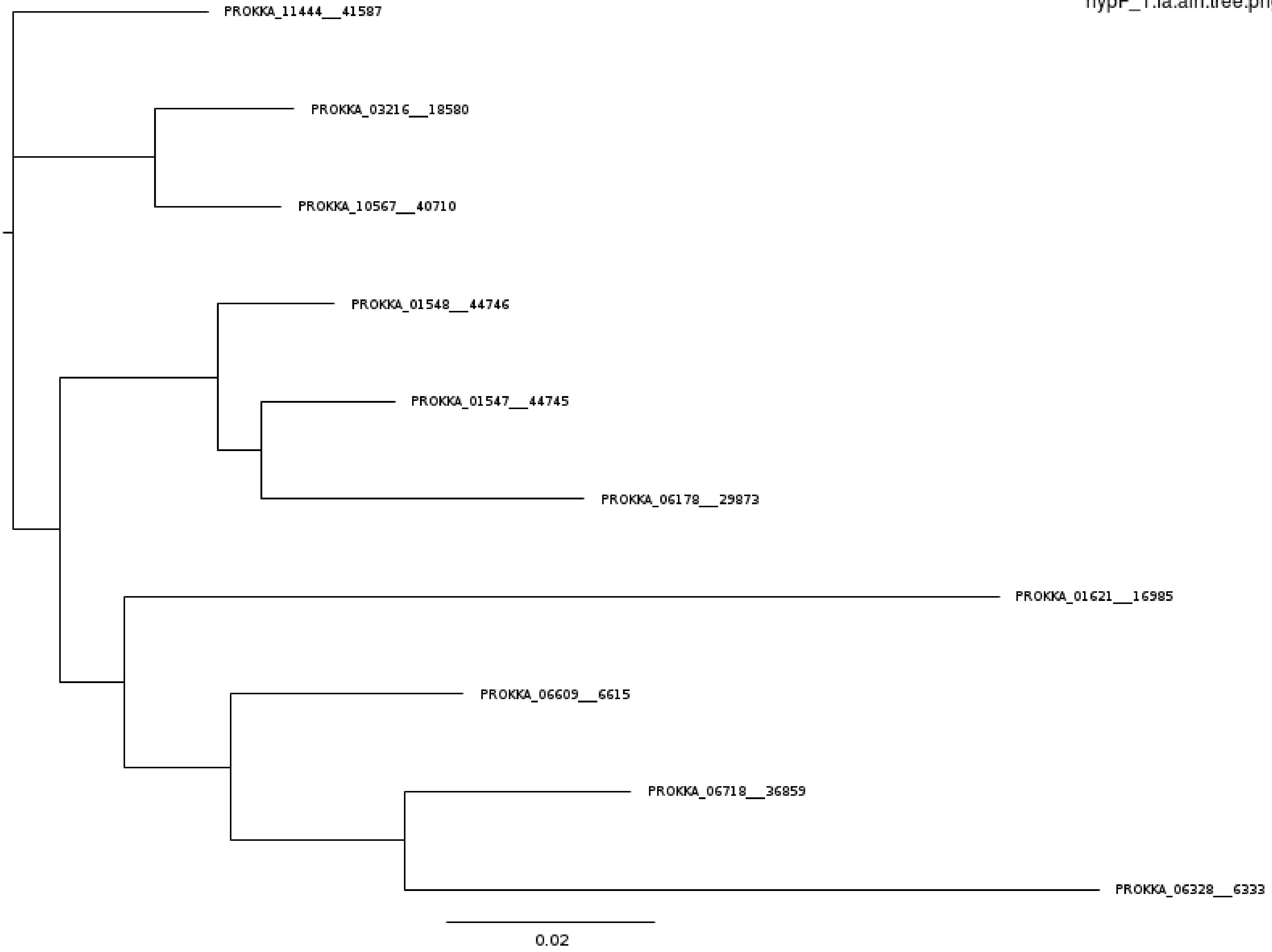

hypF_2.fa.aln.tree.png

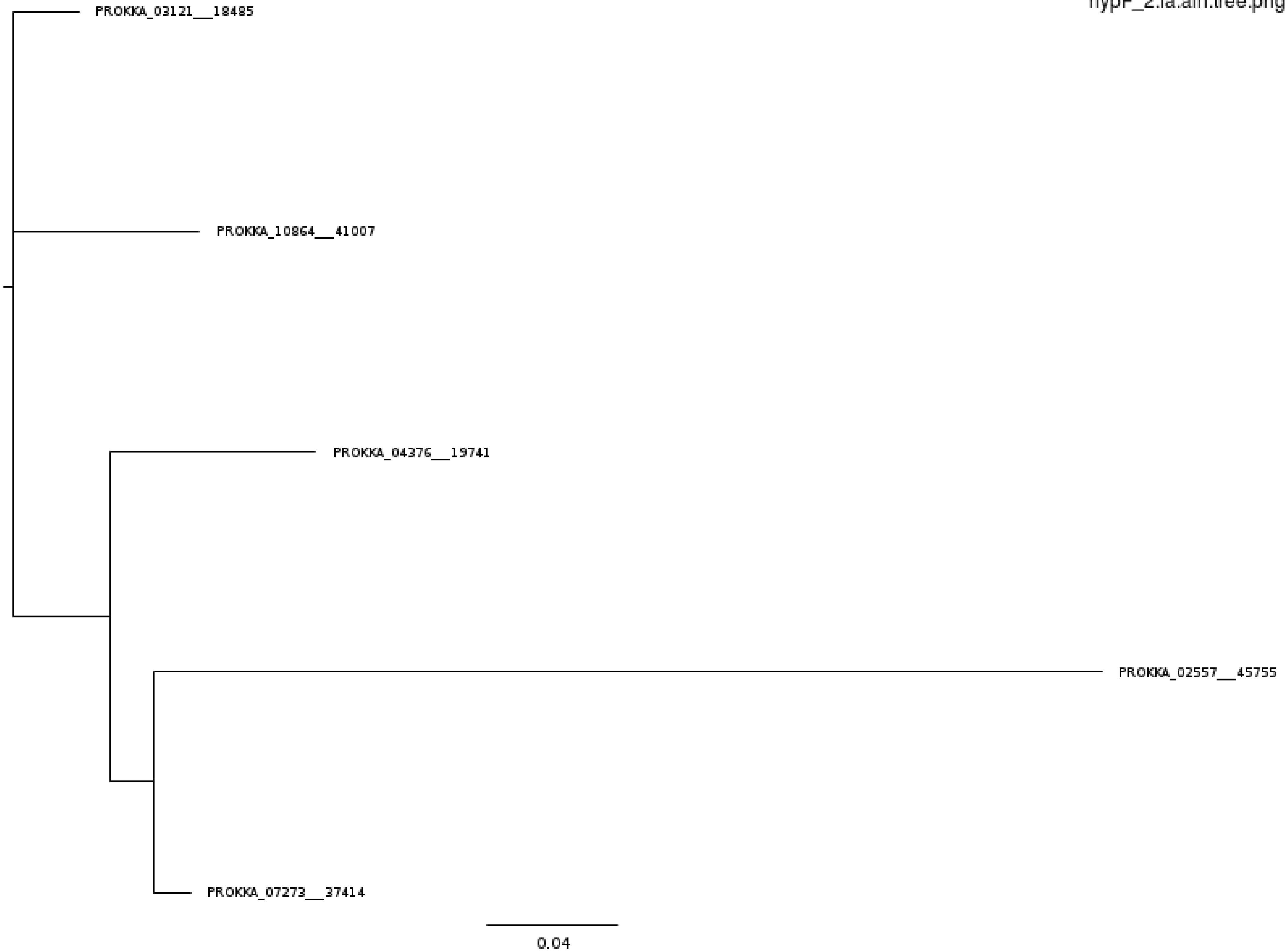

icaA_2.fa.aln.tree.png

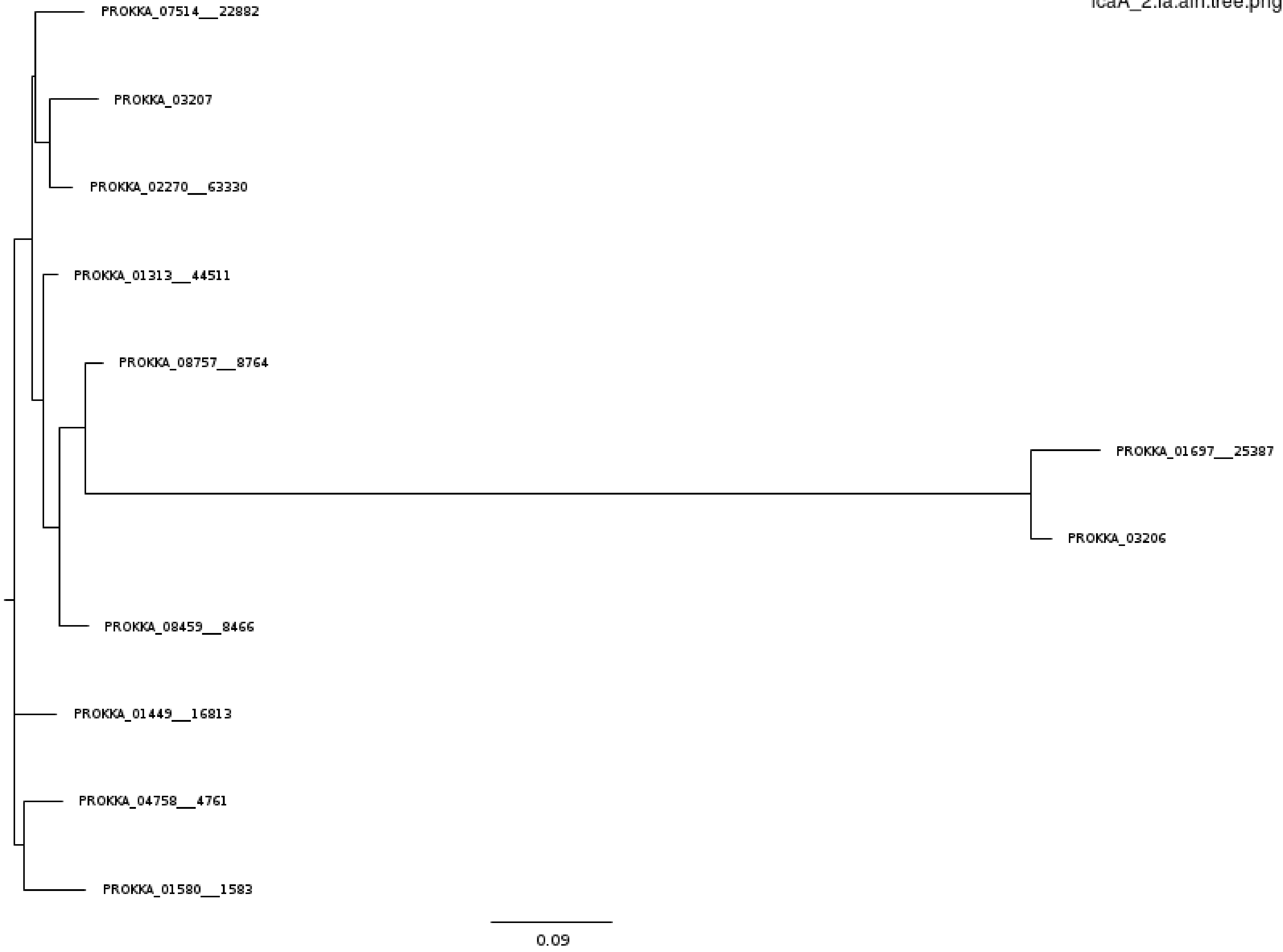

iga_2.fa.aln.tree.png

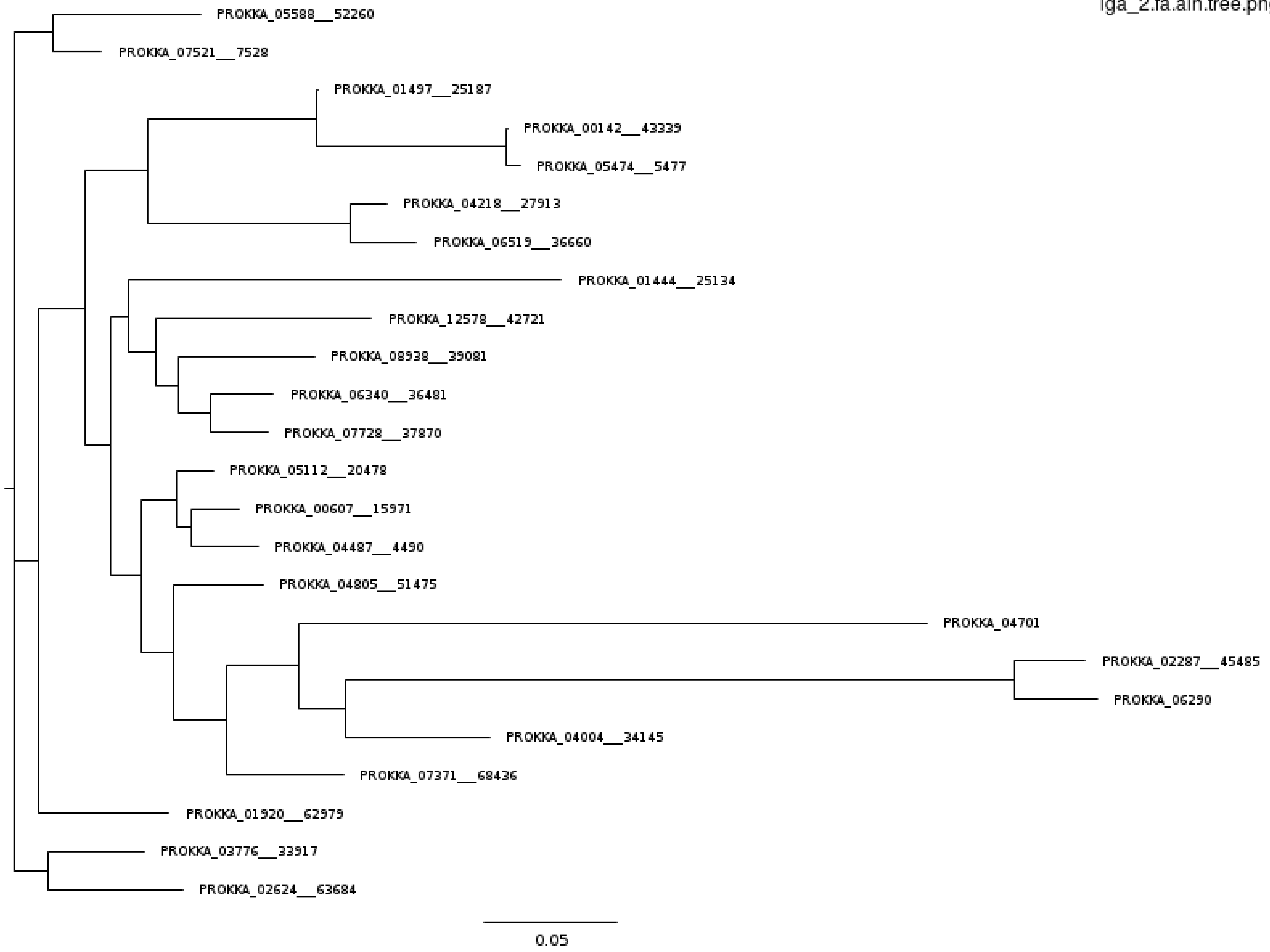

ihfB_2.fa.aln.tree.png

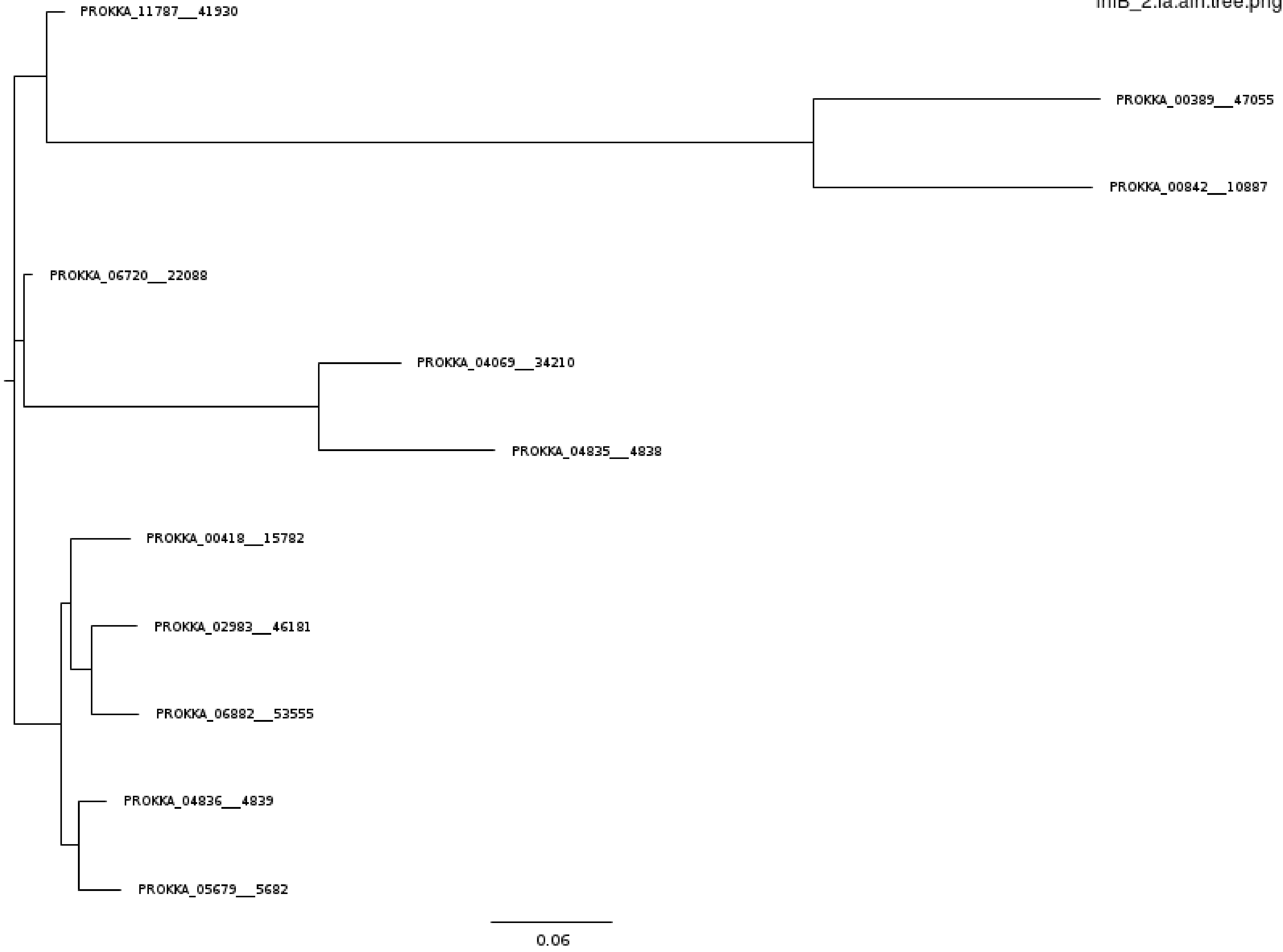

ileS_3.fa.aln.tree.png

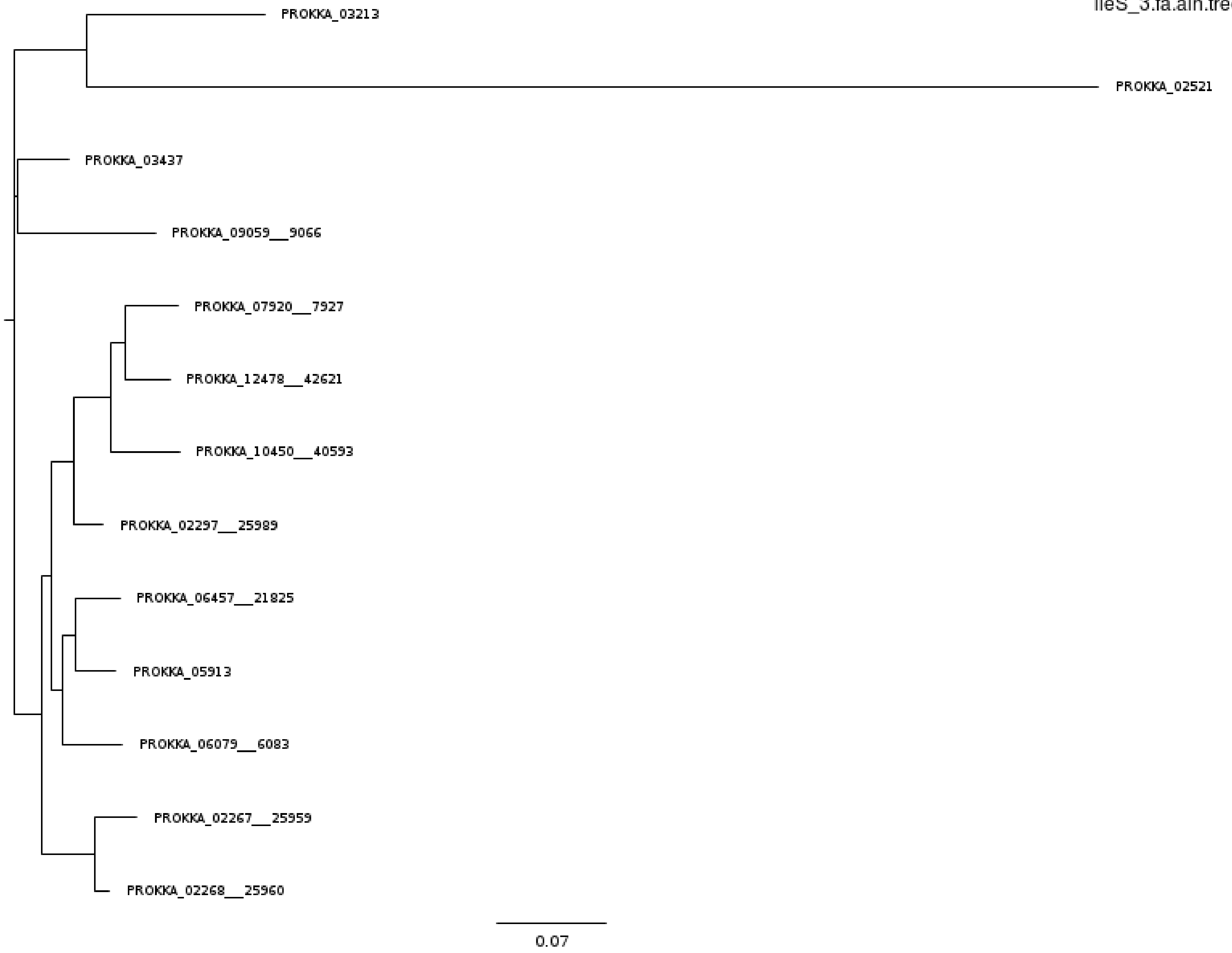

ilvC_2.fa.aln.tree.png

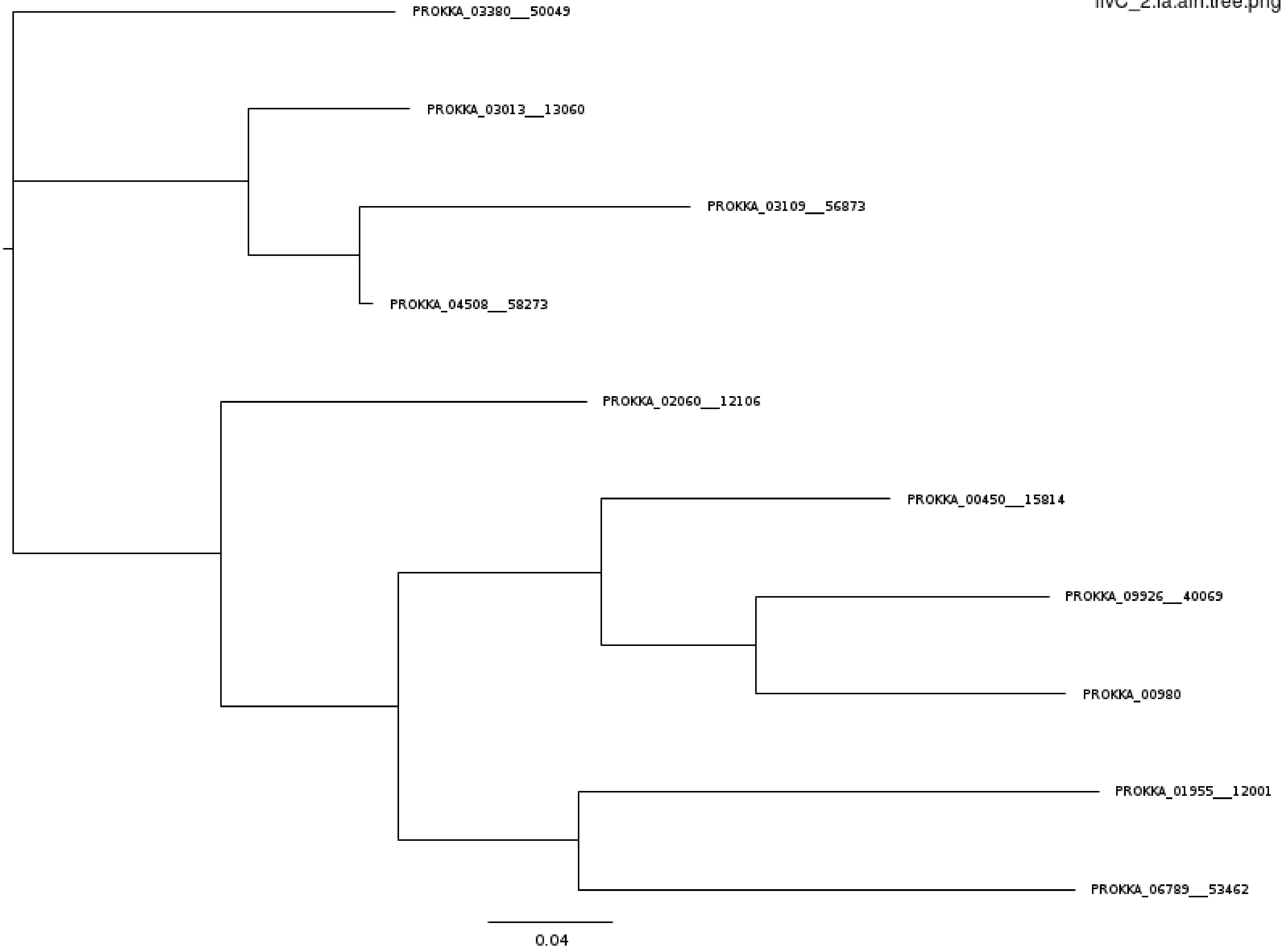

ilvD_3.fa.aln.tree.png

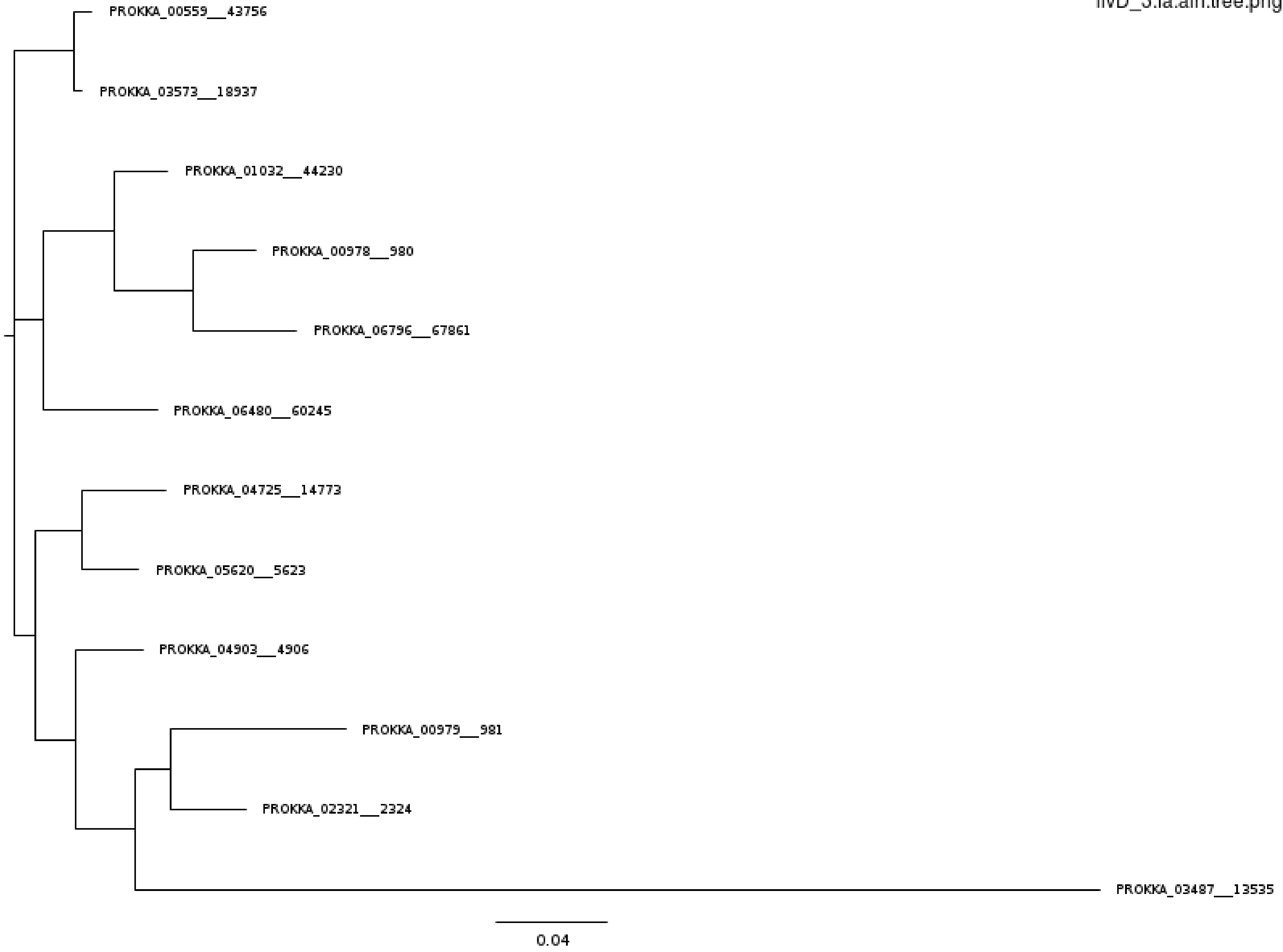

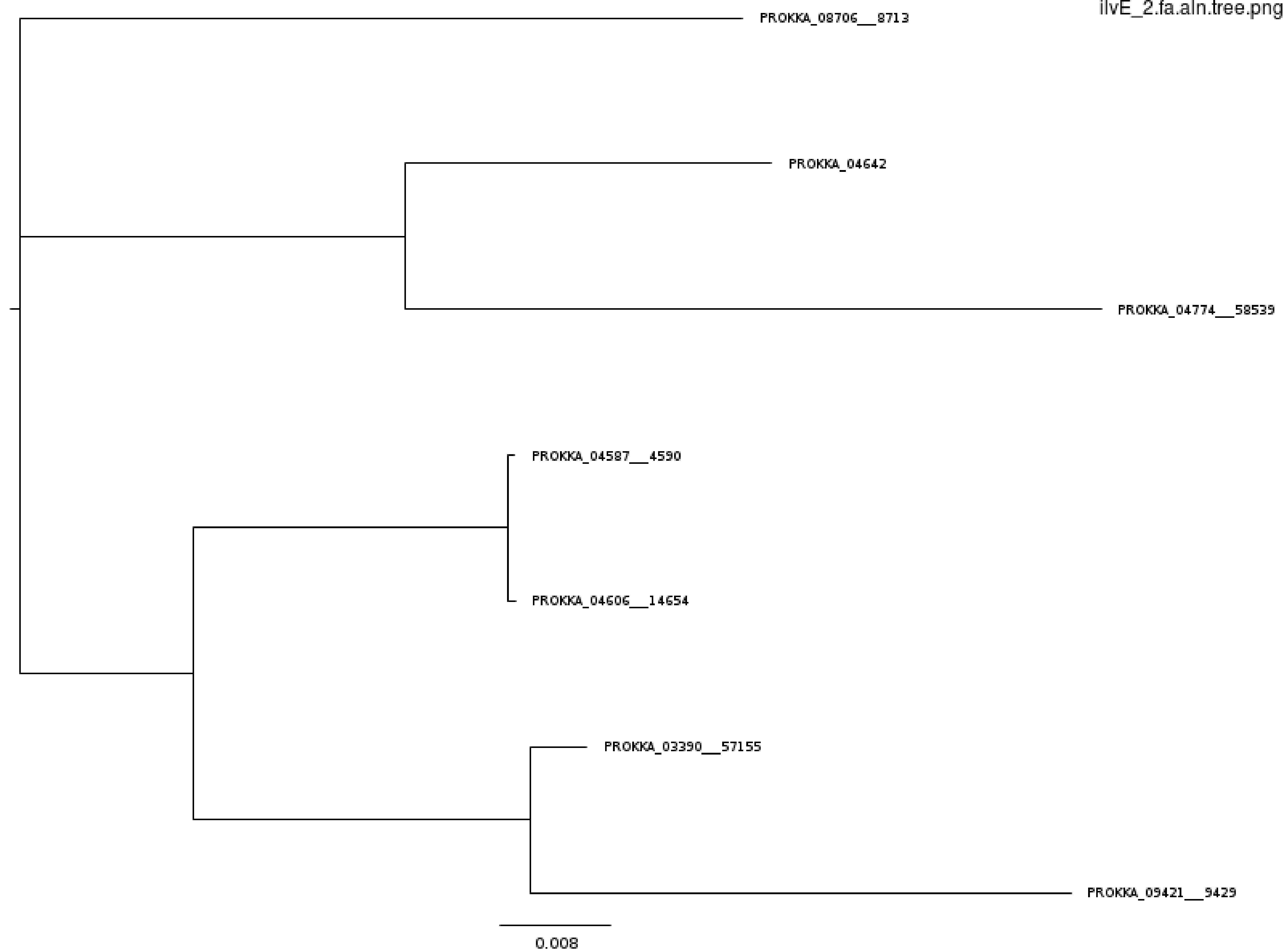
ilvE_2.fa.aln.tree.png
PROKKA_08706___8713
PROKKA_04642
PROKKA_04774___58539
PROKKA_04587___4590
PROKKA_04606___14654
PROKKA_03390___57155
PROKKA_09421___9429
0.008

ilvI.fa.aln.tree.png

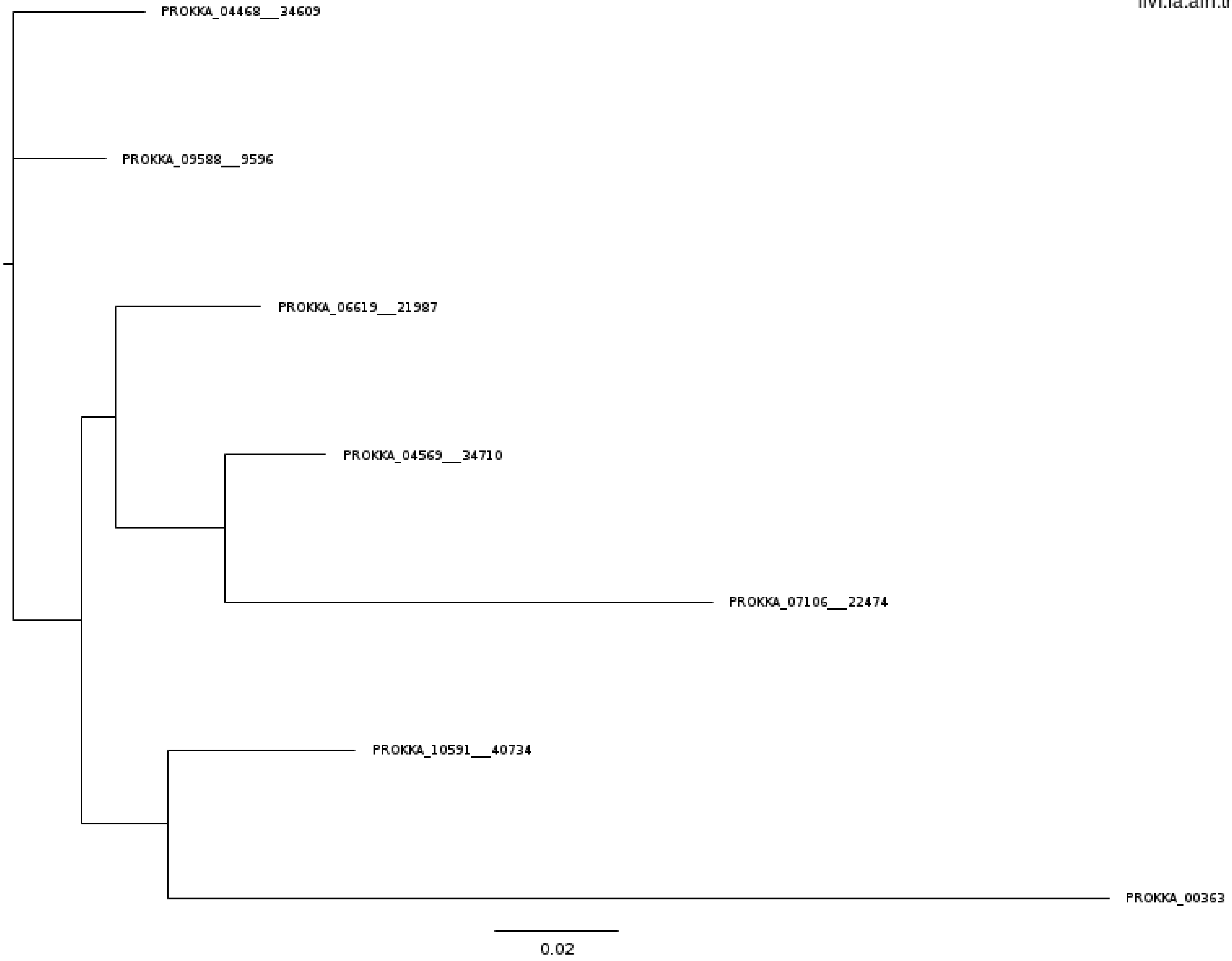

infA_1.fa.aln.tree.png

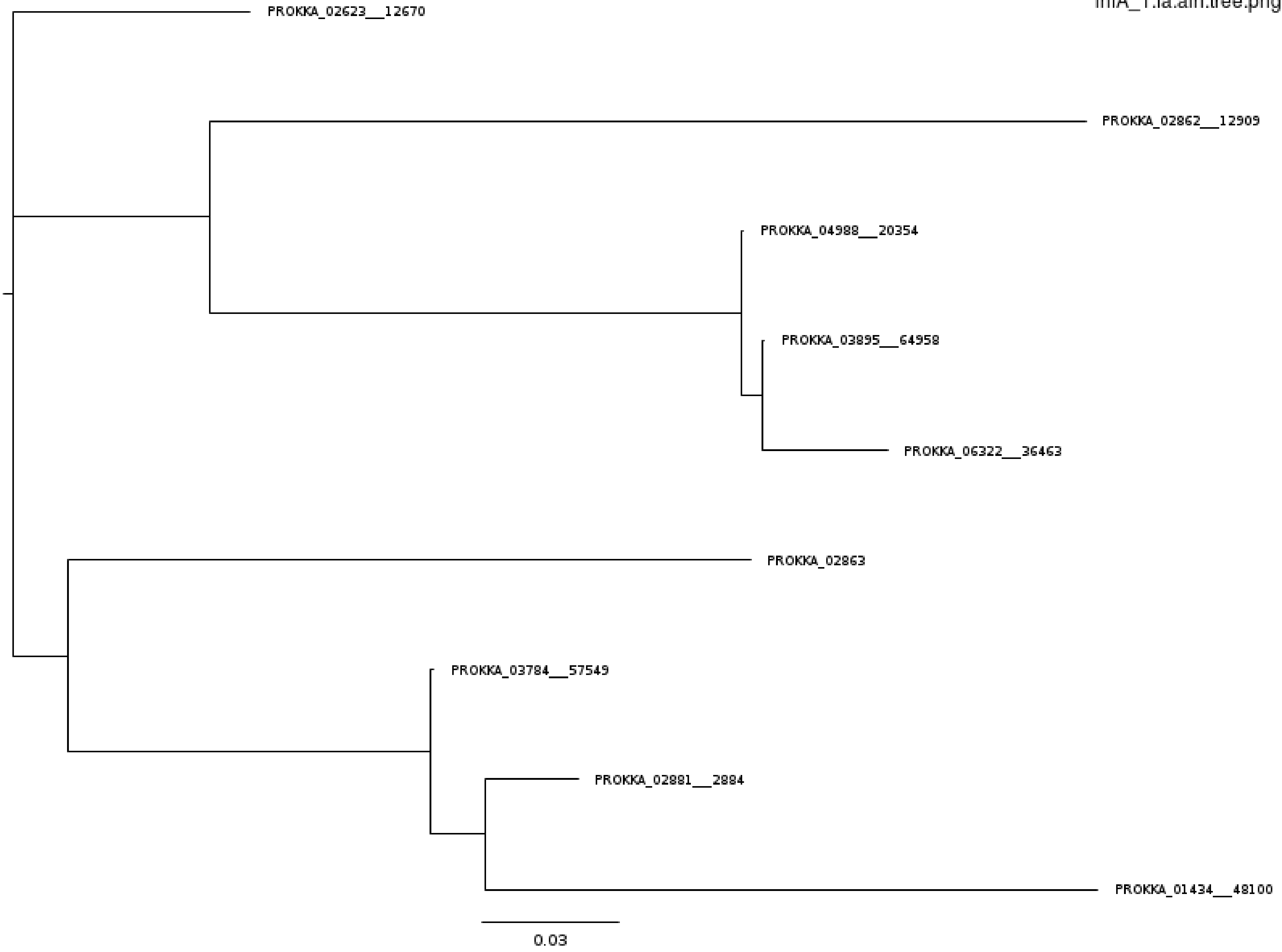

infC.fa.aln.tree.png

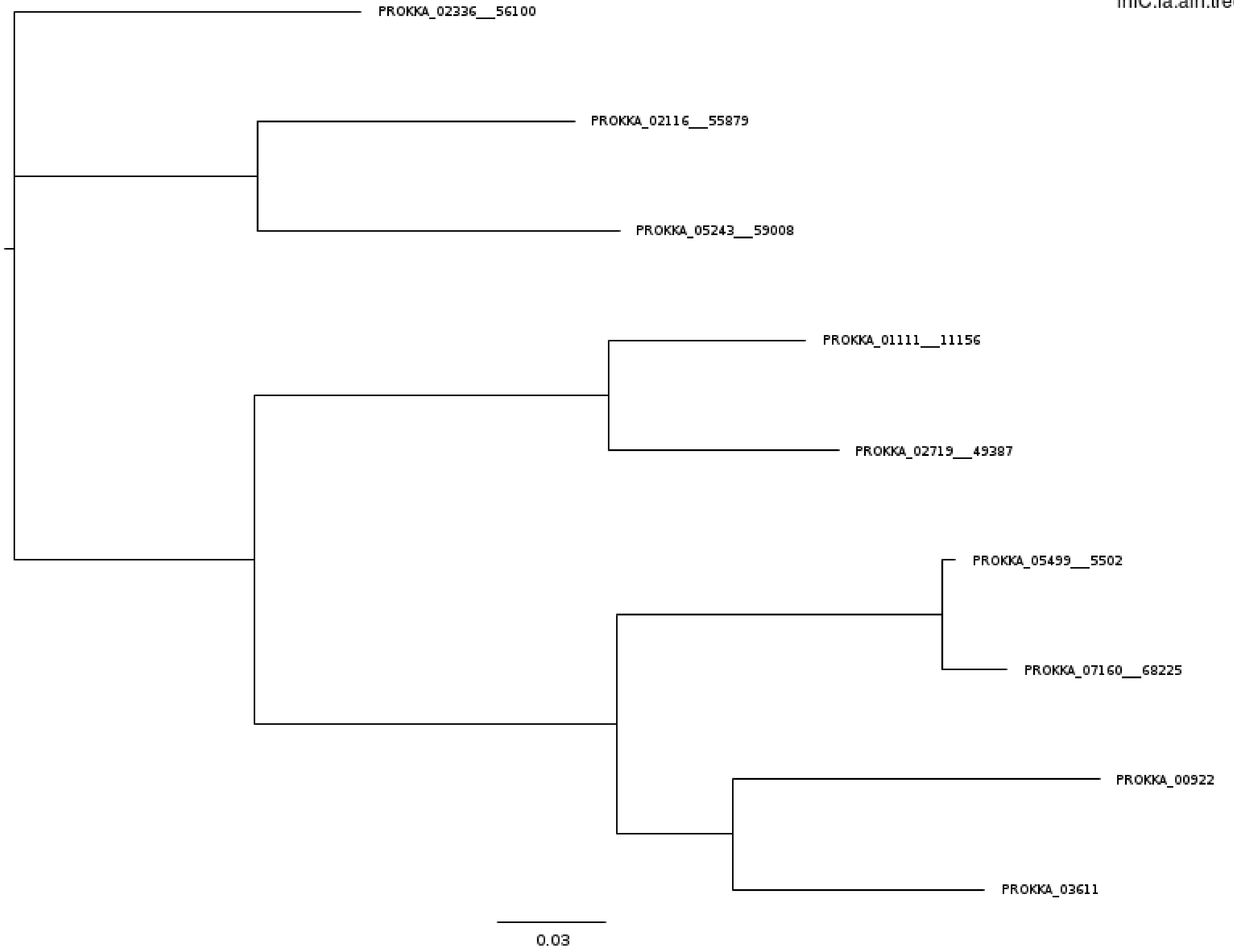

iolG.fa.aln.tree.png

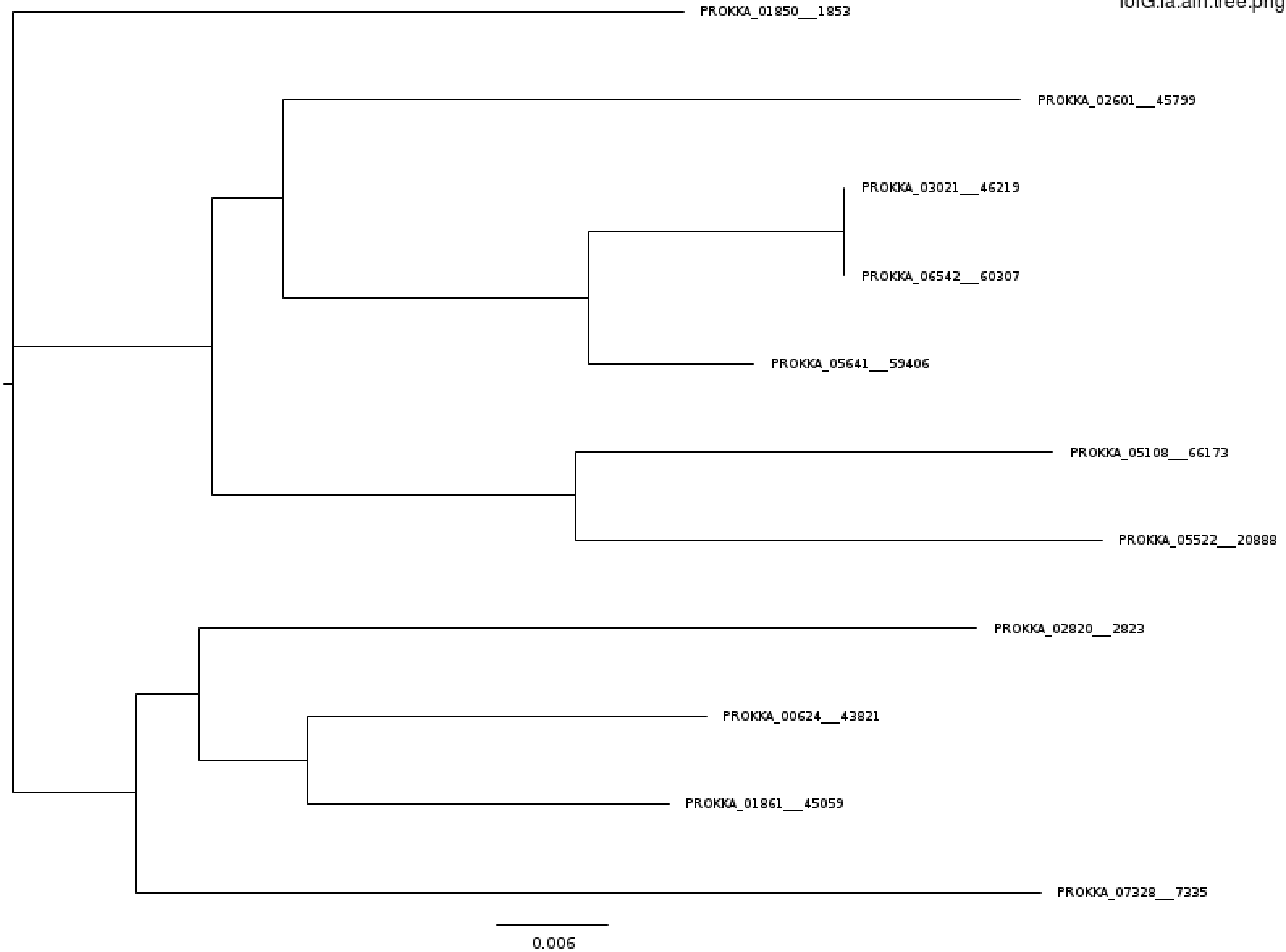

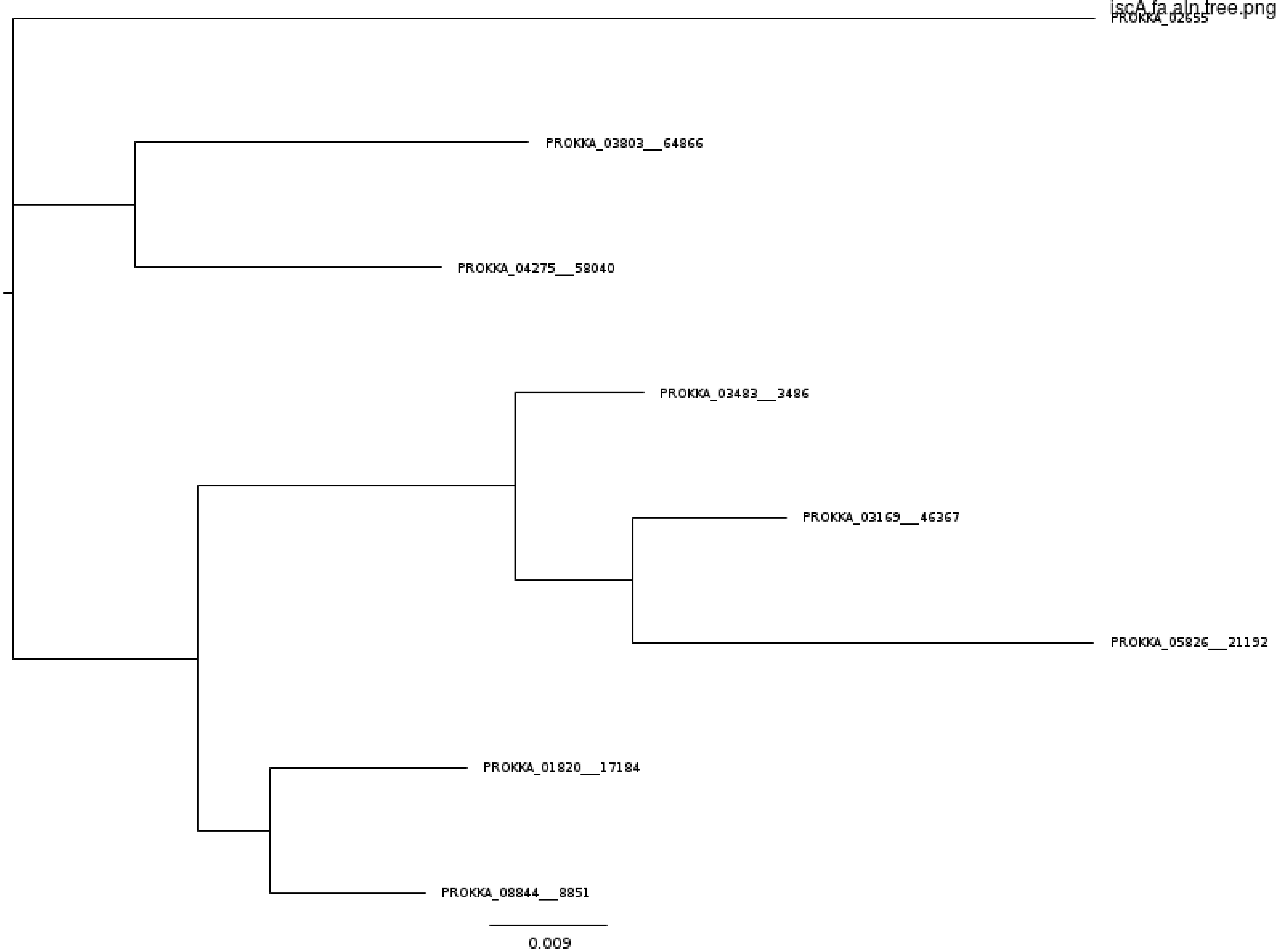
iscA.fa.aln.tree.png
PROKKA_02655
PROKKA_03803___64866
PROKKA_04275___58040
PROKKA_03483___3486
PROKKA_03169___46367
PROKKA_05826___21192
PROKKA_01820___17184
PROKKA_08844___8851
0.009

iscS_2.fa.aln.tree.png

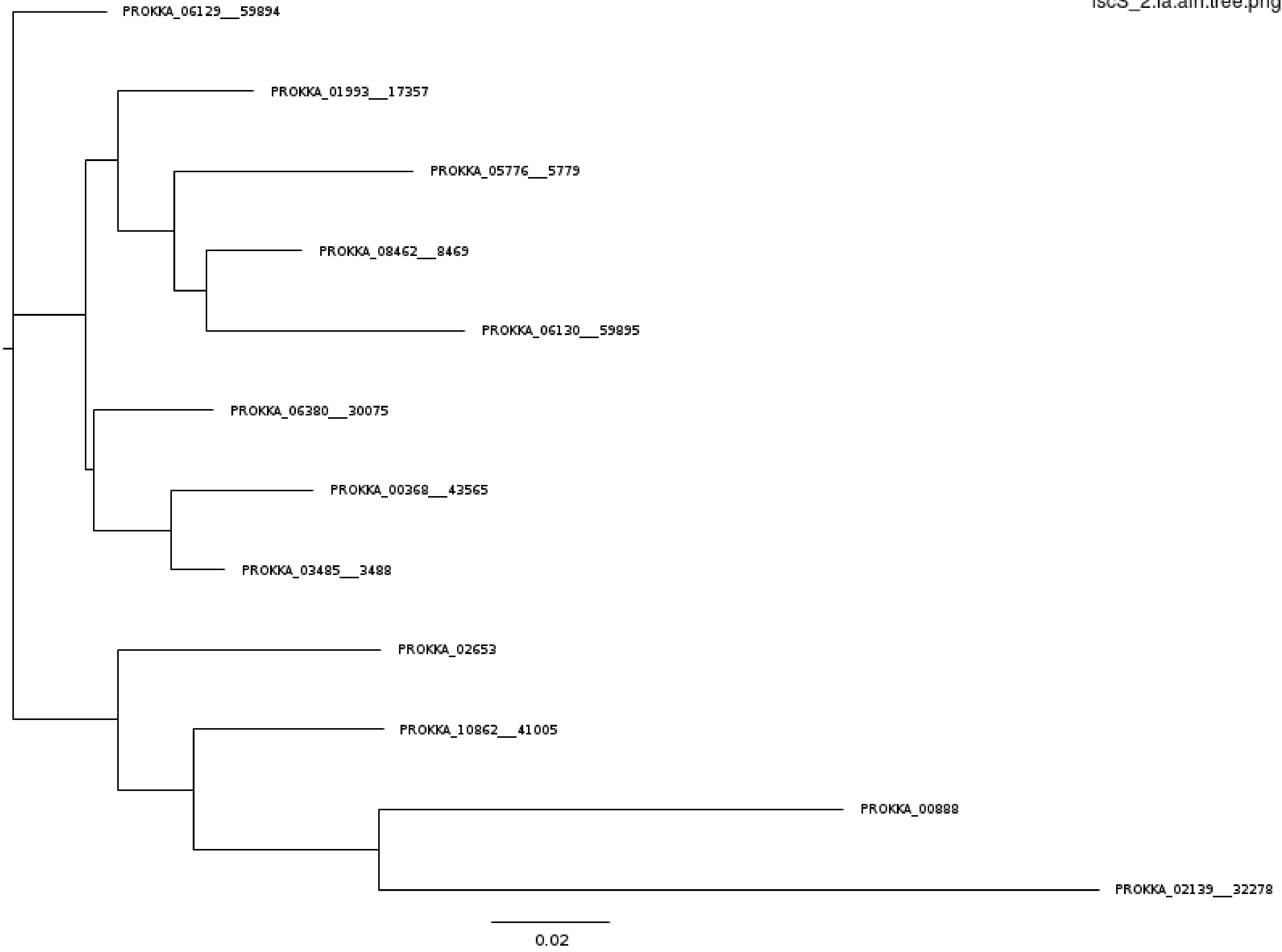

iscU.fa.aln.tree.png

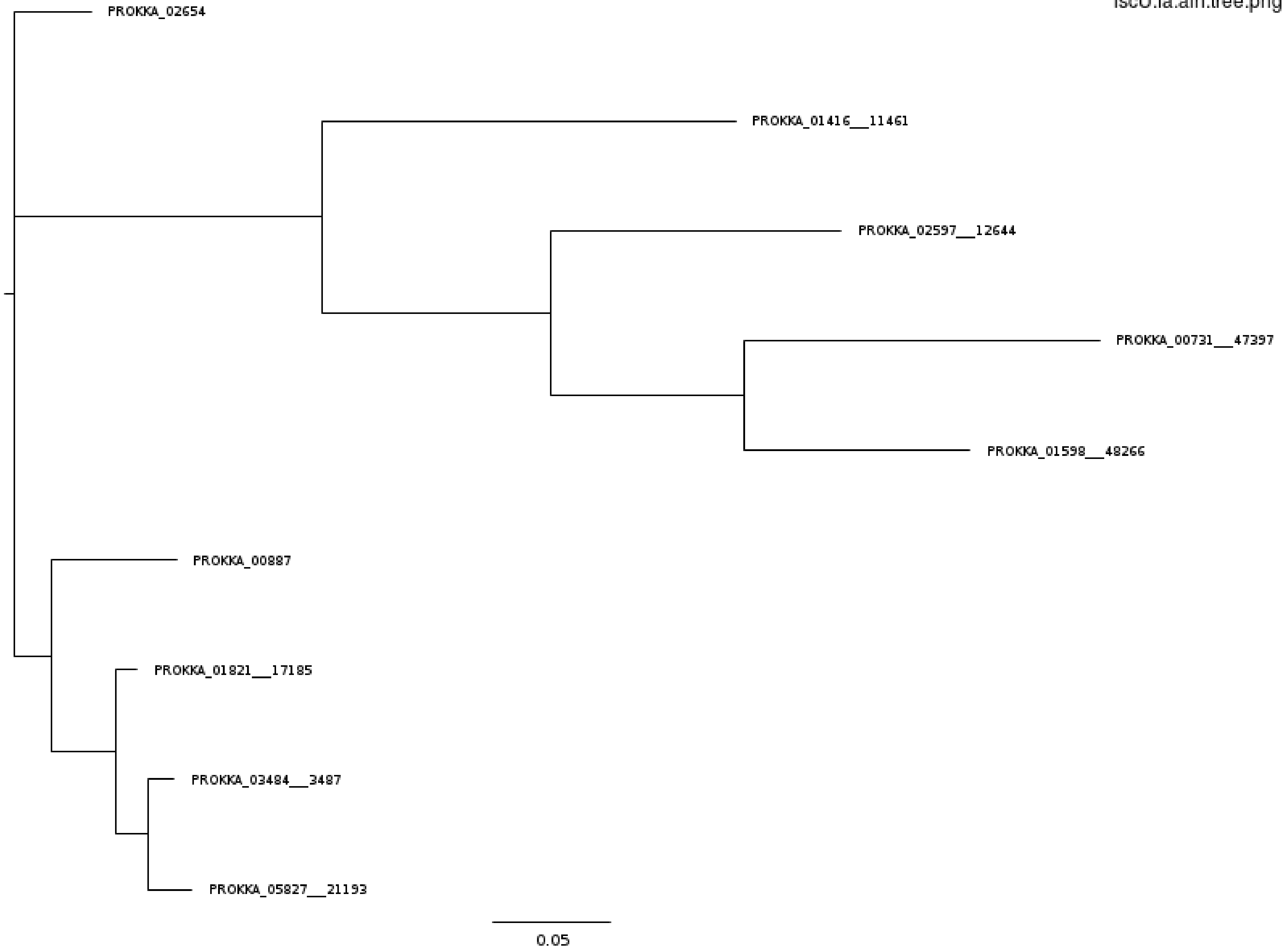

ispD_3.fa.aln.tree.png

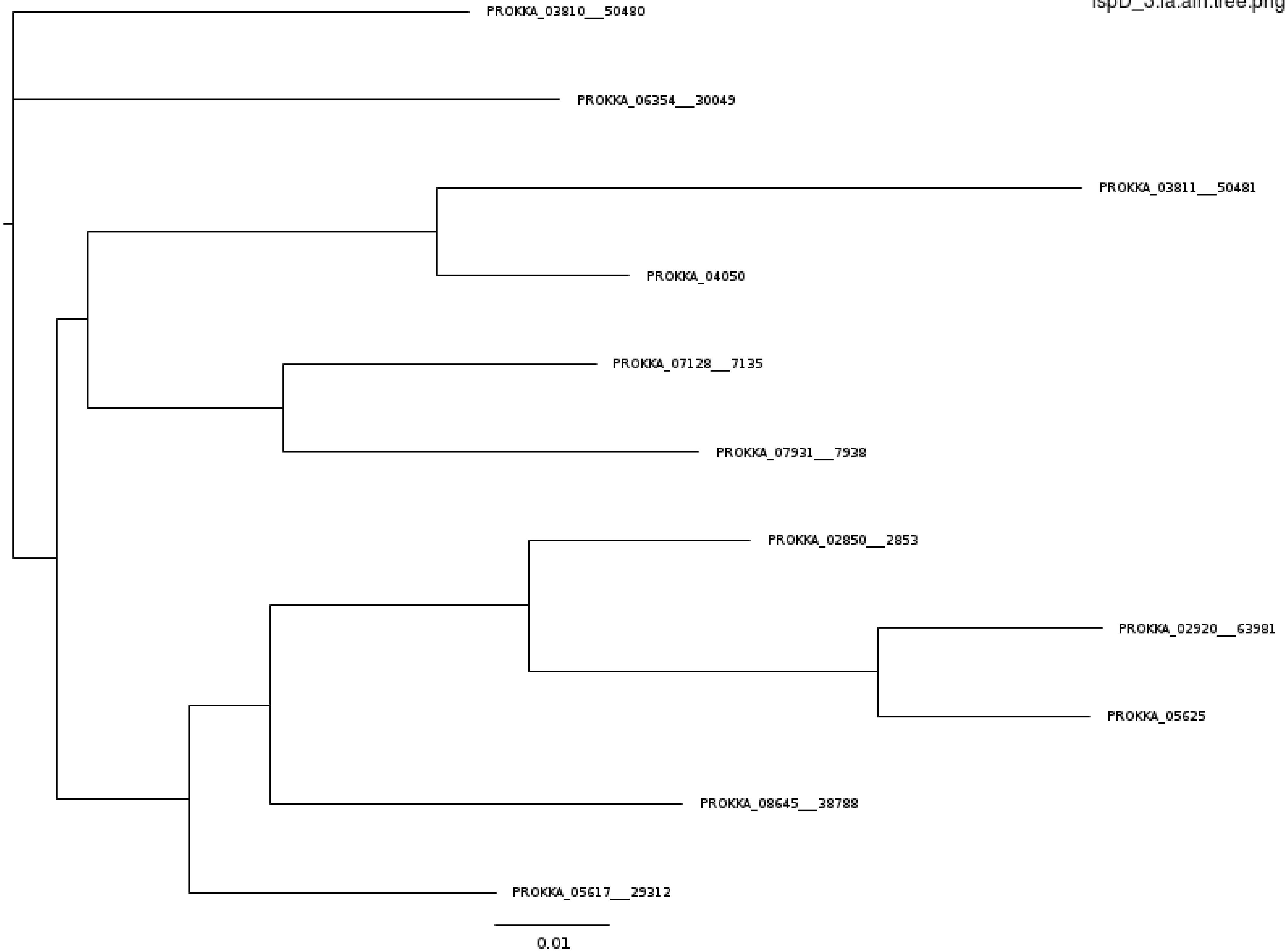

ispE_2.fa.aln.tree.png

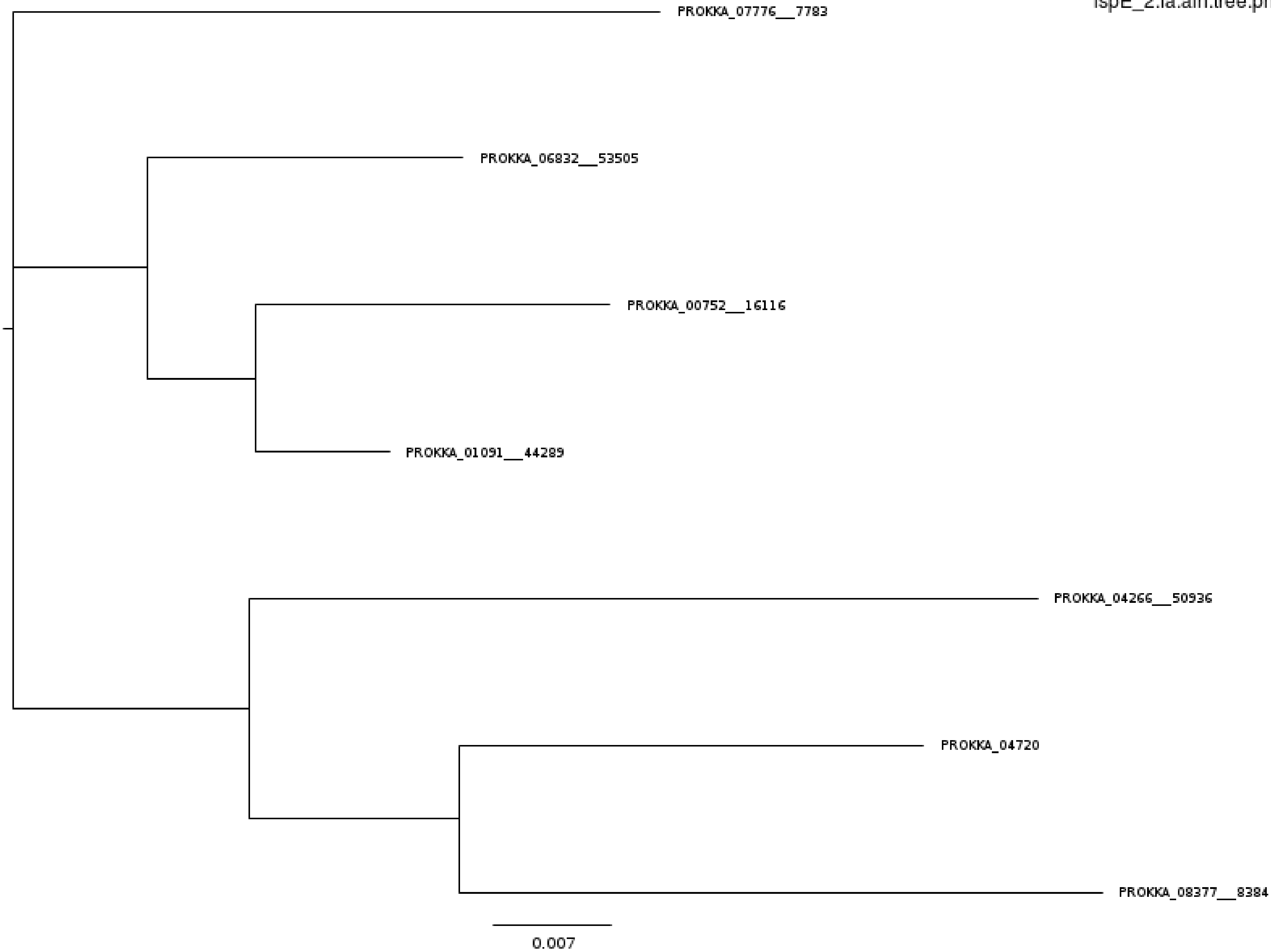

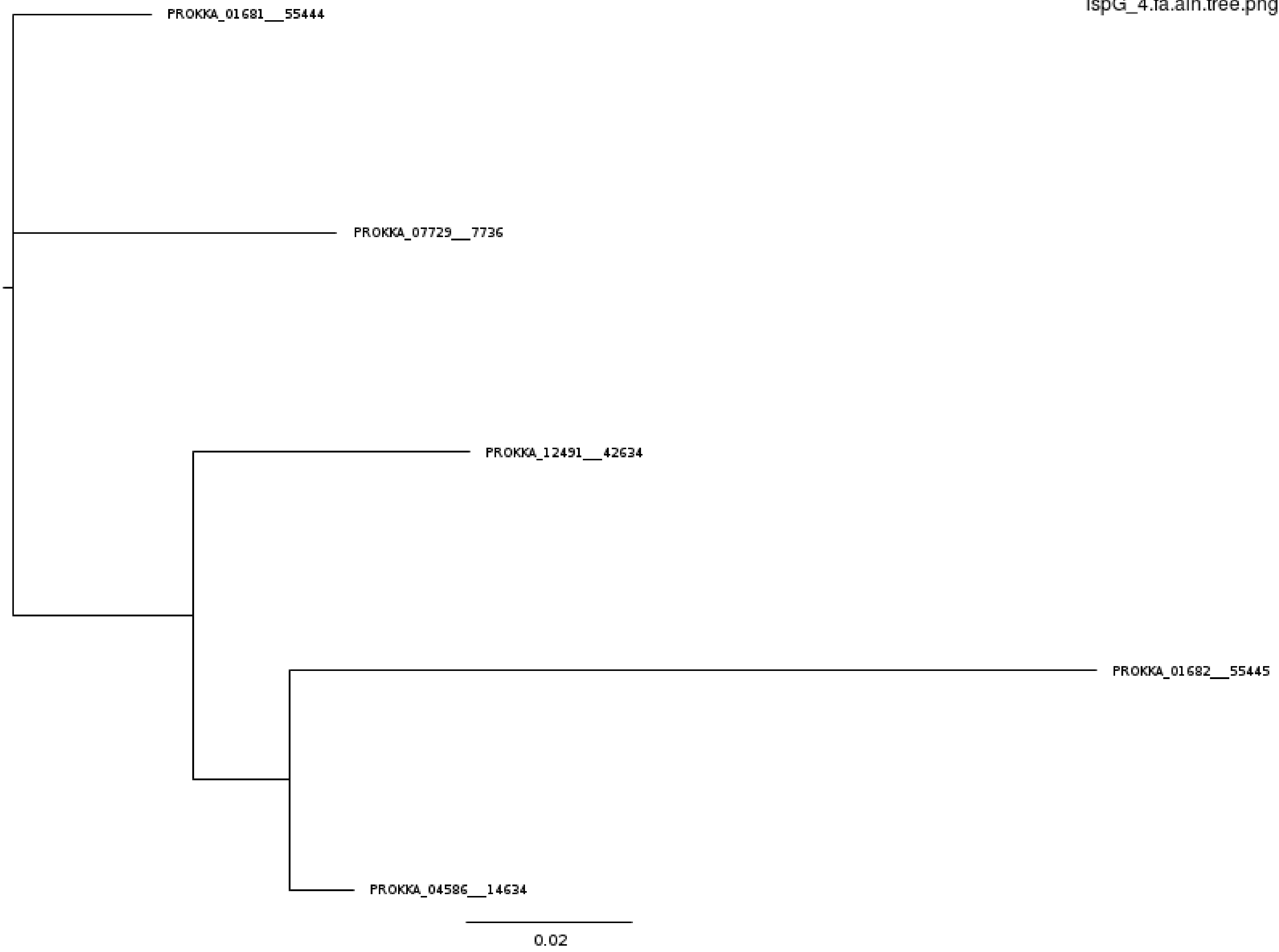
PROKKA_01681___55444
PROKKA_07729___7736
PROKKA_12491___42634
PROKKA_01682___55445
PROKKA_04586___14634
0.02

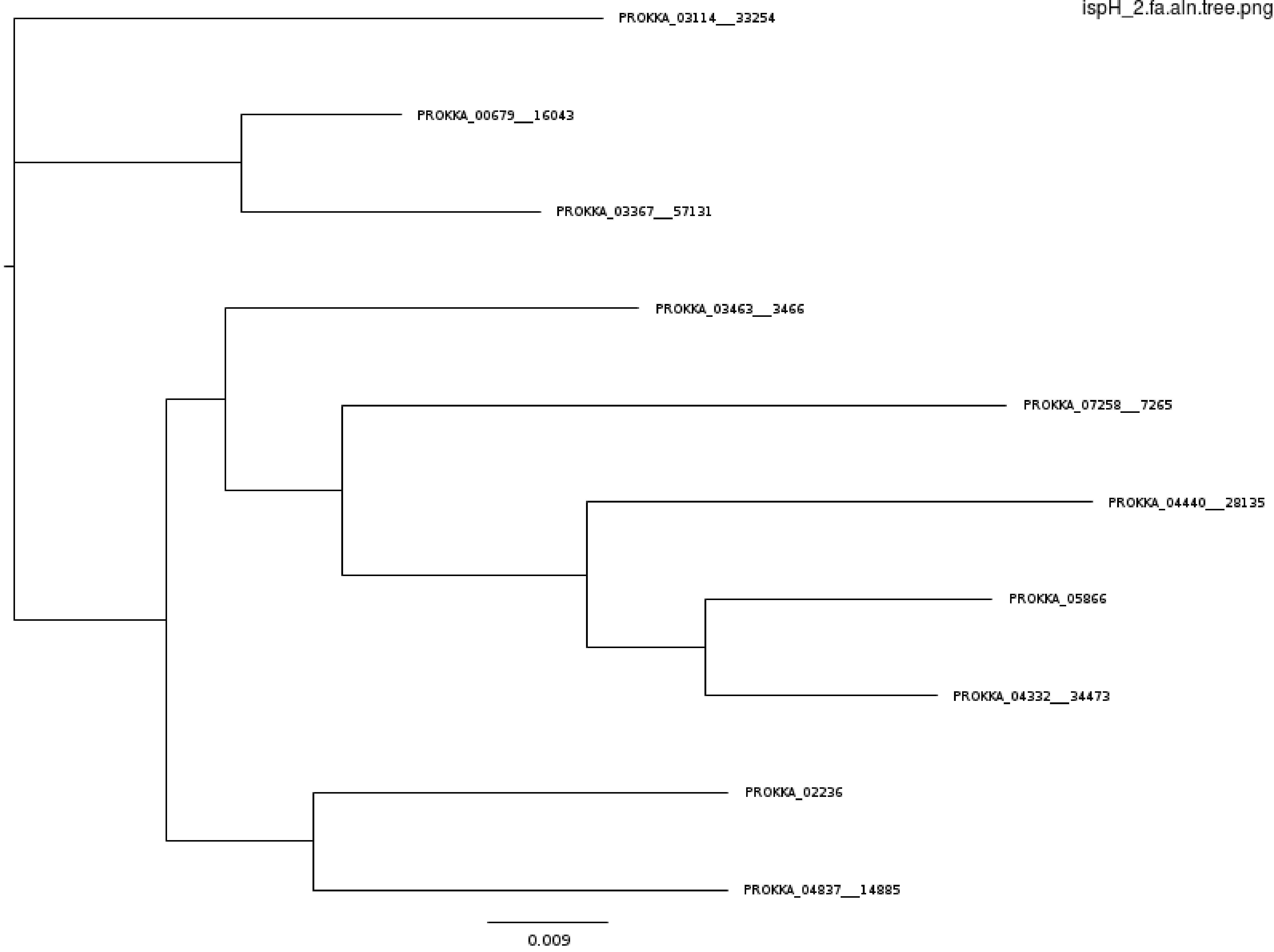
ispH_2.fa.aln.tree.png
PROKKA_03114___33254
PROKKA_00679___16043
PROKKA_03367___57131
PROKKA_03463___3466
PROKKA_07258___7265
PROKKA_04440___28135
PROKKA_05866
PROKKA_04332___34473
PROKKA_02236
PROKKA_04837___14885
0.009

ispU.fa.aln.tree.png

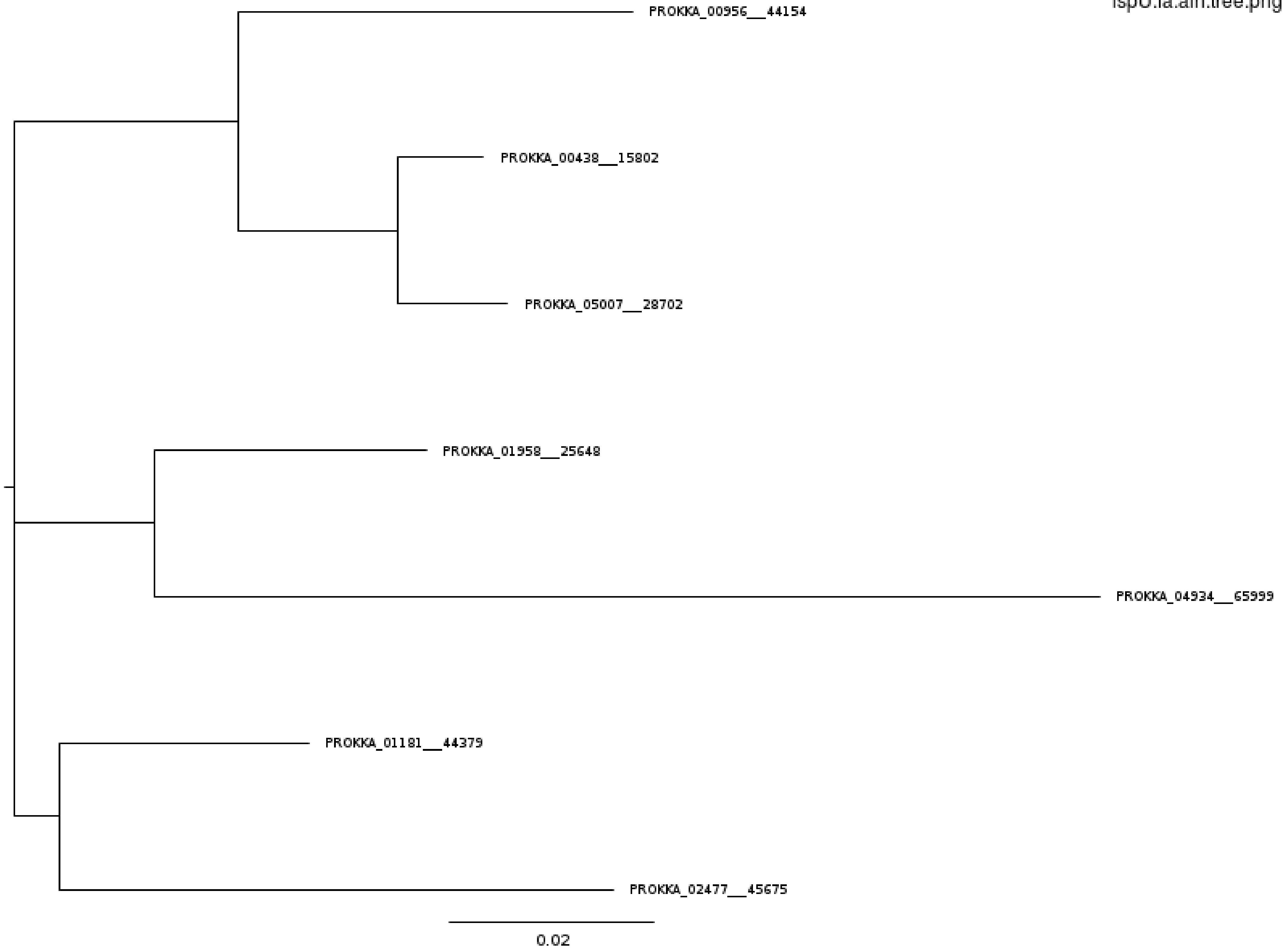

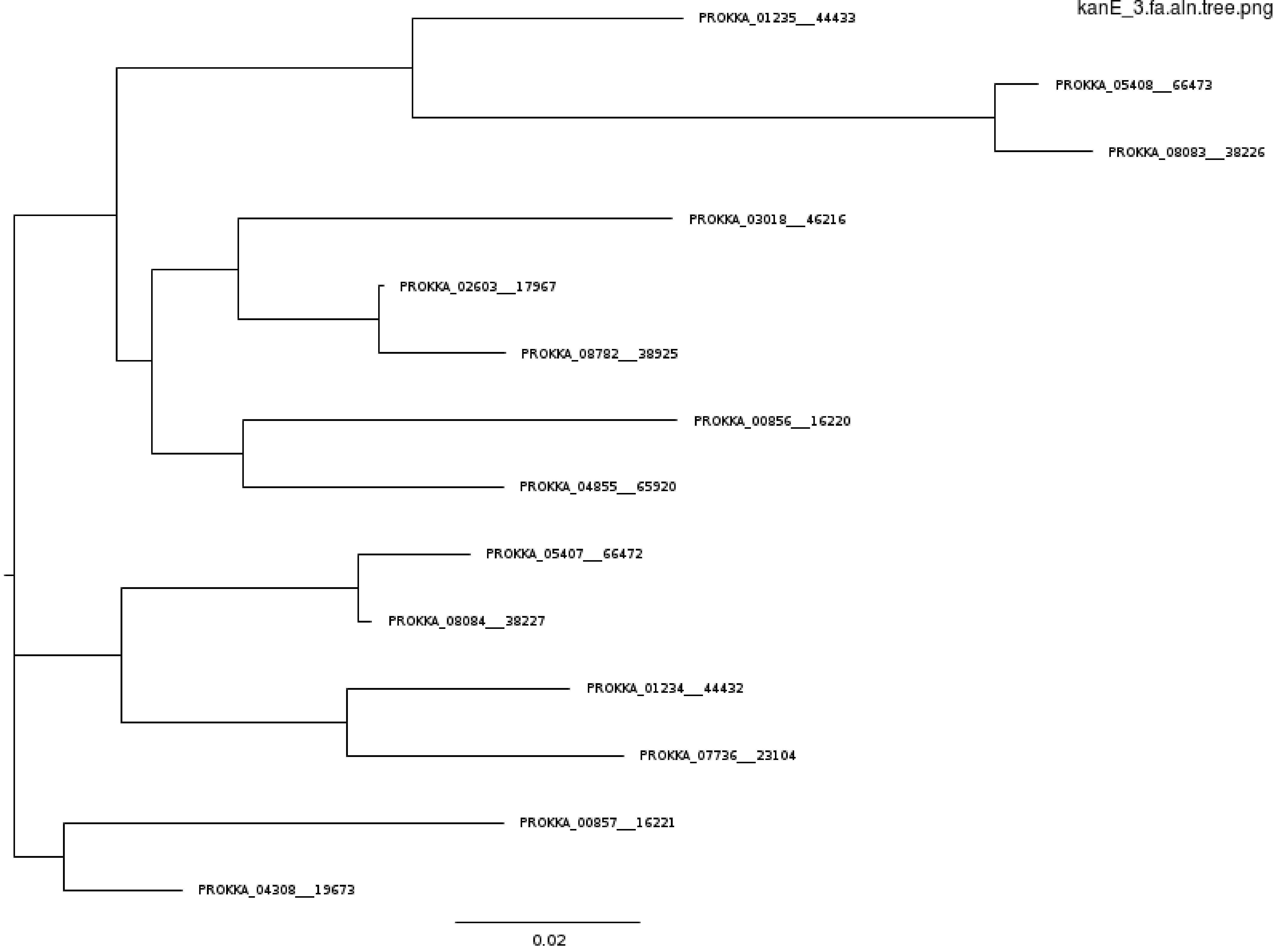
kanE_3.fa.aln.tree.png
PROKKA_01235___44433
PROKKA_05408___66473
PROKKA_08083___38226
PROKKA_03018___46216
PROKKA_02603___17967
PROKKA_08782___38925
PROKKA_00856___16220
PROKKA_04855___65920
PROKKA_05407___66472
PROKKA_08084___38227
PROKKA_01234___44432
PROKKA_07736___23104
PROKKA_00857___16221
PROKKA_04308___19673
0.02

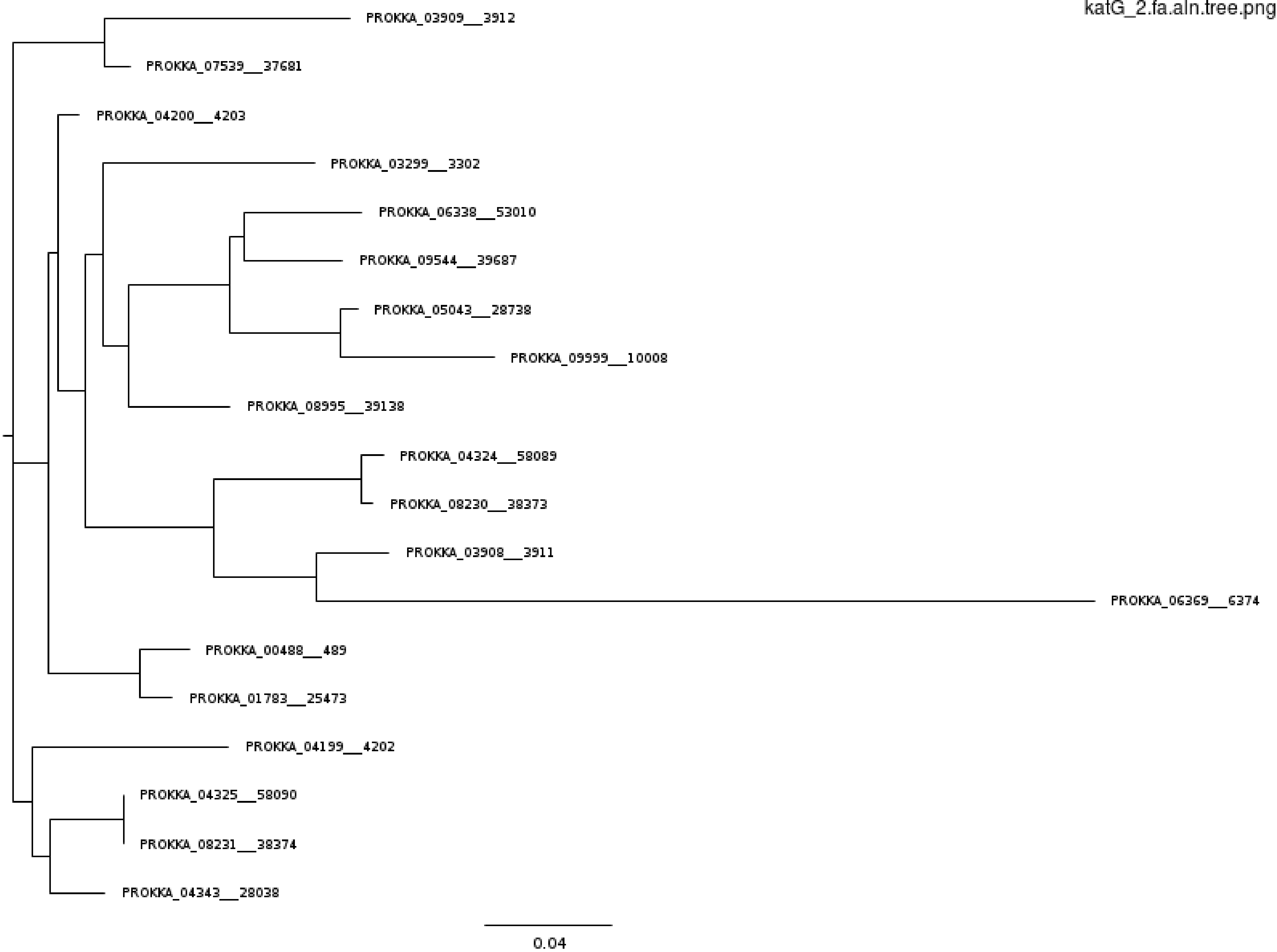
katG_2.fa.aln.tree.png
PROKKA_03909___3912
PROKKA_07539___37681
PROKKA_04200___4203
PROKKA_03299___3302
PROKKA_06338___53010
PROKKA_09544___39687
PROKKA_05043___28738
PROKKA_09999___10008
PROKKA_08995___39138
PROKKA_04324___58089
PROKKA_08230___38373
PROKKA_03908___3911
PROKKA_06369___6374
PROKKA_00488___489
PROKKA_01783___25473
PROKKA_04199___4202
PROKKA_04325___58090
PROKKA_08231___38374
PROKKA_04343___28038
0.04

kdpA_1.fa.aln.tree.png

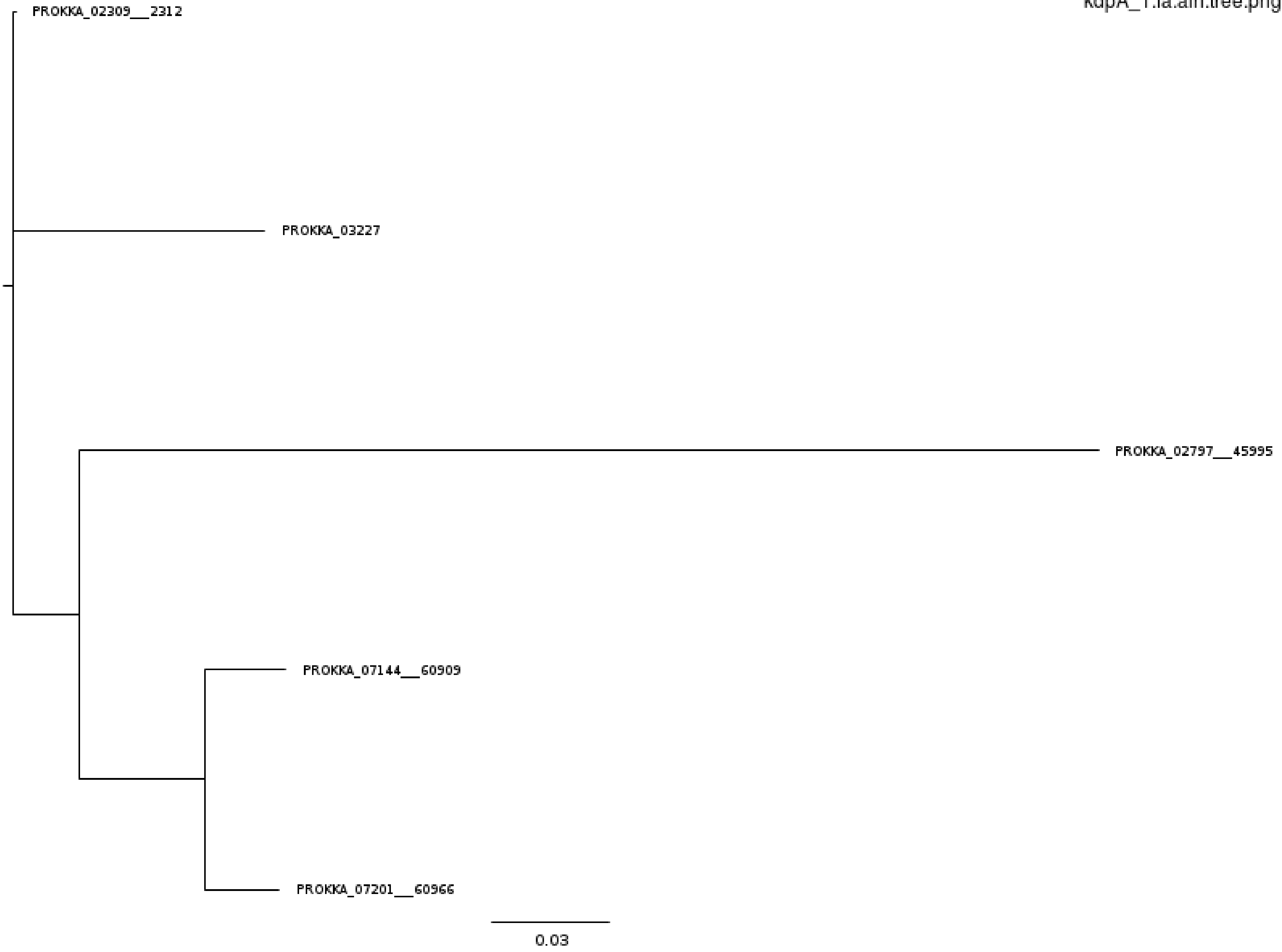

kdpB.fa.aln.tree.png

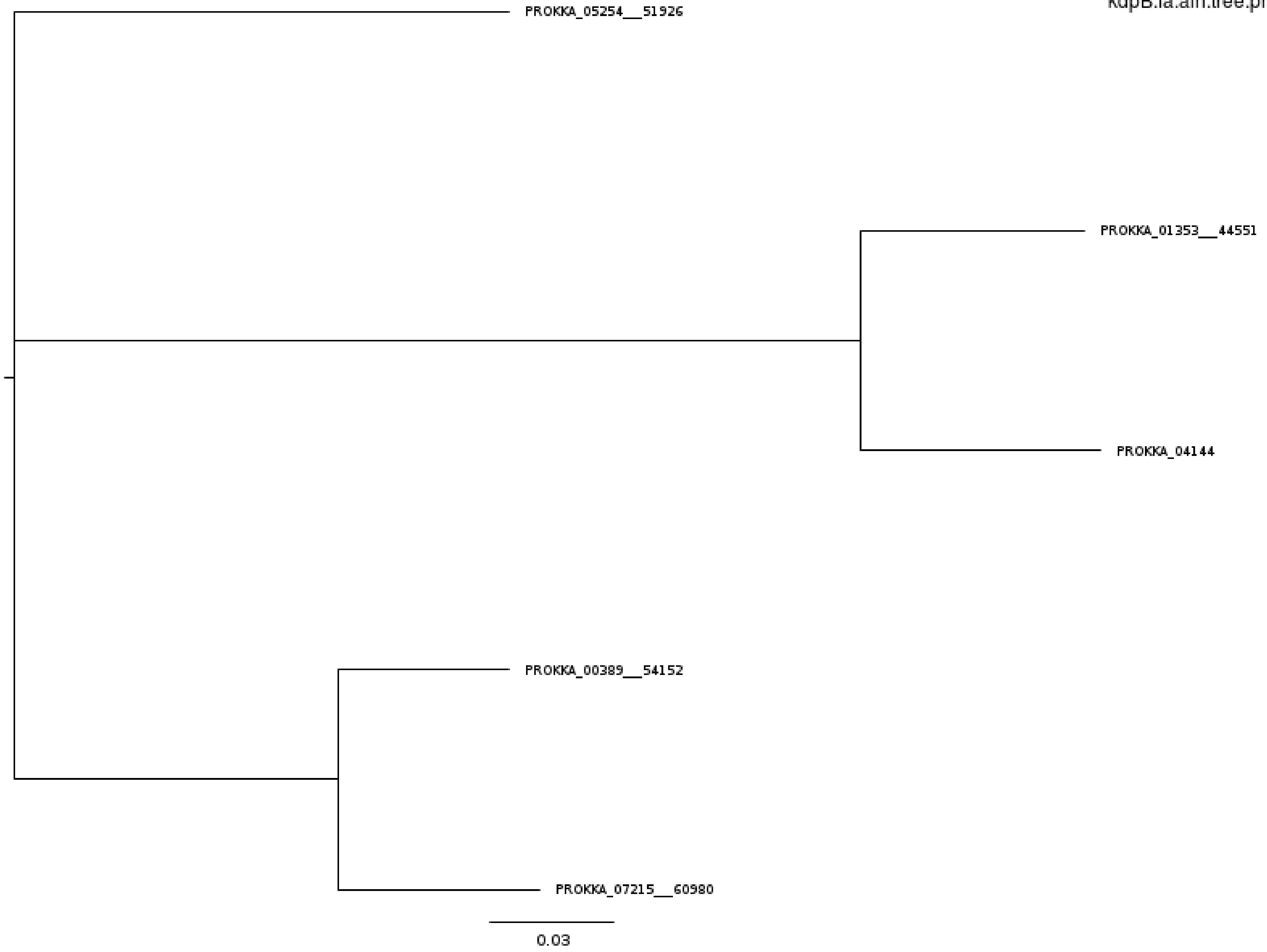

kdpC_2.fa.aln.tree.png

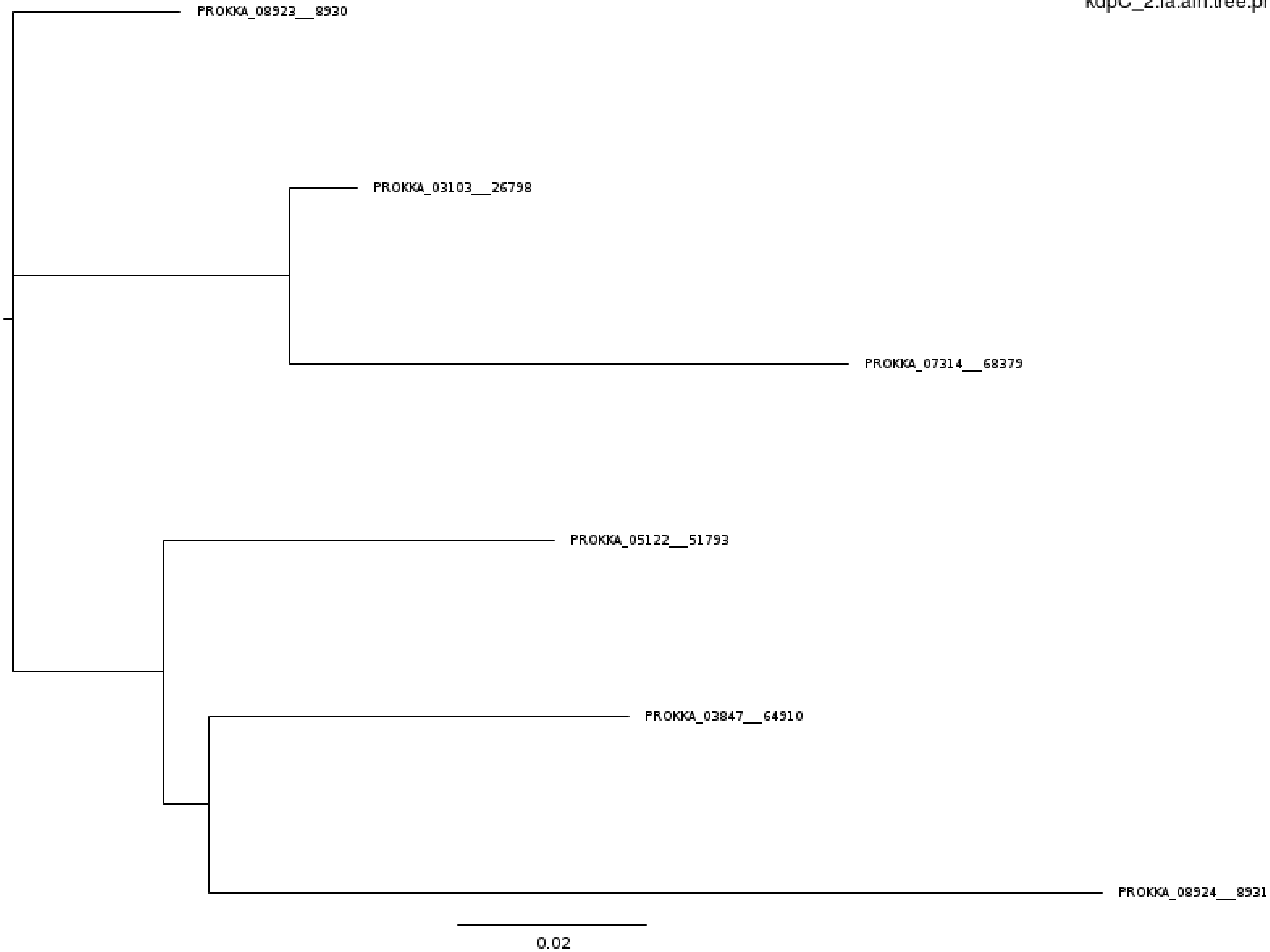

kdpD_3.fa.aln.tree.png

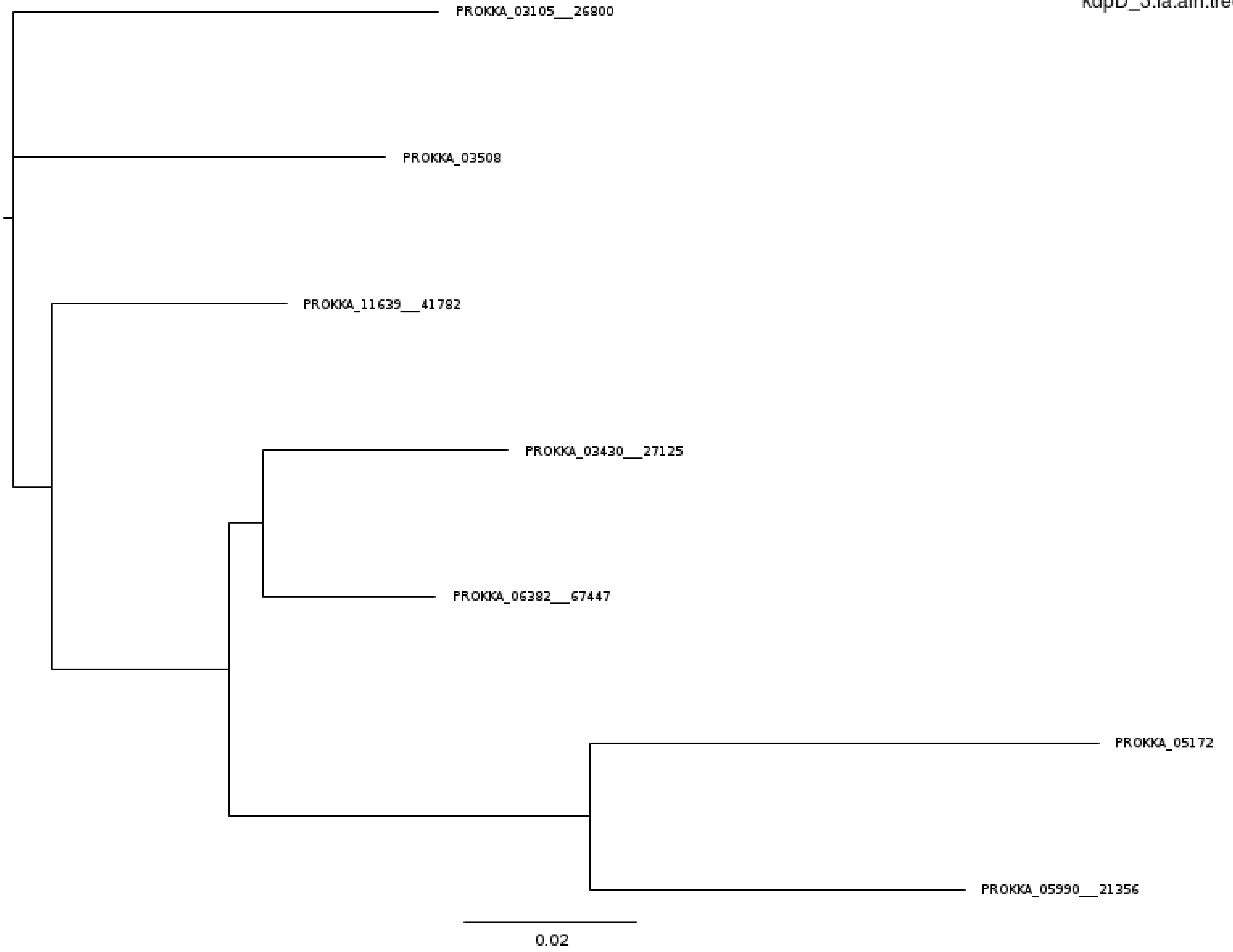

kdsA_2.fa.aln.tree.png

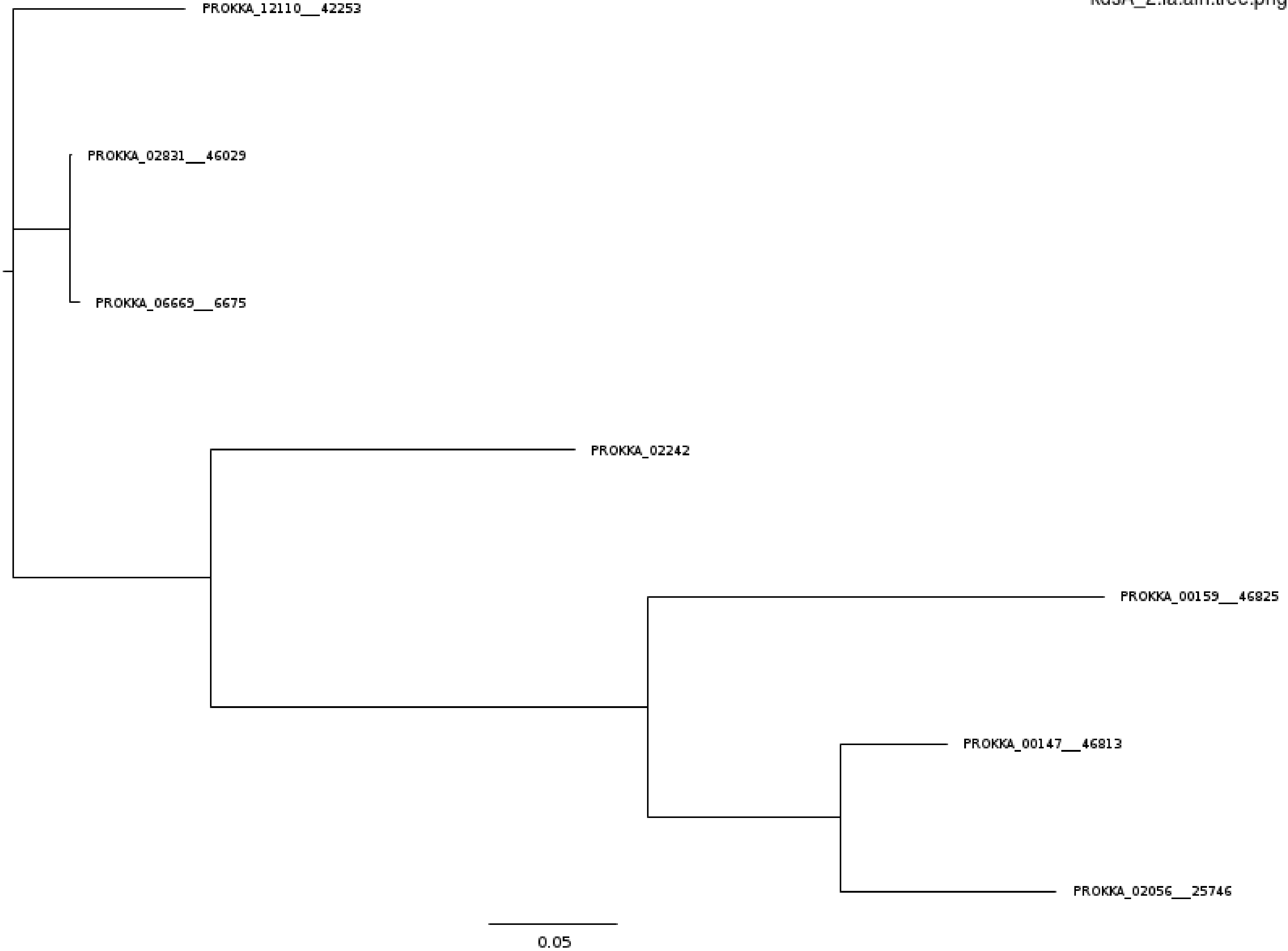

kdsB_2.fa.aln.tree.png

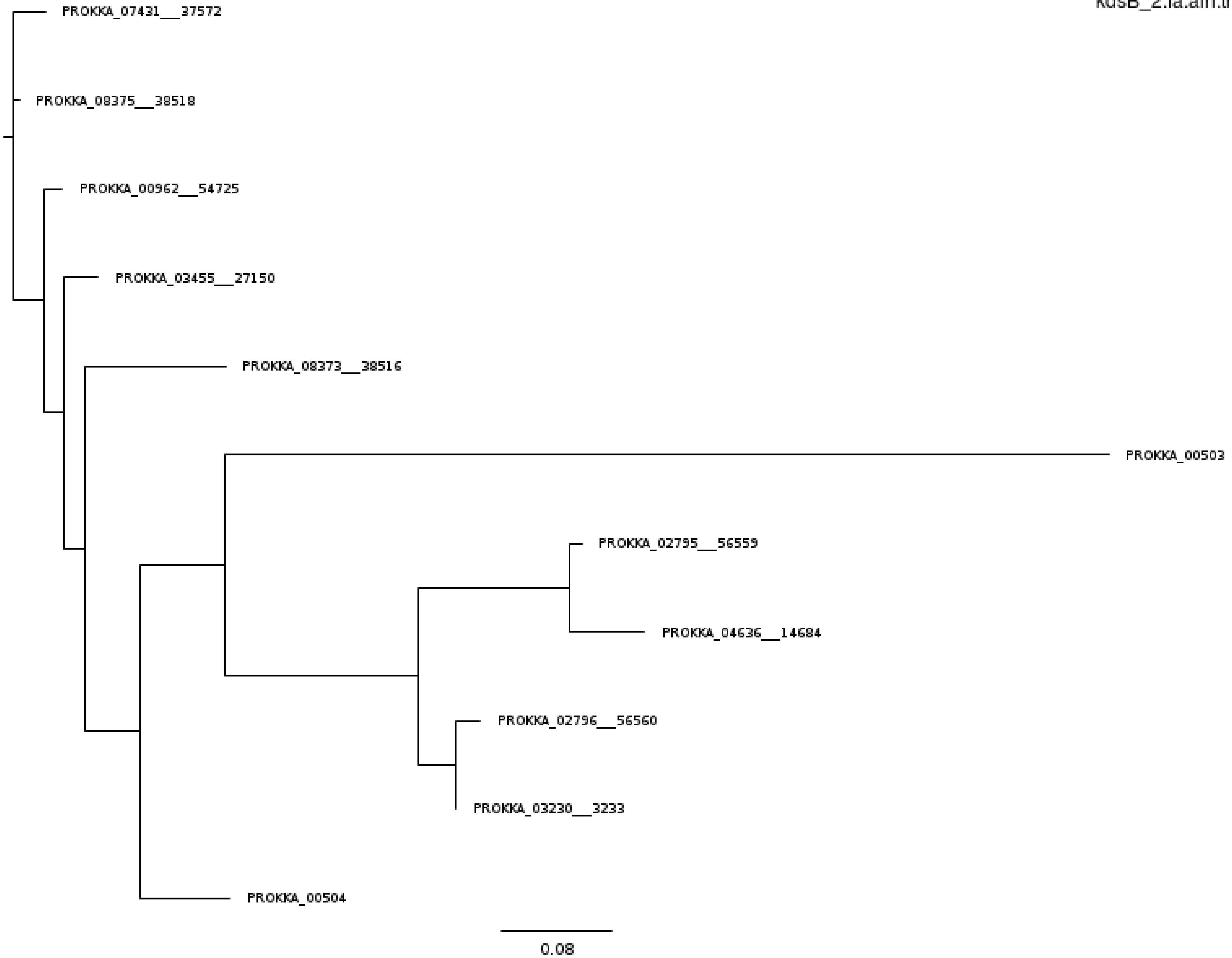

kdsC_2.fa.aln.tree.png

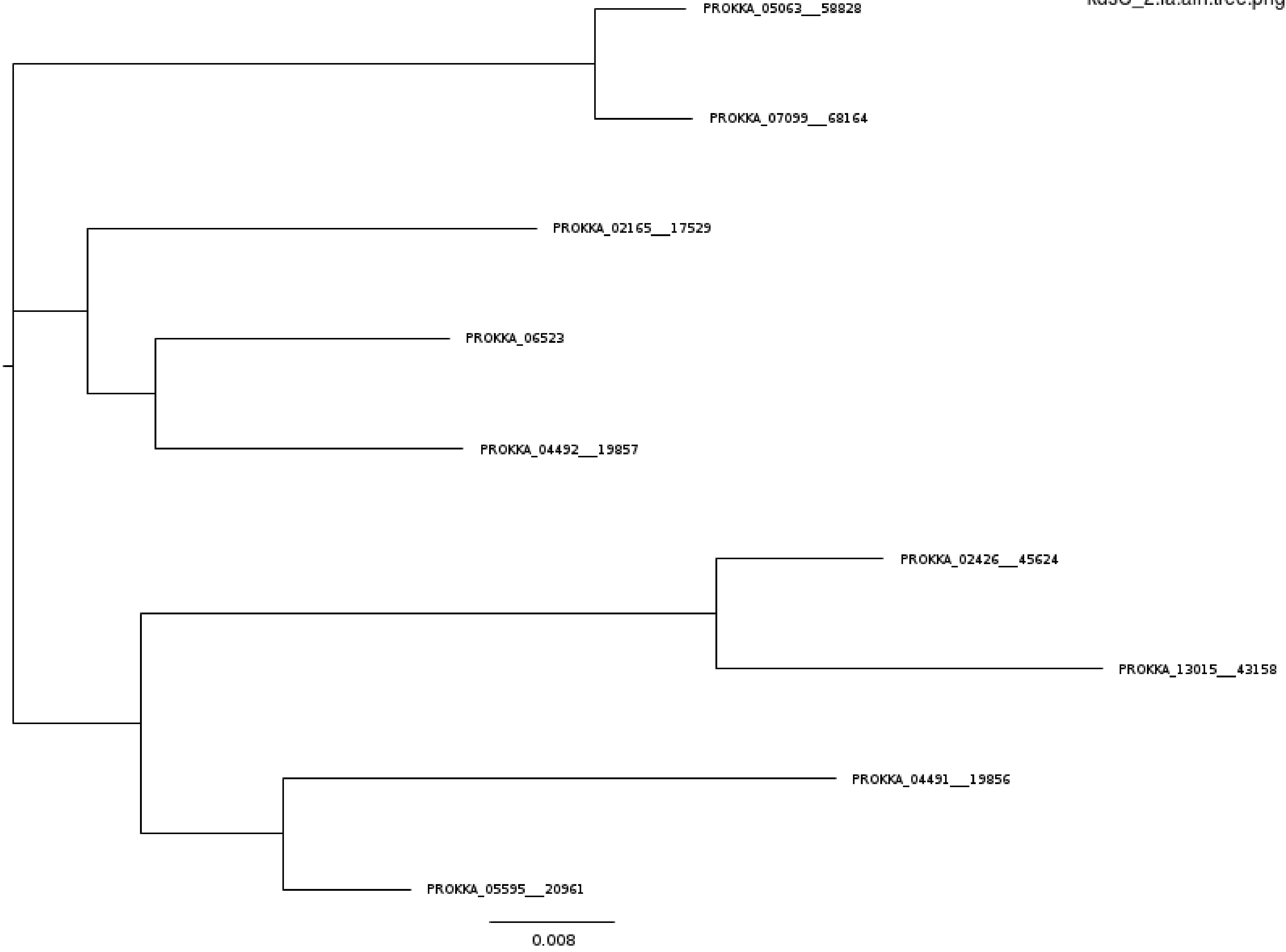

kdsD_5.fa.aln.tree.png

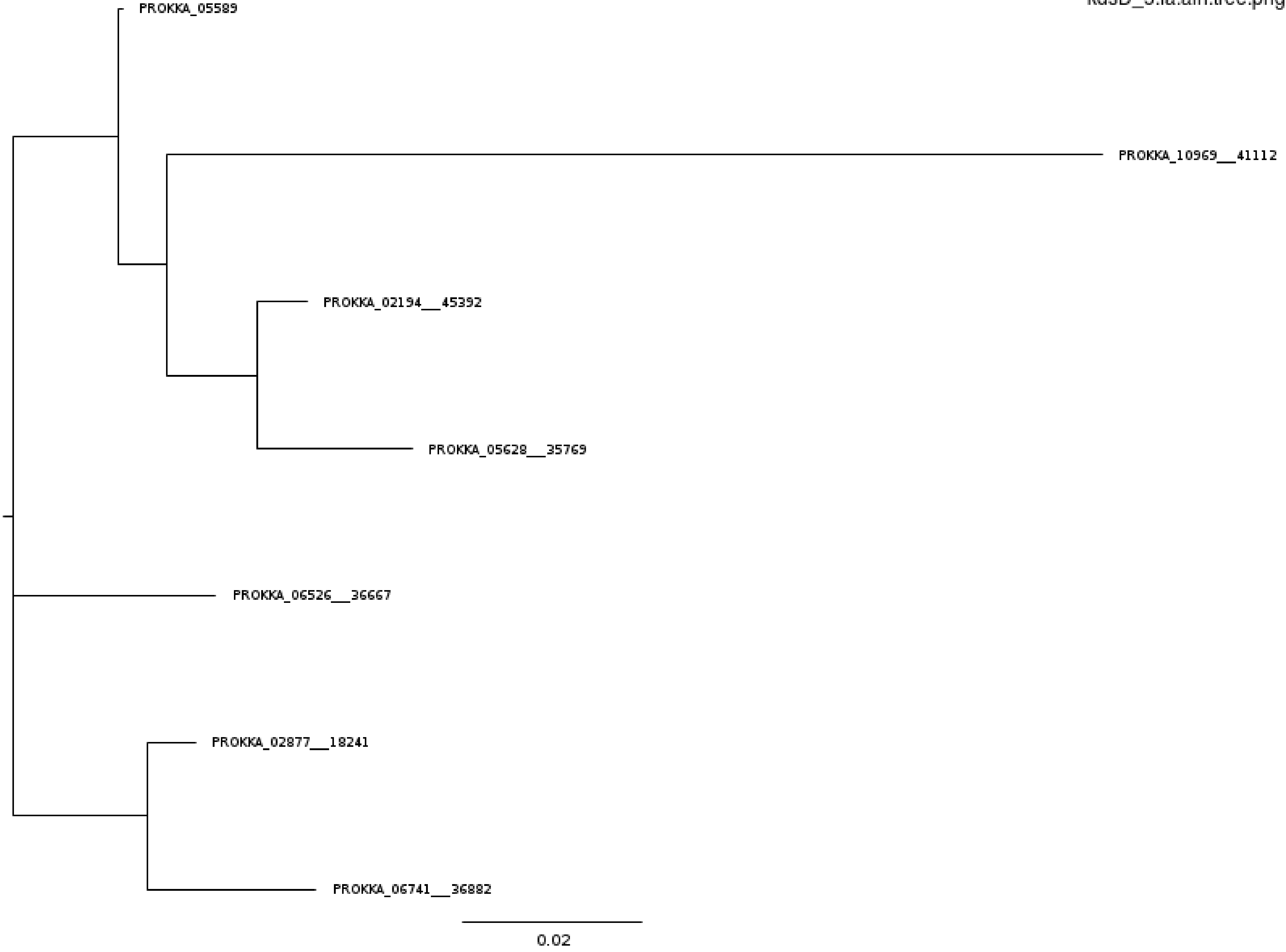

kefC_1.fa.aln.tree.png

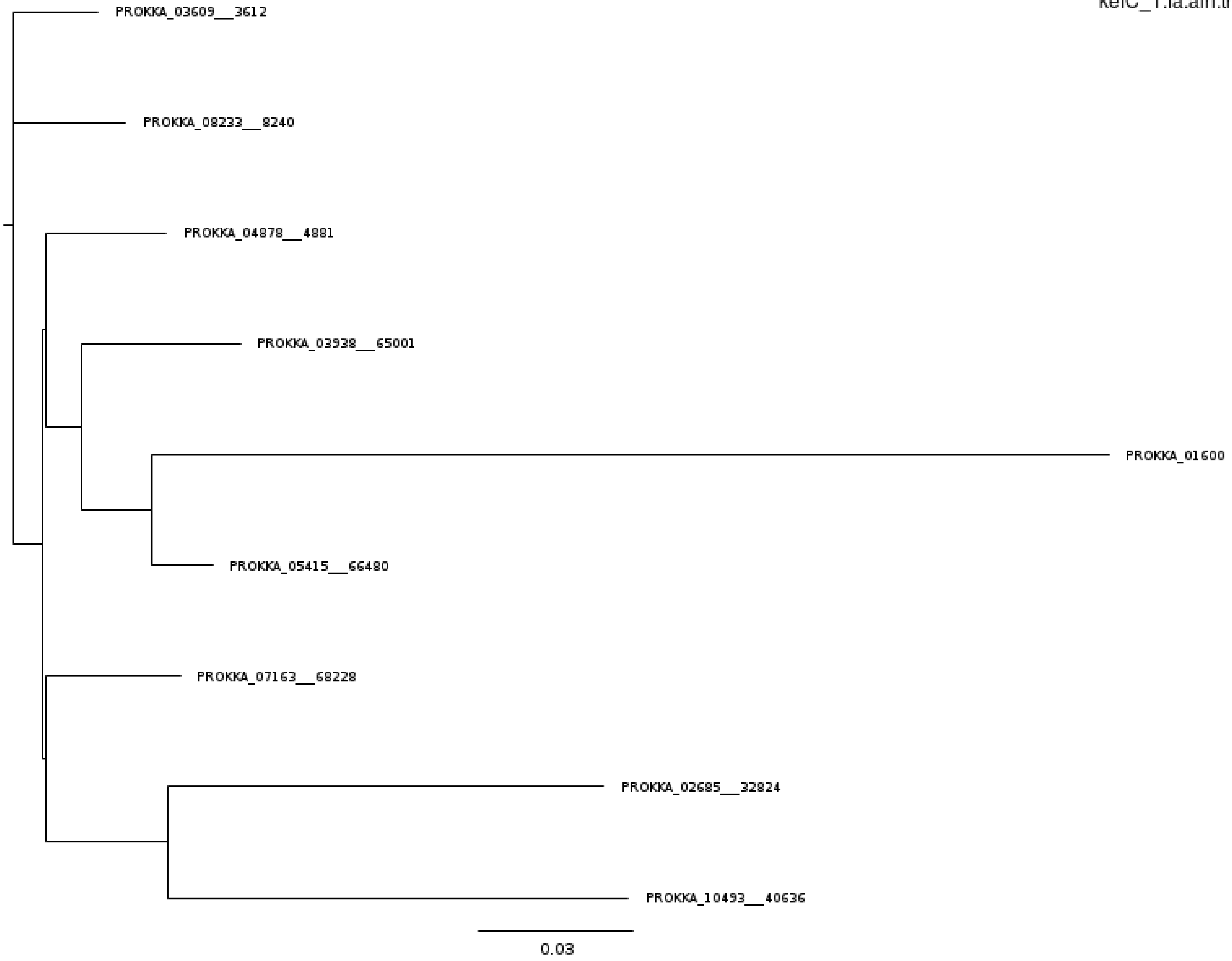

korA_3.fa.aln.tree.png

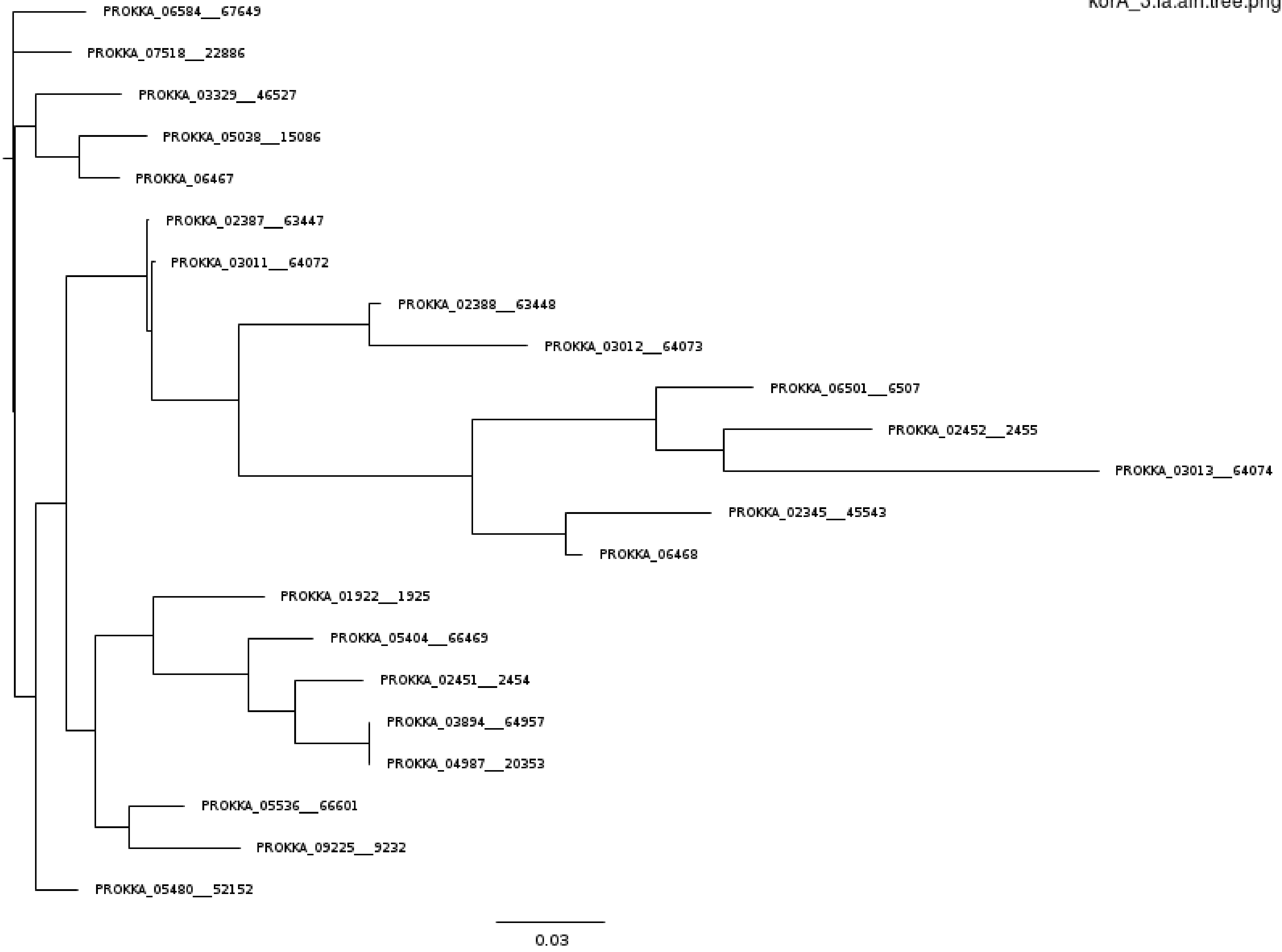

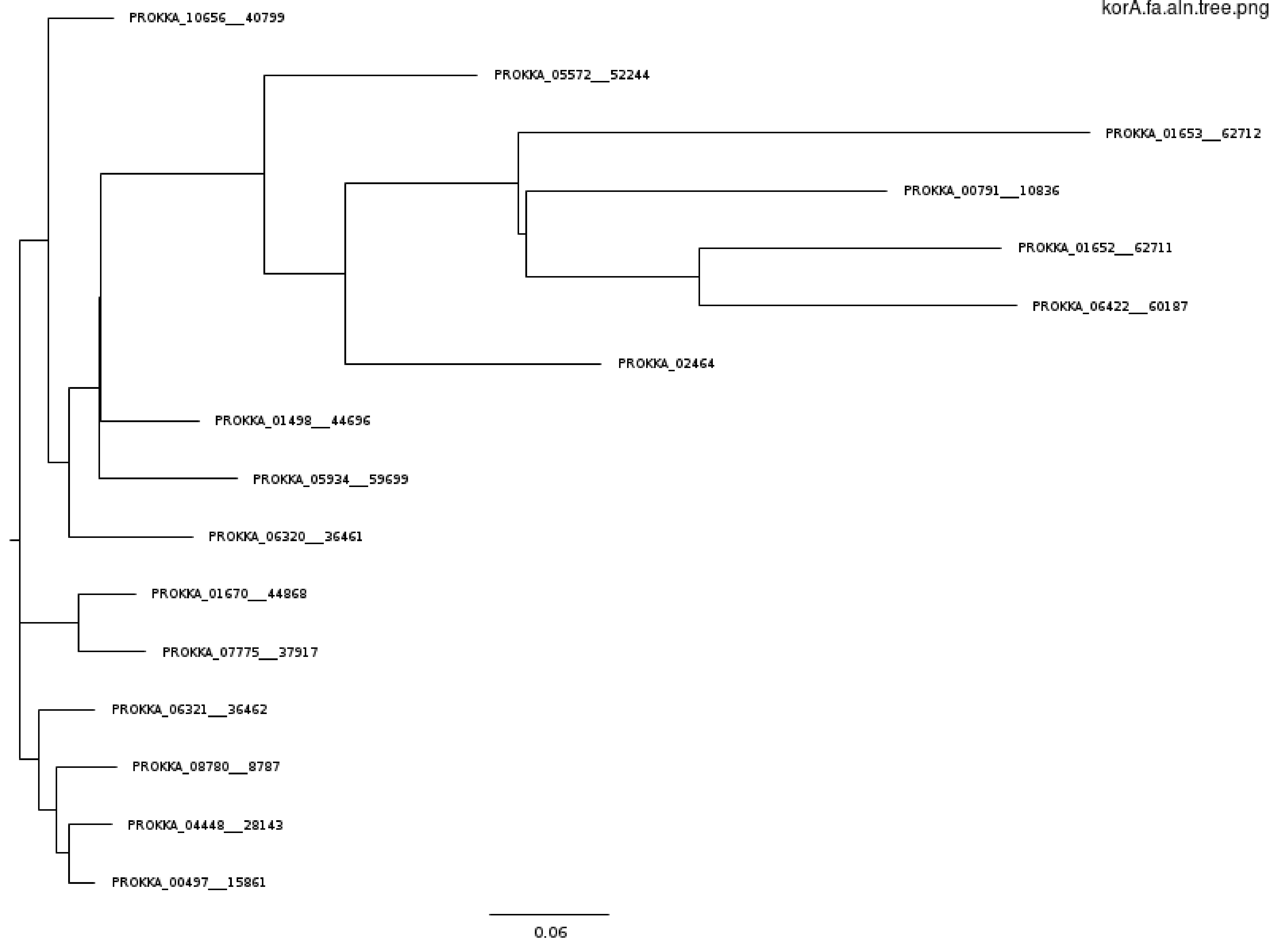

korA.fa.aln.tree.png
PROKKA_10656___40799
PROKKA_05572___52244
PROKKA_01653___62712
PROKKA_00791___10836
PROKKA_01652___62711
PROKKA_06422___60187
PROKKA_02464
PROKKA_01498___44696
PROKKA_05934___59699
PROKKA_06320___36461
PROKKA_01670___44868
PROKKA_07775___37917
PROKKA_06321___36462
PROKKA_08780___8787
PROKKA_04448___28143
PROKKA_00497___15861
0.06

korB.fa.aln.tree.png

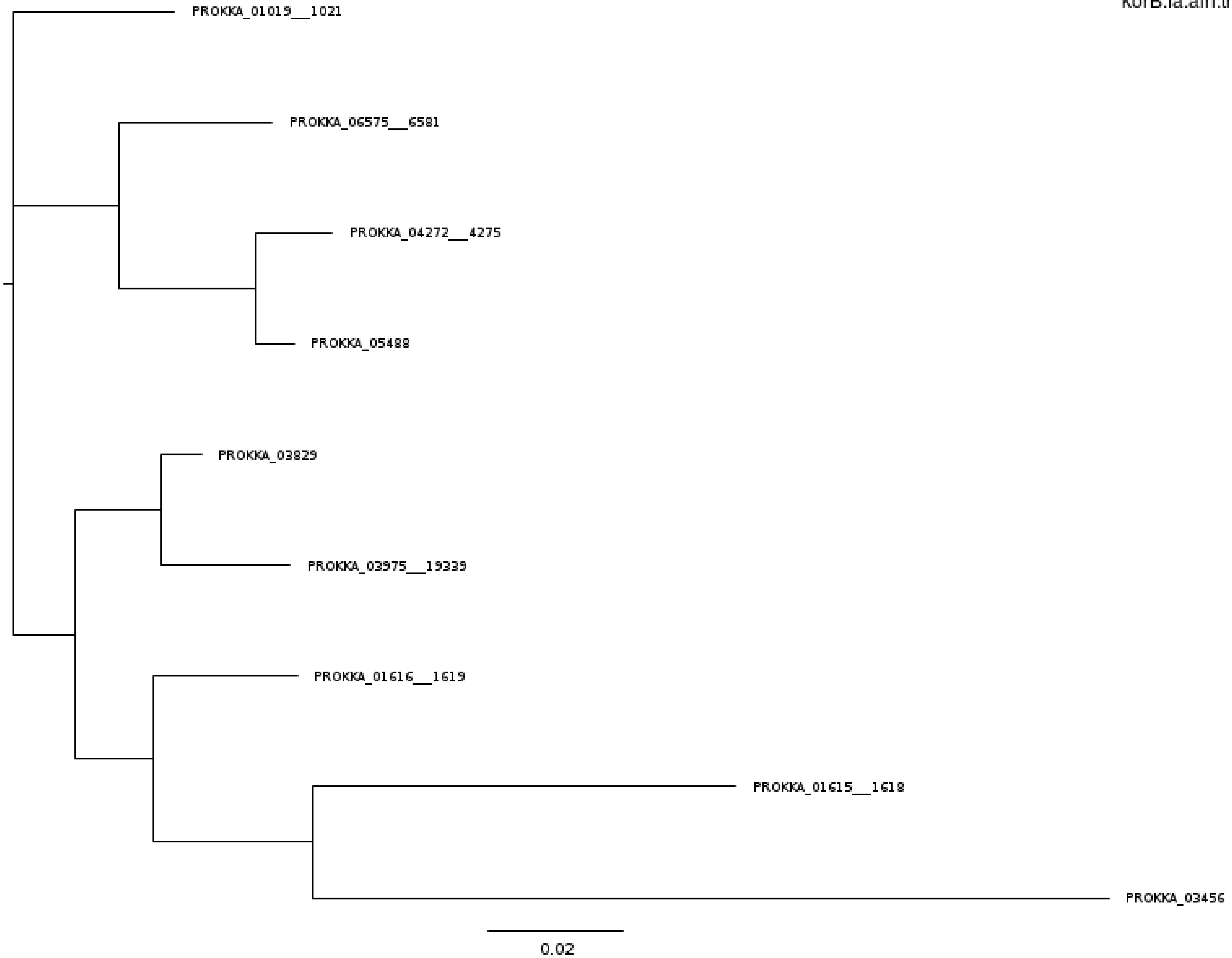

ldc.fa.aln.tree.png

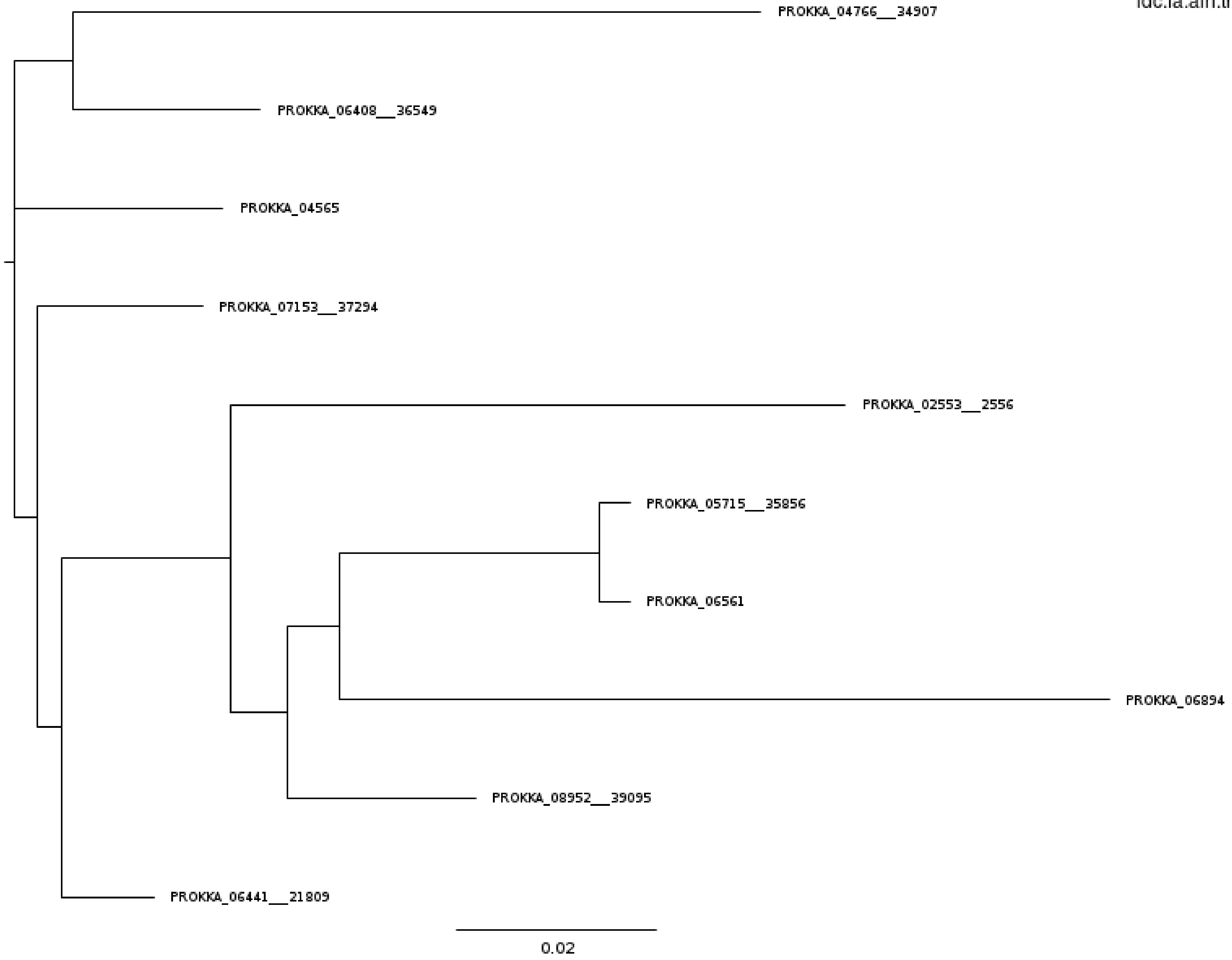

lepA_2.fa.aln.tree.png

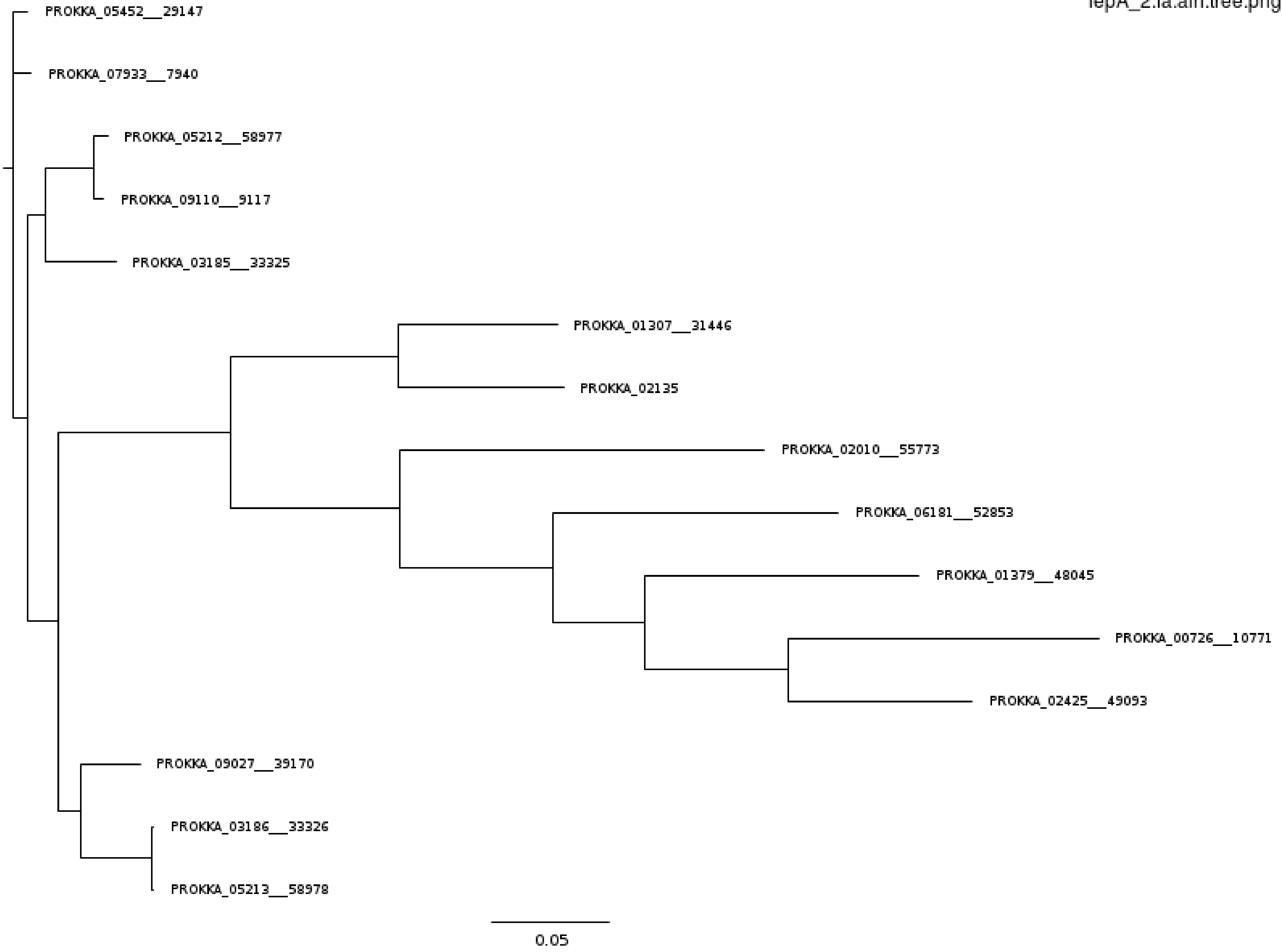

leuA_1.fa.aln.tree.png

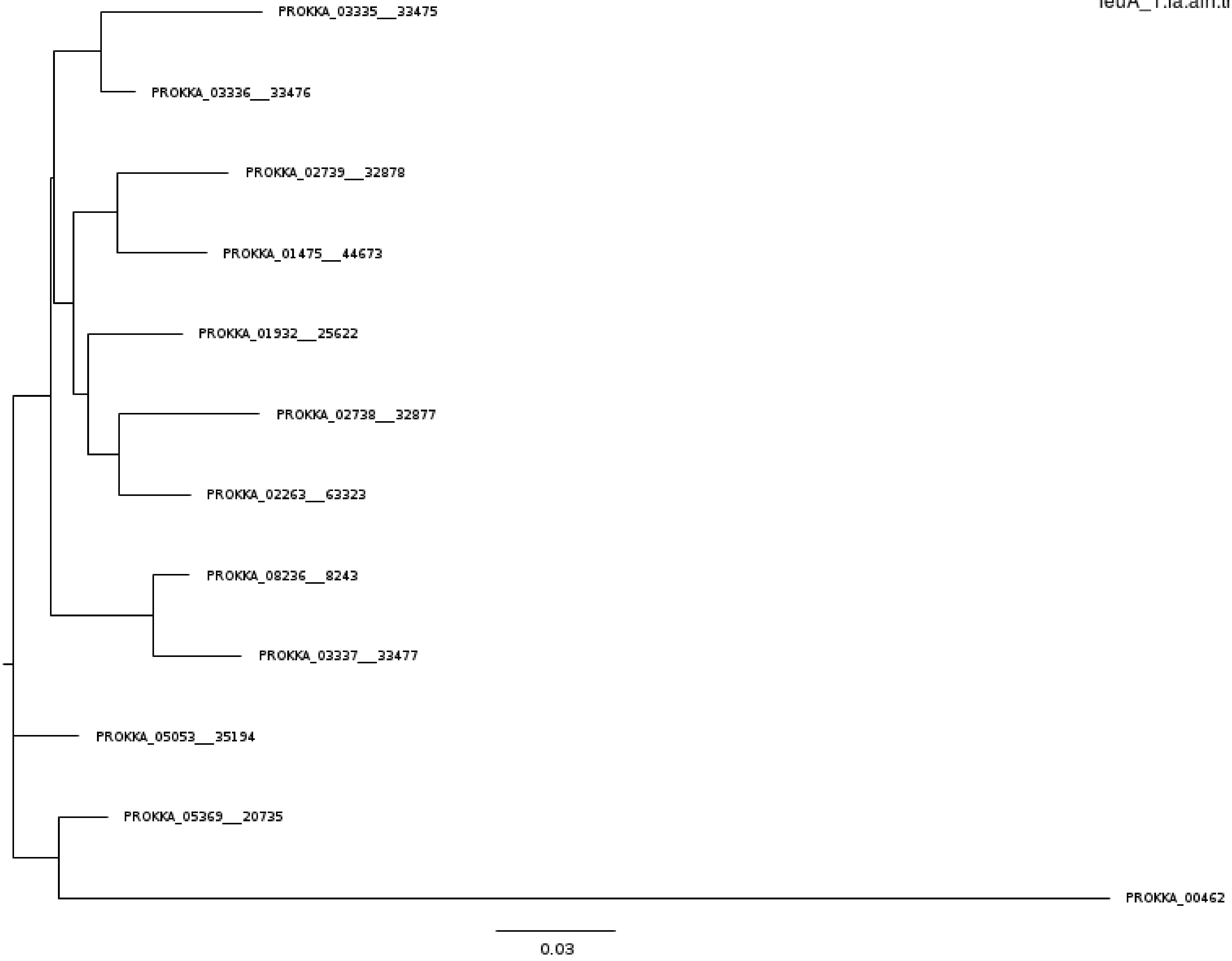

leuA_2.fa.aln.tree.png

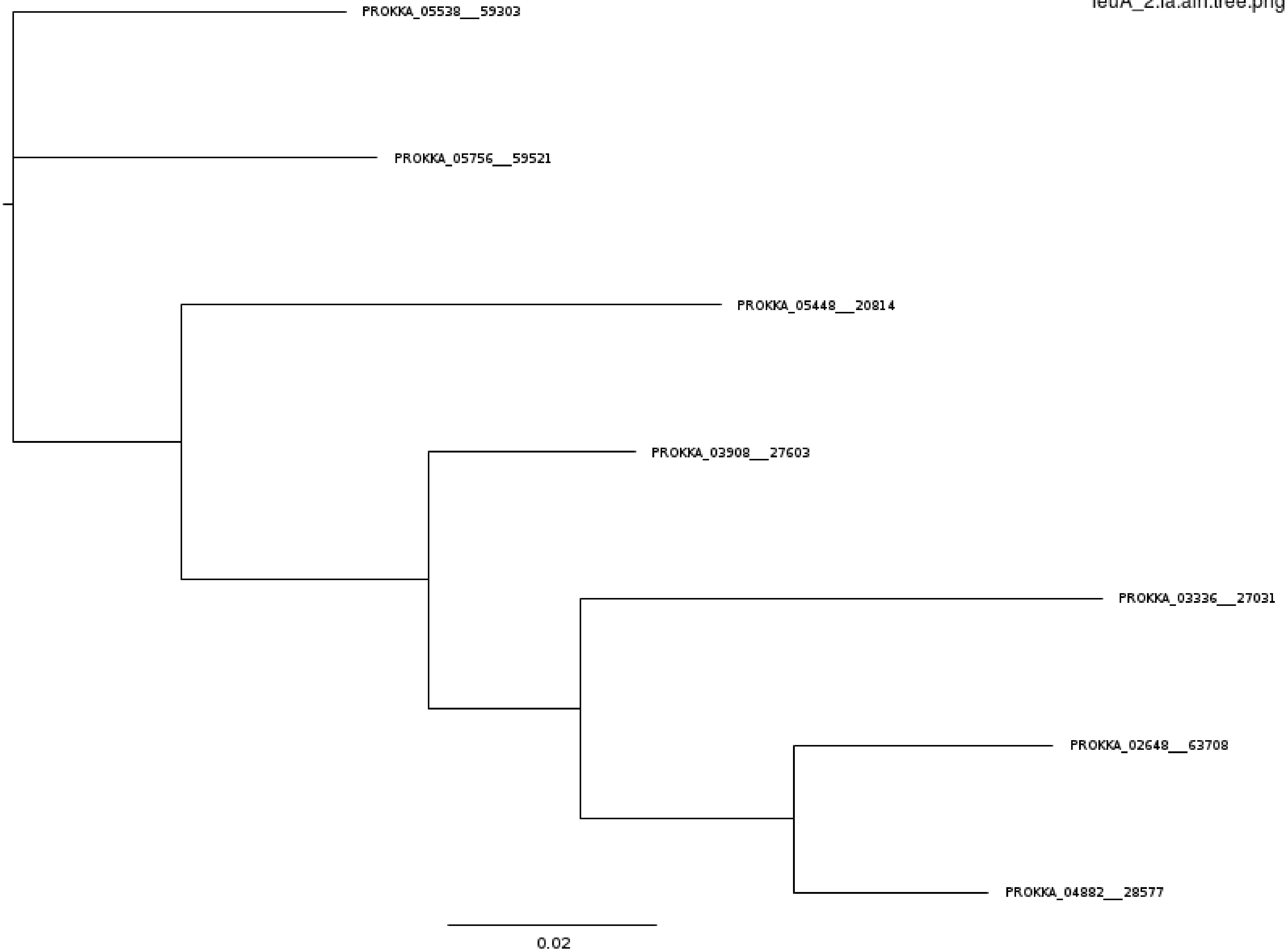

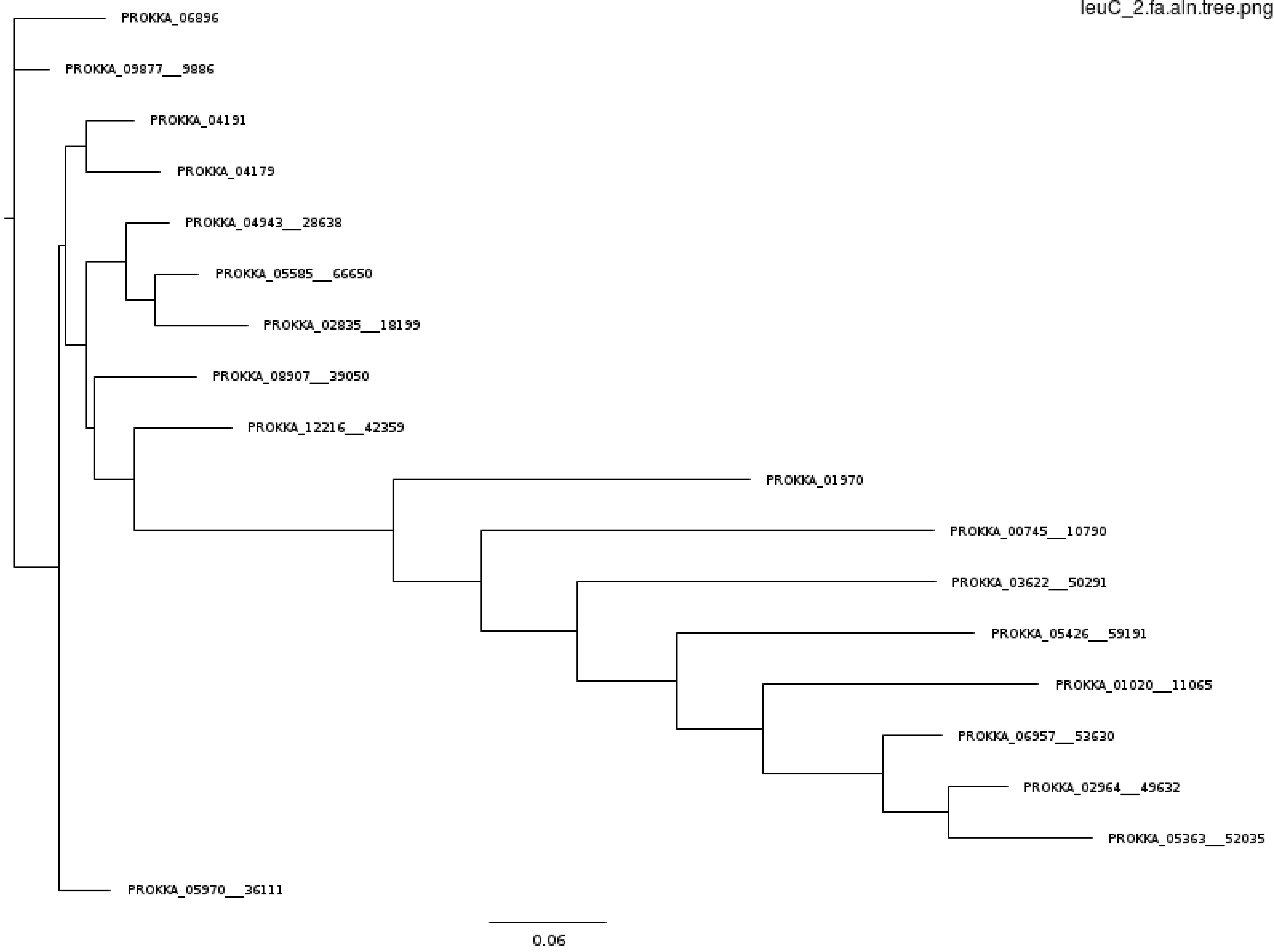
leuC_2.fa.aln.tree.png
PROKKA_06896
PROKKA_09877__9886
PROKKA_04191
PROKKA_04179
PROKKA_04943__28638
PROKKA_05585__66650
PROKKA_02835__18199
PROKKA_08907__39050
PROKKA_12216__42359
PROKKA_01970
PROKKA_00745__10790
PROKKA_03622__50291
PROKKA_05426__59191
PROKKA_01020__11065
PROKKA_06957__53630
PROKKA_02964__49632
PROKKA_05363__52035
PROKKA_05970__36111
0.06

leuD_1.fa.aln.tree.png

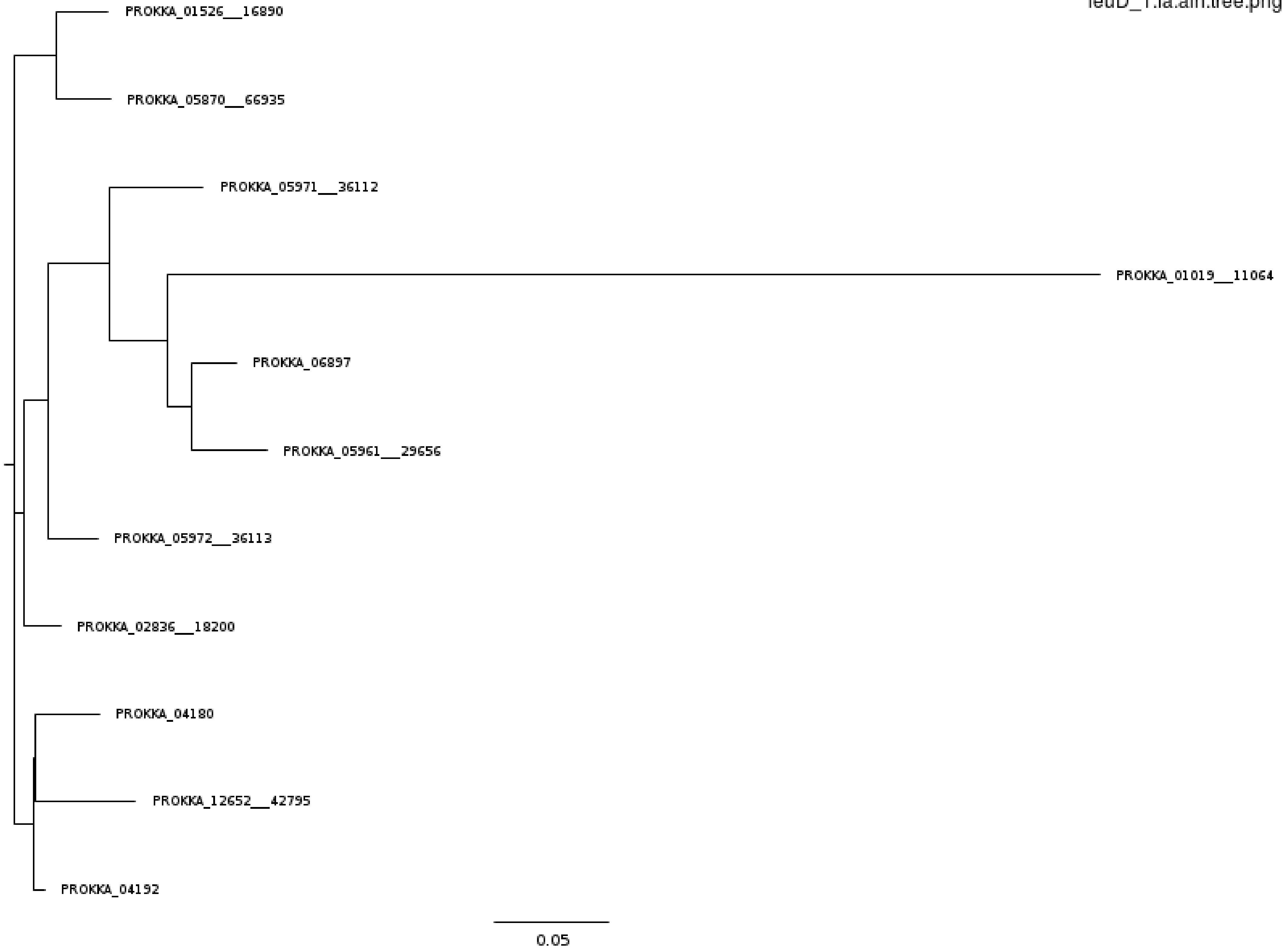

leuS_2.fa.aln.tree.png

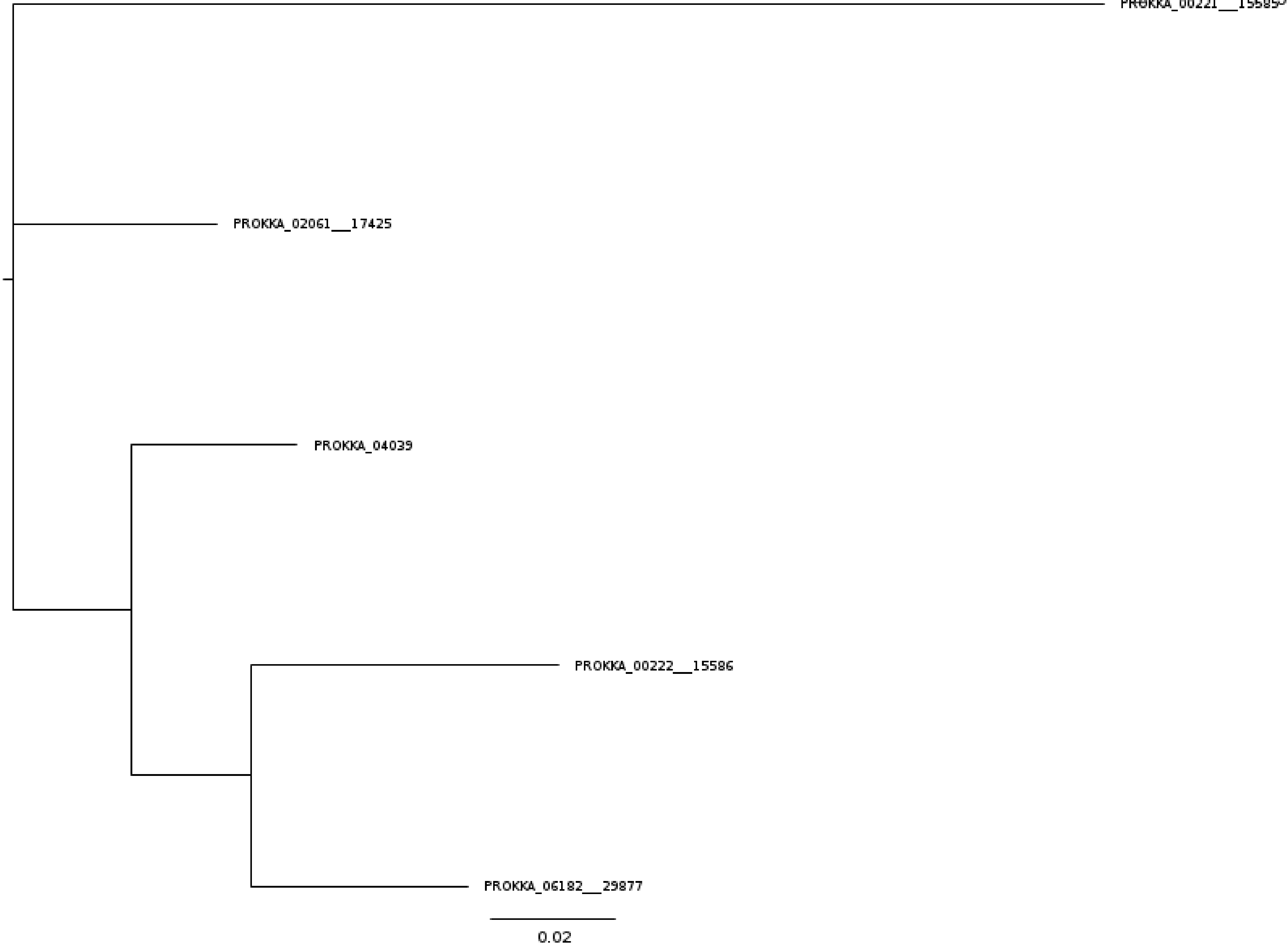

lgt_3.fa.aln.tree.png

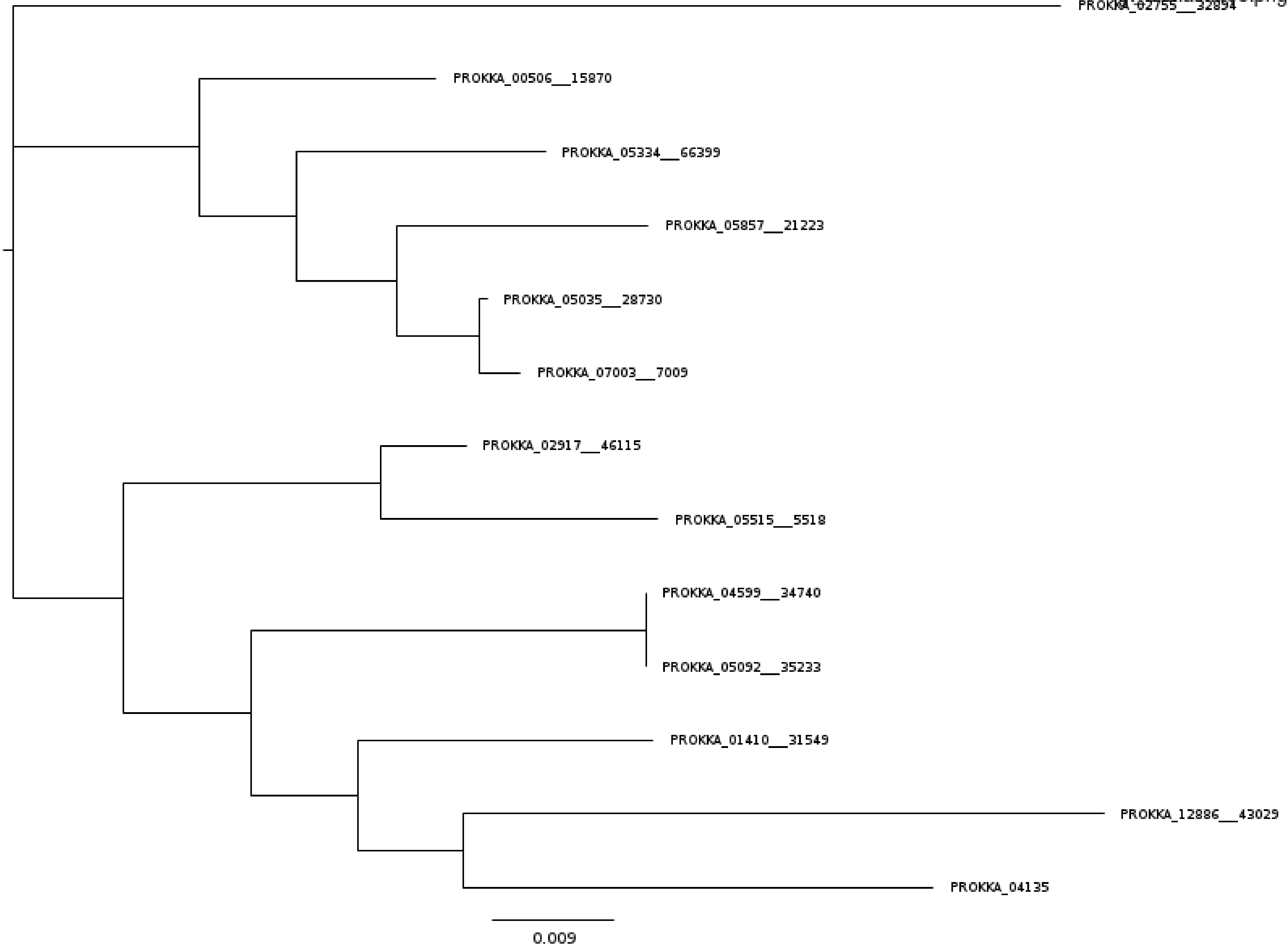

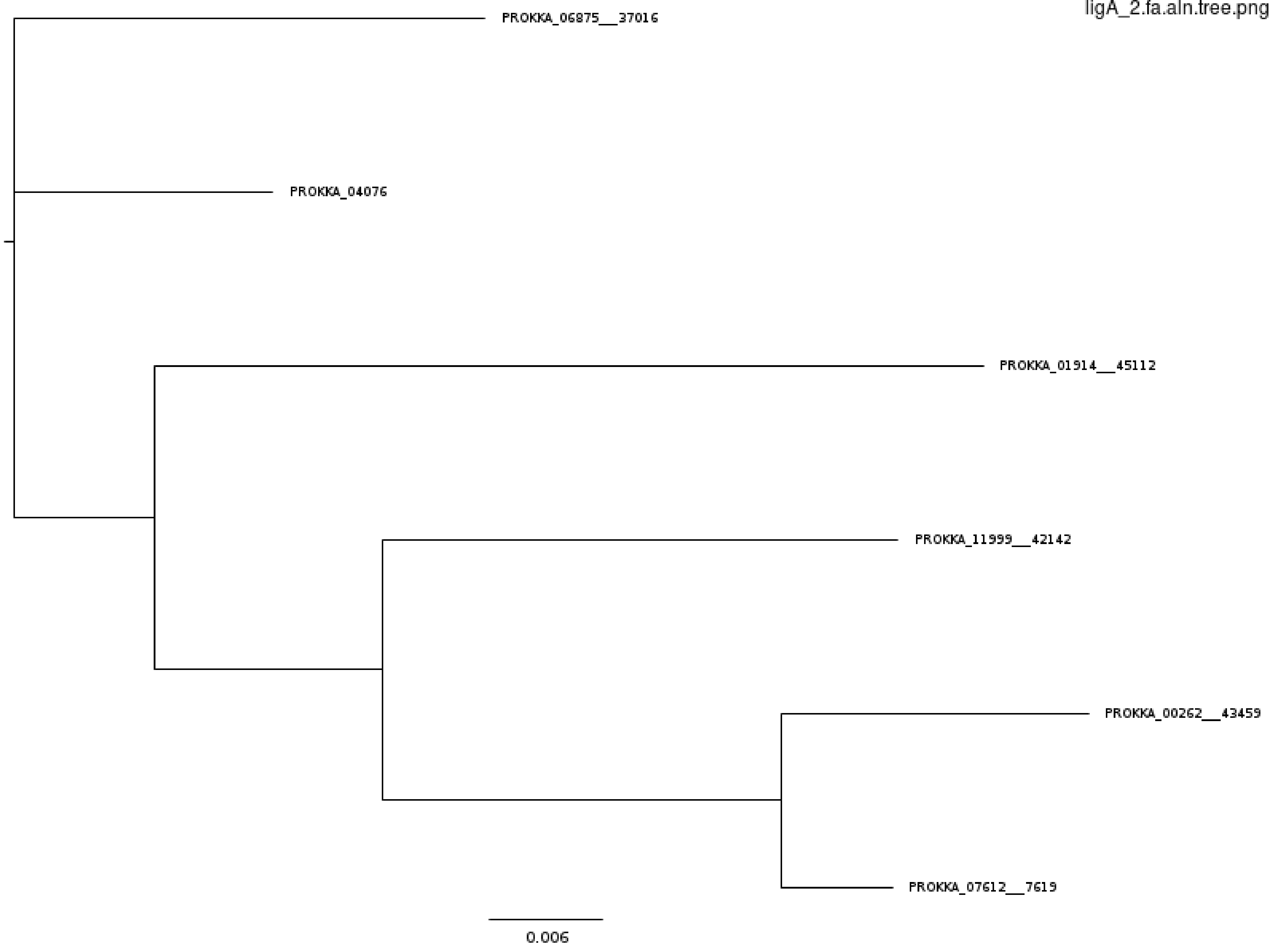
ligA_2.fa.aln.tree.png
PROKKA_06875___37016
PROKKA_04076
PROKKA_01914___45112
PROKKA_11999___42142
PROKKA_00262___43459
PROKKA_07612___7619
0.006

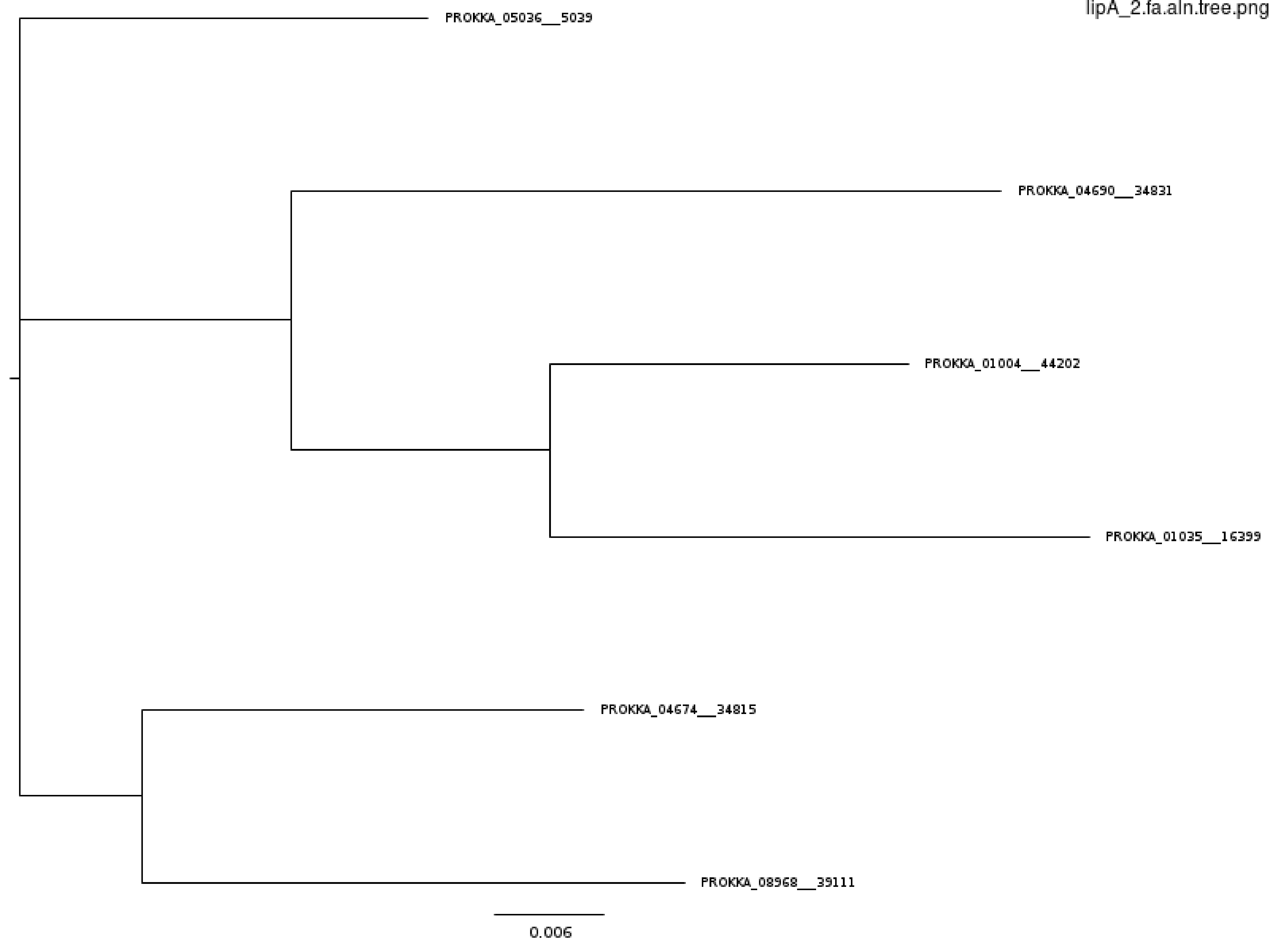
lipA_2.fa.aln.tree.png
PROKKA_05036___5039
PROKKA_04690___34831
PROKKA_01004___44202
PROKKA_01035___16399
PROKKA_04674___34815
PROKKA_08968___39111
0.006

lipB.fa.aln.tree.png

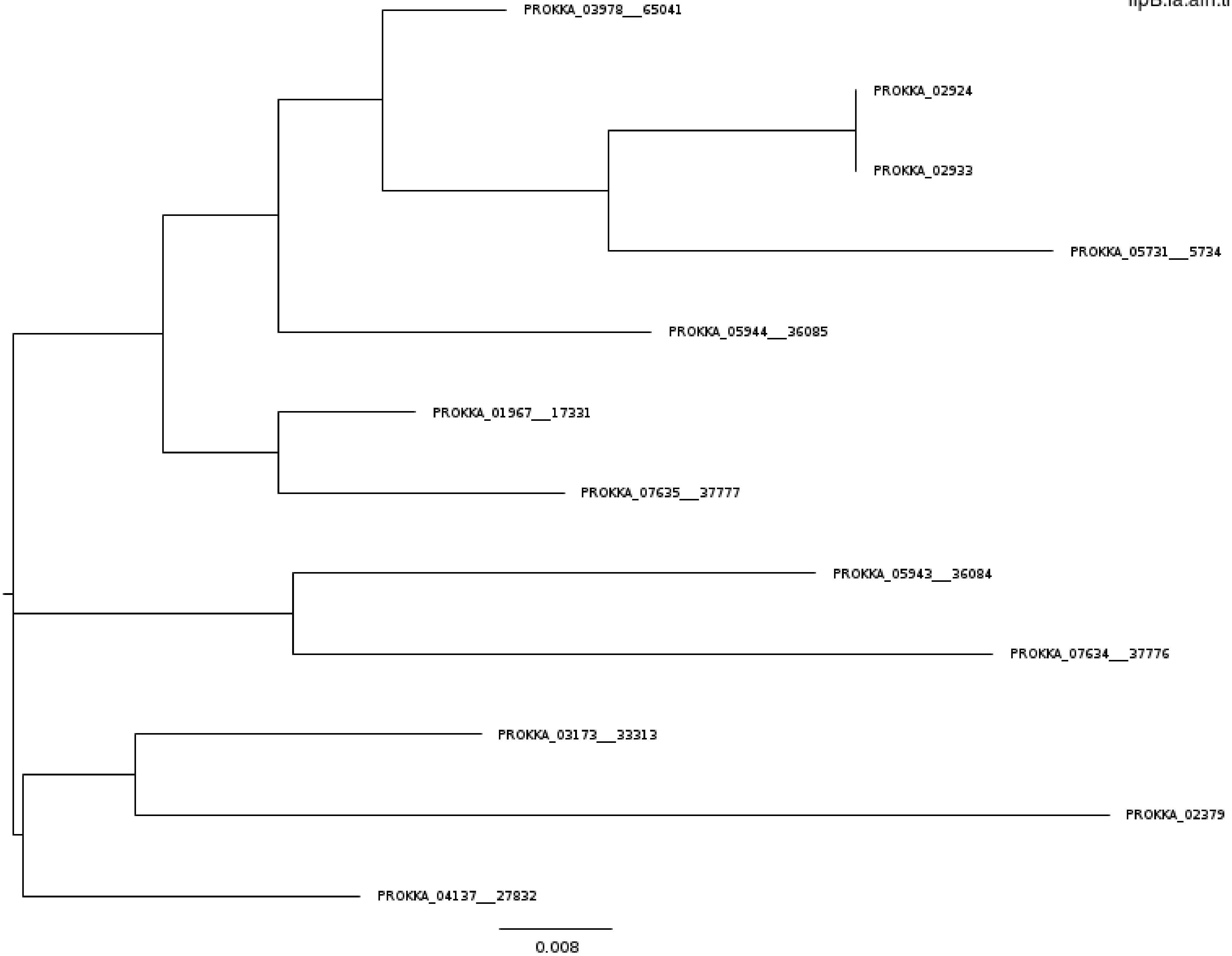

livF_1.fa.aln.tree.png

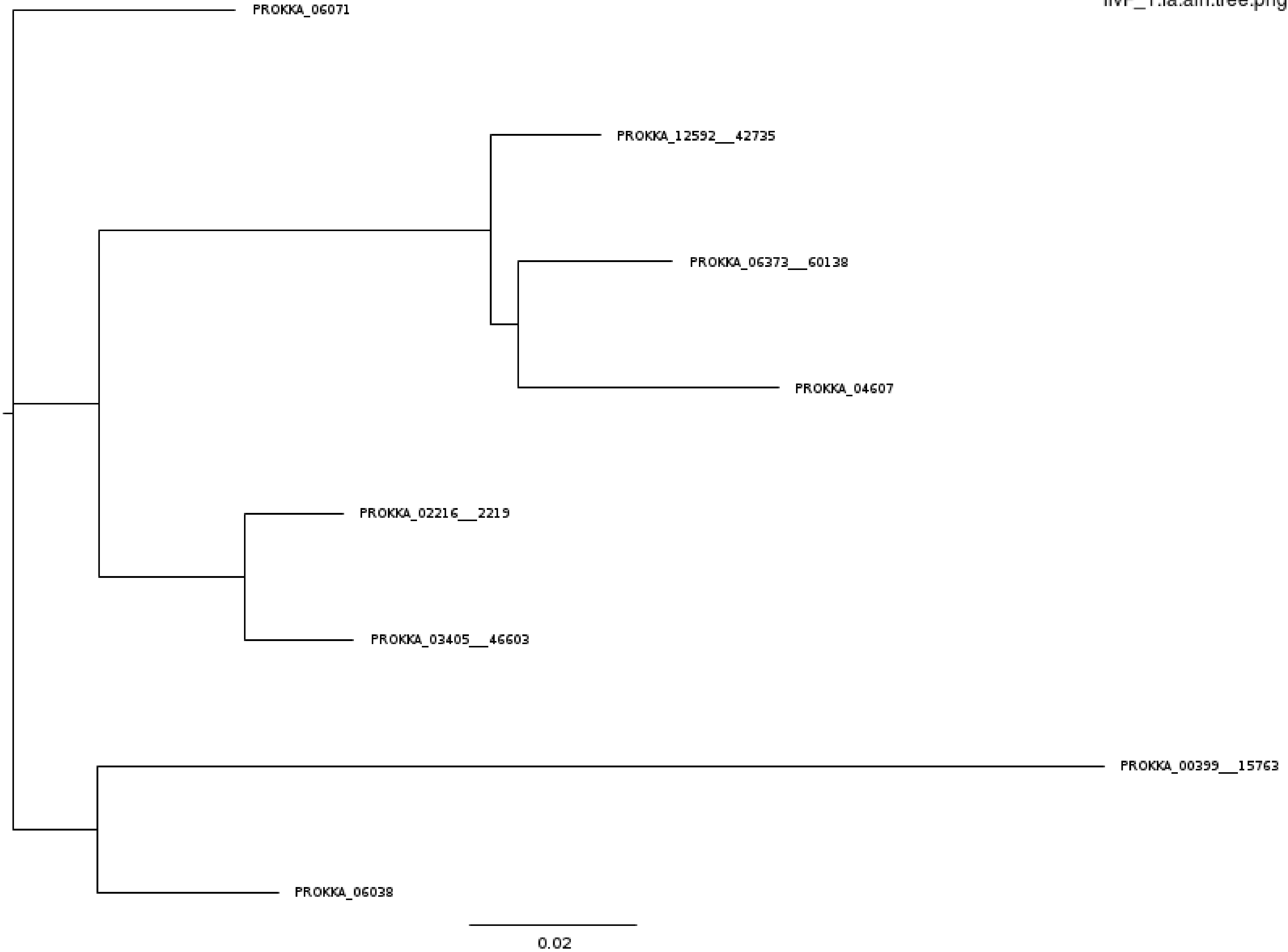

livF_2.fa.aln.tree.png

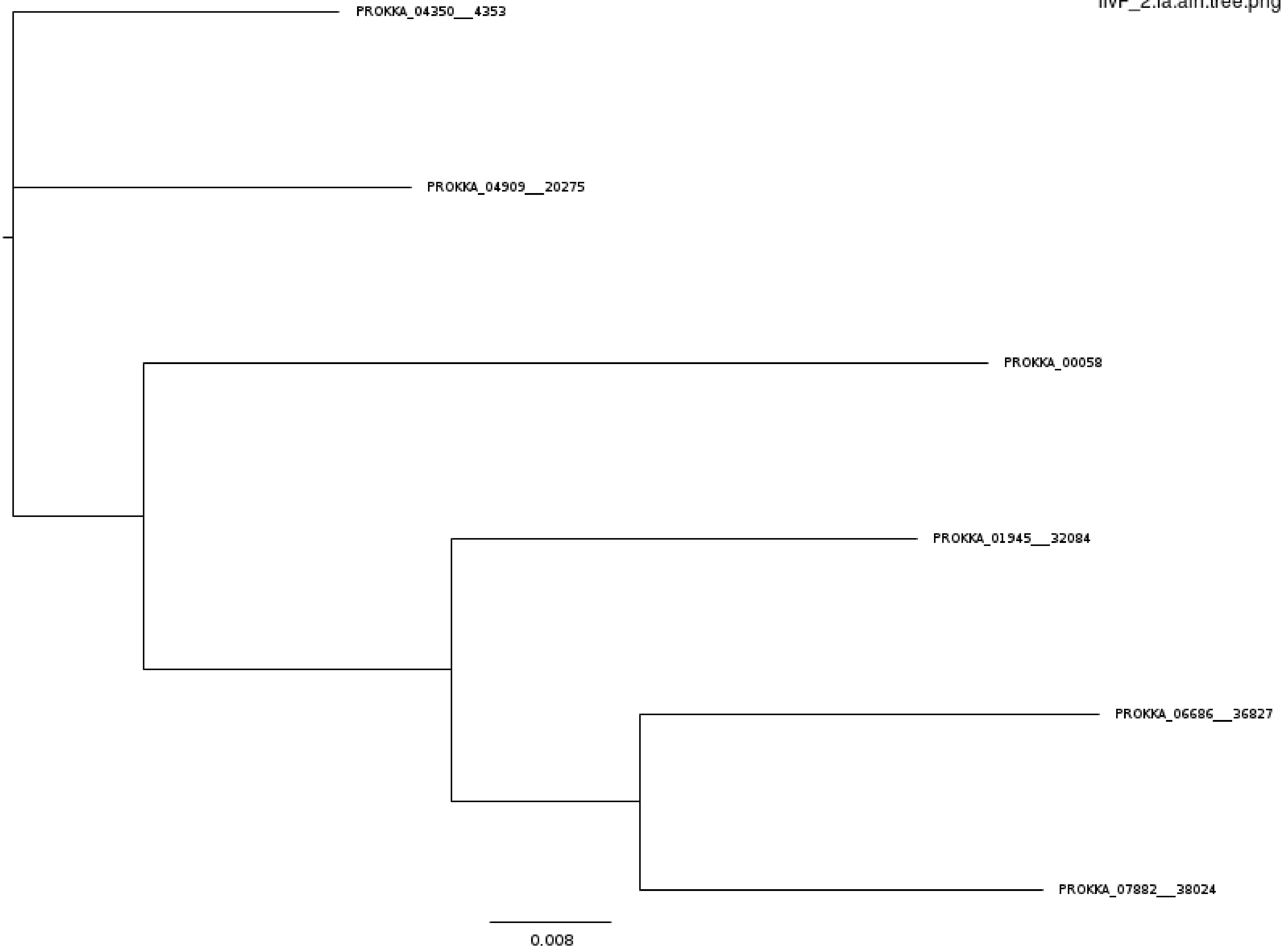

livH_2.fa.aln.tree.png

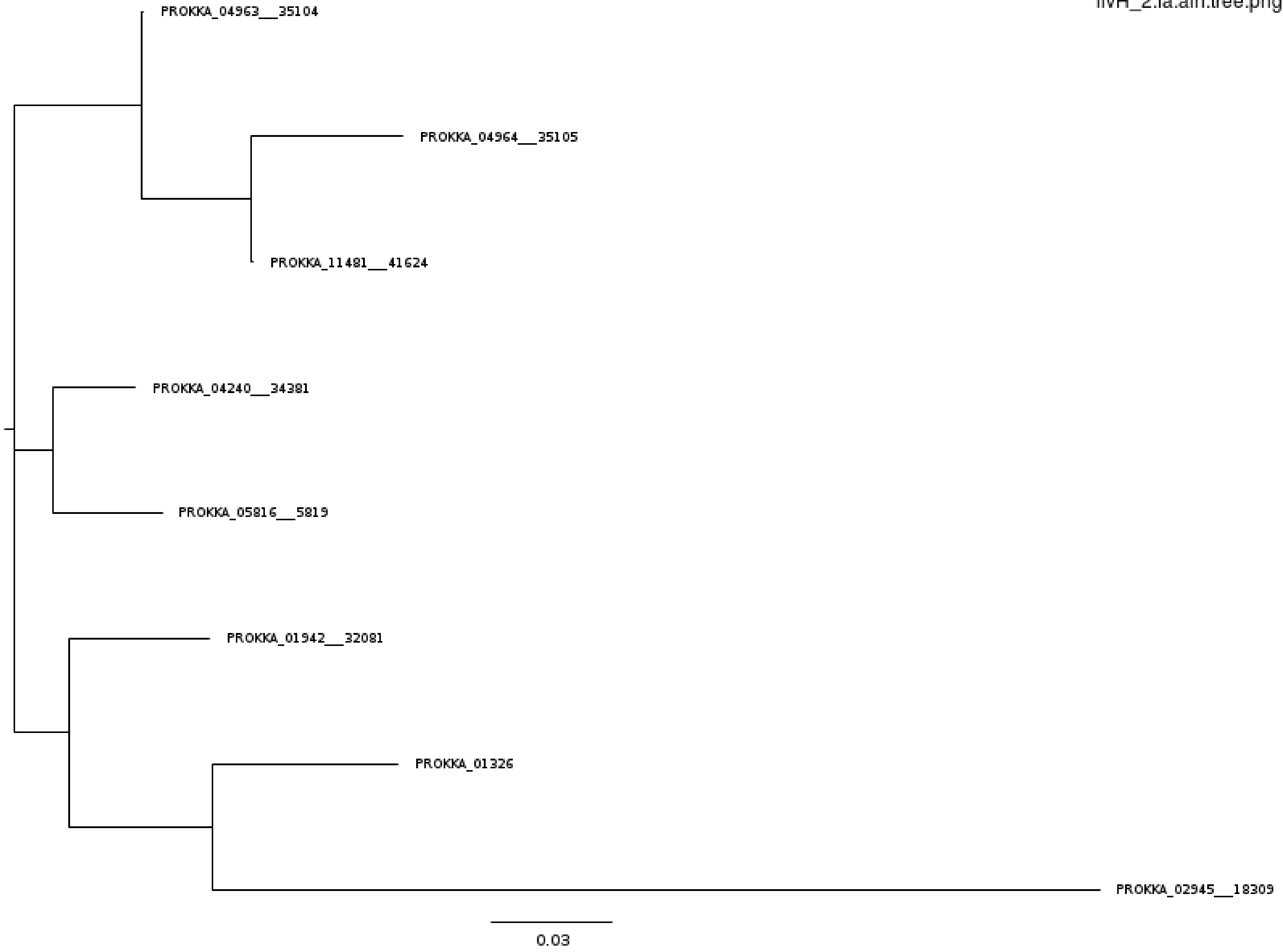

livH_3.fa.aln.tree.png

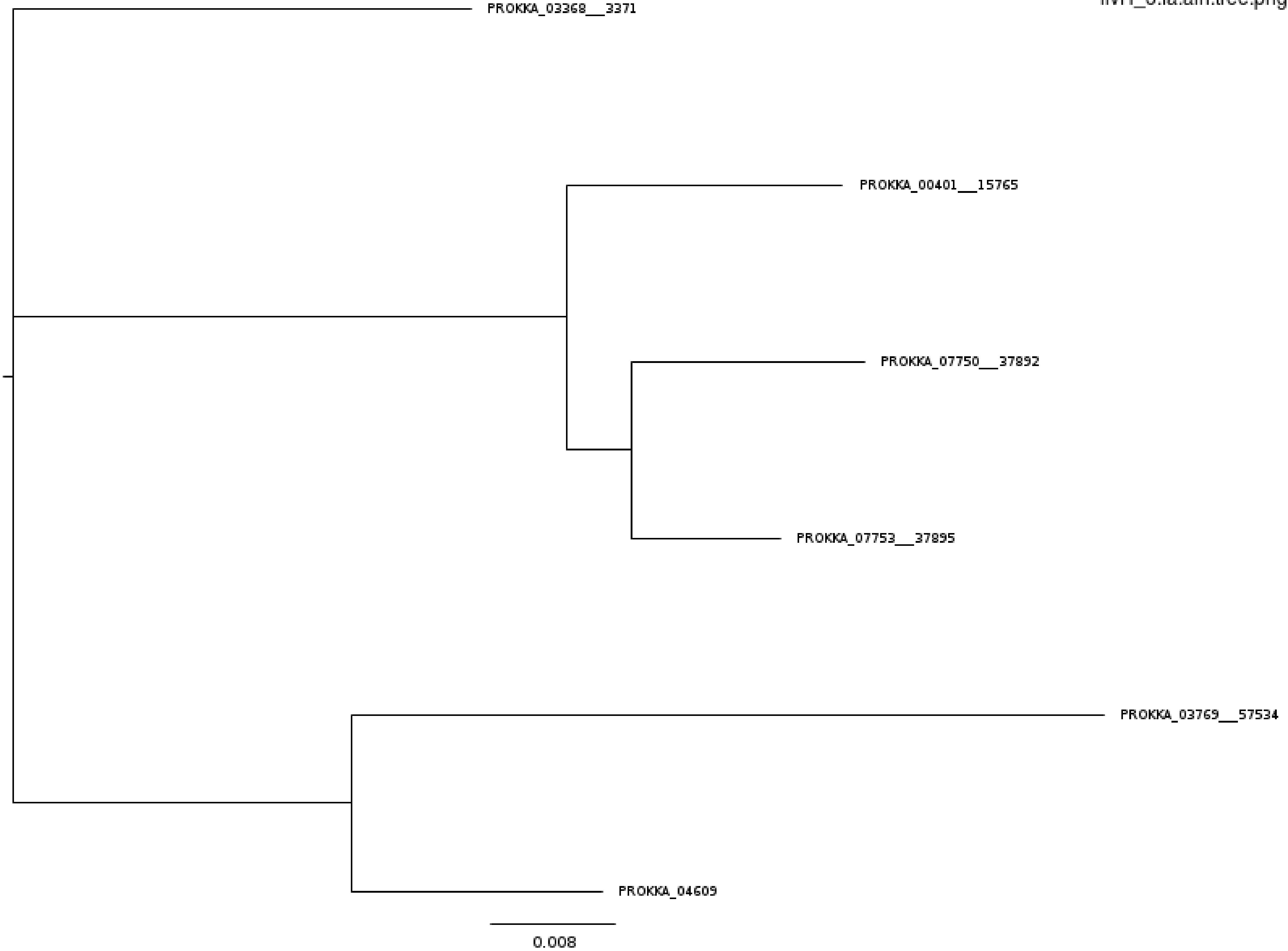

livK_2.fa.aln.tree.png

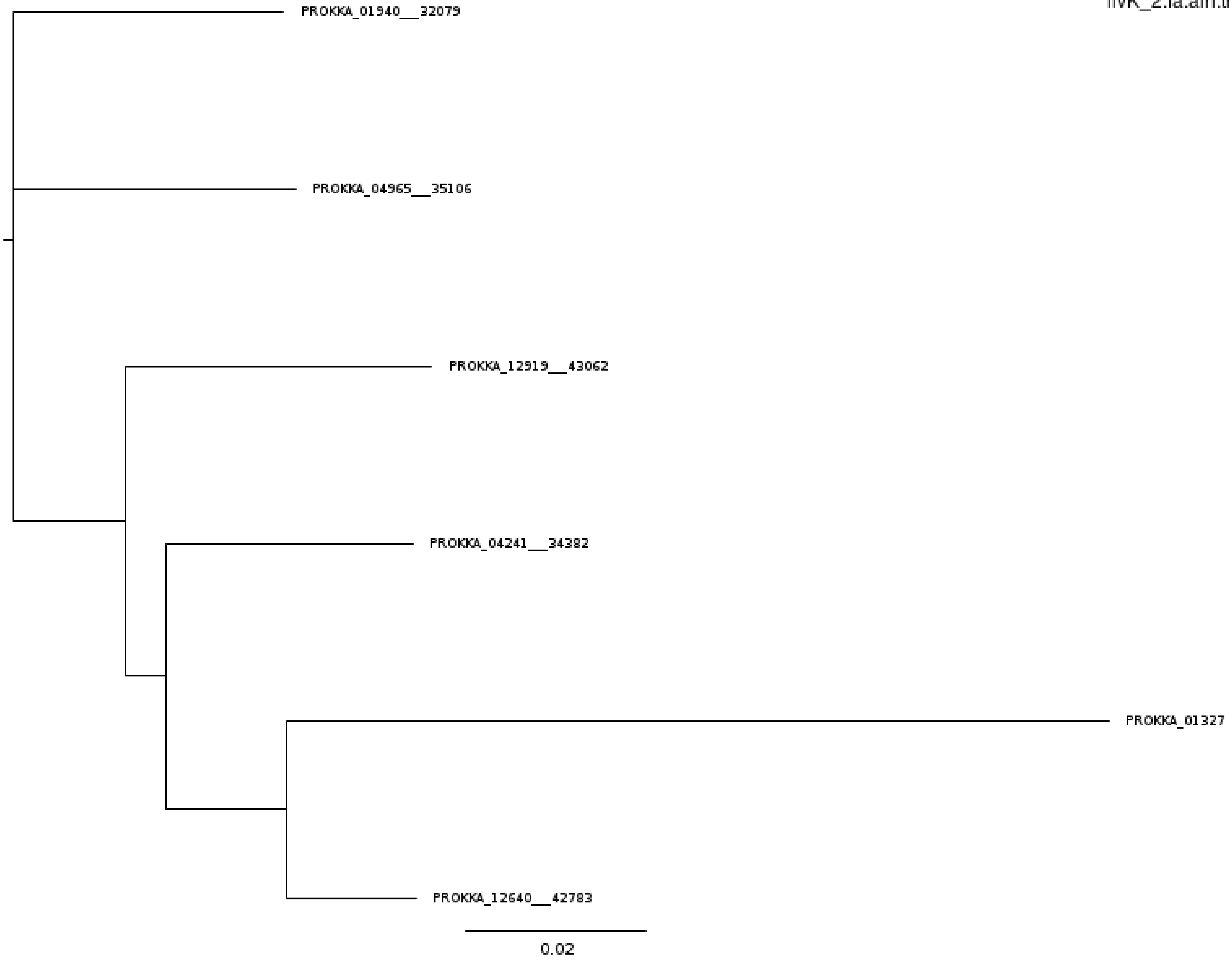

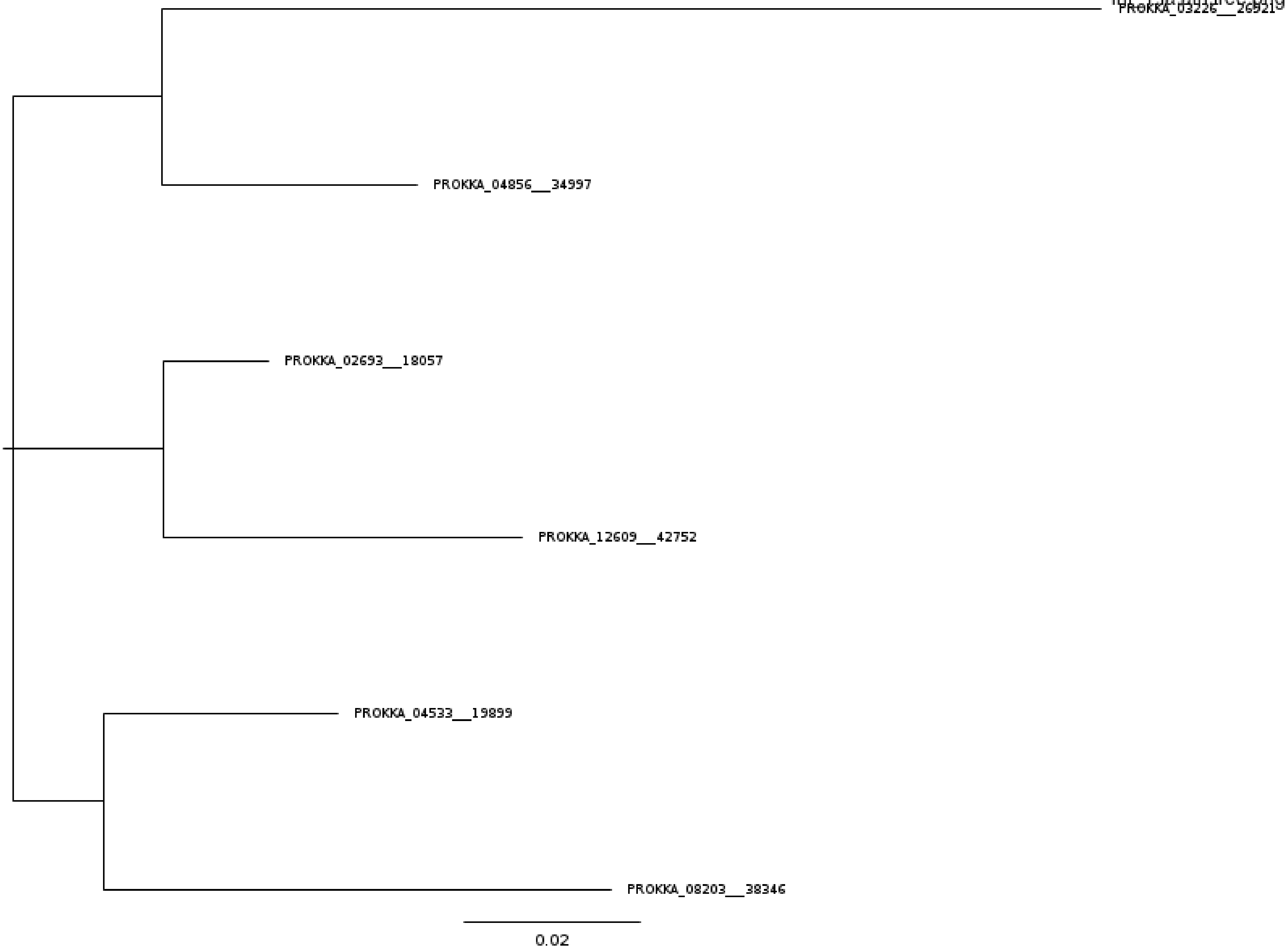
PROKKA_03226___26921
PROKKA_04856___34997
PROKKA_02693___18057
PROKKA_12609___42752
PROKKA_04533___19899
PROKKA_08203___38346
0.02

lolA_2.fa.aln.tree.png

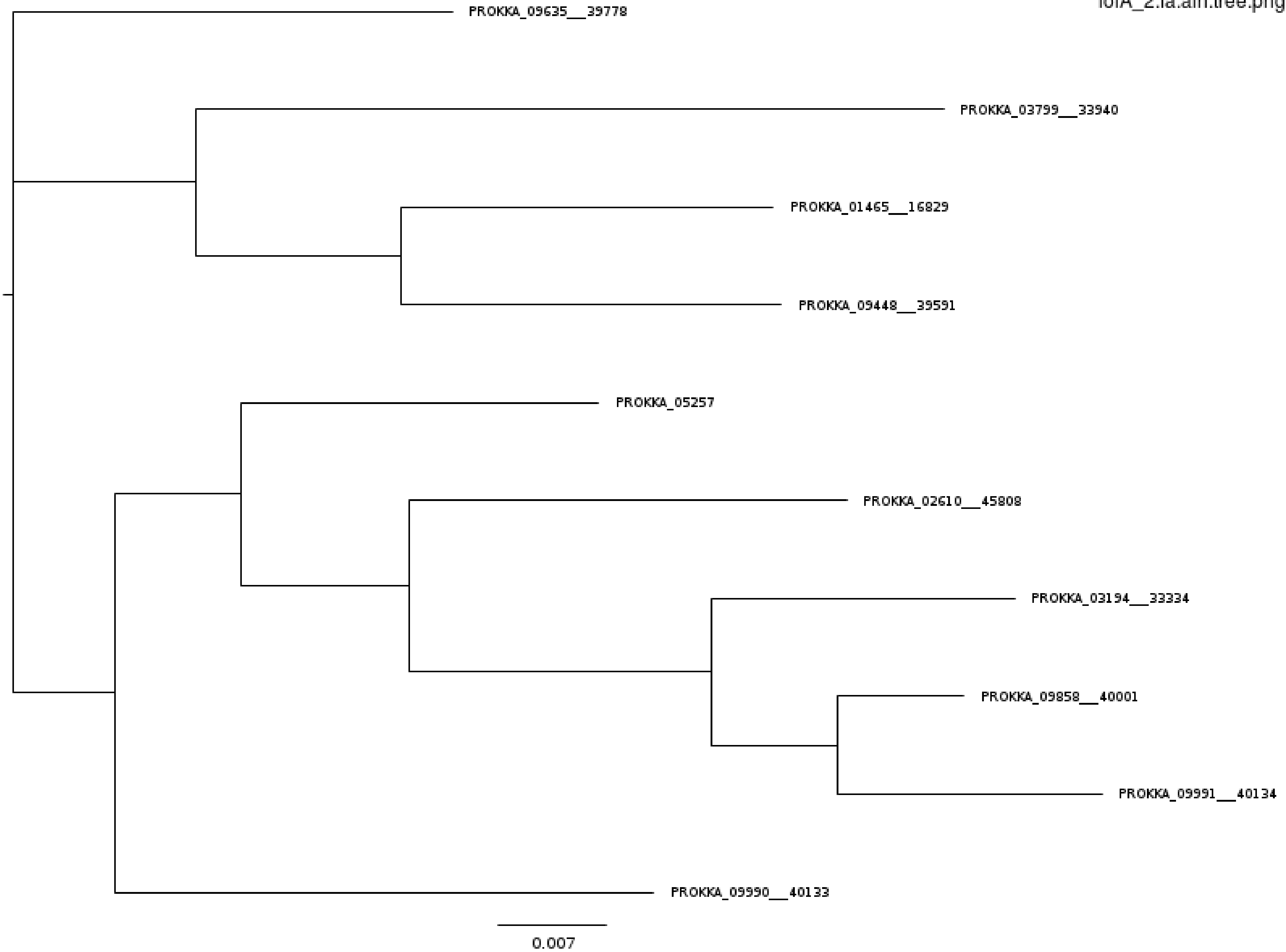

lolD_6.fa.aln.tree.png

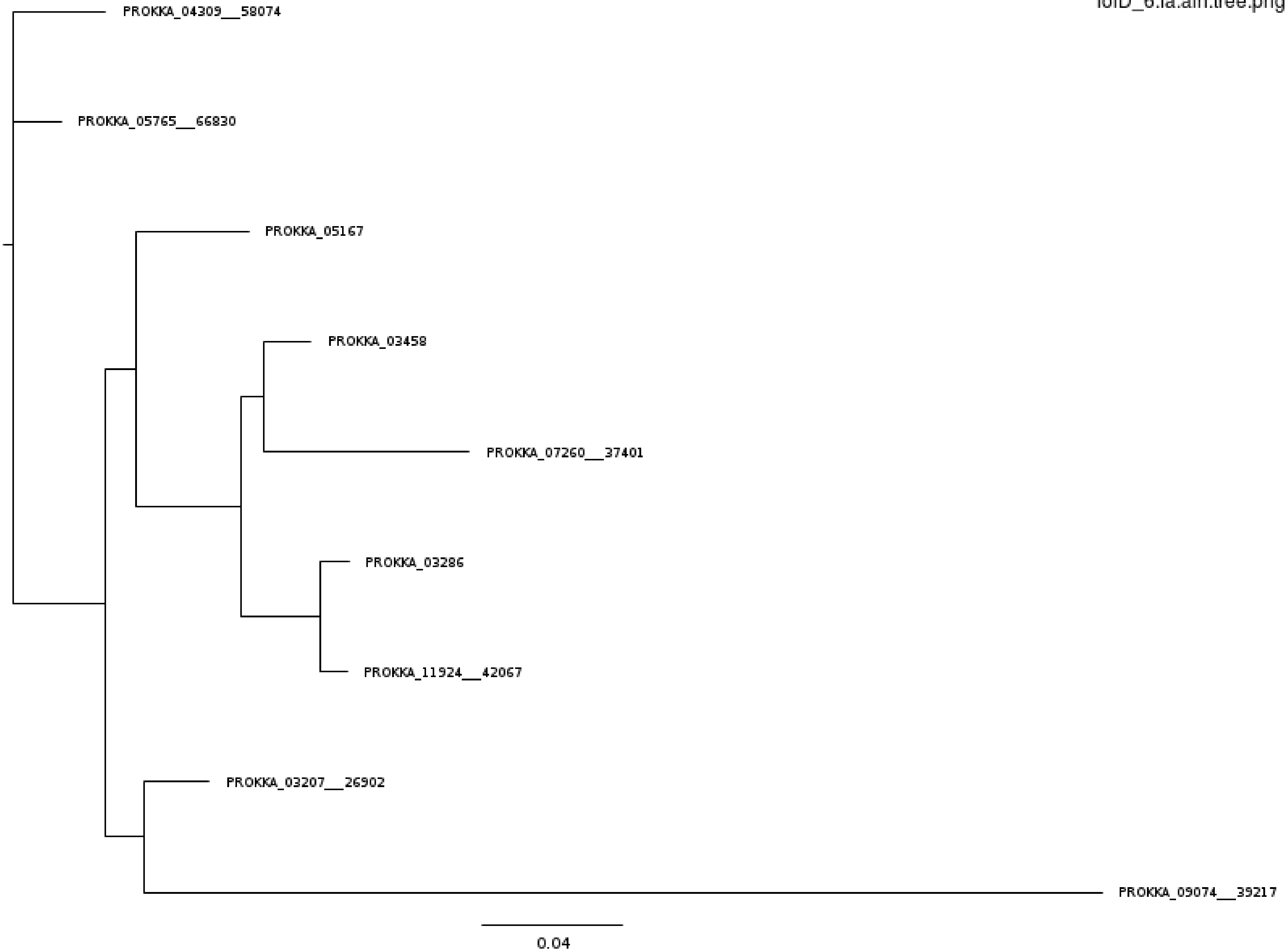

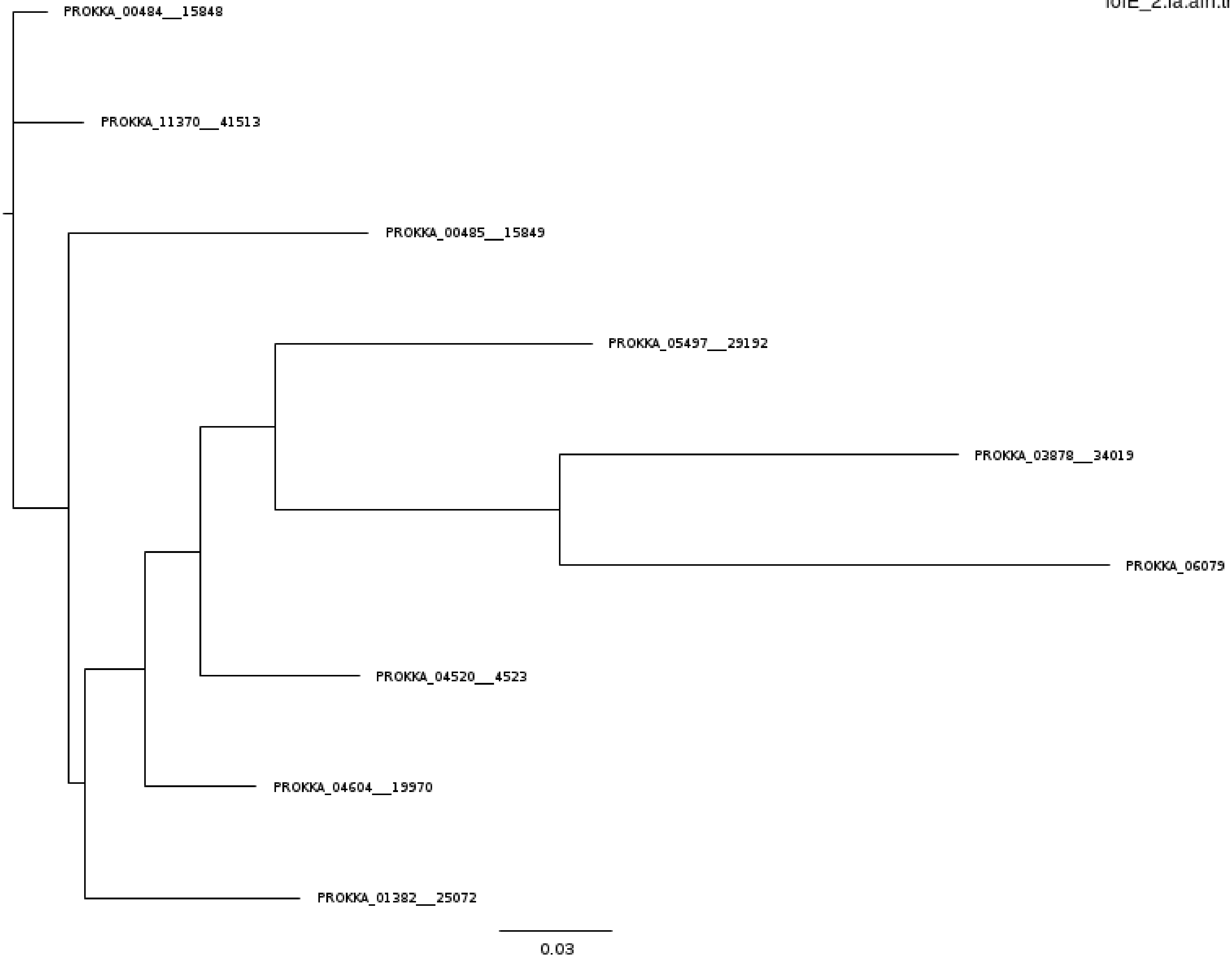
PROKKA_00484___15848
PROKKA_11370___41513
PROKKA_00485___15849
PROKKA_05497___29192
PROKKA_03878___34019
PROKKA_06079
PROKKA_04520___4523
PROKKA_04604___19970
PROKKA_01382___25072
0.03

lon_1.fa.aln.tree.png

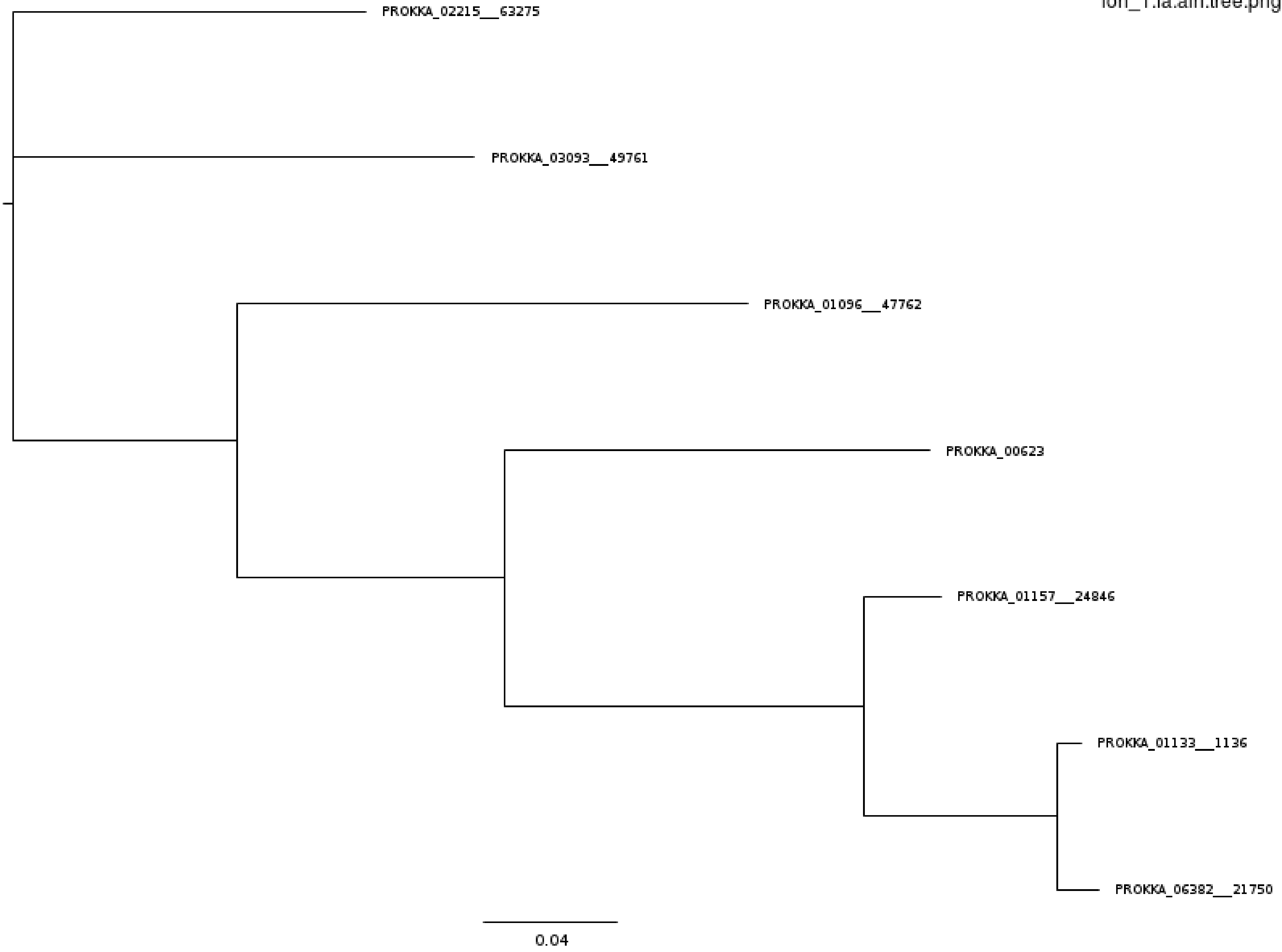

lon_2.fa.aln.tree.png

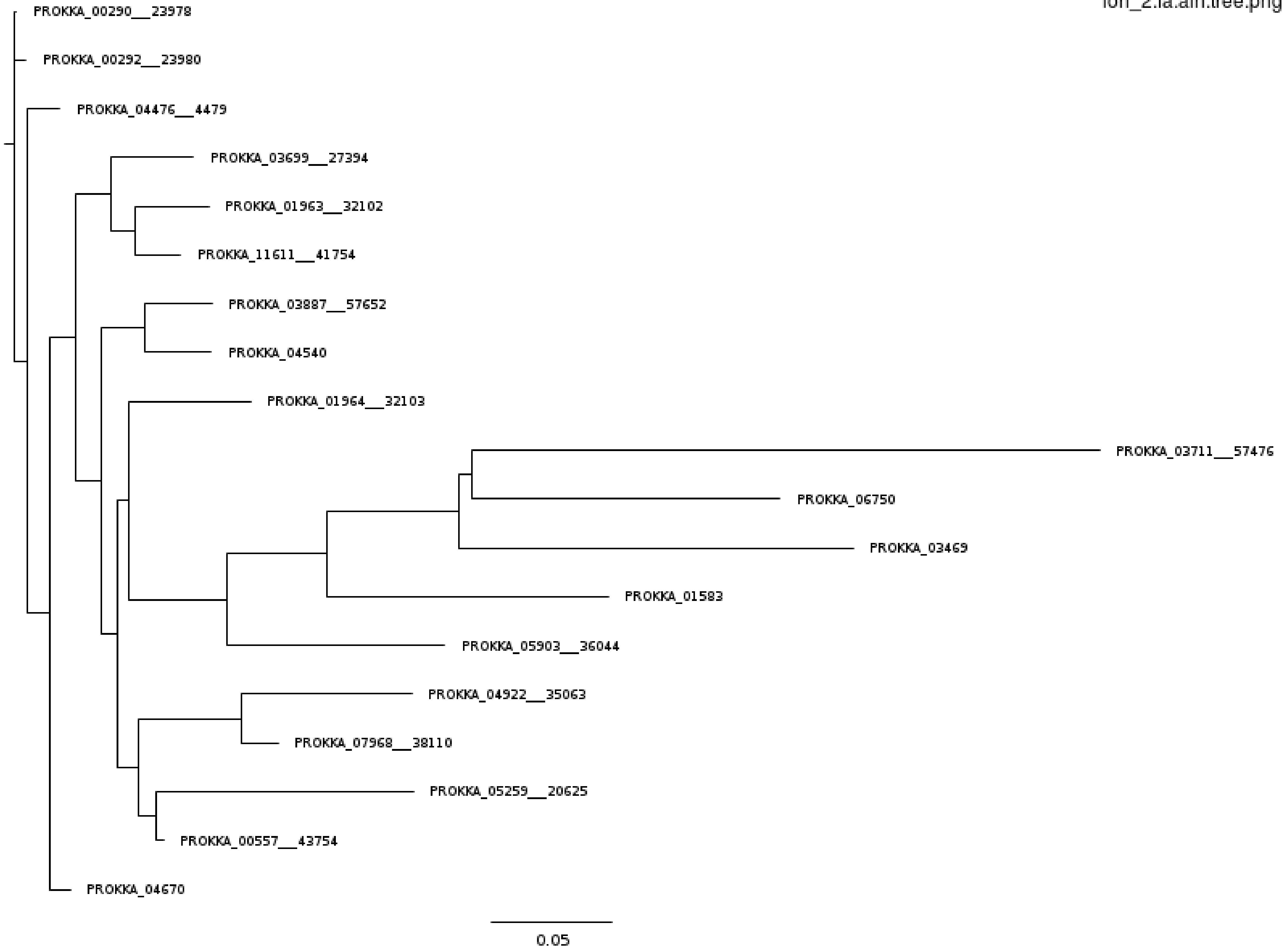

lon_3.fa.aln.tree.png

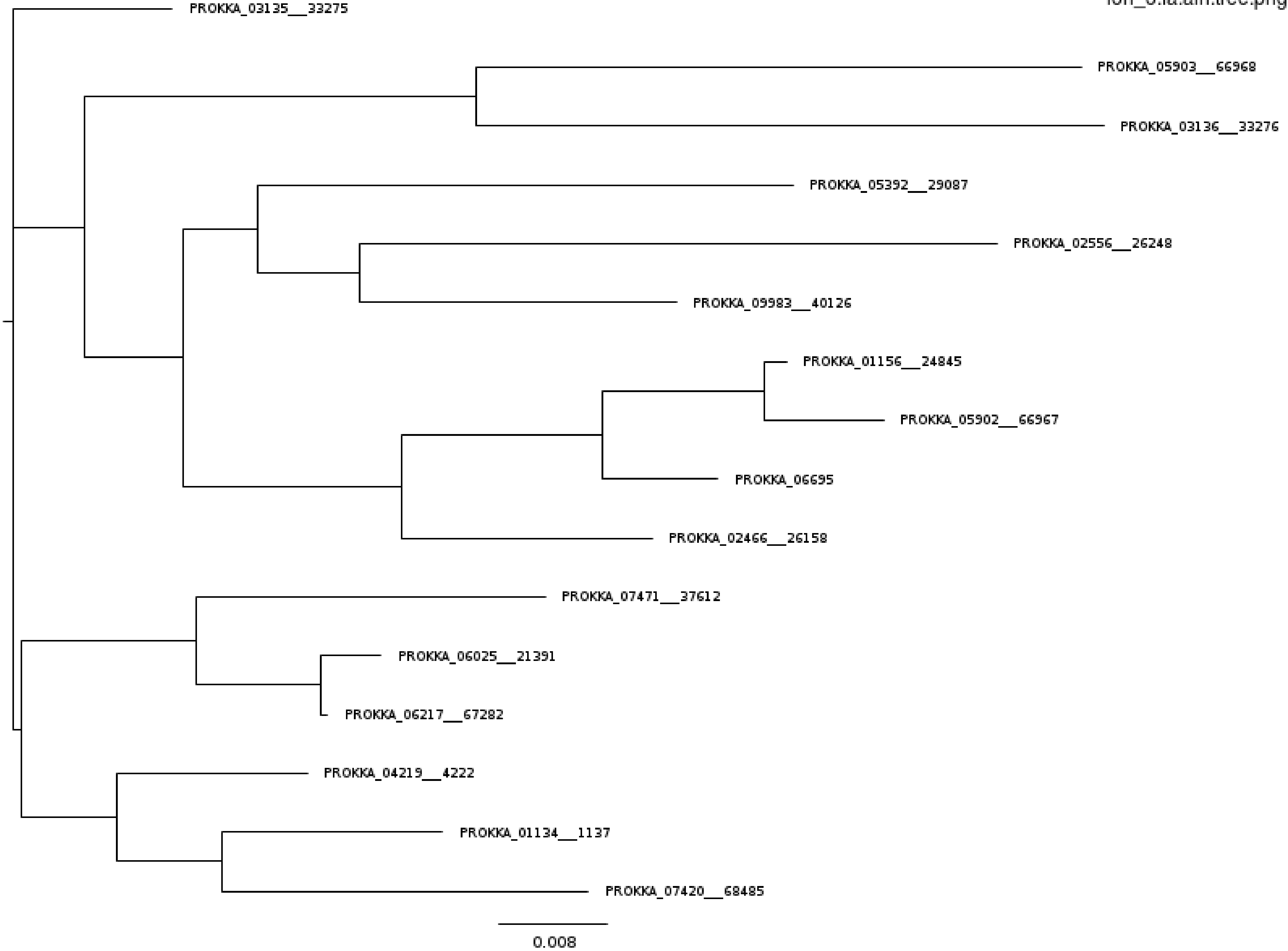

lpdA_2.fa.aln.tree.png

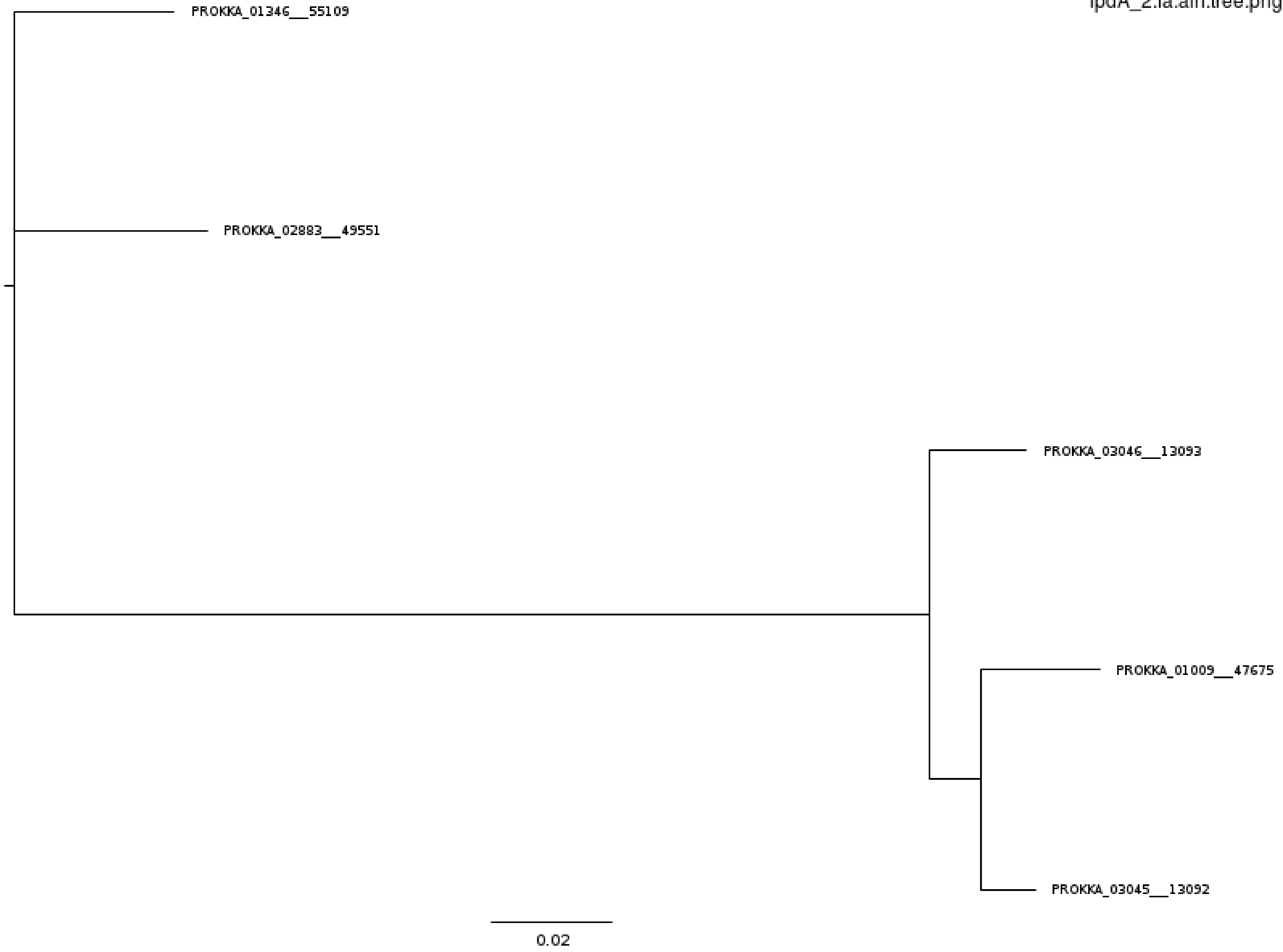

lplT.fa.aln.tree.png

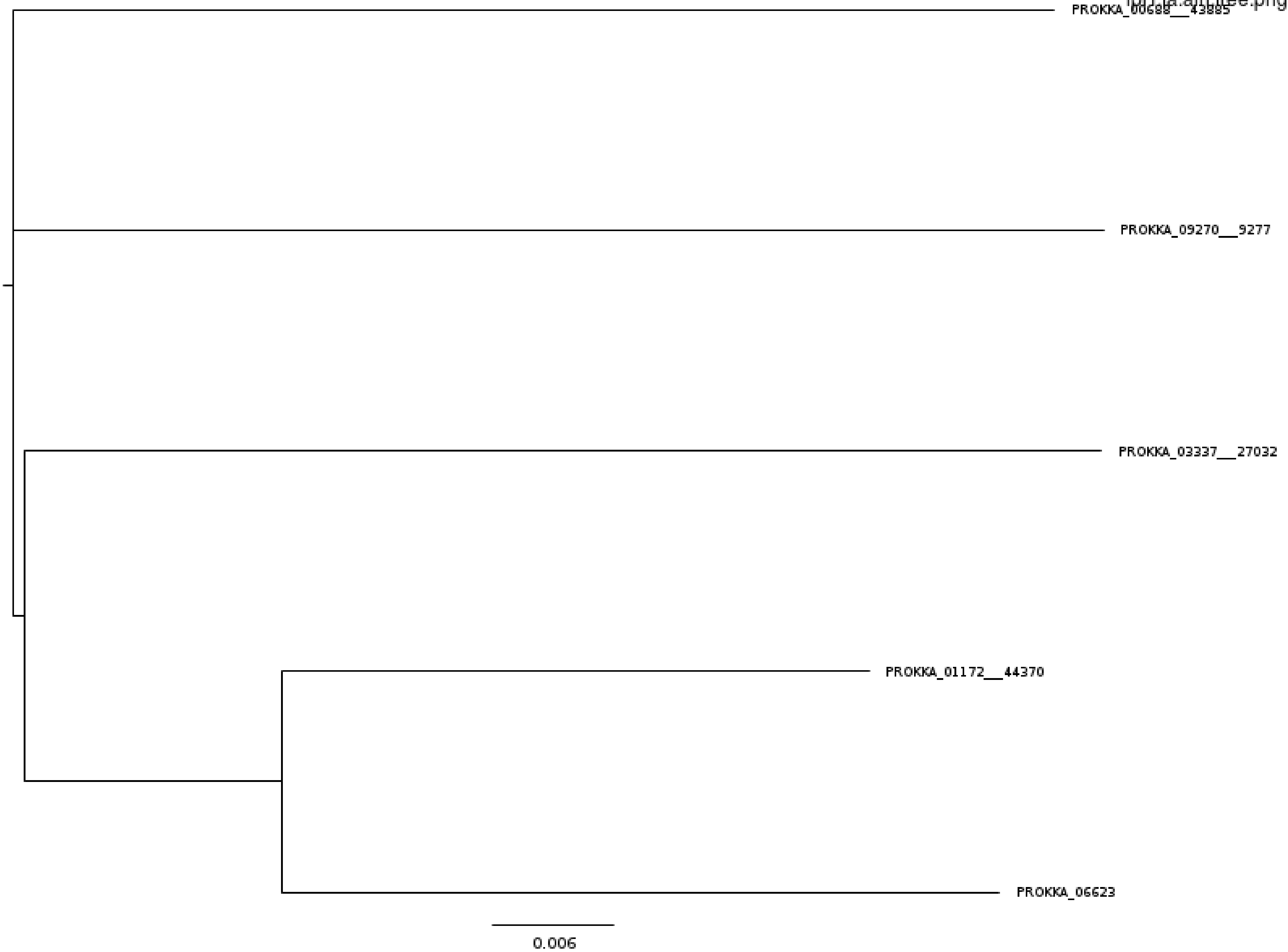

lptA_2.fa.aln.tree.png

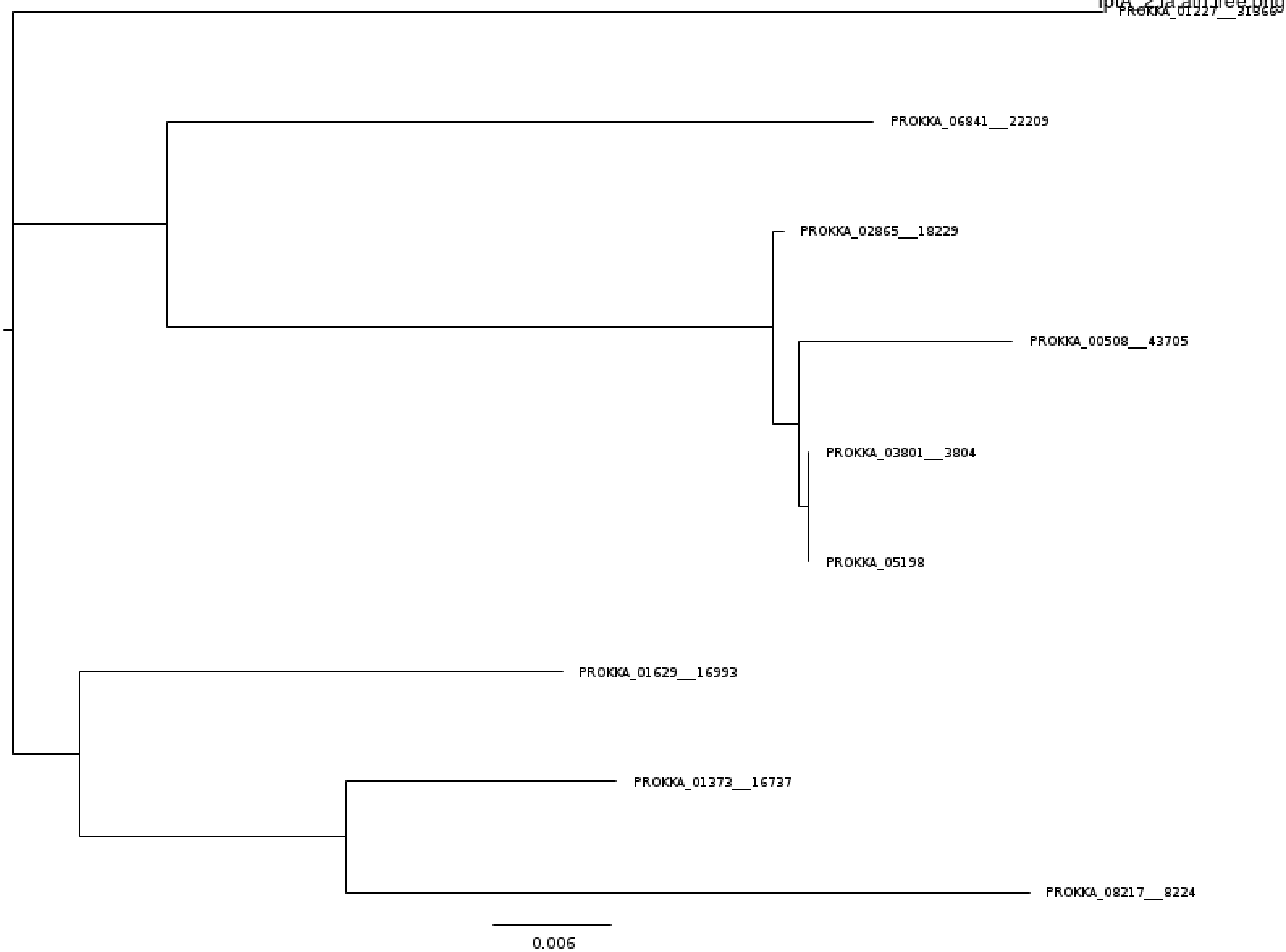

lptB_2.fa.aln.tree.png

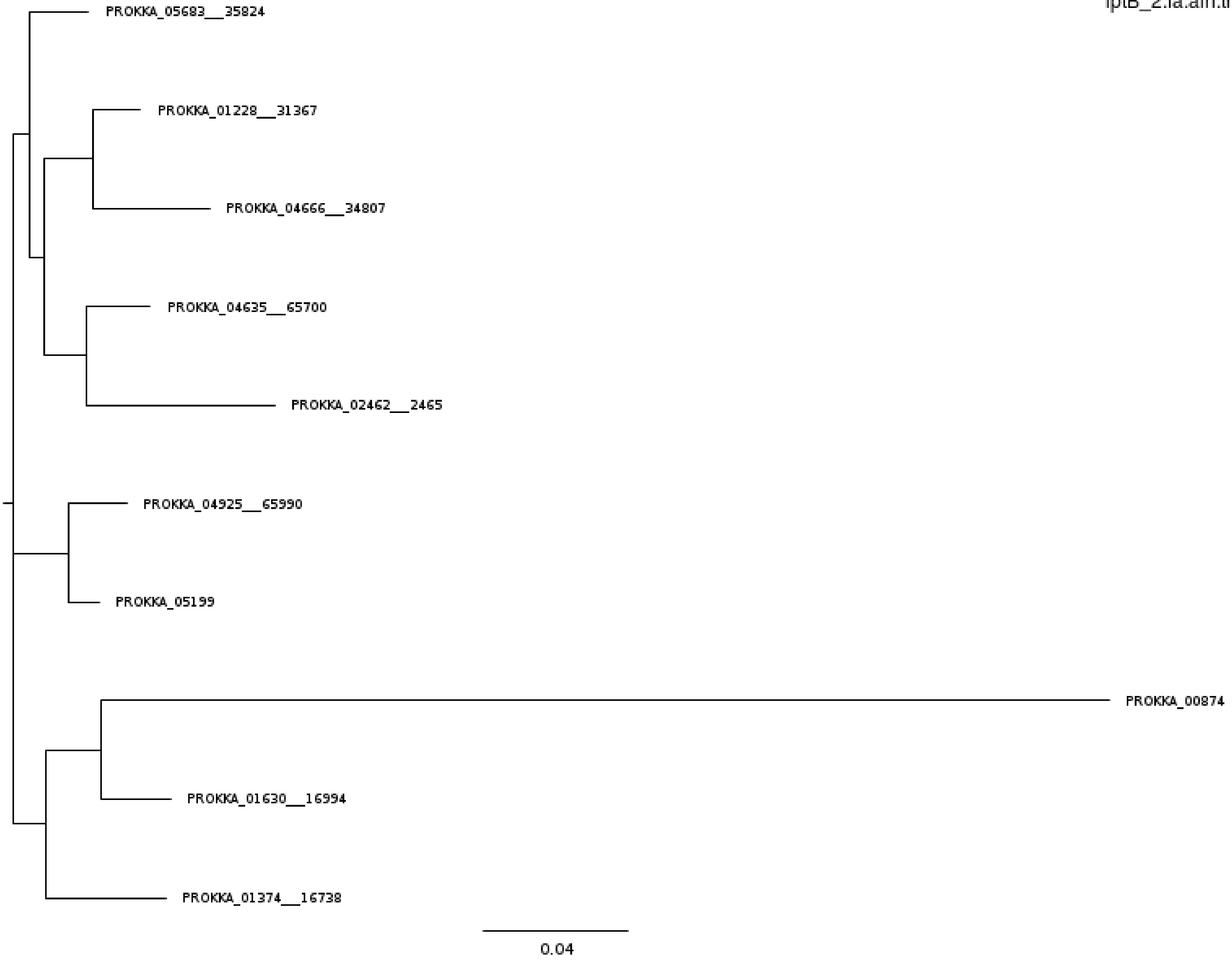

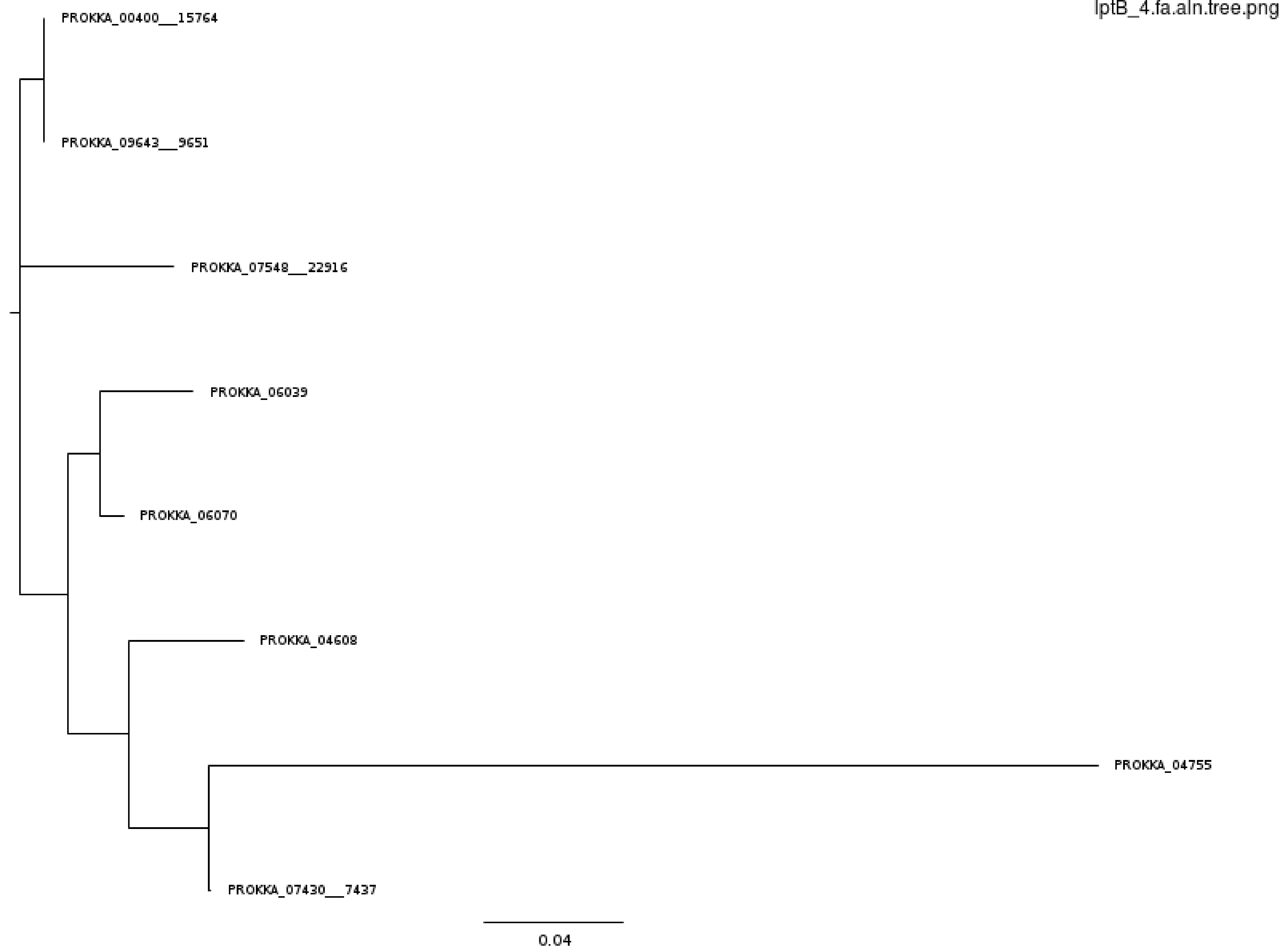
lptB_4.fa.aln.tree.png
PROKKA_00400___15764
PROKKA_09643___9651
PROKKA_07548___22916
PROKKA_06039
PROKKA_06070
PROKKA_04608
PROKKA_04755
PROKKA_07430___7437
0.04

lptB_5.fa.aln.tree.png

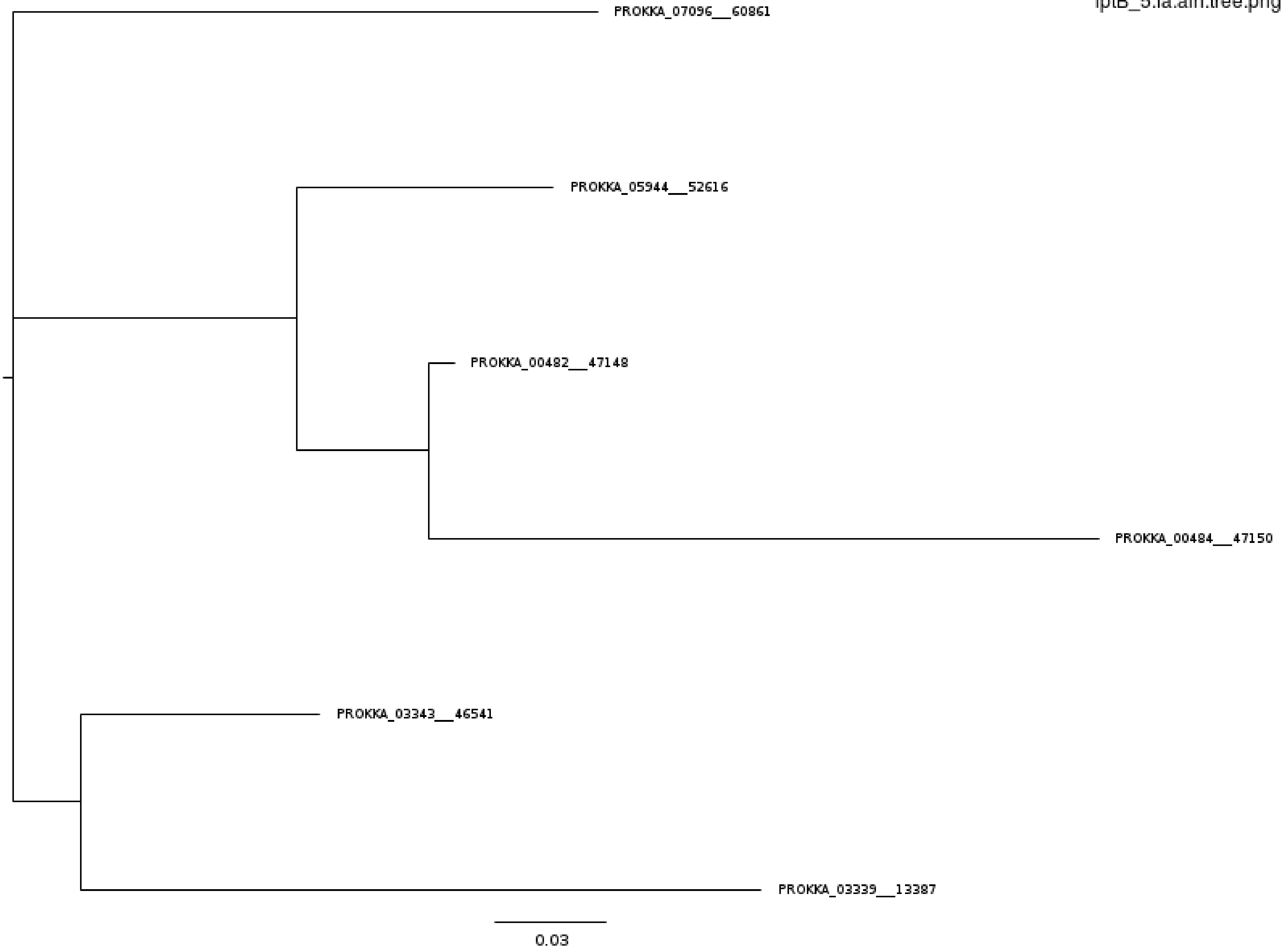

lptD_3.fa.aln.tree.png

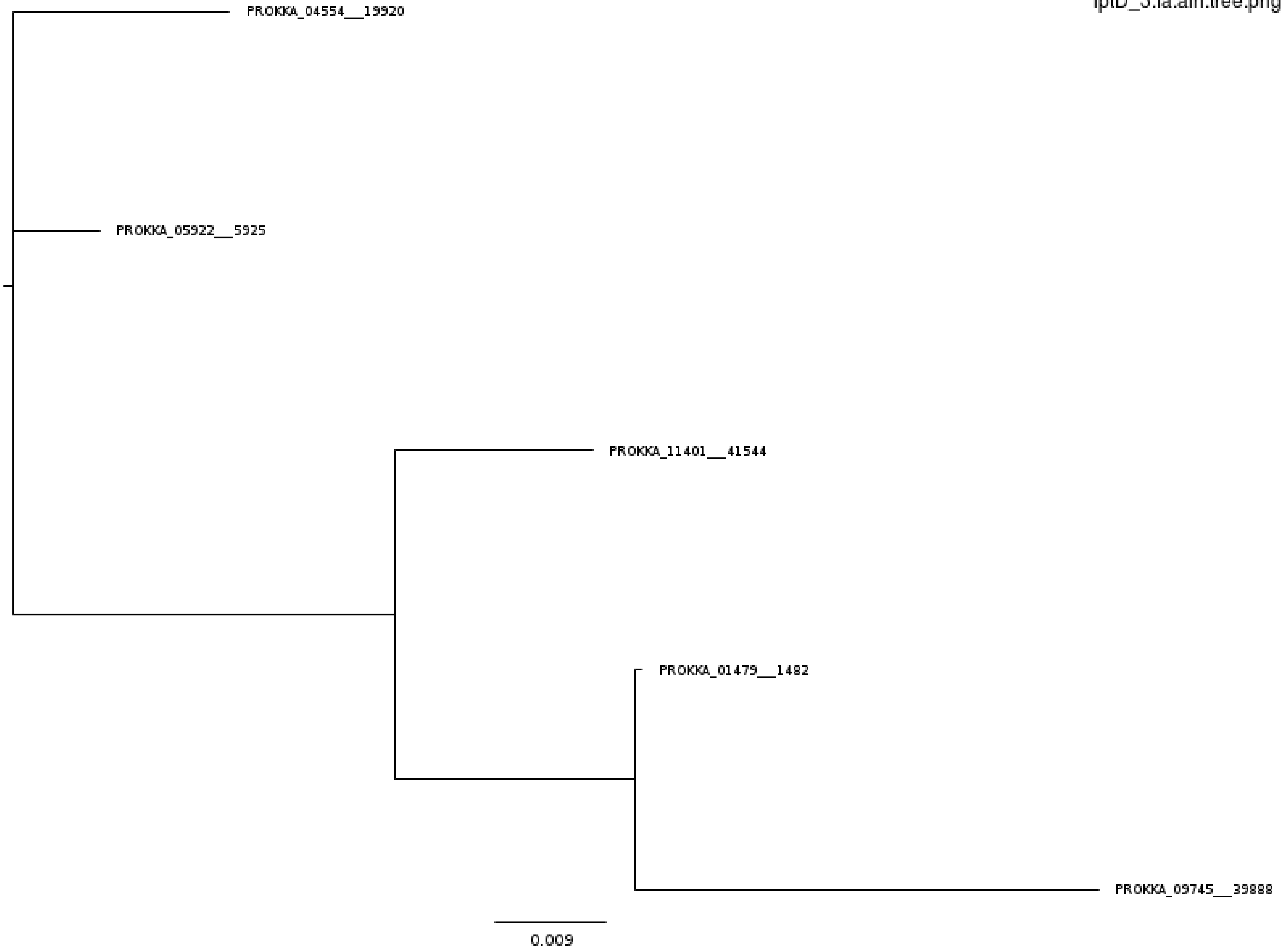

lptF_2.fa.aln.tree.png

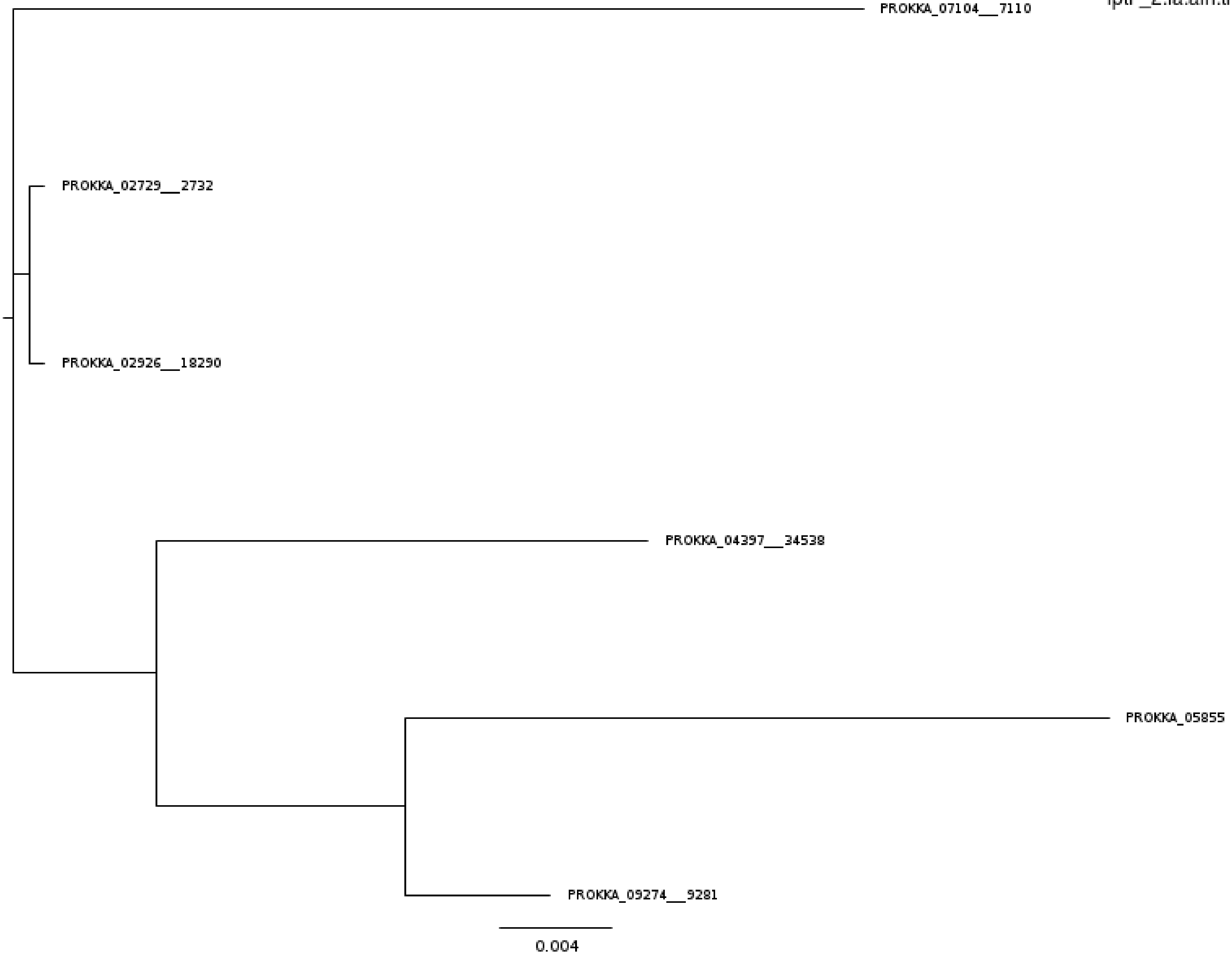

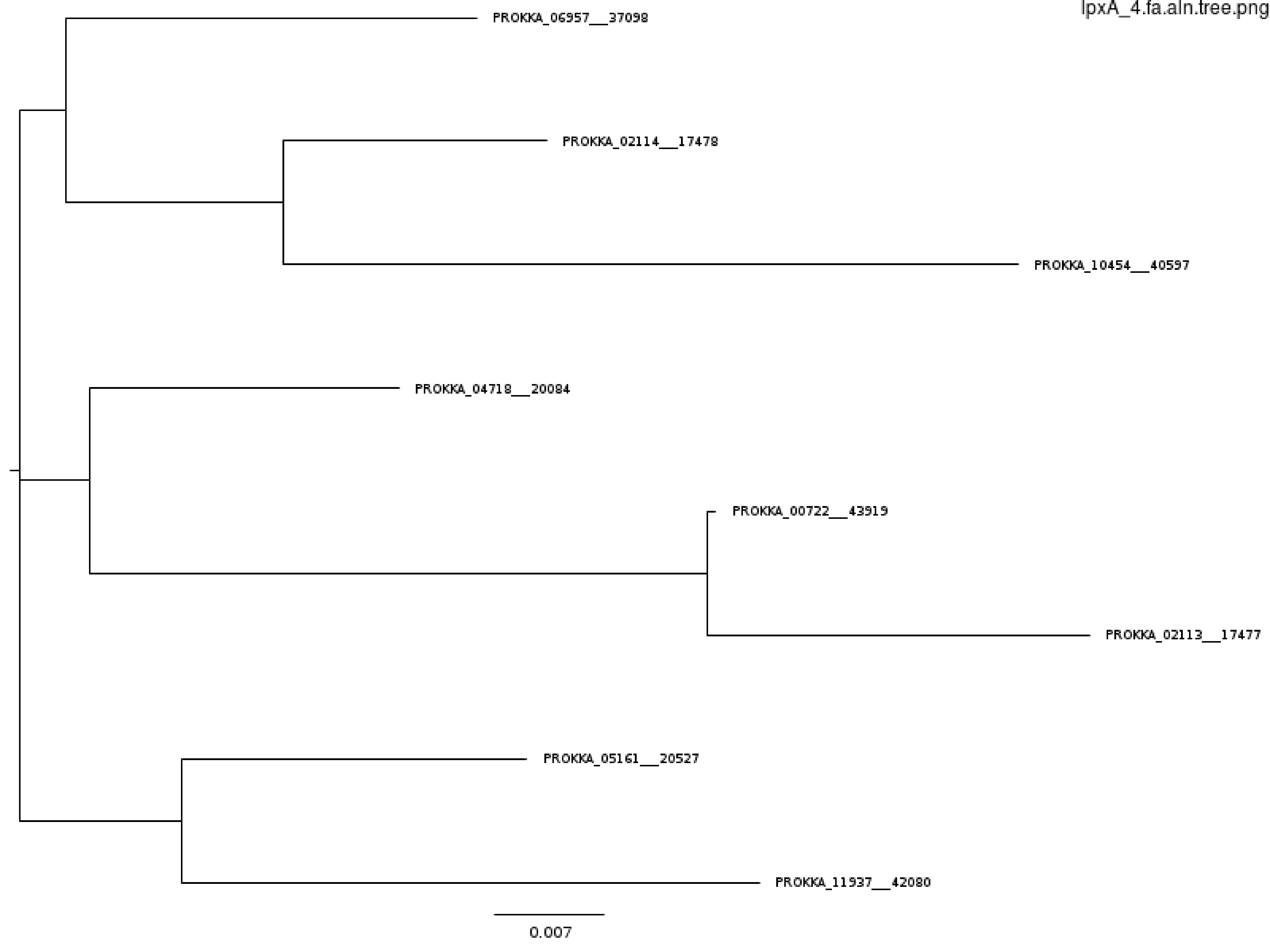
lpxA_4.fa.aln.tree.png
PROKKA_06957___37098
PROKKA_02114___17478
PROKKA_10454___40597
PROKKA_04718___20084
PROKKA_00722___43919
PROKKA_02113___17477
PROKKA_05161___20527
PROKKA_11937___42080
0.007

lpxA_7.fa.aln.tree.png

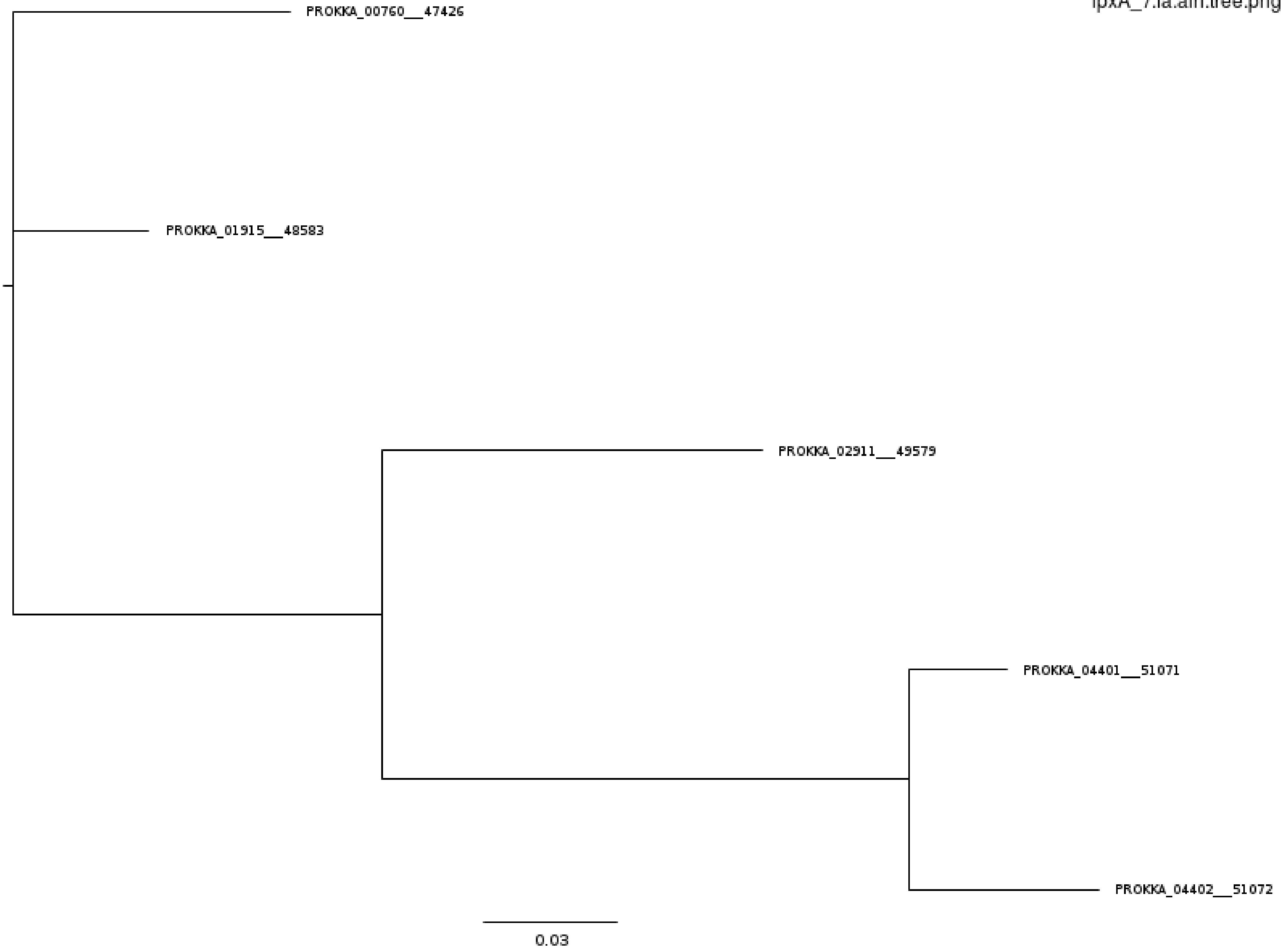

lpxB_2.fa.aln.tree.png

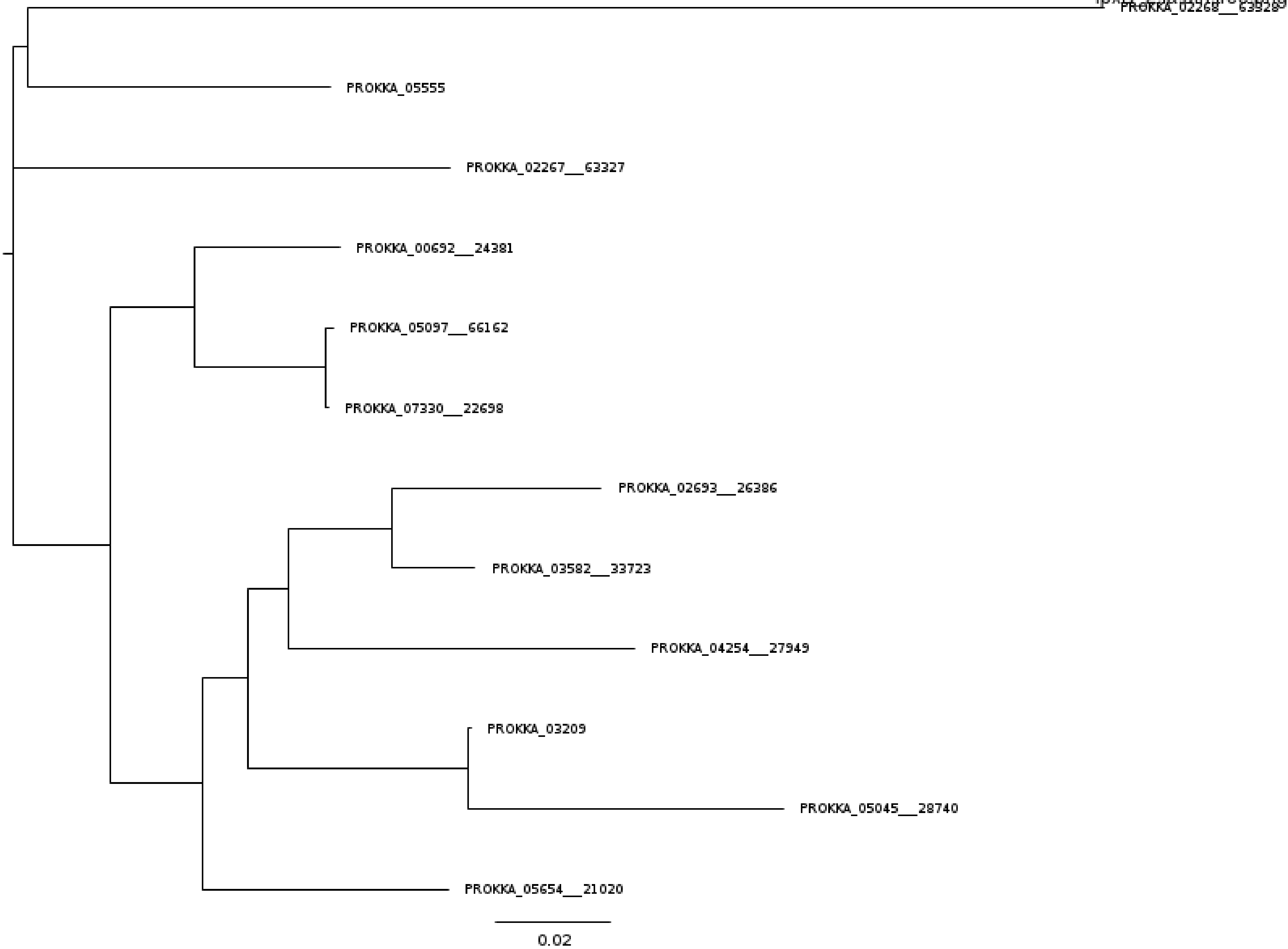

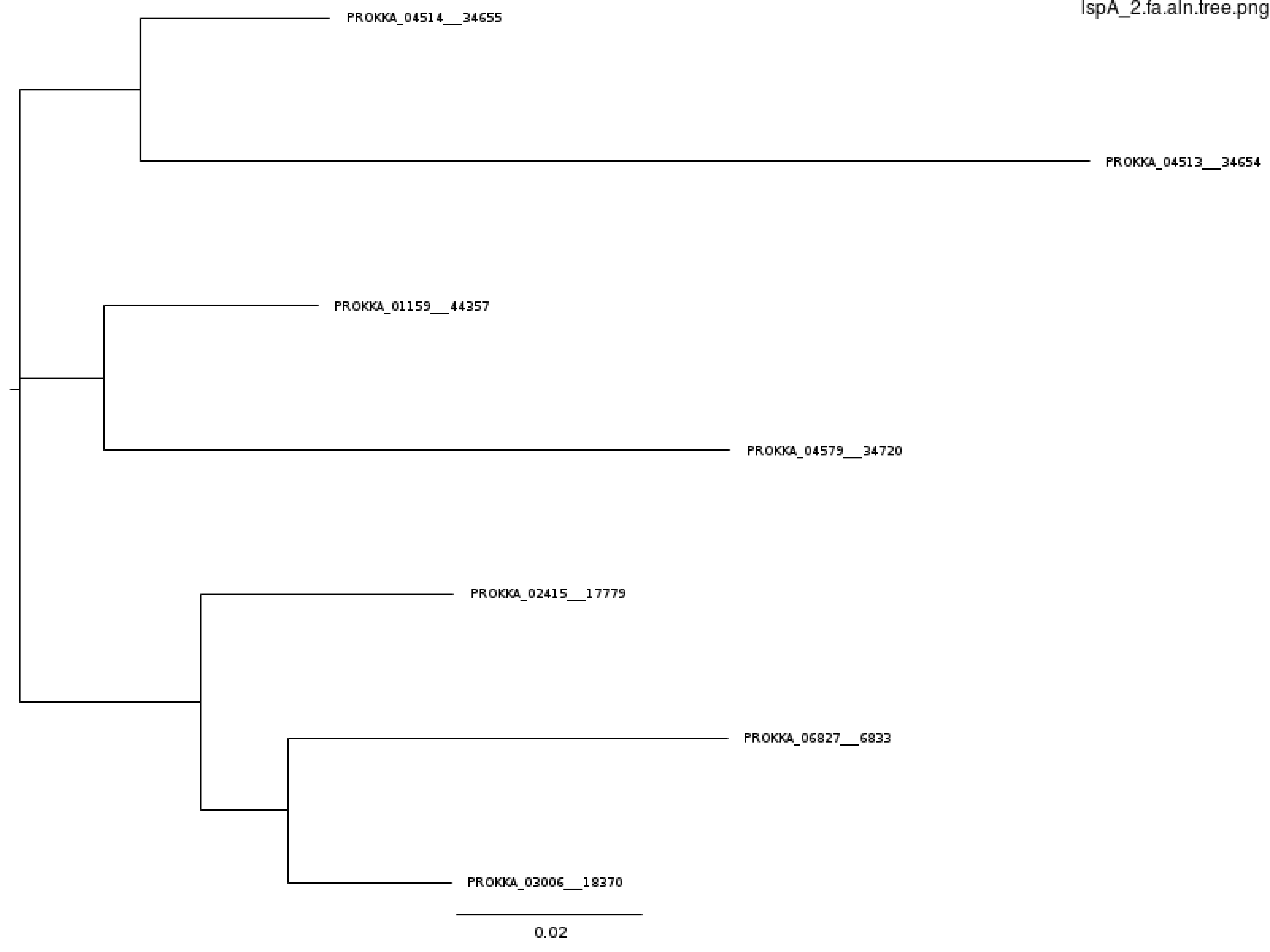
lspA_2.fa.aln.tree.png
PROKKA_04514___34655
PROKKA_04513___34654
PROKKA_01159___44357
PROKKA_04579___34720
PROKKA_02415___17779
PROKKA_06827___6833
PROKKA_03006___18370
0.02

lutA.fa.aln.tree.png

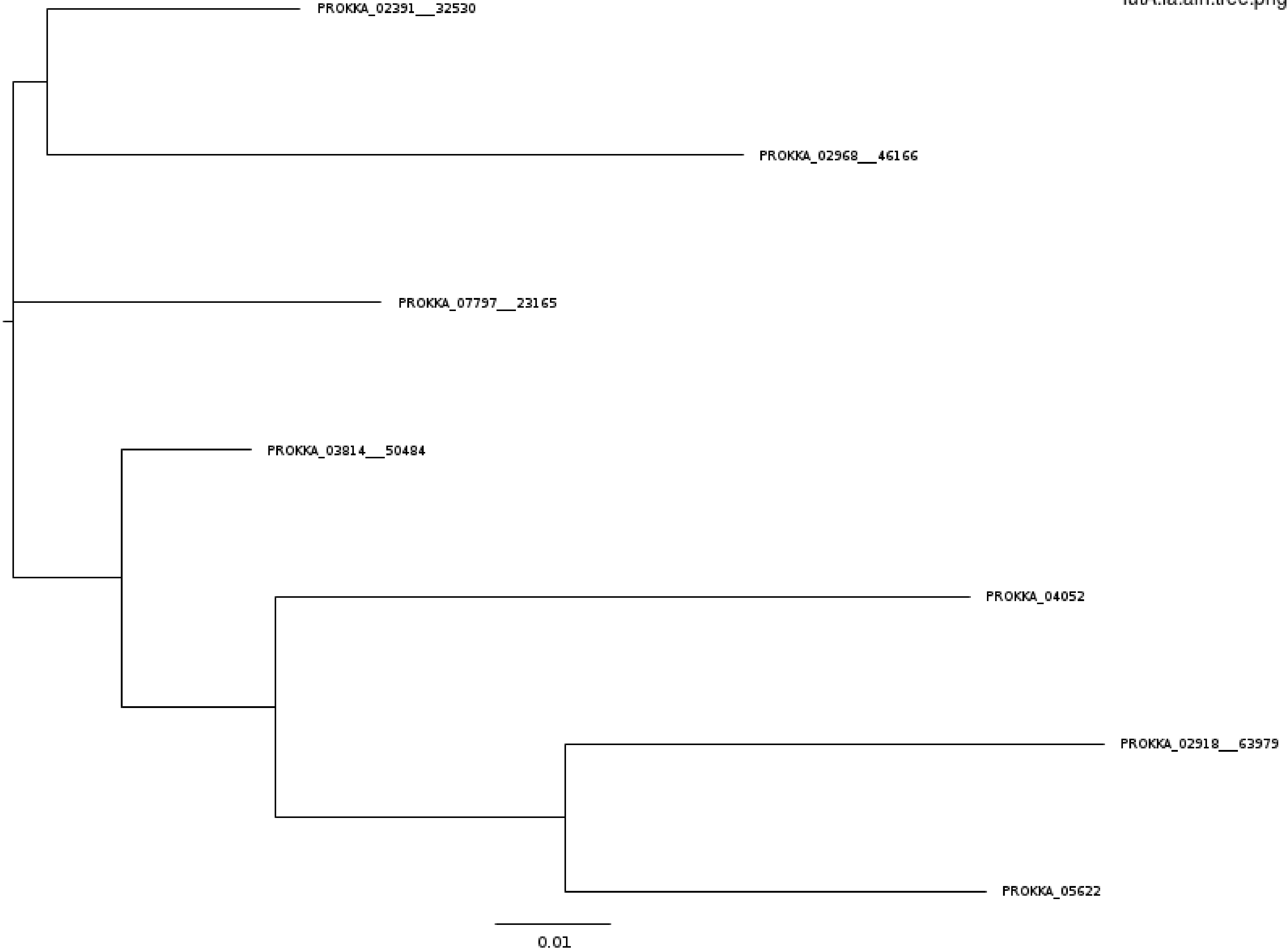

luxQ_1.fa.aln.tree.png

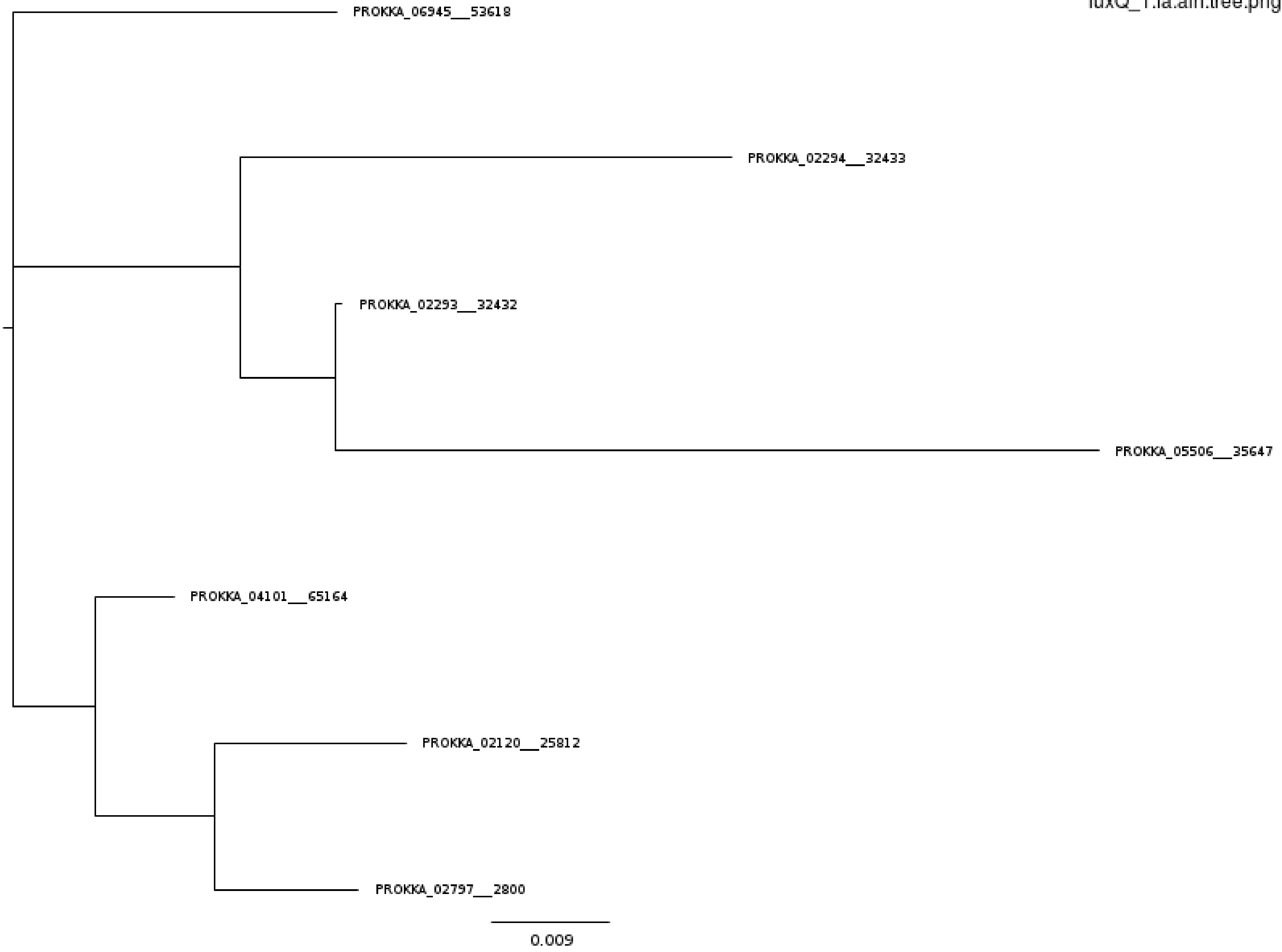

lysA_2.fa.aln.tree.png

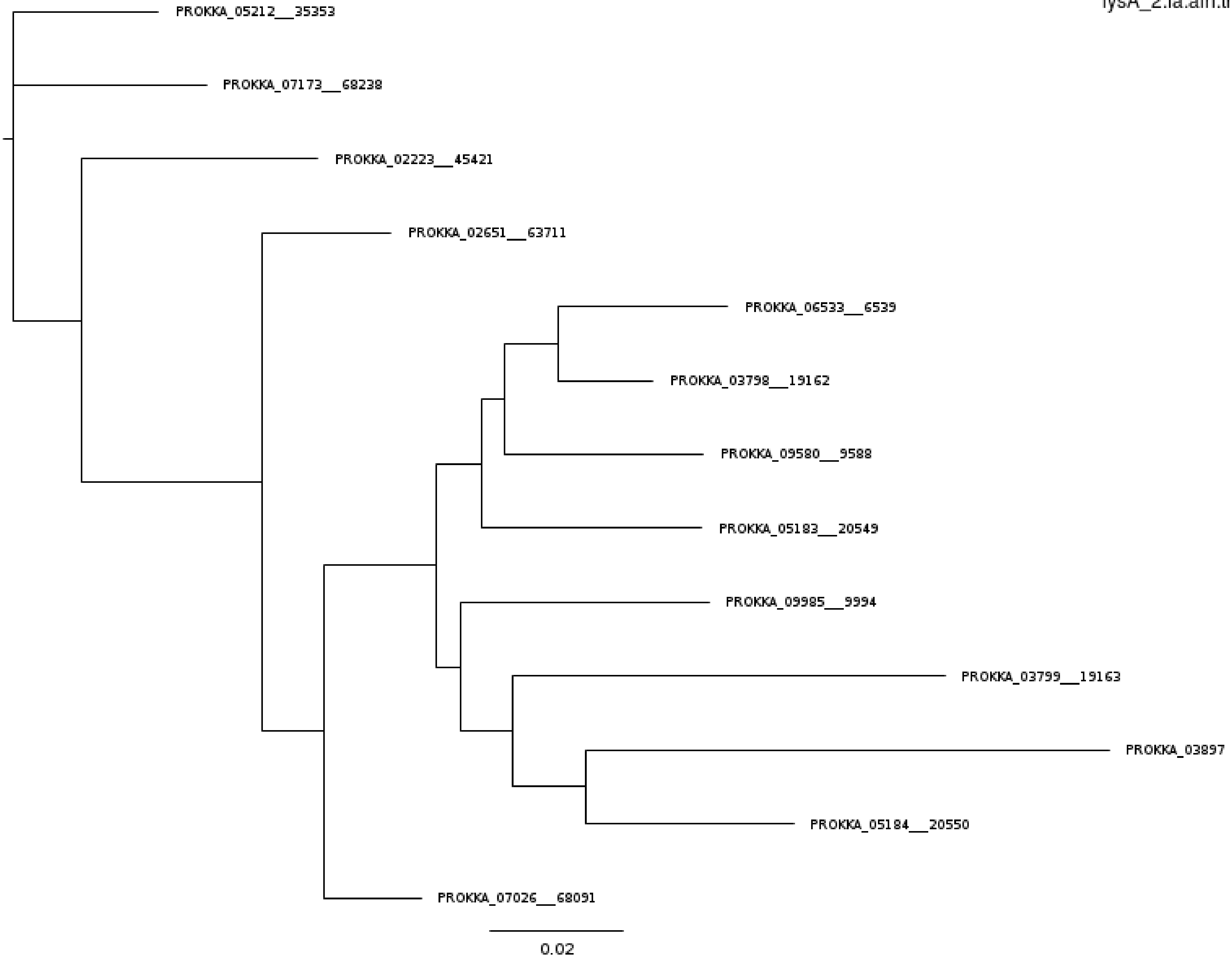

lysA_6.fa.aln.tree.png

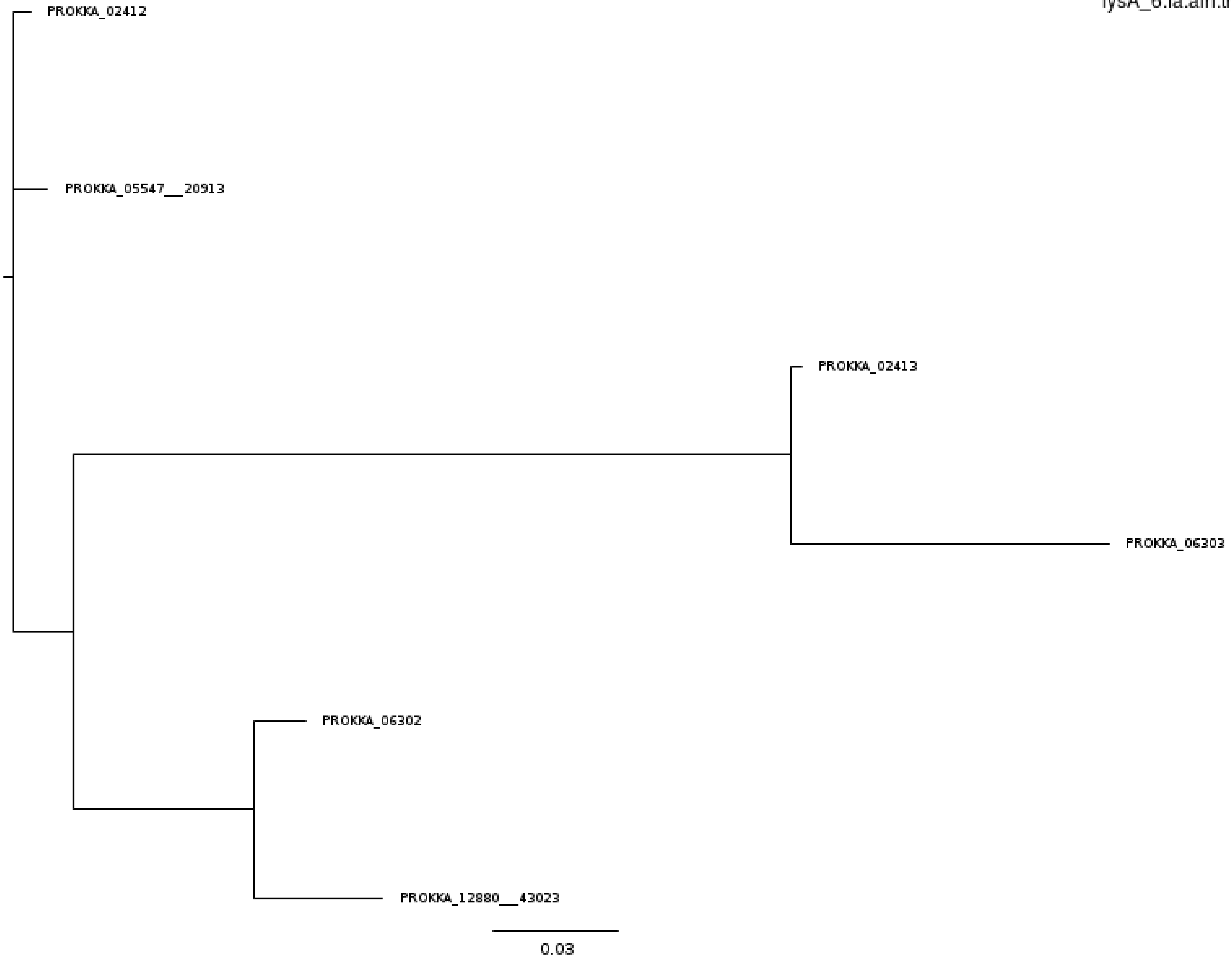

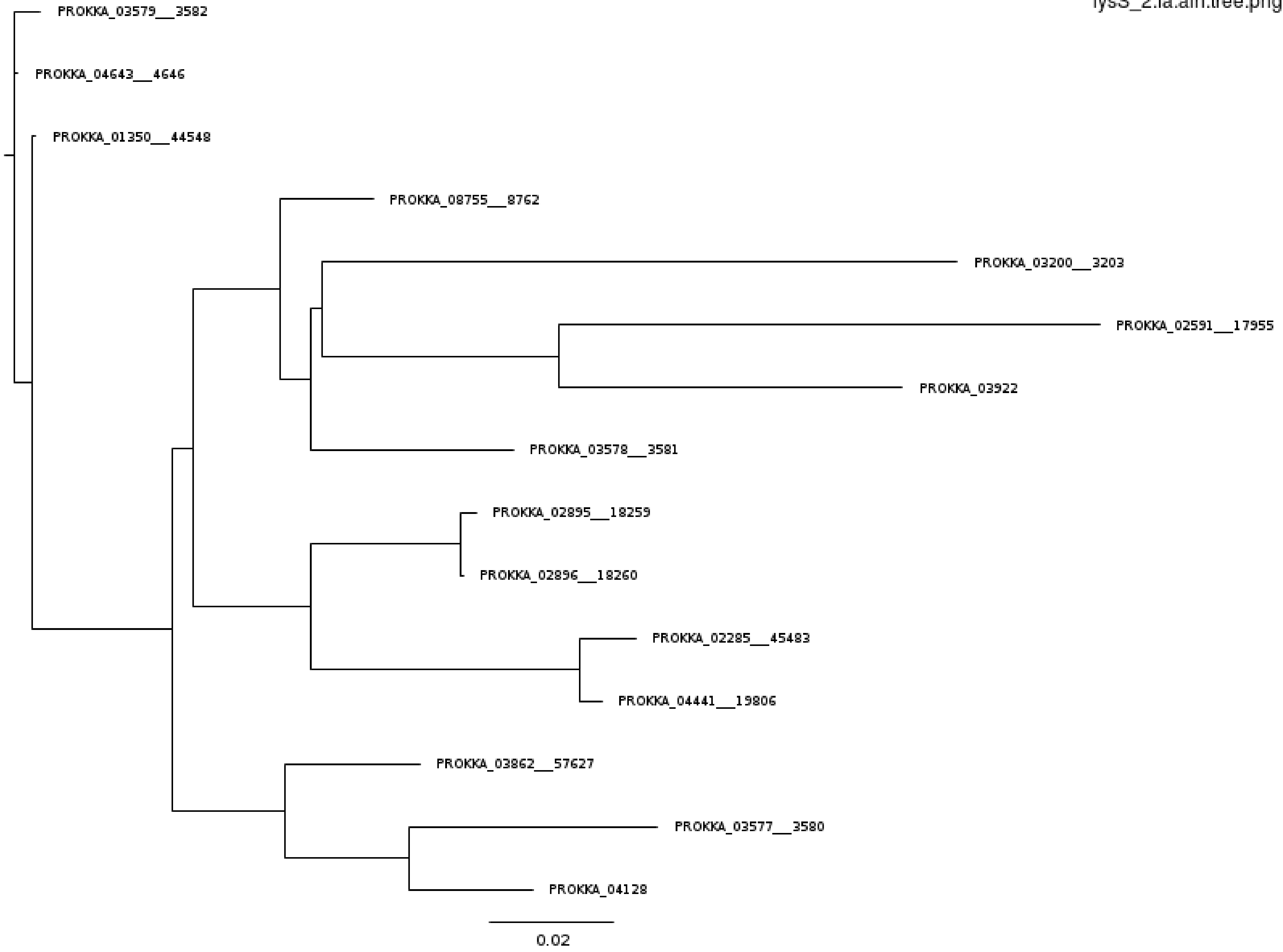
PROKKA_03579___3582
PROKKA_04643___4646
PROKKA_01350___44548
PROKKA_08755___8762
PROKKA_03200___3203
PROKKA_02591___17955
PROKKA_03922
PROKKA_03578___3581
PROKKA_02895___18259
PROKKA_02896___18260
PROKKA_02285___45483
PROKKA_04441___19806
PROKKA_03862___57627
PROKKA_03577___3580
PROKKA_04128
0.02

lysS_3.fa.aln.tree.png

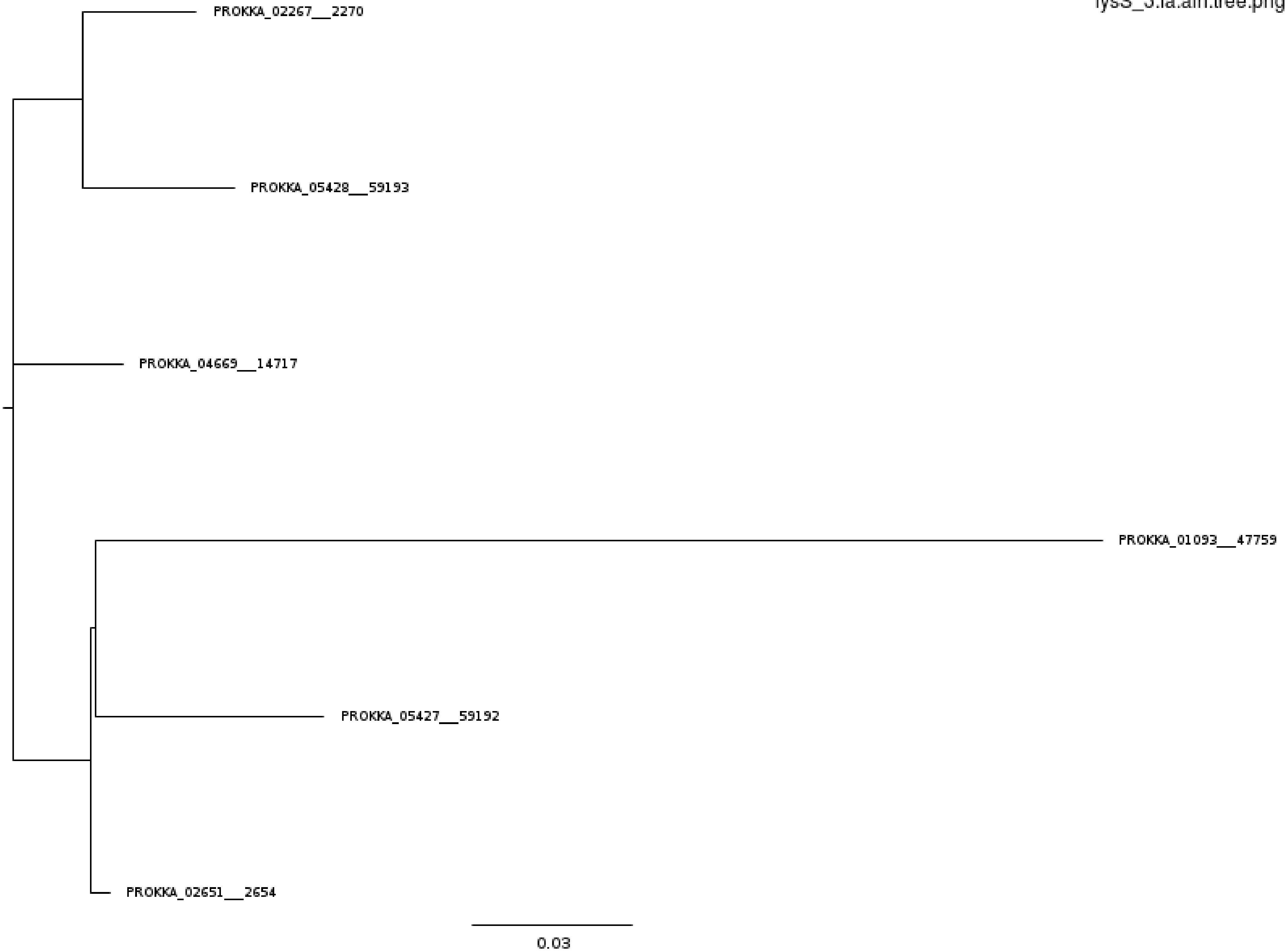

lytB_3.fa.aln.tree.png

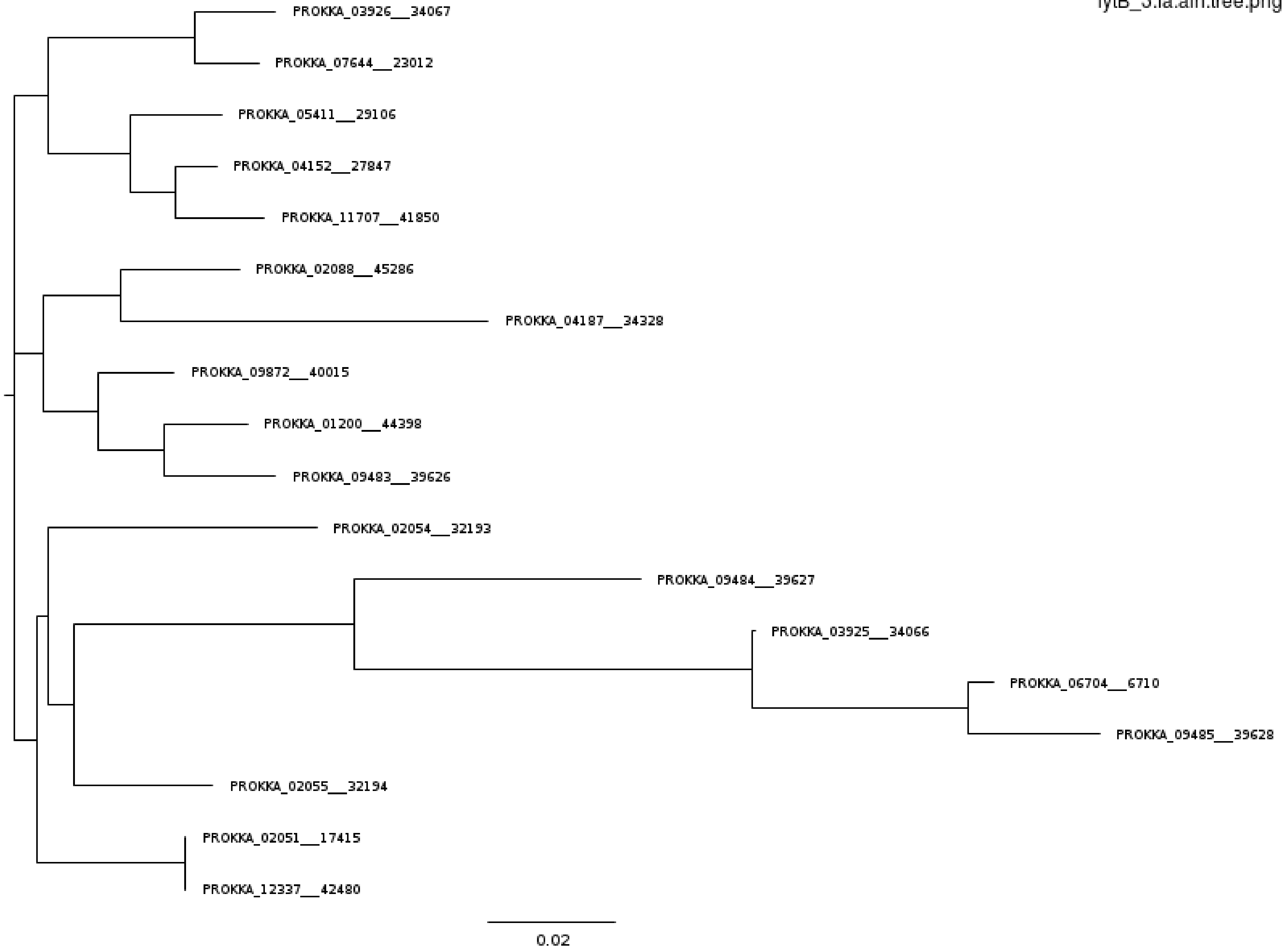

macA_2.fa.aln.tree.png

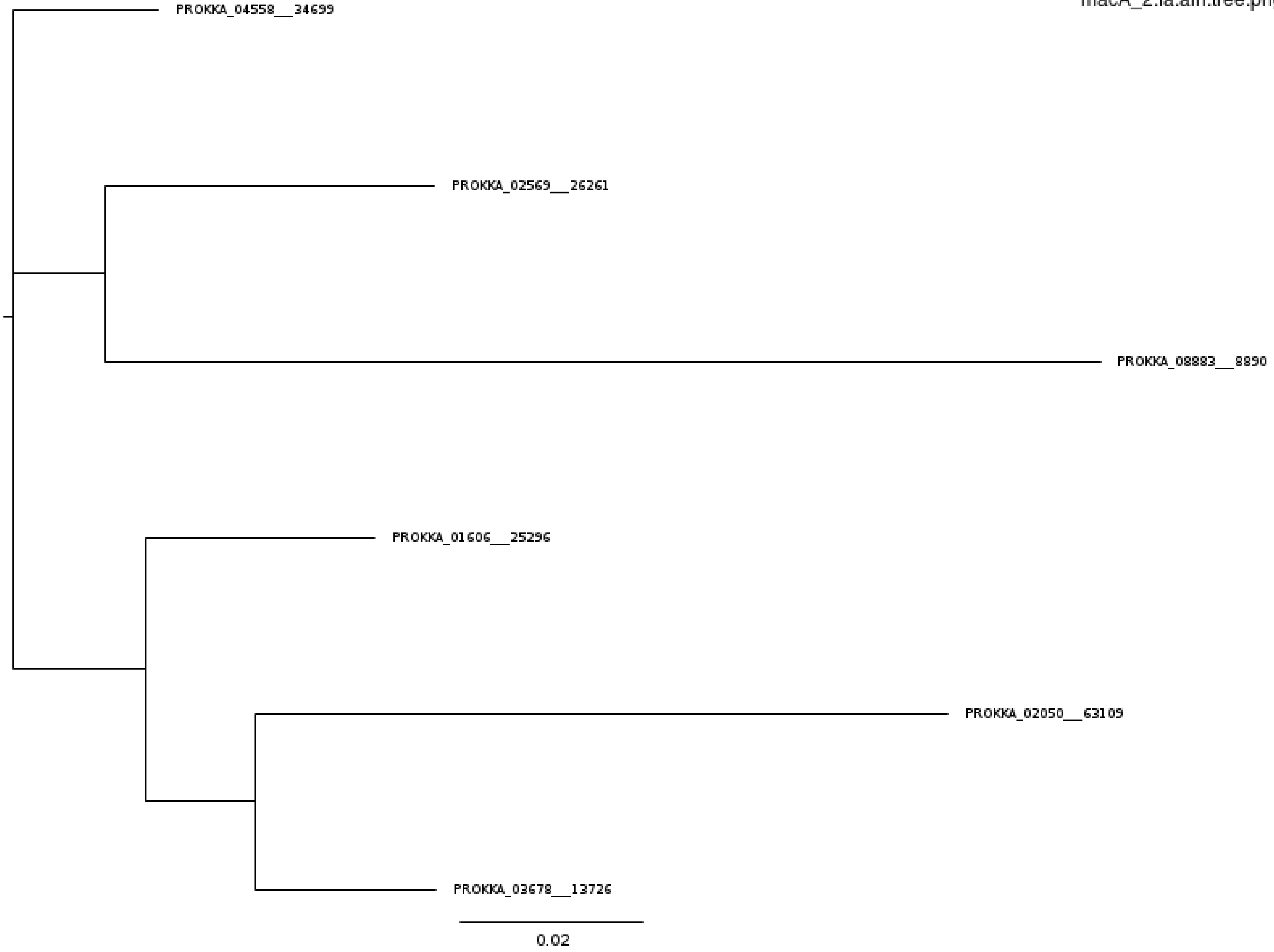

macB_1.fa.aln.tree.png

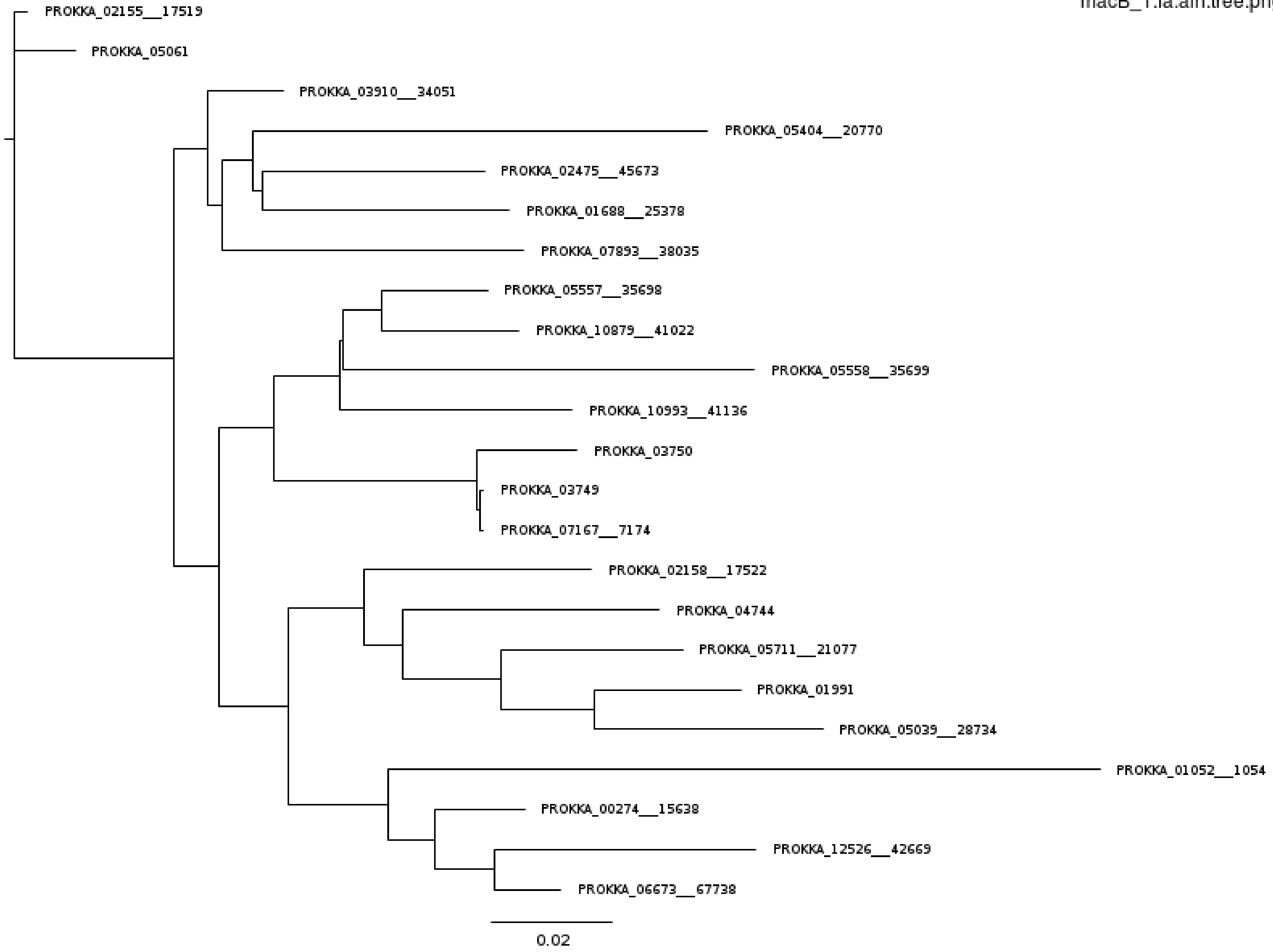

macB_2.fa.aln.tree.png

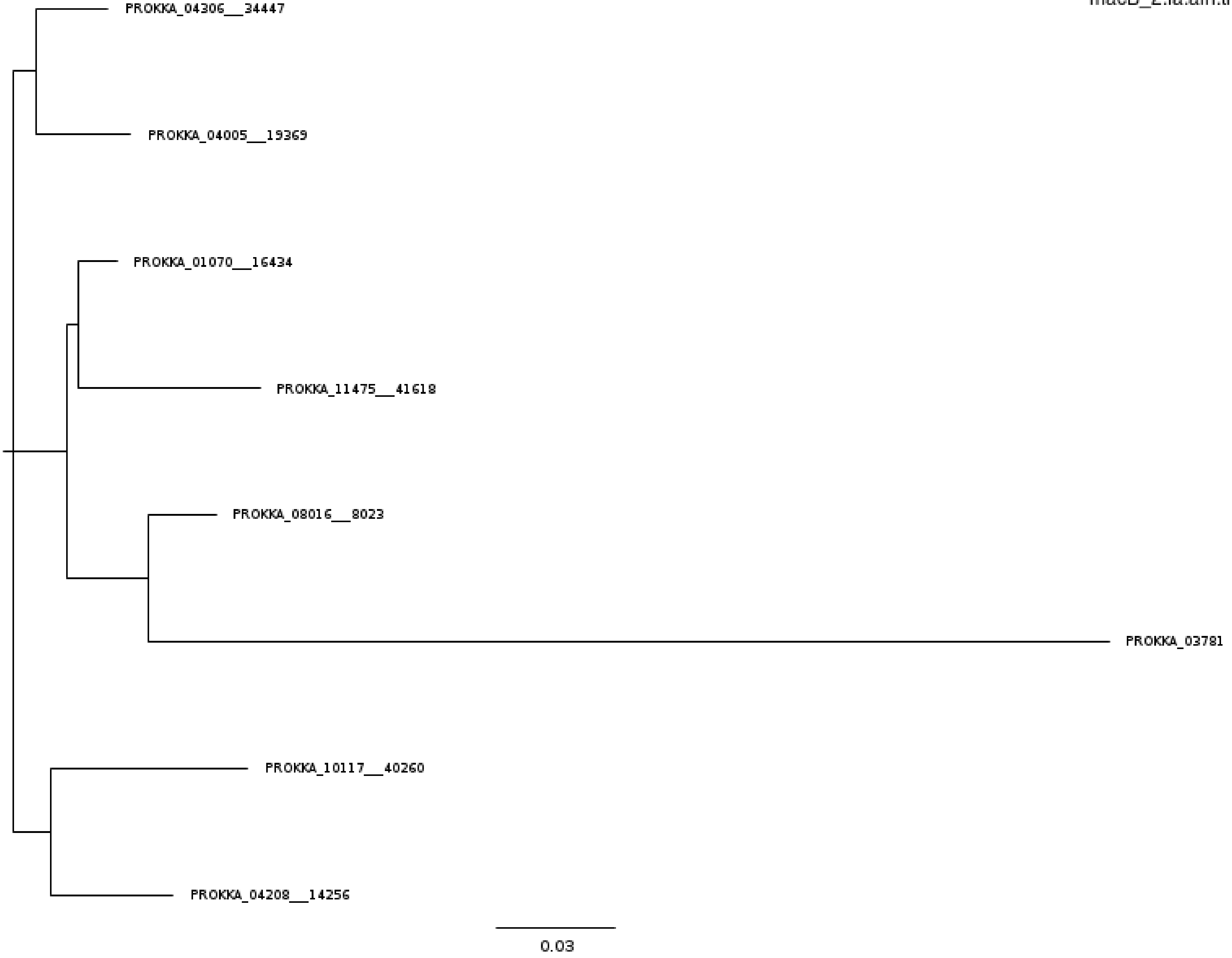

maeA_2.fa.aln.tree.png

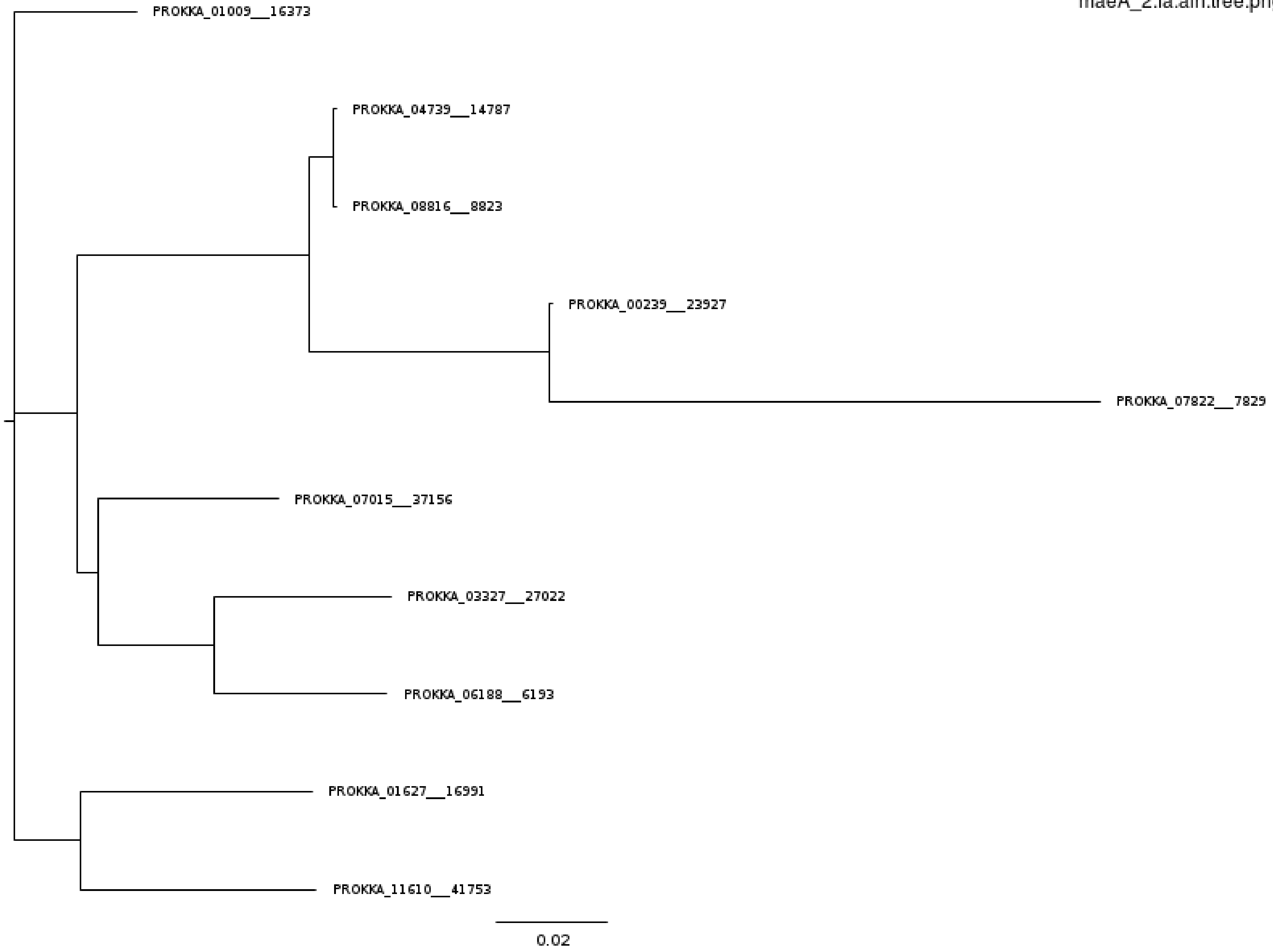

malP_1.fa.aln.tree.png

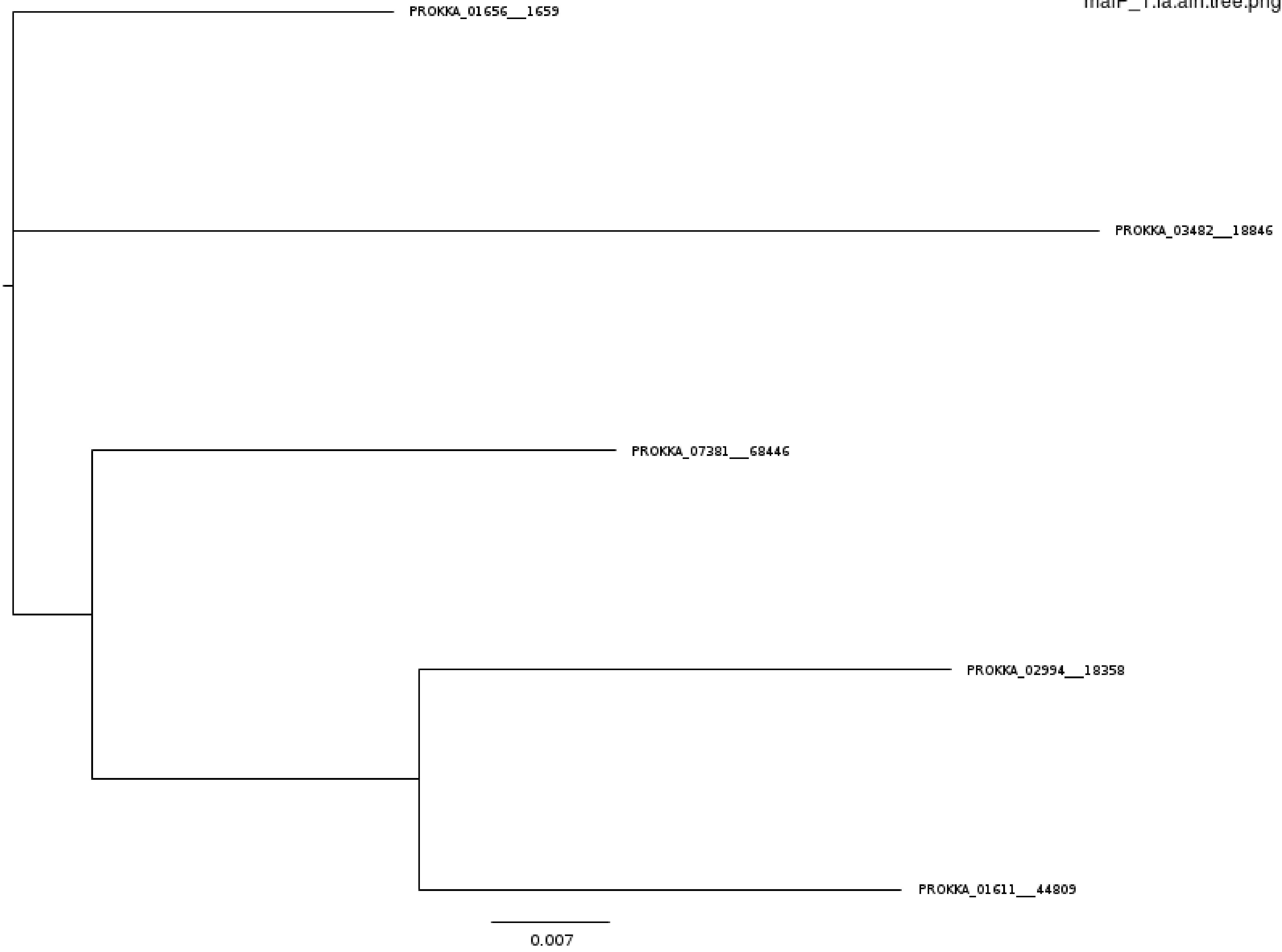

malP_2.fa.aln.tree.png

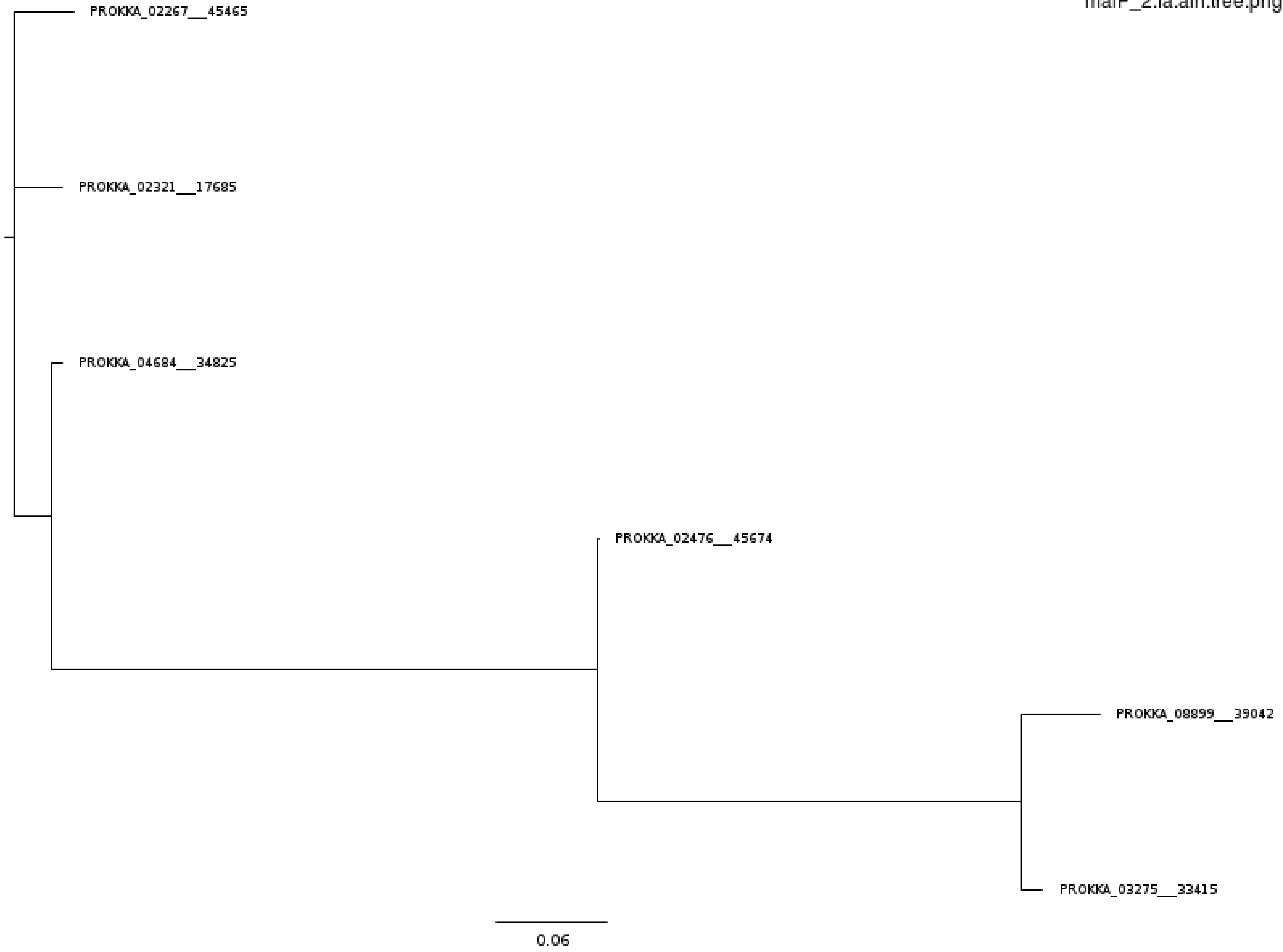

malP_3.fa.aln.tree.png

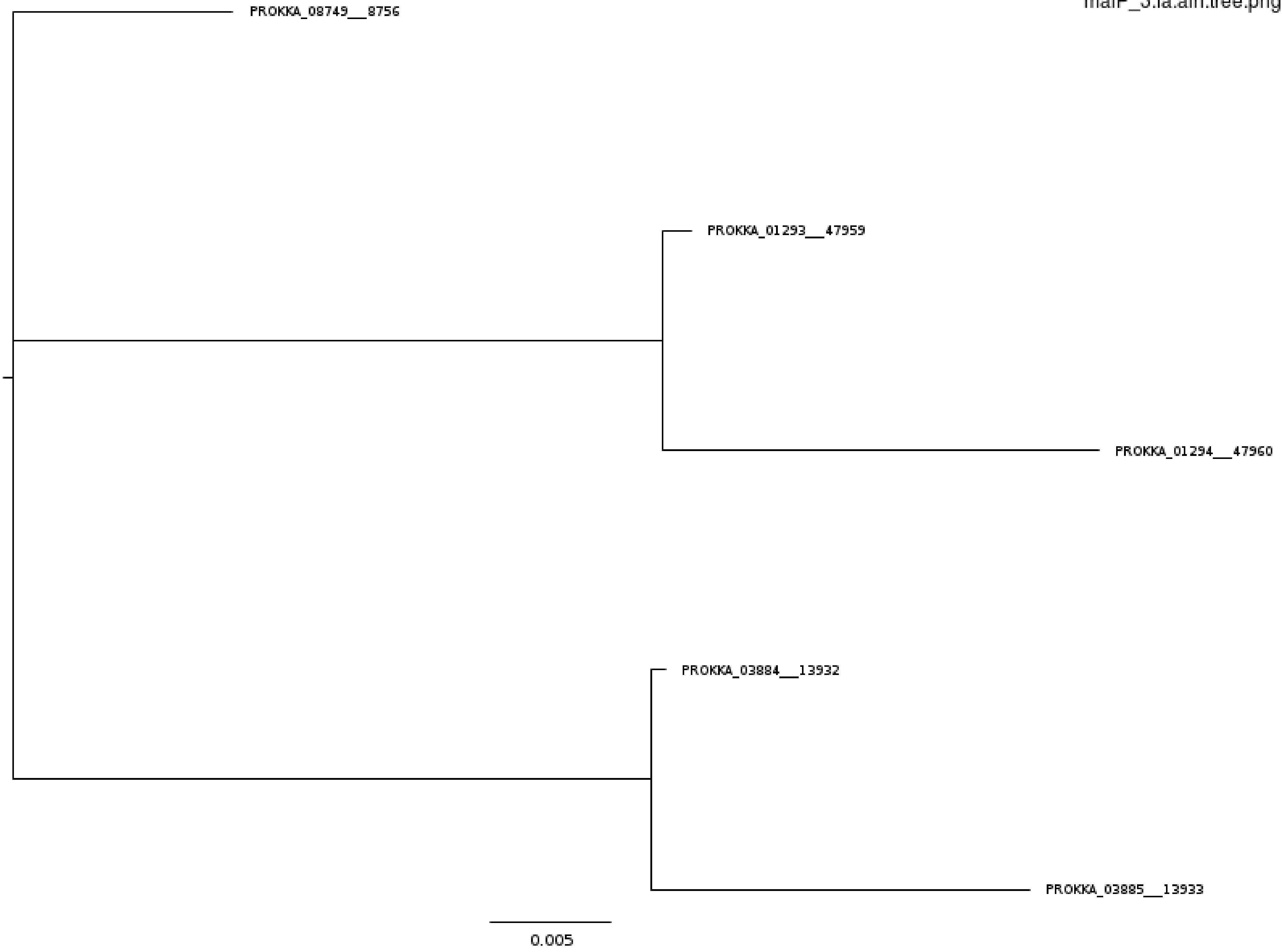

malQ_2.fa.aln.tree.png

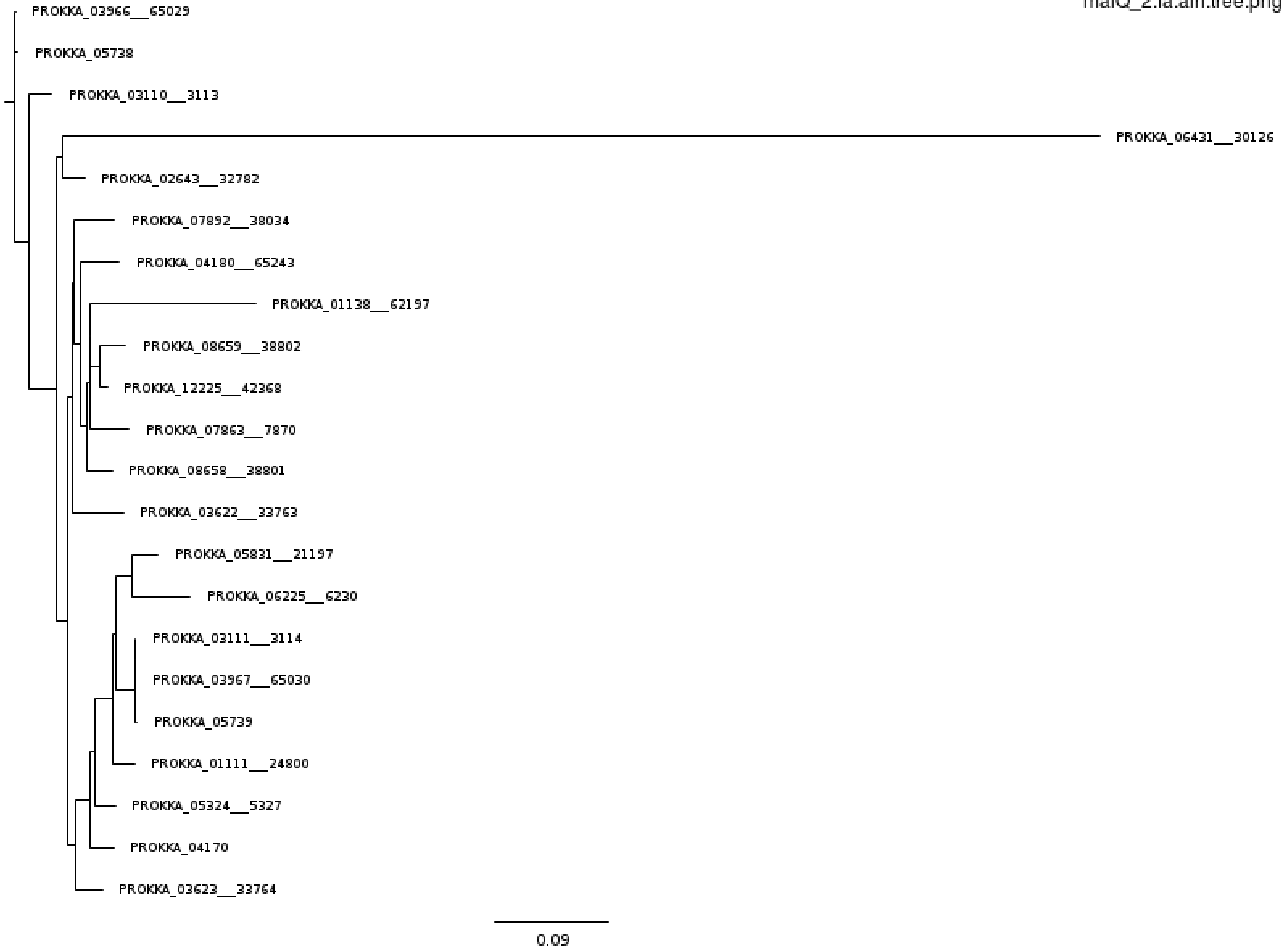

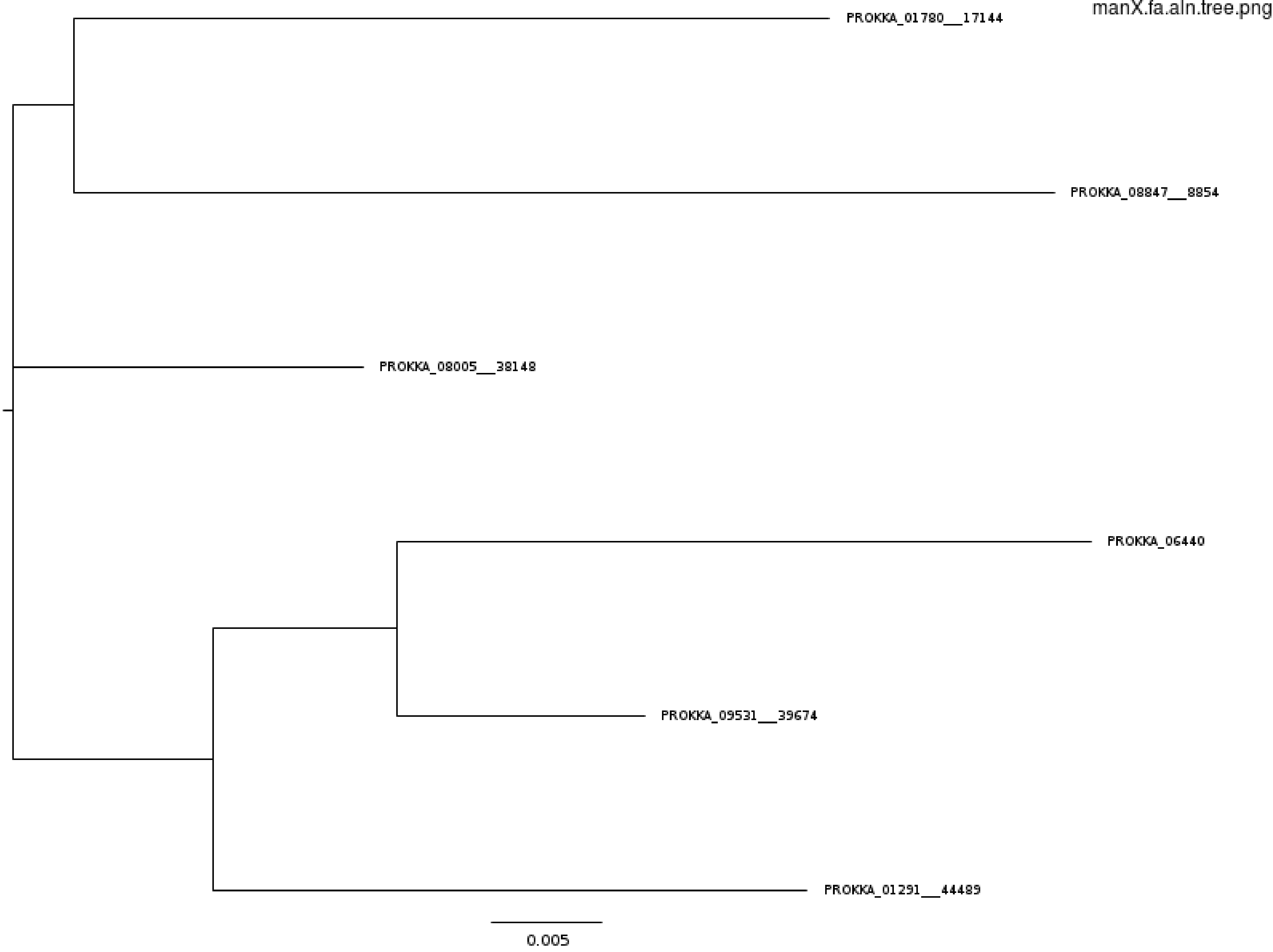
manX.fa.aln.tree.png
PROKKA_01780__17144
PROKKA_08847__8854
PROKKA_08005__38148
PROKKA_06440
PROKKA_09531__39674
PROKKA_01291__44489
0.005

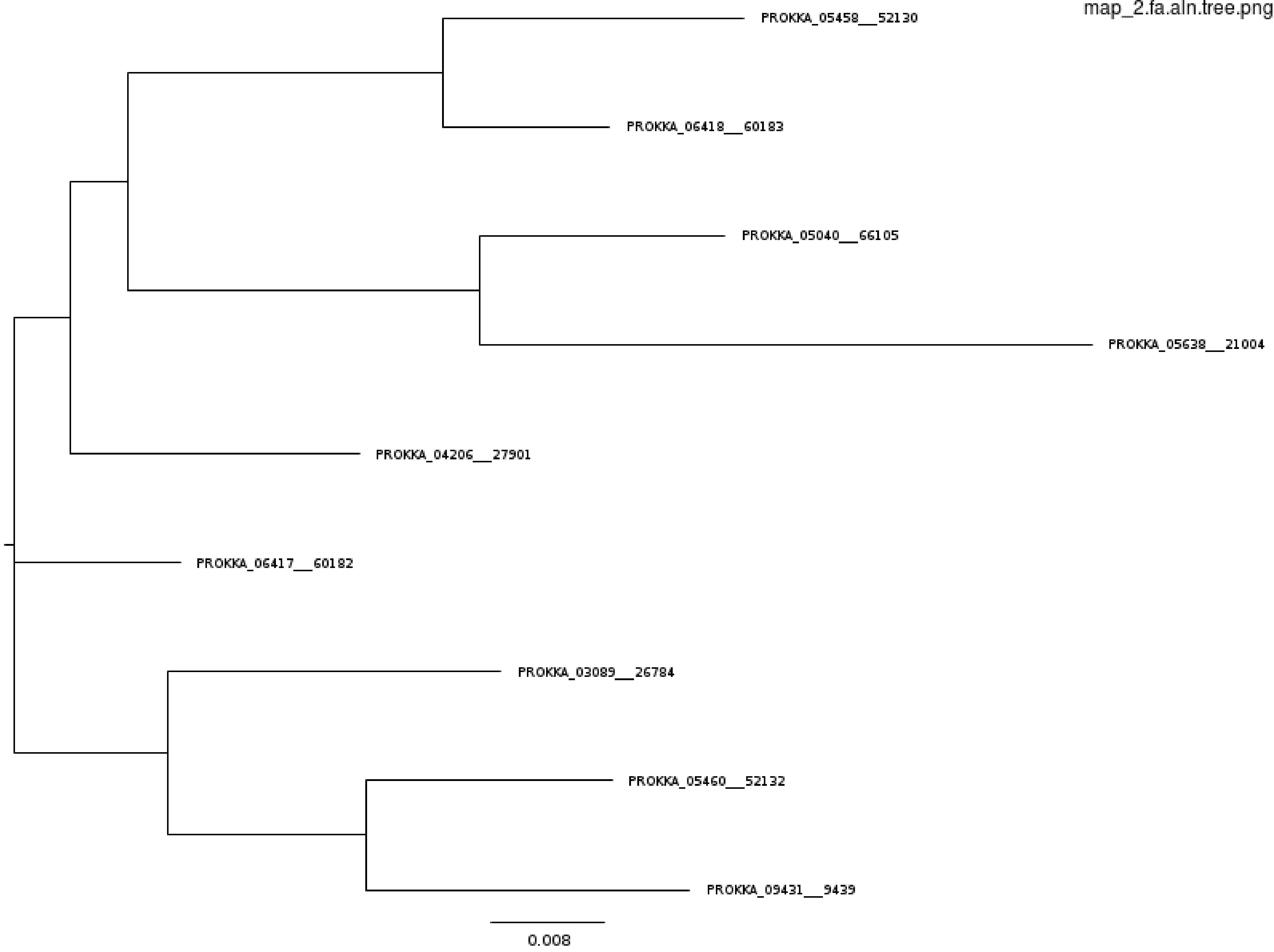
map_2.fa.aln.tree.png
PROKKA_05458___52130
PROKKA_06418___60183
PROKKA_05040___66105
PROKKA_05638___21004
PROKKA_04206___27901
PROKKA_06417___60182
PROKKA_03089___26784
PROKKA_05460___52132
PROKKA_09431___9439
0.008

mauG_2.fa.aln.tree.png

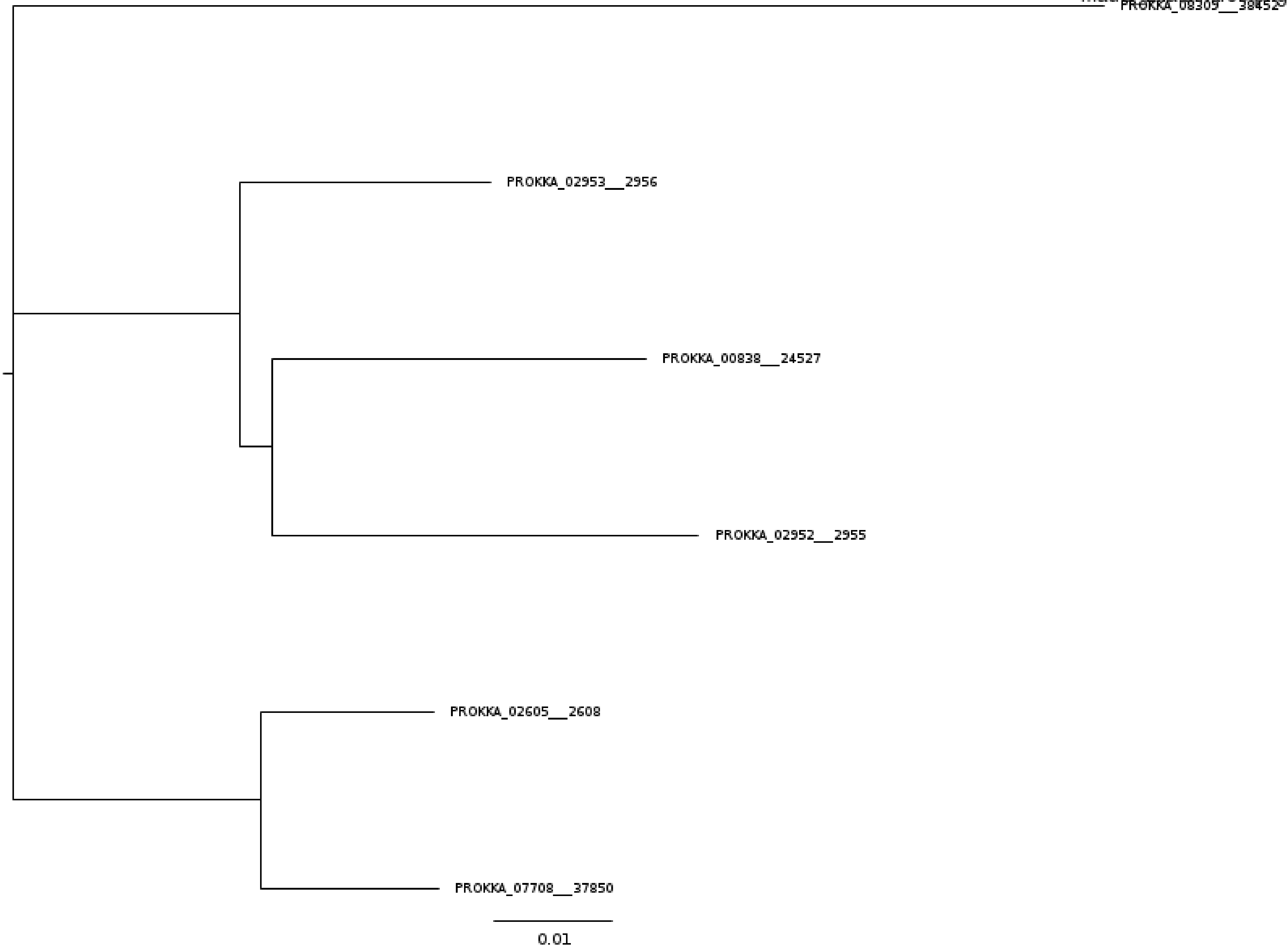

mazF_2.fa.aln.tree.png

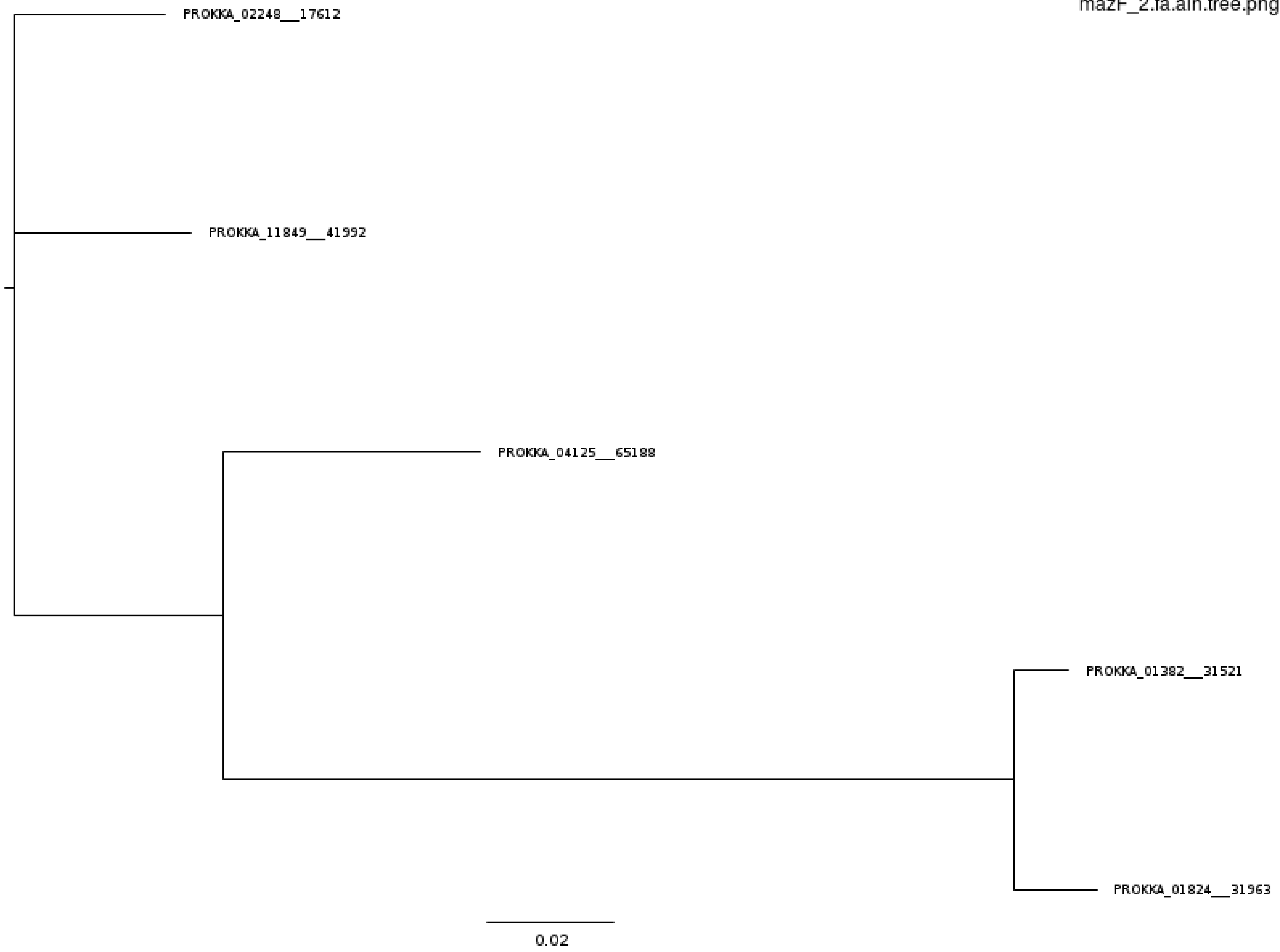

mcl2_2.fa.aln.tree.png

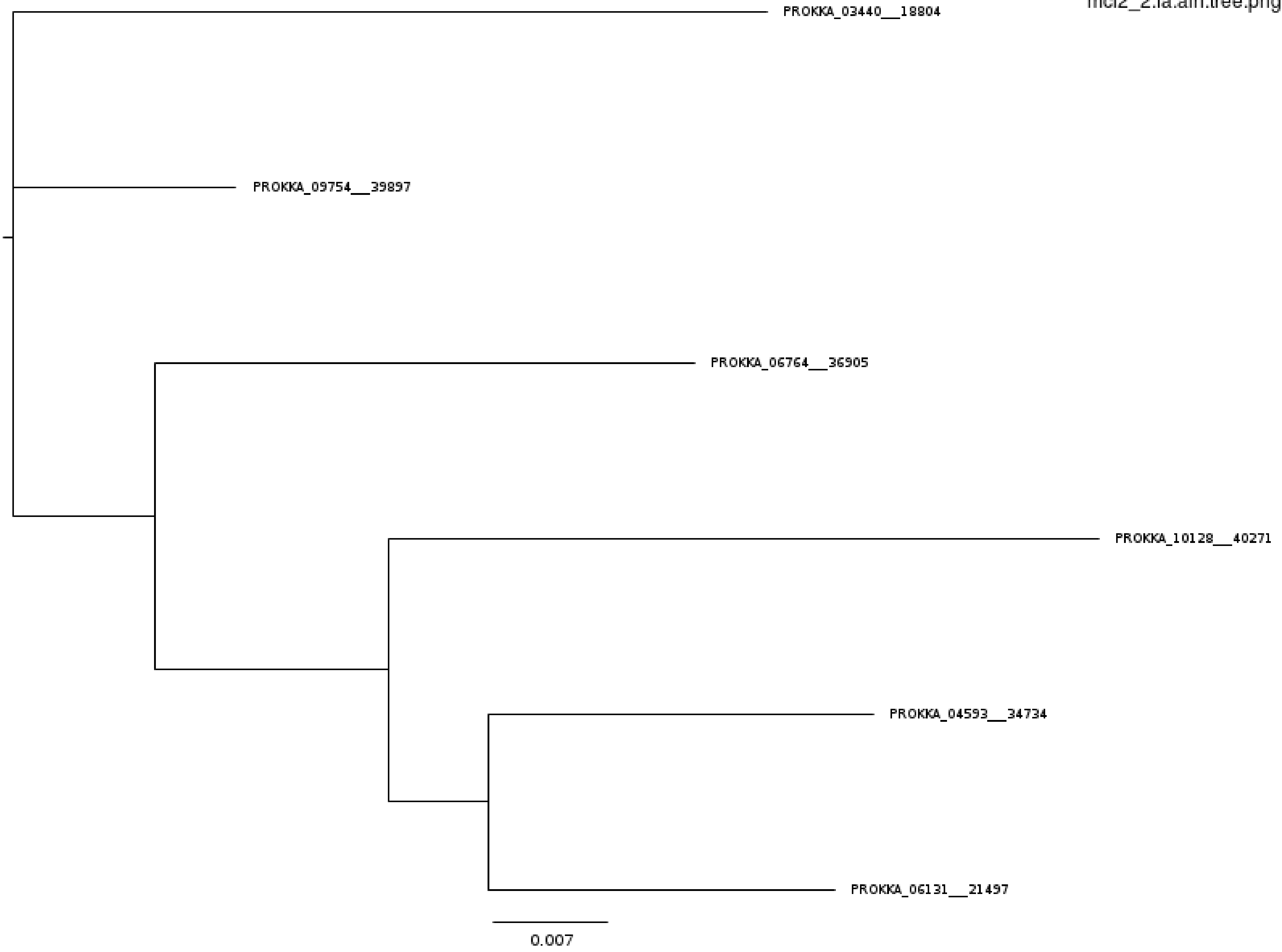

mcp2_1.fa.aln.tree.png

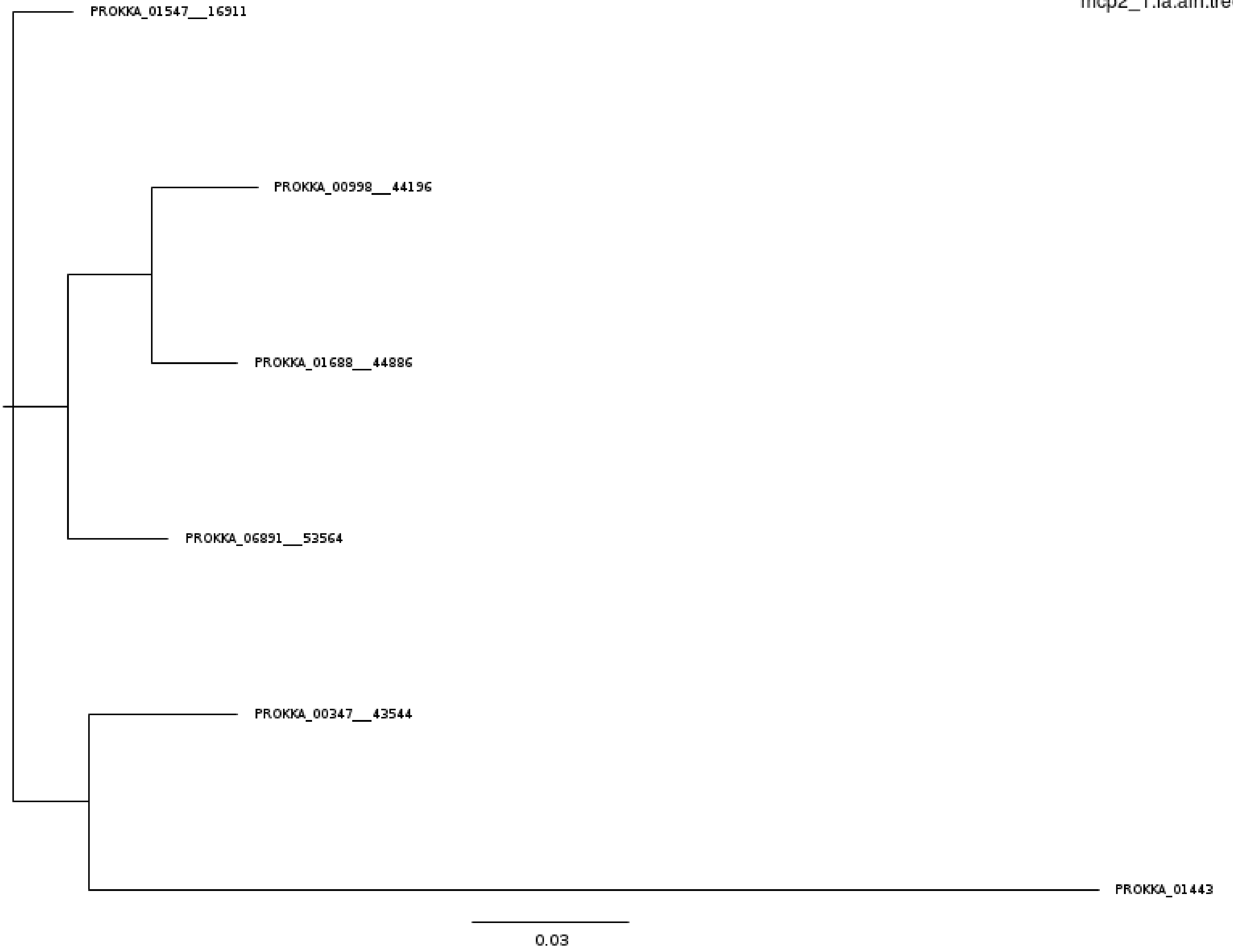

mdeA_1.fa.aln.tree.png

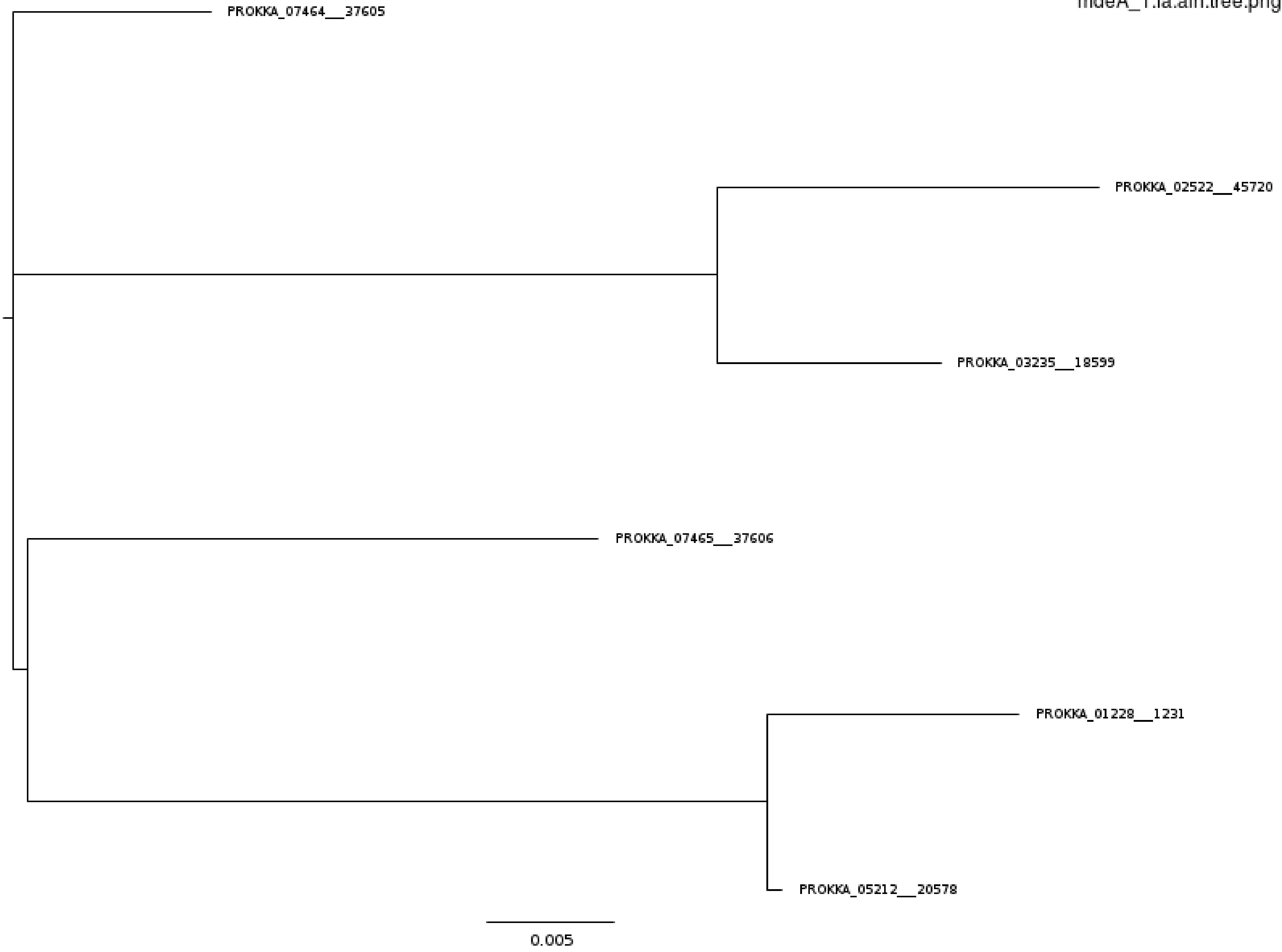

mdeA_2.fa.aln.tree.png

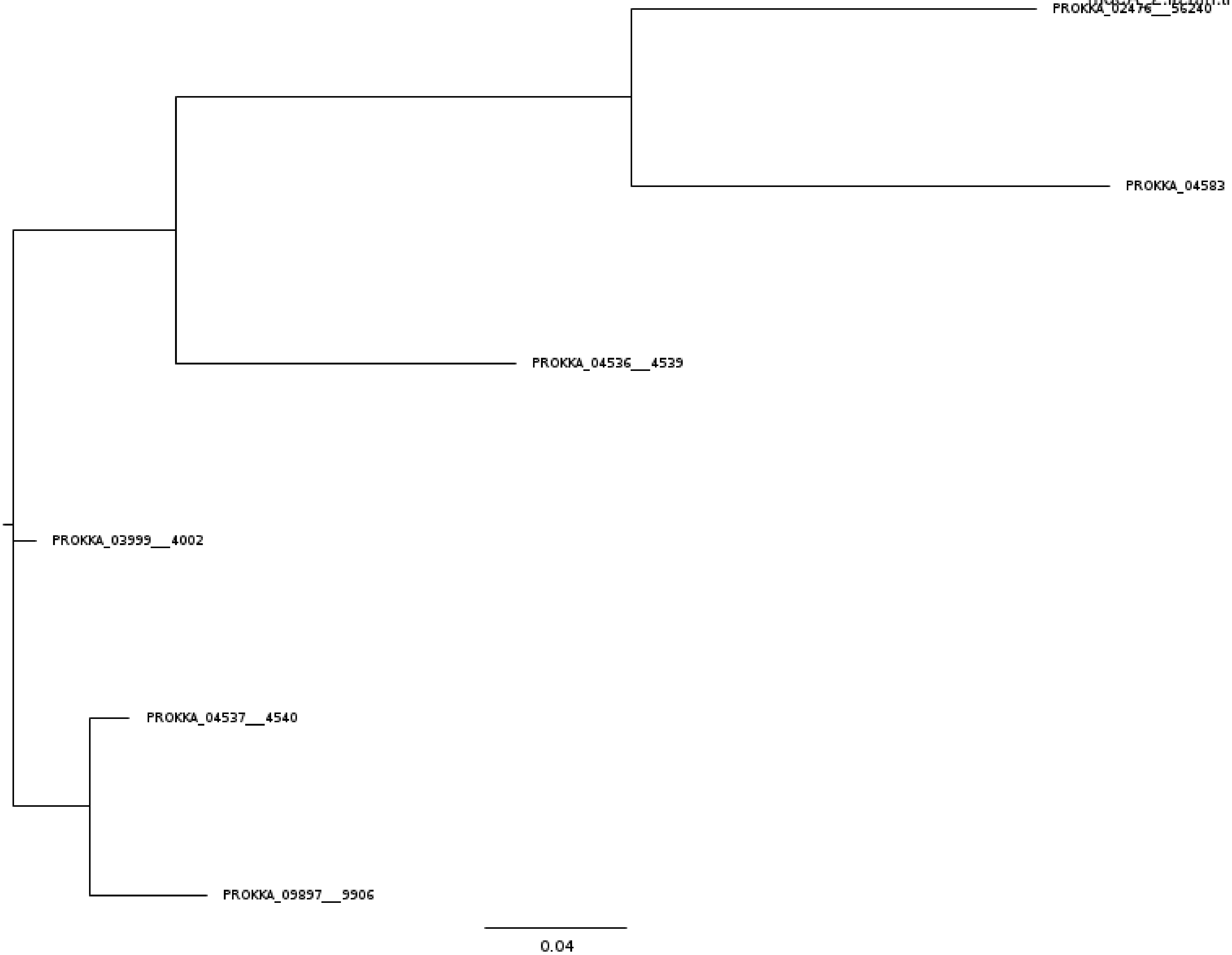

mdtC_5.fa.aln.tree.png

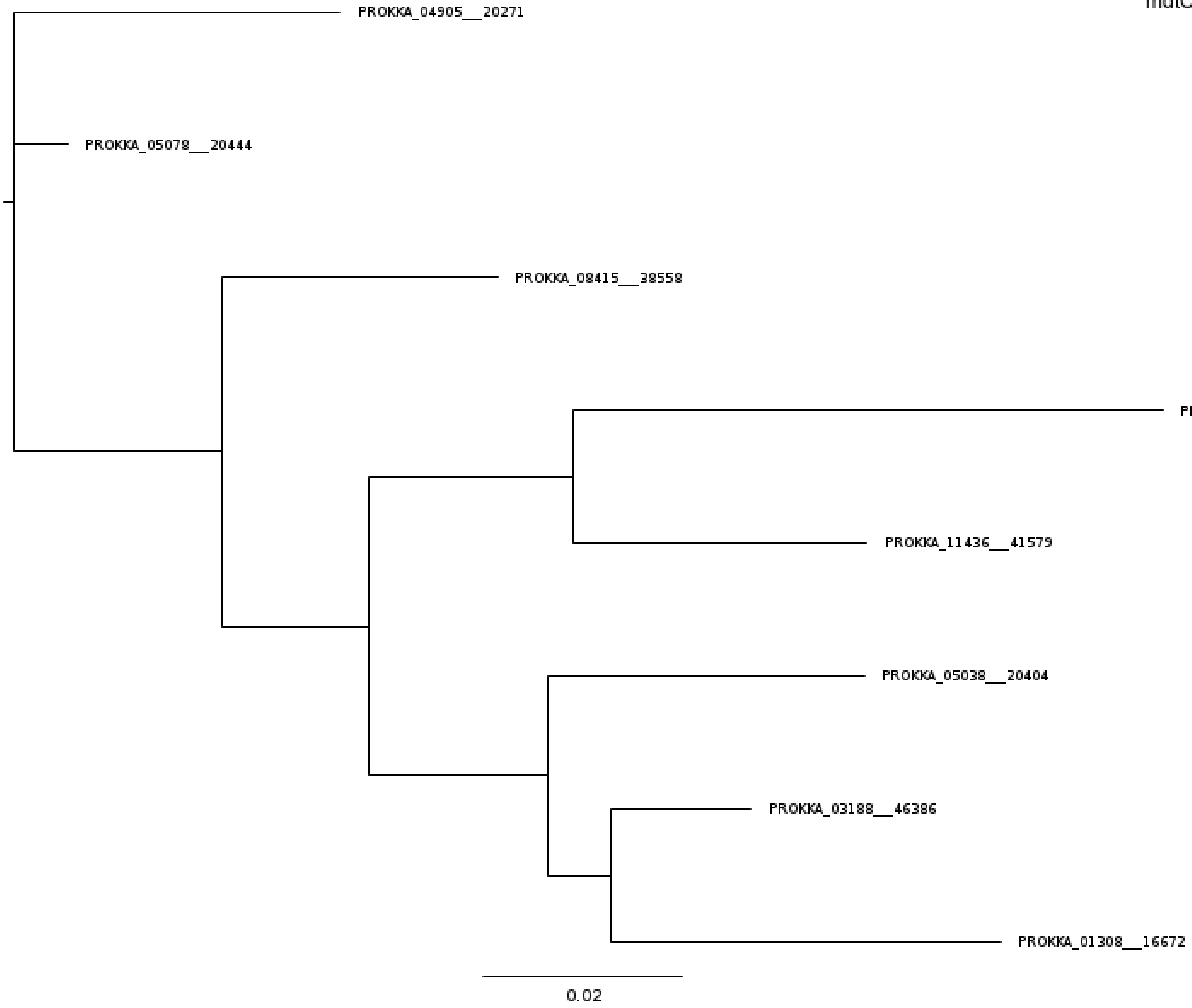

mdtE.fa.aln.tree.png

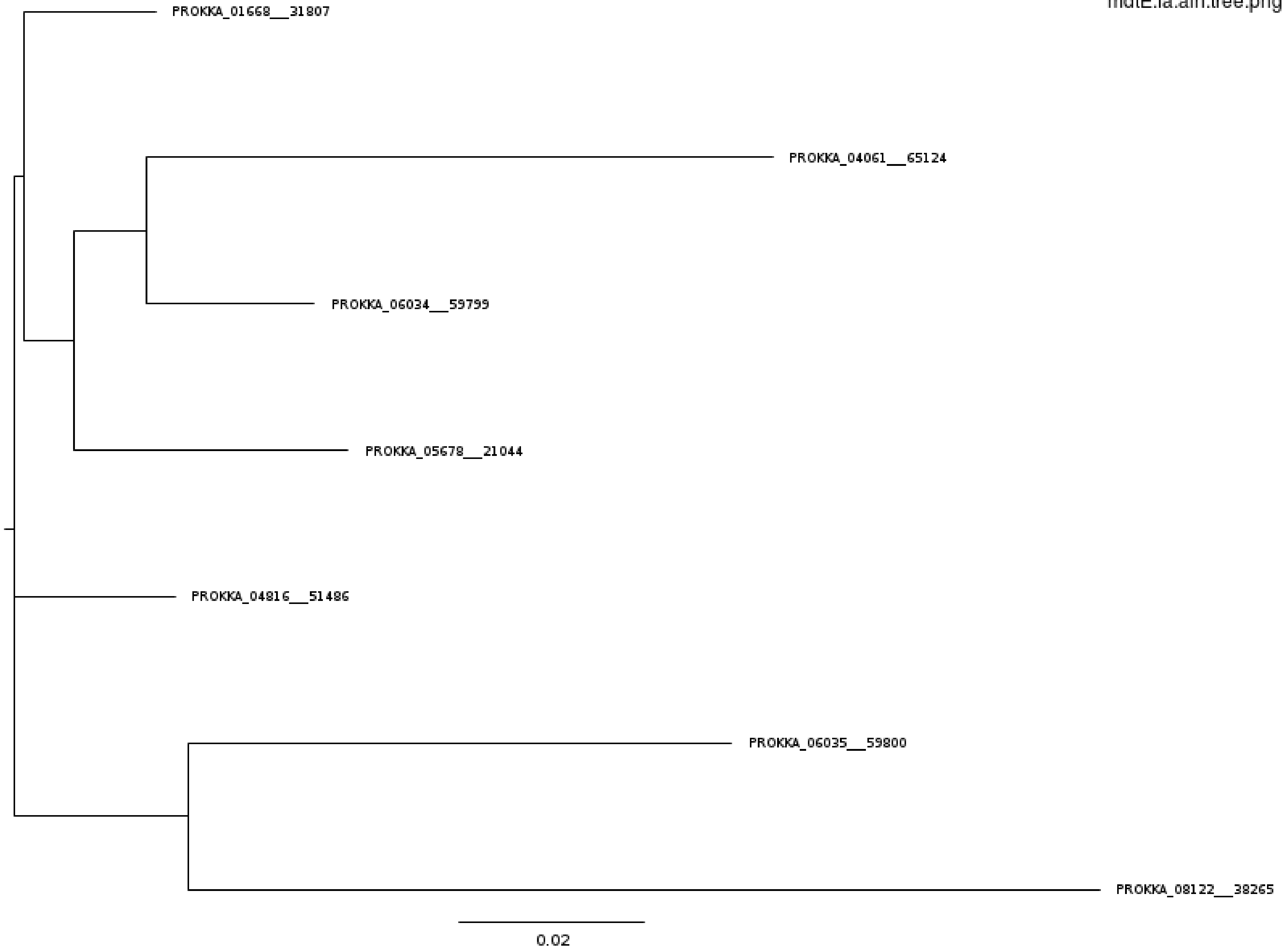

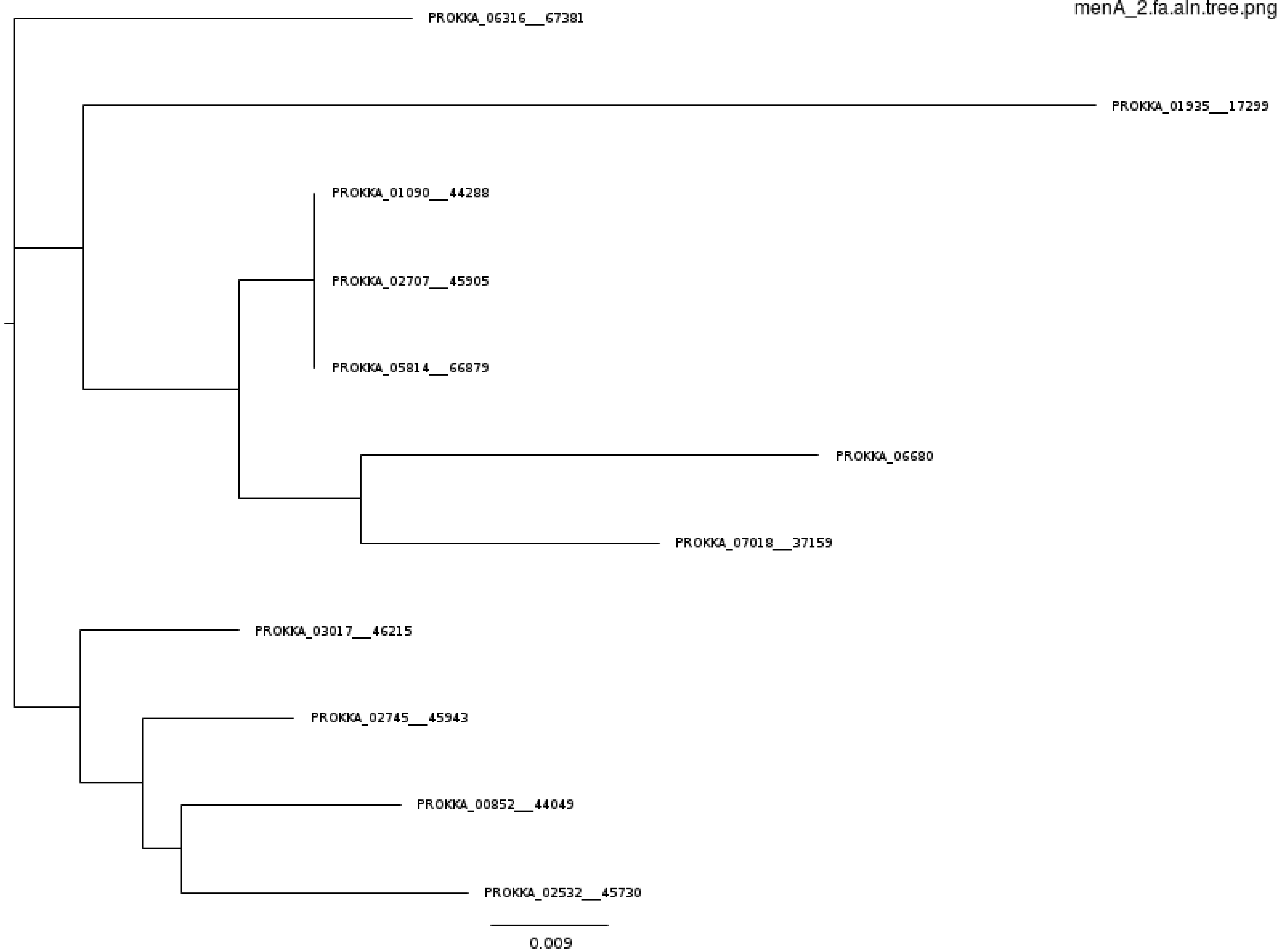

menA_2.fa.aln.tree.png
PROKKA_06316___67381
PROKKA_01935___17299
PROKKA_01090___44288
PROKKA_02707___45905
PROKKA_05814___66879
PROKKA_06680
PROKKA_07018___37159
PROKKA_03017___46215
PROKKA_02745___45943
PROKKA_00852___44049
PROKKA_02532___45730
0.009

menB_1.fa.aln.tree.png

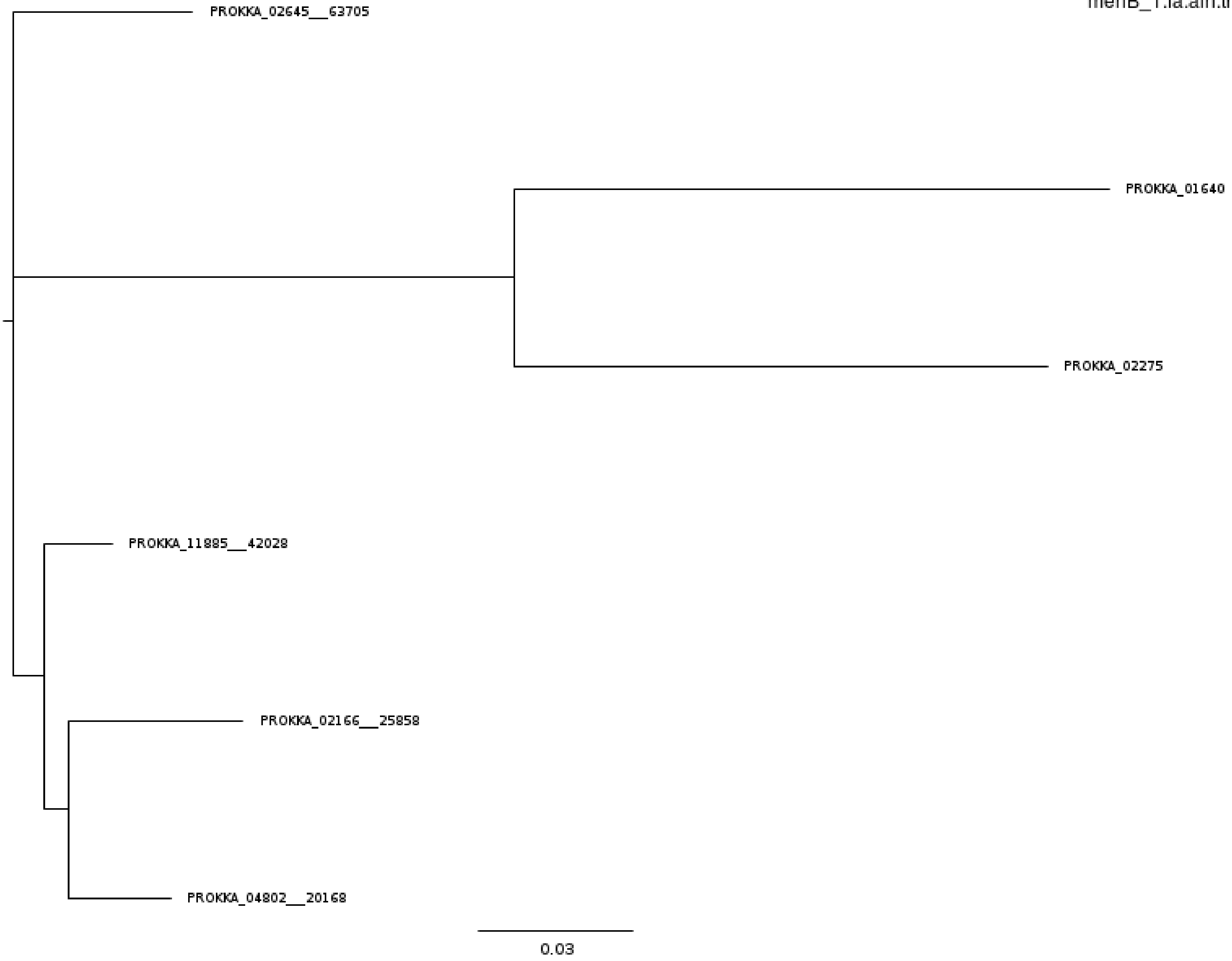

menD_3.fa.aln.tree.png

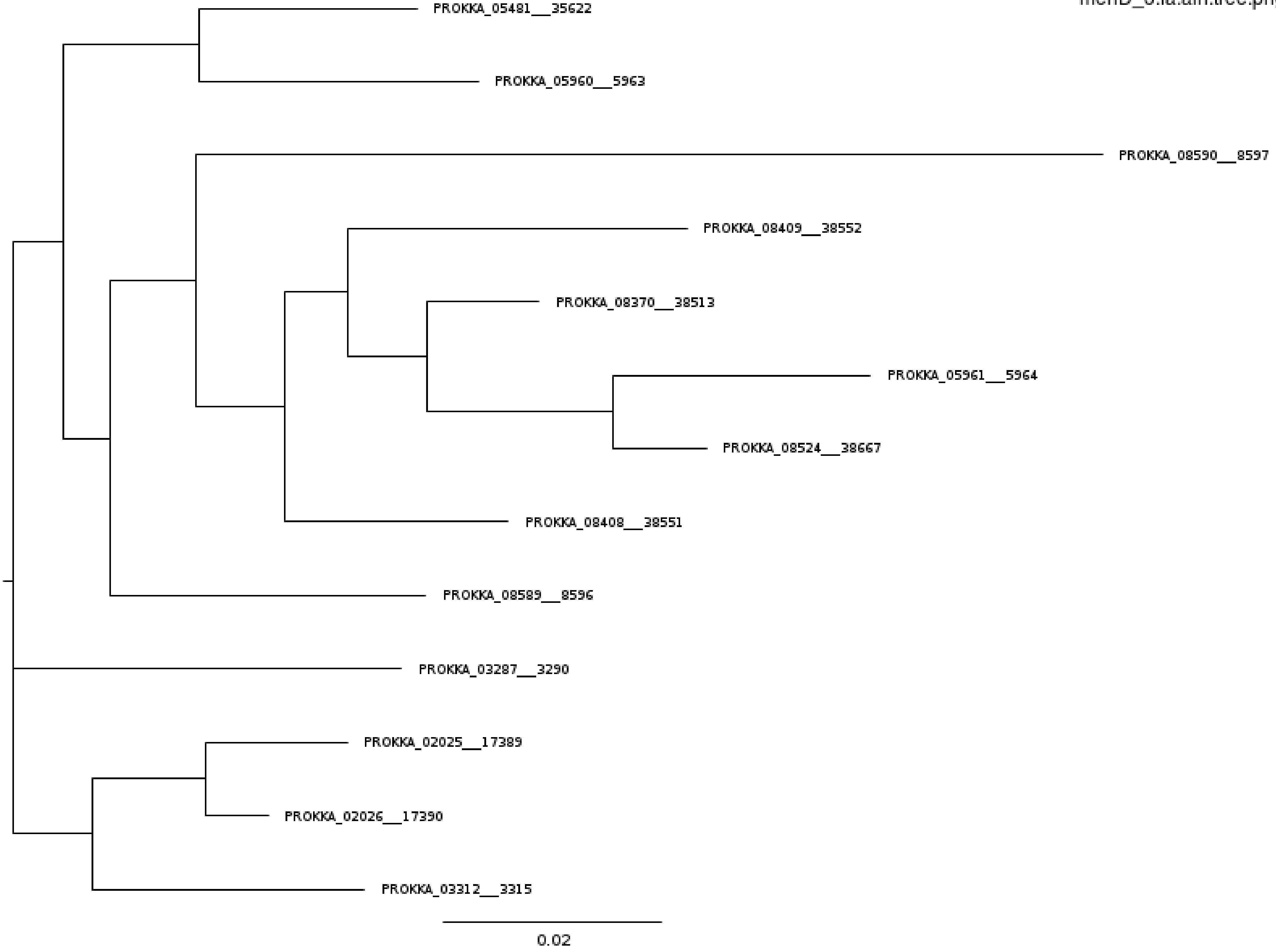

menH.fa.aln.tree.png

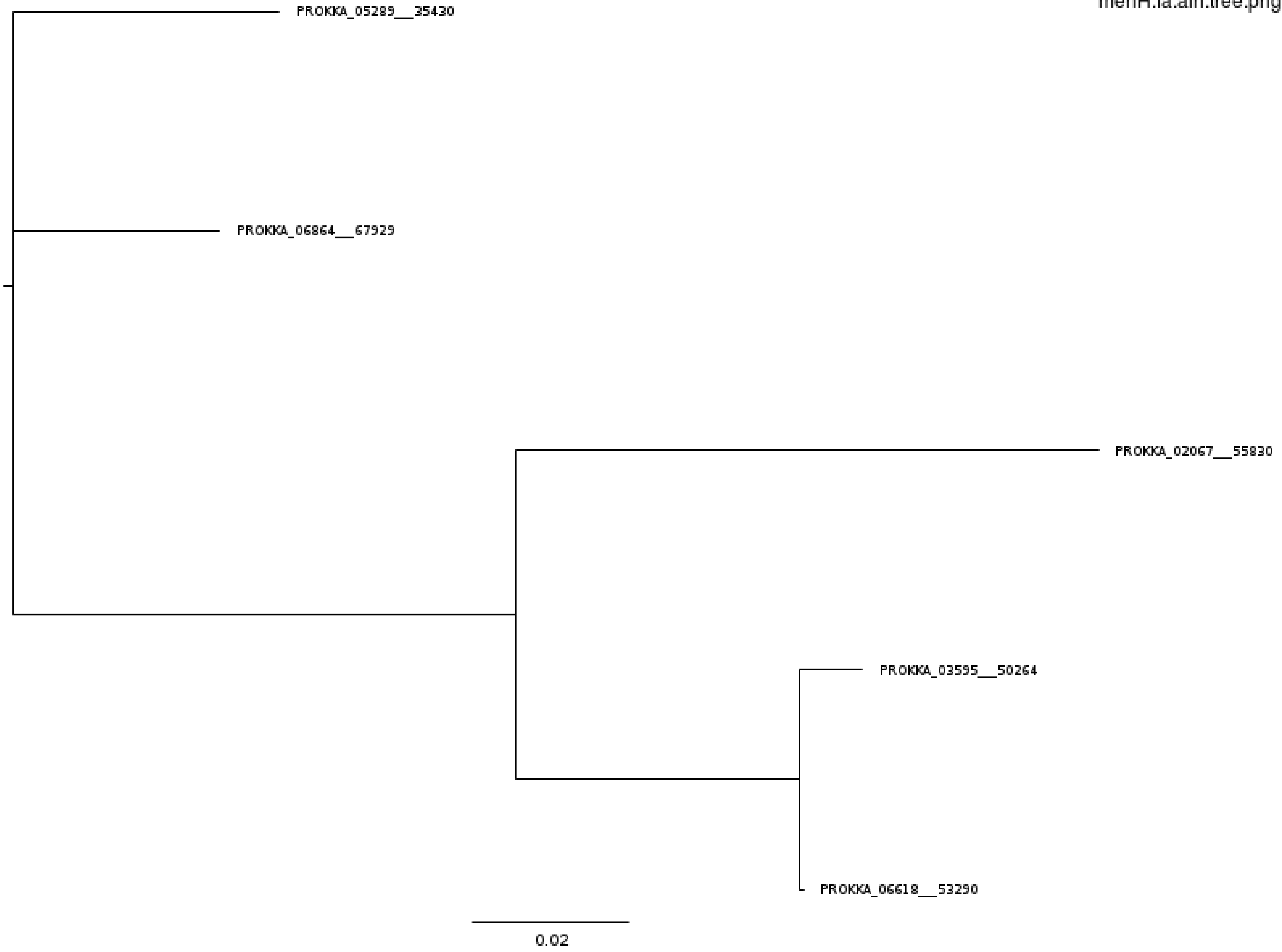

mepA_2.fa.aln.tree.png

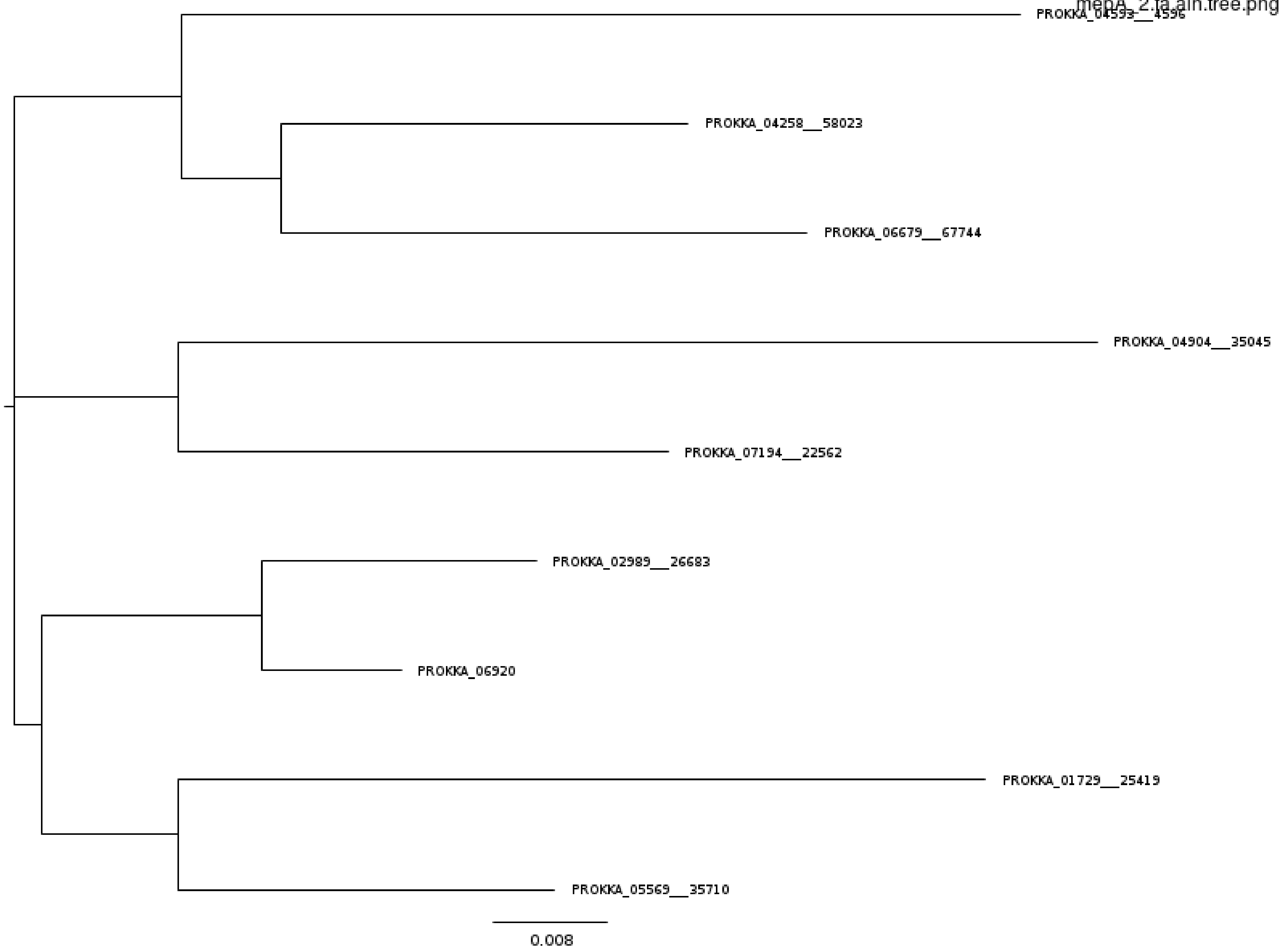

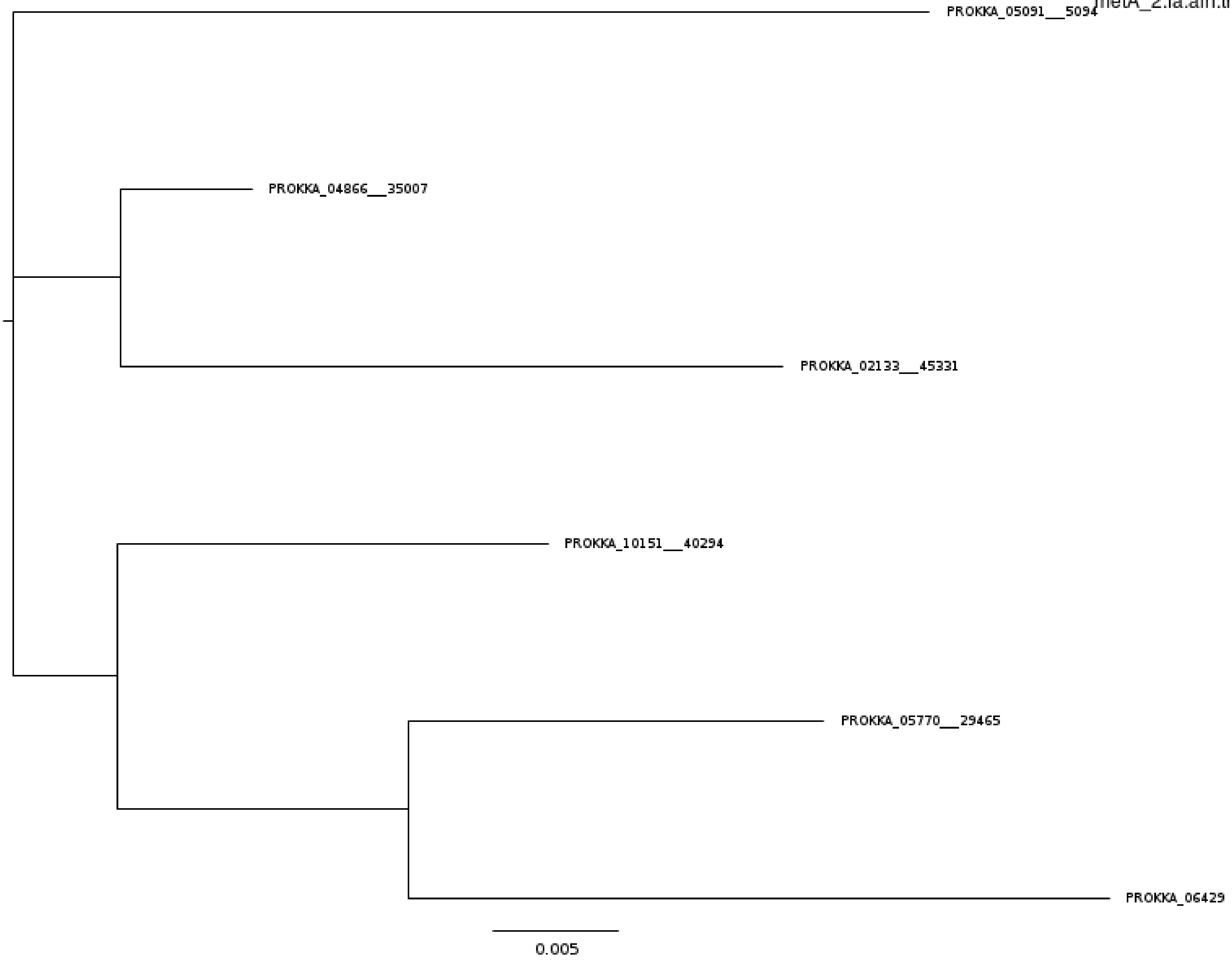
PROKKA_05091___5094
PROKKA_04866___35007
PROKKA_02133___45331
PROKKA_10151___40294
PROKKA_05770___29465
PROKKA_06429
0.005

metA.fa.aln.tree.png

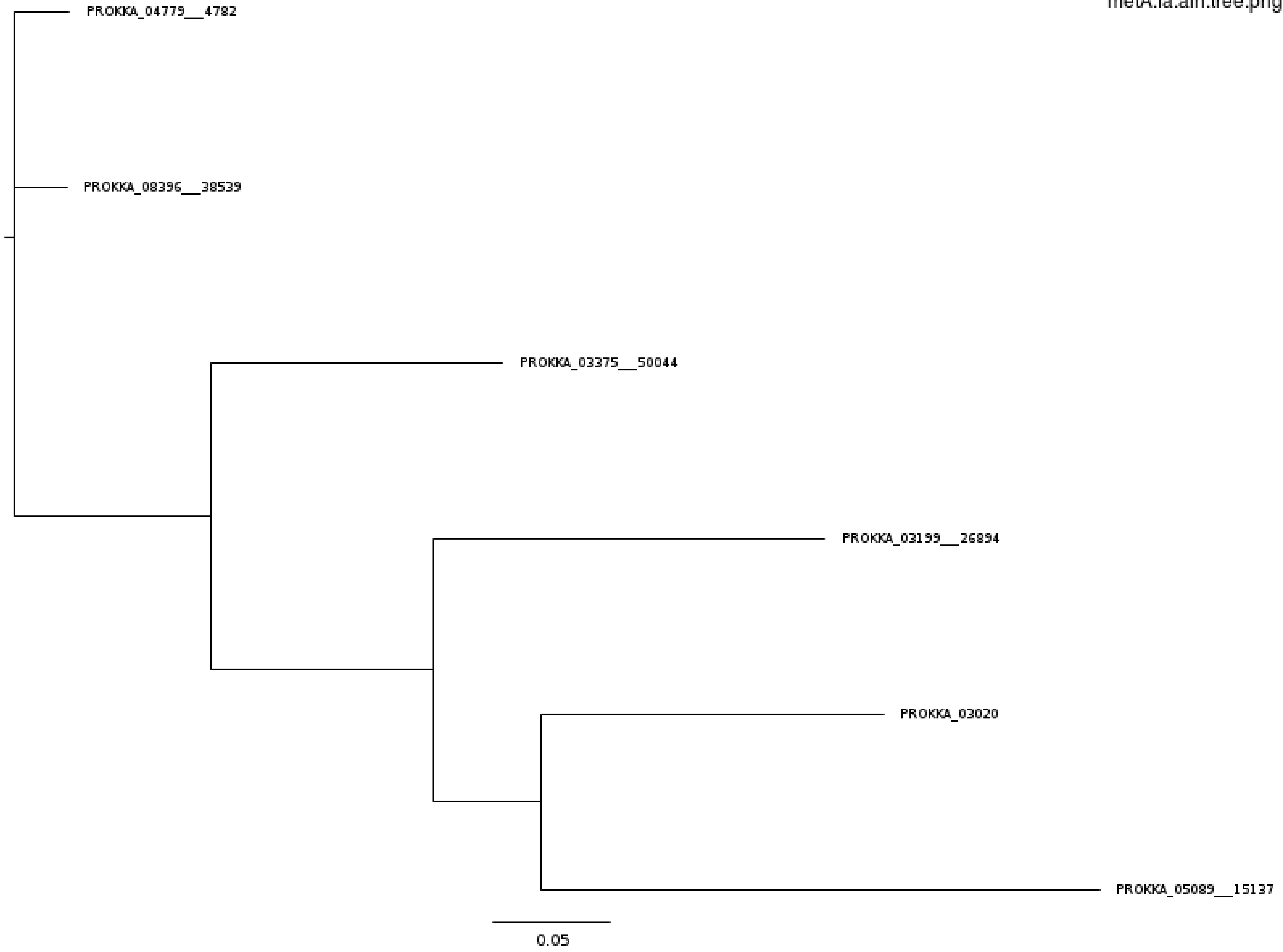

metB.fa.aln.tree.png

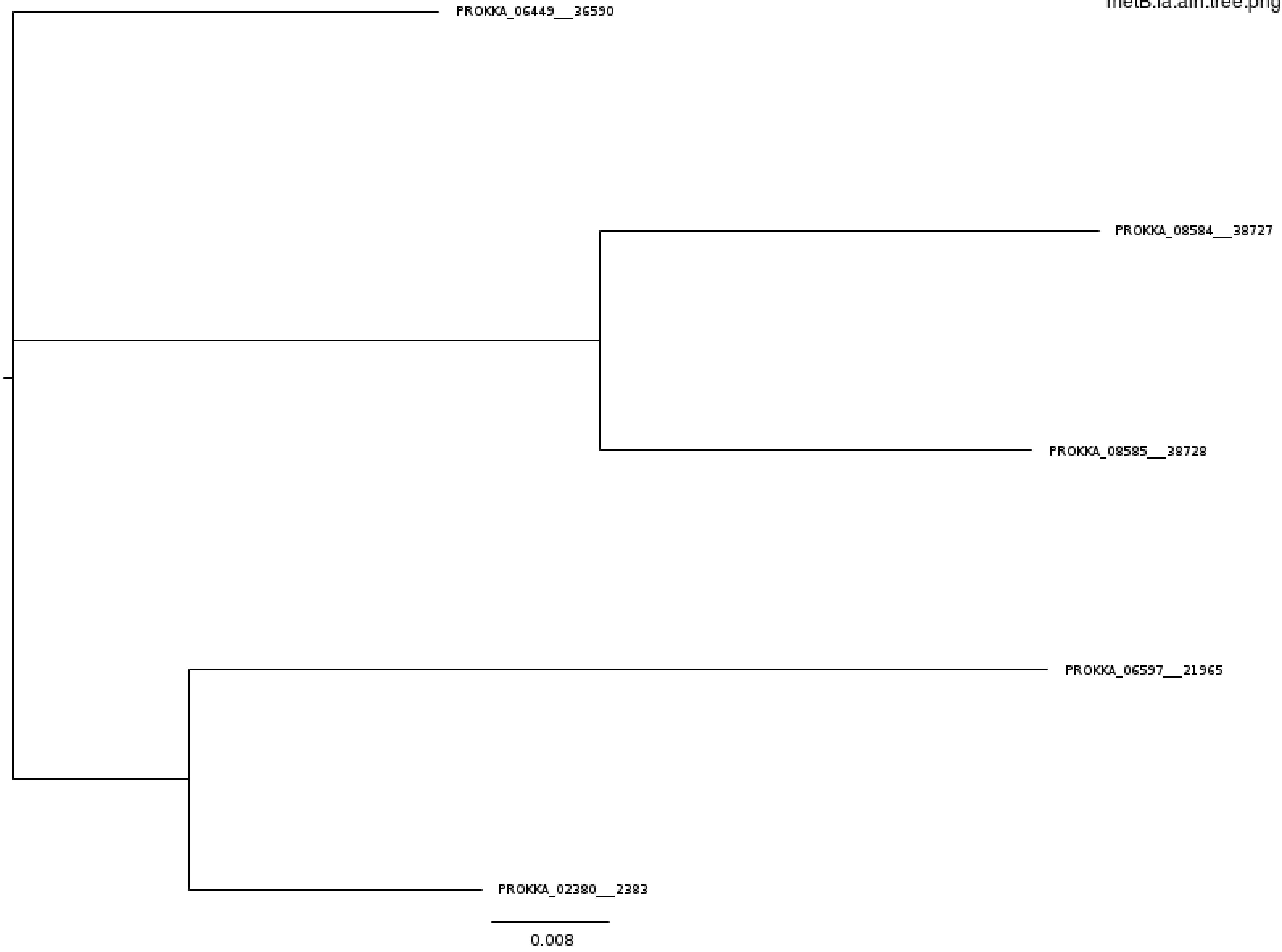

metC.fa.aln.tree.png

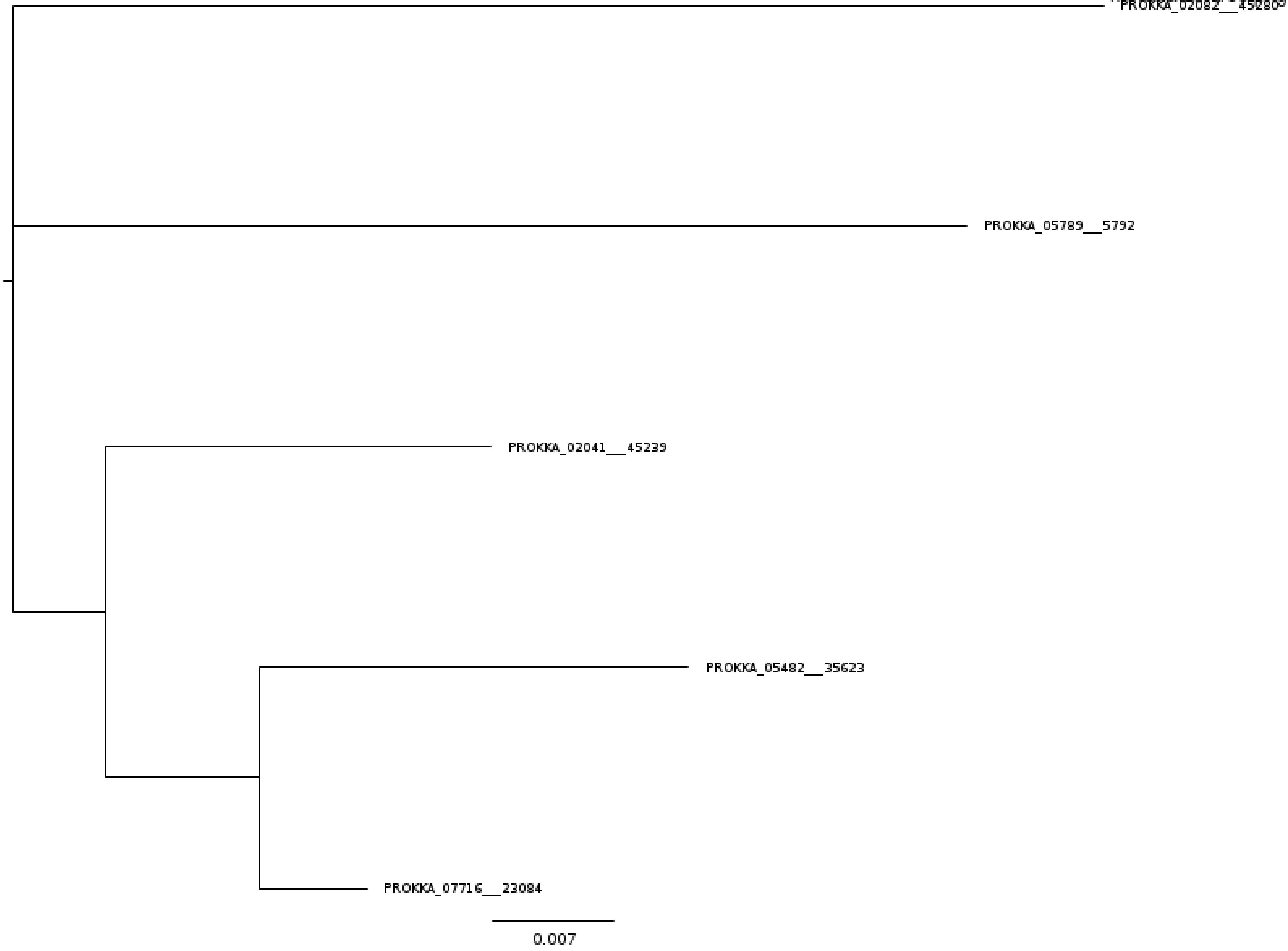

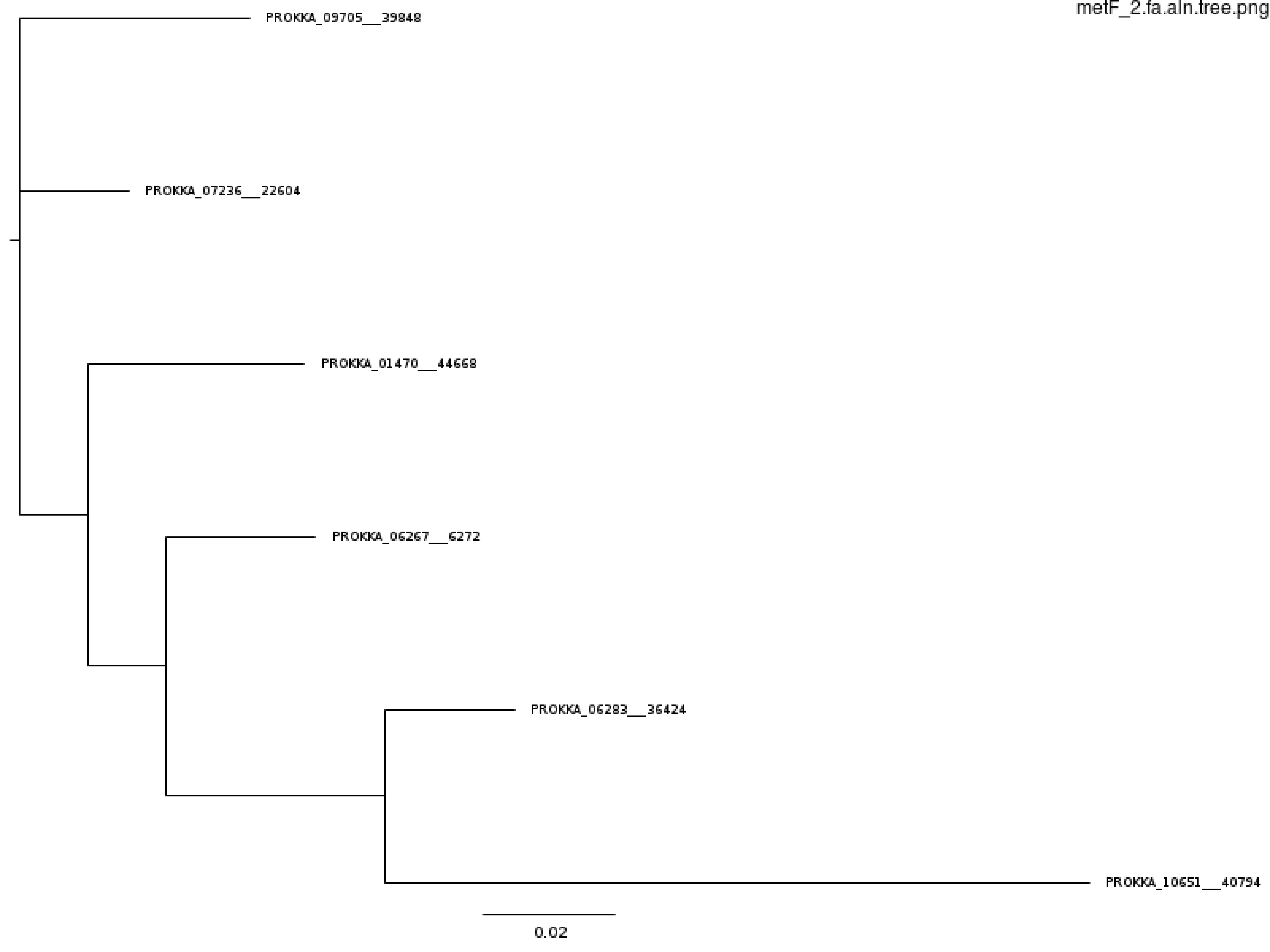
metF_2.fa.aln.tree.png
PROKKA_09705___39848
PROKKA_07236___22604
PROKKA_01470___44668
PROKKA_06267___6272
PROKKA_06283___36424
PROKKA_10651___40794
0.02

metG_2.fa.aln.tree.png

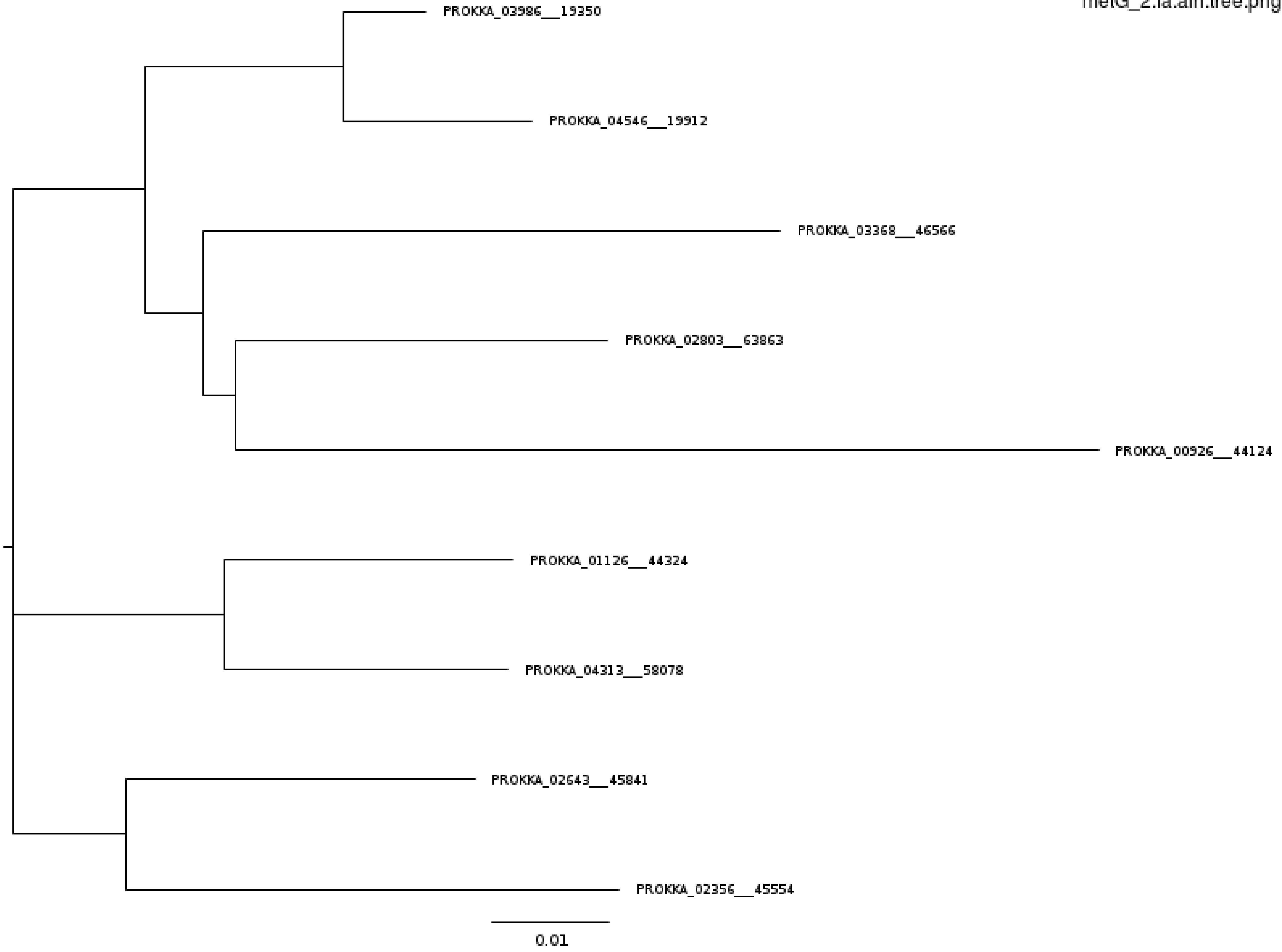

metK_2.fa.aln.tree.png

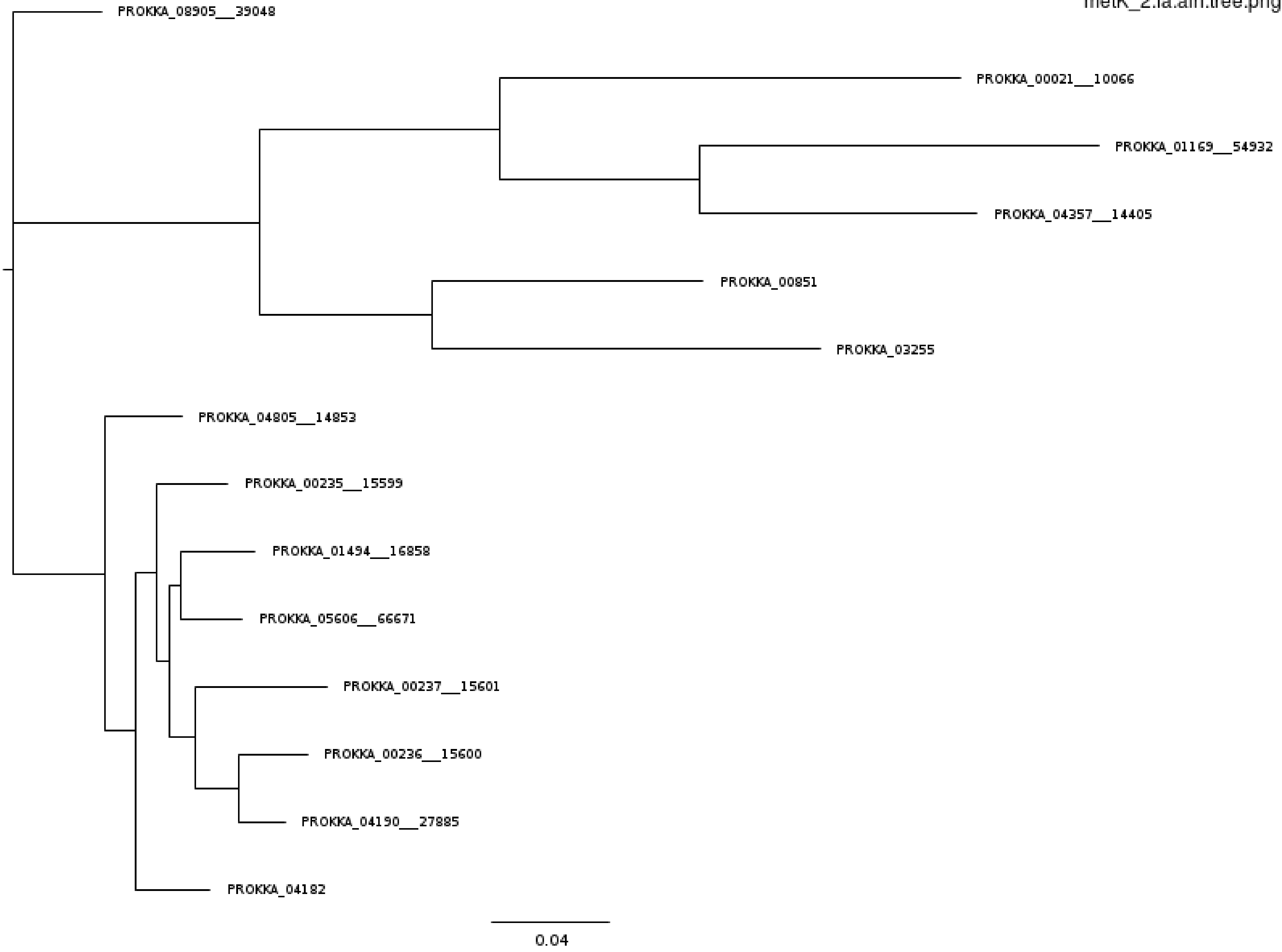

metZ_2.fa.aln.tree.png

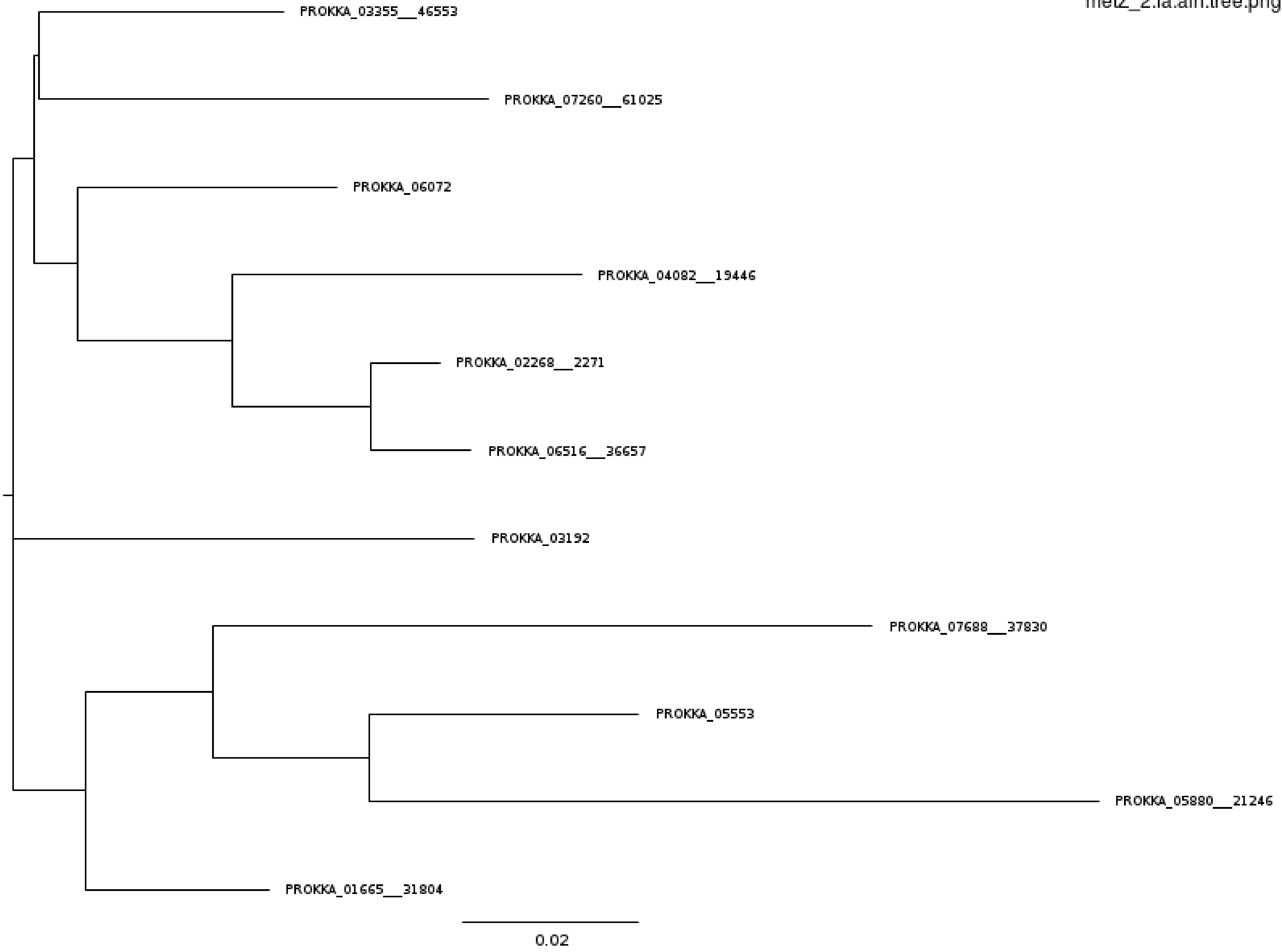

mfd_4.fa.aln.tree.png

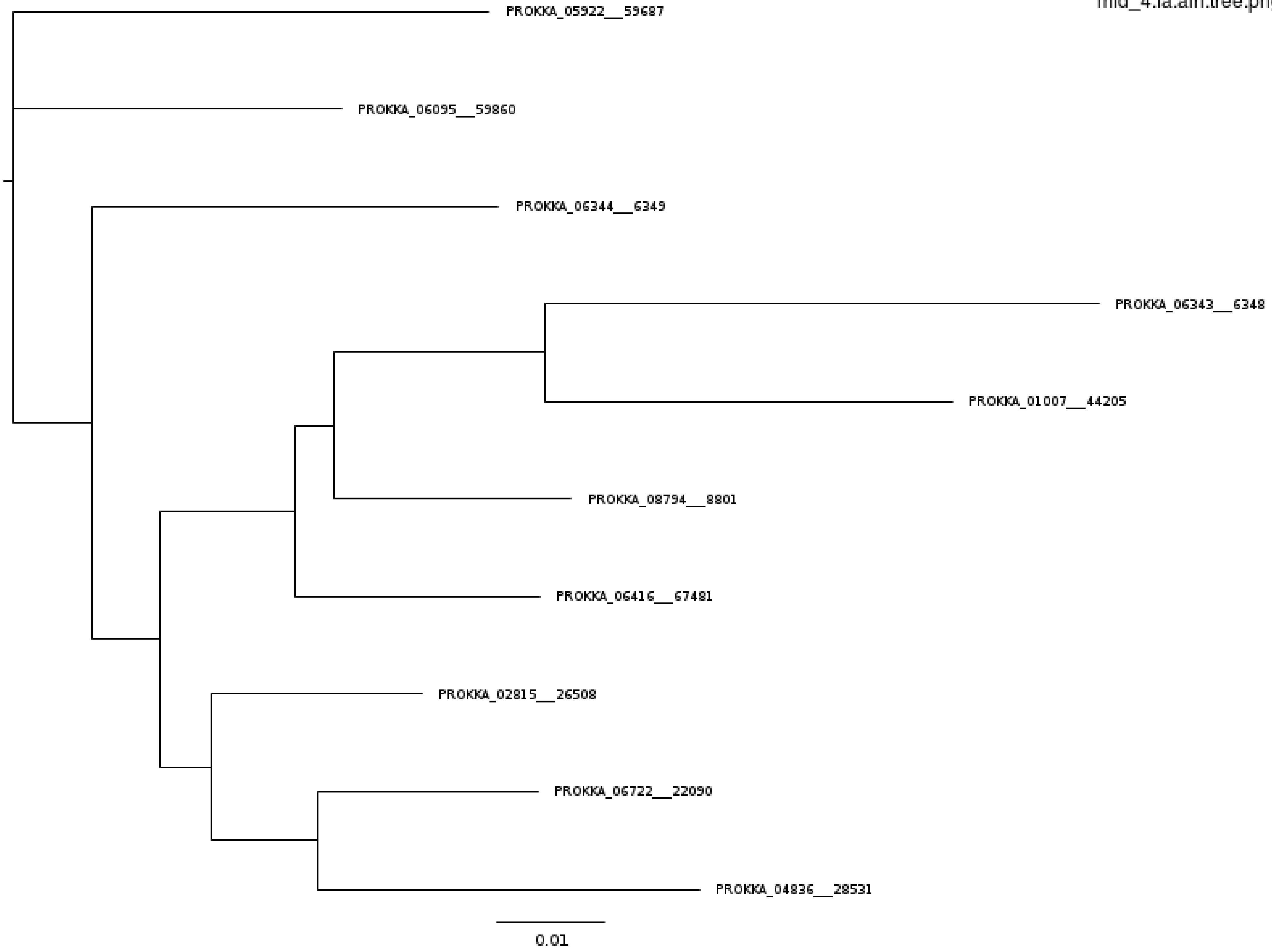

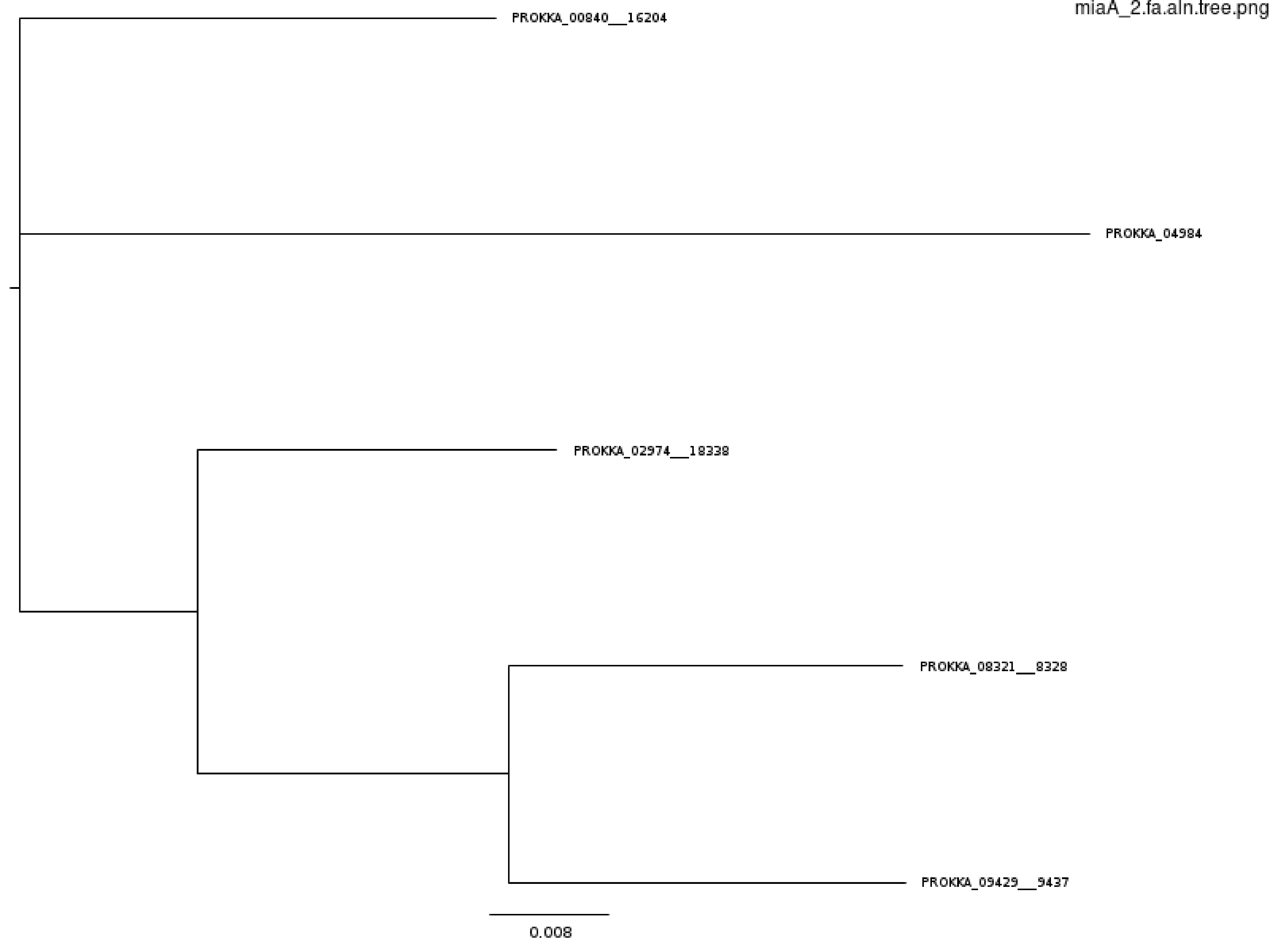

miaA_2.fa.aln.tree.png
PROKKA_00840___16204
PROKKA_04984
PROKKA_02974___18338
PROKKA_08321___8328
PROKKA_09429___9437
0.008

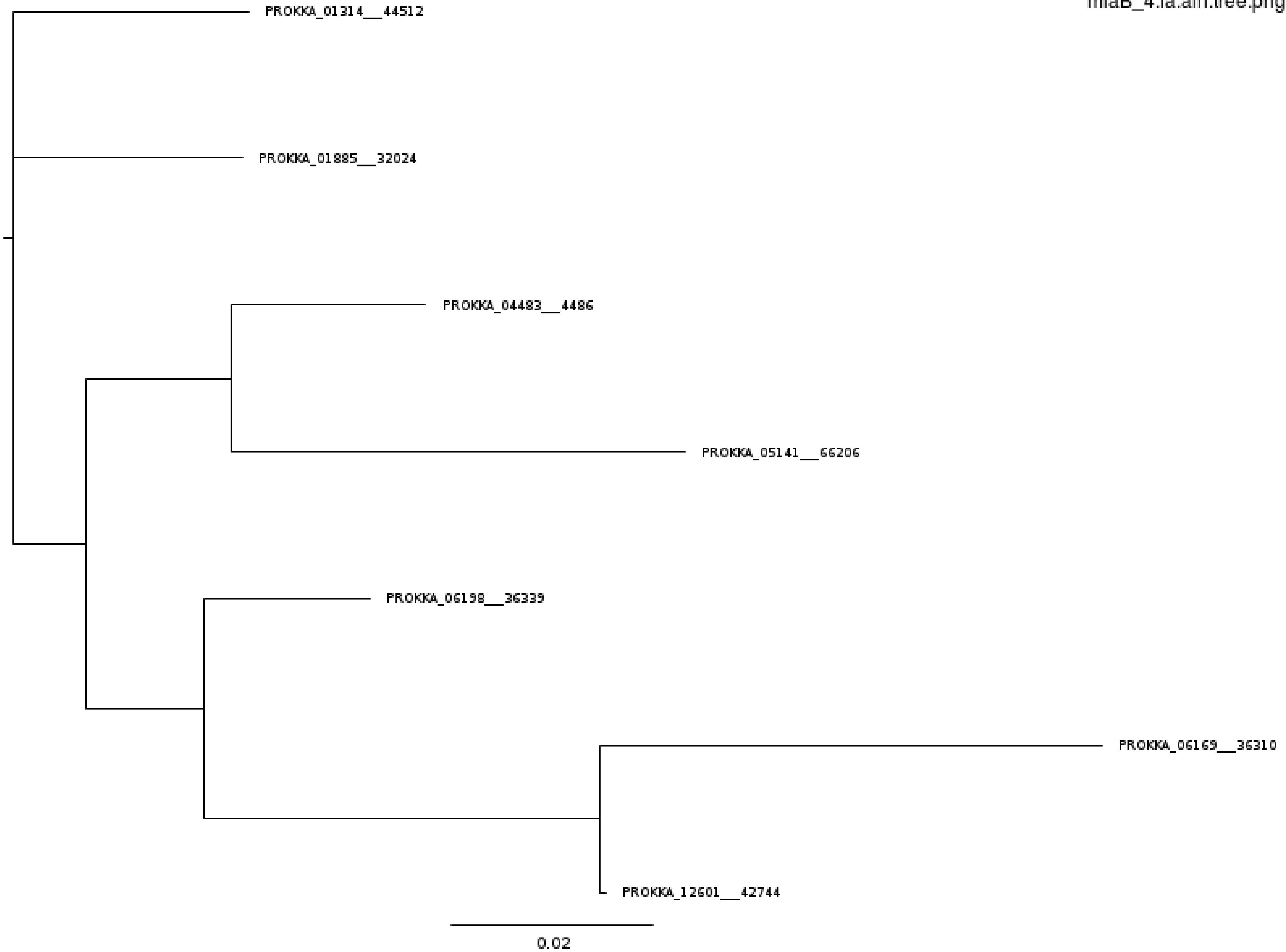
PROKKA_01314__44512
PROKKA_01885__32024
PROKKA_04483__4486
PROKKA_05141__66206
PROKKA_06198__36339
PROKKA_06169__36310
PROKKA_12601__42744
0.02

minC_1.fa.aln.tree.png

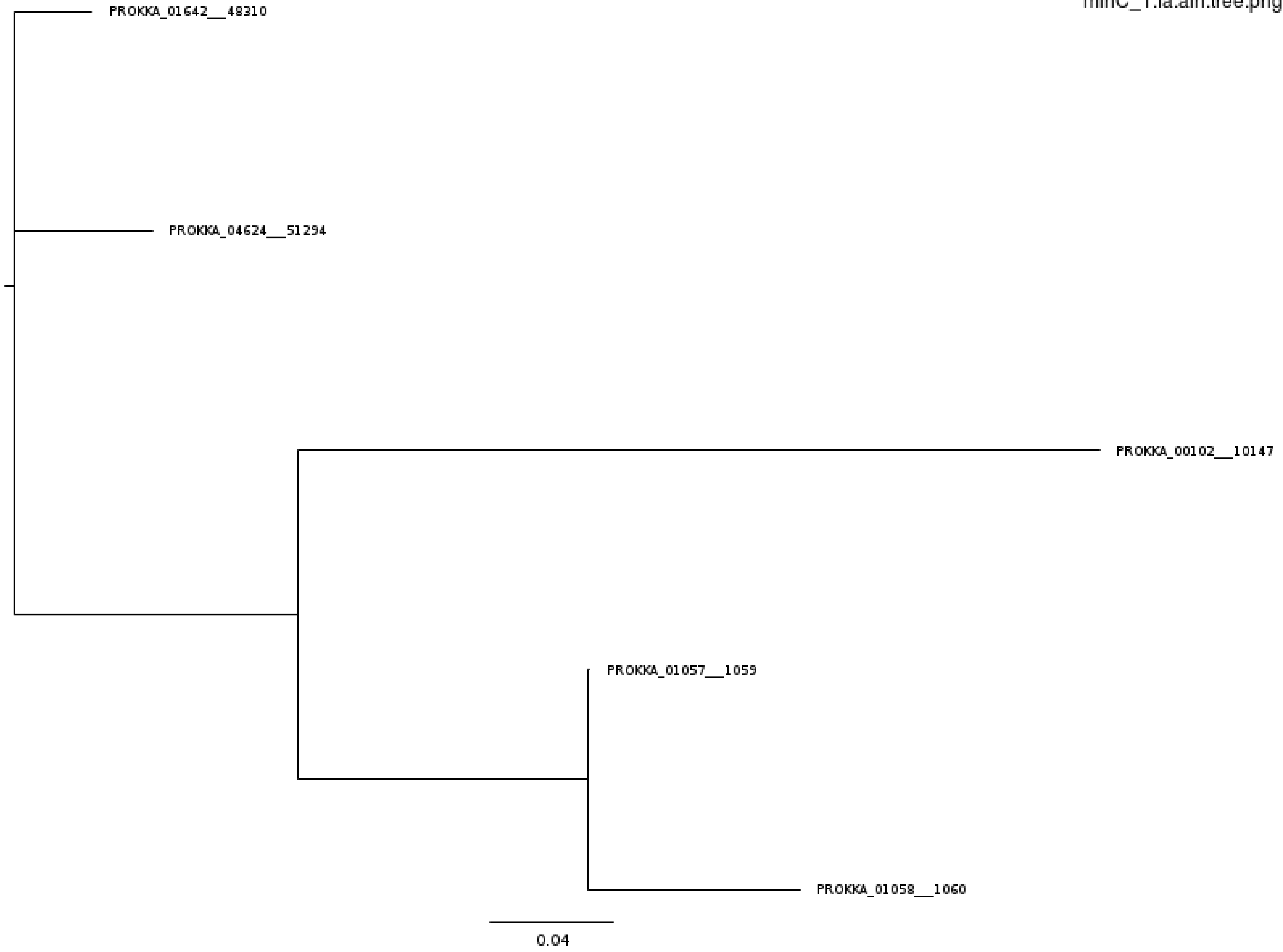

minD_1.fa.aln.tree.png

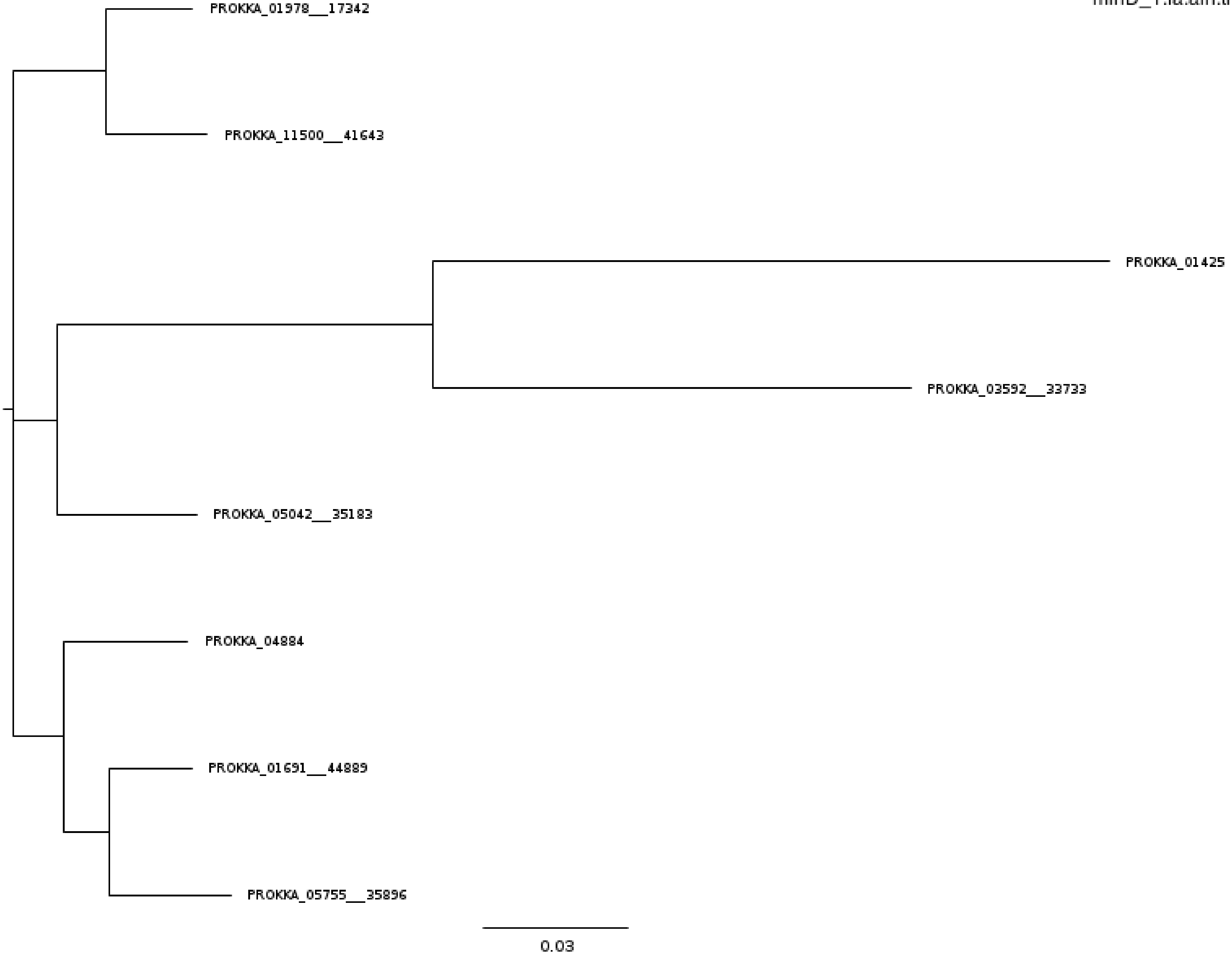

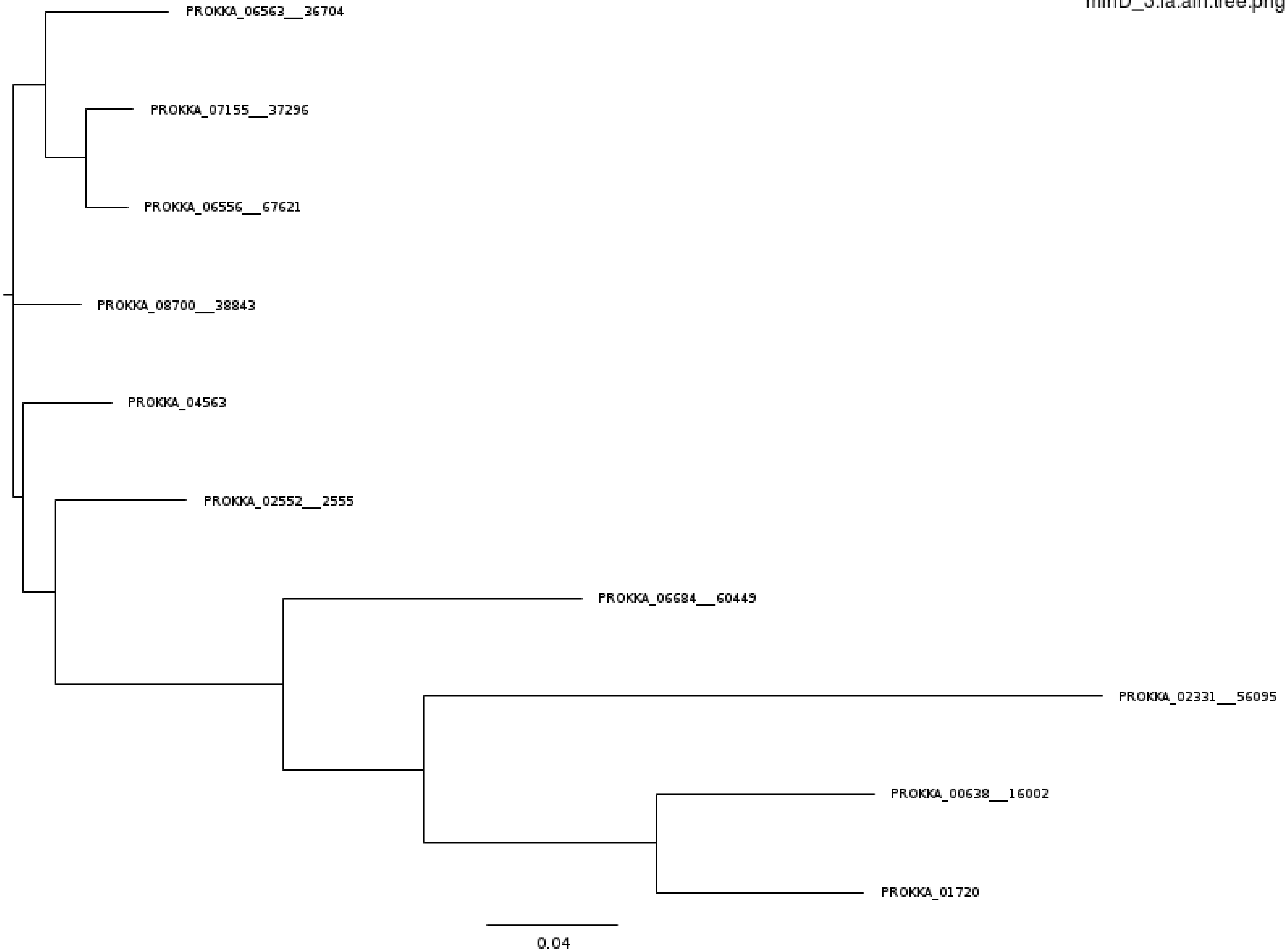
PROKKA_06563___36704
PROKKA_07155___37296
PROKKA_06556___67621
PROKKA_08700___38843
PROKKA_04563
PROKKA_02552___2555
PROKKA_06684___60449
PROKKA_02331___56095
PROKKA_00638___16002
PROKKA_01720
0.04

mlaA_2.fa.aln.tree.png

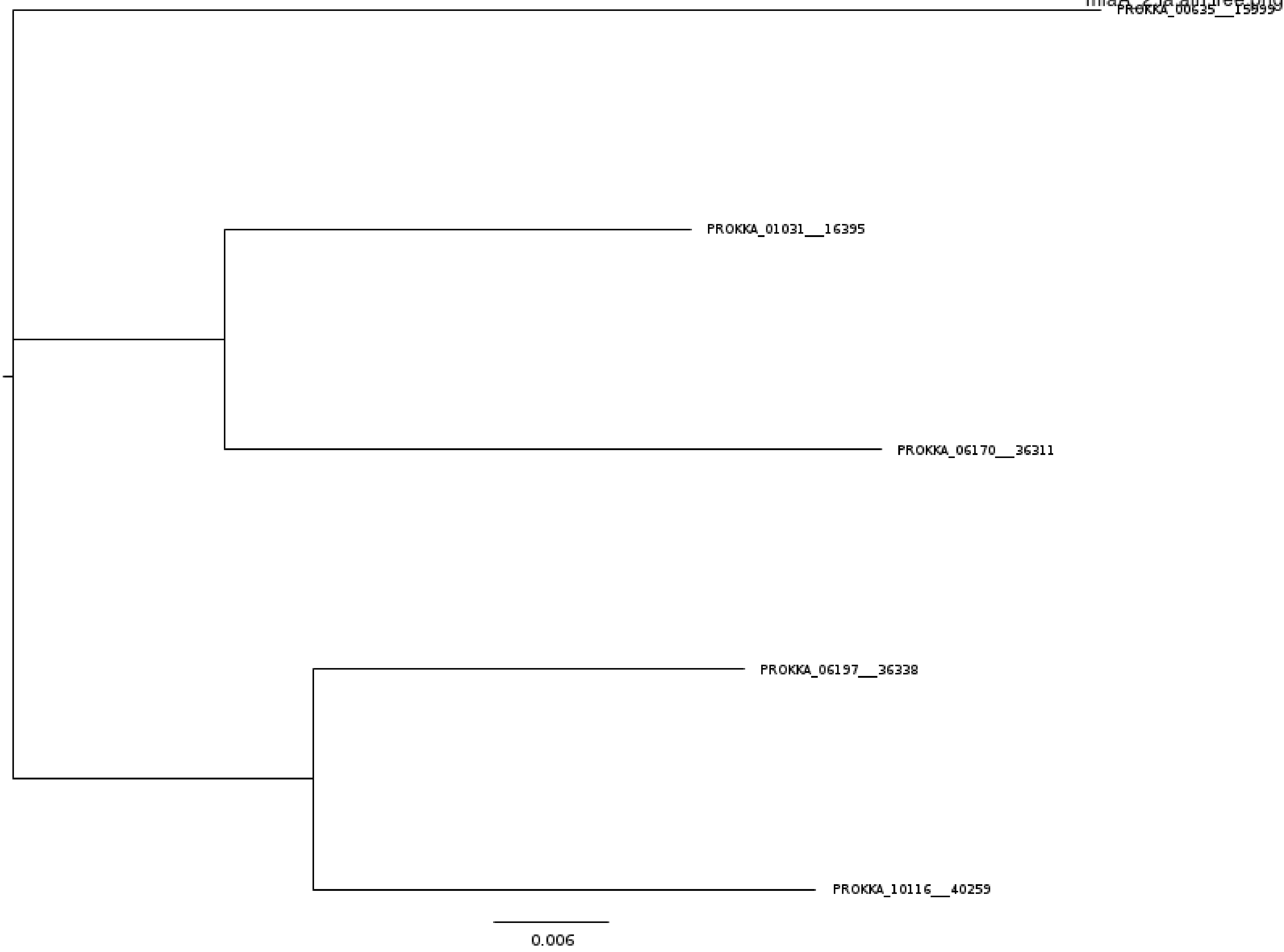

mlaC.fa.aln.tree.png

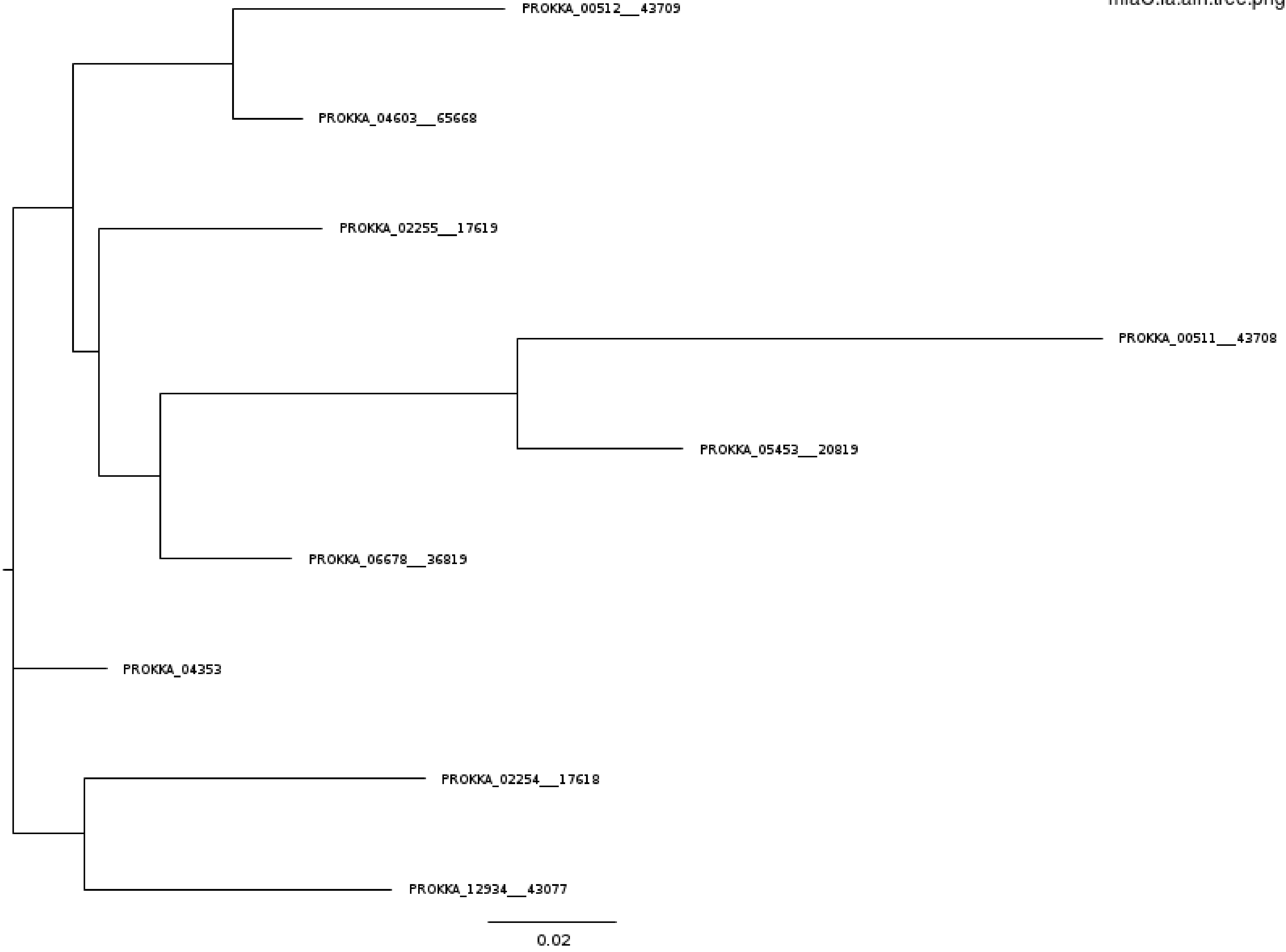

mltD_2.fa.aln.tree.png

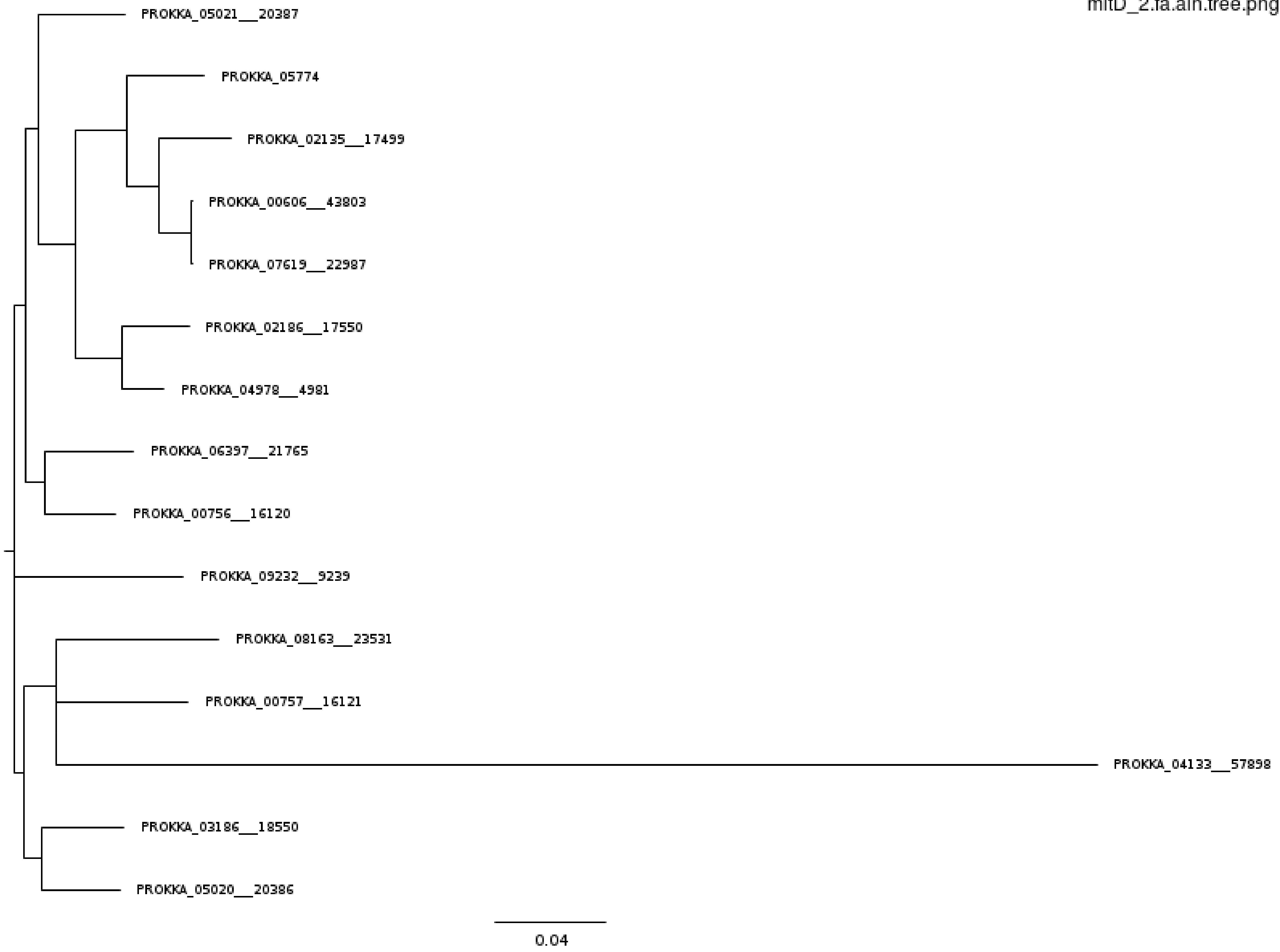

mnmA_3.fa.aln.tree.png

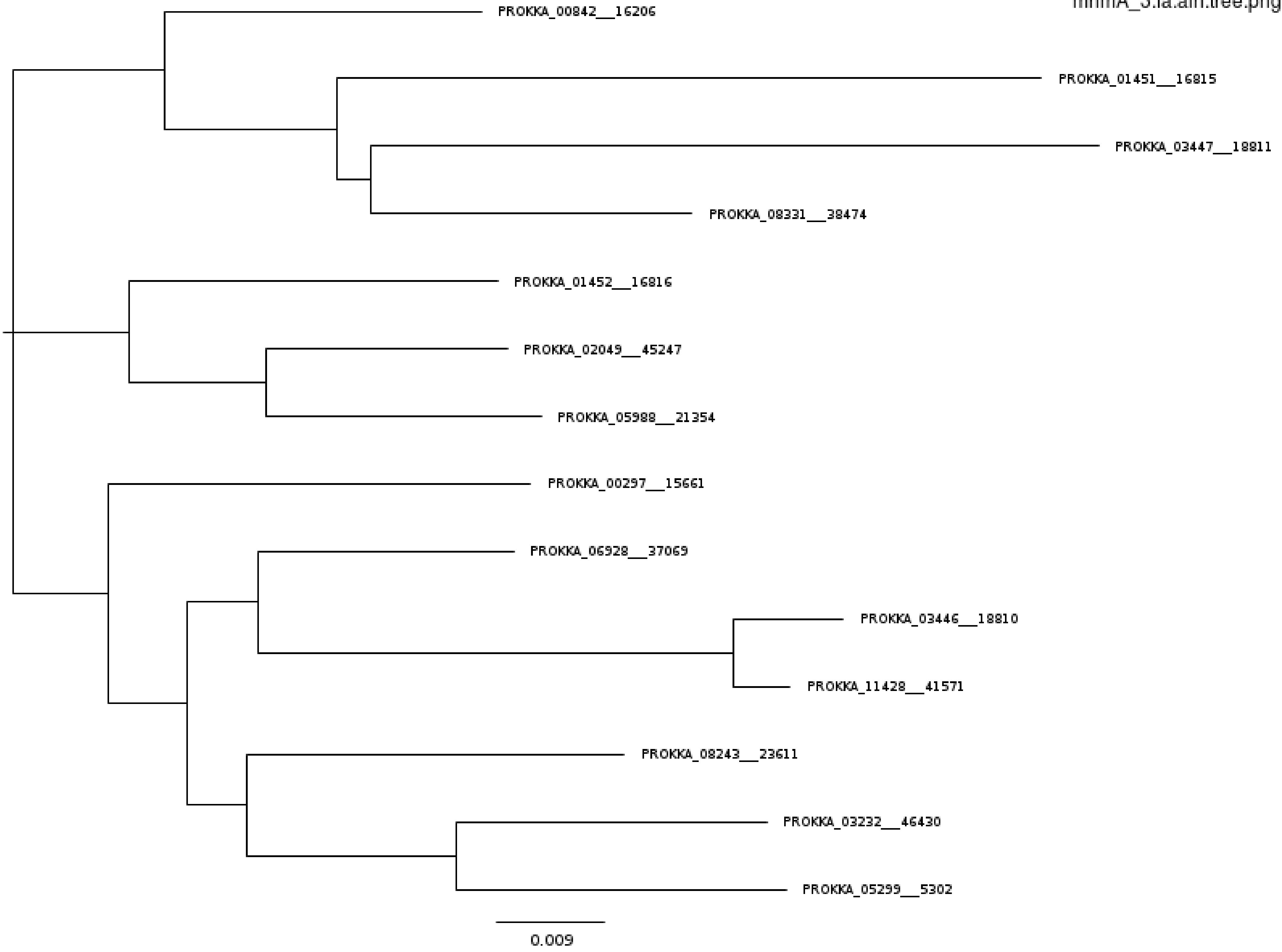

mnmE_2.fa.aln.tree.png

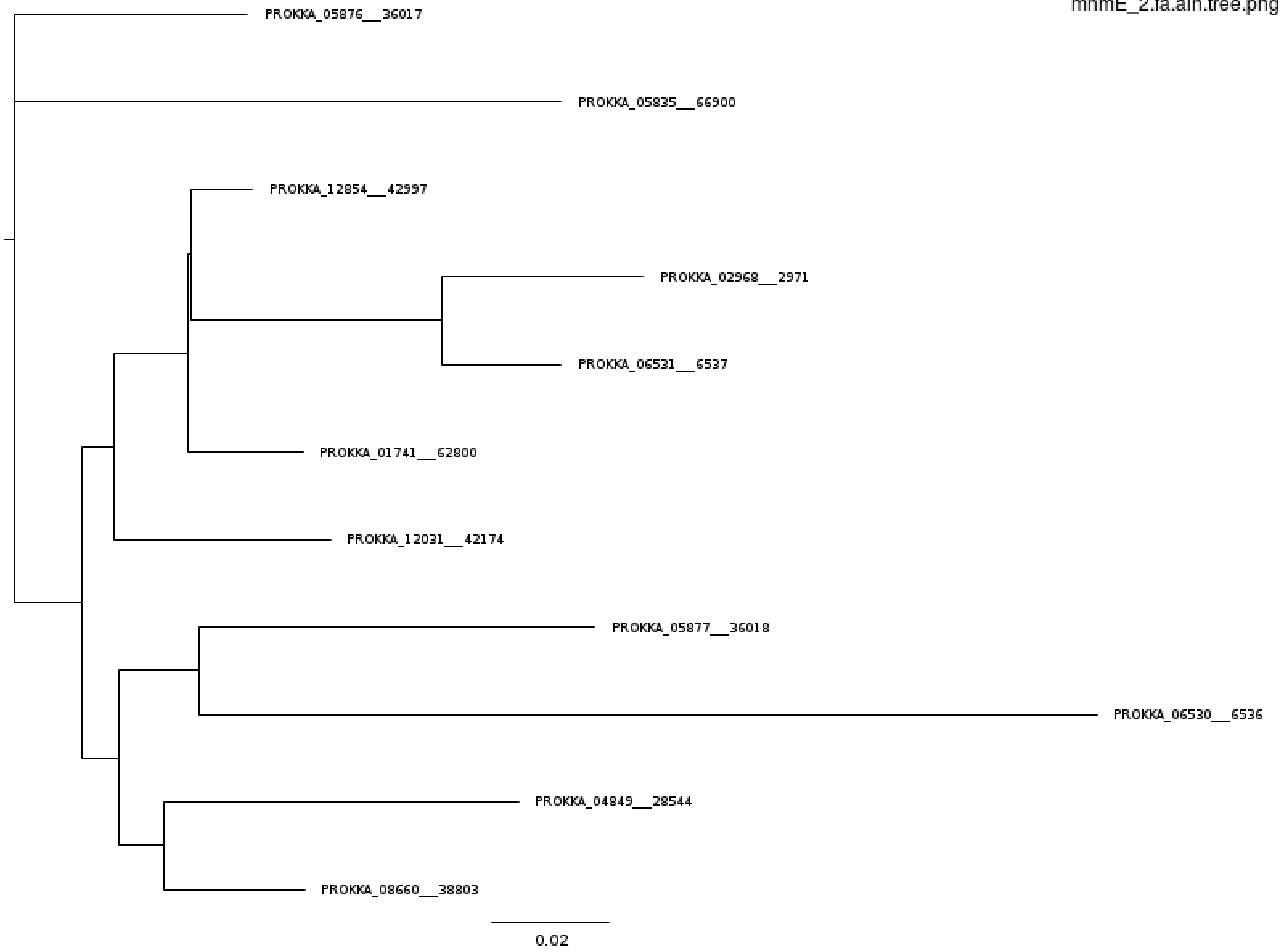

moaA_2.fa.aln.tree.png

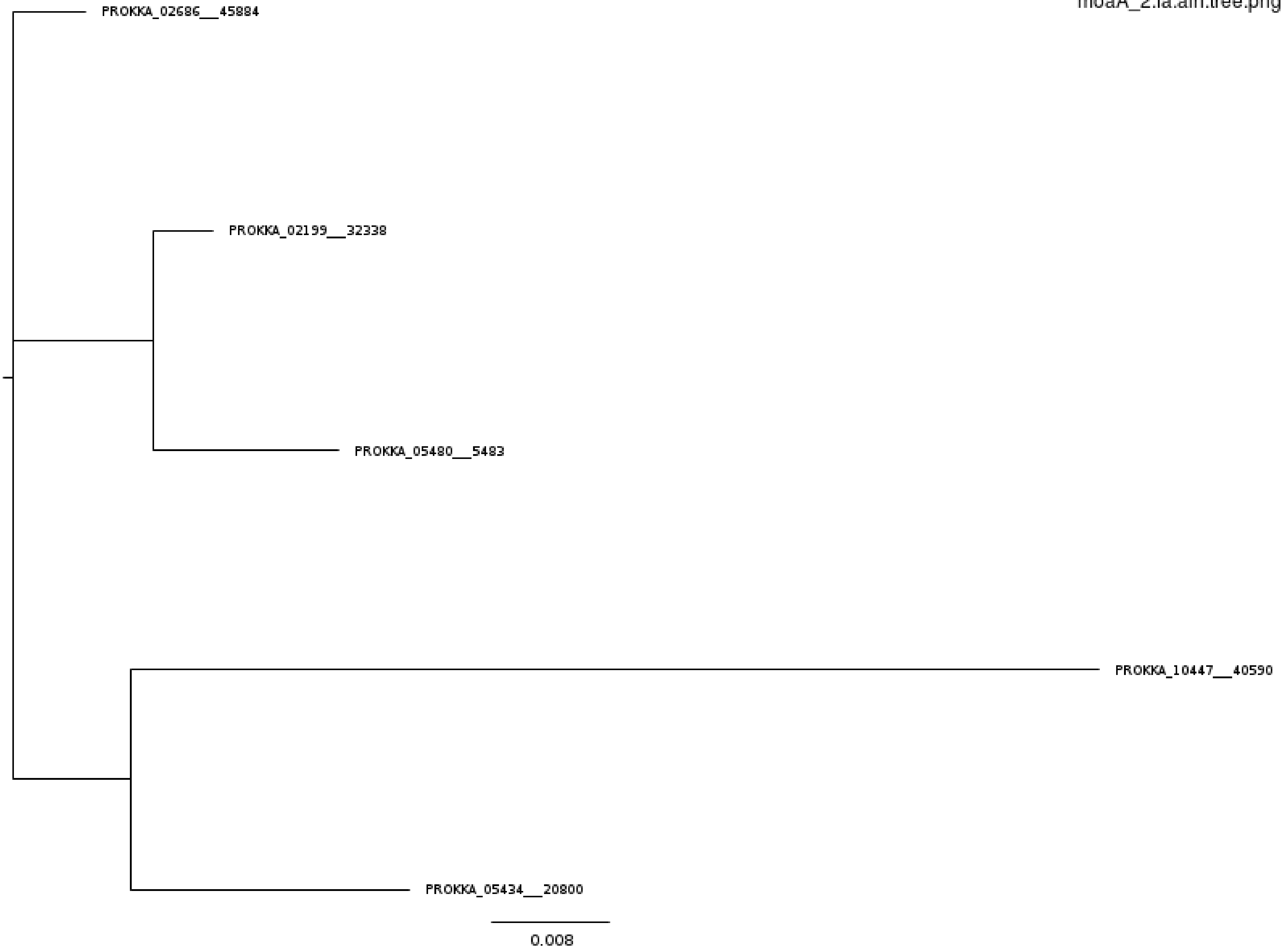

moeZ_2.fa.aln.tree.png

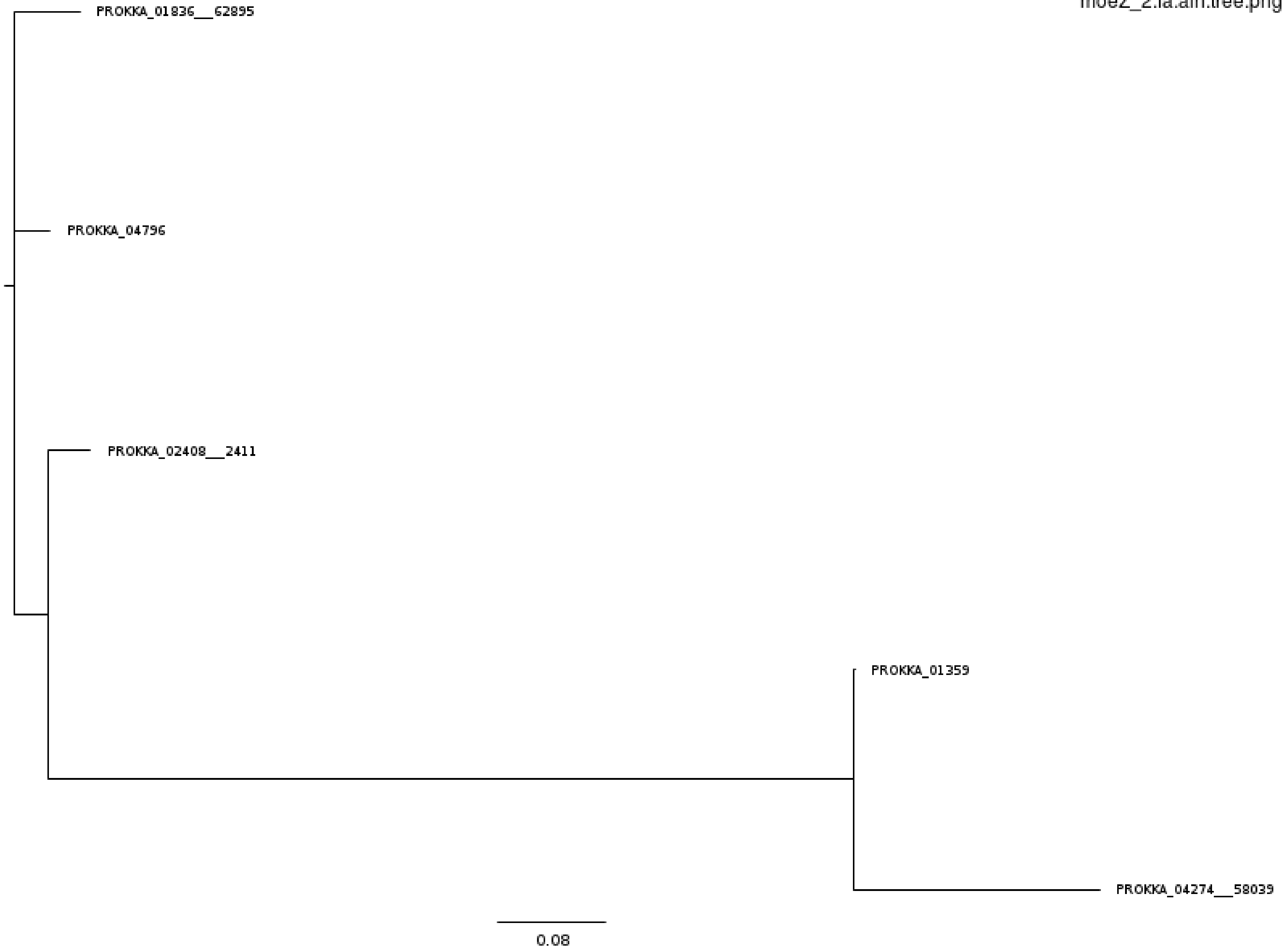

moeZ_6.fa.aln.tree.png

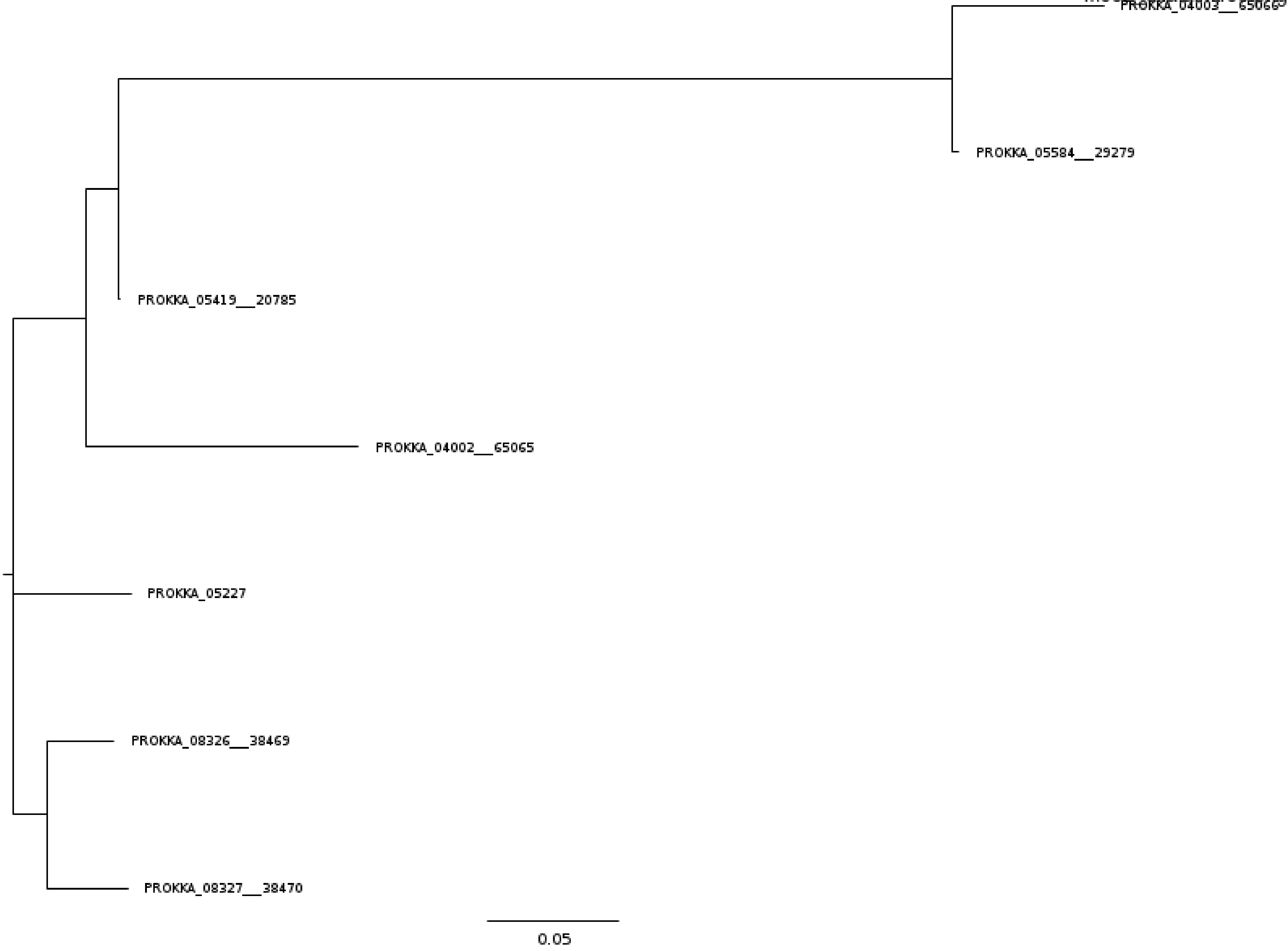

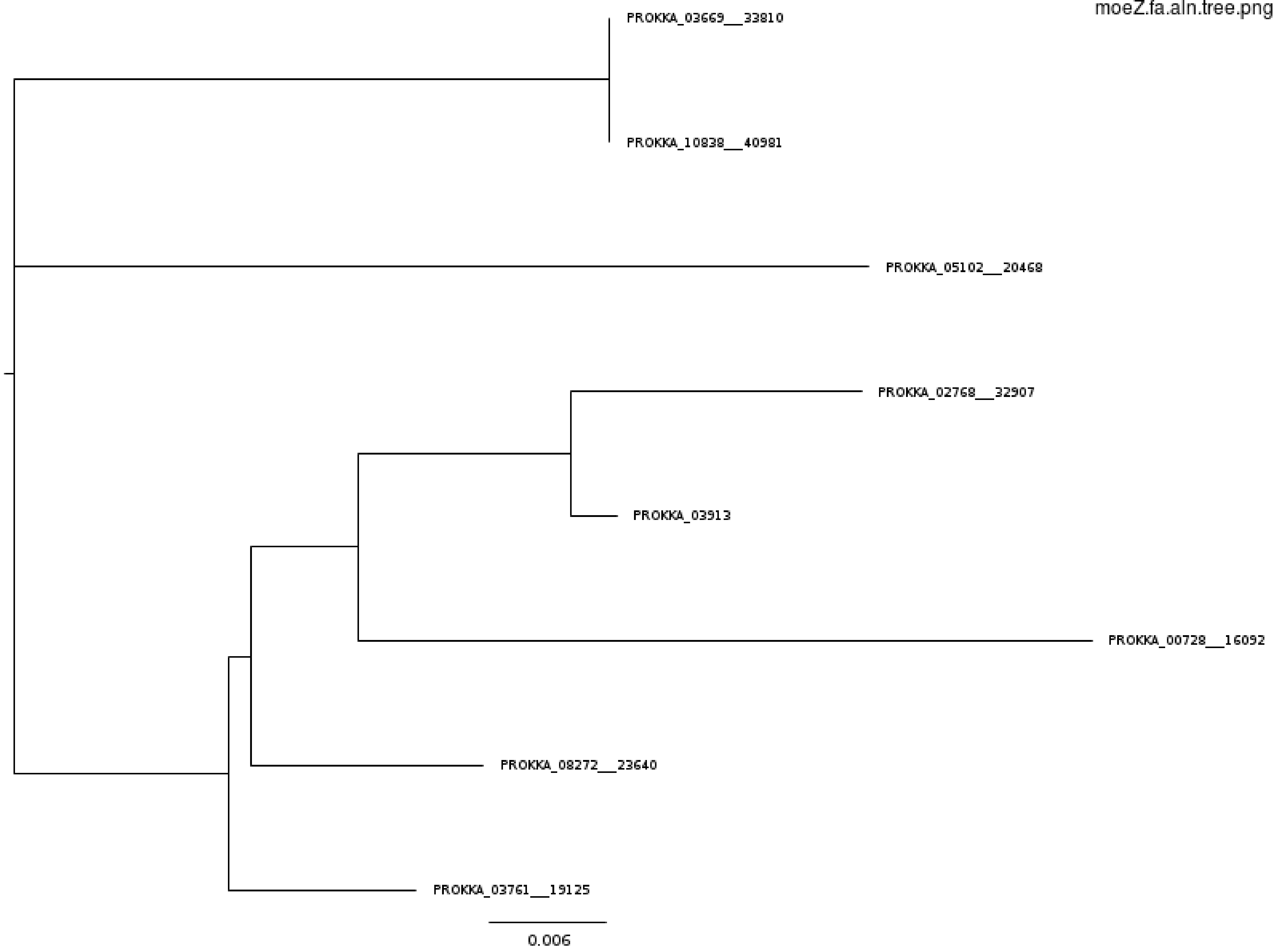
moeZ.fa.aln.tree.png
PROKKA_03669___33810
PROKKA_10838___40981
PROKKA_05102___20468
PROKKA_02768___32907
PROKKA_03913
PROKKA_00728___16092
PROKKA_08272___23640
PROKKA_03761___19125
0.006

mprA_3.fa.aln.tree.png

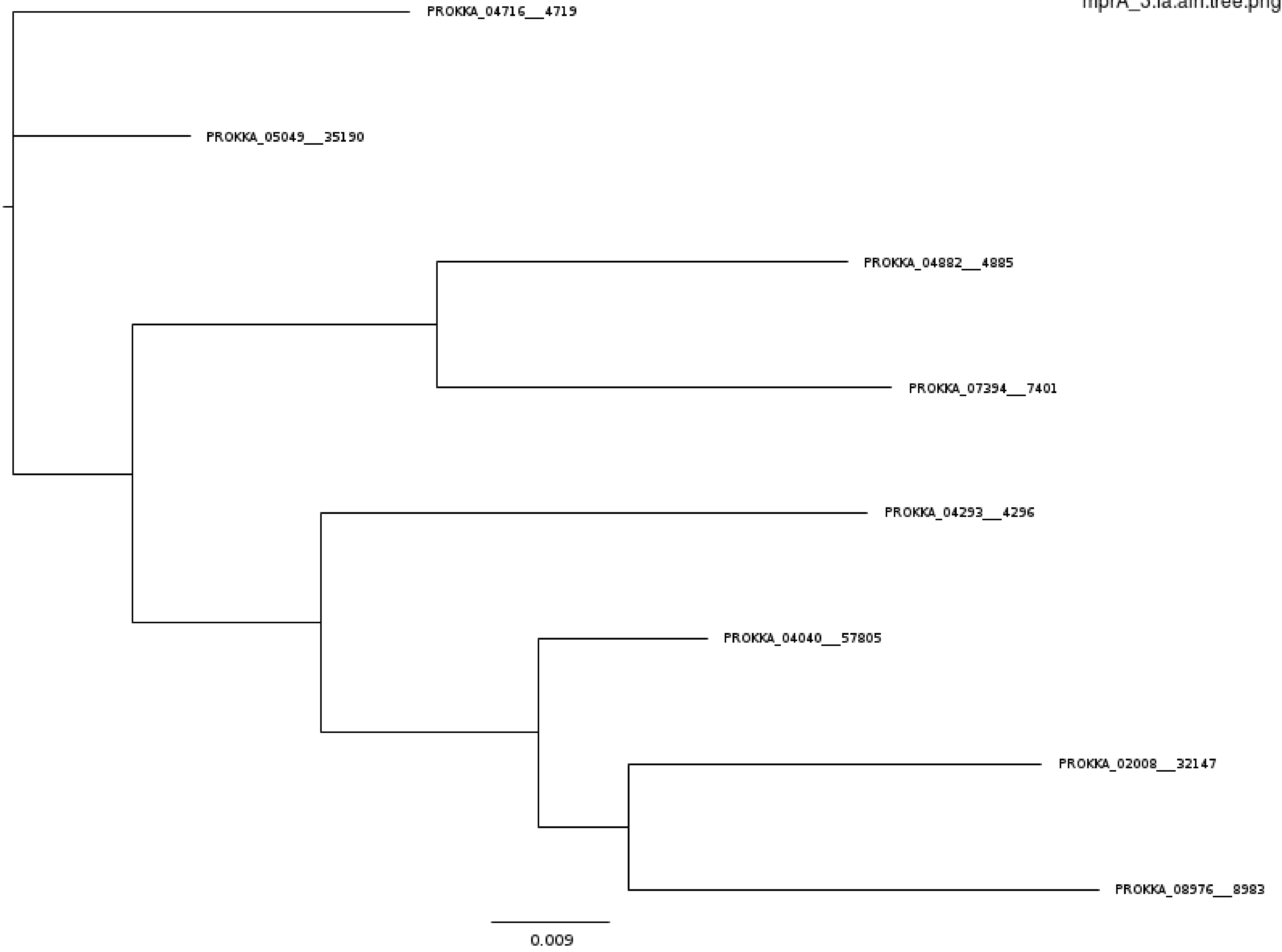

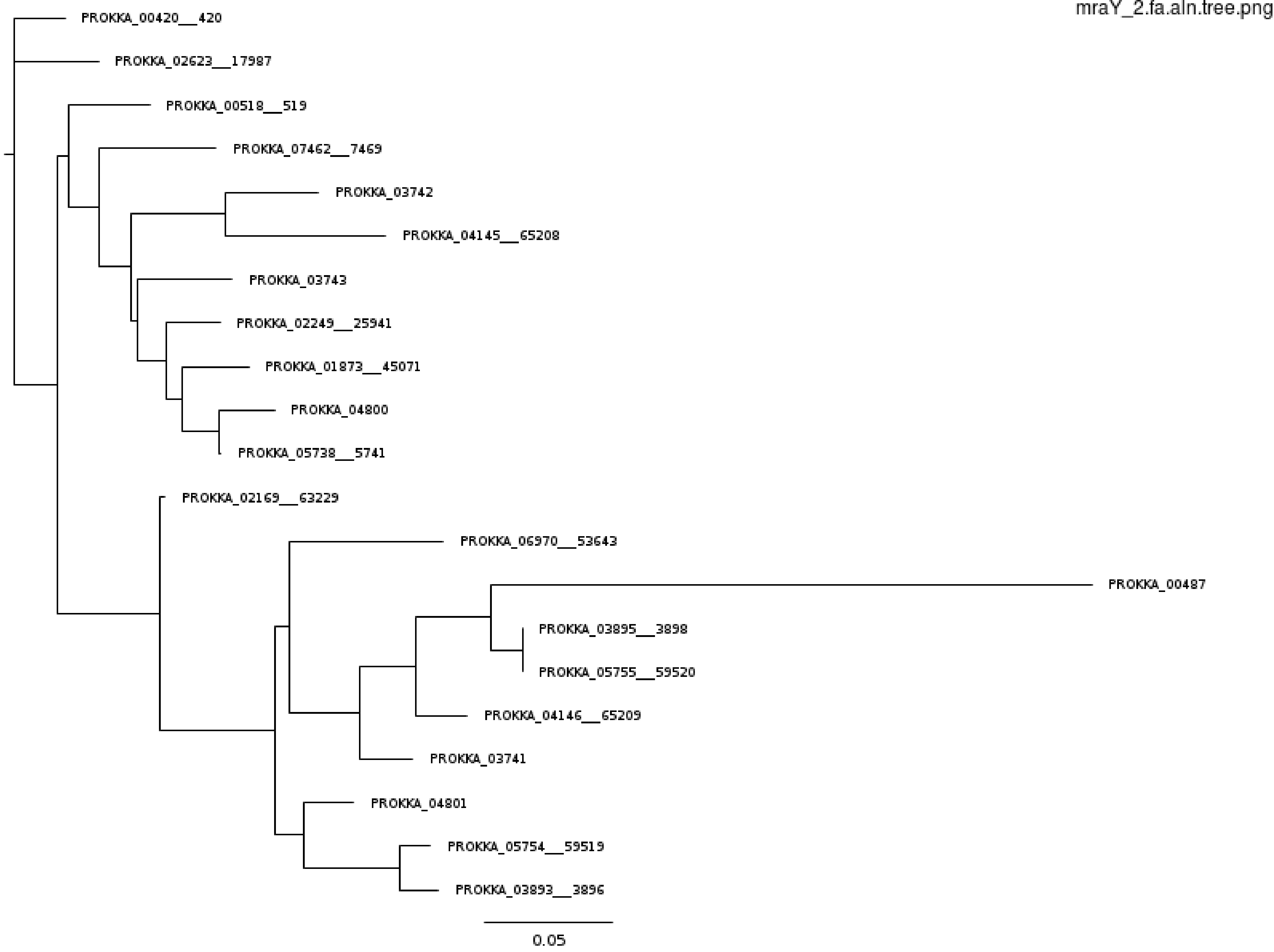

mraY_2.fa.aln.tree.png
PROKKA_00420___420
PROKKA_02623___17987
PROKKA_00518___519
PROKKA_07462___7469
PROKKA_03742
PROKKA_04145___65208
PROKKA_03743
PROKKA_02249___25941
PROKKA_01873___45071
PROKKA_04800
PROKKA_05738___5741
PROKKA_02169___63229
PROKKA_06970___53643
PROKKA_00487
PROKKA_03895___3898
PROKKA_05755___59520
PROKKA_04146___65209
PROKKA_03741
PROKKA_04801
PROKKA_05754___59519
PROKKA_03893___3896
0.05

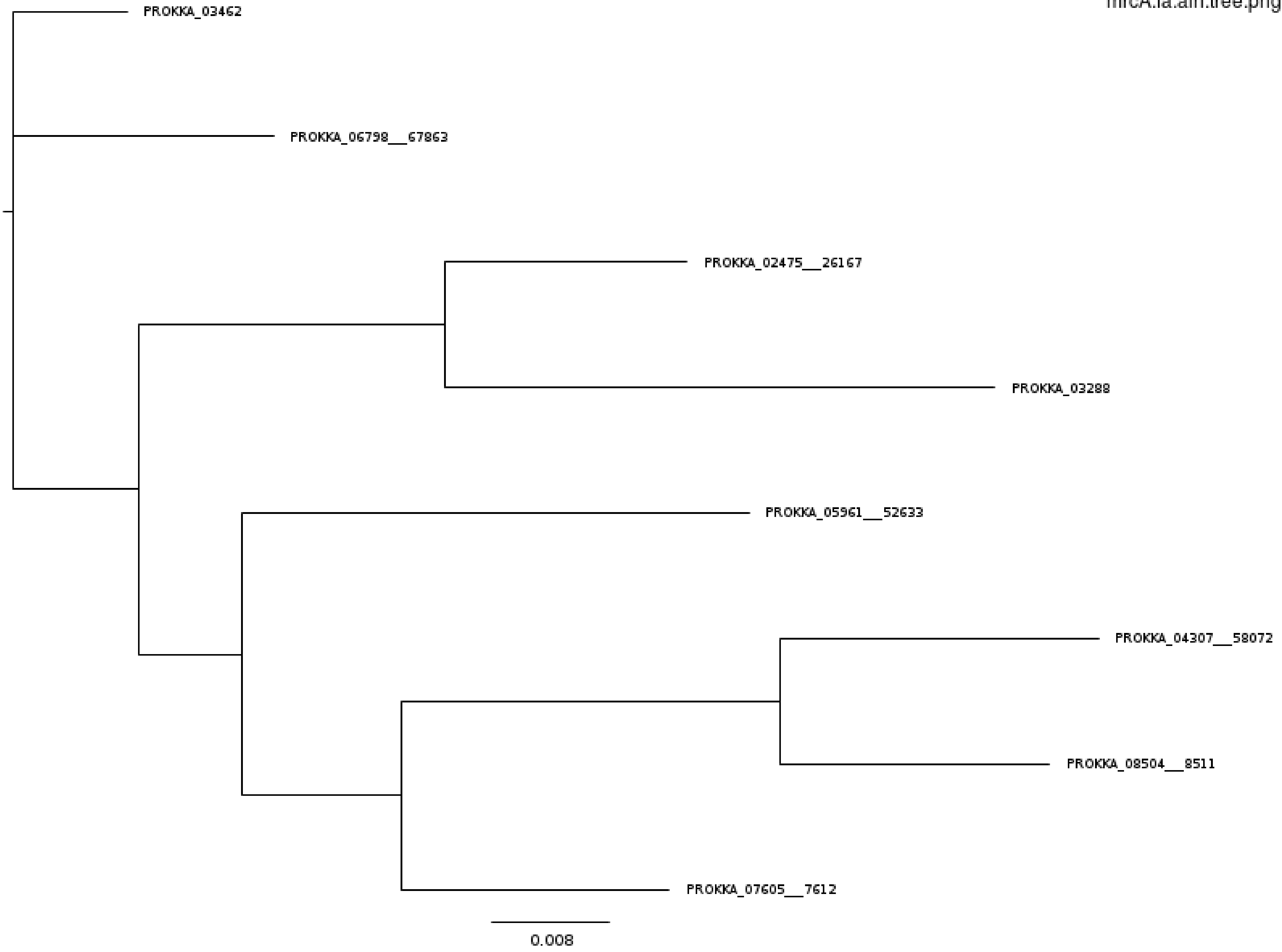
PROKKA_03462
PROKKA_06798__67863
PROKKA_02475__26167
PROKKA_03288
PROKKA_05961__52633
PROKKA_04307__58072
PROKKA_08504__8511
PROKKA_07605__7612
0.008

mrcB_1.fa.aln.tree.png

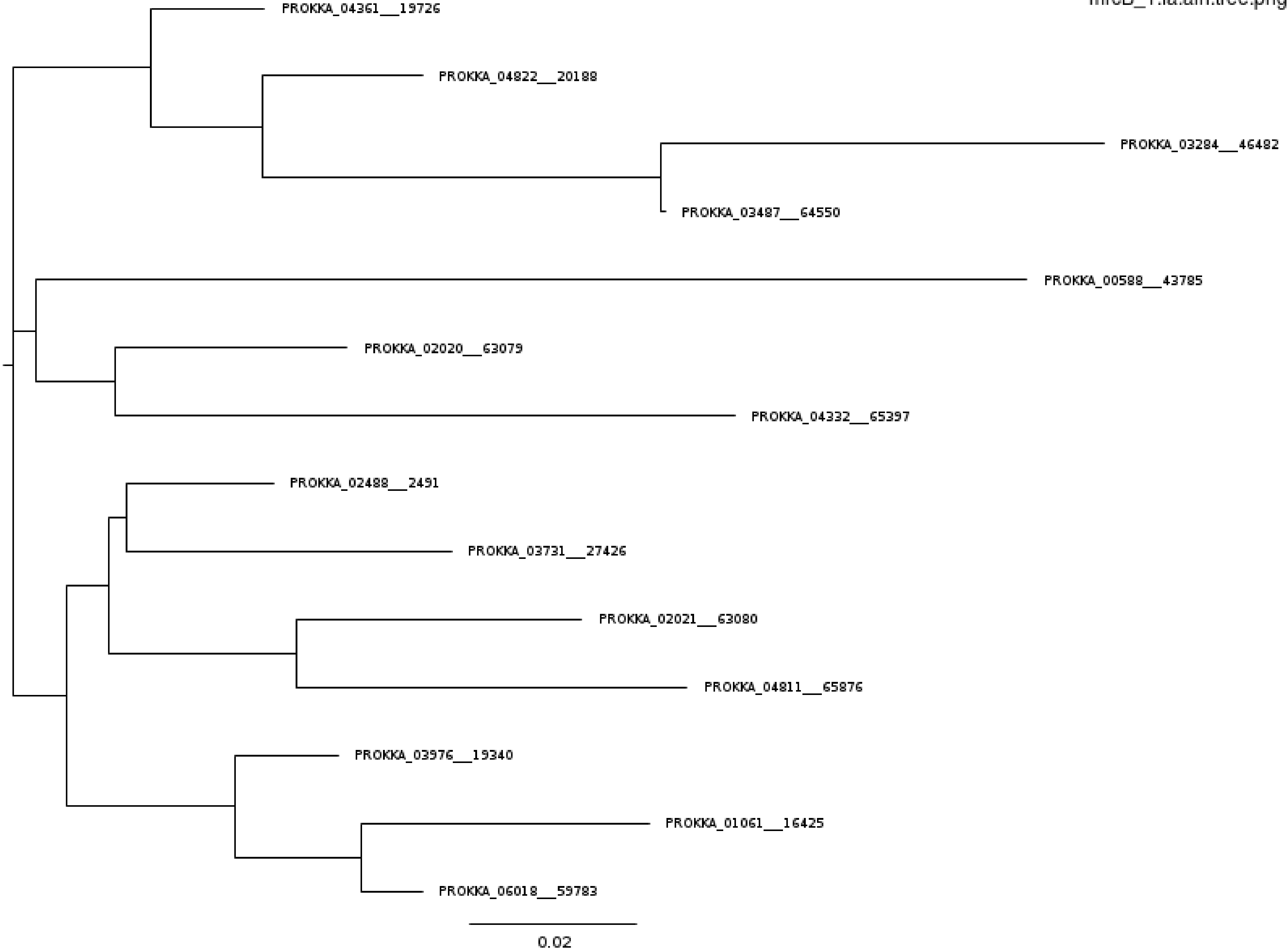

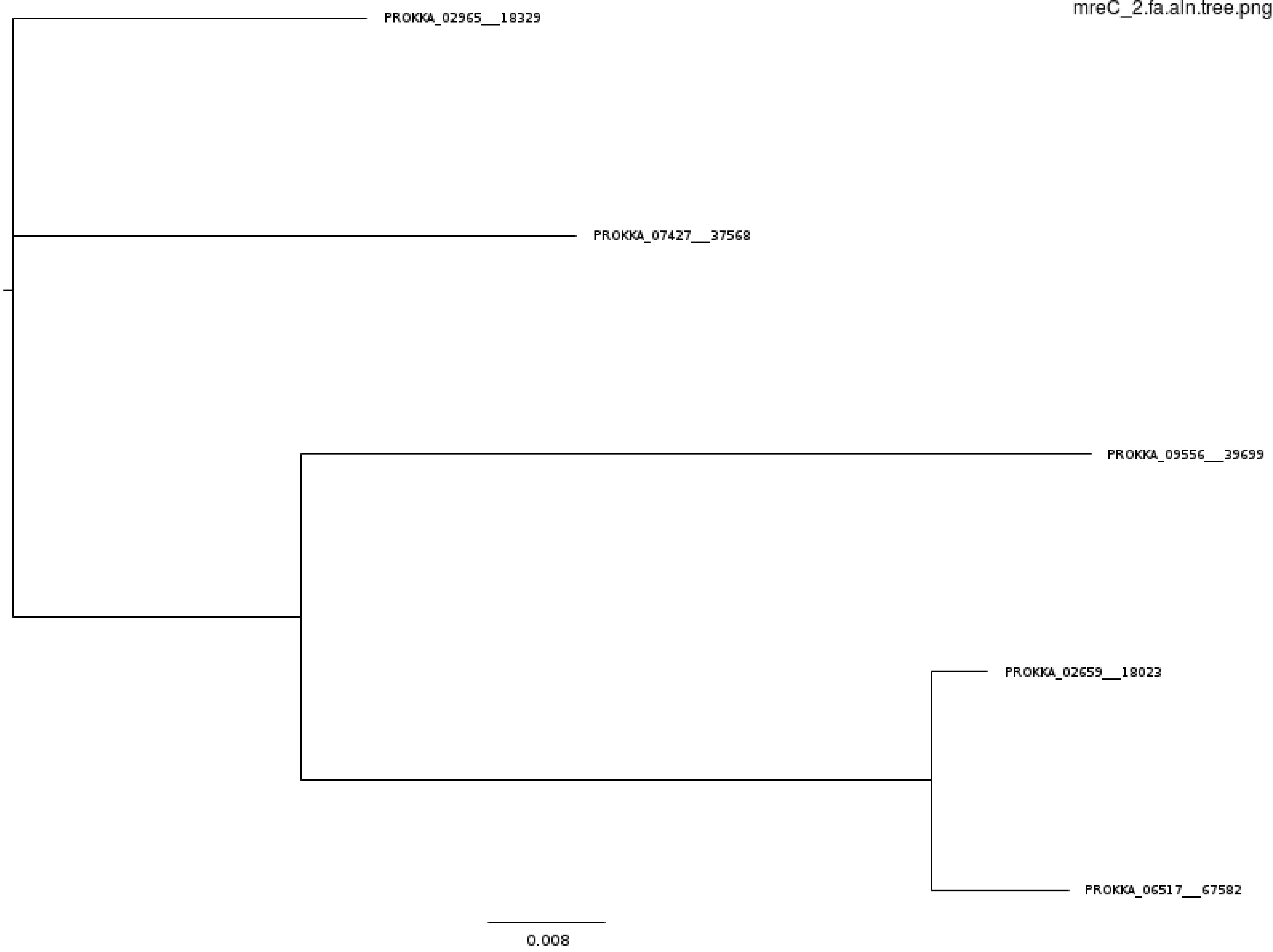
mreC_2.fa.aln.tree.png
PROKKA_02965__18329
PROKKA_07427__37568
PROKKA_09556__39699
PROKKA_02659__18023
PROKKA_06517__67582
0.008

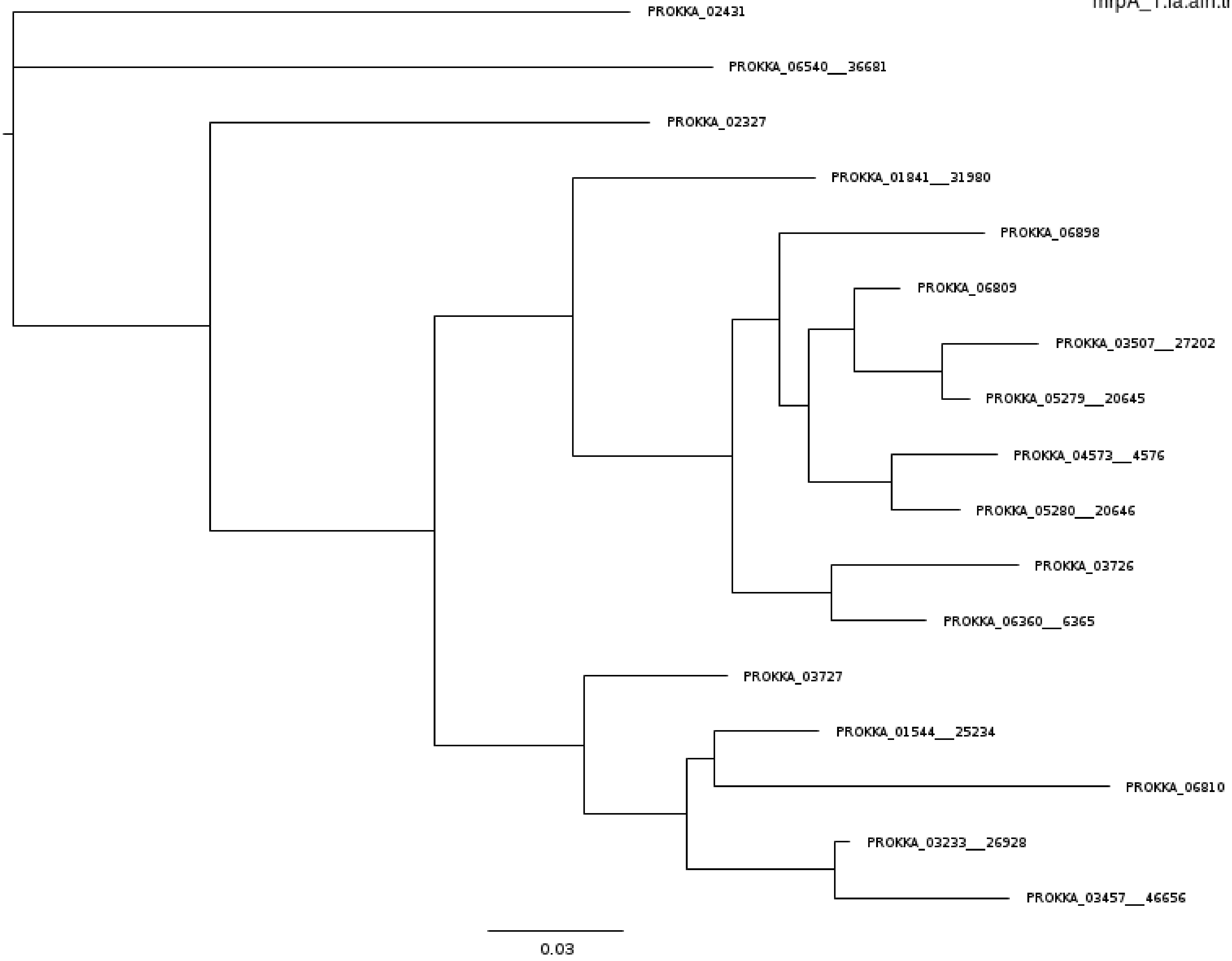
PROKKA_02431
PROKKA_06540___36681
PROKKA_02327
PROKKA_01841___31980
PROKKA_06898
PROKKA_06809
PROKKA_03507___27202
PROKKA_05279___20645
PROKKA_04573___4576
PROKKA_05280___20646
PROKKA_03726
PROKKA_06360___6365
PROKKA_03727
PROKKA_01544___25234
PROKKA_06810
PROKKA_03233___26928
PROKKA_03457___46656
0.03

mrpB_2.fa.aln.tree.png

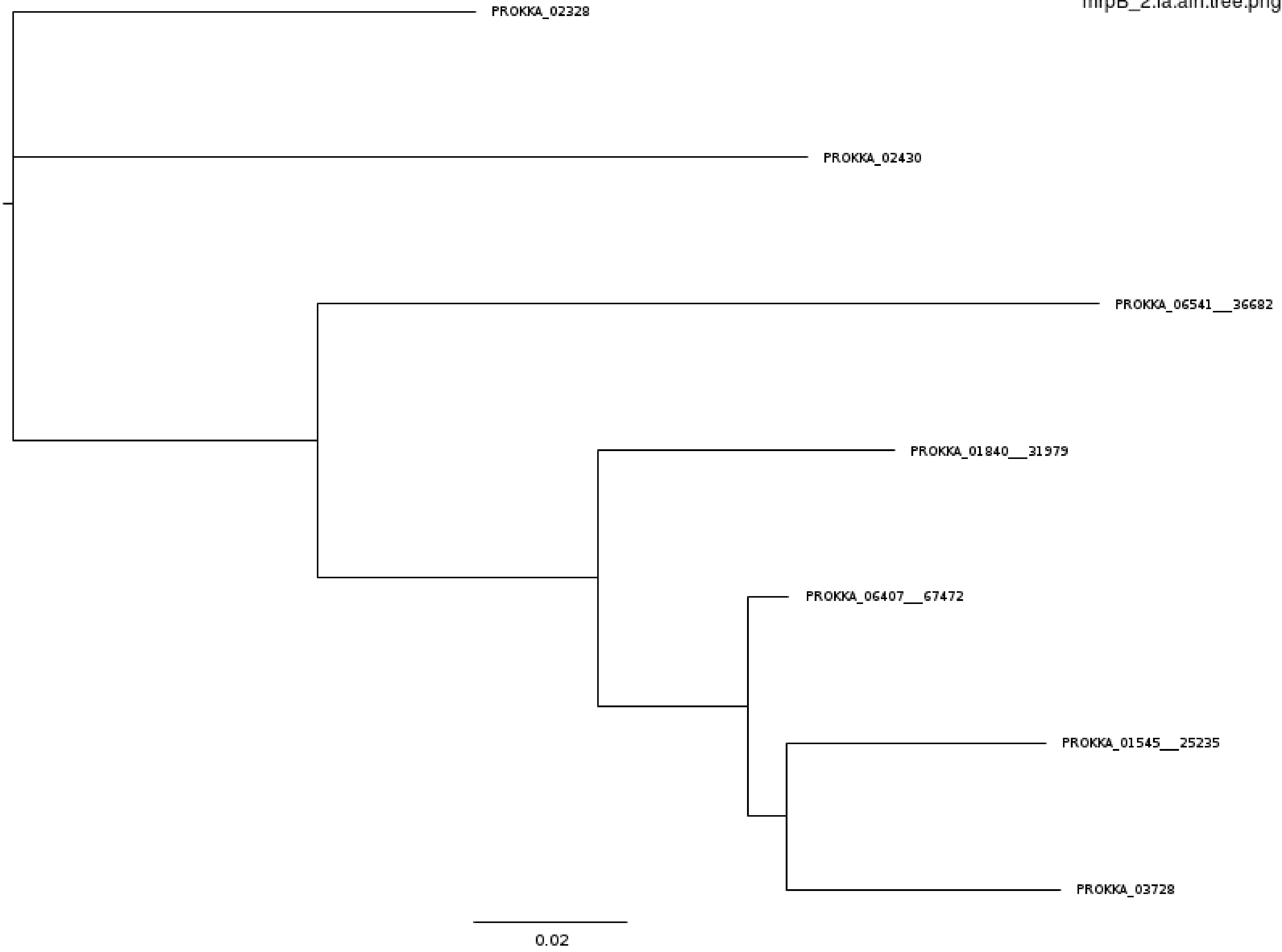

mrpC_2.fa.aln.tree.png

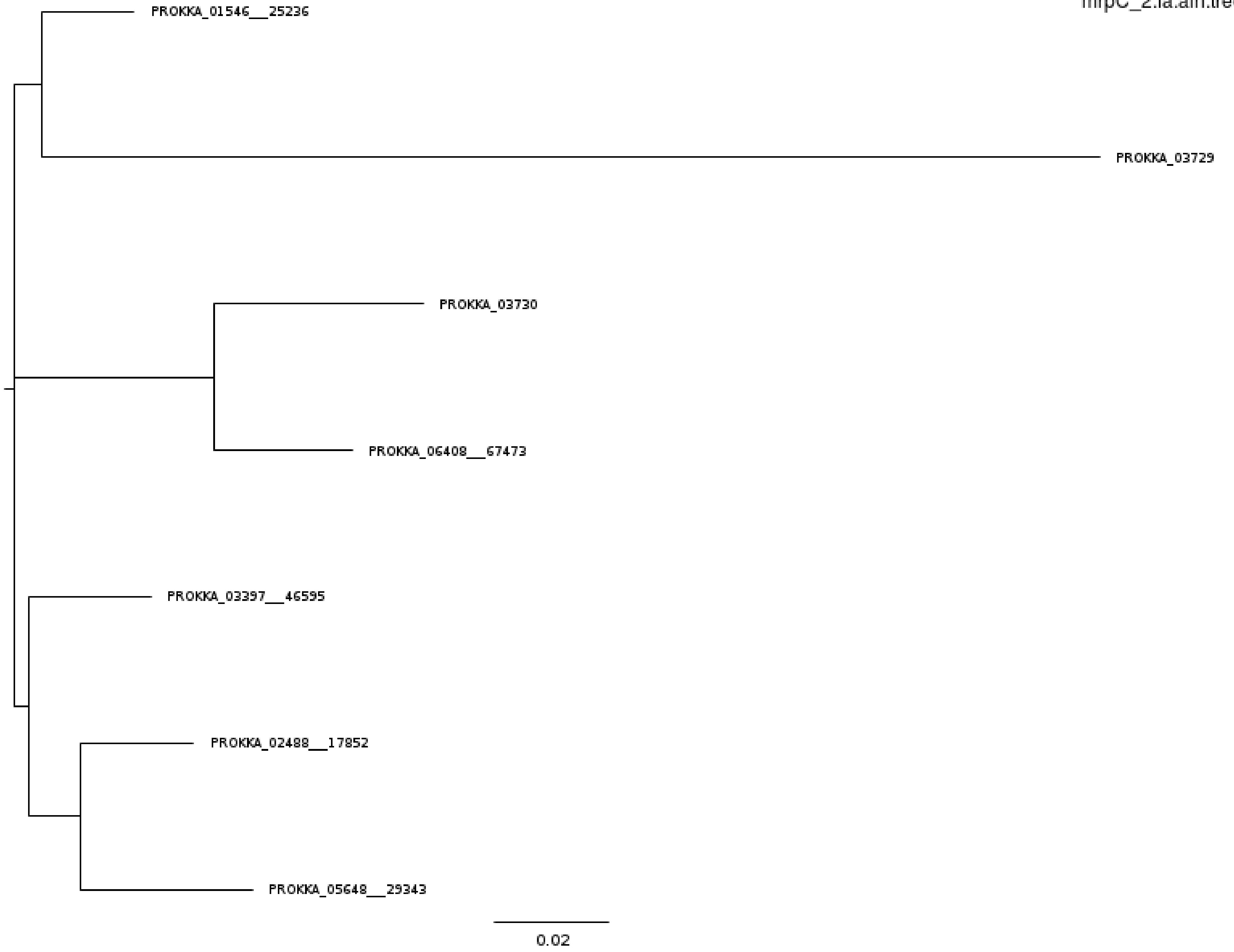

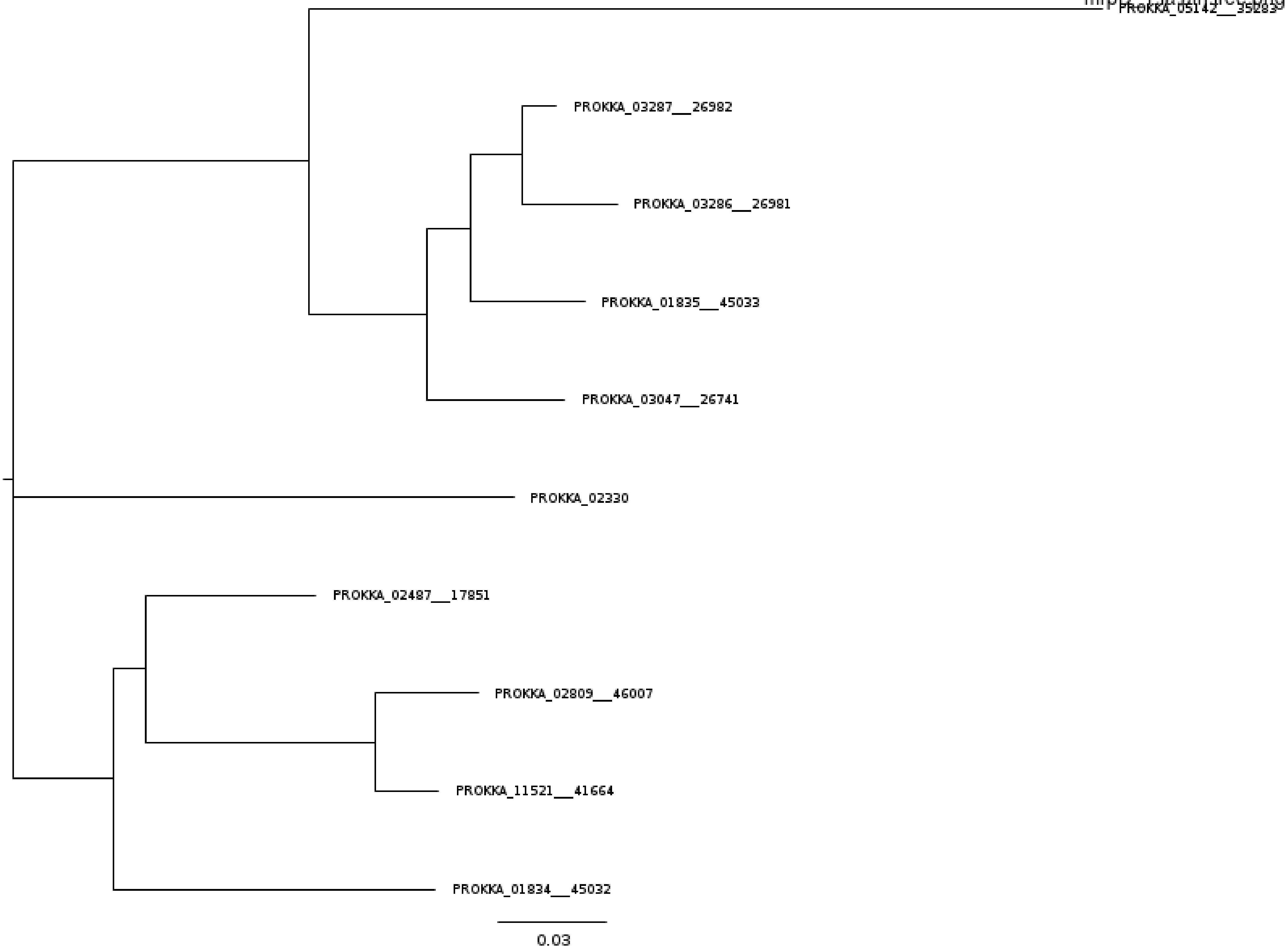
PROKKA_05142___35283
PROKKA_03287___26982
PROKKA_03286___26981
PROKKA_01835___45033
PROKKA_03047___26741
PROKKA_02330
PROKKA_02487___17851
PROKKA_02809___46007
PROKKA_11521___41664
PROKKA_01834___45032
0.03

mrpE.fa.aln.tree.png

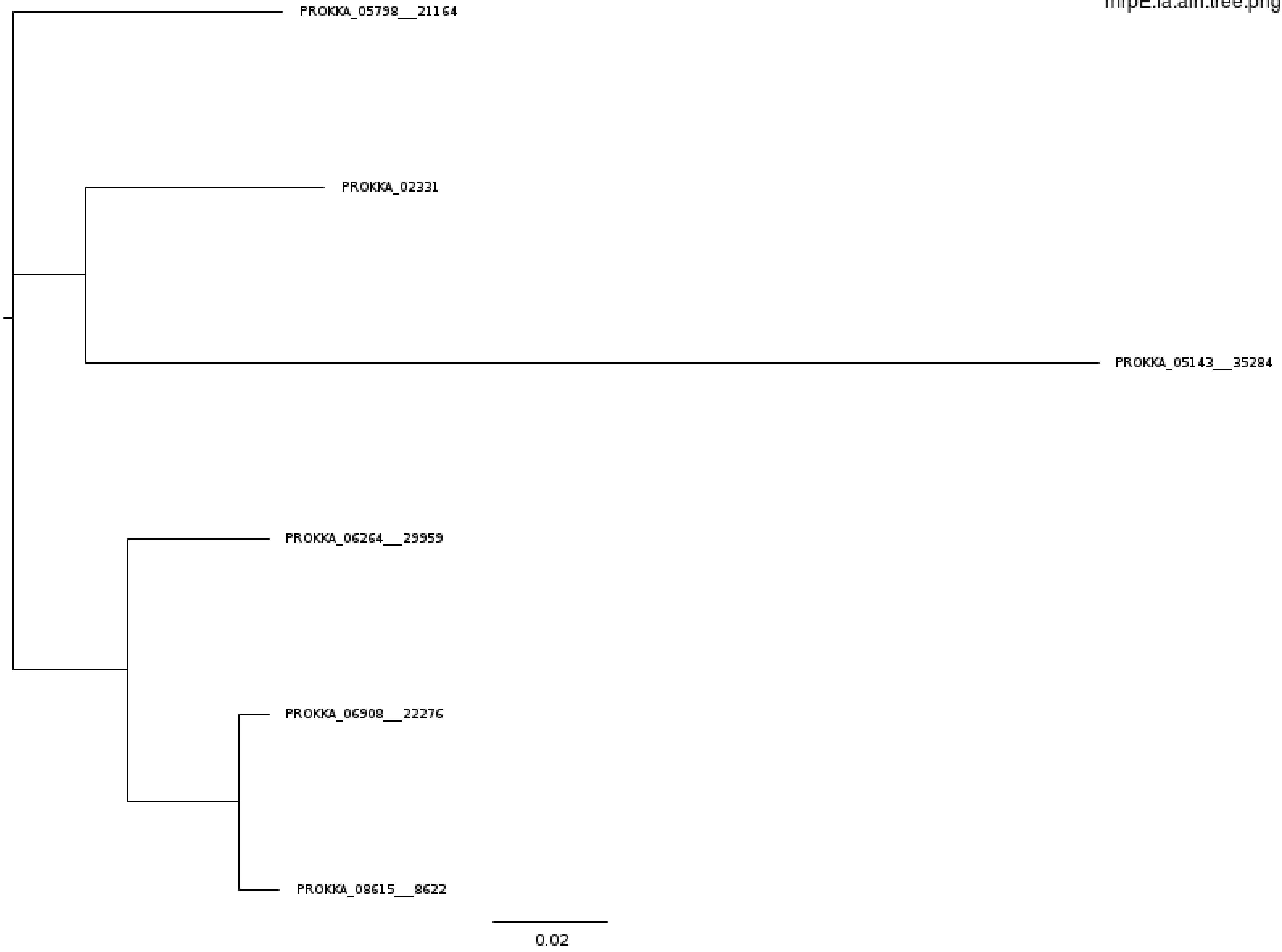

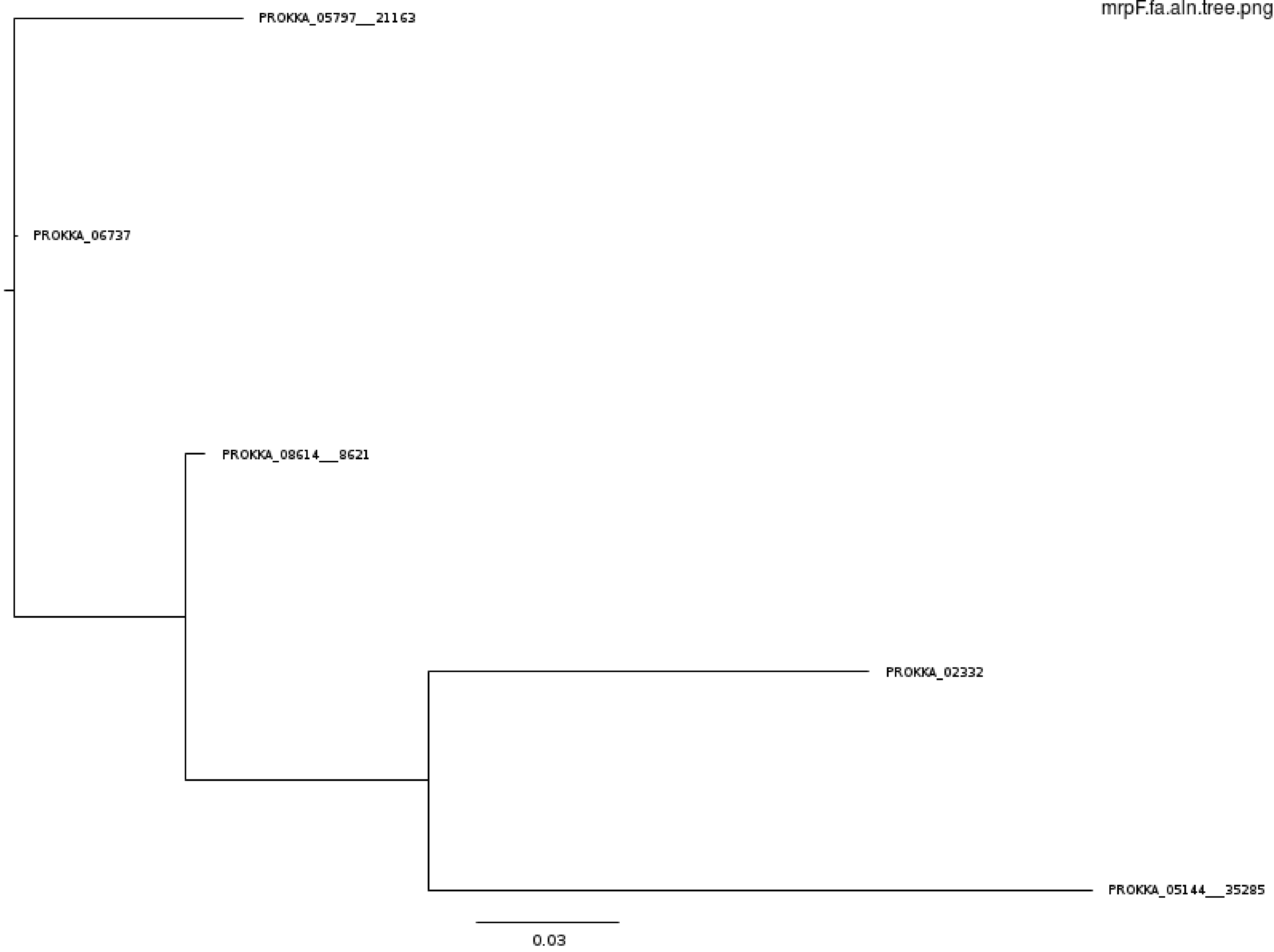
mrpF.fa.aln.tree.png
PROKKA_05797___21163
PROKKA_06737
PROKKA_08614___8621
PROKKA_02332
PROKKA_05144___35285
0.03

mrpG_2.fa.aln.tree.png

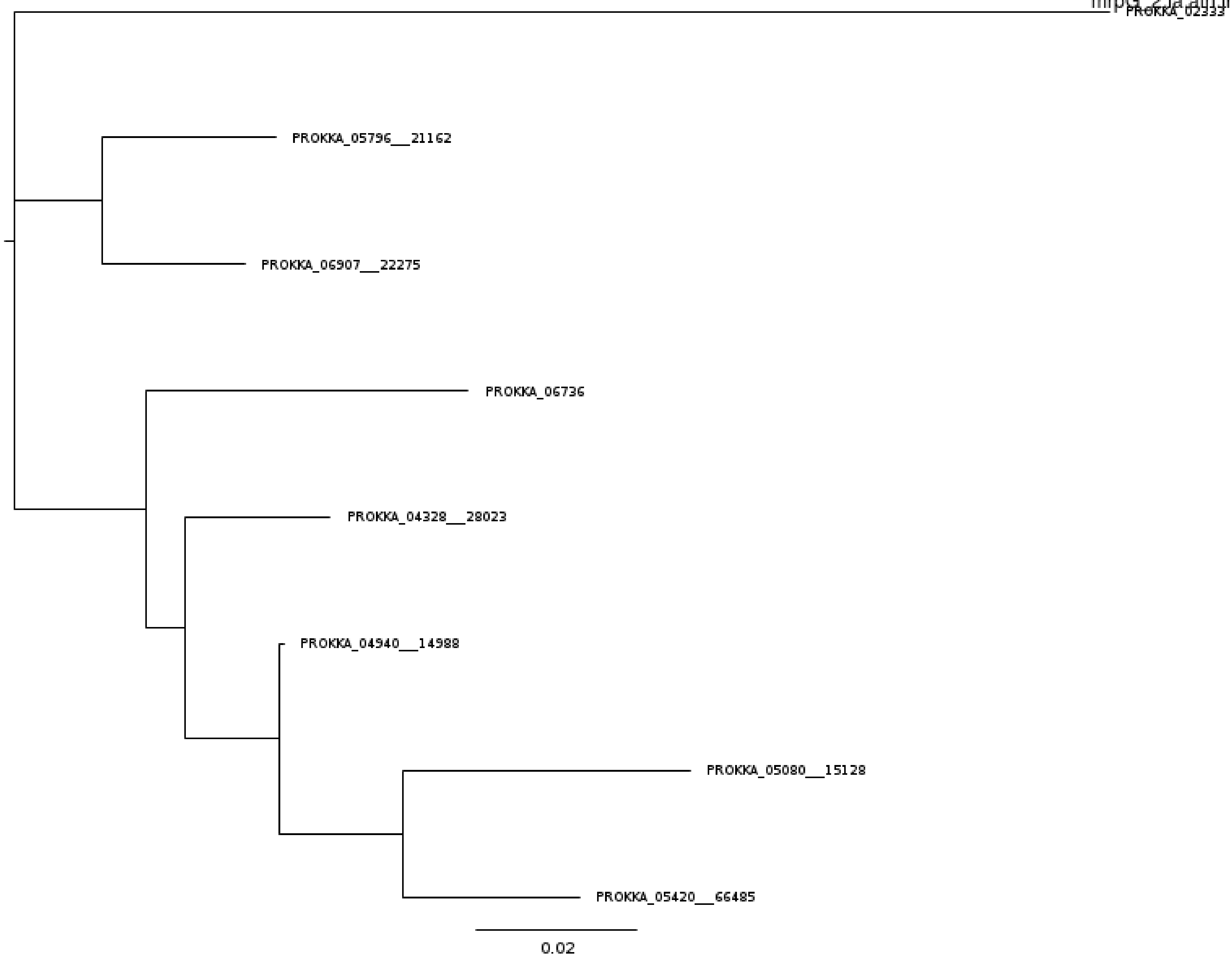

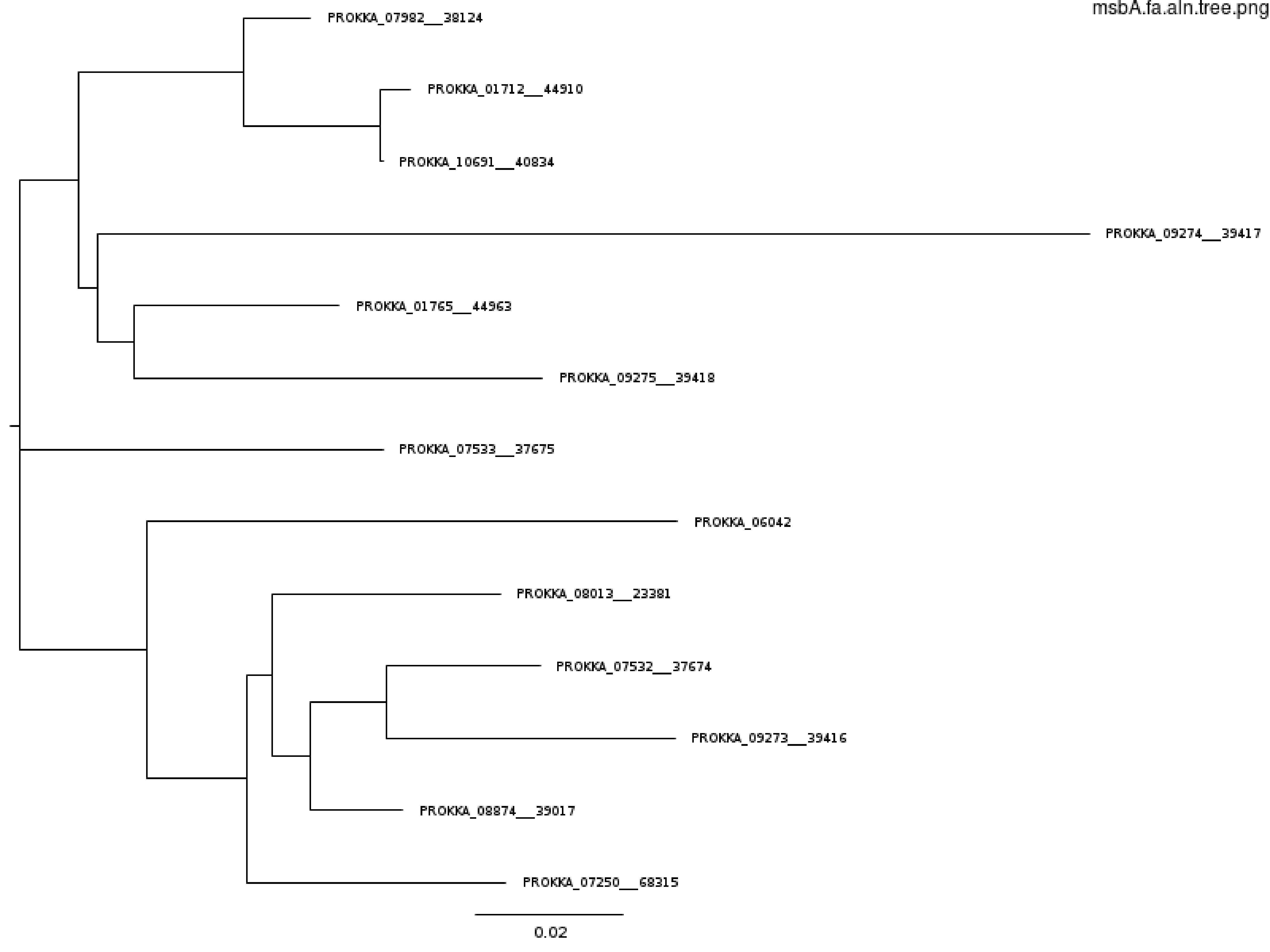
msbA.fa.aln.tree.png
PROKKA_07982___38124
PROKKA_01712___44910
PROKKA_10691___40834
PROKKA_09274___39417
PROKKA_01765___44963
PROKKA_09275___39418
PROKKA_07533___37675
PROKKA_06042
PROKKA_08013___23381
PROKKA_07532___37674
PROKKA_09273___39416
PROKKA_08874___39017
PROKKA_07250___68315
0.02

mshA_5.fa.aln.tree.png

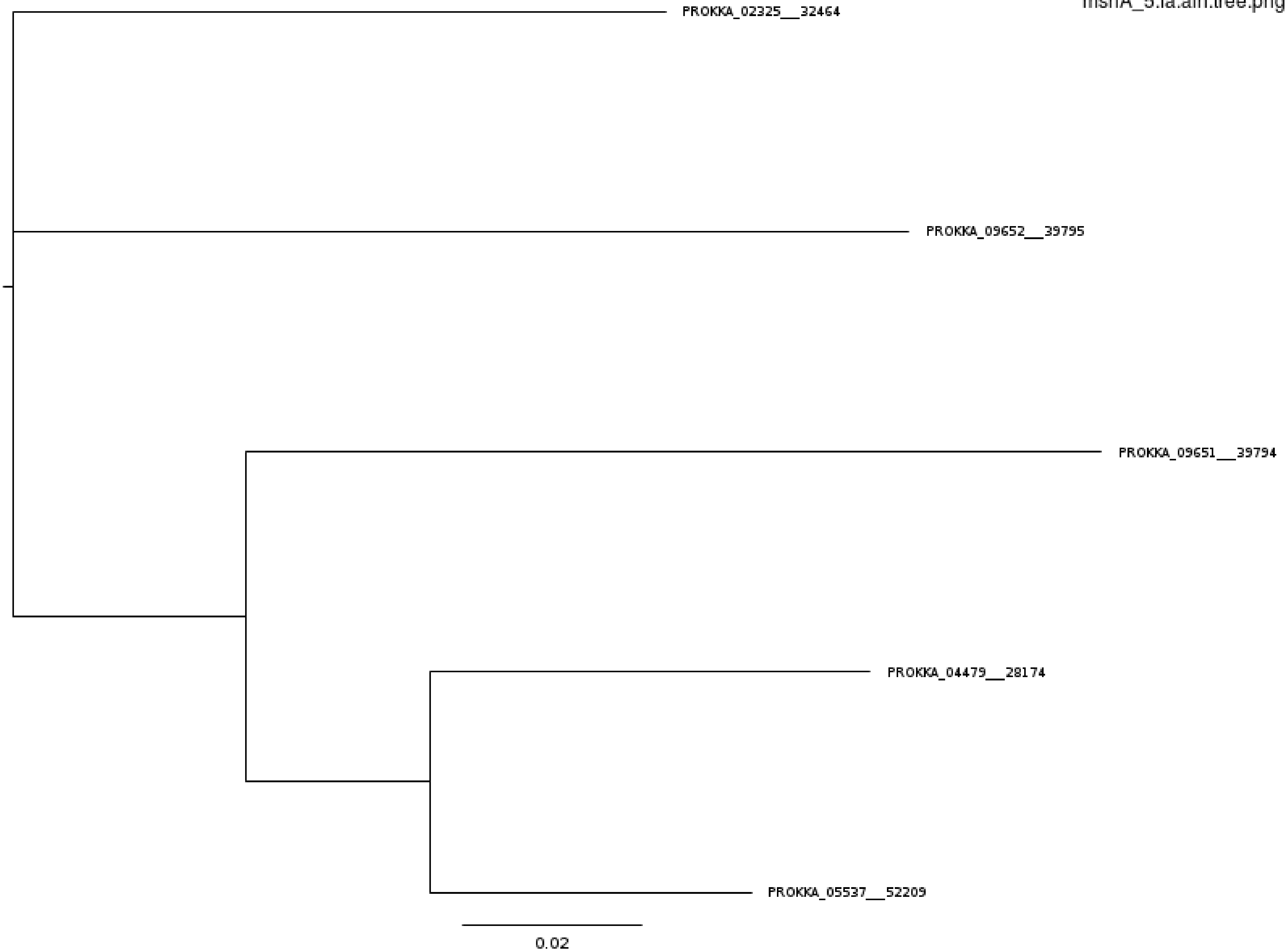

mshA.fa.aln.tree.png

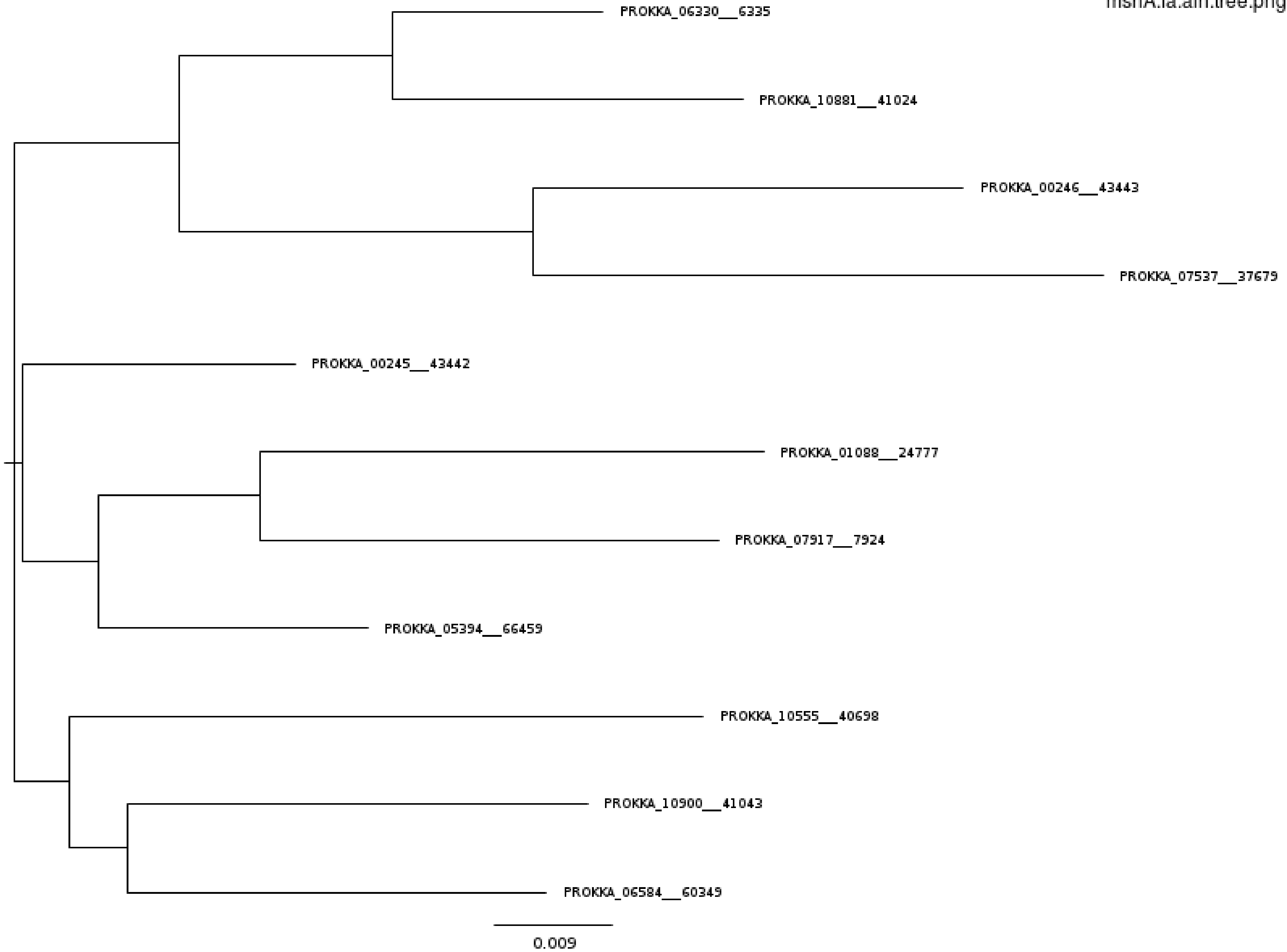

mshB.fa.aln.tree.png

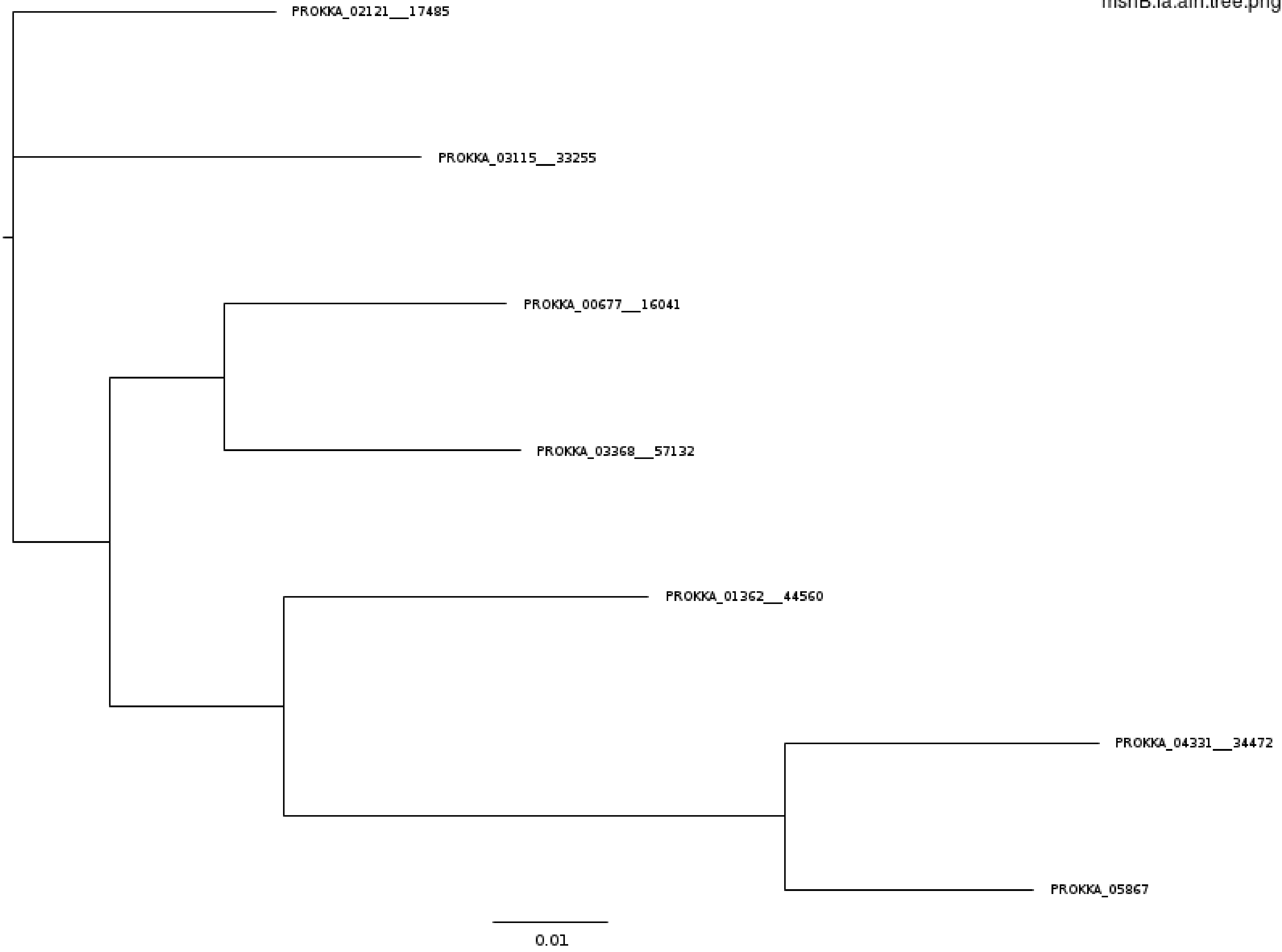

msrB_1.fa.aln.tree.png

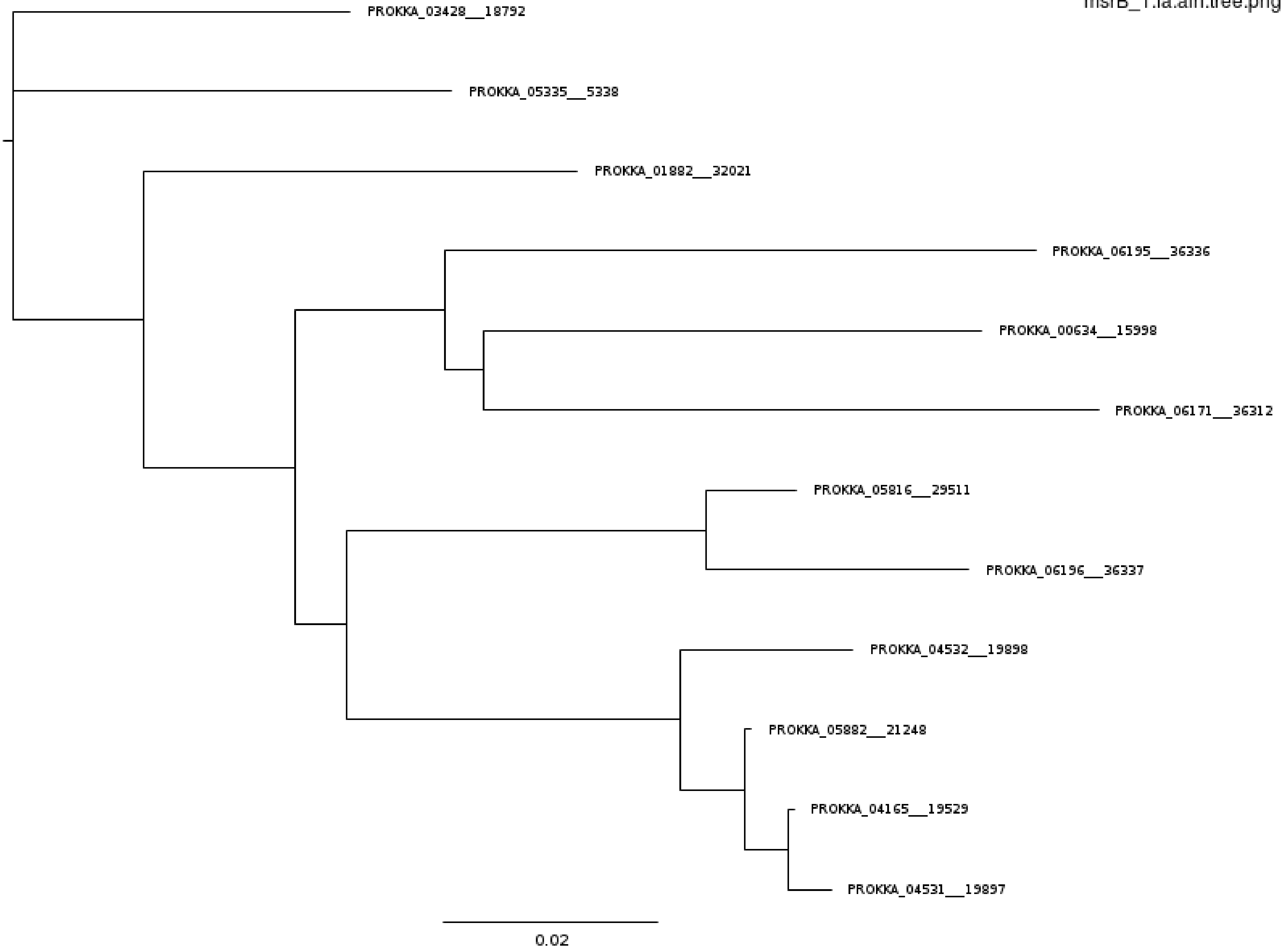

mtaD_2.fa.aln.tree.png

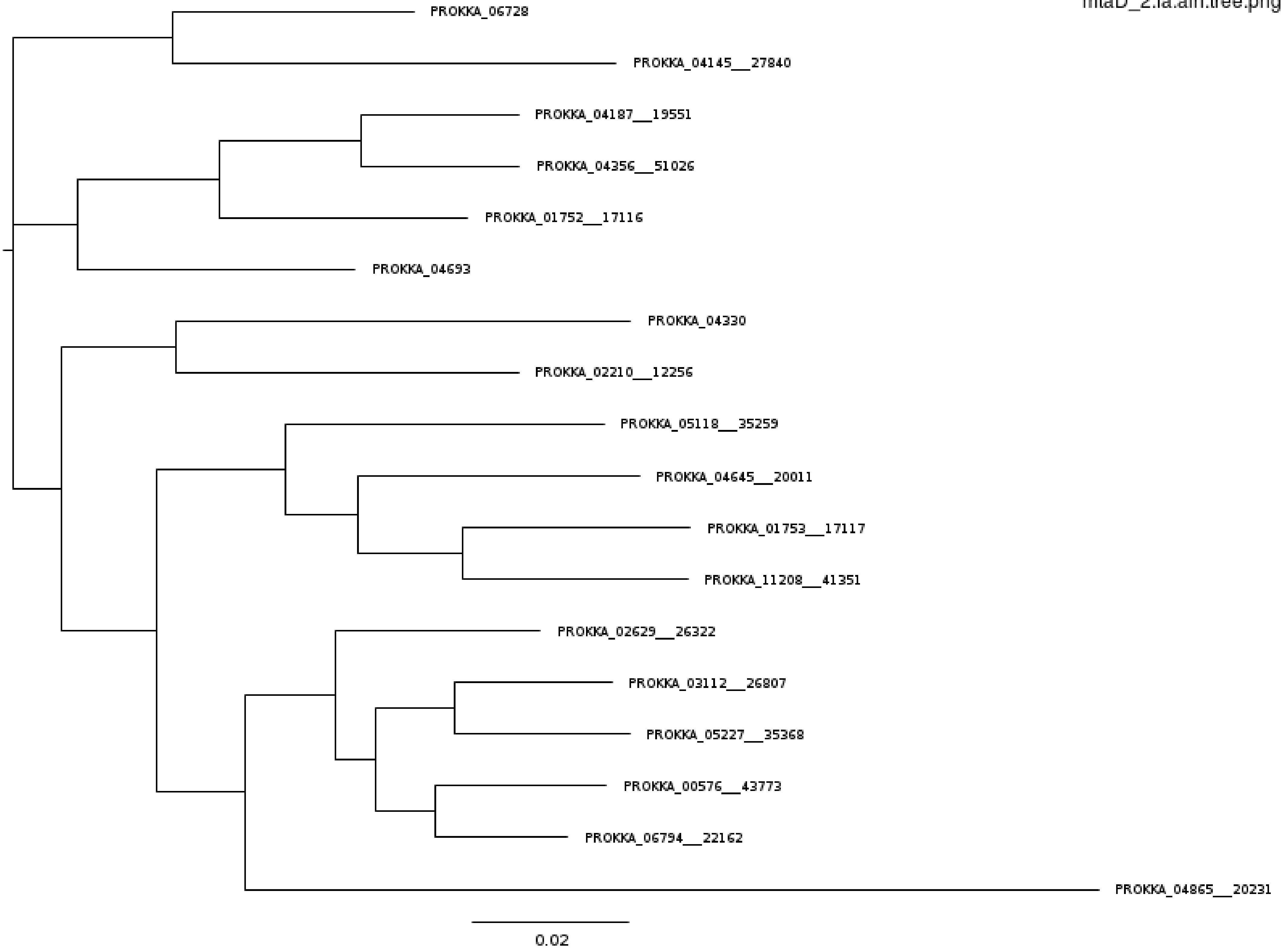

mtnA.fa.aln.tree.png

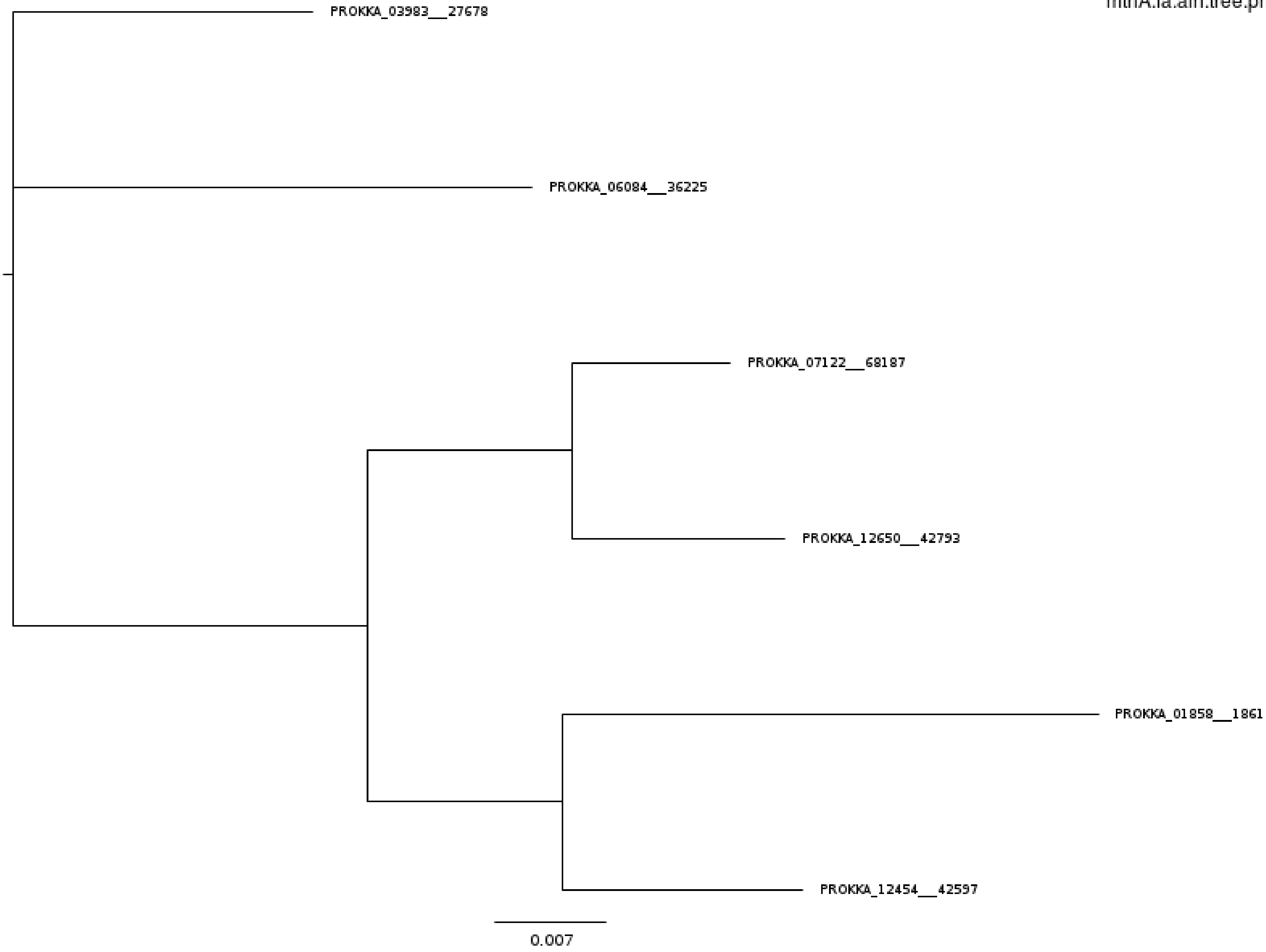

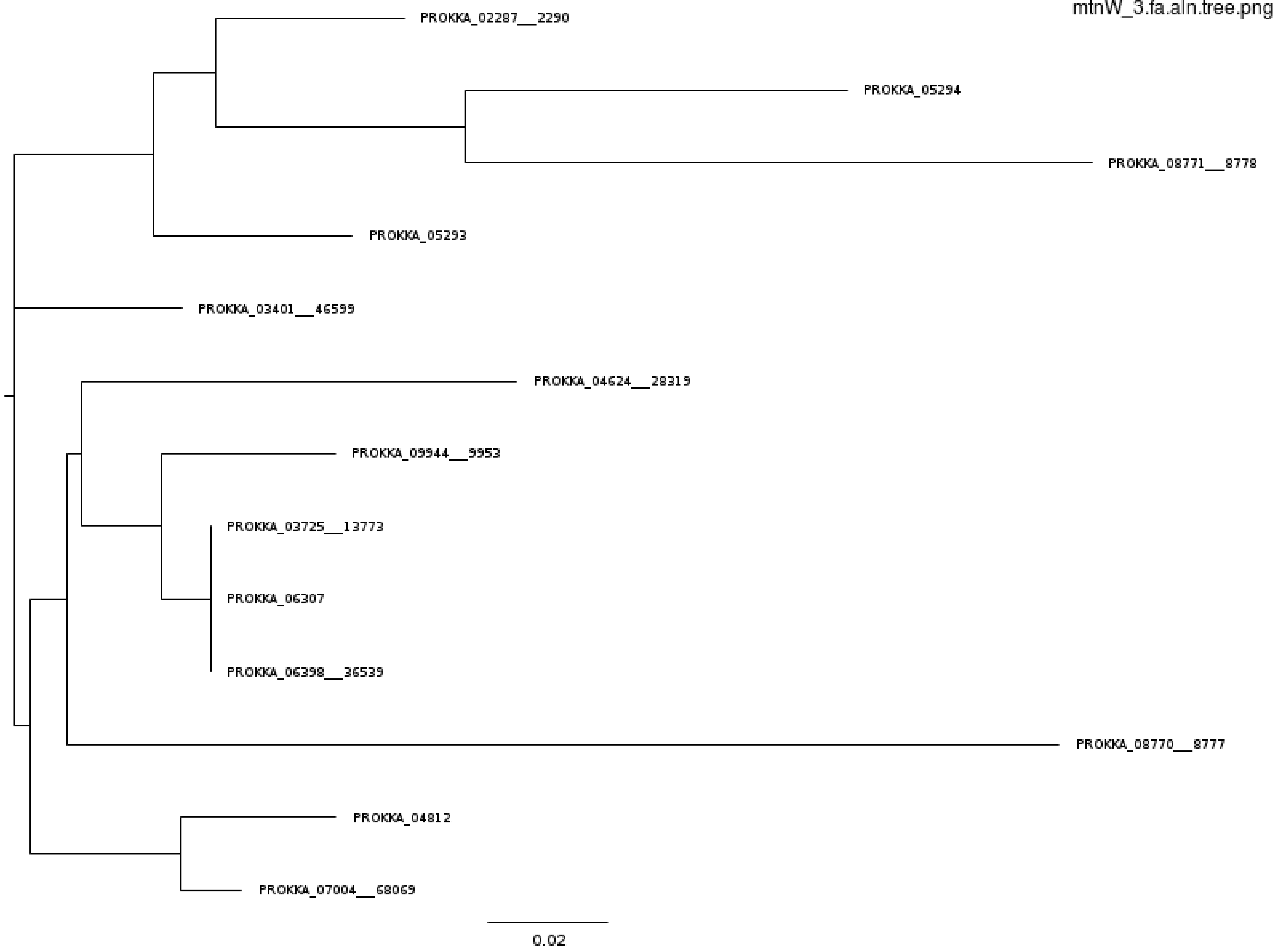
mtnW_3.fa.aln.tree.png
PROKKA_02287___2290
PROKKA_05294
PROKKA_08771___8778
PROKKA_05293
PROKKA_03401___46599
PROKKA_04624___28319
PROKKA_09944___9953
PROKKA_03725___13773
PROKKA_06307
PROKKA_06398___36539
PROKKA_08770___8777
PROKKA_04812
PROKKA_07004___68069
0.02

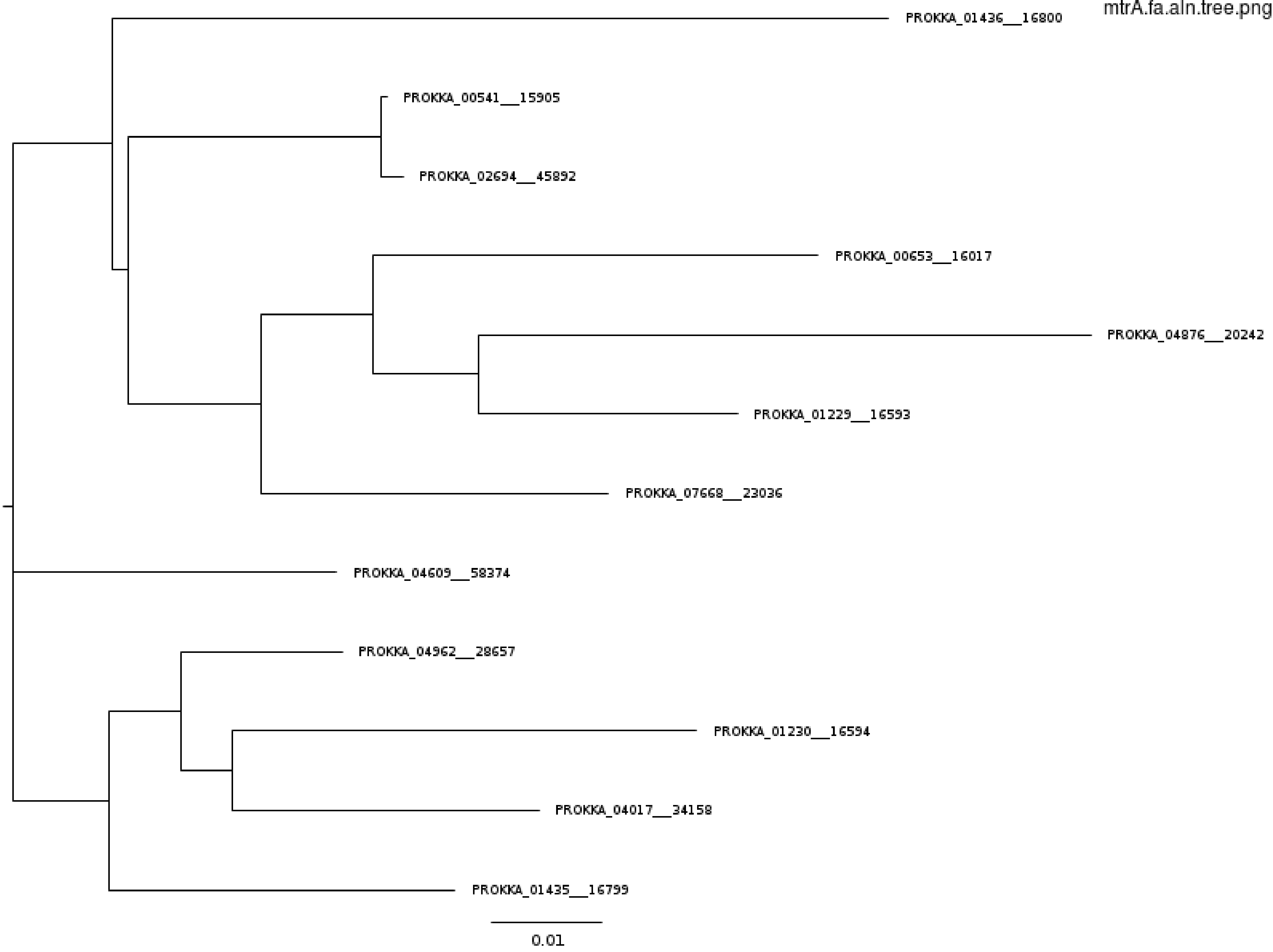

mtrA.fa.aln.tree.png
PROKKA_01436___16800
PROKKA_00541___15905
PROKKA_02694___45892
PROKKA_00653___16017
PROKKA_04876___20242
PROKKA_01229___16593
PROKKA_07668___23036
PROKKA_04609___58374
PROKKA_04962___28657
PROKKA_01230___16594
PROKKA_04017___34158
PROKKA_01435___16799
0.01

mucD_1.fa.aln.tree.png

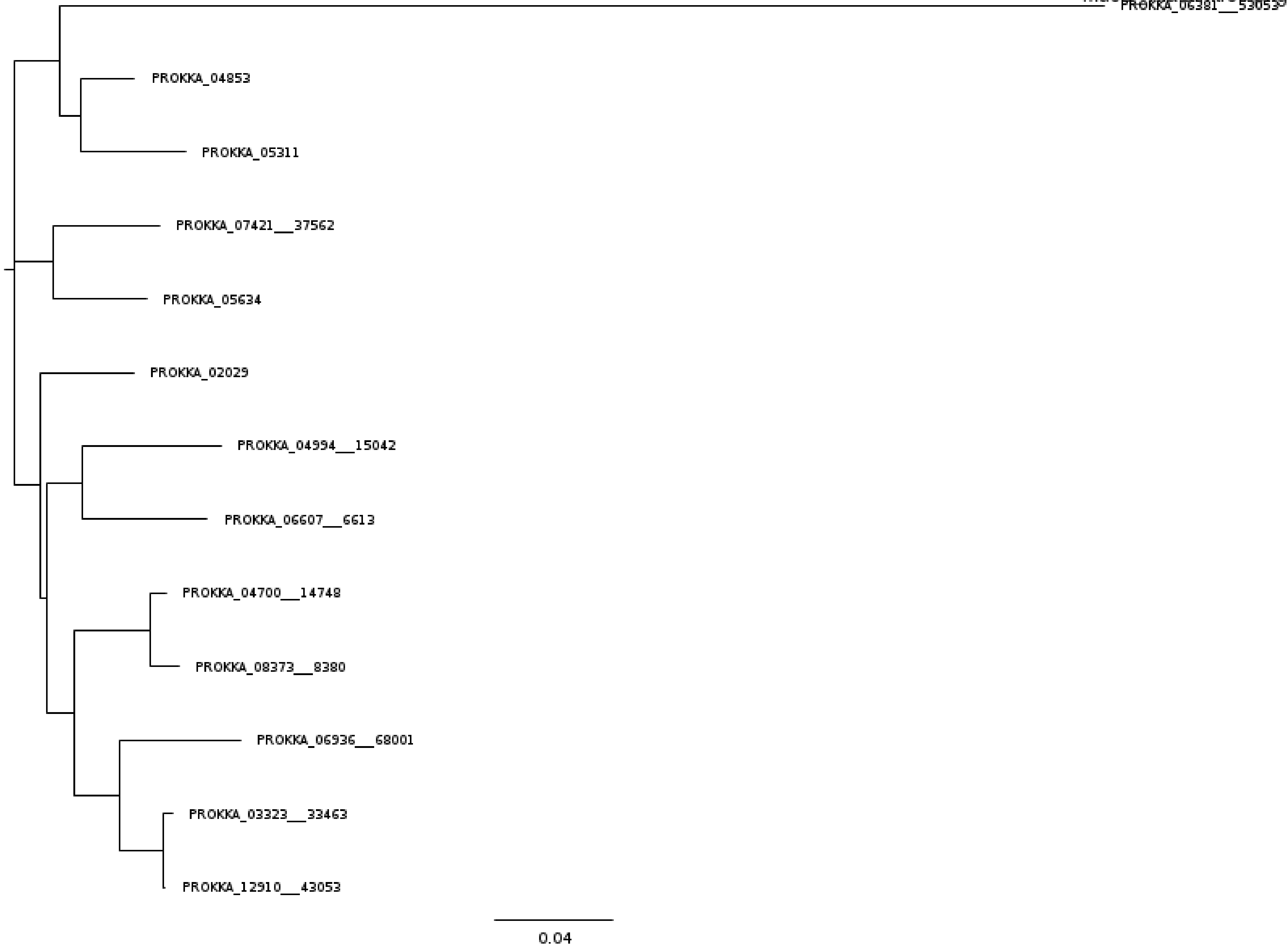

murA_3.fa.aln.tree.png

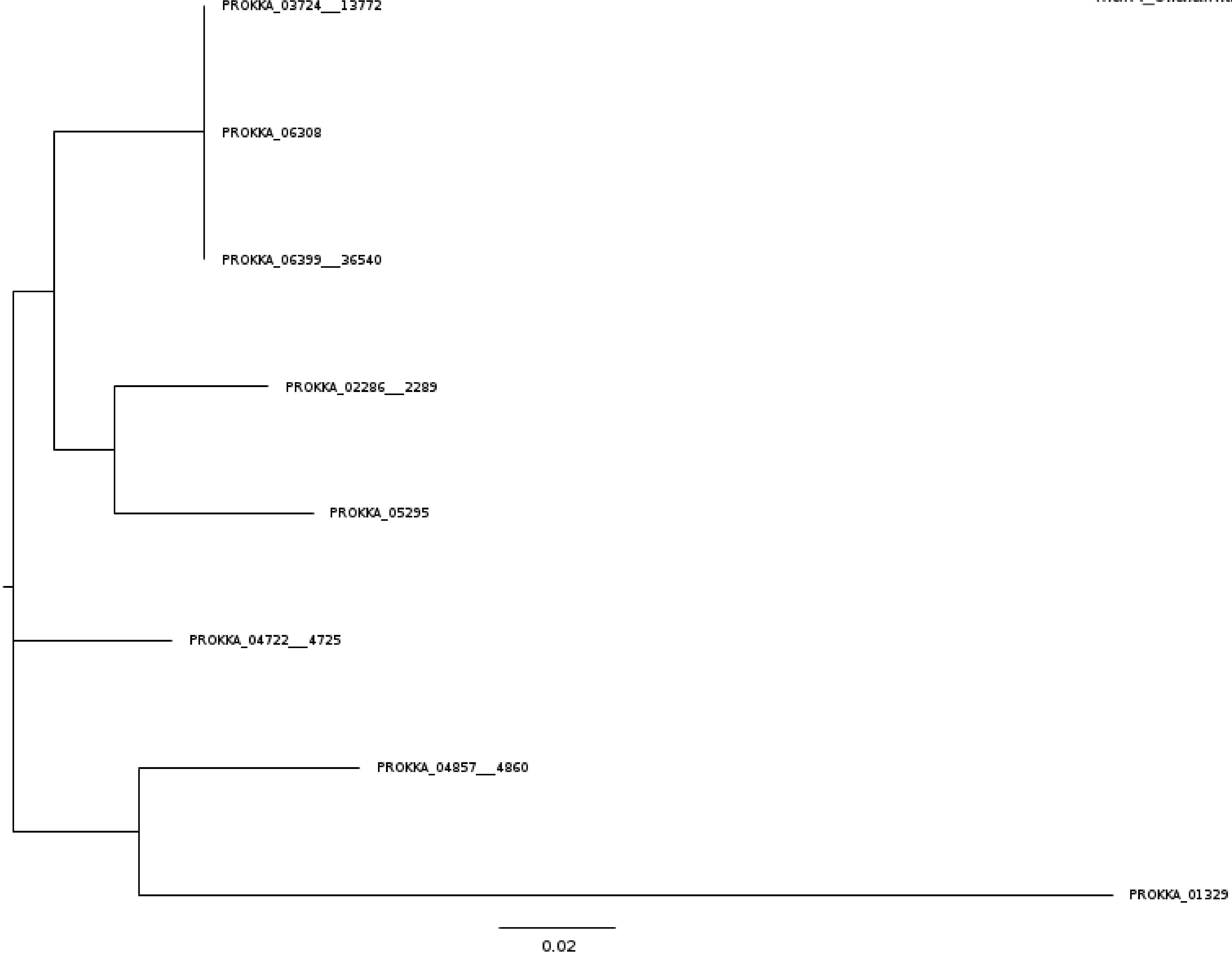

murC_2.fa.aln.tree.png

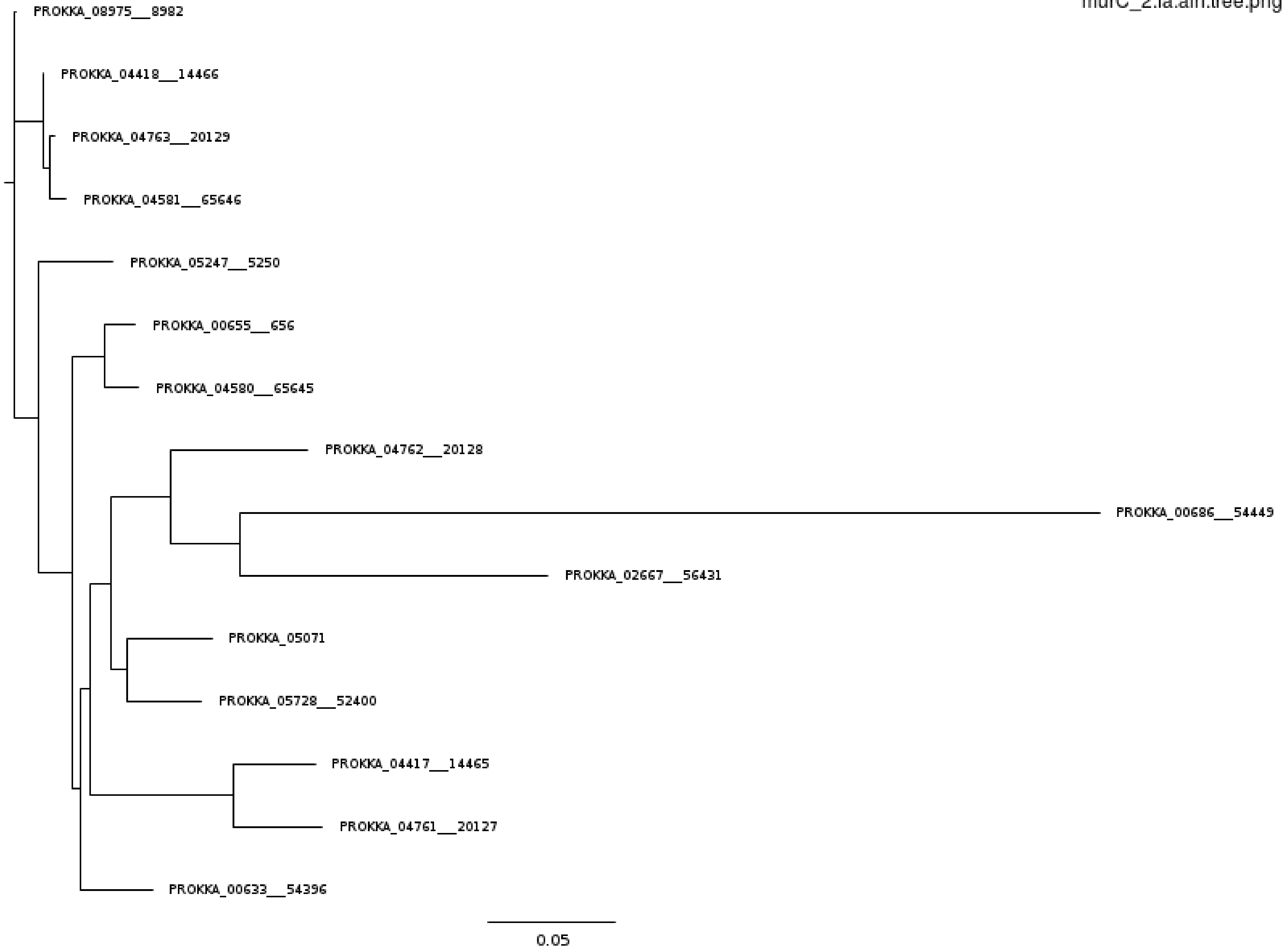

murE_3.fa.aln.tree.png

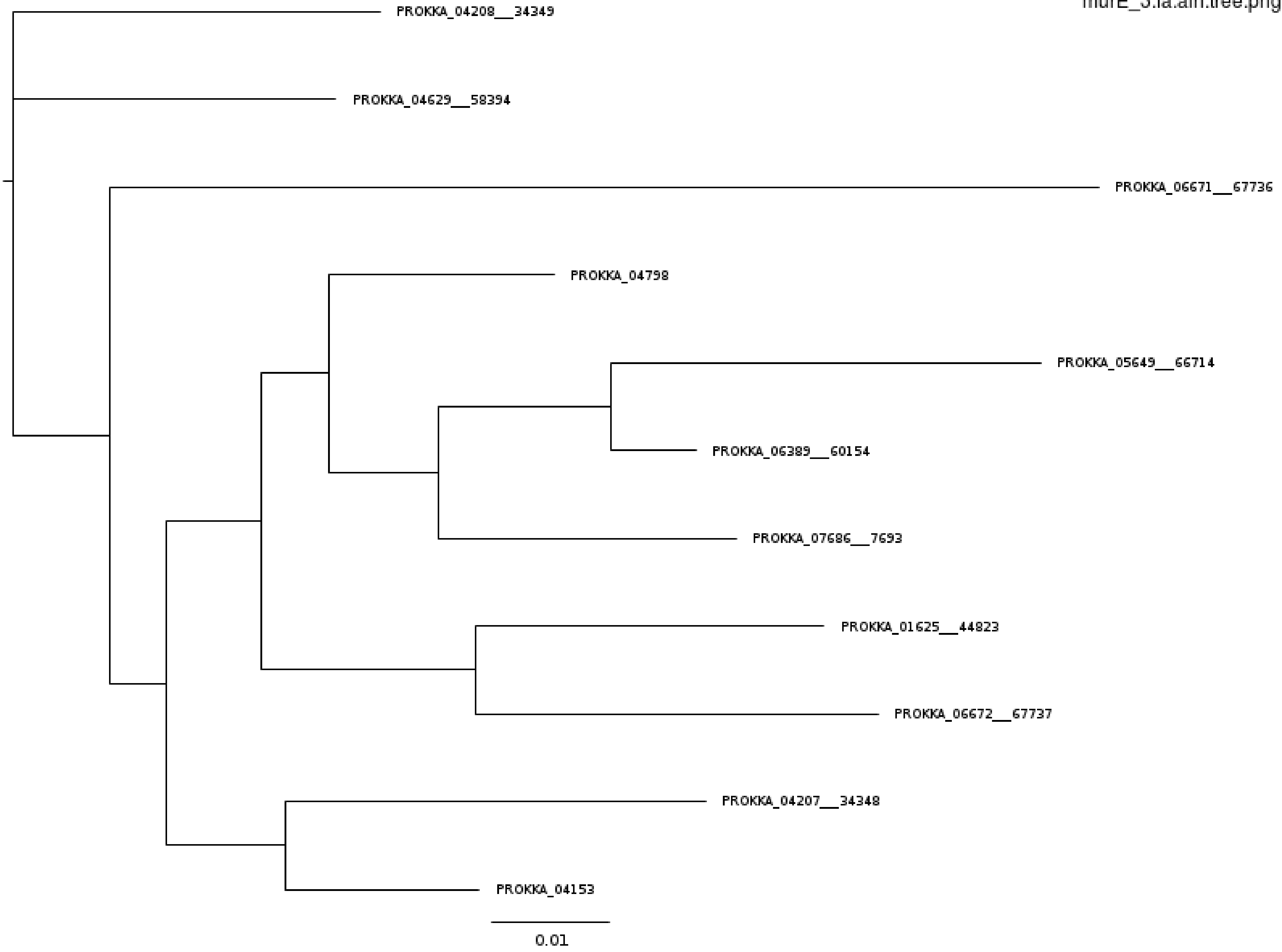

murE.fa.aln.tree.png

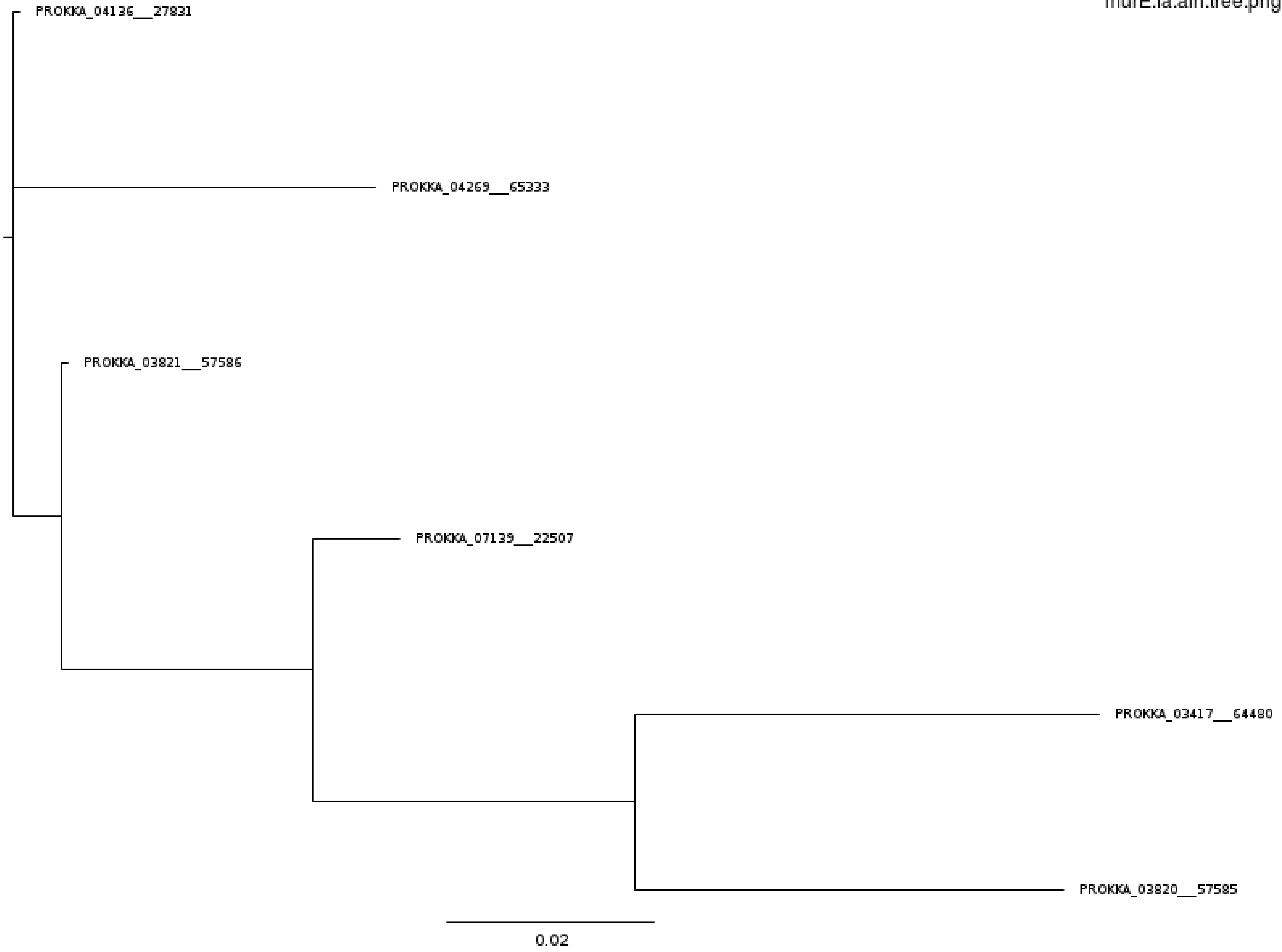

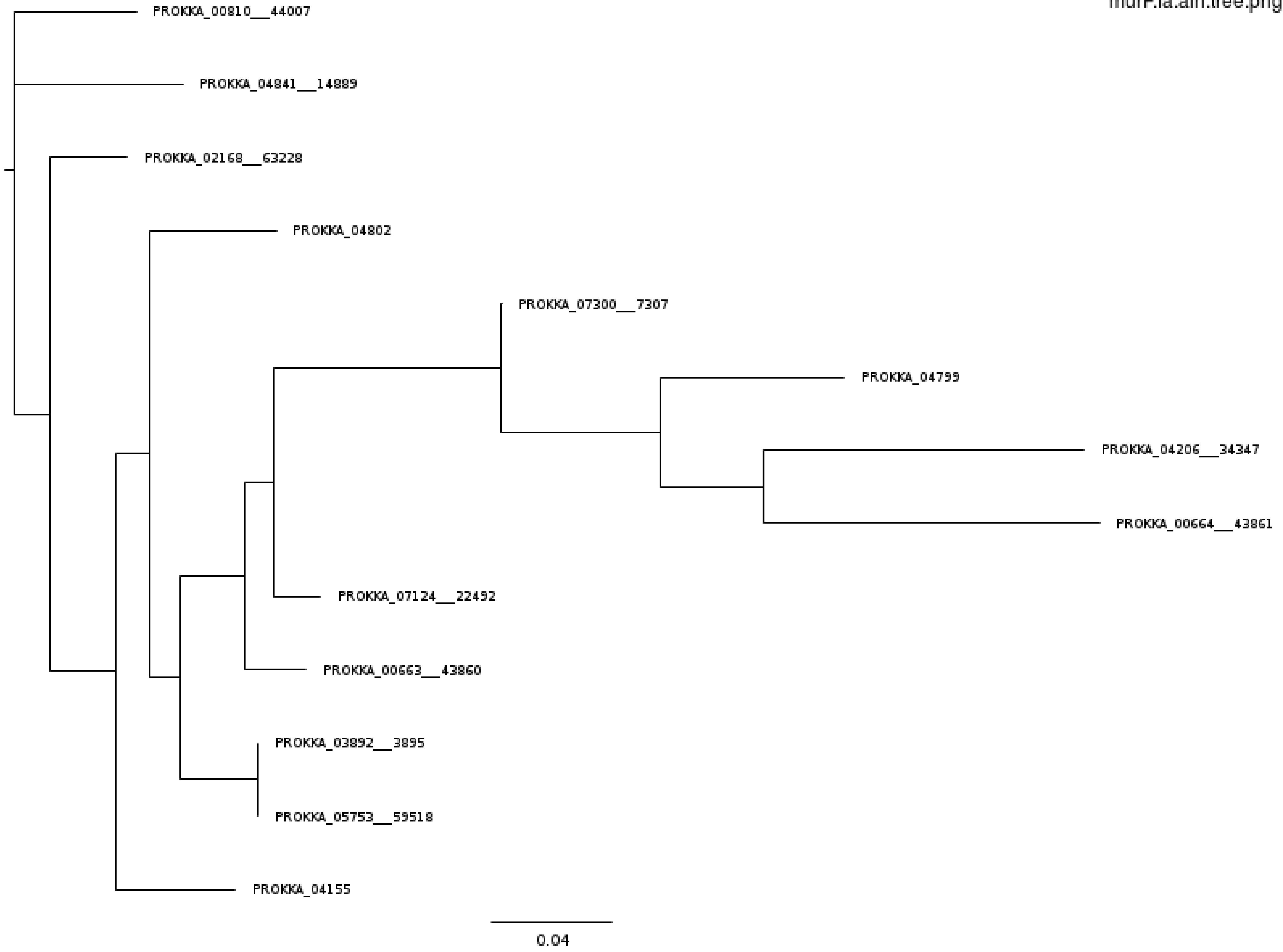
PROKKA_00810___44007
PROKKA_04841___14889
PROKKA_02168___63228
PROKKA_04802
PROKKA_07300___7307
PROKKA_04799
PROKKA_04206___34347
PROKKA_00664___43861
PROKKA_07124___22492
PROKKA_00663___43860
PROKKA_03892___3895
PROKKA_05753___59518
PROKKA_04155
0.04

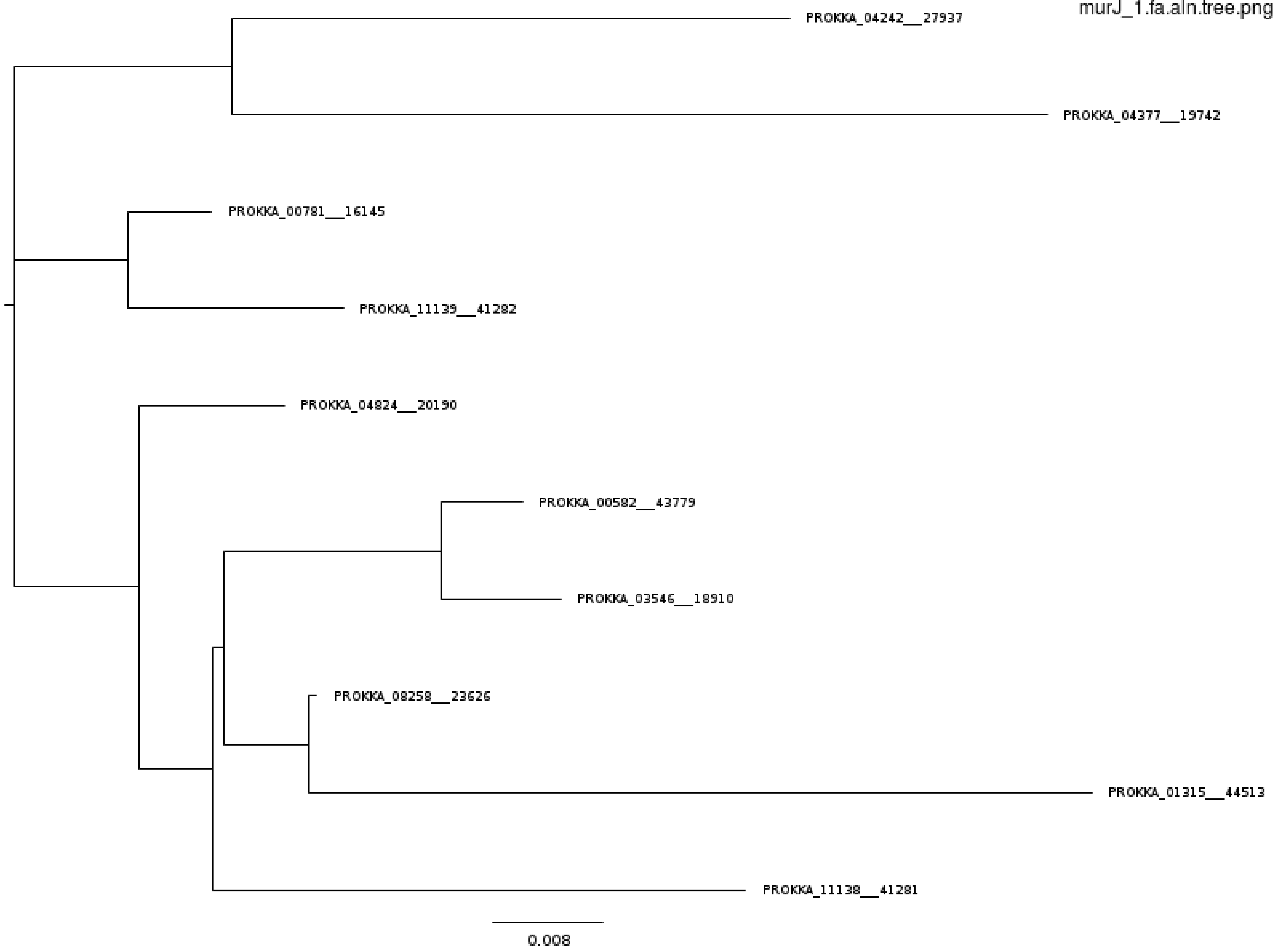
murJ_1.fa.aln.tree.png
PROKKA_04242__27937
PROKKA_04377__19742
PROKKA_00781__16145
PROKKA_11139__41282
PROKKA_04824__20190
PROKKA_00582__43779
PROKKA_03546__18910
PROKKA_08258__23626
PROKKA_01315__44513
PROKKA_11138__41281
0.008

murJ_3.fa.aln.tree.png

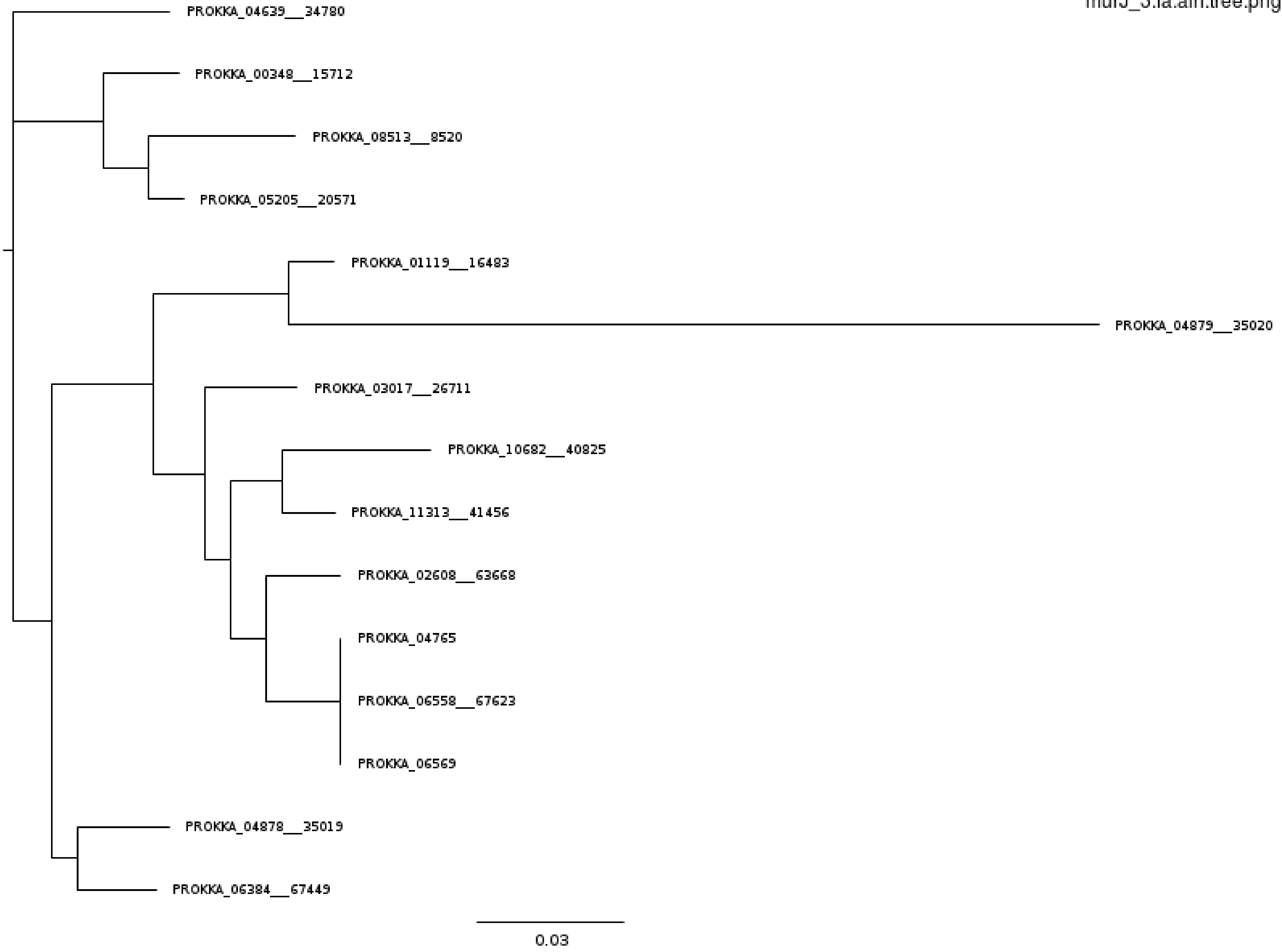

mutL_2.fa.aln.tree.png

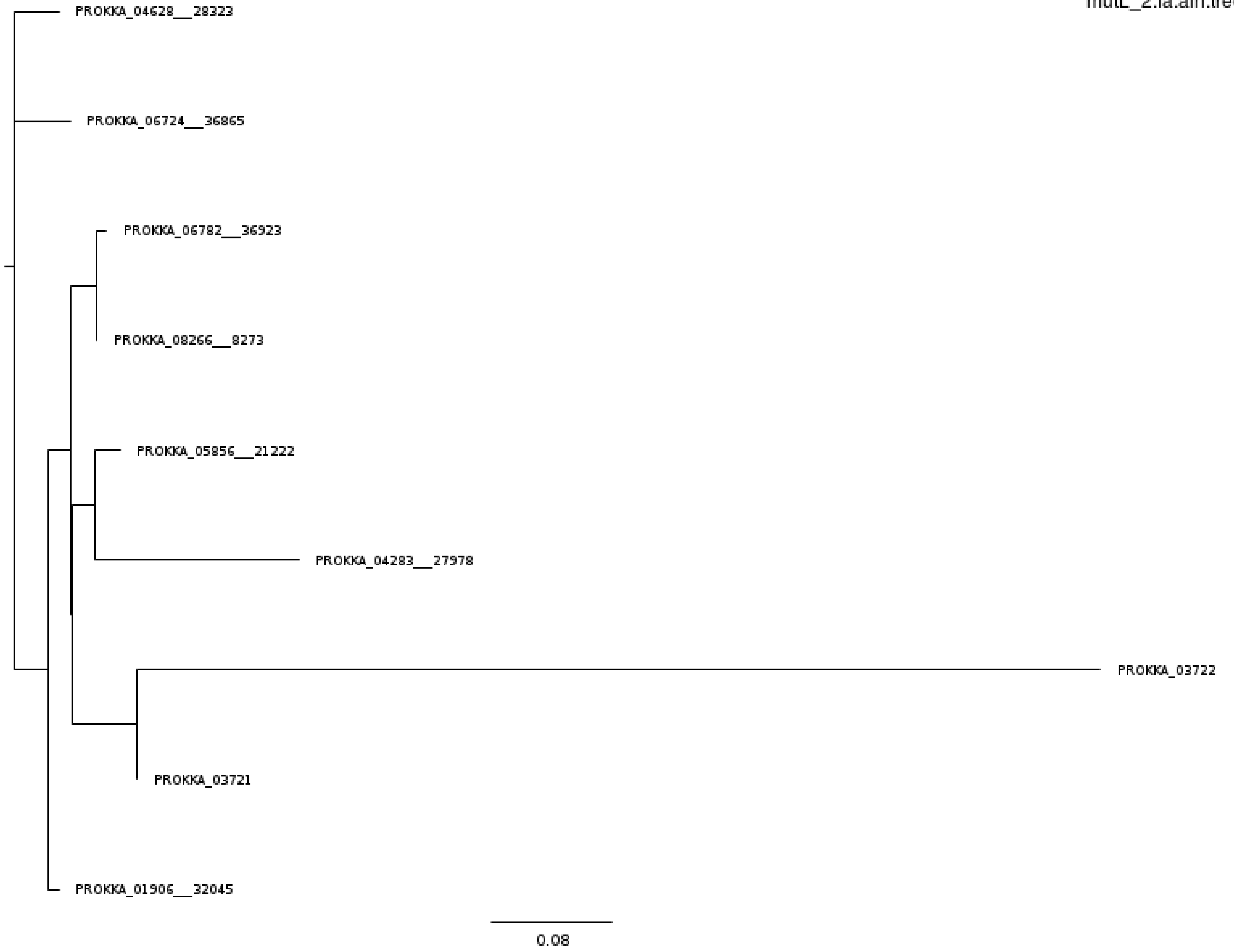

mutM_3.fa.aln.tree.png

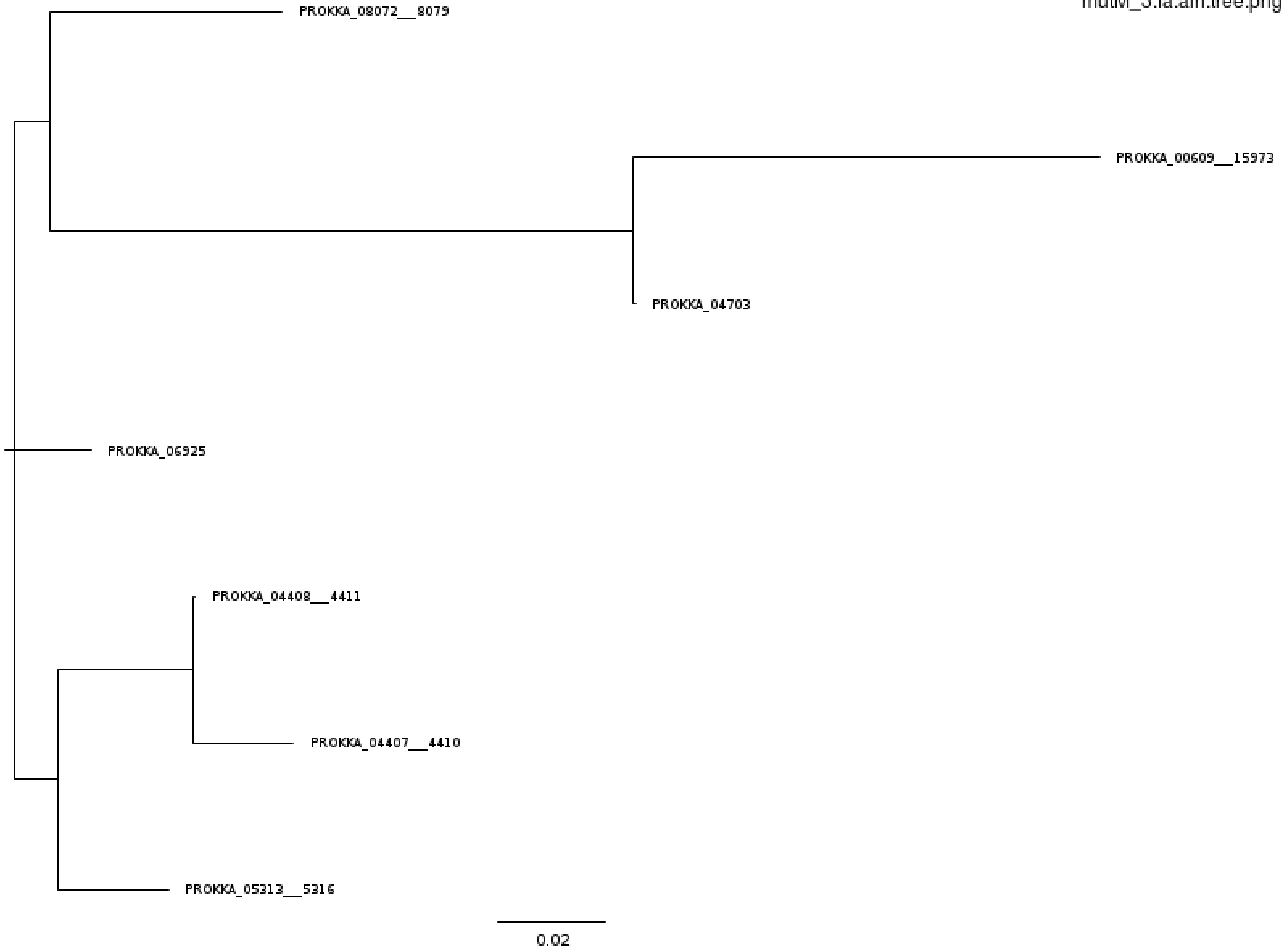

mutS_2.fa.aln.tree.png

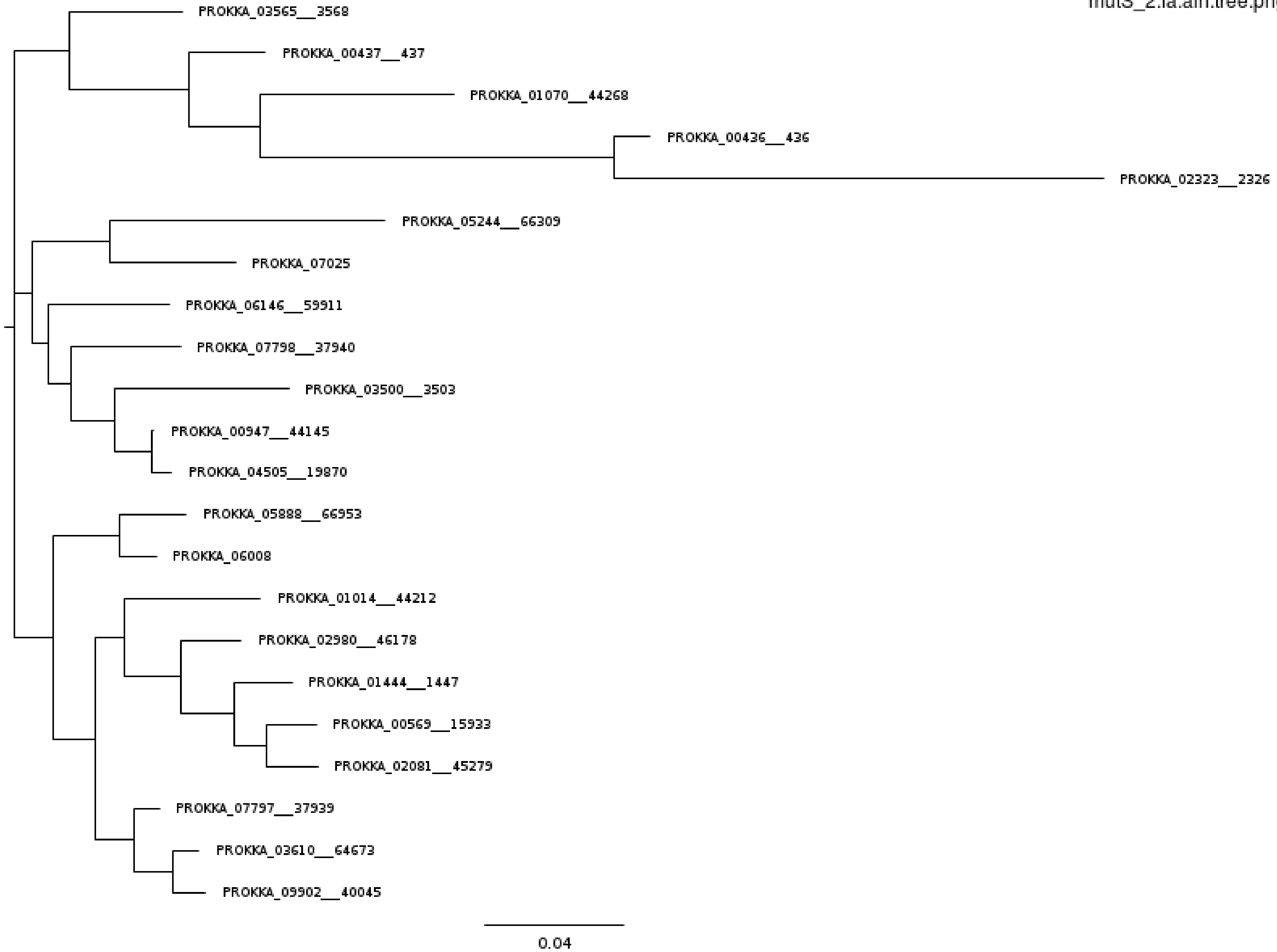

nadA.fa.aln.tree.png

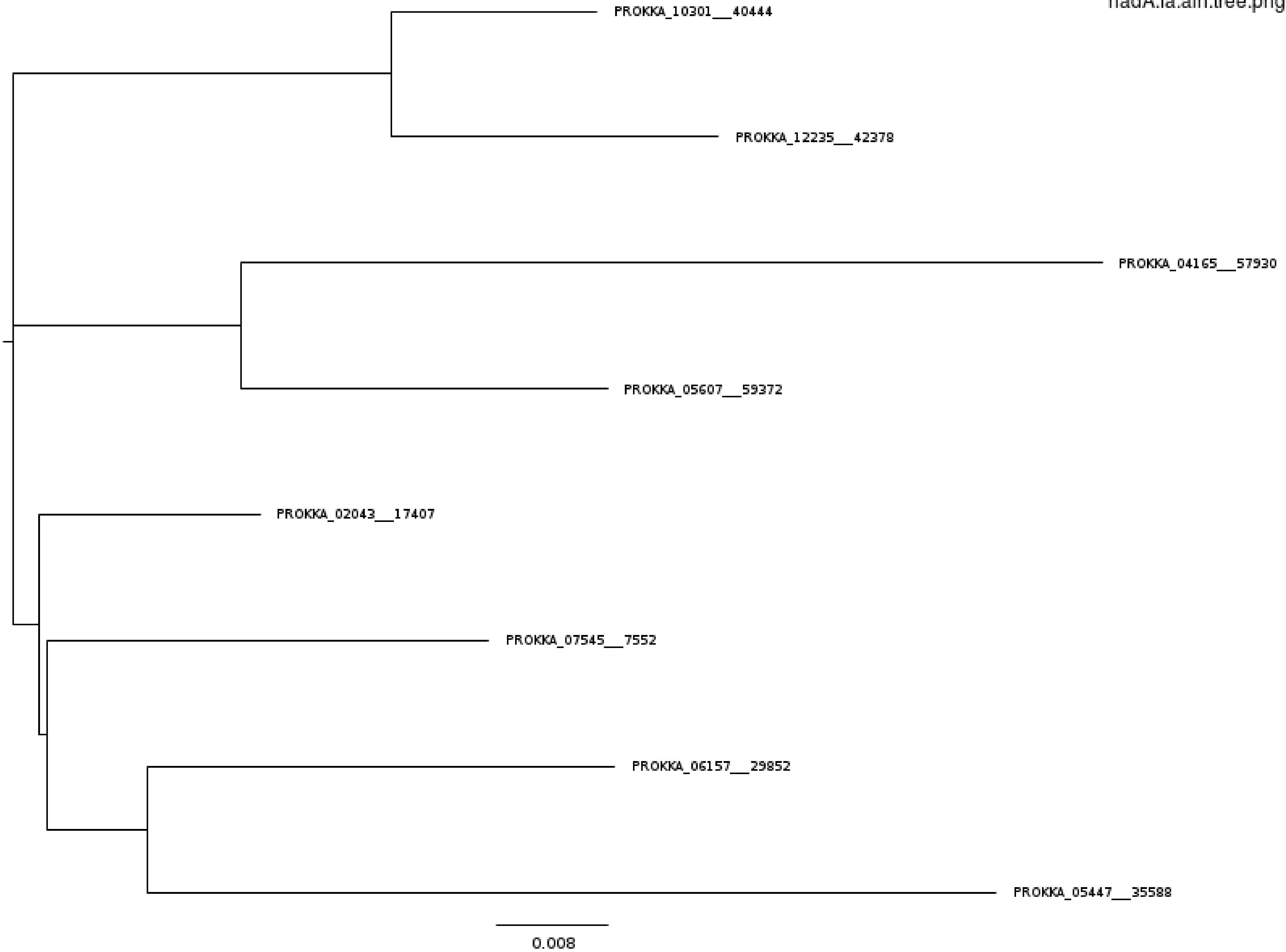

nadB_2.fa.aln.tree.png

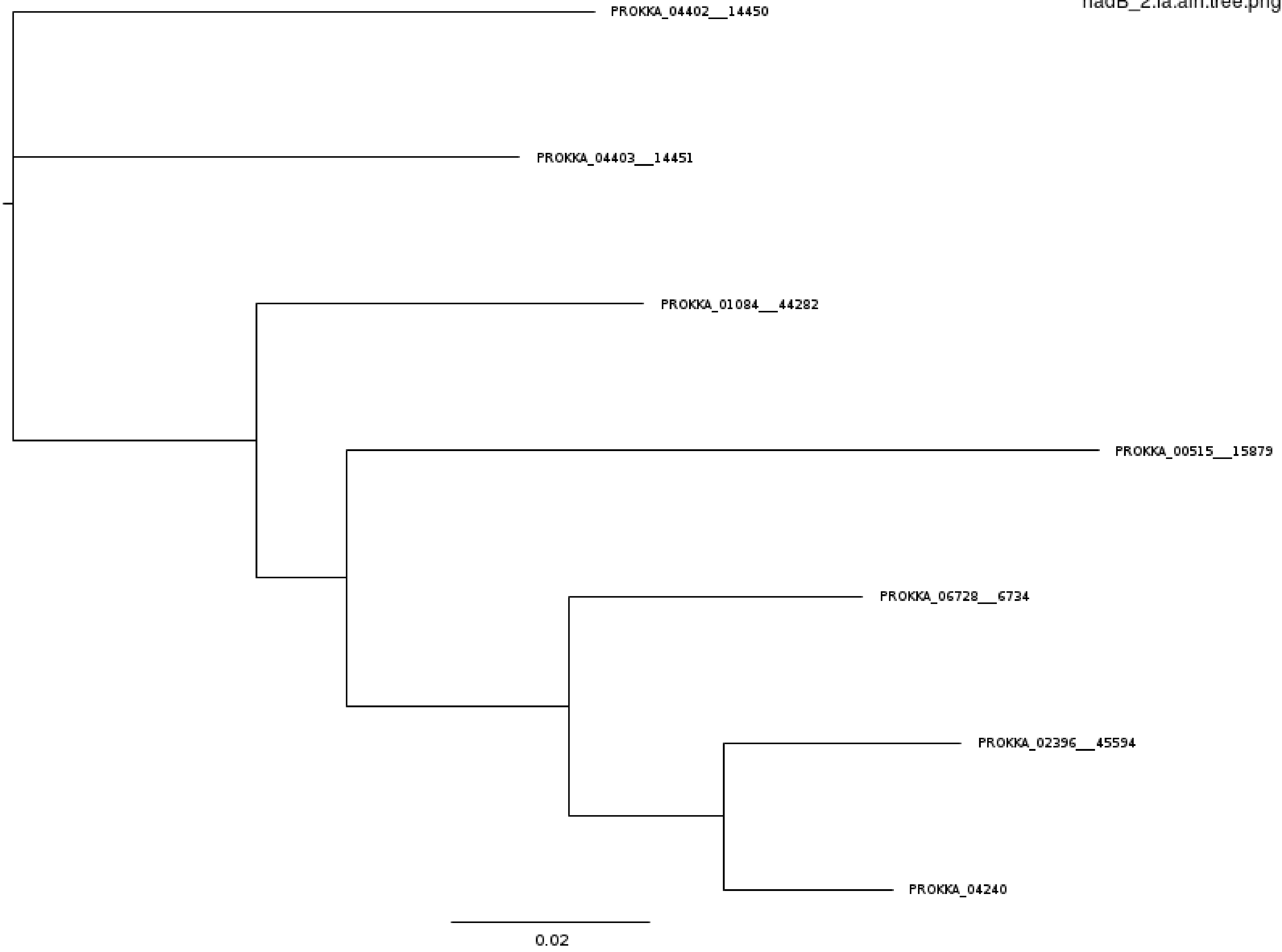

nadC_3.fa.aln.tree.png

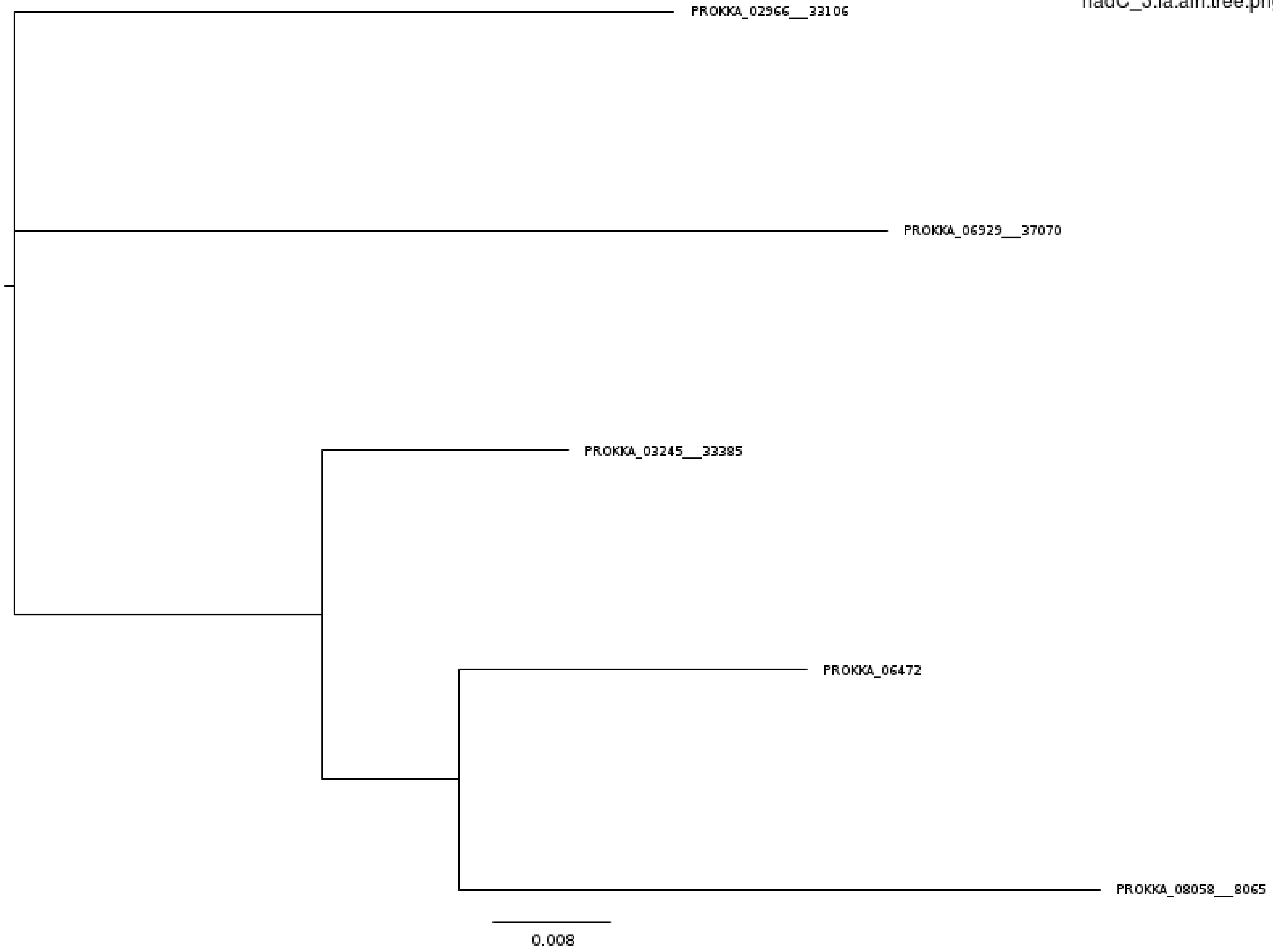

nadD_2.fa.aln.tree.png

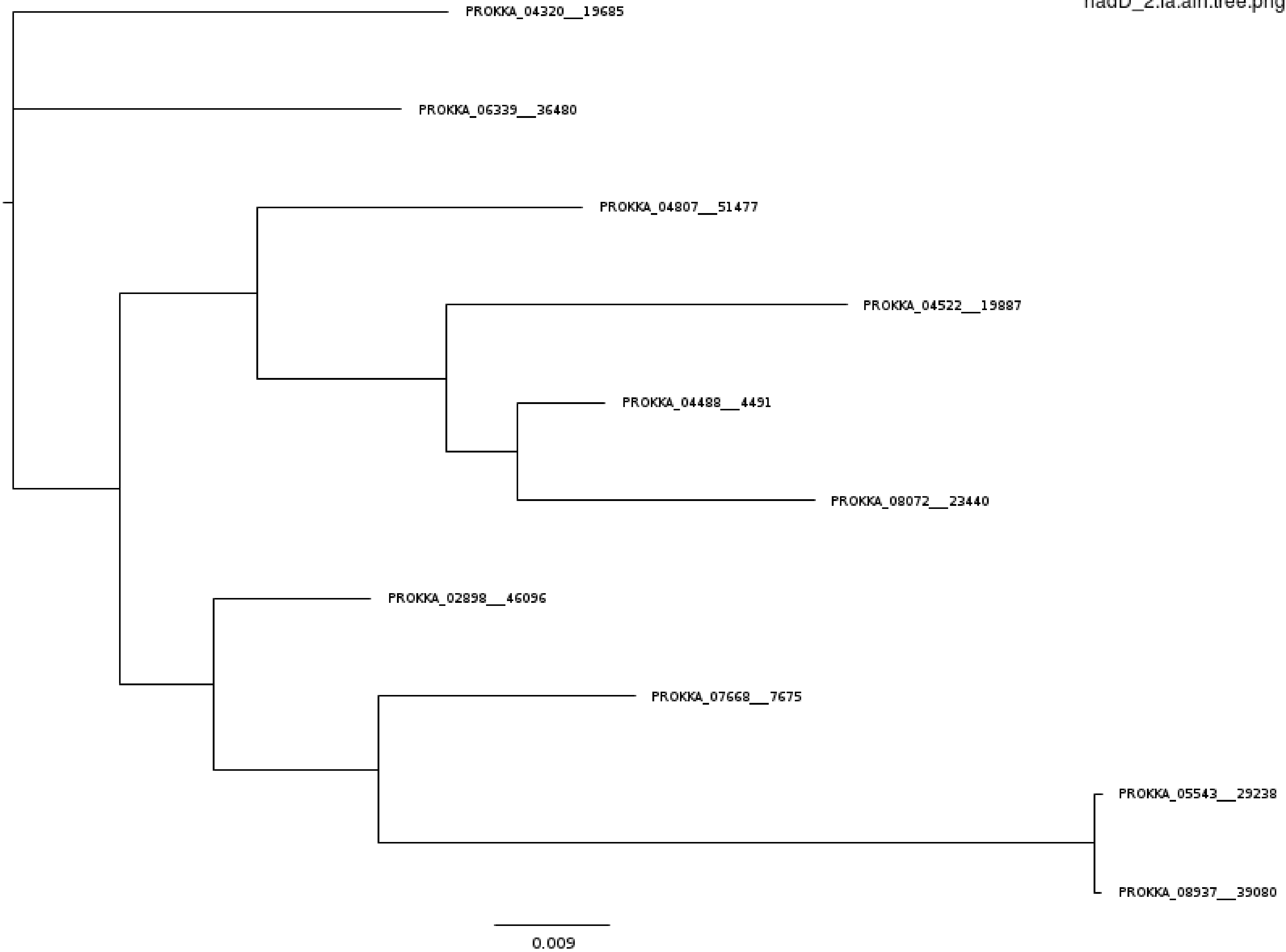

nadE_3.fa.aln.tree.png

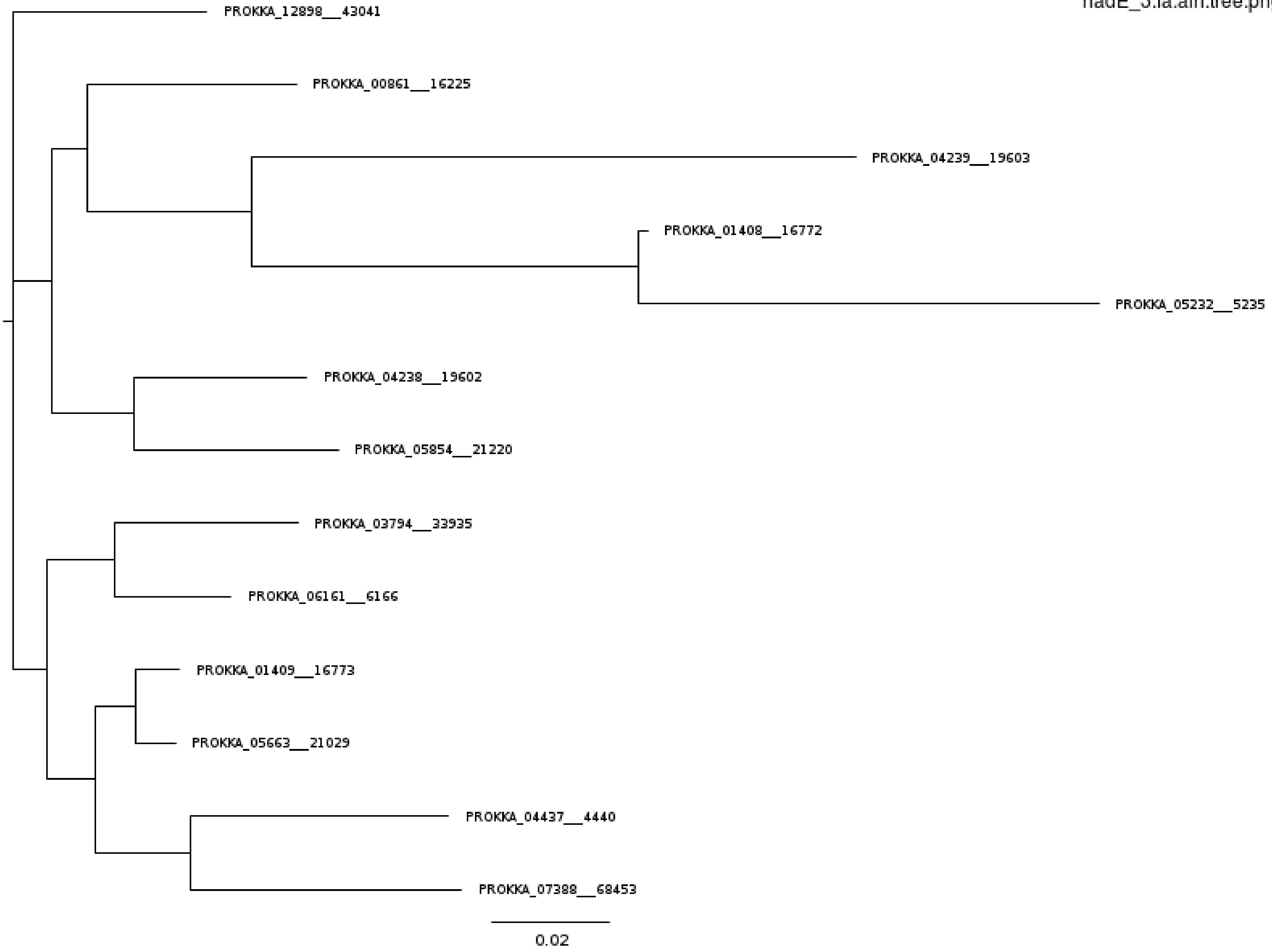

nagAb.fa.aln.tree.png

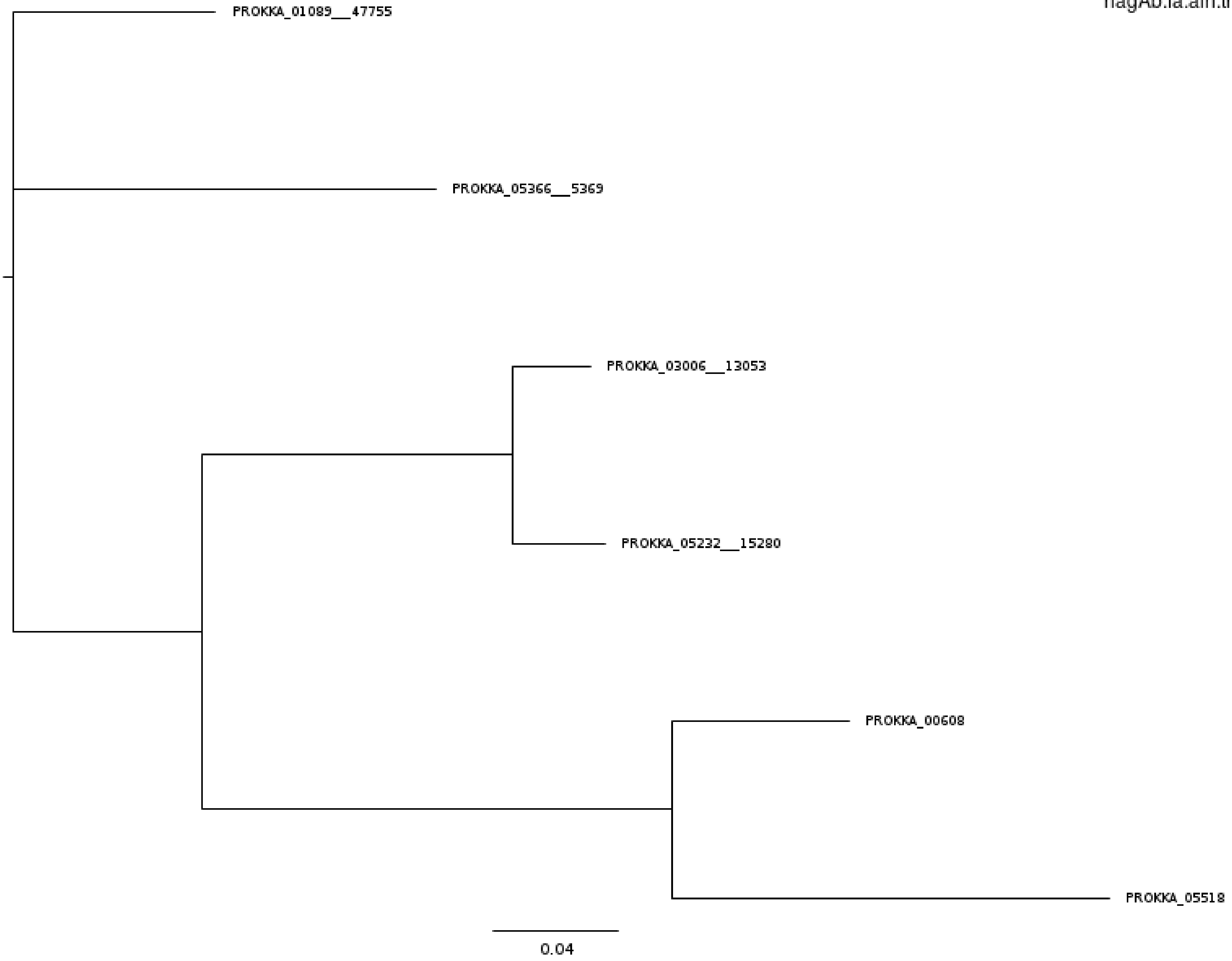

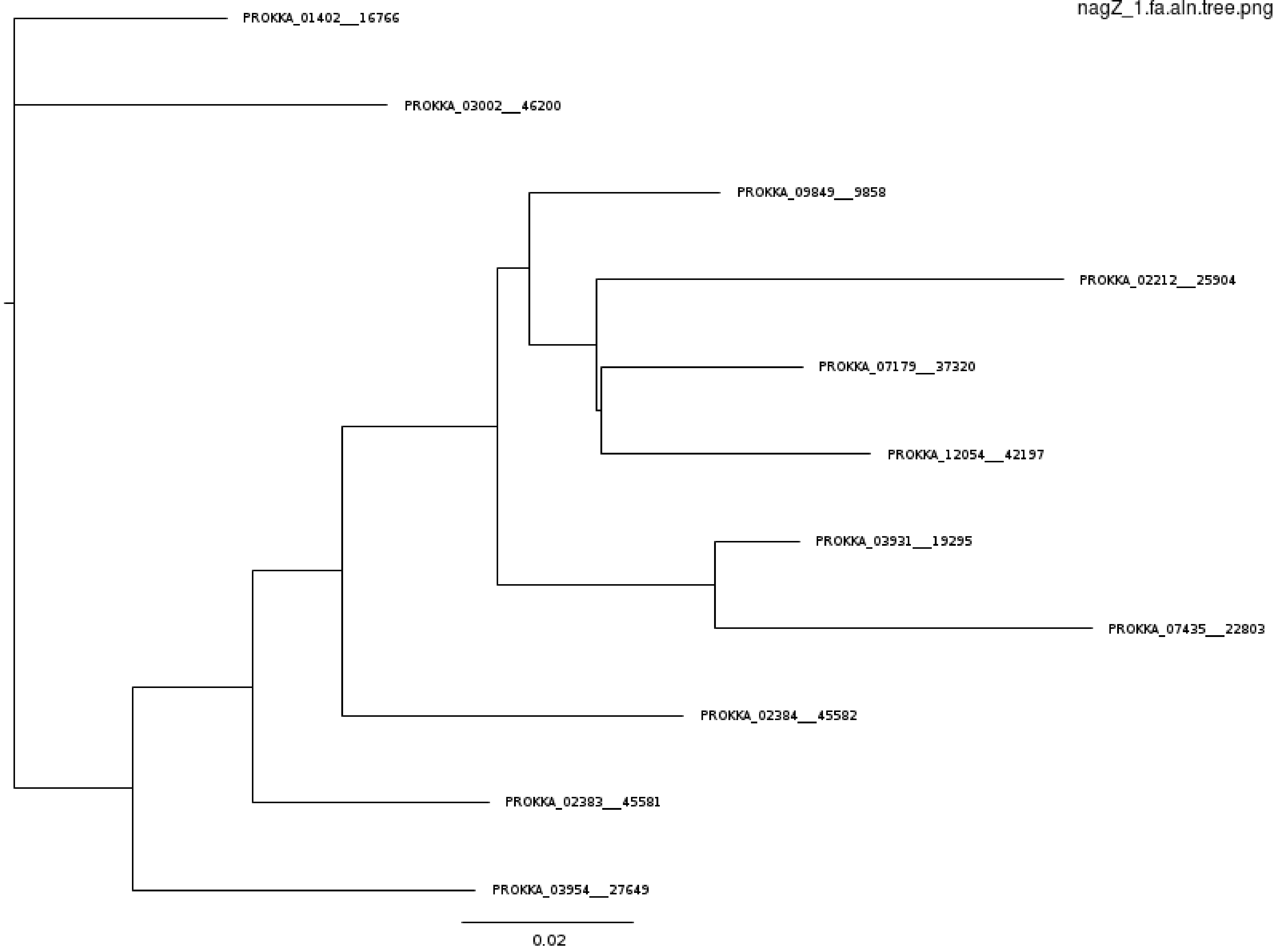
nagZ_1.fa.aln.tree.png
PROKKA_01402___16766
PROKKA_03002___46200
PROKKA_09849___9858
PROKKA_02212___25904
PROKKA_07179___37320
PROKKA_12054___42197
PROKKA_03931___19295
PROKKA_07435___22803
PROKKA_02384___45582
PROKKA_02383___45581
PROKKA_03954___27649
0.02

nahK.fa.aln.tree.png

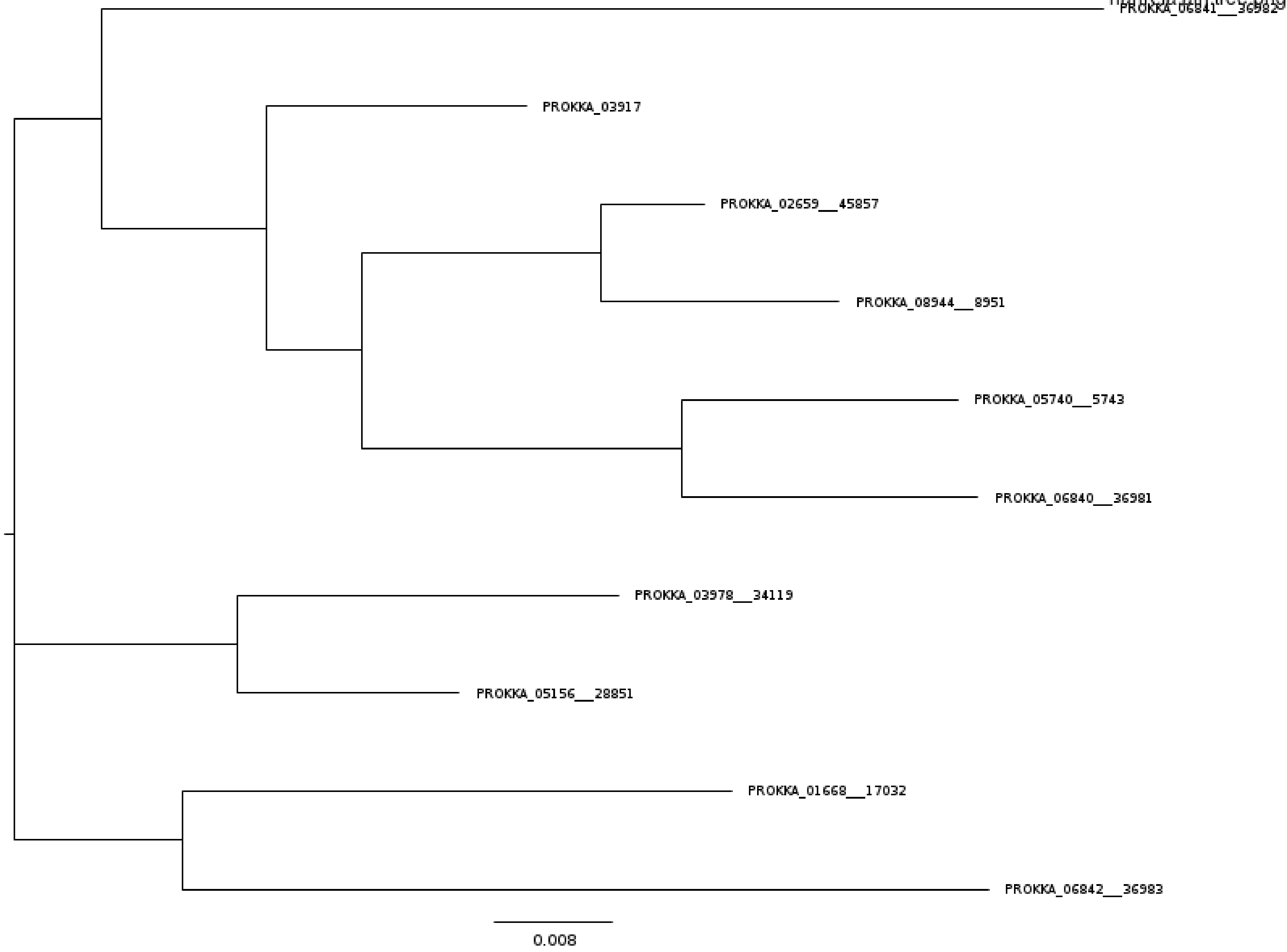

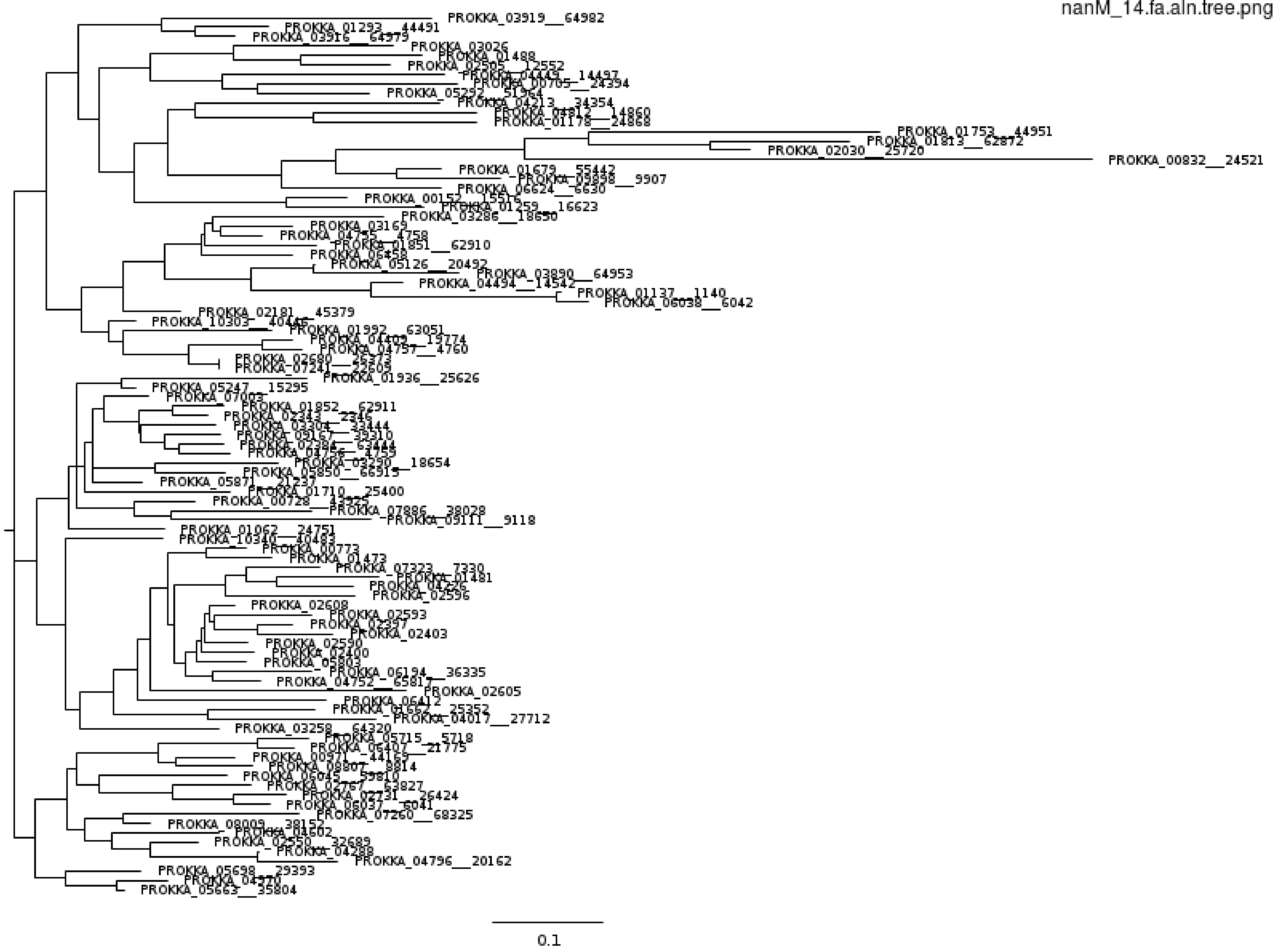
nanM_14.fa.aln.tree.png
PROKKA_03919___64982
PROKKA_01293___44491
PROKKA_03916___64979
PROKKA_03026
PROKKA_01488
PROKKA_02505___12552
PROKKA_04449___14497
PROKKA_00705___24394
PROKKA_05292___51964
PROKKA_04213___34354
PROKKA_04812___14860
PROKKA_01178___24868
PROKKA_01753___44951
PROKKA_01813___62872
PROKKA_02030___25720
PROKKA_00832___24521
PROKKA_01679___55442
PROKKA_09898___9907
PROKKA_06624___6630
PROKKA_00152___15516
PROKKA_01259___16623
PROKKA_03286___18650
PROKKA_03169
PROKKA_04755___4758
PROKKA_01851___62910
PROKKA_06458
PROKKA_05126___20492
PROKKA_03890___64953
PROKKA_04494___14542
PROKKA_01137___1140
PROKKA_06038___6042
PROKKA_02181___45379
PROKKA_10303___40446
PROKKA_01992___63051
PROKKA_04409___19774
PROKKA_04757___4760
PROKKA_02680___25373
PROKKA_07241___22609
PROKKA_01936___25626
PROKKA_05247___15295
PROKKA_07003
PROKKA_01852___62911
PROKKA_02343___2346
PROKKA_03304___33444
PROKKA_09167___39310
PROKKA_03290___18654
PROKKA_05850___66915
PROKKA_05871___21237
PROKKA_01710___25400
PROKKA_00728
PROKKA_07886___38028
PROKKA_09111___9118
PROKKA_01062___24751
PROKKA_10340___40483
PROKKA_00773
PROKKA_01473
PROKKA_07323___7330
PROKKA_01481
PROKKA_04226
PROKKA_02596
PROKKA_02608
PROKKA_02593
PROKKA_02397
PROKKA_02403
PROKKA_02590
PROKKA_02400
PROKKA_05803
PROKKA_06194___36335
PROKKA_04752___65817
PROKKA_02605
PROKKA_06412
PROKKA_01662___25352
PROKKA_04017___27712
PROKKA_03258___64320
PROKKA_05715___5718
PROKKA_06407___21775
PROKKA_00971___44169
PROKKA_08807___8814
PROKKA_06045___59810
PROKKA_02767___63827
PROKKA_02731___26424
PROKKA_06037___6041
PROKKA_07260___68325
PROKKA_08009___38152
PROKKA_04602
PROKKA_02550___32689
PROKKA_04289
PROKKA_04796___20162
PROKKA_05698___29393
PROKKA_04370
PROKKA_05663___35804
0.1

nanM_14.tree.png

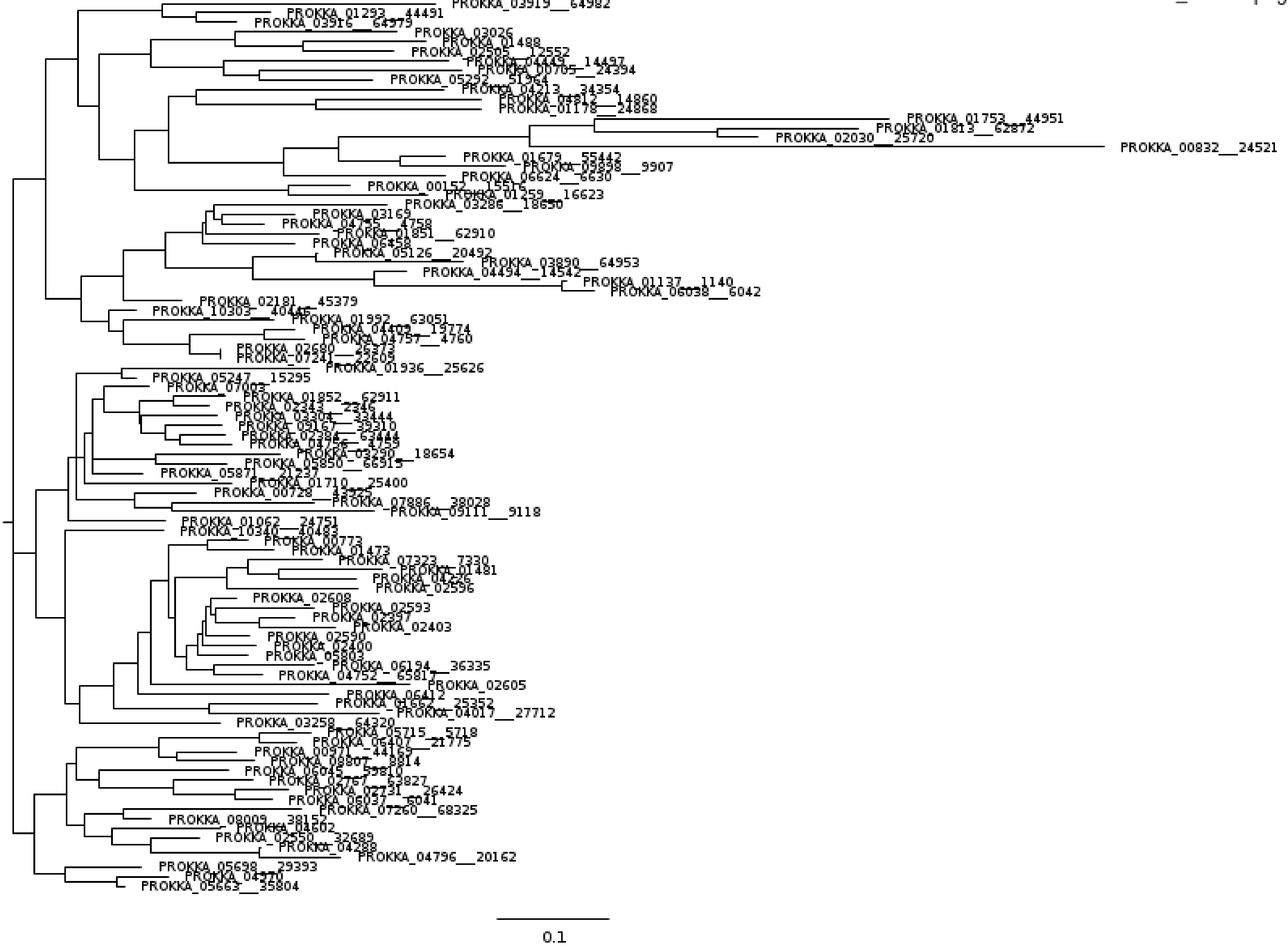

nanM_1.fa.aln.tree.png

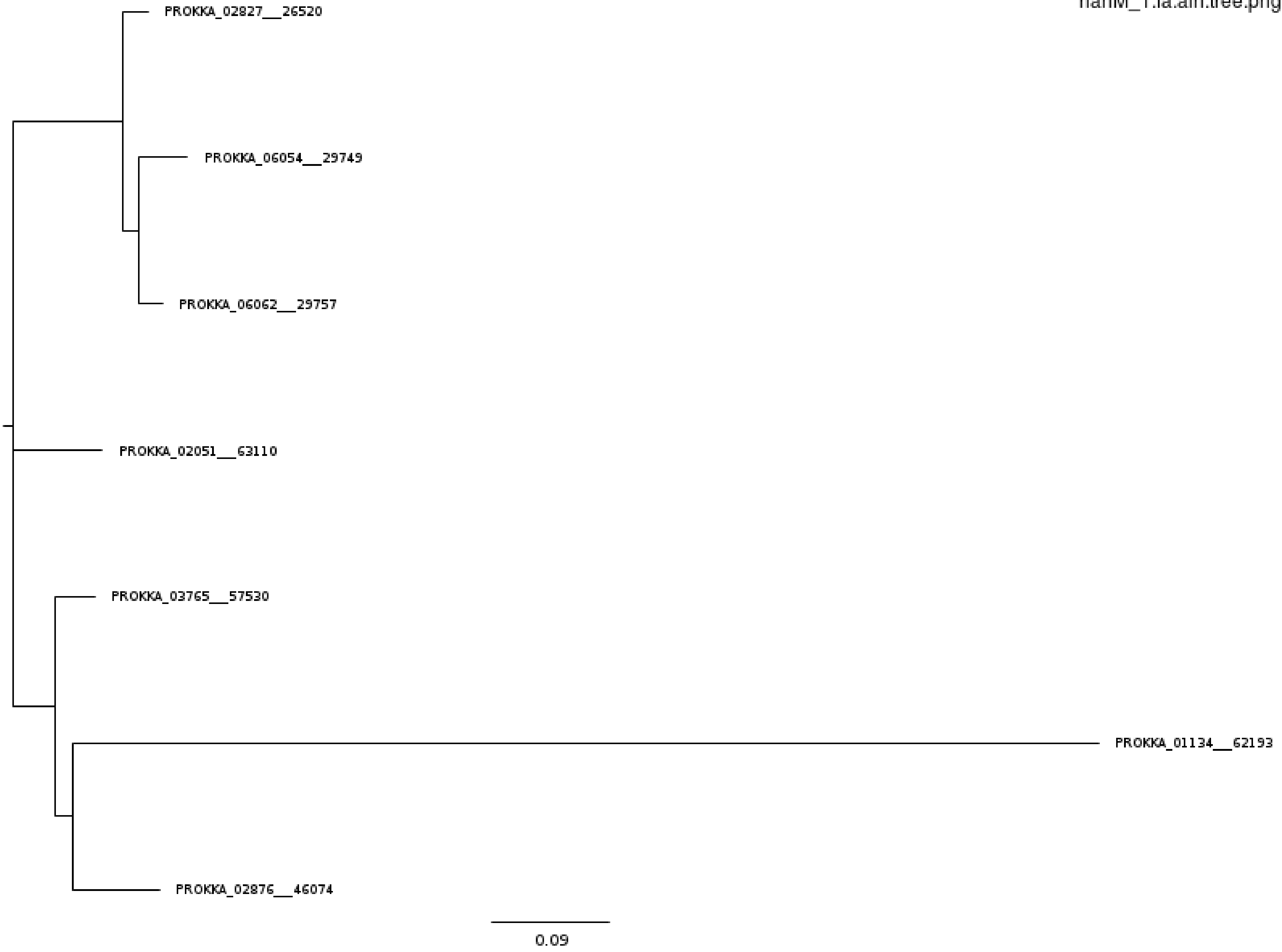

nanM_6.fa.aln.tree.png

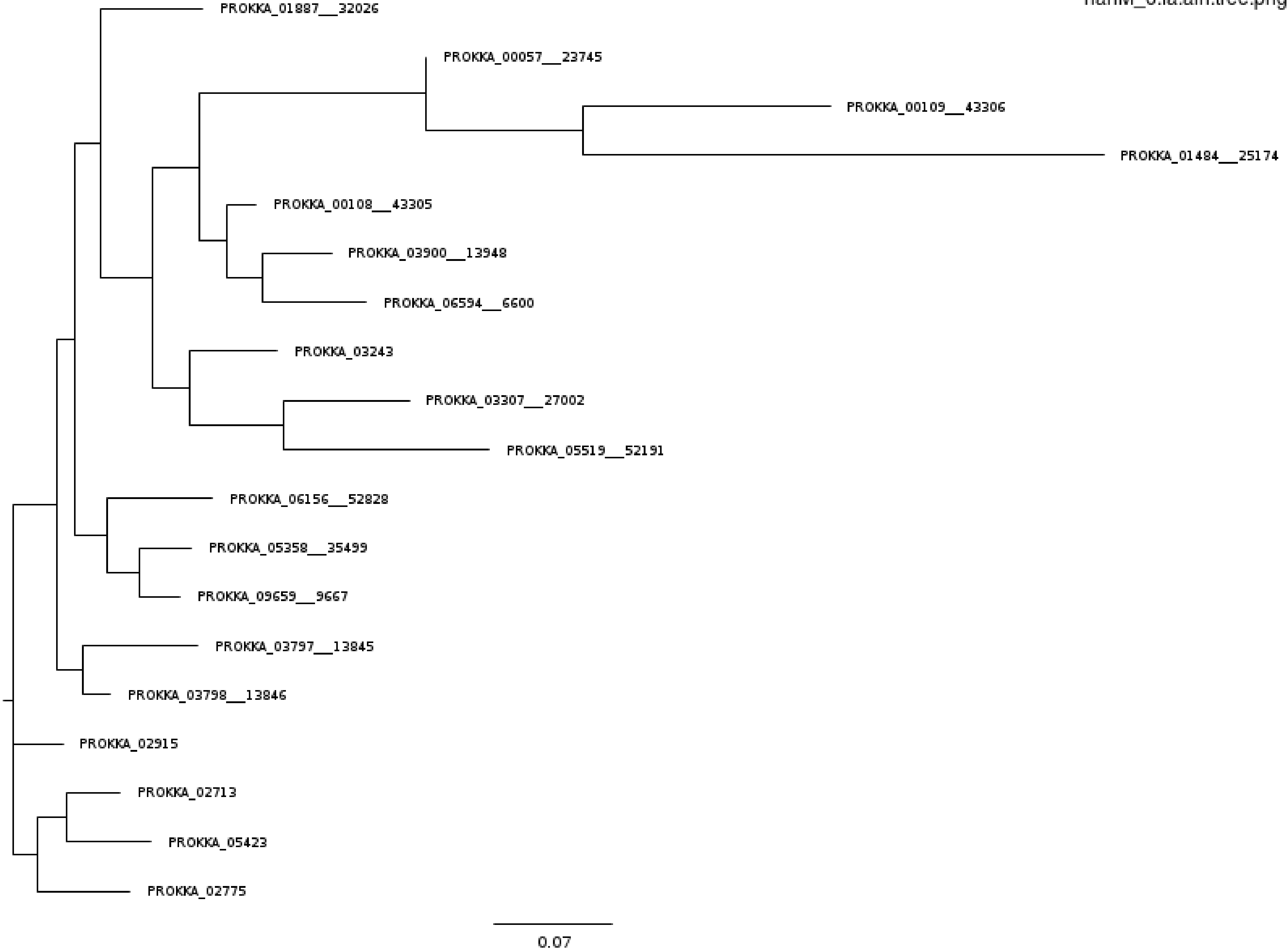

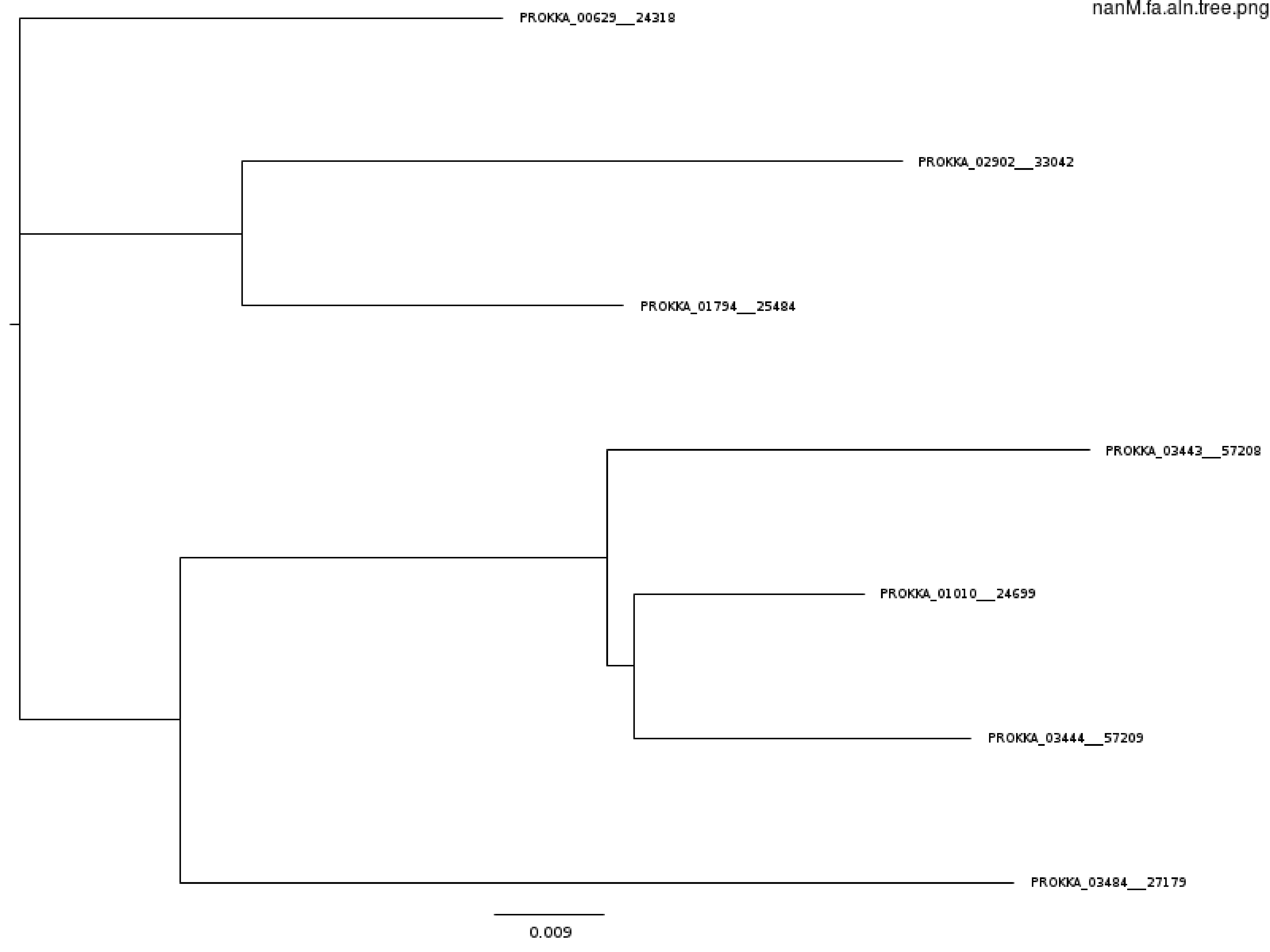
nanM.fa.aln.tree.png
PROKKA_00629___24318
PROKKA_02902___33042
PROKKA_01794___25484
PROKKA_03443___57208
PROKKA_01010___24699
PROKKA_03444___57209
PROKKA_03484___27179
0.009

nemA_2.fa.aln.tree.png

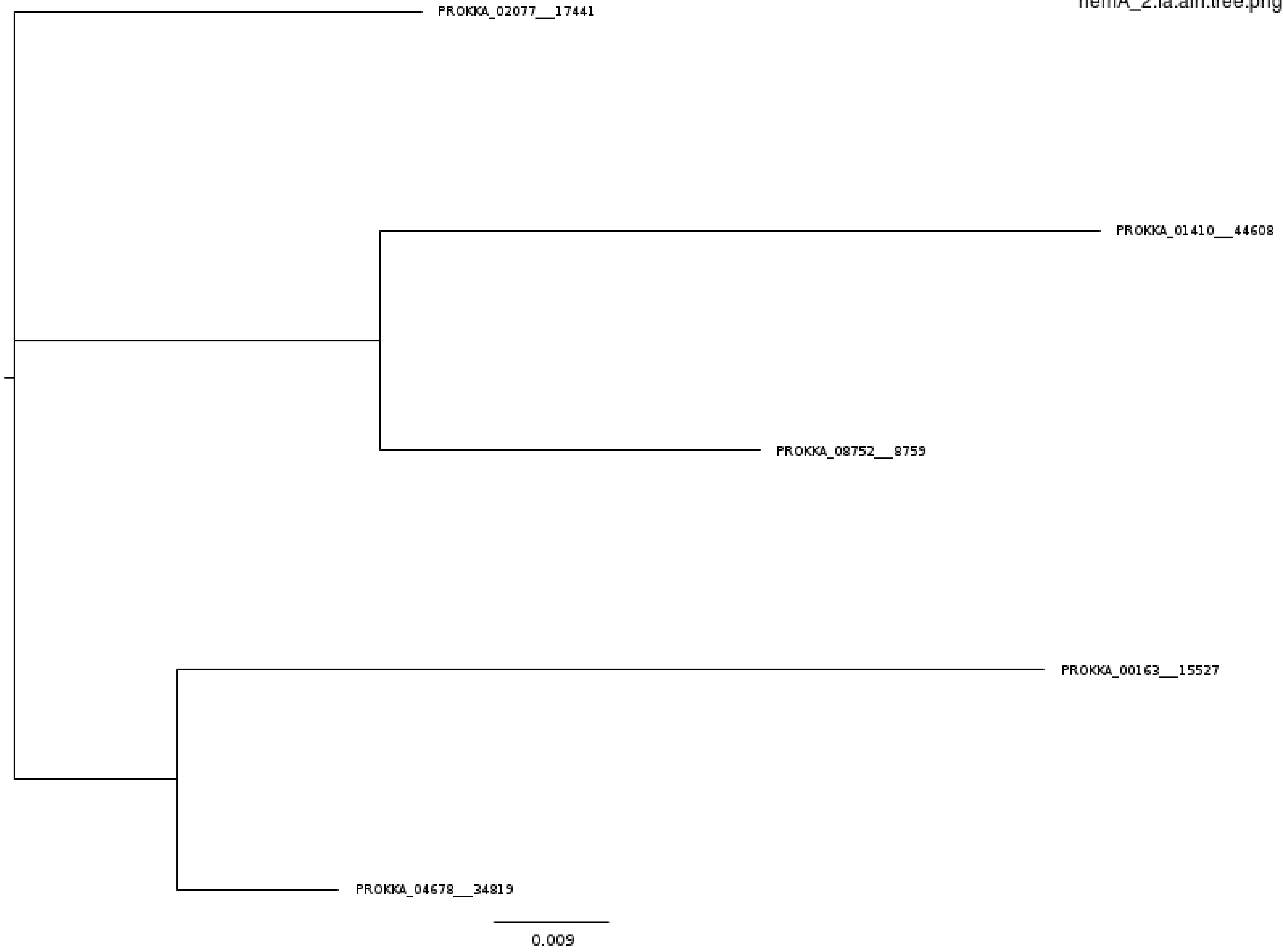

nifB_2.fa.aln.tree.png

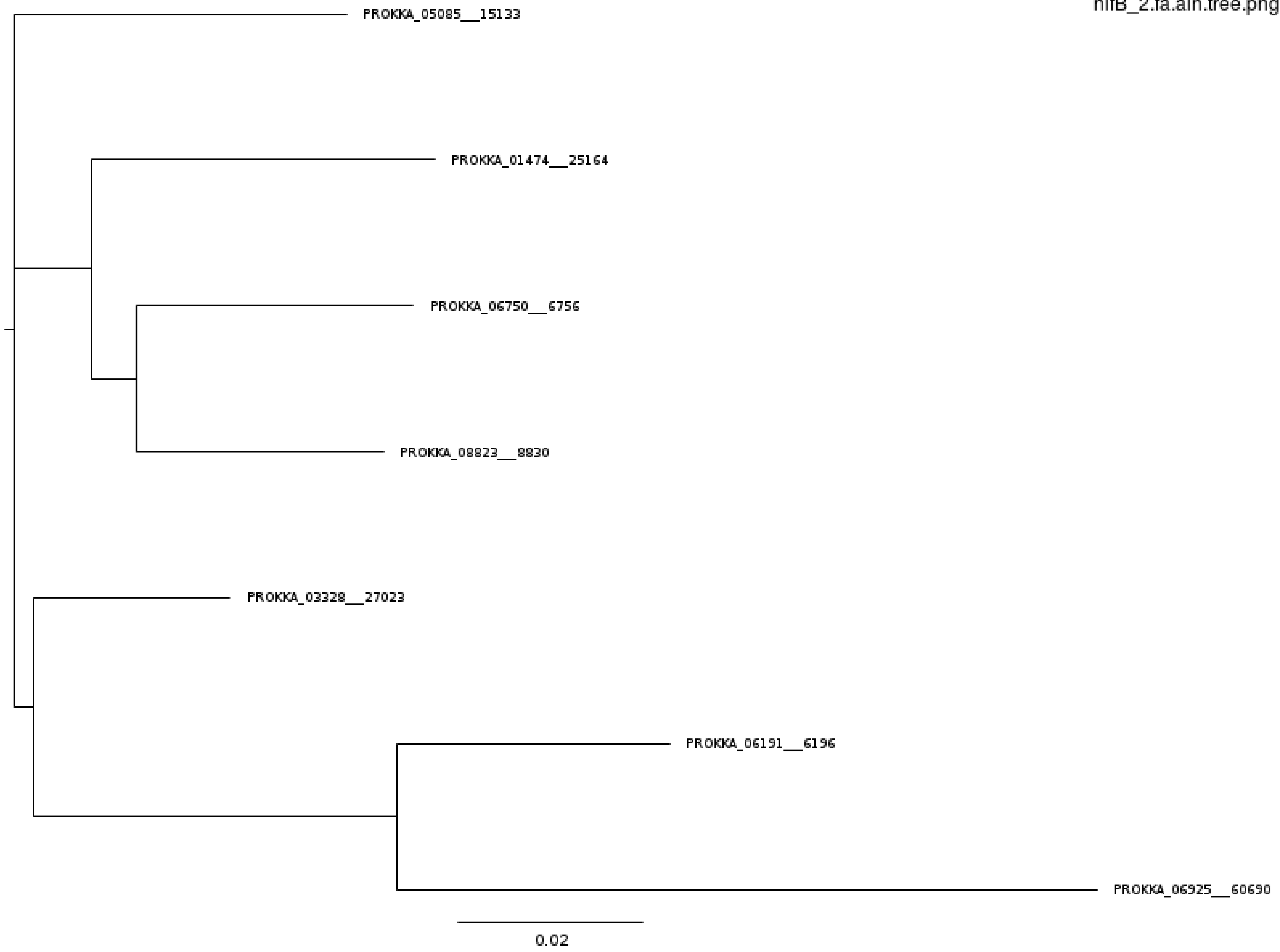

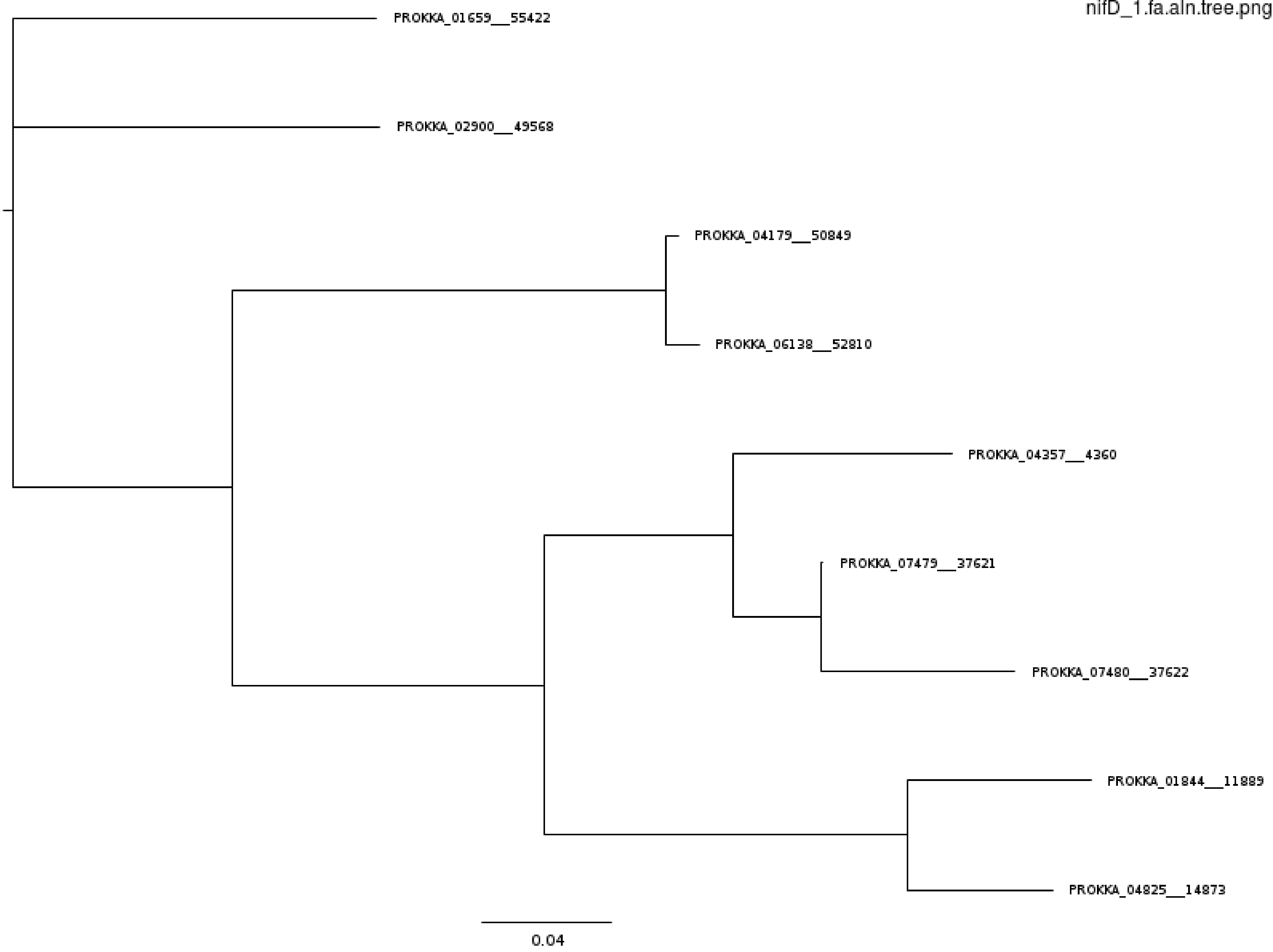
nifD_1.fa.aln.tree.png
PROKKA_01659__55422
PROKKA_02900__49568
PROKKA_04179__50849
PROKKA_06138__52810
PROKKA_04357__4360
PROKKA_07479__37621
PROKKA_07480__37622
PROKKA_01844__11889
PROKKA_04825__14873
0.04

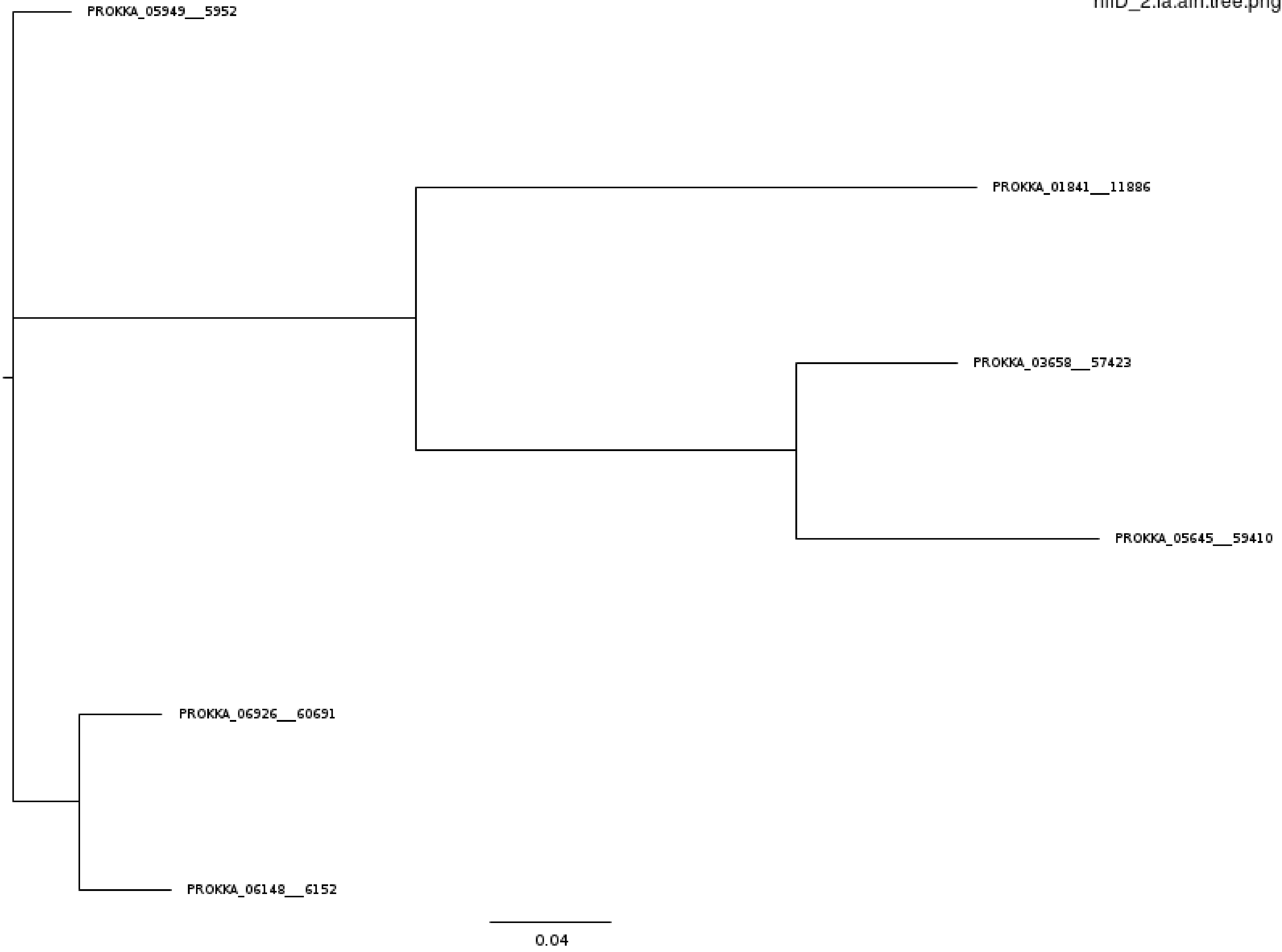
PROKKA_05949___5952
PROKKA_01841___11886
PROKKA_03658___57423
PROKKA_05645___59410
PROKKA_06926___60691
PROKKA_06148___6152
0.04

nifH1.fa.aln.tree.png

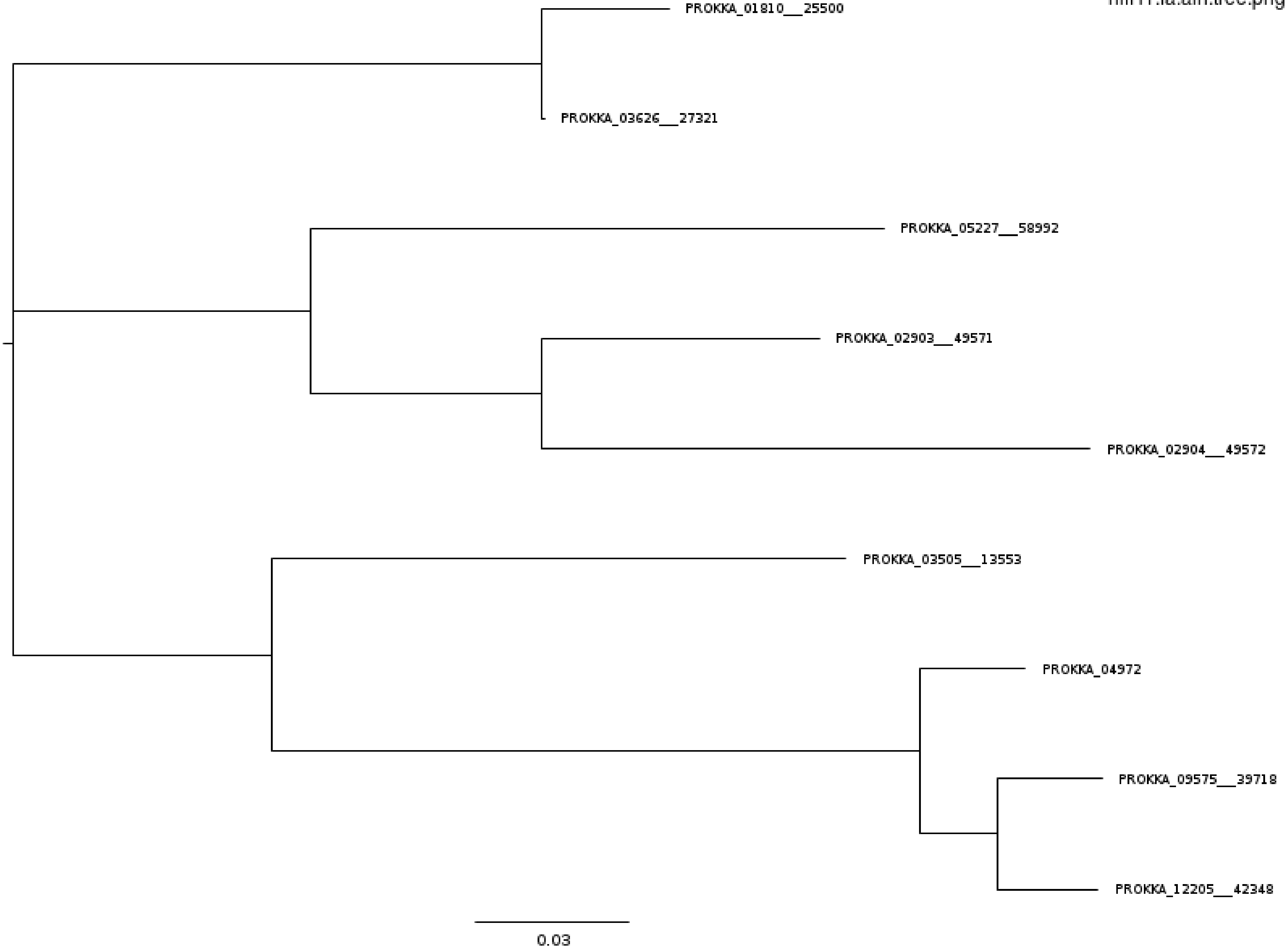

nifK_3.fa.aln.tree.png

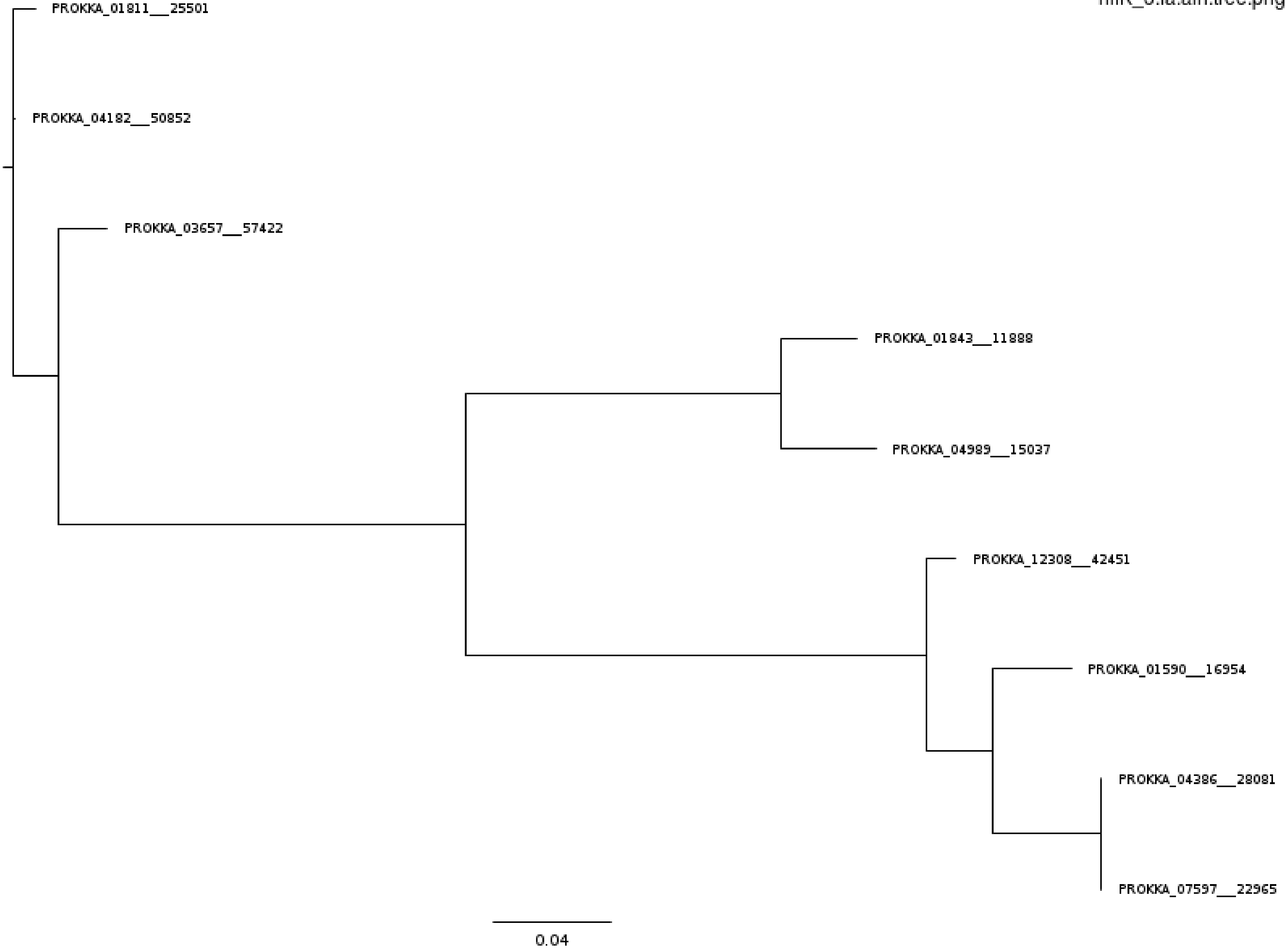

nikC.fa.aln.tree.png

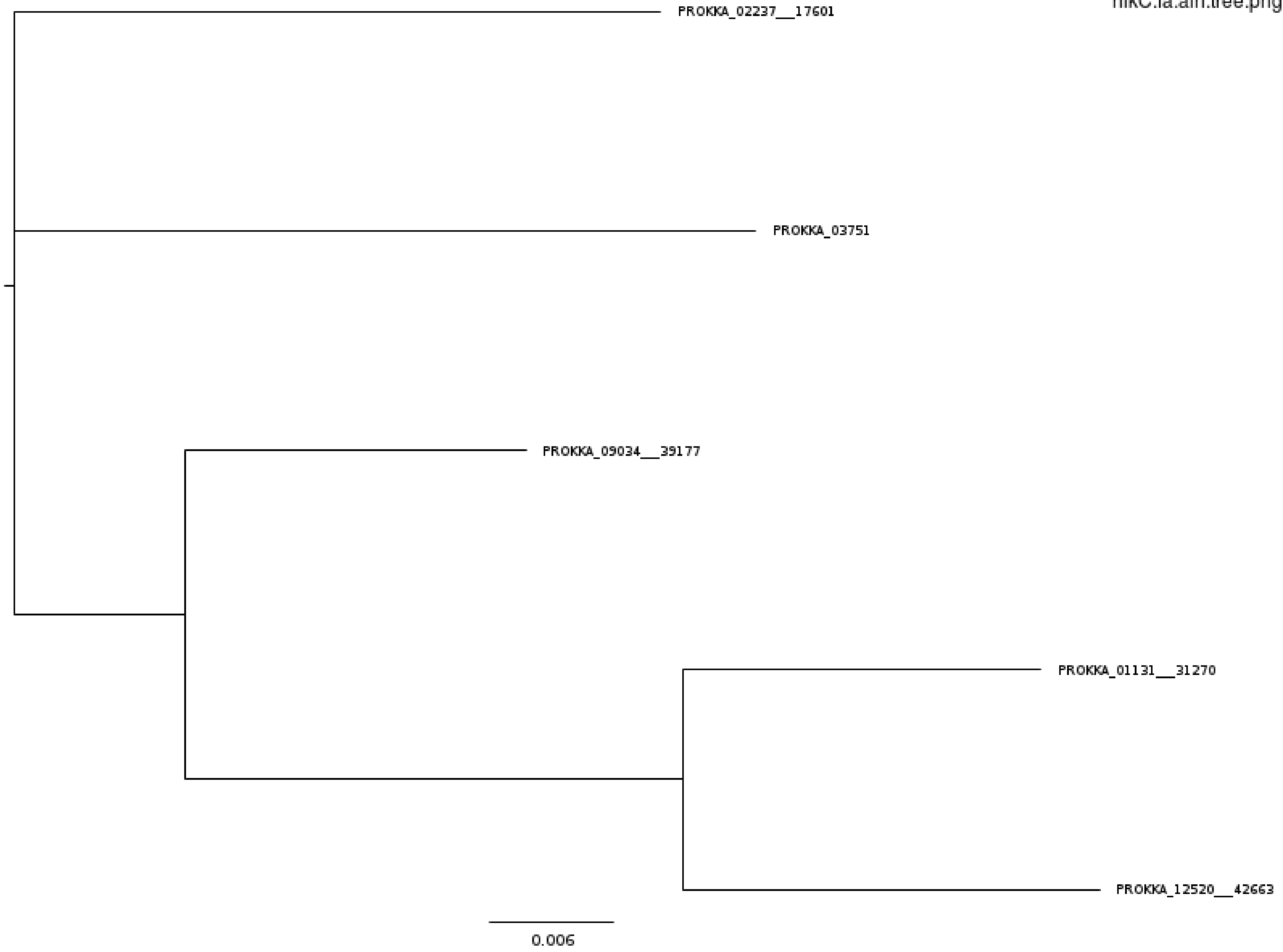

nirQ_2.fa.aln.tree.png

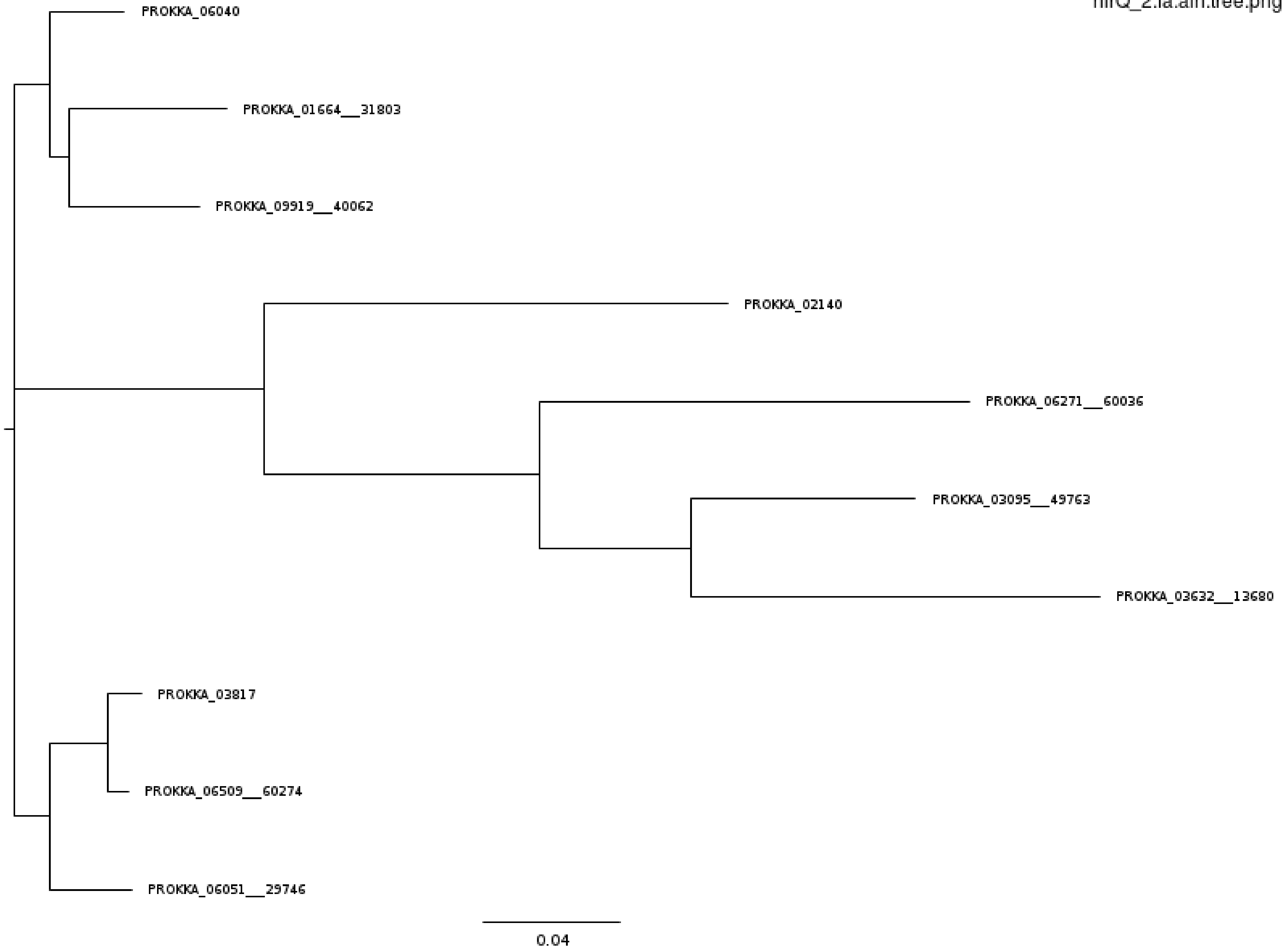

nlpD_1.fa.aln.tree.png

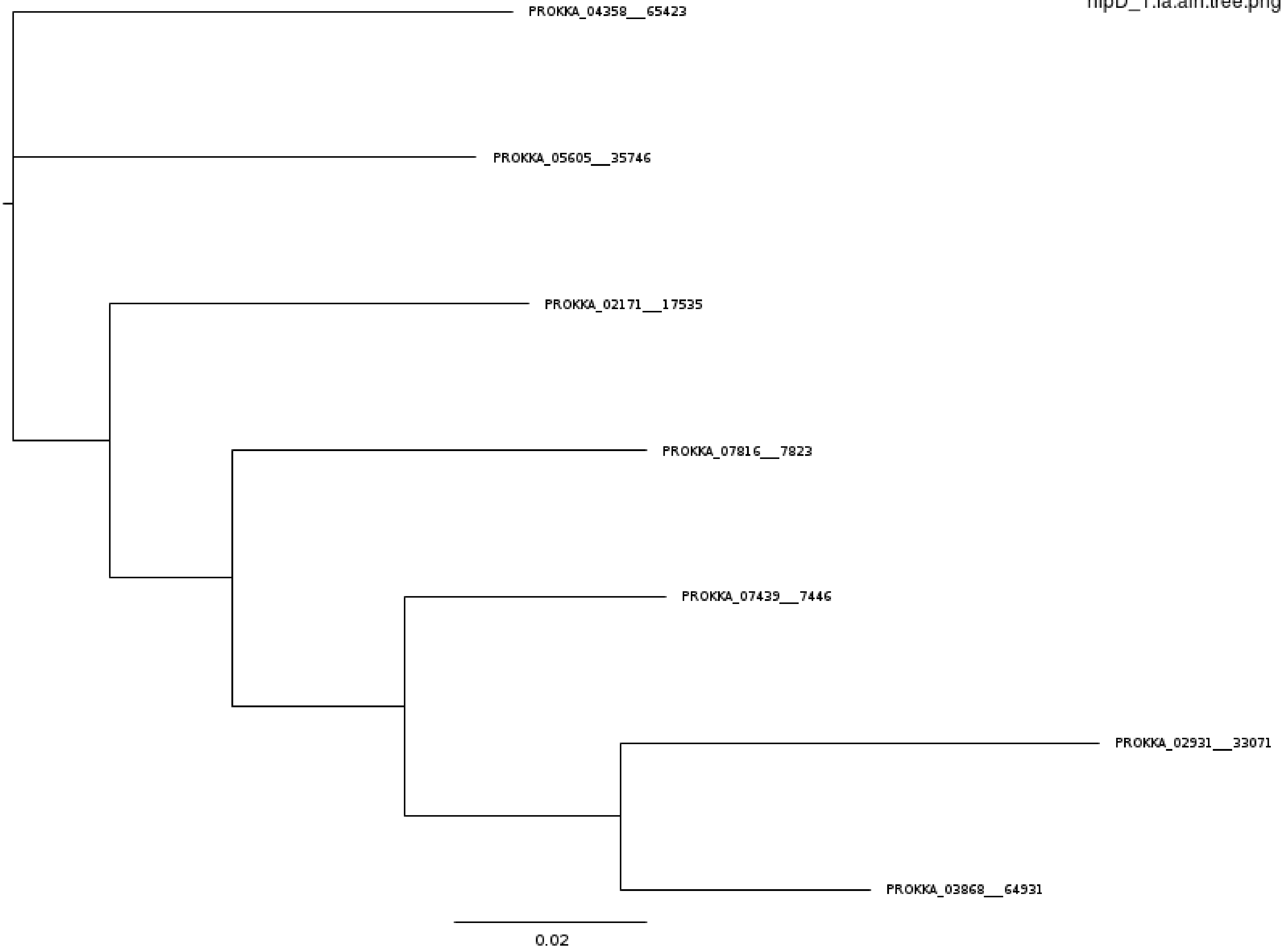

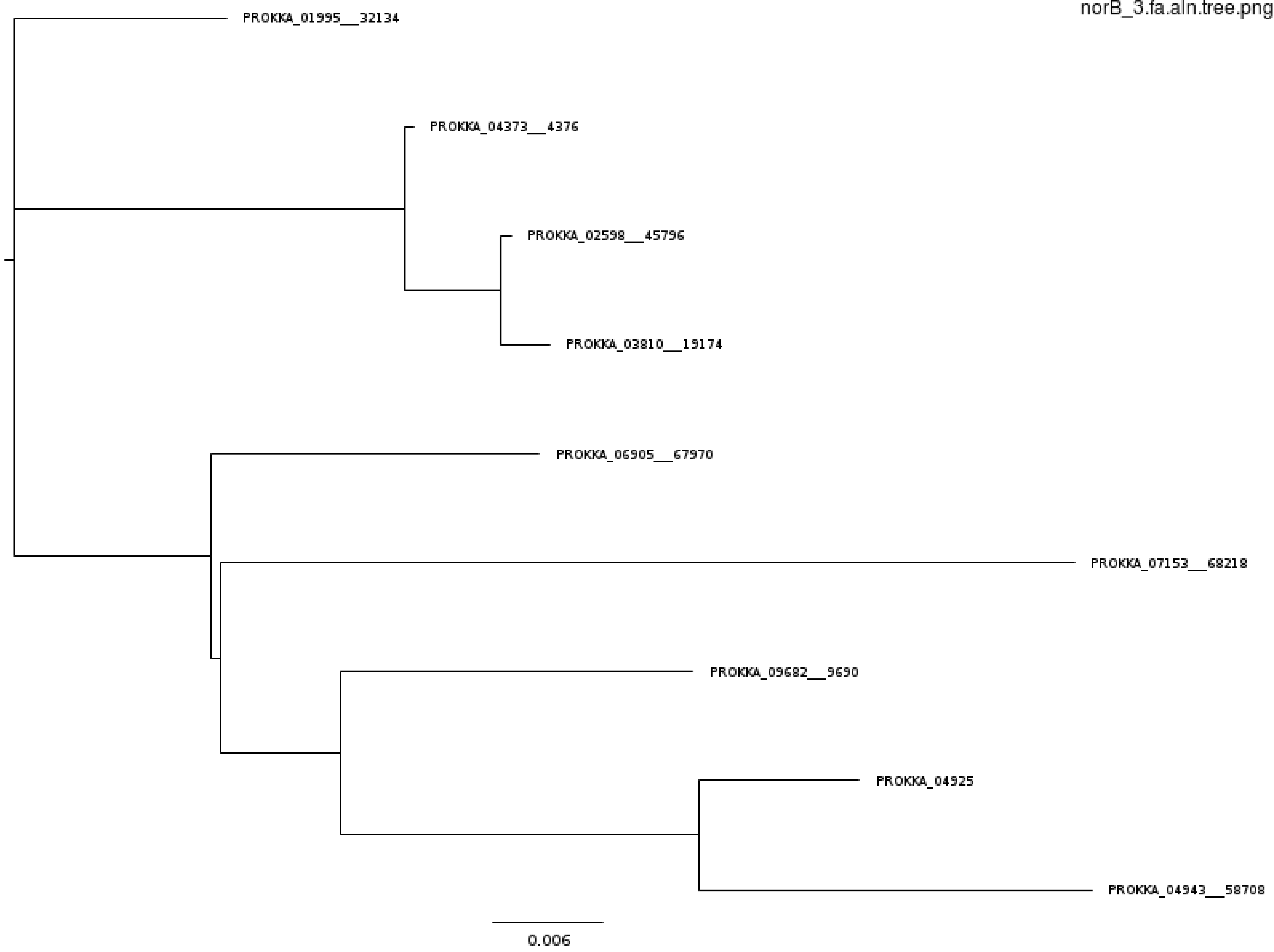

norB_3.fa.aln.tree.png
PROKKA_01995___32134
PROKKA_04373___4376
PROKKA_02598___45796
PROKKA_03810___19174
PROKKA_06905___67970
PROKKA_07153___68218
PROKKA_09682___9690
PROKKA_04925
PROKKA_04943___58708
0.006

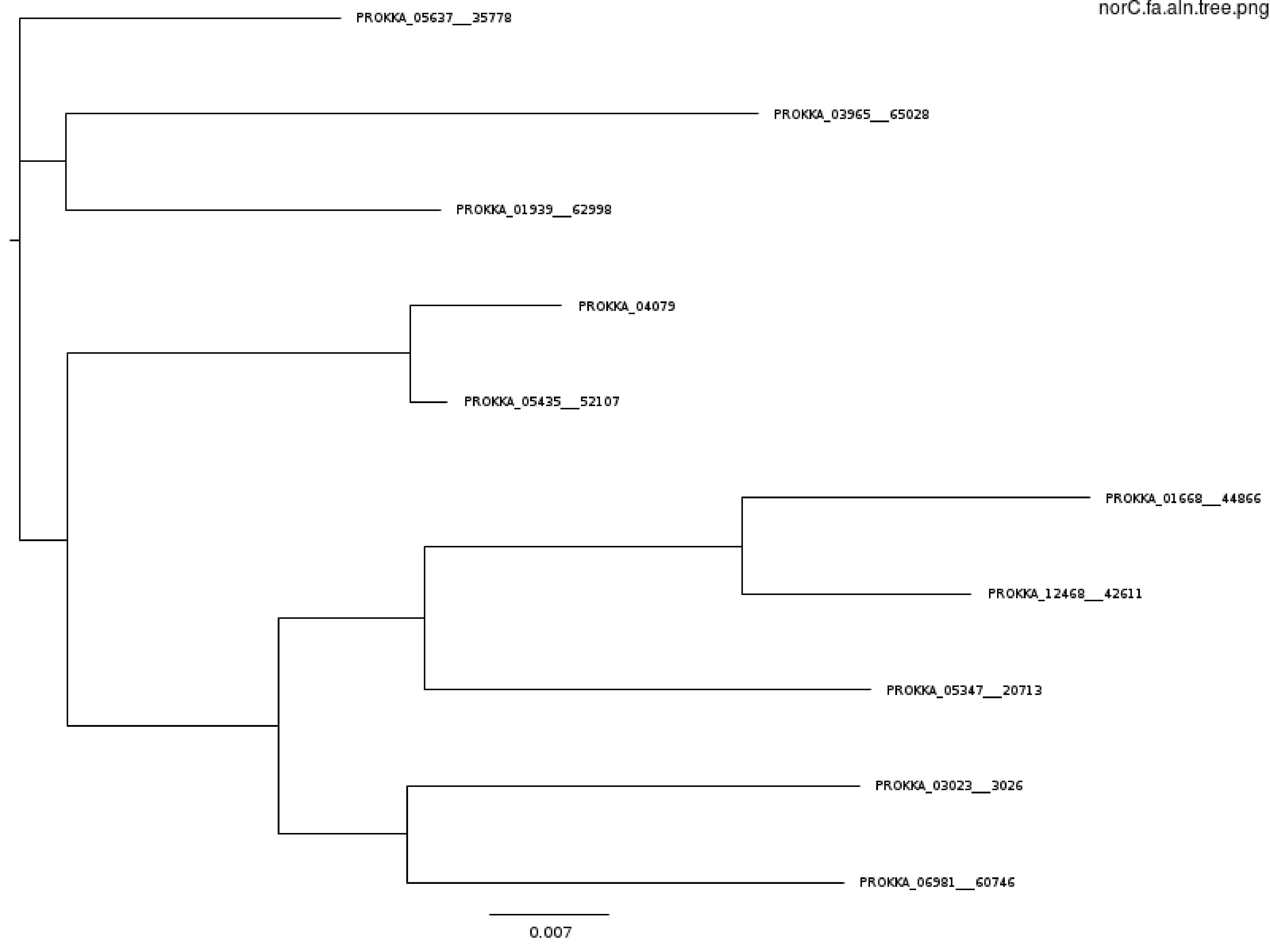

norC.fa.aln.tree.png
PROKKA_05637__35778
PROKKA_03965__65028
PROKKA_01939__62998
PROKKA_04079
PROKKA_05435__52107
PROKKA_01668__44866
PROKKA_12468__42611
PROKKA_05347__20713
PROKKA_03023__3026
PROKKA_06981__60746
0.007

novN.fa.aln.tree.png

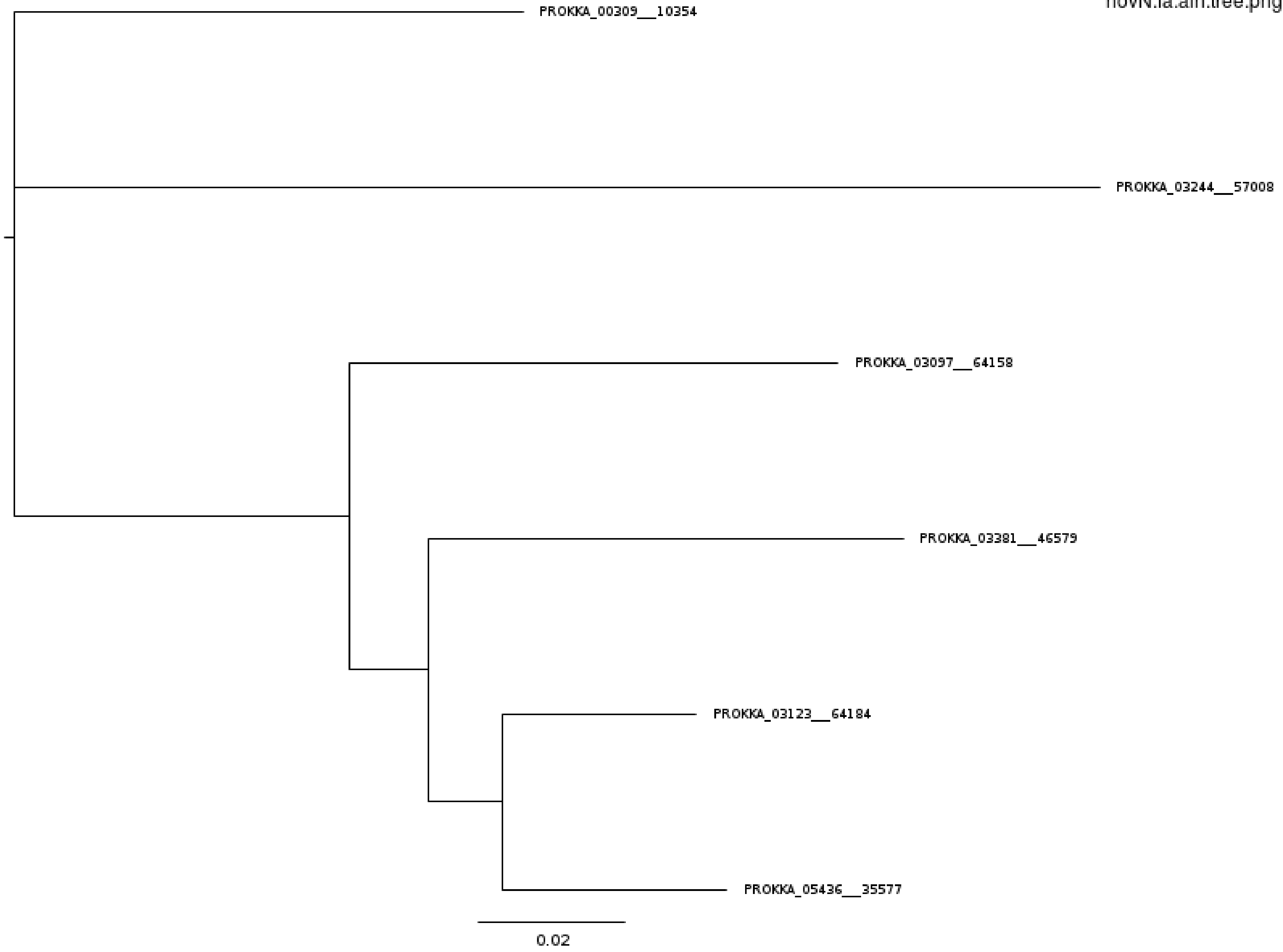

nqrA.fa.aln.tree.png

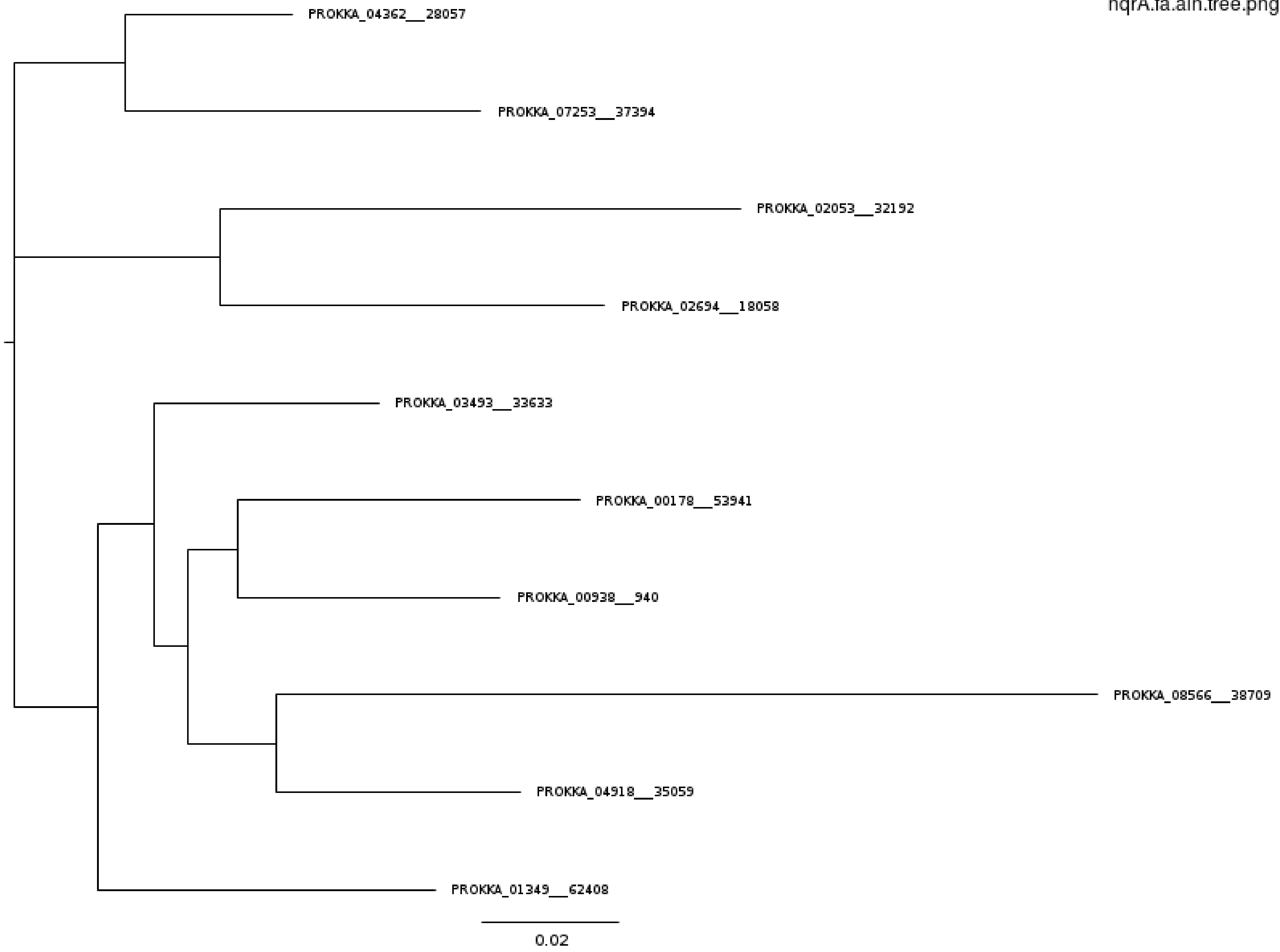

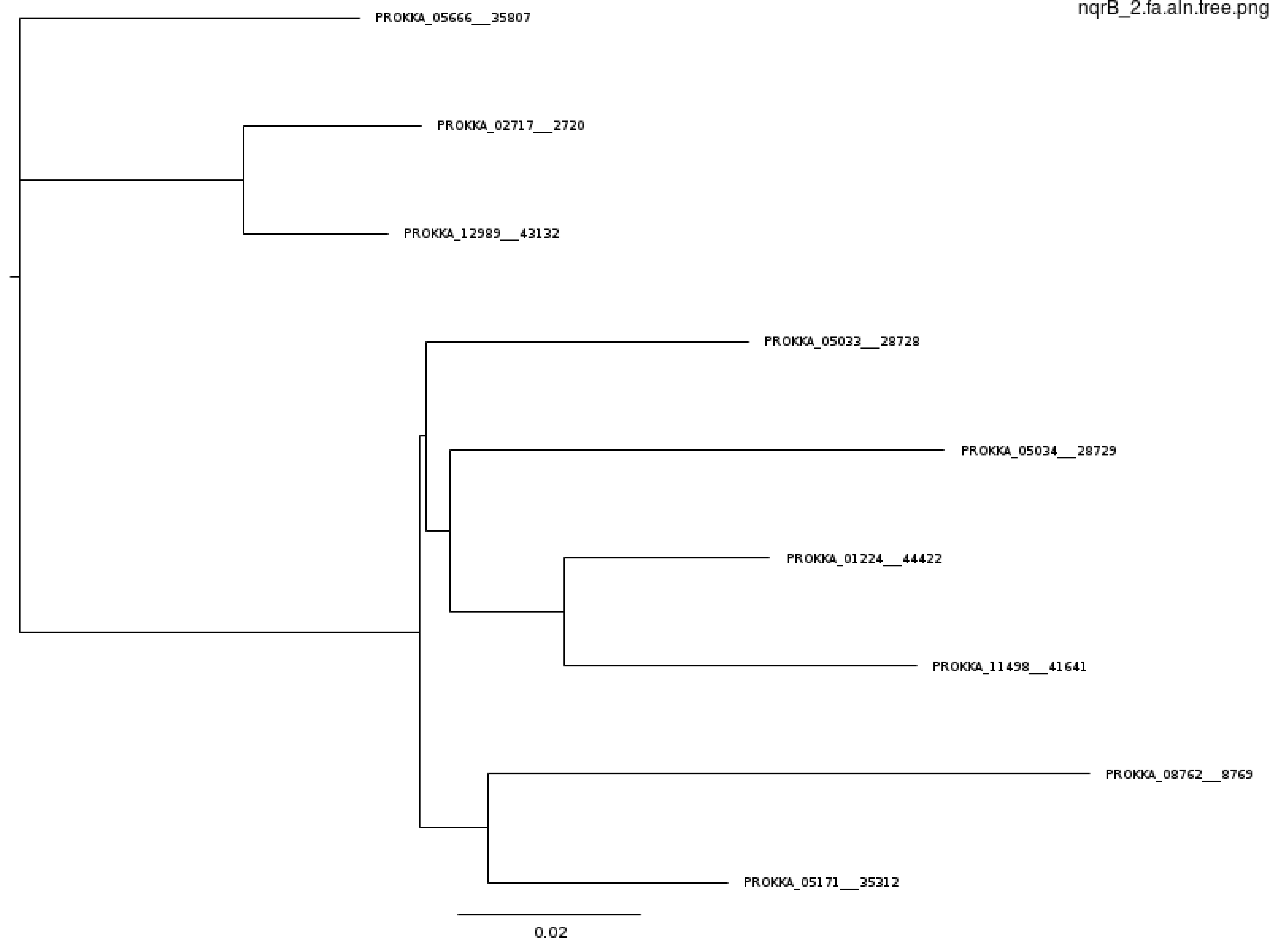

nqrB_2.fa.aln.tree.png
PROKKA_05666___35807
PROKKA_02717___2720
PROKKA_12989___43132
PROKKA_05033___28728
PROKKA_05034___28729
PROKKA_01224___44422
PROKKA_11498___41641
PROKKA_08762___8769
PROKKA_05171___35312
0.02

nqrB_3.fa.aln.tree.png

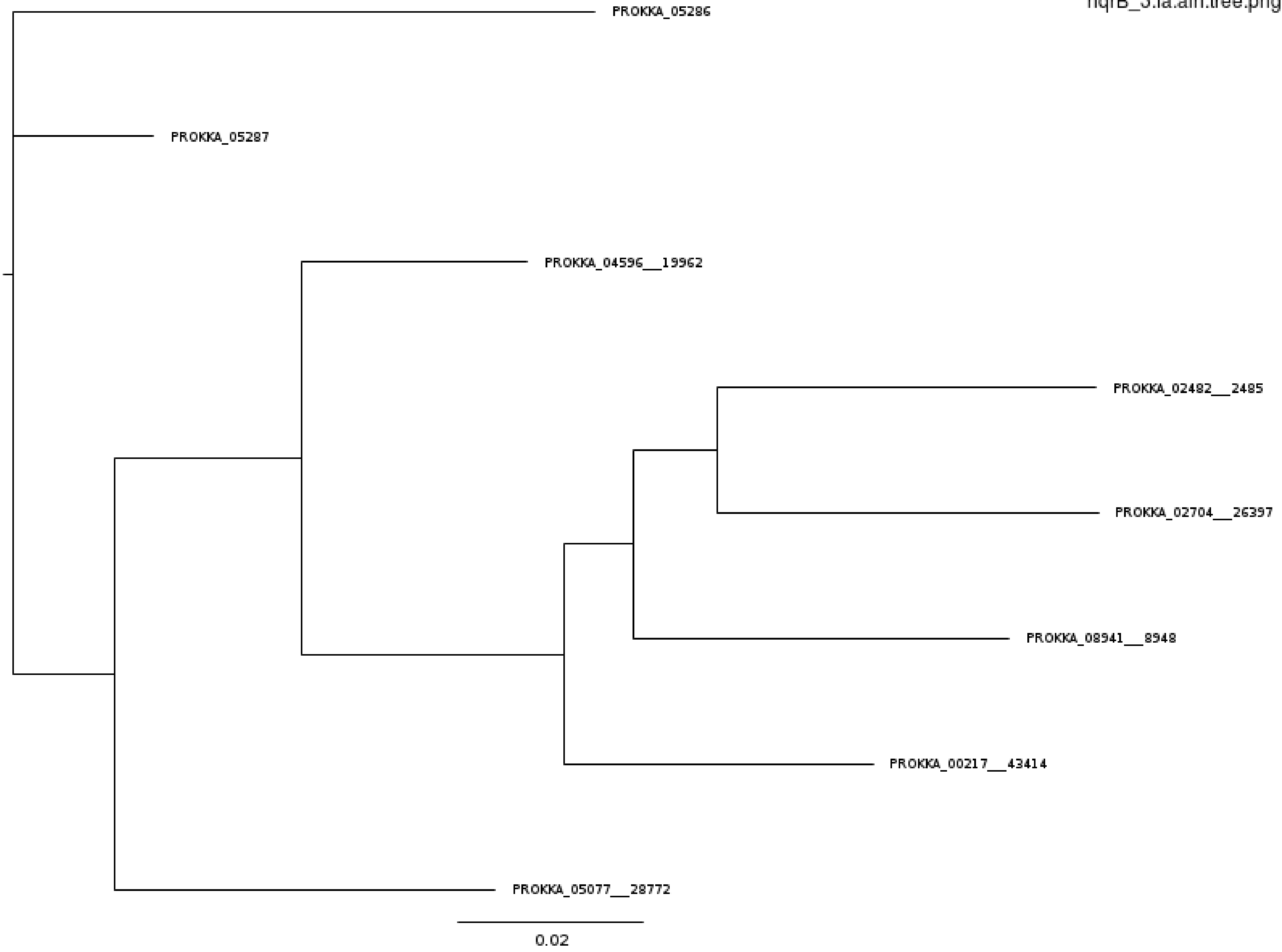

nqrC_2.fa.aln.tree.png

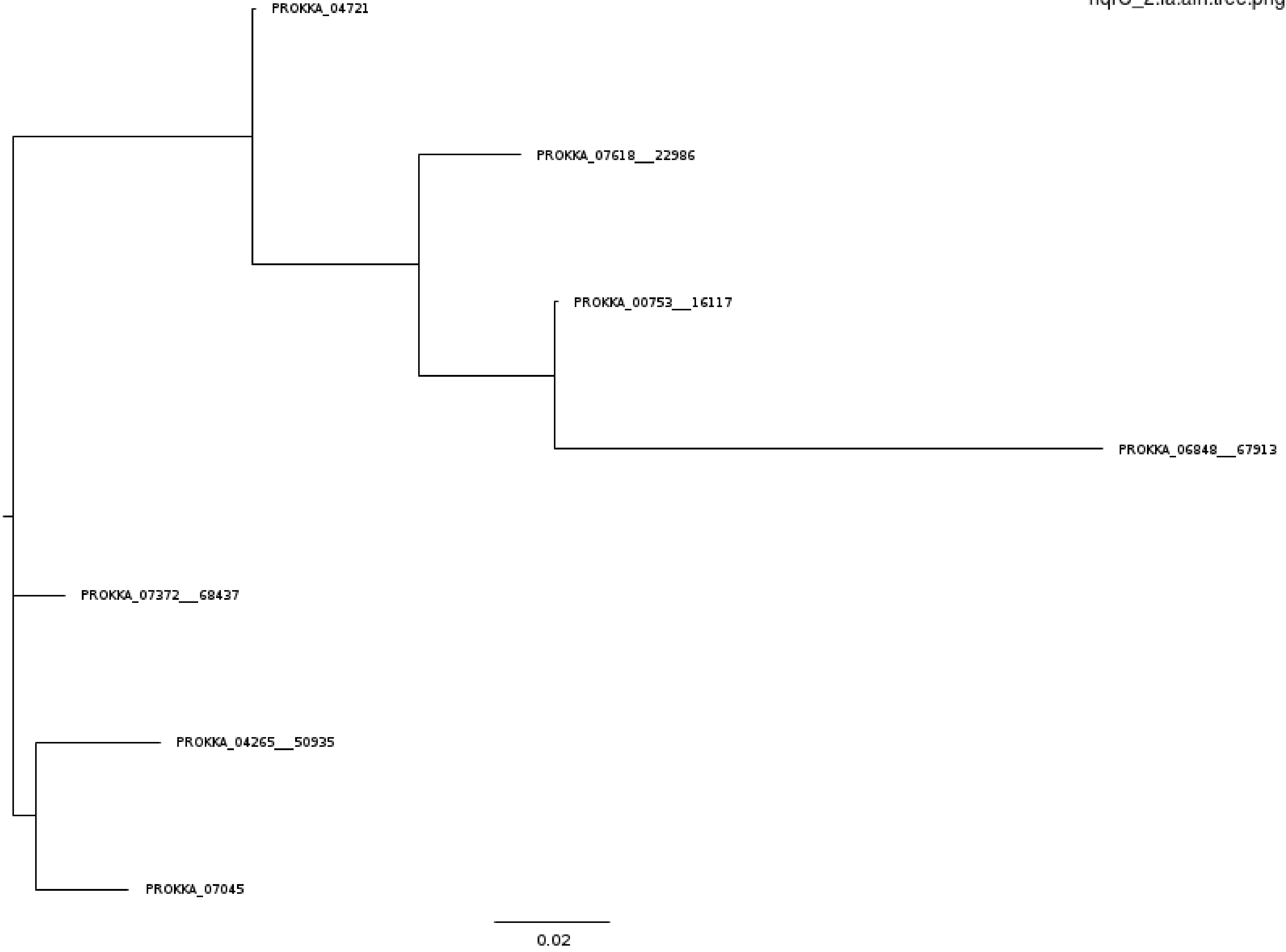

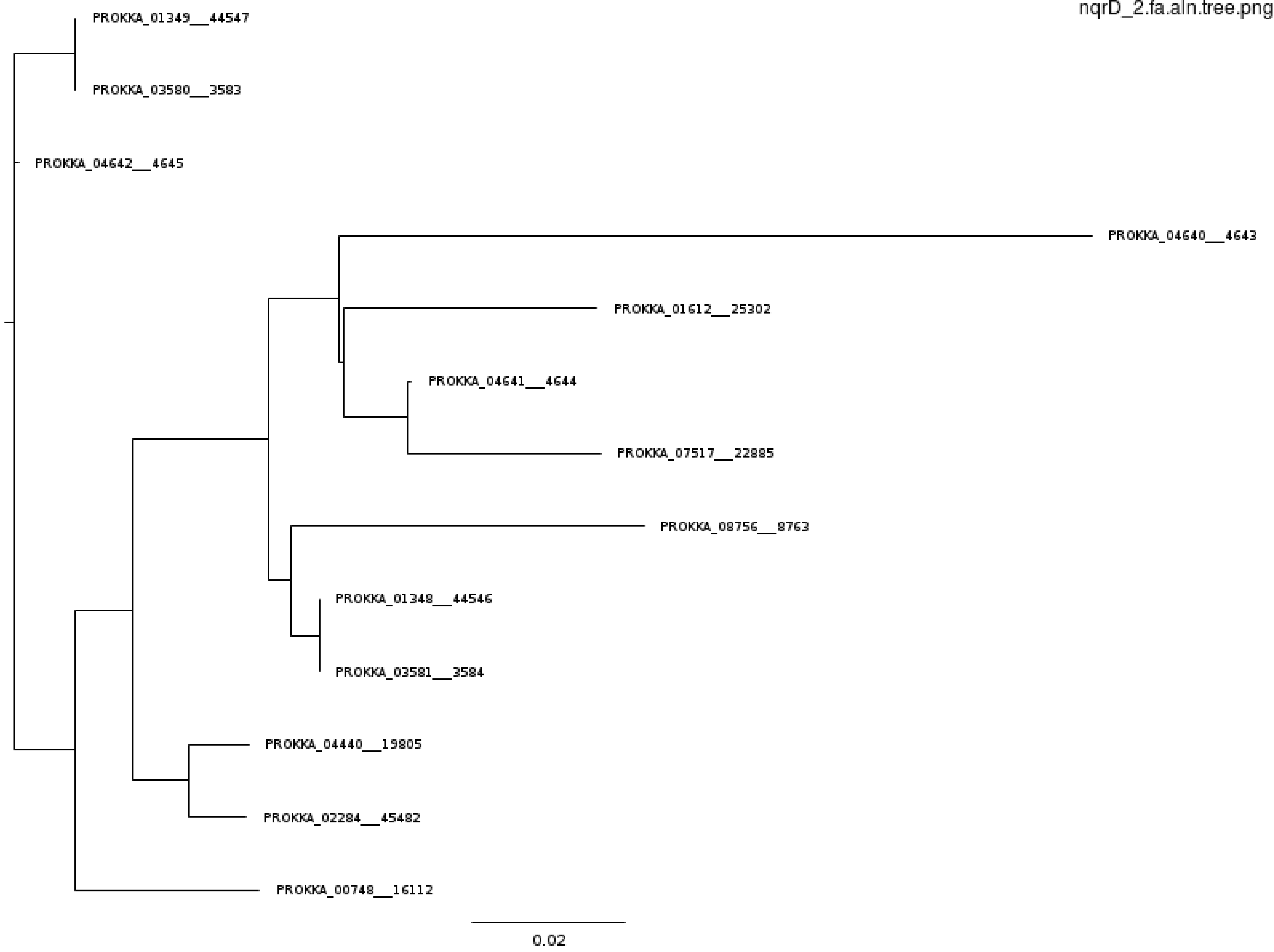
nqrD_2.fa.aln.tree.png
PROKKA_01349___44547
PROKKA_03580___3583
PROKKA_04642___4645
PROKKA_04640___4643
PROKKA_01612___25302
PROKKA_04641___4644
PROKKA_07517___22885
PROKKA_08756___8763
PROKKA_01348___44546
PROKKA_03581___3584
PROKKA_04440___19805
PROKKA_02284___45482
PROKKA_00748___16112
0.02

nqrE_1.fa.aln.tree.png

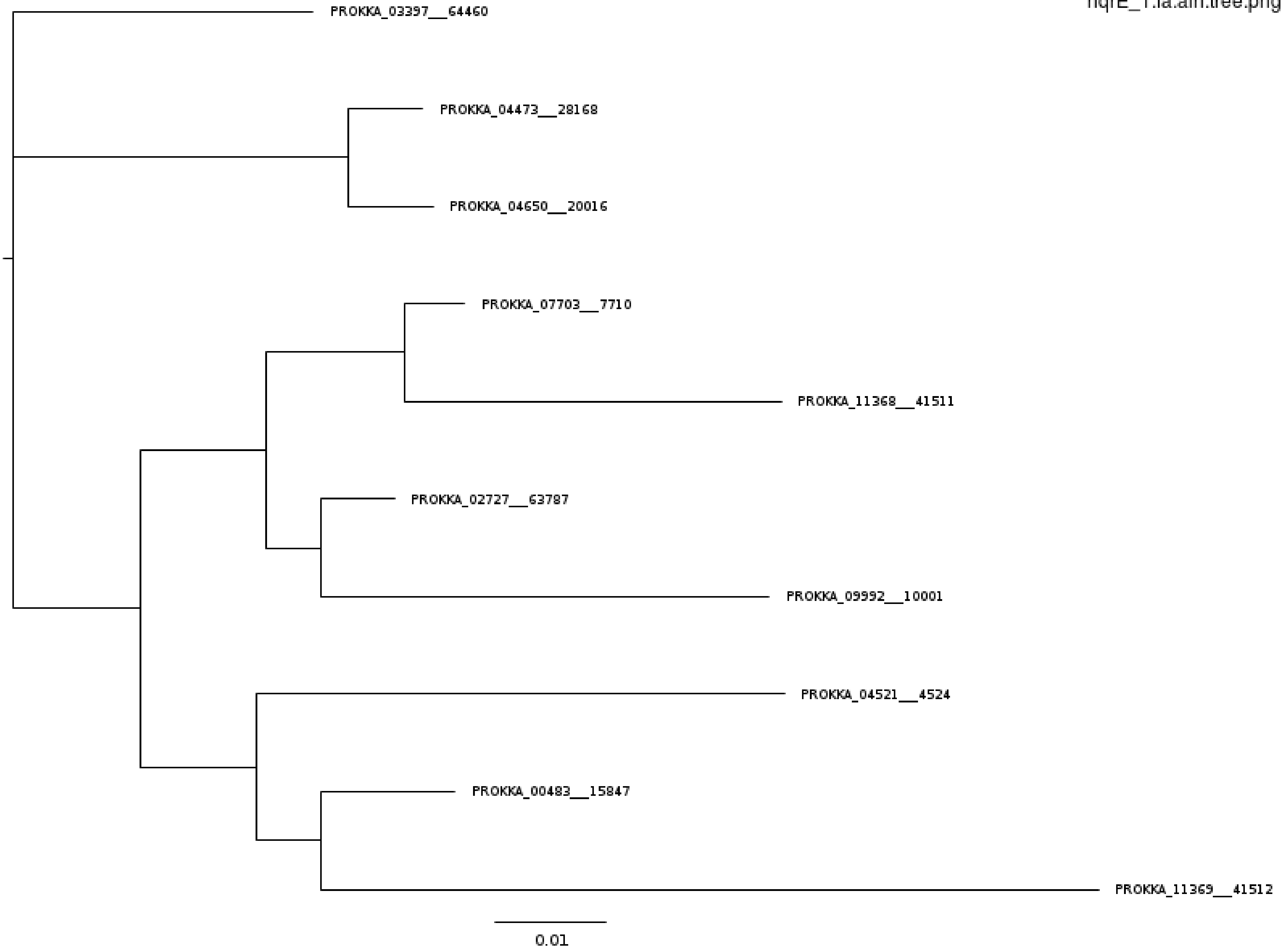

nqrF.fa.aln.tree.png

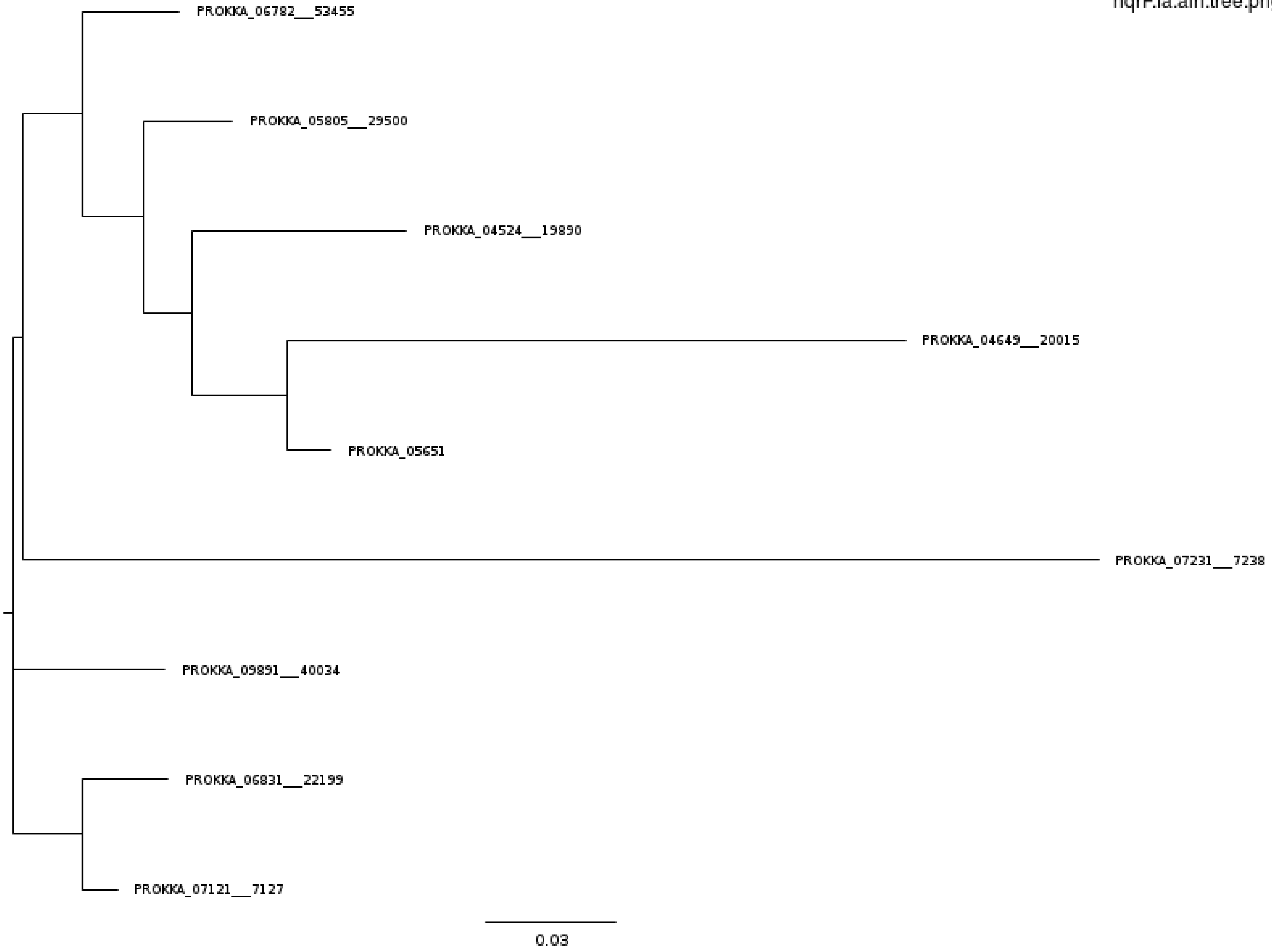

nspC_1.fa.aln.tree.png

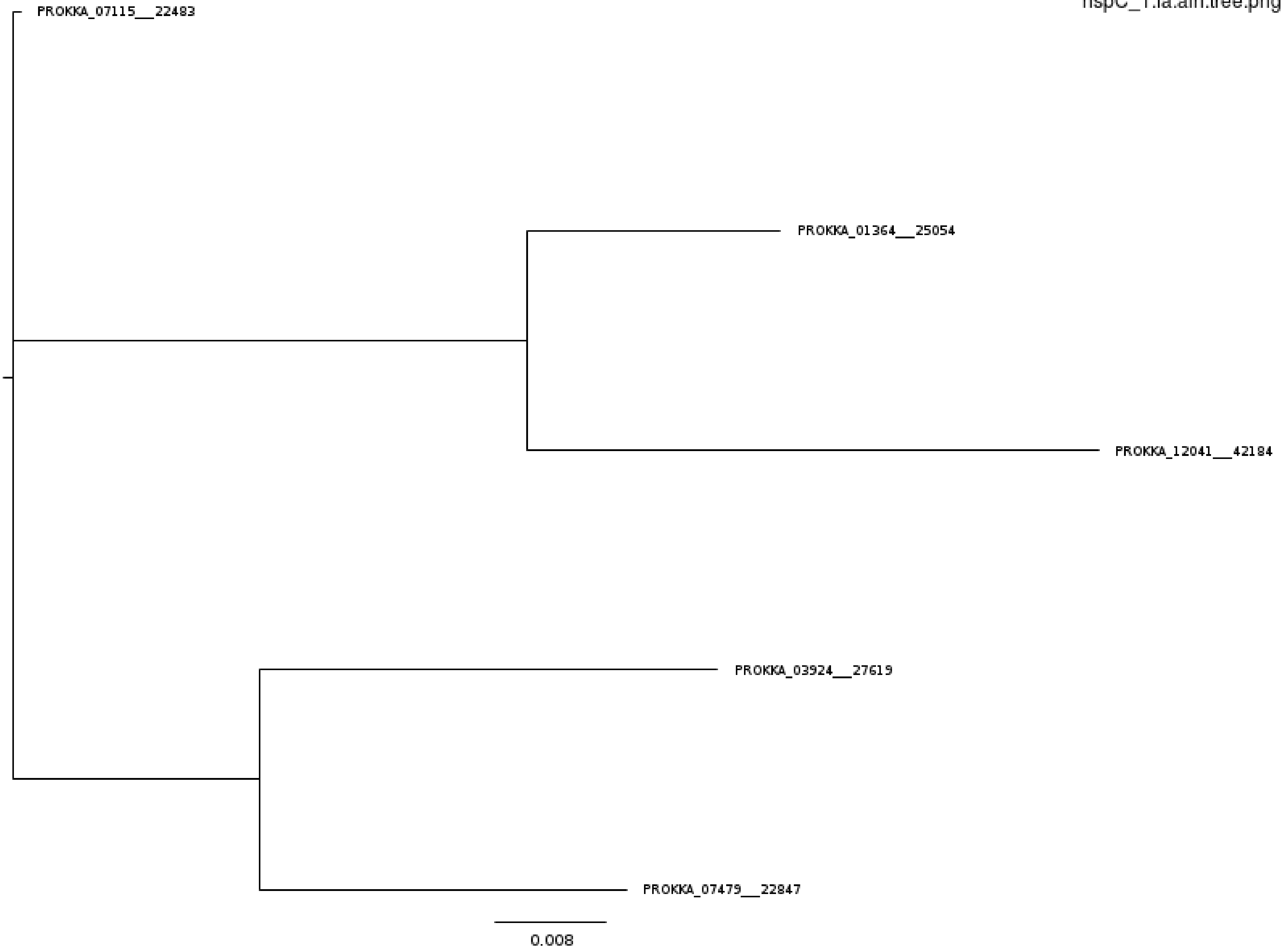

nspC_2.fa.aln.tree.png

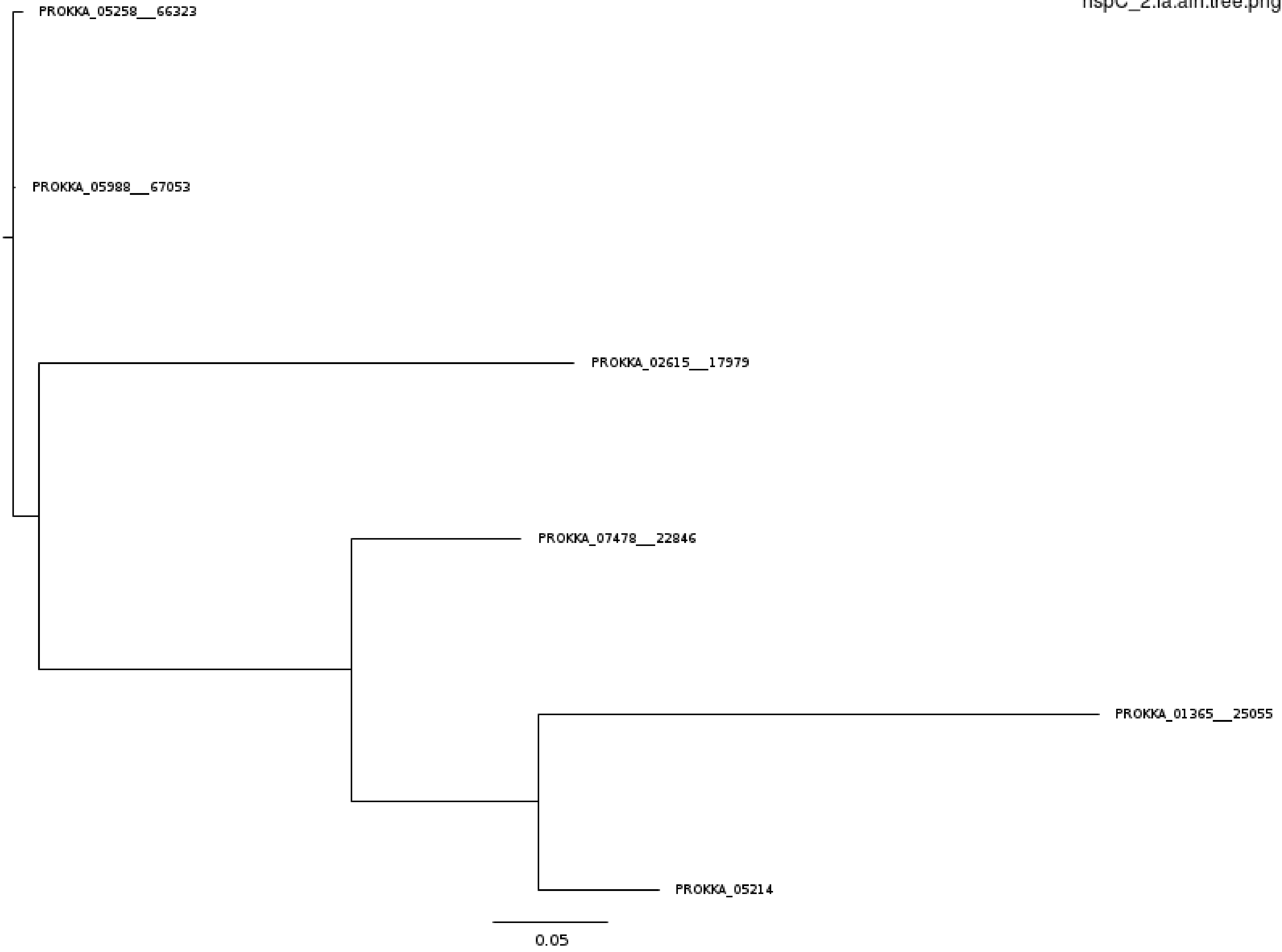

nth_2.fa.aln.tree.png

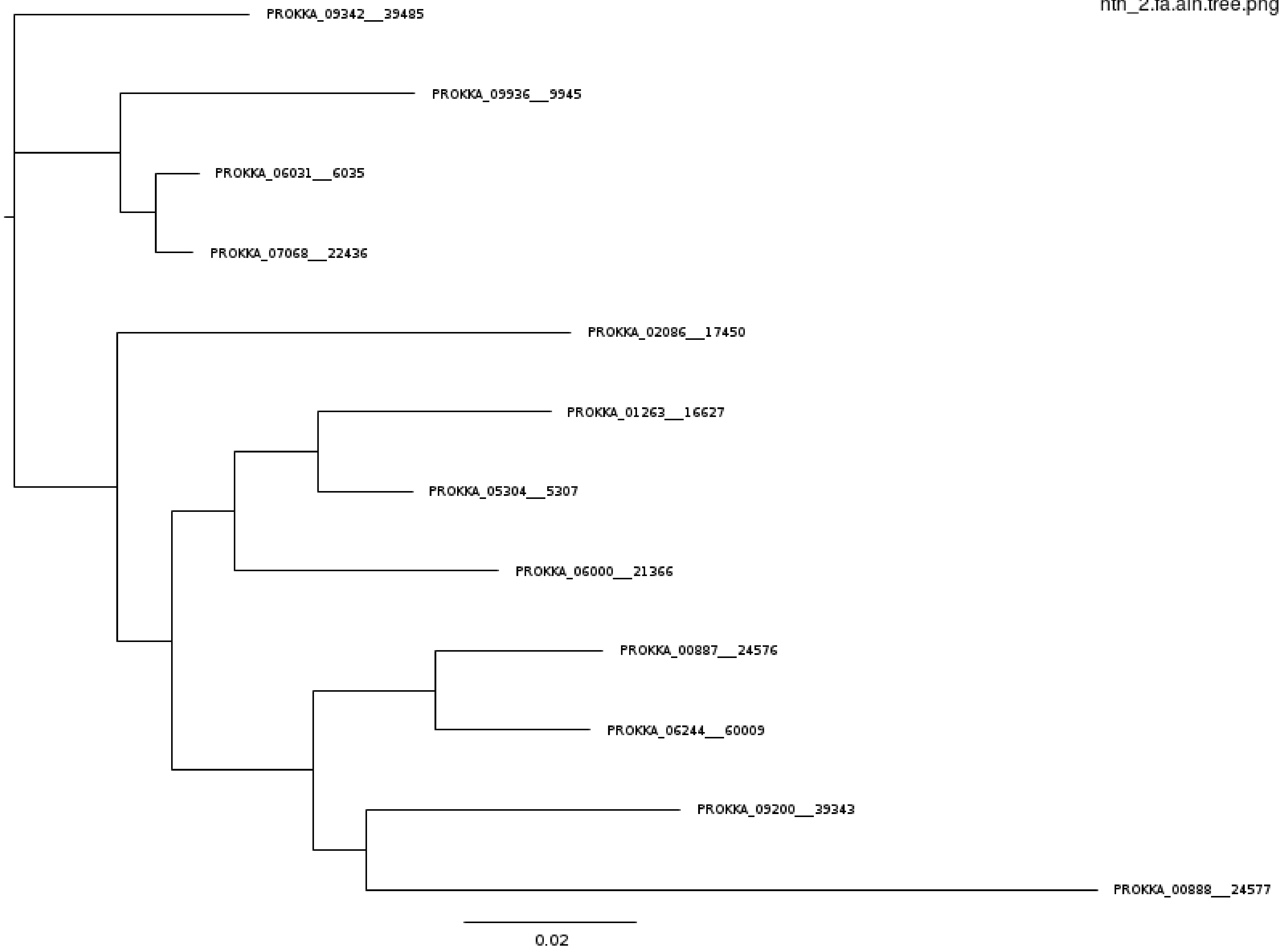

ntpB.fa.aln.tree.png

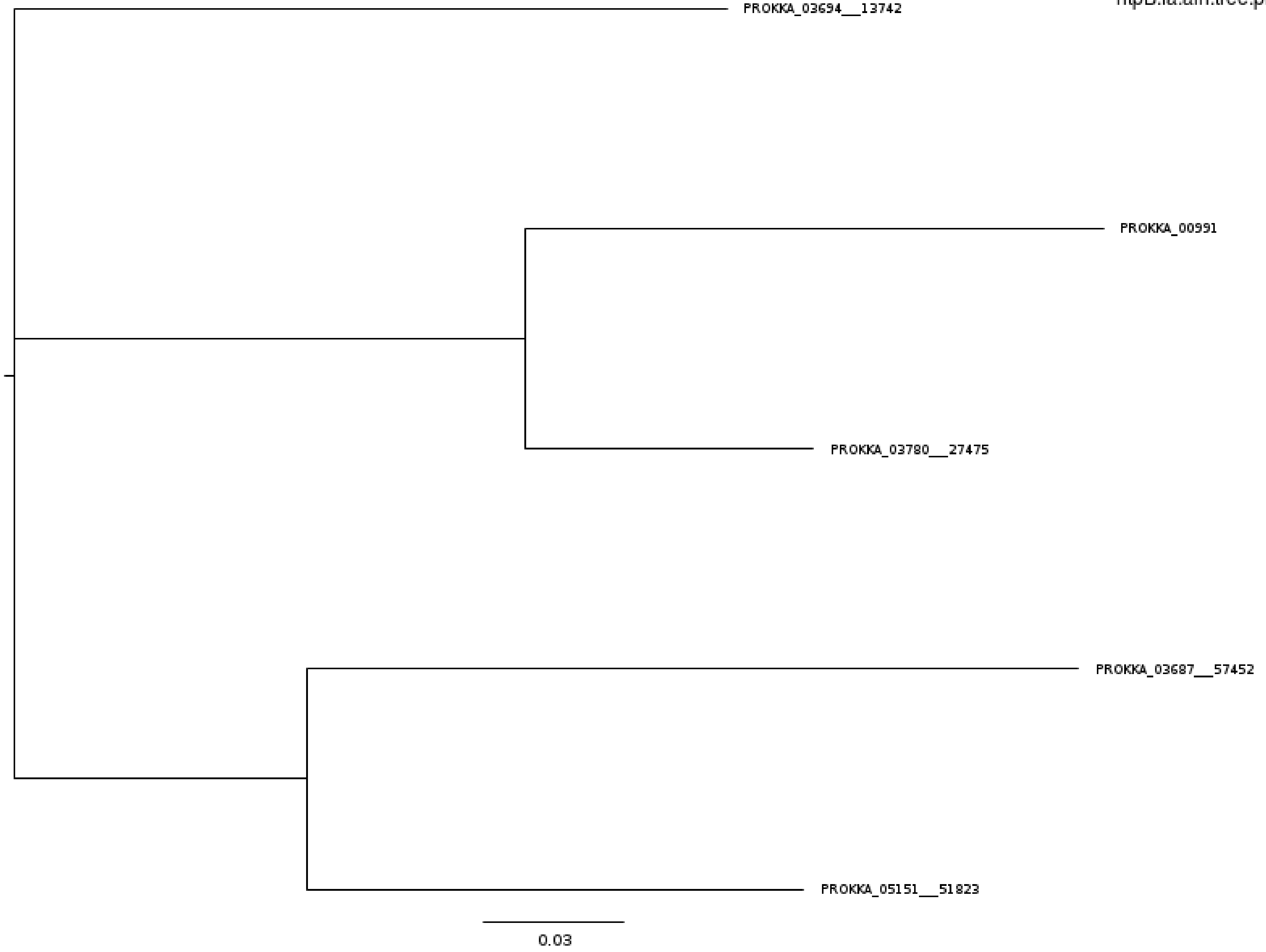

ntpD.fa.aln.tree.png

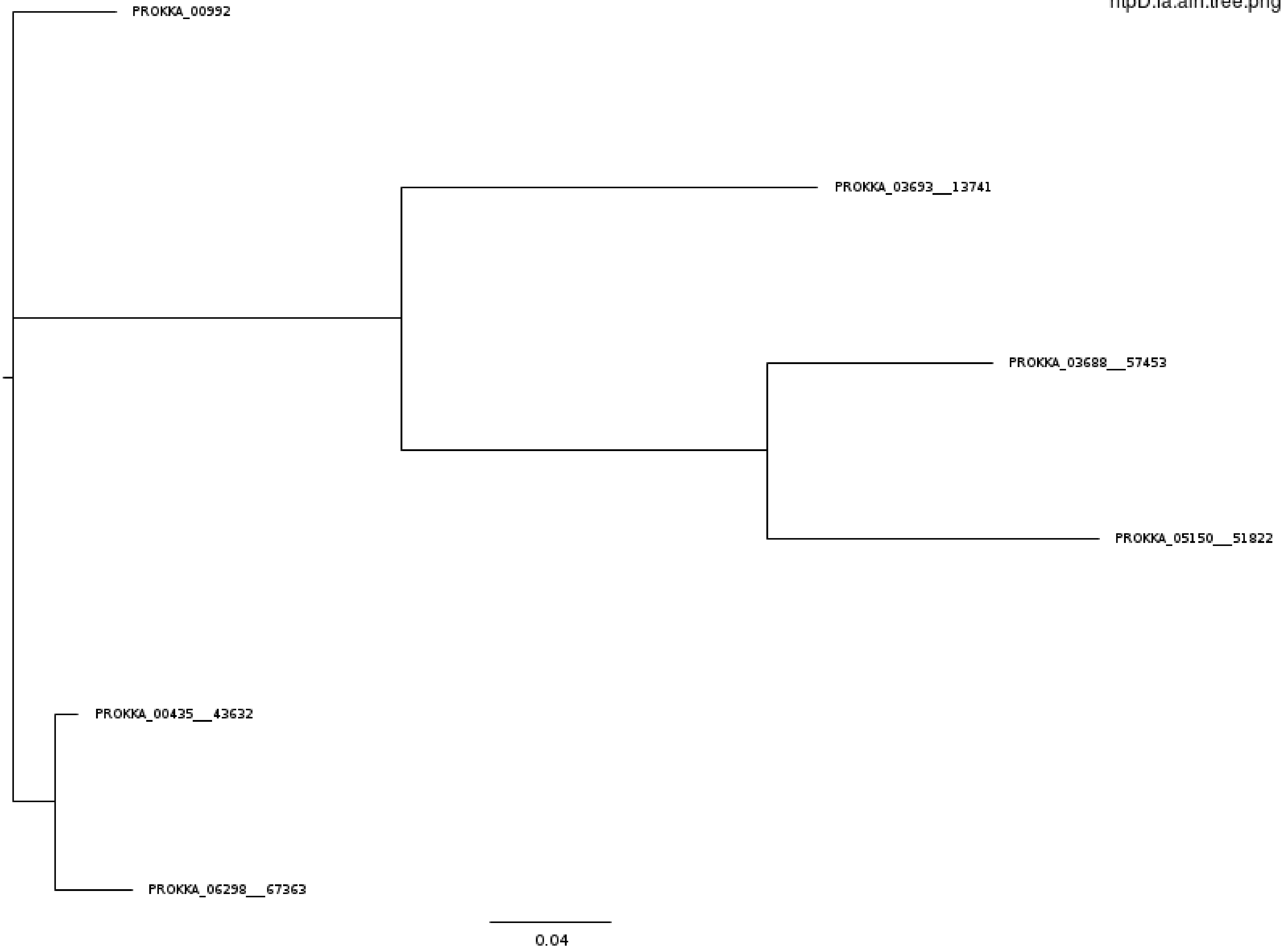

nuoB_2.fa.aln.tree.png

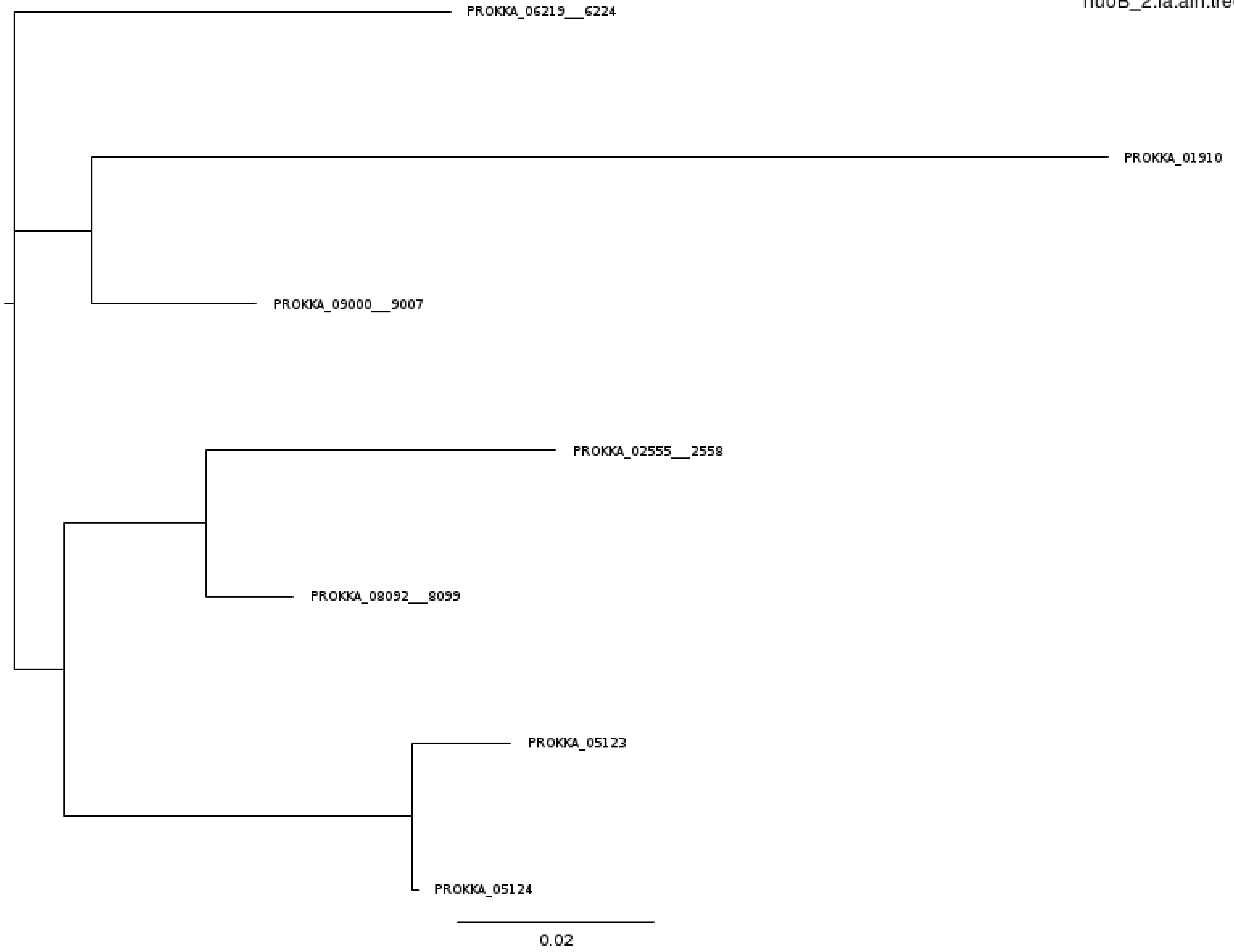

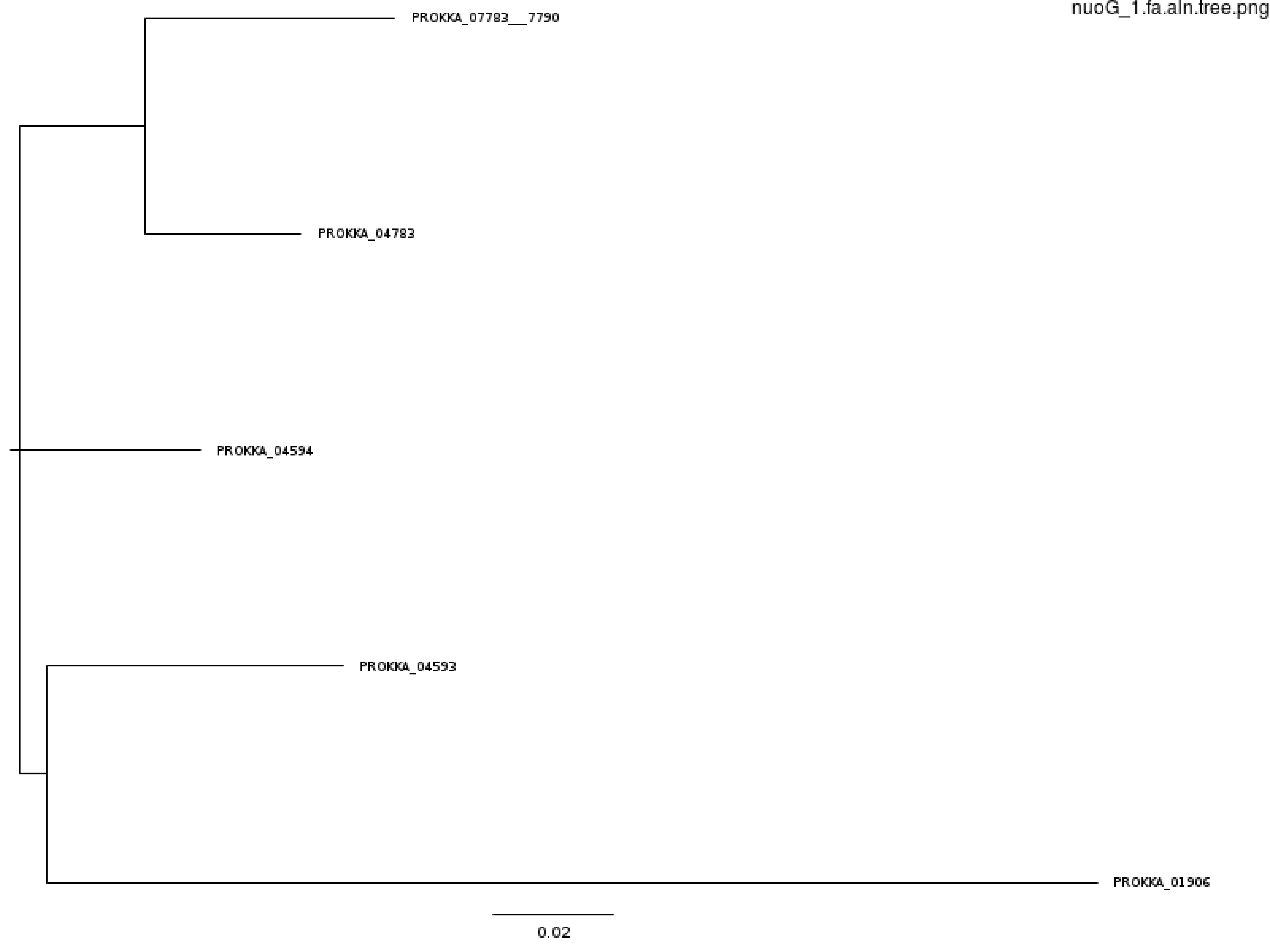

nuoG_1.fa.aln.tree.png
PROKKA_07783__7790
PROKKA_04783
PROKKA_04594
PROKKA_04593
PROKKA_01906
0.02

nuoH_1.fa.aln.tree.png

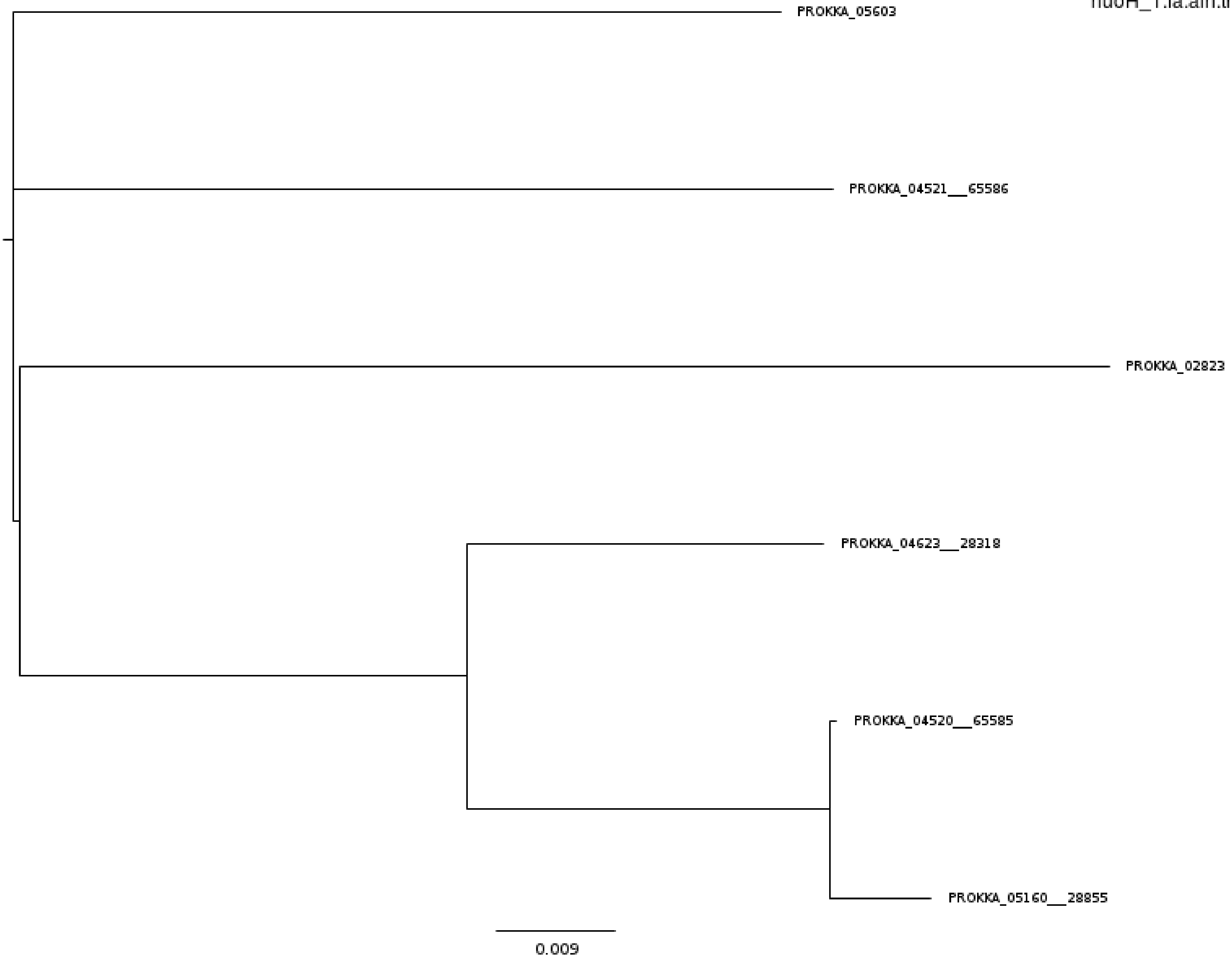

nuoJ_1.fa.aln.tree.png

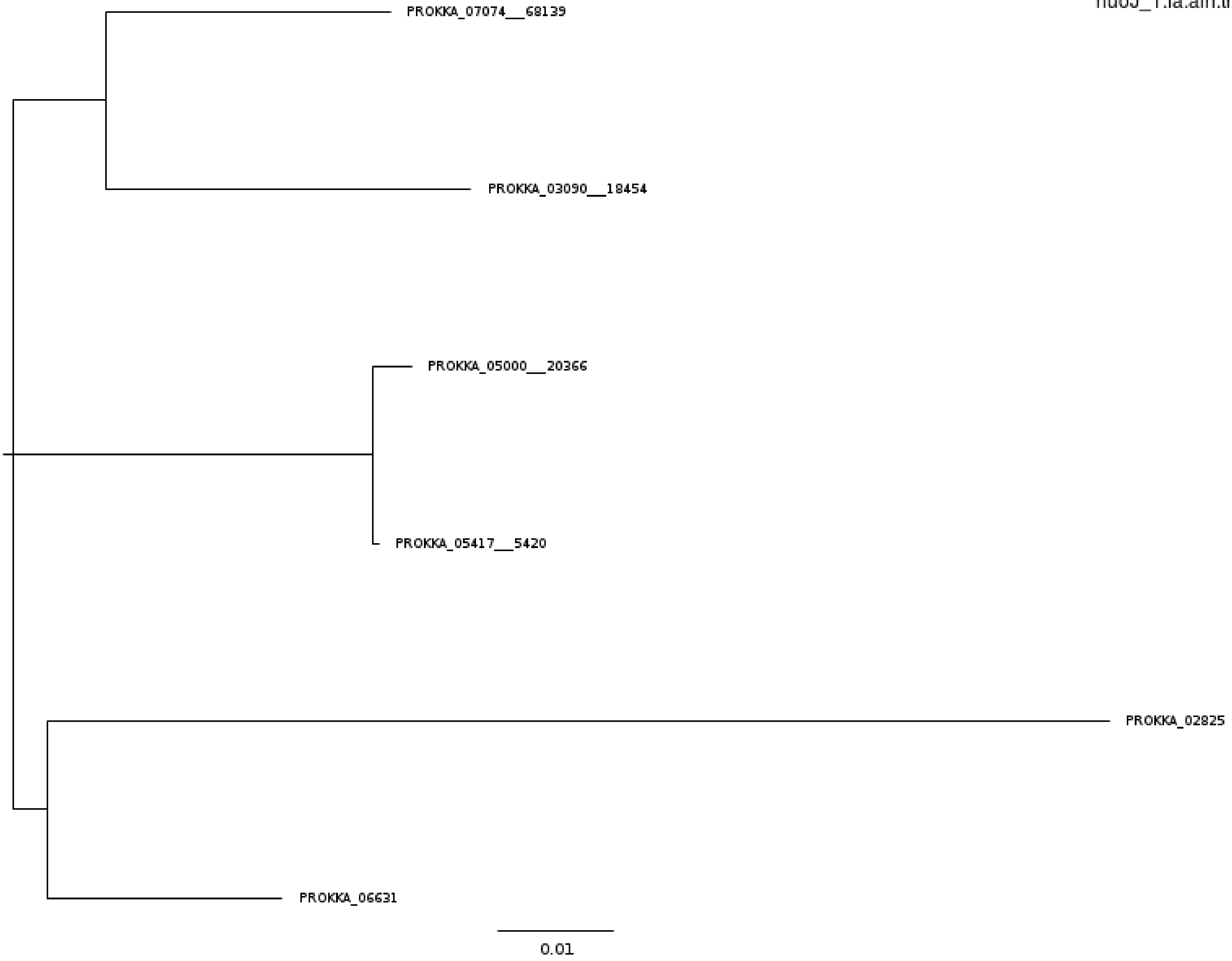

nuoM.fa.aln.tree.png

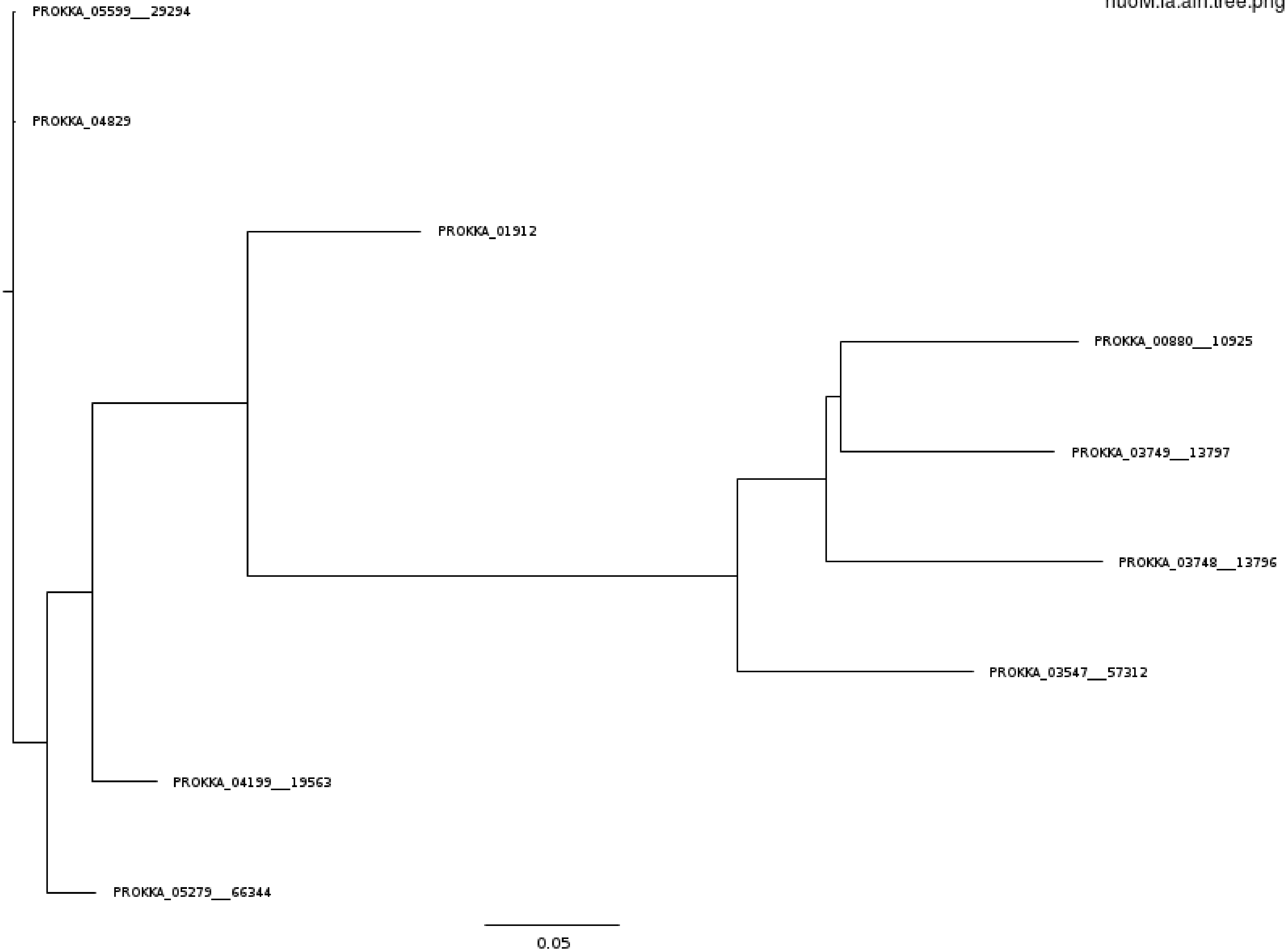

nuoN_1.fa.aln.tree.png

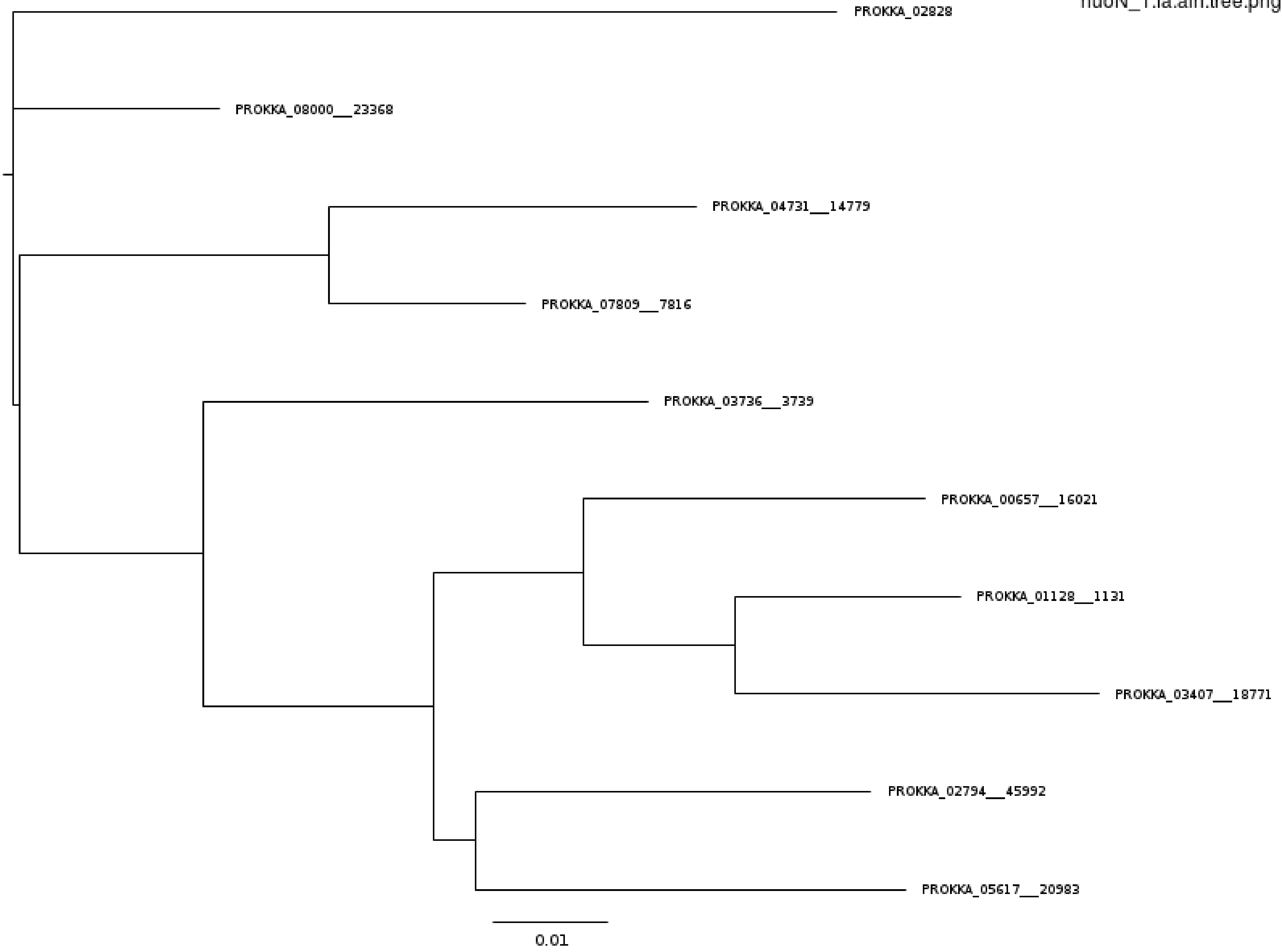

nupX.fa.aln.tree.png

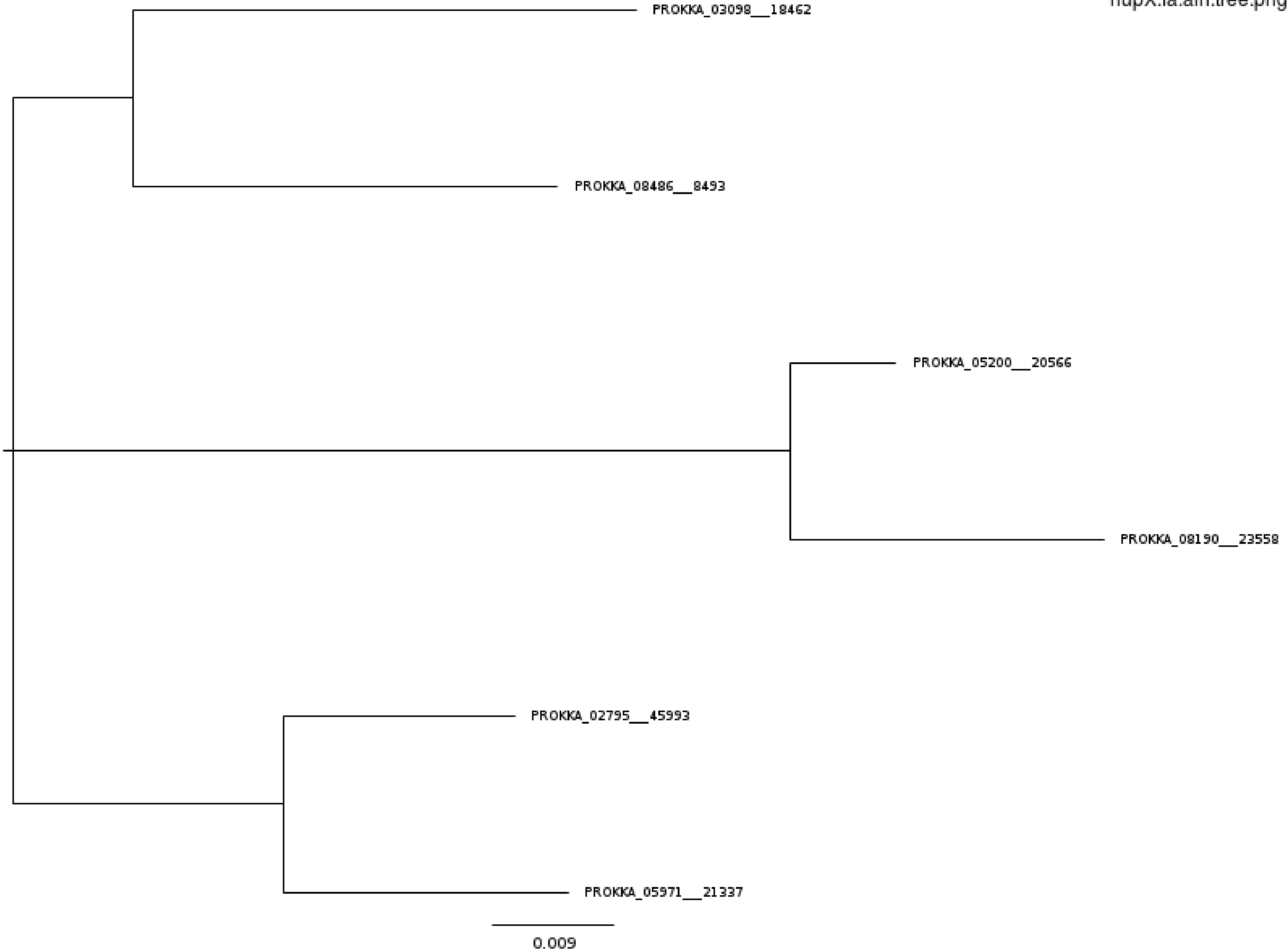

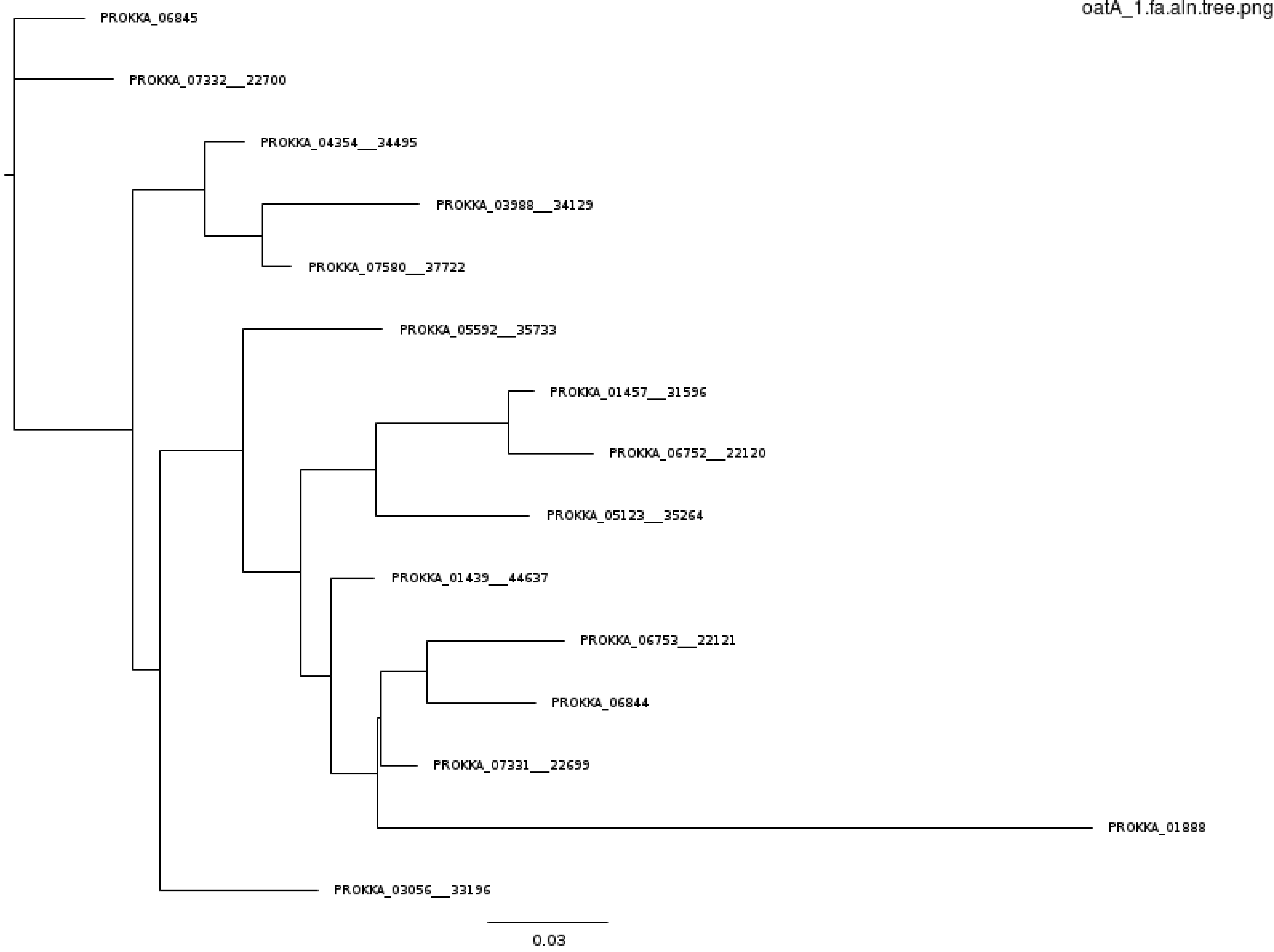

oatA_1.fa.aln.tree.png
PROKKA_06845
PROKKA_07332___22700
PROKKA_04354___34495
PROKKA_03988___34129
PROKKA_07580___37722
PROKKA_05592___35733
PROKKA_01457___31596
PROKKA_06752___22120
PROKKA_05123___35264
PROKKA_01439___44637
PROKKA_06753___22121
PROKKA_06844
PROKKA_07331___22699
PROKKA_01888
PROKKA_03056___33196
0.03

obg_1.fa.aln.tree.png

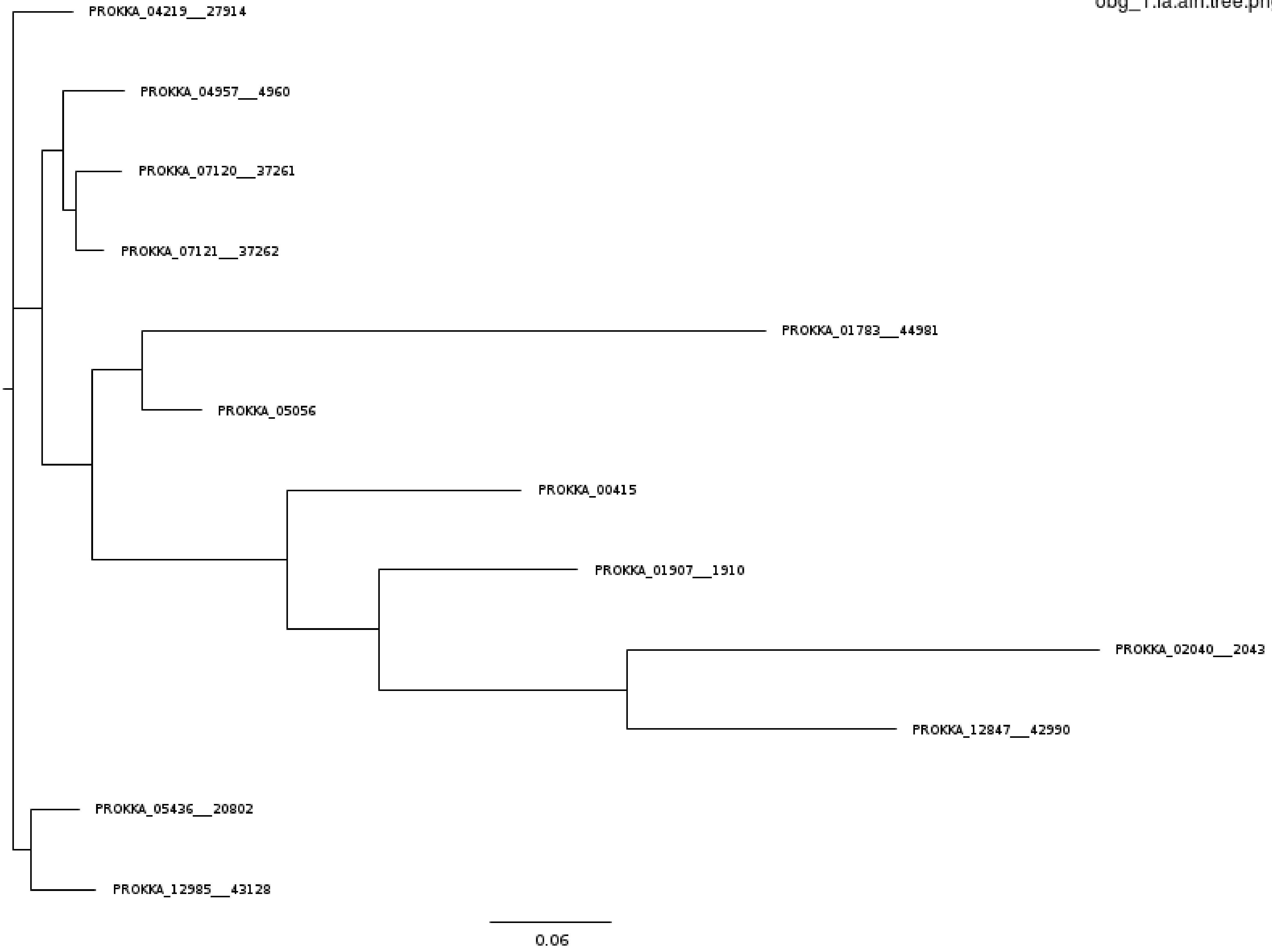

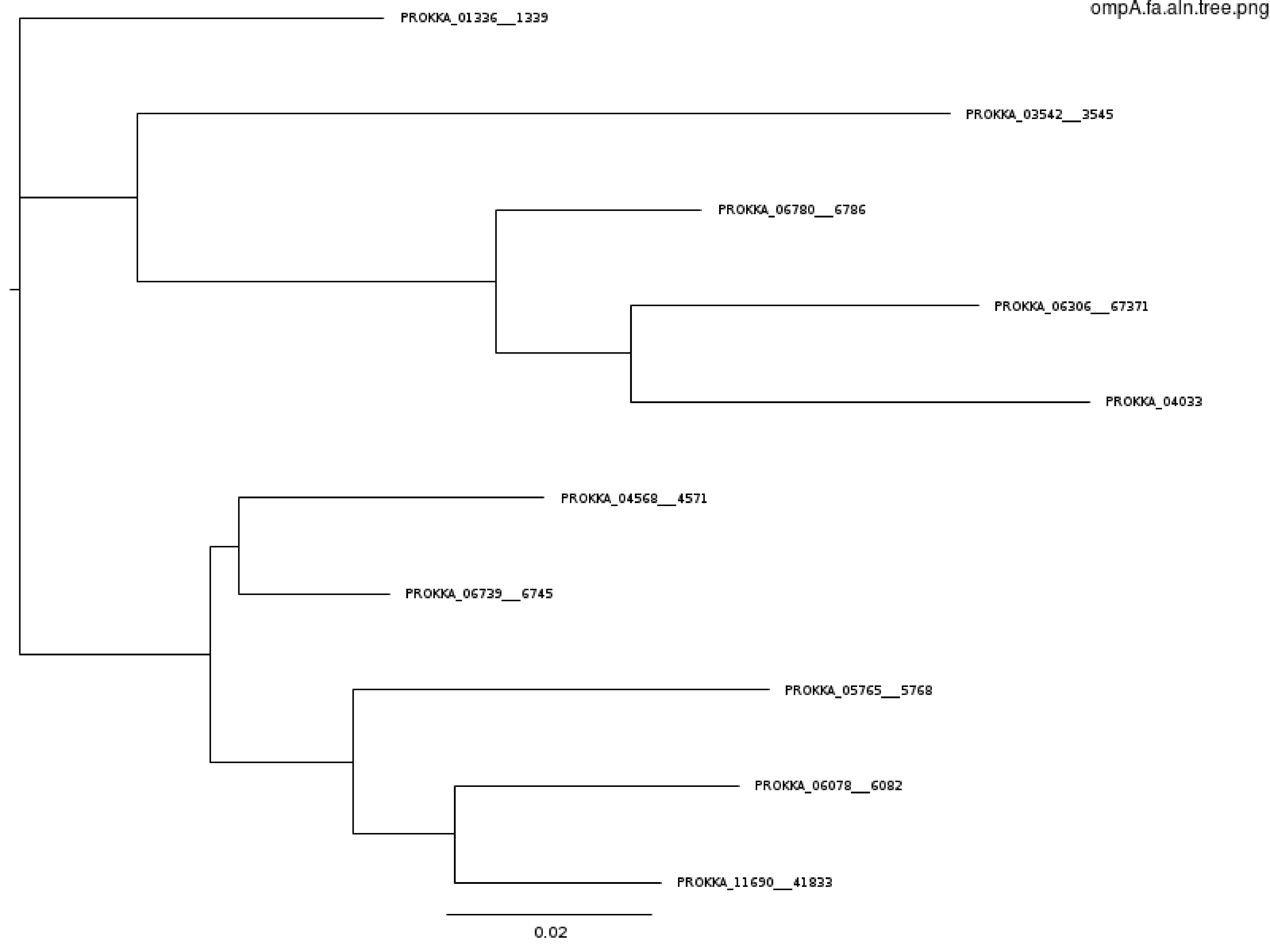
ompA.fa.aln.tree.png
PROKKA_01336__1339
PROKKA_03542__3545
PROKKA_06780__6786
PROKKA_06306__67371
PROKKA_04033
PROKKA_04568__4571
PROKKA_06739__6745
PROKKA_05765__5768
PROKKA_06078__6082
PROKKA_11690__41833
0.02

oprF_6.fa.aln.tree.png

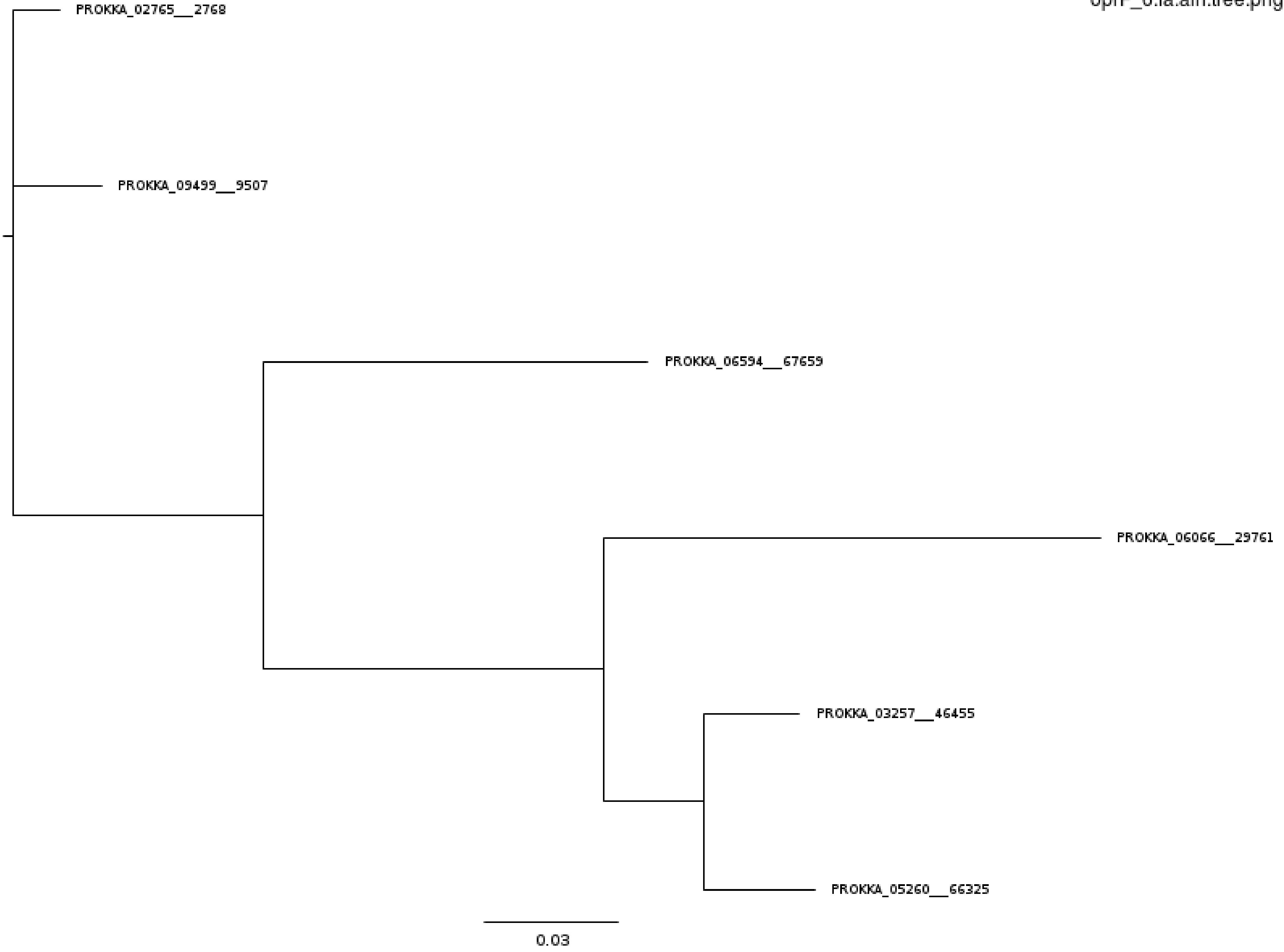

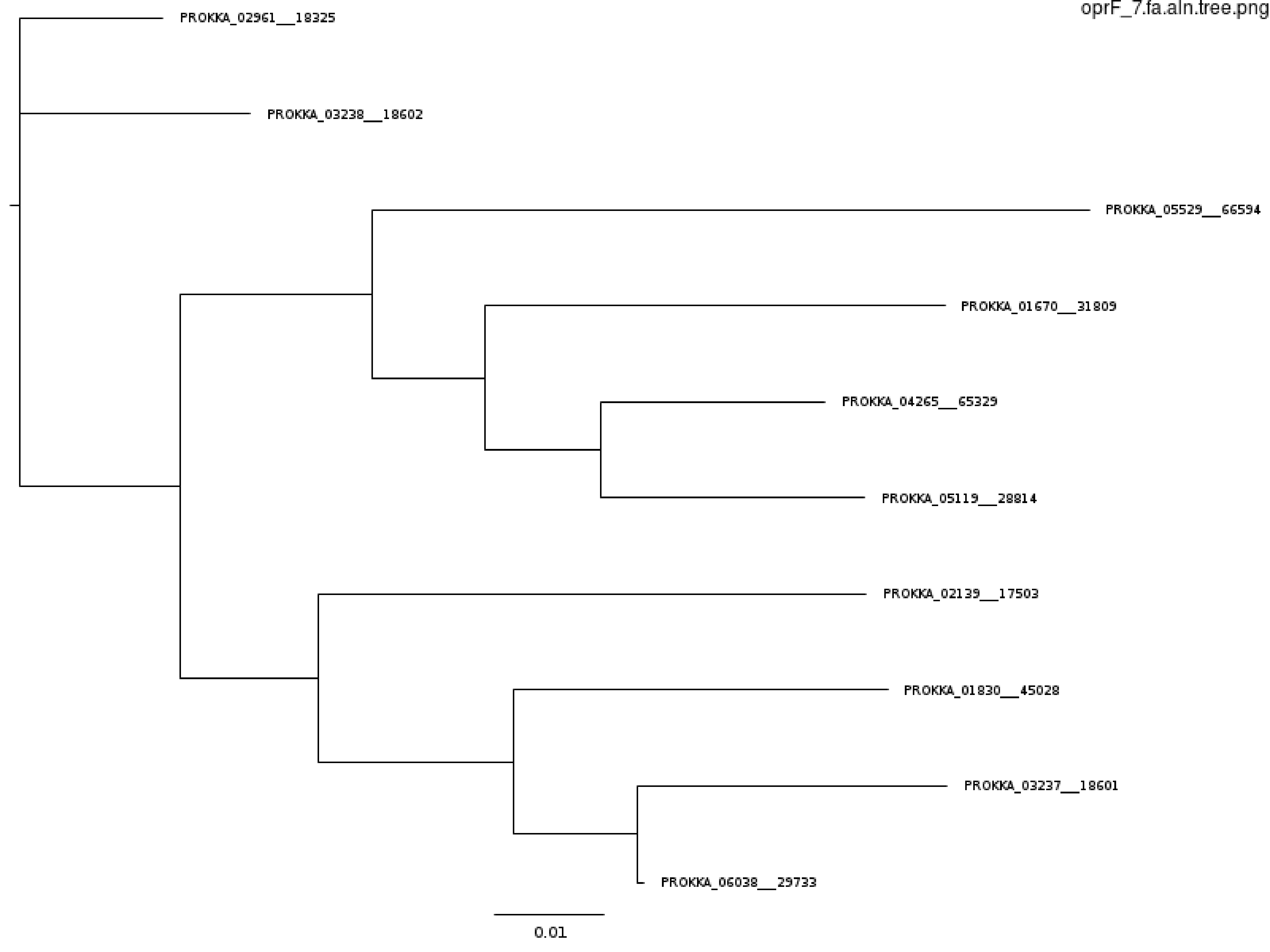

oprF_7.fa.aln.tree.png
PROKKA_02961___18325
PROKKA_03238___18602
PROKKA_05529___66594
PROKKA_01670___31809
PROKKA_04265___65329
PROKKA_05119___28814
PROKKA_02139___17503
PROKKA_01830___45028
PROKKA_03237___18601
PROKKA_06038___29733
0.01

outO_2.fa.aln.tree.png

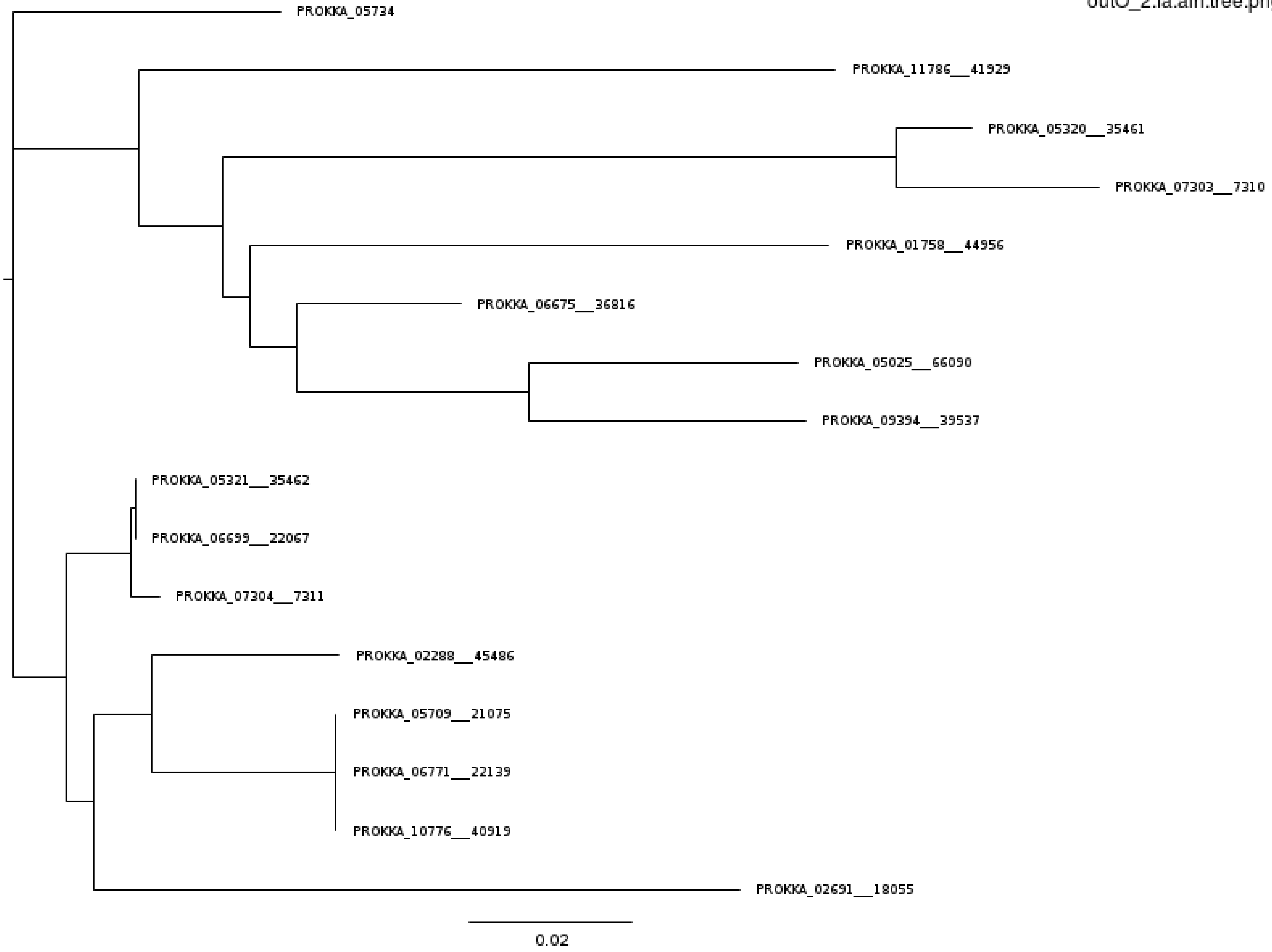

pabB_2.fa.aln.tree.png

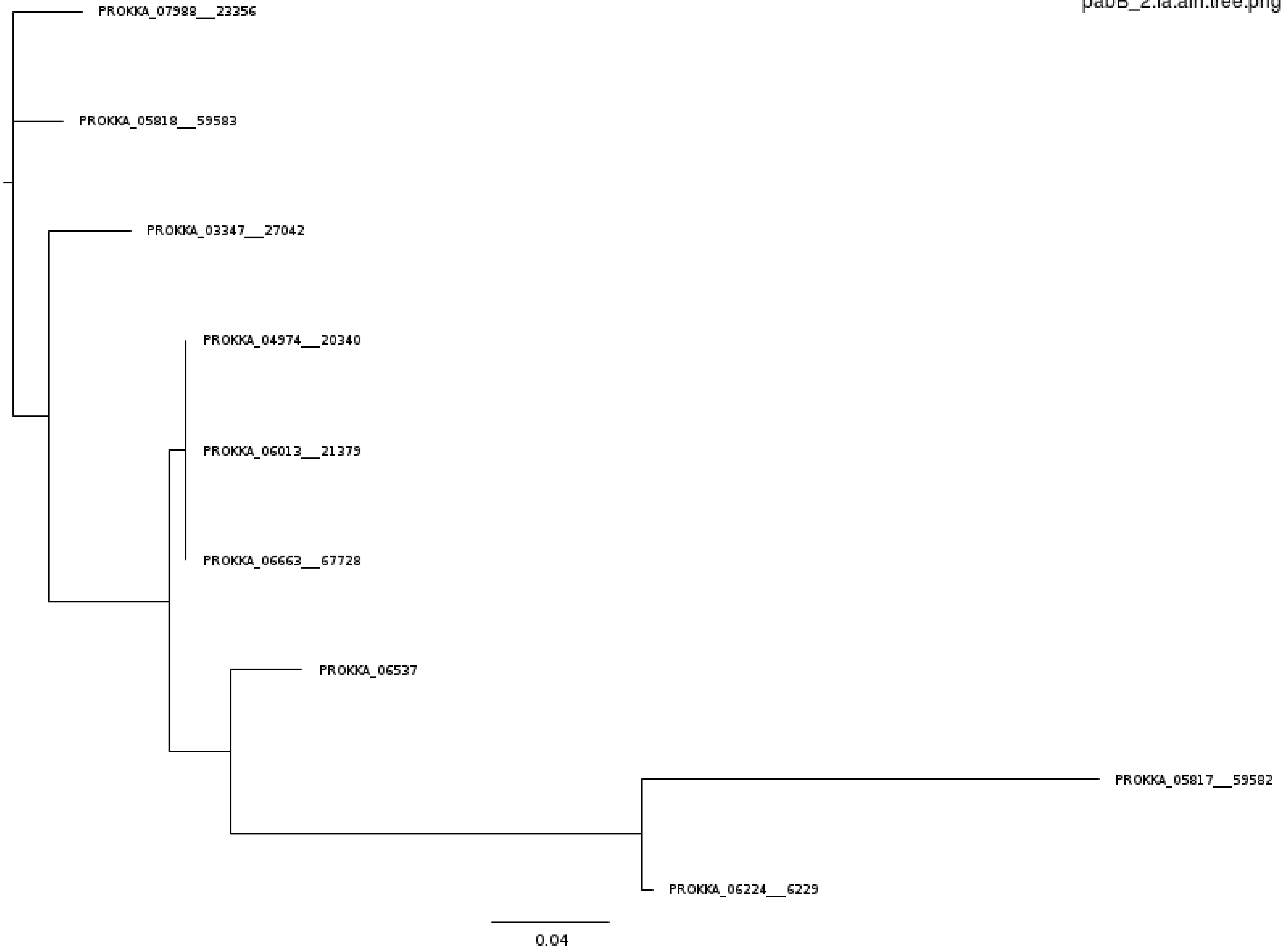

pabC_2.fa.aln.tree.png

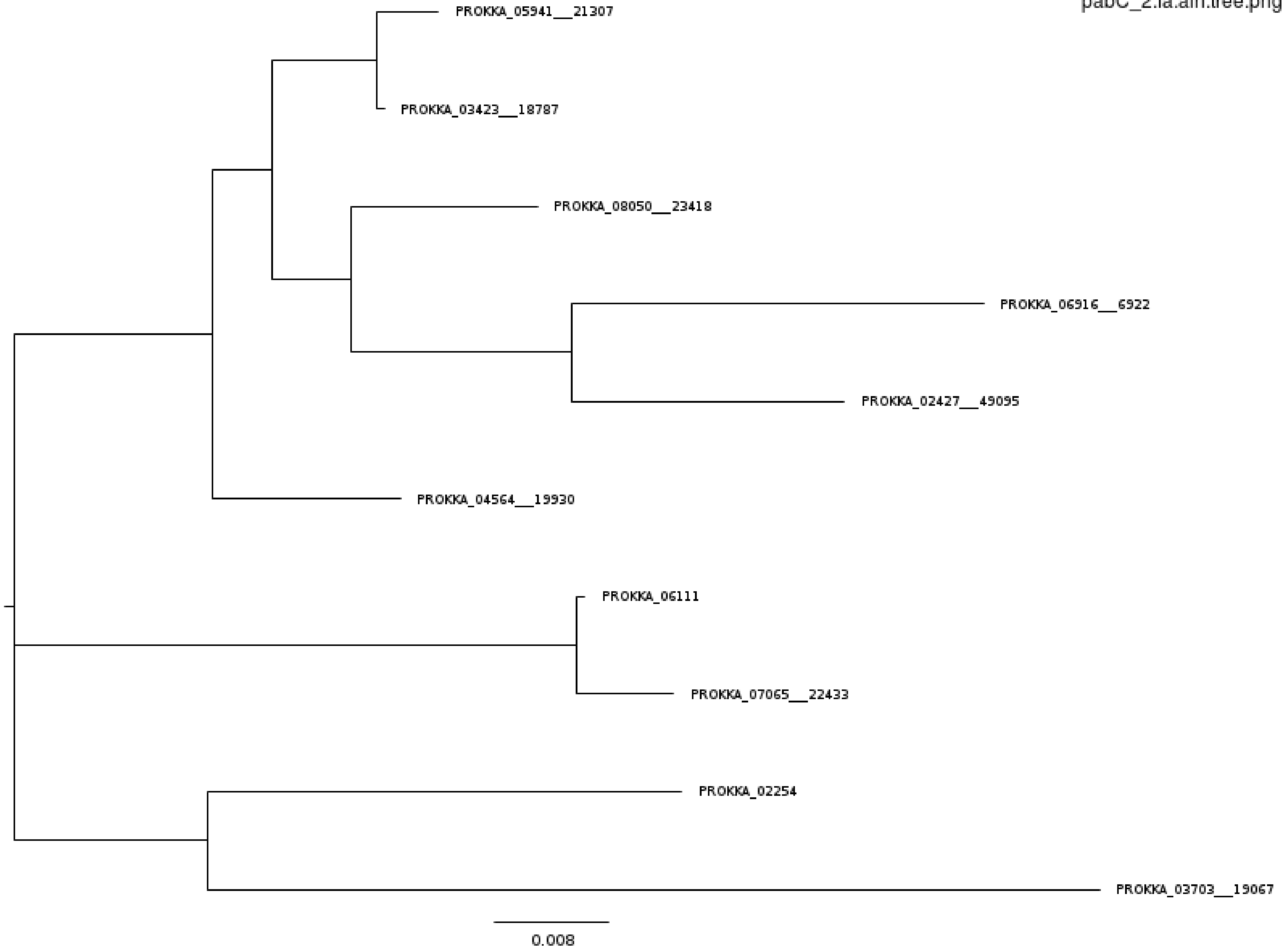

padE.fa.aln.tree.png

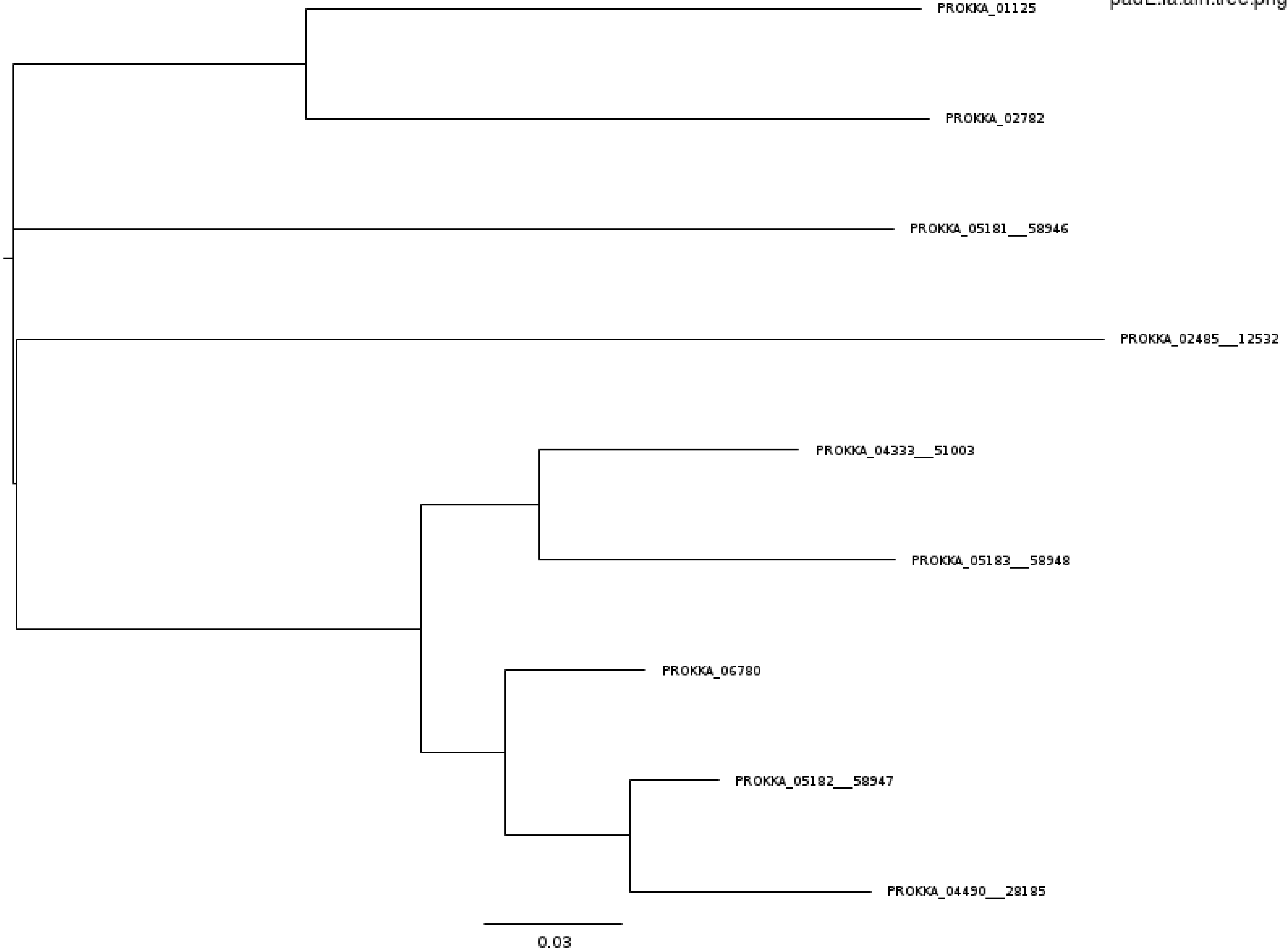

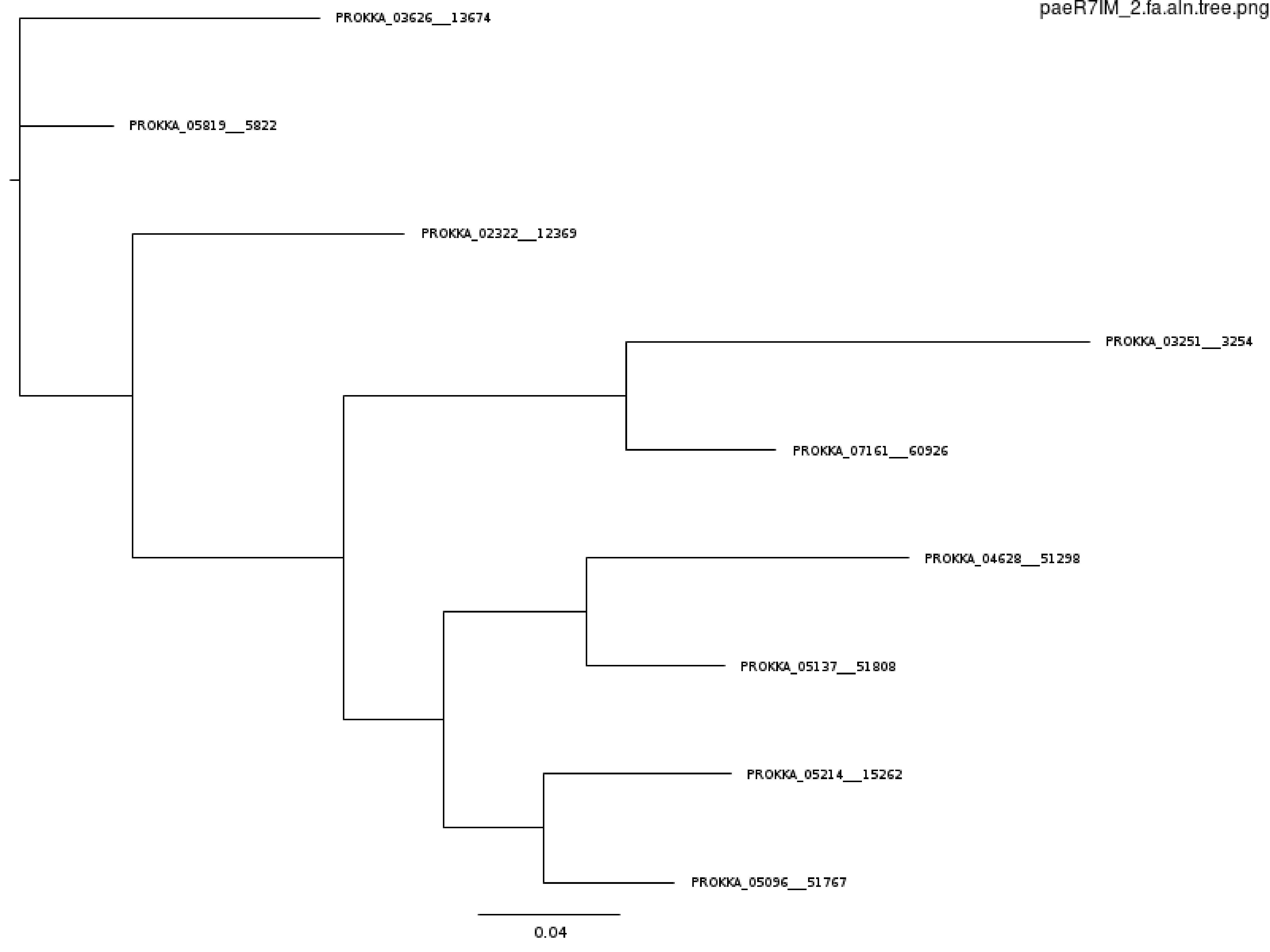

paeR7IM_2.fa.aln.tree.png
PROKKA_03626__13674
PROKKA_05819__5822
PROKKA_02322__12369
PROKKA_03251__3254
PROKKA_07161__60926
PROKKA_04628__51298
PROKKA_05137__51808
PROKKA_05214__15262
PROKKA_05096__51767
0.04

pal.fa.aln.tree.png

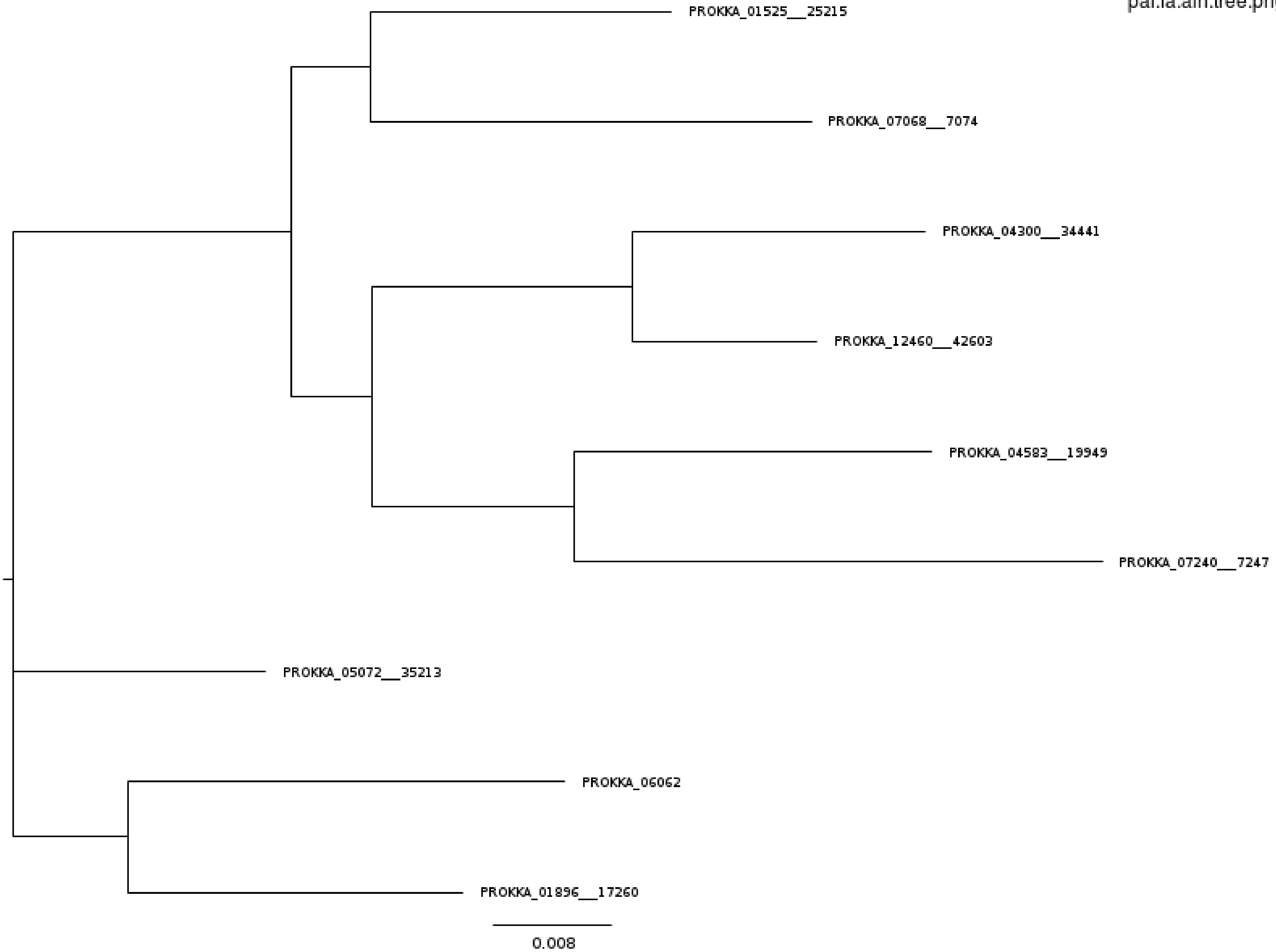

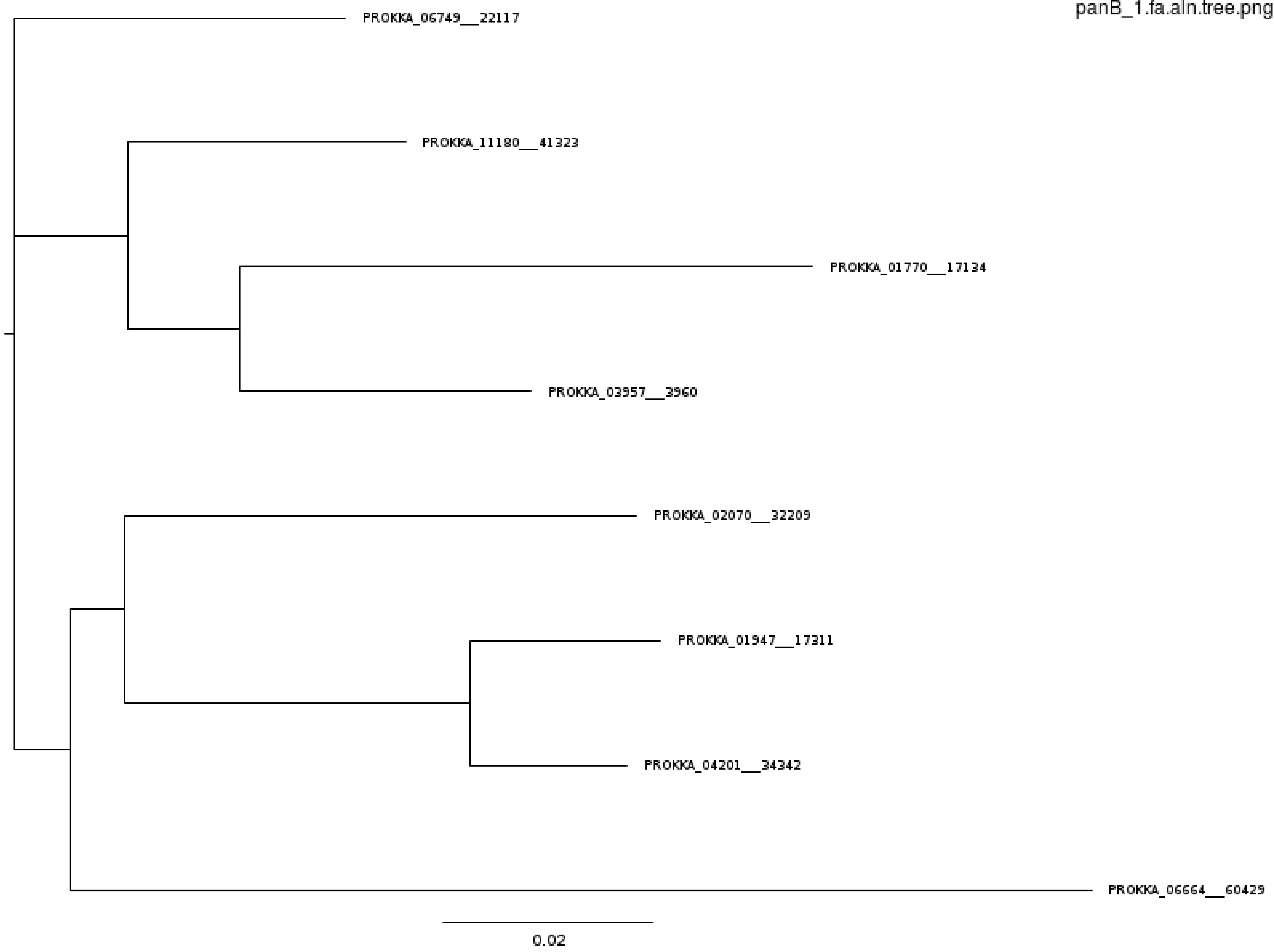

panB_1.fa.aln.tree.png
PROKKA_06749__22117
PROKKA_11180__41323
PROKKA_01770__17134
PROKKA_03957__3960
PROKKA_02070__32209
PROKKA_01947__17311
PROKKA_04201__34342
PROKKA_06664__60429
0.02

panB_2.fa.aln.tree.png

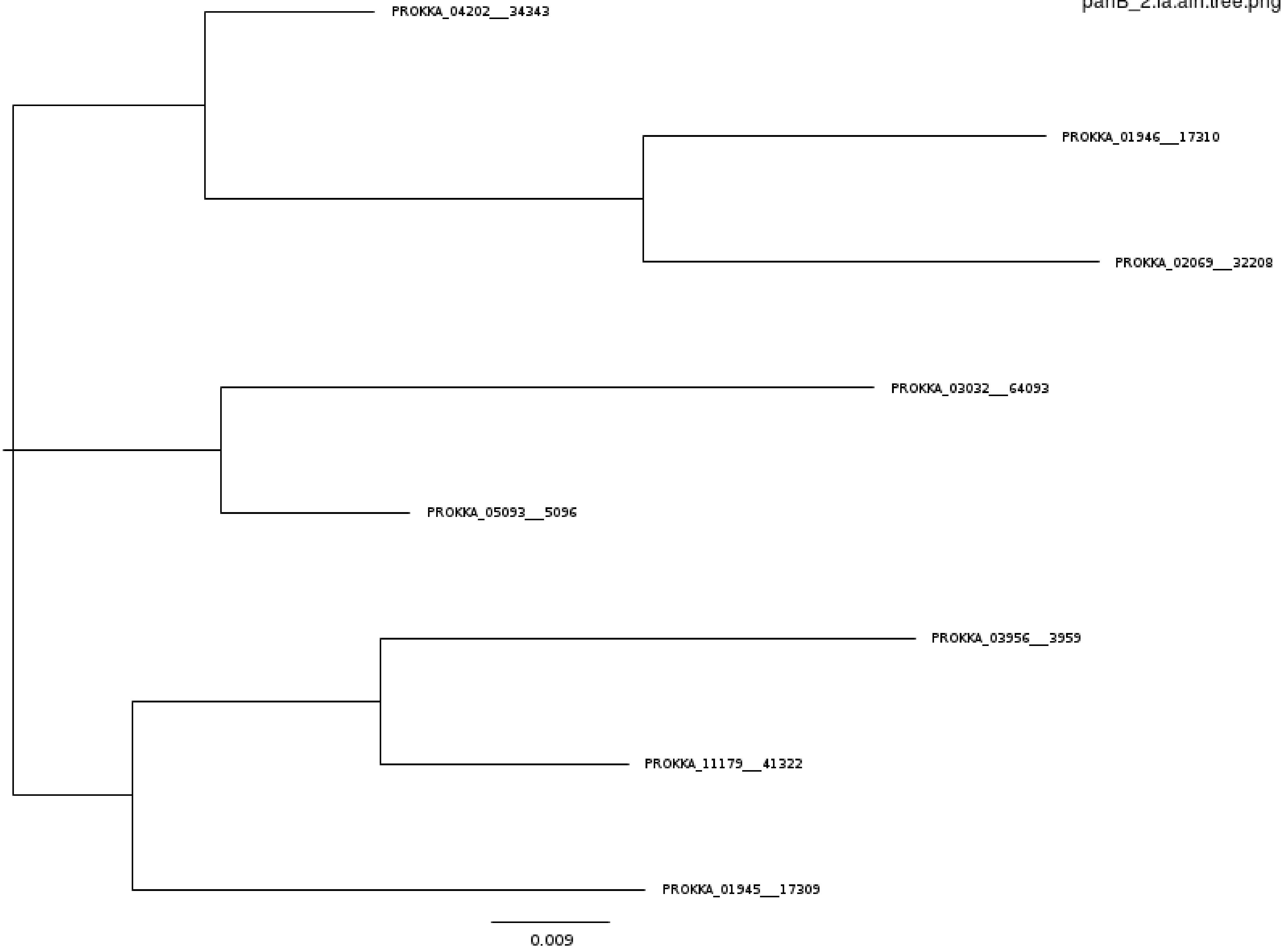

panC_1.fa.aln.tree.png

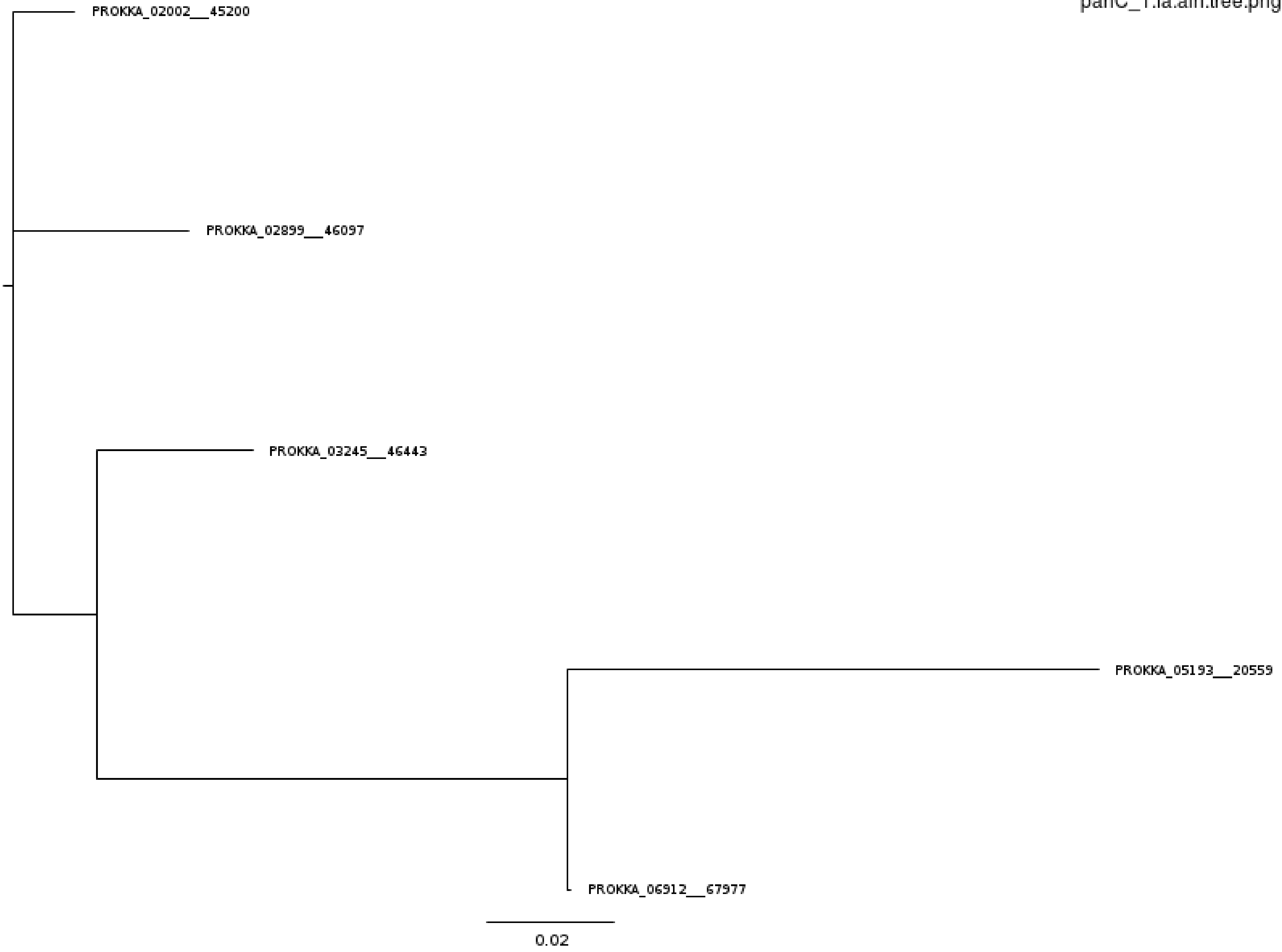

panD_2.fa.aln.tree.png

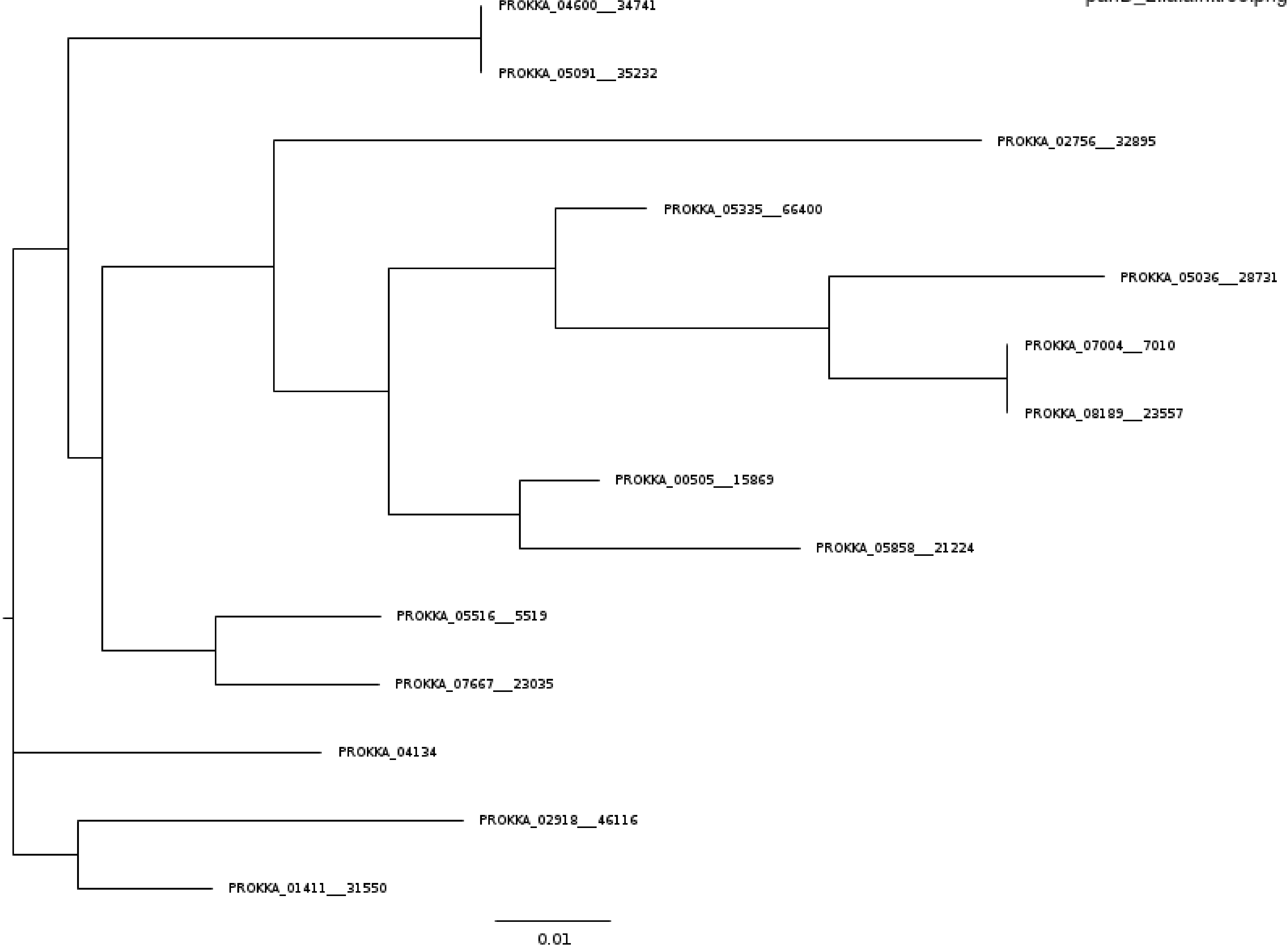

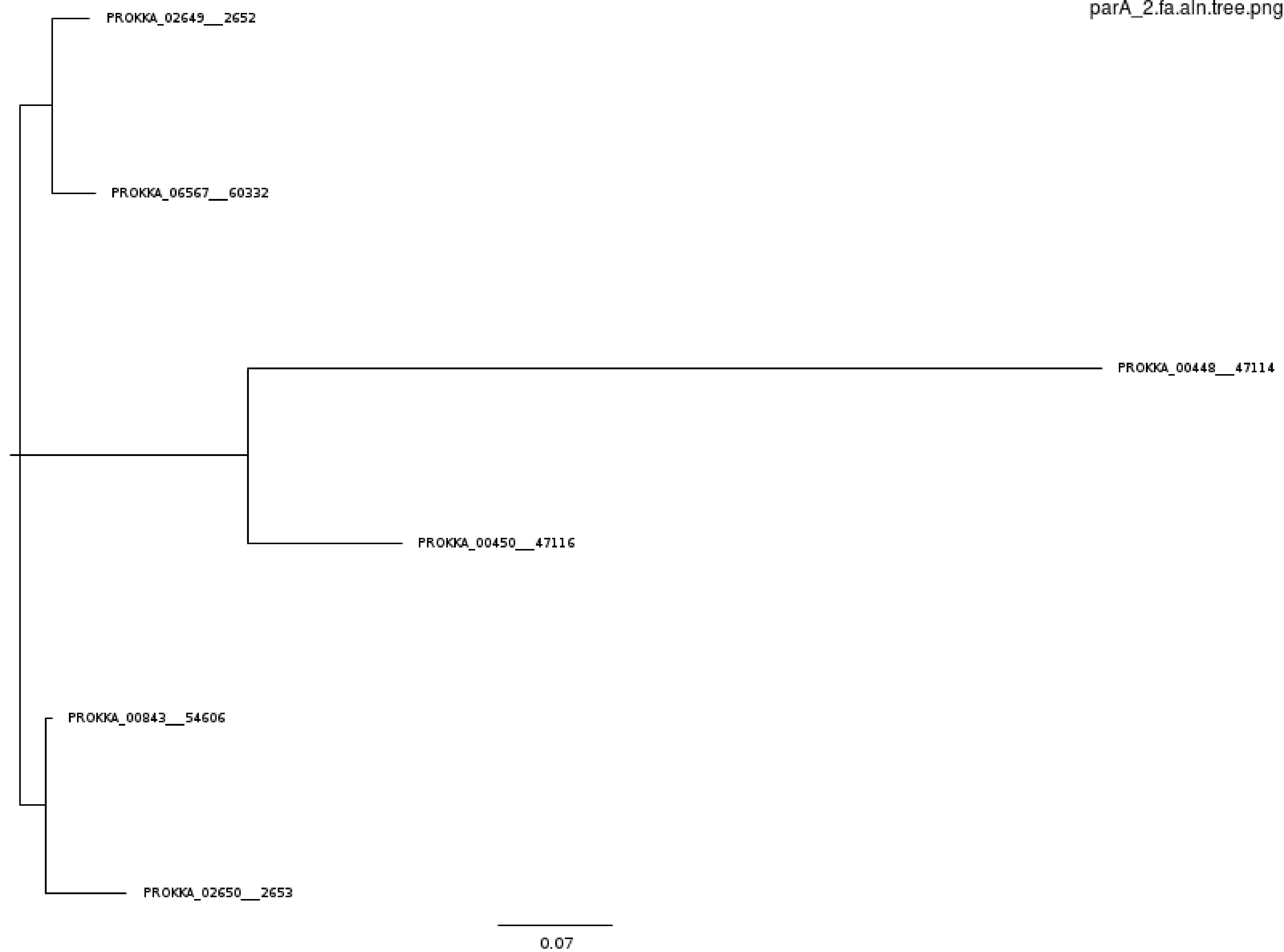
parA_2.fa.aln.tree.png
PROKKA_02649___2652
PROKKA_06567___60332
PROKKA_00448___47114
PROKKA_00450___47116
PROKKA_00843___54606
PROKKA_02650___2653
0.07

pasL_2.fa.aln.tree.png

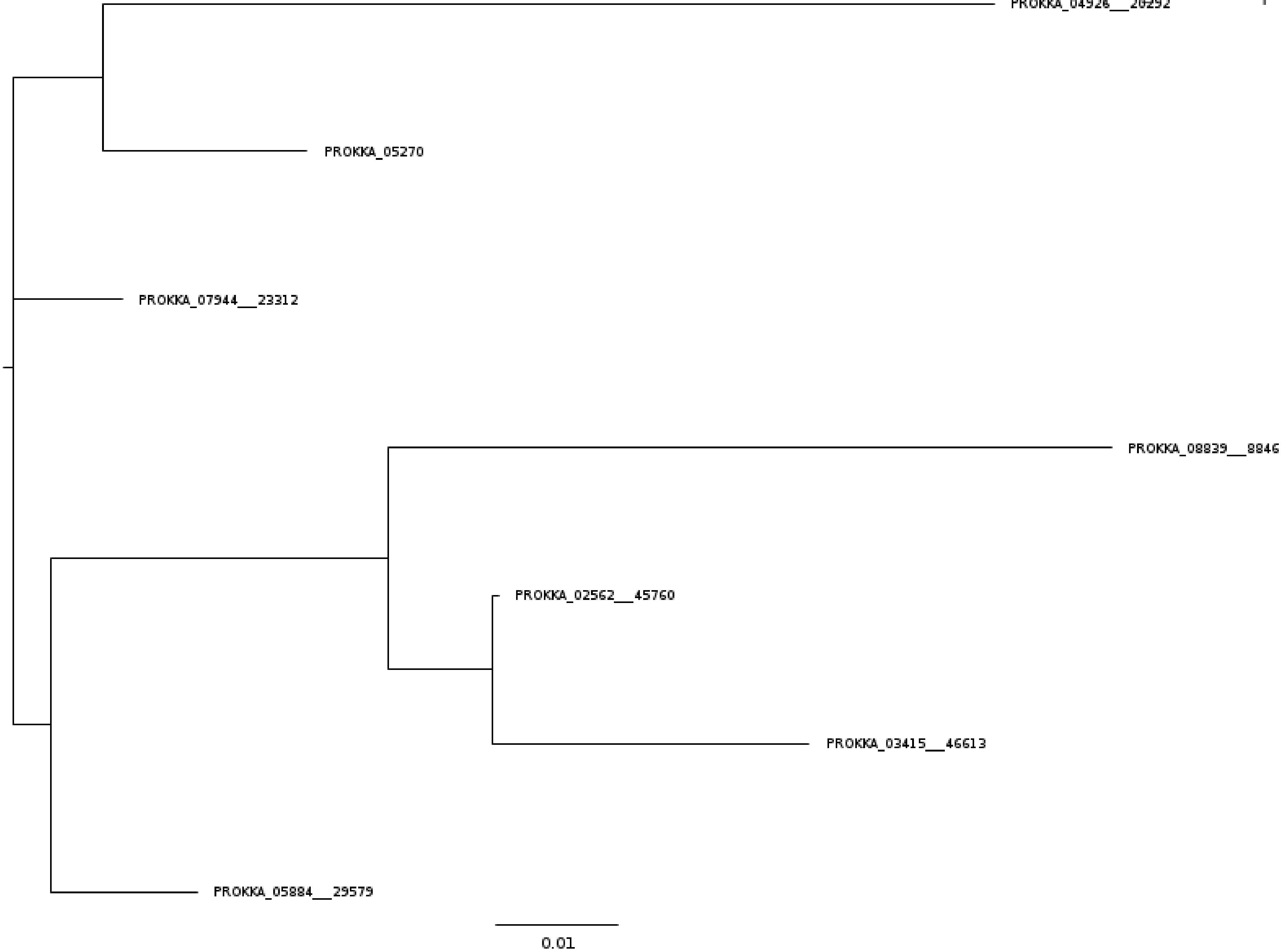

pasT_2.fa.aln.tree.png

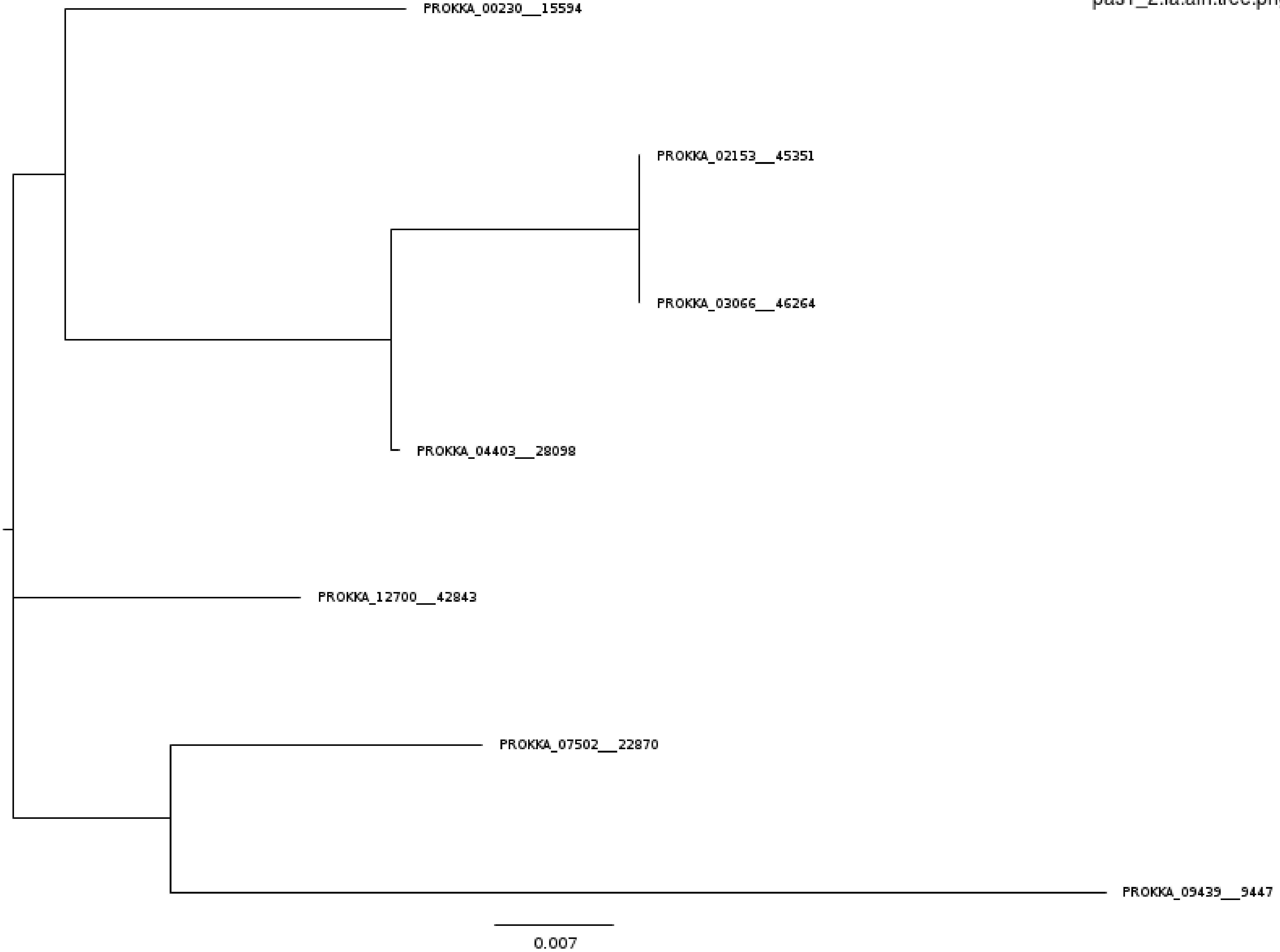

pbpF_1.fa.aln.tree.png

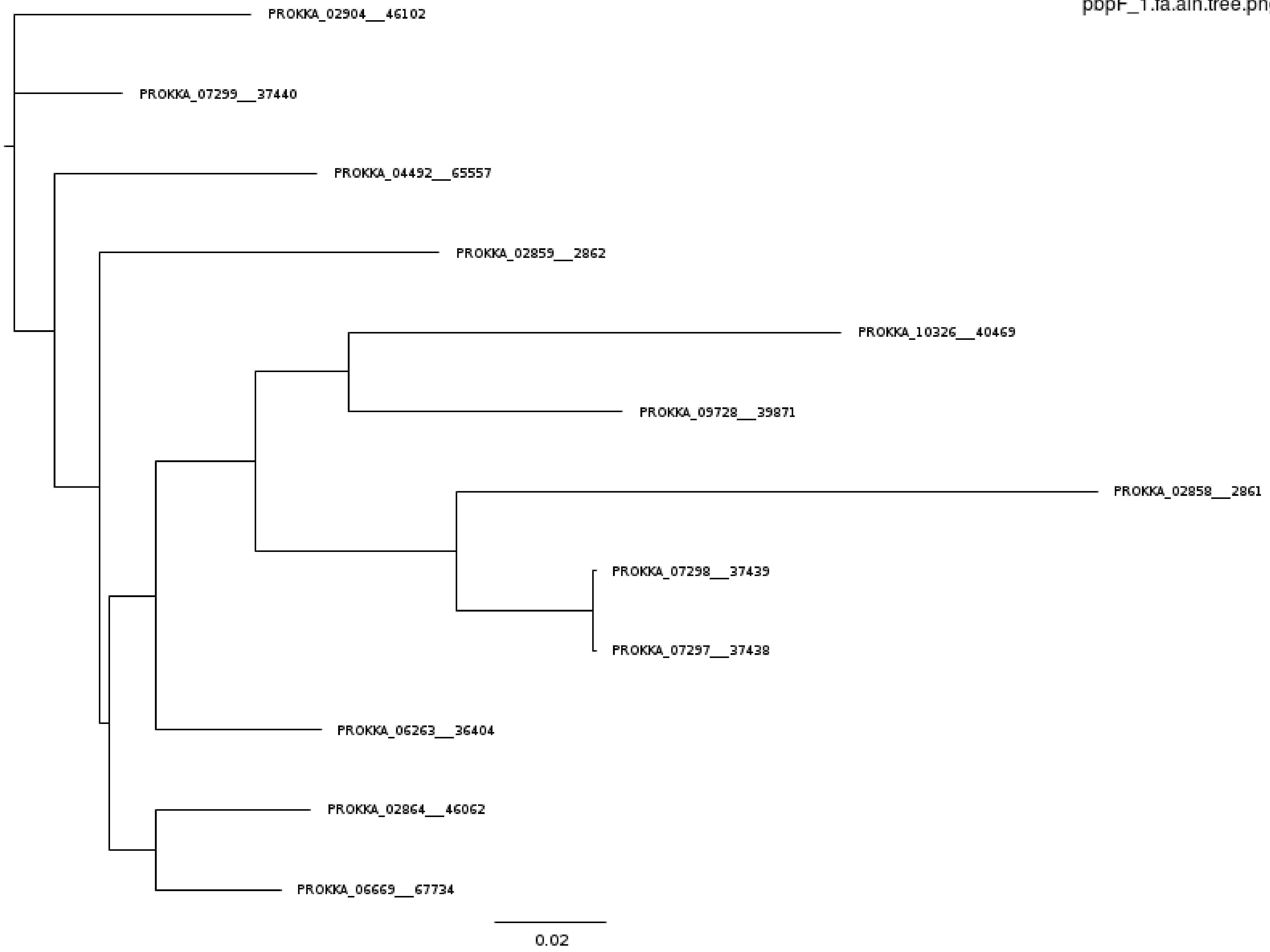

pbpG_1.fa.aln.tree.png

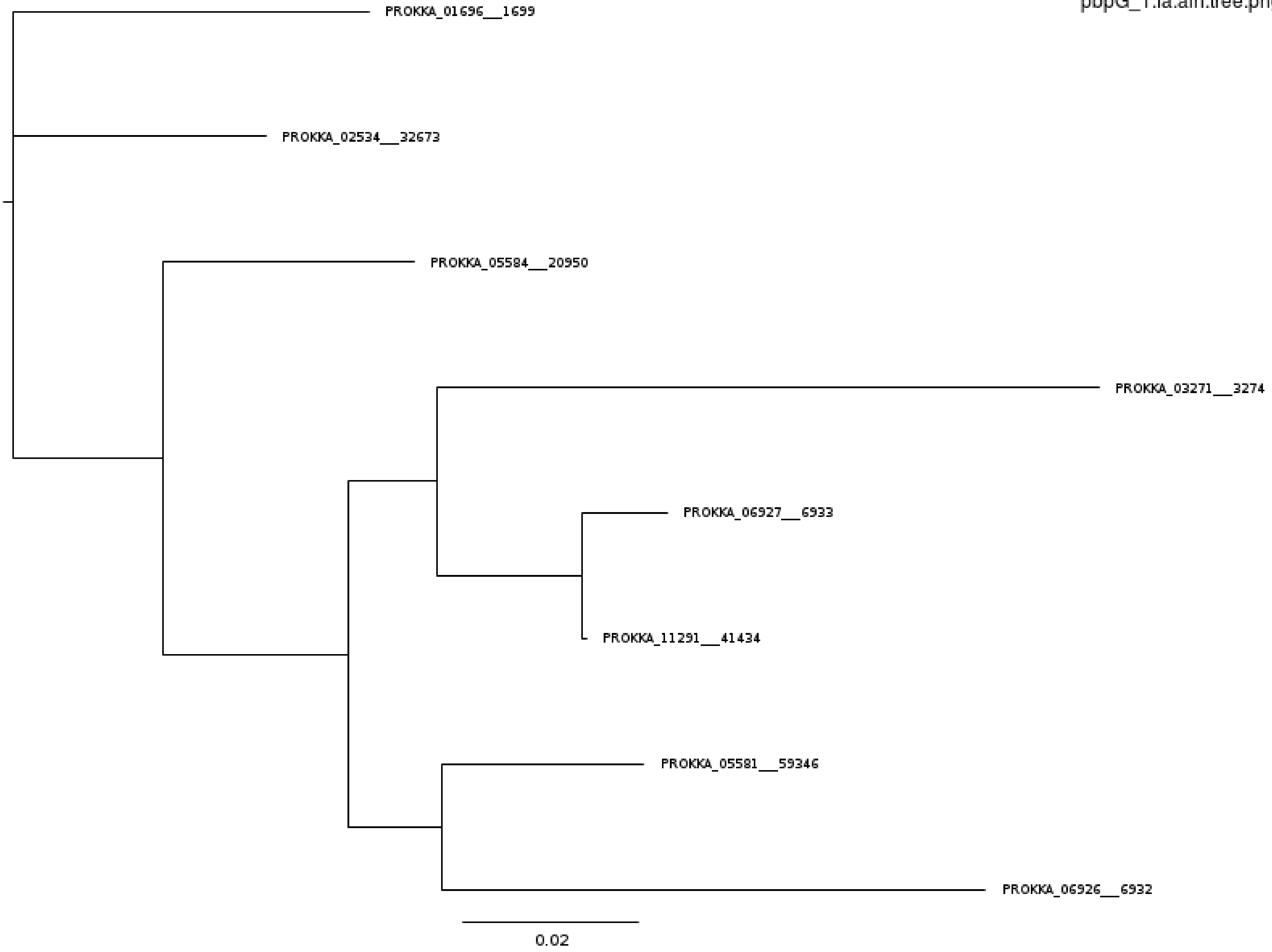

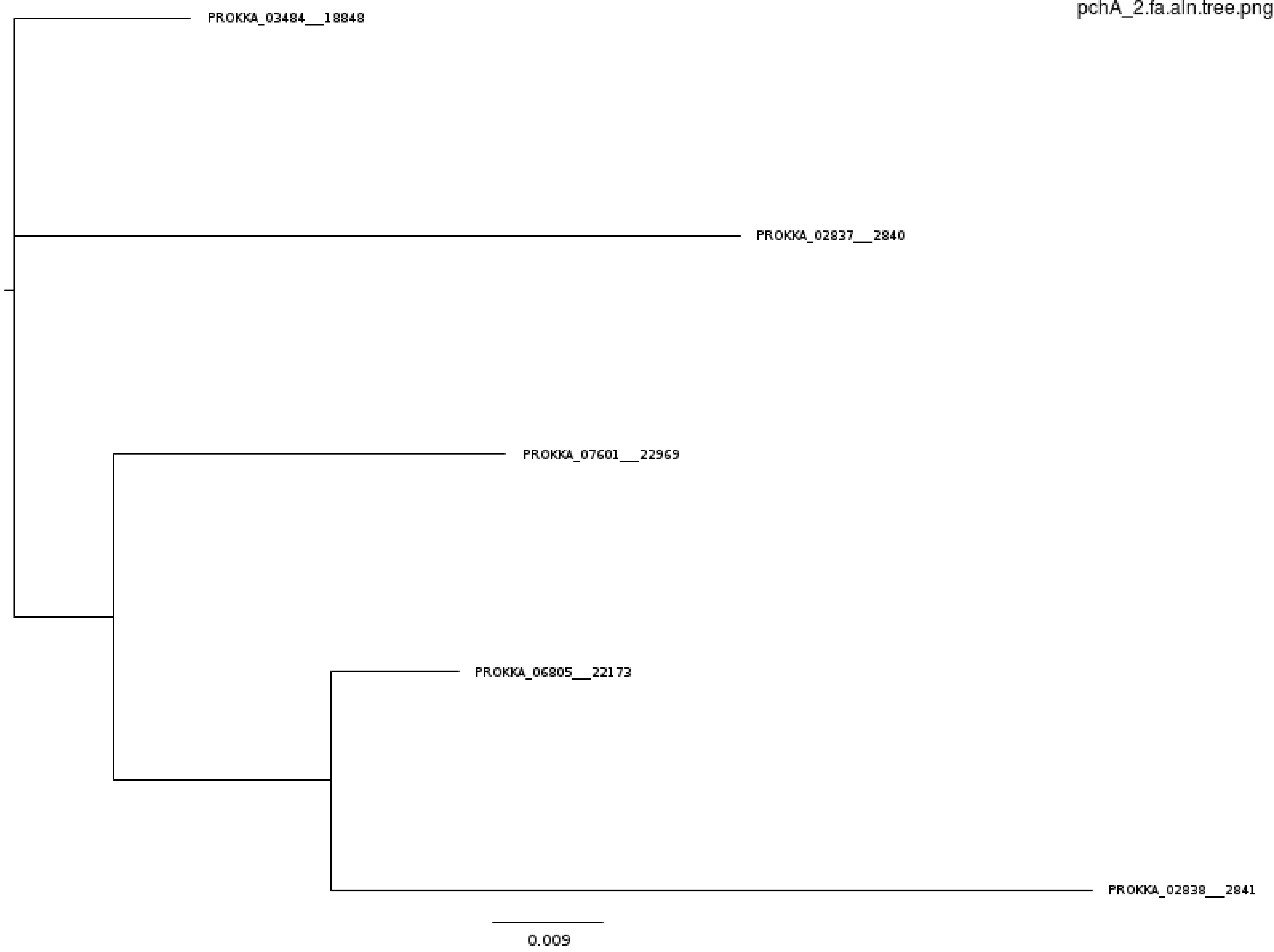
pchA_2.fa.aln.tree.png
PROKKA_03484___18848
PROKKA_02837___2840
PROKKA_07601___22969
PROKKA_06805___22173
PROKKA_02838___2841
0.009

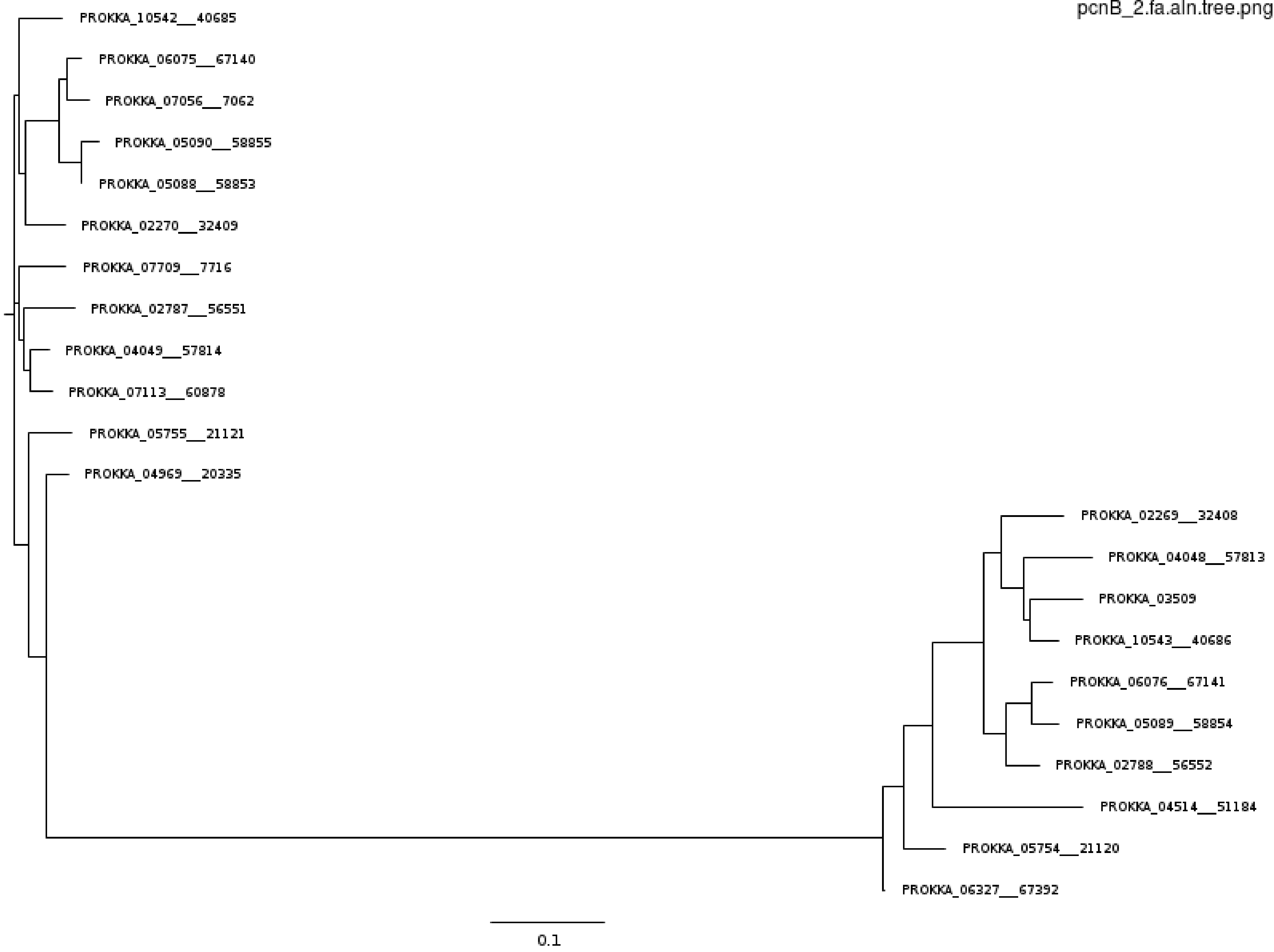
pcnB_2.fa.aln.tree.png
PROKKA_10542___40685
PROKKA_06075___67140
PROKKA_07056___7062
PROKKA_05090___58855
PROKKA_05088___58853
PROKKA_02270___32409
PROKKA_07709___7716
PROKKA_02787___56551
PROKKA_04049___57814
PROKKA_07113___60878
PROKKA_05755___21121
PROKKA_04969___20335
PROKKA_02269___32408
PROKKA_04048___57813
PROKKA_03509
PROKKA_10543___40686
PROKKA_06076___67141
PROKKA_05089___58854
PROKKA_02788___56552
PROKKA_04514___51184
PROKKA_05754___21120
PROKKA_06327___67392
0.1

pctA_3.fa.aln.tree.png

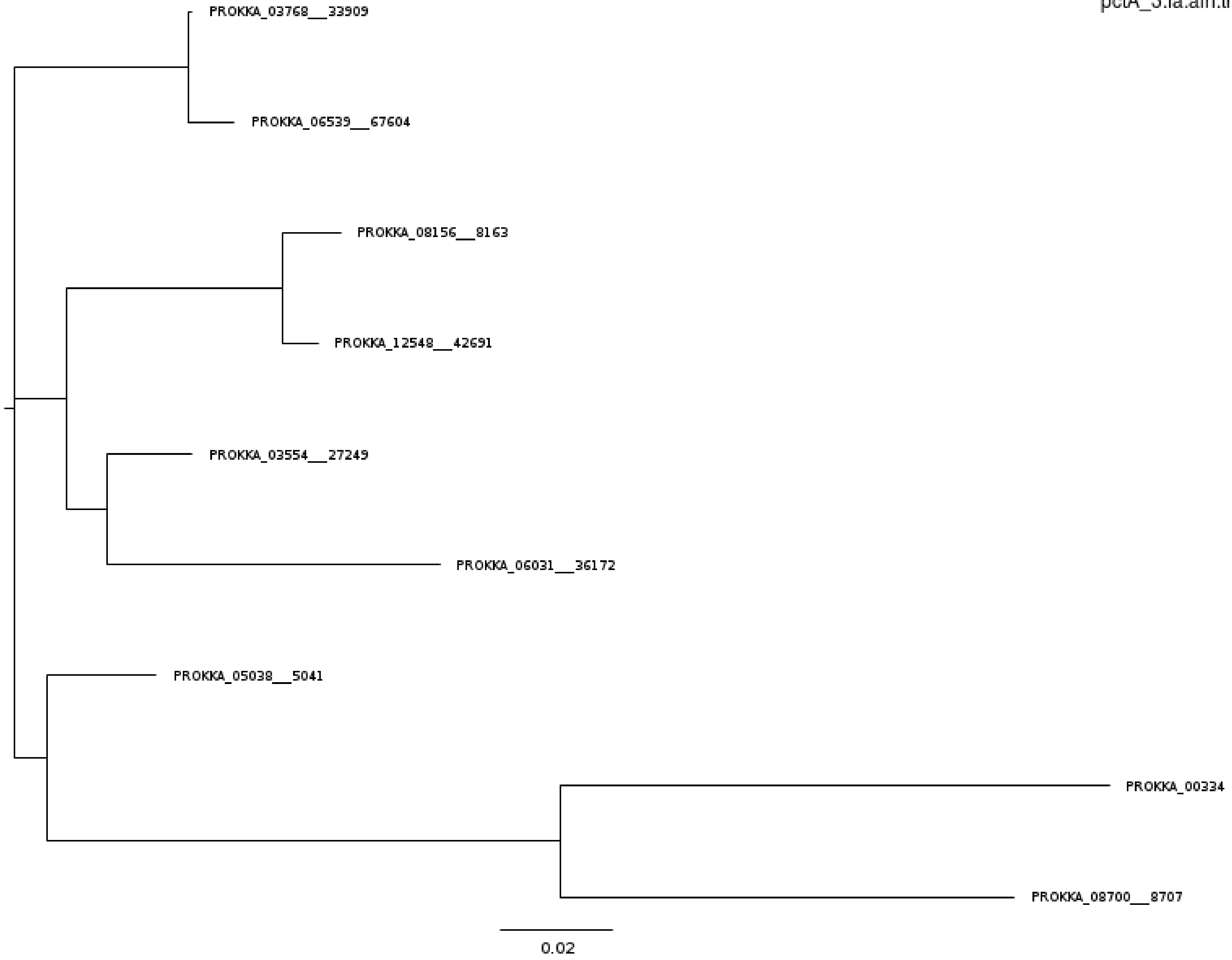

pctA.fa.aln.tree.png

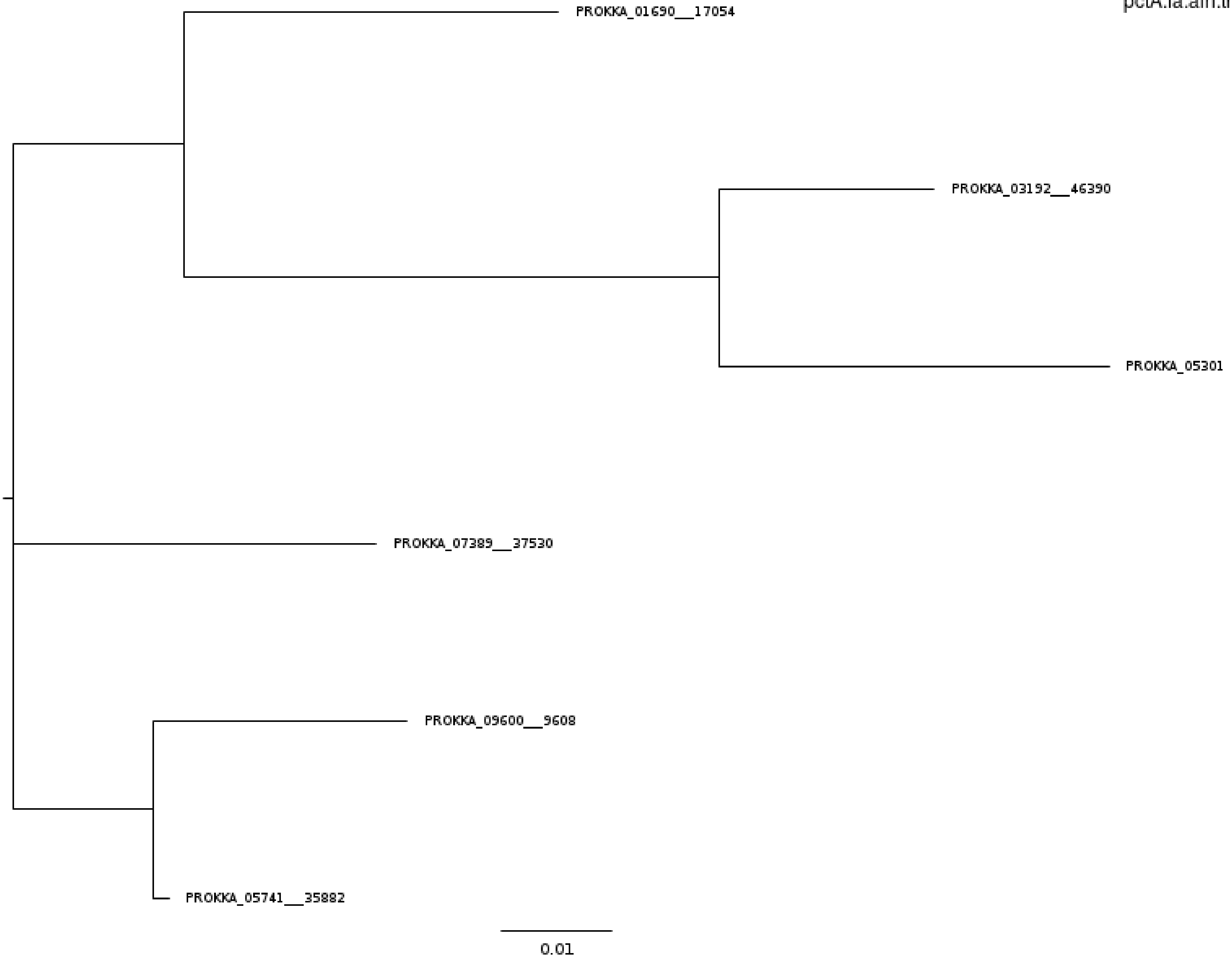

pds.fa.aln.tree.png

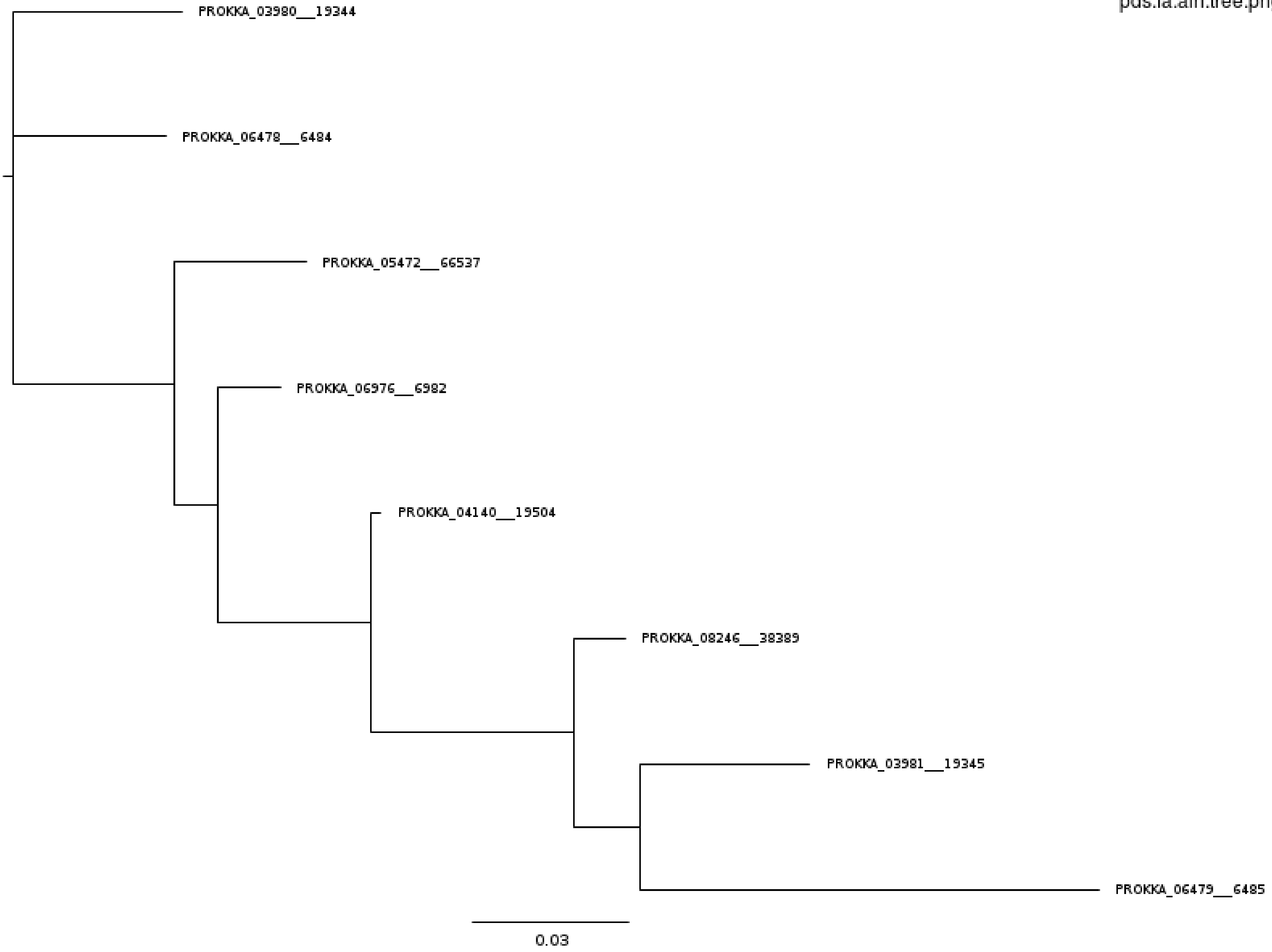

pdxA1_1.fa.aln.tree.png

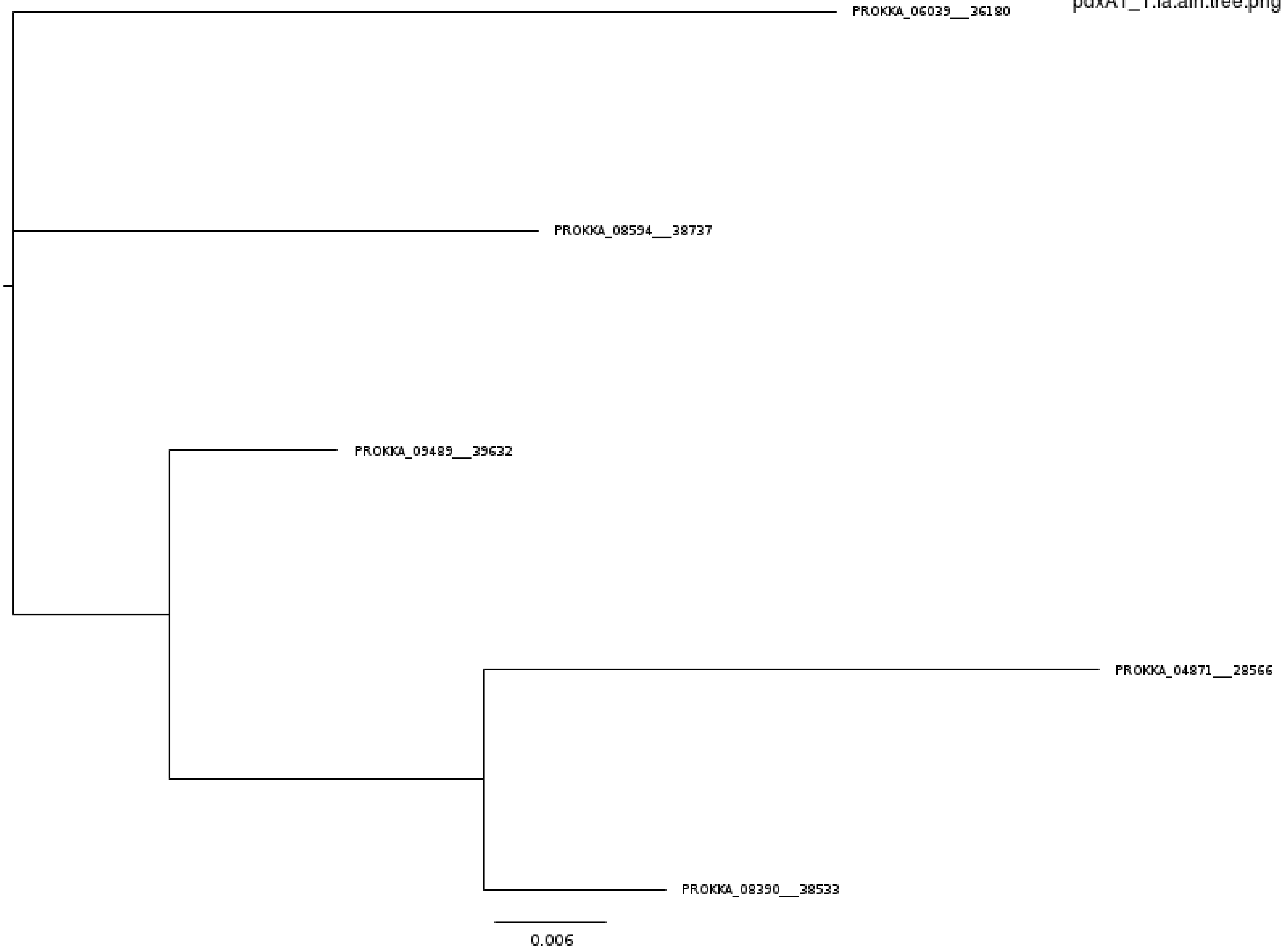

pepA_1.fa.aln.tree.png

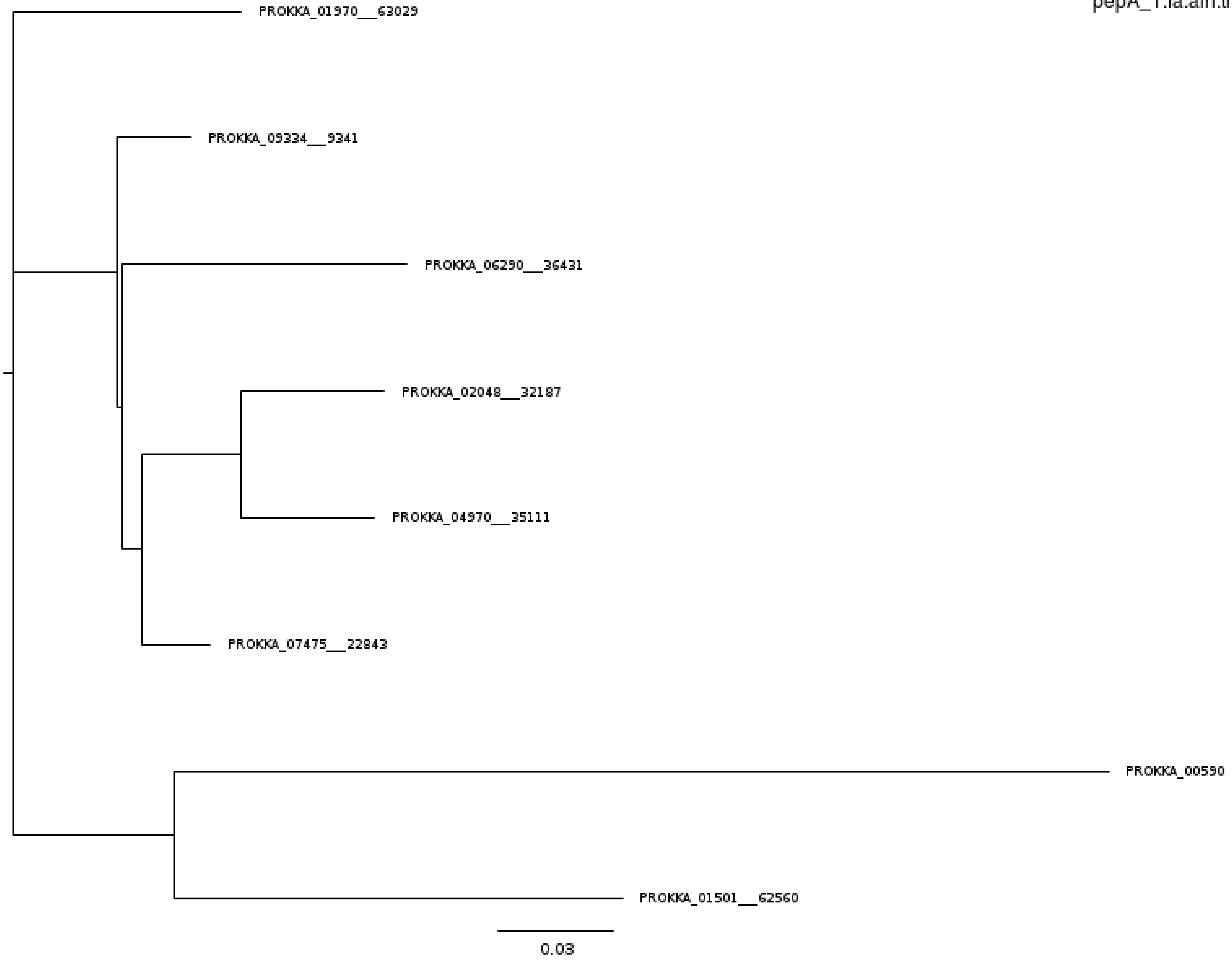

pepP_3.fa.aln.tree.png

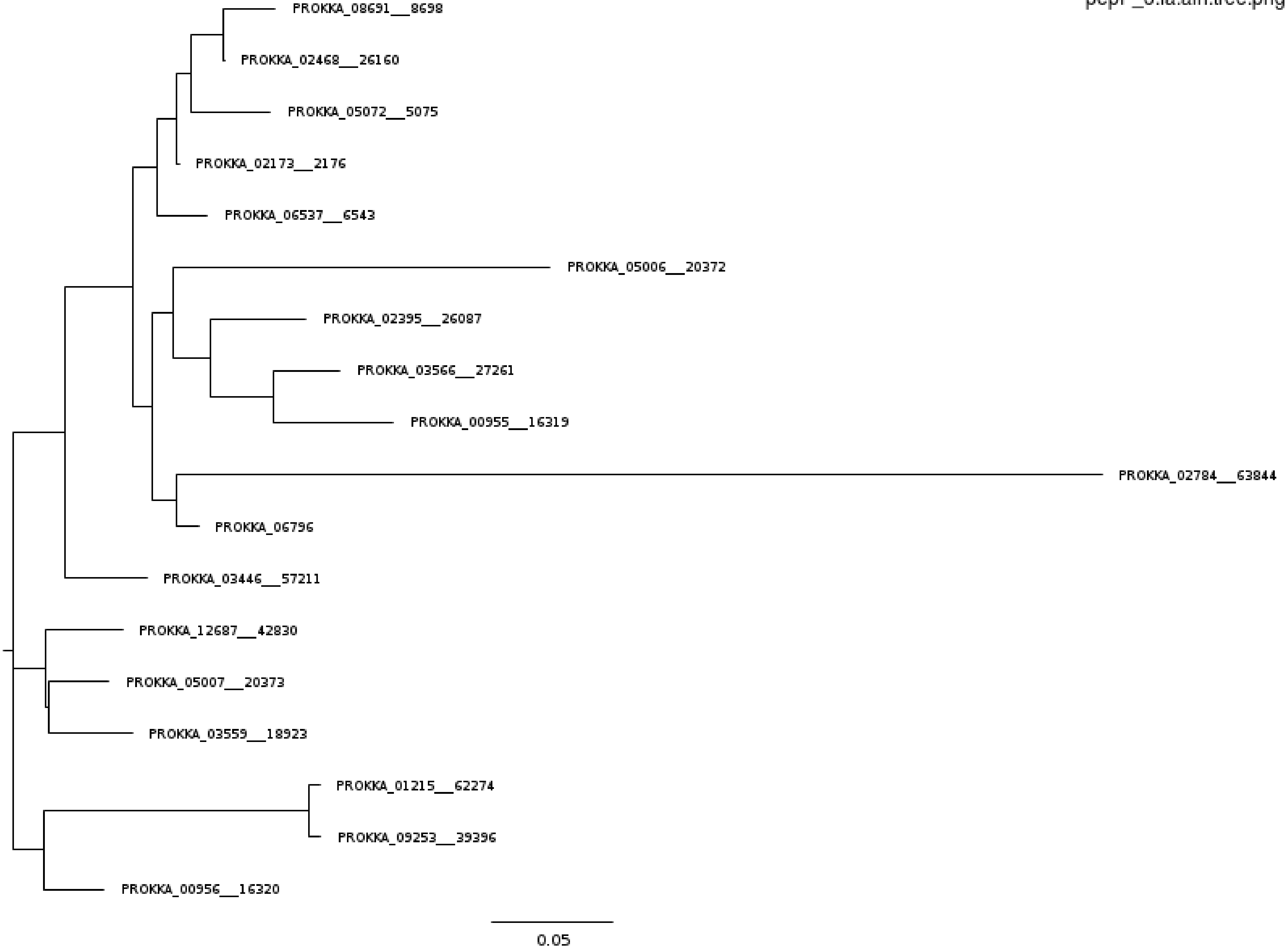

petH.fa.aln.tree.png

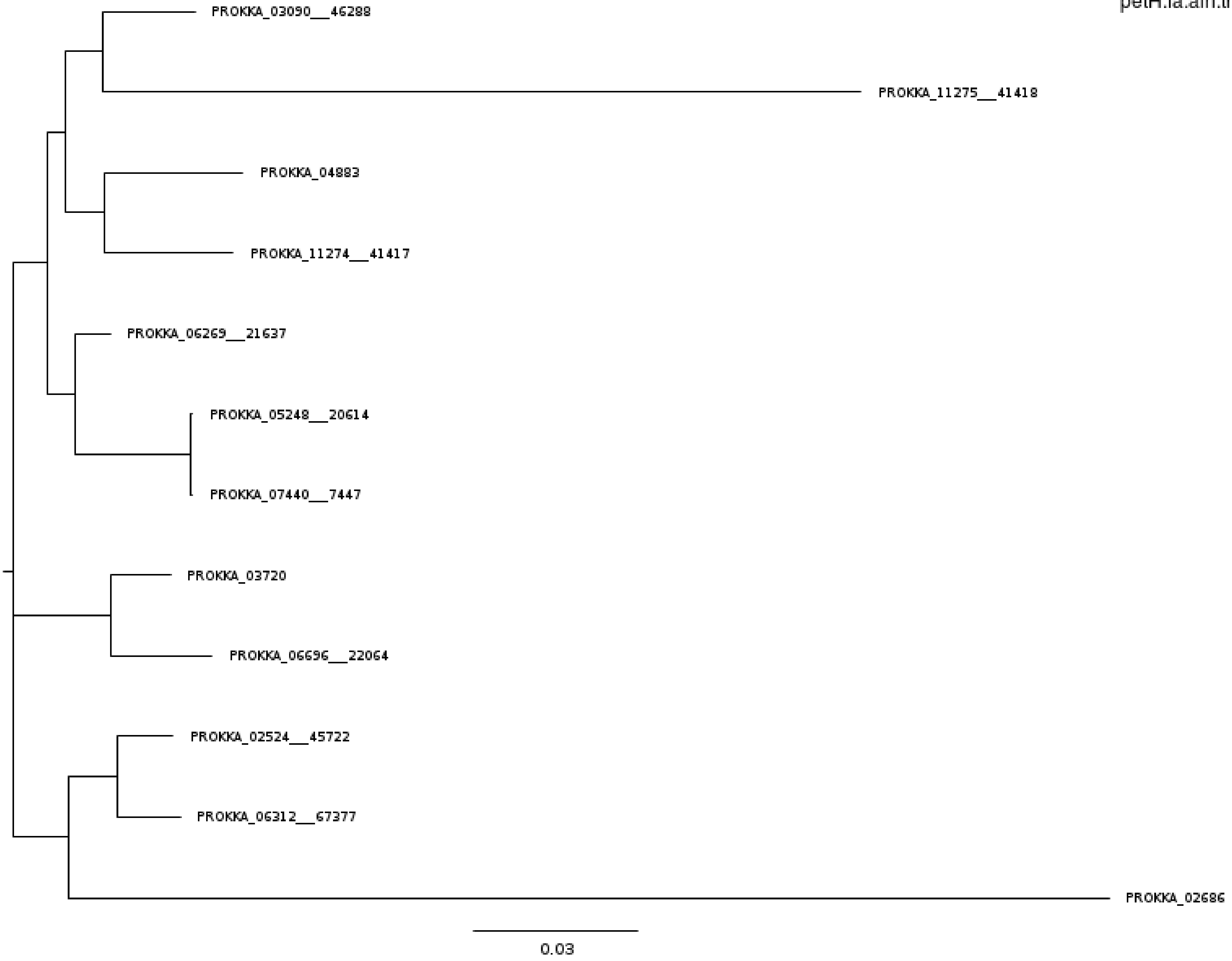

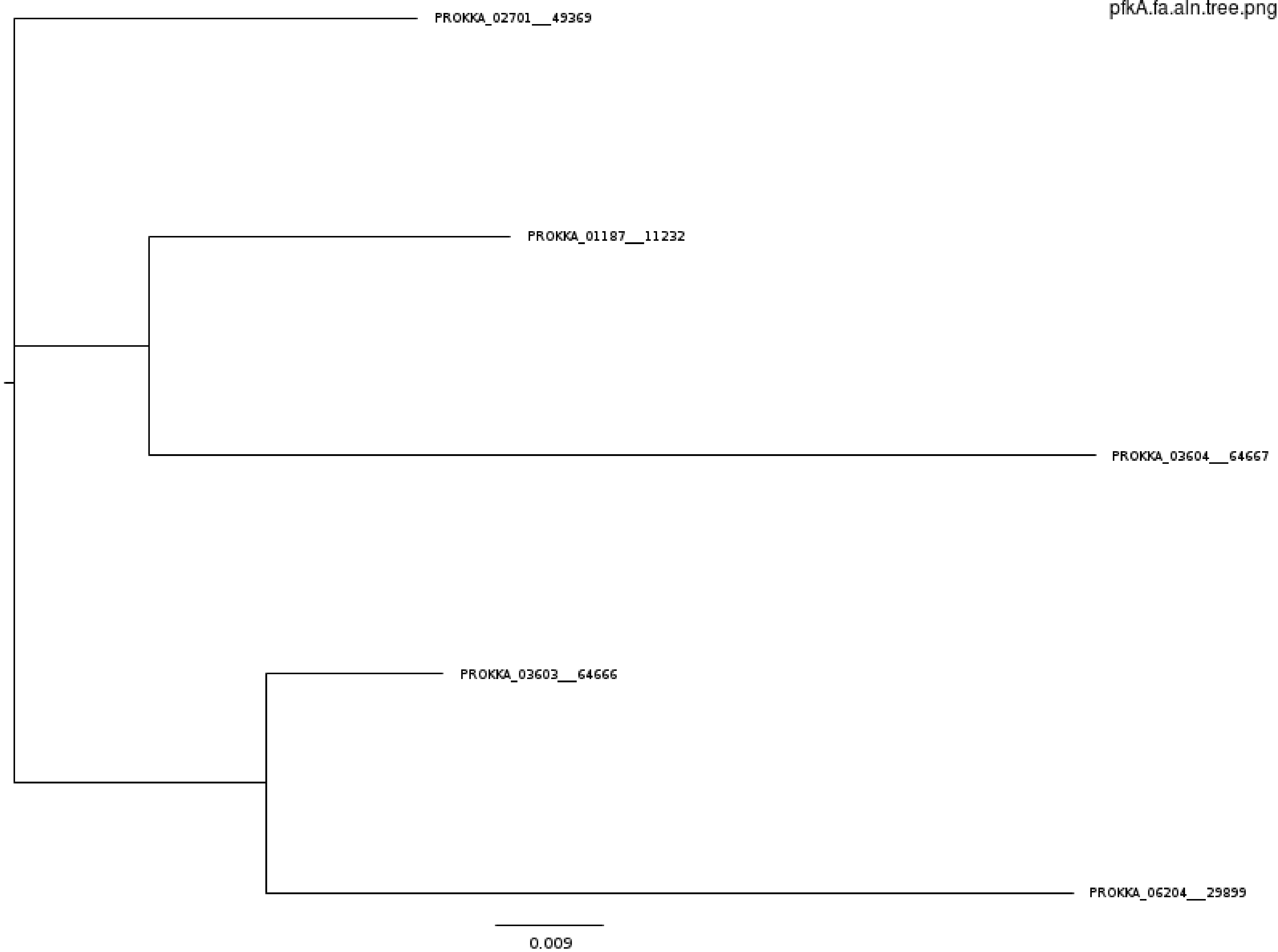
pfkA.fa.aln.tree.png
PROKKA_02701___49369
PROKKA_01187___11232
PROKKA_03604___64667
PROKKA_03603___64666
PROKKA_06204___29899
0.009

pflA_1.fa.aln.tree.png

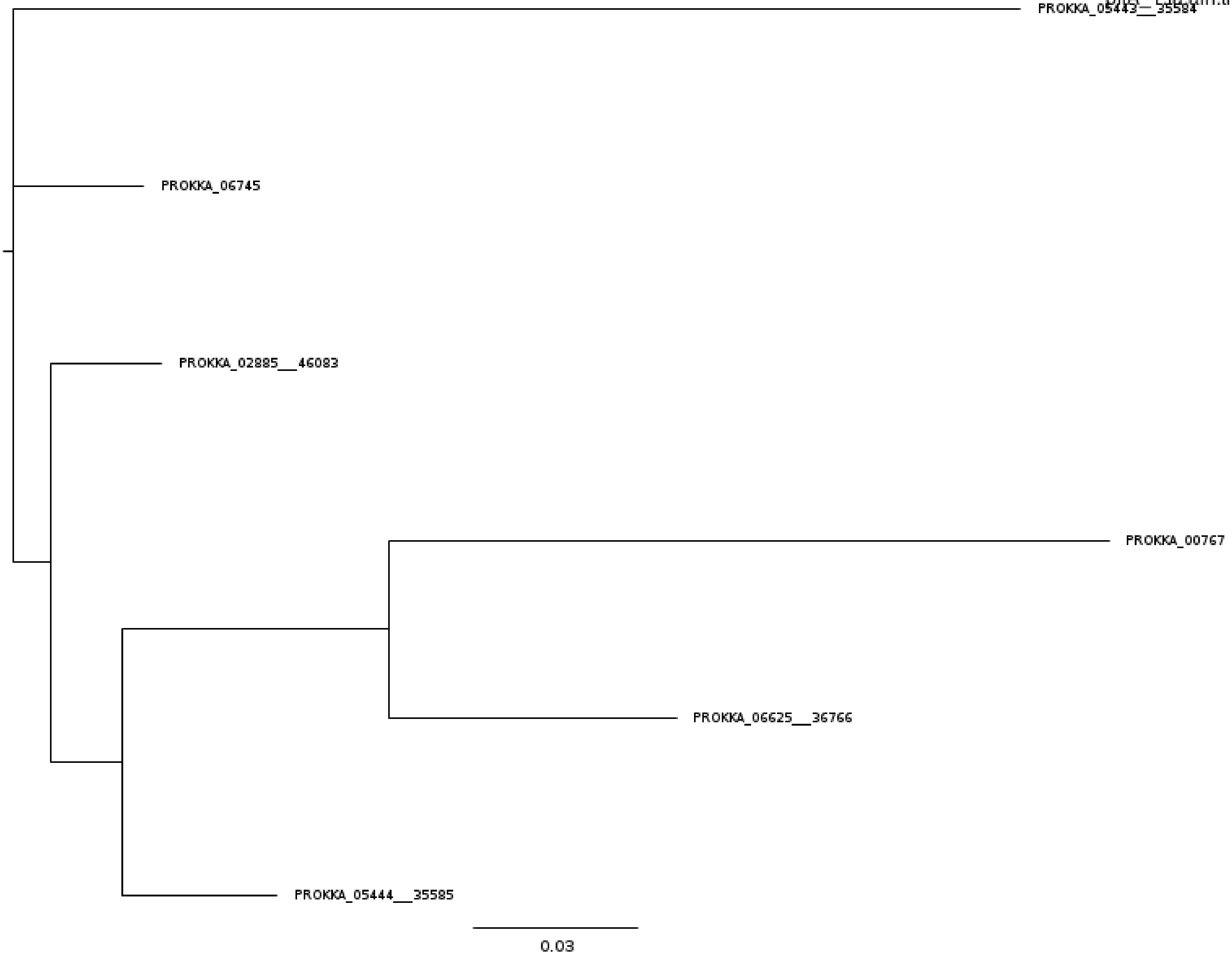

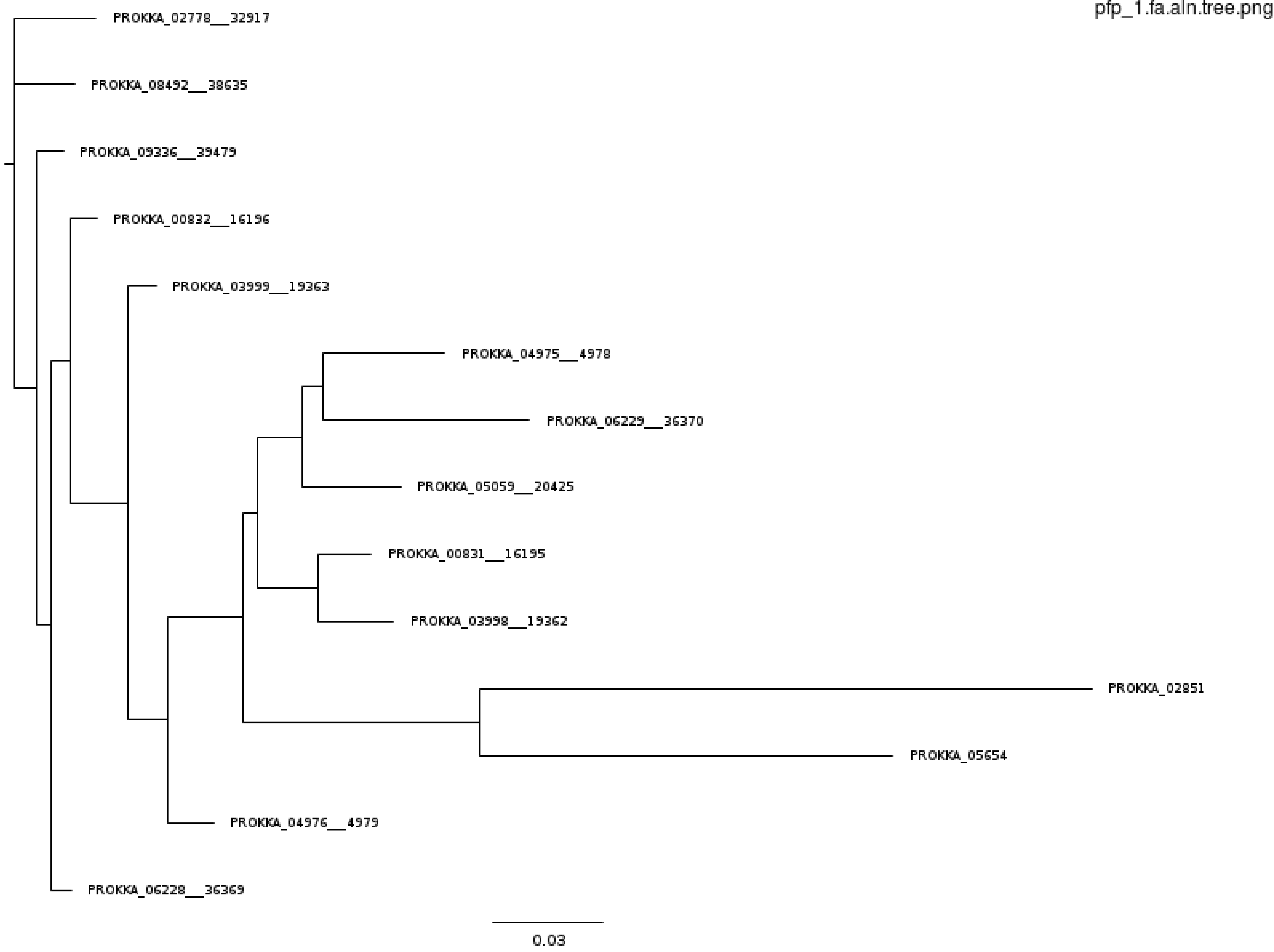

pfp_1.fa.aln.tree.png
PROKKA_02778___32917
PROKKA_08492___38635
PROKKA_09336___39479
PROKKA_00832___16196
PROKKA_03999___19363
PROKKA_04975___4978
PROKKA_06229___36370
PROKKA_05059___20425
PROKKA_00831___16195
PROKKA_03998___19362
PROKKA_02851
PROKKA_05654
PROKKA_04976___4979
PROKKA_06228___36369
0.03

pgi_4.fa.aln.tree.png

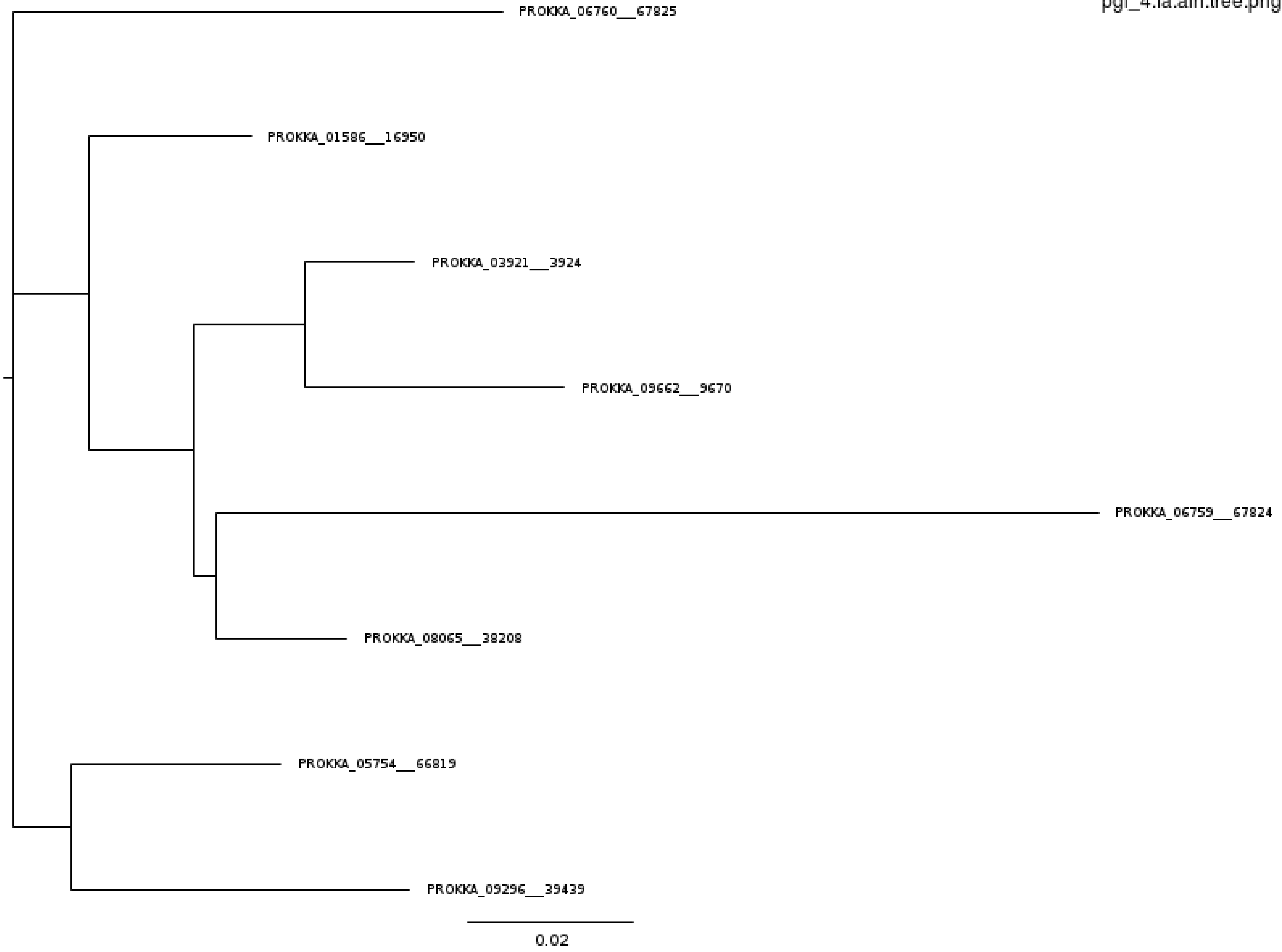

pgk_3.fa.aln.tree.png

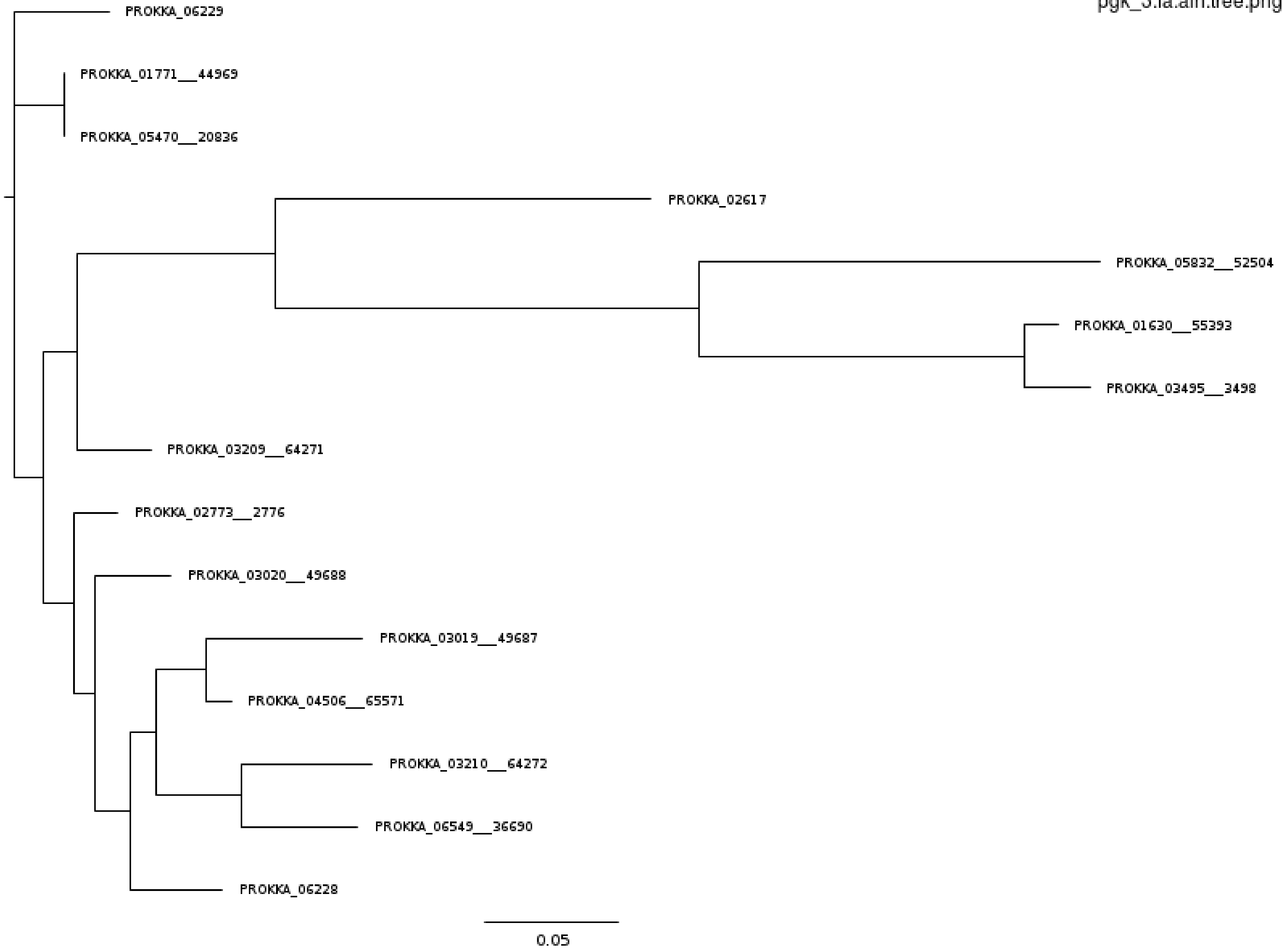

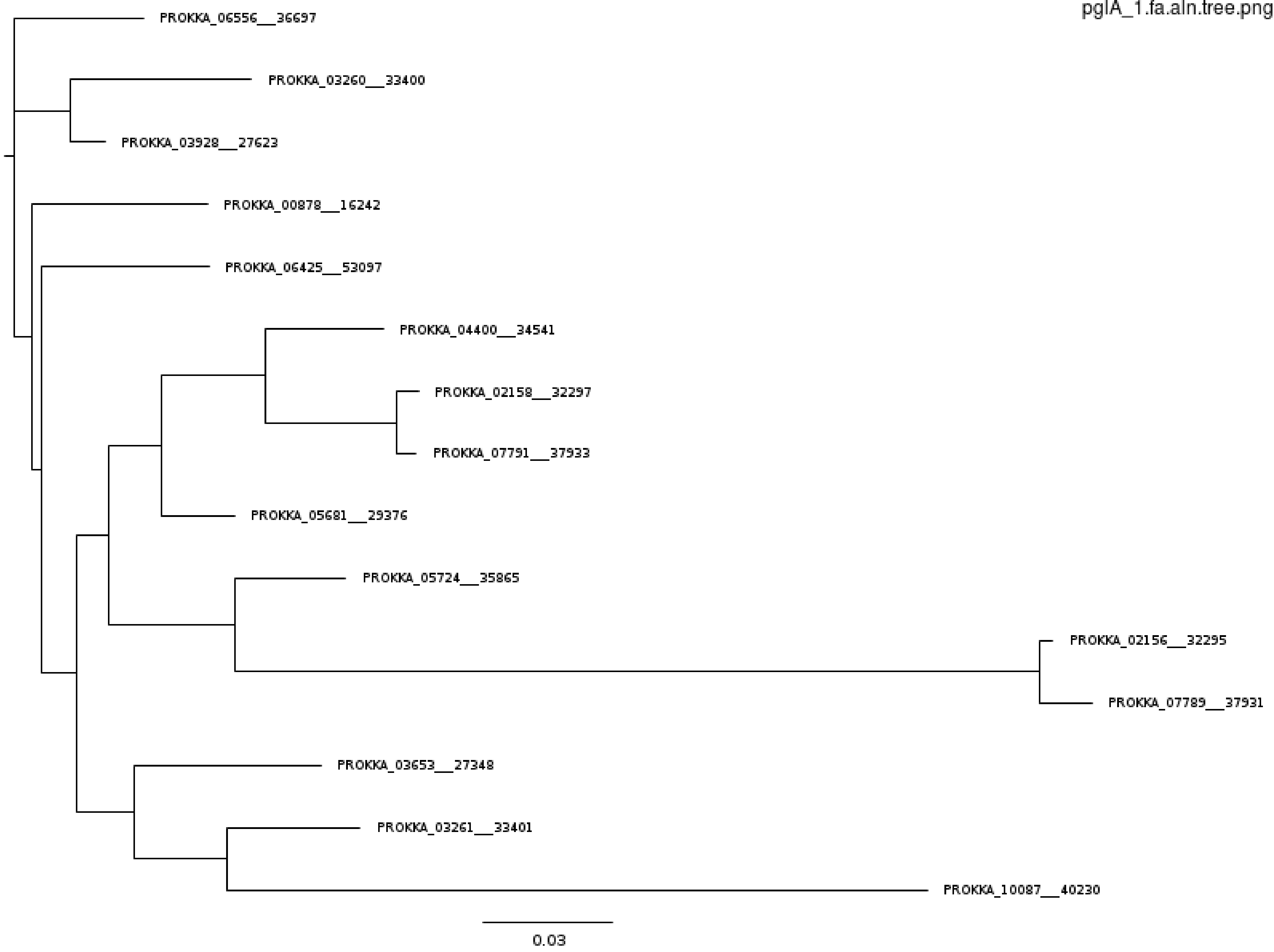
pglA_1.fa.aln.tree.png
PROKKA_06556___36697
PROKKA_03260___33400
PROKKA_03928___27623
PROKKA_00878___16242
PROKKA_06425___53097
PROKKA_04400___34541
PROKKA_02158___32297
PROKKA_07791___37933
PROKKA_05681___29376
PROKKA_05724___35865
PROKKA_02156___32295
PROKKA_07789___37931
PROKKA_03653___27348
PROKKA_03261___33401
PROKKA_10087___40230
0.03

pglF_3.fa.aln.tree.png

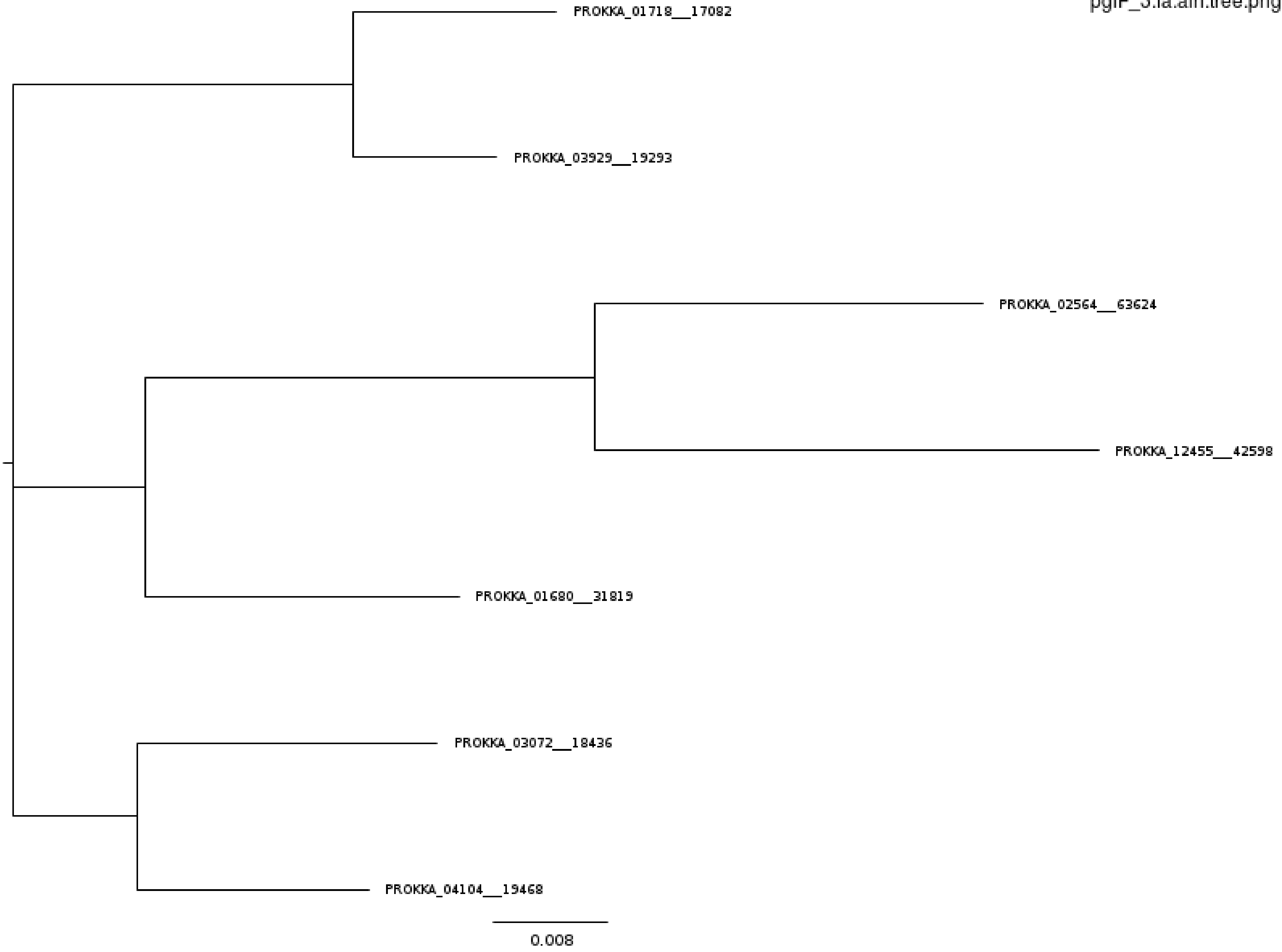

pglJ_2.fa.aln.tree.png

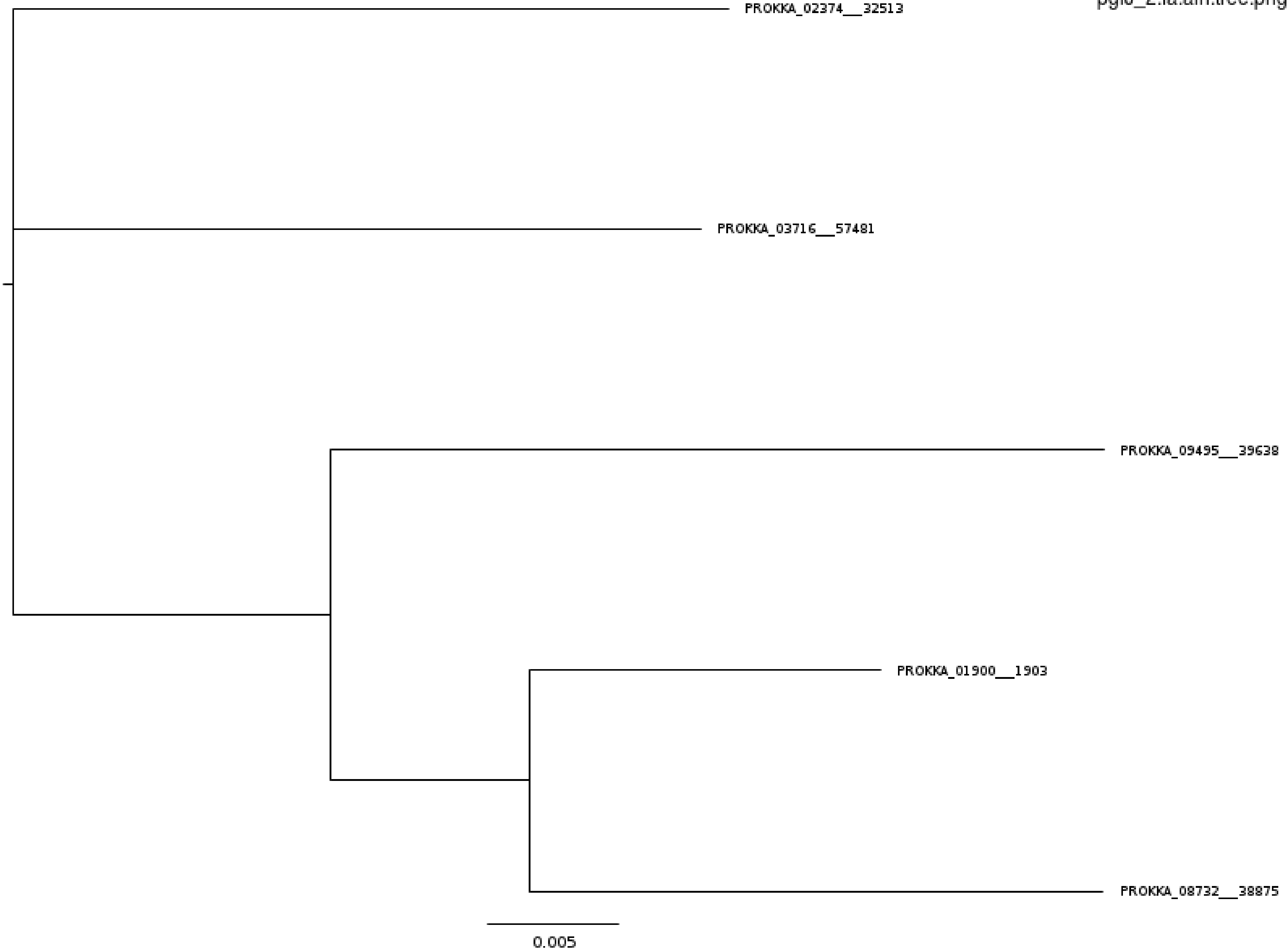

pglJ_3.fa.aln.tree.png

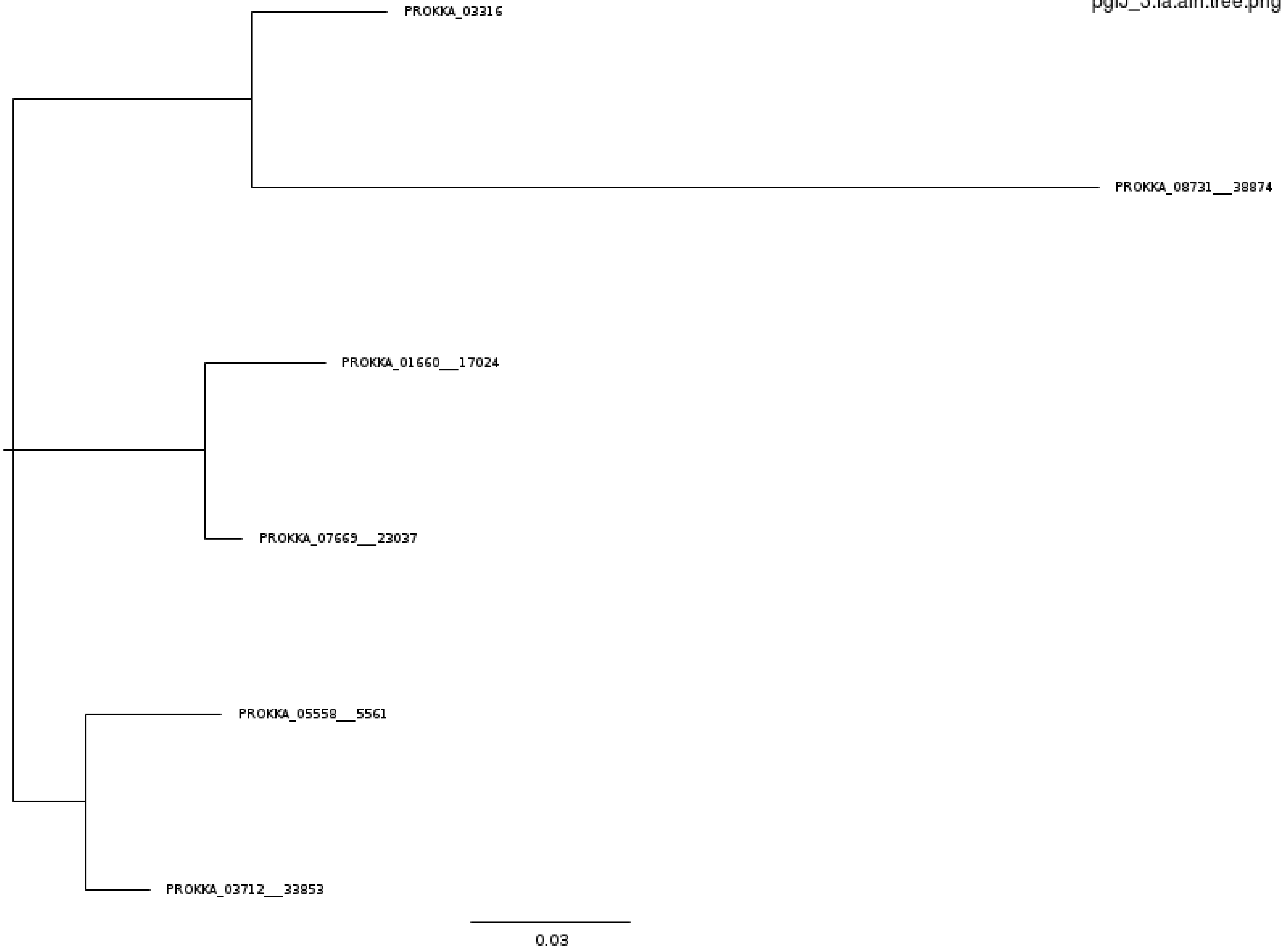

pgm_2.fa.aln.tree.png

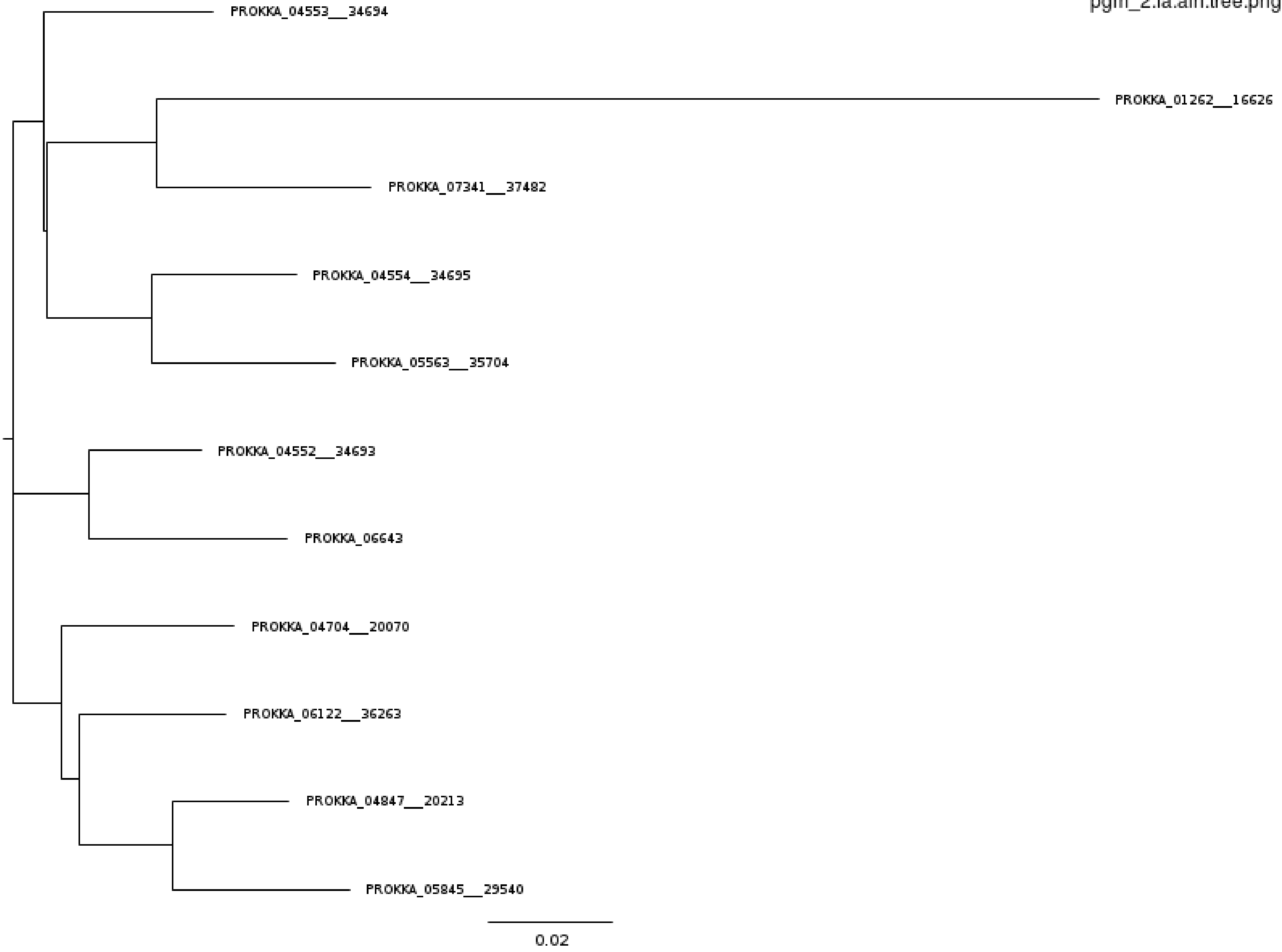

pgsA2.fa.aln.tree.png

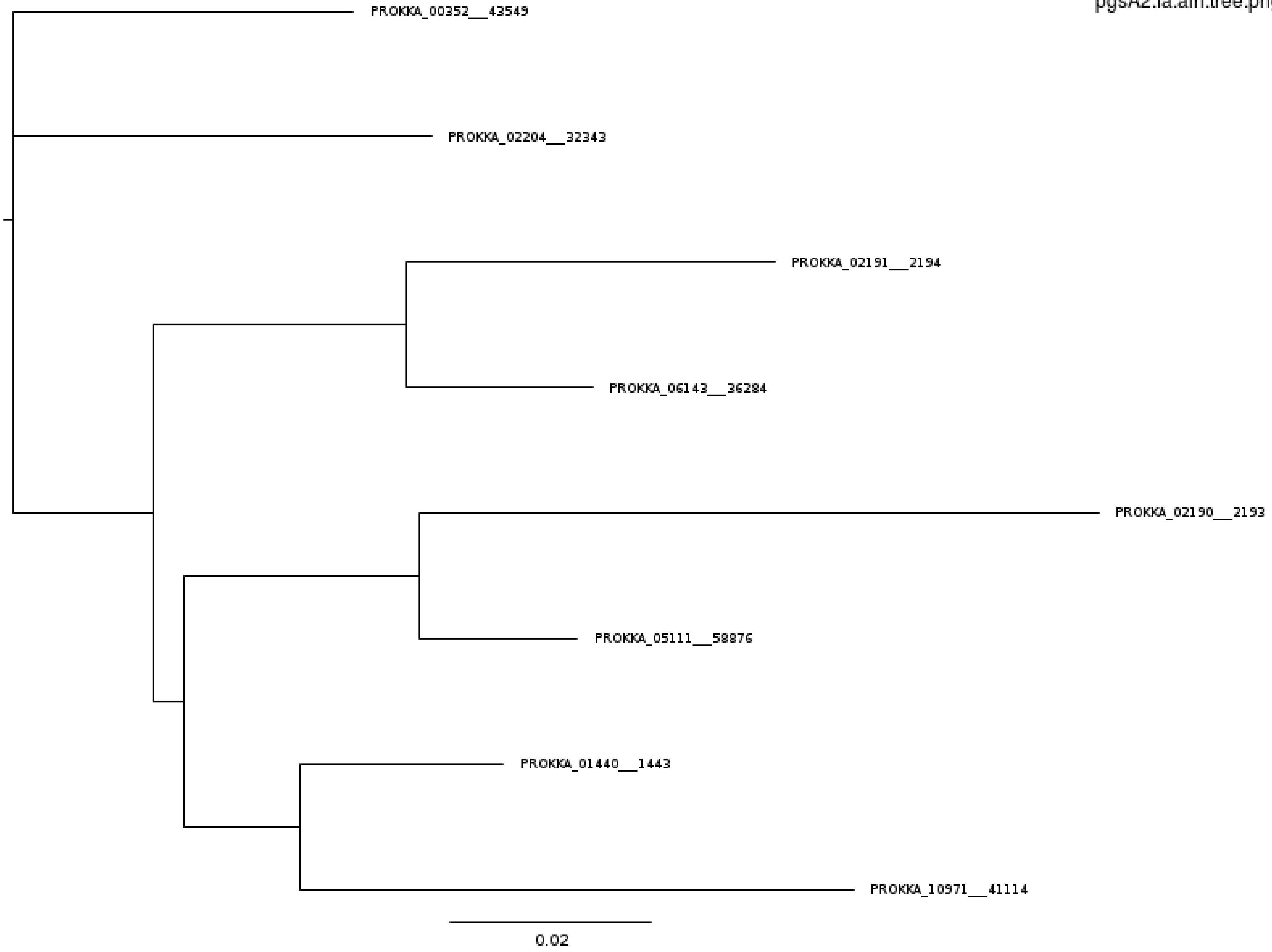

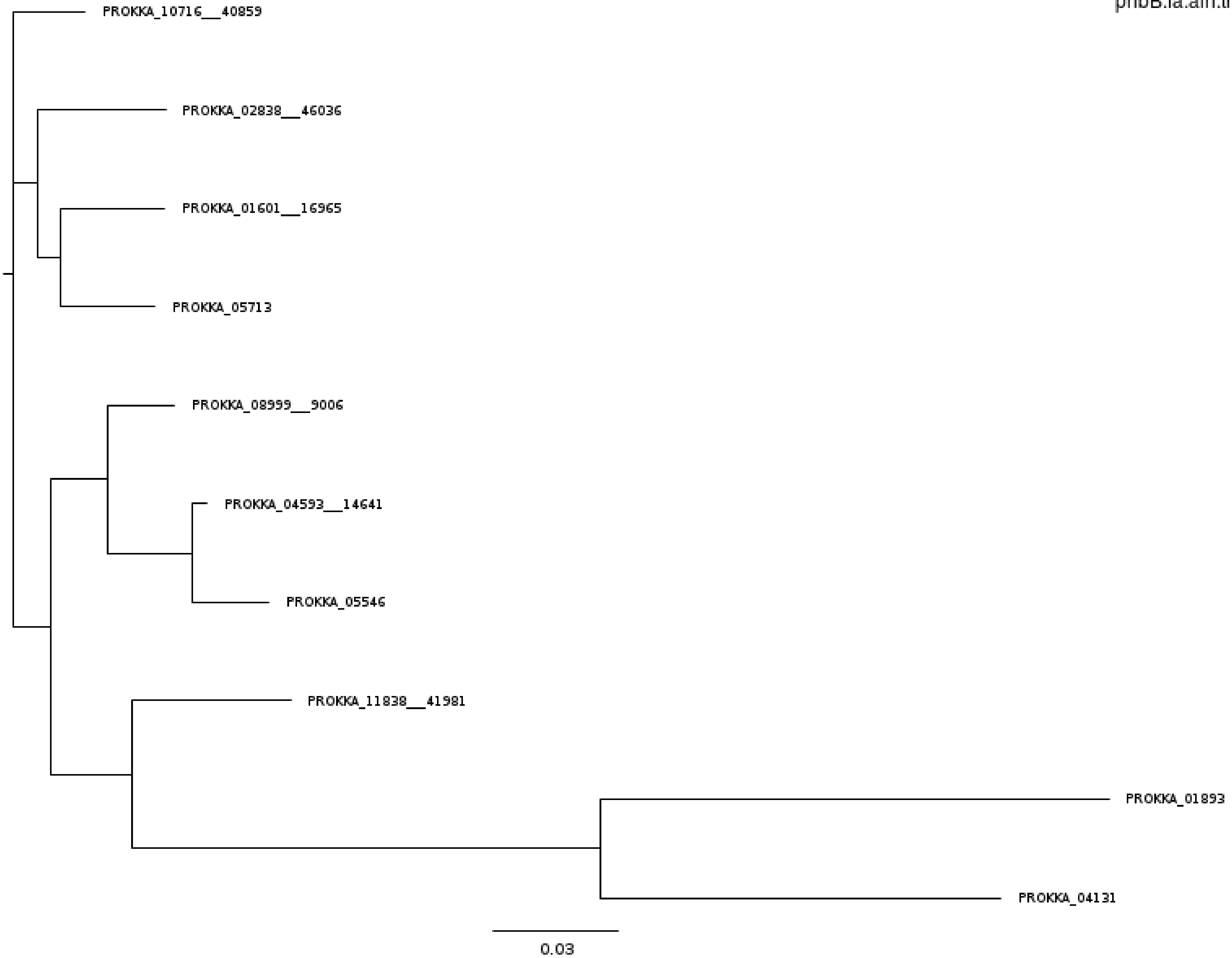
PROKKA_10716__40859
PROKKA_02838__46036
PROKKA_01601__16965
PROKKA_05713
PROKKA_08999__9006
PROKKA_04593__14641
PROKKA_05546
PROKKA_11838__41981
PROKKA_01893
PROKKA_04131
0.03

phbC_1.fa.aln.tree.png

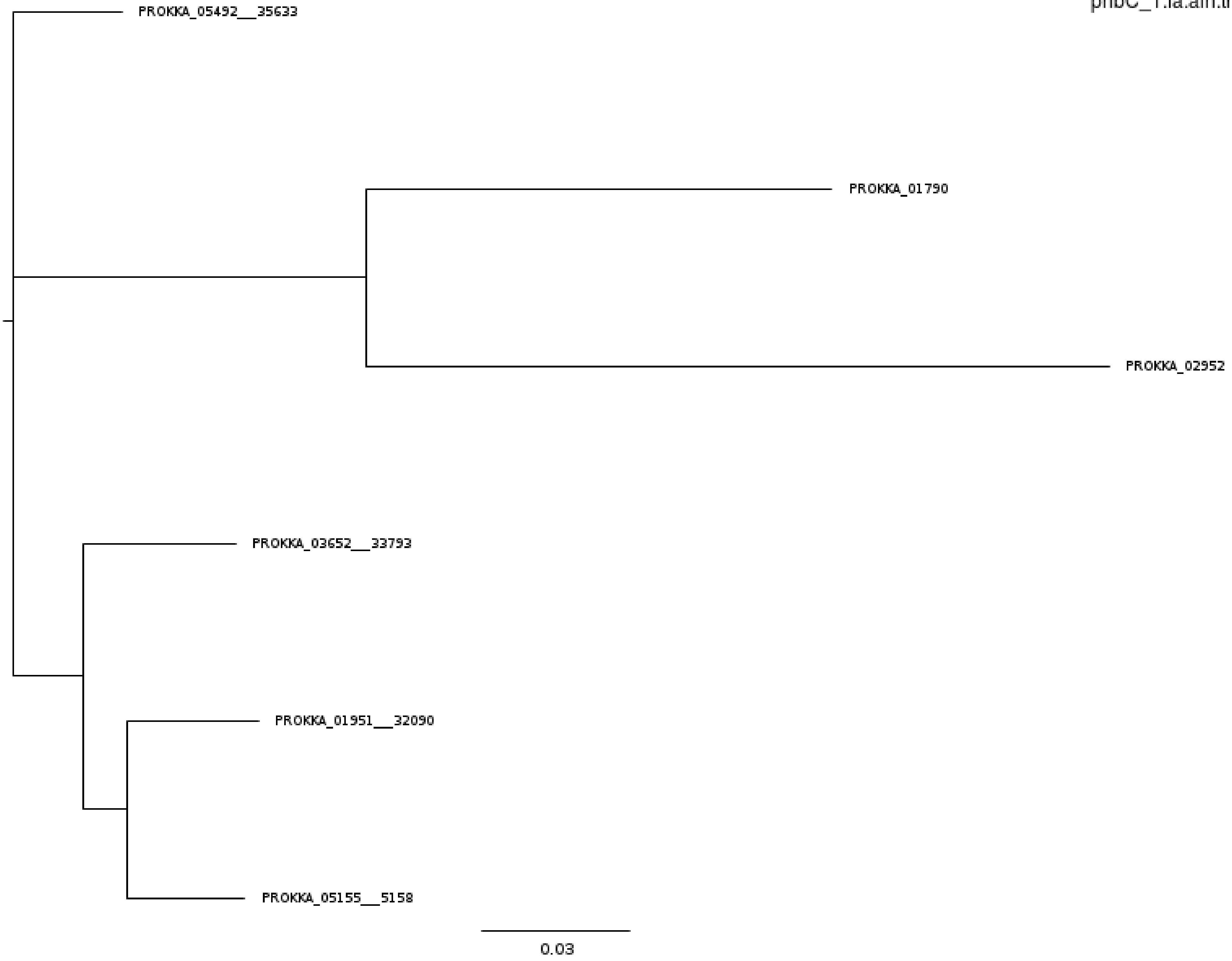

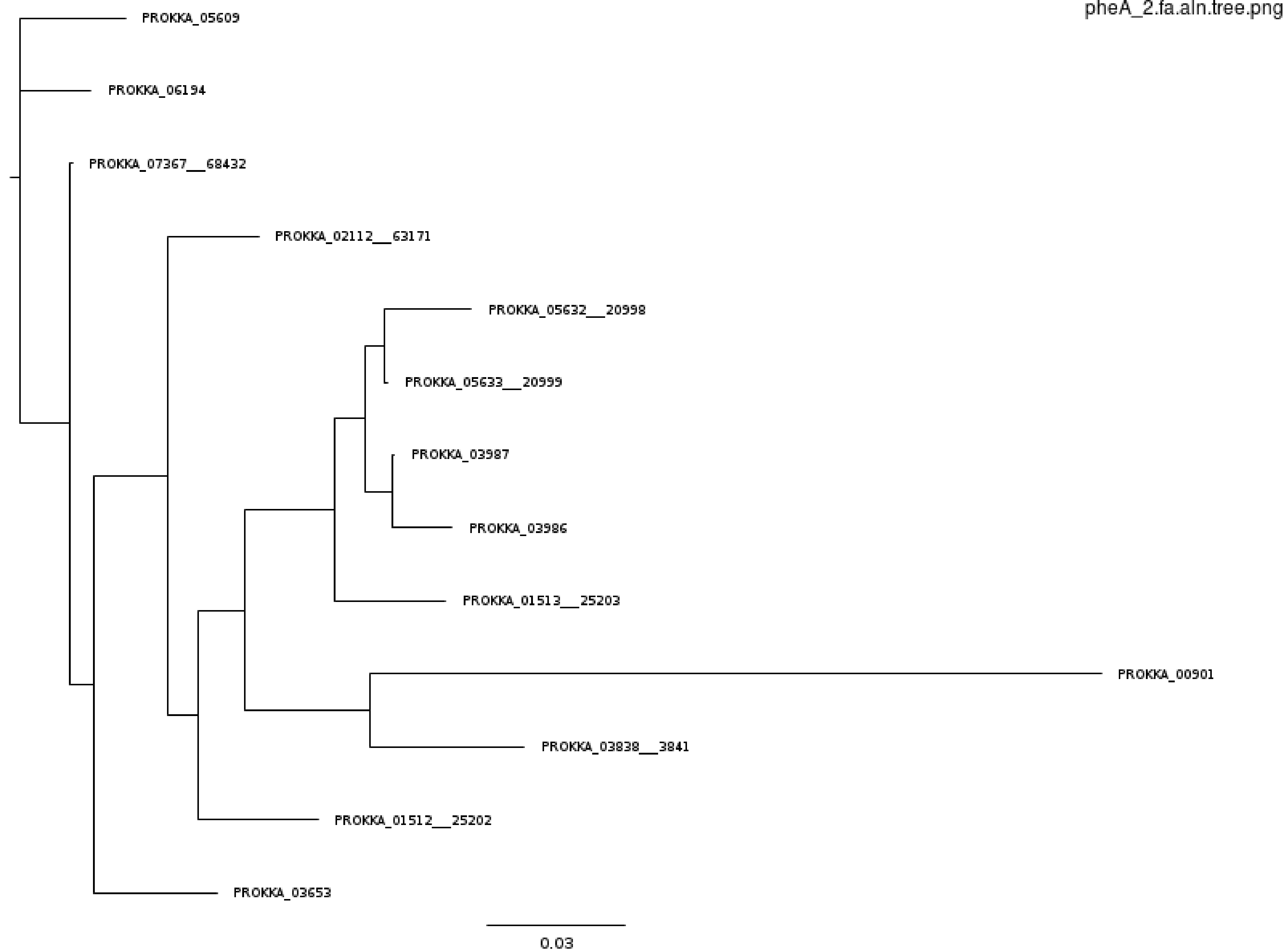
pheA_2.fa.aln.tree.png
PROKKA_05609
PROKKA_06194
PROKKA_07367___68432
PROKKA_02112___63171
PROKKA_05632___20998
PROKKA_05633___20999
PROKKA_03987
PROKKA_03986
PROKKA_01513___25203
PROKKA_00901
PROKKA_03838___3841
PROKKA_01512___25202
PROKKA_03653
0.03

pheS_2.fa.aln.tree.png

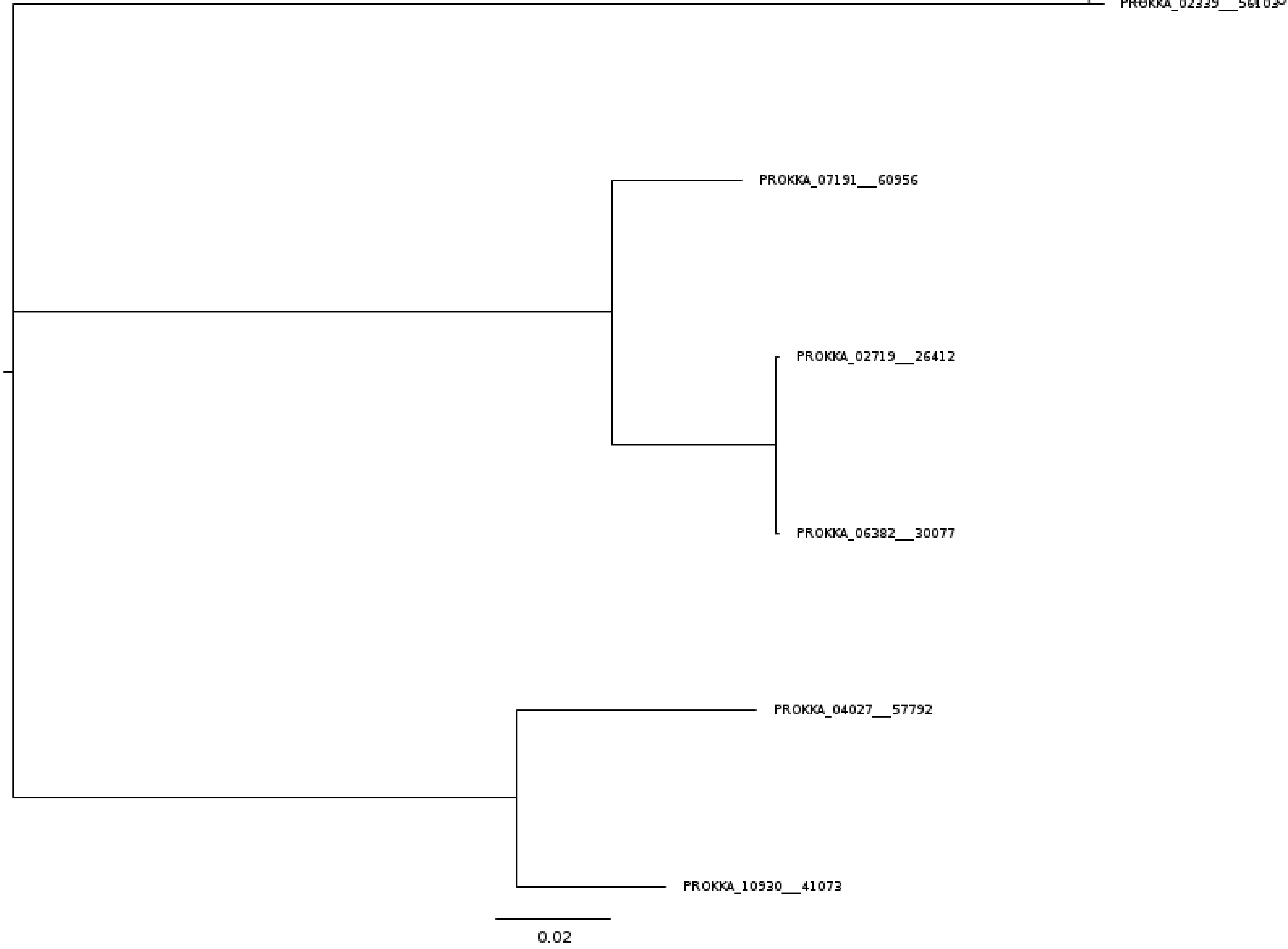

phoB_1.fa.aln.tree.png

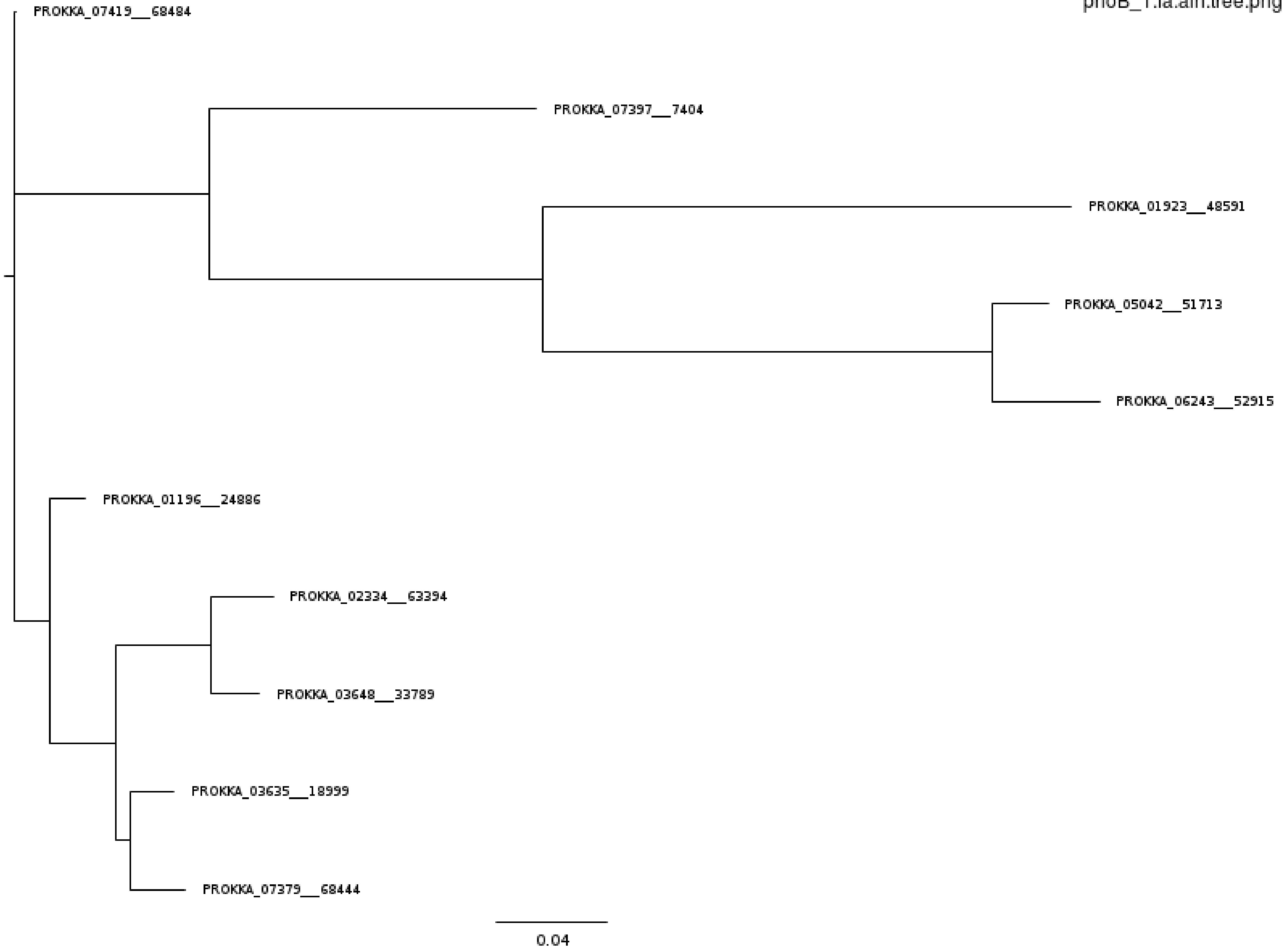

phoB_4.fa.aln.tree.png

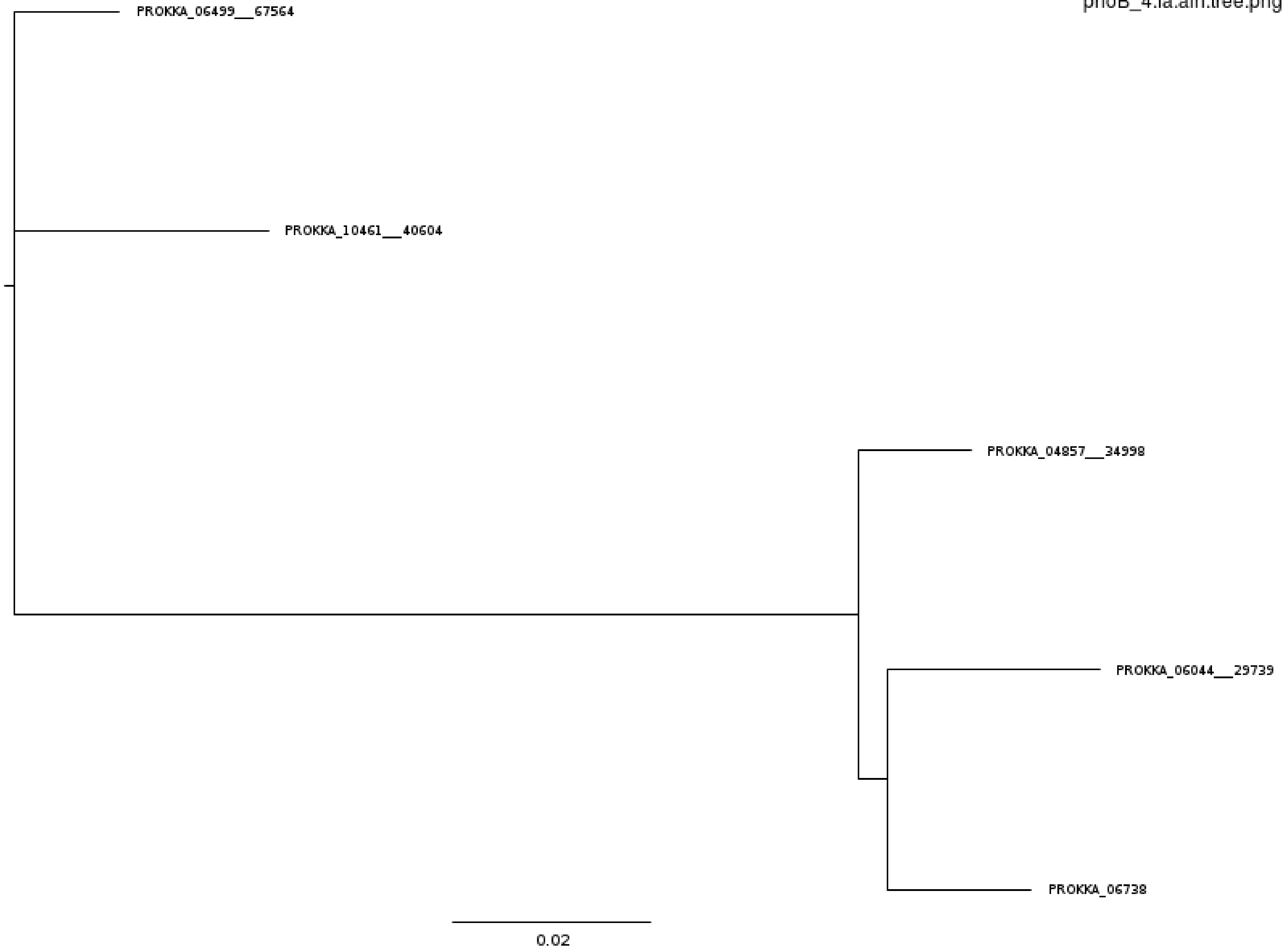

phoP_1.fa.aln.tree.png

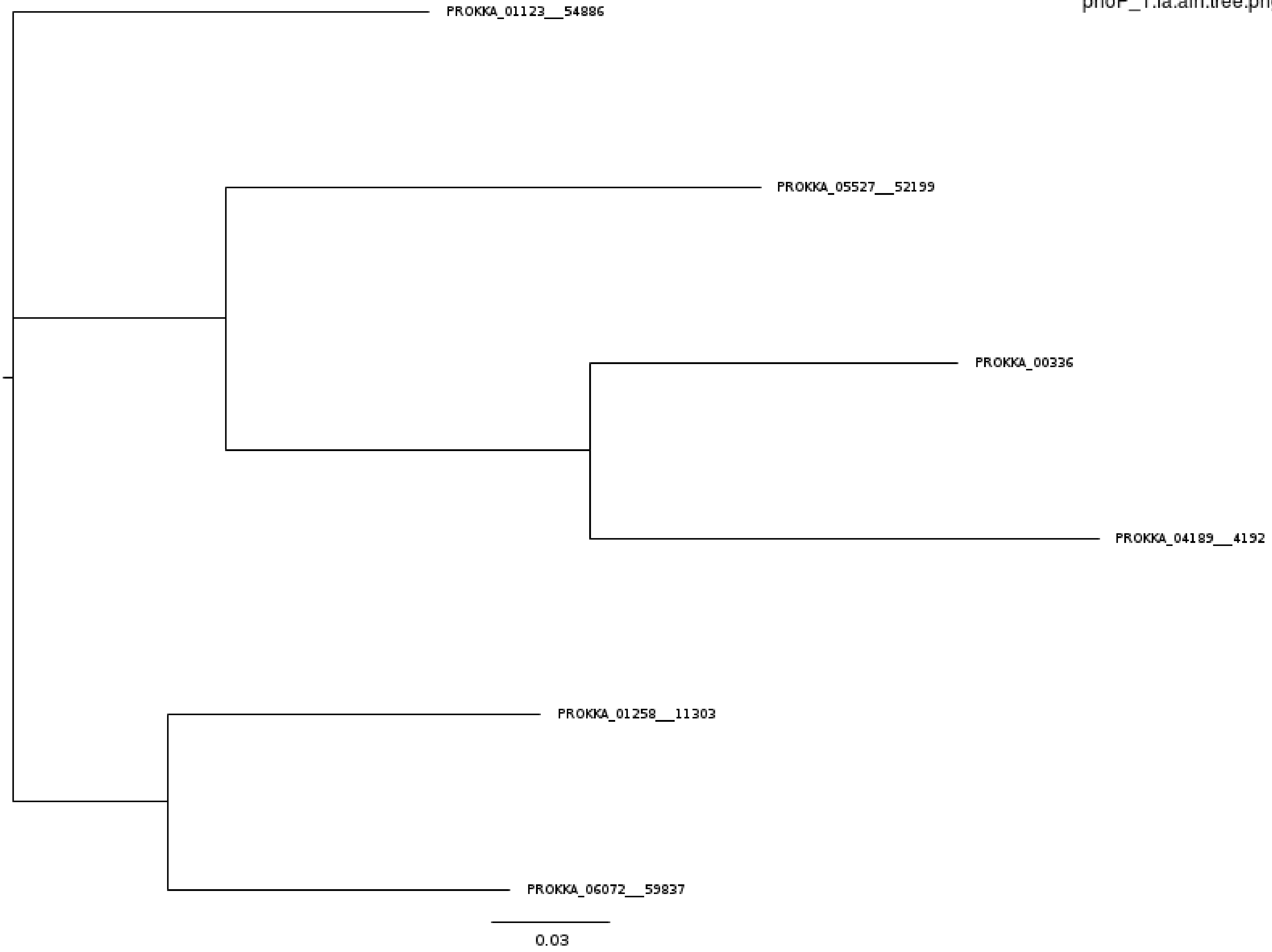

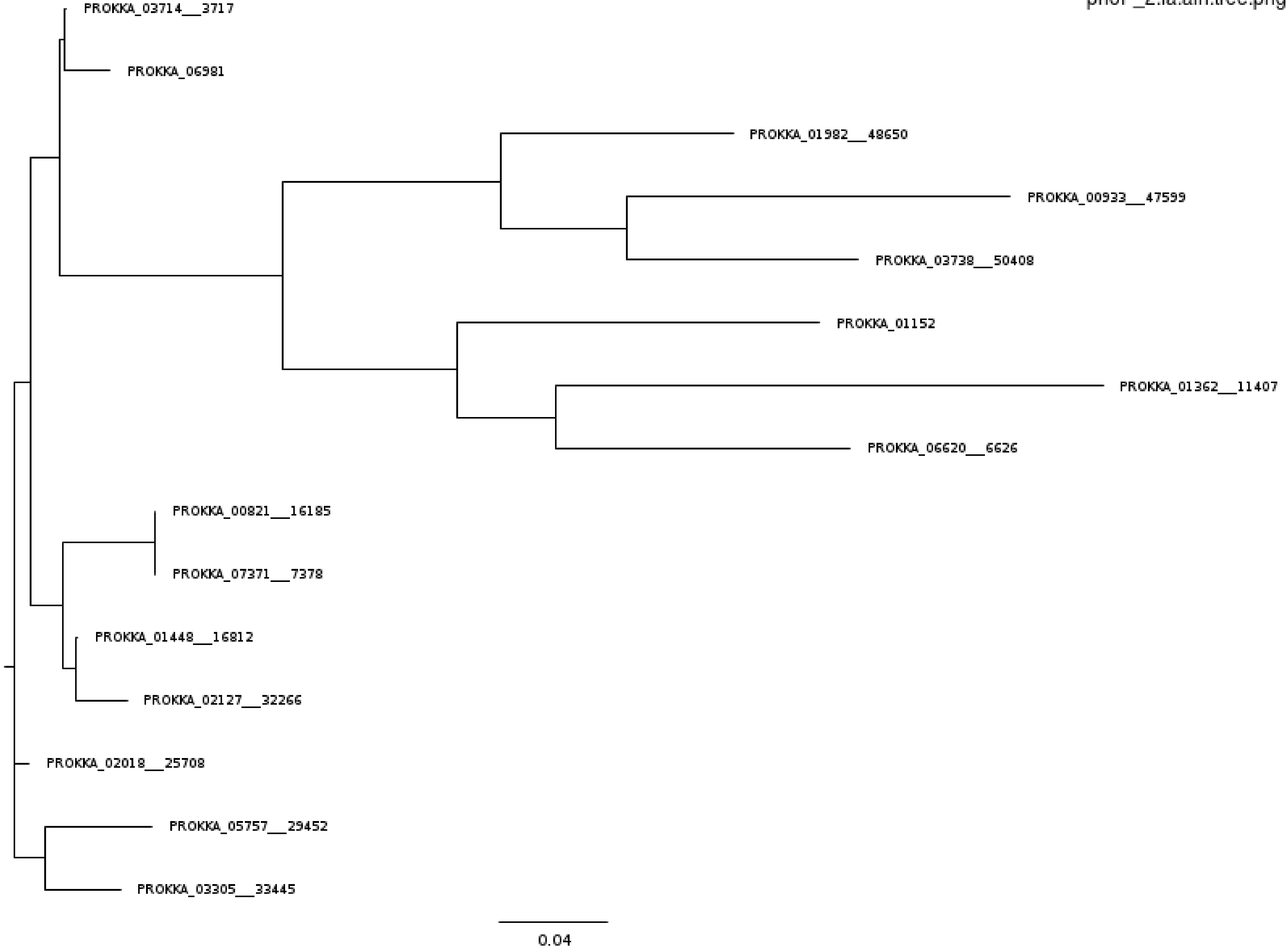
PROKKA_03714___3717
PROKKA_06981
PROKKA_01982___48650
PROKKA_00933___47599
PROKKA_03738___50408
PROKKA_01152
PROKKA_01362___11407
PROKKA_06620___6626
PROKKA_00821___16185
PROKKA_07371___7378
PROKKA_01448___16812
PROKKA_02127___32266
PROKKA_02018___25708
PROKKA_05757___29452
PROKKA_03305___33445
0.04

phoP_3.fa.aln.tree.png

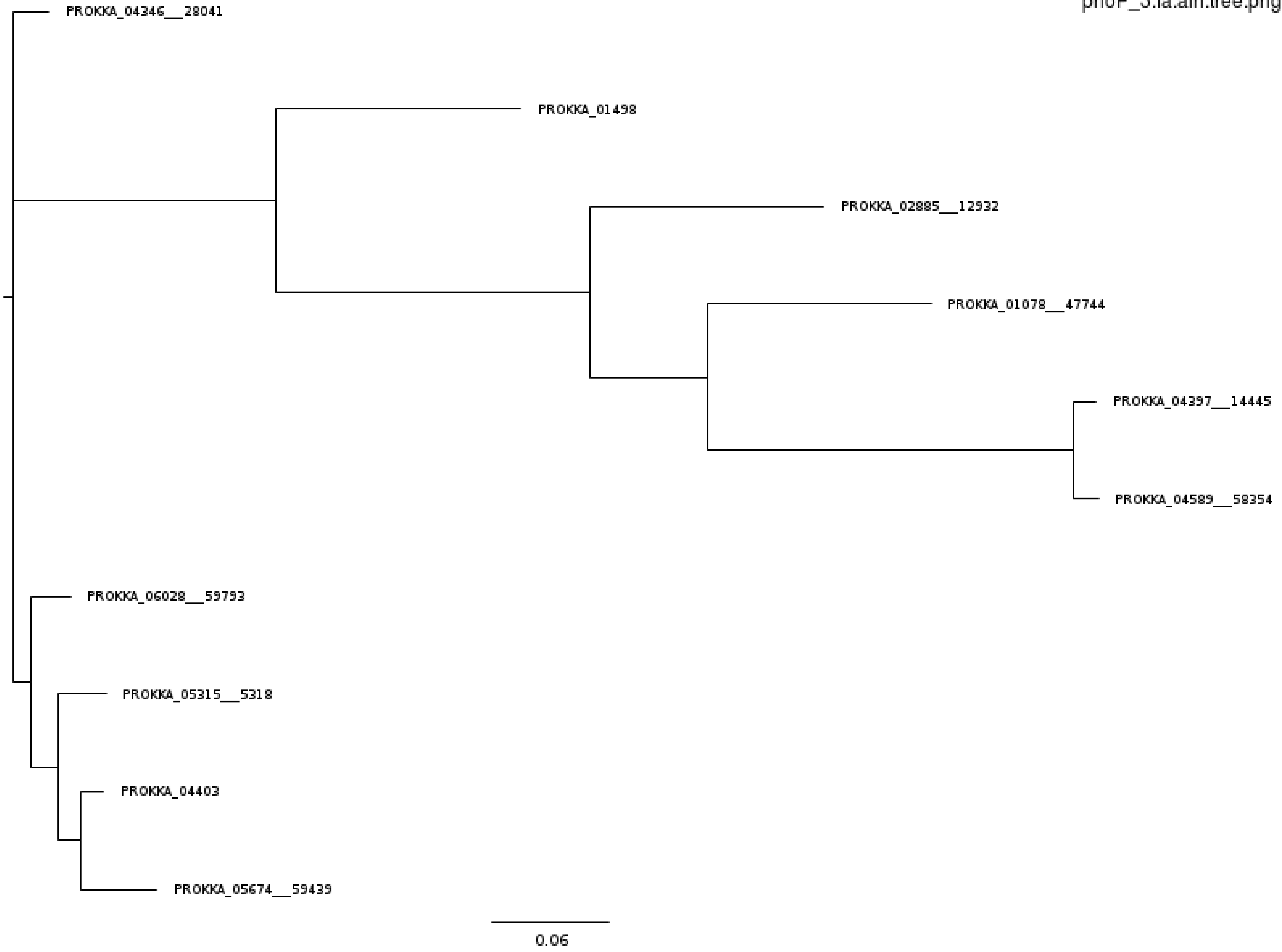

phoQ_2.fa.aln.tree.png

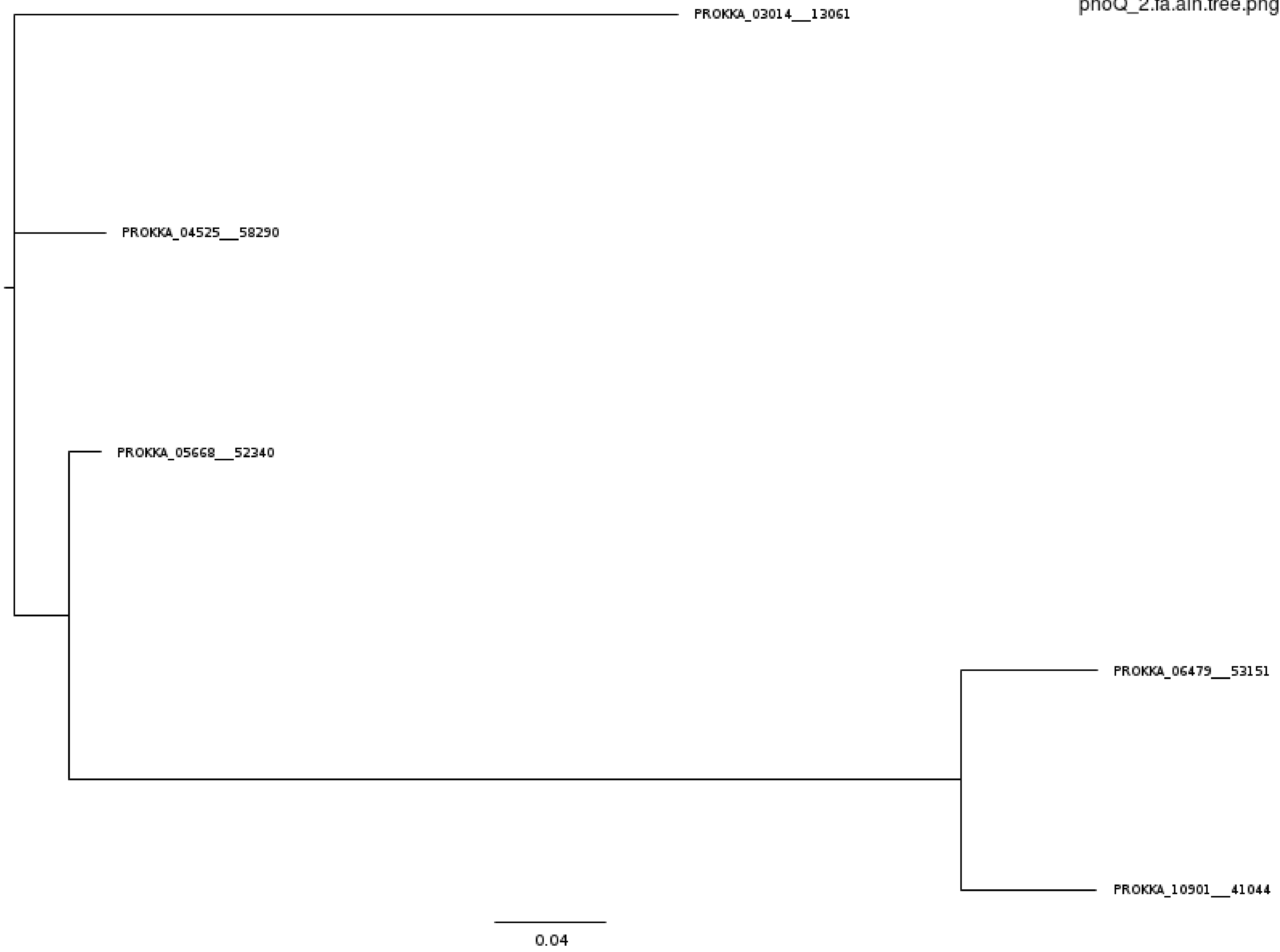

phoQ_4.fa.aln.tree.png

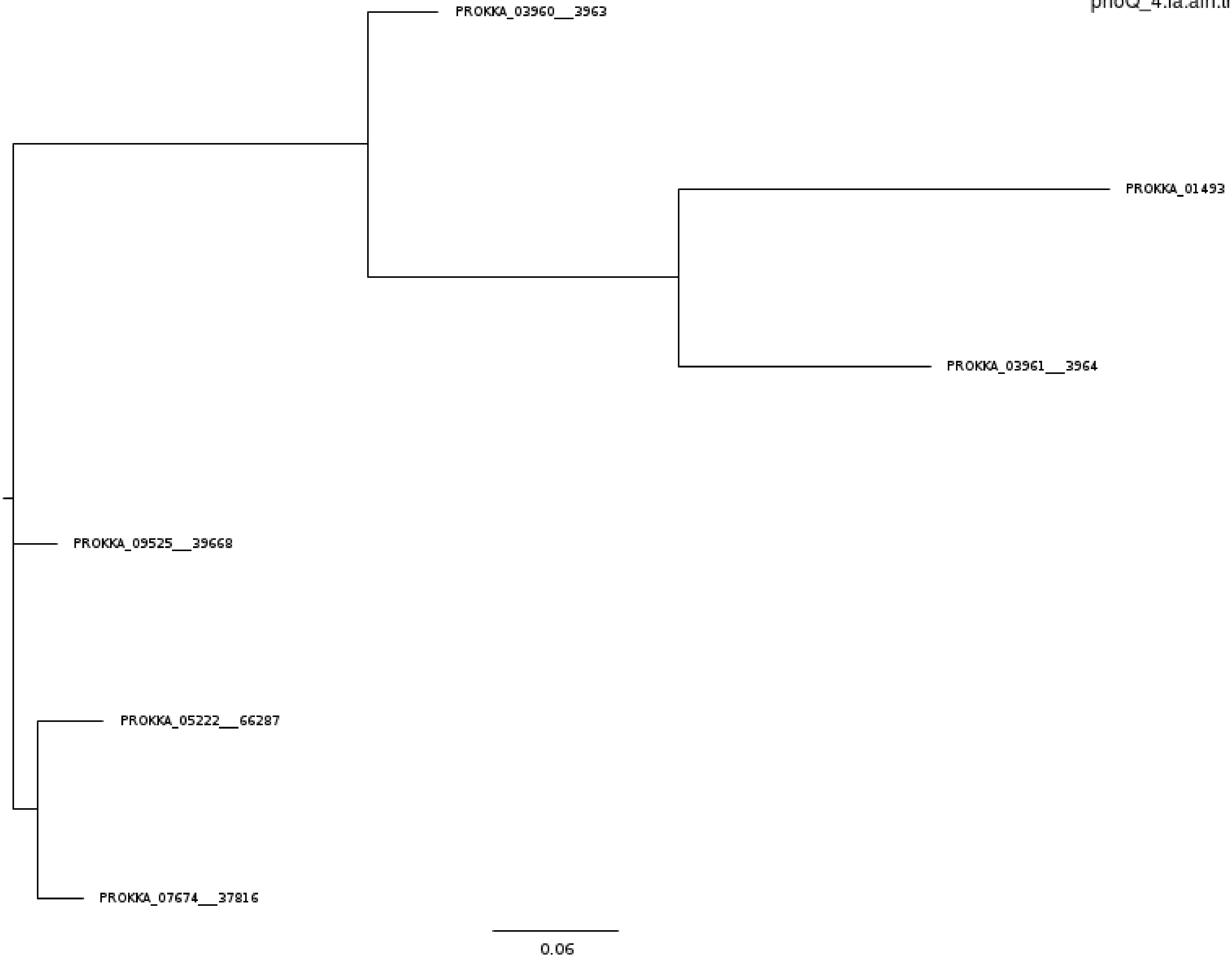

phoR_3.fa.aln.tree.png

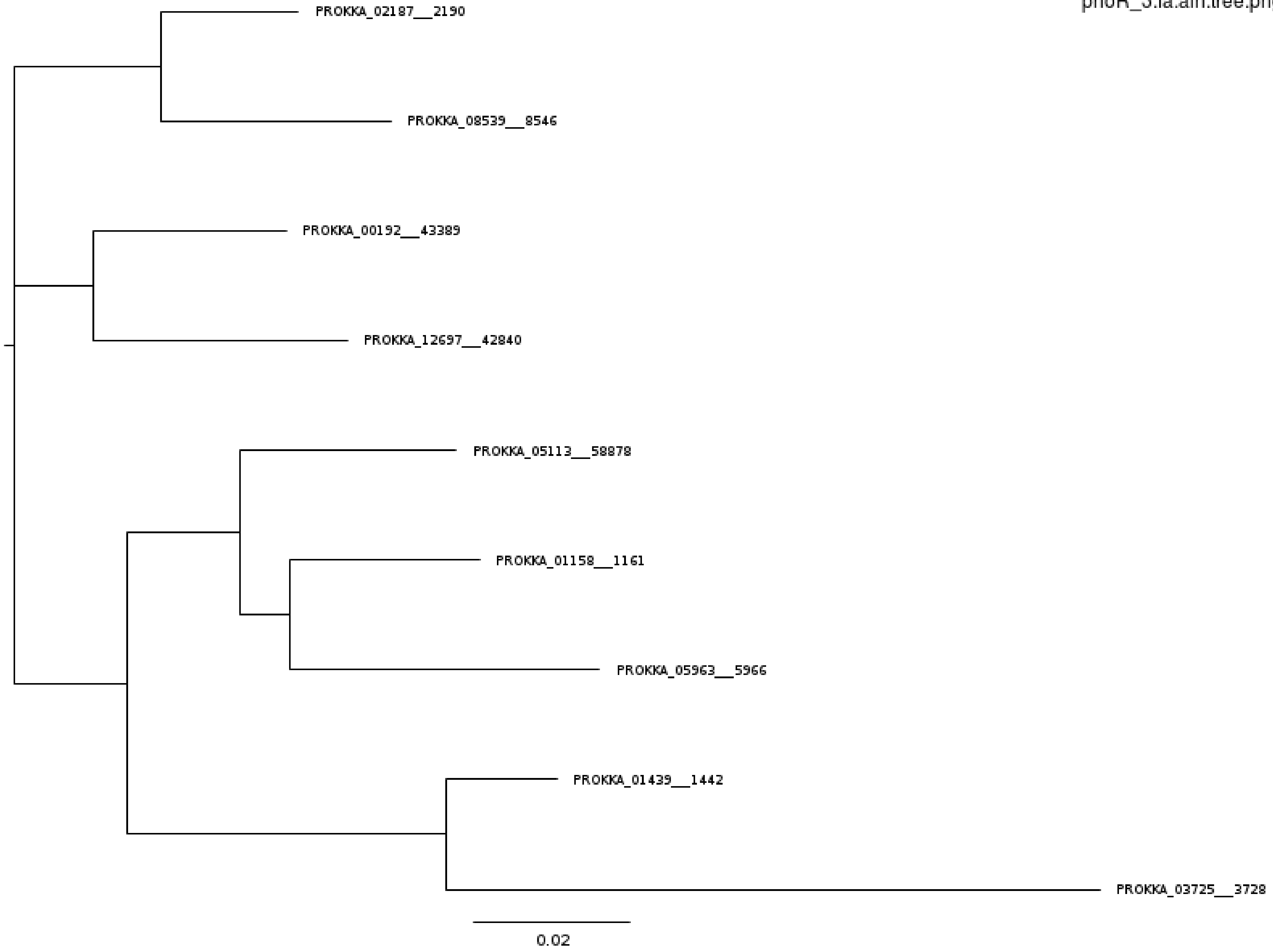

phoU_2.fa.aln.tree.png

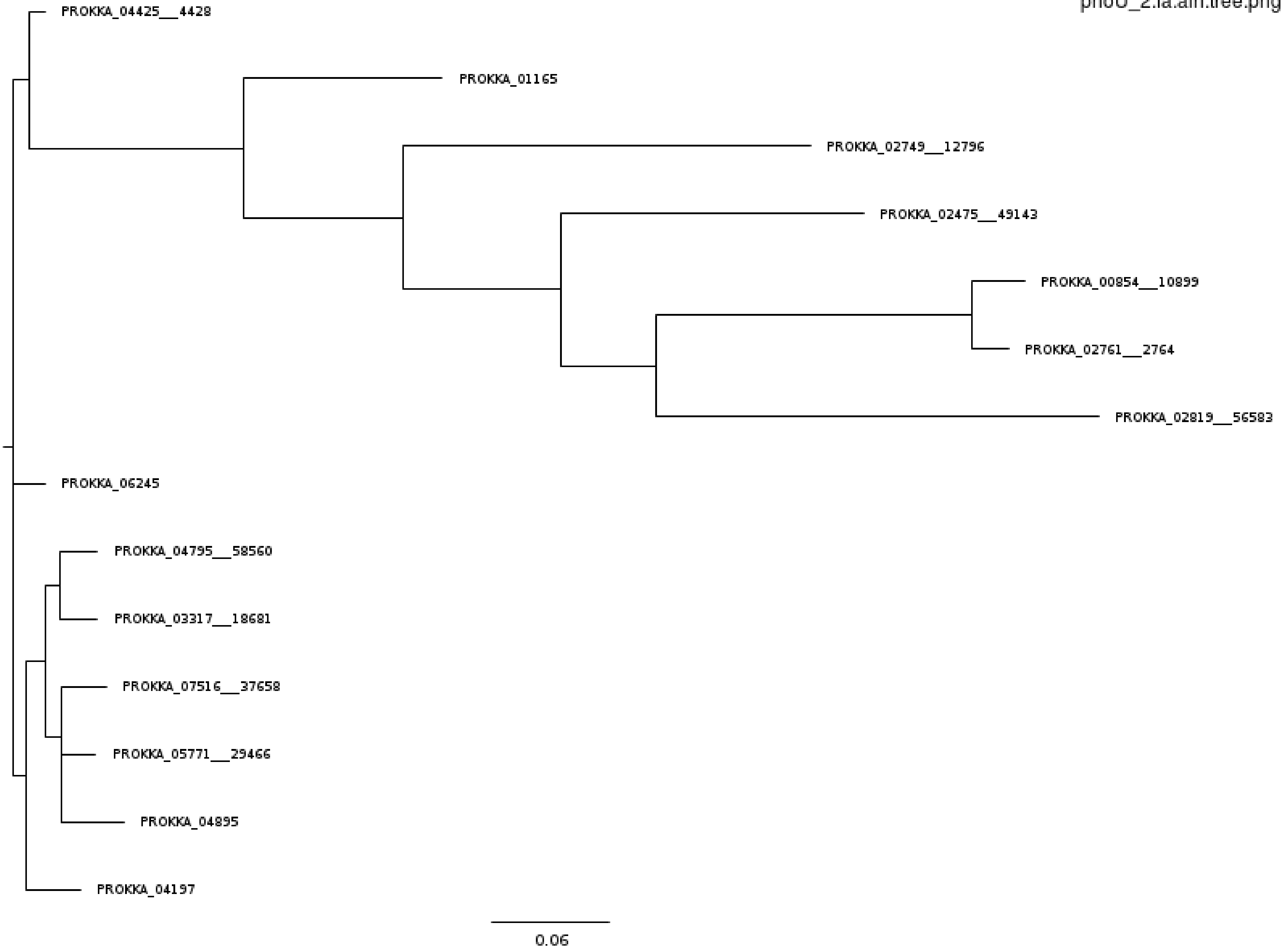

pilQ_8.fa.aln.tree.png

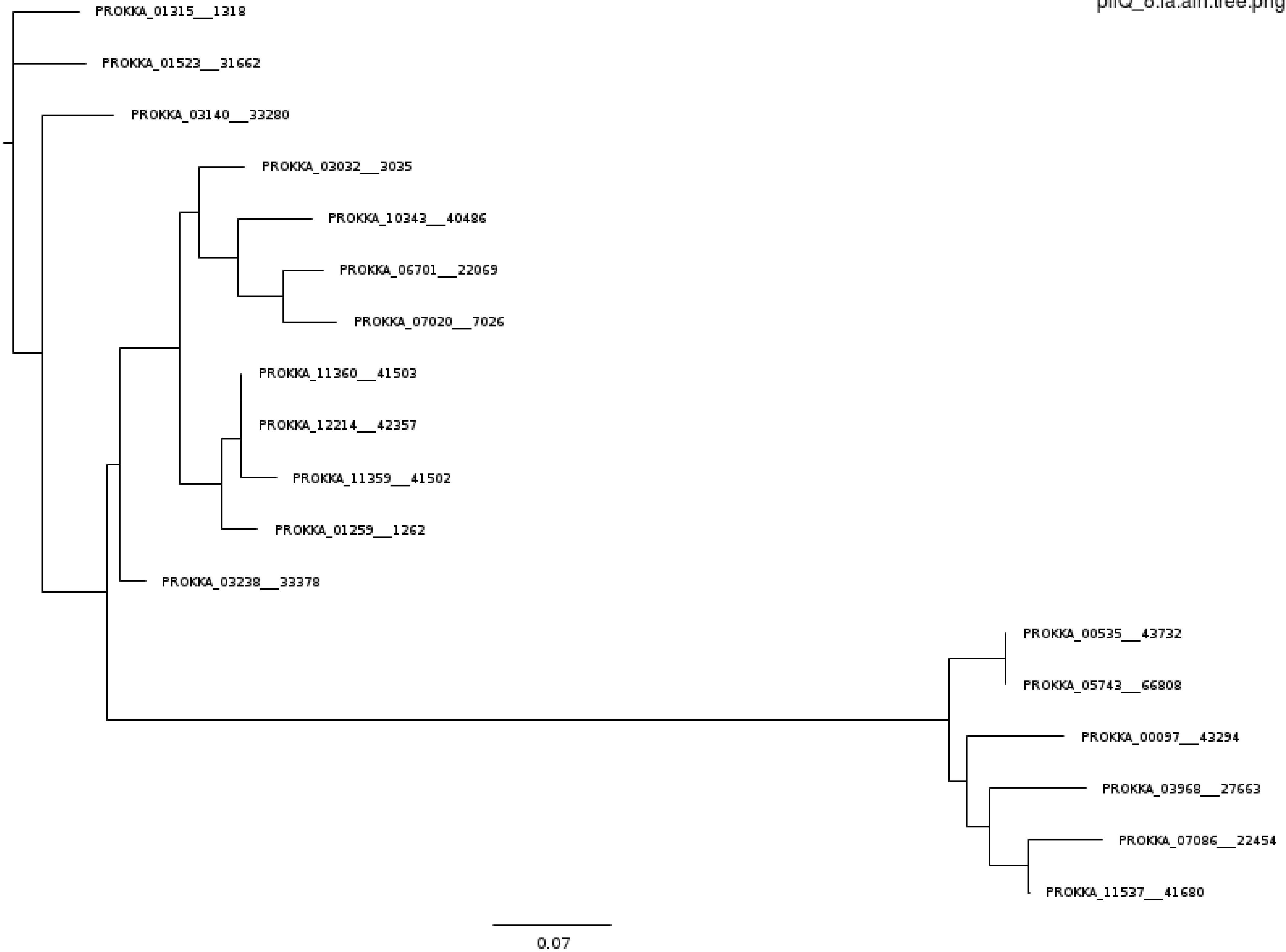

pilT_11.fa.aln.tree.png

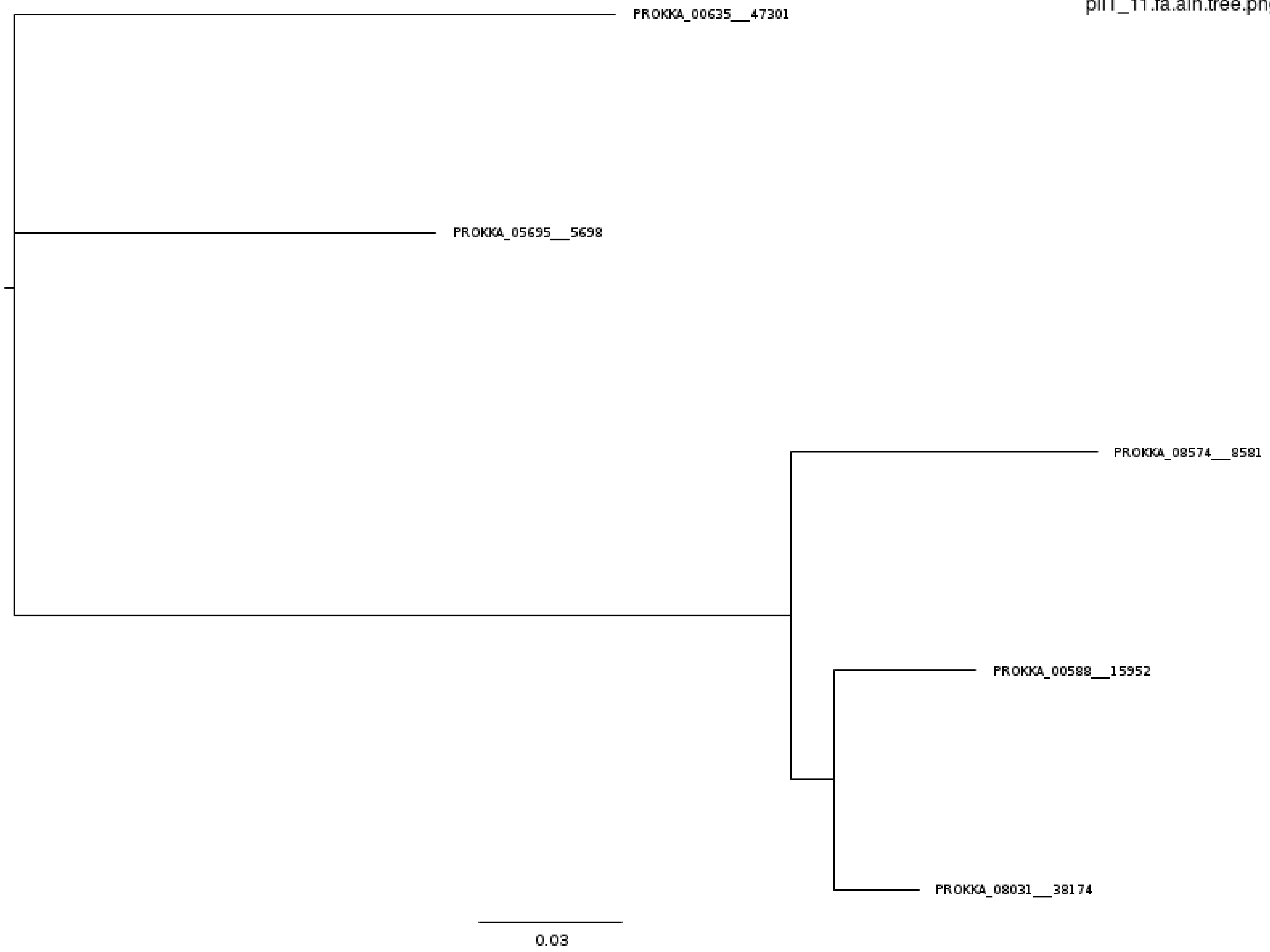

pilT_2.fa.aln.tree.png

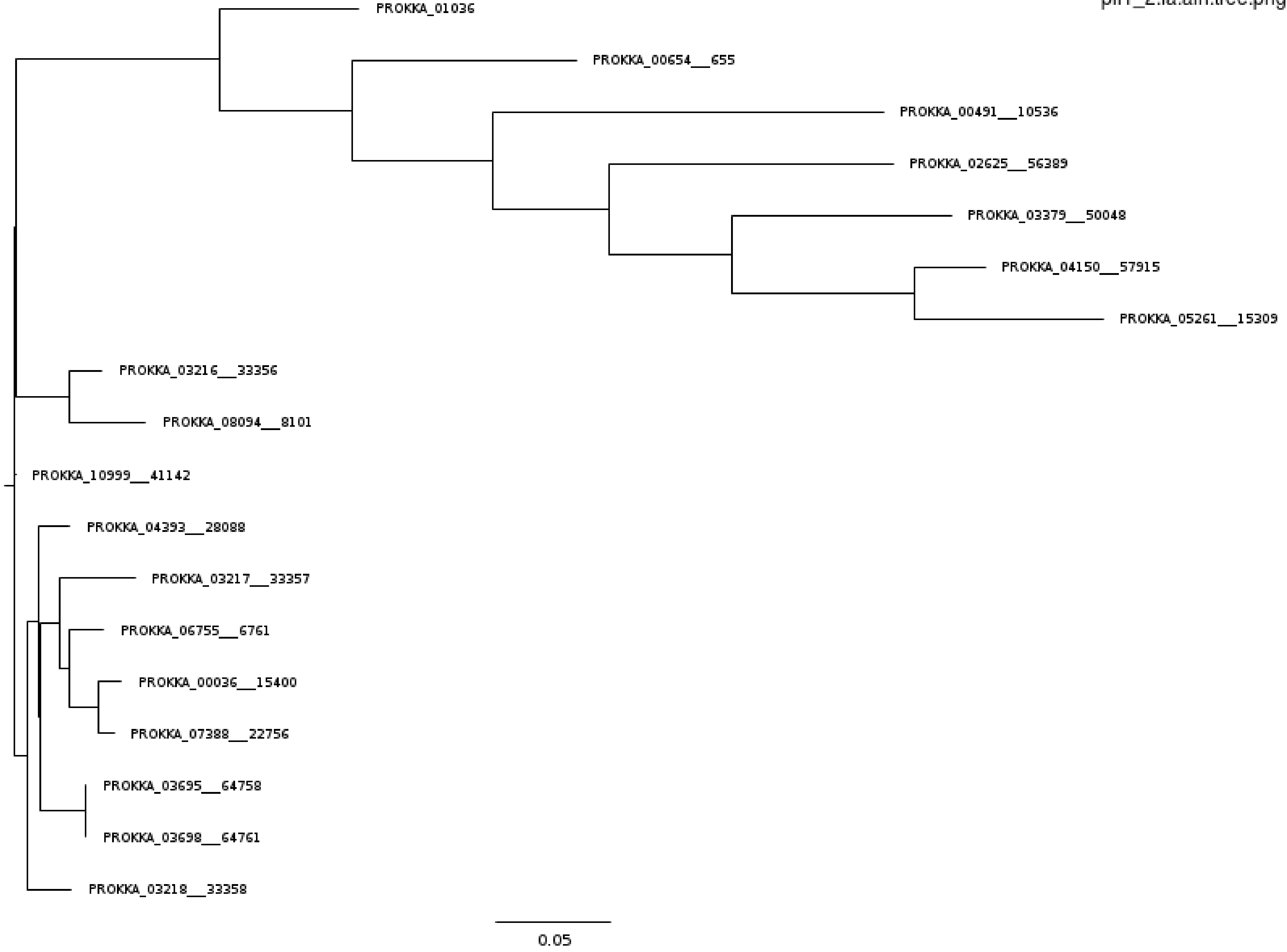

pilT_5.fa.aln.tree.png

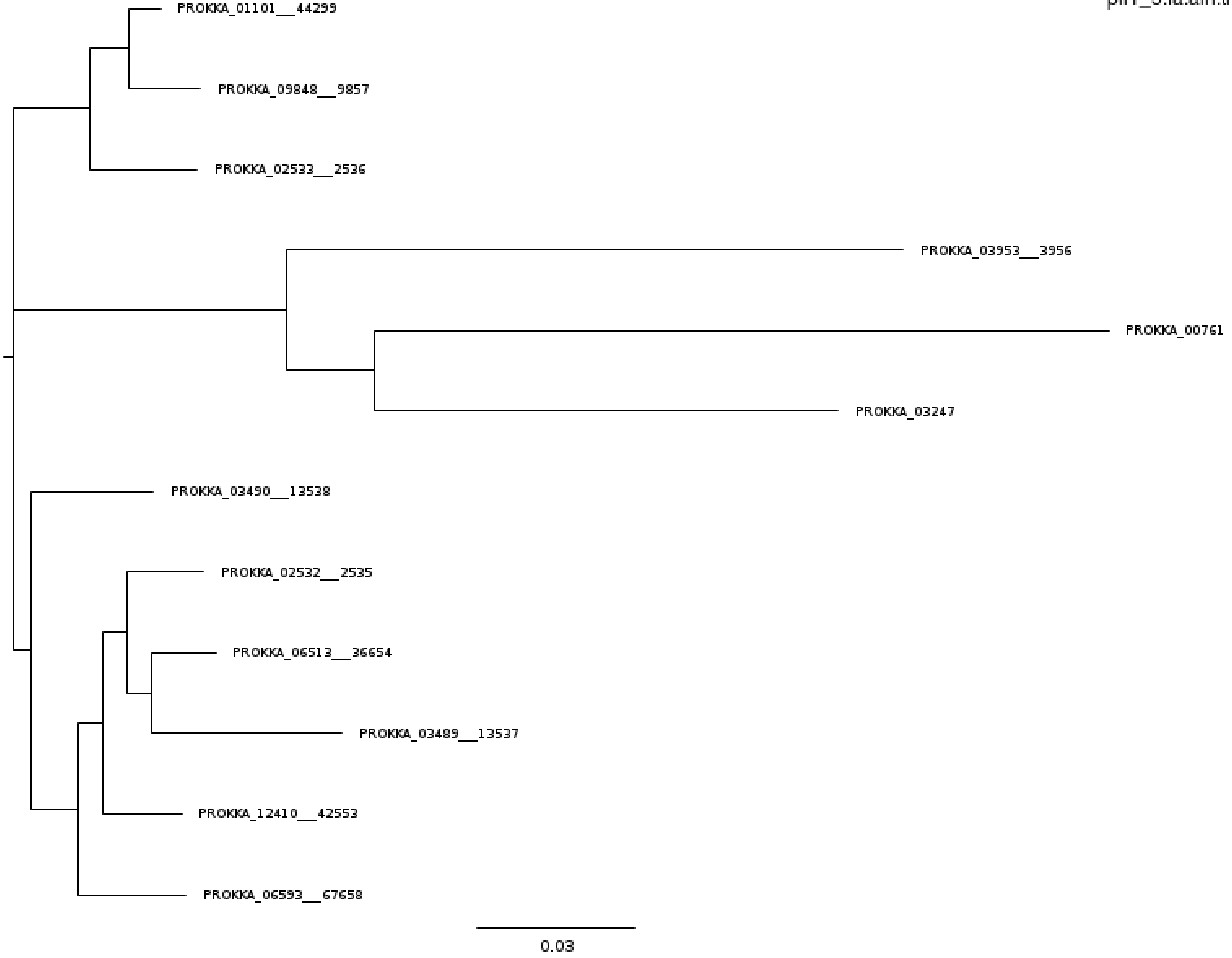

pilT_7.fa.aln.tree.png

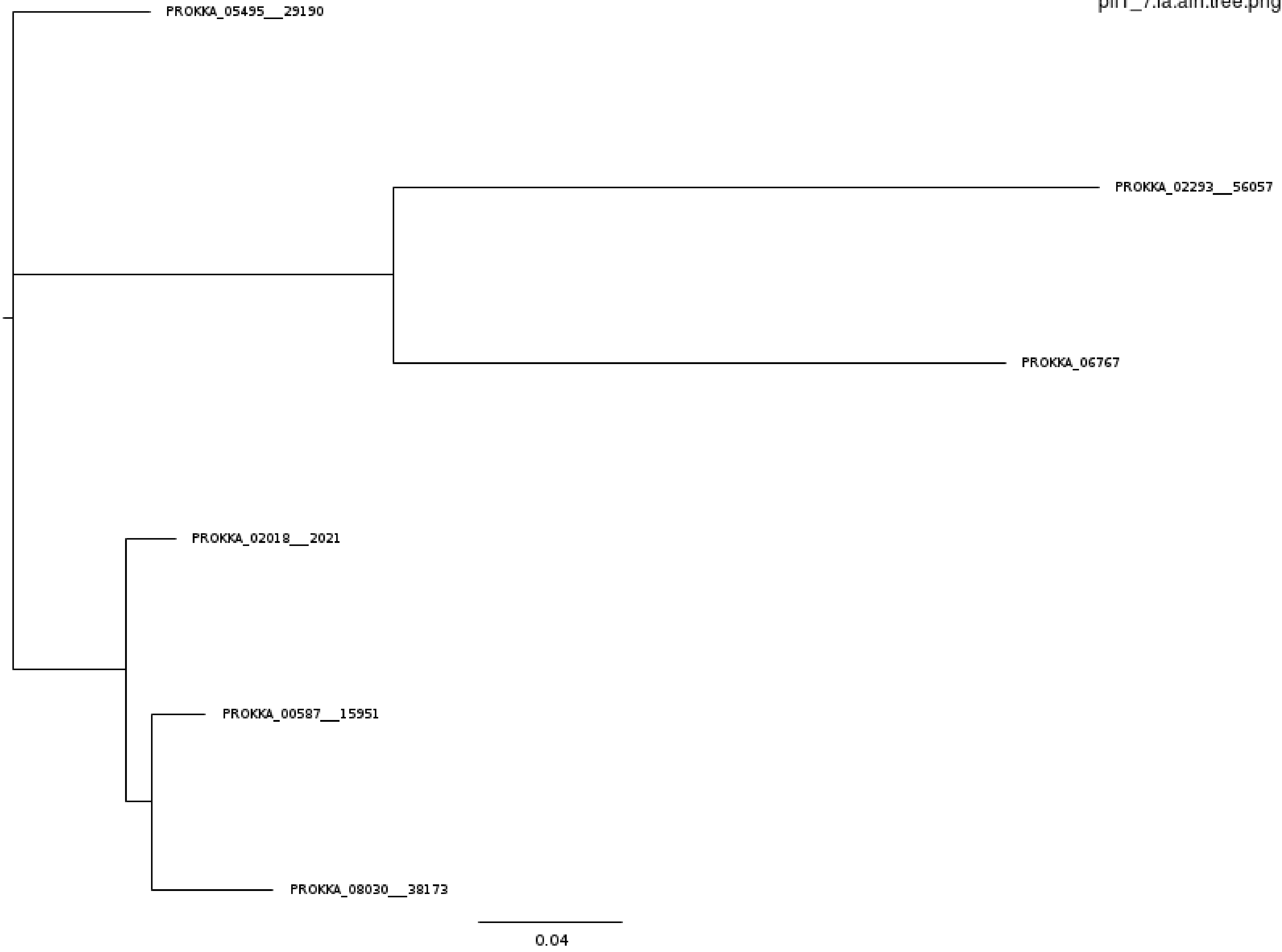

pipB2.fa.aln.tree.png

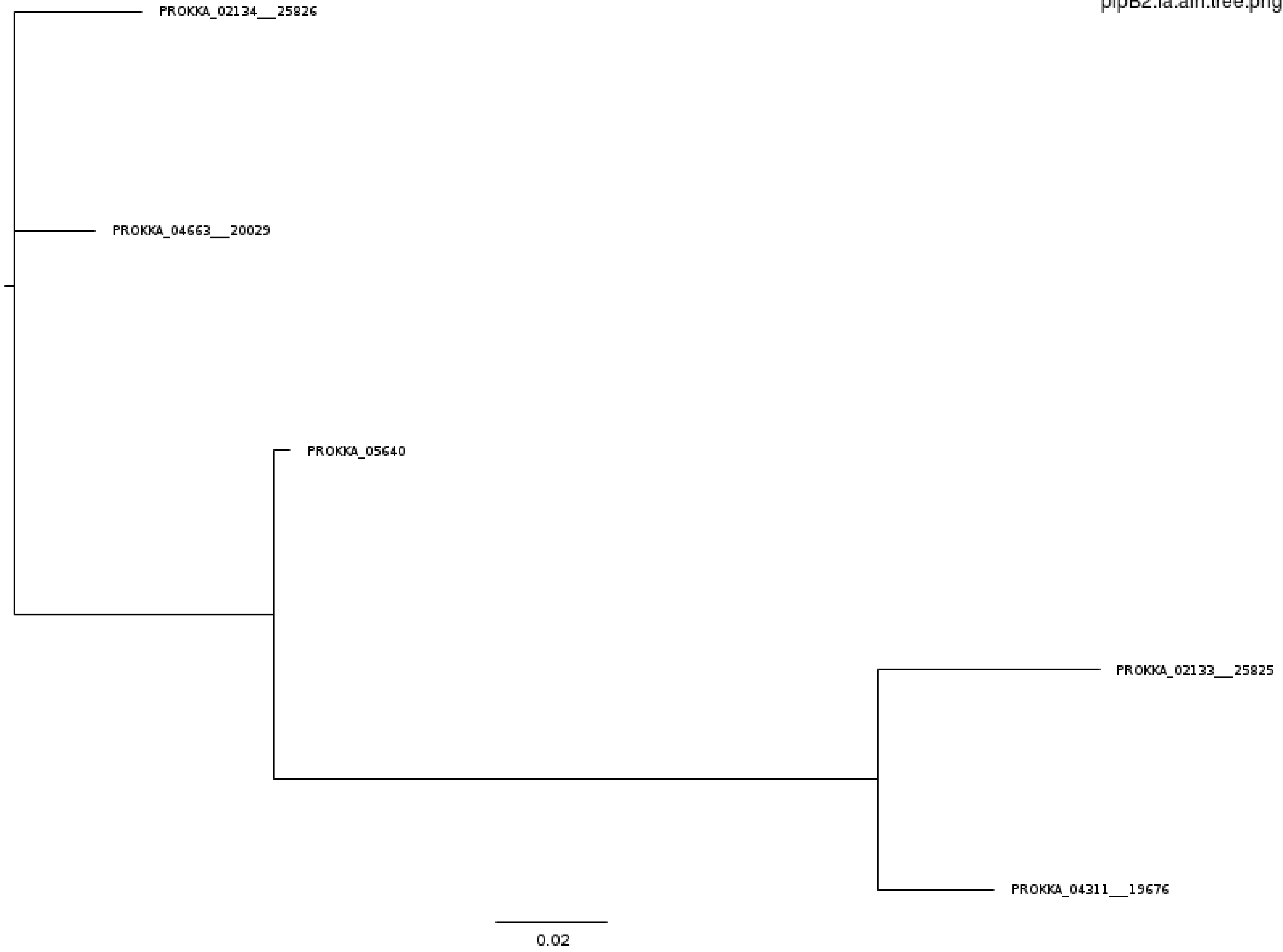

pkn1_11.fa.aln.tree.png

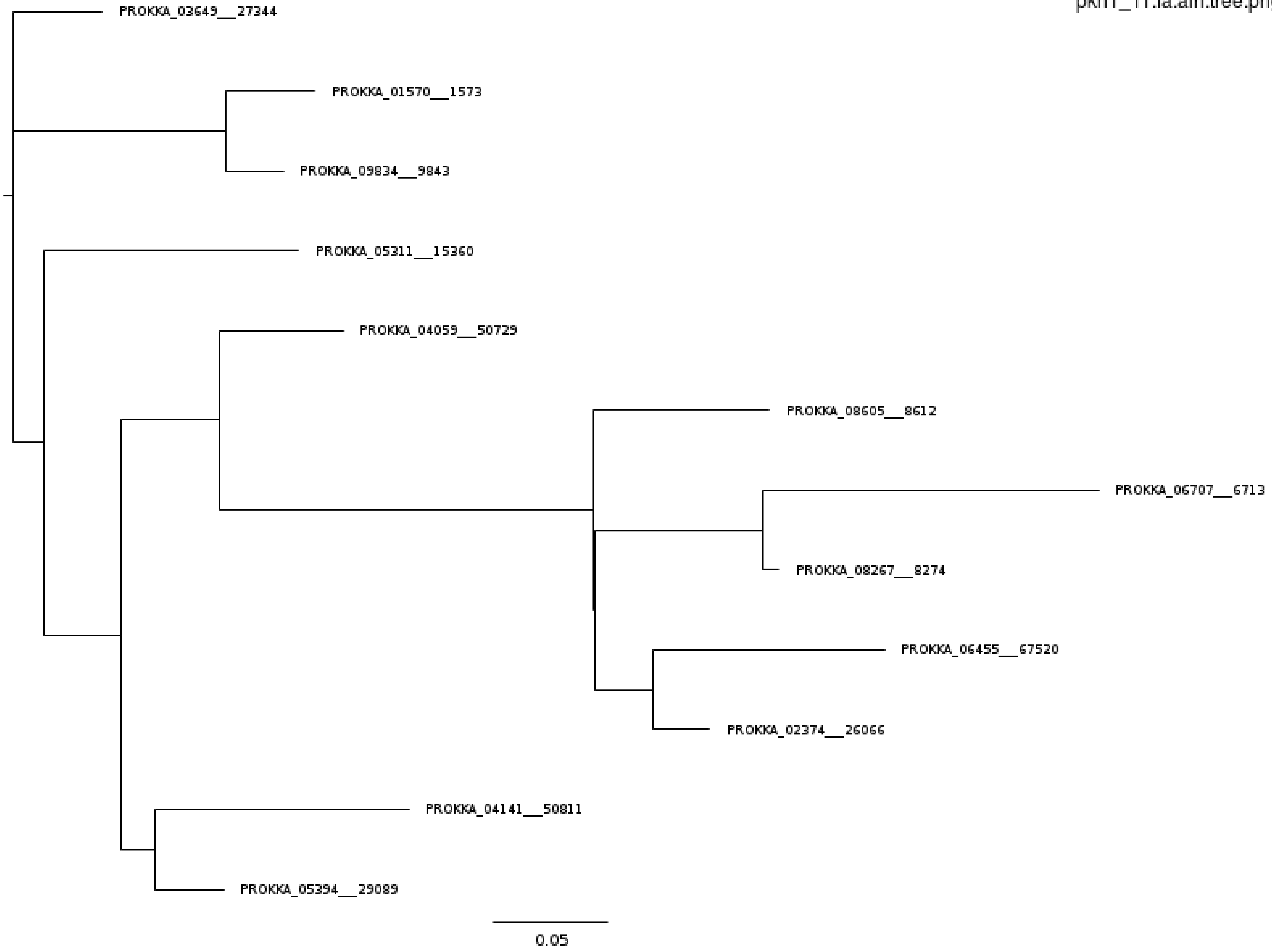

pkn1_13.fa.aln.tree.png

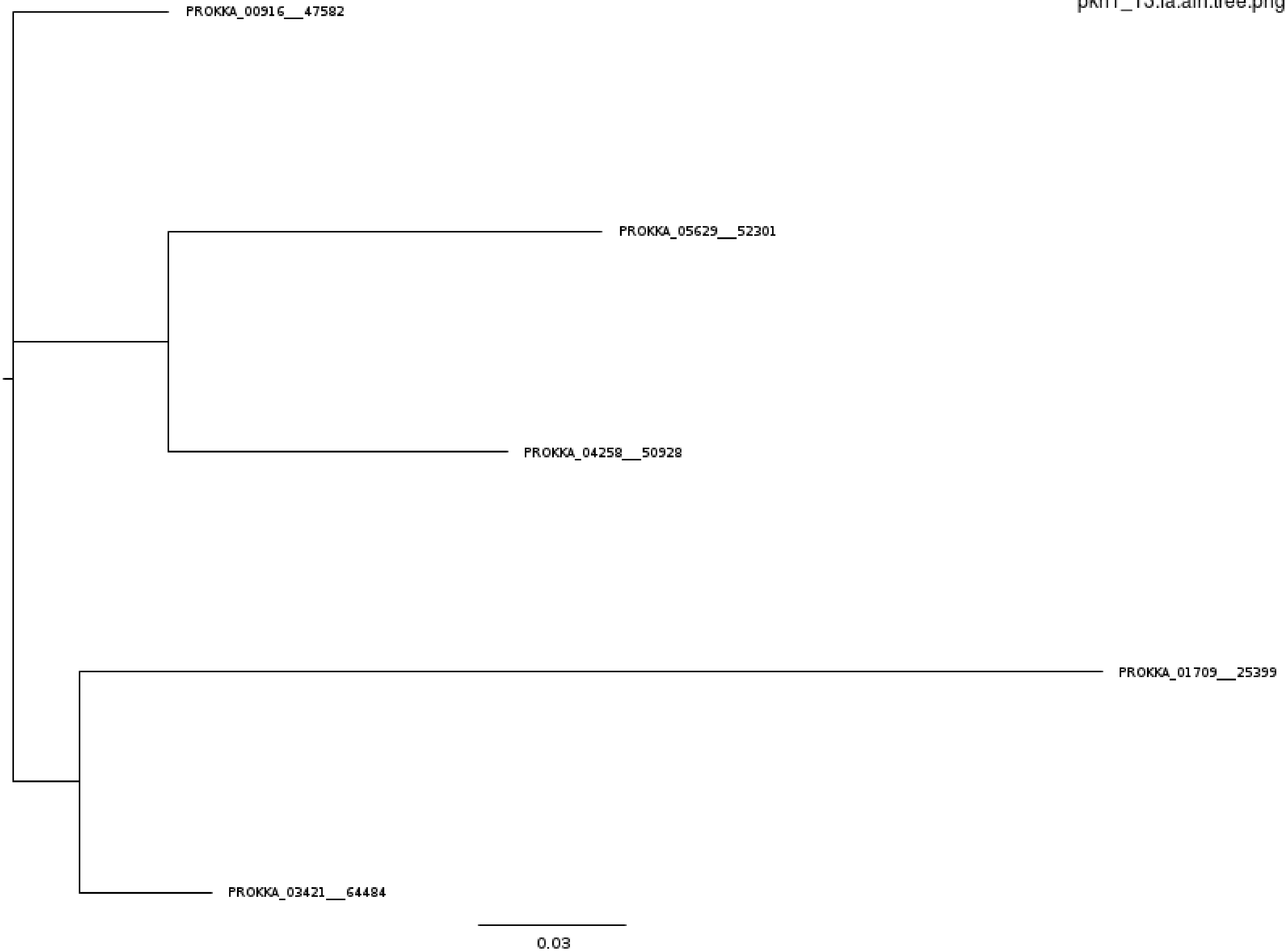

pkn1_15.fa.aln.tree.png

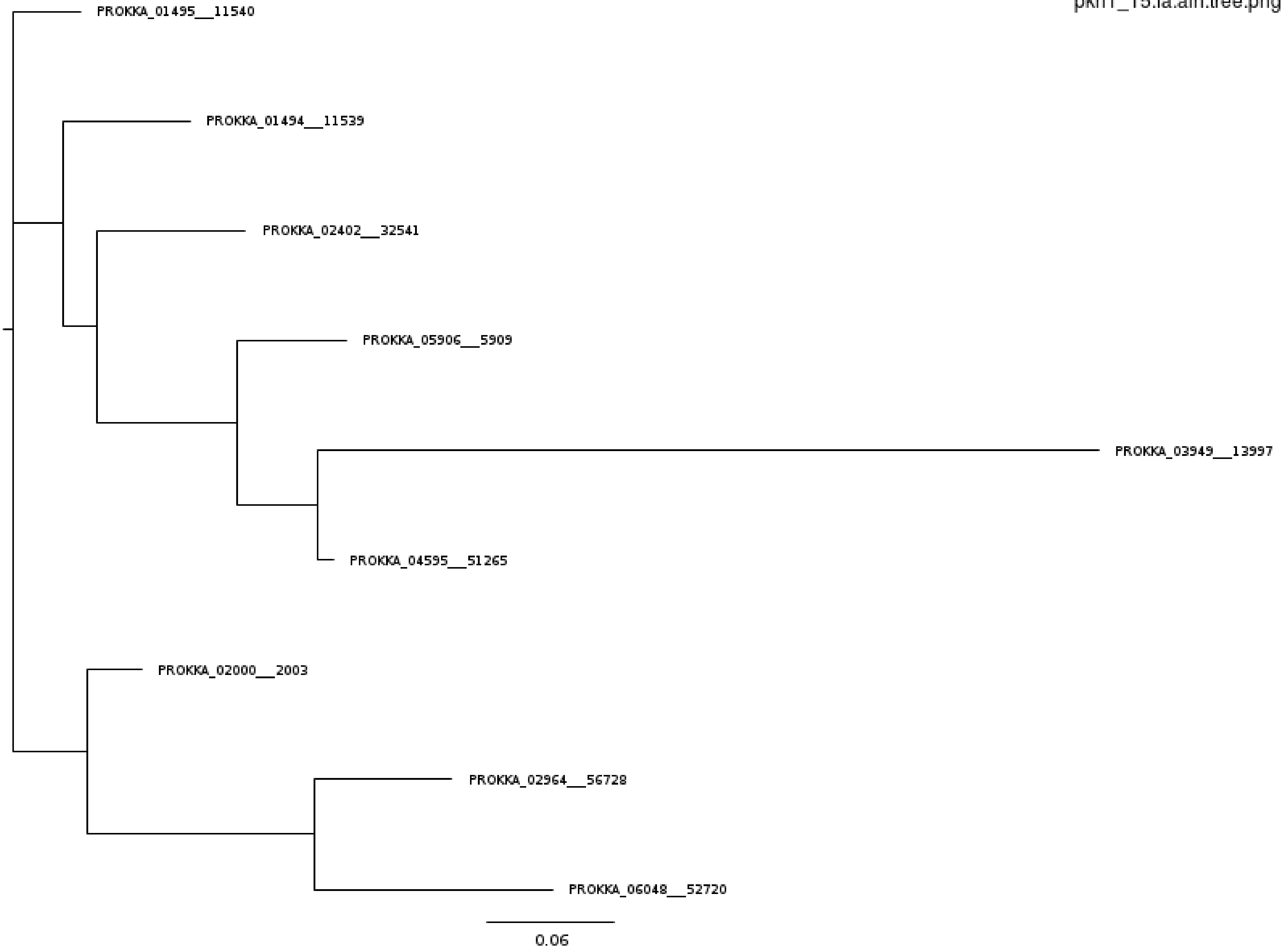

pkn1_16.fa.aln.tree.png

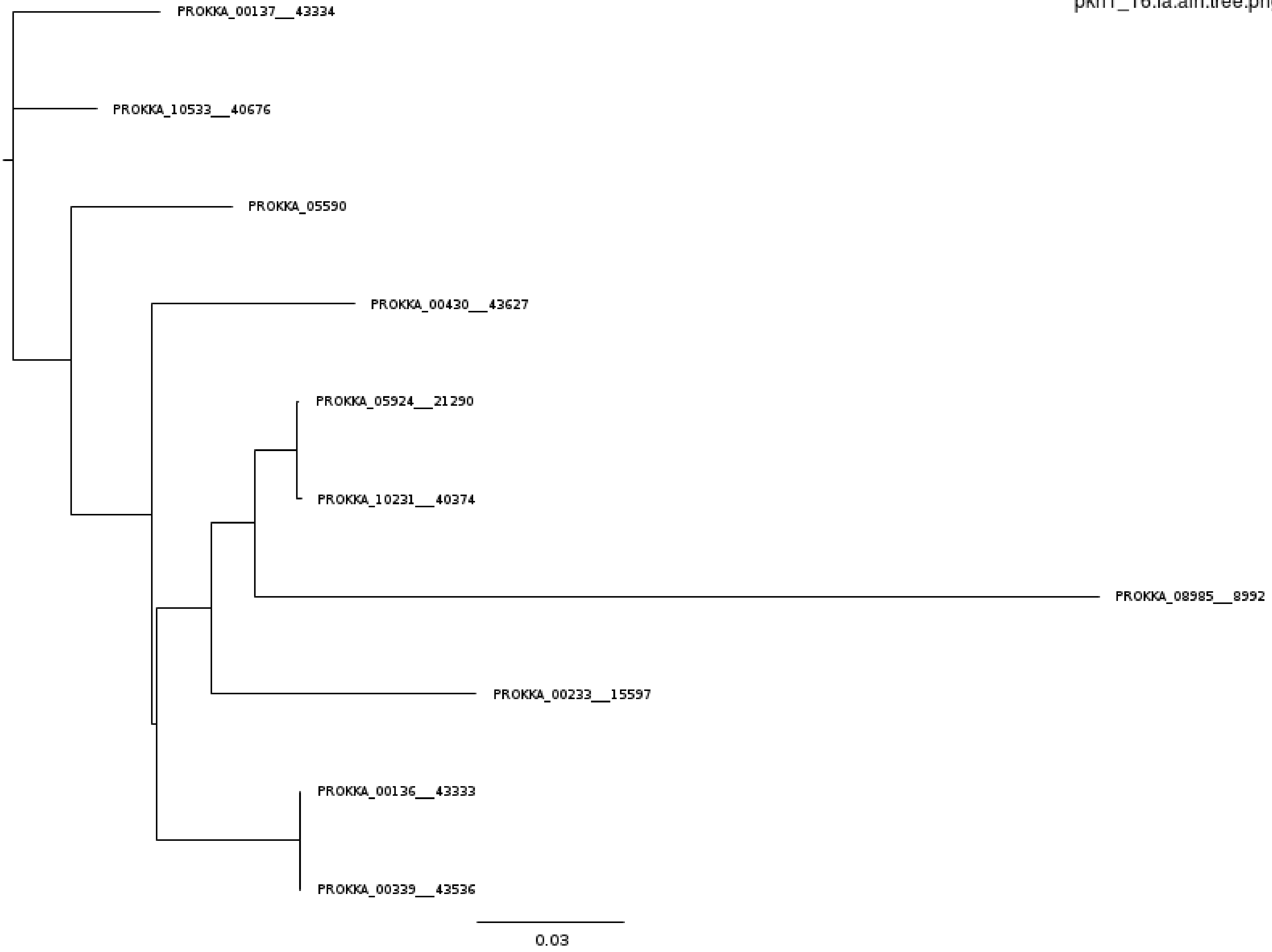

pkn1_17.fa.aln.tree.png

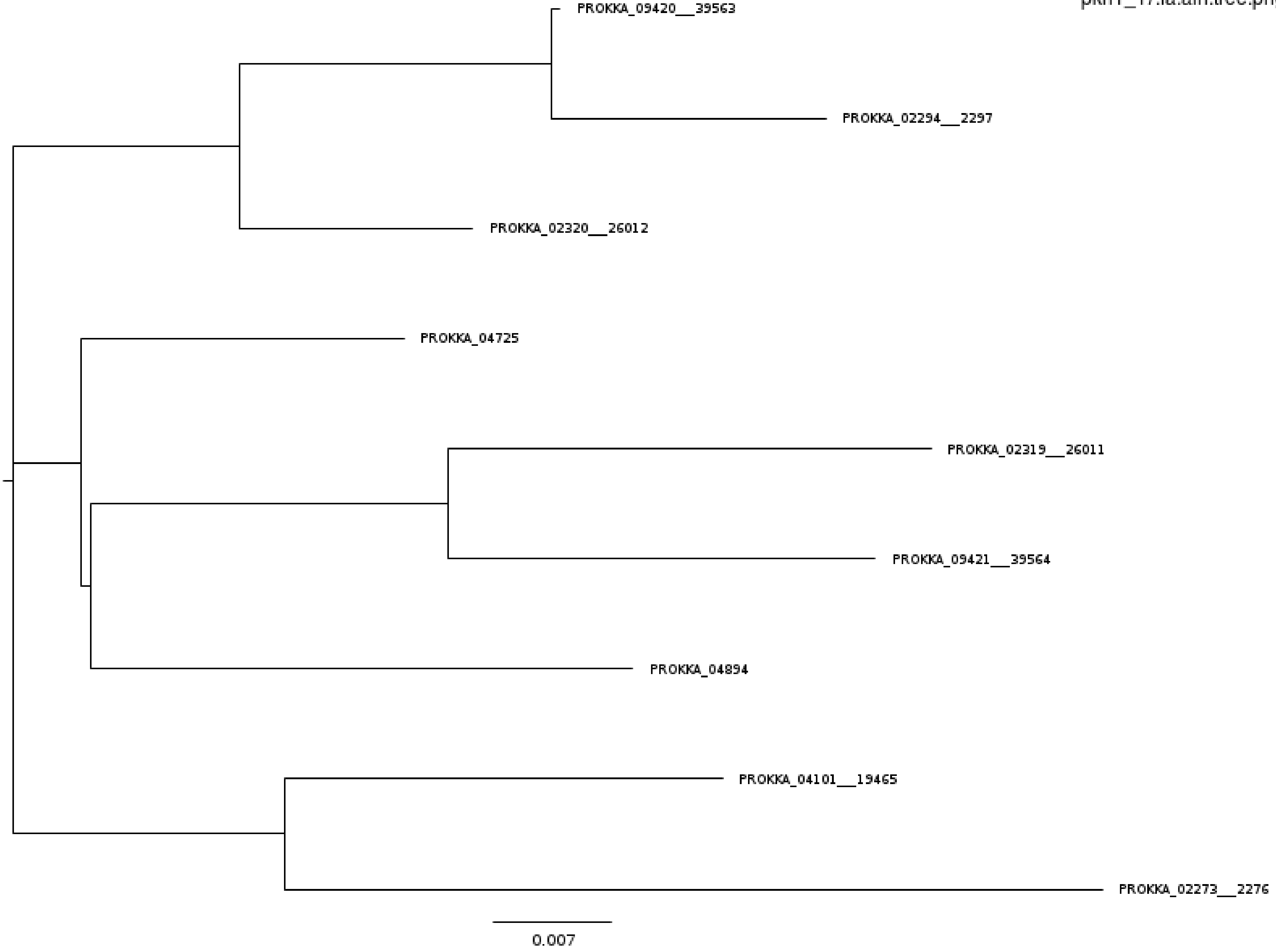

pkn1_1.fa.aln.tree.png

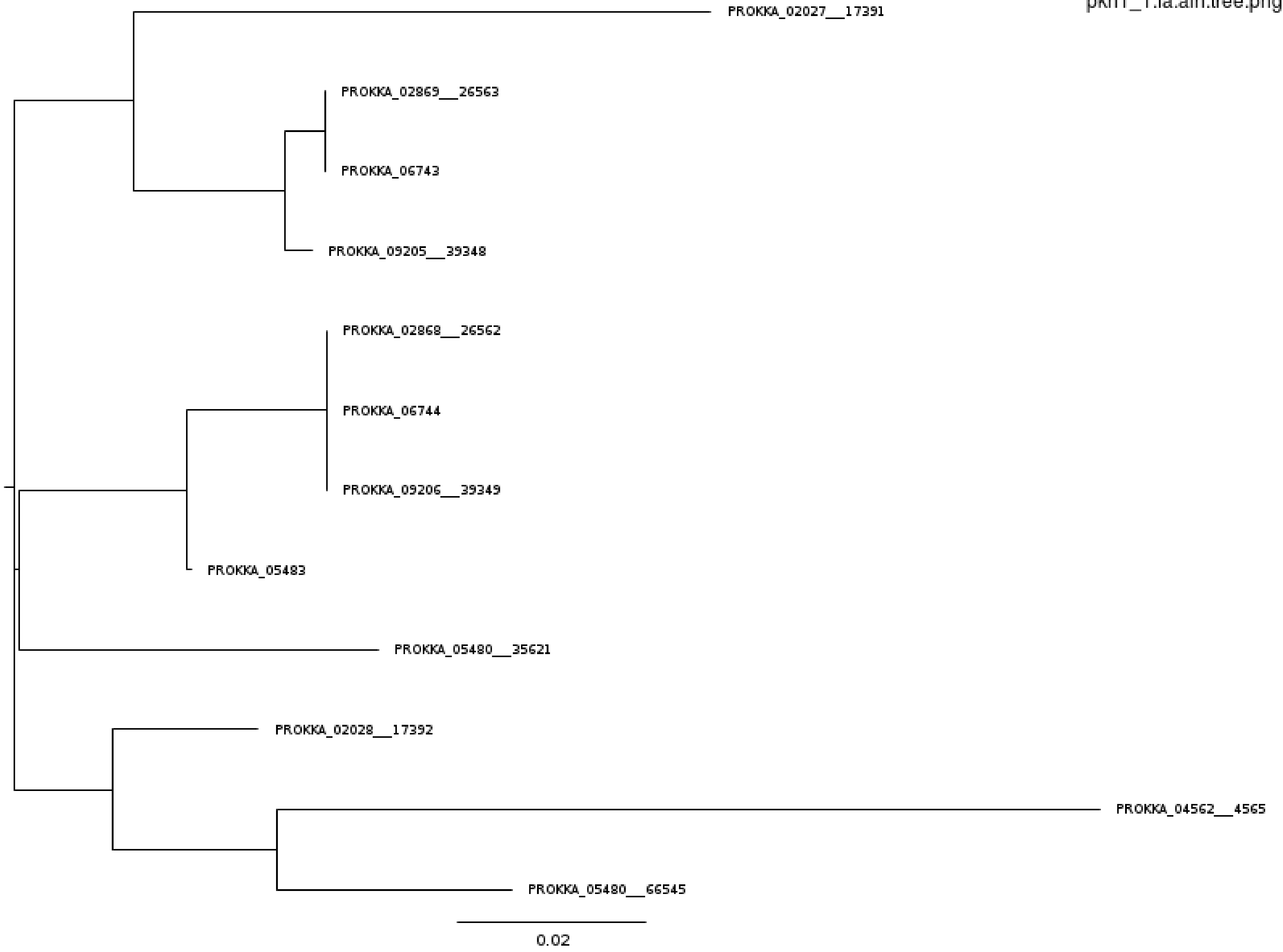

pkn1_20.fa.aln.tree.png

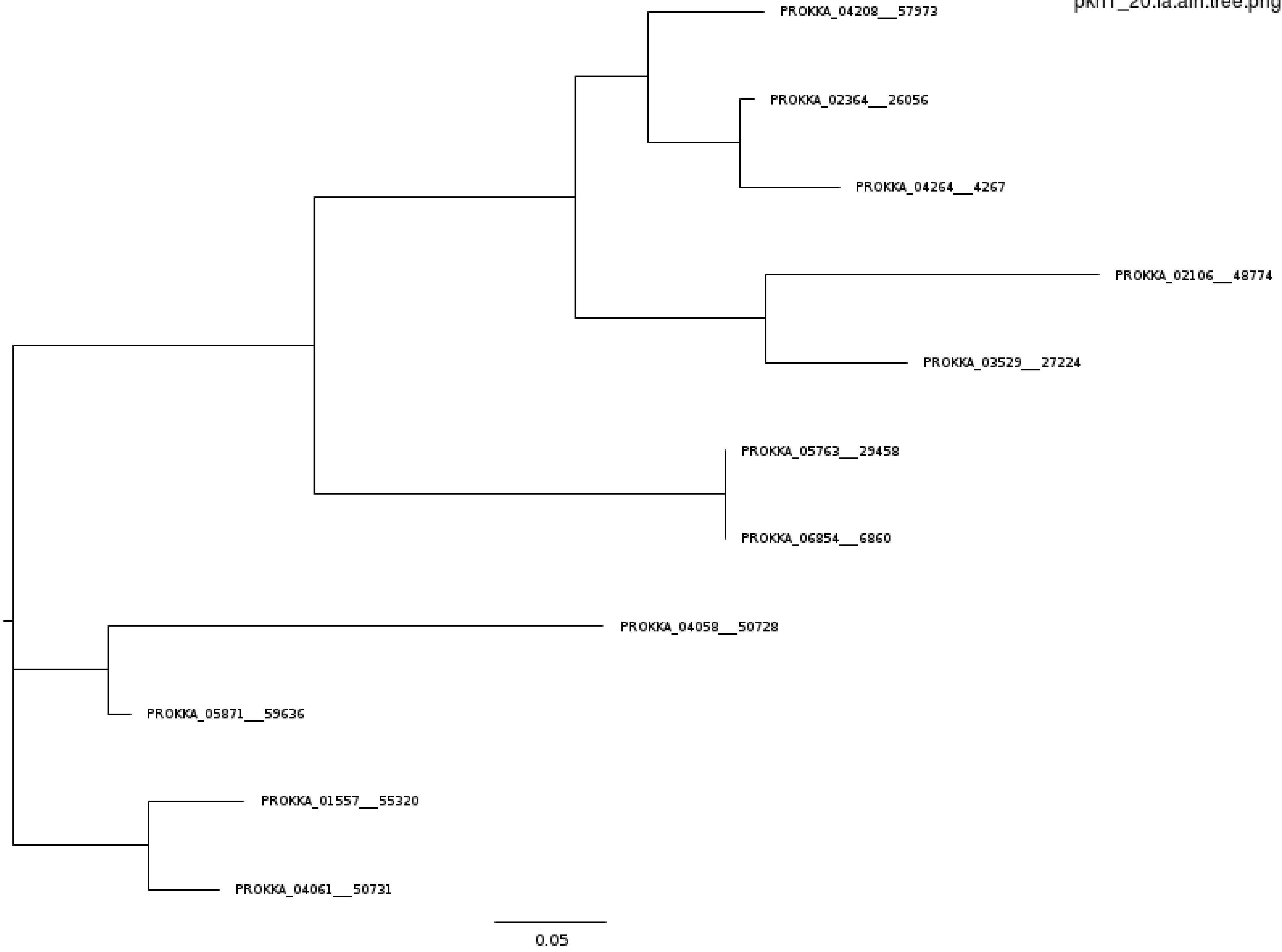

pkn1_24.fa.aln.tree.png

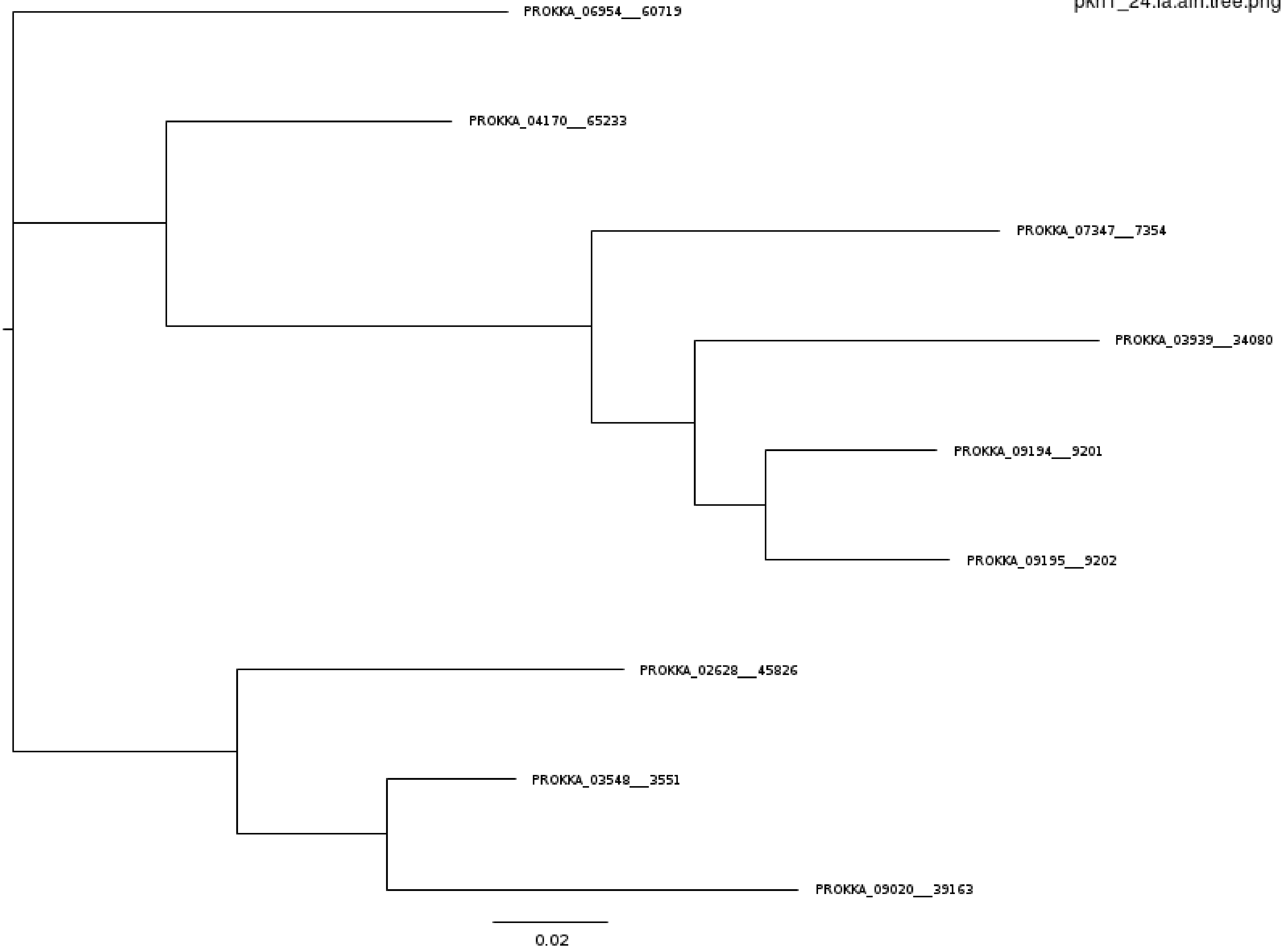

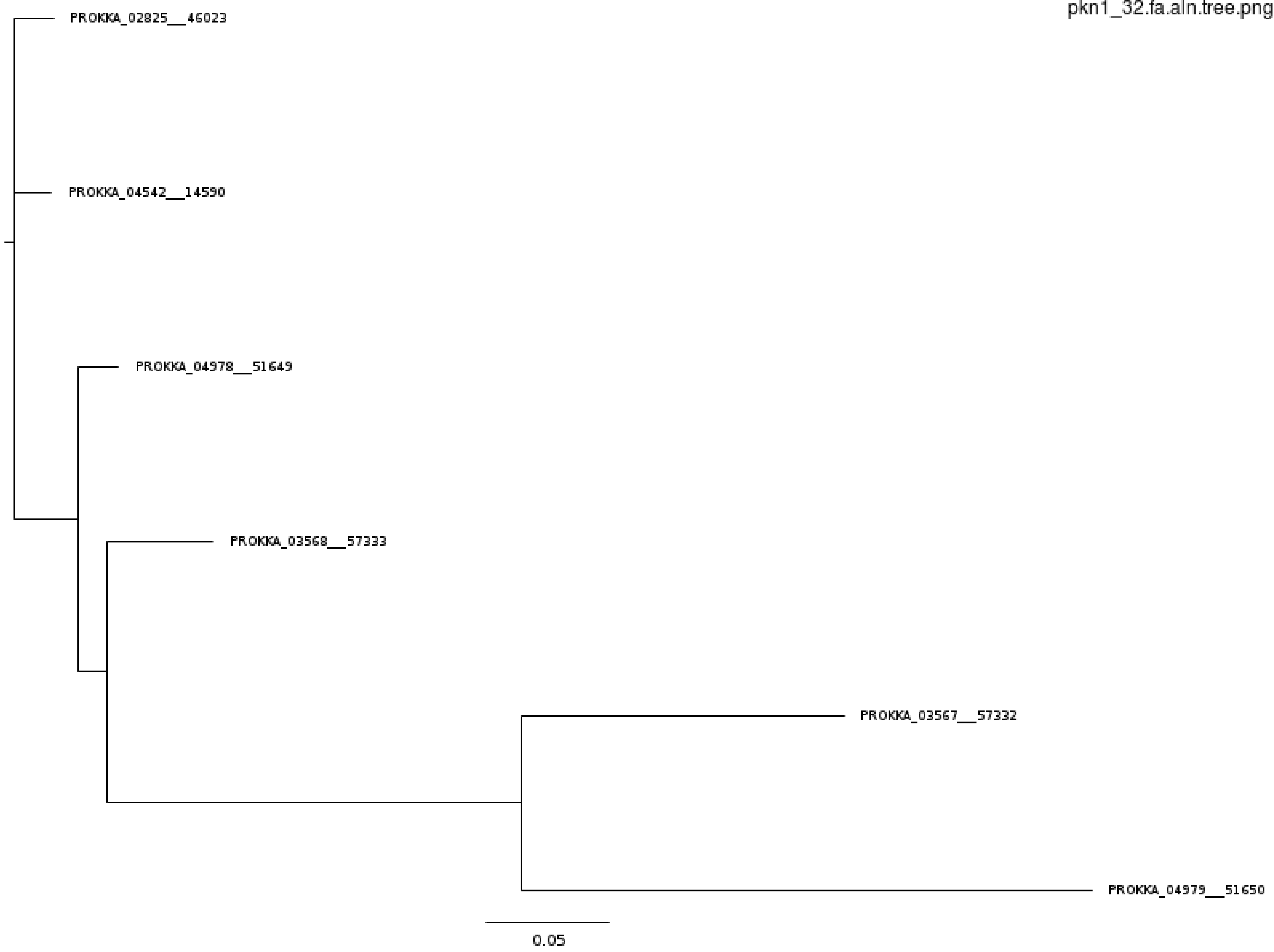
pkn1_32.fa.aln.tree.png
PROKKA_02825___46023
PROKKA_04542___14590
PROKKA_04978___51649
PROKKA_03568___57333
PROKKA_03567___57332
PROKKA_04979___51650
0.05

pkn1_8.fa.aln.tree.png

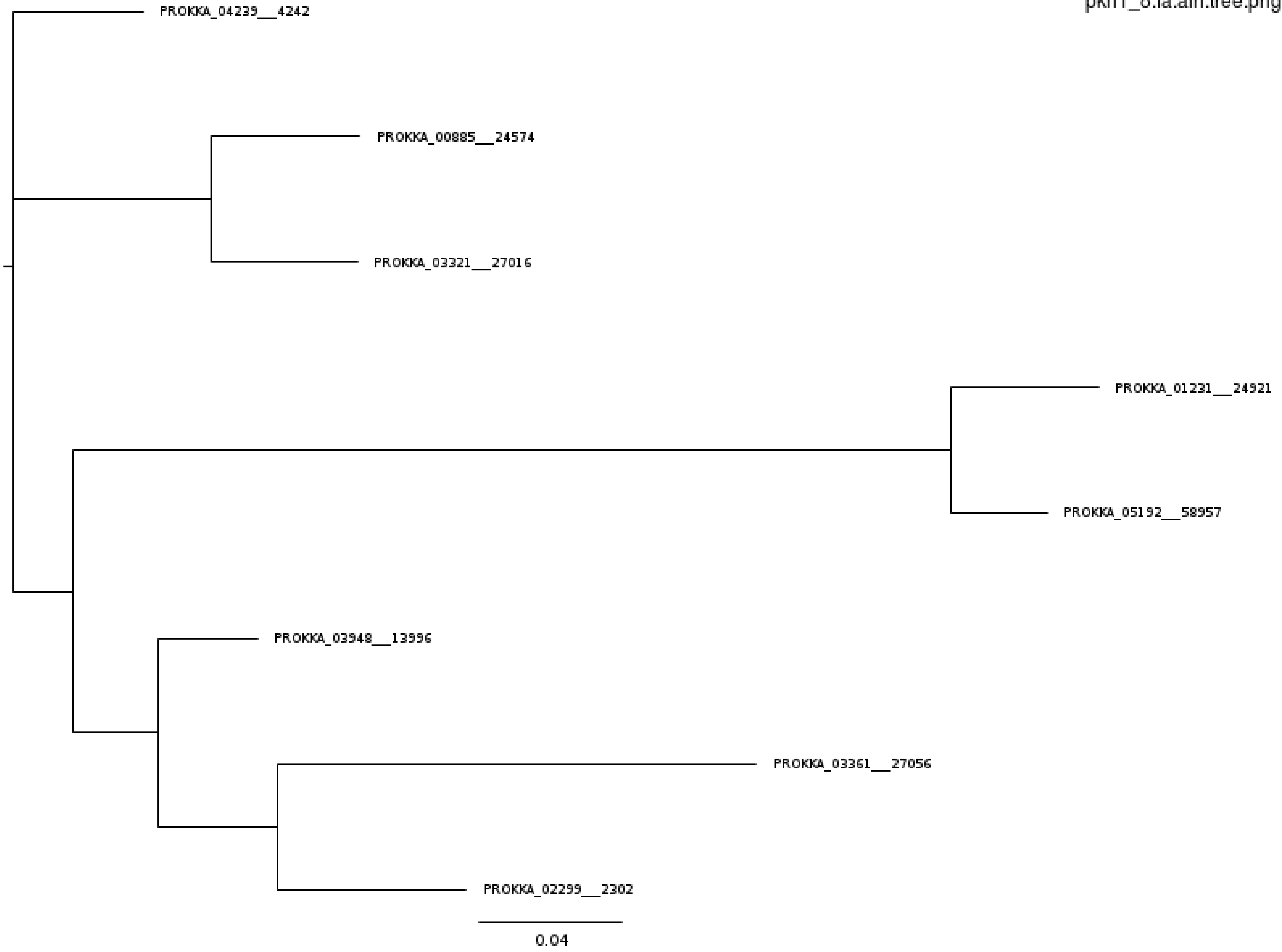

pkn1_9.fa.aln.tree.png

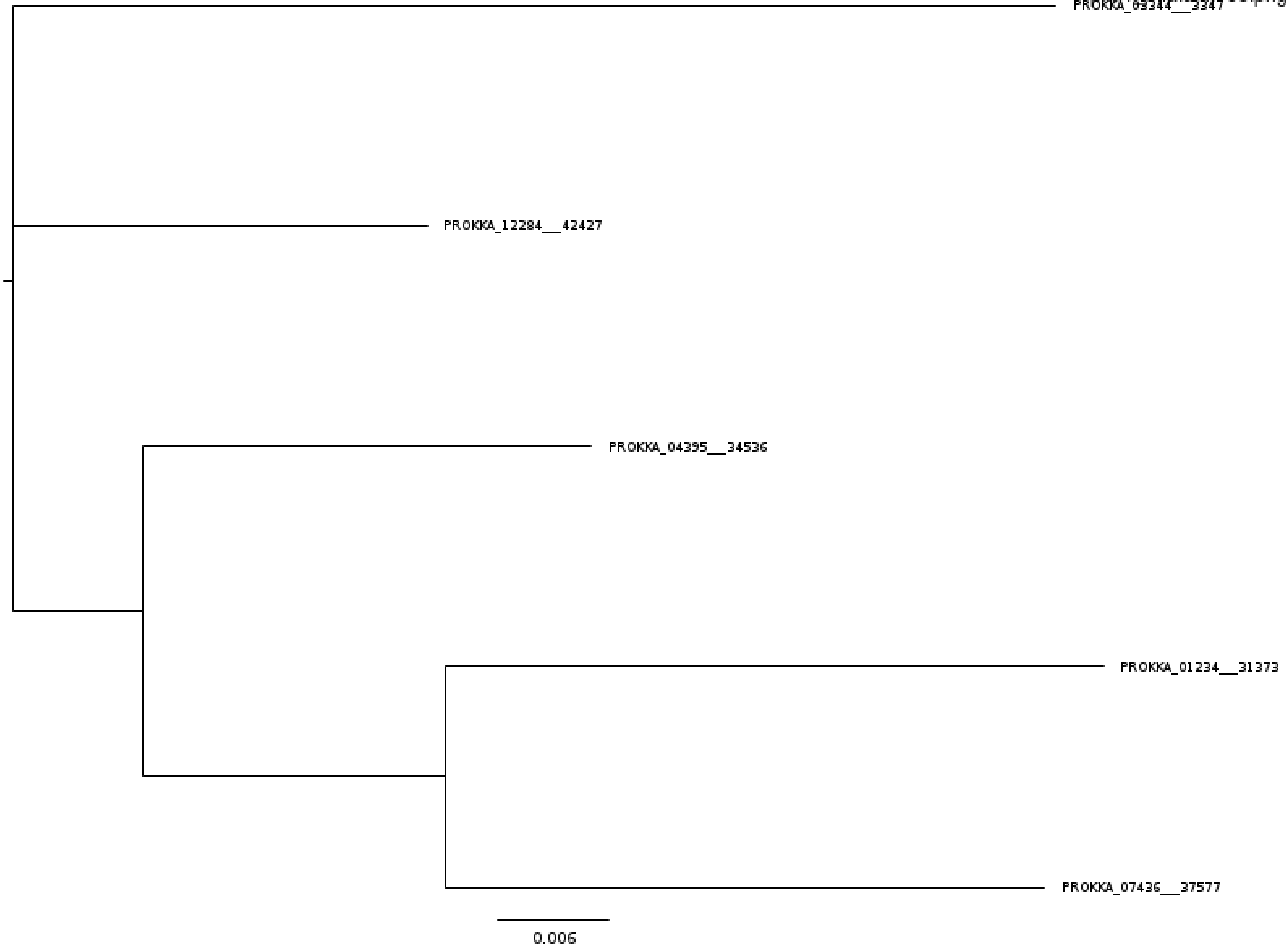

pknB_2.fa.aln.tree.png

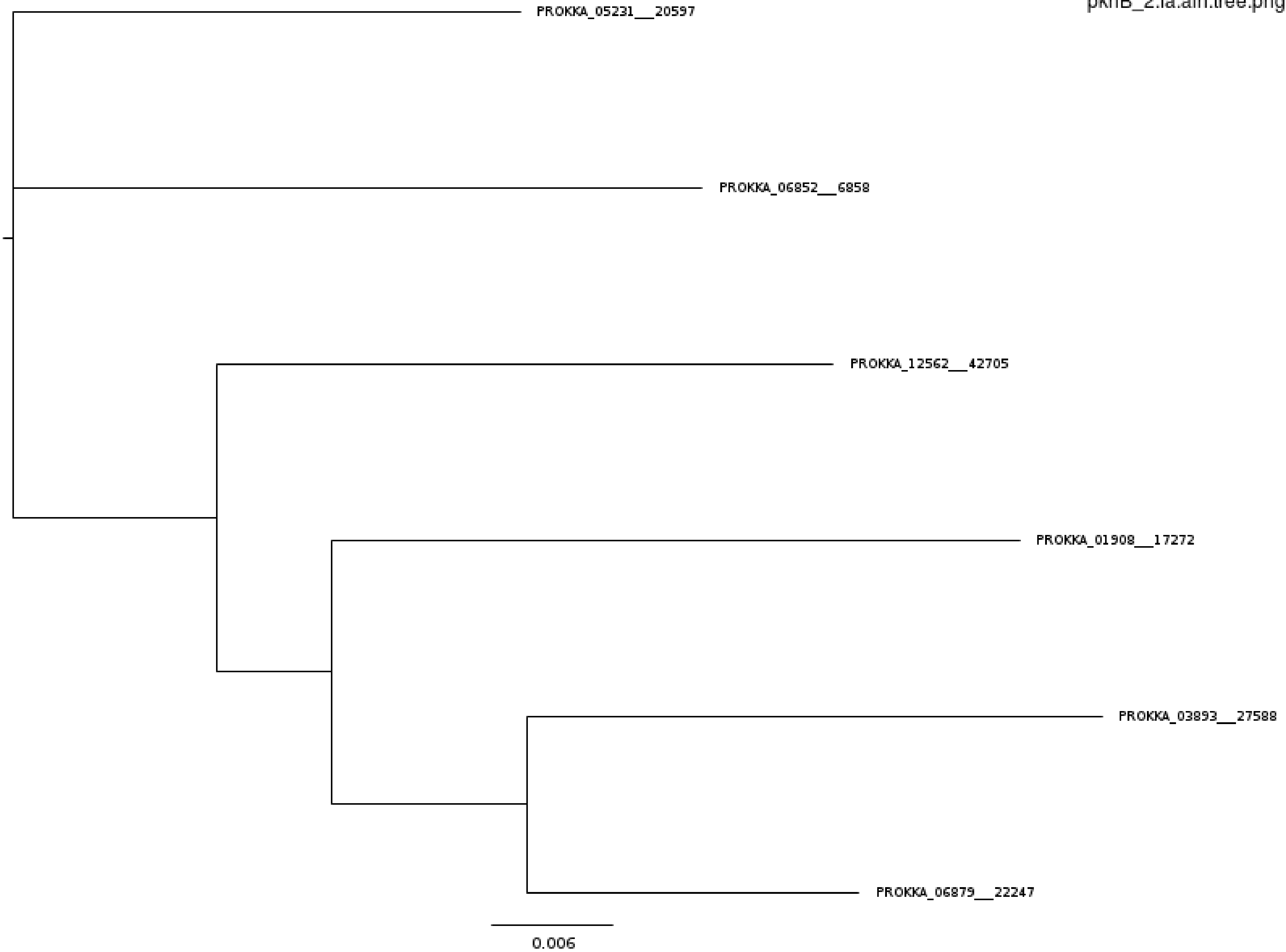

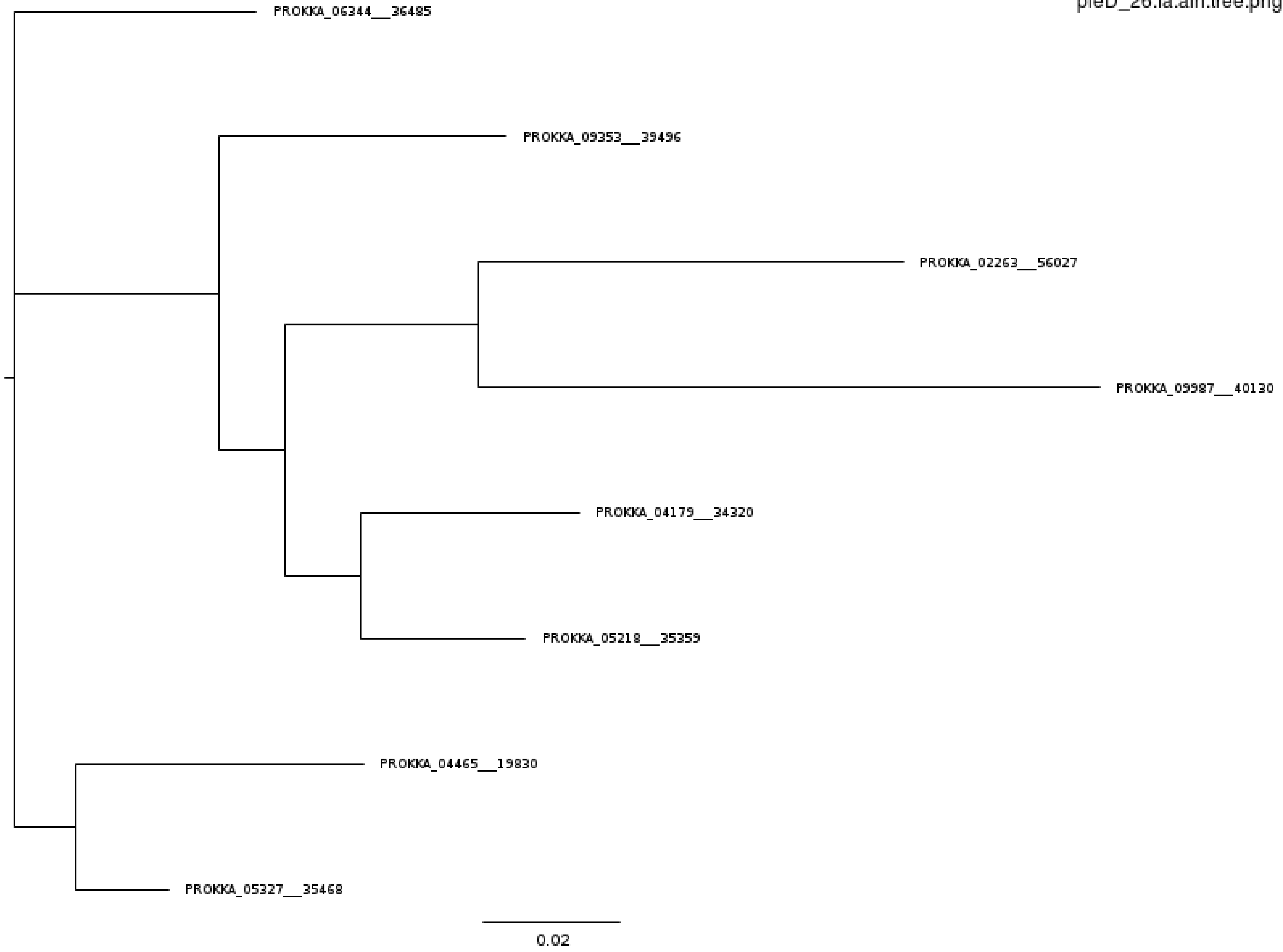
PROKKA_06344__36485
PROKKA_09353__39496
PROKKA_02263__56027
PROKKA_09987__40130
PROKKA_04179__34320
PROKKA_05218__35359
PROKKA_04465__19830
PROKKA_05327__35468
0.02

pleD_5.fa.aln.tree.png

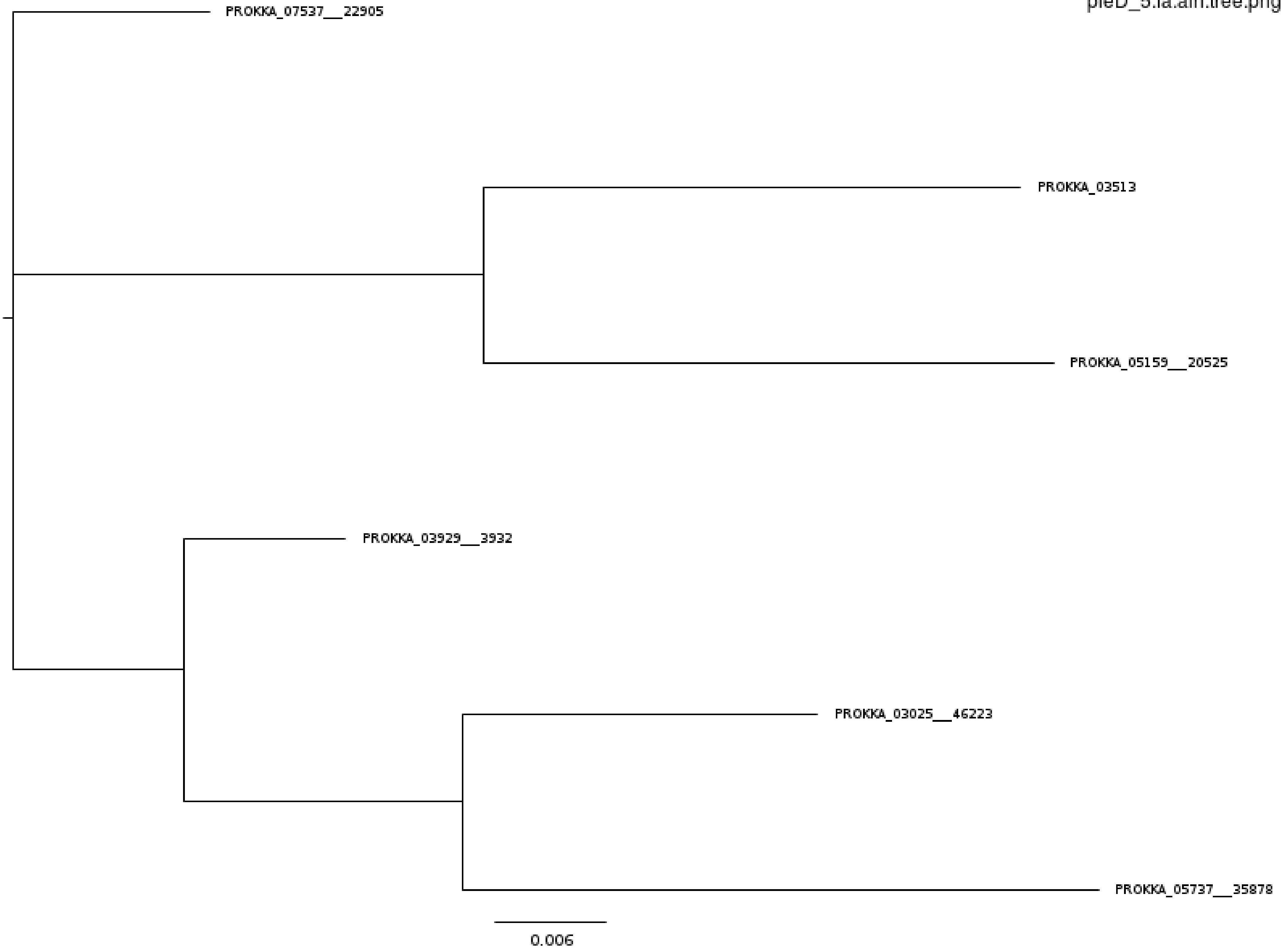

plsC_2.fa.aln.tree.png

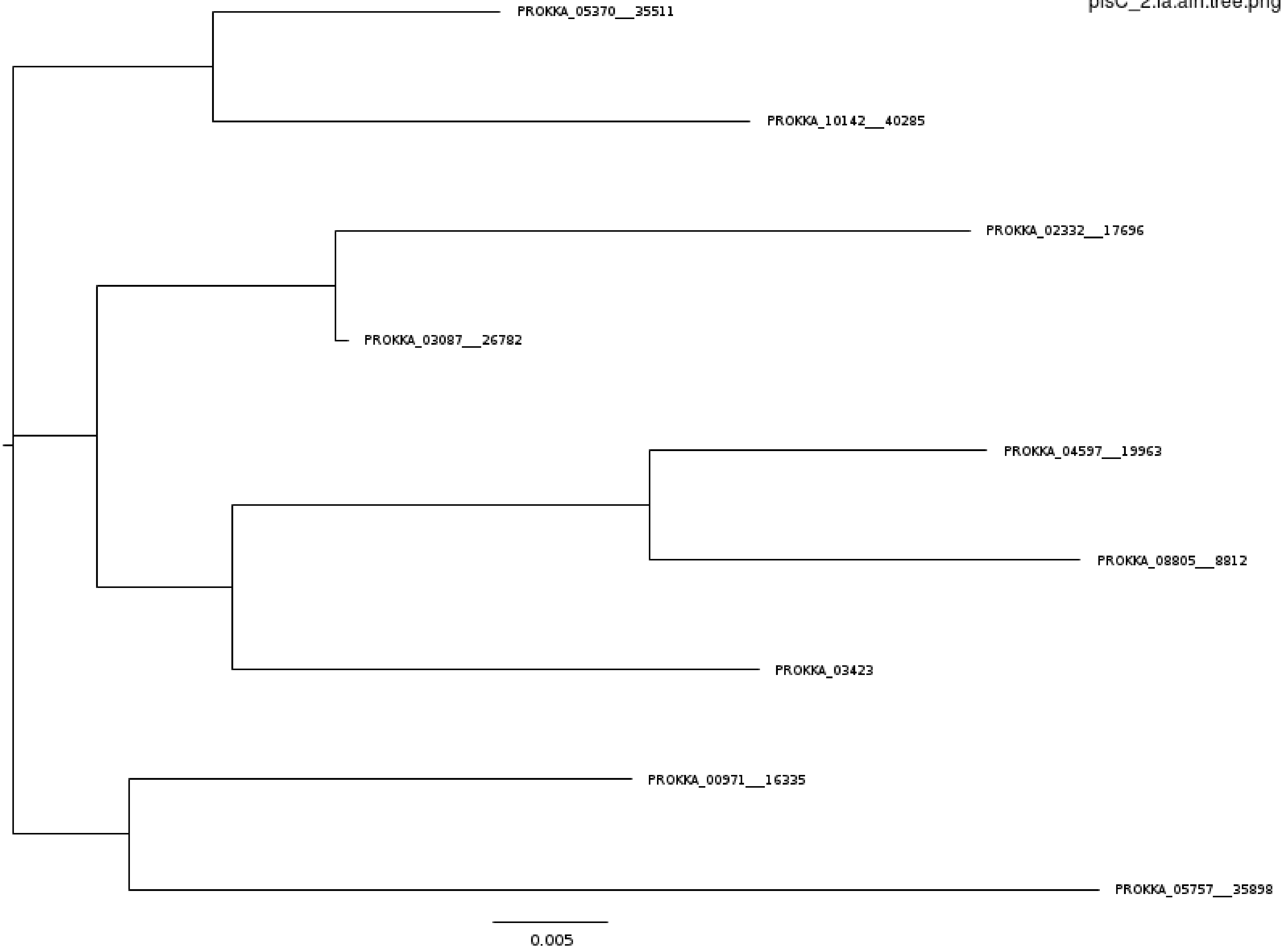

plsC_4.fa.aln.tree.png

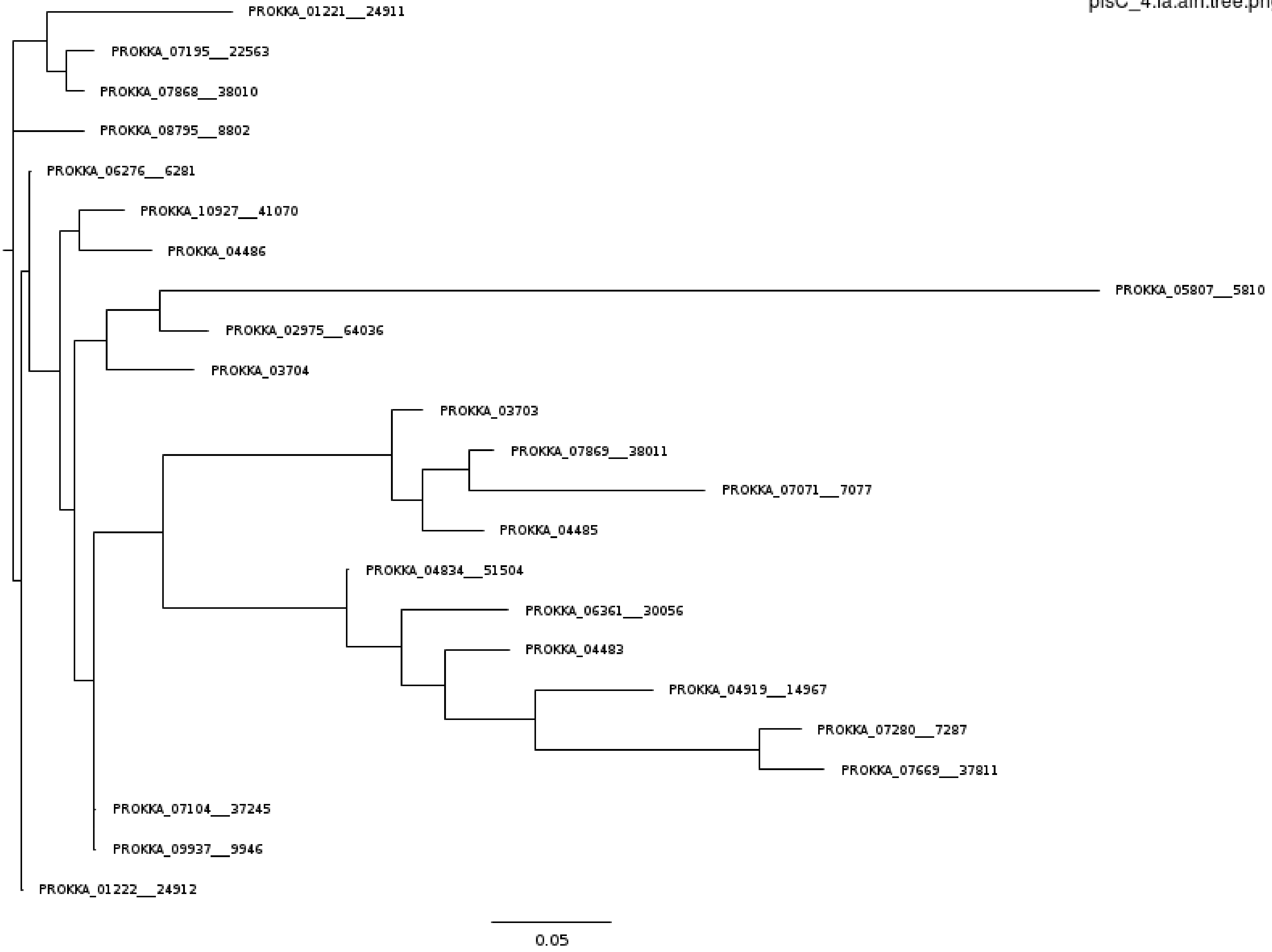

plsX_1.fa.aln.tree.png

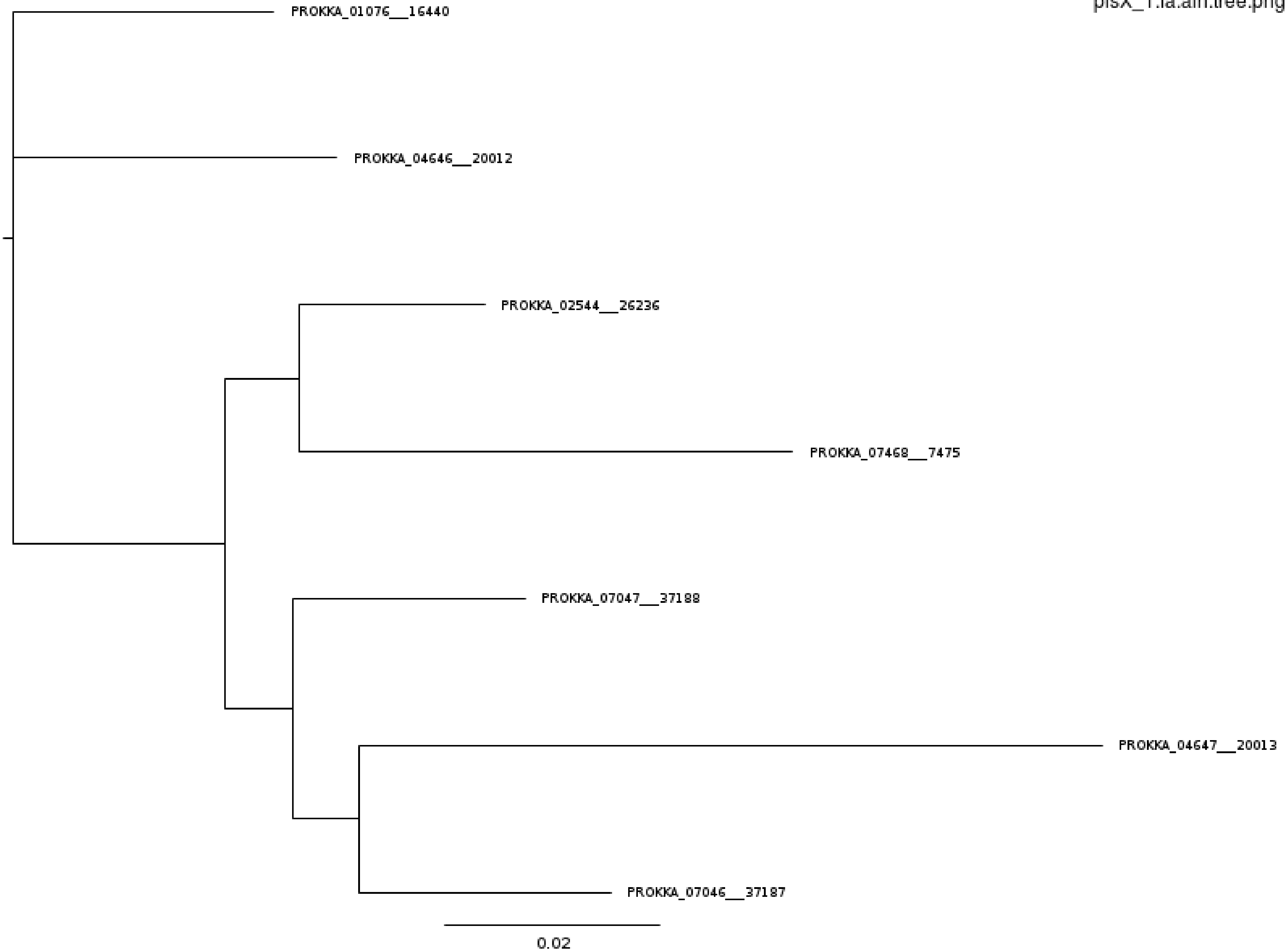

plsY_3.fa.aln.tree.png

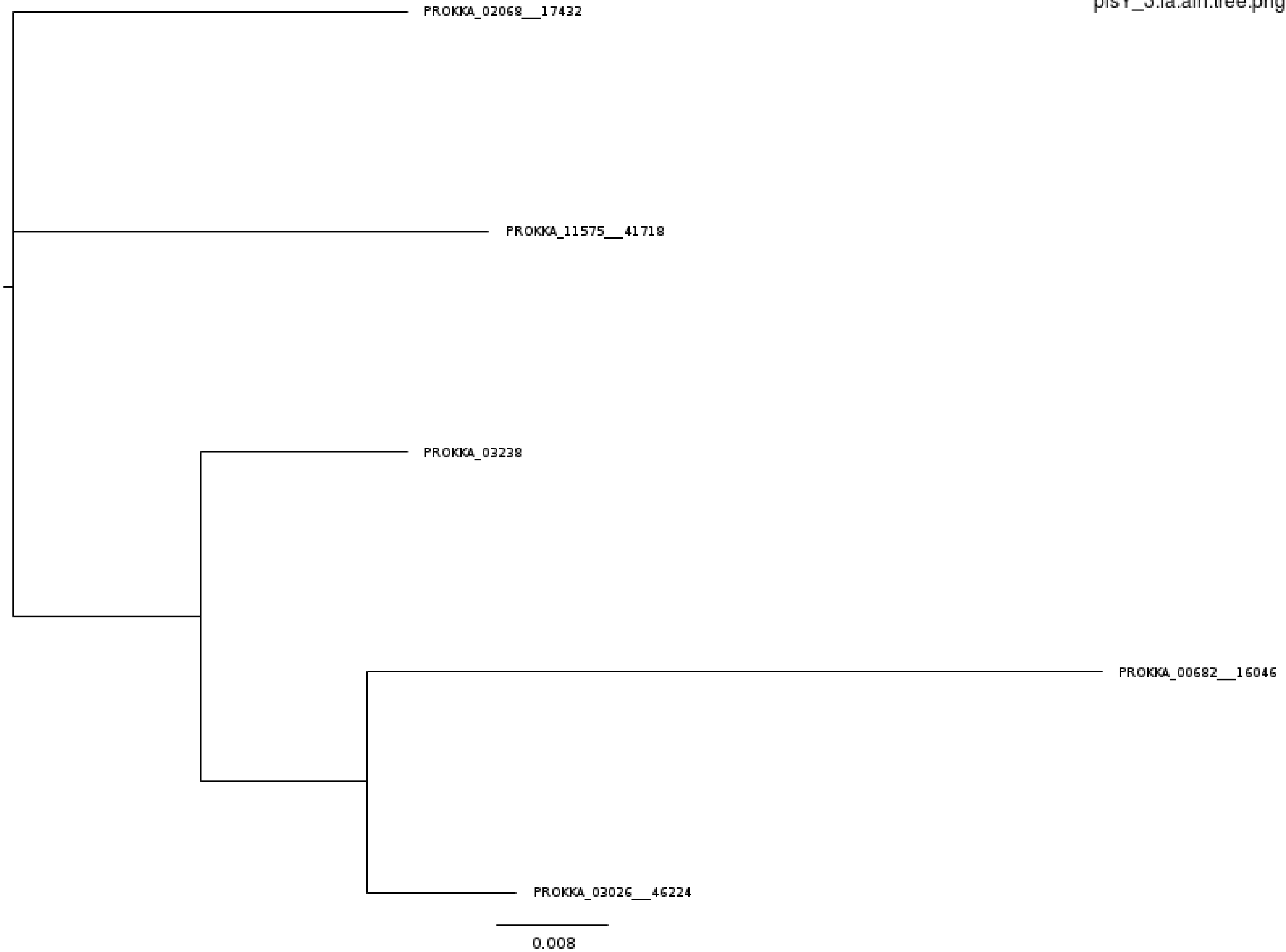

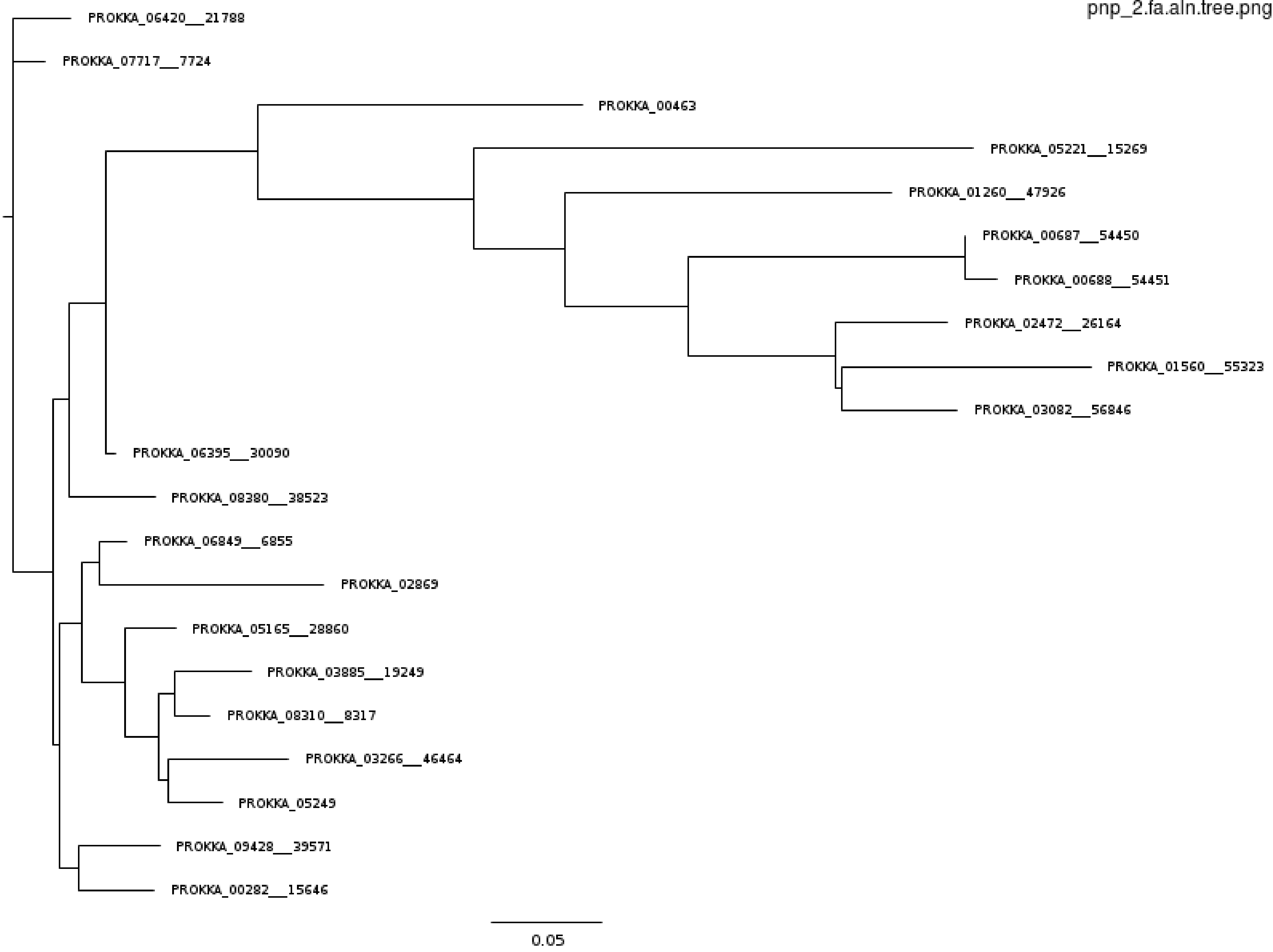
pnp_2.fa.aln.tree.png
PROKKA_06420___21788
PROKKA_07717___7724
PROKKA_00463
PROKKA_05221___15269
PROKKA_01260___47926
PROKKA_00687___54450
PROKKA_00688___54451
PROKKA_02472___26164
PROKKA_01560___55323
PROKKA_03082___56846
PROKKA_06395___30090
PROKKA_08380___38523
PROKKA_06849___6855
PROKKA_02869
PROKKA_05165___28860
PROKKA_03885___19249
PROKKA_08310___8317
PROKKA_03266___46464
PROKKA_05249
PROKKA_09428___39571
PROKKA_00282___15646
0.05

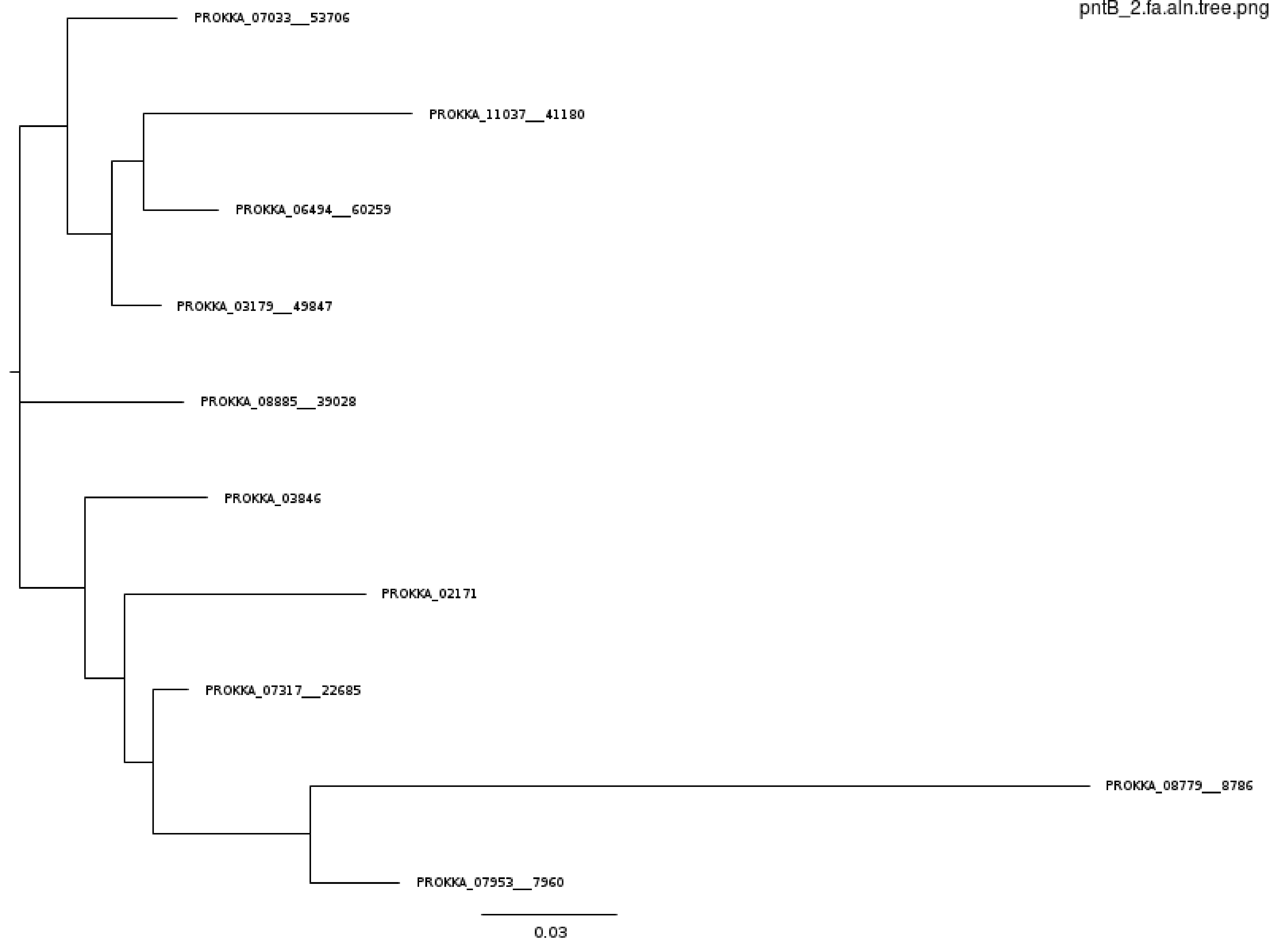
pntB_2.fa.aln.tree.png
PROKKA_07033___53706
PROKKA_11037___41180
PROKKA_06494___60259
PROKKA_03179___49847
PROKKA_08885___39028
PROKKA_03846
PROKKA_02171
PROKKA_07317___22685
PROKKA_08779___8786
PROKKA_07953___7960
0.03

podJ_2.fa.aln.tree.png

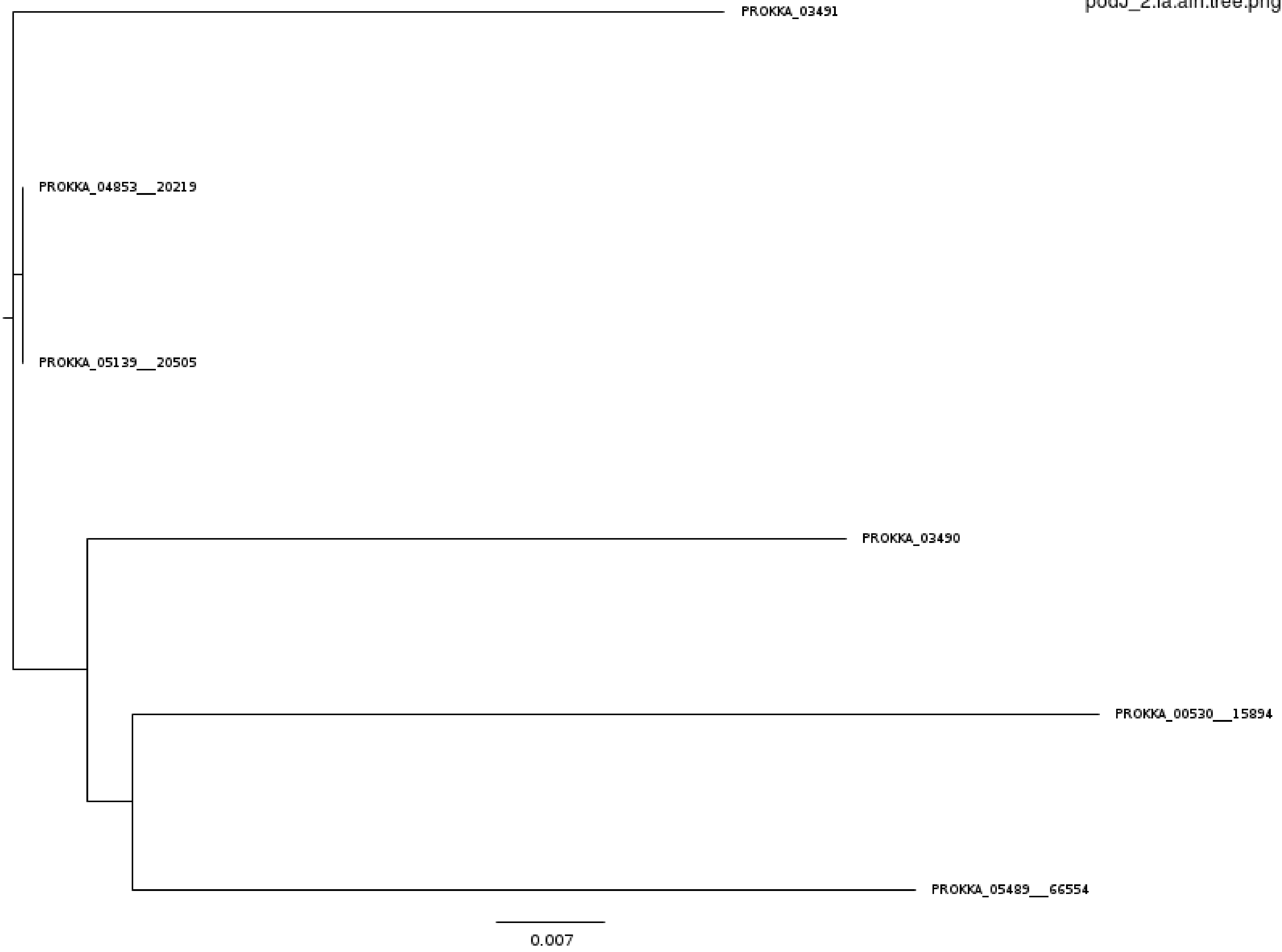

polA_4.fa.aln.tree.png

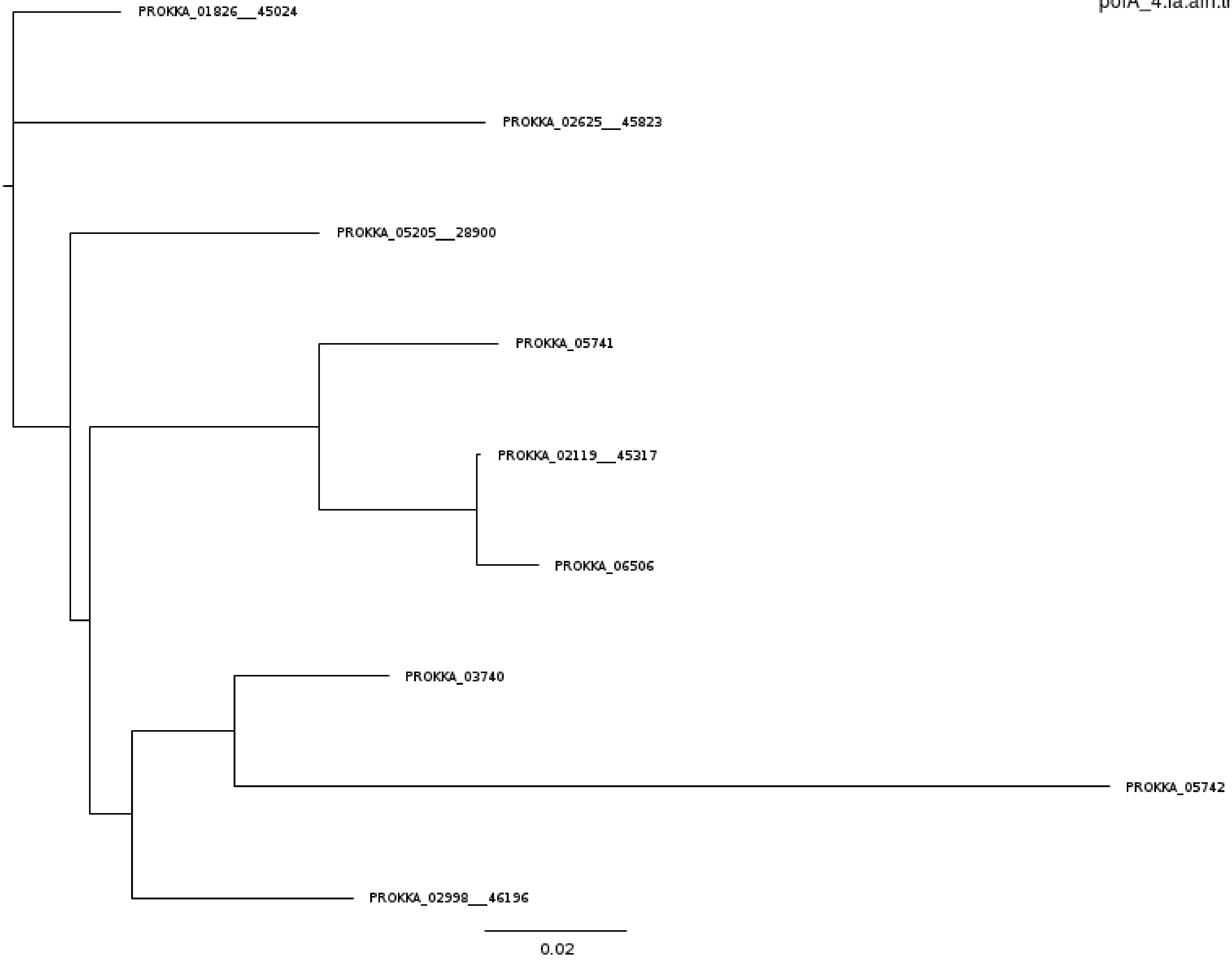

porB_1.fa.aln.tree.png

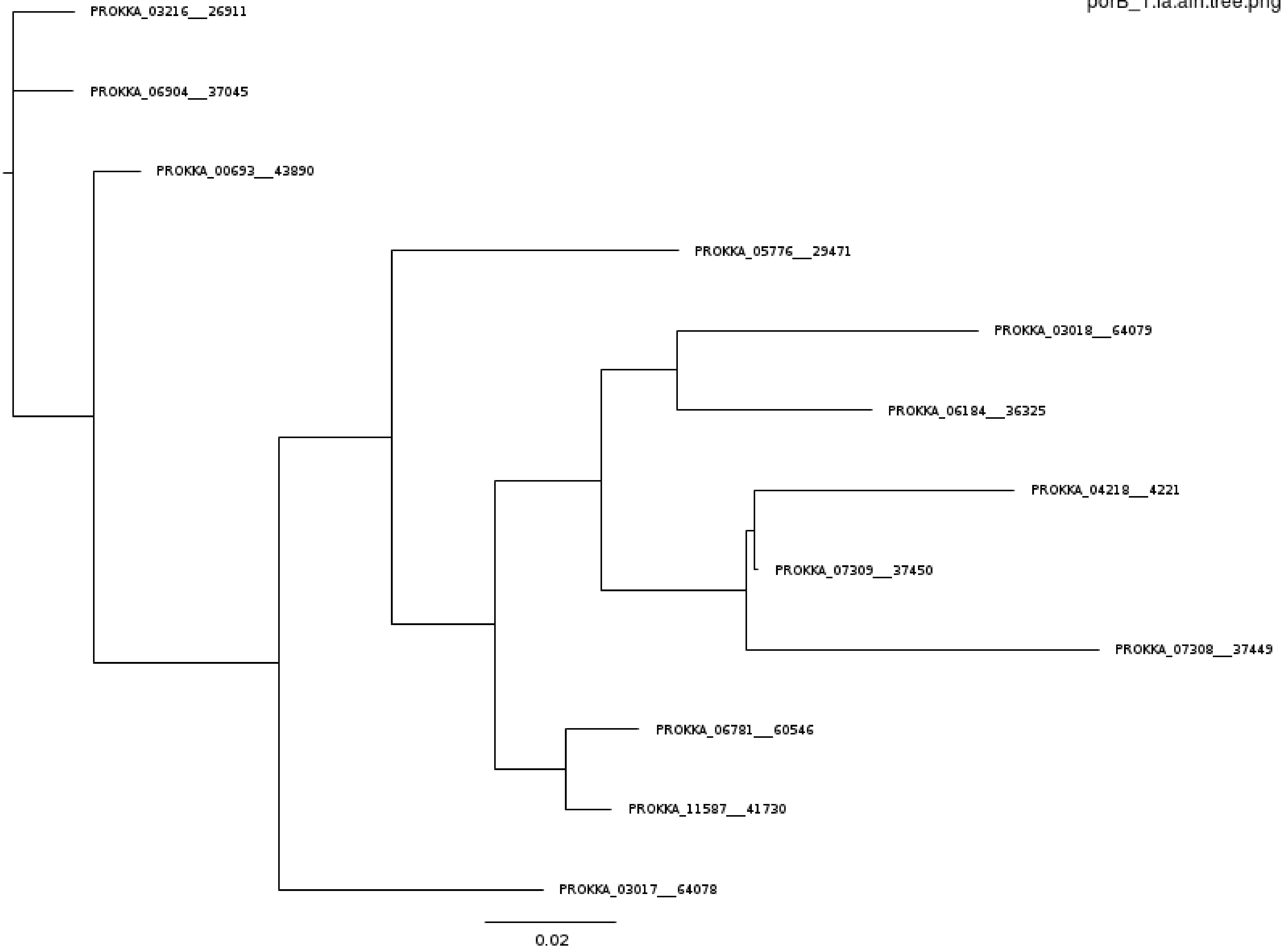

ppdK_3.fa.aln.tree.png

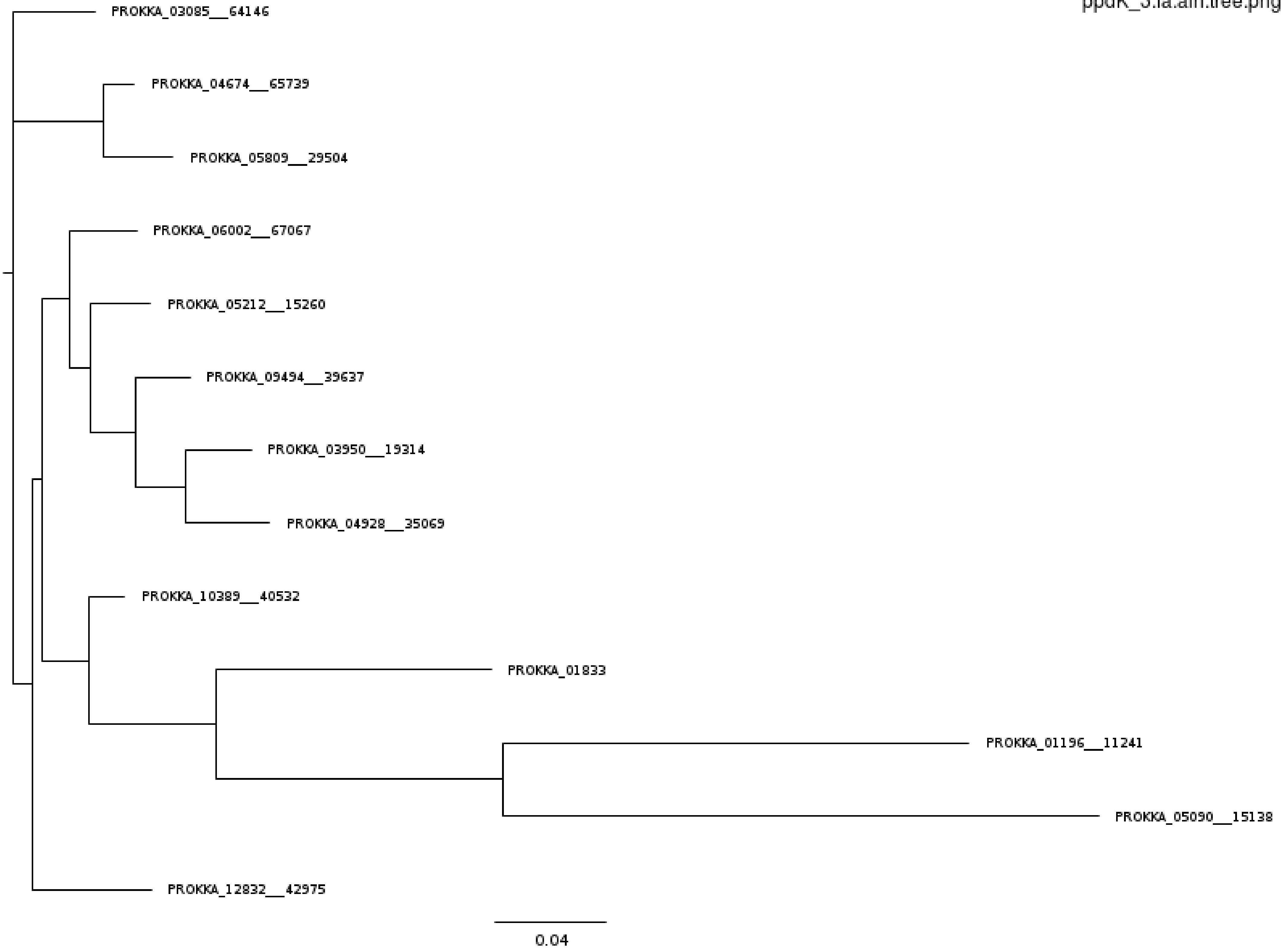

ppiB_1.fa.aln.tree.png

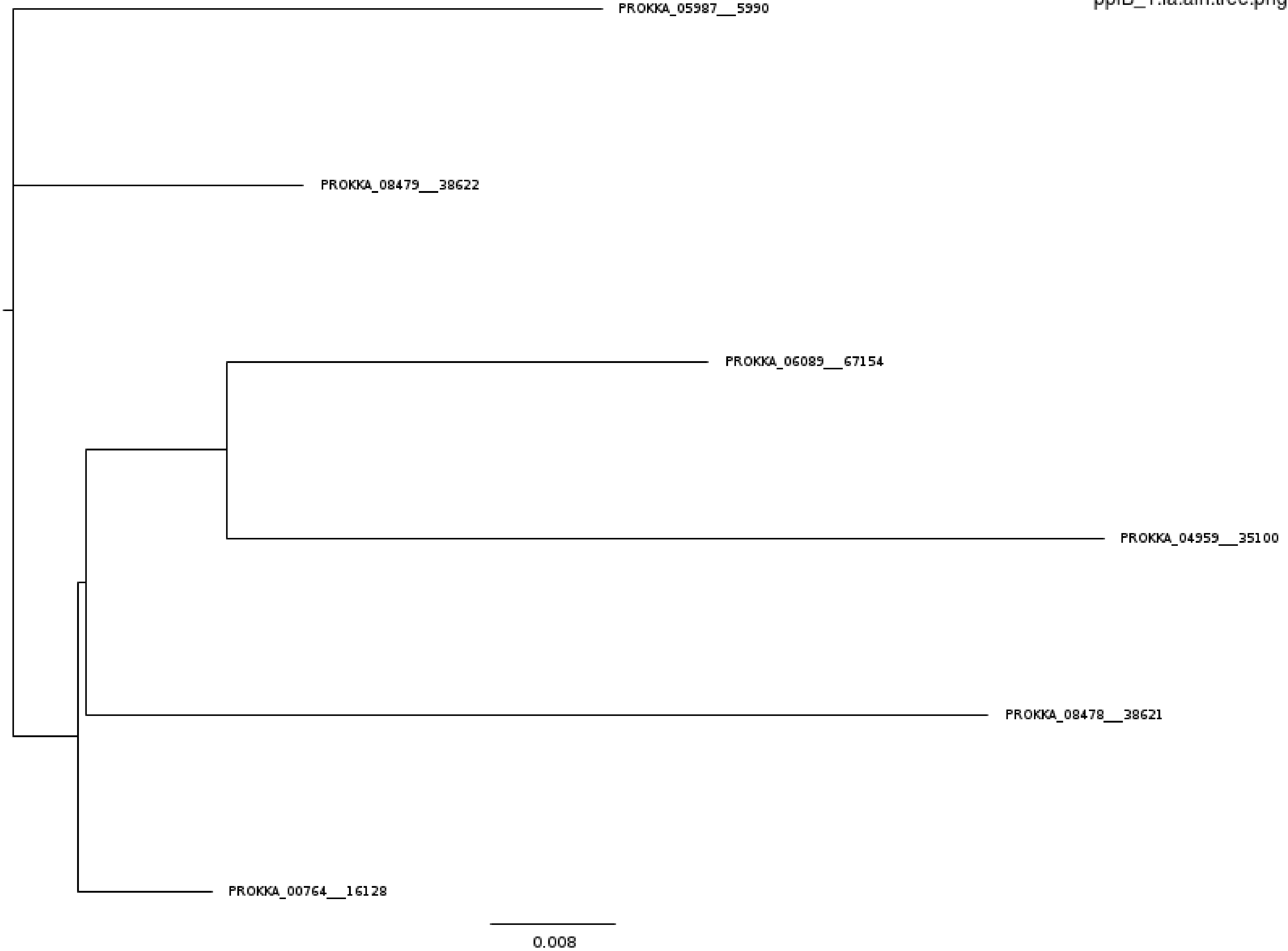

ppiB_2.fa.aln.tree.png

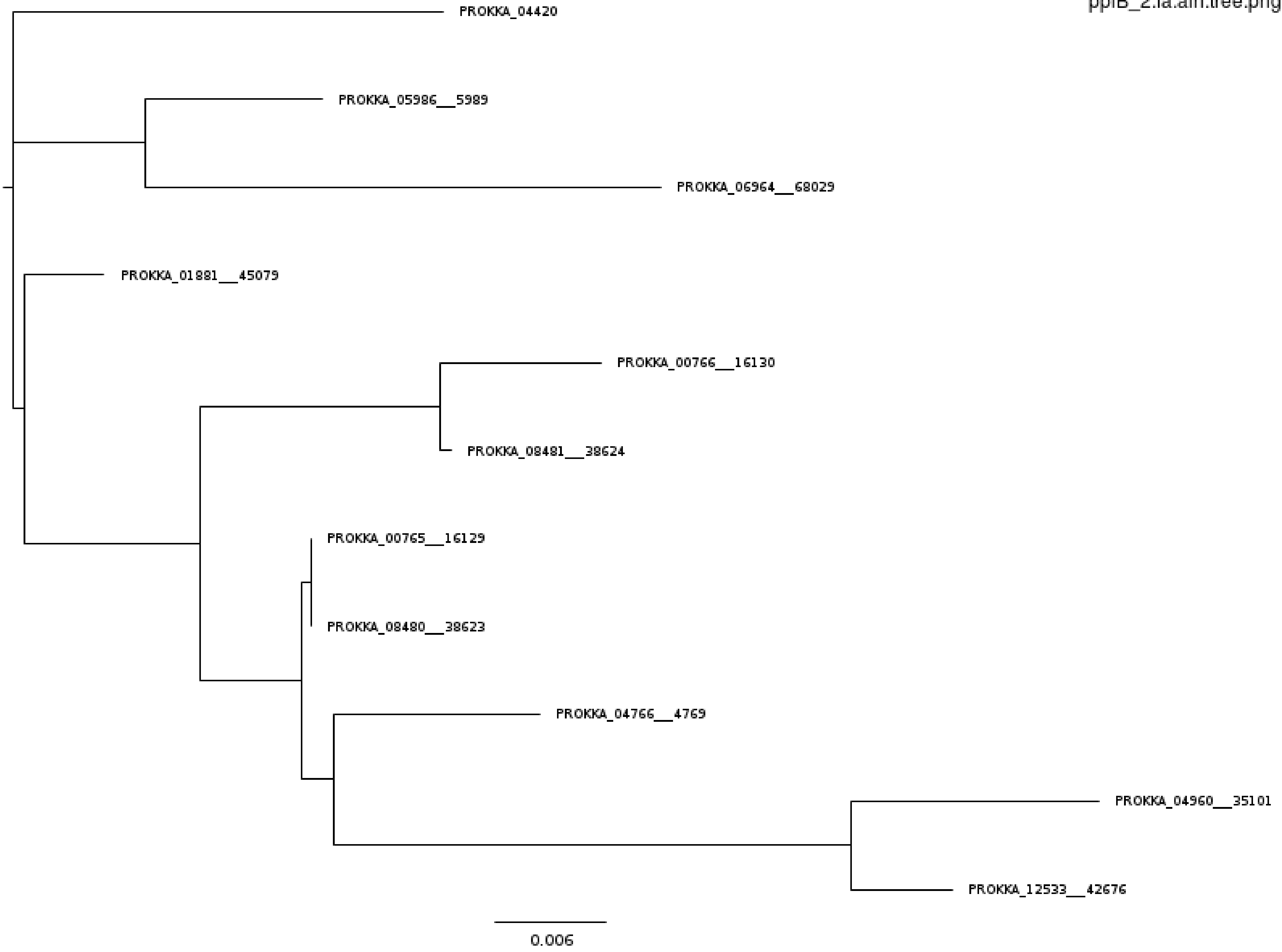

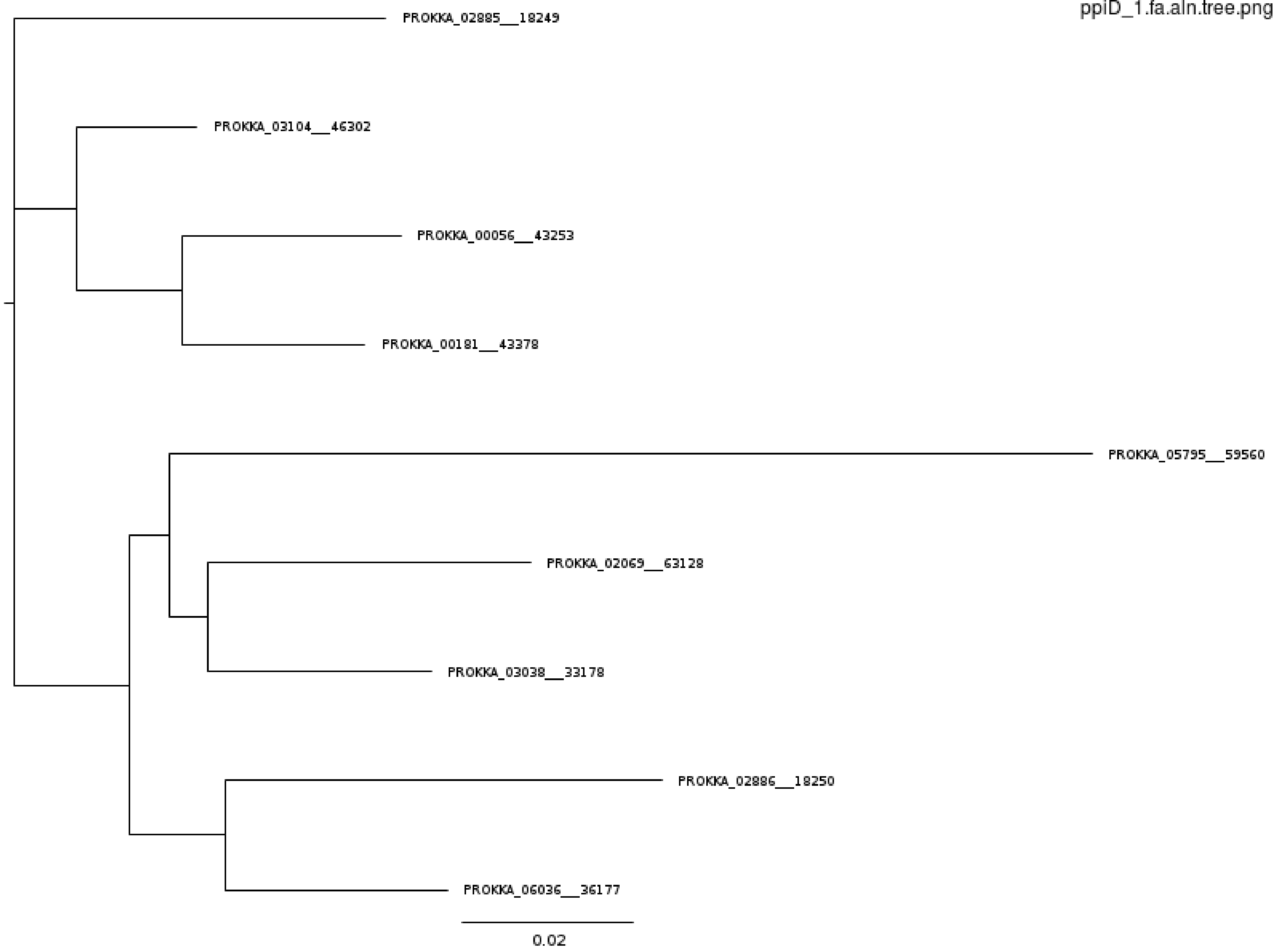
ppiD_1.fa.aln.tree.png
PROKKA_02885__18249
PROKKA_03104__46302
PROKKA_00056__43253
PROKKA_00181__43378
PROKKA_05795__59560
PROKKA_02069__63128
PROKKA_03038__33178
PROKKA_02886__18250
PROKKA_06036__36177
0.02

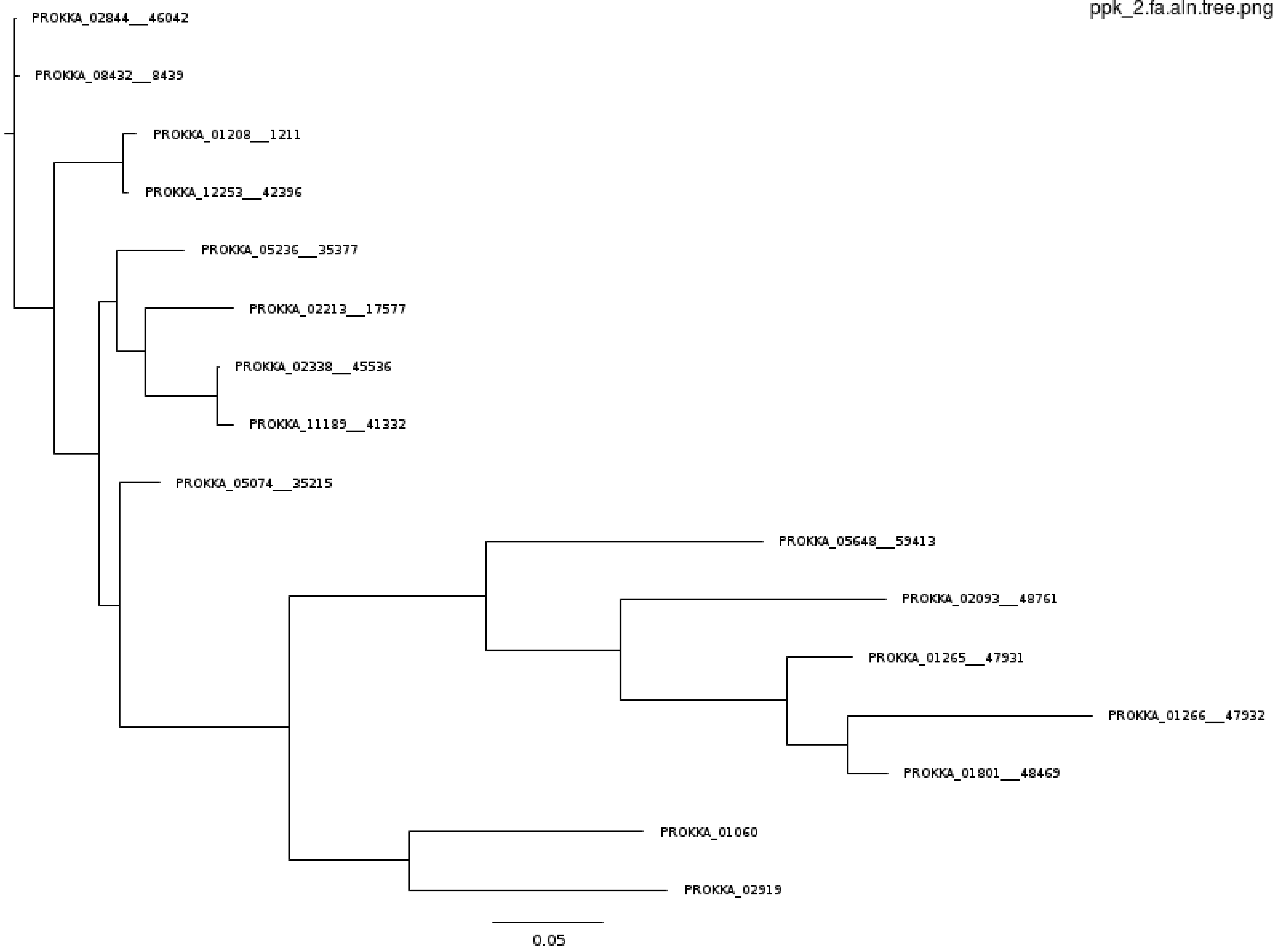

ppk_2.fa.aln.tree.png
PROKKA_02844___46042
PROKKA_08432___8439
PROKKA_01208___1211
PROKKA_12253___42396
PROKKA_05236___35377
PROKKA_02213___17577
PROKKA_02338___45536
PROKKA_11189___41332
PROKKA_05074___35215
PROKKA_05648___59413
PROKKA_02093___48761
PROKKA_01265___47931
PROKKA_01266___47932
PROKKA_01801___48469
PROKKA_01060
PROKKA_02919
0.05

prc_2.fa.aln.tree.png

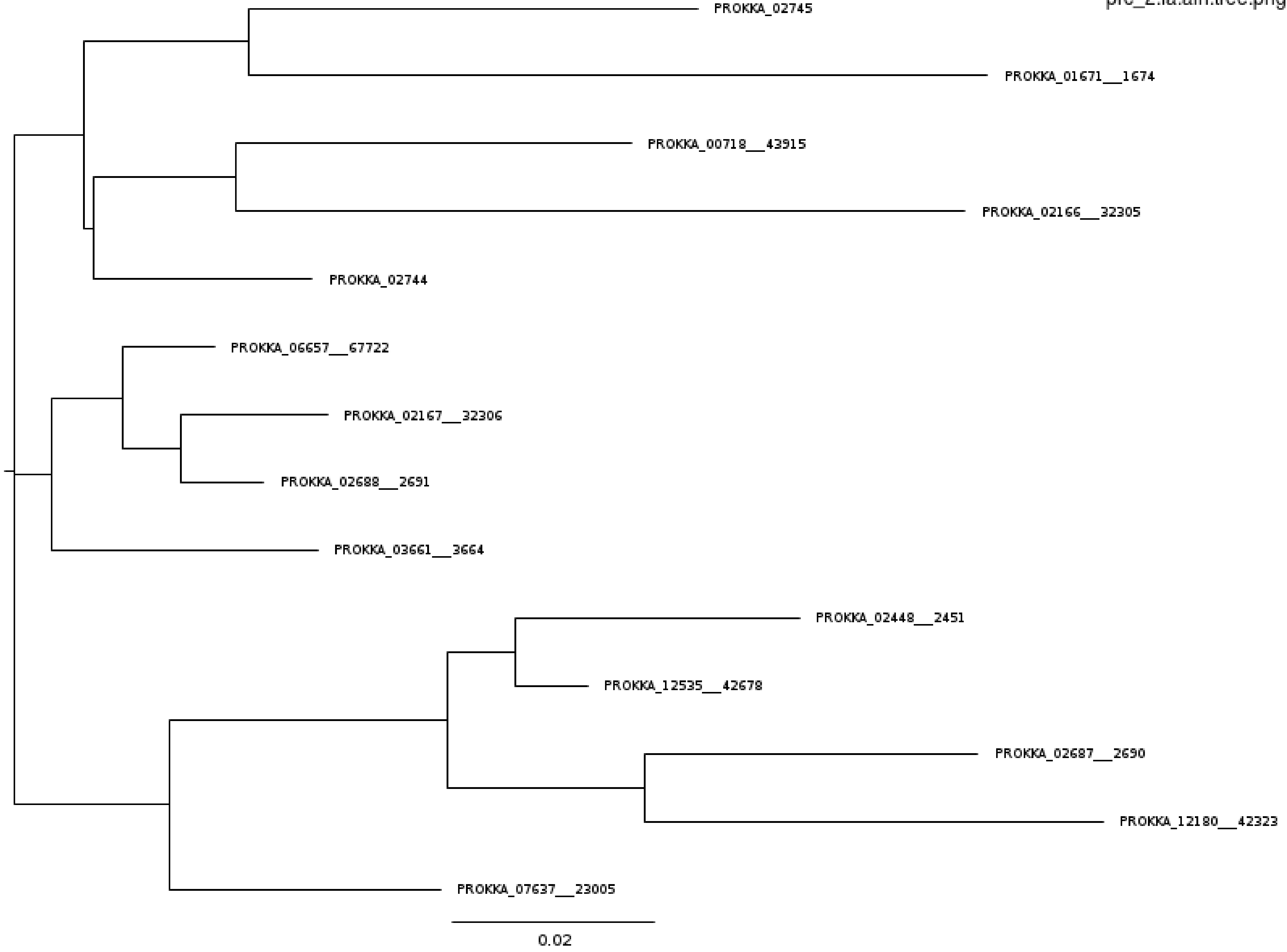

prfA_2.fa.aln.tree.png

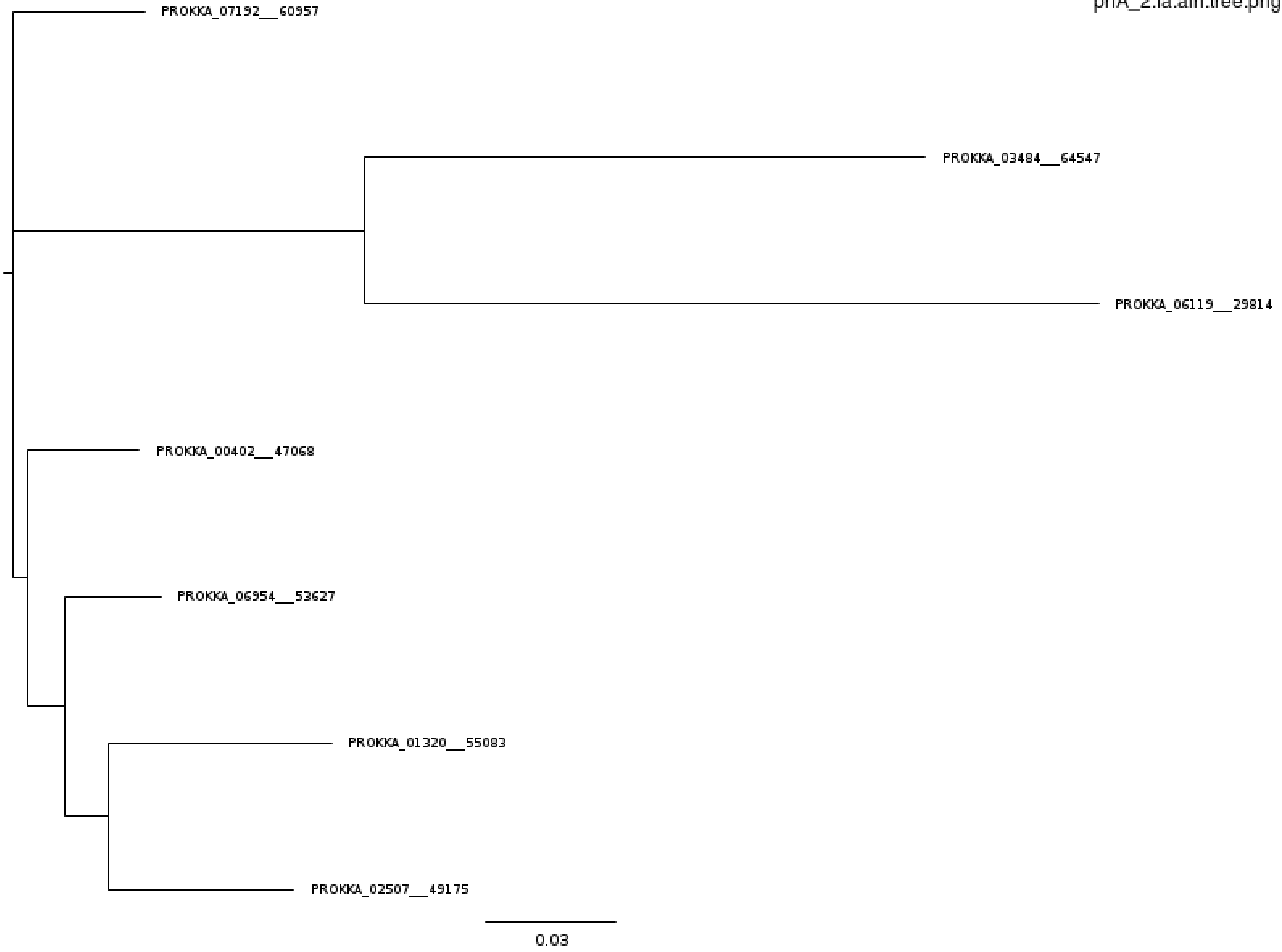

prfB_2.fa.aln.tree.png

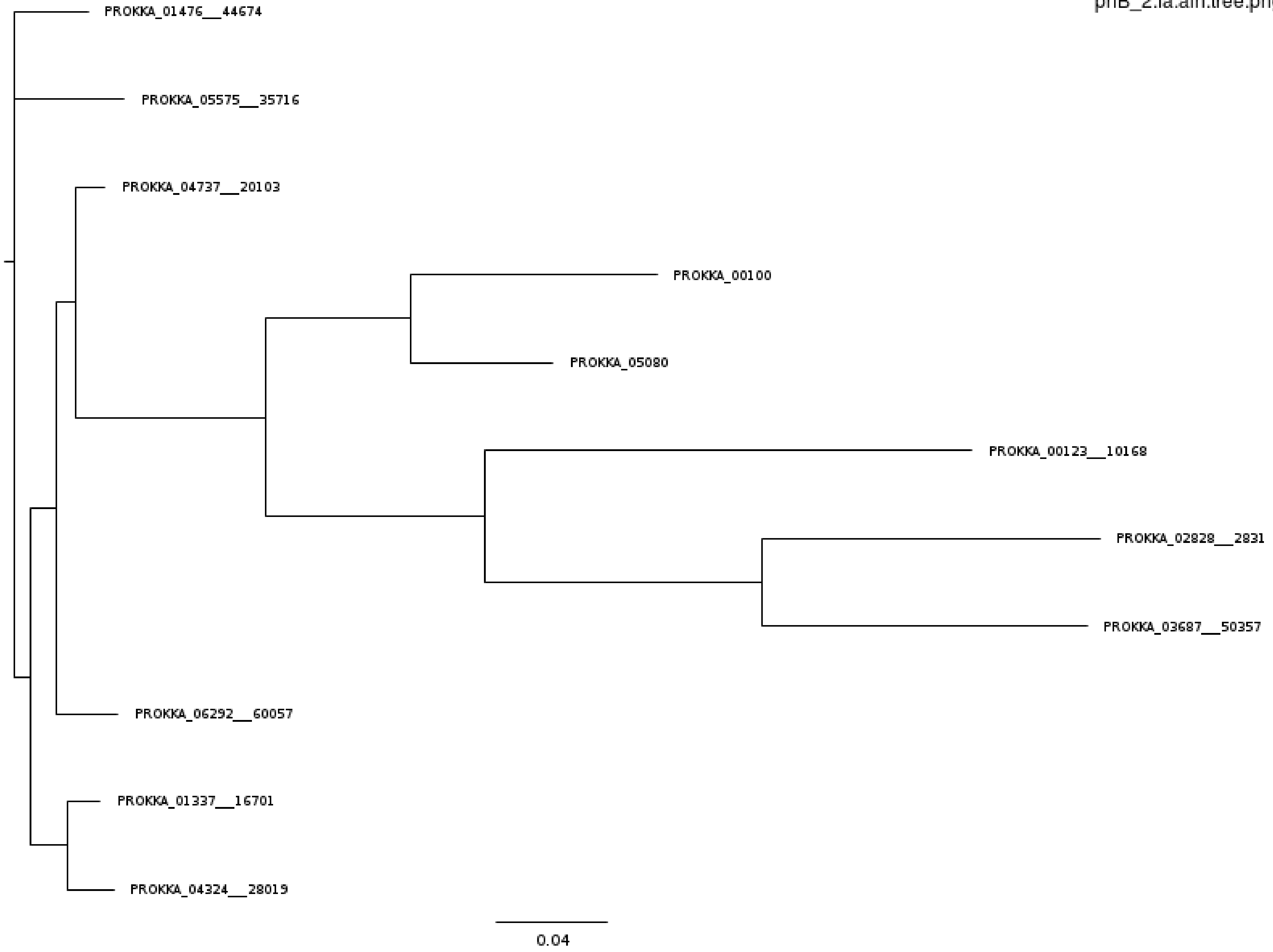

prfC_2.fa.aln.tree.png

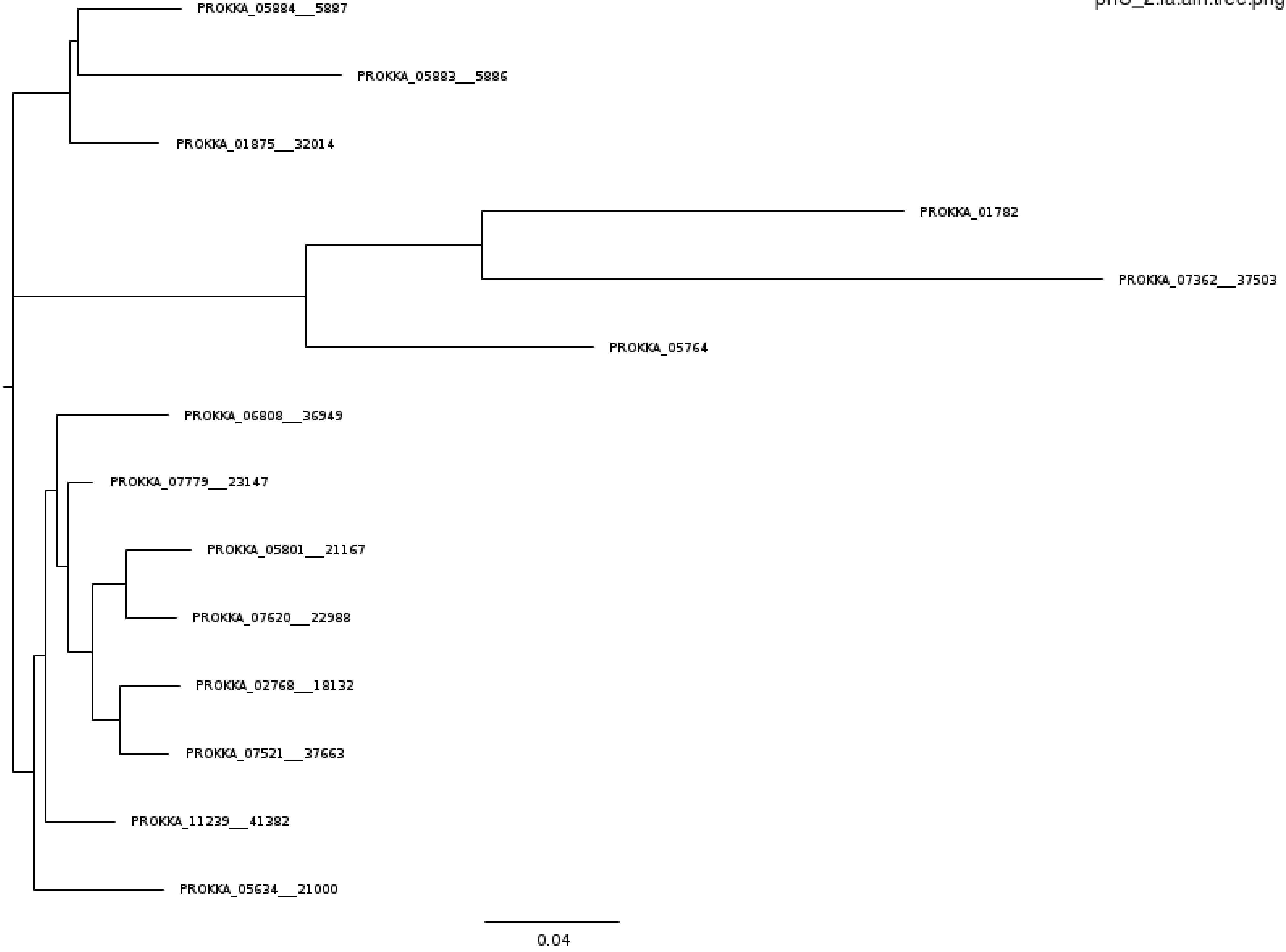

priA_2.fa.aln.tree.png

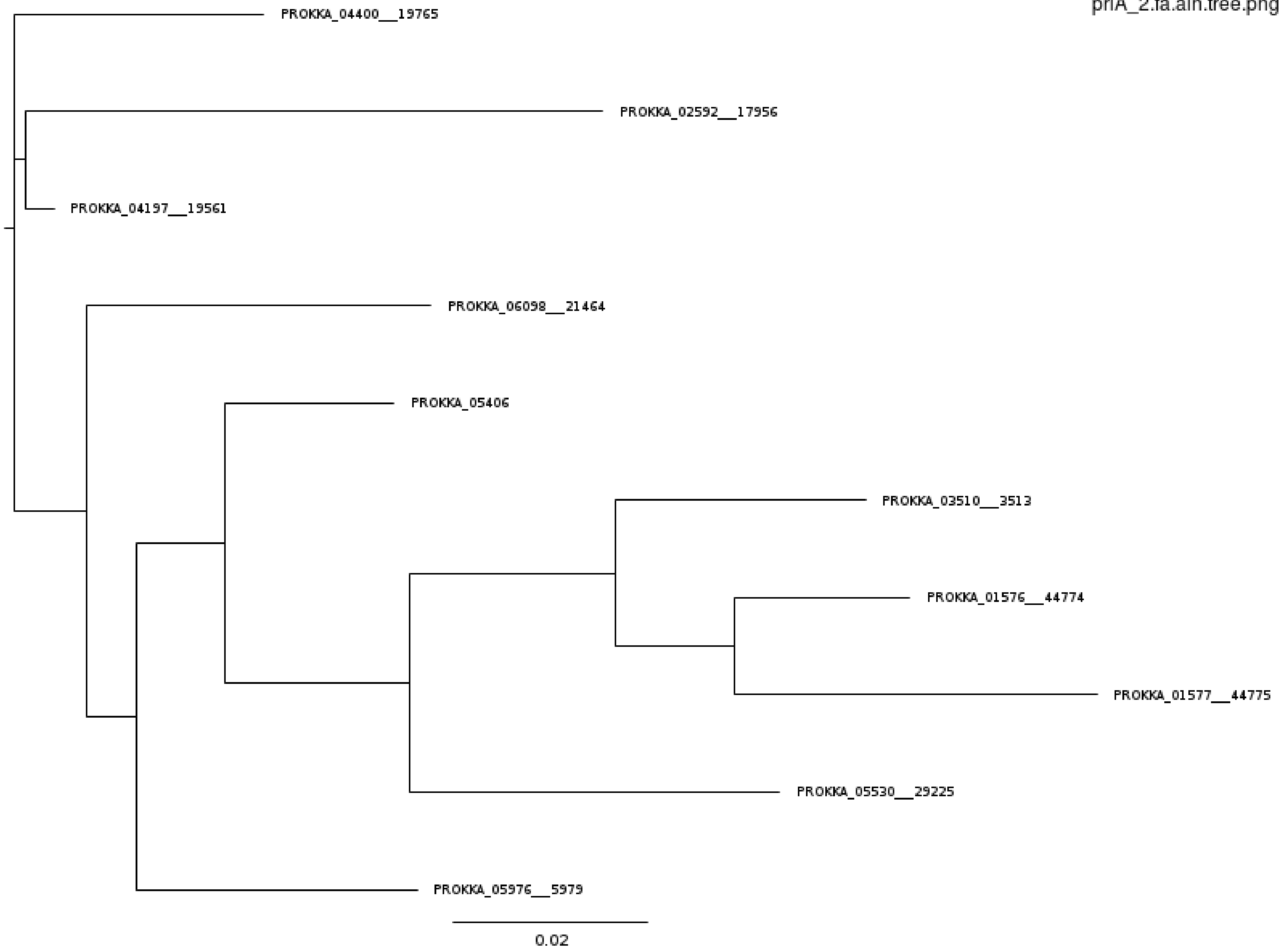

prkC_10.fa.aln.tree.png

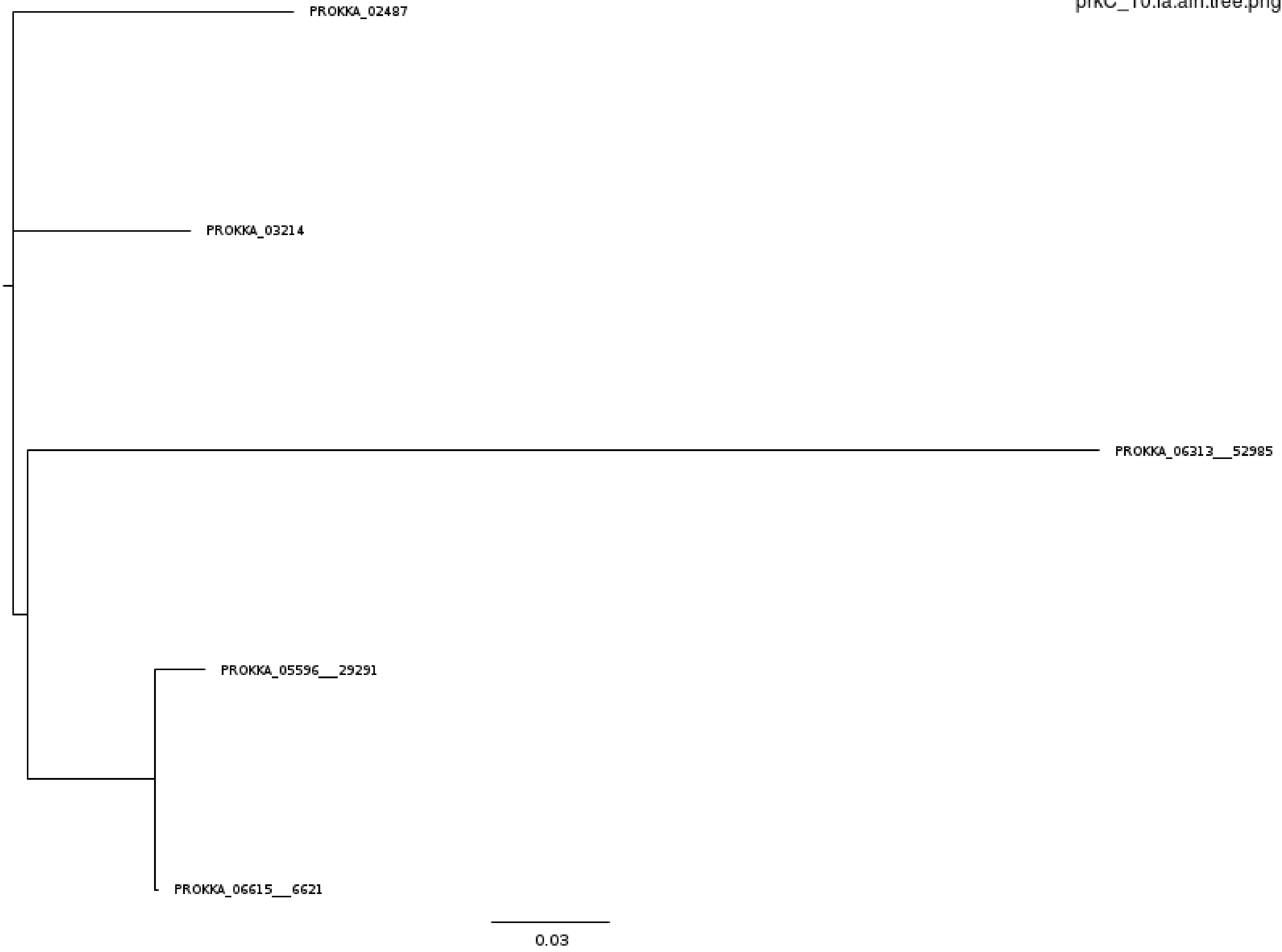

prkC_11.fa.aln.tree.png

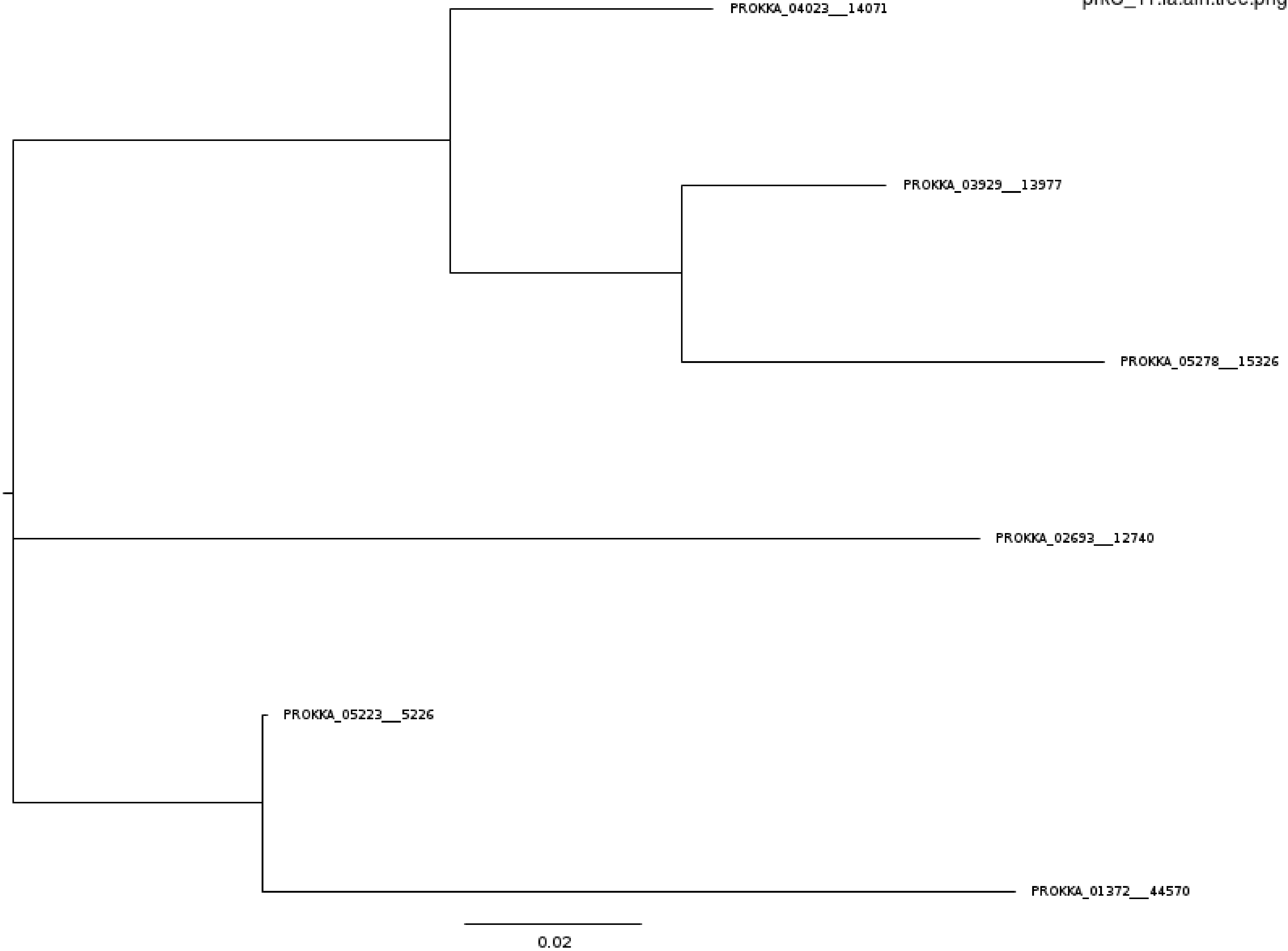

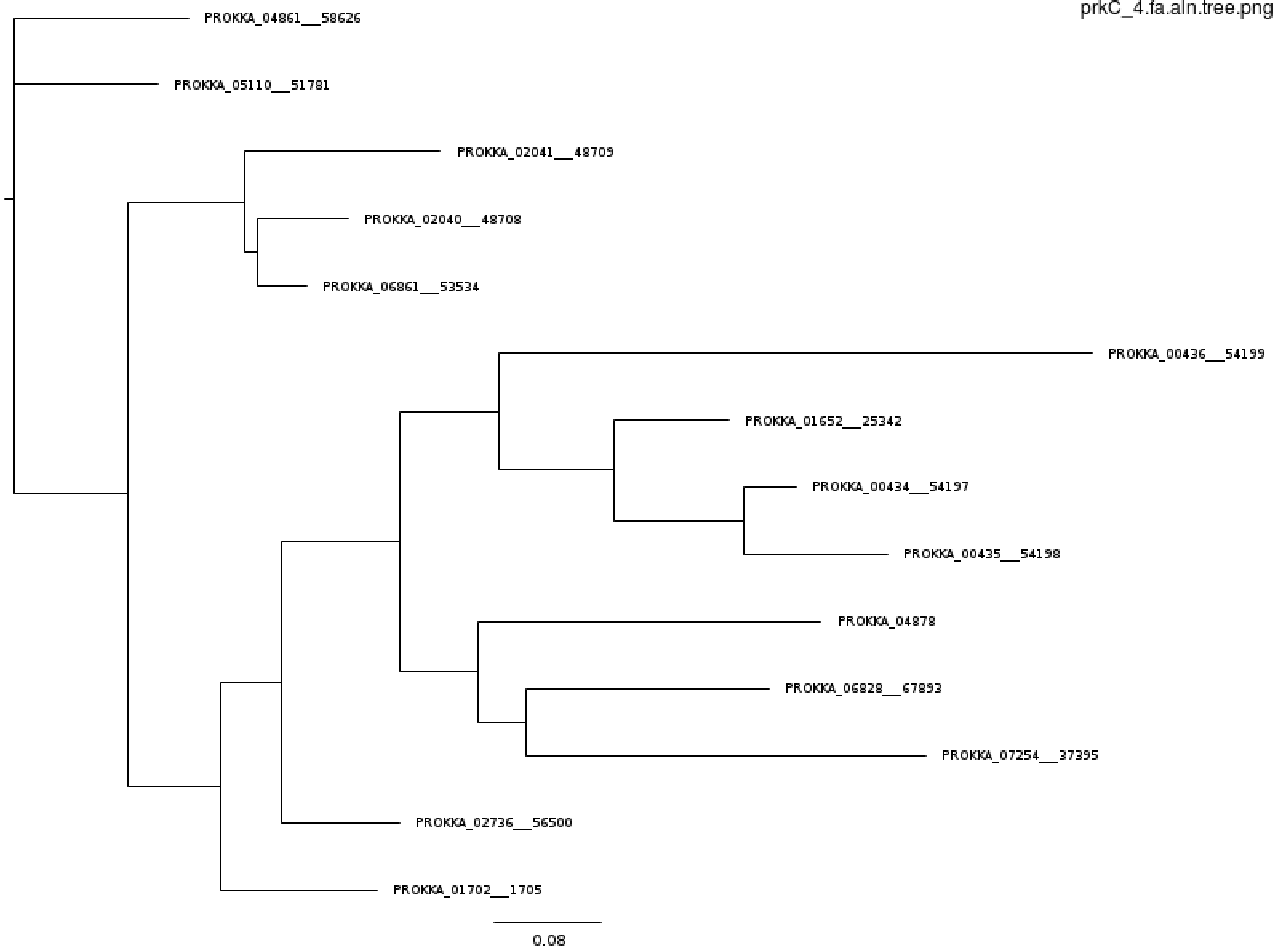
prkC_4.fa.aln.tree.png
PROKKA_04861___58626
PROKKA_05110___51781
PROKKA_02041___48709
PROKKA_02040___48708
PROKKA_06861___53534
PROKKA_00436___54199
PROKKA_01652___25342
PROKKA_00434___54197
PROKKA_00435___54198
PROKKA_04878
PROKKA_06828___67893
PROKKA_07254___37395
PROKKA_02736___56500
PROKKA_01702___1705
0.08

prkC_9.fa.aln.tree.png

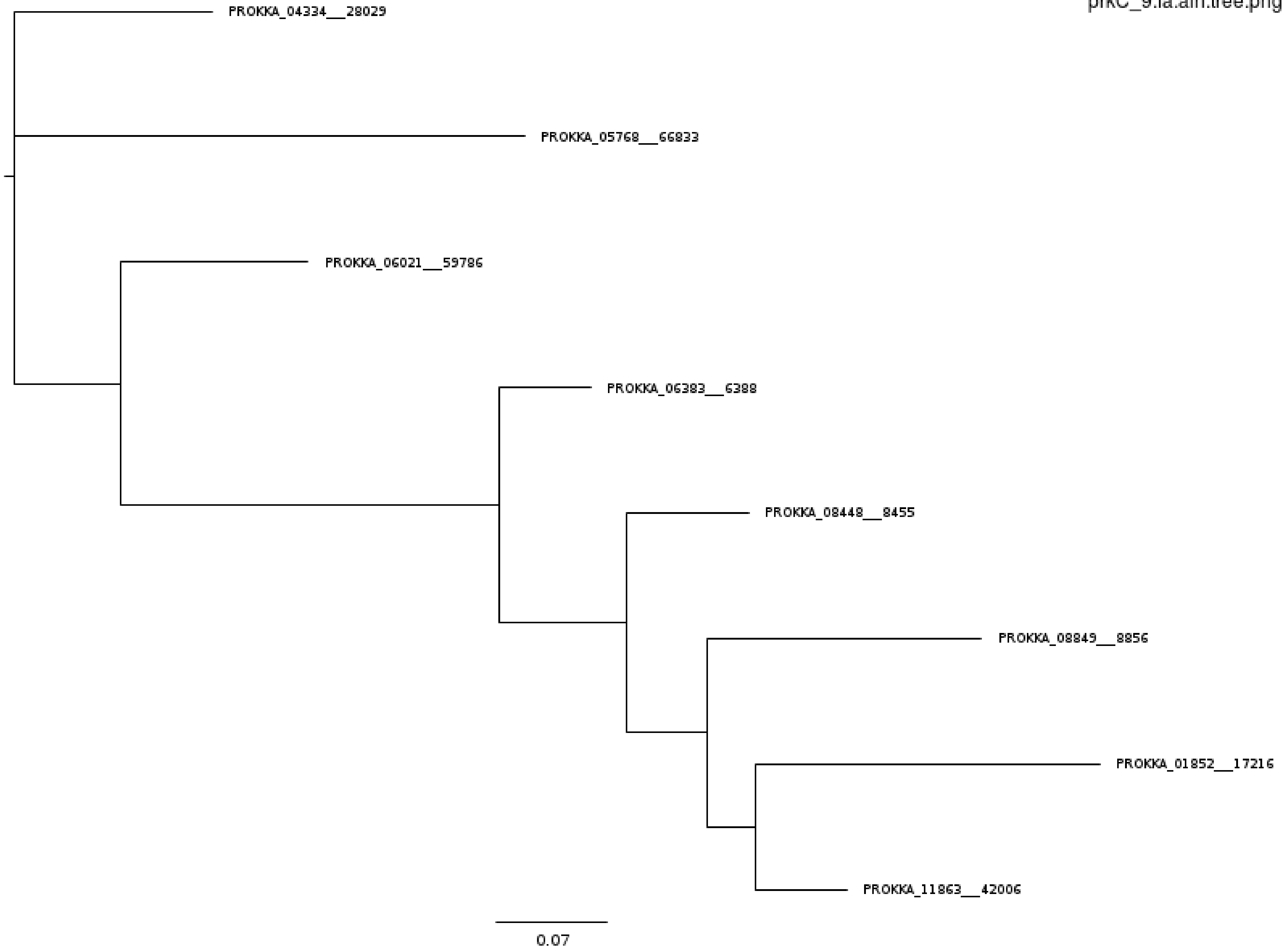

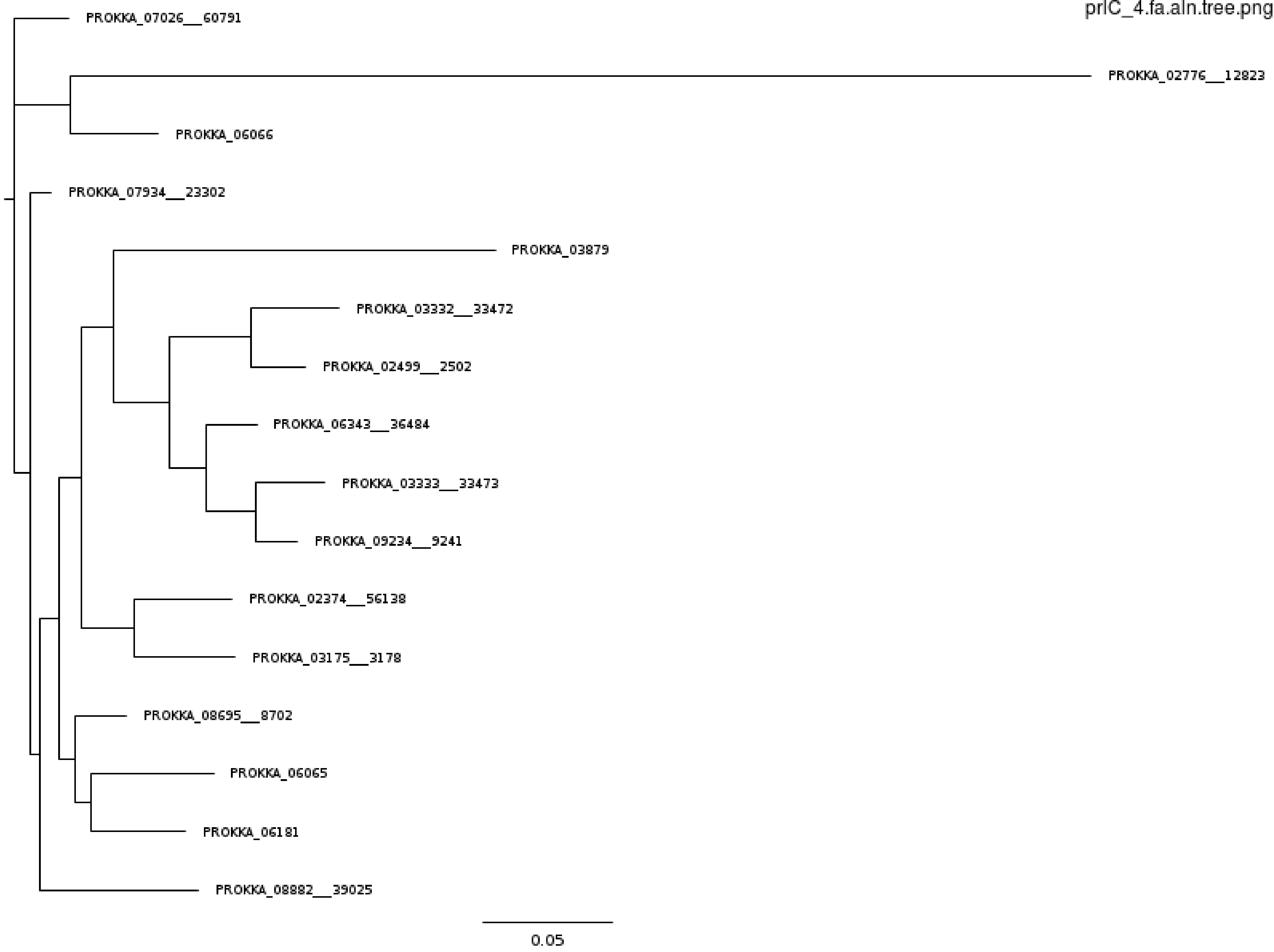
prlC_4.fa.aln.tree.png
PROKKA_07026___60791
PROKKA_02776___12823
PROKKA_06066
PROKKA_07934___23302
PROKKA_03879
PROKKA_03332___33472
PROKKA_02499___2502
PROKKA_06343___36484
PROKKA_03333___33473
PROKKA_09234___9241
PROKKA_02374___56138
PROKKA_03175___3178
PROKKA_08695___8702
PROKKA_06065
PROKKA_06181
PROKKA_08882___39025
0.05

prmC.fa.aln.tree.png

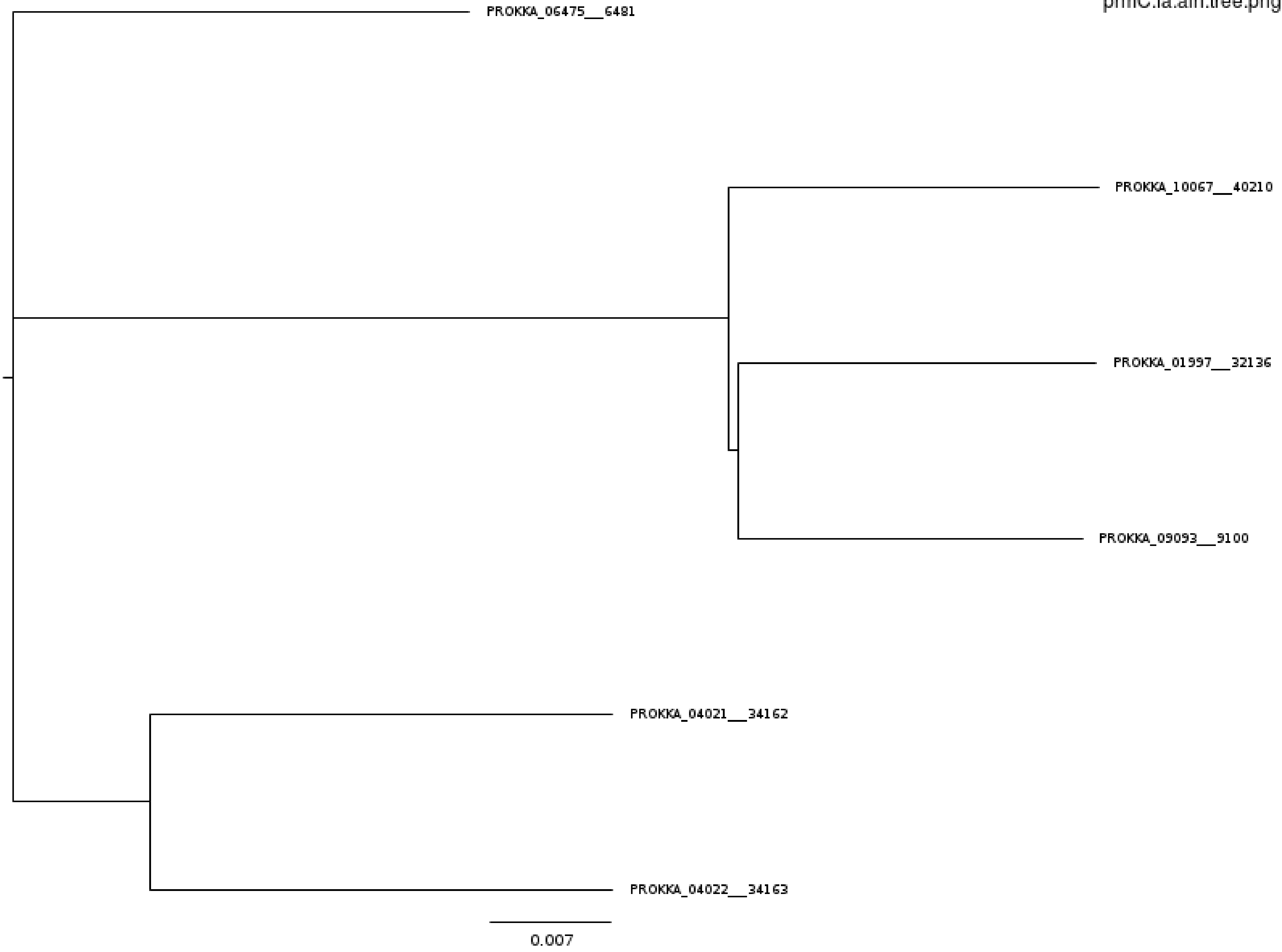

proA_1.fa.aln.tree.png

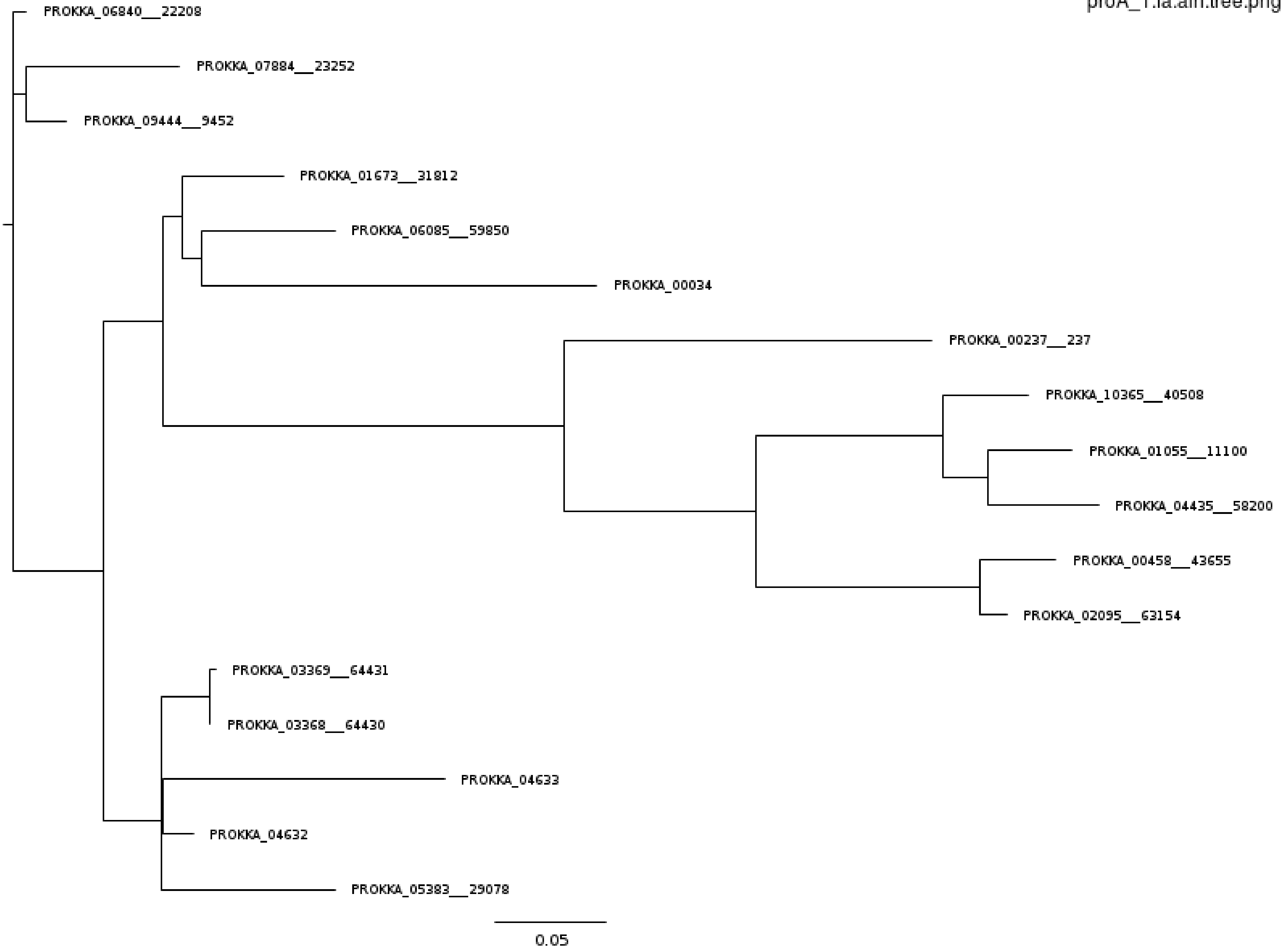

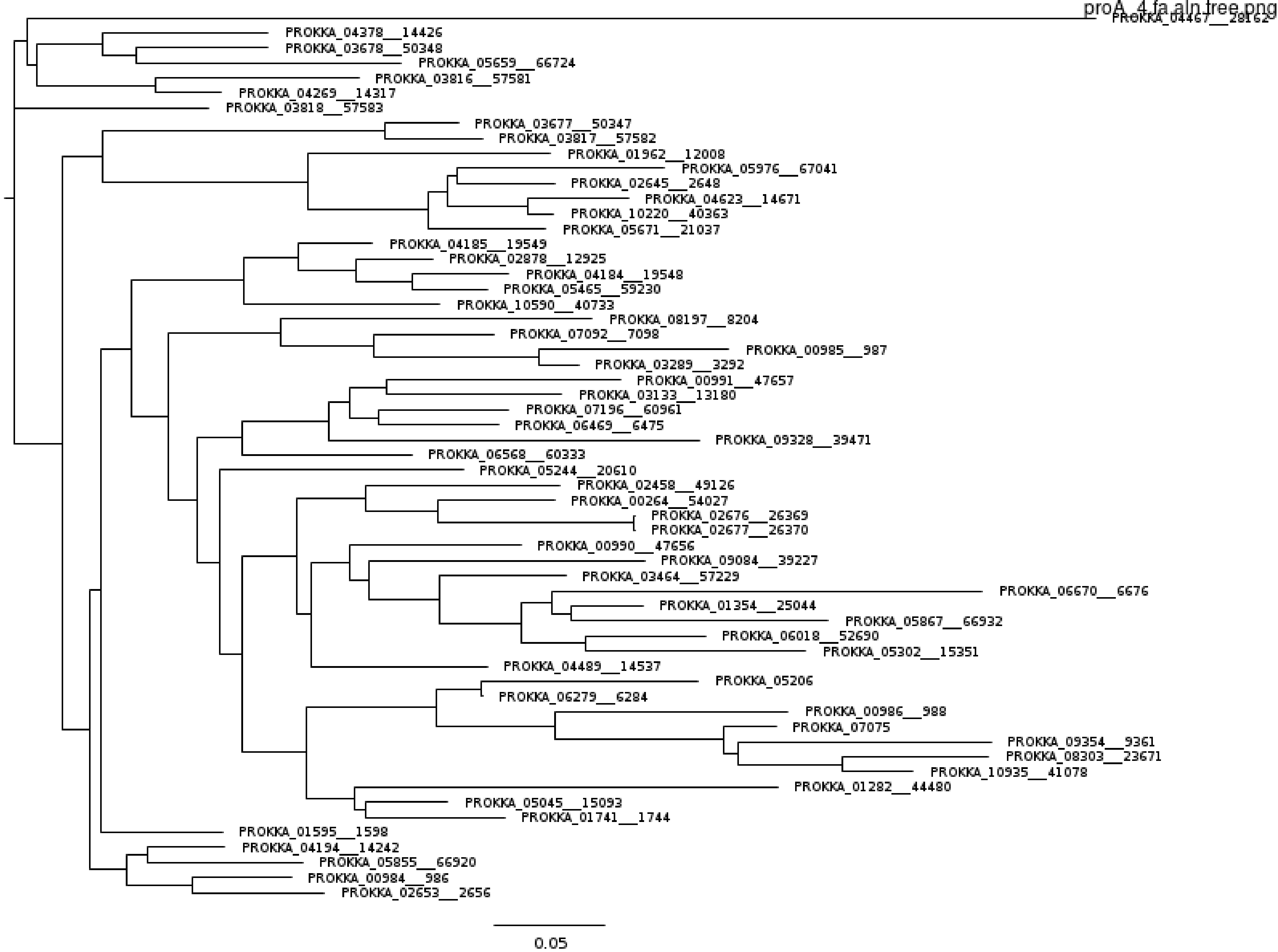
proA_4.fa.aln.tree.png
PROKKA_04467___28162
PROKKA_04378___14426
PROKKA_03678___50348
PROKKA_05659___66724
PROKKA_03816___57581
PROKKA_04269___14317
PROKKA_03818___57583
PROKKA_03677___50347
PROKKA_03817___57582
PROKKA_01962___12008
PROKKA_05976___67041
PROKKA_02645___2648
PROKKA_04623___14671
PROKKA_10220___40363
PROKKA_05671___21037
PROKKA_04185___19549
PROKKA_02878___12925
PROKKA_04184___19548
PROKKA_05465___59230
PROKKA_10590___40733
PROKKA_08197___8204
PROKKA_07092___7098
PROKKA_00985___987
PROKKA_03289___3292
PROKKA_00991___47657
PROKKA_03133___13180
PROKKA_07196___60961
PROKKA_06469___6475
PROKKA_09328___39471
PROKKA_06568___60333
PROKKA_05244___20610
PROKKA_02458___49126
PROKKA_00264___54027
PROKKA_02676___26369
PROKKA_02677___26370
PROKKA_00990___47656
PROKKA_09084___39227
PROKKA_03464___57229
PROKKA_06670___6676
PROKKA_01354___25044
PROKKA_05867___66932
PROKKA_06018___52690
PROKKA_05302___15351
PROKKA_04489___14537
PROKKA_05206
PROKKA_06279___6284
PROKKA_00986___988
PROKKA_07075
PROKKA_09354___9361
PROKKA_08303___23671
PROKKA_10935___41078
PROKKA_01282___44480
PROKKA_05045___15093
PROKKA_01741___1744
PROKKA_01595___1598
PROKKA_04194___14242
PROKKA_05855___66920
PROKKA_00984___986
PROKKA_02653___2656
0.05

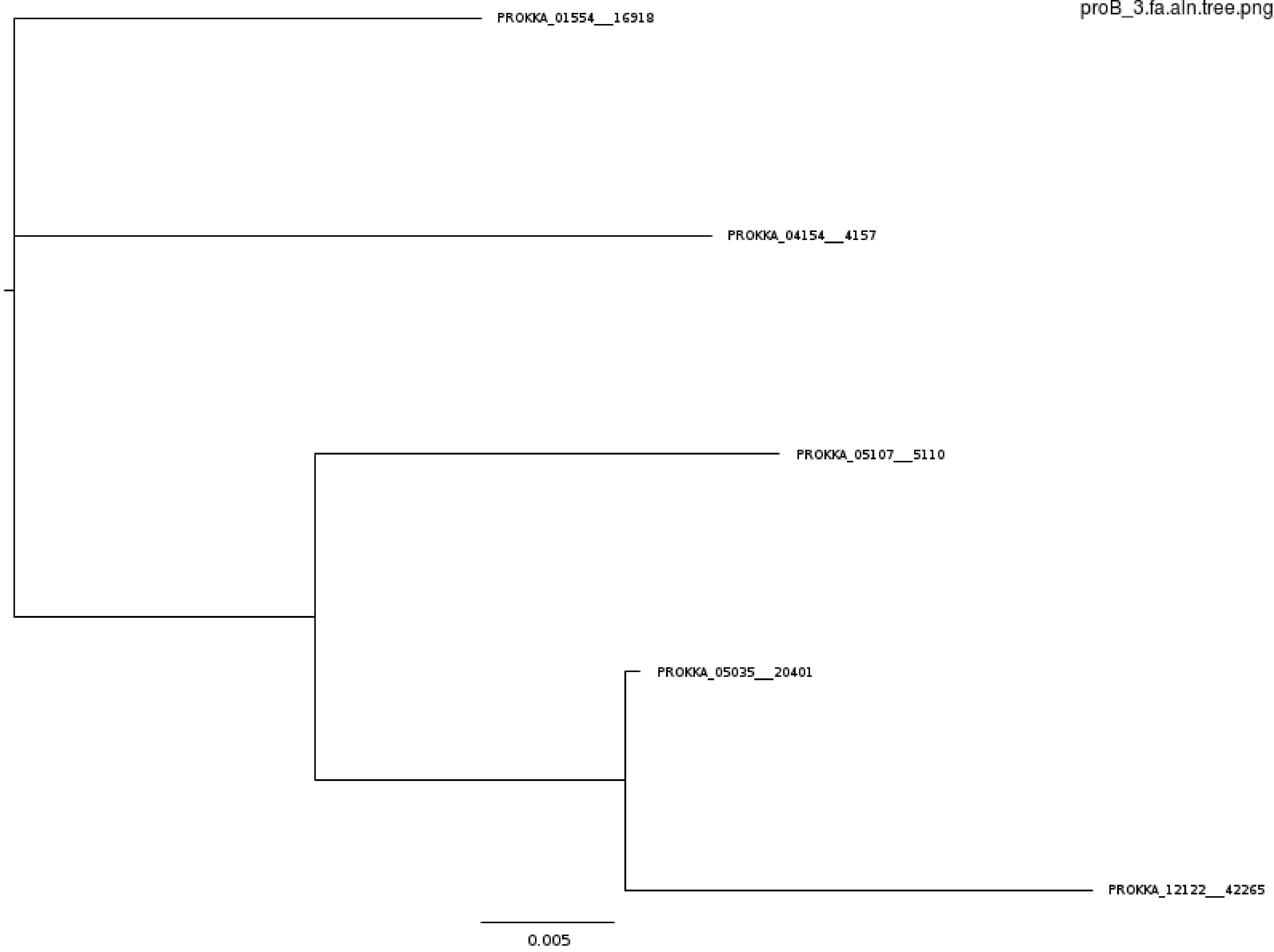
proB_3.fa.aln.tree.png
PROKKA_01554__16918
PROKKA_04154__4157
PROKKA_05107__5110
PROKKA_05035__20401
PROKKA_12122__42265
0.005

proC_2.fa.aln.tree.png

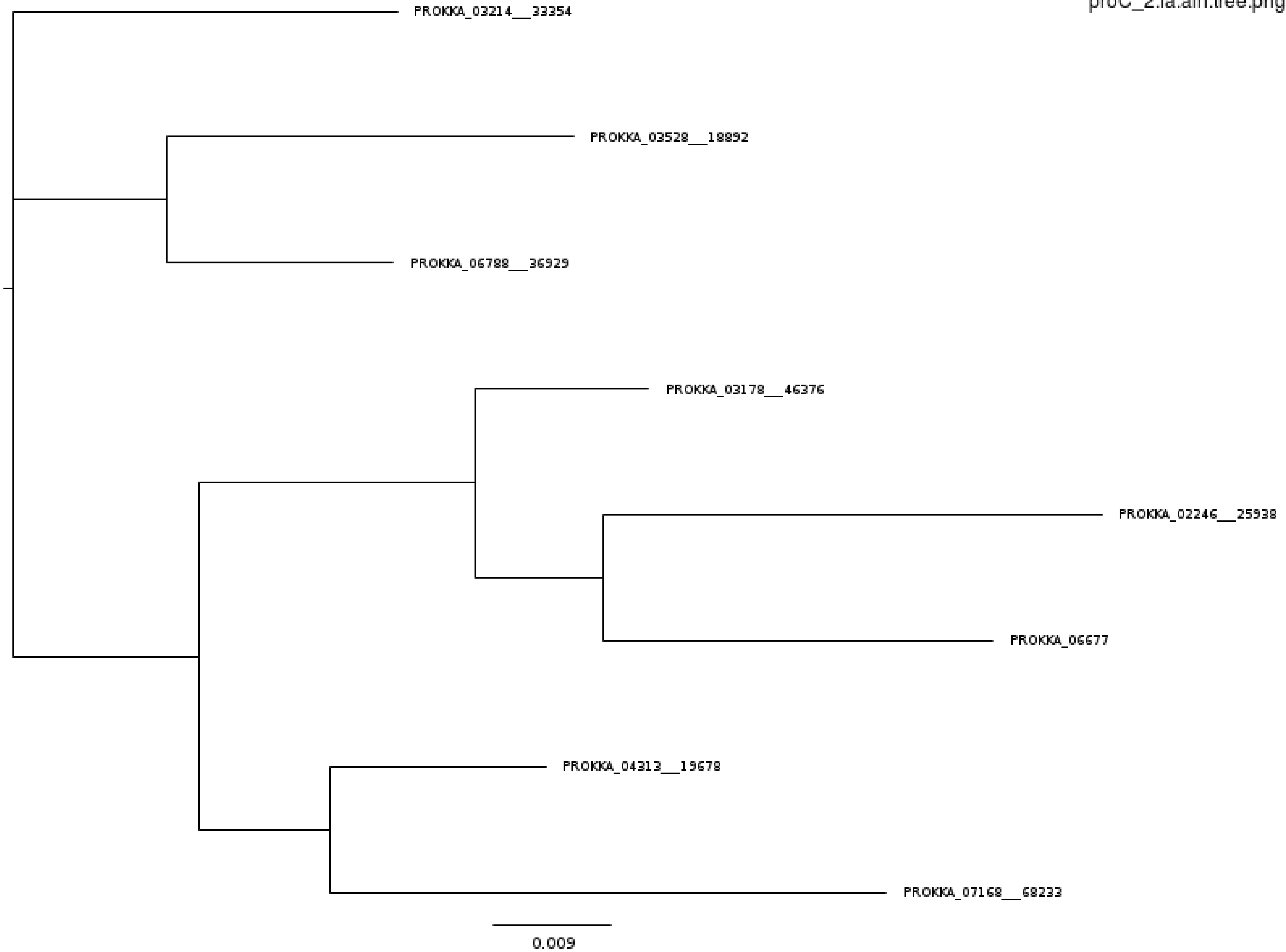

proP_2.fa.aln.tree.png

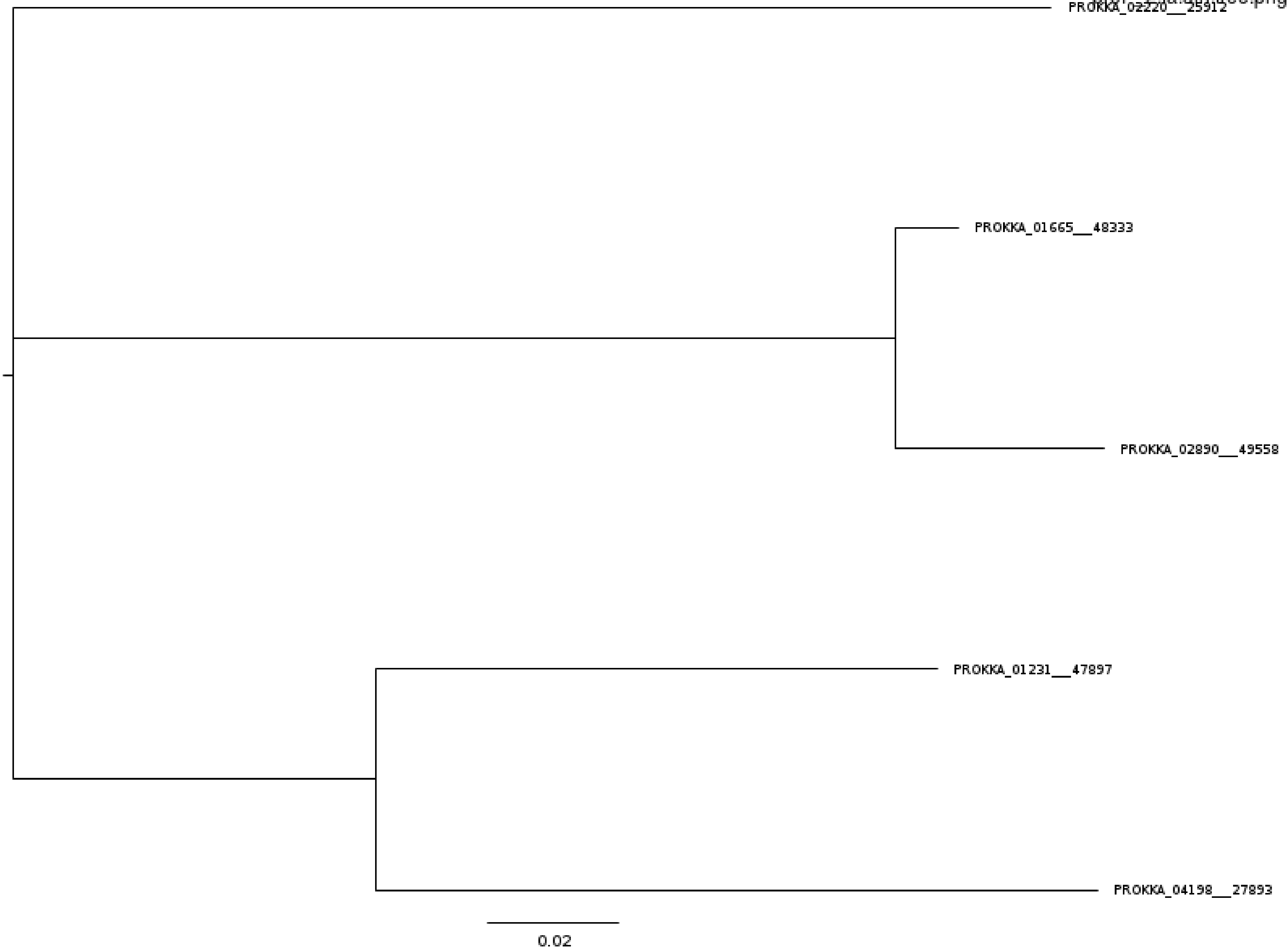

proP_4.fa.aln.tree.png

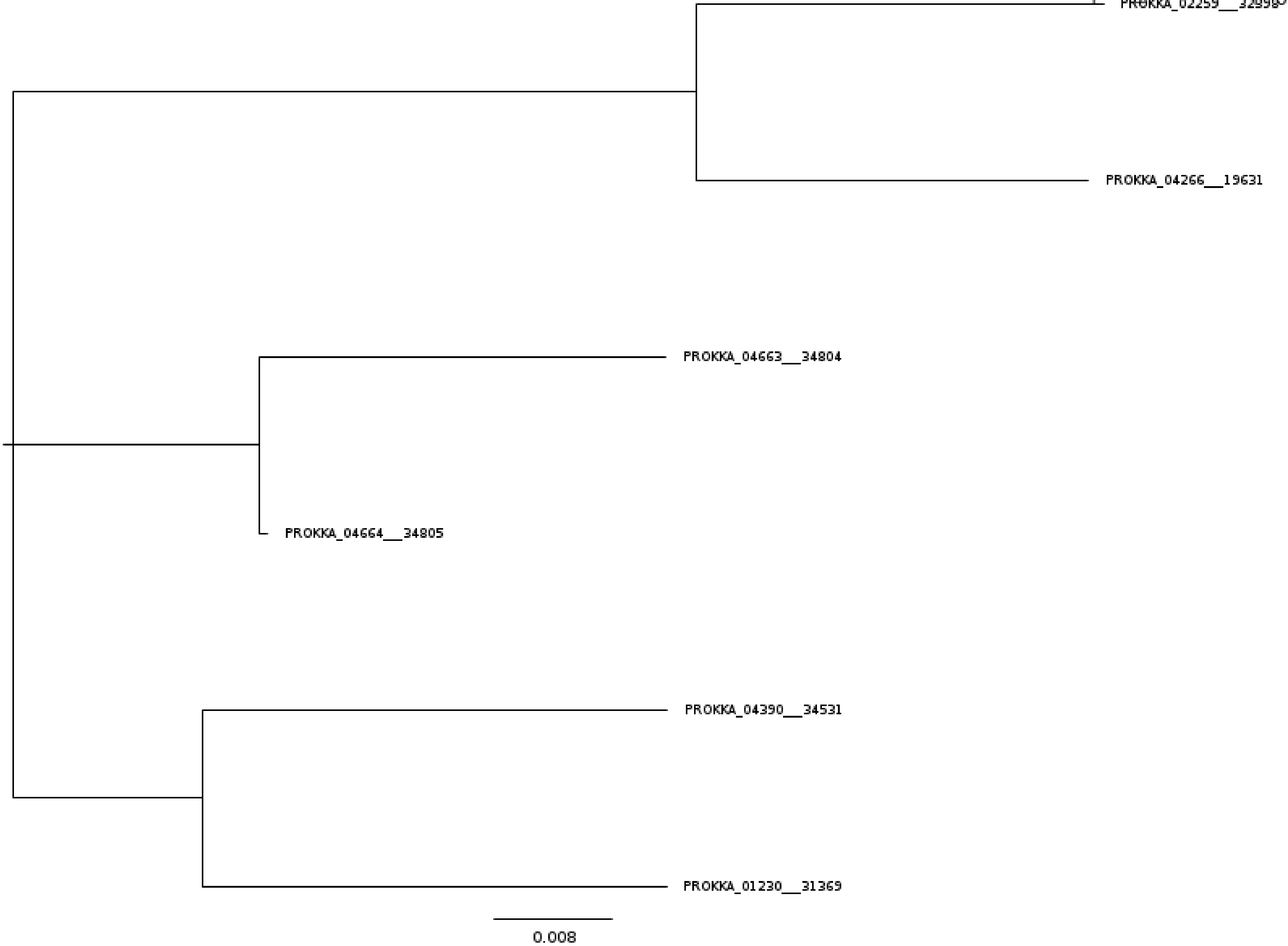

proP.fa.aln.tree.png

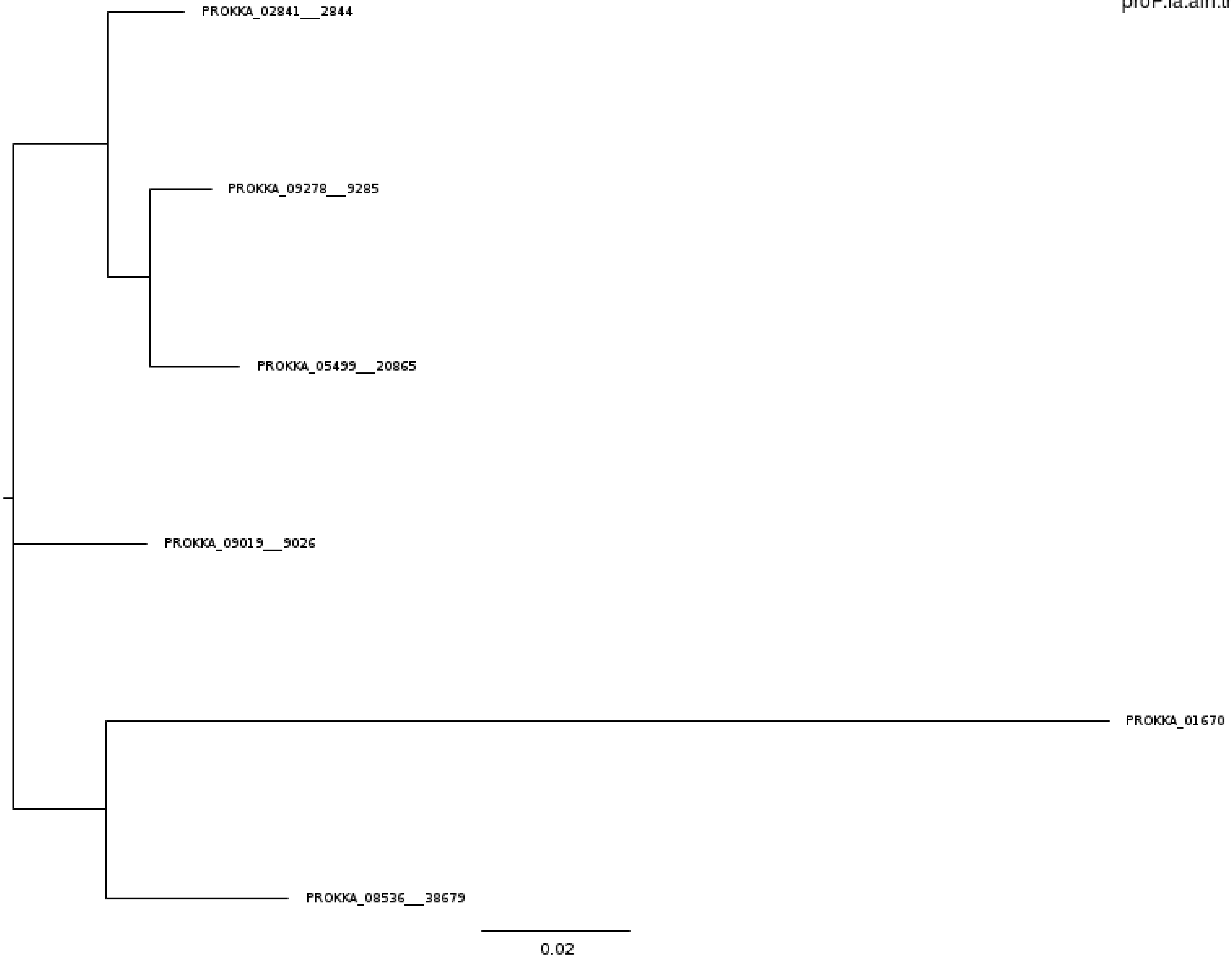

proS_2.fa.aln.tree.png

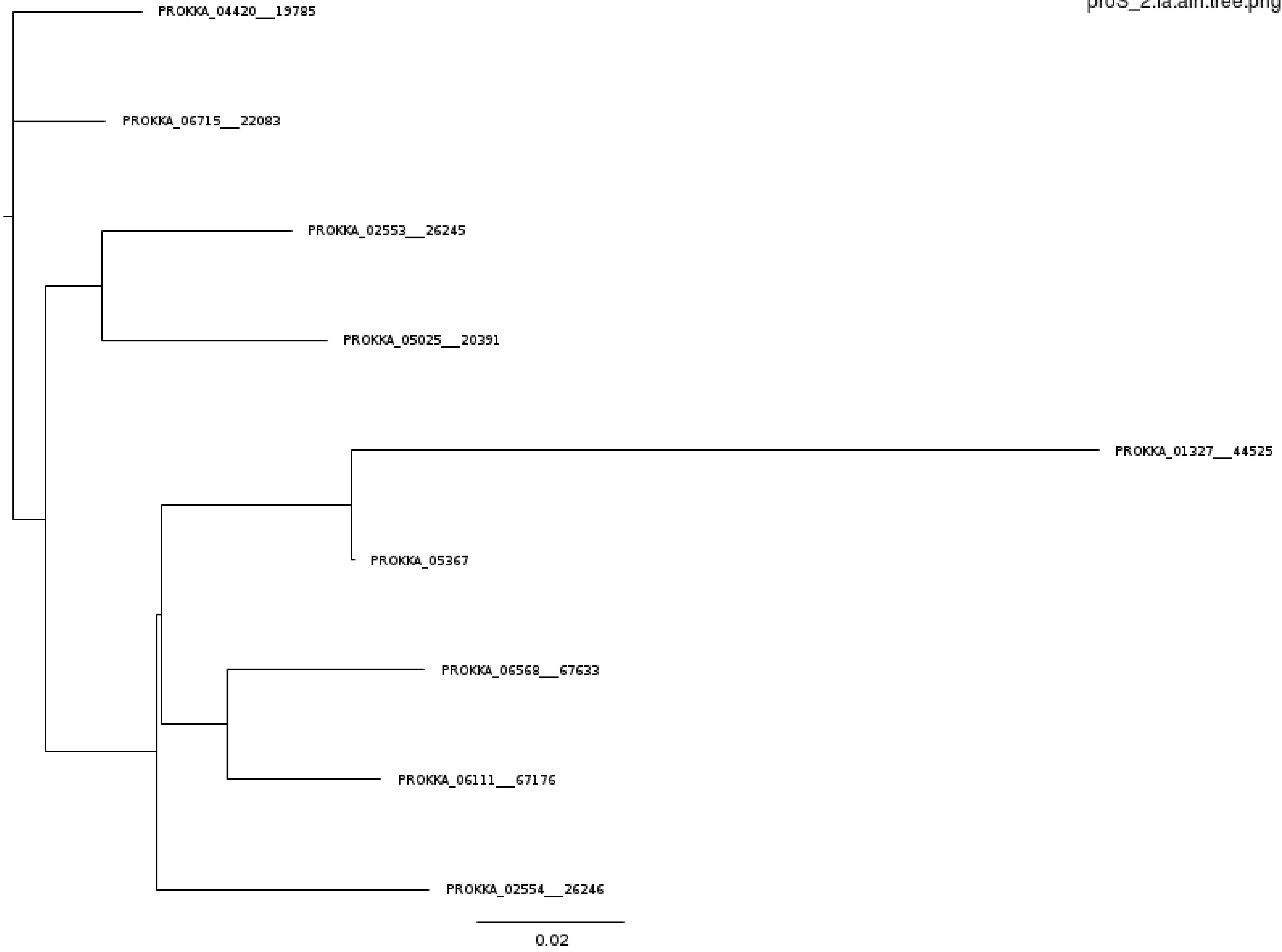

prpD.fa.aln.tree.png

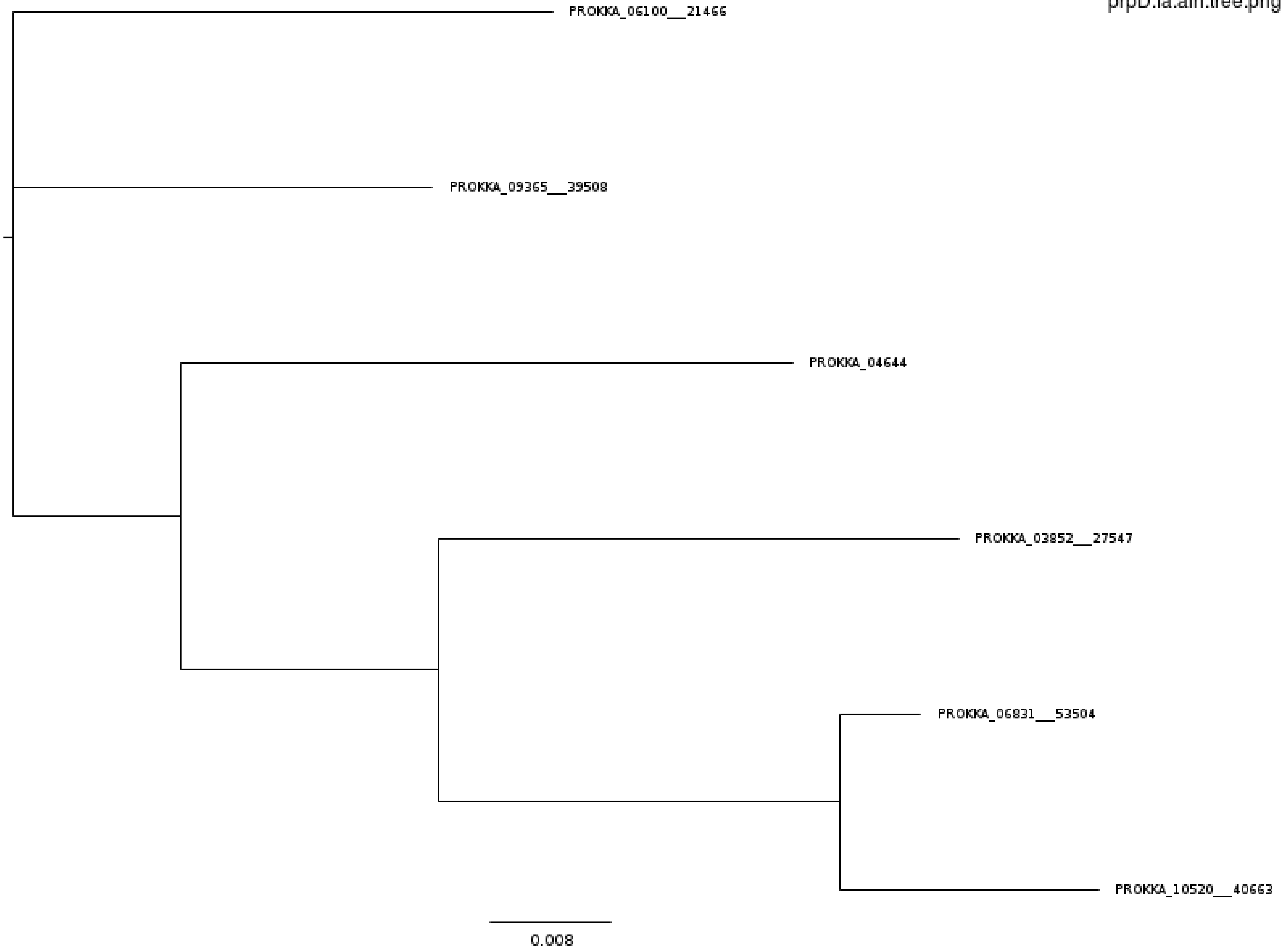

prpL_3.fa.aln.tree.png

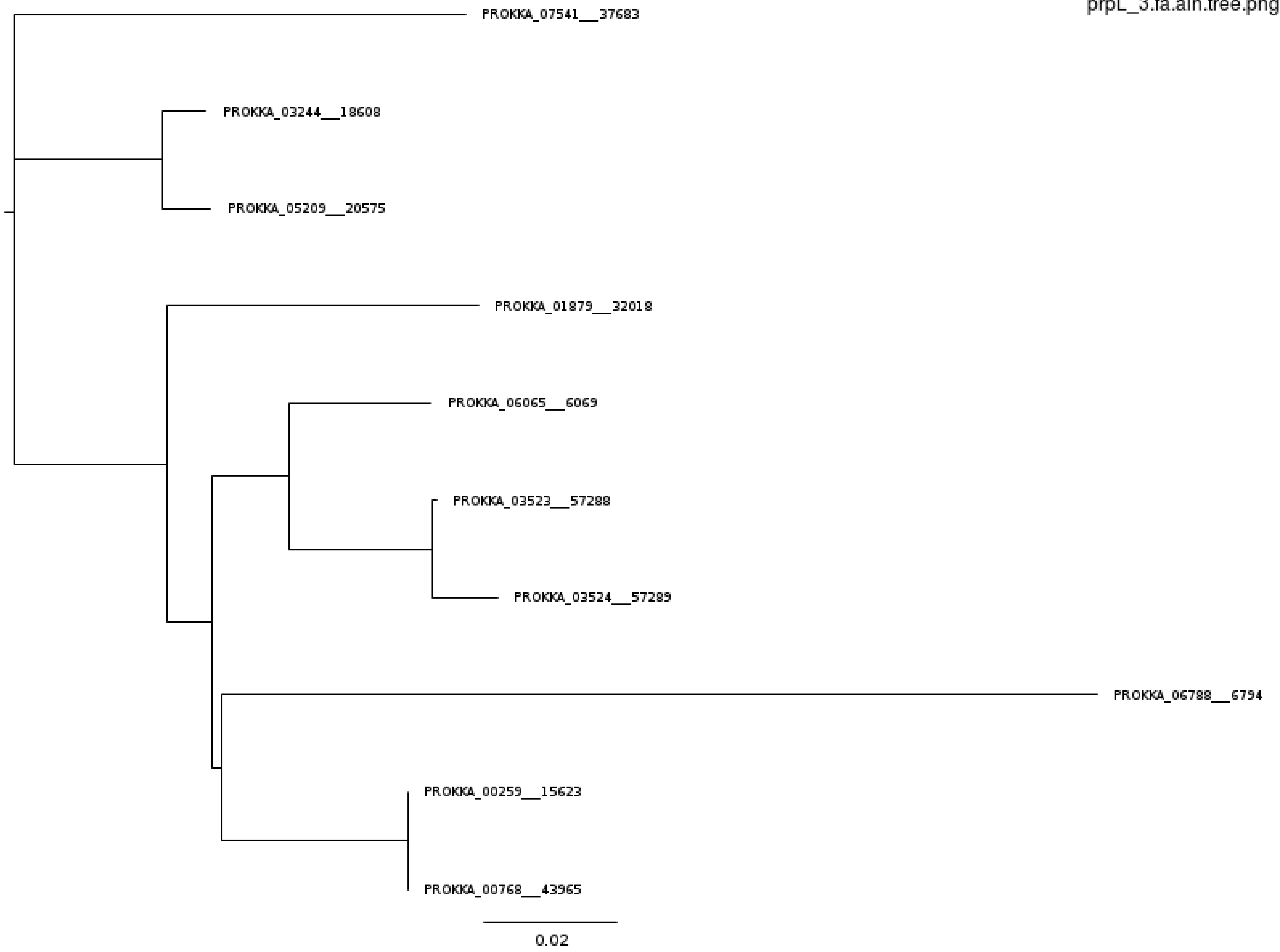

prs_3.fa.aln.tree.png

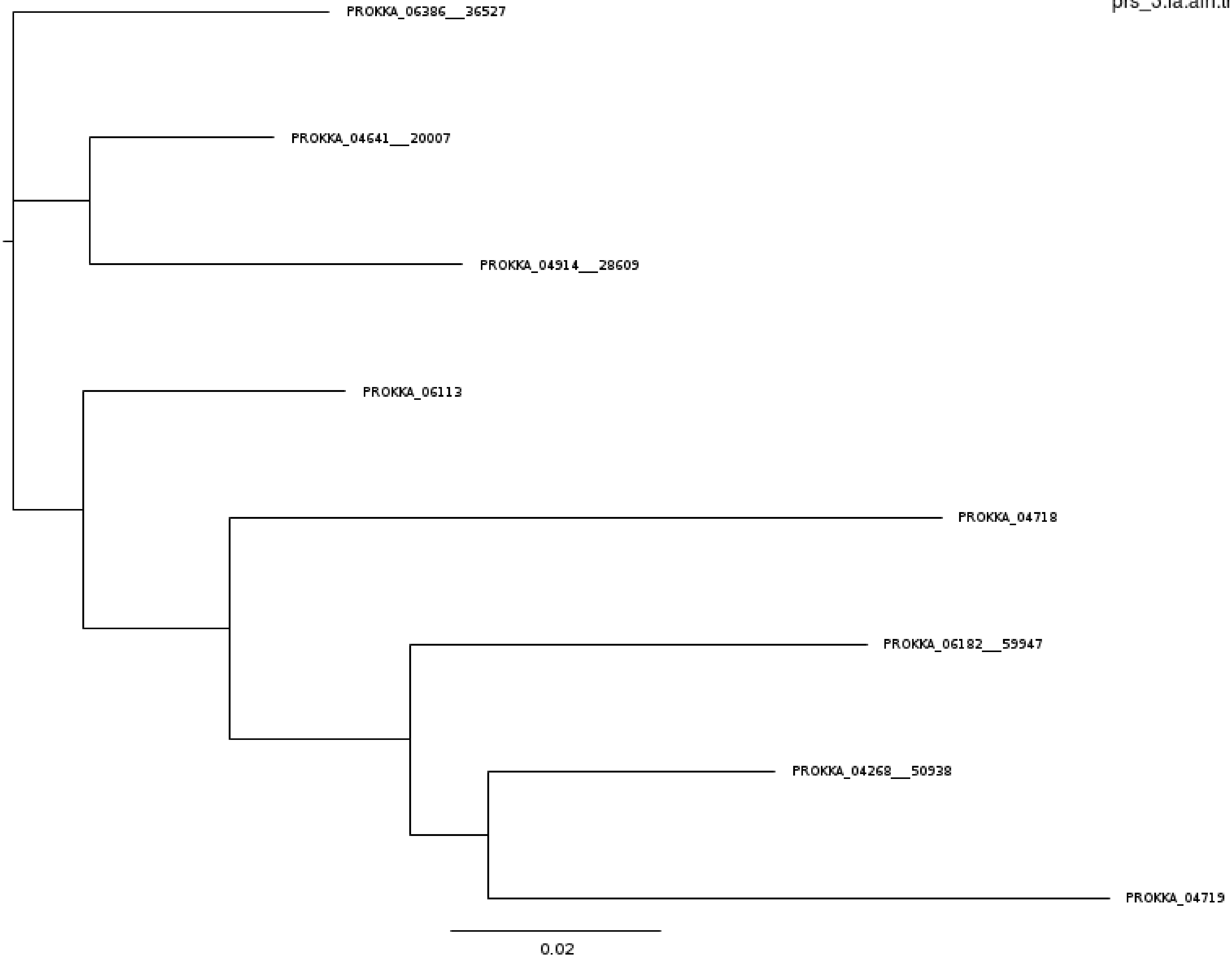

psd_2.fa.aln.tree.png

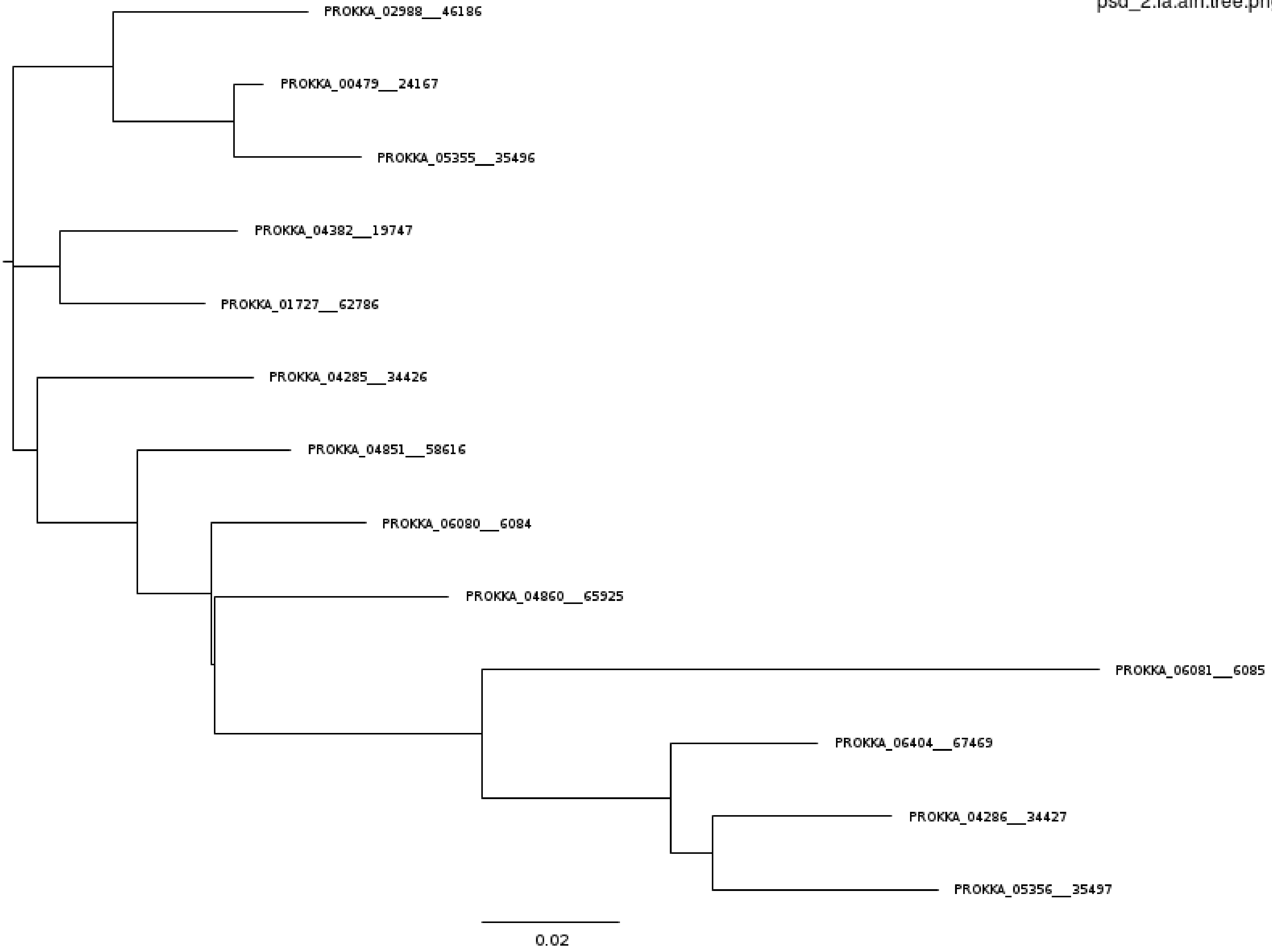

pstA_2.fa.aln.tree.png

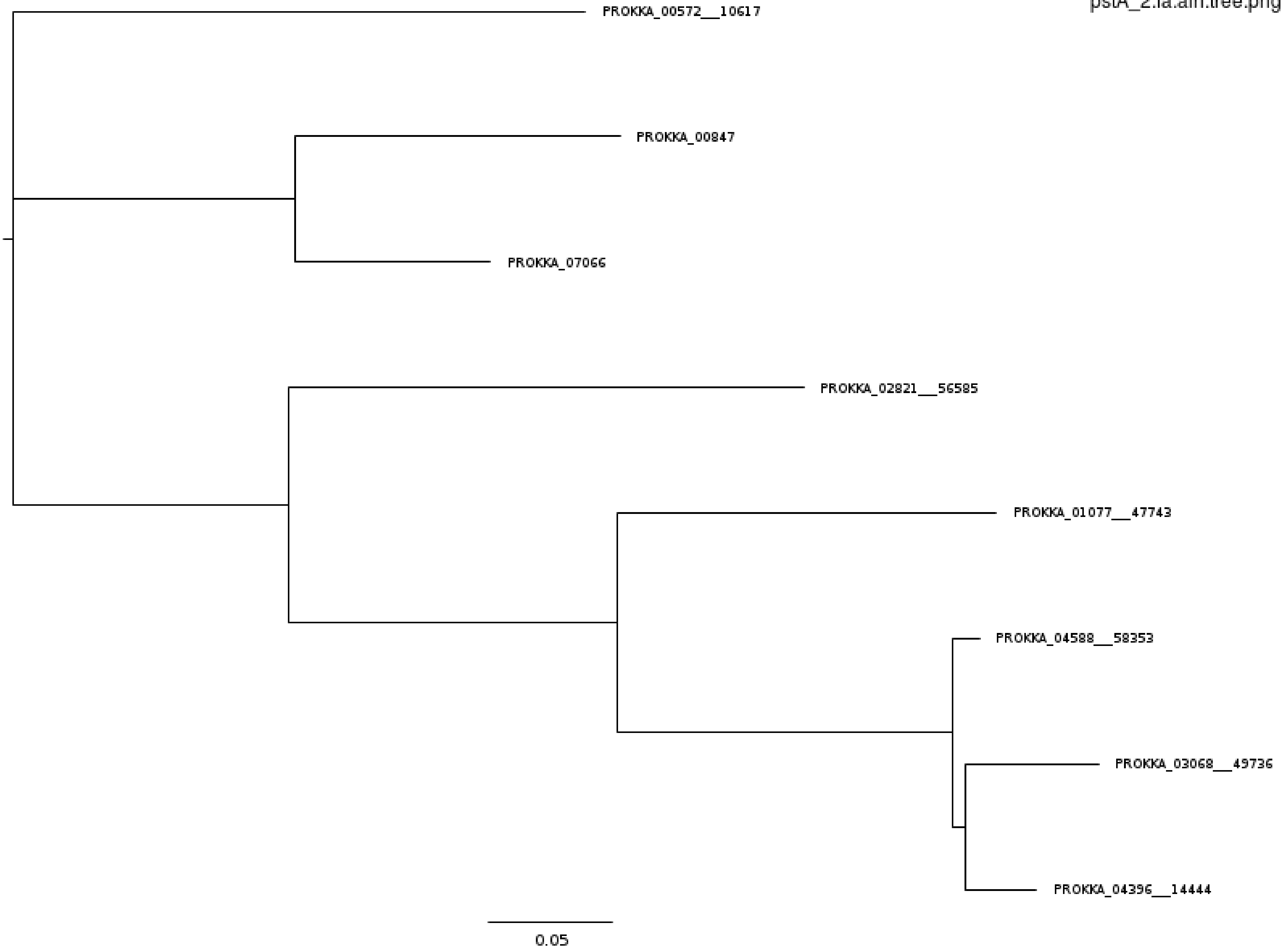

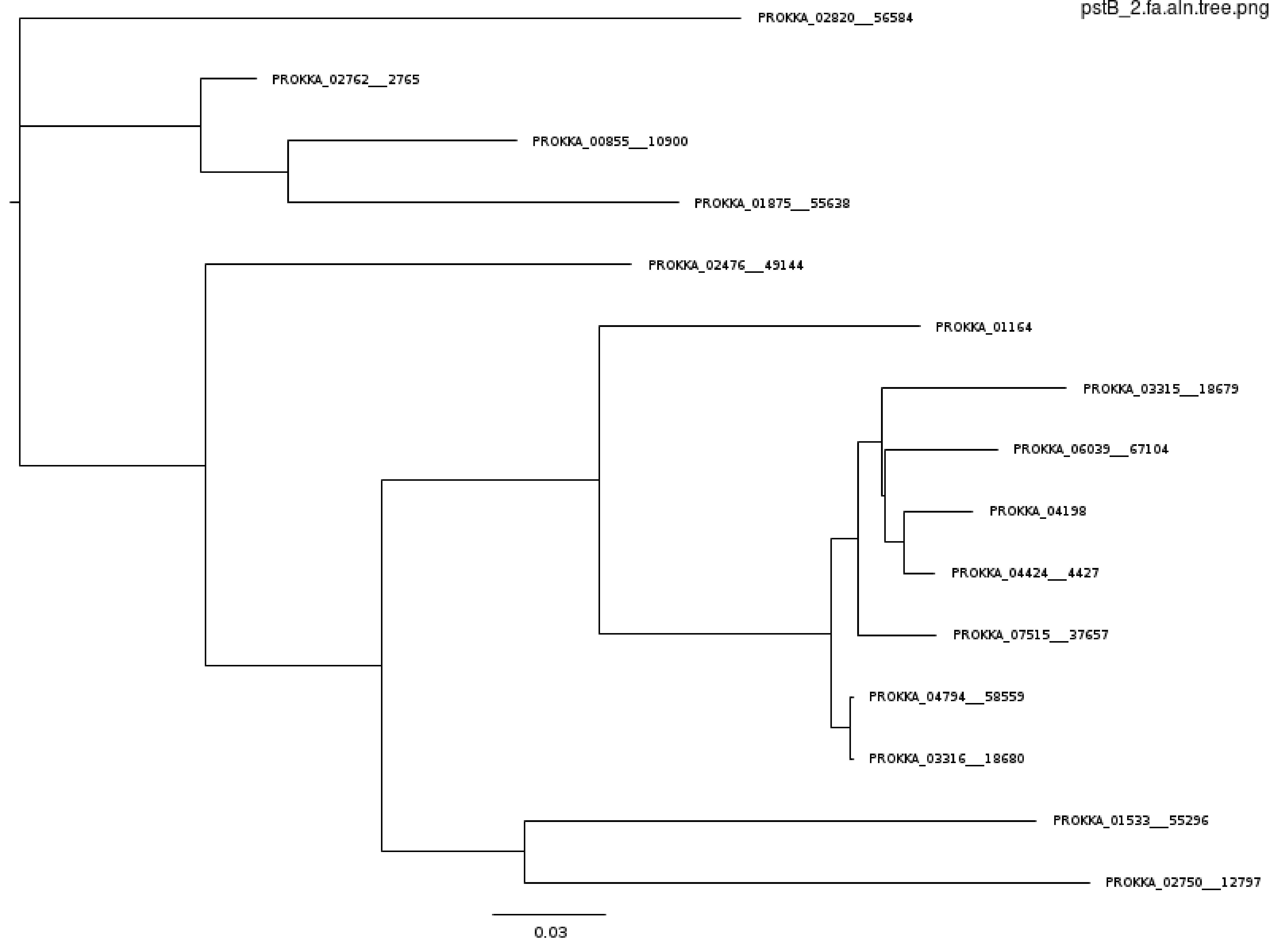
pstB_2.fa.aln.tree.png
PROKKA_02820__56584
PROKKA_02762__2765
PROKKA_00855__10900
PROKKA_01875__55638
PROKKA_02476__49144
PROKKA_01164
PROKKA_03315__18679
PROKKA_06039__67104
PROKKA_04198
PROKKA_04424__4427
PROKKA_07515__37657
PROKKA_04794__58559
PROKKA_03316__18680
PROKKA_01533__55296
PROKKA_02750__12797
0.03

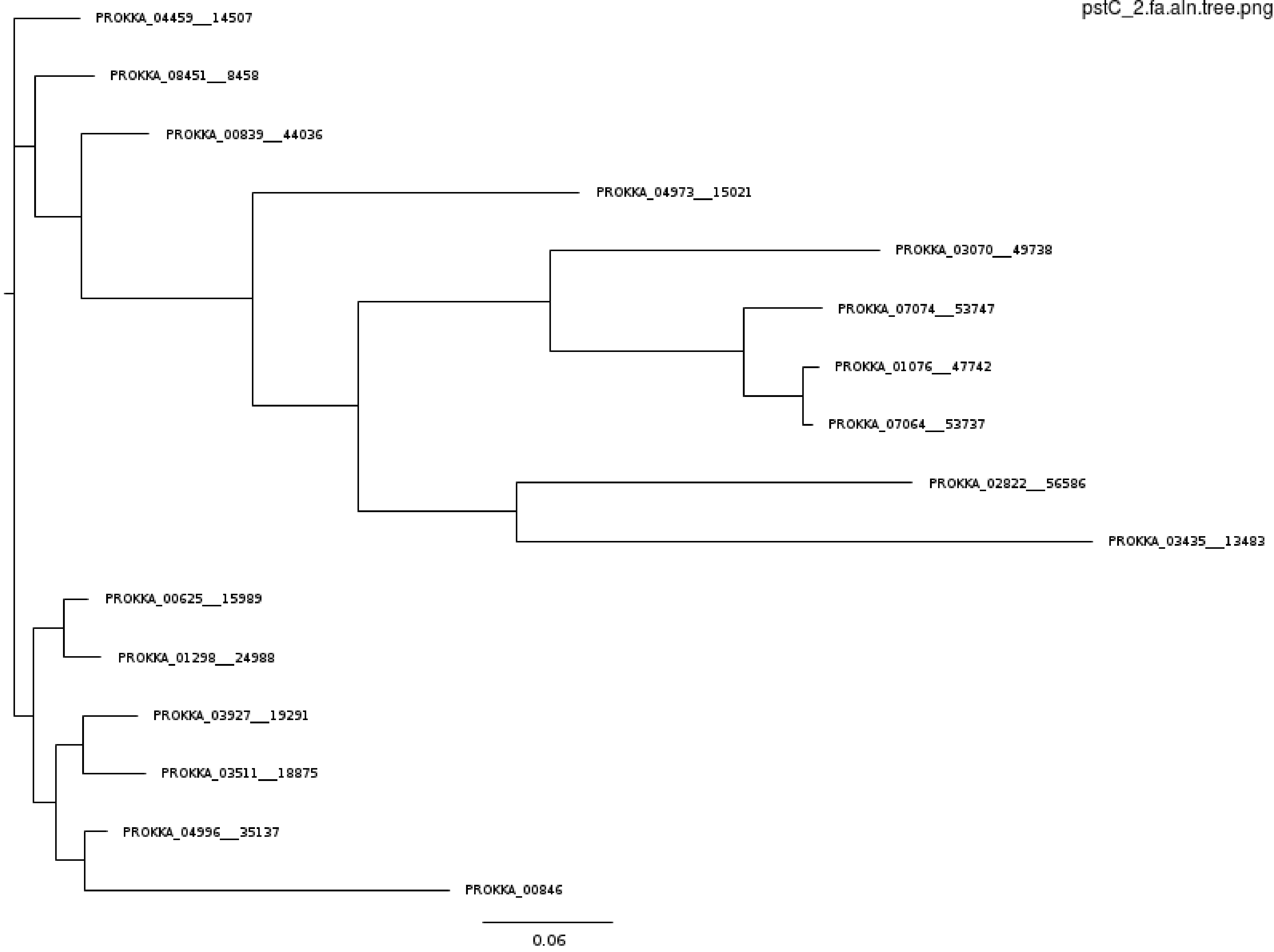
pstC_2.fa.aln.tree.png
PROKKA_04459___14507
PROKKA_08451___8458
PROKKA_00839___44036
PROKKA_04973___15021
PROKKA_03070___49738
PROKKA_07074___53747
PROKKA_01076___47742
PROKKA_07064___53737
PROKKA_02822___56586
PROKKA_03435___13483
PROKKA_00625___15989
PROKKA_01298___24988
PROKKA_03927___19291
PROKKA_03511___18875
PROKKA_04996___35137
PROKKA_00846
0.06

pstS_4.fa.aln.tree.png

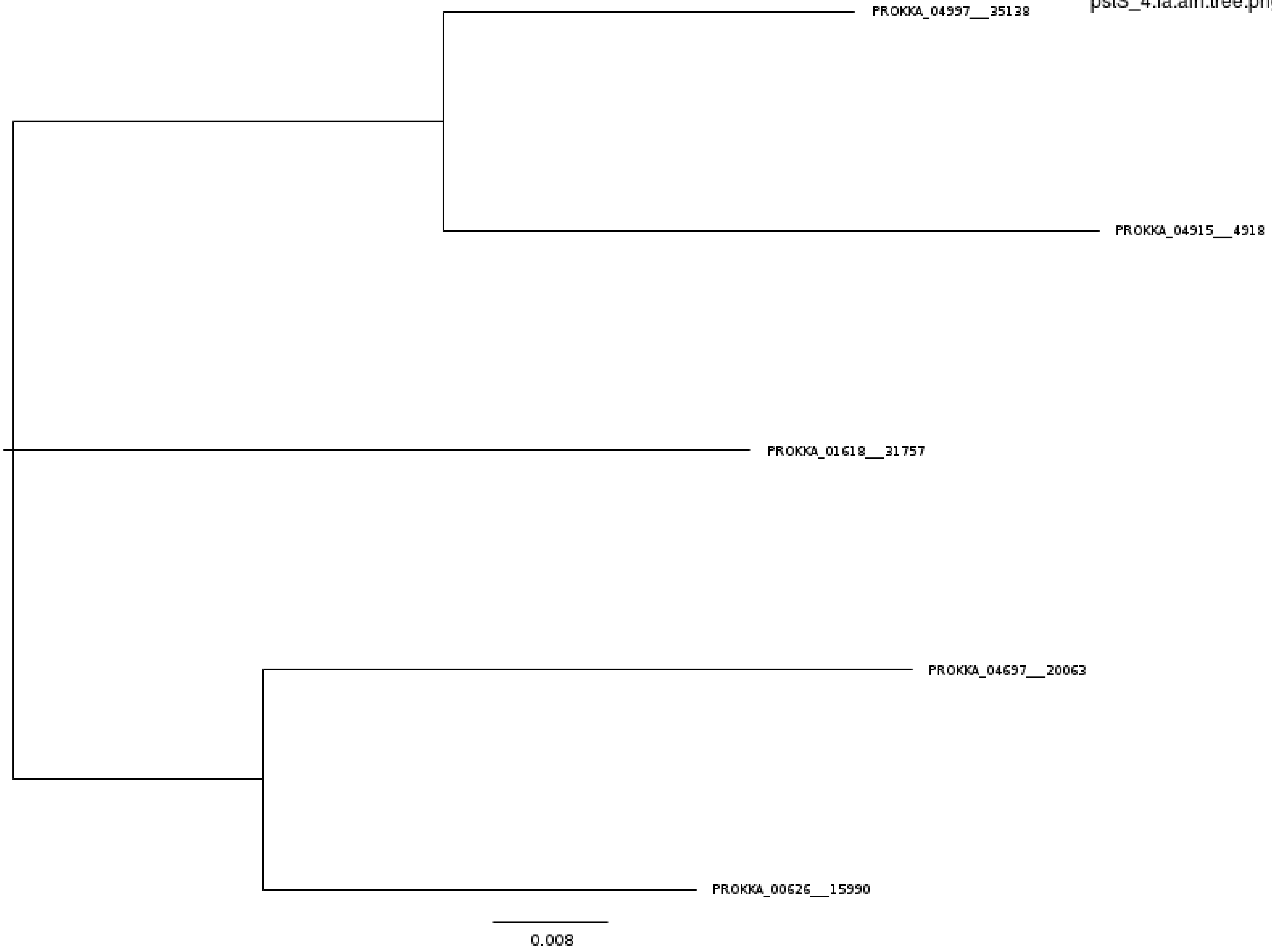

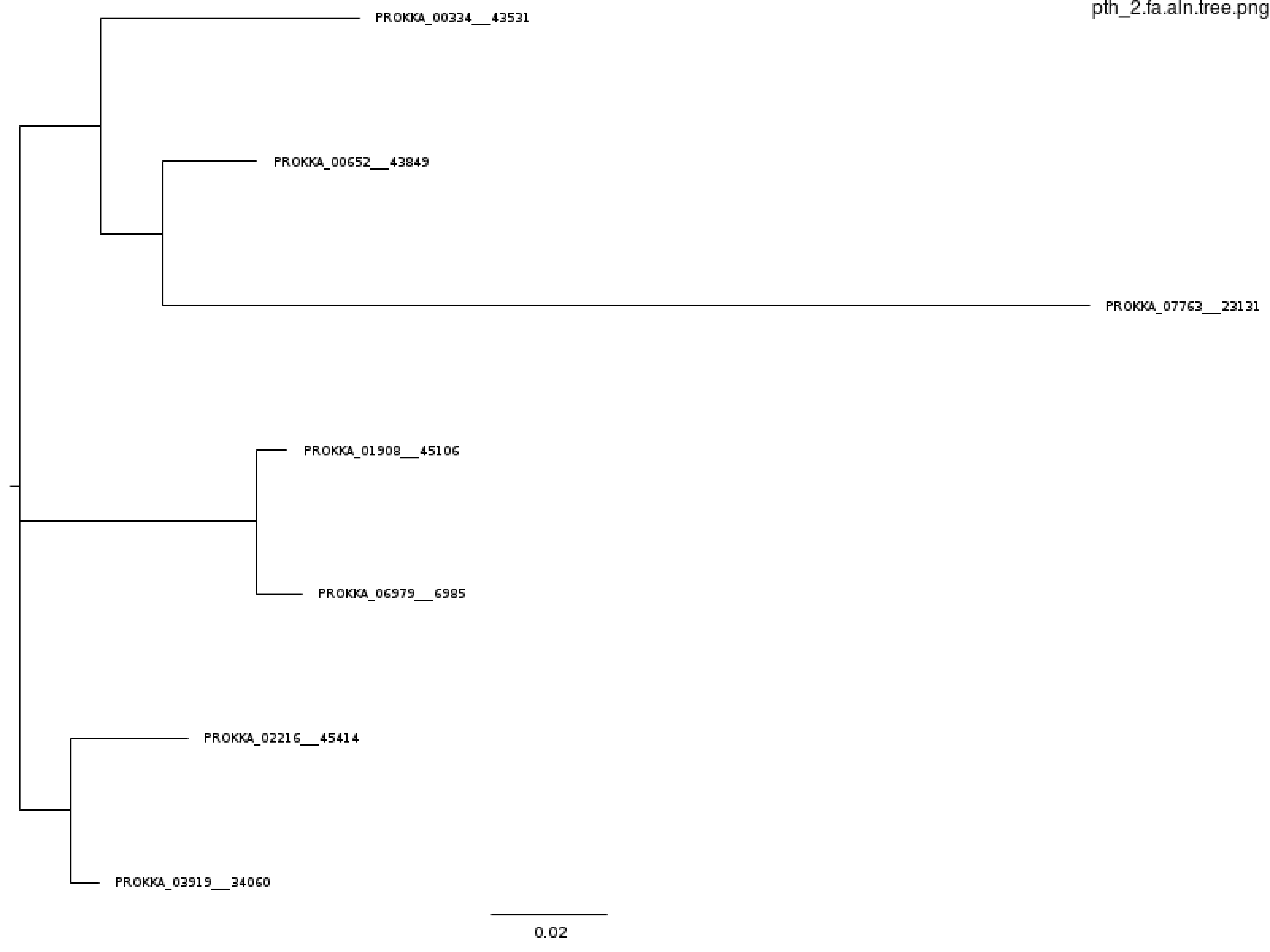
pth_2.fa.aln.tree.png
PROKKA_00334___43531
PROKKA_00652___43849
PROKKA_07763___23131
PROKKA_01908___45106
PROKKA_06979___6985
PROKKA_02216___45414
PROKKA_03919___34060
0.02

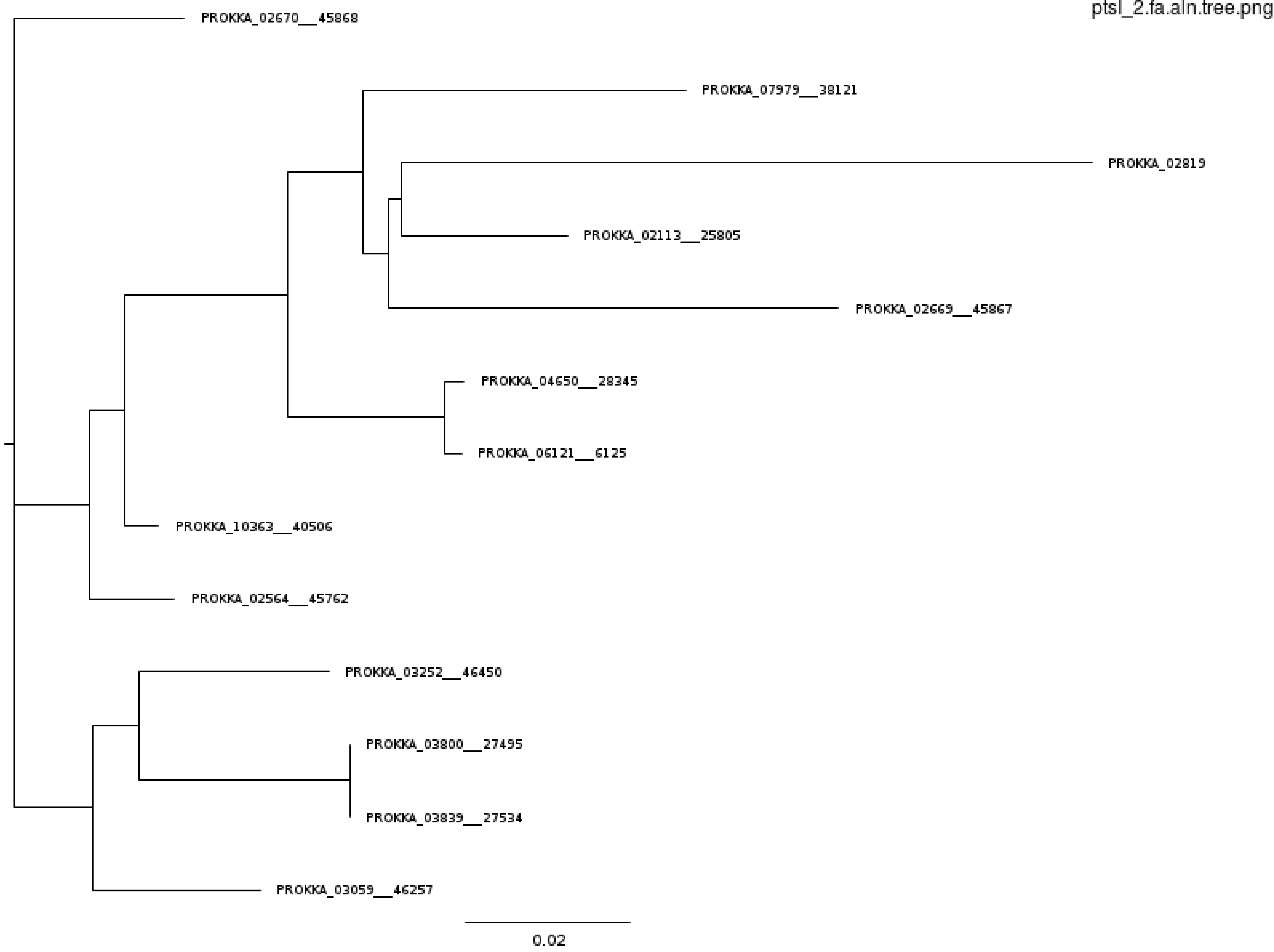
ptsI_2.fa.aln.tree.png
PROKKA_02670___45868
PROKKA_07979___38121
PROKKA_02819
PROKKA_02113___25805
PROKKA_02669___45867
PROKKA_04650___28345
PROKKA_06121___6125
PROKKA_10363___40506
PROKKA_02564___45762
PROKKA_03252___46450
PROKKA_03800___27495
PROKKA_03839___27534
PROKKA_03059___46257
0.02

ptsI_3.fa.aln.tree.png

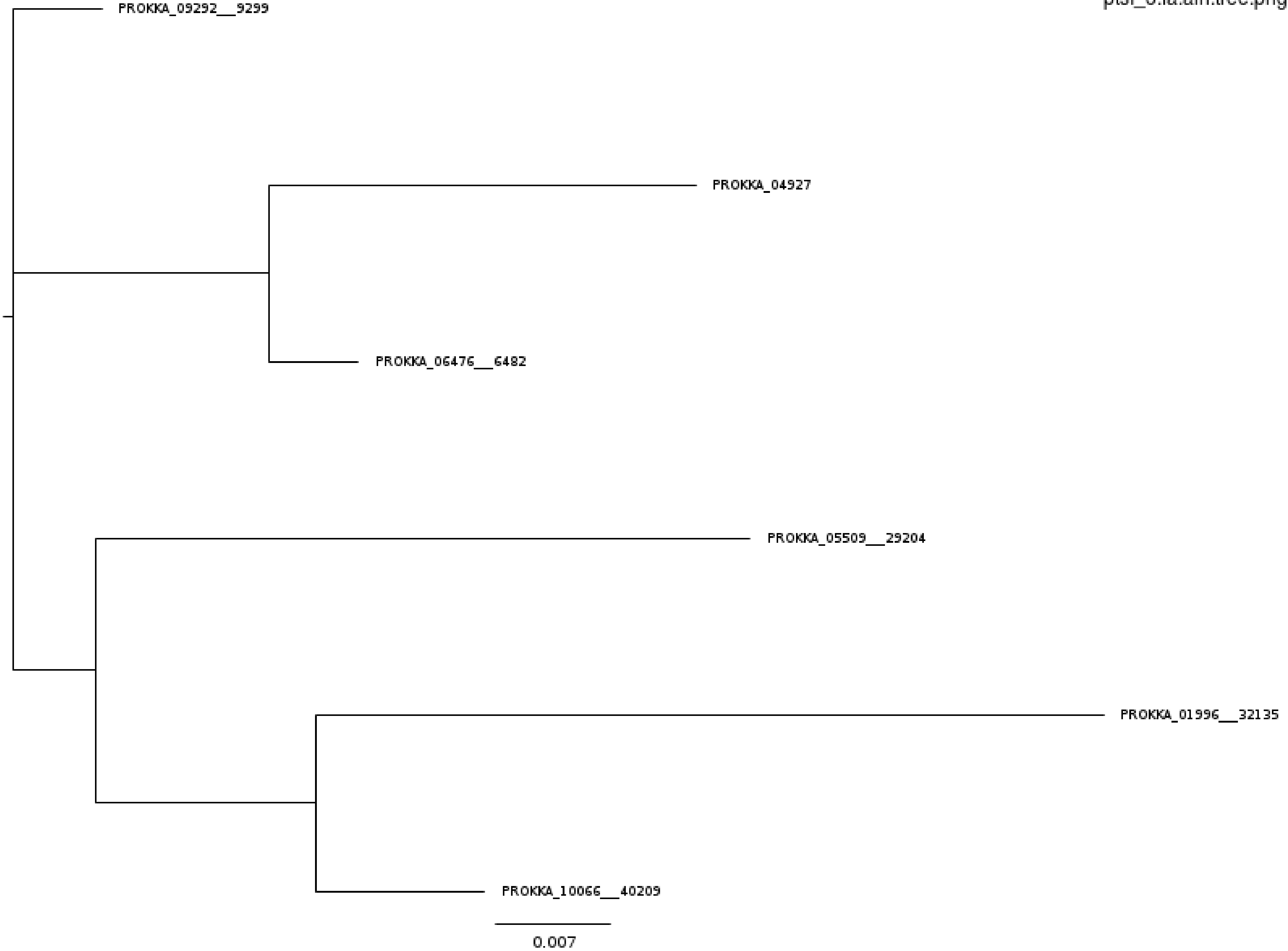

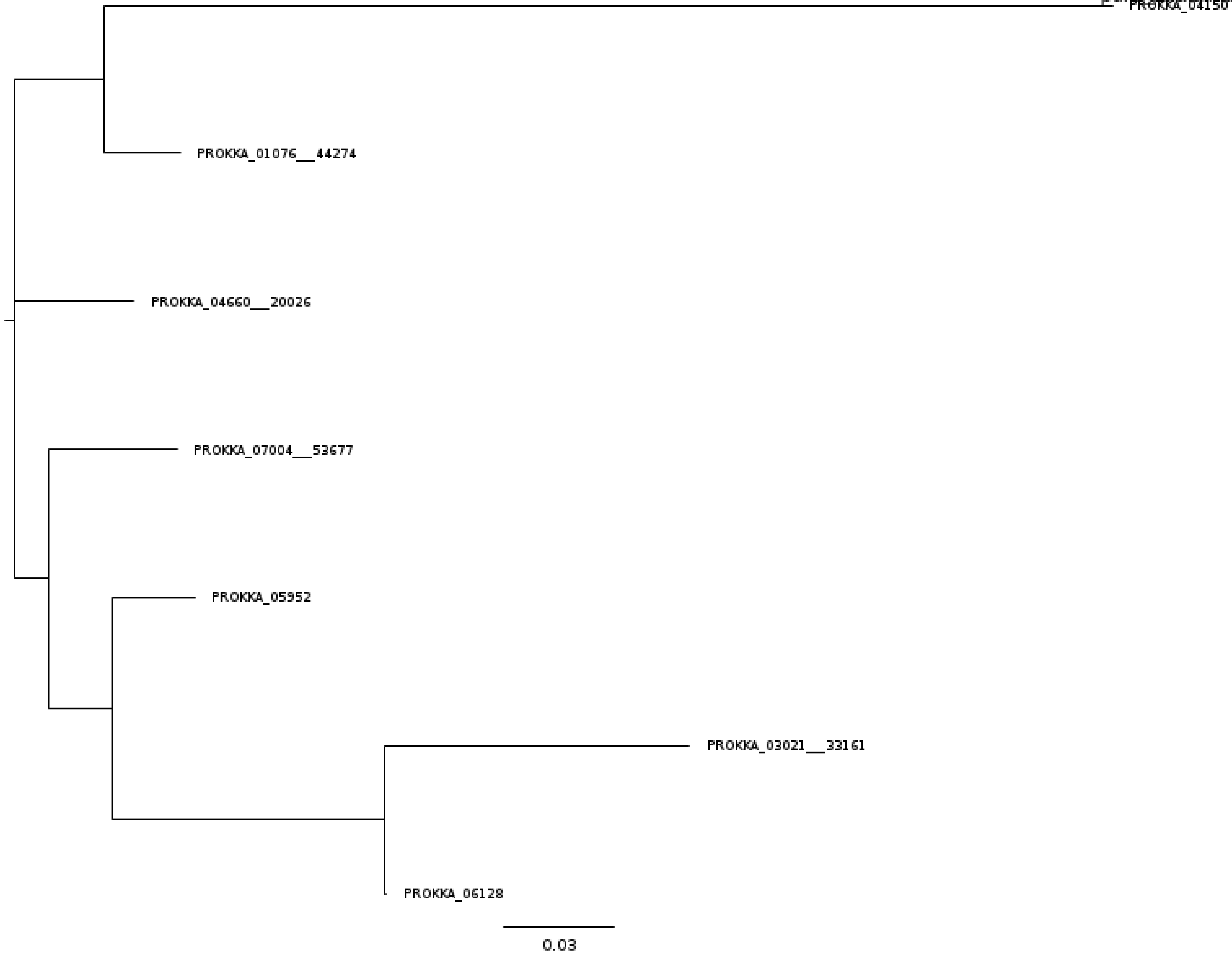
PROKKA_04150
PROKKA_01076___44274
PROKKA_04660___20026
PROKKA_07004___53677
PROKKA_05952
PROKKA_03021___33161
PROKKA_06128
0.03

purA_3.fa.aln.tree.png

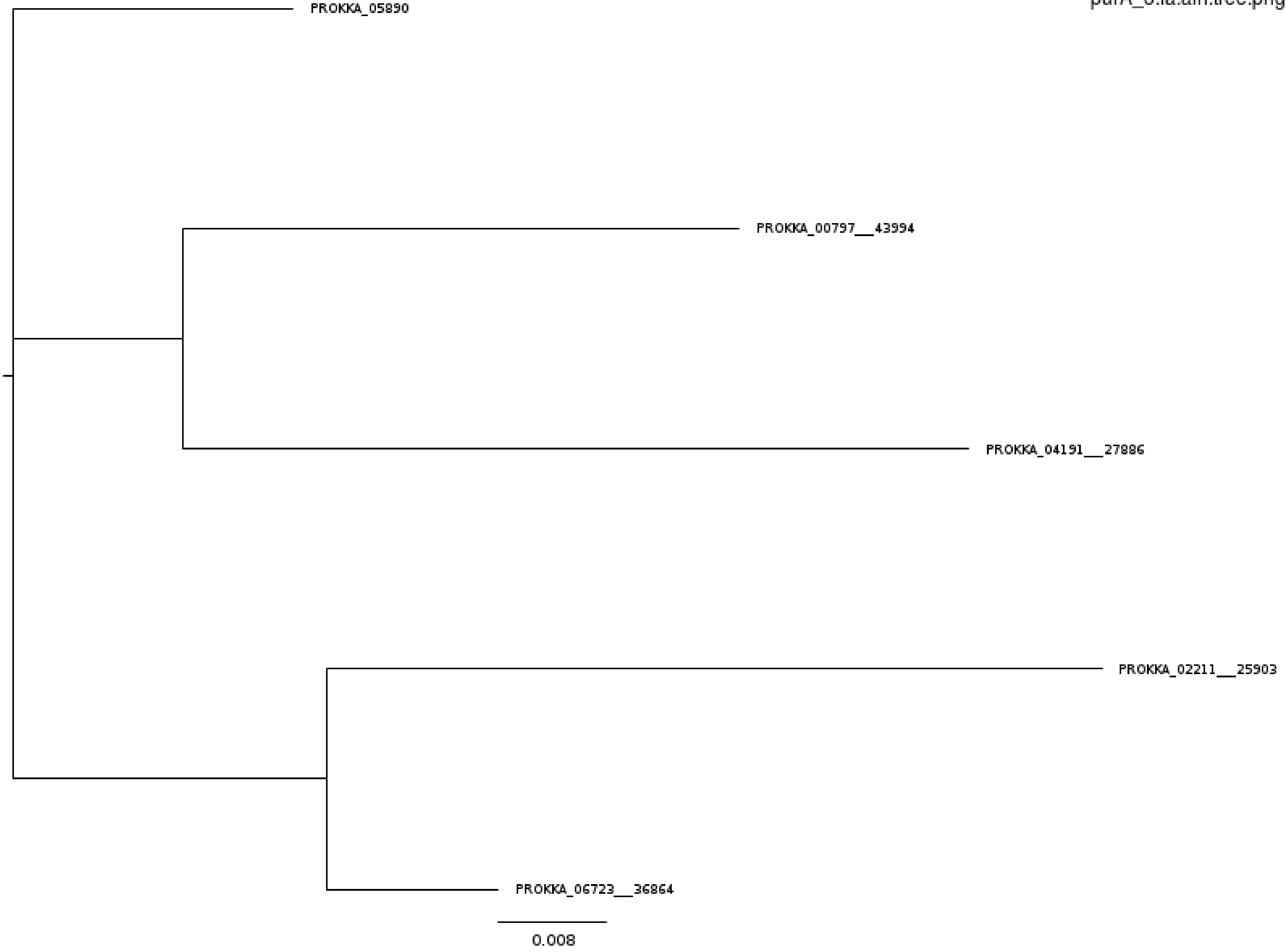

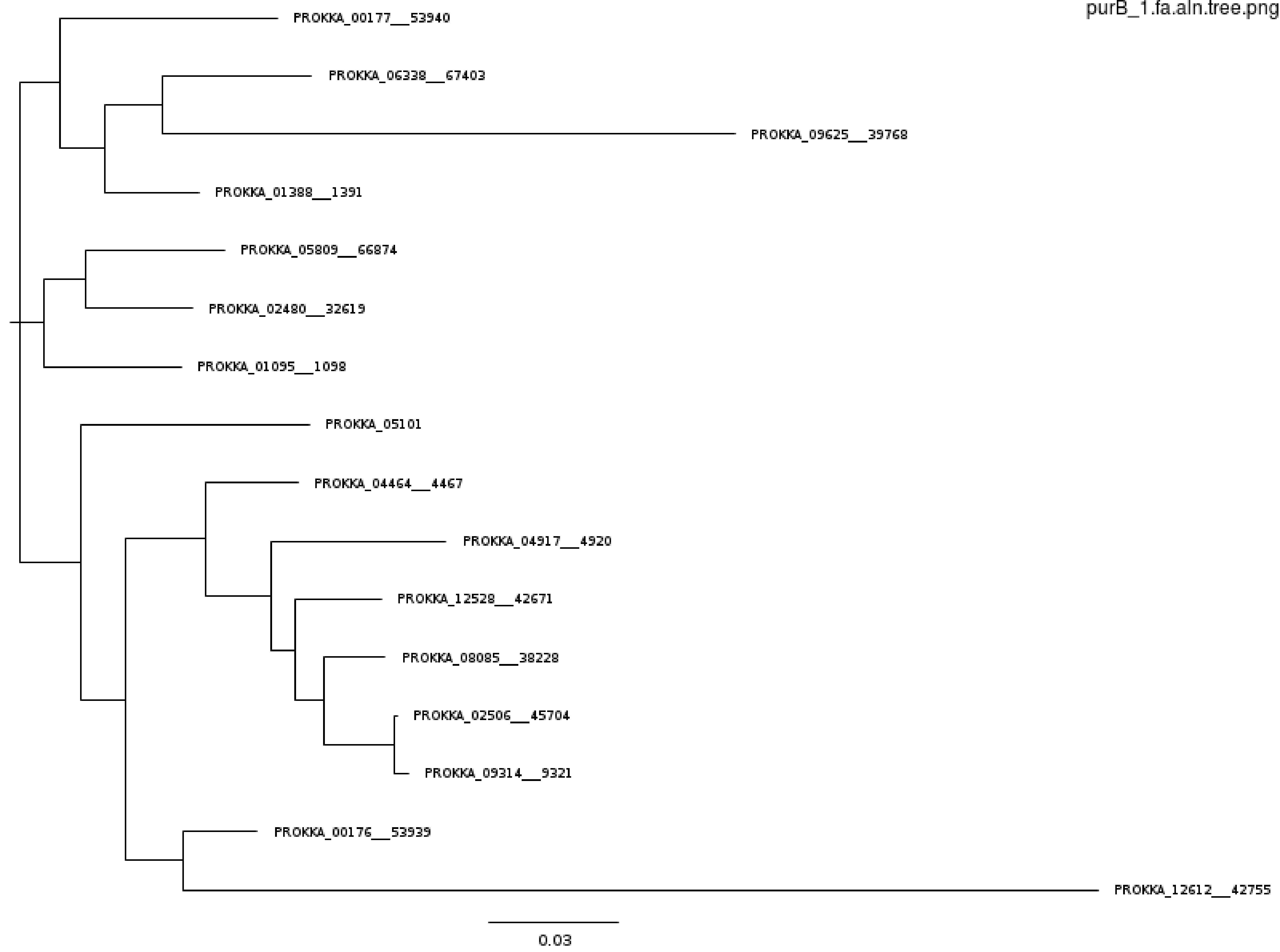
purB_1.fa.aln.tree.png
PROKKA_00177___53940
PROKKA_06338___67403
PROKKA_09625___39768
PROKKA_01388___1391
PROKKA_05809___66874
PROKKA_02480___32619
PROKKA_01095___1098
PROKKA_05101
PROKKA_04464___4467
PROKKA_04917___4920
PROKKA_12528___42671
PROKKA_08085___38228
PROKKA_02506___45704
PROKKA_09314___9321
PROKKA_00176___53939
PROKKA_12612___42755
0.03

purC_2.fa.aln.tree.png

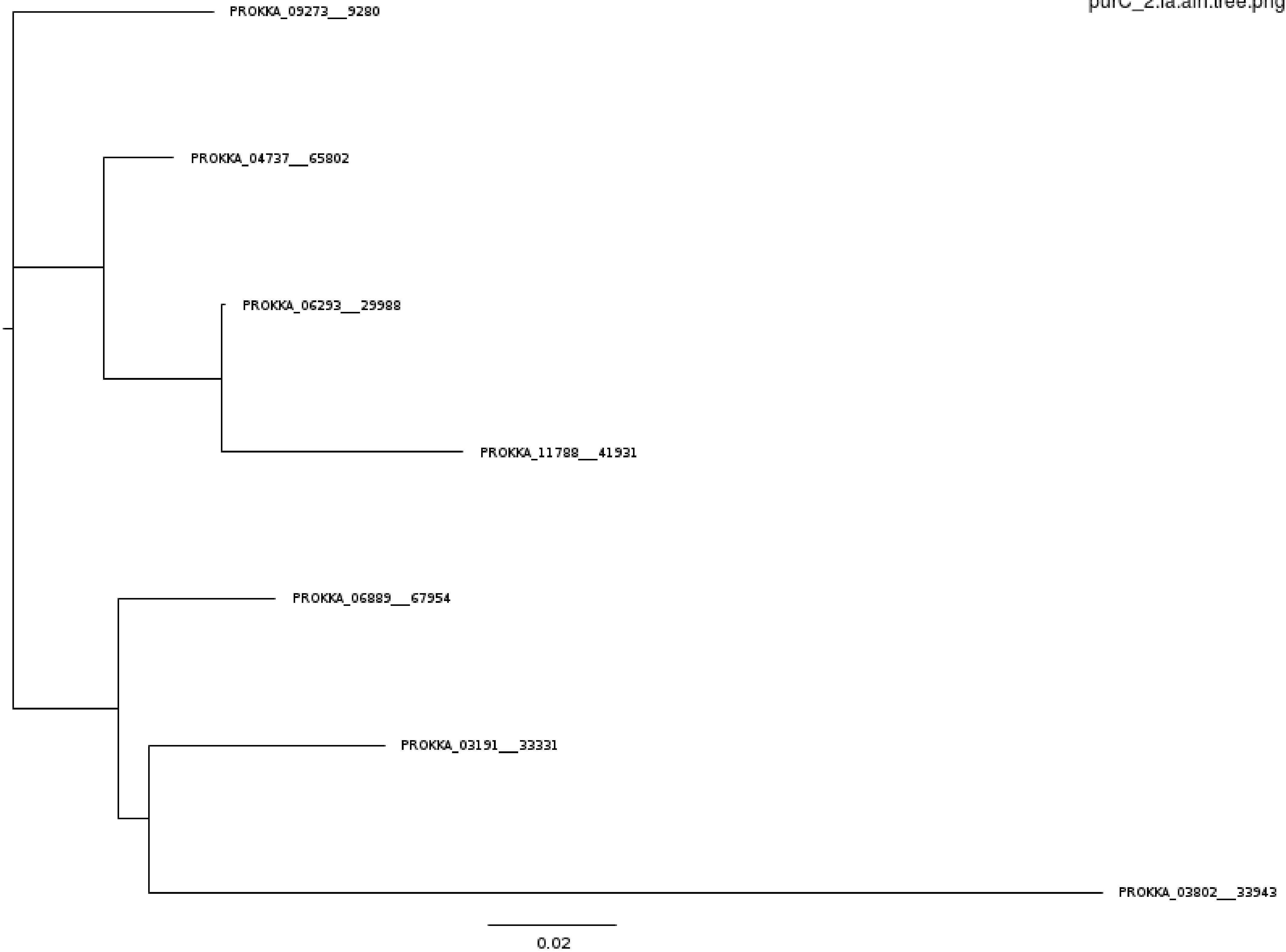

purD_3.fa.aln.tree.png

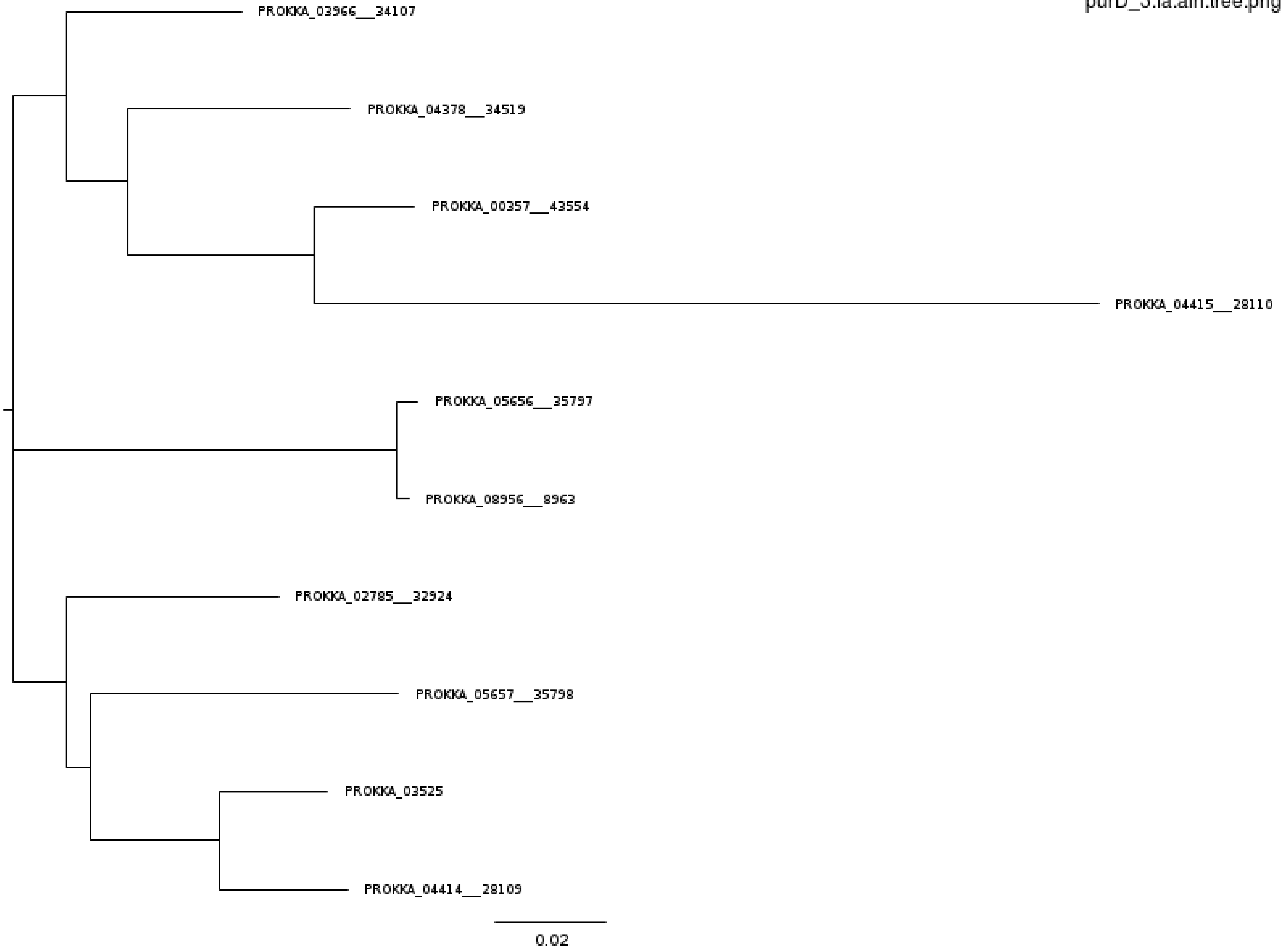

purF.fa.aln.tree.png

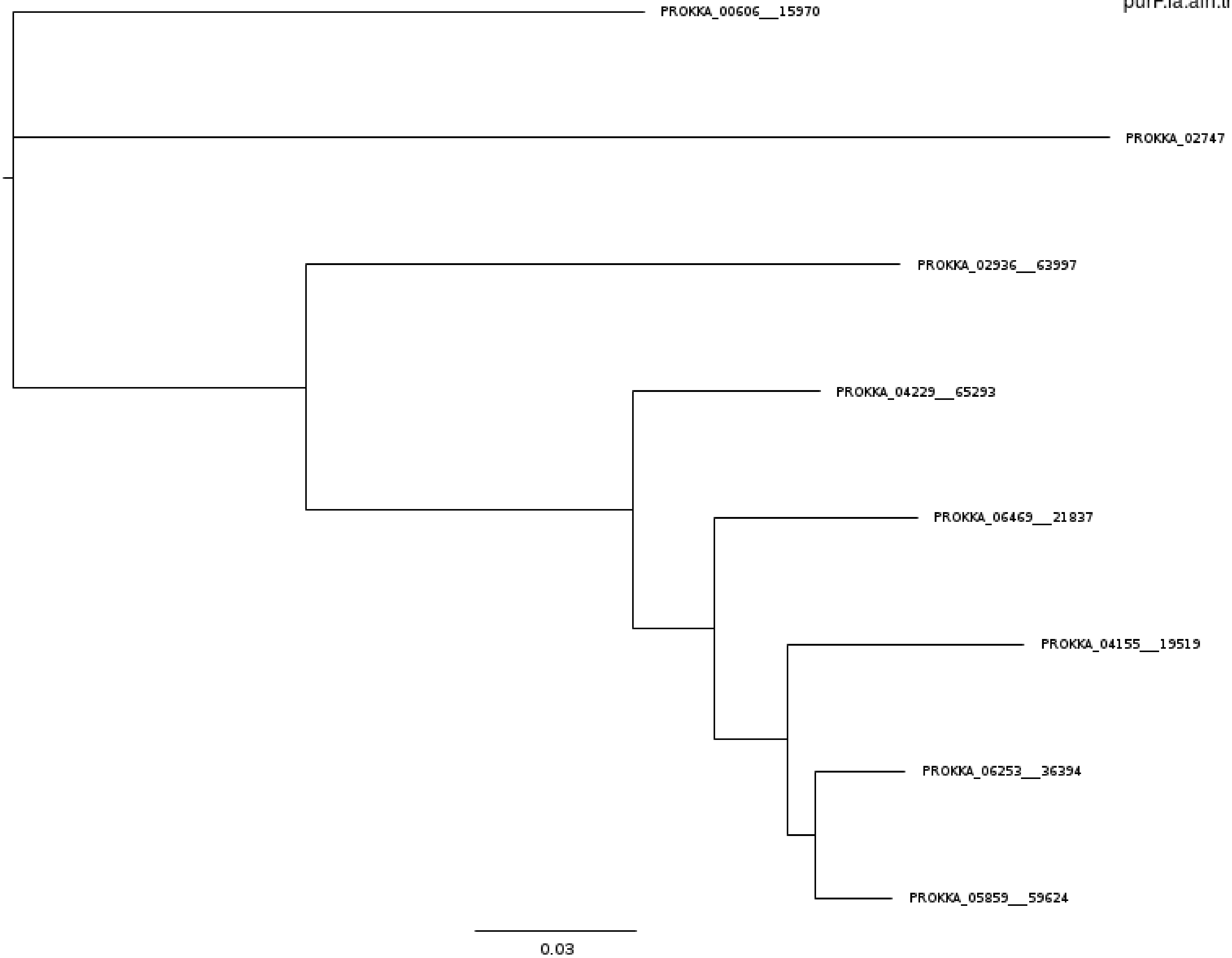

purH_2.fa.aln.tree.png

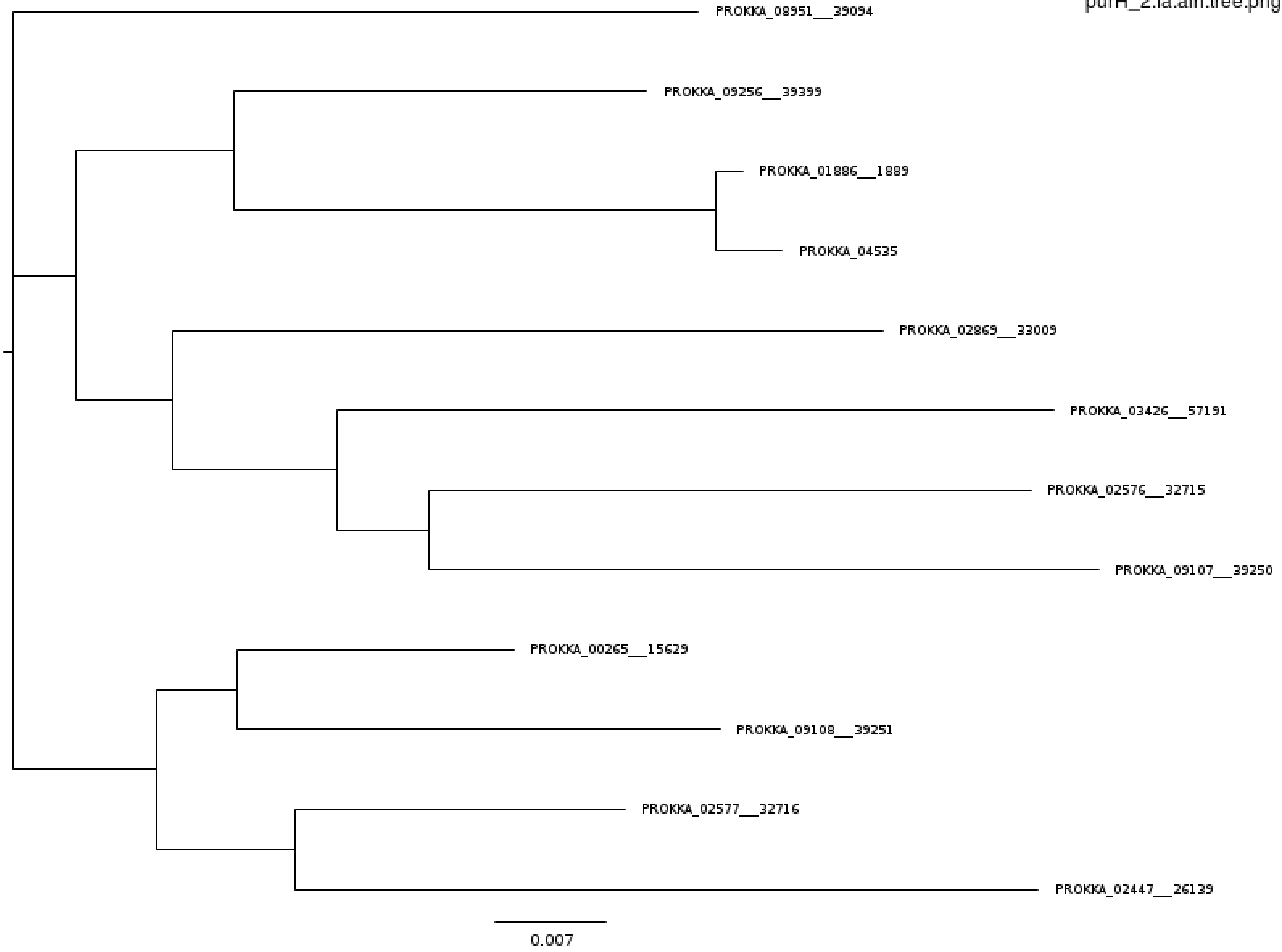

purM_2.fa.aln.tree.png

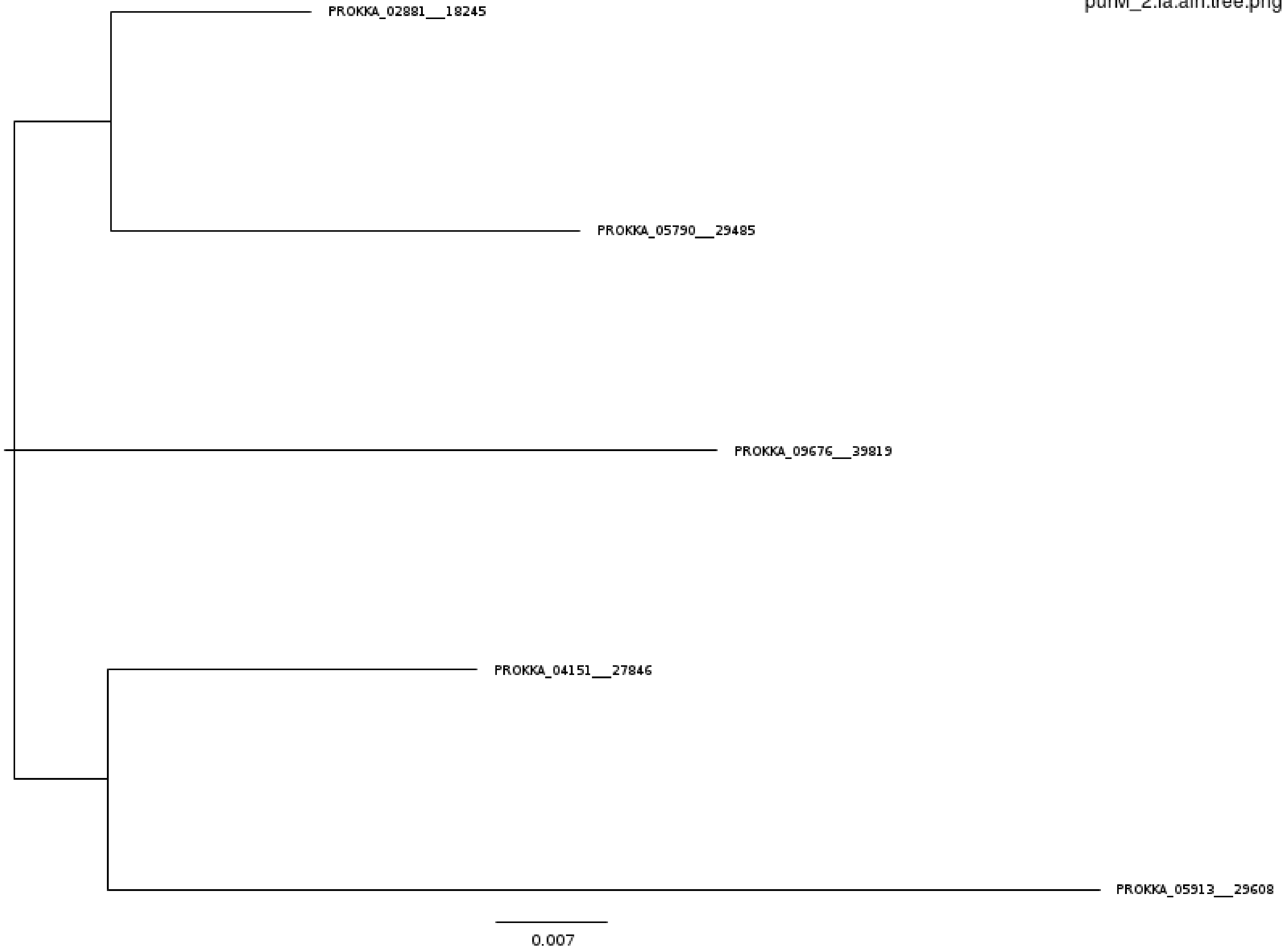

pykA_2.fa.aln.tree.png

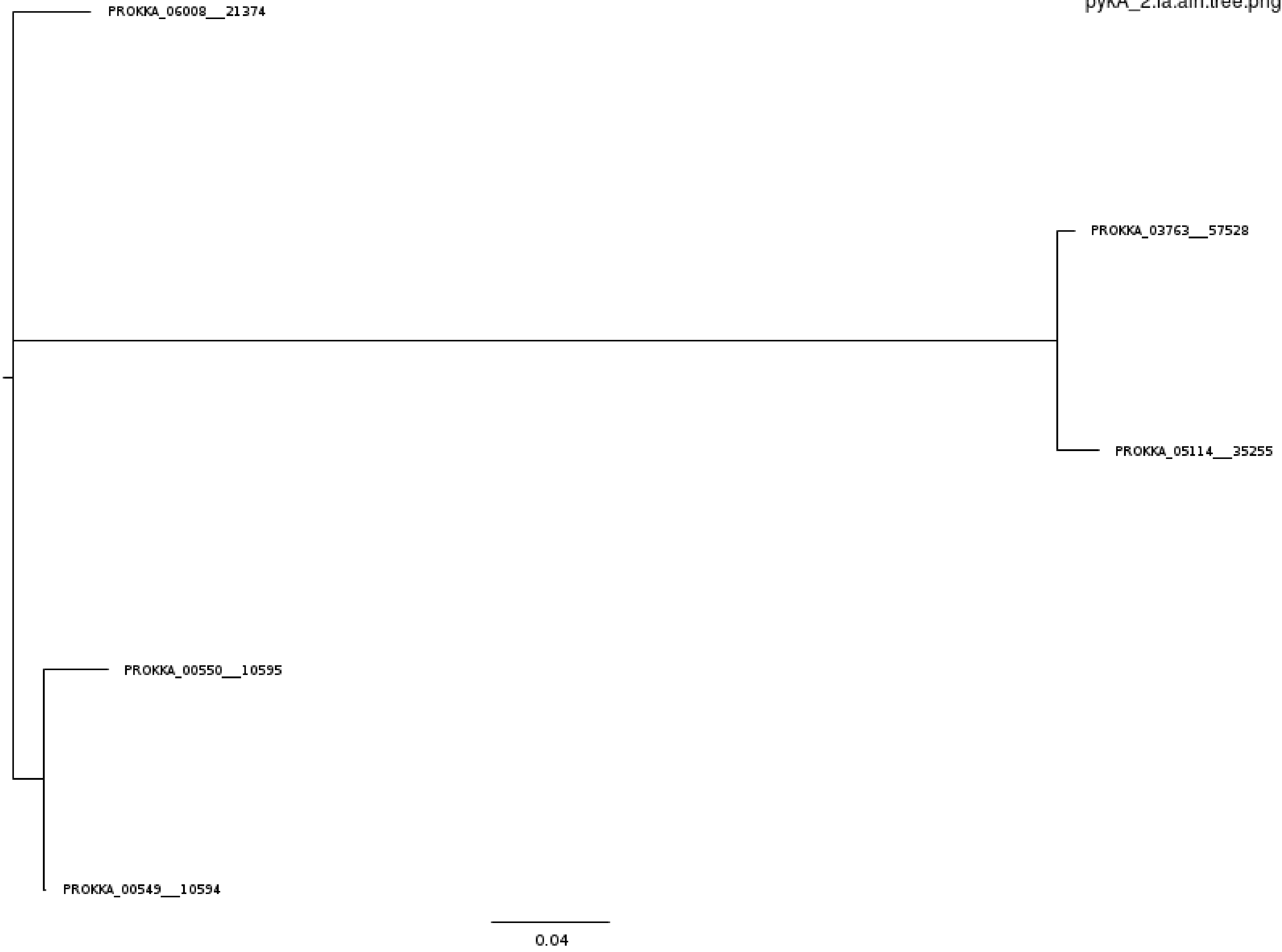

pyrB_2.fa.aln.tree.png

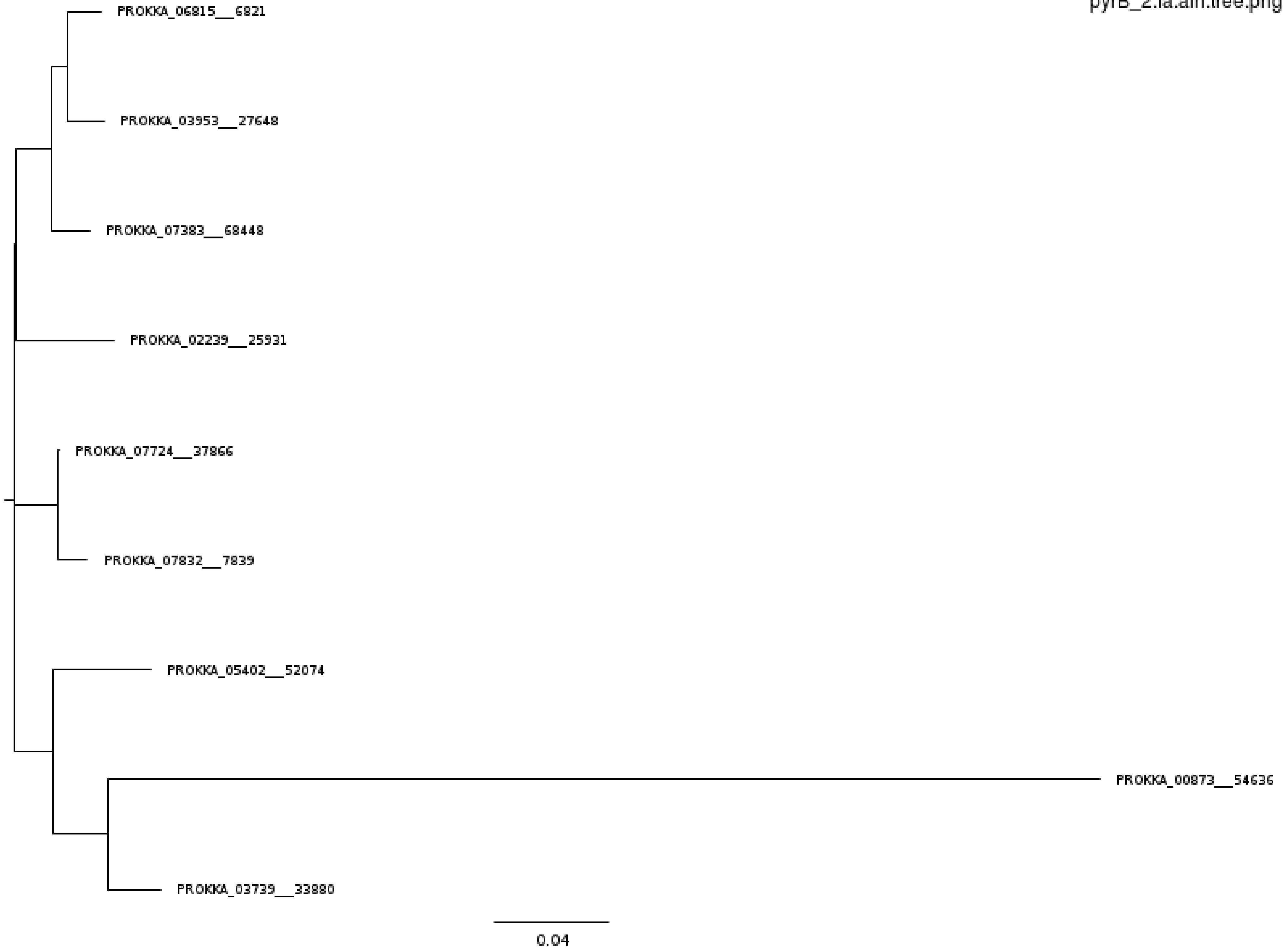

pyrC_3.fa.aln.tree.png

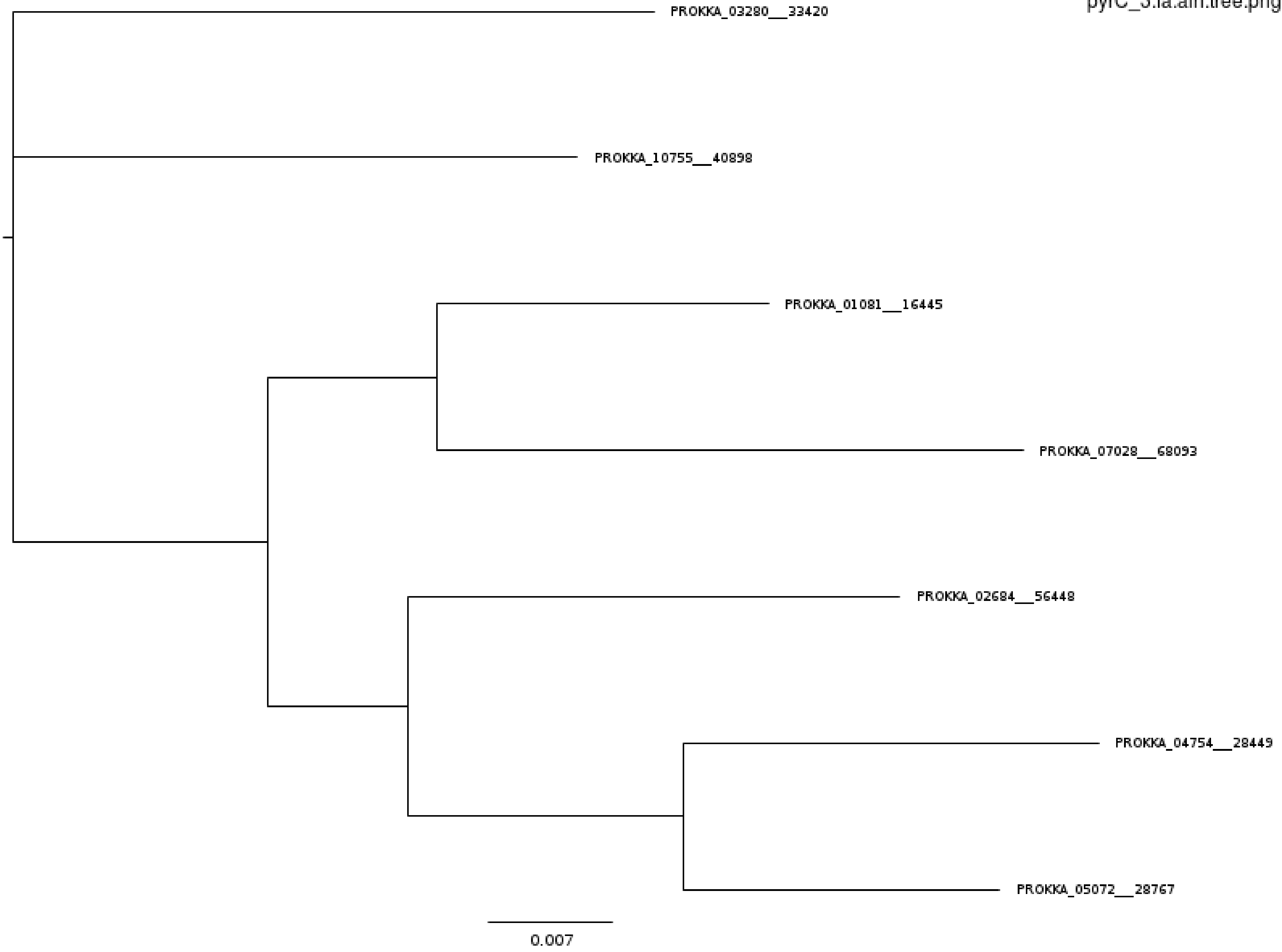

pyrC_4.fa.aln.tree.png

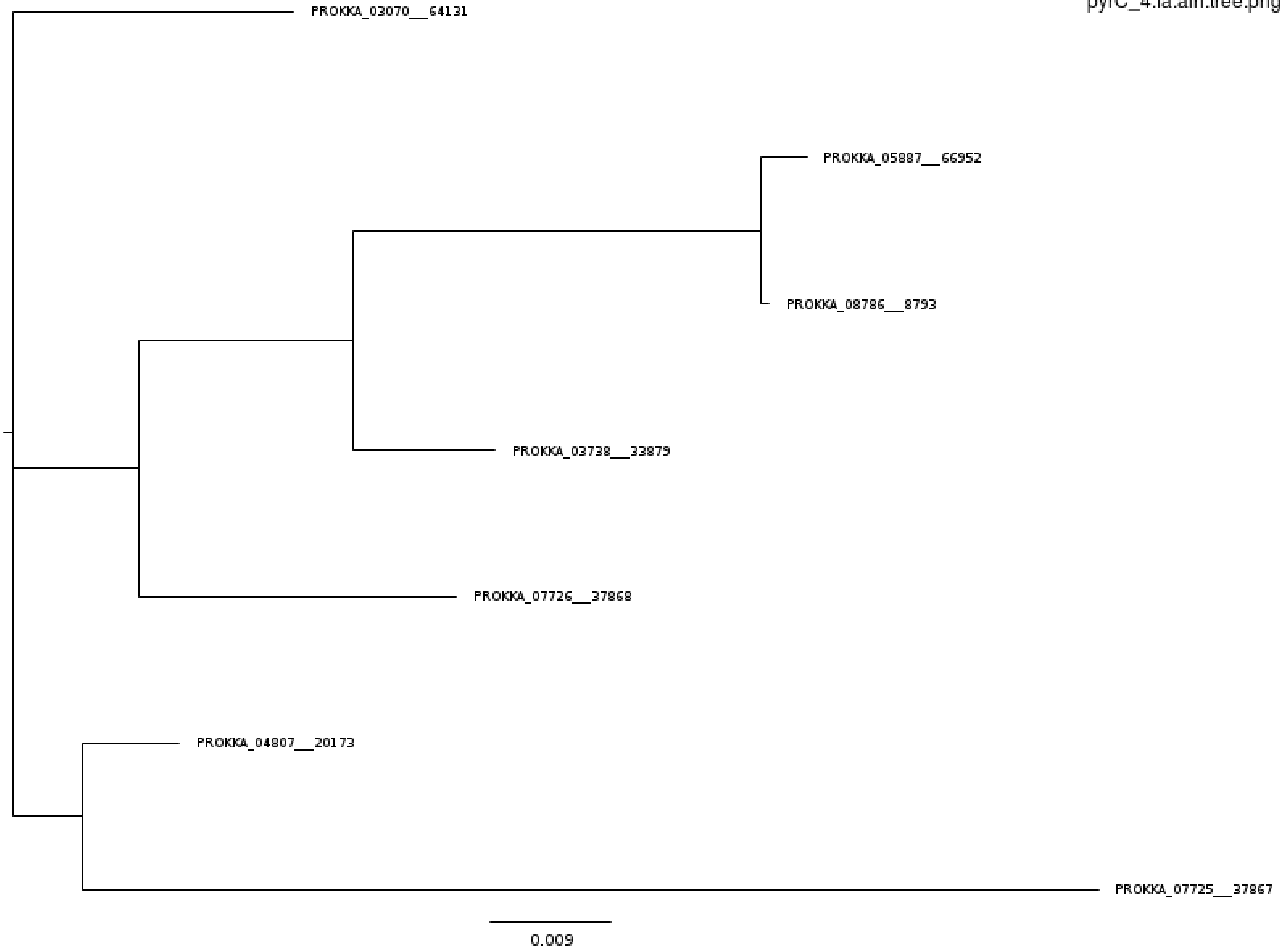

pyrD.fa.aln.tree.png

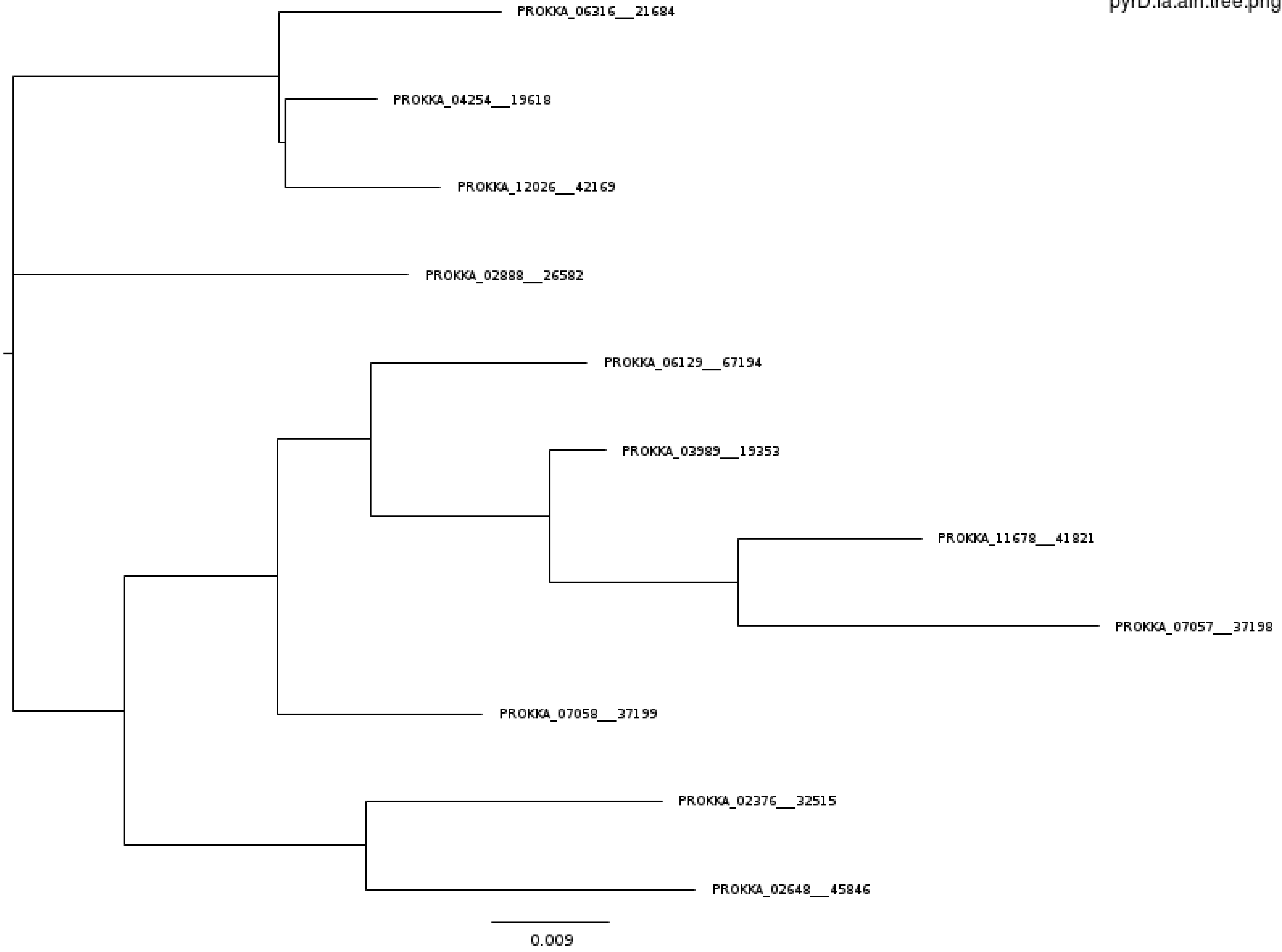

pyrE_5.fa.aln.tree.png

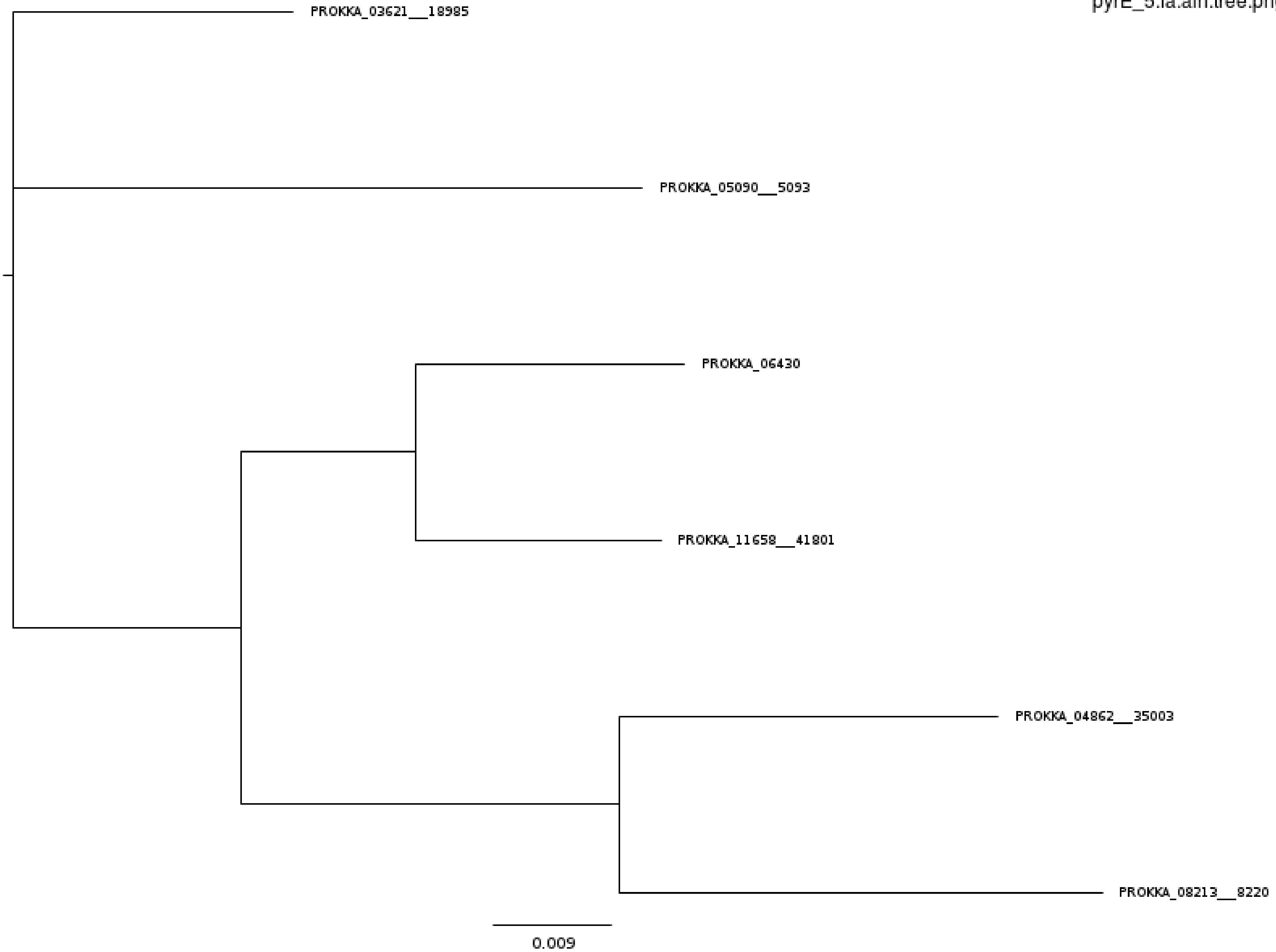

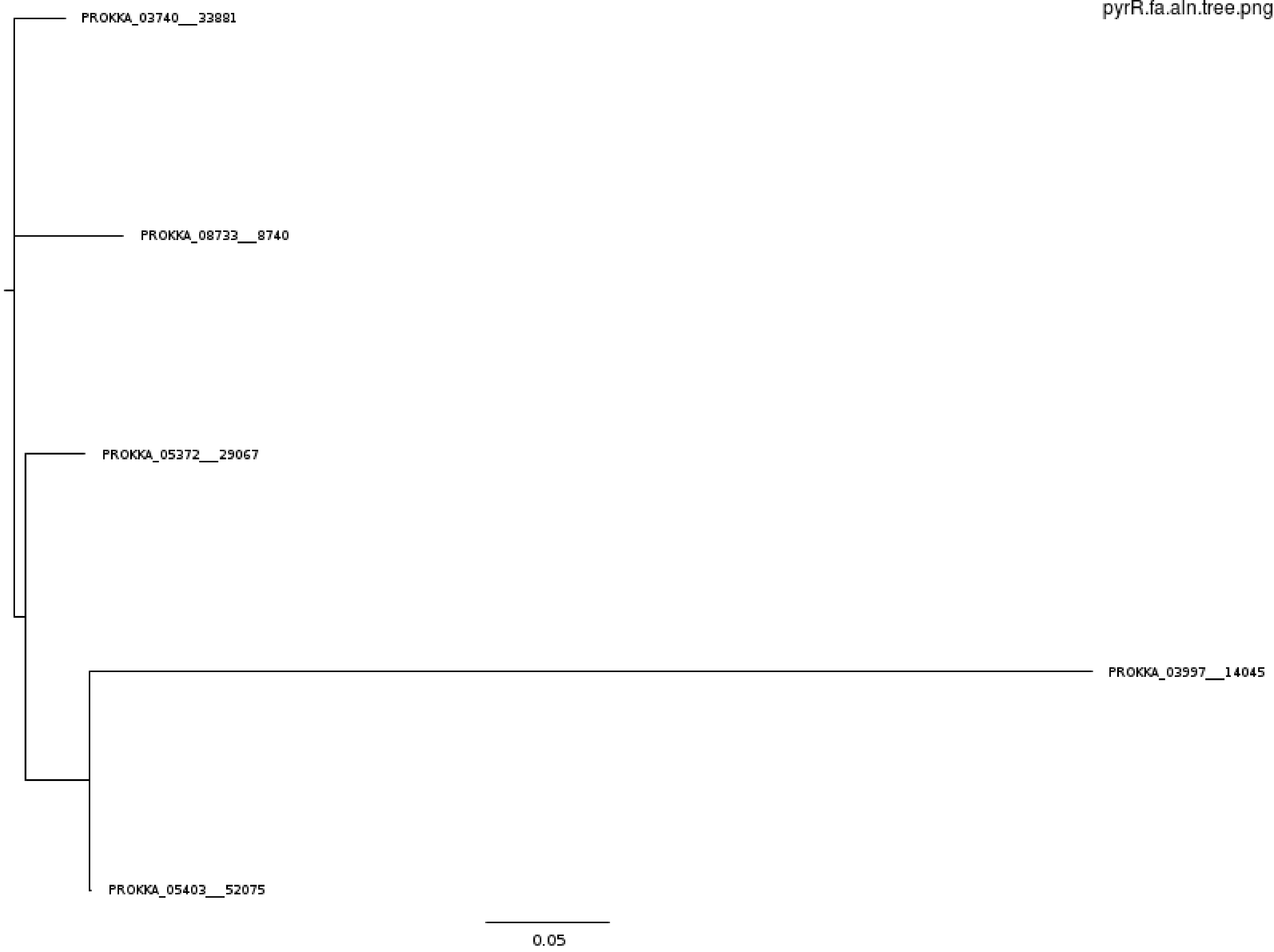
pyrR.fa.aln.tree.png
PROKKA_03740___33881
PROKKA_08733___8740
PROKKA_05372___29067
PROKKA_03997___14045
PROKKA_05403___52075
0.05

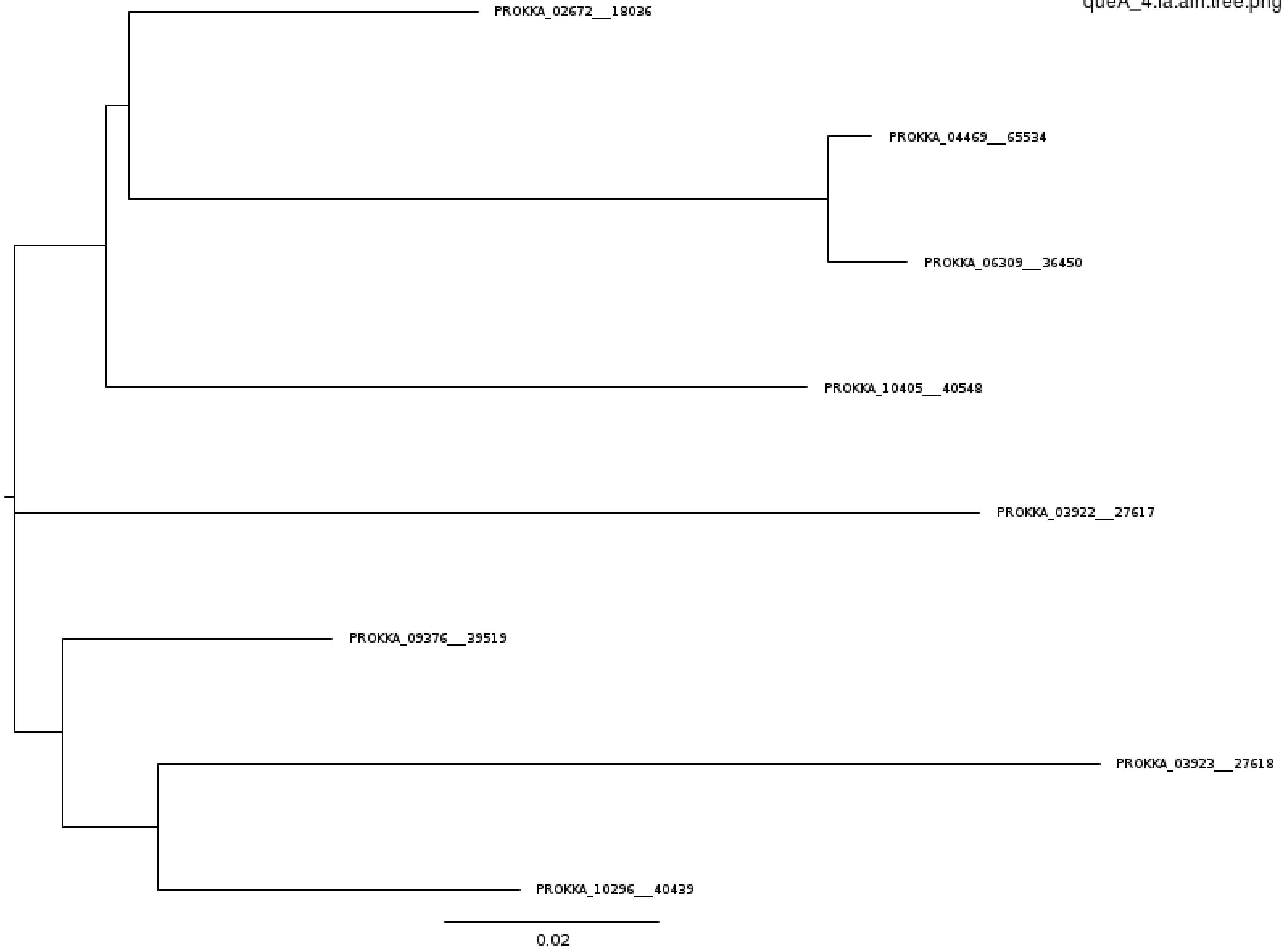

PROKKA_02672___18036
PROKKA_04469___65534
PROKKA_06309___36450
PROKKA_10405___40548
PROKKA_03922___27617
PROKKA_09376___39519
PROKKA_03923___27618
PROKKA_10296___40439
0.02

queC_2.fa.aln.tree.png

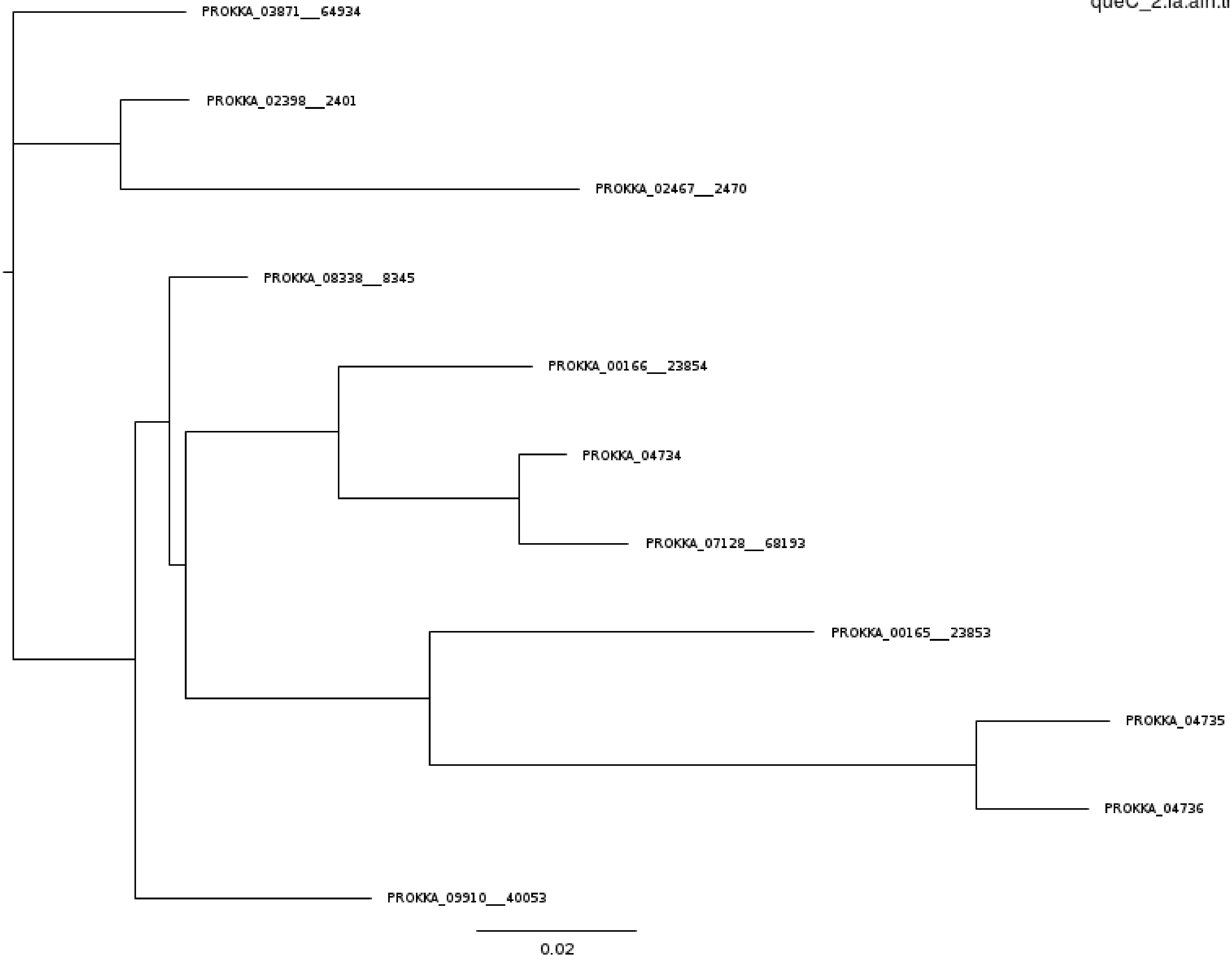

queF_2.fa.aln.tree.png

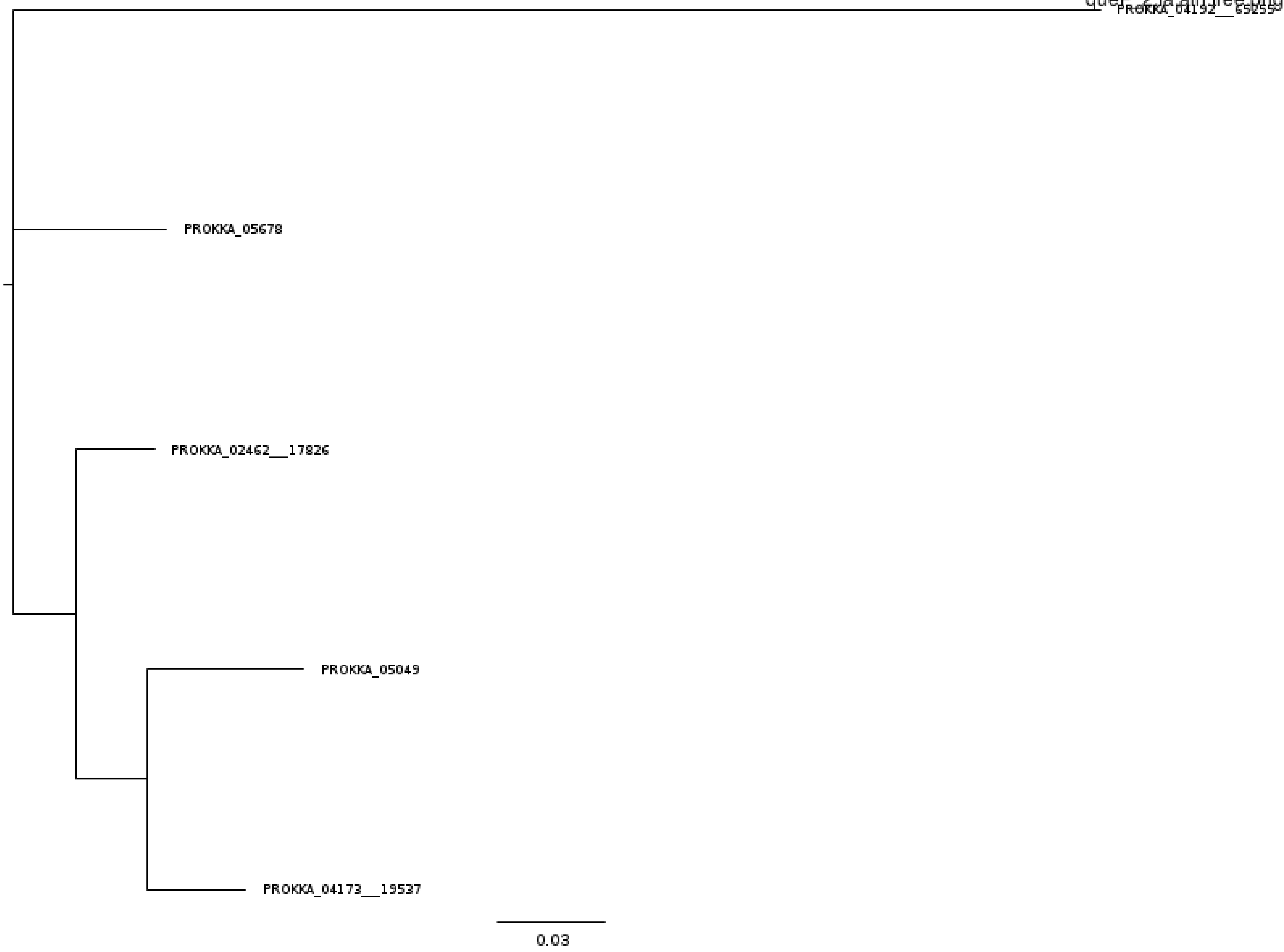

queG_1.fa.aln.tree.png

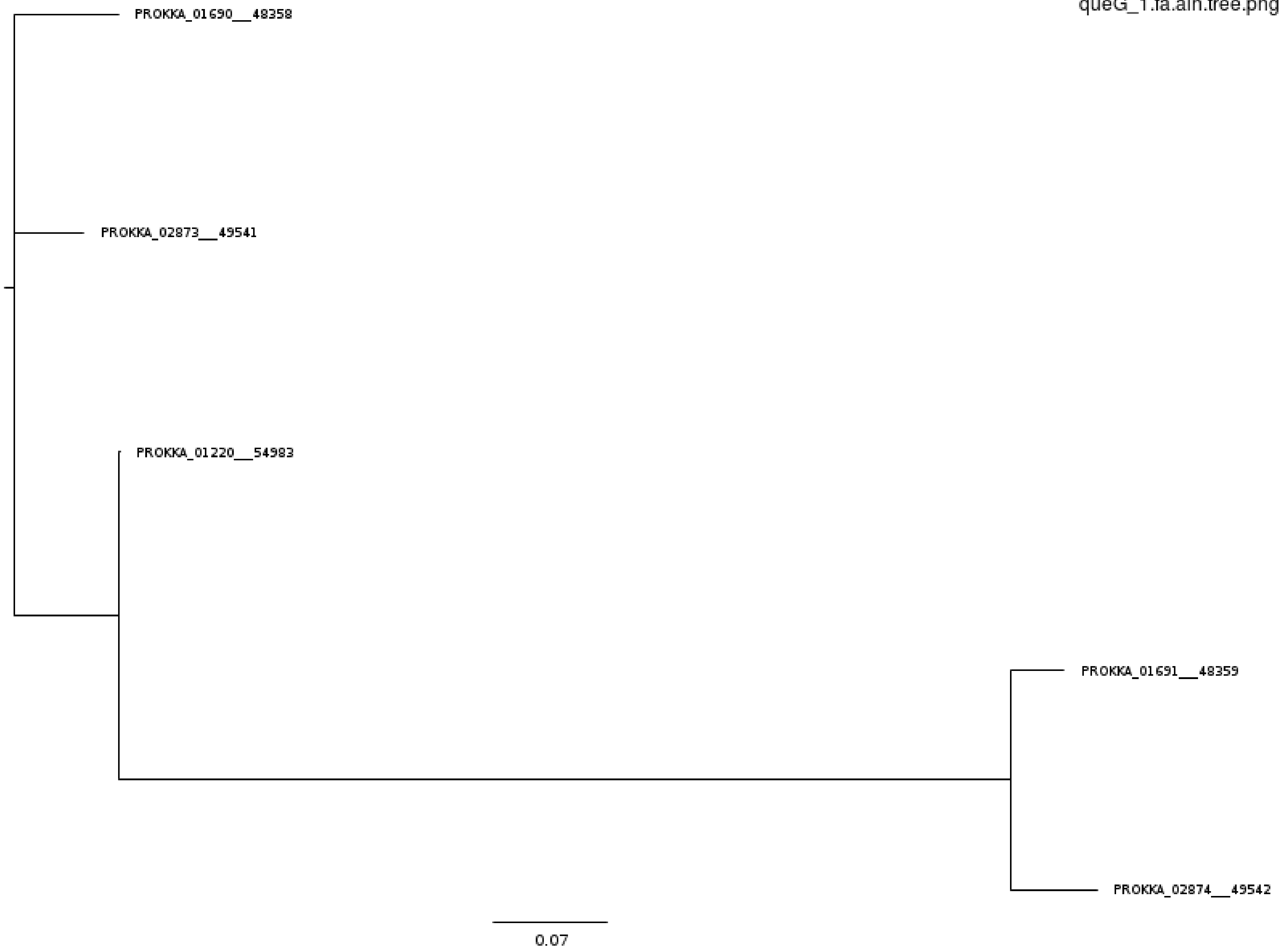

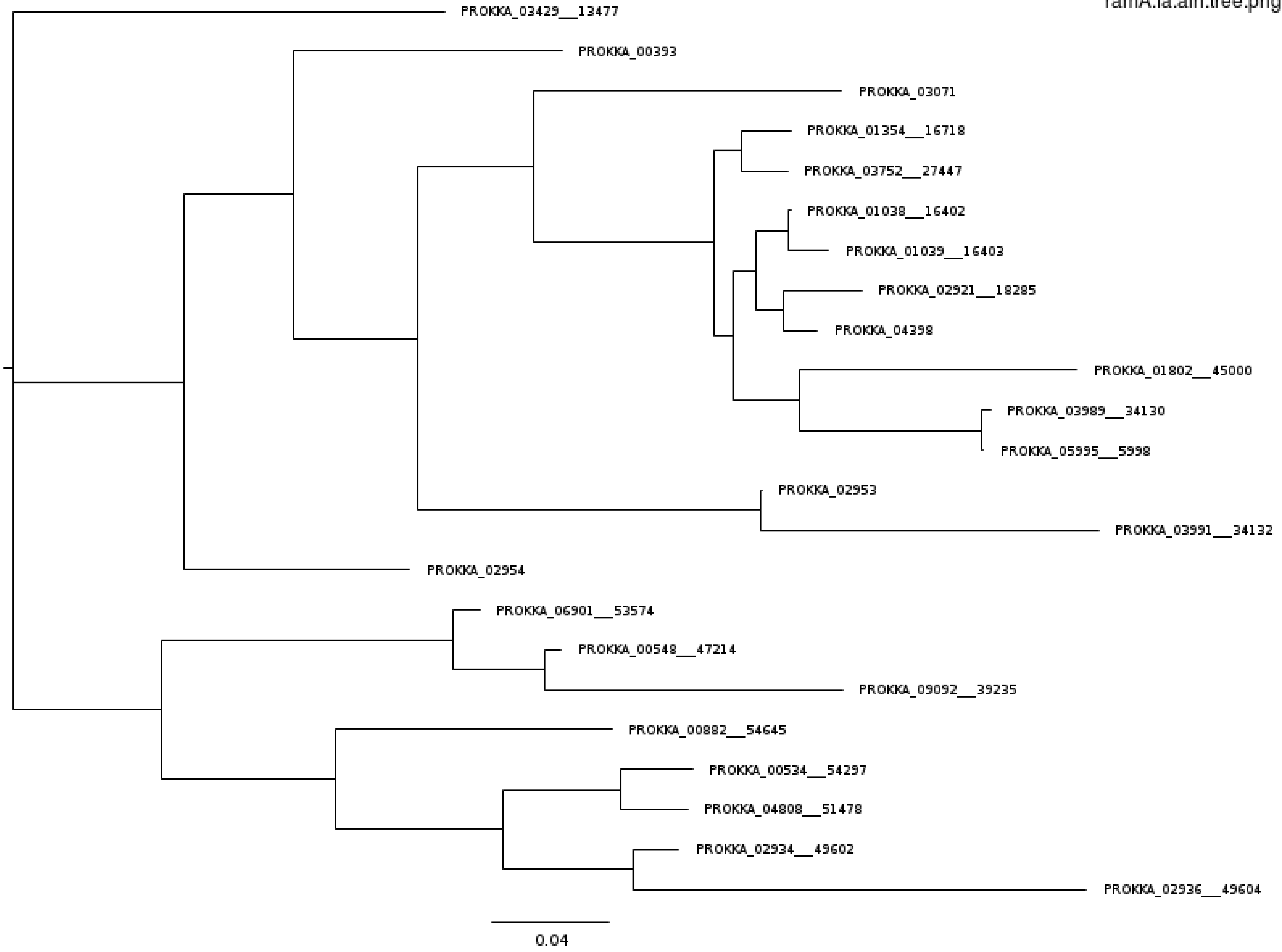

PROKKA_03429___13477
PROKKA_00393
PROKKA_03071
PROKKA_01354___16718
PROKKA_03752___27447
PROKKA_01038___16402
PROKKA_01039___16403
PROKKA_02921___18285
PROKKA_04398
PROKKA_01802___45000
PROKKA_03989___34130
PROKKA_05995___5998
PROKKA_02953
PROKKA_03991___34132
PROKKA_02954
PROKKA_06901___53574
PROKKA_00548___47214
PROKKA_09092___39235
PROKKA_00882___54645
PROKKA_00534___54297
PROKKA_04808___51478
PROKKA_02934___49602
PROKKA_02936___49604
0.04

rbr_1.fa.aln.tree.png

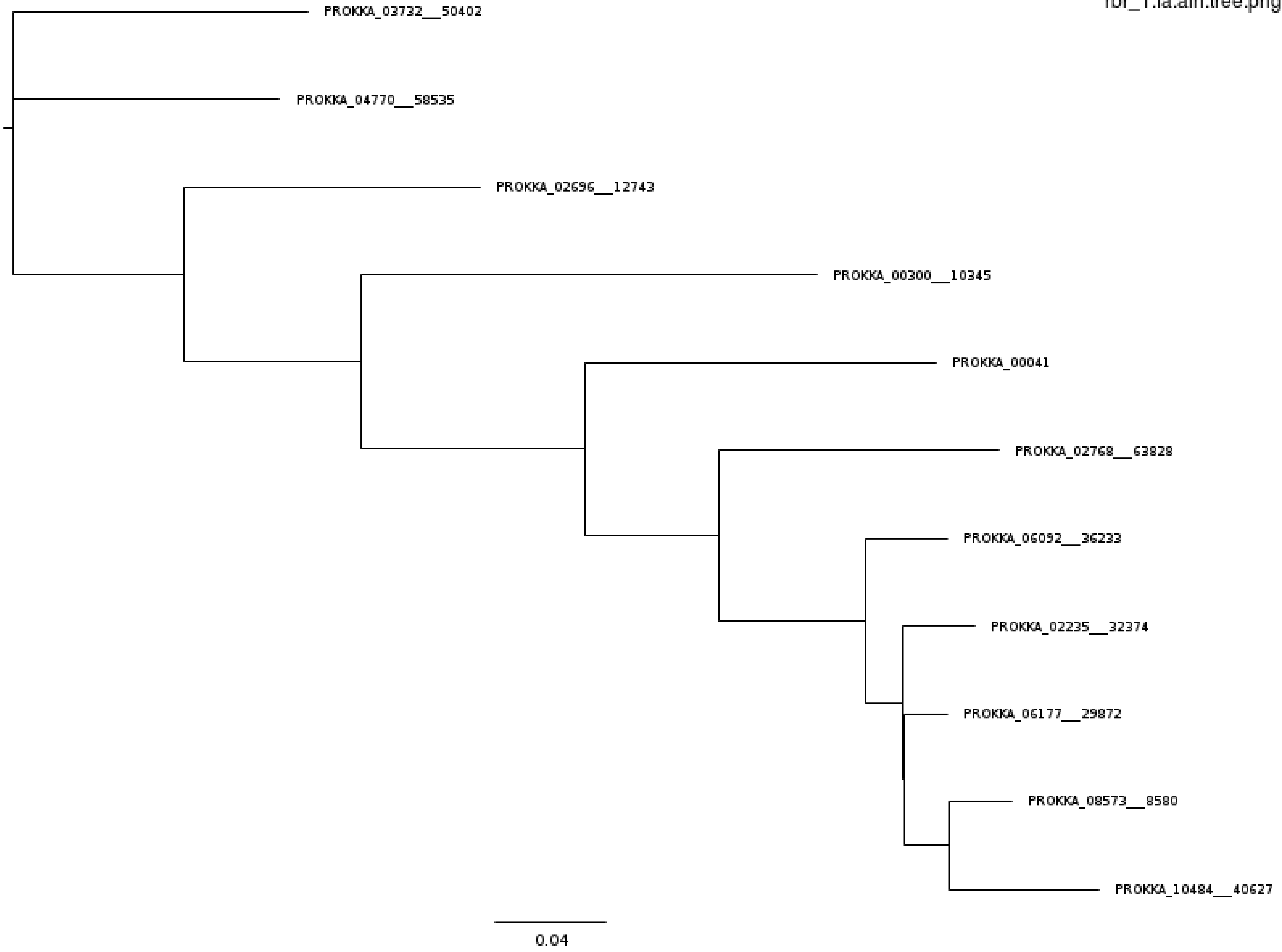

rcsC_2.fa.aln.tree.png

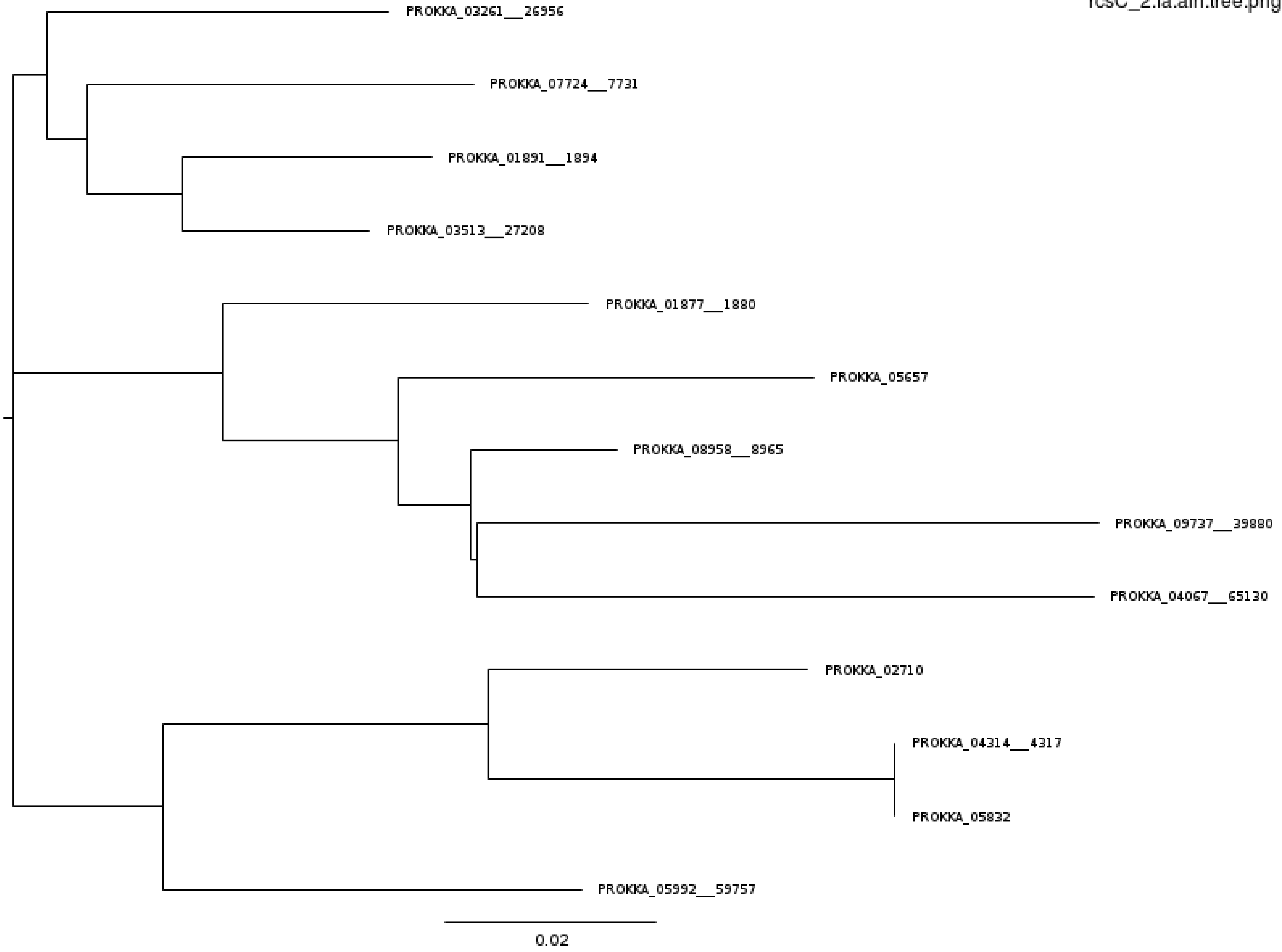

rdgB_3.fa.aln.tree.png

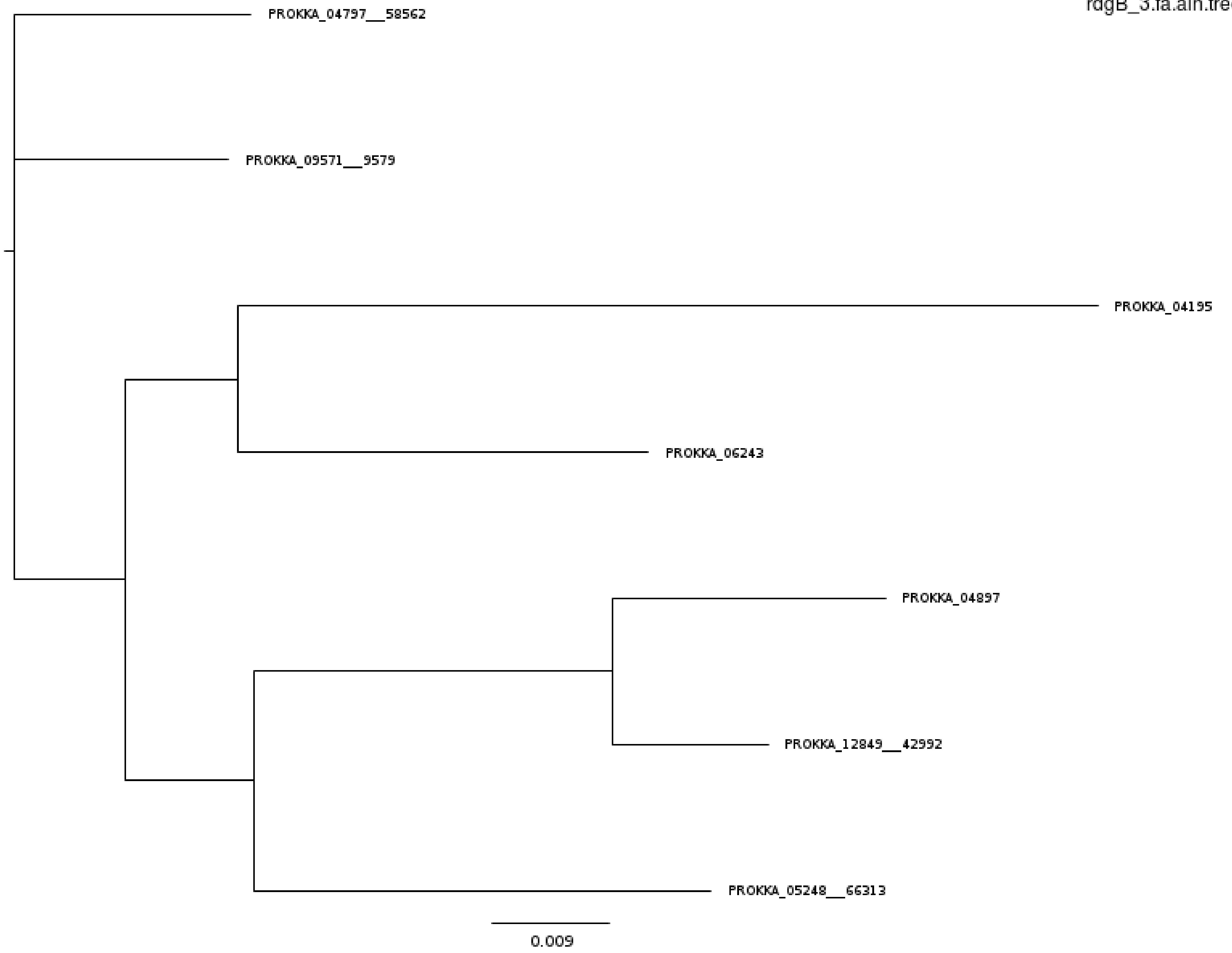

recC_2.fa.aln.tree.png

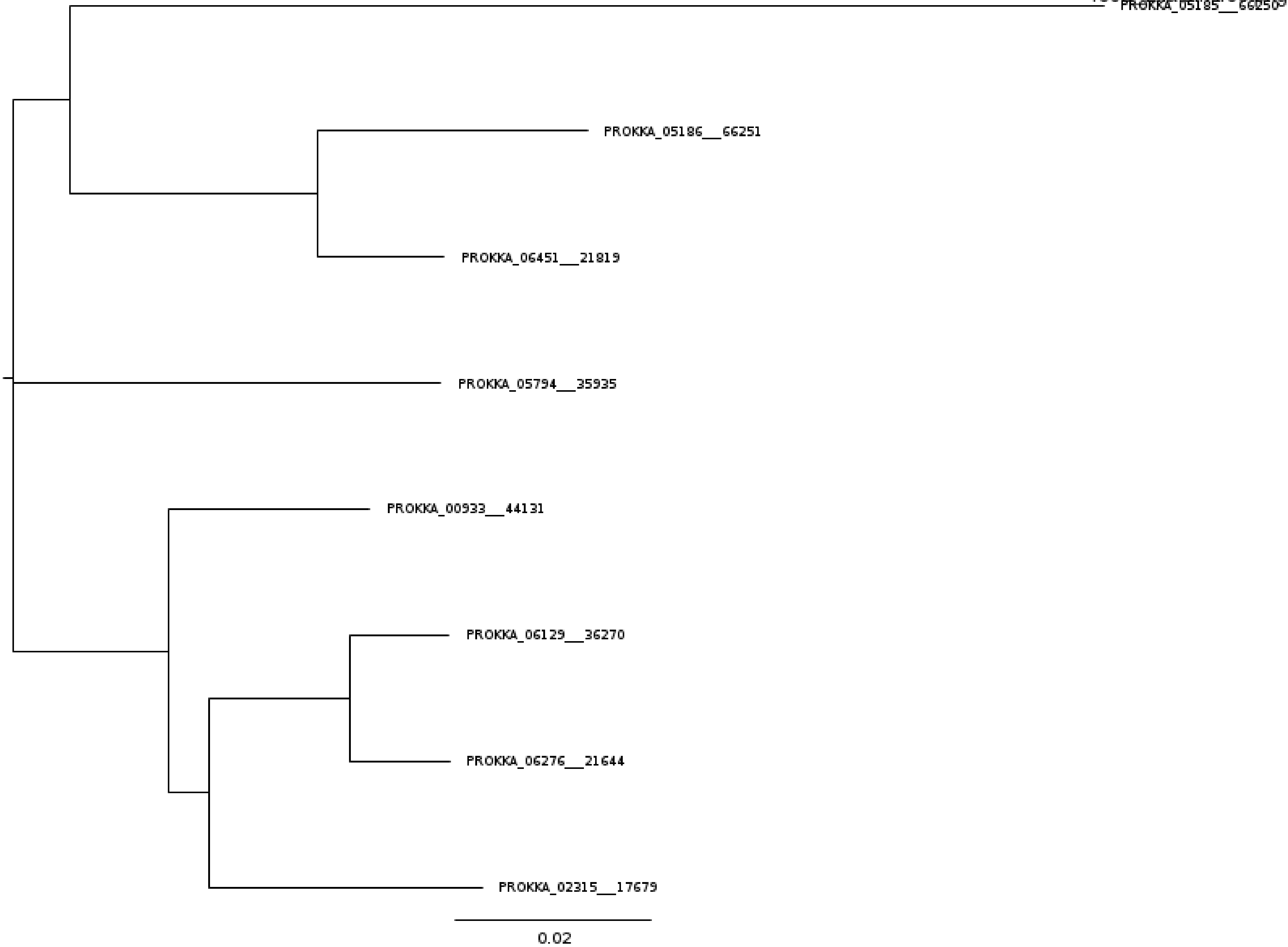

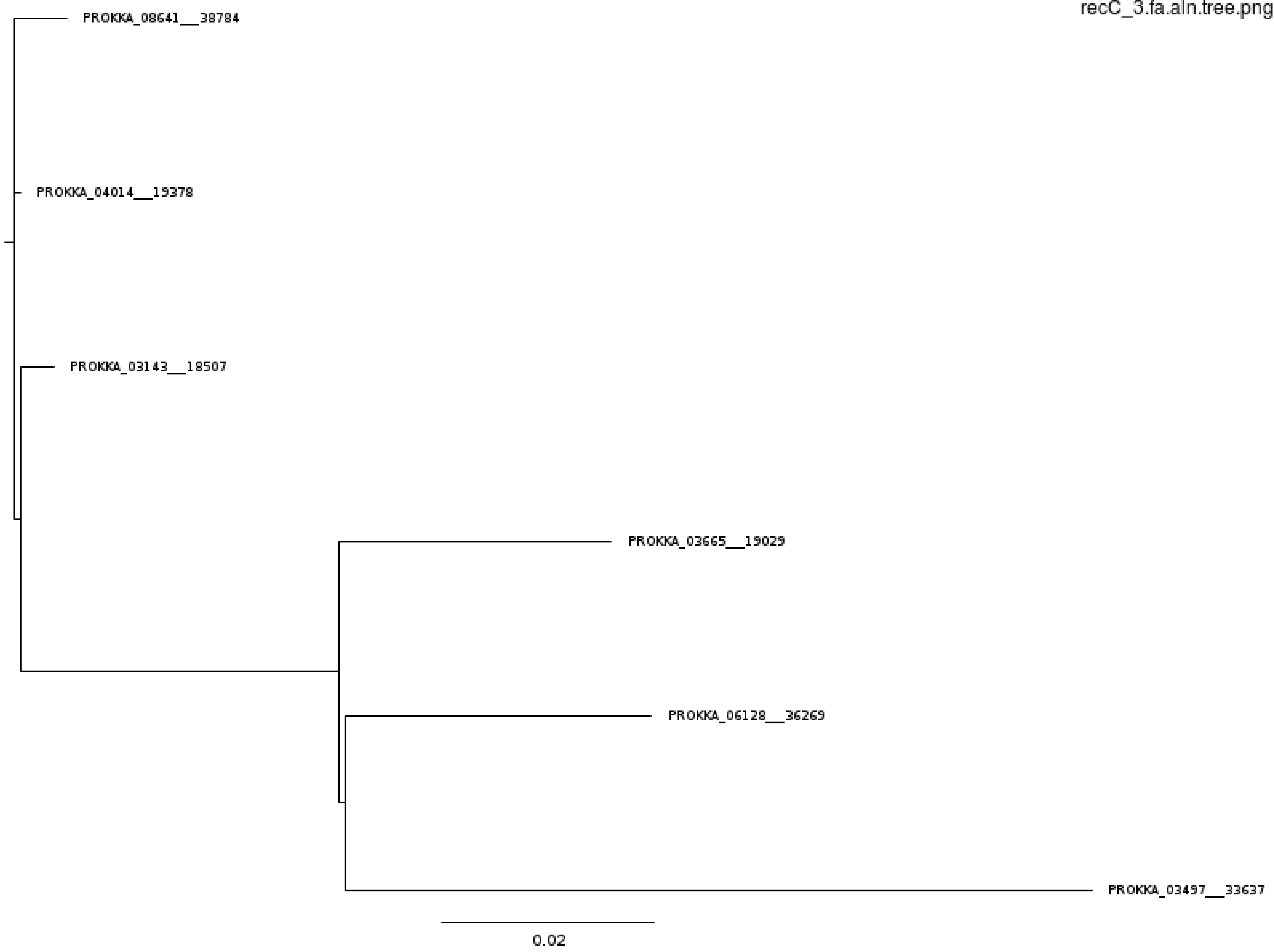
recC_3.fa.aln.tree.png
PROKKA_08641___38784
PROKKA_04014___19378
PROKKA_03143___18507
PROKKA_03665___19029
PROKKA_06128___36269
PROKKA_03497___33637
0.02

recG_4.fa.aln.tree.png

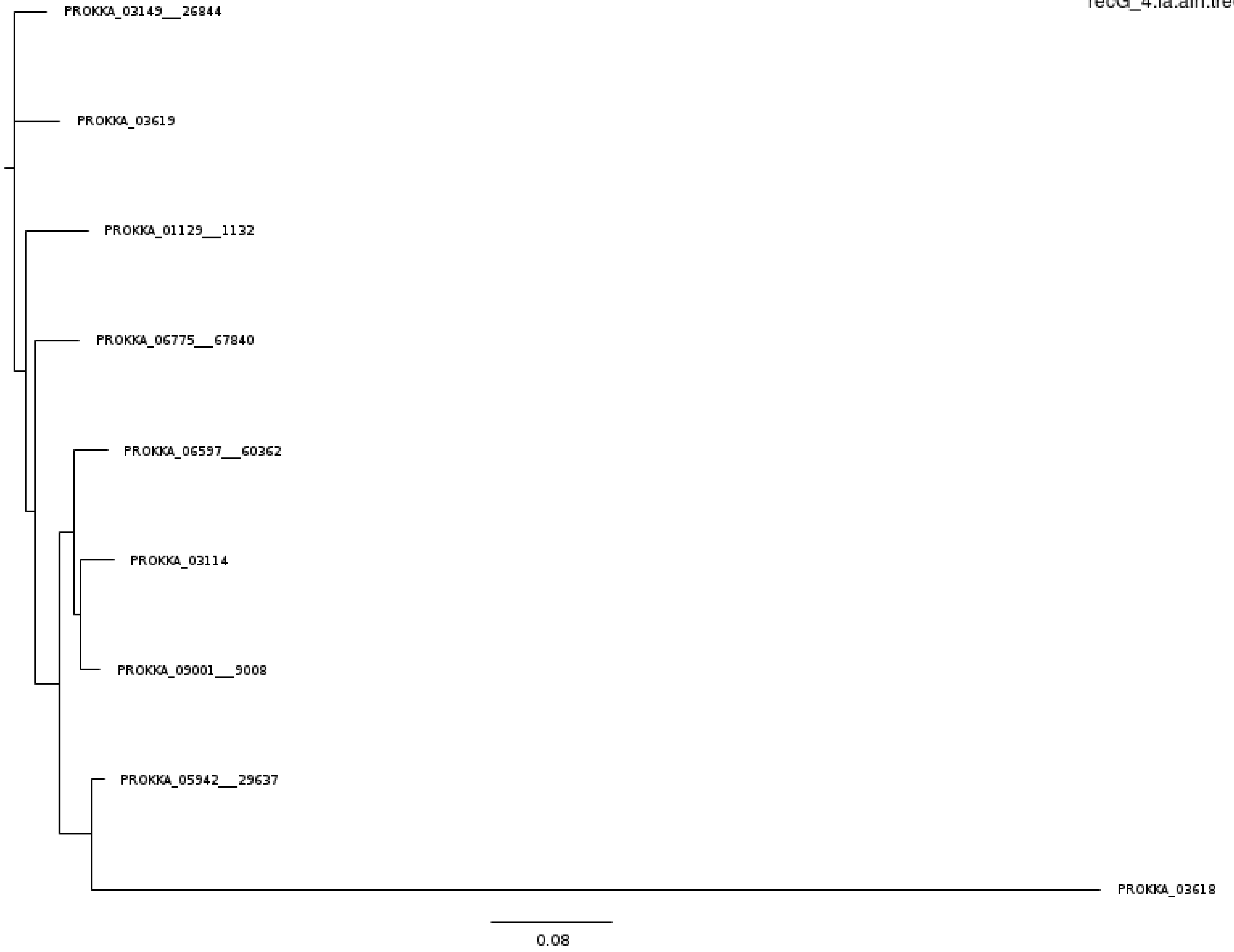

recJ.fa.aln.tree.png

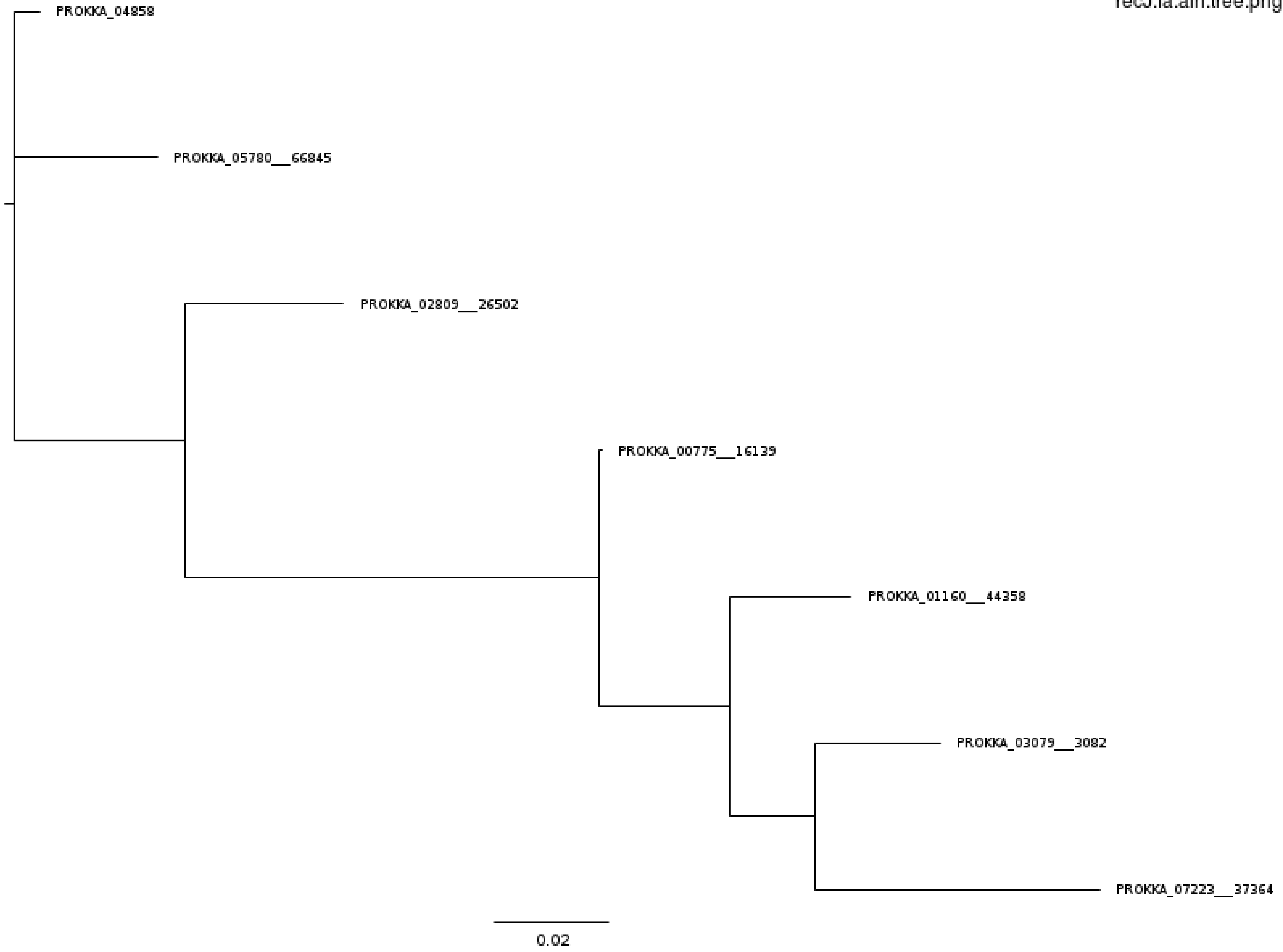

regA_2.fa.aln.tree.png

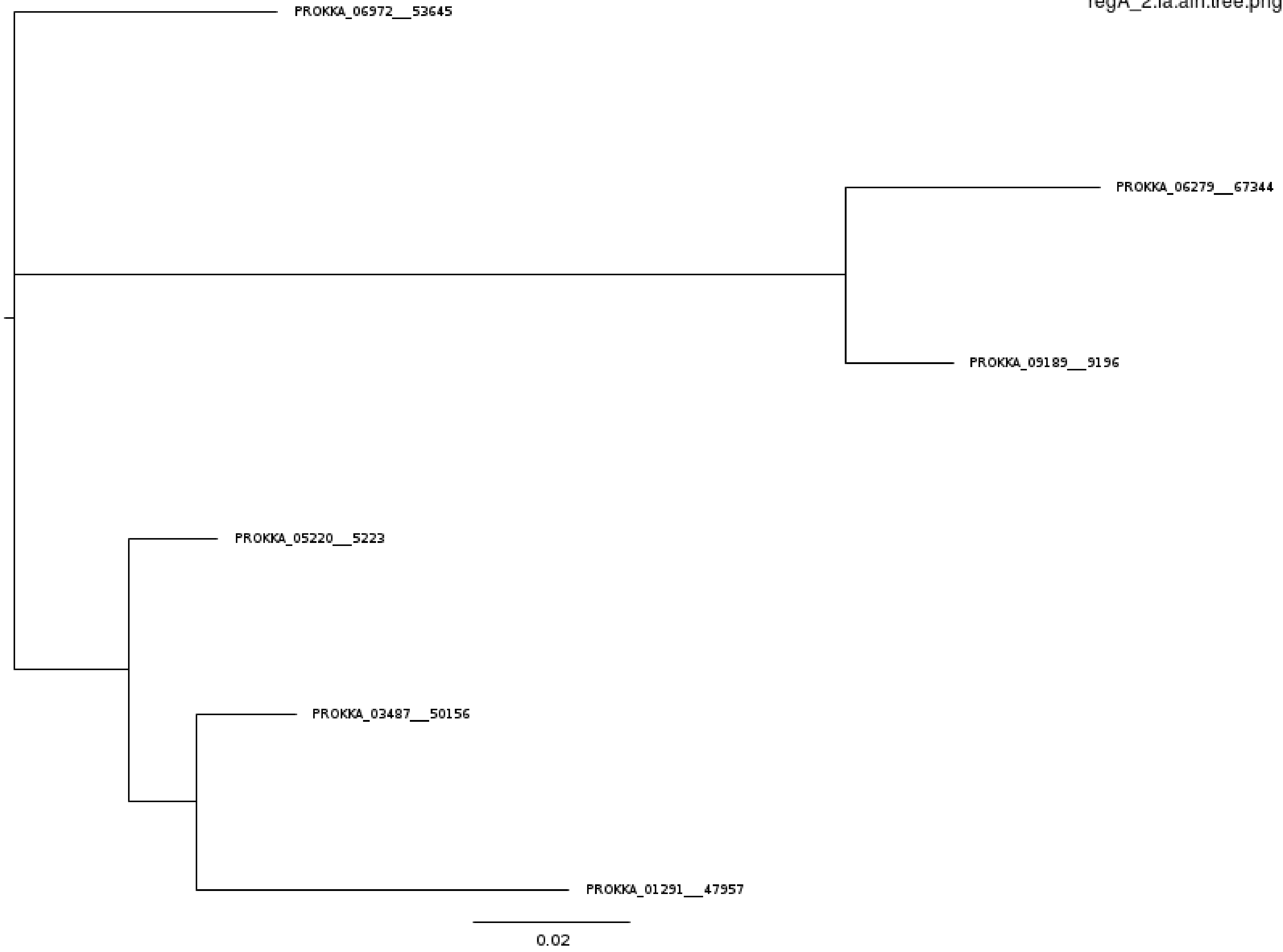

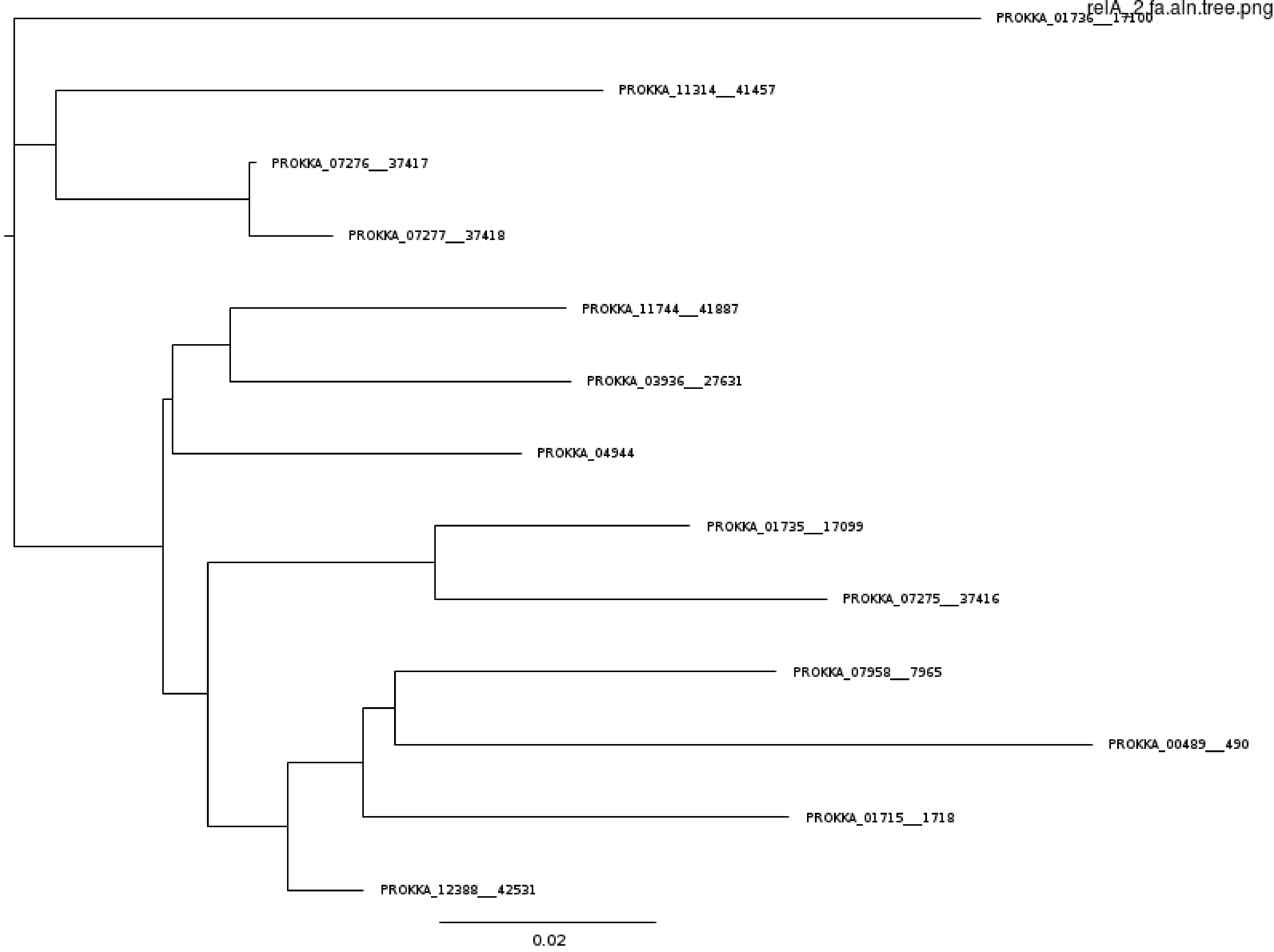

relA_2.fa.aln.tree.png
PROKKA_01736___17100
PROKKA_11314___41457
PROKKA_07276___37417
PROKKA_07277___37418
PROKKA_11744___41887
PROKKA_03936___27631
PROKKA_04944
PROKKA_01735___17099
PROKKA_07275___37416
PROKKA_07958___7965
PROKKA_00489___490
PROKKA_01715___1718
PROKKA_12388___42531
0.02

relE_2.fa.aln.tree.png

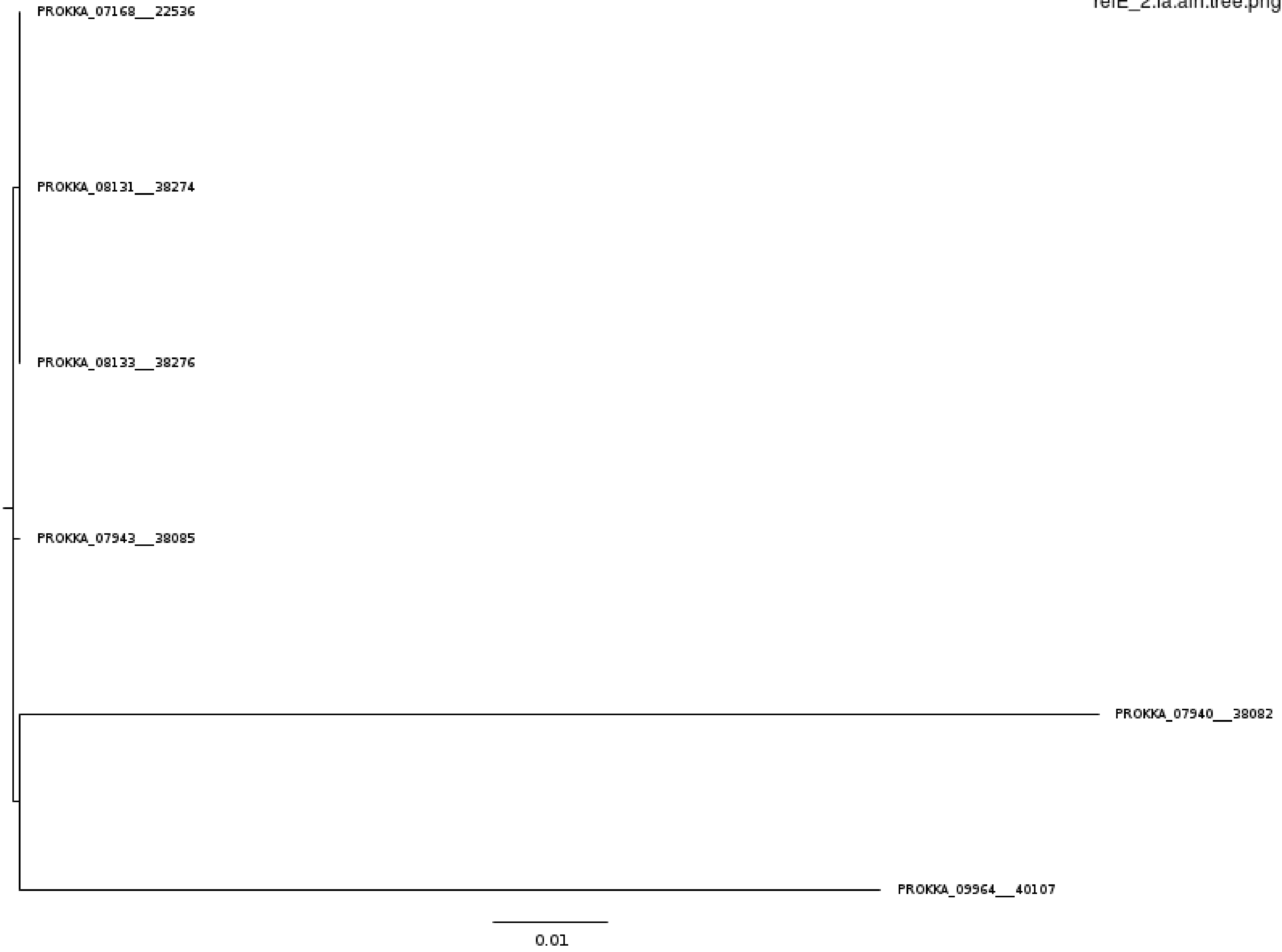

relK_1.fa.aln.tree.png

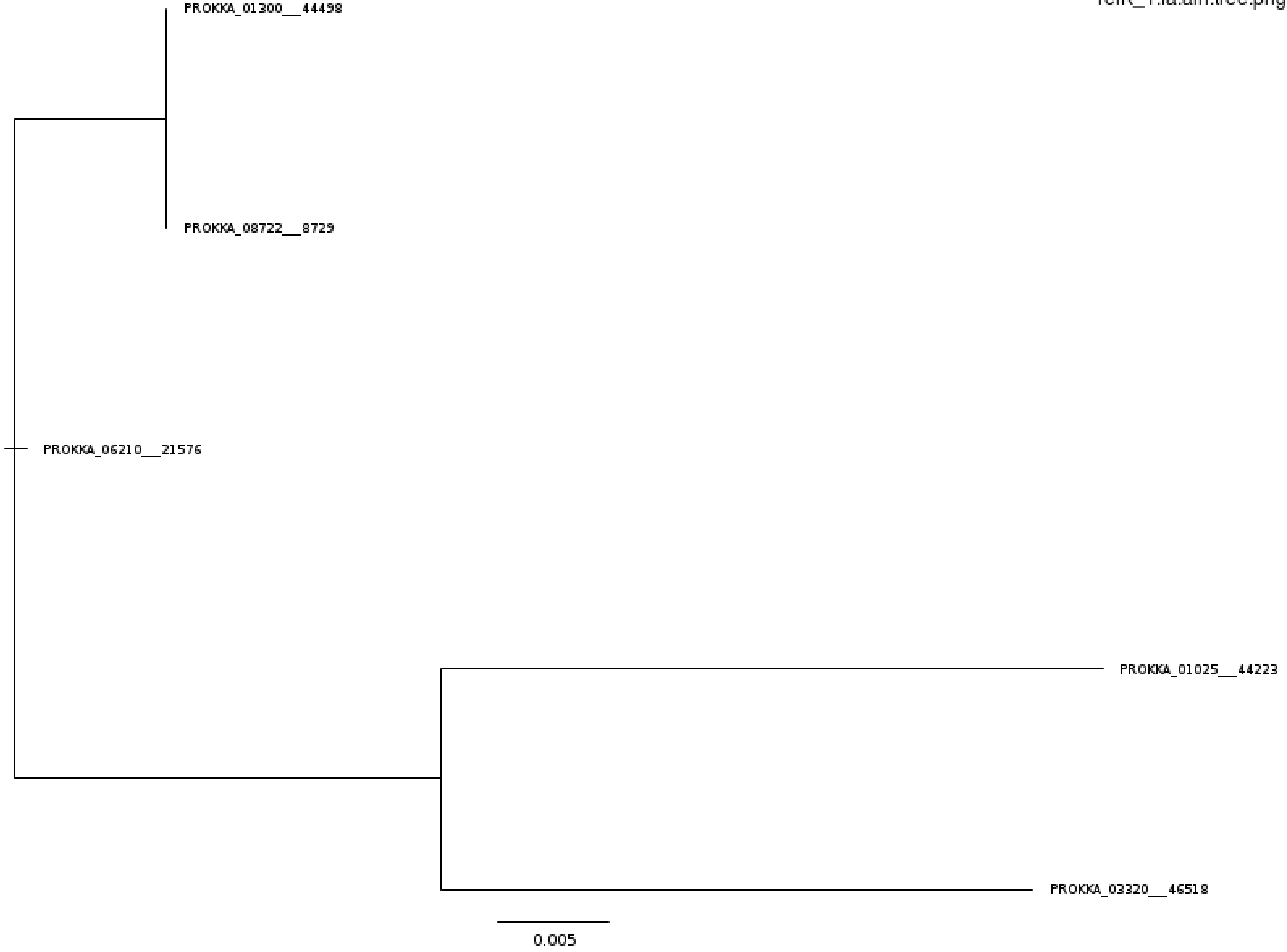

relK_2.fa.aln.tree.png

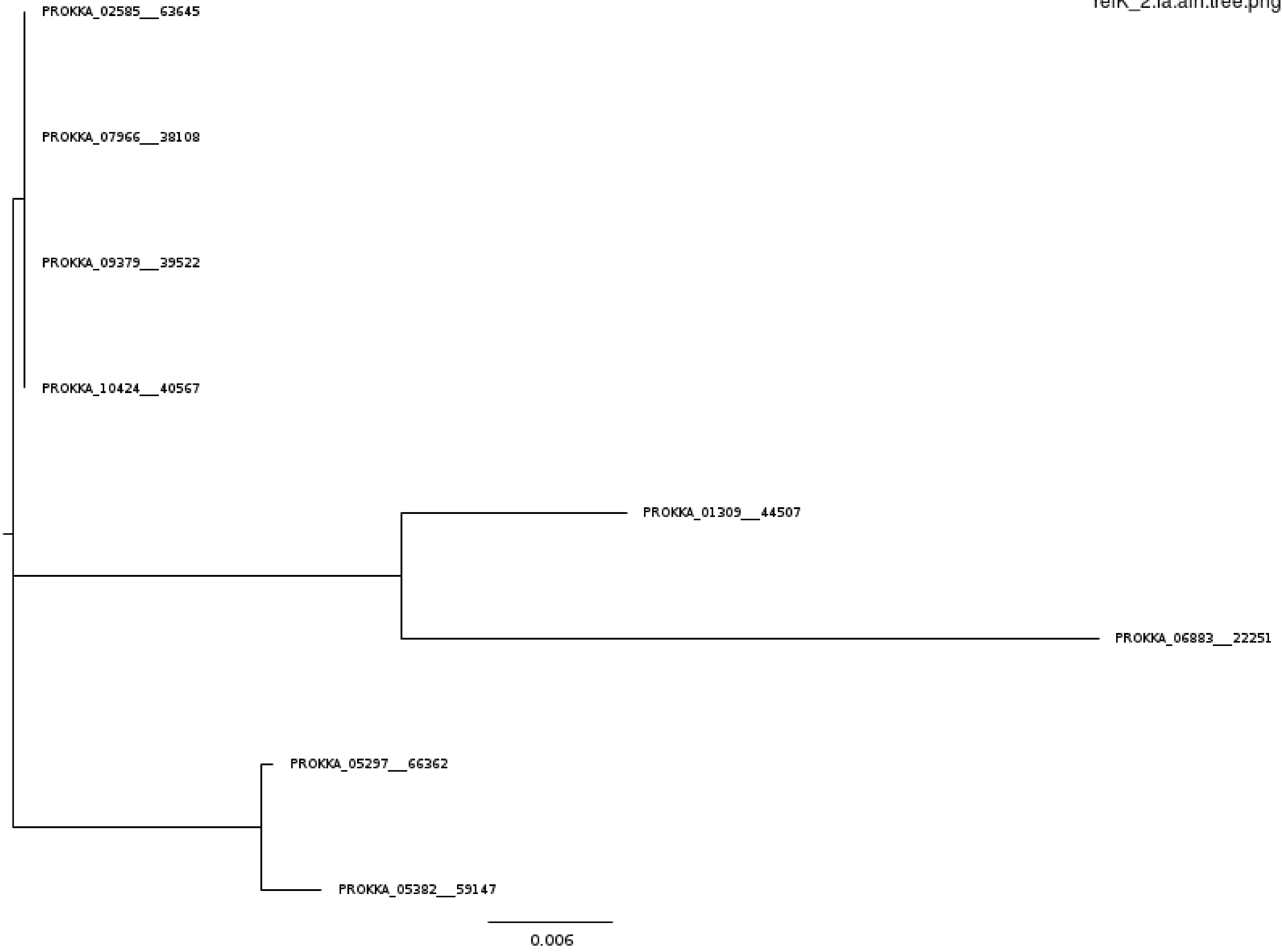

resA_1.fa.aln.tree.png

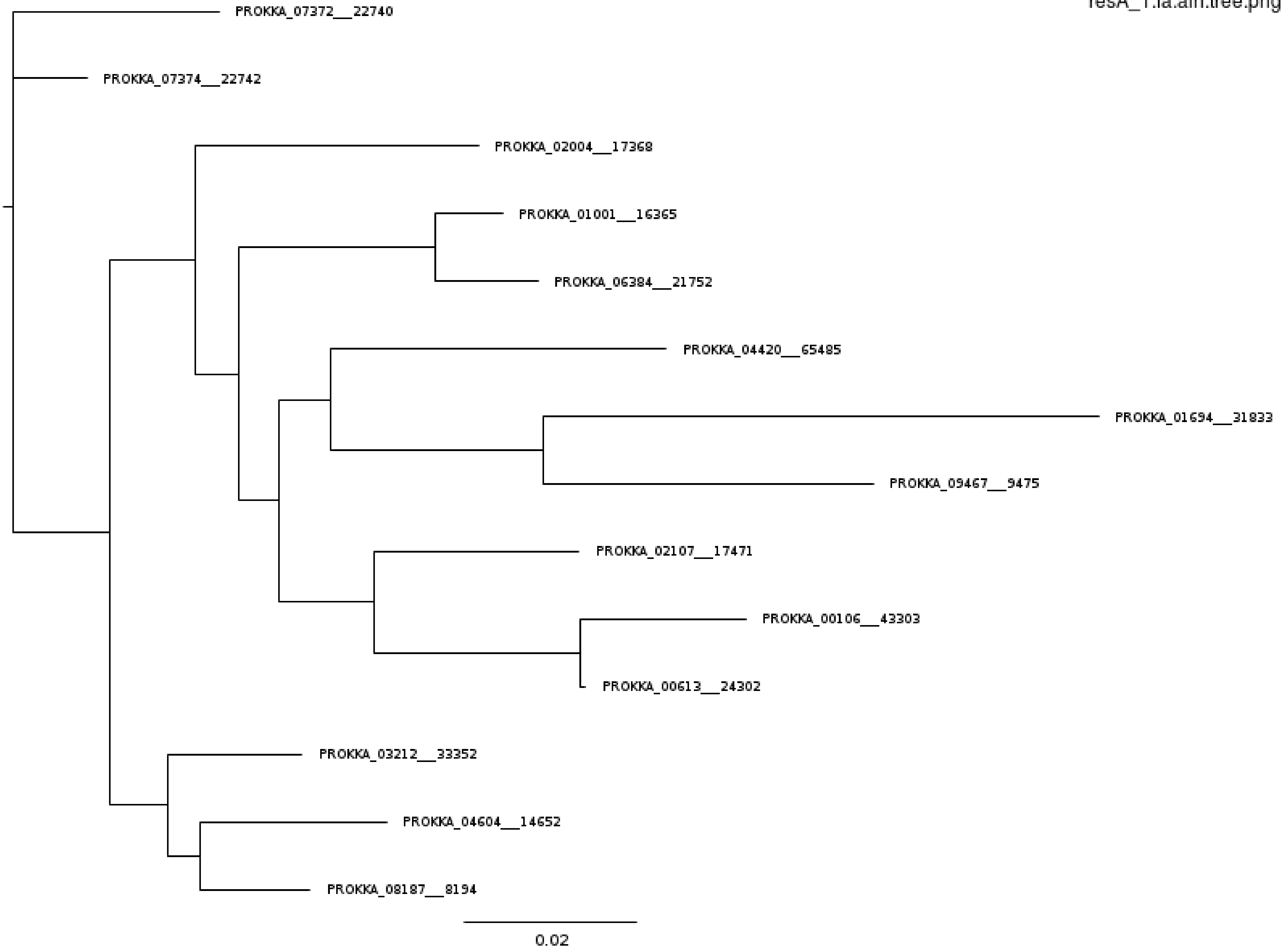

rfaC.fa.aln.tree.png

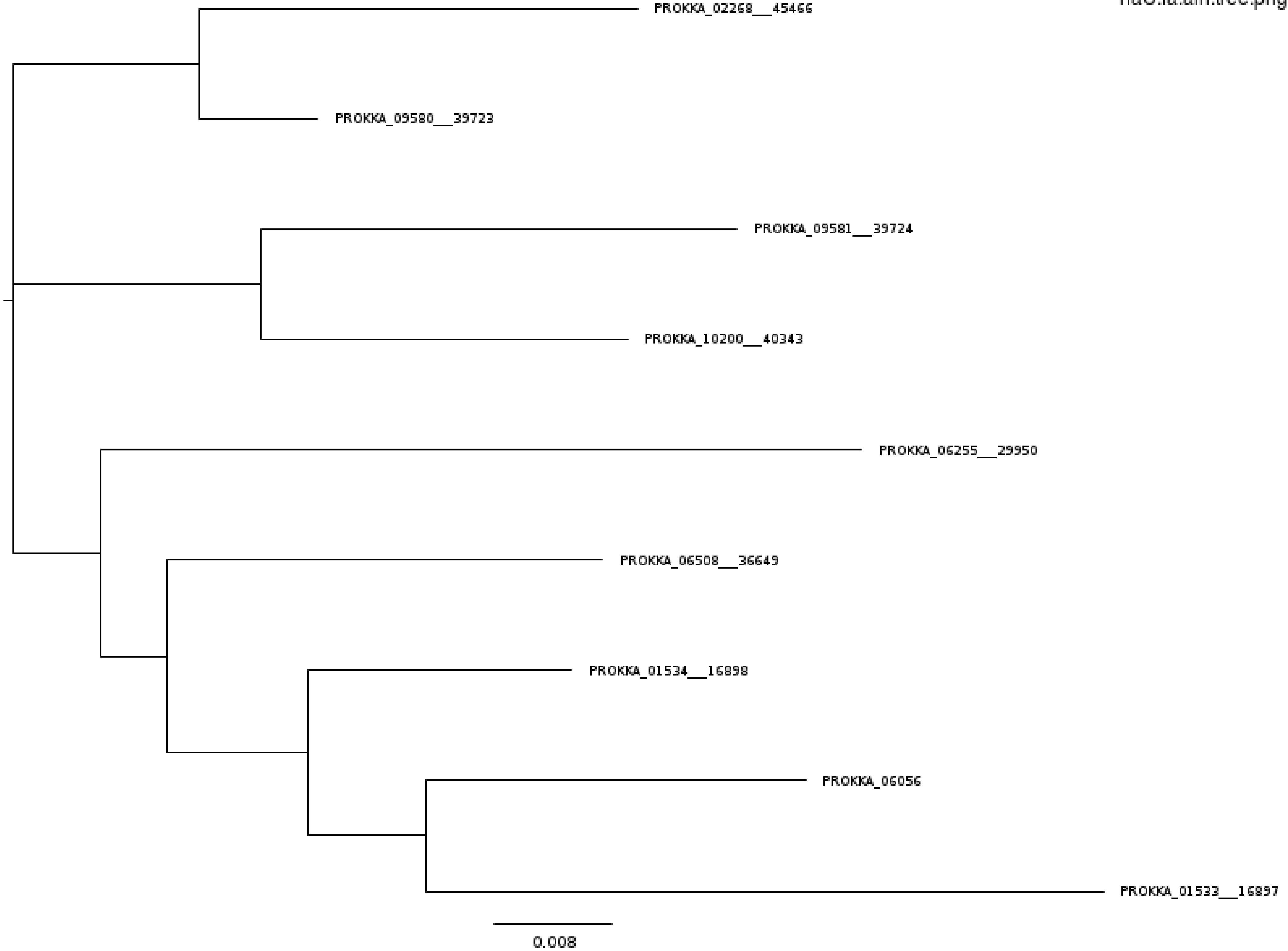

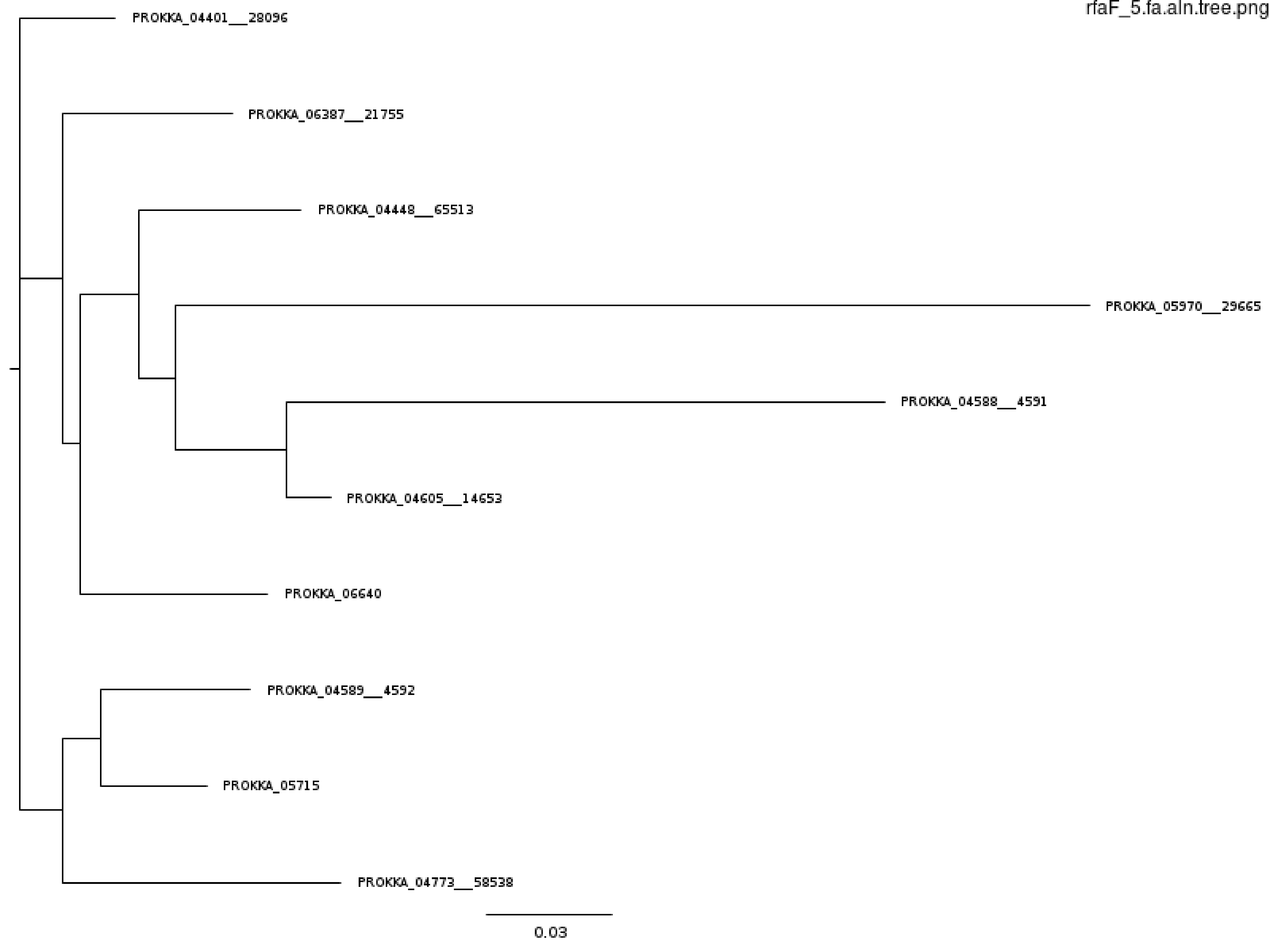
rfaF_5.fa.aln.tree.png
PROKKA_04401___28096
PROKKA_06387___21755
PROKKA_04448___65513
PROKKA_05970___29665
PROKKA_04588___4591
PROKKA_04605___14653
PROKKA_06640
PROKKA_04589___4592
PROKKA_05715
PROKKA_04773___58538
0.03

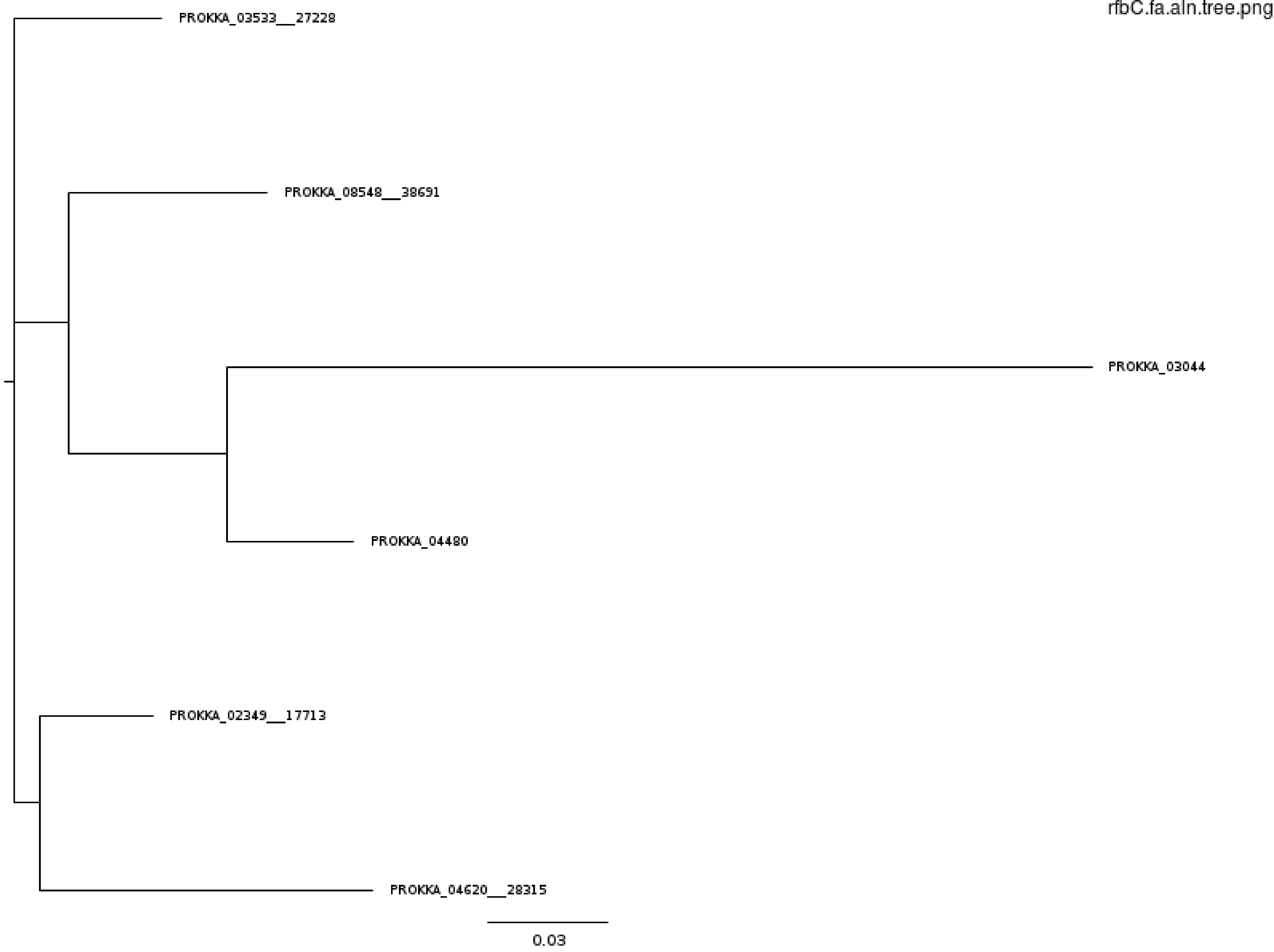
rfbC.fa.aln.tree.png
PROKKA_03533__27228
PROKKA_08548__38691
PROKKA_03044
PROKKA_04480
PROKKA_02349__17713
PROKKA_04620__28315
0.03

rfbM_1.fa.aln.tree.png

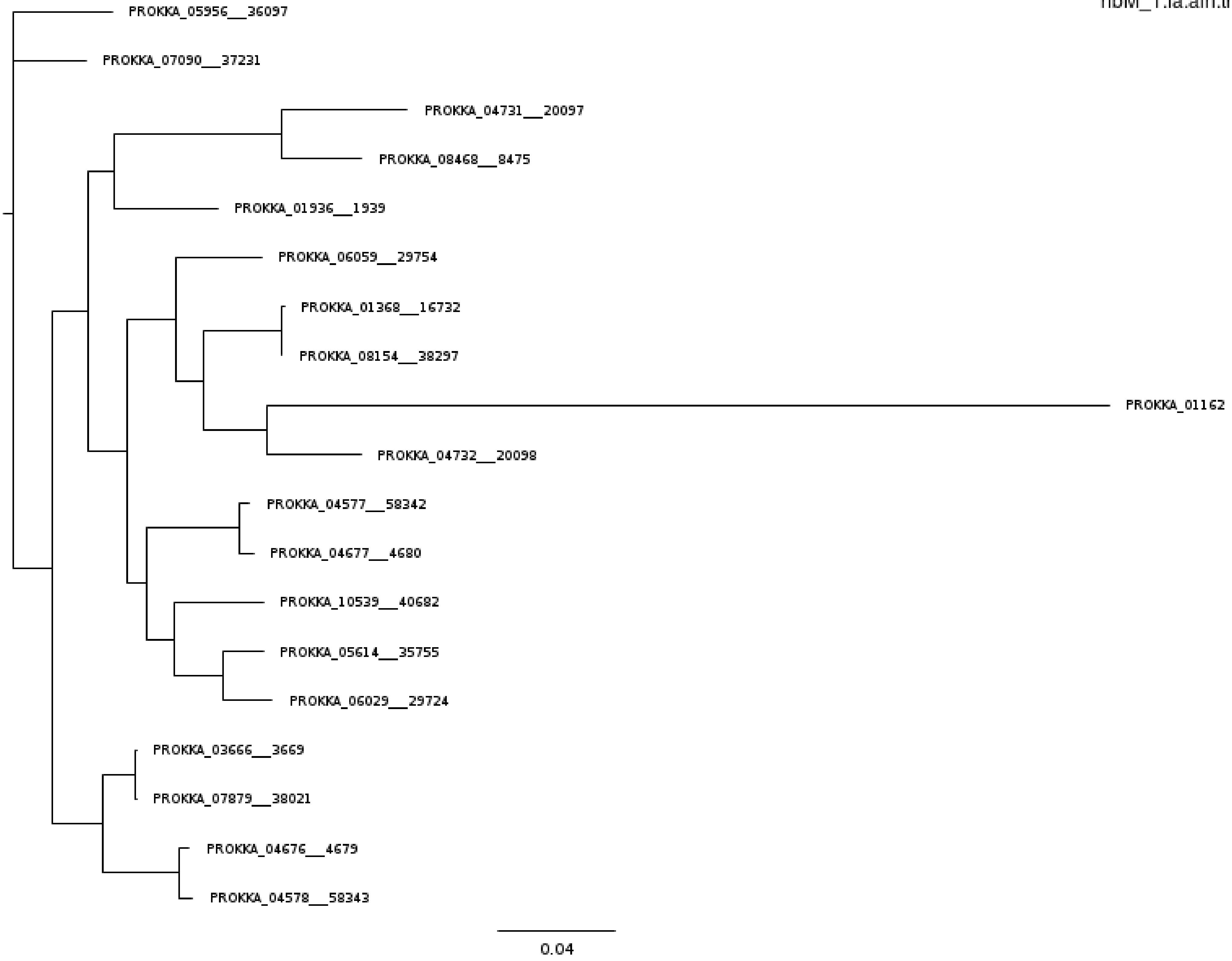

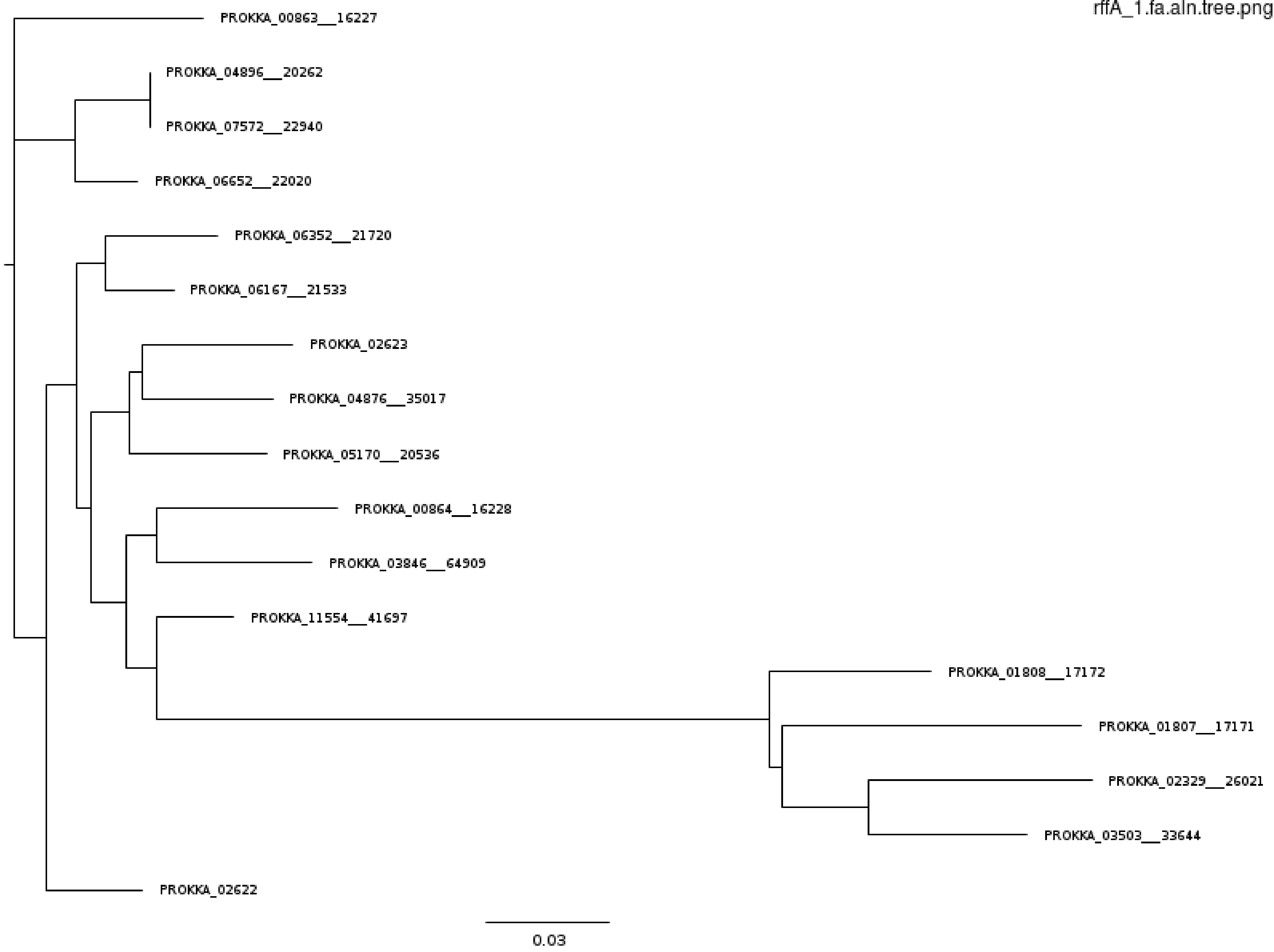
rffA_1.fa.aln.tree.png
PROKKA_00863___16227
PROKKA_04896___20262
PROKKA_07572___22940
PROKKA_06652___22020
PROKKA_06352___21720
PROKKA_06167___21533
PROKKA_02623
PROKKA_04876___35017
PROKKA_05170___20536
PROKKA_00864___16228
PROKKA_03846___64909
PROKKA_11554___41697
PROKKA_01808___17172
PROKKA_01807___17171
PROKKA_02329___26021
PROKKA_03503___33644
PROKKA_02622
0.03

ribBA_2.fa.aln.tree.png

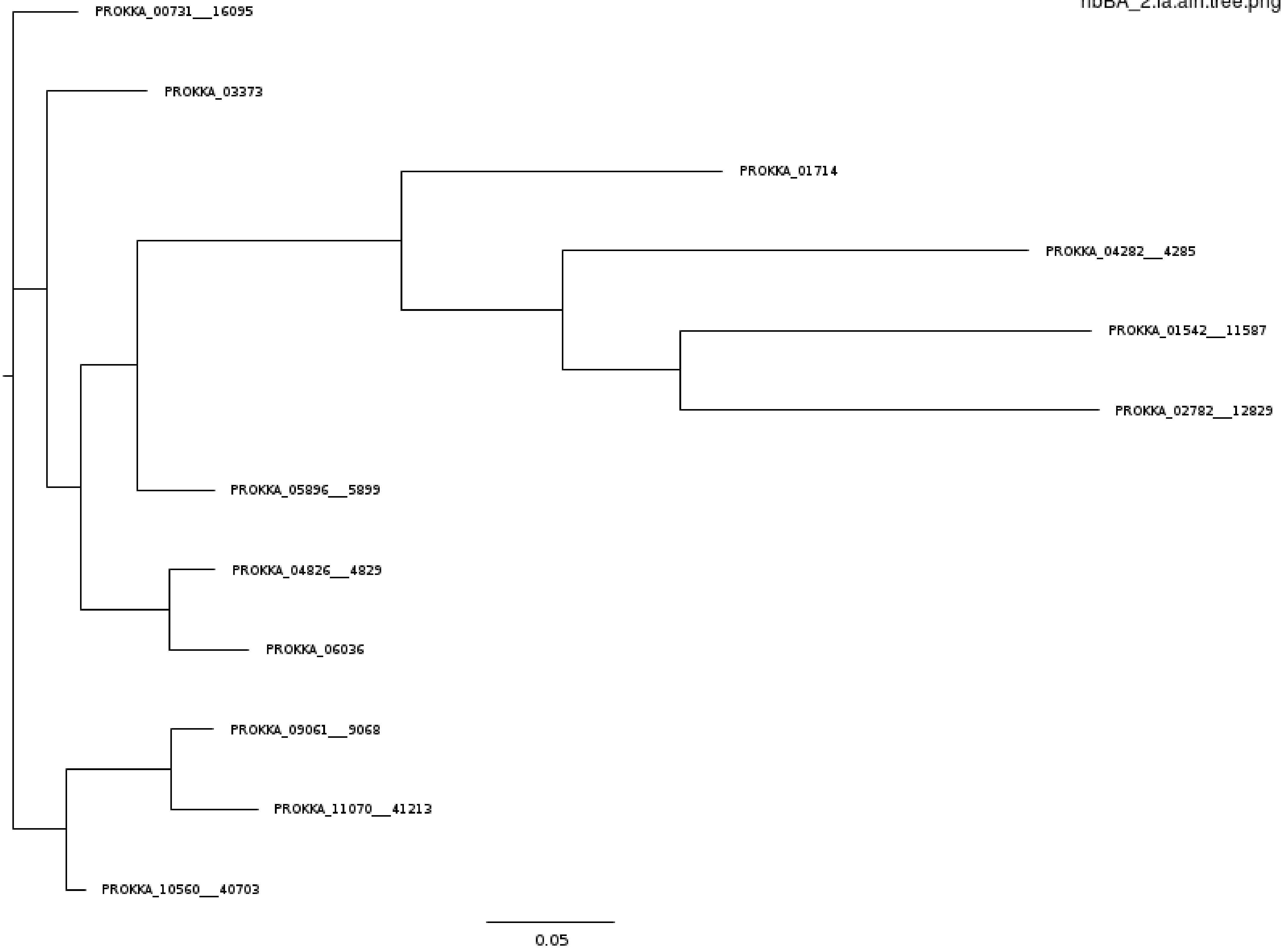

ribE_3.fa.aln.tree.png

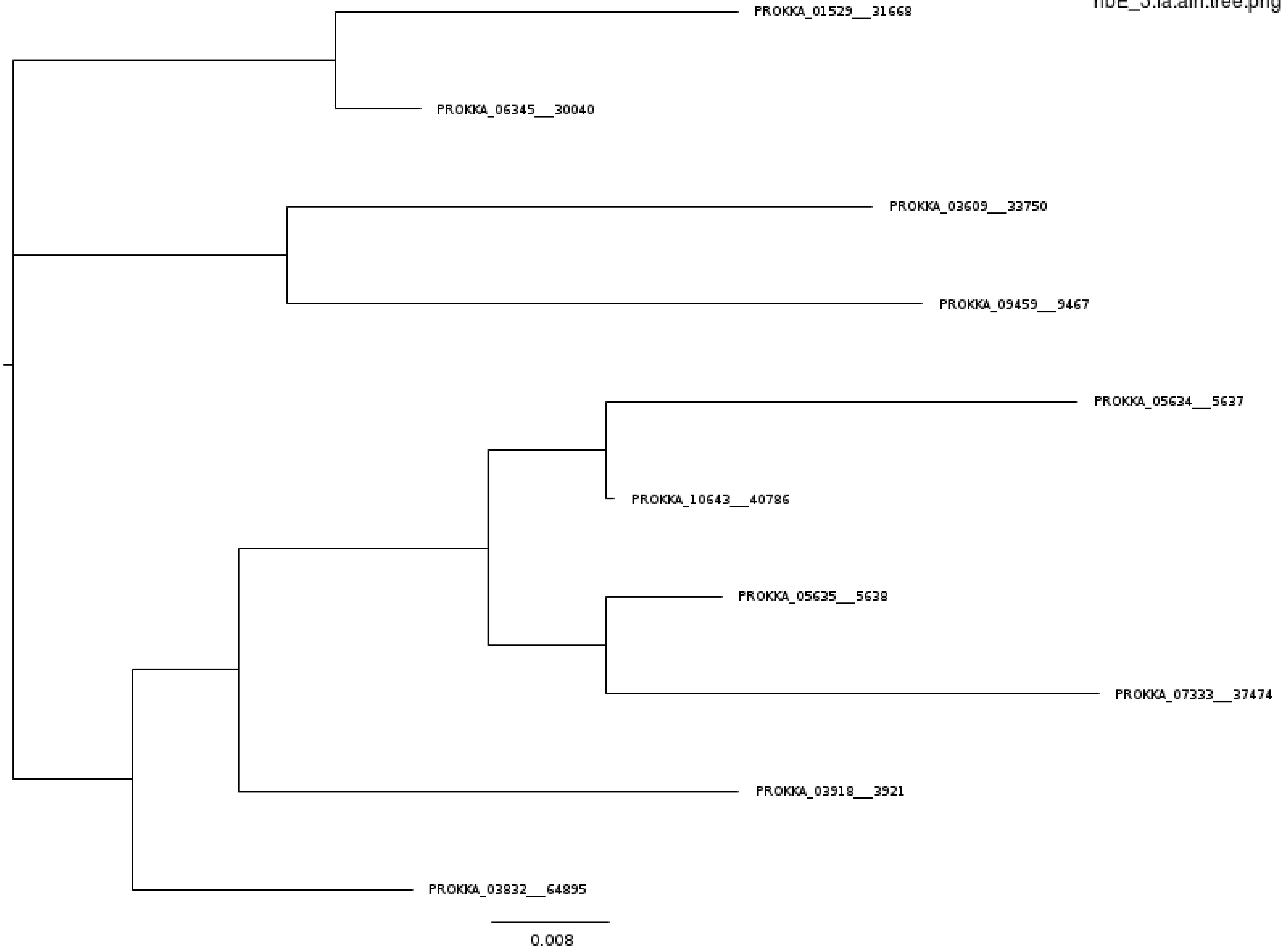

ribF_2.fa.aln.tree.png

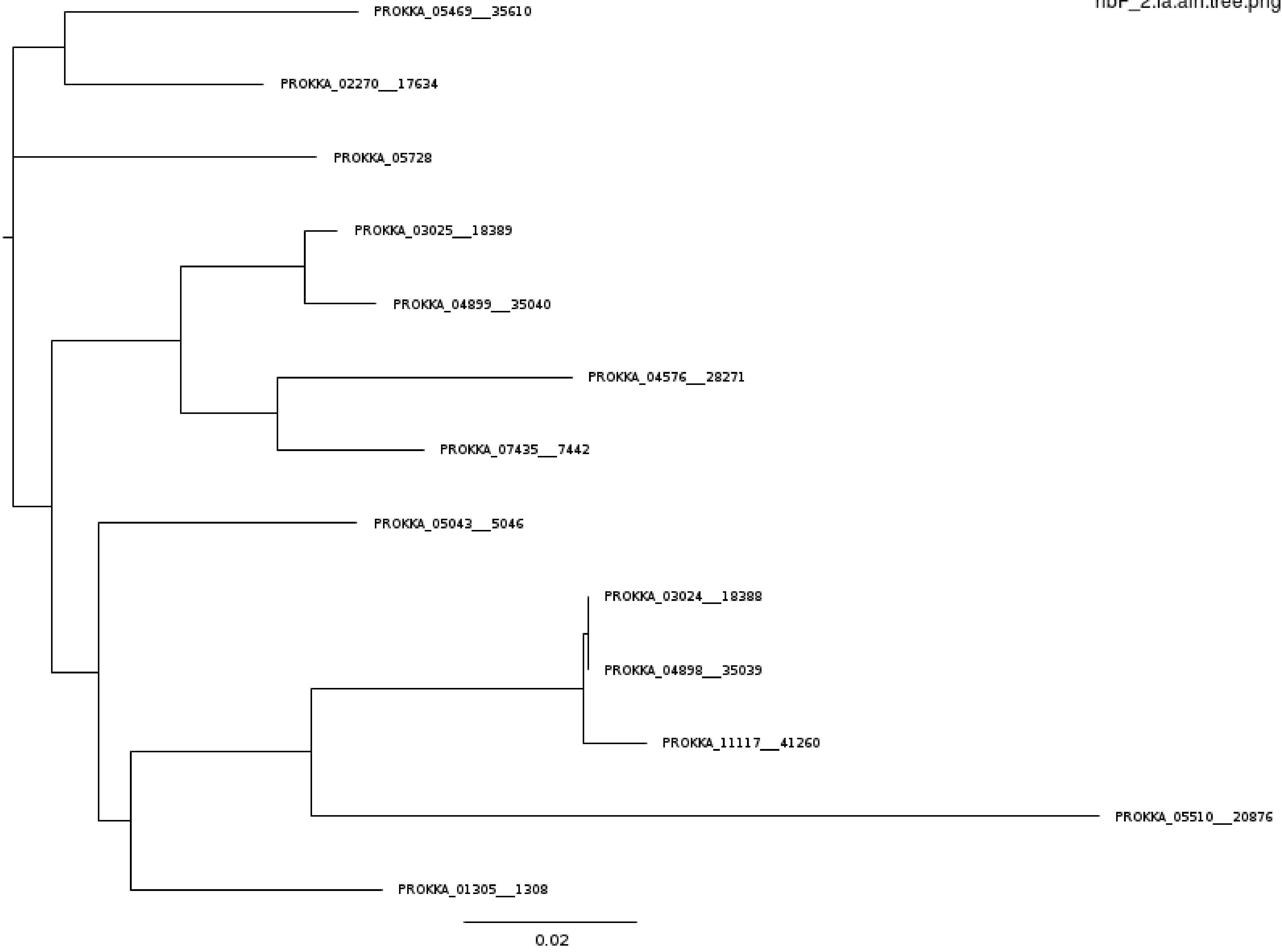

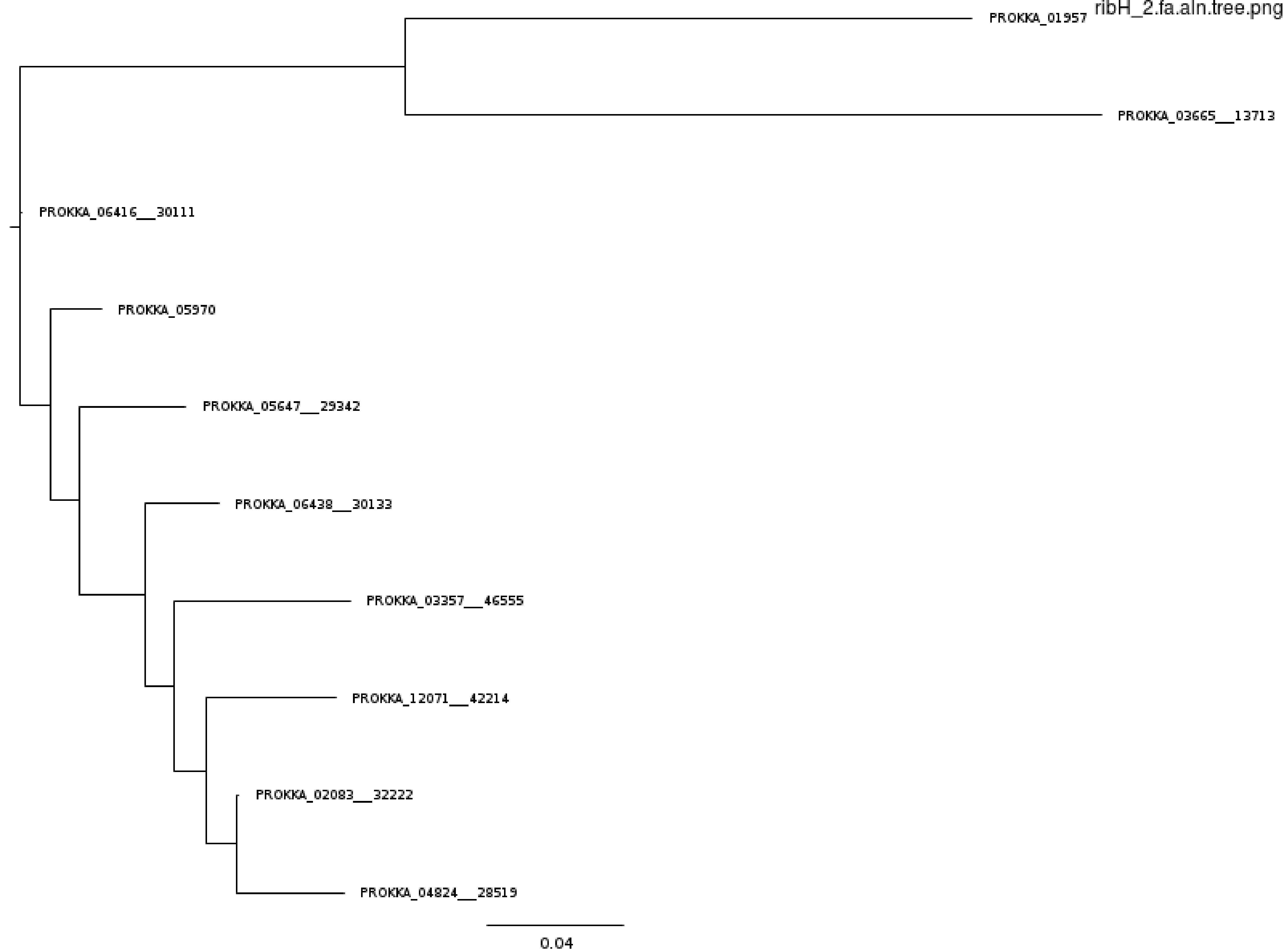
ribH_2.fa.aln.tree.png
PROKKA_01957
PROKKA_03665___13713
PROKKA_06416___30111
PROKKA_05970
PROKKA_05647___29342
PROKKA_06438___30133
PROKKA_03357___46555
PROKKA_12071___42214
PROKKA_02083___32222
PROKKA_04824___28519
0.04

rimK_1.fa.aln.tree.png

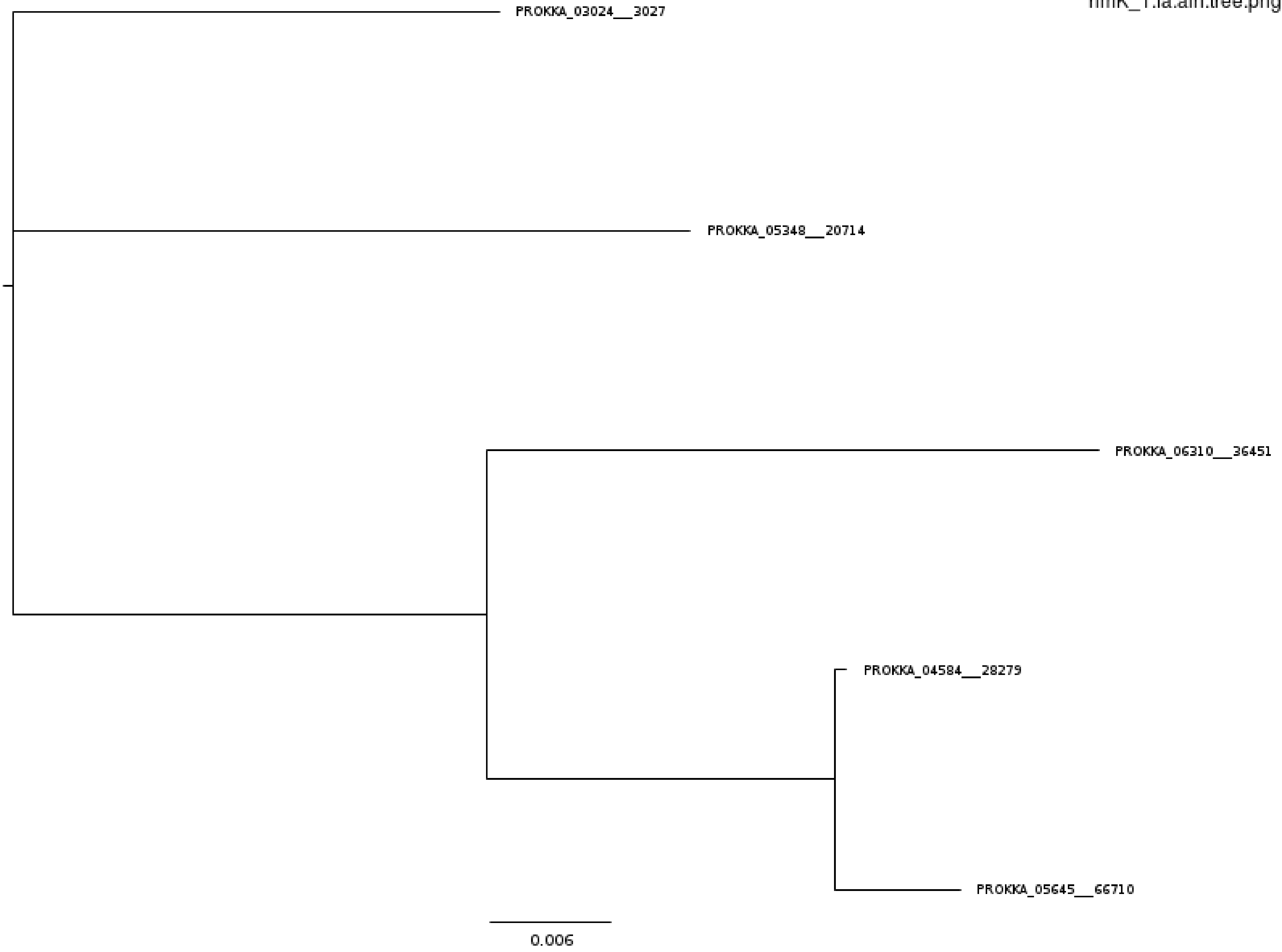

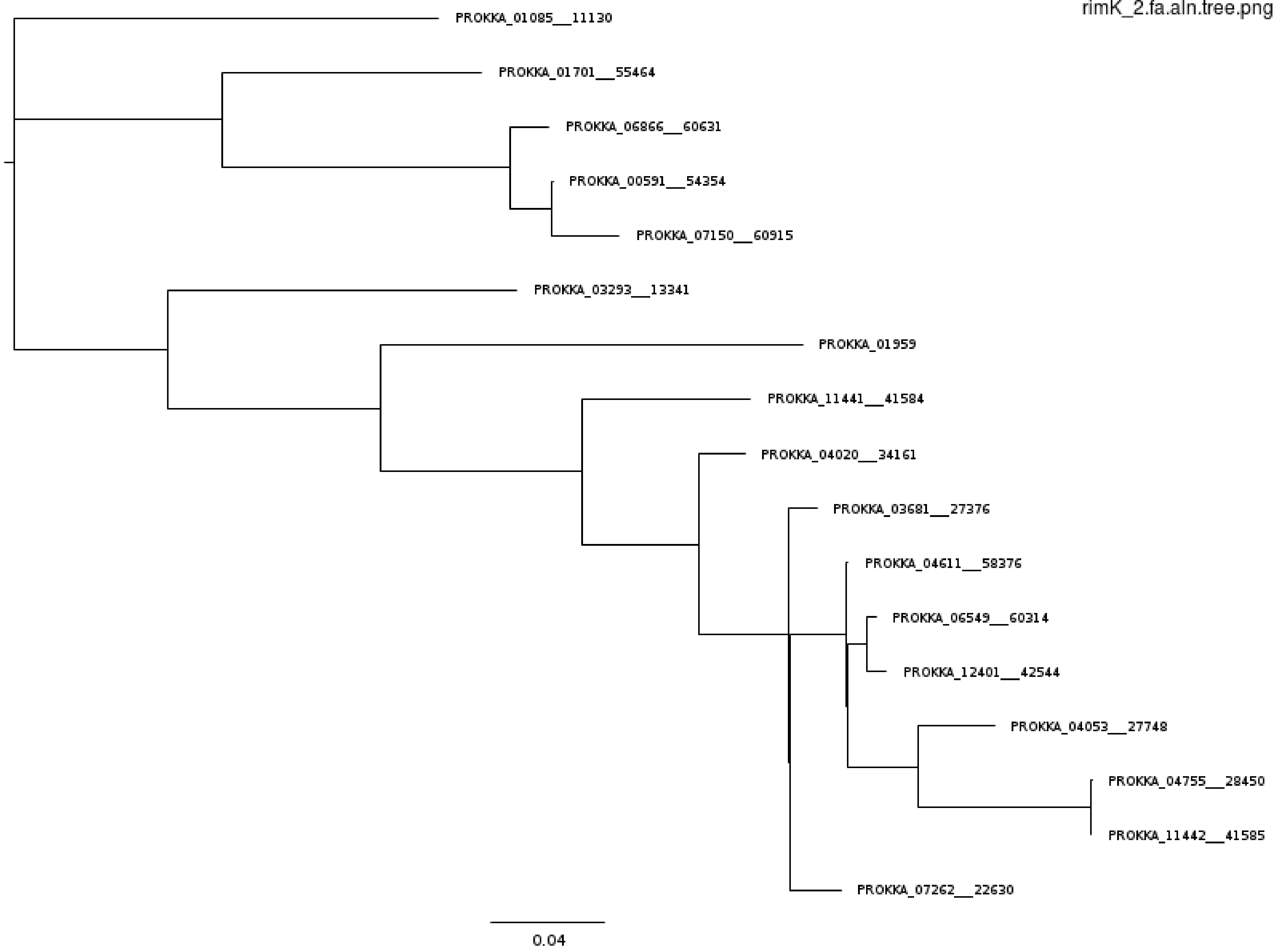
rimK_2.fa.aln.tree.png
PROKKA_01085___11130
PROKKA_01701___55464
PROKKA_06866___60631
PROKKA_00591___54354
PROKKA_07150___60915
PROKKA_03293___13341
PROKKA_01959
PROKKA_11441___41584
PROKKA_04020___34161
PROKKA_03681___27376
PROKKA_04611___58376
PROKKA_06549___60314
PROKKA_12401___42544
PROKKA_04053___27748
PROKKA_04755___28450
PROKKA_11442___41585
PROKKA_07262___22630
0.04

rimO_4.fa.aln.tree.png

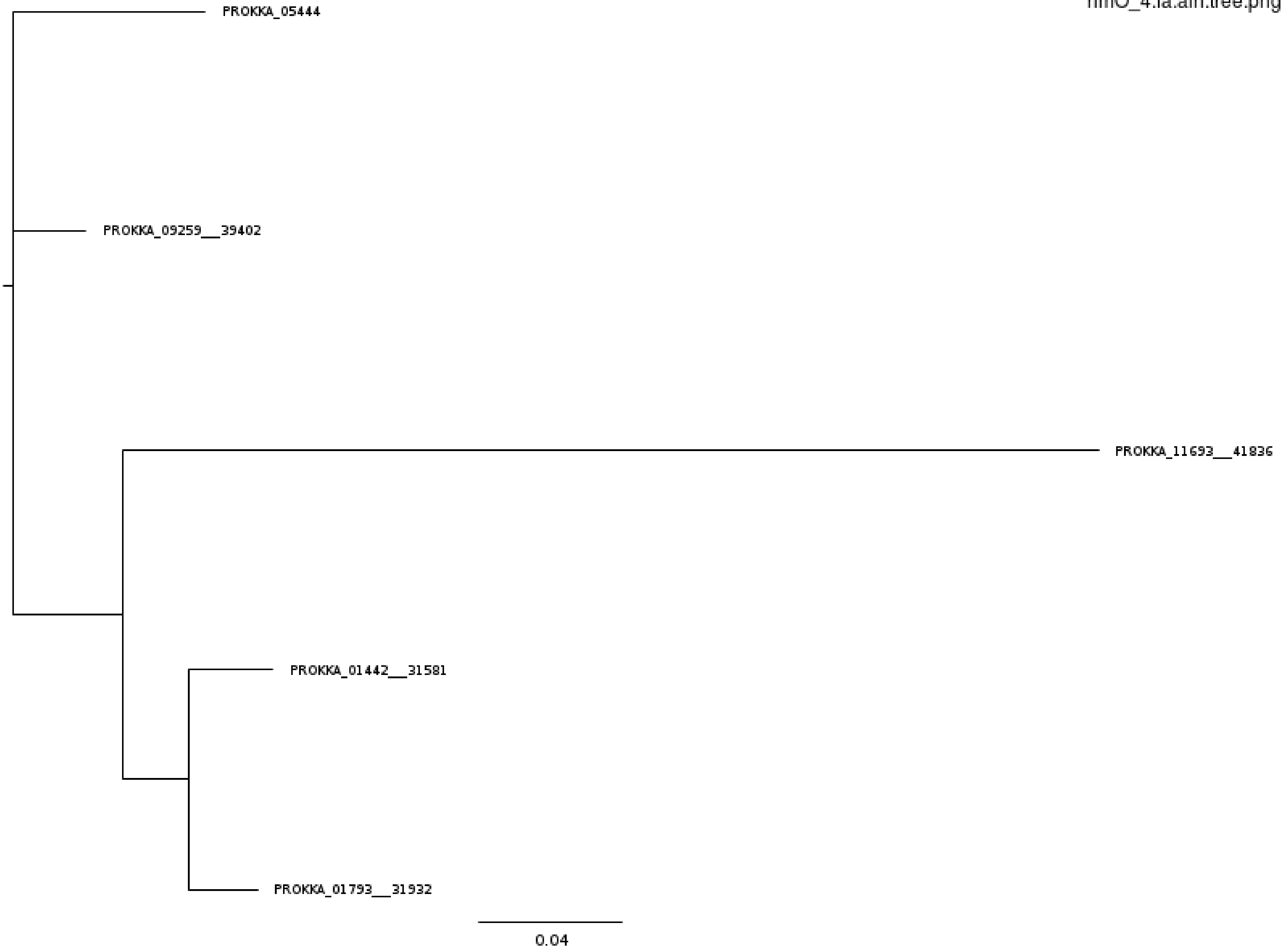

rimP.fa.aln.tree.png

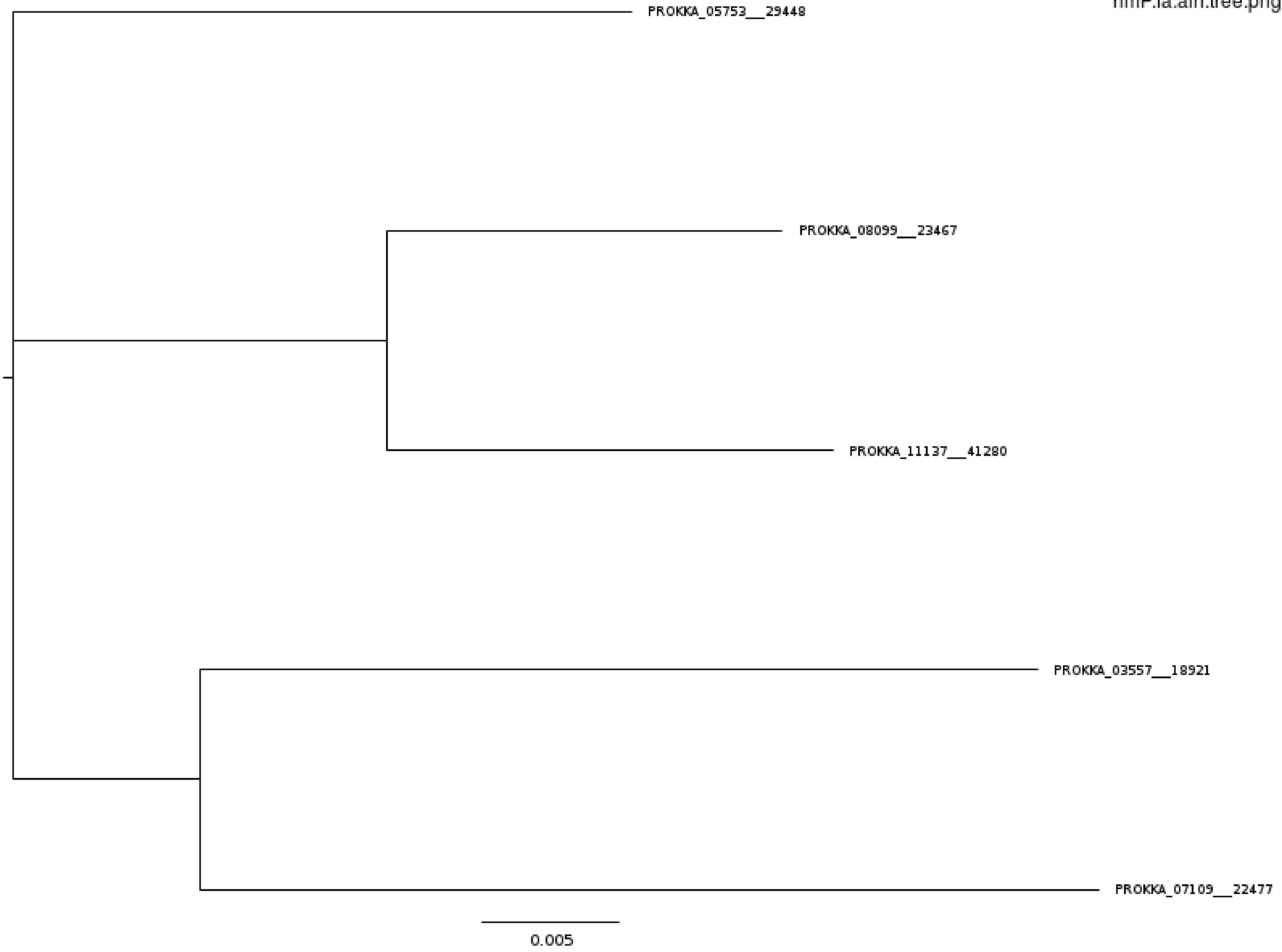

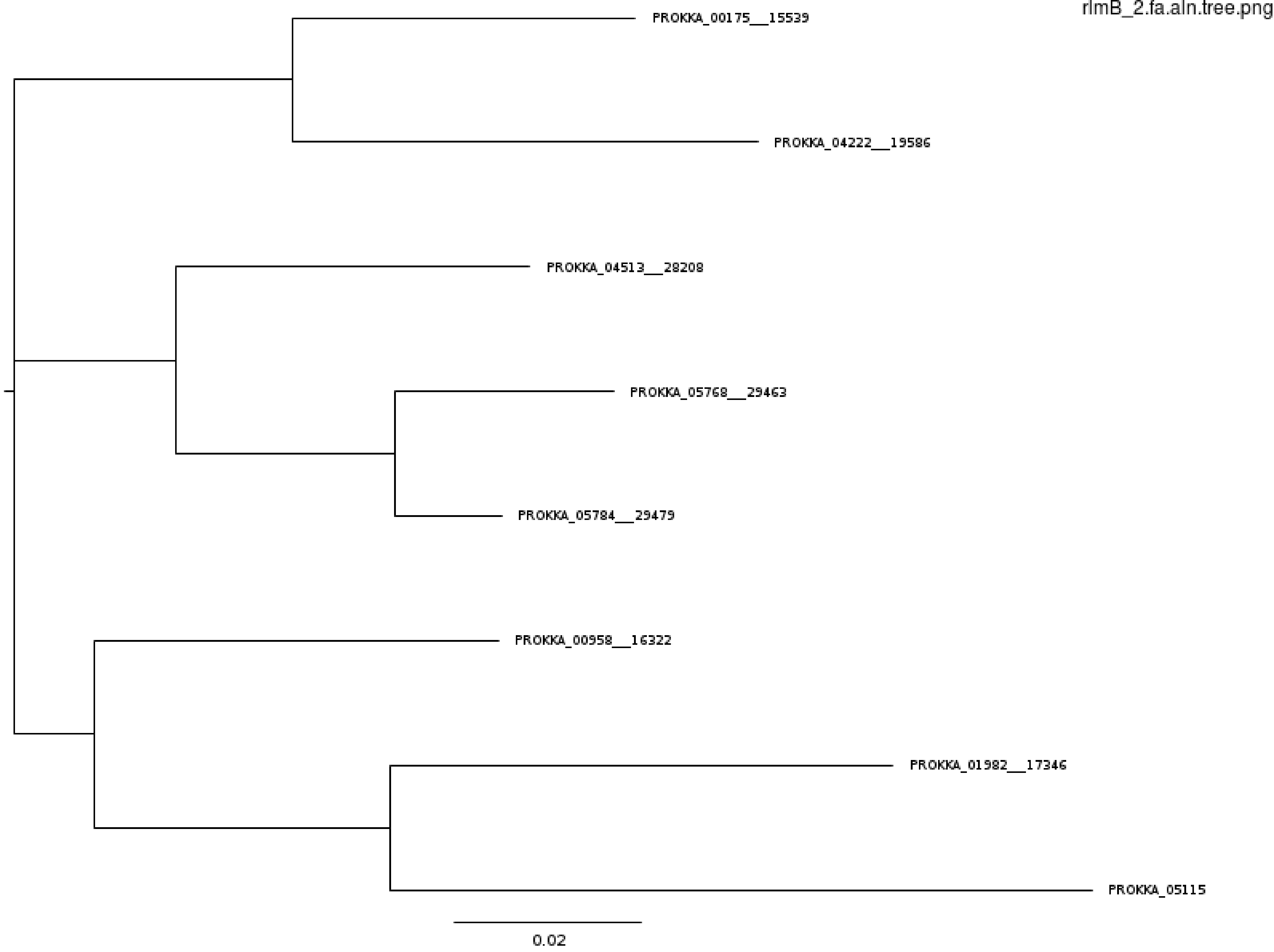
rlmB_2.fa.aln.tree.png
PROKKA_00175___15539
PROKKA_04222___19586
PROKKA_04513___28208
PROKKA_05768___29463
PROKKA_05784___29479
PROKKA_00958___16322
PROKKA_01982___17346
PROKKA_05115
0.02

rlmD_1.fa.aln.tree.png

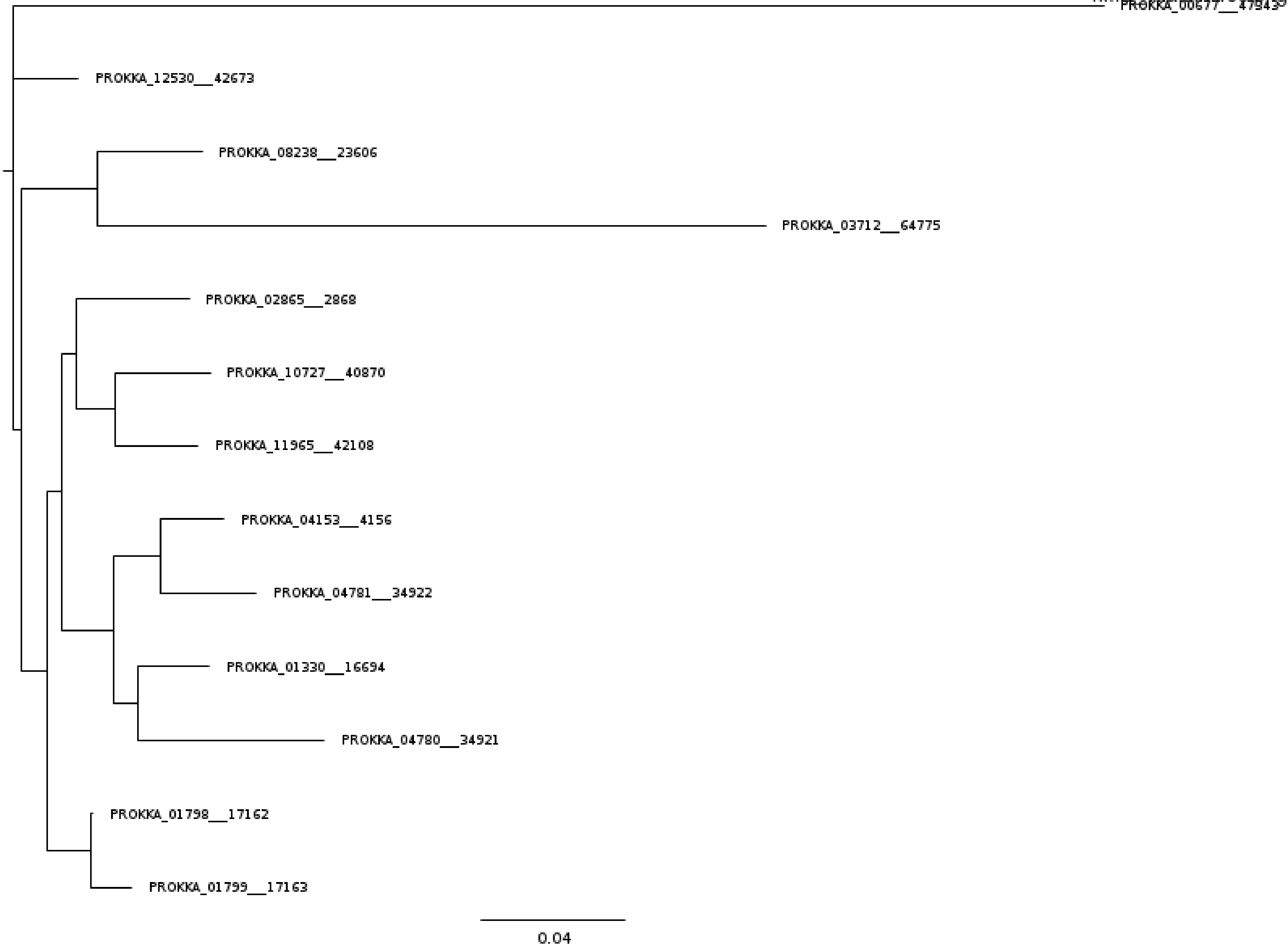

rlmE.fa.aln.tree.png

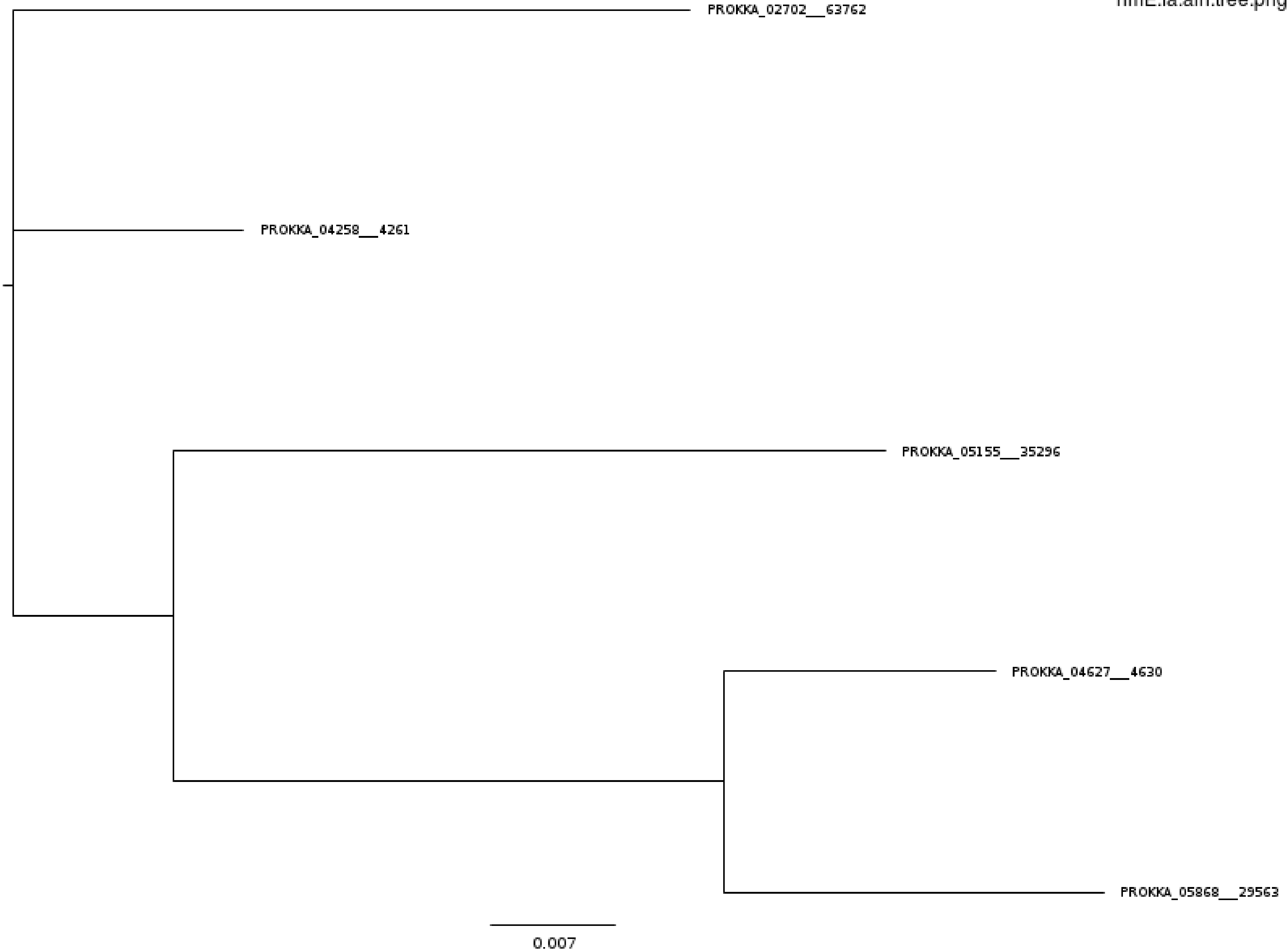

rlmH_3.fa.aln.tree.png

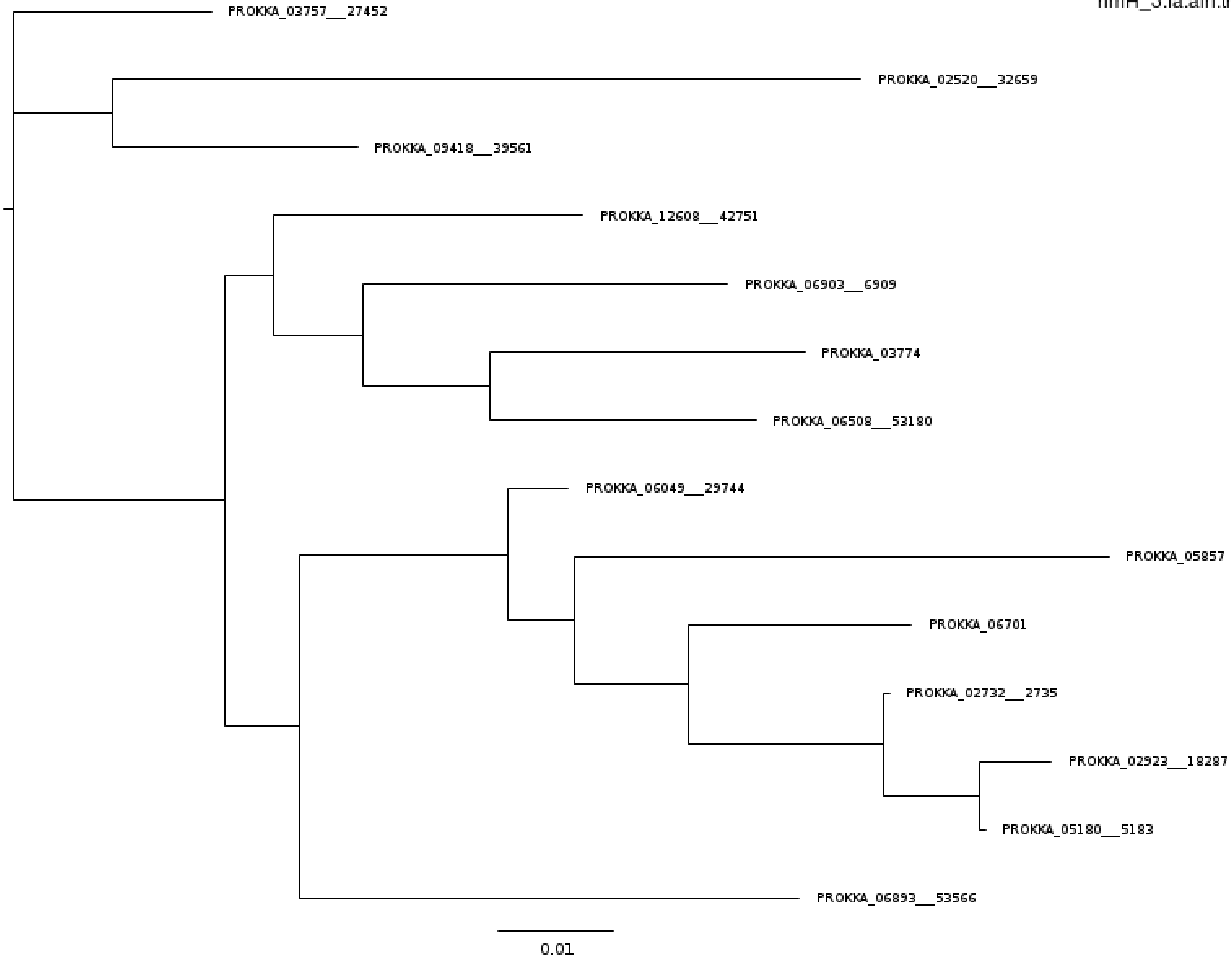

rlml_2.fa.aln.tree.png

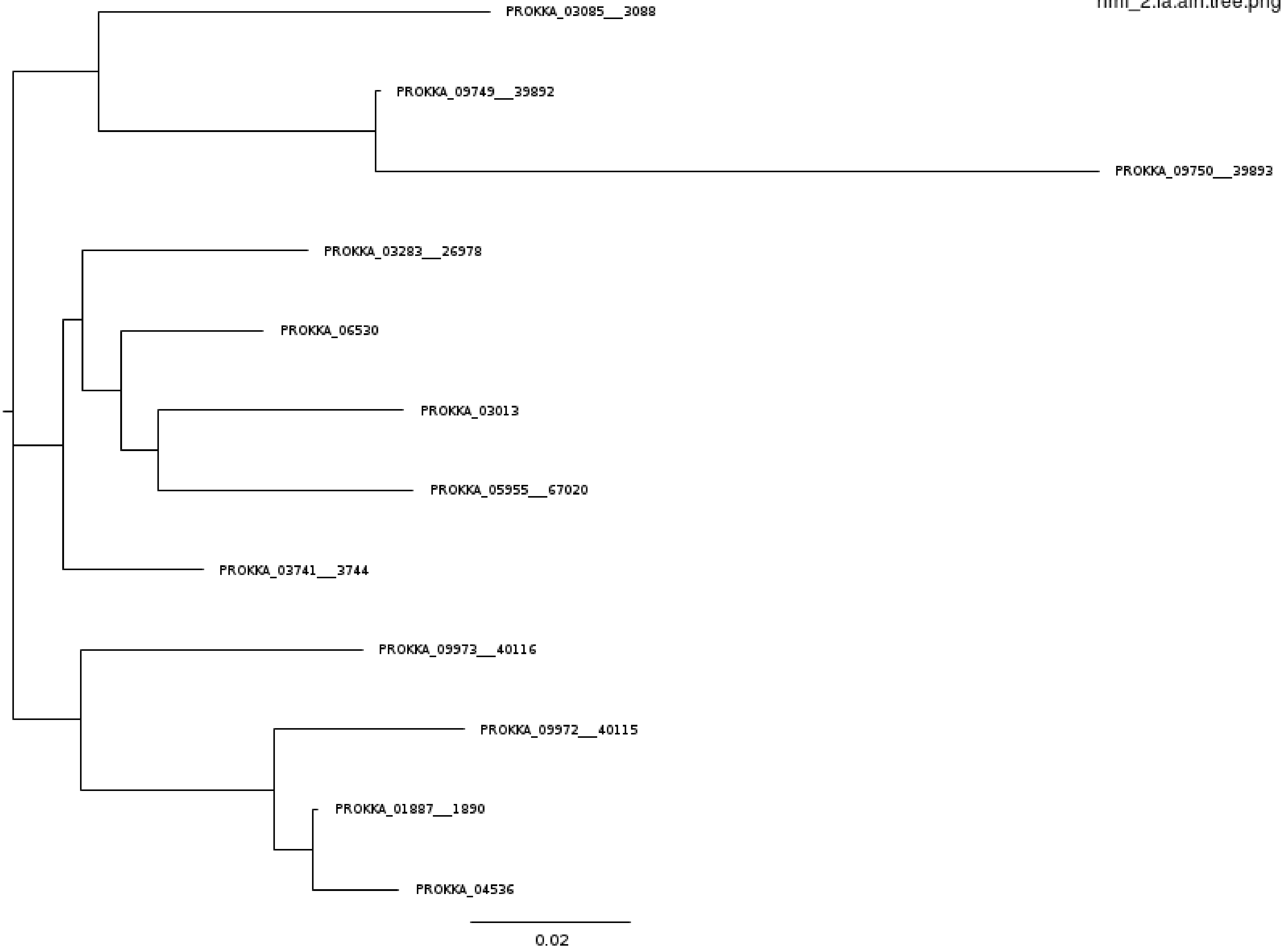

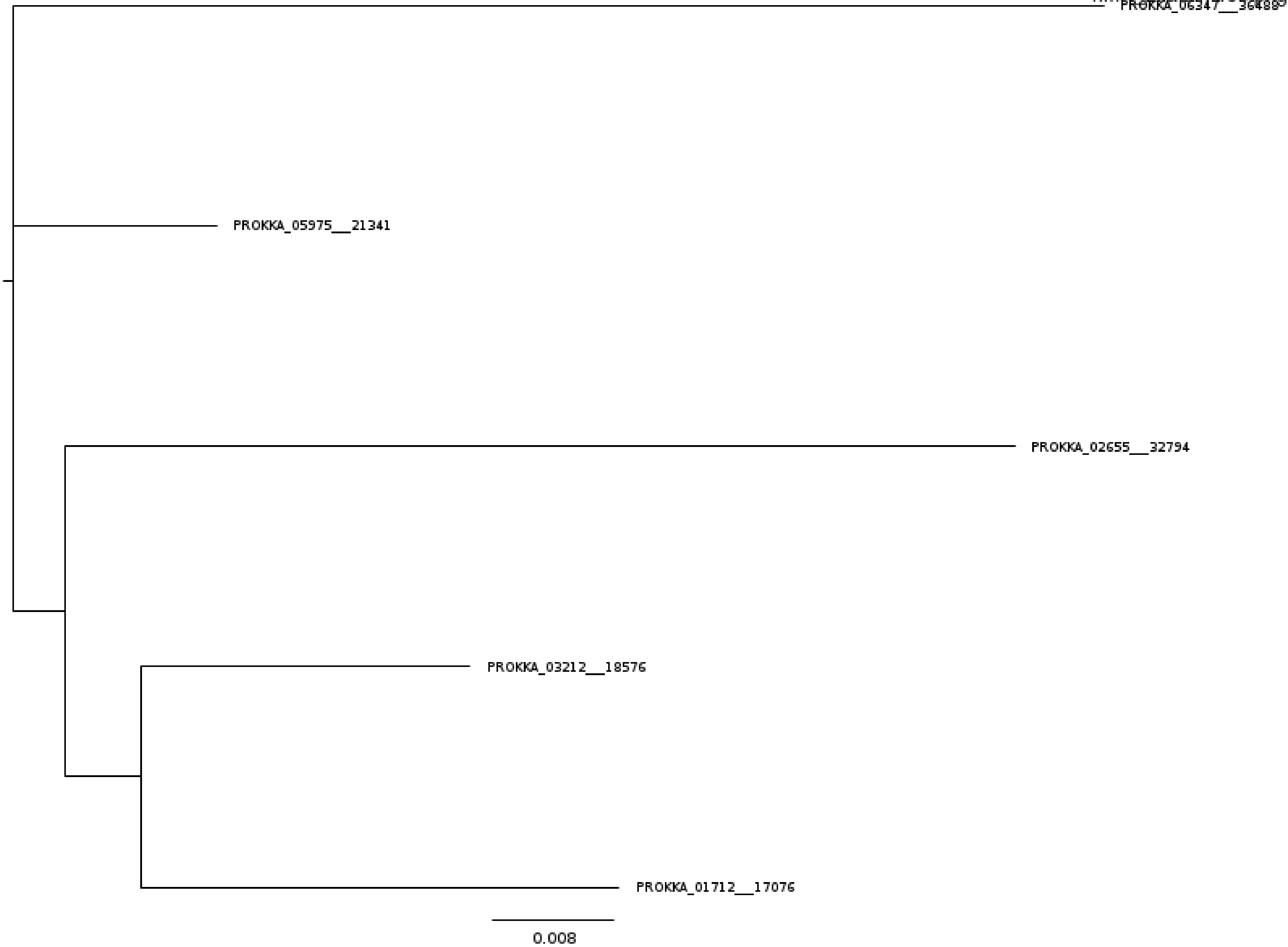
PROKKA_06347___36488
PROKKA_05975___21341
PROKKA_02655___32794
PROKKA_03212___18576
PROKKA_01712___17076
0.008

rluB.fa.aln.tree.png

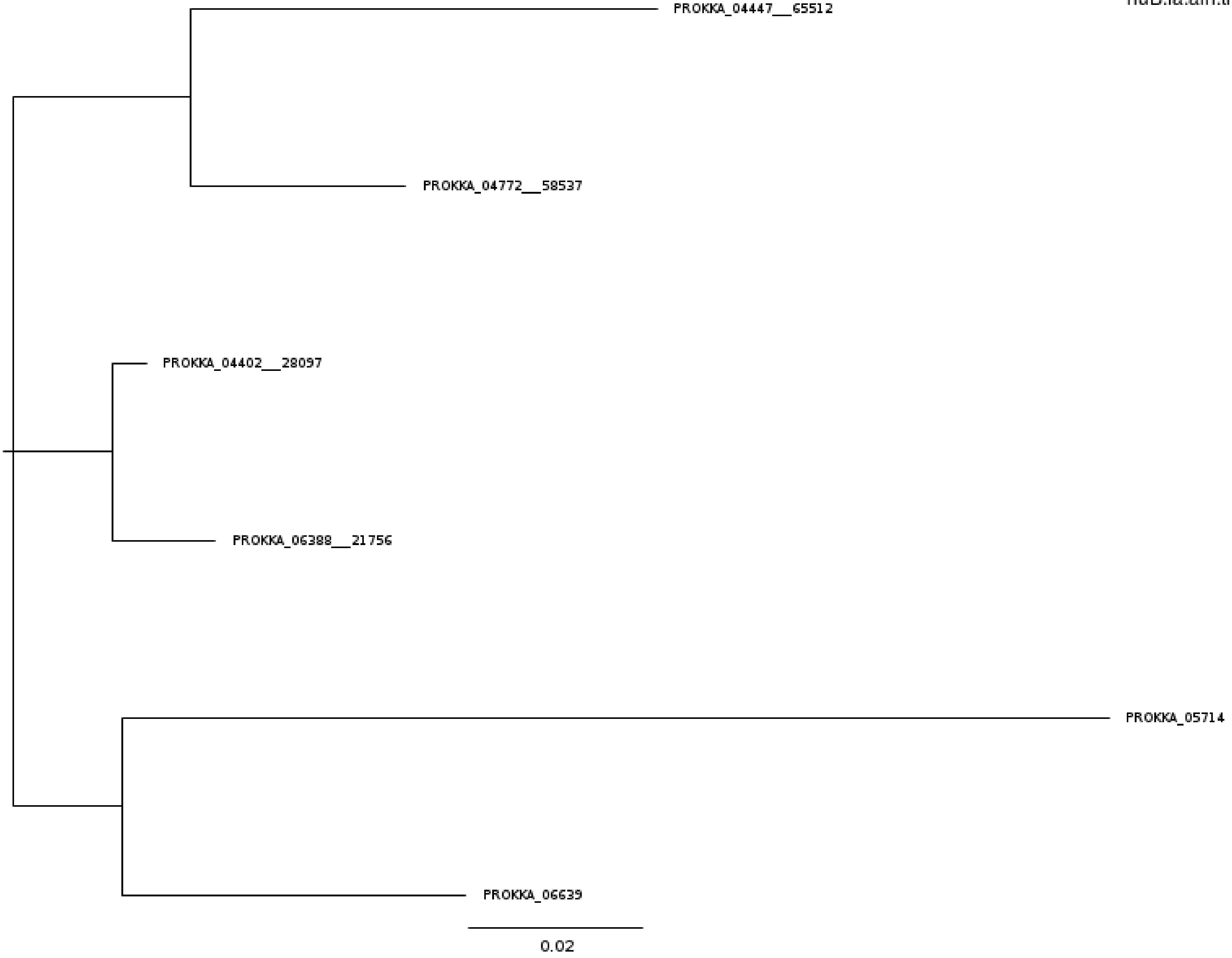

rluC_3.fa.aln.tree.png

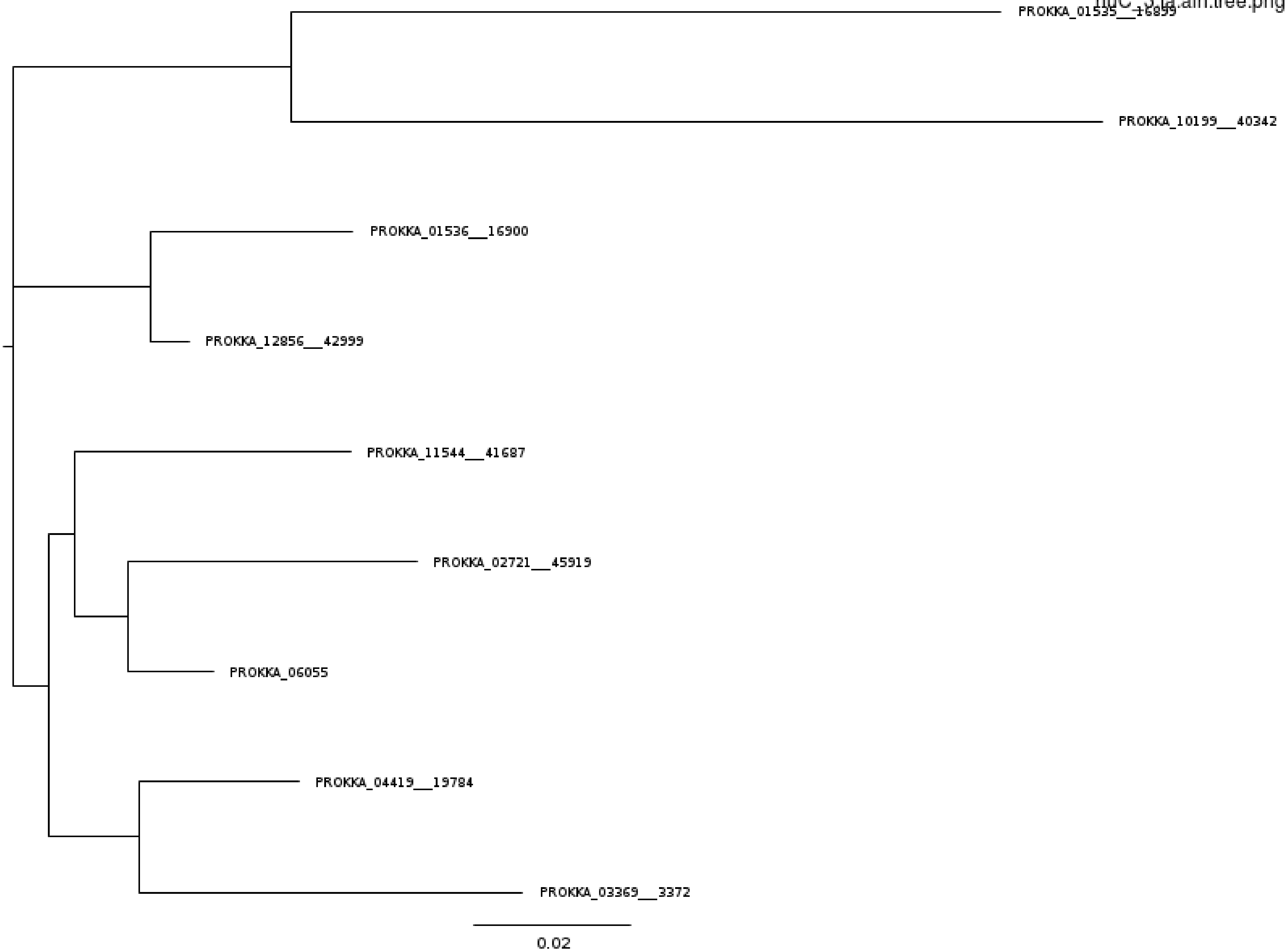

rluD_2.fa.aln.tree.png

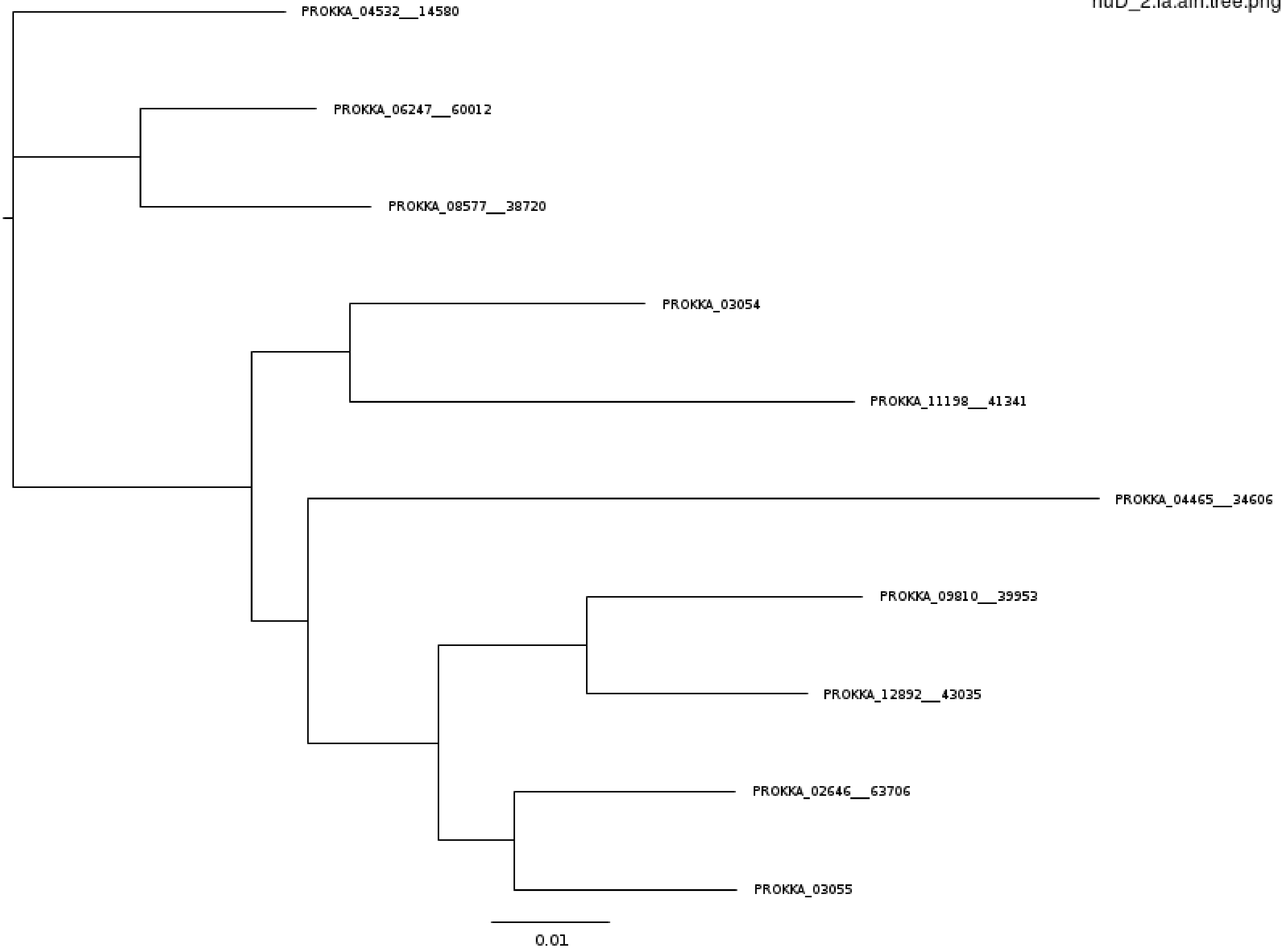

rmlA_2.fa.aln.tree.png

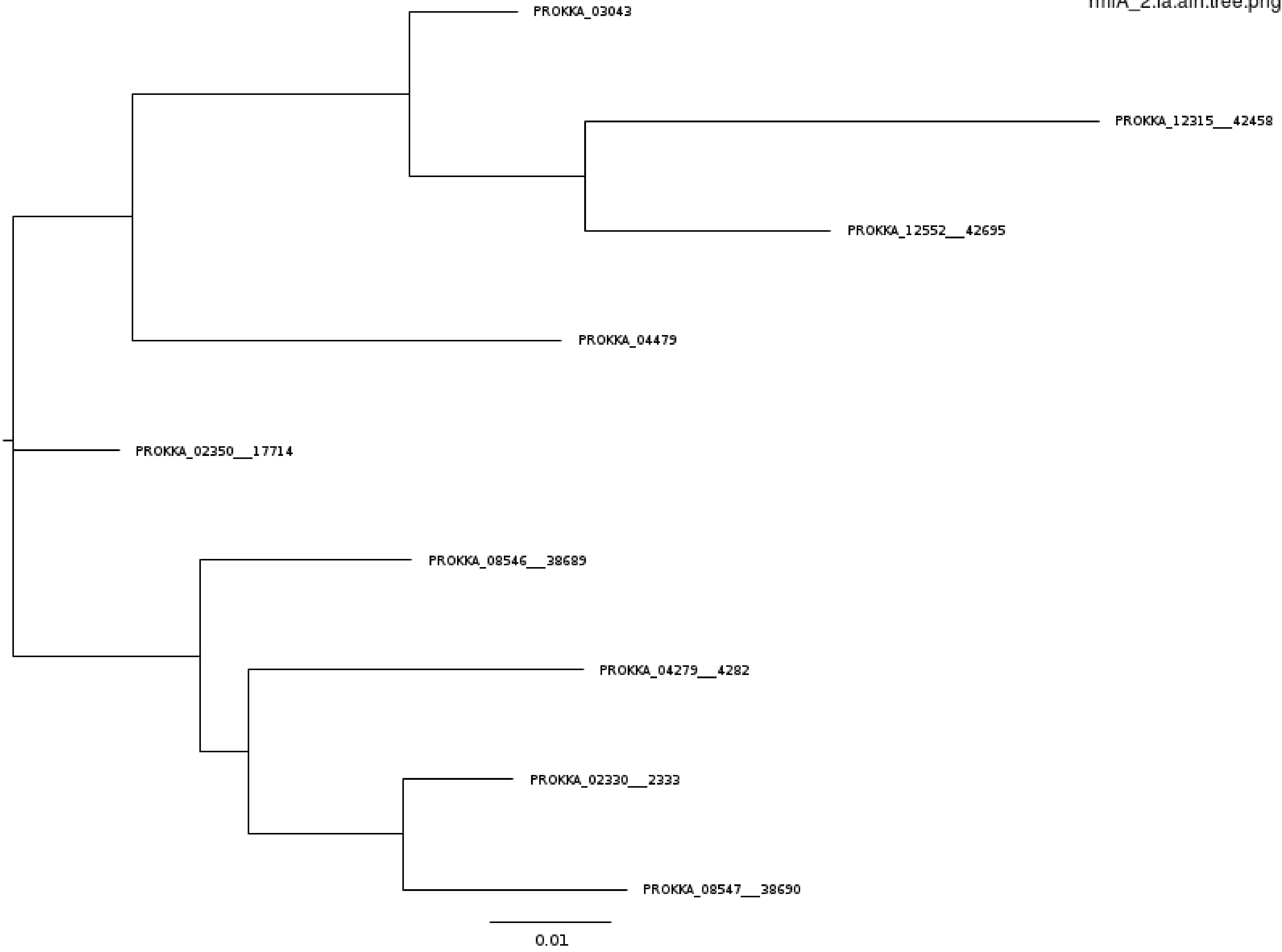

rmlD_2.fa.aln.tree.png

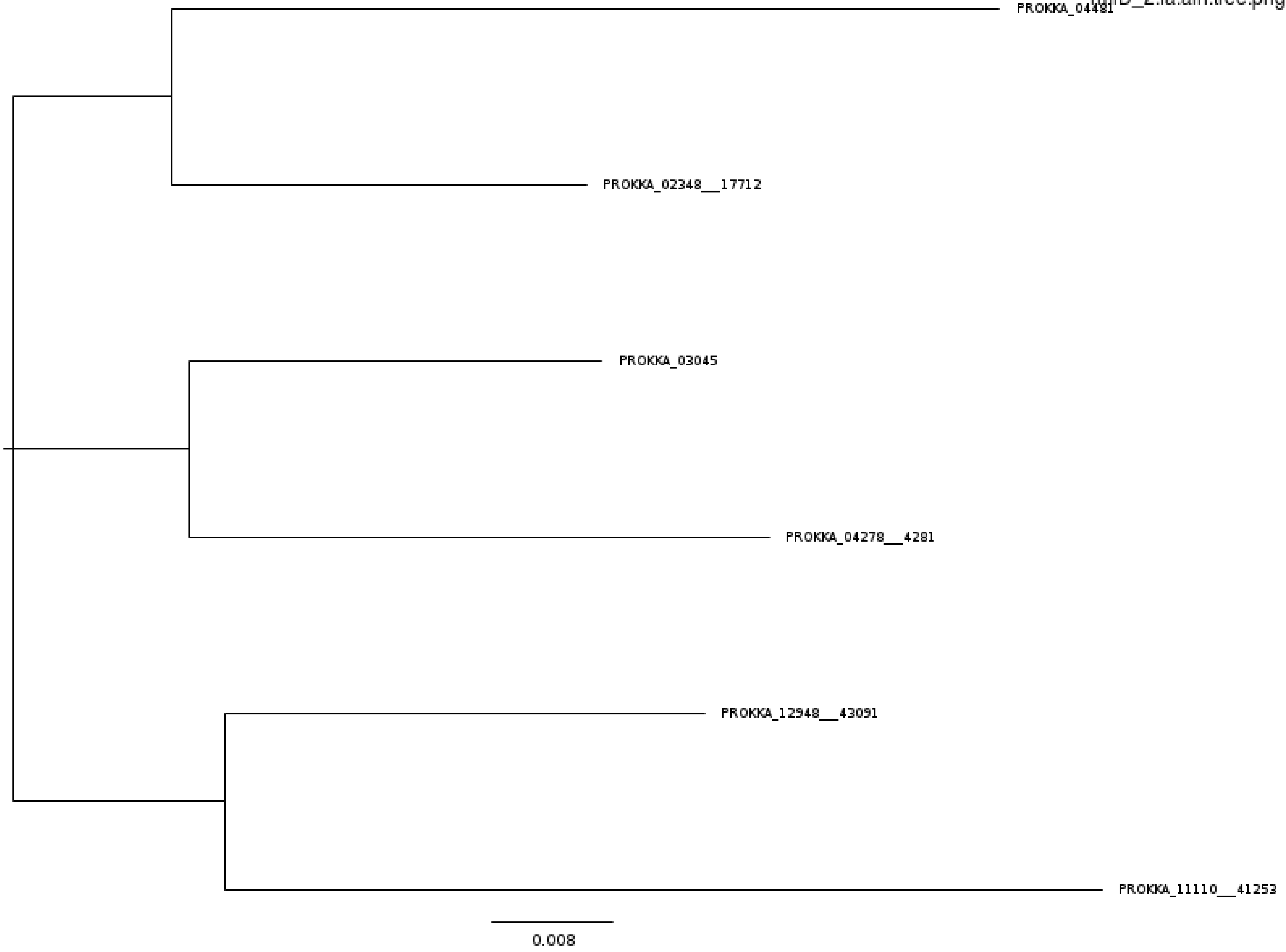

rmuC_2.fa.aln.tree.png

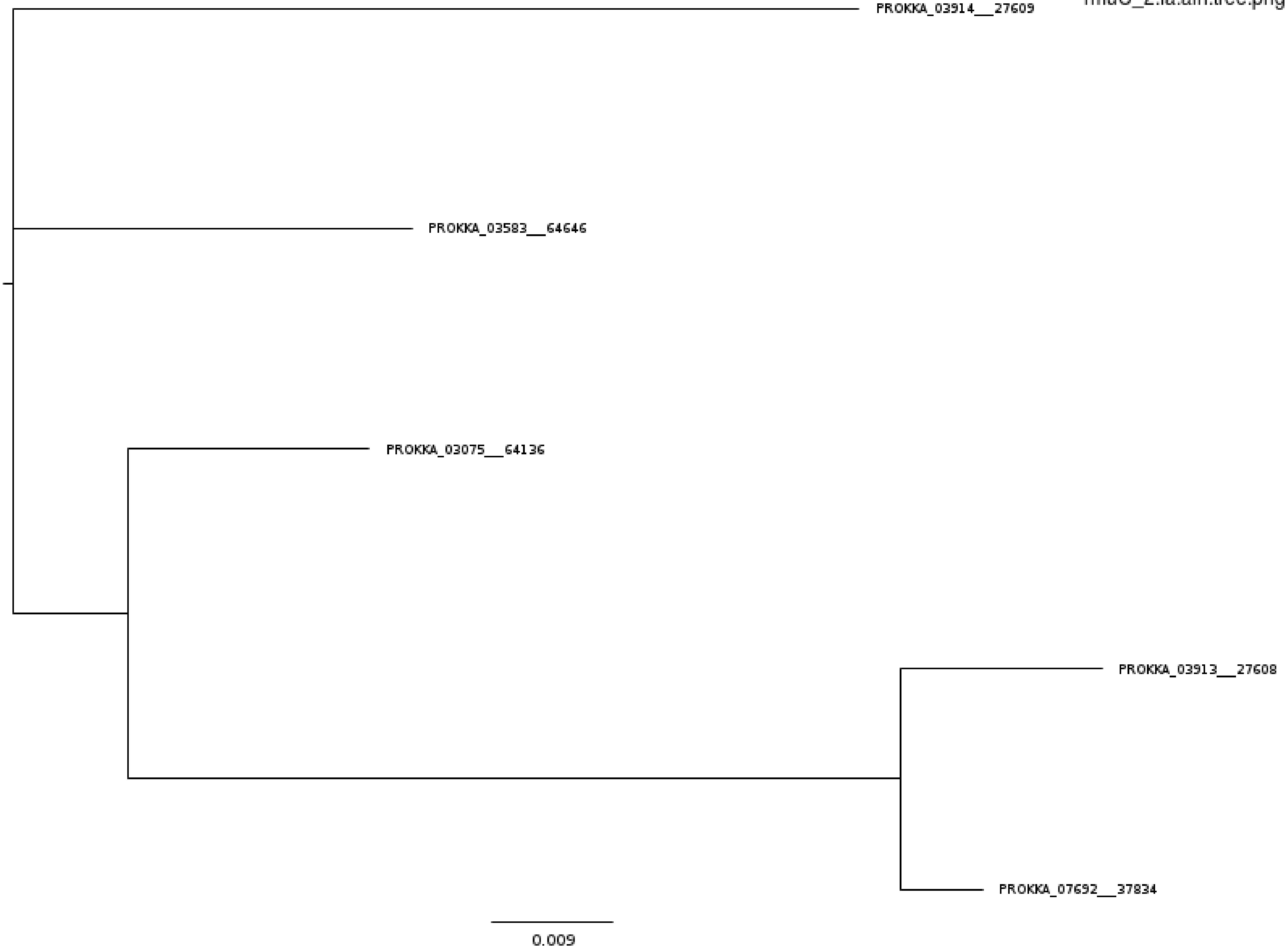

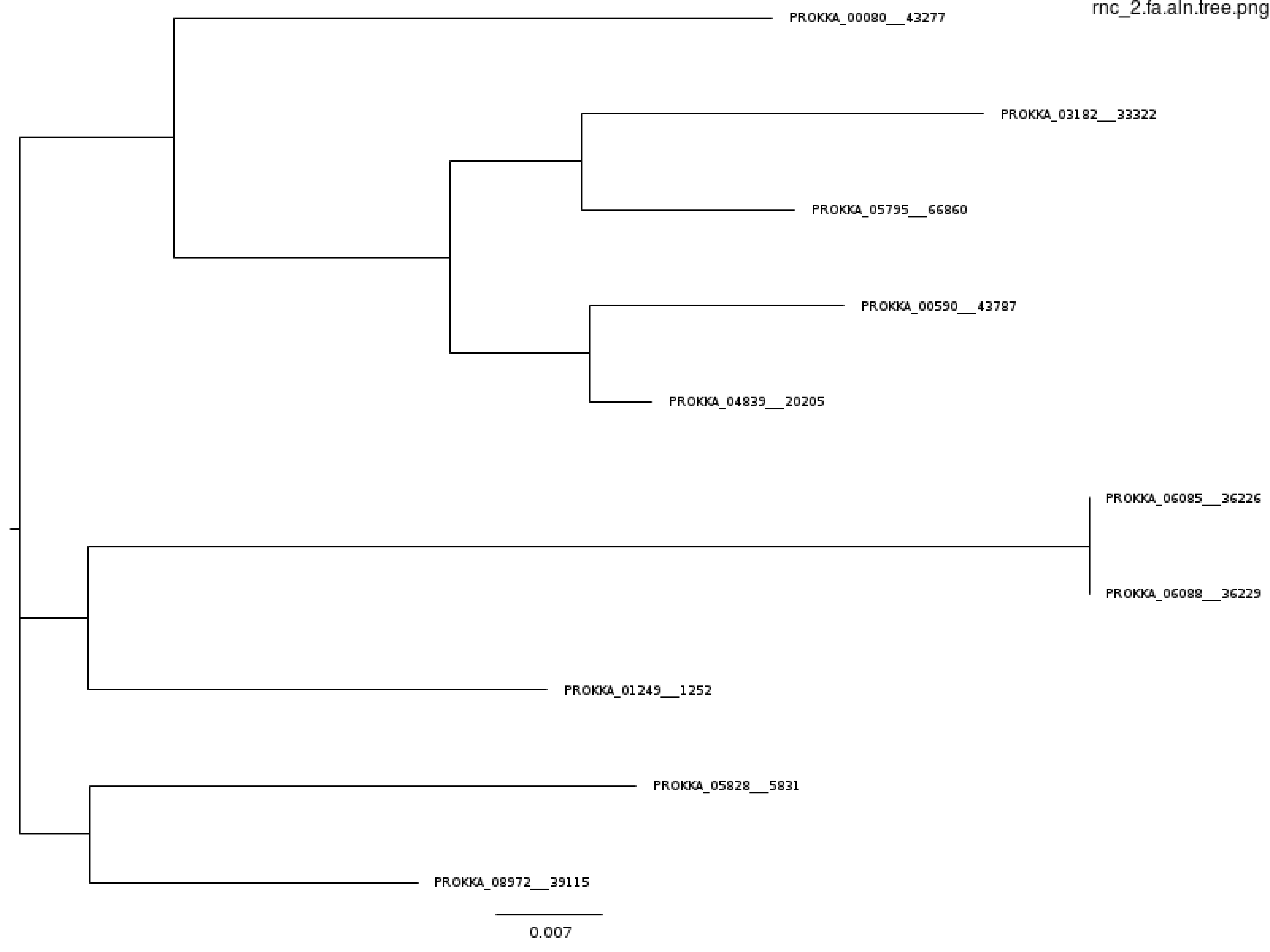

rnc_2.fa.aln.tree.png
PROKKA_00080__43277
PROKKA_03182__33322
PROKKA_05795__66860
PROKKA_00590__43787
PROKKA_04839__20205
PROKKA_06085__36226
PROKKA_06088__36229
PROKKA_01249__1252
PROKKA_05828__5831
PROKKA_08972__39115
0.007

rne_2.fa.aln.tree.png

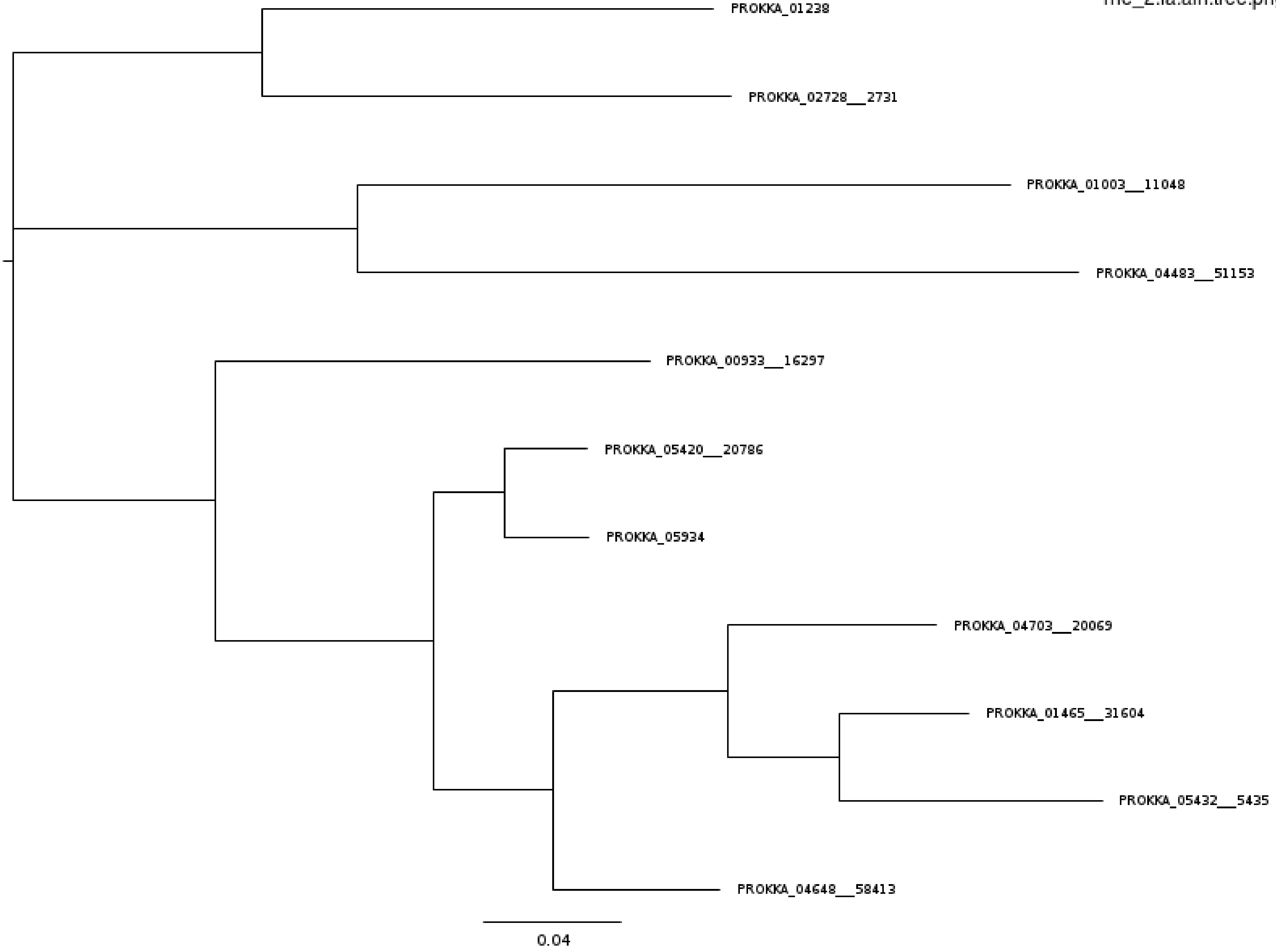

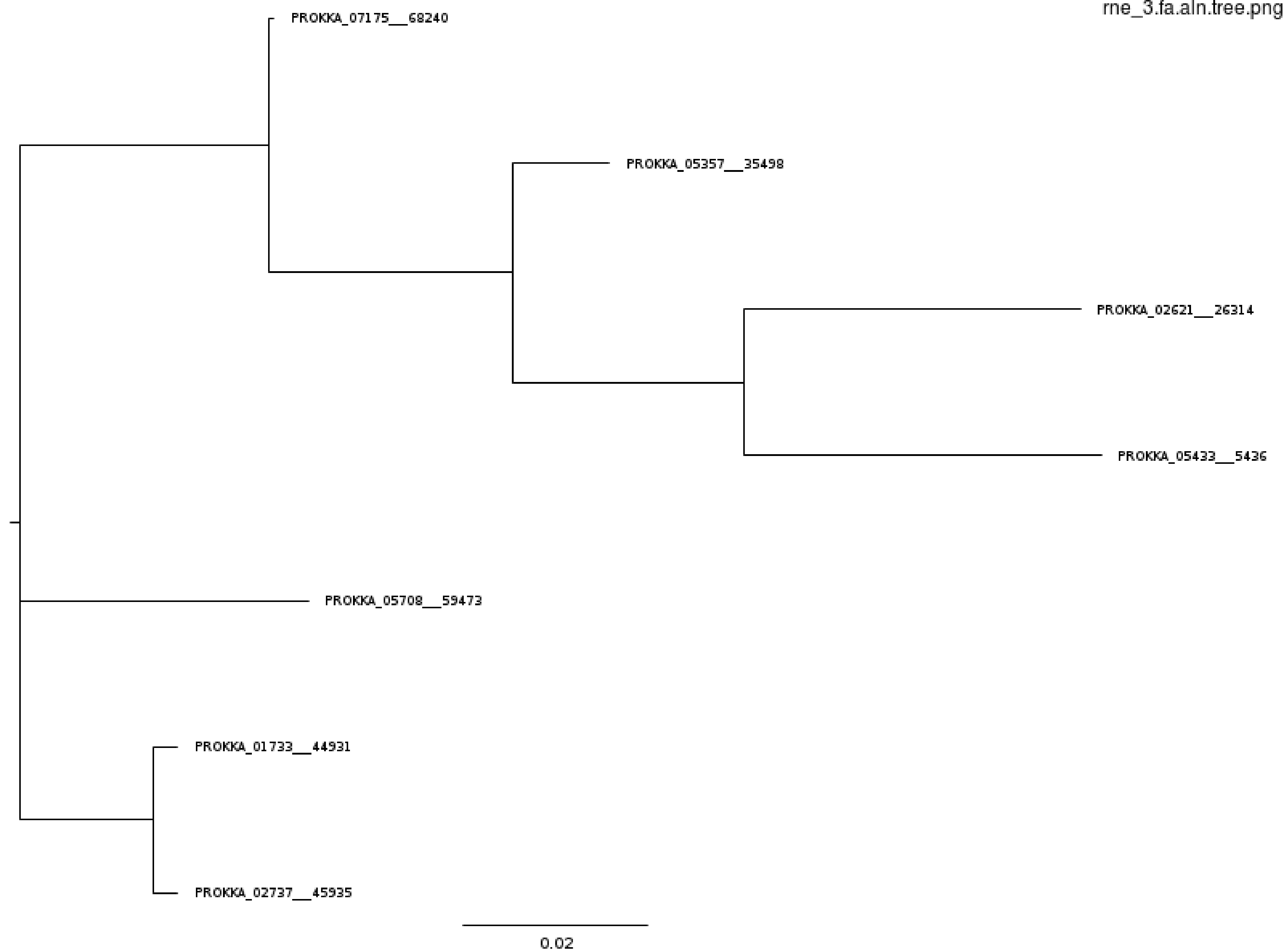
rne_3.fa.aln.tree.png
PROKKA_07175___68240
PROKKA_05357___35498
PROKKA_02621___26314
PROKKA_05433___5436
PROKKA_05708___59473
PROKKA_01733___44931
PROKKA_02737___45935
0.02

rnfA_1.fa.aln.tree.png

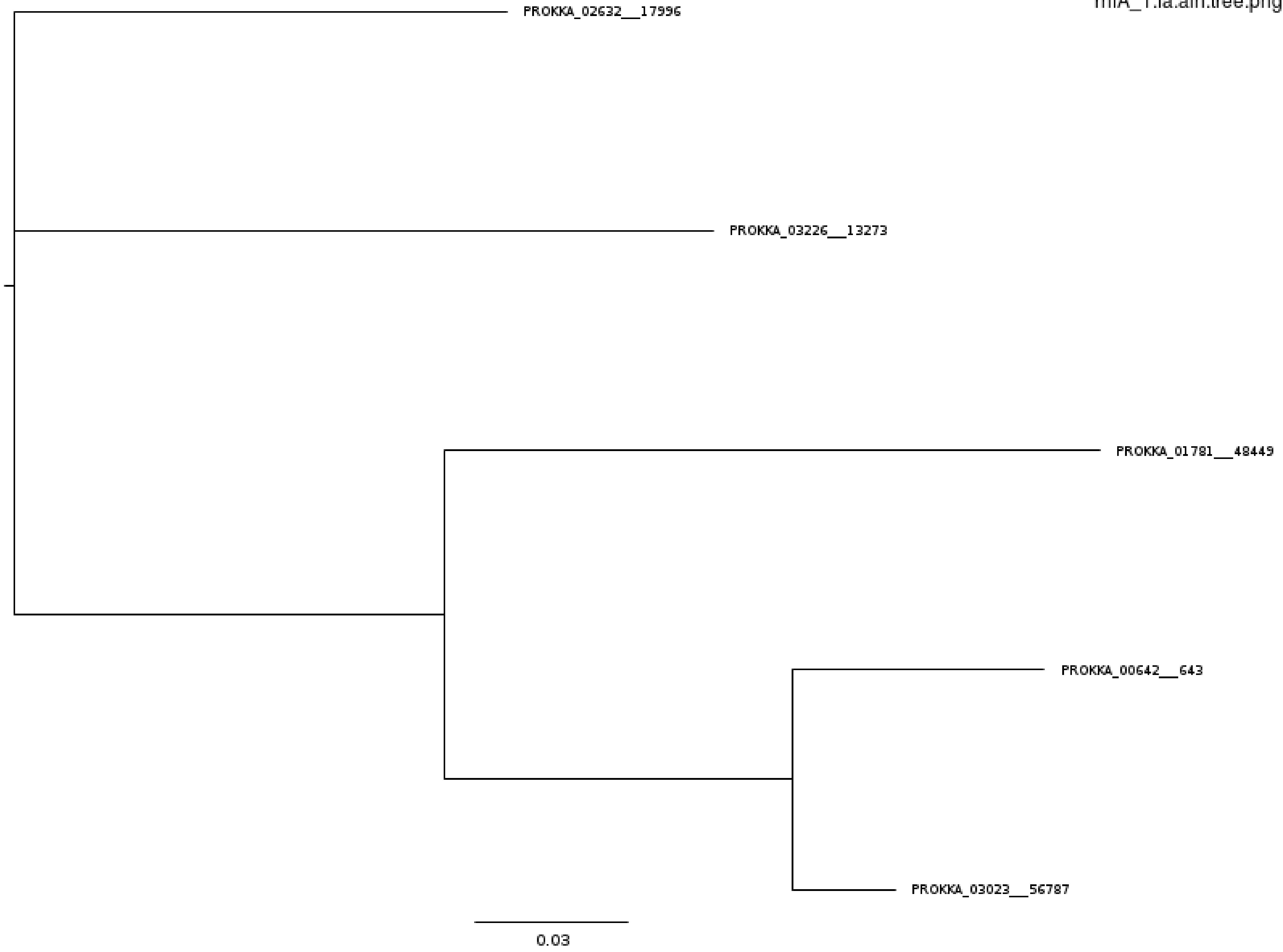

rnfA_4.fa.aln.tree.png

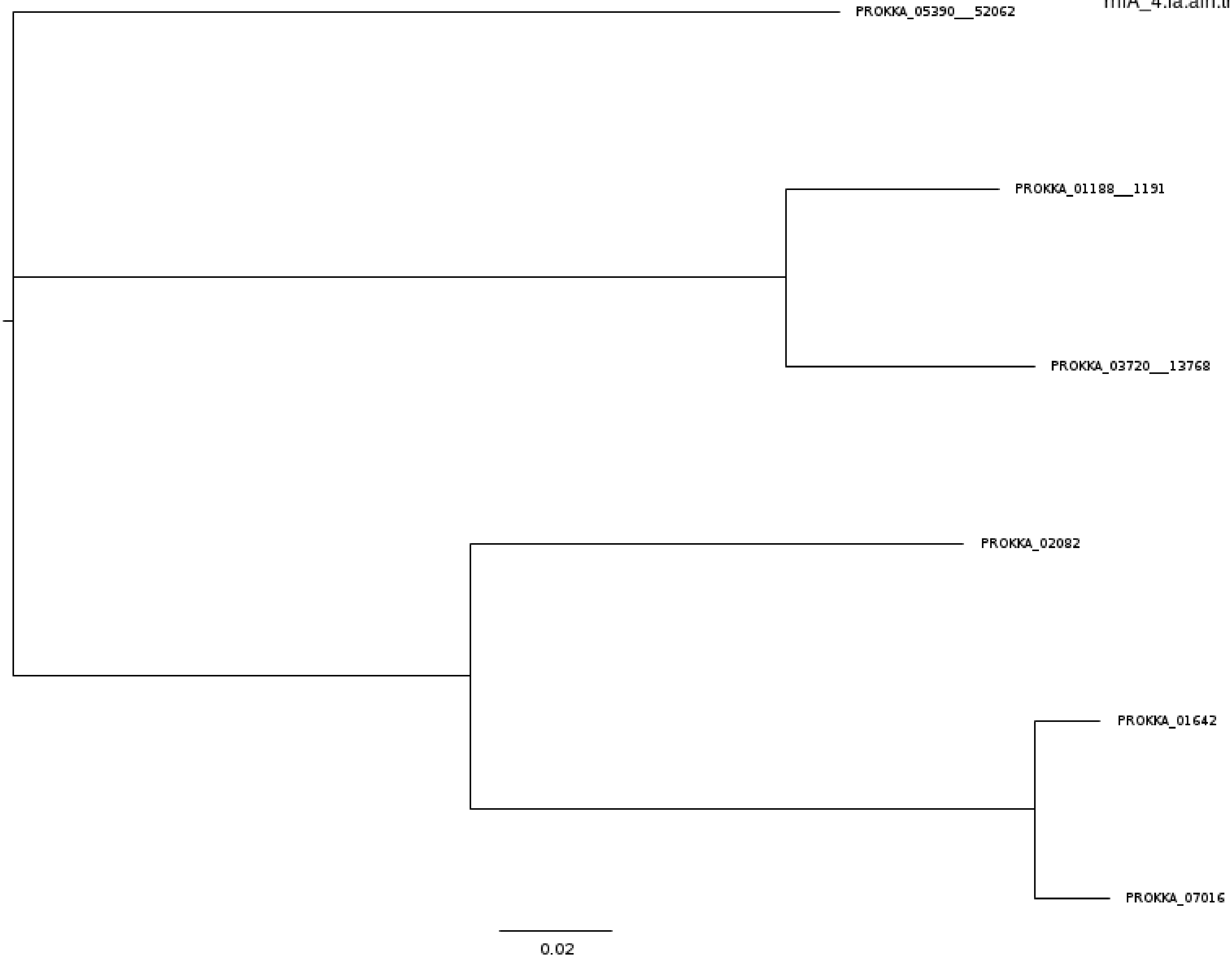

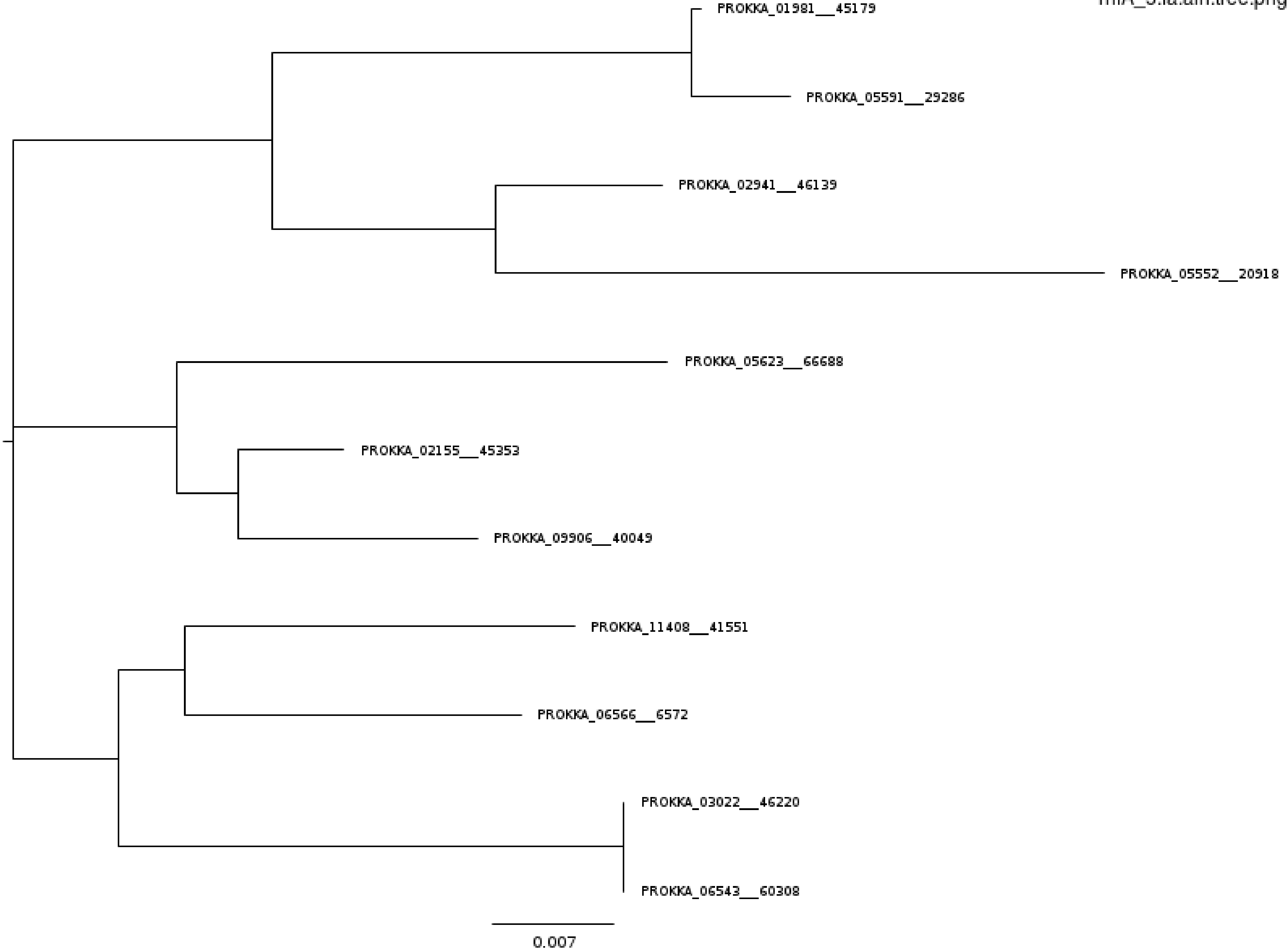
PROKKA_01981___45179
PROKKA_05591___29286
PROKKA_02941___46139
PROKKA_05552___20918
PROKKA_05623___66688
PROKKA_02155___45353
PROKKA_09906___40049
PROKKA_11408___41551
PROKKA_06566___6572
PROKKA_03022___46220
PROKKA_06543___60308
0.007

rnfA_7.fa.aln.tree.png

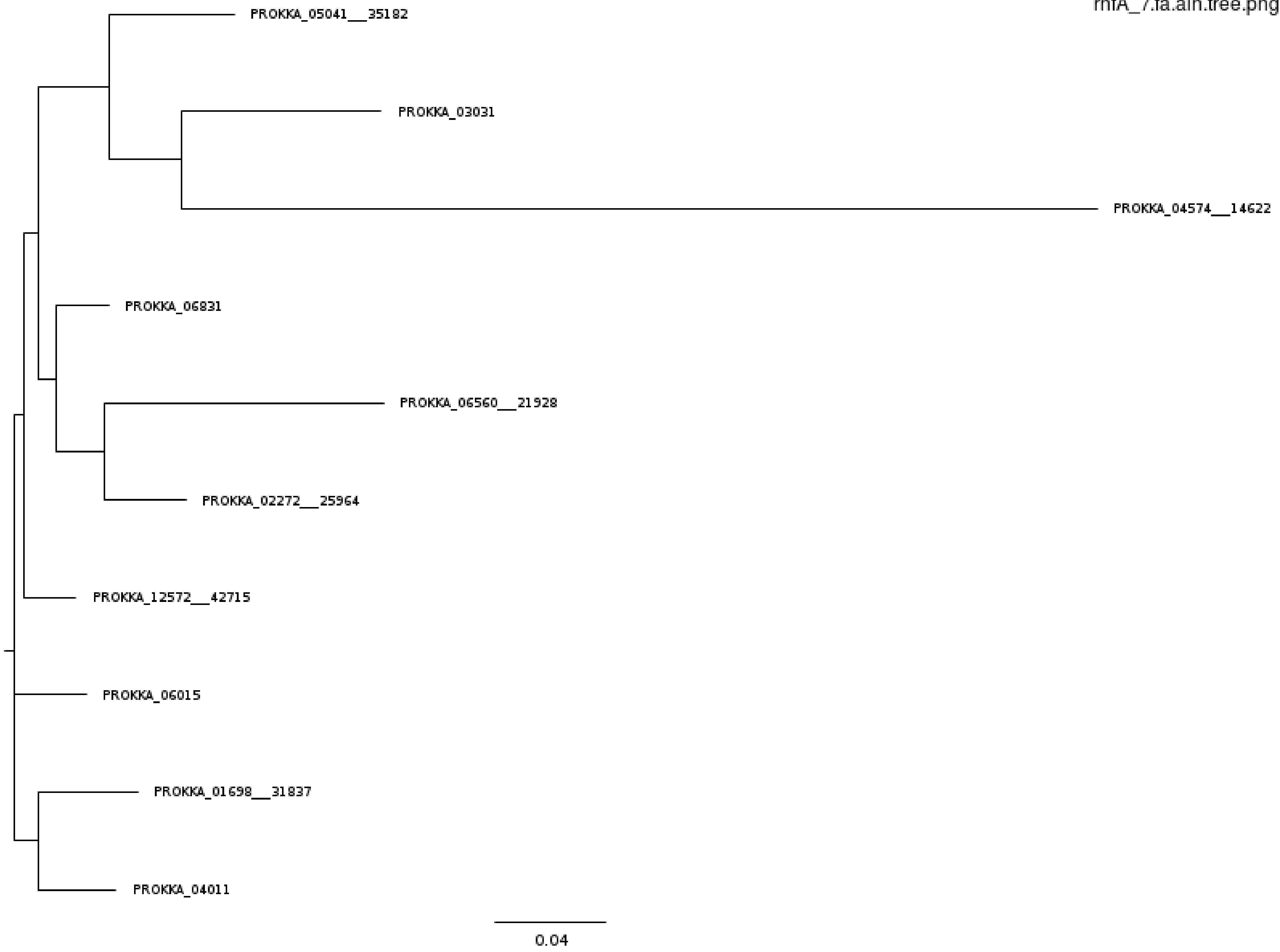

rnfB_4.fa.aln.tree.png

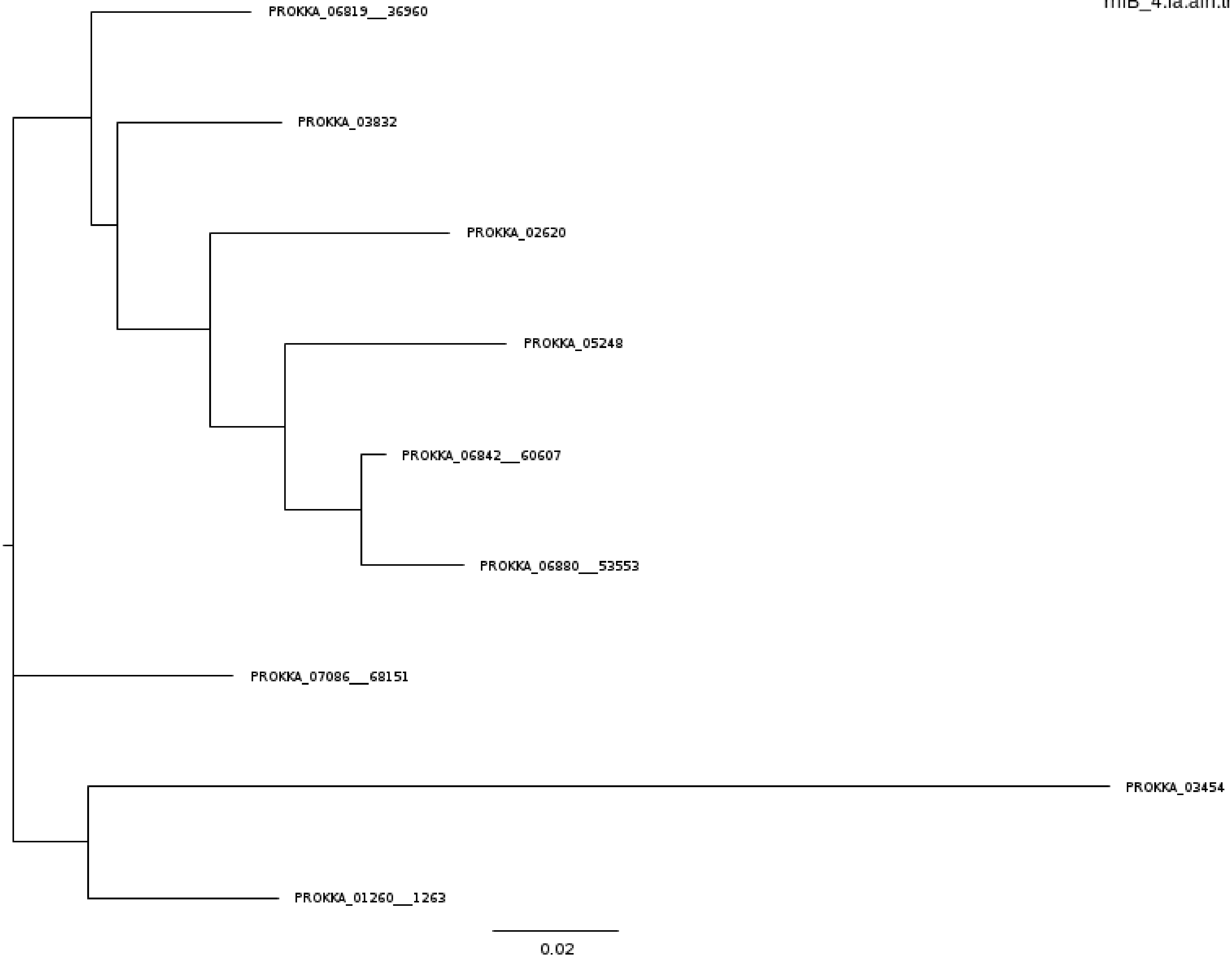

rpfB.fa.aln.tree.png

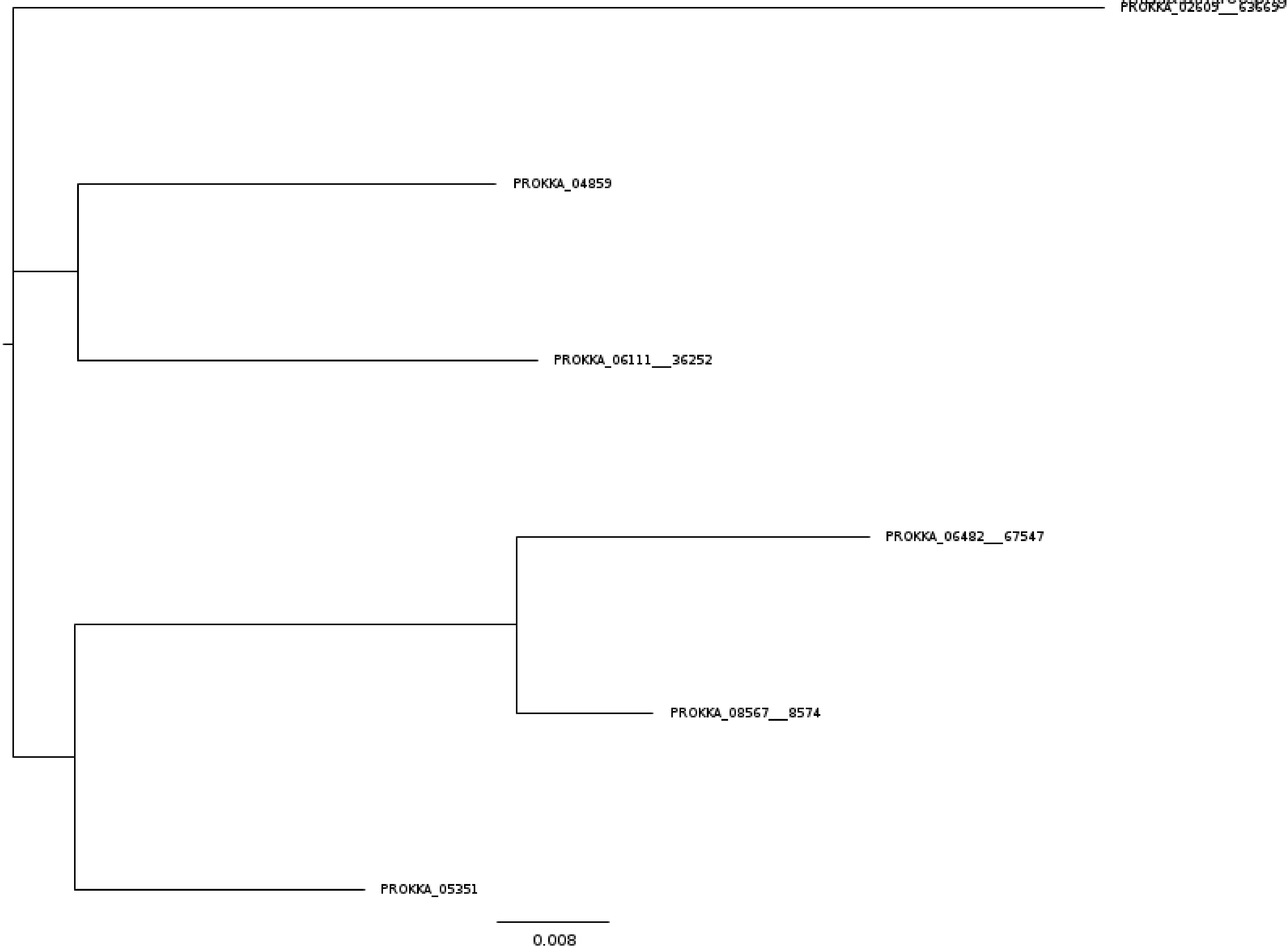

rnfC_1.fa.aln.tree.png

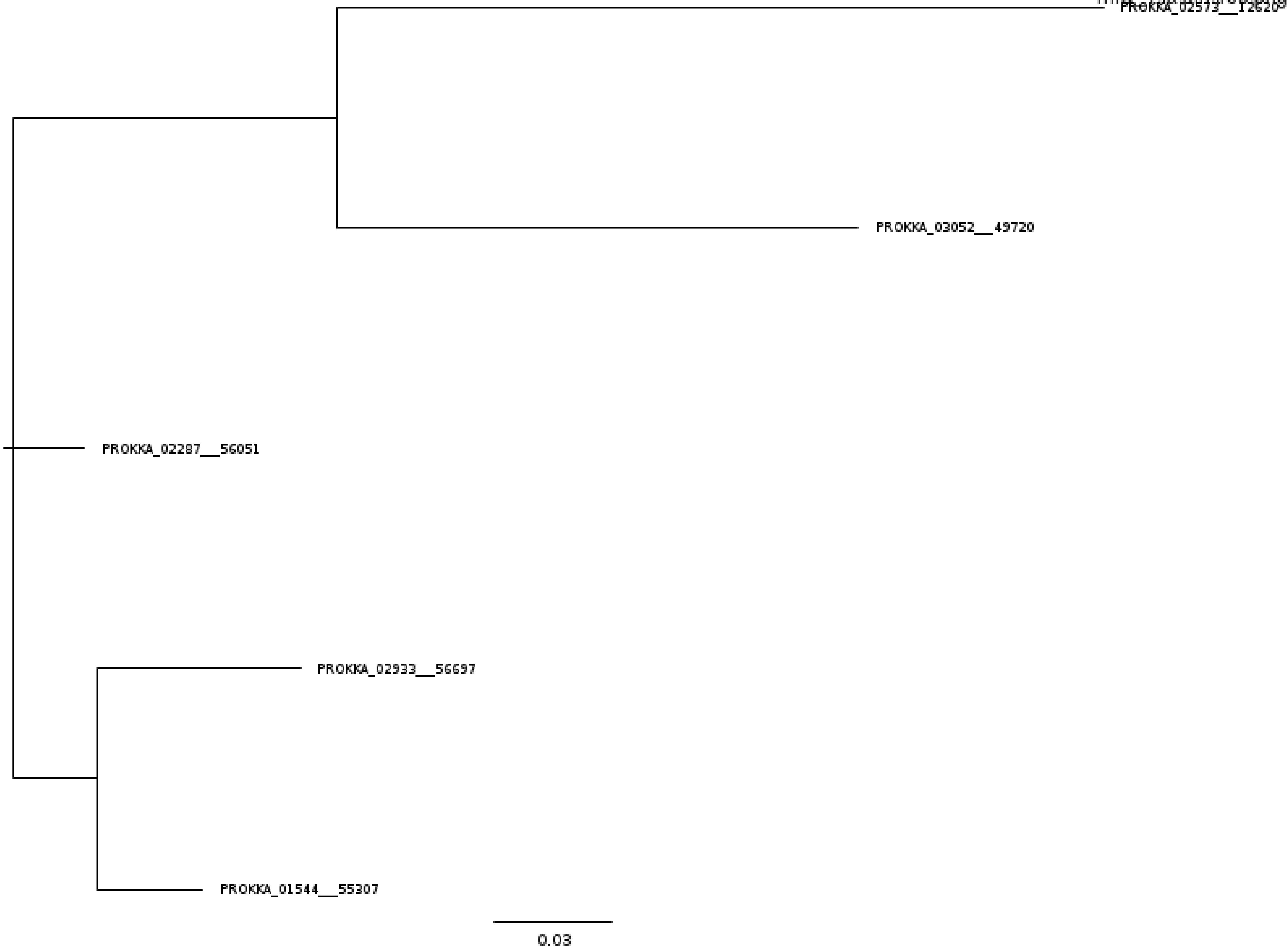

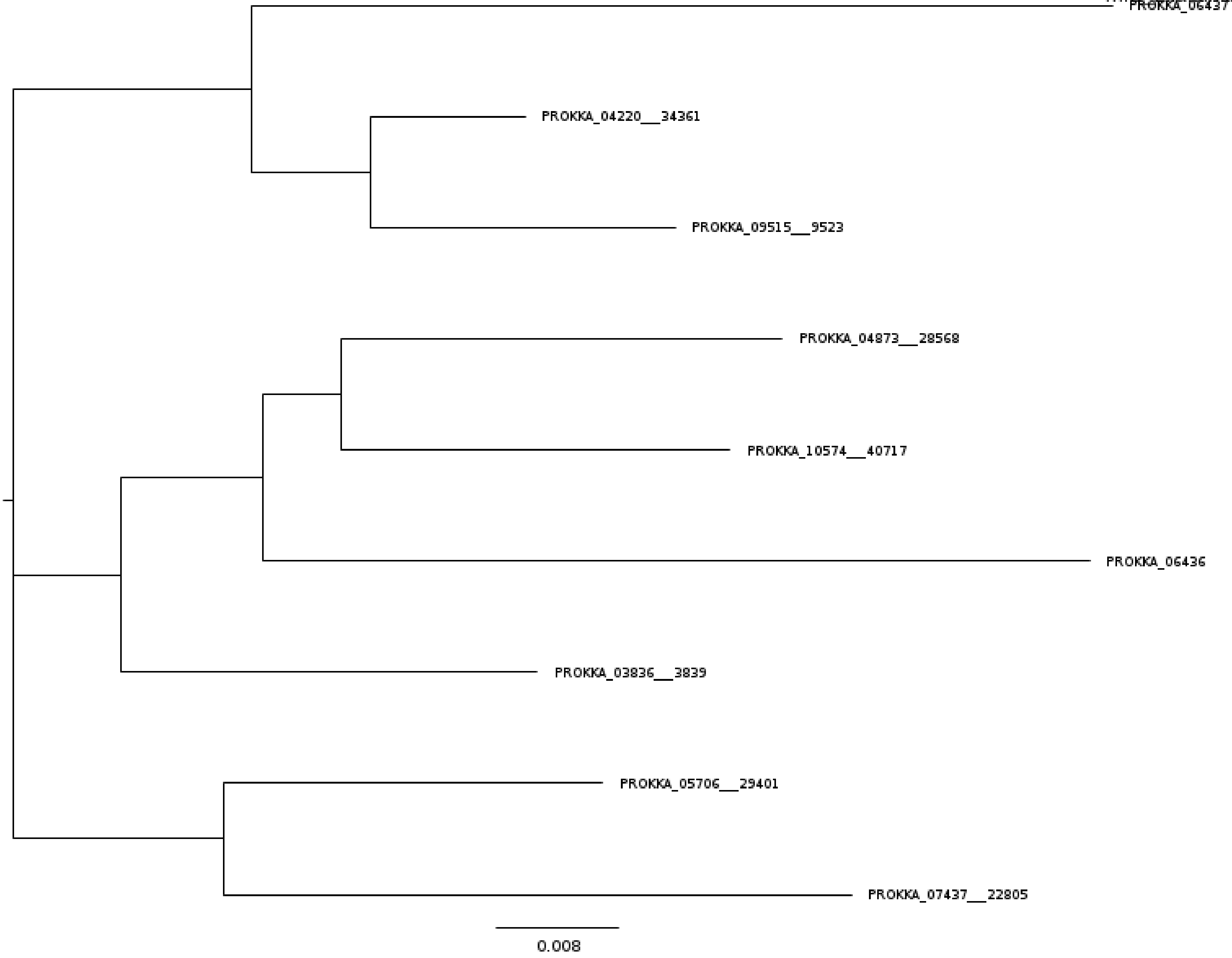
PROKKA_06437
PROKKA_04220___34361
PROKKA_09515___9523
PROKKA_04873___28568
PROKKA_10574___40717
PROKKA_06436
PROKKA_03836___3839
PROKKA_05706___29401
PROKKA_07437___22805
0.008

rnfC_3.fa.aln.tree.png

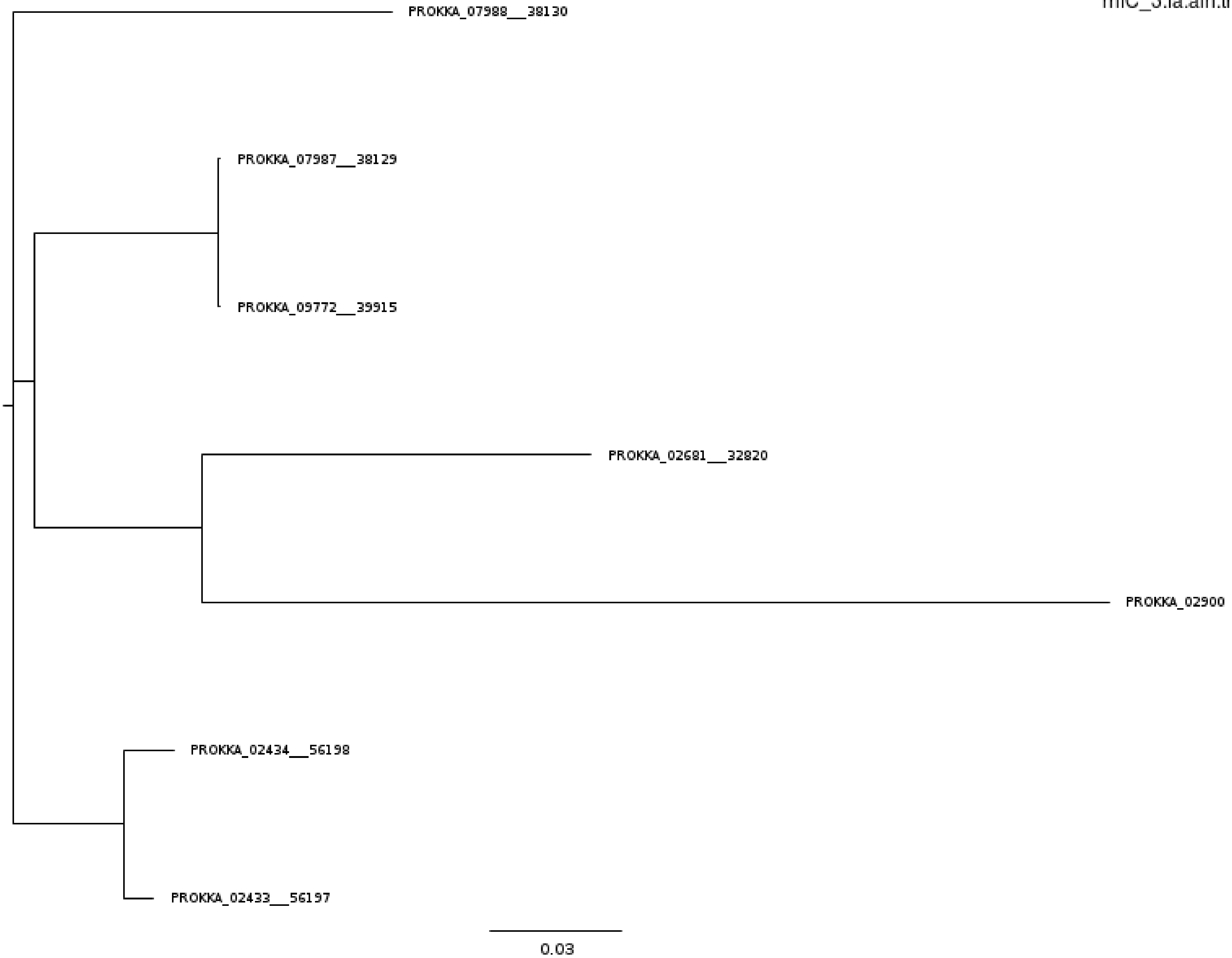

rnfD_1.fa.aln.tree.png

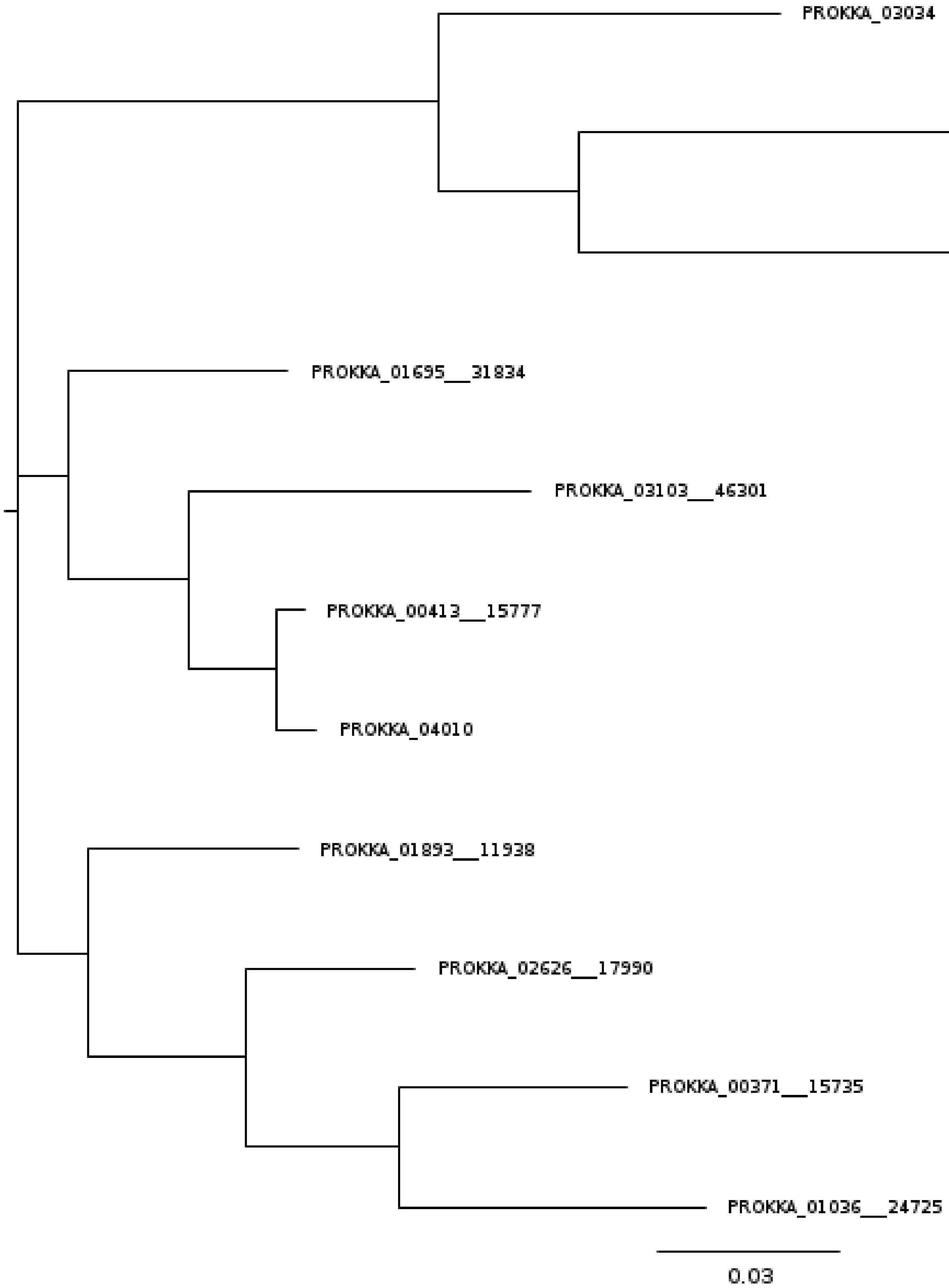

rnfE_1.fa.aln.tree.png

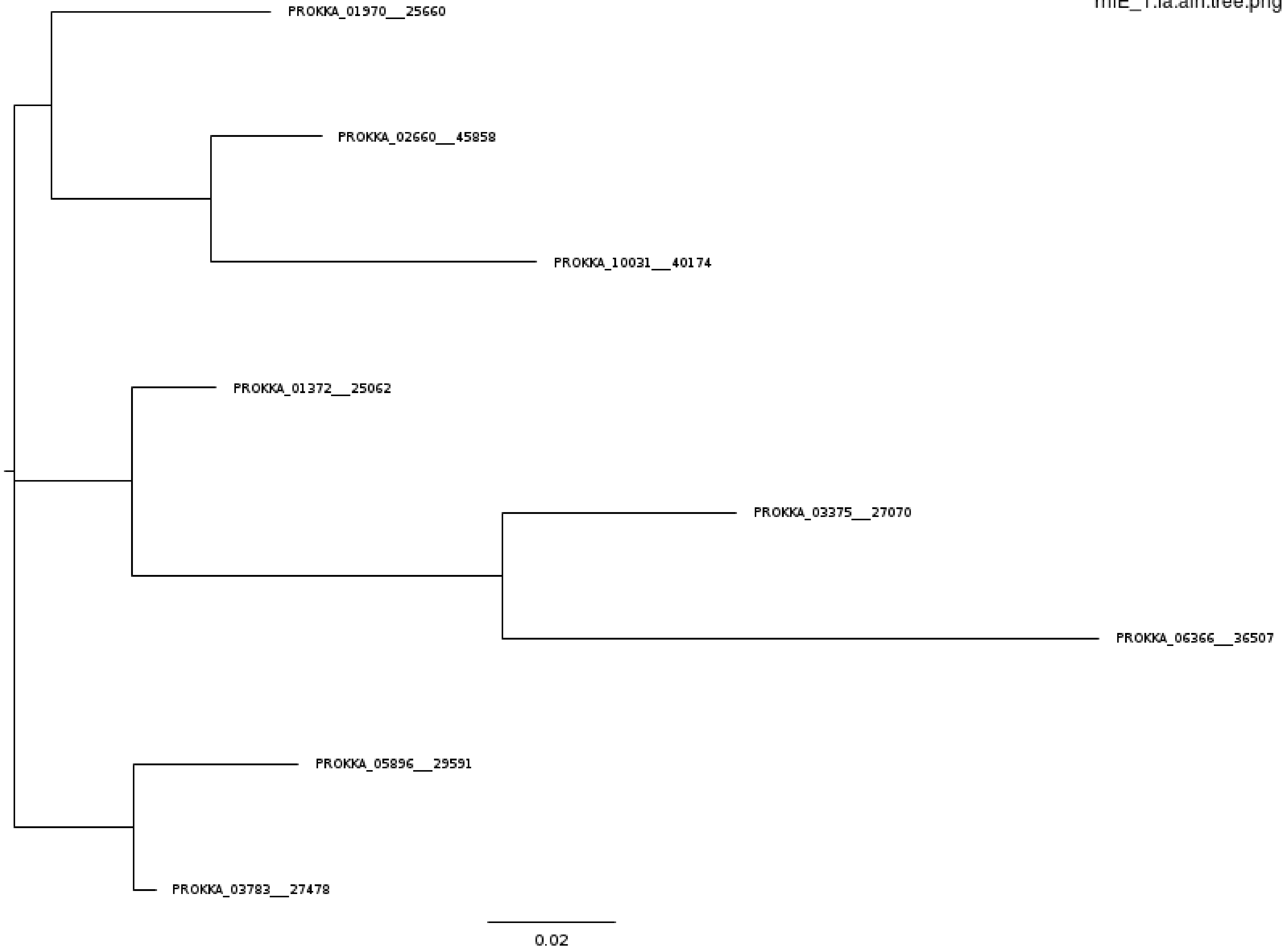

rnfE_4.fa.aln.tree.png

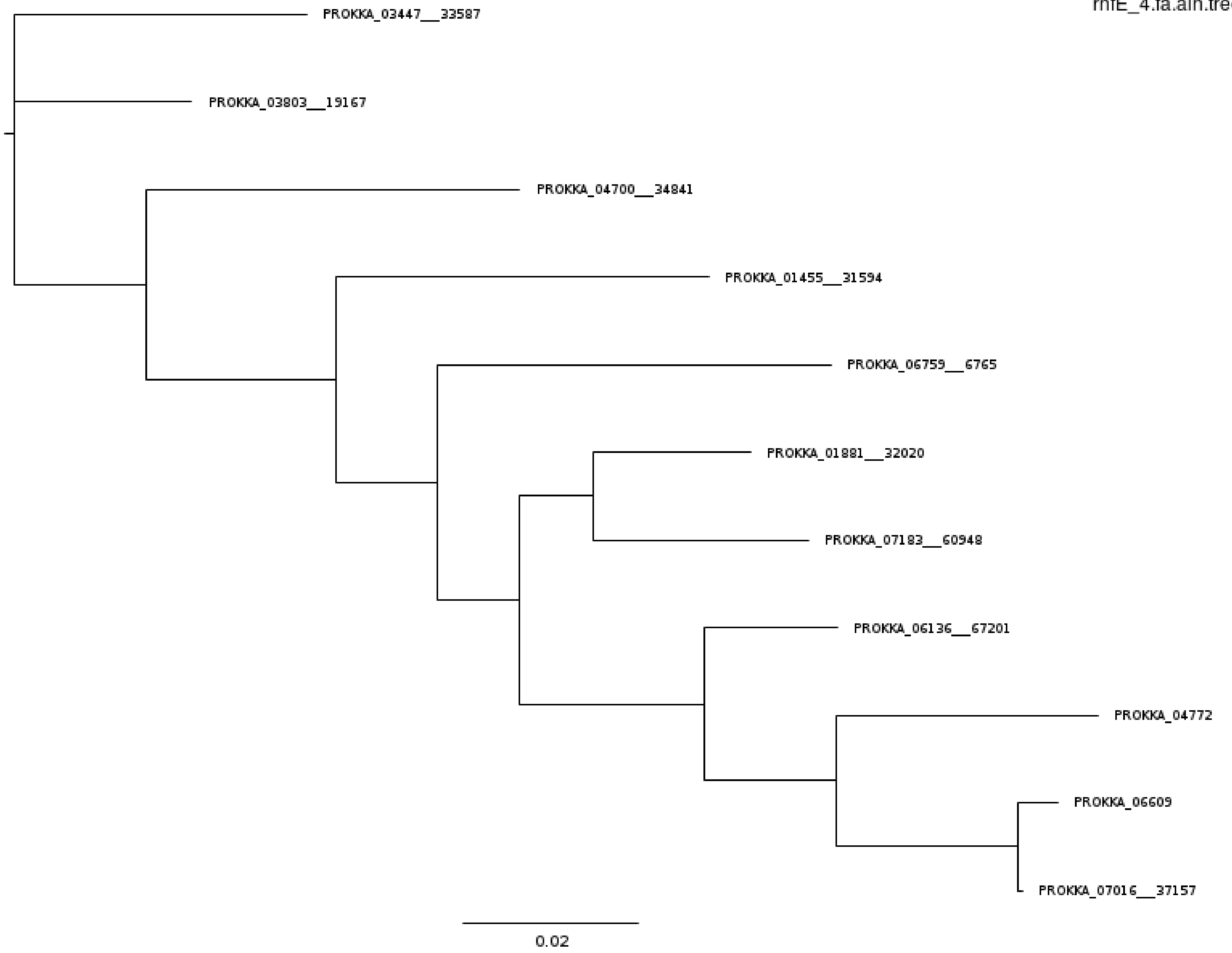

rnfE_6.fa.aln.tree.png

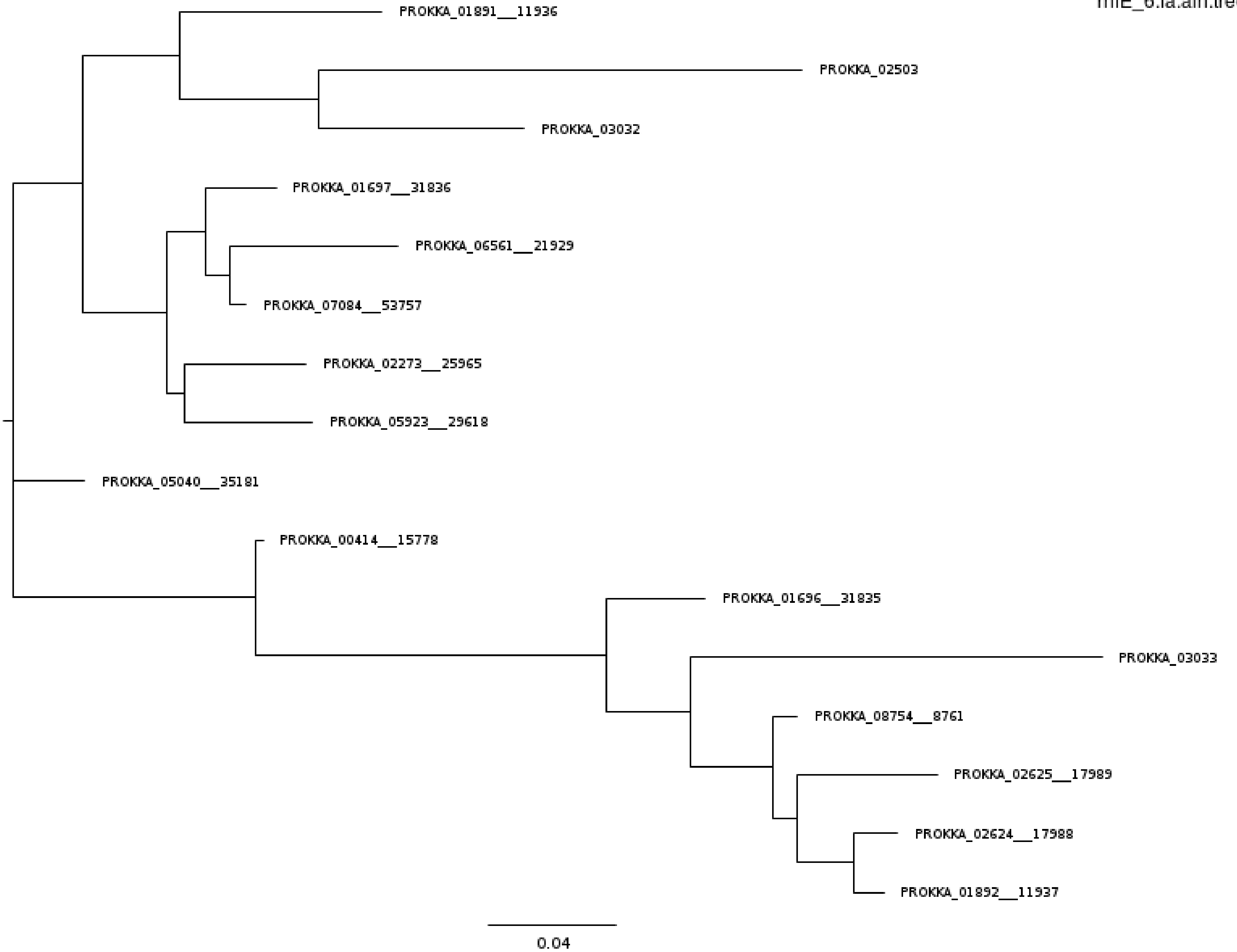

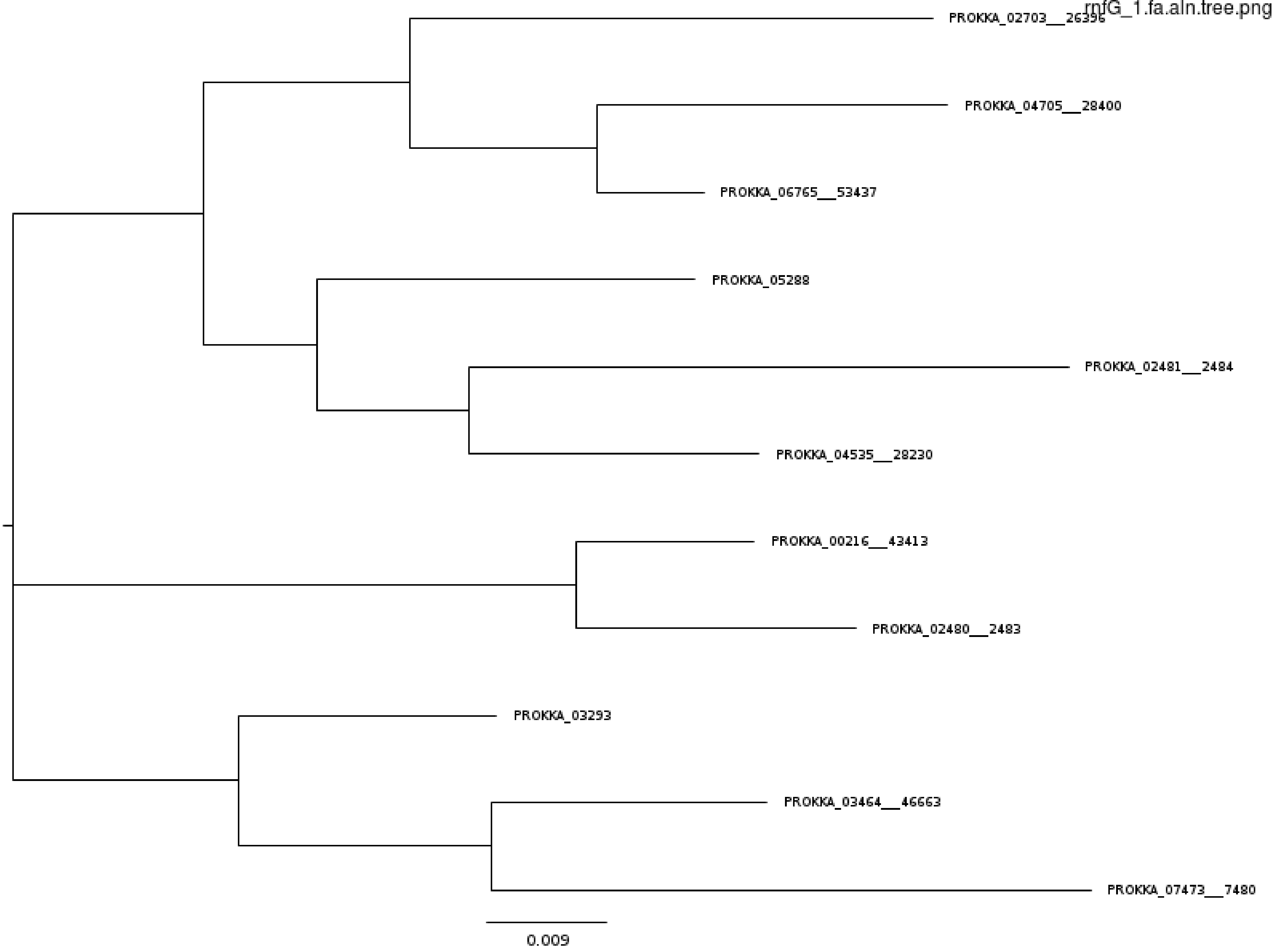
rnfG_1.fa.aln.tree.png
PROKKA_02703___26396
PROKKA_04705___28400
PROKKA_06765___53437
PROKKA_05288
PROKKA_02481___2484
PROKKA_04535___28230
PROKKA_00216___43413
PROKKA_02480___2483
PROKKA_03293
PROKKA_03464___46663
PROKKA_07473___7480
0.009

rng_1.fa.aln.tree.png

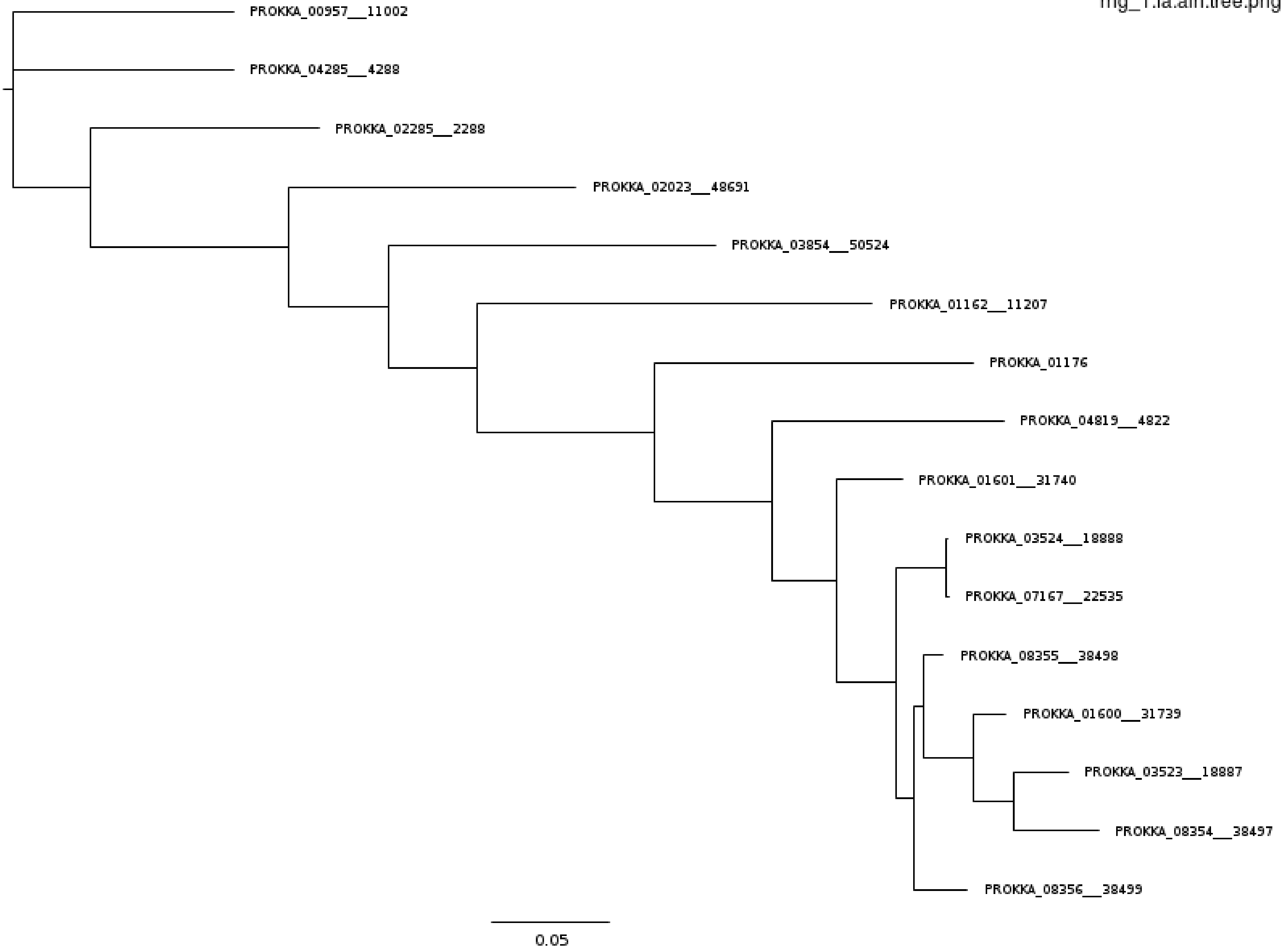

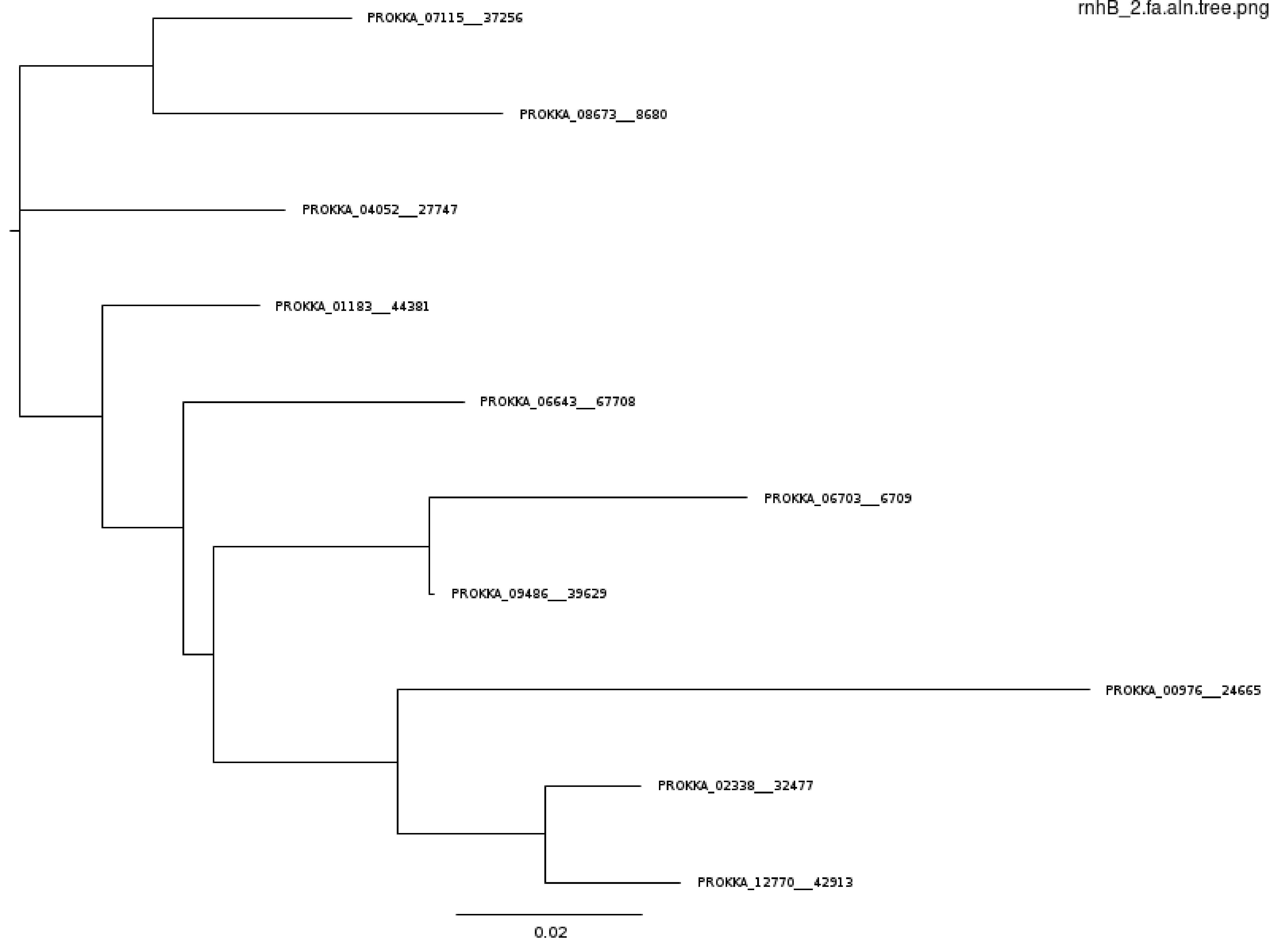
rnhB_2.fa.aln.tree.png
PROKKA_07115___37256
PROKKA_08673___8680
PROKKA_04052___27747
PROKKA_01183___44381
PROKKA_06643___67708
PROKKA_06703___6709
PROKKA_09486___39629
PROKKA_00976___24665
PROKKA_02338___32477
PROKKA_12770___42913
0.02

rnpA_2.fa.aln.tree.png

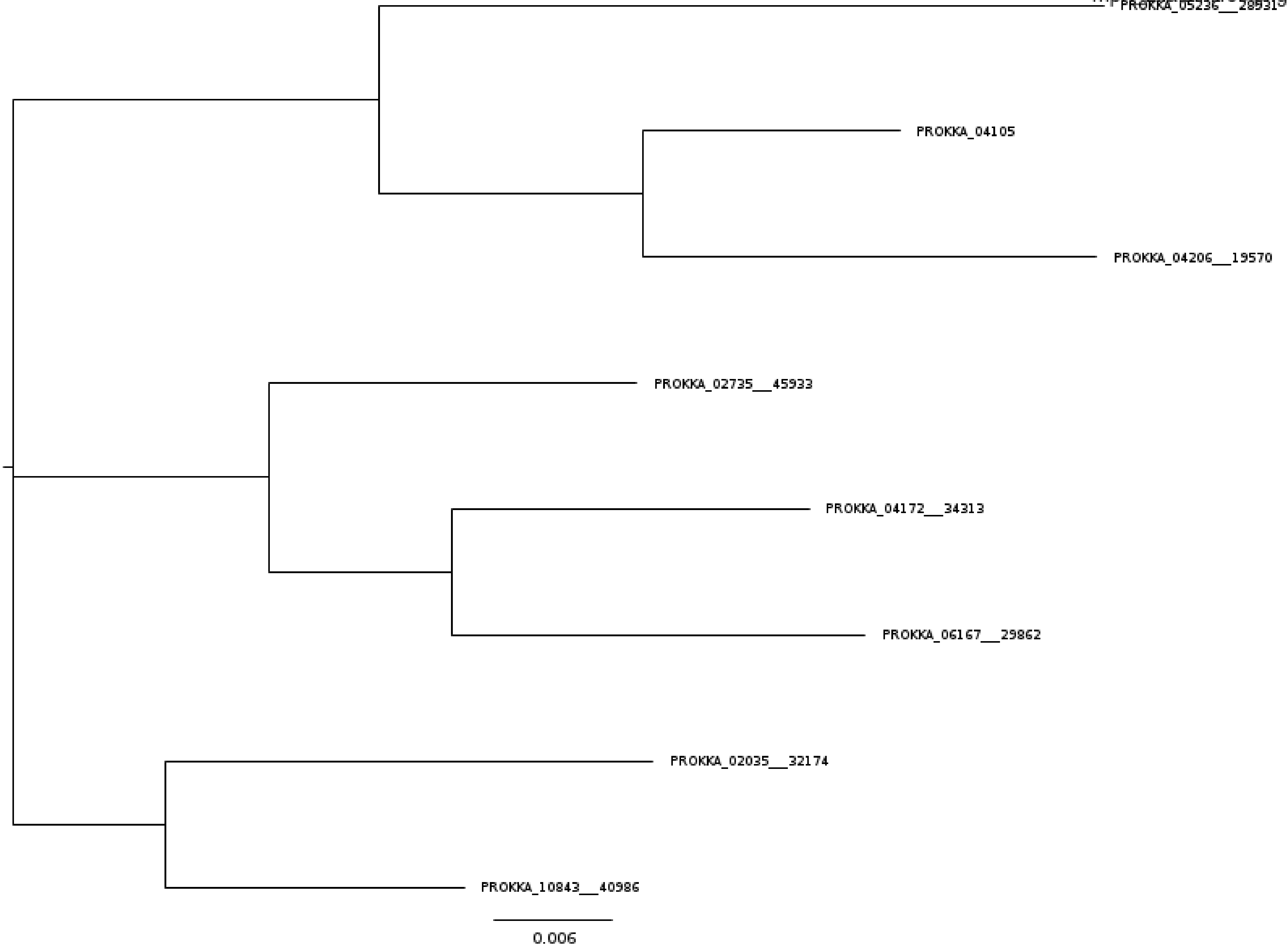

rnr_2.fa.aln.tree.png

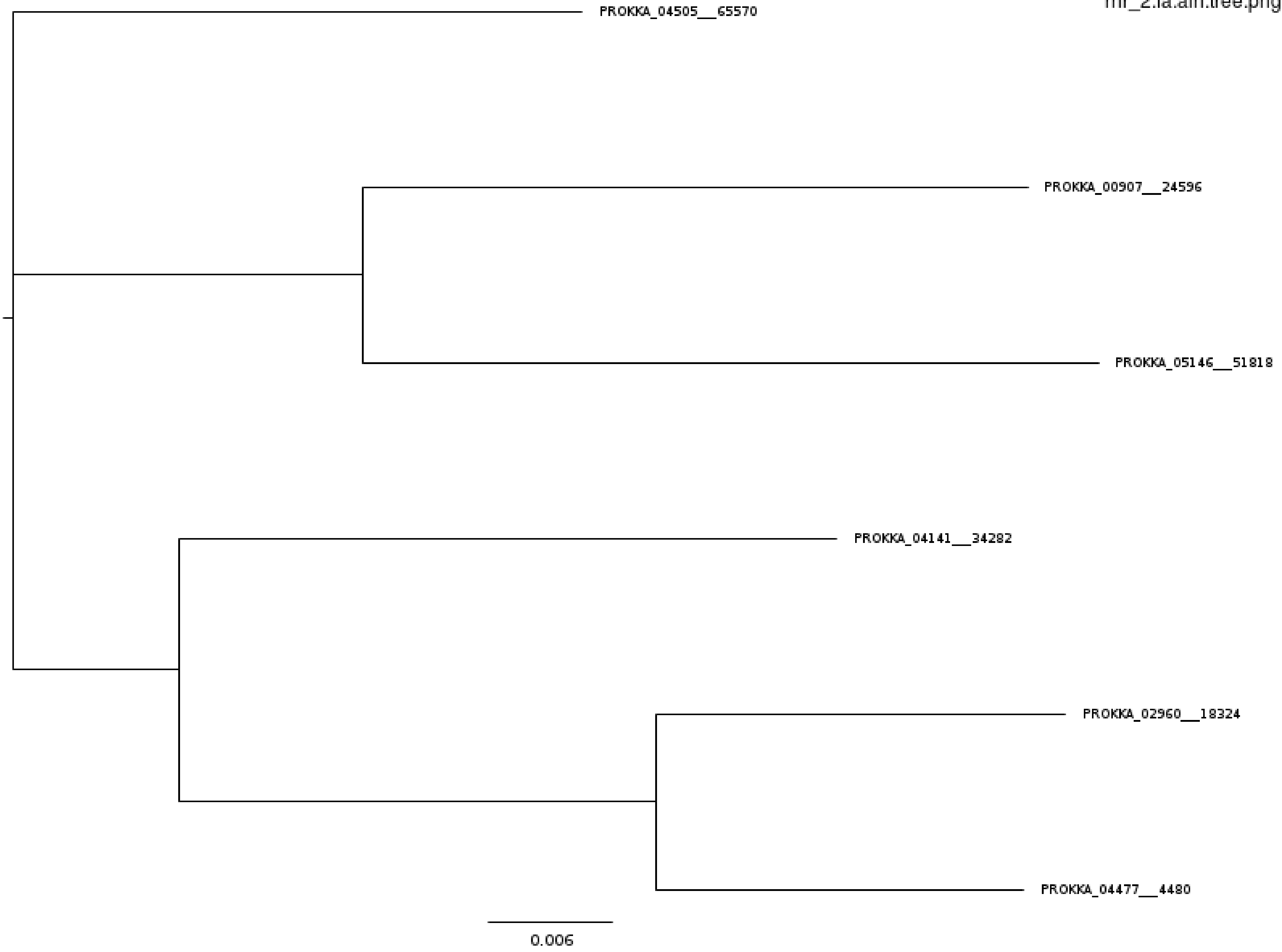

rnr_3.fa.aln.tree.png

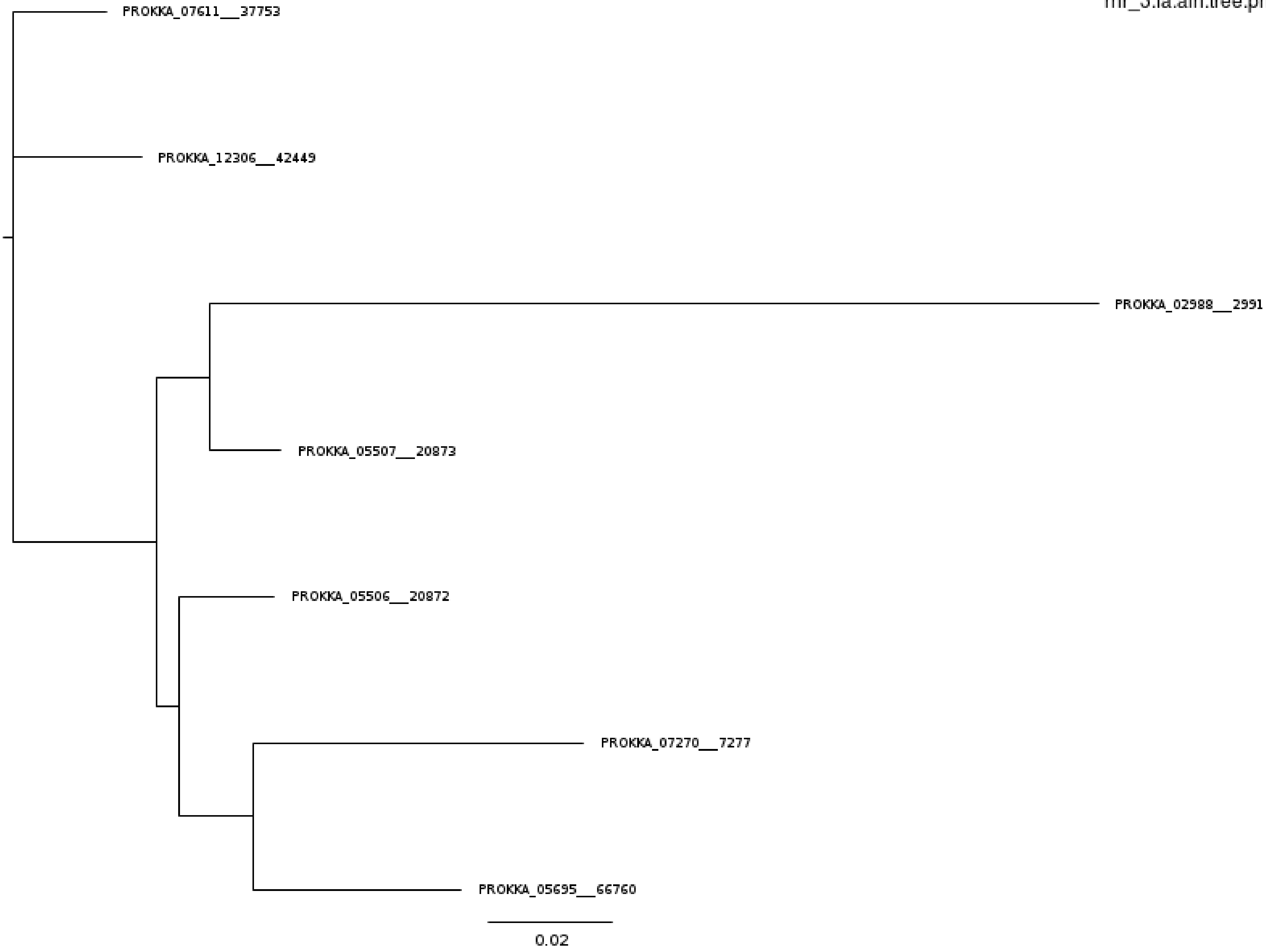

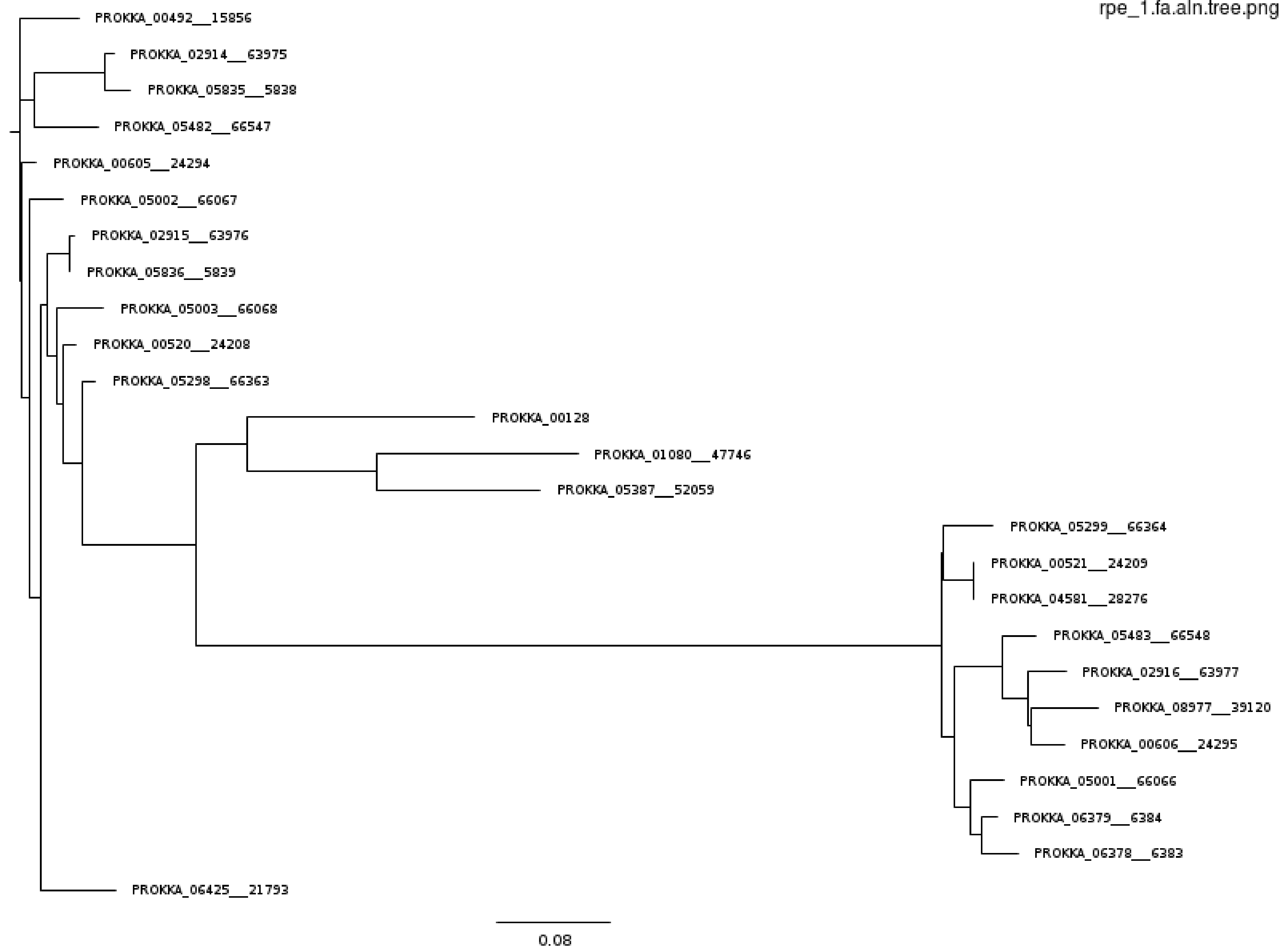

rpe_1.fa.aln.tree.png
PROKKA_00492___15856
PROKKA_02914___63975
PROKKA_05835___5838
PROKKA_05482___66547
PROKKA_00605___24294
PROKKA_05002___66067
PROKKA_02915___63976
PROKKA_05836___5839
PROKKA_05003___66068
PROKKA_00520___24208
PROKKA_05298___66363
PROKKA_00128
PROKKA_01080___47746
PROKKA_05387___52059
PROKKA_05299___66364
PROKKA_00521___24209
PROKKA_04581___28276
PROKKA_05483___66548
PROKKA_02916___63977
PROKKA_08977___39120
PROKKA_00606___24295
PROKKA_05001___66066
PROKKA_06379___6384
PROKKA_06378___6383
PROKKA_06425___21793
0.08

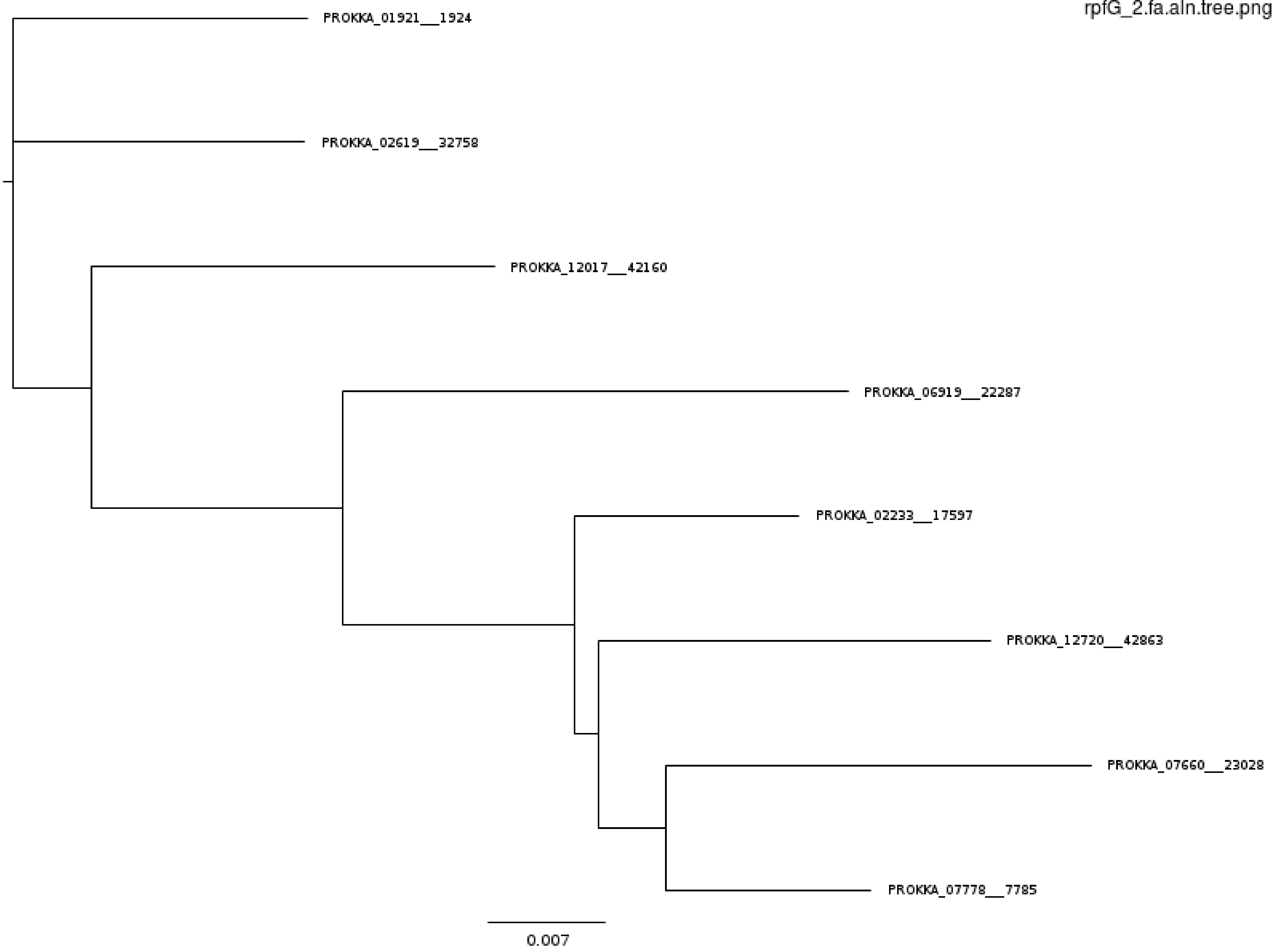
rpfG_2.fa.aln.tree.png
PROKKA_01921___1924
PROKKA_02619___32758
PROKKA_12017___42160
PROKKA_06919___22287
PROKKA_02233___17597
PROKKA_12720___42863
PROKKA_07660___23028
PROKKA_07778___7785
0.007

rpfG_4.fa.aln.tree.png

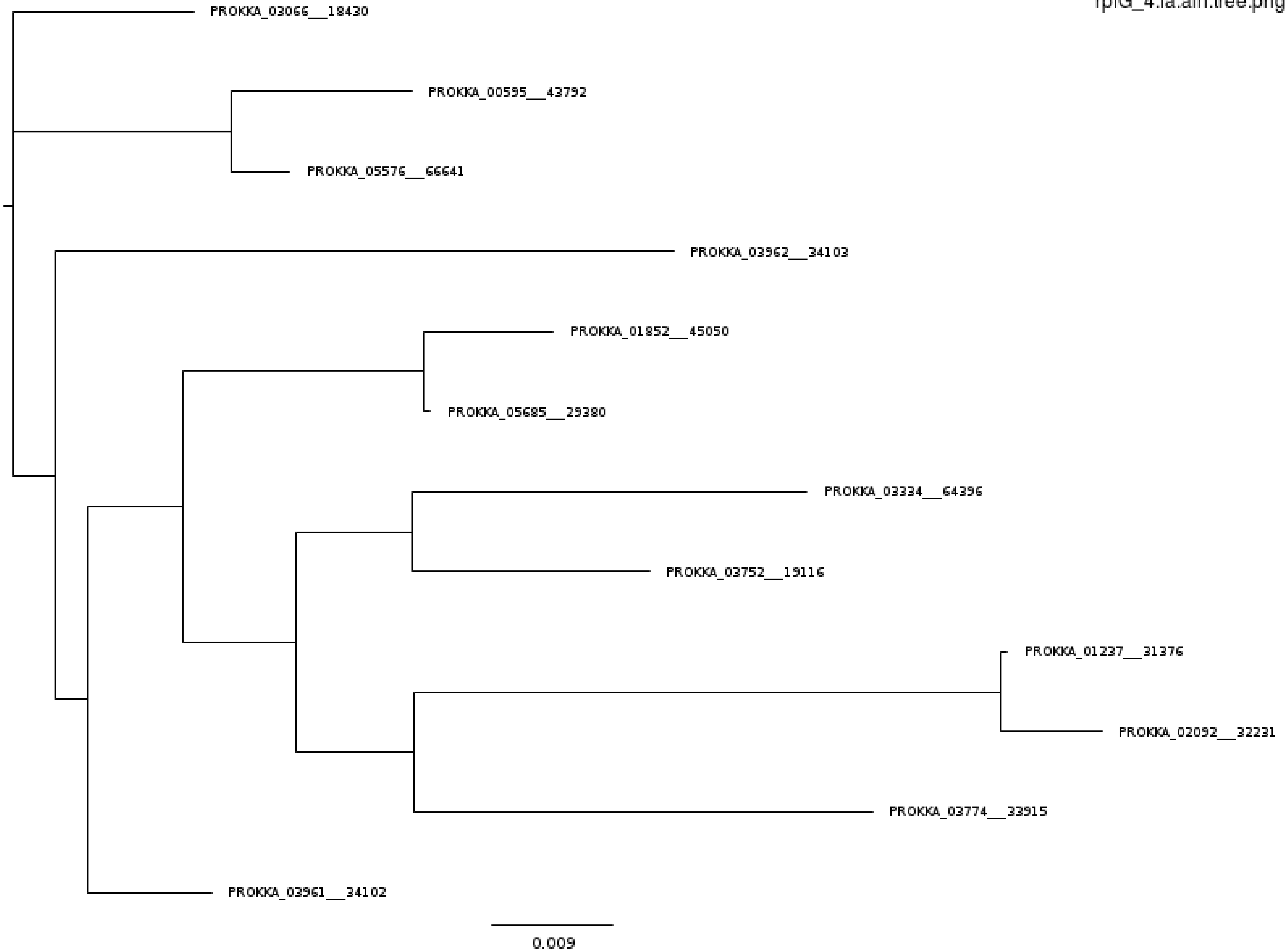

rpfG_5.fa.aln.tree.png

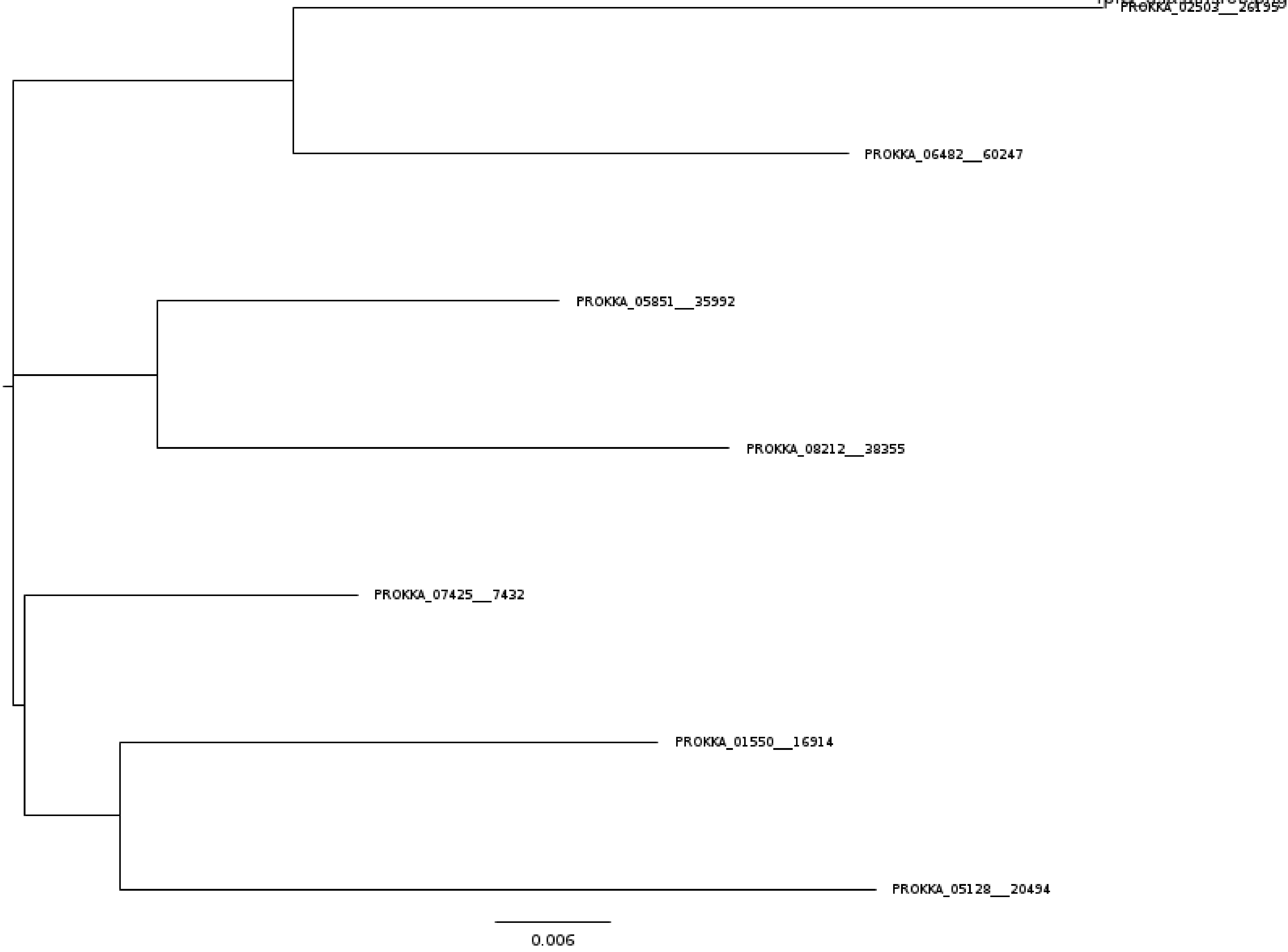

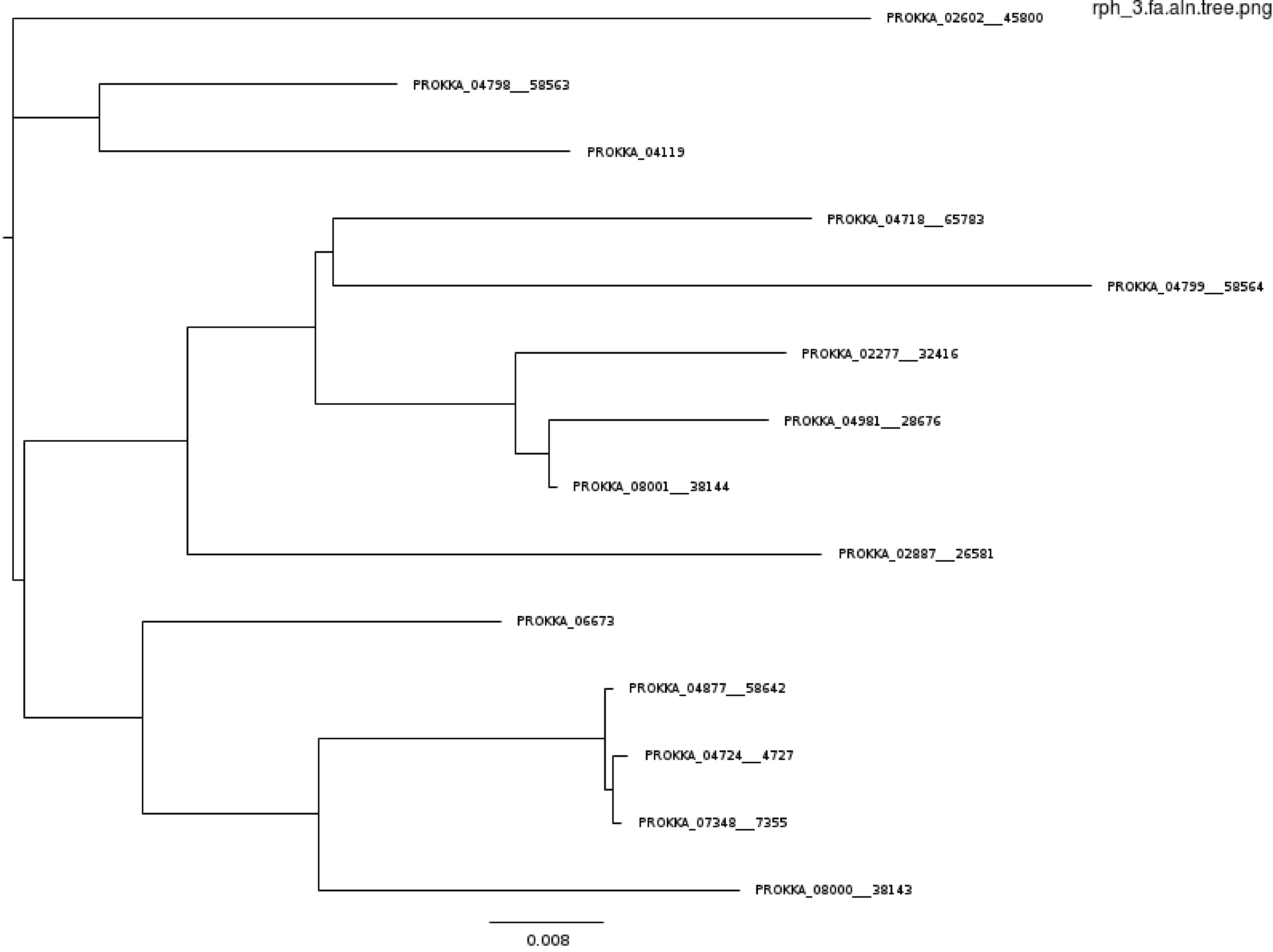

rph_3.fa.aln.tree.png
PROKKA_02602___45800
PROKKA_04798___58563
PROKKA_04119
PROKKA_04718___65783
PROKKA_04799___58564
PROKKA_02277___32416
PROKKA_04981___28676
PROKKA_08001___38144
PROKKA_02887___26581
PROKKA_06673
PROKKA_04877___58642
PROKKA_04724___4727
PROKKA_07348___7355
PROKKA_08000___38143
0.008

rpiA_3.fa.aln.tree.png

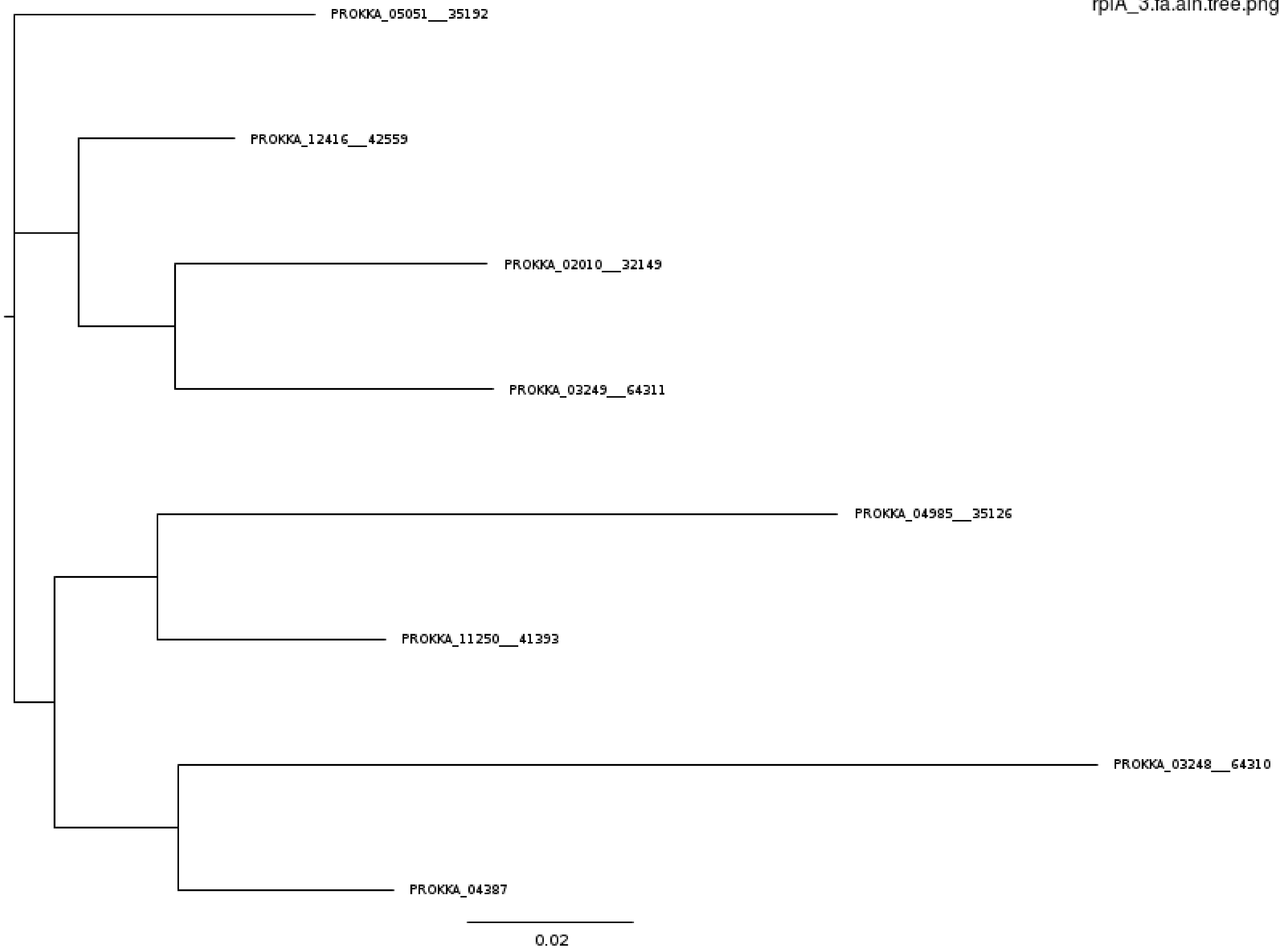

rplC.fa.aln.tree.png

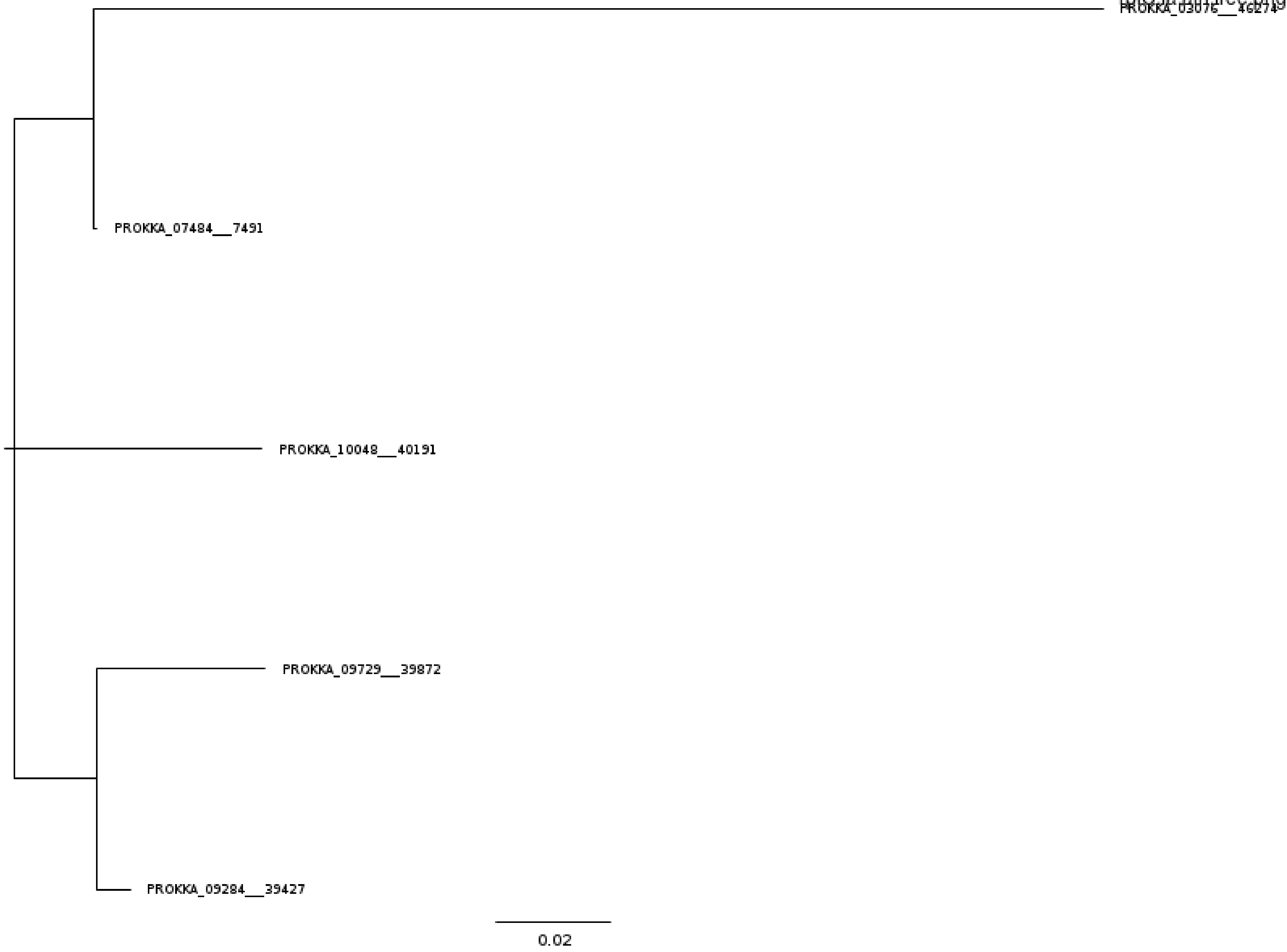

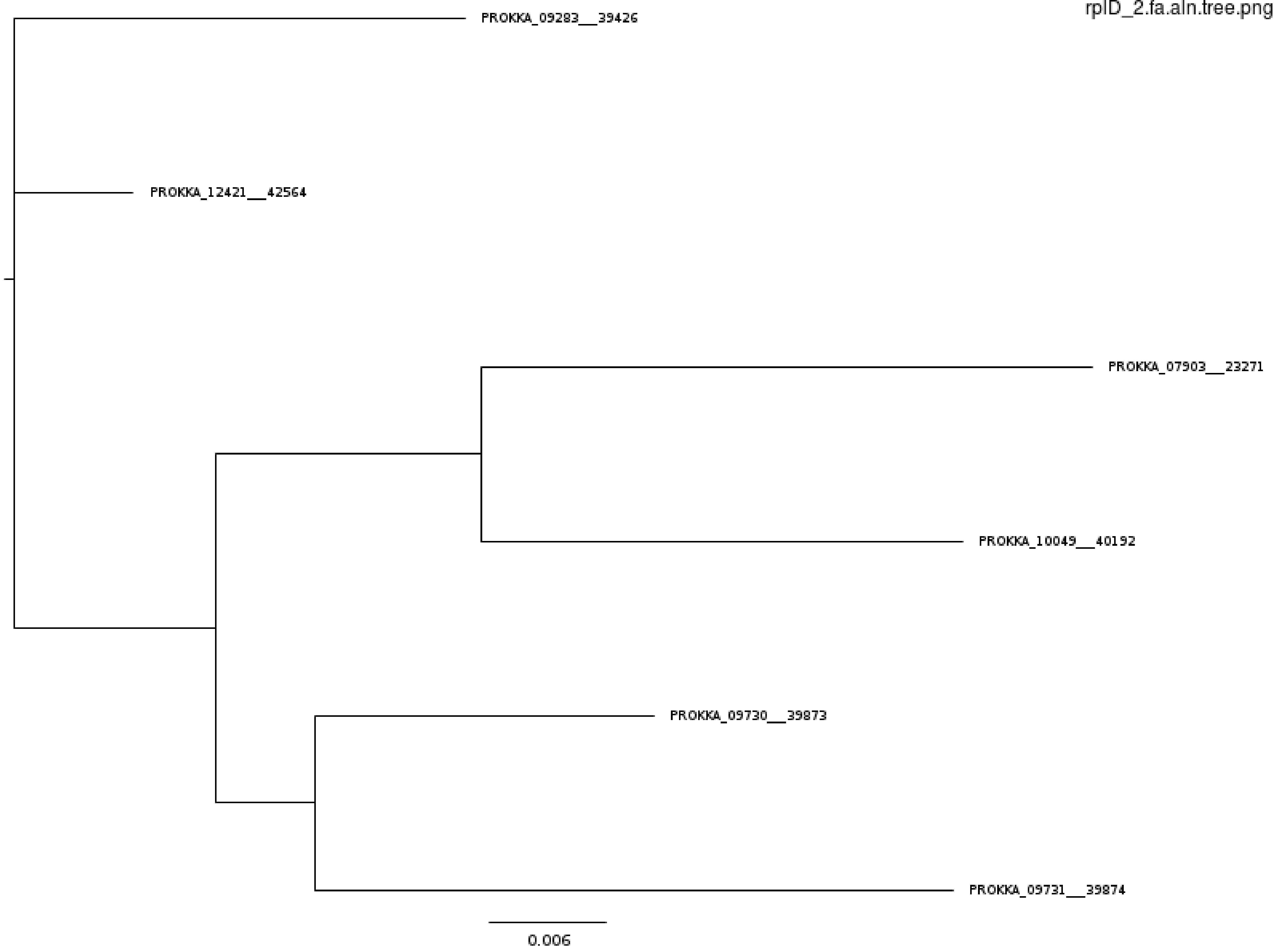
rplD_2.fa.aln.tree.png
PROKKA_09283___39426
PROKKA_12421___42564
PROKKA_07903___23271
PROKKA_10049___40192
PROKKA_09730___39873
PROKKA_09731___39874
0.006

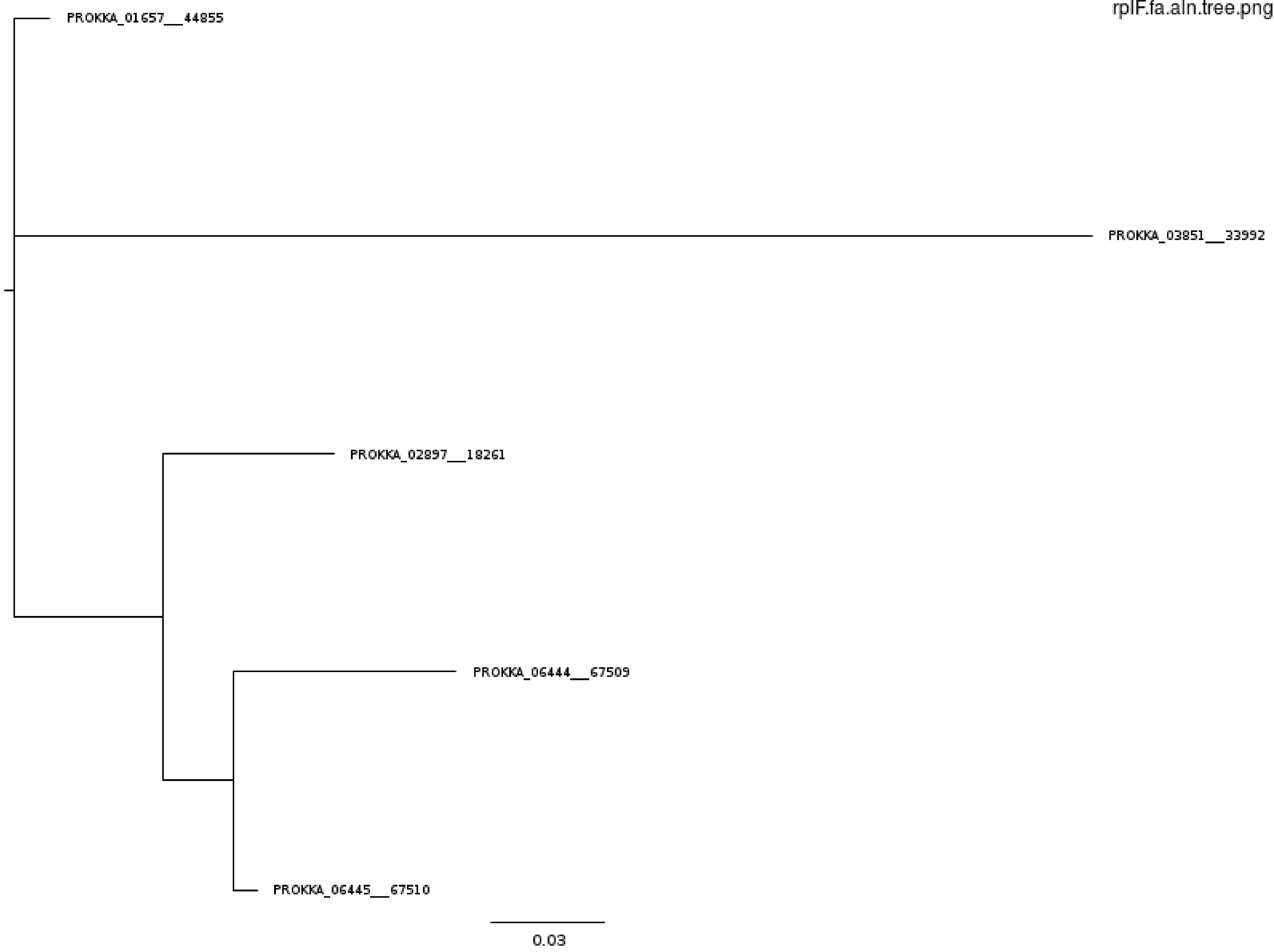
rplF.fa.aln.tree.png
PROKKA_01657___44855
PROKKA_03851___33992
PROKKA_02897___18261
PROKKA_06444___67509
PROKKA_06445___67510
0.03

rpll.fa.aln.tree.png

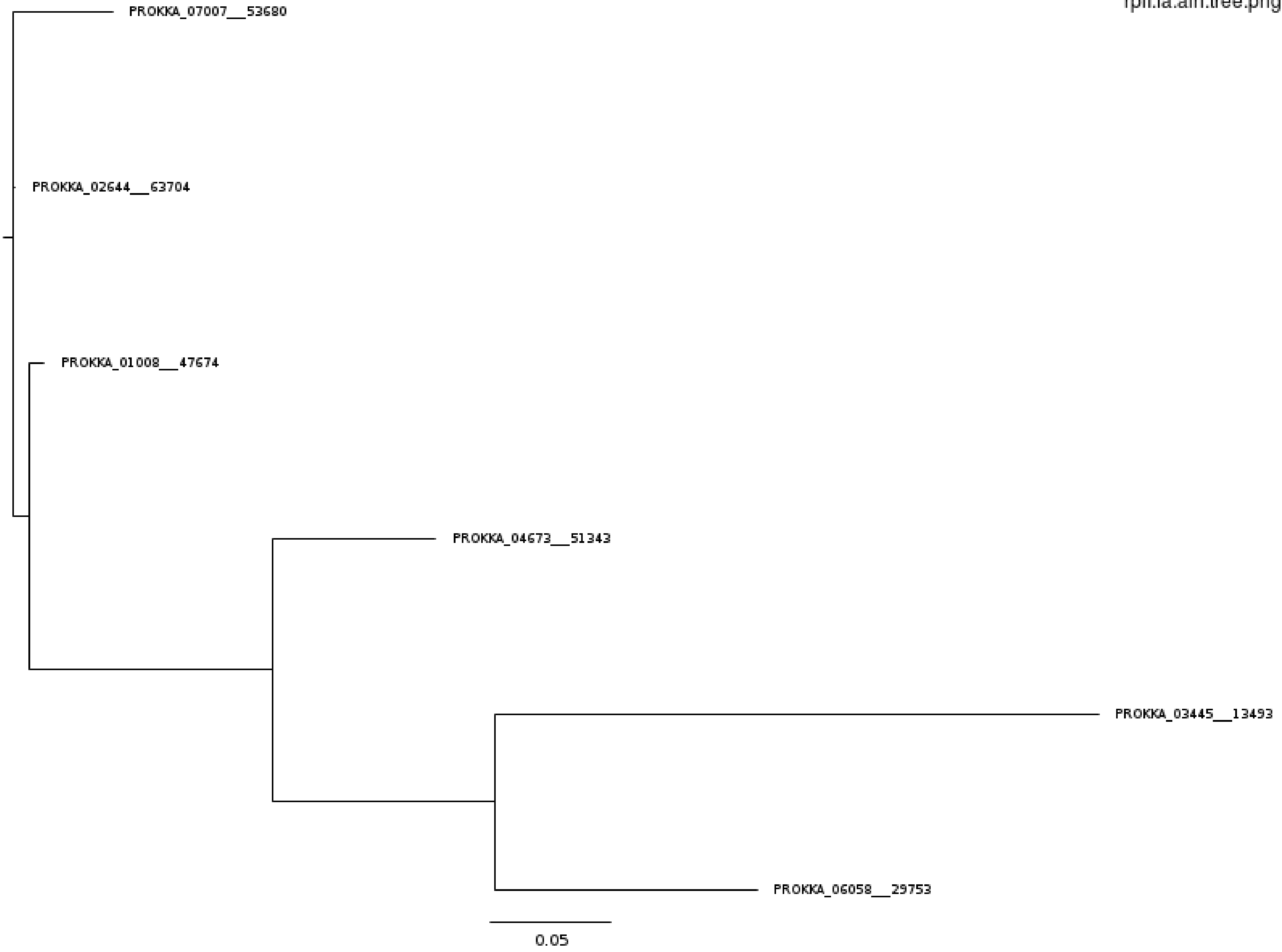

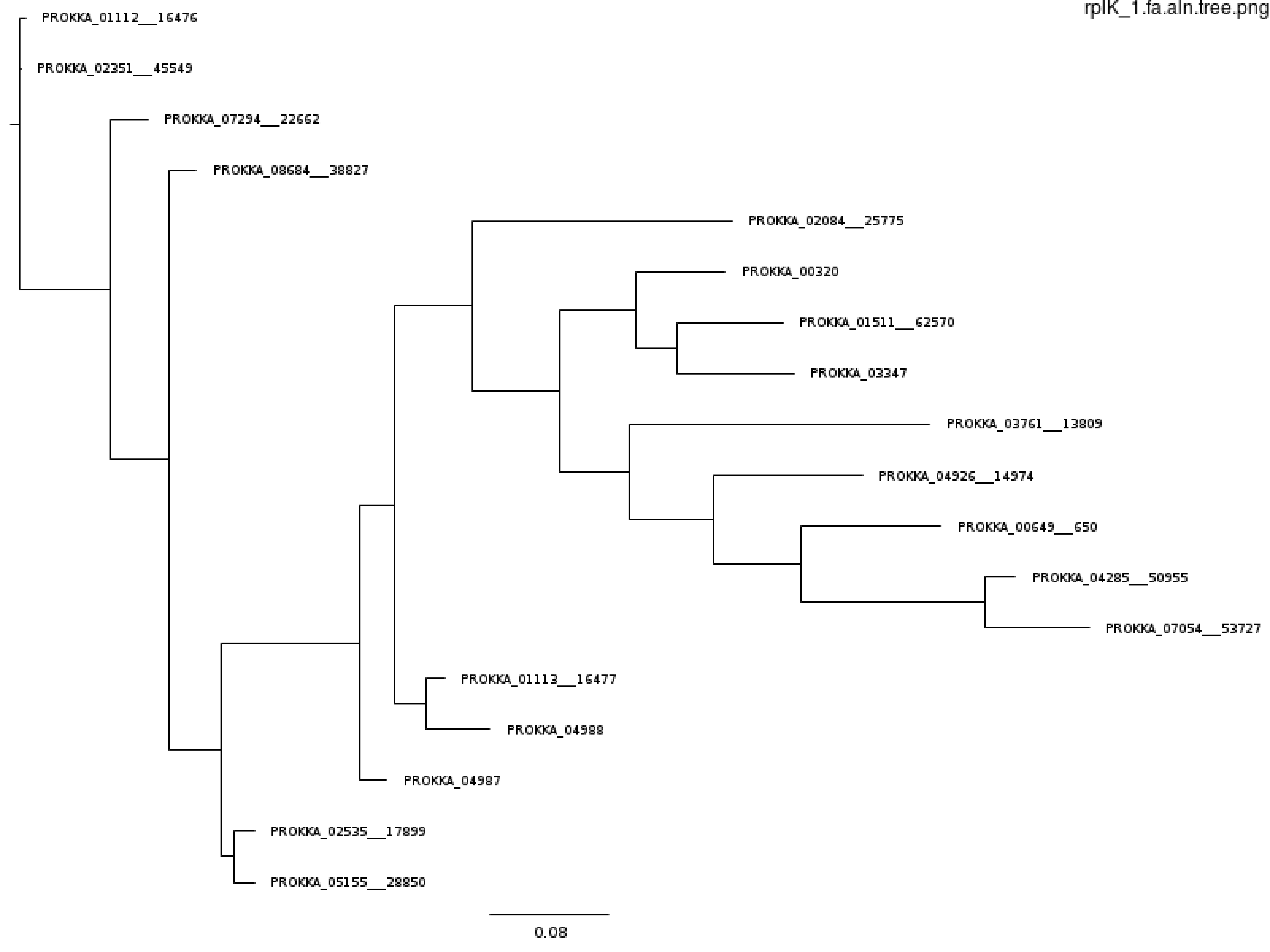

rplK_1.fa.aln.tree.png
PROKKA_01112___16476
PROKKA_02351___45549
PROKKA_07294___22662
PROKKA_08684___38827
PROKKA_02084___25775
PROKKA_00320
PROKKA_01511___62570
PROKKA_03347
PROKKA_03761___13809
PROKKA_04926___14974
PROKKA_00649___650
PROKKA_04285___50955
PROKKA_07054___53727
PROKKA_01113___16477
PROKKA_04988
PROKKA_04987
PROKKA_02535___17899
PROKKA_05155___28850
0.08

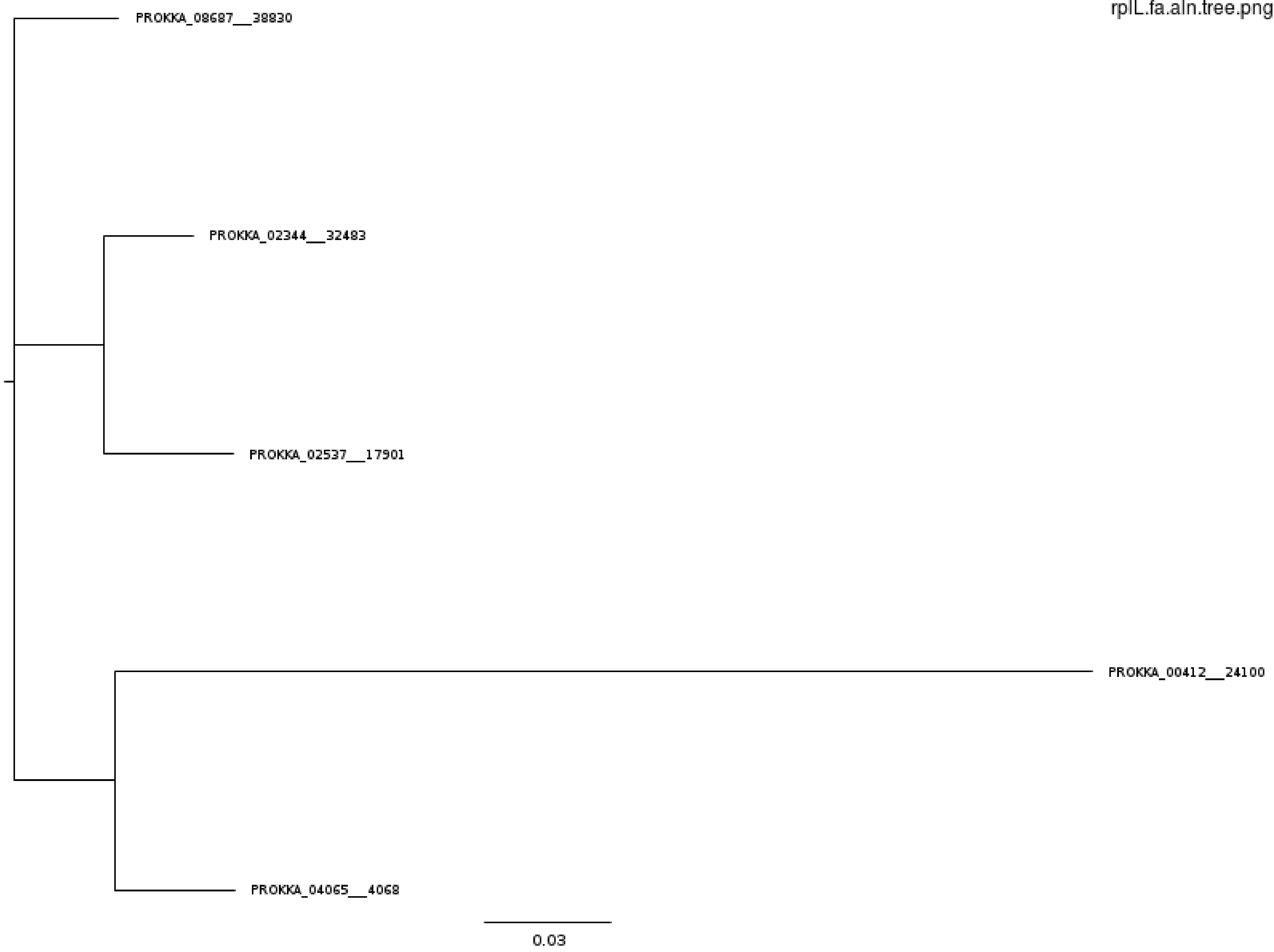
rplL.fa.aln.tree.png
PROKKA_08687___38830
PROKKA_02344___32483
PROKKA_02537___17901
PROKKA_00412___24100
PROKKA_04065___4068
0.03

rplO.fa.aln.tree.png

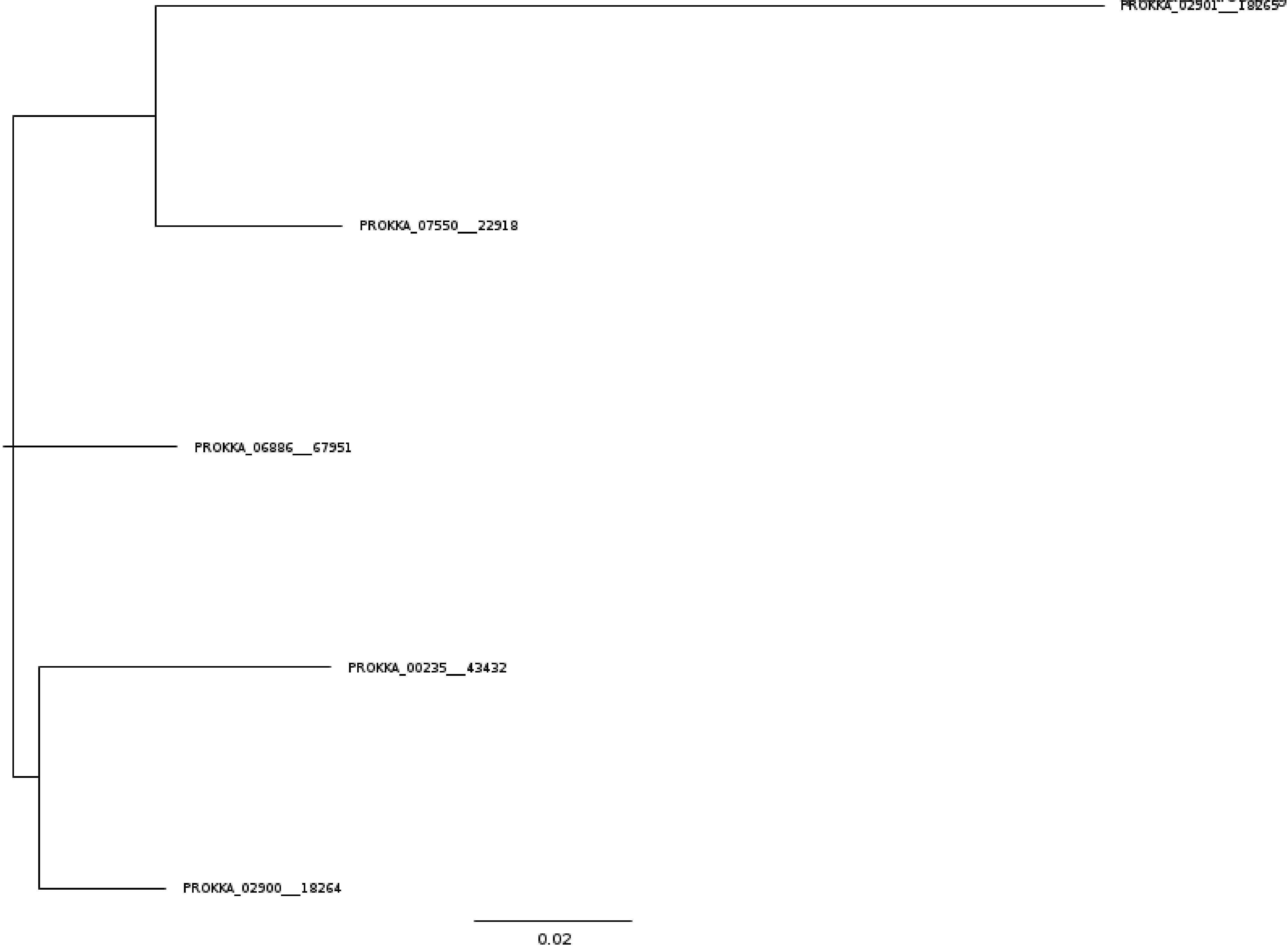

rplP_2.fa.aln.tree.png

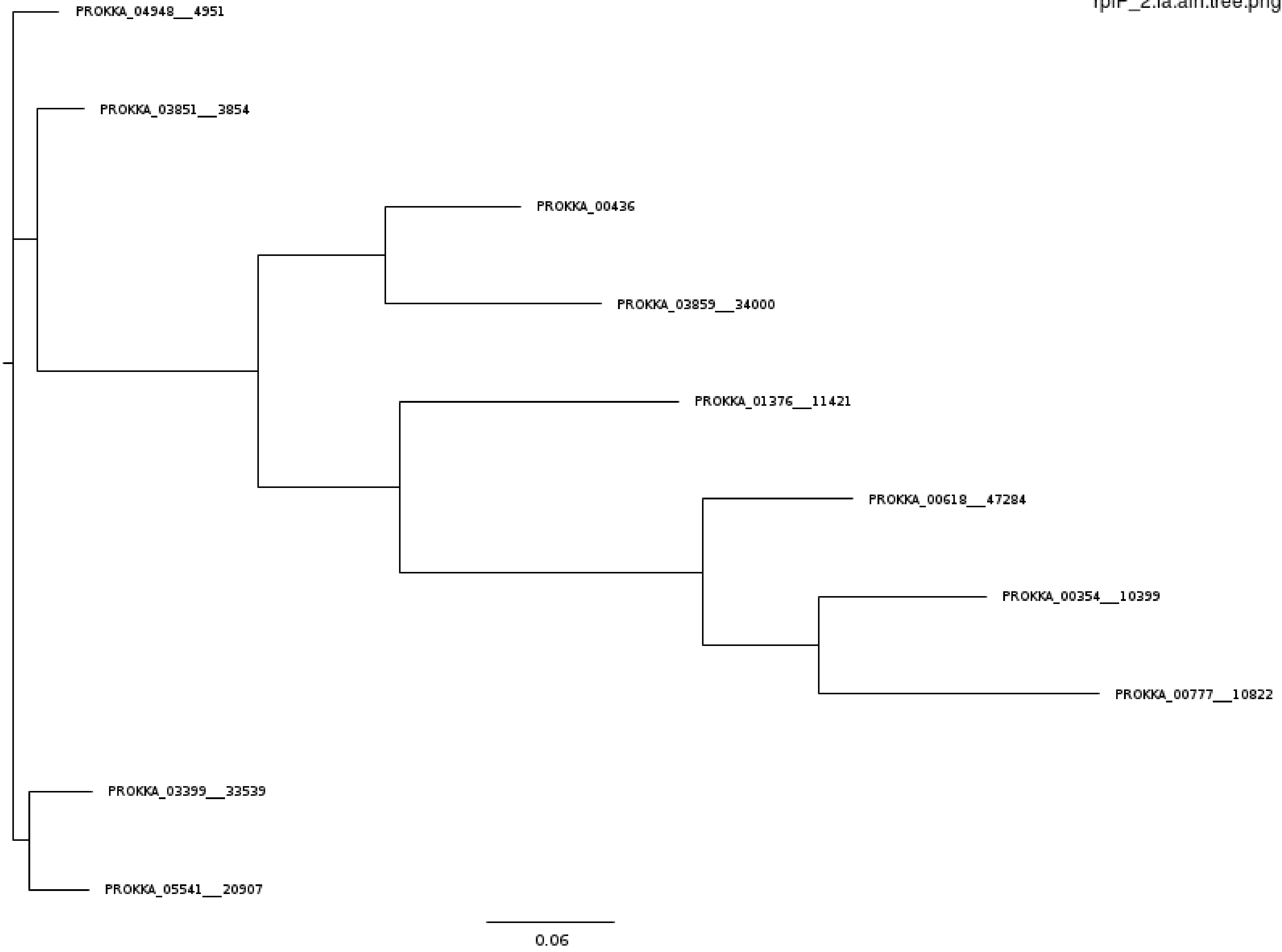

rplS_2.fa.aln.tree.png

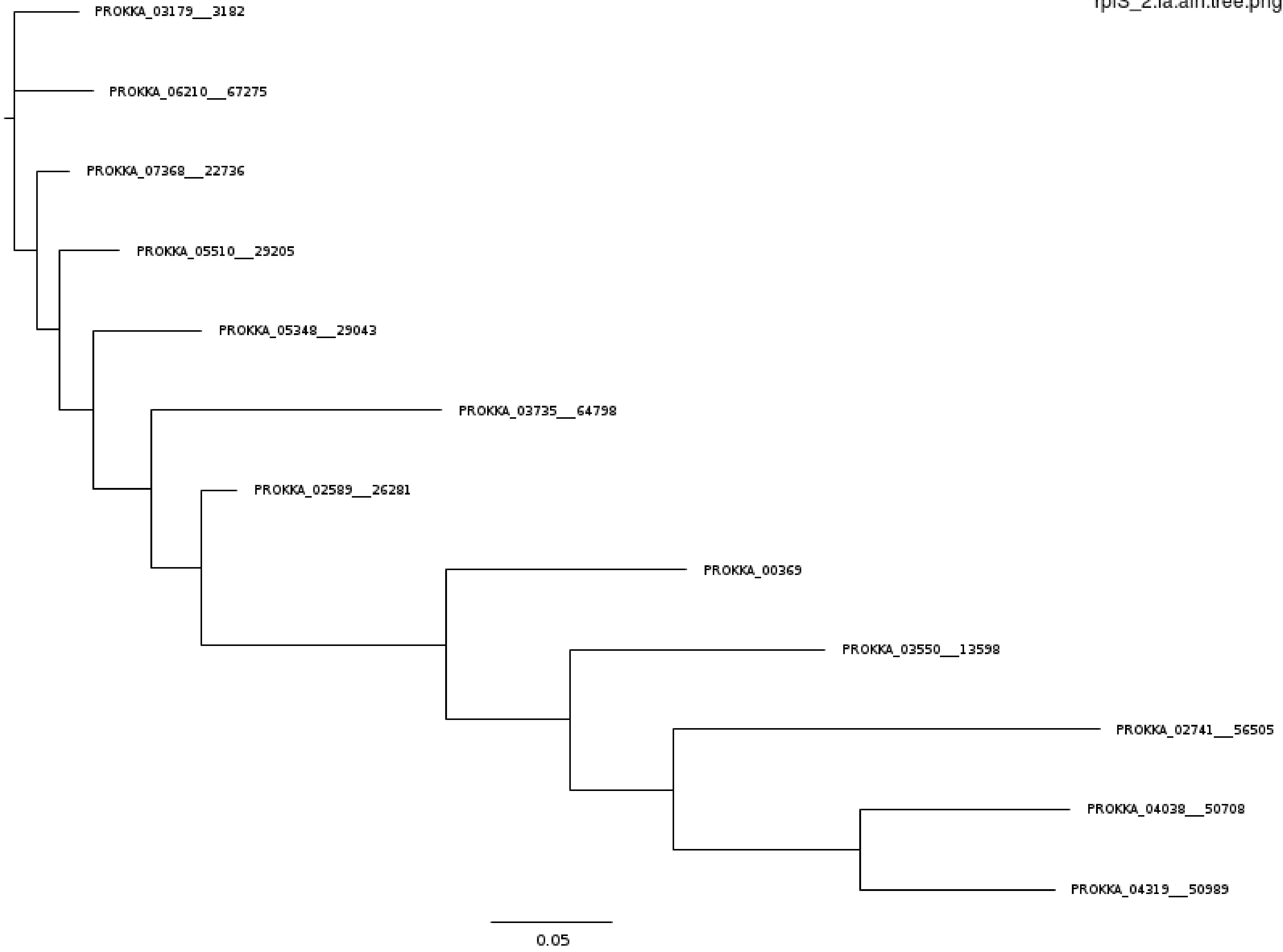

rplT_3.fa.aln.tree.png

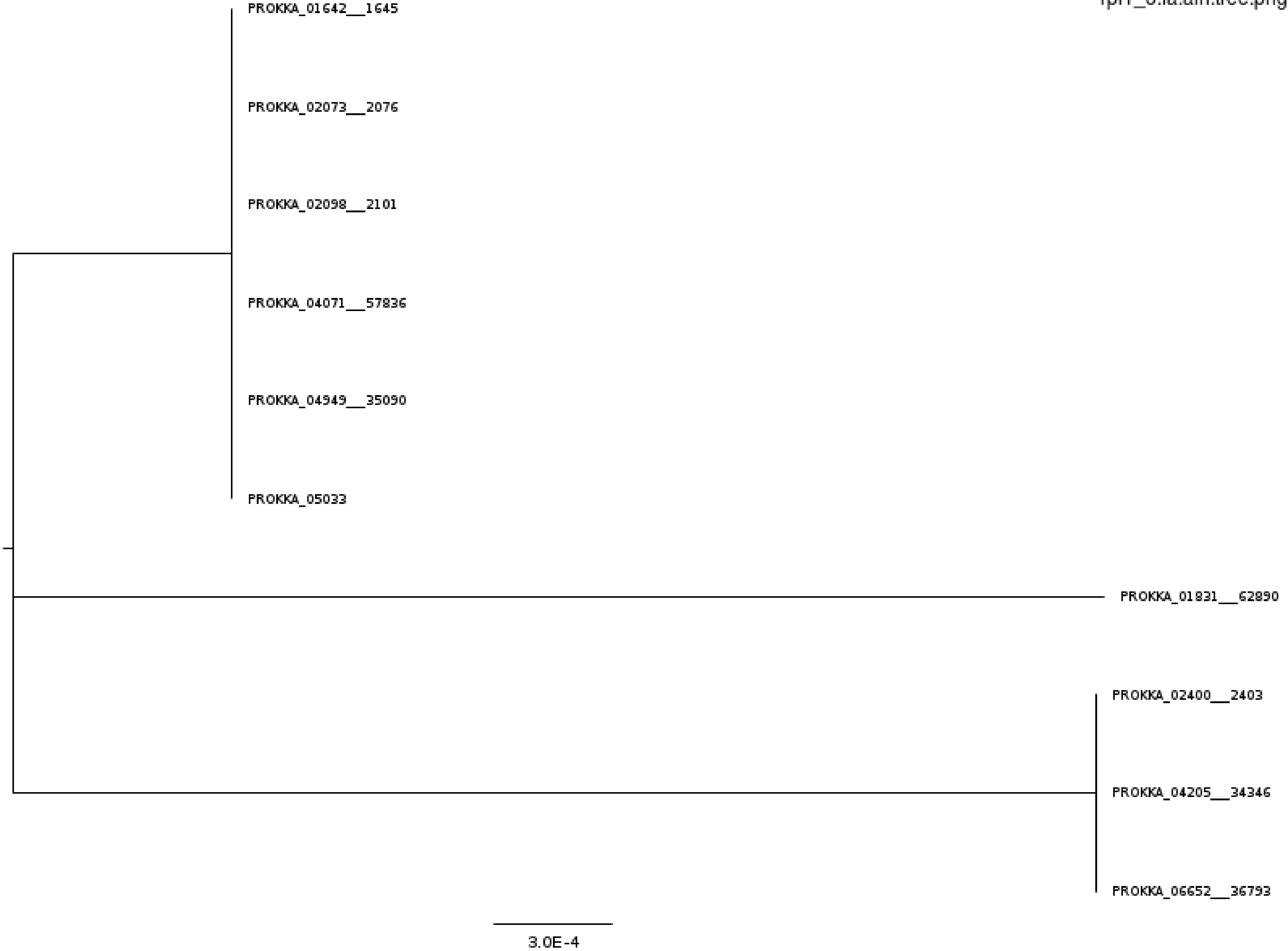

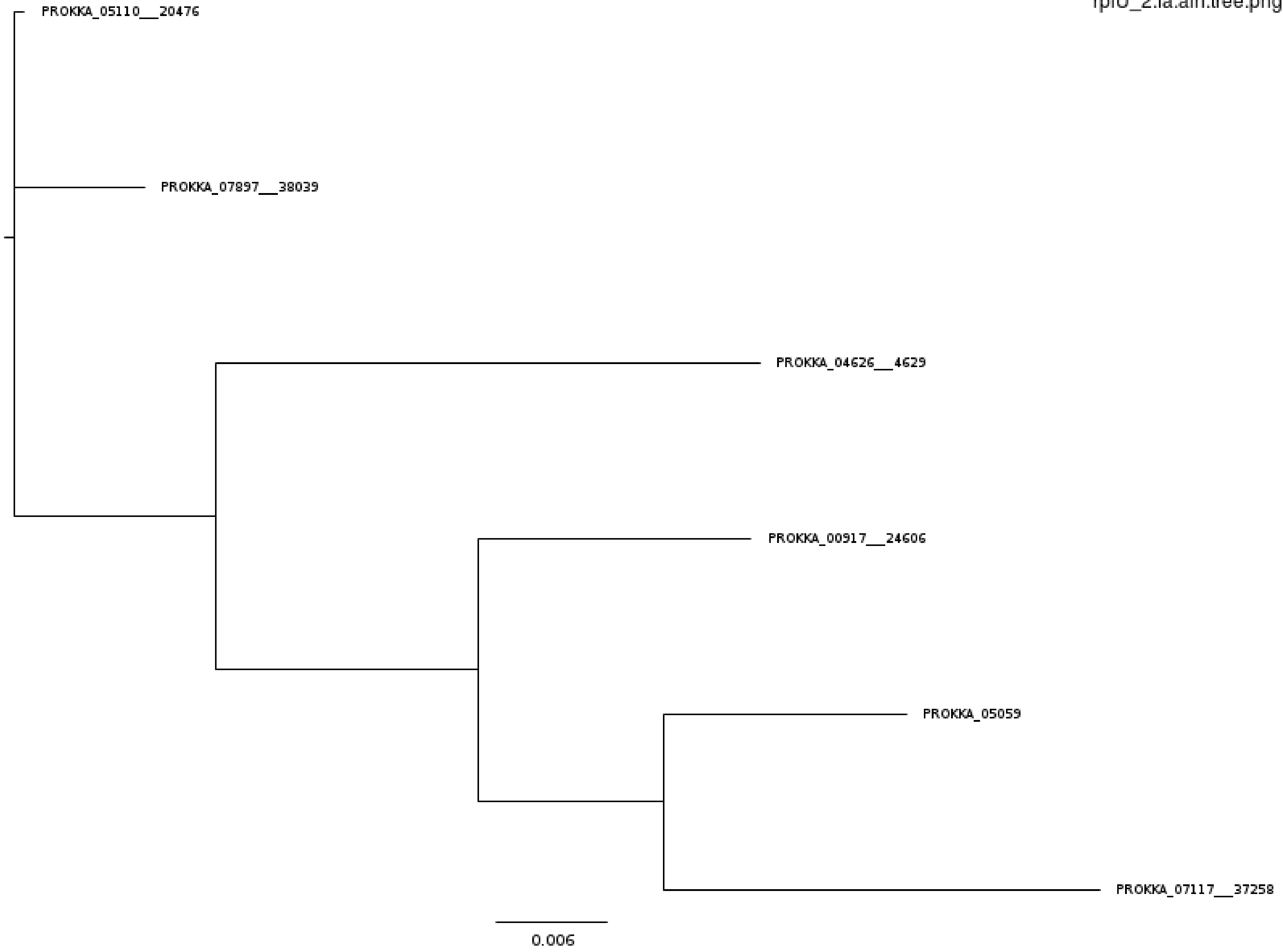
PROKKA_05110___20476
PROKKA_07897___38039
PROKKA_04626___4629
PROKKA_00917___24606
PROKKA_05059
PROKKA_07117___37258
0.006

rplY_3.fa.aln.tree.png

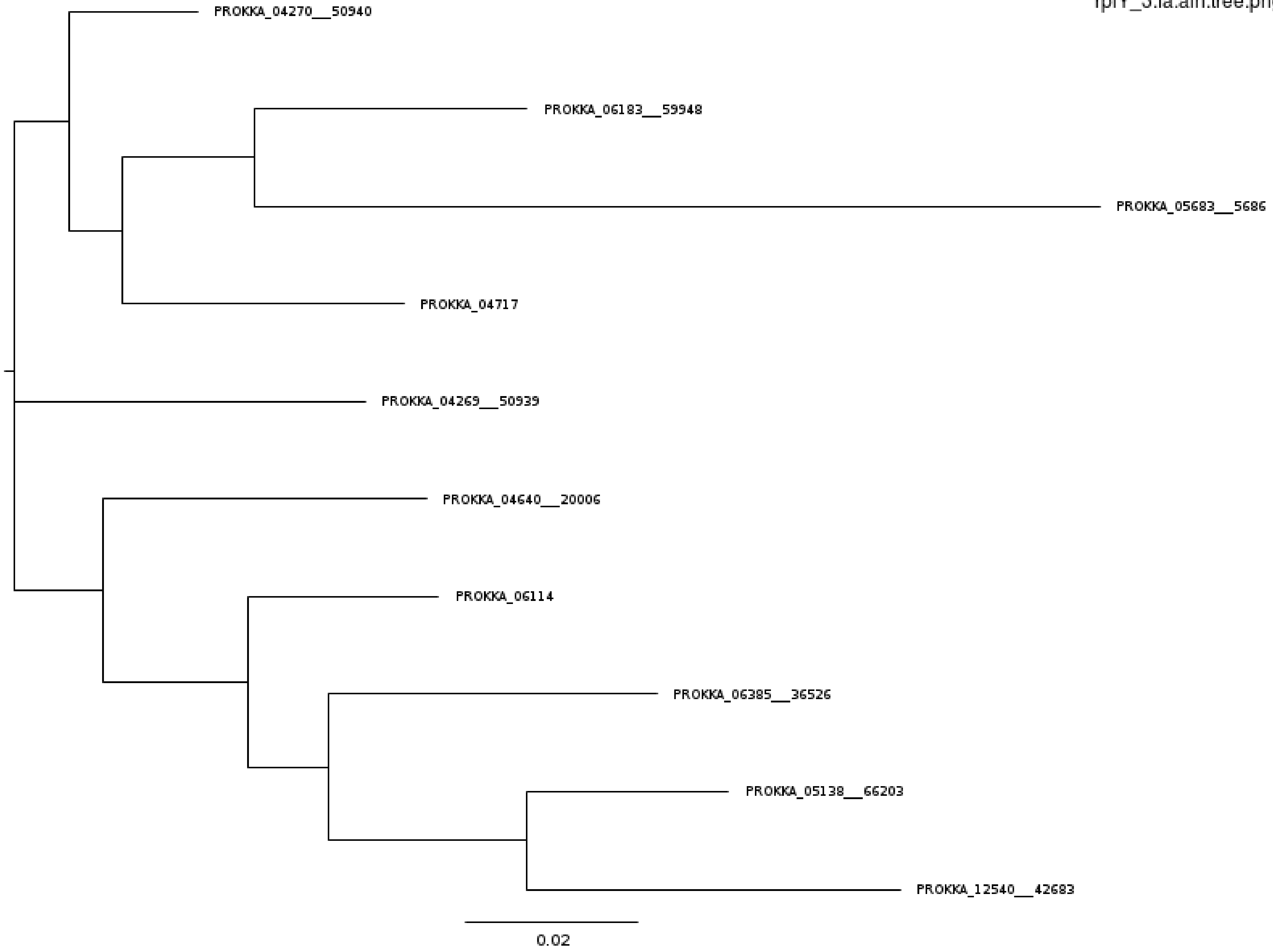

rpmB_3.fa.aln.tree.png

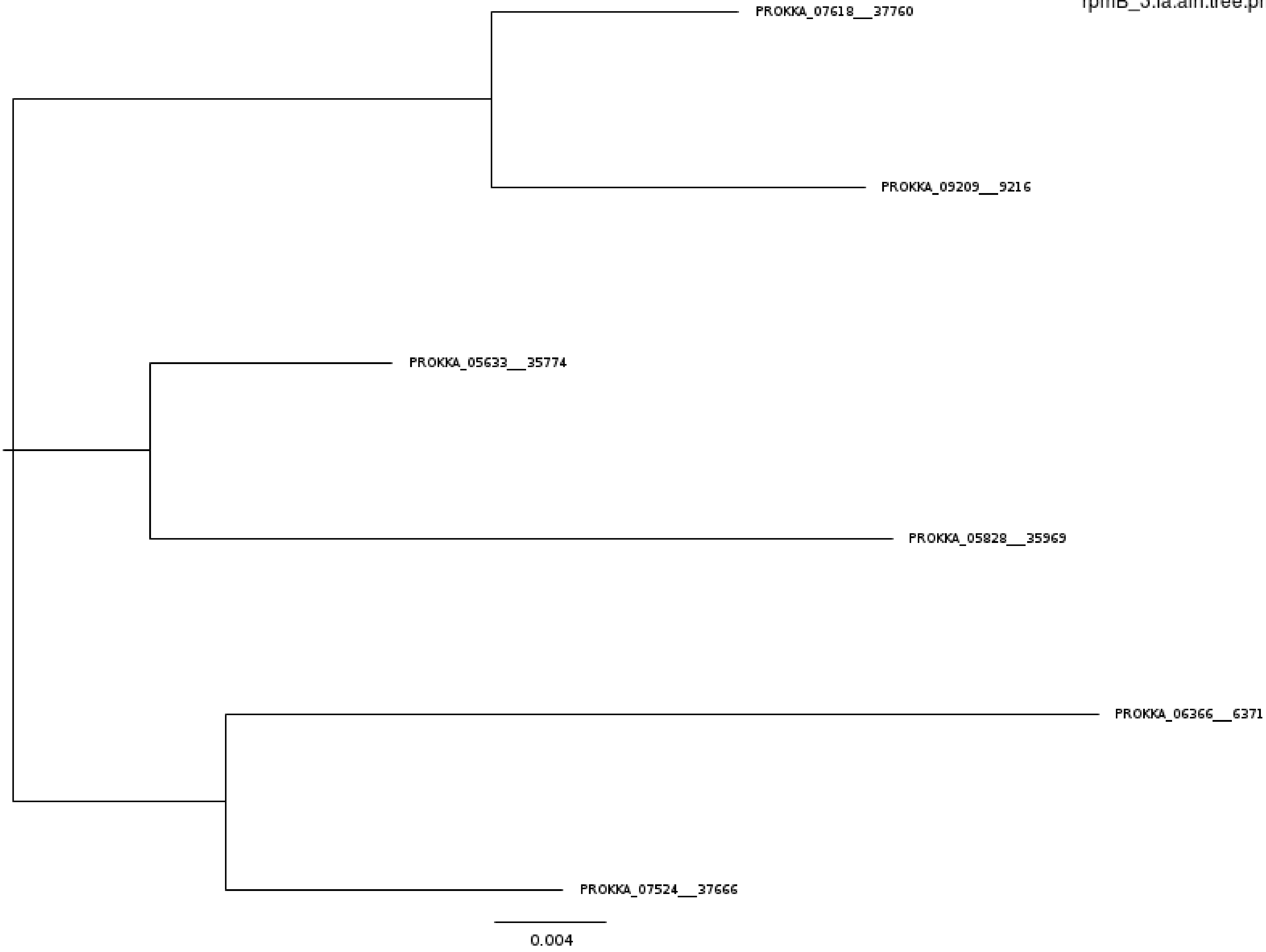

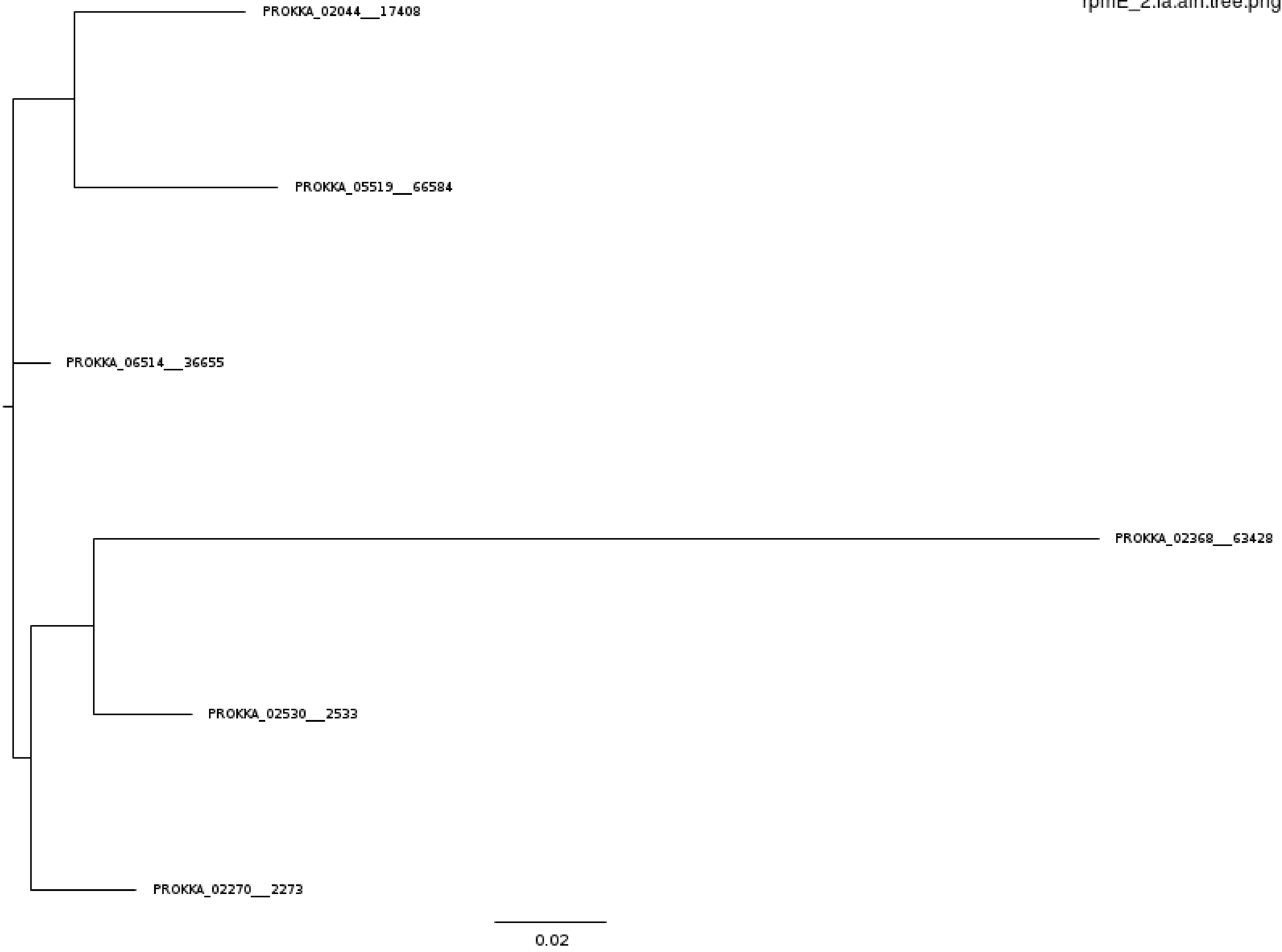
PROKKA_02044___17408
PROKKA_05519___66584
PROKKA_06514___36655
PROKKA_02368___63428
PROKKA_02530___2533
PROKKA_02270___2273
0.02

rpmG.fa.aln.tree.png

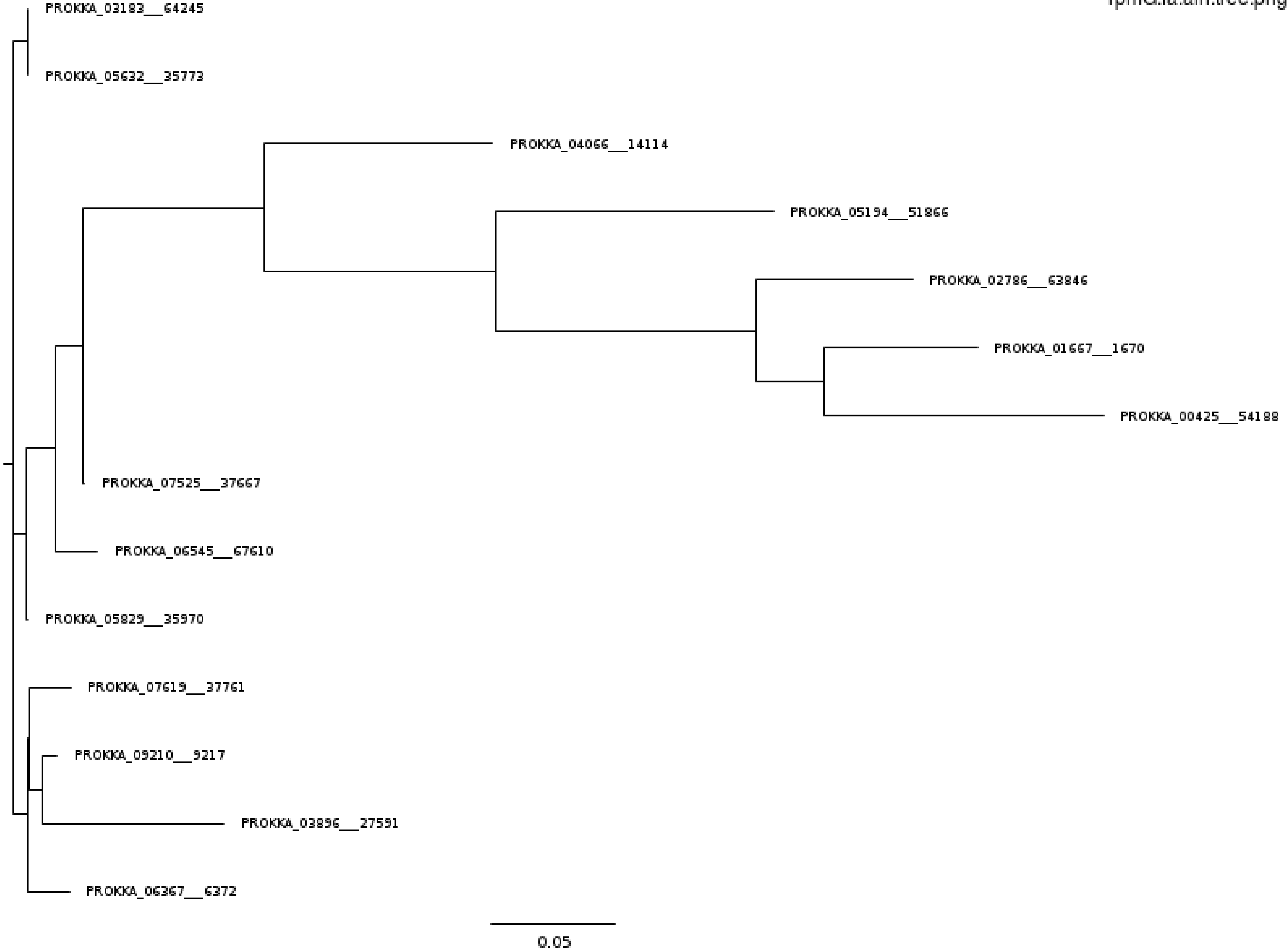

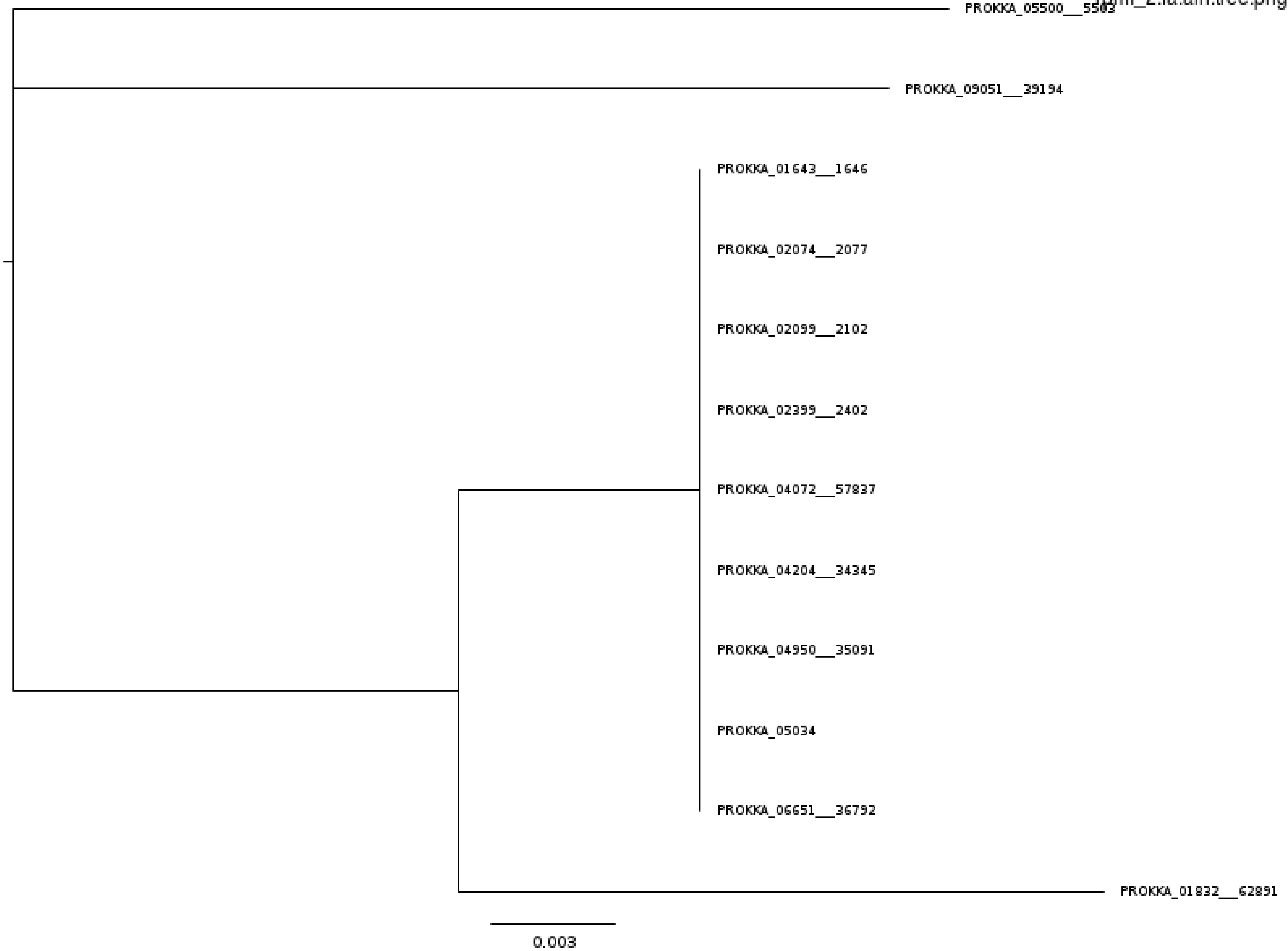
PROKKA_05500___5503
PROKKA_09051___39194
PROKKA_01643___1646
PROKKA_02074___2077
PROKKA_02099___2102
PROKKA_02399___2402
PROKKA_04072___57837
PROKKA_04204___34345
PROKKA_04950___35091
PROKKA_05034
PROKKA_06651___36792
PROKKA_01832___62891
0.003

rpoC_2.fa.aln.tree.png

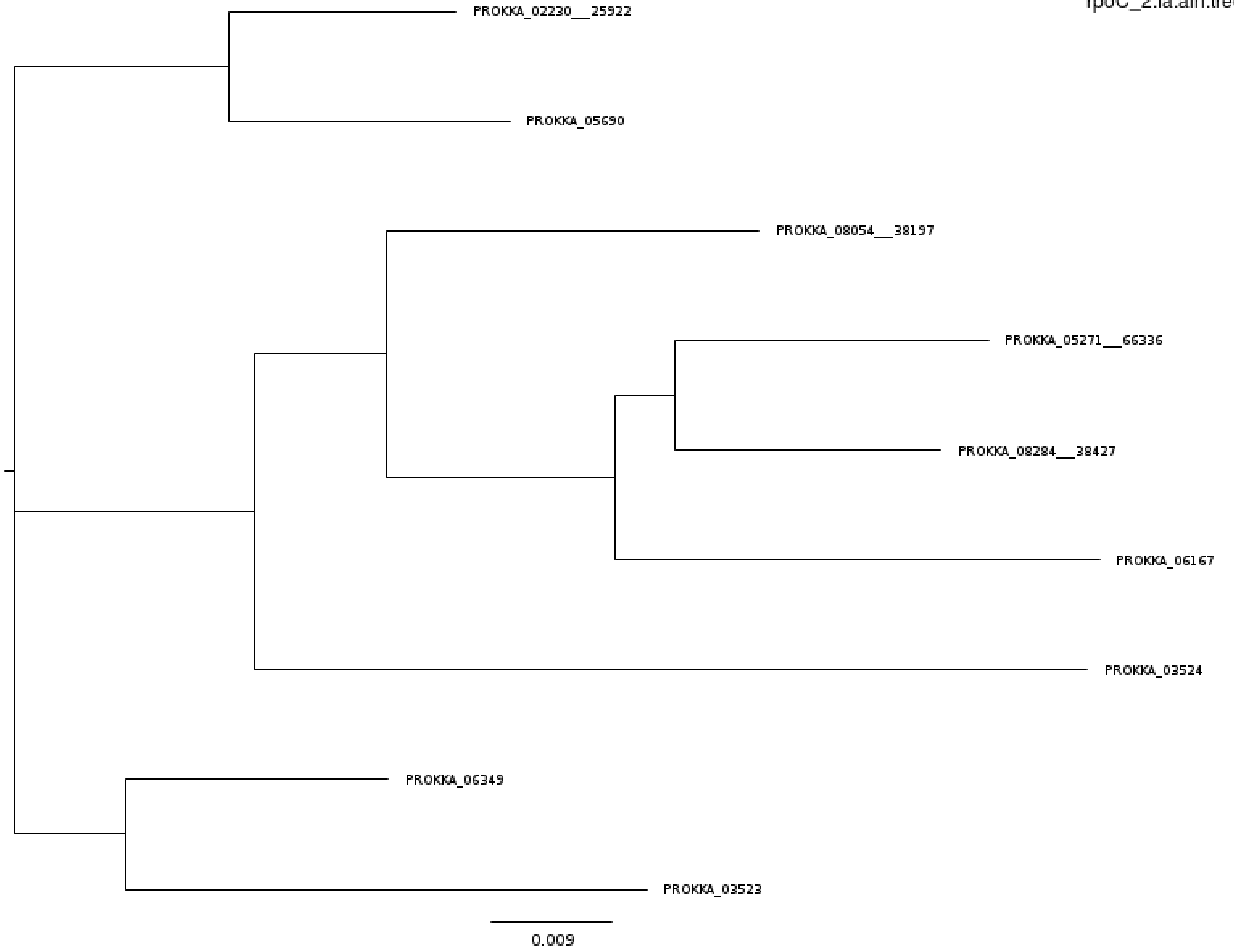

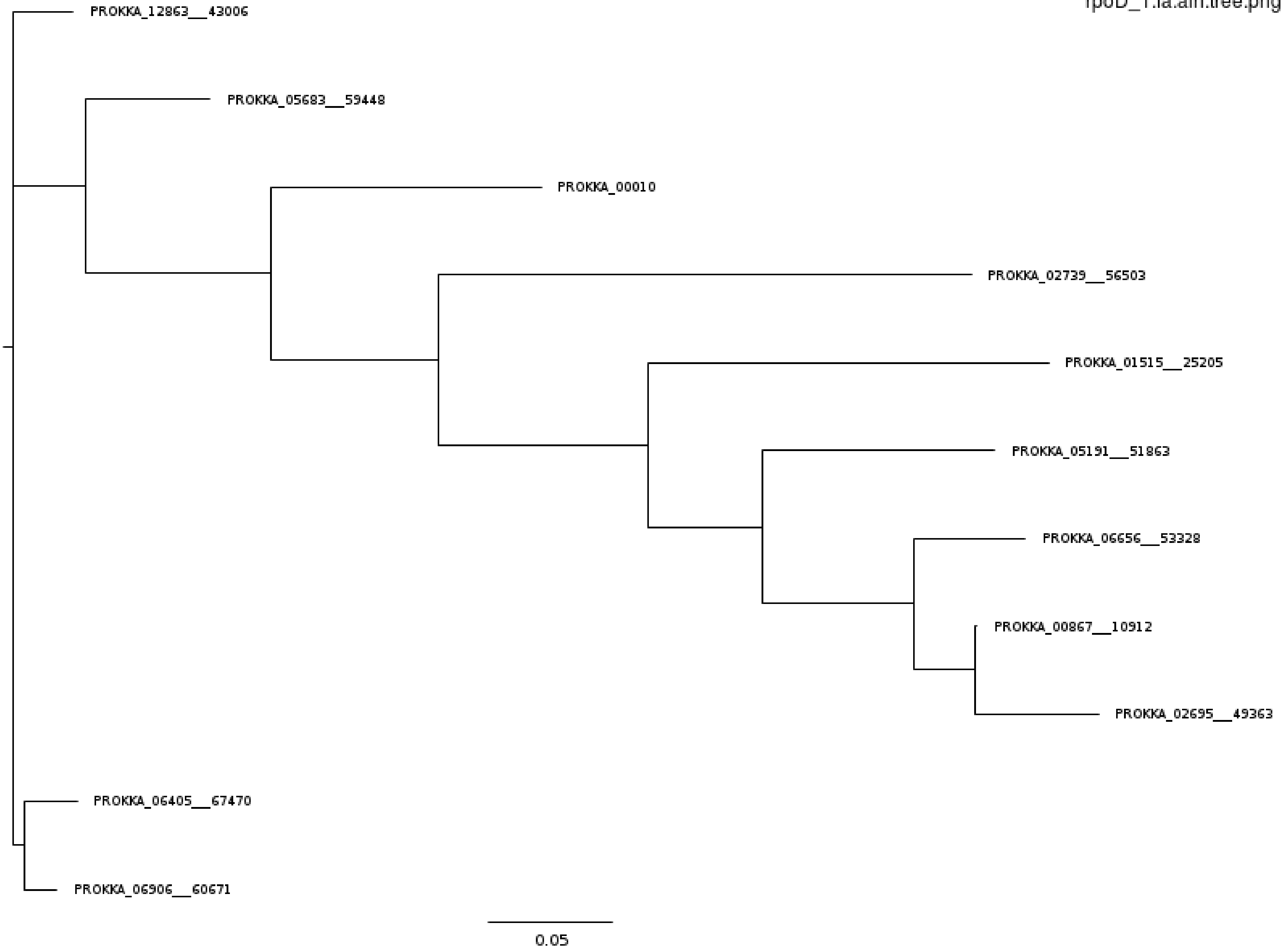
PROKKA_12863__43006
PROKKA_05683__59448
PROKKA_00010
PROKKA_02739__56503
PROKKA_01515__25205
PROKKA_05191__51863
PROKKA_06656__53328
PROKKA_00867__10912
PROKKA_02695__49363
PROKKA_06405__67470
PROKKA_06906__60671
0.05

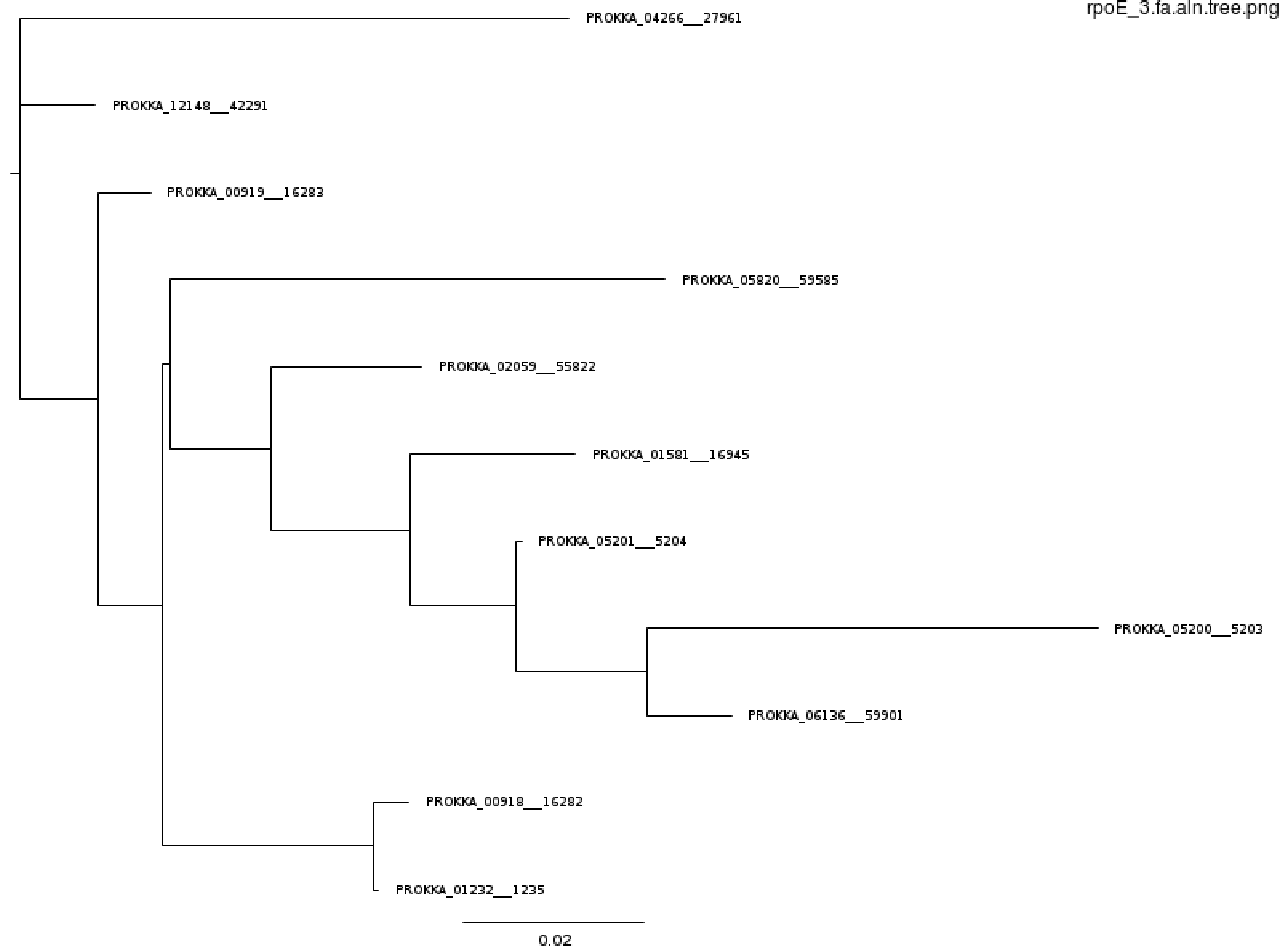
rpoE_3.fa.aln.tree.png
PROKKA_04266__27961
PROKKA_12148__42291
PROKKA_00919__16283
PROKKA_05820__59585
PROKKA_02059__55822
PROKKA_01581__16945
PROKKA_05201__5204
PROKKA_05200__5203
PROKKA_06136__59901
PROKKA_00918__16282
PROKKA_01232__1235
0.02

rpoH_3.fa.aln.tree.png

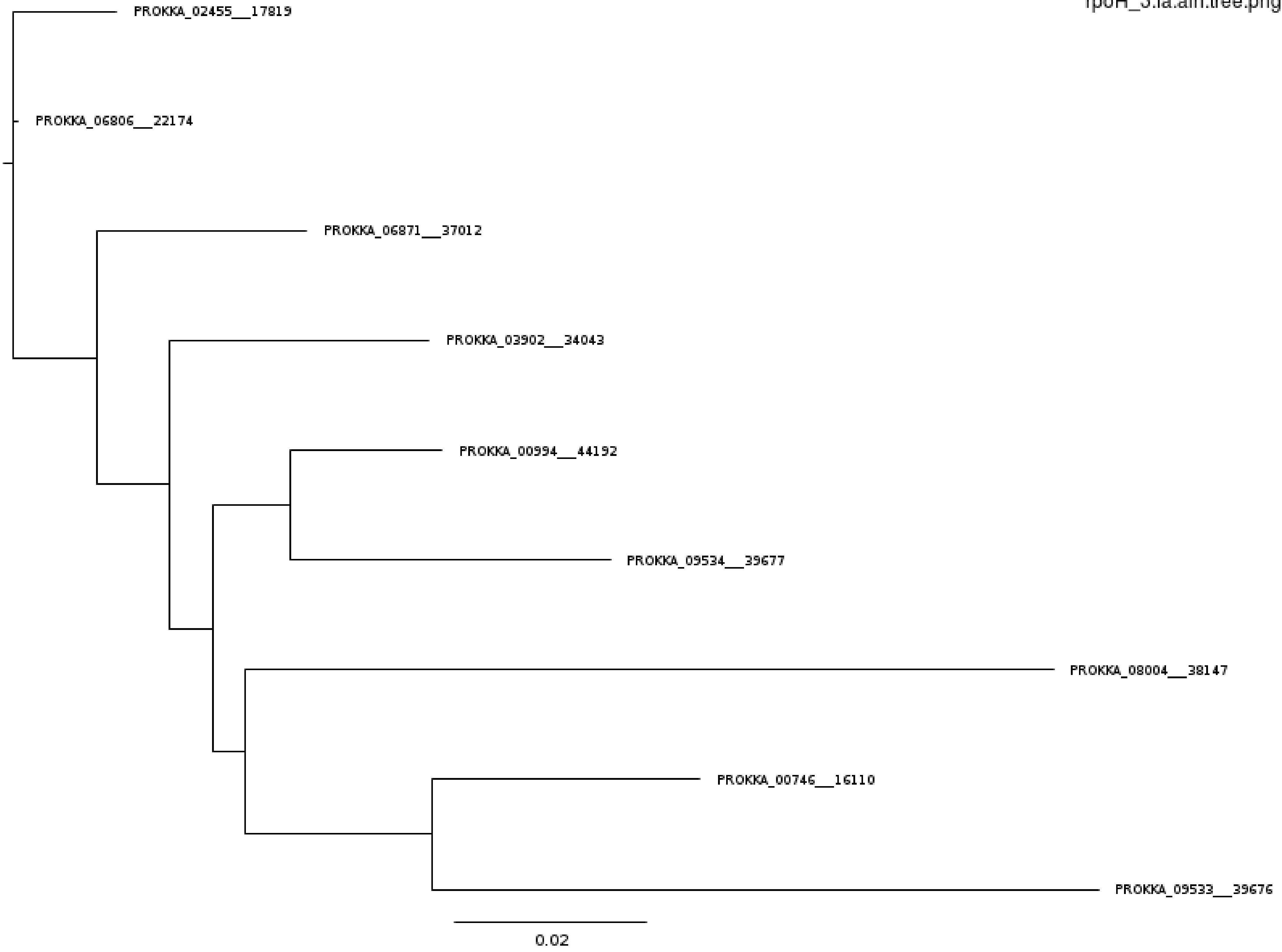

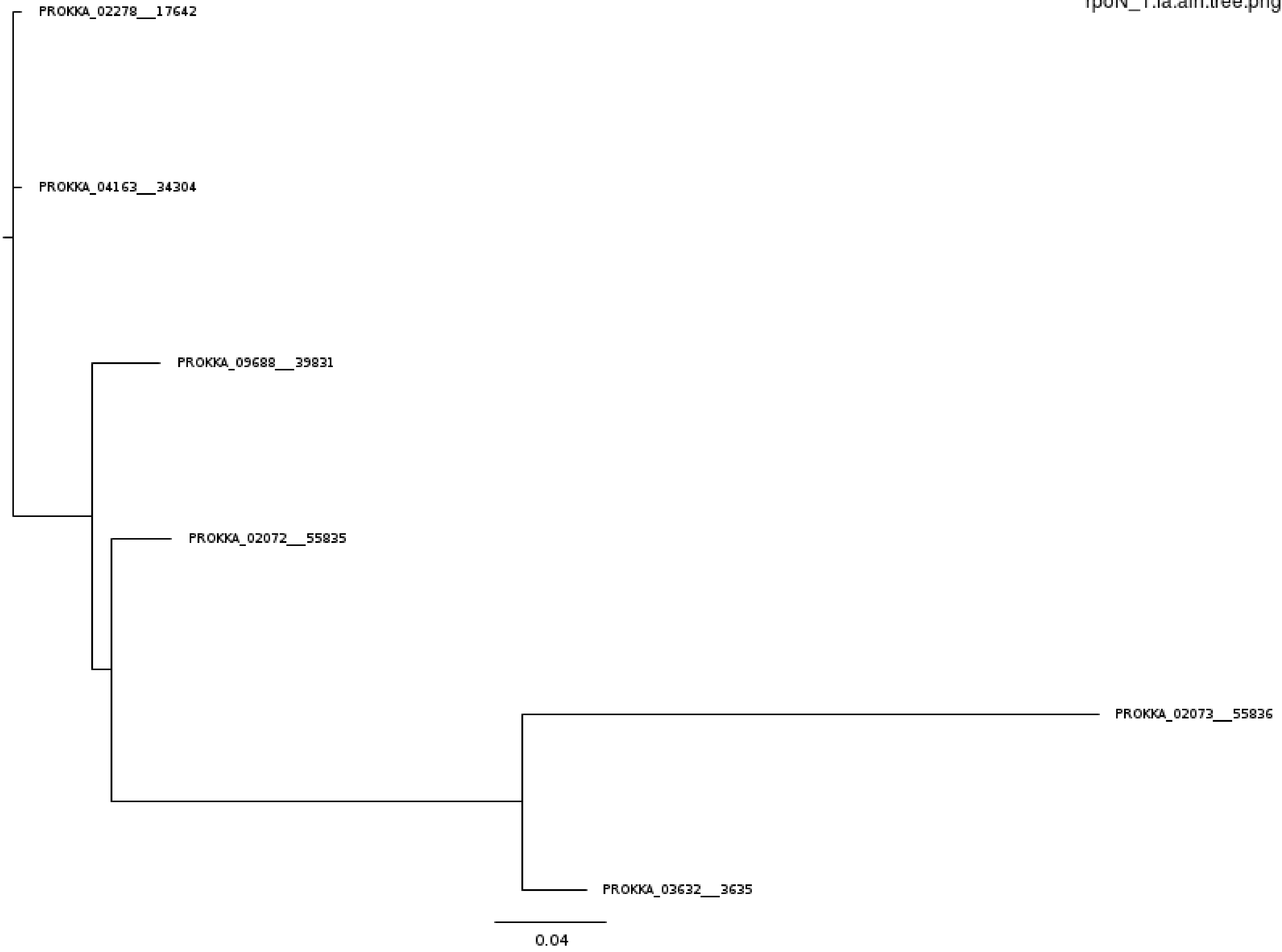
PROKKA_02278___17642
PROKKA_04163___34304
PROKKA_09688___39831
PROKKA_02072___55835
PROKKA_02073___55836
PROKKA_03632___3635
0.04

rpoZ.fa.aln.tree.png

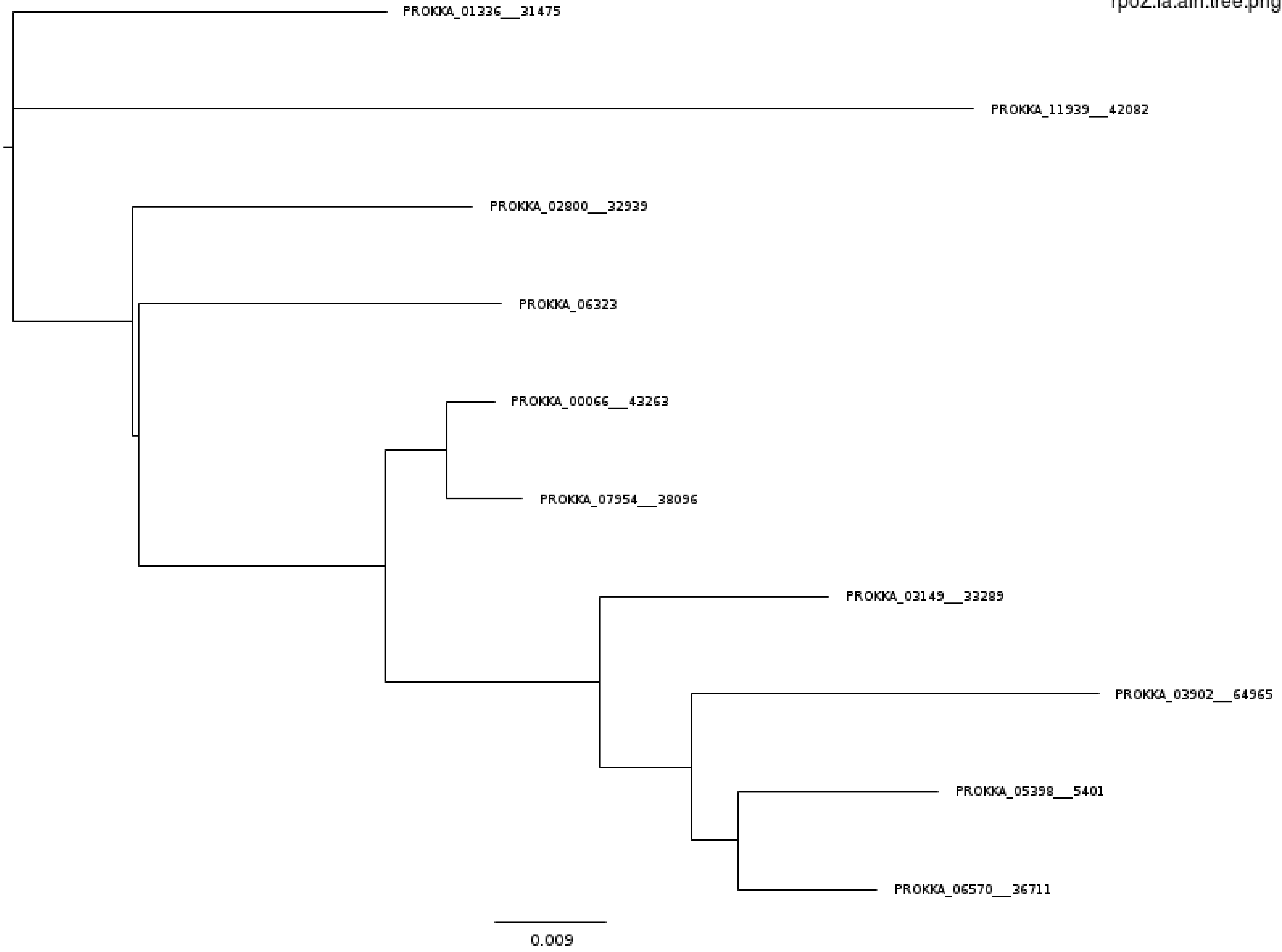

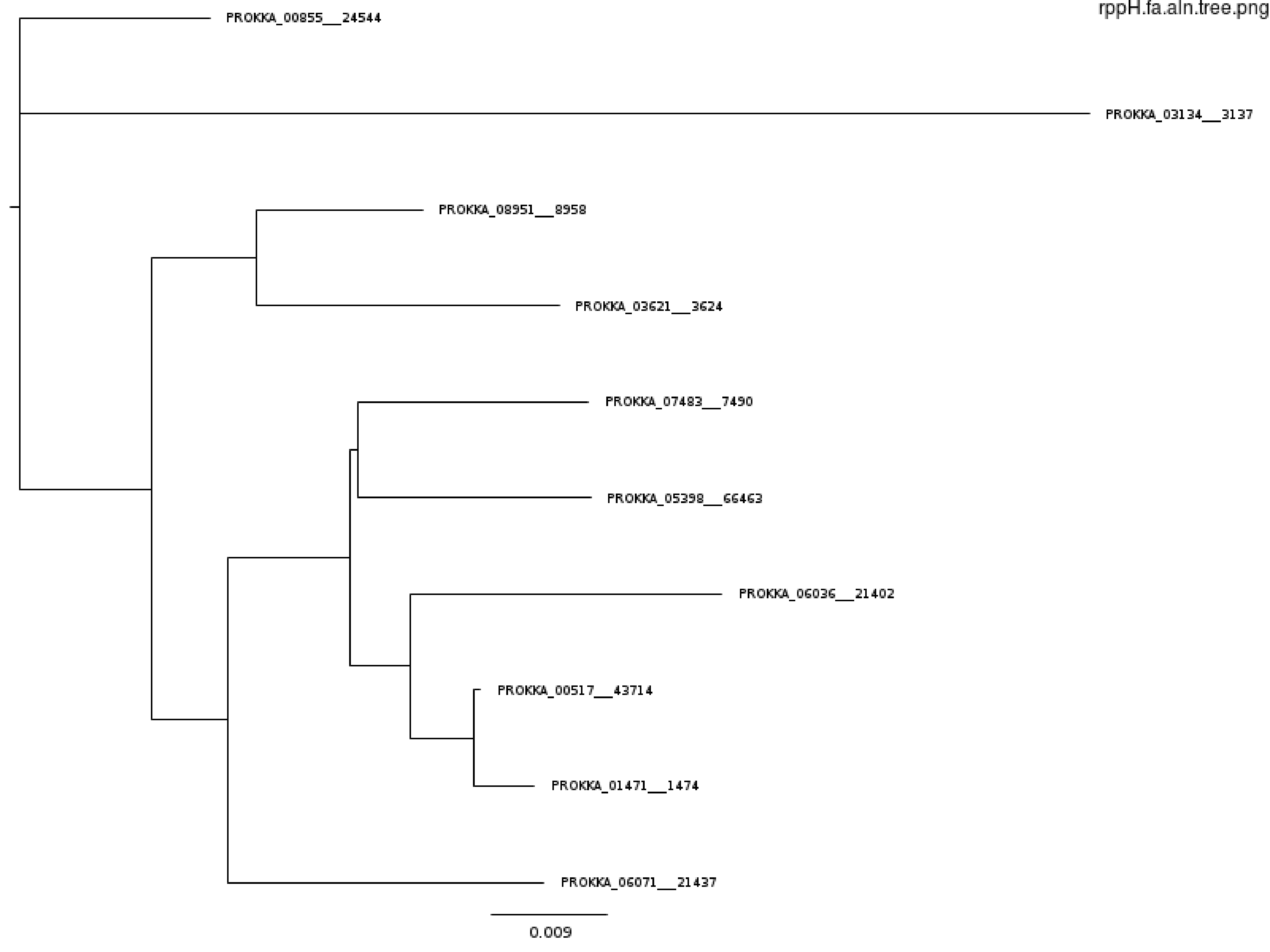
rppH.fa.aln.tree.png
PROKKA_00855___24544
PROKKA_03134___3137
PROKKA_08951___8958
PROKKA_03621___3624
PROKKA_07483___7490
PROKKA_05398___66463
PROKKA_06036___21402
PROKKA_00517___43714
PROKKA_01471___1474
PROKKA_06071___21437
0.009

rpsA_1.fa.aln.tree.png

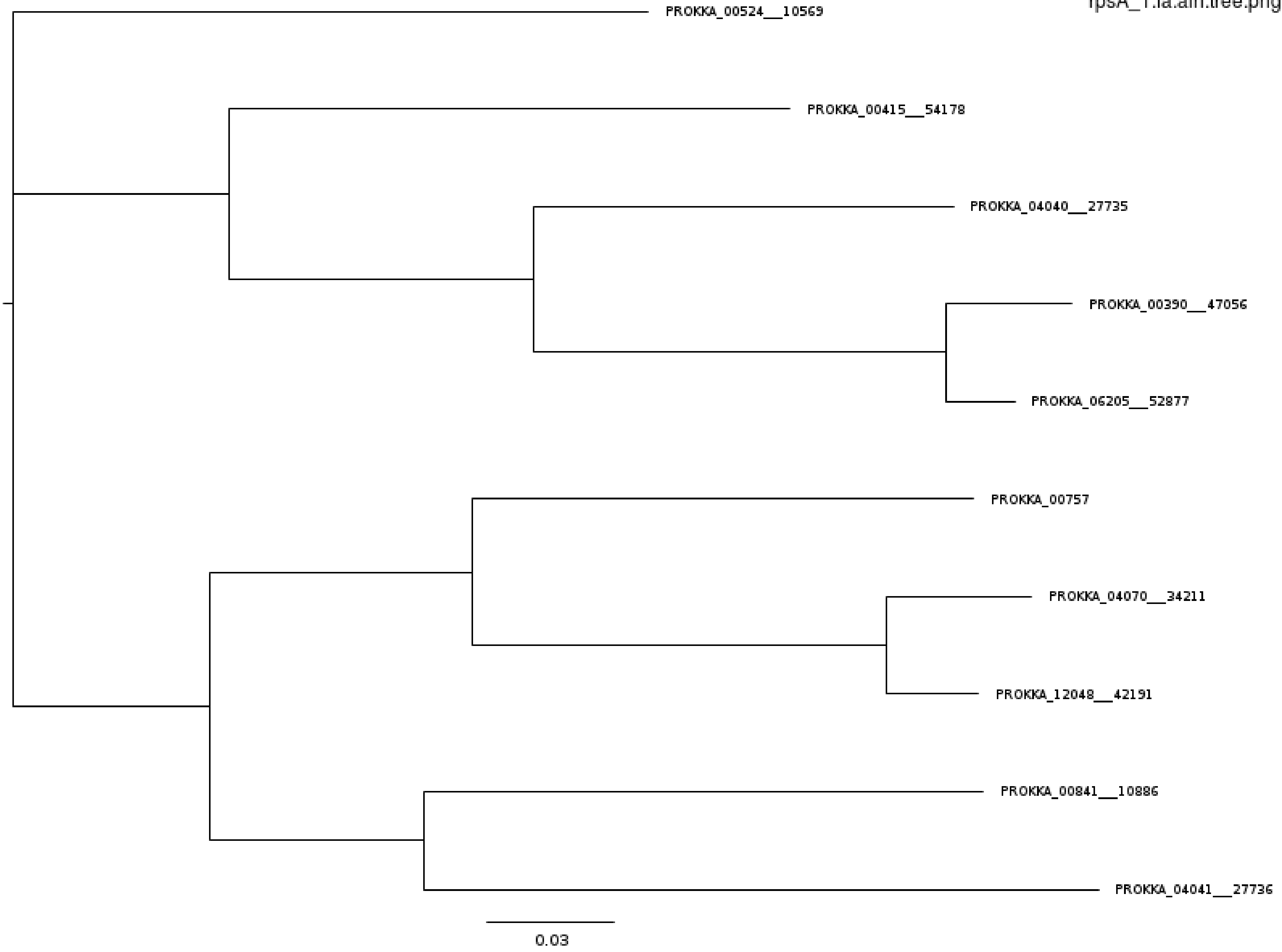

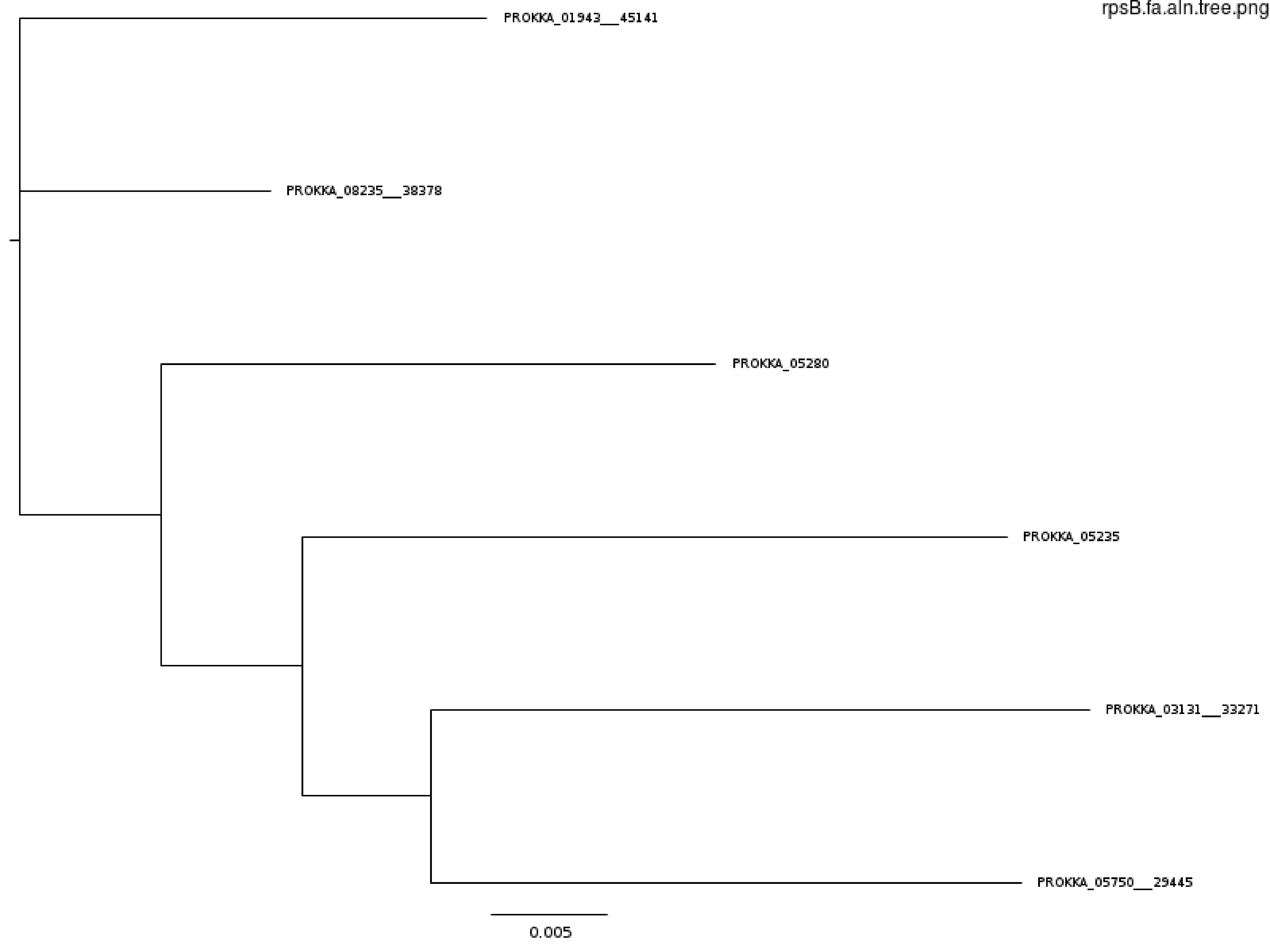
rpsB.fa.aln.tree.png
PROKKA_01943___45141
PROKKA_08235___38378
PROKKA_05280
PROKKA_05235
PROKKA_03131___33271
PROKKA_05750___29445
0.005

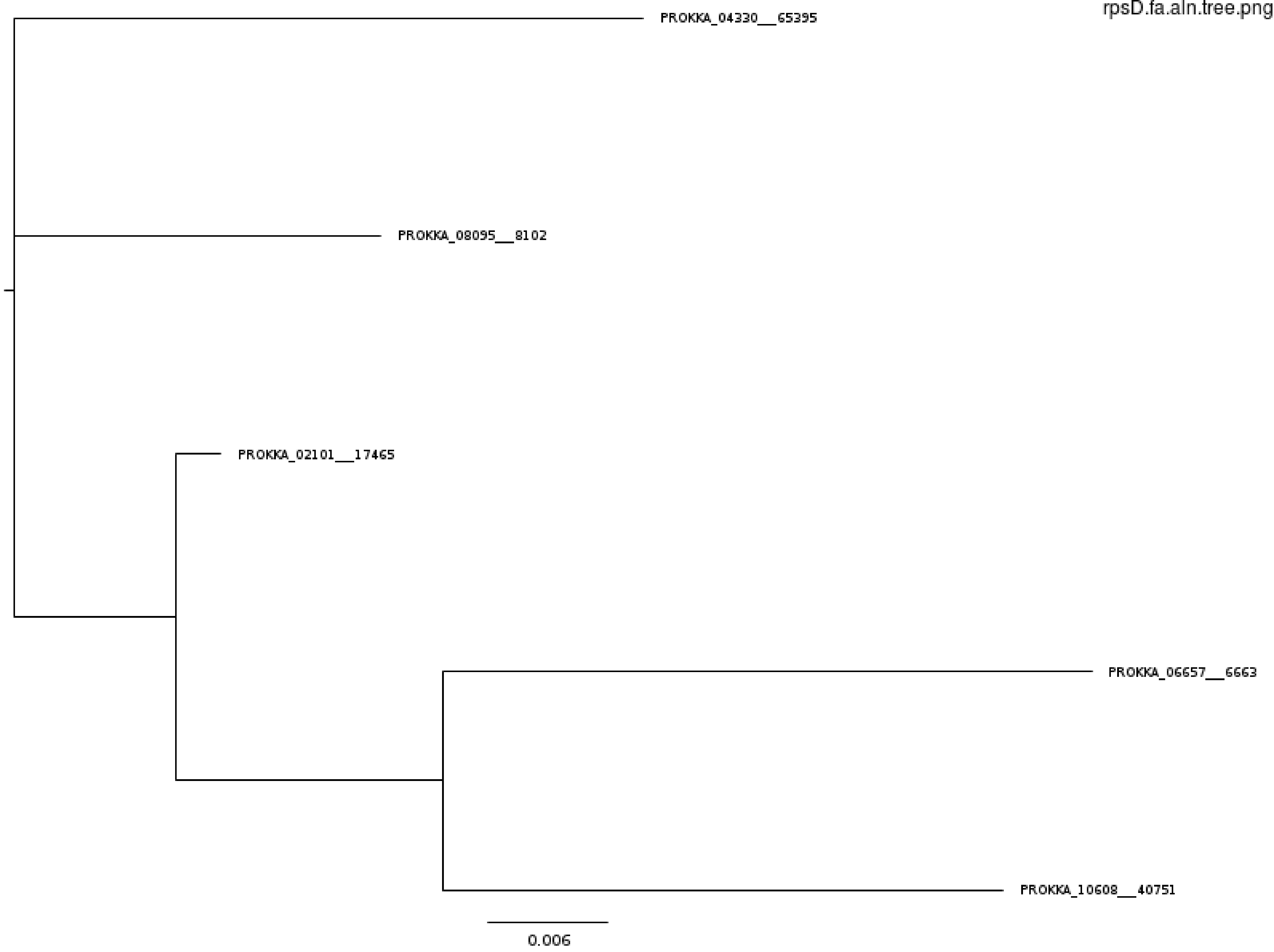
rpsD.fa.aln.tree.png
PROKKA_04330___65395
PROKKA_08095___8102
PROKKA_02101___17465
PROKKA_06657___6663
PROKKA_10608___40751
0.006

rpsL_3.fa.aln.tree.png

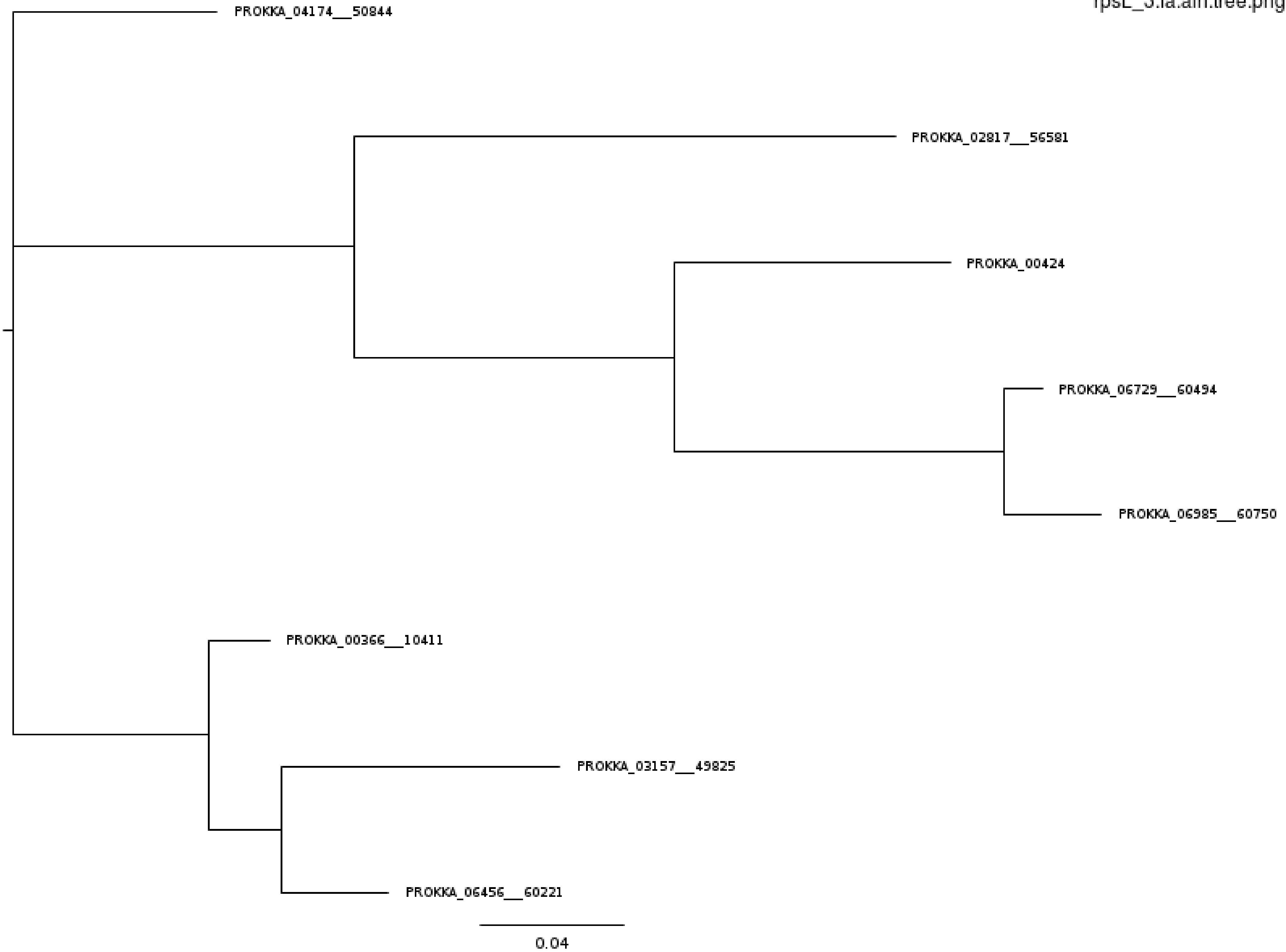

rpsR.fa.aln.tree.png

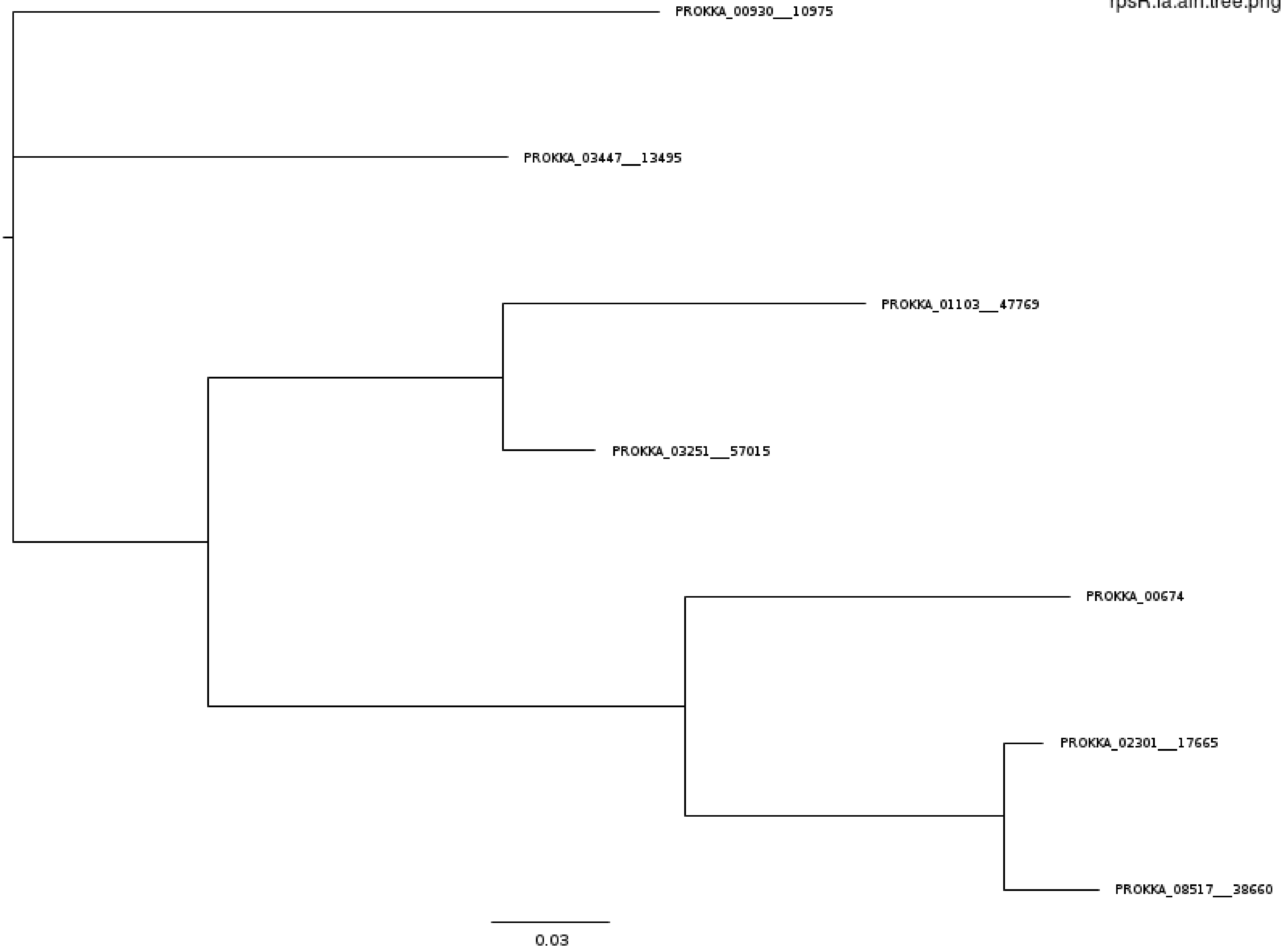

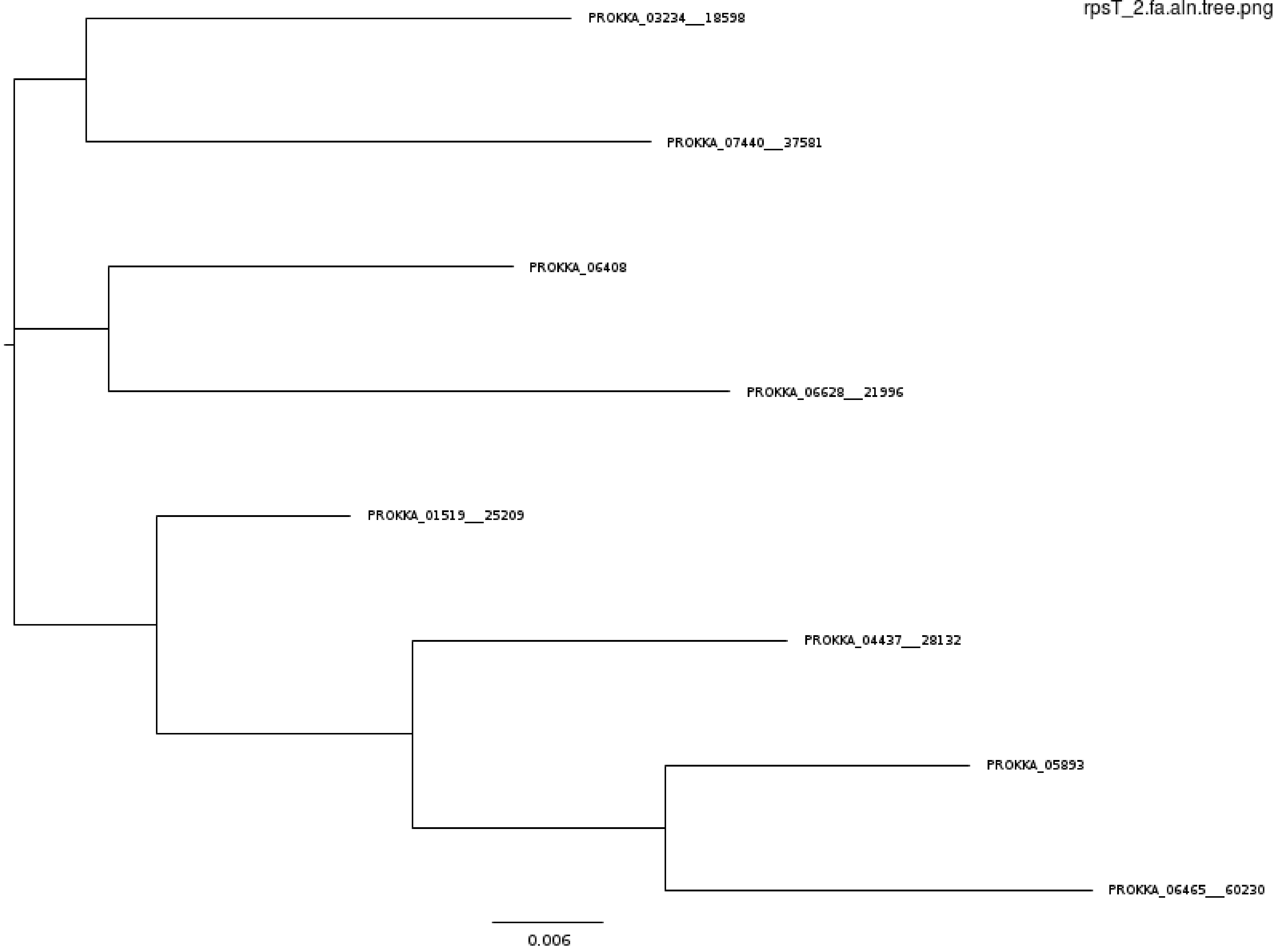
rpsT_2.fa.aln.tree.png
PROKKA_03234___18598
PROKKA_07440___37581
PROKKA_06408
PROKKA_06628___21996
PROKKA_01519___25209
PROKKA_04437___28132
PROKKA_05893
PROKKA_06465___60230
0.006

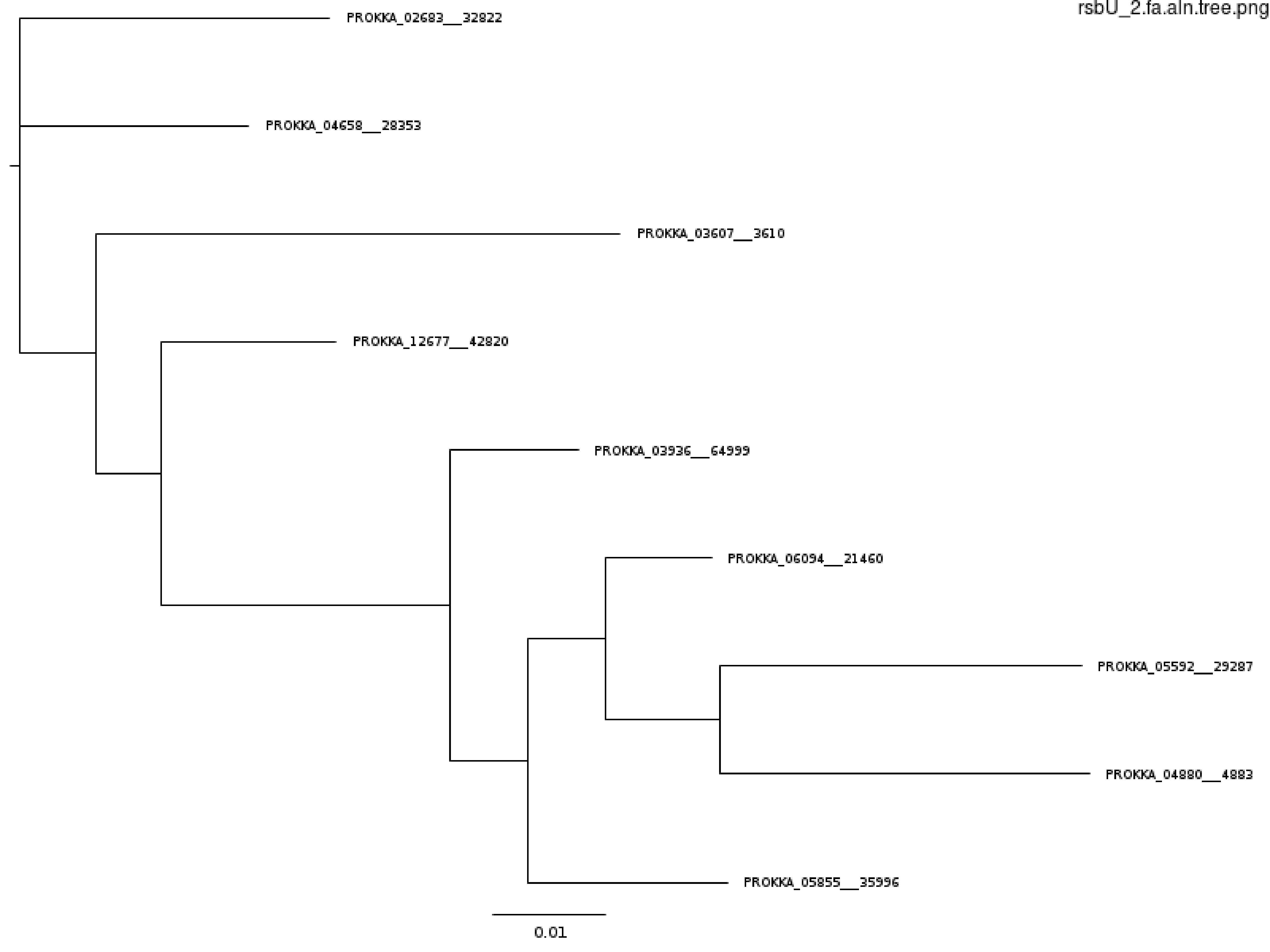
rsbU_2.fa.aln.tree.png
PROKKA_02683___32822
PROKKA_04658___28353
PROKKA_03607___3610
PROKKA_12677___42820
PROKKA_03936___64999
PROKKA_06094___21460
PROKKA_05592___29287
PROKKA_04880___4883
PROKKA_05855___35996
0.01

rsbU_4.fa.aln.tree.png

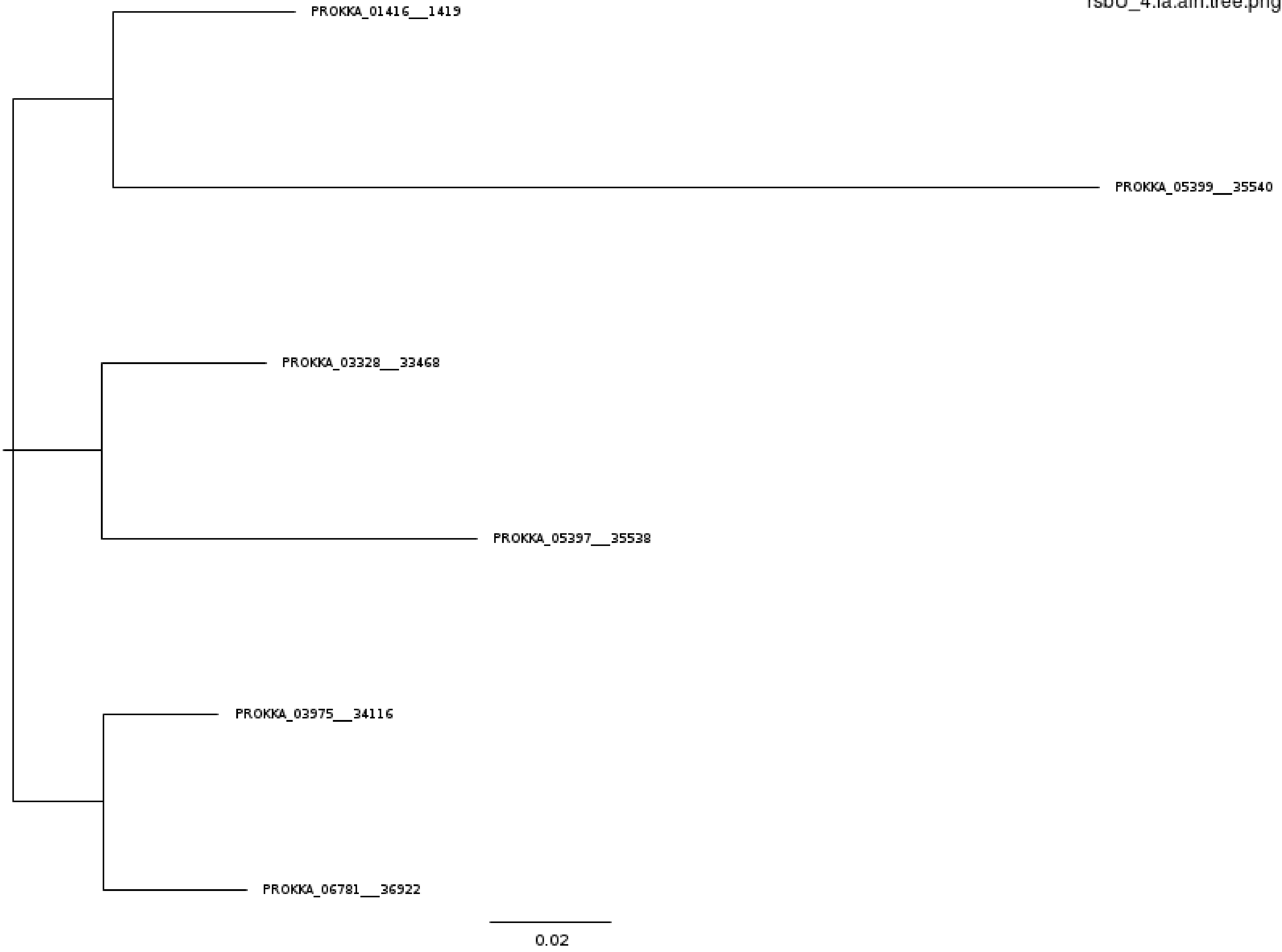

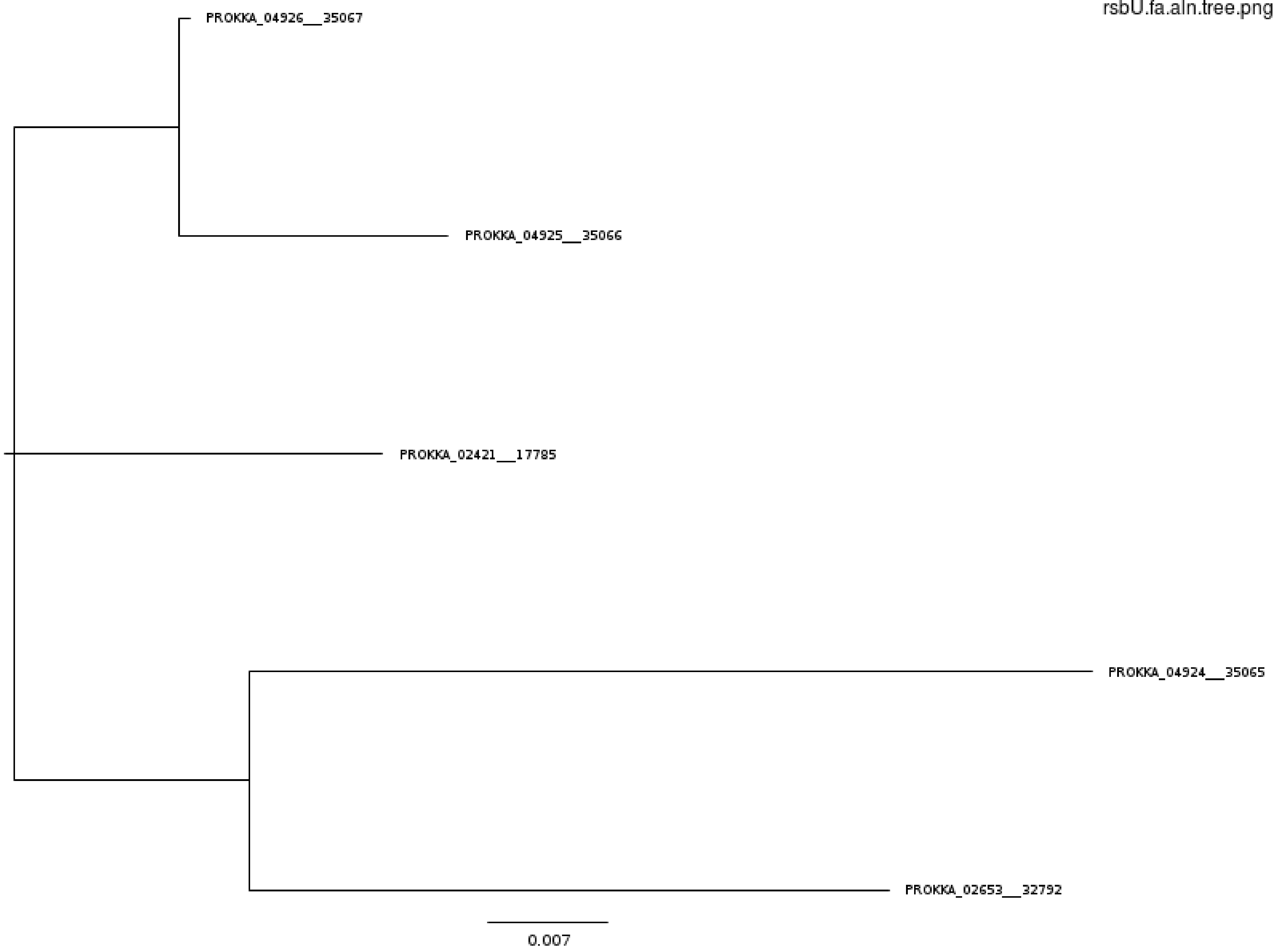
rsbU.fa.aln.tree.png
PROKKA_04926__35067
PROKKA_04925__35066
PROKKA_02421__17785
PROKKA_04924__35065
PROKKA_02653__32792
0.007

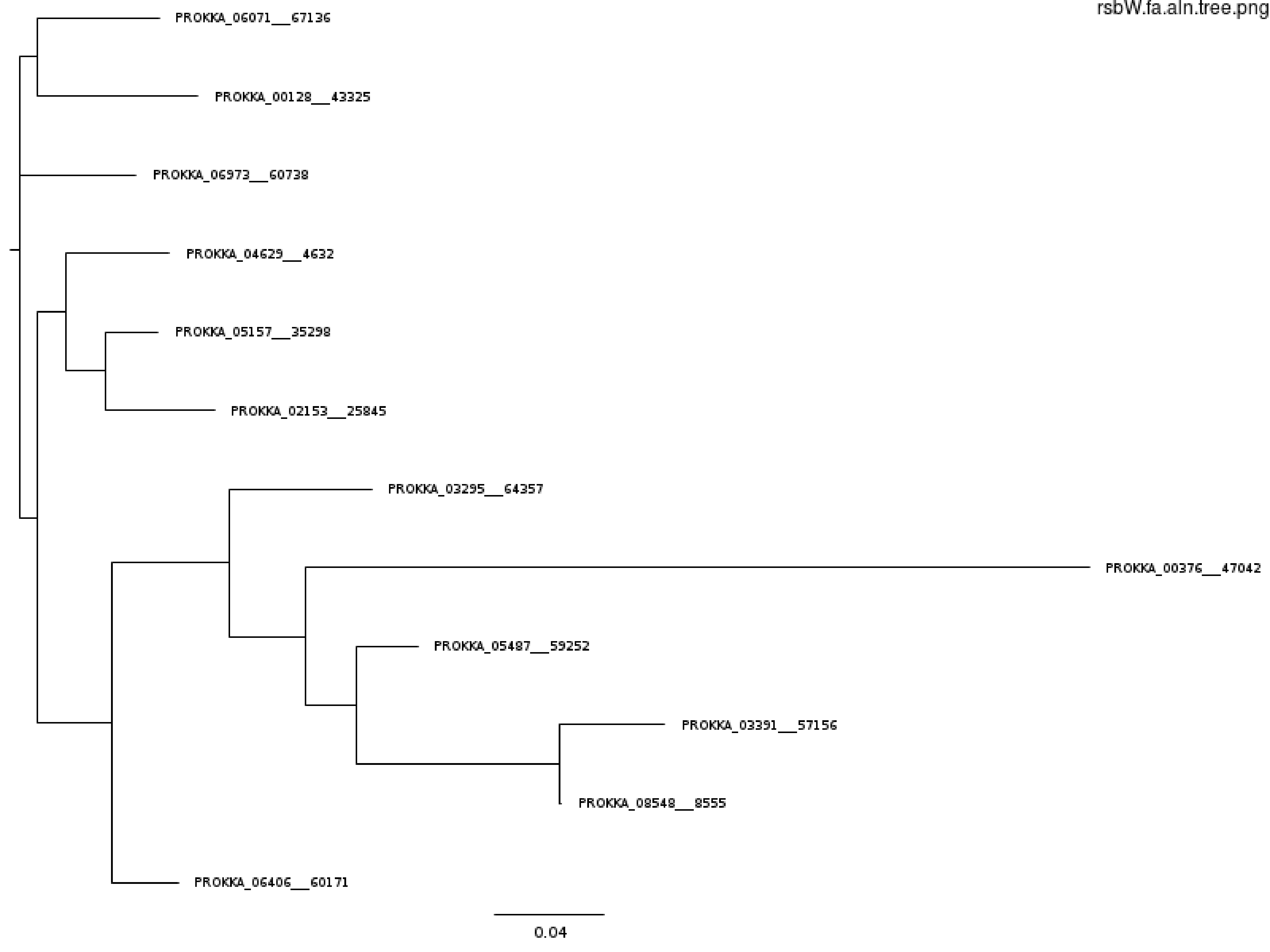
rsbW.fa.aln.tree.png
PROKKA_06071___67136
PROKKA_00128___43325
PROKKA_06973___60738
PROKKA_04629___4632
PROKKA_05157___35298
PROKKA_02153___25845
PROKKA_03295___64357
PROKKA_00376___47042
PROKKA_05487___59252
PROKKA_03391___57156
PROKKA_08548___8555
PROKKA_06406___60171
0.04

rsfS_2.fa.aln.tree.png

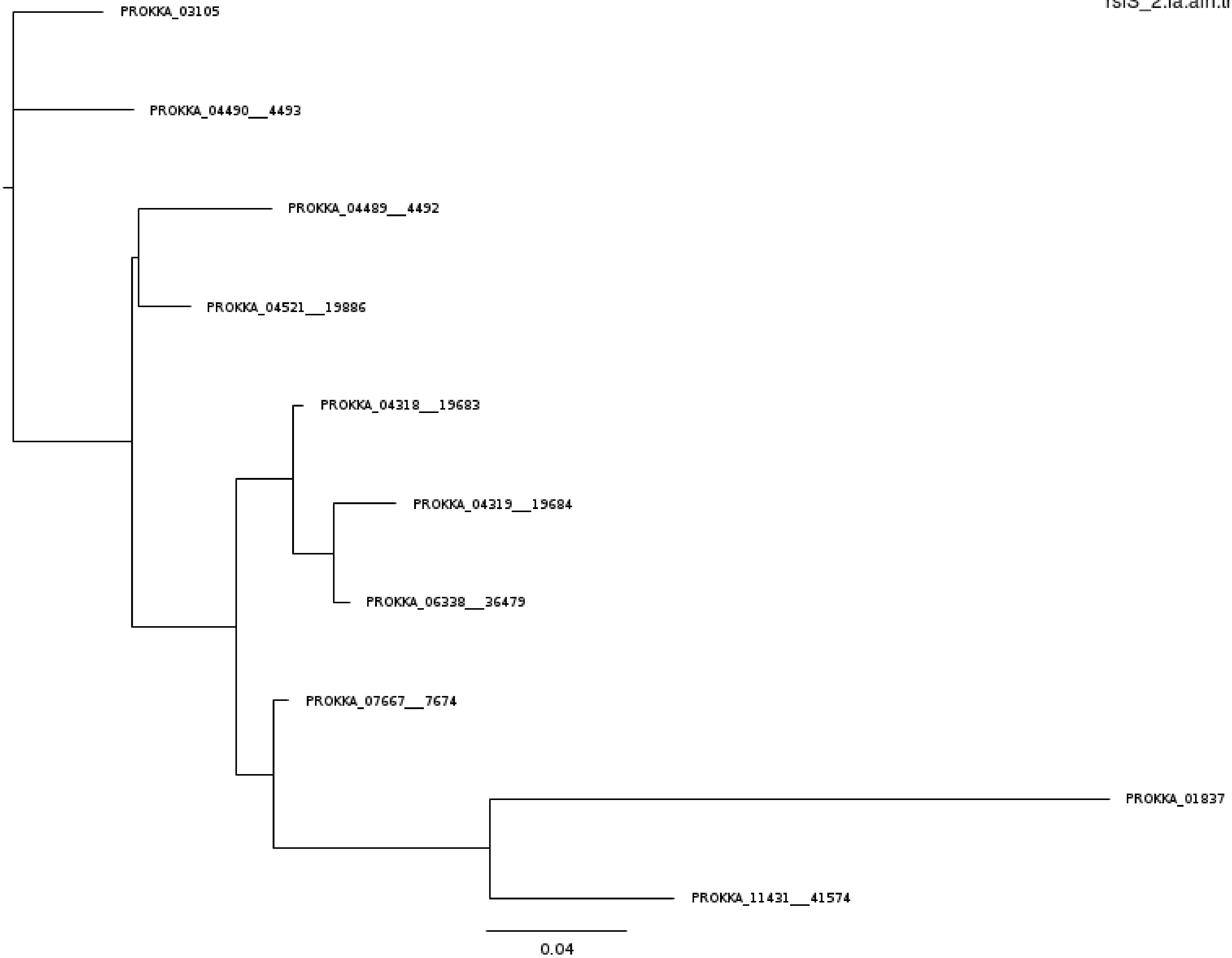

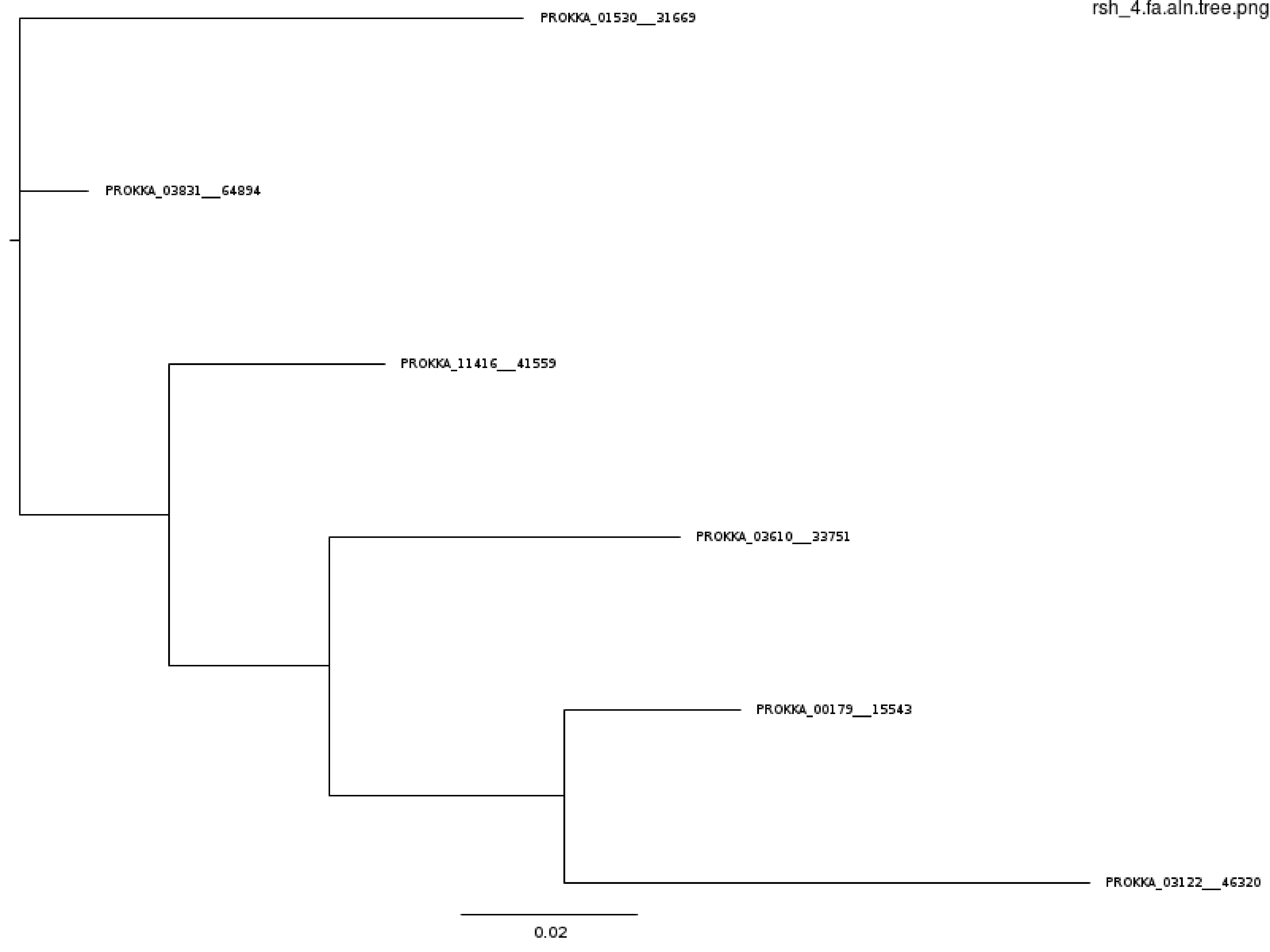
rsh_4.fa.aln.tree.png
PROKKA_01530___31669
PROKKA_03831___64894
PROKKA_11416___41559
PROKKA_03610___33751
PROKKA_00179___15543
PROKKA_03122___46320
0.02

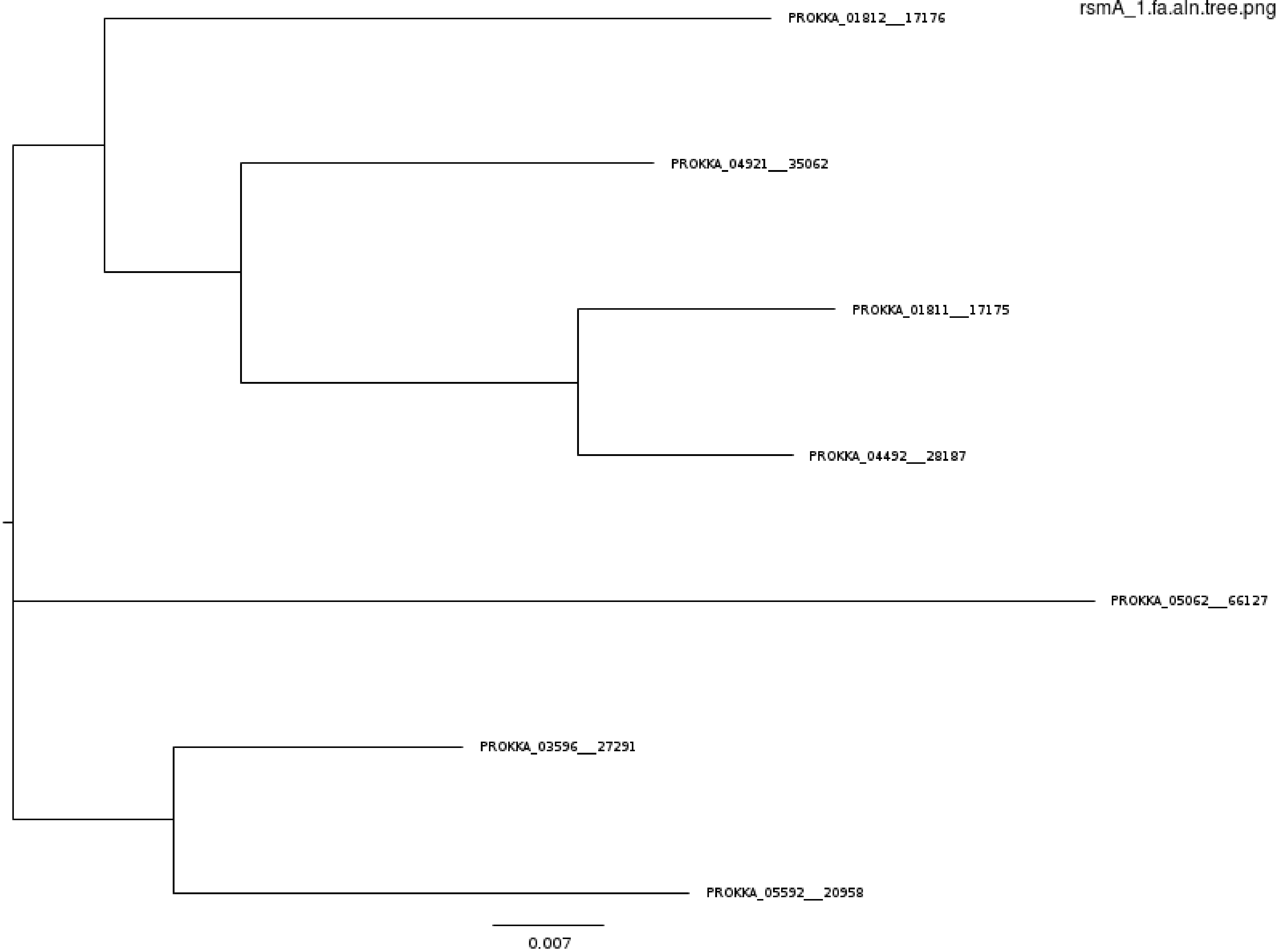
rsmA_1.fa.aln.tree.png
PROKKA_01812___17176
PROKKA_04921___35062
PROKKA_01811___17175
PROKKA_04492___28187
PROKKA_05062___66127
PROKKA_03596___27291
PROKKA_05592___20958
0.007

rsmD_2.fa.aln.tree.png

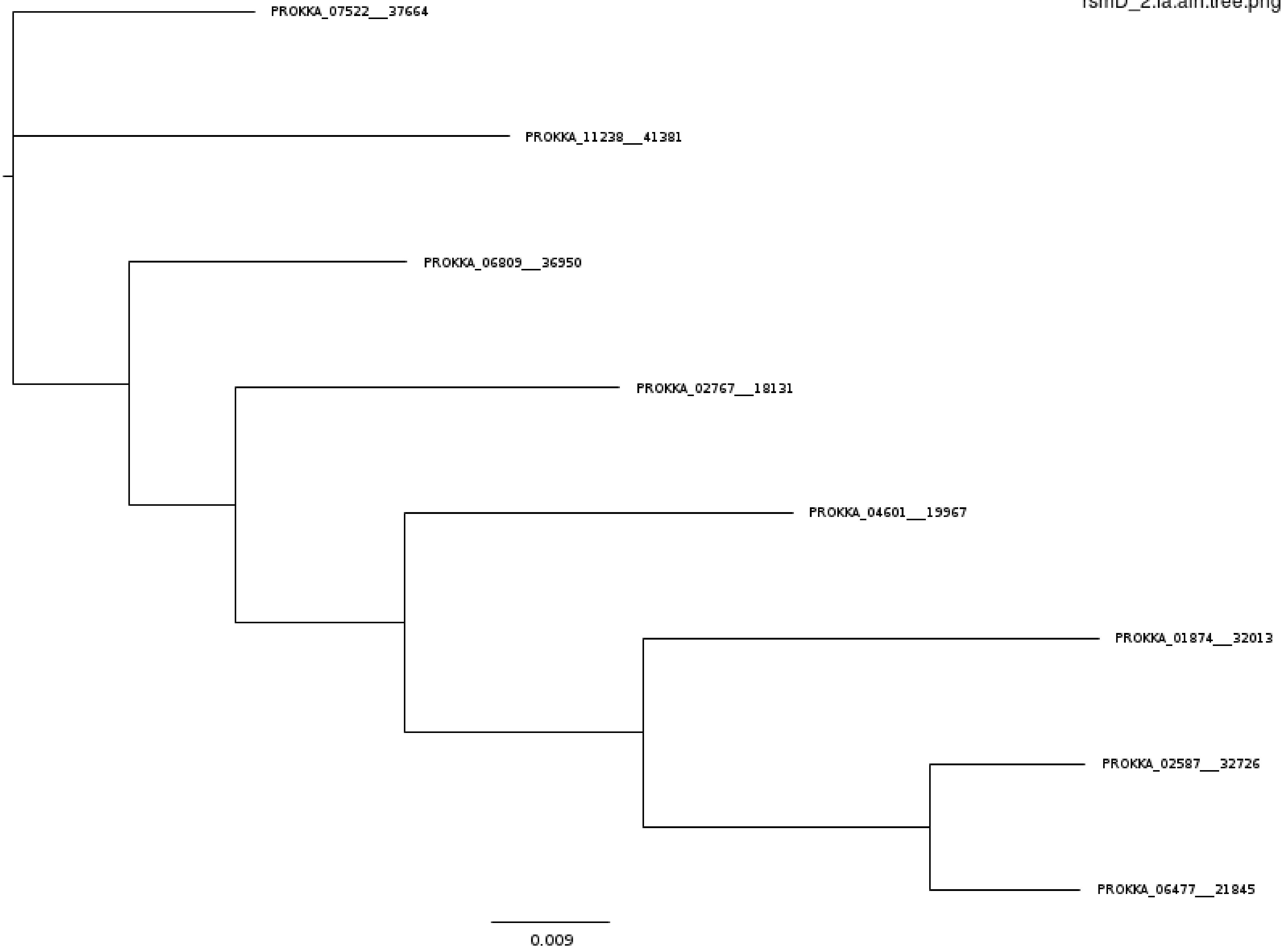

rsmE_3.fa.aln.tree.png

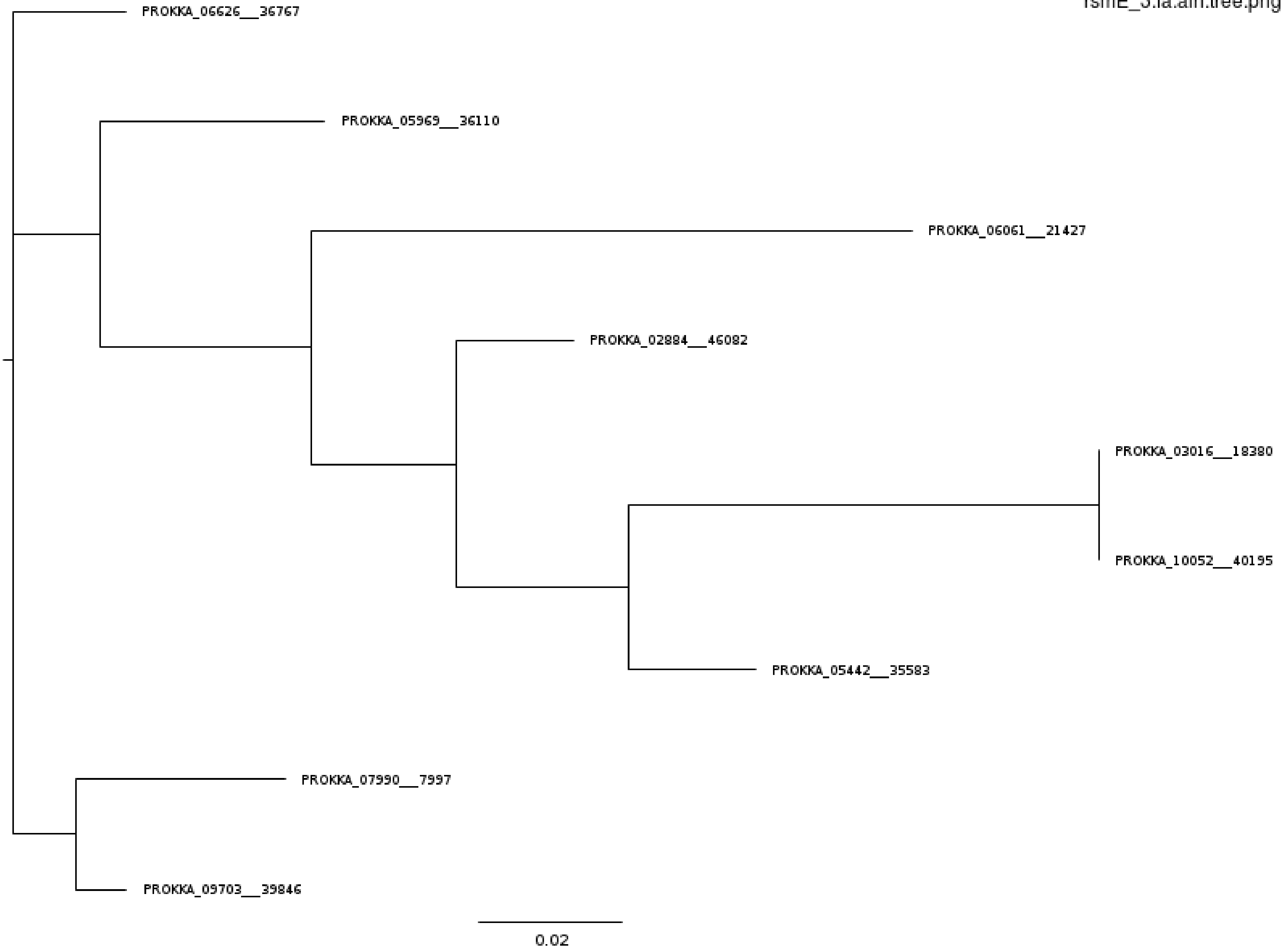

rsmH.fa.aln.tree.png

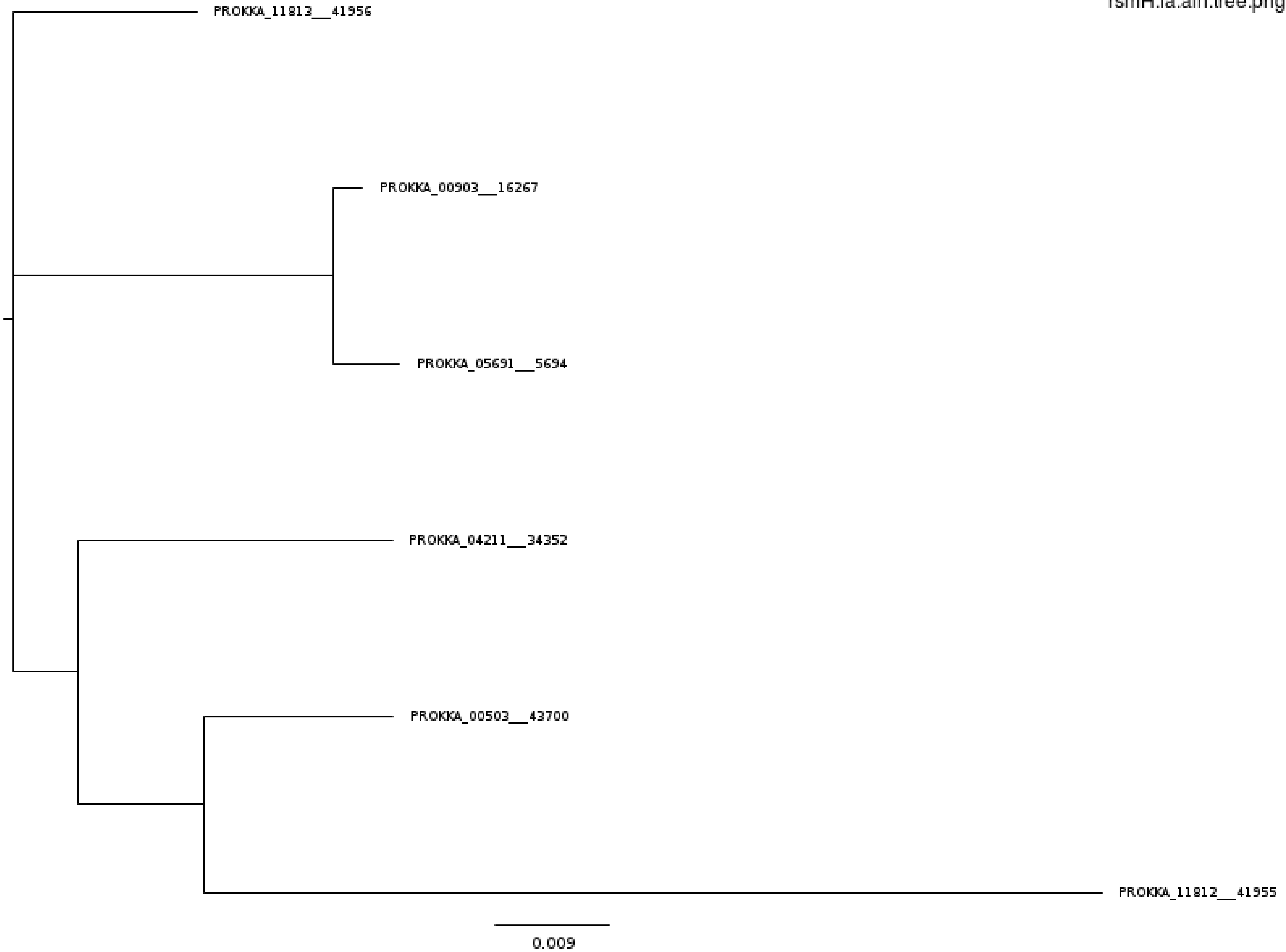

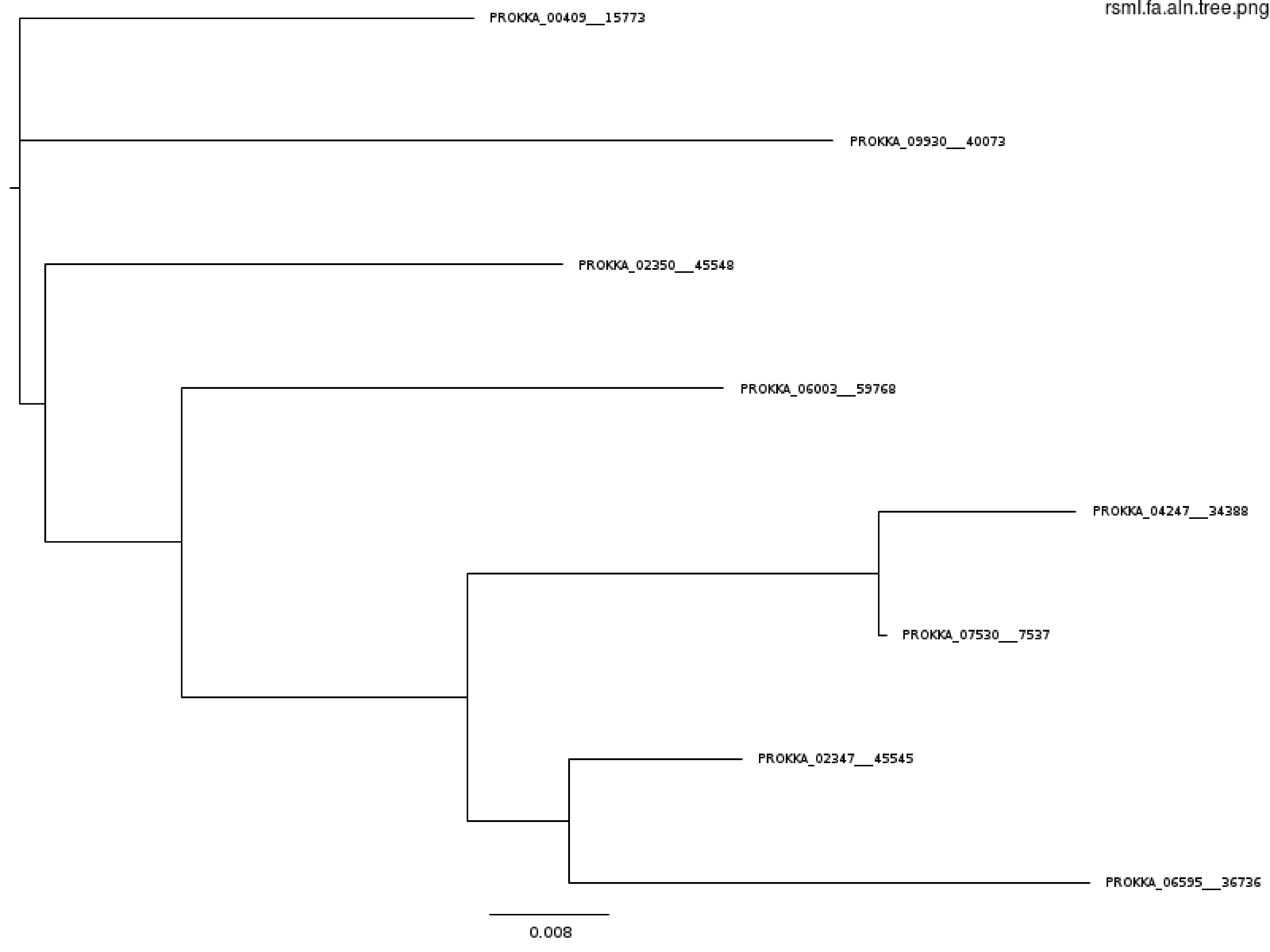

rsml.fa.aln.tree.png
PROKKA_00409___15773
PROKKA_09930___40073
PROKKA_02350___45548
PROKKA_06003___59768
PROKKA_04247___34388
PROKKA_07530___7537
PROKKA_02347___45545
PROKKA_06595___36736
0.008

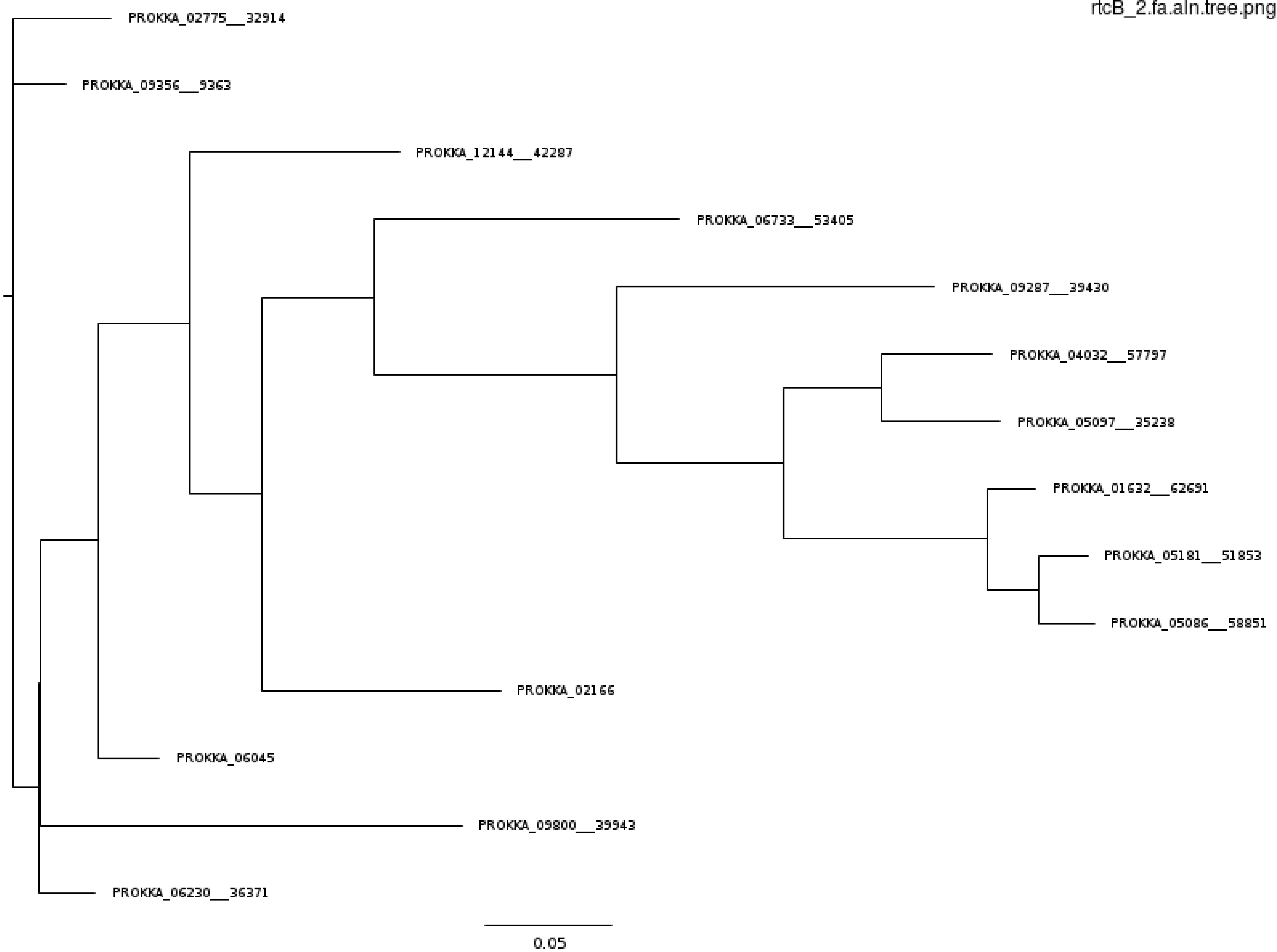

rtcB_2.fa.aln.tree.png
PROKKA_02775___32914
PROKKA_09356___9363
PROKKA_12144___42287
PROKKA_06733___53405
PROKKA_09287___39430
PROKKA_04032___57797
PROKKA_05097___35238
PROKKA_01632___62691
PROKKA_05181___51853
PROKKA_05086___58851
PROKKA_02166
PROKKA_06045
PROKKA_09800___39943
PROKKA_06230___36371
0.05

sat_cysC.fa.aln.tree.png

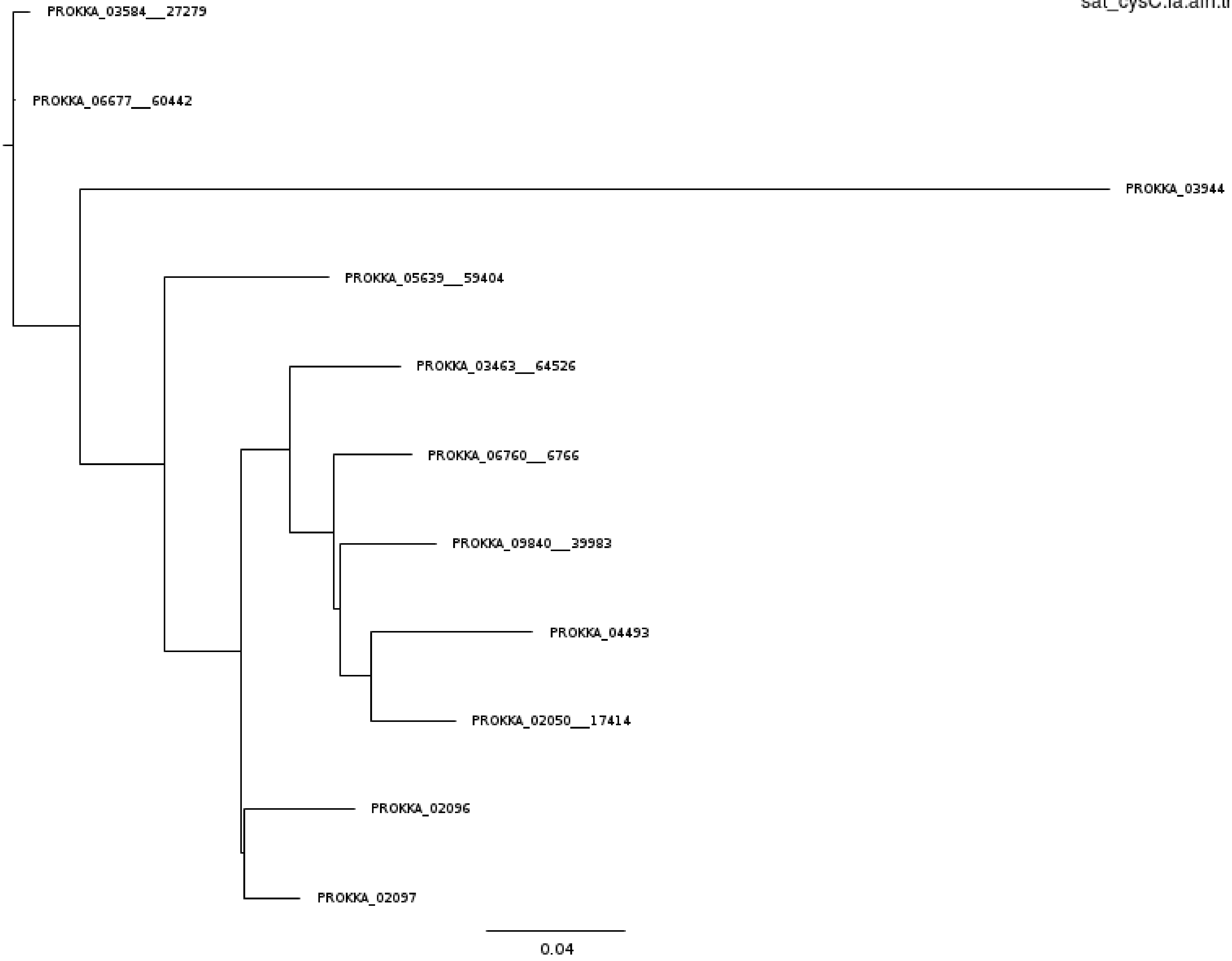

sbcB_3.fa.aln.tree.png

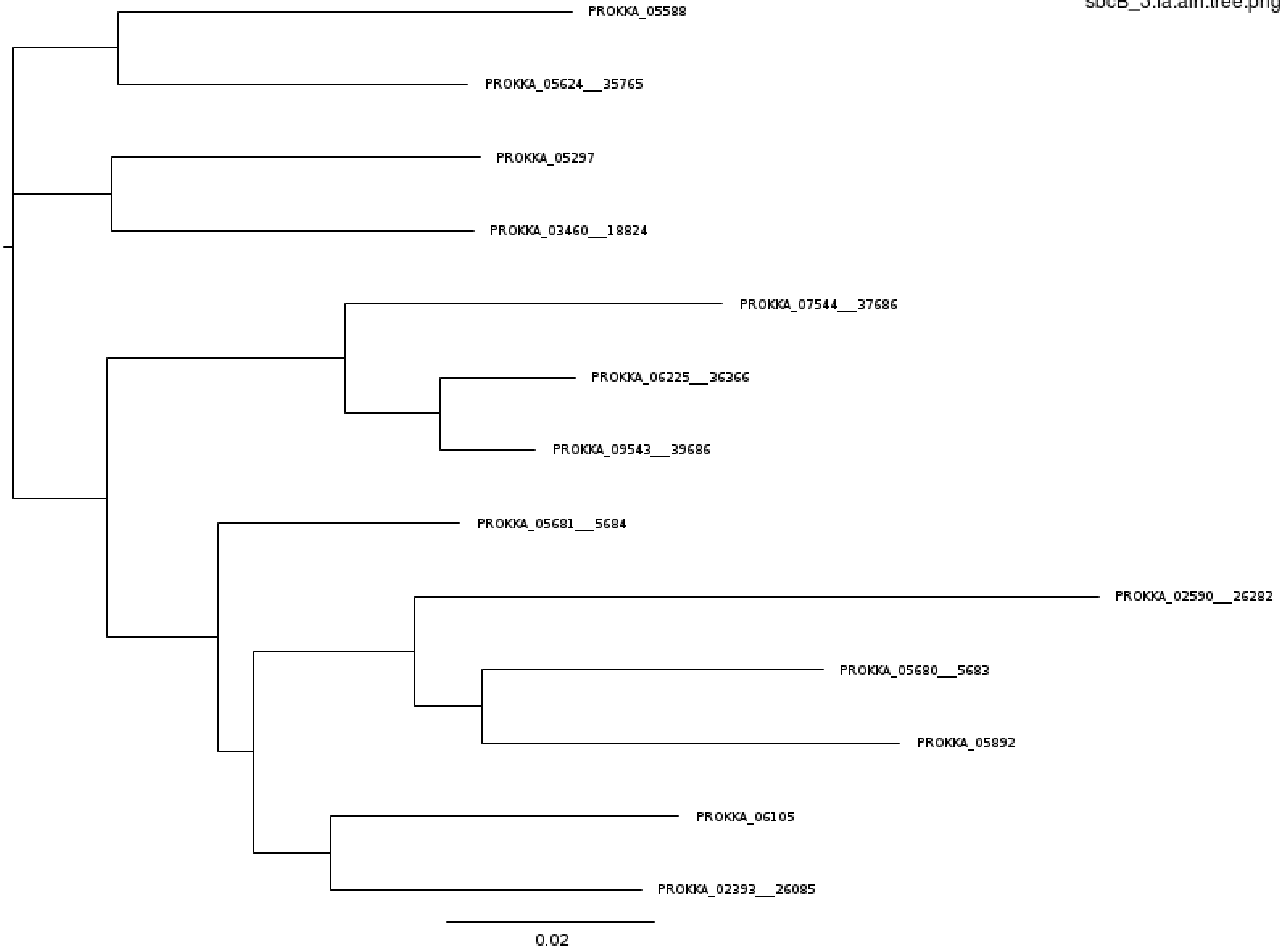

scpA.fa.aln.tree.png

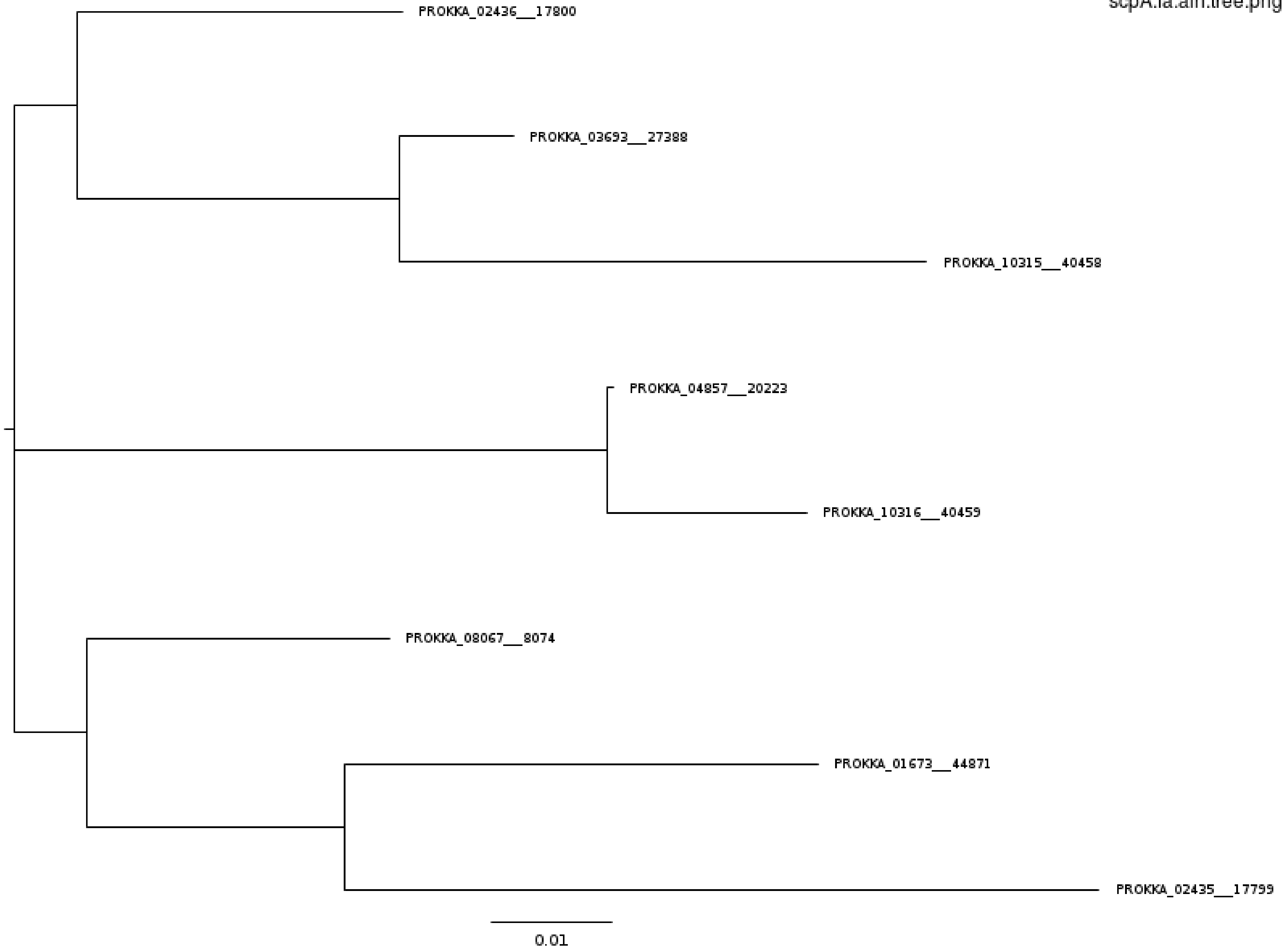

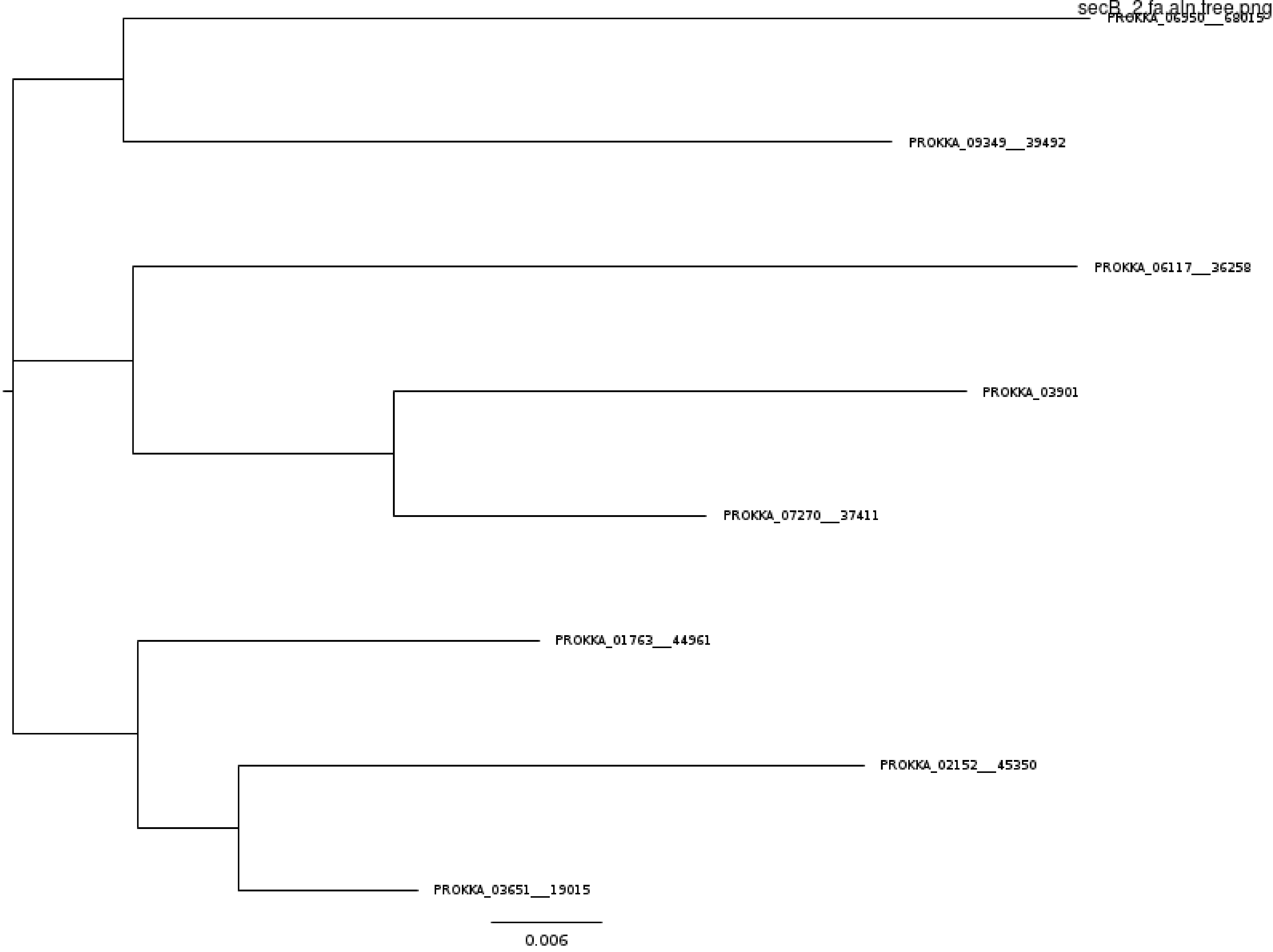
secB_2.fa.aln.tree.png
PROKKA_06950___68015
PROKKA_09349___39492
PROKKA_06117___36258
PROKKA_03901
PROKKA_07270___37411
PROKKA_01763___44961
PROKKA_02152___45350
PROKKA_03651___19015
0.006

secG.fa.aln.tree.png

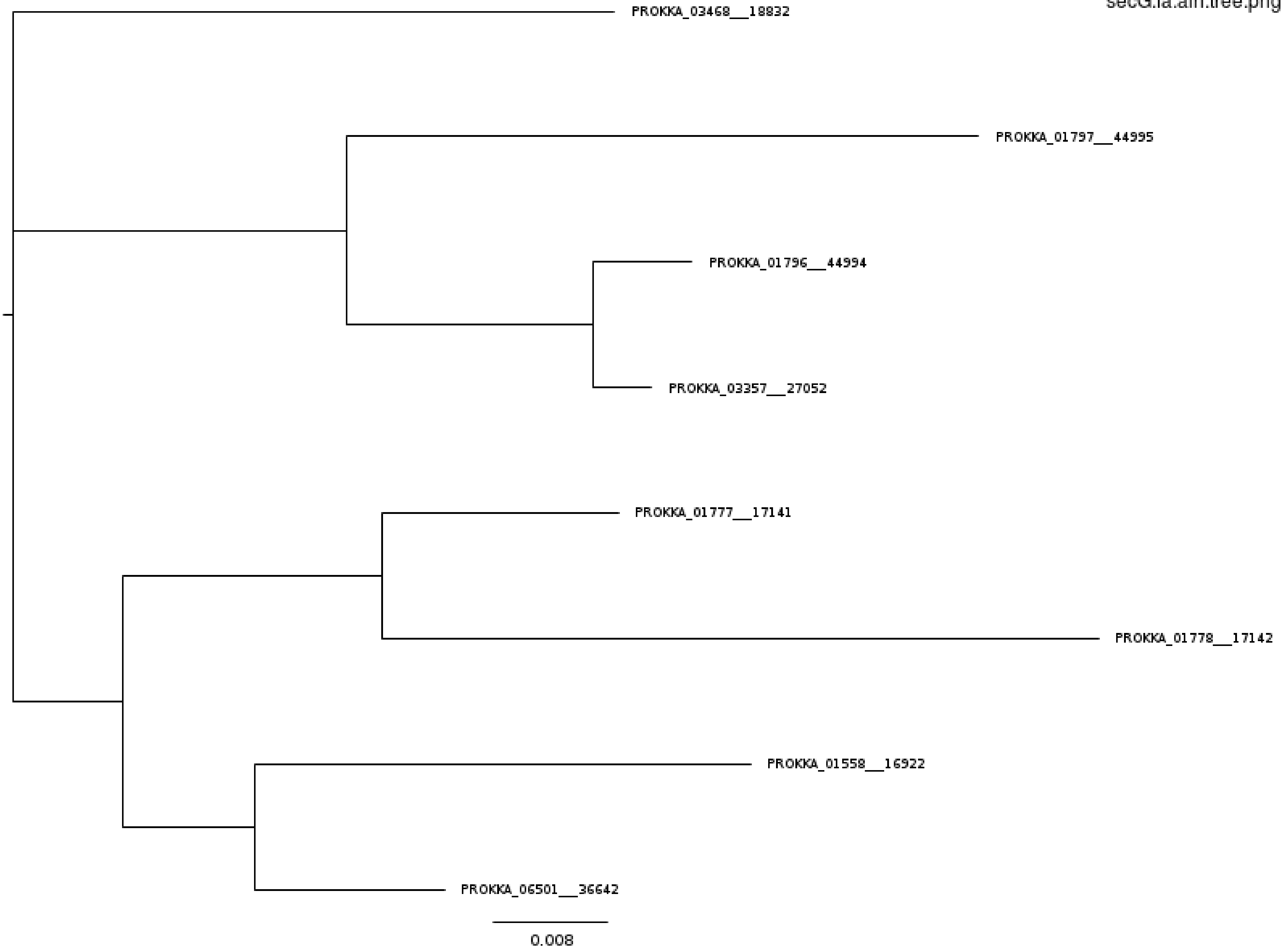

serS_1.fa.aln.tree.png

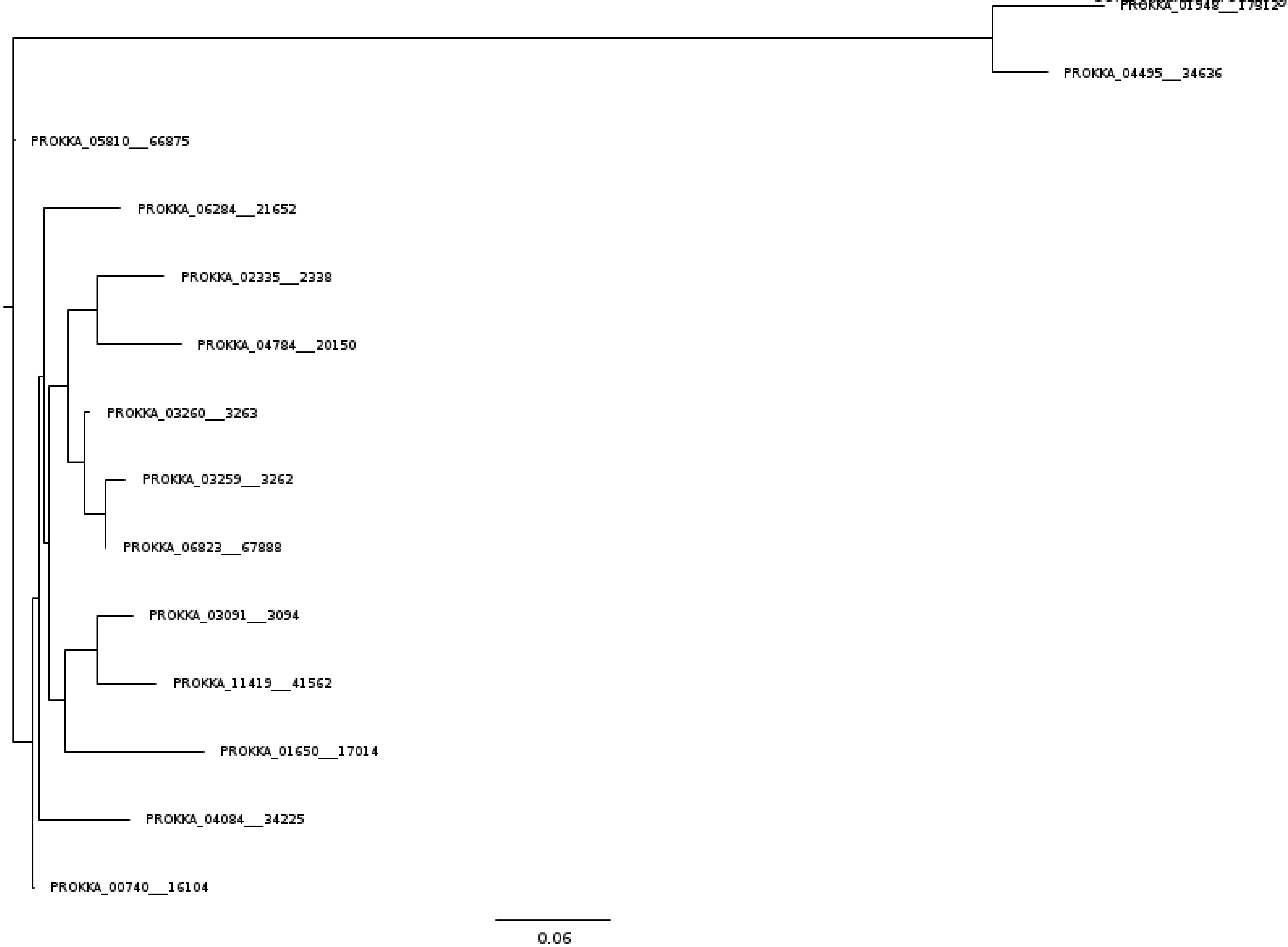

sftA.fa.aln.tree.png

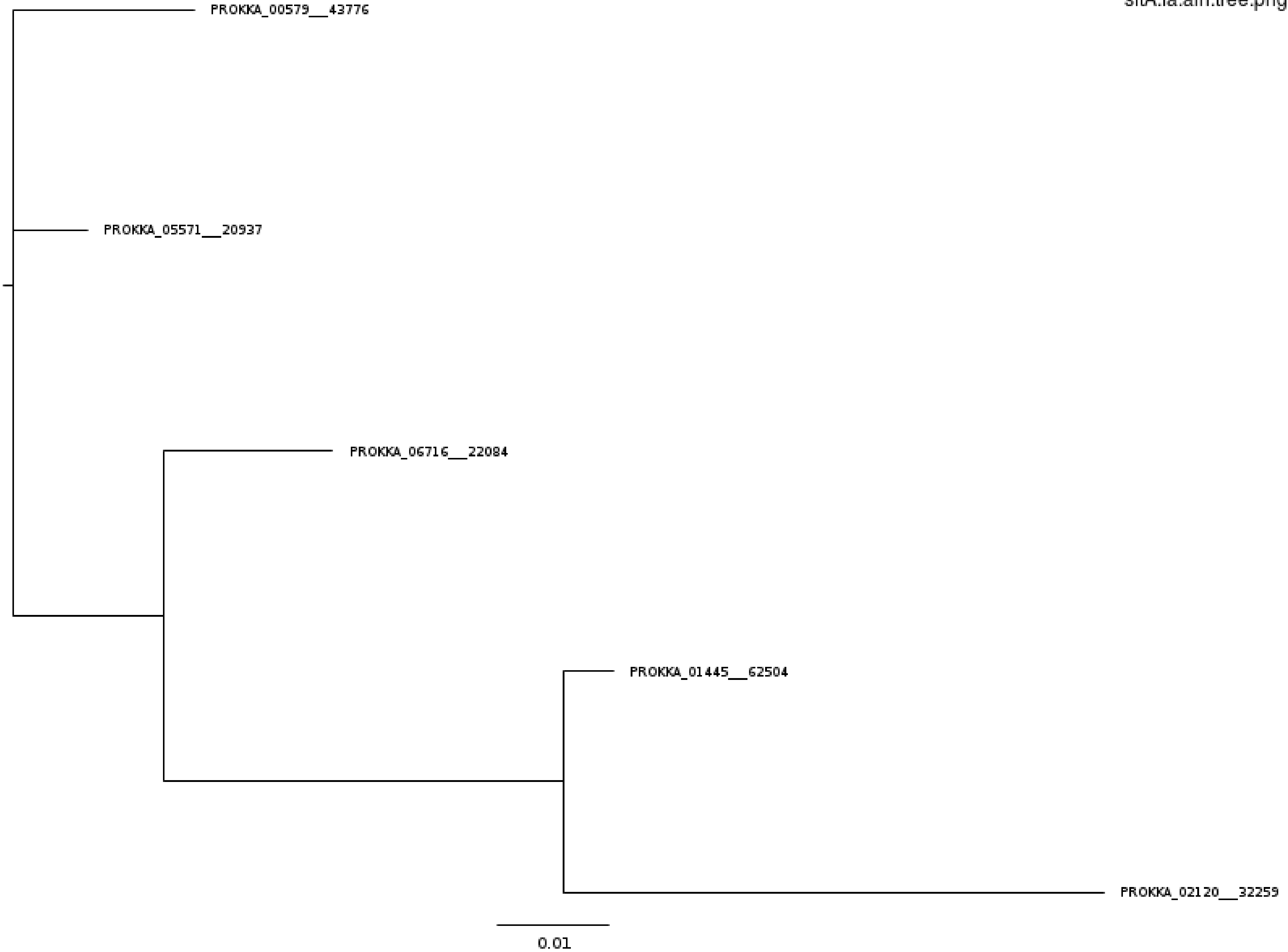

shp_2.fa.aln.tree.png

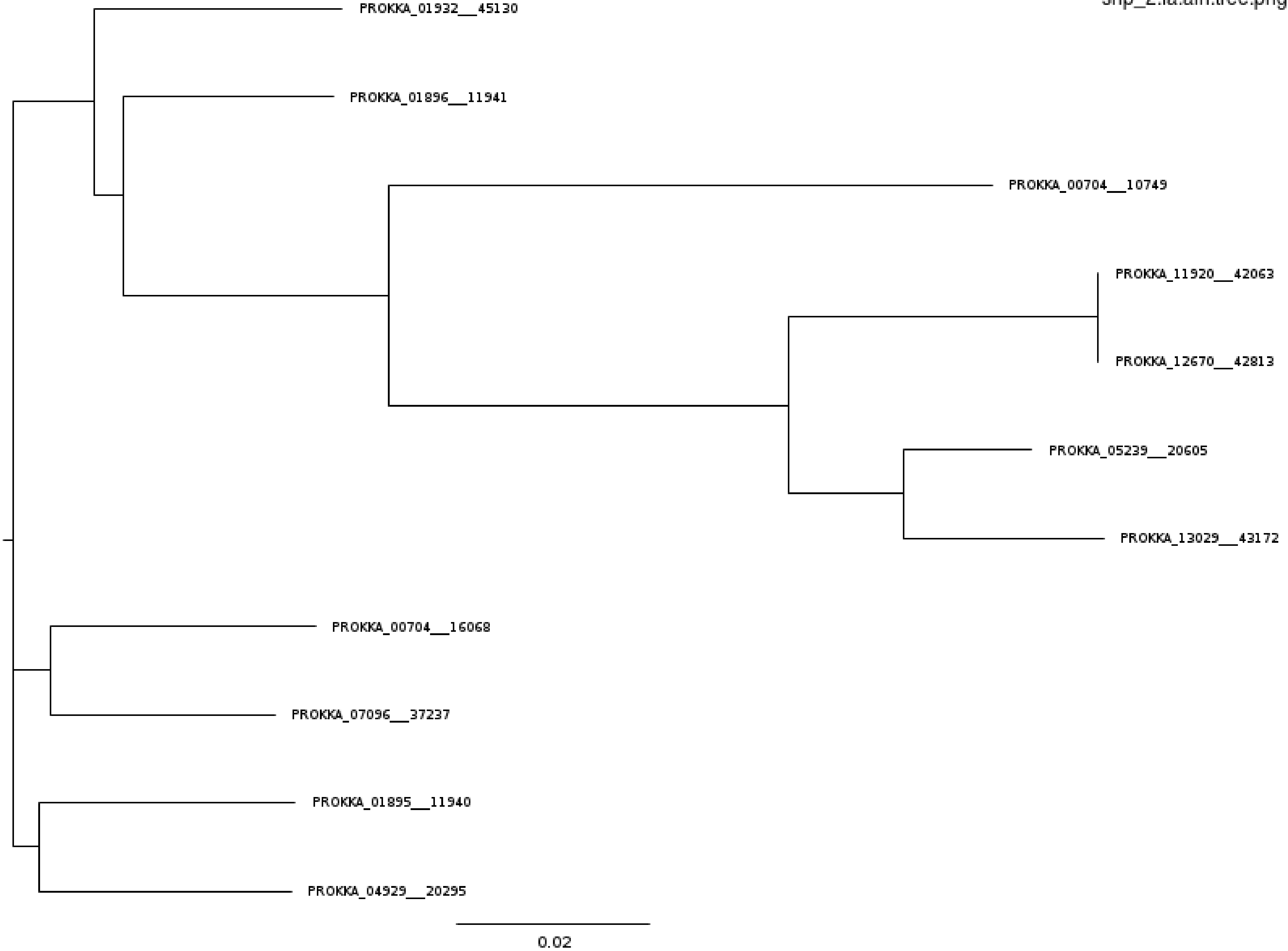

skp.fa.aln.tree.png

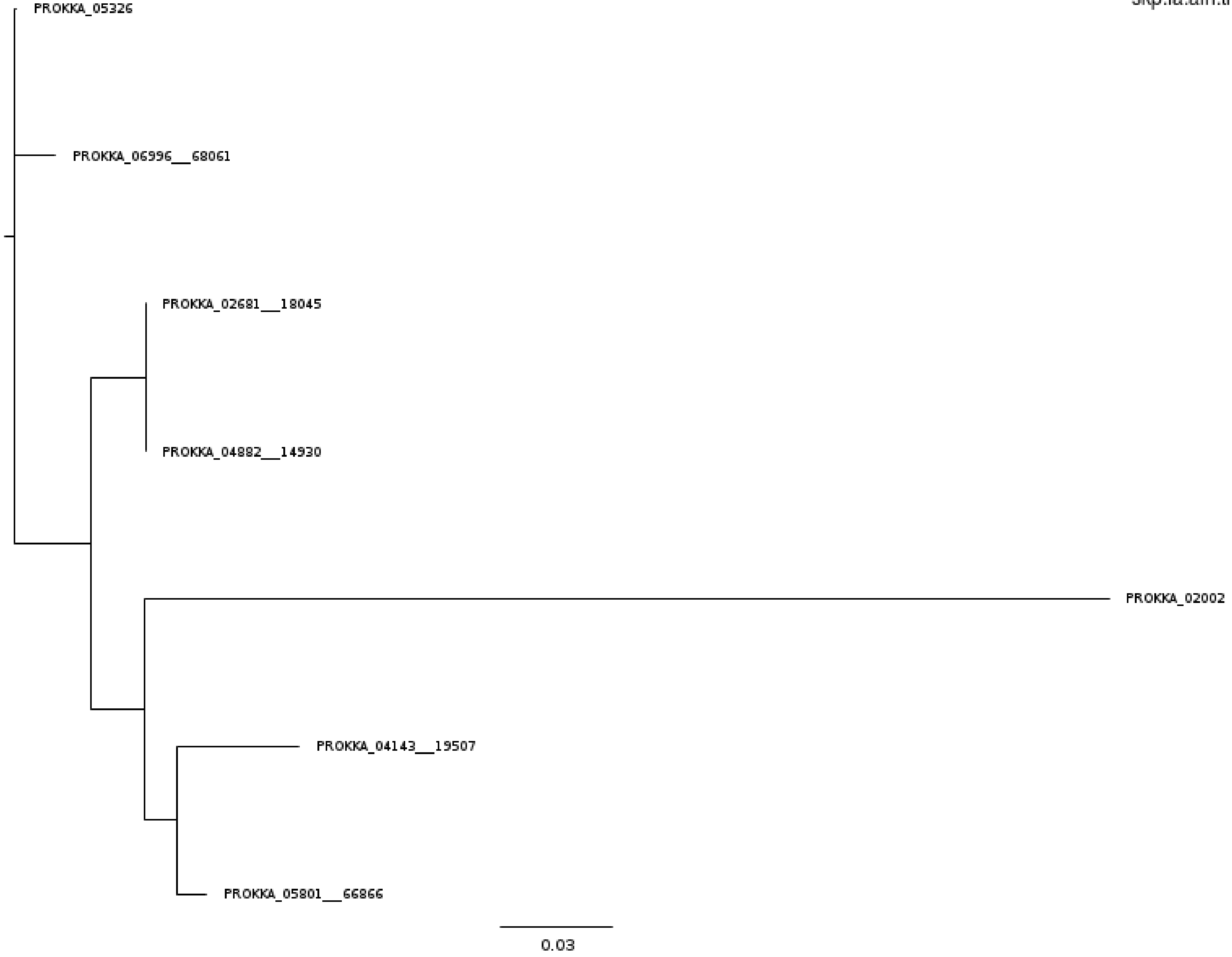

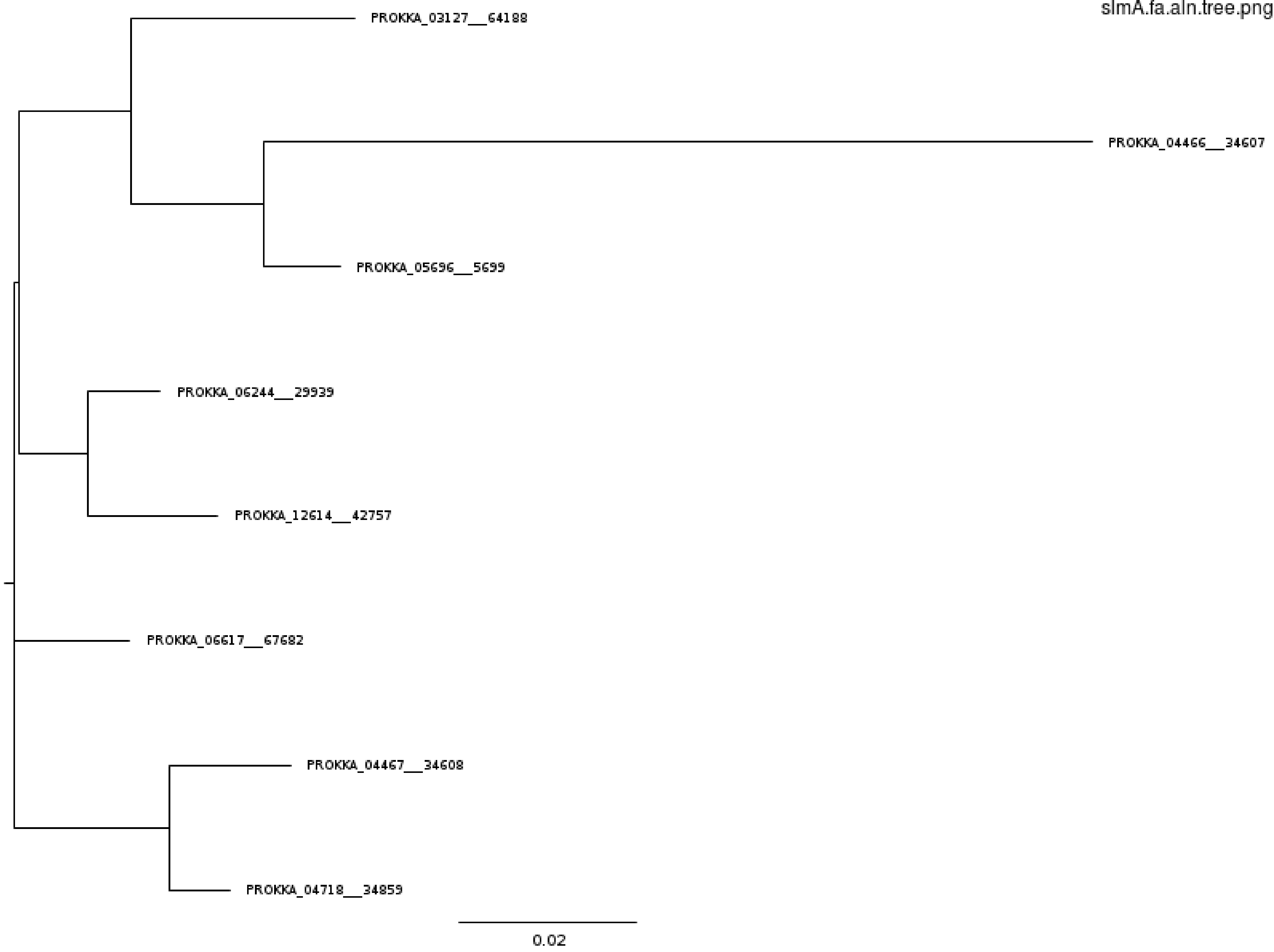

slmA.fa.aln.tree.png
PROKKA_03127___64188
PROKKA_04466___34607
PROKKA_05696___5699
PROKKA_06244___29939
PROKKA_12614___42757
PROKKA_06617___67682
PROKKA_04467___34608
PROKKA_04718___34859
0.02

slt_1.fa.aln.tree.png

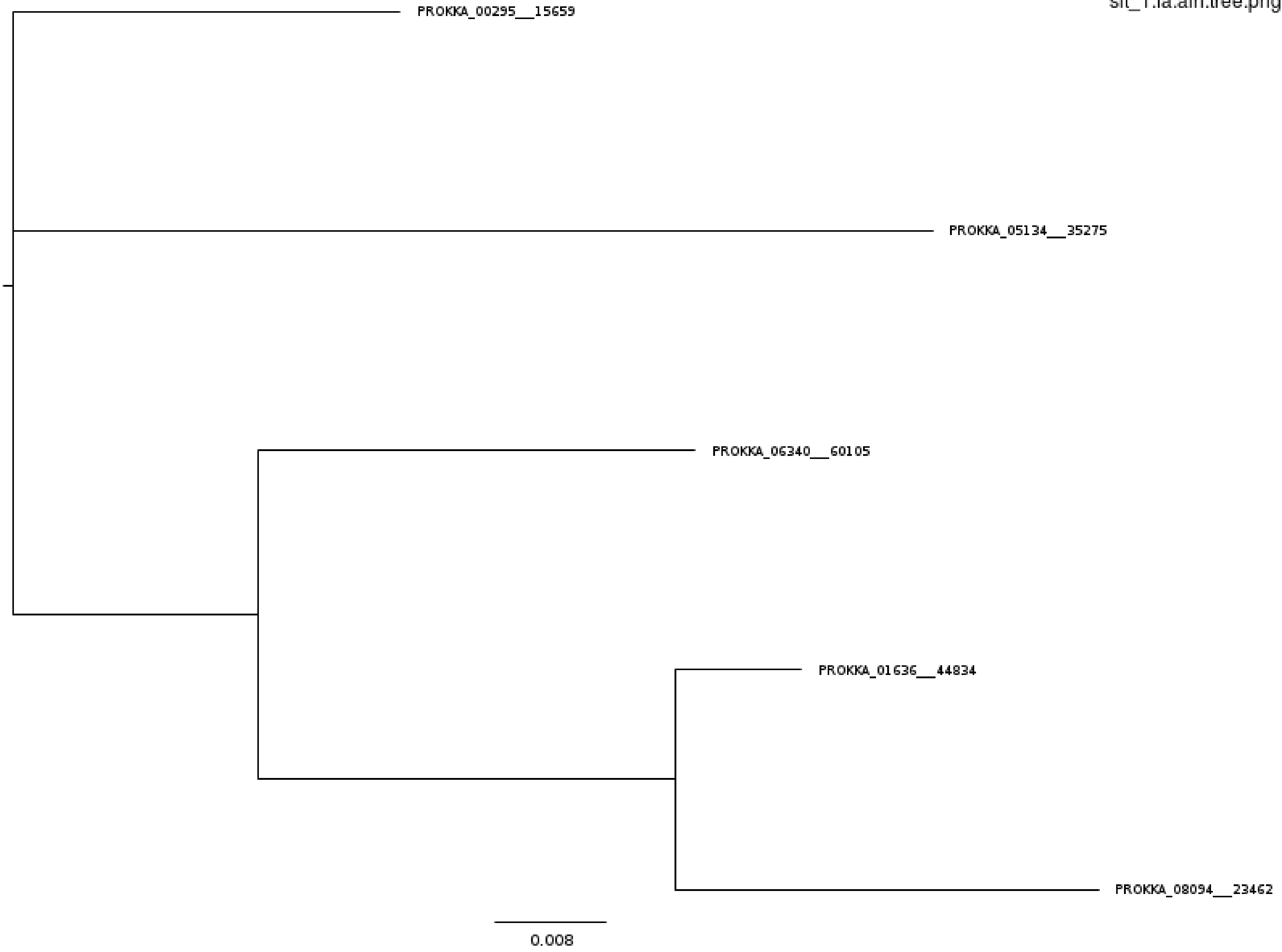

slt_2.fa.aln.tree.png

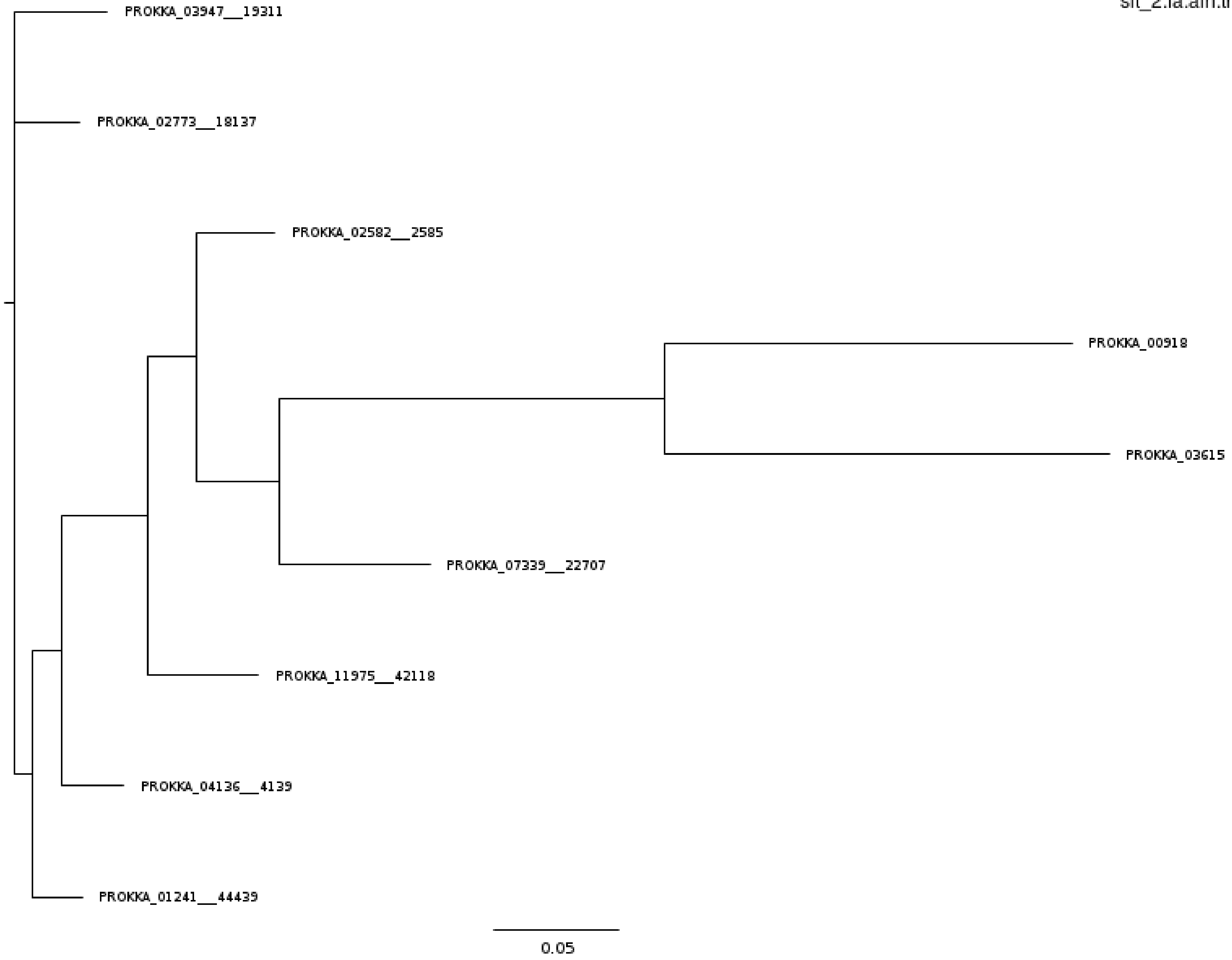

smc_3.fa.aln.tree.png

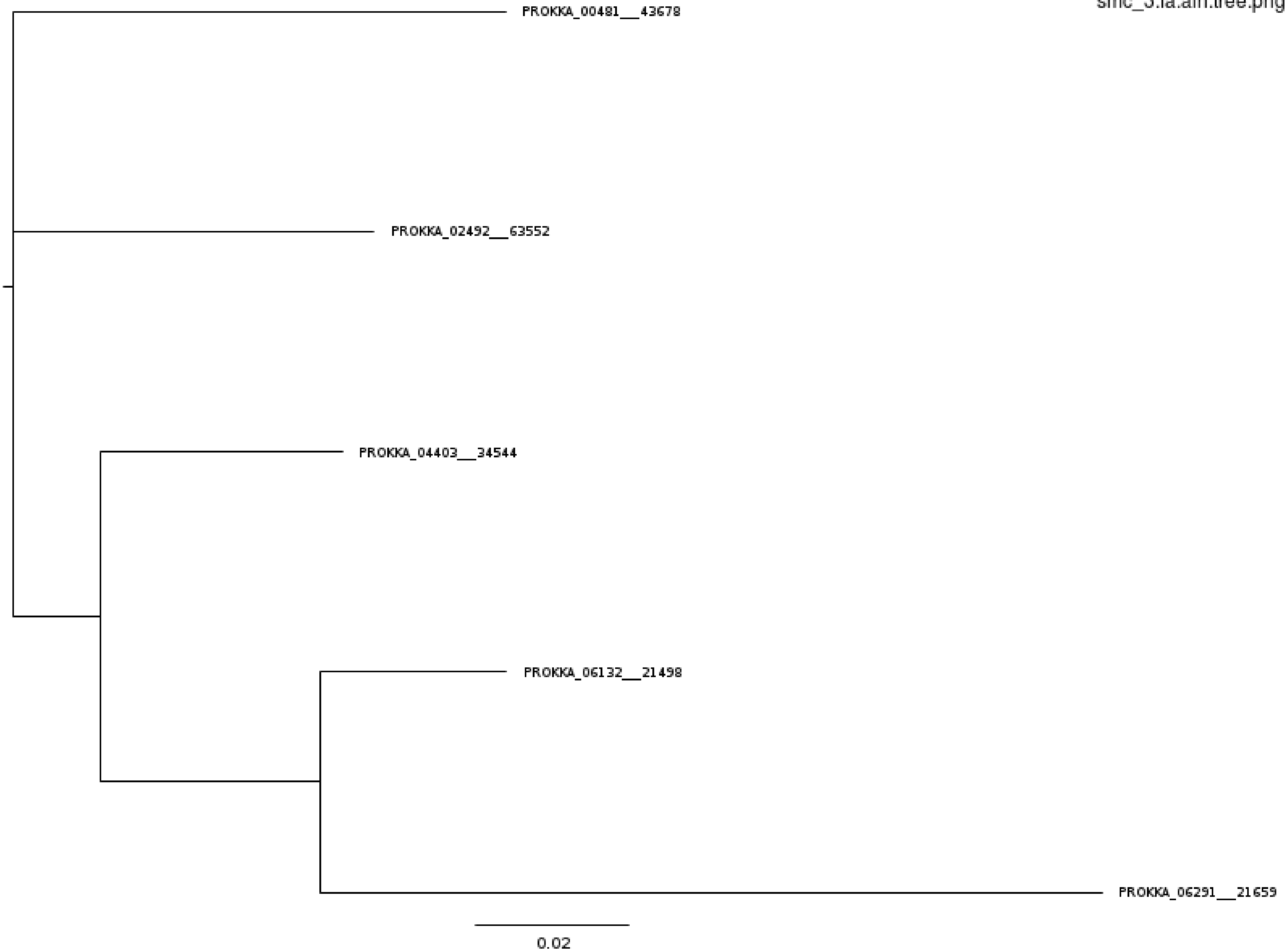

smc_5.fa.aln.tree.png

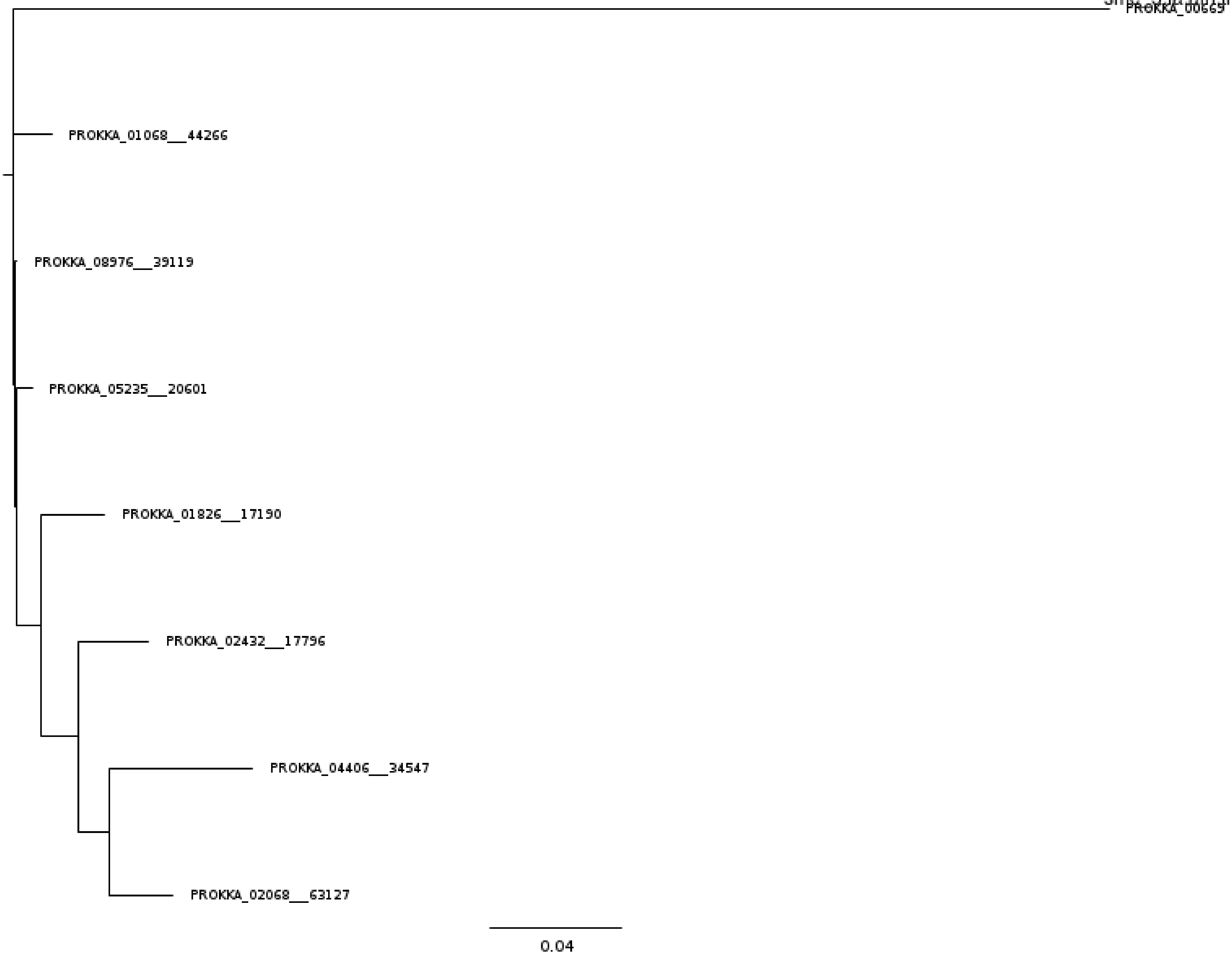

smc_6.fa.aln.tree.png

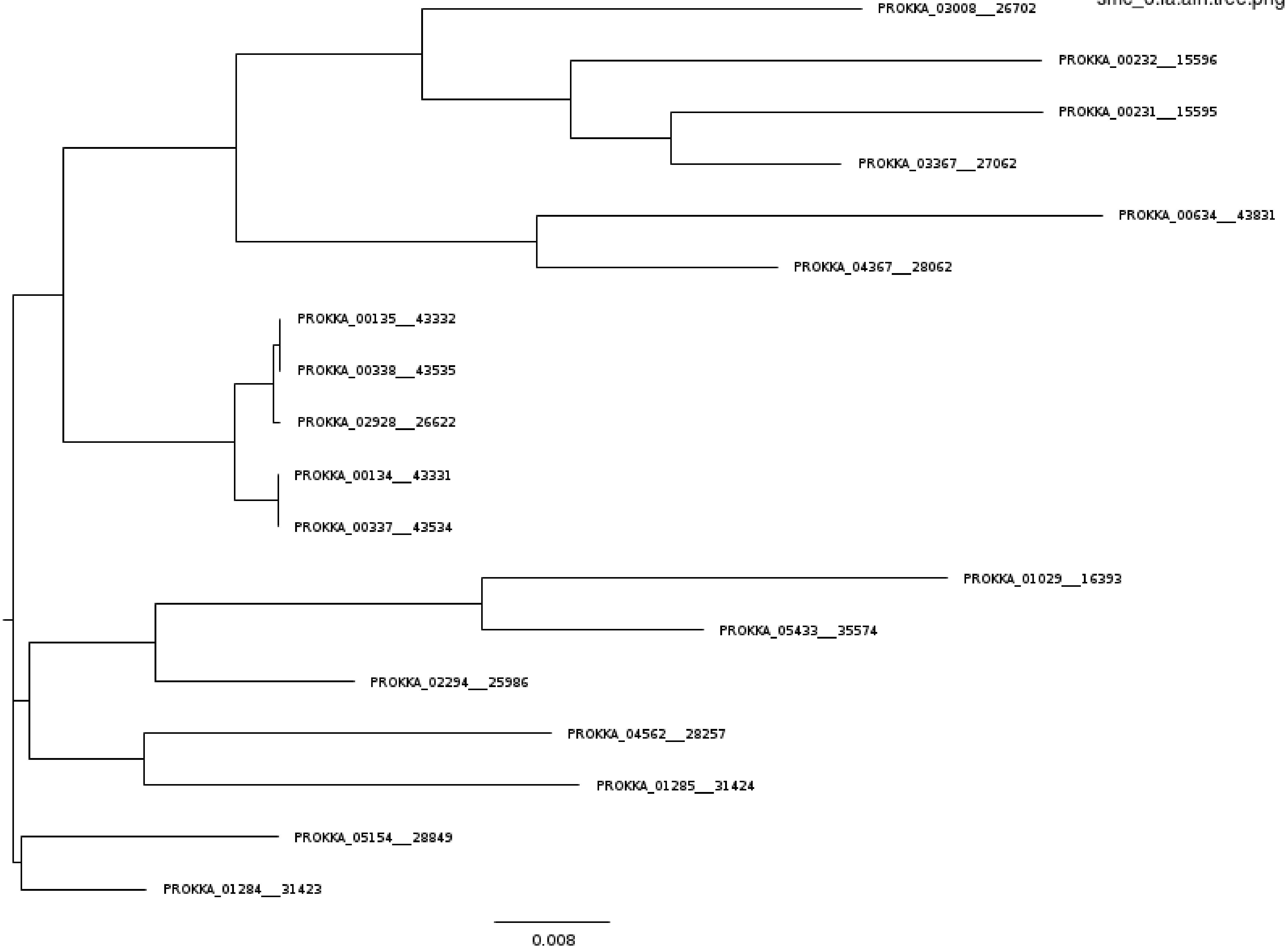

smc_7.fa.aln.tree.png

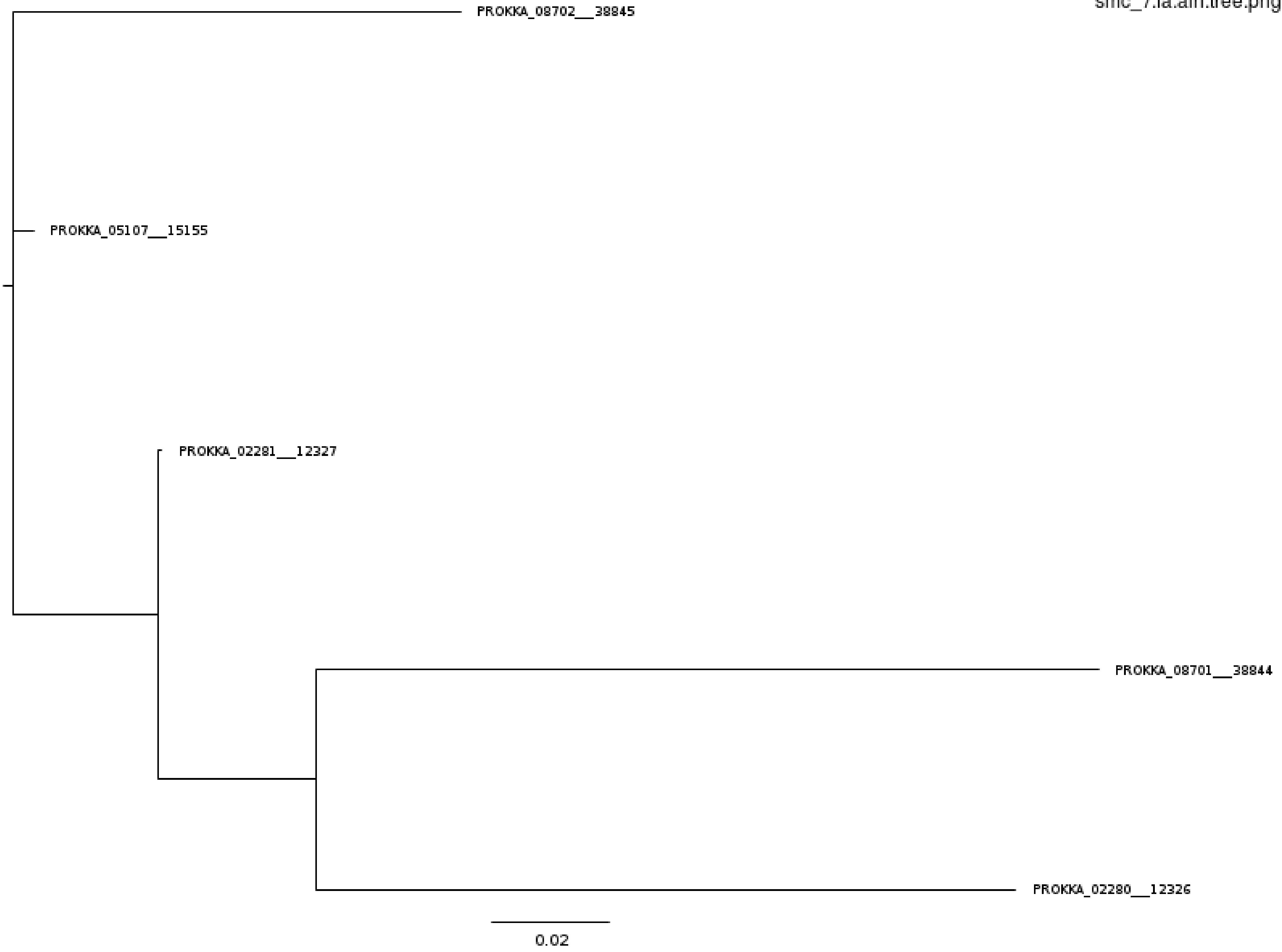

smrA_2.fa.aln.tree.png

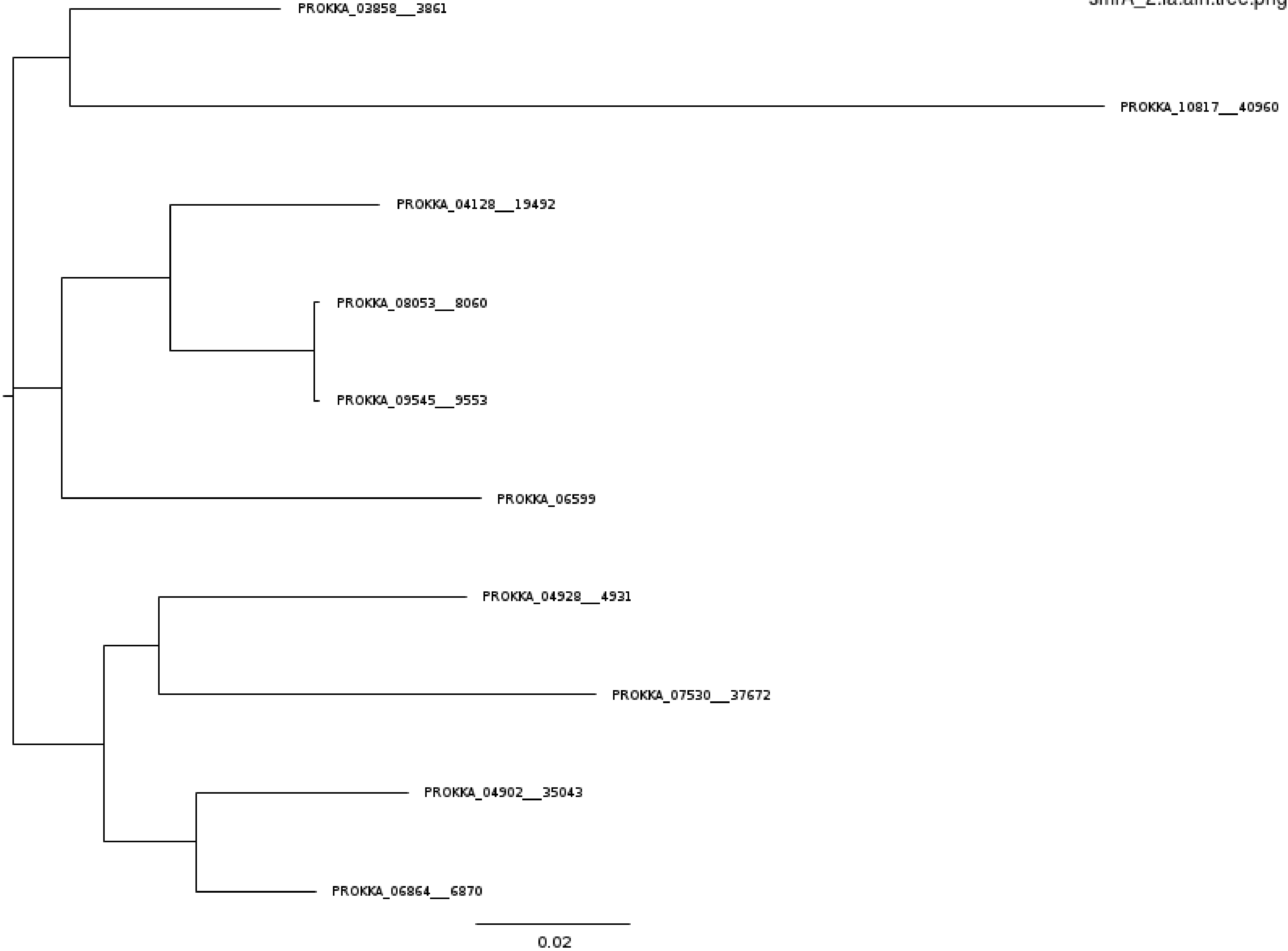

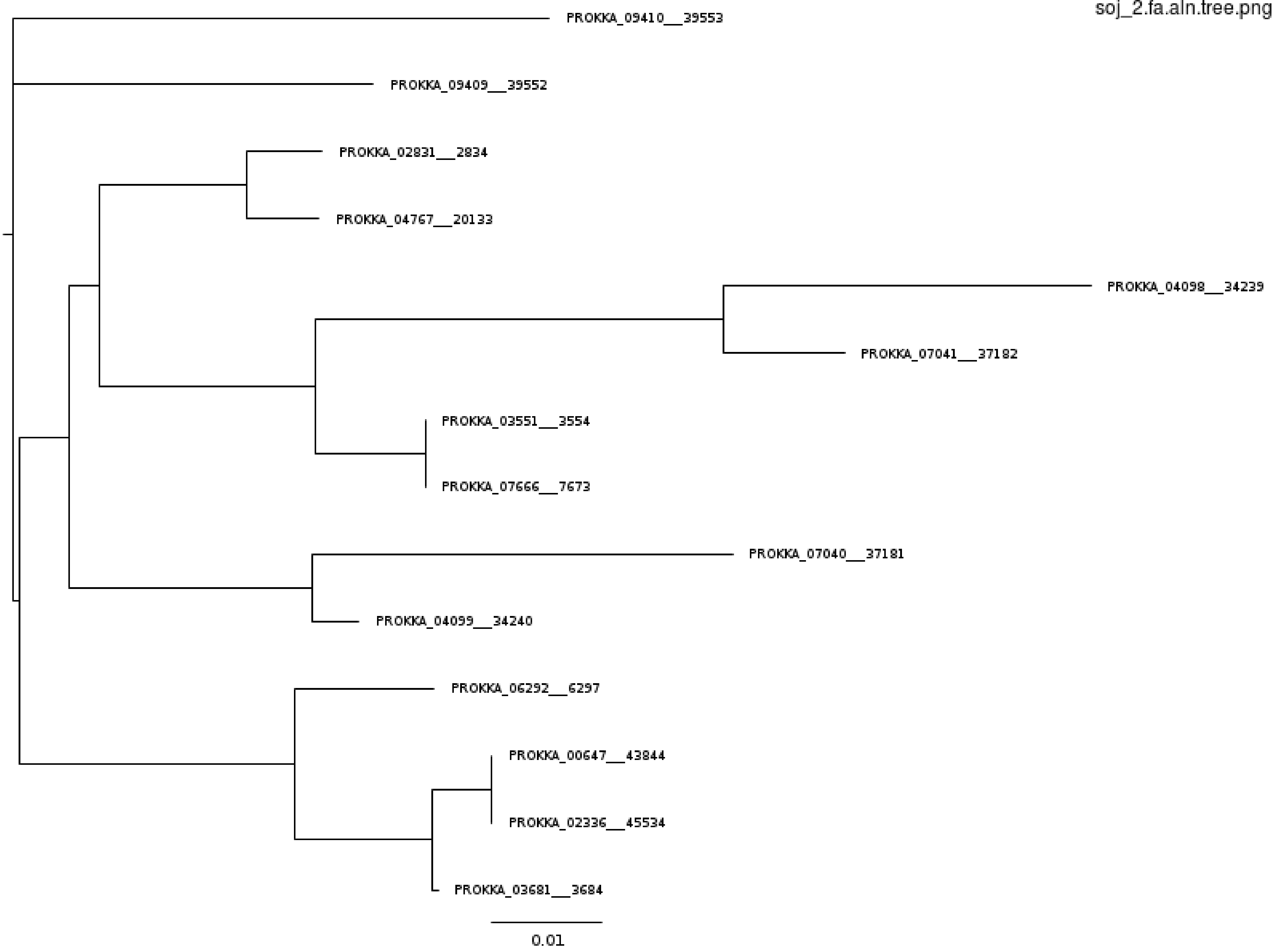
soj_2.fa.aln.tree.png
PROKKA_09410___39553
PROKKA_09409___39552
PROKKA_02831___2834
PROKKA_04767___20133
PROKKA_04098___34239
PROKKA_07041___37182
PROKKA_03551___3554
PROKKA_07666___7673
PROKKA_07040___37181
PROKKA_04099___34240
PROKKA_06292___6297
PROKKA_00647___43844
PROKKA_02336___45534
PROKKA_03681___3684
0.01

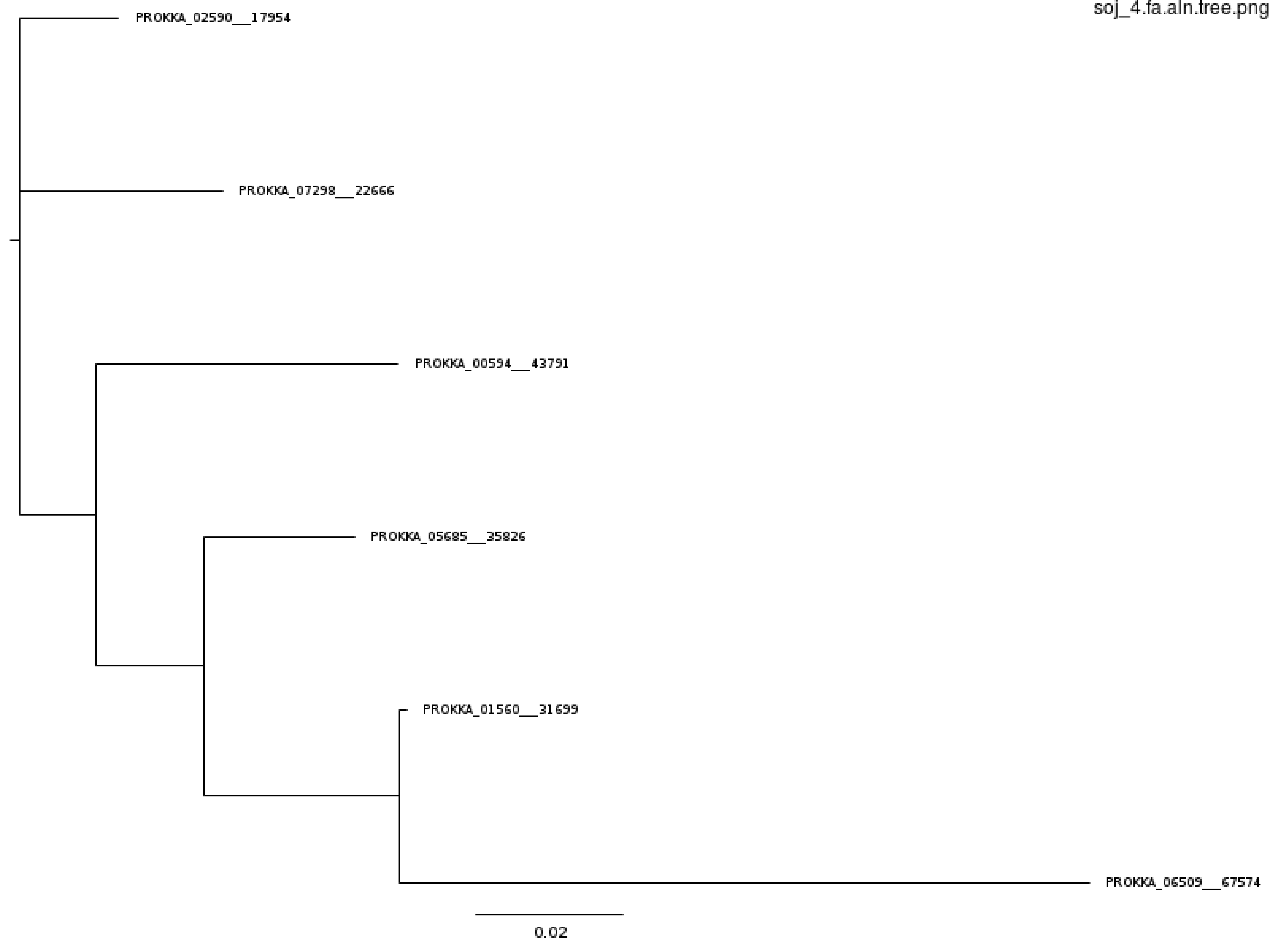
soj_4.fa.aln.tree.png
PROKKA_02590___17954
PROKKA_07298___22666
PROKKA_00594___43791
PROKKA_05685___35826
PROKKA_01560___31699
PROKKA_06509___67574
0.02

spkD.fa.aln.tree.png

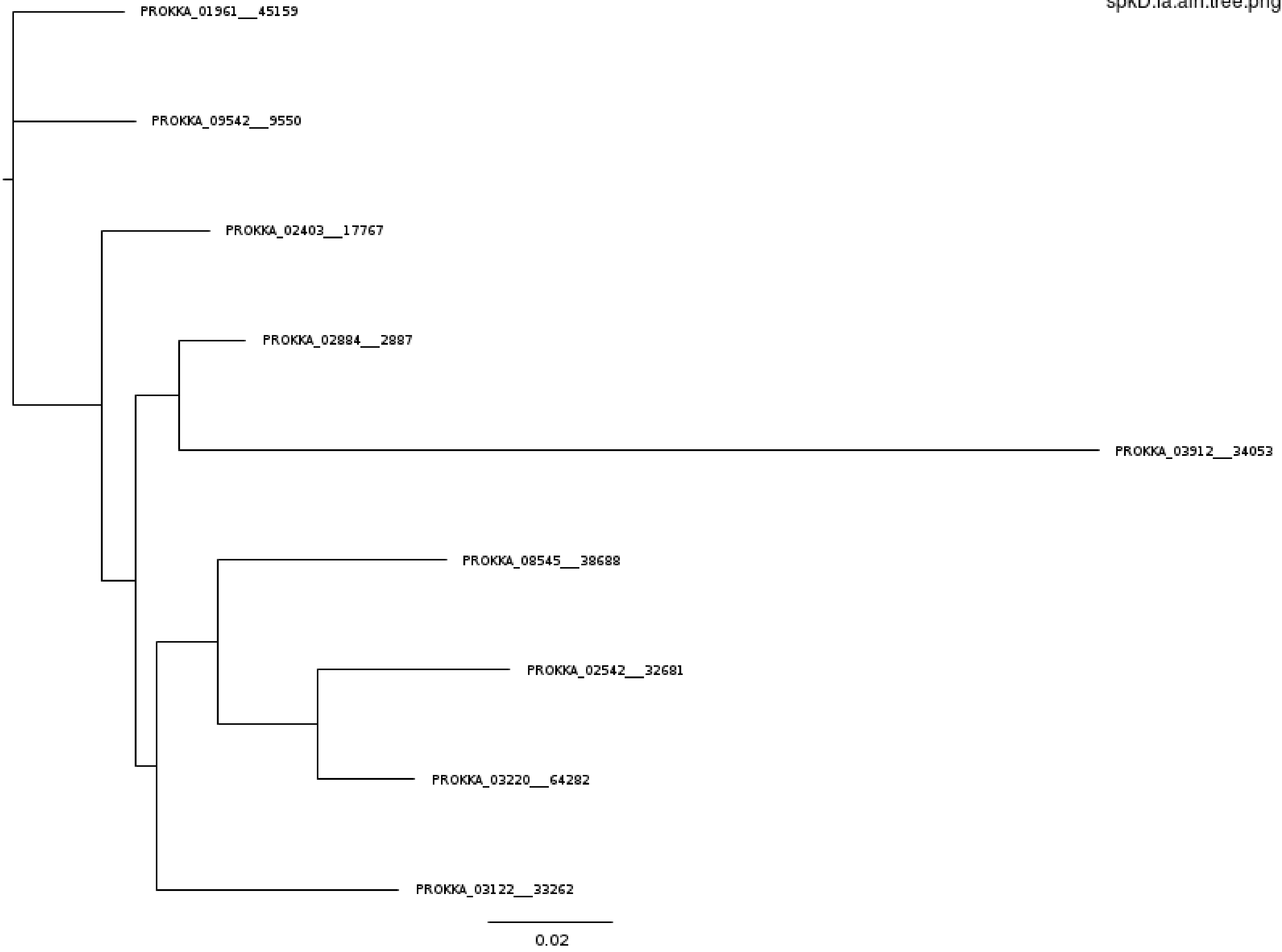

spoIIAA_2.fa.aln.tree.png

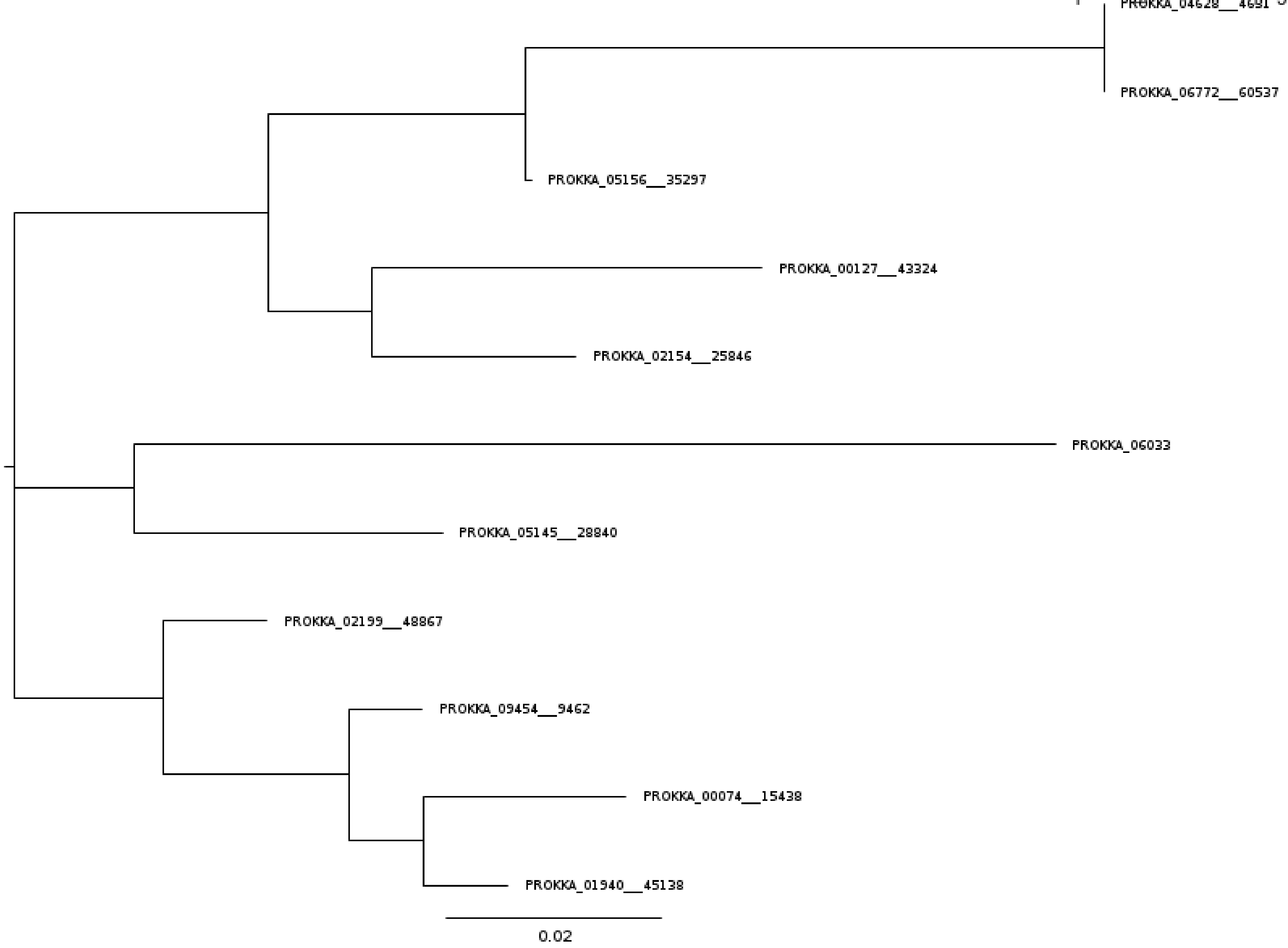

spoT_2.fa.aln.tree.png

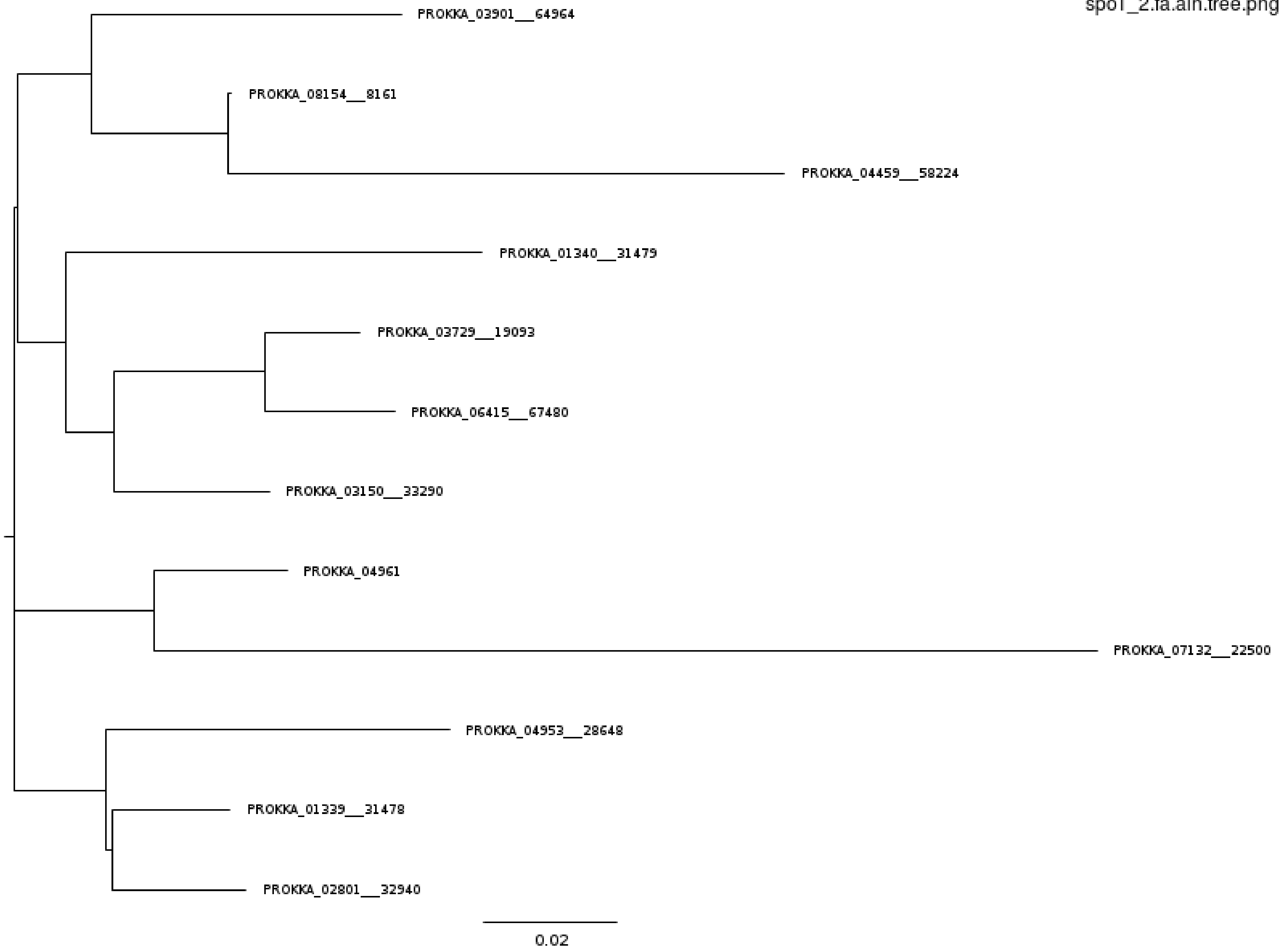

ssuA_1.fa.aln.tree.png

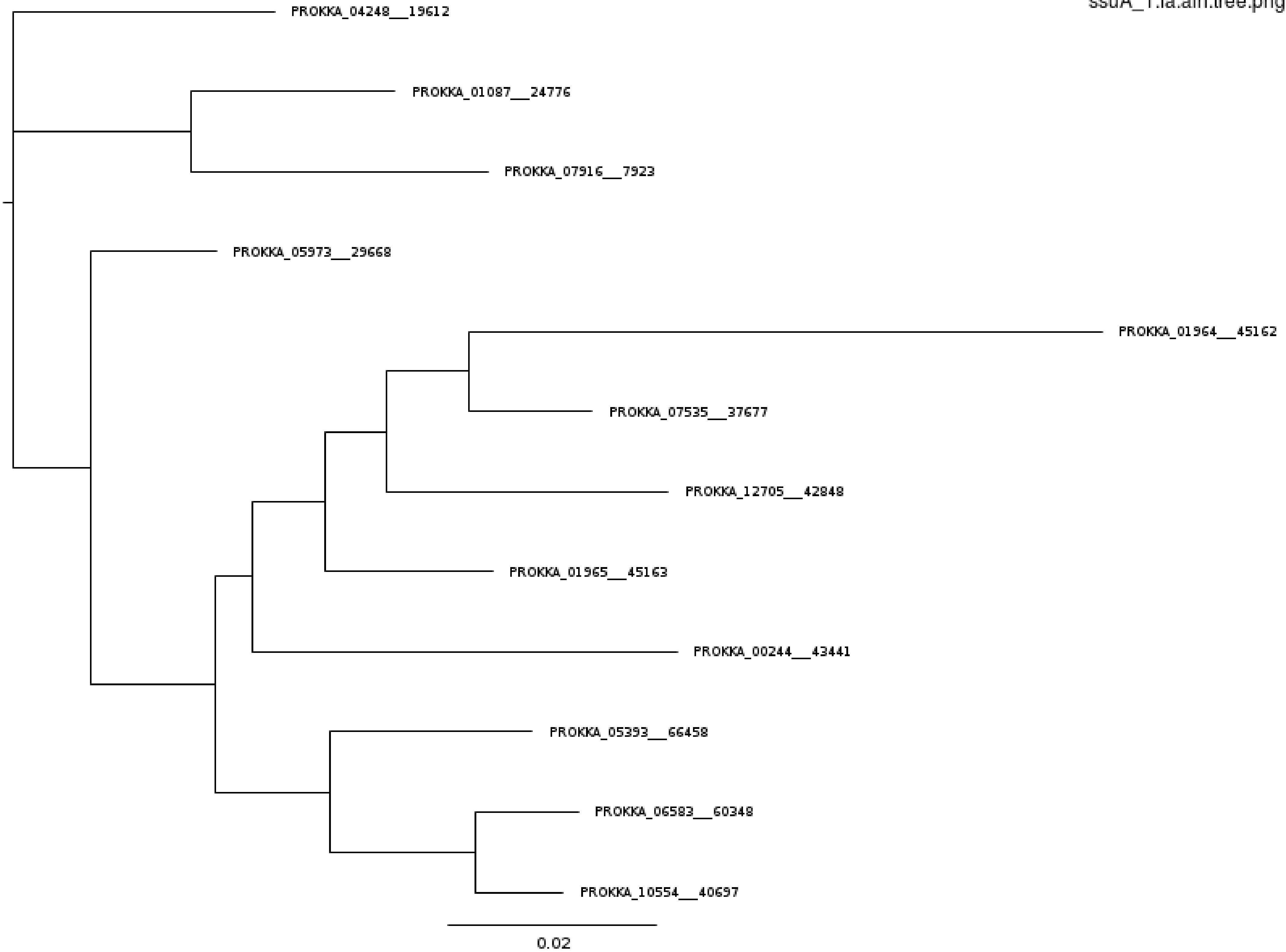

ssuB.fa.aln.tree.png

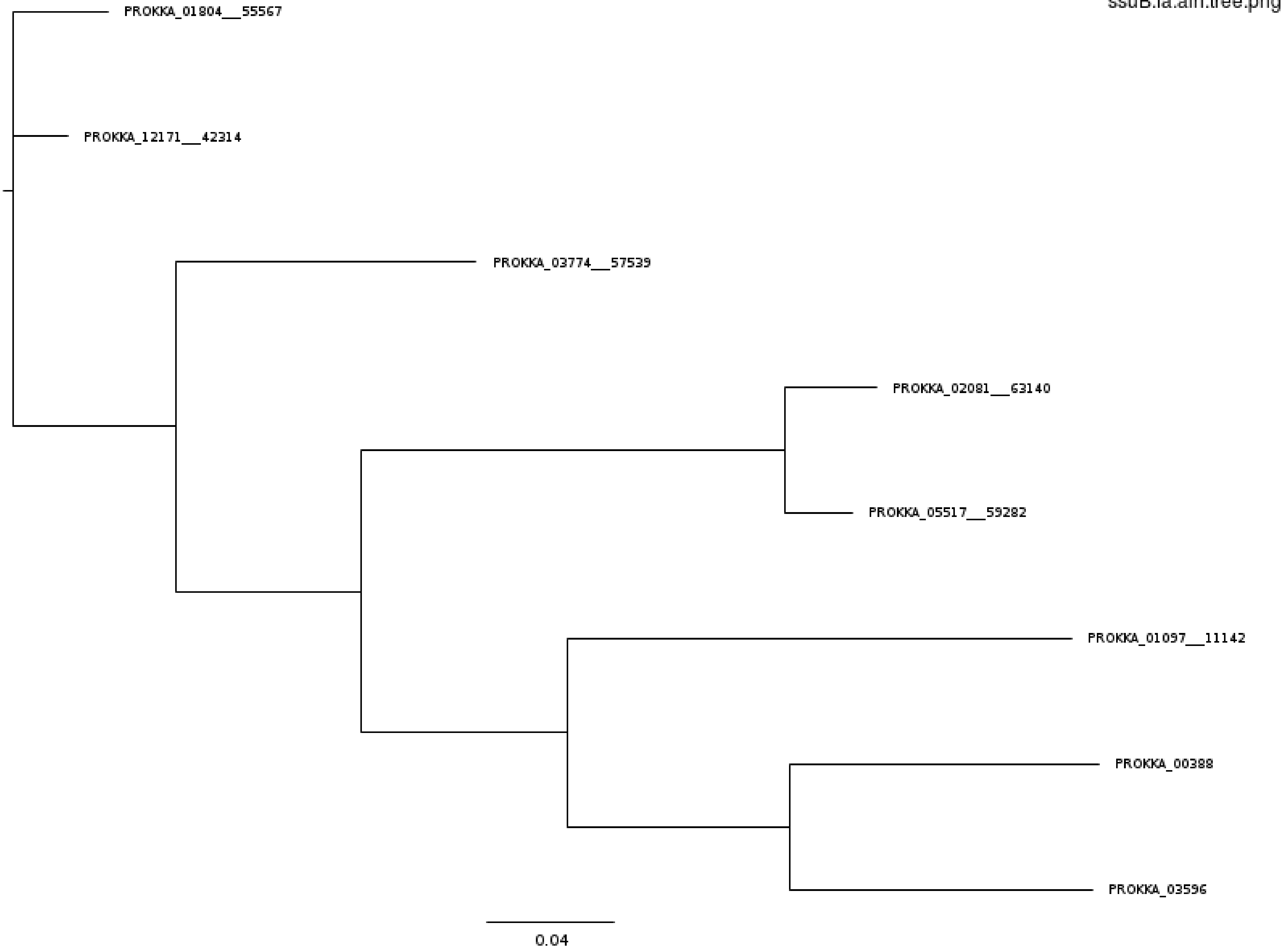

ssuC_1.fa.aln.tree.png

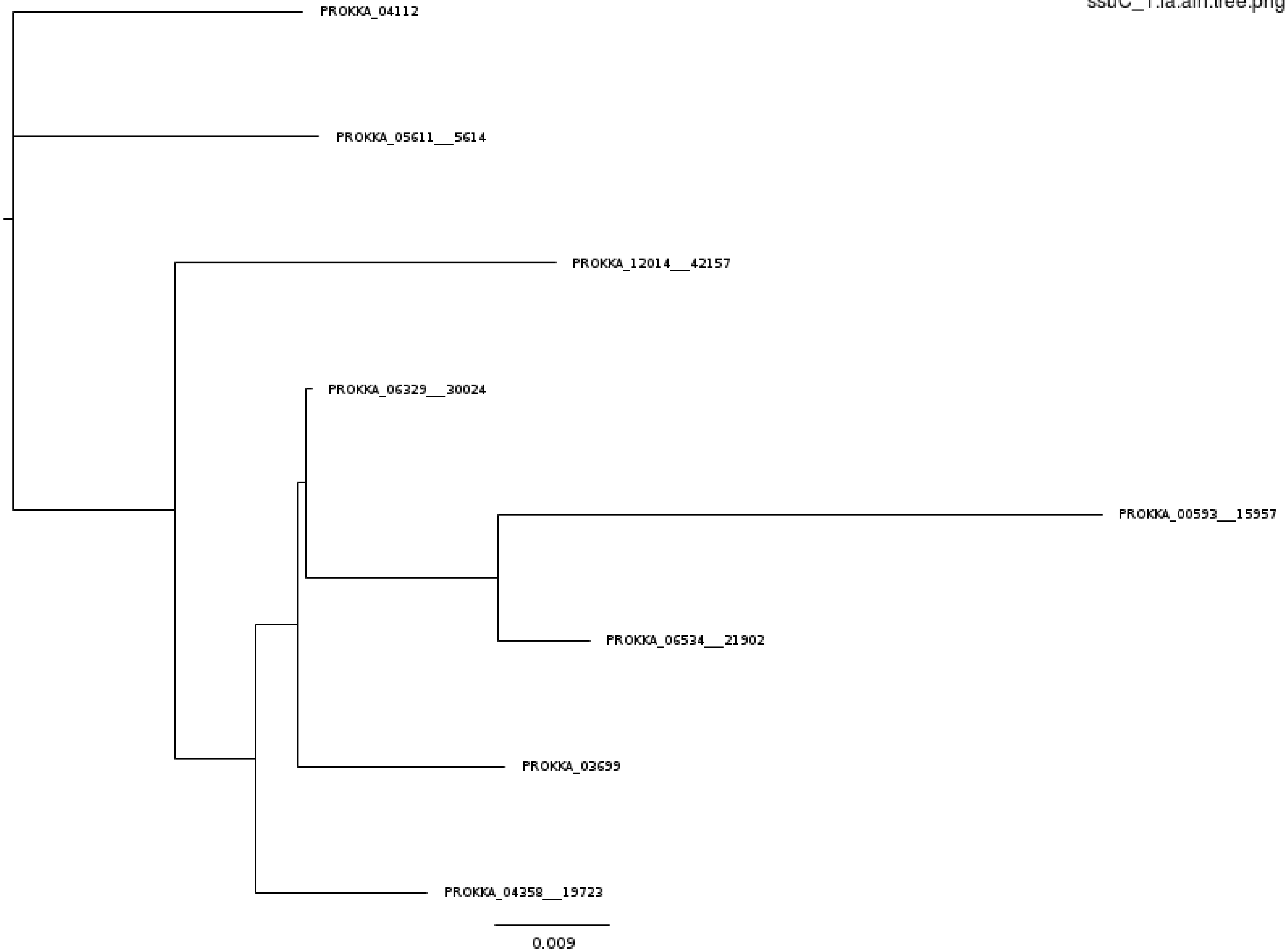

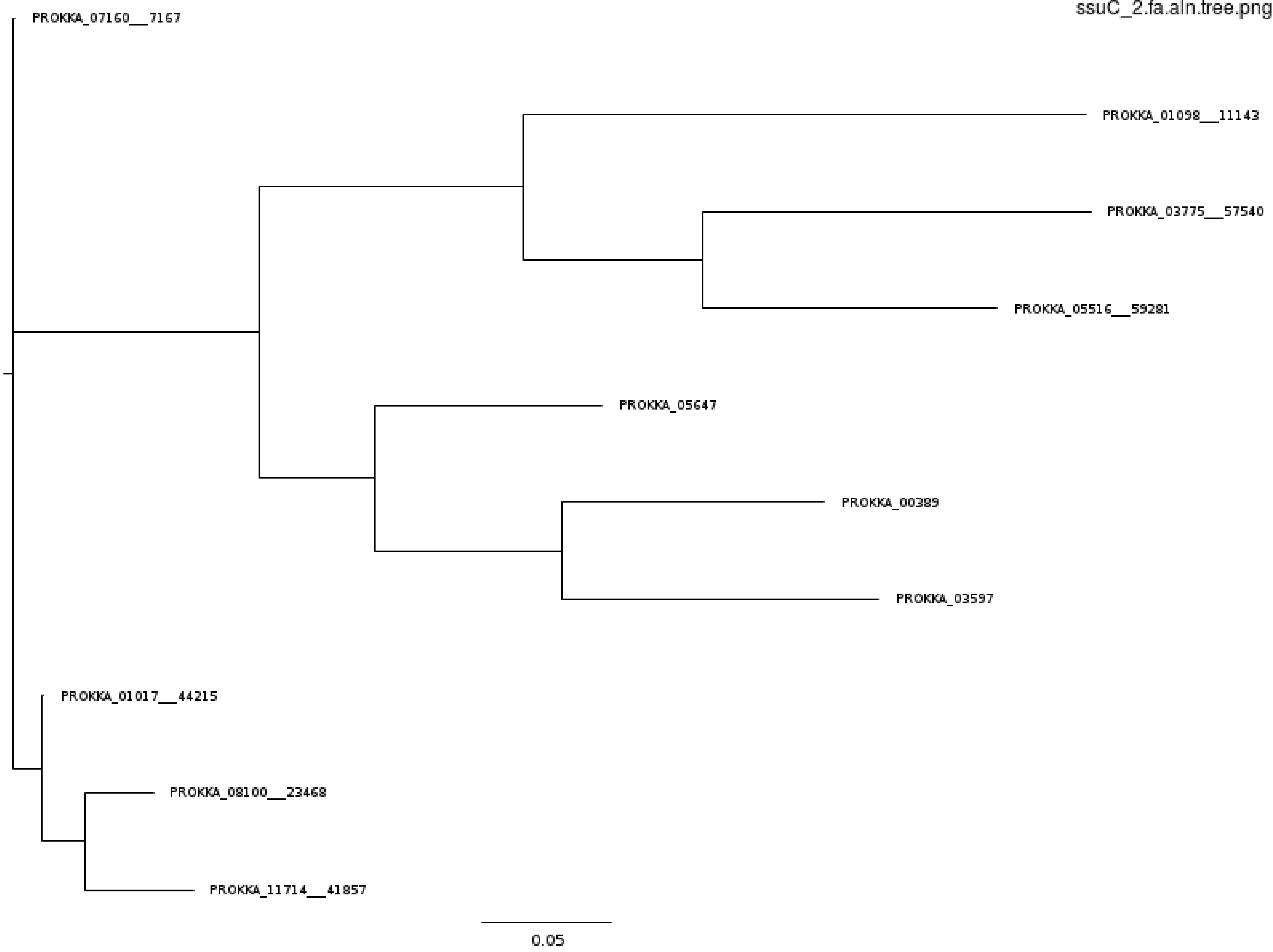
ssuC_2.fa.aln.tree.png
PROKKA_07160___7167
PROKKA_01098___11143
PROKKA_03775___57540
PROKKA_05516___59281
PROKKA_05647
PROKKA_00389
PROKKA_03597
PROKKA_01017___44215
PROKKA_08100___23468
PROKKA_11714___41857
0.05

stkP_2.fa.aln.tree.png

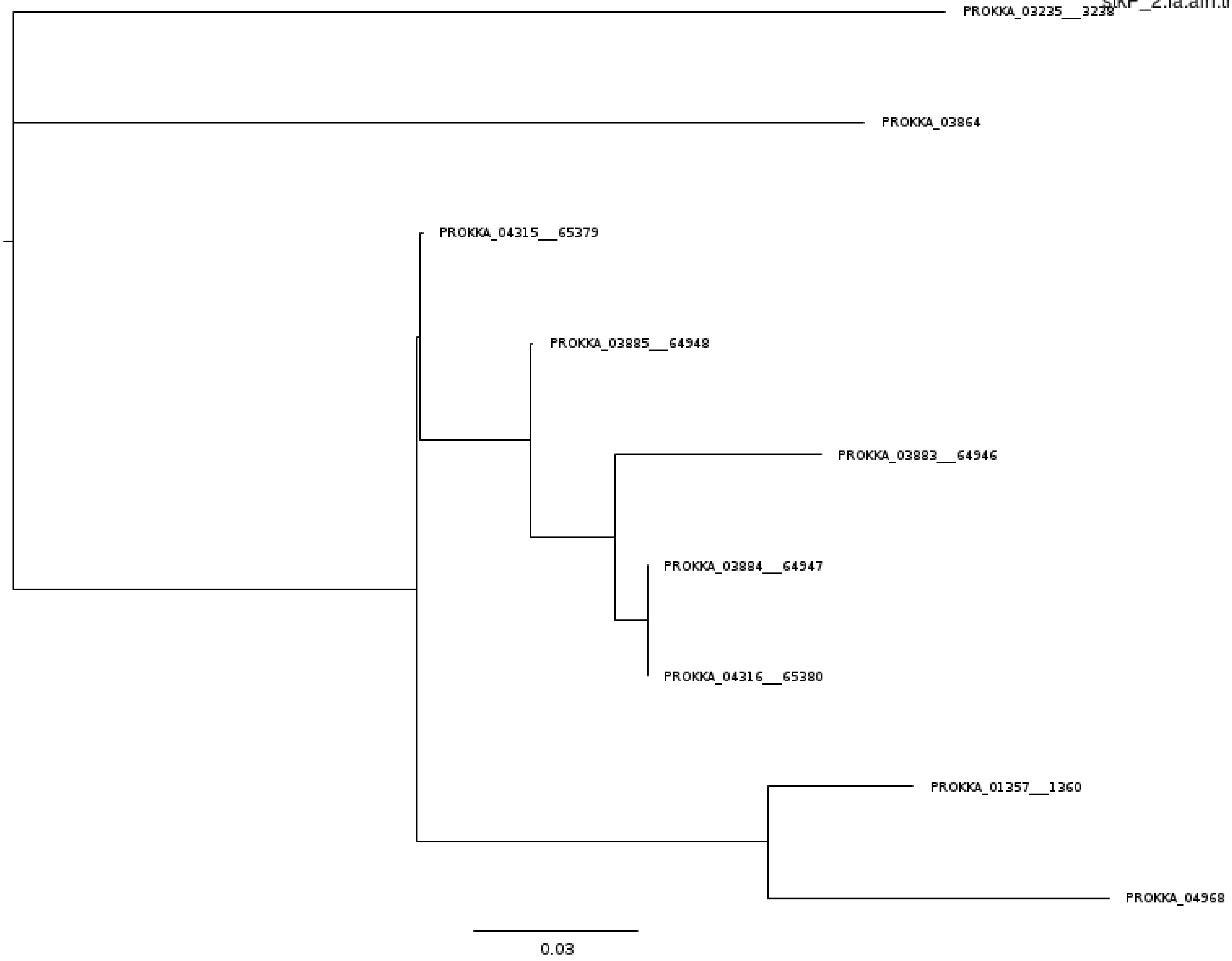

stp_4.fa.aln.tree.png

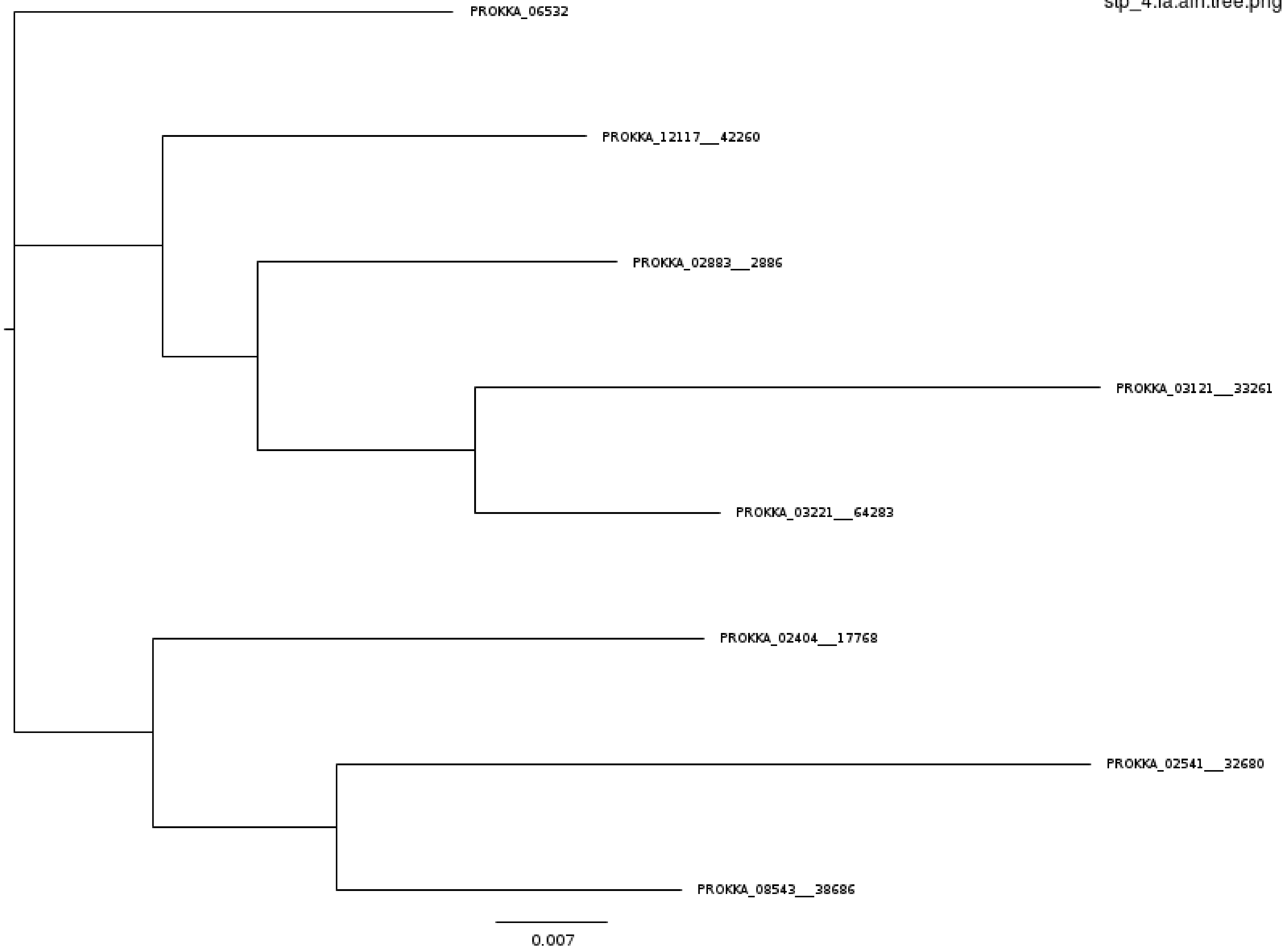

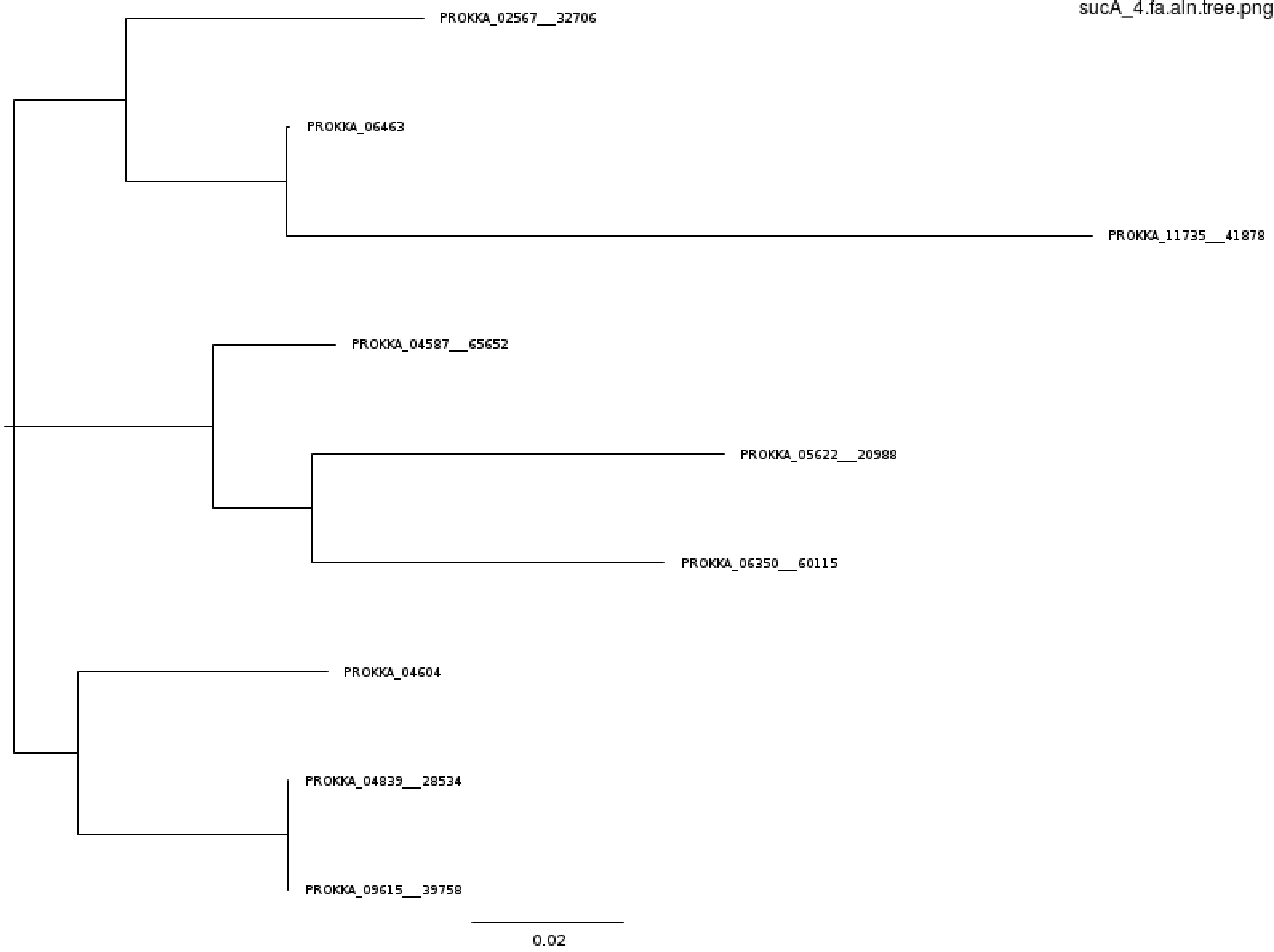
sucA_4.fa.aln.tree.png
PROKKA_02567___32706
PROKKA_06463
PROKKA_11735___41878
PROKKA_04587___65652
PROKKA_05622___20988
PROKKA_06350___60115
PROKKA_04604
PROKKA_04839___28534
PROKKA_09615___39758
0.02

sucA.fa.aln.tree.png

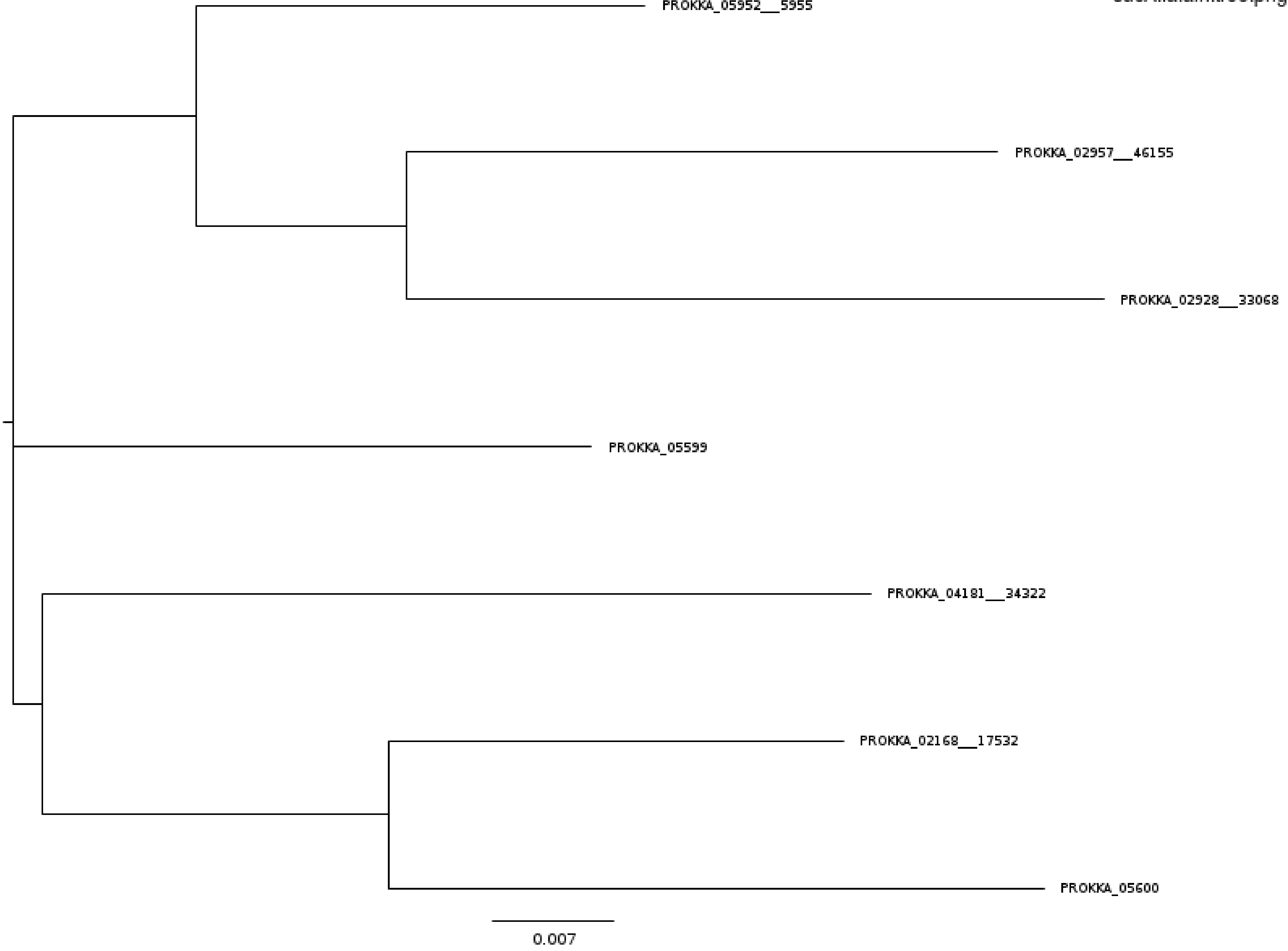

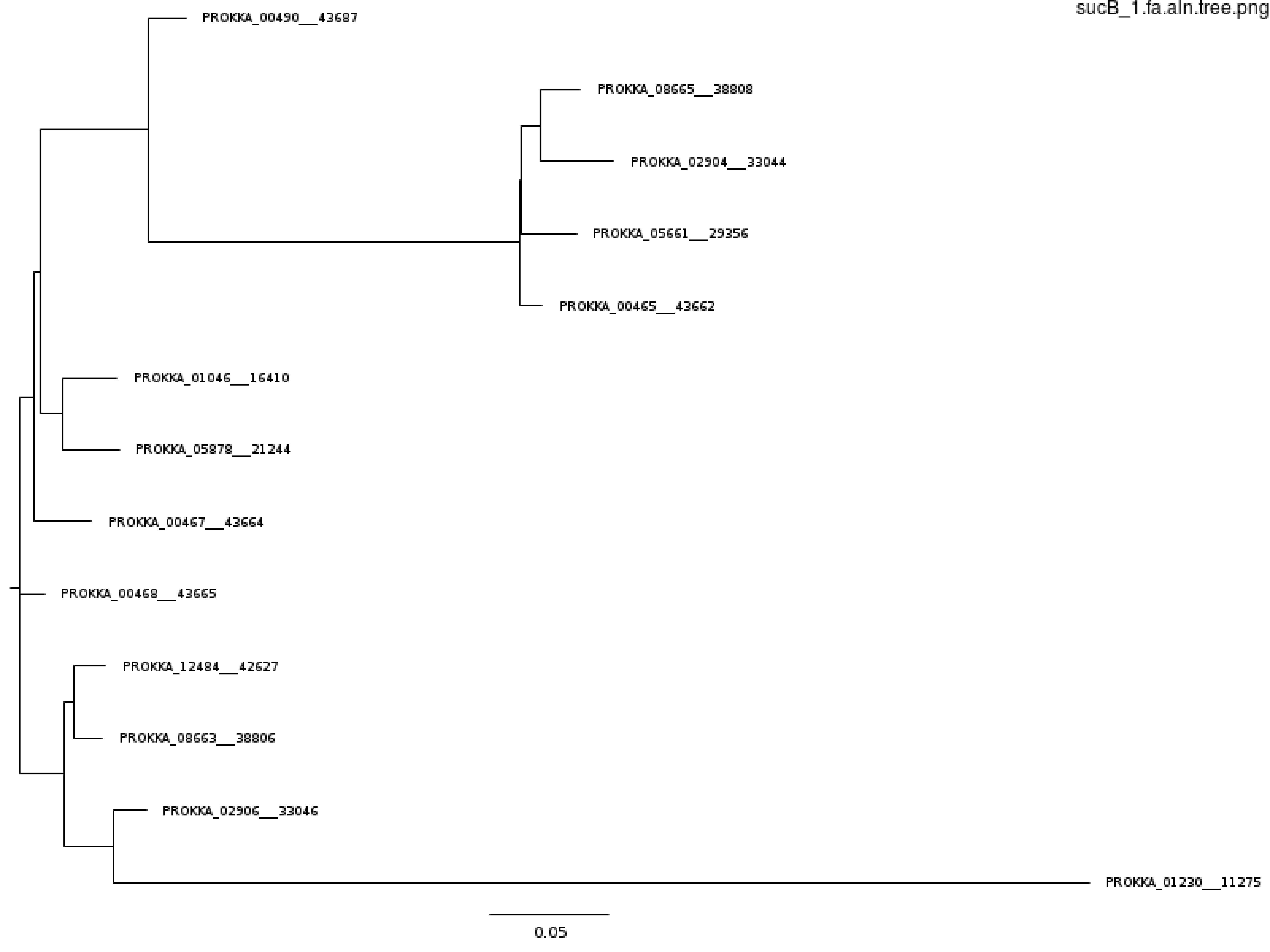
sucB_1.fa.aln.tree.png
PROKKA_00490___43687
PROKKA_08665___38808
PROKKA_02904___33044
PROKKA_05661___29356
PROKKA_00465___43662
PROKKA_01046___16410
PROKKA_05878___21244
PROKKA_00467___43664
PROKKA_00468___43665
PROKKA_12484___42627
PROKKA_08663___38806
PROKKA_02906___33046
PROKKA_01230___11275
0.05

sucC_2.fa.aln.tree.png

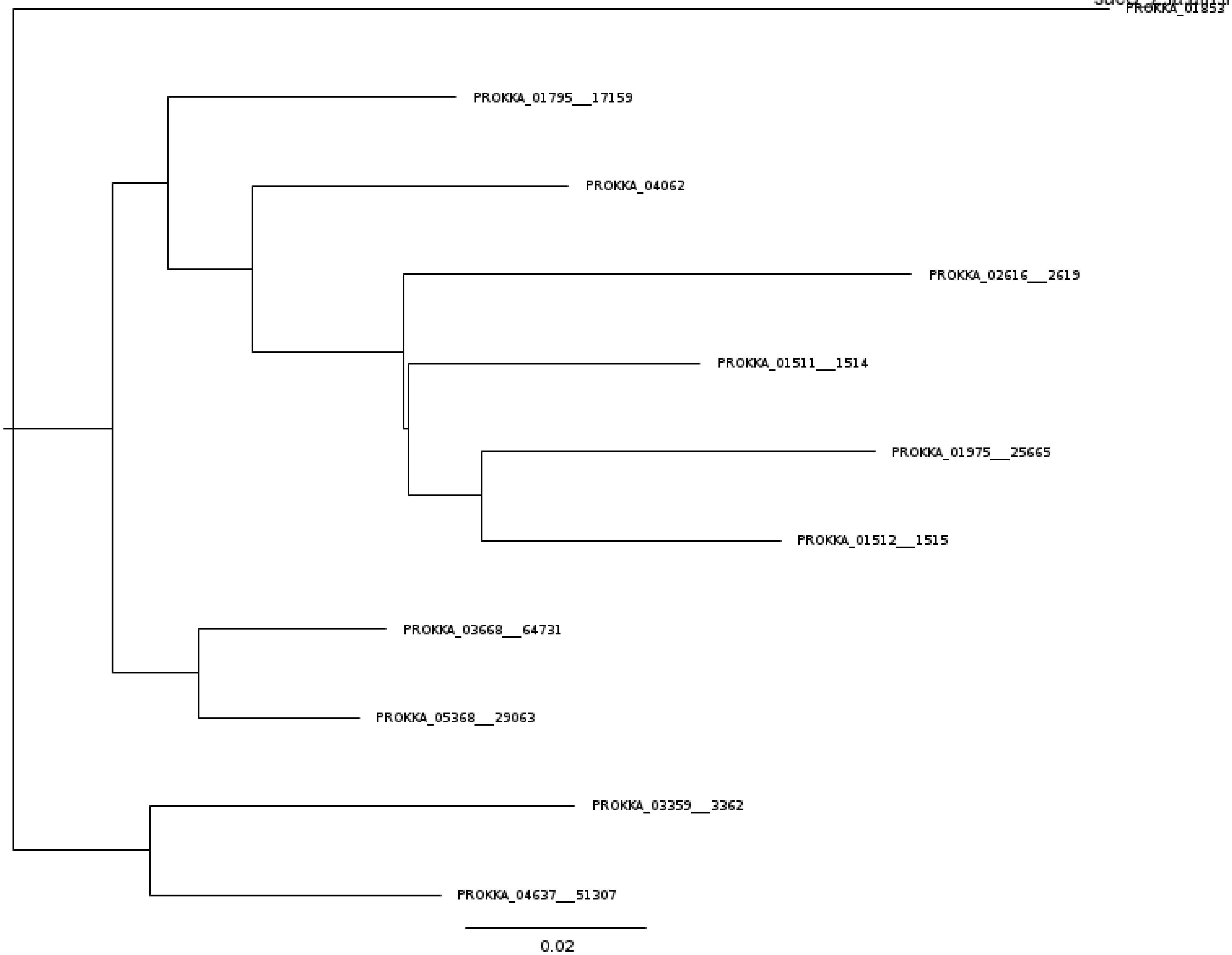

sucC_6.fa.aln.tree.png

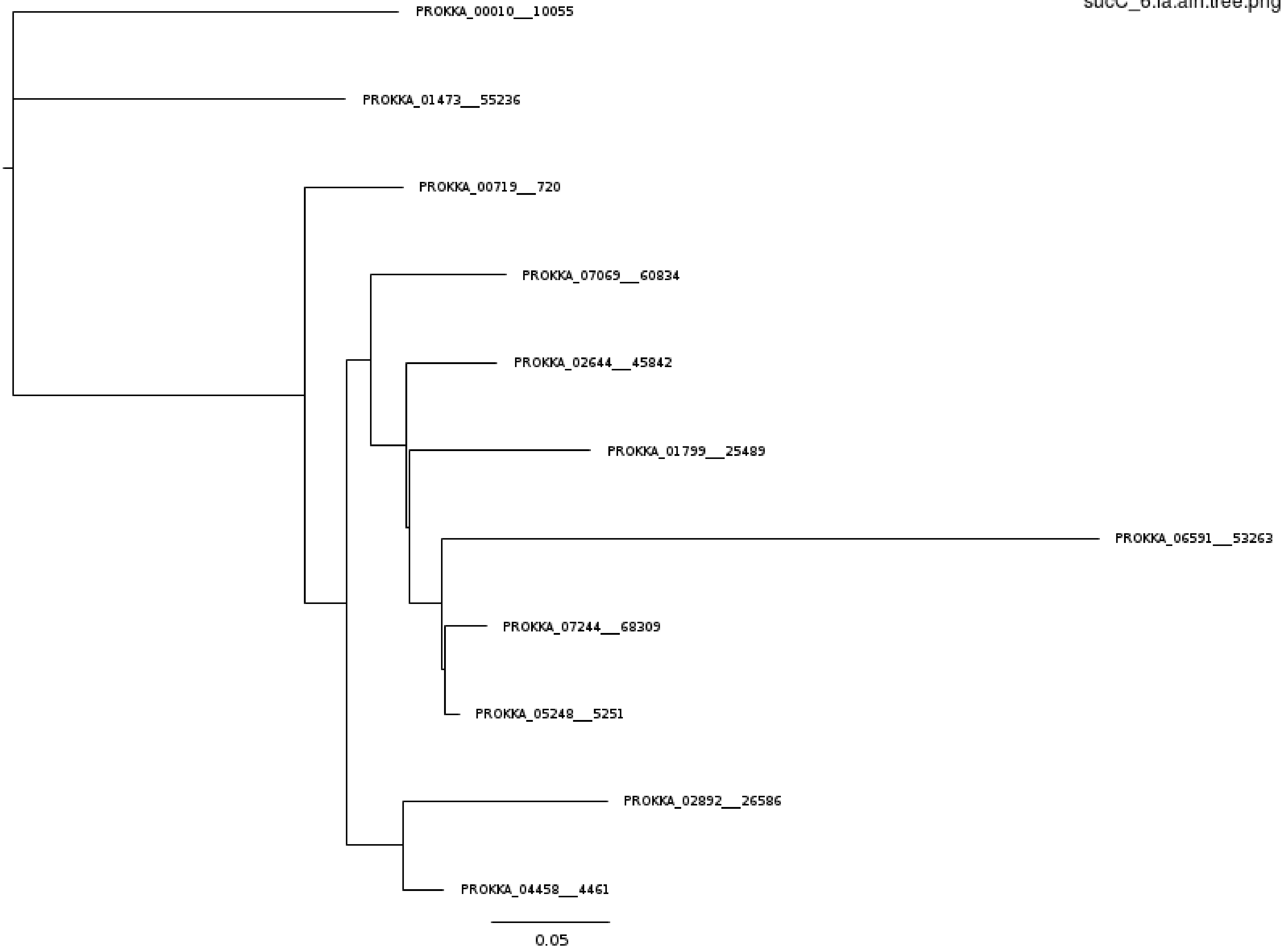

sucD_1.fa.aln.tree.png

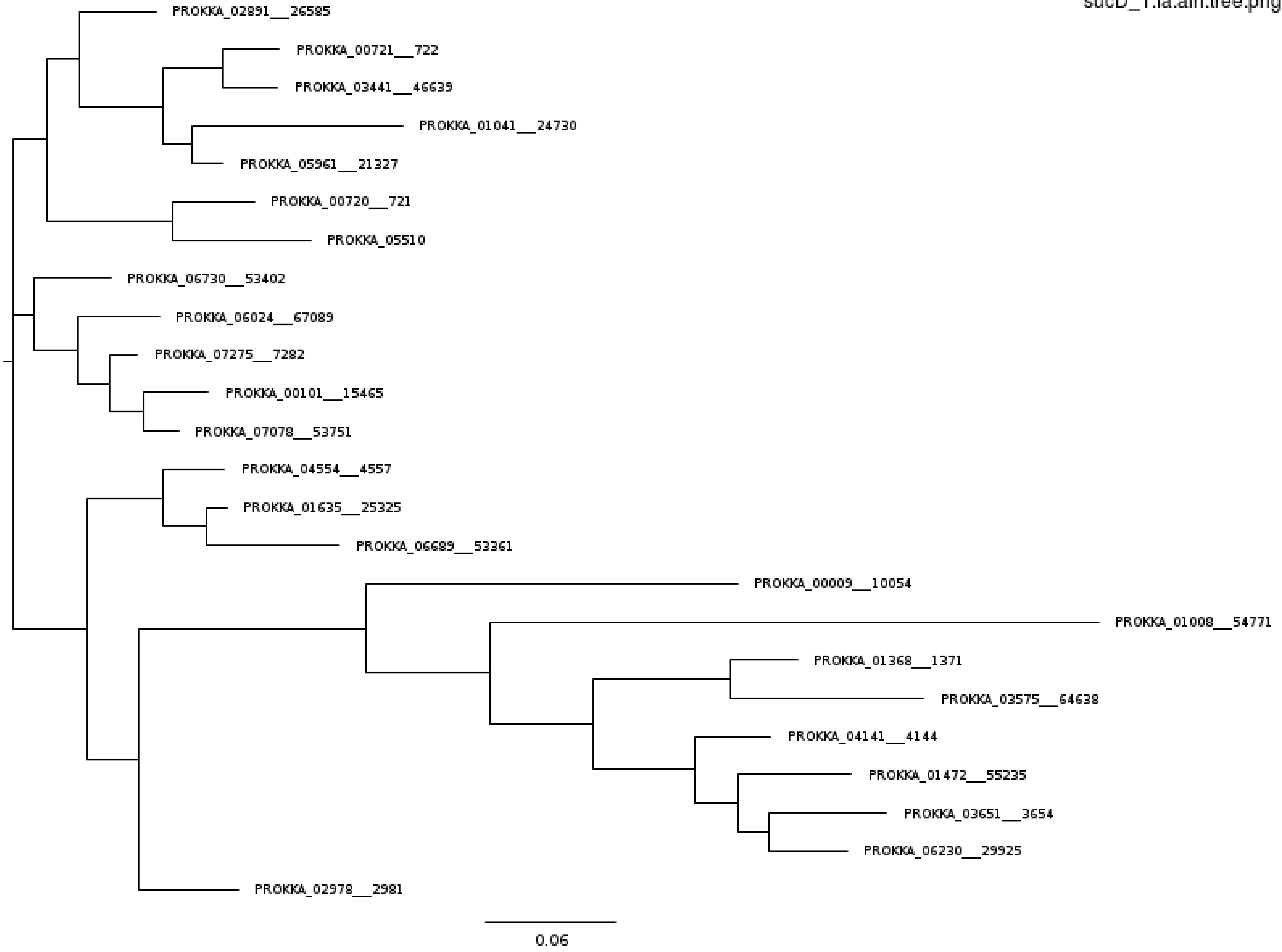

sucD_2.fa.aln.tree.png

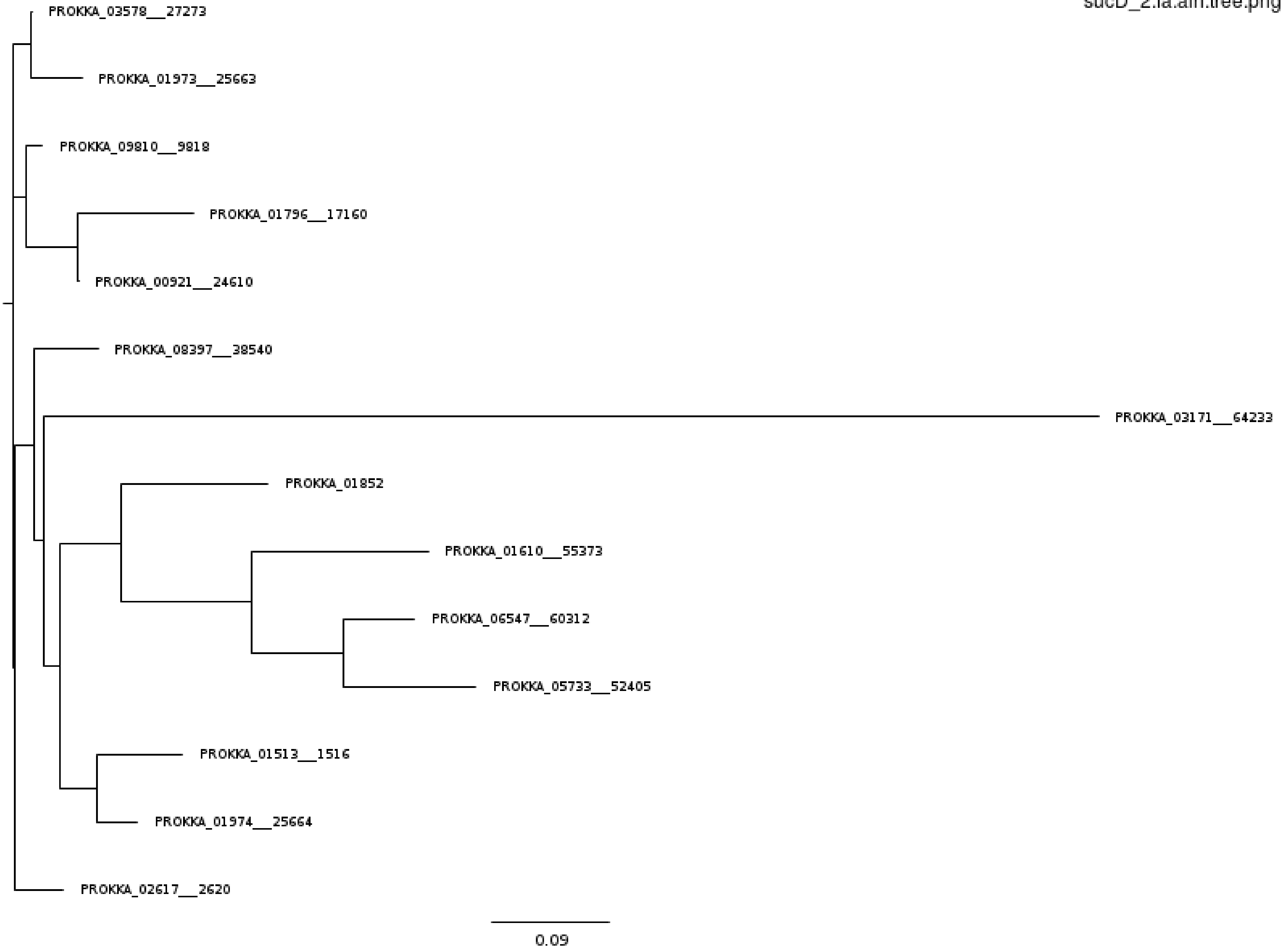

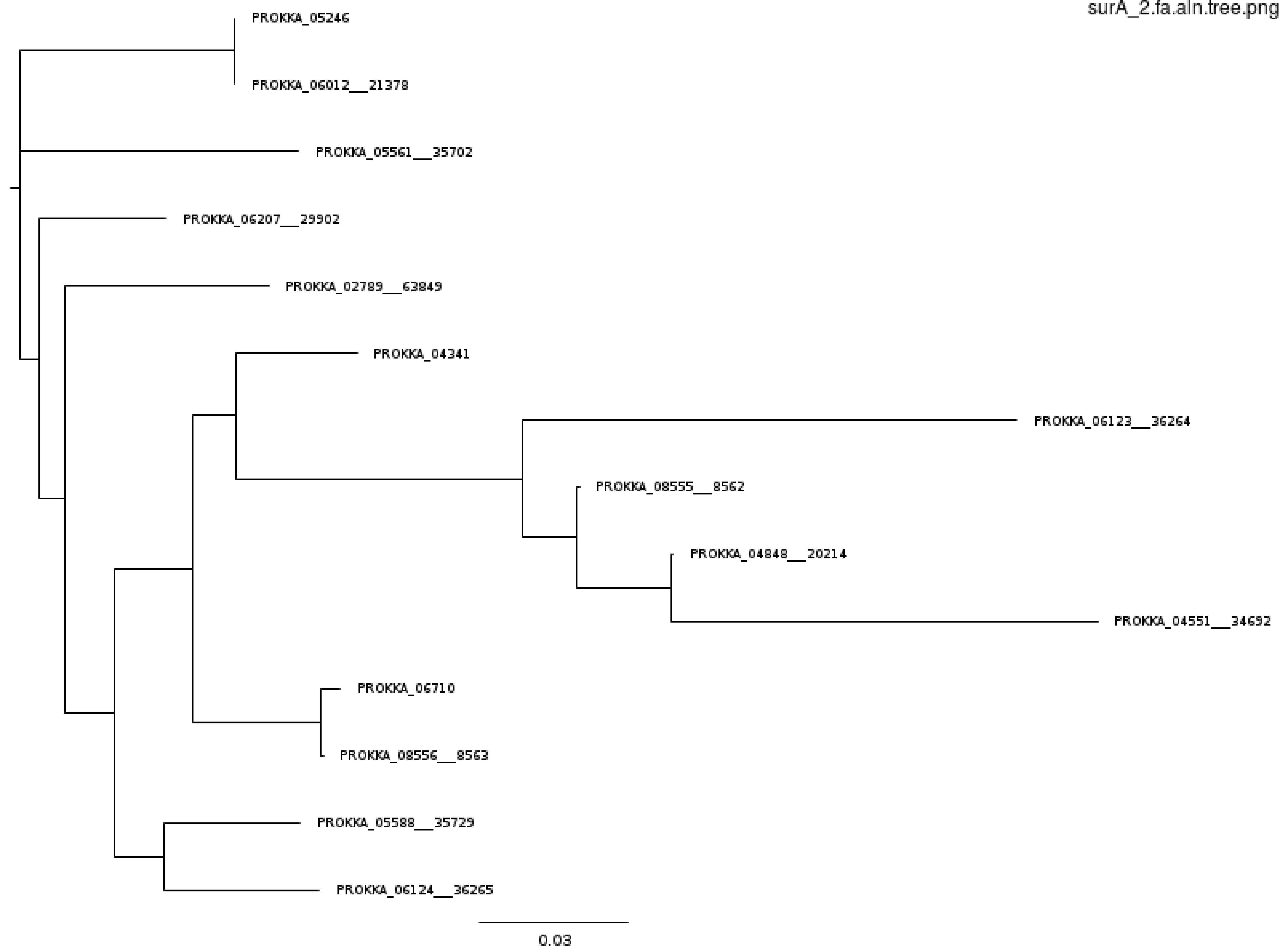
surA_2.fa.aln.tree.png
PROKKA_05246
PROKKA_06012___21378
PROKKA_05561___35702
PROKKA_06207___29902
PROKKA_02789___63849
PROKKA_04341
PROKKA_06123___36264
PROKKA_08555___8562
PROKKA_04848___20214
PROKKA_04551___34692
PROKKA_06710
PROKKA_08556___8563
PROKKA_05588___35729
PROKKA_06124___36265
0.03

surE_1.fa.aln.tree.png

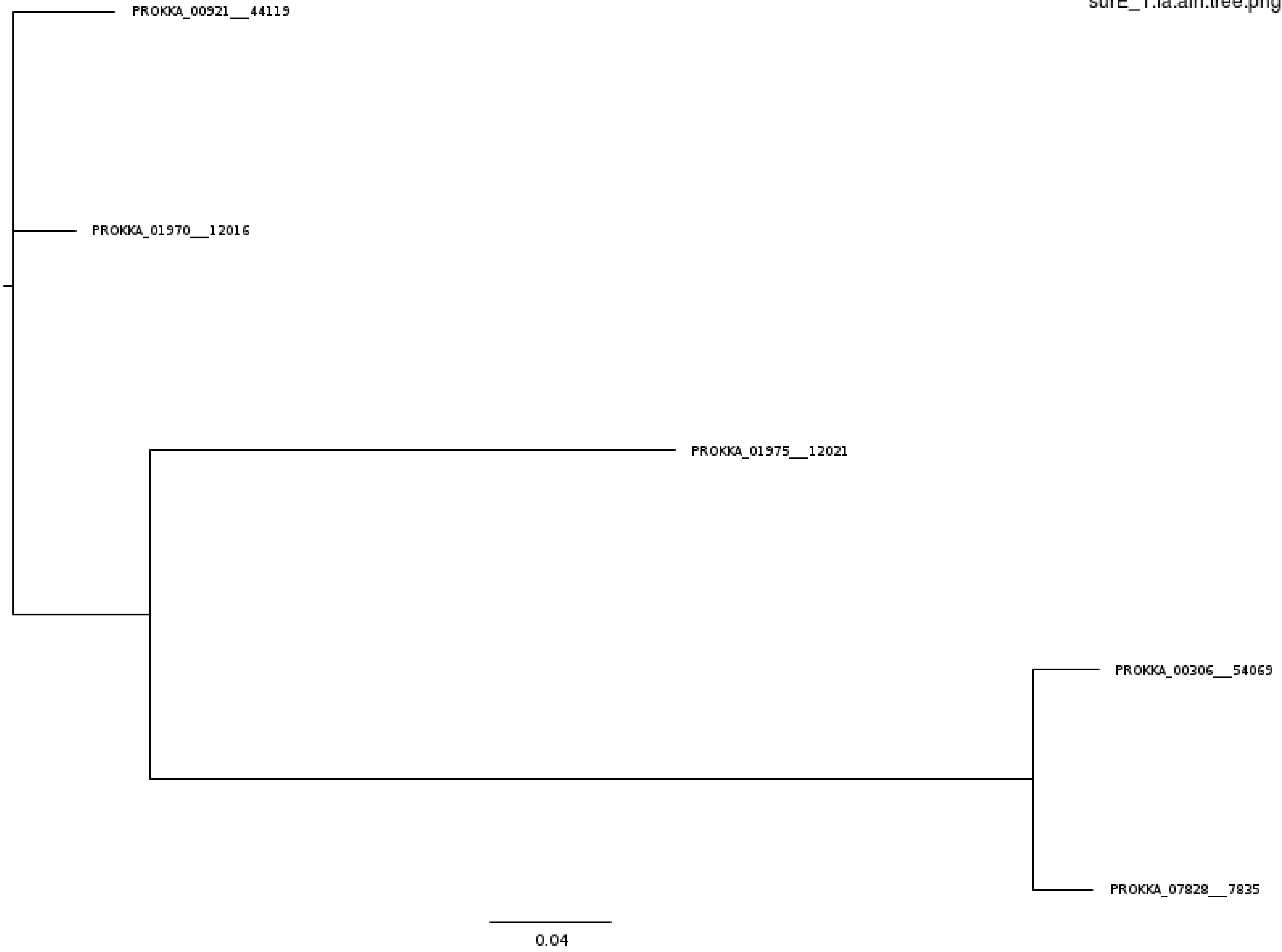

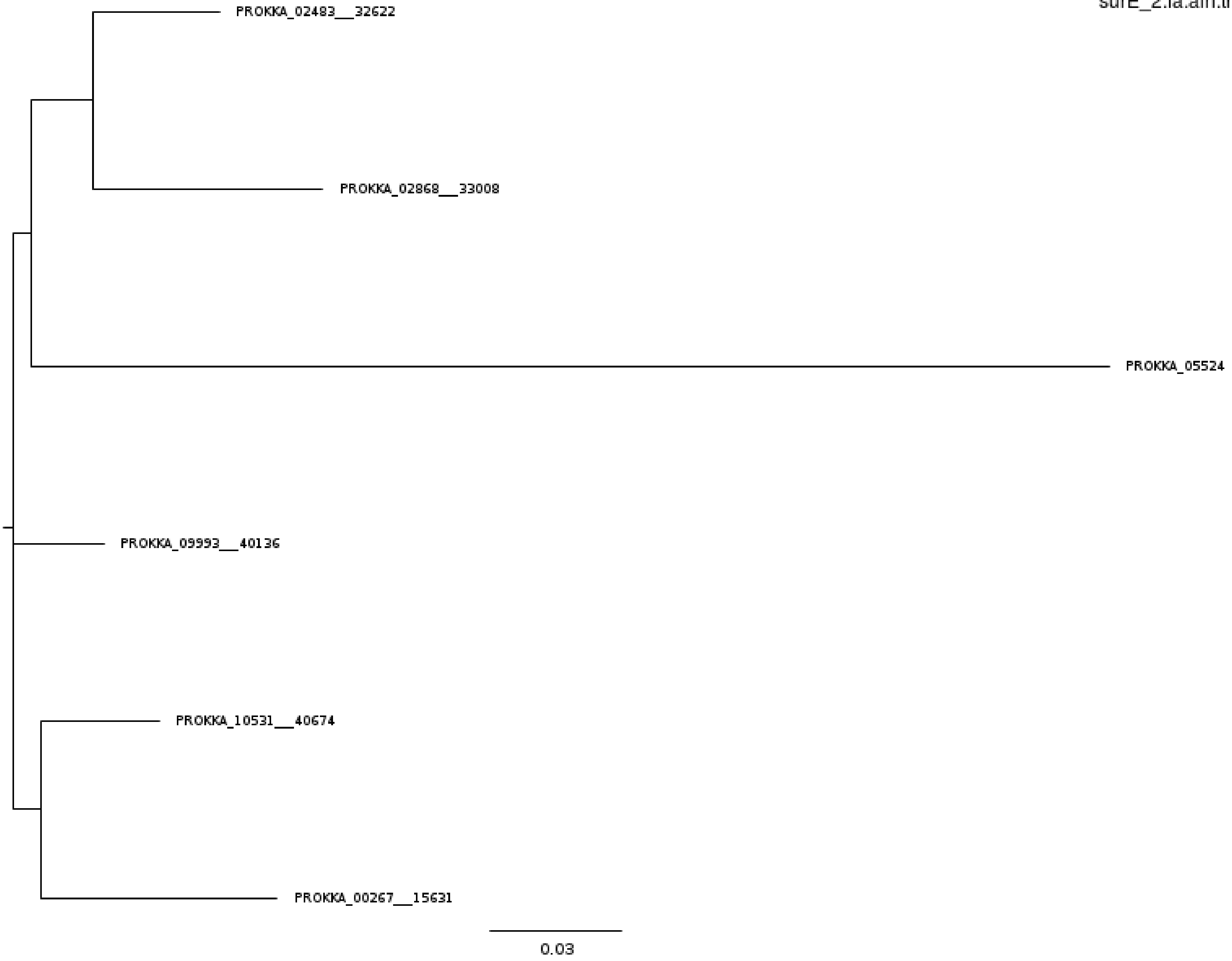
PROKKA_02483___32622
PROKKA_02868___33008
PROKKA_05524
PROKKA_09993___40136
PROKKA_10531___40674
PROKKA_00267___15631
0.03

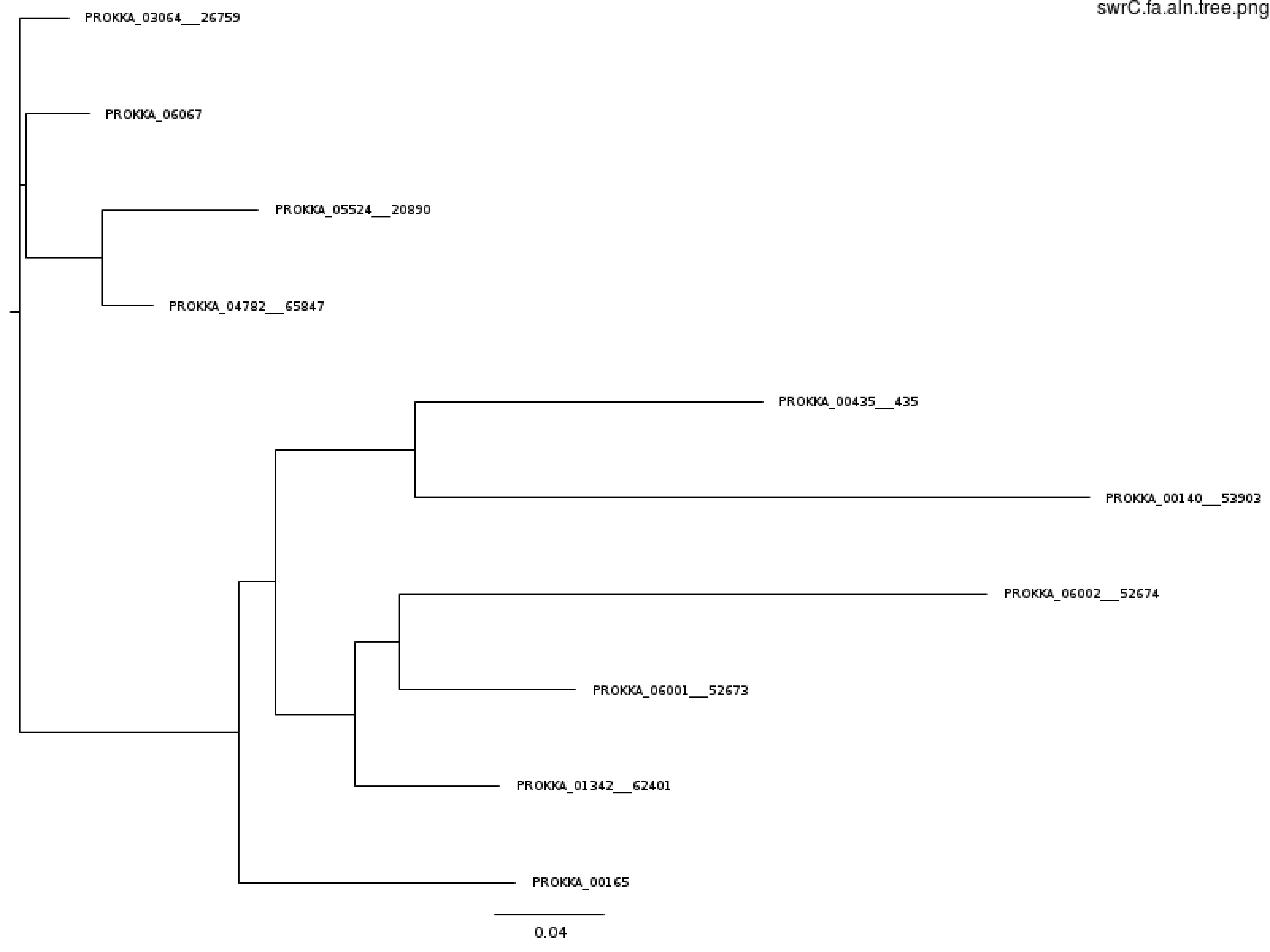

swrC.fa.aln.tree.png
PROKKA_03064__26759
PROKKA_06067
PROKKA_05524__20890
PROKKA_04782__65847
PROKKA_00435__435
PROKKA_00140__53903
PROKKA_06002__52674
PROKKA_06001__52673
PROKKA_01342__62401
PROKKA_00165
0.04

tadA.fa.aln.tree.png

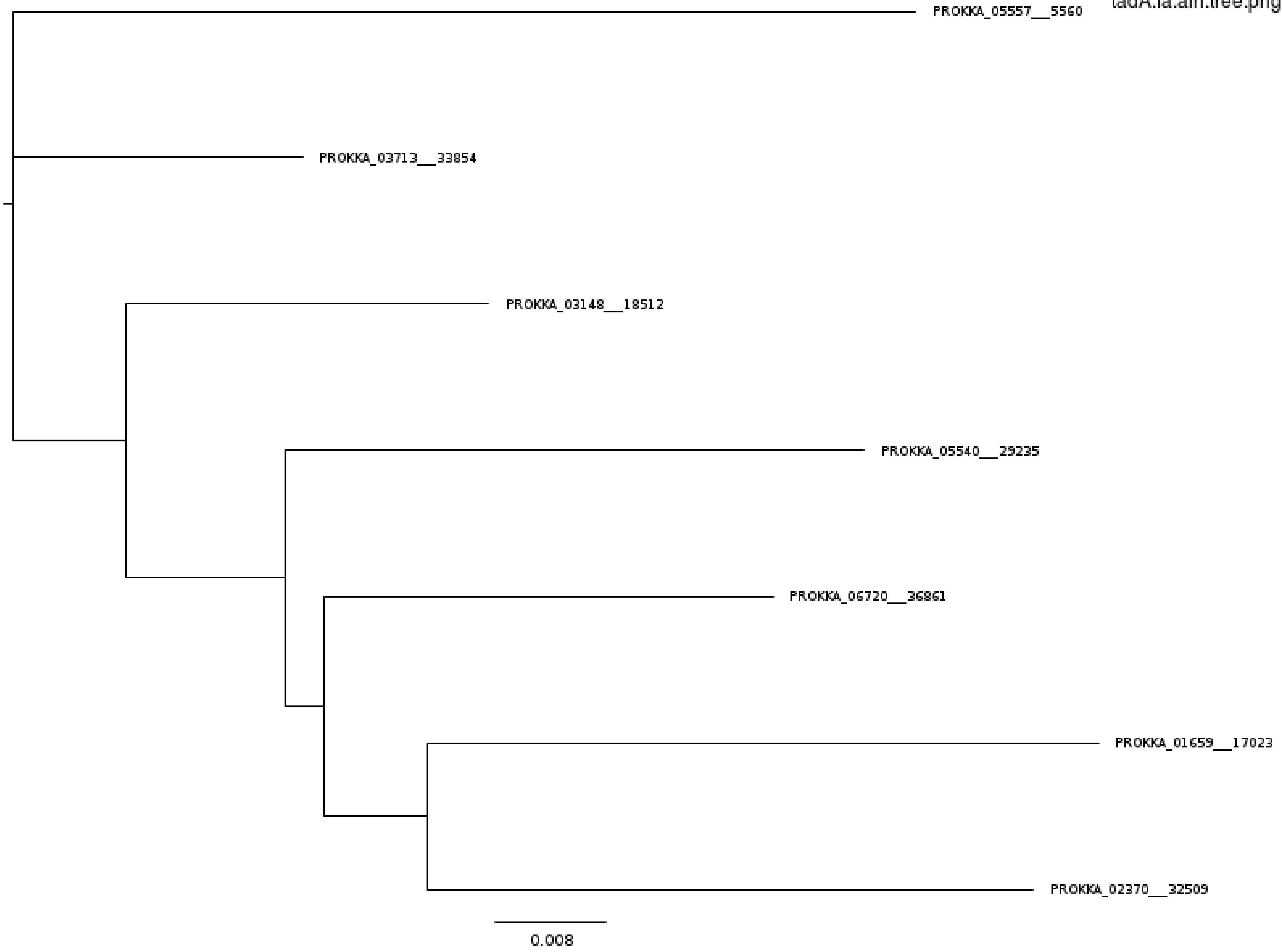

tagG_2.fa.aln.tree.png

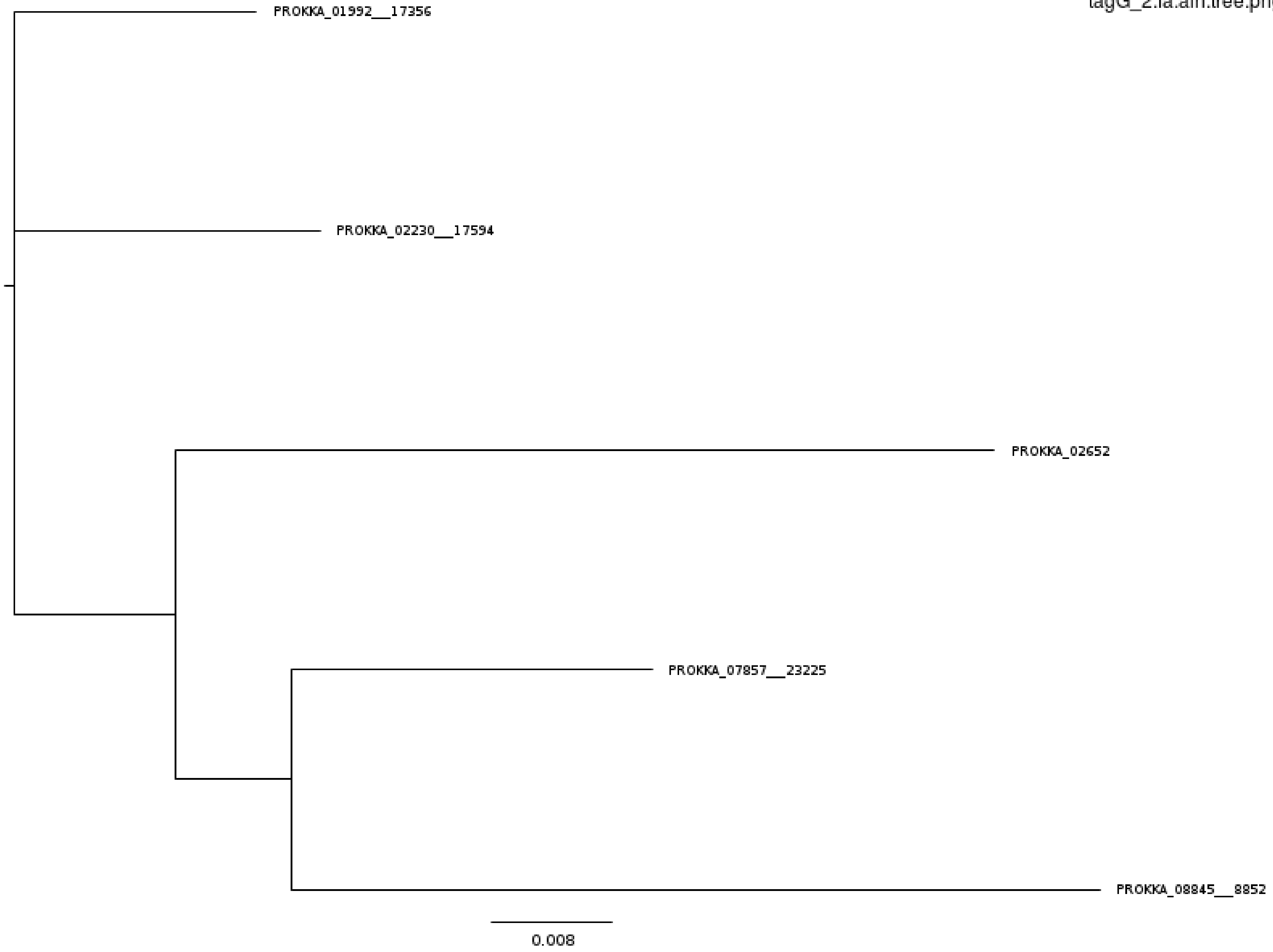

tagO_2.fa.aln.tree.png

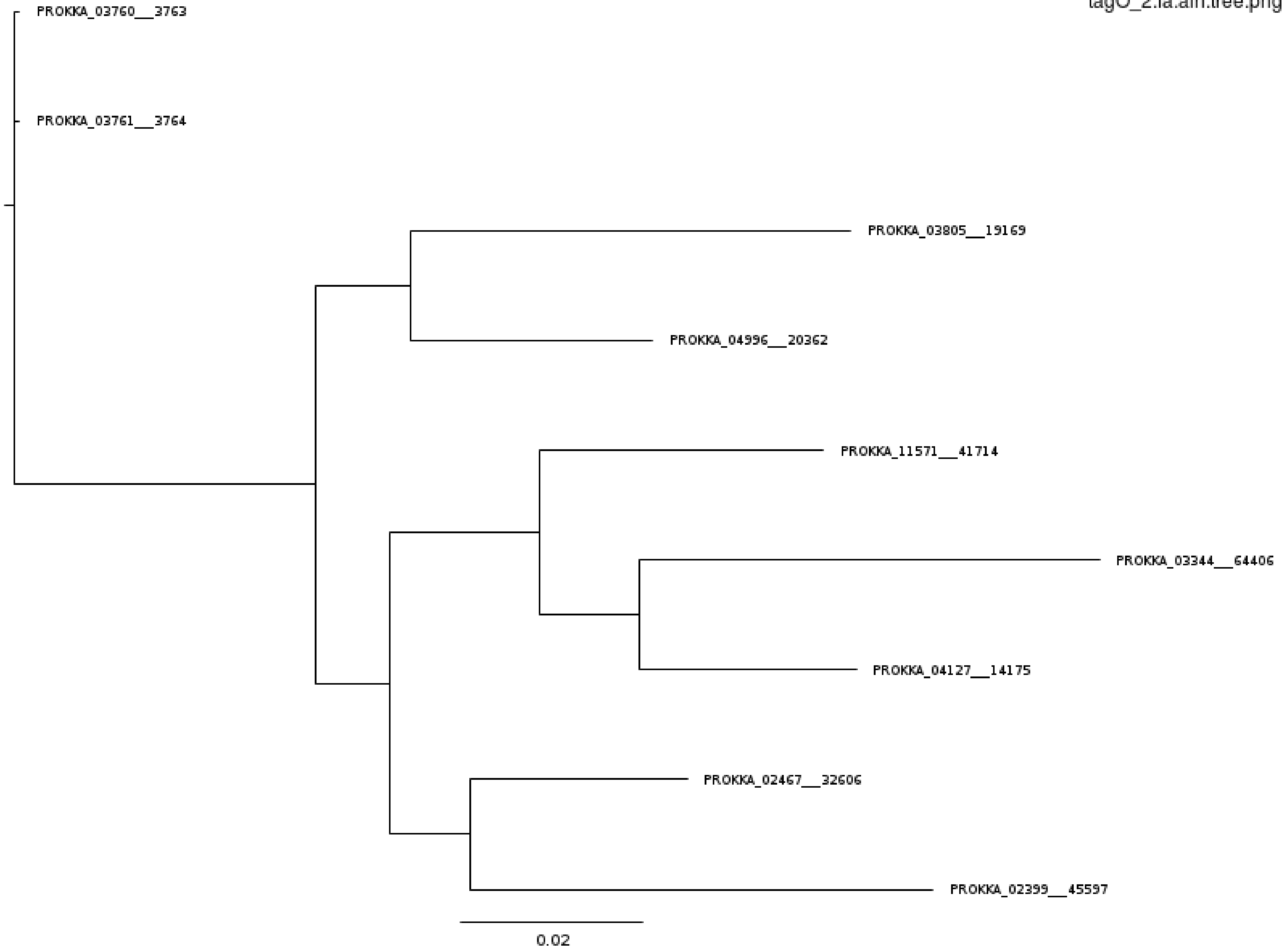

tam_3.fa.aln.tree.png

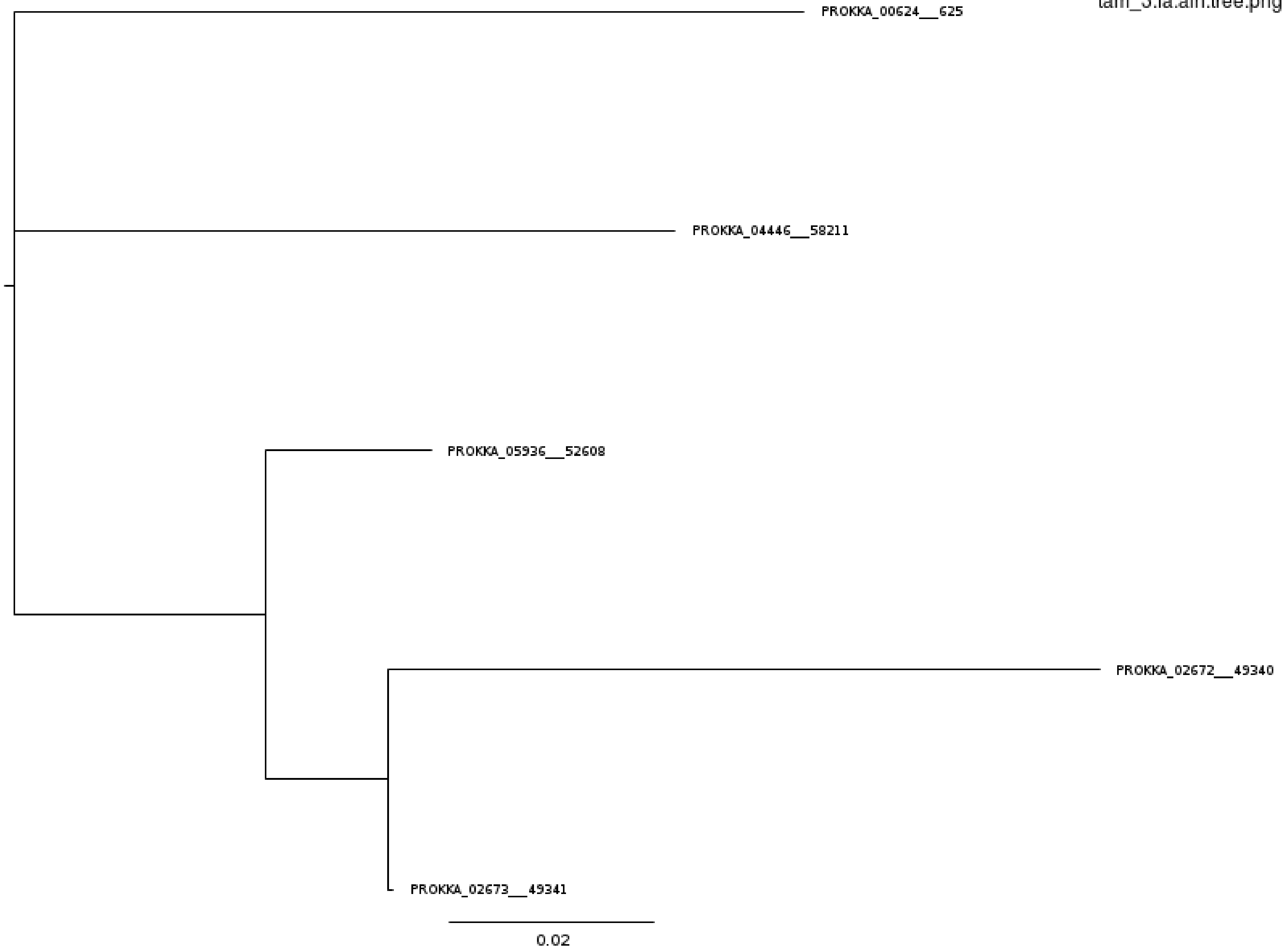

tatC_1.fa.aln.tree.png

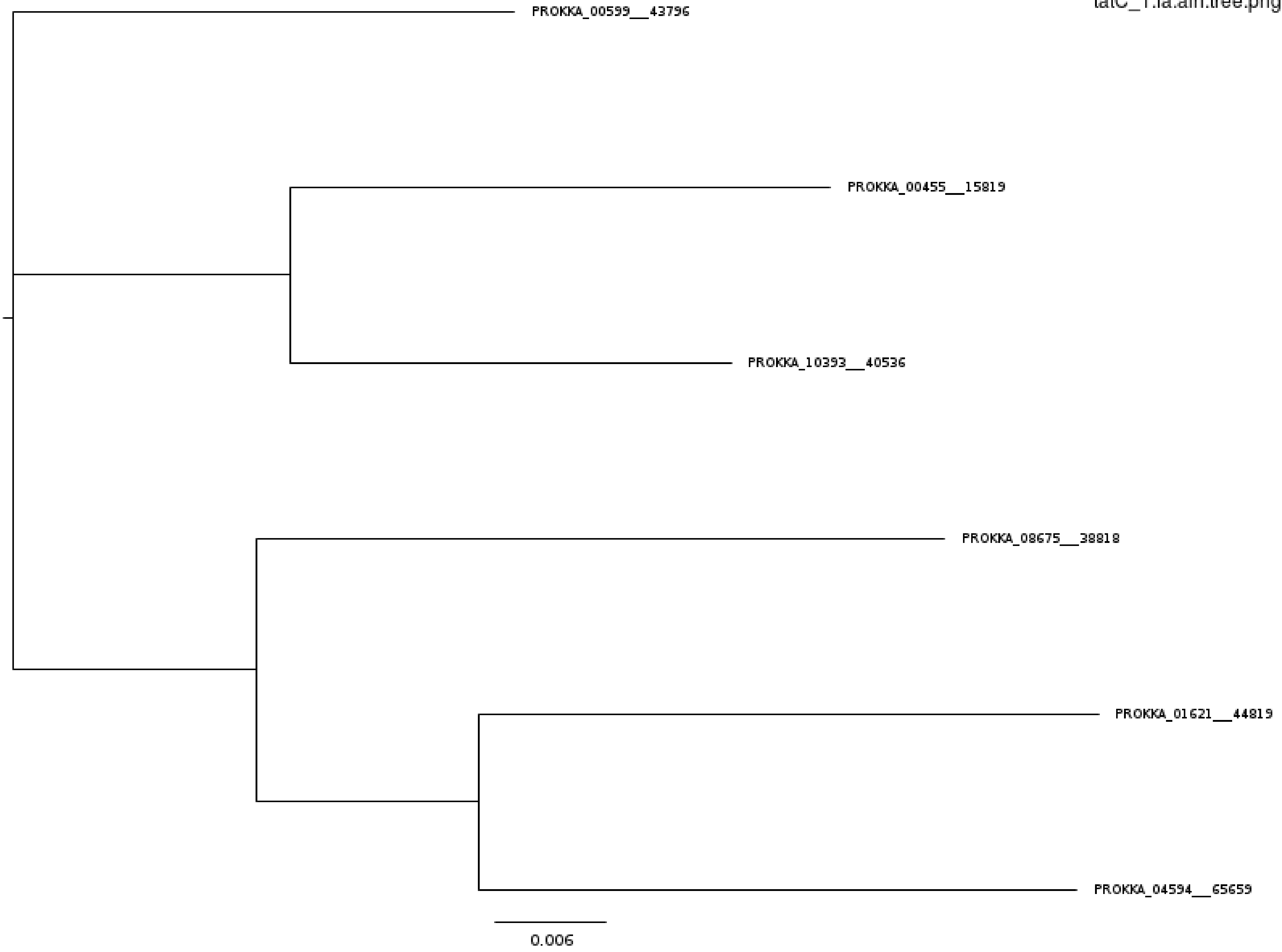

tauA.fa.aln.tree.png

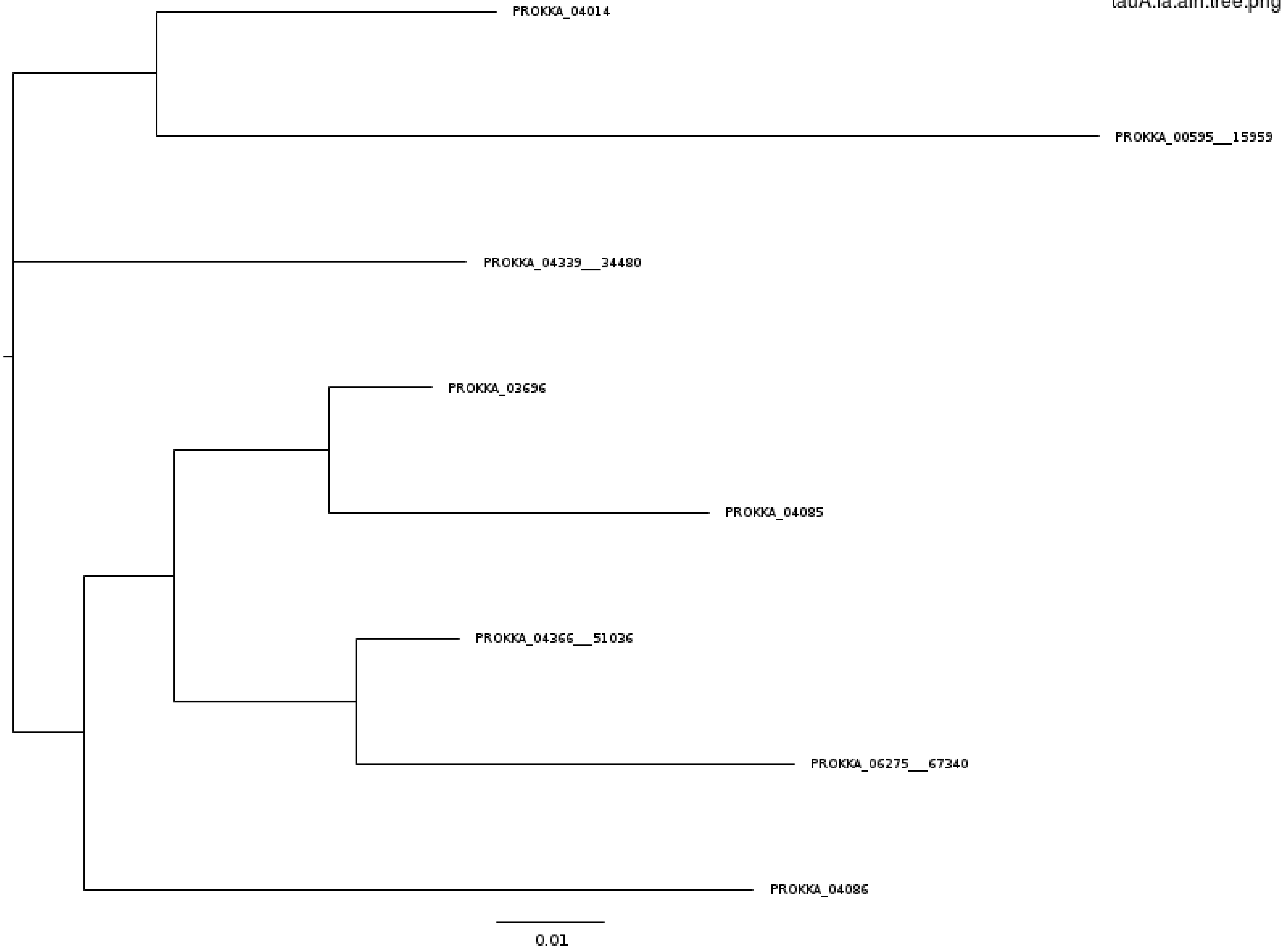

tauB_1.fa.aln.tree.png

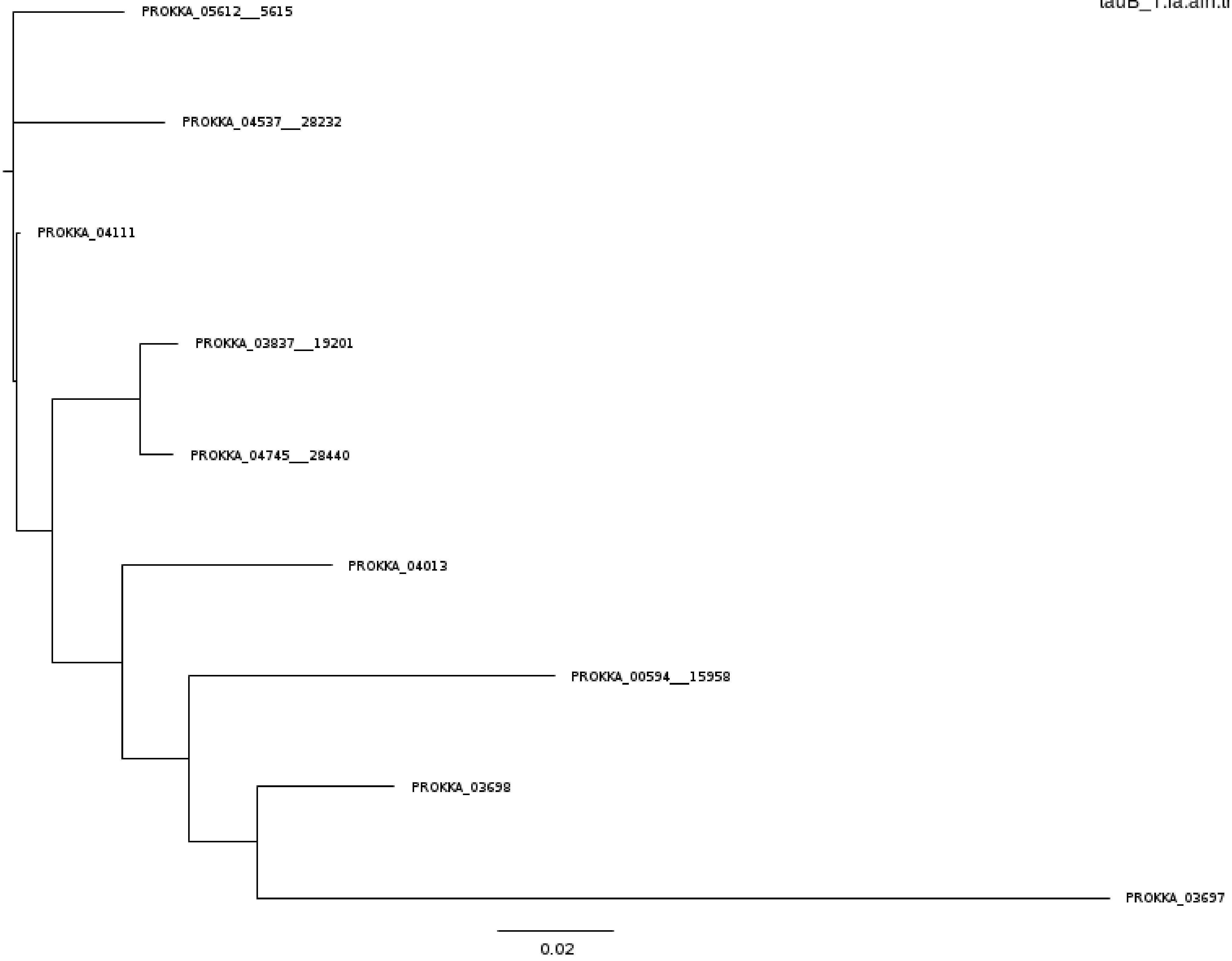

tcrA_2.fa.aln.tree.png

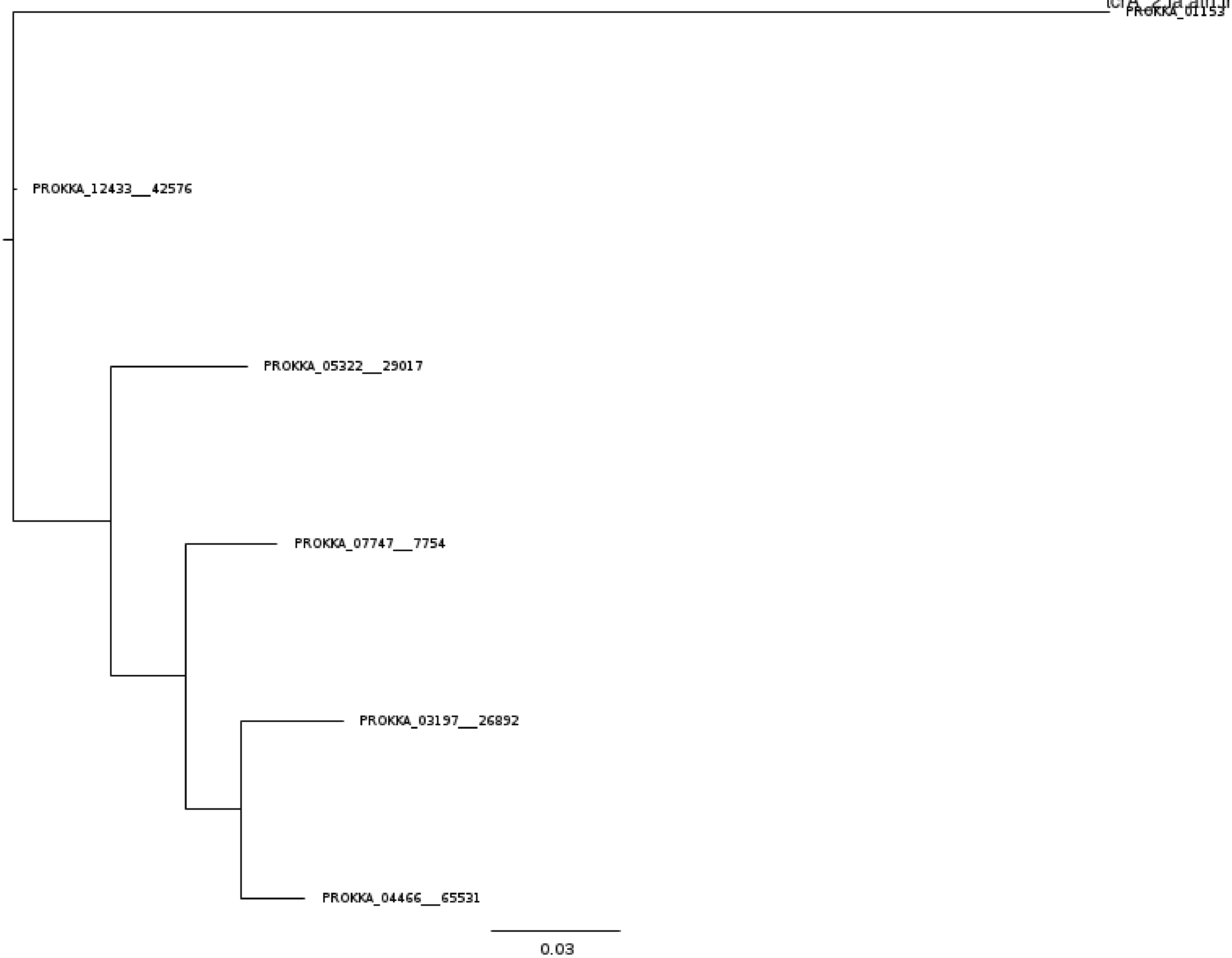

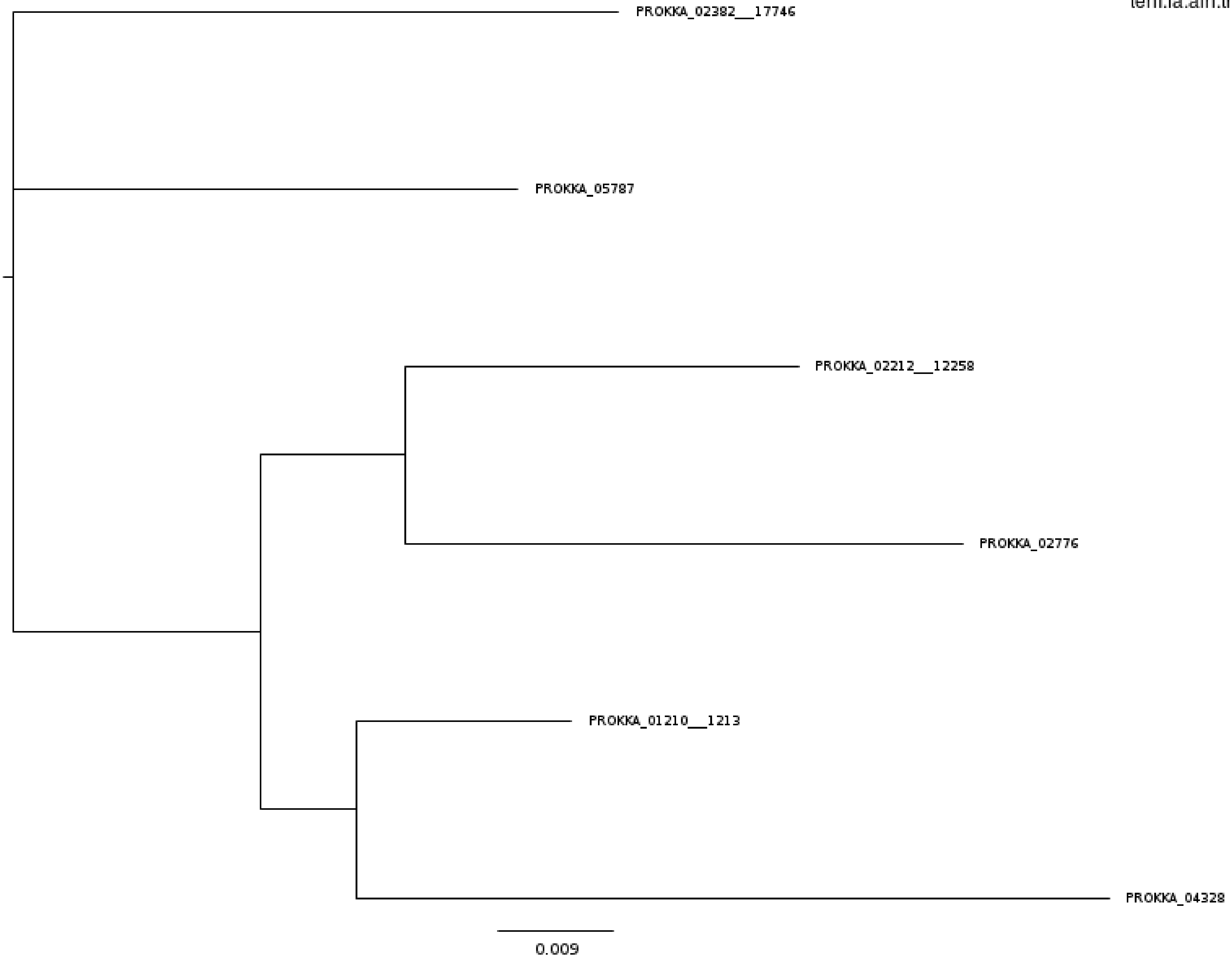
PROKKA_02382__17746
PROKKA_05787
PROKKA_02212__12258
PROKKA_02776
PROKKA_01210__1213
PROKKA_04328
0.009

tgpA_2.fa.aln.tree.png

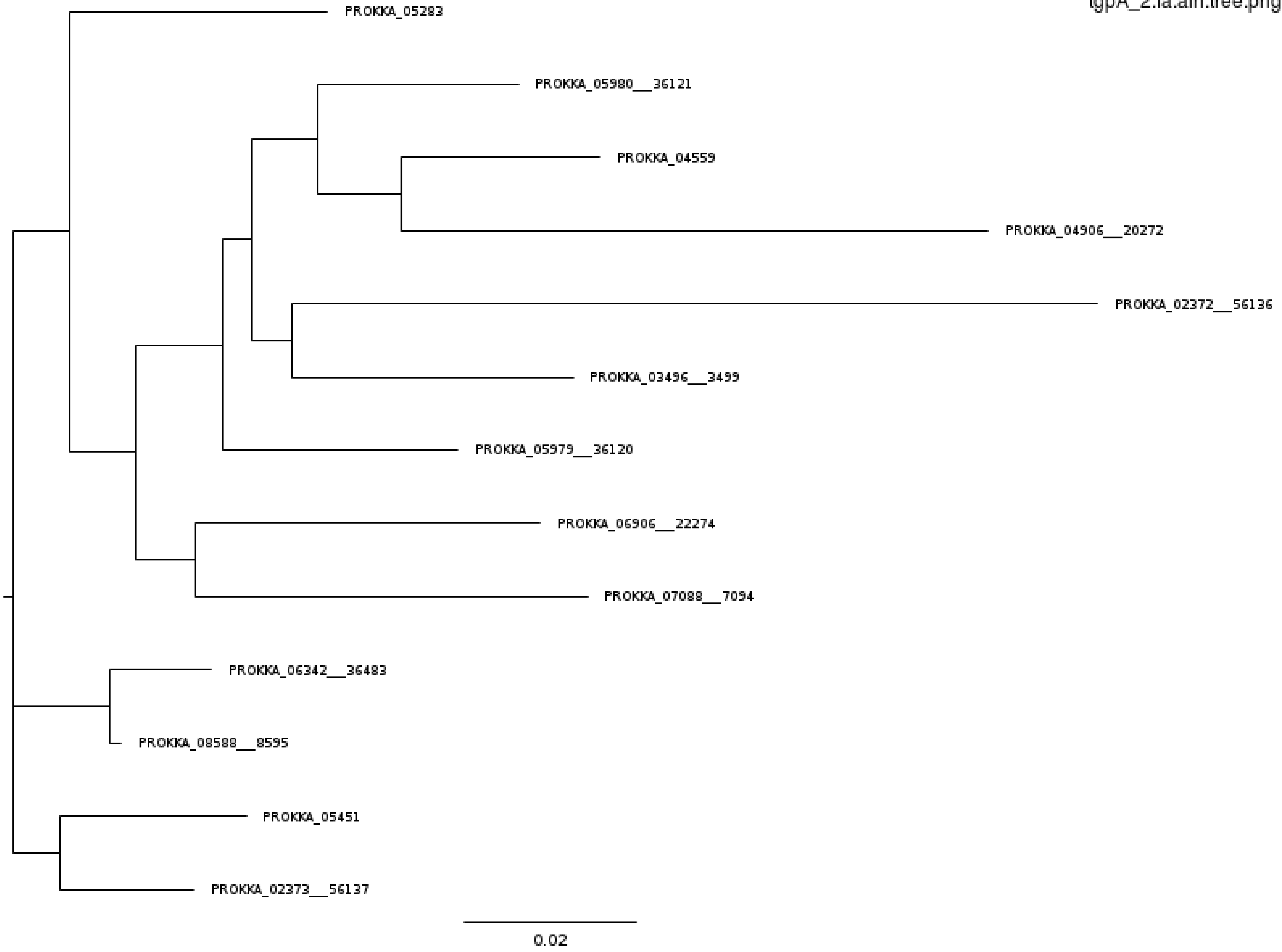

tgpA_5.fa.aln.tree.png

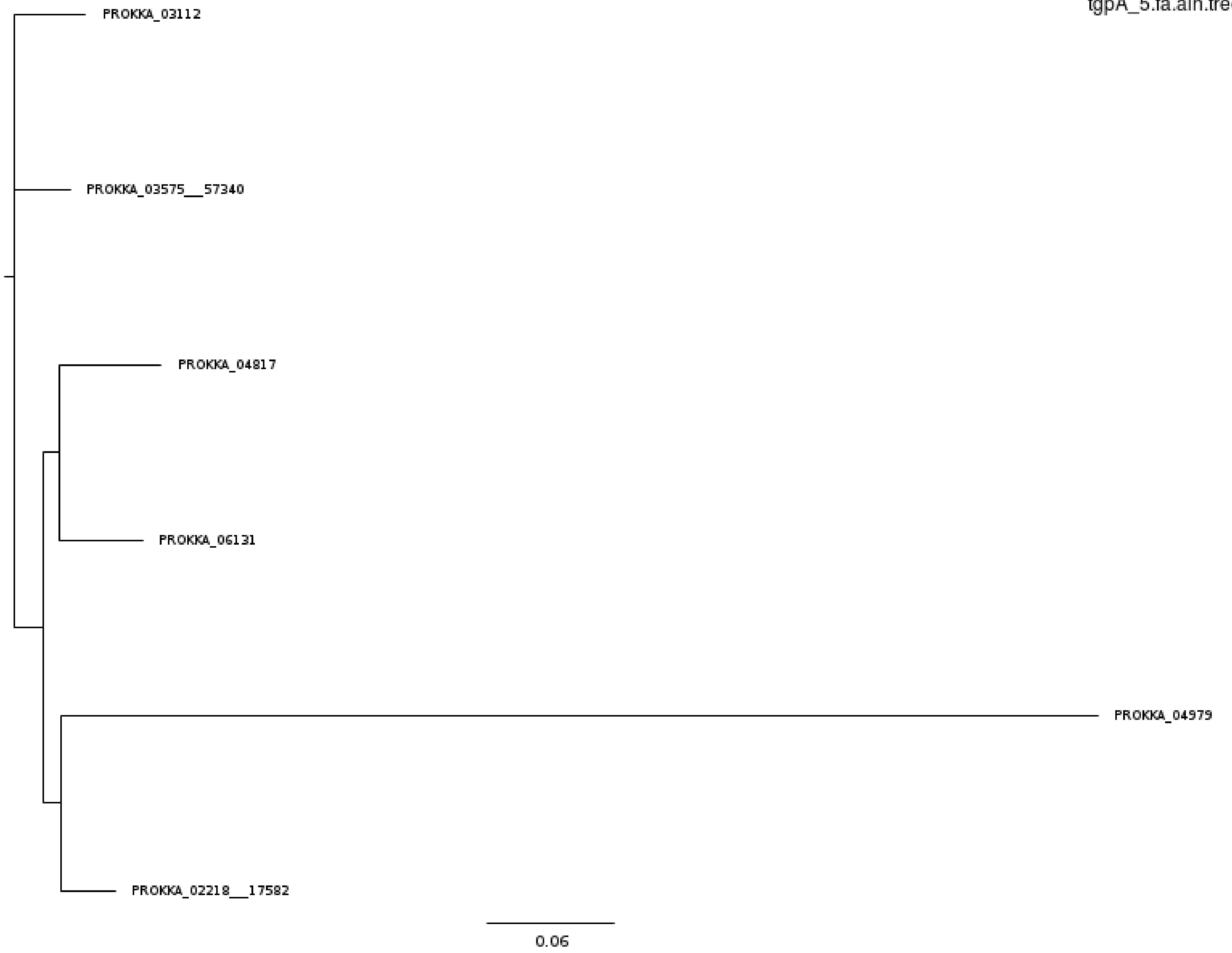

tgt.fa.aln.tree.png

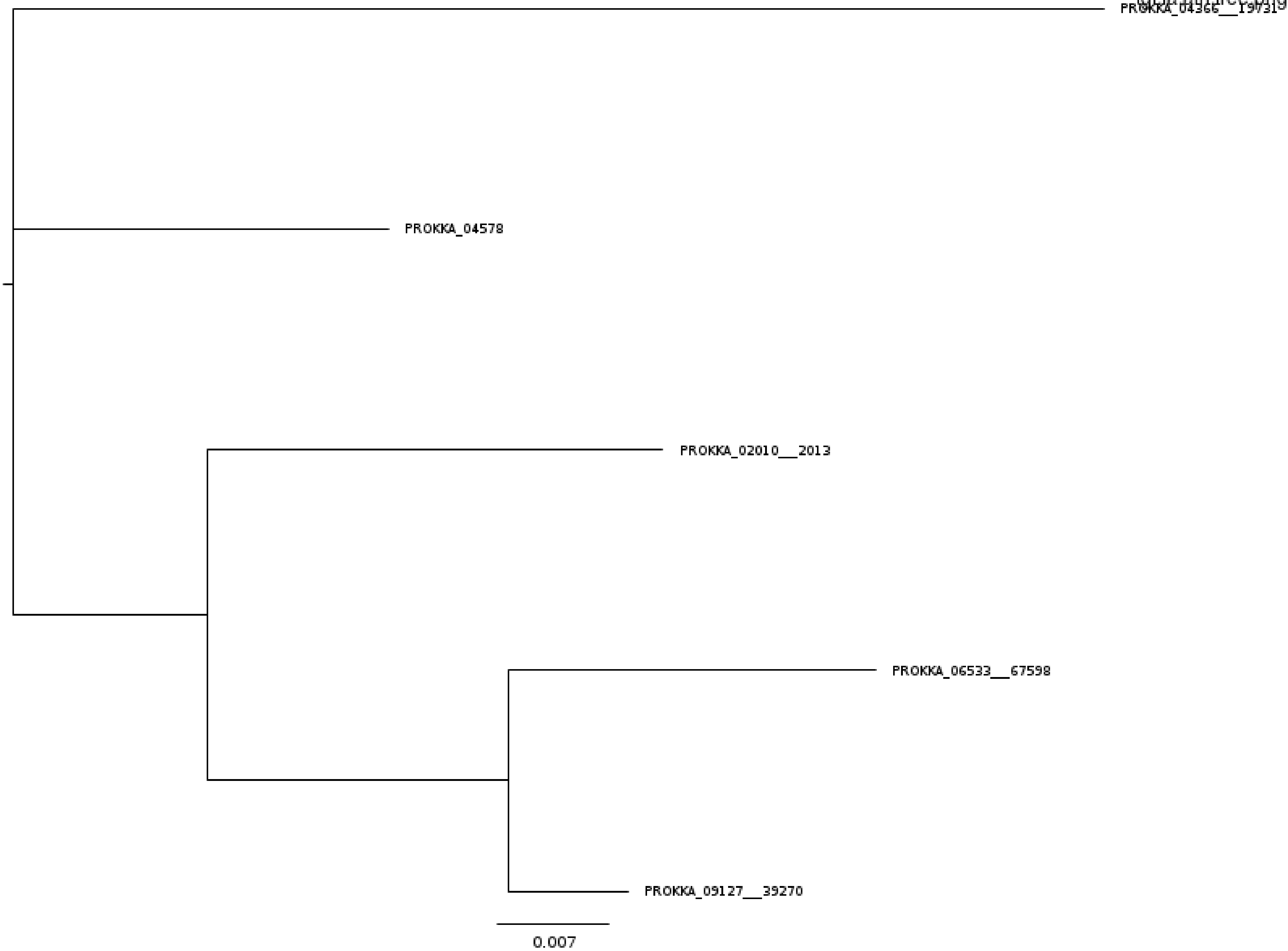

thiE_2.fa.aln.tree.png

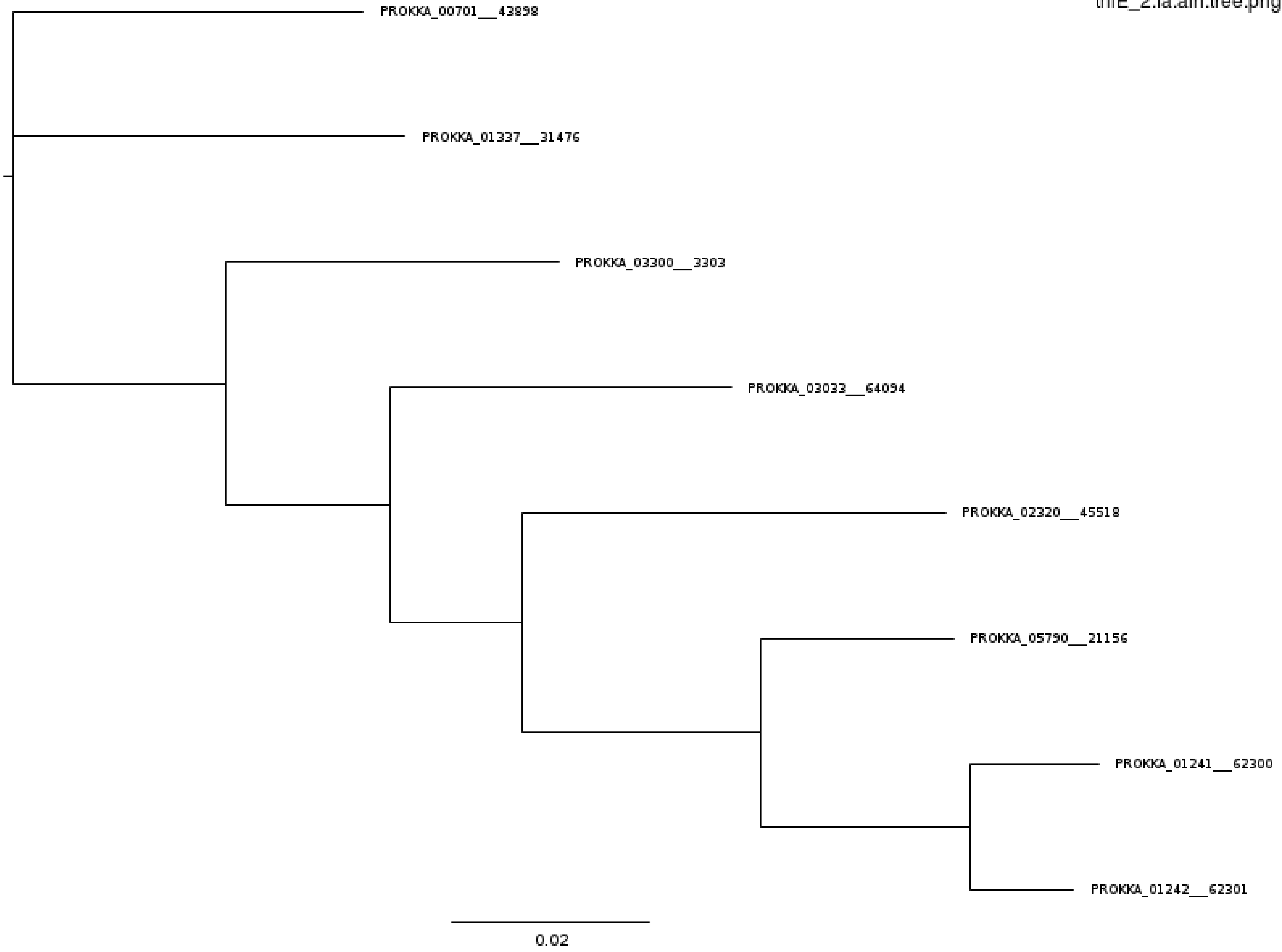

thiE.fa.aln.tree.png

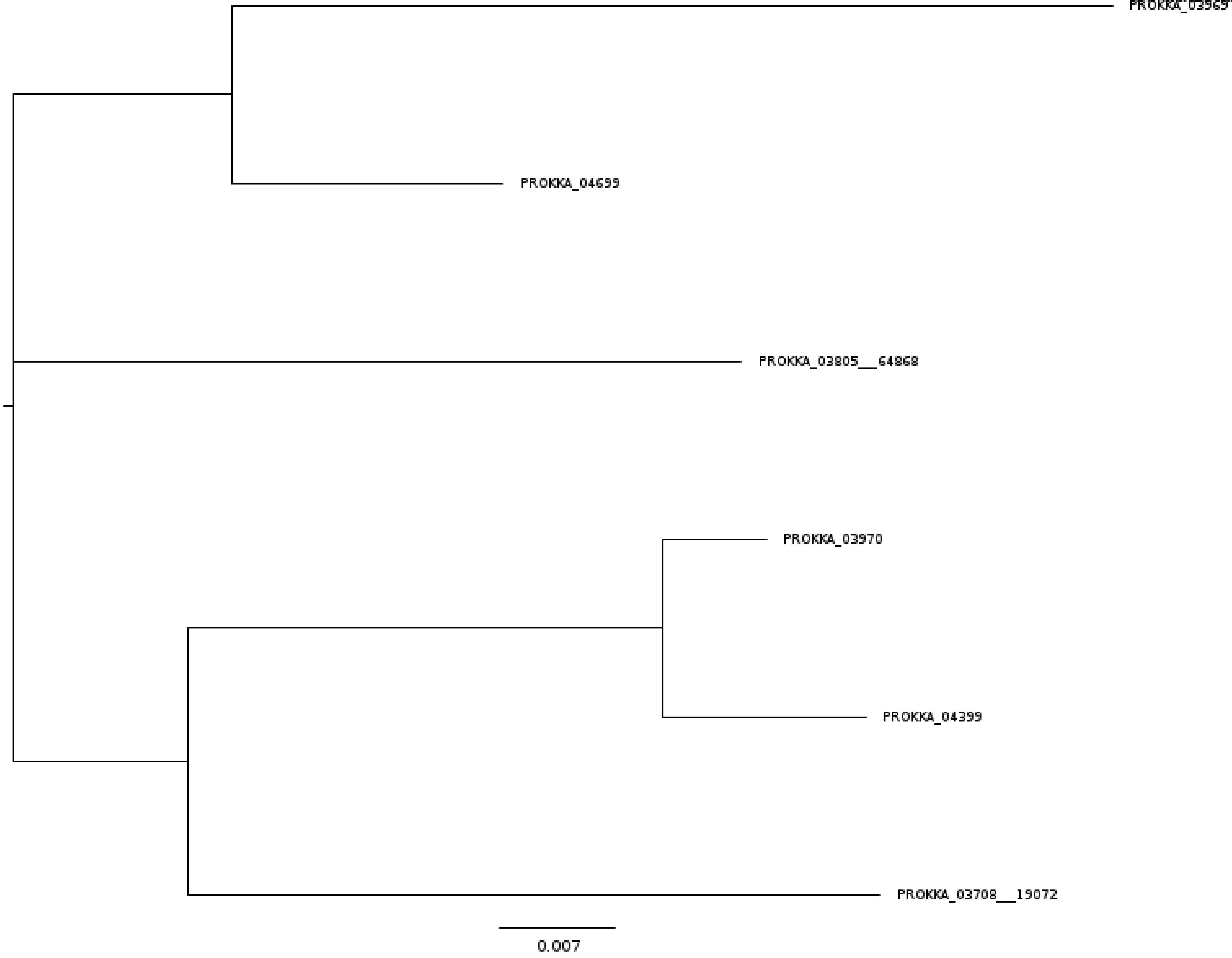

thiG_2.fa.aln.tree.png

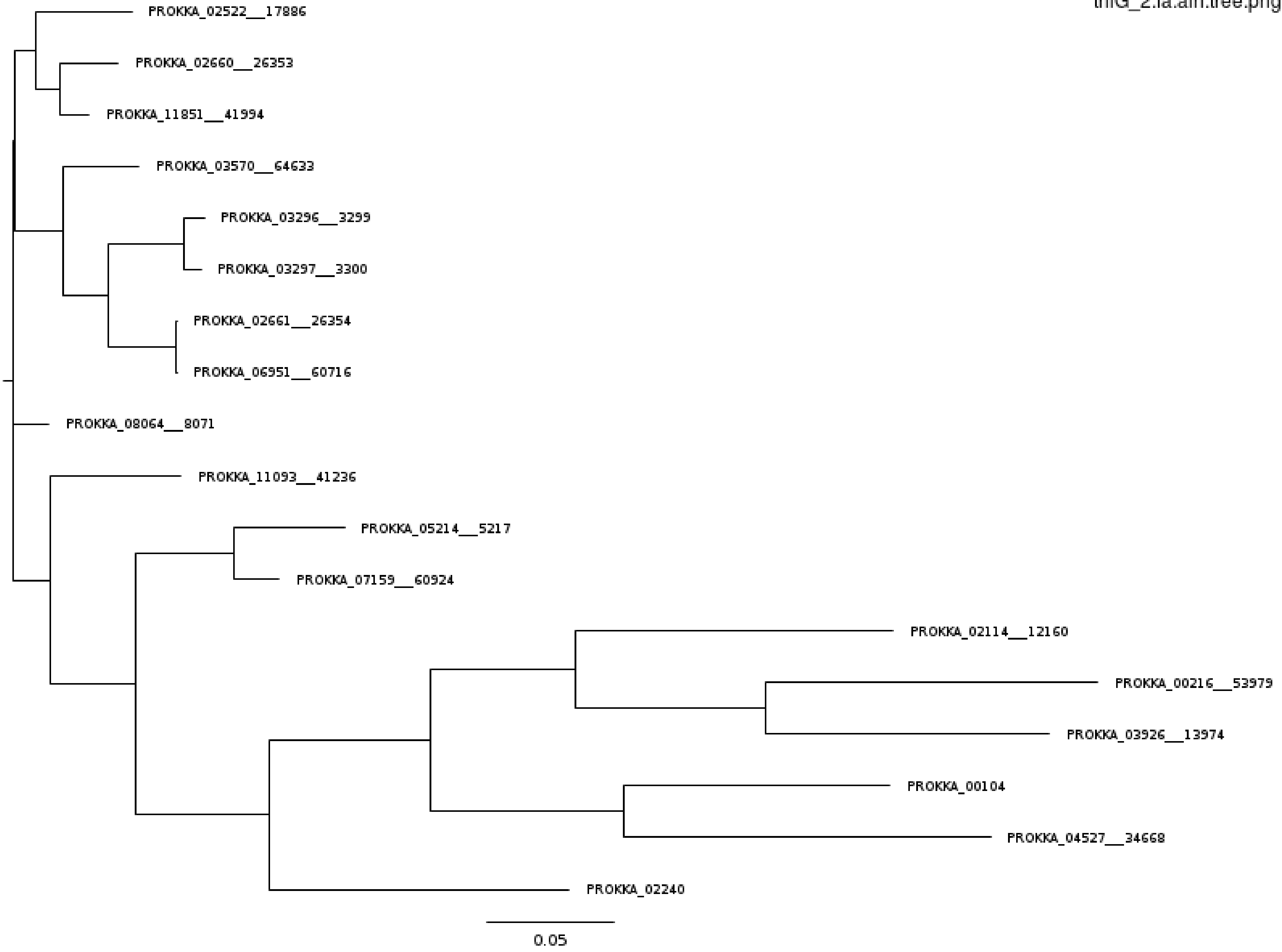

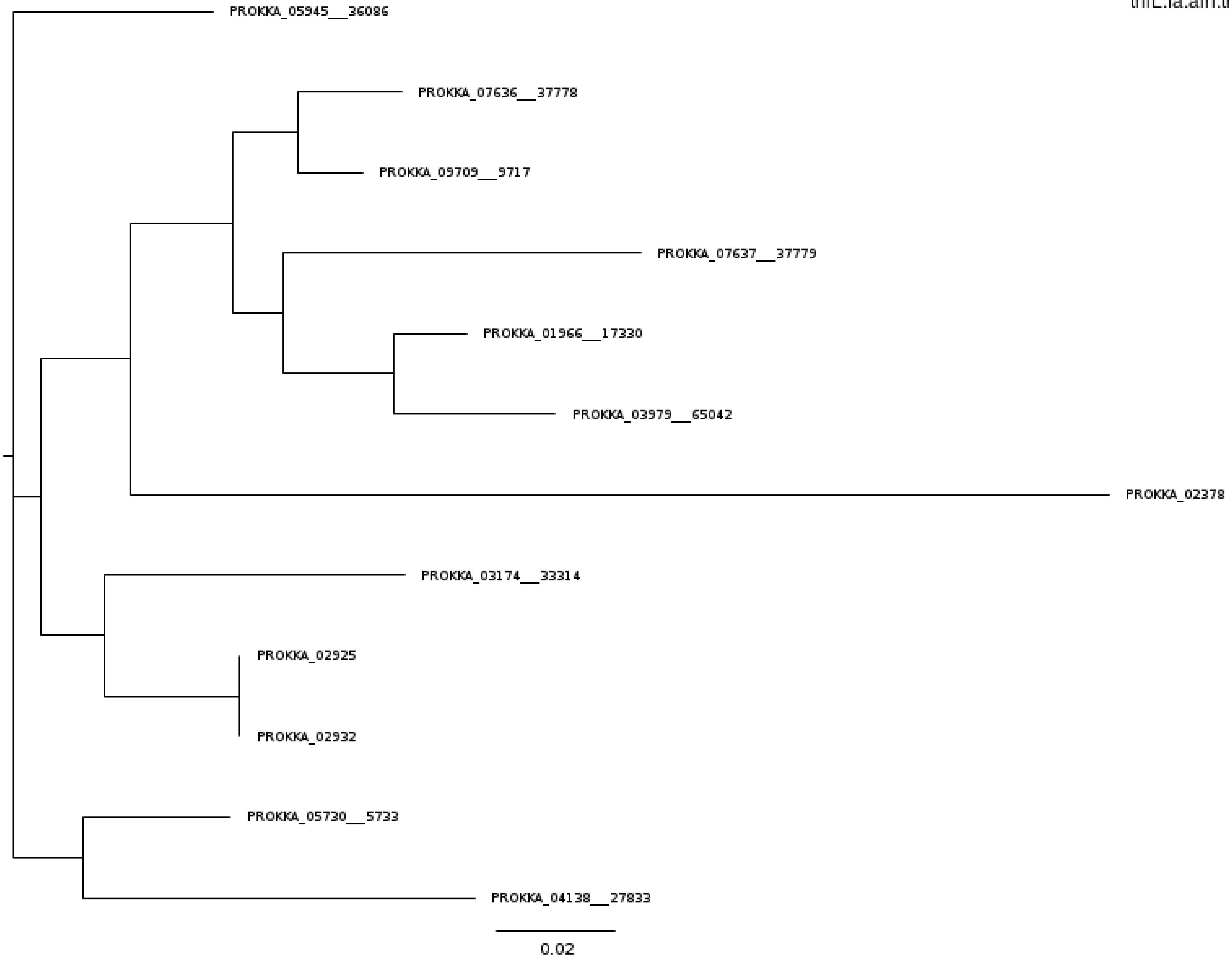
PROKKA_05945__36086
PROKKA_07636__37778
PROKKA_09709__9717
PROKKA_07637__37779
PROKKA_01966__17330
PROKKA_03979__65042
PROKKA_02378
PROKKA_03174__33314
PROKKA_02925
PROKKA_02932
PROKKA_05730__5733
PROKKA_04138__27833
0.02

thiQ.fa.aln.tree.png

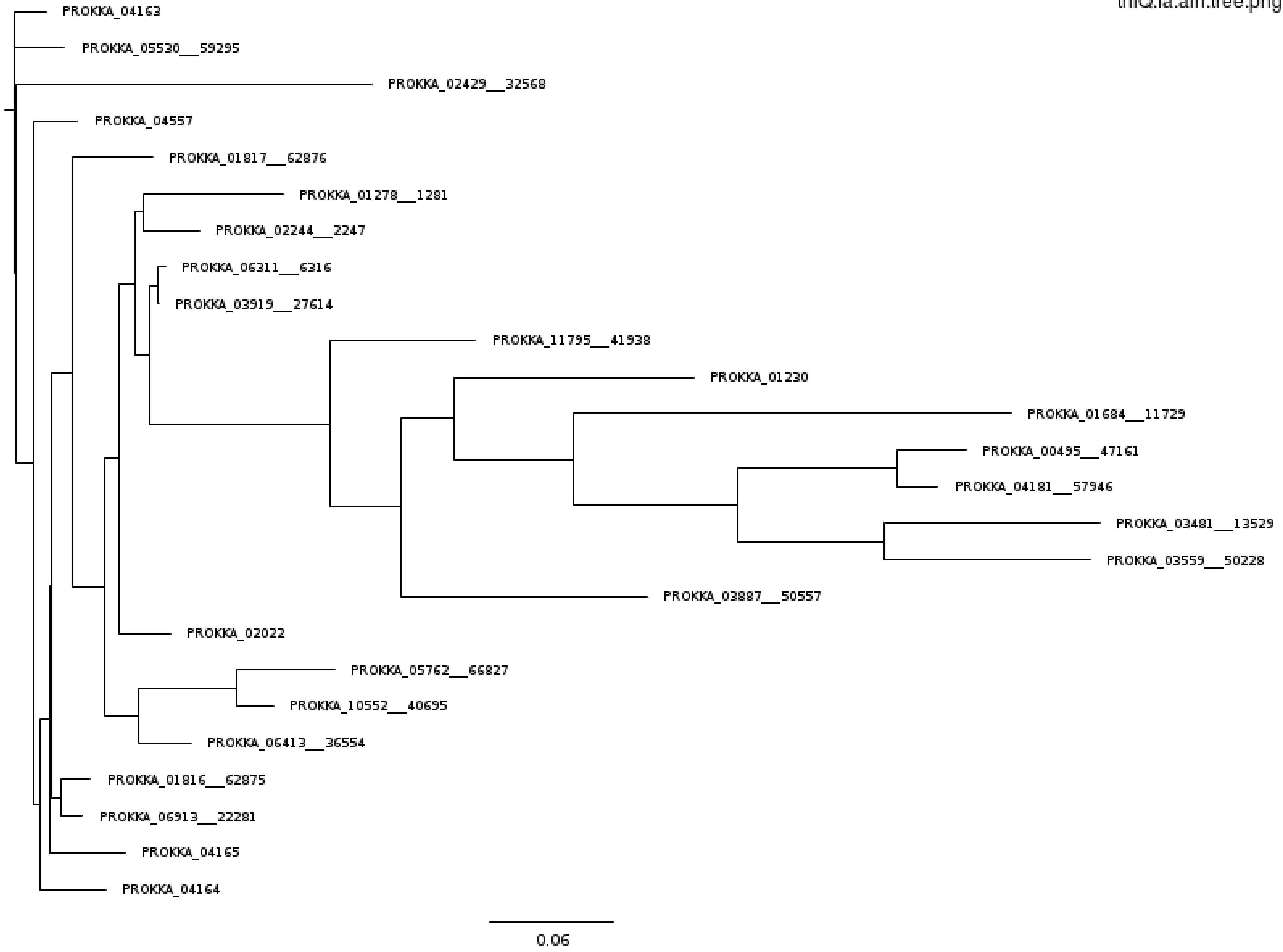

thiS_2.fa.aln.tree.png

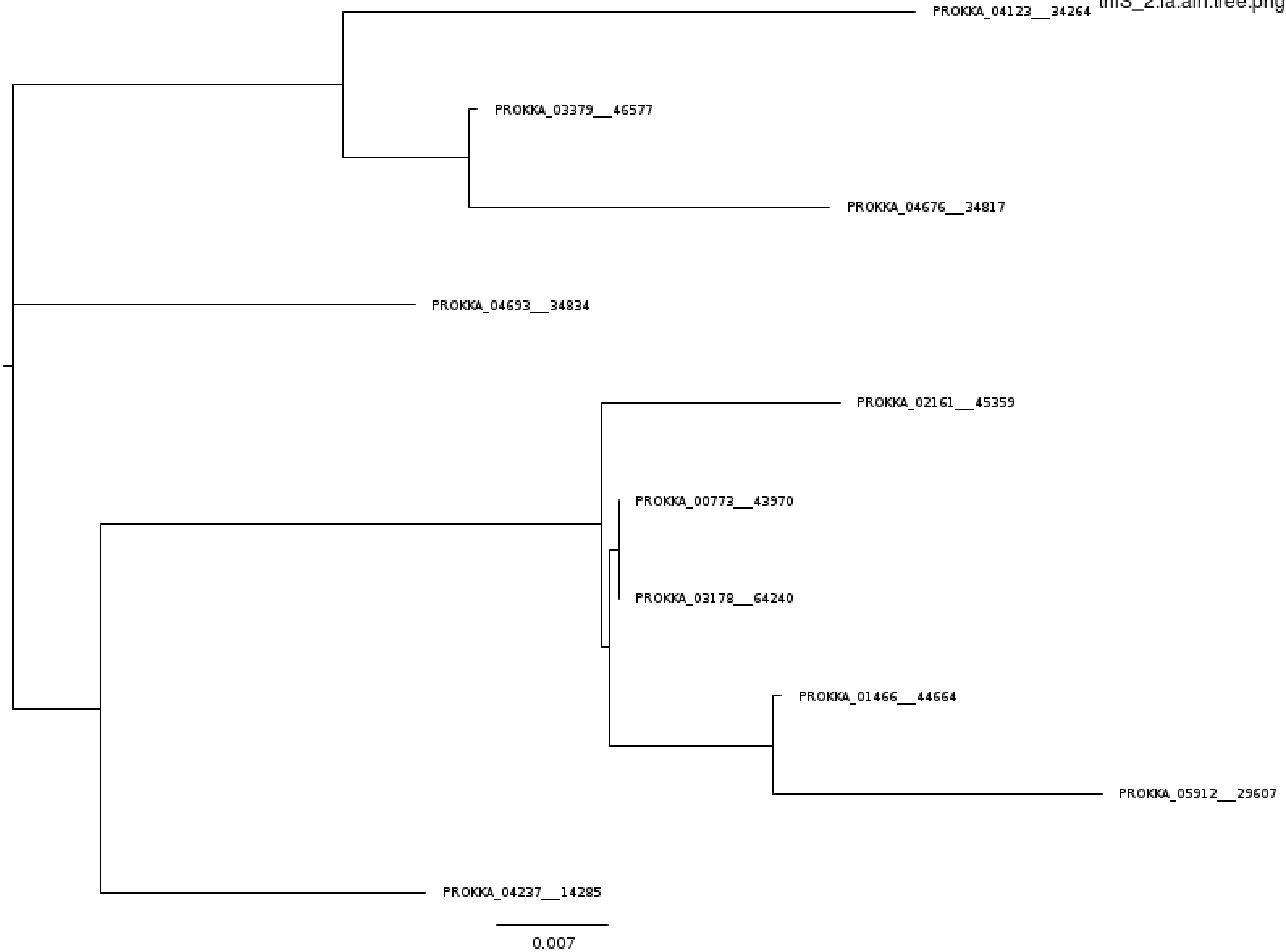

thrC_2.fa.aln.tree.png

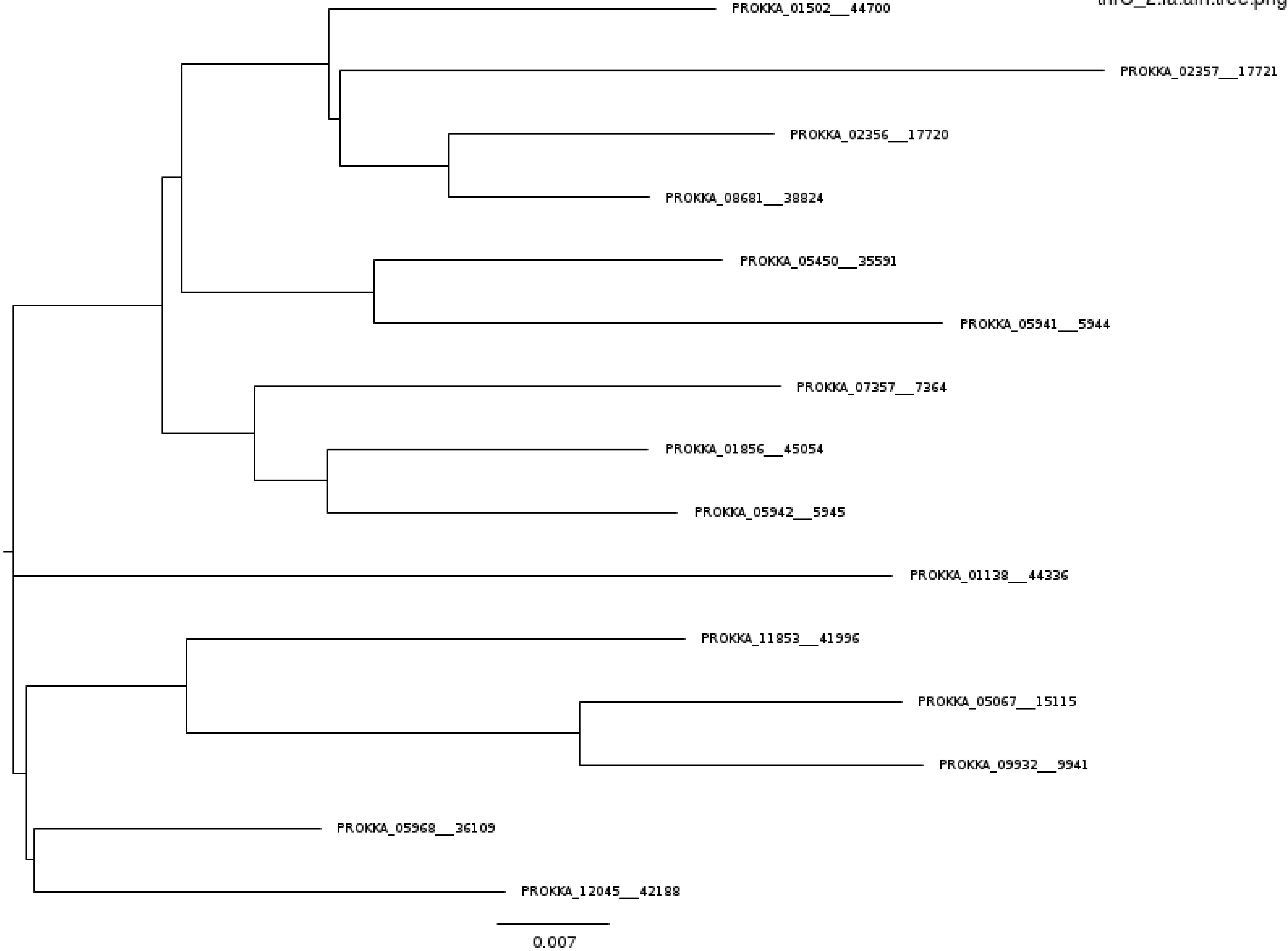

thrS_2.fa.aln.tree.png

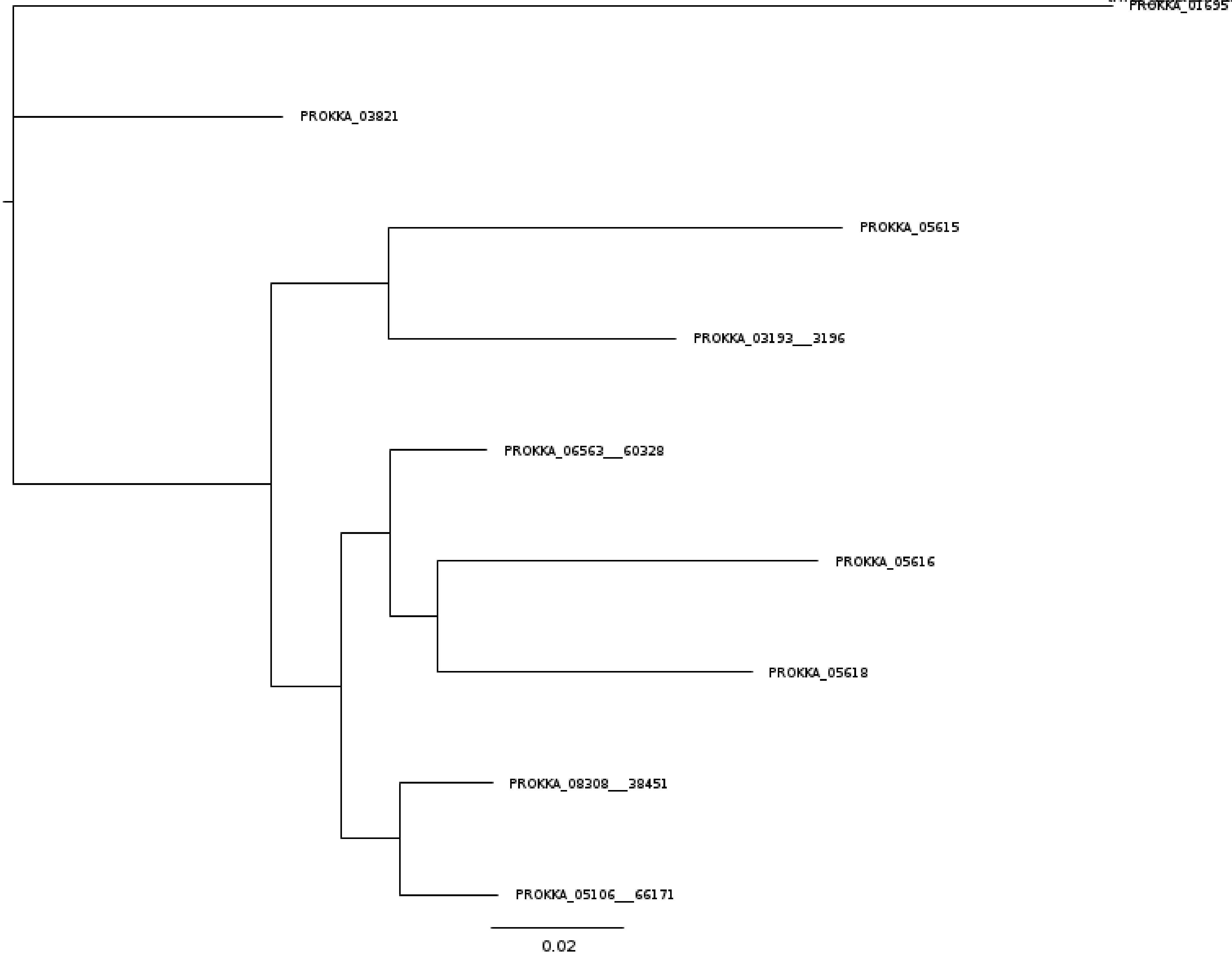

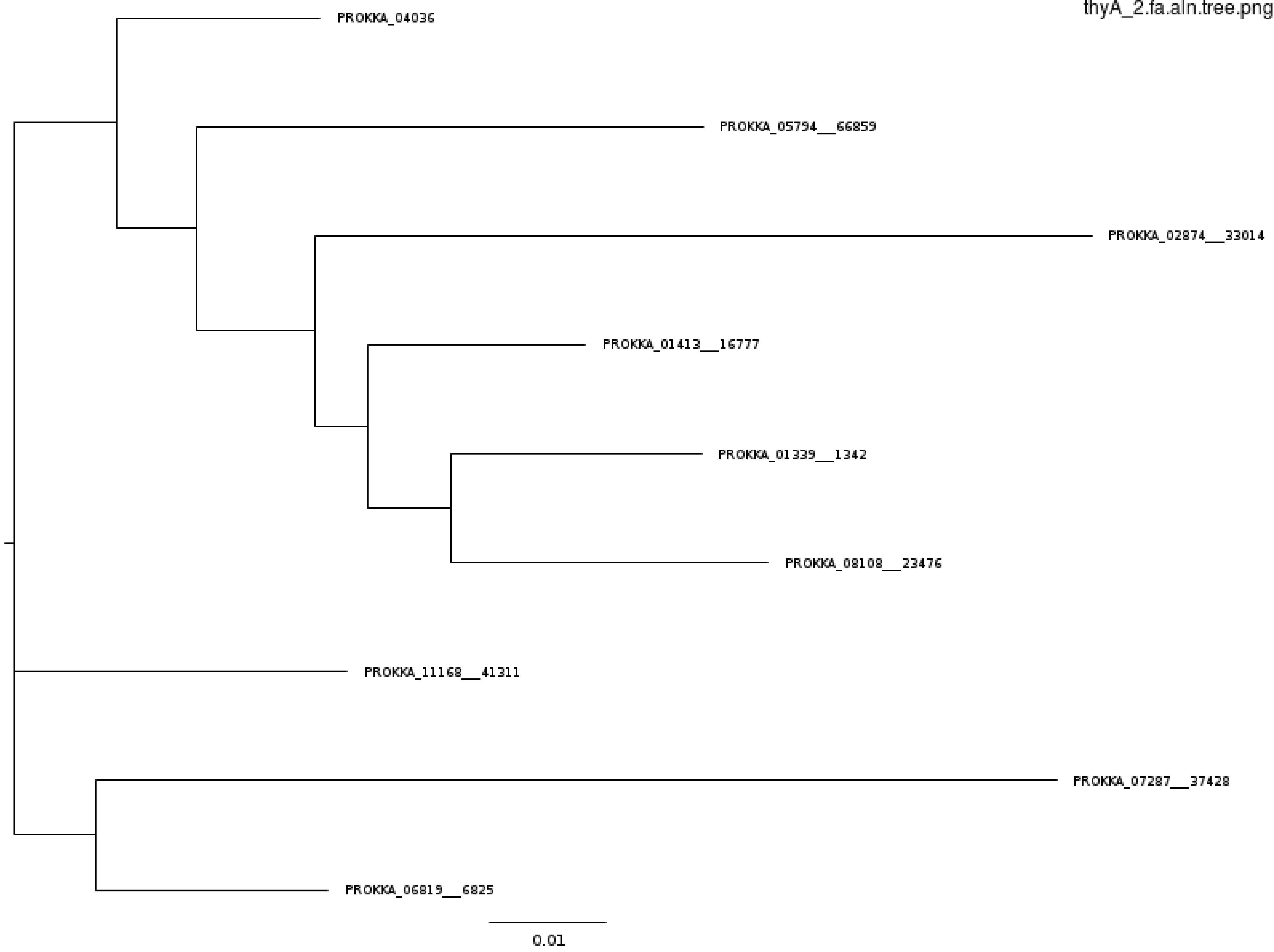
thyA_2.fa.aln.tree.png
PROKKA_04036
PROKKA_05794__66859
PROKKA_02874__33014
PROKKA_01413__16777
PROKKA_01339__1342
PROKKA_08108__23476
PROKKA_11168__41311
PROKKA_07287__37428
PROKKA_06819__6825
0.01

tig_4.fa.aln.tree.png

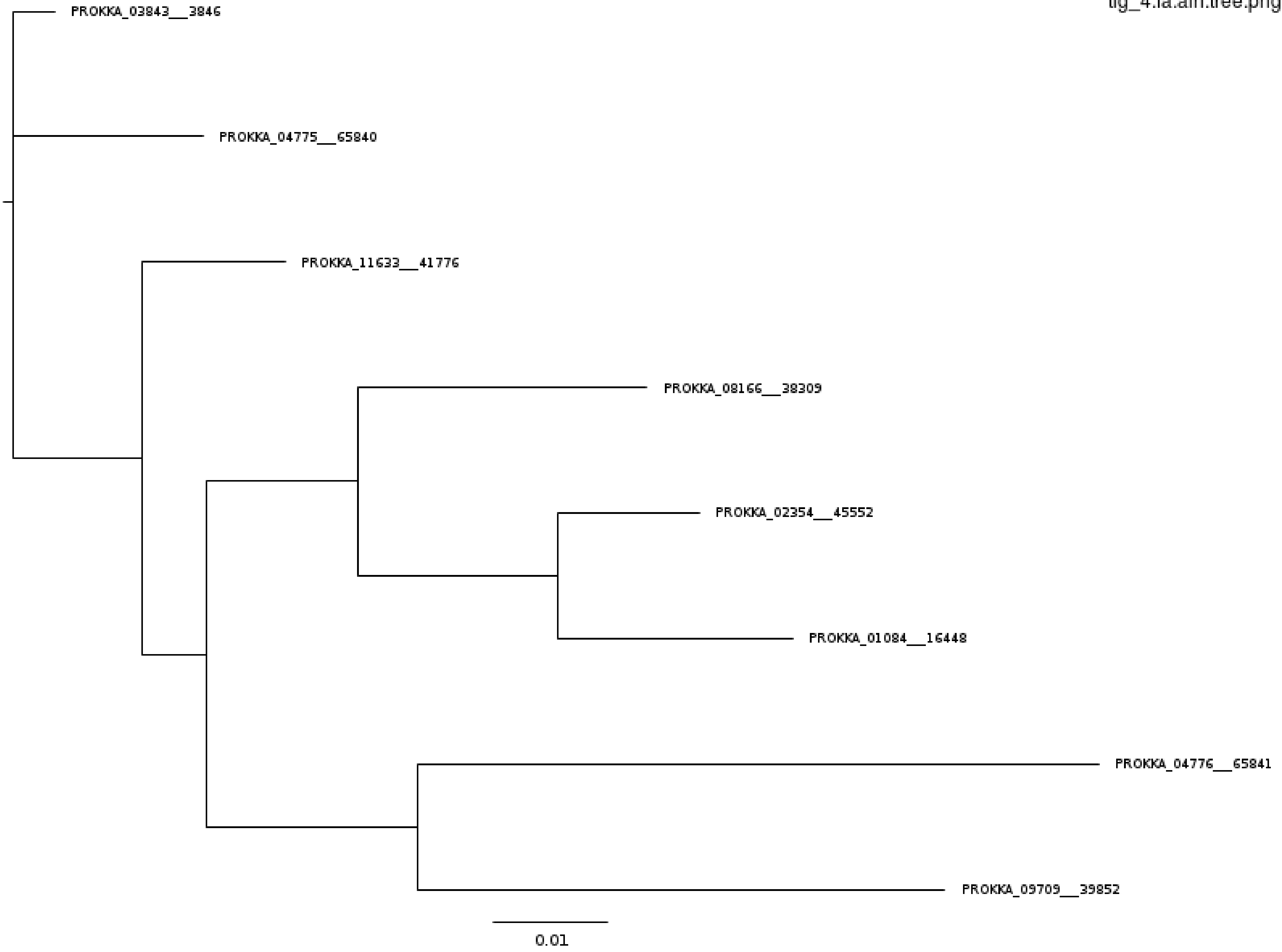

tkt_2.fa.aln.tree.png

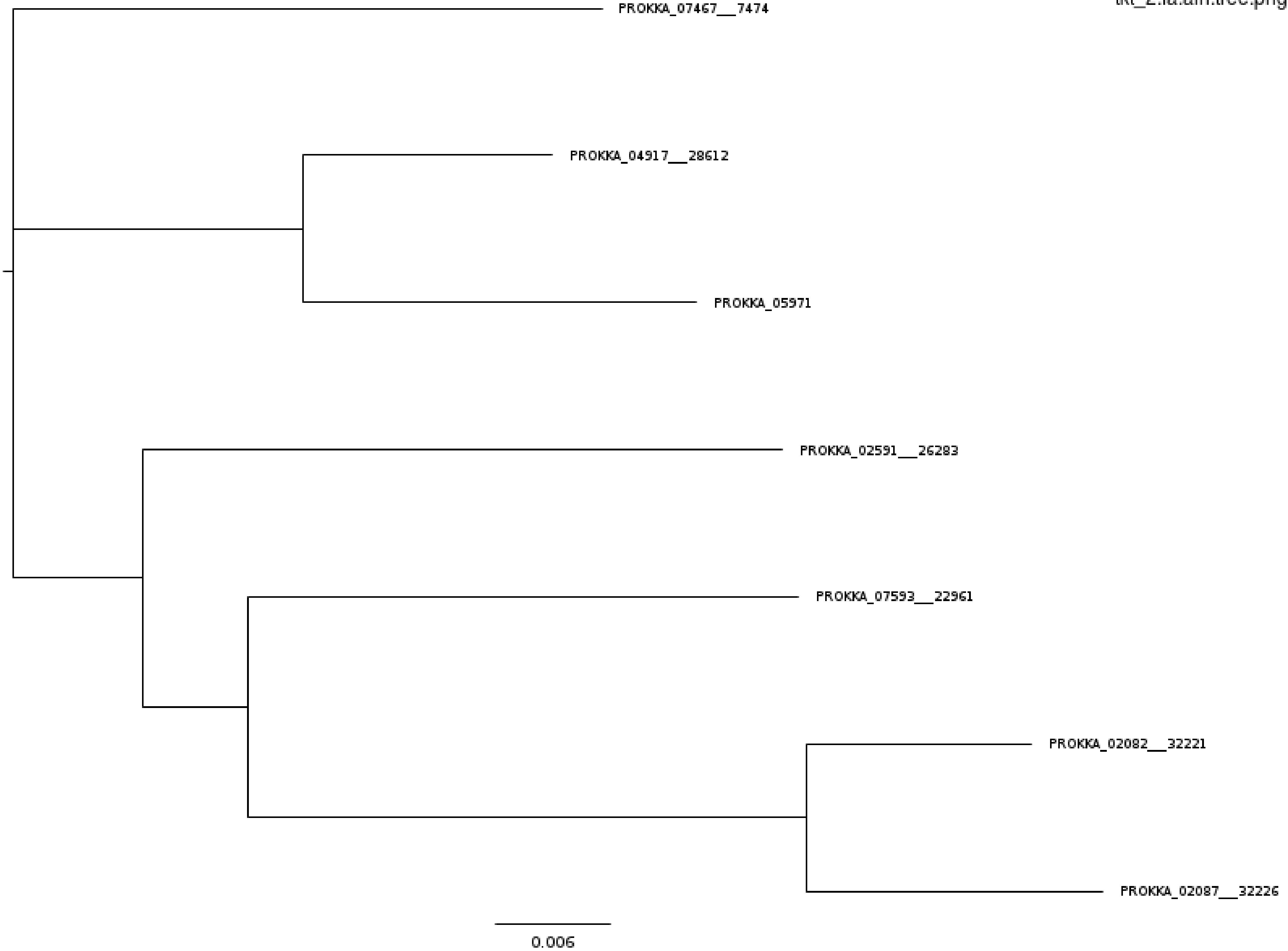

tld_1.fa.aln.tree.png

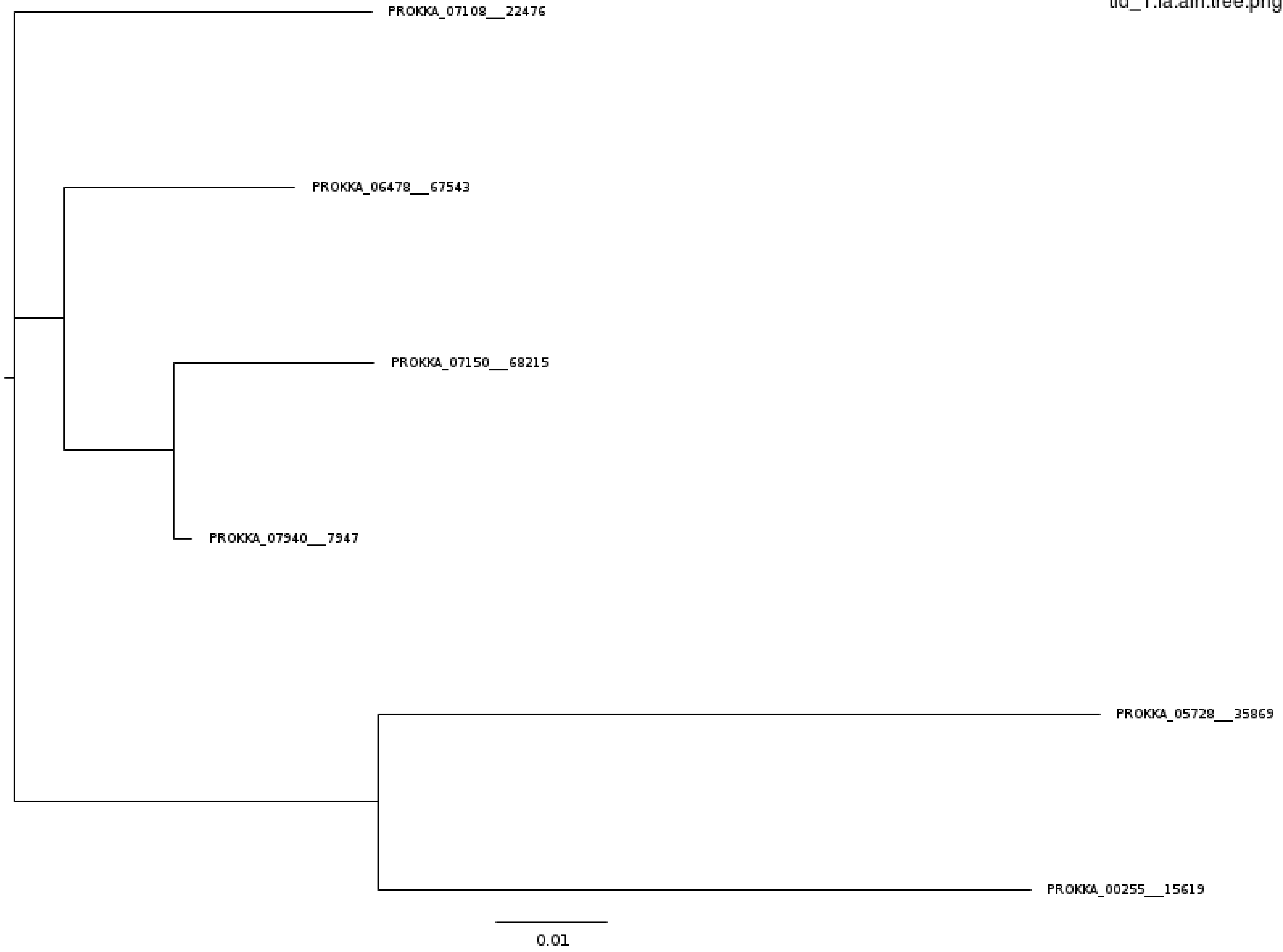

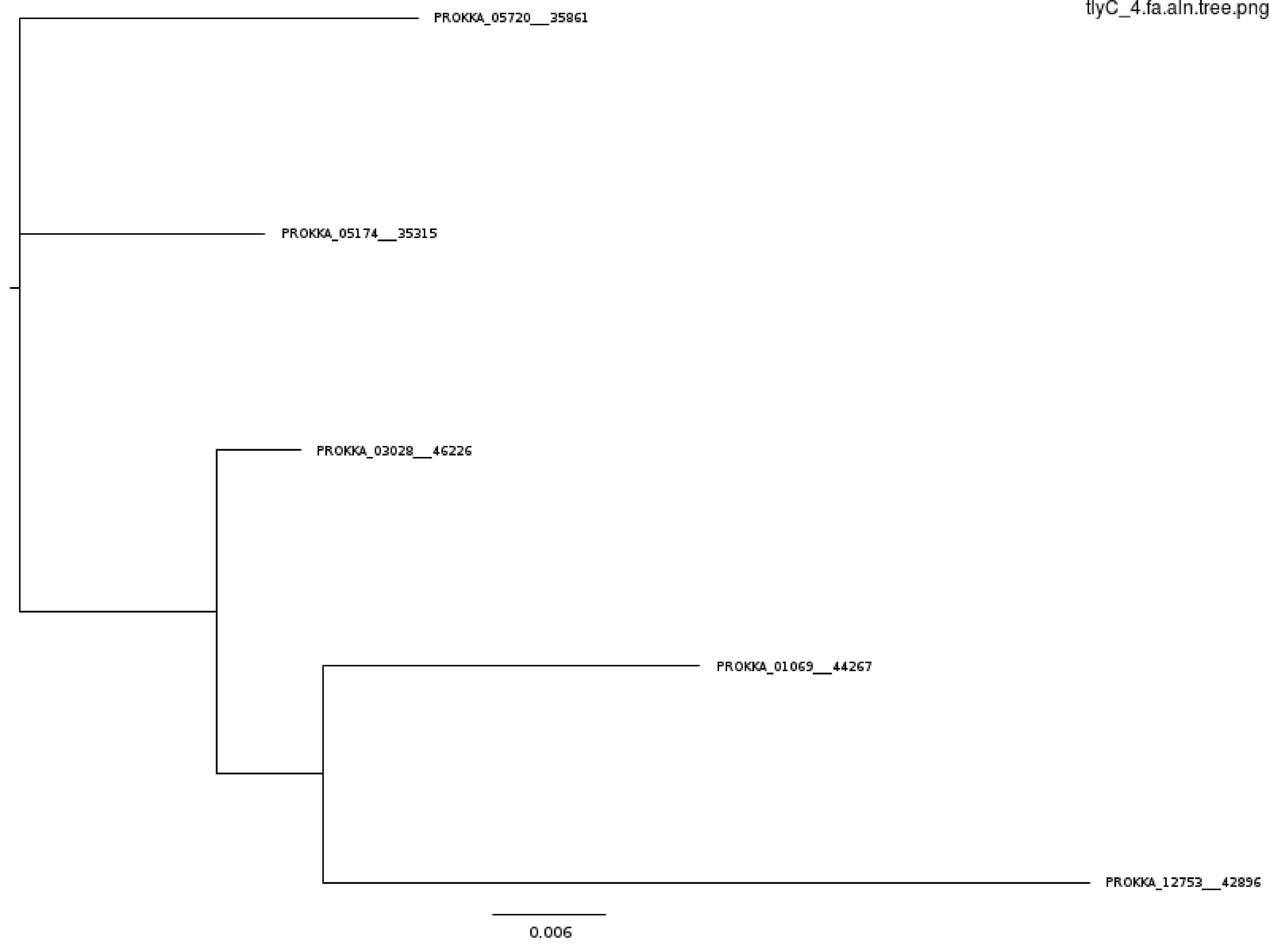
tlyC_4.fa.aln.tree.png
PROKKA_05720___35861
PROKKA_05174___35315
PROKKA_03028___46226
PROKKA_01069___44267
PROKKA_12753___42896
0.006

tolB.fa.aln.tree.png

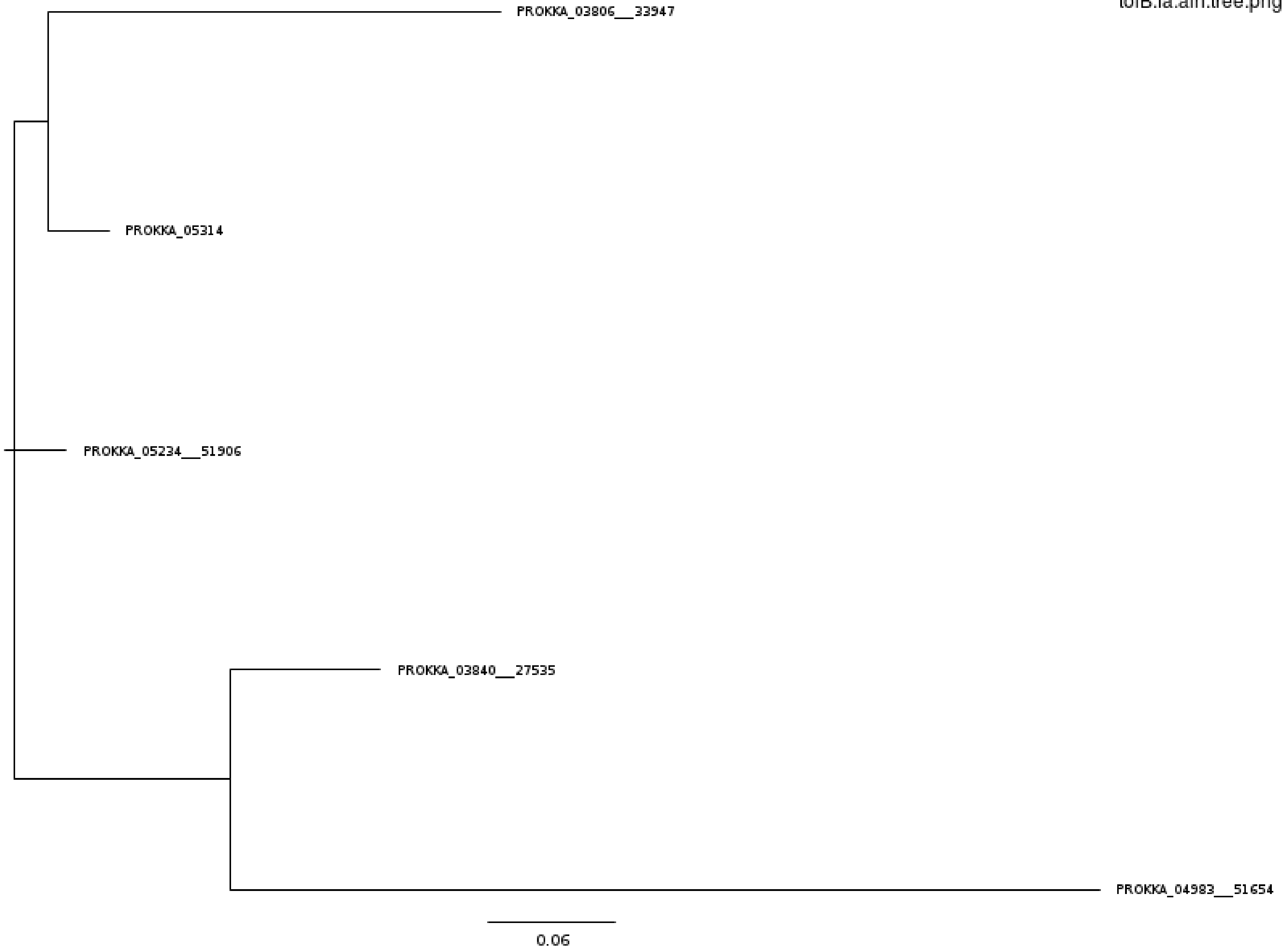

topA_2.fa.aln.tree.png

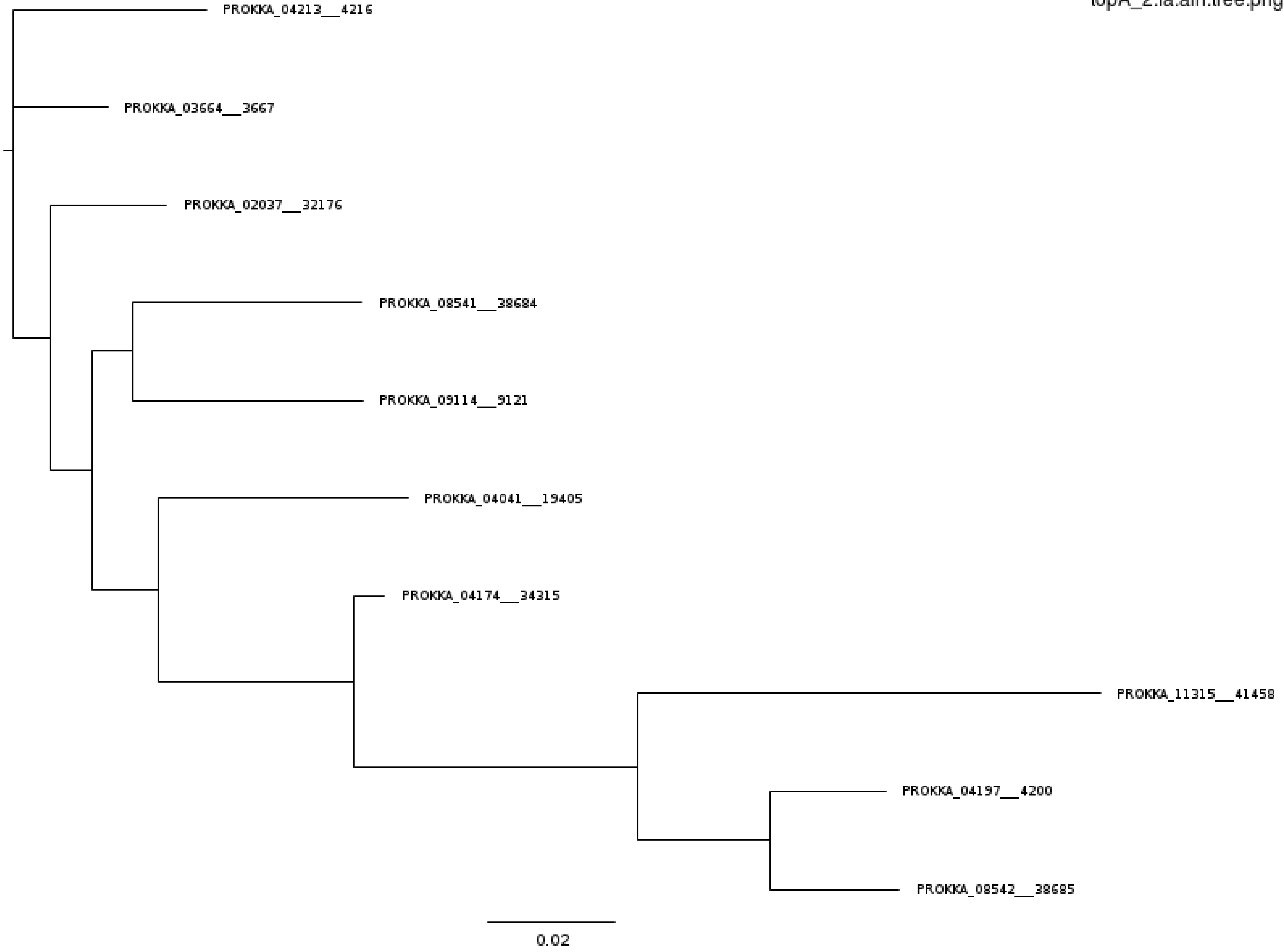

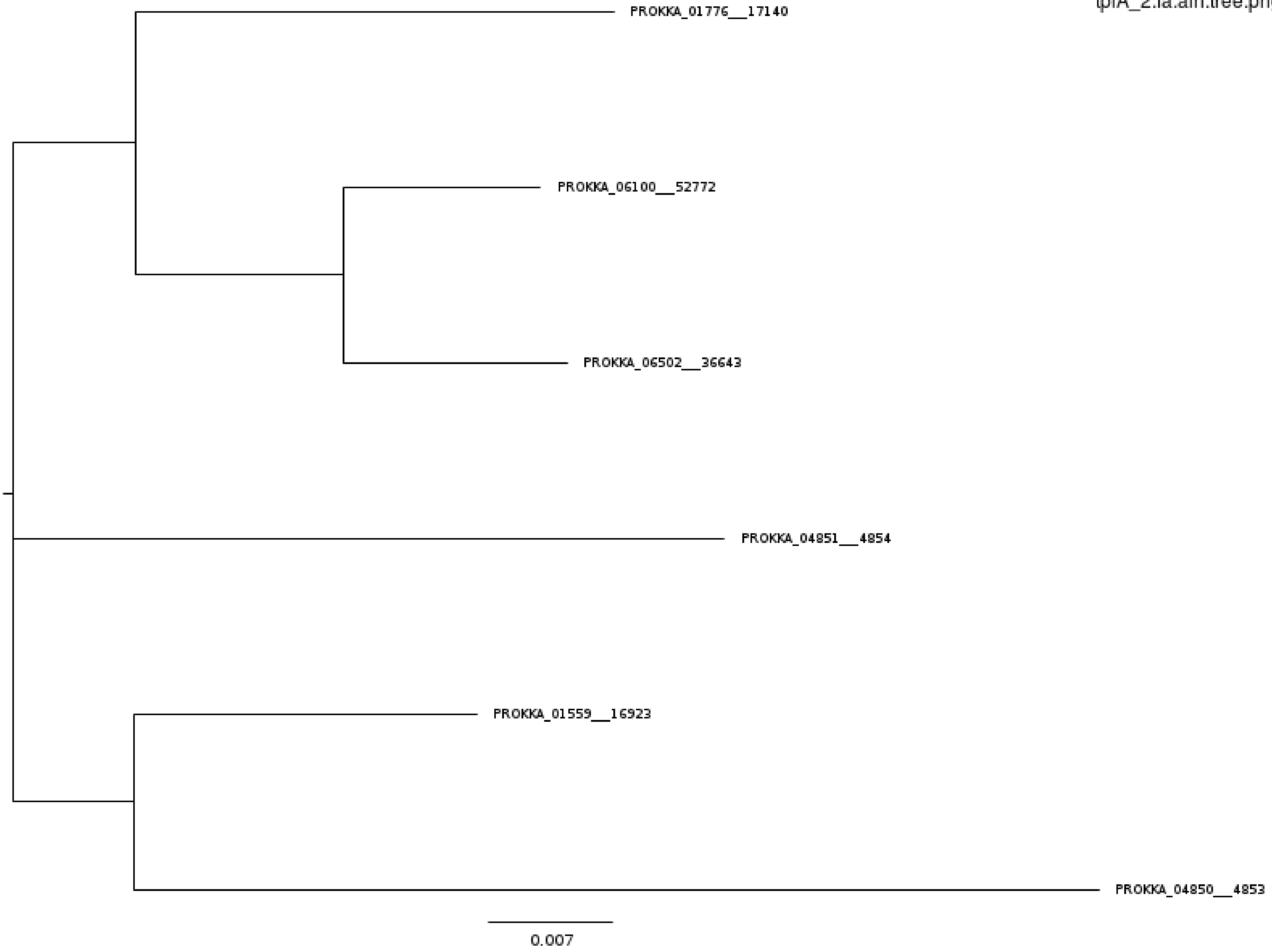
PROKKA_01776___17140
PROKKA_06100___52772
PROKKA_06502___36643
PROKKA_04851___4854
PROKKA_01559___16923
PROKKA_04850___4853
0.007

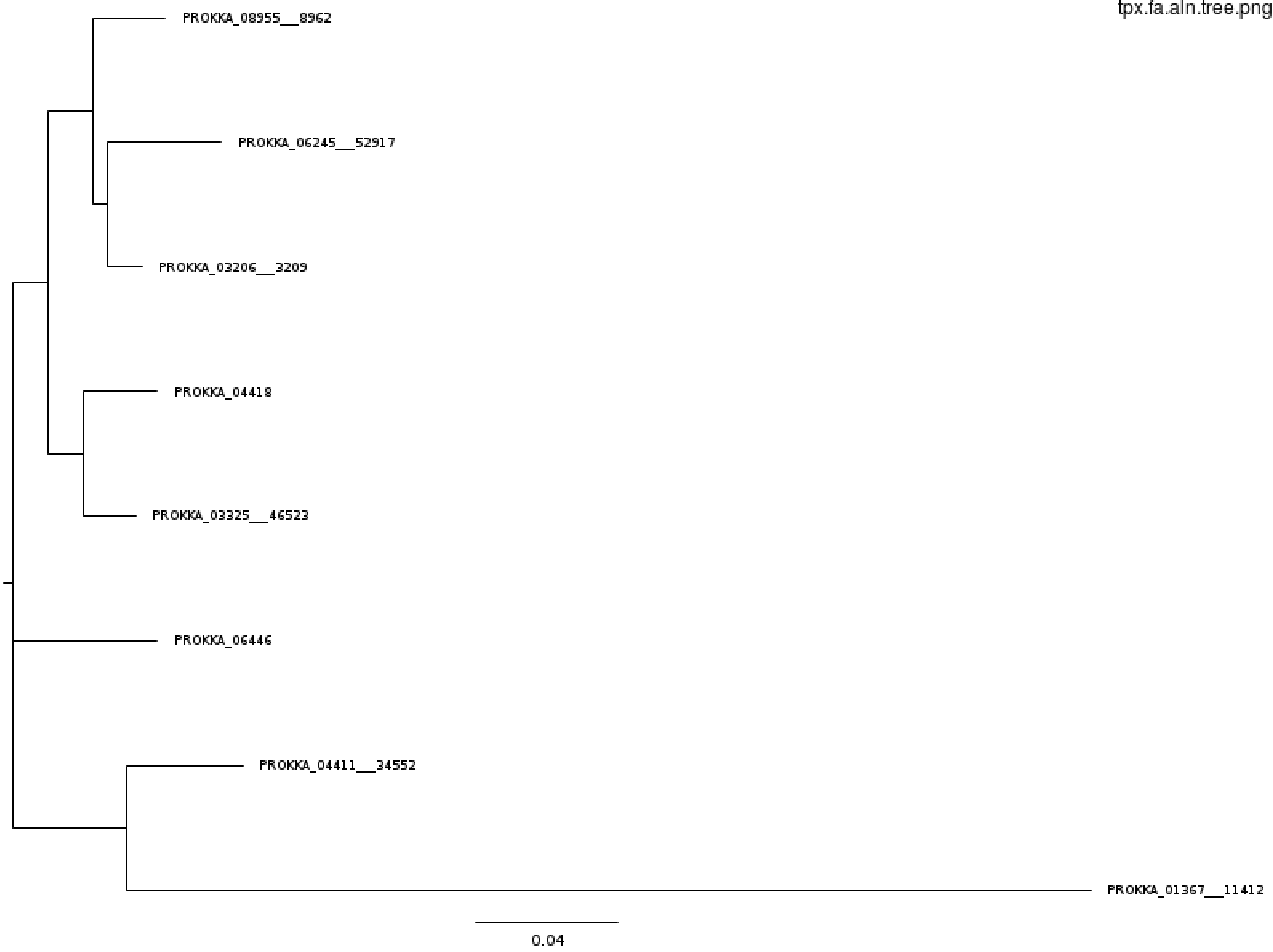

tpx.fa.aln.tree.png
PROKKA_08955___8962
PROKKA_06245___52917
PROKKA_03206___3209
PROKKA_04418
PROKKA_03325___46523
PROKKA_06446
PROKKA_04411___34552
PROKKA_01367___11412
0.04

traC_2.fa.aln.tree.png

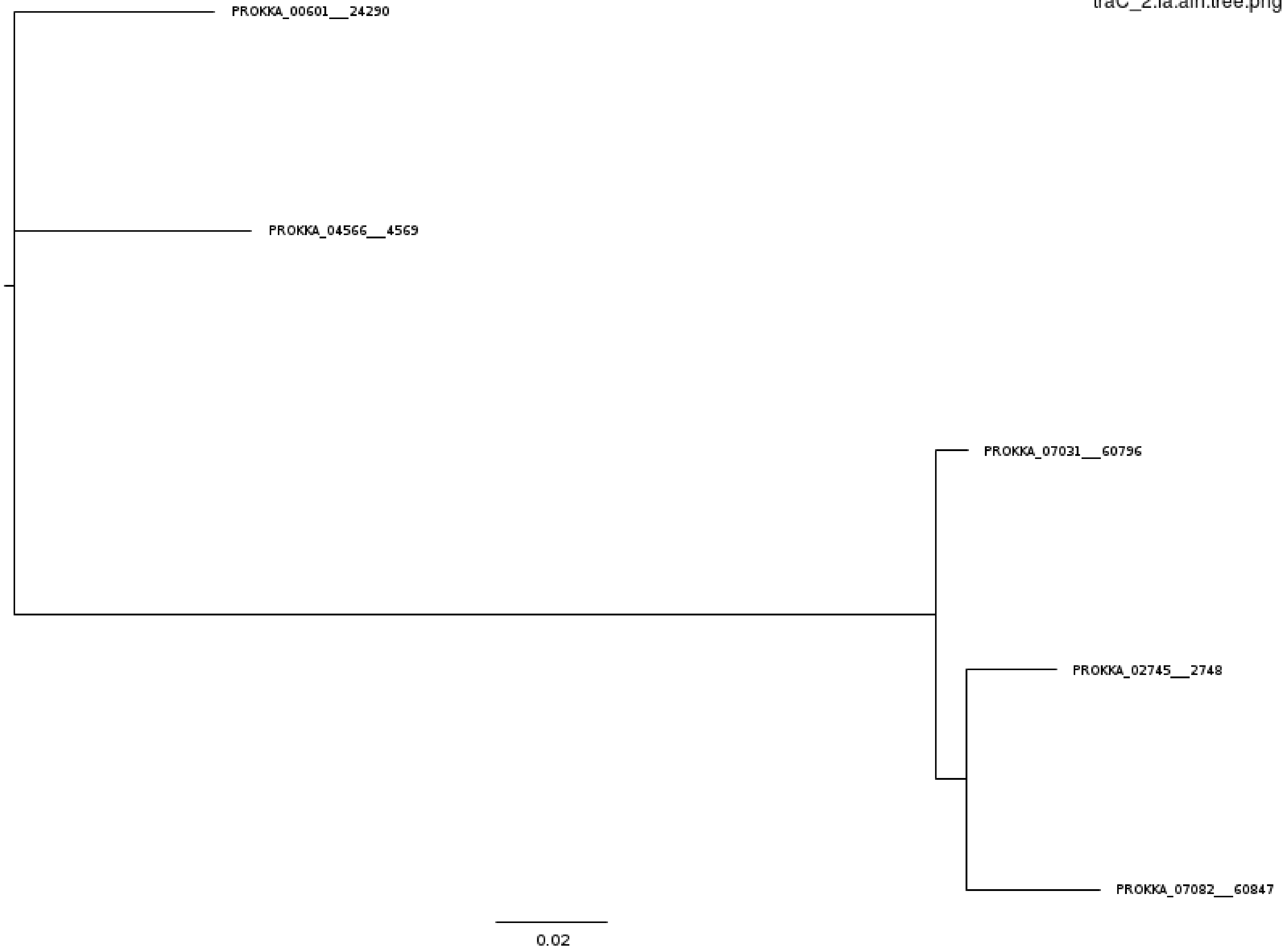

traC_5.fa.aln.tree.png

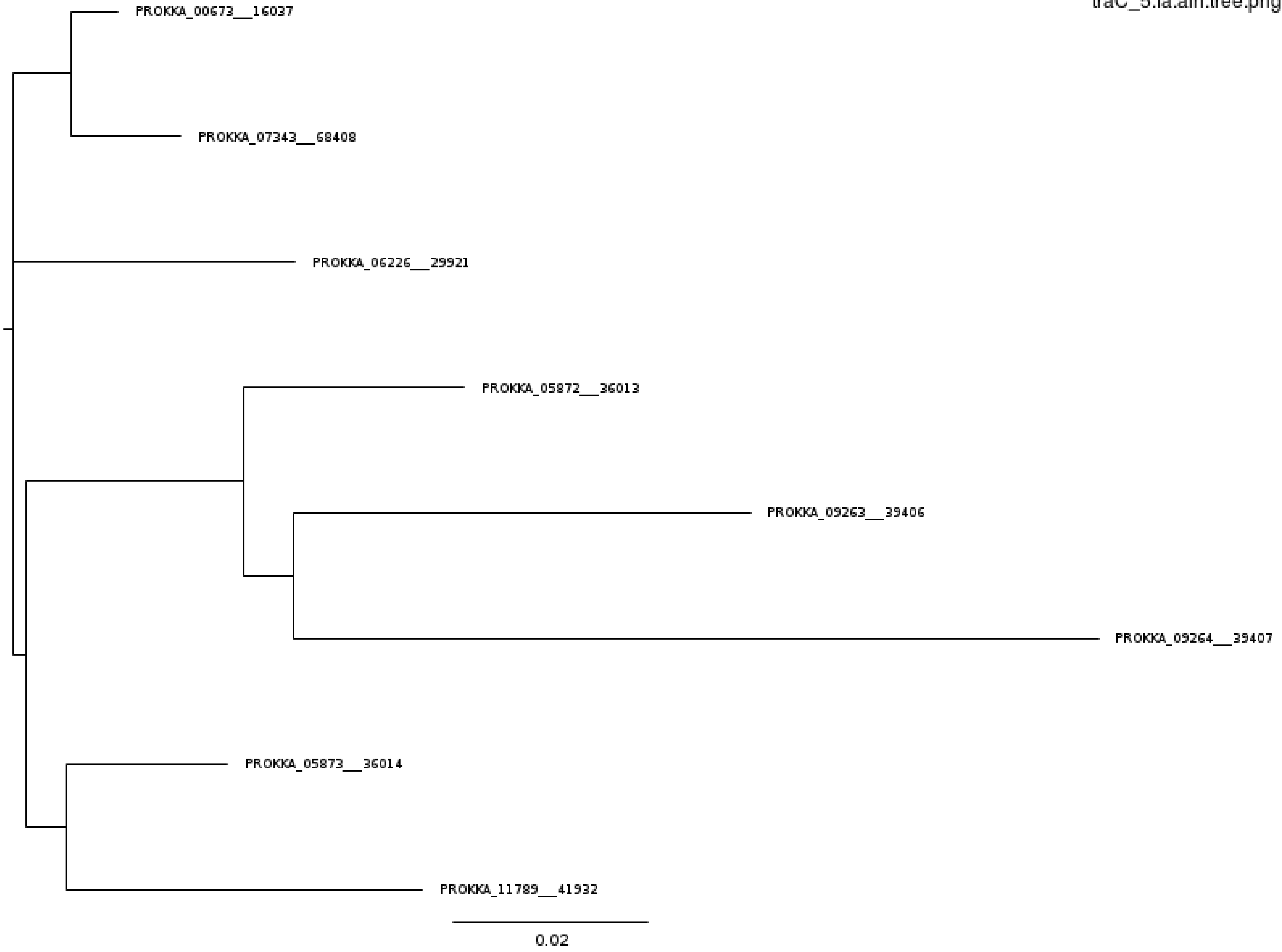

trg.fa.aln.tree.png

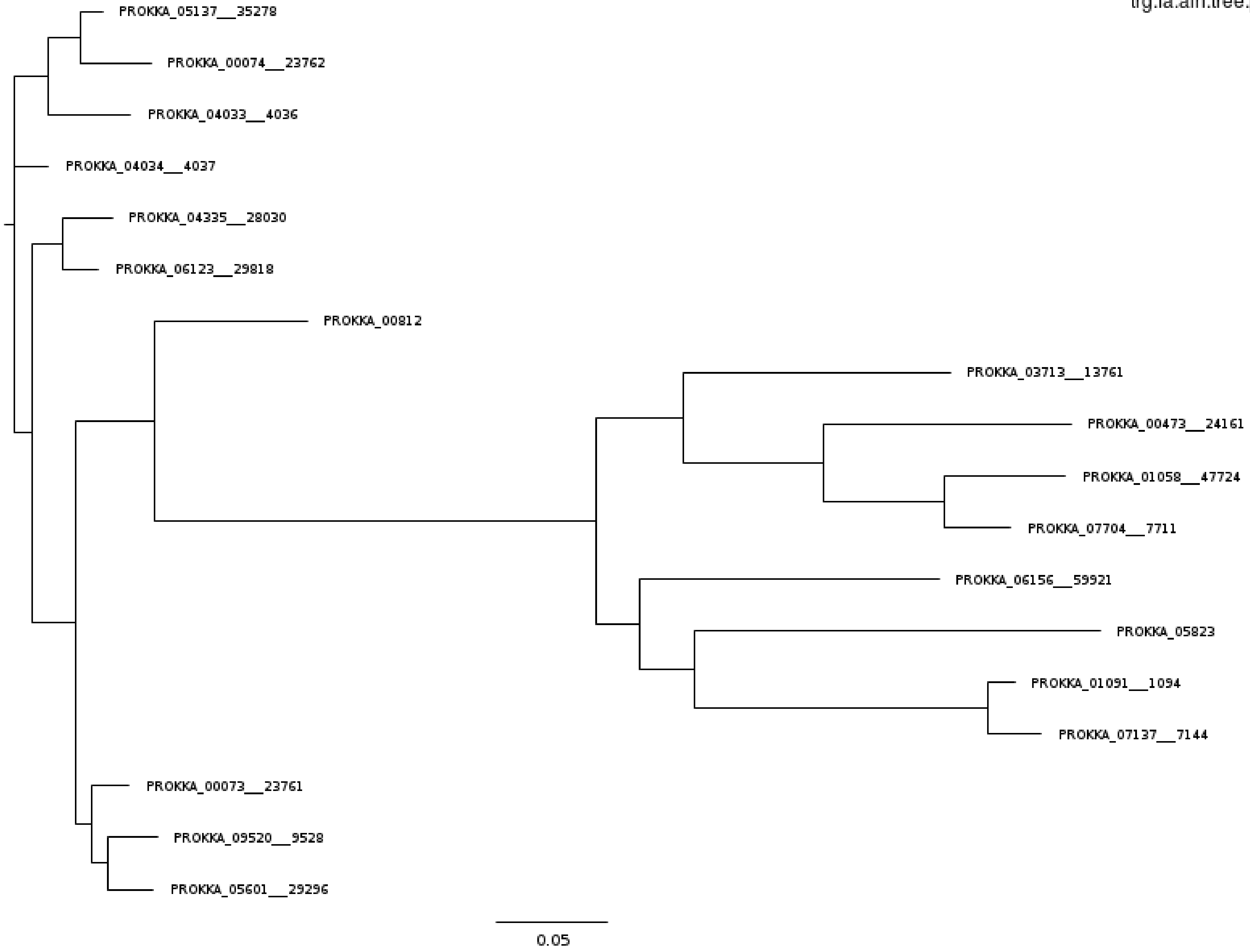

trmB_4.fa.aln.tree.png

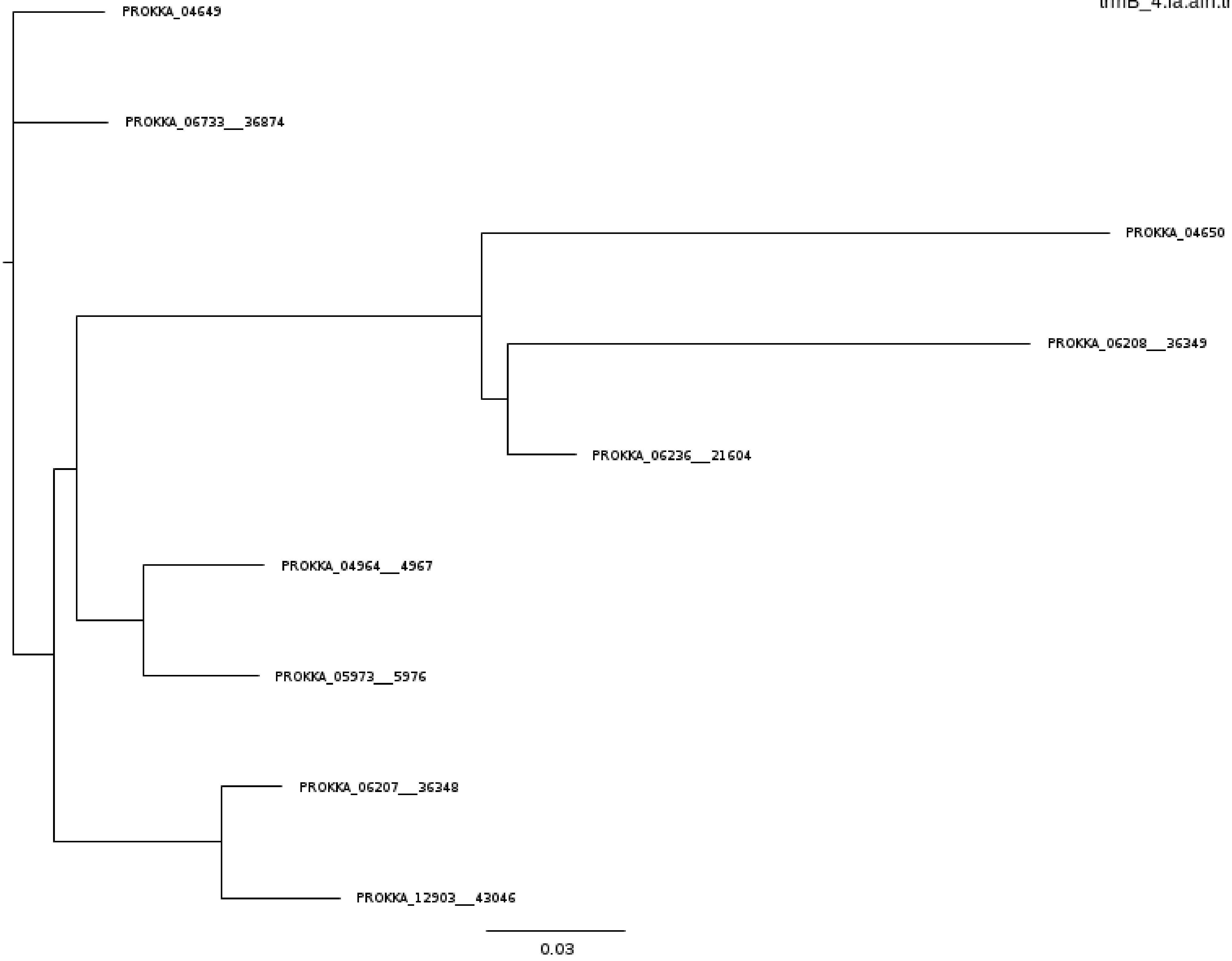

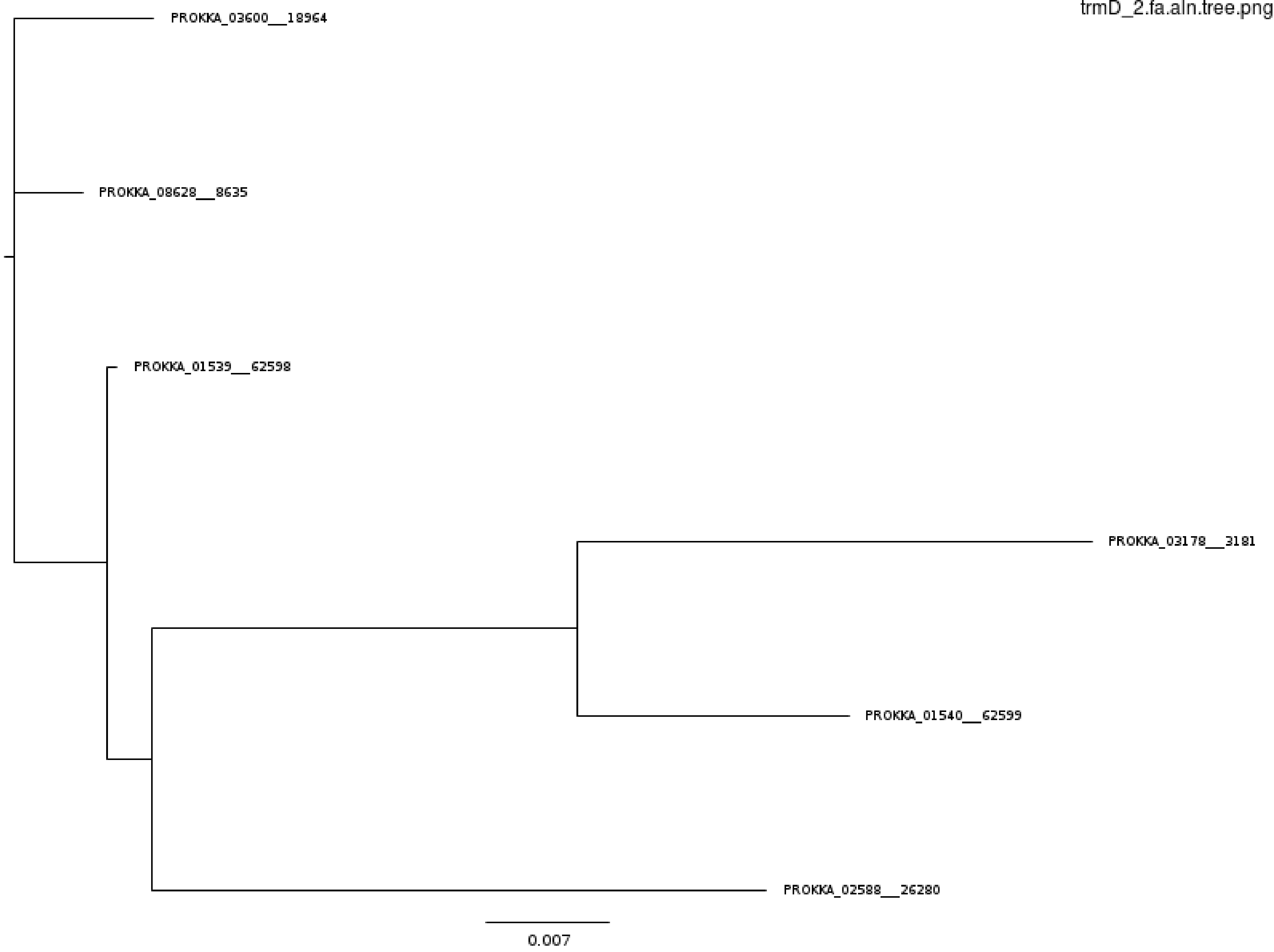
trmD_2.fa.aln.tree.png
PROKKA_03600___18964
PROKKA_08628___8635
PROKKA_01539___62598
PROKKA_03178___3181
PROKKA_01540___62599
PROKKA_02588___26280
0.007

trmJ_3.fa.aln.tree.png

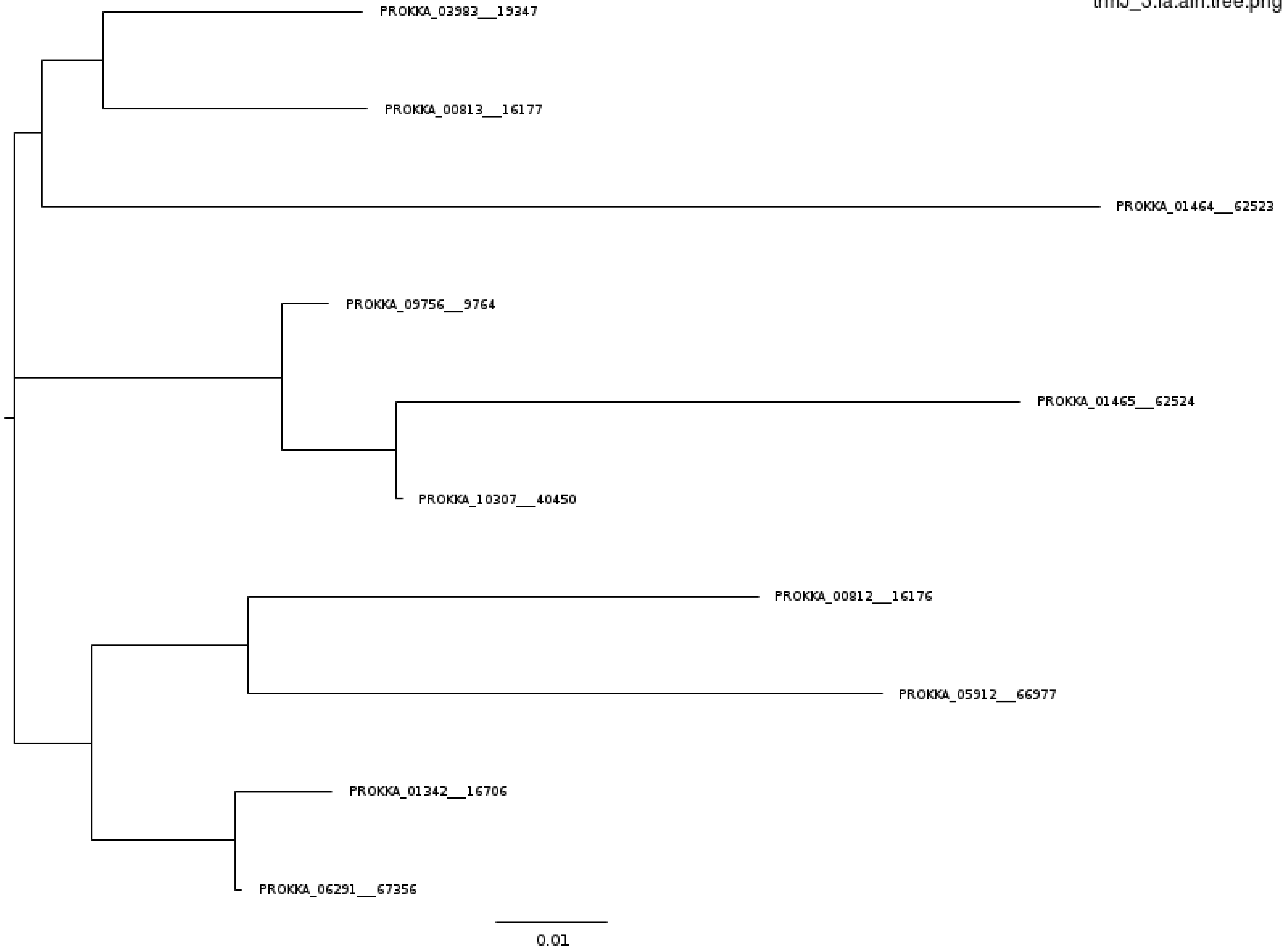

trmL_3.fa.aln.tree.png

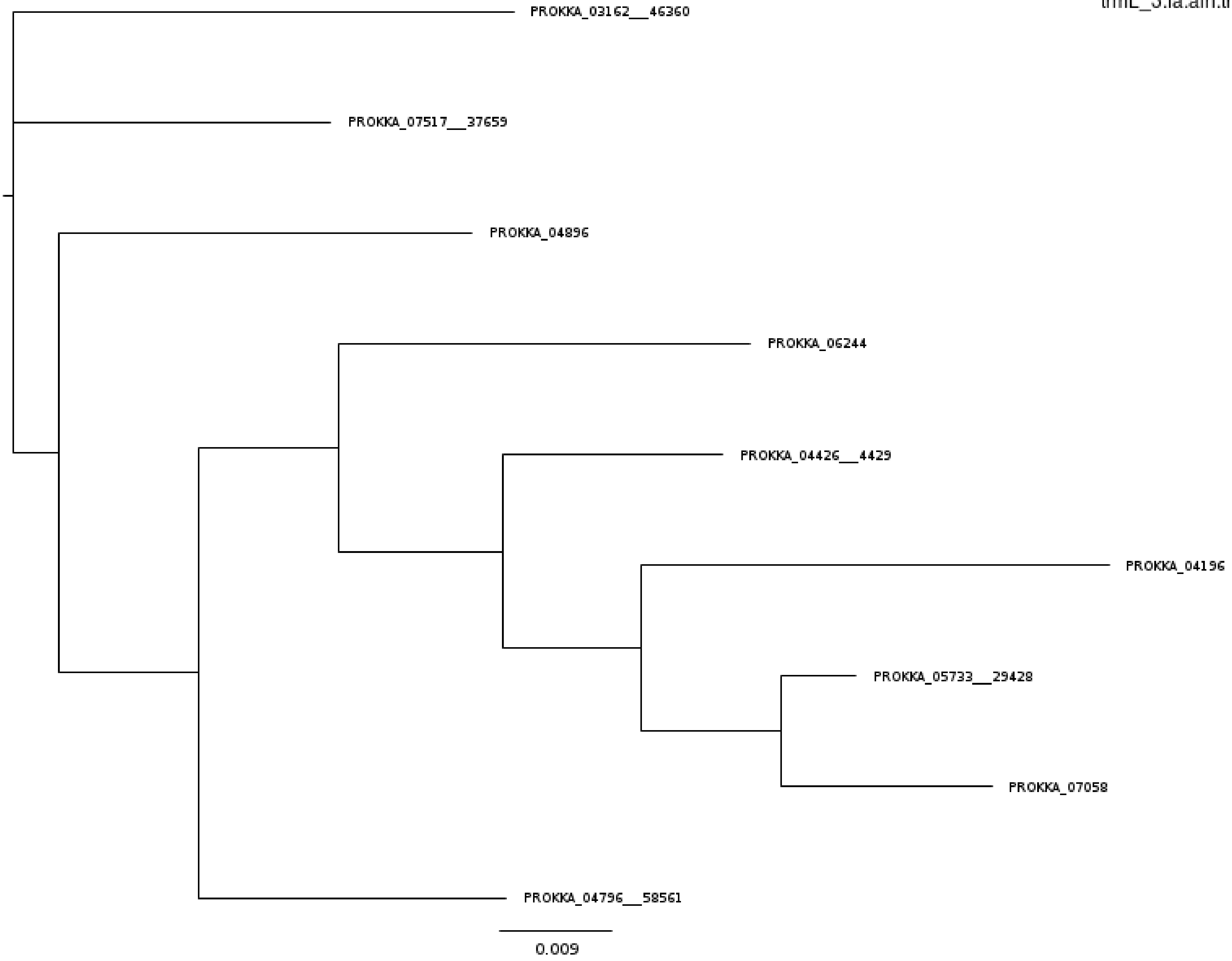

trpA_2.fa.aln.tree.png

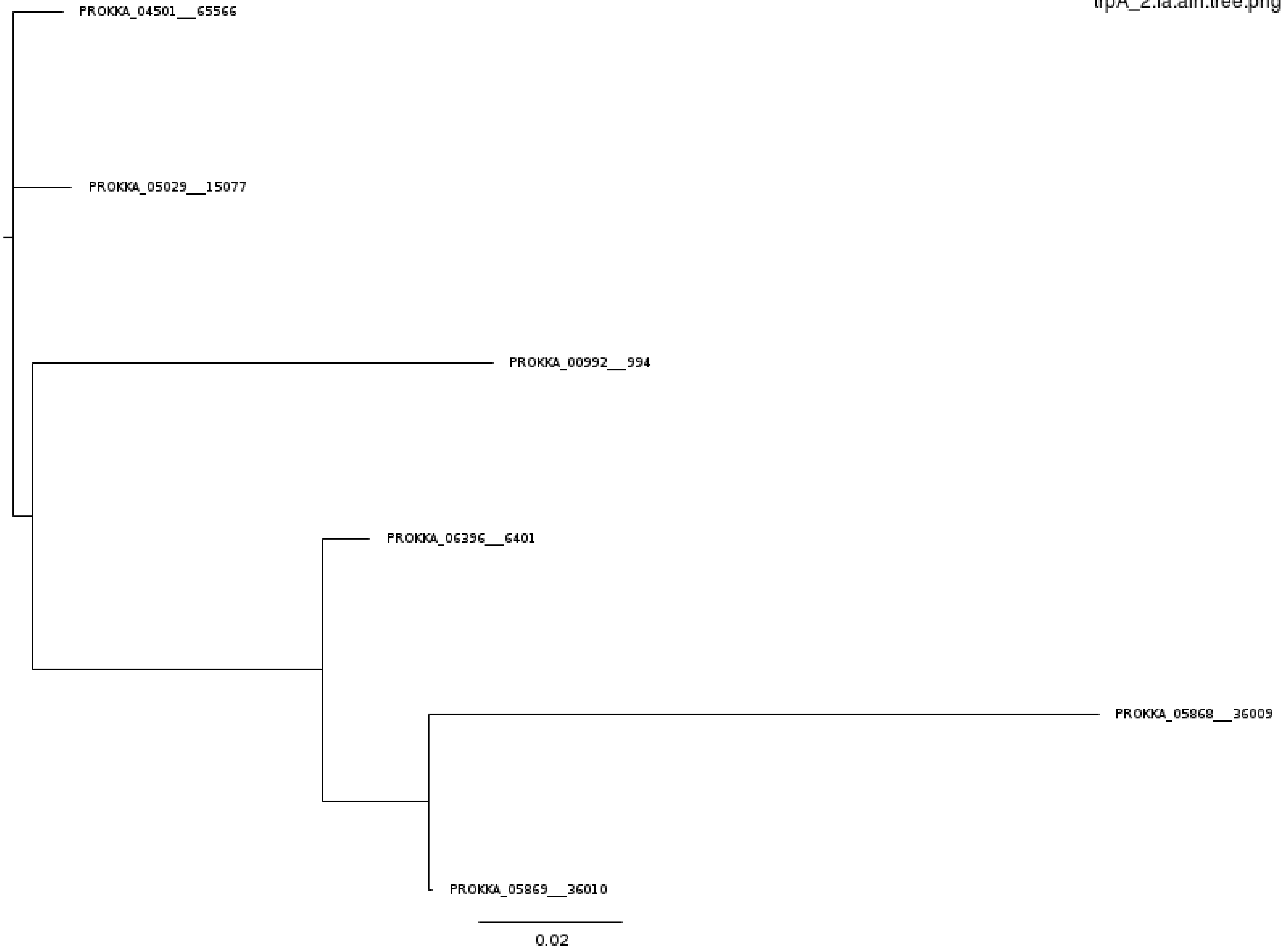

trpA.fa.aln.tree.png

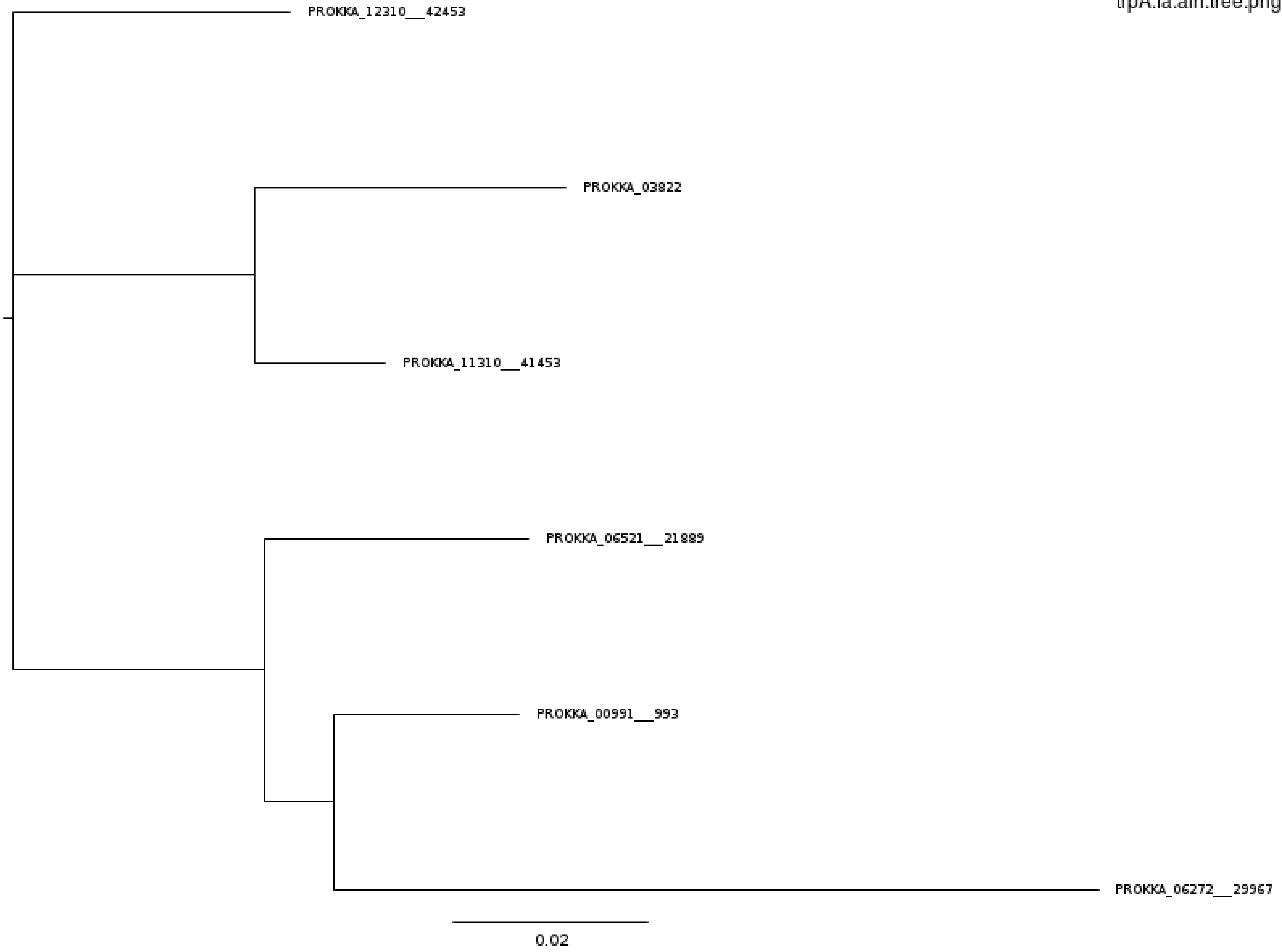

trpB_2.fa.aln.tree.png

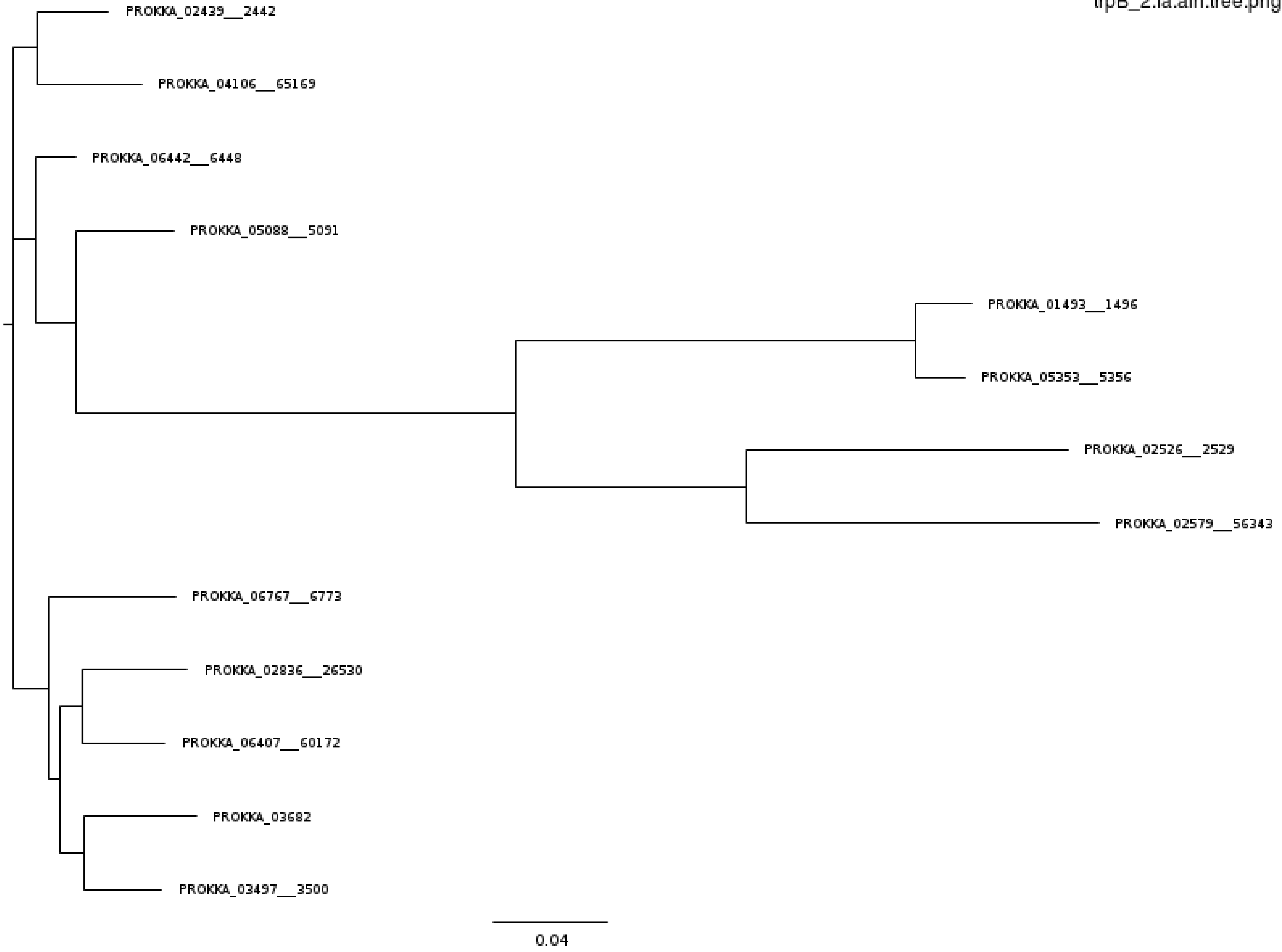

trpC_2.fa.aln.tree.png

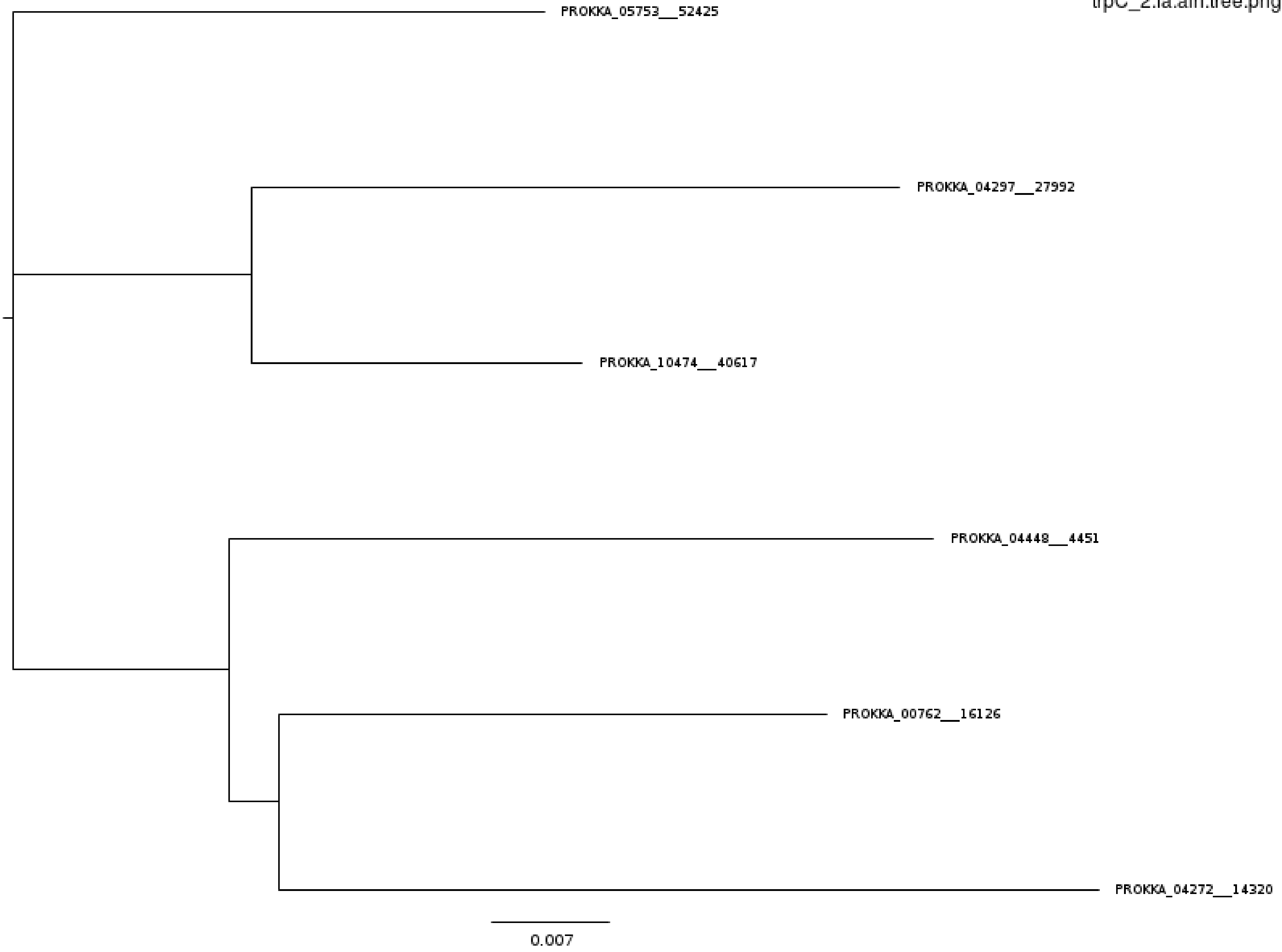

trpD_2.fa.aln.tree.png

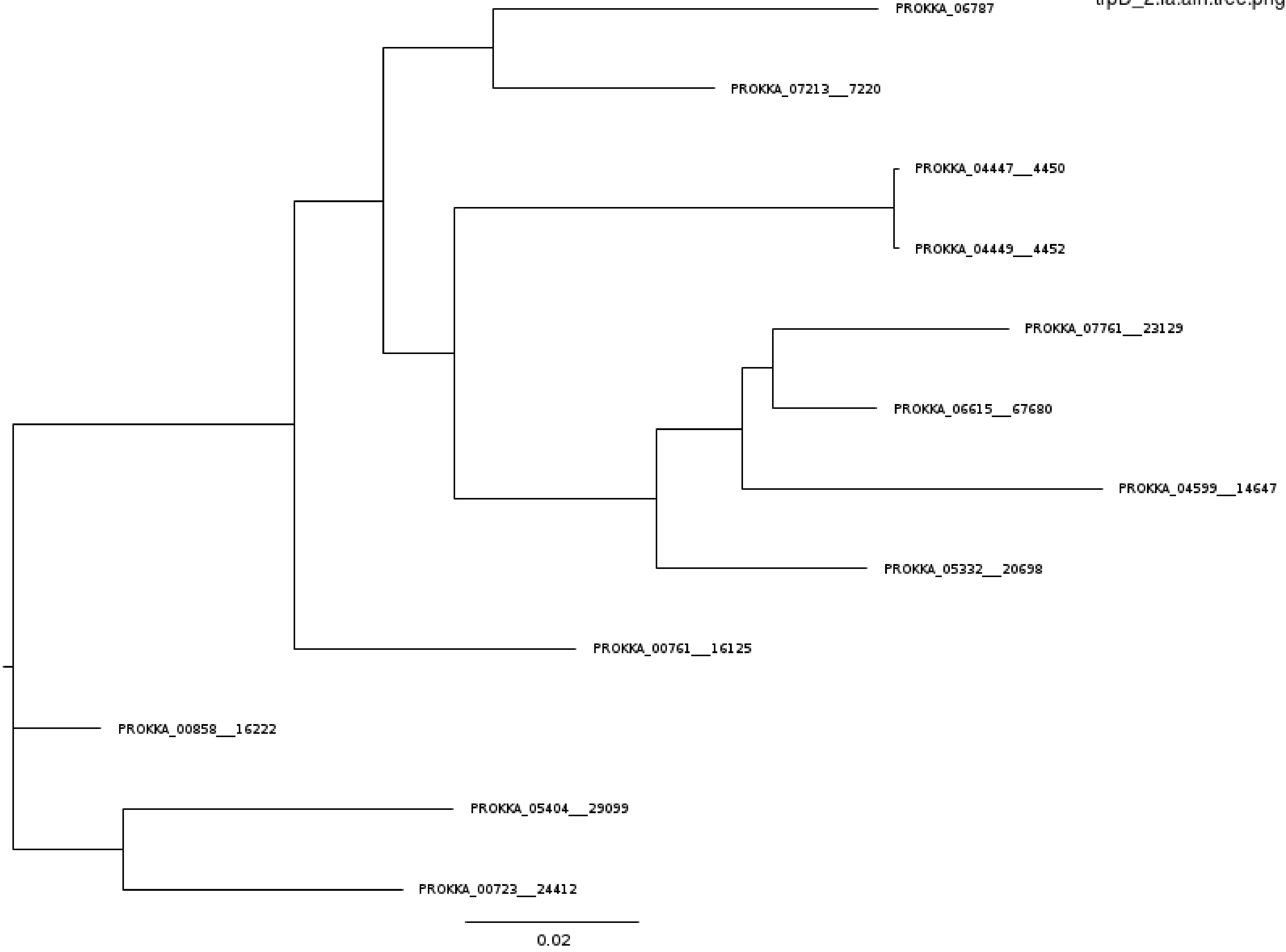

trpE_1.fa.aln.tree.png

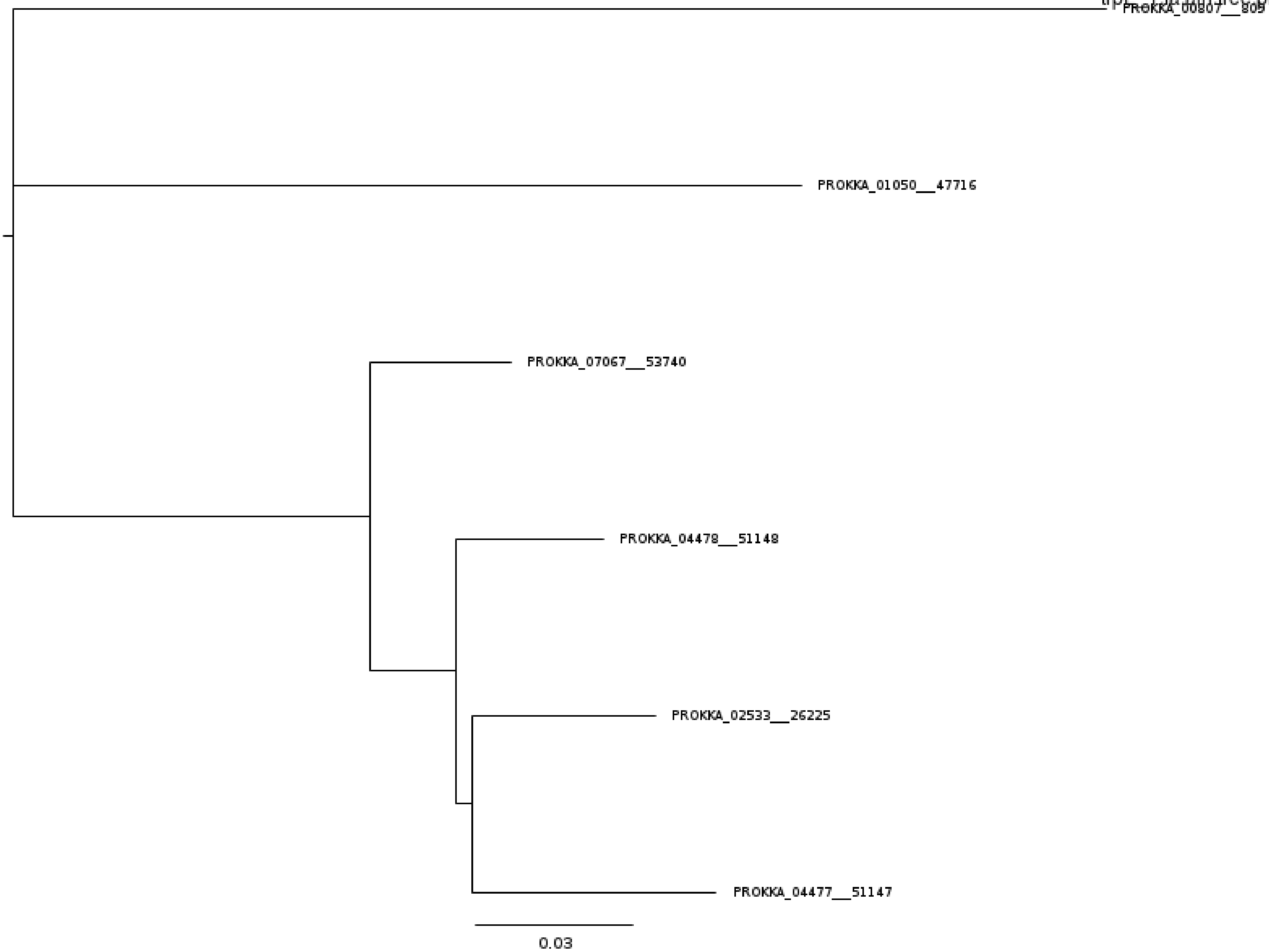

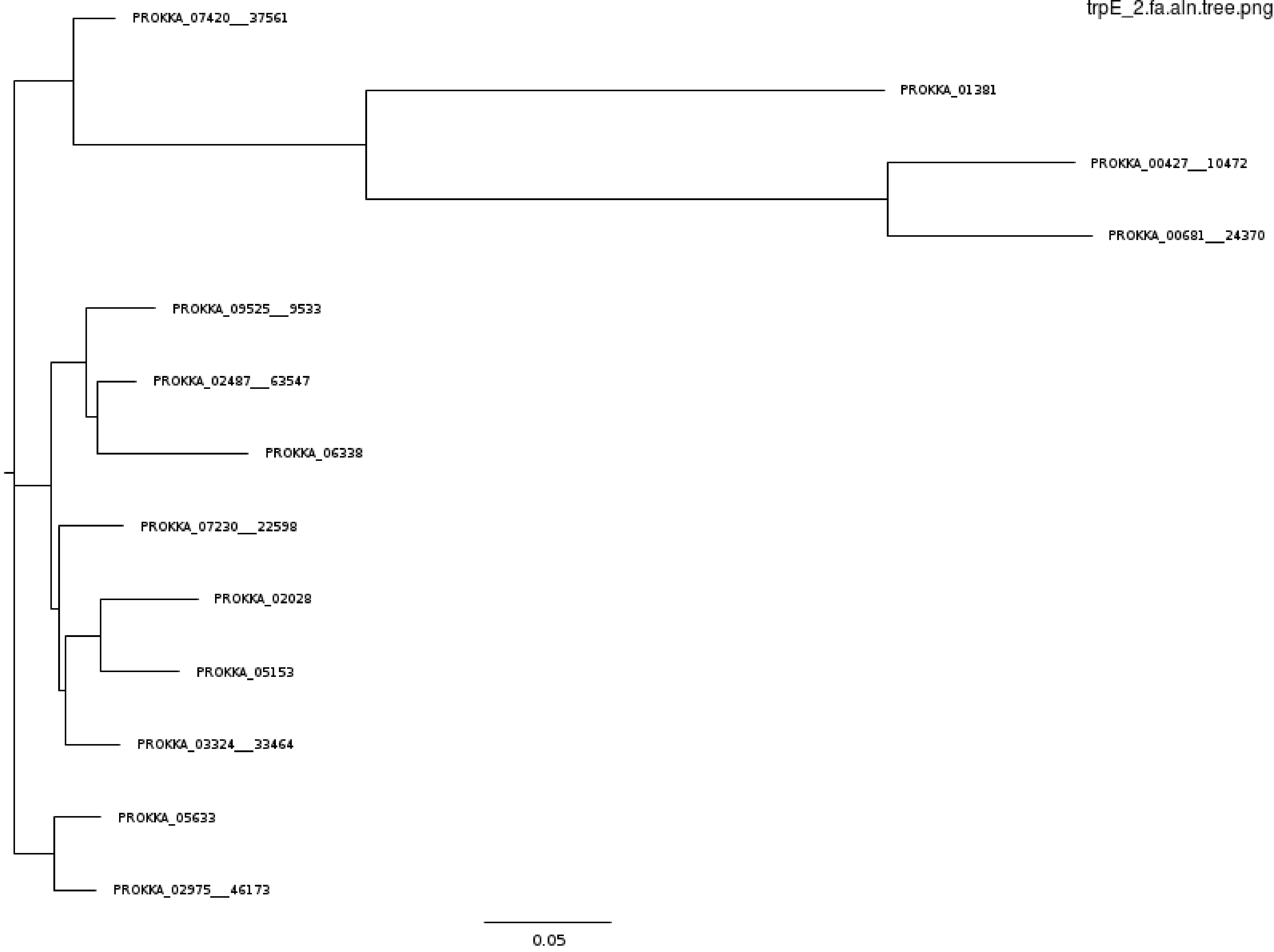
trpE_2.fa.aln.tree.png
PROKKA_07420___37561
PROKKA_01381
PROKKA_00427___10472
PROKKA_00681___24370
PROKKA_09525___9533
PROKKA_02487___63547
PROKKA_06338
PROKKA_07230___22598
PROKKA_02028
PROKKA_05153
PROKKA_03324___33464
PROKKA_05633
PROKKA_02975___46173
0.05

trpF_2.fa.aln.tree.png

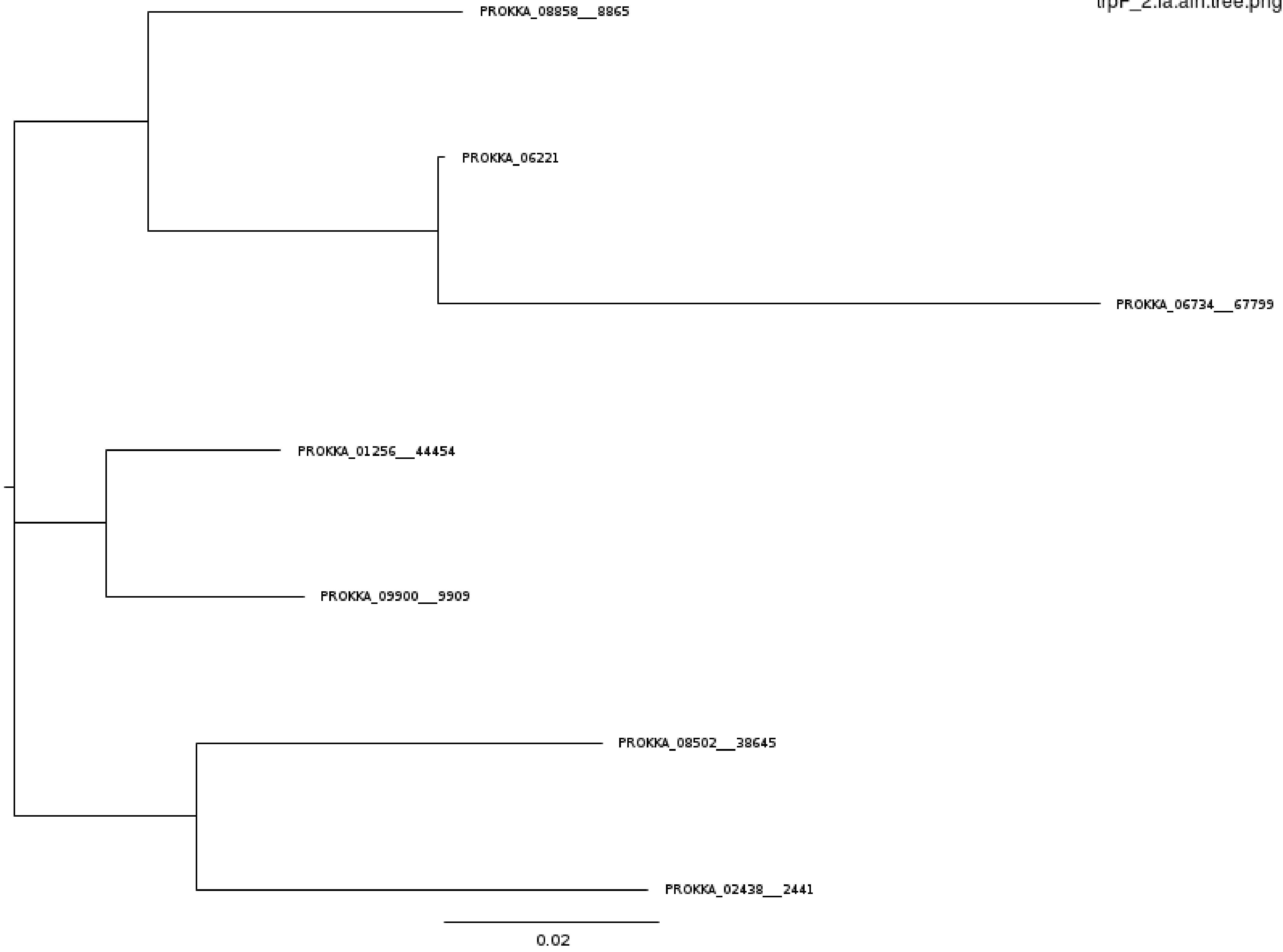

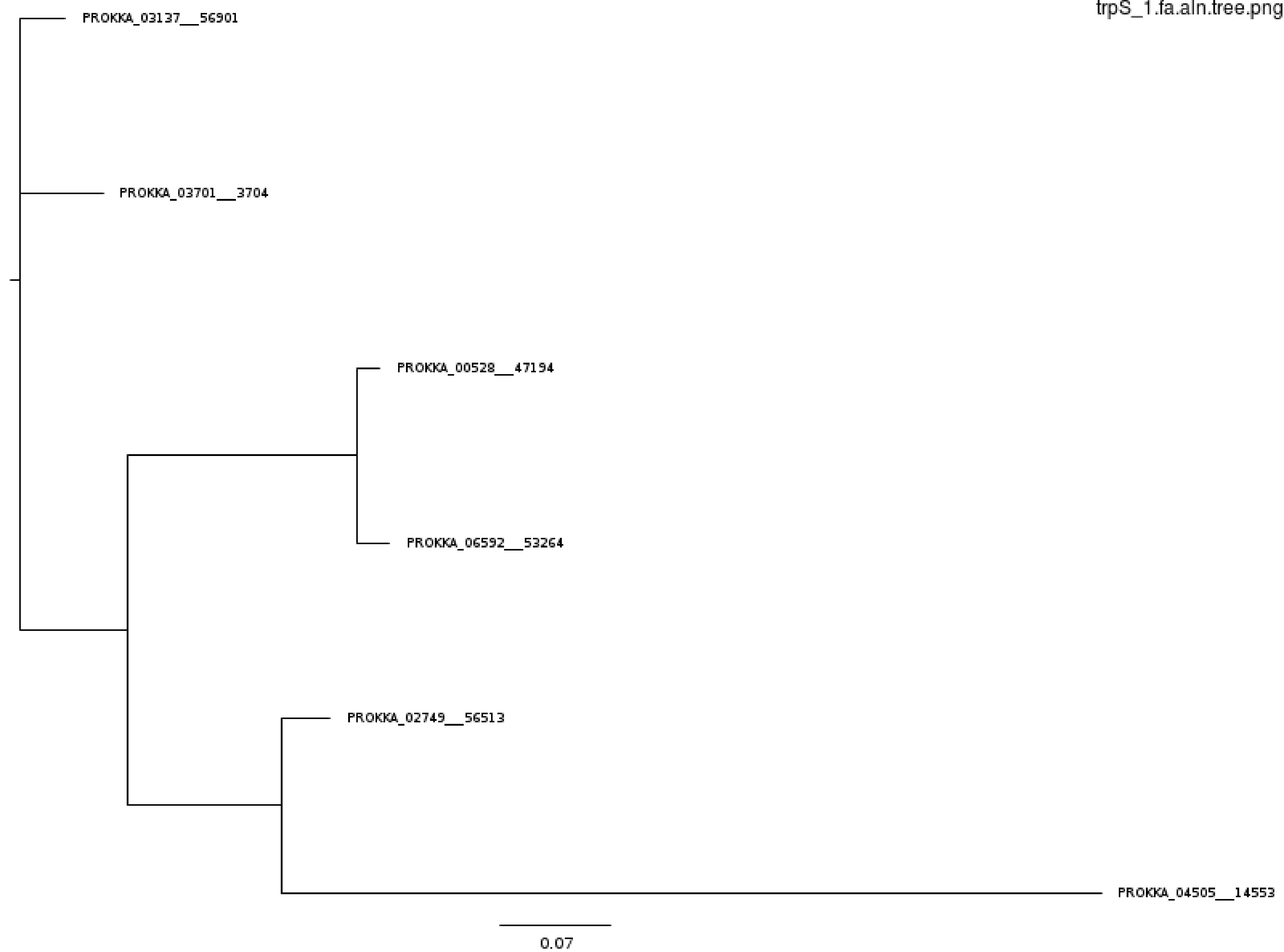
trpS_1.fa.aln.tree.png
PROKKA_03137__56901
PROKKA_03701__3704
PROKKA_00528__47194
PROKKA_06592__53264
PROKKA_02749__56513
PROKKA_04505__14553
0.07

trpS_2.fa.aln.tree.png

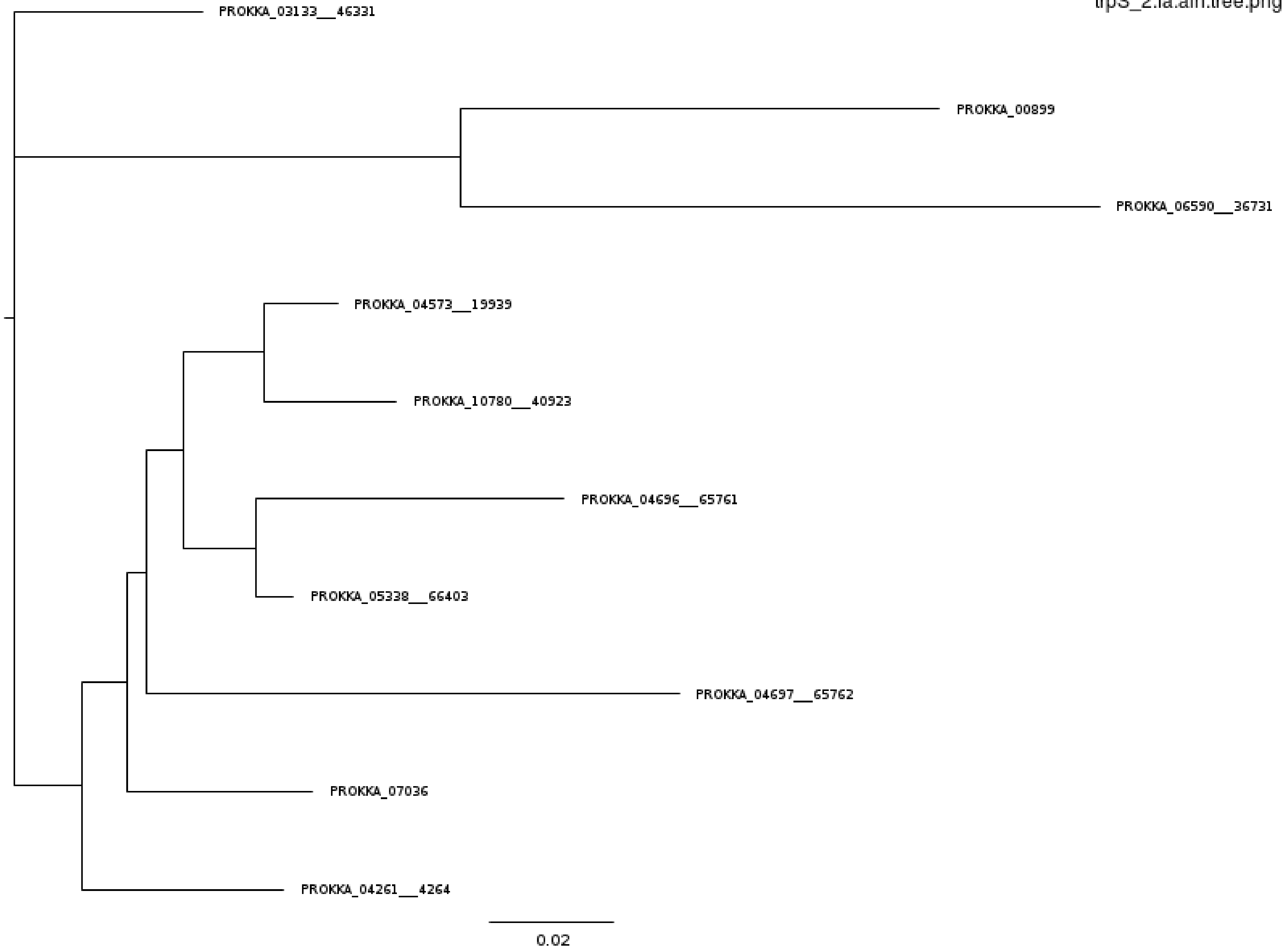

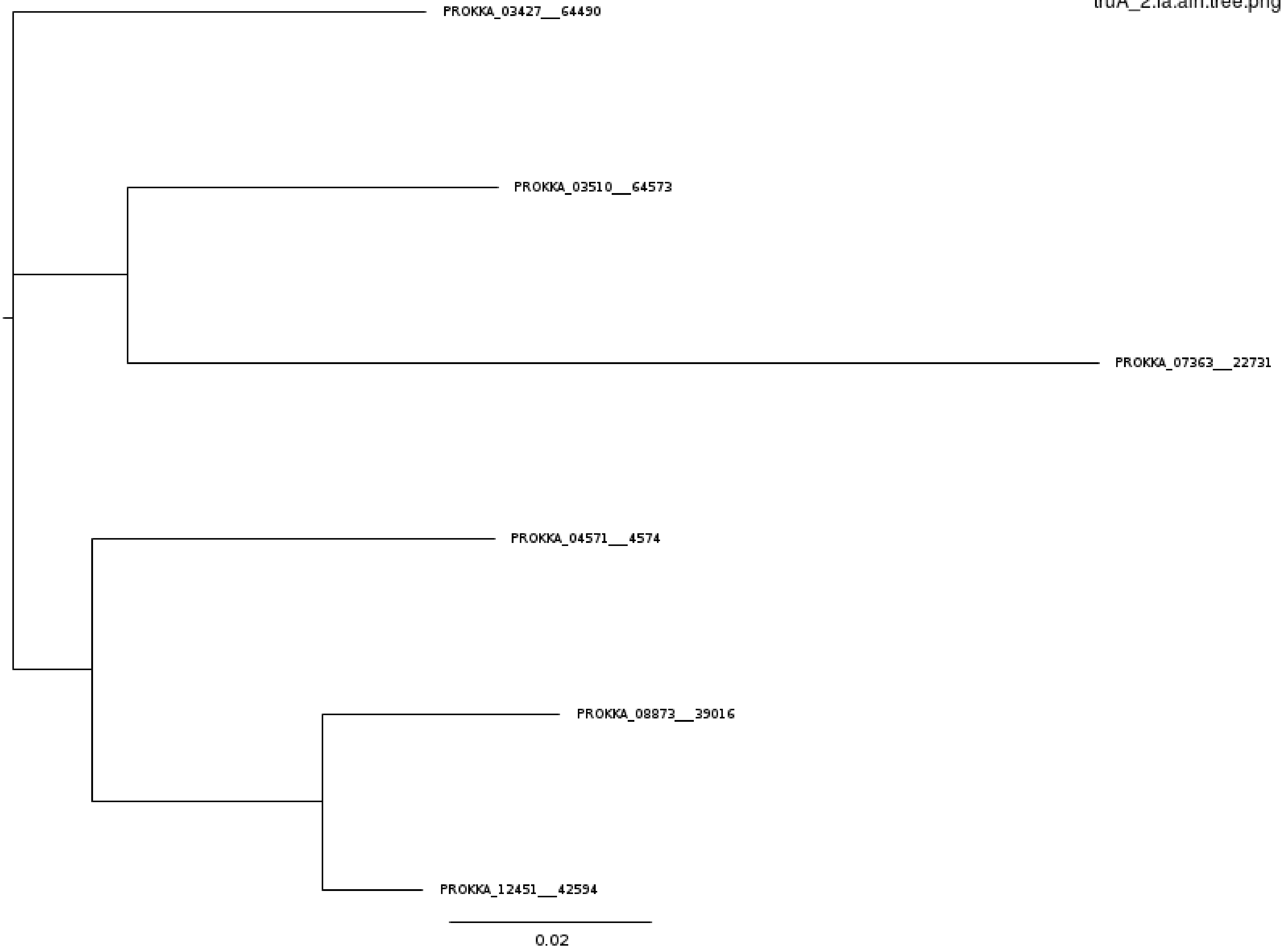
PROKKA_03427___64490
PROKKA_03510___64573
PROKKA_07363___22731
PROKKA_04571___4574
PROKKA_08873___39016
PROKKA_12451___42594
0.02

truB_2.fa.aln.tree.png

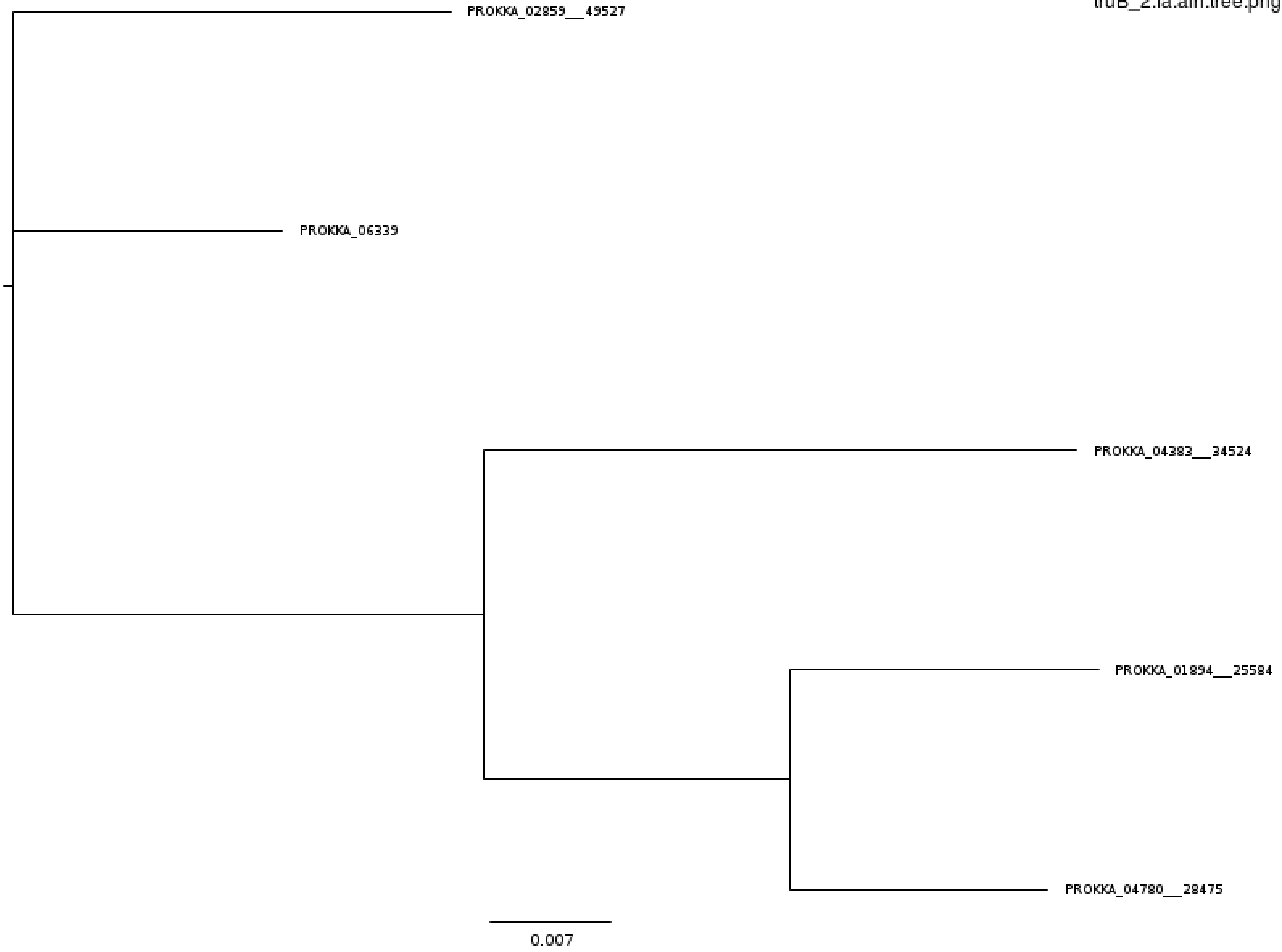

trxA_6.fa.aln.tree.png

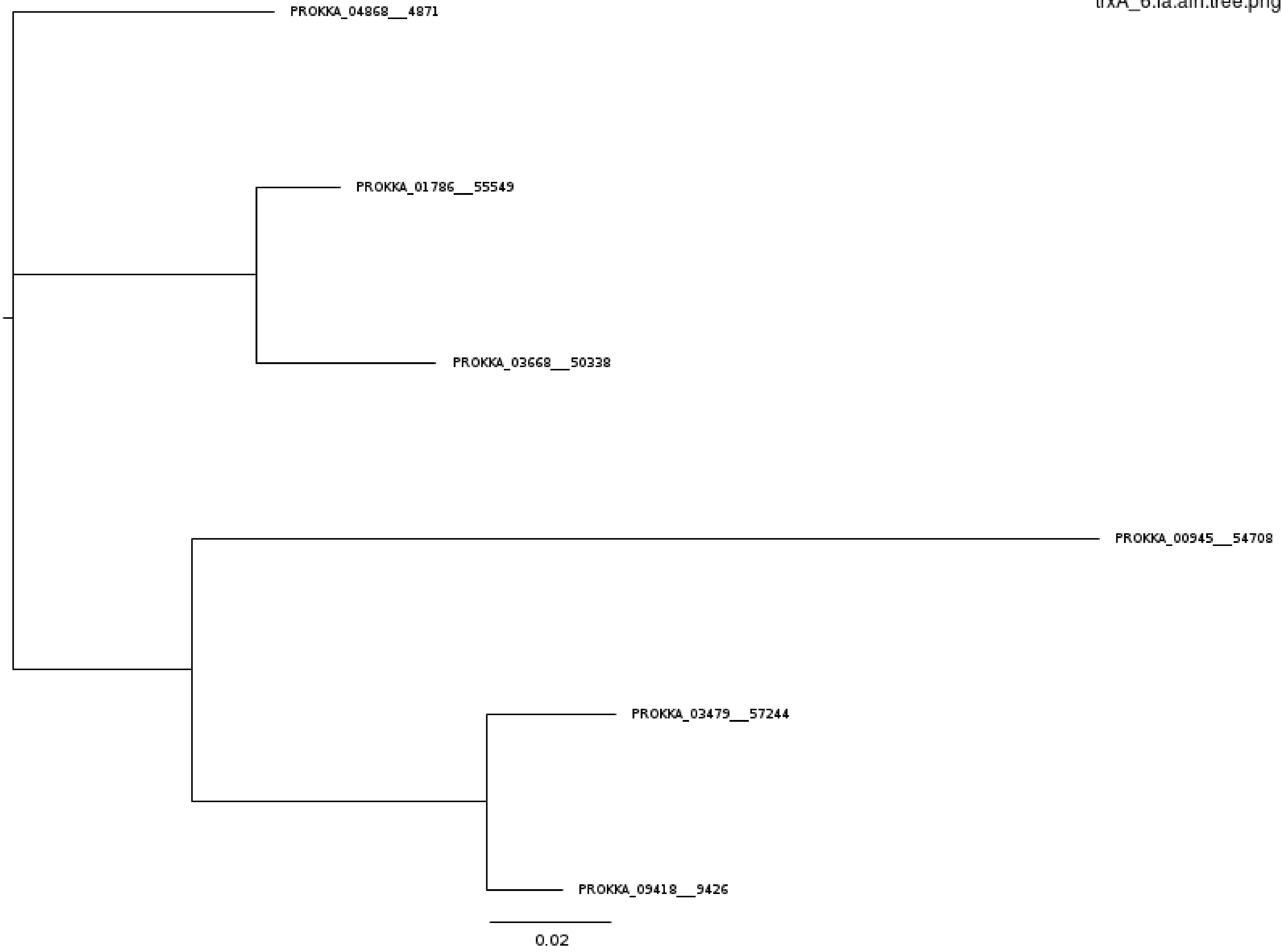

trxB_2.fa.aln.tree.png

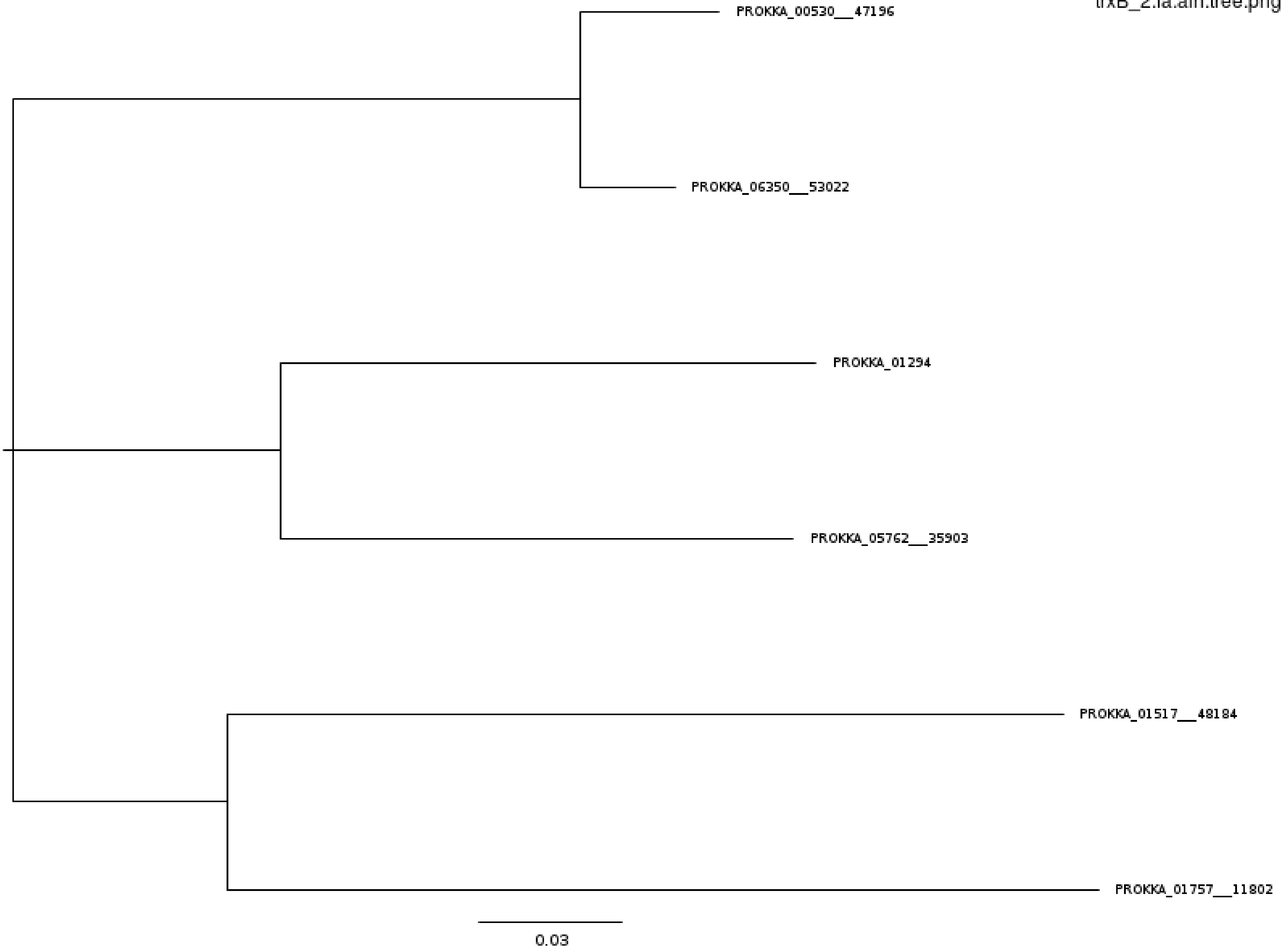

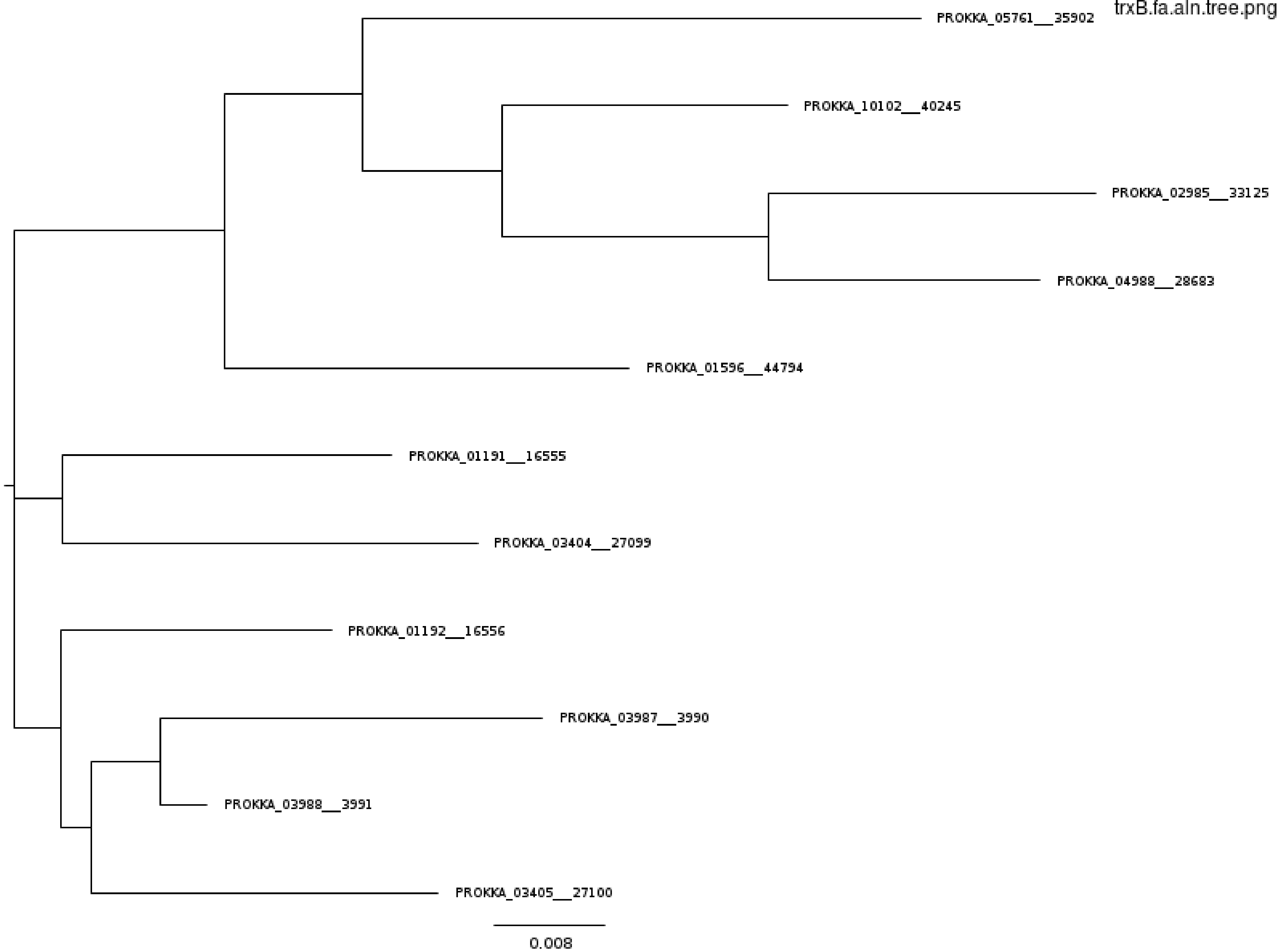
trxB.fa.aln.tree.png
PROKKA_05761___35902
PROKKA_10102___40245
PROKKA_02985___33125
PROKKA_04988___28683
PROKKA_01596___44794
PROKKA_01191___16555
PROKKA_03404___27099
PROKKA_01192___16556
PROKKA_03987___3990
PROKKA_03988___3991
PROKKA_03405___27100
0.008

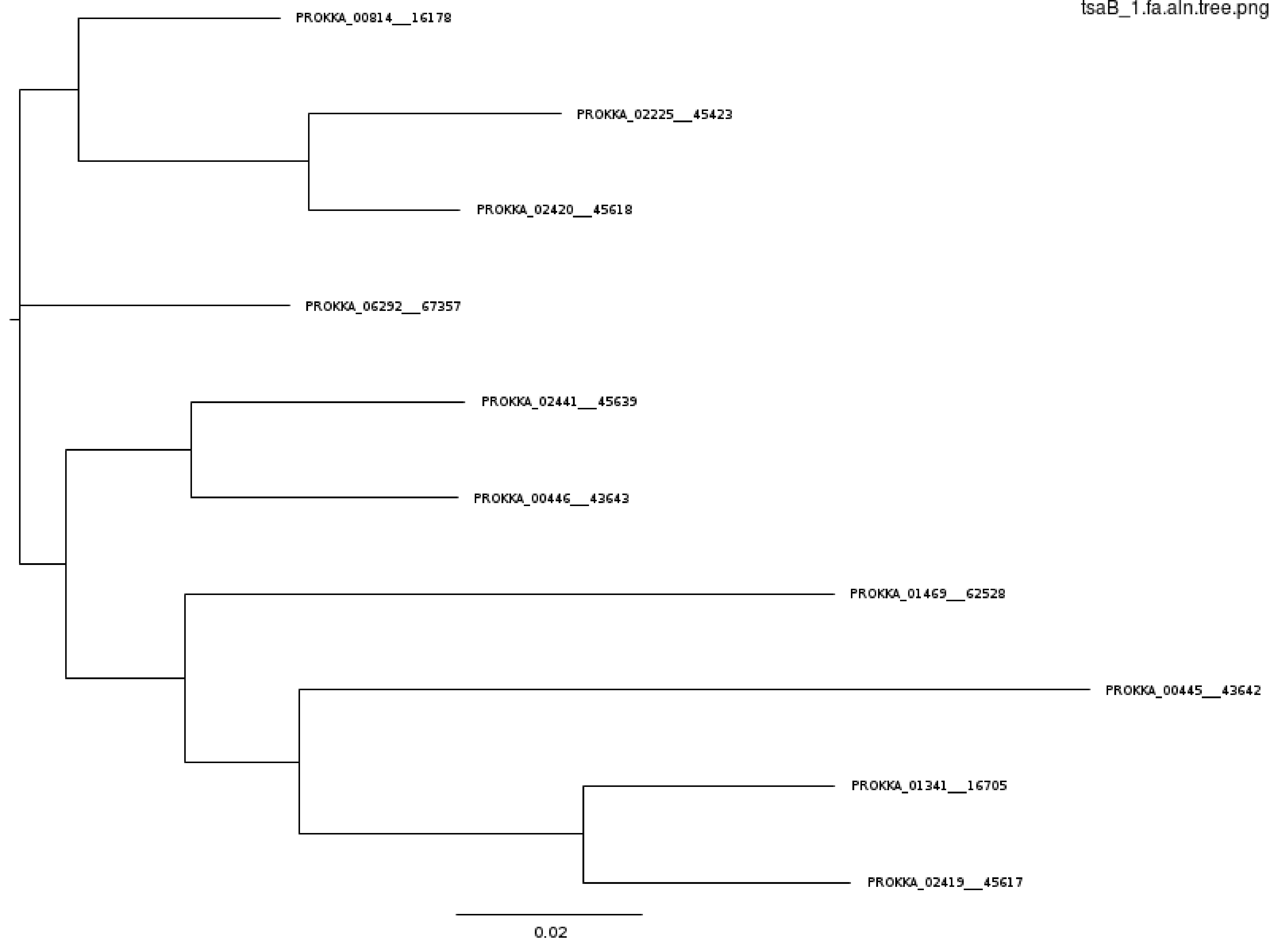
tsaB_1.fa.aln.tree.png
PROKKA_00814___16178
PROKKA_02225___45423
PROKKA_02420___45618
PROKKA_06292___67357
PROKKA_02441___45639
PROKKA_00446___43643
PROKKA_01469___62528
PROKKA_00445___43642
PROKKA_01341___16705
PROKKA_02419___45617
0.02

tsaC.fa.aln.tree.png

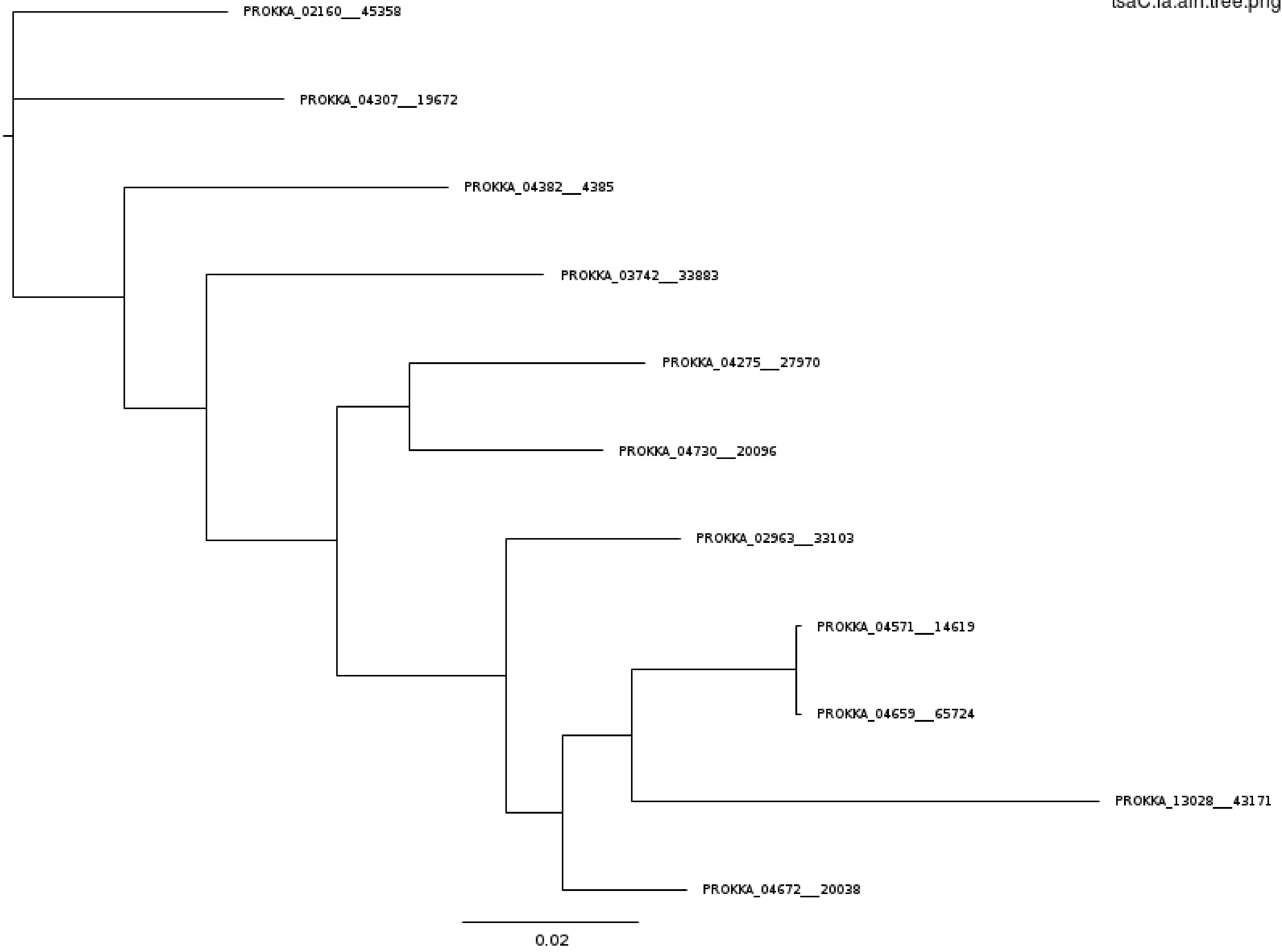

tsaD_2.fa.aln.tree.png

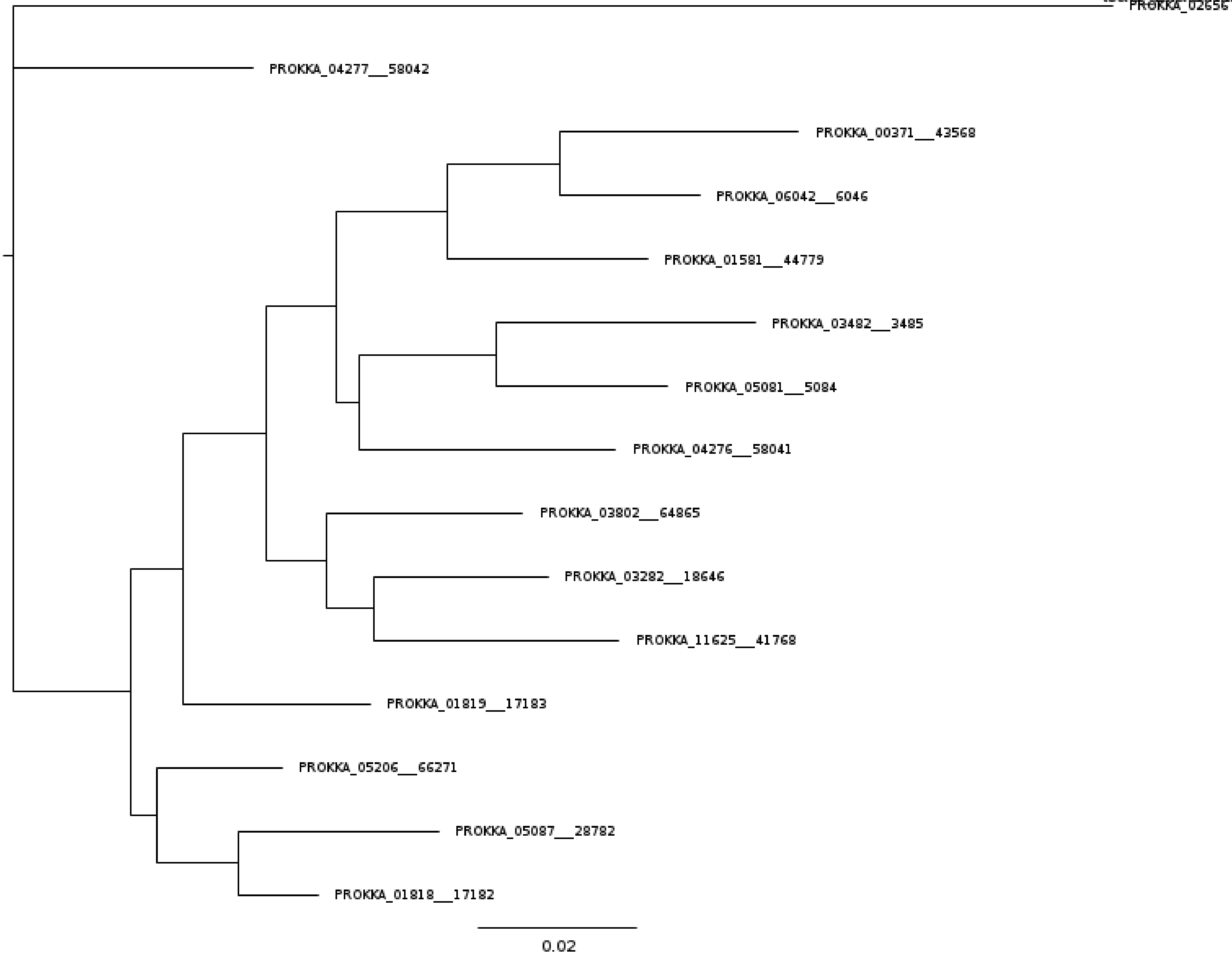

tsaE_2.fa.aln.tree.png

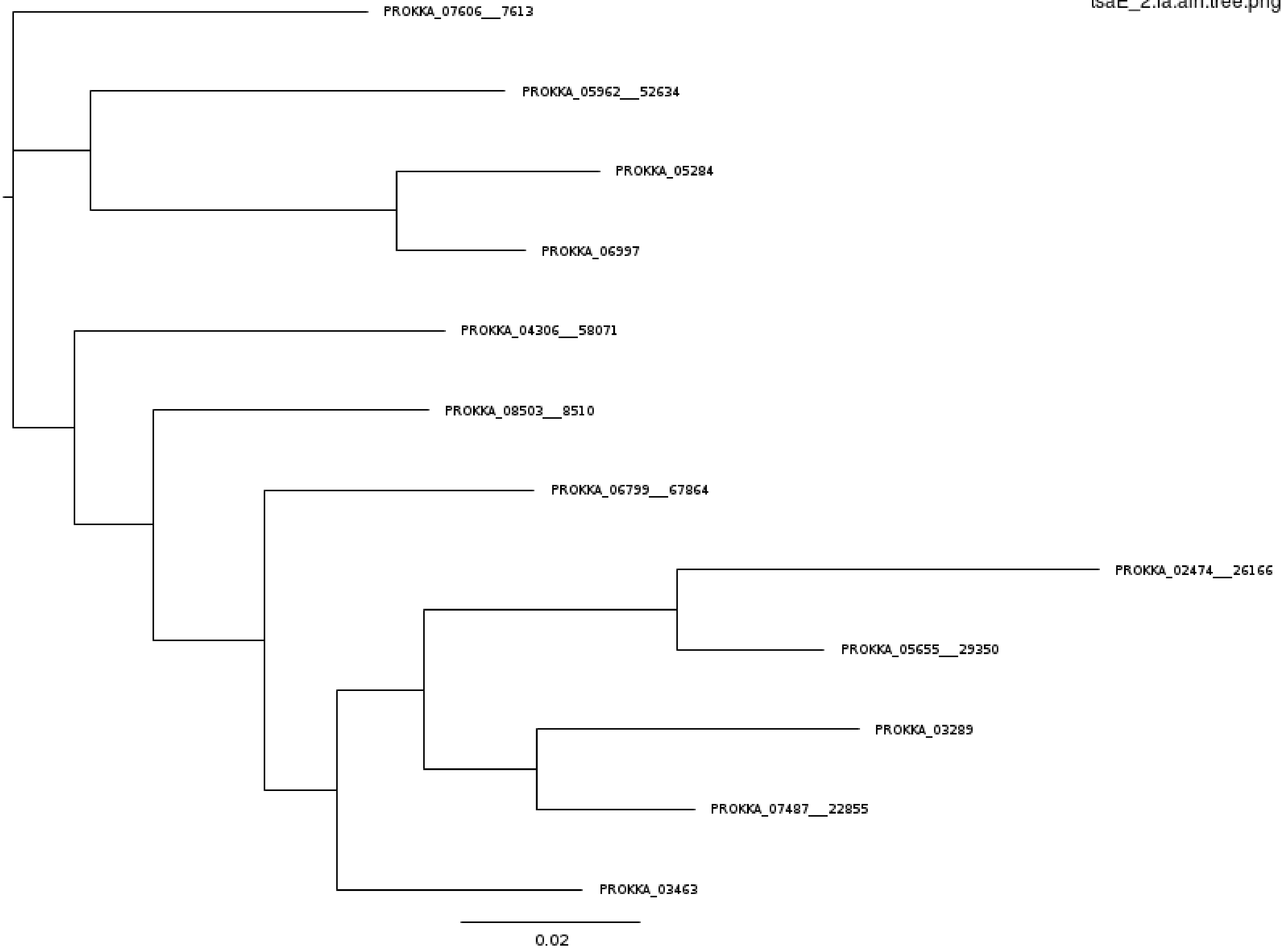

tsf.fa.aln.tree.png

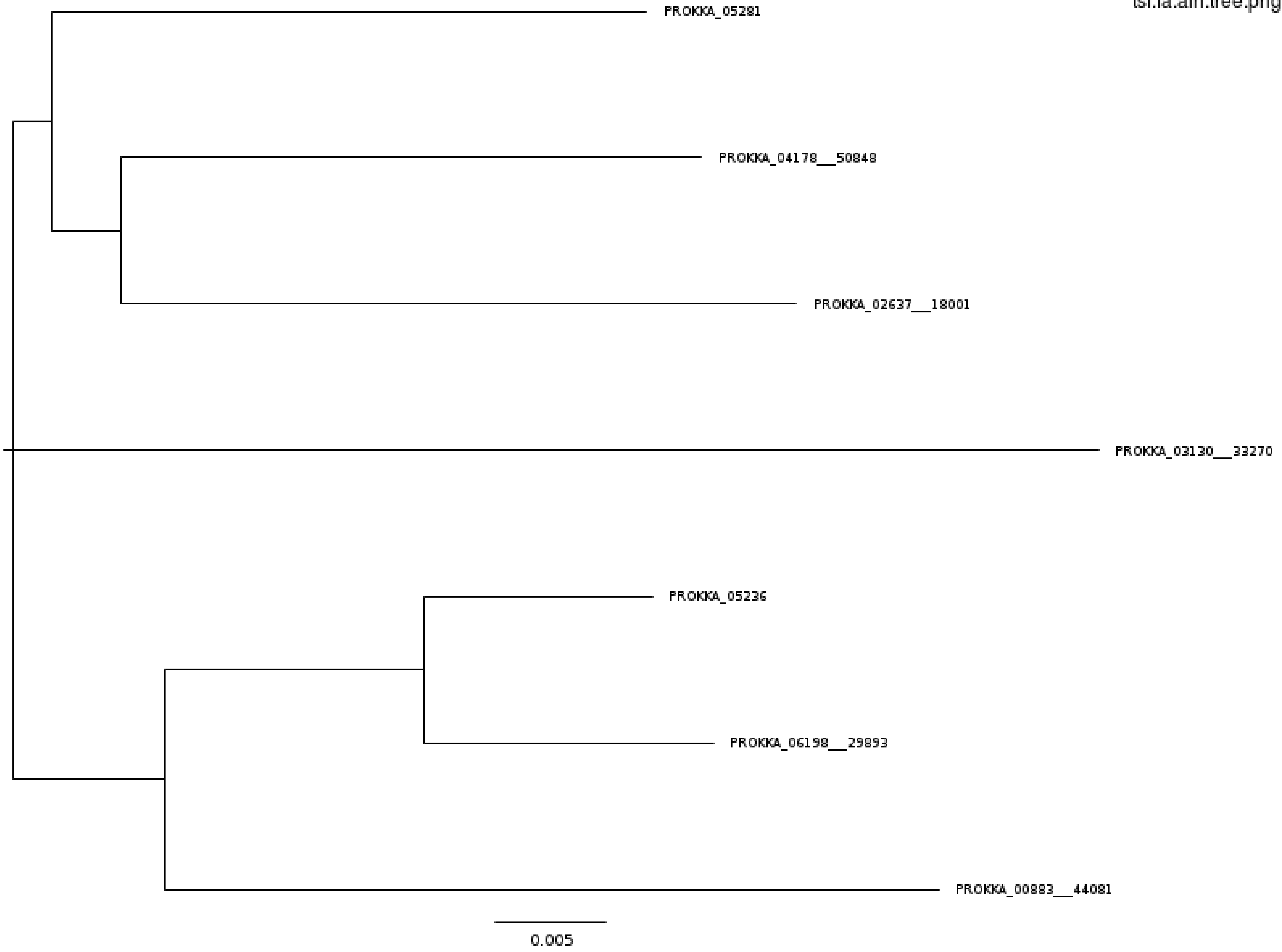

ttcA_1.fa.aln.tree.png

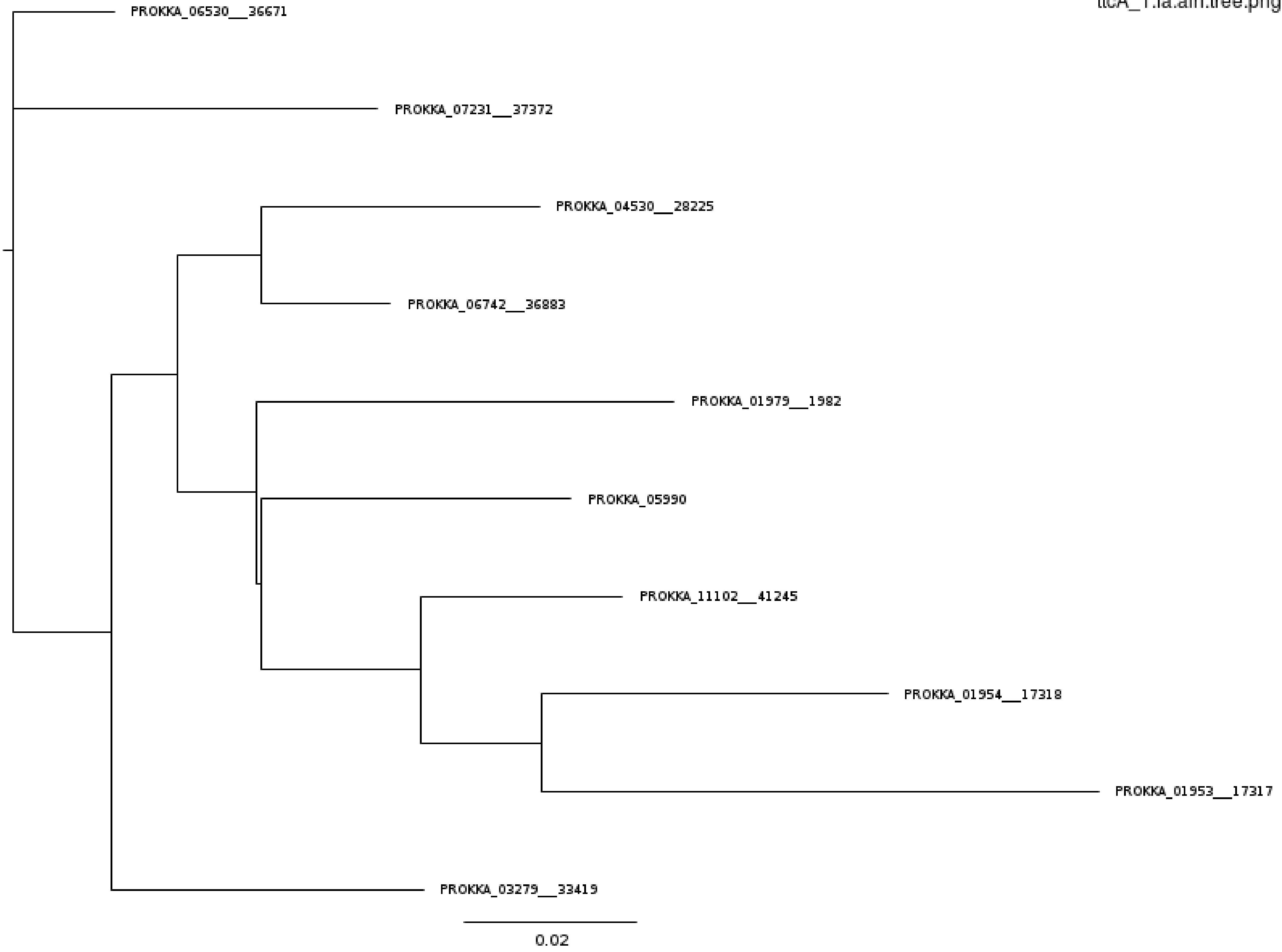

ttrB_2.fa.aln.tree.png

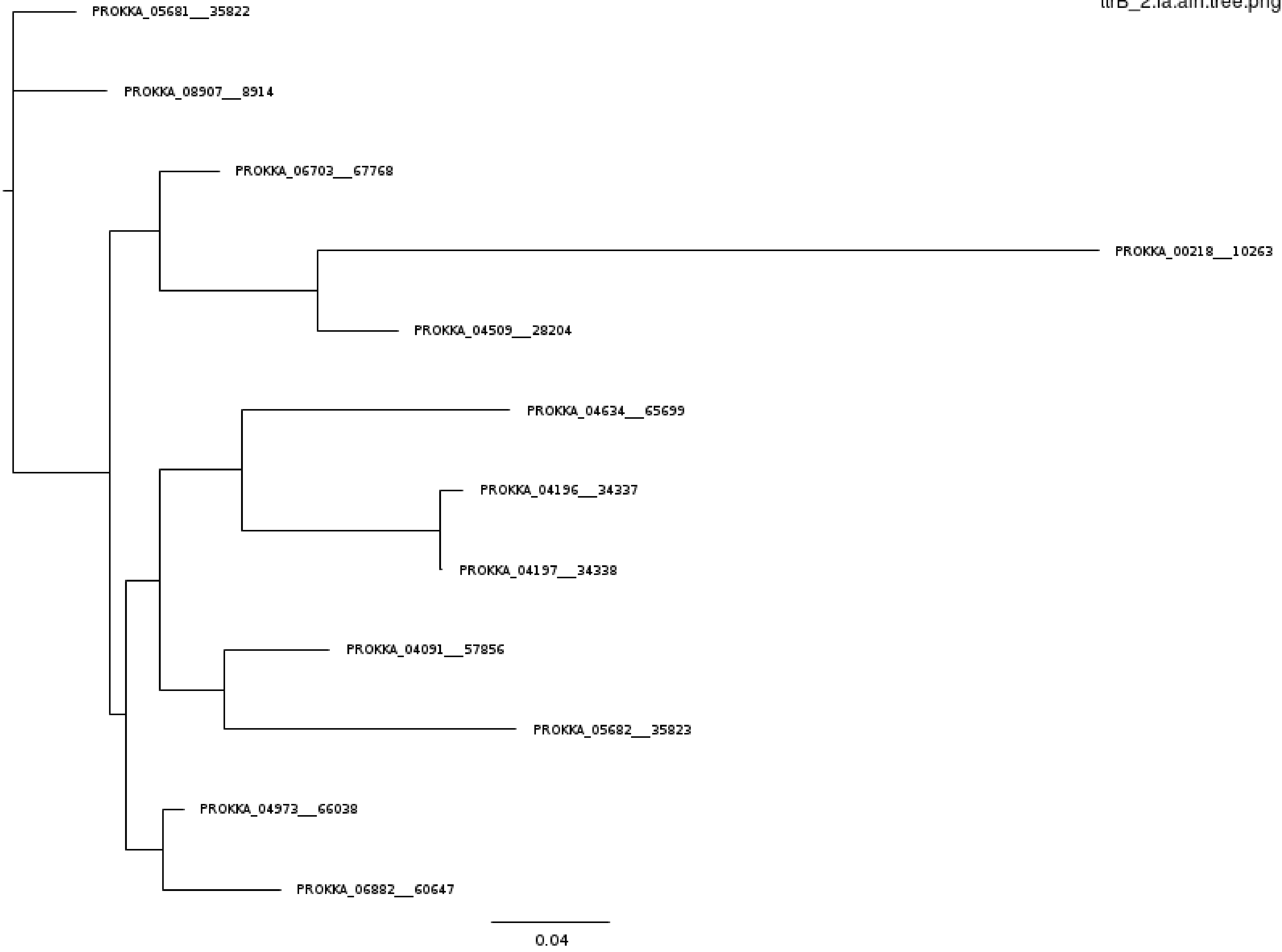

ttrB_3.fa.aln.tree.png

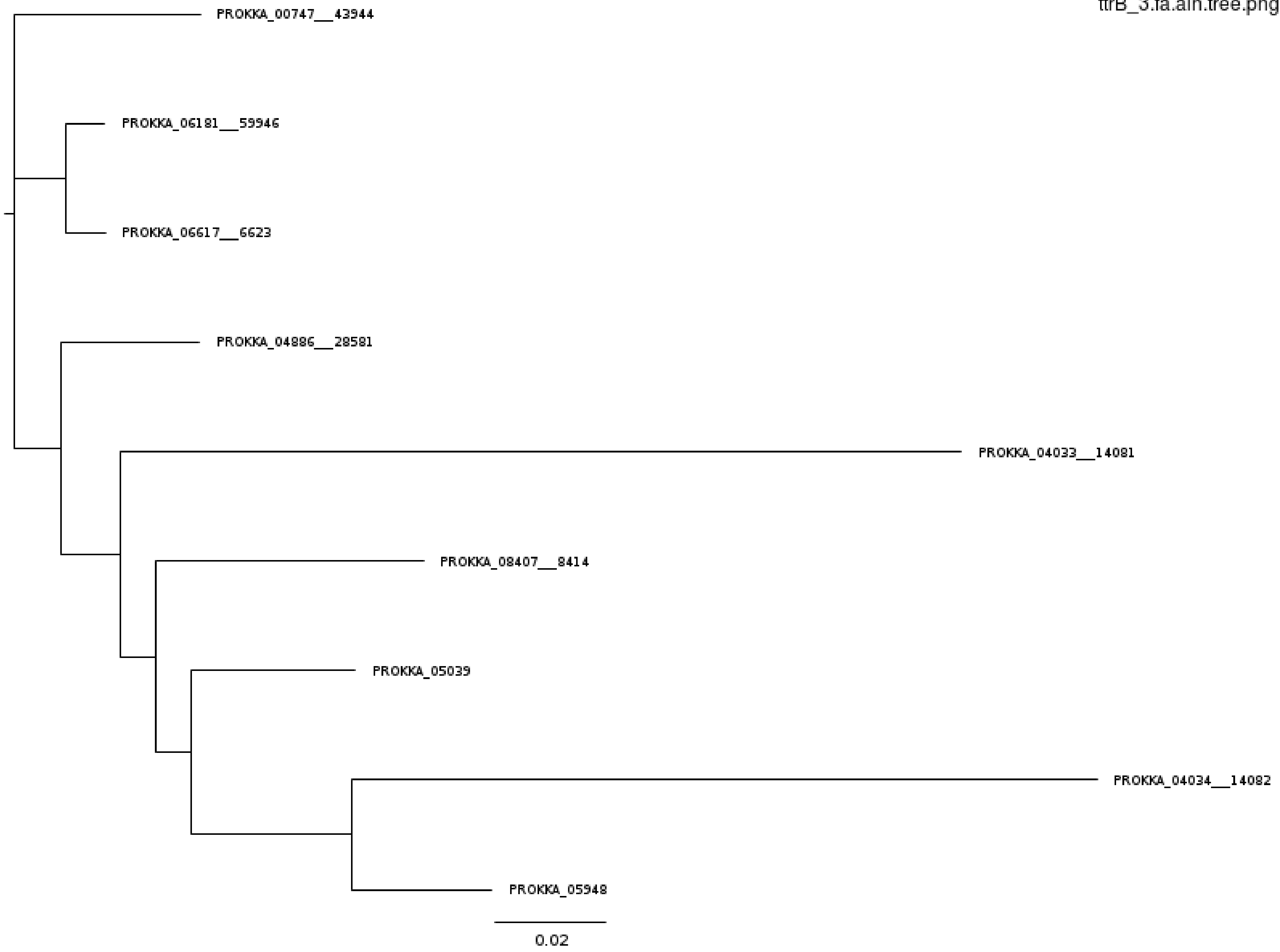

tuaD_1.fa.aln.tree.png

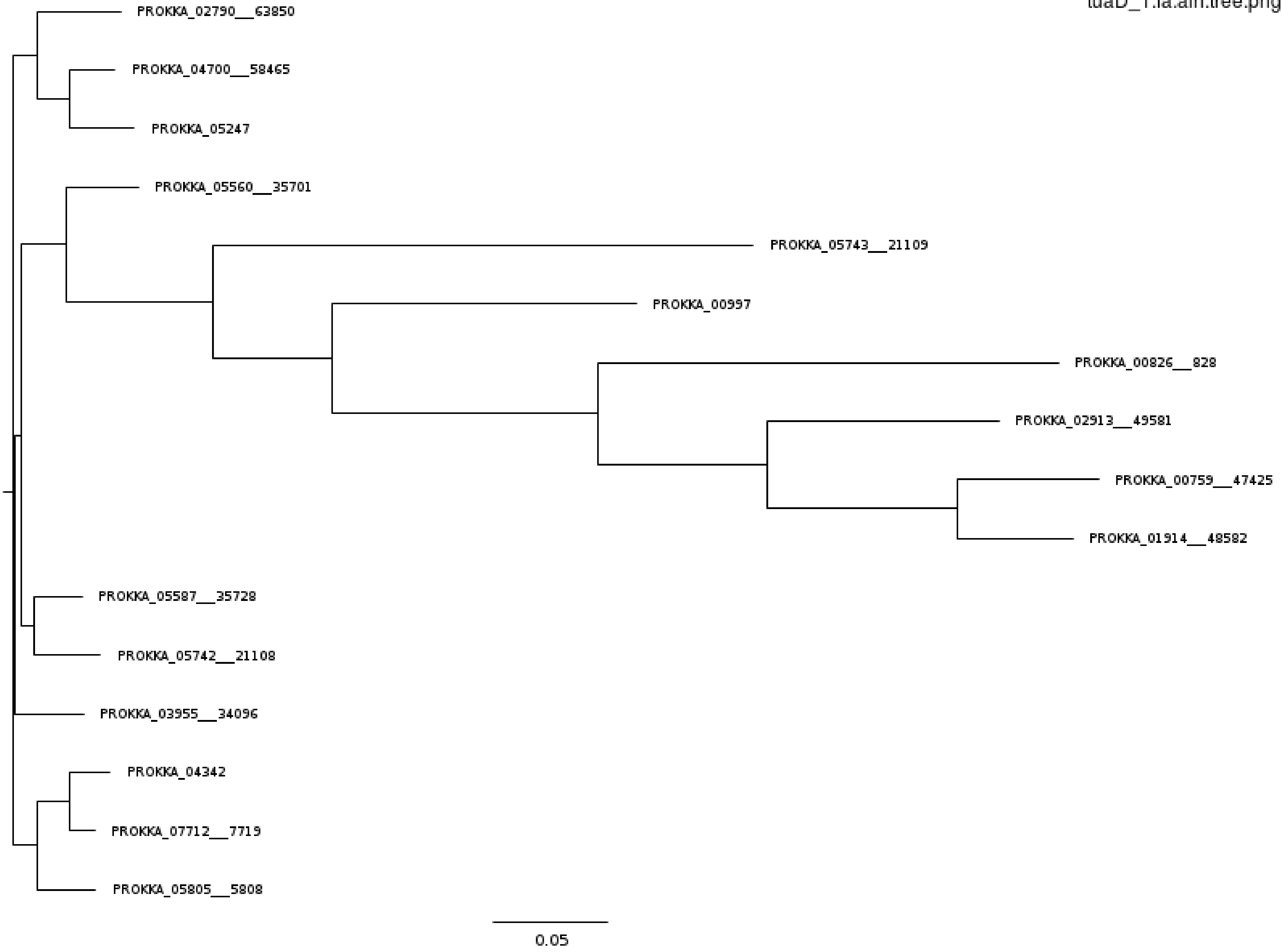

tuaG_2.fa.aln.tree.png

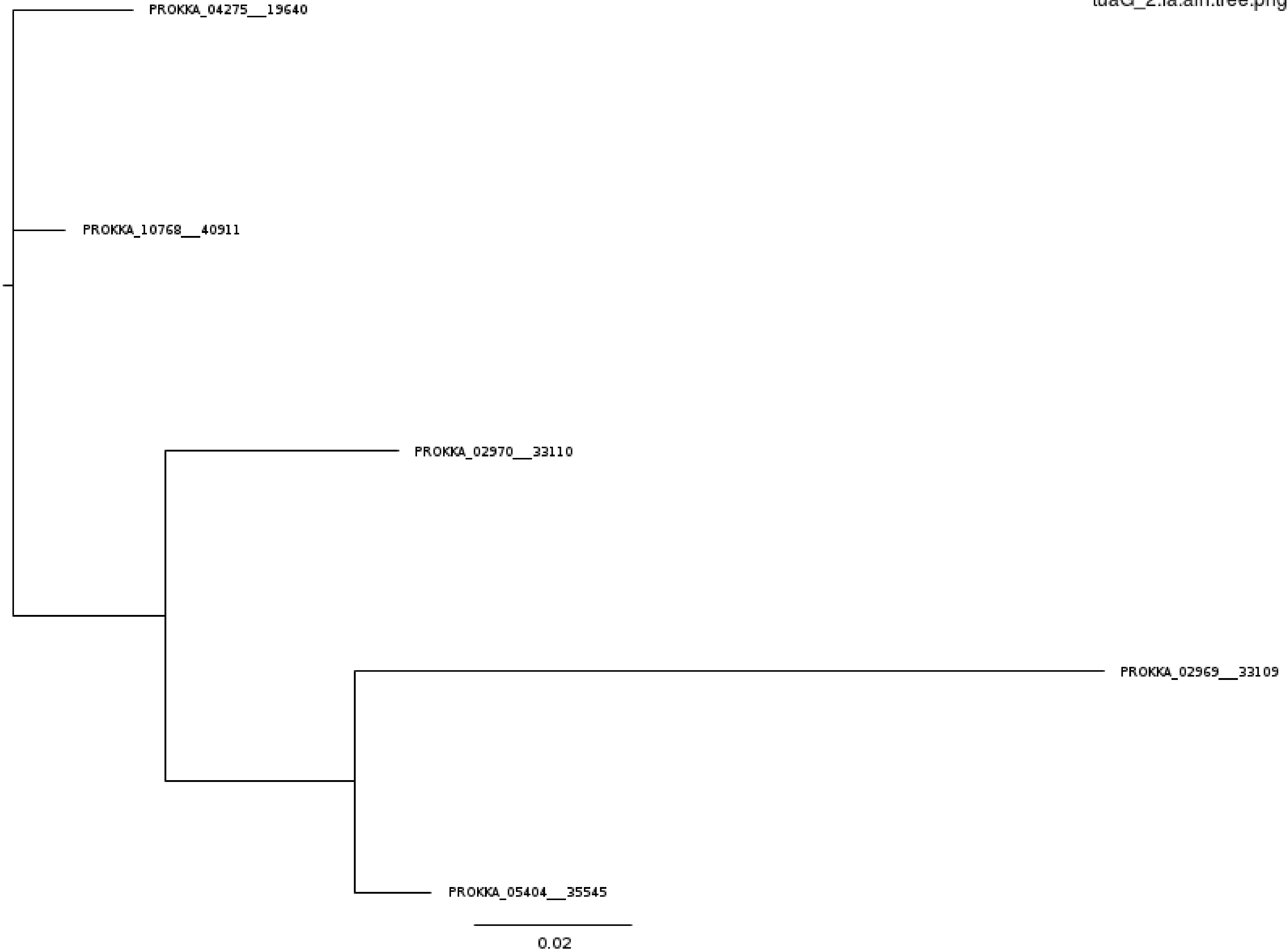

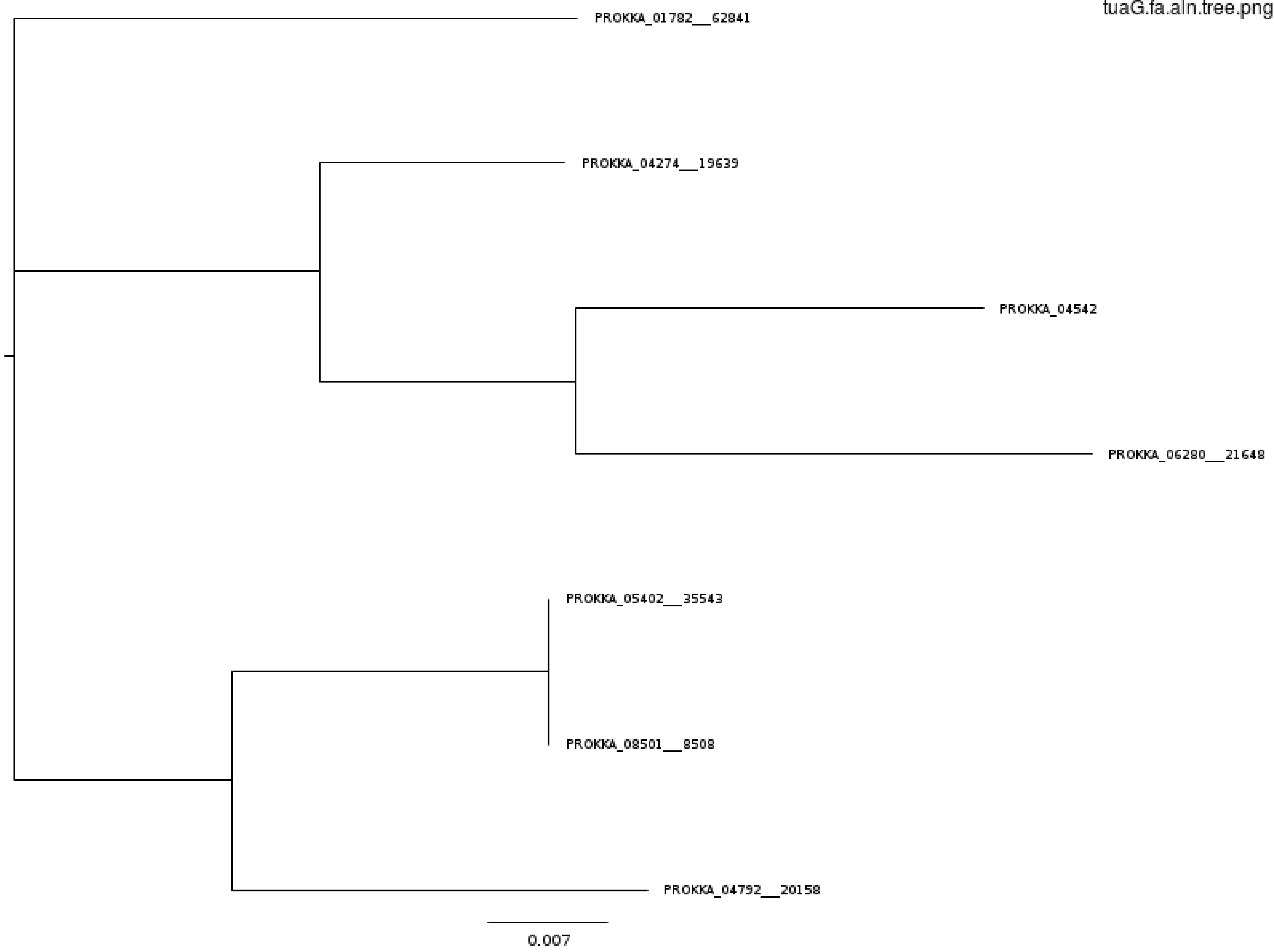
tuaG.fa.aln.tree.png
PROKKA_01782___62841
PROKKA_04274___19639
PROKKA_04542
PROKKA_06280___21648
PROKKA_05402___35543
PROKKA_08501___8508
PROKKA_04792___20158
0.007

tufA_1.fa.aln.tree.png

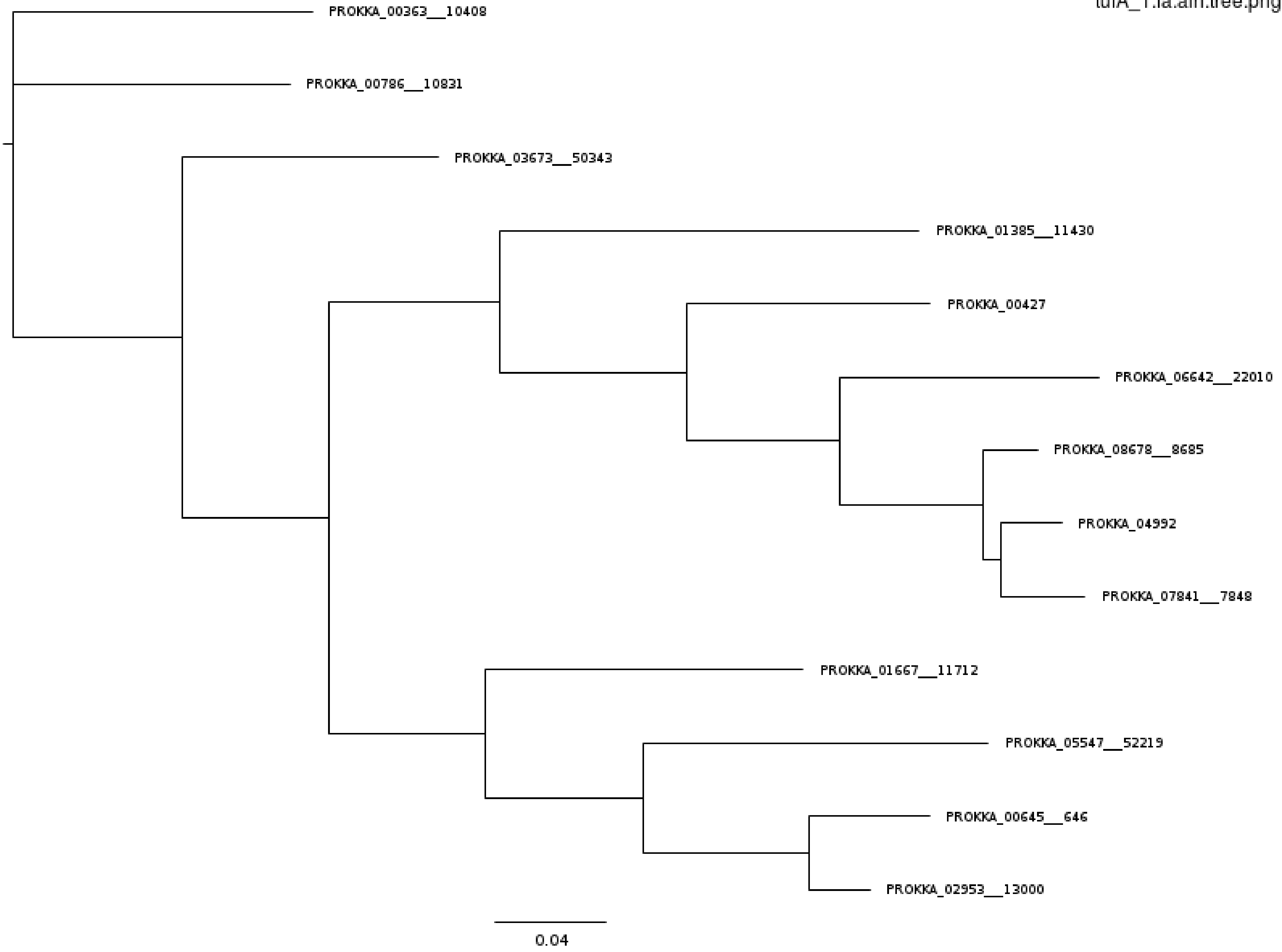

tusA_2.fa.aln.tree.png

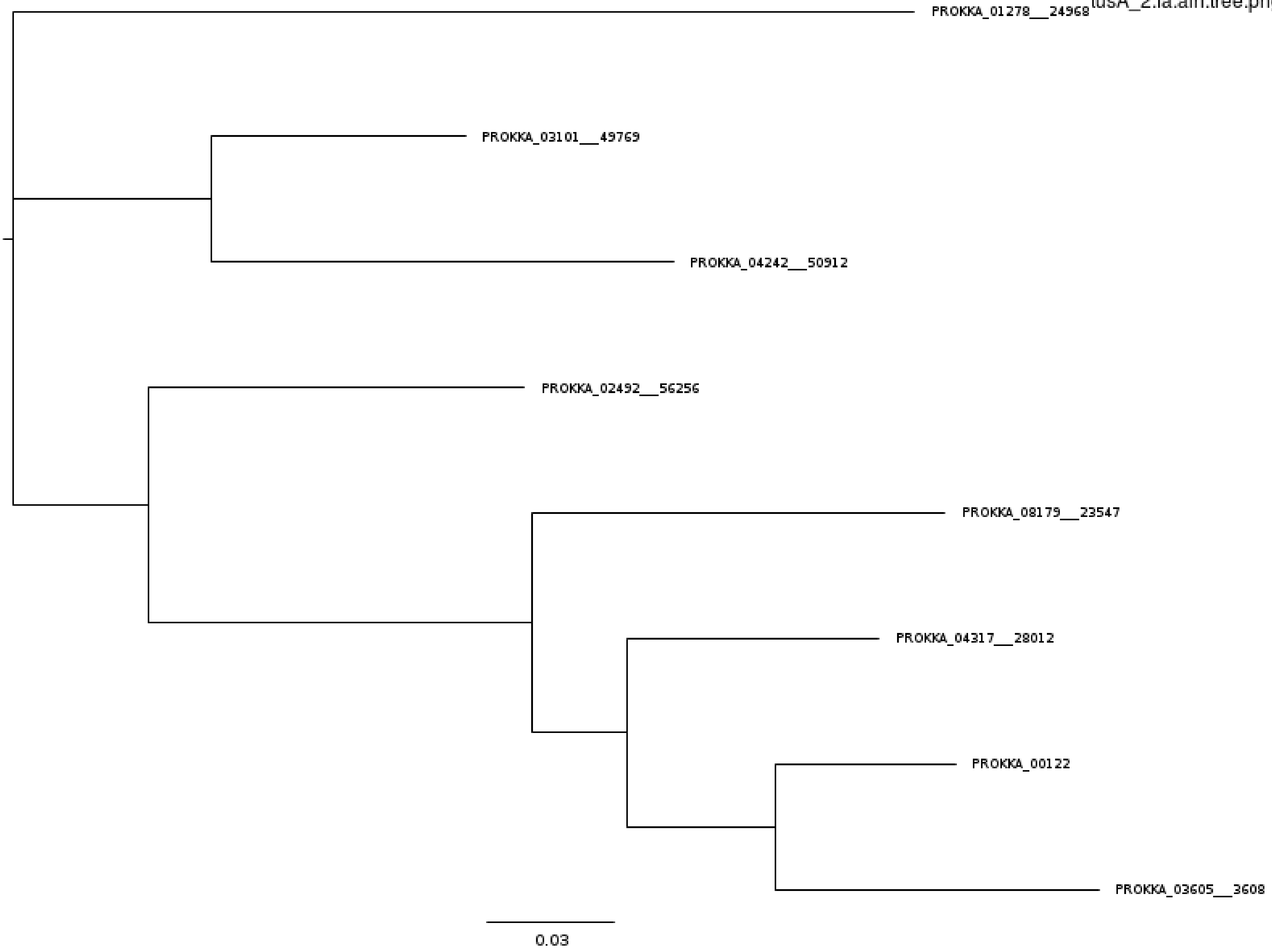

tusB.fa.aln.tree.png

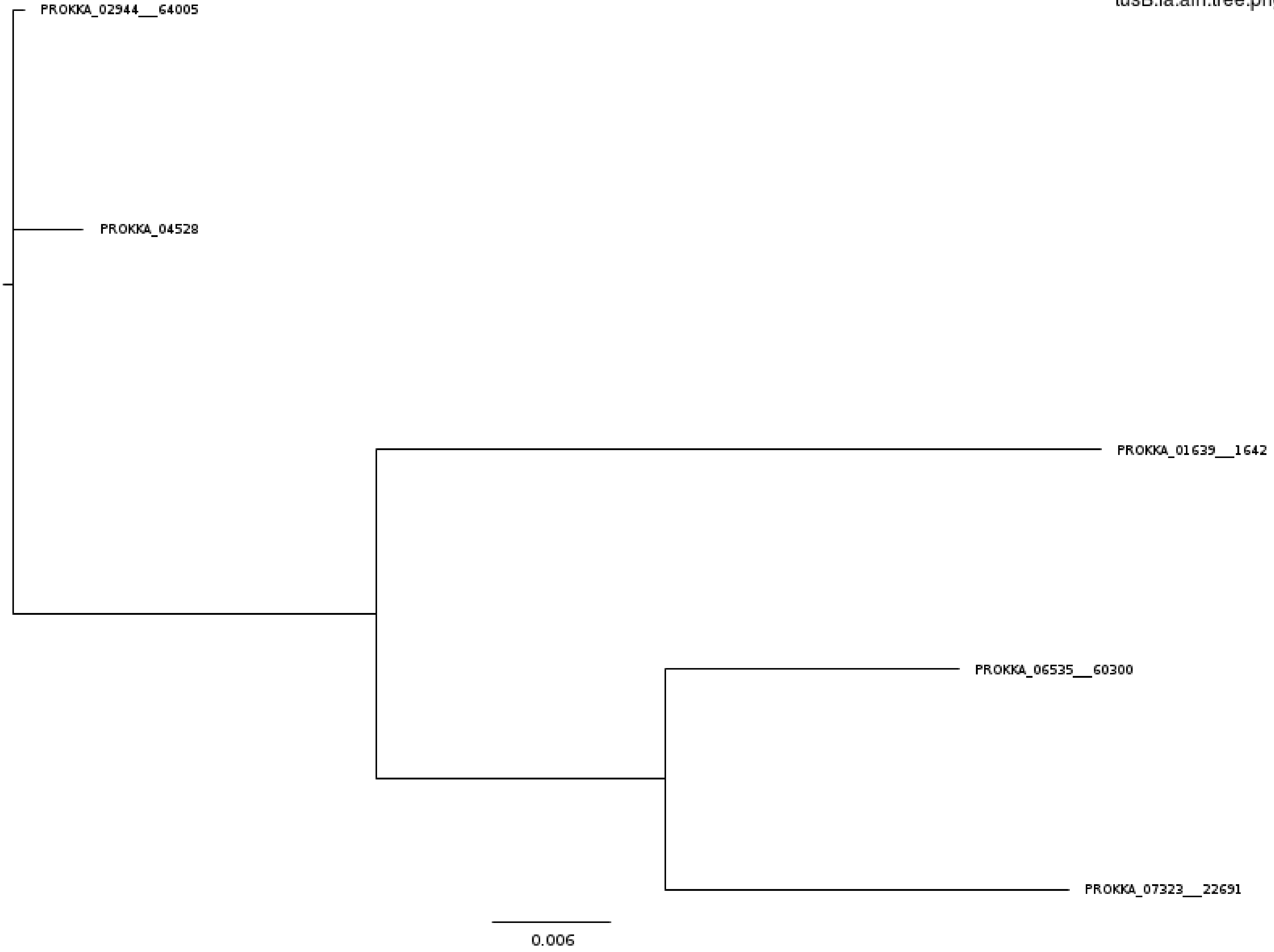

tusE_3.fa.aln.tree.png

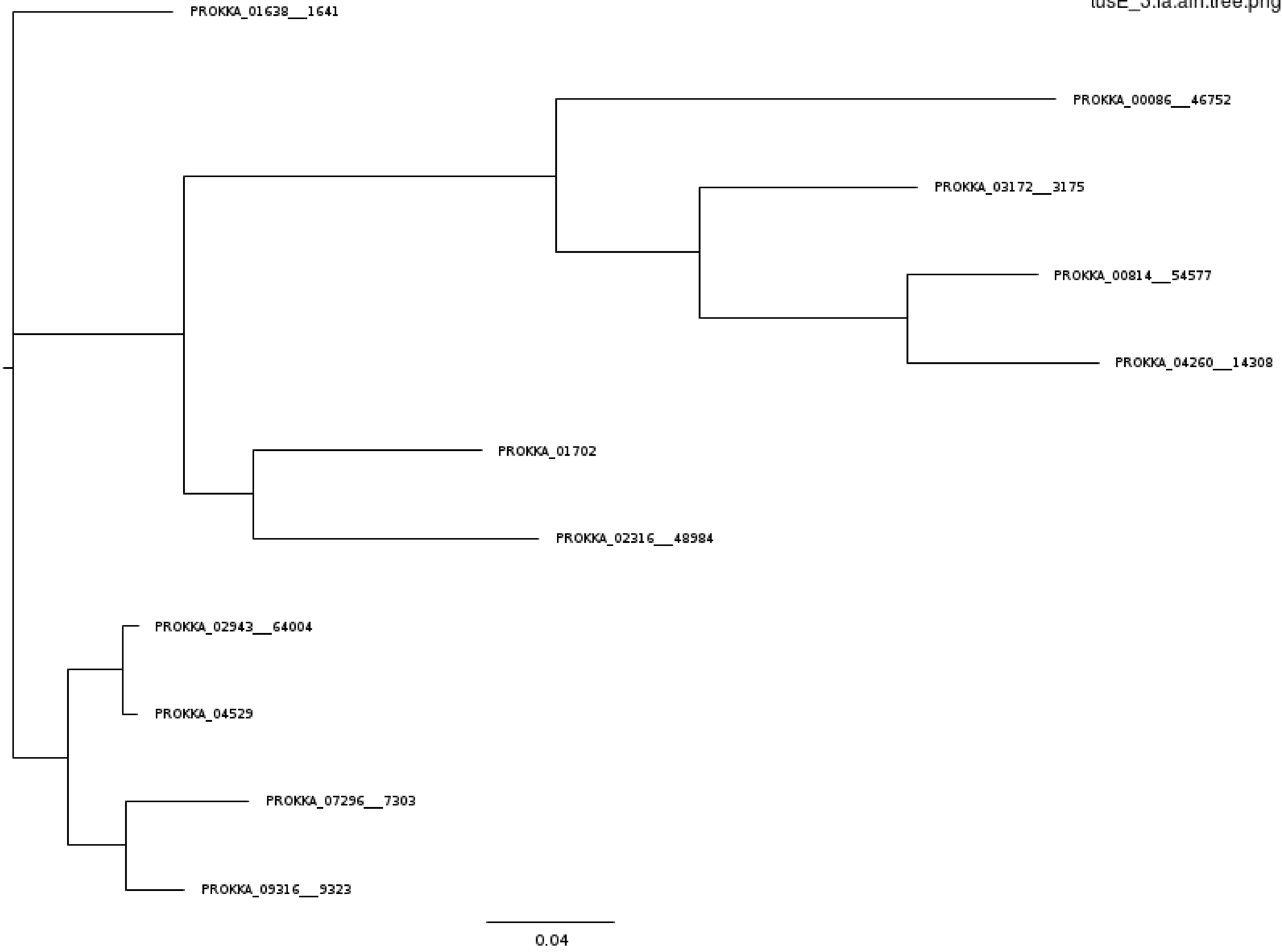

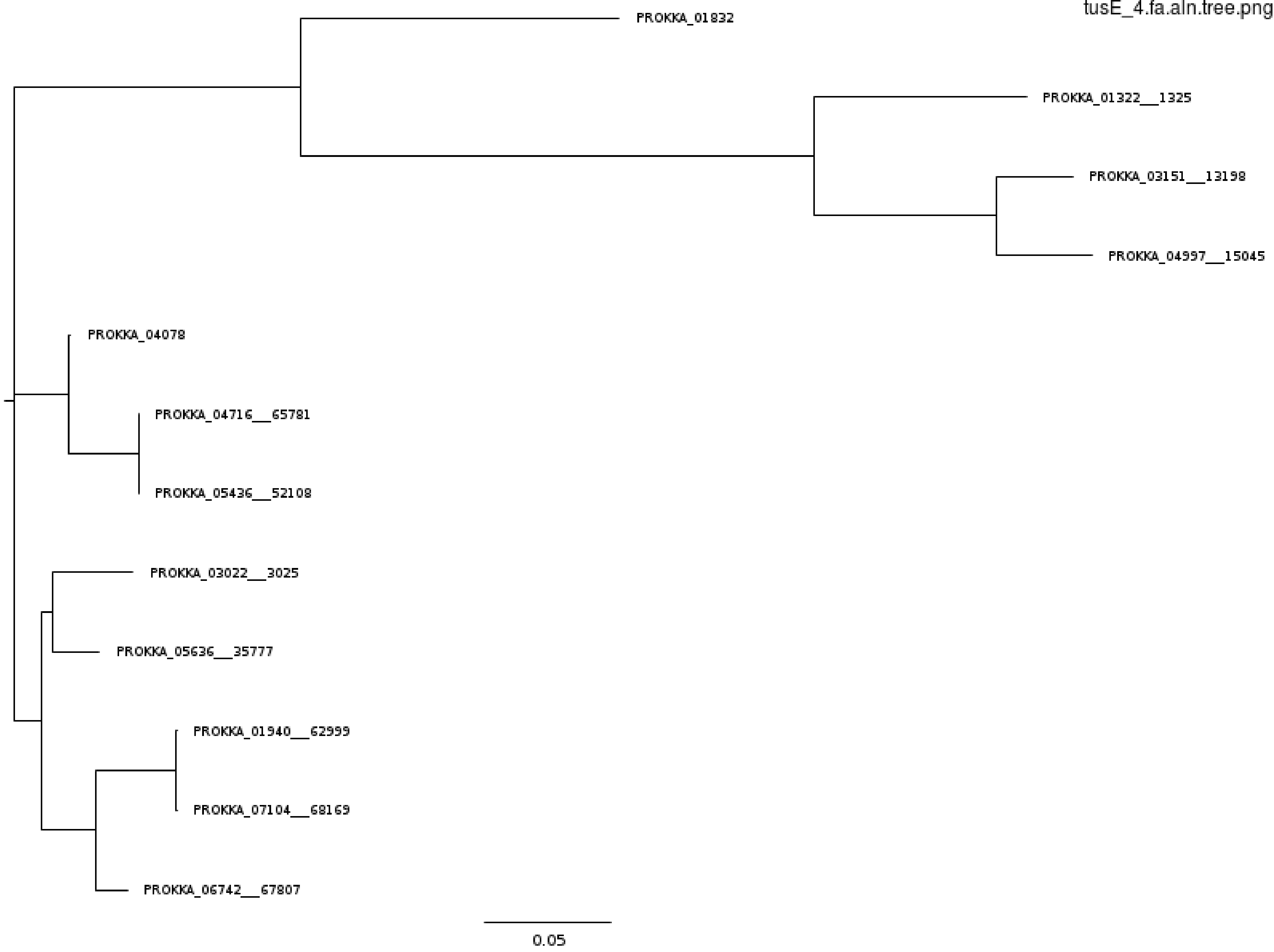
tusE_4.fa.aln.tree.png
PROKKA_01832
PROKKA_01322__1325
PROKKA_03151__13198
PROKKA_04997__15045
PROKKA_04078
PROKKA_04716__65781
PROKKA_05436__52108
PROKKA_03022__3025
PROKKA_05636__35777
PROKKA_01940__62999
PROKKA_07104__68169
PROKKA_06742__67807
0.05

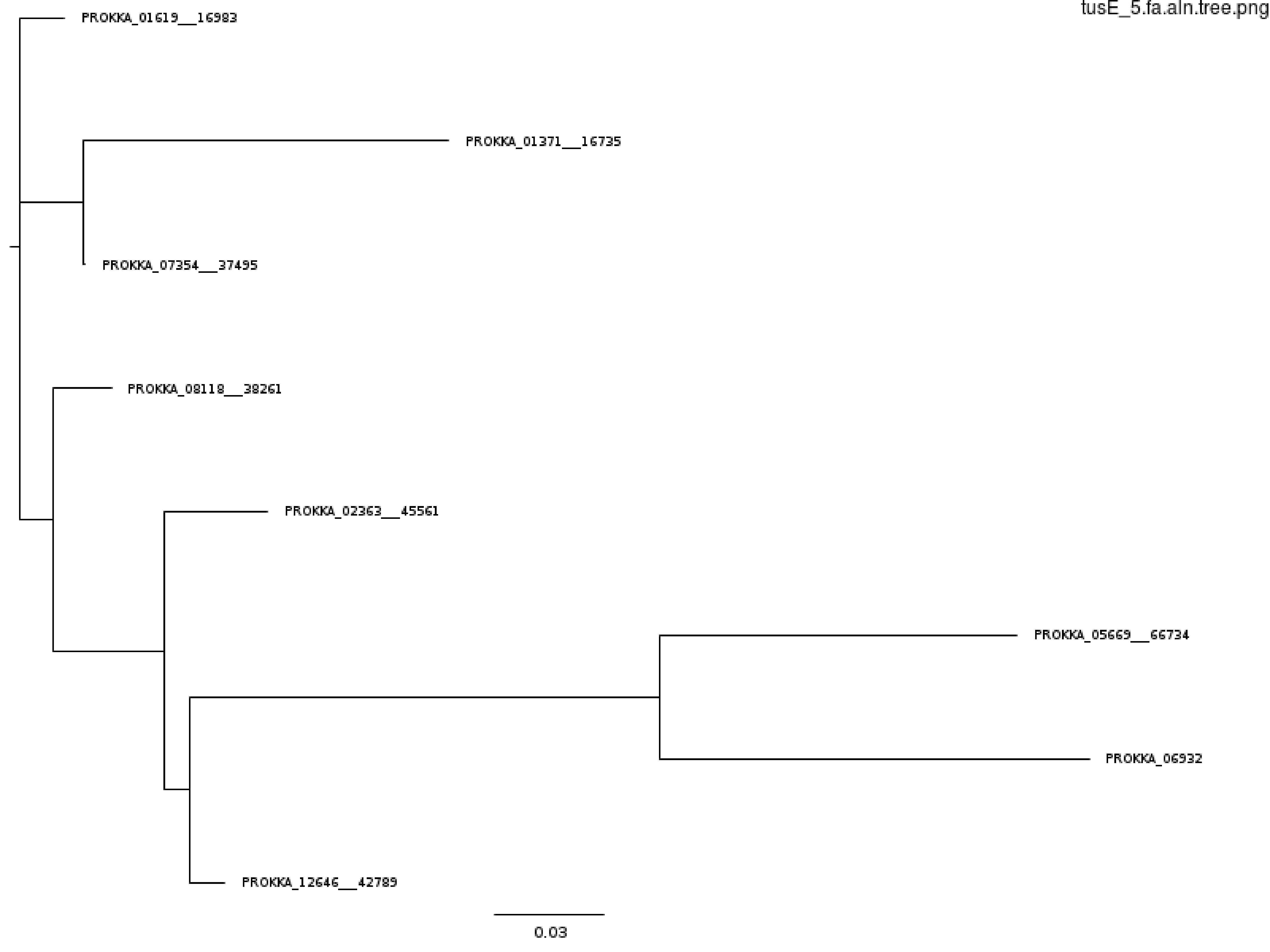
tusE_5.fa.aln.tree.png
PROKKA_01619__16983
PROKKA_01371__16735
PROKKA_07354__37495
PROKKA_08118__38261
PROKKA_02363__45561
PROKKA_05669__66734
PROKKA_06932
PROKKA_12646__42789
0.03

tusE_6.fa.aln.tree.png

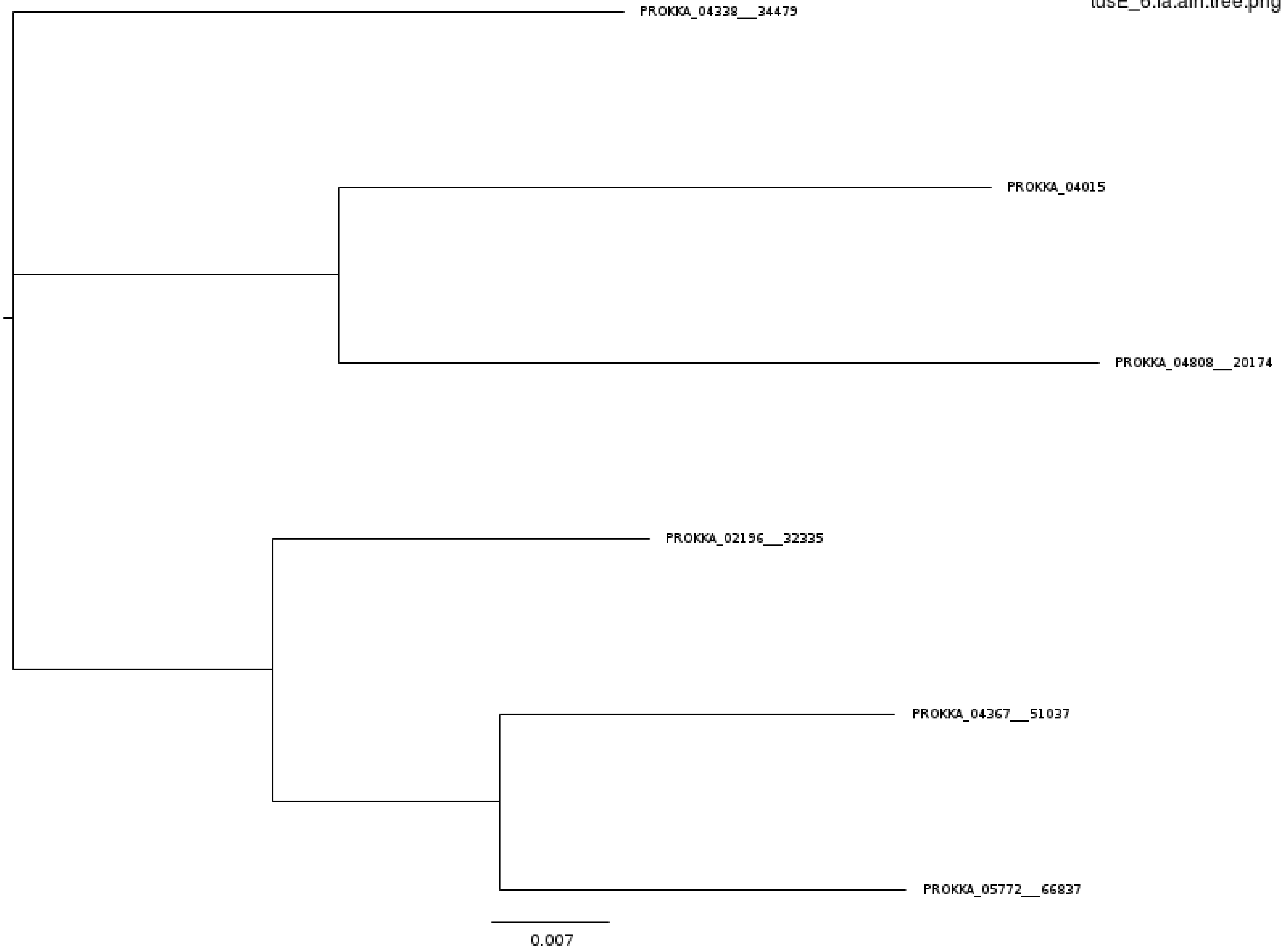

ubiB_2.fa.aln.tree.png

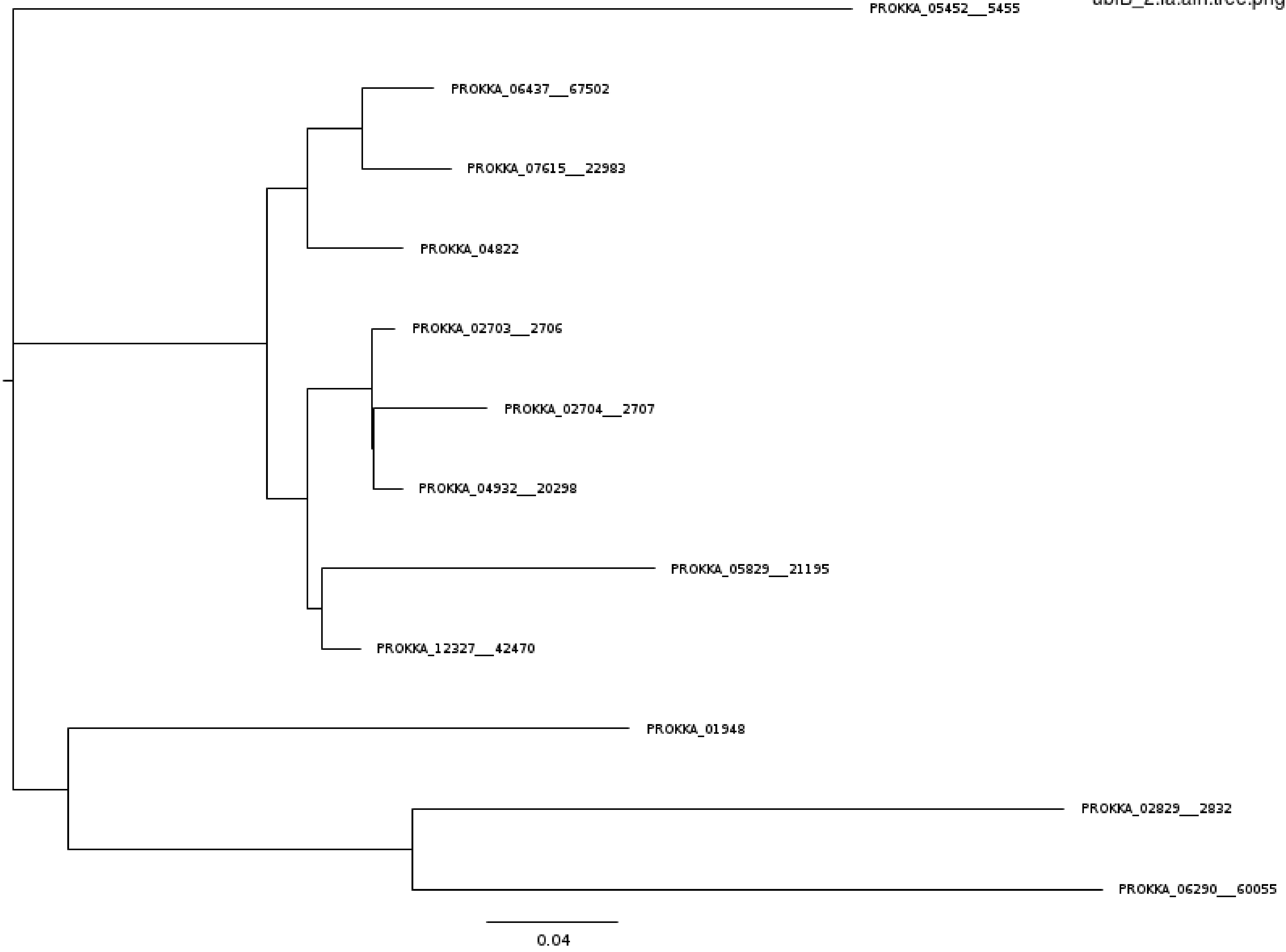

ubiD_1.fa.aln.tree.png

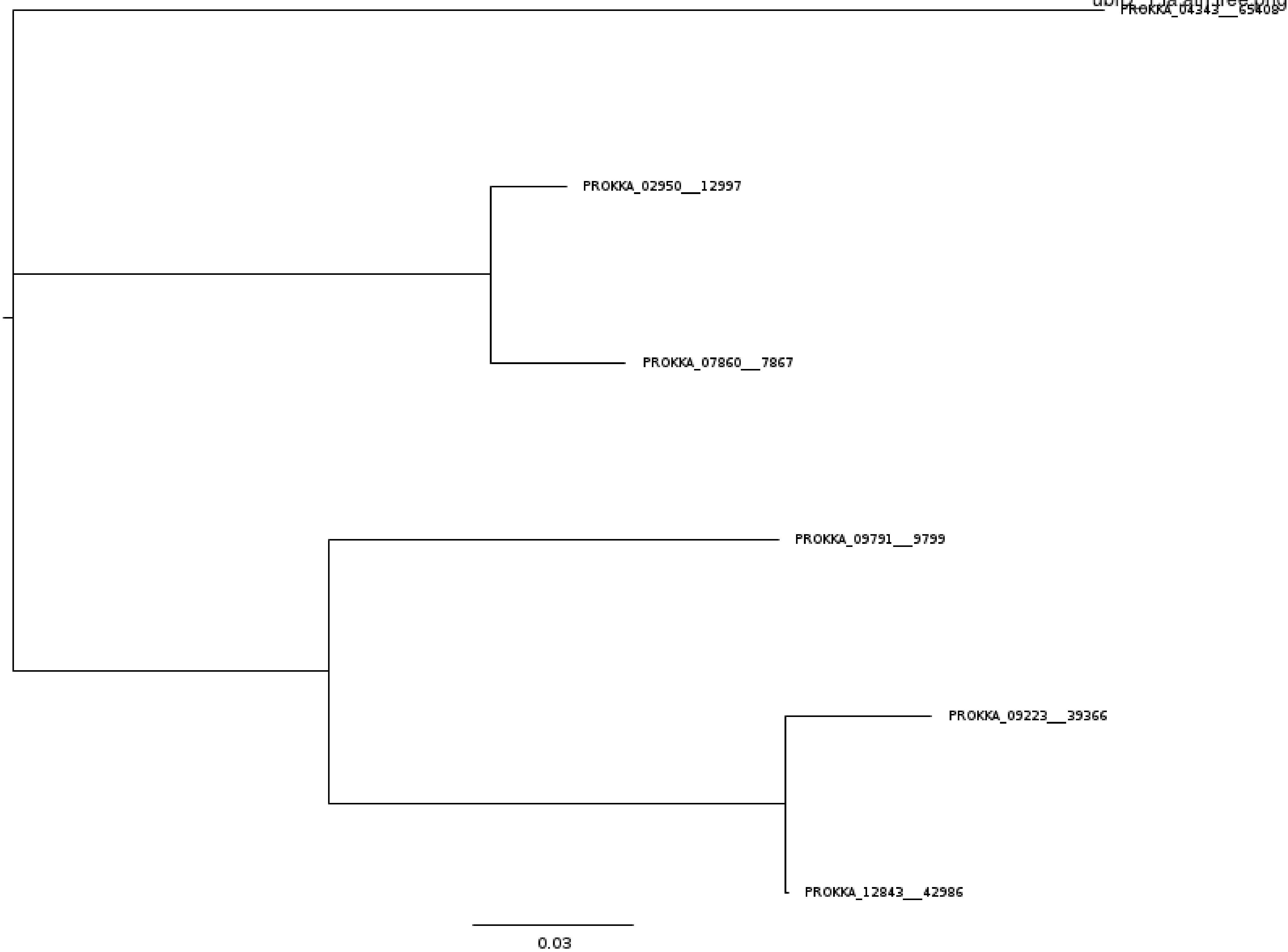

ubiE_6.fa.aln.tree.png

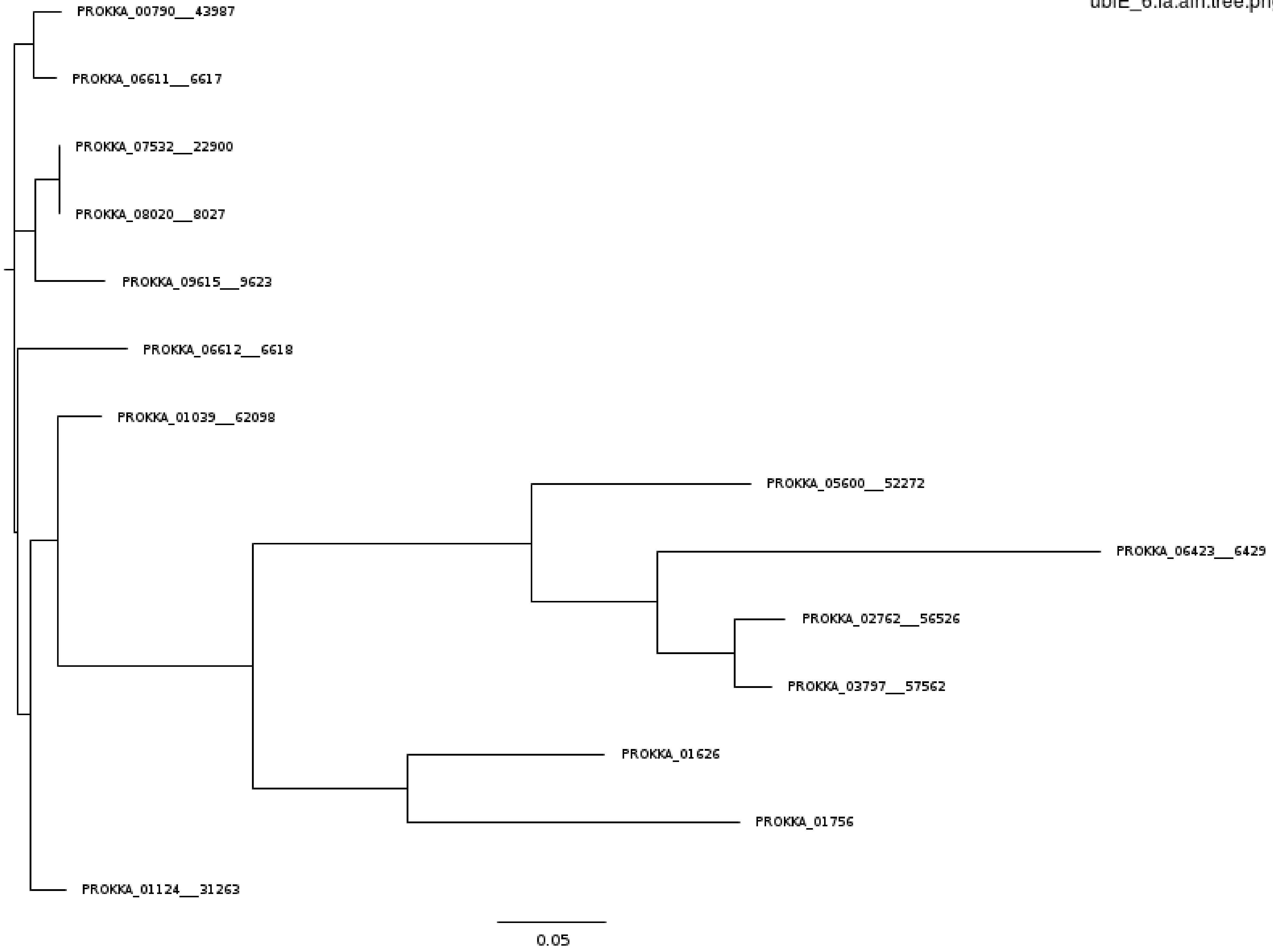

ubiE_7.fa.aln.tree.png

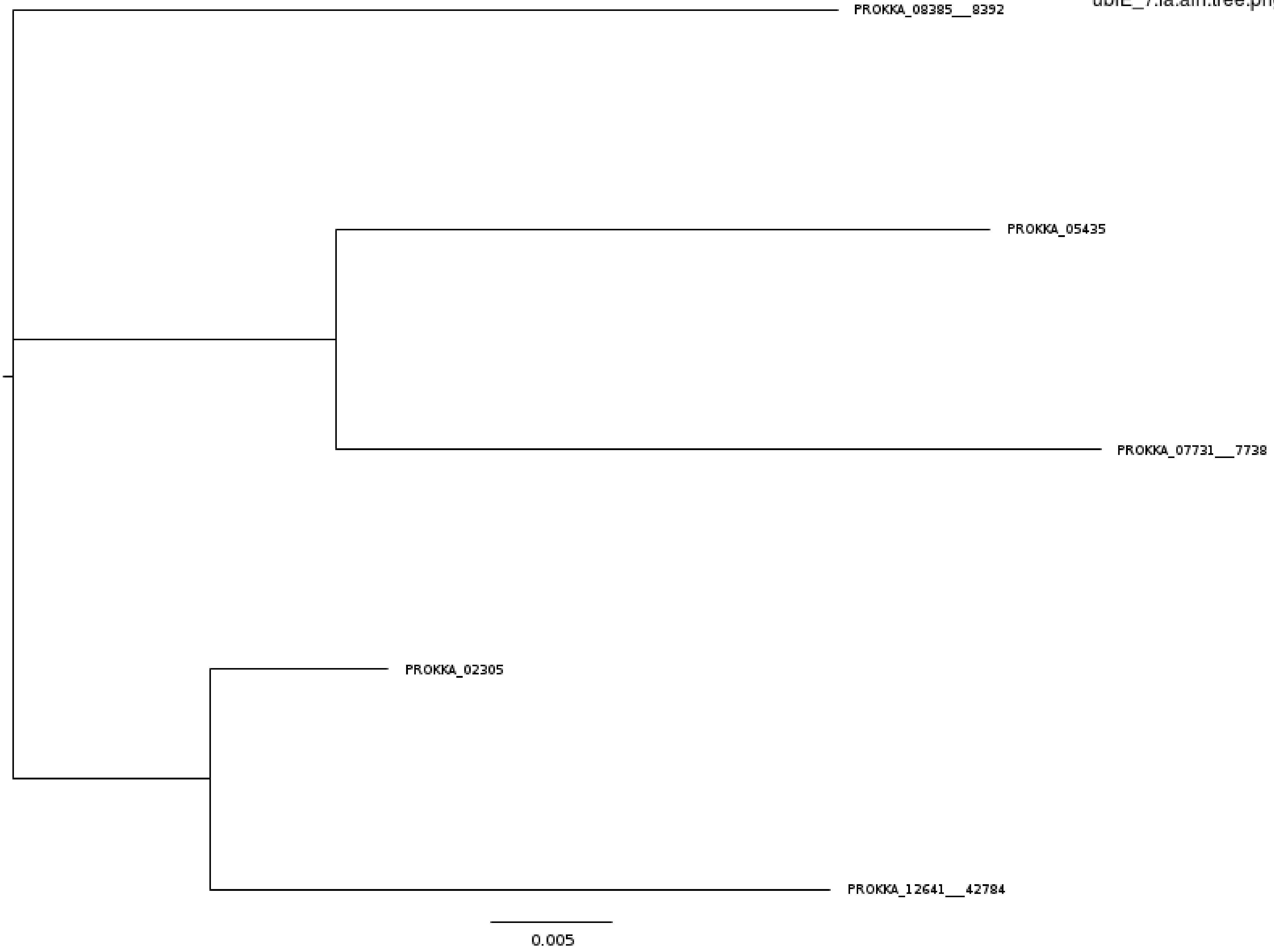

uvrA_1.fa.aln.tree.png

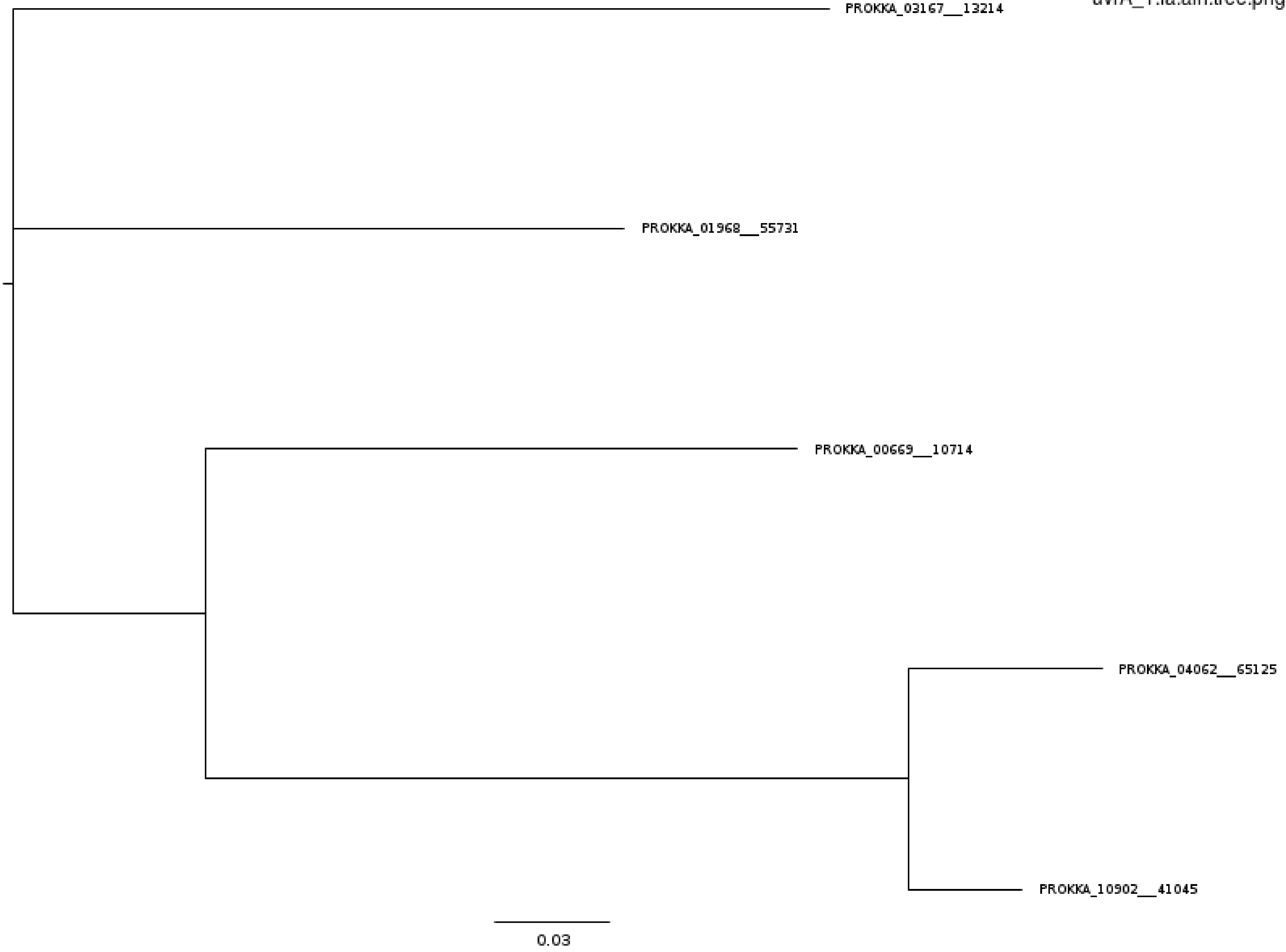

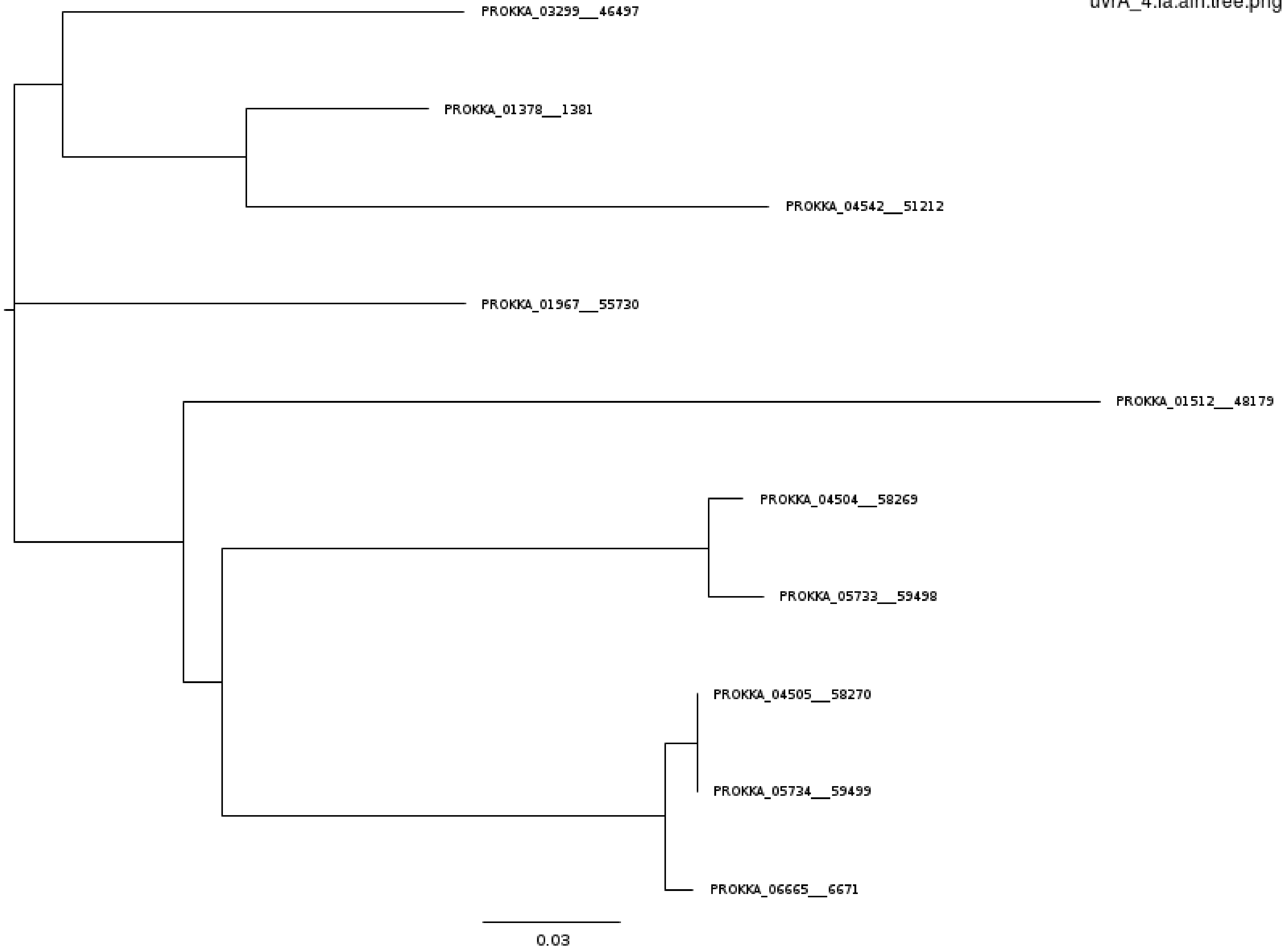
PROKKA_03299___46497
PROKKA_01378___1381
PROKKA_04542___51212
PROKKA_01967___55730
PROKKA_01512___48179
PROKKA_04504___58269
PROKKA_05733___59498
PROKKA_04505___58270
PROKKA_05734___59499
PROKKA_06665___6671
0.03

uvrB_1.fa.aln.tree.png

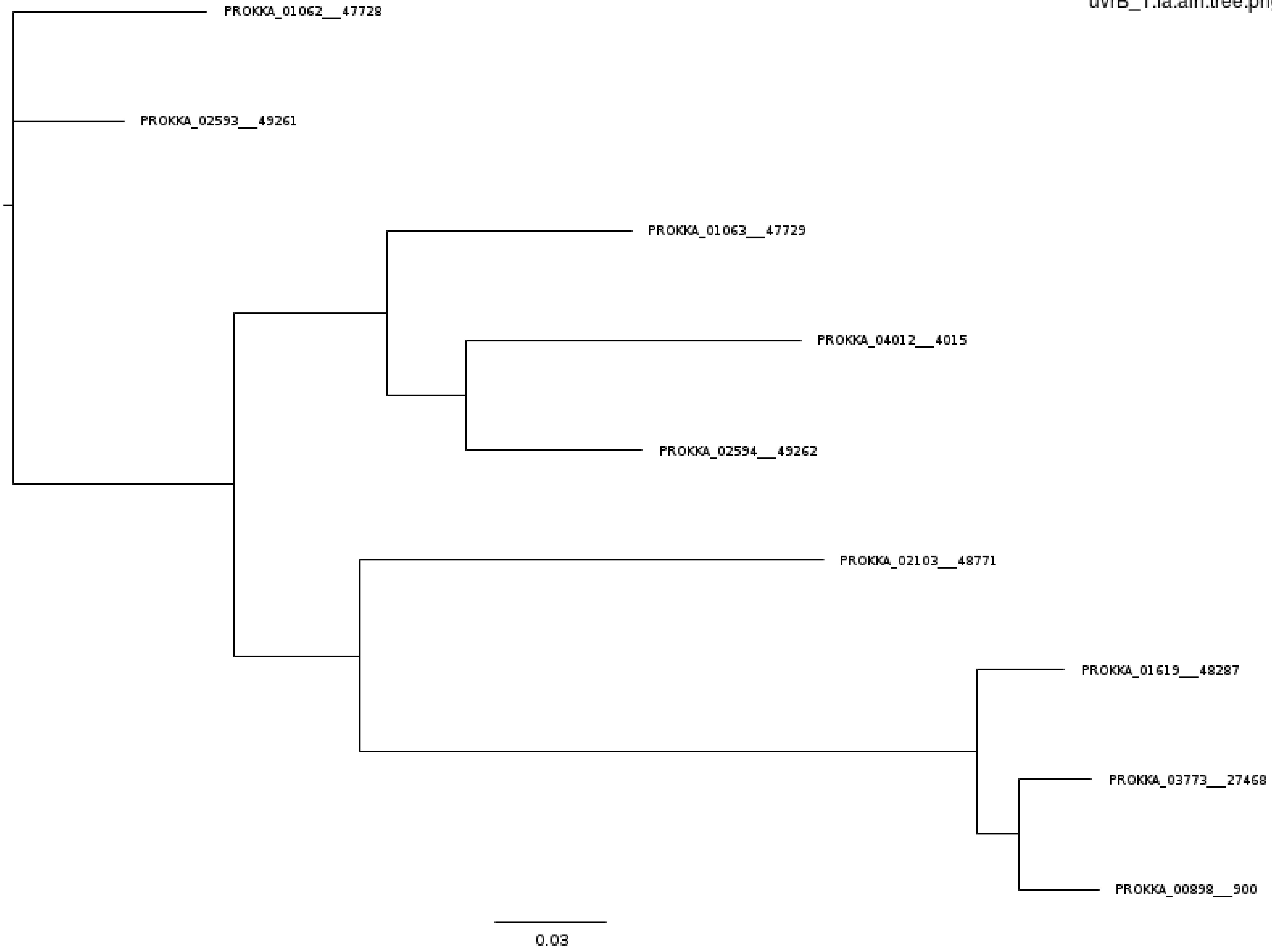

uvrC_2.fa.aln.tree.png

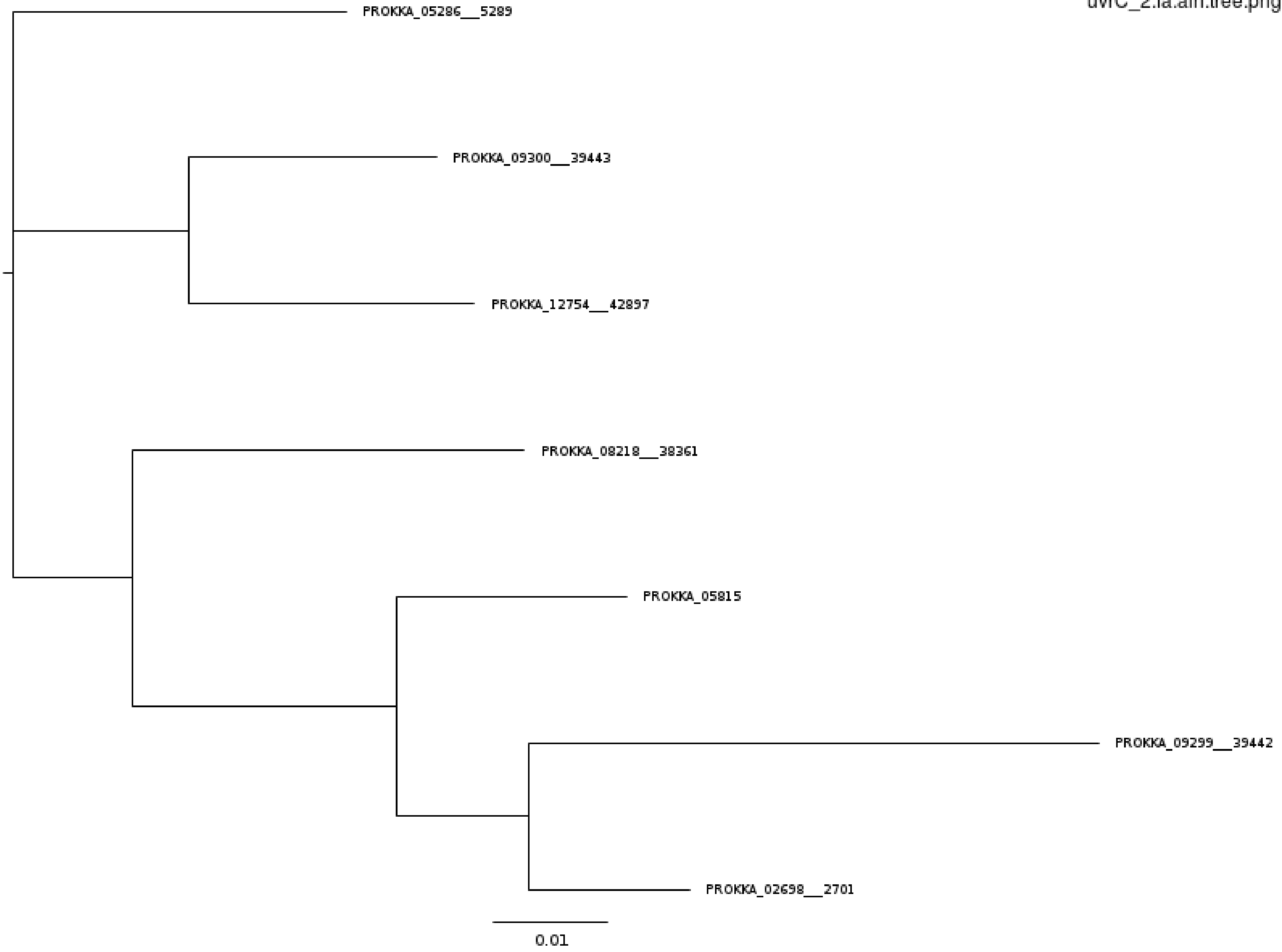

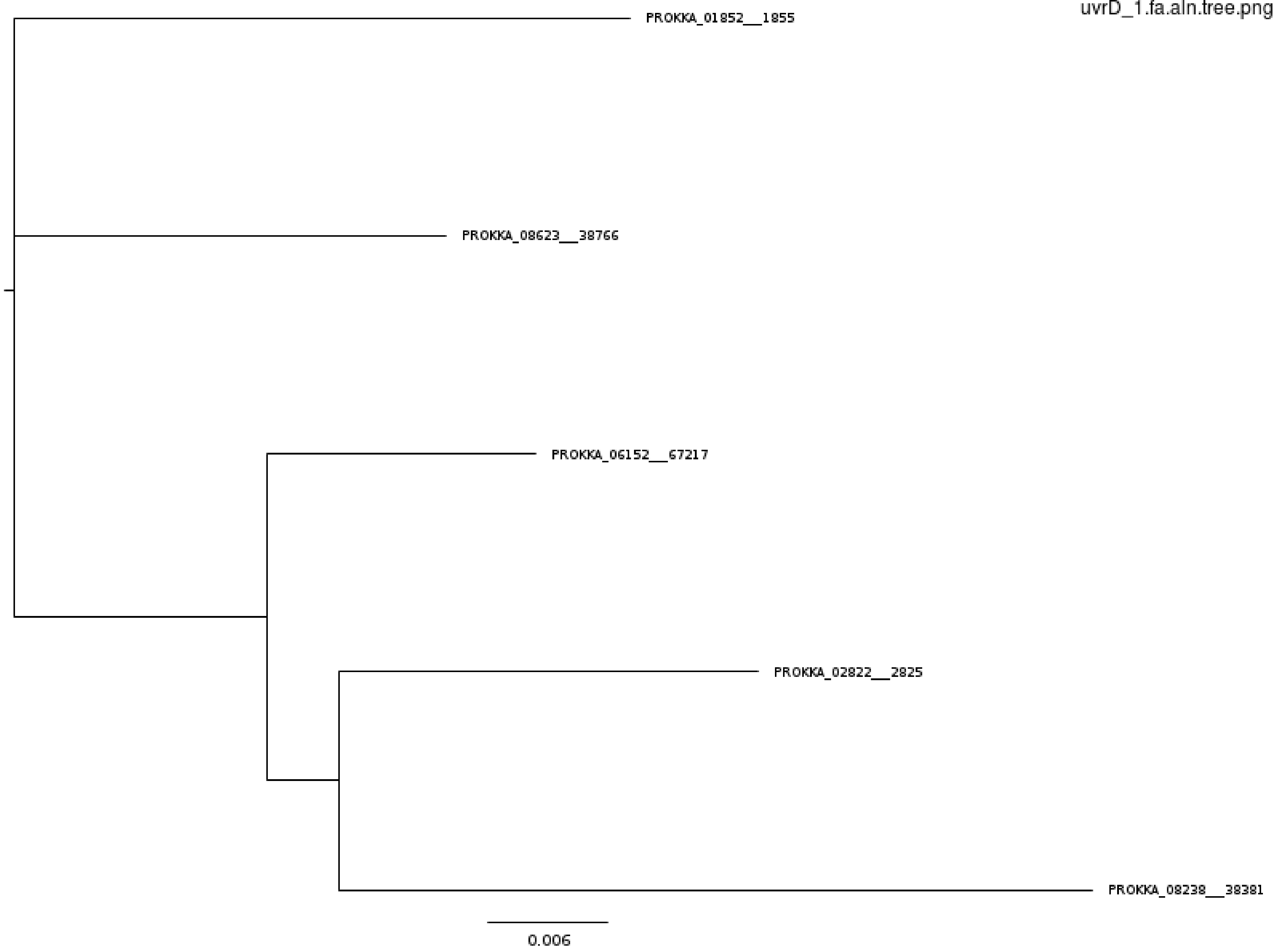
uvrD_1.fa.aln.tree.png
PROKKA_01852___1855
PROKKA_08623___38766
PROKKA_06152___67217
PROKKA_02822___2825
PROKKA_08238___38381
0.006

valS_1.fa.aln.tree.png

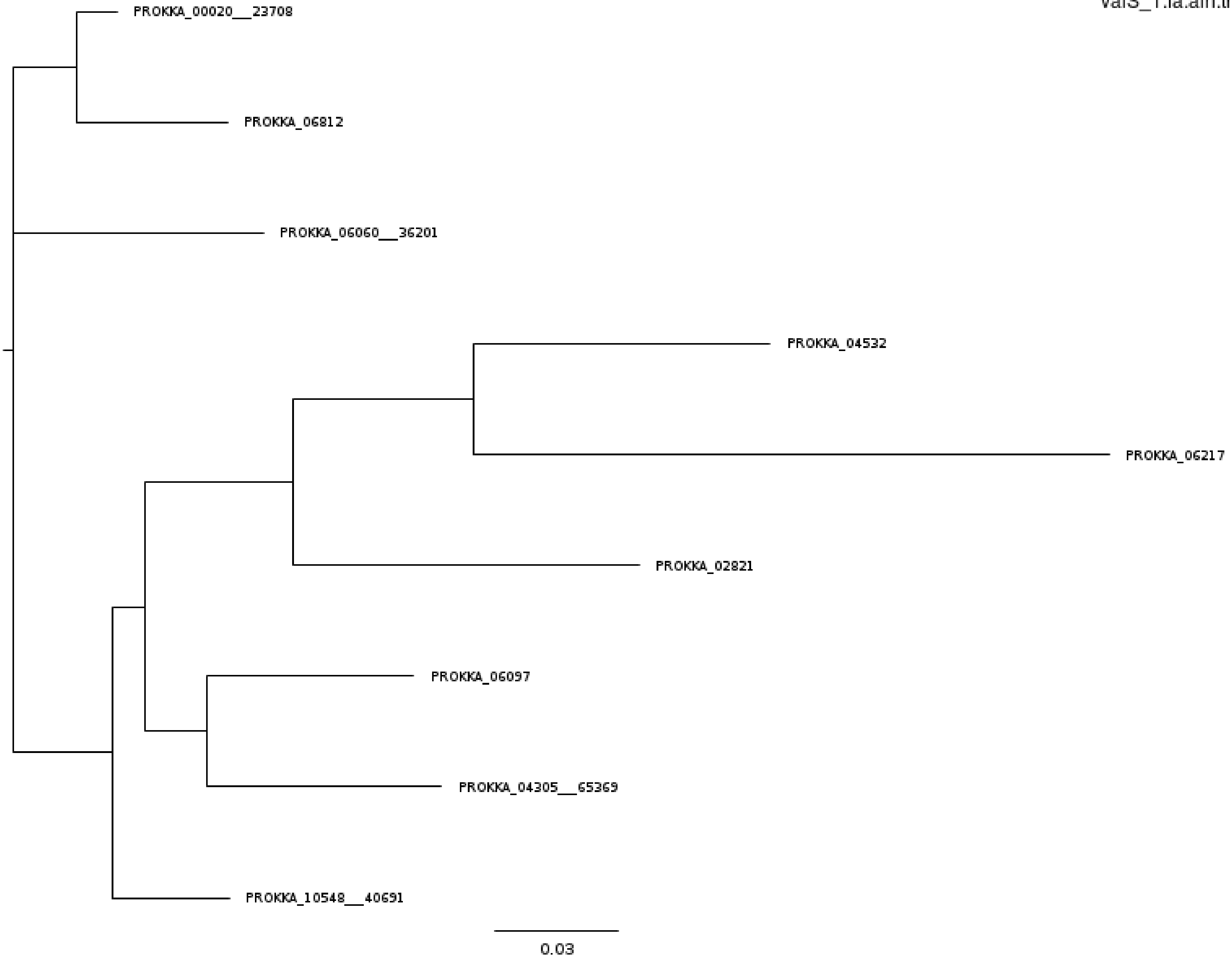

valS_2.fa.aln.tree.png

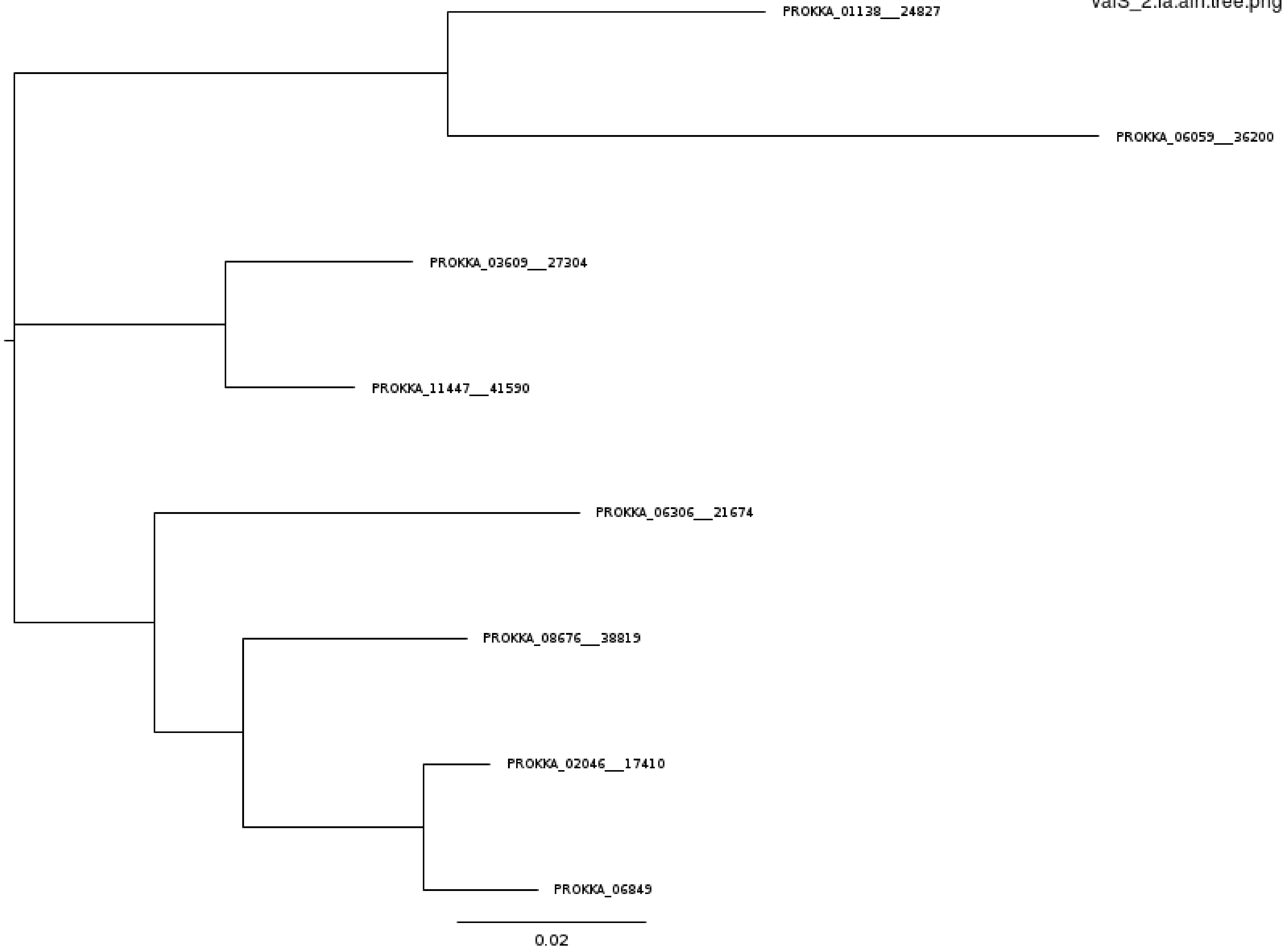

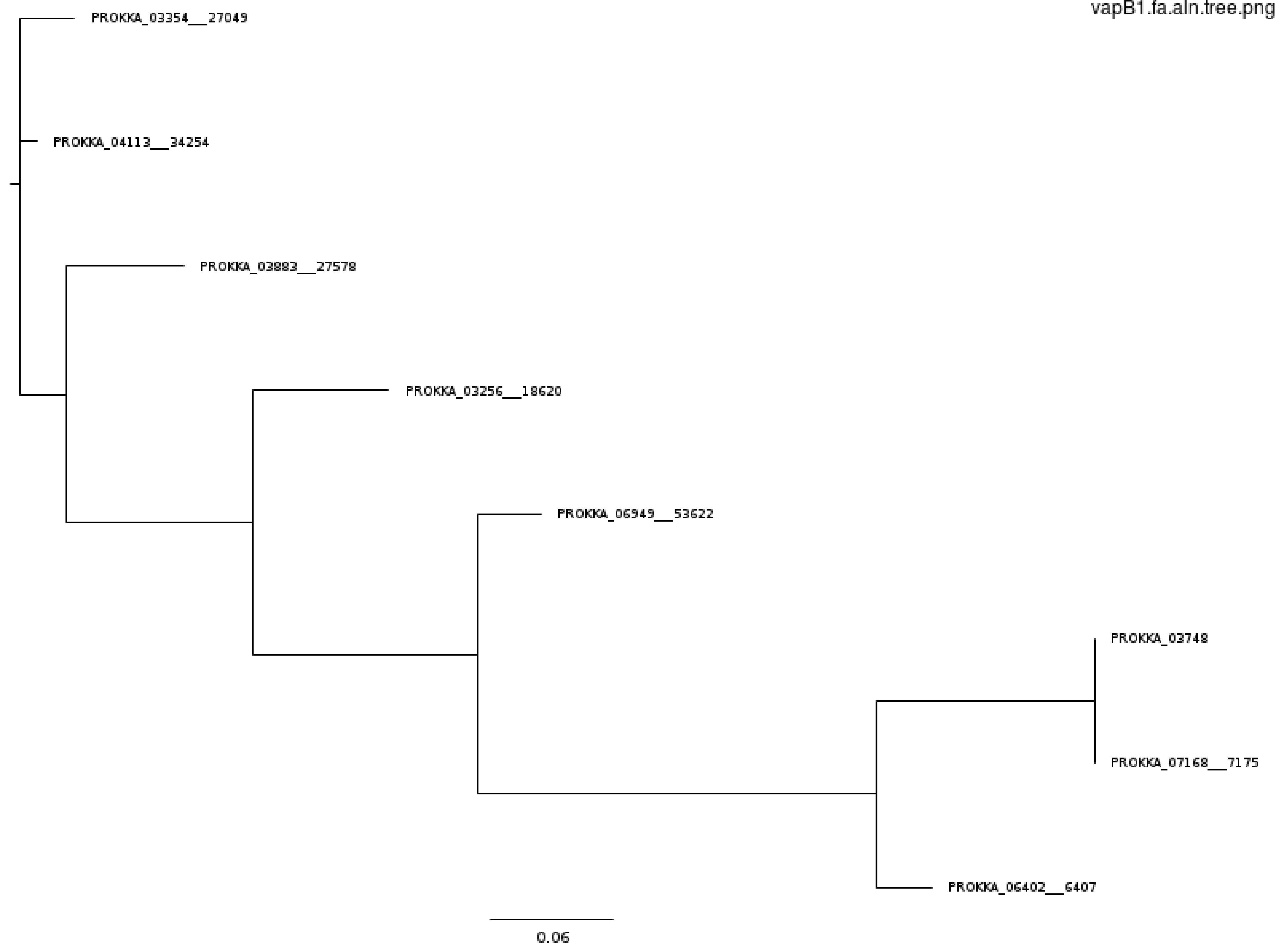
vapB1.fa.aln.tree.png
PROKKA_03354___27049
PROKKA_04113___34254
PROKKA_03883___27578
PROKKA_03256___18620
PROKKA_06949___53622
PROKKA_03748
PROKKA_07168___7175
PROKKA_06402___6407
0.06

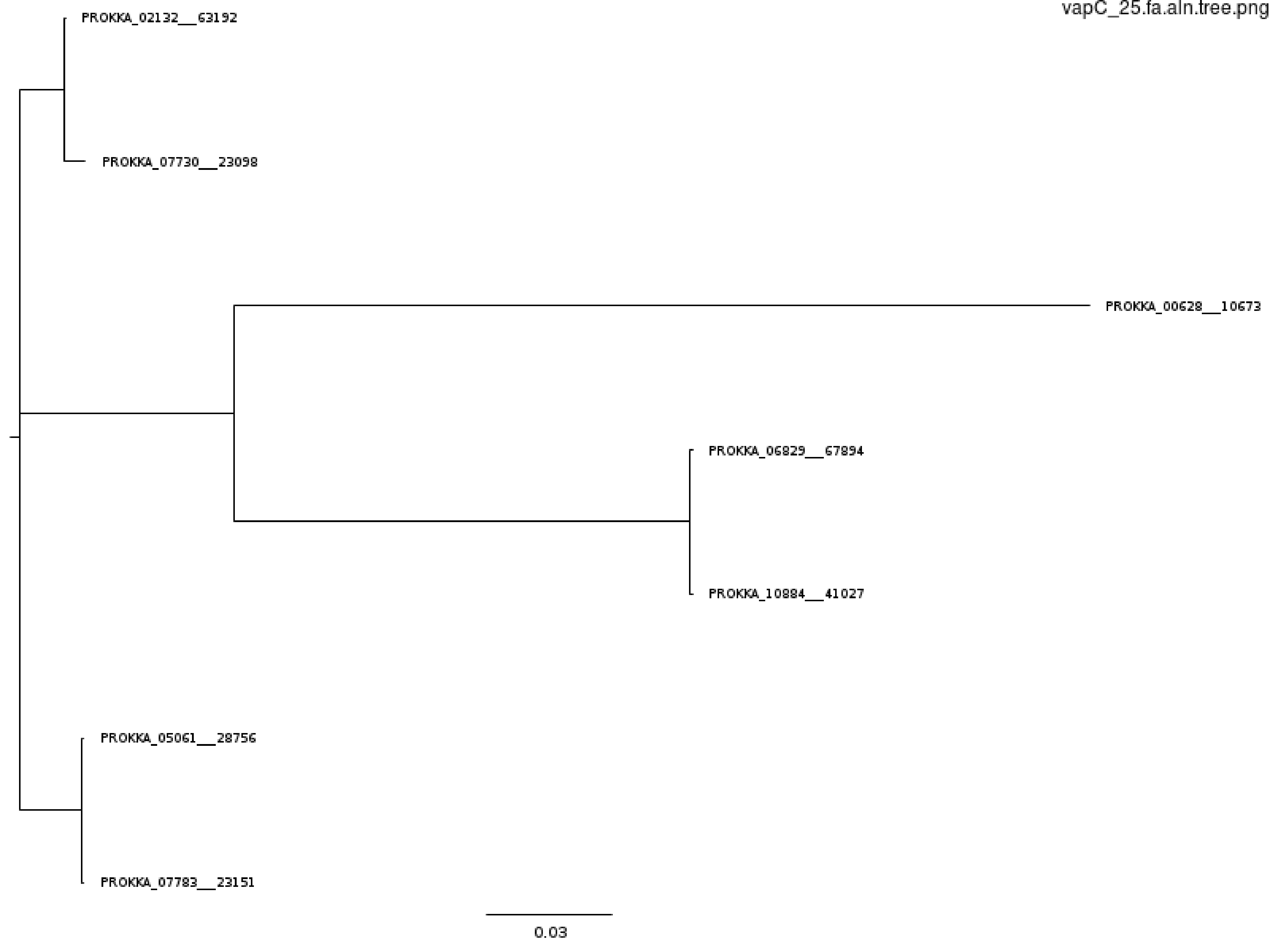
vapC_25.fa.aln.tree.png
PROKKA_02132___63192
PROKKA_07730___23098
PROKKA_00628___10673
PROKKA_06829___67894
PROKKA_10884___41027
PROKKA_05061___28756
PROKKA_07783___23151
0.03

vapC_31.fa.aln.tree.png

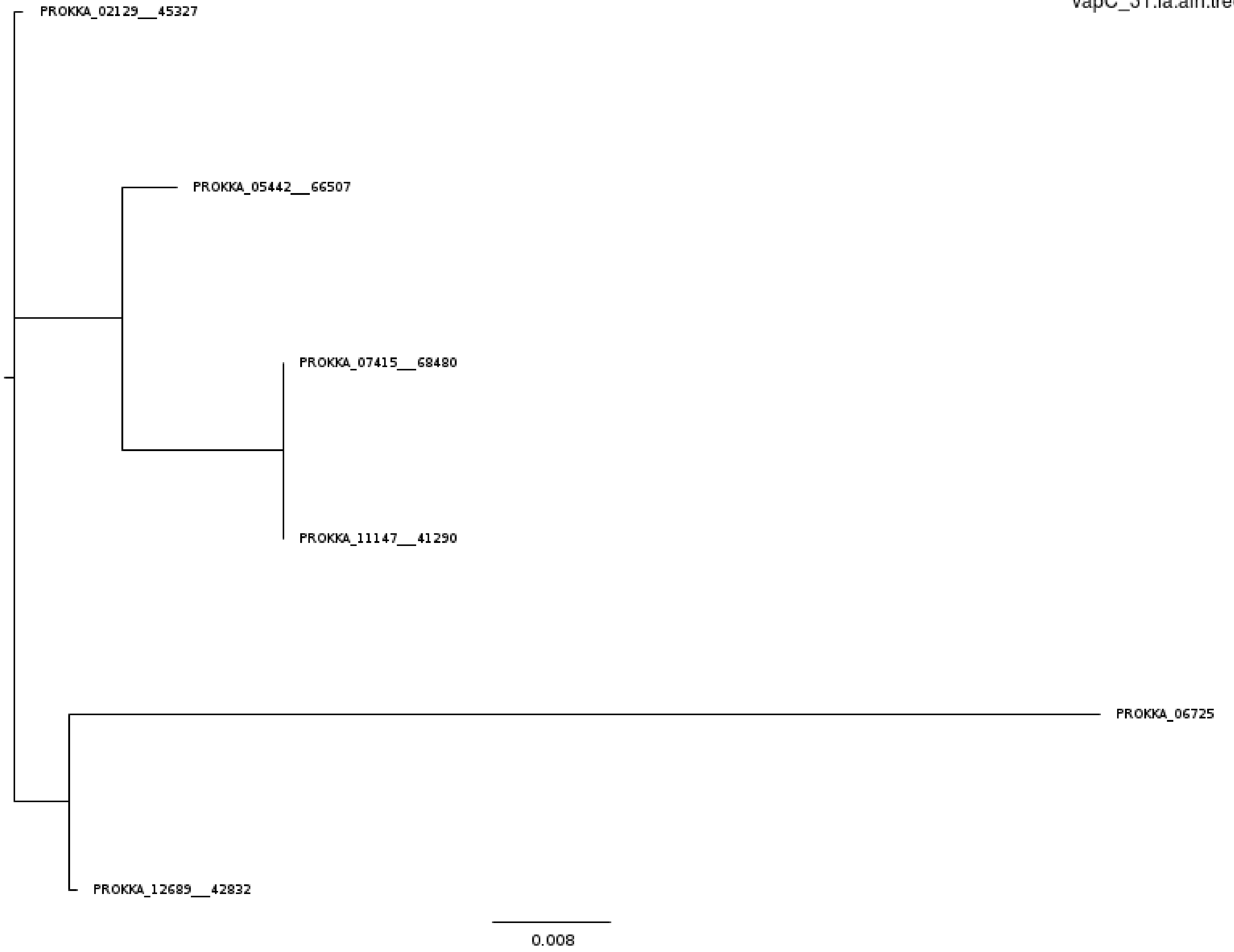

vapC_4.fa.aln.tree.png

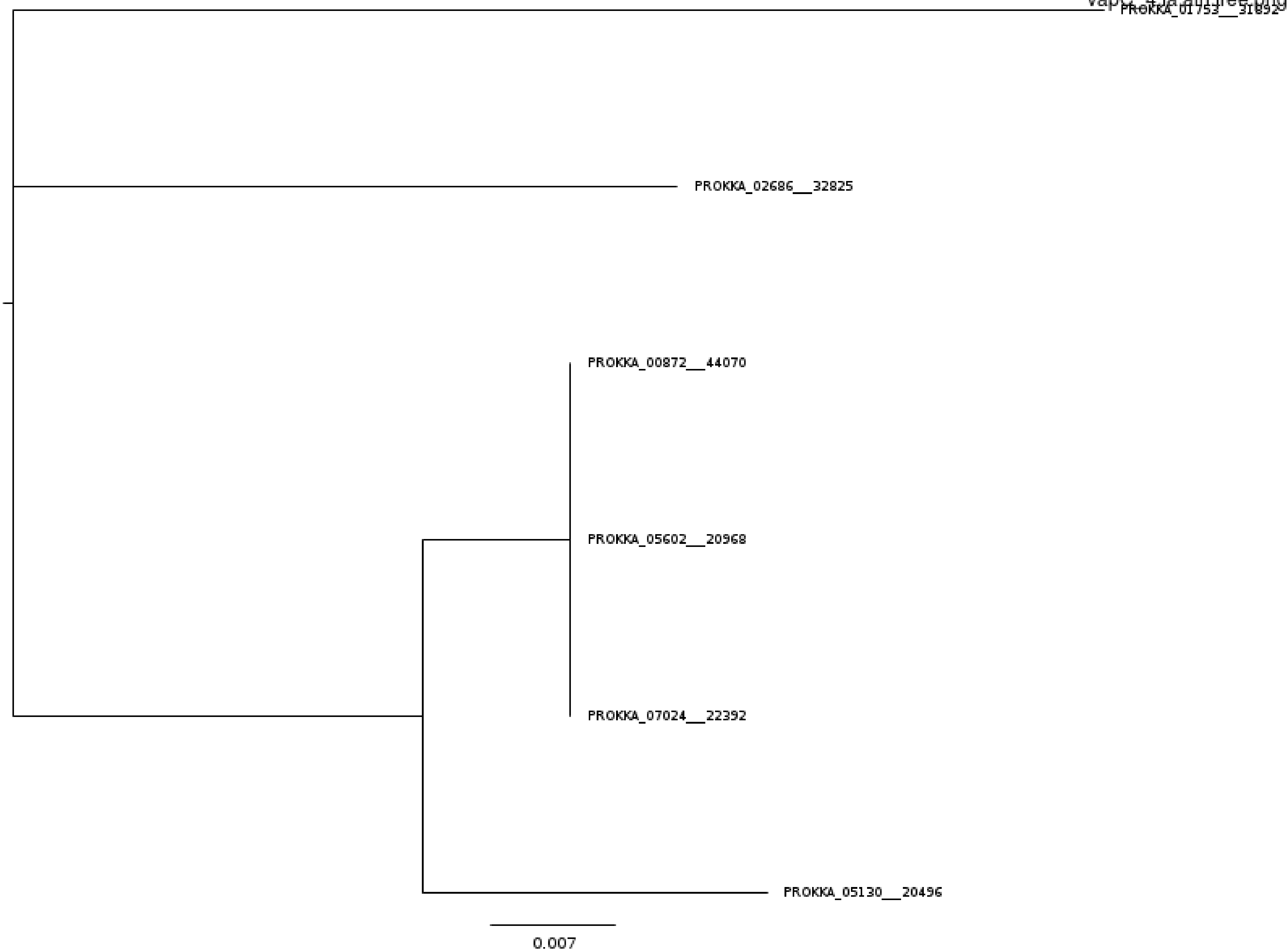

vfr.fa.aln.tree.png

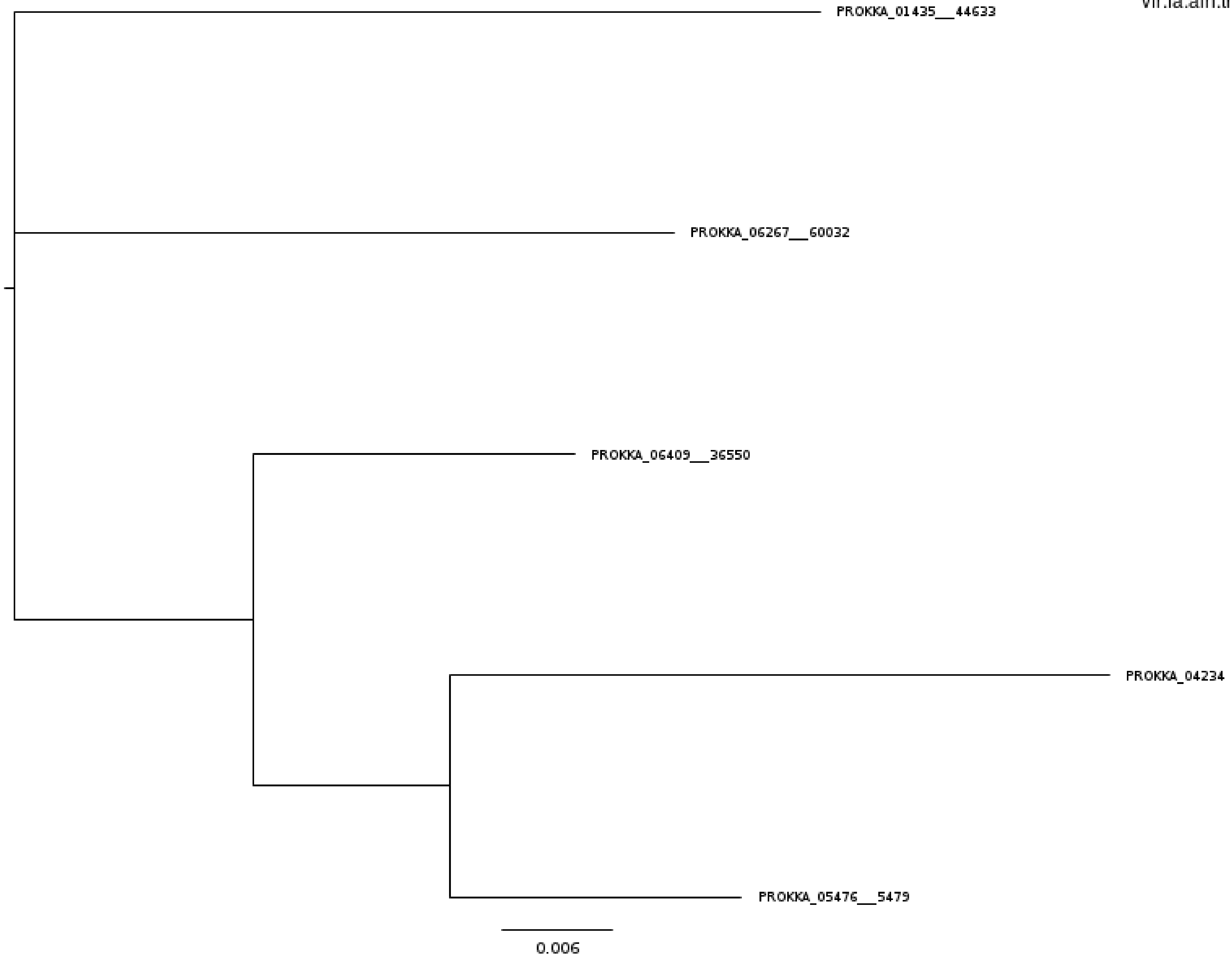

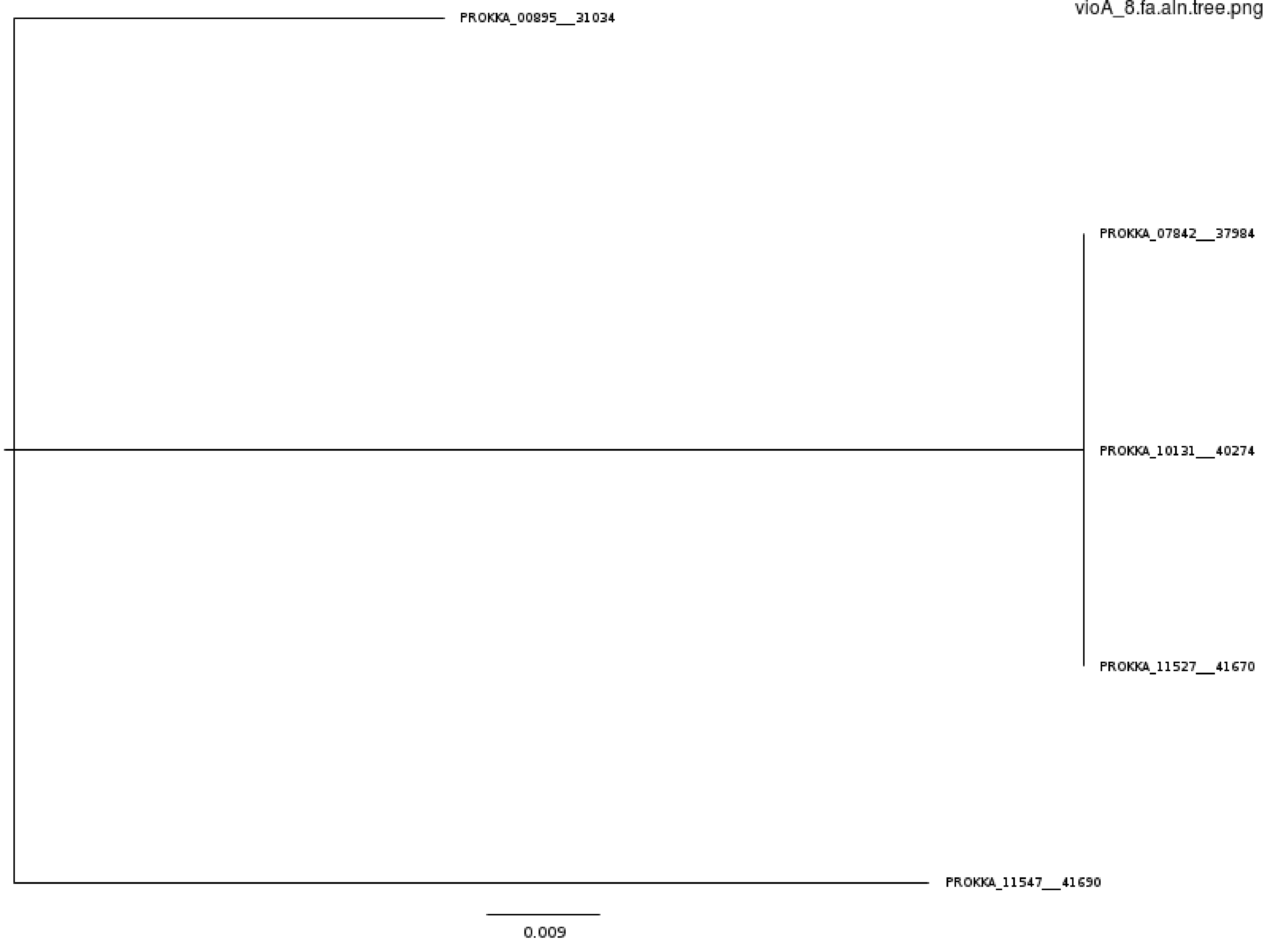
vioA_8.fa.aln.tree.png
PROKKA_00895___31034
PROKKA_07842___37984
PROKKA_10131___40274
PROKKA_11527___41670
PROKKA_11547___41690
0.009

vioB_2.fa.aln.tree.png

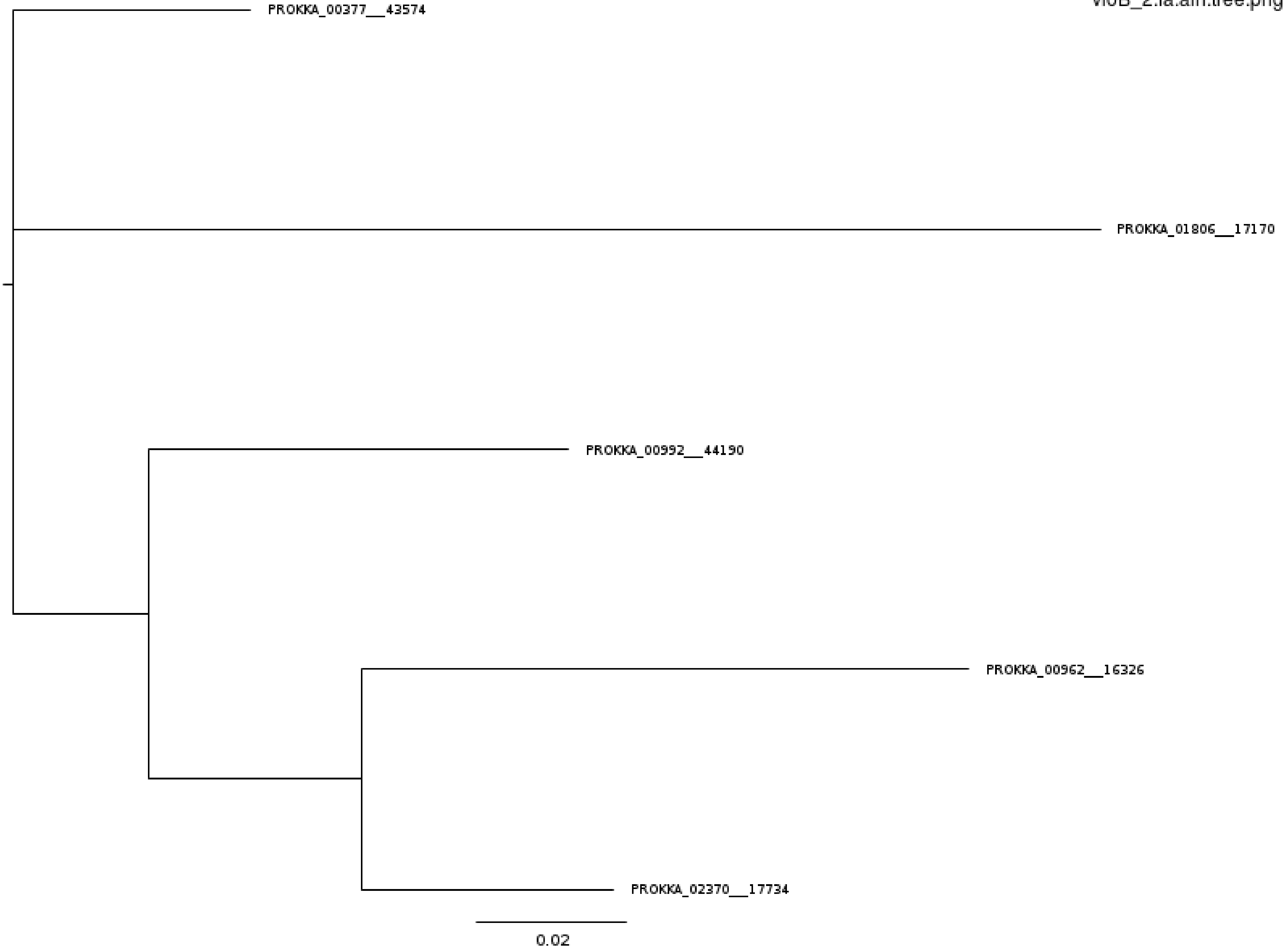

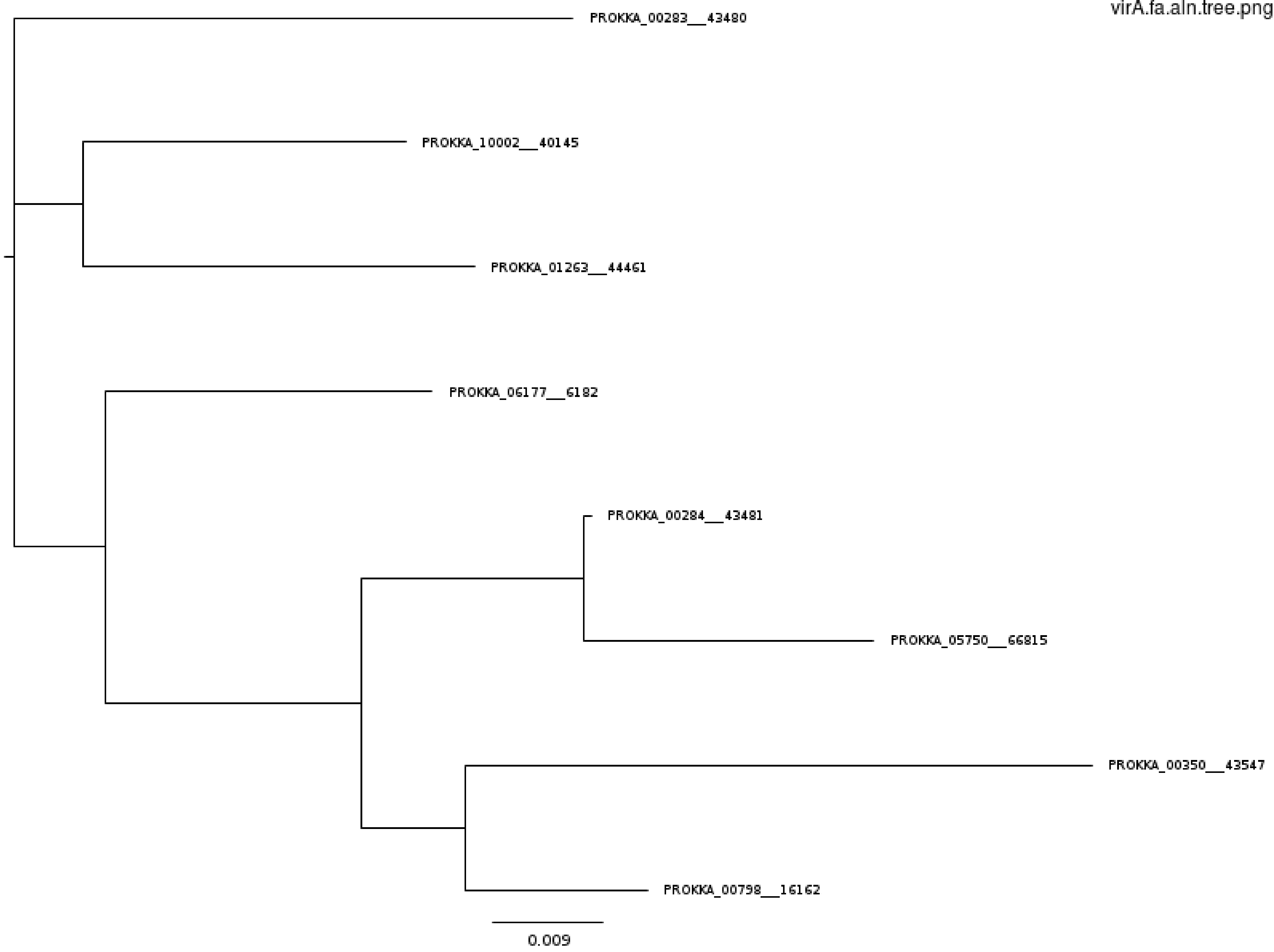
virA.fa.aln.tree.png
PROKKA_00283___43480
PROKKA_10002___40145
PROKKA_01263___44461
PROKKA_06177___6182
PROKKA_00284___43481
PROKKA_05750___66815
PROKKA_00350___43547
PROKKA_00798___16162
0.009

walR.fa.aln.tree.png

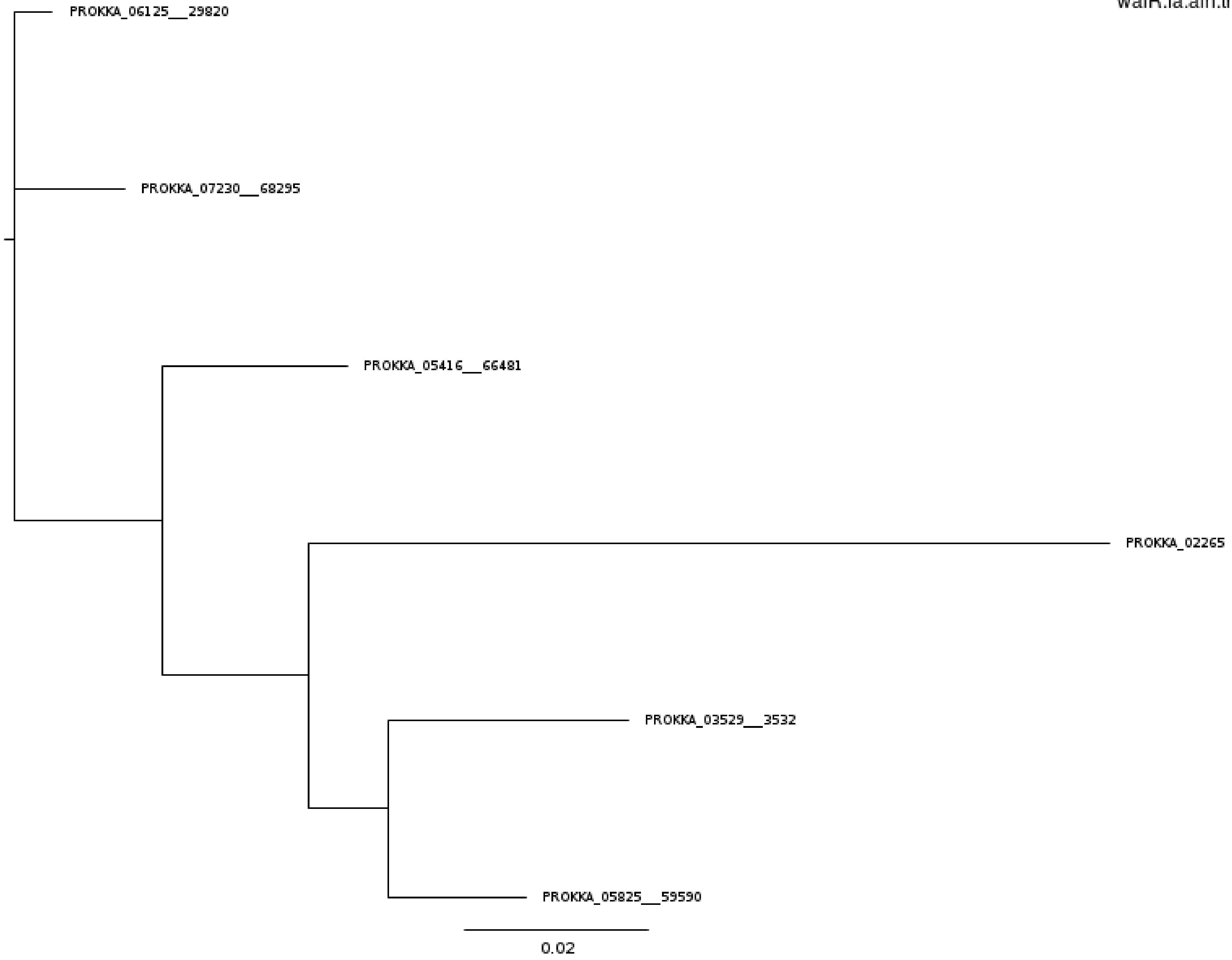

wbbL_2.fa.aln.tree.png

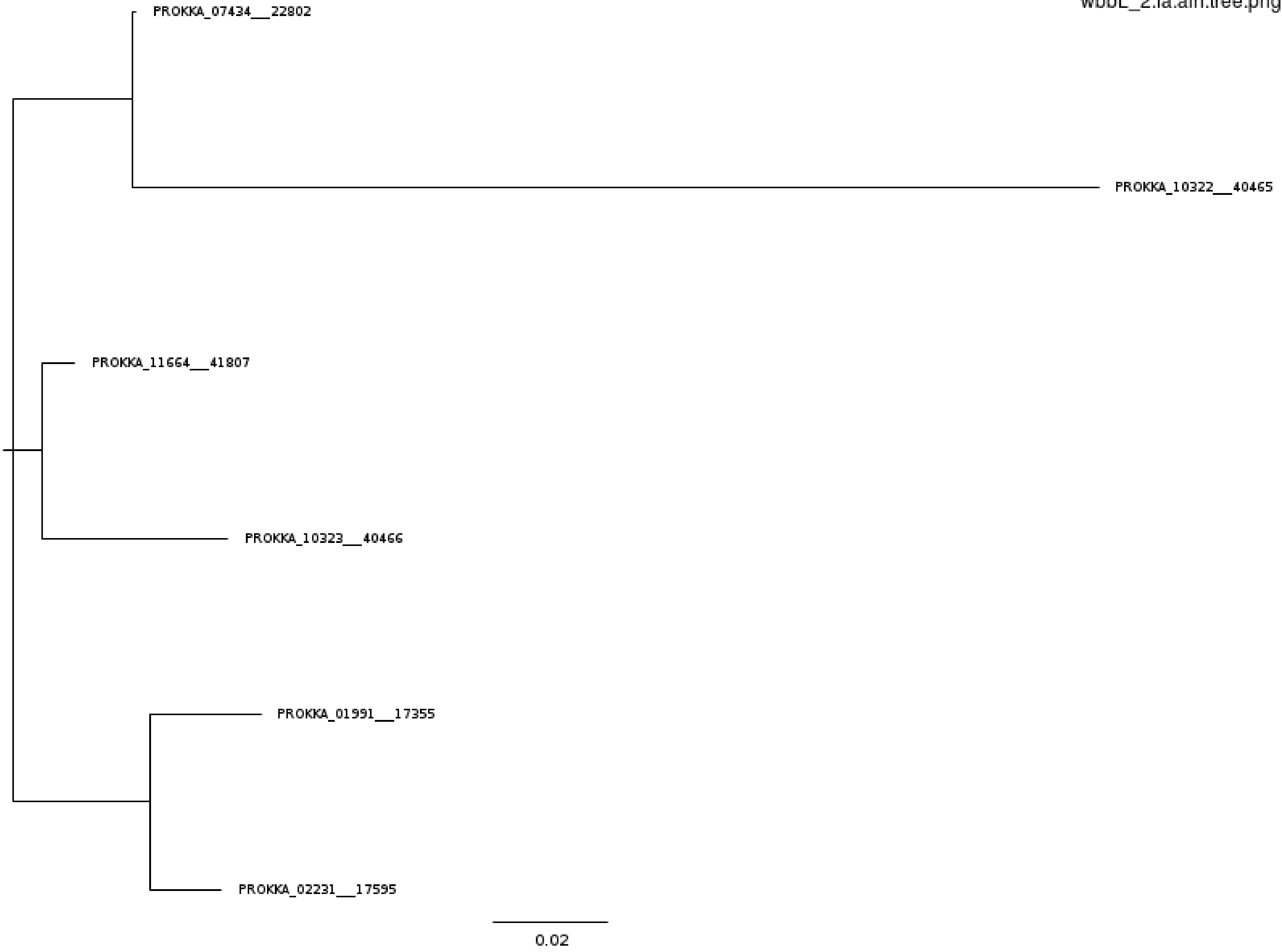

wbiB.fa.aln.tree.png

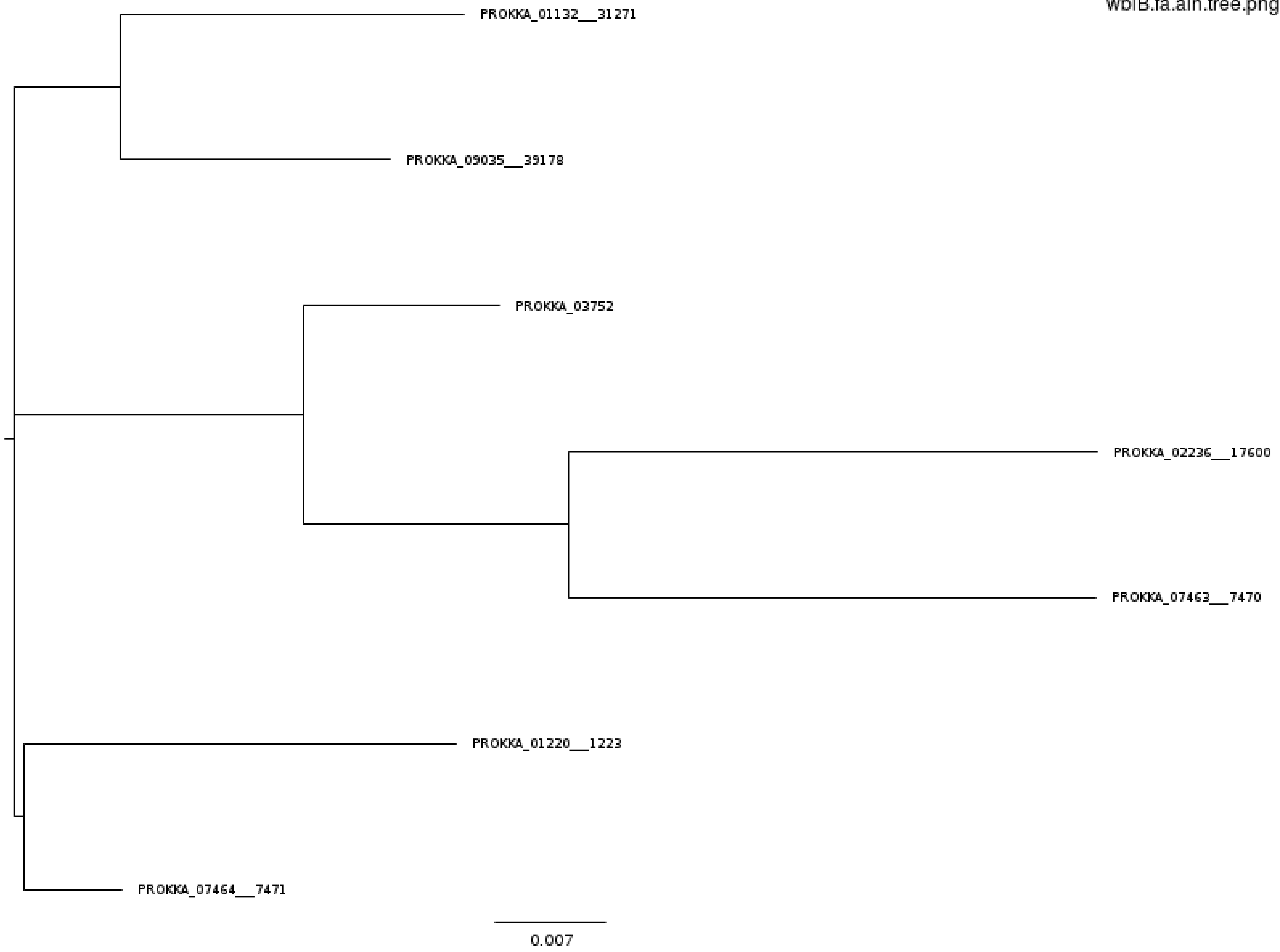

wbpA_2.fa.aln.tree.png

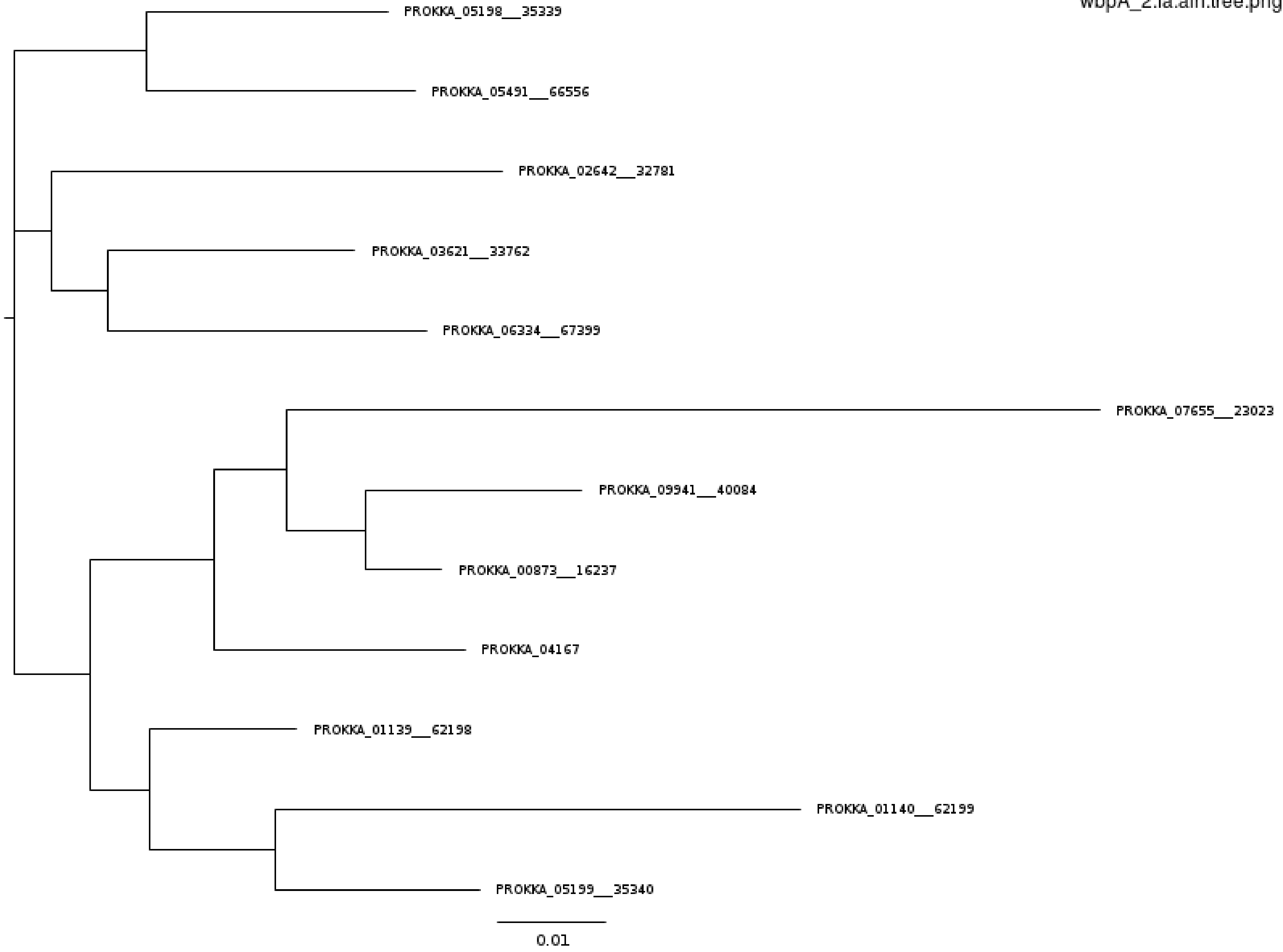

wbpE_4.fa.aln.tree.png

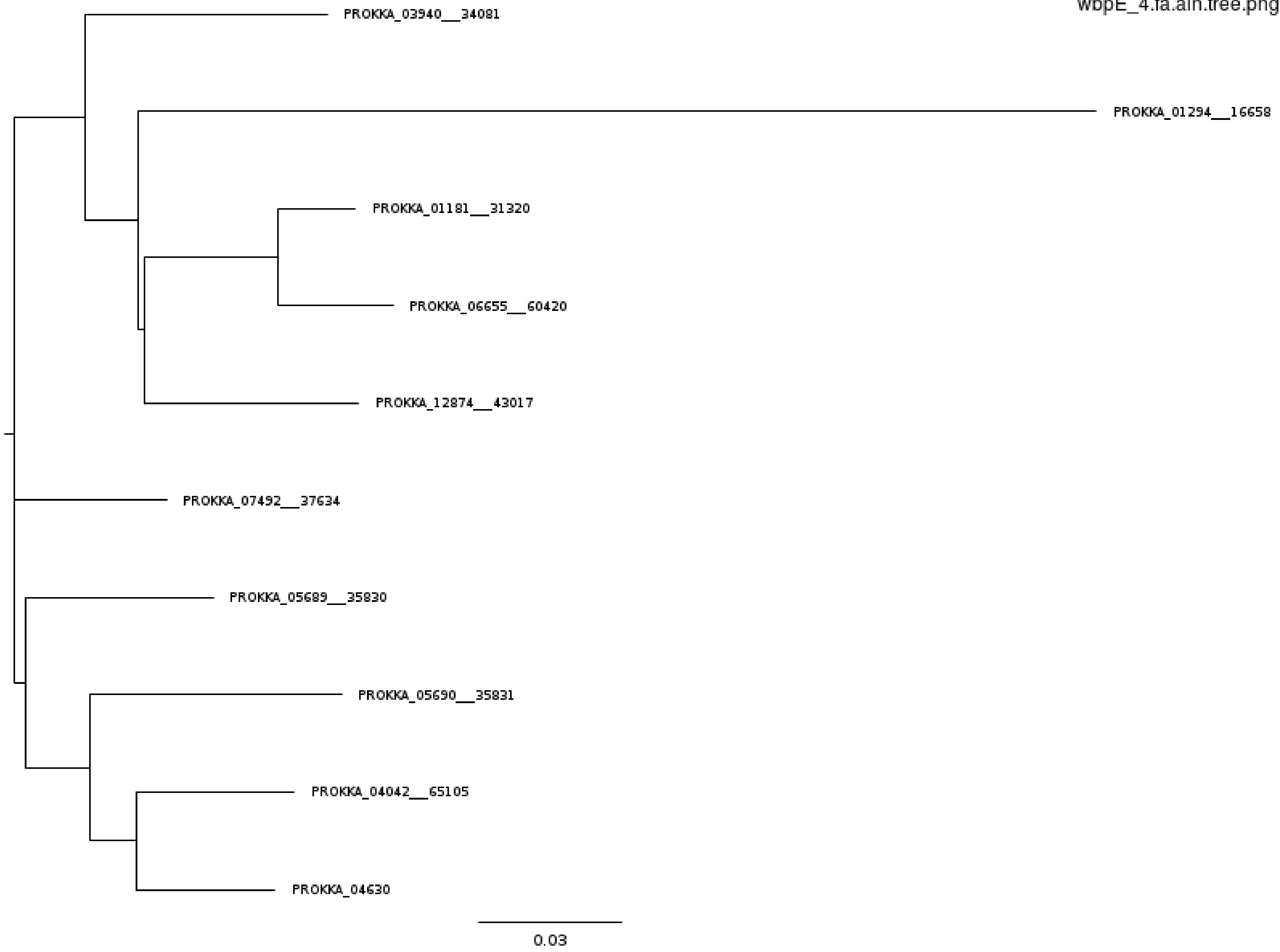

wspC_3.fa.aln.tree.png

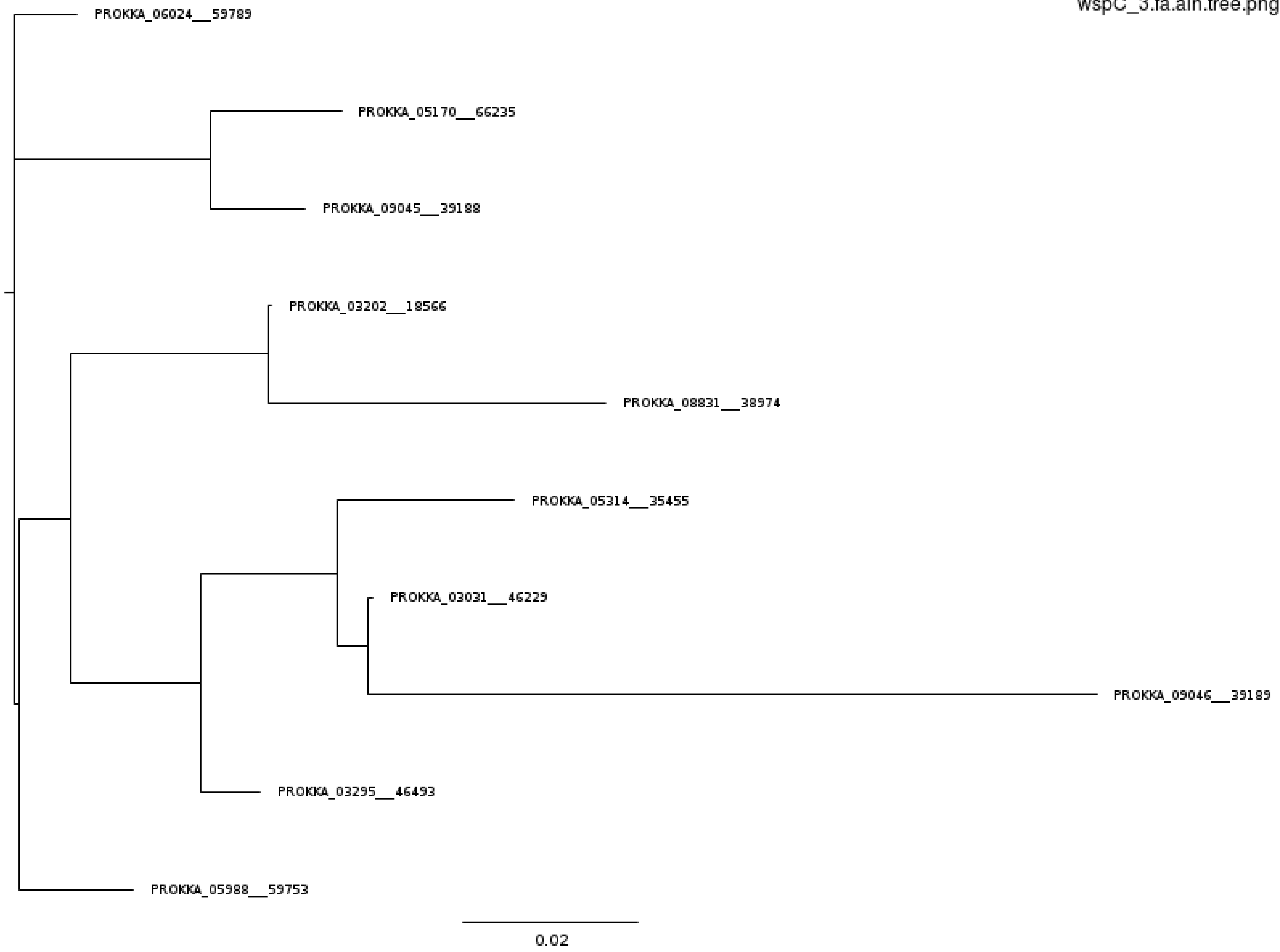

xerD.fa.aln.tree.png

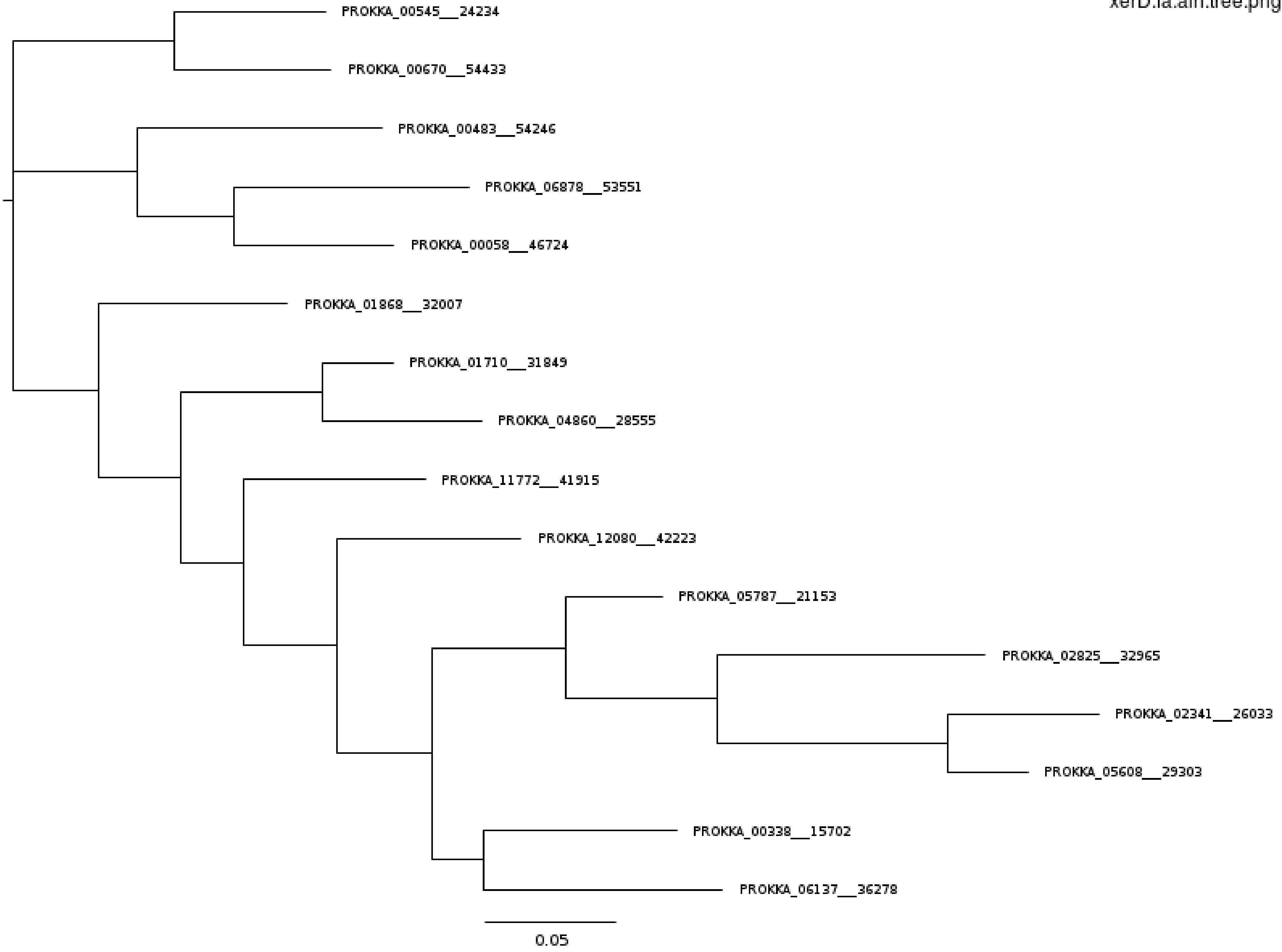

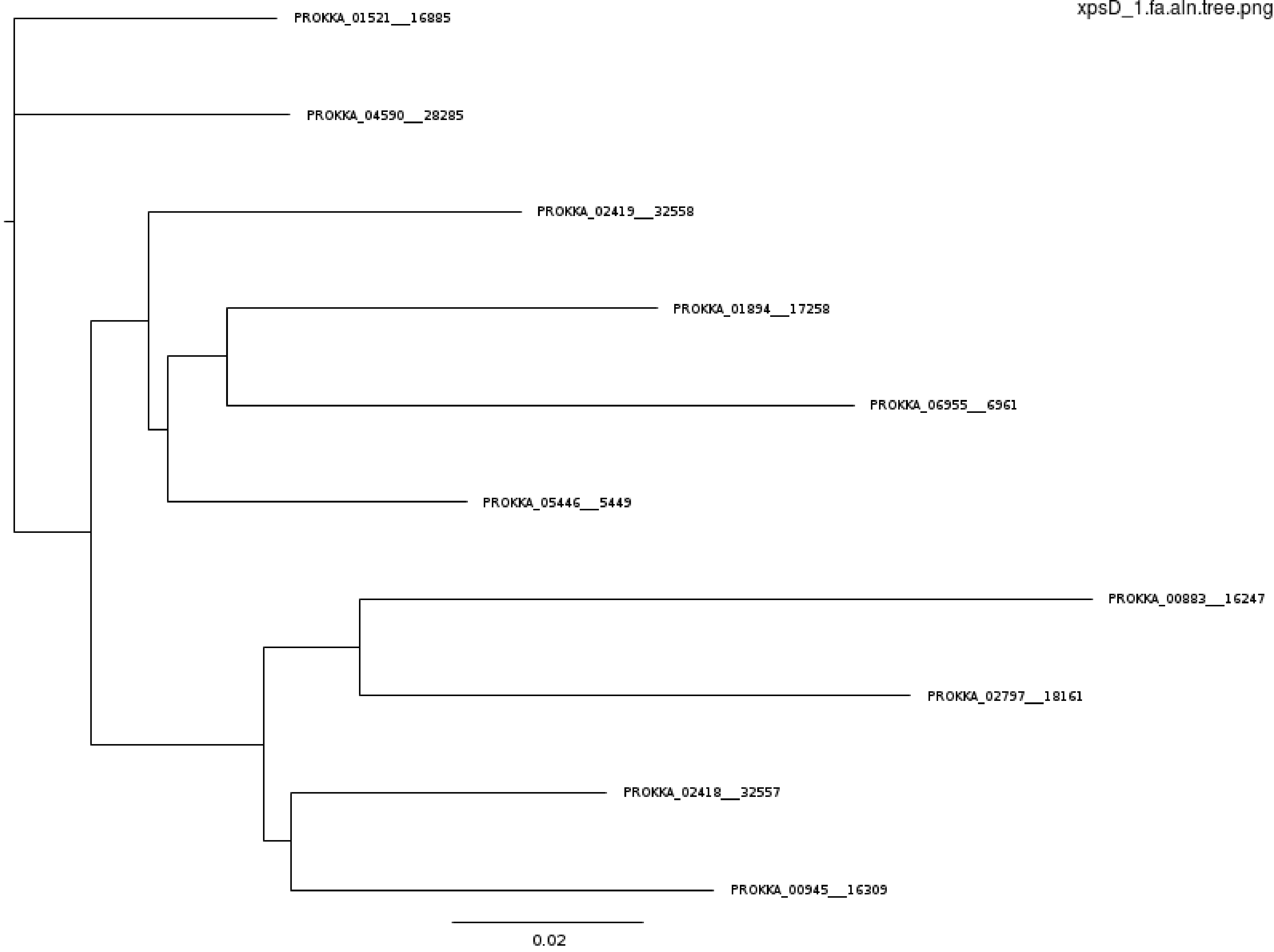
xpsD_1.fa.aln.tree.png
PROKKA_01521___16885
PROKKA_04590___28285
PROKKA_02419___32558
PROKKA_01894___17258
PROKKA_06955___6961
PROKKA_05446___5449
PROKKA_00883___16247
PROKKA_02797___18161
PROKKA_02418___32557
PROKKA_00945___16309
0.02

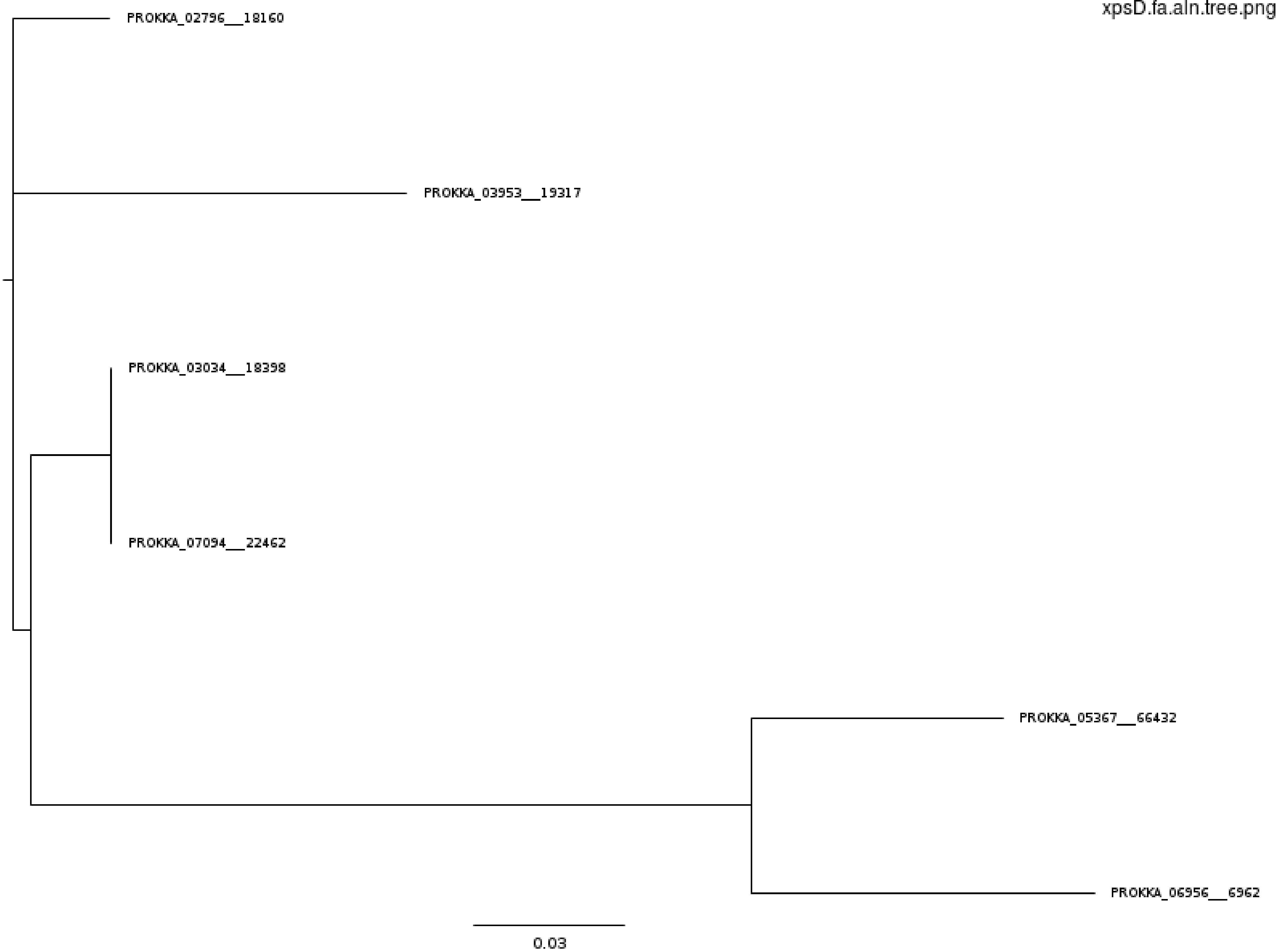

xpsD.fa.aln.tree.png
PROKKA_02796__18160
PROKKA_03953__19317
PROKKA_03034__18398
PROKKA_07094__22462
PROKKA_05367__66432
PROKKA_06956__6962
0.03

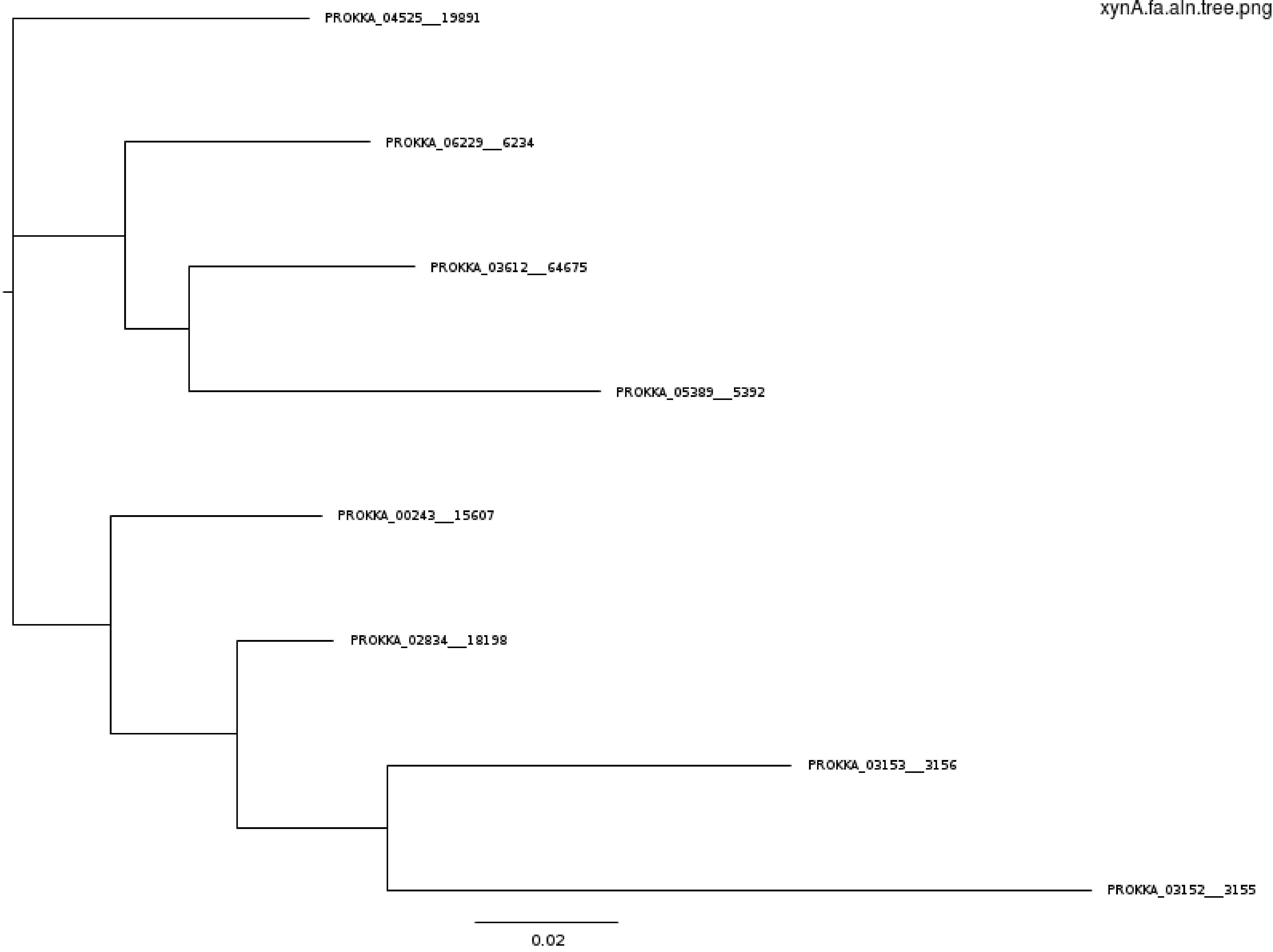
xynA.fa.aln.tree.png
PROKKA_04525___19891
PROKKA_06229___6234
PROKKA_03612___64675
PROKKA_05389___5392
PROKKA_00243___15607
PROKKA_02834___18198
PROKKA_03153___3156
PROKKA_03152___3155
0.02

yabJ_2.fa.aln.tree.png

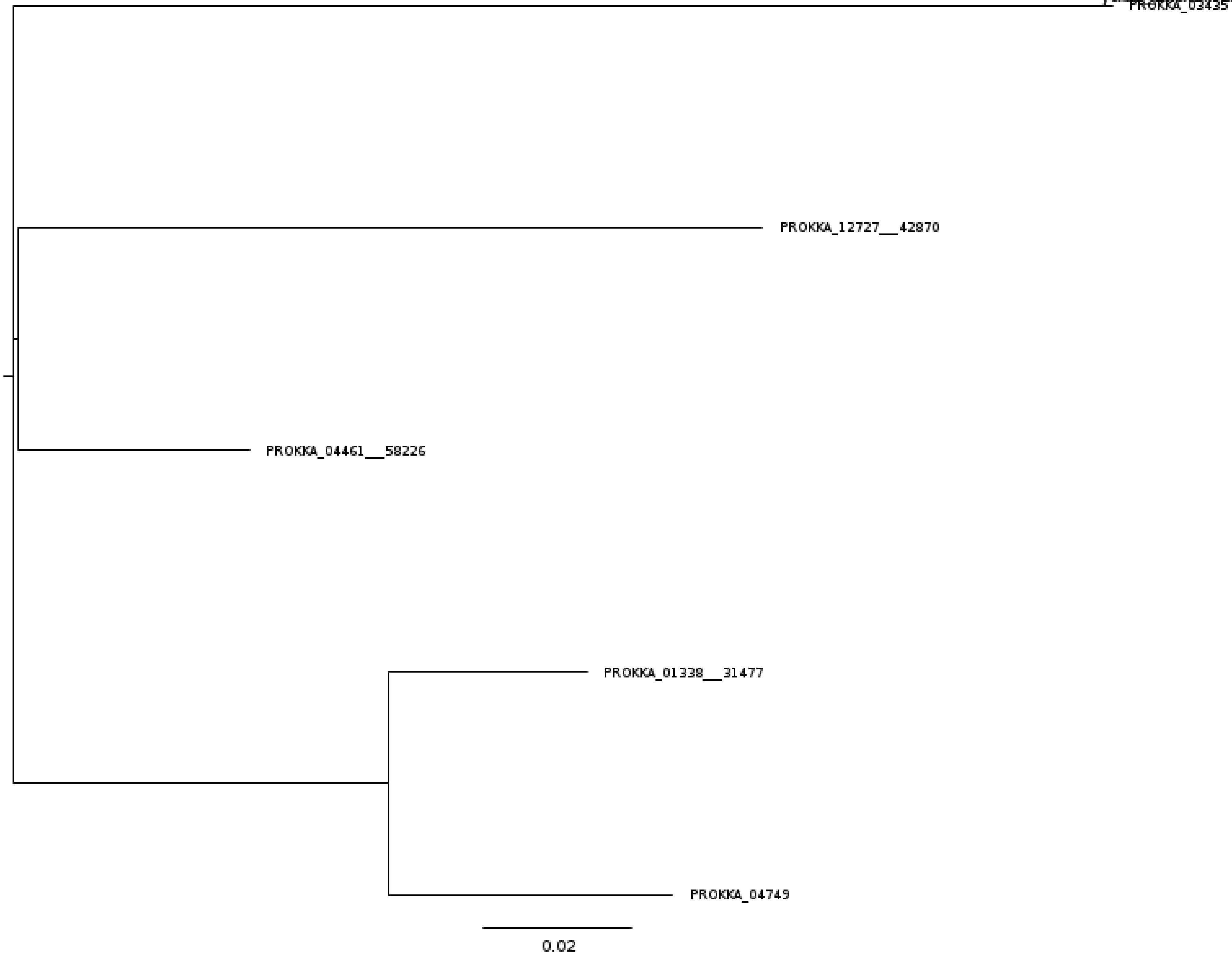

yahK_2.fa.aln.tree.png

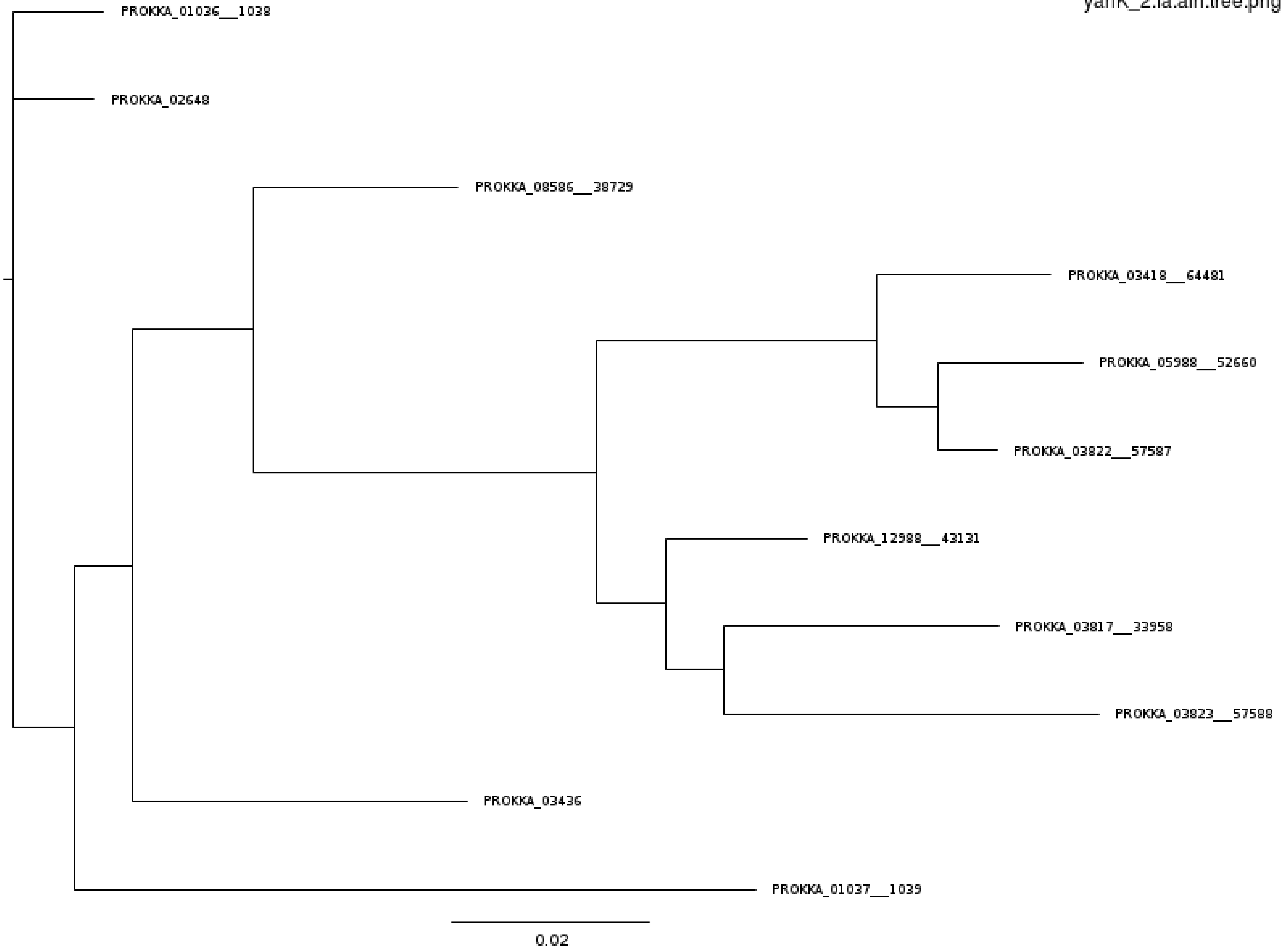

yaiL_fa.aln.tree.png

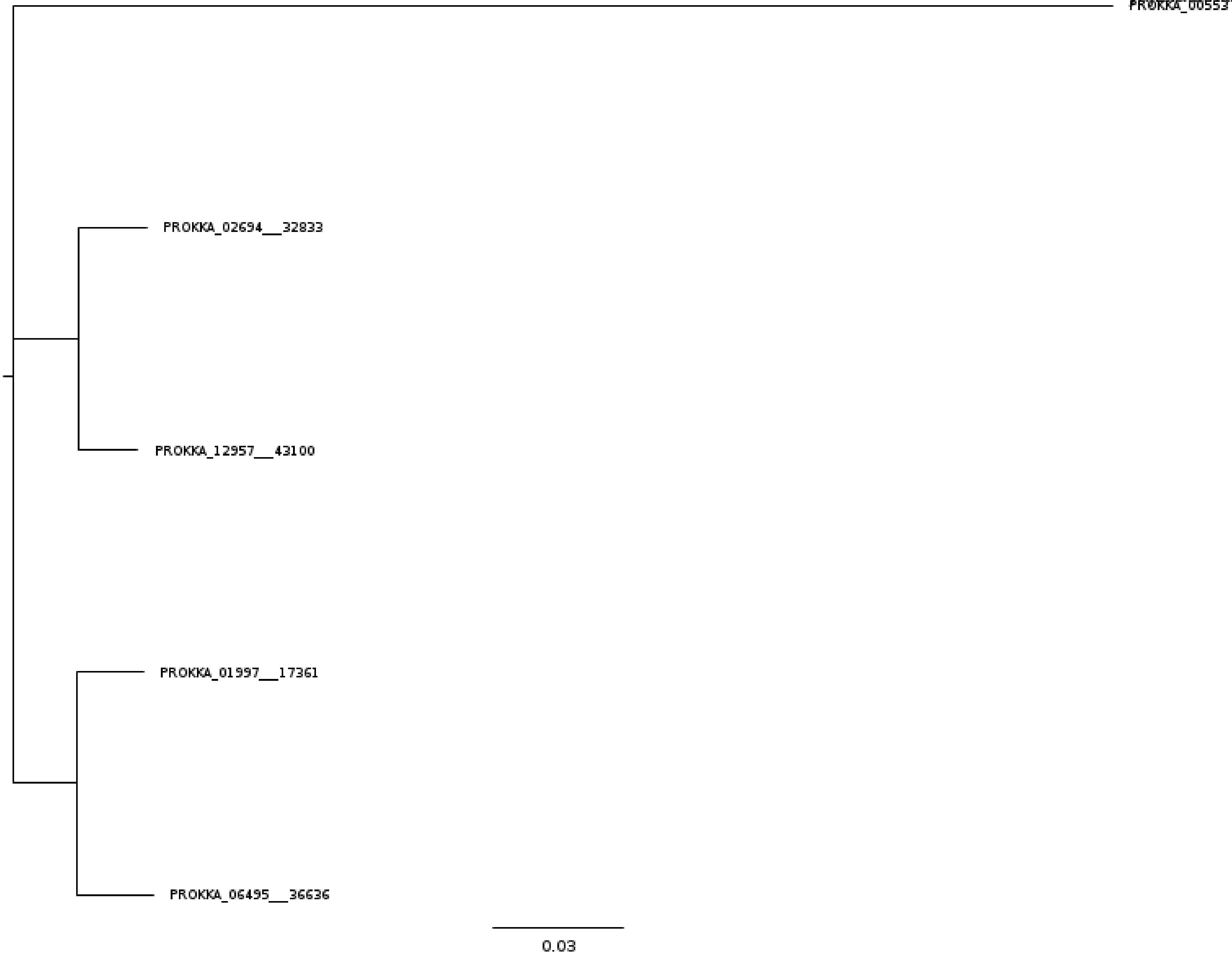

yajR_3.fa.aln.tree.png

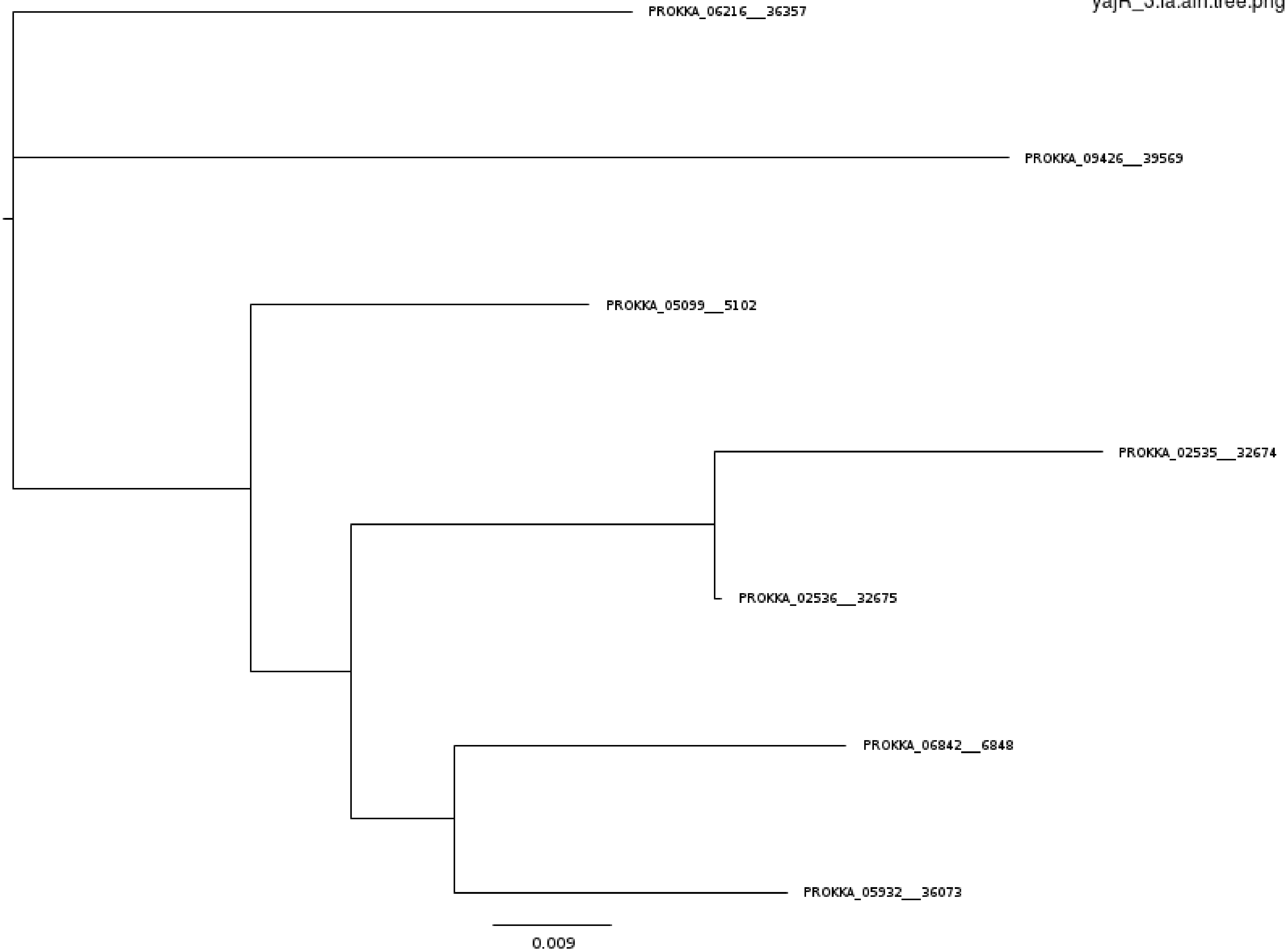

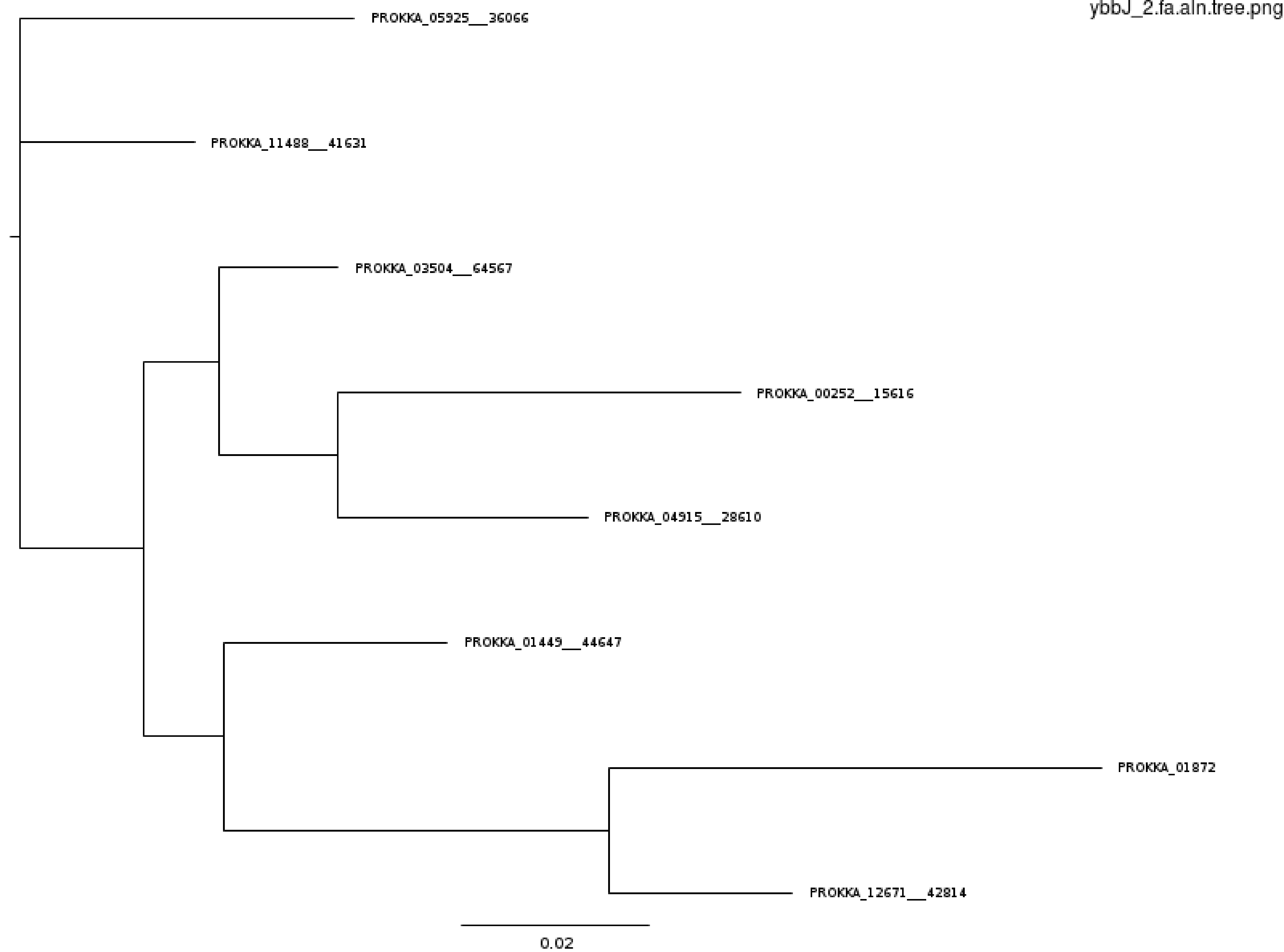
ybbJ_2.fa.aln.tree.png
PROKKA_05925__36066
PROKKA_11488__41631
PROKKA_03504__64567
PROKKA_00252__15616
PROKKA_04915__28610
PROKKA_01449__44647
PROKKA_01872
PROKKA_12671__42814
0.02

ybbJ_3.fa.aln.tree.png

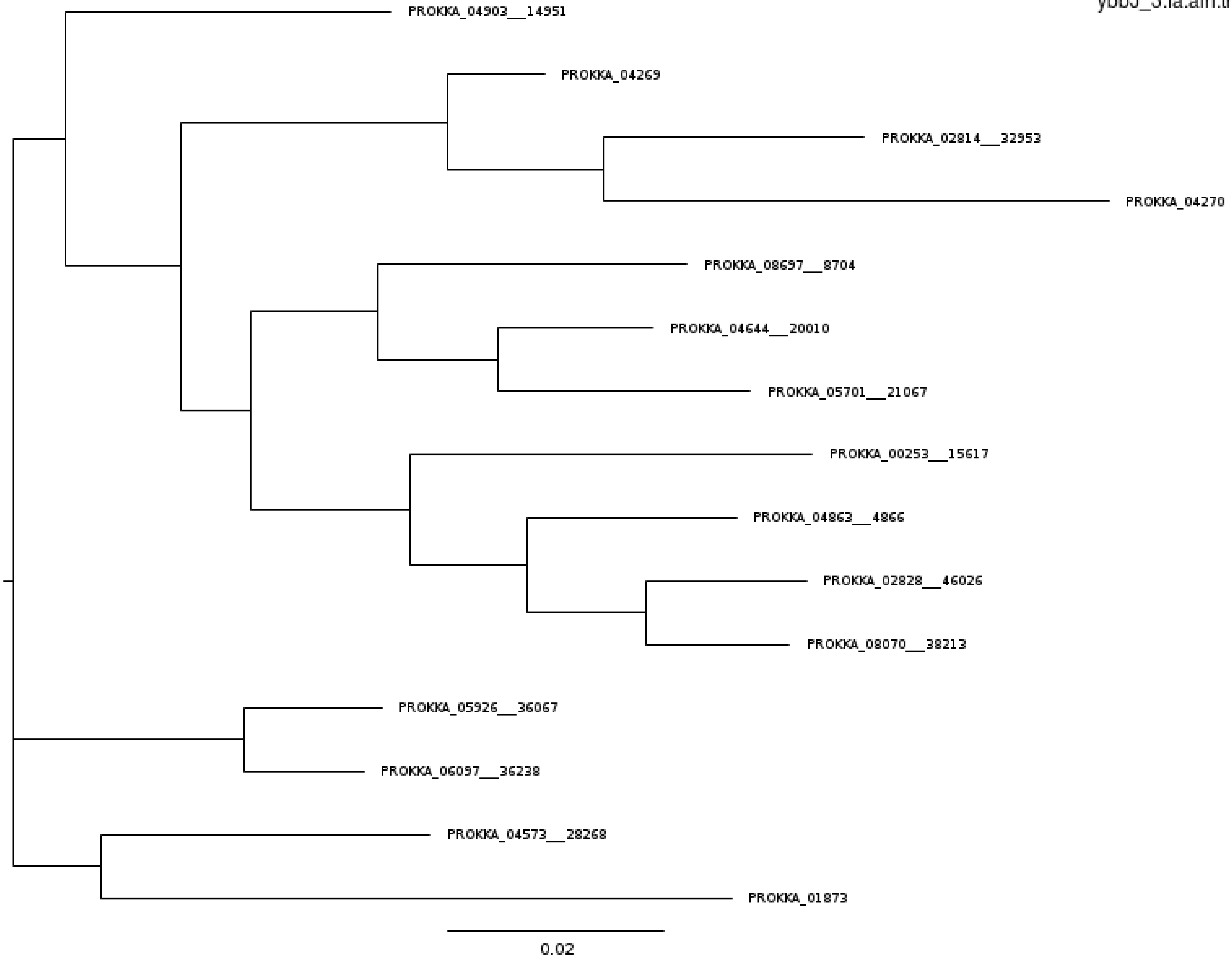

ybeY_2.fa.aln.tree.png

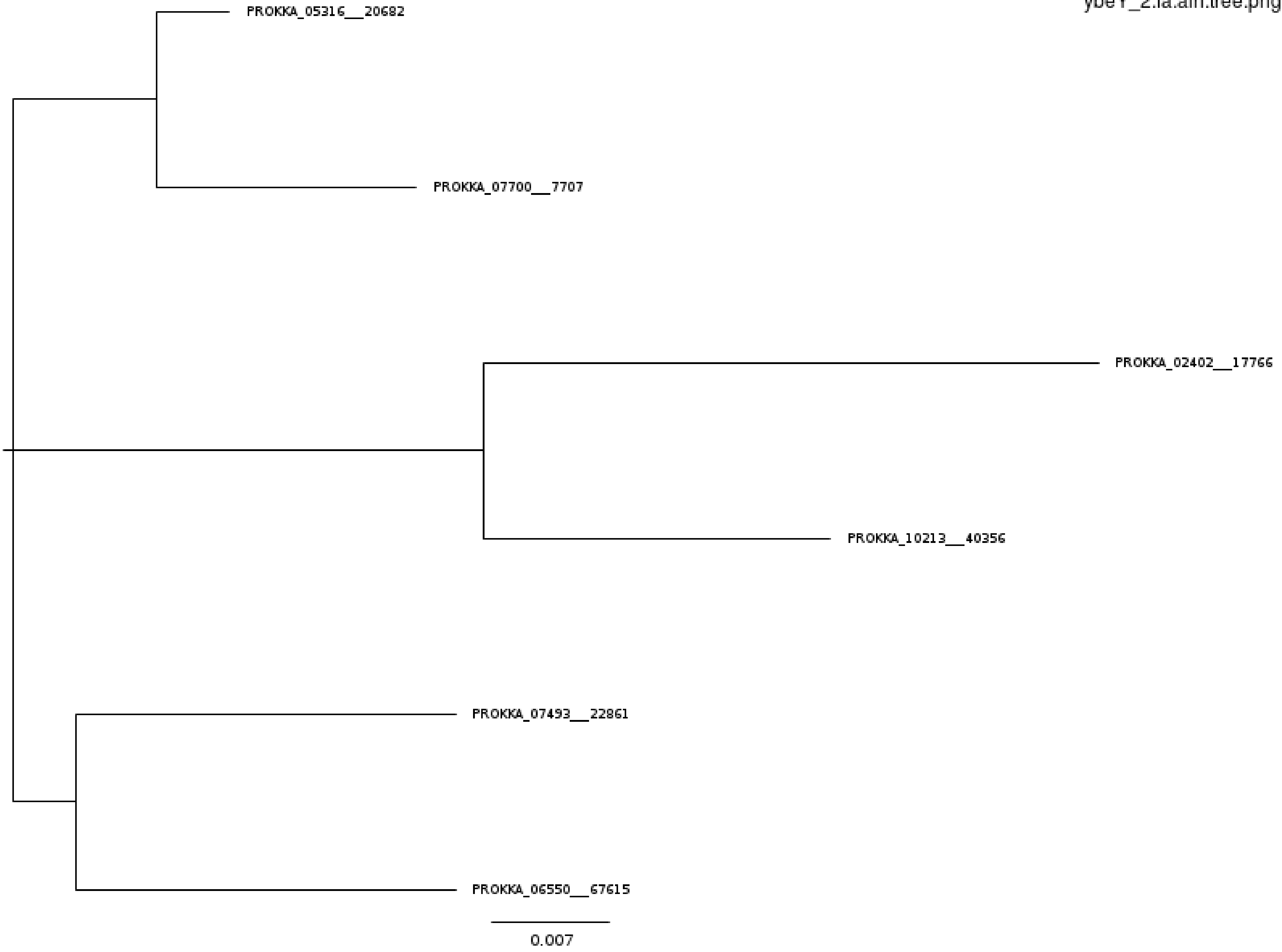

ybeZ_2.fa.aln.tree.png

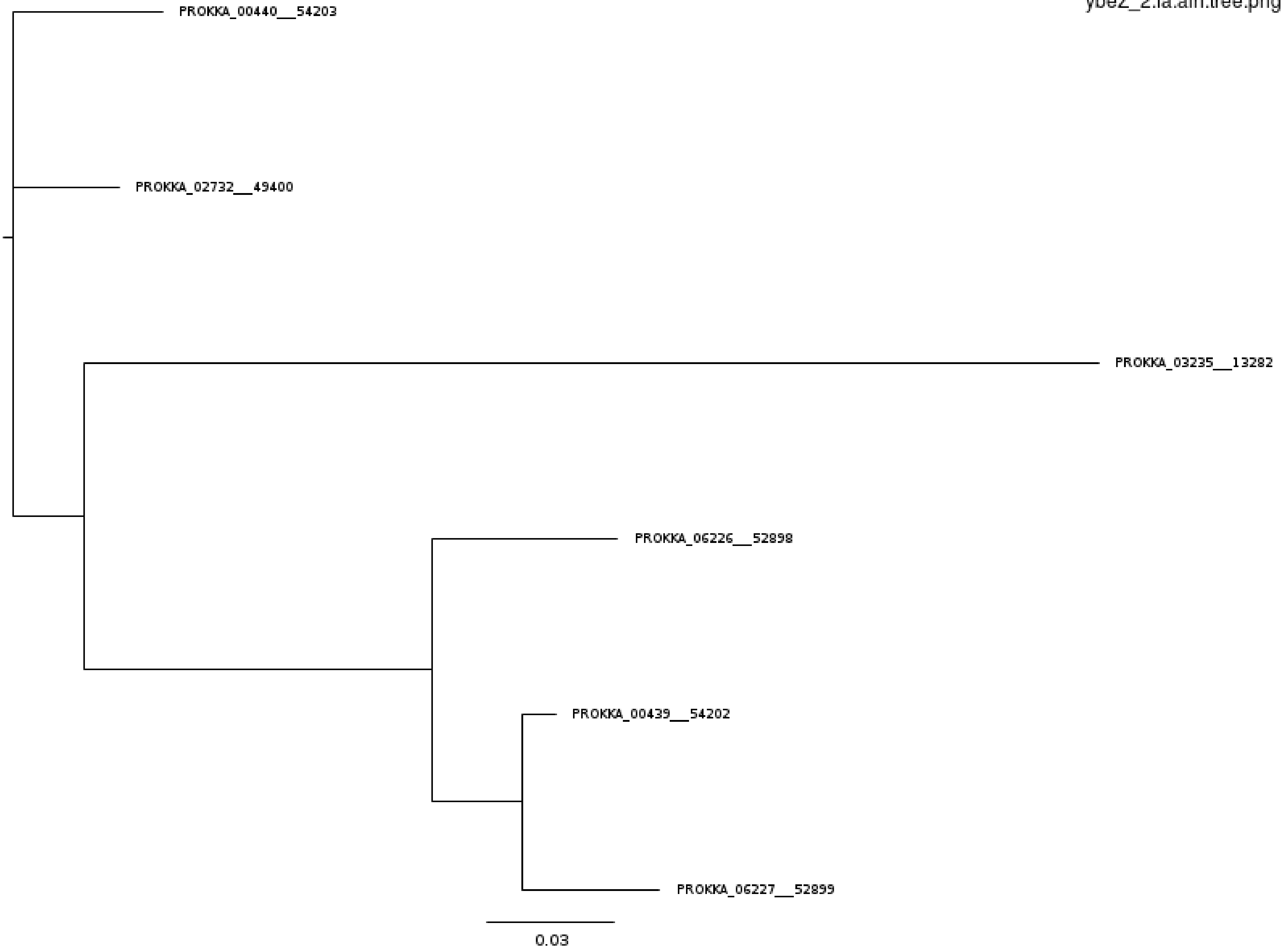

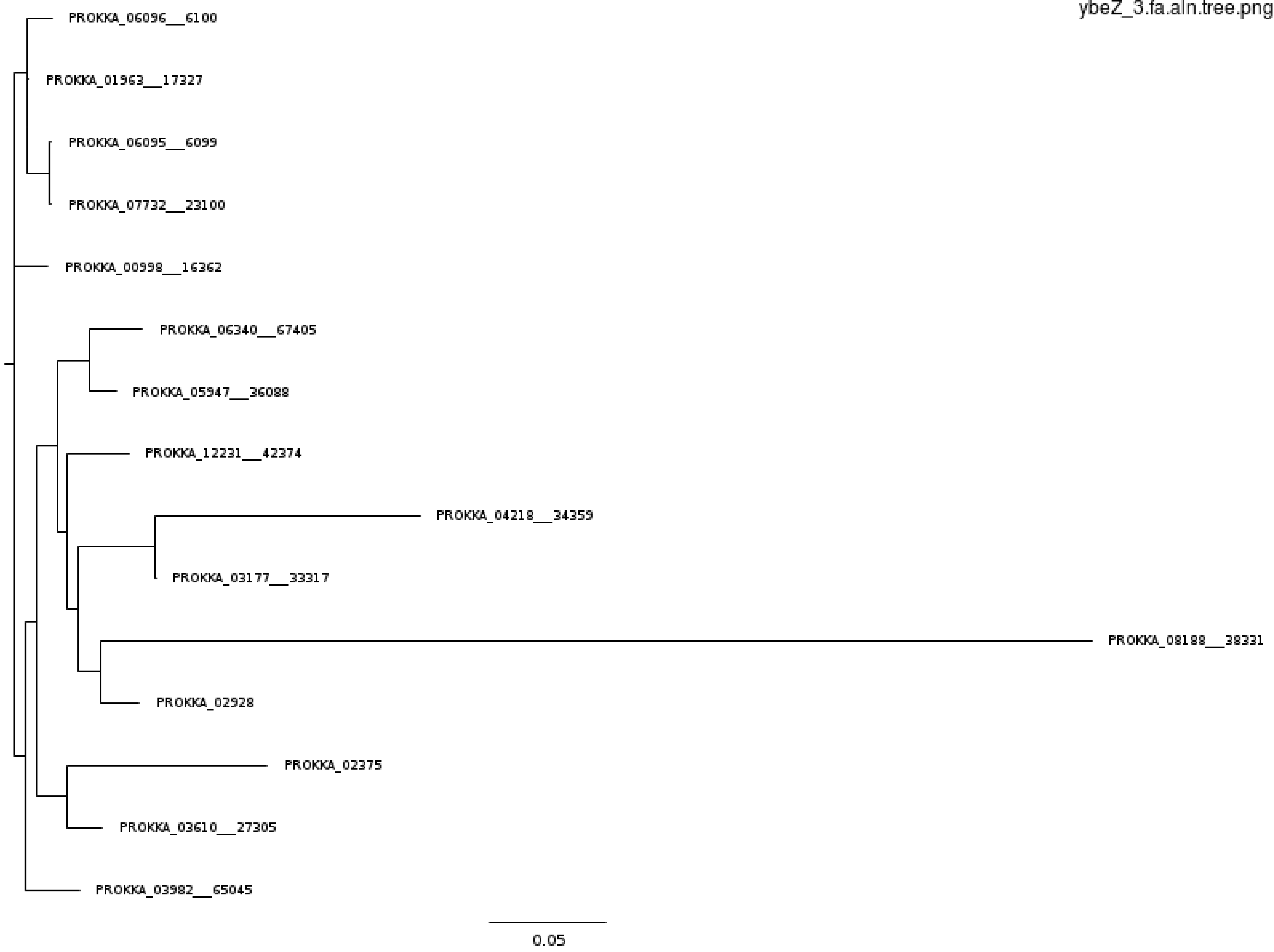
ybeZ_3.fa.aln.tree.png
PROKKA_06096___6100
PROKKA_01963___17327
PROKKA_06095___6099
PROKKA_07732___23100
PROKKA_00998___16362
PROKKA_06340___67405
PROKKA_05947___36088
PROKKA_12231___42374
PROKKA_04218___34359
PROKKA_03177___33317
PROKKA_08188___38331
PROKKA_02928
PROKKA_02375
PROKKA_03610___27305
PROKKA_03982___65045
0.05

ybeZ_4.fa.aln.tree.png

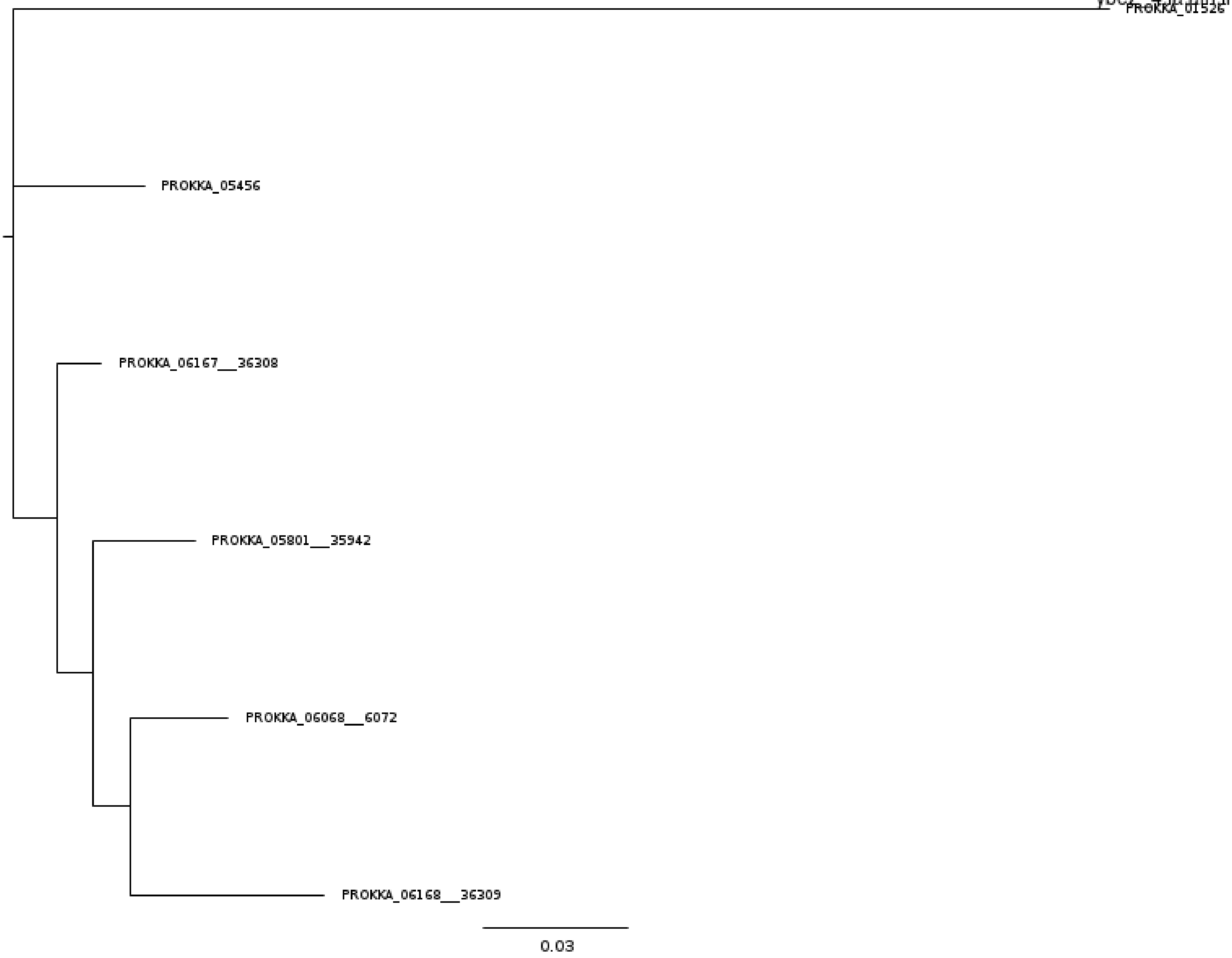

ybgC_3.fa.aln.tree.png

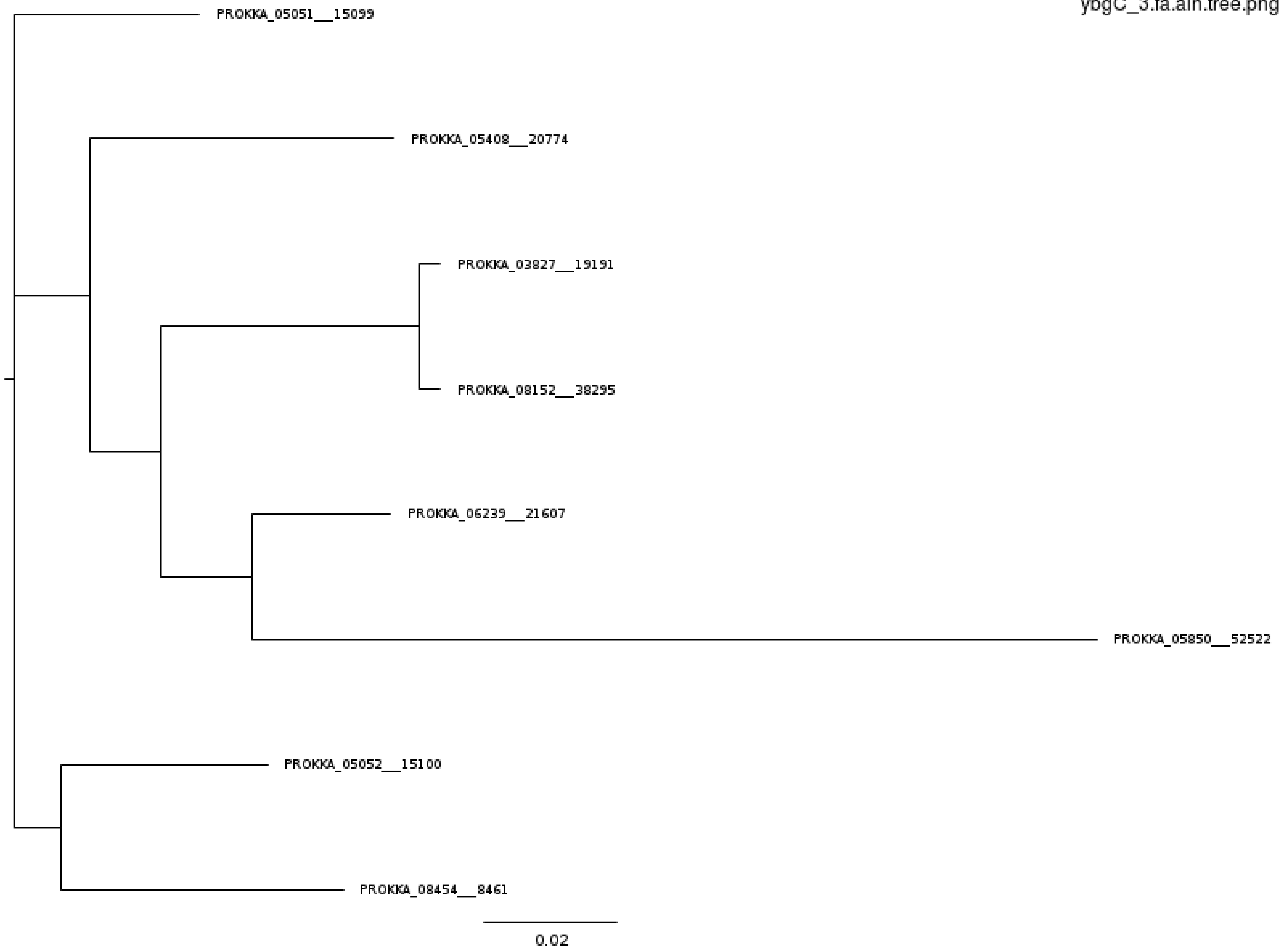

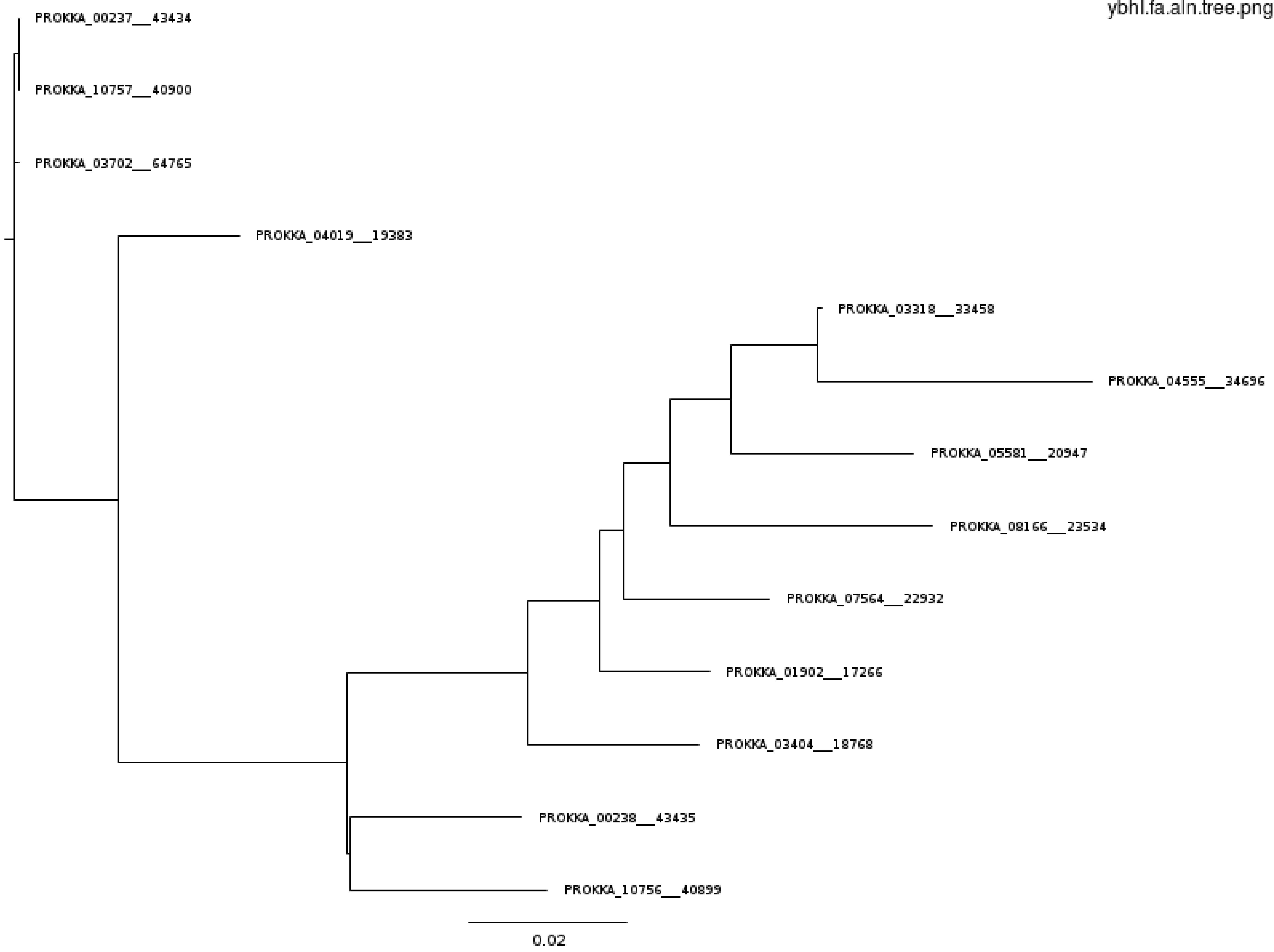
ybhI.fa.aln.tree.png
PROKKA_00237___43434
PROKKA_10757___40900
PROKKA_03702___64765
PROKKA_04019___19383
PROKKA_03318___33458
PROKKA_04555___34696
PROKKA_05581___20947
PROKKA_08166___23534
PROKKA_07564___22932
PROKKA_01902___17266
PROKKA_03404___18768
PROKKA_00238___43435
PROKKA_10756___40899
0.02

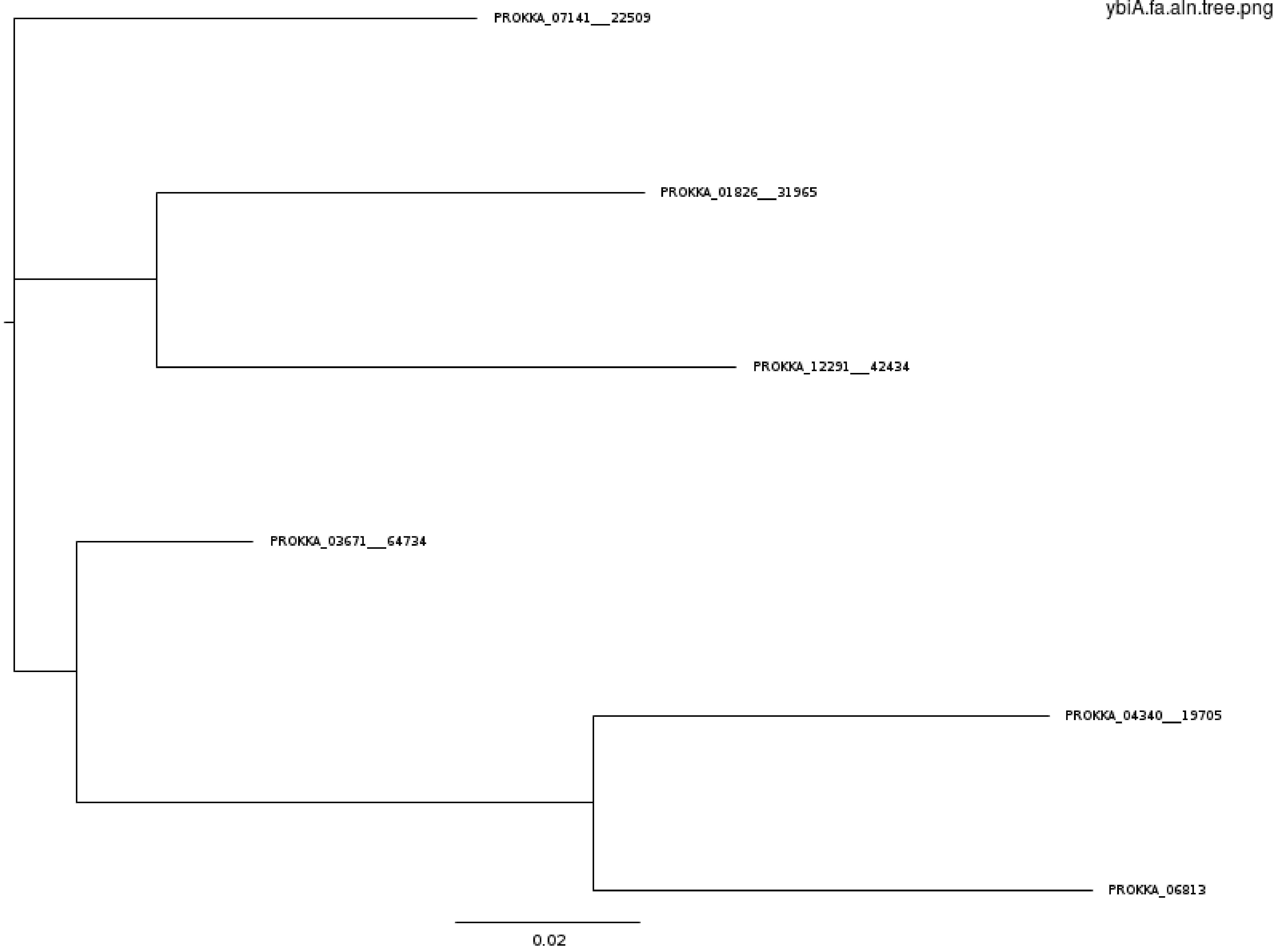

ybiA.fa.aln.tree.png
PROKKA_07141___22509
PROKKA_01826___31965
PROKKA_12291___42434
PROKKA_03671___64734
PROKKA_04340___19705
PROKKA_06813
0.02

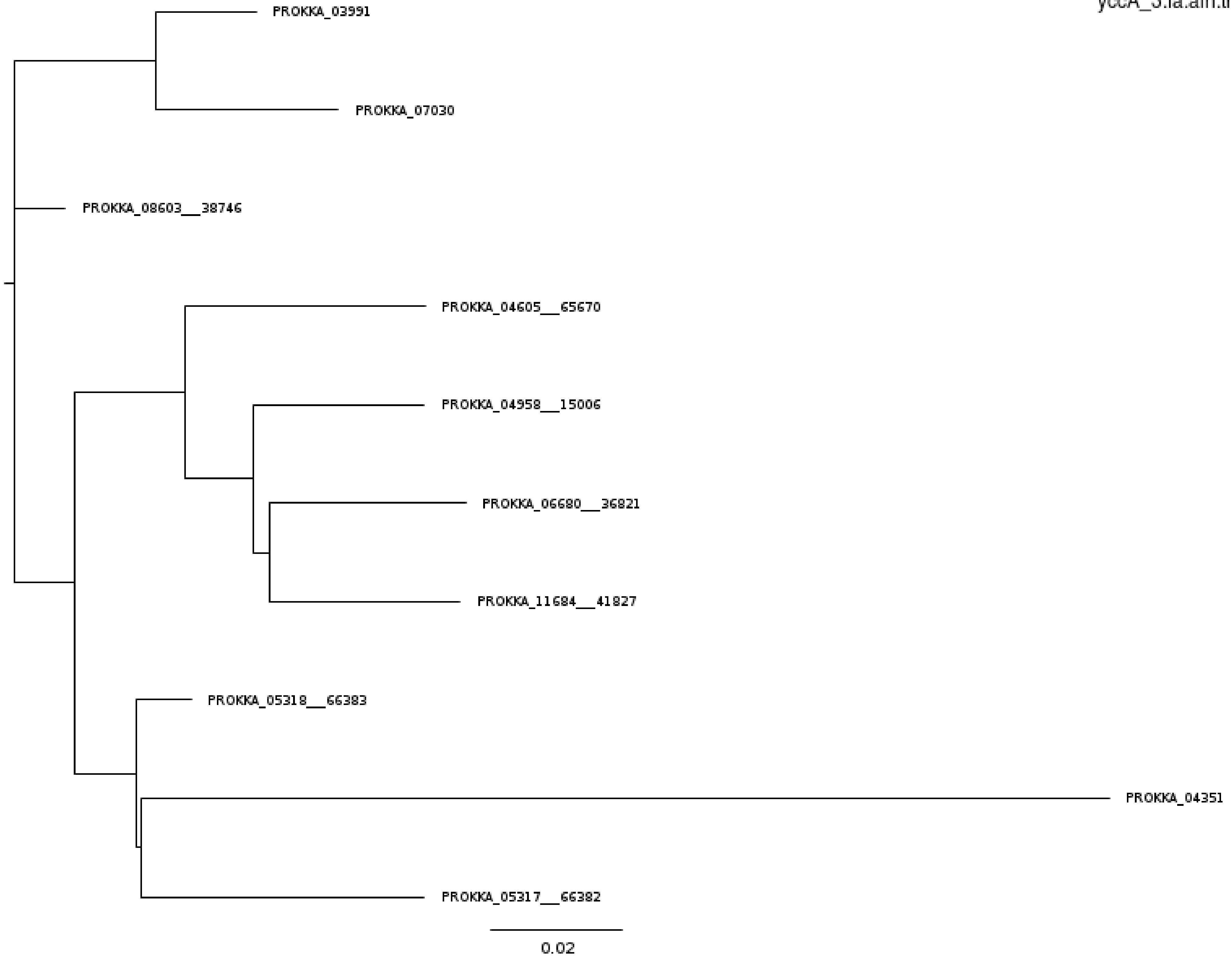
PROKKA_03991
PROKKA_07030
PROKKA_08603___38746
PROKKA_04605___65670
PROKKA_04958___15006
PROKKA_06680___36821
PROKKA_11684___41827
PROKKA_05318___66383
PROKKA_04351
PROKKA_05317___66382
0.02

ycf3.fa.aln.tree.png

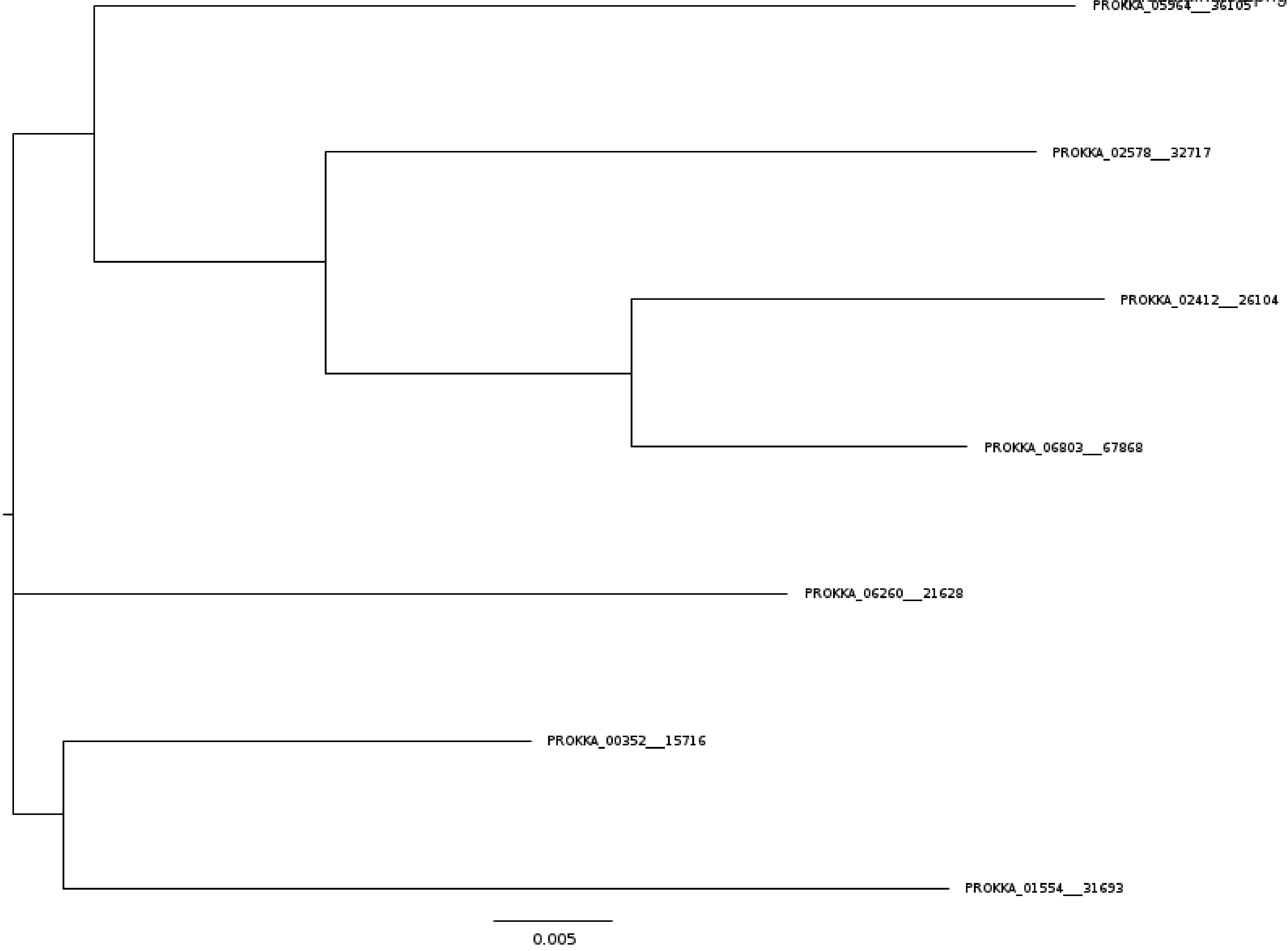

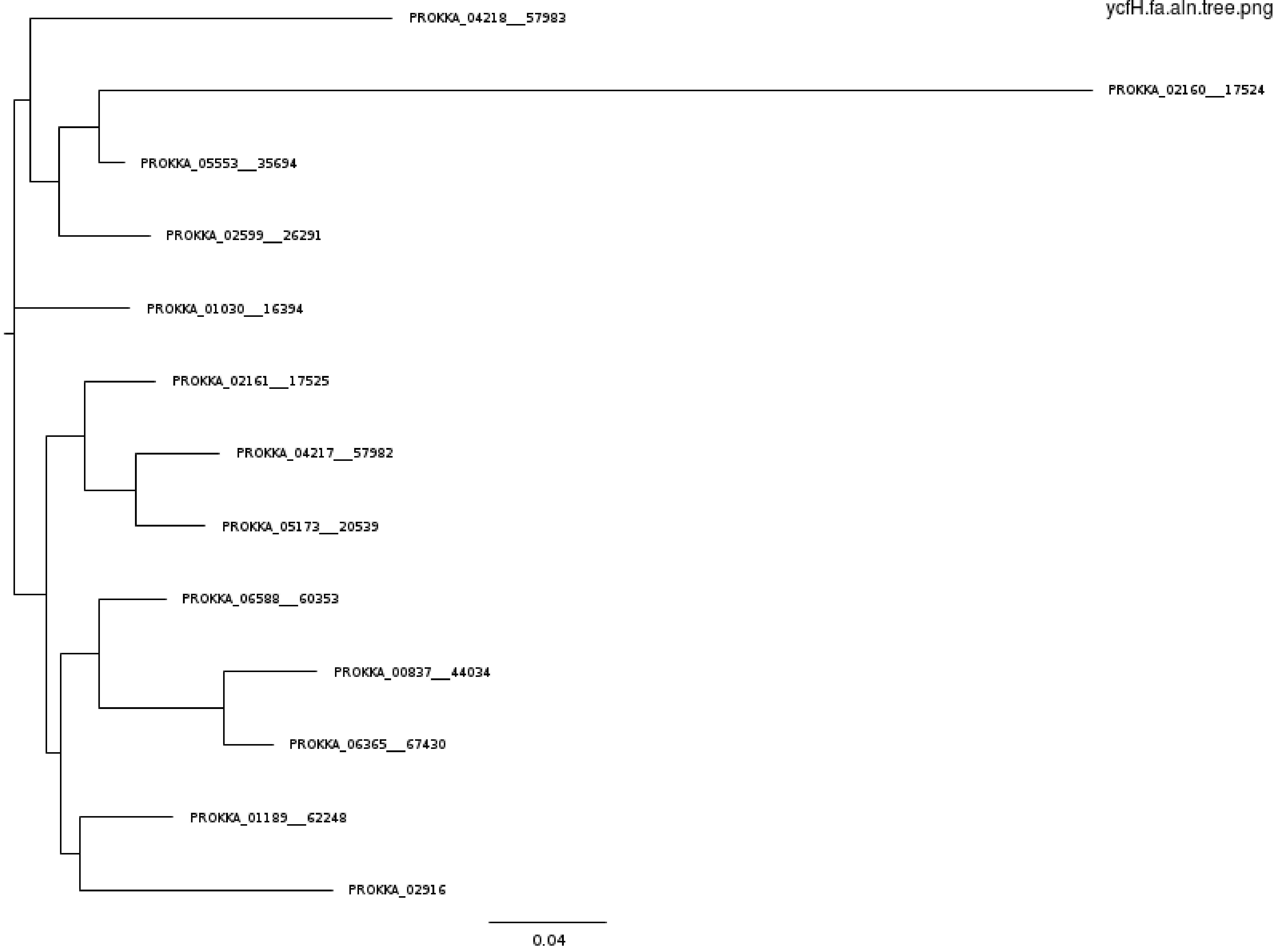

ycfH.fa.aln.tree.png
PROKKA_04218___57983
PROKKA_02160___17524
PROKKA_05553___35694
PROKKA_02599___26291
PROKKA_01030___16394
PROKKA_02161___17525
PROKKA_04217___57982
PROKKA_05173___20539
PROKKA_06588___60353
PROKKA_00837___44034
PROKKA_06365___67430
PROKKA_01189___62248
PROKKA_02916
0.04

ycfS.fa.aln.tree.png

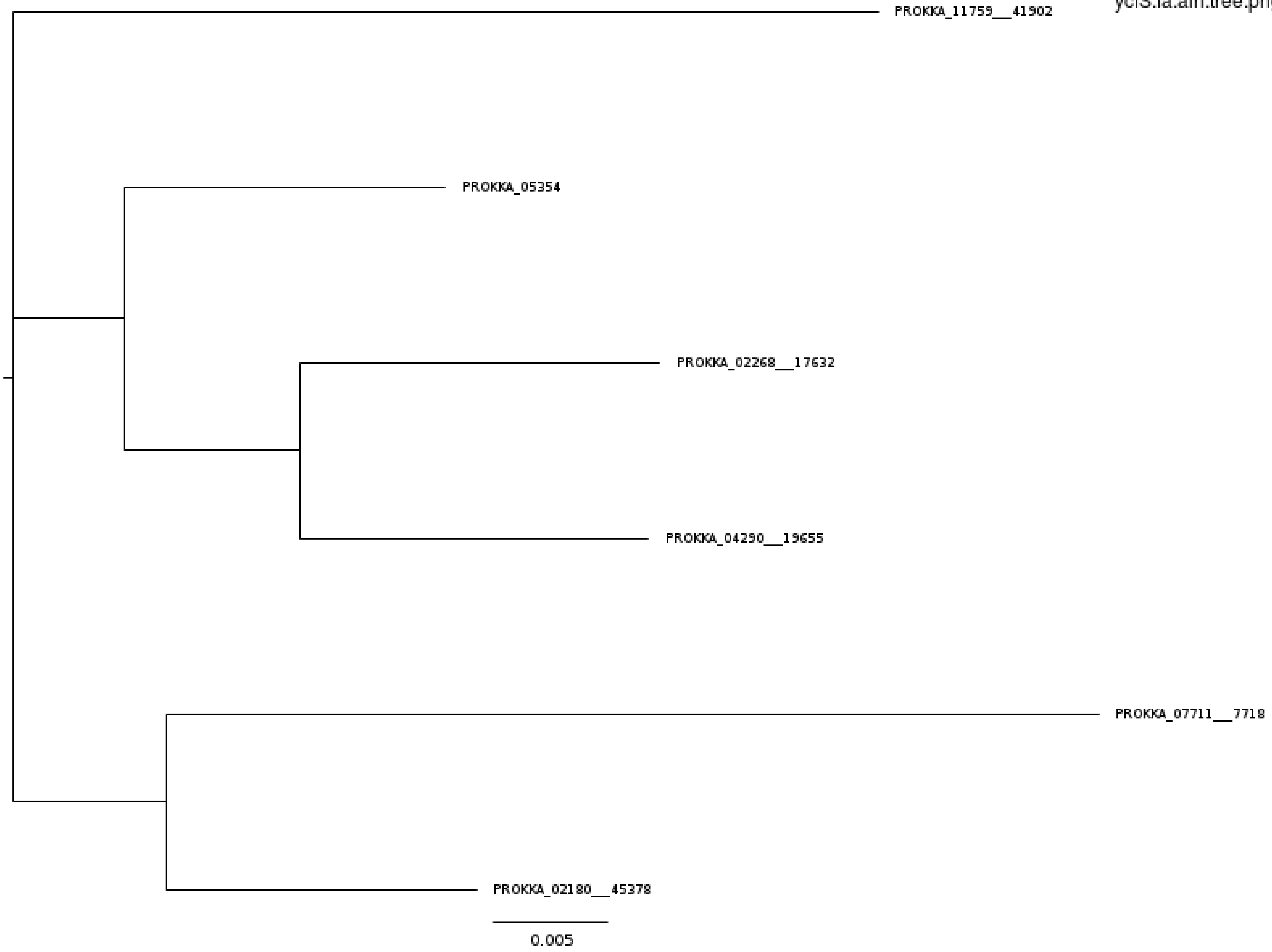

ycgF_2.fa.aln.tree.png

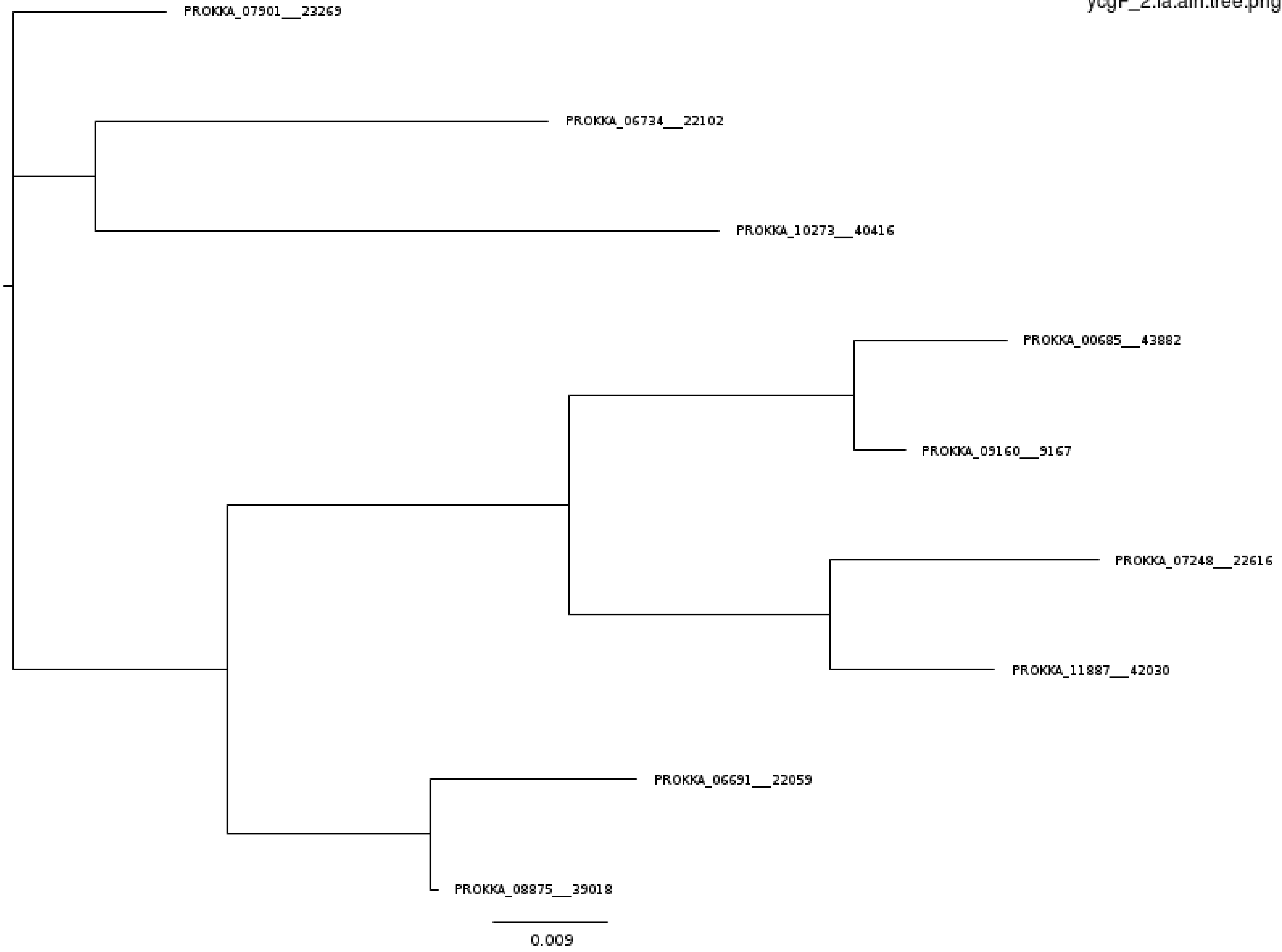

ychF.fa.aln.tree.png

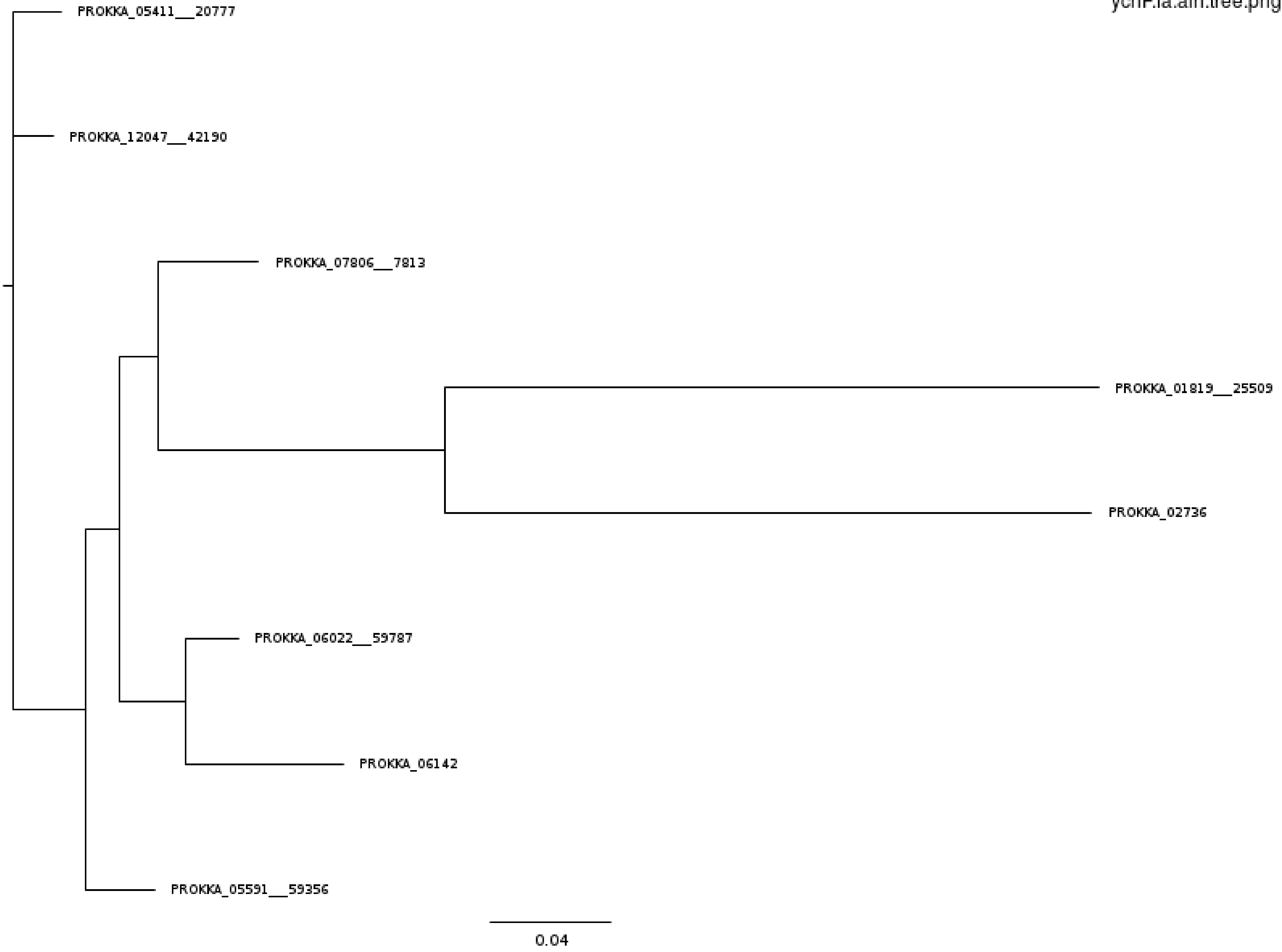

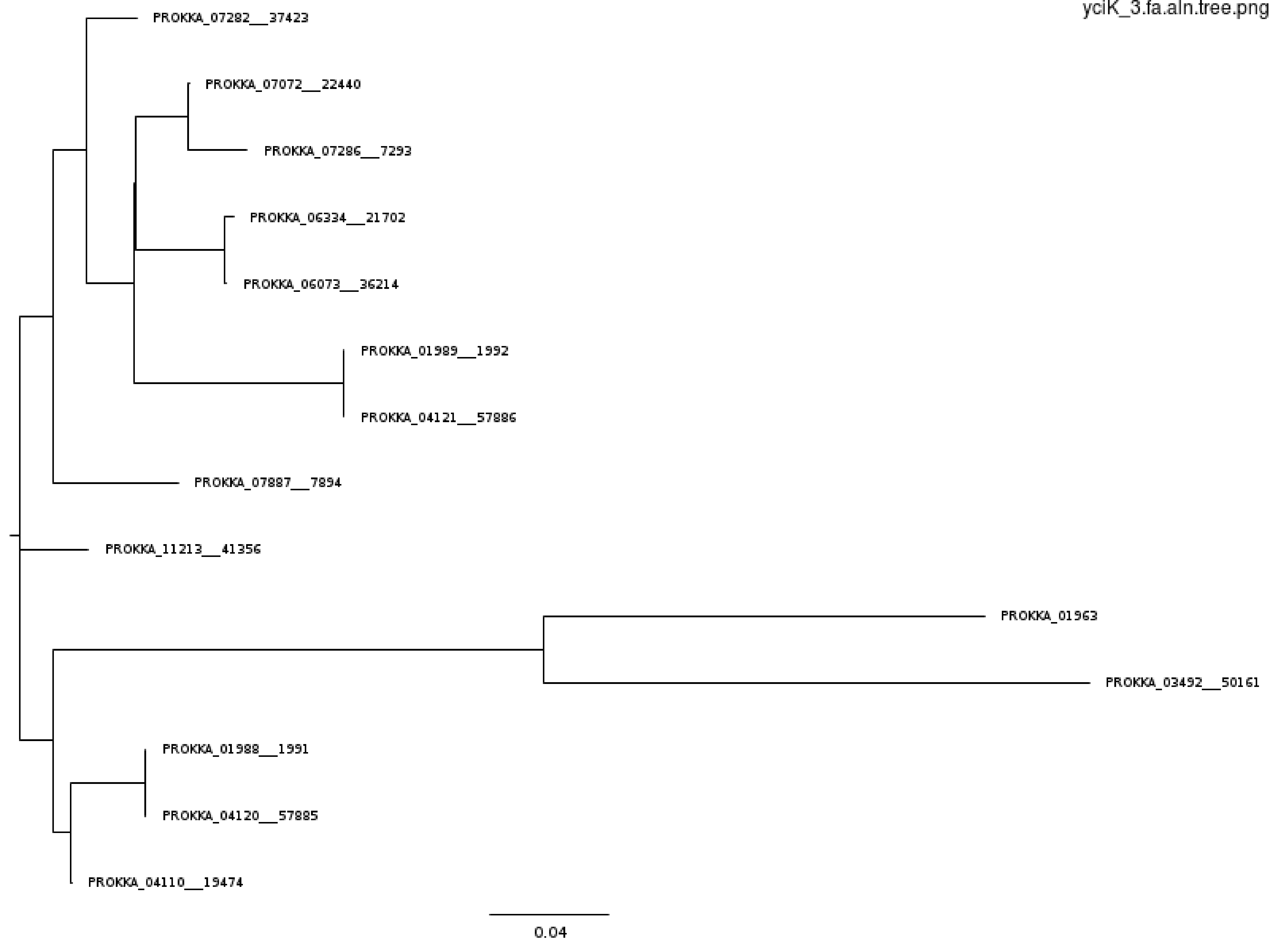
yciK_3.fa.aln.tree.png
PROKKA_07282___37423
PROKKA_07072___22440
PROKKA_07286___7293
PROKKA_06334___21702
PROKKA_06073___36214
PROKKA_01989___1992
PROKKA_04121___57886
PROKKA_07887___7894
PROKKA_11213___41356
PROKKA_01963
PROKKA_03492___50161
PROKKA_01988___1991
PROKKA_04120___57885
PROKKA_04110___19474
0.04

ydaM.fa.aln.tree.png

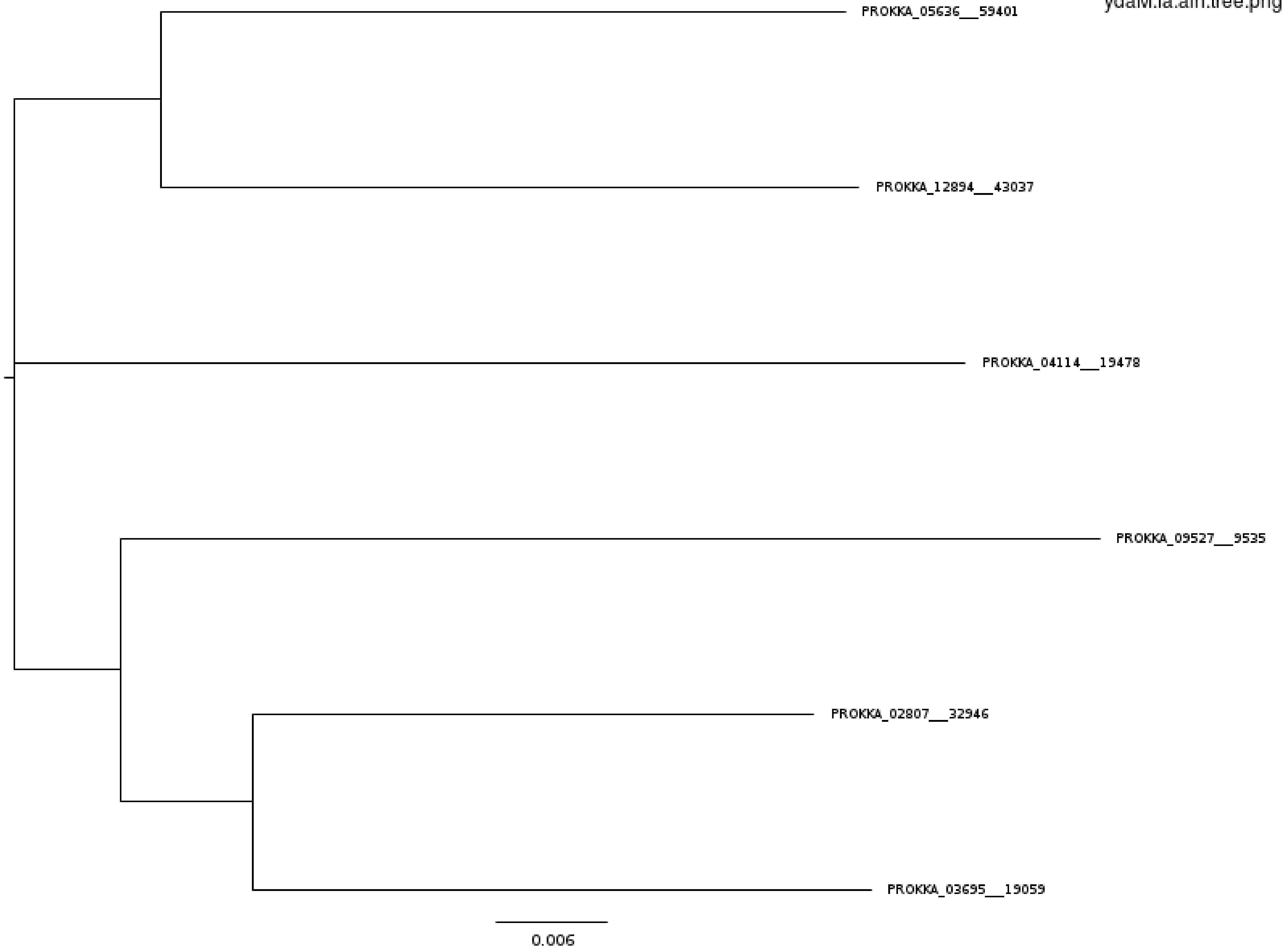

yegE_2.fa.aln.tree.png

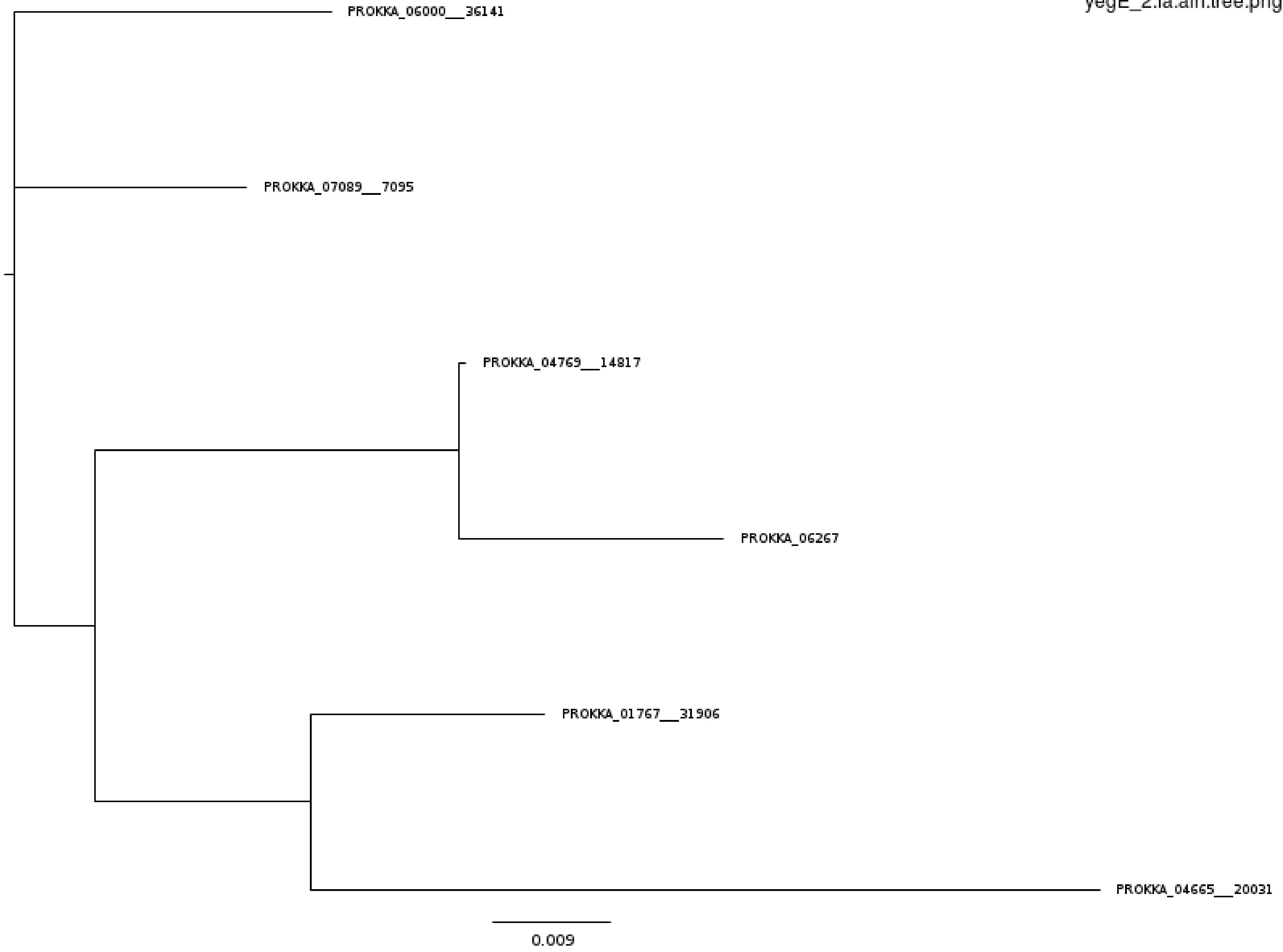

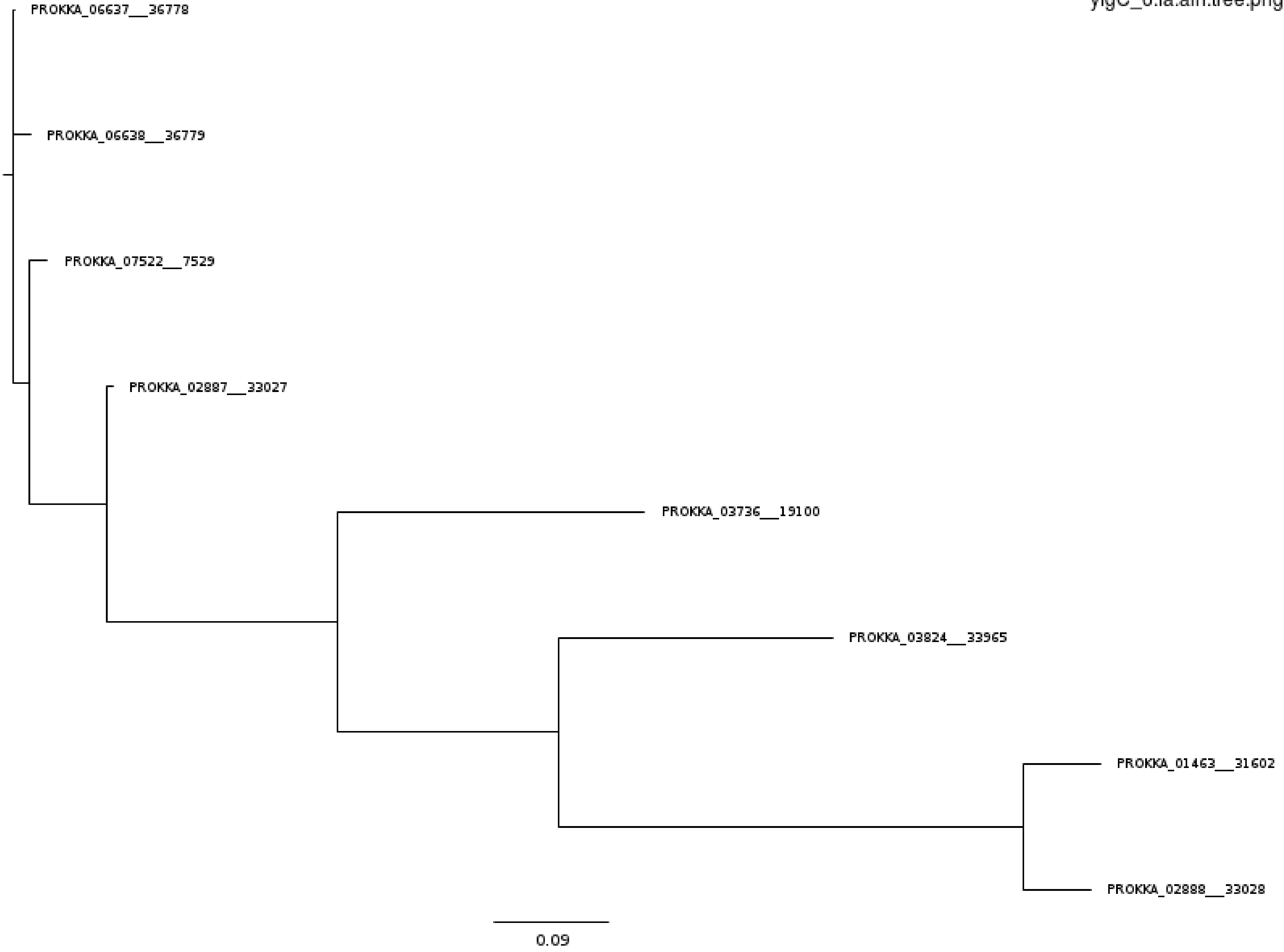
PROKKA_06637___36778
PROKKA_06638___36779
PROKKA_07522___7529
PROKKA_02887___33027
PROKKA_03736___19100
PROKKA_03824___33965
PROKKA_01463___31602
PROKKA_02888___33028
0.09

yfgF_2.fa.aln.tree.png

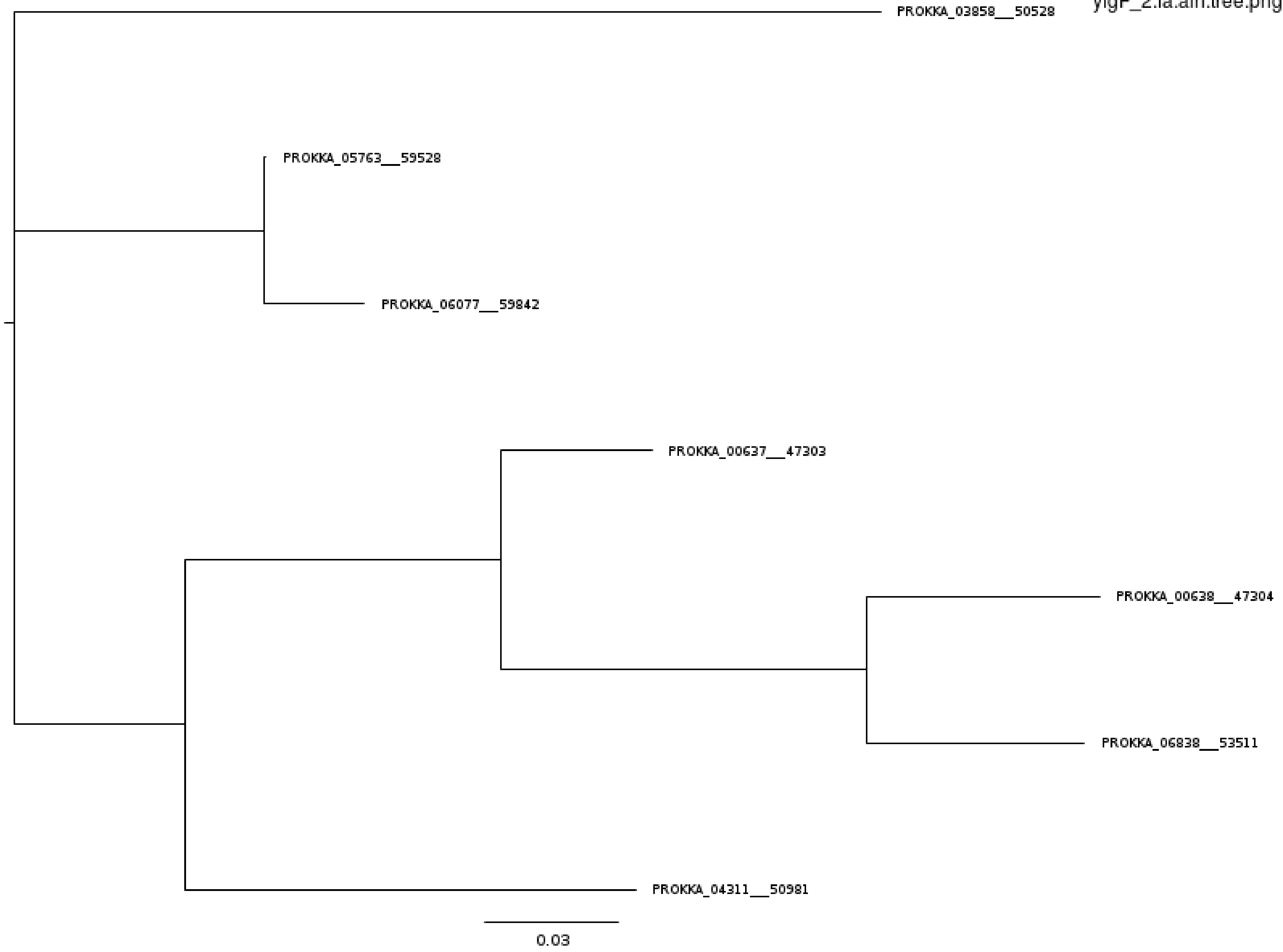

yfkN_3.fa.aln.tree.png

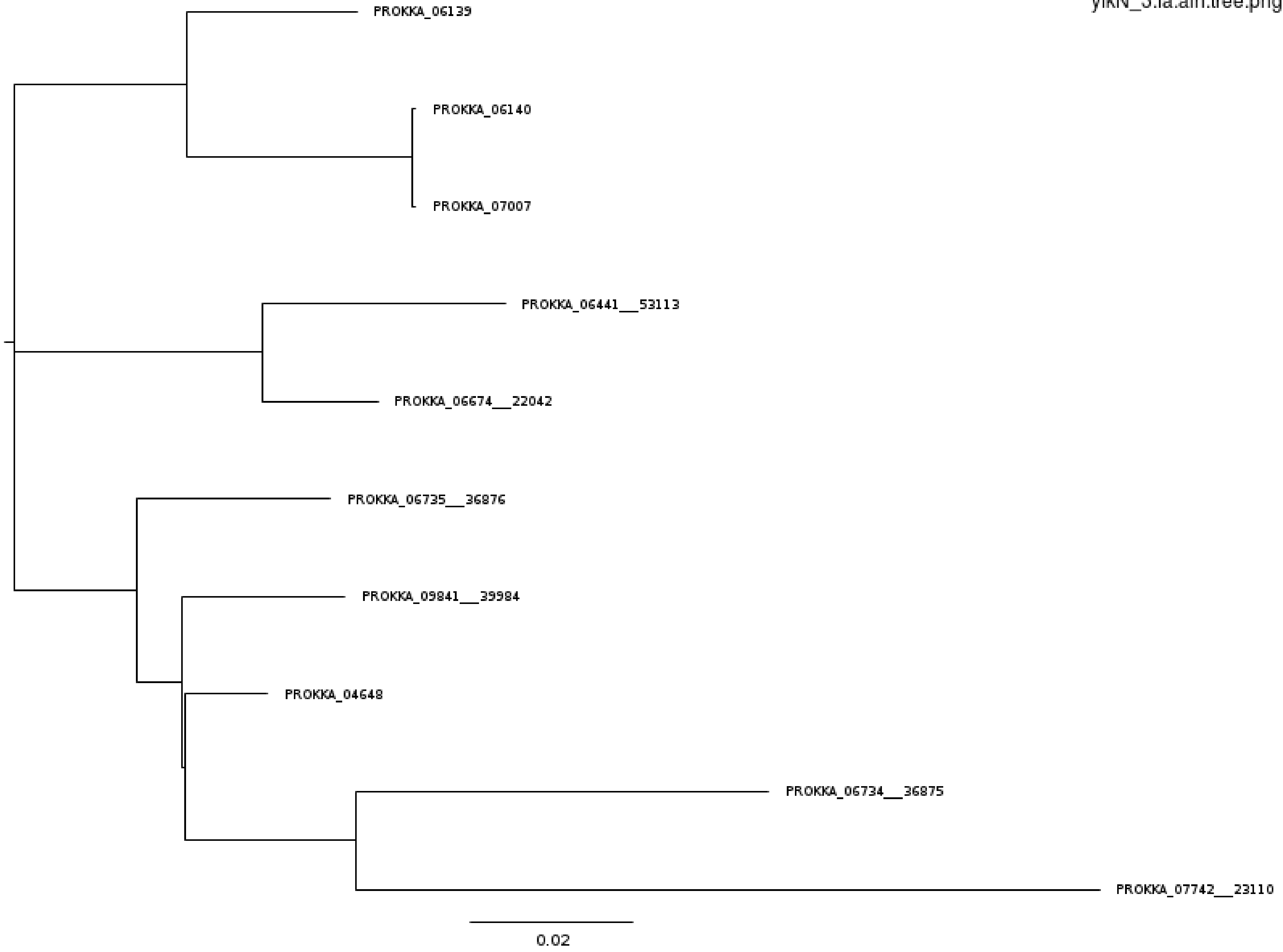

ygaV_2.fa.aln.tree.png

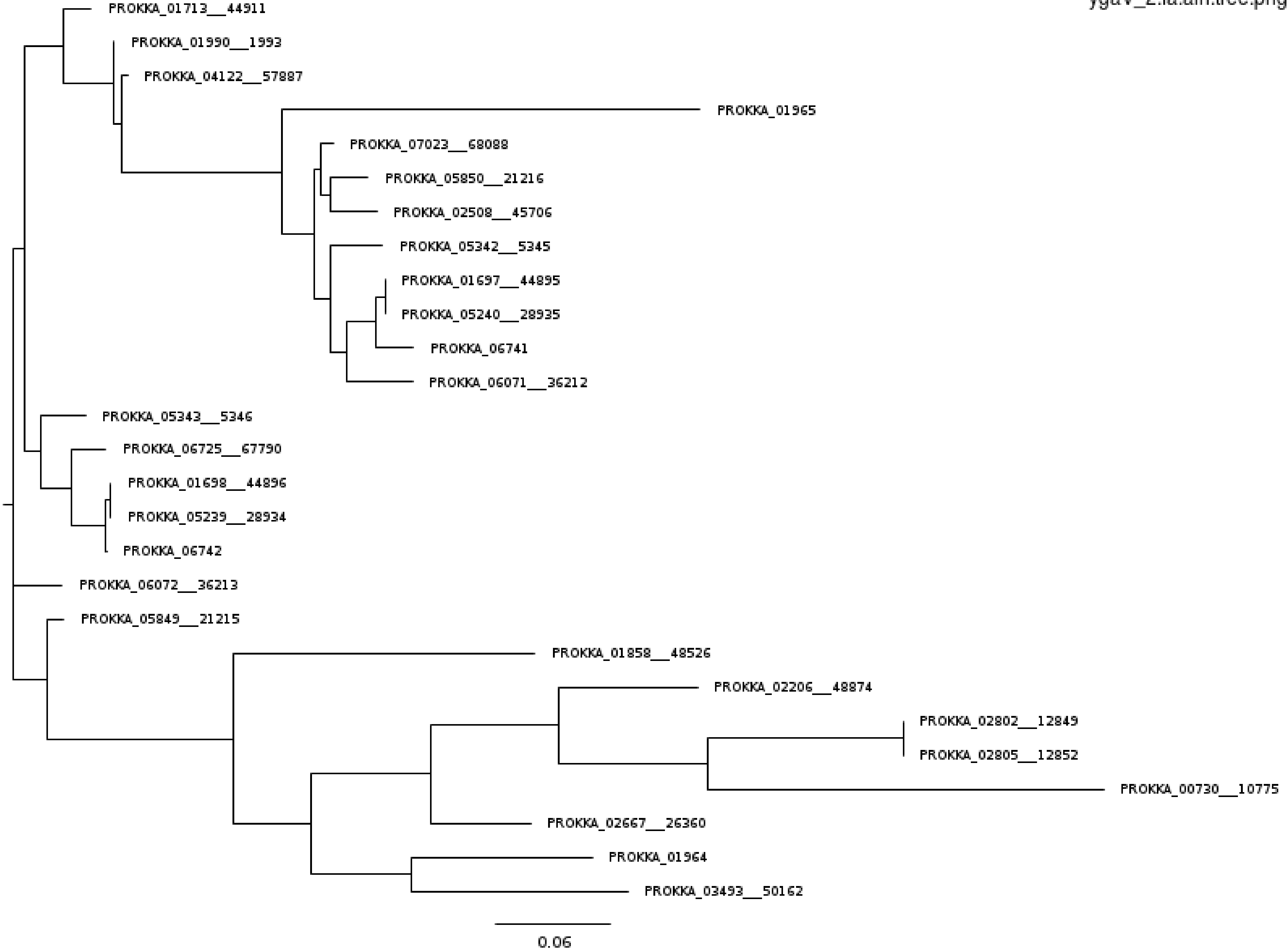

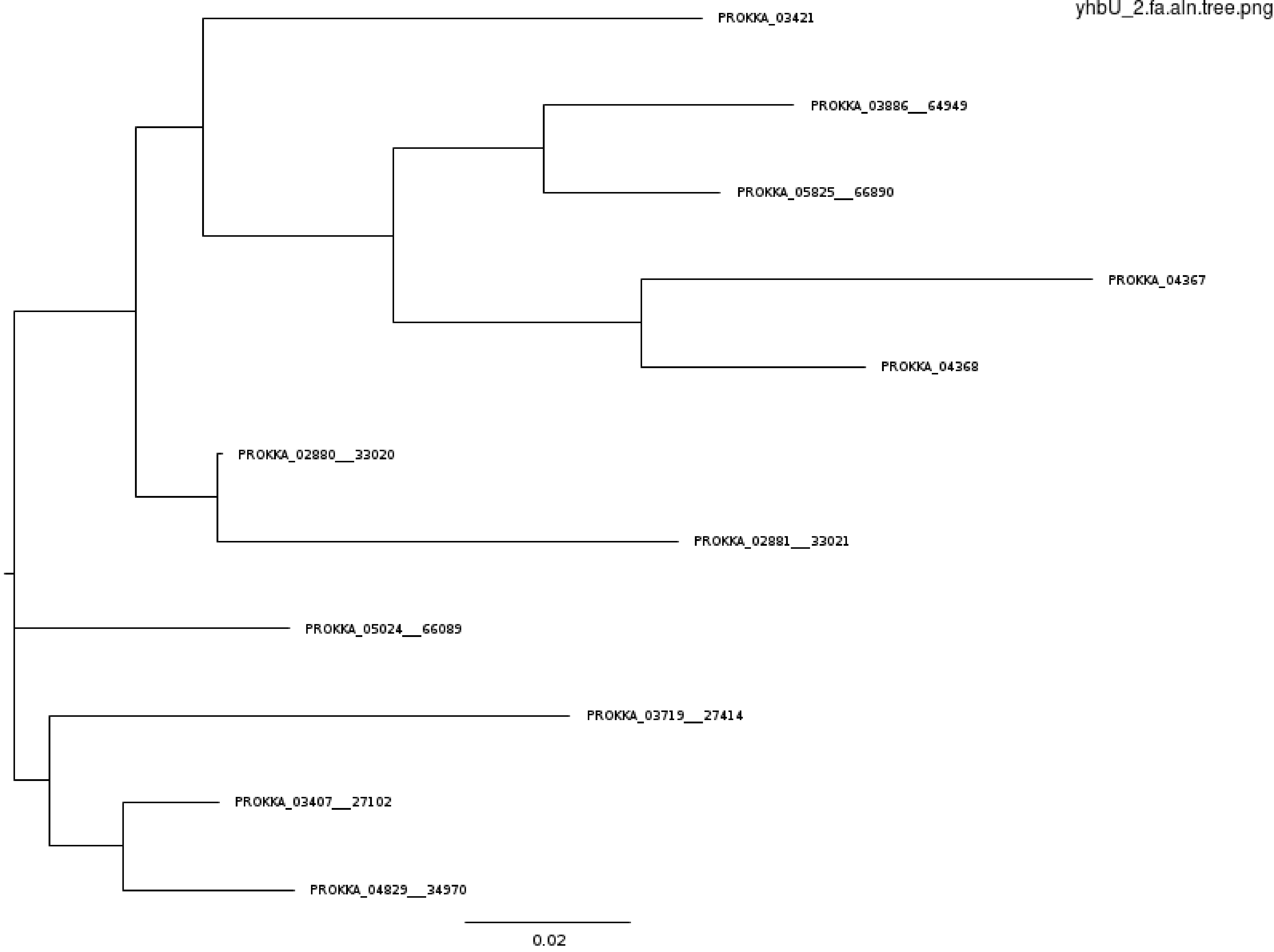

yhbU_2.fa.aln.tree.png
PROKKA_03421
PROKKA_03886___64949
PROKKA_05825___66890
PROKKA_04367
PROKKA_04368
PROKKA_02880___33020
PROKKA_02881___33021
PROKKA_05024___66089
PROKKA_03719___27414
PROKKA_03407___27102
PROKKA_04829___34970
0.02

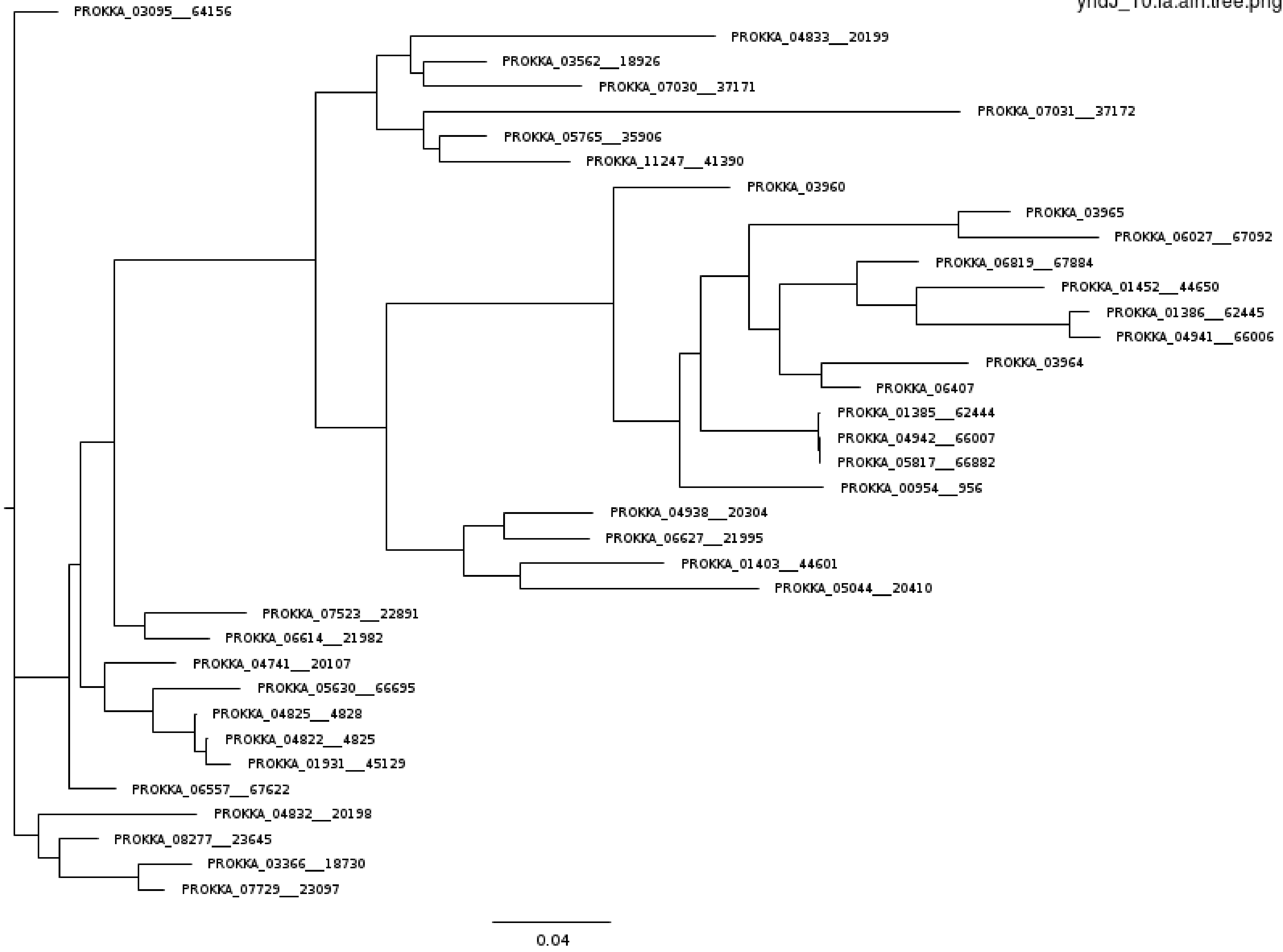
PROKKA_03095__64156
PROKKA_04833__20199
PROKKA_03562__18926
PROKKA_07030__37171
PROKKA_07031__37172
PROKKA_05765__35906
PROKKA_11247__41390
PROKKA_03960
PROKKA_03965
PROKKA_06027__67092
PROKKA_06819__67884
PROKKA_01452__44650
PROKKA_01386__62445
PROKKA_04941__66006
PROKKA_03964
PROKKA_06407
PROKKA_01385__62444
PROKKA_04942__66007
PROKKA_05817__66882
PROKKA_00954__956
PROKKA_04938__20304
PROKKA_06627__21995
PROKKA_01403__44601
PROKKA_05044__20410
PROKKA_07523__22891
PROKKA_06614__21982
PROKKA_04741__20107
PROKKA_05630__66695
PROKKA_04825__4828
PROKKA_04822__4825
PROKKA_01931__45129
PROKKA_06557__67622
PROKKA_04832__20198
PROKKA_08277__23645
PROKKA_03366__18730
PROKKA_07729__23097
0.04

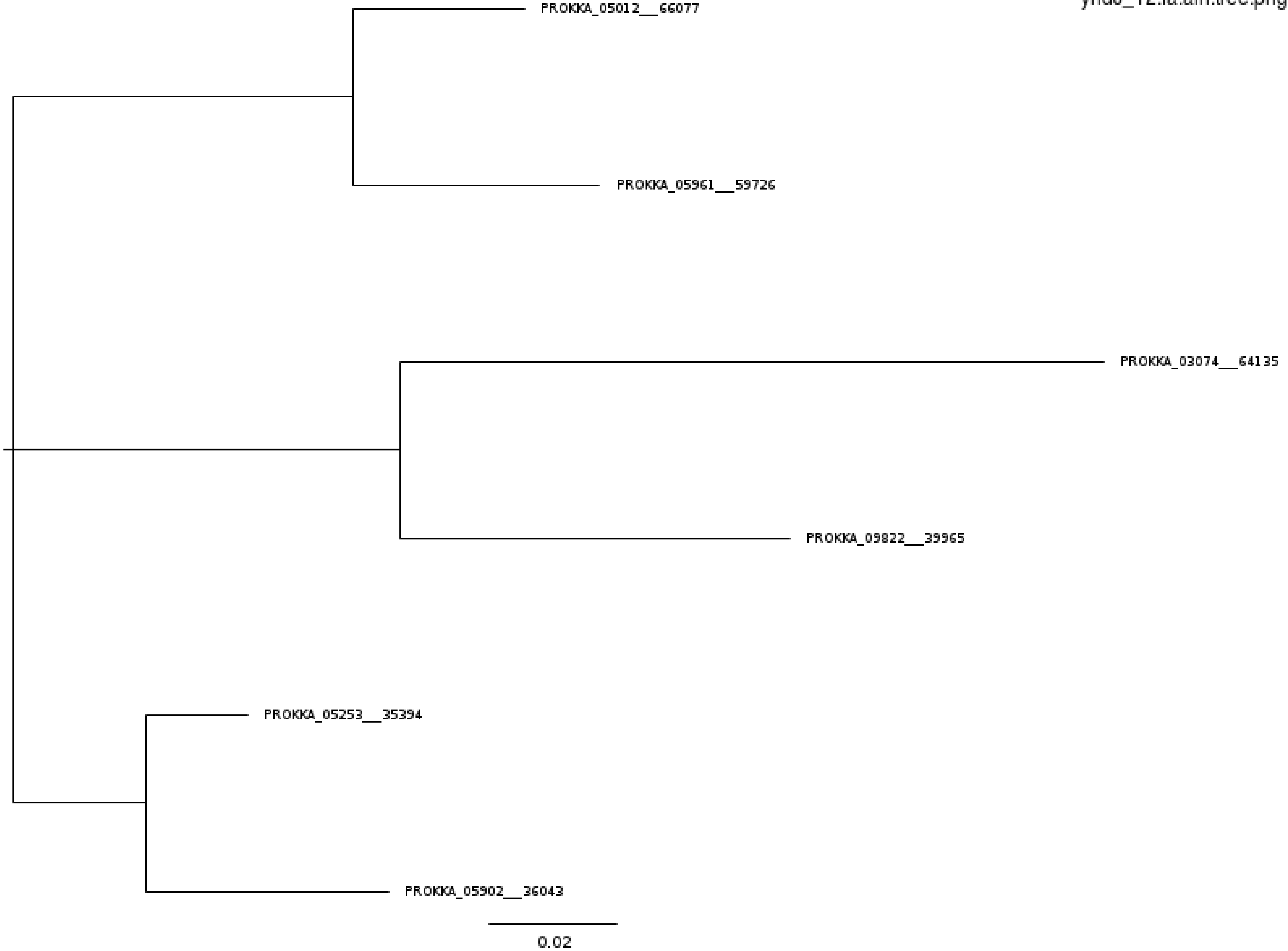
PROKKA_05012___66077
PROKKA_05961___59726
PROKKA_03074___64135
PROKKA_09822___39965
PROKKA_05253___35394
PROKKA_05902___36043
0.02

yhdJ_13.fa.aln.tree.png

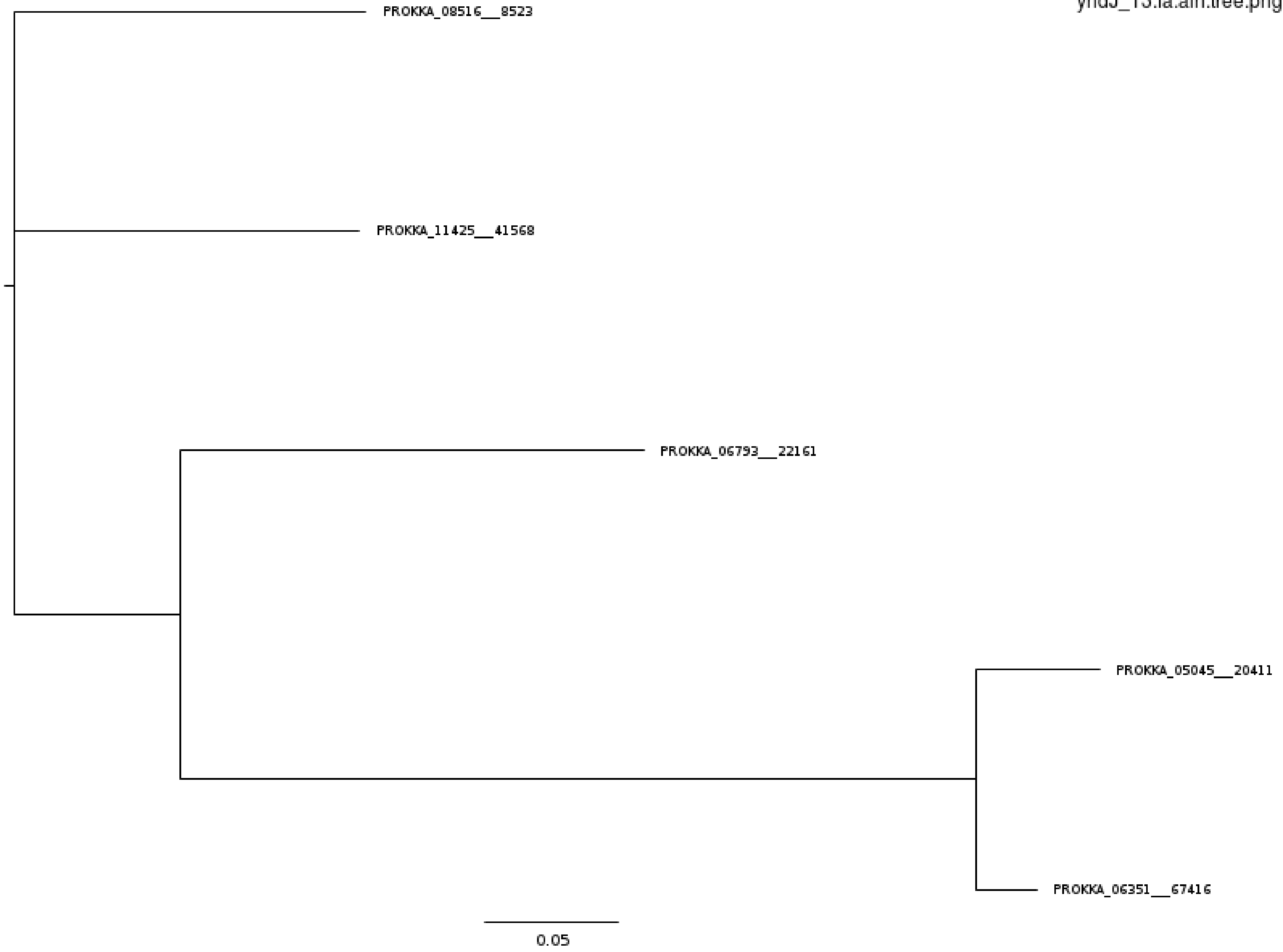

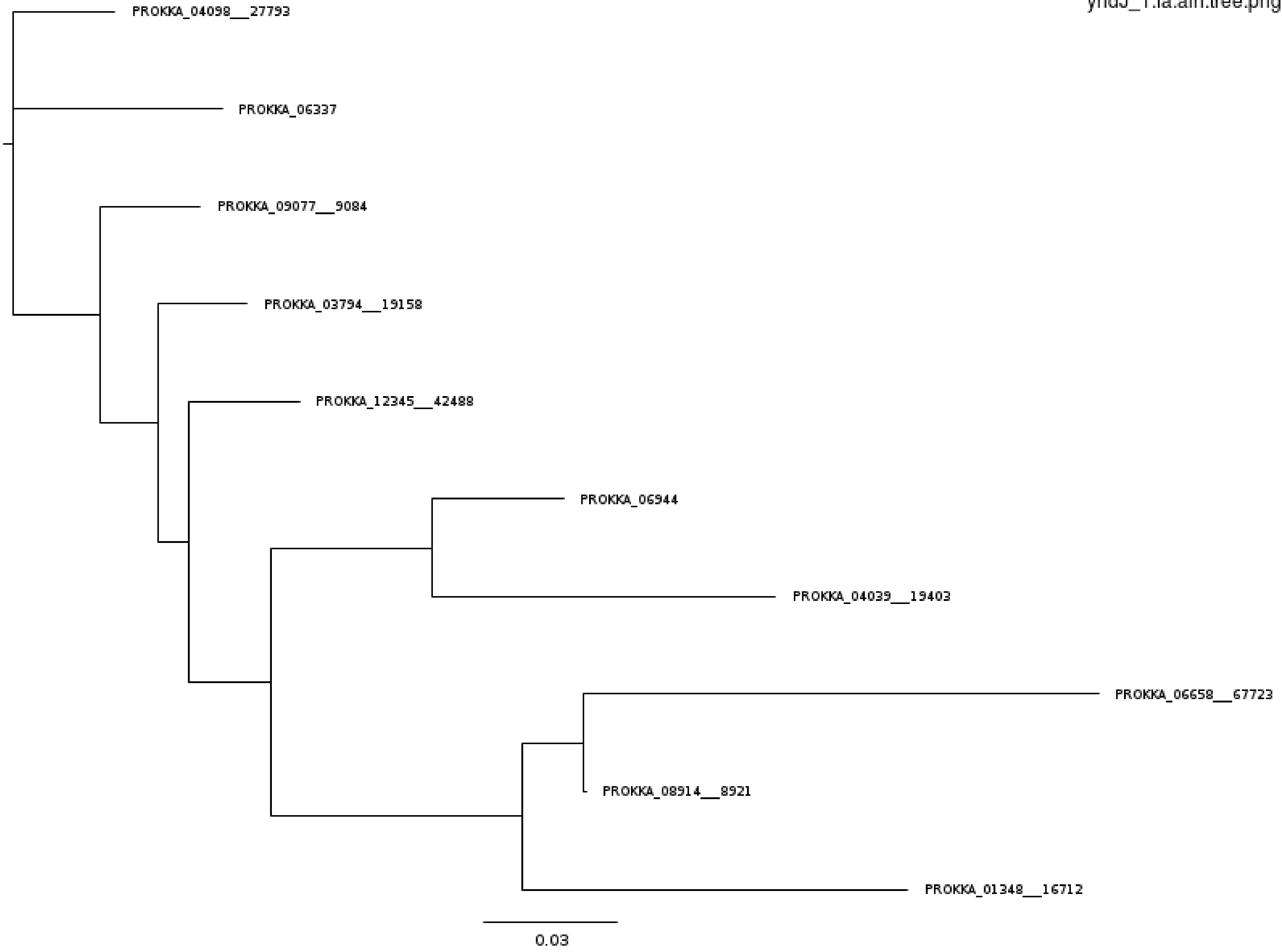
PROKKA_04098__27793
PROKKA_06337
PROKKA_09077__9084
PROKKA_03794__19158
PROKKA_12345__42488
PROKKA_06944
PROKKA_04039__19403
PROKKA_06658__67723
PROKKA_08914__8921
PROKKA_01348__16712
0.03

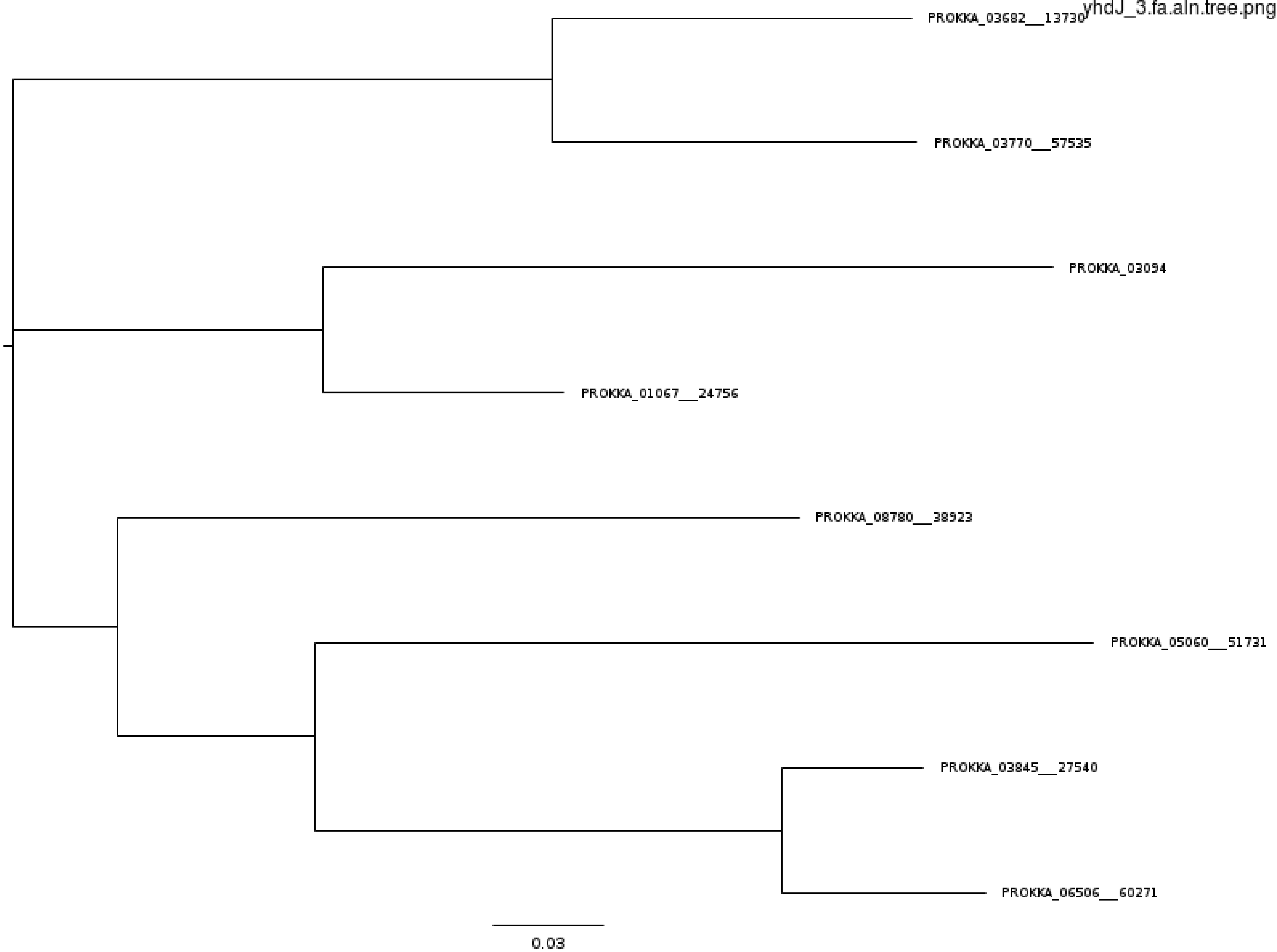
yhdJ_3.fa.aln.tree.png
PROKKA_03682___13730
PROKKA_03770___57535
PROKKA_03094
PROKKA_01067___24756
PROKKA_08780___38923
PROKKA_05060___51731
PROKKA_03845___27540
PROKKA_06506___60271
0.03

yhdJ_7.fa.aln.tree.png

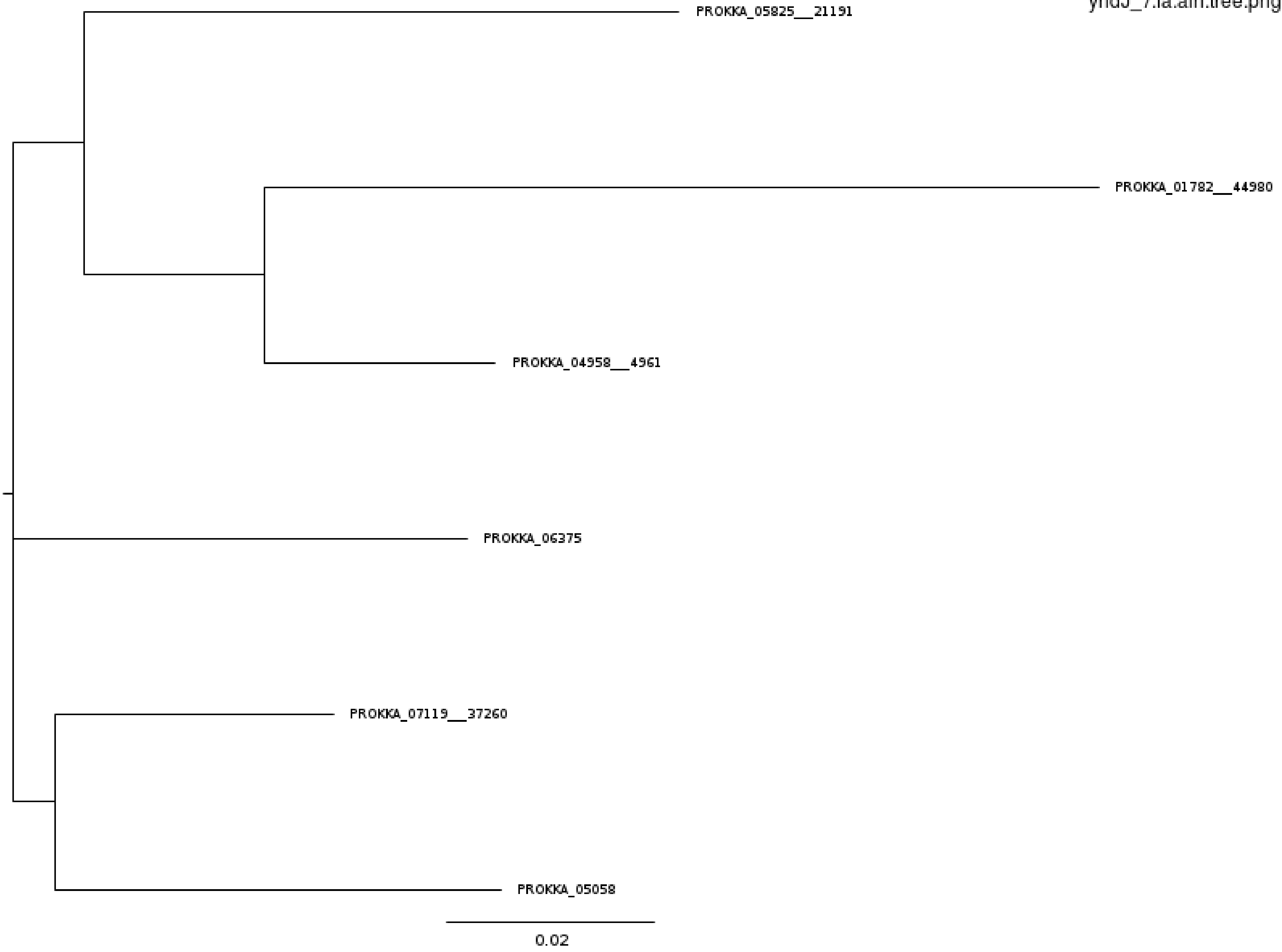

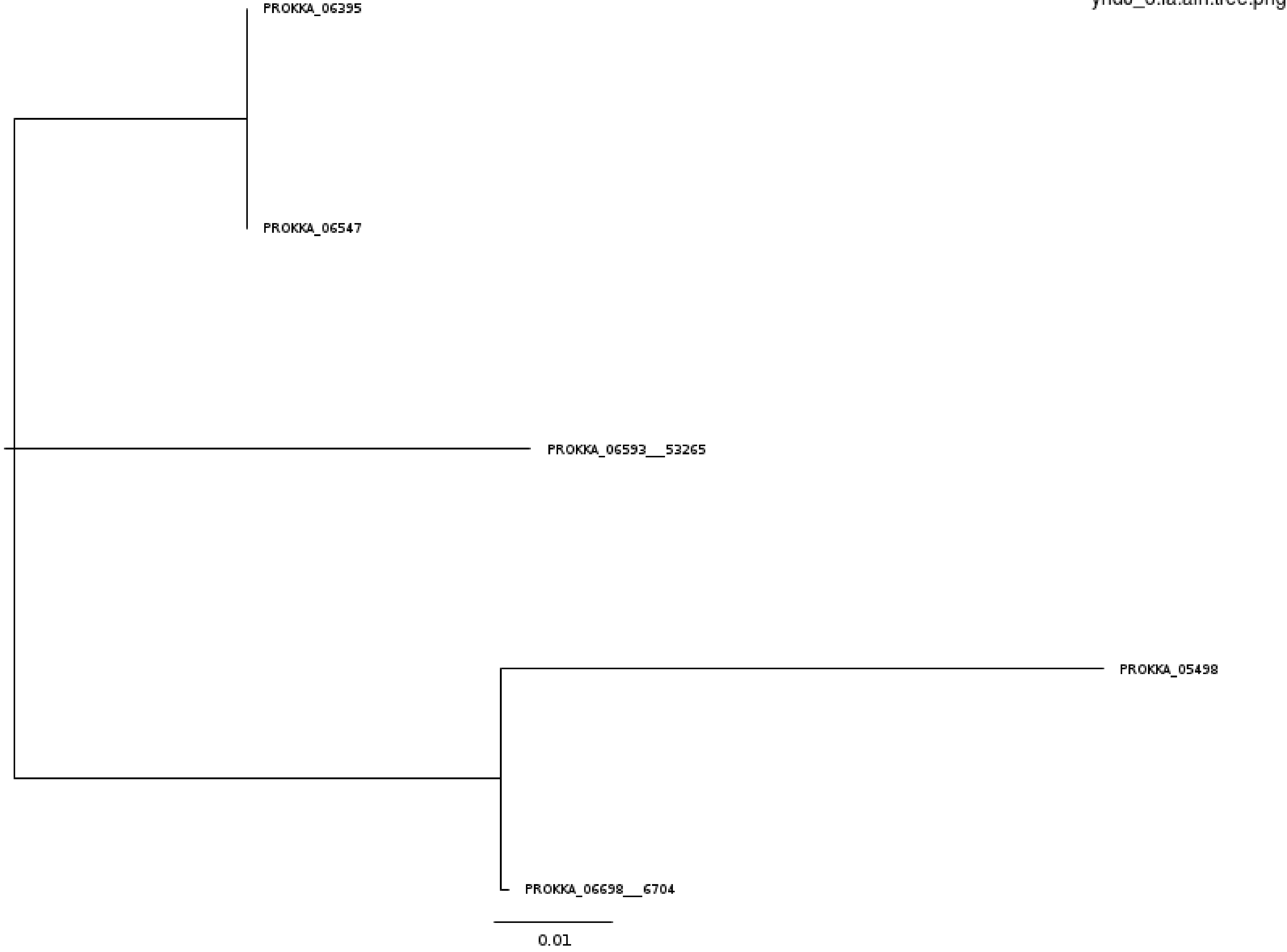
PROKKA_06395
PROKKA_06547
PROKKA_06593___53265
PROKKA_05498
PROKKA_06698___6704
0.01

yhdJ_9.fa.aln.tree.png

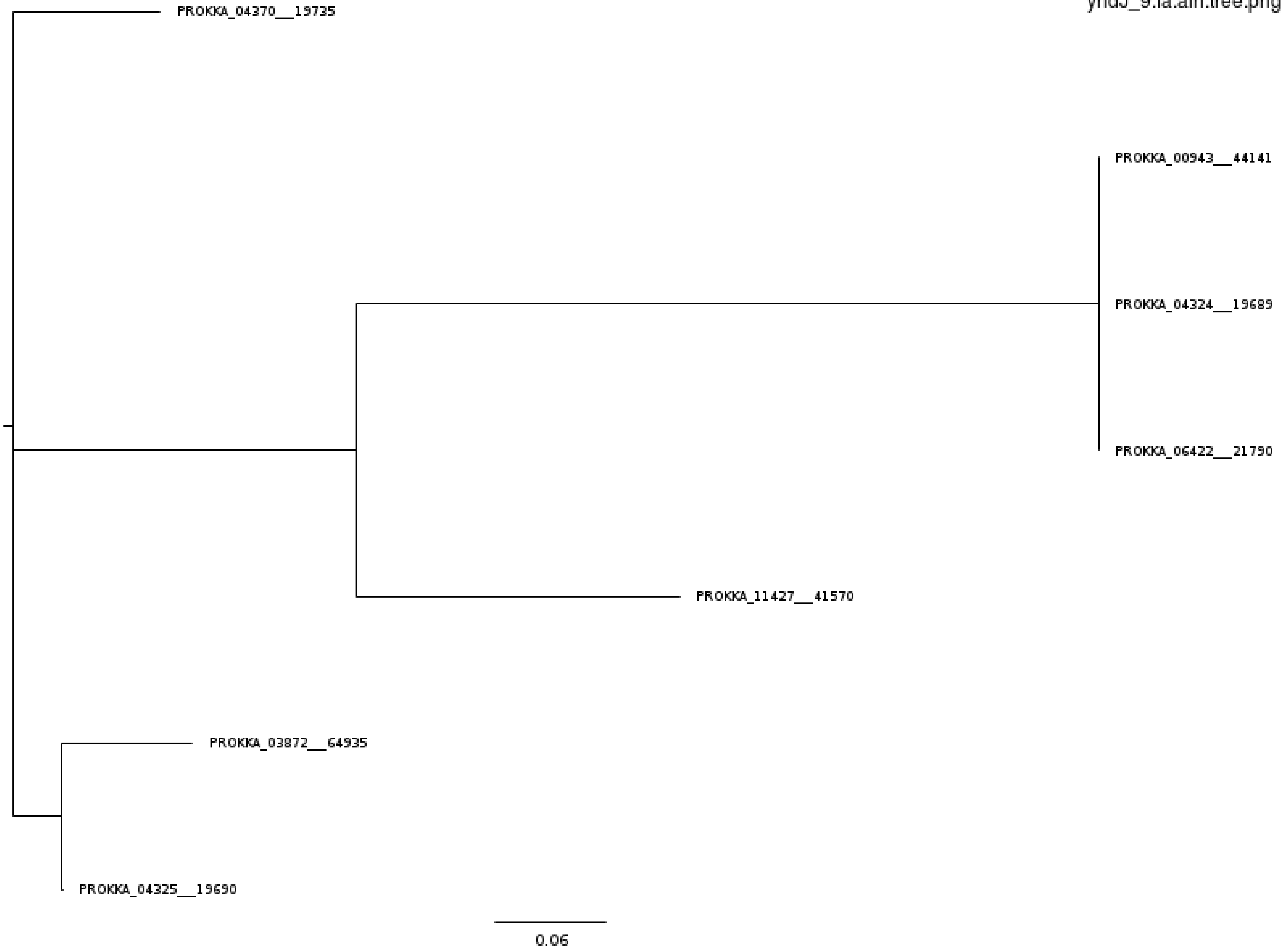

yheS_3.fa.aln.tree.png

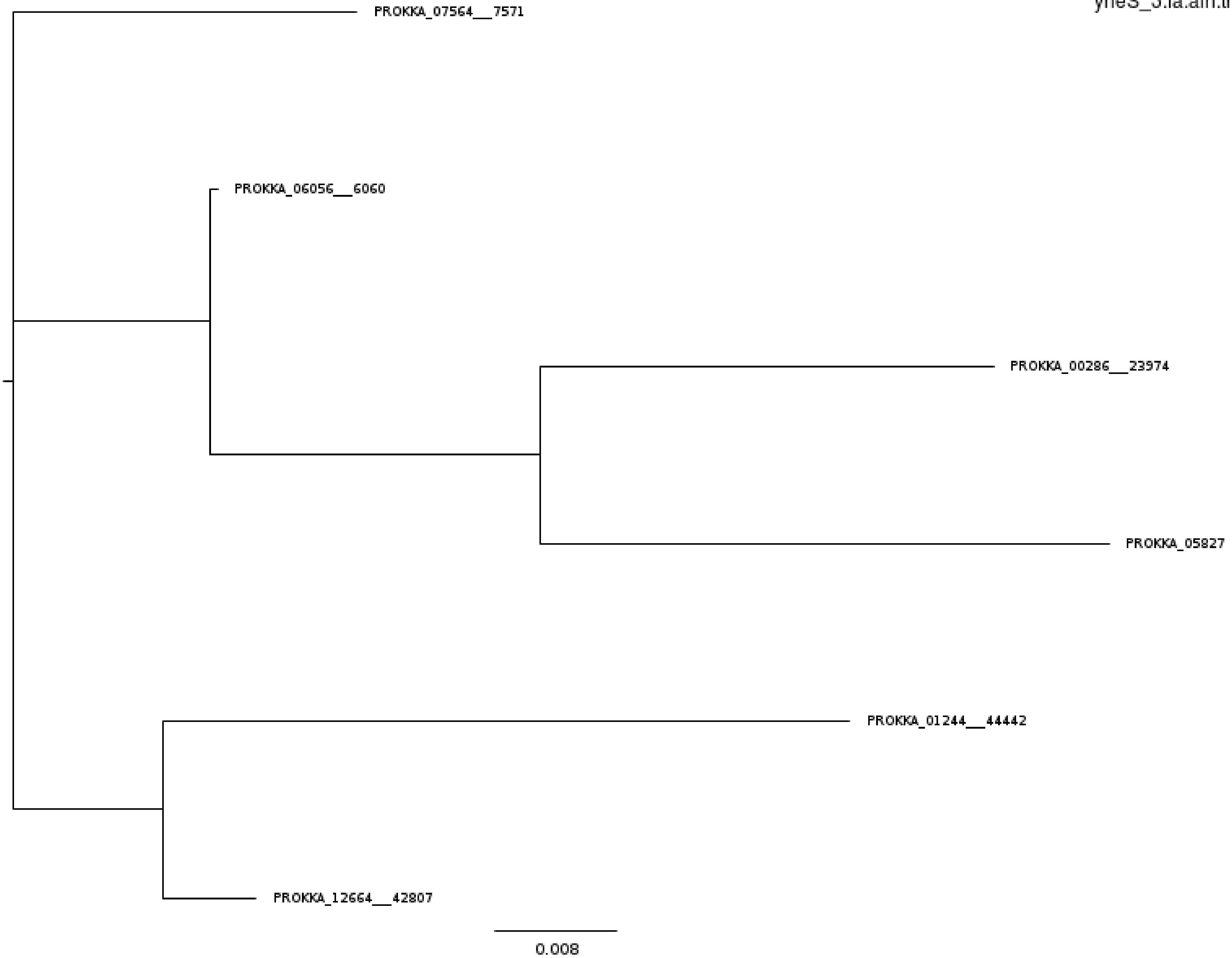

yhfT_2.fa.aln.tree.png

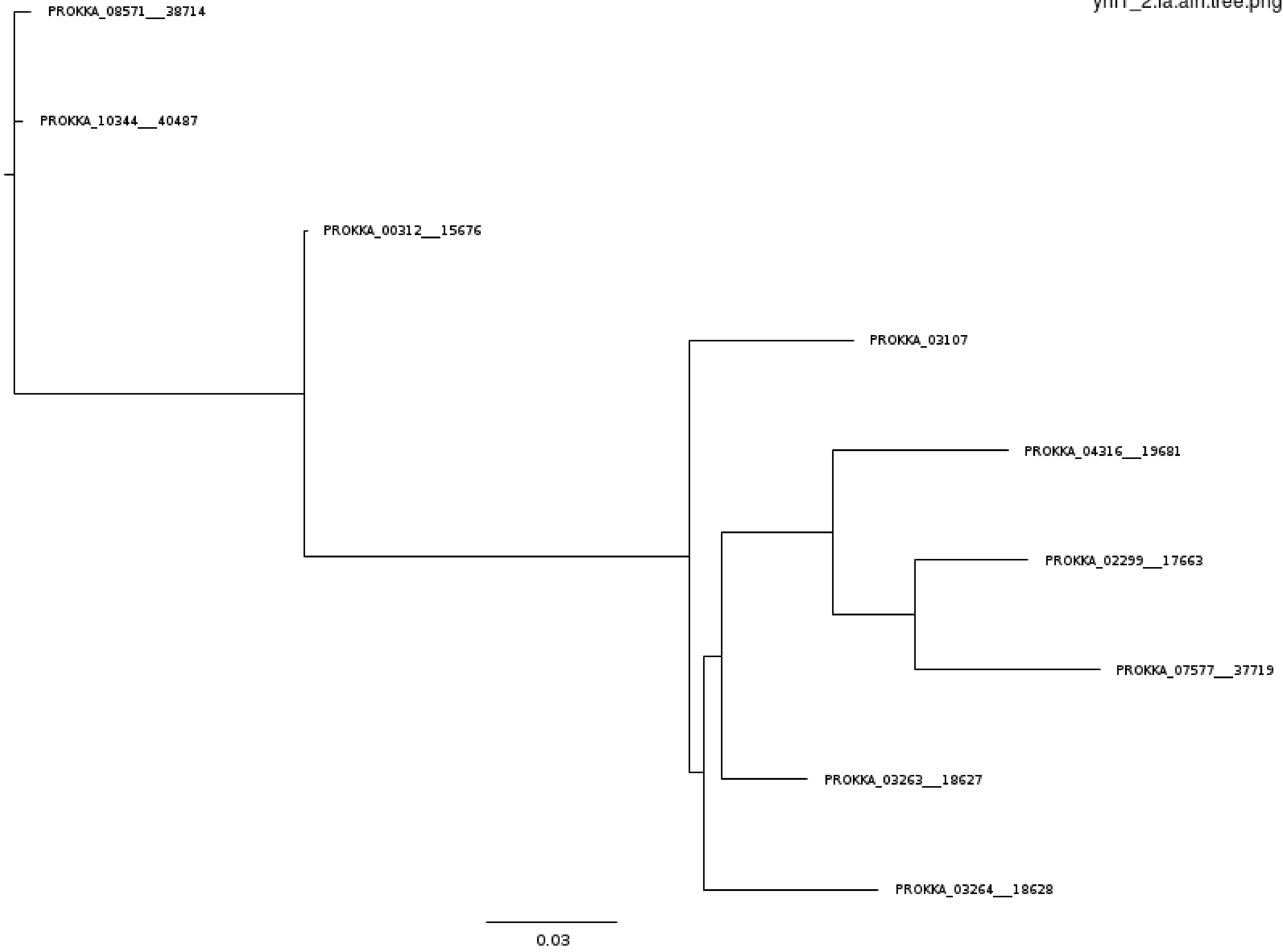

yiaD_2.fa.aln.tree.png

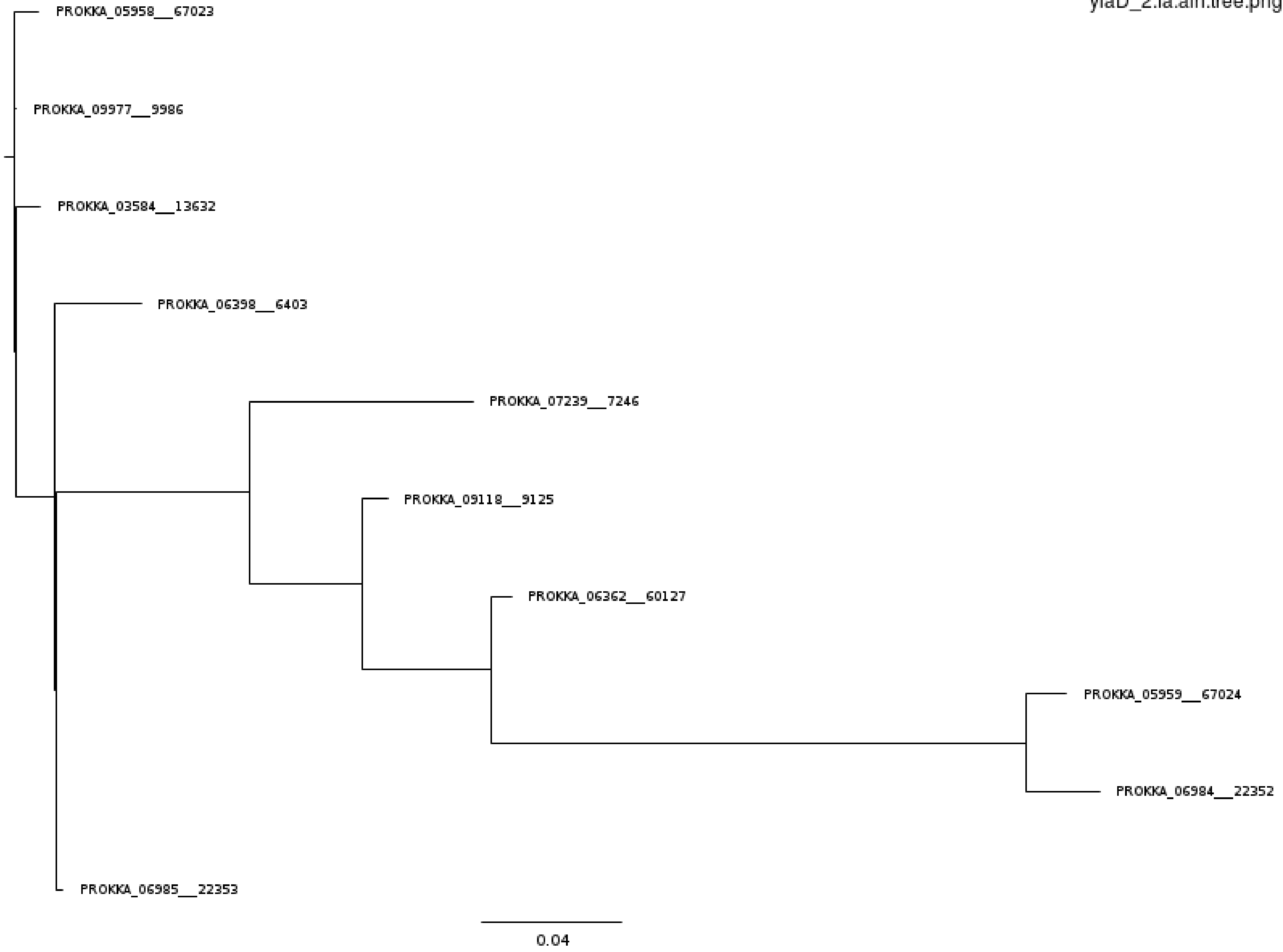

yidC.fa.aln.tree.png

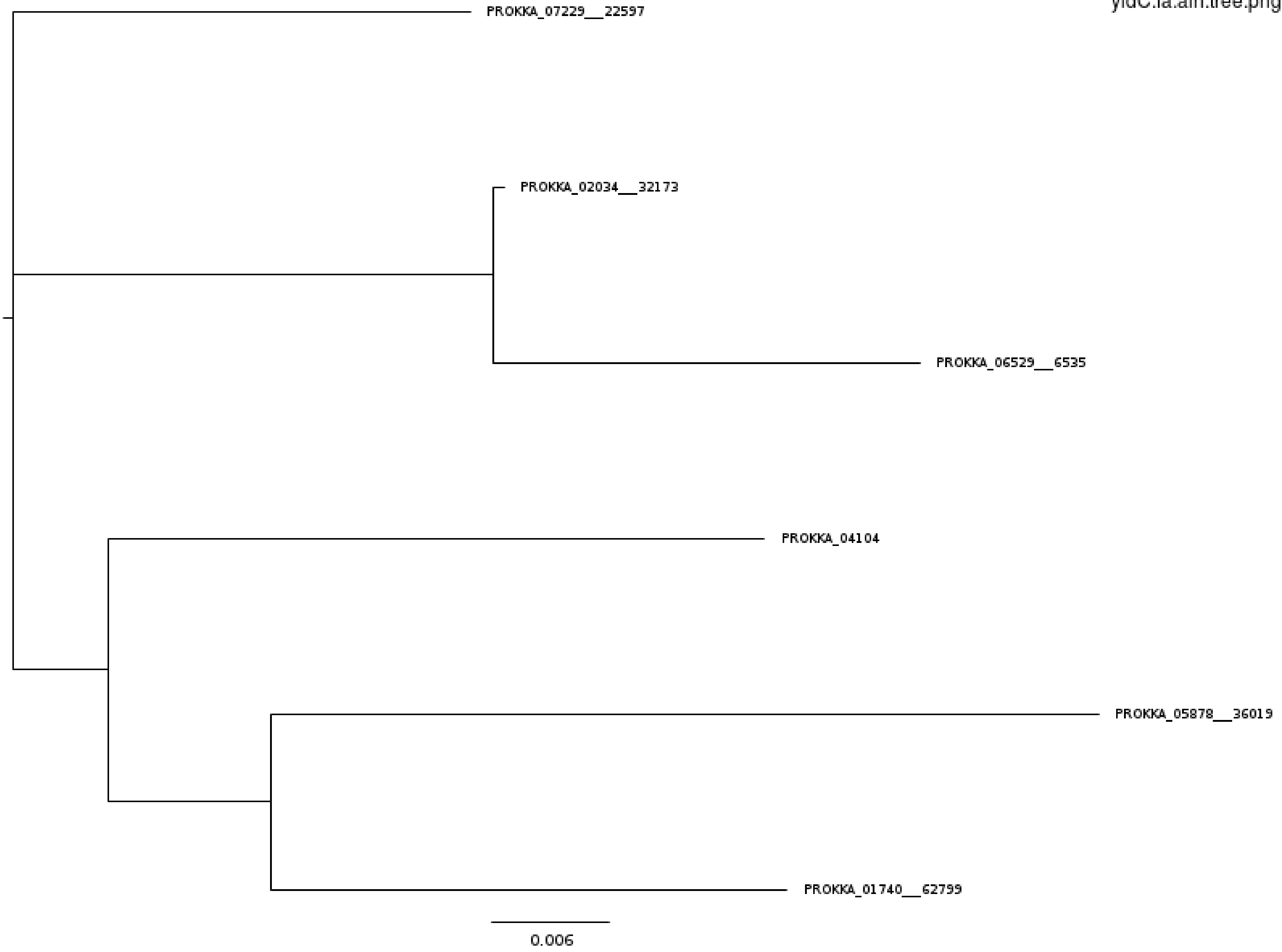

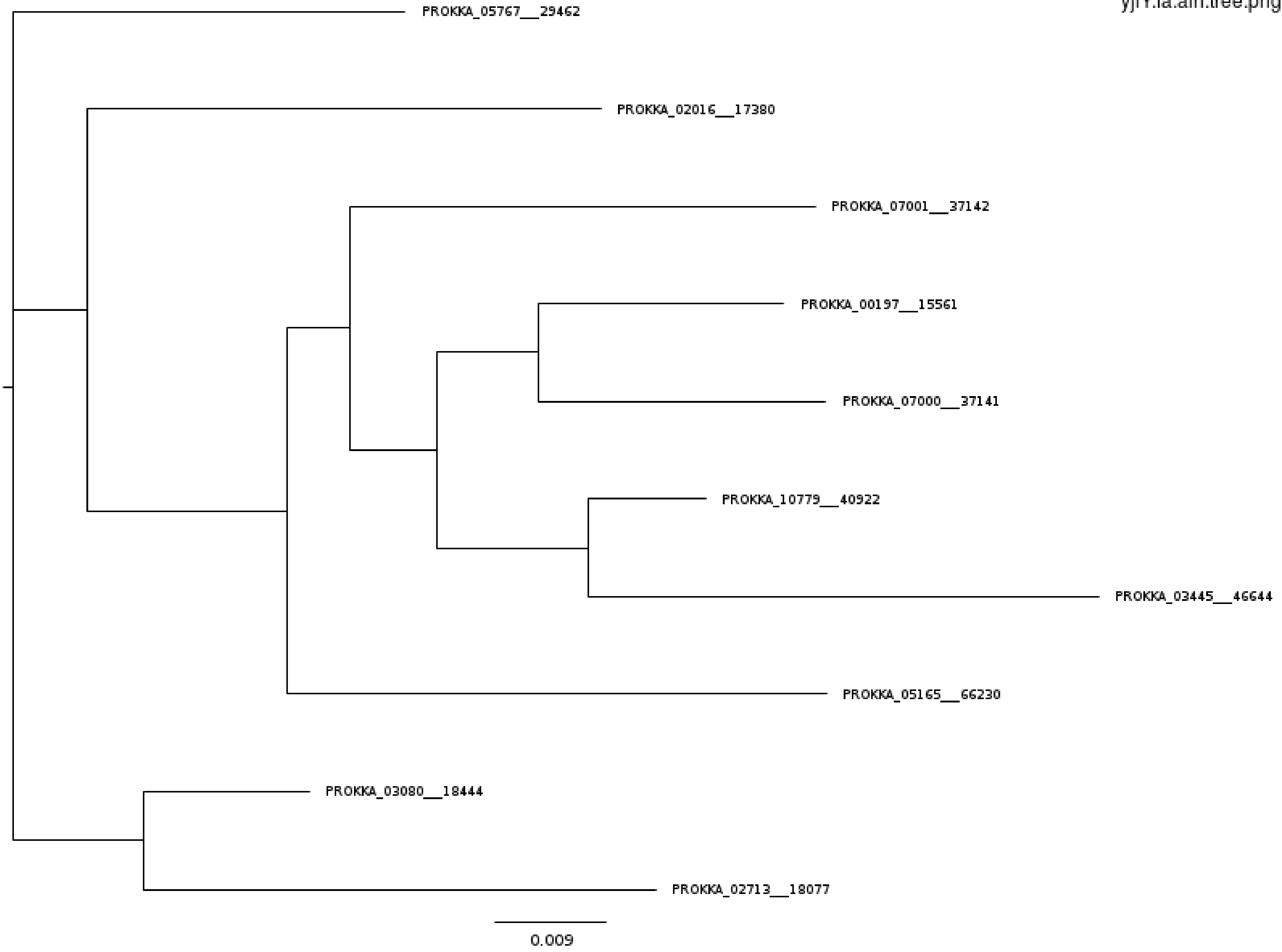
PROKKA_05767___29462
PROKKA_02016___17380
PROKKA_07001___37142
PROKKA_00197___15561
PROKKA_07000___37141
PROKKA_10779___40922
PROKKA_03445___46644
PROKKA_05165___66230
PROKKA_03080___18444
PROKKA_02713___18077
0.009

yjmD.fa.aln.tree.png

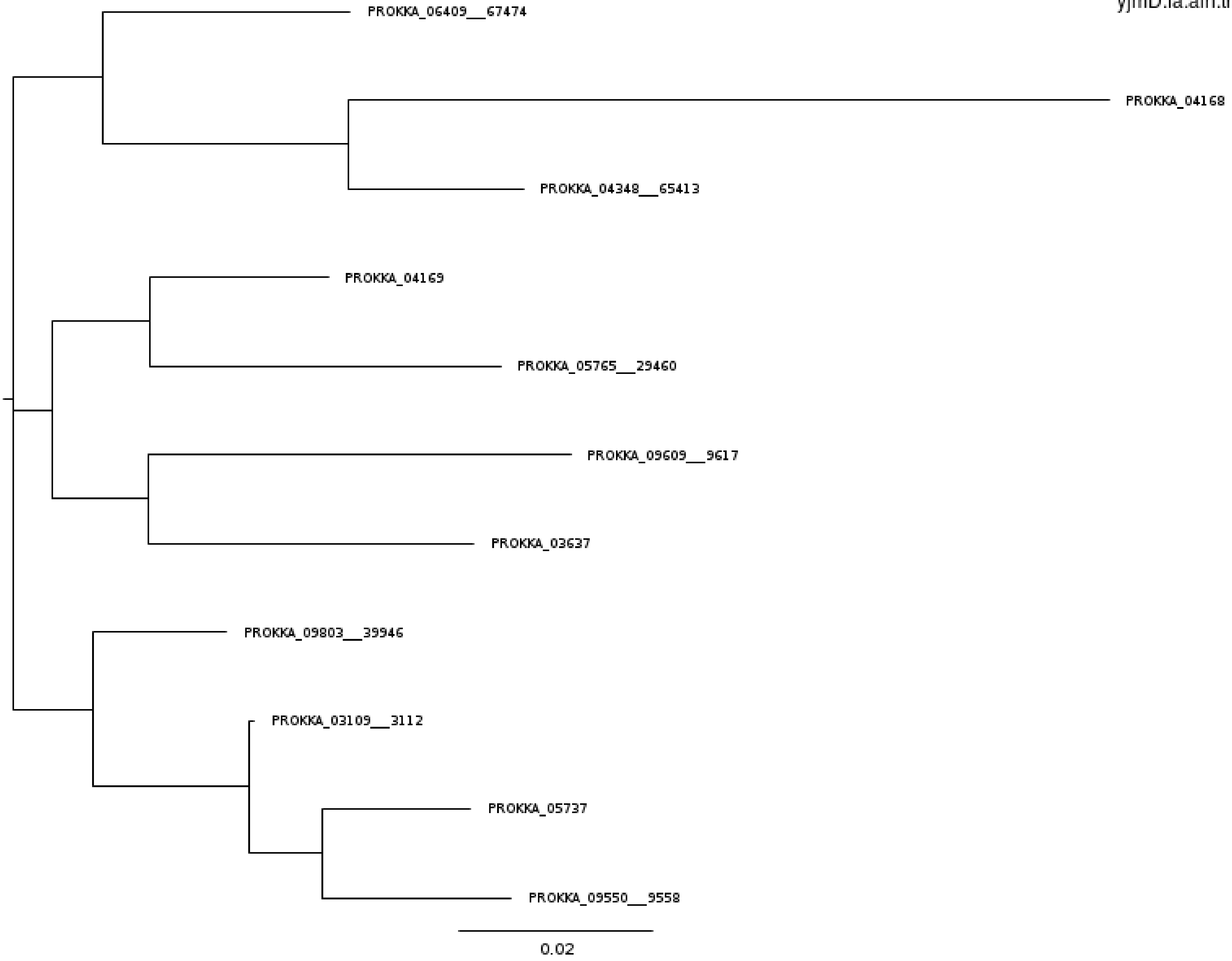

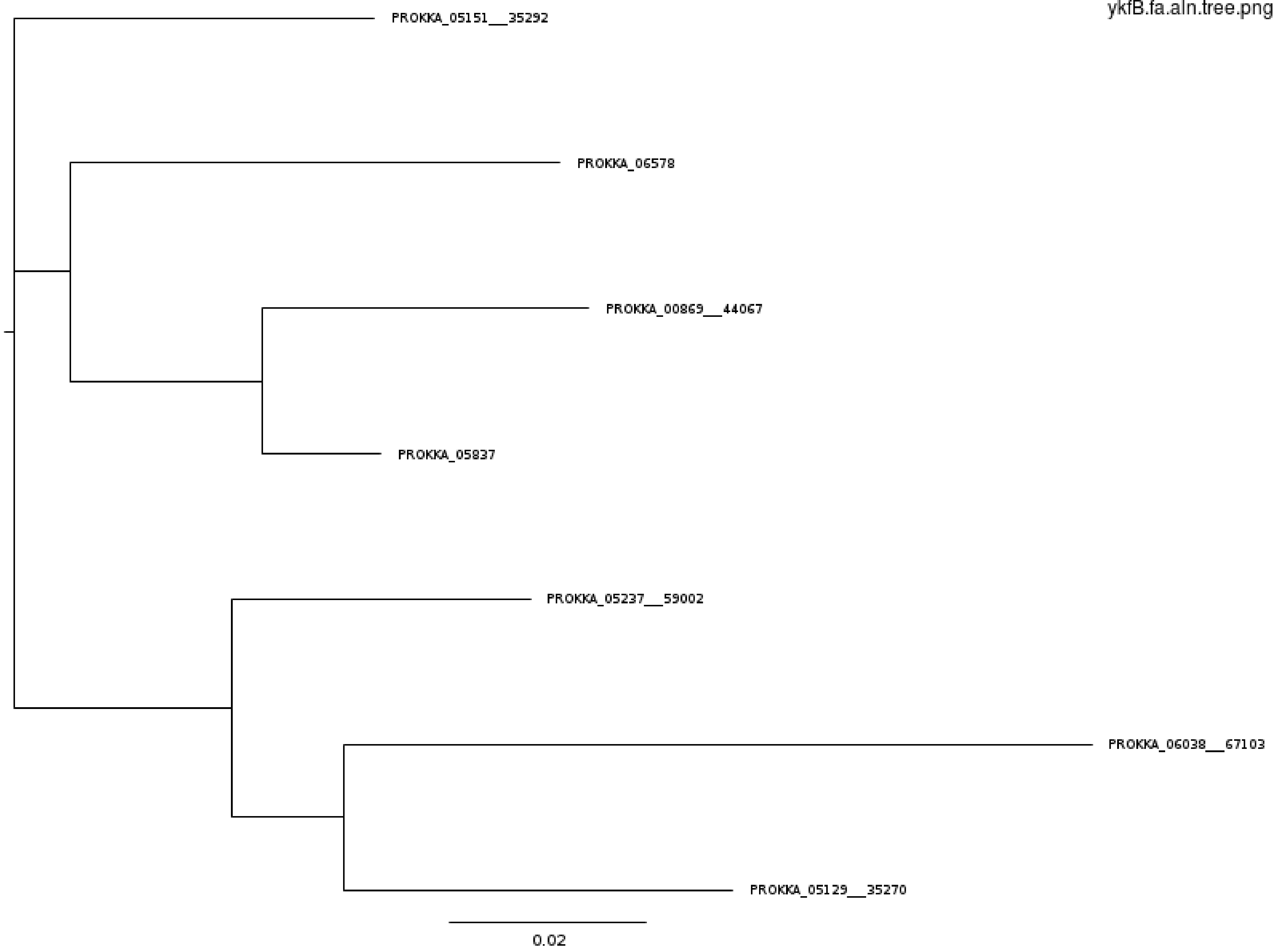

ykfB.fa.aln.tree.png
PROKKA_05151___35292
PROKKA_06578
PROKKA_00869___44067
PROKKA_05837
PROKKA_05237___59002
PROKKA_06038___67103
PROKKA_05129___35270
0.02

ykuD_2.fa.aln.tree.png

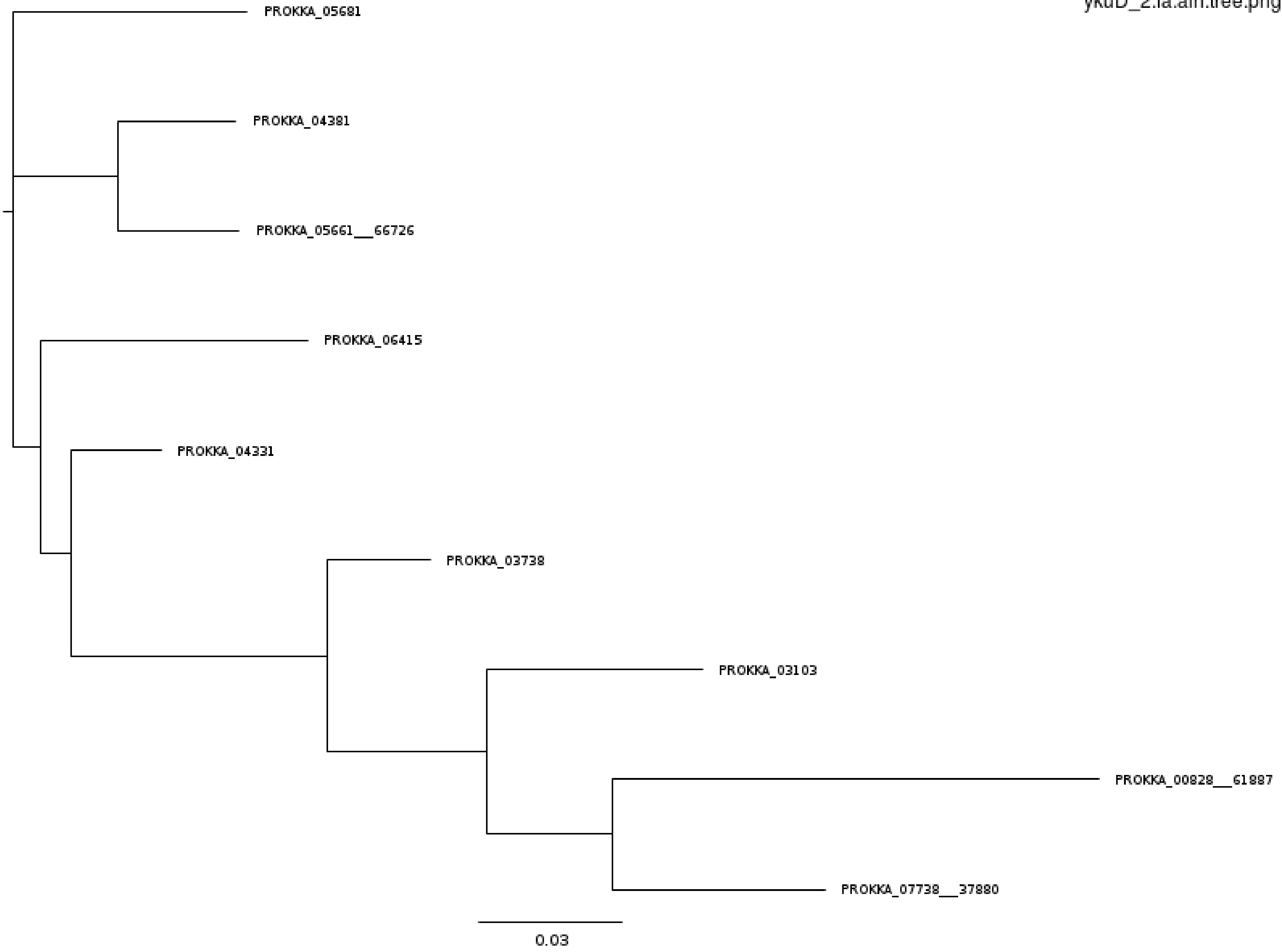

ylxH.fa.aln.tree.png

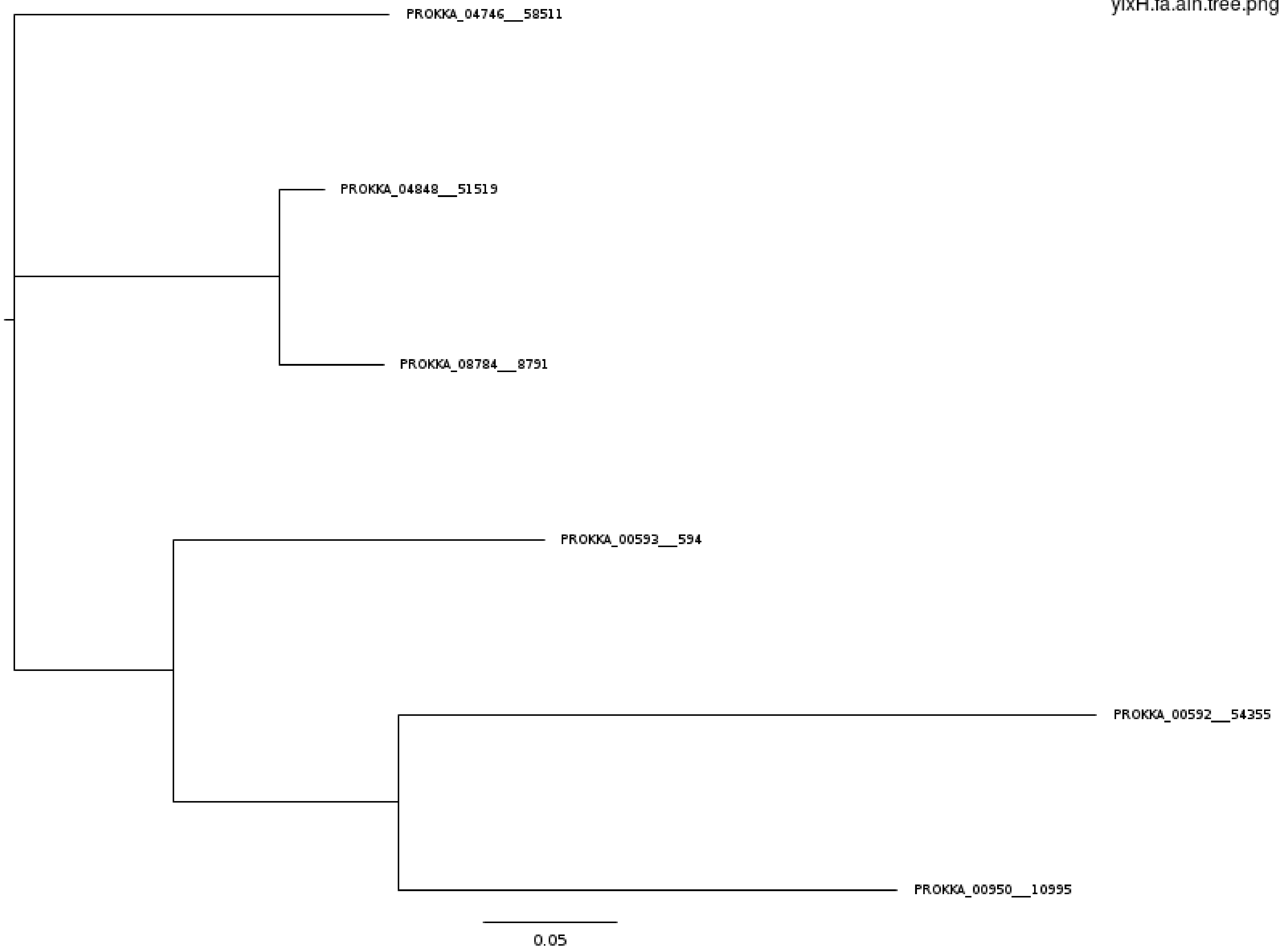

yoaH_1.fa.aln.tree.png

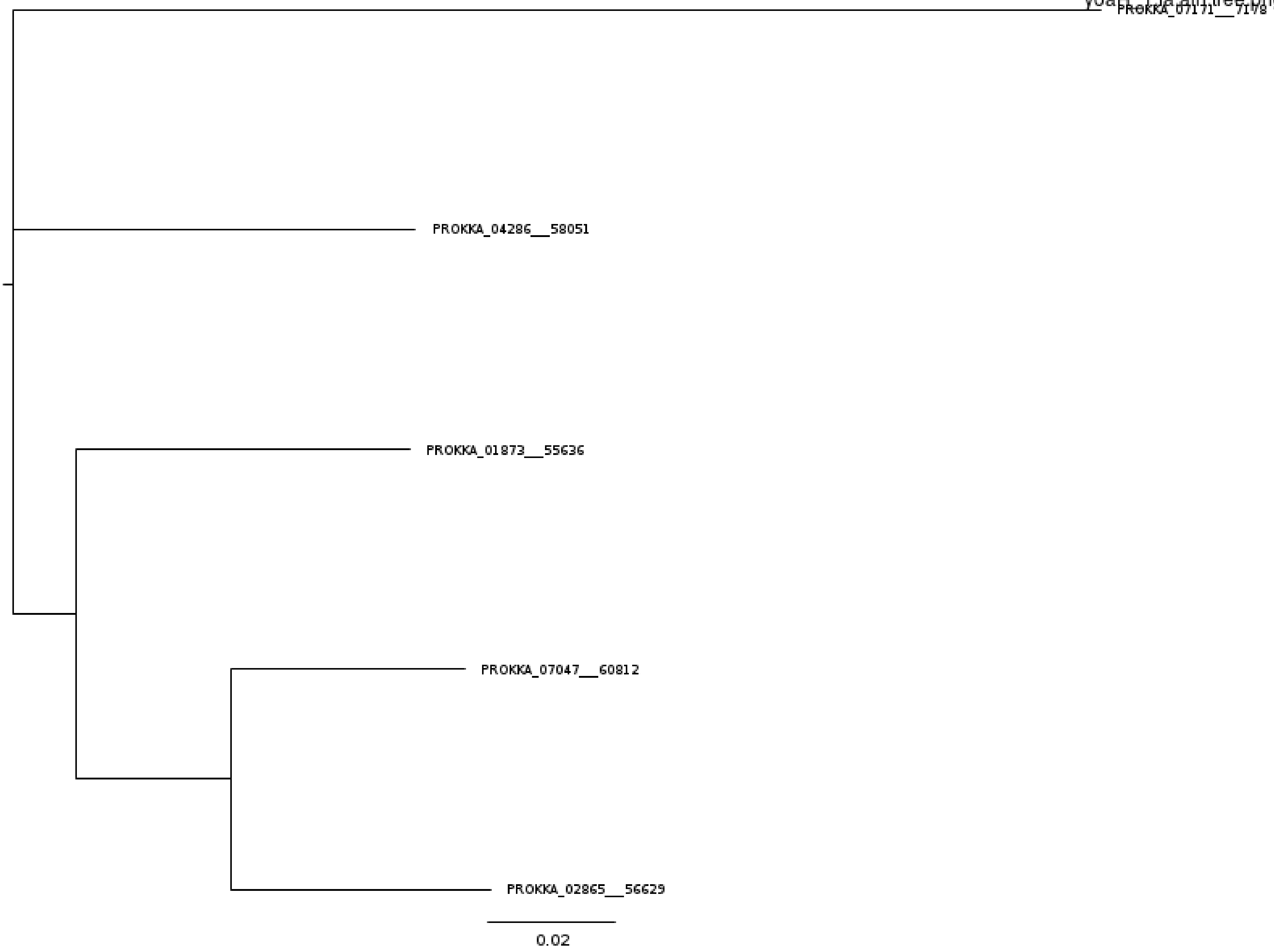

yqjA_2.fa.aln.tree.png

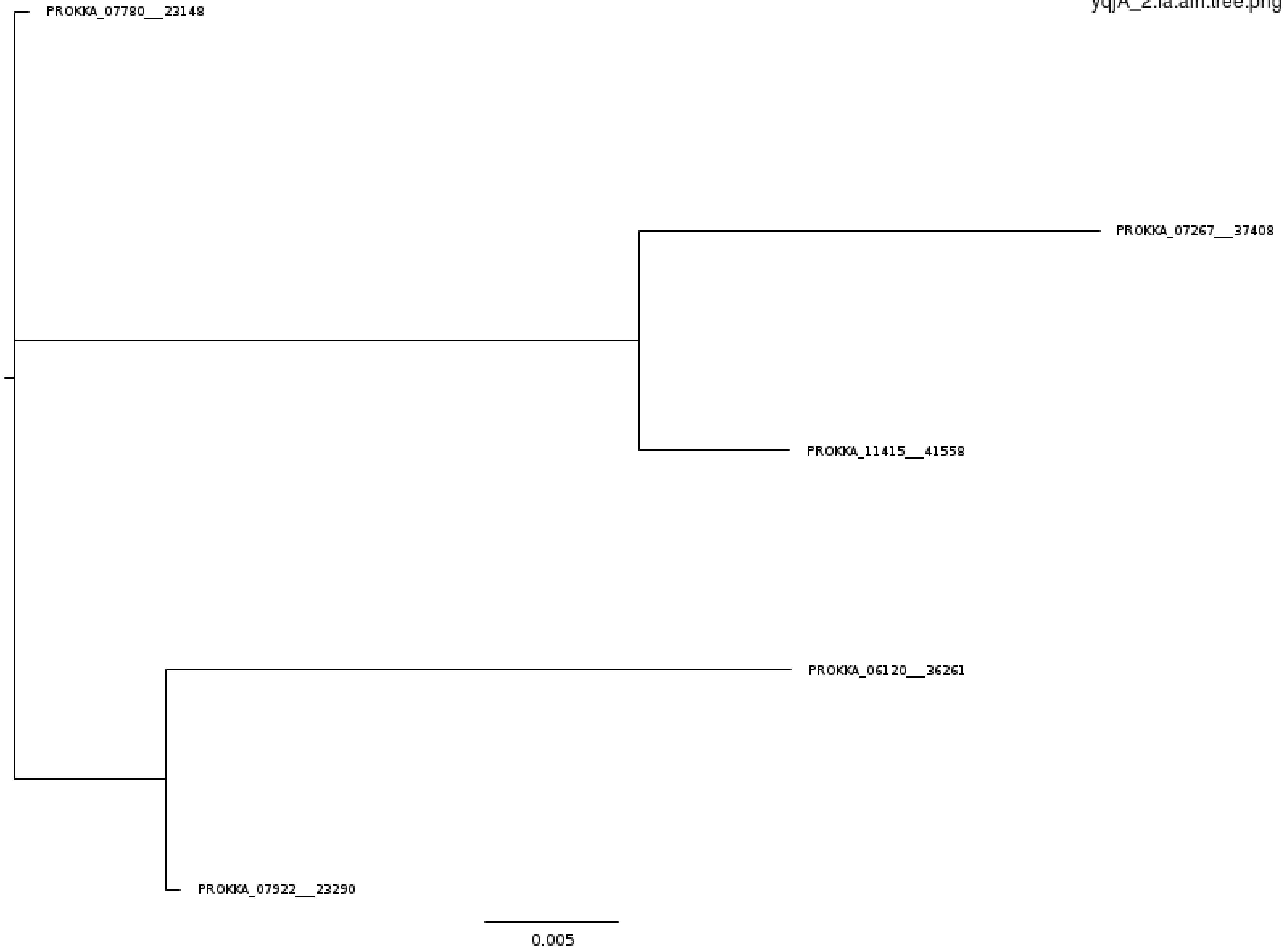

yrfG.fa.aln.tree.png

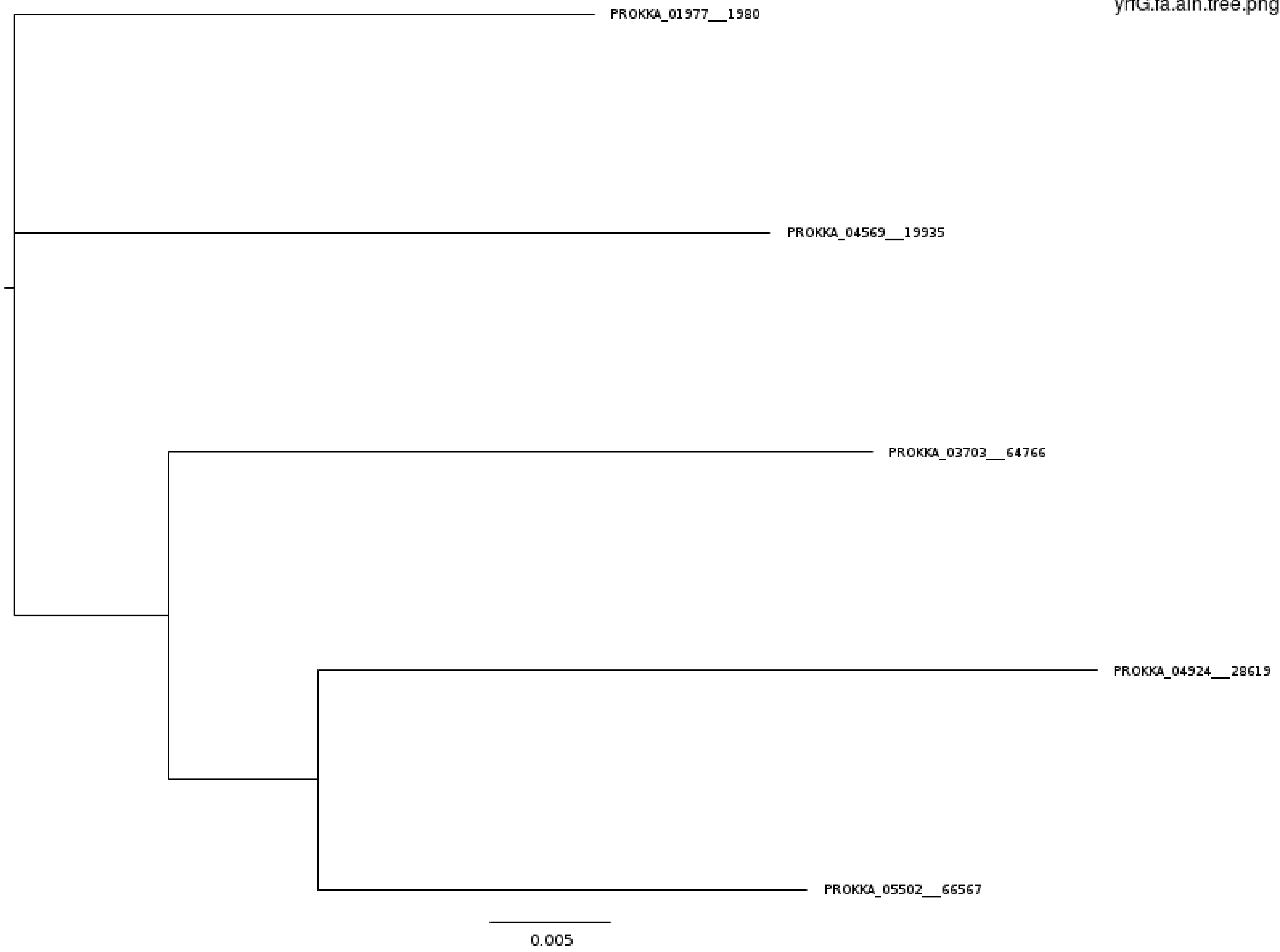

ytrE.fa.aln.tree.png

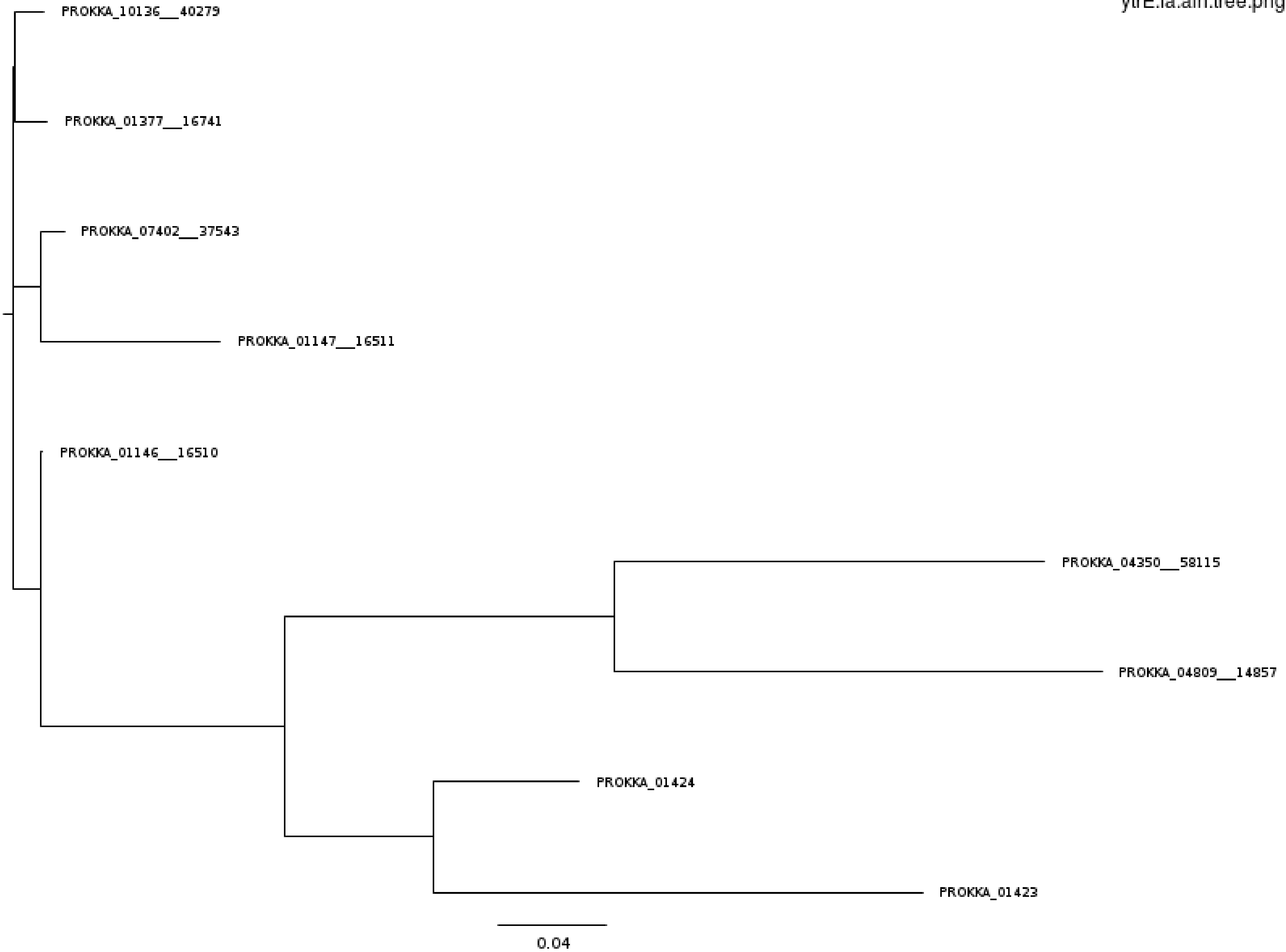

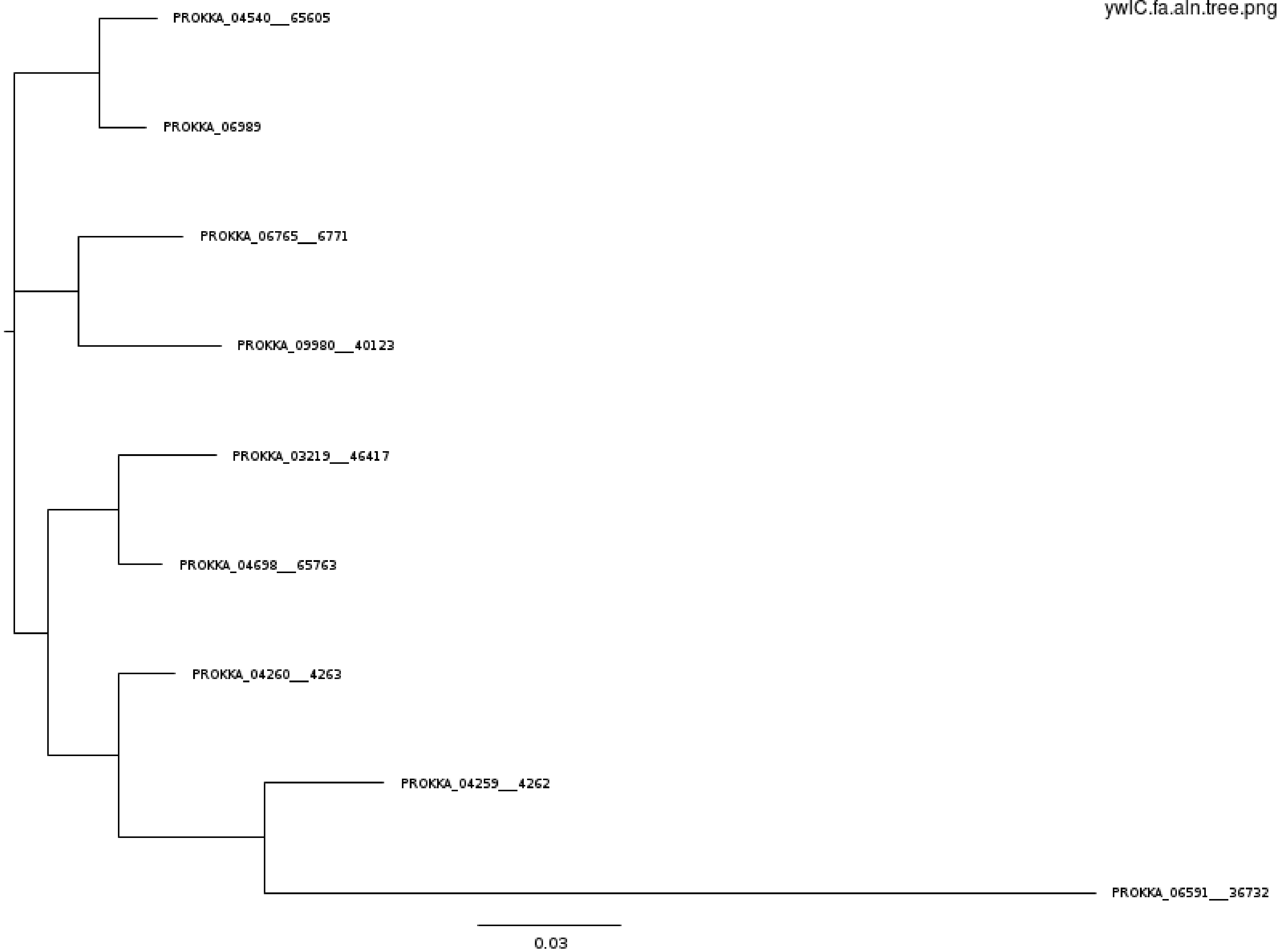
ywlC.fa.aln.tree.png
PROKKA_04540___65605
PROKKA_06989
PROKKA_06765___6771
PROKKA_09980___40123
PROKKA_03219___46417
PROKKA_04698___65763
PROKKA_04260___4263
PROKKA_04259___4262
PROKKA_06591___36732
0.03

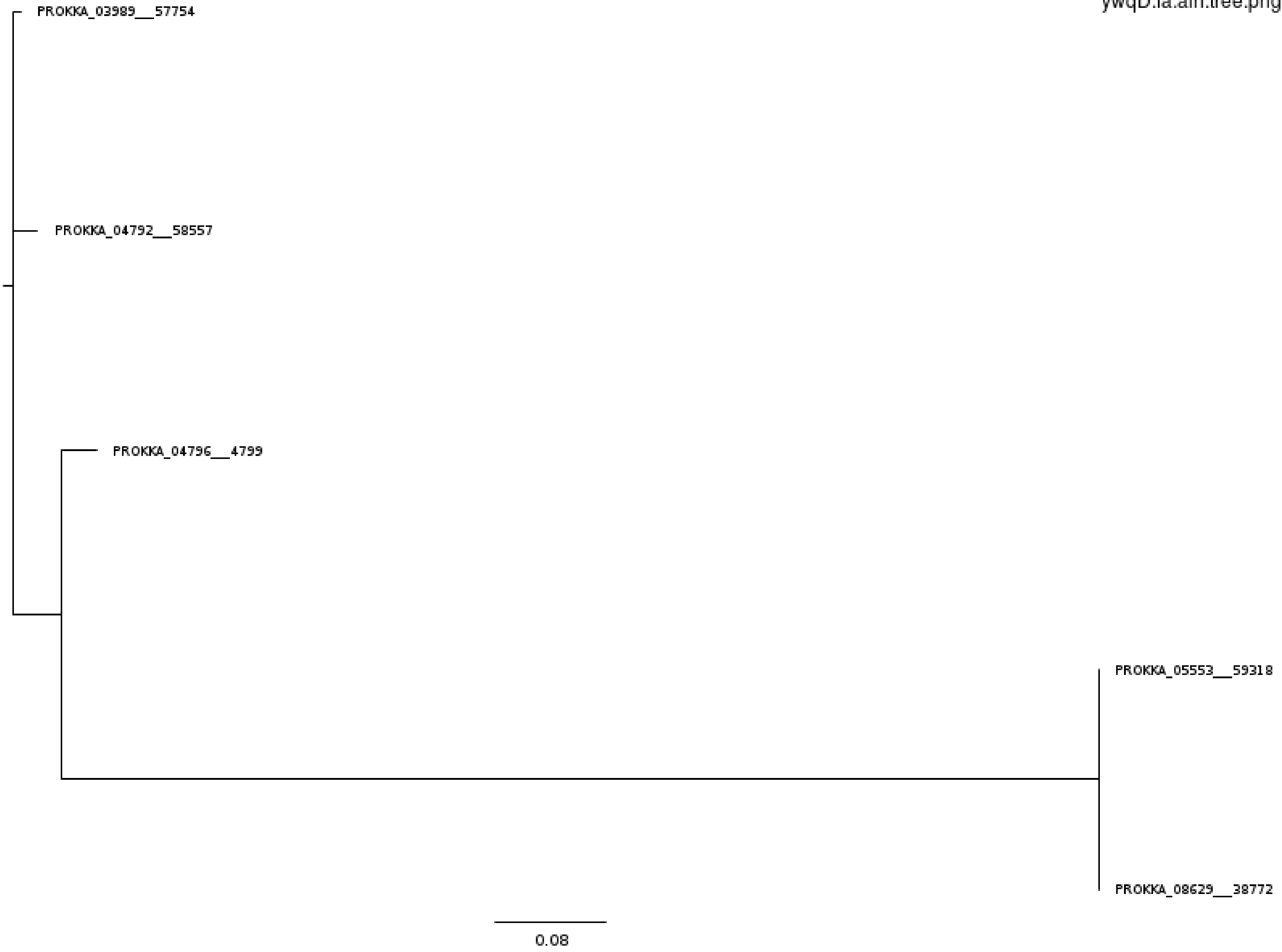
PROKKA_03989___57754
PROKKA_04792___58557
PROKKA_04796___4799
PROKKA_05553___59318
PROKKA_08629___38772
0.08

zapA.fa.aln.tree.png

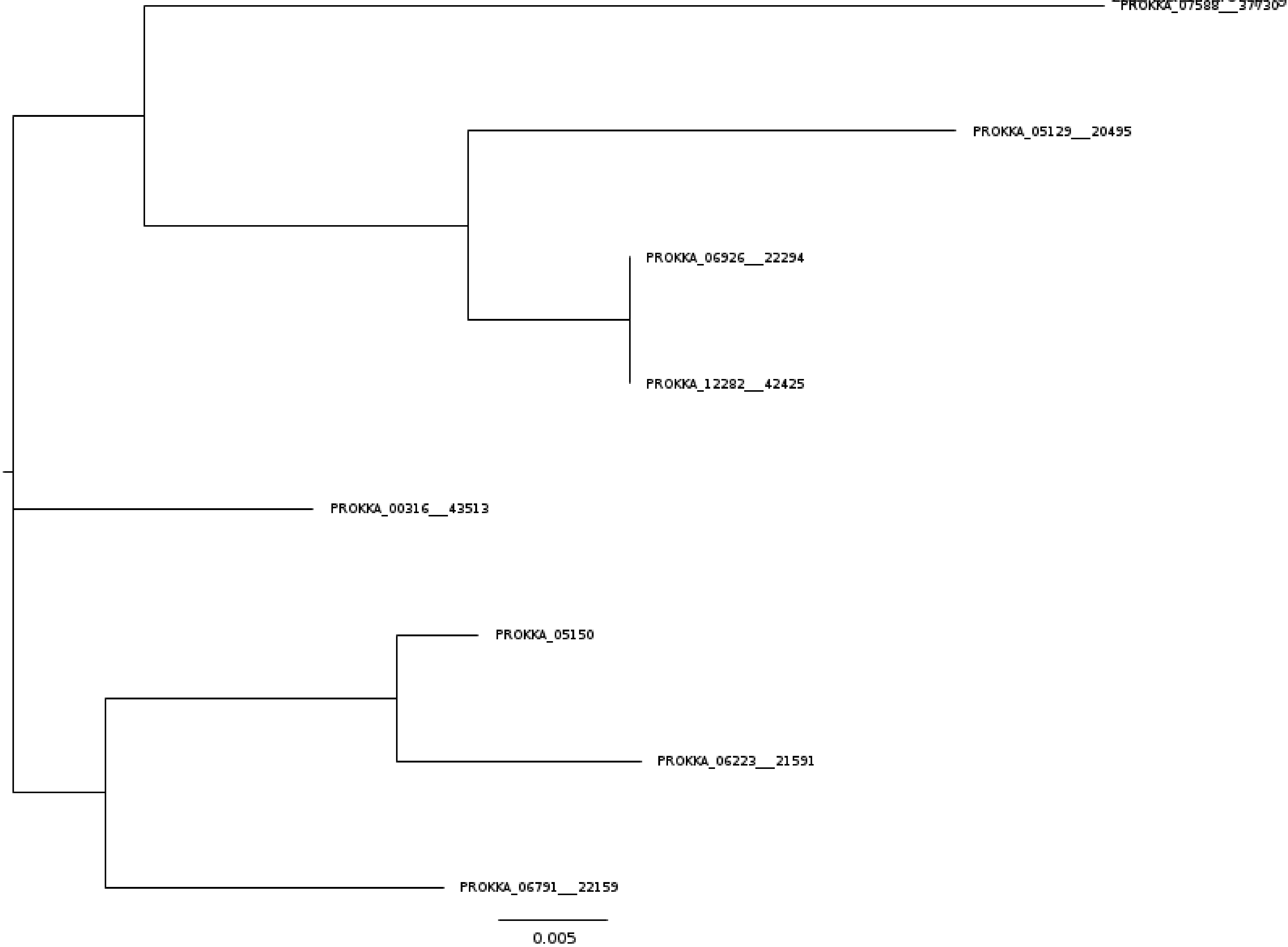

zapD.fa.aln.tree.png

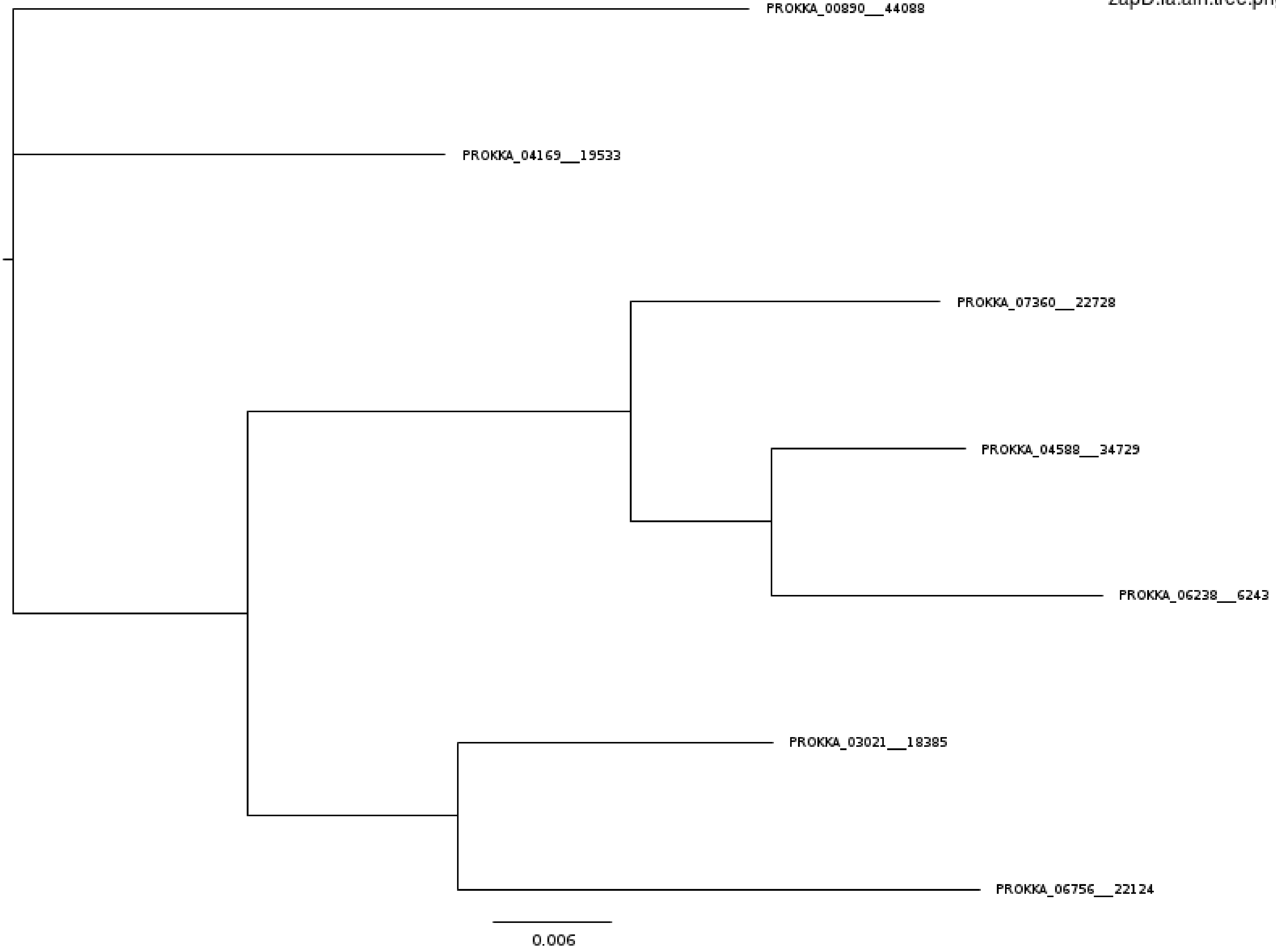

zipA_1.fa.aln.tree.png

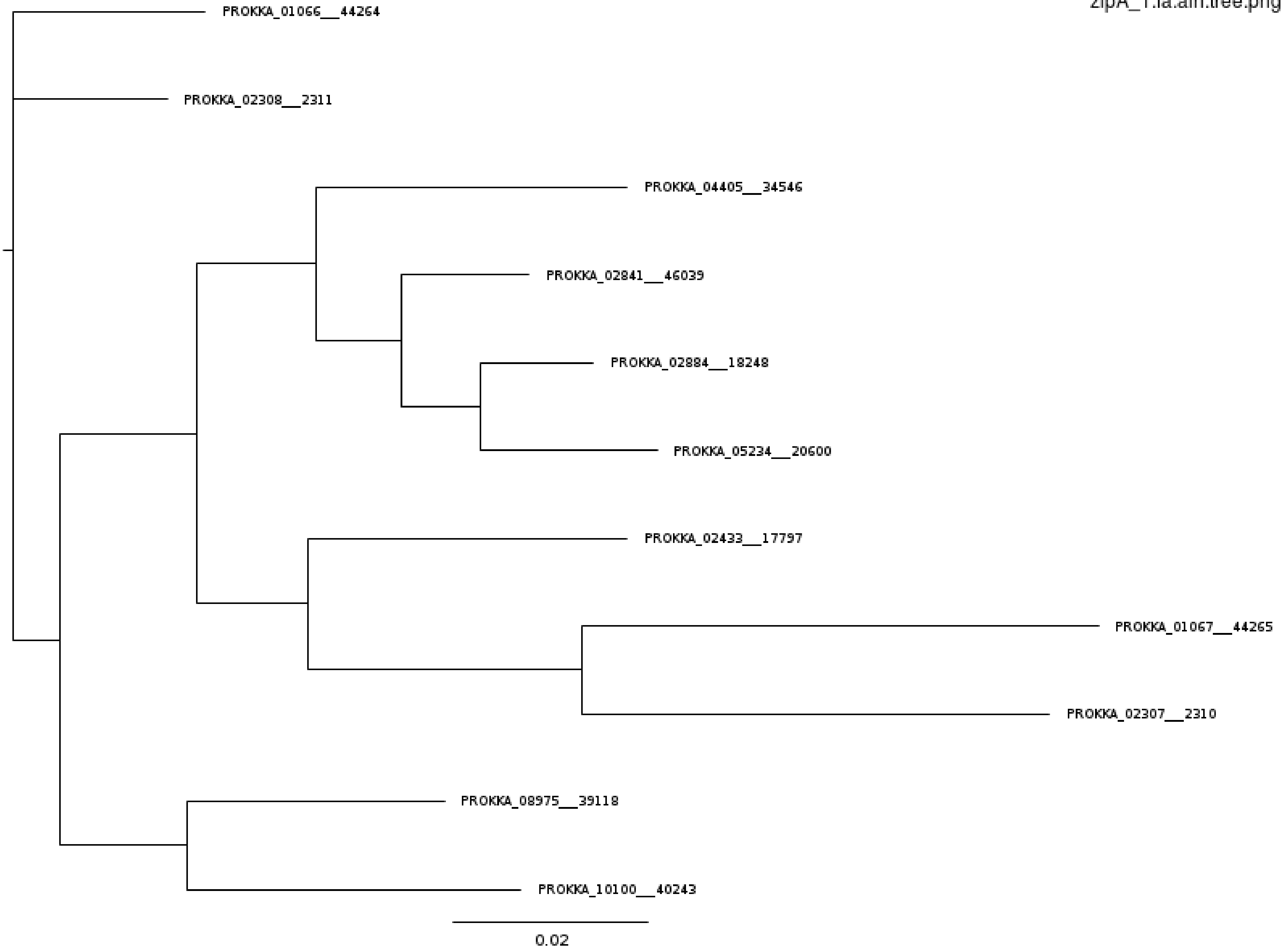

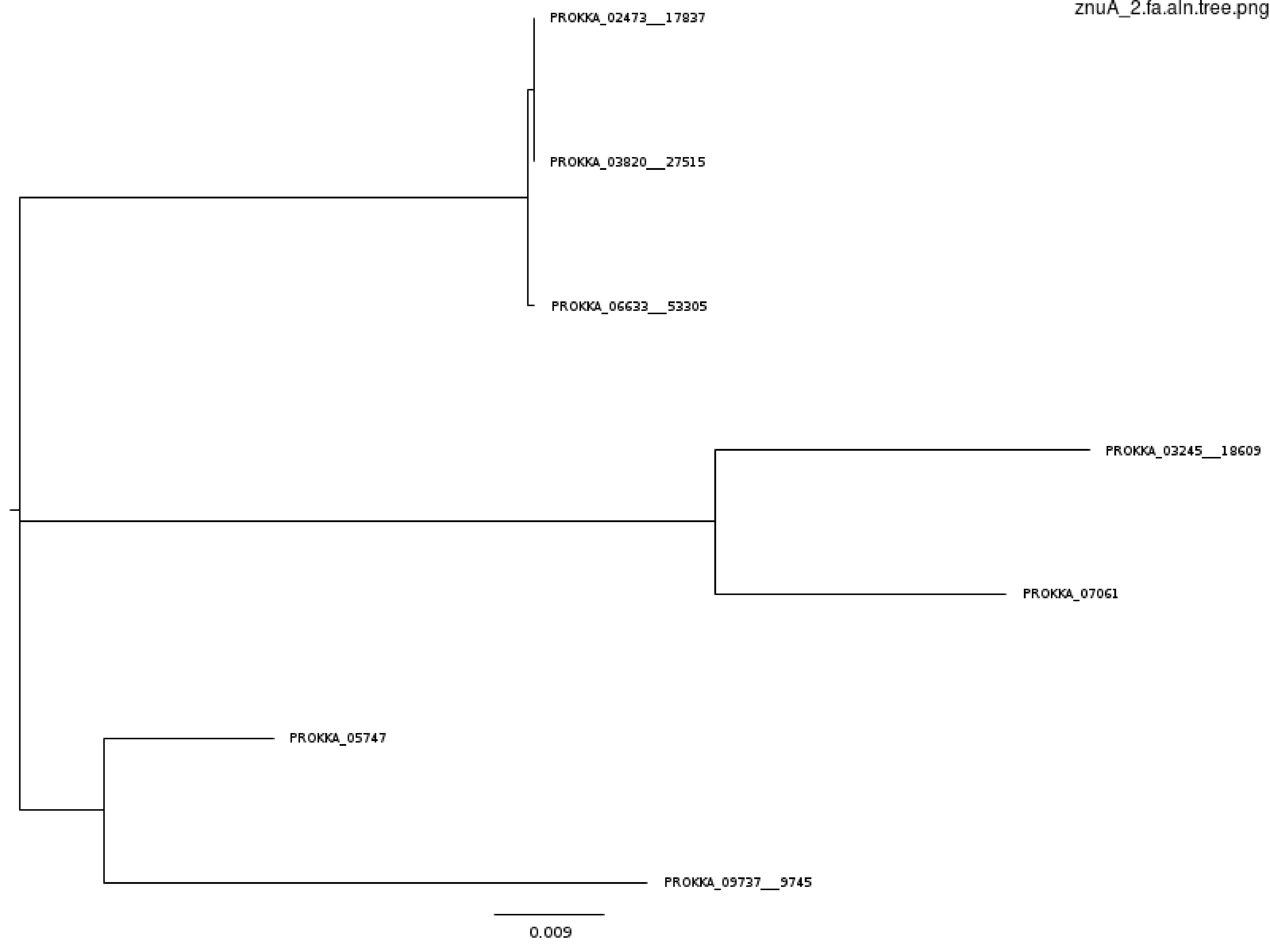
znuA_2.fa.aln.tree.png
PROKKA_02473___17837
PROKKA_03820___27515
PROKKA_06633___53305
PROKKA_03245___18609
PROKKA_07061
PROKKA_05747
PROKKA_09737___9745
0.009

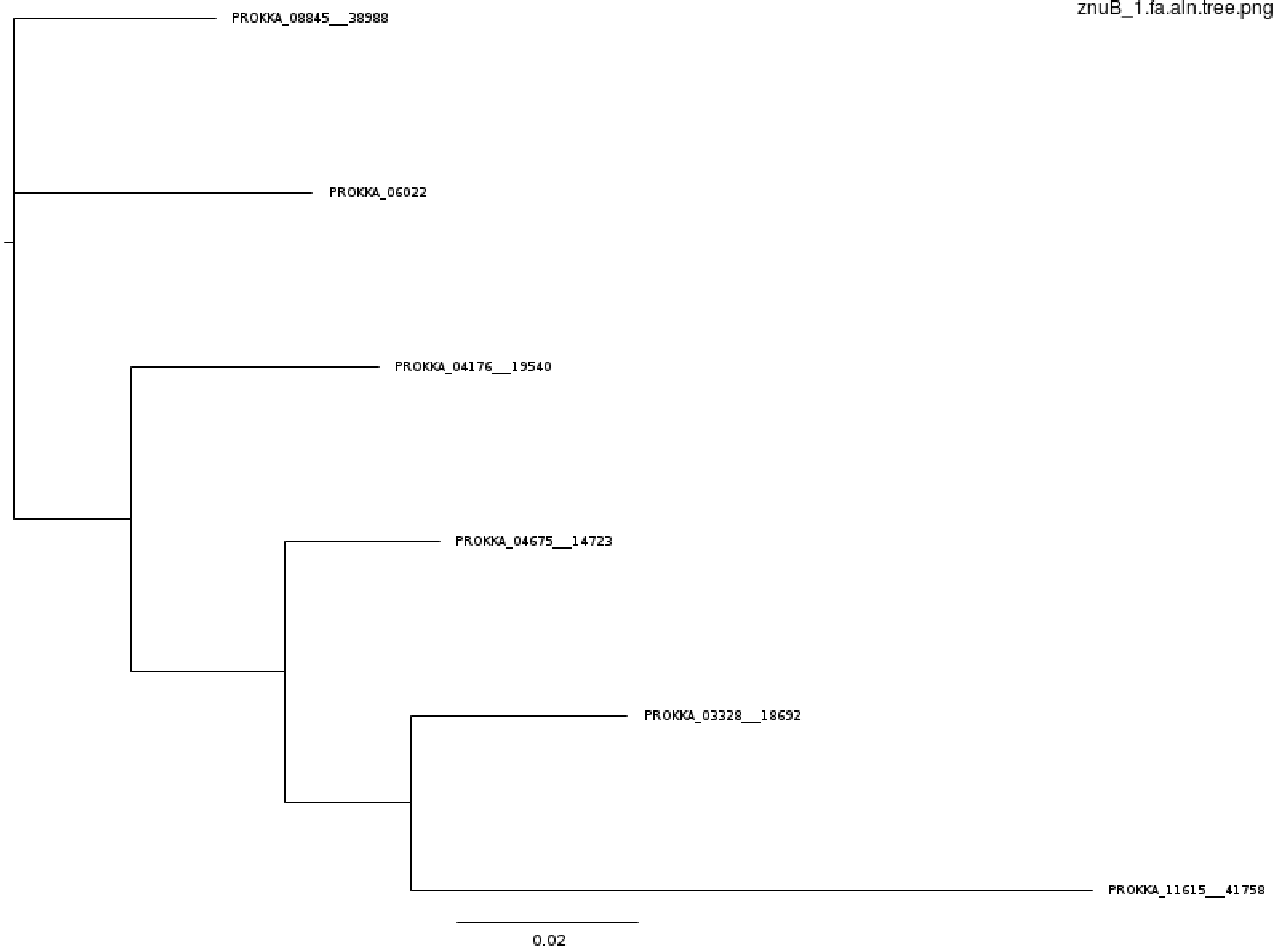

znuB_1.fa.aln.tree.png
PROKKA_08845___38988
PROKKA_06022
PROKKA_04176___19540
PROKKA_04675___14723
PROKKA_03328___18692
PROKKA_11615___41758
0.02

znuC_1.fa.aln.tree.png

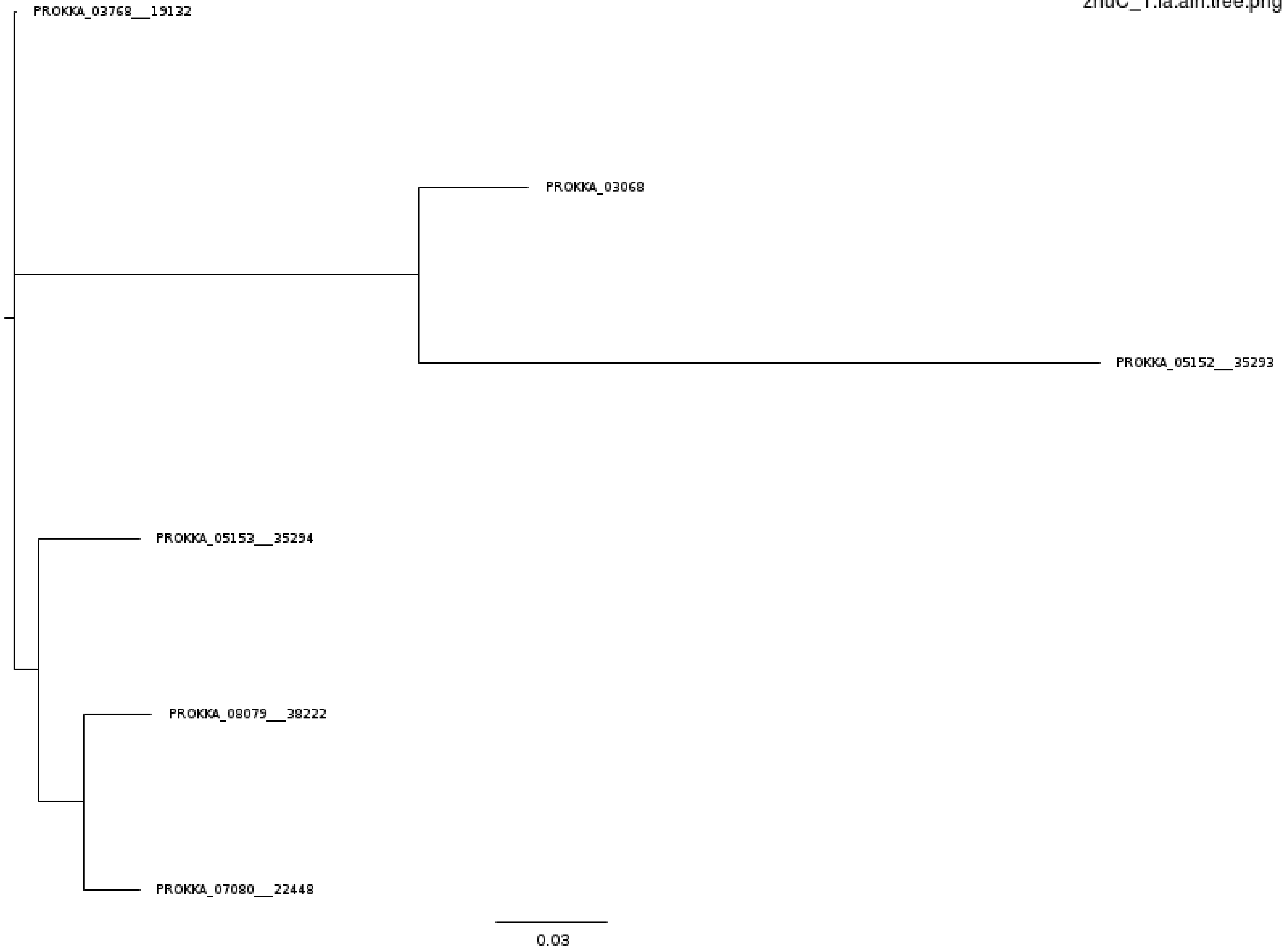

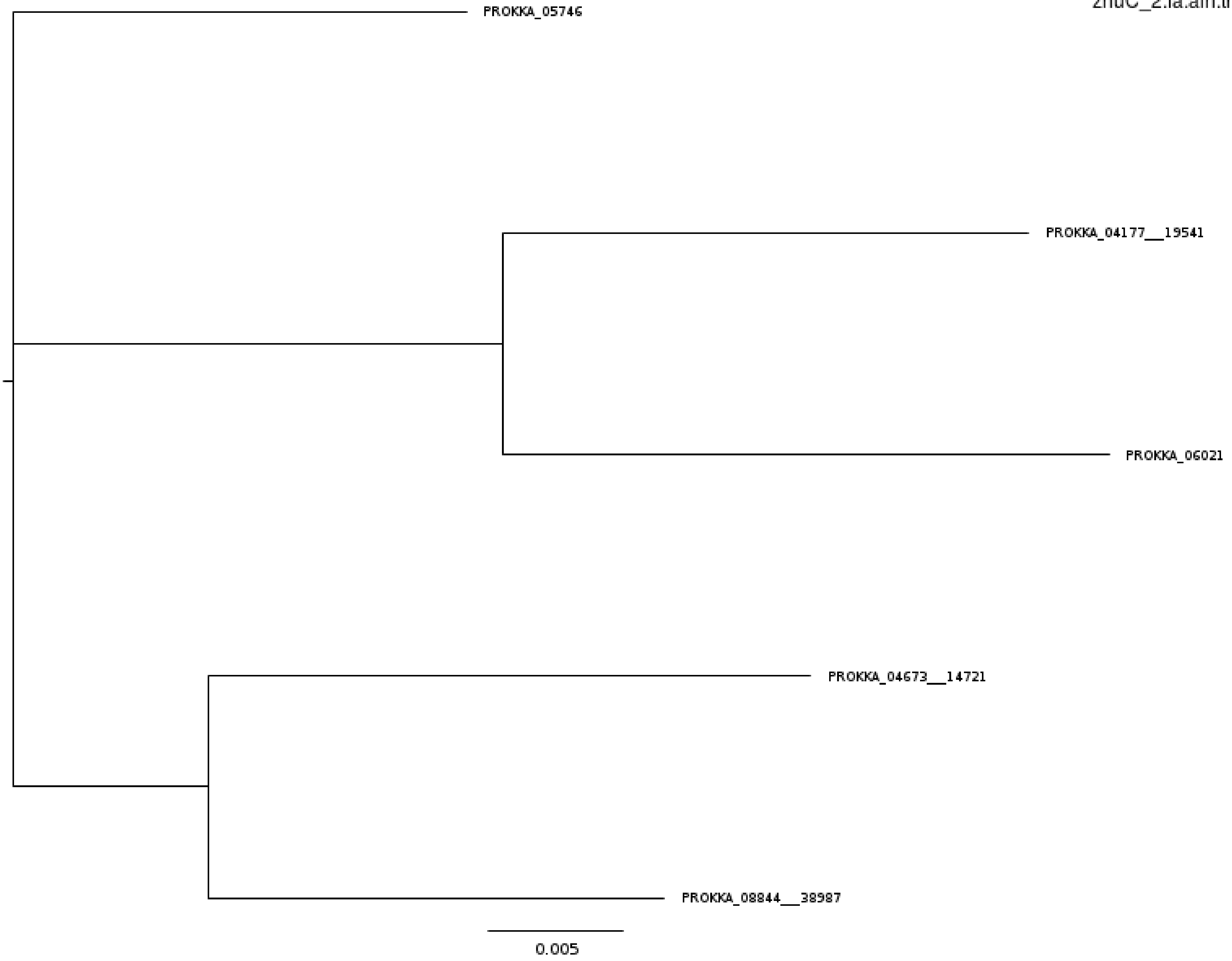
PROKKA_05746
PROKKA_04177___19541
PROKKA_06021
PROKKA_04673___14721
PROKKA_08844___38987
0.005

zraR_1.fa.aln.tree.png

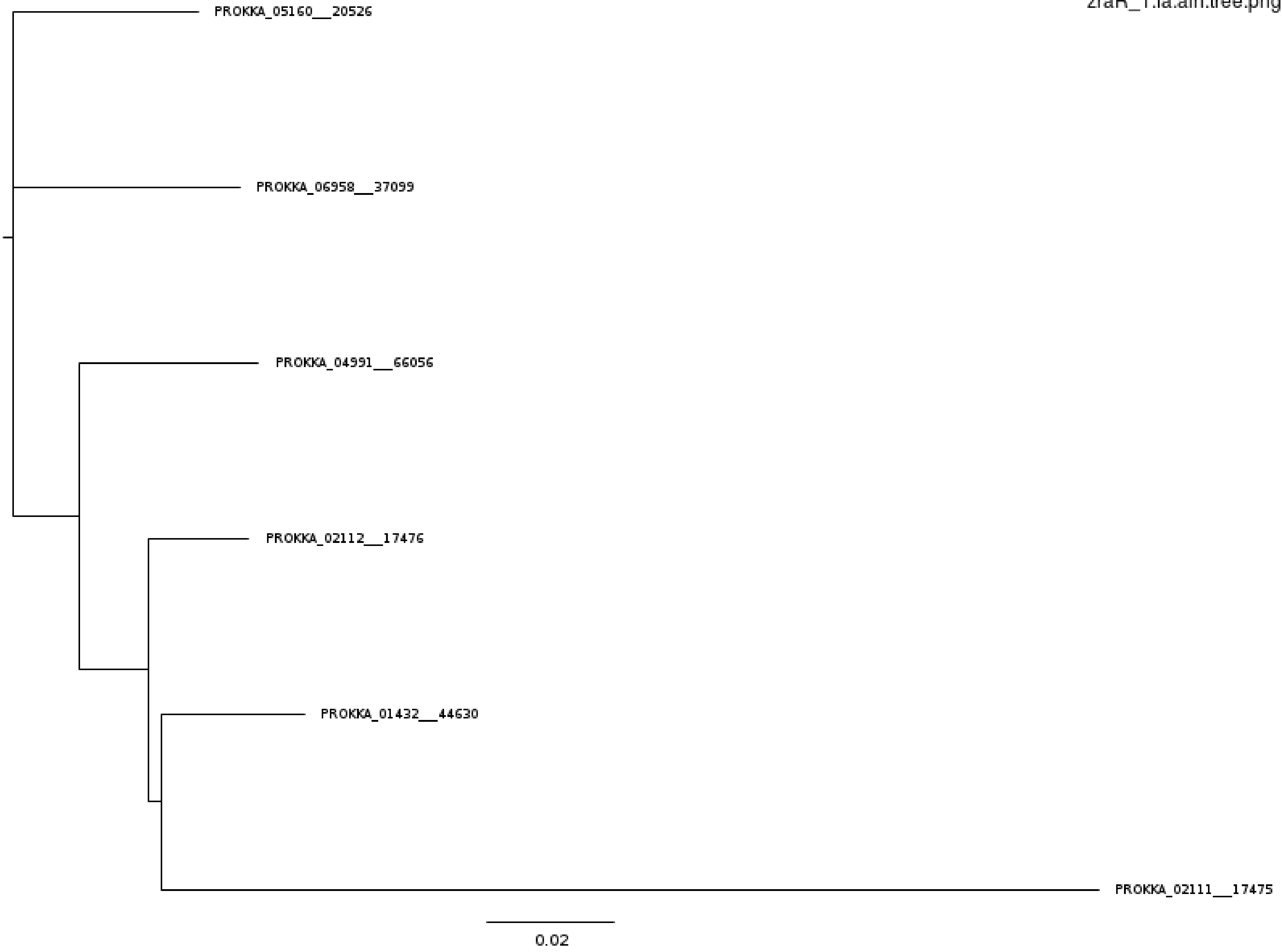

zraS_4.fa.aln.tree.png

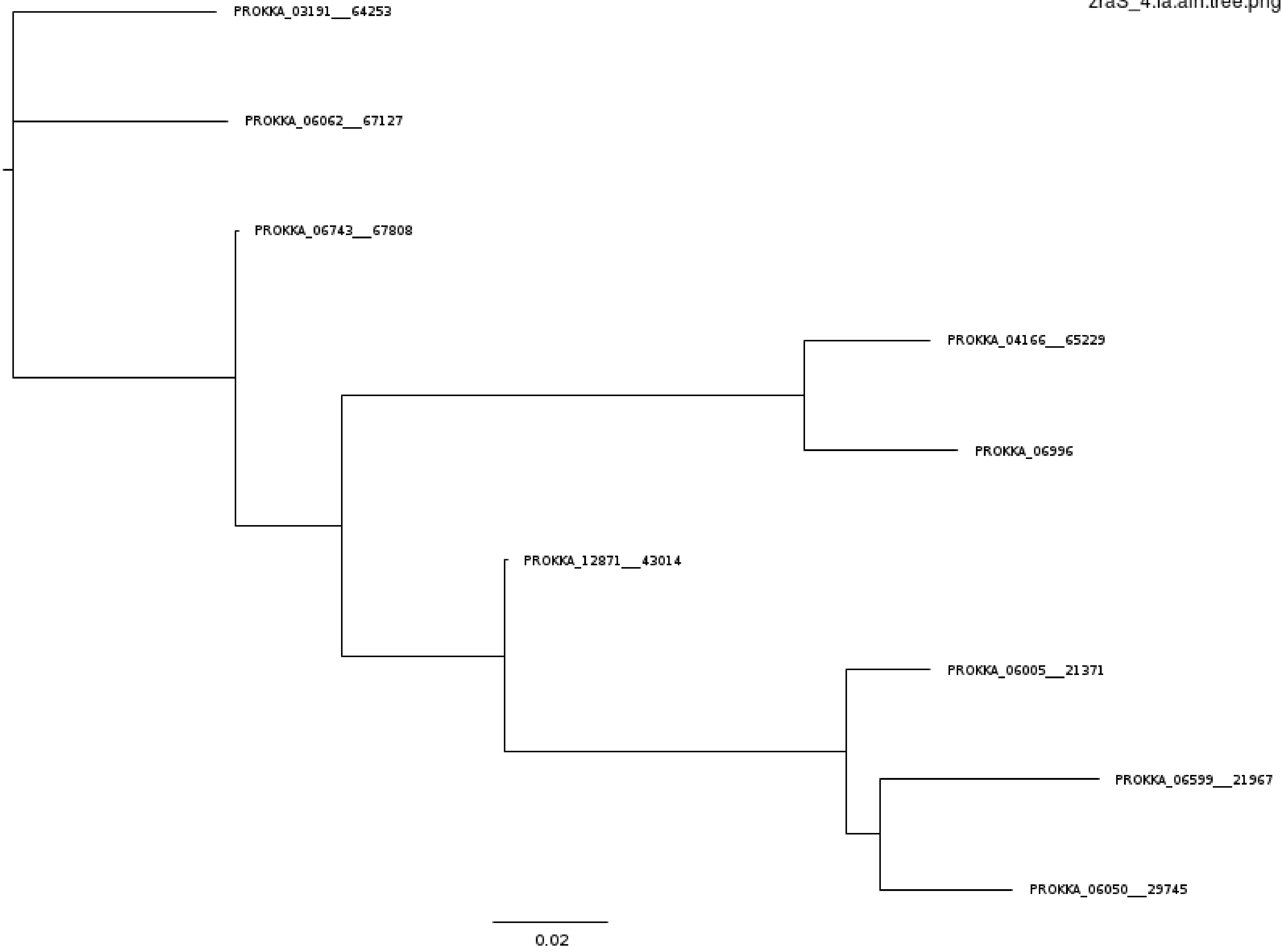

Supplement: Supplementary file 7 — Supplementary Data 6 [file 41467_2017_342_MOESM7_ESM.pdf]
